# Supplementary material for: Exploring online consumer behavior on fraudulent energy-saving products
Source: Sci Rep. 2024 Jun 21;14:14304. doi: 10.1038/s41598-024-65210-1 (PMC11192901; doi:10.1038/s41598-024-65210-1)
Supplement: Supplementary file 4 — Supplementary Table 4. [file 41598_2024_65210_MOESM4_ESM.pdf]

Store Name	Product Name	Review	Source
Xindu Flagship Store	Green Intelligent Power Saver	I've bought it for the third time and it really works, I gave one to a friend and she really liked it and said it really works, so I bought another one to give to my family.	Taobao Marketplace, a Chinese website for online shopping
Xindu Flagship Store	Green Intelligent Power Saver	Saving Electricity. Winter is more costly, the use of energy-saving appliances can save electricity. Effective. After only ten or so days of using the saver, there is a clear	Taobao Marketplace, a Chinese website for online shopping
Xindu Flagship Store	Green Intelligent Power Saver	The quality of the saver is good, the material can be well made. Easy to use.	Taobao Marketplace, a Chinese website for online shopping
Xindu Flagship Store	Green Intelligent Power Saver	Power saving is very good cost-effective good appearance good workmanship easy to use	Taobao Marketplace, a Chinese website for online shopping
Xindu Flagship Store	Green Intelligent Power Saver	Energy-saving and power-saving cost-effective good appearance and fine workmanship material is very good	Taobao Marketplace, a Chinese website for online shopping
Xindu Flagship Store	Green Intelligent Power Saver	Great power savings, very happy with the purchase!	Taobao Marketplace, a Chinese website for online shopping
Xindu Flagship Store	Green Intelligent Power Saver	Good-looking, easy to install, affordable logistics is very fast!	Taobao Marketplace, a Chinese website for online shopping
Xindu Flagship Store	Green Intelligent Power Saver	Great value for money, it does save a lot of power after using it for a while, superb!	Taobao Marketplace, a Chinese website for online shopping
Xindu Flagship Store	Green Intelligent Power Saver	It's okay, it's still working. Keep using it and see.	Taobao Marketplace, a Chinese website for online shopping
Xindu Flagship Store	Green Intelligent Power Saver	Logistics is very strong delivery speed, customer service attitude is very good to ask questions and answer praise!	Taobao Marketplace, a Chinese website for online shopping
Xindu Flagship Store	Green Intelligent Power Saver	Very good quality, fine workmanship, very much like it!	Taobao Marketplace, a Chinese website for online shopping
Xindu Flagship Store	Green Intelligent Power Saver	Received immediately plugged in, or very save electricity, outside electricity is too expensive, save a little better.	Taobao Marketplace, a Chinese website for online shopping
Xindu Flagship Store	Green Intelligent Power Saver	It really works.	Taobao Marketplace, a Chinese website for online shopping
Xindu Flagship Store	Green Intelligent Power Saver	The baby is great, and I have to say, the customer service is too good to be true!	Taobao Marketplace, a Chinese website for online shopping
Xindu Flagship Store	Green Intelligent Power Saver	It's affordable, it works, it's recommended, it works.	Taobao Marketplace, a Chinese website for online shopping
Xindu Flagship Store	Green Intelligent Power Saver	I really recommend it, the delivery was exceptionally fast, love love love it!	Taobao Marketplace, a Chinese website for online shopping
Xindu Flagship Store	Green Intelligent Power Saver	Compact appearance, the quality of workmanship is quite solid, the response is also quite sensitive, change a few sockets are very easy to unplug, quite a good product!	Taobao Marketplace, a Chinese website for online shopping
Xindu Flagship Store	Green Intelligent Power Saver	Have used it and it works well!	Taobao Marketplace, a Chinese website for online shopping
Xindu Flagship Store	Green Intelligent Power Saver	Received, and the store described the same effect is very good, the next time will patronize the	Taobao Marketplace, a Chinese website for online shopping
Xindu Flagship Store	Green Intelligent Power Saver	Good quality, good price is worth buying, much cheaper than the physical store	Taobao Marketplace, a Chinese website for online shopping
Xindu Flagship Store	Green Intelligent Power Saver	The quality is very good, customer service attitude is very, very awesome, there are problems in a timely manner to solve the problem	Taobao Marketplace, a Chinese website for online shopping
Xindu Flagship Store	Green Intelligent Power Saver	Appearance is very delicate, has been plugged into the electricity, in a few days to see the effect, the effect is good - to give everyone recommended, look forward to the effect....	Taobao Marketplace, a Chinese website for online shopping
Xindu Flagship Store	Green Intelligent Power Saver	This is really good, the power saving effect is quite good, the store shipped fast, very satisfied!	Taobao Marketplace, a Chinese website for online shopping
Xindu Flagship Store	Green Intelligent Power Saver	The quality is really good, very satisfied with it, and the customer service is also very good!	Taobao Marketplace, a Chinese website for online shopping
Xindu Flagship Store	Green Intelligent Power Saver	Fast shipping, good quality, good reviews all 5 out of 5! The	Taobao Marketplace, a Chinese website for online shopping
Xindu Flagship Store	Green Intelligent Power Saver	Power saving effect: feel the effect after using . Normally more than 6 dollars a day, after using about 5 dollars a day. Cost-effective: not expensive Appearance style: satisfied Workmanship material: satisfied Use experience: also can continue to close the check, first give five-star praise.	Taobao Marketplace, a Chinese website for online shopping
Xindu Flagship Store	Green Intelligent Power Saver	Power saving effect: 1 very good, worth commission there! Cost-effective: 1 very affordable, compared to the high cost of electricity, this is very cost-effective, and buy 2 really get 1, it is worth recommending! Appearance Style: 1 square Workmanship Material: 1 raw rubber products Experience: 1 very great 👍 I hope you buy more, I do not have a relationship with the business! Is from the heart words.	Taobao Marketplace, a Chinese website for online shopping
Xindu Flagship Store	Green Intelligent Power Saver	Power saving effect: so far it is very OK Black technology energy saving power saving, can save a little electricity cost Cost-effective: cost-effective quite high Experience: easy to use, plug and play.	Taobao Marketplace, a Chinese website for online shopping
Xindu Flagship Store	Green Intelligent Power Saver	Just received the goods, not yet used, hope not to let me disappointed!	Taobao Marketplace, a Chinese website for online shopping
Xindu Flagship Store	Green Intelligent Power Saver	The packaging is very sturdy and the stuff is well protected, so it's worth a good review	Taobao Marketplace, a Chinese website for online shopping
Xindu Flagship Store	Green Intelligent Power Saver	I just don't know where the good reviews on this come from. Do you have a conscience?	Taobao Marketplace, a Chinese website for online shopping
Xindu Flagship Store	Green Intelligent Power Saver	The voltage at home has been unstable, I heard that this can stabilize the voltage and save power, let's see the effect in the future.	Taobao Marketplace, a Chinese website for online shopping
Xindu Flagship Store	Green Intelligent Power Saver	Power-saving effect: received almost 20 days, plugged in the effect is also very good Cost-effective: compared to other styles better Appearance style: beautiful and good-looking Workmanship material: very good quality 👍	Taobao Marketplace, a Chinese website for online shopping

Xindu Flagship Store	Green Intelligent Power Saver	Honestly, I've been using it for almost a month now and I haven't seen any results yet	Taobao Marketplace, a Chinese website for online shopping
Xindu Flagship Store	Green Intelligent Power Saver	Power-saving effect is good, fast logistics, beautiful appearance, next time to buy again, ask friends to buy too.	Taobao Marketplace, a Chinese website for online shopping
Xindu Flagship Store	Green Intelligent Power Saver	It does save energy.	Taobao Marketplace, a Chinese website for online shopping
Xindu Flagship Store	Green Intelligent Power Saver	It's all a lie to the people.	Taobao Marketplace, a Chinese website for online shopping
Xindu Flagship Store	Green Intelligent Power Saver	This is my cousin has been using it, he recommended it to me, I also bought it, the effect must be good, after a month to follow up reviews	Taobao Marketplace, a Chinese website for online shopping
Xindu Flagship Store	Green Intelligent Power Saver	Appearance and style: the appearance is very exquisite, small and convenient, to experience the effect of the	Taobao Marketplace, a Chinese website for online shopping
Xindu Flagship Store	Green Intelligent Power Saver	Very nice appearance, affordable price, good quality, like friends can buy	Taobao Marketplace, a Chinese website for online shopping
Xindu Flagship Store	Green Intelligent Power Saver	This energy saver is really good, after using it, it can really save a lot of electricity!	Taobao Marketplace, a Chinese website for online shopping
Xindu Flagship Store	Green Intelligent Power Saver	Received the goods, very good installation, looking forward to the effect	Taobao Marketplace, a Chinese website for online shopping
Xindu Flagship Store	Green Intelligent Power Saver	Value for money: quite good	Taobao Marketplace, a Chinese website for online shopping
Xindu Flagship Store	Green Intelligent Power Saver	Definitely a great product, it's especially easy to use and plugs right into the plug board at home. In the past few days to turn on the air conditioning to see the meter turn a little slower than before. But the electricity consumption is a little less than before.	Taobao Marketplace, a Chinese website for online shopping
Xindu Flagship Store	Green Intelligent Power Saver	The baby has received, just plugged in, the merchant said plug about a month to have effect, we'll see how it goes.	Taobao Marketplace, a Chinese website for online shopping
Xindu Flagship Store	Green Intelligent Power Saver	It's small and compact, doesn't take up too much space, good service, and I'll be back.	Taobao Marketplace, a Chinese website for online shopping
Xindu Flagship Store	Green Intelligent Power Saver	Power saver, worth having	Taobao Marketplace, a Chinese website for online shopping
Xindu Flagship Store	Green Intelligent Power Saver	Experience: I bought it and used it. Let's see if it works and then follow up. Hope it's not bragging!	Taobao Marketplace, a Chinese website for online shopping
Xindu Flagship Store	Green Intelligent Power Saver	Tried it for a few days and the power consumption in the house became less, good, great!	Taobao Marketplace, a Chinese website for online shopping
Xindu Flagship Store	Green Intelligent Power Saver	Top notch service from the store hahaha real review! Waiting to use a stage in the follow-up review hahaha	Taobao Marketplace, a Chinese website for online shopping
Xindu Flagship Store	Green Intelligent Power Saver	It's a really nice product for the price. Affordable price and great quality!	Taobao Marketplace, a Chinese website for online shopping
Xindu Flagship Store	Green Intelligent Power Saver	Good quality, very good customer service, fast logistics	Taobao Marketplace, a Chinese website for online shopping
Xindu Flagship Store	Green Intelligent Power Saver	I don't know if it works, I don't feel it! How about a comparison?	Taobao Marketplace, a Chinese website for online shopping
Xindu Flagship Store	Green Intelligent Power Saver	Good quality, logistics is quite fast	Taobao Marketplace, a Chinese website for online shopping
Xindu Flagship Store	Green Intelligent Power Saver	The goods have been received, very power-saving	Taobao Marketplace, a Chinese website for online shopping
Xindu Flagship Store	Green Intelligent Power Saver	Baby quality is very good, energy saving and power saving, good use, good installation	Taobao Marketplace, a Chinese website for online shopping
Xindu Flagship Store	Green Intelligent Power Saver	Power-saving effect: just used, looking forward to the effect Cost-effective: the price is not expensive, it is worth recommending Appearance style: small and exquisite, easy to use, does not take up space Workmanship material: the material looks good	Taobao Marketplace, a Chinese website for online shopping
Xindu Flagship Store	Green Intelligent Power Saver	Bought to test and compare, the power saving effect is not bad, the family often use high-power appliances to buy a back, with a long time or can save a lot of money. ￼￼￼	Taobao Marketplace, a Chinese website for online shopping
Xindu Flagship Store	Green Intelligent Power Saver	Good quality cost-effective price is cheap and affordable will buy back very satisfied!	Taobao Marketplace, a Chinese website for online shopping
Xindu Flagship Store	Green Intelligent Power Saver	The stuff is even more perfect than I imagined, very powerful and worth getting!	Taobao Marketplace, a Chinese website for online shopping
Xindu Flagship Store	Green Intelligent Power Saver	Bought and used for a while before evaluating, the product is good well worth buying.	Taobao Marketplace, a Chinese website for online shopping
Xindu Flagship Store	Green Intelligent Power Saver	Tried it for a while and thought it was great, worth buying the product.	Taobao Marketplace, a Chinese website for online shopping
Xindu Flagship Store	Green Intelligent Power Saver	Used for a period of time the effect is still very not from the, is just started to use no effect of the, need to optimize, overall is still very power-saving, the	Taobao Marketplace, a Chinese website for online shopping
Xindu Flagship Store	Green Intelligent Power Saver	Try it out, the power saving effect is good oh! Pretty good 👌 very convenient	Taobao Marketplace, a Chinese website for online shopping
Xindu Flagship Store	Green Intelligent Power Saver	It doesn't work. It's a lie. I bought four of them.	Taobao Marketplace, a Chinese website for online shopping
Xindu Flagship Store	Green Intelligent Power Saver	Baby received, the appearance is very delicate, has been plugged into the electricity, in a few days to see the effect, the effect is good must give everyone recommended, look forward to the effect...	Taobao Marketplace, a Chinese website for online shopping
Xindu Flagship Store	Green Intelligent Power Saver	In use, I had experimented for an hour to do a comparison, it does work, I don't know how much electricity one saves, I haven't broken down the math yet.	Taobao Marketplace, a Chinese website for online shopping

Xindu Flagship Store	Green Intelligent Power Saver	Power-saving effect: good Cost-effective: high Appearance style: good-looking Recommended to buy, trust the merchant	Taobao Marketplace, a Chinese website for online shopping
Xindu Flagship Store	Green Intelligent Power Saver	Very good, recommended to buy, trust the merchant, will buy back, the quality is also good	Taobao Marketplace, a Chinese website for online shopping
Xindu Flagship Store	Green Intelligent Power Saver	Listen to others say is not effective, but I still insisted on buying, because the business also gives the promise of 100 days no reason to return, I did not expect to use for a period of time down really save electricity, the future also have to use their own to know!	Taobao Marketplace, a Chinese website for online shopping
Xindu Flagship Store	Green Intelligent Power Saver	Satisfied, good quality, works pretty well, good power savings	Taobao Marketplace, a Chinese website for online shopping
Xindu Flagship Store	Green Intelligent Power Saver	It's okay. A little bit.	Taobao Marketplace, a Chinese website for online shopping
Xindu Flagship Store	Green Intelligent Power Saver	Arrived quickly, good quality	Taobao Marketplace, a Chinese website for online shopping
Xindu Flagship Store	Green Intelligent Power Saver	It doesn't usually have much effect	Taobao Marketplace, a Chinese website for online shopping
Xindu Flagship Store	Green Intelligent Power Saver	Saver is indeed very useful, just started to use no effect almost returned, and then consult the business, only to know that the preliminary need to optimize, and now optimized for a period of time feel still very good, really save electricity, the product is also very good deal, buy 4 to send 2, the	Taobao Marketplace, a Chinese website for online shopping
Xindu Flagship Store	Green Intelligent Power Saver	Already used, beautiful and practical, the effect is still very good, very satisfied!	Taobao Marketplace, a Chinese website for online shopping
Xindu Flagship Store	Green Intelligent Power Saver	The power saver has been used for two days, the power saving effect is very good, plug and play, very convenient	Taobao Marketplace, a Chinese website for online shopping
Xindu Flagship Store	Green Intelligent Power Saver	Energy saver received, the quality is very good compact does not take up space, and can save electricity and environmental protection, look forward to the effect!	Taobao Marketplace, a Chinese website for online shopping
Xindu Flagship Store	Green Intelligent Power Saver	Overall it is still practical. Recommended	Taobao Marketplace, a Chinese website for online shopping
Xindu Flagship Store	Green Intelligent Power Saver	It does save power, is cost-effective and looks good	Taobao Marketplace, a Chinese website for online shopping
Xindu Flagship Store	Green Intelligent Power Saver	Easy to use, plug and play, smart power saving, very economical.	Taobao Marketplace, a Chinese website for online shopping
Xindu Flagship Store	Green Intelligent Power Saver	Power saving effect: very compact, good-looking, meticulous workmanship, easy to install, cost-effective. Recommend.	Taobao Marketplace, a Chinese website for online shopping
Xindu Flagship Store	Green Intelligent Power Saver	The seller's service is very good, ask any questions and reply quickly, logistics and shipping is also very fast. The picture matches the description.	Taobao Marketplace, a Chinese website for online shopping
Xindu Flagship Store	Green Intelligent Power Saver	Good saving appliance, plug in and go, already bought the second one, at first it is no effect, later the effect is very obvious, the	Taobao Marketplace, a Chinese website for online shopping
Xindu Flagship Store	Green Intelligent Power Saver	Just started to use the feeling is still no effect, the later the more you use the effect is better, the pre or need to optimize for a period of time, fortunately at that time did not return the goods	Taobao Marketplace, a Chinese website for online shopping
Xindu Flagship Store	Green Intelligent Power Saver	Saver received, the appearance of the workmanship material is good, sturdy and durable, affordable. Store service attitude is very good, ask questions and explain in detail. Logistics on the service is also good, the goods quickly to the hands of customers.	Taobao Marketplace, a Chinese website for online shopping
Xindu Flagship Store	Green Intelligent Power Saver	Purchased Xindu brand; power saver, used for a period of time, the effect is really good, the power receiving effect reached 30%, and the business description of the match.	Taobao Marketplace, a Chinese website for online shopping
Xindu Flagship Store	Green Intelligent Power Saver	Just took it back home and plugged it in, I don't know how the effect is, the value is not bad, and it's easy to plug it in without getting in the way, so I'm looking forward to a bit of use!	Taobao Marketplace, a Chinese website for online shopping
Xindu Flagship Store	Green Intelligent Power Saver	More satisfied, the price is not expensive, buy back to try, easy to use, in-laws are very satisfied with the	Taobao Marketplace, a Chinese website for online shopping
Xindu Flagship Store	Green Intelligent Power Saver	Good use of energy-saving appliances, the beginning of no effect, and then asked the business business told about 1 month out of the effect, and indeed this month the effect came out, and very obvious!	Taobao Marketplace, a Chinese website for online shopping
Xindu Flagship Store	Green Intelligent Power Saver	Baby received, logistics is very fast, good service attitude, worth buying!	Taobao Marketplace, a Chinese website for online shopping
Xindu Flagship Store	Green Intelligent Power Saver	The quality is very good, after using it really saves electricity, the packaging is tight no damage, worth buying.	Taobao Marketplace, a Chinese website for online shopping
Xindu Flagship Store	Green Intelligent Power Saver	Bought two and got one as a gift, used it for half a month, it's been cooler lately, still over two hundred dollars for electricity, I don't know where I got so many good reviews, just a pit. IQ tax	Taobao Marketplace, a Chinese website for online shopping
Xindu Flagship Store	Green Intelligent Power Saver	Nice and easy to use, just plug it into your own plug and it works, satisfied!	Taobao Marketplace, a Chinese website for online shopping
Xindu Flagship Store	Green Intelligent Power Saver	Power savings: f very good Price/performance ratio: t too good value	Taobao Marketplace, a Chinese website for online shopping
Xindu Flagship Store	Green Intelligent Power Saver	Energy saving is very good, save half of it, really good, truly recommended!	Taobao Marketplace, a Chinese website for online shopping
Xindu Flagship Store	Green Intelligent Power Saver	Energy saver received, very convenient, plug and play, it really saves electricity, and will come back if needed!	Taobao Marketplace, a Chinese website for online shopping
Xindu Flagship Store	Green Intelligent Power Saver	Saves electricity and money and is practical	Taobao Marketplace, a Chinese website for online shopping
Xindu Flagship Store	Green Intelligent Power Saver	I've only been using it for about 10 days, I don't know yet, I think it will save	Taobao Marketplace, a Chinese website for online shopping
Xindu Flagship Store	Green Intelligent Power Saver	Because of the home power consumption, bought 4 power saver, the business sent 2 power saver does work, just need to optimize, thought there is no effect. The day after consulting merchants, merchants let me use more time, I did not expect the effect is really obvious.	Taobao Marketplace, a Chinese website for online shopping
Xindu Flagship Store	Green Intelligent Power Saver	Power saving effect: very good Cost-effective: very good Appearance style: very good Workmanship material: very good Experience: very good Intelligent power saver has been received, exactly as described by the merchant. Very practical. Very power saving, worth buying.	Taobao Marketplace, a Chinese website for online shopping

Xindu Flagship Store	Green Intelligent Power Saver	The Smart Saver has been received and is exactly as described by the merchant. Very practical. Very affordable, very power saving, worth buying.	Taobao Marketplace, a Chinese website for online shopping
Xindu Flagship Store	Green Intelligent Power Saver	It doesn't work, no wonder the reviews are so good, just read it and you'll understand	Taobao Marketplace, a Chinese website for online shopping
Xindu Flagship Store	Green Intelligent Power Saver	Received and used a few days to evaluate, things are really good, need friends can rest assured that the purchase	Taobao Marketplace, a Chinese website for online shopping
Xindu Flagship Store	Green Intelligent Power Saver	It works.	Taobao Marketplace, a Chinese website for online shopping
Xindu Flagship Store	Green Intelligent Power Saver	I don't seem to feel anything at the moment, I'll use it for a month and then I'll comment later.	Taobao Marketplace, a Chinese website for online shopping
Xindu Flagship Store	Green Intelligent Power Saver	Why the scam, I'm still over 200 this month and over 200 last month, no point in saving electricity.	Taobao Marketplace, a Chinese website for online shopping
Xindu Flagship Store	Green Intelligent Power Saver	Baby received thanks. With the description of the same especially your store customer service attitude is very good ask a lot of questions worthy of Taobao all staff to learn.	Taobao Marketplace, a Chinese website for online shopping
Xindu Flagship Store	Green Intelligent Power Saver	It's good. I've been using it for half a month, and it's working.	Taobao Marketplace, a Chinese website for online shopping
Xindu Flagship Store	Green Intelligent Power Saver	I'll be back with a review.	Taobao Marketplace, a Chinese website for online shopping
Xindu Flagship Store	Green Intelligent Power Saver	The seller shipped very fast, good service attitude, recommend recommend recommend	Taobao Marketplace, a Chinese website for online shopping
Xindu Flagship Store	Green Intelligent Power Saver	The quality of the goods is very good, has been used, energy-saving effect is good	Taobao Marketplace, a Chinese website for online shopping
Xindu Flagship Store	Green Intelligent Power Saver	Goods received, used a few days before coming to the evaluation, the meter is very obvious go slower, really save electricity, logistics is also very fast, there want to order, really quite good.	Taobao Marketplace, a Chinese website for online shopping
Xindu Flagship Store	Green Intelligent Power Saver	Summer power consumption is relatively high, so far there is no obvious power saving effect, wait and see.	Taobao Marketplace, a Chinese website for online shopping
Xindu Flagship Store	Green Intelligent Power Saver	Use experience: the use of the effect of experience, looking forward to the later effect!	Taobao Marketplace, a Chinese website for online shopping
Xindu Flagship Store	Green Intelligent Power Saver	Good energy saving effect. Strong cost performance. Novel style. Exquisite workmanship. Satisfaction!	Taobao Marketplace, a Chinese website for online shopping
Xindu Flagship Store	Green Intelligent Power Saver	Goods received, has been installed, is it good to save electricity, the results are the test of goods, to be paid next month after the electricity bill with this month's comparison will know whether to save money, then add comments. Logistics and consulting services are very good, these two aspects of the first	Taobao Marketplace, a Chinese website for online shopping
Xindu Flagship Store	Green Intelligent Power Saver	Appearance style design novel, workmanship material is very good, next time to patronize.	Taobao Marketplace, a Chinese website for online shopping
Xindu Flagship Store	Green Intelligent Power Saver	The quality of the economizer is very good, easy to install, logistics is also very fast, satisfaction	Taobao Marketplace, a Chinese website for online shopping
Xindu Flagship Store	Green Intelligent Power Saver	Recommended by a friend, buy 2 get 1 free	Taobao Marketplace, a Chinese website for online shopping
Xindu Flagship Store	Green Intelligent Power Saver	It doesn't seem to have much effect! Personal store experience felt like nothing changed. Power saving effect: no effect	Taobao Marketplace, a Chinese website for online shopping
Xindu Flagship Store	Green Intelligent Power Saver	Just got it and ordered it for nothing	Taobao Marketplace, a Chinese website for online shopping
Xindu Flagship Store	Green Intelligent Power Saver	A very good product, the material is also very good, the future also come to buy more to introduce a few friends to buy, but also can save electricity, the	Taobao Marketplace, a Chinese website for online shopping
Xindu Flagship Store	Green Intelligent Power Saver	Very good product will come back to continue to buy in the future, but also can save electricity very comfortable, the	Taobao Marketplace, a Chinese website for online shopping
Xindu Flagship Store	Green Intelligent Power Saver	Good quality, works well	Taobao Marketplace, a Chinese website for online shopping
Xindu Flagship Store	Green Intelligent Power Saver	Brothers don't be fooled, put a few at home, the same electricity, higher than the previous month's electricity bill	Taobao Marketplace, a Chinese website for online shopping
Xindu Flagship Store	Green Intelligent Power Saver	Power saving effect: not much effect Experience: used for a few days to evaluate the	Taobao Marketplace, a Chinese website for online shopping
Xindu Flagship Store	Green Intelligent Power Saver	Satisfied, the effect is still there, will come back if needed~	Taobao Marketplace, a Chinese website for online shopping
Xindu Flagship Store	Green Intelligent Power Saver	Saver is really good to use, has been the second purchase, the last time I bought some just started to use no effect, the latter effect is very obvious, this time and purchased a few, the next time to catch up!	Taobao Marketplace, a Chinese website for online shopping
Xindu Flagship Store	Green Intelligent Power Saver	Things are very good, cost-effective electricity, the effect is very good shopping is very satisfied, logistics is also fast.	Taobao Marketplace, a Chinese website for online shopping
Xindu Flagship Store	Green Intelligent Power Saver	Things are very good, very satisfied, logistics is also fast, the main thing is really good recommended	Taobao Marketplace, a Chinese website for online shopping
Xindu Flagship Store	Green Intelligent Power Saver	This saver is compact, easy to use, save electricity after use, recommend to friends to buy buy buy buy	Taobao Marketplace, a Chinese website for online shopping
Xindu Flagship Store	Green Intelligent Power Saver	Power-saving effect: power saving is okay, anyway, the capital is deducted back in the power saving, cost-effective, it is recommended to have seen colleagues are to buy one.	Taobao Marketplace, a Chinese website for online shopping
Xindu Flagship Store	Green Intelligent Power Saver	It's genuine, power saving effect is good, it's my friend introduced me to buy it, this is my second purchase!	Taobao Marketplace, a Chinese website for online shopping
Xindu Flagship Store	Green Intelligent Power Saver	It's genuine, it saves electricity, if you need it, hurry up and buy it!	Taobao Marketplace, a Chinese website for online shopping

Xindu Flagship Store	Green Intelligent Power Saver	Baby has received, the product is really good, good, reliable yo	Taobao Marketplace, a Chinese website for online shopping
Xindu Flagship Store	Green Intelligent Power Saver	In use, looking forward to the results, follow up evaluation, hope to get satisfactory results.	Taobao Marketplace, a Chinese website for online shopping
Xindu Flagship Store	Green Intelligent Power Saver	Intelligent power saver received, the quality is not bad, good business attitude, logistics is also quite strong, after a while to see the effect of it!	Taobao Marketplace, a Chinese website for online shopping
Xindu Flagship Store	Green Intelligent Power Saver	Effective, can save one or two degrees of electricity a day, need to come back again, introduced to the basin friends!	Taobao Marketplace, a Chinese website for online shopping
Xindu Flagship Store	Green Intelligent Power Saver	Power saving effect: the effect is good, a month to save a lot of electricity bills	Taobao Marketplace, a Chinese website for online shopping
Xindu Flagship Store	Green Intelligent Power Saver	Good packaging, fast logistics, affordable, much cheaper than supermarkets	Taobao Marketplace, a Chinese website for online shopping
Xindu Flagship Store	Green Intelligent Power Saver	The logistics is very fast, came back and used, looking forward to the effect	Taobao Marketplace, a Chinese website for online shopping
Xindu Flagship Store	Green Intelligent Power Saver	I don't know if it's working or not, I won't know until a month later	Taobao Marketplace, a Chinese website for online shopping
Xindu Flagship Store	Green Intelligent Power Saver	Received it some time ago and used it and thought it was ok! Works ok especially on power saving!	Taobao Marketplace, a Chinese website for online shopping
Xindu Flagship Store	Green Intelligent Power Saver	Already evaluated, today again to follow up, the effect is really good, worth everyone to buy.	Taobao Marketplace, a Chinese website for online shopping
Xindu Flagship Store	Green Intelligent Power Saver	It works. I've been using it for a while. It works great.	Taobao Marketplace, a Chinese website for online shopping
Xindu Flagship Store	Green Intelligent Power Saver	Garbage people don't believe it, can't use it or it's good, to mess up the electric lines in the house, that's a big project	Taobao Marketplace, a Chinese website for online shopping
Xindu Flagship Store	Green Intelligent Power Saver	Power saving effect: after installing and verifying, it is still quite power saving, the effect can be.	Taobao Marketplace, a Chinese website for online shopping
Xindu Flagship Store	Green Intelligent Power Saver	Very satisfied with a shopping, merchant service is good, fast delivery speed	Taobao Marketplace, a Chinese website for online shopping
Xindu Flagship Store	Green Intelligent Power Saver	Cost-effective, value for money, satisfied	Taobao Marketplace, a Chinese website for online shopping
Xindu Flagship Store	Green Intelligent Power Saver	Baby received, only used a few days, feel pretty good, is not yet seen any effect, a month later come back to the evaluation	Taobao Marketplace, a Chinese website for online shopping
Xindu Flagship Store	Green Intelligent Power Saver	This is the second time to buy, Hainan weather is hot for a long time, worth buying	Taobao Marketplace, a Chinese website for online shopping
Xindu Flagship Store	Green Intelligent Power Saver	In the summer, with all three air conditioners on, and all the other power usage, it never goes below 30 a day, and this one worked for 16 bucks the first day, 27 bucks the next day, and it worked like a charm.	Taobao Marketplace, a Chinese website for online shopping
Xindu Flagship Store	Green Intelligent Power Saver	Just plug it into the outlet. It's convenient. Save some electricity. Buy another one.	Taobao Marketplace, a Chinese website for online shopping
Xindu Flagship Store	Green Intelligent Power Saver	Fine workmanship boss shipping speed and other effects in the follow-up evaluation, fine workmanship boss shipping speed	Taobao Marketplace, a Chinese website for online shopping
Xindu Flagship Store	Green Intelligent Power Saver	It really saves power, it doesn't even spin that fast, it's very practical, very helpful, very happy with it	Taobao Marketplace, a Chinese website for online shopping
Xindu Flagship Store	Green Intelligent Power Saver	Power-saving effect: use it and then come back to the follow-up evaluation	Taobao Marketplace, a Chinese website for online shopping
Xindu Flagship Store	Green Intelligent Power Saver	Good quality, fine workmanship, affordable, worth buying.	Taobao Marketplace, a Chinese website for online shopping
Xindu Flagship Store	Green Intelligent Power Saver	Power saving effect: really good mouth Cost-effective: really good Appearance and style: quite hello Workmanship material: quite good Use experience: quite good	Taobao Marketplace, a Chinese website for online shopping
Xindu Flagship Store	Green Intelligent Power Saver	Appearance and style: okay Cost-effective: okay Workmanship material: can Experience: quite good power-saving effect: just started to use, for the time being do not yet know	Taobao Marketplace, a Chinese website for online shopping
Xindu Flagship Store	Green Intelligent Power Saver	The saver can also be used as a small overnight light	Taobao Marketplace, a Chinese website for online shopping
Xindu Flagship Store	Green Intelligent Power Saver	Power-saving effect: originally 10 a day more than 10 degrees of electricity, now only 6 degrees of electricity a day, really can be, things are good, it is worth buying!	Taobao Marketplace, a Chinese website for online shopping
Xindu Flagship Store	Green Intelligent Power Saver	Appearance and style: compact and does not occupy space Experience: intelligent power saving Used a few days is not bad	Taobao Marketplace, a Chinese website for online shopping
Xindu Flagship Store	Green Intelligent Power Saver	Used for a period of time feeling, consistent with the store description can	Taobao Marketplace, a Chinese website for online shopping
Xindu Flagship Store	Green Intelligent Power Saver	It's a good thing to come back and install, and it's good to use.	Taobao Marketplace, a Chinese website for online shopping
Xindu Flagship Store	Green Intelligent Power Saver	I hope it works.	Taobao Marketplace, a Chinese website for online shopping
Xindu Flagship Store	Green Intelligent Power Saver	Friends used to recommend me to buy, at first I do not believe that this thing, hold the mind to try to buy 6, merchants, there are activities to buy 4 to send 2, the results are indeed unexpected, and will recommend friends to buy, now too many electrical appliances, 1 month are several hundred of the electricity bill, and now every day can save a dozen or so to the merchants praise!	Taobao Marketplace, a Chinese website for online shopping
Xindu Flagship Store	Green Intelligent Power Saver	Really too good to use , the last time to buy 4 free 2, this activity is still in, decided to buy 6 to bring back home to use Really is the national power-saving artifacts	Taobao Marketplace, a Chinese website for online shopping

Xindu Flagship Store	Green Intelligent Power Saver	Merchants buy 4 to send 2, activities are very cost-effective used for a period of time to evaluate the power-saving effect really did not let me down no longer need to worry about high-power appliances power consumption, the material is very good, the manual is simple to understand the light will look at the use of quiet use of no noise is small and exquisite inserted where it does not get in the way of a very good product! Thank you for your business!	Taobao Marketplace, a Chinese website for online shopping
Xindu Flagship Store	Green Intelligent Power Saver	Those who say no effect really do not know what mentality, they use no effect must run to my comments below to say, I use the effect is effective, and is not the first time to buy, with no effect is estimated to be their own mess buy, but also do not consult customer service.	Taobao Marketplace, a Chinese website for online shopping
Xindu Flagship Store	Green Intelligent Power Saver	Friends introduced me to buy, business activities are very cost-effective, buy 4 get 2, this power saver genuine guarantee, plug it does save a lot of electricity, my friend bought a	Taobao Marketplace, a Chinese website for online shopping
Xindu Flagship Store	Green Intelligent Power Saver	Haha really is the national power-saving artifact, now the people to make money is really too difficult, this month's electricity bill straight down 300 yuan, really too much power, 1 year to save several thousand, to chase the next Suggest that everyone buy!	Taobao Marketplace, a Chinese website for online shopping
Xindu Flagship Store	Green Intelligent Power Saver	Baby received in good condition. The seller has activities, shoot 4 sent 6 Very cost-effective, fast shipping, fine packaging, baby exquisite effect to be verified. Thank you for the integrity of the seller, thank you attentive customer service! Thank you dedicated courier! Five stars out of five!!! Bless you to not forget the beginning, move forward, business is booming! Family happiness and well-being!!! Service: service attitude service quality is awesome!!!!	Taobao Marketplace, a Chinese website for online shopping
Xindu Flagship Store	Green Intelligent Power Saver	Power saving effect: feel the effect oh. Last month's electricity bill felt less, this time another one to buy!	Taobao Marketplace, a Chinese website for online shopping
Xindu Flagship Store	Green Intelligent Power Saver	I'll continue to use it, it works, I feel like I can save money on food.	Taobao Marketplace, a Chinese website for online shopping
Xindu Flagship Store	Green Intelligent Power Saver	Goods received logistics is very fast, with customer service said the same quality, with a good use in the purchase.	Taobao Marketplace, a Chinese website for online shopping
Xindu Flagship Store	Green Intelligent Power Saver	Merchants buy 4 get 2, activities are very cost-effective, all at once purchased 6 power saver, deliberately used for a period of time to comment on this section of the power saver is indeed good to use, to a total of more than 70 blocks of electricity now, only ten days time, I believe that the future	Taobao Marketplace, a Chinese website for online shopping
Xindu Flagship Store	Green Intelligent Power Saver	I don't know when the last one broke, so I'm buying another one.	Taobao Marketplace, a Chinese website for online shopping
Xindu Flagship Store	Green Intelligent Power Saver	Last month the air conditioner was on all the time and the total electricity used in the house was 300 dollars	Taobao Marketplace, a Chinese website for online shopping
Xindu Flagship Store	Green Intelligent Power Saver	Received the meter box, good material. Already installed and used satisfied	Taobao Marketplace, a Chinese website for online shopping
Xindu Flagship Store	Green Intelligent Power Saver	Power saving is not bad, affordable, worth buying, recommended.	Taobao Marketplace, a Chinese website for online shopping
Xindu Flagship Store	Green Intelligent Power Saver	Too much electricity in the summer, a friend recommended this one and he uses it all the time. Strongly recommended.	Taobao Marketplace, a Chinese website for online shopping
Xindu Flagship Store	Green Intelligent Power Saver	Fast shipping and good quality	Taobao Marketplace, a Chinese website for online shopping
Xindu Flagship Store	Green Intelligent Power Saver	Tried it for a couple days and was really amazed at the power savings. It turns out that 24 hours a day on the air conditioning, plus the home induction cooker cooking, have to deduct 20 good, save more than 30% of the money!	Taobao Marketplace, a Chinese website for online shopping
Xindu Flagship Store	Green Intelligent Power Saver	Power saving effect: good Cost-effective: high Appearance style: good	Taobao Marketplace, a Chinese website for online shopping
Xindu Flagship Store	Green Intelligent Power Saver	Power savings: good, good, good.	Taobao Marketplace, a Chinese website for online shopping
Xindu Flagship Store	Green Intelligent Power Saver	The first time to buy this energy saver, do not know that there is this thing, hold the mind to try to buy, the business commitment to no effect of a full refund, it is very convenient, the saver is small and does not take up space, my family's electricity consumption is very large, purchased 6, the business gave away 2, the effect of the opening in the pursuit of comment!	Taobao Marketplace, a Chinese website for online shopping
Xindu Flagship Store	Green Intelligent Power Saver	Sure enough there is an effect, after 1 so long, the electricity bill decisively dropped a lot, before all to 500 more than 1 month, now are more than 300, specially to chase the next, really too good to use!	Taobao Marketplace, a Chinese website for online shopping
Xindu Flagship Store	Green Intelligent Power Saver	Friends last time to buy 4 free 2, the effect is good, every other month the electricity bill has a significant drop, this time friends recommended me to come, began to do not know that there are so good things to use, this time to buy 6, the family's self-build room is too much, it is really a one-time investment, lifelong benefit, the product of the lifelong warranty, the bad free replacement, the merchant to give the protection is very considerate. Satisfaction	Taobao Marketplace, a Chinese website for online shopping
Xindu Flagship Store	Green Intelligent Power Saver	Used for more than 1 month. It's really effective, the electricity bill has really dropped a lot, and I've already recommended it to my friends around me!	Taobao Marketplace, a Chinese website for online shopping
Xindu Flagship Store	Green Intelligent Power Saver	Power-saving effect: with super power saving, simply a blessing for the family Cost-effective: cost-effective is also very good, affordable and easy to use Appearance and style: look very advanced, the quality is okay Workmanship Material: quality is very good, very much like the use of the experience: after the use of the home than the original save a lot of electricity, really like it!	Taobao Marketplace, a Chinese website for online shopping
Xindu Flagship Store	Green Intelligent Power Saver	The king of power saving, great value for money, will continue to buy back!	Taobao Marketplace, a Chinese website for online shopping
Xindu Flagship Store	Green Intelligent Power Saver	Small, compact, easy to operate, should have bought it earlier	Taobao Marketplace, a Chinese website for online shopping
Xindu Flagship Store	Green Intelligent Power Saver	It's pretty good and easy to use	Taobao Marketplace, a Chinese website for online shopping
Xindu Flagship Store	Green Intelligent Power Saver	I used to have an old meter and the electricity was running out so fast, but I replaced it with this energy-saving one and saved a lot of money on my electricity bill!	Taobao Marketplace, a Chinese website for online shopping
Xindu Flagship Store	Green Intelligent Power Saver	The logistics is very fast, the texture is very good, very beautiful, I like it very much	Taobao Marketplace, a Chinese website for online shopping
Xindu Flagship Store	Green Intelligent Power Saver	Try it out first and come back for a few more if it works well	Taobao Marketplace, a Chinese website for online shopping
Xindu Flagship Store	Green Intelligent Power Saver	Very good quality, good use, sturdy packaging, fast logistics	Taobao Marketplace, a Chinese website for online shopping
Xindu Flagship Store	Green Intelligent Power Saver	Baby is great value for money, love it, will be back next time!	Taobao Marketplace, a Chinese website for online shopping

Xindu Flagship Store	Green Intelligent Power Saver	Power-saving effect: nothing works, it doesn't save electricity at all.	Taobao Marketplace, a Chinese website for online shopping
Xindu Flagship Store	Green Intelligent Power Saver	Power saving effect: it feels pretty good, I think the product from the merchant must be good.	Taobao Marketplace, a Chinese website for online shopping
Xindu Flagship Store	Green Intelligent Power Saver	Easy to install, good quality, very	Taobao Marketplace, a Chinese website for online shopping
Xindu Flagship Store	Green Intelligent Power Saver	No effect. Used it for five days. Averaged eight degrees of electricity per day. And the air conditioning was on for 9 - 10 hours	Taobao Marketplace, a Chinese website for online shopping
Xindu Flagship Store	Green Intelligent Power Saver	Experience: Good	Taobao Marketplace, a Chinese website for online shopping
Xindu Flagship Store	Green Intelligent Power Saver	It's very compact, fast logistics, and I'm back in use.	Taobao Marketplace, a Chinese website for online shopping
Xindu Flagship Store	Green Intelligent Power Saver	very good	Taobao Marketplace, a Chinese website for online shopping
Xindu Flagship Store	Green Intelligent Power Saver	With the power saver is indeed useful, used for a period of time to evaluate, is to see if there is no effect, I have to pay the electricity bill records, it does work, ready to buy another one!	Taobao Marketplace, a Chinese website for online shopping
Xindu Flagship Store	Green Intelligent Power Saver	Power saving effect: save several dollars a day, it smells so good! Plug it in directly and it's easy to operate	Taobao Marketplace, a Chinese website for online shopping
Xindu Flagship Store	Green Intelligent Power Saver	Purposefully used for a period of time to evaluate, quite good, power-saving effect is very good, very satisfied with the	Taobao Marketplace, a Chinese website for online shopping
Xindu Flagship Store	Green Intelligent Power Saver	Things are very good, the recent hot weather with air conditioning fans, etc. can save a lot of electricity.	Taobao Marketplace, a Chinese website for online shopping
Xindu Flagship Store	Green Intelligent Power Saver	It feels good on the body and I'm telling my friends about it.	Taobao Marketplace, a Chinese website for online shopping
Xindu Flagship Store	Green Intelligent Power Saver	Received the home saver. The store shipped quickly. Packaging is solid and delicate. Now it is plugged in and used. Everything is normal. Power saving effect. Use a period of time later to be able to know.	Taobao Marketplace, a Chinese website for online shopping
Xindu Flagship Store	Green Intelligent Power Saver	The quality is very good very power saving, affordable, logistics is also very fast, worth buying.	Taobao Marketplace, a Chinese website for online shopping
Xindu Flagship Store	Green Intelligent Power Saver	Received the appearance is very atmospheric, exquisite workmanship, try a power-saving effect is very good very obvious quite practical	Taobao Marketplace, a Chinese website for online shopping
Xindu Flagship Store	Green Intelligent Power Saver	Good material, good quality, does have power saving effect, worth buying	Taobao Marketplace, a Chinese website for online shopping
Xindu Flagship Store	Green Intelligent Power Saver	Experience: I tested it, it's useless, it's an IQ tax, I bought two models with no effect, it's not the same as the business video effect	Taobao Marketplace, a Chinese website for online shopping
Xindu Flagship Store	Green Intelligent Power Saver	Try it out first and you won't know if it works until a month later!	Taobao Marketplace, a Chinese website for online shopping
Xindu Flagship Store	Green Intelligent Power Saver	Received the goods! I don't know if it's effective or not! I'll comment later.	Taobao Marketplace, a Chinese website for online shopping
Xindu Flagship Store	Green Intelligent Power Saver	Good, like, good value for money, will come back often	Taobao Marketplace, a Chinese website for online shopping
Xindu Flagship Store	Green Intelligent Power Saver	Power saving effect: very good use Appearance style: very beautiful Workmanship material: good appearance	Taobao Marketplace, a Chinese website for online shopping
Xindu Flagship Store	Green Intelligent Power Saver	Good material, power-saving and easy to use, just great!	Taobao Marketplace, a Chinese website for online shopping
Xindu Flagship Store	Green Intelligent Power Saver	Energy saver is really good, has been the second time to buy, every month to save a lot of electricity, this time is for my relatives to buy, really good product!	Taobao Marketplace, a Chinese website for online shopping
Xindu Flagship Store	Green Intelligent Power Saver	Received the goods, look okay all said power saving, use it to see	Taobao Marketplace, a Chinese website for online shopping
Xindu Flagship Store	Green Intelligent Power Saver	Nice 👍Nice 👍King of power savers	Taobao Marketplace, a Chinese website for online shopping
Xindu Flagship Store	Green Intelligent Power Saver	Power saving effect is very good! The appearance and design is beautiful, easy to use, the effect is experiencing.	Taobao Marketplace, a Chinese website for online shopping
Xindu Flagship Store	Green Intelligent Power Saver	Boil water and feel the meter go slower!	Taobao Marketplace, a Chinese website for online shopping
Xindu Flagship Store	Green Intelligent Power Saver	Fits well and saves a bit, works well. Bought and used it myself.	Taobao Marketplace, a Chinese website for online shopping
Xindu Flagship Store	Green Intelligent Power Saver	It's a great power-saving device, so you can turn on the air conditioner in the summer without worrying.	Taobao Marketplace, a Chinese website for online shopping
Xindu Flagship Store	Green Intelligent Power Saver	It really works. Kudos.	Taobao Marketplace, a Chinese website for online shopping
Xindu Flagship Store	Green Intelligent Power Saver	It's a proper IQ tax, so don't buy it.	Taobao Marketplace, a Chinese website for online shopping
Xindu Flagship Store	Green Intelligent Power Saver	The meter's the same. It's not as good as it sounds.	Taobao Marketplace, a Chinese website for online shopping
Xindu Flagship Store	Green Intelligent Power Saver	The package wasn't delivered where it was supposed to be.	Taobao Marketplace, a Chinese website for online shopping
Xindu Flagship Store	Green Intelligent Power Saver	The goods are small and exquisite in line with the description of the platform, it is very easy to use, the effect is good. Merchant service, delivery and logistics are very fast. This Kun often satisfied.	Taobao Marketplace, a Chinese website for online shopping

Xindu Flagship Store	Green Intelligent Power Saver	Power-saving effect: it's effective, it's okay	Taobao Marketplace, a Chinese website for online shopping
Xindu Flagship Store	Green Intelligent Power Saver	It works. It's okay.	Taobao Marketplace, a Chinese website for online shopping
Xindu Flagship Store	Green Intelligent Power Saver	Used more than a month of no use at all, and customer service to communicate with only think of selling you more than one, poor service attitude, it is recommended not to buy!	Taobao Marketplace, a Chinese website for online shopping
Xindu Flagship Store	Green Intelligent Power Saver	Smart Saver received, good quality, simple and easy to operate.	Taobao Marketplace, a Chinese website for online shopping
Xindu Flagship Store	Green Intelligent Power Saver	Easy to install, very well made and practical.	Taobao Marketplace, a Chinese website for online shopping
Xindu Flagship Store	Green Intelligent Power Saver	The workmanship is quite new, I don't know the effect yet	Taobao Marketplace, a Chinese website for online shopping
Xindu Flagship Store	Green Intelligent Power Saver	It's good. It's a money saver. It's good.	Taobao Marketplace, a Chinese website for online shopping
Xindu Flagship Store	Green Intelligent Power Saver	To be verified for power savings.	Taobao Marketplace, a Chinese website for online shopping
Xindu Flagship Store	Green Intelligent Power Saver	Repurchase, not bad	Taobao Marketplace, a Chinese website for online shopping
Xindu Flagship Store	Green Intelligent Power Saver	Garbage product, no effect at all	Taobao Marketplace, a Chinese website for online shopping
Xindu Flagship Store	Green Intelligent Power Saver	Power-saving effect: power-saving effect is very good Cost-effective: the price is not too high Appearance style: very beautiful Workmanship material: workmanship material are good Experience: the use of the period is very good, it is worth recommending	Taobao Marketplace, a Chinese website for online shopping
Xindu Flagship Store	Green Intelligent Power Saver	This smart saver is a great power saver and is worth recommending to everyone!	Taobao Marketplace, a Chinese website for online shopping
Xindu Flagship Store	Green Intelligent Power Saver	Cost-effective: good, high Appearance style: appearance style is small, does not occupy space. Workmanship material: fine workmanship, good quality Easy to install	Taobao Marketplace, a Chinese website for online shopping
Xindu Flagship Store	Green Intelligent Power Saver	Saving electricity and money is green and healthy, improving the efficiency of electricity consumption and reducing the loss of electricity.	Taobao Marketplace, a Chinese website for online shopping
Xindu Flagship Store	Green Intelligent Power Saver	Power-saving effect: friends bought last year a summer save hundreds ￼ electricity Appearance style: quite good-looking than I previously bought good-looking quality is also good with the light, the	Taobao Marketplace, a Chinese website for online shopping
Xindu Flagship Store	Green Intelligent Power Saver	The use of quite simple, the socket on a plug on the line, there are lights quite good-looking, feel quite good, the effect is quite good, friends recommend quite good, power saving	Taobao Marketplace, a Chinese website for online shopping
Xindu Flagship Store	Green Intelligent Power Saver	This is my family bought the second, my family used really save electricity before buying the second to my mom's family, very good product!	Taobao Marketplace, a Chinese website for online shopping
Xindu Flagship Store	Green Intelligent Power Saver	Appearance and style: very nice appearance. Power saving effect is good!	Taobao Marketplace, a Chinese website for online shopping
Xindu Flagship Store	Green Intelligent Power Saver	This is very power-saving, and the seller's service attitude is very good! It can be purchased with confidence.	Taobao Marketplace, a Chinese website for online shopping
Xindu Flagship Store	Green Intelligent Power Saver	Online shopping for smart appliances logistics is very fast, the appearance is okay, fine workmanship, the use of good and then come back to evaluate.	Taobao Marketplace, a Chinese website for online shopping
Xindu Flagship Store	Green Intelligent Power Saver	Goods have been received, I am running to buy the favorable reviews, and now just plugged into the socket to see the effect, is the history of the use of, and so on after the use of the evaluation in the chase.	Taobao Marketplace, a Chinese website for online shopping
Xindu Flagship Store	Green Intelligent Power Saver	I just bought it, let's see how it works	Taobao Marketplace, a Chinese website for online shopping
Xindu Flagship Store	Green Intelligent Power Saver	It works well and is worth having	Taobao Marketplace, a Chinese website for online shopping
Xindu Flagship Store	Green Intelligent Power Saver	Just used it. Don't know if it's effective or not. I'll find out when I'm done.	Taobao Marketplace, a Chinese website for online shopping
Xindu Flagship Store	Green Intelligent Power Saver	Doesn't work, plugged in the meter does not respond, or the same as unplugged ! ! ! ! No friends photographed carefully photographed	Taobao Marketplace, a Chinese website for online shopping
Xindu Flagship Store	Green Intelligent Power Saver	Power-saving effect: just purchased back, wait for next month, look at the electricity bill Workmanship material: good Cost-effective: cost-effective relatively high	Taobao Marketplace, a Chinese website for online shopping
Xindu Flagship Store	Green Intelligent Power Saver	Baby received, the merchant delivery is very fast, logistics is also fast, first plugged in and so use a period of time to see the effect.	Taobao Marketplace, a Chinese website for online shopping
Xindu Flagship Store	Green Intelligent Power Saver	Used for a period of time, feel the effect of power saving, ready to get a few more, thanks to the business of inexpensive baby 😊	Taobao Marketplace, a Chinese website for online shopping
Xindu Flagship Store	Green Intelligent Power Saver	Logistics very quickly well packaged, the seller has a good service attitude	Taobao Marketplace, a Chinese website for online shopping
Xindu Flagship Store	Green Intelligent Power Saver	Used the effect is good, the quality is quite good price affordable	Taobao Marketplace, a Chinese website for online shopping
Xindu Flagship Store	Green Intelligent Power Saver	Power saving effect: Effective	Taobao Marketplace, a Chinese website for online shopping
Xindu Flagship Store	Green Intelligent Power Saver	It's good. It's worth it.	Taobao Marketplace, a Chinese website for online shopping
Xindu Flagship Store	Green Intelligent Power Saver	The stuff is very good.	Taobao Marketplace, a Chinese website for online shopping
Xindu Flagship Store	Green Intelligent Power Saver	Power-saving effect: no **, less than nine hundred rent directly to a thousand	Taobao Marketplace, a Chinese website for online shopping

Xindu Flagship Store	Green Intelligent Power Saver	The time is long, the electricity bill is not so high, very effective Oh, recommended to buy Oh ∀⊙ ⊙!	Taobao Marketplace, a Chinese website for online shopping
Xindu Flagship Store	Green Intelligent Power Saver	The saver was received, perfect for home use, satisfied!	Taobao Marketplace, a Chinese website for online shopping
Xindu Flagship Store	Green Intelligent Power Saver	Tonight received the goods, I hope there are merchants said that effect, I now have hot weather open air conditioning a month more than 300 electricity bill, tonight open with, looking forward to have a good durable and power saving. With a month to see how it first, September 3rd on the meter reading, tonight I boil water are aware of, usually burn a bucket of bath with 70 cents? Let's see what the effect will be after using this. I'll comment again next month.	Taobao Marketplace, a Chinese website for online shopping
Xindu Flagship Store	Green Intelligent Power Saver	I just tried turning on the air conditioner, and the saver doesn't respond to anything, doesn't blink that light, so I don't know if it's effective or not. Let's use it for a few days and see how it works, right?	Taobao Marketplace, a Chinese website for online shopping
Xindu Flagship Store	Green Intelligent Power Saver	Power saving effect: temporarily used is not bad, in addition, many of my friends have used the effect is good!	Taobao Marketplace, a Chinese website for online shopping
Xindu Flagship Store	Green Intelligent Power Saver	It works well and is recommended	Taobao Marketplace, a Chinese website for online shopping
Xindu Flagship Store	Green Intelligent Power Saver	Power saving effect: good Cost-effective: good Appearance and style: good Workmanship and material: good Experience: quite good	Taobao Marketplace, a Chinese website for online shopping
Xindu Flagship Store	Green Intelligent Power Saver	Power-saving effect: 0 Price-performance ratio: 0 Appearance style: 0 Workmanship material: 0 Use experience: 0 Don't gear up, everyone! This thing is a scam! Do not buy!	Taobao Marketplace, a Chinese website for online shopping
Xindu Flagship Store	Green Intelligent Power Saver	Installed it in the house, don't know if it works or not	Taobao Marketplace, a Chinese website for online shopping
Xindu Flagship Store	Green Intelligent Power Saver	Haven't used it yet, don't know how it is	Taobao Marketplace, a Chinese website for online shopping
Xindu Flagship Store	Green Intelligent Power Saver	I don't know if it works, try it first	Taobao Marketplace, a Chinese website for online shopping
Xindu Flagship Store	Green Intelligent Power Saver	Power saving effect: this is very effective and comfortable to use	Taobao Marketplace, a Chinese website for online shopping
Xindu Flagship Store	Green Intelligent Power Saver	This works very well with a very good	Taobao Marketplace, a Chinese website for online shopping
Xindu Flagship Store	Green Intelligent Power Saver	The goods have been received, well-packed, and the same as the business introduction, with instructions and warranty card, you can rest assured enough to buy.	Taobao Marketplace, a Chinese website for online shopping
Xindu Flagship Store	Green Intelligent Power Saver	The product is insured and used with confidence and results	Taobao Marketplace, a Chinese website for online shopping
Xindu Flagship Store	Green Intelligent Power Saver	It's good. It's working.	Taobao Marketplace, a Chinese website for online shopping
Xindu Flagship Store	Green Intelligent Power Saver	Power saving effect: good effect, Appearance style: good-looking Workmanship material: can Use experience: okay	Taobao Marketplace, a Chinese website for online shopping
Xindu Flagship Store	Green Intelligent Power Saver	Power saving effect: effective and good quality.	Taobao Marketplace, a Chinese website for online shopping
Xindu Flagship Store	Green Intelligent Power Saver	Power saving effect: very good, very save Cost-effective: high Experience: can be	Taobao Marketplace, a Chinese website for online shopping
Xindu Flagship Store	Green Intelligent Power Saver	I bought one before and thought it was good enough to buy another one.	Taobao Marketplace, a Chinese website for online shopping
Xindu Flagship Store	Green Intelligent Power Saver	Experience: I just used it and my friend said it works well. Immediately ordered	Taobao Marketplace, a Chinese website for online shopping
Xindu Flagship Store	Green Intelligent Power Saver	You have to use this to balance the power when the air conditioner is on a lot.	Taobao Marketplace, a Chinese website for online shopping
Xindu Flagship Store	Green Intelligent Power Saver	Good quality, works great, worth getting!	Taobao Marketplace, a Chinese website for online shopping
Xindu Flagship Store	Green Intelligent Power Saver	It's pretty good. It feels like it's turning a little slower.	Taobao Marketplace, a Chinese website for online shopping
Xindu Flagship Store	Green Intelligent Power Saver	It's pretty good. Turn slower.	Taobao Marketplace, a Chinese website for online shopping
Xindu Flagship Store	Green Intelligent Power Saver	No, it doesn't work as a power saver!	Taobao Marketplace, a Chinese website for online shopping
Xindu Flagship Store	Green Intelligent Power Saver	It doesn't work. It doesn't save electricity.	Taobao Marketplace, a Chinese website for online shopping
Xindu Flagship Store	Green Intelligent Power Saver	Cost-effective: quite cheap Appearance style: the appearance of exquisite small buy back immediately installed the use of experience: a few days back to comment oh	Taobao Marketplace, a Chinese website for online shopping
Xindu Flagship Store	Green Intelligent Power Saver	It's working great. The meter's not so fast anymore. Come on, baby, let's get it.	Taobao Marketplace, a Chinese website for online shopping
Xindu Flagship Store	Green Intelligent Power Saver	Things received, today began to experiment with the effect of workmanship is not bad, it depends on how the real-time effect, the effect is good on the follow-up. You look after the wait for good news.	Taobao Marketplace, a Chinese website for online shopping
Xindu Flagship Store	Green Intelligent Power Saver	Compact style, beautiful appearance, normal trial in the	Taobao Marketplace, a Chinese website for online shopping
Xindu Flagship Store	Green Intelligent Power Saver	Power-saving effect: is experiencing the expectation Appearance style: the overall appearance is not bad. Use experience: is experiencing the expectations Cost-effective: the price is okay Workmanship material: beautiful workmanship	Taobao Marketplace, a Chinese website for online shopping
Xindu Flagship Store	Green Intelligent Power Saver	Looking forward to the results.	Taobao Marketplace, a Chinese website for online shopping

Xindu Flagship Store	Green Intelligent Power Saver	Power saving effect: feel very good Small and simple, plugged in can be used, and then go to buy a few for the old family to use also	Taobao Marketplace, a Chinese website for online shopping
Xindu Flagship Store	Green Intelligent Power Saver	The logistics is fast, the quality is good, the price is affordable, worth buying.	Taobao Marketplace, a Chinese website for online shopping
Xindu Flagship Store	Green Intelligent Power Saver	I just started using it yesterday, and the courier broke the box, so I don't know if the stuff is good or bad	Taobao Marketplace, a Chinese website for online shopping
Xindu Flagship Store	Green Intelligent Power Saver	A month, the electricity bill a little bit not less, really false, Taobao to strictly review some companies selling fake goods ah	Taobao Marketplace, a Chinese website for online shopping
Xindu Flagship Store	Green Intelligent Power Saver	It's safe and it does save power	Taobao Marketplace, a Chinese website for online shopping
Xindu Flagship Store	Green Intelligent Power Saver	Don't fall for it. I got ripped off the first time.	Taobao Marketplace, a Chinese website for online shopping
Xindu Flagship Store	Green Intelligent Power Saver	Power saving effect: was introduced by a friend before buying. The effect is good. Appearance and style: the appearance of small and does not occupy space.	Taobao Marketplace, a Chinese website for online shopping
Xindu Flagship Store	Green Intelligent Power Saver	Workmanship material: very good workmanship,, just got back, power saving effect with a period of time, and then follow up, the courier speed is very fast, the service attitude is also very good!	Taobao Marketplace, a Chinese website for online shopping
Xindu Flagship Store	Green Intelligent Power Saver	Power savings: works great, paid for itself in just over 2 months! Value for money: better than other brands, great value for money! Using experience: feel very good	Taobao Marketplace, a Chinese website for online shopping
Xindu Flagship Store	Green Intelligent Power Saver	The third time I purchased this saver, it will pay for itself in two or three months, the power saving effect is very good, the logistics is also very fast, the cost is very high!	Taobao Marketplace, a Chinese website for online shopping
Xindu Flagship Store	Green Intelligent Power Saver	This thing is not useful, are bragging *, fake, simply does not work Power-saving effect: simply connected to the energy Cost-effective: cost-effective is also very bad	Taobao Marketplace, a Chinese website for online shopping
Xindu Flagship Store	Green Intelligent Power Saver	The stuff is still good line logistics is very fast	Taobao Marketplace, a Chinese website for online shopping
Xindu Flagship Store	Green Intelligent Power Saver	verygood	Taobao Marketplace, a Chinese website for online shopping
Xindu Flagship Store	Green Intelligent Power Saver	It's great, it has power saving effect, worth buying	Taobao Marketplace, a Chinese website for online shopping
Xindu Flagship Store	Green Intelligent Power Saver	Power savings: great service, delivery, easy to use!	Taobao Marketplace, a Chinese website for online shopping
Xindu Flagship Store	Green Intelligent Power Saver	Exactly as described by the seller 😊, the packaging is also very careful and strict, very satisfied with a shopping ✨	Taobao Marketplace, a Chinese website for online shopping
Xindu Flagship Store	Green Intelligent Power Saver	Probably tried it and left it on all night, from 9pm to 5pm the next day, it used about 3 kWh.	Taobao Marketplace, a Chinese website for online shopping
Xindu Flagship Store	Green Intelligent Power Saver	It looks good. It'll work.	Taobao Marketplace, a Chinese website for online shopping
Xindu Flagship Store	Green Intelligent Power Saver	IQ tax, hey hand*.	Taobao Marketplace, a Chinese website for online shopping
Xindu Flagship Store	Green Intelligent Power Saver	Power-saving effect: power-saving effect is strong Appearance style: the style is very good-looking, the logistics is very strong!	Taobao Marketplace, a Chinese website for online shopping
Xindu Flagship Store	Green Intelligent Power Saver	The logistics is fast, the service attitude of the merchants is very good, the power saving effect is very good	Taobao Marketplace, a Chinese website for online shopping
Xindu Flagship Store	Green Intelligent Power Saver	Appearance style: there is a certain power saving effect!	Taobao Marketplace, a Chinese website for online shopping
Xindu Flagship Store	Green Intelligent Power Saver	It's good. I'm using it. I'll comment when it's ready.	Taobao Marketplace, a Chinese website for online shopping
Xindu Flagship Store	Green Intelligent Power Saver	Power saving is very good, good quality, affordable, recommended	Taobao Marketplace, a Chinese website for online shopping
Xindu Flagship Store	Green Intelligent Power Saver	Haven't seen any results yet	Taobao Marketplace, a Chinese website for online shopping
Xindu Flagship Store	Green Intelligent Power Saver	It's okay. It's good. It saves power.	Taobao Marketplace, a Chinese website for online shopping
Xindu Flagship Store	Green Intelligent Power Saver	A friend introduced to buy, received the power saver, it really works well, now it has been used, looking forward to the next month's electricity bill	Taobao Marketplace, a Chinese website for online shopping
Xindu Flagship Store	Green Intelligent Power Saver	Used a few days it seems to have an effect, is comparing, use a few more days to come back, good use and then buy a gift for a friend, they also add a, five-star praise!	Taobao Marketplace, a Chinese website for online shopping
Xindu Flagship Store	Green Intelligent Power Saver	Started using it on July 10th, **, installing this power-saving thing, but instead, the power usage has increased greatly, pitfalls	Taobao Marketplace, a Chinese website for online shopping
Xindu Flagship Store	Green Intelligent Power Saver	Energy-saving controller is really good, good quality, easy to install, the need for pro can rest assured to buy!	Taobao Marketplace, a Chinese website for online shopping
Xindu Flagship Store	Green Intelligent Power Saver	Recommended by a friend, used it for a while, still feel quite power-saving	Taobao Marketplace, a Chinese website for online shopping
Xindu Flagship Store	Green Intelligent Power Saver	Power saving effect: very good Cost-effective: high Appearance style: very beautiful Workmanship material: very delicate	Taobao Marketplace, a Chinese website for online shopping
Xindu Flagship Store	Green Intelligent Power Saver	Saved 4 words in one day really good very good	Taobao Marketplace, a Chinese website for online shopping
Xindu Flagship Store	Green Intelligent Power Saver	Power saving effect: 1 I bought three before, this effect is good, so I bought again, useful Cost-effective: good Appearance style: can. Workmanship material: good. Experience: used.	Taobao Marketplace, a Chinese website for online shopping

Xindu Flagship Store	Green Intelligent Power Saver	Not bad. Not bad. Power saving.	Taobao Marketplace, a Chinese website for online shopping
Xindu Flagship Store	Green Intelligent Power Saver	It's really good.	Taobao Marketplace, a Chinese website for online shopping
Xindu Flagship Store	Green Intelligent Power Saver	rare	Taobao Marketplace, a Chinese website for online shopping
Xindu Flagship Store	Green Intelligent Power Saver	Very satisfied, logistics is also very fast, customer service attitude is very good	Taobao Marketplace, a Chinese website for online shopping
Xindu Flagship Store	Green Intelligent Power Saver	A very practical one, easy to install, quality is guaranteed, very satisfied!	Taobao Marketplace, a Chinese website for online shopping
Xindu Flagship Store	Green Intelligent Power Saver	Shipping was fast, the saver works great, it's small and convenient, the	Taobao Marketplace, a Chinese website for online shopping
Xindu Flagship Store	Green Intelligent Power Saver	Is in use, the effect is quite good, energy saving and simple from me!	Taobao Marketplace, a Chinese website for online shopping
Xindu Flagship Store	Green Intelligent Power Saver	Power saving effect: it does save power	Taobao Marketplace, a Chinese website for online shopping
Xindu Flagship Store	Green Intelligent Power Saver	Second time I've shopped, it's great, it saves energy, you can shop with confidence.	Taobao Marketplace, a Chinese website for online shopping
Xindu Flagship Store	Green Intelligent Power Saver	Experience: works over well. Very good. I used to pay 500 dollars for electricity at my house, but since I've been using this, I only have to pay 5 dollars a month for electricity, and I'm going to buy 10 more of them, so I'm going to use it.	Taobao Marketplace, a Chinese website for online shopping
Xindu Flagship Store	Green Intelligent Power Saver	This works really, really well.	Taobao Marketplace, a Chinese website for online shopping
Xindu Flagship Store	Green Intelligent Power Saver	The quality of the effect is particularly good, the boss service attitude is also very good, welcome to buy the product, good and affordable. Good to use very much. Power saving very next time have to come to buy this family of products.	Taobao Marketplace, a Chinese website for online shopping
Xindu Flagship Store	Green Intelligent Power Saver	The goods have been received, looks good, hope to be able to line up, looking forward to the effect!	Taobao Marketplace, a Chinese website for online shopping
Xindu Flagship Store	Green Intelligent Power Saver	After a few days of use, I feel a little bit of effect, it is worth recommending, oh.	Taobao Marketplace, a Chinese website for online shopping
Xindu Flagship Store	Green Intelligent Power Saver	Power saving effect: obvious Cost-effective: very high Appearance style: design quality is very good	Taobao Marketplace, a Chinese website for online shopping
Xindu Flagship Store	Green Intelligent Power Saver	Great value for money works very well! A great gem to get your hands on!	Taobao Marketplace, a Chinese website for online shopping
Xindu Flagship Store	Green Intelligent Power Saver	This thing is a rip off! I've been using it for two months instead my electric bill is a dollar or twenty more than it was before! I hope those of you who see this don't buy this thing!	Taobao Marketplace, a Chinese website for online shopping
Xindu Flagship Store	Green Intelligent Power Saver	Appearance and style: appearance and style is very small, does not take up space, looks also very good, experience and then evaluate ￼￼￼￼	Taobao Marketplace, a Chinese website for online shopping
Xindu Flagship Store	Green Intelligent Power Saver	The appearance looks very good, small and convenient, any corner can be put, after using to see how the effect?	Taobao Marketplace, a Chinese website for online shopping
Xindu Flagship Store	Green Intelligent Power Saver	Baby received, the material and description of the still not clear, with a period of time to follow up, logistics is very fast, the service attitude is also very good to praise.	Taobao Marketplace, a Chinese website for online shopping
Xindu Flagship Store	Green Intelligent Power Saver	Guangdong's landlord stealing electricity is too powerful, buy this no longer afraid of landlord tampering	Taobao Marketplace, a Chinese website for online shopping
Xindu Flagship Store	Green Intelligent Power Saver	Before I used it, it was about 10 kilowatts a day, but after I used it, it's about 3 kilowatts a day. It's just weird.	Taobao Marketplace, a Chinese website for online shopping
Xindu Flagship Store	Green Intelligent Power Saver	Just received the item and can't wait to use it. Looking forward to the results in a couple months 😍	Taobao Marketplace, a Chinese website for online shopping
Xindu Flagship Store	Green Intelligent Power Saver	Customer service attitude is also very good, Dad praise will buy things, hehehe, recommended!	Taobao Marketplace, a Chinese website for online shopping
Xindu Flagship Store	Green Intelligent Power Saver	Good power saving and good value for money. Fine workmanship, worth buying	Taobao Marketplace, a Chinese website for online shopping
Xindu Flagship Store	Green Intelligent Power Saver	Simple and easy to use, power saving does work, this is really a must have for the family!	Taobao Marketplace, a Chinese website for online shopping
Xindu Flagship Store	Green Intelligent Power Saver	The power saving is very good, just as described by the merchant.	Taobao Marketplace, a Chinese website for online shopping
Xindu Flagship Store	Green Intelligent Power Saver	Great value for money, easy to operate and good value for money	Taobao Marketplace, a Chinese website for online shopping
Xindu Flagship Store	Green Intelligent Power Saver	Cost-effective, very energy-efficient, workmanship materials are very good, good comments	Taobao Marketplace, a Chinese website for online shopping
Xindu Flagship Store	Green Intelligent Power Saver	I just plugged it in. I won't know if it works until a month later.	Taobao Marketplace, a Chinese website for online shopping
Xindu Flagship Store	Green Intelligent Power Saver	It didn't work at all! Brainwashed!	Taobao Marketplace, a Chinese website for online shopping
Xindu Flagship Store	Green Intelligent Power Saver	Power saving effect: good Cost-effective: high Appearance style: simple Workmanship material: high quality Experience: excellent Really value for money, technology to give power	Taobao Marketplace, a Chinese website for online shopping
Xindu Flagship Store	Green Intelligent Power Saver	It's so good, highly recommended by friends, definitely one of the best values ever bought, really amazed!	Taobao Marketplace, a Chinese website for online shopping
Xindu Flagship Store	Green Intelligent Power Saver	Power saving effect: energy-saving effect has good appearance style: appearance is good small and convenient	Taobao Marketplace, a Chinese website for online shopping

Xindu Flagship Store	Green Intelligent Power Saver	Energy saving effect a little bit, like friends can buy	Taobao Marketplace, a Chinese website for online shopping
Xindu Flagship Store	Green Intelligent Power Saver	I'm using it now to see the results, and I'll know how it works when I get my bill soon.	Taobao Marketplace, a Chinese website for online shopping
Xindu Flagship Store	Green Intelligent Power Saver	I have bought this product before and put it in my old home and now I am putting 1 more in my new home.	Taobao Marketplace, a Chinese website for online shopping
Xindu Flagship Store	Green Intelligent Power Saver	Logistics is fast, the packaging is not damaged, look good, just used, I do not know whether to save electricity, temporary praise! Use good and then follow up. When the time comes to buy another.	Taobao Marketplace, a Chinese website for online shopping
Xindu Flagship Store	Green Intelligent Power Saver	Logistics is very fast, powerful, and then the summer air conditioning electricity costs hundreds of dollars a month, with this energy-saving appliances can save some money on electricity. Use to see half a month after the evaluation.	Taobao Marketplace, a Chinese website for online shopping
Xindu Flagship Store	Green Intelligent Power Saver	I've been using it for most of the month and the air conditioner is basically on day and night, and the electric bill is fine. It saves some money.	Taobao Marketplace, a Chinese website for online shopping
Xindu Flagship Store	Green Intelligent Power Saver	Baby looks good, just use it to see how much electricity can be saved!	Taobao Marketplace, a Chinese website for online shopping
Xindu Flagship Store	Green Intelligent Power Saver	Experience: Compact and does not take up space Just received quite good Looking forward to next month's electricity costs down to follow up the evaluation	Taobao Marketplace, a Chinese website for online shopping
Xindu Flagship Store	Green Intelligent Power Saver	very happy	Taobao Marketplace, a Chinese website for online shopping
Xindu Flagship Store	Green Intelligent Power Saver	Fast shipping, well packaged, used it right after receiving it.	Taobao Marketplace, a Chinese website for online shopping
Xindu Flagship Store	Green Intelligent Power Saver	I used it right after receiving it and was able to venture into electricity.	Taobao Marketplace, a Chinese website for online shopping
Xindu Flagship Store	Green Intelligent Power Saver	Used it as soon as I received it, expect it to work well	Taobao Marketplace, a Chinese website for online shopping
Xindu Flagship Store	Green Intelligent Power Saver	I purposely used it for a while before I commented on it, the	Taobao Marketplace, a Chinese website for online shopping
Xindu Flagship Store	Green Intelligent Power Saver	It doesn't work. It's an IQ tax.	Taobao Marketplace, a Chinese website for online shopping
Xindu Flagship Store	Green Intelligent Power Saver	Summer air conditioning refrigerator power consumption is too large, friends recommended to use the effect is very good every month to save a lot of money to me also recommended to use it is very good [strong].	Taobao Marketplace, a Chinese website for online shopping
Xindu Flagship Store	Green Intelligent Power Saver	In-kind and the same as described, the quality is quite good, the seller's attitude is also good, ask questions and answer them, the speed of delivery bar, worth buying Oh!	Taobao Marketplace, a Chinese website for online shopping
Xindu Flagship Store	Green Intelligent Power Saver	I'm trying it out and I don't know if it works yet.	Taobao Marketplace, a Chinese website for online shopping
Xindu Flagship Store	Green Intelligent Power Saver	Fast shipping, good quality, as described!	Taobao Marketplace, a Chinese website for online shopping
Xindu Flagship Store	Green Intelligent Power Saver	Received, good quality, good value for money, fast shipping	Taobao Marketplace, a Chinese website for online shopping
Xindu Flagship Store	Green Intelligent Power Saver	Looking at the meter for 5 days in a row is useless.	Taobao Marketplace, a Chinese website for online shopping
Xindu Flagship Store	Green Intelligent Power Saver	Power saving effect: not bad	Taobao Marketplace, a Chinese website for online shopping
Xindu Flagship Store	Green Intelligent Power Saver	A very good power saver, I've used it for half a month before coming back to the review	Taobao Marketplace, a Chinese website for online shopping
Xindu Flagship Store	Green Intelligent Power Saver	Uses way more electricity in the summer than in the winter, so I'll get an air conditioner first. The price is not expensive, looking forward to next month's surprise. Next buy kitchen use. Trial aspect.	Taobao Marketplace, a Chinese website for online shopping
Xindu Flagship Store	Green Intelligent Power Saver	I have observed that saving electricity can have some put results through the use of power savers.	Taobao Marketplace, a Chinese website for online shopping
Xindu Flagship Store	Green Intelligent Power Saver	I just received it today, I will use it first to see the effect, if it's good, I will follow up in a few days.	Taobao Marketplace, a Chinese website for online shopping
Xindu Flagship Store	Green Intelligent Power Saver	Plug and play safety and efficiency, power saving effect is very good, cost-effective	Taobao Marketplace, a Chinese website for online shopping
Xindu Flagship Store	Green Intelligent Power Saver	Bought two more.	Taobao Marketplace, a Chinese website for online shopping
Xindu Flagship Store	Green Intelligent Power Saver	Good quality, power saving effect is very obvious, cost-effective and very satisfactory	Taobao Marketplace, a Chinese website for online shopping
Xindu Flagship Store	Green Intelligent Power Saver	The quality is very good, the power saving effect is great, save a lot of money on your electricity bill!	Taobao Marketplace, a Chinese website for online shopping
Xindu Flagship Store	Green Intelligent Power Saver	No results so far, don't know if it works or not, don't rate it as good or bad for now. Next month to see	Taobao Marketplace, a Chinese website for online shopping
Xindu Flagship Store	Green Intelligent Power Saver	Power saving effect: good Cost-effective: high	Taobao Marketplace, a Chinese website for online shopping
Xindu Flagship Store	Green Intelligent Power Saver	It is indeed very good, save a little strong effect	Taobao Marketplace, a Chinese website for online shopping
Xindu Flagship Store	Green Intelligent Power Saver	I bought four and got six free, so I received six at once, which is more than enough.	Taobao Marketplace, a Chinese website for online shopping
Xindu Flagship Store	Green Intelligent Power Saver	Very powerful, fast delivery Saver received, the appearance looks very good, with a month to see, if the effect is good, and then a positive review.	Taobao Marketplace, a Chinese website for online shopping

Xindu Flagship Store	Green Intelligent Power Saver	Merchant service is good, back to the quality is good, everyone quickly grab it, five-star praise, the courier is also very fast!	Taobao Marketplace, a Chinese website for online shopping
Xindu Flagship Store	Green Intelligent Power Saver	Workmanship material: very good, bought a previous, the effect is good, this time to buy again	Taobao Marketplace, a Chinese website for online shopping
Xindu Flagship Store	Green Intelligent Power Saver	It is the ideal product, good quality, logistics is also very fast, worthy of praise!	Taobao Marketplace, a Chinese website for online shopping
Xindu Flagship Store	Green Intelligent Power Saver	It's a good idea to buy it and save money in the long run.	Taobao Marketplace, a Chinese website for online shopping
Xindu Flagship Store	Green Intelligent Power Saver	I'm using it now and looking forward to the results 😁	Taobao Marketplace, a Chinese website for online shopping
Xindu Flagship Store	Green Intelligent Power Saver	No, it's not good, it's not good, it's a scam, it's costing me $180 more in electricity this month.	Taobao Marketplace, a Chinese website for online shopping
Xindu Flagship Store	Green Intelligent Power Saver	Didn't work well with it. Didn't feel like it was working.	Taobao Marketplace, a Chinese website for online shopping
Xindu Flagship Store	Green Intelligent Power Saver	It doesn't help at all.	Taobao Marketplace, a Chinese website for online shopping
Xindu Flagship Store	Green Intelligent Power Saver	It's a lie. It doesn't save any power.	Taobao Marketplace, a Chinese website for online shopping
Xindu Flagship Store	Green Intelligent Power Saver	It doesn't work. Don't buy it.	Taobao Marketplace, a Chinese website for online shopping
Xindu Flagship Store	Green Intelligent Power Saver	Has been the second time to buy, this power saving device is really good power, before every month to spend 300 electricity, now are about 200, the last time that 6, this device buy 4 get 2, this time to help lead the family to buy 6, the	Taobao Marketplace, a Chinese website for online shopping
Xindu Flagship Store	Green Intelligent Power Saver	Power-saving effect: especially good Cost-effective: very high Appearance style: very cute Workmanship material: very good Experience: really save electricity, a savings of 10 dollars, can not lose buy can not be fooled, it is really very suitable, the next time to come back!	Taobao Marketplace, a Chinese website for online shopping
Xindu Flagship Store	Green Intelligent Power Saver	Through a few days of testing, it's still working, so hopefully it will get better in the future.	Taobao Marketplace, a Chinese website for online shopping
Xindu Flagship Store	Green Intelligent Power Saver	Intelligent power saver received, tightly packaged, no damage, cost-effective Appearance style: look at the okay, do not know how the effect? Looking forward to the effect will be added Experience: plug in the plug can be used should not be bad	Taobao Marketplace, a Chinese website for online shopping
Xindu Flagship Store	Green Intelligent Power Saver	Didn't work, used it instead and had to have more. It's better not to buy it if you haven't, don't get ripped off	Taobao Marketplace, a Chinese website for online shopping
Xindu Flagship Store	Green Intelligent Power Saver	The energy saving is really good, a must-have first choice for families!	Taobao Marketplace, a Chinese website for online shopping
Xindu Flagship Store	Green Intelligent Power Saver	Effective and a must-have for families!	Taobao Marketplace, a Chinese website for online shopping
Xindu Flagship Store	Green Intelligent Power Saver	The first time I used it, it worked very well. It's very powerful. I didn't expect it to work so well.	Taobao Marketplace, a Chinese website for online shopping
Xindu Flagship Store	Green Intelligent Power Saver	Goods received Oh, very good use next time to buy again	Taobao Marketplace, a Chinese website for online shopping
Xindu Flagship Store	Green Intelligent Power Saver	It's really good. Get it if you need it.	Taobao Marketplace, a Chinese website for online shopping
Xindu Flagship Store	Green Intelligent Power Saver	This is a liar, just pay an IQ tax Power-saving effect: no Cost-effective: poor Appearance style: poor Workmanship material: super poor Use experience: no experience, is a liar	Taobao Marketplace, a Chinese website for online shopping
Xindu Flagship Store	Green Intelligent Power Saver	Especially satisfied, praise must praise	Taobao Marketplace, a Chinese website for online shopping
Xindu Flagship Store	Green Intelligent Power Saver	The appearance is small and compact, this is the second purchase!	Taobao Marketplace, a Chinese website for online shopping
Xindu Flagship Store	Green Intelligent Power Saver	Pretty good for saving electricity.	Taobao Marketplace, a Chinese website for online shopping
Xindu Flagship Store	Green Intelligent Power Saver	Power saving effect: very good Appearance and style: bright Workmanship and material: good Experience: like it	Taobao Marketplace, a Chinese website for online shopping
Xindu Flagship Store	Green Intelligent Power Saver	The baby has received, express delivery is very fast, the effect of the words look forward to a month after the results!	Taobao Marketplace, a Chinese website for online shopping
Xindu Flagship Store	Green Intelligent Power Saver	Together, we look forward to its results!	Taobao Marketplace, a Chinese website for online shopping
Xindu Flagship Store	Green Intelligent Power Saver	Power-saving effect: quite good Cost-effective: quite good Appearance style: very good-looking, things are good press on is power saving, good flat	Taobao Marketplace, a Chinese website for online shopping
Xindu Flagship Store	Green Intelligent Power Saver	Just used it, will see after a while.	Taobao Marketplace, a Chinese website for online shopping
Xindu Flagship Store	Green Intelligent Power Saver	Good quality, good workmanship materials, cost-effective	Taobao Marketplace, a Chinese website for online shopping
Xindu Flagship Store	Green Intelligent Power Saver	Fast shipping and used it right after receiving it.	Taobao Marketplace, a Chinese website for online shopping
Xindu Flagship Store	Green Intelligent Power Saver	Used it for a day and it's great. Ready to buy a couple more.	Taobao Marketplace, a Chinese website for online shopping
Xindu Flagship Store	Green Intelligent Power Saver	This thing is a complete fraudulent product I usually average daily electricity bill is 5 to 7 yuan plug this thing after an average of 8 to 14 yuan per day instructions and merchants say that this thing does not consume electricity, said that because of the summer heat home appliances produced by the cost of electricity, I would like to say that how do I not use your home products before the weather is not hot, how do not see the electricity bill increased so quickly, the	Taobao Marketplace, a Chinese website for online shopping

Xindu Flagship Store	Green Intelligent Power Saver	Packaged very well shipped quickly, good quality well made, easy to use really save power, the effect is ideal.	Taobao Marketplace, a Chinese website for online shopping
Xindu Flagship Store	Green Intelligent Power Saver	Practical is very simple, a plug can be, power-saving effect is very good, have a gift is very affordable	Taobao Marketplace, a Chinese website for online shopping
Xindu Flagship Store	Green Intelligent Power Saver	Works great, good results, have taken multiple shots	Taobao Marketplace, a Chinese website for online shopping
Xindu Flagship Store	Green Intelligent Power Saver	Just got back, small and cute, use it first, and then come back to the review	Taobao Marketplace, a Chinese website for online shopping
Xindu Flagship Store	Green Intelligent Power Saver	Just used it and it's fine, quite practical	Taobao Marketplace, a Chinese website for online shopping
Xindu Flagship Store	Green Intelligent Power Saver	Received fast shipping quality is quite good, the store service is very good satisfied!	Taobao Marketplace, a Chinese website for online shopping
Xindu Flagship Store	Green Intelligent Power Saver	Power-saving effect: being tried out, will comment again in a few days	Taobao Marketplace, a Chinese website for online shopping
Xindu Flagship Store	Green Intelligent Power Saver	It works well, saves energy and power	Taobao Marketplace, a Chinese website for online shopping
Xindu Flagship Store	Green Intelligent Power Saver	Power saving effect: very good	Taobao Marketplace, a Chinese website for online shopping
Xindu Flagship Store	Green Intelligent Power Saver	Express delivery was fast! The stuff is small and compact! I don't know if it works that well, well next time I'm going to buy two more! Tiny doesn't take up much space!	Taobao Marketplace, a Chinese website for online shopping
Xindu Flagship Store	Green Intelligent Power Saver	Exactly as described by the merchant, superb! Power-saving effect: good Cost-effective: high Appearance style: beautiful Workmanship material: quite good Experience: super!	Taobao Marketplace, a Chinese website for online shopping
Xindu Flagship Store	Green Intelligent Power Saver	Logistics is quite fast, in the online sales look good, I do not know how the actual effect, just arrived today to try to say!	Taobao Marketplace, a Chinese website for online shopping
Xindu Flagship Store	Green Intelligent Power Saver	Used a little, feel really have effect, but the business said that the pre to be optimized. But also long-term use of the effect will be more obvious.	Taobao Marketplace, a Chinese website for online shopping
Xindu Flagship Store	Green Intelligent Power Saver	Nice and easy to use but not sure how well it works.	Taobao Marketplace, a Chinese website for online shopping
Xindu Flagship Store	Green Intelligent Power Saver	Very good, convenient and practical, worth buying.	Taobao Marketplace, a Chinese website for online shopping
Xindu Flagship Store	Green Intelligent Power Saver	Power saving effect: not bad	Taobao Marketplace, a Chinese website for online shopping
Xindu Flagship Store	Green Intelligent Power Saver	It's good.	Taobao Marketplace, a Chinese website for online shopping
Xindu Flagship Store	Green Intelligent Power Saver	Power saving effect: nothing	Taobao Marketplace, a Chinese website for online shopping
Xindu Flagship Store	Green Intelligent Power Saver	In use	Taobao Marketplace, a Chinese website for online shopping
Xindu Flagship Store	Green Intelligent Power Saver	Just bought it back and used it, the effect is not clear, but my dad introduced that his side of the house with this, every month can save electricity 40 to 50 dollars. I hope I can do the same with this one!	Taobao Marketplace, a Chinese website for online shopping
Xindu Flagship Store	Green Intelligent Power Saver	Power saving effect: not bad Cost-effective: very high Appearance style: compact Workmanship material: good Experience: very good Worth buying	Taobao Marketplace, a Chinese website for online shopping
Xindu Flagship Store	Green Intelligent Power Saver	It's easy to use. It's great. I'll buy it again.	Taobao Marketplace, a Chinese website for online shopping
Xindu Flagship Store	Green Intelligent Power Saver	Summer is more costly electricity bought to use used to plug in the logistics is very fast simple and convenient directly plugged into the line	Taobao Marketplace, a Chinese website for online shopping
Xindu Flagship Store	Green Intelligent Power Saver	It's working well. I've recommended it to my friends. We're all saving energy together.	Taobao Marketplace, a Chinese website for online shopping
Xindu Flagship Store	Green Intelligent Power Saver	(๑•̀ㅂ•́)و✧ Super price and great performance, perfect shopping!	Taobao Marketplace, a Chinese website for online shopping
Xindu Flagship Store	Green Intelligent Power Saver	Don't know yet. I'll come back after I use it.	Taobao Marketplace, a Chinese website for online shopping
Xindu Flagship Store	Green Intelligent Power Saver	Great quality, works great, great power savings, love it!	Taobao Marketplace, a Chinese website for online shopping
Xindu Flagship Store	Green Intelligent Power Saver	No, this thing is basically not energy efficient! It's more than it used to be!	Taobao Marketplace, a Chinese website for online shopping
Xindu Flagship Store	Green Intelligent Power Saver	It doesn't help at all, I don't know where the good reviews come from	Taobao Marketplace, a Chinese website for online shopping
Xindu Flagship Store	Green Intelligent Power Saver	Power saving effect: 15%-45% power saving rate, can help me save a lot of electricity cost Cost-effective: cost-effective super high Appearance style: small and convenient, just a plug!	Taobao Marketplace, a Chinese website for online shopping
Xindu Flagship Store	Green Intelligent Power Saver	Obviously feel the meter go slower, really have the effect of saving electricity	Taobao Marketplace, a Chinese website for online shopping
Xindu Flagship Store	Green Intelligent Power Saver	Power saving effect: can Cost-effective: high Appearance style: good-looking Workmanship material: still can Use experience: good	Taobao Marketplace, a Chinese website for online shopping
Xindu Flagship Store	Green Intelligent Power Saver	Good, around a lot of friends are using the effect is obvious	Taobao Marketplace, a Chinese website for online shopping
Xindu Flagship Store	Green Intelligent Power Saver	Received the Power Saver, bought one last time and two this time. The more you use Power Saver, the more power you save. Logistics is also fast. Favorite pro can get down!	Taobao Marketplace, a Chinese website for online shopping

Xindu Flagship Store	Green Intelligent Power Saver	Power Saving King received, logistics is very fast. I bought one a few days earlier and two more in the past two days. The more you use the king of power saving, the more power saving! Favorite pro can get down!	Taobao Marketplace, a Chinese website for online shopping
Xindu Flagship Store	Green Intelligent Power Saver	The goods have been received, looking forward to the effect, first give a five-star praise!	Taobao Marketplace, a Chinese website for online shopping
Xindu Flagship Store	Green Intelligent Power Saver	Goods and the seller described the same, good service attitude, recommended to buy.	Taobao Marketplace, a Chinese website for online shopping
Xindu Flagship Store	Green Intelligent Power Saver	Works great after use, first time shopper satisfied. Recommended	Taobao Marketplace, a Chinese website for online shopping
Xindu Flagship Store	Green Intelligent Power Saver	The item was received and is quite satisfactory!	Taobao Marketplace, a Chinese website for online shopping
Xindu Flagship Store	Green Intelligent Power Saver	The stuff looks great, so I'm going to take it down and try it out.	Taobao Marketplace, a Chinese website for online shopping
Xindu Flagship Store	Green Intelligent Power Saver	Fast shipping, results to be verified.	Taobao Marketplace, a Chinese website for online shopping
Xindu Flagship Store	Green Intelligent Power Saver	Cost-effective, workmanship material is very good, with the feeling that it is saving, the effect is very good, need to get down quickly!	Taobao Marketplace, a Chinese website for online shopping
Xindu Flagship Store	Green Intelligent Power Saver	Power saving effect is good, very good, cost-effective, recommend to everyone	Taobao Marketplace, a Chinese website for online shopping
Xindu Flagship Store	Green Intelligent Power Saver	It looks OK, I don't know if it saves power or not	Taobao Marketplace, a Chinese website for online shopping
Xindu Flagship Store	Green Intelligent Power Saver	No energy saving effect at all, don't be fooled, don't buy it	Taobao Marketplace, a Chinese website for online shopping
Xindu Flagship Store	Green Intelligent Power Saver	This really saves electricity	Taobao Marketplace, a Chinese website for online shopping
Xindu Flagship Store	Green Intelligent Power Saver	Bought it to try, a friend recommended it and said it works pretty well. The material is very good.	Taobao Marketplace, a Chinese website for online shopping
Xindu Flagship Store	Green Intelligent Power Saver	Been using it for a while now overall good 👍	Taobao Marketplace, a Chinese website for online shopping
Xindu Flagship Store	Green Intelligent Power Saver	Good packaging, fast logistics, the effect is good, after a period of time to follow up!	Taobao Marketplace, a Chinese website for online shopping
Xindu Flagship Store	Green Intelligent Power Saver	The product matches the description. Doesn't take up much space. Very great product!	Taobao Marketplace, a Chinese website for online shopping
Xindu Flagship Store	Green Intelligent Power Saver	It's fast and looks good	Taobao Marketplace, a Chinese website for online shopping
Xindu Flagship Store	Green Intelligent Power Saver	Works great, saves power	Taobao Marketplace, a Chinese website for online shopping
Xindu Flagship Store	Green Intelligent Power Saver	Affordable, power-saving in place	Taobao Marketplace, a Chinese website for online shopping
Xindu Flagship Store	Green Intelligent Power Saver	The quality bar is very good and cost effective. Fine workmanship is very good, try good use	Taobao Marketplace, a Chinese website for online shopping
Xindu Flagship Store	Green Intelligent Power Saver	Shipping speed is very fast quality is very good cost-effective workmanship materials are very good power saving god	Taobao Marketplace, a Chinese website for online shopping
Xindu Flagship Store	Green Intelligent Power Saver	It's really good, will come back next time and recommend friends to buy together.	Taobao Marketplace, a Chinese website for online shopping
Xindu Flagship Store	Green Intelligent Power Saver	Power saving effect is good, the nth purchase	Taobao Marketplace, a Chinese website for online shopping
Xindu Flagship Store	Green Intelligent Power Saver	Practical and good value for money	Taobao Marketplace, a Chinese website for online shopping
Xindu Flagship Store	Green Intelligent Power Saver	Test in progress	Taobao Marketplace, a Chinese website for online shopping
Xindu Flagship Store	Green Intelligent Power Saver	It's not working.	Taobao Marketplace, a Chinese website for online shopping
Xindu Flagship Store	Green Intelligent Power Saver	I'm sure it's the same thing.	Taobao Marketplace, a Chinese website for online shopping
Xindu Flagship Store	Green Intelligent Power Saver	I've used one for myself and now I'm recommending one to my mom, it's worth it for the summer!	Taobao Marketplace, a Chinese website for online shopping
Xindu Flagship Store	Green Intelligent Power Saver	Let's buy it.	Taobao Marketplace, a Chinese website for online shopping
Xindu Flagship Store	Green Intelligent Power Saver	Power-saving effect: the effect is very good, deliberately used for a period of time to evaluate, the original electricity bill is very high, since the use of this power-saving artifact, the electricity bill saves a lot of cost-effective: too high, affordable, and save a large sum of money on electricity Appearance and style: compact, and very convenient, inserted into any socket. On the workmanship	Taobao Marketplace, a Chinese website for online shopping
Xindu Flagship Store	Green Intelligent Power Saver	Good stuff, fast logistics, trustworthy, can be purchased	Taobao Marketplace, a Chinese website for online shopping
Xindu Flagship Store	Green Intelligent Power Saver	Power saving effect: after using a few days, very satisfied with a shopping, feel the effect is obvious, it is worth buying oh, there is a need to time will patronize again.	Taobao Marketplace, a Chinese website for online shopping
Xindu Flagship Store	Green Intelligent Power Saver	Received the item and pressed it on expecting results!	Taobao Marketplace, a Chinese website for online shopping
Xindu Flagship Store	Green Intelligent Power Saver	Value for money: installed do not know the effect	Taobao Marketplace, a Chinese website for online shopping

Xindu Flagship Store	Green Intelligent Power Saver	It's working fine.	Taobao Marketplace, a Chinese website for online shopping
Xindu Flagship Store	Green Intelligent Power Saver	Express received today, opened it and used it, hope it works. Very satisfied with a shopping	Taobao Marketplace, a Chinese website for online shopping
Xindu Flagship Store	Green Intelligent Power Saver	Received the baby, exquisite packaging, beautiful. Just used, has not yet tested the effect, the first feeling should be good, to be used and then additional evaluation.	Taobao Marketplace, a Chinese website for online shopping
Xindu Flagship Store	Green Intelligent Power Saver	Worth buying, product quality is fine	Taobao Marketplace, a Chinese website for online shopping
Xindu Flagship Store	Green Intelligent Power Saver	Goods have been received, used well, will buy again!	Taobao Marketplace, a Chinese website for online shopping
Xindu Flagship Store	Green Intelligent Power Saver	I received it and I've already used it. We'll see if it works in a month.	Taobao Marketplace, a Chinese website for online shopping
Xindu Flagship Store	Green Intelligent Power Saver	Power saving effect: still in the test Cost-effective: quite high Workmanship material: not bad	Taobao Marketplace, a Chinese website for online shopping
Xindu Flagship Store	Green Intelligent Power Saver	Quality can be. Workmanship is quite fine, logistics is also very fast, overall okay	Taobao Marketplace, a Chinese website for online shopping
Xindu Flagship Store	Green Intelligent Power Saver	Hopefully this purchase will last longer, I bought 2 last July and 1 broke mid-term and had to restock 1.	Taobao Marketplace, a Chinese website for online shopping
Xindu Flagship Store	Green Intelligent Power Saver	Experience: the description does not match the real thing at all, it takes one to two months to see the effect, not at all the video said to see the effect so fast!	Taobao Marketplace, a Chinese website for online shopping
Xindu Flagship Store	Green Intelligent Power Saver	Not only fake and shoddy, we recommend that you do not fall for it!	Taobao Marketplace, a Chinese website for online shopping
Xindu Flagship Store	Green Intelligent Power Saver	It looks good, use it for a while and then come back for a review	Taobao Marketplace, a Chinese website for online shopping
Xindu Flagship Store	Green Intelligent Power Saver	This thing has been used for more than two months, useless, cheating, a month of electricity costs more than 1000	Taobao Marketplace, a Chinese website for online shopping
Xindu Flagship Store	Green Intelligent Power Saver	Expect good results.	Taobao Marketplace, a Chinese website for online shopping
Xindu Flagship Store	Green Intelligent Power Saver	**I've been searching for it in an Internet cafe.	Taobao Marketplace, a Chinese website for online shopping
Xindu Flagship Store	Green Intelligent Power Saver	It works! I don't know what makes sense, but it does save me money again on my electric bill, it's NICE!	Taobao Marketplace, a Chinese website for online shopping
Xindu Flagship Store	Green Intelligent Power Saver	It is really beneficial to the country and the people of the gods, this July to me to save half of the electricity bill, the product of the money 1 month to save back, everyone seize the time to buy it, and I do not know if I can buy it in the future!	Taobao Marketplace, a Chinese website for online shopping
Xindu Flagship Store	Green Intelligent Power Saver	The power saving is very good. Especially like 。。。。。。	Taobao Marketplace, a Chinese website for online shopping
Xindu Flagship Store	Green Intelligent Power Saver	Small, beautiful and cost-effective	Taobao Marketplace, a Chinese website for online shopping
Xindu Flagship Store	Green Intelligent Power Saver	It's not bad. It's worth having.	Taobao Marketplace, a Chinese website for online shopping
Xindu Flagship Store	Green Intelligent Power Saver	Power saving is very good	Taobao Marketplace, a Chinese website for online shopping
Xindu Flagship Store	Green Intelligent Power Saver	It's not bad.	Taobao Marketplace, a Chinese website for online shopping
Xindu Flagship Store	Green Intelligent Power Saver	Pure and simple IQ tax! Lolololololololololololololololololololololololololololololololololololololololol...	Taobao Marketplace, a Chinese website for online shopping
Xindu Flagship Store	Green Intelligent Power Saver	Guys and gals, the IQ tax should be paid, for no other reason than the general consumer...	Taobao Marketplace, a Chinese website for online shopping
Xindu Flagship Store	Green Intelligent Power Saver	Power saving effect: very good Experience: very good Three installed at home and used for a period of time before evaluation	Taobao Marketplace, a Chinese website for online shopping
Xindu Flagship Store	Green Intelligent Power Saver	Three houses, three installations, second purchase.	Taobao Marketplace, a Chinese website for online shopping
Xindu Flagship Store	Green Intelligent Power Saver	Can't wait to open it when I get it. Overall it's great!	Taobao Marketplace, a Chinese website for online shopping
Xindu Flagship Store	Green Intelligent Power Saver	It's not bad, I've been using it for a month and I've probably saved about a dozen bucks in real life.	Taobao Marketplace, a Chinese website for online shopping
Xindu Flagship Store	Green Intelligent Power Saver	Plug and play is super easy! Seller shipped quickly!	Taobao Marketplace, a Chinese website for online shopping
Xindu Flagship Store	Green Intelligent Power Saver	I just bought it, I don't know if it can save electricity, I'll comment again in a month.	Taobao Marketplace, a Chinese website for online shopping
Xindu Flagship Store	Green Intelligent Power Saver	Things are really good, very cost-effective, logistics is very fast, customer service is very patient, a very pleasant shopping, super value, like the old iron can order, very good, is genuine, like, will come to patronize your home!	Taobao Marketplace, a Chinese website for online shopping
Xindu Flagship Store	Green Intelligent Power Saver	Things are really good, very cost-effective, logistics is very fast, customer service is very patient, a very pleasant shopping, super value, like the old iron can order, very good, is genuine, like, will come to patronize your home!	Taobao Marketplace, a Chinese website for online shopping
Xindu Flagship Store	Green Intelligent Power Saver	Received, this intelligent power saver is very good to use my own is the second time to buy, save a lot of money on the electricity bill, the previous January 200 more than the electricity bill is now as long as a few dozen dollars!	Taobao Marketplace, a Chinese website for online shopping
Xindu Flagship Store	Green Intelligent Power Saver	The power saving effect is very good, is a regular customer of his family. The price is affordable, the packaging is intact logistics is also very fast, praise!	Taobao Marketplace, a Chinese website for online shopping

Xindu Flagship Store	Green Intelligent Power Saver	Easy to use, plug and play, energy-saving power-saving small hands, praise!	Taobao Marketplace, a Chinese website for online shopping
Xindu Flagship Store	Green Intelligent Power Saver	Plugged in two energy saver to see the meter speed did not change, think should not play a big role, may be the business is to see the consumer psychology exaggerated advertising Appearance is okay, slowly experience and then say chanting	Taobao Marketplace, a Chinese website for online shopping
Xindu Flagship Store	Green Intelligent Power Saver	Inserted two energy saver, table turn speed without any change, eh, difficult to really as Baidu said, the more energy saver inserted the more electricity, dozens of dollars to buy a heart	Taobao Marketplace, a Chinese website for online shopping
Xindu Flagship Store	Green Intelligent Power Saver	Power saving effect: very good, save a lot of electricity	Taobao Marketplace, a Chinese website for online shopping
Xindu Flagship Store	Green Intelligent Power Saver	Works great very power efficient	Taobao Marketplace, a Chinese website for online shopping
Xindu Flagship Store	Green Intelligent Power Saver	It's crap. It doesn't work.	Taobao Marketplace, a Chinese website for online shopping
Xindu Flagship Store	Green Intelligent Power Saver	Logistics speed, the seller's attitude is very good, always patient explanation, a very successful shopping, looking forward to good results!	Taobao Marketplace, a Chinese website for online shopping
Xindu Flagship Store	Green Intelligent Power Saver	A successful purchase, looking forward to good results!	Taobao Marketplace, a Chinese website for online shopping
Xindu Flagship Store	Green Intelligent Power Saver	Appearance and style: listen to the introduction of friends to buy, friends say especially power-saving hopefully good	Taobao Marketplace, a Chinese website for online shopping
Xindu Flagship Store	Green Intelligent Power Saver	Nice , good price, looks well made and easy to use, just plug it in and sit back and wait for the power savings.	Taobao Marketplace, a Chinese website for online shopping
Xindu Flagship Store	Green Intelligent Power Saver	Received the goods, looking forward to the effect. If there is effect will have to repurchase	Taobao Marketplace, a Chinese website for online shopping
Xindu Flagship Store	Green Intelligent Power Saver	Power saving effect: good Cost-effectiveness: good Experience: in use	Taobao Marketplace, a Chinese website for online shopping
Xindu Flagship Store	Green Intelligent Power Saver	It works! It does work.	Taobao Marketplace, a Chinese website for online shopping
Xindu Flagship Store	Green Intelligent Power Saver	Doing this **business**, ****.	Taobao Marketplace, a Chinese website for online shopping
Xindu Flagship Store	Green Intelligent Power Saver	Experience: Wan is good very good	Taobao Marketplace, a Chinese website for online shopping
Xindu Flagship Store	Green Intelligent Power Saver	I've been using it for half a month now and it's not working at all, proper IQ tax.	Taobao Marketplace, a Chinese website for online shopping
Xindu Flagship Store	Green Intelligent Power Saver	It doesn't do any good, and it increases the power consumption.	Taobao Marketplace, a Chinese website for online shopping
Xindu Flagship Store	Green Intelligent Power Saver	IQ tax, right? It's all over the TV.	Taobao Marketplace, a Chinese website for online shopping
Xindu Flagship Store	Green Intelligent Power Saver	I'll review after I use it.	Taobao Marketplace, a Chinese website for online shopping
Xindu Flagship Store	Green Intelligent Power Saver	There's no point in saving power.	Taobao Marketplace, a Chinese website for online shopping
Xindu Flagship Store	Green Intelligent Power Saver	Baby received, logistics is very fast, looks quite generous, practical, five-star praise!	Taobao Marketplace, a Chinese website for online shopping
Xindu Flagship Store	Green Intelligent Power Saver	tier	Taobao Marketplace, a Chinese website for online shopping
Xindu Flagship Store	Green Intelligent Power Saver	This is a very good product, more satisfied, will come back next time, worth recommending.	Taobao Marketplace, a Chinese website for online shopping
Xindu Flagship Store	Green Intelligent Power Saver	Power saving effect: obvious Cost-effective: good Appearance style: small and beautiful Workmanship material: excellent Overall very good in use without fever quality is very good	Taobao Marketplace, a Chinese website for online shopping
Xindu Flagship Store	Green Intelligent Power Saver	A friend recommended this and said it worked well, so I'm looking forward to the results.	Taobao Marketplace, a Chinese website for online shopping
Xindu Flagship Store	Green Intelligent Power Saver	It's genuine, I'll comment after using it	Taobao Marketplace, a Chinese website for online shopping
Xindu Flagship Store	Green Intelligent Power Saver	It's more energy efficient. The meter's spinning slower.	Taobao Marketplace, a Chinese website for online shopping
Xindu Flagship Store	Green Intelligent Power Saver	Good performance, good appearance, intelligent power saving system, very much like it.	Taobao Marketplace, a Chinese website for online shopping
Xindu Flagship Store	Green Intelligent Power Saver	Intelligent power saving, peace of mind, safe and reliable.	Taobao Marketplace, a Chinese website for online shopping
Xindu Flagship Store	Green Intelligent Power Saver	I'll have to use it for a while to evaluate whether it saves power or not!	Taobao Marketplace, a Chinese website for online shopping
Xindu Flagship Store	Green Intelligent Power Saver	Just plugged it in, let's see what happens in a month, otherwise good review!	Taobao Marketplace, a Chinese website for online shopping
Xindu Flagship Store	Green Intelligent Power Saver	The stuff doesn't work. The reviews are all swiped.	Taobao Marketplace, a Chinese website for online shopping
Xindu Flagship Store	Green Intelligent Power Saver	Works great, this has been in use for a month now and it does have less electricity	Taobao Marketplace, a Chinese website for online shopping
Xindu Flagship Store	Green Intelligent Power Saver	Bought the first one which worked well, and the second one, which I ordered additionally today, is also carrying goods	Taobao Marketplace, a Chinese website for online shopping

Xindu Flagship Store	Green Intelligent Power Saver	I've been using this type of product for years and it works great! So, buy another one to use! This one, in particular, is much better!	Taobao Marketplace, a Chinese website for online shopping
Xindu Flagship Store	Green Intelligent Power Saver	I'll follow up after two months of use!	Taobao Marketplace, a Chinese website for online shopping
Xindu Flagship Store	Green Intelligent Power Saver	Logistics is very fast, the stuff is very good, the appearance is very nice and weighty feeling	Taobao Marketplace, a Chinese website for online shopping
Xindu Flagship Store	Green Intelligent Power Saver	I'll continue to follow up after a month of use, so we'll see how it goes then	Taobao Marketplace, a Chinese website for online shopping
Xindu Flagship Store	Green Intelligent Power Saver	Intelligent economizer received, good price good workmanship material, appearance and style is also good, put on the use.	Taobao Marketplace, a Chinese website for online shopping
Xindu Flagship Store	Green Intelligent Power Saver	Rubbing the IQ of anyone who buys this shit into the ground.	Taobao Marketplace, a Chinese website for online shopping
Xindu Flagship Store	Green Intelligent Power Saver	Treating us like **	Taobao Marketplace, a Chinese website for online shopping
Xindu Flagship Store	Green Intelligent Power Saver	Second time buyer.	Taobao Marketplace, a Chinese website for online shopping
Xindu Flagship Store	Green Intelligent Power Saver	Good stuff at a great price.	Taobao Marketplace, a Chinese website for online shopping
Xindu Flagship Store	Green Intelligent Power Saver	It really works.	Taobao Marketplace, a Chinese website for online shopping
Xindu Flagship Store	Green Intelligent Power Saver	Good stuff, fast logistics, praise	Taobao Marketplace, a Chinese website for online shopping
Xindu Flagship Store	Green Intelligent Power Saver	Power saving effect: can really save section Cost-effective: "reasonable price Appearance style: \ beautiful Workmanship material: solid Experience: feel good	Taobao Marketplace, a Chinese website for online shopping
Xindu Flagship Store	Green Intelligent Power Saver	Baby received, logistics is very fast, power saving effect: very good Cost-effective: very good Appearance style: beautiful Workmanship material: very good Experience: quite good	Taobao Marketplace, a Chinese website for online shopping
Xindu Flagship Store	Green Intelligent Power Saver	Baby received, is genuine, logistics is very fast. Customer service is very careful, ask what all will answer one by one? The product must also be good, after all, it is bought inside the Tmall store. Looking forward to next month's surprise, I read the evaluation, many people say it is genuine, this product is	Taobao Marketplace, a Chinese website for online shopping
Xindu Flagship Store	Green Intelligent Power Saver	Energy saver received very quickly, the appearance of the style is very. Previously used energy-saving effect is good, exquisite workmanship is worth having.	Taobao Marketplace, a Chinese website for online shopping
Xindu Flagship Store	Green Intelligent Power Saver	Energy saver can be used correctly to save energy and save money on electricity.	Taobao Marketplace, a Chinese website for online shopping
Xindu Flagship Store	Green Intelligent Power Saver	Power-saving effect: I have tested it does go slower Cost-effective: affordable Appearance and style: I like the appearance Workmanship material: good drop-proof	Taobao Marketplace, a Chinese website for online shopping
Xindu Flagship Store	Green Intelligent Power Saver	Power saving effect: good Cost-effective: high Appearance style: beautiful Experience: really good!	Taobao Marketplace, a Chinese website for online shopping
Xindu Flagship Store	Green Intelligent Power Saver	It's really good. Tried it as soon as I got back. It's really good 😌	Taobao Marketplace, a Chinese website for online shopping
Xindu Flagship Store	Green Intelligent Power Saver	It doesn't feel like it's working.	Taobao Marketplace, a Chinese website for online shopping
Xindu Flagship Store	Green Intelligent Power Saver	Power saving effect: quite convenient, directly plugged into the line board can be used	Taobao Marketplace, a Chinese website for online shopping
Xindu Flagship Store	Green Intelligent Power Saver	Power saving effect: I don't know yet, I'll give a favorable comment next month.	Taobao Marketplace, a Chinese website for online shopping
Xindu Flagship Store	Green Intelligent Power Saver	Don't buy it. It's an IQ tax.	Taobao Marketplace, a Chinese website for online shopping
Xindu Flagship Store	Green Intelligent Power Saver	I don't know if it's real or good, so I'll take it as tuition.	Taobao Marketplace, a Chinese website for online shopping
Xindu Flagship Store	Green Intelligent Power Saver	It is authentic, the same as the physical store, the price is still favorable, decisive conversion.	Taobao Marketplace, a Chinese website for online shopping
Xindu Flagship Store	Green Intelligent Power Saver	Baby received, logistics is very fast, the description matches, more satisfied, favorable comments, recommended.	Taobao Marketplace, a Chinese website for online shopping
Xindu Flagship Store	Green Intelligent Power Saver	Power saving effect: good Cost-effective: high Appearance style: beautiful Workmanship material: fine Goods value, technology can save money	Taobao Marketplace, a Chinese website for online shopping
Xindu Flagship Store	Green Intelligent Power Saver	Technology is productivity, saving a little is a little, quality assurance	Taobao Marketplace, a Chinese website for online shopping
Xindu Flagship Store	Green Intelligent Power Saver	Energy saving and environmental protection is that we all have to do things, a small energy saver play a big role, everyone together energy saving it 👍	Taobao Marketplace, a Chinese website for online shopping
Xindu Flagship Store	Green Intelligent Power Saver	Feels good, let's see how much we can save on next month's electricity bill	Taobao Marketplace, a Chinese website for online shopping
Xindu Flagship Store	Green Intelligent Power Saver	Good product quality, very good, will come back next time	Taobao Marketplace, a Chinese website for online shopping
Xindu Flagship Store	Green Intelligent Power Saver	This little thing is so energy efficient and eco-friendly that I will push it to people around me.	Taobao Marketplace, a Chinese website for online shopping
Xindu Flagship Store	Green Intelligent Power Saver	It really saves money! It's great. I'm gonna take another one.	Taobao Marketplace, a Chinese website for online shopping
Xindu Flagship Store	Green Intelligent Power Saver	I just got it.	Taobao Marketplace, a Chinese website for online shopping

Xindu Flagship Store	Green Intelligent Power Saver	Try it this month.	Taobao Marketplace, a Chinese website for online shopping
Xindu Flagship Store	Green Intelligent Power Saver	I was worried about something being bad, tried it out and it's not bad.	Taobao Marketplace, a Chinese website for online shopping
Xindu Flagship Store	Green Intelligent Power Saver	It's in use, so I'll comment again in a month.	Taobao Marketplace, a Chinese website for online shopping
Xindu Flagship Store	Green Intelligent Power Saver	Saver does have effect, this is the second time to buy, the last time to buy 6, save a lot of electricity, and now for the stores also arranged to purchase 10, the	Taobao Marketplace, a Chinese website for online shopping
Xindu Flagship Store	Green Intelligent Power Saver	This month's electricity bill came out, as always, as good as ever, as always less, as always save, the first time to buy still really do not quite believe, can not figure out, but fortunately at that time did not give up, chose to believe in the merchant	Taobao Marketplace, a Chinese website for online shopping
Xindu Flagship Store	Green Intelligent Power Saver	This saver is really good, I heard is 2023 latest models, not only the value of good-looking, the effect is still very good, this is my fifth saver in this store, to help relatives buy a lot of it, the old customer also gave me a discount Thanks to the store!	Taobao Marketplace, a Chinese website for online shopping
Xindu Flagship Store	Green Intelligent Power Saver	The product is very formal, used with effect, and after-sales service is very good	Taobao Marketplace, a Chinese website for online shopping
Xindu Flagship Store	Green Intelligent Power Saver	Received in good condition, a friend recommended it does save some electricity. Buy a use!	Taobao Marketplace, a Chinese website for online shopping
Xindu Flagship Store	Green Intelligent Power Saver	Simple and easy to use, affordable, very good, ready to buy a few more	Taobao Marketplace, a Chinese website for online shopping
Xindu Flagship Store	Green Intelligent Power Saver	The logistics is fast, the quality is very good, since the use of this power-saving appliances since the electricity bill saved a lot, easy to use!	Taobao Marketplace, a Chinese website for online shopping
Xindu Flagship Store	Green Intelligent Power Saver	Quality is very good cheap price fine workmanship logistics very fast service attitude is very good	Taobao Marketplace, a Chinese website for online shopping
Xindu Flagship Store	Green Intelligent Power Saver	Great value for money, I didn't realize how good the value for money was until I received it, very happy with it!	Taobao Marketplace, a Chinese website for online shopping
Xindu Flagship Store	Green Intelligent Power Saver	Power saving effect is good, customer service attitude is also very good, patient answer, suggest to buy	Taobao Marketplace, a Chinese website for online shopping
Xindu Flagship Store	Green Intelligent Power Saver	Great quality and material, works great!	Taobao Marketplace, a Chinese website for online shopping
Xindu Flagship Store	Green Intelligent Power Saver	Let's use it for a while and see what happens	Taobao Marketplace, a Chinese website for online shopping
Xindu Flagship Store	Green Intelligent Power Saver	Very happy with the item very good quality well made	Taobao Marketplace, a Chinese website for online shopping
Xindu Flagship Store	Green Intelligent Power Saver	The stuff is exceptionally good, exceptionally power-saving	Taobao Marketplace, a Chinese website for online shopping
Xindu Flagship Store	Green Intelligent Power Saver	The product wasn't as good as the vendor said it would be, it didn't work at all!	Taobao Marketplace, a Chinese website for online shopping
Xindu Flagship Store	Green Intelligent Power Saver	Power saving effect: arrived just used Cost-effective: low Appearance style: beautiful Workmanship material: good Experience: the first time to use	Taobao Marketplace, a Chinese website for online shopping
Xindu Flagship Store	Green Intelligent Power Saver	Be good. Be good! Good good good! It's all good. It's all good.	Taobao Marketplace, a Chinese website for online shopping
Xindu Flagship Store	Green Intelligent Power Saver	Originally, the family's monthly electricity bill of three to four hundred dollars, after using 6 power savers the electricity bill only paid a few tens of dollars. Inadvertently saw this power saver. It is not expensive, so I bought 2 to try, I did not expect so good, and bought a few, and then do not pay the	Taobao Marketplace, a Chinese website for online shopping
Xindu Flagship Store	Green Intelligent Power Saver	Power-saving effect: effective Cost-effective: good Oh Appearance style: mini-compact, does not occupy space Experience: not bad	Taobao Marketplace, a Chinese website for online shopping
Xindu Flagship Store	Green Intelligent Power Saver	Good Oh , deliberately used a few days to evaluate.	Taobao Marketplace, a Chinese website for online shopping
Xindu Flagship Store	Green Intelligent Power Saver	Good quality and easy to use . A good product needs to be publicized	Taobao Marketplace, a Chinese website for online shopping
Xindu Flagship Store	Green Intelligent Power Saver	Power saving effect: very good Using experience: very good	Taobao Marketplace, a Chinese website for online shopping
Xindu Flagship Store	Green Intelligent Power Saver	First time to use, summer air conditioning, very energy saving electricity, worth buying	Taobao Marketplace, a Chinese website for online shopping
Xindu Flagship Store	Green Intelligent Power Saver	The first time the effect can be the second time to buy now can normally open the air conditioning ❄❄❄❄ Power-saving effect: okay Sex-price ratio: line Appearance and style: beauty Workmanship material: light Experience: the first time to buy a Now buy the second can normally open the air	Taobao Marketplace, a Chinese website for online shopping
Xindu Flagship Store	Green Intelligent Power Saver	Just received it, results to be evaluated. First give a neutral evaluation. Good use, then additional positive comments and additional orders.	Taobao Marketplace, a Chinese website for online shopping
Xindu Flagship Store	Green Intelligent Power Saver	Shipped very quickly, the installation of a do not know customer service can also be patient answer Satisfied with a shopping need to come back!	Taobao Marketplace, a Chinese website for online shopping
Xindu Flagship Store	Green Intelligent Power Saver	Express delivery is very fast, arrived the next day, power saving king wait for a period of time to use to evaluate.	Taobao Marketplace, a Chinese website for online shopping
Xindu Flagship Store	Green Intelligent Power Saver	This is the godlike power saver, really paid the IQ tax Experience: not useful at all, advise people not to buy!	Taobao Marketplace, a Chinese website for online shopping
Xindu Flagship Store	Green Intelligent Power Saver	Power saving effect: good effect	Taobao Marketplace, a Chinese website for online shopping
Xindu Flagship Store	Green Intelligent Power Saver	I don't think it's a power saver.	Taobao Marketplace, a Chinese website for online shopping
Xindu Flagship Store	Green Intelligent Power Saver	*♪ It doesn't work, it's a waste ♪	Taobao Marketplace, a Chinese website for online shopping

Xindu Flagship Store	Green Intelligent Power Saver	It's not much use.	Taobao Marketplace, a Chinese website for online shopping
Xindu Flagship Store	Green Intelligent Power Saver	Definitely a great product, it's especially easy to use and plugs right into the plug board at home. In the past few days to turn on the air conditioning to see the meter turn a little slower than before. But the electricity consumption is a little less than before. This product is trustworthy, like 💞	Taobao Marketplace, a Chinese website for online shopping
Xindu Flagship Store	Green Intelligent Power Saver	Power saving effect: good Cost-effective: high Appearance style: lightweight Workmanship material: good Experience: not bad	Taobao Marketplace, a Chinese website for online shopping
Xindu Flagship Store	Green Intelligent Power Saver	After using it for a few days I feel it does save a lot of electricity, kudos!	Taobao Marketplace, a Chinese website for online shopping
Xindu Flagship Store	Green Intelligent Power Saver	Power saving effect: l good Cost-effective: high Appearance style: \ charming Workmanship material: l good	Taobao Marketplace, a Chinese website for online shopping
Xindu Flagship Store	Green Intelligent Power Saver	a bargain	Taobao Marketplace, a Chinese website for online shopping
Xindu Flagship Store	Green Intelligent Power Saver	Baby has been received, try a period of time to see, in the end, whether it can save electricity, and then objectively to evaluation	Taobao Marketplace, a Chinese website for online shopping
Xindu Flagship Store	Green Intelligent Power Saver	That's great. Take it apart if you don't believe me.	Taobao Marketplace, a Chinese website for online shopping
Xindu Flagship Store	Green Intelligent Power Saver	Bragging It is simply useless, it seems that Taobao is also going to be **** slowly screwed up.	Taobao Marketplace, a Chinese website for online shopping
Xindu Flagship Store	Green Intelligent Power Saver	Power-saving effect: {power-saving effect is good, Cost-effective: a good 😄 Appearance style: good Workmanship material: a good	Taobao Marketplace, a Chinese website for online shopping
Xindu Flagship Store	Green Intelligent Power Saver	It's the only thing that is particularly power-saving, and cheap, and looks good, and is made of good material, too.	Taobao Marketplace, a Chinese website for online shopping
Xindu Flagship Store	Green Intelligent Power Saver	Power saving effect: good cost-effective: high Experience: bought one, it does have power saving	Taobao Marketplace, a Chinese website for online shopping
Xindu Flagship Store	Green Intelligent Power Saver	I have one at home and one at the store, and I'm very happy with it!	Taobao Marketplace, a Chinese website for online shopping
Xindu Flagship Store	Green Intelligent Power Saver	Purchased a power saver received on the put into use, rent a room with many electrical appliances, power consumption is very large, we have a piece of electricity or 1.2 dollars a time, with this power saver is a lot better, every day can save a few cents, very convenient and practical.	Taobao Marketplace, a Chinese website for online shopping
Xindu Flagship Store	Green Intelligent Power Saver	Great quality, great energy savings, great price, fast shipping!	Taobao Marketplace, a Chinese website for online shopping
Xindu Flagship Store	Green Intelligent Power Saver	Good! Very good! Good quality, very satisfied	Taobao Marketplace, a Chinese website for online shopping
Xindu Flagship Store	Green Intelligent Power Saver	Power saving effect: good Cost-effective: excellent Appearance style: good Workmanship material: exquisite Experience: good	Taobao Marketplace, a Chinese website for online shopping
Xindu Flagship Store	Green Intelligent Power Saver	Express prompt shipping arrived quickly, used the same results as said, trustworthy.	Taobao Marketplace, a Chinese website for online shopping
Xindu Flagship Store	Green Intelligent Power Saver	Received, looks very good, plugged in, hope it works	Taobao Marketplace, a Chinese website for online shopping
Xindu Flagship Store	Green Intelligent Power Saver	The effect is good, plugged in a day than before 2 degrees less, longer and longer more and more power saving, very good, recommended to get!	Taobao Marketplace, a Chinese website for online shopping
Xindu Flagship Store	Green Intelligent Power Saver	Good genuinely power-saving, the price is not expensive, the appearance of the style is exquisite, very satisfied, worth recommending!	Taobao Marketplace, a Chinese website for online shopping
Xindu Flagship Store	Green Intelligent Power Saver	The power savings are great and the exterior style is very nice.	Taobao Marketplace, a Chinese website for online shopping
Xindu Flagship Store	Green Intelligent Power Saver	Energy saving and power saving, very good quality	Taobao Marketplace, a Chinese website for online shopping
Xindu Flagship Store	Green Intelligent Power Saver	Logistics is fast, good-looking, just started to use, looking forward to the effect	Taobao Marketplace, a Chinese website for online shopping
Xindu Flagship Store	Green Intelligent Power Saver	Recommended by a coworker as effective, just used it, hope it works well.	Taobao Marketplace, a Chinese website for online shopping
Xindu Flagship Store	Green Intelligent Power Saver	Use it first to get results before you comment	Taobao Marketplace, a Chinese website for online shopping
Xindu Flagship Store	Green Intelligent Power Saver	Power saving effect: very good effect is really good Cost-effective: cost-effective very high Appearance style: good-looking style Workmanship Material: very good quality workmanship Good experience: very practical, reasonably priced, energy-saving power saving	Taobao Marketplace, a Chinese website for online shopping
Xindu Flagship Store	Green Intelligent Power Saver	Love this product and will be buying it again next time	Taobao Marketplace, a Chinese website for online shopping
Xindu Flagship Store	Green Intelligent Power Saver	Power-saving effect: the effect is very obvious, the second purchase Cost-effective: quite affordable Appearance style: occupies a small area is great Workmanship material: explanation Overall a very good shopping	Taobao Marketplace, a Chinese website for online shopping
Xindu Flagship Store	Green Intelligent Power Saver	It's very effective, saved a lot of money in a month, and the air conditioning is on at will! Happy summer!	Taobao Marketplace, a Chinese website for online shopping
Xindu Flagship Store	Green Intelligent Power Saver	Received the energy saver. Overall looks good . The price is reasonable.	Taobao Marketplace, a Chinese website for online shopping
Xindu Flagship Store	Green Intelligent Power Saver	I've been trying it for 12 hours, and I think it's pretty good.	Taobao Marketplace, a Chinese website for online shopping
Xindu Flagship Store	Green Intelligent Power Saver	Recommended by a friend, try a useful everyone can also buy a try!	Taobao Marketplace, a Chinese website for online shopping
Xindu Flagship Store	Green Intelligent Power Saver	Good and inexpensive, everyone can buy one, summer air conditioning and power consumption	Taobao Marketplace, a Chinese website for online shopping

Xindu Flagship Store	Green Intelligent Power Saver	Merchants fast delivery, logistics is also fast, energy saver compact does not take up space, energy saving effect in the trial!	Taobao Marketplace, a Chinese website for online shopping
Xindu Flagship Store	Green Intelligent Power Saver	Very good! Already started using it! Experience: expect good results!	Taobao Marketplace, a Chinese website for online shopping
Xindu Flagship Store	Green Intelligent Power Saver	This is the second time I've repurchased! Bought it for a friend!	Taobao Marketplace, a Chinese website for online shopping
Xindu Flagship Store	Green Intelligent Power Saver	This product does save power	Taobao Marketplace, a Chinese website for online shopping
Xindu Flagship Store	Green Intelligent Power Saver	Cost effective and easy to use.	Taobao Marketplace, a Chinese website for online shopping
Xindu Flagship Store	Green Intelligent Power Saver	I heard that I can save a little bit, so I bought it to try it out and hopefully I'll be pleasantly surprised	Taobao Marketplace, a Chinese website for online shopping
Xindu Flagship Store	Green Intelligent Power Saver	Shipped really fast, electricity bills are so high in the summer, this one works pretty well	Taobao Marketplace, a Chinese website for online shopping
Xindu Flagship Store	Green Intelligent Power Saver	I bought it because my friends are using it and it saves energy.	Taobao Marketplace, a Chinese website for online shopping
Xindu Flagship Store	Green Intelligent Power Saver	Beautiful appearance, very good workmanship, very good results, value for money	Taobao Marketplace, a Chinese website for online shopping
Xindu Flagship Store	Green Intelligent Power Saver	Just received the item, don't know yet!	Taobao Marketplace, a Chinese website for online shopping
Xindu Flagship Store	Green Intelligent Power Saver	Use it for a few days to see if you've thought about it.	Taobao Marketplace, a Chinese website for online shopping
Xindu Flagship Store	Green Intelligent Power Saver	I've just started using it, I don't know how effective it is, so I'll give it a good review first!	Taobao Marketplace, a Chinese website for online shopping
Xindu Flagship Store	Green Intelligent Power Saver	It doesn't work. It's fake.	Taobao Marketplace, a Chinese website for online shopping
Xindu Flagship Store	Green Intelligent Power Saver	Just received the goods, try to see how the effect of the summer power consumption hope that the effect of	Taobao Marketplace, a Chinese website for online shopping
Xindu Flagship Store	Green Intelligent Power Saver	Goods have been received, logistics and delivery very quickly, after using the power-saving effect is very good? It's the same as the store's description.	Taobao Marketplace, a Chinese website for online shopping
Xindu Flagship Store	Green Intelligent Power Saver	Power-saving effect: effective Cost-effective: high Appearance style: very beautiful Workmanship material: good Experience: less than before to spend a lot of money	Taobao Marketplace, a Chinese website for online shopping
Xindu Flagship Store	Green Intelligent Power Saver	The logistics is quite fast and well packaged! Quality is especially good! Customer service attitude is particularly good! Reasonable price is worth buying!	Taobao Marketplace, a Chinese website for online shopping
Xindu Flagship Store	Green Intelligent Power Saver	Fast shipping! Good quality oh oh (๑><๑)! Worth getting	Taobao Marketplace, a Chinese website for online shopping
Xindu Flagship Store	Green Intelligent Power Saver	Power saving effect: good, Cost-effective: good Appearance style: good Workmanship material: good	Taobao Marketplace, a Chinese website for online shopping
Xindu Flagship Store	Green Intelligent Power Saver	Good material, fine workmanship, friends recommended to buy, power saving logistics fast, to 👍	Taobao Marketplace, a Chinese website for online shopping
Xindu Flagship Store	Green Intelligent Power Saver	Good quality, thick material, fine workmanship, satisfaction	Taobao Marketplace, a Chinese website for online shopping
Xindu Flagship Store	Green Intelligent Power Saver	It's authentic, fast shipping, the price is right, it's a great value and very satisfying	Taobao Marketplace, a Chinese website for online shopping
Xindu Flagship Store	Green Intelligent Power Saver	Five stars Power saving effect: ok Experience: experiencing	Taobao Marketplace, a Chinese website for online shopping
Xindu Flagship Store	Green Intelligent Power Saver	The quality of the saver is very good, satisfied.	Taobao Marketplace, a Chinese website for online shopping
Xindu Flagship Store	Green Intelligent Power Saver	It's not much use.	Taobao Marketplace, a Chinese website for online shopping
Xindu Flagship Store	Green Intelligent Power Saver	Power saving: pretty good, just plug it in randomly	Taobao Marketplace, a Chinese website for online shopping
Xindu Flagship Store	Green Intelligent Power Saver	With it, it really saves money on electricity, it works well, it's cost-effective, and the workmanship and materials are very good	Taobao Marketplace, a Chinese website for online shopping
Xindu Flagship Store	Green Intelligent Power Saver	It simply works so well that it can only be described as perfect! It does save electricity. Satisfied!	Taobao Marketplace, a Chinese website for online shopping
Xindu Flagship Store	Green Intelligent Power Saver	Recommended by a friend, he said they use this to save a lot of electricity, I bought two to try, the use is quite simple!	Taobao Marketplace, a Chinese website for online shopping
Xindu Flagship Store	Green Intelligent Power Saver	The quality is quite good, installed and used well, the power saving is quite obvious, quite practical.	Taobao Marketplace, a Chinese website for online shopping
Xindu Flagship Store	Green Intelligent Power Saver	Just plugged in, the plug board, not much wall plug, with a month to see the effect of	Taobao Marketplace, a Chinese website for online shopping
Xindu Flagship Store	Green Intelligent Power Saver	Power-saving effect: friends introduced to say good use I bought listen to friends say can be	Taobao Marketplace, a Chinese website for online shopping
Xindu Flagship Store	Green Intelligent Power Saver	It works like a charm. I've recommended it to a friend of mine who's buying one.	Taobao Marketplace, a Chinese website for online shopping
Xindu Flagship Store	Green Intelligent Power Saver	Power-saving effect: really useful Cost-effective: really cost-effective Closed eyes into the brothers Appearance style: small	Taobao Marketplace, a Chinese website for online shopping

Xindu Flagship Store	Green Intelligent Power Saver	Appearance and style: good-looking	Taobao Marketplace, a Chinese website for online shopping
Xindu Flagship Store	Green Intelligent Power Saver	economical	Taobao Marketplace, a Chinese website for online shopping
Xindu Flagship Store	Green Intelligent Power Saver	Very good, power saving effect can be, the quality is very good, especially satisfied with the	Taobao Marketplace, a Chinese website for online shopping
Xindu Flagship Store	Green Intelligent Power Saver	Now just use, do not know the effect of saving, next month the electricity bill out will know, first praise!	Taobao Marketplace, a Chinese website for online shopping
Xindu Flagship Store	Green Intelligent Power Saver	I've got it. I'll see what I can do with it.	Taobao Marketplace, a Chinese website for online shopping
Xindu Flagship Store	Green Intelligent Power Saver	The logistics is very fast packaging intact, the quality of the bar	Taobao Marketplace, a Chinese website for online shopping
Xindu Flagship Store	Green Intelligent Power Saver	Summer is here, the air conditioning on the electricity bill can not, with a few days, it seems to really have some effect!	Taobao Marketplace, a Chinese website for online shopping
Xindu Flagship Store	Green Intelligent Power Saver	Yesterday I deliberately plugged it in and turned on the air conditioner for an hour, then unplugged it and turned it on again for an hour, and it actually saved some electricity!	Taobao Marketplace, a Chinese website for online shopping
Xindu Flagship Store	Green Intelligent Power Saver	Good very good energy saver, really effective, monthly power savings of about 30 yuan test want to buy the treasure can start!	Taobao Marketplace, a Chinese website for online shopping
Xindu Flagship Store	Green Intelligent Power Saver	Power saving effect: the goods received, quite good, the	Taobao Marketplace, a Chinese website for online shopping
Xindu Flagship Store	Green Intelligent Power Saver	The Saver is great and good quality, very happy with the purchase. Saver works great, fast delivery!	Taobao Marketplace, a Chinese website for online shopping
Xindu Flagship Store	Green Intelligent Power Saver	Very good, really save electricity, regular manufacturers, rest assured to start, recommended!	Taobao Marketplace, a Chinese website for online shopping
Xindu Flagship Store	Green Intelligent Power Saver	I'll comment after a month of use	Taobao Marketplace, a Chinese website for online shopping
Xindu Flagship Store	Green Intelligent Power Saver	It doesn't work. It's not energy efficient.	Taobao Marketplace, a Chinese website for online shopping
Xindu Flagship Store	Green Intelligent Power Saver	It didn't work. Fake. Fooled.	Taobao Marketplace, a Chinese website for online shopping
Xindu Flagship Store	Green Intelligent Power Saver	The packaging is intact, the machine is exquisite workmanship, plugged in and lit up, just used, looking forward to a power-saving effect.	Taobao Marketplace, a Chinese website for online shopping
Xindu Flagship Store	Green Intelligent Power Saver	Use a few days the machine works normally not hot, see the netizens who have used said that there is an effect, expect me to use the same power-saving effect.	Taobao Marketplace, a Chinese website for online shopping
Xindu Flagship Store	Green Intelligent Power Saver	Looking to buy 3, in the past about 100 dollars a month, two hearts in the monthly looking to record several consecutive months are about 50 dollars, note the first month the effect is not very obvious, it reduces more than 20 points, the back of basically I'm more than 50 every month, the regulation in the air conditioning is going to be used, I'm buying another one.	Taobao Marketplace, a Chinese website for online shopping
Xindu Flagship Store	Green Intelligent Power Saver	I use it next to my air conditioner, nice energy savings! It saves a lot of money on electricity in a year	Taobao Marketplace, a Chinese website for online shopping
Xindu Flagship Store	Green Intelligent Power Saver	I'm using it, I won't know until I pay my electric bill at the end of the month, then I'll come back with a follow up comment!	Taobao Marketplace, a Chinese website for online shopping
Xindu Flagship Store	Green Intelligent Power Saver	Power savings: nice worth the money! Power saving! Experience: pretty good will buy next time	Taobao Marketplace, a Chinese website for online shopping
Xindu Flagship Store	Green Intelligent Power Saver	Effective power-saving effect of the bar	Taobao Marketplace, a Chinese website for online shopping
Xindu Flagship Store	Green Intelligent Power Saver	Cost-effective: very good Appearance style: excellent, beautiful style Workmanship material: safe, easy to install	Taobao Marketplace, a Chinese website for online shopping
Xindu Flagship Store	Green Intelligent Power Saver	It feels great to use and is worth repurchasing!	Taobao Marketplace, a Chinese website for online shopping
Xindu Flagship Store	Green Intelligent Power Saver	It works very well.	Taobao Marketplace, a Chinese website for online shopping
Xindu Flagship Store	Green Intelligent Power Saver	This thing is very practical	Taobao Marketplace, a Chinese website for online shopping
Xindu Flagship Store	Green Intelligent Power Saver	It works well, this time I bought it for my sister	Taobao Marketplace, a Chinese website for online shopping
Xindu Flagship Store	Green Intelligent Power Saver	Experience: a proper IQ tax, whoever buys it will regret it!	Taobao Marketplace, a Chinese website for online shopping
Xindu Flagship Store	Green Intelligent Power Saver	Bought back and used a few days, recorded the meter, and the previous comparison, it does save electricity, because we are commercial electricity, the price of electricity is 1.31 yuan, so the power saving is especially obvious, and later consider buying for the old family as well!	Taobao Marketplace, a Chinese website for online shopping
Xindu Flagship Store	Green Intelligent Power Saver	Bought and received the goods, use should be good, looking forward to good results	Taobao Marketplace, a Chinese website for online shopping
Xindu Flagship Store	Green Intelligent Power Saver	It's good. It's worth it.	Taobao Marketplace, a Chinese website for online shopping
Xindu Flagship Store	Green Intelligent Power Saver	Really nice, just as described! Very satisfied	Taobao Marketplace, a Chinese website for online shopping
Xindu Flagship Store	Green Intelligent Power Saver	Let's just say I'm not feeling it.	Taobao Marketplace, a Chinese website for online shopping
Xindu Flagship Store	Green Intelligent Power Saver	I've been using it for two or three months and I haven't lost any electricity! It's not working!	Taobao Marketplace, a Chinese website for online shopping

Xindu Flagship Store	Green Intelligent Power Saver	The power saver was received, checked with my cell phone, it's good, it's genuine. The home uses more electricity, now, with this power saver will not have to worry, always save some electricity. Logistics is also very fast, five-star praise!	Taobao Marketplace, a Chinese website for online shopping
Xindu Flagship Store	Green Intelligent Power Saver	The power saver received, with a cell phone check, is genuine. The home uses more electricity, now there is this power saver do not have to worry about using too much electricity, this product is a good thing for the country and the people, should be vigorously promoted. Logistics is also very fast, five-star	Taobao Marketplace, a Chinese website for online shopping
Xindu Flagship Store	Green Intelligent Power Saver	I bought it and used it for a few days and it does save power compared to before I didn't use it, users who didn't buy it buy it and use it and you'll see.	Taobao Marketplace, a Chinese website for online shopping
Xindu Flagship Store	Green Intelligent Power Saver	If you want to buy it urgently, it's really genuine and works well, the power saving is really ideal! I am using a few days to compare before evaluating. I hope that want to buy assured purchase	Taobao Marketplace, a Chinese website for online shopping
Xindu Flagship Store	Green Intelligent Power Saver	It doesn't save any power at all, it's a power hog	Taobao Marketplace, a Chinese website for online shopping
Xindu Flagship Store	Green Intelligent Power Saver	Power saving effect: quite good worth buying Cost-effective: high Appearance style: beautiful Workmanship material: home fuel type	Taobao Marketplace, a Chinese website for online shopping
Xindu Flagship Store	Green Intelligent Power Saver	N times purchased the effect is very good, summer power consumption with this cost savings!	Taobao Marketplace, a Chinese website for online shopping
Xindu Flagship Store	Green Intelligent Power Saver	Appearance style: very perfect Workmanship material: workmanship is very very wow, using experience: to achieve the best results I want good	Taobao Marketplace, a Chinese website for online shopping
Xindu Flagship Store	Green Intelligent Power Saver	The effect is particularly good, the air conditioning should be open every day, every month the electricity bill is too much, friends introduced the use of this, a month down, the electricity bill than the previous month less than thirty percent or so, customer service said that the highest can be up to	Taobao Marketplace, a Chinese website for online shopping
Xindu Flagship Store	Green Intelligent Power Saver	Power saving effect: good Cost-effective: high Appearance style: compact and convenient Workmanship material: exquisite Experience: save a lot of money, easy to get through the hot summer days	Taobao Marketplace, a Chinese website for online shopping
Xindu Flagship Store	Green Intelligent Power Saver	Power-saving effect: not bad Experience: I feel that it is quite a bit of power-saving	Taobao Marketplace, a Chinese website for online shopping
Xindu Flagship Store	Green Intelligent Power Saver	I've been using it for a while and it works fine	Taobao Marketplace, a Chinese website for online shopping
Xindu Flagship Store	Green Intelligent Power Saver	It's recommended by a friend, it's good, many people use it, it's recommendable.	Taobao Marketplace, a Chinese website for online shopping
Xindu Flagship Store	Green Intelligent Power Saver	The second time I bought it, I used it myself to save electricity and bought it for my mom. Logistics is also very fast.	Taobao Marketplace, a Chinese website for online shopping
Xindu Flagship Store	Green Intelligent Power Saver	Power saving effect: good Cost-effective: high Appearance style: beautiful Workmanship material: good Use experience: good	Taobao Marketplace, a Chinese website for online shopping
Xindu Flagship Store	Green Intelligent Power Saver	Arrived, effective, logistics is very fast, the quality is very good, really save electricity, I have recommended friends have bought to use, especially the use of large amounts of electricity friends, the effect is more obvious. My home is a mahjong room, not using the power saver inches monthly electricity costs about 300, after using the power saver up to more than 200 yuan a month. The power saver is a real power saver! There is a power saver quality assurance genuine, friends can rest assured that the purchase	Taobao Marketplace, a Chinese website for online shopping
Xindu Flagship Store	Green Intelligent Power Saver	My friend recommended me to this store to buy, this store's service is very good, the product is invalid for 100 days can be refunded, my friend bought 6 last time, save a lot of electricity, now the smart meter is really too expensive, now has been used for almost half a month, really save a lot of, really	Taobao Marketplace, a Chinese website for online shopping
Xindu Flagship Store	Green Intelligent Power Saver	Electrical appliances received, immediately opened the box, is a small and delicate baby, I began to fear that occupy space due to a long time before buying. Immediately inserted in the home socket. Go to see at night, the power saver shows that it is working, do not pull out. Looks like this little baby will save me a little bit of electricity. Recommended purchase for everyone, great value for money. Five out of five for the product and five out of five for the customer service. Thank you!	Taobao Marketplace, a Chinese website for online shopping
Xindu Flagship Store	Green Intelligent Power Saver	Bought two energy-saving appliances for the family, the kitchen and bathroom water heater is too costly □Ĉ, the water heater has to be turned on every day, the monthly electricity bill is used a lot, with this, no longer afraid of the electricity bill is more than a month down the electricity bill can be less than half of the previous month it □Ħ, the effect is particularly good, the	Taobao Marketplace, a Chinese website for online shopping
Xindu Flagship Store	Green Intelligent Power Saver	Power-saving effect: power-saving effect is more obvious and easy to install does not take up space is simply a home life must have intelligent new technology Cost-effective: cost-effective more suitable for us the general public	Taobao Marketplace, a Chinese website for online shopping
Xindu Flagship Store	Green Intelligent Power Saver	Stable performance Portable and convenient There is a significant reduction in the frequency of meter jumps	Taobao Marketplace, a Chinese website for online shopping
Xindu Flagship Store	Green Intelligent Power Saver	Last time I bought 3 is still very good results, this time in the continuation of the purchase of 6, put in the store with. The store's electricity consumption is too large, the store recommended using 6, looking forward to next month's electricity bill, thank you for the patience of the store reception!	Taobao Marketplace, a Chinese website for online shopping
Xindu Flagship Store	Green Intelligent Power Saver	Power saving effect: > good Cost-effective: 丿高 Appearance style:, beautiful Workmanship material: (good Experience: - satisfied	Taobao Marketplace, a Chinese website for online shopping
Xindu Flagship Store	Green Intelligent Power Saver	This commodity is very good, can play the effect of energy saving, I open processing plant power consumption is relatively large, the effect is obvious, it is really good for the country and the people of a good product, it is worth recommending everyone to buy.	Taobao Marketplace, a Chinese website for online shopping
Xindu Flagship Store	Green Intelligent Power Saver	The actual test is effective! My family cooks by myself every day, the steam oven induction cooker almost a month to use more than 500 dollars of electricity, and now after using this, more than 400 will be enough, although I do not understand what the reasoning, but it is true to save me money to save money on	Taobao Marketplace, a Chinese website for online shopping
Xindu Flagship Store	Green Intelligent Power Saver	It's plug and play. It's convenient.	Taobao Marketplace, a Chinese website for online shopping
Xindu Flagship Store	Green Intelligent Power Saver	Saver received, the second time back to buy, before buying a installed in the old house effect is good, a month can really save a lot of electricity, the original night air conditioning power about ten degrees of electricity, now down to 4-5 degrees of electricity, and bought a put a part-time rental house, save a little salary, the quality of the material is still as good as ever, the use of a simple, randomly plugged into a socket on the line, it's very good, and is ready to buy two more to use!	Taobao Marketplace, a Chinese website for online shopping
Xindu Flagship Store	Green Intelligent Power Saver	Power savings: good, feels effective. Cost-effective: super cost-effective. Appearance and style: small, exquisite and beautiful. Workmanship material: the outer packaging is very delicate and formal. This workmanship is also beautiful and stylish. Experience: experience feels very good, used 2 months to save 200 yuan, still in the experience, ready to buy a few more to relatives and friends!	Taobao Marketplace, a Chinese website for online shopping
Xindu Flagship Store	Green Intelligent Power Saver	Things received, temporarily installed in a row plug. The appearance of small Linglong, should be good. As for whether it can save electricity, because it is living in a high floor of the district, the meter do not know where to install can not confirm, waiting for the next month to pay the electricity bill to	Taobao Marketplace, a Chinese website for online shopping
Xindu Flagship Store	Green Intelligent Power Saver	The effect is very good, deliberately used for a period of time to evaluate the original electricity bill is very high, since the use of this power-saving device, the electricity bill to save a lot of Cost-effective: too high, affordable, and save a large amount of electricity Appearance style: compact, and very convenient, inserted into any outlet on the workmanship Material: very good	Taobao Marketplace, a Chinese website for online shopping
Xindu Flagship Store	Green Intelligent Power Saver	Purchased 8 power saver received put into use, home appliances, power consumption is very large, we have a piece of electricity or 1 yuan 1 degree, with this power saver is much better, every day can save a few cents, very convenient and practical.	Taobao Marketplace, a Chinese website for online shopping

Xindu Flagship Store	Green Intelligent Power Saver	Last time I purchased 5 The effect is really good, this time in the buy, with the power saver is also more power saving than before, the power saving rate is not bad, almost a month to save more than 200 bucks it, measured effect!	Taobao Marketplace, a Chinese website for online shopping
Xindu Flagship Store	Green Intelligent Power Saver	Received the goods on the test, really can save electricity Oh!	Taobao Marketplace, a Chinese website for online shopping
Xindu Flagship Store	Green Intelligent Power Saver	I've got it! I don't know if it works.	Taobao Marketplace, a Chinese website for online shopping
Xindu Flagship Store	Green Intelligent Power Saver	There are three rooms in the house, in addition to the dining room living room, etc. I do not know where to insert the power saver I inserted it in the living room because in the living room to stay longer, the living room has air conditioning, the effect of power saving is very obvious, pay the electricity bill when you know, I have introduced it to my friends and relatives, something small and exquisite, the price is also very affordable pro-people, I'm uploading a few pictures here to show everyone, recommended to the side of the I'll upload a few pictures here to show you and recommend it to my friends and family!	Taobao Marketplace, a Chinese website for online shopping
Xindu Flagship Store	Green Intelligent Power Saver	I used to learn electricity, power factor on power saving energy consumption is really useful, although the reduction of energy consumption is limited, but can save a little is a little, but also to protect household appliances, stabilize the current and voltage, the main plug can be used. Winter home two air conditioning a refrigerator, a day down the electricity bill is almost a hundred dollars, with this after an average of two dozen dollars a day to spend less money to enjoy more, it's really good, thanks to the	Taobao Marketplace, a Chinese website for online shopping
Xindu Flagship Store	Green Intelligent Power Saver	A friend told me it saves electricity, so I came here to buy it.	Taobao Marketplace, a Chinese website for online shopping
Xindu Flagship Store	Green Intelligent Power Saver	Super fast shipping. Shipped fast too. It's only been two days. Hey, hey.	Taobao Marketplace, a Chinese website for online shopping
Xindu Flagship Store	Green Intelligent Power Saver	This economizer is really good, I heard is 2023 latest models, not only the value of good-looking, the effect is still very good, this is my fifth in this store with the economizer, help relatives buy a lot of, old customers also gave me a discount!	Taobao Marketplace, a Chinese website for online shopping
Xindu Flagship Store	Green Intelligent Power Saver	Plug and play is good. Power savings: okay.	Taobao Marketplace, a Chinese website for online shopping
Xindu Flagship Store	Green Intelligent Power Saver	effective	Taobao Marketplace, a Chinese website for online shopping
Xindu Flagship Store	Green Intelligent Power Saver	I just put it on. We'll see what happens later.	Taobao Marketplace, a Chinese website for online shopping
Xindu Flagship Store	Green Intelligent Power Saver	The second repurchase has been received, the effect is quite good, the seller shipped quickly, recommended to everyone!	Taobao Marketplace, a Chinese website for online shopping
Xindu Flagship Store	Green Intelligent Power Saver	Smart Appliance Saver received it and it feels good to use it	Taobao Marketplace, a Chinese website for online shopping
Xindu Flagship Store	Green Intelligent Power Saver	Received the goods to open the box on the installation and use, look at the appearance of small, intuitive and lightweight handwriting display clear. Power saving effect after a few days to compare out. If really so magical recommended to family and friends to use.	Taobao Marketplace, a Chinese website for online shopping
Xindu Flagship Store	Green Intelligent Power Saver	New upgraded economizer to try, I have been an old user of the economizer, from the 8 generation has been purchased, almost every generation has been bought, now it is really getting better and better, this time also a one-time purchase of 6, the effect is really nothing to say!	Taobao Marketplace, a Chinese website for online shopping
Xindu Flagship Store	Green Intelligent Power Saver	Used for a period of time does save a lot of electricity, the previous summer electricity bill is more than 600, now this saver with 6, electricity costs are more than 400 or so, appliances are still the same use. It is really a good gospel for the people, and I bought 6 more this time.	Taobao Marketplace, a Chinese website for online shopping
Xindu Flagship Store	Green Intelligent Power Saver	I pressed one in the city and one for the old house, I feel the effect is okay	Taobao Marketplace, a Chinese website for online shopping
Xindu Flagship Store	Green Intelligent Power Saver	After using it for a few days to see if there is still a change, this is something I have recognized because we also apply centralized compensation and in situ compensation in our practical work	Taobao Marketplace, a Chinese website for online shopping
Xindu Flagship Store	Green Intelligent Power Saver	Cost-effective: the best value for money Appearance and style: very good Workmanship and material: exactly the same as the picture	Taobao Marketplace, a Chinese website for online shopping
Xindu Flagship Store	Green Intelligent Power Saver	Start using it and expect the best results	Taobao Marketplace, a Chinese website for online shopping
Xindu Flagship Store	Green Intelligent Power Saver	Goods have been received, has been installed, power-saving effect to be used after the follow-up evaluation	Taobao Marketplace, a Chinese website for online shopping
Xindu Flagship Store	Green Intelligent Power Saver	I bought 2 of them, and the power savings are between thirty and forty-five percent.	Taobao Marketplace, a Chinese website for online shopping
Xindu Flagship Store	Green Intelligent Power Saver	Power saving effect: cool Workmanship material: good	Taobao Marketplace, a Chinese website for online shopping
Xindu Flagship Store	Green Intelligent Power Saver	It's good. Keep buying back.	Taobao Marketplace, a Chinese website for online shopping
Xindu Flagship Store	Green Intelligent Power Saver	Something new, try it!	Taobao Marketplace, a Chinese website for online shopping
Xindu Flagship Store	Green Intelligent Power Saver	Power-saving effect: Dad bought took a feel super good use Cost-effective: cost-effective Appearance style: exquisite appearance Experience: waiting to pay the electricity bill to see, before renting a house with this, 2 months only paid 150 electricity bill. Or winter open air conditioning heater, classmates home 2 months electricity costs more than 400 it!	Taobao Marketplace, a Chinese website for online shopping
Xindu Flagship Store	Green Intelligent Power Saver	The quality is good, also quite good, the seller also shipped very quickly, the package is very tight, no damage, the price is also cheap, the	Taobao Marketplace, a Chinese website for online shopping
Xindu Flagship Store	Green Intelligent Power Saver	I bought it from a friend's house before and said that it does save electricity, so I came to buy it with that in mind to check it out	Taobao Marketplace, a Chinese website for online shopping
Xindu Flagship Store	Green Intelligent Power Saver	Baby packaging is very good, the logistics is very fast, the boss service is thoughtful, the price is cost-effective and trustworthy.	Taobao Marketplace, a Chinese website for online shopping
Xindu Flagship Store	Green Intelligent Power Saver	Works great after installation, good quality. Save a lot of money with a large family. Very satisfied. Good value for money.	Taobao Marketplace, a Chinese website for online shopping
Xindu Flagship Store	Green Intelligent Power Saver	Controller received, good quality	Taobao Marketplace, a Chinese website for online shopping
Xindu Flagship Store	Green Intelligent Power Saver	Fast shipping and very well packaged. Good quality, good comments	Taobao Marketplace, a Chinese website for online shopping

Xindu Flagship Store	Green Intelligent Power Saver	Very satisfied with the shopping, will come back next time!	Taobao Marketplace, a Chinese website for online shopping
Xindu Flagship Store	Green Intelligent Power Saver	Save a little controller, works great and is affordable	Taobao Marketplace, a Chinese website for online shopping
Xindu Flagship Store	Green Intelligent Power Saver	Not genuine, no effect at all	Taobao Marketplace, a Chinese website for online shopping
Xindu Flagship Store	Green Intelligent Power Saver	This saver is used by a friend. It does work. When summer comes, the air conditioner can be turned on at will.	Taobao Marketplace, a Chinese website for online shopping
Xindu Flagship Store	Green Intelligent Power Saver	I bought it to try it out, the quality is still very good and I'm looking forward to the results!	Taobao Marketplace, a Chinese website for online shopping
Xindu Flagship Store	Green Intelligent Power Saver	Power-saving effect: usually a day and night with 3 degrees of electricity, the use of power-saving treasure after two days of electricity 18 degrees of electricity, too pitched Cost-effective: poor evaluation Experience: the most disappointing shopping, so that two days I have more than 16 yuan of electricity, positive feedback is the rebate to get, do not mind the pro to buy!	Taobao Marketplace, a Chinese website for online shopping
Xindu Flagship Store	Green Intelligent Power Saver	The store shipped very quickly and was well packaged with no damage. Received and installed, energy saving effect is good. Value for money.	Taobao Marketplace, a Chinese website for online shopping
Xindu Flagship Store	Green Intelligent Power Saver	This product is really good, summer electricity is very costly, with it to save a lot, good good.	Taobao Marketplace, a Chinese website for online shopping
Xindu Flagship Store	Green Intelligent Power Saver	Appearance design is reasonable, simple and convenient to use, after using it is really special power saving!	Taobao Marketplace, a Chinese website for online shopping
Xindu Flagship Store	Green Intelligent Power Saver	It's easy to use, plug and play, safe and energy saving, very good!	Taobao Marketplace, a Chinese website for online shopping
Xindu Flagship Store	Green Intelligent Power Saver	Express very quickly, just received the goods tonight to use it, I hope there is an effect, I am used in the rental room inside the four appliances, air conditioning refrigerator washing machine and water heater, I hope to save some electricity bills every month, save money for milk tea, I wish the boss a	Taobao Marketplace, a Chinese website for online shopping
Xindu Flagship Store	Green Intelligent Power Saver	Plug and play on top of the plug, works like a charm, not bad 😉	Taobao Marketplace, a Chinese website for online shopping
Xindu Flagship Store	Green Intelligent Power Saver	Fast shipping, baby fine workmanship, the material is also very good, cost-effective, worth buying	Taobao Marketplace, a Chinese website for online shopping
Xindu Flagship Store	Green Intelligent Power Saver	How to save electricity is not obvious, how to call it saving electricity?	Taobao Marketplace, a Chinese website for online shopping
Xindu Flagship Store	Green Intelligent Power Saver	Power saving effect: very good Appearance style: good Experience: very like the product. Will buy his product again next time.	Taobao Marketplace, a Chinese website for online shopping
Xindu Flagship Store	Green Intelligent Power Saver	Really like this one, it does save.	Taobao Marketplace, a Chinese website for online shopping
Xindu Flagship Store	Green Intelligent Power Saver	The display light sometimes does not light up, do not know whether it affects the use of the	Taobao Marketplace, a Chinese website for online shopping
Xindu Flagship Store	Green Intelligent Power Saver	Good quality product, good value for money and very fit for purpose.	Taobao Marketplace, a Chinese website for online shopping
Xindu Flagship Store	Green Intelligent Power Saver	Fast logistics, good quality, good power saving effect, cost-effective	Taobao Marketplace, a Chinese website for online shopping
Xindu Flagship Store	Green Intelligent Power Saver	Things received, with the page introduced the same, the effect is not yet known, good if you buy two more	Taobao Marketplace, a Chinese website for online shopping
Xindu Flagship Store	Green Intelligent Power Saver	Follow the guidance of customer service has been installed, very good installation, listening to friends say that this power-saving effect is very good, I will also try!	Taobao Marketplace, a Chinese website for online shopping
Xindu Flagship Store	Green Intelligent Power Saver	Received it, good, works well, good value for money, satisfied!	Taobao Marketplace, a Chinese website for online shopping
Xindu Flagship Store	Green Intelligent Power Saver	Experience: don't know yet.	Taobao Marketplace, a Chinese website for online shopping
Xindu Flagship Store	Green Intelligent Power Saver	Power saving effect: good Using experience: good	Taobao Marketplace, a Chinese website for online shopping
Xindu Flagship Store	Green Intelligent Power Saver	The quality is very good, the appliance stabilizes the voltage energy saving and power saving . Packaging is very good	Taobao Marketplace, a Chinese website for online shopping
Xindu Flagship Store	Green Intelligent Power Saver	Customer service is great, the delivery is not bad, I hope the boss business is getting better and better!!!!	Taobao Marketplace, a Chinese website for online shopping
Xindu Flagship Store	Green Intelligent Power Saver	Good price and good logistics, worth repurchasing!	Taobao Marketplace, a Chinese website for online shopping
Xindu Flagship Store	Green Intelligent Power Saver	The fake ones don't help at all.	Taobao Marketplace, a Chinese website for online shopping
Xindu Flagship Store	Green Intelligent Power Saver	This saver is very good, cost-effective, now it's hot, the electricity cost is very strong, specially bought this	Taobao Marketplace, a Chinese website for online shopping
Xindu Flagship Store	Green Intelligent Power Saver	The product is really good power saving forty percent, want to buy the pro can start!	Taobao Marketplace, a Chinese website for online shopping
Xindu Flagship Store	Green Intelligent Power Saver	The product is compact, good to install and does save electricity	Taobao Marketplace, a Chinese website for online shopping
Xindu Flagship Store	Green Intelligent Power Saver	Extremely bad reviews The boss will tell you it will take a month or two for it to work ...... It won't work when the statute of limitations on your reviews expires. Not a little bit of *** I bought eight of them Don't be fooled by those positive reviews	Taobao Marketplace, a Chinese website for online shopping
Xindu Flagship Store	Green Intelligent Power Saver	More power consumption! ****, does the state ignore this? It specializes in cheating the people! There's nothing inside, just two lights! Don't be fooled! You can go to Jitterbug to see how fake these are.	Taobao Marketplace, a Chinese website for online shopping
Xindu Flagship Store	Green Intelligent Power Saver	Things received, the effect is not yet seen, wait for a period of time to come back to the follow-up, first give a favorable comment on it	Taobao Marketplace, a Chinese website for online shopping

Xindu Flagship Store	Green Intelligent Power Saver	The material and workmanship are very good, the quality is very good, you can buy it.	Taobao Marketplace, a Chinese website for online shopping
Xindu Flagship Store	Green Intelligent Power Saver	Cost-effective: cheap and easy to use, appearance style: very beautiful Experience: listening to friends said that this energy-saving products are particularly good, before more than 200 a month of electricity, plugged into this energy saver, a month is only a few dozen dollars, I first use, good use, if you buy a	Taobao Marketplace, a Chinese website for online shopping
Xindu Flagship Store	Green Intelligent Power Saver	Works great, very practical, and will be back for more!	Taobao Marketplace, a Chinese website for online shopping
Xindu Flagship Store	Green Intelligent Power Saver	Properly **taxed** no.	Taobao Marketplace, a Chinese website for online shopping
Xindu Flagship Store	Green Intelligent Power Saver	It works well, is easy to use, and saves a lot of power. A must-have for your home	Taobao Marketplace, a Chinese website for online shopping
Xindu Flagship Store	Green Intelligent Power Saver	Goods have been received, has been installed, energy-saving effect to be used after evaluation, the above picture for the sign.	Taobao Marketplace, a Chinese website for online shopping
Xindu Flagship Store	Green Intelligent Power Saver	Power-saving effect: wait for the next electricity bill to come out and then comment, is it really effective good.	Taobao Marketplace, a Chinese website for online shopping
Xindu Flagship Store	Green Intelligent Power Saver	Baby received and so use a month or two to see the effect in the follow-up, looks good, the shopkeeper has a very good attitude, logistics is also very fast, thank you!	Taobao Marketplace, a Chinese website for online shopping
Xindu Flagship Store	Green Intelligent Power Saver	Alas, electricity doesn't seem to see a decrease in sensation	Taobao Marketplace, a Chinese website for online shopping
Xindu Flagship Store	Green Intelligent Power Saver	Saver just received, workmanship is still OK, the business said 100 days trial, invalid refund, really bought 3, summer power consumption is too much, looking forward to next month's electricity bill. I'll come back to the review.	Taobao Marketplace, a Chinese website for online shopping
Xindu Flagship Store	Green Intelligent Power Saver	I've been using it for a while, it's a real savings, I'm glad I tried it in the first place or I would have really missed out on something that good, the	Taobao Marketplace, a Chinese website for online shopping
Xindu Flagship Store	Green Intelligent Power Saver	Power saving effect: really save power, start to buy one to try, good good	Taobao Marketplace, a Chinese website for online shopping
Xindu Flagship Store	Green Intelligent Power Saver	Don't buy it *with* Mods!	Taobao Marketplace, a Chinese website for online shopping
Xindu Flagship Store	Green Intelligent Power Saver	It's all bullshit, it doesn't work at all, it's a great way to cheat people, I've been fooled, everyone sees it, don't buy it!	Taobao Marketplace, a Chinese website for online shopping
Xindu Flagship Store	Green Intelligent Power Saver	I've been looking at Taobao for a long time and I've been curious about this product, so I decided to buy it and try it out for myself, expecting it to work as described in the product description.	Taobao Marketplace, a Chinese website for online shopping
Xindu Flagship Store	Green Intelligent Power Saver	Plugged it in and running, waiting to see what happens.	Taobao Marketplace, a Chinese website for online shopping
Xindu Flagship Store	Green Intelligent Power Saver	It's good quality.	Taobao Marketplace, a Chinese website for online shopping
Xindu Flagship Store	Green Intelligent Power Saver	The power saving effect is very good, the package is intact, the shipment is very fast, the logistics is also very fast. Good review.	Taobao Marketplace, a Chinese website for online shopping
Xindu Flagship Store	Green Intelligent Power Saver	The product is pretty great, the power saving effect is very good, this family is trustworthy, satisfied!	Taobao Marketplace, a Chinese website for online shopping
Xindu Flagship Store	Green Intelligent Power Saver	Good quality, good power savings.	Taobao Marketplace, a Chinese website for online shopping
Xindu Flagship Store	Green Intelligent Power Saver	Useless. Garbage.	Taobao Marketplace, a Chinese website for online shopping
Xindu Flagship Store	Green Intelligent Power Saver	Good review!	Taobao Marketplace, a Chinese website for online shopping
Xindu Flagship Store	Green Intelligent Power Saver	It's expensive.	Taobao Marketplace, a Chinese website for online shopping
Xindu Flagship Store	Green Intelligent Power Saver	It looks good, it's in the same outlet as the fridge, so hopefully it'll save some power 😁	Taobao Marketplace, a Chinese website for online shopping
Xindu Flagship Store	Green Intelligent Power Saver	It's good to use it ∀⊙!	Taobao Marketplace, a Chinese website for online shopping
Xindu Flagship Store	Green Intelligent Power Saver	Received and used the effect is good sure enough the meter turn a little slower bought two at once wait for the electricity bill to come out to know whether to give power quality assured no problem	Taobao Marketplace, a Chinese website for online shopping
Xindu Flagship Store	Green Intelligent Power Saver	Power saving effect: can Cost-effective: high Appearance style: new Workmanship material: good	Taobao Marketplace, a Chinese website for online shopping
Xindu Flagship Store	Green Intelligent Power Saver	It remains to be seen if there is any effect, first favorable comments!	Taobao Marketplace, a Chinese website for online shopping
Xindu Flagship Store	Green Intelligent Power Saver	Good quality and cheap	Taobao Marketplace, a Chinese website for online shopping
Xindu Flagship Store	Green Intelligent Power Saver	Fooled, no power savings at all.	Taobao Marketplace, a Chinese website for online shopping
Xindu Flagship Store	Green Intelligent Power Saver	Power saving effect can be, you can turn off the main power to try. Cost-effective: cost-effective or very high	Taobao Marketplace, a Chinese website for online shopping
Xindu Flagship Store	Green Intelligent Power Saver	Power saving effect: very obvious Cost-effective: very high Appearance style: simple and generous Experience: energy saving can be seen	Taobao Marketplace, a Chinese website for online shopping
Xindu Flagship Store	Green Intelligent Power Saver	My friend introduced it and said it works well, I installed it when I received it and waited for next month to see the effect 😁	Taobao Marketplace, a Chinese website for online shopping
Xindu Flagship Store	Green Intelligent Power Saver	The saver is really good. Very energy efficient. And it's not expensive.	Taobao Marketplace, a Chinese website for online shopping

Xindu Flagship Store	Green Intelligent Power Saver	The courier didn't deliver at the address.	Taobao Marketplace, a Chinese website for online shopping
Xindu Flagship Store	Green Intelligent Power Saver	Works really well, saves a lot of electricity, fast shipping	Taobao Marketplace, a Chinese website for online shopping
Xindu Flagship Store	Green Intelligent Power Saver	Power saving effect: did not play any role	Taobao Marketplace, a Chinese website for online shopping
Xindu Flagship Store	Green Intelligent Power Saver	Power saving effect: not yet not yet sure if it works or not	Taobao Marketplace, a Chinese website for online shopping
Xindu Flagship Store	Green Intelligent Power Saver	Simple and easy to use, power saving effect is really quite good, recommended.	Taobao Marketplace, a Chinese website for online shopping
Xindu Flagship Store	Green Intelligent Power Saver	Express delivery is very fast, customer service attitude is quite good, indeed the effect is good, the fourth repurchase, good value for money, praise!	Taobao Marketplace, a Chinese website for online shopping
Xindu Flagship Store	Green Intelligent Power Saver	It feels great and perfect to use, really good value for money and will need to keep repurchasing in the future.	Taobao Marketplace, a Chinese website for online shopping
Xindu Flagship Store	Green Intelligent Power Saver	It works and will be used all the time. The logistics is also fast and worth the kudos!	Taobao Marketplace, a Chinese website for online shopping
Xindu Flagship Store	Green Intelligent Power Saver	Almost a month, gross useless, paid two IQ tax, but also bought two, what principle meter can turn slowly? If it really turns slow that's not stealing electricity? ☠☠	Taobao Marketplace, a Chinese website for online shopping
Xindu Flagship Store	Green Intelligent Power Saver	Exterior Styles:	Taobao Marketplace, a Chinese website for online shopping
Xindu Flagship Store	Green Intelligent Power Saver	Power saving effect: very good Workmanship material: very good , good price, fast delivery	Taobao Marketplace, a Chinese website for online shopping
Xindu Flagship Store	Green Intelligent Power Saver	Good quality and workmanship	Taobao Marketplace, a Chinese website for online shopping
Xindu Flagship Store	Green Intelligent Power Saver	Cost-effective and effective. Logistics is also very fast, satisfied	Taobao Marketplace, a Chinese website for online shopping
Xindu Flagship Store	Green Intelligent Power Saver	The power saving effect are good, workmanship material is also good, use is very great!	Taobao Marketplace, a Chinese website for online shopping
Xindu Flagship Store	Green Intelligent Power Saver	Very practical. Great value for money and good quality.	Taobao Marketplace, a Chinese website for online shopping
Xindu Flagship Store	Green Intelligent Power Saver	Not at all**. Garbage.	Taobao Marketplace, a Chinese website for online shopping
Xindu Flagship Store	Green Intelligent Power Saver	Very easy to use, inserted in the plug can be used, I used in the water heater plug next to the test, has been using this jump speed is slightly slower, a month to see the effect 😄	Taobao Marketplace, a Chinese website for online shopping
Xindu Flagship Store	Green Intelligent Power Saver	Quality is very good, fine workmanship, friends introduced this artifact, truly good, power saving effect is very obvious, love love love! 😁😁😁😁😁😁	Taobao Marketplace, a Chinese website for online shopping
Xindu Flagship Store	Green Intelligent Power Saver	Power saving effect: good Cost-effective: high Appearance style: beautiful Workmanship material: exquisite Experience: the meter obviously turned slower	Taobao Marketplace, a Chinese website for online shopping
Xindu Flagship Store	Green Intelligent Power Saver	I'm using it. I'll wait a month and see.	Taobao Marketplace, a Chinese website for online shopping
Xindu Flagship Store	Green Intelligent Power Saver	Buy it and try to save power	Taobao Marketplace, a Chinese website for online shopping
Xindu Flagship Store	Green Intelligent Power Saver	Power saving effect: very good	Taobao Marketplace, a Chinese website for online shopping
Xindu Flagship Store	Green Intelligent Power Saver	What kind of product? It doesn't save electricity at all	Taobao Marketplace, a Chinese website for online shopping
Xindu Flagship Store	Green Intelligent Power Saver	Power saving effect: <Good Price-performance ratio: `Good quality and fast logistics	Taobao Marketplace, a Chinese website for online shopping
Xindu Flagship Store	Green Intelligent Power Saver	The quality is too bad, the indicator light on a broken, broken two contact merchants on a replacement, the quality is really bad!	Taobao Marketplace, a Chinese website for online shopping
Xindu Flagship Store	Green Intelligent Power Saver	Good use of real power saving, the price is not expensive, the appearance of the style is exquisite, more satisfactory	Taobao Marketplace, a Chinese website for online shopping
Xindu Flagship Store	Green Intelligent Power Saver	Convenient and easy to use	Taobao Marketplace, a Chinese website for online shopping
Xindu Flagship Store	Green Intelligent Power Saver	Activity to buy a very good deal, bought two also send a, all-round use, look very good 👍	Taobao Marketplace, a Chinese website for online shopping
Xindu Flagship Store	Green Intelligent Power Saver	Use it all the time at home! The effect is very good! Bought for a friend this time, hope it works well as always! Logistics is very fast!	Taobao Marketplace, a Chinese website for online shopping
Xindu Flagship Store	Green Intelligent Power Saver	Very satisfied 👍, compared to a few days but actually have results	Taobao Marketplace, a Chinese website for online shopping
Xindu Flagship Store	Green Intelligent Power Saver	Power saving effect: looking forward to ...... Cost-effective: high very high Appearance style: nice nice Use experience: looking forward to surprise	Taobao Marketplace, a Chinese website for online shopping
Xindu Flagship Store	Green Intelligent Power Saver	Expect to be surprised	Taobao Marketplace, a Chinese website for online shopping
Xindu Flagship Store	Green Intelligent Power Saver	Well packaged, affordable, works well	Taobao Marketplace, a Chinese website for online shopping
Xindu Flagship Store	Green Intelligent Power Saver	Power-saving effect: not used, just received the goods Cost-effective: good Appearance style: green and black, workmanship material: good Experience: a week back before evaluation	Taobao Marketplace, a Chinese website for online shopping

Xindu Flagship Store	Green Intelligent Power Saver	I've been using it for a few months now. It's useless. The electricity bill is so high.	Taobao Marketplace, a Chinese website for online shopping
Xindu Flagship Store	Green Intelligent Power Saver	It's not working well?	Taobao Marketplace, a Chinese website for online shopping
Xindu Flagship Store	Green Intelligent Power Saver	Power saving effect: very real Cost-effective: very high Appearance style: very trendy Workmanship material: very good Experience: very satisfied!	Taobao Marketplace, a Chinese website for online shopping
Xindu Flagship Store	Green Intelligent Power Saver	This energy-saving device is too good to use, energy saving and environmental protection and does not take up space, power saving to the power	Taobao Marketplace, a Chinese website for online shopping
Xindu Flagship Store	Green Intelligent Power Saver	Saver is very good, easy to install, see the effect after a while	Taobao Marketplace, a Chinese website for online shopping
Xindu Flagship Store	Green Intelligent Power Saver	Goods received, logistics is very fast, packaging is also very delicate, the boss also sent a, plugged in the normal display, the neighbor recommended, her family used quite a long time, there are power saving, looking forward to!	Taobao Marketplace, a Chinese website for online shopping
Xindu Flagship Store	Green Intelligent Power Saver	Power-saving effect: should be very good, a few dozen dollars a month is there Cost-effective: good value for money Appearance and style: small and lovely Workmanship material: okay Experience: good	Taobao Marketplace, a Chinese website for online shopping
Xindu Flagship Store	Green Intelligent Power Saver	Appearance looks good! Need to use it for a while to know the exact power savings.	Taobao Marketplace, a Chinese website for online shopping
Xindu Flagship Store	Green Intelligent Power Saver	Received the goods very satisfied with the quality is very good workmanship next time will definitely come back to the store!	Taobao Marketplace, a Chinese website for online shopping
Xindu Flagship Store	Green Intelligent Power Saver	Workmanship material: baby received, good workmanship, do not know how the power-saving effect, later evaluation	Taobao Marketplace, a Chinese website for online shopping
Xindu Flagship Store	Green Intelligent Power Saver	Shipping was prompt and the saver is working fine with results	Taobao Marketplace, a Chinese website for online shopping
Xindu Flagship Store	Green Intelligent Power Saver	Recommended by a friend, this is a very good cost-effective, inexpensive, power-saving effect is very good, recommended!	Taobao Marketplace, a Chinese website for online shopping
Xindu Flagship Store	Green Intelligent Power Saver	Too fake, nothing useful, see people do not buy on the file to be cheated!	Taobao Marketplace, a Chinese website for online shopping
Xindu Flagship Store	Green Intelligent Power Saver	The quality of the economizer is good and thick, and the energy-saving effect is also good, satisfied!	Taobao Marketplace, a Chinese website for online shopping
Xindu Flagship Store	Green Intelligent Power Saver	I've been using it for a while now, and the power saving effect is very obvious, it's very lightweight and doesn't take up much space. Quite good. You can rest assured to buy.	Taobao Marketplace, a Chinese website for online shopping
Xindu Flagship Store	Green Intelligent Power Saver	The saver works great, good results, very happy with it	Taobao Marketplace, a Chinese website for online shopping
Xindu Flagship Store	Green Intelligent Power Saver	This business is a scam no power saving at all don't buy it!	Taobao Marketplace, a Chinese website for online shopping
Xindu Flagship Store	Green Intelligent Power Saver	Save appliances received and installed and used for a few days, feel pretty good, satisfied.	Taobao Marketplace, a Chinese website for online shopping
Xindu Flagship Store	Green Intelligent Power Saver	Experience: Trial stage	Taobao Marketplace, a Chinese website for online shopping
Xindu Flagship Store	Green Intelligent Power Saver	The saver is very good, good quality, fine workmanship, very good materials	Taobao Marketplace, a Chinese website for online shopping
Xindu Flagship Store	Green Intelligent Power Saver	The regulator has been used for a few days and is of good quality and works great	Taobao Marketplace, a Chinese website for online shopping
Xindu Flagship Store	Green Intelligent Power Saver	Used for a few days, it works quite well to save power, the quality is also good cost-effective	Taobao Marketplace, a Chinese website for online shopping
Xindu Flagship Store	Green Intelligent Power Saver	Works great, great value for money and just the right size!	Taobao Marketplace, a Chinese website for online shopping
Xindu Flagship Store	Green Intelligent Power Saver	The boss shipped quickly, the price is reasonable and affordable, has been plugged in on, looking forward to power-saving effect, worth buying!	Taobao Marketplace, a Chinese website for online shopping
Xindu Flagship Store	Green Intelligent Power Saver	Value for money: good Appearance and style: good Workmanship and material: good	Taobao Marketplace, a Chinese website for online shopping
Xindu Flagship Store	Green Intelligent Power Saver	High safety, power saving is very good, cost-effective, satisfactory	Taobao Marketplace, a Chinese website for online shopping
Xindu Flagship Store	Green Intelligent Power Saver	Good quality, power saving, good value for money.	Taobao Marketplace, a Chinese website for online shopping
Xindu Flagship Store	Green Intelligent Power Saver	Good logistics, good workmanship, good material, good power saving effect, very satisfied.	Taobao Marketplace, a Chinese website for online shopping
Xindu Flagship Store	Green Intelligent Power Saver	It's really working.	Taobao Marketplace, a Chinese website for online shopping
Xindu Flagship Store	Green Intelligent Power Saver	It's a real power saver, like 20 or 30 percent of your electricity, so you're saving a lot of money for a little money.	Taobao Marketplace, a Chinese website for online shopping
Xindu Flagship Store	Green Intelligent Power Saver	Just plugged it in, don't know the effect yet, use it for a while and then follow up!	Taobao Marketplace, a Chinese website for online shopping
Xindu Flagship Store	Green Intelligent Power Saver	Baby received, the appearance of luscious ah, ready to try, use the good, and then additional positive feedback!	Taobao Marketplace, a Chinese website for online shopping
Xindu Flagship Store	Green Intelligent Power Saver	Power saving effect: good Cost-effective: high Appearance style: good design Workmanship material: good material Experience: good effect	Taobao Marketplace, a Chinese website for online shopping
Xindu Flagship Store	Green Intelligent Power Saver	Money saver. Good for you. Easy to use.	Taobao Marketplace, a Chinese website for online shopping

Xindu Flagship Store	Green Intelligent Power Saver	The quality of the stuff is very good, exactly the same as the seller's description, very satisfied!	Taobao Marketplace, a Chinese website for online shopping
Xindu Flagship Store	Green Intelligent Power Saver	Affordable and satisfactory value for money	Taobao Marketplace, a Chinese website for online shopping
Xindu Flagship Store	Green Intelligent Power Saver	It works, good quality, fast logistics.	Taobao Marketplace, a Chinese website for online shopping
Xindu Flagship Store	Green Intelligent Power Saver	Saver is really good to use, and now use a period of time down to save a lot of business and activities buy 2 get 1 free, or very affordable, the future need will buy!	Taobao Marketplace, a Chinese website for online shopping
Xindu Flagship Store	Green Intelligent Power Saver	Fast shipping, good quality, very satisfied, trustworthy.	Taobao Marketplace, a Chinese website for online shopping
Xindu Flagship Store	Green Intelligent Power Saver	Responsive, doesn't take up much space, very power efficient	Taobao Marketplace, a Chinese website for online shopping
Xindu Flagship Store	a power saver (electronics)	Fast shipping and good quality. Measured this does save a lot of electricity, like the baby can feel free to enter.	Taobao Marketplace, a Chinese website for online shopping
Xindu Flagship Store	a power saver (electronics)	Good logistics and product, good value for money, kudos👏👏👏👏👏👏	Taobao Marketplace, a Chinese website for online shopping
Xindu Flagship Store	a power saver (electronics)	It feels good,just received it and put it on. I hope the after-sales service is as good as ever	Taobao Marketplace, a Chinese website for online shopping
Xindu Flagship Store	a power saver (electronics)	's great. It's a lot easier to get out of a contract	Taobao Marketplace, a Chinese website for online shopping
Xindu Flagship Store	a power saver (electronics)	Just started using it. See the effect!	Taobao Marketplace, a Chinese website for online shopping
Xindu Flagship Store	a power saver (electronics)	Received the baby, simple and easy to install, solid appearance, listening to neighbors said it really works ah, looking forward to the results of the next month.	Taobao Marketplace, a Chinese website for online shopping
Xindu Flagship Store	a power saver (electronics)	Appearance design perfect quality good boss service attitude is very good first use good use then come back to the follow-up evaluation	Taobao Marketplace, a Chinese website for online shopping
Xindu Flagship Store	a power saver (electronics)	Electricity received, good value for money, works well	Taobao Marketplace, a Chinese website for online shopping
Xindu Flagship Store	a power saver (electronics)	Can't tell if it saves power or not, used it for a few days before I commented on it	Taobao Marketplace, a Chinese website for online shopping
Xindu Flagship Store	a power saver (electronics)	Power saving effect: okay Cost-effective: with okay Appearance style: can Workmanship material: good Using experience: good	Taobao Marketplace, a Chinese website for online shopping
Xindu Flagship Store	a power saver (electronics)	It's working just fine.	Taobao Marketplace, a Chinese website for online shopping
Xindu Flagship Store	a power saver (electronics)	The power saving effect is still there, so this time I decided to buy a little more, otherwise this long summer is too much electricity and silver.	Taobao Marketplace, a Chinese website for online shopping
Xindu Flagship Store	a power saver (electronics)	Now do not know the effect of power saving, but it looks good, use it first, use the effect is good and then a wave of follow-up comments.	Taobao Marketplace, a Chinese website for online shopping
Xindu Flagship Store	a power saver (electronics)	It's effective, beautiful, easy to use, convenient, and power-saving.	Taobao Marketplace, a Chinese website for online shopping
Xindu Flagship Store	a power saver (electronics)	The power saver is on, the house usually uses quite a lot of electricity, I hope to use it to save some money on the electricity bill!	Taobao Marketplace, a Chinese website for online shopping
Xindu Flagship Store	a power saver (electronics)	Energy saver received, the appearance of a solid and durable, long use does not heat, very easy to use!	Taobao Marketplace, a Chinese website for online shopping
Xindu Flagship Store	a power saver (electronics)	I came back and used it for a few days and it feels great, just as described and worth having.	Taobao Marketplace, a Chinese website for online shopping
Xindu Flagship Store	a power saver (electronics)	It's the ideal product💖, good quality and fast logistics, worth the kudos!	Taobao Marketplace, a Chinese website for online shopping
Xindu Flagship Store	a power saver (electronics)	The workmanship and materials are good for the price.	Taobao Marketplace, a Chinese website for online shopping
Xindu Flagship Store	a power saver (electronics)	Received, good quality, cost-effective, very satisfied	Taobao Marketplace, a Chinese website for online shopping
Xindu Flagship Store	a power saver (electronics)	It works well and is good value for money	Taobao Marketplace, a Chinese website for online shopping
Xindu Flagship Store	a power saver (electronics)	Cost-effective: high price, the quality is also good, the overall effect is good, power saving used in the evaluation	Taobao Marketplace, a Chinese website for online shopping
Xindu Flagship Store	a power saver (electronics)	Good quality, great customer service	Taobao Marketplace, a Chinese website for online shopping
Xindu Flagship Store	a power saver (electronics)	I don't know about the energy saving effect yet, I'm using it now, let's see how it goes after some time.	Taobao Marketplace, a Chinese website for online shopping
Xindu Flagship Store	a power saver (electronics)	Very satisfied, thanks to the seller last time I bought a save me60bucks on the electricity bill, this time re-buy another one this thing is really good ah! Continue to buy back in the future.	Taobao Marketplace, a Chinese website for online shopping
Xindu Flagship Store	a power saver (electronics)	The owner of the fast delivery, has been an old customer, bought a lot of, home companies are using this it, very good to use to save money on electricity.	Taobao Marketplace, a Chinese website for online shopping
Xindu Flagship Store	a power saver (electronics)	The product was received, the quality is very good, and the seller described exactly the same	Taobao Marketplace, a Chinese website for online shopping
Xindu Flagship Store	a power saver (electronics)	It works really well and matches the seller's description, and it's quite a power saver.	Taobao Marketplace, a Chinese website for online shopping

Xindu Flagship Store	a power saver (electronics)	Baby received not bad, the effect is quite good, very satisfied.	Taobao Marketplace, a Chinese website for online shopping
Xindu Flagship Store	a power saver (electronics)	Workmanship: very good workmanship power saving effect to try a period of time to evaluate again	Taobao Marketplace, a Chinese website for online shopping
Xindu Flagship Store	a power saver (electronics)	Thruster received, very good quality, good material, plug and play, convenient and quick, long time use does not get hot!	Taobao Marketplace, a Chinese website for online shopping
Xindu Flagship Store	a power saver (electronics)	Good quality, easy to operate, as described by the merchant	Taobao Marketplace, a Chinese website for online shopping
Xindu Flagship Store	a power saver (electronics)	Easy to use, very good power saving, exquisite workmanship	Taobao Marketplace, a Chinese website for online shopping
Xindu Flagship Store	a power saver (electronics)	It doesn't get hot after a long time of use, very happy with it. Cost-effective.	Taobao Marketplace, a Chinese website for online shopping
Xindu Flagship Store	a power saver (electronics)	Good quality, satisfied	Taobao Marketplace, a Chinese website for online shopping
Xindu Flagship Store	a power saver (electronics)	Power Saving King has received, really good, thank you boss, I wish the boss a prosperous business, prosperity next time to visit the store!	Taobao Marketplace, a Chinese website for online shopping
Xindu Flagship Store	a power saver (electronics)	Things arrived in good results, cost-effective, beautiful appearance, and then follow up later evaluation	Taobao Marketplace, a Chinese website for online shopping
Xindu Flagship Store	a power saver (electronics)	I used it. It worked.	Taobao Marketplace, a Chinese website for online shopping
Xindu Flagship Store	a power saver (electronics)	At first sight, the baby is particularly fond of	Taobao Marketplace, a Chinese website for online shopping
Xindu Flagship Store	a power saver (electronics)	Energy saving effect is very good, used than the original number of electricity down a lot, highly recommended.	Taobao Marketplace, a Chinese website for online shopping
Xindu Flagship Store	a power saver (electronics)	This compact is beautiful and easy to use!	Taobao Marketplace, a Chinese website for online shopping
Xindu Flagship Store	a power saver (electronics)	Looking forward to the results	Taobao Marketplace, a Chinese website for online shopping
Xindu Flagship Store	a power saver (electronics)	Well, it is true that the receipt has been two or three days I see that the instructions say that the first five days are part of some kind of power-clearing period or something like that, andafter15days,best results come after30days, so I hope that it still works Well, the appearance of a very small one. He does not go on other machines, put him this machine does not get hot very well	Taobao Marketplace, a Chinese website for online shopping
Xindu Flagship Store	a power saver (electronics)	Power saving effect: very power saving, it's ok, second time I bought it. I only come here because of other people's referrals	Taobao Marketplace, a Chinese website for online shopping
Xindu Flagship Store	a power saver (electronics)	Super super bargain, great quality, still fast, definitely recommend, great style too, happy with it!	Taobao Marketplace, a Chinese website for online shopping
Xindu Flagship Store	a power saver (electronics)	The quality of the saver is good, the power saving effect is very good, will introduce friends to buy again, full five points! Good review!	Taobao Marketplace, a Chinese website for online shopping
Xindu Flagship Store	a power saver (electronics)	No visible results, as evidenced by screenshots of the bill.	Taobao Marketplace, a Chinese website for online shopping
Xindu Flagship Store	a power saver (electronics)	Fast shipping, good quality for the price Recommended!	Taobao Marketplace, a Chinese website for online shopping
Xindu Flagship Store	a power saver (electronics)	Fast shipping good stuff 5 stars next time buy a few more	Taobao Marketplace, a Chinese website for online shopping
Xindu Flagship Store	a power saver (electronics)	Looking forward to the results, received.	Taobao Marketplace, a Chinese website for online shopping
Xindu Flagship Store	a power saver (electronics)	I've received it, how does it work? Time will tell, but I hope there will be a surprise when I pay my bill next month.	Taobao Marketplace, a Chinese website for online shopping
Xindu Flagship Store	a power saver (electronics)	Fast shipping, good quality, easy to use, not hot to use.	Taobao Marketplace, a Chinese website for online shopping
Xindu Flagship Store	a power saver (electronics)	Power saving effect: Effective Cost-effective: High Using experience: Very satisfactory	Taobao Marketplace, a Chinese website for online shopping
Xindu Flagship Store	a power saver (electronics)	Nice Plugged it in and. Very handy	Taobao Marketplace, a Chinese website for online shopping
Xindu Flagship Store	a power saver (electronics)	Very good quality, cost-effective, good-looking and versatile	Taobao Marketplace, a Chinese website for online shopping
Xindu Flagship Store	a power saver (electronics)	Power saving effect: nothing useful, IQ tax, don't fall for it.	Taobao Marketplace, a Chinese website for online shopping
Xindu Flagship Store	a power saver (electronics)	Saver is indeed still very good to use, the beginning of the use of not much effect and role, but the latter effect is particularly obvious, thanks to the merchant	Taobao Marketplace, a Chinese website for online shopping
Xindu Flagship Store	a power saver (electronics)	Logistics is strong, customer service attitude is good, baby quality is good, the appearance of the design is novel, the power is big, save electricity and money, lasting products, with cost-effective.	Taobao Marketplace, a Chinese website for online shopping
Xindu Flagship Store	a power saver (electronics)	Sturdy and durable appearance, easy to use, not hot for a long time, the quality is quite good	Taobao Marketplace, a Chinese website for online shopping
Xindu Flagship Store	a power saver (electronics)	The quality of the saver is very good, stable to use, affordable, and I hope it saves electricity!	Taobao Marketplace, a Chinese website for online shopping
Xindu Flagship Store	a power saver (electronics)	Works great, really saved a lot of store after using it, customer service suggested just the right amount, material workmanship is good, satisfied.	Taobao Marketplace, a Chinese website for online shopping
Xindu Flagship Store	a power saver (electronics)	Logistics is fast Material is good Uses well Convenient	Taobao Marketplace, a Chinese website for online shopping

Xindu Flagship Store	a power saver (electronics)	It's pretty good, it works well, and it's such a good buy for the price	Taobao Marketplace, a Chinese website for online shopping
Xindu Flagship Store	a power saver (electronics)	Appearance and style: energy-saving effect is very good, but also very beautiful	Taobao Marketplace, a Chinese website for online shopping
Xindu Flagship Store	a power saver (electronics)	It's good. It works great. It's a power saver.	Taobao Marketplace, a Chinese website for online shopping
Xindu Flagship Store	a power saver (electronics)	Power saving effect: ∴ very good	Taobao Marketplace, a Chinese website for online shopping
Xindu Flagship Store	a power saver (electronics)	The last time I bought a nothing to feel but the power supply bureau often come to check the meter said that there is power loss, this time to buy another try	Taobao Marketplace, a Chinese website for online shopping
Xindu Flagship Store	a power saver (electronics)	Like we know when we live in self-built houses, we have electricians checking the damage every day.	Taobao Marketplace, a Chinese website for online shopping
Xindu Flagship Store	a power saver (electronics)	Great item recommended Experience: save40%electricity	Taobao Marketplace, a Chinese website for online shopping
Xindu Flagship Store	a power saver (electronics)	It's cost effective, doesn't get hot, and the shipping was fast.	Taobao Marketplace, a Chinese website for online shopping
Xindu Flagship Store	a power saver (electronics)	Logistics is very fast, received the next day, the quality is very good	Taobao Marketplace, a Chinese website for online shopping
Xindu Flagship Store	a power saver (electronics)	I can't see any power savings after a few days of use, so I'll see what I can say after a few more days?	Taobao Marketplace, a Chinese website for online shopping
Xindu Flagship Store	a power saver (electronics)	Power saving effect: very satisfied Workmanship material: can be	Taobao Marketplace, a Chinese website for online shopping
Xindu Flagship Store	a power saver (electronics)	It's great, it's nice and saves electricity, I buy from this one often.	Taobao Marketplace, a Chinese website for online shopping
Xindu Flagship Store	a power saver (electronics)	Effective and useful, I feel quite power-saving with it.	Taobao Marketplace, a Chinese website for online shopping
Xindu Flagship Store	a power saver (electronics)	Energy saver is nice and practical	Taobao Marketplace, a Chinese website for online shopping
Xindu Flagship Store	a power saver (electronics)	Power saving effect: no use at all	Taobao Marketplace, a Chinese website for online shopping
Xindu Flagship Store	a power saver (electronics)	It doesn't help at all.	Taobao Marketplace, a Chinese website for online shopping
Xindu Flagship Store	a power saver (electronics)	Appearance is good, not like some of the flashing eyes, the effect to be verified	Taobao Marketplace, a Chinese website for online shopping
Xindu Flagship Store	a power saver (electronics)	Come back when you're ready.	Taobao Marketplace, a Chinese website for online shopping
Xindu Flagship Store	a power saver (electronics)	Looks like the stuff is as good as the buyer said, plugged it in now and will follow up if it works out well	Taobao Marketplace, a Chinese website for online shopping
Xindu Flagship Store	a power saver (electronics)	Just received it today and used it as soon as I received it. Use it for a while and see how it works. Watch this meter. More pitiful. No way, a person a light, a refrigerator, a rice cooker, an electric kettle. Gives me out of200per month🤬used to goabout250bucksthree months. Moving here gives me out three months worth of electricity every month.	Taobao Marketplace, a Chinese website for online shopping
Xindu Flagship Store	a power saver (electronics)	Power saving effect:Energy saving effect is better, very power saving, before the electricity bill is more, after using this product, the electricity bill save a lot, really too good!	Taobao Marketplace, a Chinese website for online shopping
Xindu Flagship Store	a power saver (electronics)	I. It's pretty good	Taobao Marketplace, a Chinese website for online shopping
Xindu Flagship Store	a power saver (electronics)	Logistics is quite fast, energy-saving appliances are quite good, really save a lot of electricity	Taobao Marketplace, a Chinese website for online shopping
Xindu Flagship Store	a power saver (electronics)	Business buy4get2, really too cost-effective, because it is the second time to buy, but also know that this effect is very good, pre-optimization is no effect, the later effect came out, so it is still necessary to be patient and wait for the optimization of the power saver	Taobao Marketplace, a Chinese website for online shopping
Xindu Flagship Store	a power saver (electronics)	Good quality, good power saving, cost-effective, very satisfactory	Taobao Marketplace, a Chinese website for online shopping
Xindu Flagship Store	a power saver (electronics)	Used it for a while! The power saving is great! Good quality! Likes!	Taobao Marketplace, a Chinese website for online shopping
Xindu Flagship Store	a power saver (electronics)	Power saving effect: used it, it works really well, the	Taobao Marketplace, a Chinese website for online shopping
Xindu Flagship Store	a power saver (electronics)	It's working fine.	Taobao Marketplace, a Chinese website for online shopping
Xindu Flagship Store	a power saver (electronics)	Fast logistics and great customer service. First time buyer, looking forward to it	Taobao Marketplace, a Chinese website for online shopping
Xindu Flagship Store	a power saver (electronics)	Good customer service and fast logistics	Taobao Marketplace, a Chinese website for online shopping
Xindu Flagship Store	a power saver (electronics)	Logistics is very fast, the power saver got today, the product appearance is very good, immediately plugged in to try, there is no heat and current sound. The power saving effect will be known only after use.	Taobao Marketplace, a Chinese website for online shopping
Xindu Flagship Store	a power saver (electronics)	Baby received, good, not hot, logistics fast.	Taobao Marketplace, a Chinese website for online shopping
Xindu Flagship Store	a power saver (electronics)	I used it for the first time tonight, so we'll see if it works or not depending on what the electric bill is this time next month.	Taobao Marketplace, a Chinese website for online shopping
Xindu Flagship Store	a power saver (electronics)	The quality is terrible, it broke in two days	Taobao Marketplace, a Chinese website for online shopping

Xindu Flagship Store	a power saver (electronics)	The store shipped quickly, the baby received in good condition. Carton packaging instructions in English, open the Chinese manual and company profile. Baby appearance design is beautiful, good materials, just use, power saving effect to be observed.	Taobao Marketplace, a Chinese website for online shopping
Xindu Flagship Store	a power saver (electronics)	After observing the meter light, it blinks a little slower when using this baby than when not.	Taobao Marketplace, a Chinese website for online shopping
Xindu Flagship Store	a power saver (electronics)	Power saving effect: very good Cost-effective: very high Appearance style: beautiful and durable	Taobao Marketplace, a Chinese website for online shopping
Xindu Flagship Store	a power saver (electronics)	The quality of the merchandise is excellent and the results are great	Taobao Marketplace, a Chinese website for online shopping
Xindu Flagship Store	a power saver (electronics)	Great quality, affordable price, looked at a lot of homes this one was cost effective!	Taobao Marketplace, a Chinese website for online shopping
Xindu Flagship Store	a power saver (electronics)	's very practical It doesn't feel hot even after a long time The quality is very good	Taobao Marketplace, a Chinese website for online shopping
Xindu Flagship Store	a power saver (electronics)	This time to buy a super value, good quality price very satisfactory	Taobao Marketplace, a Chinese website for online shopping
Xindu Flagship Store	a power saver (electronics)	Things received, the quality is very good, easy to use, the effect is good	Taobao Marketplace, a Chinese website for online shopping
Xindu Flagship Store	a power saver (electronics)	It's good. I'm happy with it.	Taobao Marketplace, a Chinese website for online shopping
Xindu Flagship Store	a power saver (electronics)	Merchants buy4get2free, it happens to be my home power consumption, you need6, Saver pre-effective, but the merchants have100days to try the after-sales service, very good, so do not hesitate to decide to try	Taobao Marketplace, a Chinese website for online shopping
Xindu Flagship Store	a power saver (electronics)	Good to use, the only bad thing is that the first need to optimize, at first thought no effect it, and then read the instructions also know to optimize, the second month the effect will be good	Taobao Marketplace, a Chinese website for online shopping
Xindu Flagship Store	a power saver (electronics)	Power-saving effect: logistics is very fast, received the baby plugged into the electricity is being used, after a period of time will be able to see the effect of the	Taobao Marketplace, a Chinese website for online shopping
Xindu Flagship Store	a power saver (electronics)	Now to receive the baby, is powered to test the process, power saving next time in a single order!	Taobao Marketplace, a Chinese website for online shopping
Xindu Flagship Store	a power saver (electronics)	Saving appliances really save electricity, there is a little bad The first1month is not effective, it takes the second month to have an effect, the overall still good reviews	Taobao Marketplace, a Chinese website for online shopping
Xindu Flagship Store	a power saver (electronics)	Power saving effect: it works very well Appearance style: the style is also very good-looking	Taobao Marketplace, a Chinese website for online shopping
Xindu Flagship Store	a power saver (electronics)	power saving device is really good, at first it did not work all feel ineffective, and then asked the business, the business let me use for a period of time, and now the effect is also very good, the	Taobao Marketplace, a Chinese website for online shopping
Xindu Flagship Store	a power saver (electronics)	Almost missed this saving appliances, just started to use nothing, almost thought it did not work, and then asked the business to know that the original is pre-needed to optimize, and now used for a period of time down indeed also save electricity.	Taobao Marketplace, a Chinese website for online shopping
Xindu Flagship Store	a power saver (electronics)	Saving appliances have been received, the appearance is beautiful, logistics is also fast, the effect does not know, with a period of time in the comment!	Taobao Marketplace, a Chinese website for online shopping
Xindu Flagship Store	a power saver (electronics)	Power-saving effect: once plugged in can feel the meter obviously slowed down Cost-effective: afford Experience: not bad Appearance style: quack new Workmanship material: quack line	Taobao Marketplace, a Chinese website for online shopping
Xindu Flagship Store	a power saver (electronics)	After using it for a while, the electricity bill has dropped significantly.	Taobao Marketplace, a Chinese website for online shopping
Xindu Flagship Store	a power saver (electronics)	Use one saver for150kWha month,two for300kWha month, andthreefor500kWh Listening to the store ; Early in the year in the use of Use it is better than not using it Try it again	Taobao Marketplace, a Chinese website for online shopping
Xindu Flagship Store	a power saver (electronics)	I bought one before and felt it worked, so I bought two more, which my neighbor asked for	Taobao Marketplace, a Chinese website for online shopping
Xindu Flagship Store	a power saver (electronics)	Good price and good logistics, worth repurchasing!	Taobao Marketplace, a Chinese website for online shopping
Xindu Flagship Store	a power saver (electronics)	Used it, don't know the effects yet!	Taobao Marketplace, a Chinese website for online shopping
Xindu Flagship Store	a power saver (electronics)	This2023energy-saving king uhfrom Xin Du flagship storeis very convenient for home use, power-saving and energy-saving and space-saving, with stylish appearance and well-made materials, don't believe that you also buy it to try.	Taobao Marketplace, a Chinese website for online shopping
Xindu Flagship Store	a power saver (electronics)	Power-saving effect: have bought before, the effect can be Cost-effective: cost-effective👌 Appearance style: compact Workmanship material: strong and durable Experience: the effect is good, worth buying	Taobao Marketplace, a Chinese website for online shopping
Xindu Flagship Store	a power saver (electronics)	I've used it before and it does have a power saving effect, a couple of bucks a month, it's really worth getting it	Taobao Marketplace, a Chinese website for online shopping
Xindu Flagship Store	a power saver (electronics)	Better value for money, very good quality of workmanship, easy to use, have used it again	Taobao Marketplace, a Chinese website for online shopping
Xindu Flagship Store	a power saver (electronics)	Power savings: great, works great, love it.	Taobao Marketplace, a Chinese website for online shopping
Xindu Flagship Store	a power saver (electronics)	Works great, been using it for a while, great power saver. Kudos and recommendations	Taobao Marketplace, a Chinese website for online shopping
Xindu Flagship Store	a power saver (electronics)	This power saver power saving king does not have any power saving effect, not a little power saving effect, selling fake goods, cheating consumers!	Taobao Marketplace, a Chinese website for online shopping
Xindu Flagship Store	a power saver (electronics)	Fast shipping, already received, looks good, try it for a while to know the result, very much looking forward to it!	Taobao Marketplace, a Chinese website for online shopping
Xindu Flagship Store	a power saver (electronics)	Listening to people introduce the effect is good on the decisive to buy, the appearance of the packaging is better than expected. Home high-power electrical appliances more, after receiving the goods directly plugged in and used, I hope there will be a surprise!	Taobao Marketplace, a Chinese website for online shopping
Xindu Flagship Store	a power saver (electronics)	Power saving: good Price/performance ratio: good Exterior style: good looking	Taobao Marketplace, a Chinese website for online shopping

Xindu Flagship Store	a power saver (electronics)	Very good tech products	Taobao Marketplace, a Chinese website for online shopping
Xindu Flagship Store	a power saver (electronics)	I've been using it for a month or so now, and the results are really good. Monthly power savingsabout10degrees	Taobao Marketplace, a Chinese website for online shopping
Xindu Flagship Store	a power saver (electronics)	using it2months now and it's a very good saver. Savesabout10kWha month	Taobao Marketplace, a Chinese website for online shopping
Xindu Flagship Store	a power saver (electronics)	It should work because there are a lot of this type of product and we use a lot of electricity in our house so it's good to try this out and see if we can save a kilowatt hour!	Taobao Marketplace, a Chinese website for online shopping
Xindu Flagship Store	a power saver (electronics)	there's nothing wrong with it	Taobao Marketplace, a Chinese website for online shopping
Xindu Flagship Store	a power saver (electronics)	Received yesterday, is in use, customer service too much is very good, the courier timely delivery, and so on with a month and then follow up, really is able to save electricity, I will feedback to more people to buy!	Taobao Marketplace, a Chinese website for online shopping
Xindu Flagship Store	a power saver (electronics)	Good value for money, simple style, good quality, good effect	Taobao Marketplace, a Chinese website for online shopping
Xindu Flagship Store	a power saver (electronics)	Merchants have activities are very cost-effective, buy4get2, this time to buy6, get back to use the first period is no effect, the second month the effect is very obvious, but also have to insist with the use of	Taobao Marketplace, a Chinese website for online shopping
Xindu Flagship Store	a power saver (electronics)	This saver is very good to use, saving electricity to save money, I bought two, one put upstairs, one put downstairs, feel very save, have effect, today received another put the store, friends can rest assured that the purchase use.	Taobao Marketplace, a Chinese website for online shopping
Xindu Flagship Store	a power saver (electronics)	The quality is very good, rest assured that the purchase, I bought two, with a good and bought another, very save electricity, friends rest assured that the purchase and use.	Taobao Marketplace, a Chinese website for online shopping
Xindu Flagship Store	a power saver (electronics)	Received the baby, feel good, with a month to see how the effect of power saving!	Taobao Marketplace, a Chinese website for online shopping
Xindu Flagship Store	a power saver (electronics)	Power savings: there should be power savings.	Taobao Marketplace, a Chinese website for online shopping
Xindu Flagship Store	a power saver (electronics)	I've been using it for a few days now, and there's noticeably less of it than before.	Taobao Marketplace, a Chinese website for online shopping
Xindu Flagship Store	a power saver (electronics)	Workmanship material: all can	Taobao Marketplace, a Chinese website for online shopping
Xindu Flagship Store	a power saver (electronics)	Used it for a month and it didn't work! The electricity bill is not reduced at all	Taobao Marketplace, a Chinese website for online shopping
Xindu Flagship Store	a power saver (electronics)	Baby received, the appearance is also beautiful very like, really have power saving effect, worth buying.	Taobao Marketplace, a Chinese website for online shopping
Xindu Flagship Store	a power saver (electronics)	Very nice👍	Taobao Marketplace, a Chinese website for online shopping
Xindu Flagship Store	a power saver (electronics)	Previously have bought, unique design, powerful, power savingmore than40%, the test is very good, my family uses a lot of electricity, half a month can save a hundred dollars. Summer open air conditioning more, already very satisfied	Taobao Marketplace, a Chinese website for online shopping
Xindu Flagship Store	a power saver (electronics)	Saver good use, at first no effect almost lost, but fortunately their own in the use of the next, the effect of this month is very good, a lot less electricity, business welfare is also very good Buy4Get2free	Taobao Marketplace, a Chinese website for online shopping
Xindu Flagship Store	a power saver (electronics)	The item was received, it looks beautiful and is a great value for the money. Merchants shipment is also very fast. Customer service personnel also has a good attitude. Started to shoot3, did not see the merchant's promotional activities, and merchants to communicate with the merchant to shoot another, the merchant still sent another. Thanks to the merchants. Logistics is also very fast! Give praise!	Taobao Marketplace, a Chinese website for online shopping
Xindu Flagship Store	a power saver (electronics)	The voltage is quite stable, works quite well, good quality	Taobao Marketplace, a Chinese website for online shopping
Xindu Flagship Store	a power saver (electronics)	Already using it, works great, worth the money!	Taobao Marketplace, a Chinese website for online shopping
Xindu Flagship Store	a power saver (electronics)	There's no savings. Maybe I'm not wired right.	Taobao Marketplace, a Chinese website for online shopping
Xindu Flagship Store	a power saver (electronics)	Definitely a good product, it is particularly convenient to use, directly inserted into the plug board at home. This month to open the air conditioning to see the meter than before turned slower, the power consumption is also less, the effect is particularly good, this product is trustworthy.	Taobao Marketplace, a Chinese website for online shopping
Xindu Flagship Store	a power saver (electronics)	Boss replenish one Thank you boss, I hope it works!	Taobao Marketplace, a Chinese website for online shopping
Xindu Flagship Store	a power saver (electronics)	Just received the goods today, use it for a while and then evaluate it	Taobao Marketplace, a Chinese website for online shopping
Xindu Flagship Store	a power saver (electronics)	Very practical and affordable, very Nessy!	Taobao Marketplace, a Chinese website for online shopping
Xindu Flagship Store	a power saver (electronics)	Beautiful and inexpensive, good suburban fruit	Taobao Marketplace, a Chinese website for online shopping
Xindu Flagship Store	a power saver (electronics)	Power savings: not good after trying for days!	Taobao Marketplace, a Chinese website for online shopping
Xindu Flagship Store	a power saver (electronics)	Looks more advanced than previous generations, very lightweight, easy to use, great!	Taobao Marketplace, a Chinese website for online shopping
Xindu Flagship Store	a power saver (electronics)	I can't wait to use it, I feel like the meter is spinning a little slower than before!	Taobao Marketplace, a Chinese website for online shopping
Xindu Flagship Store	a power saver (electronics)	Energy saver received already is the second time to buy. Previously, the electricity bill is156yuan after buying two air conditioning power only a few dozen dollars more energy-saving suburb fruit is very good and more practical. Now buy a second hope energy saving more electricity.	Taobao Marketplace, a Chinese website for online shopping
Xindu Flagship Store	a power saver (electronics)	Is really energy-saving summer hit the air conditioning is no longer afraid of the electricity bill more, need to hurry to order it!	Taobao Marketplace, a Chinese website for online shopping
Xindu Flagship Store	a power saver (electronics)	The appearance of simple and good-looking, the plug is also very stable, the instructions are also very clear relatives recommended to buy, said it is useful	Taobao Marketplace, a Chinese website for online shopping

Xindu Flagship Store	a power saver (electronics)	It doesn't get hot after using it for a while and you don't have to worry about it blowing up. Recommend to buy	Taobao Marketplace, a Chinese website for online shopping
Xindu Flagship Store	a power saver (electronics)	Baby has received, the appearance of good-looking delicate, look forward to using the effect of it!	Taobao Marketplace, a Chinese website for online shopping
Xindu Flagship Store	a power saver (electronics)	Experience: the first time to buy are said to be quite good friends recommended to experience this magic weapon.	Taobao Marketplace, a Chinese website for online shopping
Xindu Flagship Store	a power saver (electronics)	It's ok, fast shipping, hopefully the product does the trick!	Taobao Marketplace, a Chinese website for online shopping
Xindu Flagship Store	a power saver (electronics)	To be honest, bought back two months, can only say child exemption, not a bit of use, which commented that the super power saving, save hair line, I more than a hundred degrees of electricity every month, used two months or more than a hundred degrees of electricity. Personal advice or do not buy, the only power saving is to find a professional electrician to move the meter.	Taobao Marketplace, a Chinese website for online shopping
Xindu Flagship Store	a power saver (electronics)	Power saving effect: paper is good and worth buying	Taobao Marketplace, a Chinese website for online shopping
Xindu Flagship Store	a power saver (electronics)	Haven't gotten the results yet won't know until the end of the month to add a comment then.	Taobao Marketplace, a Chinese website for online shopping
Xindu Flagship Store	a power saver (electronics)	Received, very good quality and value for money	Taobao Marketplace, a Chinese website for online shopping
Xindu Flagship Store	a power saver (electronics)	Friends recommended this power saver, just received the goods today, immediately use, friends have used the effect, I believe it is true that will save electricity.	Taobao Marketplace, a Chinese website for online shopping
Xindu Flagship Store	a power saver (electronics)	Things are especially good, the old man and children like, next time to buy, the quality of things is too good!	Taobao Marketplace, a Chinese website for online shopping
Xindu Flagship Store	a power saver (electronics)	It didn't work, used it as soon as I received it, didn't save reverse power, waste of my money	Taobao Marketplace, a Chinese website for online shopping
Xindu Flagship Store	a power saver (electronics)	Let's not talk about anything else, but let's praise the customer service first	Taobao Marketplace, a Chinese website for online shopping
Xindu Flagship Store	a power saver (electronics)	This installation can't be photographed anywhere else, a friend recommended it and bought it twice.	Taobao Marketplace, a Chinese website for online shopping
Xindu Flagship Store	a power saver (electronics)	ve been using it for a month and my electric bill is still$20more expensive😓😓😓really an IQ tax	Taobao Marketplace, a Chinese website for online shopping
Xindu Flagship Store	a power saver (electronics)	Logistics quickly received: the item is lightweight and good to use, the shape is good recommended, good customer service.	Taobao Marketplace, a Chinese website for online shopping
Xindu Flagship Store	a power saver (electronics)	It's okay. Come on over and use it. It's great.	Taobao Marketplace, a Chinese website for online shopping
Xindu Flagship Store	a power saver (electronics)	Appearance style: modern fashion Use experience: before the family has bought, or effective, buy a few more better results!	Taobao Marketplace, a Chinese website for online shopping
Xindu Flagship Store	a power saver (electronics)	Getting ready to buy another one, genuinely nice!	Taobao Marketplace, a Chinese website for online shopping
Xindu Flagship Store	a power saver (electronics)	Appearance style: exquisite	Taobao Marketplace, a Chinese website for online shopping
Xindu Flagship Store	a power saver (electronics)	It's a good thing it worked.	Taobao Marketplace, a Chinese website for online shopping
Xindu Flagship Store	a power saver (electronics)	Appearance style: a few received goods style and picture description consistent. Workmanship: exquisite workmanship, the material is very good.	Taobao Marketplace, a Chinese website for online shopping
Xindu Flagship Store	a power saver (electronics)	I haven't found any product quality problems so far, and I'll comment again after using it for a while.	Taobao Marketplace, a Chinese website for online shopping
Xindu Flagship Store	a power saver (electronics)	I don't know how it is, I won't know until a month down the road. I'll give it a good review first.	Taobao Marketplace, a Chinese website for online shopping
Xindu Flagship Store	a power saver (electronics)	Energy saver received, the quality is exceptionally good, very satisfied with a shopping.	Taobao Marketplace, a Chinese website for online shopping
Xindu Flagship Store	a power saver (electronics)	A very nice product.	Taobao Marketplace, a Chinese website for online shopping
Xindu Flagship Store	a power saver (electronics)	Hopefully, it will, with noticeable results.	Taobao Marketplace, a Chinese website for online shopping
Xindu Flagship Store	a power saver (electronics)	Power-saving effect: power-saving effect is good Appearance style: looks small Cost-effective: price point is not high Using experience: welcome to come to purchase	Taobao Marketplace, a Chinese website for online shopping
Xindu Flagship Store	a power saver (electronics)	Overall I think it's very affordable and the power saving effect is fine, next time I will come back to this store to buy	Taobao Marketplace, a Chinese website for online shopping
Xindu Flagship Store	a power saver (electronics)	The saver works well and saves a lot of money over the course of a year, it's easy to use	Taobao Marketplace, a Chinese website for online shopping
Xindu Flagship Store	a power saver (electronics)	Power-saving effect:👌 Price-performance ratio:👌 Appearance style:👌 Workmanship material:👌 Using experience:👌	Taobao Marketplace, a Chinese website for online shopping
Xindu Flagship Store	a power saver (electronics)	Received, easy to install, energy saving effect is very good, good workmanship materials	Taobao Marketplace, a Chinese website for online shopping
Xindu Flagship Store	a power saver (electronics)	Works great, well made, good quality, fast shipping.	Taobao Marketplace, a Chinese website for online shopping
Xindu Flagship Store	a power saver (electronics)	The quality is great, and using it saves a lot of electronics in the house	Taobao Marketplace, a Chinese website for online shopping
Xindu Flagship Store	a power saver (electronics)	I'm very happy with the goods.	Taobao Marketplace, a Chinese website for online shopping

Xindu Flagship Store	a power saver (electronics)	The power saver received, the appearance of rugged, durable, long use does not heat, very easy to use, power saving effect to know after a period of time.	Taobao Marketplace, a Chinese website for online shopping
Xindu Flagship Store	a power saver (electronics)	Baby works great, great value for money	Taobao Marketplace, a Chinese website for online shopping
Xindu Flagship Store	a power saver (electronics)	I've been using it for a while now and it works great	Taobao Marketplace, a Chinese website for online shopping
Xindu Flagship Store	a power saver (electronics)	The stuff is even more perfect than I imagined, very powerful and worth getting!	Taobao Marketplace, a Chinese website for online shopping
Xindu Flagship Store	a power saver (electronics)	I've been using it for a while now, it's good, it saves energy.	Taobao Marketplace, a Chinese website for online shopping
Xindu Flagship Store	a power saver (electronics)	Friends recommended that is very good, I also bought a, workmanship material is very good, used a few days, check the meter, really effective obviously, the price is very affordable, this time the power saving down can be saved some electricity bills.	Taobao Marketplace, a Chinese website for online shopping
Xindu Flagship Store	a power saver (electronics)	The product received, used the effect is good, long time will not be hot, you can see	Taobao Marketplace, a Chinese website for online shopping
Xindu Flagship Store	a power saver (electronics)	The product received, the use of the heat does not, try to have a power-saving effect is good and very satisfied!	Taobao Marketplace, a Chinese website for online shopping
Xindu Flagship Store	a power saver (electronics)	Shipping is fast. Good quality, good value for money, satisfied	Taobao Marketplace, a Chinese website for online shopping
Xindu Flagship Store	a power saver (electronics)	Reasonably priced, not sure of the effectiveness!	Taobao Marketplace, a Chinese website for online shopping
Xindu Flagship Store	a power saver (electronics)	Not as good as the seller said, this month used more electricity than last month	Taobao Marketplace, a Chinese website for online shopping
Xindu Flagship Store	a power saver (electronics)	Power savings: not much Experience: not as good as stated	Taobao Marketplace, a Chinese website for online shopping
Xindu Flagship Store	a power saver (electronics)	Power saving effect: a little	Taobao Marketplace, a Chinese website for online shopping
Xindu Flagship Store	a power saver (electronics)	Power saving effect: one move	Taobao Marketplace, a Chinese website for online shopping
Xindu Flagship Store	a power saver (electronics)	Energy saving effect is good, very satisfied	Taobao Marketplace, a Chinese website for online shopping
Xindu Flagship Store	a power saver (electronics)	IQ tax. It doesn't help at all.	Taobao Marketplace, a Chinese website for online shopping
Xindu Flagship Store	a power saver (electronics)	Personally, I don't feel there is much difference!	Taobao Marketplace, a Chinese website for online shopping
Xindu Flagship Store	a power saver (electronics)	Is a friend's home to buy, go to do guests when the grass to come, listen to friends say really save electricity, buy back to try it, very small one, lazy people money-saving artifacts!	Taobao Marketplace, a Chinese website for online shopping
Xindu Flagship Store	a power saver (electronics)	This really saves electricity, my family used for a while, the electricity bill is really less, summer air conditioning is very costly, this month the electricity bill is really less, it's really good!	Taobao Marketplace, a Chinese website for online shopping
Xindu Flagship Store	a power saver (electronics)	Plugged in anywhere can be used, I hope to save electricity! Logistics is very fast, customer service small sister attitude is very good	Taobao Marketplace, a Chinese website for online shopping
Xindu Flagship Store	a power saver (electronics)	Appearance is my favorite look, used for ten days or so feel the effect of power saving, cost-effective, ready to get another one with my family.	Taobao Marketplace, a Chinese website for online shopping
Xindu Flagship Store	a power saver (electronics)	Received the item, this product is still appropriate compared to its price point	Taobao Marketplace, a Chinese website for online shopping
Xindu Flagship Store	a power saver (electronics)	Observed it for a day and it feels fine with the power saving feature	Taobao Marketplace, a Chinese website for online shopping
Xindu Flagship Store	a power saver (electronics)	Power saving effect: after using it saved ten dollars from last month It is the second time to buy it It works very well	Taobao Marketplace, a Chinese website for online shopping
Xindu Flagship Store	a power saver (electronics)	Purchased for the second time It really saves a lot of money on your electric bill	Taobao Marketplace, a Chinese website for online shopping
Xindu Flagship Store	a power saver (electronics)	Received! Don't know the effect yet. I'll tell you later!	Taobao Marketplace, a Chinese website for online shopping
Xindu Flagship Store	a power saver (electronics)	Received the goods, but I don't know how well it works.	Taobao Marketplace, a Chinese website for online shopping
Xindu Flagship Store	a power saver (electronics)	It didn't work. It wasn't even as good as it said it would be.	Taobao Marketplace, a Chinese website for online shopping
Xindu Flagship Store	a power saver (electronics)	I don't know if it really saves power, I'll comment after I use it.	Taobao Marketplace, a Chinese website for online shopping
Xindu Flagship Store	a power saver (electronics)	The baby has been received, I tried it quite good ah	Taobao Marketplace, a Chinese website for online shopping
Xindu Flagship Store	a power saver (electronics)	Hahaha, it's really good	Taobao Marketplace, a Chinese website for online shopping
Xindu Flagship Store	a power saver (electronics)	The effect is very good, plugged in to see the meter is more obviously slow, the appearance of the style is also very good-looking, workmanship is also very good, the use of the second day to come very satisfied	Taobao Marketplace, a Chinese website for online shopping
Xindu Flagship Store	a power saver (electronics)	Power saving effect: good Workmanship material: fine Good quality, thick material Using experience: good	Taobao Marketplace, a Chinese website for online shopping
Xindu Flagship Store	a power saver (electronics)	The white color of the shape is also very beautiful, and the power saving effect is good, it works very well	Taobao Marketplace, a Chinese website for online shopping

Xindu Flagship Store	a power saver (electronics)	The goods have been received and I hope it will be the same product as described. In use	Taobao Marketplace, a Chinese website for online shopping
Xindu Flagship Store	a power saver (electronics)	Started to consult the business power consumption, the business told the larger the power consumption the more you want the saver, the more this time to buy3, power-saving effect: power-saving effect is good, compared with the previous month to savemore than200 Cost-effective: cheap and beautiful, activities to buy, buy2to sendavery cost-effective Appearance style: simple and generous, very beautiful Workmanship material: fine workmanship, genuine quality Experience: plug and play, easy to install The first time I saw this, I was able to plug it in and it worked.	Taobao Marketplace, a Chinese website for online shopping
Xindu Flagship Store	a power saver (electronics)	Lifetime product warranty, the business is very thoughtful, I purchased5 also gave me some freebies, and commitment to invalid full refund, now used for a period of time down, the feeling that the electricity bill is less, and so fully optimized after the belief that the effect will be even better, to the merchant five-star praise , recommended to everyone, this sales is also the best	Taobao Marketplace, a Chinese website for online shopping
Xindu Flagship Store	a power saver (electronics)	Experience: I am an electrician, this is the real use of electronic waves automatically connected to track the decomposition of ionized electrons, in situ compensation of reactive power, reduce effective power loss and waste, stabilize the voltage, to achieve energy saving and power saving, the overall effect is good, trustworthy, has been purchased, specially run to evaluate, will buy back, thanks to the	Taobao Marketplace, a Chinese website for online shopping
Xindu Flagship Store	a power saver (electronics)	Works well! I've been using it for three months, and I've seen results since the second month. Last month$192. This month it's only$93.75. Of course, after being out5days without power, I'm guessing the minimum savings of30%is still there.	Taobao Marketplace, a Chinese website for online shopping
Xindu Flagship Store	a power saver (electronics)	Power saving effect: packaging intact, customer service patience to answer questions is a big brand of energy-saving appliances worth buying, cost-effective, and the introduction of publicity consistent recommended that everyone buy the set cost-effective: really have a power-saving mode. Workmanship material: the latest production date Plug and play Super power-saving	Taobao Marketplace, a Chinese website for online shopping
Xindu Flagship Store	a power saver (electronics)	Received, used, regardless of whether something can save electricity, with a period of time to know, anyway, the business service is very good, delivery is fast, I bought2business also sent a, indeed have the effect Oh .	Taobao Marketplace, a Chinese website for online shopping
Xindu Flagship Store	a power saver (electronics)	Friends recommended that I buy, the last time he bought4business gift2, indeed save a lot of power, this time I bought my own goods received.High-end packaging, can not wait to plug in to save electricity to go.	Taobao Marketplace, a Chinese website for online shopping
Xindu Flagship Store	a power saver (electronics)	consult a business, the business told the more power consumption, the use of the saver will be more, the effect is very good , last month's electricity bill is 178 yuan , after using this saver is 92 yuan , ready to buy two more back home , I was introduced by a friend to buy , just at first did not believe that , the [illegible]	Taobao Marketplace, a Chinese website for online shopping
Xindu Flagship Store	a power saver (electronics)	Do not blow not black, this really has the effect, you do not say in my comments below the lack of effect and so on, you use the lack of effect is also your mess, or suggest that before you buy to consult the customer service, the	Taobao Marketplace, a Chinese website for online shopping
Xindu Flagship Store	a power saver (electronics)	My son bought it for me, my son used it for his own home, the more electricity you use the more energy savers you need, and this month paying the electricity bill is really a lot less than the previous month. Nowadays, technology is really great, and invent such a good thing, you can save a lot of money a month. I've already asked my son to buy a few more, and the whole family has started to use them, so I can save	Taobao Marketplace, a Chinese website for online shopping
Xindu Flagship Store	a power saver (electronics)	this saver is really good, the product works quietly, does not affect normal life at all, very good, now the weather is getting colder and colder, the home air conditioning costs electricity, after the installation does save some. The product can be used when plugged in, it is really good. The price is [illegible]	Taobao Marketplace, a Chinese website for online shopping
Xindu Flagship Store	a power saver (electronics)	The saver was also introduced to me by a friend, his family used4of them and saved a lot of electricity every month, this time I also purchased4of them, but this time there is an event. The merchant buy4get2free, it's a good deal.	Taobao Marketplace, a Chinese website for online shopping
Xindu Flagship Store	a power saver (electronics)	Received and used, the appearance of the style positive, practical	Taobao Marketplace, a Chinese website for online shopping
Xindu Flagship Store	a power saver (electronics)	Works great, plugged it in and went to see how slow the meter did turn. Quite a power saver! Trustworthy	Taobao Marketplace, a Chinese website for online shopping
Xindu Flagship Store	a power saver (electronics)	It's good stuff.	Taobao Marketplace, a Chinese website for online shopping
Xindu Flagship Store	a power saver (electronics)	Friends recommended to me to use, energy saver received to try two days, really useful, haha great! After that, I want to buy back!	Taobao Marketplace, a Chinese website for online shopping
Xindu Flagship Store	a power saver (electronics)	Currently using it and will post later as to whether it saves electricity or not	Taobao Marketplace, a Chinese website for online shopping
Xindu Flagship Store	a power saver (electronics)	Used for a period of time to evaluate, the quality is really quite good, the next time there will be a need to continue to buy.	Taobao Marketplace, a Chinese website for online shopping
Xindu Flagship Store	a power saver (electronics)	The baby has received it The quality is very good It is just as described by the seller	Taobao Marketplace, a Chinese website for online shopping
Xindu Flagship Store	a power saver (electronics)	The family bought two, the material is good, the value is very high, energy-saving effect wait a few days will know	Taobao Marketplace, a Chinese website for online shopping
Xindu Flagship Store	a power saver (electronics)	The saver is of good quality, well made and easy to install	Taobao Marketplace, a Chinese website for online shopping
Xindu Flagship Store	a power saver (electronics)	Very good material, fine workmanship, very satisfied	Taobao Marketplace, a Chinese website for online shopping
Xindu Flagship Store	a power saver (electronics)	Have been using it for a while now and it's great quality and very powerful	Taobao Marketplace, a Chinese website for online shopping
Xindu Flagship Store	a power saver (electronics)	I'll comment again when I've used it and experienced it	Taobao Marketplace, a Chinese website for online shopping
Xindu Flagship Store	a power saver (electronics)	Power saving effect: every night can saveabout2degrees of electricity Cost-effective: very good Appearance and style: compact and convenient Workmanship and materials: very good Experience: very good Overall indeed than not using a power saver to save electricity, it is also easy to use	Taobao Marketplace, a Chinese website for online shopping
Xindu Flagship Store	a power saver (electronics)	Is also holding a try mentality for the first time to use, have not seen this thing before, do not know how the effect, used for a period of time, it is really quite convenient to use, plugged in can be used! The key every day can save so2°electricity or so, all five points! Favorable reviews!	Taobao Marketplace, a Chinese website for online shopping
Xindu Flagship Store	a power saver (electronics)	Fake products, internal on a light, a diode, a large capacitor, capacitor is not connected to any circuit. Then got a plastic coating coated with a piece of imitation plastic chip, in fact, is a fake thing, with a multimeter amount is equivalent to a wire. No real effect Power-saving effect: no effect Experience: plugged into the socket, the socket is instead made black.	Taobao Marketplace, a Chinese website for online shopping
Xindu Flagship Store	a power saver (electronics)	Counterfeit products, communicated with the product merchant that his stuff was fake, and kept on not answering	Taobao Marketplace, a Chinese website for online shopping
Xindu Flagship Store	a power saver (electronics)	The product packaging is intact, looks very formal, I hope that the use of the effect of the due Sin Sang, when the time will be back to buy.	Taobao Marketplace, a Chinese website for online shopping
Xindu Flagship Store	a power saver (electronics)	Packaging is intact, the quality is also very good, has been used, look at this month's electricity bill to know that save some electricity does not save	Taobao Marketplace, a Chinese website for online shopping

Xindu Flagship Store	a power saver (electronics)	Power saving effect: photographed the same day delivery, packaging intact, customer service patience to answer questions is a big brand of energy-saving appliances worth buying, cost-effective, and the introduction of the publicity consistent recommend that you buy the set of Cost-effective: really have a power-saving mode. Workmanship material: the latest production date Plug and play Super power saving	Taobao Marketplace, a Chinese website for online shopping
Xindu Flagship Store	a power saver (electronics)	Power saving effect: good Appearance and style: good-looking Workmanship and material: good Cost-effective: good	Taobao Marketplace, a Chinese website for online shopping
Xindu Flagship Store	a power saver (electronics)	The product is small, beautiful, and well made at a good price, so I'll buy it again.	Taobao Marketplace, a Chinese website for online shopping
Xindu Flagship Store	a power saver (electronics)	Power saving effect: plugged in, worth looking forward to Cost-effective: high Appearance style: fashionable and beautiful Workmanship material: fine workmanship Experience: fashionable and beautiful appearance, looking forward to the subsequent power saving effect	Taobao Marketplace, a Chinese website for online shopping
Xindu Flagship Store	a power saver (electronics)	It's the ideal product💗, good quality and fast logistics, worth the kudos! It feels like the meter is going slow!	Taobao Marketplace, a Chinese website for online shopping
Xindu Flagship Store	a power saver (electronics)	Power-saving effect:'to be tested in Cost-effective:, afford Appearance style:_beautiful Workmanship material: a good	Taobao Marketplace, a Chinese website for online shopping
Xindu Flagship Store	a power saver (electronics)	Appearance style fashionable atmosphere, the material is very good, is using the experience, logistics is very fast👍	Taobao Marketplace, a Chinese website for online shopping
Xindu Flagship Store	a power saver (electronics)	Power-saving effect: has been used for half a month, save a lot of electricity, rent a house so expensive electricity, can save a little is a little	Taobao Marketplace, a Chinese website for online shopping
Xindu Flagship Store	a power saver (electronics)	It's pretty, just used it and will follow up after it's been used for a longer period of time.	Taobao Marketplace, a Chinese website for online shopping
Xindu Flagship Store	a power saver (electronics)	It wasmid to lateJulyI got the product, and after using it10days, I noticed thatmy electric bill was indeed less in July,about18%lessthanJune. Keep watching again!	Taobao Marketplace, a Chinese website for online shopping
Xindu Flagship Store	a power saver (electronics)	I've been using it for a few days and I don't know how it is yet, so I'll follow up in a few days	Taobao Marketplace, a Chinese website for online shopping
Xindu Flagship Store	a power saver (electronics)	Packaging is good, logistics is fast, looking forward to using the effect.	Taobao Marketplace, a Chinese website for online shopping
Xindu Flagship Store	a power saver (electronics)	Got it installed looking forward to the post results!	Taobao Marketplace, a Chinese website for online shopping
Xindu Flagship Store	a power saver (electronics)	Power saving effect:	Taobao Marketplace, a Chinese website for online shopping
Xindu Flagship Store	a power saver (electronics)	IQ tax!	Taobao Marketplace, a Chinese website for online shopping
Xindu Flagship Store	a power saver (electronics)	No effect at all It's a scam Customer service said it takes a month Then what's the point of buying it Don't be fooled Power-saving effect: no effect at all	Taobao Marketplace, a Chinese website for online shopping
Xindu Flagship Store	a power saver (electronics)	Logistics is very fast, the price is also affordable, received directly used, I do not know how the effect, if it is good will be back to buy!	Taobao Marketplace, a Chinese website for online shopping
Xindu Flagship Store	a power saver (electronics)	I'm just using it to follow up on my review, and it's a great one.	Taobao Marketplace, a Chinese website for online shopping
Xindu Flagship Store	a power saver (electronics)	Second time I bought it, it's very useful and effective, I hope it lasts longer.	Taobao Marketplace, a Chinese website for online shopping
Xindu Flagship Store	a power saver (electronics)	I used it myself with good results and bought another one for my elderly mother.	Taobao Marketplace, a Chinese website for online shopping
Xindu Flagship Store	a power saver (electronics)	It still feels fine, you won't know until the end of the month if it saves on your electric bill. It's still worth trying! Personally, I still feel good! Saving a little is a little	Taobao Marketplace, a Chinese website for online shopping
Xindu Flagship Store	a power saver (electronics)	Finally got the baby I needed, great stuff, great price!	Taobao Marketplace, a Chinese website for online shopping
Xindu Flagship Store	a power saver (electronics)	Very good power saving effect is also very good, a month and can save a few hundred, hahaha	Taobao Marketplace, a Chinese website for online shopping
Xindu Flagship Store	a power saver (electronics)	The seller shipped the product very quickly, the Power Saver received it, it works well, and the price is not too expensive.	Taobao Marketplace, a Chinese website for online shopping
Xindu Flagship Store	a power saver (electronics)	Fast shipping, use the effect of follow-up evaluation	Taobao Marketplace, a Chinese website for online shopping
Xindu Flagship Store	a power saver (electronics)	I've been using it for a while now and I feel good about it, very happy with it.	Taobao Marketplace, a Chinese website for online shopping
Xindu Flagship Store	a power saver (electronics)	Power saving effect: about25%, not too bad	Taobao Marketplace, a Chinese website for online shopping
Xindu Flagship Store	a power saver (electronics)	Pit, pit, pit, don't know where all the positive reviews come from, proper IQ tax	Taobao Marketplace, a Chinese website for online shopping
Xindu Flagship Store	a power saver (electronics)	Received the goods, look at the appearance or good, are genuine products have product manuals, the effect of waiting to have used and then comment on it!	Taobao Marketplace, a Chinese website for online shopping
Xindu Flagship Store	a power saver (electronics)	A friend recommended a power saver and I'm waiting to see how much I can save on my electricity bill next month!	Taobao Marketplace, a Chinese website for online shopping
Xindu Flagship Store	a power saver (electronics)	style looks good👍pretty good	Taobao Marketplace, a Chinese website for online shopping
Xindu Flagship Store	a power saver (electronics)	Power-saving effect: stable, with obvious effect Cost-effective: perfect Appearance style: very beautiful Workmanship material: high-quality materials Using experience: the effect is just like the description	Taobao Marketplace, a Chinese website for online shopping
Xindu Flagship Store	a power saver (electronics)	The quality is very good, use only after the evaluation, logistics is also very strong, satisfied with Oh!	Taobao Marketplace, a Chinese website for online shopping
Xindu Flagship Store	a power saver (electronics)	This is particularly good ah, cost-effective, with good results, especially great oh!	Taobao Marketplace, a Chinese website for online shopping

Xindu Flagship Store	a power saver (electronics)	Appearance is quite small, energy-saving a little effective	Taobao Marketplace, a Chinese website for online shopping
Xindu Flagship Store	a power saver (electronics)	Baby is good and fast	Taobao Marketplace, a Chinese website for online shopping
Xindu Flagship Store	a power saver (electronics)	Power-saving effect: very good Price-performance ratio: very good Appearance and style: beautiful Workmanship and material: exquisite Experience: very worth buying	Taobao Marketplace, a Chinese website for online shopping
Xindu Flagship Store	a power saver (electronics)	It's good, get one and use it.	Taobao Marketplace, a Chinese website for online shopping
Xindu Flagship Store	a power saver (electronics)	I've already installed many of them at home, and I heard from my friends that they work well, so I'll give them a try.	Taobao Marketplace, a Chinese website for online shopping
Xindu Flagship Store	a power saver (electronics)	After using it for a while, it really saves a bit oops, ready to buy a few more	Taobao Marketplace, a Chinese website for online shopping
Xindu Flagship Store	a power saver (electronics)	Baby received, look good quality, home on the use of the appearance is quite beautiful, the effect of verification in the	Taobao Marketplace, a Chinese website for online shopping
Xindu Flagship Store	a power saver (electronics)	The effect is still in the test, good use will come back to buy again	Taobao Marketplace, a Chinese website for online shopping
Xindu Flagship Store	a power saver (electronics)	The product has been received, the courier is really fast, to be plugged into the power-saving treasure, looking forward to the era of saving money!	Taobao Marketplace, a Chinese website for online shopping
Xindu Flagship Store	a power saver (electronics)	The product is effective and the quality is not unreal. It is a great energy saving product. You can buy it with confidence.	Taobao Marketplace, a Chinese website for online shopping
Xindu Flagship Store	a power saver (electronics)	This saver, bought one the month before last, there is a power saving, this time I bought two more, that one has been evaluated in detail, I won't repeat it here, courier delivery	Taobao Marketplace, a Chinese website for online shopping
Xindu Flagship Store	a power saver (electronics)	Things also look past not bad, small not big, use a few days feel still have effect, a month later to see the next	Taobao Marketplace, a Chinese website for online shopping
Xindu Flagship Store	a power saver (electronics)	Works exceptionally well, fast shipping, recommended	Taobao Marketplace, a Chinese website for online shopping
Xindu Flagship Store	a power saver (electronics)	It's good. It's worth it.	Taobao Marketplace, a Chinese website for online shopping
Xindu Flagship Store	a power saver (electronics)	Doesn't work well at all. Not as power efficient as it says it is	Taobao Marketplace, a Chinese website for online shopping
Xindu Flagship Store	a power saver (electronics)	I don't know how effective it is, I used it for half a month and returned it	Taobao Marketplace, a Chinese website for online shopping
Xindu Flagship Store	a power saver (electronics)	Power saving effect: not so good	Taobao Marketplace, a Chinese website for online shopping
Xindu Flagship Store	a power saver (electronics)	Power saving effect: the product has arrived, the appearance of small and exquisite, easy to use, business service is very good, looking forward to using the energy-saving effect.	Taobao Marketplace, a Chinese website for online shopping
Xindu Flagship Store	a power saver (electronics)	The saver has been used and plugged in for so many days without getting hot and steamy, let's see how much less the electric bill will be this month, overall it's not bad.	Taobao Marketplace, a Chinese website for online shopping
Xindu Flagship Store	a power saver (electronics)	Thank you boss goods received power-saving effect: quite good I'm very satisfied with the installation is also very simple express delivery is also quite fast customer service too much is very good to give you a point of praise praise praise	Taobao Marketplace, a Chinese website for online shopping
Xindu Flagship Store	a power saver (electronics)	This province electrical appliances, last month has bought a, with nearly20days, the electricity bill than last year in the same month paid nearly110yuanless, last year in the same month is423yuan, this year in the same month is312yuan, to witness the effect of pseudo-scientific, immediately ordered to buy two, look at the next month, the bank deducts how much the electricity bill, and then compared with last year in the same month, such as the effect of buying a few more, sent to friends and relatives to use, so that they can also save some money, save electrical appliances! Express delivery	Taobao Marketplace, a Chinese website for online shopping
Xindu Flagship Store	a power saver (electronics)	Look okay, the service attitude is also very good, with a period of time to see how the effect, and then tell you later, the	Taobao Marketplace, a Chinese website for online shopping
Xindu Flagship Store	a power saver (electronics)	Power-saving effect: effective, not a waste of electricity consumption, obviously in the rental landlord to change the number of tables have been effective, the overall power down some. Appearance and style: new appearance, beautiful. Fine workmanship. Use experience: enough to buy two weeks before giving evaluation of the use experience, feel the effect before giving evaluation. Worth recommending and	Taobao Marketplace, a Chinese website for online shopping
Xindu Flagship Store	a power saver (electronics)	Bought two in use, small and exquisite quite good-looking, do not know if there is no role, first use to see this month's electricity bill to see. Favorable reviews	Taobao Marketplace, a Chinese website for online shopping
Xindu Flagship Store	a power saver (electronics)	I've been using it for a while now and I don't see any results, the review section says it's so good that there's no difference between plugging it in and not plugging it in.	Taobao Marketplace, a Chinese website for online shopping
Xindu Flagship Store	a power saver (electronics)	Power saving effect: good Cost-effective: ✓ high Appearance style: a atmosphere Workmanship material: good Experience: good	Taobao Marketplace, a Chinese website for online shopping
Xindu Flagship Store	a power saver (electronics)	quality-price ratio	Taobao Marketplace, a Chinese website for online shopping
Xindu Flagship Store	a power saver (electronics)	Energy saver is really good, has been the second time to buy, every month to save a lot of electricity, this time is for my relatives to buy, really good product!	Taobao Marketplace, a Chinese website for online shopping
Xindu Flagship Store	a power saver (electronics)	Used a month before coming to evaluate, did not work at all, last month's electricity bill210yuan, after using for230yuan, this money is even for the scam bosses****, by the way, the cremation fee.	Taobao Marketplace, a Chinese website for online shopping
Xindu Flagship Store	a power saver (electronics)	The quality is particularly good price cheap affordable atmosphere upscale worth buying!	Taobao Marketplace, a Chinese website for online shopping
Xindu Flagship Store	a power saver (electronics)	I purposely used it for a few days before commenting, it really saves electricity!	Taobao Marketplace, a Chinese website for online shopping
Xindu Flagship Store	a power saver (electronics)	Not bad, good price, feel pretty good and satisfied	Taobao Marketplace, a Chinese website for online shopping
Xindu Flagship Store	a power saver (electronics)	See results in a month	Taobao Marketplace, a Chinese website for online shopping

Xindu Flagship Store	a power saver (electronics)	Installed In use	Taobao Marketplace, a Chinese website for online shopping
Xindu Flagship Store	a power saver (electronics)	Power saving effect: no energy saving effect, not much use	Taobao Marketplace, a Chinese website for online shopping
Xindu Flagship Store	a power saver (electronics)	Power-saving effect: very good Cost-effective: high Appearance and style: beautiful and beautiful Workmanship and material: very fine workmanship, put on the look is also very beautiful	Taobao Marketplace, a Chinese website for online shopping
Xindu Flagship Store	a power saver (electronics)	The light is very dim, does not affect the rest at night, power saving effect is good, the appearance of small beautiful beautiful, cost-effective	Taobao Marketplace, a Chinese website for online shopping
Xindu Flagship Store	a power saver (electronics)	Experience: good-looking very good hope to bring me surprise immediately home to try go	Taobao Marketplace, a Chinese website for online shopping
Xindu Flagship Store	a power saver (electronics)	Looking forward to expecting good results Looks good I'll seehappenswhen I use it	Taobao Marketplace, a Chinese website for online shopping
Xindu Flagship Store	a power saver (electronics)	Product appearance is particularly good-looking, power-saving effect is very satisfactory, the price is perfect, the material is very good at first glance is a regular product, and will come back for more!	Taobao Marketplace, a Chinese website for online shopping
Xindu Flagship Store	a power saver (electronics)	The more you look at the baby, the more you like it, it was a successful purchase!	Taobao Marketplace, a Chinese website for online shopping
Xindu Flagship Store	a power saver (electronics)	Power saving effect: very good Cost-effective: cheap and good Appearance and style: good-looking Workmanship and material: exquisite	Taobao Marketplace, a Chinese website for online shopping
Xindu Flagship Store	a power saver (electronics)	Appearance and style: the product design looks good, just started to use, do not know the effect, I hope to save power. Later see the effect and then comment, logistics is also very fast!	Taobao Marketplace, a Chinese website for online shopping
Xindu Flagship Store	a power saver (electronics)	using it for a while and then I'm going to comment, it does work and saves money💰	Taobao Marketplace, a Chinese website for online shopping
Xindu Flagship Store	a power saver (electronics)	Logistics is fast, looks good	Taobao Marketplace, a Chinese website for online shopping
Xindu Flagship Store	a power saver (electronics)	Bought2, one for the bedroom and one for the living room, looks good, I'll use it first!	Taobao Marketplace, a Chinese website for online shopping
Xindu Flagship Store	a power saver (electronics)	Power saving effect: good	Taobao Marketplace, a Chinese website for online shopping
Xindu Flagship Store	a power saver (electronics)	Power saving effect: it is really power saving oh, the effect is not bad, affordable price can also save electricity. Satisfaction.	Taobao Marketplace, a Chinese website for online shopping
Xindu Flagship Store	a power saver (electronics)	Baby received, go home and use it immediately, see how much to pay next month, next month in the follow-up review	Taobao Marketplace, a Chinese website for online shopping
Xindu Flagship Store	a power saver (electronics)	Tested. IQ tax. Avoid the pit, people.	Taobao Marketplace, a Chinese website for online shopping
Xindu Flagship Store	a power saver (electronics)	The saver works very well, has a clean and generous appearance and is of very good quality	Taobao Marketplace, a Chinese website for online shopping
Xindu Flagship Store	a power saver (electronics)	Good quality, love it, good review!	Taobao Marketplace, a Chinese website for online shopping
Xindu Flagship Store	a power saver (electronics)	Good quality, convenient and easy to use	Taobao Marketplace, a Chinese website for online shopping
Xindu Flagship Store	a power saver (electronics)	Itdoesn't work. It uses more electricity	Taobao Marketplace, a Chinese website for online shopping
Xindu Flagship Store	a power saver (electronics)	The effect is not too obvious now, let's see after a while	Taobao Marketplace, a Chinese website for online shopping
Xindu Flagship Store	a power saver (electronics)	It's useless.	Taobao Marketplace, a Chinese website for online shopping
Xindu Flagship Store	a power saver (electronics)	Power-saving effect: energy-saving effect can not be said to have bought the second Cost-effective: looked and looked and finally chose this Appearance style: feel especially comfortable appearance simple atmosphere Workmanship material: quality of the hand does not seem to be the quality of shoddy goods Experience: at first held a try to buy the mind with the use of the feeling that can be bought a second	Taobao Marketplace, a Chinese website for online shopping
Xindu Flagship Store	a power saver (electronics)	Already the second especially good use want to save electricity family members hurry to chop hands to buy🛍bar	Taobao Marketplace, a Chinese website for online shopping
Xindu Flagship Store	a power saver (electronics)	Power saving effect: has been the second time to buy, save a lot of electricity cost-effective: good value experience: the first time to buy after using more than a month, than the same period last year less than quite a lot, so have bought a	Taobao Marketplace, a Chinese website for online shopping
Xindu Flagship Store	a power saver (electronics)	This saver is really good, it's the second time I've purchased it,...	Taobao Marketplace, a Chinese website for online shopping
Xindu Flagship Store	a power saver (electronics)	Purchased2, the effect is good, every other month the electricity bill has dropped significantly, this time to help friends to buy, the beginning did not know that there is so good use of things, this time to buy6, the family's self-build room too much, it is really a one-time investment, lifelong benefit, the product of the lifelong warranty, the bad free replacement of the new, the merchant to give the protection is very thoughtful, and the business is very	Taobao Marketplace, a Chinese website for online shopping
Xindu Flagship Store	a power saver (electronics)	I saw a friend's home to buy a power saver, he said it does have effect. This time I also bought my own, now has been used for a period of time, really save some electricity!	Taobao Marketplace, a Chinese website for online shopping
Xindu Flagship Store	a power saver (electronics)	Merchant service attitude is also very good, the quality of goods is good, try to see how the effect. If good next time you need to come back. This shopping is very satisfied with the praise.	Taobao Marketplace, a Chinese website for online shopping
Xindu Flagship Store	a power saver (electronics)	The product is very good, played a role in saving electricity, next time will be back to enough. We are confident enough to buy, to save some of their own money.	Taobao Marketplace, a Chinese website for online shopping
Xindu Flagship Store	a power saver (electronics)	Power saving effect: haven't experienced it yet Value for money: very good value for money👍a Appearance and style: beautiful and a Workmanship and material: a workmanship is okay👌	Taobao Marketplace, a Chinese website for online shopping
Xindu Flagship Store	a power saver (electronics)	Has not been the first time to buy, old customers, the business directly to me to buy4send6, said I introduced them to a lot of customers, but also to the return of old customers,	Taobao Marketplace, a Chinese website for online shopping

Xindu Flagship Store	a power saver (electronics)	It's really good. It's great.	Taobao Marketplace, a Chinese website for online shopping
Xindu Flagship Store	a power saver (electronics)	ll buy it again It's really good It does work	Taobao Marketplace, a Chinese website for online shopping
Xindu Flagship Store	a power saver (electronics)	The product If you want to get it, don't hesitate Get it now	Taobao Marketplace, a Chinese website for online shopping
Xindu Flagship Store	a power saver (electronics)	s so handy Great value for Get it	Taobao Marketplace, a Chinese website for online shopping
Xindu Flagship Store	a power saver (electronics)	Power-saving effect: my brother, recommended to me, he used two years Cost-effective: cost-effective is also very high Appearance style: floating bright	Taobao Marketplace, a Chinese website for online shopping
Xindu Flagship Store	a power saver (electronics)	Good quality, business service is very attentive, worth having	Taobao Marketplace, a Chinese website for online shopping
Xindu Flagship Store	a power saver (electronics)	It's also very affordable, so come back when you're ready!	Taobao Marketplace, a Chinese website for online shopping
Xindu Flagship Store	a power saver (electronics)	It's working fine so far.	Taobao Marketplace, a Chinese website for online shopping
Xindu Flagship Store	a power saver (electronics)	Power saving effect: good Cost-effective: high Appearance and style: good-looking Workmanship and material: good	Taobao Marketplace, a Chinese website for online shopping
Xindu Flagship Store	a power saver (electronics)	Previously used another good results, now change this try, beautiful appearance, next month can compare the effect, praise!	Taobao Marketplace, a Chinese website for online shopping
Xindu Flagship Store	a power saver (electronics)	Honestly, after using it for more than a month, the electricity is not saved at all, but consumes more, IQ tax, proper IQ tax, don't buy it🤬🤬🤬🤬🤬🤬🤬	Taobao Marketplace, a Chinese website for online shopping
Xindu Flagship Store	a power saver (electronics)	It's good. It works. It's worth it.	Taobao Marketplace, a Chinese website for online shopping
Xindu Flagship Store	a power saver (electronics)	I've been using it for almost a month and I don't see the power saving effect	Taobao Marketplace, a Chinese website for online shopping
Xindu Flagship Store	a power saver (electronics)	Power saving effect: just bought it, hope it works, Appearance and style: overall good	Taobao Marketplace, a Chinese website for online shopping
Xindu Flagship Store	a power saver (electronics)	Power saving effect: energy-saving Cost-effective: very good value for money Appearance style: stylish appearance Experience: very good	Taobao Marketplace, a Chinese website for online shopping
Xindu Flagship Store	a power saver (electronics)	The exterior design feels niche and is environmentally friendly and energy efficient.	Taobao Marketplace, a Chinese website for online shopping
Xindu Flagship Store	a power saver (electronics)	Received on the start of use, with two days a little power-saving effect, customer service said that after a period of time will have a significant effect, home commercial can be, very good to use plug-in can be used.	Taobao Marketplace, a Chinese website for online shopping
Xindu Flagship Store	a power saver (electronics)	You say you do business, can not be more serious when shipping ah? Repeatedly emphasized to your customer service before the order to send two plugs! Order notes also wrote! As a result, after the delivery of the plug is the British standard😭. Even if you send a domestic general three-head also forget! I didn't realize you guys were going so far. Since I'm abroad, shipped to my side of the world, in the middle of the transfer of sea freight, waiting for more than a month, after receiving the goods is surprisingly so! If I return the item, the shipping cost alone is several times the cost of the item! What can I say about you?customer service about the problem and they just ignored it.If you want to buy this item, I advise	Taobao Marketplace, a Chinese website for online shopping
Xindu Flagship Store	a power saver (electronics)	The product received immediately used beautiful design big summer of too much electricity bought this still have a role need pro can start	Taobao Marketplace, a Chinese website for online shopping
Xindu Flagship Store	a power saver (electronics)	Power saving effect: very obvious	Taobao Marketplace, a Chinese website for online shopping
Xindu Flagship Store	a power saver (electronics)	The energy saving of the equipment is especially obvious, and the air conditioner in the house has never been turned off.	Taobao Marketplace, a Chinese website for online shopping
Xindu Flagship Store	a power saver (electronics)	Cost-effective: quite high Appearance and style: the size is just right, ready to get two more Workmanship and material: looks good, is in use Experience: quite good	Taobao Marketplace, a Chinese website for online shopping
Xindu Flagship Store	a power saver (electronics)	After using it for a while, I think it's good, and I'm going to get two more to save electricity.	Taobao Marketplace, a Chinese website for online shopping
Xindu Flagship Store	a power saver (electronics)	The price is affordable, I do not know how the quality, use it first, use good next time to come back.	Taobao Marketplace, a Chinese website for online shopping
Xindu Flagship Store	a power saver (electronics)	Good quality, fast logistics, good seller service	Taobao Marketplace, a Chinese website for online shopping
Xindu Flagship Store	a power saver (electronics)	It's okay.	Taobao Marketplace, a Chinese website for online shopping
Xindu Flagship Store	a power saver (electronics)	Baby received good, the boss service attitude is also very good, received the goods on the use, should save the electricity bill.	Taobao Marketplace, a Chinese website for online shopping
Xindu Flagship Store	a power saver (electronics)	I've been using it for two days. It's good.	Taobao Marketplace, a Chinese website for online shopping
Xindu Flagship Store	a power saver (electronics)	It's okay. I've used it before.	Taobao Marketplace, a Chinese website for online shopping
Xindu Flagship Store	a power saver (electronics)	Good quality goods, very good	Taobao Marketplace, a Chinese website for online shopping
Xindu Flagship Store	a power saver (electronics)	The product has been used and is really quite good	Taobao Marketplace, a Chinese website for online shopping
Xindu Flagship Store	a power saver (electronics)	The goods have been received, the first time to open the power to see the effect, really too good. Thank you！！！！ Logistics is very fast and thoughtful service, modified the shipping address can still be received in a timely manner, thanks to the business also thank the logistics brother.	Taobao Marketplace, a Chinese website for online shopping
Xindu Flagship Store	a power saver (electronics)	Good performance. It works like	Taobao Marketplace, a Chinese website for online shopping

Xindu Flagship Store	a power saver (electronics)	The stuff is even more perfect than I imagined, very powerful and worth getting!	Taobao Marketplace, a Chinese website for online shopping
Xindu Flagship Store	a power saver (electronics)	Simple and beautiful, not sure if it works	Taobao Marketplace, a Chinese website for online shopping
Xindu Flagship Store	a power saver (electronics)	ve been using it20days or so and it does save	Taobao Marketplace, a Chinese website for online shopping
Xindu Flagship Store	a power saver (electronics)	The product is not as described, does not save power, never give bad reviews, this one is really a big pitfall	Taobao Marketplace, a Chinese website for online shopping
Xindu Flagship Store	a power saver (electronics)	It's not even close to saving power yet, let's comment after a month, it should be an IQ tax	Taobao Marketplace, a Chinese website for online shopping
Xindu Flagship Store	a power saver (electronics)	There are no results so far, I hope tosee results1to2months ofuse like customer service said.	Taobao Marketplace, a Chinese website for online shopping
Xindu Flagship Store	a power saver (electronics)	Appearance and home surroundings to harmonize, a few days to observe the power-saving effect is visible, cost-effective. Appearance design, style, etc. is my favorite.	Taobao Marketplace, a Chinese website for online shopping
Xindu Flagship Store	a power saver (electronics)	Power saving effect: very good, second repurchase	Taobao Marketplace, a Chinese website for online shopping
Xindu Flagship Store	a power saver (electronics)	Really save half of the power, the effect is very good awesome after a period of time to repurchase!	Taobao Marketplace, a Chinese website for online shopping
Xindu Flagship Store	a power saver (electronics)	Product packaging, product appearance are very good, small and exquisite, cost-effective, worth recommending the purchase of	Taobao Marketplace, a Chinese website for online shopping
Xindu Flagship Store	a power saver (electronics)	Appearance is very good, but also with a night light, small and exquisite does not occupy space, worth buying!	Taobao Marketplace, a Chinese website for online shopping
Xindu Flagship Store	a power saver (electronics)	Recommended by a friend. Supposed to work well.	Taobao Marketplace, a Chinese website for online shopping
Xindu Flagship Store	a power saver (electronics)	Cost-effective: not bad, save electricity next time to pay the electricity bill to know	Taobao Marketplace, a Chinese website for online shopping
Xindu Flagship Store	a power saver (electronics)	I hope the effective fruit is really as good as it says it is.	Taobao Marketplace, a Chinese website for online shopping
Xindu Flagship Store	a power saver (electronics)	Don't see any effect with more to go more electricity is not so think about it before you make a decision you guys!	Taobao Marketplace, a Chinese website for online shopping
Xindu Flagship Store	a power saver (electronics)	Exterior style: compact and easy to use.	Taobao Marketplace, a Chinese website for online shopping
Xindu Flagship Store	a power saver (electronics)	Very good quality, worth buying	Taobao Marketplace, a Chinese website for online shopping
Xindu Flagship Store	a power saver (electronics)	It doesn't help. It doesn't save any electricity.	Taobao Marketplace, a Chinese website for online shopping
Xindu Flagship Store	a power saver (electronics)	It's useless. Don't fall for it.	Taobao Marketplace, a Chinese website for online shopping
Xindu Flagship Store	a power saver (electronics)	The product has been received, the appearance looks very good, try2days will not start the heat, power-saving effect to be verified by time, I hope I will not be disappointed, the seller's attitude is very good, logistics speed is also very fast.	Taobao Marketplace, a Chinese website for online shopping
Xindu Flagship Store	a power saver (electronics)	Received the goods, immediately used very much like, hope to have the effect, beautiful look good quality, praise!	Taobao Marketplace, a Chinese website for online shopping
Xindu Flagship Store	a power saver (electronics)	This saver is quite good, after using it. Power consumption is significantly reduced, is a good helper to save money	Taobao Marketplace, a Chinese website for online shopping
Xindu Flagship Store	a power saver (electronics)	Power-saving effect: do not know yet, should be effective, try again Cost-effective: very good Appearance and style: very good Workmanship material: small and exquisite Experience: bought and immediately plugged in, feel pretty good	Taobao Marketplace, a Chinese website for online shopping
Xindu Flagship Store	a power saver (electronics)	Merchants send SF to come, fast, the goods seem to be very good, used to follow up on the evaluation	Taobao Marketplace, a Chinese website for online shopping
Xindu Flagship Store	a power saver (electronics)	It doesn't work, it's all an IQ tax, the positive reviews are all toys, for the sake of $10 cashback, don't fall for it!	Taobao Marketplace, a Chinese website for online shopping
Xindu Flagship Store	a power saver (electronics)	Friends recommend this product is really good, energy-saving effect is very good, can save a lot of electricity, cost-effective, has been recommended to my sister!	Taobao Marketplace, a Chinese website for online shopping
Xindu Flagship Store	a power saver (electronics)	Used for a period of time, the node effect is very good, the office is also quite atmospheric, the exposure is also very nice.	Taobao Marketplace, a Chinese website for online shopping
Xindu Flagship Store	a power saver (electronics)	This is the second time I've purchased it, it's good	Taobao Marketplace, a Chinese website for online shopping
Xindu Flagship Store	a power saver (electronics)	At first glance is a regular manufacturer of products, fine workmanship, easy to use, observed the effect is obvious, because I have more household appliances, ready to add another.	Taobao Marketplace, a Chinese website for online shopping
Xindu Flagship Store	a power saver (electronics)	Appearance and style: the appearance of a generous and simple, moderate size, better materials, and then evaluate the power saving after use.	Taobao Marketplace, a Chinese website for online shopping
Xindu Flagship Store	a power saver (electronics)	I will comment after using it, the product is good and as advertised by the manufacturer.	Taobao Marketplace, a Chinese website for online shopping
Xindu Flagship Store	a power saver (electronics)	Power-saving effect: a little effect Cost-effective: okay Appearance and style: like Workmanship material: can Experience: just use a little feeling	Taobao Marketplace, a Chinese website for online shopping
Xindu Flagship Store	a power saver (electronics)	Great customer service and fast shipping.	Taobao Marketplace, a Chinese website for online shopping
Xindu Flagship Store	a power saver (electronics)	Looks good, reasonably priced, am experiencing it and believe it will work well!	Taobao Marketplace, a Chinese website for online shopping

Xindu Flagship Store	a power saver (electronics)	Power saving effect: quite good, sister's family bought a I also come to a	Taobao Marketplace, a Chinese website for online shopping
Xindu Flagship Store	a power saver (electronics)	Fast logistics and good service!!! Atmospheric appearance	Taobao Marketplace, a Chinese website for online shopping
Xindu Flagship Store	a power saver (electronics)	Received the goods, do not know the effect yet, later review it	Taobao Marketplace, a Chinese website for online shopping
Xindu Flagship Store	a power saver (electronics)	I don't know if it's good or not, but I'll comment after I use it.	Taobao Marketplace, a Chinese website for online shopping
Xindu Flagship Store	a power saver (electronics)	Just received the goods and plugged it in, feel pretty good, a little use, like to hurry to order!	Taobao Marketplace, a Chinese website for online shopping
Xindu Flagship Store	a power saver (electronics)	Works great,100%satisfaction	Taobao Marketplace, a Chinese website for online shopping
Xindu Flagship Store	a power saver (electronics)	The appearance of the power saver is small and exquisite, the price is not expensive, received and plugged in, look forward to the effect!	Taobao Marketplace, a Chinese website for online shopping
Xindu Flagship Store	a power saver (electronics)	The quality is durable, the material is sturdy, and also the warranty, the power saving is very good.	Taobao Marketplace, a Chinese website for online shopping
Xindu Flagship Store	a power saver (electronics)	Good quality, very beautiful installation, energy saving effect is very good, exquisite workmanship, logistics is very fast	Taobao Marketplace, a Chinese website for online shopping
Xindu Flagship Store	a power saver (electronics)	Works great, convenient, affordable and quality.	Taobao Marketplace, a Chinese website for online shopping
Xindu Flagship Store	a power saver (electronics)	Good review! Good quality, well made, worth buying.	Taobao Marketplace, a Chinese website for online shopping
Xindu Flagship Store	a power saver (electronics)	The goods have been received Use next month to follow up the review	Taobao Marketplace, a Chinese website for online shopping
Xindu Flagship Store	a power saver (electronics)	Baby has received, quite good, recommended to friends!	Taobao Marketplace, a Chinese website for online shopping
Xindu Flagship Store	a power saver (electronics)	I came back and installed it, looking forward to your good results	Taobao Marketplace, a Chinese website for online shopping
Xindu Flagship Store	a power saver (electronics)	Power-saving effect: just received today, in the end how effective, yet to be time to prove, but hope that next month to pay the electricity bill when there is a surprise!	Taobao Marketplace, a Chinese website for online shopping
Xindu Flagship Store	a power saver (electronics)	It is very comfortable and very handsome to wear! Sent glasses case is also very large and sturdy, cost-effective. Truth be told, this price this quality, very good, the delivery speed is particularly fast, sunglasses are very good,, the quality of the glasses is still good, the clarity of the lenses is very high, the frame is also very comfortable to wear.Cost-effective, is a good choice to wear a good degree of comfort, good shading effect, daily use is no problem at all, the appearance is also very fashionable. Packaging is very beautiful, will come back to patronize the store.	Taobao Marketplace, a Chinese website for online shopping
Xindu Flagship Store	a power saver (electronics)	Goods energy saving is very good boredom is also very fast price is very afford	Taobao Marketplace, a Chinese website for online shopping
Xindu Flagship Store	a power saver (electronics)	Power saving effect: use it to know Cost-effective: high Appearance and style: beautiful Workmanship and material: good Experience: universal power saving, I hope it is useful	Taobao Marketplace, a Chinese website for online shopping
Xindu Flagship Store	a power saver (electronics)	Power saving effect: effective Value for money: Very good Appearance and style: good-looking Workmanship and material: very good Experience of use: very good and effective.	Taobao Marketplace, a Chinese website for online shopping
Xindu Flagship Store	a power saver (electronics)	Appearance and style: high-end atmosphere upscale Workmanship material: fine workmanship Use experience: use good	Taobao Marketplace, a Chinese website for online shopping
Xindu Flagship Store	a power saver (electronics)	Fast logistics, good service, worth buying, will recommend to friends	Taobao Marketplace, a Chinese website for online shopping
Xindu Flagship Store	a power saver (electronics)	Good quality and low price, worth buying Already recommended it to my close friends	Taobao Marketplace, a Chinese website for online shopping
Xindu Flagship Store	a power saver (electronics)	Just received it today and couldn't wait to use it.	Taobao Marketplace, a Chinese website for online shopping
Xindu Flagship Store	a power saver (electronics)	Look good, logistics is also fast, used in the follow-up comment!	Taobao Marketplace, a Chinese website for online shopping
Xindu Flagship Store	a power saver (electronics)	Great, looking forward to the power saving and energy saving effect	Taobao Marketplace, a Chinese website for online shopping
Xindu Flagship Store	a power saver (electronics)	Power saving effect: nearly half a kilowatt-hour less a day, very good.	Taobao Marketplace, a Chinese website for online shopping
Xindu Flagship Store	a power saver (electronics)	Used it, felt pretty good, power saving effect is good, worth getting.	Taobao Marketplace, a Chinese website for online shopping
Xindu Flagship Store	a power saver (electronics)	The price of this saver can be accepted, used a few days to feel that it can save electricity, wait for a full month to come back to the feedback to see exactly how much a month can save!	Taobao Marketplace, a Chinese website for online shopping
Xindu Flagship Store	a power saver (electronics)	I've bought two of them before and they do save power, this is the second purchase, I'd like to declare that I'm not a trustee, I've experienced it so I'm buying it for the second time.	Taobao Marketplace, a Chinese website for online shopping
Xindu Flagship Store	a power saver (electronics)	Before the electric bill was in the$300 range, now that I've put in a third one,s only$239for the month ofJuneair conditioner on.	Taobao Marketplace, a Chinese website for online shopping
Xindu Flagship Store	a power saver (electronics)	Very good quality, energy saving is good after a while let a friend also go to order.	Taobao Marketplace, a Chinese website for online shopping
Xindu Flagship Store	a power saver (electronics)	Cheap and affordable really good, must repurchase next time!	Taobao Marketplace, a Chinese website for online shopping
Xindu Flagship Store	a power saver (electronics)	Power-saving effect: still under observation Appearance style: beautiful Workmanship material: very good Using experience: okay	Taobao Marketplace, a Chinese website for online shopping

Xindu Flagship Store	a power saver (electronics)	Workmanship Material: good workmanship Appearance Style: quite good-looking I hope it can save power, use a month to know	Taobao Marketplace, a Chinese website for online shopping
Xindu Flagship Store	a power saver (electronics)	I couldn't wait to use it when I got home, but I don't know how many points I can save yet, I'll comment again after using it for a while	Taobao Marketplace, a Chinese website for online shopping
Xindu Flagship Store	a power saver (electronics)	The logistics is super super fast! The quality is also very good, indeed value for money	Taobao Marketplace, a Chinese website for online shopping
Xindu Flagship Store	a power saver (electronics)	using it for a while now, and it's still quite effective compared to the previous one, saving15-20points ofelectricityright	Taobao Marketplace, a Chinese website for online shopping
Xindu Flagship Store	a power saver (electronics)	Goods have been received quality is very good, thanks to the merchant logistics is very fast, the courier has a good service attitude	Taobao Marketplace, a Chinese website for online shopping
Xindu Flagship Store	a power saver (electronics)	Power-saving effect: quite good effect Cost-effective: the quality is quite good Workmanship material: solid quality	Taobao Marketplace, a Chinese website for online shopping
Xindu Flagship Store	a power saver (electronics)	Power savings are obvious, not an IQ tax Buy with confidence! The shipment is also very fast	Taobao Marketplace, a Chinese website for online shopping
Xindu Flagship Store	a power saver (electronics)	I just used it and will recommend it to my friends and family if it works well.	Taobao Marketplace, a Chinese website for online shopping
Xindu Flagship Store	a power saver (electronics)	Power saving effect: very good Appearance and style: also good, small and lovely	Taobao Marketplace, a Chinese website for online shopping
Xindu Flagship Store	a power saver (electronics)	It really works.	Taobao Marketplace, a Chinese website for online shopping
Xindu Flagship Store	a power saver (electronics)	Really good, recommend friends will buy, have a very good power saving effect, save a lot of electricity.	Taobao Marketplace, a Chinese website for online shopping
Xindu Flagship Store	a power saver (electronics)	Power saving effect: received, more or less effective	Taobao Marketplace, a Chinese website for online shopping
Xindu Flagship Store	a power saver (electronics)	It's no fun if you don't save power, so just watch and order.	Taobao Marketplace, a Chinese website for online shopping
Xindu Flagship Store	a power saver (electronics)	Try it out just bought it back, it should be fine, additional comments when the time comes	Taobao Marketplace, a Chinese website for online shopping
Xindu Flagship Store	a power saver (electronics)	Received, let the effect speak…… Appearance style: good Workmanship material: good	Taobao Marketplace, a Chinese website for online shopping
Xindu Flagship Store	a power saver (electronics)	I just started using it, I'll add more comments after using it	Taobao Marketplace, a Chinese website for online shopping
Xindu Flagship Store	a power saver (electronics)	very happy	Taobao Marketplace, a Chinese website for online shopping
Xindu Flagship Store	a power saver (electronics)	It does work, very satisfied,'、、、、、、、、、	Taobao Marketplace, a Chinese website for online shopping
Xindu Flagship Store	a power saver (electronics)	Already plugged in, just waiting for the results, will follow up later	Taobao Marketplace, a Chinese website for online shopping
Xindu Flagship Store	a power saver (electronics)	Baby received, used a few days has not yet obvious effect, wait for a good use and then follow up.	Taobao Marketplace, a Chinese website for online shopping
Xindu Flagship Store	a power saver (electronics)	I've been using it for a while and it's still working and saving a little bit.	Taobao Marketplace, a Chinese website for online shopping
Xindu Flagship Store	a power saver (electronics)	The appearance is quite nice, the service is very good, patiently explain the usage. Use a period of time to evaluate ah	Taobao Marketplace, a Chinese website for online shopping
Xindu Flagship Store	a power saver (electronics)	Power-saving effect: just received the goods, use a period of time to evaluate	Taobao Marketplace, a Chinese website for online shopping
Xindu Flagship Store	a power saver (electronics)	Bao only received many days, has not come to evaluate, I use a period of1time decidedly good to evaluate, especially hot weather air conditioning24a dayopen especially costly electricity, since the use of this power saving treasure every day can save more than ten dollars, really good.	Taobao Marketplace, a Chinese website for online shopping
Xindu Flagship Store	a power saver (electronics)	I just bought, suburban fruit temporarily do not know, said to be two months after the suburban fruit and then tell you, outside the present style see picture, logistics is also very fast.	Taobao Marketplace, a Chinese website for online shopping
Xindu Flagship Store	a power saver (electronics)	Intelligent housekeeper received, for the time being do not know how the suburban fruit, said the suburban fruit only after two months, try and then reply to everyone, the clerk service attitude is very good, logistics is also very fast.	Taobao Marketplace, a Chinese website for online shopping
Xindu Flagship Store	a power saver (electronics)	It should save power, last time I bought it it was cheaper than this one and I tried	Taobao Marketplace, a Chinese website for online shopping
Xindu Flagship Store	a power saver (electronics)	The stuff is pretty good, not too noticeable at the moment, hopefully it will work, after a while of using it.	Taobao Marketplace, a Chinese website for online shopping
Xindu Flagship Store	a power saver (electronics)	It's not bad.	Taobao Marketplace, a Chinese website for online shopping
Xindu Flagship Store	a power saver (electronics)	Power-saving effect:(good, power-saving Cost-effective:1cost-effective Appearance style:夕beautiful appearance Workmanship material:'good workmanship	Taobao Marketplace, a Chinese website for online shopping
Xindu Flagship Store	a power saver (electronics)	It works well and saves power.	Taobao Marketplace, a Chinese website for online shopping
Xindu Flagship Store	a power saver (electronics)	Bought three more, one for each room and one for the kitchen and living room, great customer service, will come back if needed!	Taobao Marketplace, a Chinese website for online shopping
Xindu Flagship Store	a power saver (electronics)	I bought two at the beginning of July and started them up, and when my electric bill came out at the beginning of August, my usage was252kWhJuneand379kWhJuly, an increase127kWh. I used more electricity in July, but it wasn't that much more. What is the reason for this?	Taobao Marketplace, a Chinese website for online shopping
Xindu Flagship Store	a power saver (electronics)	It's pretty good, moderately priced, and the quality looks good!	Taobao Marketplace, a Chinese website for online shopping

Xindu Flagship Store	a power saver (electronics)	It's safe, plug and play, and really saves power.👍	Taobao Marketplace, a Chinese website for online shopping
Xindu Flagship Store	a power saver (electronics)	Expect to see power savings in the next month	Taobao Marketplace, a Chinese website for online shopping
Xindu Flagship Store	a power saver (electronics)	Effective and reasonable price, service attitude is very good, recommended praise	Taobao Marketplace, a Chinese website for online shopping
Xindu Flagship Store	a power saver (electronics)	After using it for a month, it didn't save any electricity and also instead cost a hundred dollars more than the last one. It didn't work.	Taobao Marketplace, a Chinese website for online shopping
Xindu Flagship Store	a power saver (electronics)	Baby received, the appearance of a perfect no bump, have not yet used to expect the effect, use and then come over to evaluate.	Taobao Marketplace, a Chinese website for online shopping
Xindu Flagship Store	a power saver (electronics)	It's very compact. It works great.	Taobao Marketplace, a Chinese website for online shopping
Xindu Flagship Store	a power saver (electronics)	It's good. You can get it.	Taobao Marketplace, a Chinese website for online shopping
Xindu Flagship Store	a power saver (electronics)	Received the goods on the use of the appearance of good quality, waiting for energy-saving effect.	Taobao Marketplace, a Chinese website for online shopping
Xindu Flagship Store	a power saver (electronics)	ve been using it for a while and I feel that it's quite energy efficient because the air conditioner ison24hoursa daythis hot day	Taobao Marketplace, a Chinese website for online shopping
Xindu Flagship Store	a power saver (electronics)	Power saving effect: very good Price/performance ratio: good Workmanship and material: good	Taobao Marketplace, a Chinese website for online shopping
Xindu Flagship Store	a power saver (electronics)	The quality is great! Really saves electricity!	Taobao Marketplace, a Chinese website for online shopping
Xindu Flagship Store	a power saver (electronics)	Used for a month before coming to the follow-up review, before not bought when the electricity bill320 blocks, after using the electricity bill380,****pits	Taobao Marketplace, a Chinese website for online shopping
Xindu Flagship Store	a power saver (electronics)	Tried it for one night, the power-saving effect is good, in general, the cost-effective can be, the material is also thick	Taobao Marketplace, a Chinese website for online shopping
Xindu Flagship Store	a power saver (electronics)	Product received do not know how to use a month to try to see.	Taobao Marketplace, a Chinese website for online shopping
Xindu Flagship Store	a power saver (electronics)	I feel like the meter isn't jumping as fast after using it, so I hope it works.	Taobao Marketplace, a Chinese website for online shopping
Xindu Flagship Store	a power saver (electronics)	Works great. Works great.	Taobao Marketplace, a Chinese website for online shopping
Xindu Flagship Store	a power saver (electronics)	Commodities received Oh, look pretty good, home power saving device, is the way I want, the need for the treasure can start Oh, for you tried!	Taobao Marketplace, a Chinese website for online shopping
Xindu Flagship Store	a power saver (electronics)	Power Saving King has a delicate and beautiful appearance.I also like this product,and very save electricity, need to buy friends hurry to order.	Taobao Marketplace, a Chinese website for online shopping
Xindu Flagship Store	a power saver (electronics)	Power-saving effect: use for a period of time, the power-saving effect is very good Cost-effective: better than similar products Appearance style: appearance Workmanship material: workmanship material is very good Experience: power-saving effect is great Oh!	Taobao Marketplace, a Chinese website for online shopping
Xindu Flagship Store	a power saver (electronics)	I've been using it for almost a month and the results are amazing!	Taobao Marketplace, a Chinese website for online shopping
Xindu Flagship Store	a power saver (electronics)	Only installed two days, it's okay, logistics is also very fast, business service is good.	Taobao Marketplace, a Chinese website for online shopping
Xindu Flagship Store	a power saver (electronics)	original seller's positive feedback are so come ah, no wonder I said how so many positive feedback , because the positive feedback more thought really useful , did not think that a little use did not use and not use is the same return but also shipping costs	Taobao Marketplace, a Chinese website for online shopping
Xindu Flagship Store	a power saver (electronics)	Still experiencing it, should be fine.	Taobao Marketplace, a Chinese website for online shopping
Xindu Flagship Store	a power saver (electronics)	That's good. That's good.	Taobao Marketplace, a Chinese website for online shopping
Xindu Flagship Store	a power saver (electronics)	Experience: Use it, observe for a month to see the effect	Taobao Marketplace, a Chinese website for online shopping
Xindu Flagship Store	a power saver (electronics)	I love it and the delivery was fast, 5 stars!	Taobao Marketplace, a Chinese website for online shopping
Xindu Flagship Store	a power saver (electronics)	Received the goods, feel pretty good! Don't know how effective it is, it'll take a while!	Taobao Marketplace, a Chinese website for online shopping
Xindu Flagship Store	a power saver (electronics)	It works just fine and is worth the price.	Taobao Marketplace, a Chinese website for online shopping
Xindu Flagship Store	a power saver (electronics)	Goods are good, good choice, please feel free to buy!	Taobao Marketplace, a Chinese website for online shopping
Xindu Flagship Store	a power saver (electronics)	Haven't used it yet, don't know the effect	Taobao Marketplace, a Chinese website for online shopping
Xindu Flagship Store	a power saver (electronics)	Appearance and style: not bad, good workmanship, welcome the effect of physical examination, I hope it is to save electricity, the next time also back to buy!	Taobao Marketplace, a Chinese website for online shopping
Xindu Flagship Store	a power saver (electronics)	Express is very powerful! Customer service attitude is very satisfied, small things look pretty good, have not used it yet! But still five-star praise.	Taobao Marketplace, a Chinese website for online shopping
Xindu Flagship Store	a power saver (electronics)	If the quality is good, I will continue to buy it, and my friends and family will want it.	Taobao Marketplace, a Chinese website for online shopping
Xindu Flagship Store	a power saver (electronics)	It's all about results and quality and long term use	Taobao Marketplace, a Chinese website for online shopping

Xindu Flagship Store	a power saver (electronics)	Power-saving effect: have the effect are assured to buy, I have to buy a	Taobao Marketplace, a Chinese website for online shopping
Xindu Flagship Store	a power saver (electronics)	I got it. It looks great. It's a power saver.	Taobao Marketplace, a Chinese website for online shopping
Xindu Flagship Store	a power saver (electronics)	Good stuff. Saves power.	Taobao Marketplace, a Chinese website for online shopping
Xindu Flagship Store	a power saver (electronics)	Received the goods immediately used, workmanship is quite good, use a period of time to evaluate, look forward to the effect of	Taobao Marketplace, a Chinese website for online shopping
Xindu Flagship Store	a power saver (electronics)	Value for money: so good, neighbors recommended to use1this really save some Experience: good use	Taobao Marketplace, a Chinese website for online shopping
Xindu Flagship Store	a power saver (electronics)	Customer service was great and prompt in every response. Shipping was also prompt and seemed to work.	Taobao Marketplace, a Chinese website for online shopping
Xindu Flagship Store	a power saver (electronics)	Baby received, immediately use, looking forward to the effect, feel the meter go slower.	Taobao Marketplace, a Chinese website for online shopping
Xindu Flagship Store	a power saver (electronics)	Good quality, fast logistics, the product is the same as described by the seller	Taobao Marketplace, a Chinese website for online shopping
Xindu Flagship Store	a power saver (electronics)	Received the goods packaging is very good, immediately tried, than the previous purchase of the appearance of beautiful, quality is also very good, it is worth having a, recommended to share!	Taobao Marketplace, a Chinese website for online shopping
Xindu Flagship Store	a power saver (electronics)	The product has just been used do not yet know the effect, after the evaluation, the appearance of the overall feeling is not bad.	Taobao Marketplace, a Chinese website for online shopping
Xindu Flagship Store	a power saver (electronics)	High-tech products, really useful, in previous years the high cost of electricity, with this product, the electricity bill came down. Praise a, hope that the research and development of this product of science and technology to continue to work hard, research out better products to.	Taobao Marketplace, a Chinese website for online shopping
Xindu Flagship Store	a power saver (electronics)	So useful and worth it!	Taobao Marketplace, a Chinese website for online shopping
Xindu Flagship Store	a power saver (electronics)	Baby received, logistics is also very fast, received on the installation, sit and wait for the effect, I hope it is possible to save electricity, satisfied!	Taobao Marketplace, a Chinese website for online shopping
Xindu Flagship Store	a power saver (electronics)	Don't believe it, what Power Saving King? Plugging in the Power Saving King has an extra29dollars in electricity costs, a person at home, and not using the electric water heater, super big liar.	Taobao Marketplace, a Chinese website for online shopping
Xindu Flagship Store	a power saver (electronics)	Power saving effect: in use Price/performance ratio: good Appearance and style: good Experience: very good	Taobao Marketplace, a Chinese website for online shopping
Xindu Flagship Store	a power saver (electronics)	It's okay. It should be good.	Taobao Marketplace, a Chinese website for online shopping
Xindu Flagship Store	a power saver (electronics)	Immediately after receipt, plugged in, in order to test the power saving intentionally made a delayed receipt, to today's test10days,24hours open6hp one tow five central air conditioning with a total of218degrees of electricity, than usually do not use the saver to save about20%. Still have effect.	Taobao Marketplace, a Chinese website for online shopping
Xindu Flagship Store	a power saver (electronics)	The product has been received, from the outside like on the look pretty good, should work well, there is a formal manufacturer, address, phone number, instructions.	Taobao Marketplace, a Chinese website for online shopping
Xindu Flagship Store	a power saver (electronics)	Received, basically the same as the description, fast shipping, worth a buy!	Taobao Marketplace, a Chinese website for online shopping
Xindu Flagship Store	a power saver (electronics)	Experience: in doing experiments later to evaluate	Taobao Marketplace, a Chinese website for online shopping
Xindu Flagship Store	a power saver (electronics)	Good quality. Cheap price. Being used. Looking forward to the effect	Taobao Marketplace, a Chinese website for online shopping
Xindu Flagship Store	a power saver (electronics)	Not bad for a power saver. Works very well. Satisfied!	Taobao Marketplace, a Chinese website for online shopping
Xindu Flagship Store	a power saver (electronics)	The express delivery is very powerful and fast! Very good quality and cost-effective	Taobao Marketplace, a Chinese website for online shopping
Xindu Flagship Store	a power saver (electronics)	Only installed soon, the seller said it would take a month to optimize the circuit, looking forward to the results!	Taobao Marketplace, a Chinese website for online shopping
Xindu Flagship Store	a power saver (electronics)	Has been thethirdpurchase, this power saving device is really useful, this time is also to help my friends to buy, the business is very good, buy3to give me a gift of1, a total of4received, I wish the business business prosperity, power saving device is really a blessing for the people	Taobao Marketplace, a Chinese website for online shopping
Xindu Flagship Store	a power saver (electronics)	can	Taobao Marketplace, a Chinese website for online shopping
Xindu Flagship Store	a power saver (electronics)	It's a little slow to plug in. It's pretty good.	Taobao Marketplace, a Chinese website for online shopping
Xindu Flagship Store	a power saver (electronics)	I don't know if it will save electricity at the moment, I'll come back to review it later	Taobao Marketplace, a Chinese website for online shopping
Xindu Flagship Store	a power saver (electronics)	Power saving effect: friends recommended to buy, good use of power saving Appearance style: easy to carry	Taobao Marketplace, a Chinese website for online shopping
Xindu Flagship Store	a power saver (electronics)	Tested it really quite save electricity do not have to worry about turning on the air conditioning costs electricity	Taobao Marketplace, a Chinese website for online shopping
Xindu Flagship Store	a power saver (electronics)	It's really good and trustworthy	Taobao Marketplace, a Chinese website for online shopping
Xindu Flagship Store	a power saver (electronics)	It just works.	Taobao Marketplace, a Chinese website for online shopping
Xindu Flagship Store	a power saver (electronics)	Just started using it, looking forward to the results, recommend buying it.	Taobao Marketplace, a Chinese website for online shopping
Xindu Flagship Store	a power saver (electronics)	It works well, really saves power, and is recommended!	Taobao Marketplace, a Chinese website for online shopping

Xindu Flagship Store	a power saver (electronics)	(be) worth	Taobao Marketplace, a Chinese website for online shopping
Xindu Flagship Store	a power saver (electronics)	Express received, very power-saving good use	Taobao Marketplace, a Chinese website for online shopping
Xindu Flagship Store	a power saver (electronics)	It really saves electricity, and I really recommend buying it!	Taobao Marketplace, a Chinese website for online shopping
Xindu Flagship Store	a power saver (electronics)	Shipping was pretty fast, and the energy saving effect is good after using it, yo. Satisfied.	Taobao Marketplace, a Chinese website for online shopping
Xindu Flagship Store	a power saver (electronics)	I bought it to try, it still works a little, for the price. The appearance is quite good.	Taobao Marketplace, a Chinese website for online shopping
Xindu Flagship Store	a power saver (electronics)	Saver has been the second purchase, last time my husband bought3, put at home with very convenient, really save a lot of electricity, this time to buy again, this time to buy up in their own store, because the power consumption is very large, bought8, summer electricity bills really can not afford to suffer.	Taobao Marketplace, a Chinese website for online shopping
Xindu Flagship Store	a power saver (electronics)	Very happy with the workmanship and looks good.	Taobao Marketplace, a Chinese website for online shopping
Xindu Flagship Store	a power saver (electronics)	The stuff is pretty good. It does improve. Purposefully observed. I hope this conscientious item can be recognized	Taobao Marketplace, a Chinese website for online shopping
Xindu Flagship Store	a power saver (electronics)	Not bad, not bad.	Taobao Marketplace, a Chinese website for online shopping
Xindu Flagship Store	a power saver (electronics)	Recommended by a friend, worth buying with remarkable results. Fine workmanship.	Taobao Marketplace, a Chinese website for online shopping
Xindu Flagship Store	a power saver (electronics)	My friend bought it for half a month with remarkable results. So I also purchased	Taobao Marketplace, a Chinese website for online shopping
Xindu Flagship Store	a power saver (electronics)	Just received, not yet used, after the use of evaluation	Taobao Marketplace, a Chinese website for online shopping
Xindu Flagship Store	a power saver (electronics)	I've been using it for many days now, I don't feel the power saving, let's use it for some more time to see	Taobao Marketplace, a Chinese website for online shopping
Xindu Flagship Store	a power saver (electronics)	It's ok, fast logistics, hope it brings results!	Taobao Marketplace, a Chinese website for online shopping
Xindu Flagship Store	a power saver (electronics)	Very good, worth buying, recommended.	Taobao Marketplace, a Chinese website for online shopping
Xindu Flagship Store	a power saver (electronics)	Very nice, good quality, 5 stars	Taobao Marketplace, a Chinese website for online shopping
Xindu Flagship Store	a power saver (electronics)	Power saving effect: just used it for a week, haven't seen the effect yet.	Taobao Marketplace, a Chinese website for online shopping
Xindu Flagship Store	a power saver (electronics)	Power saving effect: used a week to comment, before and now record the meter comparison, my one rental room can save two degrees of electricity per day. Cost-effective. Quite worth it.	Taobao Marketplace, a Chinese website for online shopping
Xindu Flagship Store	a power saver (electronics)	Experience: the thing received, quite good cost-effective, ready to get another one	Taobao Marketplace, a Chinese website for online shopping
Xindu Flagship Store	a power saver (electronics)	This item is connected to the power saving good, and the description of the goods, worth your money!	Taobao Marketplace, a Chinese website for online shopping
Xindu Flagship Store	a power saver (electronics)	Power-saving effect: effective Cost-effective: high Appearance and style: good Workmanship and material: first-class Experience: good	Taobao Marketplace, a Chinese website for online shopping
Xindu Flagship Store	a power saver (electronics)	This is the second time I've repurchased it and it works well	Taobao Marketplace, a Chinese website for online shopping
Xindu Flagship Store	a power saver (electronics)	Good quality product, good comments!	Taobao Marketplace, a Chinese website for online shopping
Xindu Flagship Store	a power saver (electronics)	It works exceptionally well. Great. Saves a lot of money.	Taobao Marketplace, a Chinese website for online shopping
Xindu Flagship Store	a power saver (electronics)	Used to be a couple hundred bucks a month. With him, it's a big savings. It's great.	Taobao Marketplace, a Chinese website for online shopping
Xindu Flagship Store	a power saver (electronics)	Power saving effect: good Price/performance ratio: highest Appearance and style: very clean Workmanship and material: good Using experience: very good	Taobao Marketplace, a Chinese website for online shopping
Xindu Flagship Store	a power saver (electronics)	This thing really works, I've been using it for a month and I've got about a dozen kWh left😅I don't know if it would work better if I plugged in more than one in the room	Taobao Marketplace, a Chinese website for online shopping
Xindu Flagship Store	a power saver (electronics)	Workmanship is fine, the appearance is also good-looking, hot days use too much electricity, bought this back to use, really save a lot of electricity, it is worth buying!	Taobao Marketplace, a Chinese website for online shopping
Xindu Flagship Store	a power saver (electronics)	Appearance and style: quite good Experience: quite good	Taobao Marketplace, a Chinese website for online shopping
Xindu Flagship Store	a power saver (electronics)	Exterior style: looking forward to it😉	Taobao Marketplace, a Chinese website for online shopping
Xindu Flagship Store	a power saver (electronics)	It looks good. It's good.	Taobao Marketplace, a Chinese website for online shopping
Xindu Flagship Store	a power saver (electronics)	Quality is quite good, power saving effect is good, plug and play is very convenient, safety, workmanship material are no say!	Taobao Marketplace, a Chinese website for online shopping
Xindu Flagship Store	a power saver (electronics)	The effect is not bad quite good it logistics shipping is also very fast	Taobao Marketplace, a Chinese website for online shopping
Xindu Flagship Store	a power saver (electronics)	Fake, almost the same electric bill this month as last month. I hope people don't buy it.	Taobao Marketplace, a Chinese website for online shopping

Xindu Flagship Store	a power saver (electronics)	Just received the goods, first use to see. Said there is a verification code, looked for it, could not find it, the heart is not in the right place. Let's use it first!	Taobao Marketplace, a Chinese website for online shopping
Xindu Flagship Store	a power saver (electronics)	Received the goods well packaged, open the quality is very good, installed on the more beautiful, effective!	Taobao Marketplace, a Chinese website for online shopping
Xindu Flagship Store	a power saver (electronics)	It's very light, the shape of the white is also very beautiful, matchy-matchy color, and there is no sound at all when you use it in the evening	Taobao Marketplace, a Chinese website for online shopping
Xindu Flagship Store	a power saver (electronics)	Value for money: / very good. Appearance and style: very trendy. Workmanship material: good. Experience: in use.	Taobao Marketplace, a Chinese website for online shopping
Xindu Flagship Store	a power saver (electronics)	It's in use and hopefully I'll be paying less on my electric bill in a few months!	Taobao Marketplace, a Chinese website for online shopping
Xindu Flagship Store	a power saver (electronics)	I got it back yesterday afternoon and plugged it in right then and there, and I just ran my hand over it and it didn't heat up at all. Really, really like the small, delicate look. Took a few photos for your reference.	Taobao Marketplace, a Chinese website for online shopping
Xindu Flagship Store	a power saver (electronics)	Received the saver. It looks okay. Plug it in and see how it works.	Taobao Marketplace, a Chinese website for online shopping
Xindu Flagship Store	a power saver (electronics)	Used it for a while.electricitythan with than before. Next time come back to buy	Taobao Marketplace, a Chinese website for online shopping
Xindu Flagship Store	a power saver (electronics)	Power saving effect: good Cost-effective: cheap Appearance and style: good-looking Workmanship and material: excellent very good Using experience: very good	Taobao Marketplace, a Chinese website for online shopping
Xindu Flagship Store	a power saver (electronics)	It's still being tested, it looks ok, hope it works, good service from the store.	Taobao Marketplace, a Chinese website for online shopping
Xindu Flagship Store	a power saver (electronics)	Through a few days of testing is still effective, the store said it will take one or two months to achieve the desired effect, looking forward to.	Taobao Marketplace, a Chinese website for online shopping
Xindu Flagship Store	a power saver (electronics)	Received the goods used a few days, every day to see the power is indeedmuchmore thanbefore to save a lot, it is worth buying a good thing	Taobao Marketplace, a Chinese website for online shopping
Xindu Flagship Store	a power saver (electronics)	Buy two more and put one in each room, it's too much electricity in the summer!	Taobao Marketplace, a Chinese website for online shopping
Xindu Flagship Store	a power saver (electronics)	Dad recommended immediately arranged, it is indeed convenient and energy-saving, next time to buy a few more	Taobao Marketplace, a Chinese website for online shopping
Xindu Flagship Store	a power saver (electronics)	This stuff is good, just good!	Taobao Marketplace, a Chinese website for online shopping
Xindu Flagship Store	a power saver (electronics)	Power saving effect: very good Cost-effective: high Appearance and style: beautiful Workmanship and material: very good Using experience: under experience	Taobao Marketplace, a Chinese website for online shopping
Xindu Flagship Store	a power saver (electronics)	With this power saver, power saving is economical and trustworthy!	Taobao Marketplace, a Chinese website for online shopping
Xindu Flagship Store	a power saver (electronics)	The owner**excuses too much awesome! Fast forward to two months of no power savings being duped and I spent$400charging.	Taobao Marketplace, a Chinese website for online shopping
Xindu Flagship Store	a power saver (electronics)	Experience: just received the goods look pretty good good if you buy another next time	Taobao Marketplace, a Chinese website for online shopping
Xindu Flagship Store	a power saver (electronics)	How come it's not working well after a month or two of use?	Taobao Marketplace, a Chinese website for online shopping
Xindu Flagship Store	a power saver (electronics)	There is no power saving, with and without the daily go south onlineappcomparison it's all the same	Taobao Marketplace, a Chinese website for online shopping
Xindu Flagship Store	a power saver (electronics)	Reactive power compensation really works	Taobao Marketplace, a Chinese website for online shopping
Xindu Flagship Store	a power saver (electronics)	The air conditioner is awesome in the summer, so it's so useful to buy this energy saver!	Taobao Marketplace, a Chinese website for online shopping
Xindu Flagship Store	a power saver (electronics)	Received the goods, the effect can only be evaluated after a certain amount of time of use. Thank you.	Taobao Marketplace, a Chinese website for online shopping
Xindu Flagship Store	a power saver (electronics)	Great value for money, great power savings, a recommended store!	Taobao Marketplace, a Chinese website for online shopping
Xindu Flagship Store	a power saver (electronics)	is responsible 👌 Just got it Will review when it works Thank you But Bought a few with a broken one inside Seller had a backorder Backordered Another broken one inside Weird!	Taobao Marketplace, a Chinese website for online shopping
Xindu Flagship Store	a power saver (electronics)	Exterior style: very satisfied.	Taobao Marketplace, a Chinese website for online shopping
Xindu Flagship Store	a power saver (electronics)	Baby works.	Taobao Marketplace, a Chinese website for online shopping
Xindu Flagship Store	a power saver (electronics)	Value for money: good Workmanship material: good	Taobao Marketplace, a Chinese website for online shopping
Xindu Flagship Store	a power saver (electronics)	Saving appliances have been received, than I bought a few years ago, the design is more convenient, the technology should be more mature, it has been plugged in and used, to the end of the month to see the electricity bill will know the effect of power saving, then come back to the follow-up!	Taobao Marketplace, a Chinese website for online shopping
Xindu Flagship Store	a power saver (electronics)	This saver is great will buy another one in a while	Taobao Marketplace, a Chinese website for online shopping
Xindu Flagship Store	a power saver (electronics)	The generics are fine, as soon as I got it back, I plugged it in immediately and it felt good.	Taobao Marketplace, a Chinese website for online shopping
Xindu Flagship Store	a power saver (electronics)	IQ tax, positive reviews arebrushed outby returning$10 Power-saving effect: useless	Taobao Marketplace, a Chinese website for online shopping
Xindu Flagship Store	a power saver (electronics)	Very satisfied, the effect is very good, customer service attitude is really good, there is a need will come back!	Taobao Marketplace, a Chinese website for online shopping

Xindu Flagship Store	a power saver (electronics)	I received the stuff, I don't know if it works or not, use it for a while and then we'll see	Taobao Marketplace, a Chinese website for online shopping
Xindu Flagship Store	a power saver (electronics)	With the intention of trying, the purchase of the effect is still very good, compact does not take up space, very satisfied with the	Taobao Marketplace, a Chinese website for online shopping
Xindu Flagship Store	a power saver (electronics)	Usage experience: didn't feel the power saving	Taobao Marketplace, a Chinese website for online shopping
Xindu Flagship Store	a power saver (electronics)	It's working, install two more and hopefully they will work better.	Taobao Marketplace, a Chinese website for online shopping
Xindu Flagship Store	a power saver (electronics)	After a month of testing, instead of using more power, it's a proper IQ tax to buy this product.	Taobao Marketplace, a Chinese website for online shopping
Xindu Flagship Store	a power saver (electronics)	Power saving effect: there is no such thing as power saving Instead, it consumes more power	Taobao Marketplace, a Chinese website for online shopping
Xindu Flagship Store	a power saver (electronics)	Don't fall for it, people. It's a bluff and a lie. It says it saves electricity, but it actually costs more than last month's electricity bill.	Taobao Marketplace, a Chinese website for online shopping
Xindu Flagship Store	a power saver (electronics)	Used it and waited to see the results!	Taobao Marketplace, a Chinese website for online shopping
Xindu Flagship Store	a power saver (electronics)	Power saving effect: listen to friends say there is a kind of power saver can save electricity, in the Internet to find this model, fast delivery, power saver small wonderful, fine workmanship, received on the use, looking forward to power saving effect, to be used in the evaluation.	Taobao Marketplace, a Chinese website for online shopping
Xindu Flagship Store	a power saver (electronics)	Received the stuff Looking forward to the results	Taobao Marketplace, a Chinese website for online shopping
Xindu Flagship Store	a power saver (electronics)	Used a little, indeed can. Quite save electricity, the effect is not exaggerated, wait for a period of time to see how much can actually save, then certainly have to buy back, thanks to the seller at the time of the sale of the recommendation, to give a positive feedback!	Taobao Marketplace, a Chinese website for online shopping
Xindu Flagship Store	a power saver (electronics)	The stuff is great. Very power saving. Next time I will buy back Power saving: very good	Taobao Marketplace, a Chinese website for online shopping
Xindu Flagship Store	a power saver (electronics)	Used it for a few days before commenting. There are noticeable savings. Recommend good stuff for everyone	Taobao Marketplace, a Chinese website for online shopping
Xindu Flagship Store	a power saver (electronics)	Very good, with him very power saving, especially good, recommended to buy!	Taobao Marketplace, a Chinese website for online shopping
Xindu Flagship Store	a power saver (electronics)	Received the goods, but the effect of using it is not easy to say for the time being! Only to be tried and then come back to comment on the effect!	Taobao Marketplace, a Chinese website for online shopping
Xindu Flagship Store	a power saver (electronics)	It works. , quite aesthetically pleasing. Will repurchase after use. Looks beautiful again.	Taobao Marketplace, a Chinese website for online shopping
Xindu Flagship Store	a power saver (electronics)	Your product is very good power saving is also good	Taobao Marketplace, a Chinese website for online shopping
Xindu Flagship Store	a power saver (electronics)	Small and compact, does not take up space, easy to install	Taobao Marketplace, a Chinese website for online shopping
Xindu Flagship Store	a power saver (electronics)	Energy saver received only a few days to use can not see the very obvious effect, to a month later to know whether it is effective!	Taobao Marketplace, a Chinese website for online shopping
Xindu Flagship Store	a power saver (electronics)	Received shipment, satisfied.	Taobao Marketplace, a Chinese website for online shopping
Xindu Flagship Store	a power saver (electronics)	Cost-effective: this product is very effective , but also I like the product, Appearance style: the appearance is quite good Workmanship material: fine workmanship🌷 Use experience: it is very easy to use, it is the pit of a good product, which can allow us to these hit workers👷‍♀save electricity, the	Taobao Marketplace, a Chinese website for online shopping
Xindu Flagship Store	a power saver (electronics)	Used this smart energy saver for two days! The effect is not bad the manual says it works better after a month! Summer air conditioning uses a lot of electricity every month to save a little bit of electricity is really good! It's easy to use!	Taobao Marketplace, a Chinese website for online shopping
Xindu Flagship Store	a power saver (electronics)	Used for a period of time, deliberately to follow-up comments, this economizer is really good, but also save electricity, last month the electricity bill onmore than200this time is also ready to buy some to give friends.	Taobao Marketplace, a Chinese website for online shopping
Xindu Flagship Store	a power saver (electronics)	Have received the goods, the quality is very good👌, very satisfied !	Taobao Marketplace, a Chinese website for online shopping
Xindu Flagship Store	a power saver (electronics)	Baby received and will keep an eye on it! Customer service is also strong!	Taobao Marketplace, a Chinese website for online shopping
Xindu Flagship Store	a power saver (electronics)	The instructions are very detailed and are tamper proof! Everyone can consider buying with confidence!	Taobao Marketplace, a Chinese website for online shopping
Xindu Flagship Store	a power saver (electronics)	Overall it's good, already using it, customer service is great and very patient. Answered a lot of questions, recommend buying!	Taobao Marketplace, a Chinese website for online shopping
Xindu Flagship Store	a power saver (electronics)	Planning to buy a couple more and put them in every room!	Taobao Marketplace, a Chinese website for online shopping
Xindu Flagship Store	a power saver (electronics)	Ongoing use Experience: is in use, seems to be a little different, ongoing follow-up , wait a month in the see	Taobao Marketplace, a Chinese website for online shopping
Xindu Flagship Store	a power saver (electronics)	It's useless, it's buying IQ tax again, there's no certificate of conformity, no3Ccertification	Taobao Marketplace, a Chinese website for online shopping
Xindu Flagship Store	a power saver (electronics)	Shipping speed is very fast, the seller's service is also good, something wrong, customer service immediately to deal with a good, very satisfied!	Taobao Marketplace, a Chinese website for online shopping
Xindu Flagship Store	a power saver (electronics)	Appearance can be, just do not know whether it can achieve the effect of power saving, use for the time being, when the time to follow up the evaluation!	Taobao Marketplace, a Chinese website for online shopping
Xindu Flagship Store	a power saver (electronics)	Just received the item to evaluate in a few days.	Taobao Marketplace, a Chinese website for online shopping
Xindu Flagship Store	a power saver (electronics)	Power saving effect: a few dollars of toys. This owner sells it for30.***No use, good reviews are***.	Taobao Marketplace, a Chinese website for online shopping

Xindu Flagship Store	a power saver (electronics)	Used this to save the appliance more than the original use, fraudulent business, no morals at all	Taobao Marketplace, a Chinese website for online shopping
Xindu Flagship Store	a power saver (electronics)	Very good, good review!	Taobao Marketplace, a Chinese website for online shopping
Xindu Flagship Store	a power saver (electronics)	Hopefully there will be power savings.	Taobao Marketplace, a Chinese website for online shopping
Xindu Flagship Store	a power saver (electronics)	Baby received, small and exquisite, very beautiful, as described, next time to come back to patronize. Power saving effect: very good Cost-effective:.High Appearance style: beautiful Workmanship material: excellent Use experience: good	Taobao Marketplace, a Chinese website for online shopping
Xindu Flagship Store	a power saver (electronics)	Second time I've bought it, the previous one I pulled off when I was packing and didn't know where I put it, works great.	Taobao Marketplace, a Chinese website for online shopping
Xindu Flagship Store	a power saver (electronics)	The real thing is quite good-looking, with it home can also save electricity, not bad!	Taobao Marketplace, a Chinese website for online shopping
Xindu Flagship Store	a power saver (electronics)	Received the goods on the use of, to see the effect of the future stay in the follow-up evaluation!	Taobao Marketplace, a Chinese website for online shopping
Xindu Flagship Store	a power saver (electronics)	Appearance and style: the appearance of simple and generous, fine workmanship, has not been used after the use of additional	Taobao Marketplace, a Chinese website for online shopping
Xindu Flagship Store	a power saver (electronics)	Value for money: high Exterior style: as	Taobao Marketplace, a Chinese website for online shopping
Xindu Flagship Store	a power saver (electronics)	Saving appliances bought back now used for two days, feel very good, look at the meter plugged in will turn slower, immediately go to the next order to get down to buy two more.	Taobao Marketplace, a Chinese website for online shopping
Xindu Flagship Store	a power saver (electronics)	Goods are good, first use it to see, in the future have friends need to come to this store to buy!	Taobao Marketplace, a Chinese website for online shopping
Xindu Flagship Store	a power saver (electronics)	Received in the use of a few days to see, in the evaluation	Taobao Marketplace, a Chinese website for online shopping
Xindu Flagship Store	a power saver (electronics)	Looks pretty good and can be used as a night light.	Taobao Marketplace, a Chinese website for online shopping
Xindu Flagship Store	a power saver (electronics)	Estimated that this time and was fooled, received the goods to see what positive feedback cashback5yuan, sun picture cashback5yuan, a good product is also afraid that no one praise it,, forget about spending a small amount of money to buy a lesson	Taobao Marketplace, a Chinese website for online shopping
Xindu Flagship Store	a power saver (electronics)	instructions say it is8%for7daysandup to15%after15days, I would also like to measure the average, savings after two months. If it works well, I will recommend more friends to buy this one.	Taobao Marketplace, a Chinese website for online shopping
Xindu Flagship Store	a power saver (electronics)	Works great I bought it for the second time	Taobao Marketplace, a Chinese website for online shopping
Xindu Flagship Store	a power saver (electronics)	I'll follow up with a review when I've used it for two months to see if it saves electricity.	Taobao Marketplace, a Chinese website for online shopping
Xindu Flagship Store	a power saver (electronics)	Just used it, don't know if it saves power.	Taobao Marketplace, a Chinese website for online shopping
Xindu Flagship Store	a power saver (electronics)	Very good, all five.	Taobao Marketplace, a Chinese website for online shopping
Xindu Flagship Store	a power saver (electronics)	I haven't used it yet. I won't know until I do.	Taobao Marketplace, a Chinese website for online shopping
Xindu Flagship Store	a power saver (electronics)	Workmanship material: solid material Appearance style: beautiful appearance Cost-effective: higher	Taobao Marketplace, a Chinese website for online shopping
Xindu Flagship Store	a power saver (electronics)	Workmanship material: workmanship material is very good.	Taobao Marketplace, a Chinese website for online shopping
Xindu Flagship Store	a power saver (electronics)	Workmanship appearance is beautiful, genuine is very good, especially used for a few days, really save power saving.	Taobao Marketplace, a Chinese website for online shopping
Xindu Flagship Store	a power saver (electronics)	The logistics is very fast, the quality is very good, quite energy-saving, very satisfactory	Taobao Marketplace, a Chinese website for online shopping
Xindu Flagship Store	a power saver (electronics)	The effect is really nothing to say, specially to chase down, good things we have to share, this month's list out really save30percentof the power, good stuff	Taobao Marketplace, a Chinese website for online shopping
Xindu Flagship Store	a power saver (electronics)	power saving effect is very good The cost-effective The appearance is beautiful and well made The experience of using it is also very good	Taobao Marketplace, a Chinese website for online shopping
Xindu Flagship Store	a power saver (electronics)	Good product Very convenient Plug it in and use it	Taobao Marketplace, a Chinese website for online shopping
Xindu Flagship Store	a power saver (electronics)	Power saving effect: it is indeed more power saving! Appearance and style: it looks very beautiful! Use experience: received in the morning, tried two hours! Can save60%	Taobao Marketplace, a Chinese website for online shopping
Xindu Flagship Store	a power saver (electronics)	After a month of testing, and comparison, this month does save a lot of electricity than last month a, basically every day can save nearly1degree of electricity, this shopping is very satisfied, before adding comments.	Taobao Marketplace, a Chinese website for online shopping
Xindu Flagship Store	a power saver (electronics)	You must be careful when you buy! This merchant sells products that have no effect at all, in addition to the product or three-free! No manufacturer, date! Factory address and so on! There is no quality standard certification and no3Cinferior products! After-sales attitude is also very poor! Fraudulent consumers, the obvious IQ tax, encountered such a shopping experience, really intolerable! Buy back said no effect, customer service actually let me have to buy a few more before! I said that only have effect! I said that your products are fake and false propaganda, they have been published on the situation perfunctory treatment attitude! I have complained to the local market supervision authority, such as not in accordance with the requirements of the investigation! I must be together with the Supreme Court to sue the local market supervision authority harboring merchants! As well as complaints about the Tmall platform, I said above is the truth! The truth, but also willing to take all the responsibility! Just to take a breath, so big a platform to buy fake products I do not believe there is no law of the king!	Taobao Marketplace, a Chinese website for online shopping
Xindu Flagship Store	a power saver (electronics)	propaganda Three-no products No3C No quality standard certification, no effect! Fraudulent consumers!	Taobao Marketplace, a Chinese website for online shopping

Xindu Flagship Store	a power saver (electronics)	Cost-effective, good material and power saving effect	Taobao Marketplace, a Chinese website for online shopping
Xindu Flagship Store	a power saver (electronics)	Power saving effect: general, the effect is not very obvious Using experience: general, the effect is not very obvious	Taobao Marketplace, a Chinese website for online shopping
Xindu Flagship Store	a power saver (electronics)	I'm barely using it, and I'm not returning it	Taobao Marketplace, a Chinese website for online shopping
Xindu Flagship Store	a power saver (electronics)	I'm looking forward to it, I can't wait to open it when I bought it, it's quite nice, my friend recommended to buy it, I hope it can save electricity	Taobao Marketplace, a Chinese website for online shopping
Xindu Flagship Store	a power saver (electronics)	worth buying	Taobao Marketplace, a Chinese website for online shopping
Xindu Flagship Store	a power saver (electronics)	Good appearance, fast logistics, good workmanship and materials	Taobao Marketplace, a Chinese website for online shopping
Xindu Flagship Store	a power saver (electronics)	The stuff works great, bought a couple more so the family can use more of them	Taobao Marketplace, a Chinese website for online shopping
Xindu Flagship Store	a power saver (electronics)	Received a broken, the store promptly sent a replacement, just started to use, look forward to the effect.	Taobao Marketplace, a Chinese website for online shopping
Xindu Flagship Store	a power saver (electronics)	After a month of use the electricity bill has not decreased but increased, a waste of my money, not recommended to buy	Taobao Marketplace, a Chinese website for online shopping
Xindu Flagship Store	a power saver (electronics)	Dad saw this at someone else's house and asked me to buy several of them, saying they have some effect haha!	Taobao Marketplace, a Chinese website for online shopping
Xindu Flagship Store	a power saver (electronics)	Power saving effect: very good straight to buy buy buy buy, will buy in the future	Taobao Marketplace, a Chinese website for online shopping
Xindu Flagship Store	a power saver (electronics)	Received the Saver and used it right away. See how much you can save on your electricity bill next month, last time I bought one.Every month to save more than twenty thirty dollars or so, I hope that the quality of this Taobao will be better, and its to be effective!	Taobao Marketplace, a Chinese website for online shopping
Xindu Flagship Store	a power saver (electronics)	I've been using it for a while now, and it's really great to see that the meter light doesn't really seem to be flashing as fast as it should.	Taobao Marketplace, a Chinese website for online shopping
Xindu Flagship Store	a power saver (electronics)	Baby is very good to use Power-saving effect: power-saving effect is very good like the baby hurry to go to the order, now the weather is hot with more electricity, with this saving appliances after much better	Taobao Marketplace, a Chinese website for online shopping
Xindu Flagship Store	a power saver (electronics)	Received the power saver, rushed to use it, hope it can save power	Taobao Marketplace, a Chinese website for online shopping
Xindu Flagship Store	a power saver (electronics)	It's good, I've been using it for two days now, and the meter is slowing down again, so I'll see in a few days.	Taobao Marketplace, a Chinese website for online shopping
Xindu Flagship Store	a power saver (electronics)	Power saving effect: no use at all I tried Plugged in the same rotation speed No wonder there are so many positive reviews You can cash back ten dollars False propaganda Another day of stepping on the pit A thing is only twenty or thirty Platform counter ten dollars This is the original of so many positive reviews Use cash back to blog positive reviews Stepping on the pit of the increasing number of people	Taobao Marketplace, a Chinese website for online shopping
Xindu Flagship Store	a power saver (electronics)	Works pretty well, saves electricity, looks good, just plug it into any plug.	Taobao Marketplace, a Chinese website for online shopping
Xindu Flagship Store	a power saver (electronics)	It saves a lot of electricity over the course of a day and it really works. It's worth buying.	Taobao Marketplace, a Chinese website for online shopping
Xindu Flagship Store	a power saver (electronics)	Power-saving effect: introduced by neighbors Cost-effective: the price is very cost Appearance and style: small and exquisite Workmanship and material: exquisite details	Taobao Marketplace, a Chinese website for online shopping
Xindu Flagship Store	a power saver (electronics)	Summer every day to open the air conditioning can not top the electricity bill ah, with this will solve the problem	Taobao Marketplace, a Chinese website for online shopping
Xindu Flagship Store	a power saver (electronics)	Power saving effect: very strong Price/performance ratio: very good Appearance style: simple beauty Experience: super power saving! Love it!	Taobao Marketplace, a Chinese website for online shopping
Xindu Flagship Store	a power saver (electronics)	I already bought one last time, this is an add-on and it works really well!	Taobao Marketplace, a Chinese website for online shopping
Xindu Flagship Store	a power saver (electronics)	Merchant service is very good, there are problems are the first time to solve the problem, buy back and plugged in, read a lot of reviews say it works, just hold the attitude of trying, a month later to see the results, so far all aspects are still very satisfied!	Taobao Marketplace, a Chinese website for online shopping
Xindu Flagship Store	a power saver (electronics)	Power saving effect: It is estimated that the effect is more obvious after optimization. At present the effect is not very obvious oh. Only a day or two. Normal. Cost-effective: can. Appearance and style: good-looking, fresh. Workmanship material: can. Use experience: can.	Taobao Marketplace, a Chinese website for online shopping
Xindu Flagship Store	a power saver (electronics)	Power saving effect: don't know yet Price-performance ratio: to be yet Appearance and style: compact Workmanship and material: can be Experience: in use	Taobao Marketplace, a Chinese website for online shopping
Xindu Flagship Store	a power saver (electronics)	The product received the goods, the courier is very fast, use and then follow up!	Taobao Marketplace, a Chinese website for online shopping
Xindu Flagship Store	a power saver (electronics)	It doesn't seem to be working,	Taobao Marketplace, a Chinese website for online shopping
Xindu Flagship Store	a power saver (electronics)	Absolute fools hope that people do not fall for it 100% fooled	Taobao Marketplace, a Chinese website for online shopping
Xindu Flagship Store	a power saver (electronics)	The product used to save energy not many degrees of electricity, eight o'clock in the evening to start turning on the air conditioning to six thirty in the morning is also used about eight degrees of electricity	Taobao Marketplace, a Chinese website for online shopping
Xindu Flagship Store	a power saver (electronics)	Try it.	Taobao Marketplace, a Chinese website for online shopping
Xindu Flagship Store	a power saver (electronics)	Looking forward to being useful	Taobao Marketplace, a Chinese website for online shopping
Xindu Flagship Store	a power saver (electronics)	It's really good bought and used for a while and found that it really saves electricity.	Taobao Marketplace, a Chinese website for online shopping
Xindu Flagship Store	a power saver (electronics)	I've been using it for a while and I feel like it has a power saving feature	Taobao Marketplace, a Chinese website for online shopping

Xindu Flagship Store	a power saver (electronics)	It's a good thing. You can buy another one later.	Taobao Marketplace, a Chinese website for online shopping
Xindu Flagship Store	a power saver (electronics)	Don't fall for it, it doesn't work, biggest failure of a purchase in history	Taobao Marketplace, a Chinese website for online shopping
Xindu Flagship Store	a power saver (electronics)	I just used it. I don't know if it's any good.	Taobao Marketplace, a Chinese website for online shopping
Xindu Flagship Store	a power saver (electronics)	Really good, power saving and energy saving	Taobao Marketplace, a Chinese website for online shopping
Xindu Flagship Store	a power saver (electronics)	Experience: Just used it for a few days	Taobao Marketplace, a Chinese website for online shopping
Xindu Flagship Store	a power saver (electronics)	Started using it last night and it's relatively good.has some effect!The style is also very nice, so if you need it you can just order it!	Taobao Marketplace, a Chinese website for online shopping
Xindu Flagship Store	a power saver (electronics)	Cost-effective: the price of this power saver is super value Appearance style: the appearance of the design is good-looking, Workmanship material: the material is very good Experience: is in use, followed by additional evaluation.	Taobao Marketplace, a Chinese website for online shopping
Xindu Flagship Store	a power saver (electronics)	Power-saving effect:(power-saving) Price-performance ratio: ' high Appearance style:\beautiful) Workmanship material:_plastic Use experience: a can	Taobao Marketplace, a Chinese website for online shopping
Xindu Flagship Store	a power saver (electronics)	To a friend's home to see this product, my friend recommended that I buy, he bought5ofhis own last timeto use at home, this month's electricity bill is really a lot less, so good to use things must be arranged for their own homes also, the summer power consumption is too powerful, the next purchase of6, looking forward to next month's electricity bill	Taobao Marketplace, a Chinese website for online shopping
Xindu Flagship Store	a power saver (electronics)	Power-saving effect: this power-saving effect is really good, I use all the household appliances, after using the power saver, the speed of the meter is really slow turn, in a few days I have to buy again!	Taobao Marketplace, a Chinese website for online shopping
Xindu Flagship Store	a power saver (electronics)	This economizer is very good, my two air conditioners are on, with the economizer and not use the economizer meter indicator within the number of times significantly lower, give the boss a praise, conscientious businessman!	Taobao Marketplace, a Chinese website for online shopping
Xindu Flagship Store	a power saver (electronics)	Just used, the energy saver has a light to prove that it has begun to work, the effect will be known later.	Taobao Marketplace, a Chinese website for online shopping
Xindu Flagship Store	a power saver (electronics)	I received the stuff, summer heat to open several air conditioning, so I bought this, already plugged in!	Taobao Marketplace, a Chinese website for online shopping
Xindu Flagship Store	a power saver (electronics)	Have you used it for a few days to see, hopefully it's not an IQ tax	Taobao Marketplace, a Chinese website for online shopping
Xindu Flagship Store	a power saver (electronics)	What a bargain! Bought one received opened it as three! Just doesn't work! Returns returns returns returns returns!!!	Taobao Marketplace, a Chinese website for online shopping
Xindu Flagship Store	a power saver (electronics)	Nowadays, all kinds of electrical appliances at home will cause the line power factor to be abnormal, which in turn will cause the meter to run shape too fast and the electricity bill to rise, and it is believed that this thing can improve the quality of electricity consumption, which will lead to energy	Taobao Marketplace, a Chinese website for online shopping
Xindu Flagship Store	a power saver (electronics)	Beautiful style, well made, looking forward to the results!	Taobao Marketplace, a Chinese website for online shopping
Xindu Flagship Store	a power saver (electronics)	I've had it for a while, looked at the meter when I put it on, and these days I have to look at the meter, and it does seem to be less nice than the front.	Taobao Marketplace, a Chinese website for online shopping
Xindu Flagship Store	a power saver (electronics)	Looks very nice nice exterior design	Taobao Marketplace, a Chinese website for online shopping
Xindu Flagship Store	a power saver (electronics)	Good quality of goods, details, quality, workmanship are very good, shipping thief, express delivery,~	Taobao Marketplace, a Chinese website for online shopping
Xindu Flagship Store	a power saver (electronics)	Buy back the energy saver after the field test results, no effect, pure bragging products!	Taobao Marketplace, a Chinese website for online shopping
Xindu Flagship Store	a power saver (electronics)	Power saving effect: I feel pretty good use it for a while to see	Taobao Marketplace, a Chinese website for online shopping
Xindu Flagship Store	a power saver (electronics)	It's not bad. You can buy it.	Taobao Marketplace, a Chinese website for online shopping
Xindu Flagship Store	a power saver (electronics)	It's working. I just started using it. I don't know if it'll work.	Taobao Marketplace, a Chinese website for online shopping
Xindu Flagship Store	a power saver (electronics)	It works pretty well.	Taobao Marketplace, a Chinese website for online shopping
Xindu Flagship Store	a power saver (electronics)	Logistics quickly has received, cost-effective, energy-saving effect is very good!	Taobao Marketplace, a Chinese website for online shopping
Xindu Flagship Store	a power saver (electronics)	Whether the power saving effect is useful or not will be evaluated after two months.	Taobao Marketplace, a Chinese website for online shopping
Xindu Flagship Store	a power saver (electronics)	I'll comment when I've used it	Taobao Marketplace, a Chinese website for online shopping
Xindu Flagship Store	a power saver (electronics)	Power saving effect: very good	Taobao Marketplace, a Chinese website for online shopping
Xindu Flagship Store	a power saver (electronics)	beautifully craft.looks authentic and very good.Start using it immediately.See the effect	Taobao Marketplace, a Chinese website for online shopping
Xindu Flagship Store	a power saver (electronics)	Just received the words in the morning immediately use, should be effective to save a little it! First use a period of time to say, overall to words favorable.	Taobao Marketplace, a Chinese website for online shopping
Xindu Flagship Store	a power saver (electronics)	Power-saving effect: used a few days before coming to the evaluation, things are not bad, quite useful, summer air conditioning can be opened casually, haha, I'm glad to use this baby!	Taobao Marketplace, a Chinese website for online shopping
Xindu Flagship Store	a power saver (electronics)	Power saving effect:' very good Price-performance ratio:' very high Appearance style:(white color is better Workmanship material: okay Experience:' great	Taobao Marketplace, a Chinese website for online shopping
Xindu Flagship Store	a power saver (electronics)	Customer service attitude is very good, logistics is very fast, East looks good, fine workmanship, I do not know how the effect is, after the use of follow-up comments.	Taobao Marketplace, a Chinese website for online shopping

Xindu Flagship Store	a power saver (electronics)	I don't know if it saves money on the electricity bill yet, I'll evaluate it after using it for a while	Taobao Marketplace, a Chinese website for online shopping
Xindu Flagship Store	a power saver (electronics)	This user thinks it's pretty good It works very well Just came to rate it Gave it a 5-star review	Taobao Marketplace, a Chinese website for online shopping
Xindu Flagship Store	a power saver (electronics)	I plugged it into an outlet and used it when I received it, I don't know how well it worked.	Taobao Marketplace, a Chinese website for online shopping
Xindu Flagship Store	a power saver (electronics)	Power saving effect: good Price-performance ratio: high Appearance style: good	Taobao Marketplace, a Chinese website for online shopping
Xindu Flagship Store	a power saver (electronics)	Tried it, the light is on, very good, satisfied	Taobao Marketplace, a Chinese website for online shopping
Xindu Flagship Store	a power saver (electronics)	Great looking compact stabilizer, looking forward to the results!	Taobao Marketplace, a Chinese website for online shopping
Xindu Flagship Store	a power saver (electronics)	Great love it	Taobao Marketplace, a Chinese website for online shopping
Xindu Flagship Store	a power saver (electronics)	Power saving effect: good Cost-effective: high Appearance and style: beautiful Workmanship and material: good Using experience: feel pretty good	Taobao Marketplace, a Chinese website for online shopping
Xindu Flagship Store	a power saver (electronics)	The quality of the economizer is good, the workmanship and material are very good, has been plugged in and used, I hope to have good results._x0001_ Packaging is tight, logistics is fast.	Taobao Marketplace, a Chinese website for online shopping
Xindu Flagship Store	a power saver (electronics)	Power saving effect: good Cost-effective: high Appearance and style: beautiful Workmanship and material: solid	Taobao Marketplace, a Chinese website for online shopping
Xindu Flagship Store	a power saver (electronics)	It feels good It's very delicate	Taobao Marketplace, a Chinese website for online shopping
Xindu Flagship Store	a power saver (electronics)	Installed It was easy	Taobao Marketplace, a Chinese website for online shopping
Xindu Flagship Store	a power saver (electronics)	I don't know if it's good or not, but the service is good.	Taobao Marketplace, a Chinese website for online shopping
Xindu Flagship Store	a power saver (electronics)	The energy saving effect is obvious, very good, very satisfied.	Taobao Marketplace, a Chinese website for online shopping
Xindu Flagship Store	a power saver (electronics)	Thought I'd give it a try, but it turned out to be a much lower electric bill this month. It's hot, I run the air conditioner every day, and this thing works!	Taobao Marketplace, a Chinese website for online shopping
Xindu Flagship Store	a power saver (electronics)	It doesn't feel like a power saver!	Taobao Marketplace, a Chinese website for online shopping
Xindu Flagship Store	a power saver (electronics)	I can't feel the power saving. I bought two of them and I can't feel the power saving! It's a fool's errand!	Taobao Marketplace, a Chinese website for online shopping
Xindu Flagship Store	a power saver (electronics)	Power-saving effect: just use the effect do not know it, according to the instructions marked with a month effect of the most short-tailed, to see if it is true or false!	Taobao Marketplace, a Chinese website for online shopping
Xindu Flagship Store	a power saver (electronics)	Let's use it first and see how it works	Taobao Marketplace, a Chinese website for online shopping
Xindu Flagship Store	a power saver (electronics)	Sturdy products, energy-saving and cost-effective	Taobao Marketplace, a Chinese website for online shopping
Xindu Flagship Store	a power saver (electronics)	A relative used it very well and recommended it to me!	Taobao Marketplace, a Chinese website for online shopping
Xindu Flagship Store	a power saver (electronics)	This baby is really high	Taobao Marketplace, a Chinese website for online shopping
Xindu Flagship Store	a power saver (electronics)	Power saving effect: good Appearance and style: beautiful Experience: just received, after using it, then follow up the evaluation	Taobao Marketplace, a Chinese website for online shopping
Xindu Flagship Store	a power saver (electronics)	Value for money: great value Appearance and style: very convenient and clean Experience: works well	Taobao Marketplace, a Chinese website for online shopping
Xindu Flagship Store	a power saver (electronics)	Really super like it, very good quality, as described by the seller!	Taobao Marketplace, a Chinese website for online shopping
Xindu Flagship Store	a power saver (electronics)	Finally received the baby I need, the stuff is very good, the price is good, thanks to the shopkeeper!Honestly, this is my Taobao shopping to make me the most satisfied with a shopping. Whether it is the attitude of the shopkeeper or the items, I am very satisfied. The palm of the cabinet attitude is very professional and enthusiastic, there are questions and answers, the response is also very fast, I asked a lot of questions, he does not feel annoyed, will seriously answer me, this point I express my heartfelt respect to the palm of the cabinet, such a good cabinet can not be more.	Taobao Marketplace, a Chinese website for online shopping
Xindu Flagship Store	a power saver (electronics)	Baby and the description of the same, tube, power saving effect is obvious, have bought two, good quality!	Taobao Marketplace, a Chinese website for online shopping
Xindu Flagship Store	a power saver (electronics)	I've been using it for a day and it feels like it really works, plugging in the meter really slows it down	Taobao Marketplace, a Chinese website for online shopping
Xindu Flagship Store	a power saver (electronics)	hard tofind the merchandise There has been no text message or phone call to notify that the merchandise has arrived. Unintentionally see has been signed for themselves to find.......Until now there has not been any notification of goods logistics. There is no way to score Zhongtong once half a month do not deliver goods I took the initiative to call to find courier said you check yourself......_x0001_ customer service is quite good suggest changing the courier company!	Taobao Marketplace, a Chinese website for online shopping
Xindu Flagship Store	a power saver (electronics)	dissatisfied	Taobao Marketplace, a Chinese website for online shopping
Xindu Flagship Store	a power saver (electronics)	It's good. It's very good.	Taobao Marketplace, a Chinese website for online shopping
Xindu Flagship Store	a power saver (electronics)	Power saving is very good	Taobao Marketplace, a Chinese website for online shopping
Xindu Flagship Store	a power saver (electronics)	Good quality, buy with confidence	Taobao Marketplace, a Chinese website for online shopping

Xindu Flagship Store	a power saver (electronics)	Didn't feel like it, still using as much power as I do every day. 5 out of 5	Taobao Marketplace, a Chinese website for online shopping
Xindu Flagship Store	a power saver (electronics)	Awesome. Very happy with 👍👍👍🌹	Taobao Marketplace, a Chinese website for online shopping
Xindu Flagship Store	a power saver (electronics)	Let's use it first and see how it works before evaluating	Taobao Marketplace, a Chinese website for online shopping
Xindu Flagship Store	a power saver (electronics)	I'm used to good reviews, I'll follow up after a month of use	Taobao Marketplace, a Chinese website for online shopping
Xindu Flagship Store	a power saver (electronics)	Hope it works. Good review.	Taobao Marketplace, a Chinese website for online shopping
Xindu Flagship Store	a power saver (electronics)	Very convenient, plug and play	Taobao Marketplace, a Chinese website for online shopping
Xindu Flagship Store	a power saver (electronics)	Cost-effective, just received the goods today on the use of the home appliances are many, looking forward to a good effect, temporary so, with a period of time to say, the effect is good must follow up!	Taobao Marketplace, a Chinese website for online shopping
Xindu Flagship Store	a power saver (electronics)	Power-saving effect: into a good used this machine is to save electricity Cost-effective:, high than the physical store of the good value Appearance style:'do good unlike other machines like a small processing plant Workmanship material: a material is not the same as high-tech factory products should be the material Experience:1back to the rest of the test of the power-saving	Taobao Marketplace, a Chinese website for online shopping
Xindu Flagship Store	a power saver (electronics)	Saver smart home power saving king used quite good, summer power consumption, saving electricity. Five points praise!	Taobao Marketplace, a Chinese website for online shopping
Xindu Flagship Store	a power saver (electronics)	It's working well and the family is ready to buy a few more to use. All five points praise!	Taobao Marketplace, a Chinese website for online shopping
Xindu Flagship Store	a power saver (electronics)	Express quite fast, the effect will have to wait and see. Merchants say that after a month the effect will be better, anyway, is now looking for a socket plugged in, later to see the effect.	Taobao Marketplace, a Chinese website for online shopping
Xindu Flagship Store	a power saver (electronics)	use it for a while and see how it works Plug it in and have a very dim night light I hope it really saves electricity Otherwise the electricity bill at home is quite high	Taobao Marketplace, a Chinese website for online shopping
Xindu Flagship Store	a power saver (electronics)	Nice stuff, this is2ndonebought, I didn't think it would do much, but I've used it and found that it does save electricity.	Taobao Marketplace, a Chinese website for online shopping
Xindu Flagship Store	a power saver (electronics)	This device really saves electricity and it's worth getting one	Taobao Marketplace, a Chinese website for online shopping
Xindu Flagship Store	a power saver (electronics)	The energy saver has been received and is quite good……	Taobao Marketplace, a Chinese website for online shopping
Xindu Flagship Store	a power saver (electronics)	Power saving effect: the appearance of the design is very good, small and convenient!□	Taobao Marketplace, a Chinese website for online shopping
Xindu Flagship Store	a power saver (electronics)	It's worth the money, feel free to use it, it's small and compact.	Taobao Marketplace, a Chinese website for online shopping
Xindu Flagship Store	a power saver (electronics)	The baby has been received, small and beautiful, I believe the effect is also good. Kudos!	Taobao Marketplace, a Chinese website for online shopping
Xindu Flagship Store	a power saver (electronics)	Very satisfied with the purchase have recommended to friends	Taobao Marketplace, a Chinese website for online shopping
Xindu Flagship Store	a power saver (electronics)	Value for money: high Appearance and style: good-looking Workmanship and materials: good Experience: already used	Taobao Marketplace, a Chinese website for online shopping
Xindu Flagship Store	a power saver (electronics)	Power saving effect: good Appearance style: novel Workmanship material: good	Taobao Marketplace, a Chinese website for online shopping
Xindu Flagship Store	a power saver (electronics)	The goods have been received and the same as described. Just plugged it in and used it, don't know how effective it is, but I hope it's perfect, and will come back afterward for a follow-up review!	Taobao Marketplace, a Chinese website for online shopping
Xindu Flagship Store	a power saver (electronics)	Baby received, do not know how effective? Look at the instructions said to be a month to have the effect of waiting for a month and then follow up on it!	Taobao Marketplace, a Chinese website for online shopping
Xindu Flagship Store	a power saver (electronics)	Things are not bad, really can save electricity, probably save a 20% 30%, a better product, quite satisfied with the	Taobao Marketplace, a Chinese website for online shopping
Xindu Flagship Store	a power saver (electronics)	Things can still be, can save electricity 20% to 30%, the price is not expensive, quite satisfied with the	Taobao Marketplace, a Chinese website for online shopping
Xindu Flagship Store	a power saver (electronics)	I've bought dozens of them for my friends, and I've been saving a few bucks a day, so I recommend it.	Taobao Marketplace, a Chinese website for online shopping
Xindu Flagship Store	a power saver (electronics)	Received the stuff, logistics fast, first use a night to talk about it	Taobao Marketplace, a Chinese website for online shopping
Xindu Flagship Store	a power saver (electronics)	Just got it. It works great. It's great.	Taobao Marketplace, a Chinese website for online shopping
Xindu Flagship Store	a power saver (electronics)	I just put it on and the meter seems to be slowing down, it's really good for saving power	Taobao Marketplace, a Chinese website for online shopping
Xindu Flagship Store	a power saver (electronics)	I'm happy with it.	Taobao Marketplace, a Chinese website for online shopping
Xindu Flagship Store	a power saver (electronics)	Just tried it, looking forward to the results	Taobao Marketplace, a Chinese website for online shopping
Xindu Flagship Store	a power saver (electronics)	It is indeed very effective, save a lot, has been recommended to friends.	Taobao Marketplace, a Chinese website for online shopping
Xindu Flagship Store	a power saver (electronics)	Exceeded expectations, great quality, still feel like you can save power, satisfaction	Taobao Marketplace, a Chinese website for online shopping
Xindu Flagship Store	a power saver (electronics)	Hello goods have been received, your goods are very good, quality first, reputation first, express god._x0001_good	Taobao Marketplace, a Chinese website for online shopping

Xindu Flagship Store	a power saver (electronics)	Power-saving and beautiful	Taobao Marketplace, a Chinese website for online shopping
Xindu Flagship Store	a power saver (electronics)	I really did not think it can really save me a lot of electricity, small body big energy!	Taobao Marketplace, a Chinese website for online shopping
Xindu Flagship Store	a power saver (electronics)	The seller shipped promptly, it is indeed very power-saving, recommended by a friend, very satisfied	Taobao Marketplace, a Chinese website for online shopping
Xindu Flagship Store	a power saver (electronics)	Experience: Use it for a while and then say.	Taobao Marketplace, a Chinese website for online shopping
Xindu Flagship Store	a power saver (electronics)	The packaging is very sturdy and the stuff is well protected, so it's worth a good review	Taobao Marketplace, a Chinese website for online shopping
Xindu Flagship Store	a power saver (electronics)	Good quality, fast logistics and delivery, easy to use, very energy efficient	Taobao Marketplace, a Chinese website for online shopping
Xindu Flagship Store	a power saver (electronics)	Good quality, very satisfied, everyone rest assured to buy!	Taobao Marketplace, a Chinese website for online shopping
Xindu Flagship Store	a power saver (electronics)	Good quality, convenient and practical, fast delivery, satisfied with satisfaction	Taobao Marketplace, a Chinese website for online shopping
Xindu Flagship Store	a power saver (electronics)	Power-saving effect: use a period of time to add comments Cost-effective: cost-effective, Appearance and style: good-looking style Workmanship and material: very good quality	Taobao Marketplace, a Chinese website for online shopping
Xindu Flagship Store	a power saver (electronics)	ve been using it for two months It really makes a difference. Saves a lot of electricity. Good choice. Energy Saving Specialist.	Taobao Marketplace, a Chinese website for online shopping
Xindu Flagship Store	a power saver (electronics)	Received the item, logistics was fast, can't wait to hook up the socket. Waiting for next month's electric bill, hope to save a lot!	Taobao Marketplace, a Chinese website for online shopping
Xindu Flagship Store	a power saver (electronics)	The store shipped quickly, the energy saver received hopefully useful, summer air conditioning power consumption is more powerful.	Taobao Marketplace, a Chinese website for online shopping
Xindu Flagship Store	a power saver (electronics)	Just started using it, will check back in a month to see if I can hopefully save some money on my electric bill.	Taobao Marketplace, a Chinese website for online shopping
Xindu Flagship Store	a power saver (electronics)	Power saving effect: can Appearance style: very good-looking Using experience: the effect is obviously useful	Taobao Marketplace, a Chinese website for online shopping
Xindu Flagship Store	a power saver (electronics)	Cost-effective: cost-effective boss is good, logistics is also very fast, praise	Taobao Marketplace, a Chinese website for online shopping
Xindu Flagship Store	a power saver (electronics)	Power saving effect: very good, see the effect	Taobao Marketplace, a Chinese website for online shopping
Xindu Flagship Store	a power saver (electronics)	Value for money: cost-effective Appearance and style: simple and good-looking Workmanship and material: meticulous and not sloppy Experience: good for purchase	Taobao Marketplace, a Chinese website for online shopping
Xindu Flagship Store	a power saver (electronics)	Overall use down the feeling is good, in terms of power saving is quite useful, the specific look at the future use of feeling	Taobao Marketplace, a Chinese website for online shopping
Xindu Flagship Store	a power saver (electronics)	Things received, small and exquisite, easy to install, logistics is very fast, the seller's service attitude is very good!	Taobao Marketplace, a Chinese website for online shopping
Xindu Flagship Store	a power saver (electronics)	Power saving effect: evaluate after two months Appearance style: simple atmosphere	Taobao Marketplace, a Chinese website for online shopping
Xindu Flagship Store	a power saver (electronics)	Good stuff, worth the money.	Taobao Marketplace, a Chinese website for online shopping
Xindu Flagship Store	a power saver (electronics)	Quality is not bad, recommended by friends, affordable, first use to see	Taobao Marketplace, a Chinese website for online shopping
Xindu Flagship Store	a power saver (electronics)	Fake , tried a little bit of no effect , so many positive reviews are from . Just for10dollars? , Waste of more than thirty dollars Don't be fool bad reviews !	Taobao Marketplace, a Chinese website for online shopping
Xindu Flagship Store	a power saver (electronics)	Appearance and style: exquisite, small, high	Taobao Marketplace, a Chinese website for online shopping
Xindu Flagship Store	a power saver (electronics)	Good quality, fast shipping, satisfied	Taobao Marketplace, a Chinese website for online shopping
Xindu Flagship Store	a power saver (electronics)	Good quality, it works really well	Taobao Marketplace, a Chinese website for online shopping
Xindu Flagship Store	a power saver (electronics)	Good cattle an this thing, bought4, received the goods on the rush to try, originally do not believe that this, the business said that can be returned, the results I used after this thing is really magical, just plugged in a split second the meter does not jump, I turn on the high-power appliances, the meter jumped very slowly, a day down to3degrees of electricity, recommended that relatives and friends to buy, really [illegible]	Taobao Marketplace, a Chinese website for online shopping
Xindu Flagship Store	a power saver (electronics)	The old customer repurchase pull, really save heart and effort to save money, before the greedy cheap in other stores to buy, not only no effect, but also do not give the return, rip, this product is really effective, and the service attitude is quite good, after-sales do a very good job.	Taobao Marketplace, a Chinese website for online shopping
Xindu Flagship Store	a power saver (electronics)	Appearance and style: compact and convenient Workmanship and material: very good quality Using experience: quite convenient, see the final result	Taobao Marketplace, a Chinese website for online shopping
Xindu Flagship Store	a power saver (electronics)	A friend gave one to me, and I felt that it really saved electricity, so I bought another one for my mother's family, and many people around me are using it.	Taobao Marketplace, a Chinese website for online shopping
Xindu Flagship Store	a power saver (electronics)	What a surprise, of a friend recommended, there are actually so good things, before wasting too much electricity, friends used5effect is very obvious, this time I consulted the business, because our home power consumption is very large used7, looking forward to the next month's effect, the effect is good or [illegible]	Taobao Marketplace, a Chinese website for online shopping
Xindu Flagship Store	a power saver (electronics)	Intelligent province received, well-packed, fast order, logistics is also fast service attitude is really good, not yet used, to be used a month after the effect of how, and then follow up, thank you boss.	Taobao Marketplace, a Chinese website for online shopping
Xindu Flagship Store	a power saver (electronics)	Well made, nice and compact.	Taobao Marketplace, a Chinese website for online shopping
Xindu Flagship Store	a power saver (electronics)	The logistics service is so fast, the power saver is well made, the appearance is very beautiful, I used it when I arrived home, in short, I am very satisfied.	Taobao Marketplace, a Chinese website for online shopping

Xindu Flagship Store	a power saver (electronics)	Just bought a semester, feel good, buy3at once, business service attitude is very good! Worth buying!	Taobao Marketplace, a Chinese website for online shopping
Xindu Flagship Store	a power saver (electronics)	Power saving effect: effective. Value for money: high Appearance and style: beautiful Experience: feels good.	Taobao Marketplace, a Chinese website for online shopping
Xindu Flagship Store	a power saver (electronics)	Small, compact and well made.	Taobao Marketplace, a Chinese website for online shopping
Xindu Flagship Store	a power saver (electronics)	Power savings: okay Exterior style: simple, clean, beautiful	Taobao Marketplace, a Chinese website for online shopping
Xindu Flagship Store	a power saver (electronics)	Just received it today😘looking forward to the results in a few days	Taobao Marketplace, a Chinese website for online shopping
Xindu Flagship Store	a power saver (electronics)	Experience: the style or appearance of the model is still very good, just look at this cost-effective, looking forward to a month of test results.	Taobao Marketplace, a Chinese website for online shopping
Xindu Flagship Store	a power saver (electronics)	Logistics is very fast, very satisfied with the goods received	Taobao Marketplace, a Chinese website for online shopping
Xindu Flagship Store	a power saver (electronics)	It's not bad, it's delicate and compact, and it saves power.	Taobao Marketplace, a Chinese website for online shopping
Xindu Flagship Store	a power saver (electronics)	It's very nice, it saves money and electricity, so I'm sure it's a good idea.	Taobao Marketplace, a Chinese website for online shopping
Xindu Flagship Store	a power saver (electronics)	Power saving effect: especially good Cost-effective: the price is very cheap, a month to save half of the electricity bill Appearance and style: white high fashion Workmanship material: fine workmanship Using experience: especially good	Taobao Marketplace, a Chinese website for online shopping
Xindu Flagship Store	a power saver (electronics)	I've used this one. It's fine.	Taobao Marketplace, a Chinese website for online shopping
Xindu Flagship Store	a power saver (electronics)	Not bad.	Taobao Marketplace, a Chinese website for online shopping
Xindu Flagship Store	a power saver (electronics)	Power-saving effect:(good Price-performance ratio: bac strong Appearance style:, beautiful Workmanship material: great Use experience:1le	Taobao Marketplace, a Chinese website for online shopping
Xindu Flagship Store	a power saver (electronics)	Good quality products, cost-effective, beautiful and durable, high power saving rate. We are looking forward to your purchase!	Taobao Marketplace, a Chinese website for online shopping
Xindu Flagship Store	a power saver (electronics)	Great for home use, saves electricity	Taobao Marketplace, a Chinese website for online shopping
Xindu Flagship Store	a power saver (electronics)	I hope it saves electricity.	Taobao Marketplace, a Chinese website for online shopping
Xindu Flagship Store	a power saver (electronics)	The power saver received, the outer packaging are in English, looks small and tasty, power saving effect to be examined. According to the technical indicators and consulting customer service, can be used in110Vcircuit, so this is ready to take to Japan, Japan's electricity is too expensive. First give five-	Taobao Marketplace, a Chinese website for online shopping
Xindu Flagship Store	a power saver (electronics)	first held the attitude of trying to buy, buy back to open to see the product is very good to use, business warranty5years, after the bad at any time can be replaced with a new one, looking forward to the next month's electricity bill,. By the time in the additional	Taobao Marketplace, a Chinese website for online shopping
Xindu Flagship Store	a power saver (electronics)	Power-saving effect:, the effect is very good👌👌 Cost-effective:'good price Appearance style: beautifully crafted	Taobao Marketplace, a Chinese website for online shopping
Xindu Flagship Store	a power saver (electronics)	Works great, saves a lot of money, very affordable, great energy savings.	Taobao Marketplace, a Chinese website for online shopping
Xindu Flagship Store	a power saver (electronics)	Love it, works exceptionally well	Taobao Marketplace, a Chinese website for online shopping
Xindu Flagship Store	a power saver (electronics)	Works great, already repurchased once	Taobao Marketplace, a Chinese website for online shopping
Xindu Flagship Store	a power saver (electronics)	Good to go. Good quality.	Taobao Marketplace, a Chinese website for online shopping
Xindu Flagship Store	a power saver (electronics)	I've been using it for a few days.	Taobao Marketplace, a Chinese website for online shopping
Xindu Flagship Store	a power saver (electronics)	I received it and used it, I hope it can save electricity like the merchant said, use it for a while and see what I can say again!	Taobao Marketplace, a Chinese website for online shopping
Xindu Flagship Store	a power saver (electronics)	It's working fine and I'm not sure if it will actually save power, so I'm happy with it.	Taobao Marketplace, a Chinese website for online shopping
Xindu Flagship Store	a power saver (electronics)	Fast shipping, easy to use, affordable, good reviews!	Taobao Marketplace, a Chinese website for online shopping
Xindu Flagship Store	a power saver (electronics)	Trying it out and it always feels full of IQ tax😂	Taobao Marketplace, a Chinese website for online shopping
Xindu Flagship Store	a power saver (electronics)	Nice and good quality, recommendable	Taobao Marketplace, a Chinese website for online shopping
Xindu Flagship Store	a power saver (electronics)	I don't know the effect yet, let's see how it turns out in a month or two.	Taobao Marketplace, a Chinese website for online shopping
Xindu Flagship Store	a power saver (electronics)	Appearance is the same, effects to be seen.	Taobao Marketplace, a Chinese website for online shopping
Xindu Flagship Store	a power saver (electronics)	As soon as the saver was received, it was used, this summer with the air conditioning does not hurt the power consumption, but also to shoot two.	Taobao Marketplace, a Chinese website for online shopping
Xindu Flagship Store	a power saver (electronics)	I've always wanted to take a picture of a genuine power saver, and this time I finally found a merchant with a branded logo for guaranteed power savings.	Taobao Marketplace, a Chinese website for online shopping
Xindu Flagship Store	a power saver (electronics)	Recommended by a friend, let's give it a try	Taobao Marketplace, a Chinese website for online shopping

Xindu Flagship Store	a power saver (electronics)	I bought it for my mom and dad and told me today that it's not bad, it's a little bit effective, so I'll comment on it	Taobao Marketplace, a Chinese website for online shopping
Xindu Flagship Store	a power saver (electronics)	The product just received, can not wait to open the box to see, aregfirst give a five-star praise	Taobao Marketplace, a Chinese website for online shopping
Xindu Flagship Store	a power saver (electronics)	Workmanship material: very good	Taobao Marketplace, a Chinese website for online shopping
Xindu Flagship Store	a power saver (electronics)	A very good product Oh, the need for partners to join together to buy it!	Taobao Marketplace, a Chinese website for online shopping
Xindu Flagship Store	a power saver (electronics)	Power saving effect: very good in very good use, not bad, this is the second time to buy, the effect is very good	Taobao Marketplace, a Chinese website for online shopping
Xindu Flagship Store	a power saver (electronics)	Good quality, actually feels great, does save power	Taobao Marketplace, a Chinese website for online shopping
Xindu Flagship Store	a power saver (electronics)	Cost-effective and very effective in saving electricity👍👍👍👍	Taobao Marketplace, a Chinese website for online shopping
Xindu Flagship Store	a power saver (electronics)	It's really good👍	Taobao Marketplace, a Chinese website for online shopping
Xindu Flagship Store	a power saver (electronics)	Power saving effect: very good Price/performance ratio: not bad Experience: plugged in and left alone	Taobao Marketplace, a Chinese website for online shopping
Xindu Flagship Store	a power saver (electronics)	The stuff is great. Worth buying.	Taobao Marketplace, a Chinese website for online shopping
Xindu Flagship Store	a power saver (electronics)	Power-saving effect: effective Cost-effective: very high Appearance style: moderate size does not take up space Workmanship material: good, not completely that kind of plastic feeling	Taobao Marketplace, a Chinese website for online shopping
Xindu Flagship Store	a power saver (electronics)	Workmanship material: very good workmanship, very good use	Taobao Marketplace, a Chinese website for online shopping
Xindu Flagship Store	a power saver (electronics)	Compact to use	Taobao Marketplace, a Chinese website for online shopping
Xindu Flagship Store	a power saver (electronics)	Used a few days, the effect is very good, I am opening a store, cooking and eating, refrigerator, air conditioning, power consumption is very large, with this intelligent province electrical appliances, save a lot of electricity Fo, friends, what are you waiting for, hurry up and use it!	Taobao Marketplace, a Chinese website for online shopping
Xindu Flagship Store	a power saver (electronics)	Use experience: is experiencing the use of	Taobao Marketplace, a Chinese website for online shopping
Xindu Flagship Store	a power saver (electronics)	It's pretty good, not too expensive, and the logistics are pretty good.	Taobao Marketplace, a Chinese website for online shopping
Xindu Flagship Store	a power saver (electronics)	I don't know if others save, but my last electric bill380dollars! After using this product for a month my electric bill went to480dollarsthis month. I don't know if it's saving or using electricity!***. I took it straight to the drop!	Taobao Marketplace, a Chinese website for online shopping
Xindu Flagship Store	a power saver (electronics)	I hope it works.	Taobao Marketplace, a Chinese website for online shopping
Xindu Flagship Store	a power saver (electronics)	Not bad. Keep up the good work.	Taobao Marketplace, a Chinese website for online shopping
Xindu Flagship Store	a power saver (electronics)	I've used it for a few days before commenting, and to be honest it's really, really working!	Taobao Marketplace, a Chinese website for online shopping
Xindu Flagship Store	a power saver (electronics)	Already received, plugged in to try, I do not know whether there is a power-saving effect	Taobao Marketplace, a Chinese website for online shopping
Xindu Flagship Store	a power saver (electronics)	Fast shipping, rush, great service.	Taobao Marketplace, a Chinese website for online shopping
Xindu Flagship Store	a power saver (electronics)	Received the item, use it and add comments	Taobao Marketplace, a Chinese website for online shopping
Xindu Flagship Store	a power saver (electronics)	Expecting good results. It's too much electricity to run the AC in the summer.	Taobao Marketplace, a Chinese website for online shopping
Xindu Flagship Store	a power saver (electronics)	Express very quickly, as soon as it arrived and power plugged in, the original bought a, now add another. Looking forward to better results, with a section of the elbow and then follow up.	Taobao Marketplace, a Chinese website for online shopping
Xindu Flagship Store	a power saver (electronics)	Small and exquisite, just received today on the use, I hope there is an effect, and so use a period of time, give the electricity bill to know the power saving is not, if the power saving, but also to buy a few, because my home electricity consumption is high.	Taobao Marketplace, a Chinese website for online shopping
Xindu Flagship Store	a power saver (electronics)	Power-saving effect: not bad Cost-effective: good Appearance style: can Experience: in use, and then observe a period of time	Taobao Marketplace, a Chinese website for online shopping
Xindu Flagship Store	a power saver (electronics)	is in use...... concrete effect will be evaluated at a later stage	Taobao Marketplace, a Chinese website for online shopping
Xindu Flagship Store	a power saver (electronics)	It does save power and will be back next time	Taobao Marketplace, a Chinese website for online shopping
Xindu Flagship Store	a power saver (electronics)	I'm just trying it out for now and I won't know exactly how much electricity I'll save until I settle my bill this month.	Taobao Marketplace, a Chinese website for online shopping
Xindu Flagship Store	a power saver (electronics)	Well-packed without damage, beautifully designed appearance, fine workmanship, good power-saving effect	Taobao Marketplace, a Chinese website for online shopping
Xindu Flagship Store	a power saver (electronics)	The saver was received, good quality, shipped so fast, already installed.	Taobao Marketplace, a Chinese website for online shopping
Xindu Flagship Store	a power saver (electronics)	It is the ideal product, good quality, logistics is also very fast, worthy of praise!	Taobao Marketplace, a Chinese website for online shopping
Xindu Flagship Store	a power saver (electronics)	I haven't used it yet. I'll follow up after I use it.	Taobao Marketplace, a Chinese website for online shopping

Xindu Flagship Store	a power saver (electronics)	Good quality, fast shipping and tightly packaged.	Taobao Marketplace, a Chinese website for online shopping
Xindu Flagship Store	a power saver (electronics)	Power-saving effect: power-saving effect is okay Appearance and style: appearance and style is also quite good-looking Workmanship and material: the material is also quite good	Taobao Marketplace, a Chinese website for online shopping
Xindu Flagship Store	a power saver (electronics)	Power saving effect: good effect, worth recommending	Taobao Marketplace, a Chinese website for online shopping
Xindu Flagship Store	a power saver (electronics)	It's good, it's beautiful, I've used it since I got it.	Taobao Marketplace, a Chinese website for online shopping
Xindu Flagship Store	a power saver (electronics)	Fine workmanship, very good, very energy-saving, very satisfactory	Taobao Marketplace, a Chinese website for online shopping
Xindu Flagship Store	a power saver (electronics)	Not good, no effect, used for five days, the daily electricity consumption and not used before the same, no change	Taobao Marketplace, a Chinese website for online shopping
Xindu Flagship Store	a power saver (electronics)	This item has been used for a month now, the electricity bill has not been reduced, but increased,270degreesbefore using it, after using it became340degrees, this thing is too garbage, proper IQ tax.	Taobao Marketplace, a Chinese website for online shopping
Xindu Flagship Store	a power saver (electronics)	Very good product.	Taobao Marketplace, a Chinese website for online shopping
Xindu Flagship Store	a power saver (electronics)	Very good, just as described by the merchant	Taobao Marketplace, a Chinese website for online shopping
Xindu Flagship Store	a power saver (electronics)	The stuff works great, and the seller has great service, and saves a lot of electricity.	Taobao Marketplace, a Chinese website for online shopping
Xindu Flagship Store	a power saver (electronics)	Good looking very happy with the appearance	Taobao Marketplace, a Chinese website for online shopping
Xindu Flagship Store	a power saver (electronics)	Good product, good quality, next time continue to buy!	Taobao Marketplace, a Chinese website for online shopping
Xindu Flagship Store	a power saver (electronics)	Power-saving effect: looking forward to the effect Appearance style: simple and fashionable Workmanship material: fine workmanship	Taobao Marketplace, a Chinese website for online shopping
Xindu Flagship Store	a power saver (electronics)	Power saving effect: better Workmanship material: very good Cost-effective: very high Recommended	Taobao Marketplace, a Chinese website for online shopping
Xindu Flagship Store	a power saver (electronics)	using it and it's saving me a lot of power. It's obvious	Taobao Marketplace, a Chinese website for online shopping
Xindu Flagship Store	a power saver (electronics)	It's as good as the seller's description.	Taobao Marketplace, a Chinese website for online shopping
Xindu Flagship Store	a power saver (electronics)	Just as described by the seller, plug and play convenience.	Taobao Marketplace, a Chinese website for online shopping
Xindu Flagship Store	a power saver (electronics)	Already received the goods good quality price is not high worth starting hope that the future durable	Taobao Marketplace, a Chinese website for online shopping
Xindu Flagship Store	a power saver (electronics)	This product can really save electricity, the appearance of the body set of small does not take up space, when boiling water, plugged in the meter really slowed down a lot, I have to buy a few more, to plug in each room!	Taobao Marketplace, a Chinese website for online shopping
Xindu Flagship Store	a power saver (electronics)	The product is very cost-effective, the appearance of the look is very delicate, done material is very good, logistics and distribution of strong, a very good shopping experience!	Taobao Marketplace, a Chinese website for online shopping
Xindu Flagship Store	a power saver (electronics)	Received it and it feels fine, small and convenient! Will be in to give feedback after use!	Taobao Marketplace, a Chinese website for online shopping
Xindu Flagship Store	a power saver (electronics)	It's easy to use.	Taobao Marketplace, a Chinese website for online shopping
Xindu Flagship Store	a power saver (electronics)	The thing has been pressed on and it still looks OK	Taobao Marketplace, a Chinese website for online shopping
Xindu Flagship Store	a power saver (electronics)	for money: super cheap if it works Appearance and style: feels a bit reliable Workmanship and material: very good quality Experience: not yet used	Taobao Marketplace, a Chinese website for online shopping
Xindu Flagship Store	a power saver (electronics)	This stuff doesn't work. Been using it for a month with no effect at all. Waste of money. Deceiving consumers can not save save, but also to say that a little bit of good!	Taobao Marketplace, a Chinese website for online shopping
Xindu Flagship Store	a power saver (electronics)	The energy saving is very good, also does not take up space, wait a few days to place another order	Taobao Marketplace, a Chinese website for online shopping
Xindu Flagship Store	a power saver (electronics)	Energy saving is very good, does not take up space, ready to place another order	Taobao Marketplace, a Chinese website for online shopping
Xindu Flagship Store	a power saver (electronics)	Good use of the product now specially to comment on to share with you, power-saving effect really did not say, really do not blow not black evaluation, the second purchase, the first time to buy no evaluation, this time must give a favorable review	Taobao Marketplace, a Chinese website for online shopping
Xindu Flagship Store	a power saver (electronics)	It's great quality, beautiful to look at, and most of all it saves electricity! Awesome.	Taobao Marketplace, a Chinese website for online shopping
Xindu Flagship Store	a power saver (electronics)	If you want to buy it, buy it now.	Taobao Marketplace, a Chinese website for online shopping
Xindu Flagship Store	a power saver (electronics)	Just received the item, hope it works as described!	Taobao Marketplace, a Chinese website for online shopping
Xindu Flagship Store	a power saver (electronics)	properly	Taobao Marketplace, a Chinese website for online shopping
Xindu Flagship Store	a power saver (electronics)	The saver has been received and is straight to purchase	Taobao Marketplace, a Chinese website for online shopping
Xindu Flagship Store	a power saver (electronics)	Plug and play, quite convenient, power saving effect is not bad. The price-performance ratio is quite high	Taobao Marketplace, a Chinese website for online shopping

Xindu Flagship Store	a power saver (electronics)	Appearance and style: very good very like Experience: just arrived today tomorrow morning to see the effect of the	Taobao Marketplace, a Chinese website for online shopping
Xindu Flagship Store	a power saver (electronics)	The quality is very good, the design is very good, very satisfied with a shopping, worth buying!	Taobao Marketplace, a Chinese website for online shopping
Xindu Flagship Store	a power saver (electronics)	Just arrived today first use it to say, first give a favorable comment	Taobao Marketplace, a Chinese website for online shopping
Xindu Flagship Store	a power saver (electronics)	Power-saving effect: used two months of electricity costs significantly lower, inconspicuous little things really have the effect of saving about 30% or 40%! Thank you for the business! Must praise!	Taobao Marketplace, a Chinese website for online shopping
Xindu Flagship Store	a power saver (electronics)	Workmanship material: things received, very satisfied, very good	Taobao Marketplace, a Chinese website for online shopping
Xindu Flagship Store	a power saver (electronics)	The product is good and you can get it if you need it.	Taobao Marketplace, a Chinese website for online shopping
Xindu Flagship Store	a power saver (electronics)	It's okay, the packaging is quite nice and well-made	Taobao Marketplace, a Chinese website for online shopping
Xindu Flagship Store	a power saver (electronics)	Easy to install, has some effect, can be purchased	Taobao Marketplace, a Chinese website for online shopping
Xindu Flagship Store	a power saver (electronics)	Fast delivery, already plugged in and started usingit_x0001_Customers are enthusiastic	Taobao Marketplace, a Chinese website for online shopping
Xindu Flagship Store	a power saver (electronics)	Experience: I heard from a friend that this is good to use, the first time to experience Appearance style: the appearance of a very small	Taobao Marketplace, a Chinese website for online shopping
Xindu Flagship Store	a power saver (electronics)	A friend recommended it as a good power saver and it really saves a lot in a month	Taobao Marketplace, a Chinese website for online shopping
Xindu Flagship Store	a power saver (electronics)	first time to buy this power saver, have not heard of before, really surprised that there is this product, after consulting the merchant, the merchant let me buy6, because my home power is too big, hold a try to buy under the mindset of the business to give the after-sales is very enough, the product can be tried for100days,100days within the invalid refund 👍	Taobao Marketplace, a Chinese website for online shopping
Xindu Flagship Store	a power saver (electronics)	Easy to install	Taobao Marketplace, a Chinese website for online shopping
Xindu Flagship Store	a power saver (electronics)	Arrived quickly, works very well and is of good quality	Taobao Marketplace, a Chinese website for online shopping
Xindu Flagship Store	a power saver (electronics)	I'm in Taiwan, but has been using the Tmall platform to buy things, this time found that there is this power saver, asked a number of no Taiwan's plug, the whole network only this store only, but also believe that the products of this store, because the foreign trade to sell more a lot more!	Taobao Marketplace, a Chinese website for online shopping
Xindu Flagship Store	a power saver (electronics)	This is indeed very professional, this time I bought for relatives far abroad, this Malaysia, Hong Kong, the United States have plugs, this time to buy is the British standard, very convenient, never use a converter!	Taobao Marketplace, a Chinese website for online shopping
Xindu Flagship Store	a power saver (electronics)	Energy saver is really good to save electricity, has been the third time back to buy, now the summer power consumption is too large, really do not use this can not eat,1month thousands of dollars of electricity, this product is really the gospel of the people	Taobao Marketplace, a Chinese website for online shopping
Xindu Flagship Store	a power saver (electronics)	Previously, I have always bought from other stores, all domestic plugs. Also need a converter, then see this store sales are very good, inadvertently consult the next, only to know that this store, the UK plug is also available, decisively ordered to buy, and then never need to use a converter!	Taobao Marketplace, a Chinese website for online shopping
Xindu Flagship Store	a power saver (electronics)	I did not expect that this also has a U.S. gauge socket, I am doing shopping on behalf of the previous has been a piece of hair, each time the customer has to find me to send the adapter, now well, which country you need to say directly to the seller, the seller directly shipped, it's very convenient!	Taobao Marketplace, a Chinese website for online shopping
Xindu Flagship Store	a power saver (electronics)	After receiving the goods overall evaluation of the appearance of delicate and compact, fine workmanship, does not take up space, affordable price	Taobao Marketplace, a Chinese website for online shopping
Xindu Flagship Store	a power saver (electronics)	After plugging it in, the meter slows down noticeably, worth the price!	Taobao Marketplace, a Chinese website for online shopping
Xindu Flagship Store	a power saver (electronics)	Baby received, already in use, in the end can not save electricity? We'll have to use it for a while to see the effect.	Taobao Marketplace, a Chinese website for online shopping
Xindu Flagship Store	a power saver (electronics)	Energy-saving effect is good, very practical, affordable service is also good, express also fast!	Taobao Marketplace, a Chinese website for online shopping
Xindu Flagship Store	a power saver (electronics)	Genuine quality is very good, energy-saving gods, reasonable price, overall very satisfied	Taobao Marketplace, a Chinese website for online shopping
Xindu Flagship Store	a power saver (electronics)	Power saving effect: It is known to save energy when plugged in, buy and try it first!	Taobao Marketplace, a Chinese website for online shopping
Xindu Flagship Store	a power saver (electronics)	Received, overall very satisfied, praise	Taobao Marketplace, a Chinese website for online shopping
Xindu Flagship Store	a power saver (electronics)	Yes, it saves me a lot in a month	Taobao Marketplace, a Chinese website for online shopping
Xindu Flagship Store	a power saver (electronics)	Last time I purchased6with my wife's numberand it sure saved a lot of money in the house, I came back to purchase them again this time, the store uses even more electricity, they have arrived now, looking forward to next month's electric bill coming out,	Taobao Marketplace, a Chinese website for online shopping
Xindu Flagship Store	a power saver (electronics)	Looks great and is well made.	Taobao Marketplace, a Chinese website for online shopping
Xindu Flagship Store	a power saver (electronics)	Energy saver received, easy to install, energy saving effect is quite good, cost-effective, high praise recommended!	Taobao Marketplace, a Chinese website for online shopping
Xindu Flagship Store	a power saver (electronics)	Great energy savings, easy to use, cost effective	Taobao Marketplace, a Chinese website for online shopping
Xindu Flagship Store	a power saver (electronics)	The packaging is intact, the machine is exquisite workmanship, plugged in the light, just used, power saving effect look forward to later effective. Favorable comments!	Taobao Marketplace, a Chinese website for online shopping
Xindu Flagship Store	a power saver (electronics)	Used a few days, the machine works normally, not hot, see used the netizen said have power-saving effect, looking forward to my use can also save power.	Taobao Marketplace, a Chinese website for online shopping
Xindu Flagship Store	a power saver (electronics)	Power saving effect: logistics is very fast, energy saving and environmental protection, the appearance of the shape is very good-looking, the quality of workmanship can be	Taobao Marketplace, a Chinese website for online shopping

Xindu Flagship Store	a power saver (electronics)	The logistics is fast, the stuff is fine, it can save electricity	Taobao Marketplace, a Chinese website for online shopping
Xindu Flagship Store	a power saver (electronics)	The stuff is great.	Taobao Marketplace, a Chinese website for online shopping
Xindu Flagship Store	a power saver (electronics)	Power saving effect: quite good, the effect of power connection can be. Logistics is very fast ah. Now you can save a lot of electricity in a month. Especially when the weather is hot and the air conditioner uses a lot of electricity, the power saving is very obvious!	Taobao Marketplace, a Chinese website for online shopping
Xindu Flagship Store	a power saver (electronics)	The goods are well-made, reasonably priced small and exquisite is very suitable for family use, I just received today is very eager to plug it up, and so on over a few days to see how the results	Taobao Marketplace, a Chinese website for online shopping
Xindu Flagship Store	a power saver (electronics)	Very good👌very good	Taobao Marketplace, a Chinese website for online shopping
Xindu Flagship Store	a power saver (electronics)	It's very well made and should work.	Taobao Marketplace, a Chinese website for online shopping
Xindu Flagship Store	a power saver (electronics)	Received the product, use it for a while to see	Taobao Marketplace, a Chinese website for online shopping
Xindu Flagship Store	a power saver (electronics)	Got it and started using it, looking forward to the results, beautiful style!	Taobao Marketplace, a Chinese website for online shopping
Xindu Flagship Store	a power saver (electronics)	It seems to have a power saving effect, watch it for a few days.	Taobao Marketplace, a Chinese website for online shopping
Xindu Flagship Store	a power saver (electronics)	Easy to use Just plug it into a free outlet Energy saving is pretty good Good review	Taobao Marketplace, a Chinese website for online shopping
Xindu Flagship Store	a power saver (electronics)	As soon as I received immediately installed, feel good, worth buying. The style looks good and the workmanship is perfect.	Taobao Marketplace, a Chinese website for online shopping
Xindu Flagship Store	a power saver (electronics)	Good workmanship and fast logistics!	Taobao Marketplace, a Chinese website for online shopping
Xindu Flagship Store	a power saver (electronics)	Experience: Average results in use	Taobao Marketplace, a Chinese website for online shopping
Xindu Flagship Store	a power saver (electronics)	ineffective	Taobao Marketplace, a Chinese website for online shopping
Xindu Flagship Store	a power saver (electronics)	Very practical energy saving effect is obvious very satisfied	Taobao Marketplace, a Chinese website for online shopping
Xindu Flagship Store	a power saver (electronics)	Excellent quality, convenient very good, worth buying.	Taobao Marketplace, a Chinese website for online shopping
Xindu Flagship Store	a power saver (electronics)	Plug and play, easy to use, energy saving and power saving, satisfactory	Taobao Marketplace, a Chinese website for online shopping
Xindu Flagship Store	a power saver (electronics)	Cheap, recommended by friends, first use a period of time to see how the effect, so far did not see any difference	Taobao Marketplace, a Chinese website for online shopping
Xindu Flagship Store	a power saver (electronics)	I just got it to try it out, it looks okay, I'll follow up after I use it.	Taobao Marketplace, a Chinese website for online shopping
Xindu Flagship Store	a power saver (electronics)	Keep trying to see if it's a real power saver later on	Taobao Marketplace, a Chinese website for online shopping
Xindu Flagship Store	a power saver (electronics)	Used, the meter obviously jumped slower, first use a month to follow up on the results	Taobao Marketplace, a Chinese website for online shopping
Xindu Flagship Store	a power saver (electronics)	The saver is lightweight in appearance, easy to use, plugged in and ready to go, thick material	Taobao Marketplace, a Chinese website for online shopping
Xindu Flagship Store	a power saver (electronics)	The saver was received, good quality and easy to install.	Taobao Marketplace, a Chinese website for online shopping
Xindu Flagship Store	a power saver (electronics)	Energy efficient, compact and does not take up much space, fast shipping and good service!	Taobao Marketplace, a Chinese website for online shopping
Xindu Flagship Store	a power saver (electronics)	Also recommended by a friend to buy bought at home, the store also bought, the effect is good	Taobao Marketplace, a Chinese website for online shopping
Xindu Flagship Store	a power saver (electronics)	Power-saving effect: power-saving effect is particularly obvious Cost-effective: cost-effective Appearance style: compact appearance Workmanship material: fine workmanship Experience: very good use, very convenient	Taobao Marketplace, a Chinese website for online shopping
Xindu Flagship Store	a power saver (electronics)	Power-saving effect: after use in the evaluation Cost-effective: good Appearance style: new Workmanship material: good Experience: after use in the evaluation	Taobao Marketplace, a Chinese website for online shopping
Xindu Flagship Store	a power saver (electronics)	Highly designed exterior and in perfect working order	Taobao Marketplace, a Chinese website for online shopping
Xindu Flagship Store	a power saver (electronics)	Friends recommended to this store to buy, used today, hope there is an effect!	Taobao Marketplace, a Chinese website for online shopping
Xindu Flagship Store	a power saver (electronics)	It feels okay, I just don't know how effective it is	Taobao Marketplace, a Chinese website for online shopping
Xindu Flagship Store	a power saver (electronics)	Appearance and style:'The appearance of small and exquisite, and power saving, worth getting.	Taobao Marketplace, a Chinese website for online shopping
Xindu Flagship Store	a power saver (electronics)	It does get better the more you look at it.☺	Taobao Marketplace, a Chinese website for online shopping
Xindu Flagship Store	a power saver (electronics)	Read the introductory note to achieve15%savings.	Taobao Marketplace, a Chinese website for online shopping
Xindu Flagship Store	a power saver (electronics)	Just plugged it in, we'll see how it works after a while.	Taobao Marketplace, a Chinese website for online shopping

Xindu Flagship Store	a power saver (electronics)	Received the goods and used it, power saving effect is not bad, the speed is obviously slower	Taobao Marketplace, a Chinese website for online shopping
Xindu Flagship Store	a power saver (electronics)	Not bad good price/performance ratio is good, power saving is ok. Good to use.	Taobao Marketplace, a Chinese website for online shopping
Xindu Flagship Store	a power saver (electronics)	Energy-saving effect is good, small small space, very convenient	Taobao Marketplace, a Chinese website for online shopping
Xindu Flagship Store	a power saver (electronics)	Look good, try to use it first, if good then buy two more, praise!	Taobao Marketplace, a Chinese website for online shopping
Xindu Flagship Store	a power saver (electronics)	Especially good, the physical also very small does not take up space, the quality is also very good, very satisfied!	Taobao Marketplace, a Chinese website for online shopping
Xindu Flagship Store	a power saver (electronics)	Power saving effect: just received the goods, has been used, the use of the effect is looking forward to. The next step to give further evaluation	Taobao Marketplace, a Chinese website for online shopping
Xindu Flagship Store	a power saver (electronics)	Product packaging is complete, the description of the introduction is detailed, the product is in use, the effectis OK	Taobao Marketplace, a Chinese website for online shopping
Xindu Flagship Store	a power saver (electronics)	Appearance and style: appearance of small, easy to use, the socket on that you can plug in!	Taobao Marketplace, a Chinese website for online shopping
Xindu Flagship Store	a power saver (electronics)	Come back with your feedback after the experience	Taobao Marketplace, a Chinese website for online shopping
Xindu Flagship Store	a power saver (electronics)	I haven't used it yet. I don't know how it works.	Taobao Marketplace, a Chinese website for online shopping
Xindu Flagship Store	a power saver (electronics)	It looks okay, but I won't know the results until I've used it for a month. Looking forward to getting good results.	Taobao Marketplace, a Chinese website for online shopping
Xindu Flagship Store	a power saver (electronics)	Power saving effect: it does work well and saves electricity. Cost-effective, inexpensive, and the material is quite good. It is worth buying, and will buy back next time.	Taobao Marketplace, a Chinese website for online shopping
Xindu Flagship Store	a power saver (electronics)	Noticeable power savings, pretty affordable.	Taobao Marketplace, a Chinese website for online shopping
Xindu Flagship Store	a power saver (electronics)	Friends used to recommend me over to buy, at first I do not believe this thing, hold the mind to try to buy3, the results are indeed unexpected, and will recommend friends to buy, now too many electrical appliances,1are several hundred of the electricity bill, and now every day can save a dozen or so to the merchant praise, and so on the future of the new store renovation is good also continue to purchase	Taobao Marketplace, a Chinese website for online shopping
Xindu Flagship Store	a power saver (electronics)	Value for money: just received, then checked on the plug board, looked at the pulse meter, with the same appliances, with and without a saver. There is a real difference in the blinking frequency of the pulse meter light. With the saver plugged in, the meter pulse light blinks much slower.really good5starsto the seller, also introduced to relatives to use	Taobao Marketplace, a Chinese website for online shopping
Xindu Flagship Store	a power saver (electronics)	Logistics is very fast, plugged in with a very dark night light, the instructions read and feel that it makes sense, I hope it can really save electricity, otherwise the home electricity bill is really quite high, especially in the winter, the first praise, and so on a month after the follow-up!	Taobao Marketplace, a Chinese website for online shopping
Xindu Flagship Store	a power saver (electronics)	Power saving effect: good, Cost-effective: very cost-effective. Appearance and style: very compact, does not take up space. Use experience: good.	Taobao Marketplace, a Chinese website for online shopping
Xindu Flagship Store	a power saver (electronics)	Baby received in good condition.packaging is fine, baby beautiful effect to be verified. Thank you for the honest seller, thank you attentive customer service! Thanks to the dedicated courier! Five stars out of five!!! Wish you not forget the beginning, move forward, business is prosperous! Service: service attitude service quality is awesome!!!!	Taobao Marketplace, a Chinese website for online shopping
Xindu Flagship Store	a power saver (electronics)	Used for a period of time to evaluate, power-saving effect really did not let me down no longer need to worry about the power consumption of high-power appliances, the material is very good, the manual is simple and easy to understand there are lights to see will be used, the quiet use of no noise, small and exquisite inserted where it does not get in the way of a very good product! Thanks to the seller, this	Taobao Marketplace, a Chinese website for online shopping
Xindu Flagship Store	a power saver (electronics)	Bought5gave one away to me, will repurchase if it works well, watch the electric bill next month	Taobao Marketplace, a Chinese website for online shopping
Xindu Flagship Store	a power saver (electronics)	It works well, and the store has good service	Taobao Marketplace, a Chinese website for online shopping
Xindu Flagship Store	a power saver (electronics)	Cost-effective, energy-saving small and exquisite, compared a lot of stores before buying, use a period of time to see the effect.	Taobao Marketplace, a Chinese website for online shopping
Xindu Flagship Store	a power saver (electronics)	I've been using it for a while with remarkable results and will repurchase.	Taobao Marketplace, a Chinese website for online shopping
Xindu Flagship Store	a power saver (electronics)	Bought Getting used to it When the time comes Check it out It should feel good Customer service is also good	Taobao Marketplace, a Chinese website for online shopping
Xindu Flagship Store	a power saver (electronics)	Very good, more cost-effective, beautiful material workmanship	Taobao Marketplace, a Chinese website for online shopping
Xindu Flagship Store	a power saver (electronics)	The stuff is very good used a few days to confirm a lot less power very good refund purchase	Taobao Marketplace, a Chinese website for online shopping
Xindu Flagship Store	a power saver (electronics)	Very good stuff recommended to buy	Taobao Marketplace, a Chinese website for online shopping
Xindu Flagship Store	a power saver (electronics)	The quality of the stuff is very good, exactly as described by the seller, very satisfied.	Taobao Marketplace, a Chinese website for online shopping
Xindu Flagship Store	a power saver (electronics)	Power saving effect: good Cost-effective: high Appearance and style: handsome Workmanship and material: perfect Using experience: good very	Taobao Marketplace, a Chinese website for online shopping
Xindu Flagship Store	a power saver (electronics)	first time to buy this energy saver, do not know that there is this thing, hold a try to buy the mentality, the business commitment to no effect full refund, after receiving the product there is a manual, it is very convenient, the saver is compact and does not take up space, my family's power consumption is very large, the purchase of6, the effect of the open after the chase comment	Taobao Marketplace, a Chinese website for online shopping
Xindu Flagship Store	a power saver (electronics)	Used for a period of time does produce the effect, our home3air conditioners are now almost every day24hoursday, the child summer vacation at home have not heard, the electricity bill is still almost the same as last month, that this device works	Taobao Marketplace, a Chinese website for online shopping
Xindu Flagship Store	a power saver (electronics)	Friends introduced me to buy, the store's power saver genuine guarantee, plug up really save a lot of electricity, my friends bought5, I also did not hesitate to buy5, looking forward to next month's electricity bill	Taobao Marketplace, a Chinese website for online shopping

Xindu Flagship Store	a power saver (electronics)	Power saving effect:, used ten years feel very power saving, ten years after the buy in your store, the volume is small and exquisite, and crystal page, buy back and plug in, ten years ago can save electricity, ten years after the digitized, certainly can save electricity. Next month in the follow-up	Taobao Marketplace, a Chinese website for online shopping
Xindu Flagship Store	a power saver (electronics)	I am a person monthly electricity bill to more than a hundred, is depressed to see this new technology, without hesitation to buy, I hope to be able to save me a little bit of expenditure, and so on the next month have the effect of the additional comments in the	Taobao Marketplace, a Chinese website for online shopping
Xindu Flagship Store	a power saver (electronics)	used for a period of time to evaluate, indeed useful, began to listen to people say is the IQ tax, and now they use it does have an effect, fortunately they try, or else miss the good stuff, day after day can save a lot of business informed that the greater the power consumption needs to be the more energy-saving appliances to be more and more this time to buy6. I believe that the effect of this time must also be	Taobao Marketplace, a Chinese website for online shopping
Xindu Flagship Store	a power saver (electronics)	Some people in the comment section say I have no brain, I just want to say that what works is what works, I do use it and it's not the first time I've purchased it, and none of you who haven't bought it have any right to say I have no brain, do you.	Taobao Marketplace, a Chinese website for online shopping
Xindu Flagship Store	a power saver (electronics)	Garbage products, really every day on the pawn, when the pawn is not the same, and was cheated, I hope that consumers do not believe too much in this kind of thing, be careful consumption do not be pitched again Workmanship material: garbage	Taobao Marketplace, a Chinese website for online shopping
Xindu Flagship Store	a power saver (electronics)	Received the goods look quite good, the use of the effect of later follow-up evaluation	Taobao Marketplace, a Chinese website for online shopping
Xindu Flagship Store	a power saver (electronics)	Energy saver is really good if it is open store in January can save hundreds of good service attitude, logistics fast!	Taobao Marketplace, a Chinese website for online shopping
Xindu Flagship Store	a power saver (electronics)	The energy saver does save electricity hot days open air conditioning effect is very obvious monthly savings of several hundred, household less say also save eight, ninety power consumption of more than a hundred savings, but please recognize the brand, other products I can not say.	Taobao Marketplace, a Chinese website for online shopping
Xindu Flagship Store	a power saver (electronics)	Power-saving effect is very obvious, the family is to open the supermarket has a few refrigerators and a few freezers with electricity is very large, every month the electricity bill to pay the headache, friends gave me a recommendation of this power-saving appliances, I bought a few to try a month feel good electricity is obviously reduced, and now a month of electricity, offset by a year of electricity, back to buy some of the preparation to send friends	Taobao Marketplace, a Chinese website for online shopping
Xindu Flagship Store	a power saver (electronics)	previous month's electricity bill is178yuan, with two power saver is now110yuan, the effect is too good, ready to buy two will be the old family, I was introduced by a friend to buy, just started to not believe, but also really good results, otherwise so much electricity every month, now have a power saver, or really good, the effect is fantastic, must be five-star praise! Recommended for everyone to buy	Taobao Marketplace, a Chinese website for online shopping
Xindu Flagship Store	a power saver (electronics)	Do not blow not black, the real experience down, the power saver still has a certain effect, with2months, before a month of electricity is810dollars, now680a month, the power saving effect is still visible, like our home power consumption is relatively large, a year down to save1,000dollars should not be too big a problem, small things useful! Thanks to the seller!	Taobao Marketplace, a Chinese website for online shopping
Xindu Flagship Store	a power saver (electronics)	Power-saving effect: save some good use Cost-effective: afford Appearance style: good-looking fashion Workmanship material: very good	Taobao Marketplace, a Chinese website for online shopping
Xindu Flagship Store	a power saver (electronics)	The product is very energy efficient and I feel no pressure to turn on the air conditioner. Quite good for another order	Taobao Marketplace, a Chinese website for online shopping
Xindu Flagship Store	a power saver (electronics)	After getting it, I tested it myself with a night, and it saved a little bit of electricity, not much power saving is not a day or two days, it is a month of cumulative, and it is quite good to use it at the moment.	Taobao Marketplace, a Chinese website for online shopping
Xindu Flagship Store	a power saver (electronics)	I did not think there are so magical things, before has been a waste of a lot of money, this time to buy2, plugged in after a smart display shows that the machine is working, good to use in the recommended side friends, the shopkeeper service is also very good, give5stars!	Taobao Marketplace, a Chinese website for online shopping
Xindu Flagship Store	a power saver (electronics)	It looks better It's pretty	Taobao Marketplace, a Chinese website for online shopping
Xindu Flagship Store	a power saver (electronics)	Safe and does not take up space, energy saving and low power consumption, cost-effective, good reviews	Taobao Marketplace, a Chinese website for online shopping
Xindu Flagship Store	a power saver (electronics)	Power saving effect: energy saving effect is very good Cost-effective: high price Workmanship material: quality workmanship is very good	Taobao Marketplace, a Chinese website for online shopping
Xindu Flagship Store	a power saver (electronics)	Received, and customer service said the same, save trouble and electricity, the material is made of good, logistics and delivery fast!	Taobao Marketplace, a Chinese website for online shopping
Xindu Flagship Store	a power saver (electronics)	A very energy efficient product, looking forward to its results.	Taobao Marketplace, a Chinese website for online shopping
Xindu Flagship Store	a power saver (electronics)	Plugging in the meter will really slow things down.	Taobao Marketplace, a Chinese website for online shopping
Xindu Flagship Store	a power saver (electronics)	This is a first-class energy-saving product with a simple design, fine workmanship and good value for money.	Taobao Marketplace, a Chinese website for online shopping
Xindu Flagship Store	a power saver (electronics)	This is the second time I have purchased it and the energy saving effect is really first class. The electricity bill is obviously much less than before, the effect is really good.	Taobao Marketplace, a Chinese website for online shopping
Xindu Flagship Store	a power saver (electronics)	I've been using it for a few days before commenting on it, and it does have a power saving effect and stabilizes the voltage.	Taobao Marketplace, a Chinese website for online shopping
Xindu Flagship Store	a power saver (electronics)	I do my own foreign trade platform, has been purchased many times, has always recognized this genuine power saver, and this store has Taiwan, Malaysia plug, very convenient , foreign friends do not need to convert the head, power saving effect is also quite good	Taobao Marketplace, a Chinese website for online shopping
Xindu Flagship Store	a power saver (electronics)	Things well-packaged, logistics is also fast, the appearance and description of the match, workmanship is okay, and so on the use of the effect and then add comments!	Taobao Marketplace, a Chinese website for online shopping
Xindu Flagship Store	a power saver (electronics)	Power saving effect: better Cost-effectiveness: better Appearance style: more fashionable Workmanship material: fine Experience: very good	Taobao Marketplace, a Chinese website for online shopping
Xindu Flagship Store	a power saver (electronics)	I've had a drop in power since I've been using it, it's very good and it's dropping my electric bill every month	Taobao Marketplace, a Chinese website for online shopping
Xindu Flagship Store	a power saver (electronics)	Love it, has a power saving effect, would recommend to friends to buy it	Taobao Marketplace, a Chinese website for online shopping
Xindu Flagship Store	a power saver (electronics)	It does have a power saving effect	Taobao Marketplace, a Chinese website for online shopping
Xindu Flagship Store	a power saver (electronics)	Goods just received, before I bought does save electricity, this time again to find the same brand to help a friend to buy, he was very happy, but also said to use it and then tell me how effective.	Taobao Marketplace, a Chinese website for online shopping
Xindu Flagship Store	a power saver (electronics)	The product is great and the results are only known after use.	Taobao Marketplace, a Chinese website for online shopping

Xindu Flagship Store	a power saver (electronics)	Already purchased for the second time, it is indeed real and effective, I am also a repeat customer, good stuff must be praised. Give everyone to share share	Taobao Marketplace, a Chinese website for online shopping
Xindu Flagship Store	a power saver (electronics)	I've been told I advertise for him! What a**! 我自己用的好我都我都用，我用的不好了我都退！ Anyway, there is shipping insurance! I use Taobaofor more than10, there is no need to advertise for anyone, or for anyone that to do publicity! I'm a personal that account I'm not talking about advertising that kind of publicity! I say some boring people are too boring!	Taobao Marketplace, a Chinese website for online shopping
Xindu Flagship Store	a power saver (electronics)	Appearance can be Workmanship material: looks good. Good comment.	Taobao Marketplace, a Chinese website for online shopping
Xindu Flagship Store	a power saver (electronics)	It seems to work pretty well.	Taobao Marketplace, a Chinese website for online shopping
Xindu Flagship Store	a power saver (electronics)	Since there is no one to manage, said the3-monthtrial, useless can be returned, and now are ignoring people, really convinced, it seems to be directly looking for the official website betting on the	Taobao Marketplace, a Chinese website for online shopping
Xindu Flagship Store	a power saver (electronics)	Power saving effect: very good Cost-effective: very high Appearance style: simple and fashionable Workmanship material: very good workmanship Using experience: very good	Taobao Marketplace, a Chinese website for online shopping
Xindu Flagship Store	a power saver (electronics)	Power saving effect: a better, not noticeable at first Value for money:'high quality at a low price User experience:'easy to use.	Taobao Marketplace, a Chinese website for online shopping
Xindu Flagship Store	a power saver (electronics)	Power-saving effect: the second time to buy back, the effect is very good Cost-effective: better than the other effect, can be recommended to family and friends to use	Taobao Marketplace, a Chinese website for online shopping
Xindu Flagship Store	a power saver (electronics)	Power saving effect: very good, very good Cost-effective: good, very good, power saving Appearance and style: good, very good Workmanship and material: very good Using experience: very good, power saving	Taobao Marketplace, a Chinese website for online shopping
Xindu Flagship Store	a power saver (electronics)	Experience: very nice appearance, small and does not take up much space	Taobao Marketplace, a Chinese website for online shopping
Xindu Flagship Store	a power saver (electronics)	I received the goods yesterday and plugged it in, it feels pretty good and effective	Taobao Marketplace, a Chinese website for online shopping
Xindu Flagship Store	a power saver (electronics)	Goods intact, has been plugged in to use, hope to have imagined good results	Taobao Marketplace, a Chinese website for online shopping
Xindu Flagship Store	a power saver (electronics)	Baby received, is a colleague recommended, said the use of good results, I also buy a save a little electricity, colleagues said that this should be inserted for a period of time before the effect will be more pronounced, as soon as I received today immediately inserted, look forward to the effect!	Taobao Marketplace, a Chinese website for online shopping
Xindu Flagship Store	a power saver (electronics)	Power saving effect: use good convenient, very like, save a lot of power consumption	Taobao Marketplace, a Chinese website for online shopping
Xindu Flagship Store	a power saver (electronics)	Plug and play is so convenient, the power saving effect is especially great, you can get it!	Taobao Marketplace, a Chinese website for online shopping
Xindu Flagship Store	a power saver (electronics)	Energy saving very power saving, easy to use, fine workmanship material is very good!	Taobao Marketplace, a Chinese website for online shopping
Xindu Flagship Store	a power saver (electronics)	Baby received power-saving effect: have not yet started to use, after the use of follow-up evaluation appearance and style: small and exquisite workmanship material: very good	Taobao Marketplace, a Chinese website for online shopping
Xindu Flagship Store	a power saver (electronics)	Great quality, power saving, and it's ok.	Taobao Marketplace, a Chinese website for online shopping
Xindu Flagship Store	a power saver (electronics)	Boss, I have received the goods, and I am very satisfied! Next time, I hope to have good results in the future!	Taobao Marketplace, a Chinese website for online shopping
Xindu Flagship Store	a power saver (electronics)	I've come to your house for the fourth time and am very happy with it. Use it well next time.	Taobao Marketplace, a Chinese website for online shopping
Xindu Flagship Store	a power saver (electronics)	Power-saving effect: still in use, Cost-effective: Appearance and style: good-looking Workmanship and materials: meticulous workmanship Experience: looking forward to the results	Taobao Marketplace, a Chinese website for online shopping
Xindu Flagship Store	a power saver (electronics)	Compact and doesn't take up much space, expect results	Taobao Marketplace, a Chinese website for online shopping
Xindu Flagship Store	a power saver (electronics)	I don't know if it saves electricity, I'll evaluate it after using it for a while.	Taobao Marketplace, a Chinese website for online shopping
Xindu Flagship Store	a power saver (electronics)	Received it and used it, the product appearance is small and exquisite	Taobao Marketplace, a Chinese website for online shopping
Xindu Flagship Store	a power saver (electronics)	After using it, I felt that the meter was going slow, so I ordered1more, and the merchant also gaveme 1freeone	Taobao Marketplace, a Chinese website for online shopping
Xindu Flagship Store	a power saver (electronics)	The quality of the saver is very good, good-looking, plug and play is very convenient, the price is not expensive but also can save a lot of electricity, cost-effective!	Taobao Marketplace, a Chinese website for online shopping
Xindu Flagship Store	a power saver (electronics)	I've been using it for a while now, and it seems like you can indeed save two or three kilowatts per day! Use it for a while and then continue to see, it's not too expensive anyway!!!!	Taobao Marketplace, a Chinese website for online shopping
Xindu Flagship Store	a power saver (electronics)	Bought one works great Decided to buy one for my mom in the next order Works great ! really!	Taobao Marketplace, a Chinese website for online shopping
Xindu Flagship Store	a power saver (electronics)	Power-saving effect:√first use and then see Value for money:{cheap Appearance and style: beautiful Workmanship material:>good Use experience:, easy to use	Taobao Marketplace, a Chinese website for online shopping
Xindu Flagship Store	a power saver (electronics)	It's well made and hopefully energy efficient.	Taobao Marketplace, a Chinese website for online shopping
Xindu Flagship Store	a power saver (electronics)	In use and hopefully energy efficient.	Taobao Marketplace, a Chinese website for online shopping
Xindu Flagship Store	a power saver (electronics)	The new model has a smaller body than the old model fashionable and pleasing to the eye, looking forward to saving power and energy as efficient; in addition, customer service promised to change to Hong Kong with the British plug, but the delivery of the domestic use of three-pronged plug a littledisappointing😅	Taobao Marketplace, a Chinese website for online shopping
Xindu Flagship Store	a power saver (electronics)	Cost-effective: just received the goods, I do not know if it can be good to use the first time to buy power-saving energy hand everyone needs to	Taobao Marketplace, a Chinese website for online shopping
Xindu Flagship Store	a power saver (electronics)	Good to use the next time to buy mobilize around friends to use the power-saving energy hand!	Taobao Marketplace, a Chinese website for online shopping

Xindu Flagship Store	a power saver (electronics)	Power-saving effect: the effect is very good, Cost-effective: the price is cheap Appearance style: the appearance is very good Workmanship material:, the material is very good	Taobao Marketplace, a Chinese website for online shopping
Xindu Flagship Store	a power saver (electronics)	The saver works great. The home electric meter is noticeably slower.	Taobao Marketplace, a Chinese website for online shopping
Xindu Flagship Store	a power saver (electronics)	Power-saving effect: originally every night at 10:30 pm back from work to open the air conditioning electricity costsmore than12yuan, with this king of power saving a night only4dollars. Worth buying Appearance style: small and convenient	Taobao Marketplace, a Chinese website for online shopping
Xindu Flagship Store	a power saver (electronics)	Power-saving effect: not yet clear without using it for a few days Workmanship and material: good Using experience: quite convenient	Taobao Marketplace, a Chinese website for online shopping
Xindu Flagship Store	a power saver (electronics)	Power saving effect: in use Cost-effective: good Appearance style: still very good Workmanship material: quality is guaranteed Experience: subsequent energy saving good, continue to buy back!	Taobao Marketplace, a Chinese website for online shopping
Xindu Flagship Store	a power saver (electronics)	Good review, very satisfied.	Taobao Marketplace, a Chinese website for online shopping
Xindu Flagship Store	a power saver (electronics)	Power-saving effect: save half of the electricity Cost-effective: the price is right Appearance style: exquisite workmanship Workmanship material: good Oh Experience: many times repurchase, it is indeed very power-saving. Equivalent to saving money. Plug and play! Convenient!	Taobao Marketplace, a Chinese website for online shopping
Xindu Flagship Store	a power saver (electronics)	Good looking and good quality ah! Worth buying! Have purchased his products many times. Service is pretty good in all aspects. Quality merchant!	Taobao Marketplace, a Chinese website for online shopping
Xindu Flagship Store	a power saver (electronics)	favorable criticism	Taobao Marketplace, a Chinese website for online shopping
Xindu Flagship Store	a power saver (electronics)	The quality is very good, very satisfied, praise!	Taobao Marketplace, a Chinese website for online shopping
Xindu Flagship Store	a power saver (electronics)	I haven't used it yet, I'll comment later	Taobao Marketplace, a Chinese website for online shopping
Xindu Flagship Store	a power saver (electronics)	Power saving effect: observe for one day, still seems to be effective	Taobao Marketplace, a Chinese website for online shopping
Xindu Flagship Store	a power saver (electronics)	I'll comment again in a month.	Taobao Marketplace, a Chinese website for online shopping
Xindu Flagship Store	a power saver (electronics)	(of packages or shipments) arrive	Taobao Marketplace, a Chinese website for online shopping
Xindu Flagship Store	a power saver (electronics)	It's prettier and better made than the old one.	Taobao Marketplace, a Chinese website for online shopping
Xindu Flagship Store	a power saver (electronics)	Now used, waiting for results before evaluating! Quick, easy and satisfied! Give kudos!	Taobao Marketplace, a Chinese website for online shopping
Xindu Flagship Store	a power saver (electronics)	Power saving effect: good power saving effect	Taobao Marketplace, a Chinese website for online shopping
Xindu Flagship Store	a power saver (electronics)	We'll evaluate the results when they come out.	Taobao Marketplace, a Chinese website for online shopping
Xindu Flagship Store	a power saver (electronics)	It really saves electricity after using it for a while. I recommend buying it.	Taobao Marketplace, a Chinese website for online shopping
Xindu Flagship Store	a power saver (electronics)	The stuff really works and it's worth buying	Taobao Marketplace, a Chinese website for online shopping
Xindu Flagship Store	a power saver (electronics)	*It doesn't work, it doesn't save electricity at all, it's a scam, it costs more to plug it in.	Taobao Marketplace, a Chinese website for online shopping
Xindu Flagship Store	a power saver (electronics)	Things received very good, tightly packaged without damage, the boss is very good and careful, the next time will come back, I wish the store business prosperity!	Taobao Marketplace, a Chinese website for online shopping
Xindu Flagship Store	a power saver (electronics)	It's a great one. I'm very happy with it.	Taobao Marketplace, a Chinese website for online shopping
Xindu Flagship Store	a power saver (electronics)	I compared it to last year's electricity and it still saves a lot of electricity for the same usage. It's not even an IQ tax! It's worth buying.	Taobao Marketplace, a Chinese website for online shopping
Xindu Flagship Store	a power saver (electronics)	Power saving effect: see the effect after use Appearance style: good style Workmanship material: good Experience: to be used	Taobao Marketplace, a Chinese website for online shopping
Xindu Flagship Store	a power saver (electronics)	Received and used, really works, cost-effective, and simple and convenient operation	Taobao Marketplace, a Chinese website for online shopping
Xindu Flagship Store	a power saver (electronics)	It's really good, cost-effective and good quality, and the logistics is fast ah!	Taobao Marketplace, a Chinese website for online shopping
Xindu Flagship Store	a power saver (electronics)	Genuine store, very good quality, power saving effect is good, logistics is also very fast, worth buying!	Taobao Marketplace, a Chinese website for online shopping
Xindu Flagship Store	a power saver (electronics)	Energy saver received, the appearance of the style can be, very small, easy to use plug, the effect is good!	Taobao Marketplace, a Chinese website for online shopping
Xindu Flagship Store	a power saver (electronics)	This time to buy two for the family, the kitchen and bathroom water heater is too costly, the water heater to be turned on every day, quite costly, before buying a used in the air conditioning side, January down the electricity bill can be less than the previous month are quite a lot, the effect is	Taobao Marketplace, a Chinese website for online shopping
Xindu Flagship Store	a power saver (electronics)	Very easy to use, good quality	Taobao Marketplace, a Chinese website for online shopping
Xindu Flagship Store	King of Power Saving	not have	Taobao Marketplace, a Chinese website for online shopping
Xindu Flagship Store	wonderful butler	not have	Taobao Marketplace, a Chinese website for online shopping
Black Tech Electrician & Electrician	Green Intelligent Power Saver	It's too wide to battle with another port. Can't the other port be plugged in?	Taobao Marketplace, a Chinese website for online shopping

Black Tech Electrician & Electrician	Green Intelligent Power Saver	Not noticeable when used	Taobao Marketplace, a Chinese website for online shopping
Black Tech Electrician & Electrician	Green Intelligent Power Saver	ineffective	Taobao Marketplace, a Chinese website for online shopping
Black Tech Electrician & Electrician	Green Intelligent Power Saver	Goods received, logistics is also fast appearance of good power saving before using the effect do not know, with good in the repurchase	Taobao Marketplace, a Chinese website for online shopping
Black Tech Electrician & Electrician	Green Intelligent Power Saver	simulacrum	Taobao Marketplace, a Chinese website for online shopping
Black Tech Electrician & Electrician	Green Intelligent Power Saver	Energy-saving power king received 6 days ago I bought one myself, is still in use experience, feel at home refrigerator and open the air conditioning noise sound is very small almost no sound, I think the energy-saving power king in January should be able to effectively save 4 to 50 yuan of electricity, it is special for my children to buy, however, the child said that to be used for a period of time to see!	Taobao Marketplace, a Chinese website for online shopping
Black Tech Electrician & Electrician	Green Intelligent Power Saver	no good	Taobao Marketplace, a Chinese website for online shopping
Black Tech Electrician & Electrician	Green Intelligent Power Saver	Baby received, very good use and consistent with the description, and then buy a few to use in the room as well!	Taobao Marketplace, a Chinese website for online shopping
Black Tech Electrician & Electrician	Green Intelligent Power Saver	I don't know how to save power.	Taobao Marketplace, a Chinese website for online shopping
Black Tech Electrician & Electrician	Green Intelligent Power Saver	Not pitching to poor reviews.	Taobao Marketplace, a Chinese website for online shopping
Black Tech Electrician & Electrician	Green Intelligent Power Saver	Courier does not deliver to the house, bad review.	Taobao Marketplace, a Chinese website for online shopping
Black Tech Electrician & Electrician	Green Intelligent Power Saver	It doesn't work at all, it still uses electricity, it doesn't save any electricity.	Taobao Marketplace, a Chinese website for online shopping
Black Tech Electrician & Electrician	Green Intelligent Power Saver	Cheat play with the use of two did not work, people do not buy, save what electricity, I fell for it	Taobao Marketplace, a Chinese website for online shopping
Black Tech Electrician & Electrician	Green Intelligent Power Saver	System default comments	Taobao Marketplace, a Chinese website for online shopping
Black Tech Electrician & Electrician	Green Intelligent Power Saver	Received and plugged in, there is a display, quite beautiful, this price, workmanship is really good. Power-saving effect also need to use a period of time to know!	Taobao Marketplace, a Chinese website for online shopping
Black Tech Electrician & Electrician	Green Intelligent Power Saver	It's a good power saver.	Taobao Marketplace, a Chinese website for online shopping
Black Tech Electrician & Electrician	Green Intelligent Power Saver	Start using it. It should work.	Taobao Marketplace, a Chinese website for online shopping
Black Tech Electrician & Electrician	Green Intelligent Power Saver	The size is just right and the quality is very good, the packaging of the store is so attentive, it is worth recommending!	Taobao Marketplace, a Chinese website for online shopping
Black Tech Electrician & Electrician	Green Intelligent Power Saver	The logistics is also very fast service is also very good appearance looks okay hopefully good use next time to buy again	Taobao Marketplace, a Chinese website for online shopping
Black Tech Electrician & Electrician	Green Intelligent Power Saver	Logistics is also very fast service is also very good hope good use next time to repurchase!	Taobao Marketplace, a Chinese website for online shopping
Black Tech Electrician & Electrician	Green Intelligent Power Saver	Has been the fourth time to buy, is for a friend to buy the second time, is indeed power saving, before two months to 600 or so, this two months with 284 yuan, almost half of the savings, want to buy friends can order!	Taobao Marketplace, a Chinese website for online shopping
Black Tech Electrician & Electrician	Green Intelligent Power Saver	Not yet used, customary praise	Taobao Marketplace, a Chinese website for online shopping
Black Tech Electrician & Electrician	Green Intelligent Power Saver	Bad quality sells fakes and everyone who buys them is out of luck.	Taobao Marketplace, a Chinese website for online shopping
Black Tech Electrician & Electrician	Green Intelligent Power Saver	Now that I have received the goods, it just doesn't work like the video said.	Taobao Marketplace, a Chinese website for online shopping
Black Tech Electrician & Electrician	Green Intelligent Power Saver	New era, new technology, new products, energy saving, electricity saving, cash saving, you say good, I say good, she says good, three words "good product"! Support me!	Taobao Marketplace, a Chinese website for online shopping
Black Tech Electrician & Electrician	Green Intelligent Power Saver	useless	Taobao Marketplace, a Chinese website for online shopping
Black Tech Electrician & Electrician	Green Intelligent Power Saver	To be observed and then evaluated	Taobao Marketplace, a Chinese website for online shopping
Black Tech Electrician & Electrician	Green Intelligent Power Saver	Power saving effect: good Cost-effective: good Appearance style: good Workmanship material: good Experience: can be	Taobao Marketplace, a Chinese website for online shopping
Black Tech Electrician & Electrician	Green Intelligent Power Saver	This saver is great, affordable and I love it!	Taobao Marketplace, a Chinese website for online shopping
Black Tech Electrician & Electrician	Green Intelligent Power Saver	Baby received, consistent with the description, give five stars!	Taobao Marketplace, a Chinese website for online shopping
Black Tech Electrician & Electrician	Green Intelligent Power Saver	Baby received, and the description is consistent with the five-star praise!	Taobao Marketplace, a Chinese website for online shopping
Black Tech Electrician & Electrician	Green Intelligent Power Saver	Doesn't it work at all?	Taobao Marketplace, a Chinese website for online shopping
Black Tech Electrician & Electrician	Green Intelligent Power Saver	I wonder how it works?	Taobao Marketplace, a Chinese website for online shopping
Black Tech Electrician & Electrician	Green Intelligent Power Saver	Got the stuff. Hope it works well. Hopefully there is a tiny bit of use. I'll follow up after I use it.	Taobao Marketplace, a Chinese website for online shopping
Black Tech Electrician & Electrician	Green Intelligent Power Saver	to be observed	Taobao Marketplace, a Chinese website for online shopping

Black Tech Electrician & Electrician	Green Intelligent Power Saver	general	Taobao Marketplace, a Chinese website for online shopping
Black Tech Electrician & Electrician	Green Intelligent Power Saver	Received on the socket to try to see if there is no effect, the business said that the effect of a month, when the effect of additional positive feedback, and vice versa, a long bad review!	Taobao Marketplace, a Chinese website for online shopping
Black Tech Electrician & Electrician	Green Intelligent Power Saver	The quality of the product is excellent and the energy savings are great.	Taobao Marketplace, a Chinese website for online shopping
Black Tech Electrician & Electrician	Green Intelligent Power Saver	ineffective	Taobao Marketplace, a Chinese website for online shopping
Black Tech Electrician & Electrician	Green Intelligent Power Saver	The results are still being tested	Taobao Marketplace, a Chinese website for online shopping
Black Tech Electrician & Electrician	Green Intelligent Power Saver	I don't know if it's energy efficient	Taobao Marketplace, a Chinese website for online shopping
Black Tech Electrician & Electrician	Green Intelligent Power Saver	No power saving reverse consumption	Taobao Marketplace, a Chinese website for online shopping
Black Tech Electrician & Electrician	Green Intelligent Power Saver	Workmanship material: okay	Taobao Marketplace, a Chinese website for online shopping
Black Tech Electrician & Electrician	Green Intelligent Power Saver	Power saving effect: the big liar	Taobao Marketplace, a Chinese website for online shopping
Black Tech Electrician & Electrician	Green Intelligent Power Saver	Expect it to work well	Taobao Marketplace, a Chinese website for online shopping
Black Tech Electrician & Electrician	Green Intelligent Power Saver	Great. Let's see if we can save power.	Taobao Marketplace, a Chinese website for online shopping
Black Tech Electrician & Electrician	Green Intelligent Power Saver	High efficiency, power saving rate is very good, expect a month to save a hundred degrees of electricity	Taobao Marketplace, a Chinese website for online shopping
Black Tech Electrician & Electrician	Green Intelligent Power Saver	Looking good. Looking forward to good results	Taobao Marketplace, a Chinese website for online shopping
Black Tech Electrician & Electrician	Green Intelligent Power Saver	It's not as good as it says.	Taobao Marketplace, a Chinese website for online shopping
Black Tech Electrician & Electrician	Green Intelligent Power Saver	I received the product and found that the product was not the same as the picture advertising the appearance.	Taobao Marketplace, a Chinese website for online shopping
Black Tech Electrician & Electrician	Green Intelligent Power Saver	It doesn't save electricity at all. It's a scam.	Taobao Marketplace, a Chinese website for online shopping
Black Tech Electrician & Electrician	Green Intelligent Power Saver	I bought it and used it. It didn't work.	Taobao Marketplace, a Chinese website for online shopping
Black Tech Electrician & Electrician	Green Intelligent Power Saver	Power saving effect: very saving Appearance style: perfect good-looking Experience: bought last year in the home has been used, very good, next time will patronize, will recommend to my friends.	Taobao Marketplace, a Chinese website for online shopping
Black Tech Electrician & Electrician	Green Intelligent Power Saver	The stuff is great, the courier guy has great service	Taobao Marketplace, a Chinese website for online shopping
Black Tech Electrician & Electrician	Green Intelligent Power Saver	Customer service is enthusiastic, logistics is fast, good value for money.	Taobao Marketplace, a Chinese website for online shopping
Black Tech Electrician & Electrician	Green Intelligent Power Saver	Customer service is enthusiastic, logistics is fast, good value for money.	Taobao Marketplace, a Chinese website for online shopping
Black Tech Electrician & Electrician	Green Intelligent Power Saver	Experience: the feel is very light and empty box, I do not know if it can save electricity, first plugged in to see the next month's electricity bill.	Taobao Marketplace, a Chinese website for online shopping
Black Tech Electrician & Electrician	Green Intelligent Power Saver	Experience: the description matches, use it to know the effect of the	Taobao Marketplace, a Chinese website for online shopping
Black Tech Electrician & Electrician	Green Intelligent Power Saver	Baby received ten days, the use of the effect is not bad, there is a power-saving function, satisfied. I will recommend to friends.	Taobao Marketplace, a Chinese website for online shopping
Black Tech Electrician & Electrician	Green Intelligent Power Saver	Cost-effective: affordable in use do not know the effect of how to come back later to follow up. Compact and exquisite style does not take up space plant	Taobao Marketplace, a Chinese website for online shopping
Black Tech Electrician & Electrician	Green Intelligent Power Saver	The power saving effect is very good, especially now the summer power consumption peak effect is better.	Taobao Marketplace, a Chinese website for online shopping
Black Tech Electrician & Electrician	Green Intelligent Power Saver	I didn't feel any power savings after using it for so long.	Taobao Marketplace, a Chinese website for online shopping
Black Tech Electrician & Electrician	Green Intelligent Power Saver	Baby is awesome and decides to work well! Beautiful and durable!	Taobao Marketplace, a Chinese website for online shopping
Black Tech Electrician & Electrician	Green Intelligent Power Saver	Appearance and style: s outside like good-looking not yet used on but looks good must be official use	Taobao Marketplace, a Chinese website for online shopping
Black Tech Electrician & Electrician	Green Intelligent Power Saver	Cost-effective: good goods at a good price Experience: baby received the goods on the use of the real power-saving and good price is worth buying	Taobao Marketplace, a Chinese website for online shopping
Black Tech Electrician & Electrician	Green Intelligent Power Saver	In use, to have no effect on the follow-up evaluation! Satisfied with good reviews first!	Taobao Marketplace, a Chinese website for online shopping
Black Tech Electrician & Electrician	Green Intelligent Power Saver	I just received it. I haven't used it yet.	Taobao Marketplace, a Chinese website for online shopping
Black Tech Electrician & Electrician	Green Intelligent Power Saver	It's not very effective.	Taobao Marketplace, a Chinese website for online shopping
Black Tech Electrician & Electrician	Green Intelligent Power Saver	Power-saving effect: just used, should be able to Cost-effective: favorable Appearance and style: good-looking Workmanship material: very good Experience: to be followed up	Taobao Marketplace, a Chinese website for online shopping

Black Tech Electrician & Electrician	Green Intelligent Power Saver	Goods received well packaged I am very satisfied next time to buy again	Taobao Marketplace, a Chinese website for online shopping
Black Tech Electrician & Electrician	Green Intelligent Power Saver	It's not working.	Taobao Marketplace, a Chinese website for online shopping
Black Tech Electrician & Electrician	Green Intelligent Power Saver	More than ten dollars, not at all useful, customer service: also said two months later, no effect on the refund, today looked at, now people this commodity display does not exist, purely fooling people, plug in the meter go faster!	Taobao Marketplace, a Chinese website for online shopping
Black Tech Electrician & Electrician	Green Intelligent Power Saver	First bad review, scam, just a regular circuit. No power savings at all.	Taobao Marketplace, a Chinese website for online shopping
Black Tech Electrician & Electrician	Green Intelligent Power Saver	I haven't used it yet. I'll comment later.	Taobao Marketplace, a Chinese website for online shopping
Black Tech Electrician & Electrician	Green Intelligent Power Saver	Received, well-packaged logistics quickly, and will use to see how the effect of	Taobao Marketplace, a Chinese website for online shopping
Black Tech Electrician & Electrician	Green Intelligent Power Saver	Power saving effect: not so good	Taobao Marketplace, a Chinese website for online shopping
Black Tech Electrician & Electrician	Green Intelligent Power Saver	I don't know yet, but I can't say anything else about the power-saving effect until later, when I say that I'll use it for a while.	Taobao Marketplace, a Chinese website for online shopping
Black Tech Electrician & Electrician	Green Intelligent Power Saver	How to buy the goods do not have a factory address, there is no 3C certification used for a few days without effect do not know how long with the effect of the	Taobao Marketplace, a Chinese website for online shopping
Black Tech Electrician & Electrician	Green Intelligent Power Saver	We'll see what happens in a few years!	Taobao Marketplace, a Chinese website for online shopping
Black Tech Electrician & Electrician	Green Intelligent Power Saver	Power saving effect: 1 fake	Taobao Marketplace, a Chinese website for online shopping
Black Tech Electrician & Electrician	Green Intelligent Power Saver	give it a try	Taobao Marketplace, a Chinese website for online shopping
Black Tech Electrician & Electrician	Green Intelligent Power Saver	Baby received and it's great! Consistent with the description. Giving a 5 star review!	Taobao Marketplace, a Chinese website for online shopping
Black Tech Electrician & Electrician	Green Intelligent Power Saver	I haven't used it yet. I'll comment later.	Taobao Marketplace, a Chinese website for online shopping
Black Tech Electrician & Electrician	Green Intelligent Power Saver	The Smart Power Saver works great and saves power. Good use and very affordable. The seller has a good service attitude. Thanks to the seller!	Taobao Marketplace, a Chinese website for online shopping
Black Tech Electrician & Electrician	Green Intelligent Power Saver	Baby just received, it's great 👌👍 use it right away, the courier guy is great, I will continue to support you guys.	Taobao Marketplace, a Chinese website for online shopping
Black Tech Electrician & Electrician	Green Intelligent Power Saver	Sent me a defective product, looking for customer service does not answer my questions positively, can not be used, is a black business, people do not fall for the scam in the	Taobao Marketplace, a Chinese website for online shopping
Black Tech Electrician & Electrician	Green Intelligent Power Saver	It's useless.	Taobao Marketplace, a Chinese website for online shopping
Black Tech Electrician & Electrician	Green Intelligent Power Saver	I've only been using it for a few days and I don't know the results yet	Taobao Marketplace, a Chinese website for online shopping
Black Tech Electrician & Electrician	Green Intelligent Power Saver	Experience: just started to use do not know whether it is good or not, use a period of time to speak again	Taobao Marketplace, a Chinese website for online shopping
Black Tech Electrician & Electrician	Green Intelligent Power Saver	Families inserted into the power saver for half a month, electricity savings are as follows: last year, the highest monthly electricity bill of 500 yuan. Last month, i.e. July, the electricity bill was 590 yuan, and after using the energy saver for about half a month, the electricity bill was 946 yuan in August, and now I don't dare to use the energy saver	Taobao Marketplace, a Chinese website for online shopping
Black Tech Electrician & Electrician	Green Intelligent Power Saver	Just received, fast delivery, good packaging, looking forward to this baby can be value for money, have not yet used, but I think it will not let me down, to be used after a follow-up comment.	Taobao Marketplace, a Chinese website for online shopping
Black Tech Electrician & Electrician	Green Intelligent Power Saver	Doesn't work, doesn't save power just an indicator light inside, too fake. Power saving effect: no	Taobao Marketplace, a Chinese website for online shopping
Black Tech Electrician & Electrician	Green Intelligent Power Saver	Received in a timely manner, and the promotional introduction is consistent with the quality of the appearance is very formal, the material is also good, I think the effect is also good, it is worth trusting and buying.	Taobao Marketplace, a Chinese website for online shopping
Black Tech Electrician & Electrician	Green Intelligent Power Saver	Power saving effect: 1 Power saving. Cost-effective. New style. Fine workmanship. Ideal using experience.	Taobao Marketplace, a Chinese website for online shopping
Black Tech Electrician & Electrician	Green Intelligent Power Saver	Appearance and style:, logistics is very fast, fine workmanship, good service, after using the follow-up comment.	Taobao Marketplace, a Chinese website for online shopping
Black Tech Electrician & Electrician	Green Intelligent Power Saver	No national standard, no manufacturer and address, no registration by scanning QR code with WeChat and Alipay.	Taobao Marketplace, a Chinese website for online shopping
Black Tech Electrician & Electrician	Green Intelligent Power Saver	Value for money: very good Workmanship material: fine	Taobao Marketplace, a Chinese website for online shopping
Black Tech Electrician & Electrician	Green Intelligent Power Saver	Two shots in a row! Very good! Very good! A real power saver! It's worth it.	Taobao Marketplace, a Chinese website for online shopping
Black Tech Electrician & Electrician	Green Intelligent Power Saver	Haha! Fast shipping! Good service! Power Saving King is really good value for money! Hopefully effective power saving and great quality!	Taobao Marketplace, a Chinese website for online shopping
Black Tech Electrician & Electrician	Green Intelligent Power Saver	Received the product, currently in the testing phase, a month after the results can be out!	Taobao Marketplace, a Chinese website for online shopping
Black Tech Electrician & Electrician	Green Intelligent Power Saver	I haven't gotten a physical yet, so I'll comment later.	Taobao Marketplace, a Chinese website for online shopping
Black Tech Electrician & Electrician	Green Intelligent Power Saver	Power-saving effect: just received the goods, do not know how to save power, use a period of time in the follow-up comment!	Taobao Marketplace, a Chinese website for online shopping
Black Tech Electrician & Electrician	Green Intelligent Power Saver	The power saving is good and the appearance is beautiful, worth buying!	Taobao Marketplace, a Chinese website for online shopping

Black Tech Electrician & Electrician	Green Intelligent Power Saver	Very good 👍	Taobao Marketplace, a Chinese website for online shopping
Black Tech Electrician & Electrician	Green Intelligent Power Saver	Previously bought a plug in the kitchen, used for a period of time after the personal feeling is still quite effective, can save a little electricity, so this time without hesitation and bought two, has been inserted in the home next to the appliances that use a lot of electricity!	Taobao Marketplace, a Chinese website for online shopping
Black Tech Electrician & Electrician	Green Intelligent Power Saver	Power-saving effect: the power saver received, high-tech ah; too cow is very surprised, this thing can really save electricity. Before a month more than 200 electricity bill, now can save 50 dollars a month, if the home power consumption; can rest assured that the hand!	Taobao Marketplace, a Chinese website for online shopping
Black Tech Electrician & Electrician	Green Intelligent Power Saver	Buyer does not comment within 15 days	Taobao Marketplace, a Chinese website for online shopping
Black Tech Electrician & Electrician	Green Intelligent Power Saver	This fake thing, used it for a month the electricity bill is getting higher and higher, today I dismantled it and took this thing to throw it away	Taobao Marketplace, a Chinese website for online shopping
Black Tech Electrician & Electrician	Green Intelligent Power Saver	Buyer does not comment within 15 days	Taobao Marketplace, a Chinese website for online shopping
Black Tech Electrician & Electrician	Green Intelligent Power Saver	Don't fall for it. It doesn't work.	Taobao Marketplace, a Chinese website for online shopping
Black Tech Electrician & Electrician	Green Intelligent Power Saver	Buyer does not comment within 15 days	Taobao Marketplace, a Chinese website for online shopping
Black Tech Electrician & Electrician	Green Intelligent Power Saver	QR code is not recognized, one fake. Pure deception of consumers. Suggest that we do not fall for it.	Taobao Marketplace, a Chinese website for online shopping
Black Tech Electrician & Electrician	Green Intelligent Power Saver	Buyer does not comment within 15 days	Taobao Marketplace, a Chinese website for online shopping
Black Tech Electrician & Electrician	Green Intelligent Power Saver	It doesn't work. Don't buy it. It's a rip-off.	Taobao Marketplace, a Chinese website for online shopping
Black Tech Electrician & Electrician	Green Intelligent Power Saver	Buyer does not comment within 15 days	Taobao Marketplace, a Chinese website for online shopping
Black Tech Electrician & Electrician	Green Intelligent Power Saver	cheat sb.	Taobao Marketplace, a Chinese website for online shopping
Black Tech Electrician & Electrician	Green Intelligent Power Saver	Buyer does not comment within 15 days	Taobao Marketplace, a Chinese website for online shopping
Black Tech Electrician & Electrician	Green Intelligent Power Saver	System default comments	Taobao Marketplace, a Chinese website for online shopping
Black Tech Electrician & Electrician	Fully Automatic Intelligent Power Saver	It's too wide to battle with another port. Can't the other port be plugged in?	Taobao Marketplace, a Chinese website for online shopping
Black Tech Electrician & Electrician	Fully Automatic Intelligent Power Saver	Not noticeable when used	Taobao Marketplace, a Chinese website for online shopping
Black Tech Electrician & Electrician	Fully Automatic Intelligent Power Saver	ineffective	Taobao Marketplace, a Chinese website for online shopping
Black Tech Electrician & Electrician	Fully Automatic Intelligent Power Saver	Goods received, logistics is also fast appearance of good power saving before using the effect do not know, with good in the repurchase	Taobao Marketplace, a Chinese website for online shopping
Black Tech Electrician & Electrician	Fully Automatic Intelligent Power Saver	simulacrum	Taobao Marketplace, a Chinese website for online shopping
Black Tech Electrician & Electrician	Fully Automatic Intelligent Power Saver	Energy-saving power king received 6 days ago I bought one myself, is still in use experience, feel at home refrigerator and open the air conditioning noise sound is very small almost no sound, I think the energy-saving power king in January should be able to effectively save 4 to 50 yuan of electricity, it is special for my children to buy, however, the child said that to be used for a period of time to see!	Taobao Marketplace, a Chinese website for online shopping
Black Tech Electrician & Electrician	Fully Automatic Intelligent Power Saver	no good	Taobao Marketplace, a Chinese website for online shopping
Black Tech Electrician & Electrician	Fully Automatic Intelligent Power Saver	Baby received, very good use and consistent with the description, and then buy a few to use in the room as well!	Taobao Marketplace, a Chinese website for online shopping
Black Tech Electrician & Electrician	Fully Automatic Intelligent Power Saver	I don't know how to save power.	Taobao Marketplace, a Chinese website for online shopping
Black Tech Electrician & Electrician	Fully Automatic Intelligent Power Saver	Not pitching to poor reviews.	Taobao Marketplace, a Chinese website for online shopping
Black Tech Electrician & Electrician	Fully Automatic Intelligent Power Saver	Courier does not deliver to the house, bad review.	Taobao Marketplace, a Chinese website for online shopping
Black Tech Electrician & Electrician	Fully Automatic Intelligent Power Saver	It doesn't work at all, it still uses electricity, it doesn't save any electricity.	Taobao Marketplace, a Chinese website for online shopping
Black Tech Electrician & Electrician	Fully Automatic Intelligent Power Saver	Cheat play with the use of two did not work, people do not buy, save what electricity, I fell for it	Taobao Marketplace, a Chinese website for online shopping
Black Tech Electrician & Electrician	Fully Automatic Intelligent Power Saver	System default comments	Taobao Marketplace, a Chinese website for online shopping
Black Tech Electrician & Electrician	Fully Automatic Intelligent Power Saver	Received and plugged in, there is a display, quite beautiful, this price, workmanship is really good. Power-saving effect also need to use a period of time to know!	Taobao Marketplace, a Chinese website for online shopping
Black Tech Electrician & Electrician	Fully Automatic Intelligent Power Saver	It's a good power saver.	Taobao Marketplace, a Chinese website for online shopping
Black Tech Electrician & Electrician	Fully Automatic Intelligent Power Saver	Start using it. It should work.	Taobao Marketplace, a Chinese website for online shopping
Black Tech Electrician & Electrician	Fully Automatic Intelligent Power Saver	The size is just right and the quality is very good, the packaging of the store is so attentive, it is worth recommending!	Taobao Marketplace, a Chinese website for online shopping
Black Tech Electrician & Electrician	Fully Automatic Intelligent Power Saver	The logistics is also very fast service is also very good appearance looks okay hopefully good use next time to buy again	Taobao Marketplace, a Chinese website for online shopping

Black Tech Electrician & Electrician	Fully Automatic Intelligent Power Saver	Logistics is also very fast service is also very good hope good use next time to repurchase!	Taobao Marketplace, a Chinese website for online shopping
Black Tech Electrician & Electrician	Fully Automatic Intelligent Power Saver	Has been the fourth time to buy, is for a friend to buy the second time, is indeed power saving, before two months to 600 or so, this two months with 284 yuan, almost half of the savings, want to buy friends can order!	Taobao Marketplace, a Chinese website for online shopping
Black Tech Electrician & Electrician	Fully Automatic Intelligent Power Saver	Not yet used, customary praise	Taobao Marketplace, a Chinese website for online shopping
Black Tech Electrician & Electrician	Fully Automatic Intelligent Power Saver	Bad quality sells fakes and everyone who buys them is out of luck.	Taobao Marketplace, a Chinese website for online shopping
Black Tech Electrician & Electrician	Fully Automatic Intelligent Power Saver	Now that I have received the goods, it just doesn't work like the video said.	Taobao Marketplace, a Chinese website for online shopping
Black Tech Electrician & Electrician	Fully Automatic Intelligent Power Saver	New era, new technology, new products, energy saving, electricity saving, cash saving, you say good, I say good, she says good, three words "good product"! Support me!	Taobao Marketplace, a Chinese website for online shopping
Black Tech Electrician & Electrician	Fully Automatic Intelligent Power Saver	useless	Taobao Marketplace, a Chinese website for online shopping
Black Tech Electrician & Electrician	Fully Automatic Intelligent Power Saver	To be observed and then evaluated	Taobao Marketplace, a Chinese website for online shopping
Black Tech Electrician & Electrician	Fully Automatic Intelligent Power Saver	Power saving effect: good Cost-effective: good Appearance style: good Workmanship material: good Experience: can be	Taobao Marketplace, a Chinese website for online shopping
Black Tech Electrician & Electrician	Fully Automatic Intelligent Power Saver	This saver is great, affordable and I love it!	Taobao Marketplace, a Chinese website for online shopping
Black Tech Electrician & Electrician	Fully Automatic Intelligent Power Saver	Baby received, consistent with the description, give five stars!	Taobao Marketplace, a Chinese website for online shopping
Black Tech Electrician & Electrician	Fully Automatic Intelligent Power Saver	Baby received, and the description is consistent with the five-star praise!	Taobao Marketplace, a Chinese website for online shopping
Black Tech Electrician & Electrician	Fully Automatic Intelligent Power Saver	Doesn't it work at all?	Taobao Marketplace, a Chinese website for online shopping
Black Tech Electrician & Electrician	Fully Automatic Intelligent Power Saver	I wonder how it works?	Taobao Marketplace, a Chinese website for online shopping
Black Tech Electrician & Electrician	Fully Automatic Intelligent Power Saver	Got the stuff. Hope it works well. Hopefully there is a tiny bit of use. I'll follow up after I use it.	Taobao Marketplace, a Chinese website for online shopping
Black Tech Electrician & Electrician	Fully Automatic Intelligent Power Saver	to be observed	Taobao Marketplace, a Chinese website for online shopping
Black Tech Electrician & Electrician	Fully Automatic Intelligent Power Saver	general	Taobao Marketplace, a Chinese website for online shopping
Black Tech Electrician & Electrician	Fully Automatic Intelligent Power Saver	Received on the socket to try to see if there is no effect, the business said that the effect of a month, when the effect of additional positive feedback, and vice versa, a long bad review!	Taobao Marketplace, a Chinese website for online shopping
Black Tech Electrician & Electrician	Fully Automatic Intelligent Power Saver	The quality of the product is excellent and the energy savings are great.	Taobao Marketplace, a Chinese website for online shopping
Black Tech Electrician & Electrician	Fully Automatic Intelligent Power Saver	ineffective	Taobao Marketplace, a Chinese website for online shopping
Black Tech Electrician & Electrician	Fully Automatic Intelligent Power Saver	The results are still being tested	Taobao Marketplace, a Chinese website for online shopping
Black Tech Electrician & Electrician	Fully Automatic Intelligent Power Saver	I don't know if it's energy efficient	Taobao Marketplace, a Chinese website for online shopping
Black Tech Electrician & Electrician	Fully Automatic Intelligent Power Saver	No power saving reverse consumption	Taobao Marketplace, a Chinese website for online shopping
Black Tech Electrician & Electrician	Fully Automatic Intelligent Power Saver	Workmanship material: okay	Taobao Marketplace, a Chinese website for online shopping
Black Tech Electrician & Electrician	Fully Automatic Intelligent Power Saver	Power saving effect: the big liar	Taobao Marketplace, a Chinese website for online shopping
Black Tech Electrician & Electrician	Fully Automatic Intelligent Power Saver	Expect it to work well	Taobao Marketplace, a Chinese website for online shopping
Black Tech Electrician & Electrician	Fully Automatic Intelligent Power Saver	Great. Let's see if we can save power.	Taobao Marketplace, a Chinese website for online shopping
Black Tech Electrician & Electrician	Fully Automatic Intelligent Power Saver	High efficiency, power saving rate is very good, expect a month to save a hundred degrees of electricity	Taobao Marketplace, a Chinese website for online shopping
Black Tech Electrician & Electrician	Fully Automatic Intelligent Power Saver	Looking good. Looking forward to good results	Taobao Marketplace, a Chinese website for online shopping
Black Tech Electrician & Electrician	Fully Automatic Intelligent Power Saver	It's not as good as it says.	Taobao Marketplace, a Chinese website for online shopping
Black Tech Electrician & Electrician	Fully Automatic Intelligent Power Saver	I received the product and found that the product was not the same as the picture advertising the appearance.	Taobao Marketplace, a Chinese website for online shopping
Black Tech Electrician & Electrician	Fully Automatic Intelligent Power Saver	It doesn't save electricity at all. It's a scam.	Taobao Marketplace, a Chinese website for online shopping
Black Tech Electrician & Electrician	Fully Automatic Intelligent Power Saver	I bought it and used it. It didn't work.	Taobao Marketplace, a Chinese website for online shopping
Black Tech Electrician & Electrician	Fully Automatic Intelligent Power Saver	Power saving effect: very saving Appearance style: perfect good-looking Experience: bought last year in the home has been used, very good, next time will patronize, will recommend to my friends.	Taobao Marketplace, a Chinese website for online shopping

Black Tech Electrician & Electrician	Fully Automatic Intelligent Power Saver	The stuff is great, the courier guy has great service	Taobao Marketplace, a Chinese website for online shopping
Black Tech Electrician & Electrician	Fully Automatic Intelligent Power Saver	Customer service is enthusiastic, logistics is fast, good value for money.	Taobao Marketplace, a Chinese website for online shopping
Black Tech Electrician & Electrician	Fully Automatic Intelligent Power Saver	Customer service is enthusiastic, logistics is fast, good value for money.	Taobao Marketplace, a Chinese website for online shopping
Black Tech Electrician & Electrician	Fully Automatic Intelligent Power Saver	Experience: the feel is very light and empty box, I do not know if it can save electricity, first plugged in to see the next month's electricity bill.	Taobao Marketplace, a Chinese website for online shopping
Black Tech Electrician & Electrician	Fully Automatic Intelligent Power Saver	Experience: the description matches, use it to know the effect of the	Taobao Marketplace, a Chinese website for online shopping
Black Tech Electrician & Electrician	Fully Automatic Intelligent Power Saver	Baby received ten days, the use of the effect is not bad, there is a power-saving function, satisfied. I will recommend to friends.	Taobao Marketplace, a Chinese website for online shopping
Black Tech Electrician & Electrician	Fully Automatic Intelligent Power Saver	Cost-effective: affordable in use do not know the effect of how to come back later to follow up. Compact and exquisite style does not take up space plant	Taobao Marketplace, a Chinese website for online shopping
Black Tech Electrician & Electrician	Fully Automatic Intelligent Power Saver	The power saving effect is very good, especially now the summer power consumption peak effect is better.	Taobao Marketplace, a Chinese website for online shopping
Black Tech Electrician & Electrician	Fully Automatic Intelligent Power Saver	I didn't feel any power savings after using it for so long.	Taobao Marketplace, a Chinese website for online shopping
Black Tech Electrician & Electrician	Fully Automatic Intelligent Power Saver	Baby is awesome and decides to work well! Beautiful and durable!	Taobao Marketplace, a Chinese website for online shopping
Black Tech Electrician & Electrician	Fully Automatic Intelligent Power Saver	Appearance and style: s outside like good-looking not yet used on but looks good must be official use	Taobao Marketplace, a Chinese website for online shopping
Black Tech Electrician & Electrician	Fully Automatic Intelligent Power Saver	Cost-effective: good goods at a good price Experience: baby received the goods on the use of the real power-saving and good price is worth buying	Taobao Marketplace, a Chinese website for online shopping
Black Tech Electrician & Electrician	Fully Automatic Intelligent Power Saver	In use, to have no effect on the follow-up evaluation! Satisfied with good reviews first!	Taobao Marketplace, a Chinese website for online shopping
Black Tech Electrician & Electrician	Fully Automatic Intelligent Power Saver	I just received it. I haven't used it yet.	Taobao Marketplace, a Chinese website for online shopping
Black Tech Electrician & Electrician	Fully Automatic Intelligent Power Saver	It's not very effective.	Taobao Marketplace, a Chinese website for online shopping
Black Tech Electrician & Electrician	Fully Automatic Intelligent Power Saver	Power-saving effect: just used, should be able to Cost-effective: favorable Appearance and style: good-looking Workmanship material: very good Experience: to be followed up	Taobao Marketplace, a Chinese website for online shopping
Black Tech Electrician & Electrician	Fully Automatic Intelligent Power Saver	Goods received well packaged I am very satisfied next time to buy again	Taobao Marketplace, a Chinese website for online shopping
Black Tech Electrician & Electrician	Fully Automatic Intelligent Power Saver	It's not working.	Taobao Marketplace, a Chinese website for online shopping
Black Tech Electrician & Electrician	Fully Automatic Intelligent Power Saver	More than ten dollars, not at all useful, customer service: also said two months later, no effect on the refund, today looked at, now people this commodity display does not exist, purely fooling people, plug in the meter go faster!	Taobao Marketplace, a Chinese website for online shopping
Black Tech Electrician & Electrician	Fully Automatic Intelligent Power Saver	First bad review, scam, just a regular circuit. No power savings at all.	Taobao Marketplace, a Chinese website for online shopping
Black Tech Electrician & Electrician	Fully Automatic Intelligent Power Saver	I haven't used it yet. I'll comment later.	Taobao Marketplace, a Chinese website for online shopping
Black Tech Electrician & Electrician	Fully Automatic Intelligent Power Saver	Received, well-packaged logistics quickly, and will use to see how the effect of	Taobao Marketplace, a Chinese website for online shopping
Black Tech Electrician & Electrician	Fully Automatic Intelligent Power Saver	Power saving effect: not so good	Taobao Marketplace, a Chinese website for online shopping
Black Tech Electrician & Electrician	Fully Automatic Intelligent Power Saver	I don't know yet, but I can't say anything else about the power-saving effect until later, when I say that I'll use it for a while.	Taobao Marketplace, a Chinese website for online shopping
Black Tech Electrician & Electrician	Fully Automatic Intelligent Power Saver	How to buy the goods do not have a factory address, there is no 3C certification used for a few days without effect do not know how long with the effect of the	Taobao Marketplace, a Chinese website for online shopping
Black Tech Electrician & Electrician	Fully Automatic Intelligent Power Saver	We'll see what happens in a few years!	Taobao Marketplace, a Chinese website for online shopping
Black Tech Electrician & Electrician	Fully Automatic Intelligent Power Saver	Power saving effect: 1 fake	Taobao Marketplace, a Chinese website for online shopping
Black Tech Electrician & Electrician	Fully Automatic Intelligent Power Saver	give it a try	Taobao Marketplace, a Chinese website for online shopping
Black Tech Electrician & Electrician	Fully Automatic Intelligent Power Saver	Baby received and it's great! Consistent with the description. Giving a 5 star review!	Taobao Marketplace, a Chinese website for online shopping
Black Tech Electrician & Electrician	Fully Automatic Intelligent Power Saver	I haven't used it yet. I'll comment later.	Taobao Marketplace, a Chinese website for online shopping
Black Tech Electrician & Electrician	Fully Automatic Intelligent Power Saver	The Smart Power Saver works great and saves power. Good use and very affordable. The seller has a good service attitude. Thanks to the seller!	Taobao Marketplace, a Chinese website for online shopping
Black Tech Electrician & Electrician	Fully Automatic Intelligent Power Saver	Baby just received, it's great 👌👍 use it right away, the courier guy is great, I will continue to support you guys.	Taobao Marketplace, a Chinese website for online shopping
Black Tech Electrician & Electrician	Fully Automatic Intelligent Power Saver	Sent me a defective product, looking for customer service does not answer my questions positively, can not be used, is a black business, people do not fall for the scam in the	Taobao Marketplace, a Chinese website for online shopping
Black Tech Electrician & Electrician	Fully Automatic Intelligent Power Saver	It's useless.	Taobao Marketplace, a Chinese website for online shopping

Black Tech Electrician & Electrician	Fully Automatic Intelligent Power Saver	I've only been using it for a few days and I don't know the results yet	Taobao Marketplace, a Chinese website for online shopping
Black Tech Electrician & Electrician	Fully Automatic Intelligent Power Saver	Experience: just started to use do not know whether it is good or not, use a period of time to speak again	Taobao Marketplace, a Chinese website for online shopping
Black Tech Electrician & Electrician	Fully Automatic Intelligent Power Saver	Families inserted into the power saver for half a month, electricity savings are as follows: last year, the highest monthly electricity bill of 500 yuan. Last month, i.e. July, the electricity bill was 590 yuan, and after using the energy saver for about half a month, the electricity bill was 946 yuan in August, and now I don't dare to use the energy saver	Taobao Marketplace, a Chinese website for online shopping
Black Tech Electrician & Electrician	Fully Automatic Intelligent Power Saver	Just received, fast delivery, good packaging, looking forward to this baby can be value for money, have not yet used, but I think it will not let me down, to be used after a follow-up comment.	Taobao Marketplace, a Chinese website for online shopping
Black Tech Electrician & Electrician	Fully Automatic Intelligent Power Saver	Doesn't work, doesn't save power just an indicator light inside, too fake. Power saving effect: no	Taobao Marketplace, a Chinese website for online shopping
Black Tech Electrician & Electrician	Fully Automatic Intelligent Power Saver	Received in a timely manner, and the promotional introduction is consistent with the quality of the appearance is very formal, the material is also good, I think the effect is also good, it is worth trusting and buying.	Taobao Marketplace, a Chinese website for online shopping
Black Tech Electrician & Electrician	Fully Automatic Intelligent Power Saver	Power saving effect: 1 Power saving. Cost-effective. New style. Fine workmanship. Ideal using experience.	Taobao Marketplace, a Chinese website for online shopping
Black Tech Electrician & Electrician	Fully Automatic Intelligent Power Saver	Appearance and style:, logistics is very fast, fine workmanship, good service, after using the follow-up comment.	Taobao Marketplace, a Chinese website for online shopping
Black Tech Electrician & Electrician	Fully Automatic Intelligent Power Saver	No national standard, no manufacturer and address, no registration by scanning QR code with WeChat and Alipay.	Taobao Marketplace, a Chinese website for online shopping
Black Tech Electrician & Electrician	Fully Automatic Intelligent Power Saver	Value for money: very good Workmanship material: fine	Taobao Marketplace, a Chinese website for online shopping
Black Tech Electrician & Electrician	Fully Automatic Intelligent Power Saver	Two shots in a row! Very good! Very good! A real power saver! It's worth it.	Taobao Marketplace, a Chinese website for online shopping
Black Tech Electrician & Electrician	Fully Automatic Intelligent Power Saver	Haha! Fast shipping! Good service! Power Saving King is really good value for money! Hopefully effective power saving and great quality!	Taobao Marketplace, a Chinese website for online shopping
Black Tech Electrician & Electrician	Fully Automatic Intelligent Power Saver	Received the product, currently in the testing phase, a month after the results can be out!	Taobao Marketplace, a Chinese website for online shopping
Black Tech Electrician & Electrician	Fully Automatic Intelligent Power Saver	I haven't gotten a physical yet, so I'll comment later.	Taobao Marketplace, a Chinese website for online shopping
Black Tech Electrician & Electrician	Fully Automatic Intelligent Power Saver	Power-saving effect: just received the goods, do not know how to save power, use a period of time in the follow-up comment!	Taobao Marketplace, a Chinese website for online shopping
Black Tech Electrician & Electrician	Fully Automatic Intelligent Power Saver	The power saving is good and the appearance is beautiful, worth buying!	Taobao Marketplace, a Chinese website for online shopping
Black Tech Electrician & Electrician	Fully Automatic Intelligent Power Saver	Very good 👍	Taobao Marketplace, a Chinese website for online shopping
Black Tech Electrician & Electrician	Fully Automatic Intelligent Power Saver	Previously bought a plug in the kitchen, used for a period of time after the personal feeling is still quite effective, can save a little electricity, so this time without hesitation and bought two, has been inserted in the home next to the appliances that use a lot of electricity!	Taobao Marketplace, a Chinese website for online shopping
Black Tech Electrician & Electrician	Fully Automatic Intelligent Power Saver	Power-saving effect: the power saver received, high-tech ah; too cow is very surprised, this thing can really save electricity. Before a month more than 200 electricity bill, now can save 50 dollars a month, if the home power consumption; can rest assured that the hand!	Taobao Marketplace, a Chinese website for online shopping
Black Tech Electrician & Electrician	Fully Automatic Intelligent Power Saver	Buyer does not comment within 15 days	Taobao Marketplace, a Chinese website for online shopping
Black Tech Electrician & Electrician	Fully Automatic Intelligent Power Saver	This fake thing, used it for a month the electricity bill is getting higher and higher, today I dismantled it and took this thing to throw it away	Taobao Marketplace, a Chinese website for online shopping
Black Tech Electrician & Electrician	Fully Automatic Intelligent Power Saver	Buyer does not comment within 15 days	Taobao Marketplace, a Chinese website for online shopping
Black Tech Electrician & Electrician	Fully Automatic Intelligent Power Saver	Don't fall for it. It doesn't work.	Taobao Marketplace, a Chinese website for online shopping
Black Tech Electrician & Electrician	Fully Automatic Intelligent Power Saver	Buyer does not comment within 15 days	Taobao Marketplace, a Chinese website for online shopping
Black Tech Electrician & Electrician	Fully Automatic Intelligent Power Saver	QR code is not recognized, one fake. Pure deception of consumers. Suggest that we do not fall for it.	Taobao Marketplace, a Chinese website for online shopping
Black Tech Electrician & Electrician	Fully Automatic Intelligent Power Saver	Buyer does not comment within 15 days	Taobao Marketplace, a Chinese website for online shopping
Black Tech Electrician & Electrician	Fully Automatic Intelligent Power Saver	It doesn't work. Don't buy it. It's a rip-off.	Taobao Marketplace, a Chinese website for online shopping
Black Tech Electrician & Electrician	Fully Automatic Intelligent Power Saver	Buyer does not comment within 15 days	Taobao Marketplace, a Chinese website for online shopping
Black Tech Electrician & Electrician	Fully Automatic Intelligent Power Saver	cheat sb.	Taobao Marketplace, a Chinese website for online shopping
Black Tech Electrician & Electrician	Fully Automatic Intelligent Power Saver	Buyer does not comment within 15 days	Taobao Marketplace, a Chinese website for online shopping
Black Tech Electrician & Electrician	Fully Automatic Intelligent Power Saver	System default comments	Taobao Marketplace, a Chinese website for online shopping
Black Tech Electrician & Electrician	White Intelligent Power Saver	Power-saving effect: good products can not be separated from the integrity of the business, the goods have been received, no problem, code scanning to verify that it is genuine, this product is quite a lot of friends around me to buy, but there are fakes, this store I feel reliable, has recommended friends around, there have been some people back to buy, a good product is the hard truth! Merchants do not fake	Taobao Marketplace, a Chinese website for online shopping
Black Tech Electrician & Electrician	a power saver (electronics)	Well made, no burrs, just used it, looking forward to good use.	Taobao Marketplace, a Chinese website for online shopping

Black Tech Electrician & Electrician	a power saver (electronics)	I don't know if it's energy efficient.	Taobao Marketplace, a Chinese website for online shopping
Black Tech Electrician & Electrician	a power saver (electronics)	Power-saving effect: do not blow no black, real experience down, the power saver still has a certain effect, with2, before a month of electricity is810yuan, now a month of680yuan, power-saving effect is still visible, such as our home power consumption is relatively large, a year down to save1,000should not be too big a problem, small things of great utility!	Taobao Marketplace, a Chinese website for online shopping
Jinfeng Hongyuan Flagship Store	Yellow Save Electricity	The machine was received and looks exquisite. Simple to use, has been used. The later effect, with time to test.	Taobao Marketplace, a Chinese website for online shopping
Jinfeng Hongyuan Flagship Store	Yellow Save Electricity	Fast logistics, good service attitude, product quality is very good	Taobao Marketplace, a Chinese website for online shopping
Jinfeng Hongyuan Flagship Store	Yellow Save Electricity	Thanks to the seller's careful guidance installed and deliberately observed for a period of time Indeed power saving current is also very stable value for money ah!	Taobao Marketplace, a Chinese website for online shopping
Jinfeng Hongyuan Flagship Store	Yellow Save Electricity	I'll comment again after I use it	Taobao Marketplace, a Chinese website for online shopping
Jinfeng Hongyuan Flagship Store	Yellow Save Electricity	Very good, received it and used it right away.	Taobao Marketplace, a Chinese website for online shopping
Jinfeng Hongyuan Flagship Store	Yellow Save Electricity	Practical Very energy-saving price is also suitable Suggest you know about it	Taobao Marketplace, a Chinese website for online shopping
Jinfeng Hongyuan Flagship Store	Yellow Save Electricity	Energy Saving and Power Saving King I've already used it and I'm looking forward to the effect Let's see if there's any effect after a month	Taobao Marketplace, a Chinese website for online shopping
Jinfeng Hongyuan Flagship Store	Yellow Save Electricity	The goods have just been received, connected to the normal use, compact, easy to place, the	Taobao Marketplace, a Chinese website for online shopping
Jinfeng Hongyuan Flagship Store	Yellow Save Electricity	The product is packaged exquisitely and has a beautiful appearance, very practical	Taobao Marketplace, a Chinese website for online shopping
Jinfeng Hongyuan Flagship Store	Yellow Save Electricity	The product has been received, it looks beautiful and is of good quality, I look forward to achieving the desired results.	Taobao Marketplace, a Chinese website for online shopping
Jinfeng Hongyuan Flagship Store	Yellow Save Electricity	The production is very beautiful, power saving should not talk about, at best it is a voltage digital display bar.	Taobao Marketplace, a Chinese website for online shopping
Jinfeng Hongyuan Flagship Store	Yellow Save Electricity	The stuff has been received, the store is very good, I hope it can save electricity	Taobao Marketplace, a Chinese website for online shopping
Jinfeng Hongyuan Flagship Store	Yellow Save Electricity	Stable voltage, no noise, the appliance is good, running normally, customer service is very patient, use a period of time to see the effect, now feel good!	Taobao Marketplace, a Chinese website for online shopping
Jinfeng Hongyuan Flagship Store	Yellow Save Electricity	Baby quality is very good, fine workmanship, worth buying	Taobao Marketplace, a Chinese website for online shopping
Jinfeng Hongyuan Flagship Store	Yellow Save Electricity	Very happy to receive it and the color looks great very positive The family loves it looking forward to achieving the desired results looking forward looking forward looking forward!	Taobao Marketplace, a Chinese website for online shopping
Jinfeng Hongyuan Flagship Store	Yellow Save Electricity	Received, quite satisfied, with good, bar, quite good	Taobao Marketplace, a Chinese website for online shopping
Jinfeng Hongyuan Flagship Store	Yellow Save Electricity	The product received the color blue very good looking will come again!	Taobao Marketplace, a Chinese website for online shopping
Jinfeng Hongyuan Flagship Store	Yellow Save Electricity	The stuff shipment arrived and'looked great, good quality!	Taobao Marketplace, a Chinese website for online shopping
Jinfeng Hongyuan Flagship Store	Yellow Save Electricity	Installed next to the main switch in the house a little bit of effect is not ！！！！ Don't buy it!!!	Taobao Marketplace, a Chinese website for online shopping
Jinfeng Hongyuan Flagship Store	Yellow Save Electricity	The quality of the stuff is okay and the speed of the delivery is also very powerful	Taobao Marketplace, a Chinese website for online shopping
Jinfeng Hongyuan Flagship Store	Yellow Save Electricity	Power saving treasure received, good-looking design, voltage at a glance, for power saving recommended purchase	Taobao Marketplace, a Chinese website for online shopping
Jinfeng Hongyuan Flagship Store	Yellow Save Electricity	Listening to friends, this special power saving, but the effect of how to wait until next month to know, follow-up in the feedback to you. Should be able to, friends recommended several times!	Taobao Marketplace, a Chinese website for online shopping
Jinfeng Hongyuan Flagship Store	Yellow Save Electricity	I'll comment after I use it.	Taobao Marketplace, a Chinese website for online shopping
Jinfeng Hongyuan Flagship Store	Yellow Save Electricity	The logistics were quick and easy to install, it runs fine and everything went well!	Taobao Marketplace, a Chinese website for online shopping
Jinfeng Hongyuan Flagship Store	Yellow Save Electricity	Great quality and fast shipping! Very satisfied!	Taobao Marketplace, a Chinese website for online shopping
Jinfeng Hongyuan Flagship Store	Yellow Save Electricity	Smart model, pretty good and easy to operate, good quality👌good	Taobao Marketplace, a Chinese website for online shopping
Jinfeng Hongyuan Flagship Store	Yellow Save Electricity	It didn't work at all.	Taobao Marketplace, a Chinese website for online shopping
Jinfeng Hongyuan Flagship Store	Yellow Save Electricity	It's a lie.	Taobao Marketplace, a Chinese website for online shopping
Jinfeng Hongyuan Flagship Store	Yellow Save Electricity	Purchased for the third time! Works great with it! It does stabilize the voltage current saving electricity!	Taobao Marketplace, a Chinese website for online shopping
Jinfeng Hongyuan Flagship Store	Yellow Save Electricity	This saving appliance with half a month, it seems to have a little power saving, continue to use a period of time with a month or two and then see the effect	Taobao Marketplace, a Chinese website for online shopping
Jinfeng Hongyuan Flagship Store	Yellow Save Electricity	ineffective	Taobao Marketplace, a Chinese website for online shopping
Jinfeng Hongyuan Flagship Store	Yellow Save Electricity	Don't buy it. is an IQ tax	Taobao Marketplace, a Chinese website for online shopping

Jinfeng Hongyuan Flagship Store	Yellow Save Electricity	Very like oh, get home can't wait to use look forward to the effect with a period of time to come back to review yo, good use and then order a	Taobao Marketplace, a Chinese website for online shopping
Jinfeng Hongyuan Flagship Store	Yellow Save Electricity	Baby received, used two days feel very good, feel the energy saving, the follow-up will follow up evaluation	Taobao Marketplace, a Chinese website for online shopping
Jinfeng Hongyuan Flagship Store	Yellow Save Electricity	The power saving has been received, the appearance is beautiful, I have already purchased the second time to use down the effect is very good.	Taobao Marketplace, a Chinese website for online shopping
Jinfeng Hongyuan Flagship Store	Yellow Save Electricity	So far, plugged in the voltage is more stable, as for the effect of how to quietly wait until the payment of the electricity bill to compare, the appearance of the commodity design is very good, the real thing!	Taobao Marketplace, a Chinese website for online shopping
Jinfeng Hongyuan Flagship Store	Yellow Save Electricity	I bought one before and this is the second one, I will buy it again if I need it!	Taobao Marketplace, a Chinese website for online shopping
Jinfeng Hongyuan Flagship Store	Yellow Save Electricity	The product was received and still gives a good impression. Use a period of time to see the energy saving situation and then follow up.	Taobao Marketplace, a Chinese website for online shopping
Jinfeng Hongyuan Flagship Store	Yellow Save Electricity	This power saver really works well, because I installed one at home about seven or eight years ago, which time there is no voltage display, now there is a voltage display, the appearance also looks much better, the electricity bill is also saved, the stability of the home appliances is also good, the best of both worlds, power saving and worrying about the regularity of the purchase will be installed in the store now	Taobao Marketplace, a Chinese website for online shopping
Jinfeng Hongyuan Flagship Store	Yellow Save Electricity	The physical product is consistent with the description, the product quality is very good looking forward to the effect	Taobao Marketplace, a Chinese website for online shopping
Jinfeng Hongyuan Flagship Store	Yellow Save Electricity	The product is well made and the service attitude is very good, looking forward to the effect	Taobao Marketplace, a Chinese website for online shopping
Jinfeng Hongyuan Flagship Store	Yellow Save Electricity	This is a great product, bought it and used it	Taobao Marketplace, a Chinese website for online shopping
Jinfeng Hongyuan Flagship Store	Yellow Save Electricity	Second time I bought it. That cheap one is purely unusable. Still have to buy the expensive one.	Taobao Marketplace, a Chinese website for online shopping
Jinfeng Hongyuan Flagship Store	Yellow Save Electricity	Good looking, good quality power saving bar quite large capacity nice nice nice👍	Taobao Marketplace, a Chinese website for online shopping
Jinfeng Hongyuan Flagship Store	Yellow Save Electricity	Received the goods, the use of hope can get the desired results, logistics is also very fast and powerful!	Taobao Marketplace, a Chinese website for online shopping
Jinfeng Hongyuan Flagship Store	Yellow Save Electricity	Having bought it a couple times already.	Taobao Marketplace, a Chinese website for online shopping
Jinfeng Hongyuan Flagship Store	Yellow Save Electricity	The product is very practical, the power saving effect, is also very obvious. It is worth buying!	Taobao Marketplace, a Chinese website for online shopping
Jinfeng Hongyuan Flagship Store	Yellow Save Electricity	Nice and good Repeat purchase Very power efficient	Taobao Marketplace, a Chinese website for online shopping
Jinfeng Hongyuan Flagship Store	Yellow Save Electricity	Appearance is very beautiful, with the mindset of buying to try, I did not expect to really save electricity, hesitant baby can start	Taobao Marketplace, a Chinese website for online shopping
Jinfeng Hongyuan Flagship Store	Yellow Save Electricity	Power Saving Sharpener received, product certification, accessories and other complete, good quality, very good workmanship, recommended to buy.	Taobao Marketplace, a Chinese website for online shopping
Jinfeng Hongyuan Flagship Store	Yellow Save Electricity	Very good, fast logistics, good quality very practical.	Taobao Marketplace, a Chinese website for online shopping
Jinfeng Hongyuan Flagship Store	Yellow Save Electricity	rare	Taobao Marketplace, a Chinese website for online shopping
Jinfeng Hongyuan Flagship Store	Yellow Save Electricity	The product is very well packaged, timely delivery, good service, good results, it is the preferred product.	Taobao Marketplace, a Chinese website for online shopping
Jinfeng Hongyuan Flagship Store	Yellow Save Electricity	False, it doesn't save any power at all	Taobao Marketplace, a Chinese website for online shopping
Jinfeng Hongyuan Flagship Store	Yellow Save Electricity	Fake doesn't work.	Taobao Marketplace, a Chinese website for online shopping
Jinfeng Hongyuan Flagship Store	Yellow Save Electricity	Power Saving King works well	Taobao Marketplace, a Chinese website for online shopping
Jinfeng Hongyuan Flagship Store	Yellow Save Electricity	Great quality for a first time buy, a trustworthy store with quality assurance	Taobao Marketplace, a Chinese website for online shopping
Jinfeng Hongyuan Flagship Store	Yellow Save Electricity	Goods have been received, used a few days, have not seen the effect, in some time in the look, the effect is good after the follow-up comment!	Taobao Marketplace, a Chinese website for online shopping
Jinfeng Hongyuan Flagship Store	Yellow Save Electricity	This item is quality assured, effective and trustworthy.	Taobao Marketplace, a Chinese website for online shopping
Jinfeng Hongyuan Flagship Store	Yellow Save Electricity	Very fast logistics, great customer service, good quality!	Taobao Marketplace, a Chinese website for online shopping
Jinfeng Hongyuan Flagship Store	Yellow Save Electricity	Energy savers work.	Taobao Marketplace, a Chinese website for online shopping
Jinfeng Hongyuan Flagship Store	Yellow Save Electricity	Quality is good, the shell is beautiful and generous, used a few days, feel okay, more days to observe, have the effect of continue to buy	Taobao Marketplace, a Chinese website for online shopping
Jinfeng Hongyuan Flagship Store	Yellow Save Electricity	using it for days It works great Living Electricity Saves several kWh per day Worth the price	Taobao Marketplace, a Chinese website for online shopping
Jinfeng Hongyuan Flagship Store	Yellow Save Electricity	It's a scam, tried it for a month. It didn't work at all, my friend who bought it take note, don't fall for it again	Taobao Marketplace, a Chinese website for online shopping
Jinfeng Hongyuan Flagship Store	Yellow Save Electricity	Baby received, has been used for a week, quite amazing, just for summer use, open the air conditioning more, but also plan to buy more a	Taobao Marketplace, a Chinese website for online shopping
Jinfeng Hongyuan Flagship Store	Yellow Save Electricity	Great quality, very fast shipping, satisfied!	Taobao Marketplace, a Chinese website for online shopping

Jinfeng Hongyuan Flagship Store	Yellow Save Electricity	Quality is very good, use a period of time in the follow-up comment Express very quickly!	Taobao Marketplace, a Chinese website for online shopping
Jinfeng Hongyuan Flagship Store	Yellow Save Electricity	Looking at the packaging is exquisite, expect quality five stars oh!	Taobao Marketplace, a Chinese website for online shopping
Jinfeng Hongyuan Flagship Store	Yellow Save Electricity	I've received the baby, it's great, very well packaged, I like it very much!	Taobao Marketplace, a Chinese website for online shopping
Jinfeng Hongyuan Flagship Store	Yellow Save Electricity	This baby, very good workmanship, packaging is also good, I very much like a shopping!	Taobao Marketplace, a Chinese website for online shopping
Jinfeng Hongyuan Flagship Store	Yellow Save Electricity	I received it a few days ago It looks good too I got busy and I'm giving it a good review now	Taobao Marketplace, a Chinese website for online shopping
Jinfeng Hongyuan Flagship Store	Yellow Save Electricity	I don't know if I can save it, so let's use it and see.	Taobao Marketplace, a Chinese website for online shopping
Jinfeng Hongyuan Flagship Store	Yellow Save Electricity	Save a bunch of money....Instead of using this thing for a month the electric bill is about a hundred bucks more than usual😠😠pitiful thing😂😂😂unplug it👯	Taobao Marketplace, a Chinese website for online shopping
Jinfeng Hongyuan Flagship Store	Yellow Save Electricity	Bought it for a family member, the feedback was especially good, said it worked well, recommend🙏	Taobao Marketplace, a Chinese website for online shopping
Jinfeng Hongyuan Flagship Store	Yellow Save Electricity	Item received, lightweight and beautiful. Hopefully it will save a lot of electricity.	Taobao Marketplace, a Chinese website for online shopping
Jinfeng Hongyuan Flagship Store	Yellow Save Electricity	The quality is good, the appearance is beautiful, you can start if you like, and the energy saving effect is also good!	Taobao Marketplace, a Chinese website for online shopping
Jinfeng Hongyuan Flagship Store	Yellow Save Electricity	Indeed energy saving haha👍👍👍👍🌹🌹🌹🌹Quality first👍👍👍🌹🌹🌹🌹🌹🌹🌹🌹🌹🌹🌹🌹🌹🌹🌹🌹🌹🌹🌹🌹🌹🌹🌹🌹🌹🌹🌹🌹🌹🌹🌹🌹🌹🌹🌹🌹👍👍♡	Taobao Marketplace, a Chinese website for online shopping
Jinfeng Hongyuan Flagship Store	Yellow Save Electricity	Things are good, bought on the plug to try, really plug thick, the meter turntable slows down, good good	Taobao Marketplace, a Chinese website for online shopping
Jinfeng Hongyuan Flagship Store	Yellow Save Electricity	I just got it, it looks like it's well packaged, it's pretty good, I hope it works.	Taobao Marketplace, a Chinese website for online shopping
Jinfeng Hongyuan Flagship Store	Yellow Save Electricity	Just received the goods and rushed to try, originally did not believe in this, but ended up using it for a day, I feel that this thing is really magical!	Taobao Marketplace, a Chinese website for online shopping
Jinfeng Hongyuan Flagship Store	Yellow Save Electricity	The quality looks okay, after two days of use seems to be able to save a little bit of electricity, looking forward to better results!	Taobao Marketplace, a Chinese website for online shopping
Jinfeng Hongyuan Flagship Store	Yellow Save Electricity	It will take a few months of electricity usage to make an evaluation, and it's a little early to make that evaluation.	Taobao Marketplace, a Chinese website for online shopping
Jinfeng Hongyuan Flagship Store	Yellow Save Electricity	Does it save electricity or not?	Taobao Marketplace, a Chinese website for online shopping
Jinfeng Hongyuan Flagship Store	Yellow Save Electricity	Baby to received, workmanship is very fine, logistics is very strong.	Taobao Marketplace, a Chinese website for online shopping
Jinfeng Hongyuan Flagship Store	Yellow Save Electricity	This saver works well, quite like it, well packaged this saver is worth buying! Will buy again!	Taobao Marketplace, a Chinese website for online shopping
Jinfeng Hongyuan Flagship Store	Yellow Save Electricity	new generation of home energy saver is sure to save electricity and also play a role in stabilizing the voltage very good products	Taobao Marketplace, a Chinese website for online shopping
Jinfeng Hongyuan Flagship Store	Yellow Save Electricity	Use it, and if it doesn't work, file a complaint for selling a fraudulent product	Taobao Marketplace, a Chinese website for online shopping
Jinfeng Hongyuan Flagship Store	Yellow Save Electricity	We must not believe, are liars! Inside are resistors, junior high school students do experiments with, a few dollars to sell dozens of dollars, there is no power saving effect. Who buy who fooled!!!	Taobao Marketplace, a Chinese website for online shopping
Jinfeng Hongyuan Flagship Store	Yellow Save Electricity	Very nice and perfectly packaged, very nice	Taobao Marketplace, a Chinese website for online shopping
Jinfeng Hongyuan Flagship Store	Yellow Save Electricity	I just started using it and I don't know how it is yet, I'll follow up after ten days.	Taobao Marketplace, a Chinese website for online shopping
Jinfeng Hongyuan Flagship Store	Yellow Save Electricity	Didn't work. Voltage shows how21. useless, waste of60dollars	Taobao Marketplace, a Chinese website for online shopping
Jinfeng Hongyuan Flagship Store	Yellow Save Electricity	Genuine, on trial!	Taobao Marketplace, a Chinese website for online shopping
Jinfeng Hongyuan Flagship Store	Yellow Save Electricity	Perfectly packaged and fully equipped. Detects voltage at all times, really convenient, use it well and repurchase......	Taobao Marketplace, a Chinese website for online shopping
Jinfeng Hongyuan Flagship Store	Yellow Save Electricity	The real thing is very good, power saving does save electricity, a family uses1degree of electricitya night.	Taobao Marketplace, a Chinese website for online shopping
Jinfeng Hongyuan Flagship Store	Yellow Save Electricity	This baby is supposed to save electricity, a friend has been buying them for years and highly recommends them!	Taobao Marketplace, a Chinese website for online shopping
Jinfeng Hongyuan Flagship Store	Yellow Save Electricity	Can't save electricity. It's just flimsy.	Taobao Marketplace, a Chinese website for online shopping
Jinfeng Hongyuan Flagship Store	Yellow Save Electricity	Energy saving and power saving received, I personally feel that a small very good-looking, product power saving effect now do not know, just use, look forward to, just look at the seller's service attitude is very good, shipping very quickly, feel worth buying, try after all, the summer is still very long, air conditioning every day to open more than ten hours, look forward to power saving, I wish the seller a	Taobao Marketplace, a Chinese website for online shopping
Jinfeng Hongyuan Flagship Store	Yellow Save Electricity	Has been used for a week, very good👍👍👍👍, the quality of the weather to withstand thunderstorms, it is said to have to be used for a long time to have effect	Taobao Marketplace, a Chinese website for online shopping
Jinfeng Hongyuan Flagship Store	Yellow Save Electricity	Appearance is still quite atmospheric, small and exquisite, came back to use it, looking forward to good results, after a month of use will come to follow up!😄	Taobao Marketplace, a Chinese website for online shopping
Jinfeng Hongyuan Flagship Store	Yellow Save Electricity	We'll see how it goes.	Taobao Marketplace, a Chinese website for online shopping

Jinfeng Hongyuan Flagship Store	Yellow Save Electricity	It's not energy efficient at all.	Taobao Marketplace, a Chinese website for online shopping
Jinfeng Hongyuan Flagship Store	Yellow Save Electricity	Packaging is very good The product certificate of conformity is complete Saving electricity can save a lot👍👍👍👍	Taobao Marketplace, a Chinese website for online shopping
Jinfeng Hongyuan Flagship Store	Yellow Save Electricity	Intelligent energy saver received logistics very quickly stuff is very good worth buying goods good price	Taobao Marketplace, a Chinese website for online shopping
Jinfeng Hongyuan Flagship Store	Yellow Save Electricity	Buying two at a time does work well, can play a certain role in saving electricity, worth recommending	Taobao Marketplace, a Chinese website for online shopping
Jinfeng Hongyuan Flagship Store	Yellow Save Electricity	Received it and tried it out, it shows instantaneous voltage, whether or not it will save power depends on the later results! Can't be sure if it can save power now!	Taobao Marketplace, a Chinese website for online shopping
Jinfeng Hongyuan Flagship Store	Yellow Save Electricity	Introduced by friends, good quality, service logistics are very good, power saving	Taobao Marketplace, a Chinese website for online shopping
Jinfeng Hongyuan Flagship Store	Yellow Save Electricity	Packaging is very delicate, energy-saving devices also show high-grade, very like	Taobao Marketplace, a Chinese website for online shopping
Jinfeng Hongyuan Flagship Store	Yellow Save Electricity	Goods received, appearance na, quality ah, look good. Used two or three days. Before buying that does not work, small this looks okay. After a month, two months and then comment.	Taobao Marketplace, a Chinese website for online shopping
Jinfeng Hongyuan Flagship Store	Yellow Save Electricity	Just received the goods on the use, looking forward to good results it, first give a five-star praise, after a period of time and then follow up!	Taobao Marketplace, a Chinese website for online shopping
Jinfeng Hongyuan Flagship Store	Yellow Save Electricity	Received the goods can not wait to start using, friends recommended can save money!	Taobao Marketplace, a Chinese website for online shopping
Jinfeng Hongyuan Flagship Store	Yellow Save Electricity	I've bought it before and now I'm repurchasing and it's fine.	Taobao Marketplace, a Chinese website for online shopping
Jinfeng Hongyuan Flagship Store	Yellow Save Electricity	There's no use!	Taobao Marketplace, a Chinese website for online shopping
Jinfeng Hongyuan Flagship Store	Yellow Save Electricity	Used a few days effect Feel pretty good, packaging is very tight, logistics is also very fast.Overall feeling Pretty good.	Taobao Marketplace, a Chinese website for online shopping
Jinfeng Hongyuan Flagship Store	Yellow Save Electricity	Goods received appearance is very small and exquisite, look forward to using the effect, will repurchase.	Taobao Marketplace, a Chinese website for online shopping
Jinfeng Hongyuan Flagship Store	Yellow Save Electricity	I received the item and plugged it in immediately, I'll use it for a few months and then see the results.	Taobao Marketplace, a Chinese website for online shopping
Jinfeng Hongyuan Flagship Store	Yellow Save Electricity	Already installed, very simple, good quality, specifically to see if it can save electricity	Taobao Marketplace, a Chinese website for online shopping
Jinfeng Hongyuan Flagship Store	Yellow Save Electricity	Product workmanship is fine, the packaging is complete and undamaged, looking forward to results	Taobao Marketplace, a Chinese website for online shopping
Jinfeng Hongyuan Flagship Store	Yellow Save Electricity	Very good, good quality, try it for a few days and see the results	Taobao Marketplace, a Chinese website for online shopping
Jinfeng Hongyuan Flagship Store	Yellow Save Electricity	The stuff was received yesterday: it was also advanced by a friend: the friend said it was very good:	Taobao Marketplace, a Chinese website for online shopping
Jinfeng Hongyuan Flagship Store	Yellow Save Electricity	Received after a period of time to comment, there is indeed a power saving Oh, the weather is hot with more electricity, with it to save electricity and save money!	Taobao Marketplace, a Chinese website for online shopping
Jinfeng Hongyuan Flagship Store	Yellow Save Electricity	Looks good, uses a lot of power, hope it works, plug and play is very convenient!!!!	Taobao Marketplace, a Chinese website for online shopping
Jinfeng Hongyuan Flagship Store	Yellow Save Electricity	Saving appliances is consistent with buying goods	Taobao Marketplace, a Chinese website for online shopping
Jinfeng Hongyuan Flagship Store	Yellow Save Electricity	Half a year ago used other brands, broken, because it does save electricity, so replaced with this brand to use, the packaging is very delicate, energy-saving appliances are also upscale, very like Now is the summer, the peak of electricity consumption, save electricity can be with the same period last year to go	Taobao Marketplace, a Chinese website for online shopping
Jinfeng Hongyuan Flagship Store	Yellow Save Electricity	Need time to test the effect, two or three days can not say anything yo.	Taobao Marketplace, a Chinese website for online shopping
Jinfeng Hongyuan Flagship Store	Yellow Save Electricity	Saving power received, the quality is very good, fine workmanship beautiful style, durable shell, very satisfied with an online shopping, looking forward to power-saving effect, the back will follow up!	Taobao Marketplace, a Chinese website for online shopping
Jinfeng Hongyuan Flagship Store	Yellow Save Electricity	Perfectly packaged, well made, clear digital display, long plugged in and not hot, it does save.	Taobao Marketplace, a Chinese website for online shopping
Jinfeng Hongyuan Flagship Store	Yellow Save Electricity	The product has been received, the quality is very good, use it immediately, and then add comments after use😁	Taobao Marketplace, a Chinese website for online shopping
Jinfeng Hongyuan Flagship Store	Yellow Save Electricity	Baby exquisite workmanship, but also with LCD display, just received the goods, should be effective, with a period of time and then continue to buy!	Taobao Marketplace, a Chinese website for online shopping
Jinfeng Hongyuan Flagship Store	Yellow Save Electricity	Baby has received! This is the second time I've purchased it! Indeed power saving. Cheers good exposé!	Taobao Marketplace, a Chinese website for online shopping
Jinfeng Hongyuan Flagship Store	Yellow Save Electricity	Not bad, looks pretty good, worth the money, and saves on the electric bill	Taobao Marketplace, a Chinese website for online shopping
Jinfeng Hongyuan Flagship Store	Yellow Save Electricity	Express also mailed quite fast, mailed back to use a few days, still feel good.	Taobao Marketplace, a Chinese website for online shopping
Jinfeng Hongyuan Flagship Store	Yellow Save Electricity	No power savings in sight.	Taobao Marketplace, a Chinese website for online shopping
Jinfeng Hongyuan Flagship Store	Yellow Save Electricity	It's as good as the seller's description.	Taobao Marketplace, a Chinese website for online shopping
Jinfeng Hongyuan Flagship Store	Yellow Save Electricity	Just the right amount to use! It's cheap! And less electricity.	Taobao Marketplace, a Chinese website for online shopping

Jinfeng Hongyuan Flagship Store	Yellow Save Electricity	Smart Saver received, is the second time to buy, feel pretty good.	Taobao Marketplace, a Chinese website for online shopping
Jinfeng Hongyuan Flagship Store	Yellow Save Electricity	Very good and satisfied with the 5-star rating	Taobao Marketplace, a Chinese website for online shopping
Jinfeng Hongyuan Flagship Store	Yellow Save Electricity	Received it and just used it, expecting good results, see you in a month.	Taobao Marketplace, a Chinese website for online shopping
Jinfeng Hongyuan Flagship Store	Yellow Save Electricity	Energy Savers Work Saving Electricity vs.	Taobao Marketplace, a Chinese website for online shopping
Jinfeng Hongyuan Flagship Store	Yellow Save Electricity	Good, good experience, good practicality	Taobao Marketplace, a Chinese website for online shopping
Jinfeng Hongyuan Flagship Store	Yellow Save Electricity	The product is very good, very satisfied, have introduced to friends to buy!	Taobao Marketplace, a Chinese website for online shopping
Jinfeng Hongyuan Flagship Store	Yellow Save Electricity	Very good and satisfied to give 5 stars	Taobao Marketplace, a Chinese website for online shopping
Jinfeng Hongyuan Flagship Store	Yellow Save Electricity	It's okay, it remains to be seen if it can save electricity	Taobao Marketplace, a Chinese website for online shopping
Jinfeng Hongyuan Flagship Store	Yellow Save Electricity	Recommended by a friend, should work well, fast delivery	Taobao Marketplace, a Chinese website for online shopping
Jinfeng Hongyuan Flagship Store	Yellow Save Electricity	It's hard to tell when it's just been installed. Wait a couple months and you'll see the results.	Taobao Marketplace, a Chinese website for online shopping
Jinfeng Hongyuan Flagship Store	Yellow Save Electricity	Quality is very good, really save electricity♡👍👍👍🌹🌹🌹service to give, everyone rest assured to shoot	Taobao Marketplace, a Chinese website for online shopping
Jinfeng Hongyuan Flagship Store	Yellow Save Electricity	I've already used it, waiting for next month to know the result, I feel that there is a change, I should be repurchased!	Taobao Marketplace, a Chinese website for online shopping
Jinfeng Hongyuan Flagship Store	Yellow Save Electricity	Just received the goods, wait for a period of time to use the feedback	Taobao Marketplace, a Chinese website for online shopping
Jinfeng Hongyuan Flagship Store	Yellow Save Electricity	Arrived, eager to use, has been used for a few days, do in the meter before and after the use of the record, it does save a lot!	Taobao Marketplace, a Chinese website for online shopping
Jinfeng Hongyuan Flagship Store	Yellow Save Electricity	I just received it and unpacked it and used it right away, using it with customer service guidance and hoping for a rewarding experience!	Taobao Marketplace, a Chinese website for online shopping
Jinfeng Hongyuan Flagship Store	Yellow Save Electricity	Packaging is very good, the product is also very good, has begun to use, whether it can save electricity to see the future electricity bill.	Taobao Marketplace, a Chinese website for online shopping
Jinfeng Hongyuan Flagship Store	Yellow Save Electricity	Heard from a friend. It was so hard to find your product! I, without hesitation, bought it! Now have used it! Let's see after a while, how does it work?	Taobao Marketplace, a Chinese website for online shopping
Jinfeng Hongyuan Flagship Store	Yellow Save Electricity	Goods have been received, well-packed quality is very good very like	Taobao Marketplace, a Chinese website for online shopping
Jinfeng Hongyuan Flagship Store	Yellow Save Electricity	Use it for a while and then evaluate	Taobao Marketplace, a Chinese website for online shopping
Jinfeng Hongyuan Flagship Store	Yellow Save Electricity	It's good. If you want it, get it now.	Taobao Marketplace, a Chinese website for online shopping
Jinfeng Hongyuan Flagship Store	Yellow Save Electricity	Good workmanship, plugged in the upper power supply does not heat up. Looking forward to the power saving effect.	Taobao Marketplace, a Chinese website for online shopping
Jinfeng Hongyuan Flagship Store	Yellow Save Electricity	I don't know if it works 4star ratingfirst	Taobao Marketplace, a Chinese website for online shopping
Jinfeng Hongyuan Flagship Store	Yellow Save Electricity	It feels like a power saver,, use it for two months to be better sure	Taobao Marketplace, a Chinese website for online shopping
Jinfeng Hongyuan Flagship Store	Yellow Save Electricity	Can't see a bit of power savings in a few days of comparison	Taobao Marketplace, a Chinese website for online shopping
Jinfeng Hongyuan Flagship Store	Yellow Save Electricity	Saving electricity is really good, but also friends introduced, can not be wasted is everyone must do, the seller's service attitude is very good, logistics is also very strong!	Taobao Marketplace, a Chinese website for online shopping
Jinfeng Hongyuan Flagship Store	Yellow Save Electricity	I'll comment after I use it.	Taobao Marketplace, a Chinese website for online shopping
Jinfeng Hongyuan Flagship Store	Yellow Save Electricity	The product is beautifully packaged and is on trial……does not get hot for long periods of time.	Taobao Marketplace, a Chinese website for online shopping
Jinfeng Hongyuan Flagship Store	Yellow Save Electricity	It was recommended to me by a neighbor's family and it's nice to use it to save power.	Taobao Marketplace, a Chinese website for online shopping
Jinfeng Hongyuan Flagship Store	Yellow Save Electricity	Previously purchased with a power-saving repurchase, very satisfied with the recommended	Taobao Marketplace, a Chinese website for online shopping
Jinfeng Hongyuan Flagship Store	Yellow Save Electricity	The quality is very good, just came back to the packaging is particularly professional, and then dismantled and immediately installed, but also sent5insurance. And I bought elsewhere, really not a grade. In the future, other places need will also shoot his home.	Taobao Marketplace, a Chinese website for online shopping
Jinfeng Hongyuan Flagship Store	Yellow Save Electricity	Your products are the real deal. - Great. - Thanks.	Taobao Marketplace, a Chinese website for online shopping
Jinfeng Hongyuan Flagship Store	Yellow Save Electricity	Just bought back to test the home appliances all open and then plugged in this energy saver test a clock with this energy saver has a significant energy saving effect is still in the test medium with a month or two and then do further evaluation!	Taobao Marketplace, a Chinese website for online shopping
Jinfeng Hongyuan Flagship Store	Yellow Save Electricity	Packaging protection is very good, display is normal with voltage indication.	Taobao Marketplace, a Chinese website for online shopping
Jinfeng Hongyuan Flagship Store	Yellow Save Electricity	This plant has been in business for a long time. It should be effective in saving electricity.	Taobao Marketplace, a Chinese website for online shopping

Jinfeng Hongyuan Flagship Store	Yellow Save Electricity	It's really not a bad idea to use this.	Taobao Marketplace, a Chinese website for online shopping
Jinfeng Hongyuan Flagship Store	Yellow Save Electricity	We'll see what happens later.	Taobao Marketplace, a Chinese website for online shopping
Jinfeng Hongyuan Flagship Store	Yellow Save Electricity	The product received, the workmanship is still good, can not wait to use it, see the effect.	Taobao Marketplace, a Chinese website for online shopping
Jinfeng Hongyuan Flagship Store	Yellow Save Electricity	This is a friend tried recommended to me, I also tried really can save electricity, friends can rest assured to buy!	Taobao Marketplace, a Chinese website for online shopping
Jinfeng Hongyuan Flagship Store	Yellow Save Electricity	The first time to buy, after using to follow up, but received the goods is also the quality of the bearer, the appearance is also beautiful, express delivery is very fast! Packaging is very good, and send spare insurance5, do considerate, look forward to using the follow-up comment.☆☆☆☆☆	Taobao Marketplace, a Chinese website for online shopping
Jinfeng Hongyuan Flagship Store	Yellow Save Electricity	This power saver product is suitable for all kinds of electricity, good quality, saving electricity is worth having!	Taobao Marketplace, a Chinese website for online shopping
Jinfeng Hongyuan Flagship Store	Yellow Save Electricity	Power saver saves electricity	Taobao Marketplace, a Chinese website for online shopping
Jinfeng Hongyuan Flagship Store	Yellow Save Electricity	Nice nice cheap and inexpensive received exactly the same as the physical some time ago	Taobao Marketplace, a Chinese website for online shopping
Jinfeng Hongyuan Flagship Store	Yellow Save Electricity	Nice stuff, recommend it!	Taobao Marketplace, a Chinese website for online shopping
Jinfeng Hongyuan Flagship Store	Yellow Save Electricity	The logistics is very fast, I got it and used it, and I feel relieved to turn on the air conditioner.	Taobao Marketplace, a Chinese website for online shopping
Jinfeng Hongyuan Flagship Store	Yellow Save Electricity	I just received the goods, the product looks very formal, if it can effectively save electricity, I plan to repurchase.	Taobao Marketplace, a Chinese website for online shopping
Jinfeng Hongyuan Flagship Store	Yellow Save Electricity	Friends recommended to buy, it should be okay, friends of the family said it is very energy-saving, buy a try, the color is very nice, the arrival is also fast, the quality is also can be	Taobao Marketplace, a Chinese website for online shopping
Jinfeng Hongyuan Flagship Store	Yellow Save Electricity	Baby received. It is consistent with the description, the seller is very good, I hope it will be ideal for power saving, and I will follow up after using it for a while.	Taobao Marketplace, a Chinese website for online shopping
Jinfeng Hongyuan Flagship Store	Yellow Save Electricity	Nice and easy to use, recommended	Taobao Marketplace, a Chinese website for online shopping
Jinfeng Hongyuan Flagship Store	Yellow Save Electricity	Habit to give positive feedback. Just don't know how well it works, looking forward to	Taobao Marketplace, a Chinese website for online shopping
Jinfeng Hongyuan Flagship Store	Yellow Save Electricity	Packaging is perfect texture, looking high hopefully more power saving	Taobao Marketplace, a Chinese website for online shopping
Jinfeng Hongyuan Flagship Store	Yellow Save Electricity	The saver has been received and is already in use, looking forward to the results. Follow-up evaluation later	Taobao Marketplace, a Chinese website for online shopping
Jinfeng Hongyuan Flagship Store	Yellow Save Electricity	Charge quickly, the quality is very good Oh, give a praise!	Taobao Marketplace, a Chinese website for online shopping
Jinfeng Hongyuan Flagship Store	Yellow Save Electricity	Electricity saver.., applicable equipment: refrigerator, air conditioner, washing machine, electric fanetc. any sensible household appliances and equipment, it's very good🎉🎉🎉🎉👏👏👏👏👏👏👏👏👏👏👏👏👏👏👏👏👏👏👏👏👍👍👍👍👍👍	Taobao Marketplace, a Chinese website for online shopping
Jinfeng Hongyuan Flagship Store	Yellow Save Electricity	Not bad.	Taobao Marketplace, a Chinese website for online shopping
Jinfeng Hongyuan Flagship Store	Yellow Save Electricity	There's a screw missing.	Taobao Marketplace, a Chinese website for online shopping
Jinfeng Hongyuan Flagship Store	Yellow Save Electricity	The product is good, the packaging is very good, the quality is also good, the power saving situation after the use of a follow-up evaluation	Taobao Marketplace, a Chinese website for online shopping
Jinfeng Hongyuan Flagship Store	Yellow Save Electricity	Received immediately used, logistics is also very fast hope there is an effect. Good use next time to repurchase.	Taobao Marketplace, a Chinese website for online shopping
Jinfeng Hongyuan Flagship Store	Yellow Save Electricity	The baby is great, and I have to say, the customer service is too good to be true! The packaging of the item is very upscale and really worth every penny.	Taobao Marketplace, a Chinese website for online shopping
Jinfeng Hongyuan Flagship Store	Yellow Save Electricity	Packaging is still ok, wait time a month to see the effect	Taobao Marketplace, a Chinese website for online shopping
Jinfeng Hongyuan Flagship Store	Yellow Save Electricity	It is not the first time to buy, it works, and it is used at home in the store. Will support as always.	Taobao Marketplace, a Chinese website for online shopping
Jinfeng Hongyuan Flagship Store	Yellow Save Electricity	Baby received, well packaged, very easy to use, looking forward to good results.	Taobao Marketplace, a Chinese website for online shopping
Jinfeng Hongyuan Flagship Store	Yellow Save Electricity	Works great, very practical, and will be back for more!	Taobao Marketplace, a Chinese website for online shopping
Jinfeng Hongyuan Flagship Store	Yellow Save Electricity	I'm using this product, it's a second purchase and it's great. After three months, the power saving is more than half	Taobao Marketplace, a Chinese website for online shopping
Jinfeng Hongyuan Flagship Store	Yellow Save Electricity	Logistics block, good packaging, energy saving effect is quite good, worth the next hook buy.	Taobao Marketplace, a Chinese website for online shopping
Jinfeng Hongyuan Flagship Store	Yellow Save Electricity	Already used one downstairs, power-saving effect is obvious, a month less than before to pay30%lesselectricity, soon to use the air-conditioning season, upstairs and then install one!	Taobao Marketplace, a Chinese website for online shopping
Jinfeng Hongyuan Flagship Store	Yellow Save Electricity	Just received it today, hope it saves electricity, logistics is fast	Taobao Marketplace, a Chinese website for online shopping
Jinfeng Hongyuan Flagship Store	Yellow Save Electricity	I don't have it yet, I don't know how it works, I don't have a say, I'll comment after I use it.	Taobao Marketplace, a Chinese website for online shopping
Jinfeng Hongyuan Flagship Store	Yellow Save Electricity	Arrived, eager to use it, looking at the meter is just a lot slower	Taobao Marketplace, a Chinese website for online shopping

Jinfeng Hongyuan Flagship Store	Yellow Save Electricity	Very practical and affordable, recommended purchase!	Taobao Marketplace, a Chinese website for online shopping
Jinfeng Hongyuan Flagship Store	Yellow Save Electricity	I just received the baby and used it, hoping to save some money on my electric bill.	Taobao Marketplace, a Chinese website for online shopping
Jinfeng Hongyuan Flagship Store	Yellow Save Electricity	We'll see the results in a month's time. Looking forward to it.	Taobao Marketplace, a Chinese website for online shopping
Jinfeng Hongyuan Flagship Store	Yellow Save Electricity	Very good.	Taobao Marketplace, a Chinese website for online shopping
Jinfeng Hongyuan Flagship Store	Yellow Save Electricity	The baby has received, using quite good, power saving	Taobao Marketplace, a Chinese website for online shopping
Jinfeng Hongyuan Flagship Store	Yellow Save Electricity	I received the baby! Looks pretty good! Just used it not sure if it will save power! Good review😀😀😀😀	Taobao Marketplace, a Chinese website for online shopping
Jinfeng Hongyuan Flagship Store	Yellow Save Electricity	Used less than ten days, I record the meter5656is now5657according to this, the power company will pick check me, a little afraid of the feeling, but the heart is hot, thank you thank you	Taobao Marketplace, a Chinese website for online shopping
Jinfeng Hongyuan Flagship Store	Yellow Save Electricity	The product received, the quality of genuine, fast logistics, product packaging is very good, the product to be used to know the ah? Trustworthy!	Taobao Marketplace, a Chinese website for online shopping
Jinfeng Hongyuan Flagship Store	Yellow Save Electricity	Today received the goods immediately used, packaging.Inside the perfect box, there is also a fuse.	Taobao Marketplace, a Chinese website for online shopping
Jinfeng Hongyuan Flagship Store	Yellow Save Electricity	The saver is good, quite delicate, and a spare fuse, use it for a while and then feedback!	Taobao Marketplace, a Chinese website for online shopping
Jinfeng Hongyuan Flagship Store	Yellow Save Electricity	Energy saving and environmental protection, no longer afraid of wasting electricity resources, home must have	Taobao Marketplace, a Chinese website for online shopping
Jinfeng Hongyuan Flagship Store	Yellow Save Electricity	Express has received, indeed can save a lot of electricity, this is the second time to buy, if you need will buy back!	Taobao Marketplace, a Chinese website for online shopping
Jinfeng Hongyuan Flagship Store	Yellow Save Electricity	The quality is very good.	Taobao Marketplace, a Chinese website for online shopping
Jinfeng Hongyuan Flagship Store	Yellow Save Electricity	Saver received, the outer packaging is intact, immediately used, good use and then repurchase!	Taobao Marketplace, a Chinese website for online shopping
Jinfeng Hongyuan Flagship Store	Yellow Save Electricity	The power saver has been received The package and the real thing look quite formal The workmanship is also quite exquisite Only how effective We will see in a couple of days	Taobao Marketplace, a Chinese website for online shopping
Jinfeng Hongyuan Flagship Store	Yellow Save Electricity	The item matches the description, and the main thing is that the event is cheap, so you can save a lot of money	Taobao Marketplace, a Chinese website for online shopping
Jinfeng Hongyuan Flagship Store	Yellow Save Electricity	I originally had it at home and felt it worked before purchasing this product again.	Taobao Marketplace, a Chinese website for online shopping
Jinfeng Hongyuan Flagship Store	Yellow Save Electricity	Goods received! Quite fast, looking forward to the effect, the boss is powerful66666	Taobao Marketplace, a Chinese website for online shopping
Jinfeng Hongyuan Flagship Store	Yellow Save Electricity	The product works great, the merchant shipped quickly, order in the morning and ship at noon. Next time I need I will buy from this family.	Taobao Marketplace, a Chinese website for online shopping
Jinfeng Hongyuan Flagship Store	Yellow Save Electricity	Product quality is good, the merchant also fast delivery, order in the morning at noon delivery, next time if need I still buy here.	Taobao Marketplace, a Chinese website for online shopping
Jinfeng Hongyuan Flagship Store	Yellow Save Electricity	It works well, it helps to tie up the electricity, it's quite a good deal, and it reduces the electricity bill in the house	Taobao Marketplace, a Chinese website for online shopping
Jinfeng Hongyuan Flagship Store	Yellow Save Electricity	Installed, logistics is quite fast, do not know the effect of what, packaging is very good, logistics is also good, after using the evaluation again	Taobao Marketplace, a Chinese website for online shopping
Jinfeng Hongyuan Flagship Store	Yellow Save Electricity	Shipping speed logistics is also very strong, baby fine workmanship quality is very good, energy saving and environmental protection to save electricity, the appearance of the design is very good, it is worth buying!	Taobao Marketplace, a Chinese website for online shopping
Jinfeng Hongyuan Flagship Store	Yellow Save Electricity	I've bought this a couple of times before and the energy savings are great, so I'm buying it again.	Taobao Marketplace, a Chinese website for online shopping
Jinfeng Hongyuan Flagship Store	Yellow Save Electricity	Fast delivery, that node electricity and beautiful, will recommend friends to buy!	Taobao Marketplace, a Chinese website for online shopping
Jinfeng Hongyuan Flagship Store	Yellow Save Electricity	To be honest, it's not very good. It's not very energy efficient.	Taobao Marketplace, a Chinese website for online shopping
Jinfeng Hongyuan Flagship Store	Yellow Save Electricity	I'm already a2-time repurchaser Energy saving and power saving Nice and compact design No standing space	Taobao Marketplace, a Chinese website for online shopping
Jinfeng Hongyuan Flagship Store	Yellow Save Electricity	Electricity costs nothing at all, scammers everyone don't fall for it, disassembled inside just a resistor😠😠😠😠	Taobao Marketplace, a Chinese website for online shopping
Jinfeng Hongyuan Flagship Store	Yellow Save Electricity	According to the observation of the packaging, it should be authentic, and also sent a peg, very warm	Taobao Marketplace, a Chinese website for online shopping
Jinfeng Hongyuan Flagship Store	Yellow Save Electricity	According to the observation of the packaging, it should be genuine, and also sent a peg, very warm, hurry to plug it in to see if the electricity bill will be cheaper next month?	Taobao Marketplace, a Chinese website for online shopping
Jinfeng Hongyuan Flagship Store	Yellow Save Electricity	So far do not know whether the power saving……but the seller goods show the voltage another sent a few fuses feel very thoughtful……give good reviews	Taobao Marketplace, a Chinese website for online shopping
Jinfeng Hongyuan Flagship Store	Yellow Save Electricity	The packaging is pretty good, it's new, I'll give feedback after I use it.	Taobao Marketplace, a Chinese website for online shopping
Jinfeng Hongyuan Flagship Store	Yellow Save Electricity	Received the item, tried it and it still works well, will buy again.	Taobao Marketplace, a Chinese website for online shopping
Jinfeng Hongyuan Flagship Store	Yellow Save Electricity	Express quite fast, the packaging is quite formal, the effect of a few days before commenting on the feeling is quite good, friends say it works, first try to use it!	Taobao Marketplace, a Chinese website for online shopping

Jinfeng Hongyuan Flagship Store	Yellow Save Electricity	Scam! Big scam! Been using it for a month and the electric bill has gone up and not down!	Taobao Marketplace, a Chinese website for online shopping
Jinfeng Hongyuan Flagship Store	Yellow Save Electricity	It works well, and I feel like I'm saving a lot of electricity	Taobao Marketplace, a Chinese website for online shopping
Jinfeng Hongyuan Flagship Store	Yellow Save Electricity	I used it right after I received it, and it came with a small gift, which was nice.	Taobao Marketplace, a Chinese website for online shopping
Jinfeng Hongyuan Flagship Store	Yellow Save Electricity	It looks ok The display is jumping Use it for a while I'll follow up	Taobao Marketplace, a Chinese website for online shopping
Jinfeng Hongyuan Flagship Store	Yellow Save Electricity	Doesn't do anything only measures voltage……	Taobao Marketplace, a Chinese website for online shopping
Jinfeng Hongyuan Flagship Store	Yellow Save Electricity	Shipped quickly, has received the goods, the goods and the description is almost the same, the quality is no problem, the key price is also appropriate, buy here or very assured!	Taobao Marketplace, a Chinese website for online shopping
Jinfeng Hongyuan Flagship Store	Yellow Save Electricity	The logistics is very fast, not yet used, its waiting to be able to really save electricity costs	Taobao Marketplace, a Chinese website for online shopping
Jinfeng Hongyuan Flagship Store	Yellow Save Electricity	I'll wait for two months to comment. I'm not sure yet.	Taobao Marketplace, a Chinese website for online shopping
Jinfeng Hongyuan Flagship Store	Yellow Save Electricity	Received the goods to see very satisfied, the packaging is high, there are small gifts inside, take out immediately used. Good will repurchase, first praise.	Taobao Marketplace, a Chinese website for online shopping
Jinfeng Hongyuan Flagship Store	Yellow Save Electricity	Good value for money, recommend buying, hope the quality will be better	Taobao Marketplace, a Chinese website for online shopping
Jinfeng Hongyuan Flagship Store	Yellow Save Electricity	Very good. Very satisfied. Would recommend friends to buy	Taobao Marketplace, a Chinese website for online shopping
Jinfeng Hongyuan Flagship Store	Yellow Save Electricity	Trying to see how it works, not sure if it will save power, bought two! Looking forward to the effect!	Taobao Marketplace, a Chinese website for online shopping
Jinfeng Hongyuan Flagship Store	Yellow Save Electricity	I haven't paid my electric bill yet, so I don't know if it's working?	Taobao Marketplace, a Chinese website for online shopping
Jinfeng Hongyuan Flagship Store	Yellow Save Electricity	Works great, worth it, bought several already!	Taobao Marketplace, a Chinese website for online shopping
Jinfeng Hongyuan Flagship Store	Yellow Save Electricity	Use it for a while before commenting	Taobao Marketplace, a Chinese website for online shopping
Jinfeng Hongyuan Flagship Store	Yellow Save Electricity	Baby received, packaging is very good, has been installed, I hope it works!	Taobao Marketplace, a Chinese website for online shopping
Jinfeng Hongyuan Flagship Store	Yellow Save Electricity	Used it for most of the pon, it does work, very good product666	Taobao Marketplace, a Chinese website for online shopping
Jinfeng Hongyuan Flagship Store	Yellow Save Electricity	Very good, thanks! 5 stars for the positive feedback!	Taobao Marketplace, a Chinese website for online shopping
Jinfeng Hongyuan Flagship Store	Yellow Save Electricity	Great service, should work, can't possibly save much once it's installed? It's a lot more over time!	Taobao Marketplace, a Chinese website for online shopping
Jinfeng Hongyuan Flagship Store	Yellow Save Electricity	Fine workmanship, good quality, will come back to patronize again in the future!	Taobao Marketplace, a Chinese website for online shopping
Jinfeng Hongyuan Flagship Store	Yellow Save Electricity	New Generation Power Saver Nice model! Beautiful color! Looking forward to the effect!	Taobao Marketplace, a Chinese website for online shopping
Jinfeng Hongyuan Flagship Store	Yellow Save Electricity	Energy saver received, not yet used, used to save electricity and then add comment!	Taobao Marketplace, a Chinese website for online shopping
Jinfeng Hongyuan Flagship Store	Yellow Save Electricity	Received the goods, use it and then evaluate	Taobao Marketplace, a Chinese website for online shopping
Jinfeng Hongyuan Flagship Store	Yellow Save Electricity	According to the observation of the packaging, it should be genuine, and also sent a hanging stare, very warm ❤, hurry up to plug it in to see if there is a change in the next month's electricity bill.	Taobao Marketplace, a Chinese website for online shopping
Jinfeng Hongyuan Flagship Store	Yellow Save Electricity	Logistics is very fast, beautifully packaged, as soon as it arrived immediately installed, class, do not know the effect, after a month to come back!	Taobao Marketplace, a Chinese website for online shopping
Jinfeng Hongyuan Flagship Store	Yellow Save Electricity	The product has been installed, looking forward to the energy saving effect	Taobao Marketplace, a Chinese website for online shopping
Jinfeng Hongyuan Flagship Store	Yellow Save Electricity	Goods received, packaging intact, look at the shell workmanship is also quite good. Bought two together! High-power appliances are obviously a change in voltage after opening. I do not know if there is no effect, use and then see! First praise it! Other use and then comment!	Taobao Marketplace, a Chinese website for online shopping
Jinfeng Hongyuan Flagship Store	Yellow Save Electricity	I'll follow up after a month of use	Taobao Marketplace, a Chinese website for online shopping
Jinfeng Hongyuan Flagship Store	Yellow Save Electricity	Things received, good quality, the material looks good, plugged into the socket, the screen has a display, quite good.	Taobao Marketplace, a Chinese website for online shopping
Jinfeng Hongyuan Flagship Store	Yellow Save Electricity	After using it for two months, it does use less electricity.	Taobao Marketplace, a Chinese website for online shopping
Jinfeng Hongyuan Flagship Store	Yellow Save Electricity	Great service fast shipping, great quality,...	Taobao Marketplace, a Chinese website for online shopping
Jinfeng Hongyuan Flagship Store	Yellow Save Electricity	I'll use it for now, and then I'll follow up after a while to compare my previous electricity bill and see if it's working!	Taobao Marketplace, a Chinese website for online shopping
Jinfeng Hongyuan Flagship Store	Yellow Save Electricity	The quality is OK!	Taobao Marketplace, a Chinese website for online shopping
Jinfeng Hongyuan Flagship Store	Yellow Save Electricity	Baby received, feel okay, looking forward to good results	Taobao Marketplace, a Chinese website for online shopping

Jinfeng Hongyuan Flagship Store	Yellow Save Electricity	It feels pretty good, use it first to see if it works?	Taobao Marketplace, a Chinese website for online shopping
Jinfeng Hongyuan Flagship Store	Yellow Save Electricity	Little aunt saidDecemberlast month electricity bill700yuan for two months, in order to buy electricity to save to the little aunt, and thenMarchstrange electricity bill500yuan inversion, support, gas😍😍😍😍😍😍	Taobao Marketplace, a Chinese website for online shopping
Jinfeng Hongyuan Flagship Store	Yellow Save Electricity	I don't think it's useful to lie to people.	Taobao Marketplace, a Chinese website for online shopping
Jinfeng Hongyuan Flagship Store	Yellow Save Electricity	Fast shipping, great product quality, full certificates.	Taobao Marketplace, a Chinese website for online shopping
Jinfeng Hongyuan Flagship Store	Yellow Save Electricity	Has been installed, exquisite workmanship, customer service to introduce the use of satisfactory.	Taobao Marketplace, a Chinese website for online shopping
Jinfeng Hongyuan Flagship Store	Yellow Save Electricity	Baby received, well-packed, look at the quality is good, after a while in the follow-up evaluation	Taobao Marketplace, a Chinese website for online shopping
Jinfeng Hongyuan Flagship Store	Yellow Save Electricity	Shipping is very fast Look is quite good Use the effect will come back to buy again	Taobao Marketplace, a Chinese website for online shopping
Jinfeng Hongyuan Flagship Store	Yellow Save Electricity	Power saving is fake Please don't be like me don't be fooled Customer service will also be fooled that is plugged in don't unplug it plugged in the longer the more power saving Believe you the hell There is no point in power saving They this claim is afraid that you use a period of time without power saving effect will return Just make up a claim that the longer it is plugged in the more power saving And then a long time will be unable to return the goods they will earn	Taobao Marketplace, a Chinese website for online shopping
Jinfeng Hongyuan Flagship Store	Yellow Save Electricity	tax, used itfor2months, didn't save a single degree of electricity, plugged in as a mere decoration, reviews are all$5bonus! False	Taobao Marketplace, a Chinese website for online shopping
Jinfeng Hongyuan Flagship Store	Yellow Save Electricity	Bad review Bought it almost 3 months ago and the electricity bill hasn't gone down at all	Taobao Marketplace, a Chinese website for online shopping
Jinfeng Hongyuan Flagship Store	Yellow Save Electricity	It doesn't save electricity at all, don't buy it, it's a scam. I tried it for a couple months and my electric bill is still more, I hope people don't fall for it. Let the fraudulent company go out of business.	Taobao Marketplace, a Chinese website for online shopping
Jinfeng Hongyuan Flagship Store	Yellow Save Electricity	It's not working. It's not costing me anything.	Taobao Marketplace, a Chinese website for online shopping
Jinfeng Hongyuan Flagship Store	Yellow Save Electricity	It's not working at all.	Taobao Marketplace, a Chinese website for online shopping
Jinfeng Hongyuan Flagship Store	Yellow Save Electricity	Logistics is particularly fast, things work well, customer service attitude is also very good, you can buy.	Taobao Marketplace, a Chinese website for online shopping
Jinfeng Hongyuan Flagship Store	Yellow Save Electricity	Goods received Really good This month's electricity bill is much less The quality of the product is excellent It is worth recommending to everyone The logistics is very powerful The order arrived in two and a half days	Taobao Marketplace, a Chinese website for online shopping
Jinfeng Hongyuan Flagship Store	Yellow Save Electricity	Go pay some IQ tax if you want to buy one.	Taobao Marketplace, a Chinese website for online shopping
Jinfeng Hongyuan Flagship Store	Yellow Save Electricity	First time using this Looks good quality Plugged it in right after I bought it Hope it works well Thanks for the extra fuse	Taobao Marketplace, a Chinese website for online shopping
Jinfeng Hongyuan Flagship Store	Yellow Save Electricity	I bought one on double 11 This one is a repurchase ～it's good~	Taobao Marketplace, a Chinese website for online shopping
Jinfeng Hongyuan Flagship Store	Yellow Save Electricity	It's useless. It's a lie.	Taobao Marketplace, a Chinese website for online shopping
Jinfeng Hongyuan Flagship Store	Yellow Save Electricity	I hope it's good~ It looks good~ I heard from a friend and bought it~	Taobao Marketplace, a Chinese website for online shopping
Jinfeng Hongyuan Flagship Store	Yellow Save Electricity	The owner was really nice and shipped quickly. The quality of the item is also quite good. Love it so much, thanks!	Taobao Marketplace, a Chinese website for online shopping
Jinfeng Hongyuan Flagship Store	Yellow Save Electricity	The logistics is very fast, the baby is very good, the shopkeeperNICE!	Taobao Marketplace, a Chinese website for online shopping
Jinfeng Hongyuan Flagship Store	Yellow Save Electricity	My friends say it works and I bought another one.	Taobao Marketplace, a Chinese website for online shopping
Jinfeng Hongyuan Flagship Store	Yellow Save Electricity	Baby works, looks good, bought a second one.	Taobao Marketplace, a Chinese website for online shopping
Jinfeng Hongyuan Flagship Store	Yellow Save Electricity	Not bad, regular manufacturer production, real power-saving function	Taobao Marketplace, a Chinese website for online shopping
Jinfeng Hongyuan Flagship Store	Yellow Save Electricity	Good, can save electricity, the effect is good, late observation	Taobao Marketplace, a Chinese website for online shopping
Jinfeng Hongyuan Flagship Store	Yellow Save Electricity	Nice baby, beautifully packaged. Express speed is fast.	Taobao Marketplace, a Chinese website for online shopping
Jinfeng Hongyuan Flagship Store	Yellow Save Electricity	Nice product, looks pretty good. Also comes with a fuse, safety saver.	Taobao Marketplace, a Chinese website for online shopping
Jinfeng Hongyuan Flagship Store	Yellow Save Electricity	Just arrived Look at the shape is very good A month. just evaluating the effect and I'm not sure I'm wrong. It's worth having	Taobao Marketplace, a Chinese website for online shopping
Jinfeng Hongyuan Flagship Store	Yellow Save Electricity	The saver works great, looks beautiful, good quality, and the portions are generous.	Taobao Marketplace, a Chinese website for online shopping
Jinfeng Hongyuan Flagship Store	Yellow Save Electricity	Fast shipping, tight packaging, the quality of goods is very good, much cheaper than the counter, customer service attitude is also particularly good, praise!	Taobao Marketplace, a Chinese website for online shopping
Jinfeng Hongyuan Flagship Store	Yellow Save Electricity	Affordable price, great quality, very good shopping, next time come back!	Taobao Marketplace, a Chinese website for online shopping
Jinfeng Hongyuan Flagship Store	Yellow Save Electricity	Very good very good. Good review.	Taobao Marketplace, a Chinese website for online shopping

Jinfeng Hongyuan Flagship Store	Yellow Save Electricity	666	Taobao Marketplace, a Chinese website for online shopping
Jinfeng Hongyuan Flagship Store	Yellow Save Electricity	😌😌😌	Taobao Marketplace, a Chinese website for online shopping
Jinfeng Hongyuan Flagship Store	Yellow Save Electricity	😌😌😌😌	Taobao Marketplace, a Chinese website for online shopping
Jinfeng Hongyuan Flagship Store	Yellow Save Electricity	Things received, look really quite noble, a look is a good baby, really useful!	Taobao Marketplace, a Chinese website for online shopping
Jinfeng Hongyuan Flagship Store	Yellow Save Electricity	I've been using it for a few days now and it does have some effect, I hope it lasts longer!	Taobao Marketplace, a Chinese website for online shopping
Jinfeng Hongyuan Flagship Store	Yellow Save Electricity	I have received the baby and can't wait to use it and look forward to the results!	Taobao Marketplace, a Chinese website for online shopping
Jinfeng Hongyuan Flagship Store	Yellow Save Electricity	Received the goods, beautifully packaged, high-end atmospheric upscale, has begun to use, such as the use of a month and then evaluate, look forward to using the effect!	Taobao Marketplace, a Chinese website for online shopping
Jinfeng Hongyuan Flagship Store	Yellow Save Electricity	pretty good	Taobao Marketplace, a Chinese website for online shopping
Jinfeng Hongyuan Flagship Store	Yellow Save Electricity	Inexpensive, great quality, easy to use	Taobao Marketplace, a Chinese website for online shopping
Jinfeng Hongyuan Flagship Store	Yellow Save Electricity	Inexpensive and easy to use	Taobao Marketplace, a Chinese website for online shopping
Jinfeng Hongyuan Flagship Store	Yellow Save Electricity	Works pretty well, saves a lot of electricity in a month, truly recommended.	Taobao Marketplace, a Chinese website for online shopping
Jinfeng Hongyuan Flagship Store	Yellow Save Electricity	I've been using it for a while now, it works really well and I truly recommend it.	Taobao Marketplace, a Chinese website for online shopping
Jinfeng Hongyuan Flagship Store	Yellow Save Electricity	Intelligent energy saver received, is to buy for friends, because friends see me buy, with quite good, with the intelligent energy saver is than not to use to save some electricity, praise!	Taobao Marketplace, a Chinese website for online shopping
Jinfeng Hongyuan Flagship Store	Yellow Save Electricity	Intelligent energy saver, bought for a friend, because I use a good, so my friend let me buy one for me, my friend said that it is quite good to use, can save some electricity, praise!	Taobao Marketplace, a Chinese website for online shopping
Jinfeng Hongyuan Flagship Store	Yellow Save Electricity	Baby received, looks authentic, wait to see how much I can save!	Taobao Marketplace, a Chinese website for online shopping
Jinfeng Hongyuan Flagship Store	Yellow Save Electricity	I've received it and it looks genuine, so I'll see how much I can save.	Taobao Marketplace, a Chinese website for online shopping
Jinfeng Hongyuan Flagship Store	Yellow Save Electricity	The seller's service is very good,and the economizer also works	Taobao Marketplace, a Chinese website for online shopping
Jinfeng Hongyuan Flagship Store	Yellow Save Electricity	Got it and plugged it in Looks great Waiting to see how it works next month	Taobao Marketplace, a Chinese website for online shopping
Jinfeng Hongyuan Flagship Store	Yellow Save Electricity	I've been using it for a while now and I'm very happy with the quality of the product	Taobao Marketplace, a Chinese website for online shopping
Jinfeng Hongyuan Flagship Store	Yellow Save Electricity	Works well Repurchased several times Recommended	Taobao Marketplace, a Chinese website for online shopping
Jinfeng Hongyuan Flagship Store	Yellow Save Electricity	This is the second one I have purchased, the product is really good, good review.	Taobao Marketplace, a Chinese website for online shopping
Jinfeng Hongyuan Flagship Store	Yellow Save Electricity	That's true.	Taobao Marketplace, a Chinese website for online shopping
Jinfeng Hongyuan Flagship Store	Yellow Save Electricity	handy	Taobao Marketplace, a Chinese website for online shopping
Jinfeng Hongyuan Flagship Store	Yellow Save Electricity	Fast delivery, very cheap, saved a lot of money on my electric bill by using it.	Taobao Marketplace, a Chinese website for online shopping
Jinfeng Hongyuan Flagship Store	Yellow Save Electricity	Fast delivery, very cheap, saved a lot of money on my electric bill by using it.	Taobao Marketplace, a Chinese website for online shopping
Jinfeng Hongyuan Flagship Store	Light Blue Saving Electricity	Very good quality, good packaging, buy three at once, only de recommend.	Taobao Marketplace, a Chinese website for online shopping
Jinfeng Hongyuan Flagship Store	Light Blue Saving Electricity	Received the baby to be used after the review	Taobao Marketplace, a Chinese website for online shopping
Jinfeng Hongyuan Flagship Store	Light Blue Saving Electricity	This color is very like, feel the texture is good, versatile ~ packaging is also very atmospheric, used to gift is also a good choice!	Taobao Marketplace, a Chinese website for online shopping
Jinfeng Hongyuan Flagship Store	Light Blue Saving Electricity	Business service is very enthusiastic, the explanation is also in place, the same day delivery, packaging is also veryOK, the product workmanship is also very exquisite, looking forward to power-saving effect, use for a period of time to come back to the follow-up evaluation.	Taobao Marketplace, a Chinese website for online shopping
Jinfeng Hongyuan Flagship Store	Light Blue Saving Electricity	I'll use it for a while and then I'll comment.	Taobao Marketplace, a Chinese website for online shopping
Jinfeng Hongyuan Flagship Store	Light Blue Saving Electricity	Looked at the quality is good, the body is compact, hope will have energy saving effect, five praise!	Taobao Marketplace, a Chinese website for online shopping
Jinfeng Hongyuan Flagship Store	Light Blue Saving Electricity	The saver is not big but exquisitely packaged, sturdy material, original and genuine!	Taobao Marketplace, a Chinese website for online shopping
Jinfeng Hongyuan Flagship Store	Light Blue Saving Electricity	It's very small and convenient, can be placed in any position without affecting the aesthetics! The numbers are clear, hope it works!	Taobao Marketplace, a Chinese website for online shopping
Jinfeng Hongyuan Flagship Store	Light Blue Saving Electricity	Well packaged and in use.	Taobao Marketplace, a Chinese website for online shopping

Jinfeng Hongyuan Flagship Store	Light Blue Saving Electricity	Very good workmanship The principle is also clearly written, very simple to use The display voltage is accurate I hope it will be of good effect	Taobao Marketplace, a Chinese website for online shopping
Jinfeng Hongyuan Flagship Store	Light Blue Saving Electricity	Logistics is fast, Power Saver is in perfect condition and works great!	Taobao Marketplace, a Chinese website for online shopping
Jinfeng Hongyuan Flagship Store	Light Blue Saving Electricity	It works really well, very happy with the item, it is an infinite repurchase model, recommended!	Taobao Marketplace, a Chinese website for online shopping
Jinfeng Hongyuan Flagship Store	Light Blue Saving Electricity	Effective bar, or good, supported. Good use ah	Taobao Marketplace, a Chinese website for online shopping
Jinfeng Hongyuan Flagship Store	Light Blue Saving Electricity	Big brand, with a very assured, very good quality, catching up with the activities of the purchase is very cost-effective!	Taobao Marketplace, a Chinese website for online shopping
Jinfeng Hongyuan Flagship Store	Light Blue Saving Electricity	I got it back and used it, it feels quite convenient and good to use quite good.	Taobao Marketplace, a Chinese website for online shopping
Jinfeng Hongyuan Flagship Store	Light Blue Saving Electricity	This baby is good, very practical original has bought a, due to the very save electricity this time to buy again!	Taobao Marketplace, a Chinese website for online shopping
Jinfeng Hongyuan Flagship Store	Light Blue Saving Electricity	This item is the best product I have ever used and I will definitely refer my friends and family to buy here.	Taobao Marketplace, a Chinese website for online shopping
Jinfeng Hongyuan Flagship Store	Light Blue Saving Electricity	Good product, very satisfied with a shopping, need to come back!	Taobao Marketplace, a Chinese website for online shopping
Jinfeng Hongyuan Flagship Store	Light Blue Saving Electricity	The quality is very good, praise	Taobao Marketplace, a Chinese website for online shopping
Jinfeng Hongyuan Flagship Store	Light Blue Saving Electricity	Fast shipping, inexpensive, works well, regular manufacturer	Taobao Marketplace, a Chinese website for online shopping
Jinfeng Hongyuan Flagship Store	Light Blue Saving Electricity	The machine works great Have bought several of them already	Taobao Marketplace, a Chinese website for online shopping
Jinfeng Hongyuan Flagship Store	Light Blue Saving Electricity	Baby packaging is very good, the product is well made, beautiful appearance, very satisfied.	Taobao Marketplace, a Chinese website for online shopping
Jinfeng Hongyuan Flagship Store	Light Blue Saving Electricity	Very satisfied👍, very good workmanship, fast logistics, great customer service, kudos	Taobao Marketplace, a Chinese website for online shopping
Jinfeng Hongyuan Flagship Store	Light Blue Saving Electricity	Power Saver Works	Taobao Marketplace, a Chinese website for online shopping
Jinfeng Hongyuan Flagship Store	Light Blue Saving Electricity	The quality is the same as the owner said, fast shipping, received the package in perfect condition	Taobao Marketplace, a Chinese website for online shopping
Jinfeng Hongyuan Flagship Store	Light Blue Saving Electricity	I've been using it for a while and it's working.	Taobao Marketplace, a Chinese website for online shopping
Jinfeng Hongyuan Flagship Store	Light Blue Saving Electricity	Easy to use plug and play, really effective and worth the money!	Taobao Marketplace, a Chinese website for online shopping
Jinfeng Hongyuan Flagship Store	Light Blue Saving Electricity	It's not working at all.	Taobao Marketplace, a Chinese website for online shopping
Jinfeng Hongyuan Flagship Store	Light Blue Saving Electricity	Baby received, look very practical, good quality!	Taobao Marketplace, a Chinese website for online shopping
Jinfeng Hongyuan Flagship Store	Light Blue Saving Electricity	First time to buy, feel good, quality is quite good, use for a period of time to see if the power saving!	Taobao Marketplace, a Chinese website for online shopping
Jinfeng Hongyuan Flagship Store	Light Blue Saving Electricity	Very beautiful and generous upscale, the appearance of all such beautiful products, a month to say the least to save two hundred should not be a problem.	Taobao Marketplace, a Chinese website for online shopping
Jinfeng Hongyuan Flagship Store	Light Blue Saving Electricity	Let's use it first.	Taobao Marketplace, a Chinese website for online shopping
Jinfeng Hongyuan Flagship Store	Light Blue Saving Electricity	Received the goods to use, customer service, said in detail can not be inserted into the hot water or air conditioning and other high-power appliances next to the hope that it can save twenty percent of the electricity	Taobao Marketplace, a Chinese website for online shopping
Jinfeng Hongyuan Flagship Store	Light Blue Saving Electricity	I used it for a month after I bought it before I commented on it, and it did save a lot of power, so I bought another one back.	Taobao Marketplace, a Chinese website for online shopping
Jinfeng Hongyuan Flagship Store	Light Blue Saving Electricity	didn't get the goods but the courier paid the claim The customer service is very good	Taobao Marketplace, a Chinese website for online shopping
Jinfeng Hongyuan Flagship Store	Light Blue Saving Electricity	Received the goods, beautifully packaged, product workmanship is fine, very satisfactory	Taobao Marketplace, a Chinese website for online shopping
Jinfeng Hongyuan Flagship Store	Light Blue Saving Electricity	Just got the baby, don't know how it is yet, hope it works, if it works will come again	Taobao Marketplace, a Chinese website for online shopping
Jinfeng Hongyuan Flagship Store	Light Blue Saving Electricity	Baby received, complete packaging, power saving effect to be confirmed.	Taobao Marketplace, a Chinese website for online shopping
Jinfeng Hongyuan Flagship Store	Light Blue Saving Electricity	Baby received, well packaged, results to be confirmed.	Taobao Marketplace, a Chinese website for online shopping
Jinfeng Hongyuan Flagship Store	Light Blue Saving Electricity	Use it for a while, then evaluate	Taobao Marketplace, a Chinese website for online shopping
Jinfeng Hongyuan Flagship Store	Light Blue Saving Electricity	Received it, hope it works well, see the results in a month ah. Support	Taobao Marketplace, a Chinese website for online shopping
Jinfeng Hongyuan Flagship Store	Light Blue Saving Electricity	It's good quality.	Taobao Marketplace, a Chinese website for online shopping
Jinfeng Hongyuan Flagship Store	Light Blue Saving Electricity	The stuff is very substantial, have been using it for a week now, I've bought it before and it works well	Taobao Marketplace, a Chinese website for online shopping

Jinfeng Hongyuan Flagship Store	Light Blue Saving Electricity	Looking forward to using the effect	Taobao Marketplace, a Chinese website for online shopping
Jinfeng Hongyuan Flagship Store	Light Blue Saving Electricity	The stuff is pretty good, trying it out, looking forward to it	Taobao Marketplace, a Chinese website for online shopping
Jinfeng Hongyuan Flagship Store	Light Blue Saving Electricity	Looks great, pretty good quality, use it and see how it works!	Taobao Marketplace, a Chinese website for online shopping
Jinfeng Hongyuan Flagship Store	Light Blue Saving Electricity	The courier is fast.	Taobao Marketplace, a Chinese website for online shopping
Jinfeng Hongyuan Flagship Store	Light Blue Saving Electricity	It's fast.	Taobao Marketplace, a Chinese website for online shopping
Jinfeng Hongyuan Flagship Store	Light Blue Saving Electricity	Very good, introduced several friends to use, all said good, save a lot of electricity.	Taobao Marketplace, a Chinese website for online shopping
Jinfeng Hongyuan Flagship Store	Light Blue Saving Electricity	**Merchants, no integrity to speak of, or multiple purchases, can be such a brush with赖, really speechless, and resolutely will not be patronized in the future.	Taobao Marketplace, a Chinese website for online shopping
Jinfeng Hongyuan Flagship Store	Light Blue Saving Electricity	Customer service was unfamiliar and got it wrong, have apologized, still recommend!	Taobao Marketplace, a Chinese website for online shopping
Jinfeng Hongyuan Flagship Store	Light Blue Saving Electricity	Good review!	Taobao Marketplace, a Chinese website for online shopping
Jinfeng Hongyuan Flagship Store	Light Blue Saving Electricity	IQ tax, capacitor reactive compensation is only effective before the compensation point and does not affect the power factor of the load circuit after the compensation point.	Taobao Marketplace, a Chinese website for online shopping
Jinfeng Hongyuan Flagship Store	Light Blue Saving Electricity	It is genuine, and the same as the physical store, the price is also favorable, decisively turn powder	Taobao Marketplace, a Chinese website for online shopping
Jinfeng Hongyuan Flagship Store	Light Blue Saving Electricity	Received the goods, immediately try, really good	Taobao Marketplace, a Chinese website for online shopping
Jinfeng Hongyuan Flagship Store	Light Blue Saving Electricity	It's recommended by others. Try it first.	Taobao Marketplace, a Chinese website for online shopping
Jinfeng Hongyuan Flagship Store	Light Blue Saving Electricity	Power Saver Works	Taobao Marketplace, a Chinese website for online shopping
Jinfeng Hongyuan Flagship Store	Light Blue Saving Electricity	Good to use, the display voltage217v, look high, the manufacturer is also equipped with a fuse, the service is really sweet, thank you business will buy back,	Taobao Marketplace, a Chinese website for online shopping
Jinfeng Hongyuan Flagship Store	Light Blue Saving Electricity	Power Saving King has received the packaging is very good, just dismantled plugged in to see very good to have a display digital, and also with five fuses. Next time also want to buy a	Taobao Marketplace, a Chinese website for online shopping
Jinfeng Hongyuan Flagship Store	Light Blue Saving Electricity	I've been using it myself for two days now, and I can see that it's okay, but I haven't seen how much I can save.	Taobao Marketplace, a Chinese website for online shopping
Jinfeng Hongyuan Flagship Store	Light Blue Saving Electricity	The product is great, I received it, the meter turned fast before I plugged it in, the meter turned slower when I plugged in the saver.	Taobao Marketplace, a Chinese website for online shopping
Jinfeng Hongyuan Flagship Store	Light Blue Saving Electricity	The quality is very good and the logistics is very fast! And the picture is the same, saving electricity and money, but also to introduce relatives and friends to buy, regret not buying earlier.	Taobao Marketplace, a Chinese website for online shopping
Jinfeng Hongyuan Flagship Store	Light Blue Saving Electricity	please rewardmewithfivedollars in red packets	Taobao Marketplace, a Chinese website for online shopping
Jinfeng Hongyuan Flagship Store	Light Blue Saving Electricity	Quality is very over the top, quality is guaranteed, quite a good choice, a lot of friends to see me use all want to buy, I recommend it!	Taobao Marketplace, a Chinese website for online shopping
Jinfeng Hongyuan Flagship Store	Light Blue Saving Electricity	Very nice.	Taobao Marketplace, a Chinese website for online shopping
Jinfeng Hongyuan Flagship Store	Light Blue Saving Electricity	I heard my friend was using it so I bought one for the store.	Taobao Marketplace, a Chinese website for online shopping
Jinfeng Hongyuan Flagship Store	Light Blue Saving Electricity	Things are really great, good quality, fine workmanship, affordable price, logistics is also very fast, praise.	Taobao Marketplace, a Chinese website for online shopping
Jinfeng Hongyuan Flagship Store	Light Blue Saving Electricity	The stuff is good and there are discounts, so you can save money and buy without hesitation	Taobao Marketplace, a Chinese website for online shopping
Jinfeng Hongyuan Flagship Store	Light Blue Saving Electricity	I've been using it for a while and it feels good.	Taobao Marketplace, a Chinese website for online shopping
Jinfeng Hongyuan Flagship Store	Light Blue Saving Electricity	A very good shopping experience, attentive service, fast logistics, delivery is also very thoughtful, attitude is also very good, value for money ah, decisive praise!	Taobao Marketplace, a Chinese website for online shopping
Jinfeng Hongyuan Flagship Store	Light Blue Saving Electricity	Logistics is very fast, received well-packaged, the product is exquisite, the use of the effect of use after evaluation!	Taobao Marketplace, a Chinese website for online shopping
Jinfeng Hongyuan Flagship Store	Light Blue Saving Electricity	Good quality and fast logistics is worth buying friends who like to buy rest assured!	Taobao Marketplace, a Chinese website for online shopping
Jinfeng Hongyuan Flagship Store	Light Blue Saving Electricity	The product is great, I received it, the meter turned fast before I plugged it in, the meter turned slower when I plugged in the saver, it was great.	Taobao Marketplace, a Chinese website for online shopping
Jinfeng Hongyuan Flagship Store	Light Blue Saving Electricity	Looked like the picture packaging is quite exquisite look forward to using the surprise!	Taobao Marketplace, a Chinese website for online shopping
Jinfeng Hongyuan Flagship Store	Light Blue Saving Electricity	Fast logistics, beautifully packaged, customer service is very satisfied with the service, really good, with a use and then come back to the feedback effect!	Taobao Marketplace, a Chinese website for online shopping
Jinfeng Hongyuan Flagship Store	Light Blue Saving Electricity	Good quality, just as described by the merchant	Taobao Marketplace, a Chinese website for online shopping
Jinfeng Hongyuan Flagship Store	Light Blue Saving Electricity	Service attitude is very good, logistics is also fast, baby I started to use today, should be good	Taobao Marketplace, a Chinese website for online shopping

Jinfeng Hongyuan Flagship Store	Light Blue Saving Electricity	The stuff is very good, very power saving beautiful and durable.	Taobao Marketplace, a Chinese website for online shopping
Jinfeng Hongyuan Flagship Store	Light Blue Saving Electricity	Great value, looking forward to the performance in a month, good reviews first!	Taobao Marketplace, a Chinese website for online shopping
Jinfeng Hongyuan Flagship Store	Light Blue Saving Electricity	Installed,I't know how it works	Taobao Marketplace, a Chinese website for online shopping
Jinfeng Hongyuan Flagship Store	Light Blue Saving Electricity	The saver is metallic and much better than previous purchases, which were tens of dollars of plastic shells. There is a power saving effect	Taobao Marketplace, a Chinese website for online shopping
Jinfeng Hongyuan Flagship Store	Light Blue Saving Electricity	On use, normal operation, expect efficacy._x005f_x005f_x0001_ good service attitude	Taobao Marketplace, a Chinese website for online shopping
Jinfeng Hongyuan Flagship Store	Light Blue Saving Electricity	Baby received, well packaged, exquisite workmanship, trustworthy.	Taobao Marketplace, a Chinese website for online shopping
Jinfeng Hongyuan Flagship Store	Light Blue Saving Electricity	It's not working well, it doesn't save any points, it's a waste of money and it's so expensive	Taobao Marketplace, a Chinese website for online shopping
Jinfeng Hongyuan Flagship Store	Light Blue Saving Electricity	I received the baby, looking forward to saving part of my electricity bill, use a period of time to come over to the follow-up review!	Taobao Marketplace, a Chinese website for online shopping
Jinfeng Hongyuan Flagship Store	Light Blue Saving Electricity	I just received it, I also heard people introduce the product, wait for a period of time to use before evaluating!	Taobao Marketplace, a Chinese website for online shopping
Jinfeng Hongyuan Flagship Store	Light Blue Saving Electricity	It's pretty good. I recommend it.	Taobao Marketplace, a Chinese website for online shopping
Jinfeng Hongyuan Flagship Store	Light Blue Saving Electricity	Great stuff, second purchase already.	Taobao Marketplace, a Chinese website for online shopping
Jinfeng Hongyuan Flagship Store	Light Blue Saving Electricity	Nice and practical	Taobao Marketplace, a Chinese website for online shopping
Jinfeng Hongyuan Flagship Store	Light Blue Saving Electricity	Installed it and it looks exquisite, we'll see how it looks in a month.	Taobao Marketplace, a Chinese website for online shopping
Jinfeng Hongyuan Flagship Store	Light Blue Saving Electricity	Great service, fast shipping, no problems with quality.	Taobao Marketplace, a Chinese website for online shopping
Jinfeng Hongyuan Flagship Store	Light Blue Saving Electricity	Works great , I've purchased so many brands All of them made me return them because they really didn't work This brand I've been using regularly2months It really lets me see the results I like it I recommend It's the only one I've ever used that works the best.	Taobao Marketplace, a Chinese website for online shopping
Jinfeng Hongyuan Flagship Store	Light Blue Saving Electricity	Received the goods the first time to plug in, feel very good to use, good, powerful	Taobao Marketplace, a Chinese website for online shopping
Jinfeng Hongyuan Flagship Store	Light Blue Saving Electricity	Baby has been quickly received, the packaging is also very delicate, try a period of time to come back to the review, first praise!	Taobao Marketplace, a Chinese website for online shopping
Jinfeng Hongyuan Flagship Store	Light Blue Saving Electricity	Baby just received on the use, good packaging, fine workmanship feel authentic products, look forward to having the effect of the	Taobao Marketplace, a Chinese website for online shopping
Jinfeng Hongyuan Flagship Store	Light Blue Saving Electricity	It's very affordable and cost-effective, it's kind of affordable and good, I will repurchase it	Taobao Marketplace, a Chinese website for online shopping
Jinfeng Hongyuan Flagship Store	Light Blue Saving Electricity	Good logistics and product, good value for money, kudos👏👏👏👏👏👏	Taobao Marketplace, a Chinese website for online shopping
Jinfeng Hongyuan Flagship Store	Light Blue Saving Electricity	So far everything is working fine, how well the power saving works, we have to wait until the end of next January, then we will follow up!	Taobao Marketplace, a Chinese website for online shopping
Jinfeng Hongyuan Flagship Store	Light Blue Saving Electricity	Get another one.	Taobao Marketplace, a Chinese website for online shopping
Jinfeng Hongyuan Flagship Store	Light Blue Saving Electricity	Saving appliances received to open the Hopper Certificate of Conformity manual are regular products immediately installed to the main switch under the socket is very beautiful depends on the future energy-saving effect!	Taobao Marketplace, a Chinese website for online shopping
Jinfeng Hongyuan Flagship Store	Light Blue Saving Electricity	Looks good, just used it and will comment later	Taobao Marketplace, a Chinese website for online shopping
Jinfeng Hongyuan Flagship Store	Light Blue Saving Electricity	This packaging is really too advanced, at first glance is a good thing, has been plugged into the power supply, good use will continue to buy back, ready to give parents and the store also buy a try!	Taobao Marketplace, a Chinese website for online shopping
Jinfeng Hongyuan Flagship Store	Light Blue Saving Electricity	I'm using it. I don't know if it's working.	Taobao Marketplace, a Chinese website for online shopping
Jinfeng Hongyuan Flagship Store	Light Blue Saving Electricity	Has received the packaging is intact, packaging is also very good, the product quality is also good, the voltage display is clear, still trying, looking forward to the effect of it, and then use a period of time to see, the service attitude is good!	Taobao Marketplace, a Chinese website for online shopping
Jinfeng Hongyuan Flagship Store	Light Blue Saving Electricity	Low less weak power at night, normal220~230powerduring daytime	Taobao Marketplace, a Chinese website for online shopping
Jinfeng Hongyuan Flagship Store	Light Blue Saving Electricity	Baby is really good, do really careful, worth to have, fast delivery!	Taobao Marketplace, a Chinese website for online shopping
Jinfeng Hongyuan Flagship Store	Light Blue Saving Electricity	Logistics is very fast, the product is beautiful, texture, installation is very convenient	Taobao Marketplace, a Chinese website for online shopping
Jinfeng Hongyuan Flagship Store	Light Blue Saving Electricity	Exquisite appearance Details are on point Bought two Looking forward to the results	Taobao Marketplace, a Chinese website for online shopping
Jinfeng Hongyuan Flagship Store	Light Blue Saving Electricity	The power saver is good, the value is very high, aluminum alloy shell, LCD display, this workmanship, bar. I hope to be able to save a lot of electricity ah	Taobao Marketplace, a Chinese website for online shopping
Jinfeng Hongyuan Flagship Store	Light Blue Saving Electricity	Very good quality, well made, very satisfied.	Taobao Marketplace, a Chinese website for online shopping
Jinfeng Hongyuan Flagship Store	Light Blue Saving Electricity	The saver is plug-and-play, very convenient, and saves electricity very well	Taobao Marketplace, a Chinese website for online shopping

Jinfeng Hongyuan Flagship Store	Light Blue Saving Electricity	We'll see how it works in a while.	Taobao Marketplace, a Chinese website for online shopping
Jinfeng Hongyuan Flagship Store	Light Blue Saving Electricity	Baby received it👌, really great ah!🌈The quality can't be beat, very satisfied!🆗Happy happy happy😃	Taobao Marketplace, a Chinese website for online shopping
Jinfeng Hongyuan Flagship Store	Light Blue Saving Electricity	I don't know how it is yet, I'll use it first, I guess it still works	Taobao Marketplace, a Chinese website for online shopping
Jinfeng Hongyuan Flagship Store	Light Blue Saving Electricity	Have not yet seen the effect, things look good, quite heavy, is genuine	Taobao Marketplace, a Chinese website for online shopping
Jinfeng Hongyuan Flagship Store	Light Blue Saving Electricity	Baby received and used it, need time to verify. First praise five points. Believe in big brands, quality is guaranteed.	Taobao Marketplace, a Chinese website for online shopping
Jinfeng Hongyuan Flagship Store	Light Blue Saving Electricity	Bought and tested the device, it's good and as described	Taobao Marketplace, a Chinese website for online shopping
Jinfeng Hongyuan Flagship Store	Light Blue Saving Electricity	Products can	Taobao Marketplace, a Chinese website for online shopping
Jinfeng Hongyuan Flagship Store	Light Blue Saving Electricity	The description matches the logistics very quickly customer service is very enthusiastic	Taobao Marketplace, a Chinese website for online shopping
Jinfeng Hongyuan Flagship Store	Light Blue Saving Electricity	Baby received, the packaging is very fine to, and the seller describes the same, use a period of time to know the effect, and then follow up.	Taobao Marketplace, a Chinese website for online shopping
Jinfeng Hongyuan Flagship Store	Light Blue Saving Electricity	Packaging intact, the first2months of relatives bought a power-saving effect is obvious, introduced me to buy another one, the business is trustworthy basket color, LCD display upgraded version	Taobao Marketplace, a Chinese website for online shopping
Jinfeng Hongyuan Flagship Store	Light Blue Saving Electricity	"one" radical in Chinese characters (Kangxi radical 1)	Taobao Marketplace, a Chinese website for online shopping
Jinfeng Hongyuan Flagship Store	Light Blue Saving Electricity	High-powered, nice, pretty good quality	Taobao Marketplace, a Chinese website for online shopping
Jinfeng Hongyuan Flagship Store	Light Blue Saving Electricity	It's still good! Looking forward to future affordable use ! ! ! !	Taobao Marketplace, a Chinese website for online shopping
Jinfeng Hongyuan Flagship Store	Light Blue Saving Electricity	Baby beautiful, stable performance .	Taobao Marketplace, a Chinese website for online shopping
Jinfeng Hongyuan Flagship Store	Light Blue Saving Electricity	Power saving treasure design unique exquisite beautiful, voltage display clearly, plugged in that is good to use, I observed the meter, after using the meter is gone slow.	Taobao Marketplace, a Chinese website for online shopping
Jinfeng Hongyuan Flagship Store	Light Blue Saving Electricity	It's working and shipping was quick and easy.	Taobao Marketplace, a Chinese website for online shopping
Jinfeng Hongyuan Flagship Store	Light Blue Saving Electricity	Just received the goods immediately disassembled and plugged up to use, very satisfied, ready to buy back again!	Taobao Marketplace, a Chinese website for online shopping
Jinfeng Hongyuan Flagship Store	Light Blue Saving Electricity	Appearance:Fine workmanship, feel good, the description is consistent with the object Function:Just started using3days, as for whether there is a power-saving effect, as well as the power-saving rate still need time and equipment to verify, I hope to meet the expected effect.	Taobao Marketplace, a Chinese website for online shopping
Jinfeng Hongyuan Flagship Store	Light Blue Saving Electricity	Power Saving King works well	Taobao Marketplace, a Chinese website for online shopping
Jinfeng Hongyuan Flagship Store	Light Blue Saving Electricity	Looks good, looking forward to the results	Taobao Marketplace, a Chinese website for online shopping
Jinfeng Hongyuan Flagship Store	Light Blue Saving Electricity	Just received the baby, has been plugged in the test to observe for a period of time, to see how it is in the back to comment.	Taobao Marketplace, a Chinese website for online shopping
Jinfeng Hongyuan Flagship Store	Light Blue Saving Electricity	Got it, plugging it in now to see if it's as good as I hoped it would be.	Taobao Marketplace, a Chinese website for online shopping
Jinfeng Hongyuan Flagship Store	Light Blue Saving Electricity	rare	Taobao Marketplace, a Chinese website for online shopping
Jinfeng Hongyuan Flagship Store	Light Blue Saving Electricity	Goods are very good, packaging is also very good, product quality is also good, see how to use the effect?It looks good.	Taobao Marketplace, a Chinese website for online shopping
Jinfeng Hongyuan Flagship Store	Light Blue Saving Electricity	Pretty good, very satisfied with the purchase	Taobao Marketplace, a Chinese website for online shopping
Jinfeng Hongyuan Flagship Store	Light Blue Saving Electricity	Baby received and very satisfied. The quality is very good.	Taobao Marketplace, a Chinese website for online shopping
Jinfeng Hongyuan Flagship Store	Light Blue Saving Electricity	Perfect, the product is amazing, the price/performance ratio is right, I hope it lasts a long time! Logistics is super super fast! The quality is also very good, indeed value for money	Taobao Marketplace, a Chinese website for online shopping
Jinfeng Hongyuan Flagship Store	Light Blue Saving Electricity	It's okay hahahahahahahahahahahahahahahahahahaha	Taobao Marketplace, a Chinese website for online shopping
Jinfeng Hongyuan Flagship Store	Light Blue Saving Electricity	I've been using it for a while, and it's not bad, the power is really less!	Taobao Marketplace, a Chinese website for online shopping
Jinfeng Hongyuan Flagship Store	Light Blue Saving Electricity	The last time I bought this kind of bad in another store and changed to another one to experience it first and then talk about it later.	Taobao Marketplace, a Chinese website for online shopping
Jinfeng Hongyuan Flagship Store	Light Blue Saving Electricity	Received,, super quality, looking forward to the results!	Taobao Marketplace, a Chinese website for online shopping
Jinfeng Hongyuan Flagship Store	Light Blue Saving Electricity	I used it as soon as I received it, and it really saves power!	Taobao Marketplace, a Chinese website for online shopping
Jinfeng Hongyuan Flagship Store	Light Blue Saving Electricity	Bought it and raved about Looking forward to the results	Taobao Marketplace, a Chinese website for online shopping
Jinfeng Hongyuan Flagship Store	Light Blue Saving Electricity	Power Saver works great!	Taobao Marketplace, a Chinese website for online shopping

Jinfeng Hongyuan Flagship Store	Light Blue Saving Electricity	It works and I'm happy with Bought two Planning to buy two more	Taobao Marketplace, a Chinese website for online shopping
Jinfeng Hongyuan Flagship Store	Light Blue Saving Electricity	The goods have arrived, energy saving electricity do not know if the business said good	Taobao Marketplace, a Chinese website for online shopping
Jinfeng Hongyuan Flagship Store	Light Blue Saving Electricity	The appearance of the sky blue beautiful and classy. There are fuse buttons to adjust the use of the LCD display merchants think really thoughtful, this is a monthly power within 500 degrees of electricity. Merchants recommended, good business service, courier logistics to give three days less than to the goods. Thank you for the merchants and logistics support, with good will recommend to relatives and [illegible]	Taobao Marketplace, a Chinese website for online shopping
Jinfeng Hongyuan Flagship Store	Light Blue Saving Electricity	Already added to cart for easy re-purchase in the future, thanks to the merchant for the great support. Many😁	Taobao Marketplace, a Chinese website for online shopping
Jinfeng Hongyuan Flagship Store	Light Blue Saving Electricity	Energy saver is beautiful, affordable, good, you can buy one to use it	Taobao Marketplace, a Chinese website for online shopping
Jinfeng Hongyuan Flagship Store	Light Blue Saving Electricity	I've been using it for almost a month now, and I don't know if it works or not, but the daily electricity bill isn't much less anyway.	Taobao Marketplace, a Chinese website for online shopping
Jinfeng Hongyuan Flagship Store	Light Blue Saving Electricity	Not bad worth buying trust good stuff	Taobao Marketplace, a Chinese website for online shopping
Jinfeng Hongyuan Flagship Store	Light Blue Saving Electricity	Not bad worth buying trust good stuff	Taobao Marketplace, a Chinese website for online shopping
Jinfeng Hongyuan Flagship Store	Light Blue Saving Electricity	Good quality, metal shell is drop-proof, good design can be hung up to use, worth buying.	Taobao Marketplace, a Chinese website for online shopping
Jinfeng Hongyuan Flagship Store	Light Blue Saving Electricity	I won't know if it will save power until I use it for a while.	Taobao Marketplace, a Chinese website for online shopping
Jinfeng Hongyuan Flagship Store	Light Blue Saving Electricity	Baby is very good!	Taobao Marketplace, a Chinese website for online shopping
Jinfeng Hongyuan Flagship Store	Light Blue Saving Electricity	Packaging is good also works properly, use it first to see how it works.	Taobao Marketplace, a Chinese website for online shopping
Jinfeng Hongyuan Flagship Store	Light Blue Saving Electricity	First time buyer, good quality, look forward to the effect	Taobao Marketplace, a Chinese website for online shopping
Jinfeng Hongyuan Flagship Store	Light Blue Saving Electricity	Plugged in it's okay the more power saving the better, love it!	Taobao Marketplace, a Chinese website for online shopping
Jinfeng Hongyuan Flagship Store	Light Blue Saving Electricity	Baby received, fine workmanship, installed a few days feel good power saving. Also sent five fuse, with a period of time and then follow up!	Taobao Marketplace, a Chinese website for online shopping
Jinfeng Hongyuan Flagship Store	Light Blue Saving Electricity	The product received, have not yet used, do not know the effect of how, after using and then follow up the evaluation	Taobao Marketplace, a Chinese website for online shopping
Jinfeng Hongyuan Flagship Store	Light Blue Saving Electricity	Reduce line losses and use for long periods of time to achieve power savings.	Taobao Marketplace, a Chinese website for online shopping
Jinfeng Hongyuan Flagship Store	Light Blue Saving Electricity	It's not bad.	Taobao Marketplace, a Chinese website for online shopping
Jinfeng Hongyuan Flagship Store	Light Blue Saving Electricity	Come back to install and use, can be good, next time to come back to purchase	Taobao Marketplace, a Chinese website for online shopping
Jinfeng Hongyuan Flagship Store	Light Blue Saving Electricity	Baby to receive, the quality is very good.	Taobao Marketplace, a Chinese website for online shopping
Jinfeng Hongyuan Flagship Store	Light Blue Saving Electricity	Power saver to mounted	Taobao Marketplace, a Chinese website for online shopping
Jinfeng Hongyuan Flagship Store	Light Blue Saving Electricity	Baby received can not wait to plug in the power supply, used for a period of time to come over to evaluate, can really save electricity, the girl used very good, this is not I bought another👍👍👍👍👍	Taobao Marketplace, a Chinese website for online shopping
Jinfeng Hongyuan Flagship Store	Light Blue Saving Electricity	Power Saver is useful	Taobao Marketplace, a Chinese website for online shopping
Jinfeng Hongyuan Flagship Store	Light Blue Saving Electricity	Goods received, material bar, good value for money, worth buying!	Taobao Marketplace, a Chinese website for online shopping
Jinfeng Hongyuan Flagship Store	Light Blue Saving Electricity	Bought 3 received Liquid display voltage varies How do you guys explain Effectiveness how to use and then evaluate	Taobao Marketplace, a Chinese website for online shopping
Jinfeng Hongyuan Flagship Store	Light Blue Saving Electricity	(of an unmarried couple) be close	Taobao Marketplace, a Chinese website for online shopping
Jinfeng Hongyuan Flagship Store	Light Blue Saving Electricity	Small size, easy to use, long-term use can really save energy, digital display is very convenient to use ah!	Taobao Marketplace, a Chinese website for online shopping
Jinfeng Hongyuan Flagship Store	Light Blue Saving Electricity	Things just arrived have not yet seen the effect, customer service attitude is very good, if the effect is good will continue to buy!	Taobao Marketplace, a Chinese website for online shopping
Jinfeng Hongyuan Flagship Store	Light Blue Saving Electricity	Save a lot of volume, really save, recommended for public use	Taobao Marketplace, a Chinese website for online shopping
Jinfeng Hongyuan Flagship Store	Light Blue Saving Electricity	I really recommend it, the delivery was exceptionally fast, love love love it!	Taobao Marketplace, a Chinese website for online shopping
Jinfeng Hongyuan Flagship Store	Light Blue Saving Electricity	I have received it today and will review after using it.	Taobao Marketplace, a Chinese website for online shopping
Jinfeng Hongyuan Flagship Store	Light Blue Saving Electricity	Used it for a while and it works well	Taobao Marketplace, a Chinese website for online shopping
Jinfeng Hongyuan Flagship Store	Light Blue Saving Electricity	Fourth purchase, which is a great testament to the product.	Taobao Marketplace, a Chinese website for online shopping
Jinfeng Hongyuan Flagship Store	Light Blue Saving Electricity	I checked it every day, but it didn't help.	Taobao Marketplace, a Chinese website for online shopping

Jinfeng Hongyuan Flagship Store	Light Blue Saving Electricity	Compact size, does not take up space, digital display looks more convenient, the shell of the iron is safer, not fragile	Taobao Marketplace, a Chinese website for online shopping
Jinfeng Hongyuan Flagship Store	Light Blue Saving Electricity	This is the second time I've bought it.	Taobao Marketplace, a Chinese website for online shopping
Jinfeng Hongyuan Flagship Store	Light Blue Saving Electricity	Received, look at the goods are more satisfied, use a period of time to follow up the evaluation	Taobao Marketplace, a Chinese website for online shopping
Jinfeng Hongyuan Flagship Store	Light Blue Saving Electricity	The saver is so god, specially tested it for one day and one night, the effect is so awesome, the same electricity consumption really saves electricity. Worth recommending.	Taobao Marketplace, a Chinese website for online shopping
Jinfeng Hongyuan Flagship Store	Light Blue Saving Electricity	Logistics is very fast, the seller's service attitude is very good, tried, no problems	Taobao Marketplace, a Chinese website for online shopping
Jinfeng Hongyuan Flagship Store	Light Blue Saving Electricity	Good quality, as described, easy to use, recommended, affordable price	Taobao Marketplace, a Chinese website for online shopping
Jinfeng Hongyuan Flagship Store	Light Blue Saving Electricity	Bought nearly a month today just started to review, because at that time, I bought do not know good and bad, more do not know in the end to save electricity, after this period of time to test, about a month to save30bucks it. The quality is also beautiful, I recommend him to my friends.	Taobao Marketplace, a Chinese website for online shopping
Jinfeng Hongyuan Flagship Store	Light Blue Saving Electricity	The saver was received and I am very happy with it!	Taobao Marketplace, a Chinese website for online shopping
Jinfeng Hongyuan Flagship Store	Light Blue Saving Electricity	I just received it and opened it, everyone said it was a good idea to buy one and try it out.	Taobao Marketplace, a Chinese website for online shopping
Jinfeng Hongyuan Flagship Store	Light Blue Saving Electricity	I just used it to hit the first positive feedback.	Taobao Marketplace, a Chinese website for online shopping
Jinfeng Hongyuan Flagship Store	Light Blue Saving Electricity	The packaging is very high-grade, the effect should be right, the quality is also very good, looking forward to the effect	Taobao Marketplace, a Chinese website for online shopping
Jinfeng Hongyuan Flagship Store	Light Blue Saving Electricity	Arrived very quickly, the quality can be customer service is very professional service attitude is great, praise👍	Taobao Marketplace, a Chinese website for online shopping
Jinfeng Hongyuan Flagship Store	Light Blue Saving Electricity	Great power savings after two months or so Very happy with	Taobao Marketplace, a Chinese website for online shopping
Jinfeng Hongyuan Flagship Store	Light Blue Saving Electricity	The quality is good and it does save electricity in the long run, it is a reactive power compensator.	Taobao Marketplace, a Chinese website for online shopping
Jinfeng Hongyuan Flagship Store	Light Blue Saving Electricity	Commercial use can be used, power saving, customer service is very serious recommended, will be back to buy praise!	Taobao Marketplace, a Chinese website for online shopping
Jinfeng Hongyuan Flagship Store	Light Blue Saving Electricity	This baby is quite good, bought about three days before coming to comment, the effect is quite good	Taobao Marketplace, a Chinese website for online shopping
Jinfeng Hongyuan Flagship Store	Light Blue Saving Electricity	Second one, works great, saves$40+a monthelectric bill	Taobao Marketplace, a Chinese website for online shopping
Jinfeng Hongyuan Flagship Store	Light Blue Saving Electricity	Good value for money worth getting	Taobao Marketplace, a Chinese website for online shopping
Jinfeng Hongyuan Flagship Store	Light Blue Saving Electricity	Report received look forward to the effect, very satisfied with the trustworthy merchants	Taobao Marketplace, a Chinese website for online shopping
Jinfeng Hongyuan Flagship Store	Light Blue Saving Electricity	Logistics delivery fast, the product is easy to use, just use do not know how the effect, wait two months after the effect is good, will come back to buy one.	Taobao Marketplace, a Chinese website for online shopping
Jinfeng Hongyuan Flagship Store	Light Blue Saving Electricity	Third purchase is an absolute endorsement of the product	Taobao Marketplace, a Chinese website for online shopping
Jinfeng Hongyuan Flagship Store	Light Blue Saving Electricity	It's very energy efficient. It'll save you in a month.	Taobao Marketplace, a Chinese website for online shopping
Jinfeng Hongyuan Flagship Store	Light Blue Saving Electricity	Good quality, works fine	Taobao Marketplace, a Chinese website for online shopping
Jinfeng Hongyuan Flagship Store	Light Blue Saving Electricity	The quality is very good, the packaging is very good! You will be surprised after using it.	Taobao Marketplace, a Chinese website for online shopping
Jinfeng Hongyuan Flagship Store	Light Blue Saving Electricity	Baby is basically the same as the description, fast shipping, worth it!	Taobao Marketplace, a Chinese website for online shopping
Jinfeng Hongyuan Flagship Store	Light Blue Saving Electricity	It works.	Taobao Marketplace, a Chinese website for online shopping
Jinfeng Hongyuan Flagship Store	Light Blue Saving Electricity	I have been using this brand of power saver for many years and this time I saw this upgraded version and got it again. The main purpose of this product is to reduce the loss of electricity for a long time, so as to achieve the purpose of saving electricity.	Taobao Marketplace, a Chinese website for online shopping
Jinfeng Hongyuan Flagship Store	Light Blue Saving Electricity	The product is very good, the appearance and quality is also very good, it is very easy to use and very stable	Taobao Marketplace, a Chinese website for online shopping
Jinfeng Hongyuan Flagship Store	Light Blue Saving Electricity	Received it and tried it out, it's great, great quality and worth having! Five stars praise!	Taobao Marketplace, a Chinese website for online shopping
Jinfeng Hongyuan Flagship Store	Light Blue Saving Electricity	worth buying	Taobao Marketplace, a Chinese website for online shopping
Jinfeng Hongyuan Flagship Store	Light Blue Saving Electricity	Power Saving King power-saving effect is obvious very good with a product, my neighbor recommended her home for more than a year to save a lot of electricity it!	Taobao Marketplace, a Chinese website for online shopping
Jinfeng Hongyuan Flagship Store	Light Blue Saving Electricity	It's the real deal. It's well packaged. I like it.	Taobao Marketplace, a Chinese website for online shopping
Jinfeng Hongyuan Flagship Store	Light Blue Saving Electricity	Just received the goods and opened it, the bag is very delicate, the product is the same as the picture! Honest merchants! Worth having to buy!	Taobao Marketplace, a Chinese website for online shopping
Jinfeng Hongyuan Flagship Store	Light Blue Saving Electricity	The quality is pretty good, it works fine, it does work	Taobao Marketplace, a Chinese website for online shopping

Jinfeng Hongyuan Flagship Store	Light Blue Saving Electricity	The effect is good, the fuse is also very conscientious installation and replacement is simple, much better than the plastic models I bought before, and LCD current display and stabilize the voltage function security is also guaranteed, the electricity meter has just come out to send to a colleague, before talking to him still do not believe that he believes he is also going to get two, conscientious	Taobao Marketplace, a Chinese website for online shopping
Jinfeng Hongyuan Flagship Store	Light Blue Saving Electricity	Howdy first and second purchase. Meticulous workmanship	Taobao Marketplace, a Chinese website for online shopping
Jinfeng Hongyuan Flagship Store	Light Blue Saving Electricity	Baby is very satisfied, packaging is also very good, have used, will introduce friends to buy, logistics is very fast.👍	Taobao Marketplace, a Chinese website for online shopping
Jinfeng Hongyuan Flagship Store	Light Blue Saving Electricity	Baby is very satisfied, packaging is also very good. Has been used, will introduce friends to buy, logistics is very fast.👍	Taobao Marketplace, a Chinese website for online shopping
Jinfeng Hongyuan Flagship Store	Light Blue Saving Electricity	Match the description of the merchant, it can indeed save electricity, it takes a long time to save electricity, this thing is also very small, where to put it will not be in the way, very satisfied, especially good	Taobao Marketplace, a Chinese website for online shopping
Jinfeng Hongyuan Flagship Store	Light Blue Saving Electricity	One day down the road, comparing the electricity bills saved. Kudos.	Taobao Marketplace, a Chinese website for online shopping
Jinfeng Hongyuan Flagship Store	Light Blue Saving Electricity	Item has been received, blue in color, looks good, just plugged it in and used it.	Taobao Marketplace, a Chinese website for online shopping
Jinfeng Hongyuan Flagship Store	Light Blue Saving Electricity	Nice color, compact, convenient, gave it away!	Taobao Marketplace, a Chinese website for online shopping
Jinfeng Hongyuan Flagship Store	Light Blue Saving Electricity	The power saving effect is really good, several hundred dollars a month of electricity, I bought2with the intention of trying, the saver arrived I plugged it in, looking forward to good results	Taobao Marketplace, a Chinese website for online shopping
Jinfeng Hongyuan Flagship Store	Light Blue Saving Electricity	Recommended by a friend, looks ok, hopefully it will save power	Taobao Marketplace, a Chinese website for online shopping
Jinfeng Hongyuan Flagship Store	Light Blue Saving Electricity	Friends link to order, friends say good before ordering, received immediately use	Taobao Marketplace, a Chinese website for online shopping
Jinfeng Hongyuan Flagship Store	Light Blue Saving Electricity	Shopping here is safe, convenient and fast💗,courier guy is very nice, this price is very powerful💪, very recommended, will continue to buy back	Taobao Marketplace, a Chinese website for online shopping
Jinfeng Hongyuan Flagship Store	Light Blue Saving Electricity	It's good. It's worth it.	Taobao Marketplace, a Chinese website for online shopping
Jinfeng Hongyuan Flagship Store	Light Blue Saving Electricity	Good quality goods, affordable price, good business service!	Taobao Marketplace, a Chinese website for online shopping
Jinfeng Hongyuan Flagship Store	Light Blue Saving Electricity	Great store service, very happy with receipt.	Taobao Marketplace, a Chinese website for online shopping
Jinfeng Hongyuan Flagship Store	Light Blue Saving Electricity	Really super like it, very good quality, as described by the seller!	Taobao Marketplace, a Chinese website for online shopping
Jinfeng Hongyuan Flagship Store	Light Blue Saving Electricity	This baby works really well and I recommend it!	Taobao Marketplace, a Chinese website for online shopping
Jinfeng Hongyuan Flagship Store	Light Blue Saving Electricity	Thank you to the merchants! Received the baby at a good price, easy to operate, fast logistics, praise👍, do not hesitate to get down if you need it!	Taobao Marketplace, a Chinese website for online shopping
Jinfeng Hongyuan Flagship Store	Light Blue Saving Electricity	The power saving is really good	Taobao Marketplace, a Chinese website for online shopping
Jinfeng Hongyuan Flagship Store	Light Blue Saving Electricity	Still trying it out, looking forward to good results	Taobao Marketplace, a Chinese website for online shopping
Jinfeng Hongyuan Flagship Store	Light Blue Saving Electricity	Very good quality, good material, fast delivery from the merchant, recommendable!	Taobao Marketplace, a Chinese website for online shopping
Jinfeng Hongyuan Flagship Store	Light Blue Saving Electricity	Fast shipping, referred to me by a coworker, looking forward to saving some pocket change😄	Taobao Marketplace, a Chinese website for online shopping
Jinfeng Hongyuan Flagship Store	Light Blue Saving Electricity	Used itmore than60days, good power saving, buy another one	Taobao Marketplace, a Chinese website for online shopping
Jinfeng Hongyuan Flagship Store	Light Blue Saving Electricity	Great quality, perfect design, very durable	Taobao Marketplace, a Chinese website for online shopping
Jinfeng Hongyuan Flagship Store	Light Blue Saving Electricity	ibid	Taobao Marketplace, a Chinese website for online shopping
Jinfeng Hongyuan Flagship Store	Light Blue Saving Electricity	Good review. It's great.	Taobao Marketplace, a Chinese website for online shopping
Jinfeng Hongyuan Flagship Store	Light Blue Saving Electricity	The product is well made and the blue color is not harsh. Energy saving to be used after. It should be the same reason as the reactive power compensation of industrial electricity.	Taobao Marketplace, a Chinese website for online shopping
Jinfeng Hongyuan Flagship Store	Light Blue Saving Electricity	Comparison of several online, before choosing this one, with LCD display, easy to use, and then use a period of time to see if it is able to save electricity	Taobao Marketplace, a Chinese website for online shopping
Jinfeng Hongyuan Flagship Store	Light Blue Saving Electricity	Yes, you can.	Taobao Marketplace, a Chinese website for online shopping
Jinfeng Hongyuan Flagship Store	Light Blue Saving Electricity	This businessman's customer-oriented service is of high quality, leaving a good impression on customers! Must be able to business is booming, eight sources of wealth rolling. Returns and refunds are extremely fast, and shipping insurance is also extraordinarily fast with the merchant's refund.	Taobao Marketplace, a Chinese website for online shopping
Jinfeng Hongyuan Flagship Store	Light Blue Saving Electricity	workmanship is exquisite, the quality is great, the installation is simple and convenient, the effect is very good after use.	Taobao Marketplace, a Chinese website for online shopping
Jinfeng Hongyuan Flagship Store	Light Blue Saving Electricity	Intelligent power saver received, immediately opened to see, the packaging is very good, the power saver is small and exquisite. Merchants fast delivery, wait for the use of a follow-up comment, give full marks, wish the boss a prosperous business!	Taobao Marketplace, a Chinese website for online shopping
Jinfeng Hongyuan Flagship Store	Light Blue Saving Electricity	The goods have been received, beautiful appearance, in use	Taobao Marketplace, a Chinese website for online shopping
Jinfeng Hongyuan Flagship Store	Light Blue Saving Electricity	Baby has received, with the buyer described the same, the quality is very good, is genuine licensed goods, the price is also more reasonable, the main thing is to save electricity costs. Next time to buy👍👍👍👍👍👍	Taobao Marketplace, a Chinese website for online shopping

Jinfeng Hongyuan Flagship Store	Light Blue Saving Electricity	Baby used for a period of time here to evaluate, indeed have power-saving function is very good!	Taobao Marketplace, a Chinese website for online shopping
Jinfeng Hongyuan Flagship Store	Light Blue Saving Electricity	Saver received, good quality, good effect, easy to install, simple to use	Taobao Marketplace, a Chinese website for online shopping
Jinfeng Hongyuan Flagship Store	Light Blue Saving Electricity	The packaging is intact and the price is more affordable than others	Taobao Marketplace, a Chinese website for online shopping
Jinfeng Hongyuan Flagship Store	Light Blue Saving Electricity	Power-saving effect: it just so happens that the weather is very hot these days, every day there is open air conditioning, as if the power meter goes really slower, it should be effective. Workmanship material: the material is okay.	Taobao Marketplace, a Chinese website for online shopping
Jinfeng Hongyuan Flagship Store	Light Blue Saving Electricity	Second time I bought this product. The first one was used for a couple of months at home, and it saves a little bit of money every month compared to the previous one.	Taobao Marketplace, a Chinese website for online shopping
Jinfeng Hongyuan Flagship Store	Light Blue Saving Electricity	Saving number received, just started to use today, the packaging is very noble, looking forward to the effect	Taobao Marketplace, a Chinese website for online shopping
Jinfeng Hongyuan Flagship Store	Light Blue Saving Electricity	Good quality!	Taobao Marketplace, a Chinese website for online shopping
Jinfeng Hongyuan Flagship Store	Light Blue Saving Electricity	Good quality, works well	Taobao Marketplace, a Chinese website for online shopping
Jinfeng Hongyuan Flagship Store	Light Blue Saving Electricity	It's working great.	Taobao Marketplace, a Chinese website for online shopping
Jinfeng Hongyuan Flagship Store	Light Blue Saving Electricity	It also feels like it's using more power than before	Taobao Marketplace, a Chinese website for online shopping
Jinfeng Hongyuan Flagship Store	Light Blue Saving Electricity	Let's use it first and see how it works.	Taobao Marketplace, a Chinese website for online shopping
Jinfeng Hongyuan Flagship Store	Light Blue Saving Electricity	Looks good.	Taobao Marketplace, a Chinese website for online shopping
Jinfeng Hongyuan Flagship Store	Light Blue Saving Electricity	Intelligent power saver logistics is very fast, small and exquisite, looking forward to next month's meter degrees have gained.	Taobao Marketplace, a Chinese website for online shopping
Jinfeng Hongyuan Flagship Store	Light Blue Saving Electricity	Baby just received, packaging is perfect, baby exquisite workmanship, economizer just connected, a month later to see the effect and then comment.	Taobao Marketplace, a Chinese website for online shopping
Jinfeng Hongyuan Flagship Store	Light Blue Saving Electricity	Store service is good, fast delivery, received the baby installed, skewer to the spare socket on the line, very simple, to be used for a period of time to know how much power savings.	Taobao Marketplace, a Chinese website for online shopping
Jinfeng Hongyuan Flagship Store	Light Blue Saving Electricity	Tried itfor more than2months, the overall power saving is good and	Taobao Marketplace, a Chinese website for online shopping
Jinfeng Hongyuan Flagship Store	Light Blue Saving Electricity	I've been using it for a month before commenting, it worked well after installing it a few days ago. Electricity bill downabout16%per month	Taobao Marketplace, a Chinese website for online shopping
Jinfeng Hongyuan Flagship Store	Light Blue Saving Electricity	Recommended by a friend, it does save a lot of electricity	Taobao Marketplace, a Chinese website for online shopping
Jinfeng Hongyuan Flagship Store	Light Blue Saving Electricity	Received, as described, well packaged, looks good, is using it, don't know how effective it is, but hopefully it works.	Taobao Marketplace, a Chinese website for online shopping
Jinfeng Hongyuan Flagship Store	Light Blue Saving Electricity	The product is particularly formal, at first glance is the goods of a large manufacturer. Currently has been used for a period of time to follow up.	Taobao Marketplace, a Chinese website for online shopping
Jinfeng Hongyuan Flagship Store	Light Blue Saving Electricity	The power saver received, logistics fast, complete packaging, good, powerful	Taobao Marketplace, a Chinese website for online shopping
Jinfeng Hongyuan Flagship Store	Light Blue Saving Electricity	It's great to save on electricity.	Taobao Marketplace, a Chinese website for online shopping
Jinfeng Hongyuan Flagship Store	Light Blue Saving Electricity	The service attitude is very good, and the effect is very good, you can save2-3degrees of electricitya day	Taobao Marketplace, a Chinese website for online shopping
Jinfeng Hongyuan Flagship Store	Light Blue Saving Electricity	(of an unmarried couple) be close	Taobao Marketplace, a Chinese website for online shopping
Jinfeng Hongyuan Flagship Store	Light Blue Saving Electricity	(of an unmarried couple) be close	Taobao Marketplace, a Chinese website for online shopping
Jinfeng Hongyuan Flagship Store	Light Blue Saving Electricity	Review after use.	Taobao Marketplace, a Chinese website for online shopping
Jinfeng Hongyuan Flagship Store	Light Blue Saving Electricity	Great value for money, I love it and will be back next time!	Taobao Marketplace, a Chinese website for online shopping
Jinfeng Hongyuan Flagship Store	Light Blue Saving Electricity	Used itover100days, all summer long, compared to having node power	Taobao Marketplace, a Chinese website for online shopping
Jinfeng Hongyuan Flagship Store	Light Blue Saving Electricity	Very advanced, simple and good-looking appearance, packaging is also very good, easy to use and power saving, recommend👍👍👍👍	Taobao Marketplace, a Chinese website for online shopping
Jinfeng Hongyuan Flagship Store	Light Blue Saving Electricity	The quality is very good, completely exceeded the expectations, the delivery speed is very fast, the packaging is very careful and strict, very satisfied with a shopping	Taobao Marketplace, a Chinese website for online shopping
Jinfeng Hongyuan Flagship Store	Light Blue Saving Electricity	Good power saving	Taobao Marketplace, a Chinese website for online shopping
Jinfeng Hongyuan Flagship Store	Light Blue Saving Electricity	pretty good	Taobao Marketplace, a Chinese website for online shopping
Jinfeng Hongyuan Flagship Store	Light Blue Saving Electricity	Fine workmanship, the quality looks good, use it first to see if it can save power before commenting.	Taobao Marketplace, a Chinese website for online shopping
Jinfeng Hongyuan Flagship Store	Light Blue Saving Electricity	useless	Taobao Marketplace, a Chinese website for online shopping

Jinfeng Hongyuan Flagship Store	Light Blue Saving Electricity	Get a special hand and texture, in order to press on, observe a period of time to see the effect of	Taobao Marketplace, a Chinese website for online shopping
Jinfeng Hongyuan Flagship Store	Light Blue Saving Electricity	This is a scam thing, we do not just look at the surface,more than100dollars or heavy electricity is more fragrant, just sent to plug in the electricity, display386v, this return is very troublesome	Taobao Marketplace, a Chinese website for online shopping
Jinfeng Hongyuan Flagship Store	Light Blue Saving Electricity	The product is as described, works very well and is very effective	Taobao Marketplace, a Chinese website for online shopping
Jinfeng Hongyuan Flagship Store	Light Blue Saving Electricity	Express delivery is very fast, the energy saver is very good, powerful, I believe it can save electricity, after a while to follow up on the evaluation	Taobao Marketplace, a Chinese website for online shopping
Jinfeng Hongyuan Flagship Store	Light Blue Saving Electricity	This product is very good, power-saving effect is obvious, have bought three, were sent to relatives and friends!	Taobao Marketplace, a Chinese website for online shopping
Jinfeng Hongyuan Flagship Store	Light Blue Saving Electricity	I hope it works.	Taobao Marketplace, a Chinese website for online shopping
Jinfeng Hongyuan Flagship Store	Light Blue Saving Electricity	I don't know if it works yet.	Taobao Marketplace, a Chinese website for online shopping
Jinfeng Hongyuan Flagship Store	Light Blue Saving Electricity	Just tried it for a few days and it's obviously working	Taobao Marketplace, a Chinese website for online shopping
Jinfeng Hongyuan Flagship Store	Light Blue Saving Electricity	No significant reduction in electricity bills so far, feel screwed	Taobao Marketplace, a Chinese website for online shopping
Jinfeng Hongyuan Flagship Store	Light Blue Saving Electricity	Baby received, very simple to plug in and can be used directly, looking forward to saving more power!	Taobao Marketplace, a Chinese website for online shopping
Jinfeng Hongyuan Flagship Store	Light Blue Saving Electricity	I've been using it for a while and it does work	Taobao Marketplace, a Chinese website for online shopping
Jinfeng Hongyuan Flagship Store	Light Blue Saving Electricity	Received the baby, can not wait open the box plugged into the socket, the packaging is very fine, the product appearance and quality is also very good, the effect is looking forward to, on the face value of this estimate will not be poor👍👍👍👍	Taobao Marketplace, a Chinese website for online shopping
Jinfeng Hongyuan Flagship Store	Light Blue Saving Electricity	Powerful, under observation, logistics is also very fast, customer service is very good! Will come back again!	Taobao Marketplace, a Chinese website for online shopping
Jinfeng Hongyuan Flagship Store	Light Blue Saving Electricity	It savesmore than20% ofelectricity, which is very satisfying	Taobao Marketplace, a Chinese website for online shopping
Jinfeng Hongyuan Flagship Store	Light Blue Saving Electricity	The product is exquisite and beautiful, and the manufacturer gives five spare safe tubes, which deserves some praise.	Taobao Marketplace, a Chinese website for online shopping
Jinfeng Hongyuan Flagship Store	Light Blue Saving Electricity	not bad, you can save3kilowatts of electricitya day, it's still quite good, I recommend it	Taobao Marketplace, a Chinese website for online shopping
Jinfeng Hongyuan Flagship Store	Light Blue Saving Electricity	Fast logistics, good service and intact packaging! Already used it for a day, let's look forward to its effect together😃	Taobao Marketplace, a Chinese website for online shopping
Jinfeng Hongyuan Flagship Store	Light Blue Saving Electricity	Good quality.	Taobao Marketplace, a Chinese website for online shopping
Jinfeng Hongyuan Flagship Store	Light Blue Saving Electricity	Goods received, used to save electricity after evaluation	Taobao Marketplace, a Chinese website for online shopping
Jinfeng Hongyuan Flagship Store	Light Blue Saving Electricity	Already the fifth time to buy, this time is also to help others to buy, power saving	Taobao Marketplace, a Chinese website for online shopping
Jinfeng Hongyuan Flagship Store	Light Blue Saving Electricity	I've been using it for a few months, but I don't cook at home very often, so I feel it's okay.	Taobao Marketplace, a Chinese website for online shopping
Jinfeng Hongyuan Flagship Store	Light Blue Saving Electricity	It's necessary for the display to be on all the time, right?	Taobao Marketplace, a Chinese website for online shopping
Jinfeng Hongyuan Flagship Store	Light Blue Saving Electricity	Baby's on trial.	Taobao Marketplace, a Chinese website for online shopping
Jinfeng Hongyuan Flagship Store	Light Blue Saving Electricity	Commodities have been received, and business description of a to, good packaging, logistics, fast, after the use of follow-up comments.	Taobao Marketplace, a Chinese website for online shopping
Jinfeng Hongyuan Flagship Store	Light Blue Saving Electricity	Exquisite appearance and atmosphere, easy to use, plug and play.	Taobao Marketplace, a Chinese website for online shopping
Jinfeng Hongyuan Flagship Store	Light Blue Saving Electricity	In use.	Taobao Marketplace, a Chinese website for online shopping
Jinfeng Hongyuan Flagship Store	Light Blue Saving Electricity	Very good, very satisfied!	Taobao Marketplace, a Chinese website for online shopping
Jinfeng Hongyuan Flagship Store	Light Blue Saving Electricity	The product has been used, personally I think it should be pretty good, looking forward to two months to see how the effect is, if it's good, I will buy two more!	Taobao Marketplace, a Chinese website for online shopping
Jinfeng Hongyuan Flagship Store	Light Blue Saving Electricity	dissatisfied	Taobao Marketplace, a Chinese website for online shopping
Jinfeng Hongyuan Flagship Store	Light Blue Saving Electricity	After-sales service: very good service attitude, very patient, very satisfied.	Taobao Marketplace, a Chinese website for online shopping
Jinfeng Hongyuan Flagship Store	Light Blue Saving Electricity	Only after the use of evaluation, a day under to be able to save electricity2-3degrees of electricity, a month downabout60degrees of electricity, more good value for money saving. Now the summer is coming to open the air conditioning friends can buy. Than more afford than the average energy-saving power saving a lot appearance small and exquisite exquisite	Taobao Marketplace, a Chinese website for online shopping
Jinfeng Hongyuan Flagship Store	Light Blue Saving Electricity	Baby received, the old wrench service enthusiasm, logistics is very fast, the product workmanship is fine, power saving effect is obvious, this is the second time to relatives to buy.	Taobao Marketplace, a Chinese website for online shopping
Jinfeng Hongyuan Flagship Store	Light Blue Saving Electricity	The Saver is a great power saving device, it's the second time I've purchased it, it works exceptionally well and is worth the money.	Taobao Marketplace, a Chinese website for online shopping
Jinfeng Hongyuan Flagship Store	Light Blue Saving Electricity	The stuff received, logistics is very fast, but hopefully the effect is very good!	Taobao Marketplace, a Chinese website for online shopping

Jinfeng Hongyuan Flagship Store	Light Blue Saving Electricity	Okay, that worked out well.	Taobao Marketplace, a Chinese website for online shopping
Jinfeng Hongyuan Flagship Store	Light Blue Saving Electricity	Started using it, looking forward to the results	Taobao Marketplace, a Chinese website for online shopping
Jinfeng Hongyuan Flagship Store	Light Blue Saving Electricity	Can't wait to use it when it arrives, looking forward to saving money on my electric bill this month!	Taobao Marketplace, a Chinese website for online shopping
Jinfeng Hongyuan Flagship Store	Light Blue Saving Electricity	The stuff is very good, very practical, hurry up and order!	Taobao Marketplace, a Chinese website for online shopping
Jinfeng Hongyuan Flagship Store	Light Blue Saving Electricity	Item received, well packaged and undamaged	Taobao Marketplace, a Chinese website for online shopping
Jinfeng Hongyuan Flagship Store	Light Blue Saving Electricity	Saver received, fast logistics, exquisite packaging, product workmanship is very fine, I believe that there will be results, it is necessary to recommend to friends around.	Taobao Marketplace, a Chinese website for online shopping
Jinfeng Hongyuan Flagship Store	Light Blue Saving Electricity	Everything is fine! The packaging is quite exquisite! Looking forward to using it!	Taobao Marketplace, a Chinese website for online shopping
Jinfeng Hongyuan Flagship Store	Light Blue Saving Electricity	Sorry, wrong you guys. At first I thought it was immediate, adjusting the meter does not go, but fortunately Xiao-Ting patiently explained that the general use of two months to compare the cost reduction. It is not illegal to adjust the meter.two months, a comparison of electricity costs, indeed, than before the use ofabout30degreesless, which also added an air conditioner, it seems that it does play a role in energy saving. This time I want a pair of combinations again.	Taobao Marketplace, a Chinese website for online shopping
Jinfeng Hongyuan Flagship Store	Light Blue Saving Electricity	Baby received, fast logistics, easy to use, five-star praise!	Taobao Marketplace, a Chinese website for online shopping
Jinfeng Hongyuan Flagship Store	Light Blue Saving Electricity	This is the second one and I feel ok about it! I'll follow up with a review after another two months.	Taobao Marketplace, a Chinese website for online shopping
Jinfeng Hongyuan Flagship Store	Light Blue Saving Electricity	Nice, these kinda work, worth having for everyone! Great gain for the buyer!	Taobao Marketplace, a Chinese website for online shopping
Jinfeng Hongyuan Flagship Store	Light Blue Saving Electricity	Purposefully used for a period of time to evaluate, this device is somewhat unreliable, plugged in, the voltage showsmore than250v, up to270v, the boss said to change a place to plug in, after changing a few times to showbetween230-240v, the businessman said it is normal. Speaking of power saving: used for half a month, really did not find power saving, it seems to be more than before, of course, this is a personal feeling. But really did not save electricity is true, did not buy on the abandonment of it, more than a hundred dollars to stay to pay the electricity bill is not fragrant.	Taobao Marketplace, a Chinese website for online shopping
Jinfeng Hongyuan Flagship Store	Light Blue Saving Electricity	Can't wait to use it when it arrives, looking forward to saving on my electric bill this month!	Taobao Marketplace, a Chinese website for online shopping
Jinfeng Hongyuan Flagship Store	Light Blue Saving Electricity	Not a few days things received, look okay, open on the plug, but also with a few insurance, is not relatively easy to short-circuit fried insurance do not know, hope to be able to use, don't save electricity is also busy changing the fuse on the annoying😓😓😓😓	Taobao Marketplace, a Chinese website for online shopping
Jinfeng Hongyuan Flagship Store	Light Blue Saving Electricity	It's good, my friend used it and said it works well. I just bought it.	Taobao Marketplace, a Chinese website for online shopping
Jinfeng Hongyuan Flagship Store	Light Blue Saving Electricity	Good quality and cost-effective	Taobao Marketplace, a Chinese website for online shopping
Jinfeng Hongyuan Flagship Store	Light Blue Saving Electricity	Just received the goods and already used it, looking forward to saving power!	Taobao Marketplace, a Chinese website for online shopping
Jinfeng Hongyuan Flagship Store	Light Blue Saving Electricity	After using itmore than60days, the power saving effect isabout20%, ready to buy another one	Taobao Marketplace, a Chinese website for online shopping
Jinfeng Hongyuan Flagship Store	Light Blue Saving Electricity	Item received, well packaged, repeat purchase! Thanks!	Taobao Marketplace, a Chinese website for online shopping
Jinfeng Hongyuan Flagship Store	Light Blue Saving Electricity	The quality is first-class, the packaging is good, the logistics is fast, and the price is reasonable and very affordable	Taobao Marketplace, a Chinese website for online shopping
Jinfeng Hongyuan Flagship Store	Light Blue Saving Electricity	The stuff is very good and stable, looking forward to the results of the electricity bill in a month.	Taobao Marketplace, a Chinese website for online shopping
Jinfeng Hongyuan Flagship Store	Light Blue Saving Electricity	As soon as I got home, I plugged it into the socket as required and used it for two months to see the results	Taobao Marketplace, a Chinese website for online shopping
Jinfeng Hongyuan Flagship Store	Light Blue Saving Electricity	Having bought it a couple times now.	Taobao Marketplace, a Chinese website for online shopping
Jinfeng Hongyuan Flagship Store	Light Blue Saving Electricity	Things are genuine packaging perfect, the closest outlet to the empty open place is the network, it is plugged in there, do not know if it works yet. A month in the look	Taobao Marketplace, a Chinese website for online shopping
Jinfeng Hongyuan Flagship Store	Light Blue Saving Electricity	I've received the goods, it's good.	Taobao Marketplace, a Chinese website for online shopping
Jinfeng Hongyuan Flagship Store	Light Blue Saving Electricity	Listening to a friend, I bought a reactive power compensator, just installed it, and look at the effect later. In principle, it can improve power factor and reduce reactive power loss.	Taobao Marketplace, a Chinese website for online shopping
Jinfeng Hongyuan Flagship Store	Light Blue Saving Electricity	Just got it I'll talk more about whether it has a power saving effect after I use it.	Taobao Marketplace, a Chinese website for online shopping
Jinfeng Hongyuan Flagship Store	Light Blue Saving Electricity	Baby has received, the packaging is very delicate product and the introduction of the same, look forward to the effect, use a few days to add comments.	Taobao Marketplace, a Chinese website for online shopping
Jinfeng Hongyuan Flagship Store	Light Blue Saving Electricity	The baby has been received! Looks good the business even delivered insurance! Can't wait to get it working and will follow up next month.	Taobao Marketplace, a Chinese website for online shopping
Jinfeng Hongyuan Flagship Store	Light Blue Saving Electricity	I used it as soon as it came in and look forward to using it.	Taobao Marketplace, a Chinese website for online shopping
Jinfeng Hongyuan Flagship Store	Light Blue Saving Electricity	The quality is fine, I'll comment after using it for two months.	Taobao Marketplace, a Chinese website for online shopping
Jinfeng Hongyuan Flagship Store	Light Blue Saving Electricity	Nice stuff, good power savings, let's see what the quality is like in the back	Taobao Marketplace, a Chinese website for online shopping

Jinfeng Hongyuan Flagship Store	Light Blue Saving Electricity	Great packaging and product with5, now plugged in and looking forward to energy saving results.	Taobao Marketplace, a Chinese website for online shopping
Jinfeng Hongyuan Flagship Store	Light Blue Saving Electricity	I just used it for a few days, and it is quite energy efficient with significantly less power than the previous days! Worth buying	Taobao Marketplace, a Chinese website for online shopping
Jinfeng Hongyuan Flagship Store	Light Blue Saving Electricity	I hope you won't be fooled. I've been using it for two months and it's not working at all.	Taobao Marketplace, a Chinese website for online shopping
Jinfeng Hongyuan Flagship Store	Light Blue Saving Electricity	Item received, delivered and well packaged, thanks!	Taobao Marketplace, a Chinese website for online shopping
Jinfeng Hongyuan Flagship Store	Light Blue Saving Electricity	Big brand, with a very reassuring, the quality is very good, very cost,customer service is really very good Oh ~	Taobao Marketplace, a Chinese website for online shopping
Jinfeng Hongyuan Flagship Store	Light Blue Saving Electricity	Very Good Very Good Practical	Taobao Marketplace, a Chinese website for online shopping
Jinfeng Hongyuan Flagship Store	Light Blue Saving Electricity	Purchased again.	Taobao Marketplace, a Chinese website for online shopping
Jinfeng Hongyuan Flagship Store	Light Blue Saving Electricity	The quality is very good, especially meet my requirements, will repurchase!	Taobao Marketplace, a Chinese website for online shopping
Jinfeng Hongyuan Flagship Store	Light Blue Saving Electricity	I just received it, the packaging is very nice, the product looks good, I'm looking forward to its effect!	Taobao Marketplace, a Chinese website for online shopping
Jinfeng Hongyuan Flagship Store	Light Blue Saving Electricity	Very good. Very good. Very good.	Taobao Marketplace, a Chinese website for online shopping
Jinfeng Hongyuan Flagship Store	Light Blue Saving Electricity	Used it for a while to comment, it's pretty good. Place another order. Recommend to everyone. Praise praise praise praise praise.	Taobao Marketplace, a Chinese website for online shopping
Jinfeng Hongyuan Flagship Store	Light Blue Saving Electricity	Kudos to you, Kudos to you, Kudos to you, Kudos to you.	Taobao Marketplace, a Chinese website for online shopping
Jinfeng Hongyuan Flagship Store	Light Blue Saving Electricity	My neighbor's family bought one, he recommended me to buy it, said it can save electricity, it looks like the quality is okay, the packaging is also good, after a month we will know if it can save electricity	Taobao Marketplace, a Chinese website for online shopping
Jinfeng Hongyuan Flagship Store	Light Blue Saving Electricity	Baby received, as described, recommended	Taobao Marketplace, a Chinese website for online shopping
Jinfeng Hongyuan Flagship Store	Light Blue Saving Electricity	The quality of the stuff is very good, exactly the same as the seller's description, very satisfied!	Taobao Marketplace, a Chinese website for online shopping
Jinfeng Hongyuan Flagship Store	Light Blue Saving Electricity	I've been using it for a while now, and I feel like it's working okay, and I'm still using it consistently	Taobao Marketplace, a Chinese website for online shopping
Jinfeng Hongyuan Flagship Store	Light Blue Saving Electricity	It was easy to install!	Taobao Marketplace, a Chinese website for online shopping
Jinfeng Hongyuan Flagship Store	Light Blue Saving Electricity	Got the baby! Looks pretty delicate!	Taobao Marketplace, a Chinese website for online shopping
Jinfeng Hongyuan Flagship Store	Light Blue Saving Electricity	Just received.	Taobao Marketplace, a Chinese website for online shopping
Jinfeng Hongyuan Flagship Store	Light Blue Saving Electricity	Good, looking forward to the results.	Taobao Marketplace, a Chinese website for online shopping
Jinfeng Hongyuan Flagship Store	Light Blue Saving Electricity	Has been used for a few days, looks good, after a month in to see how much money to save, affordable price, recommend!	Taobao Marketplace, a Chinese website for online shopping
Jinfeng Hongyuan Flagship Store	Light Blue Saving Electricity	Summer with more than three months, the air conditioning open for a long time, the overall and last year's comparison of electricity consumption is also a little less, power-saving effect is good!	Taobao Marketplace, a Chinese website for online shopping
Jinfeng Hongyuan Flagship Store	Light Blue Saving Electricity	Whether it can save electricity is not known at the moment, we will know after using it. Service attitude is very good	Taobao Marketplace, a Chinese website for online shopping
Jinfeng Hongyuan Flagship Store	Light Blue Saving Electricity	Friends introduced, not bad, the price, not expensive, can be accepted	Taobao Marketplace, a Chinese website for online shopping
Jinfeng Hongyuan Flagship Store	Light Blue Saving Electricity	s been more than2.lot of air conditioning in the summer,soI'll just forget about it. It has an energy-saving effect ofabout20%.	Taobao Marketplace, a Chinese website for online shopping
Jinfeng Hongyuan Flagship Store	Light Blue Saving Electricity	We'll see what happens. Baby looks good.	Taobao Marketplace, a Chinese website for online shopping
Jinfeng Hongyuan Flagship Store	Light Blue Saving Electricity	Energy saver quality is very good, Jinfeng Hongyuan brand energy saver in the industry is considered the best, the price is also affordable, worth buying, praise!	Taobao Marketplace, a Chinese website for online shopping
Jinfeng Hongyuan Flagship Store	Light Blue Saving Electricity	Saving power received, small and exquisite, not yet used, first give a favorable comment on it	Taobao Marketplace, a Chinese website for online shopping
Jinfeng Hongyuan Flagship Store	Light Blue Saving Electricity	The goods have been received, very good	Taobao Marketplace, a Chinese website for online shopping
Jinfeng Hongyuan Flagship Store	Light Blue Saving Electricity	This is a repurchase I bought one last year It works well It may not be very effective in the short term But I've been using it for a year It really works It saves electricity If you use a lot of electricity in your home It's highly recommended to buy one	Taobao Marketplace, a Chinese website for online shopping
Jinfeng Hongyuan Flagship Store	Light Blue Saving Electricity	arrived quickly, brand new and authentic[illegible], packaged carefully, no problems	Taobao Marketplace, a Chinese website for online shopping
Jinfeng Hongyuan Flagship Store	Light Blue Saving Electricity	Good quality.	Taobao Marketplace, a Chinese website for online shopping
Jinfeng Hongyuan Flagship Store	Light Blue Saving Electricity	Baby has received, is what I want, feel very good, the quality is also quite good, there are problems customer service is also quickly resolved, the attitude is very good, must praise!	Taobao Marketplace, a Chinese website for online shopping
Jinfeng Hongyuan Flagship Store	Light Blue Saving Electricity	Just arrived a little weight, can give me more power savings, support genuine in the open air-conditioning experiments	Taobao Marketplace, a Chinese website for online shopping

Jinfeng Hongyuan Flagship Store	Light Blue Saving Electricity	False, totally just not useful at all, pure and simple IQ tax	Taobao Marketplace, a Chinese website for online shopping
Jinfeng Hongyuan Flagship Store	Light Blue Saving Electricity	dissatisfied	Taobao Marketplace, a Chinese website for online shopping
Jinfeng Hongyuan Flagship Store	Light Blue Saving Electricity	The packaging is nice and upscale, I've had a good one before.	Taobao Marketplace, a Chinese website for online shopping
Jinfeng Hongyuan Flagship Store	Light Blue Saving Electricity	Baby received, packaging is very good, two or three layers of packaging, things look like it should not be wrong, and so used for a period of time to come back to the comments	Taobao Marketplace, a Chinese website for online shopping
Jinfeng Hongyuan Flagship Store	Light Blue Saving Electricity	This is good, I bought almost10of them, personally I think it's really good, buy it for home.	Taobao Marketplace, a Chinese website for online shopping
Jinfeng Hongyuan Flagship Store	Light Blue Saving Electricity	It looks exquisite, and I'll follow up in a month.	Taobao Marketplace, a Chinese website for online shopping
Jinfeng Hongyuan Flagship Store	Light Blue Saving Electricity	It's pretty good, very practical and good quality!	Taobao Marketplace, a Chinese website for online shopping
Jinfeng Hongyuan Flagship Store	Light Blue Saving Electricity	Looks pretty good, bought it and plugged it in. I've been using it for a while now hoping to save more electricity, and I've introduced it to a friend who bought it.	Taobao Marketplace, a Chinese website for online shopping
Jinfeng Hongyuan Flagship Store	Light Blue Saving Electricity	Received the baby and it was fast, kudos to the store!	Taobao Marketplace, a Chinese website for online shopping
Jinfeng Hongyuan Flagship Store	Light Blue Saving Electricity	Just as the seller said. Packaged very nicely and beautifully. Can't wait to use it. Looking forward to the effect.	Taobao Marketplace, a Chinese website for online shopping
Jinfeng Hongyuan Flagship Store	Light Blue Saving Electricity	The power saver is installed, very satisfied, worth buying, friends hurry to get Oh! Should save electricity effect is good	Taobao Marketplace, a Chinese website for online shopping
Jinfeng Hongyuan Flagship Store	Light Blue Saving Electricity	Very good operation, easy to learn. High quality and low price, power saving expert, small store with conscience, honest business.	Taobao Marketplace, a Chinese website for online shopping
Jinfeng Hongyuan Flagship Store	Light Blue Saving Electricity	Been using it for a few days now, looks good, will see how much I save in a month!	Taobao Marketplace, a Chinese website for online shopping
Jinfeng Hongyuan Flagship Store	Light Blue Saving Electricity	It is genuine, and the same as the physical store, the price is also favorable, decisively turn powder	Taobao Marketplace, a Chinese website for online shopping
Jinfeng Hongyuan Flagship Store	Light Blue Saving Electricity	It looks beautiful. I'm looking forward to saving power.	Taobao Marketplace, a Chinese website for online shopping
Jinfeng Hongyuan Flagship Store	Light Blue Saving Electricity	Purchased several times, very satisfied	Taobao Marketplace, a Chinese website for online shopping
Jinfeng Hongyuan Flagship Store	Light Blue Saving Electricity	Maybought Jinfeng Hongyuan power saver, used almost2, indeed very save electricity, than not installed before the power saver to savemore than100yuanper month, value for money customer service attitude!	Taobao Marketplace, a Chinese website for online shopping
Jinfeng Hongyuan Flagship Store	Light Blue Saving Electricity	The quality is great and it is very energy efficient, indeed it is the perfect product for me!	Taobao Marketplace, a Chinese website for online shopping
Jinfeng Hongyuan Flagship Store	Light Blue Saving Electricity	I've received it, I don't know if it can save electricity, I'll evaluate it after using it for a few days.	Taobao Marketplace, a Chinese website for online shopping
Jinfeng Hongyuan Flagship Store	Light Blue Saving Electricity	I received the item and have been using it for a month. It doesn't save any power at all.	Taobao Marketplace, a Chinese website for online shopping
Jinfeng Hongyuan Flagship Store	Light Blue Saving Electricity	Fast shipping and high power. Works well and is genuine.	Taobao Marketplace, a Chinese website for online shopping
Jinfeng Hongyuan Flagship Store	Light Blue Saving Electricity	Received the goods, the quality is very good, trustworthy	Taobao Marketplace, a Chinese website for online shopping
Jinfeng Hongyuan Flagship Store	Light Blue Saving Electricity	Fast courier, good quality goods.	Taobao Marketplace, a Chinese website for online shopping
Jinfeng Hongyuan Flagship Store	Light Blue Saving Electricity	received goods	Taobao Marketplace, a Chinese website for online shopping
Jinfeng Hongyuan Flagship Store	Light Blue Saving Electricity	Received the goods, the quality is very good	Taobao Marketplace, a Chinese website for online shopping
Jinfeng Hongyuan Flagship Store	Light Blue Saving Electricity	I got it. It works great.	Taobao Marketplace, a Chinese website for online shopping
Jinfeng Hongyuan Flagship Store	Light Blue Saving Electricity	Arrived today, touched the quality is still good, can not wait to plug in, looking forward to the back of the power-saving effect.	Taobao Marketplace, a Chinese website for online shopping
Jinfeng Hongyuan Flagship Store	Light Blue Saving Electricity	Received the item, very satisfied	Taobao Marketplace, a Chinese website for online shopping
Jinfeng Hongyuan Flagship Store	Light Blue Saving Electricity	The packaging is too skimpy.	Taobao Marketplace, a Chinese website for online shopping
Jinfeng Hongyuan Flagship Store	Light Blue Saving Electricity	In use	Taobao Marketplace, a Chinese website for online shopping
Jinfeng Hongyuan Flagship Store	Light Blue Saving Electricity	The goods have been received, well packaged, already installed, waiting for the results.	Taobao Marketplace, a Chinese website for online shopping
Jinfeng Hongyuan Flagship Store	Light Blue Saving Electricity	Received the goods, the first feeling is quite good, look at the later power saving effect	Taobao Marketplace, a Chinese website for online shopping
Jinfeng Hongyuan Flagship Store	Light Blue Saving Electricity	Just received the courier, quite fast. Opened it and looked at it, it's beautiful and small. Use and then evaluate.	Taobao Marketplace, a Chinese website for online shopping
Jinfeng Hongyuan Flagship Store	Light Blue Saving Electricity	Bought at the beginning ofApril, used a month to compare really can save electricity, the texture is quite good, and it is very convenient to plug in the socket to leave it alone	Taobao Marketplace, a Chinese website for online shopping

Jinfeng Hongyuan Flagship Store	Light Blue Saving Electricity	Good quality.	Taobao Marketplace, a Chinese website for online shopping
Jinfeng Hongyuan Flagship Store	Light Blue Saving Electricity	Purchased again, good review!_x005f_x005f_x0001_ Good review!	Taobao Marketplace, a Chinese website for online shopping
Jinfeng Hongyuan Flagship Store	Light Blue Saving Electricity	It works great, and it's even more convenient with the LCD display.	Taobao Marketplace, a Chinese website for online shopping
Jinfeng Hongyuan Flagship Store	Light Blue Saving Electricity	Used more than three months, the summer air conditioning open more, and the same period last year compared to the power consumption or less less, the overall effect is good!	Taobao Marketplace, a Chinese website for online shopping
Jinfeng Hongyuan Flagship Store	Light Blue Saving Electricity	Saving power received, the quality is still the same very good, before I bought a, used for a long time, there is indeed a power-saving effect, and now the family added a few more electrical appliances, so this time to buy another, the shopkeeper's service attitude is also very good, this is also with a display,	Taobao Marketplace, a Chinese website for online shopping
Jinfeng Hongyuan Flagship Store	Light Blue Saving Electricity	Checked the meter and it did saveover40compared to last month	Taobao Marketplace, a Chinese website for online shopping
Jinfeng Hongyuan Flagship Store	Light Blue Saving Electricity	Cost-effective, comparison shopping before buying, the store owner is very good, answering questions is very patient and detailed, there is a need to come back!	Taobao Marketplace, a Chinese website for online shopping
Jinfeng Hongyuan Flagship Store	Light Blue Saving Electricity	Previously went to Hong Kong to buy a, work, this time deliberately bought the most expensive, looks to be a lot higher than the previous, the home central air conditioning every day, compared to the effect is quite satisfactory, a day less7degrees or so, the overall is still more recommended this	Taobao Marketplace, a Chinese website for online shopping
Jinfeng Hongyuan Flagship Store	Light Blue Saving Electricity	More than three months, the air conditioning is on a lot in the summer, comparing the electricity saving of15%	Taobao Marketplace, a Chinese website for online shopping
Jinfeng Hongyuan Flagship Store	Light Blue Saving Electricity	quality is good The logistics is also very fast, too much cheaper than the physical store, use	Taobao Marketplace, a Chinese website for online shopping
Jinfeng Hongyuan Flagship Store	Light Blue Saving Electricity	ve been using it for a few days now and I don't know how much it saves electricity yet, Ill evaluate2months.	Taobao Marketplace, a Chinese website for online shopping
Jinfeng Hongyuan Flagship Store	Light Blue Saving Electricity	Introduced by a friend to buy, the electricity bill saves a lot, it's worth buying	Taobao Marketplace, a Chinese website for online shopping
Jinfeng Hongyuan Flagship Store	Light Blue Saving Electricity	It's easy to use, I've been using it for a few days now and it really saves power and is safe to use	Taobao Marketplace, a Chinese website for online shopping
Jinfeng Hongyuan Flagship Store	Light Blue Saving Electricity	Must buy if you have a lot of electrical appliances at home, important thing to say three times, must buy, must buy!with Pacific Insurance and is just a perfect match for the Bull High Power S!	Taobao Marketplace, a Chinese website for online shopping
Jinfeng Hongyuan Flagship Store	Light Blue Saving Electricity	I've been using it for more than three months, and the air conditioner is on a lot in the summer, so it's still a good power saver	Taobao Marketplace, a Chinese website for online shopping
Jinfeng Hongyuan Flagship Store	Light Blue Saving Electricity	I bought it inMarch, and after using it for so long, it consumes less power compared to last summer, there is a power saving effect	Taobao Marketplace, a Chinese website for online shopping
Jinfeng Hongyuan Flagship Store	Light Blue Saving Electricity	Third purchase.	Taobao Marketplace, a Chinese website for online shopping
Jinfeng Hongyuan Flagship Store	Light Blue Saving Electricity	It's no use at all.	Taobao Marketplace, a Chinese website for online shopping
Jinfeng Hongyuan Flagship Store	Light Blue Saving Electricity	Good, good, very good! Yeah!	Taobao Marketplace, a Chinese website for online shopping
Jinfeng Hongyuan Flagship Store	Light Blue Saving Electricity	Genuine guarantee, good value for money, conscientious business, power saving effect, everyone rest assured to buy!	Taobao Marketplace, a Chinese website for online shopping
Jinfeng Hongyuan Flagship Store	Light Blue Saving Electricity	By checking the principle, there is indeed a power-saving function, there is also a display voltage function, the home's voltage in mind, check the meter does savemore than20degrees of electricity than last month.	Taobao Marketplace, a Chinese website for online shopping
Jinfeng Hongyuan Flagship Store	Light Blue Saving Electricity	Last month120kWh, this month also did not turn on the air-conditioning only22days to260kWh, but so much more electricity	Taobao Marketplace, a Chinese website for online shopping
Jinfeng Hongyuan Flagship Store	Light Blue Saving Electricity	It's a piece of junk, it doesn't work, it doesn't even show the voltage correctly.	Taobao Marketplace, a Chinese website for online shopping
Jinfeng Hongyuan Flagship Store	Light Blue Saving Electricity	I've been using it for two months now, and it really saves electricity, a couple dozen dollars a month less.	Taobao Marketplace, a Chinese website for online shopping
Jinfeng Hongyuan Flagship Store	Light Blue Saving Electricity	Just received the goods, very exquisite energy saver, and five fuses. I hope the store is doing great business!!!	Taobao Marketplace, a Chinese website for online shopping
Jinfeng Hongyuan Flagship Store	Light Blue Saving Electricity	The store shipped quickly, well packaged, plugged in to have a role in order to recommend friends to buy.	Taobao Marketplace, a Chinese website for online shopping
Jinfeng Hongyuan Flagship Store	Light Blue Saving Electricity	Jinfeng Hongyuan power saver received, fast delivery, good packaging, plug a plug on the line, convenient and practical.	Taobao Marketplace, a Chinese website for online shopping
Jinfeng Hongyuan Flagship Store	Light Blue Saving Electricity	Just turned it on, plugged it into the pod and it showed voltage, great stability, insurance company covered product, worth having.	Taobao Marketplace, a Chinese website for online shopping
Jinfeng Hongyuan Flagship Store	Light Blue Saving Electricity	Jinfeng Hongyuan power saver household high-power220vintelligent air conditioning power saving energy saving expert enhanced version of the king of power saving, the material is very good just used looking forward to the effect!	Taobao Marketplace, a Chinese website for online shopping
Jinfeng Hongyuan Flagship Store	Light Blue Saving Electricity	The baby was received exactly as presented and has been plugged in and used.	Taobao Marketplace, a Chinese website for online shopping
Jinfeng Hongyuan Flagship Store	Light Blue Saving Electricity	Just started using, do not know how the effect, the effect in the follow-up, logistics is quite fast!	Taobao Marketplace, a Chinese website for online shopping
Jinfeng Hongyuan Flagship Store	Light Blue Saving Electricity	not use it and throw it away, after two months do not give a refund, I give forget it is2months, does not work at all, if you want to buy it, you do not have to buy it, I have tried this5months, white bullshit.	Taobao Marketplace, a Chinese website for online shopping
Jinfeng Hongyuan Flagship Store	Light Blue Saving Electricity	Friends introduced, said the use of really can save, I deliberately bought a few units, the old family with the2sisters also sent each one, it seems to be good quality, the color is very nice, at present do not know how much can be saved, just hurry up the dining room lights before a flicker, and now there is no longer, there should be a certain effect of the	Taobao Marketplace, a Chinese website for online shopping
Jinfeng Hongyuan Flagship Store	Light Blue Saving Electricity	Branded energy saver, alloy material, exquisite workmanship, efficacy to be verified.	Taobao Marketplace, a Chinese website for online shopping

Jinfeng Hongyuan Flagship Store	Light Blue Saving Electricity	Baby received, used for two days is quite good, hope to save more electricity!	Taobao Marketplace, a Chinese website for online shopping
Jinfeng Hongyuan Flagship Store	Light Blue Saving Electricity	I don't know if it's working.	Taobao Marketplace, a Chinese website for online shopping
Jinfeng Hongyuan Flagship Store	Light Blue Saving Electricity	It's not working.	Taobao Marketplace, a Chinese website for online shopping
Jinfeng Hongyuan Flagship Store	Light Blue Saving Electricity	Don't buy it Pay the IQ tax Seriously bad review	Taobao Marketplace, a Chinese website for online shopping
Jinfeng Hongyuan Flagship Store	Light Blue Saving Electricity	It has been used for a while and feels good, so if you haven't done it yet, you can get it.	Taobao Marketplace, a Chinese website for online shopping
Jinfeng Hongyuan Flagship Store	Light Blue Saving Electricity	ve been using it for a little over two months and it's not comparable to what I used to do, maybe......	Taobao Marketplace, a Chinese website for online shopping
Jinfeng Hongyuan Flagship Store	Light Blue Saving Electricity	The economizer received, the workmanship is small and exquisite, looks very delicate and delicate! It is better than the one bought in the physical store, and it is worth recommending!	Taobao Marketplace, a Chinese website for online shopping
Jinfeng Hongyuan Flagship Store	Light Blue Saving Electricity	The saver is well made and the voltage is stable when plugged in.	Taobao Marketplace, a Chinese website for online shopping
Jinfeng Hongyuan Flagship Store	Light Blue Saving Electricity	It looks like good quality, quite delicate, worthy of users' trust and purchase. Recommended for users to use with confidence	Taobao Marketplace, a Chinese website for online shopping
Jinfeng Hongyuan Flagship Store	Light Blue Saving Electricity	Packaging is complete, item matches, small and lightweight, good looking, good quality at a good price, great,👍👍👍	Taobao Marketplace, a Chinese website for online shopping
Jinfeng Hongyuan Flagship Store	Light Blue Saving Electricity	Baby has been received, packaging is very delicate, looks good, the business also gave5.	Taobao Marketplace, a Chinese website for online shopping
Jinfeng Hongyuan Flagship Store	Light Blue Saving Electricity	Quality is very good, with the seller's description of the exact same, very satisfied, really like it, completely exceeded expectations, the delivery speed is very fast, the packaging is very careful and strict, the logistics company has a very good service attitude, the delivery speed is very fast, very	Taobao Marketplace, a Chinese website for online shopping
Jinfeng Hongyuan Flagship Store	Light Blue Saving Electricity	Started trying it out and looking forward to the power savings.	Taobao Marketplace, a Chinese website for online shopping
Jinfeng Hongyuan Flagship Store	Light Blue Saving Electricity	How anxious to wait is to see the results now, pro praise for the store is so important, it is the affirmation of the store's services, but also for the store's silent support of the work, it is not only inspired by the store's potential to pursue a higher standard, but also for the store's biggest reward, so that the store feels that all the effort is so worthwhile, thank you for the support of the pro, I believe that the store will do a better job, because there are pro. Also hope that the pro always remember that there is a store such as an expectant looking forward to pro's visit again!	Taobao Marketplace, a Chinese website for online shopping
Jinfeng Hongyuan Flagship Store	Light Blue Saving Electricity	I've already used it, it looks good quality and delicate, I'm looking forward to the power saving effect after a month,...	Taobao Marketplace, a Chinese website for online shopping
Jinfeng Hongyuan Flagship Store	Light Blue Saving Electricity	Baby received, received the goods on the use, now do not know how the effect? The effect is obvious to come back to the evaluation	Taobao Marketplace, a Chinese website for online shopping
Jinfeng Hongyuan Flagship Store	Light Blue Saving Electricity	The saver is great.	Taobao Marketplace, a Chinese website for online shopping
Jinfeng Hongyuan Flagship Store	Light Blue Saving Electricity	It seems to be exceptionally good quality and well made. Use it for a while and then follow up!	Taobao Marketplace, a Chinese website for online shopping
Jinfeng Hongyuan Flagship Store	Light Blue Saving Electricity	Already returned. Don't be fooled, the positive reviews are given to red packets to brush out.	Taobao Marketplace, a Chinese website for online shopping
Jinfeng Hongyuan Flagship Store	Light Blue Saving Electricity	Things are very good to use, before the family has bought two a month down, save a lot of electricity it, now buy the store with the	Taobao Marketplace, a Chinese website for online shopping
Jinfeng Hongyuan Flagship Store	Light Blue Saving Electricity	Good quality products and logistics service is also fast and kind attitude	Taobao Marketplace, a Chinese website for online shopping
Jinfeng Hongyuan Flagship Store	Light Blue Saving Electricity	It looks delicate, good packaging, use it first and then evaluate it	Taobao Marketplace, a Chinese website for online shopping
Jinfeng Hongyuan Flagship Store	Light Blue Saving Electricity	Fast shipping and good seller service, very happy so far!	Taobao Marketplace, a Chinese website for online shopping
Jinfeng Hongyuan Flagship Store	Light Blue Saving Electricity	I received the saver, I have plugged it in and hopefully it will save electricity	Taobao Marketplace, a Chinese website for online shopping
Jinfeng Hongyuan Flagship Store	Light Blue Saving Electricity	It looks like good quality now, still looking forward to good results, the	Taobao Marketplace, a Chinese website for online shopping
Jinfeng Hongyuan Flagship Store	Light Blue Saving Electricity	Goods received, very satisfied, next time recommended to friends.	Taobao Marketplace, a Chinese website for online shopping
Jinfeng Hongyuan Flagship Store	Light Blue Saving Electricity	Great, peace of mind with electricity. You can save some electricity.	Taobao Marketplace, a Chinese website for online shopping
Jinfeng Hongyuan Flagship Store	Light Blue Saving Electricity	Unpacking the package, the saver is well packaged and the saver looks great.	Taobao Marketplace, a Chinese website for online shopping
Jinfeng Hongyuan Flagship Store	Light Blue Saving Electricity	Quality is guaranteed, fine workmanship, service attitude is not bad, worth having	Taobao Marketplace, a Chinese website for online shopping
Jinfeng Hongyuan Flagship Store	Light Blue Saving Electricity	The home electricity bill is high. I hope the saver is effective.	Taobao Marketplace, a Chinese website for online shopping
Jinfeng Hongyuan Flagship Store	Light Blue Saving Electricity	Second time repurchase, satisfied with the product, the boss is rich, business is booming!	Taobao Marketplace, a Chinese website for online shopping
Jinfeng Hongyuan Flagship Store	Light Blue Saving Electricity	Look really good, the shell printed can last5-8years of time use, manufactured quite exquisite, plugged in screen voltage no longer frequent changes, I do not know whether it is not a power saver itself plays a role, the home or need to have a current modulation protection things!	Taobao Marketplace, a Chinese website for online shopping
Jinfeng Hongyuan Flagship Store	Light Blue Saving Electricity	Exquisite workmanship, looks high-end atmosphere, should be good, began to try, have a period of time to evaluate!_x005f_x005f_x0001_good	Taobao Marketplace, a Chinese website for online shopping

Jinfeng Hongyuan Flagship Store	Light Blue Saving Electricity	Received the goods quickly, used the effect is not bad, thanks to the merchant product service.	Taobao Marketplace, a Chinese website for online shopping
Jinfeng Hongyuan Flagship Store	Light Blue Saving Electricity	Baby just received, the appearance is quite exquisite, looking forward to saving electricity, to use for a period of time and then follow up. Thanks to the merchant also sent5.	Taobao Marketplace, a Chinese website for online shopping
Jinfeng Hongyuan Flagship Store	Light Blue Saving Electricity	The stuff was received, the delivery was fast, the logistics was fast, the stuff looks pretty good, the	Taobao Marketplace, a Chinese website for online shopping
Jinfeng Hongyuan Flagship Store	Light Blue Saving Electricity	Beautiful appearance very high time effect wait to use to see then have a follow-up review	Taobao Marketplace, a Chinese website for online shopping
Jinfeng Hongyuan Flagship Store	Light Blue Saving Electricity	Just bought Haven't tried it yet	Taobao Marketplace, a Chinese website for online shopping
Jinfeng Hongyuan Flagship Store	Light Blue Saving Electricity	pretty good	Taobao Marketplace, a Chinese website for online shopping
Jinfeng Hongyuan Flagship Store	Light Blue Saving Electricity	It feels like it's working.	Taobao Marketplace, a Chinese website for online shopping
Jinfeng Hongyuan Flagship Store	Light Blue Saving Electricity	Purchase caution, Jinfeng Hongyuan node device is not good to make, please do not be fooled, the business has no trust, said their own products are very good to use, do not believe that I used less than two months feel a little role did not contact the business, the business said that the general are good to let me pick up the use of the let used60days will not be given to the refund, but also do not care, after60days in contact with the business, the business said that60-day accident is not a refund,**business, in order to make theThe people used60days do not refund regardless, buy with caution	Taobao Marketplace, a Chinese website for online shopping
Jinfeng Hongyuan Flagship Store	Light Blue Saving Electricity	Power Saving King is useful, the New Year's electricity consumption is much higher, the actual electricity bill is not much higher	Taobao Marketplace, a Chinese website for online shopping
Jinfeng Hongyuan Flagship Store	Light Blue Saving Electricity	Used it2to3months! No effect, used it2to3!No effect, used2to3!No effect,2to3!No effect,2to3! No effect.	Taobao Marketplace, a Chinese website for online shopping
Jinfeng Hongyuan Flagship Store	Light Blue Saving Electricity	Aluminum alloy shell, overall very texture, more satisfied.	Taobao Marketplace, a Chinese website for online shopping
Jinfeng Hongyuan Flagship Store	Light Blue Saving Electricity	Baby, received today, looks very delicate, easy to use, next time continue to buy, express delivery is also very fast.	Taobao Marketplace, a Chinese website for online shopping
Jinfeng Hongyuan Flagship Store	Light Blue Saving Electricity	Saving appliances are effective, satisfied	Taobao Marketplace, a Chinese website for online shopping
Jinfeng Hongyuan Flagship Store	Light Blue Saving Electricity	Received, shipping quickly, the quality is also quite good, very satisfied, there is a need to buy back!	Taobao Marketplace, a Chinese website for online shopping
Jinfeng Hongyuan Flagship Store	Light Blue Saving Electricity	How anxious to wait is to see the results now, pro praise for the store is so important, it is the affirmation of the store's services, but also for the store's silent support of the work, it is not only inspired by the store's potential to pursue a higher standard, but also for the store's biggest reward, so that the store feels that all the effort is so worthwhile, thank you for the support of the pro, I believe that the store will do a better job, because there are pro. Also hope that the pro always remember that there is a store such as an expectant looking forward to pro's visit again!	Taobao Marketplace, a Chinese website for online shopping
Jinfeng Hongyuan Flagship Store	Light Blue Saving Electricity	Beautifully packaged, more formal than other manufacturers, because of the cold winter days in the south, air conditioning is used frequently, buy one to see if you can save electricity, use a period of time and then follow up.	Taobao Marketplace, a Chinese website for online shopping
Jinfeng Hongyuan Flagship Store	Light Blue Saving Electricity	I received it and plugged it in to see if I could save power and see the results after using it.	Taobao Marketplace, a Chinese website for online shopping
Jinfeng Hongyuan Flagship Store	Light Blue Saving Electricity	I just forgot to put up a picture.	Taobao Marketplace, a Chinese website for online shopping
Jinfeng Hongyuan Flagship Store	Light Blue Saving Electricity	The productdidn't work at all60days, it's a scam, I hope you don't fall for it!	Taobao Marketplace, a Chinese website for online shopping
Jinfeng Hongyuan Flagship Store	Light Blue Saving Electricity	Use it after the New Year and look forward to the results	Taobao Marketplace, a Chinese website for online shopping
Jinfeng Hongyuan Flagship Store	Light Blue Saving Electricity	's working Hope it saves power Good review	Taobao Marketplace, a Chinese website for online shopping
Jinfeng Hongyuan Flagship Store	Light Blue Saving Electricity	The product used60days, not a little effect, but more power than usual, was going to return the product, the result was busy and forgot the time, more than a few days can not return the product, in Taobao shopping very few bad reviews, really quite unhappy, but also bought two	Taobao Marketplace, a Chinese website for online shopping
Jinfeng Hongyuan Flagship Store	Light Blue Saving Electricity	Very small Very good Good quality Very suitable for family use Customer service is very good Recommended purchase Oh	Taobao Marketplace, a Chinese website for online shopping
Jinfeng Hongyuan Flagship Store	Light Blue Saving Electricity	Just received, see the quality of the product is very good, immediately plugged it in, hope it will save electricity, looking forward to the effect! After a period of time to see the effect and then follow up!	Taobao Marketplace, a Chinese website for online shopping
Jinfeng Hongyuan Flagship Store	Light Blue Saving Electricity	Jinfeng Hongyuan power saver household high power220vintelligent power saver, energy saving expert enhanced version of the king of power saving good quality, power saving intelligent LCD display, saving electricity convenient to use. The People's Insurance Company underwriting is guaranteed.	Taobao Marketplace, a Chinese website for online shopping
Jinfeng Hongyuan Flagship Store	Light Blue Saving Electricity	Every month the home electricity bill is really much, with this same can give the home to save electricity, with a good and then come back to the review, currently using the	Taobao Marketplace, a Chinese website for online shopping
Jinfeng Hongyuan Flagship Store	Light Blue Saving Electricity	I haven't used it yet, I don't know how it is	Taobao Marketplace, a Chinese website for online shopping
Jinfeng Hongyuan Flagship Store	Light Blue Saving Electricity	Baby quality is very good, look forward to using the effect, after a while to follow up the review!	Taobao Marketplace, a Chinese website for online shopping
Jinfeng Hongyuan Flagship Store	Light Blue Saving Electricity	Goods have been received! It feels good, I hope it can help my family, and I will continue to buy it if it is good! Hope your company can help more families Oh!	Taobao Marketplace, a Chinese website for online shopping
Jinfeng Hongyuan Flagship Store	Light Blue Saving Electricity	I just used it and will be back in a while to see how it works.	Taobao Marketplace, a Chinese website for online shopping
Jinfeng Hongyuan Flagship Store	Light Blue Saving Electricity	Haven't used this stuff, is it energy efficient, buy a swab and try it out.	Taobao Marketplace, a Chinese website for online shopping
Jinfeng Hongyuan Flagship Store	Light Blue Saving Electricity	Already the second time to buy in his home, my family used this after I saved a lot of electricity😄this time is for my family to buy, the quality is really good , stabilized voltage balanced current.home essential	Taobao Marketplace, a Chinese website for online shopping

Jinfeng Hongyuan Flagship Store	Light Blue Saving Electricity	(of an unmarried couple) be close	Taobao Marketplace, a Chinese website for online shopping
Jinfeng Hongyuan Flagship Store	Light Blue Saving Electricity	Smart Saver is very convenient and practical, the LCD numbers are very clear, very satisfactory	Taobao Marketplace, a Chinese website for online shopping
Jinfeng Hongyuan Flagship Store	Light Blue Saving Electricity	It's a piece of crap that doesn't work.	Taobao Marketplace, a Chinese website for online shopping
Jinfeng Hongyuan Flagship Store	Light Blue Saving Electricity	The appearance of fine workmanship, very texture, plug and play, looking forward to the effect.	Taobao Marketplace, a Chinese website for online shopping
Jinfeng Hongyuan Flagship Store	Light Blue Saving Electricity	Nice customer service patience	Taobao Marketplace, a Chinese website for online shopping
Jinfeng Hongyuan Flagship Store	Light Blue Saving Electricity	Things received, eager to use, but do not know how the effect, and so use a period of time to see the effect and then additional comments	Taobao Marketplace, a Chinese website for online shopping
Jinfeng Hongyuan Flagship Store	Light Blue Saving Electricity	I've been using it for a while now, and it doesn't work at all, and it consumes even more power!	Taobao Marketplace, a Chinese website for online shopping
Jinfeng Hongyuan Flagship Store	Light Blue Saving Electricity	I've been using it for three months now, and I haven't seen any results. I've been fooled.	Taobao Marketplace, a Chinese website for online shopping
Jinfeng Hongyuan Flagship Store	Light Blue Saving Electricity	Second time I bought it, it works great, so if you want to buy it, buy it with confidence!	Taobao Marketplace, a Chinese website for online shopping
Jinfeng Hongyuan Flagship Store	Light Blue Saving Electricity	Friends use good introduced me to use, high power, easy to use and convenient, quite practical, I hope to save more electricity.	Taobao Marketplace, a Chinese website for online shopping
Jinfeng Hongyuan Flagship Store	Light Blue Saving Electricity	It looks good.	Taobao Marketplace, a Chinese website for online shopping
Jinfeng Hongyuan Flagship Store	Light Blue Saving Electricity	The stuff looks good.	Taobao Marketplace, a Chinese website for online shopping
Jinfeng Hongyuan Flagship Store	Light Blue Saving Electricity	Always use this product, there are so good, want to continue to support the	Taobao Marketplace, a Chinese website for online shopping
Jinfeng Hongyuan Flagship Store	Light Blue Saving Electricity	A lot of people say it's great, so I'm going to get one, and when it's good, I'm going to get a couple more.	Taobao Marketplace, a Chinese website for online shopping
Jinfeng Hongyuan Flagship Store	Light Blue Saving Electricity	I bought one for my home two years ago, and I bought another one for this store and just plugged it in. Great customer service and questions answered.	Taobao Marketplace, a Chinese website for online shopping
Jinfeng Hongyuan Flagship Store	Light Blue Saving Electricity	The first one is the voltage shown when I just bought it, and the latter one is the voltage after using it for a week, the voltage is down, and I don't know if the power is less, the store said that the effect will come out after using it for a long time, so I hope it will.	Taobao Marketplace, a Chinese website for online shopping
Jinfeng Hongyuan Flagship Store	Light Blue Saving Electricity	I am using it and will only comment on how it works. Good reviews first.	Taobao Marketplace, a Chinese website for online shopping
Jinfeng Hongyuan Flagship Store	Light Blue Saving Electricity	I don't know if it's good yet, I'm looking forward to it.	Taobao Marketplace, a Chinese website for online shopping
Jinfeng Hongyuan Flagship Store	Light Blue Saving Electricity	Well packaged, clear display, and5insurances, not bad	Taobao Marketplace, a Chinese website for online shopping
Jinfeng Hongyuan Flagship Store	Light Blue Saving Electricity	The stuff is great, fully documented and security marked, kudos👍	Taobao Marketplace, a Chinese website for online shopping
Jinfeng Hongyuan Flagship Store	Light Blue Saving Electricity	Energy saver received today, beautifully packaged, genuine, plugged in to see the meter, indeed, the meter goes slow, I hope the end of the month the electricity bill down!	Taobao Marketplace, a Chinese website for online shopping
Jinfeng Hongyuan Flagship Store	Light Blue Saving Electricity	Repurchased for the second time It works wellI have recommended it to my friends	Taobao Marketplace, a Chinese website for online shopping
Jinfeng Hongyuan Flagship Store	Light Blue Saving Electricity	Recommended by a friend, very well packaged, used it when it arrived	Taobao Marketplace, a Chinese website for online shopping
Jinfeng Hongyuan Flagship Store	Light Blue Saving Electricity	Not bad quality very good use a period of time in the evaluation.	Taobao Marketplace, a Chinese website for online shopping
Jinfeng Hongyuan Flagship Store	Light Blue Saving Electricity	Genuine product, will follow up after use.	Taobao Marketplace, a Chinese website for online shopping
Jinfeng Hongyuan Flagship Store	Light Blue Saving Electricity	Put in the home can save a lot of electricity, very practical, with no trouble simple and convenient, this store's energy-saving appliances are quite good, satisfied with the	Taobao Marketplace, a Chinese website for online shopping
Jinfeng Hongyuan Flagship Store	Light Blue Saving Electricity	store shipped quickly Very good workmanship Already in use Exact results to be evaluated.	Taobao Marketplace, a Chinese website for online shopping
Jinfeng Hongyuan Flagship Store	Light Blue Saving Electricity	: beautifully made, from today with watching the meter, there is a certain effect, because I'm raising fish to be, heating, the heating rod is800watts, after plugging in the power saver, I went to observe the meter, the total number of meters to be slower	Taobao Marketplace, a Chinese website for online shopping
Jinfeng Hongyuan Flagship Store	Light Blue Saving Electricity	Good quality, authentic, worth buying the product	Taobao Marketplace, a Chinese website for online shopping
Jinfeng Hongyuan Flagship Store	Light Blue Saving Electricity	Baby received, power plugged in, works well, can save money with this!	Taobao Marketplace, a Chinese website for online shopping
Jinfeng Hongyuan Flagship Store	Light Blue Saving Electricity	Bought it for a family member!!! Use it to follow up, well well well well well well	Taobao Marketplace, a Chinese website for online shopping
Jinfeng Hongyuan Flagship Store	Light Blue Saving Electricity	Use it and then evaluate.	Taobao Marketplace, a Chinese website for online shopping
Jinfeng Hongyuan Flagship Store	Light Blue Saving Electricity	Packaging is very good, there are test certificates and other accessories of the regular company, power-saving effect used after a follow-up evaluation	Taobao Marketplace, a Chinese website for online shopping
Jinfeng Hongyuan Flagship Store	Light Blue Saving Electricity	Jinfeng Hongyuan energy-saving appliances look beautiful, the quality is very good, both power-saving and stabilizing pressure, is a must-have for the family, how much power can be saved to be next month's collection to know, then follow up.	Taobao Marketplace, a Chinese website for online shopping

Jinfeng Hongyuan Flagship Store	Light Blue Saving Electricity	Looks good, used it, waiting for results	Taobao Marketplace, a Chinese website for online shopping
Jinfeng Hongyuan Flagship Store	Light Blue Saving Electricity	The quality is very good, it also shows how many volts the voltage is, it's quite good! Very satisfied, specially to evaluate, recommended to buy.	Taobao Marketplace, a Chinese website for online shopping
Jinfeng Hongyuan Flagship Store	Light Blue Saving Electricity	Purchased again, very well made, this time got a high power one.	Taobao Marketplace, a Chinese website for online shopping
Jinfeng Hongyuan Flagship Store	Light Blue Saving Electricity	I'll comment when I've used it.	Taobao Marketplace, a Chinese website for online shopping
Jinfeng Hongyuan Flagship Store	Light Blue Saving Electricity	Received the stuff, feel the quality is good, in the trial	Taobao Marketplace, a Chinese website for online shopping
Jinfeng Hongyuan Flagship Store	Light Blue Saving Electricity	Fine workmanship, good quality, next time again, the boss is good	Taobao Marketplace, a Chinese website for online shopping
Jinfeng Hongyuan Flagship Store	Light Blue Saving Electricity	favorable criticism	Taobao Marketplace, a Chinese website for online shopping
Jinfeng Hongyuan Flagship Store	Light Blue Saving Electricity	My friend's family has used it for more than half a year and said it saves a lot, highly recommended to me, I believe the quality is not a problem!	Taobao Marketplace, a Chinese website for online shopping
Jinfeng Hongyuan Flagship Store	Light Blue Saving Electricity	Finally received the baby I need, the stuff is very good, the price is good, thanks to the shopkeeper!!Honestly, this is my Taobao shopping to make me the most satisfied with a shopping. Whether it is the attitude of the shopkeeper or the items, I am very satisfied. The palm of the cabinet attitude is very professional and enthusiastic, there are questions and answers, the response is also very fast, I asked a lot of questions, he does not feel annoyed, will seriously answer me, this point I express my heartfelt respect to the palm of the cabinet, such a good cabinet can not be more.	Taobao Marketplace, a Chinese website for online shopping
Jinfeng Hongyuan Flagship Store	Light Blue Saving Electricity	Currently using normal, is not power saving, power saving how much still need time to verify, nowabout350degreesper month, see the next few months of power consumption and then follow up!	Taobao Marketplace, a Chinese website for online shopping
Jinfeng Hongyuan Flagship Store	Light Blue Saving Electricity	No change in habits, electricity bills rising month after month, useless, identified!	Taobao Marketplace, a Chinese website for online shopping
Jinfeng Hongyuan Flagship Store	Light Blue Saving Electricity	Received the goods! In use	Taobao Marketplace, a Chinese website for online shopping
Jinfeng Hongyuan Flagship Store	Light Blue Saving Electricity	Quality is very good, is genuine, seller service attitude is very good, next time will patronize, praise!	Taobao Marketplace, a Chinese website for online shopping
Jinfeng Hongyuan Flagship Store	Light Blue Saving Electricity	Very easy to use, display voltage is accurate, hopefully it will work well, will follow up later!	Taobao Marketplace, a Chinese website for online shopping
Jinfeng Hongyuan Flagship Store	Light Blue Saving Electricity	Workmanship material is very good, durable high-power, delivery is also very fast, rest assured that the purchase of	Taobao Marketplace, a Chinese website for online shopping
Jinfeng Hongyuan Flagship Store	Light Blue Saving Electricity	By checking the principle, there is indeed a power-saving function, and there is also a function to display the voltage, so that you have a good idea of the voltage in your own home.	Taobao Marketplace, a Chinese website for online shopping
Jinfeng Hongyuan Flagship Store	Light Blue Saving Electricity	Good price, fast shipping, smart electricity, save resources, save money, will buy again if needed!	Taobao Marketplace, a Chinese website for online shopping
Jinfeng Hongyuan Flagship Store	Light Blue Saving Electricity	I've been using it for a month before commenting, it did generate almost half of the power last month, we'll see next month	Taobao Marketplace, a Chinese website for online shopping
Jinfeng Hongyuan Flagship Store	Light Blue Saving Electricity	Can't see the effect	Taobao Marketplace, a Chinese website for online shopping
Jinfeng Hongyuan Flagship Store	Light Blue Saving Electricity	Saving appliances received, the packaging is particularly good, the quality is particularly smooth, looking particularly high-grade, I sent a circle of friends, so that relatives and friends also go to enjoy the joy of saving electricity, I wish the boss what I seek full of wishes, day to day things	Taobao Marketplace, a Chinese website for online shopping
Jinfeng Hongyuan Flagship Store	Light Blue Saving Electricity	Saving appliances have been received, open a look, the product is very exquisite, feel very worthy, must praise, and customer service attitude is very good, I shot wrong, very carefully help me analyze and change back. Use a period of time to follow up.	Taobao Marketplace, a Chinese website for online shopping
Jinfeng Hongyuan Flagship Store	Light Blue Saving Electricity	I just received the goods and used it when I got home I hope it can save electricity	Taobao Marketplace, a Chinese website for online shopping
Jinfeng Hongyuan Flagship Store	Light Blue Saving Electricity	I just received it and installed it, how does it work to see if there is any reduction in the electricity bill in the next few months.	Taobao Marketplace, a Chinese website for online shopping
Jinfeng Hongyuan Flagship Store	Light Blue Saving Electricity	A power saver that works well!	Taobao Marketplace, a Chinese website for online shopping
Jinfeng Hongyuan Flagship Store	Light Blue Saving Electricity	Saver is good quality and works great second time I bought it	Taobao Marketplace, a Chinese website for online shopping
Jinfeng Hongyuan Flagship Store	Light Blue Saving Electricity	After using it for more than three months, the power saving effect is still very good, it really saves electricity	Taobao Marketplace, a Chinese website for online shopping
Jinfeng Hongyuan Flagship Store	Light Blue Saving Electricity	The power saver was installed as soon as I bought it. It will take a while to see if this one works	Taobao Marketplace, a Chinese website for online shopping
Jinfeng Hongyuan Flagship Store	Light Blue Saving Electricity	Great appearance, good price, looking forward to the results.	Taobao Marketplace, a Chinese website for online shopping
Jinfeng Hongyuan Flagship Store	Light Blue Saving Electricity	Bought it and plugged it in today. It's pretty small. Haha my mom bought one too. It works great.	Taobao Marketplace, a Chinese website for online shopping
Jinfeng Hongyuan Flagship Store	Light Blue Saving Electricity	Looks good, don't know how well it works, will be back in a few days with a follow up review.	Taobao Marketplace, a Chinese website for online shopping
Jinfeng Hongyuan Flagship Store	Light Blue Saving Electricity	I got it, it's great, it's worth buying!	Taobao Marketplace, a Chinese website for online shopping
Jinfeng Hongyuan Flagship Store	Light Blue Saving Electricity	Just used it, not bad workmanship, electric bill in the store is too high at the moment, will come back for more if it works!	Taobao Marketplace, a Chinese website for online shopping
Jinfeng Hongyuan Flagship Store	Light Blue Saving Electricity	Half a month of use, the effect is very obvious, according to the same period of use than in the past to pay the electricity bill of the number of kWh reduced	Taobao Marketplace, a Chinese website for online shopping

Jinfeng Hongyuan Flagship Store	Light Blue Saving Electricity	Bought it for a friend, first praise	Taobao Marketplace, a Chinese website for online shopping
Jinfeng Hongyuan Flagship Store	Light Blue Saving Electricity	East is very good, very satisfied with a shopping, next time will come back!	Taobao Marketplace, a Chinese website for online shopping
Jinfeng Hongyuan Flagship Store	Light Blue Saving Electricity	Baby value is very high, easy to operate, plug and play, the elderly can also be fully operated, energy-saving also into the insurance, double eleven value for money, looking forward to a good power-saving effect!	Taobao Marketplace, a Chinese website for online shopping
Jinfeng Hongyuan Flagship Store	Light Blue Saving Electricity	High value, easy to operate, double eleven value for money, the family very much like, will introduce to friends, there is a need to come back, looking forward to a good power saving effect!	Taobao Marketplace, a Chinese website for online shopping
Jinfeng Hongyuan Flagship Store	Light Blue Saving Electricity	Easy to use, the effect is very obvious, the price is affordable quality assurance, worth having!	Taobao Marketplace, a Chinese website for online shopping
Jinfeng Hongyuan Flagship Store	Light Blue Saving Electricity	Nice and stable. Looks good and should work well!	Taobao Marketplace, a Chinese website for online shopping
Jinfeng Hongyuan Flagship Store	Light Blue Saving Electricity	Packed tightly, high, look good, satisfied.	Taobao Marketplace, a Chinese website for online shopping
Jinfeng Hongyuan Flagship Store	Light Blue Saving Electricity	Used more than three months, winter open air conditioning, the overall comparison with the power-saving effect	Taobao Marketplace, a Chinese website for online shopping
Jinfeng Hongyuan Flagship Store	Light Blue Saving Electricity	Comparison before coming here to evaluate The power saving effect really can't be described	Taobao Marketplace, a Chinese website for online shopping
Jinfeng Hongyuan Flagship Store	Light Blue Saving Electricity	Baby quality is good, just pressed today, after a month to see the effect of additional comments in the	Taobao Marketplace, a Chinese website for online shopping
Jinfeng Hongyuan Flagship Store	Light Blue Saving Electricity	Things received, well-packaged, business service attitude is also very good, energy saver has been installed with, to be used for a period of time and then make a follow-up evaluation.	Taobao Marketplace, a Chinese website for online shopping
Jinfeng Hongyuan Flagship Store	Light Blue Saving Electricity	Comparison of1month then evaluation, the beginning of the day a little less, the back of the stable, the original20degreesa dayto the current15degrees or so, the effect is very good	Taobao Marketplace, a Chinese website for online shopping
Jinfeng Hongyuan Flagship Store	Light Blue Saving Electricity	The power saver is great, it's been plugged in for a month or so and it's saving power!	Taobao Marketplace, a Chinese website for online shopping
Jinfeng Hongyuan Flagship Store	Light Blue Saving Electricity	Very good energy saving appliances	Taobao Marketplace, a Chinese website for online shopping
Jinfeng Hongyuan Flagship Store	Light Blue Saving Electricity	Fine workmanship, easy to operate.price.Worth buying.Looking forward to the effect	Taobao Marketplace, a Chinese website for online shopping
Jinfeng Hongyuan Flagship Store	Light Blue Saving Electricity	The appearance looks quite exquisite, first praise it, after a few months to see the results	Taobao Marketplace, a Chinese website for online shopping
Jinfeng Hongyuan Flagship Store	Light Blue Saving Electricity	It's really good.	Taobao Marketplace, a Chinese website for online shopping
Jinfeng Hongyuan Flagship Store	Light Blue Saving Electricity	Very fast shipping and worth the money! Came back and installed it looking forward to the results!	Taobao Marketplace, a Chinese website for online shopping
Jinfeng Hongyuan Flagship Store	Light Blue Saving Electricity	I hope it works.	Taobao Marketplace, a Chinese website for online shopping
Jinfeng Hongyuan Flagship Store	Light Blue Saving Electricity	Used itfor over2months with power savings and bought another one	Taobao Marketplace, a Chinese website for online shopping
Jinfeng Hongyuan Flagship Store	Light Blue Saving Electricity	The outer packaging is exquisite, the product appearance is also sturdy, feel good, has been plugged in, to be effect.	Taobao Marketplace, a Chinese website for online shopping
Jinfeng Hongyuan Flagship Store	Light Blue Saving Electricity	(*^_^*)Very satisfied with a shopping, value for money, much more affordable than the physical store!	Taobao Marketplace, a Chinese website for online shopping
Jinfeng Hongyuan Flagship Store	Light Blue Saving Electricity	It was well packaged and the item was exquisite! A very pleasant shopping	Taobao Marketplace, a Chinese website for online shopping
Jinfeng Hongyuan Flagship Store	Light Blue Saving Electricity	The product is really super great, the next time you will buy, may the more you do better and better, very good shopping experience very good buying platform, good quality, especially with the brand, trustworthy shopkeeper shipping very fast, good service attitude, it should be genuine, a very pleasant shopping logistics is very fast,👍product was received very quickly, better than imagined, good good! I hope it can be durable has been received, it is very good, there are freebies, cost-effective too high, love love love baby received, the packaging is very strong, it should be a good thing, the first to give a good review of the quality is very good, completely exceeded expectations, the shipping speed is very fast, the packaging is very careful, strict, very satisfied with a shopping really good, very satisfied with the goods, belong to the unlimited repurchase models, recommended! Things have been received! Hand feel good! Solid material! Quality is very good! Usually use basically no problem! Baby is very great, is the kind of style they want, the arrival is also very fast, thanks to the boss, the service five stars Baby is very great, is the kind of style they want, the arrival is also very fast, thanks to the boss, the service five stars Very delicate, the quality is very good, very satisfied with a shopping. Thanks to the store! Take advantage of the offer, hurry to get it, it is genuine, the packaging is also very good, [illegible]	Taobao Marketplace, a Chinese website for online shopping
Jinfeng Hongyuan Flagship Store	Light Blue Saving Electricity	(of an unmarried couple) be close	Taobao Marketplace, a Chinese website for online shopping
Jinfeng Hongyuan Flagship Store	Light Blue Saving Electricity	The product received, the quality looks good, the seller service, next time again!	Taobao Marketplace, a Chinese website for online shopping
Jinfeng Hongyuan Flagship Store	Light Blue Saving Electricity	Very nice energy efficient appliance, looks great and worth the money!	Taobao Marketplace, a Chinese website for online shopping
Jinfeng Hongyuan Flagship Store	Light Blue Saving Electricity	It's a bit skimpy and tiny packaging. Not sure how effective it is. Already used it. Looking forward to the effect.	Taobao Marketplace, a Chinese website for online shopping
Jinfeng Hongyuan Flagship Store	Light Blue Saving Electricity	Power saving clamor received, logistics is very fast, the packaging is perfect, as for whether it can save electricity to be a period of time to come back to the follow-up evaluation.	Taobao Marketplace, a Chinese website for online shopping
Jinfeng Hongyuan Flagship Store	Light Blue Saving Electricity	The product received is practical and beautiful, and the power saving effect is good!	Taobao Marketplace, a Chinese website for online shopping

Jinfeng Hongyuan Flagship Store	Light Blue Saving Electricity	Saving appliances quality is very good, two layers of packaging texture, used for a month can save electricity	Taobao Marketplace, a Chinese website for online shopping
Jinfeng Hongyuan Flagship Store	Light Blue Saving Electricity	ve been using it for two months and it doesn't do anything😓 No comment 😓	Taobao Marketplace, a Chinese website for online shopping
Jinfeng Hongyuan Flagship Store	Light Blue Saving Electricity	Small and lightweight, plug it in and use it, compare for a while, there are power saving	Taobao Marketplace, a Chinese website for online shopping
Jinfeng Hongyuan Flagship Store	Light Blue Saving Electricity	Everyday on Taobao, life is no trouble!	Taobao Marketplace, a Chinese website for online shopping
Jinfeng Hongyuan Flagship Store	Light Blue Saving Electricity	Saving electricity received the logistics very quickly and good quality saves a lot of electricity very satisfied with the friends who did not start to go away immediately into it!	Taobao Marketplace, a Chinese website for online shopping
Jinfeng Hongyuan Flagship Store	Light Blue Saving Electricity	Got it and rushed it back, dad said this would work! Worth recommending!	Taobao Marketplace, a Chinese website for online shopping
Jinfeng Hongyuan Flagship Store	Light Blue Saving Electricity	A very good energy-saving appliance, beautiful appearance, worth buying, praise!	Taobao Marketplace, a Chinese website for online shopping
Jinfeng Hongyuan Flagship Store	Light Blue Saving Electricity	Packaging is very good, immediately after receipt of hanging, the store is also careful to explain the store, but also sent some gifts, must praise, as for power saving, have to be some days to say, is a friend recommended to come!	Taobao Marketplace, a Chinese website for online shopping
Jinfeng Hongyuan Flagship Store	Light Blue Saving Electricity	Compact appearance does not take up much space, tried it out and it works well, also got a couple extra fuses. Recommended!	Taobao Marketplace, a Chinese website for online shopping
Jinfeng Hongyuan Flagship Store	Light Blue Saving Electricity	I bought it in late October andused it for a full month in November and figured I saved like a hundred and fifty.	Taobao Marketplace, a Chinese website for online shopping
Jinfeng Hongyuan Flagship Store	Light Blue Saving Electricity	Home Power Saver has been used for more than a month, and the comparison with last month has effect	Taobao Marketplace, a Chinese website for online shopping
Jinfeng Hongyuan Flagship Store	Light Blue Saving Electricity	Just received the goods! Looking forward to good use ！！！！	Taobao Marketplace, a Chinese website for online shopping
Jinfeng Hongyuan Flagship Store	Light Blue Saving Electricity	I don't see any power savings. That's a lie.	Taobao Marketplace, a Chinese website for online shopping
Jinfeng Hongyuan Flagship Store	Light Blue Saving Electricity	Good product, tamper proof, the rest is to see how well it saves power!	Taobao Marketplace, a Chinese website for online shopping
Jinfeng Hongyuan Flagship Store	Light Blue Saving Electricity	IQ tax. Whoever buys it must regret it.	Taobao Marketplace, a Chinese website for online shopping
Jinfeng Hongyuan Flagship Store	Light Blue Saving Electricity	I just used it to see the effect later, and it looks beautiful	Taobao Marketplace, a Chinese website for online shopping
Jinfeng Hongyuan Flagship Store	Light Blue Saving Electricity	Feel pretty good, have not yet used, wait to see the effect after use and then follow up!	Taobao Marketplace, a Chinese website for online shopping
Jinfeng Hongyuan Flagship Store	Light Blue Saving Electricity	Power Saver is useful.	Taobao Marketplace, a Chinese website for online shopping
Jinfeng Hongyuan Flagship Store	Light Blue Saving Electricity	I've used it, I'll know if it's good or not after a while, I'll comment then.	Taobao Marketplace, a Chinese website for online shopping
Jinfeng Hongyuan Flagship Store	Light Blue Saving Electricity	inexpensive	Taobao Marketplace, a Chinese website for online shopping
Jinfeng Hongyuan Flagship Store	Light Blue Saving Electricity	Saves electricity.	Taobao Marketplace, a Chinese website for online shopping
Jinfeng Hongyuan Flagship Store	Light Blue Saving Electricity	Save energy, technology for a better life! Green electricity use! Easy to use! Insurance Coverage	Taobao Marketplace, a Chinese website for online shopping
Jinfeng Hongyuan Flagship Store	Light Blue Saving Electricity	On my second week of use, follow up at the end of the month.....	Taobao Marketplace, a Chinese website for online shopping
Jinfeng Hongyuan Flagship Store	Light Blue Saving Electricity	It works great.	Taobao Marketplace, a Chinese website for online shopping
Jinfeng Hongyuan Flagship Store	Light Blue Saving Electricity	I used it for a few months and then I didn't lose a penny on my electricity bill, don't believe it!	Taobao Marketplace, a Chinese website for online shopping
Jinfeng Hongyuan Flagship Store	Light Blue Saving Electricity	This saver is beautiful plug and play, warranty two years, cheap price, worth having oh! Recommended for everyone.	Taobao Marketplace, a Chinese website for online shopping
Jinfeng Hongyuan Flagship Store	Light Blue Saving Electricity	Let's see if we can reduce our electricity bill next month.	Taobao Marketplace, a Chinese website for online shopping
Jinfeng Hongyuan Flagship Store	Light Blue Saving Electricity	It's not much use.	Taobao Marketplace, a Chinese website for online shopping
Jinfeng Hongyuan Flagship Store	Light Blue Saving Electricity	Received,	Taobao Marketplace, a Chinese website for online shopping
Jinfeng Hongyuan Flagship Store	Light Blue Saving Electricity	The baby has been received very energy-saving if necessary will buy again	Taobao Marketplace, a Chinese website for online shopping
Jinfeng Hongyuan Flagship Store	Light Blue Saving Electricity	I just received the goods; I don't know how effective the power-saving effect is; I have to use it for a while and then make a follow-up comment!	Taobao Marketplace, a Chinese website for online shopping
Jinfeng Hongyuan Flagship Store	Light Blue Saving Electricity	I guess it's a joke to buy a console like one for hundreds of dollars, I guess it's a joke to savepower** 🤗	Taobao Marketplace, a Chinese website for online shopping
Jinfeng Hongyuan Flagship Store	Light Blue Saving Electricity	Baby received, indeed power saving you can rest assured to buy!	Taobao Marketplace, a Chinese website for online shopping
Jinfeng Hongyuan Flagship Store	Light Blue Saving Electricity	Very good energy saving appliance, I can only say really good. The energy saving effect is still very obvious, I'm using this for half a month before I come to evaluate.	Taobao Marketplace, a Chinese website for online shopping

Jinfeng Hongyuan Flagship Store	Light Blue Saving Electricity	I don't know exactly whether it works or not. I've been using it as recommended, so I'm looking forward to the results.	Taobao Marketplace, a Chinese website for online shopping
Jinfeng Hongyuan Flagship Store	Light Blue Saving Electricity	Received the power saver. It's very delicate, plug it in and use it for a while to see if it can save electricity! Good review!	Taobao Marketplace, a Chinese website for online shopping
Jinfeng Hongyuan Flagship Store	Light Blue Saving Electricity	Too many appliances at home, bought it hoping it will save electricity. The product is pretty well packaged and started using it.	Taobao Marketplace, a Chinese website for online shopping
Jinfeng Hongyuan Flagship Store	Light Blue Saving Electricity	Pretty good! Well ヽ(○^㉨^)ノ♪	Taobao Marketplace, a Chinese website for online shopping
Jinfeng Hongyuan Flagship Store	Light Blue Saving Electricity	Quite good. I hope it works and support support support support support support support!!!!	Taobao Marketplace, a Chinese website for online shopping
Jinfeng Hongyuan Flagship Store	Light Blue Saving Electricity	Support support support support support support. I hope it works.	Taobao Marketplace, a Chinese website for online shopping
Jinfeng Hongyuan Flagship Store	Light Blue Saving Electricity	Used it for two months to save power, not returning it	Taobao Marketplace, a Chinese website for online shopping
Jinfeng Hongyuan Flagship Store	Light Blue Saving Electricity	The goods have been received and I look forward to the results!	Taobao Marketplace, a Chinese website for online shopping
Jinfeng Hongyuan Flagship Store	Light Blue Saving Electricity	Intelligent energy saver received, I was also introduced to buy, very good, wait a few days to try and then come back to the follow-up comment!	Taobao Marketplace, a Chinese website for online shopping
Jinfeng Hongyuan Flagship Store	Light Blue Saving Electricity	Logistics is particularly fast! Good quality, worth buying.	Taobao Marketplace, a Chinese website for online shopping
Jinfeng Hongyuan Flagship Store	Light Blue Saving Electricity	Received the product, don't know how much electricity can be saved	Taobao Marketplace, a Chinese website for online shopping
Jinfeng Hongyuan Flagship Store	Light Blue Saving Electricity	To have used two months, there is no effect, did not use the power saver before my monthly electricity bill in120yuan or so, and now after using now170, the seller said that a short period of time can not see the effect, and now with two months, no effect does not say that you can not return the goods, it is very speechless😔, we must be careful to shoot ah, a true tip, I hope to give everyone a reference	Taobao Marketplace, a Chinese website for online shopping
Jinfeng Hongyuan Flagship Store	Light Blue Saving Electricity	Baby received, immediately installed to enable, look forward to power-saving effect, customer service is very good, give five-star praise, two months and then follow up!	Taobao Marketplace, a Chinese website for online shopping
Jinfeng Hongyuan Flagship Store	Light Blue Saving Electricity	Looking forward to the resultsif it works well buy a few more all set up😀 😀😀	Taobao Marketplace, a Chinese website for online shopping
Jinfeng Hongyuan Flagship Store	Light Blue Saving Electricity	I saw it at a friend's place and bought this one decisively, if it can save electricity, it's amazing, dripping water, I look forward to the results and will buy it again.	Taobao Marketplace, a Chinese website for online shopping
Jinfeng Hongyuan Flagship Store	Light Blue Saving Electricity	Received the company's products, the appearance of the design is safe and reliable, well-packed. Technology upgrade technology can bring better protection to life. Let the customer more peace of mind, more worry. Happier!	Taobao Marketplace, a Chinese website for online shopping
Jinfeng Hongyuan Flagship Store	Light Blue Saving Electricity	According to the current evaluation within the time rules can not wait for the effect out, wait for a follow-up review later!	Taobao Marketplace, a Chinese website for online shopping
Jinfeng Hongyuan Flagship Store	Light Blue Saving Electricity	Let's see one later.	Taobao Marketplace, a Chinese website for online shopping
Jinfeng Hongyuan Flagship Store	Light Blue Saving Electricity	No power saving, still the same as before, using it for a few days, not working then return it	Taobao Marketplace, a Chinese website for online shopping
Jinfeng Hongyuan Flagship Store	Light Blue Saving Electricity	Just received, installed a month to see the results of the suburb and then add comment!	Taobao Marketplace, a Chinese website for online shopping
Jinfeng Hongyuan Flagship Store	Light Blue Saving Electricity	The quality of the stuff is great!	Taobao Marketplace, a Chinese website for online shopping
Jinfeng Hongyuan Flagship Store	Light Blue Saving Electricity	an excellent	Taobao Marketplace, a Chinese website for online shopping
Jinfeng Hongyuan Flagship Store	Light Blue Saving Electricity	an excellent	Taobao Marketplace, a Chinese website for online shopping
Jinfeng Hongyuan Flagship Store	Light Blue Saving Electricity	Good workmanship, the principle is also written more clearly, reactive power compensation to save electricity, that is to say, electrical appliances with motors, pumps more power saving is more obvious, look forward to the effect!	Taobao Marketplace, a Chinese website for online shopping
Jinfeng Hongyuan Flagship Store	Light Blue Saving Electricity	The power saver is great, it gives you5fuses and really saves power	Taobao Marketplace, a Chinese website for online shopping
Jinfeng Hongyuan Flagship Store	Light Blue Saving Electricity	Connecting or not connecting has no1%effecton power factor	Taobao Marketplace, a Chinese website for online shopping
Jinfeng Hongyuan Flagship Store	Light Blue Saving Electricity	I thought it was doing capacitor compensation. Chihuahua meters these days have power factor displays that are easy to measure. IQ has to be used in the right way, otherwise.	Taobao Marketplace, a Chinese website for online shopping
Jinfeng Hongyuan Flagship Store	Light Blue Saving Electricity	Just received plugged in to try, the quality is very formal manufacturers, logistics is also very fast, customer service attitude is very good!	Taobao Marketplace, a Chinese website for online shopping
Jinfeng Hongyuan Flagship Store	Light Blue Saving Electricity	I've been using it for a while and now I'm commenting on it. It's good, it does save energy.	Taobao Marketplace, a Chinese website for online shopping
Jinfeng Hongyuan Flagship Store	Light Blue Saving Electricity	The stuff is great, the owner is enthusiastic, positive feedback!	Taobao Marketplace, a Chinese website for online shopping
Jinfeng Hongyuan Flagship Store	Light Blue Saving Electricity	Smart saver with flawless packaging! Great quality!	Taobao Marketplace, a Chinese website for online shopping
Jinfeng Hongyuan Flagship Store	Light Blue Saving Electricity	Received! I don't know if it saves electricity or not, add it after using it, I was speechless when I read the reviews that it was well packaged, it was just packed up in a thin plastic shell! Look at the picture.	Taobao Marketplace, a Chinese website for online shopping
Jinfeng Hongyuan Flagship Store	Light Blue Saving Electricity	Another thing to scam the people!!!!	Taobao Marketplace, a Chinese website for online shopping
Jinfeng Hongyuan Flagship Store	Light Blue Saving Electricity	Very good. Satisfied.	Taobao Marketplace, a Chinese website for online shopping

Jinfeng Hongyuan Flagship Store	Light Blue Saving Electricity	satisfied	Taobao Marketplace, a Chinese website for online shopping
Jinfeng Hongyuan Flagship Store	Light Blue Saving Electricity	Let's see if we can get the electric bill down.	Taobao Marketplace, a Chinese website for online shopping
Jinfeng Hongyuan Flagship Store	Light Blue Saving Electricity	dissatisfied	Taobao Marketplace, a Chinese website for online shopping
Jinfeng Hongyuan Flagship Store	Light Blue Saving Electricity	Friends recommended that this product is still very effective, this time also deliberately bought a back to try to use, use a period of time to evaluate, the effect is good!	Taobao Marketplace, a Chinese website for online shopping
Jinfeng Hongyuan Flagship Store	Light Blue Saving Electricity	Exquisite and durable, power saving effect is obvious, value for money	Taobao Marketplace, a Chinese website for online shopping
Jinfeng Hongyuan Flagship Store	Light Blue Saving Electricity	Packaging is sturdy! Easy to use, compact and lightweight, recommended by a friend! So looking forward to the results!	Taobao Marketplace, a Chinese website for online shopping
Jinfeng Hongyuan Flagship Store	Light Blue Saving Electricity	Baby with a very good,plugged in after the meter ran slower obviously	Taobao Marketplace, a Chinese website for online shopping
Jinfeng Hongyuan Flagship Store	Light Blue Saving Electricity	That's good. That's good.	Taobao Marketplace, a Chinese website for online shopping
Jinfeng Hongyuan Flagship Store	Light Blue Saving Electricity	The product is very good and safe to use	Taobao Marketplace, a Chinese website for online shopping
Jinfeng Hongyuan Flagship Store	Light Blue Saving Electricity	Energy saver received, packaging is very good, logistics is very fast, the goods look very delicate, my family uses a lot of electricity, a few hundred dollars a month, I hope this product can reduce some for me.	Taobao Marketplace, a Chinese website for online shopping
Jinfeng Hongyuan Flagship Store	Shamrock Saves Electricity	Baby to receive the appearance of good workmanship to open the power-saving mode, waiting to verify the first to give a favorable review	Taobao Marketplace, a Chinese website for online shopping
Jinfeng Hongyuan Flagship Store	Shamrock Saves Electricity	Currently used has not seen the effect, if you use another month no effect and then return the product	Taobao Marketplace, a Chinese website for online shopping
Jinfeng Hongyuan Flagship Store	Shamrock Saves Electricity	Still using it, not sure if it will work or not, this is only clear to the seller.	Taobao Marketplace, a Chinese website for online shopping
Jinfeng Hongyuan Flagship Store	Shamrock Saves Electricity	Nice packaging, good workmanship, runs pretty smooth	Taobao Marketplace, a Chinese website for online shopping
Jinfeng Hongyuan Flagship Store	Shamrock Saves Electricity	ll review theproductafter2months, but the merchant's math doesn't work, and I can't understand the difference in price calculation	Taobao Marketplace, a Chinese website for online shopping
Jinfeng Hongyuan Flagship Store	Shamrock Saves Electricity	I heard that this thing works very well, the goods received, look at the quality is quite good, immediately plugged in, after a month and then come over to the level to see how much electricity is saved!	Taobao Marketplace, a Chinese website for online shopping
Jinfeng Hongyuan Flagship Store	Shamrock Saves Electricity	Baby received, shipping fast, packaging is also very good, open to see the workmanship is also powerful, now installed, with a period of time to see the effect of the most to evaluate.	Taobao Marketplace, a Chinese website for online shopping
Jinfeng Hongyuan Flagship Store	Shamrock Saves Electricity	The company's products and services are designed to meet the needs of the market, and the company's products and services are designed to meet the needs of the market, and the company's products and services are designed to meet the needs of the market!	Taobao Marketplace, a Chinese website for online shopping
Jinfeng Hongyuan Flagship Store	Shamrock Saves Electricity	Good attitude, fast, I'm very satisfied, worth 5 stars!	Taobao Marketplace, a Chinese website for online shopping
Jinfeng Hongyuan Flagship Store	Shamrock Saves Electricity	Things arrived, asked customer service, do not have to assemble and install anything, plug in the electricity on the line, ready to plug into the electric boiler next to the plug row, the family bought an electric boiler, more than 1,000 degrees of electricity per month, too costly, listening to friends say that this can save some electricity, then try it, if it is good to use and then come back to follow	Taobao Marketplace, a Chinese website for online shopping
Jinfeng Hongyuan Flagship Store	Shamrock Saves Electricity	Express delivery is very fast, use a period of time to see how the effect	Taobao Marketplace, a Chinese website for online shopping
Jinfeng Hongyuan Flagship Store	Shamrock Saves Electricity	Todaythe6th, just received it and used it, won't know the effect until next month's electric bill comes.	Taobao Marketplace, a Chinese website for online shopping
Jinfeng Hongyuan Flagship Store	Shamrock Saves Electricity	thankfully	Taobao Marketplace, a Chinese website for online shopping
Jinfeng Hongyuan Flagship Store	Shamrock Saves Electricity	Great value for money, I love it and have purchased it for the third time.	Taobao Marketplace, a Chinese website for online shopping
Jinfeng Hongyuan Flagship Store	Shamrock Saves Electricity	Baby received, the quality is very good, exquisite packaging, excellent material, better than expected, gift for self-use is very suitable, next time continue to buy now is the second purchase.	Taobao Marketplace, a Chinese website for online shopping
Jinfeng Hongyuan Flagship Store	Shamrock Saves Electricity	Received it by courier this afternoon, opened it up and it looks good. Did not break it!!!	Taobao Marketplace, a Chinese website for online shopping
Jinfeng Hongyuan Flagship Store	Shamrock Saves Electricity	good-looking	Taobao Marketplace, a Chinese website for online shopping
Jinfeng Hongyuan Flagship Store	Shamrock Saves Electricity	Very good, good description, save your heart ❤	Taobao Marketplace, a Chinese website for online shopping
Jinfeng Hongyuan Flagship Store	Shamrock Saves Electricity	ibid	Taobao Marketplace, a Chinese website for online shopping
Jinfeng Hongyuan Flagship Store	Shamrock Saves Electricity	Used Effect unknown	Taobao Marketplace, a Chinese website for online shopping
Jinfeng Hongyuan Flagship Store	Shamrock Saves Electricity	Received the saver2days, it can be, the boss said to be more than a month, the longer the more power saving, then come back to follow up, the saver is very delicate, the boss has a very good service attitude, ask questions and answer them, tomorrow is ready to buy another one	Taobao Marketplace, a Chinese website for online shopping
Jinfeng Hongyuan Flagship Store	Shamrock Saves Electricity	I don't know if it can save electricity, I'll use it for two months and then comment again	Taobao Marketplace, a Chinese website for online shopping
Jinfeng Hongyuan Flagship Store	Shamrock Saves Electricity	Just arrived immediately use, according to the principle should be able to save electricity, big brand, enough weight!	Taobao Marketplace, a Chinese website for online shopping
Jinfeng Hongyuan Flagship Store	Shamrock Saves Electricity	Received! Fast logistics! Good stuff, very good!	Taobao Marketplace, a Chinese website for online shopping

Jinfeng Hongyuan Flagship Store	Shamrock Saves Electricity	Packaging is intact. At a glance, we know is a regular product, qualified record, product manual complete. Merchants are very sharecropping also sent a fuse.	Taobao Marketplace, a Chinese website for online shopping
Jinfeng Hongyuan Flagship Store	Shamrock Saves Electricity	Second time I repurchased, trust the product, it works great!	Taobao Marketplace, a Chinese website for online shopping
Jinfeng Hongyuan Flagship Store	Shamrock Saves Electricity	From the outside, the quality is very good, in the end, how much electricity can be saved, use a month later to know!	Taobao Marketplace, a Chinese website for online shopping
Jinfeng Hongyuan Flagship Store	Shamrock Saves Electricity	Energy saver just received, looks good, use it and then come back to evaluate!	Taobao Marketplace, a Chinese website for online shopping
Jinfeng Hongyuan Flagship Store	Shamrock Saves Electricity	Very good, compact, does not take up much space, great power saver!	Taobao Marketplace, a Chinese website for online shopping
Jinfeng Hongyuan Flagship Store	Shamrock Saves Electricity	Workmanship is very fine, it seems to be quite formal manufacturers, as soon as it arrived on the use, after a period of time to see the effect.	Taobao Marketplace, a Chinese website for online shopping
Jinfeng Hongyuan Flagship Store	Shamrock Saves Electricity	effective	Taobao Marketplace, a Chinese website for online shopping
Jinfeng Hongyuan Flagship Store	Shamrock Saves Electricity	Already installed, directly connected to an outlet. Easy to install, power saving effect and other time.	Taobao Marketplace, a Chinese website for online shopping
Jinfeng Hongyuan Flagship Store	Shamrock Saves Electricity	The logistics arrived quickly, used a few hours to experience once, not bad, very satisfactory	Taobao Marketplace, a Chinese website for online shopping
Jinfeng Hongyuan Flagship Store	Shamrock Saves Electricity	Baby looks great, love it, and hopefully it will be a power saver!	Taobao Marketplace, a Chinese website for online shopping
Jinfeng Hongyuan Flagship Store	Shamrock Saves Electricity	The logistics arrived quite quickly, the packaging is very good, first rubbed on to try!	Taobao Marketplace, a Chinese website for online shopping
Jinfeng Hongyuan Flagship Store	Shamrock Saves Electricity	Things are very satisfied Oh, beautiful and stylish atmosphere, customer service is super patient, service is meticulous and attentive, the big brands are not the same!	Taobao Marketplace, a Chinese website for online shopping
Jinfeng Hongyuan Flagship Store	Shamrock Saves Electricity	No**.	Taobao Marketplace, a Chinese website for online shopping
Jinfeng Hongyuan Flagship Store	Shamrock Saves Electricity	Baby received it and plugged it in and it feels pretty good!	Taobao Marketplace, a Chinese website for online shopping
Jinfeng Hongyuan Flagship Store	Shamrock Saves Electricity	Did you just receive the next one to find out?	Taobao Marketplace, a Chinese website for online shopping
Jinfeng Hongyuan Flagship Store	Shamrock Saves Electricity	Jinfenghong quality is very good, power saving and durable, just received the goods to start saving money!	Taobao Marketplace, a Chinese website for online shopping
Jinfeng Hongyuan Flagship Store	Shamrock Saves Electricity	Logistics is quite fast, looks quite exquisite, has been installed, try for a period of time, really save electricity before evaluating.	Taobao Marketplace, a Chinese website for online shopping
Jinfeng Hongyuan Flagship Store	Blue Saving Electricity	It's super bad, even if it didn't save electricity, it also broke my meter! Really don't buy this, it doesn't help at all!	Taobao Marketplace, a Chinese website for online shopping
Jinfeng Hongyuan Flagship Store	Blue Saving Electricity	Goods received, the product is the same as the merchant description, very good and trustworthy products, the	Taobao Marketplace, a Chinese website for online shopping
Jinfeng Hongyuan Flagship Store	Blue Saving Electricity	Baby has been received. Logistics brother is very powerful. Merchant service is also very good. Can't wait to install it. Energy saver, good quality. With the merchant description, has been. I hope it can really protect appliances or save electricity. Use a period of time to follow up.	Taobao Marketplace, a Chinese website for online shopping
Jinfeng Hongyuan Flagship Store	Blue Saving Electricity	It should be energy efficient, I've only had it for a few days and didn't try it out, I'll follow up with an evaluation later!	Taobao Marketplace, a Chinese website for online shopping
Jinfeng Hongyuan Flagship Store	Blue Saving Electricity	Effective, used for more than a month with power saving	Taobao Marketplace, a Chinese website for online shopping
Jinfeng Hongyuan Flagship Store	Blue Saving Electricity	Although the model sent me the wrong, but the store has a good service attitude, explained the model is not the same but the same parameters as the same effect, if you want to change it to me, I think the same parameters as the same effect is not changed also line, save the store and I have trouble.	Taobao Marketplace, a Chinese website for online shopping
Jinfeng Hongyuan Flagship Store	Blue Saving Electricity	I just tried it, I haven't seen the effect yet, let's give it 5 stars first!	Taobao Marketplace, a Chinese website for online shopping
Jinfeng Hongyuan Flagship Store	Blue Saving Electricity	Thanks to the owner's home, my friend has received the baby, the logistics is very fast! I hope the little one can be satisfied, thanks!	Taobao Marketplace, a Chinese website for online shopping
Jinfeng Hongyuan Flagship Store	Copper saves electricity	Fast shipping, nice texture. Effective with follow-up comments.	Taobao Marketplace, a Chinese website for online shopping
Jinfeng Hongyuan Flagship Store	Red Save Electricity	can save electricity	Taobao Marketplace, a Chinese website for online shopping
Jinfeng Hongyuan Flagship Store	Shamrock Saves Electricity	The goods have been received! I don't know if it will work as a power saver, I'll comment after a few months!	Taobao Marketplace, a Chinese website for online shopping
Jinfeng Hongyuan Flagship Store	Shamrock Saves Electricity	As always, the owner was very nice and answered all questions, second purchase already.	Taobao Marketplace, a Chinese website for online shopping
Jinfeng Hongyuan Flagship Store	Shamrock Saves Electricity	Fast shipping, has been received, looking at the quality can be, looking forward to the effect Oh!	Taobao Marketplace, a Chinese website for online shopping
Jinfeng Hongyuan Flagship Store	Shamrock Saves Electricity	I've heard that the product is a fake, there's no way it will save electricity	Taobao Marketplace, a Chinese website for online shopping
Jinfeng Hongyuan Flagship Store	Shamrock Saves Electricity	First time to buy, feel good, quality is quite good, use for a period of time to see if the power saving!	Taobao Marketplace, a Chinese website for online shopping
Jinfeng Hongyuan Flagship Store	Shamrock Saves Electricity	A bit pricey, I went to try it and it didn't save as much power as they advertised as noticeable, I felt like I couldn't tell that the meter would slow down, and I bought two more.	Taobao Marketplace, a Chinese website for online shopping
Jinfeng Hongyuan Flagship Store	Shamrock Saves Electricity	It's a bit pricey, I went to try it and it didn't save as much power as they advertised as being noticeable, and I didn't feel like I could tell that the meter was going to slow down.	Taobao Marketplace, a Chinese website for online shopping

Jinfeng Hongyuan Flagship Store	Shamrock Saves Electricity	No evaluation for the time being	Taobao Marketplace, a Chinese website for online shopping
Jinfeng Hongyuan Flagship Store	Shamrock Saves Electricity	I'm not sure if it's a good idea to buy the best one, I picked it out for a long time, my mom said it works, a lot of people don't know about this good stuff hahaha	Taobao Marketplace, a Chinese website for online shopping
Jinfeng Hongyuan Flagship Store	Shamrock Saves Electricity	Good quality product, fast shipping, all 5 stars.	Taobao Marketplace, a Chinese website for online shopping
Jinfeng Hongyuan Flagship Store	Shamrock Saves Electricity	It really works.	Taobao Marketplace, a Chinese website for online shopping
Jinfeng Hongyuan Flagship Store	Shamrock Saves Electricity	It's basically a power saver. It doesn't work.	Taobao Marketplace, a Chinese website for online shopping
Jinfeng Hongyuan Flagship Store	Shamrock Saves Electricity	The baby is received, looks good and works great!	Taobao Marketplace, a Chinese website for online shopping
Jinfeng Hongyuan Flagship Store	Shamrock Saves Electricity	Looks great, good quality, we'll see how it works after a while	Taobao Marketplace, a Chinese website for online shopping
Jinfeng Hongyuan Flagship Store	Shamrock Saves Electricity	It seems to be okay.	Taobao Marketplace, a Chinese website for online shopping
Jinfeng Hongyuan Flagship Store	Shamrock Saves Electricity	Plugged it in as soon as I received it, and it came with a fuse, which is nice. In principle it should work, hopefully it will last a long time	Taobao Marketplace, a Chinese website for online shopping
Jinfeng Hongyuan Flagship Store	Shamrock Saves Electricity	Very good, good review!	Taobao Marketplace, a Chinese website for online shopping
Jinfeng Hongyuan Flagship Store	Shamrock Saves Electricity	It looks great 👍 and the quality is fantastic	Taobao Marketplace, a Chinese website for online shopping
Jinfeng Hongyuan Flagship Store	Shamrock Saves Electricity	I'm using it and I hope it works well	Taobao Marketplace, a Chinese website for online shopping
Jinfeng Hongyuan Flagship Store	Shamrock Saves Electricity	That's good.	Taobao Marketplace, a Chinese website for online shopping
Jinfeng Hongyuan Flagship Store	Shamrock Saves Electricity	I'm on my second purchase. It works.	Taobao Marketplace, a Chinese website for online shopping
Jinfeng Hongyuan Flagship Store	Shamrock Saves Electricity	First of all, I'd like to give you a good review.	Taobao Marketplace, a Chinese website for online shopping
Jinfeng Hongyuan Flagship Store	Shamrock Saves Electricity	I've just started using it. I'll comment later.	Taobao Marketplace, a Chinese website for online shopping
Jinfeng Hongyuan Flagship Store	Shamrock Saves Electricity	in use	Taobao Marketplace, a Chinese website for online shopping
Jinfeng Hongyuan Flagship Store	Shamrock Saves Electricity	Goods have been received, well-packed, home immediately installed on the display voltage, the effect of the use of after seeing the follow-up comment!	Taobao Marketplace, a Chinese website for online shopping
Jinfeng Hongyuan Flagship Store	Shamrock Saves Electricity	quality-price ratio	Taobao Marketplace, a Chinese website for online shopping
Jinfeng Hongyuan Flagship Store	Shamrock Saves Electricity	The first time to buy, feel good, the main business service attitude is very good, do not understand they will teach you 😉😉😉	Taobao Marketplace, a Chinese website for online shopping
Jinfeng Hongyuan Flagship Store	Shamrock Saves Electricity	Has received the goods, the product looks exquisite, use for a period of time to see if the power saving	Taobao Marketplace, a Chinese website for online shopping
Jinfeng Hongyuan Flagship Store	Shamrock Saves Electricity	Home electricity bill several hundred a month, simply can not afford the number, buy this try, I hope it works well, good use in the pursuit of peace!	Taobao Marketplace, a Chinese website for online shopping
Jinfeng Hongyuan Flagship Store	Shamrock Saves Electricity	I'll use it for a month, see if it works, and then I'll comment again.	Taobao Marketplace, a Chinese website for online shopping
Jinfeng Hongyuan Flagship Store	Shamrock Saves Electricity	Power consumption spikes in the winter and it has been used up to third gear plus price electricity this November. The blue metal case is beautiful and quality, accessed immediately upon arrival to see if it will reduce the loss next year...	Taobao Marketplace, a Chinese website for online shopping
Jinfeng Hongyuan Flagship Store	Shamrock Saves Electricity	Baby received, this is the second one bought	Taobao Marketplace, a Chinese website for online shopping
Jinfeng Hongyuan Flagship Store	Shamrock Saves Electricity	After using it for a month, I found that the electricity bill is about twenty dollars cheaper than before, and it's worth getting it for the large amount of electricity used in the home	Taobao Marketplace, a Chinese website for online shopping
Jinfeng Hongyuan Flagship Store	Shamrock Saves Electricity	A month's worth of comparisons do show power savings	Taobao Marketplace, a Chinese website for online shopping
Jinfeng Hongyuan Flagship Store	Shamrock Saves Electricity	Can be shipped fast works well second purchase	Taobao Marketplace, a Chinese website for online shopping
Jinfeng Hongyuan Flagship Store	Shamrock Saves Electricity	Exquisite workmanship, beautiful atmosphere upscale. First from the sense of giving favorable comments, the use of the effect of waiting for a period of time to follow up!	Taobao Marketplace, a Chinese website for online shopping
Jinfeng Hongyuan Flagship Store	Shamrock Saves Electricity	I've bought one before and it really saves power, so I bought another one this time	Taobao Marketplace, a Chinese website for online shopping
Jinfeng Hongyuan Flagship Store	Shamrock Saves Electricity	The battery is good, the installer said it is genuine. Packaging is also good, no bad	Taobao Marketplace, a Chinese website for online shopping
Jinfeng Hongyuan Flagship Store	Shamrock Saves Electricity	The logistics is fast, the baby has been received, this stuff really works well, this is the second one I bought. .	Taobao Marketplace, a Chinese website for online shopping
Jinfeng Hongyuan Flagship Store	Shamrock Saves Electricity	Initial metering can save about 1 kWh per day and 7 kWh per day of original use.	Taobao Marketplace, a Chinese website for online shopping
Jinfeng Hongyuan Flagship Store	Shamrock Saves Electricity	Works pretty well! Satisfied!	Taobao Marketplace, a Chinese website for online shopping

Jinfeng Hongyuan Flagship Store	Shamrock Saves Electricity	I don't know how well it works, but it looks okay	Taobao Marketplace, a Chinese website for online shopping
Jinfeng Hongyuan Flagship Store	Shamrock Saves Electricity	I'll wait a month to comment.	Taobao Marketplace, a Chinese website for online shopping
Jinfeng Hongyuan Flagship Store	Shamrock Saves Electricity	Things received, has been used for a few days, summer with the most energy-saving, worth buying!	Taobao Marketplace, a Chinese website for online shopping
Jinfeng Hongyuan Flagship Store	Shamrock Saves Electricity	Plugged it in right after I received it and ran it for a couple days to see that the voltage was relatively smooth. I wonder what the power saving effect will be?	Taobao Marketplace, a Chinese website for online shopping
Jinfeng Hongyuan Flagship Store	Shamrock Saves Electricity	As described and runs smoothly.	Taobao Marketplace, a Chinese website for online shopping
Jinfeng Hongyuan Flagship Store	Shamrock Saves Electricity	It seems to be okay.	Taobao Marketplace, a Chinese website for online shopping
Jinfeng Hongyuan Flagship Store	Shamrock Saves Electricity	The item was received, well packaged, and the logistics was fast! Thanks!	Taobao Marketplace, a Chinese website for online shopping
Jinfeng Hongyuan Flagship Store	Shamrock Saves Electricity	I haven't used it yet.	Taobao Marketplace, a Chinese website for online shopping
Jinfeng Hongyuan Flagship Store	Shamrock Saves Electricity	It's good, it saves power, and it ships quickly.	Taobao Marketplace, a Chinese website for online shopping
Jinfeng Hongyuan Flagship Store	Shamrock Saves Electricity	It's very good. It's the real deal.	Taobao Marketplace, a Chinese website for online shopping
Jinfeng Hongyuan Flagship Store	Shamrock Saves Electricity	This is the fourth time I've purchased it and it works great.	Taobao Marketplace, a Chinese website for online shopping
Jinfeng Hongyuan Flagship Store	Shamrock Saves Electricity	It is genuine, the material is superior, just inserted the effect to be verified. First of all, I would like to praise one.	Taobao Marketplace, a Chinese website for online shopping
Jinfeng Hongyuan Flagship Store	Shamrock Saves Electricity	Great quality and affordable price! Works great!	Taobao Marketplace, a Chinese website for online shopping
Jinfeng Hongyuan Flagship Store	Shamrock Saves Electricity	After receiving it, I checked the manufacturer, product certificate and manual, and I feel good. As for the power saving, after the use of follow-up evaluation	Taobao Marketplace, a Chinese website for online shopping
Jinfeng Hongyuan Flagship Store	Shamrock Saves Electricity	Works great, worth the money	Taobao Marketplace, a Chinese website for online shopping
Jinfeng Hongyuan Flagship Store	Shamrock Saves Electricity	Second time I've purchased it, this time for my parents' side of the family, and it's generally fine.	Taobao Marketplace, a Chinese website for online shopping
Jinfeng Hongyuan Flagship Store	Shamrock Saves Electricity	This product is lightweight and stylish, and the price is affordable, packaging is exquisite, not bad!	Taobao Marketplace, a Chinese website for online shopping
Jinfeng Hongyuan Flagship Store	Shamrock Saves Electricity	In the mouth of a friend learned that there are saving appliances this thing, buy a try, compared to many homes, or choose to buy more expensive, the quality looks good, there are 60 days of trial can experience the effect of	Taobao Marketplace, a Chinese website for online shopping
Jinfeng Hongyuan Flagship Store	Shamrock Saves Electricity	Worthy of the top ten brands of the first each of the power saver, good workmanship good quality	Taobao Marketplace, a Chinese website for online shopping
Jinfeng Hongyuan Flagship Store	Shamrock Saves Electricity	At present, there is not much feeling, see how the effect after using a month, the business guarantees 60 days to try unsatisfied at any time you can return.	Taobao Marketplace, a Chinese website for online shopping
Jinfeng Hongyuan Flagship Store	Shamrock Saves Electricity	Can't tell if it saves power or not	Taobao Marketplace, a Chinese website for online shopping
Jinfeng Hongyuan Flagship Store	Shamrock Saves Electricity	I just got it. I'll use it and comment later.	Taobao Marketplace, a Chinese website for online shopping
Jinfeng Hongyuan Flagship Store	Shamrock Saves Electricity	People don't buy this power saver, it doesn't save any power at all, it's a rip off.	Taobao Marketplace, a Chinese website for online shopping
Jinfeng Hongyuan Flagship Store	Shamrock Saves Electricity	Looks good, talked to the clerk about my home's electrical usage to help me recommend it. I hope to have good results. Received the goods immediately plugged in the power	Taobao Marketplace, a Chinese website for online shopping
Jinfeng Hongyuan Flagship Store	Shamrock Saves Electricity	I've been using it since I got back.	Taobao Marketplace, a Chinese website for online shopping
Jinfeng Hongyuan Flagship Store	Shamrock Saves Electricity	I bought one a few days ago and this is a repeat purchase, mounted upstairs!	Taobao Marketplace, a Chinese website for online shopping
Jinfeng Hongyuan Flagship Store	Shamrock Saves Electricity	The delivery arrived in the evening and when I opened it, I thought the outer packaging and appearance were quite nice!	Taobao Marketplace, a Chinese website for online shopping
Jinfeng Hongyuan Flagship Store	Shamrock Saves Electricity	It really works.	Taobao Marketplace, a Chinese website for online shopping
Jinfeng Hongyuan Flagship Store	Shamrock Saves Electricity	227V is lower, usually 240V in Malaysia, this is very light and hopefully effective.	Taobao Marketplace, a Chinese website for online shopping
Jinfeng Hongyuan Flagship Store	Shamrock Saves Electricity	Baby received, the product is very good, worth the next purchase!	Taobao Marketplace, a Chinese website for online shopping
Jinfeng Hongyuan Flagship Store	Shamrock Saves Electricity	It's a real savings. It's really good. I saved half my money.	Taobao Marketplace, a Chinese website for online shopping
Jinfeng Hongyuan Flagship Store	Shamrock Saves Electricity	Good cosmetic quality, in use...	Taobao Marketplace, a Chinese website for online shopping
Jinfeng Hongyuan Flagship Store	Shamrock Saves Electricity	Logistics is fast, packaging is good, looks like it should be genuine.	Taobao Marketplace, a Chinese website for online shopping
Jinfeng Hongyuan Flagship Store	Shamrock Saves Electricity	This I just used, just now I evaluated, said five-star praise,, but customer service for various reasons, refused to return cash, so I will once again evaluate, honestly, with power and without power are the same, can not see where it will save power, may be long-term use, should consume power, because it has	Taobao Marketplace, a Chinese website for online shopping

Jinfeng Hongyuan Flagship Store	Shamrock Saves Electricity	Power Saving. Good to use, good to save housekeeping	Taobao Marketplace, a Chinese website for online shopping
Jinfeng Hongyuan Flagship Store	Shamrock Saves Electricity	This is good quality. High quality product. The effect is also good. My brother bought it for the second time	Taobao Marketplace, a Chinese website for online shopping
Jinfeng Hongyuan Flagship Store	Shamrock Saves Electricity	The goods have been received very well, thank you, with good use will buy again!	Taobao Marketplace, a Chinese website for online shopping
Jinfeng Hongyuan Flagship Store	Shamrock Saves Electricity	I've got this baby, and it's really, really good.	Taobao Marketplace, a Chinese website for online shopping
Jinfeng Hongyuan Flagship Store	Shamrock Saves Electricity	I got it.	Taobao Marketplace, a Chinese website for online shopping
Jinfeng Hongyuan Flagship Store	Shamrock Saves Electricity	My home open air conditioning when a month of electricity four or five hundred degrees, according to customer service advice to choose this, 800 degrees or less can meet the requirements of power saving is certain, no need to doubt.	Taobao Marketplace, a Chinese website for online shopping
Jinfeng Hongyuan Flagship Store	Shamrock Saves Electricity	I'll use it for a while to see how it works and then I'll give you a report on how it goes!	Taobao Marketplace, a Chinese website for online shopping
Jinfeng Hongyuan Flagship Store	Shamrock Saves Electricity	The goods have been received, beautiful workmanship, I do not know if it is useful	Taobao Marketplace, a Chinese website for online shopping
Jinfeng Hongyuan Flagship Store	Shamrock Saves Electricity	The thing is undamaged, looks neat and complete, there are no errors in the wires, there are no errors in the display, it feels like it should work well	Taobao Marketplace, a Chinese website for online shopping
Jinfeng Hongyuan Flagship Store	Shamrock Saves Electricity	Installed, whether it can save electricity to be observed, better workmanship	Taobao Marketplace, a Chinese website for online shopping
Jinfeng Hongyuan Flagship Store	Shamrock Saves Electricity	The first time to buy power saving treasure, deliberately selected the quality of a good order, after all, it needs to be inserted in the socket all the time, safety is the most important, received the goods in front of the eyes, I feel that value for money, power-saving treasure is beautifully packaged, the body of aluminum-magnesium alloy material, workmanship sharp edges, dark blue feel stable and technology sense of surplus, hold in the hand heavy very texture, anti-counterfeiting code, insurance companies to underwrite, and a gift of five tubes. After powering up the red value keeps changing, about two hours later stabilized, asked customer service to know is the voltage floating value. Currently used for two days is not clear how much specific power saving, wait for next month's electricity bill down and then follow up, customer service is professional and careful, ask questions and answer seconds to reply,	Taobao Marketplace, a Chinese website for online shopping
Jinfeng Hongyuan Flagship Store	Shamrock Saves Electricity	Craftsmanship is well done, the quality is quite good, very practical, and save money on electricity.	Taobao Marketplace, a Chinese website for online shopping
Jinfeng Hongyuan Flagship Store	Shamrock Saves Electricity	It's good. I'll try it out.	Taobao Marketplace, a Chinese website for online shopping
Jinfeng Hongyuan Flagship Store	Shamrock Saves Electricity	The appearance is clean and beautiful, the workmanship is very good, I did not find any flaws, I personally feel that the size is also quite suitable, I have been looking for a long time to choose this one, I love it! Really recommend ah, express speed especially fast, love love love things like, quality is also very good, this price can buy is really good value!	Taobao Marketplace, a Chinese website for online shopping
Jinfeng Hongyuan Flagship Store	Shamrock Saves Electricity	It's really good and recommended	Taobao Marketplace, a Chinese website for online shopping
Jinfeng Hongyuan Flagship Store	Shamrock Saves Electricity	It's from a regular manufacturer and it's really good.	Taobao Marketplace, a Chinese website for online shopping
Jinfeng Hongyuan Flagship Store	Shamrock Saves Electricity	Baby received, started to use again, and will tell you how much I can save when I use it!	Taobao Marketplace, a Chinese website for online shopping
Jinfeng Hongyuan Flagship Store	Shamrock Saves Electricity	It's not bad. It saves a little.	Taobao Marketplace, a Chinese website for online shopping
Jinfeng Hongyuan Flagship Store	Shamrock Saves Electricity	The product is very good, energy-saving, energy-saving, super good, recommend that you folks can buy it!	Taobao Marketplace, a Chinese website for online shopping
Jinfeng Hongyuan Flagship Store	Shamrock Saves Electricity	Saving appliances received, the packaging is intact, logistics is very fast, the quality of business description in line with the third time to buy, the first time to buy has been used for two months, the electricity bill and the first few months have declined, about ten percent **, a little bit of effect.	Taobao Marketplace, a Chinese website for online shopping
Jinfeng Hongyuan Flagship Store	Shamrock Saves Electricity	The quality of the product is good and worth having.	Taobao Marketplace, a Chinese website for online shopping
Jinfeng Hongyuan Flagship Store	Shamrock Saves Electricity	I received it. I'll try it for a month and see how it works.	Taobao Marketplace, a Chinese website for online shopping
Jinfeng Hongyuan Flagship Store	Shamrock Saves Electricity	Great platform to buy from, good quality, especially with a brand name, trustworthy	Taobao Marketplace, a Chinese website for online shopping
Jinfeng Hongyuan Flagship Store	Shamrock Saves Electricity	Packaging is very good, and the workmanship is exquisite. Already used, wait for a period of time to use the additional evaluation of the use of the effect.	Taobao Marketplace, a Chinese website for online shopping
Jinfeng Hongyuan Flagship Store	Shamrock Saves Electricity	Items received, packaging is very good, just received not yet used, I hope it is good!	Taobao Marketplace, a Chinese website for online shopping
Jinfeng Hongyuan Flagship Store	Shamrock Saves Electricity	Already used it, looking forward to the results	Taobao Marketplace, a Chinese website for online shopping
Jinfeng Hongyuan Flagship Store	Shamrock Saves Electricity	Energy saver quality is very good, fine workmanship logistics is very fast, and the store described the same use for a period of time really save electricity	Taobao Marketplace, a Chinese website for online shopping
Jinfeng Hongyuan Flagship Store	Shamrock Saves Electricity	I just got it back, I'll use it for a while and then I'll comment	Taobao Marketplace, a Chinese website for online shopping
Jinfeng Hongyuan Flagship Store	Shamrock Saves Electricity	Very good baby, perfect packaging and kind owner! Worth recommending!	Taobao Marketplace, a Chinese website for online shopping
Jinfeng Hongyuan Flagship Store	Shamrock Saves Electricity	Baby received very happy shopping very happy online shopping.	Taobao Marketplace, a Chinese website for online shopping
Jinfeng Hongyuan Flagship Store	Shamrock Saves Electricity	Goldwind Hongyuan power saver, very good 👍, query is genuine, fine workmanship, stable work, packaging is quite good, low noise, it is a good product, power saving situation to be used in the evaluation, I believe it will not be wrong.	Taobao Marketplace, a Chinese website for online shopping
Jinfeng Hongyuan Flagship Store	Shamrock Saves Electricity	Plugged it in and used it for a bit, didn't hear any noise and it's the real deal. Worth having.	Taobao Marketplace, a Chinese website for online shopping

Jinfeng Hongyuan Flagship Store	Shamrock Saves Electricity	Received the baby, the stuff is exquisite and well made, looking forward to saving electricity 😊	Taobao Marketplace, a Chinese website for online shopping
Jinfeng Hongyuan Flagship Store	Shamrock Saves Electricity	Good for waiting a month to know the power savings	Taobao Marketplace, a Chinese website for online shopping
Jinfeng Hongyuan Flagship Store	Shamrock Saves Electricity	Shipping was prompt, received it quickly, the item is the right size, looking forward to doing its job and seeing the results after a while.	Taobao Marketplace, a Chinese website for online shopping
Jinfeng Hongyuan Flagship Store	Shamrock Saves Electricity	Perfect, the product is amazing, the price is right for the money and I hope it lasts a long time!	Taobao Marketplace, a Chinese website for online shopping
Jinfeng Hongyuan Flagship Store	Shamrock Saves Electricity	The quality of the machine is very good, very classy feeling ^_^.	Taobao Marketplace, a Chinese website for online shopping
Jinfeng Hongyuan Flagship Store	Shamrock Saves Electricity	On Trial	Taobao Marketplace, a Chinese website for online shopping
Jinfeng Hongyuan Flagship Store	Shamrock Saves Electricity	Used a multimeter to measure the current and it is working, slightly less current. It saves power.	Taobao Marketplace, a Chinese website for online shopping
Jinfeng Hongyuan Flagship Store	Shamrock Saves Electricity	Good quality, looking forward to the effect. Seller has great service and fast shipping.	Taobao Marketplace, a Chinese website for online shopping
Jinfeng Hongyuan Flagship Store	Platinum Power Saving Specialist	Worthy of a big brand, the texture is super good, texture color are no words, customer service attitude is good heart fine, packaging exquisite, experience the high quality of the service	Taobao Marketplace, a Chinese website for online shopping
Jinfeng Hongyuan Flagship Store	Platinum Power Saving Specialist	point (in space or time)	Taobao Marketplace, a Chinese website for online shopping
Jinfeng Hongyuan Flagship Store	Platinum Power Saving Specialist	Works great, got an extra one, third one now	Taobao Marketplace, a Chinese website for online shopping
Jinfeng Hongyuan Flagship Store	Platinum Power Saving Specialist	Just received the goods are in use, praise	Taobao Marketplace, a Chinese website for online shopping
Jinfeng Hongyuan Flagship Store	Platinum Power Saving Specialist	Baby received!	Taobao Marketplace, a Chinese website for online shopping
Jinfeng Hongyuan Flagship Store	Platinum Power Saving Specialist	Looked like it was made by the regular manufacturer, good service, use it and then comment on the results.	Taobao Marketplace, a Chinese website for online shopping
Jinfeng Hongyuan Flagship Store	Platinum Power Saving Specialist	I'm just using it. I'll comment on it later.	Taobao Marketplace, a Chinese website for online shopping
Jinfeng Hongyuan Flagship Store	Platinum Power Saving Specialist	Expect power savings	Taobao Marketplace, a Chinese website for online shopping
Jinfeng Hongyuan Flagship Store	Platinum Power Saving Specialist	I just received it and am using it to see how much I can save on my electric bill next month before I comment. Good review!	Taobao Marketplace, a Chinese website for online shopping
Jinfeng Hongyuan Flagship Store	Purple Power Saving Specialist	The goods have been received! I don't know if it will work as a power saver, I'll comment after a few months!	Taobao Marketplace, a Chinese website for online shopping
Jinfeng Hongyuan Flagship Store	Purple Power Saving Specialist	As always, the owner was very nice and answered all questions, second purchase already.	Taobao Marketplace, a Chinese website for online shopping
Jinfeng Hongyuan Flagship Store	Purple Power Saving Specialist	Fast shipping, has been received, looking at the quality can be, looking forward to the effect Oh!	Taobao Marketplace, a Chinese website for online shopping
Jinfeng Hongyuan Flagship Store	Purple Power Saving Specialist	I've heard that the product is a fake, there's no way it will save electricity	Taobao Marketplace, a Chinese website for online shopping
Jinfeng Hongyuan Flagship Store	Purple Power Saving Specialist	First time to buy, feel good, quality is quite good, use for a period of time to see if the power saving!	Taobao Marketplace, a Chinese website for online shopping
Jinfeng Hongyuan Flagship Store	Purple Power Saving Specialist	A bit pricey, I went to try it and it didn't save as much power as they advertised as noticeable, I felt like I couldn't tell that the meter would slow down, and I bought two more.	Taobao Marketplace, a Chinese website for online shopping
Jinfeng Hongyuan Flagship Store	Purple Power Saving Specialist	It's a bit pricey, I went to try it and it didn't save as much power as they advertised as being noticeable, and I didn't feel like I could tell that the meter was going to slow down.	Taobao Marketplace, a Chinese website for online shopping
Jinfeng Hongyuan Flagship Store	Purple Power Saving Specialist	No evaluation for the time being	Taobao Marketplace, a Chinese website for online shopping
Jinfeng Hongyuan Flagship Store	Purple Power Saving Specialist	I'm not sure if it's a good idea to buy the best one, I picked it out for a long time, my mom said it works, a lot of people don't know about this good stuff hahaha	Taobao Marketplace, a Chinese website for online shopping
Jinfeng Hongyuan Flagship Store	Purple Power Saving Specialist	Good quality product, fast shipping, all 5 stars.	Taobao Marketplace, a Chinese website for online shopping
Jinfeng Hongyuan Flagship Store	Purple Power Saving Specialist	It really works.	Taobao Marketplace, a Chinese website for online shopping
Jinfeng Hongyuan Flagship Store	Purple Power Saving Specialist	It's basically a power saver. It doesn't work.	Taobao Marketplace, a Chinese website for online shopping
Jinfeng Hongyuan Flagship Store	Purple Power Saving Specialist	The baby is received, looks good and works great!	Taobao Marketplace, a Chinese website for online shopping
Jinfeng Hongyuan Flagship Store	Purple Power Saving Specialist	Looks great, good quality, we'll see how it works after a while	Taobao Marketplace, a Chinese website for online shopping
Jinfeng Hongyuan Flagship Store	Purple Power Saving Specialist	It seems to be okay.	Taobao Marketplace, a Chinese website for online shopping
Jinfeng Hongyuan Flagship Store	Purple Power Saving Specialist	Plugged it in as soon as I received it, and it came with a fuse, which is nice. In principle it should work, hopefully it will last a long time	Taobao Marketplace, a Chinese website for online shopping
Jinfeng Hongyuan Flagship Store	Purple Power Saving Specialist	Very good, good review!	Taobao Marketplace, a Chinese website for online shopping

Jinfeng Hongyuan Flagship Store	Purple Power Saving Specialist	It looks great 👍 and the quality is fantastic	Taobao Marketplace, a Chinese website for online shopping
Jinfeng Hongyuan Flagship Store	Purple Power Saving Specialist	I'm using it and I hope it works well	Taobao Marketplace, a Chinese website for online shopping
Jinfeng Hongyuan Flagship Store	Purple Power Saving Specialist	That's good.	Taobao Marketplace, a Chinese website for online shopping
Jinfeng Hongyuan Flagship Store	Purple Power Saving Specialist	I'm on my second purchase. It works.	Taobao Marketplace, a Chinese website for online shopping
Jinfeng Hongyuan Flagship Store	Purple Power Saving Specialist	First of all, I'd like to give you a good review.	Taobao Marketplace, a Chinese website for online shopping
Jinfeng Hongyuan Flagship Store	Purple Power Saving Specialist	I've just started using it. I'll comment later.	Taobao Marketplace, a Chinese website for online shopping
Jinfeng Hongyuan Flagship Store	Purple Power Saving Specialist	in use	Taobao Marketplace, a Chinese website for online shopping
Jinfeng Hongyuan Flagship Store	Purple Power Saving Specialist	Goods have been received, well-packed, home immediately installed on the display voltage, the effect of the use of after seeing the follow-up comment!	Taobao Marketplace, a Chinese website for online shopping
Jinfeng Hongyuan Flagship Store	Purple Power Saving Specialist	quality-price ratio	Taobao Marketplace, a Chinese website for online shopping
Jinfeng Hongyuan Flagship Store	Purple Power Saving Specialist	The first time to buy, feel good, the main business service attitude is very good, do not understand they will teach you 😉😉😉	Taobao Marketplace, a Chinese website for online shopping
Jinfeng Hongyuan Flagship Store	Purple Power Saving Specialist	Has received the goods, the product looks exquisite, use for a period of time to see if the power saving	Taobao Marketplace, a Chinese website for online shopping
Jinfeng Hongyuan Flagship Store	Purple Power Saving Specialist	Home electricity bill several hundred a month, simply can not afford the number, buy this try, I hope it works well, good use in the pursuit of peace!	Taobao Marketplace, a Chinese website for online shopping
Jinfeng Hongyuan Flagship Store	Purple Power Saving Specialist	I'll use it for a month, see if it works, and then I'll comment again.	Taobao Marketplace, a Chinese website for online shopping
Jinfeng Hongyuan Flagship Store	Purple Power Saving Specialist	Power consumption spikes in the winter and it has been used up to third gear plus price electricity this November. The blue metal case is beautiful and quality, accessed immediately upon arrival to see if it will reduce the loss next year...	Taobao Marketplace, a Chinese website for online shopping
Jinfeng Hongyuan Flagship Store	Purple Power Saving Specialist	Baby received, this is the second one bought	Taobao Marketplace, a Chinese website for online shopping
Jinfeng Hongyuan Flagship Store	Purple Power Saving Specialist	After using it for a month, I found that the electricity bill is about twenty dollars cheaper than before, and it's worth getting it for the large amount of electricity used in the home	Taobao Marketplace, a Chinese website for online shopping
Jinfeng Hongyuan Flagship Store	Purple Power Saving Specialist	A month's worth of comparisons do show power savings	Taobao Marketplace, a Chinese website for online shopping
Jinfeng Hongyuan Flagship Store	Purple Power Saving Specialist	Can be shipped fast works well second purchase	Taobao Marketplace, a Chinese website for online shopping
Jinfeng Hongyuan Flagship Store	Purple Power Saving Specialist	Exquisite workmanship, beautiful atmosphere upscale. First from the sense of giving favorable comments, the use of the effect of waiting for a period of time to follow up!	Taobao Marketplace, a Chinese website for online shopping
Jinfeng Hongyuan Flagship Store	Purple Power Saving Specialist	I've bought one before and it really saves power, so I bought another one this time	Taobao Marketplace, a Chinese website for online shopping
Jinfeng Hongyuan Flagship Store	Purple Power Saving Specialist	The battery is good, the installer said it is genuine. Packaging is also good, no bad	Taobao Marketplace, a Chinese website for online shopping
Jinfeng Hongyuan Flagship Store	Purple Power Saving Specialist	The logistics is fast, the baby has been received, this stuff really works well, this is the second one I bought. .	Taobao Marketplace, a Chinese website for online shopping
Jinfeng Hongyuan Flagship Store	Purple Power Saving Specialist	Initial metering can save about 1 kWh per day and 7 kWh per day of original use.	Taobao Marketplace, a Chinese website for online shopping
Jinfeng Hongyuan Flagship Store	Purple Power Saving Specialist	Works pretty well! Satisfied!	Taobao Marketplace, a Chinese website for online shopping
Jinfeng Hongyuan Flagship Store	Purple Power Saving Specialist	I don't know how well it works, but it looks okay	Taobao Marketplace, a Chinese website for online shopping
Jinfeng Hongyuan Flagship Store	Purple Power Saving Specialist	I'll wait a month to comment.	Taobao Marketplace, a Chinese website for online shopping
Jinfeng Hongyuan Flagship Store	Purple Power Saving Specialist	Things received, has been used for a few days, summer with the most energy-saving, worth buying!	Taobao Marketplace, a Chinese website for online shopping
Jinfeng Hongyuan Flagship Store	Purple Power Saving Specialist	Plugged it in right after I received it and ran it for a couple days to see that the voltage was relatively smooth. I wonder what the power saving effect will be?	Taobao Marketplace, a Chinese website for online shopping
Jinfeng Hongyuan Flagship Store	Purple Power Saving Specialist	As described and runs smoothly.	Taobao Marketplace, a Chinese website for online shopping
Jinfeng Hongyuan Flagship Store	Purple Power Saving Specialist	It seems to be okay.	Taobao Marketplace, a Chinese website for online shopping
Jinfeng Hongyuan Flagship Store	Purple Power Saving Specialist	The item was received, well packaged, and the logistics was fast! Thanks!	Taobao Marketplace, a Chinese website for online shopping
Jinfeng Hongyuan Flagship Store	Purple Power Saving Specialist	I haven't used it yet.	Taobao Marketplace, a Chinese website for online shopping
Jinfeng Hongyuan Flagship Store	Purple Power Saving Specialist	It's good, it saves power, and it ships quickly.	Taobao Marketplace, a Chinese website for online shopping
Jinfeng Hongyuan Flagship Store	Purple Power Saving Specialist	It's very good. It's the real deal.	Taobao Marketplace, a Chinese website for online shopping

Jinfeng Hongyuan Flagship Store	Purple Power Saving Specialist	This is the fourth time I've purchased it and it works great.	Taobao Marketplace, a Chinese website for online shopping
Jinfeng Hongyuan Flagship Store	Purple Power Saving Specialist	It is genuine, the material is superior, just inserted the effect to be verified. First of all, I would like to praise one.	Taobao Marketplace, a Chinese website for online shopping
Jinfeng Hongyuan Flagship Store	Purple Power Saving Specialist	Great quality and affordable price! Works great!	Taobao Marketplace, a Chinese website for online shopping
Jinfeng Hongyuan Flagship Store	Purple Power Saving Specialist	After receiving it, I checked the manufacturer, product certificate and manual, and I feel good. As for the power saving, after the use of follow-up evaluation	Taobao Marketplace, a Chinese website for online shopping
Jinfeng Hongyuan Flagship Store	Purple Power Saving Specialist	Works great, worth the money	Taobao Marketplace, a Chinese website for online shopping
Jinfeng Hongyuan Flagship Store	Purple Power Saving Specialist	Second time I've purchased it, this time for my parents' side of the family, and it's generally fine.	Taobao Marketplace, a Chinese website for online shopping
Jinfeng Hongyuan Flagship Store	Purple Power Saving Specialist	This product is lightweight and stylish, and the price is affordable, packaging is exquisite, not bad!	Taobao Marketplace, a Chinese website for online shopping
Jinfeng Hongyuan Flagship Store	Purple Power Saving Specialist	In the mouth of a friend learned that there are saving appliances this thing, buy a try, compared to many homes, or choose to buy more expensive, the quality looks good, there are 60 days of trial can experience the effect of	Taobao Marketplace, a Chinese website for online shopping
Jinfeng Hongyuan Flagship Store	Purple Power Saving Specialist	Worthy of the top ten brands of the first each of the power saver, good workmanship good quality	Taobao Marketplace, a Chinese website for online shopping
Jinfeng Hongyuan Flagship Store	Purple Power Saving Specialist	At present, there is not much feeling, see how the effect after using a month, the business guarantees 60 days to try unsatisfied at any time you can return.	Taobao Marketplace, a Chinese website for online shopping
Jinfeng Hongyuan Flagship Store	Purple Power Saving Specialist	Can't tell if it saves power or not	Taobao Marketplace, a Chinese website for online shopping
Jinfeng Hongyuan Flagship Store	Purple Power Saving Specialist	I just got it. I'll use it and comment later.	Taobao Marketplace, a Chinese website for online shopping
Jinfeng Hongyuan Flagship Store	Purple Power Saving Specialist	People don't buy this power saver, it doesn't save any power at all, it's a rip off.	Taobao Marketplace, a Chinese website for online shopping
Jinfeng Hongyuan Flagship Store	Purple Power Saving Specialist	Looks good, talked to the clerk about my home's electrical usage to help me recommend it. I hope to have good results. Received the goods immediately plugged in the power	Taobao Marketplace, a Chinese website for online shopping
Jinfeng Hongyuan Flagship Store	Purple Power Saving Specialist	I've been using it since I got back.	Taobao Marketplace, a Chinese website for online shopping
Jinfeng Hongyuan Flagship Store	Purple Power Saving Specialist	I bought one a few days ago and this is a repeat purchase, mounted upstairs!	Taobao Marketplace, a Chinese website for online shopping
Jinfeng Hongyuan Flagship Store	Purple Power Saving Specialist	The delivery arrived in the evening and when I opened it, I thought the outer packaging and appearance were quite nice!	Taobao Marketplace, a Chinese website for online shopping
Jinfeng Hongyuan Flagship Store	Purple Power Saving Specialist	It really works.	Taobao Marketplace, a Chinese website for online shopping
Jinfeng Hongyuan Flagship Store	Purple Power Saving Specialist	227V is lower, usually 240V in Malaysia, this is very light and hopefully effective.	Taobao Marketplace, a Chinese website for online shopping
Jinfeng Hongyuan Flagship Store	Purple Power Saving Specialist	Baby received, the product is very good, worth the next purchase!	Taobao Marketplace, a Chinese website for online shopping
Jinfeng Hongyuan Flagship Store	Purple Power Saving Specialist	It's a real savings. It's really good. I saved half my money.	Taobao Marketplace, a Chinese website for online shopping
Jinfeng Hongyuan Flagship Store	Purple Power Saving Specialist	Good cosmetic quality, in use...	Taobao Marketplace, a Chinese website for online shopping
Jinfeng Hongyuan Flagship Store	Purple Power Saving Specialist	Logistics is fast, packaging is good, looks like it should be genuine.	Taobao Marketplace, a Chinese website for online shopping
Jinfeng Hongyuan Flagship Store	Purple Power Saving Specialist	This I just used, just now I evaluated, said five-star praise,, but customer service for various reasons, refused to return cash, so I will once again evaluate, honestly, with power and without power are the same, can not see where it will save power, may be long-term use, should consume power, because it has	Taobao Marketplace, a Chinese website for online shopping
Jinfeng Hongyuan Flagship Store	Purple Power Saving Specialist	Power Saving. Good to use, good to save housekeeping	Taobao Marketplace, a Chinese website for online shopping
Jinfeng Hongyuan Flagship Store	Purple Power Saving Specialist	This is good quality. High quality product. The effect is also good. My brother bought it for the second time	Taobao Marketplace, a Chinese website for online shopping
Jinfeng Hongyuan Flagship Store	Purple Power Saving Specialist	The goods have been received very well, thank you, with good use will buy again!	Taobao Marketplace, a Chinese website for online shopping
Jinfeng Hongyuan Flagship Store	Purple Power Saving Specialist	I've got this baby, and it's really, really good.	Taobao Marketplace, a Chinese website for online shopping
Jinfeng Hongyuan Flagship Store	Purple Power Saving Specialist	I got it.	Taobao Marketplace, a Chinese website for online shopping
Jinfeng Hongyuan Flagship Store	Purple Power Saving Specialist	My home open air conditioning when a month of electricity four or five hundred degrees, according to customer service advice to choose this, 800 degrees or less can meet the requirements of power saving is certain, no need to doubt.	Taobao Marketplace, a Chinese website for online shopping
Jinfeng Hongyuan Flagship Store	Purple Power Saving Specialist	I'll use it for a while to see how it works and then I'll give you a report on how it goes!	Taobao Marketplace, a Chinese website for online shopping
Jinfeng Hongyuan Flagship Store	Purple Power Saving Specialist	The goods have been received, beautiful workmanship, I do not know if it is useful	Taobao Marketplace, a Chinese website for online shopping
Jinfeng Hongyuan Flagship Store	Purple Power Saving Specialist	The thing is undamaged, looks neat and complete, there are no errors in the wires, there are no errors in the display, it feels like it should work well	Taobao Marketplace, a Chinese website for online shopping
Jinfeng Hongyuan Flagship Store	Purple Power Saving Specialist	Installed, whether it can save electricity to be observed, better workmanship	Taobao Marketplace, a Chinese website for online shopping

Jinfeng Hongyuan Flagship Store	Purple Power Saving Specialist	The first time to buy power saving treasure, deliberately selected the quality of a good order, after all, it needs to be inserted in the socket all the time, safety is the most important, received the goods in front of the eyes, I feel that value for money, power-saving treasure is beautifully packaged, the body of aluminum-magnesium alloy material, workmanship sharp edges, dark blue feel stable and technology sense of surplus, hold in the hand heavy very texture, anti-counterfeiting code, insurance companies to underwrite, and a gift of five tubes. After powering up the red value keeps changing, about two hours later stabilized, asked customer service to know is the voltage floating value. Currently used for two days is not clear how much specific power saving, wait for next month's electricity bill down and then follow up, customer service is professional and careful, ask questions and answer seconds to reply,	Taobao Marketplace, a Chinese website for online shopping
Jinfeng Hongyuan Flagship Store	Purple Power Saving Specialist	Craftsmanship is well done, the quality is quite good, very practical, and save money on electricity.	Taobao Marketplace, a Chinese website for online shopping
Jinfeng Hongyuan Flagship Store	Purple Power Saving Specialist	It's good. I'll try it out.	Taobao Marketplace, a Chinese website for online shopping
Jinfeng Hongyuan Flagship Store	Purple Power Saving Specialist	The appearance is clean and beautiful, the workmanship is very good, I did not find any flaws, I personally feel that the size is also quite suitable, I have been looking for a long time to choose this one, I love it! Really recommend ah, express speed especially fast, love love love things like, quality is also very good, this price can buy is really good value!	Taobao Marketplace, a Chinese website for online shopping
Jinfeng Hongyuan Flagship Store	Purple Power Saving Specialist	It's really good and recommended	Taobao Marketplace, a Chinese website for online shopping
Jinfeng Hongyuan Flagship Store	Purple Power Saving Specialist	It's from a regular manufacturer and it's really good.	Taobao Marketplace, a Chinese website for online shopping
Jinfeng Hongyuan Flagship Store	Purple Power Saving Specialist	Baby received, started to use again, and will tell you how much I can save when I use it!	Taobao Marketplace, a Chinese website for online shopping
Jinfeng Hongyuan Flagship Store	Purple Power Saving Specialist	It's not bad. It saves a little.	Taobao Marketplace, a Chinese website for online shopping
Jinfeng Hongyuan Flagship Store	Purple Power Saving Specialist	The product is very good, energy-saving, energy-saving, super good, recommend that you folks can buy it!	Taobao Marketplace, a Chinese website for online shopping
Jinfeng Hongyuan Flagship Store	Purple Power Saving Specialist	Saving appliances received, the packaging is intact, logistics is very fast, the quality of business description in line with the third time to buy, the first time to buy has been used for two months, the electricity bill and the first few months have declined, about ten percent **, a little bit of effect.	Taobao Marketplace, a Chinese website for online shopping
Jinfeng Hongyuan Flagship Store	Purple Power Saving Specialist	The quality of the product is good and worth having.	Taobao Marketplace, a Chinese website for online shopping
Jinfeng Hongyuan Flagship Store	Purple Power Saving Specialist	I received it. I'll try it for a month and see how it works.	Taobao Marketplace, a Chinese website for online shopping
Jinfeng Hongyuan Flagship Store	Purple Power Saving Specialist	Great platform to buy from, good quality, especially with a brand name, trustworthy	Taobao Marketplace, a Chinese website for online shopping
Jinfeng Hongyuan Flagship Store	Purple Power Saving Specialist	Packaging is very good, and the workmanship is exquisite. Already used, wait for a period of time to use the additional evaluation of the use of the effect.	Taobao Marketplace, a Chinese website for online shopping
Jinfeng Hongyuan Flagship Store	Purple Power Saving Specialist	Items received, packaging is very good, just received not yet used, I hope it is good!	Taobao Marketplace, a Chinese website for online shopping
Jinfeng Hongyuan Flagship Store	Purple Power Saving Specialist	Already used it, looking forward to the results	Taobao Marketplace, a Chinese website for online shopping
Jinfeng Hongyuan Flagship Store	Purple Power Saving Specialist	Energy saver quality is very good, fine workmanship logistics is very fast, and the store described the same use for a period of time really save electricity	Taobao Marketplace, a Chinese website for online shopping
Jinfeng Hongyuan Flagship Store	Purple Power Saving Specialist	I just got it back, I'll use it for a while and then I'll comment	Taobao Marketplace, a Chinese website for online shopping
Jinfeng Hongyuan Flagship Store	Purple Power Saving Specialist	Very good baby, perfect packaging and kind owner! Worth recommending!	Taobao Marketplace, a Chinese website for online shopping
Jinfeng Hongyuan Flagship Store	Purple Power Saving Specialist	Baby received very happy shopping very happy online shopping.	Taobao Marketplace, a Chinese website for online shopping
Jinfeng Hongyuan Flagship Store	Purple Power Saving Specialist	Goldwind Hongyuan power saver, very good 👍, query is genuine, fine workmanship, stable work, packaging is quite good, low noise, it is a good product, power saving situation to be used in the evaluation, I believe it will not be wrong.	Taobao Marketplace, a Chinese website for online shopping
Jinfeng Hongyuan Flagship Store	Purple Power Saving Specialist	Plugged it in and used it for a bit, didn't hear any noise and it's the real deal. Worth having.	Taobao Marketplace, a Chinese website for online shopping
Jinfeng Hongyuan Flagship Store	Purple Power Saving Specialist	Received the baby, the stuff is exquisite and well made, looking forward to saving electricity 😊	Taobao Marketplace, a Chinese website for online shopping
Jinfeng Hongyuan Flagship Store	Purple Power Saving Specialist	Good for waiting a month to know the power savings	Taobao Marketplace, a Chinese website for online shopping
Jinfeng Hongyuan Flagship Store	Purple Power Saving Specialist	Shipping was prompt, received it quickly, the item is the right size, looking forward to doing its job and seeing the results after a while.	Taobao Marketplace, a Chinese website for online shopping
Jinfeng Hongyuan Flagship Store	Purple Power Saving Specialist	Perfect, the product is amazing, the price is right for the money and I hope it lasts a long time!	Taobao Marketplace, a Chinese website for online shopping
Jinfeng Hongyuan Flagship Store	Purple Power Saving Specialist	The quality of the machine is very good, very classy feeling ^_^.	Taobao Marketplace, a Chinese website for online shopping
Jinfeng Hongyuan Flagship Store	Purple Power Saving Specialist	On Trial	Taobao Marketplace, a Chinese website for online shopping
Jinfeng Hongyuan Flagship Store	Purple Power Saving Specialist	Used a multimeter to measure the current and it is working, slightly less current. It saves power.	Taobao Marketplace, a Chinese website for online shopping
Jinfeng Hongyuan Flagship Store	Purple Power Saving Specialist	Good quality, looking forward to the effect. Seller has great service and fast shipping.	Taobao Marketplace, a Chinese website for online shopping
Jinfeng Hongyuan Flagship Store	Gold Power Saving Specialist	Fast shipping, good product quality, really effective, can save power real test full five-star praise.	Taobao Marketplace, a Chinese website for online shopping
Jinfeng Hongyuan Flagship Store	Gold Power Saving Specialist	I just bought it, I don't know if it's good yet, but it's a nice texture	Taobao Marketplace, a Chinese website for online shopping

Jinfeng Hongyuan Flagship Store	Gold Power Saving Specialist	Great stuff, this is a second purchase! Ordered three at once this time!	Taobao Marketplace, a Chinese website for online shopping
Jinfeng Hongyuan Flagship Store	Gold Power Saving Specialist	I've received it! Use it for a while and see how it works! I'll add more! I think it's good!	Taobao Marketplace, a Chinese website for online shopping
Jinfeng Hongyuan Flagship Store	Gold Power Saving Specialist	It doesn't help at all.	Taobao Marketplace, a Chinese website for online shopping
Jinfeng Hongyuan Flagship Store	Gold Power Saving Specialist	First time using it, it feels good too, I won't know if it works until after a while	Taobao Marketplace, a Chinese website for online shopping
Jinfeng Hongyuan Flagship Store	Gold Power Saving Specialist	The saver was shipped promptly and packaged safely and securely, so kudos in advance.	Taobao Marketplace, a Chinese website for online shopping
Jinfeng Hongyuan Flagship Store	Gold Power Saving Specialist	After a few months of using the effect proved that this power saver has no effect at all, not yet purchased to consider carefully.	Taobao Marketplace, a Chinese website for online shopping
Jinfeng Hongyuan Flagship Store	Gold Power Saving Specialist	The service attitude is very good! Logistics is fast!	Taobao Marketplace, a Chinese website for online shopping
Jinfeng Hongyuan Flagship Store	Gold Power Saving Specialist	Have received the item is really good, display real-time voltage, I believe that certainly can be left for my electricity bill. The main thing is that the customer service is very good and the logistics is fast.	Taobao Marketplace, a Chinese website for online shopping
Jinfeng Hongyuan Flagship Store	Gold Power Saving Specialist	Baby is very good, customer service is particularly enthusiastic to explain the particularly clear, but also a warranty of 2 years, straight to 👍!	Taobao Marketplace, a Chinese website for online shopping
Jinfeng Hongyuan Flagship Store	Lake Blue Power Saving Specialist	Doesn't save power ****ing rip off	Taobao Marketplace, a Chinese website for online shopping
Jinfeng Hongyuan Flagship Store	Lake Blue Power Saving Specialist	Baby to receive, logistics is quite fast, cost-effective is quite good, praise praise praise!	Taobao Marketplace, a Chinese website for online shopping
Jinfeng Hongyuan Flagship Store	Lake Blue Power Saving Specialist	Fast logistics, nice seller. Product design is beautiful, looking forward to the effect after use.	Taobao Marketplace, a Chinese website for online shopping
Jinfeng Hongyuan Flagship Store	Lake Blue Power Saving Specialist	Pretty good.	Taobao Marketplace, a Chinese website for online shopping
Jinfeng Hongyuan Flagship Store	Lake Blue Power Saving Specialist	Already bought is the third one, I think it works um, quite good, you can buy a try	Taobao Marketplace, a Chinese website for online shopping
Jinfeng Hongyuan Flagship Store	Lake Blue Power Saving Specialist	I've only just started using it, so I don't know if it's working.	Taobao Marketplace, a Chinese website for online shopping
Jinfeng Hongyuan Flagship Store	Red Power Saving Specialist	Goods have been received, easy to install, the effect of waiting for a period of time and then evaluate Oh!	Taobao Marketplace, a Chinese website for online shopping
Jinfeng Hongyuan Flagship Store	Red Power Saving Specialist	This stuff is great ah, everyone use it if you like.	Taobao Marketplace, a Chinese website for online shopping
Jinfeng Hongyuan Flagship Store	Red Power Saving Specialist	This stuff is great ah, everyone use it if you like.	Taobao Marketplace, a Chinese website for online shopping
Jinfeng Hongyuan Flagship Store	High-power energy-saving specialists	* Use are not, but also power consumption, the above said 60 articles package return, enough to buy into the male after becoming 7 days. Also a fool you that it takes two months to have effect.	Taobao Marketplace, a Chinese website for online shopping
Jinfeng Hongyuan Flagship Store	High-power energy-saving specialists	First of all, I'd like to give you a good review! Try it for 2 months, if it doesn't work get a refund, if it works get another one!	Taobao Marketplace, a Chinese website for online shopping
Jinfeng Hongyuan Flagship Store	High-power energy-saving specialists	Don't buy fake goods that cheat people out of their money	Taobao Marketplace, a Chinese website for online shopping
Jinfeng Hongyuan Flagship Store	High-power energy-saving specialists	Boss this kind of energy-saving appliances is a high-quality brand; my home to open a large hotel; very good use; want to hurry to order; you want to buy, do not worry; is good use of the	Taobao Marketplace, a Chinese website for online shopping
Jinfeng Hongyuan Flagship Store	High-power energy-saving specialists	(of an unmarried couple) be close	Taobao Marketplace, a Chinese website for online shopping
Jinfeng Hongyuan Flagship Store	High-power energy-saving specialists	It's too big. I've returned it. It was a great delivery.	Taobao Marketplace, a Chinese website for online shopping
Jinfeng Hongyuan Flagship Store	Three-phase stabilized power saver	not have	Taobao Marketplace, a Chinese website for online shopping
Jinfeng Hongyuan Flagship Store	High-power energy-saving specialists	Has been installed and completed, sitting and waiting for the effect, the business commitment of 2 months unlimited dissatisfaction can be returned, give a 👍	Taobao Marketplace, a Chinese website for online shopping
Jinfeng Hongyuan Flagship Store	High-power energy-saving specialists	Used a few months did not help at all, electricity is not more, we must not believe his bullshit!	Taobao Marketplace, a Chinese website for online shopping
Jinfeng Hongyuan Flagship Store	High-power energy-saving specialists	Haven't installed it yet, looks like it should be ok	Taobao Marketplace, a Chinese website for online shopping
Jinfeng Hongyuan Flagship Store	High-power energy-saving specialists	I haven't installed it yet, let's see when it's done	Taobao Marketplace, a Chinese website for online shopping
Jinfeng Hongyuan Flagship Store	High-power energy-saving specialists	Just received the goods, wait for the trial and then follow up the evaluation	Taobao Marketplace, a Chinese website for online shopping
Jinfeng Hongyuan Flagship Store	High-power energy-saving specialists	I've been using it for a while, and the effect is about 12%	Taobao Marketplace, a Chinese website for online shopping
Jinfeng Hongyuan Flagship Store	High-power energy-saving specialists	Recommended by the electrician, already installed, looking forward to the results!	Taobao Marketplace, a Chinese website for online shopping
Jinfeng Hongyuan Flagship Store	High-power energy-saving specialists	Installed on a month, power saving effect of a little more than 10%, the overall good	Taobao Marketplace, a Chinese website for online shopping
Jinfeng Hongyuan Flagship Store	High-power energy-saving specialists	Third purchase for a friend!	Taobao Marketplace, a Chinese website for online shopping

Jinfeng Hongyuan Flagship Store	High-power energy-saving specialists	I've been using it for a while, and the power saving is about 8%, which is not bad overall	Taobao Marketplace, a Chinese website for online shopping
Jinfeng Hongyuan Flagship Store	High-power energy-saving specialists	Logistics is very fast, the packaging is also very good, the second unit, as always, looking forward to the results	Taobao Marketplace, a Chinese website for online shopping
Jinfeng Hongyuan Flagship Store	High-power energy-saving specialists	Friends say they are factories, in this buy have effect, also buy a try to see, epidemic at the moment, open the store is really not easy, hope to save some more!	Taobao Marketplace, a Chinese website for online shopping
Jinfeng Hongyuan Flagship Store	High-power energy-saving specialists	I installed it yesterday and will comment again after using it for a while to see if it saves energy.	Taobao Marketplace, a Chinese website for online shopping
Jinfeng Hongyuan Flagship Store	High-power energy-saving specialists	I installed it yesterday and will comment again after using it for a while to see if it saves energy.	Taobao Marketplace, a Chinese website for online shopping
Jinfeng Hongyuan Flagship Store	High-power energy-saving specialists	The second time back to buy, according to customer service taught to connect in the main gate below, the effect is good worth buying!	Taobao Marketplace, a Chinese website for online shopping
Jinfeng Hongyuan Flagship Store	High-power energy-saving specialists	Baby received, second purchase for the company!	Taobao Marketplace, a Chinese website for online shopping
Jinfeng Hongyuan Flagship Store	High-power energy-saving specialists	Friends with good results recommended to me, look at the appearance of a very beautiful, very texture, packaging is very good, it seems to be a large factory production, in accordance with customer service taught to find an electrician to install in my cafeteria below the main gate, look forward to the effect!	Taobao Marketplace, a Chinese website for online shopping
Jinfeng Hongyuan Flagship Store	High-power energy-saving specialists	Already received it, very good, there are freebies, cost-effective too high ah, love love, affordable price, the quality of the bar, a very good shopping, the next time to come back!	Taobao Marketplace, a Chinese website for online shopping
Jinfeng Hongyuan Flagship Store	High-power energy-saving specialists	Received the goods, bought a household effect is obvious, immediately hot, to the hotel also installed a power saver, can save a lot of money!	Taobao Marketplace, a Chinese website for online shopping
Jinfeng Hongyuan Flagship Store	High-power energy-saving specialists	Baby received! Installed it myself! How well it works, to be reviewed after use!	Taobao Marketplace, a Chinese website for online shopping
Jinfeng Hongyuan Flagship Store	High-power energy-saving specialists	Since I bought this saver, it really saves quite a bit of electricity in the middle of winter.	Taobao Marketplace, a Chinese website for online shopping
Jinfeng Hongyuan Flagship Store	High-power energy-saving specialists	Packaging, quality aspects are done in place, and compensation cabinets, the same principle of reactive power compensation, much cheaper than buying compensation cabinets, the effect is the same, the test, you can save 15% or so, to a friend of the factory is also recommended!	Taobao Marketplace, a Chinese website for online shopping
Jinfeng Hongyuan Flagship Store	High-power energy-saving specialists	Purchased for the company, tested, can improve the power factor, reduce the current, can really save electricity, the follow-up intends to take a bulk!	Taobao Marketplace, a Chinese website for online shopping
Jinfeng Hongyuan Flagship Store	High-power energy-saving specialists	Factory electrician master installed, the price is very affordable, the effect of waiting for next month to see	Taobao Marketplace, a Chinese website for online shopping
Jinfeng Hongyuan Flagship Store	High-power energy-saving specialists	It's ok, looks delicate! Use it for a month and see the results!	Taobao Marketplace, a Chinese website for online shopping
Jinfeng Hongyuan Flagship Store	High-power energy-saving specialists	Some effect, not bad 👍	Taobao Marketplace, a Chinese website for online shopping
Jinfeng Hongyuan Flagship Store	Green Intelligent Power Saver	not have	Taobao Marketplace, a Chinese website for online shopping
Jinfeng Hongyuan Flagship Store	King of Power Saving	This really saves electricity, compared to the recent electricity bill, it really works, before half a month on the 50 gone, now half a month only use 30, really buy the right one!	Taobao Marketplace, a Chinese website for online shopping
Jinfeng Hongyuan Flagship Store	King of Power Saving	Very convenient. No need for complicated wiring, directly plugged into the place where electricity is concentrated, the effect is good. The effect is really obvious, before 197 dollars, now 130!	Taobao Marketplace, a Chinese website for online shopping
Jinfeng Hongyuan Flagship Store	King of Power Saving	Definitely a great product, it's especially easy to use and plugs directly into the plug board at home. In the past few days to turn on the air conditioning to see the meter turn a little slower than before. But the electricity consumption is a little less than before.	Taobao Marketplace, a Chinese website for online shopping
Jinfeng Hongyuan Flagship Store	King of Power Saving	Good packaging, fast logistics, affordable, much cheaper than outside things are very good quality, and the seller described exactly the same, very satisfied	Taobao Marketplace, a Chinese website for online shopping
Jinfeng Hongyuan Flagship Store	a power saver (electronics)	It's not working at all.	Taobao Marketplace, a Chinese website for online shopping
Jinfeng Hongyuan Flagship Store	a power saver (electronics)	The stuff is good and there are discounts, so you can save money and buy without hesitation	Taobao Marketplace, a Chinese website for online shopping
Jinfeng Hongyuan Flagship Store	a power saver (electronics)	Recommended by the owner of the store next door, looked at his home does have to save electricity, decided to get. Indeed quite can, the voltage is much more stable, there is a reduction in electricity consumption.	Taobao Marketplace, a Chinese website for online shopping
Jinfeng Hongyuan Flagship Store	a power saver (electronics)	Arrived, open and merchants described the same, satisfied with the	Taobao Marketplace, a Chinese website for online shopping
Jinfeng Hongyuan Flagship Store	a power saver (electronics)	Previously used another of his home economizer, the effect is very good, this time to see another upgrade product can not wait to order, his products do not have to worry too much, workmanship as well as quality is very good.	Taobao Marketplace, a Chinese website for online shopping
Jinfeng Hongyuan Flagship Store	a power saver (electronics)	Really effective, not an IQ tax, with a month down the electricity bill to save a few dozen dollars, the meter also slows down, a good product is worth recommending!	Taobao Marketplace, a Chinese website for online shopping
Jinfeng Hongyuan Flagship Store	a power saver (electronics)	The logistics is super super fast! The quality is also very good, indeed value for money	Taobao Marketplace, a Chinese website for online shopping
Jinfeng Hongyuan Flagship Store	a power saver (electronics)	Previously also bought a, is indeed a power-saving effect, used for many years, this time to move to a new home, the recent days and hot, electrical appliances and air conditioning with a lot of purpose and bought 2, inserted in a different location, with the same as the previous purchase, good use of	Taobao Marketplace, a Chinese website for online shopping
Jinfeng Hongyuan Flagship Store	a power saver (electronics)	Consulted with customer service, who answered my questions promptly! Saver is very easy to use, plug and play, the current experience of a week is still effective!	Taobao Marketplace, a Chinese website for online shopping
Jinfeng Hongyuan Flagship Store	a power saver (electronics)	Pretty good, affordable, quality looks good	Taobao Marketplace, a Chinese website for online shopping
Jinfeng Hongyuan Flagship Store	a power saver (electronics)	The old family bought a last time, a month to save tens of dollars it, this new home bought two, inserted in different locations, two together with the effect will certainly be better. The seller's attitude is very good, the commitment to 90 days invalid refund, really not bad!	Taobao Marketplace, a Chinese website for online shopping
Centauri medicine	intelligent saving	The original also bought a few, now shot this from the appearance of the original few exquisite feasible, I believe that will save electricity effect, wait for a period of time to use it will be clear.	Taobao Marketplace, a Chinese website for online shopping

Centauri medicine	intelligent saving	Referred a friend to buy it	Taobao Marketplace, a Chinese website for online shopping
Centauri medicine	intelligent saving	Grabbed it. Love the mamacita.	Taobao Marketplace, a Chinese website for online shopping
Centauri medicine	intelligent saving	What a great price. What a bargain.	Taobao Marketplace, a Chinese website for online shopping
Centauri medicine	intelligent saving	I've been using it for a while, it's still good, and I'm still happy with it overall	Taobao Marketplace, a Chinese website for online shopping
Centauri medicine	intelligent saving	Cost-effective: the price is relatively cheap, appearance and style: small and exquisite quite good-looking power saving effect: just received today has not yet been used	Taobao Marketplace, a Chinese website for online shopping
Centauri medicine	intelligent saving	Average power saving effect	Taobao Marketplace, a Chinese website for online shopping
Centauri medicine	intelligent saving	Another IQ tax Value for money: not recommended Appearance and style: rough and crude Workmanship and material: poor to the extreme	Taobao Marketplace, a Chinese website for online shopping
Centauri medicine	intelligent saving	It hasn't worked yet.	Taobao Marketplace, a Chinese website for online shopping
Centauri medicine	intelligent saving	This energy saver does save electricity after comparison, kudos 👍	Taobao Marketplace, a Chinese website for online shopping
Centauri medicine	intelligent saving	This is good. It saves electricity.	Taobao Marketplace, a Chinese website for online shopping
Centauri medicine	intelligent saving	Goods received, logistics is very fast, not expensive and not troublesome, the effect is also great, praise!	Taobao Marketplace, a Chinese website for online shopping
Centauri medicine	intelligent saving	The product was received and the merchant described the same use of the effect is very good very satisfied	Taobao Marketplace, a Chinese website for online shopping
Centauri medicine	intelligent saving	Logistics is very fast, the quality is very good, easy to use, very satisfied with a shopping	Taobao Marketplace, a Chinese website for online shopping
Centauri medicine	intelligent saving	It's really good, cost-effective and good quality, and the logistics are fast! It arrived in one day! A very good shopping experience, attentive service, fast logistics, delivery is also thoughtful, attitude is also very good, value for money ah, decisive praise!	Taobao Marketplace, a Chinese website for online shopping
Centauri medicine	intelligent saving	Power saving effect: good Cost-effective: good Appearance and style: good Workmanship and material: lightweight Experience: good	Taobao Marketplace, a Chinese website for online shopping
Centauri medicine	intelligent saving	Baby received today, get back to the power to try, look good, use is also good, customer service attitude is very good full five points praise!	Taobao Marketplace, a Chinese website for online shopping
Centauri medicine	intelligent saving	The power saving effect is superb, cost-effective, and the appearance and style are normal and new, and the experience is great.	Taobao Marketplace, a Chinese website for online shopping
Centauri medicine	intelligent saving	It's very convenient to use, so it's also quite power-saving very good Oh, it's still quite good it	Taobao Marketplace, a Chinese website for online shopping
Centauri medicine	intelligent saving	Since using the Saver, our home's electric bill is less and we can save a good amount of money. After installing the saver, the electrics became that way, I feel that it should extend the life of the store's products, and the saver is very durable!	Taobao Marketplace, a Chinese website for online shopping
Centauri medicine	intelligent saving	Good-looking workmanship is also good to use and affordable to buy can not lose buy can not be fooled the price is also cheap power-saving effect is very good logistics very quickly very satisfied with the store manager's attitude is also good to give five-star praise 😌😌😌	Taobao Marketplace, a Chinese website for online shopping
Centauri medicine	intelligent saving	As described, the power saving effect is quite good	Taobao Marketplace, a Chinese website for online shopping
Centauri medicine	intelligent saving	The description matches, has been used, the price is not expensive, value for money. Shipping is fast, logistics is fast, packaging is very good. Boss service attitude is also very good. Give full five-star praise!	Taobao Marketplace, a Chinese website for online shopping
Centauri medicine	intelligent saving	Really effective ah family, I went to pay the electricity bill this time really less, this time the use of a few days the electricity bill is less than last month, recommended recommended, I bought two more	Taobao Marketplace, a Chinese website for online shopping
Centauri medicine	intelligent saving	Rental room. Summer air conditioning refrigerator power consumption is too large, friends recommend using the effect is very good every month to save a lot of money to me also recommend using it is very good!	Taobao Marketplace, a Chinese website for online shopping
Centauri medicine	intelligent saving	Intelligent is convenient, really like, need the treasure do not hesitate, grab take try bla!	Taobao Marketplace, a Chinese website for online shopping
Centauri medicine	intelligent saving	Particularly great, comparison shopping before buying, the store host is very good, answering questions is very patient and detailed, there is a need to come back again, things used to feel very good, the quality of the goods is good, the shipment is also quite fast, in short, it's awesome!	Taobao Marketplace, a Chinese website for online shopping
Centauri medicine	intelligent saving	I am very satisfied with this product can be, save electricity, the product works very well!	Taobao Marketplace, a Chinese website for online shopping
Centauri medicine	intelligent saving	Good use this month than last month 20 or 30 dollars less, very satisfied, the quality is also good, and the seller's description is the same	Taobao Marketplace, a Chinese website for online shopping
Centauri medicine	intelligent saving	I've been using it for a while now, and I can indeed save 5 or 6 degrees of electricity per day! Use a period of time and then continue to see it, anyway, the price is not expensive!!!!	Taobao Marketplace, a Chinese website for online shopping
Centauri medicine	intelligent saving	Consistent with the description of the business, logistics is very fast, the quality is very good, as soon as it arrives to start using the energy-saving power saving, if the effect is good then will continue to buy back!	Taobao Marketplace, a Chinese website for online shopping
Centauri medicine	King of Power Saving	It's off the shelves.	Taobao Marketplace, a Chinese website for online shopping
Slu King Flagship Store	Green Intelligent Power Saver	The product is good small and convenient, the product introduction is detailed, there is a corporate culture construction, especially that sentence is very pattern, so that energy saving into thousands of households, 💡 support for national products to support national brands	Taobao Marketplace, a Chinese website for online shopping
Slu King Flagship Store	Green Intelligent Power Saver	Support the national brand, support the national product CNCN, so that energy saving into thousands of households, this sentence has a national bear, corporate self-confidence, and more social responsibility. 👍👍👍	Taobao Marketplace, a Chinese website for online shopping
Slu King Flagship Store	Green Intelligent Power Saver	Baby received, as described	Taobao Marketplace, a Chinese website for online shopping

Slu King Flagship Store	Green Intelligent Power Saver	The price is pretty affordable and the customer service is great.	Taobao Marketplace, a Chinese website for online shopping
Slu King Flagship Store	Green Intelligent Power Saver	Appearance and style: good-looking and does not take up space, simple and clear I believe that the power-saving effect will be good!	Taobao Marketplace, a Chinese website for online shopping
Slu King Flagship Store	Green Intelligent Power Saver	Power saving effect: the product is very good, cost-effective, looking forward to better	Taobao Marketplace, a Chinese website for online shopping
Slu King Flagship Store	Green Intelligent Power Saver	Power-saving effect: good Cost-effective: 〉high Next time will come back to buy Appearance style: good Workmanship material: good Use experience: good	Taobao Marketplace, a Chinese website for online shopping
Slu King Flagship Store	Green Intelligent Power Saver	It's good. Good review.	Taobao Marketplace, a Chinese website for online shopping
Slu King Flagship Store	Green Intelligent Power Saver	The second repurchase, the effect is okay, really effective oh	Taobao Marketplace, a Chinese website for online shopping
Slu King Flagship Store	Green Intelligent Power Saver	Third time repurchasing it works fine	Taobao Marketplace, a Chinese website for online shopping
Slu King Flagship Store	Green Intelligent Power Saver	This product is really good I've bought one and used it for a year it really saves a lot of electricity.	Taobao Marketplace, a Chinese website for online shopping
Slu King Flagship Store	Green Intelligent Power Saver	Energy-saving effect is very good, compact and practical, but also to buy two home use	Taobao Marketplace, a Chinese website for online shopping
Slu King Flagship Store	Green Intelligent Power Saver	Just received the goods on the use of experience, workmanship material is not bad	Taobao Marketplace, a Chinese website for online shopping
Slu King Flagship Store	Green Intelligent Power Saver	Very good quality Power saving effect: very good quality Appearance style: the effect should be okay	Taobao Marketplace, a Chinese website for online shopping
Slu King Flagship Store	Green Intelligent Power Saver	I bought it and tried the results, it works quite well and is worth buying.	Taobao Marketplace, a Chinese website for online shopping
Slu King Flagship Store	Green Intelligent Power Saver	Used for a period of time feel is more than before to save electricity, than the same period in previous years, less electricity, and then plan to purchase more than one to try!	Taobao Marketplace, a Chinese website for online shopping
Slu King Flagship Store	Green Intelligent Power Saver	I bought one last time, and this time I bought another one indeed quite good, and the service is also very good	Taobao Marketplace, a Chinese website for online shopping
Slu King Flagship Store	Green Intelligent Power Saver	Experience: it's okay Price-performance ratio: 👍👍👍👍	Taobao Marketplace, a Chinese website for online shopping
Slu King Flagship Store	Green Intelligent Power Saver	The object is exactly as described, the logistics is also very fast, wait for a period of time and then follow up. Good reviews!	Taobao Marketplace, a Chinese website for online shopping
Slu King Flagship Store	Green Intelligent Power Saver	How come it doesn't save electricity? It's a scam！！！！！！	Taobao Marketplace, a Chinese website for online shopping
Slu King Flagship Store	Green Intelligent Power Saver	Received the energy saver! It's still good! The packaging is also very formal	Taobao Marketplace, a Chinese website for online shopping
Slu King Flagship Store	Green Intelligent Power Saver	After using it feels very good, cost-effective, small and exquisite value is also very high, it is worth buying!	Taobao Marketplace, a Chinese website for online shopping
Slu King Flagship Store	Green Intelligent Power Saver	Keep buying next time! Not bad!	Taobao Marketplace, a Chinese website for online shopping
Slu King Flagship Store	Green Intelligent Power Saver	Power saving effect: quality is very good Cost-effective: very good Appearance style: simple good-looking Workmanship material: exquisite Experience: power saving	Taobao Marketplace, a Chinese website for online shopping
Slu King Flagship Store	Green Intelligent Power Saver	Experience: This is my second time to buy, it can be more energy-saving	Taobao Marketplace, a Chinese website for online shopping
Slu King Flagship Store	Green Intelligent Power Saver	Pretty good, affordable, quality looks good	Taobao Marketplace, a Chinese website for online shopping
Slu King Flagship Store	Green Intelligent Power Saver	Goods received, the quality is very good, next time to buy back!	Taobao Marketplace, a Chinese website for online shopping
Slu King Flagship Store	Green Intelligent Power Saver	Just received the goods, experience, additional comments to follow!	Taobao Marketplace, a Chinese website for online shopping
Slu King Flagship Store	Green Intelligent Power Saver	I'm just using it, and I'm looking forward to the power savings.	Taobao Marketplace, a Chinese website for online shopping
Slu King Flagship Store	Green Intelligent Power Saver	Power-saving effect: power-saving effect is good Cost-effective: cost-effective super high Appearance style: the appearance of the shape of the design style is very satisfied Experience: worth having something Workmanship material: workmanship material nothing to say	Taobao Marketplace, a Chinese website for online shopping
Slu King Flagship Store	Green Intelligent Power Saver	Power-saving effect: without this when I was fifty a month, with this ninety a month of electricity, the meter can not be seen on the effect of a little, liar business, liar liar, their own commitment will be realized is just a matter of time Use experience: can only say that the dupe	Taobao Marketplace, a Chinese website for online shopping
Slu King Flagship Store	Green Intelligent Power Saver	Review after use.	Taobao Marketplace, a Chinese website for online shopping
Slu King Flagship Store	Green Intelligent Power Saver	I don't know if I can use it.	Taobao Marketplace, a Chinese website for online shopping
Slu King Flagship Store	Green Intelligent Power Saver	After two days of testing. The effect is good! The first day is about 1/4, the second day is 1/3, and is not "stealing electricity", is through the intelligent integrated circuit, automatically reduce the loss of electrical appliances to achieve the purpose of saving electricity! Recommended!	Taobao Marketplace, a Chinese website for online shopping
Slu King Flagship Store	Green Intelligent Power Saver	Things received, look very good, look forward to good use, good use will buy back!	Taobao Marketplace, a Chinese website for online shopping
Slu King Flagship Store	Green Intelligent Power Saver	Experience: to be a month to test the effect before evaluation	Taobao Marketplace, a Chinese website for online shopping
Slu King Flagship Store	Green Intelligent Power Saver	In use, looking forward to the results	Taobao Marketplace, a Chinese website for online shopping

Slu King Flagship Store	Green Intelligent Power Saver	It's not much use.	Taobao Marketplace, a Chinese website for online shopping
Slu King Flagship Store	Green Intelligent Power Saver	Logistics is quite fast, packaging is very good, the appearance of small style, lightweight materials, power-saving effect with the aftermath of a follow-up evaluation!	Taobao Marketplace, a Chinese website for online shopping
Slu King Flagship Store	Green Intelligent Power Saver	Garbage courier, charge for picking up	Taobao Marketplace, a Chinese website for online shopping
Slu King Flagship Store	Green Intelligent Power Saver	Workmanship material: the goods got, get back and plugged in to the payment of the electricity bill will know that the	Taobao Marketplace, a Chinese website for online shopping
Slu King Flagship Store	Green Intelligent Power Saver	Used it for a week, the power saving is fake, change as much or as little as you want, it doesn't feel effective at all	Taobao Marketplace, a Chinese website for online shopping
Slu King Flagship Store	Green Intelligent Power Saver	Received the product, it looks good, try it and then come back to evaluate!	Taobao Marketplace, a Chinese website for online shopping
Slu King Flagship Store	Green Intelligent Power Saver	Very satisfied. Quality is very good. Power saving is good. Logistics is fast.	Taobao Marketplace, a Chinese website for online shopping
Slu King Flagship Store	Green Intelligent Power Saver	I'm using it, I don't know the results yet, but I'll give you a good review.	Taobao Marketplace, a Chinese website for online shopping
Slu King Flagship Store	Green Intelligent Power Saver	Good reviews, good detail just bought back to try to see it, may save the effect can be, and so on good I will do a follow-up review, the manufacturer is very polite, very good 。。。。。	Taobao Marketplace, a Chinese website for online shopping
Slu King Flagship Store	Green Intelligent Power Saver	Express delivery is very fast, the energy saver is small and exquisite does not take up space, easy to operate	Taobao Marketplace, a Chinese website for online shopping
Slu King Flagship Store	Green Intelligent Power Saver	Power-saving effect: good Cost-effective: high Appearance style: can also be used as a night light Workmanship material: can be oh	Taobao Marketplace, a Chinese website for online shopping
Slu King Flagship Store	Green Intelligent Power Saver	Can't tell if it's saving power yet	Taobao Marketplace, a Chinese website for online shopping
Slu King Flagship Store	Green Intelligent Power Saver	All three products, bought back and used for a week, now the lights do not light up, please ask the business what is the situation? Where is the problem? I think it is a counterfeit product, we need to be careful when buying, all the fraud, where is this so much praise it?	Taobao Marketplace, a Chinese website for online shopping
Slu King Flagship Store	Green Intelligent Power Saver	Used it, don't know how it works	Taobao Marketplace, a Chinese website for online shopping
Slu King Flagship Store	Green Intelligent Power Saver	Experience: good results, beautiful packaging, fast logistics	Taobao Marketplace, a Chinese website for online shopping
Slu King Flagship Store	Green Intelligent Power Saver	Power saving effect: very good effect	Taobao Marketplace, a Chinese website for online shopping
Slu King Flagship Store	Green Intelligent Power Saver	Second time I bought it, it doesn't get hot, so feel free to use it!	Taobao Marketplace, a Chinese website for online shopping
Slu King Flagship Store	Green Intelligent Power Saver	Great quality, great service, received in two days.	Taobao Marketplace, a Chinese website for online shopping
Slu King Flagship Store	Green Intelligent Power Saver	Very satisfied, ordered two Power-saving effect: really power-saving Cost-effective: very high Appearance style: very beautiful Workmanship material: the quality of no problem	Taobao Marketplace, a Chinese website for online shopping
Slu King Flagship Store	Green Intelligent Power Saver	I don't know if it saves money or not, I'll use it first, it's in my mind.	Taobao Marketplace, a Chinese website for online shopping
Slu King Flagship Store	Green Intelligent Power Saver	I'm using it every day.	Taobao Marketplace, a Chinese website for online shopping
Slu King Flagship Store	Green Intelligent Power Saver	I'll comment after using it.	Taobao Marketplace, a Chinese website for online shopping
Slu King Flagship Store	Green Intelligent Power Saver	Power-saving effect: can Cost-effective: high Appearance and style: just the right size, quite can Workmanship material: delicate	Taobao Marketplace, a Chinese website for online shopping
Slu King Flagship Store	Green Intelligent Power Saver	I've been using it for almost a month now, it doesn't work, it doesn't save any power, it's a scam Experience: scam, it doesn't save any power	Taobao Marketplace, a Chinese website for online shopping
Slu King Flagship Store	Green Intelligent Power Saver	Power-saving effect: feel useless, plugged in or not plugged in all the same bird	Taobao Marketplace, a Chinese website for online shopping
Slu King Flagship Store	Green Intelligent Power Saver	Energy-saving effect of good cost-effective, small and exquisite appearance, flame-retardant material, save point	Taobao Marketplace, a Chinese website for online shopping
Slu King Flagship Store	Green Intelligent Power Saver	Still in the process of using it, not sure if it works.	Taobao Marketplace, a Chinese website for online shopping
Slu King Flagship Store	Green Intelligent Power Saver	Power-saving effect: can, have the effect of repurchase	Taobao Marketplace, a Chinese website for online shopping
Slu King Flagship Store	Green Intelligent Power Saver	Before buying more than 10 degrees of electricity a day, after using no more than 8 degrees a day, a little effect oh, and ordered a, a month can also save a little bit of	Taobao Marketplace, a Chinese website for online shopping
Slu King Flagship Store	Green Intelligent Power Saver	The product really works in order to have a friend look at it and say buy.	Taobao Marketplace, a Chinese website for online shopping
Slu King Flagship Store	Green Intelligent Power Saver	Baby received, quite good, used, give five points, after three months in the follow-up evaluation Workmanship material: quite detailed, not for nothing!	Taobao Marketplace, a Chinese website for online shopping
Slu King Flagship Store	Green Intelligent Power Saver	Fast shipping, exquisite appearance, enthusiastic service, due to the large amount of electricity to buy two, the boss sent a, three at the same time installed in the home should be more effective in saving electricity. To be used after the follow-up evaluation.	Taobao Marketplace, a Chinese website for online shopping
Slu King Flagship Store	Green Intelligent Power Saver	Power-saving effect: the effect is very obvious Cost-effective: relatively high Appearance style: new beautiful Workmanship material: fine Experience: worth buying	Taobao Marketplace, a Chinese website for online shopping
Slu King Flagship Store	Green Intelligent Power Saver	Appearance and style: looks exquisite, has installed 3, waiting for the arrival of power saving!	Taobao Marketplace, a Chinese website for online shopping

Slu King Flagship Store	Green Intelligent Power Saver	Bought three of them, looking forward to the results, follow up reviews	Taobao Marketplace, a Chinese website for online shopping
Slu King Flagship Store	Green Intelligent Power Saver	Second purchase, still feel save some electricity bill, this time to help a friend to buy, looking forward to friends favorable comments	Taobao Marketplace, a Chinese website for online shopping
Slu King Flagship Store	Green Intelligent Power Saver	Power saving effect can be, cost-effective, good appearance, good workmanship ! It is convenient to use, after using continue to observe, good live and then purchase.	Taobao Marketplace, a Chinese website for online shopping
Slu King Flagship Store	Green Intelligent Power Saver	Looks good , good quality, the air conditioner can be turned on without worry ! ! ! !	Taobao Marketplace, a Chinese website for online shopping
Slu King Flagship Store	Green Intelligent Power Saver	Just identify the receipt, look very beautiful, the results of large units of research is certainly excellent quality, the effect of trying to know, if you can I will recommend to friends in Hebei, good service, logistics is also very fast.	Taobao Marketplace, a Chinese website for online shopping
Slu King Flagship Store	Green Intelligent Power Saver	Power saving effect: power saving Cost-effective: affordable Great quality, superb service, received in two days	Taobao Marketplace, a Chinese website for online shopping
Slu King Flagship Store	Green Intelligent Power Saver	I'm using it now, and I hope it's as power-saving as it's described by the merchant.	Taobao Marketplace, a Chinese website for online shopping
Slu King Flagship Store	Green Intelligent Power Saver	I used to buy it from another company, it didn't work for long, the light didn't work, it must be broken, it's cheaper than this one, this one is much more expensive, let's see how long it lasts, let's wait and see how long it lasts.	Taobao Marketplace, a Chinese website for online shopping
Slu King Flagship Store	Green Intelligent Power Saver	Just received the goods, look at the appearance is very good, is a regular manufacturer production, service is very good, logistics is very fast, to be inserted into the effect after I recommended to friends in the Hebei region.	Taobao Marketplace, a Chinese website for online shopping
Slu King Flagship Store	Green Intelligent Power Saver	It works very well and the price is very good.	Taobao Marketplace, a Chinese website for online shopping
Slu King Flagship Store	Green Intelligent Power Saver	Power-saving effect: not bad Cost-effective: also can Use experience: just started using, after a period of time to add	Taobao Marketplace, a Chinese website for online shopping
Slu King Flagship Store	Green Intelligent Power Saver	Plugged in, looking forward to next month's electric bill to see the results	Taobao Marketplace, a Chinese website for online shopping
Slu King Flagship Store	Green Intelligent Power Saver	It's not bad, it's affordable, try using it first	Taobao Marketplace, a Chinese website for online shopping
Slu King Flagship Store	Green Intelligent Power Saver	Value for money: good stuff, bought several, fast delivery	Taobao Marketplace, a Chinese website for online shopping
Slu King Flagship Store	Green Intelligent Power Saver	Experience: positive feedback very good use in the future that in the purchase of	Taobao Marketplace, a Chinese website for online shopping
Slu King Flagship Store	Green Intelligent Power Saver	(euphemism) go to the toilet	Taobao Marketplace, a Chinese website for online shopping
Slu King Flagship Store	Green Intelligent Power Saver	Good energy saving effect. Strong cost performance. Novel style. Exquisite workmanship.	Taobao Marketplace, a Chinese website for online shopping
Slu King Flagship Store	Green Intelligent Power Saver	Appearance and style: look good, friends introduced to buy	Taobao Marketplace, a Chinese website for online shopping
Slu King Flagship Store	Green Intelligent Power Saver	Fast express shipping, better appearance and workmanship, second time enough to buy.	Taobao Marketplace, a Chinese website for online shopping
Slu King Flagship Store	Green Intelligent Power Saver	Really save a lot, the meter turns much slower, want to save electricity, choose this energy king, too powerful!	Taobao Marketplace, a Chinese website for online shopping
Slu King Flagship Store	Green Intelligent Power Saver	Nonsense and propaganda. The more you use the meter, the faster it spins, and there are records to prove it! In order to reflect the real situation, I from the date of ordering every morning to shoot the meter for record. And when I returned the product, the attitude was extremely bad! I suspect that they and the power supply system are teaming up to cheat consumers. If it's really what they say, it's a wonder that the National Grid doesn't penalize them!	Taobao Marketplace, a Chinese website for online shopping
Slu King Flagship Store	Green Intelligent Power Saver	I just tried it, I don't know how it is, I'll comment after a while.	Taobao Marketplace, a Chinese website for online shopping
Slu King Flagship Store	Green Intelligent Power Saver	There is a certain power-saving effect, cost-effective can be, the appearance is okay, the workmanship material top say past, continue to experience, will follow up later.	Taobao Marketplace, a Chinese website for online shopping
Slu King Flagship Store	Green Intelligent Power Saver	Baby received the appearance of okay, in the experience of looking forward to good results	Taobao Marketplace, a Chinese website for online shopping
Slu King Flagship Store	Green Intelligent Power Saver	Let's try it out first, then we'll give it a good review.	Taobao Marketplace, a Chinese website for online shopping
Slu King Flagship Store	Green Intelligent Power Saver	Power saving effect: look at the meter is slowed down, cost-effective: can Appearance style: lightweight Workmanship material: very good Experience: good	Taobao Marketplace, a Chinese website for online shopping
Slu King Flagship Store	Green Intelligent Power Saver	Power-saving effect: very good Cost-effective: high Appearance style: very beautiful Workmanship material: very good Experience: good effect	Taobao Marketplace, a Chinese website for online shopping
Slu King Flagship Store	Green Intelligent Power Saver	Power saving effect: power saving Cost-effective: high Appearance style: good-looking	Taobao Marketplace, a Chinese website for online shopping
Slu King Flagship Store	Green Intelligent Power Saver	The power saver I was waiting for arrived and I'm very happy with it!	Taobao Marketplace, a Chinese website for online shopping
Slu King Flagship Store	Green Intelligent Power Saver	Baby received the logistics very quickly, now plugged into the power outlet on the meter than before turning half slow very good to use convenient	Taobao Marketplace, a Chinese website for online shopping
Slu King Flagship Store	Green Intelligent Power Saver	The product works great, recommend it 😁😁😁😁😁😁	Taobao Marketplace, a Chinese website for online shopping
Slu King Flagship Store	Green Intelligent Power Saver	Good product, very satisfied with the logistics is also fast	Taobao Marketplace, a Chinese website for online shopping
Slu King Flagship Store	Green Intelligent Power Saver	Power-saving effect: power-saving effect is very good Cost-effective: cost-effective Friends who want to seize the hand!	Taobao Marketplace, a Chinese website for online shopping

Slu King Flagship Store	Green Intelligent Power Saver	Power-saving effect: just bought back in a hurry to use a month after the follow-up evaluation	Taobao Marketplace, a Chinese website for online shopping
Slu King Flagship Store	Green Intelligent Power Saver	Just received the goods, easy to use looks good	Taobao Marketplace, a Chinese website for online shopping
Slu King Flagship Store	Green Intelligent Power Saver	It's good. Try it out.	Taobao Marketplace, a Chinese website for online shopping
Slu King Flagship Store	Green Intelligent Power Saver	No effect at all, junk product	Taobao Marketplace, a Chinese website for online shopping
Slu King Flagship Store	Green Intelligent Power Saver	Power saving effect: poor	Taobao Marketplace, a Chinese website for online shopping
Slu King Flagship Store	Green Intelligent Power Saver	Expecting better results, nothing unusual about rubbing it on	Taobao Marketplace, a Chinese website for online shopping
Slu King Flagship Store	Green Intelligent Power Saver	Good quality product worth buying can order	Taobao Marketplace, a Chinese website for online shopping
Slu King Flagship Store	Green Intelligent Power Saver	Just received it, looks like good quality, use it for a month and see how much you can save!	Taobao Marketplace, a Chinese website for online shopping
Slu King Flagship Store	Green Intelligent Power Saver	It works pretty well and is worth buying! Try it works well before you buy it! Power saving effect: pretty good	Taobao Marketplace, a Chinese website for online shopping
Slu King Flagship Store	Green Intelligent Power Saver	Very happy with the sub!	Taobao Marketplace, a Chinese website for online shopping
Slu King Flagship Store	Green Intelligent Power Saver	Power savings: hmmm	Taobao Marketplace, a Chinese website for online shopping
Slu King Flagship Store	Green Intelligent Power Saver	Received the item, very satisfied	Taobao Marketplace, a Chinese website for online shopping
Slu King Flagship Store	Green Intelligent Power Saver	It doesn't save power at all. It doesn't work at all.	Taobao Marketplace, a Chinese website for online shopping
Slu King Flagship Store	Green Intelligent Power Saver	The goods have been received and used for two days feel very good quality 👍	Taobao Marketplace, a Chinese website for online shopping
Slu King Flagship Store	Green Intelligent Power Saver	Baby received, the seller shipped in a timely manner logistics is very fast, the seller's service is very good, quality assurance, praise 👍	Taobao Marketplace, a Chinese website for online shopping
Slu King Flagship Store	Green Intelligent Power Saver	Workmanship material: very good, bought two and sent one, easy to install	Taobao Marketplace, a Chinese website for online shopping
Slu King Flagship Store	Green Intelligent Power Saver	Just got back and used it immediately, I hope it can help me save some money on electricity, my mother's family bought one and used it with good results, so I got one for myself, I hope it will be effective!	Taobao Marketplace, a Chinese website for online shopping
Slu King Flagship Store	Green Intelligent Power Saver	Experience: online pure bragging * hope the majority of users do not believe that my electricity costs this product higher everyone see and July I have half a month not at home	Taobao Marketplace, a Chinese website for online shopping
Slu King Flagship Store	Green Intelligent Power Saver	** Merchants are ** Merchants **	Taobao Marketplace, a Chinese website for online shopping
Slu King Flagship Store	Green Intelligent Power Saver	I'm looking forward to the results.	Taobao Marketplace, a Chinese website for online shopping
Slu King Flagship Store	Green Intelligent Power Saver	Energy-saving appliances received the goods, the use of experience: 1 with energy-saving appliances to save money and save time, good will come back in the order!	Taobao Marketplace, a Chinese website for online shopping
Slu King Flagship Store	Green Intelligent Power Saver	Power saving energy device received the goods, the packaging is very carefully packaged. Small and exquisite and power saving good use, want to order one at home, praise.	Taobao Marketplace, a Chinese website for online shopping
Slu King Flagship Store	Green Intelligent Power Saver	Has been used to evaluate, the quality is quite good, the appearance of the design is beautiful, and also energy-saving!	Taobao Marketplace, a Chinese website for online shopping
Slu King Flagship Store	Green Intelligent Power Saver	Summer is here to turn on the air conditioning costs electricity, bought to save electricity to use, I hope the effect is good!	Taobao Marketplace, a Chinese website for online shopping
Slu King Flagship Store	Green Intelligent Power Saver	Cost-effective: perfect Appearance and style: very good-looking Workmanship and material: fine Worth buying	Taobao Marketplace, a Chinese website for online shopping
Slu King Flagship Store	Green Intelligent Power Saver	Power saving effect: very good use of cost-effective Recommended to buy Fine workmanship	Taobao Marketplace, a Chinese website for online shopping
Slu King Flagship Store	Green Intelligent Power Saver	Now do not know how effective, to be used - months later, to be sent by the power supply unit information on how much electricity costs this month to know how much energy savings in electricity costs, is the energy-saving effect of this product.	Taobao Marketplace, a Chinese website for online shopping
Slu King Flagship Store	Green Intelligent Power Saver	Value for money: just received the goods today, a friend recommended to me, I heard it was very good	Taobao Marketplace, a Chinese website for online shopping
Slu King Flagship Store	Green Intelligent Power Saver	It's no use.	Taobao Marketplace, a Chinese website for online shopping
Slu King Flagship Store	Green Intelligent Power Saver	Black tech product, waiting for the results and follow up to share with you.	Taobao Marketplace, a Chinese website for online shopping
Slu King Flagship Store	Green Intelligent Power Saver	Whoever buys it is fooled. ***	Taobao Marketplace, a Chinese website for online shopping
Slu King Flagship Store	Green Intelligent Power Saver	Power saving effect: very good Cost-effective: cheap Appearance style: perfect Workmanship material: good Experience: very power-saving	Taobao Marketplace, a Chinese website for online shopping
Slu King Flagship Store	Green Intelligent Power Saver	Just received the goods, and so on for a period of time and then comment, the manufacturer is very timely delivery, customer service attitude is also very good, if the effect is good, but also can be recommended to friends!	Taobao Marketplace, a Chinese website for online shopping
Slu King Flagship Store	Green Intelligent Power Saver	Express delivery is very fast, the first day of purchase is not yet used, save electricity if you continue to shop two more frames	Taobao Marketplace, a Chinese website for online shopping

Slu King Flagship Store	Green Intelligent Power Saver	I won't know until I've used it for a while.	Taobao Marketplace, a Chinese website for online shopping
Slu King Flagship Store	Green Intelligent Power Saver	This is too fake, it's a scam, the more you use this the more your electricity bill increases	Taobao Marketplace, a Chinese website for online shopping
Slu King Flagship Store	Green Intelligent Power Saver	It's good, it's practical, it's real.	Taobao Marketplace, a Chinese website for online shopping
Slu King Flagship Store	Green Intelligent Power Saver	It's useless. Not only does it not save electricity, it costs it.	Taobao Marketplace, a Chinese website for online shopping
Slu King Flagship Store	Green Intelligent Power Saver	Energy saver received, not used before, I hope to realize the effect of power saving, logistics is very fast five-star praise!	Taobao Marketplace, a Chinese website for online shopping
Slu King Flagship Store	Green Intelligent Power Saver	The quality is very good, put in the corner plug does not take up space, a month to calculate can be left more than 100 yuan, a very cost-effective product	Taobao Marketplace, a Chinese website for online shopping
Slu King Flagship Store	Green Intelligent Power Saver	I have received the goods and they are good value for money.	Taobao Marketplace, a Chinese website for online shopping
Slu King Flagship Store	Green Intelligent Power Saver	Online reviews, true or false, we recommend that you do not go cheap, a penny for a penny. See through not to say.	Taobao Marketplace, a Chinese website for online shopping
Slu King Flagship Store	Green Intelligent Power Saver	It's super good. It's really good for my family.	Taobao Marketplace, a Chinese website for online shopping
Slu King Flagship Store	Green Intelligent Power Saver	Experience: a received the goods, the feel is very light, plug in will not light up bad directly returned.	Taobao Marketplace, a Chinese website for online shopping
Slu King Flagship Store	Green Intelligent Power Saver	Don't believe what they say. Ideal is beautiful, reality is cruel.	Taobao Marketplace, a Chinese website for online shopping
Slu King Flagship Store	Green Intelligent Power Saver	Power saving effect: good Cost performance: good Appearance style: showy	Taobao Marketplace, a Chinese website for online shopping
Slu King Flagship Store	Green Intelligent Power Saver	Power-saving effect: the quality of the effect is really good, introduced several friends over to buy	Taobao Marketplace, a Chinese website for online shopping
Slu King Flagship Store	Green Intelligent Power Saver	I've been using it for a week and it still feels like it's having some effect, kudos!	Taobao Marketplace, a Chinese website for online shopping
Slu King Flagship Store	Green Intelligent Power Saver	Need to use it for a while to see the effect before evaluating	Taobao Marketplace, a Chinese website for online shopping
Slu King Flagship Store	Green Intelligent Power Saver	It's a lie. It's a fake. It's plugged in like it's not plugged in, and the meter turns the same.	Taobao Marketplace, a Chinese website for online shopping
Slu King Flagship Store	Green Intelligent Power Saver	Power saving effect: not significant.	Taobao Marketplace, a Chinese website for online shopping
Slu King Flagship Store	Green Intelligent Power Saver	Fake and deluded.	Taobao Marketplace, a Chinese website for online shopping
Slu King Flagship Store	Green Intelligent Power Saver	Power-saving effect: it looks good, just arrived, not yet used, after use and then additional praise Workmanship material: quality is very good, the appearance looks genuine	Taobao Marketplace, a Chinese website for online shopping
Slu King Flagship Store	Green Intelligent Power Saver	Baby received, just used it on, next month to have results, will repurchase one more, the	Taobao Marketplace, a Chinese website for online shopping
Slu King Flagship Store	Green Intelligent Power Saver	It's good, it really saves electricity 👍👍👍👍	Taobao Marketplace, a Chinese website for online shopping
Slu King Flagship Store	Green Intelligent Power Saver	The energy saver has been received and has just been used, not sure how effective it is	Taobao Marketplace, a Chinese website for online shopping
Slu King Flagship Store	Green Intelligent Power Saver	Power-saving effect: very poor Cost-effective: a lie Workmanship material: poor Experience: no worst, only worse	Taobao Marketplace, a Chinese website for online shopping
Slu King Flagship Store	Green Intelligent Power Saver	Received, looking forward to testing results	Taobao Marketplace, a Chinese website for online shopping
Slu King Flagship Store	Green Intelligent Power Saver	I don't know how it works.	Taobao Marketplace, a Chinese website for online shopping
Slu King Flagship Store	Green Intelligent Power Saver	It's a well-made smart energy saver, and it's covered by Pacific Product Liability Insurance.	Taobao Marketplace, a Chinese website for online shopping
Slu King Flagship Store	Green Intelligent Power Saver	Just got it and used it, looking forward to the energy savings, good customer service.	Taobao Marketplace, a Chinese website for online shopping
Slu King Flagship Store	Green Intelligent Power Saver	Baby received, and the business description of the same, well-packed, reasonably priced, fast delivery, after using the follow-up comment.	Taobao Marketplace, a Chinese website for online shopping
Slu King Flagship Store	Green Intelligent Power Saver	Used very power-saving, worth buying	Taobao Marketplace, a Chinese website for online shopping
Slu King Flagship Store	Green Intelligent Power Saver	Works very well especially like it. Smart energy saver Very energy efficient	Taobao Marketplace, a Chinese website for online shopping
Slu King Flagship Store	Green Intelligent Power Saver	Really save electricity, all used a few days before coming to comment, worth buying, recommend everyone to buy, the	Taobao Marketplace, a Chinese website for online shopping
Slu King Flagship Store	Green Intelligent Power Saver	I haven't seen any results yet, but I'll give you a good review first.	Taobao Marketplace, a Chinese website for online shopping
Slu King Flagship Store	Green Intelligent Power Saver	Power-saving effect: no effect at all, fooling people's ghost tricks, we do not fall for it again.	Taobao Marketplace, a Chinese website for online shopping
Slu King Flagship Store	Green Intelligent Power Saver	Just received the goods today, and so on for a period of time and then follow up, the manufacturer's delivery is very timely, customer service attitude is also very good, if the effect is good, you can also recommend to friends!	Taobao Marketplace, a Chinese website for online shopping

Slu King Flagship Store	Green Intelligent Power Saver	Power saving effect: first bought two trial two months feel still useful, so now buy another one. Five stars praise	Taobao Marketplace, a Chinese website for online shopping
Slu King Flagship Store	Green Intelligent Power Saver	Very happy with the goods received, good quality.	Taobao Marketplace, a Chinese website for online shopping
Slu King Flagship Store	Green Intelligent Power Saver	Very good, inexpensive, energy efficient	Taobao Marketplace, a Chinese website for online shopping
Slu King Flagship Store	Green Intelligent Power Saver	No publicity for it	Taobao Marketplace, a Chinese website for online shopping
Slu King Flagship Store	Green Intelligent Power Saver	It works, this is the second time I've purchased it. It actually really works.	Taobao Marketplace, a Chinese website for online shopping
Slu King Flagship Store	Green Intelligent Power Saver	The stuff is great, it really saves electricity and my friend asked me to buy it for him. This is the third time I bought it.	Taobao Marketplace, a Chinese website for online shopping
Slu King Flagship Store	Green Intelligent Power Saver	Got it, going to general time to see if I can save a little on the electric bill	Taobao Marketplace, a Chinese website for online shopping
Slu King Flagship Store	Green Intelligent Power Saver	It's not bad after a couple days of use! Thanks boss haha! It matches the picture.	Taobao Marketplace, a Chinese website for online shopping
Slu King Flagship Store	Green Intelligent Power Saver	Fake fake fake! Used more than last month's electricity bill	Taobao Marketplace, a Chinese website for online shopping
Slu King Flagship Store	Green Intelligent Power Saver	It looks good, expect to save power, use it and come back with additional comments, you can try the new technology!	Taobao Marketplace, a Chinese website for online shopping
Slu King Flagship Store	Green Intelligent Power Saver	It feels good, since I bought the energy saver, I've saved a lot of electricity, it's really a blessing in the age of air conditioning.	Taobao Marketplace, a Chinese website for online shopping
Slu King Flagship Store	Green Intelligent Power Saver	Power Saving Effect: I actually purchased one of these babies online a year ago, and according to usage observations, it does save power. Just the original one accidentally fell in the water when engaged in sanitation, so it immediately shot one. This baby is genuine, beautiful appearance, product certification, warranty card, genuine logo and other certificates, trustworthy.	Taobao Marketplace, a Chinese website for online shopping
Slu King Flagship Store	Green Intelligent Power Saver	Power-saving effect: the effect is okay Appearance style: appearance of compact does not take up space Experience: okay	Taobao Marketplace, a Chinese website for online shopping
Slu King Flagship Store	Green Intelligent Power Saver	Didn't feel any significant power savings, don't know if it will work over time.	Taobao Marketplace, a Chinese website for online shopping
Slu King Flagship Store	Green Intelligent Power Saver	normal	Taobao Marketplace, a Chinese website for online shopping
Slu King Flagship Store	Green Intelligent Power Saver	I've used the power saver since I bought it, and I'm looking forward to the power saving effect!	Taobao Marketplace, a Chinese website for online shopping
Slu King Flagship Store	Green Intelligent Power Saver	Just received the goods, try the effect	Taobao Marketplace, a Chinese website for online shopping
Slu King Flagship Store	Green Intelligent Power Saver	Energy saving is pretty good, I bought it for the third time	Taobao Marketplace, a Chinese website for online shopping
Slu King Flagship Store	Green Intelligent Power Saver	*Nothing works, it's all a scam, I ***** just bought this thing.	Taobao Marketplace, a Chinese website for online shopping
Slu King Flagship Store	Green Intelligent Power Saver	No effect was seen!	Taobao Marketplace, a Chinese website for online shopping
Slu King Flagship Store	Green Intelligent Power Saver	I just received it and used it, baby it's good, I'll comment after using it.	Taobao Marketplace, a Chinese website for online shopping
Slu King Flagship Store	Green Intelligent Power Saver	After using it for a few days, it's really energy-saving. It's worth every penny. I'll recommend it to my friends.	Taobao Marketplace, a Chinese website for online shopping
Slu King Flagship Store	Green Intelligent Power Saver	Experience: has been used for three months, power saving obvious, electricity and not open the same electricity cost	Taobao Marketplace, a Chinese website for online shopping
Slu King Flagship Store	Green Intelligent Power Saver	It takes two to know the effect.	Taobao Marketplace, a Chinese website for online shopping
Slu King Flagship Store	Green Intelligent Power Saver	Power saving effect: energy saving power supply	Taobao Marketplace, a Chinese website for online shopping
Slu King Flagship Store	Green Intelligent Power Saver	Satisfied, need to repurchase again	Taobao Marketplace, a Chinese website for online shopping
Slu King Flagship Store	Green Intelligent Power Saver	Just used it and don't know the effect yet	Taobao Marketplace, a Chinese website for online shopping
Slu King Flagship Store	Green Intelligent Power Saver	None of it works. Plugging it in takes more power.	Taobao Marketplace, a Chinese website for online shopping
Slu King Flagship Store	Green Intelligent Power Saver	Power-saving effect: the effect is strong, Cost-effective: really good Appearance style: very good Experience: I personally use the power savings of 50%, I really think it can be, and then the next order to send back to their homes!	Taobao Marketplace, a Chinese website for online shopping
Slu King Flagship Store	Green Intelligent Power Saver	Experience: Just received the goods Feel pretty good Try it first I really hope it works	Taobao Marketplace, a Chinese website for online shopping
Slu King Flagship Store	Green Intelligent Power Saver	Still in the process of using it, don't know if it's fast yet	Taobao Marketplace, a Chinese website for online shopping
Slu King Flagship Store	Green Intelligent Power Saver	I think I've been duped.	Taobao Marketplace, a Chinese website for online shopping
Slu King Flagship Store	Green Intelligent Power Saver	Intelligent energy saver has just arrived, opened to look at the commodity anti-counterfeiting inquiry center, there is a genuine logo, the original genuine, logistics is very fast, I hope that the power-saving effect is good!	Taobao Marketplace, a Chinese website for online shopping
Slu King Flagship Store	Green Intelligent Power Saver	Power-saving effect: used a few days before evaluation, the home is mainly air conditioning costs electricity, plugged in the meter obviously slowed down, waiting for next month's electricity bill out to know how much can be saved! Looking forward to it!	Taobao Marketplace, a Chinese website for online shopping

Slu King Flagship Store	Green Intelligent Power Saver	Power saving effect: works great, repurchased many times. Introduced friends to buy too	Taobao Marketplace, a Chinese website for online shopping
Slu King Flagship Store	Green Intelligent Power Saver	Quality is very good, use to see what the effect is, just installed, the effect is good will also buy back, logistics is also very fast, thanks to the seller carefully packaged! I hope the effect is good!	Taobao Marketplace, a Chinese website for online shopping
Slu King Flagship Store	Green Intelligent Power Saver	Installed and hopefully achieving the desired results! Looking forward to it!!!	Taobao Marketplace, a Chinese website for online shopping
Slu King Flagship Store	Green Intelligent Power Saver	It's good. Today used tomorrow feel right power saving! Power saving effect: great Cost-effective: 6 Appearance style: okay Experience: 666	Taobao Marketplace, a Chinese website for online shopping
Slu King Flagship Store	Green Intelligent Power Saver	Goods received, easy to use, really good	Taobao Marketplace, a Chinese website for online shopping
Slu King Flagship Store	Green Intelligent Power Saver	I'll tell you when I use it.	Taobao Marketplace, a Chinese website for online shopping
Slu King Flagship Store	Green Intelligent Power Saver	Power saving effect: can have a certain effect, Cost-effective: high Appearance style: beautiful and generous Workmanship material: good Experience: have a certain energy-saving effect	Taobao Marketplace, a Chinese website for online shopping
Slu King Flagship Store	Green Intelligent Power Saver	Friends with a good use recommended the purchase, down after the customer service attitude and timely, logistics is very fast, after the arrival of the plug to try, really good, like, can pay less for electricity later, want to save money friends can start to buy.	Taobao Marketplace, a Chinese website for online shopping
Slu King Flagship Store	Green Intelligent Power Saver	Energy saving is pretty good quality	Taobao Marketplace, a Chinese website for online shopping
Slu King Flagship Store	Green Intelligent Power Saver	Families, don't be fooled, this saver doesn't work and it's still the same power consumption, a scam, I talked to the merchant and the merchant won't give a refund.	Taobao Marketplace, a Chinese website for online shopping
Slu King Flagship Store	Green Intelligent Power Saver	I used it before coming to comment, this product is of good quality and it does work.	Taobao Marketplace, a Chinese website for online shopping
Slu King Flagship Store	Green Intelligent Power Saver	The goods have been received, look and feel good, just do not know if it can save electricity.	Taobao Marketplace, a Chinese website for online shopping
Slu King Flagship Store	Green Intelligent Power Saver	Not absolutely good to use, the business in order to get good reviews to five yuan red packets, many consumers, in order to get five yuan red packets, conscience all to the business of good reviews, you see good reviews are not necessarily really good to use, one of my friends is an electrician he said it is impossible to use, I am ready to return the product!	Taobao Marketplace, a Chinese website for online shopping
Slu King Flagship Store	Green Intelligent Power Saver	The power saving effect is still a good product, worth buying	Taobao Marketplace, a Chinese website for online shopping
Slu King Flagship Store	Green Intelligent Power Saver	Power-saving effect: just started to use, plugged in the meter rotation speed turned much slower, should be able to save electricity Appearance style: good-looking Workmanship material: delicate	Taobao Marketplace, a Chinese website for online shopping
Slu King Flagship Store	Green Intelligent Power Saver	Very good very works as pictured	Taobao Marketplace, a Chinese website for online shopping
Slu King Flagship Store	Green Intelligent Power Saver	Power saving effect: very save electricity Cost-effective: economic and affordable Experience: very good use	Taobao Marketplace, a Chinese website for online shopping
Slu King Flagship Store	Green Intelligent Power Saver	I just got the goods, this product of your manufacturer is not bad	Taobao Marketplace, a Chinese website for online shopping
Slu King Flagship Store	Green Intelligent Power Saver	Value for money: fairly inexpensive, looking forward to using it	Taobao Marketplace, a Chinese website for online shopping
Slu King Flagship Store	Green Intelligent Power Saver	It's really good, and the power usage has increased	Taobao Marketplace, a Chinese website for online shopping
Slu King Flagship Store	Green Intelligent Power Saver	I'm using it, it should save electricity, my friend introduced me to buy it, she said it works quite well, so I bought it!	Taobao Marketplace, a Chinese website for online shopping
Slu King Flagship Store	Green Intelligent Power Saver	It worked last year when I bought it and used it, and it does save electricity. My neighbor and friend asked me to buy it and then asked me to buy it again.	Taobao Marketplace, a Chinese website for online shopping
Slu King Flagship Store	Green Intelligent Power Saver	Shipping and delivery is fast, the power-saving effect has a comparison before commenting.	Taobao Marketplace, a Chinese website for online shopping
Slu King Flagship Store	Green Intelligent Power Saver	This product is a scam and does not work.	Taobao Marketplace, a Chinese website for online shopping
Slu King Flagship Store	Green Intelligent Power Saver	Haven't seen any results yet.	Taobao Marketplace, a Chinese website for online shopping
Slu King Flagship Store	Green Intelligent Power Saver	Don't be fooled people, this does absolutely nothing at all. It's not worth buying, it's a waste of money to buy it, it's pressurized and doesn't work at all	Taobao Marketplace, a Chinese website for online shopping
Slu King Flagship Store	Green Intelligent Power Saver	Goods quickly received well-packaged very satisfied with the workmanship is also very delicate power-saving effect is also very good straight in the have to buy!	Taobao Marketplace, a Chinese website for online shopping
Slu King Flagship Store	Green Intelligent Power Saver	Energy saver received, looks small and convenient, use to see the effect of it!	Taobao Marketplace, a Chinese website for online shopping
Slu King Flagship Store	Green Intelligent Power Saver	Small and exquisite, energy saving, no problem, cost-effective Welcome to buy!	Taobao Marketplace, a Chinese website for online shopping
Slu King Flagship Store	Green Intelligent Power Saver	Power saving king is indeed very save electricity, plug up the meter will go slow, workmanship material is exquisite, no longer need to worry about the electricity bill is too high, can give the family to save a big expense, did not buy the user can rest assured that the purchase of the purchase! Express very fast service satisfaction! All five-star praise!	Taobao Marketplace, a Chinese website for online shopping
Slu King Flagship Store	Green Intelligent Power Saver	False Experience: {false only two months to save electricity, to that time I buy can not be returned	Taobao Marketplace, a Chinese website for online shopping
Slu King Flagship Store	Green Intelligent Power Saver	Power saving effect: looking forward to the effect Cost-effectiveness: very good Appearance style: small and exquisite Workmanship material: very good 👍	Taobao Marketplace, a Chinese website for online shopping
Slu King Flagship Store	Green Intelligent Power Saver	I'll give you a good review and come back after I've used it.	Taobao Marketplace, a Chinese website for online shopping

Slu King Flagship Store	Green Intelligent Power Saver	Power saving effect: not good, not much use Workmanship material: poor workmanship quality	Taobao Marketplace, a Chinese website for online shopping
Slu King Flagship Store	Green Intelligent Power Saver	I'll comment after I've used it	Taobao Marketplace, a Chinese website for online shopping
Slu King Flagship Store	Green Intelligent Power Saver	Use it and see how it works then follow up	Taobao Marketplace, a Chinese website for online shopping
Slu King Flagship Store	Green Intelligent Power Saver	It's good quality.	Taobao Marketplace, a Chinese website for online shopping
Slu King Flagship Store	Green Intelligent Power Saver	Experience: average	Taobao Marketplace, a Chinese website for online shopping
Slu King Flagship Store	Green Intelligent Power Saver	It's not working.	Taobao Marketplace, a Chinese website for online shopping
Slu King Flagship Store	Green Intelligent Power Saver	Power-saving effect: to be observed, because just retrieved from the courier point, according to your video and instructions to explain the need for three months to see the effect Cost-effective: high, more than the same type of Appearance style: beautiful, generous Workmanship Material: in fine, feel good	Taobao Marketplace, a Chinese website for online shopping
Slu King Flagship Store	Green Intelligent Power Saver	Packaging is intact, is in use, looking quite good high, and the seller describes the same satisfaction	Taobao Marketplace, a Chinese website for online shopping
Slu King Flagship Store	Green Intelligent Power Saver	Power-saving effect: effective Cost-effective: OK Appearance style: good Workmanship material: good	Taobao Marketplace, a Chinese website for online shopping
Slu King Flagship Store	Green Intelligent Power Saver	The goods have been received, but I do not know whether it can really save electricity Oh? I also see others bought, I also try to see, how about the effect of power saving? We three people cooperate to buy six, was to send us three, only send us two, so the business is too buckle, we also help you save the	Taobao Marketplace, a Chinese website for online shopping
Slu King Flagship Store	Green Intelligent Power Saver	Received quickly, small size has big energy, good value for money, punch 😆	Taobao Marketplace, a Chinese website for online shopping
Slu King Flagship Store	Green Intelligent Power Saver	Experience: Tried it for a week, and it worked wonders. The original meter went 8 words per day before the energy saver was installed, but now it goes 4 words. I will continue to use it to see if there is a bigger change.	Taobao Marketplace, a Chinese website for online shopping
Slu King Flagship Store	Green Intelligent Power Saver	It really saves power after using it and it's genuine. It works well.	Taobao Marketplace, a Chinese website for online shopping
Slu King Flagship Store	Green Intelligent Power Saver	I just used it. The product is affordable and compact.	Taobao Marketplace, a Chinese website for online shopping
Slu King Flagship Store	Green Intelligent Power Saver	Workmanship material: workmanship is not bad, the goods arrived quickly, just started to use, friends said the effect is not bad!	Taobao Marketplace, a Chinese website for online shopping
Slu King Flagship Store	Green Intelligent Power Saver	Cost-effective: cheap and good energy saving and environmental protection, I hope to save me a lot of electricity	Taobao Marketplace, a Chinese website for online shopping
Slu King Flagship Store	Green Intelligent Power Saver	The quality is great and I will be back next time.	Taobao Marketplace, a Chinese website for online shopping
Slu King Flagship Store	Green Intelligent Power Saver	The most failed a shopping, video ads say what, instant results, huh, customer service actually let me wait 2 to 3 months to see the results, completely and the business ads do not serve ah, when they buy a lesson!	Taobao Marketplace, a Chinese website for online shopping
Slu King Flagship Store	Green Intelligent Power Saver	have nothing to say	Taobao Marketplace, a Chinese website for online shopping
Slu King Flagship Store	Green Intelligent Power Saver	Great quality, effective and durable.	Taobao Marketplace, a Chinese website for online shopping
Slu King Flagship Store	Green Intelligent Power Saver	Power saving effect:, This power saving plug saves energy and power. Beneficial.	Taobao Marketplace, a Chinese website for online shopping
Slu King Flagship Store	Green Intelligent Power Saver	Works really well. Convenient and smart.	Taobao Marketplace, a Chinese website for online shopping
Slu King Flagship Store	Green Intelligent Power Saver	This stuff doesn't work. It doesn't work at all.	Taobao Marketplace, a Chinese website for online shopping
Slu King Flagship Store	Green Intelligent Power Saver	It's very easy to use, plug it in and don't worry about it, electrical appliances can be used at will, and the air conditioner can be turned on at will.	Taobao Marketplace, a Chinese website for online shopping
Slu King Flagship Store	Green Intelligent Power Saver	Scanned the QR code, checked the security code, and confirmed that it is original and authentic. The labeling is complete. Small and exquisite, very beautiful, as for the power-saving effect, another day to comment.	Taobao Marketplace, a Chinese website for online shopping
Slu King Flagship Store	Green Intelligent Power Saver	Received the goods for a period of time and look forward to the effect	Taobao Marketplace, a Chinese website for online shopping
Slu King Flagship Store	Green Intelligent Power Saver	Bought three, broke one.	Taobao Marketplace, a Chinese website for online shopping
Slu King Flagship Store	Green Intelligent Power Saver	Energy efficient and very good	Taobao Marketplace, a Chinese website for online shopping
Slu King Flagship Store	Green Intelligent Power Saver	Second purchase, works really well	Taobao Marketplace, a Chinese website for online shopping
Slu King Flagship Store	Green Intelligent Power Saver	I just plugged it in, I don't know how effective it is, I'll come back with a follow-up comment when it's working!	Taobao Marketplace, a Chinese website for online shopping
Slu King Flagship Store	Green Intelligent Power Saver	It's purely an IQ tax. Don't buy it, people.	Taobao Marketplace, a Chinese website for online shopping
Slu King Flagship Store	Green Intelligent Power Saver	Cost-effective: very affordable buy two get one free, better than others! Appearance and style: well packaged and shipped fast! Received before the weekend! Workmanship material: the appearance of the look should be good, to wait for the results of the experiment, after all, the summer air conditioning power is too strong, such as the results of the experiment to come out and then evaluate the repurchase, we recommend that you buy this store, I also compared a number of people before choosing!	Taobao Marketplace, a Chinese website for online shopping
Slu King Flagship Store	Green Intelligent Power Saver	Customer service is patient and meticulous, the appearance of the design is exquisite, looking forward to working together again.	Taobao Marketplace, a Chinese website for online shopping

Slu King Flagship Store	Green Intelligent Power Saver	I just received it today, I'm using it, I'll see if there's any effect after a while	Taobao Marketplace, a Chinese website for online shopping
Slu King Flagship Store	Green Intelligent Power Saver	The power savings are noticeable, cost effective and the material is very good!	Taobao Marketplace, a Chinese website for online shopping
Slu King Flagship Store	Green Intelligent Power Saver	My friend used it and said it can really save some electricity every month, she introduced it to me, I expect it to be effective, next time I will buy a few more!	Taobao Marketplace, a Chinese website for online shopping
Slu King Flagship Store	Green Intelligent Power Saver	Very good value for money, works very well, very power-saving Recommended for everyone!	Taobao Marketplace, a Chinese website for online shopping
Slu King Flagship Store	Green Intelligent Power Saver	Intelligent energy-saving king, it is true to its name, both power saving and convenient, need friends can order, the next time there will be a need to buy back.	Taobao Marketplace, a Chinese website for online shopping
Slu King Flagship Store	Green Intelligent Power Saver	The logistics is very fast, the material feels thin and lightweight, the quality is not bad! Can hold a lot of things! Too convenient!	Taobao Marketplace, a Chinese website for online shopping
Slu King Flagship Store	Green Intelligent Power Saver	This energy saver is really good, plug in the power meter is much slower, really feel good, plug in this energy saver can boldly rest assured that the power no longer worry about it, it is worth buying, must praise praise 👍	Taobao Marketplace, a Chinese website for online shopping
Slu King Flagship Store	Green Intelligent Power Saver	Fast logistics, match the description, now I do not know whether it is good or not, use it and then come back to the follow-up evaluation	Taobao Marketplace, a Chinese website for online shopping
Slu King Flagship Store	Green Intelligent Power Saver	Power-saving effect: only plugged in, looking forward to the effect. Appearance and style: simple Workmanship material: can Use experience: simple	Taobao Marketplace, a Chinese website for online shopping
Slu King Flagship Store	Green Intelligent Power Saver	Experience: being used Appearance style: good quality Cost-effective: similar products are more satisfactory	Taobao Marketplace, a Chinese website for online shopping
Slu King Flagship Store	Green Intelligent Power Saver	It was recommended by a friend. Says it works.	Taobao Marketplace, a Chinese website for online shopping
Slu King Flagship Store	Green Intelligent Power Saver	Power saving effect: a real power saving Appearance style: beautiful appearance Experience: after the use of the effect is very good	Taobao Marketplace, a Chinese website for online shopping
Slu King Flagship Store	Green Intelligent Power Saver	Baby received, has not yet been used, after using it will be and everyone common Heng! Look at the appearance:Feeling can't be wrong!	Taobao Marketplace, a Chinese website for online shopping
Slu King Flagship Store	Green Intelligent Power Saver	It only saves one kilowatt hour a night to run the air conditioner	Taobao Marketplace, a Chinese website for online shopping
Slu King Flagship Store	Green Intelligent Power Saver	Received a number of times to buy the use of easy to plug in and save electricity to save a lot of money, electricity is also assured. Store activities also sent a give a favorable review!	Taobao Marketplace, a Chinese website for online shopping
Slu King Flagship Store	Green Intelligent Power Saver	Power saving effect: very good Cost-effective: high Appearance style: simple Very good, continue to repurchase. Courier service is good. Kudos 👍	Taobao Marketplace, a Chinese website for online shopping
Slu King Flagship Store	Green Intelligent Power Saver	Power saving effect: very good Appearance and style: general Workmanship and material: very durable	Taobao Marketplace, a Chinese website for online shopping
Slu King Flagship Store	Green Intelligent Power Saver	Personally, I think it works very well and I've purchased it for the second time already	Taobao Marketplace, a Chinese website for online shopping
Slu King Flagship Store	Green Intelligent Power Saver	Power saving effect: very good	Taobao Marketplace, a Chinese website for online shopping
Slu King Flagship Store	Green Intelligent Power Saver	Appearance and style: fashionable appearance, soft light display	Taobao Marketplace, a Chinese website for online shopping
Slu King Flagship Store	Green Intelligent Power Saver	I've used this before with good results and proceeded to buy it. Let's see how much we can save next month!	Taobao Marketplace, a Chinese website for online shopping
Slu King Flagship Store	Green Intelligent Power Saver	Feels very very good and worth the money 🤗🤗🤗🤗	Taobao Marketplace, a Chinese website for online shopping
Slu King Flagship Store	Green Intelligent Power Saver	I've just received it, and I've tried it out, and the number of revolutions on the meter doesn't seem to have changed, just like it did before, so I won't be able to comment on the savings until I've had some time.	Taobao Marketplace, a Chinese website for online shopping
Slu King Flagship Store	Green Intelligent Power Saver	Power saving effect: very can be very satisfied Cost-effective: super high very good	Taobao Marketplace, a Chinese website for online shopping
Slu King Flagship Store	Green Intelligent Power Saver	Just received it and looking forward to the results! Will continue to repurchase if it's good!	Taobao Marketplace, a Chinese website for online shopping
Slu King Flagship Store	Green Intelligent Power Saver	Power-saving effect: energy-saving good, we can rest assured that the purchase, I tried Cost-effective: high Workmanship material: good quality	Taobao Marketplace, a Chinese website for online shopping
Slu King Flagship Store	Green Intelligent Power Saver	I bought the product a long time ago, and today I bought another one that works well	Taobao Marketplace, a Chinese website for online shopping
Slu King Flagship Store	Green Intelligent Power Saver	Power-saving effect: merchants fast delivery, received the goods can not wait to plug in the power to try, I hope the effect of bar ￼!	Taobao Marketplace, a Chinese website for online shopping
Slu King Flagship Store	Green Intelligent Power Saver	Just received, packaging is very good, workmanship material is good, service received, logistics is very fast, straight to buy.	Taobao Marketplace, a Chinese website for online shopping
Slu King Flagship Store	Green Intelligent Power Saver	Power saving effect: cheating doesn't help at all!	Taobao Marketplace, a Chinese website for online shopping
Slu King Flagship Store	Green Intelligent Power Saver	It is reasonable that can not be around the speed of the meter, otherwise the relevant departments have outlawed the product. Product packaging products exposed, no protection.	Taobao Marketplace, a Chinese website for online shopping
Slu King Flagship Store	Green Intelligent Power Saver	It worked well, couldn't pass it up, guess I'll have to buy another one	Taobao Marketplace, a Chinese website for online shopping
Slu King Flagship Store	Green Intelligent Power Saver	There is a need for the old iron straight purchase, because I never tell lies Wish the boss a prosperous business!	Taobao Marketplace, a Chinese website for online shopping
Slu King Flagship Store	Green Intelligent Power Saver	I will not how to comment, it is authentic and straight to buy the old ironmen believe me	Taobao Marketplace, a Chinese website for online shopping

Slu King Flagship Store	Green Intelligent Power Saver	Power saving effect: can Cost-effective: good Appearance style: can Workmanship material: okay Experience: energy-saving effect is okay	Taobao Marketplace, a Chinese website for online shopping
Slu King Flagship Store	Green Intelligent Power Saver	Cost-effective: ready to use, like can start. Appearance and style: looks good, the first time to buy, do not know what kind of	Taobao Marketplace, a Chinese website for online shopping
Slu King Flagship Store	Green Intelligent Power Saver	It's a total scam, and should be held criminally liable for cheating so many people	Taobao Marketplace, a Chinese website for online shopping
Slu King Flagship Store	Green Intelligent Power Saver	Not interested in purchasing, it's a scam, throw it away.	Taobao Marketplace, a Chinese website for online shopping
Slu King Flagship Store	Green Intelligent Power Saver	Power saving effect: an ineffective product	Taobao Marketplace, a Chinese website for online shopping
Slu King Flagship Store	Green Intelligent Power Saver	Power-saving effect: satisfied 👍 Cost-effective: high Experience: good, the second time back to buy have to buy hurry up!	Taobao Marketplace, a Chinese website for online shopping
Slu King Flagship Store	Green Intelligent Power Saver	Experience: felt good enough to purchase one again.	Taobao Marketplace, a Chinese website for online shopping
Slu King Flagship Store	Green Intelligent Power Saver	Power saving effect: very good have obvious effect. Appearance style quality is very good. ￼	Taobao Marketplace, a Chinese website for online shopping
Slu King Flagship Store	Green Intelligent Power Saver	I have not used it yet, it takes time to prove whether it works or not. Evaluation after use.	Taobao Marketplace, a Chinese website for online shopping
Slu King Flagship Store	Green Intelligent Power Saver	Not only does it not save electricity, but it ****ing costs it.	Taobao Marketplace, a Chinese website for online shopping
Slu King Flagship Store	Green Intelligent Power Saver	I'm using 7 or 8 more kilowatts than I did before I didn't use it, and I'm using less electricity than the last 月's	Taobao Marketplace, a Chinese website for online shopping
Slu King Flagship Store	Green Intelligent Power Saver	It doesn't work, don't be fooled	Taobao Marketplace, a Chinese website for online shopping
Slu King Flagship Store	Green Intelligent Power Saver	Power-saving effect: no effect Experience: garbage product, no use at all, want to return it, it is troublesome.	Taobao Marketplace, a Chinese website for online shopping
Slu King Flagship Store	Green Intelligent Power Saver	The product can be quite good to use and save money, good quality	Taobao Marketplace, a Chinese website for online shopping
Slu King Flagship Store	Green Intelligent Power Saver	Power-saving effect: used to save electricity and energy, unexpected, Cost-effective: high Appearance style: compact and practical	Taobao Marketplace, a Chinese website for online shopping
Slu King Flagship Store	Green Intelligent Power Saver	It's so fake, it doesn't work, don't buy it, don't be fooled by him I've been using it for more than two months and it doesn't work at all.	Taobao Marketplace, a Chinese website for online shopping
Slu King Flagship Store	Green Intelligent Power Saver	It doesn't feel like it's working.	Taobao Marketplace, a Chinese website for online shopping
Slu King Flagship Store	Green Intelligent Power Saver	Already is the second purchase, the first is to my mom's home to buy, with 2 months really good, a month of 70 or 80 electricity bill to save more than 20, I bought another for their own home! Really good, will recommend friends and family! Praise 👍!	Taobao Marketplace, a Chinese website for online shopping
Slu King Flagship Store	Green Intelligent Power Saver	Every day at work a little busy, did not care about timely evaluation, try a few days really good it! There is a power-saving effect, the summer electricity costs rack, almost catch up with the rent, decisive praise, the need for friends to hurry down! Value for money	Taobao Marketplace, a Chinese website for online shopping
Slu King Flagship Store	Green Intelligent Power Saver	Experience: just got it back and used it.	Taobao Marketplace, a Chinese website for online shopping
Slu King Flagship Store	Green Intelligent Power Saver	Not much use, waste of money, turned on the air conditioner for four hours, spent almost 10 dollars a day on electricity, positive reviews don't know where to come from	Taobao Marketplace, a Chinese website for online shopping
Slu King Flagship Store	Green Intelligent Power Saver	Has been the second purchase, a small and exquisite saving appliances, very good, friends also want to buy link has been recommended, the next time will also after the sale.	Taobao Marketplace, a Chinese website for online shopping
Slu King Flagship Store	Green Intelligent Power Saver	Power-saving effect: feel can save electricity, looking forward to the effect of a month Cost-effective: cost-effective quite high Appearance style: green quite good-looking Workmanship material: the quality is quite good Experience: experience quite good	Taobao Marketplace, a Chinese website for online shopping
Slu King Flagship Store	Green Intelligent Power Saver	May began to use the first, the electricity bill really cheaper, and then bought two, to see if you can save, the house 140 square feet, air conditioning, refrigerators, water heaters are high energy consumption appliances, every little bit of money ah!	Taobao Marketplace, a Chinese website for online shopping
Slu King Flagship Store	Green Intelligent Power Saver	I've just used it for a few days, so I'm looking forward to good results!	Taobao Marketplace, a Chinese website for online shopping
Slu King Flagship Store	Green Intelligent Power Saver	Power-saving effect: good Cost-effective: high Appearance style: novel Workmanship material: good Experience: very good, worth buying, recommended	Taobao Marketplace, a Chinese website for online shopping
Slu King Flagship Store	Green Intelligent Power Saver	Power saving effect is very good, much better than other power savers, beautiful appearance, fine workmanship, with the previous comparison to save a lot of electricity costs	Taobao Marketplace, a Chinese website for online shopping
Slu King Flagship Store	Green Intelligent Power Saver	Cost-effective: just used a few days, looking forward to saving electricity The effect of saving money, the boss said that the use of half a year does not save electricity free refund, everyone rushed duck ...	Taobao Marketplace, a Chinese website for online shopping
Slu King Flagship Store	Green Intelligent Power Saver	Appearance style: fashionable appearance design, soft light display. Workmanship material: fine workmanship on, practical and save money.	Taobao Marketplace, a Chinese website for online shopping
Slu King Flagship Store	Green Intelligent Power Saver	I've bought a few power savers, too cheap to buy, not long to use the bad. This product feels good, the quality of workmanship is very good, look at the effect and then follow up ha!	Taobao Marketplace, a Chinese website for online shopping
Slu King Flagship Store	Green Intelligent Power Saver	Very lightweight! I hope it works well!	Taobao Marketplace, a Chinese website for online shopping
Slu King Flagship Store	Green Intelligent Power Saver	Experience: baby is very good worth buying, good value for money	Taobao Marketplace, a Chinese website for online shopping
Slu King Flagship Store	Green Intelligent Power Saver	The bag received disassembled and installed, and business description of the same, inexpensive, over a period of time to follow up on the evaluation	Taobao Marketplace, a Chinese website for online shopping
Slu King Flagship Store	Green Intelligent Power Saver	Light and fluffy doesn't help at all, I've been using it for over a month and it doesn't save any electricity, it's a rip off 😂	Taobao Marketplace, a Chinese website for online shopping

Slu King Flagship Store	Green Intelligent Power Saver	I just used it and couldn't see any results.	Taobao Marketplace, a Chinese website for online shopping
Slu King Flagship Store	Green Intelligent Power Saver	Power saving effect: the effect is not at all, this thing is an IQ tax, very, very not recommended to buy!	Taobao Marketplace, a Chinese website for online shopping
Slu King Flagship Store	Green Intelligent Power Saver	Power saving effect: save about 20 dollars, cost-effective: price general appearance style: beautiful, workmanship material: can.	Taobao Marketplace, a Chinese website for online shopping
Slu King Flagship Store	Green Intelligent Power Saver	Power saving effect: recommended by friends, use good and then continue to recommend Appearance style:. Good workmanship	Taobao Marketplace, a Chinese website for online shopping
Slu King Flagship Store	Green Intelligent Power Saver	The appearance is small and compact easy to use hopefully it will save me more money, the	Taobao Marketplace, a Chinese website for online shopping
Slu King Flagship Store	Green Intelligent Power Saver	Received it, looking forward to saving electricity at home, next time there is a purchase	Taobao Marketplace, a Chinese website for online shopping
Slu King Flagship Store	Green Intelligent Power Saver	I bought this thing, used two days no effect, inserted a little response is not, Ben did not have what he said, I just asked the customer service, customer service told me that, to use 1 to 2 months to have effect, I want to ask is, with 1 to 2 months, if said no effect, can still be refunded? I think this is	Taobao Marketplace, a Chinese website for online shopping
Slu King Flagship Store	Green Intelligent Power Saver	Received the baby a couple of months, deliberately pay attention to, feel good, Z is not bad, can save a power, but it is very good.	Taobao Marketplace, a Chinese website for online shopping
Slu King Flagship Store	Green Intelligent Power Saver	Just received the goods today, looks good, good workmanship material	Taobao Marketplace, a Chinese website for online shopping
Slu King Flagship Store	Green Intelligent Power Saver	Energy saving is really good, the price is not expensive, the appearance of fashionable atmosphere, fire-retardant material is safer, is experiencing the	Taobao Marketplace, a Chinese website for online shopping
Slu King Flagship Store	Green Intelligent Power Saver	Arrived on the use, do not know how the effect, with good in the follow-up comment.	Taobao Marketplace, a Chinese website for online shopping
Slu King Flagship Store	Green Intelligent Power Saver	Power saving effect: very satisfied Cost performance: very satisfied Appearance style: very satisfied Workmanship material: very satisfied	Taobao Marketplace, a Chinese website for online shopping
Slu King Flagship Store	Green Intelligent Power Saver	It's a genius to invent this. The country should put it to good use, otherwise it's a waste of talent.	Taobao Marketplace, a Chinese website for online shopping
Slu King Flagship Store	Green Intelligent Power Saver	Shot after the logistics is very fast, the boss has a very good service attitude, the appearance of the packaging fine workmanship power-saving effect should also be very good, will not affect the use of home appliances	Taobao Marketplace, a Chinese website for online shopping
Slu King Flagship Store	Green Intelligent Power Saver	Appearance and style: quite exquisite Workmanship and material: good quality	Taobao Marketplace, a Chinese website for online shopping
Slu King Flagship Store	Green Intelligent Power Saver	It's useless. IQ tax. Big loser.	Taobao Marketplace, a Chinese website for online shopping
Slu King Flagship Store	Green Intelligent Power Saver	Received the goods, can not wait to plug into the socket to let it work, the next waiting for a miracle, just recorded the meter degrees, and so on the end of the month to see if there is no effect, if there are a few right back to buy!	Taobao Marketplace, a Chinese website for online shopping
Slu King Flagship Store	Green Intelligent Power Saver	Great as always, great results as always, great support as always, thanks as always!	Taobao Marketplace, a Chinese website for online shopping
Slu King Flagship Store	Green Intelligent Power Saver	Power savings: pretty good. Cost-effective: quite good. Appearance and style: fine workmanship. Workmanship material: material is good. Use experience: very good.	Taobao Marketplace, a Chinese website for online shopping
Slu King Flagship Store	Green Intelligent Power Saver	The product is compact and does not take up space, before and after the use of the contrast has obvious results, but also affordable and worth buying 👍	Taobao Marketplace, a Chinese website for online shopping
Slu King Flagship Store	Green Intelligent Power Saver	Received the saver, it's the second time I bought it, it should work!	Taobao Marketplace, a Chinese website for online shopping
Slu King Flagship Store	Green Intelligent Power Saver	Not bad, mom says she pays less for electricity than before, and now that the air conditioner is on, I recommend using it!	Taobao Marketplace, a Chinese website for online shopping
Slu King Flagship Store	Green Intelligent Power Saver	Power saving effect: received, Cost-effective: acceptable Appearance style: quite good Workmanship material: workmanship looks good	Taobao Marketplace, a Chinese website for online shopping
Slu King Flagship Store	Green Intelligent Power Saver	Great customer service and great product quality!	Taobao Marketplace, a Chinese website for online shopping
Slu King Flagship Store	Green Intelligent Power Saver	Appearance and style: very good, good workmanship Workmanship material: can be Usage experience: just bought, I heard it is very good	Taobao Marketplace, a Chinese website for online shopping
Slu King Flagship Store	Green Intelligent Power Saver	The goods have been received, look good and work well, the quality is exceptionally good.	Taobao Marketplace, a Chinese website for online shopping
Slu King Flagship Store	Green Intelligent Power Saver	Baby's fine. I hope it gets more energy efficient. It's great.	Taobao Marketplace, a Chinese website for online shopping
Slu King Flagship Store	Green Intelligent Power Saver	Things received, also used a few days, have not looked at the meter there is no obvious effect, and so on the end of the month will know, I hope the effect can be	Taobao Marketplace, a Chinese website for online shopping
Slu King Flagship Store	Green Intelligent Power Saver	Power-saving effect: just received a few days hope to have the effect Cost-effective: the price is very affordable Appearance style: style is very exquisite	Taobao Marketplace, a Chinese website for online shopping
Slu King Flagship Store	Green Intelligent Power Saver	Good. I hope it gets better.	Taobao Marketplace, a Chinese website for online shopping
Slu King Flagship Store	Green Intelligent Power Saver	Smart saver, saves electricity, next time you need to buy in.	Taobao Marketplace, a Chinese website for online shopping
Slu King Flagship Store	Green Intelligent Power Saver	The quality is quite good, the logistics is also very fast, praise	Taobao Marketplace, a Chinese website for online shopping
Slu King Flagship Store	Green Intelligent Power Saver	This intelligent energy saver is especially good to use and save electricity hope everyone will buy it!	Taobao Marketplace, a Chinese website for online shopping
Slu King Flagship Store	Green Intelligent Power Saver	Power saving effect: very good effect, lightweight and beautiful, feel good	Taobao Marketplace, a Chinese website for online shopping

Slu King Flagship Store	Green Intelligent Power Saver	Save electricity and moneyGreen and healthy.	Taobao Marketplace, a Chinese website for online shopping
Slu King Flagship Store	Green Intelligent Power Saver	A tiny circuit board saves power? Don't be fooled. The tiny piece of circuit board inside is just to mention the common display light function.	Taobao Marketplace, a Chinese website for online shopping
Slu King Flagship Store	Green Intelligent Power Saver	Goods received to the description of the arrival of the clean use look forward to	Taobao Marketplace, a Chinese website for online shopping
Slu King Flagship Store	Green Intelligent Power Saver	I just used it and hope it is energy efficient.	Taobao Marketplace, a Chinese website for online shopping
Slu King Flagship Store	Green Intelligent Power Saver	Back to plug in and use for a few days, the effect is okay, easy to use, worth buying!	Taobao Marketplace, a Chinese website for online shopping
Slu King Flagship Store	Green Intelligent Power Saver	The saver was received, as described, it is genuine and trustworthy.	Taobao Marketplace, a Chinese website for online shopping
Slu King Flagship Store	Green Intelligent Power Saver	Power savings: very much looking forward to the power savings. Saving electricity is the best way to go in the summer. Thanks to the owner for the recommendation. Value for money: good value for money Experience: it does save electricity throughout the day	Taobao Marketplace, a Chinese website for online shopping
Slu King Flagship Store	Green Intelligent Power Saver	Power-saving effect: for the time being, it is not yet used, it should not be disappointed! Cost-effective: there is no comparison of goods, directly trust the store Workmanship material: the first sense is good.	Taobao Marketplace, a Chinese website for online shopping
Slu King Flagship Store	Green Intelligent Power Saver	Power saving effect: very good Cost-effective: very high Appearance style: very beautiful Workmanship material: very good Experience: very good	Taobao Marketplace, a Chinese website for online shopping
Slu King Flagship Store	Green Intelligent Power Saver	The logistics is very fast, I'll come back to buy it when I have time.	Taobao Marketplace, a Chinese website for online shopping
Slu King Flagship Store	Green Intelligent Power Saver	It's a piece of junk, it raises your electric bill instead of reducing it, don't buy it!	Taobao Marketplace, a Chinese website for online shopping
Slu King Flagship Store	Green Intelligent Power Saver	I got it back and I'm using it. I hope it works.	Taobao Marketplace, a Chinese website for online shopping
Slu King Flagship Store	Green Intelligent Power Saver	I've used it and it feels fine, so grab it quickly!	Taobao Marketplace, a Chinese website for online shopping
Slu King Flagship Store	Green Intelligent Power Saver	Good quality.	Taobao Marketplace, a Chinese website for online shopping
Slu King Flagship Store	Green Intelligent Power Saver	The product has been received, looking forward to satisfactory results	Taobao Marketplace, a Chinese website for online shopping
Slu King Flagship Store	Green Intelligent Power Saver	Just as good as last time.	Taobao Marketplace, a Chinese website for online shopping
Slu King Flagship Store	Green Intelligent Power Saver	Power-saving effect: very good Cost-effective: high, and activities, buy two get one free Appearance style: a new generation of more energy-saving Workmanship material: fine quality Experience: very good, the second time back to buy.	Taobao Marketplace, a Chinese website for online shopping
Slu King Flagship Store	Green Intelligent Power Saver	Experience: I finally found it, great value for money, saves electricity, kudos 👍	Taobao Marketplace, a Chinese website for online shopping
Slu King Flagship Store	Green Intelligent Power Saver	Power saving effect: is experiencing, turn on the air conditioning with a night is okay, worth buying!	Taobao Marketplace, a Chinese website for online shopping
Slu King Flagship Store	Green Intelligent Power Saver	This is the third time I've purchased this product, it's easy to use, practical, and the power saving effect is obvious, I would recommend it.	Taobao Marketplace, a Chinese website for online shopping
Slu King Flagship Store	Green Intelligent Power Saver	Things received, before looking at the evaluation of the buy, after using to see the effect and then additional evaluation, the price is moderate, looking forward to the results	Taobao Marketplace, a Chinese website for online shopping
Slu King Flagship Store	Green Intelligent Power Saver	I've bought the stuff, I'll wait for two months and come back to review it.	Taobao Marketplace, a Chinese website for online shopping
Slu King Flagship Store	Green Intelligent Power Saver	Works great, easy to use, looking forward to getting started!	Taobao Marketplace, a Chinese website for online shopping
Slu King Flagship Store	Green Intelligent Power Saver	I've only just used it, I'll wait a while to see how it works and follow up if it's good.	Taobao Marketplace, a Chinese website for online shopping
Slu King Flagship Store	Green Intelligent Power Saver	Received the goods, looking forward to the effect	Taobao Marketplace, a Chinese website for online shopping
Slu King Flagship Store	Green Intelligent Power Saver	I used it for one night, and I feel that the power saving effect is OK.	Taobao Marketplace, a Chinese website for online shopping
Slu King Flagship Store	Green Intelligent Power Saver	Express to the home immediately used up, feel quite mini quite convenient to the	Taobao Marketplace, a Chinese website for online shopping
Slu King Flagship Store	Green Intelligent Power Saver	Photographed things received, logistics is very fast. Used for a period of time, the saver does work, save electricity than before. Praise!	Taobao Marketplace, a Chinese website for online shopping
Slu King Flagship Store	Green Intelligent Power Saver	Workmanship material: fine workmanship, original genuine, there are genuine logo look good, after using the follow-up evaluation	Taobao Marketplace, a Chinese website for online shopping
Slu King Flagship Store	Green Intelligent Power Saver	Worth buying a new product economizer expect good results logistics quickly good attitude good choice.	Taobao Marketplace, a Chinese website for online shopping
Slu King Flagship Store	Green Intelligent Power Saver	Works well! Still a bit of a power saver!	Taobao Marketplace, a Chinese website for online shopping
Slu King Flagship Store	Green Intelligent Power Saver	Power saving effect: already used it, looking forward to the effect. Fine workmanship, it should work.	Taobao Marketplace, a Chinese website for online shopping
Slu King Flagship Store	Green Intelligent Power Saver	Very [illegible] satisfied!	Taobao Marketplace, a Chinese website for online shopping
Slu King Flagship Store	Green Intelligent Power Saver	Power saving effect: just used, see the effect in two months	Taobao Marketplace, a Chinese website for online shopping

Slu King Flagship Store	Green Intelligent Power Saver	Save electricity and money (intelligent power saving system) applicable equipment: refrigerators, air conditioners, washing machines, electric fans, ... and any other sensible household appliances and equipment, are good ah 💐💐💐👏👏👏👏👏👏👏👏👏👏👏👏👍👍👍	Taobao Marketplace, a Chinese website for online shopping
Slu King Flagship Store	Green Intelligent Power Saver	Not bad.	Taobao Marketplace, a Chinese website for online shopping
Slu King Flagship Store	Green Intelligent Power Saver	Power saving effect: good Cost-effective: good Appearance and style: good Workmanship and material: good Experience: good	Taobao Marketplace, a Chinese website for online shopping
Slu King Flagship Store	Green Intelligent Power Saver	I'm used to giving good reviews, I just don't know how the results will be expected	Taobao Marketplace, a Chinese website for online shopping
Slu King Flagship Store	Green Intelligent Power Saver	Bought one, it worked, bought another.	Taobao Marketplace, a Chinese website for online shopping
Slu King Flagship Store	Green Intelligent Power Saver	It's really great, it does save electricity, bought three of them!	Taobao Marketplace, a Chinese website for online shopping
Slu King Flagship Store	Green Intelligent Power Saver	Works great and does have a power saving effect!!!!	Taobao Marketplace, a Chinese website for online shopping
Slu King Flagship Store	Green Intelligent Power Saver	It's true that the electricity bill is lower than before.	Taobao Marketplace, a Chinese website for online shopping
Slu King Flagship Store	Green Intelligent Power Saver	Very good, good review!	Taobao Marketplace, a Chinese website for online shopping
Slu King Flagship Store	Green Intelligent Power Saver	Experience: Duck	Taobao Marketplace, a Chinese website for online shopping
Slu King Flagship Store	Green Intelligent Power Saver	Just received the goods today, use a few days in the evaluation ! ! ! ! !	Taobao Marketplace, a Chinese website for online shopping
Slu King Flagship Store	Green Intelligent Power Saver	Power saving effect: good oh! Recommended by friends Cost-effective: cost-effective Appearance and style: simple and compact style Workmanship Material: durable	Taobao Marketplace, a Chinese website for online shopping
Slu King Flagship Store	Green Intelligent Power Saver	Power-saving effect: the effect is very good, can save a few dozen dollars a month	Taobao Marketplace, a Chinese website for online shopping
Slu King Flagship Store	Green Intelligent Power Saver	It works well, so feel free to buy it.	Taobao Marketplace, a Chinese website for online shopping
Slu King Flagship Store	Green Intelligent Power Saver	Looks good. Hope it saves power.	Taobao Marketplace, a Chinese website for online shopping
Slu King Flagship Store	Green Intelligent Power Saver	Baby received, it works great, hubby says it's especially good!	Taobao Marketplace, a Chinese website for online shopping
Slu King Flagship Store	Green Intelligent Power Saver	It looks good. We'll see how it goes in a month.	Taobao Marketplace, a Chinese website for online shopping
Slu King Flagship Store	Green Intelligent Power Saver	I heard my friend say that it can save electricity, so I bought one to try, and it can really save electricity, good!	Taobao Marketplace, a Chinese website for online shopping
Slu King Flagship Store	Green Intelligent Power Saver	It's very convenient and easy to use, I'll buy one for upstairs some other time	Taobao Marketplace, a Chinese website for online shopping
Slu King Flagship Store	Green Intelligent Power Saver	Goods have been received well-packaged logistics fast, I do not know how the effect of the use of post-evaluation	Taobao Marketplace, a Chinese website for online shopping
Slu King Flagship Store	Green Intelligent Power Saver	So far so good, expect it to get better later.	Taobao Marketplace, a Chinese website for online shopping
Slu King Flagship Store	Green Intelligent Power Saver	Already in use, looking forward to good results	Taobao Marketplace, a Chinese website for online shopping
Slu King Flagship Store	Green Intelligent Power Saver	I got one after hearing my coworker say this saves electricity, and as the product description says plug it in the meter is a little slower, looking forward to how much I can save next month 😀 😀 😀 .	Taobao Marketplace, a Chinese website for online shopping
Slu King Flagship Store	Green Intelligent Power Saver	The effect is not yet known, but the logistics is very fast and the delivery is very good, the good use will come to the follow-up evaluation	Taobao Marketplace, a Chinese website for online shopping
Slu King Flagship Store	Green Intelligent Power Saver	Experience: very good, a little effect, after a month to see, in the purchase of a	Taobao Marketplace, a Chinese website for online shopping
Slu King Flagship Store	Green Intelligent Power Saver	Don't know about possible savings, just used	Taobao Marketplace, a Chinese website for online shopping
Slu King Flagship Store	Green Intelligent Power Saver	Appearance and style: exquisite appearance Workmanship Material: the material looks pretty good Experience: just said just received the goods, immediately experience up	Taobao Marketplace, a Chinese website for online shopping
Slu King Flagship Store	Green Intelligent Power Saver	Packaging is very good logistics very fast service attitude is very good price affordable than the entity store is much cheaper, value for money is worth buying!	Taobao Marketplace, a Chinese website for online shopping
Slu King Flagship Store	Green Intelligent Power Saver	Saver received today, which is already bought the fourth, last year has bought a, after two with a significant effect, especially air conditioning, water heater, battery furnace most practical, effective, affordable, appearance and material is also okay! Really ~ worth having. Because it is evaluated in the garage, or home appliances with the scene to shoot a few, forgive me.	Taobao Marketplace, a Chinese website for online shopping
Slu King Flagship Store	Green Intelligent Power Saver	Experience: I feel good and recommend it if I use it well.	Taobao Marketplace, a Chinese website for online shopping
Slu King Flagship Store	Green Intelligent Power Saver	Neighbors bought and recommended this one, works well!	Taobao Marketplace, a Chinese website for online shopping
Slu King Flagship Store	Green Intelligent Power Saver	Power-saving effect: very power-saving, has been the second purchase	Taobao Marketplace, a Chinese website for online shopping
Slu King Flagship Store	Green Intelligent Power Saver	Not bad, power saving effect: looks like a genuine product, expensive has the reason, look forward to saving power	Taobao Marketplace, a Chinese website for online shopping
Slu King Flagship Store	Green Intelligent Power Saver	Power saving effect: can Price performance: very good Appearance style: can	Taobao Marketplace, a Chinese website for online shopping

Slu King Flagship Store	Green Intelligent Power Saver	Power-saving effect: very good Cost-effective: very high Appearance style: perfect Workmanship material: the quality is very good, I like it very much, is genuine, good value for money, worth buying Experience: a pleasant shopping	Taobao Marketplace, a Chinese website for online shopping
Slu King Flagship Store	Green Intelligent Power Saver	Power-saving effect: have bought a second time, really effective Cost-effective: not bad Appearance style: roughly the same A few months home electricity is indeed less, this time is a re-purchase of three, send friends two, keep one for yourself	Taobao Marketplace, a Chinese website for online shopping
Slu King Flagship Store	Green Intelligent Power Saver	Experience: just received it and used it, good	Taobao Marketplace, a Chinese website for online shopping
Slu King Flagship Store	Green Intelligent Power Saver	It's a scam, don't believe it, I've been using it for two months and it's the same tone as before, it doesn't *work*.	Taobao Marketplace, a Chinese website for online shopping
Slu King Flagship Store	Green Intelligent Power Saver	Power Saving Effect: I have purchased a couple of these before than and they do save power. I hope this one is the same.	Taobao Marketplace, a Chinese website for online shopping
Slu King Flagship Store	Green Intelligent Power Saver	Baby has been received, looking forward to the power saving effect	Taobao Marketplace, a Chinese website for online shopping
Slu King Flagship Store	Green Intelligent Power Saver	The saver was received with exquisite craftsmanship and some power savings.	Taobao Marketplace, a Chinese website for online shopping
Slu King Flagship Store	Green Intelligent Power Saver	Merchant service is very good, and go out of their way to make me moved, thank you!	Taobao Marketplace, a Chinese website for online shopping
Slu King Flagship Store	Green Intelligent Power Saver	This thing will save power is really a scam, buy back to use two or three months of power does not reduce but increase a lot, this is a scam trick, we must not buy!	Taobao Marketplace, a Chinese website for online shopping
Slu King Flagship Store	Green Intelligent Power Saver	Appearance and style: just arrived, get back can not wait to plug in, the appearance of small and exquisite, look forward to the effect of	Taobao Marketplace, a Chinese website for online shopping
Slu King Flagship Store	Green Intelligent Power Saver	Very good product, can save electricity, longer time more can see the effect, summer is coming, the air conditioning use up, praise praise praise praise praise	Taobao Marketplace, a Chinese website for online shopping
Slu King Flagship Store	Green Intelligent Power Saver	The second time I bought, pro-tested effective, thinking that immediately heat up, the use of electricity, so I bought Power-saving effect: useful Cost-effective: can Use experience: yes	Taobao Marketplace, a Chinese website for online shopping
Slu King Flagship Store	Green Intelligent Power Saver	Express delivery has been received, second time I've bought it and I'm very happy with it!	Taobao Marketplace, a Chinese website for online shopping
Slu King Flagship Store	Green Intelligent Power Saver	Power saving effect: indeed quite like, quite good, workmanship material: meticulous craftsmanship, using experience: indeed also save	Taobao Marketplace, a Chinese website for online shopping
Slu King Flagship Store	Green Intelligent Power Saver	I've been using it for a month and it doesn't work, I feel like I might as well leave it unplugged, and my electricity bill is even more than it was before, so I don't think it's a good idea.	Taobao Marketplace, a Chinese website for online shopping
Slu King Flagship Store	Green Intelligent Power Saver	Power saving effect: is using, looking forward to more power saving, worth buying! Cost-effective: better than other models, appearance style: very fine workmanship, workmanship material: material is very good! Experience: is experiencing!	Taobao Marketplace, a Chinese website for online shopping
Slu King Flagship Store	Green Intelligent Power Saver	Power-saving effect: very power-saving Cost-effective: very high Appearance style: quite good-looking Workmanship material: very good Experience: both convenient and practical	Taobao Marketplace, a Chinese website for online shopping
Slu King Flagship Store	Green Intelligent Power Saver	I do know that this thing is simple on the inside and doesn't work at all. I bought it just to do a professional test, and the result is also very obvious, this thing can not save electricity at all. Power saving effect: not useful at all Workmanship material: extremely rough	Taobao Marketplace, a Chinese website for online shopping
Slu King Flagship Store	Green Intelligent Power Saver	Just received it today and used it immediately, looking forward to	Taobao Marketplace, a Chinese website for online shopping
Slu King Flagship Store	Green Intelligent Power Saver	Just used it and will comment again after some time.	Taobao Marketplace, a Chinese website for online shopping
Slu King Flagship Store	Green Intelligent Power Saver	Merchants lied to say with three months to return and do not give back, cheating merchants such type of sellers are closed to him to be	Taobao Marketplace, a Chinese website for online shopping
Slu King Flagship Store	Green Intelligent Power Saver	Power saving effect: quite good cost-effective: very high appearance and style: good good workmanship material: exquisite experience: like	Taobao Marketplace, a Chinese website for online shopping
Slu King Flagship Store	Green Intelligent Power Saver	serviceable	Taobao Marketplace, a Chinese website for online shopping
Slu King Flagship Store	Green Intelligent Power Saver	Listened to customer service Bought three in total. Looking forward to good results with them.	Taobao Marketplace, a Chinese website for online shopping
Slu King Flagship Store	Green Intelligent Power Saver	The appearance is small and lightweight, and it doesn't take up much space, which is nice.	Taobao Marketplace, a Chinese website for online shopping
Slu King Flagship Store	Green Intelligent Power Saver	Received the goods quickly to grab it purchase friends friends this can also be very satisfied!	Taobao Marketplace, a Chinese website for online shopping
Slu King Flagship Store	Green Intelligent Power Saver	Nice stuff, I bought it after seeing someone else's say it was good	Taobao Marketplace, a Chinese website for online shopping
Slu King Flagship Store	Green Intelligent Power Saver	First time buyer, looks good quality 😄 can save money, give the merchant 5 stars!	Taobao Marketplace, a Chinese website for online shopping
Slu King Flagship Store	Green Intelligent Power Saver	Wait a month and see how much power you save.	Taobao Marketplace, a Chinese website for online shopping
Slu King Flagship Store	Green Intelligent Power Saver	Baby received, just used to feel very good, such as after the use of a follow-up comment, customer service attitude is quite good, 🈶 ask and answer.	Taobao Marketplace, a Chinese website for online shopping
Slu King Flagship Store	Green Intelligent Power Saver	Nice stuff, looks good, feels ok	Taobao Marketplace, a Chinese website for online shopping
Slu King Flagship Store	Green Intelligent Power Saver	I just received my shipment today, it's very well made and I'm looking forward to better results.	Taobao Marketplace, a Chinese website for online shopping
Slu King Flagship Store	Green Intelligent Power Saver	Just received the goods today, the appearance looks good, hope to use can save some electricity 😄 Appearance style: look good	Taobao Marketplace, a Chinese website for online shopping
Slu King Flagship Store	Green Intelligent Power Saver	Power-saving effect: quite good Used a day seems to be able to save a bit of electricity Power meter than about 2 kWh less than every day This month's household can save down dozens of electric bills Appearance style: there is anti-counterfeiting marking at first glance is the genuine good evaluation!	Taobao Marketplace, a Chinese website for online shopping

Slu King Flagship Store	Green Intelligent Power Saver	Cost-effective: high Appearance style: beautiful Workmanship material: fine Use experience: convenient	Taobao Marketplace, a Chinese website for online shopping
Slu King Flagship Store	Green Intelligent Power Saver	I'm using it and will follow up later!	Taobao Marketplace, a Chinese website for online shopping
Slu King Flagship Store	Green Intelligent Power Saver	Simple and convenient, good and inexpensive, is the first choice for home or store	Taobao Marketplace, a Chinese website for online shopping
Slu King Flagship Store	Green Intelligent Power Saver	It looks good, I bought two and got one free, I'll comment on the results after I've used it!	Taobao Marketplace, a Chinese website for online shopping
Slu King Flagship Store	Green Intelligent Power Saver	I just got it back and used it right away, looking forward to the results!	Taobao Marketplace, a Chinese website for online shopping
Slu King Flagship Store	Green Intelligent Power Saver	The power saver works quite well to save electricity	Taobao Marketplace, a Chinese website for online shopping
Slu King Flagship Store	Green Intelligent Power Saver	Received the goods today, looking forward to the power saving effect	Taobao Marketplace, a Chinese website for online shopping
Slu King Flagship Store	Green Intelligent Power Saver	It's not bad to use, quite power-saving, and the price is favorable, you can order if you need it!	Taobao Marketplace, a Chinese website for online shopping
Slu King Flagship Store	Green Intelligent Power Saver	The power saver received, plugged in and used quite good, wait a month to see the power saving situation and then follow up.	Taobao Marketplace, a Chinese website for online shopping
Slu King Flagship Store	Green Intelligent Power Saver	Power saving effect: good Cost-effective: high Appearance style: novel Workmanship material: very good	Taobao Marketplace, a Chinese website for online shopping
Slu King Flagship Store	Green Intelligent Power Saver	Expect a good performance Follow up review after using hopefully good results	Taobao Marketplace, a Chinese website for online shopping
Slu King Flagship Store	Green Intelligent Power Saver	I heard from a friend that it can save a lot of electricity, so I bought it, and it was very convenient to use it directly after it arrived.	Taobao Marketplace, a Chinese website for online shopping
Slu King Flagship Store	Green Intelligent Power Saver	Looks good, logistics is also very fast, courier boy service attitude is quite good, thank you boss!	Taobao Marketplace, a Chinese website for online shopping
Slu King Flagship Store	Green Intelligent Power Saver	Experience: Received the power saving king, wait for a month to see the effect!	Taobao Marketplace, a Chinese website for online shopping
Slu King Flagship Store	Green Intelligent Power Saver	Just used it, looking forward to the results	Taobao Marketplace, a Chinese website for online shopping
Slu King Flagship Store	Green Intelligent Power Saver	Power saving effect: the effect is very good, the use of experience: than before to save electricity costs, trustworthy	Taobao Marketplace, a Chinese website for online shopping
Slu King Flagship Store	Green Intelligent Power Saver	Power-saving, cost-effective and economical to use, it's worth having.	Taobao Marketplace, a Chinese website for online shopping
Slu King Flagship Store	Green Intelligent Power Saver	I bought one now, if the effect is good, I will continue to buy a few, logistics is very fast, good service! Good review!	Taobao Marketplace, a Chinese website for online shopping
Slu King Flagship Store	Green Intelligent Power Saver	Of course it is true that you can save electricity, but it is not possible to save 100%, but at least you can save one or two dollars compared to the usual. But already very good. A year down can save hundreds of dollars, so it is a very good choice.	Taobao Marketplace, a Chinese website for online shopping
Slu King Flagship Store	Green Intelligent Power Saver	I used it right after I bought it and it started showing right away Buy two and get one free, it's really a great deal!	Taobao Marketplace, a Chinese website for online shopping
Slu King Flagship Store	Green Intelligent Power Saver	Recommended by a coworker, it's compact and works well, so it should be fine!	Taobao Marketplace, a Chinese website for online shopping
Slu King Flagship Store	Green Intelligent Power Saver	Third purchase, bought for my old family and they all say it works great!	Taobao Marketplace, a Chinese website for online shopping
Slu King Flagship Store	Green Intelligent Power Saver	Fourth purchase, works exceptionally well, bought for all the family	Taobao Marketplace, a Chinese website for online shopping
Slu King Flagship Store	Green Intelligent Power Saver	Second time repurchase, bought two for my sister too, it really works!	Taobao Marketplace, a Chinese website for online shopping
Slu King Flagship Store	Green Intelligent Power Saver	Use it for a while and then evaluate	Taobao Marketplace, a Chinese website for online shopping
Slu King Flagship Store	Green Intelligent Power Saver	For the time being, I don't know if there is any effect, so I'll add a comment on the effect after using it for two months!	Taobao Marketplace, a Chinese website for online shopping
Slu King Flagship Store	Green Intelligent Power Saver	Haven't used it yet don't know if it saves power	Taobao Marketplace, a Chinese website for online shopping
Slu King Flagship Store	Green Intelligent Power Saver	Good packaging, good quality, after using the follow-up evaluation	Taobao Marketplace, a Chinese website for online shopping
Slu King Flagship Store	Green Intelligent Power Saver	Power-saving effect: just used, looking forward to the desired effect Cost-effective: as long as the effect is good, it is not expensive Appearance style: a small palm size, quite good-looking Workmanship material: good workmanship, and genuine logo Experience: logistics is quite fast, feel quite good	Taobao Marketplace, a Chinese website for online shopping
Slu King Flagship Store	Green Intelligent Power Saver	Small and lightweight, listened to customer service bought three pieces, already plugged in, looking forward to using the effect of	Taobao Marketplace, a Chinese website for online shopping
Slu King Flagship Store	Green Intelligent Power Saver	Fast logistics, good quality, power saving I like it	Taobao Marketplace, a Chinese website for online shopping
Slu King Flagship Store	Green Intelligent Power Saver	Power saving effect: praise Cost-effective: praise Appearance style: good, worth buying a new product economizer, has been the second purchase, will continue to pay attention to the workmanship material: praise	Taobao Marketplace, a Chinese website for online shopping
Slu King Flagship Store	Green Intelligent Power Saver	recommend	Taobao Marketplace, a Chinese website for online shopping
Slu King Flagship Store	Green Intelligent Power Saver	The exterior design is beautiful and doesn't stand out of place.	Taobao Marketplace, a Chinese website for online shopping

Slu King Flagship Store	Green Intelligent Power Saver	Goods authentic, well packaged, good results	Taobao Marketplace, a Chinese website for online shopping
Slu King Flagship Store	Green Intelligent Power Saver	Energy saving and power saving, very like Power saving effect: good Cost-effective: good Appearance style: good Workmanship material: good Use experience: good	Taobao Marketplace, a Chinese website for online shopping
Slu King Flagship Store	Green Intelligent Power Saver	Power-saving effect:, before buying a, power-saving effect is not bad, this just received the goods, not yet used, wait for the use of a follow-up evaluation.	Taobao Marketplace, a Chinese website for online shopping
Slu King Flagship Store	Green Intelligent Power Saver	Energy-saving effect is very good, the appearance is very good-looking, I should be able to it, use a period of time and then come back to comment on the complementary price. Wish the boss a prosperous business and prosperity.	Taobao Marketplace, a Chinese website for online shopping
Slu King Flagship Store	Green Intelligent Power Saver	Originally bought for home use, used for two months, the electricity bill from more than 2,00 yuan per month, down to more than 100 yuan, there is indeed a power-saving effect, this time and sold two, the unit with. Merchants also gave away a.	Taobao Marketplace, a Chinese website for online shopping
Slu King Flagship Store	Green Intelligent Power Saver	Power saving effect: not bad, something good, workmanship material: good quality	Taobao Marketplace, a Chinese website for online shopping
Slu King Flagship Store	Green Intelligent Power Saver	It's already plugged in. It doesn't take up much space. It's very convenient.	Taobao Marketplace, a Chinese website for online shopping
Slu King Flagship Store	Green Intelligent Power Saver	I've used it before it's very energy efficient, thanks for the energy savings, used electricity is so expensive!	Taobao Marketplace, a Chinese website for online shopping
Slu King Flagship Store	Green Intelligent Power Saver	Power-saving effect: effective Cost-effective: good-looking and good use Experience: used for a week, it really saves	Taobao Marketplace, a Chinese website for online shopping
Slu King Flagship Store	Green Intelligent Power Saver	Power saving effect: the effect is good, indeed less than last month's electricity bill	Taobao Marketplace, a Chinese website for online shopping
Slu King Flagship Store	Green Intelligent Power Saver	Power saving effect: just plugged in, wait for the effect after a month. Appearance and style: this is more convenient, plug and play.	Taobao Marketplace, a Chinese website for online shopping
Slu King Flagship Store	Green Intelligent Power Saver	Already installed, don't know the results yet, looking forward to it!	Taobao Marketplace, a Chinese website for online shopping
Slu King Flagship Store	Green Intelligent Power Saver	I just used it to turn on the air conditioner and saw the meter turn a little slower.	Taobao Marketplace, a Chinese website for online shopping
Slu King Flagship Store	Green Intelligent Power Saver	Comparatively speaking, it's really okay, use it for a while and then see	Taobao Marketplace, a Chinese website for online shopping
Slu King Flagship Store	Green Intelligent Power Saver	This really saves power.	Taobao Marketplace, a Chinese website for online shopping
Slu King Flagship Store	Green Intelligent Power Saver	Received the goods, is using	Taobao Marketplace, a Chinese website for online shopping
Slu King Flagship Store	Green Intelligent Power Saver	Baby has been received, the store service enthusiasm, fast logistics, I am the second purchase, this thing really save, the future friends need I will tell him also to this appliance to buy!	Taobao Marketplace, a Chinese website for online shopping
Slu King Flagship Store	Green Intelligent Power Saver	I just plugged it in. I don't know how it's gonna turn out. I hope it's really good.	Taobao Marketplace, a Chinese website for online shopping
Slu King Flagship Store	Green Intelligent Power Saver	Power-saving effect: use it and then evaluate it Cost-effective: unknown Appearance style: general Workmanship material: a penny for a penny Use experience: use it and then evaluate it	Taobao Marketplace, a Chinese website for online shopping
Slu King Flagship Store	Green Intelligent Power Saver	Power saving effect: good effect	Taobao Marketplace, a Chinese website for online shopping
Slu King Flagship Store	Green Intelligent Power Saver	I just bought it and haven't used it yet I think it should work well I'm on my second one now	Taobao Marketplace, a Chinese website for online shopping
Slu King Flagship Store	Green Intelligent Power Saver	The energy savings are great, just plug it in and go!	Taobao Marketplace, a Chinese website for online shopping
Slu King Flagship Store	Green Intelligent Power Saver	I just received it I don't know if it will work yet, it looks good	Taobao Marketplace, a Chinese website for online shopping
Slu King Flagship Store	Green Intelligent Power Saver	Cost-effective:, inexpensive Appearance and style: beautiful appearance Workmanship Material: fine workmanship	Taobao Marketplace, a Chinese website for online shopping
Slu King Flagship Store	Green Intelligent Power Saver	Good. Sell it again.	Taobao Marketplace, a Chinese website for online shopping
Slu King Flagship Store	Green Intelligent Power Saver	I saw my friend buy it to save energy, so I decided to buy 2 of them and look forward to good results in my house as well.	Taobao Marketplace, a Chinese website for online shopping
Slu King Flagship Store	Green Intelligent Power Saver	Do not be deceived by the advertising language, said no return for a refund, where there is so easy, with a month when said short time, I was afraid that she would not be refunded at that time, because there is really no effect, non let in a month, and a month past la, and said 100 days, really let people speechless, said to return for a refund is so not dry, have the effect that I have nothing to do, looking for you to return, their own things they are not clear? Still looking for a variety of reasons, but also non-provide me with the home electricity bill, there is no mistake, friends bought a direct refund, but in his home a variety of reasons and excuses, not refund, no effect of the talent to refund, right, the effect of what I also buy a few more it, said what reason, in the use of a month, we must not be fooled, don't buy, really really have no effect at all, to put it bluntly, is to deceive you to buy a rubbing of the head, can not be used to set up, and then watch Shake it and friends said, "I'm not sure if I can do this, I'm not sure if I can do this. At that time to see the jitterbug said and friends to buy together, friends in other people's homes to buy, but also no effect, people directly back, which like this, but	Taobao Marketplace, a Chinese website for online shopping
Slu King Flagship Store	Green Intelligent Power Saver	This talk can save electricity, say plugged in 100 days after the effect of power saving, that are deceptive, do not listen to them chewing well batch, do not save electricity talk full refund, all fooling people's hand guts!	Taobao Marketplace, a Chinese website for online shopping
Slu King Flagship Store	Green Intelligent Power Saver	Power saving effect: energy saving effect is very good. Cost-effective: very good Appearance style: beautiful Workmanship material: fine workmanship. Use experience: is experiencing	Taobao Marketplace, a Chinese website for online shopping
Slu King Flagship Store	Green Intelligent Power Saver	Still in use, don't know the results yet	Taobao Marketplace, a Chinese website for online shopping
Slu King Flagship Store	Green Intelligent Power Saver	This product, I plugged in the power used, even pointing to the city lights are not, the meter as usual turn, a little role has not, I hope that the enterprise a product do not cheat people!	Taobao Marketplace, a Chinese website for online shopping

Slu King Flagship Store	Green Intelligent Power Saver	Merchants are particularly good, the front that encountered a small problem, the merchants and gave me a complementary one, the service is really very good, try to use for a period of time to see, good will introduce friends to buy!	Taobao Marketplace, a Chinese website for online shopping
Slu King Flagship Store	Green Intelligent Power Saver	Logistics is very fast, things and online consistent, just use the effect to be seen after the effect.	Taobao Marketplace, a Chinese website for online shopping
Slu King Flagship Store	Green Intelligent Power Saver	Great power savings, second purchase and cost effective.	Taobao Marketplace, a Chinese website for online shopping
Slu King Flagship Store	Green Intelligent Power Saver	Genuine, buy with confidence, the price is fair, satisfied!	Taobao Marketplace, a Chinese website for online shopping
Slu King Flagship Store	Green Intelligent Power Saver	Bought 2 months later to evaluate! Really good to use, the electricity bill normal seventy or eighty, experience 2 months, only use more than forty each month, a year down to save a lot! Super awesome!	Taobao Marketplace, a Chinese website for online shopping
Slu King Flagship Store	Green Intelligent Power Saver	Express arrived, fast logistics, packaging is very good, it is worth buying!	Taobao Marketplace, a Chinese website for online shopping
Slu King Flagship Store	Green Intelligent Power Saver	Appearance and style: convenient and compact. Easy to carry. Looking forward to the real effect, will repurchase again	Taobao Marketplace, a Chinese website for online shopping
Slu King Flagship Store	Green Intelligent Power Saver	Already plugged into the plug, waiting to see the meter count speak. Good results will definitely repurchase.	Taobao Marketplace, a Chinese website for online shopping
Slu King Flagship Store	Green Intelligent Power Saver	Used for two days, recorded the electricity bill, than the usual to save a little bit some, quite good	Taobao Marketplace, a Chinese website for online shopping
Slu King Flagship Store	Green Intelligent Power Saver	Well made, nice style, easy to use!	Taobao Marketplace, a Chinese website for online shopping
Slu King Flagship Store	Green Intelligent Power Saver	Very good quality and very good value for money!	Taobao Marketplace, a Chinese website for online shopping
Slu King Flagship Store	Green Intelligent Power Saver	I'll give you feedback after using it, it works quite well and it's a good deal for the price!	Taobao Marketplace, a Chinese website for online shopping
Slu King Flagship Store	Green Intelligent Power Saver	The stuff arrived very quickly, and I'll follow up with a review when it works out later!	Taobao Marketplace, a Chinese website for online shopping
Slu King Flagship Store	Green Intelligent Power Saver	Buy one for each of the three rooms that use a lot of electricity, and see how much is left after a month.	Taobao Marketplace, a Chinese website for online shopping
Slu King Flagship Store	Green Intelligent Power Saver	Power-saving effect: just received the goods before using, quite good compact does not take up space	Taobao Marketplace, a Chinese website for online shopping
Slu King Flagship Store	Green Intelligent Power Saver	Power saving effect: used a few days Feel left a little, quite good, recommended to buy. Shipping is also very fast.	Taobao Marketplace, a Chinese website for online shopping
Slu King Flagship Store	Green Intelligent Power Saver	How does it work? You'll know when you use it.	Taobao Marketplace, a Chinese website for online shopping
Slu King Flagship Store	Green Intelligent Power Saver	I got it. I haven't used it yet.	Taobao Marketplace, a Chinese website for online shopping
Slu King Flagship Store	Green Intelligent Power Saver	Power-saving effect: just used is not yet obvious Cost-effective: affordable Appearance style: small and exquisite Workmanship material: high-quality insulation plastic Experience: use a period of time to evaluate	Taobao Marketplace, a Chinese website for online shopping
Slu King Flagship Store	Green Intelligent Power Saver	Cost-effective: good, online than down, the reputation of this is very good, the price is not expensive Workmanship material: still look very reliable, more solid than expected at this price.	Taobao Marketplace, a Chinese website for online shopping
Slu King Flagship Store	Green Intelligent Power Saver	Power saving is great and matches the store's description	Taobao Marketplace, a Chinese website for online shopping
Slu King Flagship Store	Green Intelligent Power Saver	I'm just starting to do it to see how it works later on	Taobao Marketplace, a Chinese website for online shopping
Slu King Flagship Store	Green Intelligent Power Saver	I don't know yet after using it for a few days, I won't know until next month	Taobao Marketplace, a Chinese website for online shopping
Slu King Flagship Store	Green Intelligent Power Saver	Power-saving effect: power-saving effect is good, especially compact, recommendable.	Taobao Marketplace, a Chinese website for online shopping
Slu King Flagship Store	Green Intelligent Power Saver	I heard from a friend that it is quite energy efficient. It's small and delicate, I'll buy one to try first. Next month to see the results and then follow up.	Taobao Marketplace, a Chinese website for online shopping
Slu King Flagship Store	Green Intelligent Power Saver	Baby received, as described, love it!	Taobao Marketplace, a Chinese website for online shopping
Slu King Flagship Store	Green Intelligent Power Saver	The small and handy saver was received, easy to use, and I'm glad to be able to get what I want without having to pay for unnecessary money. Thank you for the patience of the businessman's explanation, I wish your store a prosperous business!	Taobao Marketplace, a Chinese website for online shopping
Slu King Flagship Store	Green Intelligent Power Saver	I just used it, I don't know if it works yet	Taobao Marketplace, a Chinese website for online shopping
Slu King Flagship Store	Green Intelligent Power Saver	Power saving effect is not yet viewed, it should be effective, first praise. Waiting for a month to pay the electricity bill and then add comments!	Taobao Marketplace, a Chinese website for online shopping
Slu King Flagship Store	Green Intelligent Power Saver	Just received plugged in, look at the effect of a month, summer home monthly electricity bill of about 600, if it is really introduced, it will really save a lot of money it!	Taobao Marketplace, a Chinese website for online shopping
Slu King Flagship Store	Green Intelligent Power Saver	Convenient and durable, really save electricity, so practical	Taobao Marketplace, a Chinese website for online shopping
Slu King Flagship Store	Green Intelligent Power Saver	Appearance style: very like	Taobao Marketplace, a Chinese website for online shopping
Slu King Flagship Store	Green Intelligent Power Saver	very happy	Taobao Marketplace, a Chinese website for online shopping
Slu King Flagship Store	Green Intelligent Power Saver	I don't see any effect from plugging it in.	Taobao Marketplace, a Chinese website for online shopping

Slu King Flagship Store	Green Intelligent Power Saver	Power saving effect: good Cost performance: cheap Appearance style: beautiful	Taobao Marketplace, a Chinese website for online shopping
Slu King Flagship Store	Green Intelligent Power Saver	Power-saving effect: used before the effect is not bad Oh!	Taobao Marketplace, a Chinese website for online shopping
Slu King Flagship Store	Green Intelligent Power Saver	Experience: I heard from a friend that it works well! Just bought it.	Taobao Marketplace, a Chinese website for online shopping
Slu King Flagship Store	Green Intelligent Power Saver	Power saving effect: power saving Cost-effective: cheap Appearance style: good-looking Workmanship material: excellent Experience: normal	Taobao Marketplace, a Chinese website for online shopping
Slu King Flagship Store	Green Intelligent Power Saver	Power saving effect: the effect is very good, highly recommended	Taobao Marketplace, a Chinese website for online shopping
Slu King Flagship Store	Green Intelligent Power Saver	Power on, can be, power saving can be, the meter goes slower, can be, later to buy again	Taobao Marketplace, a Chinese website for online shopping
Slu King Flagship Store	Green Intelligent Power Saver	I just used it, I'll wait for two months and then I'll tell you more about it.	Taobao Marketplace, a Chinese website for online shopping
Slu King Flagship Store	Green Intelligent Power Saver	Looking good, hopefully I can save more on my electric bill next month	Taobao Marketplace, a Chinese website for online shopping
Slu King Flagship Store	Green Intelligent Power Saver	Appearance and style: compact and convenient Use experience: waiting for 2 months	Taobao Marketplace, a Chinese website for online shopping
Slu King Flagship Store	Green Intelligent Power Saver	Already the second purchase, the product is very good, the effect is obvious, worth recommending!	Taobao Marketplace, a Chinese website for online shopping
Slu King Flagship Store	Green Intelligent Power Saver	First praise, power-saving effect to be used to know, logistics fast!	Taobao Marketplace, a Chinese website for online shopping
Slu King Flagship Store	Green Intelligent Power Saver	Workmanship material: very good, worth buying good Use experience: very good, really can that	Taobao Marketplace, a Chinese website for online shopping
Slu King Flagship Store	Green Intelligent Power Saver	Bought 9 at once, received the goods in accordance with the introduction of the manual immediately used, looking forward to the effect, if good, other stores and then buy some.	Taobao Marketplace, a Chinese website for online shopping
Slu King Flagship Store	Green Intelligent Power Saver	Power saving effect: 丨非常满意，效果好 Appearance and style: ＜very small, recommended to everyone yo	Taobao Marketplace, a Chinese website for online shopping
Slu King Flagship Store	Green Intelligent Power Saver	Five stars for Oh!	Taobao Marketplace, a Chinese website for online shopping
Slu King Flagship Store	Green Intelligent Power Saver	There is no obvious effect yet, I'm looking forward to the later effect, then come back to review!	Taobao Marketplace, a Chinese website for online shopping
Slu King Flagship Store	Green Intelligent Power Saver	Power saving effect: looks very good Cost-effectiveness: good Appearance style: good-looking Workmanship material: good quality Experience: plugged in for a period of time does save electricity	Taobao Marketplace, a Chinese website for online shopping
Slu King Flagship Store	Green Intelligent Power Saver	Shipped quickly, good stuff, already bought a few to try, works great!	Taobao Marketplace, a Chinese website for online shopping
Slu King Flagship Store	Green Intelligent Power Saver	Recommended by a friend that can save electricity, buy to try, really good will come back!	Taobao Marketplace, a Chinese website for online shopping
Slu King Flagship Store	Green Intelligent Power Saver	Things received plugged in the power supply with a multimeter to try the current, it does save a little. Effective, everyone can buy	Taobao Marketplace, a Chinese website for online shopping
Slu King Flagship Store	Green Intelligent Power Saver	Just received the goods yesterday, use a month to know the effect	Taobao Marketplace, a Chinese website for online shopping
Slu King Flagship Store	Green Intelligent Power Saver	Very good, try a month is really good, really evaluation, you can rest assured that the purchase of	Taobao Marketplace, a Chinese website for online shopping
Slu King Flagship Store	Green Intelligent Power Saver	Packaging is very complete power saving especially strong a month down to save a lot of electricity	Taobao Marketplace, a Chinese website for online shopping
Slu King Flagship Store	Green Intelligent Power Saver	Cost-effective: cost-effective, a start to buy 2, with a free gift, looking forward to power-saving effect in the good Xu!	Taobao Marketplace, a Chinese website for online shopping
Slu King Flagship Store	Green Intelligent Power Saver	I bought one and it worked well, so I bought another one to see how it works.	Taobao Marketplace, a Chinese website for online shopping
Slu King Flagship Store	Green Intelligent Power Saver	The logistics were fast and the power savings were good. Worth buying	Taobao Marketplace, a Chinese website for online shopping
Slu King Flagship Store	Green Intelligent Power Saver	Easy to use plug in the power supply can be used, does not take up space, there are detailed instructions inside, looking forward to power saving effect.	Taobao Marketplace, a Chinese website for online shopping
Slu King Flagship Store	Green Intelligent Power Saver	Last time I bought one, the power saving effect is very obvious, this time without hesitation to buy three more, the merchant also gave a free one, it's really great!	Taobao Marketplace, a Chinese website for online shopping
Slu King Flagship Store	Green Intelligent Power Saver	Last time I bought and used it, it really saves electricity this time I bought 3 two and got 3 one for free The power saving effect:	Taobao Marketplace, a Chinese website for online shopping
Slu King Flagship Store	Green Intelligent Power Saver	Power-saving effect: good Cost-effective: excellent Appearance style: good Workmanship material: good	Taobao Marketplace, a Chinese website for online shopping
Slu King Flagship Store	Green Intelligent Power Saver	Received it, waiting for results, feels good!	Taobao Marketplace, a Chinese website for online shopping
Slu King Flagship Store	Green Intelligent Power Saver	I just received my shipment today, so I'll use it for now.	Taobao Marketplace, a Chinese website for online shopping
Slu King Flagship Store	Green Intelligent Power Saver	I bought one for myself, it's good, I bought another one for my mom.	Taobao Marketplace, a Chinese website for online shopping
Slu King Flagship Store	Green Intelligent Power Saver	Power saving effect: the goods just arrived, looking forward to the effect!	Taobao Marketplace, a Chinese website for online shopping

Slu King Flagship Store	Green Intelligent Power Saver	Have been using it for a few days, very good very good very good	Taobao Marketplace, a Chinese website for online shopping
Slu King Flagship Store	Green Intelligent Power Saver	Third time buyer,, bought 8 of them. It works.	Taobao Marketplace, a Chinese website for online shopping
Slu King Flagship Store	Green Intelligent Power Saver	I just started using it for a couple of months and then I'll come back to it.	Taobao Marketplace, a Chinese website for online shopping
Slu King Flagship Store	Green Intelligent Power Saver	Value for money: high Experience: good Quite good good value for money good	Taobao Marketplace, a Chinese website for online shopping
Slu King Flagship Store	Green Intelligent Power Saver	Logistics is very fast, three or four days to receive the delivery, the quality is very good, very satisfied	Taobao Marketplace, a Chinese website for online shopping
Slu King Flagship Store	Green Intelligent Power Saver	Very good, very nice, very satisfied.	Taobao Marketplace, a Chinese website for online shopping
Slu King Flagship Store	Green Intelligent Power Saver	Experience: fast logistics, good product	Taobao Marketplace, a Chinese website for online shopping
Slu King Flagship Store	Green Intelligent Power Saver	Power saving effect: actual, good	Taobao Marketplace, a Chinese website for online shopping
Slu King Flagship Store	Green Intelligent Power Saver	Has been plugged in to use, the effect of how to wait for time to test whether it is effective. Customer service attitude is still quite good, careful to help customers explain how to use the method, thank you very much! So first praise!	Taobao Marketplace, a Chinese website for online shopping
Slu King Flagship Store	Green Intelligent Power Saver	Got the stuff, looks good. Use this and see.	Taobao Marketplace, a Chinese website for online shopping
Slu King Flagship Store	Green Intelligent Power Saver	It's no use.	Taobao Marketplace, a Chinese website for online shopping
Slu King Flagship Store	Green Intelligent Power Saver	Appearance and style: the feeling is not bad, simple and beautiful, how about the effect with a month to see the results. Overall not bad!	Taobao Marketplace, a Chinese website for online shopping
Slu King Flagship Store	Green Intelligent Power Saver	The goods have been received, the courier is too powerful, really fast, the goods are very good.	Taobao Marketplace, a Chinese website for online shopping
Slu King Flagship Store	Green Intelligent Power Saver	Logistics is very fast, immediately try the power-saving effect, the appearance of the shape of the workmanship is very delicate, to be followed up later evaluation!	Taobao Marketplace, a Chinese website for online shopping
Slu King Flagship Store	Green Intelligent Power Saver	Already plugged in and looking forward to the power savings!	Taobao Marketplace, a Chinese website for online shopping
Slu King Flagship Store	Green Intelligent Power Saver	Received the item and feel the power saving effect is very obvious 👏	Taobao Marketplace, a Chinese website for online shopping
Slu King Flagship Store	Green Intelligent Power Saver	The stuff is beautifully made, I don't know exactly how effective it is, if you look at the principle, it's the right one	Taobao Marketplace, a Chinese website for online shopping
Slu King Flagship Store	Green Intelligent Power Saver	. The delivery was fast, the guy was polite and didn't mind waiting a few extra minutes for me. Packaging is complete, no damage. Appearance style: solid appearance	Taobao Marketplace, a Chinese website for online shopping
Slu King Flagship Store	Green Intelligent Power Saver	I don't know if it's working or not, I'm waiting for next month's bill to see if there's any surprises	Taobao Marketplace, a Chinese website for online shopping
Slu King Flagship Store	Green Intelligent Power Saver	It's the real deal, it's cost effective, and it's power efficient!	Taobao Marketplace, a Chinese website for online shopping
Slu King Flagship Store	Green Intelligent Power Saver	The goods have been received, I do not know whether the power-saving effect is effective, with a month to compare to know, later reply to the use of feeling. Very satisfied!	Taobao Marketplace, a Chinese website for online shopping
Slu King Flagship Store	Green Intelligent Power Saver	Goods received the quality of the very I used five years really very power-saving, thanks to the merchant business is booming, next time continue to cooperate.	Taobao Marketplace, a Chinese website for online shopping
Slu King Flagship Store	Green Intelligent Power Saver	Referred by others, will comment after a while	Taobao Marketplace, a Chinese website for online shopping
Slu King Flagship Store	Green Intelligent Power Saver	Merchants are very good, friends introduced to buy, logistics is also very fast, look forward to good results!	Taobao Marketplace, a Chinese website for online shopping
Slu King Flagship Store	Green Intelligent Power Saver	It works and is more noticeable! I added another order of two, as my house uses around 250 kWh per month!	Taobao Marketplace, a Chinese website for online shopping
Slu King Flagship Store	Green Intelligent Power Saver	It looks good, it has a genuine label, so hopefully it will achieve the savings that the	Taobao Marketplace, a Chinese website for online shopping
Slu King Flagship Store	Green Intelligent Power Saver	Power saving effect: good Worth having	Taobao Marketplace, a Chinese website for online shopping
Slu King Flagship Store	Green Intelligent Power Saver	Appearance looks good, is used for almost a month than before the old power consumption, feel a little pitiful!	Taobao Marketplace, a Chinese website for online shopping
Slu King Flagship Store	Green Intelligent Power Saver	Using the effect is quite good, indeed as the seller said, the effect is obvious! Power-saving effect: through the use of power-saving more than 20 no problem!	Taobao Marketplace, a Chinese website for online shopping
Slu King Flagship Store	Green Intelligent Power Saver	Power-saving effect: the effect is quite good Cost-effective: can Appearance style: good Workmanship material: bar Use experience: can	Taobao Marketplace, a Chinese website for online shopping
Slu King Flagship Store	Green Intelligent Power Saver	It does save a lot, I'm on my third repurchase	Taobao Marketplace, a Chinese website for online shopping
Slu King Flagship Store	Green Intelligent Power Saver	The quality is pretty good, others have used it and it's quite energy efficient!	Taobao Marketplace, a Chinese website for online shopping
Slu King Flagship Store	Green Intelligent Power Saver	Goods received, buy back I used, there are activities, buy two free a quite good, I hope to achieve good results, quality appearance no problem. Home with a lot of electricity, you can try, it should be good. Looking forward to good results. Appearance style: beautiful	Taobao Marketplace, a Chinese website for online shopping
Slu King Flagship Store	Green Intelligent Power Saver	Bought to put in the home, very suitable, the family's small children love it, the quality is also very good!	Taobao Marketplace, a Chinese website for online shopping

Slu King Flagship Store	Green Intelligent Power Saver	Works great, waiting a month before adding comments.	Taobao Marketplace, a Chinese website for online shopping
Slu King Flagship Store	Green Intelligent Power Saver	Power saving effect: usually more than 300 at the end of each month merchants, with the monthly 270 or so, very energy-saving, save about 800 per year, very happy!	Taobao Marketplace, a Chinese website for online shopping
Slu King Flagship Store	Green Intelligent Power Saver	Use a month to save 30 dollars, good, recommended, the original home refrigerator, water heater and other appliances, the monthly electricity bill of more than 300, using this 240 dollars a month is enough, so go on and on to save!	Taobao Marketplace, a Chinese website for online shopping
Slu King Flagship Store	Green Intelligent Power Saver	Power-saving effect: power-saving effect is quite good, previously bought a store, the store's electricity costs before every month need 1000 yuan, with a month after a basic month of about 1000 yuan of electricity will be enough, so shoot 2 stores in the store in the addition of a, placed in the home a	Taobao Marketplace, a Chinese website for online shopping
Slu King Flagship Store	Green Intelligent Power Saver	Baby has received the goods, small and exquisite, does not take up space at all, very good, convenient!	Taobao Marketplace, a Chinese website for online shopping
Slu King Flagship Store	Green Intelligent Power Saver	This smart power saving system is as described, it works really well, I took three of them, one on each plug, it's great, five stars!	Taobao Marketplace, a Chinese website for online shopping
Slu King Flagship Store	Green Intelligent Power Saver	Workmanship Material: Good Experience: Good Appearance Style: Good Cost-effective: Good	Taobao Marketplace, a Chinese website for online shopping
Slu King Flagship Store	Green Intelligent Power Saver	The item is as described, and it does have a slow meter!	Taobao Marketplace, a Chinese website for online shopping
Slu King Flagship Store	Green Intelligent Power Saver	Using experience: cool Cost-effective: high Power-saving effect: great Workmanship material: good	Taobao Marketplace, a Chinese website for online shopping
Slu King Flagship Store	Green Intelligent Power Saver	The stuff is pretty good ah! It's as good as the owner said it would be	Taobao Marketplace, a Chinese website for online shopping
Slu King Flagship Store	Green Intelligent Power Saver	Appearance and style: the design is quite compact, no need to wire around and around the convenience. Using experience: just got it and opened it to look quite good.	Taobao Marketplace, a Chinese website for online shopping
Slu King Flagship Store	Green Intelligent Power Saver	I've been using it for a while now, the power saving effect is very obvious, it's very lightweight and doesn't take up much space.	Taobao Marketplace, a Chinese website for online shopping
Slu King Flagship Store	Green Intelligent Power Saver	This energy-saving device is particularly good, really can save a lot of electricity, before selling one, and then buy a back!	Taobao Marketplace, a Chinese website for online shopping
Slu King Flagship Store	Green Intelligent Power Saver	Nice stuff. Worth the money.	Taobao Marketplace, a Chinese website for online shopping
Slu King Flagship Store	Green Intelligent Power Saver	Received the goods and used the same on the feeling is okay	Taobao Marketplace, a Chinese website for online shopping
Slu King Flagship Store	Green Intelligent Power Saver	The saver was received and shipped pretty fast, used it right away looks good!	Taobao Marketplace, a Chinese website for online shopping
Slu King Flagship Store	Green Intelligent Power Saver	Power saving effect: received the goods, very satisfied. Will take it from your house next time.	Taobao Marketplace, a Chinese website for online shopping
Slu King Flagship Store	Green Intelligent Power Saver	I love it. I'll buy from you again.	Taobao Marketplace, a Chinese website for online shopping
Slu King Flagship Store	Green Intelligent Power Saver	I just received the goods and immediately put it on, looking forward to the effect	Taobao Marketplace, a Chinese website for online shopping
Slu King Flagship Store	Green Intelligent Power Saver	Simply the top pyrometer sizzling wind test sister this one in a one in a one in a one in a one in a glory not a one you have have have not a one in a this this one in a one	Taobao Marketplace, a Chinese website for online shopping
Slu King Flagship Store	Green Intelligent Power Saver	Other people introduced to buy, quite good, really did save electricity, the	Taobao Marketplace, a Chinese website for online shopping
Slu King Flagship Store	Green Intelligent Power Saver	That's good. I'll have to get another one.	Taobao Marketplace, a Chinese website for online shopping
Slu King Flagship Store	Green Intelligent Power Saver	Experience: really good, use can, quality is also good, energy saving and power saving	Taobao Marketplace, a Chinese website for online shopping
Slu King Flagship Store	Green Intelligent Power Saver	Power saving effect: energy saving effect is good, five-star praise	Taobao Marketplace, a Chinese website for online shopping
Slu King Flagship Store	Green Intelligent Power Saver	Power saving effect: quite good Cost-effective: can Appearance style: can Workmanship material: can Use experience: can	Taobao Marketplace, a Chinese website for online shopping
Slu King Flagship Store	Green Intelligent Power Saver	Energy saver received, energy saving effect is good. The seller has a good service attitude, very patient to communicate the use of methods. Very good shopping experience, five stars!	Taobao Marketplace, a Chinese website for online shopping
Slu King Flagship Store	Green Intelligent Power Saver	Power-saving effect: very good Cost-effective: good Appearance and style: overall can Workmanship material: satisfied Experience: very satisfied	Taobao Marketplace, a Chinese website for online shopping
Slu King Flagship Store	Green Intelligent Power Saver	Friends introduced to, said a month to save a lot of electricity it, can save half of the electricity bill it, energy saver is very lightweight, all five-star praise.	Taobao Marketplace, a Chinese website for online shopping
Slu King Flagship Store	Green Intelligent Power Saver	Workmanship material: very good good, your smart saver has arrived at home, thank you pro	Taobao Marketplace, a Chinese website for online shopping
Slu King Flagship Store	Green Intelligent Power Saver	Feels good, power saving effect: very good	Taobao Marketplace, a Chinese website for online shopping
Slu King Flagship Store	Green Intelligent Power Saver	The meter isn't moving hahaha	Taobao Marketplace, a Chinese website for online shopping
Slu King Flagship Store	Green Intelligent Power Saver	Power-saving effect: after receiving the goods today to take home in accordance with the instructions to plug in the socket, look forward to the effect!	Taobao Marketplace, a Chinese website for online shopping
Slu King Flagship Store	Green Intelligent Power Saver	Received, a lot of baby, logistics is fast	Taobao Marketplace, a Chinese website for online shopping
Slu King Flagship Store	Green Intelligent Power Saver	Shipped very quickly arrived at the goods opened and used to observe the meter went a little slower looking forward to a month after the effect!	Taobao Marketplace, a Chinese website for online shopping

Slu King Flagship Store	Green Intelligent Power Saver	Small and compact, good value for money, reliable quality	Taobao Marketplace, a Chinese website for online shopping
Slu King Flagship Store	Green Intelligent Power Saver	I just got it, I don't know if it's any good, I'll give it a good review in a couple months.	Taobao Marketplace, a Chinese website for online shopping
Slu King Flagship Store	Green Intelligent Power Saver	Energy saver energy saving king baby has received, are using to experience this power-saving effect, especially the price is affordable, quality assurance, it is worth buying!	Taobao Marketplace, a Chinese website for online shopping
Slu King Flagship Store	Green Intelligent Power Saver	Appearance is very good, compact size, is my favorite, power-saving effect after a period of time will come to follow up the review oh!	Taobao Marketplace, a Chinese website for online shopping
Slu King Flagship Store	Green Intelligent Power Saver	Good power saving, cost-effective, good looking	Taobao Marketplace, a Chinese website for online shopping
Slu King Flagship Store	Green Intelligent Power Saver	Compact appearance, easy to use, fine workmanship is a good power-saving products	Taobao Marketplace, a Chinese website for online shopping
Slu King Flagship Store	Green Intelligent Power Saver	Appearance and style: appearance of fine workmanship, logistics is quite fast! Workmanship: fine workmanship, just received the goods with a try!	Taobao Marketplace, a Chinese website for online shopping
Slu King Flagship Store	Green Intelligent Power Saver	Received goods quite good, good quality, energy saving and power saving praise.	Taobao Marketplace, a Chinese website for online shopping
Slu King Flagship Store	Green Intelligent Power Saver	This stuff is great! The key is to save money, so I recommend it.	Taobao Marketplace, a Chinese website for online shopping
Slu King Flagship Store	Green Intelligent Power Saver	Power saving effect: good Cost-effective: high Appearance style: small and compact, does not affect the use of other appliances. I have used it, there is a power saving effect, repurchase.	Taobao Marketplace, a Chinese website for online shopping
Slu King Flagship Store	Green Intelligent Power Saver	The saver has been received, it is the second time to buy, very useful!	Taobao Marketplace, a Chinese website for online shopping
Slu King Flagship Store	Green Intelligent Power Saver	Highly recommended purchase, works really well	Taobao Marketplace, a Chinese website for online shopping
Slu King Flagship Store	Green Intelligent Power Saver	Received the item, proceeded to plug it in and the meter slowed down significantly. The effect is very good. Merchant service is also good, logistics is very fast.	Taobao Marketplace, a Chinese website for online shopping
Slu King Flagship Store	Intelligent power saver	I haven't tested it yet to see if it saves money or not	Taobao Marketplace, a Chinese website for online shopping
Slu King Flagship Store	Intelligent power saver	It looks good, I've been using it for a few days and I'm waiting for the next month to pay my electric bill.	Taobao Marketplace, a Chinese website for online shopping
Slu King Flagship Store	Intelligent power saver	This power saver promises not to achieve the effect within six months, the company package refund and replacement. Factory date: 2023, very good saver.	Taobao Marketplace, a Chinese website for online shopping
Slu King Flagship Store	Intelligent power saver	Power-saving effect: the effect is great Cost-effective: good Appearance style: high-end atmospheric upscale Workmanship material: more durable Experience: see the effect on the can	Taobao Marketplace, a Chinese website for online shopping
Slu King Flagship Store	Intelligent power saver	I don't know how it works. I've only had it for a week.	Taobao Marketplace, a Chinese website for online shopping
Slu King Flagship Store	Intelligent power saver	Power saving effect: didn't feel the electricity bill where energy saving, after using it didn't feel power saving! Use experience: bought can not wait to use, almost no power saving, people do not buy, do not be fooled! Don't be fooled! Don't be fooled! Cheat! Scam! Scam! Value for money: there would be no value for money! Fake is fake! Whoever buys it will be fooled! Consider it as paying the IQ tax! Packaging is removed and gone, and not returned (bought 2) really crying and laughing!	Taobao Marketplace, a Chinese website for online shopping
Slu King Flagship Store	Intelligent power saver	It's back and already plugged in, not sure how well it works? It looks fine on the outside.	Taobao Marketplace, a Chinese website for online shopping
Slu King Flagship Store	Intelligent power saver	It's been a month and I feel like it's still so much IQ tax to not use the electricity bill	Taobao Marketplace, a Chinese website for online shopping
Slu King Flagship Store	Intelligent power saver	I went to buy it in the mood to try it out, and after using it for a month, it was obvious that my electricity bill had dropped.	Taobao Marketplace, a Chinese website for online shopping
Slu King Flagship Store	Intelligent power saver	Appearance is beautiful, used a few days to evaluate, especially good, intend to buy another, affordable service attitude is very good 👌	Taobao Marketplace, a Chinese website for online shopping
Slu King Flagship Store	Intelligent power saver	Expected results	Taobao Marketplace, a Chinese website for online shopping
Slu King Flagship Store	Intelligent power saver	Too fake, no effect at all. Power saving effect: as good as useless.	Taobao Marketplace, a Chinese website for online shopping
Slu King Flagship Store	Intelligent power saver	It doesn't work. It's a fraud. It's a rip-off.	Taobao Marketplace, a Chinese website for online shopping
Slu King Flagship Store	Intelligent power saver	Power-saving effect: just plugged in still do not know can not save electricity Cost-effective: okay, acceptable Appearance and style: delicate and beautiful Workmanship material: okay Experience: still using	Taobao Marketplace, a Chinese website for online shopping
Slu King Flagship Store	Intelligent power saver	What do you call a power saving king, it's all a scam, I've been using it for a month and it's costing me more than a hundred bucks.	Taobao Marketplace, a Chinese website for online shopping
Slu King Flagship Store	Intelligent power saver	Power saving effect: four days of continuous use does not see the slightest power saving effect	Taobao Marketplace, a Chinese website for online shopping
Slu King Flagship Store	Intelligent power saver	Power saving effect: no power saving effect at all	Taobao Marketplace, a Chinese website for online shopping
Slu King Flagship Store	Intelligent power saver	Fast delivery, as soon as I received it, I plugged it in, I believe it should work, thanks to the seller!	Taobao Marketplace, a Chinese website for online shopping
Slu King Flagship Store	Intelligent power saver	Appearance is beautiful, small and exquisite, once the power on a flash work, a look is the quality of the bar, worth buying!	Taobao Marketplace, a Chinese website for online shopping
Slu King Flagship Store	Intelligent power saver	Power saving effect: okay Appearance style: exquisite Workmanship material: exquisite Cost-effective: very high Experience: there is a difference	Taobao Marketplace, a Chinese website for online shopping

Slu King Flagship Store	Intelligent power saver	I've been using one for over a month now and it doesn't work well it doesn't work like they said it would, it doesn't save any power at all! I bought two and got one as a gift, a total of 3 I have used it does not work!	Taobao Marketplace, a Chinese website for online shopping
Slu King Flagship Store	Intelligent power saver	Goods have been received, look out quite good-looking, very satisfied with a shopping, customer service is particularly patient!	Taobao Marketplace, a Chinese website for online shopping
Slu King Flagship Store	Intelligent power saver	I love this smart energy saver. It's great.	Taobao Marketplace, a Chinese website for online shopping
Slu King Flagship Store	Intelligent power saver	I love this smart energy saver. - Uh, that too. That's great.	Taobao Marketplace, a Chinese website for online shopping
Slu King Flagship Store	Intelligent power saver	Nothing works, I feel a bit cheated and I don't see any power saving	Taobao Marketplace, a Chinese website for online shopping
Slu King Flagship Store	Intelligent power saver	This thing is pure IQ tax. Don't buy it. It's a waste of money. It's useless.	Taobao Marketplace, a Chinese website for online shopping
Slu King Flagship Store	Intelligent power saver	Don't be fooled, it doesn't work, it's pure IQ tax, and you'll have to go through a lot of trouble to return it.	Taobao Marketplace, a Chinese website for online shopping
Slu King Flagship Store	Intelligent power saver	Received the goods and used it, do not know how effective	Taobao Marketplace, a Chinese website for online shopping
Slu King Flagship Store	Intelligent power saver	Appearance and style: not bad first use a few months to try to see	Taobao Marketplace, a Chinese website for online shopping
Slu King Flagship Store	Intelligent power saver	Saver quality workmanship is very good, affordable, has been installed waiting for the effect, praise!	Taobao Marketplace, a Chinese website for online shopping
Slu King Flagship Store	Intelligent power saver	Looking forward to the results	Taobao Marketplace, a Chinese website for online shopping
Slu King Flagship Store	Intelligent power saver	Got it, set it up, looks good, waiting for the results.	Taobao Marketplace, a Chinese website for online shopping
Slu King Flagship Store	Intelligent power saver	This product has been used for a while! It's not that it's saving power! Rather, the power consumption has doubled! Experience: the seller said that you have to use it for a few months before you get results! Drunk!	Taobao Marketplace, a Chinese website for online shopping
Slu King Flagship Store	Intelligent power saver	The goods have been received but I don't know how effective it is? I guess I'll have to wait until my electric bill comes out next month to compare!	Taobao Marketplace, a Chinese website for online shopping
Slu King Flagship Store	Intelligent power saver	I haven't had time to open it yet, I hope the product quality is good, Leman!	Taobao Marketplace, a Chinese website for online shopping
Slu King Flagship Store	Intelligent power saver	Power saving effect: no savings of one degree, and more costly	Taobao Marketplace, a Chinese website for online shopping
Slu King Flagship Store	Intelligent power saver	It's so good, this stuff is honestly so good.	Taobao Marketplace, a Chinese website for online shopping
Slu King Flagship Store	Intelligent power saver	This stuff really works so well.	Taobao Marketplace, a Chinese website for online shopping
Slu King Flagship Store	Intelligent power saver	Nice. Love it.	Taobao Marketplace, a Chinese website for online shopping
Slu King Flagship Store	Intelligent power saver	Received the goods, fine quality and compact, energy saving cost-effective!	Taobao Marketplace, a Chinese website for online shopping
Slu King Flagship Store	Intelligent power saver	Very good□very good	Taobao Marketplace, a Chinese website for online shopping
Slu King Flagship Store	Intelligent power saver	Logistics is very fast, intelligent energy saver power saving effect is very good, this energy saver is genuine, the appearance of the style is very beautiful, with my previous use of the same power saving, will recommend to others.	Taobao Marketplace, a Chinese website for online shopping
Slu King Flagship Store	Intelligent power saver	Power saving effect: good Cost-effective: good Appearance and style: good Workmanship and material: good Experience: good	Taobao Marketplace, a Chinese website for online shopping
Slu King Flagship Store	Intelligent power saver	Appearance and style: very good-looking Experience: should be good power saving effect, worth recommending!	Taobao Marketplace, a Chinese website for online shopping
Slu King Flagship Store	Intelligent power saver	Looks good, hopefully it will be effective in saving electricity. Good review.	Taobao Marketplace, a Chinese website for online shopping
Slu King Flagship Store	Intelligent power saver	Fine workmanship, praise praise praise praise	Taobao Marketplace, a Chinese website for online shopping
Slu King Flagship Store	Intelligent power saver	No power savings at all. Not very effective and expensive. Poorly evaluated poorly evaluated poorly evaluated poorly evaluated poorly evaluated poorly evaluated	Taobao Marketplace, a Chinese website for online shopping
Slu King Flagship Store	Intelligent power saver	I guess it doesn't work, but I can't resist the urge to try it, so let's give it a try first! If it works, I'll share it with you. But it's still quite pretty!	Taobao Marketplace, a Chinese website for online shopping
Slu King Flagship Store	Intelligent power saver	serviceable	Taobao Marketplace, a Chinese website for online shopping
Slu King Flagship Store	Intelligent power saver	I just got it and plugged it in. I'm looking forward to a lower electric bill next month.	Taobao Marketplace, a Chinese website for online shopping
Slu King Flagship Store	Intelligent power saver	Performance testing in progress, hopefully it works.	Taobao Marketplace, a Chinese website for online shopping
Slu King Flagship Store	Intelligent power saver	Got the smart energy saver and plugged it in, looking forward to the results, the	Taobao Marketplace, a Chinese website for online shopping
Slu King Flagship Store	Intelligent power saver	Third purchase, bought for a friend, cost effective and power saving.	Taobao Marketplace, a Chinese website for online shopping
Slu King Flagship Store	Intelligent power saver	It feels like it's working.	Taobao Marketplace, a Chinese website for online shopping

Slu King Flagship Store	Intelligent power saver	The baby has been received and is recommended for those who need it!	Taobao Marketplace, a Chinese website for online shopping
Slu King Flagship Store	Intelligent power saver	Looks good, well packaged, hope it works	Taobao Marketplace, a Chinese website for online shopping
Slu King Flagship Store	Intelligent power saver	Energy efficient, inexpensive, beautiful appearance, fine workmanship, very good！！！！！！	Taobao Marketplace, a Chinese website for online shopping
Slu King Flagship Store	Intelligent power saver	Exquisite appearance, looking forward to the energy saving effect.	Taobao Marketplace, a Chinese website for online shopping
Slu King Flagship Store	Intelligent power saver	Plugged it in, try it first, 5 stars!	Taobao Marketplace, a Chinese website for online shopping
Slu King Flagship Store	Intelligent power saver	Just got home and set it up to see if I can save some money on my electric bill.	Taobao Marketplace, a Chinese website for online shopping
Slu King Flagship Store	Intelligent power saver	There is no instruction manual, asked customer service, patient answer. First favorable comments, after use and then follow up.	Taobao Marketplace, a Chinese website for online shopping
Slu King Flagship Store	Intelligent power saver	The first time I confirmed receipt, I hope it works and I'm not paying an IQ tax!	Taobao Marketplace, a Chinese website for online shopping
Slu King Flagship Store	Intelligent power saver	Workmanship material: packaging is good, look okay, use it, see the effect in the future!	Taobao Marketplace, a Chinese website for online shopping
Slu King Flagship Store	Intelligent power saver	Merchants fast delivery, good quality, lightweight and good-looking, worth using and recommending 👍	Taobao Marketplace, a Chinese website for online shopping
Slu King Flagship Store	Intelligent power saver	favorite	Taobao Marketplace, a Chinese website for online shopping
Slu King Flagship Store	Intelligent power saver	Goods received logistics is very strong, the service is very good only to use and so on the next look!	Taobao Marketplace, a Chinese website for online shopping
Slu King Flagship Store	Intelligent power saver	I don't know how well it works, and I look forward to paying less for electricity in the future.	Taobao Marketplace, a Chinese website for online shopping
Slu King Flagship Store	Intelligent power saver	Shipping was fast! Works very well with it!	Taobao Marketplace, a Chinese website for online shopping
Slu King Flagship Store	Intelligent power saver	Exquisite and compact, as described, trustworthy!	Taobao Marketplace, a Chinese website for online shopping
Slu King Flagship Store	Intelligent power saver	Appearance and style: small and exquisite, beautiful appearance. Just received the goods today, look go feel good, after using it to follow up the power saving effect, looking forward to!	Taobao Marketplace, a Chinese website for online shopping
Slu King Flagship Store	Intelligent power saver	Beautiful appearance, have used, feel the effect is obvious	Taobao Marketplace, a Chinese website for online shopping
Slu King Flagship Store	Intelligent power saver	Power saving effect: good Cost-effective: high Appearance style: good Workmanship material: good Use experience: good	Taobao Marketplace, a Chinese website for online shopping
Slu King Flagship Store	Intelligent power saver	Shipping is fast, two days to receive the goods, looks like the quality to carry, customer service, service attitude is also good.	Taobao Marketplace, a Chinese website for online shopping
Slu King Flagship Store	Intelligent power saver	Power saving effect: very good, too cool Cost-effective: not bad Appearance style: simple and generous Workmanship material: not cheap Experience: not bad	Taobao Marketplace, a Chinese website for online shopping
Slu King Flagship Store	Intelligent power saver	Power-saving effect: very good Cost-effective: high Appearance style: beautiful Workmanship material: good Experience: has been used for three days, the feeling is OK, it is indeed energy-saving power saving.	Taobao Marketplace, a Chinese website for online shopping
Slu King Flagship Store	Intelligent power saver	Just received, indeed beautiful, do not know whether the power saving, use a few days to talk about it	Taobao Marketplace, a Chinese website for online shopping
Slu King Flagship Store	Intelligent power saver	This item is indeed nice and delicate. I am very satisfied! Also introduce, to friends.	Taobao Marketplace, a Chinese website for online shopping
Slu King Flagship Store	Intelligent power saver	It doesn't work at all. We'll see after a while.	Taobao Marketplace, a Chinese website for online shopping
Slu King Flagship Store	Intelligent power saver	Power-saving effect: I bought a very good use of this cost-effective: also cheap Appearance style: outside off very beautiful Workmanship material: material is good Experience: very power-saving	Taobao Marketplace, a Chinese website for online shopping
Slu King Flagship Store	Intelligent power saver	It really saves power, is great quality and works wonderfully.	Taobao Marketplace, a Chinese website for online shopping
Slu King Flagship Store	Intelligent power saver	Exterior style: very bad not good at all	Taobao Marketplace, a Chinese website for online shopping
Slu King Flagship Store	Intelligent power saver	There's for you to lie to me. Power-saving effect: one.	Taobao Marketplace, a Chinese website for online shopping
Slu King Flagship Store	Intelligent power saver	Intelligent energy saver received, shipping fast packaging is very good! Buy two get one free high price, the outside like a good-looking indicator light, can identify whether the power or energy saver condition is good or bad, good workmanship materials, looking forward to the effect. Customer service is good. All	Taobao Marketplace, a Chinese website for online shopping
Slu King Flagship Store	Intelligent power saver	Power saving effect: I hope it will be effective Price/performance ratio: not expensive	Taobao Marketplace, a Chinese website for online shopping
Slu King Flagship Store	Intelligent power saver	Ok.	Taobao Marketplace, a Chinese website for online shopping
Slu King Flagship Store	Intelligent power saver	I haven't used it yet. I'll comment afterward.	Taobao Marketplace, a Chinese website for online shopping
Slu King Flagship Store	Intelligent power saver	Today's baby has received, plugged in to try the lights are bright, customer service said that the power-saving appliances have to have a process of power saving, there is a 100-day trial period, after the trial period to come back to the follow-up!	Taobao Marketplace, a Chinese website for online shopping
Slu King Flagship Store	Intelligent power saver	Power saving effect: good Cost-effective: high Appearance style: good Experience: simple and good use	Taobao Marketplace, a Chinese website for online shopping

Slu King Flagship Store	Intelligent power saver	Power saving effect: baby received superb workmanship, trustworthy	Taobao Marketplace, a Chinese website for online shopping
Slu King Flagship Store	Intelligent power saver	Yes, the quality is quite good.	Taobao Marketplace, a Chinese website for online shopping
Slu King Flagship Store	Intelligent power saver	Power-saving effect: I was introduced by a friend who said that her family's original 160 more than the electricity bill is now as long as more than 80, with the mindset of trying to buy, with down really save a lot!	Taobao Marketplace, a Chinese website for online shopping
Slu King Flagship Store	Intelligent power saver	Appearance and style: appearance and design is quite good, fine workmanship, energy saving effect is good	Taobao Marketplace, a Chinese website for online shopping
Slu King Flagship Store	Intelligent power saver	Not bad very good, leftover electricity, logistics is very fast	Taobao Marketplace, a Chinese website for online shopping
Slu King Flagship Store	Intelligent power saver	The appearance of small and exquisite, the price is not expensive, received and plugged in, but I hope there is an effect!	Taobao Marketplace, a Chinese website for online shopping
Slu King Flagship Store	Intelligent power saver	The product has been received, the logistics is very fast, as described, in the end, whether there can be power-saving effect, the trial in January and then comment!	Taobao Marketplace, a Chinese website for online shopping
Slu King Flagship Store	Intelligent power saver	Effective, well made, genuine, German brand.	Taobao Marketplace, a Chinese website for online shopping
Slu King Flagship Store	Intelligent power saver	As soon as it arrived on the check, I hope to turn on the air conditioning meter runs slow, this month when you pay the bill will know the results. Good results and two more to come.	Taobao Marketplace, a Chinese website for online shopping
Slu King Flagship Store	Intelligent power saver	Good workmanship and materials, the appearance of novel blue light beautiful, pleasing to the eye	Taobao Marketplace, a Chinese website for online shopping
Slu King Flagship Store	Intelligent power saver	Good-looking, meticulous workmanship, just use a week temporarily do not know how the effect?	Taobao Marketplace, a Chinese website for online shopping
Slu King Flagship Store	Intelligent power saver	I received the goods and I'm satisfied.	Taobao Marketplace, a Chinese website for online shopping
Slu King Flagship Store	Intelligent power saver	The baby is well made and looks great, so expect the desired results!	Taobao Marketplace, a Chinese website for online shopping
Slu King Flagship Store	Intelligent power saver	Baby received, logistics is very fast, can not wait to plug in, customer service attitude is very good and patient answer, something small does not take up space, the appearance of the quality of the look is very good, but first give a favorable review, follow-up to see the effect and then follow up! Thank you for the store! Business is booming! 😀	Taobao Marketplace, a Chinese website for online shopping
Slu King Flagship Store	Intelligent power saver	Nice looking and well made. Hope it can save electricity. Experiencing.	Taobao Marketplace, a Chinese website for online shopping
Slu King Flagship Store	Intelligent power saver	Received the first time to plug in, good quality, looks like it should work, looks good, well packaged	Taobao Marketplace, a Chinese website for online shopping
Slu King Flagship Store	Intelligent power saver	Already used it, don't know how it works yet	Taobao Marketplace, a Chinese website for online shopping
Slu King Flagship Store	Intelligent power saver	I received the item, but I don't understand how it works yet.	Taobao Marketplace, a Chinese website for online shopping
Slu King Flagship Store	Intelligent power saver	Appearance looks okay, is not sure whether energy-saving, with a period of time to add, like the pro can rest assured that you can buy.	Taobao Marketplace, a Chinese website for online shopping
Slu King Flagship Store	Intelligent power saver	The boss said it was 300 degrees of electricity can be used as a result of a month bad once, the boss also let me change the big, small can not guarantee the quality, spend hundreds of dollars to buy a big one is not it, the network products are not guaranteed to buy be careful!	Taobao Marketplace, a Chinese website for online shopping
Slu King Flagship Store	Intelligent power saver	Turning on the air conditioner for a night only cost two dollars in electricity, it should be very energy efficient, not an IQ tax, it is recommended to buy. Experience: great!	Taobao Marketplace, a Chinese website for online shopping
Slu King Flagship Store	Intelligent power saver	Cost-effective, beautiful appearance, the effect is obvious, favorable comments	Taobao Marketplace, a Chinese website for online shopping
Slu King Flagship Store	Intelligent power saver	It looks ok, looking forward to what the merchant said it would do!	Taobao Marketplace, a Chinese website for online shopping
Slu King Flagship Store	Intelligent power saver	be of no use at all	Taobao Marketplace, a Chinese website for online shopping
Slu King Flagship Store	Intelligent power saver	Power saving effect: ' The product is very good and has a power saving effect	Taobao Marketplace, a Chinese website for online shopping
Slu King Flagship Store	Intelligent power saver	Value for money:' cheap and significant power savings	Taobao Marketplace, a Chinese website for online shopping
Slu King Flagship Store	Intelligent power saver	Experience: this product is worth having	Taobao Marketplace, a Chinese website for online shopping
Slu King Flagship Store	Intelligent power saver	Power saving effect: It works really well! Very power saving! This is the second one I've repurchased! Works great!	Taobao Marketplace, a Chinese website for online shopping
Slu King Flagship Store	Intelligent power saver	It works well worth getting, mainly to save money	Taobao Marketplace, a Chinese website for online shopping
Slu King Flagship Store	Intelligent power saver	It's already working. Just plug it in. It works.	Taobao Marketplace, a Chinese website for online shopping
Slu King Flagship Store	Intelligent power saver	It's good quality.	Taobao Marketplace, a Chinese website for online shopping
Slu King Flagship Store	Intelligent power saver	Very good, the baby is very high, the quality is very good, it looks like it gives a sense of high class, the price is also suitable, this online shopping is very pleasant, big praise 👍	Taobao Marketplace, a Chinese website for online shopping
Slu King Flagship Store	Intelligent power saver	The logistics is quite fast, and the goods are cost-effective and well-made.	Taobao Marketplace, a Chinese website for online shopping
Slu King Flagship Store	Intelligent power saver	It doesn't feel very effective, like I've paid an IQ tax.	Taobao Marketplace, a Chinese website for online shopping

Slu King Flagship Store	Intelligent power saver	Plugged it in as soon as I received it, we'll see how it works after a while	Taobao Marketplace, a Chinese website for online shopping
Slu King Flagship Store	Intelligent power saver	Friends recommended to buy, bought three at a time, the home store are used up!	Taobao Marketplace, a Chinese website for online shopping
Slu King Flagship Store	Intelligent power saver	Nothing works.	Taobao Marketplace, a Chinese website for online shopping
Slu King Flagship Store	Intelligent power saver	The customer didn't say a word about the return.	Taobao Marketplace, a Chinese website for online shopping
Slu King Flagship Store	Intelligent power saver	Power saving effect: good Cost performance: good Appearance style: good Workmanship material: good	Taobao Marketplace, a Chinese website for online shopping
Slu King Flagship Store	Intelligent power saver	Junk products,****, used with no effect, the promise of ineffective refunds simply do not honor, and you have been dragging, dragged to the end of the ignore. ** merchants, strongly recommended not to buy, who bought who fooled!	Taobao Marketplace, a Chinese website for online shopping
Slu King Flagship Store	Intelligent power saver	It just arrived, I don't know if it's good yet, I'll talk about it in a month.	Taobao Marketplace, a Chinese website for online shopping
Slu King Flagship Store	Intelligent power saver	I don't know, I'll comment when it's ready.	Taobao Marketplace, a Chinese website for online shopping
Slu King Flagship Store	Intelligent power saver	Nothing works after using it, and when I asked the merchant, he said it takes three months for the results to come out. False advertising, pure bullshit!	Taobao Marketplace, a Chinese website for online shopping
Slu King Flagship Store	Intelligent power saver	Don't be fooled into buying it, it's really bad	Taobao Marketplace, a Chinese website for online shopping
Slu King Flagship Store	Intelligent power saver	Cost-effective: buy over the rush to install up, home electricity costs feel saved, January electricity costs are too high, save some electricity ah. It is worth buying.	Taobao Marketplace, a Chinese website for online shopping
Slu King Flagship Store	Intelligent power saver	Second purchase, good quality and value for money	Taobao Marketplace, a Chinese website for online shopping
Slu King Flagship Store	Intelligent power saver	I've been using it for five days and it's really saving power compared to before.	Taobao Marketplace, a Chinese website for online shopping
Slu King Flagship Store	Intelligent power saver	This baby is great, good value for money and worth having!	Taobao Marketplace, a Chinese website for online shopping
Slu King Flagship Store	Intelligent power saver	The stuff arrived pretty good, compact and easy to see. See how the rear effect	Taobao Marketplace, a Chinese website for online shopping
Slu King Flagship Store	Intelligent power saver	Baby received, look very good, high power saving effect: not bad Experience: just received is experiencing	Taobao Marketplace, a Chinese website for online shopping
Slu King Flagship Store	Intelligent power saver	Logistics is very fast, the workmanship of the power saver is also very delicate, the specific effect of waiting for the use of the	Taobao Marketplace, a Chinese website for online shopping
Slu King Flagship Store	Intelligent power saver	Proper IQ tax, not good at all, it's the same money as before, no less, it seems to be more than last month	Taobao Marketplace, a Chinese website for online shopping
Slu King Flagship Store	Intelligent power saver	I just started using it, I don't know what it looks like, it's not bad, I'll come back and comment on it.	Taobao Marketplace, a Chinese website for online shopping
Slu King Flagship Store	Intelligent power saver	The product is well-packaged, excellent quality, buy two get one free, very cost-effective, waiting to save the effect, worth having, five-star praise!	Taobao Marketplace, a Chinese website for online shopping
Slu King Flagship Store	Intelligent power saver	Appearance and style: bought two and gave one as a gift don't know how the energy saving effect is already plugged in looking forward to next month's electricity bill 😉	Taobao Marketplace, a Chinese website for online shopping
Slu King Flagship Store	Intelligent power saver	I don't know if it works or not, just plug it in for now. I'm used to good reviews.	Taobao Marketplace, a Chinese website for online shopping
Slu King Flagship Store	Intelligent power saver	That's the way it is after the sale. No one cares anymore.	Taobao Marketplace, a Chinese website for online shopping
Slu King Flagship Store	Intelligent power saver	Let's not talk about the experience of using it, it's this logistics, he delivers the latest every time, I see it has been dispatched in the morning, and then it's delivered in the evening, I don't call when it's delivered, I don't send a message! Poor 👎	Taobao Marketplace, a Chinese website for online shopping
Slu King Flagship Store	Intelligent power saver	Throttle Saver King is a scam product.	Taobao Marketplace, a Chinese website for online shopping
Slu King Flagship Store	Intelligent power saver	Power saving effect: received a period of time, the use of the effect is very good, the power is really less than last month	Taobao Marketplace, a Chinese website for online shopping
Slu King Flagship Store	Intelligent power saver	The exterior design is beautiful and the logistics are fast.	Taobao Marketplace, a Chinese website for online shopping
Slu King Flagship Store	Intelligent power saver	The goods have been received, wait to try in the evaluation.	Taobao Marketplace, a Chinese website for online shopping
Slu King Flagship Store	Intelligent power saver	After months of testing, I'm responsible for telling those who haven't bought it yet, don't buy it, it doesn't do a thing and it's a waste of money.	Taobao Marketplace, a Chinese website for online shopping
Slu King Flagship Store	Intelligent power saver	It's all a lie.	Taobao Marketplace, a Chinese website for online shopping
Slu King Flagship Store	Intelligent power saver	ineffective	Taobao Marketplace, a Chinese website for online shopping
Slu King Flagship Store	Intelligent power saver	Good workmanship, quality is guaranteed, effective, very satisfied	Taobao Marketplace, a Chinese website for online shopping
Slu King Flagship Store	Intelligent power saver	I'm using it. I won't know until I've had it for a month.	Taobao Marketplace, a Chinese website for online shopping
Slu King Flagship Store	Intelligent power saver	I've got it. I won't know until I use it. I'll check the meter in a few days.	Taobao Marketplace, a Chinese website for online shopping

Slu King Flagship Store	Intelligent power saver	are obscurantist this conscience to speak, does not work does not work, do not let more people on the file, shoot with caution no scientific basis, * with no	Taobao Marketplace, a Chinese website for online shopping
Slu King Flagship Store	Intelligent power saver	This is totally fake, I've been using it for a month or two and it doesn't help at all	Taobao Marketplace, a Chinese website for online shopping
Slu King Flagship Store	Intelligent power saver	high color value	Taobao Marketplace, a Chinese website for online shopping
Slu King Flagship Store	Intelligent power saver	Baby quality is very good, the appearance of the design is very delicate, especially satisfied! Back in to buy a few for my mom.	Taobao Marketplace, a Chinese website for online shopping
Slu King Flagship Store	Intelligent power saver	The quality of the stuff is very good Oh and the description of the same good and sturdy price concessions cost-effective recommended to buy look forward to the effect of using a good in the buy!	Taobao Marketplace, a Chinese website for online shopping
Slu King Flagship Store	Intelligent power saver	The logistics is fast, the package is complete, and I look forward to the energy-saving effect appearing.	Taobao Marketplace, a Chinese website for online shopping
Slu King Flagship Store	Intelligent power saver	Power saving effect: good	Taobao Marketplace, a Chinese website for online shopping
Slu King Flagship Store	Intelligent power saver	Appearance looks OK, the quality is also OK, is only used, do not know if there is no effect yet!	Taobao Marketplace, a Chinese website for online shopping
Slu King Flagship Store	Intelligent power saver	Just used it, should be fine. Looking forward to the results.	Taobao Marketplace, a Chinese website for online shopping
Slu King Flagship Store	Intelligent power saver	It's not working, it's been over a month and I don't see a change in the meter count.	Taobao Marketplace, a Chinese website for online shopping
Slu King Flagship Store	Intelligent power saver	Just received the baby today, open a very delicate, look at the quality is okay, I hope to the businessman said can save electricity, after a while to come back to evaluate	Taobao Marketplace, a Chinese website for online shopping
Slu King Flagship Store	Intelligent power saver	Power saving effect: it does save power	Taobao Marketplace, a Chinese website for online shopping
Slu King Flagship Store	Intelligent power saver	Experience: began to try, as if there is a power-saving effect, but also use a period of time to try to wait!	Taobao Marketplace, a Chinese website for online shopping
Slu King Flagship Store	Intelligent power saver	The hand knife feels pretty good. Packaging material. Consistent with the description. Let's follow up after the experience.	Taobao Marketplace, a Chinese website for online shopping
Slu King Flagship Store	Intelligent power saver	Workmanship material: kung fu Use experience: real and effective	Taobao Marketplace, a Chinese website for online shopping
Slu King Flagship Store	Intelligent power saver	I can't wait to use it. They say it saves power. Try it.	Taobao Marketplace, a Chinese website for online shopping
Slu King Flagship Store	Intelligent power saver	Looks good, haven't seen any results yet, going to use it for a while to see, hope it works haha	Taobao Marketplace, a Chinese website for online shopping
Slu King Flagship Store	Intelligent power saver	It doesn't work. Don't be fooled.	Taobao Marketplace, a Chinese website for online shopping
Slu King Flagship Store	Intelligent power saver	The quality is very good, the service attitude is first-class, the product is well-made and trustworthy!	Taobao Marketplace, a Chinese website for online shopping
Slu King Flagship Store	Intelligent power saver	Very fine workmanship, better than expected, see the comments are good, try it first, I hope there is an effect!	Taobao Marketplace, a Chinese website for online shopping
Slu King Flagship Store	Intelligent power saver	IQ tax! I've been using it for a week now, using the National Grid's energy analysis and plugging it in, and I can't believe my bill's gone up. See for yourself, you're paying the IQ tax again.	Taobao Marketplace, a Chinese website for online shopping
Slu King Flagship Store	Intelligent power saver	Doesn't work at all. Used it for a month, normal use. Electricity bill is not less but more. Beyond the return period, don't trust these things are not reliable	Taobao Marketplace, a Chinese website for online shopping
Slu King Flagship Store	Intelligent power saver	The product quality is very good and works very well	Taobao Marketplace, a Chinese website for online shopping
Slu King Flagship Store	Intelligent power saver	I don't know about the power saving effect, I'll evaluate it after using it for a while	Taobao Marketplace, a Chinese website for online shopping
Slu King Flagship Store	Intelligent power saver	Received, the quality is not bad delivery is also timely, use two days and then follow up!	Taobao Marketplace, a Chinese website for online shopping
Slu King Flagship Store	Intelligent power saver	Cost-effective: the goods received, very good, good-looking	Taobao Marketplace, a Chinese website for online shopping
Slu King Flagship Store	Intelligent power saver	Power-saving effect: look forward to Cost-effective: okay Appearance style: very beautiful Workmanship material: not bad Experience: look forward to the effect, good use of follow-up comments!	Taobao Marketplace, a Chinese website for online shopping
Slu King Flagship Store	Intelligent power saver	666	Taobao Marketplace, a Chinese website for online shopping
Slu King Flagship Store	Intelligent power saver	It doesn't work at all, it doesn't work at all, it's a scam, don't fall for it.	Taobao Marketplace, a Chinese website for online shopping
Slu King Flagship Store	Intelligent power saver	Already used it, if it works well, recommend it to everyone!	Taobao Marketplace, a Chinese website for online shopping
Slu King Flagship Store	Intelligent power saver	No effect at all, is a scam please do not buy!	Taobao Marketplace, a Chinese website for online shopping
Slu King Flagship Store	Intelligent power saver	The goods have been received, well made, beautiful appearance, the price is not high, the	Taobao Marketplace, a Chinese website for online shopping
Slu King Flagship Store	Intelligent power saver	Already used it, looking forward to good use	Taobao Marketplace, a Chinese website for online shopping
Slu King Flagship Store	Intelligent power saver	It feels effective.	Taobao Marketplace, a Chinese website for online shopping

Slu King Flagship Store	Intelligent power saver	Shipped quickly, very nice looking design and great material. Continue to follow up after use.	Taobao Marketplace, a Chinese website for online shopping
Slu King Flagship Store	Intelligent power saver	It feels like it's working a little bit, keep watching the use.	Taobao Marketplace, a Chinese website for online shopping
Slu King Flagship Store	Intelligent power saver	Power-saving effect: used for a period of time, I feel that the time to buy electricity is a few days later than the last time, it should be some effect, and then observe, time is the only way to test the truth.	Taobao Marketplace, a Chinese website for online shopping
Slu King Flagship Store	Intelligent power saver	Just received the goods, although not yet used but feel pretty good if you use a good, will recommend to friends to buy!	Taobao Marketplace, a Chinese website for online shopping
Slu King Flagship Store	Intelligent power saver	In use It will take some time to know if it works well or not. Appearance to is good, the green band light on the unit can be used as a ground light at night.	Taobao Marketplace, a Chinese website for online shopping
Slu King Flagship Store	Intelligent power saver	I just got it today and I'm just using it. I'll see how much electricity it uses next month and then I'll make a review.	Taobao Marketplace, a Chinese website for online shopping
Slu King Flagship Store	Intelligent power saver	Second purchase, works fine, fast shipping, good quality.	Taobao Marketplace, a Chinese website for online shopping
Slu King Flagship Store	Intelligent power saver	No effect, the plastic shell bread a small component out to fool people, three products, fooled!	Taobao Marketplace, a Chinese website for online shopping
Slu King Flagship Store	Intelligent power saver	Just received.	Taobao Marketplace, a Chinese website for online shopping
Slu King Flagship Store	Intelligent power saver	The saver was received, thanks to the seller shipping really fast. Can let us experience early. The effect will wait for a follow-up review and repurchase after use.	Taobao Marketplace, a Chinese website for online shopping
Slu King Flagship Store	Intelligent power saver	It works really well and saves kings Power savings: very good 👍	Taobao Marketplace, a Chinese website for online shopping
Slu King Flagship Store	Intelligent power saver	Goods received, the quality is very good, cost-effective, the style is also very beautiful, strong power-saving effect	Taobao Marketplace, a Chinese website for online shopping
Slu King Flagship Store	Intelligent power saver	The product is easy to use, safe, effective, and better when placed on lines that use a lot of electricity. I will introduce friends to buy.	Taobao Marketplace, a Chinese website for online shopping
Slu King Flagship Store	Intelligent power saver	Experience: logistics, packaging, product quality, really quite wow Recommended to buy!	Taobao Marketplace, a Chinese website for online shopping
Slu King Flagship Store	Intelligent power saver	Good review!	Taobao Marketplace, a Chinese website for online shopping
Slu King Flagship Store	Intelligent power saver	expectation	Taobao Marketplace, a Chinese website for online shopping
Slu King Flagship Store	Intelligent power saver	Exact results to be seen and commented on later?	Taobao Marketplace, a Chinese website for online shopping
Slu King Flagship Store	Intelligent power saver	Try it before you judge.	Taobao Marketplace, a Chinese website for online shopping
Slu King Flagship Store	Intelligent power saver	The credit has been received, I'll evaluate it later.	Taobao Marketplace, a Chinese website for online shopping
Slu King Flagship Store	Intelligent power saver	Pretty good, good price, looking forward to good results	Taobao Marketplace, a Chinese website for online shopping
Slu King Flagship Store	Intelligent power saver	Pretty good.	Taobao Marketplace, a Chinese website for online shopping
Slu King Flagship Store	Intelligent power saver	Value for money: 6	Taobao Marketplace, a Chinese website for online shopping
Slu King Flagship Store	Intelligent power saver	The goods have been received! Logistics is fast! Good service attitude! Packaging is very delicate! To be used after the follow-up evaluation!	Taobao Marketplace, a Chinese website for online shopping
Slu King Flagship Store	Intelligent power saver	Appearance looks very good, power-saving effect to know after use, after a period of time to follow up!	Taobao Marketplace, a Chinese website for online shopping
Slu King Flagship Store	Intelligent power saver	Power-saving effect: just received the evaluation after use, logistics fast! Workmanship: good workmanship	Taobao Marketplace, a Chinese website for online shopping
Slu King Flagship Store	Intelligent power saver	This product is a fake, it doesn't save any power at all.	Taobao Marketplace, a Chinese website for online shopping
Slu King Flagship Store	Intelligent power saver	Power saving effect: practical, used is able to node point. Ready to buy another one again to use.	Taobao Marketplace, a Chinese website for online shopping
Slu King Flagship Store	Intelligent power saver	Appearance and style: the appearance of the power saver is good-looking, the express delivery is very fast, just used for two days	Taobao Marketplace, a Chinese website for online shopping
Slu King Flagship Store	Intelligent power saver	Recommended it to a friend, ready to buy a few more for the hotel	Taobao Marketplace, a Chinese website for online shopping
Slu King Flagship Store	Intelligent power saver	Saving electricity is impossible, I had 16 extra kWh in the first month, what's going on? What's going on? Or is it just bad usage?	Taobao Marketplace, a Chinese website for online shopping
Slu King Flagship Store	Intelligent power saver	It doesn't work, it's a scam, you have to return it and it's gone.	Taobao Marketplace, a Chinese website for online shopping
Slu King Flagship Store	Intelligent power saver	Plugged in, have not yet observed the effect, wait for the effect in the future in the follow-up evaluation	Taobao Marketplace, a Chinese website for online shopping
Slu King Flagship Store	Intelligent power saver	Power saving effect: in use, looking forward to. Cost-effective: high Appearance style: high-end atmosphere upscale Workmanship material: good Use experience: in trial	Taobao Marketplace, a Chinese website for online shopping
Slu King Flagship Store	Intelligent power saver	The quality is quite good, use a month to try to add.	Taobao Marketplace, a Chinese website for online shopping

Slu King Flagship Store	Intelligent power saver	Workmanship material: beautiful and nice	Taobao Marketplace, a Chinese website for online shopping
Slu King Flagship Store	Intelligent power saver	It's a piece of junk! I've been using it for a month and not only did I not save any electricity, I paid a few dollars more than I did last month! Think about it, it's plugged in! How can it not cost electricity?	Taobao Marketplace, a Chinese website for online shopping
Slu King Flagship Store	Intelligent power saver	Small and compact, well made, doesn't take up much space. Looking forward to good results. Five-star praise. After the follow-up evaluation.	Taobao Marketplace, a Chinese website for online shopping
Slu King Flagship Store	Intelligent power saver	Power-saving effect: previously bought a good effect, this model is different believe that there will be better results, if good then into a look forward to first praise Appearance style: beautiful Workmanship material: better than before	Taobao Marketplace, a Chinese website for online shopping
Slu King Flagship Store	Intelligent power saver	Power-saving effect: not yet tried Appearance style: looks good Workmanship material: okay Experience: used yesterday, have not yet seen the results	Taobao Marketplace, a Chinese website for online shopping
Slu King Flagship Store	Intelligent power saver	I don't know if it saves electricity until it's time to pay the bill.	Taobao Marketplace, a Chinese website for online shopping
Slu King Flagship Store	Intelligent power saver	Workmanship material: the stuff is great	Taobao Marketplace, a Chinese website for online shopping
Slu King Flagship Store	Intelligent power saver	This is a great nodesetter, with significant power savings, highly recommended!	Taobao Marketplace, a Chinese website for online shopping
Slu King Flagship Store	Intelligent power saver	Evaluation after use	Taobao Marketplace, a Chinese website for online shopping
Slu King Flagship Store	Intelligent power saver	Compact and beautiful, just used it, I do not know if it can save electricity, looking forward to	Taobao Marketplace, a Chinese website for online shopping
Slu King Flagship Store	Intelligent power saver	The product is very formal, very good workmanship, the use of two days to evaluate, compared to a look, it is true that the daily power consumption has decreased, the long-term use of the effect must be very good.	Taobao Marketplace, a Chinese website for online shopping
Slu King Flagship Store	Intelligent power saver	After using it for two days and checking the power consumption has indeed dropped, I am sure it will save a lot of electricity in the long run.	Taobao Marketplace, a Chinese website for online shopping
Slu King Flagship Store	Intelligent power saver	Exquisite workmanship, seems ideal, workmanship at a glance particularly agreeable, because just received, the effect of how to use after a period of time and then make an evaluation	Taobao Marketplace, a Chinese website for online shopping
Slu King Flagship Store	Intelligent power saver	I received it today and plugged it in immediately, looks good, hope it works well ☺	Taobao Marketplace, a Chinese website for online shopping
Slu King Flagship Store	Intelligent power saver	Cost-effective: this energy-saving product is very good, really energy-saving and power saving, it is worth recommending to everyone!	Taobao Marketplace, a Chinese website for online shopping
Slu King Flagship Store	Intelligent power saver	inconspicuous	Taobao Marketplace, a Chinese website for online shopping
Slu King Flagship Store	Intelligent power saver	I just got it and plugged it in. They say it saves electricity. I'll give it a try.	Taobao Marketplace, a Chinese website for online shopping
Slu King Flagship Store	Intelligent power saver	Power-saving effect: not yet felt, should be good Appearance and style: beautiful	Taobao Marketplace, a Chinese website for online shopping
Slu King Flagship Store	Intelligent power saver	Baby received, whether it is useful, after use to evaluate, now being used, I believe effective.	Taobao Marketplace, a Chinese website for online shopping
Slu King Flagship Store	Intelligent power saver	Looking forward to the results	Taobao Marketplace, a Chinese website for online shopping
Slu King Flagship Store	Intelligent power saver	I hope it works.	Taobao Marketplace, a Chinese website for online shopping
Slu King Flagship Store	Intelligent power saver	I just installed it. I don't know how it works.	Taobao Marketplace, a Chinese website for online shopping
Slu King Flagship Store	Intelligent power saver	Appearance and style: good-looking, power-saving effect do not know the use of a few months to evaluate, just received today!	Taobao Marketplace, a Chinese website for online shopping
Slu King Flagship Store	Intelligent power saver	IQ tax, don't buy it, I bought 2 of them, I've been using them for 3 months and not only is my electricity bill not lower, it's even higher!	Taobao Marketplace, a Chinese website for online shopping
Slu King Flagship Store	Intelligent power saver	Being used! Quietly watching the post effects	Taobao Marketplace, a Chinese website for online shopping
Slu King Flagship Store	Intelligent power saver	I'm using it and waiting to see the later results...	Taobao Marketplace, a Chinese website for online shopping
Slu King Flagship Store	Intelligent power saver	I just started using it and I don't know how well it works, but I'll comment after I use it.	Taobao Marketplace, a Chinese website for online shopping
Slu King Flagship Store	Intelligent power saver	Don't know the effect yet	Taobao Marketplace, a Chinese website for online shopping
Slu King Flagship Store	Intelligent power saver	This thing is bought back, I do not know if it saves electricity, you say it is the optimization of the circuit or what, use a period of time after the follow-up evaluation	Taobao Marketplace, a Chinese website for online shopping
Slu King Flagship Store	Intelligent power saver	It's a rip-off. It doesn't work. It's just a display. Not recommended.	Taobao Marketplace, a Chinese website for online shopping
Slu King Flagship Store	Intelligent power saver	Workmanship material: forehead	Taobao Marketplace, a Chinese website for online shopping
Slu King Flagship Store	Intelligent power saver	Use it to see how it works. Bought it for someone else.	Taobao Marketplace, a Chinese website for online shopping
Slu King Flagship Store	Intelligent power saver	very good	Taobao Marketplace, a Chinese website for online shopping
Slu King Flagship Store	Intelligent power saver	The logistics company is very fast, the power saving effect is good, and the business service is also very satisfactory	Taobao Marketplace, a Chinese website for online shopping

Slu King Flagship Store	Intelligent power saver	Plugged it in for a couple days and it does save power	Taobao Marketplace, a Chinese website for online shopping
Slu King Flagship Store	Intelligent power saver	Good seller, good product, works very well Saves money and electricity	Taobao Marketplace, a Chinese website for online shopping
Slu King Flagship Store	Intelligent power saver	It's in use and will follow up with a review.	Taobao Marketplace, a Chinese website for online shopping
Slu King Flagship Store	Intelligent power saver	It's not working.	Taobao Marketplace, a Chinese website for online shopping
Slu King Flagship Store	Intelligent power saver	Cost-effective, stylish and beautiful appearance, fine workmanship	Taobao Marketplace, a Chinese website for online shopping
Slu King Flagship Store	Intelligent power saver	The packaging is complete and the quality is quite good. The logistics is very fast, looking forward to the energy-saving effect!	Taobao Marketplace, a Chinese website for online shopping
Slu King Flagship Store	Intelligent power saver	Appearance and style: very beautiful, and the quality is also the upper level 👍👍👍👍 Workmanship and material: speak 👍👍👍👍👍👍	Taobao Marketplace, a Chinese website for online shopping
Slu King Flagship Store	Intelligent power saver	Has received, the appearance is very beautiful, easy to use, the healing effect how to use after the evaluation.	Taobao Marketplace, a Chinese website for online shopping
Slu King Flagship Store	Intelligent power saver	Power Saver King received, just used it hope it works ❗	Taobao Marketplace, a Chinese website for online shopping
Slu King Flagship Store	Intelligent power saver	Not bad, beautiful and energy-saving	Taobao Marketplace, a Chinese website for online shopping
Slu King Flagship Store	Intelligent power saver	Good appearance and style, cost-effective, satisfactory service, fast logistics, great!	Taobao Marketplace, a Chinese website for online shopping
Slu King Flagship Store	Intelligent power saver	Just started using it to see the results	Taobao Marketplace, a Chinese website for online shopping
Slu King Flagship Store	Intelligent power saver	Get your hands immediately inserted into the kitchen outlet, the kitchen refrigerator and battery stove more power consumption, temporarily do not know how the power-saving effect, if it is good, I will get a few, summer is coming, the air conditioning is the most power-consuming!	Taobao Marketplace, a Chinese website for online shopping
Slu King Flagship Store	Intelligent power saver	It's really good. It's a power saver.	Taobao Marketplace, a Chinese website for online shopping
Slu King Flagship Store	Intelligent power saver	It's really good.	Taobao Marketplace, a Chinese website for online shopping
Slu King Flagship Store	Intelligent power saver	Received today looked good very good, with a month to see if there is no effect, look at the positive feedback buy should have effect! Follow-up in the follow-up. Appearance and style: the same as the picture.	Taobao Marketplace, a Chinese website for online shopping
Slu King Flagship Store	Intelligent power saver	May come to favorable reviews are so fooled to get, now cashback new tricks are cheating, I'm not bad these few dollars I don't want, remind everyone don't be fooled.	Taobao Marketplace, a Chinese website for online shopping
Slu King Flagship Store	Intelligent power saver	The power saver has received the goods, not too big and not too small is very beautiful, praise!	Taobao Marketplace, a Chinese website for online shopping
Slu King Flagship Store	Intelligent power saver	It's okay. It's okay!	Taobao Marketplace, a Chinese website for online shopping
Slu King Flagship Store	Intelligent power saver	Logistics is quite fast, immediately after receiving the goods to open the appearance are very good, with the effect is really obvious.	Taobao Marketplace, a Chinese website for online shopping
Slu King Flagship Store	Intelligent power saver	I am a professional electrician, this power saver is really useful, the real use of electronic waves automatically connected to track the decomposition of ionized electrons, in situ compensation, reduce the effective power loss and waste, stabilize the voltage to achieve the power connection, the overall is still quite good, it is worthwhile to buy, the next time there is a need to purchase will also be	Taobao Marketplace, a Chinese website for online shopping
Slu King Flagship Store	Intelligent power saver	Received it and started using it looking forward to the results	Taobao Marketplace, a Chinese website for online shopping
Slu King Flagship Store	Intelligent power saver	Workmanship is very fine, just received the goods on the plug, the quality should be able to, if the effect is good will also buy a second time. Power-saving effect: not yet used Appearance style: very good Workmanship material: exquisite	Taobao Marketplace, a Chinese website for online shopping
Slu King Flagship Store	Intelligent power saver	I've heard from friends that this is a good power saver, good value for money, and looks great, so it's worth looking forward to	Taobao Marketplace, a Chinese website for online shopping
Slu King Flagship Store	Intelligent power saver	Workmanship material: logistics is very fast, the shape is beautiful and generous, used two days did not feel obvious, and then use a period of time to see the effect, good to use and then get it!	Taobao Marketplace, a Chinese website for online shopping
Slu King Flagship Store	Intelligent power saver	It works. It works very well. It saves money and electricity.	Taobao Marketplace, a Chinese website for online shopping
Slu King Flagship Store	Intelligent power saver	Absolutely good stuff, with particularly convenient and simple, directly into the home plug board on it. Now look at the meter really than before turn a lot slower, I now still open the air conditioning as usual to watch TV, and the original did not change at all, but the power really less. This product effect	Taobao Marketplace, a Chinese website for online shopping
Slu King Flagship Store	Intelligent power saver	Used before, works pretty well first month electricity bill $730 down to $510, second month $482, bought two more and got one free, very well made as described	Taobao Marketplace, a Chinese website for online shopping
Slu King Flagship Store	Intelligent power saver	Office with the air heating, two months to save more than five hundred of the electricity bill, this is the effect of a, and bought two send a are plugged in!	Taobao Marketplace, a Chinese website for online shopping
Slu King Flagship Store	Intelligent power saver	I don't know how it works yet, but I'm trying to find out.	Taobao Marketplace, a Chinese website for online shopping
Slu King Flagship Store	Intelligent power saver	Goods arrived broken waiting for a replacement!	Taobao Marketplace, a Chinese website for online shopping
Slu King Flagship Store	Intelligent power saver	Received, power saving effect: plugged in the effect Appearance style: beautiful atmosphere	Taobao Marketplace, a Chinese website for online shopping
Slu King Flagship Store	Intelligent power saver	The value is very high, I got it back and used it straight away, as for how effective it is, I'll comment at the end of the month.	Taobao Marketplace, a Chinese website for online shopping
Slu King Flagship Store	Intelligent power saver	Power saving effect: good waiting for power saving effect customer service good next time to buy again	Taobao Marketplace, a Chinese website for online shopping

Slu King Flagship Store	Intelligent power saver	Power saving effect: good effect Cost-effective: cheap Appearance style: good-looking Workmanship material: very good	Taobao Marketplace, a Chinese website for online shopping
Slu King Flagship Store	Intelligent power saver	Received very well packaged, the material is very beautiful, plugged in to save a lot of electricity, scientific energy saving, it is worth buying with confidence.	Taobao Marketplace, a Chinese website for online shopping
Slu King Flagship Store	Intelligent power saver	Received the goods look forward to energy saving effect seller good service	Taobao Marketplace, a Chinese website for online shopping
Slu King Flagship Store	Intelligent power saver	Expect results, hopefully it's not an IQ tax	Taobao Marketplace, a Chinese website for online shopping
Slu King Flagship Store	Intelligent power saver	Fast shipping, packaging is very good, query, is genuine, intelligent power saver with a few days, use it to monitor the voltage is particularly good. My home because of the installation of solar power on the Internet installed is a three-phase meter, the original house appliances connected to 2-phase power, see the power saver shows the voltage is sometimes high to more than 240 volts, with this, the effect is very good, a lot of normal, very satisfied with a shopping, there is a need to continue to visit!	Taobao Marketplace, a Chinese website for online shopping
Slu King Flagship Store	Intelligent power saver	Merchants ship quickly, friends introduced, said to save a lot, I also try to see if it is not the case, so far the quality of the product is very good!	Taobao Marketplace, a Chinese website for online shopping
Slu King Flagship Store	Intelligent power saver	I bought it for my dad. I'll comment after I use it.	Taobao Marketplace, a Chinese website for online shopping
Slu King Flagship Store	Intelligent power saver	Good workmanship, power saving device, good customer service, worth buying	Taobao Marketplace, a Chinese website for online shopping
Slu King Flagship Store	Intelligent power saver	. Saver received, the quality of workmanship did not let me disappoint! With the air conditioning TV can see the change in the above figures, winter home electricity is more powerful, energy saver can save a lot of electricity, look forward to the effect!	Taobao Marketplace, a Chinese website for online shopping
Slu King Flagship Store	Intelligent power saver	Saving appliances are really good, or relatives introduced, if you know this device earlier, 1 year down really can save a lot of money, thank you for the seller's heart fine explanation!	Taobao Marketplace, a Chinese website for online shopping
Slu King Flagship Store	Intelligent power saver	Fast shipping, the quality is quite good, and the seller described the same, very satisfied as always good quality, very like a product, very great! o(*￣︶￣*)o	Taobao Marketplace, a Chinese website for online shopping
Slu King Flagship Store	Intelligent power saver	Power saving effect is good, the electricity bill is not as expensive as before. Cost-effective moderate, this price to buy such an effective power saver, quite good. The appearance of small, exquisite, portable, does not take up space.	Taobao Marketplace, a Chinese website for online shopping
Slu King Flagship Store	Intelligent power saver	Sex ratio is very cost-effective Oh Recommended use, very minimalist appearance, with a good feeling good, inserted in the home does not affect the beauty, it is worth a try.	Taobao Marketplace, a Chinese website for online shopping
Slu King Flagship Store	Intelligent power saver	Saving appliances received the packaging is very attentive, the appearance of the design is quite good, it looks quite high, friends recommended to buy than my previous good-looking, white, the color is also satisfied, and so on with a period of time to come back to the review, after all, a day or two days to see the effect of a good intention to buy for parents	Taobao Marketplace, a Chinese website for online shopping
Slu King Flagship Store	Intelligent power saver	The air conditioner, refrigerator, TV, etc. at home is particularly power consumption, so I bought this power saver, just use it and do not know the results, but this product looks very formal, the packaging is particularly good, looking forward to power-saving effect.	Taobao Marketplace, a Chinese website for online shopping
Slu King Flagship Store	Intelligent power saver	Saver has received, very satisfied, business service good shipping lightning, I was ordering two business also sent me a thank you. Whether the material or workmanship are beyond expectations, value, with a very good-looking Oh. Seller shipping is also much earlier than expected, will come again. Buy it, will not	Taobao Marketplace, a Chinese website for online shopping
Slu King Flagship Store	Intelligent power saver	Goods received, intelligent power saver appearance design is good compact, good quality, read a lot of reviews say very save electricity, I immediately ordered to try, home appliances more effective will continue to buy back.	Taobao Marketplace, a Chinese website for online shopping
Slu King Flagship Store	Intelligent power saver	Neighbors introduced, said the summer children are at home, the family day open three air conditioning a month electricity bill of five or six hundred, the second month decisively bought this, and then a comparison to save a lot of it!	Taobao Marketplace, a Chinese website for online shopping
Slu King Flagship Store	Intelligent power saver	Saver good not expensive, went to buy several, also in this store, the effect is indeed as the business said, this time to help relatives to buy, if you know earlier this magic weapon previously would not spend so much money on electricity unjustly	Taobao Marketplace, a Chinese website for online shopping
Slu King Flagship Store	Intelligent power saver	. Overall, the product is relatively simple and compact, the use is relatively simple, do not have to spend time to learn how to install how to make use of ah what this point I think it is very good. Appearance also seems to be the kind of sturdy and durable, after all, can save is to save a little is to	Taobao Marketplace, a Chinese website for online shopping
Slu King Flagship Store	Intelligent power saver	I think it will work. I just used it. I hope it works.	Taobao Marketplace, a Chinese website for online shopping
Slu King Flagship Store	Intelligent power saver	Installation of their own, very simple, logistics is also very fast, before buying a, this time and bought two to the old family inside a, this side of the store and bought a, power-saving effect is also very good, like we open the store, summer ah, to open a few refrigerators, and air conditioning, quite suitable for the purchase of two of this installed inside the store!	Taobao Marketplace, a Chinese website for online shopping
Slu King Flagship Store	Intelligent power saver	Very satisfied with a shopping, wish the store business prosperity	Taobao Marketplace, a Chinese website for online shopping
Slu King Flagship Store	Intelligent power saver	Boss service is particularly good, fast delivery, product quality is particularly good, used to save a lot of electricity, recommended to my friends, I wish the boss business is booming, prosperity, there is a need to get down quickly, early use early to save money!	Taobao Marketplace, a Chinese website for online shopping
Slu King Flagship Store	Intelligent power saver	The material is environmentally safe, silent work, the whole use of not hot, used a few days, there is indeed a saving effect. After all, do a little business outside, can save a little is a little.	Taobao Marketplace, a Chinese website for online shopping
Slu King Flagship Store	Intelligent power saver	Saving appliances received the packaging is very attentive, the appearance of the design is quite good, it looks quite high, friends recommended to buy than my previous good-looking, white, the color is also satisfied, and so on with a period of time to come back to the review, after all, a day or two days to see the effect of a good intention to buy for parents	Taobao Marketplace, a Chinese website for online shopping
Slu King Flagship Store	Intelligent power saver	Saving appliances are really too good to use, this is already the third time to buy, because the relatives of the family electricity is very expensive, after buying this device are said to be very effective, thanks to the patience of the seller's reception, and will recommend more relatives and	Taobao Marketplace, a Chinese website for online shopping
Slu King Flagship Store	Intelligent power saver	Small size, does not take up much space. It's great, it does work very effectively and uses a lot of electricity for a lot of work. It does use a lot less electricity after installing it. About 30 degrees of electricity a day, and it does work a few degrees less. Scientific energy saving, very reassuring.	Taobao Marketplace, a Chinese website for online shopping
Slu King Flagship Store	Intelligent power saver	Saving appliances are really too good to use, because the relatives of the family electricity is very expensive, after buying this device are said to be very effective, thanks to the patience of the seller's reception, and will recommend more relatives and friends!	Taobao Marketplace, a Chinese website for online shopping
Slu King Flagship Store	Intelligent power saver	Received well-packed, the merchant is very attentive service is very thoughtful. It is genuine query are big factory products, trustworthy [strong] buy assured, with a lot of peace of mind. Worthy of five-star praise	Taobao Marketplace, a Chinese website for online shopping
Slu King Flagship Store	Intelligent power saver	Nice. Satisfied!	Taobao Marketplace, a Chinese website for online shopping
Slu King Flagship Store	Intelligent power saver	Stream very quickly, a few days to arrive, hotel hotel with, winter power consumption is very costly, placed in the office under the base of the air conditioning test for a period of time, the voltage stabilized, it is estimated that this winter will save a lot of electricity!	Taobao Marketplace, a Chinese website for online shopping

Slu King Flagship Store	Intelligent power saver	Intelligent power saver received, good-looking, cost-effective, working properly, easy to use, no noise, logistics is fast, customer service attitude is very good, intact, compact, will recommend friends to buy.	Taobao Marketplace, a Chinese website for online shopping
Slu King Flagship Store	Intelligent power saver	Energy saver received, the quality of workmanship did not let me disappointed! With the air conditioning TV can see the change in the above figures, winter home electricity is more powerful, energy saver can save a lot of electricity, look forward to the effect!	Taobao Marketplace, a Chinese website for online shopping
Slu King Flagship Store	Intelligent power saver	Silent work does not affect, the main air conditioning with more buy this saver, the quality of workmanship can be, the business has a problem to follow up in place. The main long-term use of things, after-sales protection worry-free, with a very reassuring.	Taobao Marketplace, a Chinese website for online shopping
Slu King Flagship Store	Intelligent power saver	How does it work?	Taobao Marketplace, a Chinese website for online shopping
Slu King Flagship Store	Intelligent power saver	Is this stuff real yo, kinda don't believe it /:077/:077/:077	Taobao Marketplace, a Chinese website for online shopping
Slu King Flagship Store	Intelligent power saver	May I know where to plug in this Smart Saver to save electricity?	Taobao Marketplace, a Chinese website for online shopping
Slu King Flagship Store	Intelligent power saver	Is it illegal?	Taobao Marketplace, a Chinese website for online shopping
Slu King Flagship Store	Intelligent power saver	Will the power supply bureau check if it's illegal to use it?	Taobao Marketplace, a Chinese website for online shopping
Slu King Flagship Store	Intelligent power saver	IQ harvester	Taobao Marketplace, a Chinese website for online shopping
Slu King Flagship Store	Intelligent power saver	Can you really save some?	Taobao Marketplace, a Chinese website for online shopping
Slu King Flagship Store	Intelligent power saver	Is it true?	Taobao Marketplace, a Chinese website for online shopping
Slu King Flagship Store	Intelligent power saver	Is this real? Would love to buy one to plant on the Three Gorges!	Taobao Marketplace, a Chinese website for online shopping
Slu King Flagship Store	Intelligent power saver	Does this work for all air conditioning appliances when plugged into any plug in the house?	Taobao Marketplace, a Chinese website for online shopping
Slu King Flagship Store	Intelligent power saver	Does it really save electricity?	Taobao Marketplace, a Chinese website for online shopping
Slu King Flagship Store	Intelligent power saver	Can I ask if it really saves electricity?	Taobao Marketplace, a Chinese website for online shopping
Slu King Flagship Store	Intelligent power saver	Is it really possible to get the first power?	Taobao Marketplace, a Chinese website for online shopping
Slu King Flagship Store	Intelligent power saver	What's the principle of this thing?	Taobao Marketplace, a Chinese website for online shopping
Slu King Flagship Store	Intelligent power saver	Does it really work?	Taobao Marketplace, a Chinese website for online shopping
Slu King Flagship Store	Intelligent power saver	Is it stolen electricity?	Taobao Marketplace, a Chinese website for online shopping
Slu King Flagship Store	Intelligent power saver	Has it ever worked?	Taobao Marketplace, a Chinese website for online shopping
Slu King Flagship Store	Intelligent power saver	Hello everyone did you get any results from your purchases?	Taobao Marketplace, a Chinese website for online shopping
Slu King Flagship Store	Intelligent power saver	IQ tax, right?	Taobao Marketplace, a Chinese website for online shopping
Slu King Flagship Store	Intelligent power saver	Does it really work?	Taobao Marketplace, a Chinese website for online shopping
Slu King Flagship Store	Intelligent power saver	How effective is it?	Taobao Marketplace, a Chinese website for online shopping
Slu King Flagship Store	Intelligent power saver	Has anyone taken it apart?	Taobao Marketplace, a Chinese website for online shopping
Slu King Flagship Store	Intelligent power saver	Is it true? I'm asking for the truth.	Taobao Marketplace, a Chinese website for online shopping
Slu King Flagship Store	Intelligent power saver	Does it work?	Taobao Marketplace, a Chinese website for online shopping
Slu King Flagship Store	Intelligent power saver	Can I just plug it in? Should I turn it off when the power goes out?	Taobao Marketplace, a Chinese website for online shopping
Slu King Flagship Store	Intelligent power saver	Didn't Musk come to his knees begging to buy this high-tech program?	Taobao Marketplace, a Chinese website for online shopping
Slu King Flagship Store	Intelligent power saver	Plugs into outlets - cost electricity.	Taobao Marketplace, a Chinese website for online shopping
Slu King Flagship Store	Intelligent power saver	Isn't this a proper nine-year compulsory education leak?	Taobao Marketplace, a Chinese website for online shopping
Slu King Flagship Store	Intelligent power saver	Does it really work?	Taobao Marketplace, a Chinese website for online shopping
Slu King Flagship Store	Intelligent power saver	Does it really work? I'm asking for a genuine answer.	Taobao Marketplace, a Chinese website for online shopping
Slu King Flagship Store	Intelligent power saver	Did you buy it and tell me if it works?	Taobao Marketplace, a Chinese website for online shopping

Slu King Flagship Store	Intelligent power saver	Any friends who have taken it apart and looked at it? Can there be there?	Taobao Marketplace, a Chinese website for online shopping
Slu King Flagship Store	Intelligent power saver	For those of you who have bought it, is it a real power saver, please be honest	Taobao Marketplace, a Chinese website for online shopping
Slu King Flagship Store	Intelligent power saver	Is it really energy efficient?	Taobao Marketplace, a Chinese website for online shopping
Slu King Flagship Store	Intelligent power saver	Does it work?	Taobao Marketplace, a Chinese website for online shopping
Slu King Flagship Store	Intelligent power saver	You guys have a high IQ, but anyone who went to kindergarten knows this is fake, right?	Taobao Marketplace, a Chinese website for online shopping
Slu King Flagship Store	Intelligent power saver	Is it an IQ tax, please?	Taobao Marketplace, a Chinese website for online shopping
Slu King Flagship Store	Intelligent power saver	Used brothers and sisters useful, a month of civilian electricity to open the air conditioning eight hundred and ninety degrees of electricity	Taobao Marketplace, a Chinese website for online shopping
Slu King Flagship Store	Intelligent power saver	Anyone who has bought it, can you tell me if it works?	Taobao Marketplace, a Chinese website for online shopping
Slu King Flagship Store	Intelligent power saver	Can you really save if you've bought one?	Taobao Marketplace, a Chinese website for online shopping
Slu King Flagship Store	Intelligent power saver	I just have to ask, how can he save power when he's an electrical appliance that only consumes power? What principle is this? /:065	Taobao Marketplace, a Chinese website for online shopping
Slu King Flagship Store	Intelligent power saver	Does this saver really save electricity, please tell me the truth, can you tell me about it?	Taobao Marketplace, a Chinese website for online shopping
Slu King Flagship Store	Intelligent power saver	Can I ask if it really saves electricity?	Taobao Marketplace, a Chinese website for online shopping
Slu King Flagship Store	Intelligent power saver	This percentage is useless, right?	Taobao Marketplace, a Chinese website for online shopping
Slu King Flagship Store	Intelligent power saver	How's this for power savings?	Taobao Marketplace, a Chinese website for online shopping
Slu King Flagship Store	Intelligent power saver	Does this work?	Taobao Marketplace, a Chinese website for online shopping
Slu King Flagship Store	Intelligent power saver	Is it true that once the plug is removed the meter spins rapidly to the number of calls actually used or even more?	Taobao Marketplace, a Chinese website for online shopping
Slu King Flagship Store	Intelligent power saver	Do friends work? Is it an IQ tax	Taobao Marketplace, a Chinese website for online shopping
Slu King Flagship Store	Intelligent power saver	It doesn't work. It's mostly an electric starter to balance the voltage.	Taobao Marketplace, a Chinese website for online shopping
Slu King Flagship Store	Intelligent power saver	Is it safe?	Taobao Marketplace, a Chinese website for online shopping
Slu King Flagship Store	Intelligent power saver	Does it work?	Taobao Marketplace, a Chinese website for online shopping
Slu King Flagship Store	Intelligent power saver	outlet	Taobao Marketplace, a Chinese website for online shopping
Slu King Flagship Store	Intelligent power saver	I did. It worked.	Taobao Marketplace, a Chinese website for online shopping
Slu King Flagship Store	Intelligent power saver	Just any plug will do, and to be honest it doesn't actually save any power at all	Taobao Marketplace, a Chinese website for online shopping
Slu King Flagship Store	Intelligent power saver	not illegal	Taobao Marketplace, a Chinese website for online shopping
Slu King Flagship Store	Intelligent power saver	I don't know. It saves electricity.	Taobao Marketplace, a Chinese website for online shopping
Slu King Flagship Store	Intelligent power saver	It should be.	Taobao Marketplace, a Chinese website for online shopping
Slu King Flagship Store	Intelligent power saver	Just used it, don't know yet!	Taobao Marketplace, a Chinese website for online shopping
Slu King Flagship Store	Intelligent power saver	Don't buy it. You've been duped.	Taobao Marketplace, a Chinese website for online shopping
Slu King Flagship Store	Intelligent power saver	Just bought it, not sure if it will save power	Taobao Marketplace, a Chinese website for online shopping
Slu King Flagship Store	Intelligent power saver	Doesn't feel like it's working.	Taobao Marketplace, a Chinese website for online shopping
Slu King Flagship Store	Intelligent power saver	Yes, not too much. A little goes a long way.	Taobao Marketplace, a Chinese website for online shopping
Slu King Flagship Store	Intelligent power saver	I don't know if it's true or not.	Taobao Marketplace, a Chinese website for online shopping
Slu King Flagship Store	Intelligent power saver	I don't feel like saving power.	Taobao Marketplace, a Chinese website for online shopping
Slu King Flagship Store	Intelligent power saver	Just look it up online.	Taobao Marketplace, a Chinese website for online shopping

Slu King Flagship Store	Intelligent power saver	unknown	Taobao Marketplace, a Chinese website for online shopping
Slu King Flagship Store	Intelligent power saver	fault	Taobao Marketplace, a Chinese website for online shopping
Slu King Flagship Store	Intelligent power saver	Just started using it.	Taobao Marketplace, a Chinese website for online shopping
Slu King Flagship Store	Intelligent power saver	Landlords aren't losing money if it actually works.	Taobao Marketplace, a Chinese website for online shopping
Slu King Flagship Store	Intelligent power saver	We'll find out next month.	Taobao Marketplace, a Chinese website for online shopping
Slu King Flagship Store	Intelligent power saver	It's only been three days. I don't know.	Taobao Marketplace, a Chinese website for online shopping
Slu King Flagship Store	Intelligent power saver	It doesn't work.	Taobao Marketplace, a Chinese website for online shopping
Slu King Flagship Store	Intelligent power saver	It doesn't seem to be working.	Taobao Marketplace, a Chinese website for online shopping
Slu King Flagship Store	Intelligent power saver	Feelings are average.	Taobao Marketplace, a Chinese website for online shopping
Slu King Flagship Store	Intelligent power saver	There's no point!	Taobao Marketplace, a Chinese website for online shopping
Slu King Flagship Store	Intelligent power saver	No need to close	Taobao Marketplace, a Chinese website for online shopping
Slu King Flagship Store	Intelligent power saver	IQ tax	Taobao Marketplace, a Chinese website for online shopping
Slu King Flagship Store	Intelligent power saver	acceptable	Taobao Marketplace, a Chinese website for online shopping
Slu King Flagship Store	Intelligent power saver	thankfully	Taobao Marketplace, a Chinese website for online shopping
Slu King Flagship Store	Intelligent power saver	Honestly, I don't feel it.	Taobao Marketplace, a Chinese website for online shopping
Slu King Flagship Store	Intelligent power saver	Not yet.	Taobao Marketplace, a Chinese website for online shopping
Slu King Flagship Store	Intelligent power saver	Didn't find it useful, the electricity calendar says it all	Taobao Marketplace, a Chinese website for online shopping
Slu King Flagship Store	Intelligent power saver	It's a lie. It doesn't work.	Taobao Marketplace, a Chinese website for online shopping
Slu King Flagship Store	Intelligent power saver	IQ tax	Taobao Marketplace, a Chinese website for online shopping
Slu King Flagship Store	Intelligent power saver	Bought two, didn't feel energy efficient	Taobao Marketplace, a Chinese website for online shopping
Slu King Flagship Store	Intelligent power saver	Didn't seem to find any use for it /:079	Taobao Marketplace, a Chinese website for online shopping
Slu King Flagship Store	Intelligent power saver	You're right about everything, college boy.	Taobao Marketplace, a Chinese website for online shopping
Slu King Flagship Store	Intelligent power saver	What IQ tax?	Taobao Marketplace, a Chinese website for online shopping
Slu King Flagship Store	Intelligent power saver	Believe what you want, but not what you don't.	Taobao Marketplace, a Chinese website for online shopping
Slu King Flagship Store	Intelligent power saver	Bake the sweet potatoes once and they don't cook until the second time.	Taobao Marketplace, a Chinese website for online shopping
Slu King Flagship Store	Intelligent power saver	I don't think so.	Taobao Marketplace, a Chinese website for online shopping
Slu King Flagship Store	Intelligent power saver	Current Principle	Taobao Marketplace, a Chinese website for online shopping
Slu King Flagship Store	Intelligent power saver	I just bought this one as well and I don't know how well it works yet.	Taobao Marketplace, a Chinese website for online shopping
Slu King Flagship Store	Intelligent power saver	I really don't know.	Taobao Marketplace, a Chinese website for online shopping
Slu King Flagship Store	Intelligent power saver	acute (of sight and hearing)	Taobao Marketplace, a Chinese website for online shopping
Slu King Flagship Store	Intelligent power saver	IQ tax	Taobao Marketplace, a Chinese website for online shopping
Slu King Flagship Store	Intelligent power saver	It doesn't seem to be working. The previous meter was mechanical, and the current meter is electronic.	Taobao Marketplace, a Chinese website for online shopping
Slu King Flagship Store	Intelligent power saver	Probably not, think it's a bit useful, saves 2-3 kWh per day	Taobao Marketplace, a Chinese website for online shopping
Slu King Flagship Store	Intelligent power saver	incompetent	Taobao Marketplace, a Chinese website for online shopping

Slu King Flagship Store	Intelligent power saver	I don't know. I use it all the time.	Taobao Marketplace, a Chinese website for online shopping
Slu King Flagship Store	Intelligent power saver	Safe, but not much use.	Taobao Marketplace, a Chinese website for online shopping
Slu King Flagship Store	Intelligent power saver	I don't feel it yet.	Taobao Marketplace, a Chinese website for online shopping
Slu King Flagship Store	King of Power Saving	Already purchased several times! They bought and used the power saving effect is obvious, and subsequently bought a few times to send to friends and relatives. Power saving effect: the effect is obvious Cost-effective: high Appearance style: beautiful Workmanship material: good Experience: safe!	Taobao Marketplace, a Chinese website for online shopping
Slu King Flagship Store	King of Power Saving	Bought it half-heartedly and after a couple of months, it does work!!!!	Taobao Marketplace, a Chinese website for online shopping
Slu King Flagship Store	King of Power Saving	I've just started using it, and it's okay, so I'll give it some time, and then I'll tell you more about it.	Taobao Marketplace, a Chinese website for online shopping
Slu King Flagship Store	King of Power Saving	Have bought for the 2nd time. The last time was to buy their own home. The effect is still good, in the past a summer every day to open the air conditioning, electricity costs at least 4,500 or 5,600. This summer open air conditioning, electricity costs only 300 more. So I bought another one for my parents'	Taobao Marketplace, a Chinese website for online shopping
Slu King Flagship Store	King of Power Saving	The goods have been received, the effect is unknown!	Taobao Marketplace, a Chinese website for online shopping
Slu King Flagship Store	King of Power Saving	Power-saving effect: very energy-saving Experience: very easy to use Cost-effective High appearance, worth buying trustworthy, worth recommending to colleagues.	Taobao Marketplace, a Chinese website for online shopping
Slu King Flagship Store	King of Power Saving	Baby is very beautiful, very practical, very easy to use, it is worth getting everyone to buy. It is worth recommending to colleagues.	Taobao Marketplace, a Chinese website for online shopping
Slu King Flagship Store	King of Power Saving	Power-saving effect: especially good Cost-effective: very cost-effective Appearance style: good-looking Workmanship material: very good Experience: very satisfied with very good Recommended	Taobao Marketplace, a Chinese website for online shopping
Slu King Flagship Store	King of Power Saving	Just received the goods, small and exquisite, the store has a good service attitude, the quality is guaranteed, first use a month, compared to the last month's electricity costs, and then comment!	Taobao Marketplace, a Chinese website for online shopping
Slu King Flagship Store	King of Power Saving	Nice style, good workmanship, feels pretty good	Taobao Marketplace, a Chinese website for online shopping
Slu King Flagship Store	King of Power Saving	I hope it works, the house uses too much electricity.	Taobao Marketplace, a Chinese website for online shopping
Slu King Flagship Store	King of Power Saving	A little electricity can not save, said what can save fifteen percent of the electricity, the results are still higher than last month two hundred, really too false, do not buy who buy who fooled!	Taobao Marketplace, a Chinese website for online shopping
Slu King Flagship Store	King of Power Saving	Can't wait to open it when it arrives, it really didn't let me down, the quality is particularly good, the value is also quite high, satisfied with a shopping, thoughtful service!	Taobao Marketplace, a Chinese website for online shopping
Slu King Flagship Store	King of Power Saving	Baby received, logistics is very fast, thanks to the shopkeeper's very patient explanation, just tried to feel a little effect and also comes with air purification, in short, feel pretty good. First use a, late good will buy again.	Taobao Marketplace, a Chinese website for online shopping
Slu King Flagship Store	King of Power Saving	It's easy to use, trust the company, and we'll see how it works before we review it.	Taobao Marketplace, a Chinese website for online shopping
Slu King Flagship Store	King of Power Saving	Ordered fast shipping, timely shipping, not only save electricity, but also comes with purified air, the	Taobao Marketplace, a Chinese website for online shopping
Slu King Flagship Store	King of Power Saving	Power-saving effect: basically do not need any electricity fast charging, good effect after using the air is much fresher small and very convenient to put wherever possible	Taobao Marketplace, a Chinese website for online shopping
Slu King Flagship Store	King of Power Saving	Trying it out.	Taobao Marketplace, a Chinese website for online shopping
Slu King Flagship Store	King of Power Saving	Logistics is very fast, the goods have been received. Immediately plugged in and used it, the effect should be okay. Workmanship material: the appearance of the quality is very good.	Taobao Marketplace, a Chinese website for online shopping
Slu King Flagship Store	King of Power Saving	Great quality, saves electricity and money, very energy efficient. Shipping was fast, buyer service was great, and the product was well described.	Taobao Marketplace, a Chinese website for online shopping
Slu King Flagship Store	King of Power Saving	Power saving effect: just use, feel good, has been purchased many times Recommended factories and families can be used Cost-effective is also good, the customer service is great ha	Taobao Marketplace, a Chinese website for online shopping
Slu King Flagship Store	King of Power Saving	Use can be not bad, quite suitable for use	Taobao Marketplace, a Chinese website for online shopping
Slu King Flagship Store	King of Power Saving	After using it for 2 months to see the actual effect and then evaluate, point of view logistics fast!	Taobao Marketplace, a Chinese website for online shopping
Slu King Flagship Store	King of Power Saving	The goods have received a very delicate appearance, logistics is also very fast, very satisfied with a shopping	Taobao Marketplace, a Chinese website for online shopping
Slu King Flagship Store	King of Power Saving	Workmanship material: look pretty good, the seller also gave a gift, really value for money,! My own absolute product is quite good! Very satisfied with a shopping!!!	Taobao Marketplace, a Chinese website for online shopping
Slu King Flagship Store	King of Power Saving	Second time I bought it, it does save power, no fooling around	Taobao Marketplace, a Chinese website for online shopping
Slu King Flagship Store	King of Power Saving	Plugged it in as soon as it arrived, bought a total of 3, and used it for 2 months before chasing it down.	Taobao Marketplace, a Chinese website for online shopping
Slu King Flagship Store	King of Power Saving	Just received can not wait to plug in the power supply, looking forward to good results, the product is well made, looks very good, worth having!	Taobao Marketplace, a Chinese website for online shopping
Slu King Flagship Store	King of Power Saving	Not good, scam merchants, will be good to have to let people evaluate? Also not allowed to return, and then complained only to return	Taobao Marketplace, a Chinese website for online shopping
Slu King Flagship Store	King of Power Saving	Received it, it works great, it really is just like what the merchant told me. Next time will buy back again.	Taobao Marketplace, a Chinese website for online shopping
Slu King Flagship Store	King of Power Saving	Received goods and the picture in line with the logistics is very fast, the service is enthusiastic!	Taobao Marketplace, a Chinese website for online shopping

Slu King Flagship Store	King of Power Saving	Cost-effective: cost-effective Appearance and style: quite exquisite Workmanship Material: material is very solid Experience: look forward to the effect yo	Taobao Marketplace, a Chinese website for online shopping
Slu King Flagship Store	King of Power Saving	I've used it and I'll give it 5 stars for now and look forward to the results.	Taobao Marketplace, a Chinese website for online shopping
Slu King Flagship Store	King of Power Saving	Look pretty good, just used, customer service explanation is more careful	Taobao Marketplace, a Chinese website for online shopping
Slu King Flagship Store	King of Power Saving	Good customer service and fast delivery	Taobao Marketplace, a Chinese website for online shopping
Slu King Flagship Store	King of Power Saving	The stuff works super well. Just used it and it worked, and the logistics were great.	Taobao Marketplace, a Chinese website for online shopping
Slu King Flagship Store	King of Power Saving	Superb stuff.	Taobao Marketplace, a Chinese website for online shopping
Slu King Flagship Store	King of Power Saving	Good value for money, nice looking, has air purification, nice!	Taobao Marketplace, a Chinese website for online shopping
Slu King Flagship Store	King of Power Saving	It doesn't work. It doesn't work at all. Don't be fooled. I've been using it for months and it doesn't work.	Taobao Marketplace, a Chinese website for online shopping
Slu King Flagship Store	King of Power Saving	Power saving effect: used a few days, feel the effect, exquisite appearance, the most important is genuine, value for money	Taobao Marketplace, a Chinese website for online shopping
Slu King Flagship Store	King of Power Saving	Liar, liar, liar, don't step in the hole	Taobao Marketplace, a Chinese website for online shopping
Slu King Flagship Store	King of Power Saving	Packaging is intact, customer service response in a timely manner, the introduction is also very detailed! Operating in accordance with the instructions is simple and easy to understand, wait until after use to comment on the effect Oh!	Taobao Marketplace, a Chinese website for online shopping
Slu King Flagship Store	King of Power Saving	Power savings: really only and looks great. Was a good shopping trip.	Taobao Marketplace, a Chinese website for online shopping
Slu King Flagship Store	King of Power Saving	I've been using it for a while, but it's not working.	Taobao Marketplace, a Chinese website for online shopping
Slu King Flagship Store	King of Power Saving	This is really quite good. You can shoot, if you want to use it.	Taobao Marketplace, a Chinese website for online shopping
Slu King Flagship Store	King of Power Saving	It's the pits, don't buy it, it doesn't work, just ignore it and don't return it	Taobao Marketplace, a Chinese website for online shopping
Slu King Flagship Store	King of Power Saving	It's not working, the same electricity usage as last year without any reduction in electricity usage	Taobao Marketplace, a Chinese website for online shopping
Slu King Flagship Store	King of Power Saving	Small and lightweight, listened to customer service bought three pieces, already plugged in, looking forward to using the effect of	Taobao Marketplace, a Chinese website for online shopping
Slu King Flagship Store	King of Power Saving	Power-saving effect: this is the 3rd time to buy, energy-saving effect is good, every house is finished buying, the earliest has been a has been used for almost half a year, it is worth having!	Taobao Marketplace, a Chinese website for online shopping
Slu King Flagship Store	King of Power Saving	Hubby asked to buy it, don't know what the principle is, said it can save electricity, use it to see	Taobao Marketplace, a Chinese website for online shopping
Slu King Flagship Store	King of Power Saving	Overall, it's good.	Taobao Marketplace, a Chinese website for online shopping
Slu King Flagship Store	King of Power Saving	not bad	Taobao Marketplace, a Chinese website for online shopping
Slu King Flagship Store	King of Power Saving	This has been used and I don't know if it works. Looking forward to it.	Taobao Marketplace, a Chinese website for online shopping
Slu King Flagship Store	King of Power Saving	Power-saving effect: this is the second time to buy, the effect is okay Appearance style: the appearance is also good-looking Cost-effective: the price is cheap, the logistics is also very fast Workmanship Material: very fine workmanship	Taobao Marketplace, a Chinese website for online shopping
Slu King Flagship Store	King of Power Saving	Logistics is very fast, the goods are very good worth buying, five-star praise, beautiful appearance, with a short time is very good, will recommend to friends, the	Taobao Marketplace, a Chinese website for online shopping
Slu King Flagship Store	King of Power Saving	Well well well use it! It's energy efficient and environmentally friendly.	Taobao Marketplace, a Chinese website for online shopping
Slu King Flagship Store	King of Power Saving	Workmanship material: more durable Power-saving effect: looking forward to the effect of power saving Appearance style: beautiful atmosphere	Taobao Marketplace, a Chinese website for online shopping
Slu King Flagship Store	King of Power Saving	The product works great and saves 1/3 by plugging in	Taobao Marketplace, a Chinese website for online shopping
Slu King Flagship Store	King of Power Saving	Power-saving effect: looking forward to two months to see the effect Cost-effective: cost-effective Appearance style: high-grade Workmanship material: aluminum alloy shell Experience: just started to use	Taobao Marketplace, a Chinese website for online shopping
Slu King Flagship Store	King of Power Saving	Baby has received, the appearance of beautiful and generous, customer service patience and meticulous, energy-saving effect to be used and then follow up.	Taobao Marketplace, a Chinese website for online shopping
Slu King Flagship Store	King of Power Saving	After nearly four and a half months of testing, especially in the summer peak season, compared with the same period of electricity consumption in previous years, the power saving efficiency of the power saver has been converted to about 35%. In short, it is cost-effective to buy this power saver, worth buying and	Taobao Marketplace, a Chinese website for online shopping
Slu King Flagship Store	King of Power Saving	The product has been received, the quality and business description consistent with the expected results.	Taobao Marketplace, a Chinese website for online shopping
Slu King Flagship Store	King of Power Saving	Please do not be cheated, this is a ** business products with this invalid personally try 90 days without a little effect, the request to return the refund with this ignore you also do not return the message, I hope that we will not be cheated.	Taobao Marketplace, a Chinese website for online shopping
Slu King Flagship Store	King of Power Saving	Use it for a while and then comment	Taobao Marketplace, a Chinese website for online shopping
Slu King Flagship Store	King of Power Saving	Experience: I'm looking forward to the results, not bad, you can get it if you like it!	Taobao Marketplace, a Chinese website for online shopping

Slu King Flagship Store	King of Power Saving	Ugh 😔 I feel like it doesn't work at all, it uses as much power as it does, so buy with caution!	Taobao Marketplace, a Chinese website for online shopping
Slu King Flagship Store	King of Power Saving	High-end atmosphere, tested with a hair dryer. The effect is great, use it for two more months and then come back to comment. Looking forward to...	Taobao Marketplace, a Chinese website for online shopping
Slu King Flagship Store	King of Power Saving	Baby has received, the quality is very good, plug in the power can be used, power saving in use; boss service is also very good, conscientious business.	Taobao Marketplace, a Chinese website for online shopping
Slu King Flagship Store	King of Power Saving	Just received the goods have not yet used, feel okay	Taobao Marketplace, a Chinese website for online shopping
Slu King Flagship Store	King of Power Saving	Pretty good, good service! Just installed it expecting results! Have data in a few days! I'll be back to talk about it.	Taobao Marketplace, a Chinese website for online shopping
Slu King Flagship Store	King of Power Saving	Fraudulent fakes.	Taobao Marketplace, a Chinese website for online shopping
Slu King Flagship Store	King of Power Saving	Power saving effect: very good Cost-effective: high Appearance style: small, not in the way of the place Workmanship material: aluminum alloy Material Experience: power saving	Taobao Marketplace, a Chinese website for online shopping
Slu King Flagship Store	King of Power Saving	More than 100 days no effect at all, the commitment is invalid refund, contact the seller to send the monthly electricity map to the seller, the seller does not reply, playing deaf and dumb without a little integrity to speak.	Taobao Marketplace, a Chinese website for online shopping
Slu King Flagship Store	King of Power Saving	Things look good, plugged in and working properly, looking forward to energy-saving effect	Taobao Marketplace, a Chinese website for online shopping
Slu King Flagship Store	King of Power Saving	We'll send you more photos to see how it works and look forward to saving energy.	Taobao Marketplace, a Chinese website for online shopping
Slu King Flagship Store	King of Power Saving	This stuff is cheap and good.	Taobao Marketplace, a Chinese website for online shopping
Slu King Flagship Store	King of Power Saving	I've got it. I'll try it out. It works well. I'll buy it again.	Taobao Marketplace, a Chinese website for online shopping
Slu King Flagship Store	King of Power Saving	It doesn't work, it's a pure IQ tax, and anyone who buys from them will be out of luck for the rest of their lives, and their children and grandchildren as well.	Taobao Marketplace, a Chinese website for online shopping
Slu King Flagship Store	King of Power Saving	Very good use Cost-effective: cost-effective and good workmanship	Taobao Marketplace, a Chinese website for online shopping
Slu King Flagship Store	King of Power Saving	I am operating a billiard room, the monthly electricity bill up to more than 1,000 yuan, look at this product online feel can help me, the store also patiently and carefully to me one by one to explain the performance, bought to try. The product got a bright spot, packaging at a glance is very attentive, open the packaging and then look at the product, is also fine manufacturing, looking forward to good results,	Taobao Marketplace, a Chinese website for online shopping
Slu King Flagship Store	King of Power Saving	When the goods were received, it was plugged in and used, very good workmanship , good service and some fuses! Current looks much more stable!	Taobao Marketplace, a Chinese website for online shopping
Slu King Flagship Store	King of Power Saving	Power-saving effect: feel good look forward to good results high appearance	Taobao Marketplace, a Chinese website for online shopping
Slu King Flagship Store	King of Power Saving	Quite noble, used for a few days, looks good, worth buying	Taobao Marketplace, a Chinese website for online shopping
Slu King Flagship Store	King of Power Saving	I just bought half a month in the trial, do not know the effect, first use it. First praise, if the effect is not good, to return the business attitude is good, that is conscientious business, because buy this thing money is not much, are looking forward to the effect.	Taobao Marketplace, a Chinese website for online shopping
Slu King Flagship Store	King of Power Saving	I've just started using it. I'll comment after 3 months.	Taobao Marketplace, a Chinese website for online shopping
Slu King Flagship Store	King of Power Saving	Power saving effect: good Cost-effective: good Appearance style: compact and practical Workmanship material: aluminum alloy Experience: looking forward to saving electricity	Taobao Marketplace, a Chinese website for online shopping
Slu King Flagship Store	King of Power Saving	After buying and using two months really see the effect, so only to evaluate! Originally held the mindset of trying, I did not expect really effective, worth having!	Taobao Marketplace, a Chinese website for online shopping
Slu King Flagship Store	King of Power Saving	Power saving effect: very good, beautiful appearance, really save power.	Taobao Marketplace, a Chinese website for online shopping
Slu King Flagship Store	King of Power Saving	Baby has received, and the same as seen on the Internet, is the effect to be used to know, must be praise	Taobao Marketplace, a Chinese website for online shopping
Slu King Flagship Store	King of Power Saving	Power-saving effect: about power saving, still in use, I hope to save electricity, a summer air conditioning power is too powerful Cost-effective: not expensive, quite good Appearance style: compact, can be placed anywhere Experience: is trying, late after the leveling	Taobao Marketplace, a Chinese website for online shopping
Slu King Flagship Store	King of Power Saving	Power saving effect: very good. Cost-effective: very high. Appearance and style: beautiful. Workmanship material: very good quality. Using experience: very good, worth buying.	Taobao Marketplace, a Chinese website for online shopping
Slu King Flagship Store	King of Power Saving	The efficacy will be seen next month, so I'll comment then.	Taobao Marketplace, a Chinese website for online shopping
Slu King Flagship Store	King of Power Saving	Power saving effect: very good Cost-effective: no problem Appearance style: good-looking Workmanship material: absolutely good to use	Taobao Marketplace, a Chinese website for online shopping
Slu King Flagship Store	King of Power Saving	Must save electricity and worth every penny!	Taobao Marketplace, a Chinese website for online shopping
Slu King Flagship Store	King of Power Saving	This definitely works and the electricity bill is significantly lower!	Taobao Marketplace, a Chinese website for online shopping
Slu King Flagship Store	King of Power Saving	Just bought back today can't wait to install it, plug and play, power-saving effect is still unknown, the Tmall store should not have any problem, with a period of time back to evaluation!	Taobao Marketplace, a Chinese website for online shopping
Slu King Flagship Store	King of Power Saving	general	Taobao Marketplace, a Chinese website for online shopping
Slu King Flagship Store	King of Power Saving	Originally the store bought with, see the effect decisively into a few put at home, after all, can save a lot of money it.	Taobao Marketplace, a Chinese website for online shopping
Slu King Flagship Store	King of Power Saving	It's okay. I'll review it in a month. I'm looking forward to the results.	Taobao Marketplace, a Chinese website for online shopping

Slu King Flagship Store	King of Power Saving	Already in experience! Heard from a friend that it works well! Let's hope it works!	Taobao Marketplace, a Chinese website for online shopping
Slu King Flagship Store	King of Power Saving	Small, convenient and doesn't take up much space.	Taobao Marketplace, a Chinese website for online shopping
Slu King Flagship Store	King of Power Saving	Received it, don't know if I'll be able to follow up on it in two months time	Taobao Marketplace, a Chinese website for online shopping
Slu King Flagship Store	King of Power Saving	IQ tax, 😬 can't buy it!	Taobao Marketplace, a Chinese website for online shopping
Slu King Flagship Store	King of Power Saving	IQ tax has no suburban fruit	Taobao Marketplace, a Chinese website for online shopping
Slu King Flagship Store	King of Power Saving	Workmanship material: anyway, it's good, very good, very good. Praise.	Taobao Marketplace, a Chinese website for online shopping
Slu King Flagship Store	King of Power Saving	Only from having this power saver king electricity bill is getting less and less, it's so good.	Taobao Marketplace, a Chinese website for online shopping
Slu King Flagship Store	King of Power Saving	Looking forward to the results	Taobao Marketplace, a Chinese website for online shopping
Slu King Flagship Store	King of Power Saving	The goods have been received, the product appearance of the atmosphere, looking forward to the effect of the bar!	Taobao Marketplace, a Chinese website for online shopping
Slu King Flagship Store	King of Power Saving	Baby just received today, with two days to evaluate, I hope the effect bar.	Taobao Marketplace, a Chinese website for online shopping
Slu King Flagship Store	King of Power Saving	The product is great and you can order it if you like it.	Taobao Marketplace, a Chinese website for online shopping
Slu King Flagship Store	King of Power Saving	Power saving effect: good Cost-effective: a little high Appearance style: beautiful Experience: very good	Taobao Marketplace, a Chinese website for online shopping
Slu King Flagship Store	King of Power Saving	Power Saving King products are small and exquisite, well-made, work without how noise, although only save so little electricity every day, but a long time is very considerable, I think the value for money, it is worth having.	Taobao Marketplace, a Chinese website for online shopping
Slu King Flagship Store	King of Power Saving	Great product, works well, worth having, great power savings.	Taobao Marketplace, a Chinese website for online shopping
Slu King Flagship Store	King of Power Saving	Power saving effect: energy saving effect is good, our side of the village a lot of people are using this product!	Taobao Marketplace, a Chinese website for online shopping
Slu King Flagship Store	King of Power Saving	Power saving effect: just started using it, waiting for good results. Appearance and style: does not take up space, good-looking, and the function of purifying air, I hope it can really save electricity. Workmanship material: quite good, aluminum alloy surgery, strong and durable.	Taobao Marketplace, a Chinese website for online shopping
Slu King Flagship Store	King of Power Saving	The item was received, perfectly packaged and made of very good material. Already started to use it, looking forward to the effect.	Taobao Marketplace, a Chinese website for online shopping
Slu King Flagship Store	King of Power Saving	Power-saving effect: just bought, still in the experiment Cost-effective: still good Appearance style: good, quite good-looking Workmanship material: quality is trustworthy	Taobao Marketplace, a Chinese website for online shopping
Slu King Flagship Store	King of Power Saving	Very very good baby. Referring friends to use your products as well!	Taobao Marketplace, a Chinese website for online shopping
Slu King Flagship Store	King of Power Saving	Power-saving effect: strong Cost-effective: quite good Appearance style: beautiful Workmanship material: meticulous Experience: looking forward to power-saving effect	Taobao Marketplace, a Chinese website for online shopping
Slu King Flagship Store	King of Power Saving	Experience: first confirm receipt, try a month or two to see the effect, first praise	Taobao Marketplace, a Chinese website for online shopping
Slu King Flagship Store	King of Power Saving	Power saving effect: good Price-performance ratio: good Appearance style: good	Taobao Marketplace, a Chinese website for online shopping
Slu King Flagship Store	King of Power Saving	Experience: received the goods to try, feel pretty good	Taobao Marketplace, a Chinese website for online shopping
Slu King Flagship Store	King of Power Saving	Electricity saver power saving effect: used a few days a little effect, customer service said that with a month or two better results, look forward to the effect!	Taobao Marketplace, a Chinese website for online shopping
Slu King Flagship Store	King of Power Saving	Fast shipping, received it on the third day of ordering! This just got here and plugged it in! Looking forward to the power saving effect	Taobao Marketplace, a Chinese website for online shopping
Slu King Flagship Store	King of Power Saving	It is pure bragging and cheating and does not take any effect at all!	Taobao Marketplace, a Chinese website for online shopping
Slu King Flagship Store	King of Power Saving	Not great value for money, very small looking and well made	Taobao Marketplace, a Chinese website for online shopping
Slu King Flagship Store	King of Power Saving	Power-saving effect: effective Cost-effective: worth the price Appearance and style: good-looking Workmanship material: careful Use experience: convenient and simple	Taobao Marketplace, a Chinese website for online shopping
Slu King Flagship Store	King of Power Saving	Power saving effect: do not know for the time being, the appearance is good, I hope to achieve the desired effect	Taobao Marketplace, a Chinese website for online shopping
Slu King Flagship Store	King of Power Saving	Cost-effective: can Appearance style: good Workmanship material: good Power-saving effect: to use for a period of time to see, expect as described in the manual, not up to the effect can also be returned.	Taobao Marketplace, a Chinese website for online shopping
Slu King Flagship Store	King of Power Saving	Again shoot baby, used to see the affordability, thanks to the manufacturer of good products.	Taobao Marketplace, a Chinese website for online shopping
Slu King Flagship Store	King of Power Saving	Power saving effect: looking forward to saving electricity. Appearance and style: very atmospheric Workmanship material: aluminum alloy material	Taobao Marketplace, a Chinese website for online shopping
Slu King Flagship Store	King of Power Saving	Good, good.	Taobao Marketplace, a Chinese website for online shopping
Slu King Flagship Store	King of Power Saving	The results are quite noticeable The service is excellent The value of the item is beautiful!	Taobao Marketplace, a Chinese website for online shopping

Slu King Flagship Store	King of Power Saving	Started yesterday and looking forward to the power savings!	Taobao Marketplace, a Chinese website for online shopping
Slu King Flagship Store	King of Power Saving	Received the goods on the plug to use Looking forward to the later effect, the shipment is very timely, customer service to answer questions This is very good, but also with two prepared fuse 👍	Taobao Marketplace, a Chinese website for online shopping
Slu King Flagship Store	King of Power Saving	Power saving effect: just started to use, do not know if there is no effect Workmanship material: quality is okay	Taobao Marketplace, a Chinese website for online shopping
Slu King Flagship Store	King of Power Saving	Power saving effect: very good Cost-effective: more energy-saving than others Appearance style: Workmanship material: aluminum alloy material	Taobao Marketplace, a Chinese website for online shopping
Slu King Flagship Store	King of Power Saving	Friends recommended to buy this product, power saving effect is obvious, with air purification function, just put into the newly renovated house inside.	Taobao Marketplace, a Chinese website for online shopping
Slu King Flagship Store	King of Power Saving	Covered for peace of mind	Taobao Marketplace, a Chinese website for online shopping
Slu King Flagship Store	King of Power Saving	It's working. I hope it saves electricity. The electricity bill is getting higher and higher every month. I'll buy more if it's good.	Taobao Marketplace, a Chinese website for online shopping
Slu King Flagship Store	King of Power Saving	I got it right away. I hope it will save some electricity in the next few months. If it's good, I'll make some more. Let's see how amazing this thing is.	Taobao Marketplace, a Chinese website for online shopping
Slu King Flagship Store	King of Power Saving	Bought and just installed at present can not see the effect, and the picture is the same, is experimenting	Taobao Marketplace, a Chinese website for online shopping
Slu King Flagship Store	King of Power Saving	Has been the second time to buy back, energy saving effect, worth recommending, you can try, 👍👍👍👍👍👍👍 Power saving effect: very good Experience: can save more than one-third of the	Taobao Marketplace, a Chinese website for online shopping
Slu King Flagship Store	King of Power Saving	Great customer service, very patient with my questions, looking forward to the results in 3 months!	Taobao Marketplace, a Chinese website for online shopping
Slu King Flagship Store	King of Power Saving	Regardless of the deception, when I said my store electricity costs 380 to 450 costs, ask the kind of suitable they recommend 168 can, the commitment of 3 months according to the cost of electricity if not a full refund, and then how to contact also ignore, after the complaint replied that because of the winter power beyond the scope of this can only be returned to the loss of more than 60, and then I'm this 3 months of the electricity costs are in the 430 or so, did not go beyond the scope of the original given!	Taobao Marketplace, a Chinese website for online shopping
Slu King Flagship Store	King of Power Saving	Baby to receive, the appearance of exquisite and beautiful, compact. Logistics is also very fast, customer service is very good. Now on the static belt with good results	Taobao Marketplace, a Chinese website for online shopping
Slu King Flagship Store	King of Power Saving	Cost-effective: high Workmanship material: quite exquisite Experience: just used, two months after the additional comments	Taobao Marketplace, a Chinese website for online shopping
Slu King Flagship Store	King of Power Saving	Power-saving effect: after two months to see the effect of Cost-effective: can Appearance style: relatively good Experience: just arrived today feel the appearance is not bad, with a period of time to see the effect, looking forward to good results	Taobao Marketplace, a Chinese website for online shopping
Slu King Flagship Store	King of Power Saving	Very good, very satisfied!	Taobao Marketplace, a Chinese website for online shopping
Slu King Flagship Store	King of Power Saving	Goods to receive, express very quickly, the quality is okay, just do not know whether the power saving, use in the see!	Taobao Marketplace, a Chinese website for online shopping
Slu King Flagship Store	King of Power Saving	Goods received, power saving useful, feel very obvious Cost-effective: the indicator light is very obvious, the voltage is very stable	Taobao Marketplace, a Chinese website for online shopping
Slu King Flagship Store	King of Power Saving	Power-saving effect: very good praise a Cost-effective: good Appearance style: good-looking metal texture Workmanship material: very good look, the use of experience: look forward to look forward to.	Taobao Marketplace, a Chinese website for online shopping
Slu King Flagship Store	King of Power Saving	Experience: the goods to receive, plug in the power can be used, simple and easy to use!	Taobao Marketplace, a Chinese website for online shopping
Slu King Flagship Store	King of Power Saving	Has been used for more than two months, the effect only to evaluate, the first month did not see the effect, buy customer service also said so, so use two months to evaluate, really effective, this month to save quite a lot of electricity, recommend that you buy!	Taobao Marketplace, a Chinese website for online shopping
Slu King Flagship Store	King of Power Saving	Delivery was fast and packed tightly. Recommended by a friend hope it works as well as they did	Taobao Marketplace, a Chinese website for online shopping
Slu King Flagship Store	King of Power Saving	Power-saving effect: only started to use the effect of waiting for the appearance of style: does not take up space, the color is good, and the function of purifying the air, waiting to see the best power-saving effect, I hope that really can save electricity!	Taobao Marketplace, a Chinese website for online shopping
Slu King Flagship Store	King of Power Saving	Goods have been received, the express delivery is very fast, a very good product, the effect is looking forward to the time to make comments.	Taobao Marketplace, a Chinese website for online shopping
Slu King Flagship Store	King of Power Saving	Yesterday shot today arrived, really fast, immediately used, look forward to the effect!	Taobao Marketplace, a Chinese website for online shopping
Slu King Flagship Store	King of Power Saving	Power saving effect: energy-saving effect is obvious Cost-effective: cost-effective Appearance style: novel appearance Workmanship material: fine workmanship Experience: special power-saving	Taobao Marketplace, a Chinese website for online shopping
Slu King Flagship Store	King of Power Saving	Really good.	Taobao Marketplace, a Chinese website for online shopping
Slu King Flagship Store	King of Power Saving	I'll add more when I've used it.	Taobao Marketplace, a Chinese website for online shopping
Slu King Flagship Store	King of Power Saving	Truth this thing do not know whether to rely on, the business requires 3 pictures to give counter 20 yuan, I really regret the positive feedback!	Taobao Marketplace, a Chinese website for online shopping
Slu King Flagship Store	King of Power Saving	Already purchased for the second time, it works very well and will buy it again in the summer when it uses more electricity!	Taobao Marketplace, a Chinese website for online shopping
Slu King Flagship Store	King of Power Saving	Power-saving effect: under observation Cost-effectiveness: general Appearance style: formal Workmanship material: satisfied Experience: simple and convenient, I hope it can save me some electricity, and then come back to add comments after three months!	Taobao Marketplace, a Chinese website for online shopping
Slu King Flagship Store	King of Power Saving	I've seen a good one, but I don't know if it'll save power. I'll review it in a month.	Taobao Marketplace, a Chinese website for online shopping
Slu King Flagship Store	King of Power Saving	Only received the goods for two days, looking forward to the effect is very good	Taobao Marketplace, a Chinese website for online shopping

Slu King Flagship Store	King of Power Saving	Goods received, just used, looking forward to energy-saving effect is obvious	Taobao Marketplace, a Chinese website for online shopping
Slu King Flagship Store	King of Power Saving	Saving appliances have been received for a period of time, I feel that the appearance of saving appliances is very new and unique, there are additional features air purification, I feel that the use of this period of time the electricity bill seems to be less than last month, good, I hope to continue to	Taobao Marketplace, a Chinese website for online shopping
Slu King Flagship Store	King of Power Saving	Power saving effect: very good The product works very well I hope to save me more money on my electricity bill each month	Taobao Marketplace, a Chinese website for online shopping
Slu King Flagship Store	King of Power Saving	It's been a long time since I bought it and I don't feel it's useful at all. The big IQ tax is still so expensive. Month after month the electricity bill is still the same amount!	Taobao Marketplace, a Chinese website for online shopping
Slu King Flagship Store	King of Power Saving	Poorly rated, no power savings at all. Customer service is as bad as it gets.	Taobao Marketplace, a Chinese website for online shopping
Slu King Flagship Store	King of Power Saving	I don't know what effect it has either!!!	Taobao Marketplace, a Chinese website for online shopping
Slu King Flagship Store	King of Power Saving	Goods received, used for a period of time, energy-saving effect is very good, the appearance of good-looking style, fine workmanship, the family used three, than before, much more power saving. Kudos!	Taobao Marketplace, a Chinese website for online shopping
Slu King Flagship Store	King of Power Saving	Goods have been received, look at the appearance of the quality is very good, has not been used, do not know what the effect is, looking forward to.	Taobao Marketplace, a Chinese website for online shopping
Slu King Flagship Store	King of Power Saving	It's very good, plug it in and it's easy to use, and there's an extra fuse, it's very good, full five praise!	Taobao Marketplace, a Chinese website for online shopping
Slu King Flagship Store	King of Power Saving	Power-saving effect: trying Cost-effective: very good Appearance and style: atmosphere Workmanship material: good Experience: can be	Taobao Marketplace, a Chinese website for online shopping
Slu King Flagship Store	King of Power Saving	unused	Taobao Marketplace, a Chinese website for online shopping
Slu King Flagship Store	King of Power Saving	Experience: the goods arrived and rushed to use it just to save money on electricity 💰💰💰💰	Taobao Marketplace, a Chinese website for online shopping
Slu King Flagship Store	King of Power Saving	Experience: just installed soon looking forward to good earnings	Taobao Marketplace, a Chinese website for online shopping
Slu King Flagship Store	King of Power Saving	It purifies the air and feels good	Taobao Marketplace, a Chinese website for online shopping
Slu King Flagship Store	King of Power Saving	It doesn't feel good. It's not supposed to be energy efficient.	Taobao Marketplace, a Chinese website for online shopping
Slu King Flagship Store	King of Power Saving	Baby is exquisite, looks great and works well!	Taobao Marketplace, a Chinese website for online shopping
Slu King Flagship Store	King of Power Saving	Power saving effect: good Cost-effective: good Appearance style: very innovative Workmanship material: fine and beautiful Experience: excellent	Taobao Marketplace, a Chinese website for online shopping
Slu King Flagship Store	King of Power Saving	It works really well, and it really saves energy.	Taobao Marketplace, a Chinese website for online shopping
Slu King Flagship Store	King of Power Saving	Received the goods logistics very quickly can not wait to install, plug can be	Taobao Marketplace, a Chinese website for online shopping
Slu King Flagship Store	King of Power Saving	Power-saving effect: praise Cost-effective: praise Appearance style: praise Workmanship material: praise Experience: praise	Taobao Marketplace, a Chinese website for online shopping
Slu King Flagship Store	King of Power Saving	Workmanship material: have received it, feel good, customer service is also active in answering questions	Taobao Marketplace, a Chinese website for online shopping
Slu King Flagship Store	King of Power Saving	an excellent	Taobao Marketplace, a Chinese website for online shopping
Slu King Flagship Store	King of Power Saving	Baby is good, I like it, use it also can, future use, I will promptly share with you, praise!	Taobao Marketplace, a Chinese website for online shopping
Slu King Flagship Store	King of Power Saving	Power saving effect:, try a few days effect is good Cost-effective: energy-saving Appearance style: novel aluminum alloy shell Workmanship material: good quality Experience: and purification function	Taobao Marketplace, a Chinese website for online shopping
Slu King Flagship Store	King of Power Saving	There's also a purification function.	Taobao Marketplace, a Chinese website for online shopping
Slu King Flagship Store	King of Power Saving	Received the goods and used, feel pretty good, looking forward to the effect, five praise, worth getting!	Taobao Marketplace, a Chinese website for online shopping
Slu King Flagship Store	King of Power Saving	Goods received, can not wait to plug in, and the store described the same, truly good, recommended to buy!	Taobao Marketplace, a Chinese website for online shopping
Slu King Flagship Store	King of Power Saving	Things are very good, but also very energy-saving practical, has been the second time to buy!	Taobao Marketplace, a Chinese website for online shopping
Slu King Flagship Store	King of Power Saving	This quality is very good, the highest cost-effective, door and home are used, than before to save a lot of electricity, but also introduced friends to buy, the seller's attitude is good logistics very fast, the	Taobao Marketplace, a Chinese website for online shopping
Slu King Flagship Store	King of Power Saving	Received very quickly, appearance intact, effect to be seen	Taobao Marketplace, a Chinese website for online shopping
Slu King Flagship Store	King of Power Saving	Cost-effective: look forward to the effect of a month after the follow-up evaluation should be no problem also purify the air	Taobao Marketplace, a Chinese website for online shopping
Slu King Flagship Store	King of Power Saving	I received my baby and I'm very happy with it.	Taobao Marketplace, a Chinese website for online shopping
Slu King Flagship Store	King of Power Saving	Received the first time to install the feeling of good quality, good service attitude, the next time to patronize, I wish the boss a prosperous business	Taobao Marketplace, a Chinese website for online shopping
Slu King Flagship Store	King of Power Saving	Already started to use. Looking forward to the effect, the effect of how to follow up will be evaluated Appearance style: just like the picture	Taobao Marketplace, a Chinese website for online shopping

Slu King Flagship Store	King of Power Saving	Experience: just received the goods, first use a few days to see. Do not know how the effect.	Taobao Marketplace, a Chinese website for online shopping
Slu King Flagship Store	King of Power Saving	Fast, good quality, great results, recommend it to friends!	Taobao Marketplace, a Chinese website for online shopping
Slu King Flagship Store	King of Power Saving	Arrived today, used directly, waiting for the effect ...... first-class service, quality assured, give merchants praise	Taobao Marketplace, a Chinese website for online shopping
Slu King Flagship Store	King of Power Saving	Not bad. Not bad!	Taobao Marketplace, a Chinese website for online shopping
Slu King Flagship Store	King of Power Saving	The last time I bought one it was a bit obvious, this one is for my friend, I hope the effect is the same, and I will recommend it to more people in the future.	Taobao Marketplace, a Chinese website for online shopping
Slu King Flagship Store	King of Power Saving	Goods received, the quality is good Oh, intelligent power saving system can be very save, in the introduction of friends also buy this	Taobao Marketplace, a Chinese website for online shopping
Slu King Flagship Store	King of Power Saving	This is the second purchase, the last time I bought has been used for a month, less than the previous power consumption of 120 yuan, a normal month in 600 or so, genuinely save a lot of electricity, the use of large amounts of electricity friends quickly get it, five praise!	Taobao Marketplace, a Chinese website for online shopping
Slu King Flagship Store	King of Power Saving	I couldn't wait to plug in the saver once I received it and it works great. Mom and Dad said they could finally turn on the air conditioner without worry. And the customer service attitude is very good. Problems are helped to solve in a timely manner. This is affordable and energy-saving power saving, will	Taobao Marketplace, a Chinese website for online shopping
Slu King Flagship Store	King of Power Saving	And the store described the same, received the goods and plugged in, the effect is very good, really can save electricity, truly recommended to buy!	Taobao Marketplace, a Chinese website for online shopping
Slu King Flagship Store	King of Power Saving	Logistics is very fast, the courier boy service enthusiasm. Goods packaging intact, after receiving the goods will be opened to use, the reason why now comment is to use a period of time to see how the effect. The results prove that the effect is still possible, with the same description of the business, the power consumption is obviously a little reduced, conscientious business, there is a need to save electrical	Taobao Marketplace, a Chinese website for online shopping
Slu King Flagship Store	King of Power Saving	The material is environmentally friendly and safe, works quietly, and does not get hot throughout use. Indeed is genuine, put in the warehouse to use. Friends use quite good to me also recommended. Very satisfied	Taobao Marketplace, a Chinese website for online shopping
Slu King Flagship Store	King of Power Saving	Received it, opened it up to see that it was intact, plugged it into an outlet and the display light came on! Always good reviews! This time I also believe it will save electricity and give it 5 stars!	Taobao Marketplace, a Chinese website for online shopping
Slu King Flagship Store	King of Power Saving	Things received, used to save much more power cost-effective and inexpensive price is not expensive very satisfied with a shopping	Taobao Marketplace, a Chinese website for online shopping
Slu King Flagship Store	King of Power Saving	Small size, does not take up space. It's very good, it does work very effectively, the tool room uses a lot of electricity for a lot of work. After installing it it did reduce the electricity bill quite a bit. About 30 degrees of electricity a day, work down indeed a few degrees less. Scientific energy saving,	Taobao Marketplace, a Chinese website for online shopping
Slu King Flagship Store	King of Power Saving	I have never used this product, I used it for some days with the intention of trying it, but I didn't expect to really save a lot! It's very good.	Taobao Marketplace, a Chinese website for online shopping
Slu King Flagship Store	King of Power Saving	My sister recommended this place to me, and the monthly electricity bill is very expensive. She said this power saver works really well, so I'm buying one too.	Taobao Marketplace, a Chinese website for online shopping
Slu King Flagship Store	King of Power Saving	Express quite quickly arrived, things are very fine compact and convenient just used a little, great really as described! Good to use!	Taobao Marketplace, a Chinese website for online shopping
Slu King Flagship Store	King of Power Saving	Friends recommended the purchase, used for two days, feel still a little effect, and so on and then use a period of time to see	Taobao Marketplace, a Chinese website for online shopping
Slu King Flagship Store	King of Power Saving	Logistics was fast, used it on arrival, looking forward to next month	Taobao Marketplace, a Chinese website for online shopping
Slu King Flagship Store	King of Power Saving	Seems to work a bit, pretty good, satisfied, looking forward to the subsequent electricity bill now	Taobao Marketplace, a Chinese website for online shopping
Slu King Flagship Store	King of Power Saving	It's not bad, but it's good to try it out and save money.	Taobao Marketplace, a Chinese website for online shopping
Slu King Flagship Store	King of Power Saving	This thing is useless. It's costing me more every month.	Taobao Marketplace, a Chinese website for online shopping
Slu King Flagship Store	King of Power Saving	It's not useful, but rather, it uses more electricity.	Taobao Marketplace, a Chinese website for online shopping
Slu King Flagship Store	King of Power Saving	Appearance and style: just bought watching a lot of buddies in the buy this thing, buy a try.	Taobao Marketplace, a Chinese website for online shopping
Slu King Flagship Store	King of Power Saving	The stuff works very well really.	Taobao Marketplace, a Chinese website for online shopping
Slu King Flagship Store	King of Power Saving	It's useless. It's just a circuit board inside.	Taobao Marketplace, a Chinese website for online shopping
Slu King Flagship Store	King of Power Saving	Workmanship material: very good	Taobao Marketplace, a Chinese website for online shopping
Slu King Flagship Store	King of Power Saving	Just got in to see if my electric bill will be lower next month	Taobao Marketplace, a Chinese website for online shopping
Slu King Flagship Store	King of Power Saving	Very good, works without noise	Taobao Marketplace, a Chinese website for online shopping
Slu King Flagship Store	King of Power Saving	The stuff works great and it's the 2nd time I've bought it.	Taobao Marketplace, a Chinese website for online shopping
Slu King Flagship Store	King of Power Saving	You can save a lot of money in a month.	Taobao Marketplace, a Chinese website for online shopping
Slu King Flagship Store	King of Power Saving	Buy power-saving king, customer service said invalid can be returned for a refund, when this power-saving king with two months, I tested through the use of the results do not play a power-saving effect, and customer service to reflect the situation, and asked for a refund, they replied that with three months will have power-saving, and I listen to them, with the third month, power-saving king is still the same not only will not save electricity, power-saving king themselves also consume electricity, according to this situation, I contacted customer service again, there is no reply. According to this situation, I contacted customer service again, there is no reply, really bad luck, encountered bad business, purely in	Taobao Marketplace, a Chinese website for online shopping

Slu King Flagship Store	King of Power Saving	Used a few months before the evaluation, we must not be fooled to buy, used a few months, every month did not save a penny of electricity, do not know this plug in the home to play what role! Loss I initially bought four, all lies!	Taobao Marketplace, a Chinese website for online shopping
Slu King Flagship Store	King of Power Saving	Before the busy work did not have time to evaluate, the previous evaluation is the system default evaluation, I now give bad, bad, bad, whether there is no effect first, not to mention, after the purchase of inadvertently found that the exact same other sellers of this economizer only sell more than 40 dollars, and this one sells for 168 yuan, more than a hundred dollars more expensive, I was instantly very uncomfortable in the heart. So here I hope to order a small partner cautious and careful. There are pictures as evidence, I hope you can look at it with a sharp eye.	Taobao Marketplace, a Chinese website for online shopping
Slu King Flagship Store	King of Power Saving	Power saving effect: very satisfied Cost-effective: a cost-effective Appearance style: \ beautiful atmosphere	Taobao Marketplace, a Chinese website for online shopping
Slu King Flagship Store	King of Power Saving	I've been using it for a while before coming to evaluate it, and I'm especially happy with it	Taobao Marketplace, a Chinese website for online shopping
Slu King Flagship Store	King of Power Saving	I advise you not to be fooled, not at all useful, pro-measurement of a month gross did not save a little, business false propaganda, inside is a capacitor, not at all power-saving effect, fire also bought two, really **!	Taobao Marketplace, a Chinese website for online shopping
Slu King Flagship Store	King of Power Saving	Effectiveness to be verified	Taobao Marketplace, a Chinese website for online shopping
Slu King Flagship Store	King of Power Saving	Use it for a month and then comment again	Taobao Marketplace, a Chinese website for online shopping
Slu King Flagship Store	King of Power Saving	Power-saving effect is very good, the appearance of the atmosphere, fine workmanship, bought two, logistics fast!	Taobao Marketplace, a Chinese website for online shopping
Slu King Flagship Store	King of Power Saving	Pressure root did not use, buy before said no use contact customer service refund Find customer service to return, simply ignore people, do not return the information Service attitude super super bad, these positive feedback may be brushed out of it!	Taobao Marketplace, a Chinese website for online shopping
Slu King Flagship Store	King of Power Saving	Experience: is using do not know if there is no effect	Taobao Marketplace, a Chinese website for online shopping
Slu King Flagship Store	King of Power Saving	I just started using it, I'll comment after a month	Taobao Marketplace, a Chinese website for online shopping
Slu King Flagship Store	King of Power Saving	Workmanship material: should work! Looking forward to looking forward to	Taobao Marketplace, a Chinese website for online shopping
Slu King Flagship Store	King of Power Saving	I don't see any effect, but rather a few degrees more than before.	Taobao Marketplace, a Chinese website for online shopping
Slu King Flagship Store	King of Power Saving	It's okay.	Taobao Marketplace, a Chinese website for online shopping
Slu King Flagship Store	King of Power Saving	Power saving effect: good	Taobao Marketplace, a Chinese website for online shopping
Slu King Flagship Store	King of Power Saving	Very beautiful and sophisticated and so power efficient!	Taobao Marketplace, a Chinese website for online shopping
Slu King Flagship Store	King of Power Saving	Power saving effect: try to say Price: quite good Appearance style: quite lightweight Workmanship material: good Experience: look forward to saving money	Taobao Marketplace, a Chinese website for online shopping
Slu King Flagship Store	King of Power Saving	I've been using it for a while now, and I can see some results in my electricity bill.	Taobao Marketplace, a Chinese website for online shopping
Slu King Flagship Store	King of Power Saving	Special to change the distribution box, my home recently 50 degrees per day up and down, next to the electricity box to connect a, the house to connect a, the two together a day to save about 10 degrees of electricity, or 鈥挻e satisfied with two months back to the capital!	Taobao Marketplace, a Chinese website for online shopping
Slu King Flagship Store	King of Power Saving	Look good, summer air conditioning power consumption, buy plugged in the first use to see how much can save, save more will buy again!	Taobao Marketplace, a Chinese website for online shopping
Slu King Flagship Store	King of Power Saving	Started to use, indeed can connect the power saving, in the end how much still need to use more see	Taobao Marketplace, a Chinese website for online shopping
Slu King Flagship Store	King of Power Saving	This doesn't feel like it's worth it, and the power savings are unknown. Not recommended.	Taobao Marketplace, a Chinese website for online shopping
Slu King Flagship Store	King of Power Saving	The reviews are factual! First, Bright Lights is using this product. Price $168, the same incidentally, buy elsewhere $65. It's exactly the same, compared over and over again, and sure enough it's the same product, both in terms of packaging and anti-counterfeiting. Secondly, just to say, boss, can you be real? Doubling it a couple times, is that a test of our consumer IQ tax? I bought it directly because I didn't compare, because of trust, so, today I just so feel the feeling of being pitched!	Taobao Marketplace, a Chinese website for online shopping
Slu King Flagship Store	King of Power Saving	I got it right away, and I've got to certify that it looks good and that it's made of good material.	Taobao Marketplace, a Chinese website for online shopping
Slu King Flagship Store	King of Power Saving	Power saving effect:, just received the goods do not know whether the effect is good or not	Taobao Marketplace, a Chinese website for online shopping
Slu King Flagship Store	King of Power Saving	Good quality, good price, power saving effect is obvious, cost-effective, worth buying, recommend that you buy, recommend that you buy!	Taobao Marketplace, a Chinese website for online shopping
Slu King Flagship Store	King of Power Saving	Good quality, good price, power saving effect is obvious, cost-effective, worth buying, recommend that you buy, recommend that you buy!	Taobao Marketplace, a Chinese website for online shopping
Slu King Flagship Store	King of Power Saving	Customer service was very professional in their instructions. Press to use on front end outlet at home! Current through the king of power saving. Work compensation effect is indeed very powerful [strong] Comparison of previous years at this time to open the air conditioning a month are almost 600 degrees Now the same month the same use also smooth at more than 400 degrees	Taobao Marketplace, a Chinese website for online shopping
Slu King Flagship Store	King of Power Saving	Good, Appearance style: 👍👍👍👍 Workmanship material: 👍👍👍👍👍 Experience: 👍👍👍👍👍	Taobao Marketplace, a Chinese website for online shopping
Slu King Flagship Store	King of Power Saving	I've been using Save's Jedi Jedi, and I've recommended it to my friends.	Taobao Marketplace, a Chinese website for online shopping
Slu King Flagship Store	King of Power Saving	Good quality and guaranteed	Taobao Marketplace, a Chinese website for online shopping
Slu King Flagship Store	King of Power Saving	Haven't looked at it yet don't know if it saves power	Taobao Marketplace, a Chinese website for online shopping

Slu King Flagship Store	King of Power Saving	Things are very good, in the hand is quite weighty, small and exquisite night can also be a night light to make, power saving effect is very good!	Taobao Marketplace, a Chinese website for online shopping
Slu King Flagship Store	King of Power Saving	Plug it in and start the power saving mode, we'll see what happens in a month. My friend also bought Power Saver and said it works, so I bought one to try.	Taobao Marketplace, a Chinese website for online shopping
Slu King Flagship Store	King of Power Saving	Great power saver, get one if you need it haha	Taobao Marketplace, a Chinese website for online shopping
Slu King Flagship Store	King of Power Saving	Power Saving King received, fast delivery! Well packaged, good quality, good power saving effect! Will promote and recommend to friends! Five-star praise!	Taobao Marketplace, a Chinese website for online shopping
Slu King Flagship Store	King of Power Saving	This is my second purchase and the quality is excellent and exceptionally energy efficient. Keep buying and get more for your family.	Taobao Marketplace, a Chinese website for online shopping
Slu King Flagship Store	King of Power Saving	It's pretty good, saves a bit of electricity compared to others, and I'm happy with the results!	Taobao Marketplace, a Chinese website for online shopping
Slu King Flagship Store	King of Power Saving	The energy saving meter is very practical, you can save space and put so many things in it.	Taobao Marketplace, a Chinese website for online shopping
Slu King Flagship Store	King of Power Saving	Feel pretty good, too much electricity at home, get one to try, next month good to continue to buy back, praise praise praise!	Taobao Marketplace, a Chinese website for online shopping
Slu King Flagship Store	King of Power Saving	an excellent	Taobao Marketplace, a Chinese website for online shopping
Slu King Flagship Store	King of Power Saving	Small and compact feel, quite like it very good	Taobao Marketplace, a Chinese website for online shopping
Slu King Flagship Store	King of Power Saving	Fast shipping and good packaging, its description and match, has been connected to the power in use, can save power to view the effect of the product is good! Give praise.	Taobao Marketplace, a Chinese website for online shopping
Slu King Flagship Store	King of Power Saving	Fast shipping, good power savings, cost effective	Taobao Marketplace, a Chinese website for online shopping
Slu King Flagship Store	King of Power Saving	Very good, bought for the elderly use, recently looked at the bill every month to save electricity. Good product can save me electricity are worth the cost! In the repurchase a to their own work rent here to use!	Taobao Marketplace, a Chinese website for online shopping
Slu King Flagship Store	King of Power Saving	After receiving the baby with the effect is very good, after the installation does play a power saving effect, you can rest assured that the purchase of the	Taobao Marketplace, a Chinese website for online shopping
Slu King Flagship Store	King of Power Saving	Depends on the line. Before see friends use a month less more than 80 degrees, I also buy a use A month only saved more than 30 dollars Effective or effective is more points on the good!	Taobao Marketplace, a Chinese website for online shopping
Slu King Flagship Store	King of Power Saving	Received, was broken by the courier, and the business said immediately sent me a new one! Service is really good. Replacement of this is very good	Taobao Marketplace, a Chinese website for online shopping
Slu King Flagship Store	King of Power Saving	It works fine and saves electricity. The work room uses a lot of electricity machine after using the same electricity is indeed less than 60 degrees of electricity or very useful and cost-effective	Taobao Marketplace, a Chinese website for online shopping
Slu King Flagship Store	King of Power Saving	Very good, especially practical	Taobao Marketplace, a Chinese website for online shopping
Slu King Flagship Store	King of Power Saving	The appearance is clean and beautiful, the workmanship is very good, I did not find any flaws, I personally feel that the size is also quite suitable, I have been looking for a long time to choose this one, love it!	Taobao Marketplace, a Chinese website for online shopping
Slu King Flagship Store	King of Power Saving	Works great, electric bill is down a lot worth repurchasing recommend! Over 40 degrees less in a month Was over 600 degrees.	Taobao Marketplace, a Chinese website for online shopping
Slu King Flagship Store	a power saver (electronics)	Great, I'll give it a try and see.	Taobao Marketplace, a Chinese website for online shopping
Slu King Flagship Store	a power saver (electronics)	This is the second repurchase, the first time I bought one and used it for 2 months, saving more than 60 degrees of electricity	Taobao Marketplace, a Chinese website for online shopping
Slu King Flagship Store	a power saver (electronics)	No power saving features at all. Totally intellectually taxing.	Taobao Marketplace, a Chinese website for online shopping
Slu King Flagship Store	a power saver (electronics)	I've got it. I'm trying it out. It's a beautiful design.	Taobao Marketplace, a Chinese website for online shopping
Slu King Flagship Store	a power saver (electronics)	This product does not work at all, to customer service contact customer service does not talk, buy this product are fooled. There is no effect at all.	Taobao Marketplace, a Chinese website for online shopping
Slu King Flagship Store	a power saver (electronics)	I don't see any effect.	Taobao Marketplace, a Chinese website for online shopping
Slu King Flagship Store	a power saver (electronics)	It is for the good of all, I hope to save electricity, is to save energy, the desired effect of achievement	Taobao Marketplace, a Chinese website for online shopping
Slu King Flagship Store	a power saver (electronics)	goody	Taobao Marketplace, a Chinese website for online shopping
Slu King Flagship Store	a power saver (electronics)	Power-saving effect: < good	Taobao Marketplace, a Chinese website for online shopping
Slu King Flagship Store	a power saver (electronics)	Power-saving effect: read the evaluation, the effect is all very good Workmanship material: the quality is quite good Use experience: simple and easy to use	Taobao Marketplace, a Chinese website for online shopping
Slu King Flagship Store	a power saver (electronics)	Purchase with a try attitude, after using it for a while, then come back to the review	Taobao Marketplace, a Chinese website for online shopping
Slu King Flagship Store	a power saver (electronics)	Power saving effect: useless, paid the IQ tax	Taobao Marketplace, a Chinese website for online shopping
Slu King Flagship Store	a power saver (electronics)	I haven't used it yet, I don't know if it's bragging	Taobao Marketplace, a Chinese website for online shopping
Slu King Flagship Store	a power saver (electronics)	. Saver received seller shipment fast. Product workmanship fine appearance and good quality, the use of the effect of follow-up comments. Favorable comments five stars.	Taobao Marketplace, a Chinese website for online shopping

Slu King Flagship Store	a power saver (electronics)	Good baby! Good baby! Good baby! Good review!	Taobao Marketplace, a Chinese website for online shopping
Slu King Flagship Store	a power saver (electronics)	*No use, it's a lie!	Taobao Marketplace, a Chinese website for online shopping
Slu King Flagship Store	a power saver (electronics)	It works.	Taobao Marketplace, a Chinese website for online shopping
Slu King Flagship Store	a power saver (electronics)	Goods received quite good-looking, just used a few days, and so on next month's electricity bill came out to know how much the electricity bill can save next month to comment on the business service attitude is very good!	Taobao Marketplace, a Chinese website for online shopping
Slu King Flagship Store	a power saver (electronics)	Goods received, intelligent power saver appearance design is good compact, good quality, read a lot of reviews say very save electricity, I immediately ordered to try, home appliances more effective will continue to buy back.	Taobao Marketplace, a Chinese website for online shopping
Slu King Flagship Store	a power saver (electronics)	Now accommodation, usually consume more power, accidentally swiped this product, the appearance really attracted me, ordered one, as always, good quality, very like a product, very good! o(*￣︶￣*)o	Taobao Marketplace, a Chinese website for online shopping
Slu King Flagship Store	a power saver (electronics)	False advertisements at the head and the tail	Taobao Marketplace, a Chinese website for online shopping
Slu King Flagship Store	a power saver (electronics)	Saving appliances are really too good to use, because the relatives of the family electricity is very expensive, after buying this device are said to be very effective, thanks to the patience of the seller's reception, and will recommend more relatives and friends!	Taobao Marketplace, a Chinese website for online shopping
Slu King Flagship Store	a power saver (electronics)	This energy saver can save electricity is to save money plug it in the elderly will also use how convenient, the style is very like, really good! I want to buy 2 more	Taobao Marketplace, a Chinese website for online shopping
Slu King Flagship Store	a power saver (electronics)	Intelligent power saver quickly arrived, opened to see, the appearance is very beautiful it, get it plugged into the socket, before looking at a friend's home to install this, according to him, the power saving effect is quite good, so take advantage of the activities to buy one.	Taobao Marketplace, a Chinese website for online shopping
Slu King Flagship Store	a power saver (electronics)	This side of the home due to the meter power consumption is relatively large, my colleague introduced me to buy a power saver, he said his family is also using this store to buy a power saver, I want to buy the link, looking forward to power saving effect!	Taobao Marketplace, a Chinese website for online shopping
Slu King Flagship Store	a power saver (electronics)	Cold dad bought a high-power heating furnace, warm is warm, is the electricity bill a little unbearable, see the neighbors installed a power-saving appliances, I also went to buy back, the quality is very good, the appearance is very beautiful, get plugged directly into the outlet after the	Taobao Marketplace, a Chinese website for online shopping
Slu King Flagship Store	a power saver (electronics)	What a surprise, or a friend recommended, there are actually so good things, before wasting too much electricity, friends used 5 effect is very obvious, this time I consulted the business, our home power consumption is very large, looking forward to next month's results	Taobao Marketplace, a Chinese website for online shopping
Slu King Flagship Store	a power saver (electronics)	This intelligent energy saver home model is quite good, and bought another one, have thrown away the previous 16 yuan bought on Poundland, not only does not save electricity but also burned my kettle, this energy saver can stabilize the voltage, energy saving and consumption, it's very good!	Taobao Marketplace, a Chinese website for online shopping
Slu King Flagship Store	a power saver (electronics)	Power saving effect is very good, this time it is for the father-in-law's home shot, the old man likes it very much, energy saving, energy saving, compact and easy to use, protect home appliances, voltage, current stability, very good.	Taobao Marketplace, a Chinese website for online shopping
Slu King Flagship Store	a power saver (electronics)	Feel the effect is not bad, my side is living in a rental house. Originally thought that the smart meter can not be used, I did not think that the smart meter can also save electricity, consulting the store gave me a recommendation, decisive order to buy!	Taobao Marketplace, a Chinese website for online shopping
Slu King Flagship Store	a power saver (electronics)	I am out to stay outside, this side of the meter do not know how to go very fast, just started not too obvious, the appearance is very delicate, looking forward to the effect	Taobao Marketplace, a Chinese website for online shopping
Slu King Flagship Store	a power saver (electronics)	Received, looks pretty good, only a few days, the effect has not been compared out, next month the electricity bill out of the follow-up effect, the seller's attitude is very good, especially enthusiastic, I hope business is getting better and better!	Taobao Marketplace, a Chinese website for online shopping
Slu King Flagship Store	a power saver (electronics)	Intelligent compensation economizer received, looks very sturdy, like it very much. It is being used, very satisfied with a shopping, very easy to use. Buy home and plug it into the socket. Recommended to buy with confidence!	Taobao Marketplace, a Chinese website for online shopping
Slu King Flagship Store	a power saver (electronics)	Used for a week, everything is normal, and does not affect the line at home, and is very convenient, just plug in the socket can be, before a friend bought, the feedback is very good, I also asked him to buy me a, see after a month or two after the electricity bill.	Taobao Marketplace, a Chinese website for online shopping
Slu King Flagship Store	a power saver (electronics)	Energy saving effect is very good Use to save electricity and save money Can save electricity for the family, beautiful shape, exquisite workmanship worth buying.	Taobao Marketplace, a Chinese website for online shopping
Slu King Flagship Store	a power saver (electronics)	Intelligent power saver use effect is very good, can effectively stabilize the voltage, reduce circuit losses to achieve the effect of energy saving. It is also inadvertently see a friend's home has to use, by his recommendation to buy a first use.	Taobao Marketplace, a Chinese website for online shopping
Slu King Flagship Store	a power saver (electronics)	This side is to live in an apartment, this side of everything is good is expensive electricity, this side to friends as guests, friends introduced me to buy this, just a few days is not too obvious, with the effect of the feeling is quite good!	Taobao Marketplace, a Chinese website for online shopping
Slu King Flagship Store	a power saver (electronics)	I'm an electrician, was to try the psychological buy, did not expect the effect is not bad, this thing is not bad, now the science is really a generation to update, even I'm an electrician feel incredible, the effect is okay	Taobao Marketplace, a Chinese website for online shopping
Slu King Flagship Store	a power saver (electronics)	At the same time to buy three styles of power saver, this installed in the first floor of the home, the other two installed in the second floor of the home, to see which effect is better, but look at this power saver is very beautiful workmanship, first plugged into the socket to use for a period of time, to see the effect of how to come back to comment.	Taobao Marketplace, a Chinese website for online shopping
Slu King Flagship Store	a power saver (electronics)	Home appliances, electricity is very expensive, I hope that this device can save a little is a little, the quality of workmanship packaging, especially customer service attitude is really nothing to say, praise!	Taobao Marketplace, a Chinese website for online shopping
Slu King Flagship Store	a power saver (electronics)	Received, looks pretty good, only a few days, the effect has not been compared out, next month the electricity bill out of the follow-up effect, the seller's attitude is very good, especially enthusiastic, I hope business is getting better and better!	Taobao Marketplace, a Chinese website for online shopping
Slu King Flagship Store	a power saver (electronics)	Friends recommended, buy back and start using, before every month need to pay three or four hundred of the electricity bill, I have to say the effect is really very good, in a few days will continue to buy back a , this can be more energy-saving!	Taobao Marketplace, a Chinese website for online shopping
Slu King Flagship Store	a power saver (electronics)	Logistics is very fast, my son immediately disassembled to play, was I beat up, haha, product quality is really good, was he fell after a little trace is not, praise!	Taobao Marketplace, a Chinese website for online shopping
Slu King Flagship Store	a power saver (electronics)	Energy saving appliances received today and used it, the cost-effectiveness, appearance and style is very good, exquisite workmanship! Energy-saving appliances as soon as received and started to enable, looks good quality!	Taobao Marketplace, a Chinese website for online shopping
Slu King Flagship Store	a power saver (electronics)	The goods are very fast, the quality of the power saver is very good, and the customer is patient. Just used it, looking forward to using the power saving, exquisite workmanship, the material is exquisite	Taobao Marketplace, a Chinese website for online shopping
Slu King Flagship Store	a power saver (electronics)	Used more than one product, on the effect of this is the most stable, is indeed a penny a good, especially to the sales customer service point of praise, I would have liked to buy other models, said I do not use much electricity, a month on more than 80 degrees , this is the right, the gentleman to take	Taobao Marketplace, a Chinese website for online shopping
Slu King Flagship Store	a power saver (electronics)	The quality of this product is very good, excellent workmanship materials, it is worth recommending the purchase. Looking forward to everyone to see samples to buy. I score full marks.	Taobao Marketplace, a Chinese website for online shopping
Slu King Flagship Store	a power saver (electronics)	Baby received, a friend recommended the link, and was not disappointed, the instructions are very detailed, the business customer service response is very good to ask questions. The after-sales aspect is in place, Taobao platform is really trustworthy.	Taobao Marketplace, a Chinese website for online shopping

Slu King Flagship Store	Intelligent Power Saver	Fast delivery, cost-effective Power-saving effect: power-saving effect is good, the store has a good service attitude, Appearance style: fine workmanship.	Taobao Marketplace, a Chinese website for online shopping
Slu King Flagship Store	Intelligent Power Saver	Not bad. Very good.	Taobao Marketplace, a Chinese website for online shopping
Slu King Flagship Store	Intelligent Power Saver	I'll comment after I use it.	Taobao Marketplace, a Chinese website for online shopping
Slu King Flagship Store	Intelligent Power Saver	Things received, the second purchase, a one-time purchase of 2, buy two get one free, very affordable, tightly packaged, very well protected, very easy to install, fast shipping, the seller has a very good service attitude, must be five praise.	Taobao Marketplace, a Chinese website for online shopping
Slu King Flagship Store	Intelligent Power Saver	The air conditioner can be happy with this little gem.	Taobao Marketplace, a Chinese website for online shopping
Slu King Flagship Store	Intelligent Power Saver	Just received the goods and will see the effect in January	Taobao Marketplace, a Chinese website for online shopping
Slu King Flagship Store	Intelligent Power Saver	Things received, very good-looking, with a large LED LCD yo, texture is good, very high-tech, power saving is still in use, the specific effect, and then make up the comment on the use of the experience, the seller's enthusiasm for service, fast delivery, five points praise!	Taobao Marketplace, a Chinese website for online shopping
Slu King Flagship Store	Intelligent Power Saver	The picture has not been able to get on , add the picture can not evaluate, only to type, five points praise!	Taobao Marketplace, a Chinese website for online shopping
Slu King Flagship Store	Intelligent Power Saver	A proper IQ tax.	Taobao Marketplace, a Chinese website for online shopping
Slu King Flagship Store	Intelligent Power Saver	Power saving effect: (good)	Taobao Marketplace, a Chinese website for online shopping
Slu King Flagship Store	Intelligent Power Saver	Baby received well-packaged freight and fast, performance and effect is consistent with the description of the business to wait a month to see the results, the results are satisfied with continue to buy!	Taobao Marketplace, a Chinese website for online shopping
Slu King Flagship Store	Intelligent Power Saver	Baby received, just started using it hope it works!	Taobao Marketplace, a Chinese website for online shopping
Slu King Flagship Store	Intelligent Power Saver	I don't know if it works, I bought one before and it broke in less than a year.	Taobao Marketplace, a Chinese website for online shopping
Slu King Flagship Store	Intelligent Power Saver	Power-saving effect: just installed, no effect for the time being, two months later to comment again!	Taobao Marketplace, a Chinese website for online shopping
Slu King Flagship Store	Intelligent Power Saver	I won't know if it works until a month later.	Taobao Marketplace, a Chinese website for online shopping
Slu King Flagship Store	Intelligent Power Saver	Very good quality, very happy with the purchase. Customer service is very professional with questions and answers!!!!	Taobao Marketplace, a Chinese website for online shopping
Slu King Flagship Store	Intelligent Power Saver	It's not much use.	Taobao Marketplace, a Chinese website for online shopping
Slu King Flagship Store	Intelligent Power Saver	Good quality product, recommend it.	Taobao Marketplace, a Chinese website for online shopping
Slu King Flagship Store	Intelligent Power Saver	It's good, it's practical, the boss is a good talker, the delivery is fast enough.	Taobao Marketplace, a Chinese website for online shopping
Slu King Flagship Store	Intelligent Power Saver	Don't buy it, I've been using it for 2 months and it's increasing instead of decreasing, **shop**!	Taobao Marketplace, a Chinese website for online shopping
Slu King Flagship Store	Intelligent Power Saver	Not great, haven't seen any results	Taobao Marketplace, a Chinese website for online shopping
Slu King Flagship Store	Intelligent Power Saver	Received the goods, used a few days do not know the effect, first give a favorable comment	Taobao Marketplace, a Chinese website for online shopping
Slu King Flagship Store	Intelligent Power Saver	I've been using it for a while now, and it feels fine, so I'm looking forward to a month's worth of results.	Taobao Marketplace, a Chinese website for online shopping
Slu King Flagship Store	Intelligent Power Saver	Workmanship material: okay Power-saving effect: just started to use Cost-effective: still relatively affordable	Taobao Marketplace, a Chinese website for online shopping
Slu King Flagship Store	Intelligent Power Saver	Power-saving effect: the goods have been received, use to see how the effect Appearance style: the appearance is very delicate Workmanship material: the material is very strong	Taobao Marketplace, a Chinese website for online shopping
Slu King Flagship Store	Intelligent Power Saver	Baby received, have not yet begun to use, but the principle is very clear. I hope it is useful, the boss is very favorable, buy two and get one free, thank you!	Taobao Marketplace, a Chinese website for online shopping
Slu King Flagship Store	Intelligent Power Saver	The product has been received, the effect is good very good, very satisfied with the store five-star praise!	Taobao Marketplace, a Chinese website for online shopping
Slu King Flagship Store	Intelligent Power Saver	The product has been received to try a very good effect is good very good to the store follow-up comments	Taobao Marketplace, a Chinese website for online shopping
Slu King Flagship Store	Intelligent Power Saver	Baby received, charger is good now, good	Taobao Marketplace, a Chinese website for online shopping
Slu King Flagship Store	Intelligent Power Saver	It's been plugged in for over a month and it's as good as unplugged.	Taobao Marketplace, a Chinese website for online shopping
Slu King Flagship Store	Intelligent Power Saver	Used it and got the big energy one too	Taobao Marketplace, a Chinese website for online shopping
Slu King Flagship Store	Intelligent Power Saver	This thing doesn't save power at all it's useless	Taobao Marketplace, a Chinese website for online shopping
Slu King Flagship Store	Intelligent Power Saver	Energy saver fine workmanship, novel appearance, inexpensive, after the receipt of the installation, looking forward to the use of the effect, plus follow-up, customer service attitude, logistics, five-star praise!	Taobao Marketplace, a Chinese website for online shopping
Slu King Flagship Store	Intelligent Power Saver	Pretty good, try it at home and come back in a month to comment	Taobao Marketplace, a Chinese website for online shopping

Slu King Flagship Store	Intelligent Power Saver	It looks fine to me. We'll see if it works or not when we try it.	Taobao Marketplace, a Chinese website for online shopping
Slu King Flagship Store	Intelligent Power Saver	I bought one last time and it worked well, so I'm ordering another one for my family.	Taobao Marketplace, a Chinese website for online shopping
Slu King Flagship Store	Intelligent Power Saver	You can save a little bit of money on your electricity bill every month, so we recommend you to buy it.	Taobao Marketplace, a Chinese website for online shopping
Slu King Flagship Store	Intelligent Power Saver	(of an unmarried couple) be close	Taobao Marketplace, a Chinese website for online shopping
Slu King Flagship Store	Intelligent Power Saver	Used for more than a month, no effect, disassembled to see a LED light, do you believe that can have the effect of power saving? Don't be fooled again!	Taobao Marketplace, a Chinese website for online shopping
Slu King Flagship Store	Intelligent Power Saver	It's a scam. Don't buy it.	Taobao Marketplace, a Chinese website for online shopping
Slu King Flagship Store	Intelligent Power Saver	Fast shipping and good seller service, very happy so far!	Taobao Marketplace, a Chinese website for online shopping
Slu King Flagship Store	Intelligent Power Saver	Really saves money on the electric bill each month and have already recommended to friends to buy. Must praise	Taobao Marketplace, a Chinese website for online shopping
Slu King Flagship Store	Intelligent Power Saver	I'm going to buy two more for my family.	Taobao Marketplace, a Chinese website for online shopping
Slu King Flagship Store	Intelligent Power Saver	Power savings: very good, I bought one before and used it for about 4 years, moved again and bought a new one.	Taobao Marketplace, a Chinese website for online shopping
Slu King Flagship Store	Intelligent Power Saver	Cost-effective: wait to share with you Appearance style: look like high-tech, after using the follow-up comment Experience: buy back are used, look forward to the effect it	Taobao Marketplace, a Chinese website for online shopping
Slu King Flagship Store	Intelligent Power Saver	Power saving effect: very good Cost-effective: high Appearance style: beautiful appearance Workmanship material: very good workmanship	Taobao Marketplace, a Chinese website for online shopping
Slu King Flagship Store	Intelligent Power Saver	Power saving effect: a full month to compare. Appearance and style: it looks good. Use experience: quite easy to use, plug in the outlet.	Taobao Marketplace, a Chinese website for online shopping
Slu King Flagship Store	Intelligent Power Saver	The logistics were terrible and no notification was given when it arrived. If you don't check on your own, you may not know for the rest of your life that your purchase has arrived.	Taobao Marketplace, a Chinese website for online shopping
Slu King Flagship Store	Intelligent Power Saver	Satisfied Satisfied Satisfied Satisfied Satisfied Satisfied Satisfied Satisfied Satisfied Satisfied Satisfied Situation	Taobao Marketplace, a Chinese website for online shopping
Slu King Flagship Store	Intelligent Power Saver	I've seen other people buy it and use it, and I've heard great things about it, so I'm looking forward to being surprised!	Taobao Marketplace, a Chinese website for online shopping
Slu King Flagship Store	Intelligent Power Saver	Just received yesterday, do not know what the effect is, use a period of time to add.	Taobao Marketplace, a Chinese website for online shopping
Slu King Flagship Store	Intelligent Power Saver	Baby received, after arriving home to open and use, the product feel good, easy to use, good value, worth buying.	Taobao Marketplace, a Chinese website for online shopping
Slu King Flagship Store	Intelligent Power Saver	Baby has received 🎈, super like, high value and practical, super like! ❥❥❥❥ Just don't know how effective the power saving is? I'll add more comments after using it.	Taobao Marketplace, a Chinese website for online shopping
Slu King Flagship Store	Intelligent Power Saver	I've used it before and it works well, so I recommend it!	Taobao Marketplace, a Chinese website for online shopping
Slu King Flagship Store	Intelligent Power Saver	serviceable	Taobao Marketplace, a Chinese website for online shopping
Slu King Flagship Store	Intelligent Power Saver	This thing is just a plastic shell, it's useless, there's just a small light inside, it's not useful at all. He's just cheating.	Taobao Marketplace, a Chinese website for online shopping
Slu King Flagship Store	Intelligent Power Saver	There's no IQ tax. Don't be fooled.	Taobao Marketplace, a Chinese website for online shopping
Slu King Flagship Store	Intelligent Power Saver	The product has been received, compact does not take up space, power-saving effect is good, recommended to the side of the friend	Taobao Marketplace, a Chinese website for online shopping
Slu King Flagship Store	Intelligent Power Saver	The stuff has been received. Looks good. Waiting to see the results. Let's hope it works.	Taobao Marketplace, a Chinese website for online shopping
Slu King Flagship Store	Intelligent Power Saver	Just started using it, hope it works	Taobao Marketplace, a Chinese website for online shopping
Slu King Flagship Store	Intelligent Power Saver	Appearance and style: very delicate, workmanship materials: fine workmanship, power saving effect: just used, do not know the effect, after a few days to comment. Experience: now no effect, in a few days to see it, but hopefully as good as said. Cost-effective: cost-effective or good.	Taobao Marketplace, a Chinese website for online shopping
Slu King Flagship Store	Intelligent Power Saver	Power-saving effect: feel down the meter did not run so fast before, workmanship material: feel quite good	Taobao Marketplace, a Chinese website for online shopping
Slu King Flagship Store	Intelligent Power Saver	It's no use.	Taobao Marketplace, a Chinese website for online shopping
Slu King Flagship Store	Intelligent Power Saver	Power-saving effect: in the test, see the effect Cost-effective: superb Appearance and style: good Workmanship material: good Experience: good	Taobao Marketplace, a Chinese website for online shopping
Slu King Flagship Store	Intelligent Power Saver	I'll comment after using it.	Taobao Marketplace, a Chinese website for online shopping
Slu King Flagship Store	Intelligent Power Saver	Fake! Real fakes! Fake without the technicalities!	Taobao Marketplace, a Chinese website for online shopping
Slu King Flagship Store	Intelligent Power Saver	Received the goods quickly, small and compact, does not take up space, looking forward to the effect.	Taobao Marketplace, a Chinese website for online shopping
Slu King Flagship Store	Intelligent Power Saver	Did not use, proper IQ tax, completely fraudulent, from March 7 to buy to now has been four months, every month the electricity bill is the highest in the factory, with a month did not see the effect, contact customer service said to use to three months to see the effect, with four months, contact customer service, customer service are ignoring people, please do not be fooled!	Taobao Marketplace, a Chinese website for online shopping

Slu King Flagship Store	Intelligent Power Saver	Appearance and style: quite compact, plugged directly with, just received do not know how the effect.	Taobao Marketplace, a Chinese website for online shopping
Slu King Flagship Store	Intelligent Power Saver	Works great, definitely recommend to friends and family!	Taobao Marketplace, a Chinese website for online shopping
Slu King Flagship Store	Intelligent Power Saver	Received the goods, plugged it in, just waiting to see the results, with the use of if good in the chase flat!	Taobao Marketplace, a Chinese website for online shopping
Slu King Flagship Store	Intelligent Power Saver	Held try to buy the heart, the results were surprising, used for a period of time to compare does have the effect, good stuff worth having a year to save a lot of money.	Taobao Marketplace, a Chinese website for online shopping
Slu King Flagship Store	Intelligent Power Saver	Power-saving effect: the effect is very good Cost-effective: cost-effective very high Workmanship material: things are good quality, look very texture	Taobao Marketplace, a Chinese website for online shopping
Slu King Flagship Store	Intelligent Power Saver	Experience: already plugged in the first use to look forward to the effect, I hope it will be good!	Taobao Marketplace, a Chinese website for online shopping
Slu King Flagship Store	Intelligent Power Saver	Photographed 4 also sent 2, small, does not take up space, very affordable, the principle of power saving instructions are also very clear, according to customer service guidance on the use of the requirements to go! Shipping is extremely fast! There is a promise of results, you can rest assured that the purchase! Picked several before selecting, live up to expectations!	Taobao Marketplace, a Chinese website for online shopping
Slu King Flagship Store	Intelligent Power Saver	Power saving effect: good	Taobao Marketplace, a Chinese website for online shopping
Slu King Flagship Store	Intelligent Power Saver	Haven't used it yet, read the manual and customers who have already used it say the power saving effect is good, if it does save power, will definitely add two more!	Taobao Marketplace, a Chinese website for online shopping
Slu King Flagship Store	Intelligent Power Saver	It's not bad, just arrived today, use it for a while and see if it's good, I'll continue to buy it.	Taobao Marketplace, a Chinese website for online shopping
Slu King Flagship Store	Intelligent Power Saver	Looks good, I'll follow up after I use it	Taobao Marketplace, a Chinese website for online shopping
Slu King Flagship Store	Intelligent Power Saver	Here's a picture of the interior. It's plastic. It's very light.	Taobao Marketplace, a Chinese website for online shopping
Slu King Flagship Store	Intelligent Power Saver	This is what you call a super power saver. It's a big lie.	Taobao Marketplace, a Chinese website for online shopping
Slu King Flagship Store	Intelligent Power Saver	The packaging is intact, looking forward to the effect will repurchase!	Taobao Marketplace, a Chinese website for online shopping
Slu King Flagship Store	Intelligent Power Saver	Experience: just used it today don't know yet	Taobao Marketplace, a Chinese website for online shopping
Slu King Flagship Store	Intelligent Power Saver	Power saving effect: your product quality is very good, transportation is fast, service quality is very good, creditability is high, worthy of praise 👍!	Taobao Marketplace, a Chinese website for online shopping
Slu King Flagship Store	Intelligent Power Saver	In use!	Taobao Marketplace, a Chinese website for online shopping
Slu King Flagship Store	Intelligent Power Saver	Not useful at all! It doesn't save electricity! It's useless.	Taobao Marketplace, a Chinese website for online shopping
Slu King Flagship Store	Intelligent Power Saver	Good good good good	Taobao Marketplace, a Chinese website for online shopping
Slu King Flagship Store	Intelligent Power Saver	Very good stuff, saves money on your electric bill, repurchased this time!	Taobao Marketplace, a Chinese website for online shopping
Slu King Flagship Store	Intelligent Power Saver	It's not working. People didn't buy it.	Taobao Marketplace, a Chinese website for online shopping
Slu King Flagship Store	Intelligent Power Saver	The stuff is great, I used it right after I received it, good customer service, and I'll follow up next month when I see how many degrees I get on my electric bill.	Taobao Marketplace, a Chinese website for online shopping
Slu King Flagship Store	Intelligent Power Saver	Things received, packaging is very good, open a look, the quality is very good, fine workmanship	Taobao Marketplace, a Chinese website for online shopping
Slu King Flagship Store	Intelligent Power Saver	Use it one after the other, can't rate it yet	Taobao Marketplace, a Chinese website for online shopping
Slu King Flagship Store	Intelligent Power Saver	I have used it for some time and it feels fine. You can order if you want.	Taobao Marketplace, a Chinese website for online shopping
Slu King Flagship Store	Intelligent Power Saver	It basically didn't work, and not only did the electric bill stay the same, it was a couple dozen dollars more than usual	Taobao Marketplace, a Chinese website for online shopping
Slu King Flagship Store	Intelligent Power Saver	Have not used it, ask customer service home to buy two or three to be able to	Taobao Marketplace, a Chinese website for online shopping
Slu King Flagship Store	Intelligent Power Saver	There is a power saving effect so I am buying it for the second time.	Taobao Marketplace, a Chinese website for online shopping
Slu King Flagship Store	Intelligent Power Saver	Power-saving effect: just received the power-saving effect can be used for a period of time to know, if the effect is good to ask my friends to buy a Appearance style: quite good, there are attacking the display Experience: now being used	Taobao Marketplace, a Chinese website for online shopping
Slu King Flagship Store	Intelligent Power Saver	Don't know the effects yet.	Taobao Marketplace, a Chinese website for online shopping
Slu King Flagship Store	Intelligent Power Saver	It's not working. It feels like an IQ tax.	Taobao Marketplace, a Chinese website for online shopping
Slu King Flagship Store	Intelligent Power Saver	Power saving effect: no effect at all, cheating	Taobao Marketplace, a Chinese website for online shopping
Slu King Flagship Store	Intelligent Power Saver	Thank you for the service of the merchant Shentong Express Company. The epidemic was so severe that some stores did not ship and some courier companies did not deliver, but the merchant and Shentong Express Company overcame the difficulties to ship the goods in time and deliver them safely. Things also work	Taobao Marketplace, a Chinese website for online shopping
Slu King Flagship Store	Intelligent Power Saver	Power-saving effect: the use of good, a large degree of power saving problems, appearance and style: style is very satisfactory, very consistent with personal aesthetic point of view, simple and easy to understand, no trouble.	Taobao Marketplace, a Chinese website for online shopping

Slu King Flagship Store	Intelligent Power Saver	The item is compact and any place can be used node function is very satisfactory, the	Taobao Marketplace, a Chinese website for online shopping
Slu King Flagship Store	Intelligent Power Saver	I'm trying it out. I'll come back to it after I've used it for a while.	Taobao Marketplace, a Chinese website for online shopping
Slu King Flagship Store	Intelligent Power Saver	Use it for a couple months and see first! Whether it works well or not is to be determined!	Taobao Marketplace, a Chinese website for online shopping
Slu King Flagship Store	Intelligent Power Saver	Power Saving King products are small and exquisite, well-made, work without how noise, although only save so little electricity every day, but a long time is very considerable, I think the value for money, it is worth having.	Taobao Marketplace, a Chinese website for online shopping
Slu King Flagship Store	Intelligent Power Saver	High-tech stuff, energy-saving and environmentally friendly, a must-have for families, easy to use	Taobao Marketplace, a Chinese website for online shopping
Slu King Flagship Store	Intelligent Power Saver	Buy yes buy can you connect the electricity does it work, I don't know	Taobao Marketplace, a Chinese website for online shopping
Slu King Flagship Store	Intelligent Power Saver	It's just a scam! There's just two light points in there and nothing else. Just asking how you're going to save electricity?	Taobao Marketplace, a Chinese website for online shopping
Slu King Flagship Store	Intelligent Power Saver	Received the goods, do not know if there is a power-saving effect. Buy two get one free, there are three substation boxes at home, just one layer to install one.	Taobao Marketplace, a Chinese website for online shopping
Slu King Flagship Store	Intelligent Power Saver	Baby has been received, it does work, the meter is noticeably slower	Taobao Marketplace, a Chinese website for online shopping
Slu King Flagship Store	Intelligent Power Saver	The product is good value for money It works well If you want to buy it, you can try it 😌😌😌😌	Taobao Marketplace, a Chinese website for online shopping
Slu King Flagship Store	Intelligent Power Saver	I've bought other models in the past that were power saving, so I'm switching to this one this time	Taobao Marketplace, a Chinese website for online shopping
Slu King Flagship Store	Intelligent Power Saver	Appearance and style: small and exquisite, use it to see the results	Taobao Marketplace, a Chinese website for online shopping
Slu King Flagship Store	Intelligent Power Saver	Power saving effect: just installed, looking forward to good results Cost-effective: quite good Appearance style: very beautiful Experience: wow!	Taobao Marketplace, a Chinese website for online shopping
Slu King Flagship Store	Intelligent Power Saver	The goods have been received.	Taobao Marketplace, a Chinese website for online shopping
Slu King Flagship Store	Intelligent Power Saver	Appearance and style: good	Taobao Marketplace, a Chinese website for online shopping
Slu King Flagship Store	Intelligent Power Saver	The seller has a good service attitude, logistics is also strong, received the baby has also been used, and so on for a period of time to come back! Customer service attitude is very good, after communication, said that can be used for one hundred days, no effect can be returned, on the basis of the promise, believe the seller! Everyone can rest assured to buy!	Taobao Marketplace, a Chinese website for online shopping
Slu King Flagship Store	Intelligent Power Saver	Appearance of small and convenient, power-saving effect and so on after the use of evaluation, the overall okay	Taobao Marketplace, a Chinese website for online shopping
Slu King Flagship Store	Intelligent Power Saver	Just received, on the use, the boss is enough to send a more meaningful, currently do not know the effect, need a month to the effect, the boss is very good, ask questions, the boss said, you use a few months, you feel did not save a little can also be returned, really good business, more recommended!	Taobao Marketplace, a Chinese website for online shopping
Slu King Flagship Store	Intelligent Power Saver	The quality is fine, it is small and compact and can be plugged in for all queries. Looking forward to the results it will bring back to me, after all, savings create value.	Taobao Marketplace, a Chinese website for online shopping
Slu King Flagship Store	Intelligent Power Saver	I just received it and haven't used it yet, I'll comment after using it.	Taobao Marketplace, a Chinese website for online shopping
Slu King Flagship Store	Intelligent Power Saver	This is the so-called energy saver, surprise, two LED lights, 🐯, **high-tech scammer, feeling intellectually insulted	Taobao Marketplace, a Chinese website for online shopping
Slu King Flagship Store	Intelligent Power Saver	Works great, will definitely repurchase next time	Taobao Marketplace, a Chinese website for online shopping
Slu King Flagship Store	Intelligent Power Saver	Received the goods, the first time to buy, expect to be surprised!	Taobao Marketplace, a Chinese website for online shopping
Slu King Flagship Store	Intelligent Power Saver	Power saving effect: good! Price/performance ratio: nice yo! Appearance and style: very good! Using experience: good yo!	Taobao Marketplace, a Chinese website for online shopping
Slu King Flagship Store	Intelligent Power Saver	You can use it already.	Taobao Marketplace, a Chinese website for online shopping
Slu King Flagship Store	Intelligent Power Saver	Item has been received and is very nice, looking forward to buying again next time!!!!	Taobao Marketplace, a Chinese website for online shopping
Slu King Flagship Store	Intelligent Power Saver	Energy saving king received, the packaging is intact, small and exquisite lightweight, looking forward to good results good news.	Taobao Marketplace, a Chinese website for online shopping
Slu King Flagship Store	Intelligent Power Saver	Friends introduced, his own home installed 3 every month really save a lot of electricity. Very unexpected, there are power-saving appliances, usually spend a lot of money for nothing, this time decisively bought 4, the business sent 2!	Taobao Marketplace, a Chinese website for online shopping
Slu King Flagship Store	Intelligent Power Saver	Things received, tried it is okay, every month the electricity bill to several hundred dollars, can save electricity is simply great, not only to save electricity costs, but also save the national resources.	Taobao Marketplace, a Chinese website for online shopping
Slu King Flagship Store	Intelligent Power Saver	Power saving effect: I just received it and just used it, looking forward to how it works.	Taobao Marketplace, a Chinese website for online shopping
Slu King Flagship Store	Intelligent Power Saver	The quality is very good, the voltage is stable, the external appearance of the item is good, exquisite and beautiful. Packaging intact, genuine manufacturers, no noise, logistics is very fast five-star praise, good value for money!	Taobao Marketplace, a Chinese website for online shopping
Slu King Flagship Store	Intelligent Power Saver	The goods have just received, looks quite good, can connect to the electricity is good	Taobao Marketplace, a Chinese website for online shopping
Slu King Flagship Store	Intelligent Power Saver	Match the description of the merchant, can indeed save electricity, it takes a long time to save electricity, this thing is also very small, where to put it will not be in the way, very satisfied, especially good, has been inserted in each room a	Taobao Marketplace, a Chinese website for online shopping
Slu King Flagship Store	Intelligent Power Saver	Received it and installed it, exactly as pictured, the merchant is a real person. Good value for money! The most important late to bring us to save money, next time will add a few more to put the store!	Taobao Marketplace, a Chinese website for online shopping

Slu King Flagship Store	Intelligent Power Saver	Good appearance, very practical, really saves electricity, well made, good experience .	Taobao Marketplace, a Chinese website for online shopping
Slu King Flagship Store	Intelligent Power Saver	Is genuine, a variety of anti-counterfeiting mark and the Pacific Insurance logo, the quality is also good, but also with LCD display, the price is not expensive, my family uses a lot of electricity, half a month to save a hundred or ten dollars, the summer open air conditioning more, it is estimated that a month can be more to save some, already very satisfied!	Taobao Marketplace, a Chinese website for online shopping
Slu King Flagship Store	Intelligent Power Saver	Smaller and lighter than expected, received in accordance with the instructions were installed in their own rooms, fast delivery logistics. Happy shopping satisfaction praise!	Taobao Marketplace, a Chinese website for online shopping
Slu King Flagship Store	Intelligent Power Saver	Energy saver shipped quickly, the product box is beautiful, green energy saving, after plugging in, the green light is on, has begun to operate mode. You can use the appliance with peace of mind, the economizer can filter out the part of the line that jumps, and play a good energy saving effect. Store service is very good, ask questions and answer them!	Taobao Marketplace, a Chinese website for online shopping
Slu King Flagship Store	Intelligent Power Saver	Quiet work doesn't bother me, I use a lot of air conditioning mostly, and I did get a few kWh less when I put it in. Mainly good to use. Affordable	Taobao Marketplace, a Chinese website for online shopping
Slu King Flagship Store	Intelligent Power Saver	Simple to use, stable performance, used today, very convenient, a plug is good, the item appearance is good, exquisite and beautiful, affordable, according to the principle, should be able to node electricity, looking forward to the miracle, the first full 5 points praise.	Taobao Marketplace, a Chinese website for online shopping
Slu King Flagship Store	Intelligent Power Saver	Energy-saving effect is very good, plugged in to see the meter is more obviously slow, the price is also more moderate, the appearance of the style is also very good-looking, workmanship is also very fine, the use of a few days down is very satisfied!	Taobao Marketplace, a Chinese website for online shopping
Slu King Flagship Store	Intelligent Power Saver	Received the goods immediately opened, thought for a long time before ordering, no matter how good they buy a try to know, consulting the business business support 100 days free trial, very intimate service!	Taobao Marketplace, a Chinese website for online shopping
Slu King Flagship Store	Intelligent Power Saver	Last time I bought 2 from this store, I think it's good. It does save a lot, this time I bought 4 in one breath, the store is very polite, and also sent 2, so good stuff must give 5 stars praise, before really spent a lot of money in vain.	Taobao Marketplace, a Chinese website for online shopping
Slu King Flagship Store	Intelligent Power Saver	Power-saving effect is good, cost-effective, workmanship and materials are also very good, very practical, worthy of saving the family cost of living expenses of a good helper, like the quick order!	Taobao Marketplace, a Chinese website for online shopping
Slu King Flagship Store	Intelligent Power Saver	It does save electricity, the thing works well, and the customer service attitude is also very good. At first I did not know what principle, the businessman patiently explained, I downloaded the National Grid app, after the installation of the daily power consumption can be seen, it does save electricity!	Taobao Marketplace, a Chinese website for online shopping
Slu King Flagship Store	Intelligent Power Saver	Power saving effect is reduced, cost-effective, reasonable and beautiful, this power saver is very good to use, saving quite a lot of electricity, before the monthly electricity bill to 600 or so, now the two at the same time, a month to pay 420 yuan, save a lot of money, has been given to the side of the	Taobao Marketplace, a Chinese website for online shopping
Slu King Flagship Store	Intelligent Power Saver	The saver really saves electricity. I used to use over a hundred dollars a month, but with the saver, it's only sixty or seventy dollars a month!	Taobao Marketplace, a Chinese website for online shopping
Slu King Flagship Store	Intelligent Power Saver	Appearance of small and convenient, does not occupy a position, friends recommended to buy, the effect is really good, good use sincerely recommend that everyone buy.	Taobao Marketplace, a Chinese website for online shopping
Slu King Flagship Store	Intelligent Power Saver	I received this product and started using it, the little thing looks great, it saves some money on the electric bill, which is just too high every month. Very good stuff. It's only good after you use it, you don't even unplug it after plugging it in, it still saves a lot of money on your electric bill, kudos!	Taobao Marketplace, a Chinese website for online shopping
Slu King Flagship Store	Intelligent Power Saver	Previously also bought 1, is indeed a power-saving effect, used for many years, this time to move to a new home, deliberately bought a few more, received, with the same as previously purchased, good use of power back to buy one!	Taobao Marketplace, a Chinese website for online shopping
Yungtongtong Technology	King of Power Saving	I've been using it for almost a month now and I don't think it's saving any power. I don't believe you guys try it!	Taobao Marketplace, a Chinese website for online shopping
Yungtongtong Technology	King of Power Saving	Haven't used it yet.	Taobao Marketplace, a Chinese website for online shopping
Yungtongtong Technology	King of Power Saving	No results yet, use it for a few days and see what happens.	Taobao Marketplace, a Chinese website for online shopping
Yungtongtong Technology	Red Power Saving Specialist	not have	Taobao Marketplace, a Chinese website for online shopping
Yungtongtong Technology	Gold Power Saving Specialist	This saver really works well, the electricity bill is saved, and the appliances in the house are stabilized, so you get the best of both worlds, saving electricity and saving your mind.	Taobao Marketplace, a Chinese website for online shopping
Yungtongtong Technology	Gold Power Saving Specialist	Baby received, as described, love it!	Taobao Marketplace, a Chinese website for online shopping
Yungtongtong Technology	Gold Power Saving Specialist	It's not clear if you buy the same one as the platform display option, and it's not clear if it saves electricity.	Taobao Marketplace, a Chinese website for online shopping
Yungtongtong Technology	Gold Power Saving Specialist	It is indeed very good, the air conditioning is turned on hard, 3degreeslesselectricityone night, bar, very practical, friends who use a lot of electricity can get it.	Taobao Marketplace, a Chinese website for online shopping
Yungtongtong Technology	Gold Power Saving Specialist	Repurchased for the second time and have recommended it to close friends as well. Power saving ability is good. Very practical. Affordable price.	Taobao Marketplace, a Chinese website for online shopping
Yungtongtong Technology	Gold Power Saving Specialist	It works well, introduced by a friend, saves electricity and money, long-lasting products are cost-effective. Big factory production Good quality Exquisite workmanship Cost-effective	Taobao Marketplace, a Chinese website for online shopping
Yungtongtong Technology	Gold Power Saving Specialist	Easy to install, customer service is very careful guidance, pressed in the front end of the power use more, power saving effect is good. I do not understand the place is also very patient guidance. Very satisfied, value for money	Taobao Marketplace, a Chinese website for online shopping
Yungtongtong Technology	Gold Power Saving Specialist	Excellent.🌹🌹🌹🌹🌹	Taobao Marketplace, a Chinese website for online shopping
Yungtongtong Technology	Gold Power Saving Specialist	Has been used for a period of time, the summer heat using air conditioning frequency is higher, compared to last month to save a lot of Oh!	Taobao Marketplace, a Chinese website for online shopping
Yungtongtong Technology	Gold Power Saving Specialist	Good quality workmanship, service follow-up guidance is very patient, the family recommended. Said the family every month less a lot of electricity, let me store also press a. Reduce electricity costs	Taobao Marketplace, a Chinese website for online shopping
Yungtongtong Technology	Gold Power Saving Specialist	How effective it will be remains to be seen	Taobao Marketplace, a Chinese website for online shopping
Yungtongtong Technology	Gold Power Saving Specialist	You're useless.	Taobao Marketplace, a Chinese website for online shopping
Yungtongtong Technology	Gold Power Saving Specialist	Great price, so much better than brick and mortar!	Taobao Marketplace, a Chinese website for online shopping

Yungtongtong Technology	Gold Power Saving Specialist	You won't know if it's a power saver until you use it.	Taobao Marketplace, a Chinese website for online shopping
Yungtongtong Technology	Gold Power Saving Specialist	Genuine, good quality, power saving and money saving, value for money recommendation	Taobao Marketplace, a Chinese website for online shopping
Yungtongtong Technology	Gold Power Saving Specialist	Fast shipping Perfect packaging Quality packaging Great customer service	Taobao Marketplace, a Chinese website for online shopping
Yungtongtong Technology	Gold Power Saving Specialist	Logistics is very fast quality is very good price is very satisfied with a shopping!	Taobao Marketplace, a Chinese website for online shopping
Yungtongtong Technology	Gold Power Saving Specialist	Air conditioning meter quality is good, easy to install, the material is very good, workmanship is also good, the price is also affordable, logistics is also very fast, praise!	Taobao Marketplace, a Chinese website for online shopping
Yungtongtong Technology	Gold Power Saving Specialist	The effect is really good, used for a few days, obviously less power consumption	Taobao Marketplace, a Chinese website for online shopping
Yungtongtong Technology	Gold Power Saving Specialist	Use it for a month before evaluating, I don't know yet without having used it.	Taobao Marketplace, a Chinese website for online shopping
Yungtongtong Technology	Gold Power Saving Specialist	There's no publicity to see the effect of this	Taobao Marketplace, a Chinese website for online shopping
Yungtongtong Technology	Gold Power Saving Specialist	This power saver is so good, I've already bought two of them.	Taobao Marketplace, a Chinese website for online shopping
Yungtongtong Technology	Gold Power Saving Specialist	We are renting a large1hp. , air conditioning ten clock8kWh, see how the manufacturer? It's a scam.	Taobao Marketplace, a Chinese website for online shopping
Yungtongtong Technology	Gold Power Saving Specialist	It's not working.	Taobao Marketplace, a Chinese website for online shopping
Yungtongtong Technology	Gold Power Saving Specialist	Shipping was fast, stuff was well packaged, great price, I'm happy with👍and will repurchase	Taobao Marketplace, a Chinese website for online shopping
Yungtongtong Technology	Gold Power Saving Specialist	🌈Great quality, bought it to my liking💛Awesome! ✨ Buy with confidence if you need it!	Taobao Marketplace, a Chinese website for online shopping
Yungtongtong Technology	Gold Power Saving Specialist	Goods today's received to use, in the evaluation	Taobao Marketplace, a Chinese website for online shopping
Yungtongtong Technology	Gold Power Saving Specialist	Consistently good reviews I guess! Hopefully it works well!	Taobao Marketplace, a Chinese website for online shopping
Yungtongtong Technology	Gold Power Saving Specialist	I just started using it, and I won't know until the electric bill comes in until it is	Taobao Marketplace, a Chinese website for online shopping
Yungtongtong Technology	Gold Power Saving Specialist	That's good. I'll buy it next time.	Taobao Marketplace, a Chinese website for online shopping
Yungtongtong Technology	Gold Power Saving Specialist	Not much use.	Taobao Marketplace, a Chinese website for online shopping
Yungtongtong Technology	Gold Power Saving Specialist	Baby received, so surprised, the quality is very good, cost-effective, really out of my expectation!	Taobao Marketplace, a Chinese website for online shopping
Yungtongtong Technology	Gold Power Saving Specialist	Appearance is beautiful, power saving or not use after evaluation.	Taobao Marketplace, a Chinese website for online shopping
Yungtongtong Technology	Gold Power Saving Specialist	Use it and then evaluate.	Taobao Marketplace, a Chinese website for online shopping
Yungtongtong Technology	Gold Power Saving Specialist	Deceiving consumers I really don't get it Paying the IQ tax Don't believe in90dayfree trials	Taobao Marketplace, a Chinese website for online shopping
Yungtongtong Technology	Gold Power Saving Specialist	ve been using it for a few months and it hasn't worked at all****are not	Taobao Marketplace, a Chinese website for online shopping
Yungtongtong Technology	Gold Power Saving Specialist	using it for almost two months and it doesn't work Don't buy it	Taobao Marketplace, a Chinese website for online shopping
Yungtongtong Technology	Gold Power Saving Specialist	Comparison of the recent electricity bill, it does work, before half a month on the50gone, now half a month only use30, really buy the treasure, praise.	Taobao Marketplace, a Chinese website for online shopping
Yungtongtong Technology	Gold Power Saving Specialist	phoney	Taobao Marketplace, a Chinese website for online shopping
Yungtongtong Technology	Gold Power Saving Specialist	I'll try the process in the meantime to see if there are any suburbia fruits and come back with a review when I get to a month.	Taobao Marketplace, a Chinese website for online shopping
Yungtongtong Technology	Gold Power Saving Specialist	Very satisfied with the purchase, the quality is really good!	Taobao Marketplace, a Chinese website for online shopping
Yungtongtong Technology	Gold Power Saving Specialist	Logistics is very fast appearance beautiful effect is very good worth buying	Taobao Marketplace, a Chinese website for online shopping
Yungtongtong Technology	Gold Power Saving Specialist	Logistics is very fast appearance beautiful effect is very good worth buying	Taobao Marketplace, a Chinese website for online shopping
Yungtongtong Technology	Gold Power Saving Specialist	It's not much use.	Taobao Marketplace, a Chinese website for online shopping
Yungtongtong Technology	Gold Power Saving Specialist	Almost twenty days after receiving the product, I have not yet felt the advantages of the product's power savings	Taobao Marketplace, a Chinese website for online shopping
Yungtongtong Technology	Gold Power Saving Specialist	Fast logistics, good appearance	Taobao Marketplace, a Chinese website for online shopping
Yungtongtong Technology	Gold Power Saving Specialist	We are still observing the results after 2 months.	Taobao Marketplace, a Chinese website for online shopping

Yungtongtong Technology	Gold Power Saving Specialist	Just received it and don't know the effect yet	Taobao Marketplace, a Chinese website for online shopping
Yungtongtong Technology	Gold Power Saving Specialist	Look good first give a good review, the product is good	Taobao Marketplace, a Chinese website for online shopping
Yungtongtong Technology	Gold Power Saving Specialist	from9bucksa dayto7bucks👍	Taobao Marketplace, a Chinese website for online shopping
Yungtongtong Technology	Gold Power Saving Specialist	Thanks.	Taobao Marketplace, a Chinese website for online shopping
Yungtongtong Technology	Gold Power Saving Specialist	It's okay. It's good.	Taobao Marketplace, a Chinese website for online shopping
Yungtongtong Technology	Gold Power Saving Specialist	I hope there are good power savings.	Taobao Marketplace, a Chinese website for online shopping
Yungtongtong Technology	Gold Power Saving Specialist	Nowadays, fast delivery is fast, but it seems that there is not much management of buying consumers' stuff, whatever	Taobao Marketplace, a Chinese website for online shopping
Yungtongtong Technology	Gold Power Saving Specialist	Just arrived, only with the use of the effect, rift not know	Taobao Marketplace, a Chinese website for online shopping
Yungtongtong Technology	Gold Power Saving Specialist	(of an unmarried couple) be close	Taobao Marketplace, a Chinese website for online shopping
Yungtongtong Technology	Gold Power Saving Specialist	I don't know how much electricity is saved by using it	Taobao Marketplace, a Chinese website for online shopping
Yungtongtong Technology	Gold Power Saving Specialist	Never used it. Bought it for someone else. I don't know if it's good.	Taobao Marketplace, a Chinese website for online shopping
Yungtongtong Technology	Gold Power Saving Specialist	cudgel	Taobao Marketplace, a Chinese website for online shopping
Yungtongtong Technology	Gold Power Saving Specialist	Energy saver quality is very good, everyone who wants to buy can start, customer service is good!	Taobao Marketplace, a Chinese website for online shopping
Yungtongtong Technology	Gold Power Saving Specialist	Received it and used it, used it for a month to know two months to comment later	Taobao Marketplace, a Chinese website for online shopping
Yungtongtong Technology	Gold Power Saving Specialist	Received and installed, looking forward to seeing the results in a month!	Taobao Marketplace, a Chinese website for online shopping
Yungtongtong Technology	Gold Power Saving Specialist	The rattling works well. Home pressed on a lot more stable. Work does not get hot, but also can purify the air, equivalent to the practical.	Taobao Marketplace, a Chinese website for online shopping
Yungtongtong Technology	Gold Power Saving Specialist	*No use	Taobao Marketplace, a Chinese website for online shopping
Yungtongtong Technology	Gold Power Saving Specialist	Recommended by the owner of the store next door, looked at his home does have to save electricity, decided to get. Indeed quite can, the voltage is much more stable, there is a reduction in electricity consumption.	Taobao Marketplace, a Chinese website for online shopping
Yungtongtong Technology	Gold Power Saving Specialist	Installation is very easy, according to the guidance of the press in the front end of the electricity is really good, the screen can also watch the voltage fluctuations, very high-tech, the experience of full	Taobao Marketplace, a Chinese website for online shopping
Yungtongtong Technology	Gold Power Saving Specialist	Very easy to operate and install, customer service is also very enthusiastic and patient guidance, ask questions and answer them! After-sales aspect is very protection, use very assured.	Taobao Marketplace, a Chinese website for online shopping
Yungtongtong Technology	Gold Power Saving Specialist	Quality workmanship is very good, durable, the second repurchase with, good	Taobao Marketplace, a Chinese website for online shopping
Yungtongtong Technology	Gold Power Saving Specialist	Easy to use and install, customer service is very patient, there is an answer, the product is very satisfied, the store service is careful and patient, so satisfied!	Taobao Marketplace, a Chinese website for online shopping
Yungtongtong Technology	Gold Power Saving Specialist	It's great, works pretty well, fast shipping and well packaged.	Taobao Marketplace, a Chinese website for online shopping
Yungtongtong Technology	Gold Power Saving Specialist	Saving power received, logistics is very fast, the installation of the master is very professional, the current use of all normal, while the function is really powerful, really good use of intelligent saving, saving electricity and saving money is not a waste of resources, ZanZan!	Taobao Marketplace, a Chinese website for online shopping
Yungtongtong Technology	Gold Power Saving Specialist	Works well, good quality workmanship. Aluminum alloy material is safe and environmentally friendly. Looking for customer service guidance of pressing in the front part of the home with electricity, it is really good. Value for money!	Taobao Marketplace, a Chinese website for online shopping
Yungtongtong Technology	Gold Power Saving Specialist	Installed, good quality, hopefully it will save power	Taobao Marketplace, a Chinese website for online shopping
Yungtongtong Technology	Gold Power Saving Specialist	Easy to install and great value for money. Very powerful, full experience, very satisfied!	Taobao Marketplace, a Chinese website for online shopping
Yungtongtong Technology	Gold Power Saving Specialist	Exquisite packaging, good goods, good quality, recommended friends.	Taobao Marketplace, a Chinese website for online shopping
Yungtongtong Technology	Gold Power Saving Specialist	Good quality, cost effective, saves power and money, great value for a long lasting product.	Taobao Marketplace, a Chinese website for online shopping
Yungtongtong Technology	Gold Power Saving Specialist	It works well and the logistics are fast	Taobao Marketplace, a Chinese website for online shopping
Yungtongtong Technology	Gold Power Saving Specialist	Easy to install, customer service is very careful guidance, pressed in the front end of the electricity more, do not understand the place is also very patient guidance. Very satisfied, value for money	Taobao Marketplace, a Chinese website for online shopping
Yungtongtong Technology	Gold Power Saving Specialist	Is genuine, good quality, well-packed, strong delivery, fast shipping, online shopping is convenient and affordable!	Taobao Marketplace, a Chinese website for online shopping
Yungtongtong Technology	Gold Power Saving Specialist	Exquisite workmanship, fine not to take up space, the body of a small function is quite good. Also comes with air purification function. Good	Taobao Marketplace, a Chinese website for online shopping
Yungtongtong Technology	Gold Power Saving Specialist	Indeed good, friends recommended to use, the use of very safe, very satisfied!	Taobao Marketplace, a Chinese website for online shopping

Yungtongtong Technology	Gold Power Saving Specialist	Merchant good thing, product quality is hard enough, very good, will give you back to buy recommended customers[strong]	Taobao Marketplace, a Chinese website for online shopping
Yungtongtong Technology	Gold Power Saving Specialist	Received, well-packed Oh, no damage, logistics is also very fast!	Taobao Marketplace, a Chinese website for online shopping
Yungtongtong Technology	Gold Power Saving Specialist	Quite satisfied, very stable, working quietly. The quality is very good, many times back to buy, around the friends home are using, are quite good. Practical	Taobao Marketplace, a Chinese website for online shopping
Yungtongtong Technology	Gold Power Saving Specialist	The saver works great, it's easy to use and affordable!	Taobao Marketplace, a Chinese website for online shopping
Yungtongtong Technology	Gold Power Saving Specialist	Customer service is really good, the product is packed tightly	Taobao Marketplace, a Chinese website for online shopping
Yungtongtong Technology	Gold Power Saving Specialist	The baby received today, first try a month and then give a follow-up review.	Taobao Marketplace, a Chinese website for online shopping
Yungtongtong Technology	Gold Power Saving Specialist	Very good material, visual display, easy to use and peace of mind	Taobao Marketplace, a Chinese website for online shopping
Yungtongtong Technology	Gold Power Saving Specialist	Workmanship feel good, friends use quite good to me recommended. Logistics is very fast, received and installed very convenient	Taobao Marketplace, a Chinese website for online shopping
Yungtongtong Technology	Gold Power Saving Specialist	Very satisfied with a shopping, value for money, much more affordable than the physical store!	Taobao Marketplace, a Chinese website for online shopping
Yungtongtong Technology	Gold Power Saving Specialist	Very easy to operate and install, customer service is also very enthusiastic and patient guidance, ask questions and answer them! After-sales aspect is very protection, use very assured.	Taobao Marketplace, a Chinese website for online shopping
Yungtongtong Technology	Gold Power Saving Specialist	Very good quality and easy to use. It does save a lot of points, not too expensive, shipping is pretty fast, very satisfied!	Taobao Marketplace, a Chinese website for online shopping
Yungtongtong Technology	Gold Power Saving Specialist	Workmanship feel good, friends use quite good to me recommended. Logistics is very fast, received and installed very convenient	Taobao Marketplace, a Chinese website for online shopping
Yungtongtong Technology	Gold Power Saving Specialist	Logistics is very fast and well-packed customer service attitude attentive very satisfied with a shopping!	Taobao Marketplace, a Chinese website for online shopping
Yungtongtong Technology	Gold Power Saving Specialist	The merchant is reliable and the quality of the old store is guaranteed. Repurchase many times. Durable Very practical	Taobao Marketplace, a Chinese website for online shopping
Yungtongtong Technology	Gold Power Saving Specialist	Quality is very good, logistics is also very fast, fine workmanship, with good, worth buying.	Taobao Marketplace, a Chinese website for online shopping
Yungtongtong Technology	Gold Power Saving Specialist	Many times to help repurchase, really good, than not used before a lot less electricity, the main durable, value for money!	Taobao Marketplace, a Chinese website for online shopping
Yungtongtong Technology	Gold Power Saving Specialist	It works well, introduced by a friend, saves electricity and money, long-lasting products are cost-effective. Big factory production Good quality Exquisite workmanship Cost-effective	Taobao Marketplace, a Chinese website for online shopping
Yungtongtong Technology	Gold Power Saving Specialist	Good quality,saves a lot of electricity	Taobao Marketplace, a Chinese website for online shopping
Yungtongtong Technology	Gold Power Saving Specialist	I bought one after hearing a friend say it worked well, and it's a real power saver.	Taobao Marketplace, a Chinese website for online shopping
Yungtongtong Technology	Gold Power Saving Specialist	Logistics is very fast, immediately test, the quality should be good, see how the effect, and then follow up!	Taobao Marketplace, a Chinese website for online shopping
Yungtongtong Technology	Gold Power Saving Specialist	Logistics is very fast, the quality is very good, immediately test to see how the effect and then follow up!	Taobao Marketplace, a Chinese website for online shopping
Yungtongtong Technology	Gold Power Saving Specialist	Suggest not to buy it, it's a scam, it doesn't save power at all	Taobao Marketplace, a Chinese website for online shopping
Yungtongtong Technology	Gold Power Saving Specialist	I'll come back with a follow up review after I've used it!	Taobao Marketplace, a Chinese website for online shopping
Yungtongtong Technology	Gold Power Saving Specialist	Genuine, buy with confidence, the price is fair, satisfied!	Taobao Marketplace, a Chinese website for online shopping
Yungtongtong Technology	Gold Power Saving Specialist	Previously bought a small, used can this time in the purchase of a high-power, multi-functional again in the price more to buy a put on the third floor feel good can save electricity is a good product[strong] to the merchant a good comment satisfied with	Taobao Marketplace, a Chinese website for online shopping
Yungtongtong Technology	Gold Power Saving Specialist	Usually consume a large amount of electricity, this side of the friend introduced, used really good, feel very can, good use, very worthwhile, quality workmanship fine	Taobao Marketplace, a Chinese website for online shopping
Yungtongtong Technology	Gold Power Saving Specialist	It's not bad The installation is very easy, and the high power is moderately priced and inexpensive. Quite good, the home line stabilized a lot, less tripping	Taobao Marketplace, a Chinese website for online shopping
Yungtongtong Technology	Gold Power Saving Specialist	It is genuine! Quality after-sales protection is in place, a very good shopping, thanks to the platform, thanks to the merchant goodies, will help promote[strong]	Taobao Marketplace, a Chinese website for online shopping
Yungtongtong Technology	Gold Power Saving Specialist	This one is really great! Quality is very good, meticulous workmanship, the package is very tight express very quickly, business service enthusiasm, very satisfied!	Taobao Marketplace, a Chinese website for online shopping
Yungtongtong Technology	Gold Power Saving Specialist	Things received, the quality can be, very good, material workmanship is quite good. Aluminum alloy material is safe and secure. Merchant service is very enthusiastic. Patient answer guide. Very satisfied	Taobao Marketplace, a Chinese website for online shopping
Yungtongtong Technology	Gold Power Saving Specialist	District electrician specified this let me help buy, I think the quality is good, logistics is very fast!	Taobao Marketplace, a Chinese website for online shopping
Yungtongtong Technology	Gold Power Saving Specialist	Packaging is particularly exquisite, the goods are very intact. With electricity big customer service advice can be used to upgrade the model. Electrician installed, customer service guidance is very good. Asked and answered.	Taobao Marketplace, a Chinese website for online shopping
Yungtongtong Technology	Gold Power Saving Specialist	Saver battery quality is superb, high power can standby for a long time, fine workmanship, sturdy and durable, safe and convenient to use, worth buying!	Taobao Marketplace, a Chinese website for online shopping
Yungtongtong Technology	Gold Power Saving Specialist	The stuff is even more perfect than I imagined, very powerful and worth getting!	Taobao Marketplace, a Chinese website for online shopping

Yungtongtong Technology	Gold Power Saving Specialist	Baby has been received, logistics is very fast, the product is as described, good workmanship. The guidance is very detailed, good	Taobao Marketplace, a Chinese website for online shopping
Yungtongtong Technology	Gold Power Saving Specialist	Consistent in kind, the effect is very good. Logistics is fast, business service is very thoughtful. Genuine quality is very assured! Very satisfied with the practical goodies	Taobao Marketplace, a Chinese website for online shopping
Yungtongtong Technology	Gold Power Saving Specialist	Friends recommended to buy consulting customer service which is more suitable recommended this new2023upgraded version of the high power received the goods and plugged in the use of can also see the real current situation at home very good	Taobao Marketplace, a Chinese website for online shopping
Yungtongtong Technology	Gold Power Saving Specialist	Useful! Home electrical wiring is a bit messy Turning on appliances at the same time trips the circuit breaker from time to time. After using the Energy Saver King, it does reduce the phenomenon, and the effect is very good. It saves electricity when you compare the results.	Taobao Marketplace, a Chinese website for online shopping
Yungtongtong Technology	Gold Power Saving Specialist	Rental room use. Summer air conditioning refrigerator power consumption is too large, friends recommended to use the effect is very good every month to save a lot of money to me also recommended to use it is really very good[strong]	Taobao Marketplace, a Chinese website for online shopping
Yungtongtong Technology	Gold Power Saving Specialist	Goods received quality feel very good very simple and easy to plug in and use mute mode certificates is genuine can buy[strong]	Taobao Marketplace, a Chinese website for online shopping
Yungtongtong Technology	Gold Power Saving Specialist	It does work quite well. Second repurchase, this year in the purchase of a put the second floor. Summer is coming The air conditioner is going to be on too hard.	Taobao Marketplace, a Chinese website for online shopping
Yungtongtong Technology	Gold Power Saving Specialist	Installation was simple and easy. Customer service was very patient in responding to instructions on where to install. Warranty is guaranteed. Very satisfied with the purchase.	Taobao Marketplace, a Chinese website for online shopping
Yungtongtong Technology	Gold Power Saving Specialist	The material is environmentally friendly and safe, works quietly, and does not get hot throughout the use. Indeed is genuine, put in the home to use. Friends use quite good to me also recommended. Very satisfied	Taobao Marketplace, a Chinese website for online shopping
Yungtongtong Technology	Gold Power Saving Specialist	Pressed one in the warehouse a year ago and it worked well enough to arrange for the other side as well. Very practical, safe and quiet work. Friends who use a lot of electricity can start. A must have indeed!	Taobao Marketplace, a Chinese website for online shopping
Yungtongtong Technology	Gold Power Saving Specialist	Customer service guidance current foremost, in the meter to connect a socket installation effect is really good very satisfied	Taobao Marketplace, a Chinese website for online shopping
Yungtongtong Technology	King of Power Saving	Hopefully there are better power savings.	Taobao Marketplace, a Chinese website for online shopping
Yungtongtong Technology	King of Power Saving	It doesn't save any power at all.	Taobao Marketplace, a Chinese website for online shopping
Yungtongtong Technology	(Three-phase) Electrical Officer	not have	Taobao Marketplace, a Chinese website for online shopping
Yungtongtong Technology	(Electricity (three-phase) Ruyi	not have	Taobao Marketplace, a Chinese website for online shopping
Yungtongtong Technology	(Three-phase) Ant King Intelligence	Easy to install Customer service is very professional guidance Three fire wires and one zero wire can be parallel. Can be installed by yourself Product workmanship is very good is genuine. Like our store which uses a lot of electricity, you can rest assured to get it.	Taobao Marketplace, a Chinese website for online shopping
Yungtongtong Technology	(Three-phase) Ant King Intelligence	Big brand is trustworthy. Many times back to buy, friends factory also recommended to use together, it is indeed power saving. A month down much less. Installation is simple, workmanship material safety is very assured! Good quality	Taobao Marketplace, a Chinese website for online shopping
Yungtongtong Technology	(Three-phase) Ant King Intelligence	Received that is installed, customer service is very patient to follow up professional guidance. Big brand, quality workmanship guarantee. Recommended by friends. Indeed very good	Taobao Marketplace, a Chinese website for online shopping
Yungtongtong Technology	(Three-phase) Intelligent Power Saving Voltage Regulator	not have	Taobao Marketplace, a Chinese website for online shopping
Yungtongtong Technology	(Three-phase) 380V applicable economizer	not have	Taobao Marketplace, a Chinese website for online shopping
Yungtongtong Technology	(Electricity (three-phase) Ruyi	not have	Taobao Marketplace, a Chinese website for online shopping
Yungtongtong Technology	Power saving voltage stabilizing compensator	not have	Taobao Marketplace, a Chinese website for online shopping
Yungtongtong Technology	Three-phase voltage stabilizer and economizer	not have	Taobao Marketplace, a Chinese website for online shopping
Cedar Smart	Gold Power Saving Specialist	very good	Taobao Marketplace, a Chinese website for online shopping
Cedar Smart	Gold Power Saving Specialist	Fast logistics, cost-effective, very satisfied	Taobao Marketplace, a Chinese website for online shopping
Cedar Smart	Gold Power Saving Specialist	Very happy with the results, building up to reasonable and legal energy savings. Very good and practical. Very satisfied	Taobao Marketplace, a Chinese website for online shopping
Cedar Smart	Gold Power Saving Specialist	It does work quite well, and this month the electricity bill is less than usual. Good product at an affordable price	Taobao Marketplace, a Chinese website for online shopping
Cedar Smart	Gold Power Saving Specialist	It really saves electricity than before it was used, it doesn't get hot when you use it all day, it's easy to use, energy-saving and quiet.	Taobao Marketplace, a Chinese website for online shopping
Cedar Smart	Gold Power Saving Specialist	Power saving effect: very good Cost-effective: high Appearance style: exquisite Workmanship material: fine	Taobao Marketplace, a Chinese website for online shopping
Cedar Smart	Gold Power Saving Specialist	The logistics is quite fast very good their family service is also very good	Taobao Marketplace, a Chinese website for online shopping
Cedar Smart	Gold Power Saving Specialist	I'll buy three more if they're good.	Taobao Marketplace, a Chinese website for online shopping
Cedar Smart	Gold Power Saving Specialist	Experience:(seems to have no effect, I do not know if it is time short-term can not see whether it is effective	Taobao Marketplace, a Chinese website for online shopping
Cedar Smart	Gold Power Saving Specialist	Didn't see the power saving effect	Taobao Marketplace, a Chinese website for online shopping
Cedar Smart	Gold Power Saving Specialist	Workmanship material: good	Taobao Marketplace, a Chinese website for online shopping

Cedar Smart	Gold Power Saving Specialist	The power saving has not been noticed yet, I will comment again when I use it for a month.	Taobao Marketplace, a Chinese website for online shopping
Cedar Smart	Gold Power Saving Specialist	Fast logistics. Very satisfied with the goods worth buying	Taobao Marketplace, a Chinese website for online shopping
Cedar Smart	Gold Power Saving Specialist	Very easy to install and customer service was very patient in guiding the best location. Installed for a while and feels really good! Home lines are much more stable.	Taobao Marketplace, a Chinese website for online shopping
Cedar Smart	Gold Power Saving Specialist	It does work quite well, and the line in the house is stabilized.electricity bill has been reduced by less. It is very reasonable and legal, and we can use it with confidence.	Taobao Marketplace, a Chinese website for online shopping
Cedar Smart	Gold Power Saving Specialist	The saver is good and easy to use, the power saving is good and the service is good.	Taobao Marketplace, a Chinese website for online shopping
Cedar Smart	Gold Power Saving Specialist	Works great, inexpensive, worth the price	Taobao Marketplace, a Chinese website for online shopping
Cedar Smart	Gold Power Saving Specialist	Shipped quickly, well-packed, the price is particularly affordable, power-saving effect is very good, a month can save a lot of money, it is worth buying!	Taobao Marketplace, a Chinese website for online shopping
Cedar Smart	Gold Power Saving Specialist	Experience: is experiencing, the specific effect of waiting for the end of the month of the electricity bill out and then evaluate the	Taobao Marketplace, a Chinese website for online shopping
Cedar Smart	Gold Power Saving Specialist	It does work quite well and really saves points much better than before, very good.	Taobao Marketplace, a Chinese website for online shopping
Cedar Smart	Gold Power Saving Specialist	Fast shipping, good packaging, back to install and use for a period of time, energy saving and power saving effect is very good	Taobao Marketplace, a Chinese website for online shopping
Cedar Smart	Gold Power Saving Specialist	Power savings: one very good!	Taobao Marketplace, a Chinese website for online shopping
Cedar Smart	Gold Power Saving Specialist	The saver is of good quality, saves electricity well and is very satisfactory.	Taobao Marketplace, a Chinese website for online shopping
Cedar Smart	Gold Power Saving Specialist	I don't see much effect at the moment, so let's wait for it to kick in.	Taobao Marketplace, a Chinese website for online shopping
Cedar Smart	Gold Power Saving Specialist	Good quality and reasonably priced for what you get. Good value for money.	Taobao Marketplace, a Chinese website for online shopping
Cedar Smart	Gold Power Saving Specialist	The saver works great and looks great, doesn't take up much space, love it!	Taobao Marketplace, a Chinese website for online shopping
Cedar Smart	Gold Power Saving Specialist	It's not working.	Taobao Marketplace, a Chinese website for online shopping
Cedar Smart	Gold Power Saving Specialist	Energy saver received, the quality is very good, logistics is very fast	Taobao Marketplace, a Chinese website for online shopping
Cedar Smart	Gold Power Saving Specialist	Quite good, the second repurchase, the summer is here to the upstairs also set up more than one, the air conditioning all open up the electricity bill can not top, save some is some. Practical Good value	Taobao Marketplace, a Chinese website for online shopping
Cedar Smart	Gold Power Saving Specialist	It's too small, and light, so I don't know if it saves power.	Taobao Marketplace, a Chinese website for online shopping
Cedar Smart	Gold Power Saving Specialist	Very good quality, very satisfied with the purchase. Customer service is very professional with questions and answers	Taobao Marketplace, a Chinese website for online shopping
Cedar Smart	Gold Power Saving Specialist	After comparing, it is indeed a dozen dollars less. Affordable. Great product to use and save!	Taobao Marketplace, a Chinese website for online shopping
Cedar Smart	Gold Power Saving Specialist	Looked at a few to get started, his quality is good and the price point is more than appropriate	Taobao Marketplace, a Chinese website for online shopping
Cedar Smart	Gold Power Saving Specialist	Easy to install, customer service is very careful guidance, pressed in the front end of the power use more, power saving effect is good. I do not understand the place is also very patient guidance. Very satisfied, value for money	Taobao Marketplace, a Chinese website for online shopping
Cedar Smart	Gold Power Saving Specialist	That's great. Saves a lot of money on the electric bill, doesn't take up much space.	Taobao Marketplace, a Chinese website for online shopping
Cedar Smart	Gold Power Saving Specialist	Special power saving operation is simple, fine workmanship Satisfaction	Taobao Marketplace, a Chinese website for online shopping
Cedar Smart	Gold Power Saving Specialist	Looking good, hope it works well.	Taobao Marketplace, a Chinese website for online shopping
Cedar Smart	Gold Power Saving Specialist	Value for money: average	Taobao Marketplace, a Chinese website for online shopping
Cedar Smart	Gold Power Saving Specialist	Exterior Style:Thank	Taobao Marketplace, a Chinese website for online shopping
Cedar Smart	Gold Power Saving Specialist	I don't know if it works or not, I'll use it for a while and see.	Taobao Marketplace, a Chinese website for online shopping
Cedar Smart	Gold Power Saving Specialist	Energy saving and power saving with quality assurance	Taobao Marketplace, a Chinese website for online shopping
Cedar Smart	Gold Power Saving Specialist	Arrived quickly, with a good, good workmanship materials are very good, there is a need to purchase	Taobao Marketplace, a Chinese website for online shopping
Cedar Smart	Gold Power Saving Specialist	Good quality, workmanship can be, practical and convenient, the effect is good satisfaction	Taobao Marketplace, a Chinese website for online shopping
Cedar Smart	Gold Power Saving Specialist	I've bought it for a month now, it doesn't work, it's the same with him as without him, I feel duped.	Taobao Marketplace, a Chinese website for online shopping
Cedar Smart	Gold Power Saving Specialist	Power saving effect: }good	Taobao Marketplace, a Chinese website for online shopping

Cedar Smart	Gold Power Saving Specialist	Power-saving effect: for the time being, I have not found any effect, waiting for60dayscustomer service said tosee the effect, and then follow-up comments.	Taobao Marketplace, a Chinese website for online shopping
Cedar Smart	Gold Power Saving Specialist	Experience: average	Taobao Marketplace, a Chinese website for online shopping
Cedar Smart	Gold Power Saving Specialist	Meter received, exquisite workmanship, very good-looking, good material	Taobao Marketplace, a Chinese website for online shopping
Cedar Smart	Gold Power Saving Specialist	I don't know if I'm connected to the electricity or not, but if I plug this in and use the induction cooker, the induction cooker will automatically turn off the electricity.	Taobao Marketplace, a Chinese website for online shopping
Cedar Smart	Gold Power Saving Specialist	It's justover800kWhper month without using him, and when he does, he usesover100kWhmore a month... I'm really speechless...	Taobao Marketplace, a Chinese website for online shopping
Cedar Smart	Gold Power Saving Specialist	It still feels OK, you won't know until the end of the month if it saves on your electric bill. It's still worth a try	Taobao Marketplace, a Chinese website for online shopping
Cedar Smart	Gold Power Saving Specialist	Thanks.	Taobao Marketplace, a Chinese website for online shopping
Cedar Smart	Gold Power Saving Specialist	No power saving. IQ tax, not recommended!	Taobao Marketplace, a Chinese website for online shopping
Cedar Smart	Gold Power Saving Specialist	We'll follow up later.	Taobao Marketplace, a Chinese website for online shopping
Cedar Smart	Gold Power Saving Specialist	The energy saving effect is OK, and the quality is quite good, cost-effective	Taobao Marketplace, a Chinese website for online shopping
Cedar Smart	Gold Power Saving Specialist	Power-saving effect: not much effect, Workmanship material: goods quite showy Use experience: is not bought IQ tax	Taobao Marketplace, a Chinese website for online shopping
Cedar Smart	Gold Power Saving Specialist	Goods received, just plugged in a month later to evaluate, workmanship, appearance saw good!	Taobao Marketplace, a Chinese website for online shopping
Cedar Smart	Gold Power Saving Specialist	I just used it and I don't know how well it works yet, I'll follow up after I use it?	Taobao Marketplace, a Chinese website for online shopping
Cedar Smart	Gold Power Saving Specialist	Use it for a while and evaluate it objectively	Taobao Marketplace, a Chinese website for online shopping
Cedar Smart	Gold Power Saving Specialist	Has been plugged into the power supply, just so far did not see any effect, the meter flashes or about the same time, the first default positive feedback it	Taobao Marketplace, a Chinese website for online shopping
Cedar Smart	Gold Power Saving Specialist	System default comments	Taobao Marketplace, a Chinese website for online shopping
Cedar Smart	Gold Power Saving Specialist	Experience: use good use in the evaluation	Taobao Marketplace, a Chinese website for online shopping
Cedar Smart	Gold Power Saving Specialist	Let's hope the power savings are good	Taobao Marketplace, a Chinese website for online shopping
Cedar Smart	Gold Power Saving Specialist	Power saving effect: after using it for a few days, there's nothing like saving power	Taobao Marketplace, a Chinese website for online shopping
Cedar Smart	Gold Power Saving Specialist	Received it and plugged it in, looking forward to the power savings!	Taobao Marketplace, a Chinese website for online shopping
Cedar Smart	Gold Power Saving Specialist	Good thing to listen to friends to sell a friend to save more than three hundred a month electricity bill!	Taobao Marketplace, a Chinese website for online shopping
Cedar Smart	Gold Power Saving Specialist	Power-saving effect: just plugged in on the display220, I do not know whether the power saving power do not know, look at the next January will know😄😄😄😄😄😄	Taobao Marketplace, a Chinese website for online shopping
Cedar Smart	Gold Power Saving Specialist	Appearance and style: received the goods indicator light even fell inside to, can still use customer service returned three red packets	Taobao Marketplace, a Chinese website for online shopping
Cedar Smart	Gold Power Saving Specialist	I haven't seen any results yet, I'll see what happens when I use it.	Taobao Marketplace, a Chinese website for online shopping
Cedar Smart	Gold Power Saving Specialist	I don't know if it's a power saver.	Taobao Marketplace, a Chinese website for online shopping
Cedar Smart	Gold Power Saving Specialist	The goods arrived, logistics fast, see use to know whether the power saving?	Taobao Marketplace, a Chinese website for online shopping
Cedar Smart	Gold Power Saving Specialist	It's not working. It's the same as before.	Taobao Marketplace, a Chinese website for online shopping
Cedar Smart	Gold Power Saving Specialist	Power savings: not bad at all	Taobao Marketplace, a Chinese website for online shopping
Cedar Smart	Gold Power Saving Specialist	The power saving is great, it really saves electricity. Recommended to buy.	Taobao Marketplace, a Chinese website for online shopping
Cedar Smart	Gold Power Saving Specialist	Cost-effective: high, compared to the price, this is a very good deal Workmanship material: good Experience: not yet used, good use the first time to follow up the comment	Taobao Marketplace, a Chinese website for online shopping
Cedar Smart	Gold Power Saving Specialist	Saver, is in use… …effect unknown… …if you can next time continue to cooperate to buy… …workmanship aluminum material, quality is very good. The seller's service attitude is very good.	Taobao Marketplace, a Chinese website for online shopping
Cedar Smart	Gold Power Saving Specialist	Economical to use, the second repurchase, quality workmanship is good, the effect of the crunch, indeed than not on time to compare less, really good!	Taobao Marketplace, a Chinese website for online shopping
Cedar Smart	Gold Power Saving Specialist	Fast delivery, great quality, recommended!	Taobao Marketplace, a Chinese website for online shopping
Cedar Smart	Gold Power Saving Specialist	Recommended by a friend, very happy with the experience, originallyover400, paidover300when pressed. See he does work well before getting it, great workmanship. Very satisfied. Later need will continue to buy back	Taobao Marketplace, a Chinese website for online shopping

Cedar Smart	Gold Power Saving Specialist	Good quality, good customer service, logistics is fast, satisfactory	Taobao Marketplace, a Chinese website for online shopping
Cedar Smart	Gold Power Saving Specialist	It doesn't feel like there's any difference, no reduction in power usage? What's the percentage savings? It's a bit of a crap shoot.	Taobao Marketplace, a Chinese website for online shopping
Cedar Smart	Gold Power Saving Specialist	Second repurchase, power saving as always, do use it, recommend👍	Taobao Marketplace, a Chinese website for online shopping
Cedar Smart	Gold Power Saving Specialist	Already using it Very good Not too expensive Very happy with	Taobao Marketplace, a Chinese website for online shopping
Cedar Smart	Gold Power Saving Specialist	Rental room use. Summer air conditioning refrigerator power consumption is too large, friends recommended to use the effect is very good every month to save a lot of money to me also recommended to use it is really very good[strong]	Taobao Marketplace, a Chinese website for online shopping
Cedar Smart	Gold Power Saving Specialist	Baby received that is inserted and used, very good. The appearance is simple and generous, power saving effect is good. Quality bar, late electricity will continue to buy back!	Taobao Marketplace, a Chinese website for online shopping
Cedar Smart	Gold Power Saving Specialist	I've been using it for a long time and it doesn't work.	Taobao Marketplace, a Chinese website for online shopping
Cedar Smart	Gold Power Saving Specialist	Big factory brand, fine quality workmanship, installation is very convenient and simple, customer service follow up is very on point, also very patient guidance. Good review!	Taobao Marketplace, a Chinese website for online shopping
Cedar Smart	Gold Power Saving Specialist	Had a heart Also gave extra spare fuse. Installation instructions are very detailed. Product quality is very good, logistics is very fast, all aspects of five stars!	Taobao Marketplace, a Chinese website for online shopping
Cedar Smart	Gold Power Saving Specialist	Good service, fast logistics, good value, affordable, this thing I used a year ago, can play the effect of electrical protection and power-saving effect, affordable, easy to use, worth repurchasing, this is the second repurchase!	Taobao Marketplace, a Chinese website for online shopping
Cedar Smart	Gold Power Saving Specialist	Savedover 20 electric bill last month Baby works great Looking at it now I've saved 5 kWh	Taobao Marketplace, a Chinese website for online shopping
Cedar Smart	Gold Power Saving Specialist	Installation was easy and customer service was patient with one and all. Works well and saves some. Quite good to make Will recommend friends	Taobao Marketplace, a Chinese website for online shopping
Cedar Smart	Gold Power Saving Specialist	It's not working.	Taobao Marketplace, a Chinese website for online shopping
Cedar Smart	Gold Power Saving Specialist	It's a rip-off. Still the same electric bill every month! It doesn't help at all!	Taobao Marketplace, a Chinese website for online shopping
Cedar Smart	Gold Power Saving Specialist	I've been using it for half a month now, and it doesn't seem to be saving any power.	Taobao Marketplace, a Chinese website for online shopping
Cedar Smart	Gold Power Saving Specialist	I don't know. I'm just using it.	Taobao Marketplace, a Chinese website for online shopping
Cedar Smart	Gold Power Saving Specialist	Use it for a while and follow up!	Taobao Marketplace, a Chinese website for online shopping
Cedar Smart	Gold Power Saving Specialist	I just received it to try and observe it looks very good and I like it.	Taobao Marketplace, a Chinese website for online shopping
Cedar Smart	Gold Power Saving Specialist	It's plugged in now, let's see if I can get a little surprise at the end of the month	Taobao Marketplace, a Chinese website for online shopping
Cedar Smart	Gold Power Saving Specialist	Power saving effect: praise praise	Taobao Marketplace, a Chinese website for online shopping
Cedar Smart	Gold Power Saving Specialist	Workmanship material: okay. But haven't had a chance to use it yet I'll comment after I use it	Taobao Marketplace, a Chinese website for online shopping
Cedar Smart	Gold Power Saving Specialist	Just started to use! Do not know the effect yet, later review, first positive feedback	Taobao Marketplace, a Chinese website for online shopping
Cedar Smart	Gold Power Saving Specialist	Look at Taobao feel big, but bring this power saving king is a little bit small not quite the same	Taobao Marketplace, a Chinese website for online shopping
Cedar Smart	Gold Power Saving Specialist	Energy saving and power saving king received, cheap, good quality, quality is guaranteed, fast power saving, energy saving and environmental protection, logistics services are very fast, worthy of praise, I hope that everyone goes to buy!	Taobao Marketplace, a Chinese website for online shopping
Cedar Smart	Gold Power Saving Specialist	Energy saving and power saving king received, cheap, good quality, quality is guaranteed, fast power saving, energy saving and environmental protection, logistics services are very fast, worthy of praise, I hope that everyone goes to buy!	Taobao Marketplace, a Chinese website for online shopping
Cedar Smart	Gold Power Saving Specialist	Family members sign for qualityOk	Taobao Marketplace, a Chinese website for online shopping
Cedar Smart	Gold Power Saving Specialist	My family uses a lot of electricity every month, inadvertently found this good thing on the Internet, said it can save energy, customer service attitude is very good, if there is an effect will be back to buy!	Taobao Marketplace, a Chinese website for online shopping
Cedar Smart	Gold Power Saving Specialist	Has received a temporary five-star review, observe a period of time to see the energy-saving come back to the follow-up evaluation	Taobao Marketplace, a Chinese website for online shopping
Cedar Smart	Gold Power Saving Specialist	It still looks good, just see if it saves power	Taobao Marketplace, a Chinese website for online shopping
Cedar Smart	Gold Power Saving Specialist	IQ tax😂😂😂	Taobao Marketplace, a Chinese website for online shopping
Cedar Smart	Gold Power Saving Specialist	Use it for a few months to compare and contrast before evaluating	Taobao Marketplace, a Chinese website for online shopping
Cedar Smart	Gold Power Saving Specialist	(of an unmarried couple) be close	Taobao Marketplace, a Chinese website for online shopping
Cedar Smart	Gold Power Saving Specialist	Does work well, many times back to buy, this one is pressed in the home, originallymore than11degrees a day, the product after the change of9degrees, has been recommended around friends!	Taobao Marketplace, a Chinese website for online shopping
Cedar Smart	Gold Power Saving Specialist	Indeed quite good to use, many times back to buy, to friends also recommended home also saves a lot of electricity. Will come back again!	Taobao Marketplace, a Chinese website for online shopping

Cedar Smart	Gold Power Saving Specialist	Power saving effect: works great	Taobao Marketplace, a Chinese website for online shopping
Cedar Smart	Gold Power Saving Specialist	Used a few days the effect is very good easy to use simple price affordable value logistics is also very fast	Taobao Marketplace, a Chinese website for online shopping
Cedar Smart	Gold Power Saving Specialist	Not bad. A must have for the home saves power and reduces losses! Fewer trips in the house Voltage stabilization does work	Taobao Marketplace, a Chinese website for online shopping
Cedar Smart	Gold Power Saving Specialist	Very good quality Fine workmanship Great power savings Affordable too	Taobao Marketplace, a Chinese website for online shopping
Cedar Smart	Gold Power Saving Specialist	Very satisfied with the results, the accumulation of small amounts, reasonable and legal energy saving. The quality is very good to use and practical. Very satisfied	Taobao Marketplace, a Chinese website for online shopping
Cedar Smart	king of power savers	Exquisite workmanship, comfortable feel, plug and play, air purification and voltage digital display, beautiful and generous. Just did not find out, the manufacturing plant address; in addition, how to save power, after use and then come back to the review.	Taobao Marketplace, a Chinese website for online shopping
Cedar Smart	king of power savers	I've been using it for a while now and haven't found anything magical so far	Taobao Marketplace, a Chinese website for online shopping
Cedar Smart	Red Power Saving Specialist	not have	Taobao Marketplace, a Chinese website for online shopping
Cedar Smart	a power saver (electronics)	very good	Taobao Marketplace, a Chinese website for online shopping
Cedar Smart	a power saver (electronics)	Fast logistics, cost-effective, very satisfied	Taobao Marketplace, a Chinese website for online shopping
Cedar Smart	a power saver (electronics)	Very happy with the results, building up to reasonable and legal energy savings. Very good and practical. Very satisfied	Taobao Marketplace, a Chinese website for online shopping
Cedar Smart	a power saver (electronics)	It does work quite well, and this month the electricity bill is less than usual. Good product at an affordable price	Taobao Marketplace, a Chinese website for online shopping
Cedar Smart	a power saver (electronics)	It really saves electricity than before it was used, it doesn't get hot when you use it all day, it's easy to use, energy-saving and quiet.	Taobao Marketplace, a Chinese website for online shopping
Cedar Smart	a power saver (electronics)	Power saving effect: very good Cost-effective: high Appearance style: exquisite Workmanship material: fine	Taobao Marketplace, a Chinese website for online shopping
Cedar Smart	a power saver (electronics)	The logistics is quite fast very good their family service is also very good	Taobao Marketplace, a Chinese website for online shopping
Cedar Smart	a power saver (electronics)	I'll buy three more if they're good.	Taobao Marketplace, a Chinese website for online shopping
Cedar Smart	a power saver (electronics)	Experience:(seems to have no effect, I do not know if it is time short-term can not see whether it is effective	Taobao Marketplace, a Chinese website for online shopping
Cedar Smart	a power saver (electronics)	Didn't see the power saving effect	Taobao Marketplace, a Chinese website for online shopping
Cedar Smart	a power saver (electronics)	Workmanship material: good	Taobao Marketplace, a Chinese website for online shopping
Cedar Smart	a power saver (electronics)	The power saving has not been noticed yet, I will comment again when I use it for a month.	Taobao Marketplace, a Chinese website for online shopping
Cedar Smart	a power saver (electronics)	Fast logistics. Very satisfied with the goods worth buying	Taobao Marketplace, a Chinese website for online shopping
Cedar Smart	a power saver (electronics)	Very easy to install and customer service was very patient in guiding the best location. Installed for a while and feels really good! Home lines are much more stable.	Taobao Marketplace, a Chinese website for online shopping
Cedar Smart	a power saver (electronics)	It does work quite well, and the line in the house is stabilized.electricity bill has been reduced by less. It is very reasonable and legal, and we can use it with confidence.	Taobao Marketplace, a Chinese website for online shopping
Cedar Smart	a power saver (electronics)	The saver is good and easy to use, the power saving is good and the service is good.	Taobao Marketplace, a Chinese website for online shopping
Cedar Smart	a power saver (electronics)	Works great, inexpensive, worth the price	Taobao Marketplace, a Chinese website for online shopping
Cedar Smart	a power saver (electronics)	Shipped quickly, well-packed, the price is particularly affordable, power-saving effect is very good, a month can save a lot of money, it is worth buying!	Taobao Marketplace, a Chinese website for online shopping
Cedar Smart	a power saver (electronics)	Experience: is experiencing, the specific effect of waiting for the end of the month of the electricity bill out and then evaluate the	Taobao Marketplace, a Chinese website for online shopping
Cedar Smart	a power saver (electronics)	It does work quite well and really saves points much better than before, very good.	Taobao Marketplace, a Chinese website for online shopping
Cedar Smart	a power saver (electronics)	Fast shipping, good packaging, back to install and use for a period of time, energy saving and power saving effect is very good	Taobao Marketplace, a Chinese website for online shopping
Cedar Smart	a power saver (electronics)	Power savings: one very good!	Taobao Marketplace, a Chinese website for online shopping
Cedar Smart	a power saver (electronics)	The saver is of good quality, saves electricity well and is very satisfactory.	Taobao Marketplace, a Chinese website for online shopping
Cedar Smart	a power saver (electronics)	I don't see much effect at the moment, so let's wait for it to kick in.	Taobao Marketplace, a Chinese website for online shopping
Cedar Smart	a power saver (electronics)	Good quality and reasonably priced for what you get. Good value for money.	Taobao Marketplace, a Chinese website for online shopping
Cedar Smart	a power saver (electronics)	The saver works great and looks great, doesn't take up much space, love it!	Taobao Marketplace, a Chinese website for online shopping

Cedar Smart	a power saver (electronics)	It's not working.	Taobao Marketplace, a Chinese website for online shopping
Cedar Smart	a power saver (electronics)	Energy saver received, the quality is very good, logistics is very fast	Taobao Marketplace, a Chinese website for online shopping
Cedar Smart	a power saver (electronics)	Quite good, the second repurchase, the summer is here to the upstairs also set up more than one, the air conditioning all open up the electricity bill can not top, save some is some. Practical Good value	Taobao Marketplace, a Chinese website for online shopping
Cedar Smart	a power saver (electronics)	It's too small, and light, so I don't know if it saves power.	Taobao Marketplace, a Chinese website for online shopping
Cedar Smart	a power saver (electronics)	Very good quality, very satisfied with the purchase. Customer service is very professional with questions and answers	Taobao Marketplace, a Chinese website for online shopping
Cedar Smart	a power saver (electronics)	After comparing, it is indeed a dozen dollars less. Affordable. Great product to use and save!	Taobao Marketplace, a Chinese website for online shopping
Cedar Smart	a power saver (electronics)	Looked at a few to get started, his quality is good and the price point is more than appropriate	Taobao Marketplace, a Chinese website for online shopping
Cedar Smart	a power saver (electronics)	Easy to install, customer service is very careful guidance, pressed in the front end of the power use more, power saving effect is good. I do not understand the place is also very patient guidance. Very satisfied, value for money	Taobao Marketplace, a Chinese website for online shopping
Cedar Smart	a power saver (electronics)	That's great. Saves a lot of money on the electric bill, doesn't take up much space.	Taobao Marketplace, a Chinese website for online shopping
Cedar Smart	a power saver (electronics)	Special power saving operation is simple, fine workmanship Satisfaction	Taobao Marketplace, a Chinese website for online shopping
Cedar Smart	a power saver (electronics)	Looking good, hope it works well.	Taobao Marketplace, a Chinese website for online shopping
Cedar Smart	a power saver (electronics)	Value for money: average	Taobao Marketplace, a Chinese website for online shopping
Cedar Smart	a power saver (electronics)	Exterior Style:Thank	Taobao Marketplace, a Chinese website for online shopping
Cedar Smart	a power saver (electronics)	I don't know if it works or not, I'll use it for a while and see.	Taobao Marketplace, a Chinese website for online shopping
Cedar Smart	a power saver (electronics)	Energy saving and power saving with quality assurance	Taobao Marketplace, a Chinese website for online shopping
Cedar Smart	a power saver (electronics)	Arrived quickly, with a good, good workmanship materials are very good, there is a need to purchase	Taobao Marketplace, a Chinese website for online shopping
Cedar Smart	a power saver (electronics)	Good quality, workmanship can be, practical and convenient, the effect is good satisfaction	Taobao Marketplace, a Chinese website for online shopping
Cedar Smart	a power saver (electronics)	I've bought it for a month now, it doesn't work, it's the same with him as without him, I feel duped.	Taobao Marketplace, a Chinese website for online shopping
Cedar Smart	a power saver (electronics)	Power saving effect: }good	Taobao Marketplace, a Chinese website for online shopping
Cedar Smart	a power saver (electronics)	Power-saving effect: for the time being, I have not found any effect, waiting for60dayscustomer service said tosee the effect, and then follow-up comments.	Taobao Marketplace, a Chinese website for online shopping
Cedar Smart	a power saver (electronics)	Experience: average	Taobao Marketplace, a Chinese website for online shopping
Cedar Smart	a power saver (electronics)	Meter received, exquisite workmanship, very good-looking, good material	Taobao Marketplace, a Chinese website for online shopping
Cedar Smart	a power saver (electronics)	I don't know if I'm connected to the electricity or not, but if I plug this in and use the induction cooker, the induction cooker will automatically turn off the electricity.	Taobao Marketplace, a Chinese website for online shopping
Cedar Smart	a power saver (electronics)	It's justover800kWhper month without using him, and when he does, he usesover100kWhmore a month... I'm really speechless...	Taobao Marketplace, a Chinese website for online shopping
Cedar Smart	a power saver (electronics)	It still feels OK, you won't know until the end of the month if it saves on your electric bill. It's still worth a try	Taobao Marketplace, a Chinese website for online shopping
Cedar Smart	a power saver (electronics)	Thanks.	Taobao Marketplace, a Chinese website for online shopping
Cedar Smart	a power saver (electronics)	No power saving. IQ tax, not recommended!	Taobao Marketplace, a Chinese website for online shopping
Cedar Smart	a power saver (electronics)	We'll follow up later.	Taobao Marketplace, a Chinese website for online shopping
Cedar Smart	a power saver (electronics)	The energy saving effect is OK, and the quality is quite good, cost-effective	Taobao Marketplace, a Chinese website for online shopping
Cedar Smart	a power saver (electronics)	Power-saving effect: not much effect, Workmanship material: goods quite showy Use experience: is not bought IQ tax	Taobao Marketplace, a Chinese website for online shopping
Cedar Smart	a power saver (electronics)	Goods received, just plugged in a month later to evaluate, workmanship, appearance saw good!	Taobao Marketplace, a Chinese website for online shopping
Cedar Smart	a power saver (electronics)	I just used it and I don't know how well it works yet, I'll follow up after I use it?	Taobao Marketplace, a Chinese website for online shopping
Cedar Smart	a power saver (electronics)	Use it for a while and evaluate it objectively	Taobao Marketplace, a Chinese website for online shopping
Cedar Smart	a power saver (electronics)	Has been plugged into the power supply, just so far did not see any effect, the meter flashes or about the same time, the first default positive feedback it	Taobao Marketplace, a Chinese website for online shopping

Cedar Smart	a power saver (electronics)	System default comments	Taobao Marketplace, a Chinese website for online shopping
Cedar Smart	a power saver (electronics)	Experience: use good use in the evaluation	Taobao Marketplace, a Chinese website for online shopping
Cedar Smart	a power saver (electronics)	Let's hope the power savings are good	Taobao Marketplace, a Chinese website for online shopping
Cedar Smart	a power saver (electronics)	Power saving effect: after using it for a few days, there's nothing like saving power	Taobao Marketplace, a Chinese website for online shopping
Cedar Smart	a power saver (electronics)	Received it and plugged it in, looking forward to the power savings!	Taobao Marketplace, a Chinese website for online shopping
Cedar Smart	a power saver (electronics)	Good thing to listen to friends to sell a friend to save more than three hundred a month electricity bill!	Taobao Marketplace, a Chinese website for online shopping
Cedar Smart	a power saver (electronics)	Power-saving effect: just plugged in on the display220, I do not know whether the power saving power do not know, look at the next January will know😀😀😀😀😀😀	Taobao Marketplace, a Chinese website for online shopping
Cedar Smart	a power saver (electronics)	Appearance and style: received the goods indicator light even fell inside to, can still use customer service returned three red packets	Taobao Marketplace, a Chinese website for online shopping
Cedar Smart	a power saver (electronics)	I haven't seen any results yet, I'll see what happens when I use it.	Taobao Marketplace, a Chinese website for online shopping
Cedar Smart	a power saver (electronics)	I don't know if it's a power saver.	Taobao Marketplace, a Chinese website for online shopping
Cedar Smart	a power saver (electronics)	The goods arrived, logistics fast, see use to know whether the power saving?	Taobao Marketplace, a Chinese website for online shopping
Cedar Smart	a power saver (electronics)	It's not working. It's the same as before.	Taobao Marketplace, a Chinese website for online shopping
Cedar Smart	a power saver (electronics)	Power savings: not bad at all	Taobao Marketplace, a Chinese website for online shopping
Cedar Smart	a power saver (electronics)	The power saving is great, it really saves electricity. Recommended to buy.	Taobao Marketplace, a Chinese website for online shopping
Cedar Smart	a power saver (electronics)	Cost-effective: high, compared to the price, this is a very good deal Workmanship material: good Experience: not yet used, good use the first time to follow up the comment	Taobao Marketplace, a Chinese website for online shopping
Cedar Smart	a power saver (electronics)	Saver, is in use… …effect unknown… …if you can next time continue to cooperate to buy… …workmanship aluminum material, quality is very good. The seller's service attitude is very good.	Taobao Marketplace, a Chinese website for online shopping
Cedar Smart	a power saver (electronics)	Economical to use, the second repurchase, quality workmanship is good, the effect of the crunch, indeed than not on time to compare less, really good!	Taobao Marketplace, a Chinese website for online shopping
Cedar Smart	a power saver (electronics)	Fast delivery, great quality, recommended!	Taobao Marketplace, a Chinese website for online shopping
Cedar Smart	a power saver (electronics)	Recommended by a friend, very happy with the experience, originallyover400, paidover300when pressed. See he does work well before getting it, great workmanship. Very satisfied. Later need will continue to buy back	Taobao Marketplace, a Chinese website for online shopping
Cedar Smart	a power saver (electronics)	Good quality, good customer service, logistics is fast, satisfactory	Taobao Marketplace, a Chinese website for online shopping
Cedar Smart	a power saver (electronics)	It doesn't feel like there's any difference, no reduction in power usage? What's the percentage savings? It's a bit of a crap shoot.	Taobao Marketplace, a Chinese website for online shopping
Cedar Smart	a power saver (electronics)	Second repurchase, power saving as always, do use it, recommend👍	Taobao Marketplace, a Chinese website for online shopping
Cedar Smart	a power saver (electronics)	Already using it Very good Not too expensive Very happy with	Taobao Marketplace, a Chinese website for online shopping
Cedar Smart	a power saver (electronics)	Rental room use. Summer air conditioning refrigerator power consumption is too large, friends recommended to use the effect is very good every month to save a lot of money to me also recommended to use it is really very good[strong]	Taobao Marketplace, a Chinese website for online shopping
Cedar Smart	a power saver (electronics)	Baby received that is inserted and used, very good. The appearance is simple and generous, power saving effect is good. Quality bar, late electricity will continue to buy back!	Taobao Marketplace, a Chinese website for online shopping
Cedar Smart	a power saver (electronics)	I've been using it for a long time and it doesn't work.	Taobao Marketplace, a Chinese website for online shopping
Cedar Smart	a power saver (electronics)	Big factory brand, fine quality workmanship, installation is very convenient and simple, customer service follow up is very on point, also very patient guidance. Good review!	Taobao Marketplace, a Chinese website for online shopping
Cedar Smart	a power saver (electronics)	Had a heart Also gave extra spare fuse. Installation instructions are very detailed. Product quality is very good, logistics is very fast, all aspects of five stars!	Taobao Marketplace, a Chinese website for online shopping
Cedar Smart	a power saver (electronics)	Good service, fast logistics, good value, affordable, this thing I used a year ago, can play the effect of electrical protection and power-saving effect, affordable, easy to use, worth repurchasing, this is the second repurchase!	Taobao Marketplace, a Chinese website for online shopping
Cedar Smart	a power saver (electronics)	Savedover 20 electric bill last month Baby works great Looking at it now I've saved 5 kWh	Taobao Marketplace, a Chinese website for online shopping
Cedar Smart	a power saver (electronics)	Installation was easy and customer service was patient with one and all. Works well and saves some. Quite good to make Will recommend friends	Taobao Marketplace, a Chinese website for online shopping
Cedar Smart	a power saver (electronics)	It's not working.	Taobao Marketplace, a Chinese website for online shopping
Cedar Smart	a power saver (electronics)	It's a rip-off. Still the same electric bill every month! It doesn't help at all!	Taobao Marketplace, a Chinese website for online shopping
Cedar Smart	a power saver (electronics)	I've been using it for half a month now, and it doesn't seem to be saving any power.	Taobao Marketplace, a Chinese website for online shopping

Cedar Smart	a power saver (electronics)	I don't know. I'm just using it.	Taobao Marketplace, a Chinese website for online shopping
Cedar Smart	a power saver (electronics)	Use it for a while and follow up!	Taobao Marketplace, a Chinese website for online shopping
Cedar Smart	a power saver (electronics)	I just received it to try and observe it looks very good and I like it.	Taobao Marketplace, a Chinese website for online shopping
Cedar Smart	a power saver (electronics)	It's plugged in now, let's see if I can get a little surprise at the end of the month	Taobao Marketplace, a Chinese website for online shopping
Cedar Smart	a power saver (electronics)	Power saving effect: praise praise	Taobao Marketplace, a Chinese website for online shopping
Cedar Smart	a power saver (electronics)	Workmanship material: okay. But haven't had a chance to use it yet I'll comment after I use it	Taobao Marketplace, a Chinese website for online shopping
Cedar Smart	a power saver (electronics)	Just started to use! Do not know the effect yet, later review, first positive feedback	Taobao Marketplace, a Chinese website for online shopping
Cedar Smart	a power saver (electronics)	Look at Taobao feel big, but bring this power saving king is a little bit small not quite the same	Taobao Marketplace, a Chinese website for online shopping
Cedar Smart	a power saver (electronics)	Energy saving and power saving king received, cheap, good quality, quality is guaranteed, fast power saving, energy saving and environmental protection, logistics services are very fast, worthy of praise, I hope that everyone goes to buy!	Taobao Marketplace, a Chinese website for online shopping
Cedar Smart	a power saver (electronics)	Energy saving and power saving king received, cheap, good quality, quality is guaranteed, fast power saving, energy saving and environmental protection, logistics services are very fast, worthy of praise, I hope that everyone goes to buy!	Taobao Marketplace, a Chinese website for online shopping
Cedar Smart	a power saver (electronics)	Family members sign for qualityOk	Taobao Marketplace, a Chinese website for online shopping
Cedar Smart	a power saver (electronics)	My family uses a lot of electricity every month, inadvertently found this good thing on the Internet, said it can save energy, customer service attitude is very good, if there is an effect will be back to buy!	Taobao Marketplace, a Chinese website for online shopping
Cedar Smart	a power saver (electronics)	Has received a temporary five-star review, observe a period of time to see the energy-saving come back to the follow-up evaluation	Taobao Marketplace, a Chinese website for online shopping
Cedar Smart	a power saver (electronics)	It still looks good, just see if it saves power	Taobao Marketplace, a Chinese website for online shopping
Cedar Smart	a power saver (electronics)	IQ tax😂😂😂	Taobao Marketplace, a Chinese website for online shopping
Cedar Smart	a power saver (electronics)	Use it for a few months to compare and contrast before evaluating	Taobao Marketplace, a Chinese website for online shopping
Cedar Smart	a power saver (electronics)	(of an unmarried couple) be close	Taobao Marketplace, a Chinese website for online shopping
Cedar Smart	a power saver (electronics)	Does work well, many times back to buy, this one is pressed in the home, originallymore than11degrees a day, the product after the change of9degrees, has been recommended around friends!	Taobao Marketplace, a Chinese website for online shopping
Cedar Smart	a power saver (electronics)	Indeed quite good to use, many times back to buy, to friends also recommended home also saves a lot of electricity. Will come back again!	Taobao Marketplace, a Chinese website for online shopping
Cedar Smart	a power saver (electronics)	Power saving effect: works great	Taobao Marketplace, a Chinese website for online shopping
Cedar Smart	a power saver (electronics)	Used a few days the effect is very good easy to use simple price affordable value logistics is also very fast	Taobao Marketplace, a Chinese website for online shopping
Cedar Smart	a power saver (electronics)	Not bad. A must have for the home Saves electricity and reduces losses! Fewer trips in the house Voltage stabilization does work!	Taobao Marketplace, a Chinese website for online shopping
Cedar Smart	a power saver (electronics)	Very good quality, fine workmanship, good power saving and affordable price.	Taobao Marketplace, a Chinese website for online shopping
Cedar Smart	a power saver (electronics)	Very satisfied with the results, the accumulation of small amounts, reasonable and legal energy saving. The quality is very good to use and practical. Very satisfied	Taobao Marketplace, a Chinese website for online shopping
Cedar Smart	Gold Power Saving Specialist	not have	Taobao Marketplace, a Chinese website for online shopping
Cedar Smart	King of Power Saving	Baby has received good, see how the effect	Taobao Marketplace, a Chinese website for online shopping
Cedar Smart	Deep Blue Power Saving Specialist	Power saving effect: can.Very power saving, bought one earlier this year, Experience: bought before, power saving significantly over20%a30%. This is the second time to buy, An another suite.	Taobao Marketplace, a Chinese website for online shopping
Cedar Smart	Deep Blue Power Saving Specialist	Express very quickly, energy saving and power saving is very good, in the use of a period of time to see how it is	Taobao Marketplace, a Chinese website for online shopping
Cedar Smart	Deep Blue Power Saving Specialist	I got it.	Taobao Marketplace, a Chinese website for online shopping
Cedar Smart	Deep Blue Power Saving Specialist	No power saving properties! Uses more electricity than the original	Taobao Marketplace, a Chinese website for online shopping
Cedar Smart	Deep Blue Power Saving Specialist	Power-saving effect: just use can not see the effect, to see later Appearance style: can Workmanship material: good	Taobao Marketplace, a Chinese website for online shopping
Cedar Smart	Deep Blue Power Saving Specialist	I just received my shipment and immediately took a second one.	Taobao Marketplace, a Chinese website for online shopping
Cedar Smart	Deep Blue Power Saving Specialist	It's great. It's awesome! Thanks a lot for the hard work!	Taobao Marketplace, a Chinese website for online shopping
Cedar Smart	Deep Blue Power Saving Specialist	Good review!	Taobao Marketplace, a Chinese website for online shopping

Cedar Smart	Deep Blue Power Saving Specialist	Workmanship material: good	Taobao Marketplace, a Chinese website for online shopping
Cedar Smart	Deep Blue Power Saving Specialist	The goods received, the quality is really very good, very satisfied.	Taobao Marketplace, a Chinese website for online shopping
Cedar Smart	Deep Blue Power Saving Specialist	Recommended by the owner of the store next door, looked at his home does have to save electricity, decided to get. Indeed quite can, the voltage is much more stable, there is a reduction in electricity consumption.	Taobao Marketplace, a Chinese website for online shopping
Cedar Smart	Deep Blue Power Saving Specialist	The logistics is pretty fast, so it's okay	Taobao Marketplace, a Chinese website for online shopping
Cedar Smart	Deep Blue Power Saving Specialist	Summer with more electricity, pressed two, according to customer service guidance on the installation location, the front end of the surrounding one, with more electricity in one place. Work is really good, the end of the epidemic, slowly getting better, more electricity, so friends good with the introduction.	Taobao Marketplace, a Chinese website for online shopping
Cedar Smart	Deep Blue Power Saving Specialist	Baby received, also gave a multi-back with the fuse, installation instructions are very detailed, good quality, logistics is very fast, it is worth recommending!	Taobao Marketplace, a Chinese website for online shopping
Cedar Smart	Deep Blue Power Saving Specialist	I used a small one before and it worked well, so I purchased a powerful one for the store this time. Summer electricity consumption Just what we need Good to use and save	Taobao Marketplace, a Chinese website for online shopping
Cedar Smart	Deep Blue Power Saving Specialist	Durable. Very good quality. Second purchase. Very practical and cost-effective.	Taobao Marketplace, a Chinese website for online shopping
Cedar Smart	Deep Blue Power Saving Specialist	Good quality and very practical And the installation video is very detailed Good comment	Taobao Marketplace, a Chinese website for online shopping
Cedar Smart	Deep Blue Power Saving Specialist	Easy to use and install, customer service is very patient, there is an answer, the product is very satisfied, the store service is careful and patient, so satisfied!	Taobao Marketplace, a Chinese website for online shopping
Cedar Smart	Deep Blue Power Saving Specialist	Had a heart Also gave extra spare fuse. Installation instructions are very detailed. Great product quality, fast logistics, five stars all around! Would recommend	Taobao Marketplace, a Chinese website for online shopping
Cedar Smart	Deep Blue Power Saving Specialist	Not bad. A must have for the home saves power and reduces losses! Fewer trips in the house Voltage stabilization does work	Taobao Marketplace, a Chinese website for online shopping
Cedar Smart	Deep Blue Power Saving Specialist	Logistics courier very quickly, received that is installed, there is a very good answer, the product quality is very good, the service is very in place. Very satisfied	Taobao Marketplace, a Chinese website for online shopping
Cedar Smart	Deep Blue Power Saving Specialist	Received plugged in and used, easy to install, customer service is very patient guidance to answer. Good business good products! Kudos!	Taobao Marketplace, a Chinese website for online shopping
Cedar Smart	Deep Blue Power Saving Specialist	Easy to operate and install, the customer is also very patient guidance, there are questions and answers, after-sales aspects of the protection, the use of peace of mind!	Taobao Marketplace, a Chinese website for online shopping
Cedar Smart	Deep Blue Power Saving Specialist	Good quality, as described by the merchant, cost-effective and very energy efficient👍👍👍👍👍	Taobao Marketplace, a Chinese website for online shopping
Cedar Smart	Deep Blue Power Saving Specialist	The quality is pretty good and it's also practical so I'll give this a good review	Taobao Marketplace, a Chinese website for online shopping
Cedar Smart	Deep Blue Power Saving Specialist	Quite good, the second repurchase, the summer is here to the upstairs also set up more than one, the air conditioning all open up the electricity bill can not top, save some is some. Practical Good value	Taobao Marketplace, a Chinese website for online shopping
Cedar Smart	Blue Power Saving Specialist	After the follow-up observation, in the evaluation, customer service is quite good, patient answer.	Taobao Marketplace, a Chinese website for online shopping
Cedar Smart	Blue Power Saving Specialist	Power saving effect: good Cost-effective: high Appearance style: simple Workmanship material: good Using experience: power saving	Taobao Marketplace, a Chinese website for online shopping
Cedar Smart	Blue Power Saving Specialist	This thing, I don't know much about the principle, but it does hold less power than before it was used. It does work.works Good	Taobao Marketplace, a Chinese website for online shopping
Cedar Smart	Blue Power Saving Specialist	Many times to help repurchase, really good, than not used before a lot less electricity, the main durable, value for money!	Taobao Marketplace, a Chinese website for online shopping
Cedar Smart	Blue Power Saving Specialist	Power saving treasure received, the quality is very good, very satisfied with the practical and convenient	Taobao Marketplace, a Chinese website for online shopping
Cedar Smart	Blue Power Saving Specialist	It's great, this electric bill is a lot less than going to school	Taobao Marketplace, a Chinese website for online shopping
Cedar Smart	Blue Power Saving Specialist	Genuine, quality assurance, tight packaging, logistics is also very fast!	Taobao Marketplace, a Chinese website for online shopping
Cedar Smart	Blue Power Saving Specialist	Quite good, the second repurchase, the summer is here to the upstairs also set up more than one, the air conditioning all open up the electricity bill can not top, save some is some. Practical Good value	Taobao Marketplace, a Chinese website for online shopping
Cedar Smart	Blue Power Saving Specialist	Energy saver received and installed on it, a day down really save a lot of money Oh, the material is also very good.	Taobao Marketplace, a Chinese website for online shopping
Cedar Smart	Blue Power Saving Specialist	Quite good to use, node baby with a period of time home line is very stable.the purification function is also very practical, there is a smoker at home second-hand smoke flying all over the day. Press on also really great role also help purification!	Taobao Marketplace, a Chinese website for online shopping
Cedar Smart	Blue Power Saving Specialist	Very power efficient and cost effective.	Taobao Marketplace, a Chinese website for online shopping
Cedar Smart	Blue Power Saving Specialist	Baby received, the quality is very good, the logistics is very fast, very satisfied!	Taobao Marketplace, a Chinese website for online shopping
Cedar Smart	Blue Power Saving Specialist	Received the goods, the quality is very good, very good, the price is affordable, satisfactory	Taobao Marketplace, a Chinese website for online shopping
Cedar Smart	Blue Power Saving Specialist	Reliable quality, easy and convenient installation, used for a period of time electricity than before is to save some, satisfied!	Taobao Marketplace, a Chinese website for online shopping
Cedar Smart	Blue Power Saving Specialist	already installed It's still very good It's very energy efficient and environmentally friendly	Taobao Marketplace, a Chinese website for online shopping
Cedar Smart	Blue Power Saving Specialist	Energy saver power saving effect can be, cost-effective and cost-effective, after-sales time is also relatively long, rest assured that purchase	Taobao Marketplace, a Chinese website for online shopping

Cedar Smart	Blue Power Saving Specialist	Works pretty well, saves a lot of electricity in a month.performance ratio is high👍	Taobao Marketplace, a Chinese website for online shopping
Cedar Smart	Blue Power Saving Specialist	Shipped very quickly, received immediately used, looked at the meter obviously slowed down the speed, the effect is good!	Taobao Marketplace, a Chinese website for online shopping
Cedar Smart	Blue Power Saving Specialist	Easy and convenient to operate Quality👍logistics is also fast	Taobao Marketplace, a Chinese website for online shopping
Cedar Smart	Blue Power Saving Specialist	Power saving effect: very good Price/performance ratio: satisfactory Using experience: very worthwhile	Taobao Marketplace, a Chinese website for online shopping
Cedar Smart	Blue Power Saving Specialist	Great quality Great delivery👍	Taobao Marketplace, a Chinese website for online shopping
Cedar Smart	Blue Power Saving Specialist	Energy saving and power saving, easy to use, fine workmanship and good materials, cost-effective	Taobao Marketplace, a Chinese website for online shopping
Cedar Smart	Blue Power Saving Specialist	Has received the goods, delivery is very fast, logistics is also very fast, immediately after receipt of the plug in the trial, feel good, observe a month after the power saving to see the effect again.	Taobao Marketplace, a Chinese website for online shopping
Cedar Smart	Blue Power Saving Specialist	The owner shipped quickly and the quality is excellent, consistently charged, simple and easy to operate, and inexpensive.	Taobao Marketplace, a Chinese website for online shopping
Cedar Smart	Blue Power Saving Specialist	It's not working.	Taobao Marketplace, a Chinese website for online shopping
Cedar Smart	Blue Power Saving Specialist	Still pretty good, good style, quality workmanship is guaranteed, the price is affordable!	Taobao Marketplace, a Chinese website for online shopping
Cedar Smart	Blue Power Saving Specialist	Affordable and effective	Taobao Marketplace, a Chinese website for online shopping
Cedar Smart	Blue Power Saving Specialist	Easy to install, customer service is very careful guidance, pressed in the front end of the power use more, power saving effect is good. I do not understand the place is also very patient guidance. Very satisfied, value for money	Taobao Marketplace, a Chinese website for online shopping
Cedar Smart	Blue Power Saving Specialist	The quality is great, the power savings are noticeable, and the shipping is fast, so it's worth getting.	Taobao Marketplace, a Chinese website for online shopping
Cedar Smart	Blue Power Saving Specialist	I got it. It's good quality. It works.	Taobao Marketplace, a Chinese website for online shopping
Cedar Smart	Blue Power Saving Specialist	It's really good, I've been doing it for a while now, and it really saves electricity, and the quality is very good, I've had no problems with it.	Taobao Marketplace, a Chinese website for online shopping
Cedar Smart	Blue Power Saving Specialist	The quality of the saver is very good, easy to operate, the power saving effect is quite good, easy to use.	Taobao Marketplace, a Chinese website for online shopping
Cedar Smart	Blue Power Saving Specialist	Buyer does not comment within15days	Taobao Marketplace, a Chinese website for online shopping
Cedar Smart	Blue Power Saving Specialist	very practical	Taobao Marketplace, a Chinese website for online shopping
Cedar Smart	(Three-phase) 380V applicable economizer	The store has a freezer, long years open loss quite a lot, last year pressed a really few hundred degrees less! Genuine manufacturer old brand, worth good use recommended, the second repurchase!	Taobao Marketplace, a Chinese website for online shopping
Cedar Smart	(Three-phase) 381V applicable economizer	Easy to install, customer service is very professional guidance, the product is very well made is genuine. Stores like ours that use a lot of electricity can rest assured to get it!	Taobao Marketplace, a Chinese website for online shopping
Cedar Smart	(Three-phase) 382V applicable economizer	Friends of the factory is also using, he said the effect is very good, every month can save a few hundred dollars! Give him a link, I also press a, now the electricity bill shall not be! Can save is the hard truth!	Taobao Marketplace, a Chinese website for online shopping
Cedar Smart	(Three-phase) 383V applicable economizer	Easy to install, not easy to rust, really nice	Taobao Marketplace, a Chinese website for online shopping
Cedar Smart	(Three-phase) 384V applicable economizer	Uses good effect, tested the comparison energy saving up to20%. Three-phase big brand, recommended by friends. Good product is hard enough, lever drop!	Taobao Marketplace, a Chinese website for online shopping
Cedar Smart	(Three-phase) 385V applicable economizer	Good quality, good appearance, no noise, good power saving effect, satisfactory	Taobao Marketplace, a Chinese website for online shopping
Cedar Smart	(Three-phase) 386V applicable economizer	Summer is ready to come, the hotel electricity is particularly large, can not top, friends store with this brand is very good, recommended to me. Installation is very convenient, business customer service is very professional! Very enthusiastic response.	Taobao Marketplace, a Chinese website for online shopping
Cedar Smart	(Three-phase) 387V applicable economizer	Saving power saving safe and reliable, practicality is very good, praise.	Taobao Marketplace, a Chinese website for online shopping
Cedar Smart	(Three-phase) 388V Applicable Saver	Next door to the factory friends use quite good, take a link with him, ant king three-phase brand genuine. Checked really big factory factory, the quality of workmanship is very great. Installation is very convenient and simple!	Taobao Marketplace, a Chinese website for online shopping
Cedar Smart	(Three-phase) 389V applicable economizer	The quality is very good, very power-saving, very convenient to use, satisfaction	Taobao Marketplace, a Chinese website for online shopping
Cedar Smart	(Three-phase) 390V applicable economizer	The logistics is very fast It matches the picture The store owner has good service There is credit I like it very much I will buy it again next time	Taobao Marketplace, a Chinese website for online shopping
Cedar Smart	(Three-phase) 391V Applicable Saver	Three-phase economizer received, the box is perfect, complete accessories inside, it is genuine. Has been used, energized normally, customer service is very professional. Very praise.	Taobao Marketplace, a Chinese website for online shopping
Cedar Smart	(Three-phase) 392V applicable economizer	Logistics and products are good, cost-effective, kudos!	Taobao Marketplace, a Chinese website for online shopping
Cedar Smart	(Three-phase) 393V applicable economizer	Very easy to wire customer service is very detailed product quality is very good! Friends with good recommended! Very good	Taobao Marketplace, a Chinese website for online shopping
Cedar Smart	(Three-phase) 394V applicable economizer	Three-phase power saver is easy to install, safe and fast to use, saves electricity and money, as good as the seller's description.	Taobao Marketplace, a Chinese website for online shopping
Cedar Smart	(Three-phase) 395V applicable power saver	Merchant customer service is very professional. Patiently followed up with tutorial wiring verification, good product great label customer service professional. Very satisfied	Taobao Marketplace, a Chinese website for online shopping

Cedar Smart	(Three-phase) 396V applicable economizer	Power can arrive in good quality, logistics is strong no damage, the use of high efficiency	Taobao Marketplace, a Chinese website for online shopping
Cedar Smart	(Three-phase) 397V applicable economizer	Buyer does not comment within15days	Taobao Marketplace, a Chinese website for online shopping
Cedar Smart	(Three-phase) 398V applicable economizer	Bought last year, put aside and forget to install, just installed today, the store is very careful, with two months later to see the effect and then follow up! The quality of the stuff is very good, all aluminum!	Taobao Marketplace, a Chinese website for online shopping
Cedar Smart	Three-phase power saver	not have	Taobao Marketplace, a Chinese website for online shopping
Cedar Smart	economizer	not have	Taobao Marketplace, a Chinese website for online shopping
Intelligent power-saving new technology	King of Power Saving	Just used it today don't know the effect, hope it really saves power.	Taobao Marketplace, a Chinese website for online shopping
Intelligent power-saving new technology	King of Power Saving	Power-saving effect: no effect are scam Cost-effective: no quality is very good	Taobao Marketplace, a Chinese website for online shopping
Intelligent power-saving new technology	Green Intelligent Power Saver	Power saving effect: Somewhat effective	Taobao Marketplace, a Chinese website for online shopping
Intelligent power-saving new technology	Green Intelligent Power Saver	There's no way this product can save power.Forget$10I was going to refund	Taobao Marketplace, a Chinese website for online shopping
Intelligent power-saving new technology	Green Intelligent Power Saver	To be reviewed after use	Taobao Marketplace, a Chinese website for online shopping
Intelligent power-saving new technology	Green Intelligent Power Saver	ve bought them many times, and this time I bought3moreto use in areas of the house that use a lot of electricity and save as much as I can.	Taobao Marketplace, a Chinese website for online shopping
Intelligent power-saving new technology	Green Intelligent Power Saver	Power saving effect: basically useless!	Taobao Marketplace, a Chinese website for online shopping
Intelligent power-saving new technology	Green Intelligent Power Saver	Roger that.	Taobao Marketplace, a Chinese website for online shopping
Intelligent power-saving new technology	Green Intelligent Power Saver	Energy-saving effect when used singly, but less pronounced when used in multiples (more than two)	Taobao Marketplace, a Chinese website for online shopping
Intelligent power-saving new technology	Green Intelligent Power Saver	Shipped quickly, received to see the results	Taobao Marketplace, a Chinese website for online shopping
Intelligent power-saving new technology	Green Intelligent Power Saver	Power Savings: the energy saver I bought does not save power well.	Taobao Marketplace, a Chinese website for online shopping
Intelligent power-saving new technology	Green Intelligent Power Saver	Nice and as described, next time	Taobao Marketplace, a Chinese website for online shopping
Intelligent power-saving new technology	Green Intelligent Power Saver	Power saving effect: no Experience: no effect at all, cheating	Taobao Marketplace, a Chinese website for online shopping
Intelligent power-saving new technology	Green Intelligent Power Saver	Received, just used, have not yet seen the effect. Waiting to see the effect to come back to comment.	Taobao Marketplace, a Chinese website for online shopping
Intelligent power-saving new technology	Green Intelligent Power Saver	Too bad no effect at all after two days of use of the electricity bill more than 50 cents more than	Taobao Marketplace, a Chinese website for online shopping
Intelligent power-saving new technology	Green Intelligent Power Saver	Good quality! Express delivery is fast!	Taobao Marketplace, a Chinese website for online shopping
Intelligent power-saving new technology	Green Intelligent Power Saver	I don't know if it saves electricity, I'll be able to tell after a while. I hope it works. I would like to give a good review first.	Taobao Marketplace, a Chinese website for online shopping
Intelligent power-saving new technology	Green Intelligent Power Saver	I don't know how well it works.	Taobao Marketplace, a Chinese website for online shopping
Intelligent power-saving new technology	Green Intelligent Power Saver	Saver has been used, the quality is very good, exquisite workmanship, good material, beautiful appearance, affordable, worth buying.	Taobao Marketplace, a Chinese website for online shopping
Intelligent power-saving new technology	Green Intelligent Power Saver	Workmanship material: good	Taobao Marketplace, a Chinese website for online shopping
Intelligent power-saving new technology	Green Intelligent Power Saver	Price/performance ratio: the fakes don't work!	Taobao Marketplace, a Chinese website for online shopping
Intelligent power-saving new technology	Green Intelligent Power Saver	I'm using it now will review after a while.	Taobao Marketplace, a Chinese website for online shopping
Intelligent power-saving new technology	Green Intelligent Power Saver	Five out of five.	Taobao Marketplace, a Chinese website for online shopping
Intelligent power-saving new technology	Green Intelligent Power Saver	Junk product, useless! Ignore after sales.	Taobao Marketplace, a Chinese website for online shopping
Intelligent power-saving new technology	Green Intelligent Power Saver	I haven't used it yet.	Taobao Marketplace, a Chinese website for online shopping
Intelligent power-saving new technology	Green Intelligent Power Saver	Don't buy without using it, said it didn't work refunds don't answer back, not used before the electricity bill is low after using the electricity bill is even higher	Taobao Marketplace, a Chinese website for online shopping
Intelligent power-saving new technology	Green Intelligent Power Saver	No durability at all, it's only been a few days and it's not lighting up again, and they talk about energy efficiency, this energy saver's probably not even earning back the electricity it consumes. Liar!	Taobao Marketplace, a Chinese website for online shopping
Intelligent power-saving new technology	Green Intelligent Power Saver	There's just a little light bulb in there.	Taobao Marketplace, a Chinese website for online shopping
Intelligent power-saving new technology	Green Intelligent Power Saver	Power saving treasure received, as for the effect, according to the instructions written, a short period of time can not be seen, at least2-3months before the real effect, then follow up evaluation	Taobao Marketplace, a Chinese website for online shopping

Intelligent power-saving new technology	Green Intelligent Power Saver	Used it for a while and found no power savings.	Taobao Marketplace, a Chinese website for online shopping
Intelligent power-saving new technology	Green Intelligent Power Saver	It's good.	Taobao Marketplace, a Chinese website for online shopping
Intelligent power-saving new technology	Green Intelligent Power Saver	Power saving effect: no effect	Taobao Marketplace, a Chinese website for online shopping
Intelligent power-saving new technology	Green Intelligent Power Saver	Use it for a couple months and see what happens.	Taobao Marketplace, a Chinese website for online shopping
Intelligent power-saving new technology	Green Intelligent Power Saver	first started I thought it worked a little Now I don't think it works much anymore	Taobao Marketplace, a Chinese website for online shopping
Intelligent power-saving new technology	Green Intelligent Power Saver	I don't know if I can save it yet, it will take time to test!	Taobao Marketplace, a Chinese website for online shopping
Intelligent power-saving new technology	Green Intelligent Power Saver	I'll buy it again if it breaks.	Taobao Marketplace, a Chinese website for online shopping
Intelligent power-saving new technology	Green Intelligent Power Saver	It's already in use, so let's see how it compares over a few months of use before evaluating.	Taobao Marketplace, a Chinese website for online shopping
Intelligent power-saving new technology	Green Intelligent Power Saver	I've been using it for a month or so and it doesn't seem to help much.	Taobao Marketplace, a Chinese website for online shopping
Intelligent power-saving new technology	Green Intelligent Power Saver	an excellent	Taobao Marketplace, a Chinese website for online shopping
Intelligent power-saving new technology	Green Intelligent Power Saver	Said you can try100days, I'm only2months, every month the electricity bill increased bymore than30dollars, I want to return, the store directly ignored my information, this is the proper IQ tax, who buy who regret, more money casually buy	Taobao Marketplace, a Chinese website for online shopping
Intelligent power-saving new technology	Green Intelligent Power Saver	Power saving effect: average Workmanship material: average	Taobao Marketplace, a Chinese website for online shopping
Intelligent power-saving new technology	Green Intelligent Power Saver	It's full of IQ tax. One word, stupid.	Taobao Marketplace, a Chinese website for online shopping
Intelligent power-saving new technology	Green Intelligent Power Saver	Cost-effective: very cheap, do not know the quality of Experience: the first time to buy, is trying, nearly please wait for it	Taobao Marketplace, a Chinese website for online shopping
Intelligent power-saving new technology	Green Intelligent Power Saver	Just received the goods today, the goods on the expiration date does not exist, I am also curious to spend19.8to buy the shoot two send three, back I disassemble a, we see the video, so far I feel that is not the top of the matter, in any case, bought back, first plugged into the power supply on the try, can not save power on the thrown, as a lesson learned when you buy a lesson it.	Taobao Marketplace, a Chinese website for online shopping
Intelligent power-saving new technology	Green Intelligent Power Saver	Don't fall for this, people! An absolute trick!	Taobao Marketplace, a Chinese website for online shopping
Intelligent power-saving new technology	Green Intelligent Power Saver	Power saving effect: save half of electricity. Cost-effective: high. Appearance style: exquisite. Workmanship material: short and compact. Experience: second purchase, buy2get1free, good price. Last time I bought it in another store.	Taobao Marketplace, a Chinese website for online shopping
Intelligent power-saving new technology	Green Intelligent Power Saver	Appearance and style: Satisfactory	Taobao Marketplace, a Chinese website for online shopping
Intelligent power-saving new technology	Green Intelligent Power Saver	ok	Taobao Marketplace, a Chinese website for online shopping
Intelligent power-saving new technology	Green Intelligent Power Saver	Power saving effect: really good oh	Taobao Marketplace, a Chinese website for online shopping
Intelligent power-saving new technology	Green Intelligent Power Saver	I don't know if it works or not, but it's plastered anyway. Let's wait and see.	Taobao Marketplace, a Chinese website for online shopping
Intelligent power-saving new technology	Green Intelligent Power Saver	System default comments	Taobao Marketplace, a Chinese website for online shopping
Intelligent power-saving new technology	Green Intelligent Power Saver	Advise everyone not to be fooled, on the IQ tax, the business has no conscience, the zero line connected to a diode to the light-emitting diode, the firewire connected to a resistor to the light-emitting diode, which can save electricity? We don't fall. We don't fall We don't fall We don't fall We [illegible]	Taobao Marketplace, a Chinese website for online shopping
Intelligent power-saving new technology	Green Intelligent Power Saver	Not useful at all! Fooled people!!! No savings at all on the electric bill!	Taobao Marketplace, a Chinese website for online shopping
Intelligent power-saving new technology	Green Intelligent Power Saver	Shipping was fast, stuff was well packaged, great price, I'm happy with👍and will repurchase	Taobao Marketplace, a Chinese website for online shopping
Intelligent power-saving new technology	Green Intelligent Power Saver	IQ tax. Don't buy it. It doesn't work.	Taobao Marketplace, a Chinese website for online shopping
Intelligent power-saving new technology	Green Intelligent Power Saver	It doesn't work, it doesn't save electricity,………	Taobao Marketplace, a Chinese website for online shopping
Intelligent power-saving new technology	Green Intelligent Power Saver	100% IQ tax, absolute counterfeit!	Taobao Marketplace, a Chinese website for online shopping
Intelligent power-saving new technology	Green Intelligent Power Saver	tax ah How come there are so many positive reviews*****?	Taobao Marketplace, a Chinese website for online shopping
Intelligent power-saving new technology	Green Intelligent Power Saver	Results after using it for a while not so good	Taobao Marketplace, a Chinese website for online shopping
Intelligent power-saving new technology	Green Intelligent Power Saver	Liar Don't buy it	Taobao Marketplace, a Chinese website for online shopping
Intelligent power-saving new technology	Green Intelligent Power Saver	Use it for a month and see how it works before you comment	Taobao Marketplace, a Chinese website for online shopping
Intelligent power-saving new technology	Green Intelligent Power Saver	Not good.	Taobao Marketplace, a Chinese website for online shopping
Intelligent power-saving new technology	Green Intelligent Power Saver	The stuff is pretty light Looking forward to the results	Taobao Marketplace, a Chinese website for online shopping

Intelligent power-saving new technology	Green Intelligent Power Saver	Workmanship material: workmanship is not good	Taobao Marketplace, a Chinese website for online shopping
Intelligent power-saving new technology	Green Intelligent Power Saver	(of an unmarried couple) be close	Taobao Marketplace, a Chinese website for online shopping
Intelligent power-saving new technology	Green Intelligent Power Saver	System default comments	Taobao Marketplace, a Chinese website for online shopping
Intelligent power-saving new technology	Green Intelligent Power Saver	Power saving effect: good Cost-effective: good Appearance and style: good Workmanship and material: good Experience: good	Taobao Marketplace, a Chinese website for online shopping
Intelligent power-saving new technology	Green Intelligent Power Saver	Appearance and style: compact and convenient!	Taobao Marketplace, a Chinese website for online shopping
Intelligent power-saving new technology	Green Intelligent Power Saver	I hope it works.	Taobao Marketplace, a Chinese website for online shopping
Intelligent power-saving new technology	Green Intelligent Power Saver	average	Taobao Marketplace, a Chinese website for online shopping
Intelligent power-saving new technology	Green Intelligent Power Saver	I bought it back and it didn't work at all, so don't be fooled again if you want to buy it. Experience: no effect at all	Taobao Marketplace, a Chinese website for online shopping
Intelligent power-saving new technology	Green Intelligent Power Saver	I'll comment on the results later.	Taobao Marketplace, a Chinese website for online shopping
Intelligent power-saving new technology	Green Intelligent Power Saver	Baby received, looks good used up, hope it works! Good review!	Taobao Marketplace, a Chinese website for online shopping
Intelligent power-saving new technology	Green Intelligent Power Saver	I'll confirm receipt first and follow up in a couple months	Taobao Marketplace, a Chinese website for online shopping
Intelligent power-saving new technology	Green Intelligent Power Saver	I'm using it, I don't know how effective it is yet, I'll follow up later.	Taobao Marketplace, a Chinese website for online shopping
Intelligent power-saving new technology	Green Intelligent Power Saver	The style and material is average, I guess, I don't know if it works or not, use it anyway!	Taobao Marketplace, a Chinese website for online shopping
Intelligent power-saving new technology	Green Intelligent Power Saver	very good	Taobao Marketplace, a Chinese website for online shopping
Intelligent power-saving new technology	Green Intelligent Power Saver	No comment for now, follow up after use	Taobao Marketplace, a Chinese website for online shopping
Intelligent power-saving new technology	Green Intelligent Power Saver	OnTrial……	Taobao Marketplace, a Chinese website for online shopping
Intelligent power-saving new technology	Green Intelligent Power Saver	The power saver has been received, but can really save electricity, the quality is very good, I am the second time to buy!	Taobao Marketplace, a Chinese website for online shopping
Intelligent power-saving new technology	Green Intelligent Power Saver	Used the product! Power saving is good! We recommend you to buy it!	Taobao Marketplace, a Chinese website for online shopping
Intelligent power-saving new technology	Green Intelligent Power Saver	Very good Price/performance ratio: very good Appearance style: good-looking	Taobao Marketplace, a Chinese website for online shopping
Intelligent power-saving new technology	Green Intelligent Power Saver	Just used it and looking forward to the results	Taobao Marketplace, a Chinese website for online shopping
Intelligent power-saving new technology	Green Intelligent Power Saver	Power saving effect:\Poor	Taobao Marketplace, a Chinese website for online shopping
Intelligent power-saving new technology	Green Intelligent Power Saver	No effect, said no months can save electricity tens of percent, I see no savings, no use.	Taobao Marketplace, a Chinese website for online shopping
Intelligent power-saving new technology	Green Intelligent Power Saver	Baby works great, thanks to the seller, there is a real reduction in electricity consumption at home. Cost-effective: much cheaper than others	Taobao Marketplace, a Chinese website for online shopping
Intelligent power-saving new technology	Green Intelligent Power Saver	Workmanship material: only two small secondary tube to the indicator power supply what is not, is a fraud, I opened to see, friends do not fall for it, I opened to customer service, customer service a moment to ignore me.	Taobao Marketplace, a Chinese website for online shopping
Intelligent power-saving new technology	Green Intelligent Power Saver	Experience: just received, ready to use, hope the effect is good	Taobao Marketplace, a Chinese website for online shopping
Intelligent power-saving new technology	Green Intelligent Power Saver	Product quality, customer service attitude is particularly good, like, praise!	Taobao Marketplace, a Chinese website for online shopping
Intelligent power-saving new technology	Green Intelligent Power Saver	Buyer does not comment within15days	Taobao Marketplace, a Chinese website for online shopping
Intelligent power-saving new technology	Green Intelligent Power Saver	Useless, used a few times before commenting, poor results	Taobao Marketplace, a Chinese website for online shopping
Intelligent power-saving new technology	In-situ compensated economizer	not have	Taobao Marketplace, a Chinese website for online shopping
Super Digital Specialty Store	a power saver (electronics)	store closure	Taobao Marketplace, a Chinese website for online shopping
New Generation Science and Technology Museum	Dr. Electricity	Item has been received, good value for money, have not tried it yet, thanks to the owner for the warm service!👍👍	Taobao Marketplace, a Chinese website for online shopping
New Generation Science and Technology Museum	Dr. Electricity	Power saving effect: I haven't tried it yet, I don't know.	Taobao Marketplace, a Chinese website for online shopping
New Generation Science and Technology Museum	Dr. Electricity	Baby received😉, energy saving and power saving, 5 stars?	Taobao Marketplace, a Chinese website for online shopping
New Generation Science and Technology Museum	Dr. Electricity	The effect is not obvious	Taobao Marketplace, a Chinese website for online shopping

New Generation Science and Technology Museum	Dr. Electricity	have nothing to say	Taobao Marketplace, a Chinese website for online shopping
New Generation Science and Technology Museum	Dr. Electricity	I've been using it for a few days and it seems to be working a little bit	Taobao Marketplace, a Chinese website for online shopping
New Generation Science and Technology Museum	Dr. Electricity	Just use it I don't know if it saves power or not don't know at the moment	Taobao Marketplace, a Chinese website for online shopping
New Generation Science and Technology Museum	Dr. Electricity	Smart Saver is small and does not take up much space, the introduction can repel mosquitoes, cockroaches, mosquitoes and so on, very much like it. I hope it really can play a role in energy saving.	Taobao Marketplace, a Chinese website for online shopping
New Generation Science and Technology Museum	Dr. Electricity	Just as the merchant said, reasonable power saving, safe and energy saving! Thank you, I will buy again next time!	Taobao Marketplace, a Chinese website for online shopping
New Generation Science and Technology Museum	Dr. Electricity	Great quality and fast shipping from the seller. Very satisfied.	Taobao Marketplace, a Chinese website for online shopping
New Generation Science and Technology Museum	Dr. Electricity	Very good, a machine multi-function, leakage and reminders, and mosquito and insect repellent function. Very powerful and very technology!	Taobao Marketplace, a Chinese website for online shopping
New Generation Science and Technology Museum	Dr. Electricity	Received very well packaged, very easy to use, customer service is very enthusiastic, and also gave a sheet to enhance the signal. Thanks to the merchant Business	Taobao Marketplace, a Chinese website for online shopping
New Generation Science and Technology Museum	Dr. Electricity	It's very convenient It arrived and plugged it in and used it, a friend recommended it, and it does work quite well for his family.	Taobao Marketplace, a Chinese website for online shopping
New Generation Science and Technology Museum	Dr. Electricity	It is the ideal product, good quality, logistics is also very fast, worthy of praise!	Taobao Marketplace, a Chinese website for online shopping
New Generation Science and Technology Museum	Dr. Electricity	Consistent in kind, the effect is very good. Logistics is very fast, and the business service is very thoughtful. Genuine quality is very assured	Taobao Marketplace, a Chinese website for online shopping
New Generation Science and Technology Museum	Dr. Electricity	Seeing that my friend's family uses it very well, I asked him to push a link for me as well. The logistics is very fast, very satisfied	Taobao Marketplace, a Chinese website for online shopping
New Generation Science and Technology Museum	Dr. Electricity	also has a voice leakage announcement function, it's so powerful[strong] It's very functional It's good	Taobao Marketplace, a Chinese website for online shopping
New Generation Science and Technology Museum	Dr. Electricity	Logistics is very fast, received the goods Quality can be fine workmanship.	Taobao Marketplace, a Chinese website for online shopping
New Generation Science and Technology Museum	Dr. Electricity	Works great, referred by a friend. Saves electricity and money, long lasting product Good value	Taobao Marketplace, a Chinese website for online shopping
New Generation Science and Technology Museum	Dr. Electricity	Merchants are very enthusiastic, fast courier service. Installation is very easy to operate, the price is right, good to use	Taobao Marketplace, a Chinese website for online shopping
New Generation Science and Technology Museum	Dr. Electricity	A machine multi-purpose is very cost-effective, the activities of the purchase. There is a voice leakage reminder function, mosquito and rodent repellent function, and a free signal sticker[strong]	Taobao Marketplace, a Chinese website for online shopping
New Generation Science and Technology Museum	Dr. Electricity	Baby received, good price, very satisfied! The store is also super nice, the package is careful, the goods are shipped quickly, trustworthy! Baby received, good value, very satisfied! The store is also super nice, the package is fine, the goods are shipped quickly, trustworthy!	Taobao Marketplace, a Chinese website for online shopping
New Generation Science and Technology Museum	Dr. Electricity	Baby received, fast logistics, good service attitude. The quality is exquisite and trustworthy!	Taobao Marketplace, a Chinese website for online shopping
New Generation Science and Technology Museum	Dr. Electricity	A very good shopping experience, attentive service, fast logistics, quality assurance, rest assured that the purchase!	Taobao Marketplace, a Chinese website for online shopping
New Generation Science and Technology Museum	Dr. Electricity	Very well made, detailed instructions, and quite a lot of features for a small machine. Good value for money. Very good to use.	Taobao Marketplace, a Chinese website for online shopping
New Generation Science and Technology Museum	Dr. Electricity	💌been wanting it for a long time, I decided to go for it, I feel great after using it, it's genuine and the delivery is great, ititsname👍	Taobao Marketplace, a Chinese website for online shopping
New Generation Science and Technology Museum	Dr. Electricity	Good energy savings, cheap and cost effective.	Taobao Marketplace, a Chinese website for online shopping
New Generation Science and Technology Museum	Dr. Electricity	Followed customer service's instruction to press on the area that uses a lot of electricity and it does work quite well. Very patient guidance, give you guys satisfactory praise👍	Taobao Marketplace, a Chinese website for online shopping
New Generation Science and Technology Museum	Dr. Electricity	The power saving effect is good, cost-effective and inexpensive, worth buying	Taobao Marketplace, a Chinese website for online shopping
New Generation Science and Technology Museum	a power saver (electronics)	(of an unmarried couple) be close	Taobao Marketplace, a Chinese website for online shopping
New Generation Science and Technology Museum	a power saver (electronics)	Good quality. Just used it and haven't seen results yet. Will comment later!	Taobao Marketplace, a Chinese website for online shopping
New Generation Science and Technology Museum	a power saver (electronics)	Cost-effective, this summer electricity is very big, this time to take advantage of the event back to buy. It's true that the electricity bill is less than when I didn't use it.	Taobao Marketplace, a Chinese website for online shopping
New Generation Science and Technology Museum	a power saver (electronics)	It's easy to use works well and is very cost effective	Taobao Marketplace, a Chinese website for online shopping
New Generation Science and Technology Museum	a power saver (electronics)	The effect is very good, very obvious, very satisfied. It is worth buying Oh, there is a need to come back again!	Taobao Marketplace, a Chinese website for online shopping
New Generation Science and Technology Museum	a power saver (electronics)	Dad's home with a good, gave me a recommendation for the family also press a, indeed quite good, the price is favorable and practical!	Taobao Marketplace, a Chinese website for online shopping
New Generation Science and Technology Museum	a power saver (electronics)	It works pretty well and is conveniently priced. Economical and practical, good value for money!	Taobao Marketplace, a Chinese website for online shopping
New Generation Science and Technology Museum	a power saver (electronics)	Home store must-have good, economical and practical, the main work is very obvious. Friends in need can rest assured that the hand	Taobao Marketplace, a Chinese website for online shopping
New Generation Science and Technology Museum	a power saver (electronics)	Take advantage of the event and stock up fast. The store uses a lot of electricity, one unit with one. Good to use and save. Practical offers	Taobao Marketplace, a Chinese website for online shopping

New Generation Science and Technology Museum	a power saver (electronics)	Installation was easy and logistics were very fast. Worked well.	Taobao Marketplace, a Chinese website for online shopping
New Generation Science and Technology Museum	a power saver (electronics)	works very well, and has been repurchased many times, originally10degreesa day, but now it becomes6degrees of electricity, it works very well. Worth recommending	Taobao Marketplace, a Chinese website for online shopping
New Generation Science and Technology Museum	a power saver (electronics)	Around friends are also using, really quite good, the price is not expensive, the family must have new technology products.	Taobao Marketplace, a Chinese website for online shopping
New Generation Science and Technology Museum	a power saver (electronics)	Received very well packaged, product quality is very good, very well installed and works well. Satisfied	Taobao Marketplace, a Chinese website for online shopping
New Generation Science and Technology Museum	King of Power Saving	Easy to use logistics is fast! Product received intact quality is very practical. Good	Taobao Marketplace, a Chinese website for online shopping
New Generation Science and Technology Museum	King of Power Saving	I've used them before and bought a couple of them back while they were cheap for the event. Give the old family also pressed	Taobao Marketplace, a Chinese website for online shopping
New Generation Science and Technology Museum	King of Power Saving	It really saves power and line loss is reduced. I bought it again. Match all places in the house that use a lot of electricity.	Taobao Marketplace, a Chinese website for online shopping
New Generation Science and Technology Museum	King of Power Saving	I heard from a friend that it works pretty well! This summer his family used electricity during the summer, a lot less electricity, I also get into the home by one! Very affordable and practical	Taobao Marketplace, a Chinese website for online shopping
New Generation Science and Technology Museum	King of Power Saving	Received that is plugged in to use, and now still fall tigers weather is still hot, compared to the night opened the air conditioning to save 3 degrees. Good use in two days and then buy back!	Taobao Marketplace, a Chinese website for online shopping
New Generation Science and Technology Museum	King of Power Saving	Very nice, the merchant was very considerate and gave extra spare fuses. Easy to install and use, work very smoothly. Very good, good and practical	Taobao Marketplace, a Chinese website for online shopping
New Generation Science and Technology Museum	King of Power Saving	Second time repurchase, as always power saving, do use it, worth to promote [strong]	Taobao Marketplace, a Chinese website for online shopping
New Generation Science and Technology Museum	King of Power Saving	The price is affordable and the quality is very good. Instructions, security code, accessories are complete is genuine. Will recommend friends to buy	Taobao Marketplace, a Chinese website for online shopping
New Generation Science and Technology Museum	King of Power Saving	Affordable price, very fast logistics, received the goods very quickly, installation is very convenient! Merchant service attitude is very good. Very satisfied	Taobao Marketplace, a Chinese website for online shopping
New Generation Science and Technology Museum	Black Power Saving Specialist	Used for two months, the electricity bill is more than before, the second month with more than the previous month's more than a hundred dollars, do not use a month can also be more than two hundred dollars less electricity, this is not power saving, but the king of power consumption, really is	Taobao Marketplace, a Chinese website for online shopping
New Generation Science and Technology Museum	(Three-phase) 380V applicable economizer	not have	Taobao Marketplace, a Chinese website for online shopping
Jintianyuan Smart Home	Red Power Saving Specialist	not have	Taobao Marketplace, a Chinese website for online shopping
Jintianyuan Smart Home	power saving device	not have	Taobao Marketplace, a Chinese website for online shopping
Jintianyuan Smart Home	King of Power Saving	Generous and beautiful.	Taobao Marketplace, a Chinese website for online shopping
Jintianyuan Smart Home	King of Power Saving	Product quality is very good, use more than a month the effect is very good, it is worth recommending.	Taobao Marketplace, a Chinese website for online shopping
Jintianyuan Smart Home	King of Power Saving	Okay, I got it.	Taobao Marketplace, a Chinese website for online shopping
Jintianyuan Smart Home	King of Power Saving	The item was received and is as shown in the picture. Courier guy was quick to deliver. Kudos.	Taobao Marketplace, a Chinese website for online shopping
Jintianyuan Smart Home	King of Power Saving	Very good stuff.	Taobao Marketplace, a Chinese website for online shopping
Jintianyuan Smart Home	King of Power Saving	It's flimsy, like an empty shell, and I don't know what's in it, so I'm not sure if it's useful or not. If it really has a reactive power compensation function, it is still a bit useful!	Taobao Marketplace, a Chinese website for online shopping
Jintianyuan Smart Home	King of Power Saving	Out of curiosity I dismantled it, and for nothing I lost dozens of dollars, fake! Power line directly and a capacitor, it is impossible to save energy! Next to there is a way to block the capacitance circuit to buck the voltage to that small chip, so that the digital tube is lit up is an illusion, we must not buy!	Taobao Marketplace, a Chinese website for online shopping
Jintianyuan Smart Home	King of Power Saving	This is not a genuine factory and not a holiday	Taobao Marketplace, a Chinese website for online shopping
Jintianyuan Smart Home	King of Power Saving	I used to buy them from physical stores, but this time I chose to buy them online, not only are they authentic, but they are also affordable!	Taobao Marketplace, a Chinese website for online shopping
Jintianyuan Smart Home	King of Power Saving	First time using it, let's use it	Taobao Marketplace, a Chinese website for online shopping
Jintianyuan Smart Home	King of Power Saving	Good merchandise,, logistics is fast, good service attitude,, the appearance of good-looking	Taobao Marketplace, a Chinese website for online shopping
Jintianyuan Smart Home	King of Power Saving	It doesn't work. It's a waste of money.	Taobao Marketplace, a Chinese website for online shopping
Jintianyuan Smart Home	King of Power Saving	Genuine power saver, logistics is very fast, will recommend friends to buy, the quality is very good, cost-effective, inexpensive, it is worth buying the product received, the quality is very good, is genuine, the effect is good, it is worth buying Oh!	Taobao Marketplace, a Chinese website for online shopping
Jintianyuan Smart Home	King of Power Saving	Express received, especially satisfied, very good, recommended, praise.	Taobao Marketplace, a Chinese website for online shopping
Jintianyuan Smart Home	King of Power Saving	Baby received, logistics is very fast, fine workmanship, good quality, affordable price, the need can rest assured that the purchase.	Taobao Marketplace, a Chinese website for online shopping
Jintianyuan Smart Home	King of Power Saving	Logistics is relatively fast, business service attitude is good, very patient to answer questions and guidance, has been installed.	Taobao Marketplace, a Chinese website for online shopping
Jintianyuan Smart Home	King of Power Saving	Good use and power saving, good quality and fine workmanship! Will refer friends to buy.	Taobao Marketplace, a Chinese website for online shopping

Jintianyuan Smart Home	King of Power Saving	Works very well, second repurchase. The product does work in the summer with the high electricity usage. It does work well in my home, satisfied with it	Taobao Marketplace, a Chinese website for online shopping
Jintianyuan Smart Home	King of Power Saving	System default comments	Taobao Marketplace, a Chinese website for online shopping
Jintianyuan Smart Home	King of Power Saving	Good-looking, very good quality, sensitive, genuine, plugged in and energized, clearly indicate the fluctuation data of power line voltage and current in the family, overall operation is normal! Store service attitude is very good and careful guidance, very satisfied, five-star award!	Taobao Marketplace, a Chinese website for online shopping
Jintianyuan Smart Home	King of Power Saving	Not bad. A must have for the home saves power and reduces losses! Home tripping less voltage stabilization does work!	Taobao Marketplace, a Chinese website for online shopping
Jintianyuan Smart Home	King of Power Saving	The saver arrived, super like, good quality, will recommend friends, good thing practical	Taobao Marketplace, a Chinese website for online shopping
Jintianyuan Smart Home	King of Power Saving	Goods received, good quality, high quality of workmanship, the shopkeeper good service fast delivery, next time to come back to patronize!	Taobao Marketplace, a Chinese website for online shopping
Jintianyuan Smart Home	King of Power Saving	As described by the seller very good worth buying next time will come again	Taobao Marketplace, a Chinese website for online shopping
Jintianyuan Smart Home	King of Power Saving	System default comments	Taobao Marketplace, a Chinese website for online shopping
Jintianyuan Smart Home	King of Power Saving	Energy saving power saving treasure is very practical, and business description of the match, logistics is also very fast, very satisfied with a shopping	Taobao Marketplace, a Chinese website for online shopping
Jintianyuan Smart Home	King of Power Saving	Fast shipping, works great, saves power, recommended!	Taobao Marketplace, a Chinese website for online shopping
Jintianyuan Smart Home	King of Power Saving	Take it off.	Taobao Marketplace, a Chinese website for online shopping
Jintianyuan Smart Home	King of Power Saving	There is no use ah, it is not the original do not use this to save electrical appliances like power	Taobao Marketplace, a Chinese website for online shopping
Jintianyuan Smart Home	King of Power Saving	an excellent	Taobao Marketplace, a Chinese website for online shopping
Jintianyuan Smart Home	King of Power Saving	I used one before and couldn't tell what effect it had. Bought another one this time and used it in the bathroom to see again.	Taobao Marketplace, a Chinese website for online shopping
Jintianyuan Smart Home	King of Power Saving	It also saves electricity and money, after using it for a month the electricity bill is more than last month's	Taobao Marketplace, a Chinese website for online shopping
Jintianyuan Smart Home	King of Power Saving	Received shipment. In use	Taobao Marketplace, a Chinese website for online shopping
Jintianyuan Smart Home	King of Power Saving	System default comments	Taobao Marketplace, a Chinese website for online shopping
Jintianyuan Smart Home	King of Power Saving	It's just deceptive.	Taobao Marketplace, a Chinese website for online shopping
Jintianyuan Smart Home	King of Power Saving	If you can't save electricity why do you write that the meter slows down and saves electricity, fill in the quality problem and refuse to return it, what is this not cheating, please?	Taobao Marketplace, a Chinese website for online shopping
Jintianyuan Smart Home	King of Power Saving	I just received the item and will comment after using it.	Taobao Marketplace, a Chinese website for online shopping
Jintianyuan Smart Home	King of Power Saving	Received the same period of time to evaluate, I hope that really have the effect, customer service attitude is very good to ask and answer!	Taobao Marketplace, a Chinese website for online shopping
Jintianyuan Smart Home	King of Power Saving	The Power Saver King has been received and I went back to buy another one because of the significant power savings. Good review.	Taobao Marketplace, a Chinese website for online shopping
Jintianyuan Smart Home	King of Power Saving	2023New Home Saver Electricity Saver Electricity Meter Deceleration Electricity Saver Air Conditioner Appliance Refrigerator Intelligent Power Saver,energy sahas just been used, and will be evaluated again when it has an effect.	Taobao Marketplace, a Chinese website for online shopping
Jintianyuan Smart Home	King of Power Saving	First time I've used it.	Taobao Marketplace, a Chinese website for online shopping
Jintianyuan Smart Home	King of Power Saving	Customer service is very good questions answered Try for a month and then buy another one	Taobao Marketplace, a Chinese website for online shopping
Jintianyuan Smart Home	King of Power Saving	One smart power saving device, received it. It is being used and feels good.	Taobao Marketplace, a Chinese website for online shopping
Jintianyuan Smart Home	King of Power Saving	Roger that! Using it right away now! Hopefully it will be safe and effective! But what I received is a little different from the picture! Also hope the effect is the same!	Taobao Marketplace, a Chinese website for online shopping
Jintianyuan Smart Home	King of Power Saving	The packaging is very good, the delivery is also very fast, but I do not know how effective the product is.	Taobao Marketplace, a Chinese website for online shopping
Jintianyuan Smart Home	King of Power Saving	The product appearance is well-designed, the specific energy-saving effect to be observed test,,.	Taobao Marketplace, a Chinese website for online shopping
Jintianyuan Smart Home	king of power savers	It's that horse's system again.	Taobao Marketplace, a Chinese website for online shopping
Jintianyuan Smart Home	Smart Saver	useless	Taobao Marketplace, a Chinese website for online shopping
Jintianyuan Smart Home	Smart Saver	Smart Saver is a scam! Not only did it not reduce the kWh for two months, but it also increased the kWh every month.	Taobao Marketplace, a Chinese website for online shopping
Jintianyuan Smart Home	Smart Saver	It's a rip-off.	Taobao Marketplace, a Chinese website for online shopping
Jintianyuan Smart Home	Smart Saver	It's okay. It's working.	Taobao Marketplace, a Chinese website for online shopping

Jintianyuan Smart Home	Smart Saver	It does work.	Taobao Marketplace, a Chinese website for online shopping
Jintianyuan Smart Home	Smart Saver	favorable criticism	Taobao Marketplace, a Chinese website for online shopping
Jintianyuan Smart Home	Smart Saver	The product is not useful at all, can not play the effect of power saving ultra-long use of90days, but do not give a refund want to buy the need to be careful	Taobao Marketplace, a Chinese website for online shopping
Jintianyuan Smart Home	Smart Saver	Let's start with a 5-star review use it and come back afterward	Taobao Marketplace, a Chinese website for online shopping
Jintianyuan Smart Home	Smart Saver	I don't know if it works yet.	Taobao Marketplace, a Chinese website for online shopping
Jintianyuan Smart Home	Smart Saver	I got it.	Taobao Marketplace, a Chinese website for online shopping
Jintianyuan Smart Home	Smart Saver	See you next month for the results.	Taobao Marketplace, a Chinese website for online shopping
Jintianyuan Smart Home	Smart Saver	don't know how it is yet saving power or not.	Taobao Marketplace, a Chinese website for online shopping
Jintianyuan Smart Home	Smart Saver	It's good quality and it does save power after a few days of use.	Taobao Marketplace, a Chinese website for online shopping
Jintianyuan Smart Home	Smart Saver	Baby received and used it, very power-saving, workmanship is also very good, praise.	Taobao Marketplace, a Chinese website for online shopping
Jintianyuan Smart Home	Smart Saver	I've used it a couple times now and it's fine, no problems so far.	Taobao Marketplace, a Chinese website for online shopping
Jintianyuan Smart Home	Smart Saver	It shipped quickly, worked well, came back to try a little less than usual	Taobao Marketplace, a Chinese website for online shopping
Jintianyuan Smart Home	Smart Saver	Good use, durable quality, second repurchase. Indeed quite good to use, has recommended friends around.Strong]	Taobao Marketplace, a Chinese website for online shopping
Jintianyuan Smart Home	Smart Saver	The voltage of the economizer is stable, easy to use, and the power saving effect is very good	Taobao Marketplace, a Chinese website for online shopping
Jintianyuan Smart Home	Smart Saver	Very good quality, used without getting hot. Works very stable. Pretty good baby.	Taobao Marketplace, a Chinese website for online shopping
Jintianyuan Smart Home	Smart Saver	general	Taobao Marketplace, a Chinese website for online shopping
Jintianyuan Smart Home	Smart Saver	Already tried, is genuine, good use of power, and customer service is very enthusiastic!	Taobao Marketplace, a Chinese website for online shopping
Jintianyuan Smart Home	Smart Saver	I'll comment after using it.	Taobao Marketplace, a Chinese website for online shopping
Jintianyuan Smart Home	Smart Saver	Used a few days, it is indeed more energy-saving, the previous day's power, now three or four days to use up, buy the right!	Taobao Marketplace, a Chinese website for online shopping
Jintianyuan Smart Home	Smart Saver	Seeing that my friend's family is using it well, I recommended that I link to it and press one for my family as well. The quality of workmanship is very good. Customer service is very professional guide installation is very patient, very satisfied!	Taobao Marketplace, a Chinese website for online shopping
Jintianyuan Smart Home	Smart Saver	Quite good, the first floor has been pressed a, now the peak of electricity is coming, in the purchase of two units to put the upstairs use. Indeed very good	Taobao Marketplace, a Chinese website for online shopping
Jintianyuan Smart Home	Smart Saver	Arrived quickly, things are very good workmanship, power saving effect is much better than expected, the price is not expensive, recommended!	Taobao Marketplace, a Chinese website for online shopping
Jintianyuan Smart Home	Smart Saver	Customer service is very professional and patient to guide the installation, the quality of workmanship is very fine. Durable, second repurchase. Good to use and save. Value for money!	Taobao Marketplace, a Chinese website for online shopping
Jintianyuan Smart Home	Smart Saver	Baby received, logistics is very fast, very good, really good value for money, friends see also want to buy, let me send the link, it's really worth it!	Taobao Marketplace, a Chinese website for online shopping
Jintianyuan Smart Home	Smart Saver	Easy to use also saves electricity, good price, satisfied!	Taobao Marketplace, a Chinese website for online shopping
Jintianyuan Smart Home	Smart Saver	Intelligent energy-saving products received in two or three days, fast shipping, very good service, I have bought several times, really good, but also to help friends, neighbors recommended this product, all say good. Give a five-star praise	Taobao Marketplace, a Chinese website for online shopping
Jintianyuan Smart Home	Smart Saver	The baby is really great, I like it very much, my friends also say good to come, recommended to buy!	Taobao Marketplace, a Chinese website for online shopping
Jintianyuan Smart Home	Smart Saver	Quite satisfied, very stable, working quietly. The quality is very good, many times back to buy, around the friends home are using, are quite good. Practical	Taobao Marketplace, a Chinese website for online shopping
Jintianyuan Smart Home	Smart Saver	Confirmation that it is good, naturally favorable comments. Works well, saves electricity and money, cost-effective for family use, thanks to the manufacturer	Taobao Marketplace, a Chinese website for online shopping
Jintianyuan Smart Home	Smart Saver	Fast shipping, genuine, good quality, an ideal energy-saving tool, very beautiful, multi-functional, good service attitude, affordable price, logistics is also very fast, the	Taobao Marketplace, a Chinese website for online shopping
Jintianyuan Smart Home	Smart Saver	Scientific power saving, trustworthy, reasonable design, logistics is quite fast, long business warranty, excellent service, fast logistics! Description match, quality assurance, worth buying. Good service attitude.	Taobao Marketplace, a Chinese website for online shopping
Jintianyuan Smart Home	Smart Saver	Saving power received immediately used. Machine workmanship is fine, lightweight, plugged into the electricity for a few days does not heat, the voltage is also very stable, the role is good, quite good!	Taobao Marketplace, a Chinese website for online shopping
Jintianyuan Smart Home	Smart Saver	Exceeds expectations, saves electricity, very good quality, well made, satisfactory	Taobao Marketplace, a Chinese website for online shopping
Jintianyuan Smart Home	Smart Saver	Logistics is very fast, express instant, feel good quality, cost-effective, save a few dozen dollars a month, save a lot of money a year down, it is worth buying, I wish the businessmen prosperous business, financial resources!	Taobao Marketplace, a Chinese website for online shopping

Jintianyuan Smart Home	Smart Saver	Used, the logistics is particularly fast, the service is particularly good, the baby quality is also great, satisfied with a hundred percent did not say, is really good value for money an online shopping! Will buy back, thanks to meet	Taobao Marketplace, a Chinese website for online shopping
Jintianyuan Smart Home	Smart Saver	Genuine power saver, logistics is very fast, will recommend friends to buy, the quality is very good, cost-effective, inexpensive, it is worth buying the product received, the quality is very good, is genuine, the effect is good, it is worth buying Oh!	Taobao Marketplace, a Chinese website for online shopping
Jintianyuan Smart Home	Smart Saver	It does save some electricity, easy to install and convenient	Taobao Marketplace, a Chinese website for online shopping
Jintianyuan Smart Home	Smart Saver	Works well, great power saver for the price. Affordable price	Taobao Marketplace, a Chinese website for online shopping
Jintianyuan Smart Home	Smart Saver	Powerful, super practical and easy to use🌹🌹🌹🌹	Taobao Marketplace, a Chinese website for online shopping
Jintianyuan Smart Home	Smart Saver	It does have an effect electricity bills are a little less month to month.	Taobao Marketplace, a Chinese website for online shopping
Jintianyuan Smart Home	Smart Saver	It's especially good. Really saves power. Great.	Taobao Marketplace, a Chinese website for online shopping
Jintianyuan Smart Home	Smart Saver	Friends installed this at home, the effect is very good, friends introduced this so buy to try, installation is very easy, after a few days to see the effect and then follow up.	Taobao Marketplace, a Chinese website for online shopping
Jintianyuan Smart Home	Blue Power Saving Specialist	The quality of the objects alone is exquisite. However, whether the customer can save electricity, need two months of electricity bill payment comparison to know the real performance.	Taobao Marketplace, a Chinese website for online shopping
Jintianyuan Smart Home	Blue Power Saving Specialist	Seller service attitude is very good, also understand the installation of the best bit, looking forward to the later effect	Taobao Marketplace, a Chinese website for online shopping
Jintianyuan Smart Home	Blue Power Saving Specialist	(*^_^*)Very satisfied with a shopping, value for money, much more affordable than the physical store!	Taobao Marketplace, a Chinese website for online shopping
Jintianyuan Smart Home	senior official in charge of electricity	not have	Taobao Marketplace, a Chinese website for online shopping
Edison Technology Factory Store	King of Power Saving	It didn't work at all. IQ tax.	Taobao Marketplace, a Chinese website for online shopping
Edison Technology Factory Store	King of Power Saving	Changing people's doesn't help at all, never, never, never claw waste of money, platforms should approve insubstantial stuff on the shelves	Taobao Marketplace, a Chinese website for online shopping
Edison Technology Factory Store	King of Power Saving	The product opens up with really good quality, the aluminum looks and has a great texture! It's also very powerful to use! Very nice product! Already recommended it to my family!	Taobao Marketplace, a Chinese website for online shopping
Edison Technology Factory Store	King of Power Saving	This is indeed a trustworthy, my own personal real test, with more than a month, the home air conditioning two, a refrigerator, appliances and computers have, air conditioning day afternoon and night are open, this month than last month to savemore than100dollars! A good product worthy of recognition	Taobao Marketplace, a Chinese website for online shopping
Edison Technology Factory Store	King of Power Saving	Saving appliances is my uncle recommended to me, said his village in his hometown to engage in that science and technology to the countryside free of charge to send, let me take a look at, contrast to this business is still reliable, is the manufacturer directly! Decided to buy two! Very good use! Home voltage has recently been very stable power-saving effect is great!	Taobao Marketplace, a Chinese website for online shopping
Edison Technology Factory Store	King of Power Saving	The product is really good, I like it very much, my family is using a power saving device, my parents say it is quite good, let me bring one to my grandma's house! The price is so affordable, I'm sure there is no problem, conscientious businessman!	Taobao Marketplace, a Chinese website for online shopping
Edison Technology Factory Store	King of Power Saving	Quite good to use, unit colleagues recommended, said July and August electricity bill than before to save more than five hundred, our group of several colleagues have bought, I also make a fuss! Not expensive, really good	Taobao Marketplace, a Chinese website for online shopping
Edison Technology Factory Store	King of Power Saving	Bought this for a family member and it's great, very happy with the results	Taobao Marketplace, a Chinese website for online shopping
Edison Technology Factory Store	King of Power Saving	Second purchase, good stuff, worth it.	Taobao Marketplace, a Chinese website for online shopping
Edison Technology Factory Store	King of Power Saving	Today recently the air conditioning in the house has not been turned off, the product is good, very good, the elderly in the family like it, said I will know to save! I bought one for my mom and grandma's house!	Taobao Marketplace, a Chinese website for online shopping
Edison Technology Factory Store	King of Power Saving	Good products stand the test of time! This is a little more expensive however it is really good! I use it for my own company too be able to!	Taobao Marketplace, a Chinese website for online shopping
Edison Technology Factory Store	King of Power Saving	I was torn before I bought it, I've been looking at this product for days and have been comparing! Other people compared to three I compared to ten! But look and see or this store owner is real, the goods are real, the goods I received on the start of the review! Than before it can save more than twenty dollars a day! Has been a week in a row! It's so reliable.	Taobao Marketplace, a Chinese website for online shopping
Edison Technology Factory Store	King of Power Saving	System default comments	Taobao Marketplace, a Chinese website for online shopping
Edison Technology Factory Store	King of Power Saving	I have a two bedroom, one bathroom,over80square meters, two air conditioners! TV, washing machine, water heater is gas, bought a gold color, so far use feel pretty good! Use it for a few months first! I see black is also reliable, but gold color bought back, and do not want to change around! I'll buy a black	Taobao Marketplace, a Chinese website for online shopping
Edison Technology Factory Store	King of Power Saving	Three air conditioners a day have to be turned on, so I really can't do without them! No way! Electricity is too awesome, the first day I used it saved me more than a hundred dollars, the performance is too awesome, now this time this product is really good! It works great.	Taobao Marketplace, a Chinese website for online shopping
Edison Technology Factory Store	King of Power Saving	My brother-in-law asked me to buy it, he said it works very well after using it for a month! So he introduced me to buy one too! I've already received it! My brother-in-law loves me.	Taobao Marketplace, a Chinese website for online shopping
Edison Technology Factory Store	King of Power Saving	But it's not. There's a power saving feature, and the meter's still whizzing. Do I need to pay an IQ tax?	Taobao Marketplace, a Chinese website for online shopping
Edison Technology Factory Store	King of Power Saving	The saver performs well! Specialized code scanning to verify it's authentic! And exclusive insurance! Reliable.	Taobao Marketplace, a Chinese website for online shopping
Edison Technology Factory Store	King of Power Saving	My home voltage is really not very stable, high and low, there is a power saver or rest assured! At least reduce the voltage damage to electrical appliances longer life, now it is so difficult to earn money! Saving is a virtue.	Taobao Marketplace, a Chinese website for online shopping
Edison Technology Factory Store	King of Power Saving	I am a dry restaurant, the air conditioning can not be turned off in the summer, or customers can not eat! But the cost of electricity makes my head big, commercial electricity is too expensive, there is this product is really good, or my brother recommended to me, or their own people pro! Good product buy	Taobao Marketplace, a Chinese website for online shopping
Edison Technology Factory Store	King of Power Saving	The effect really can not be said, the third time to buy in his family, bought last year's old family mom and dad to use, summer this year just hot, but their electricity bill is quite save, I work in the city, and bought another! Reliable products	Taobao Marketplace, a Chinese website for online shopping

Edison Technology Factory Store	King of Power Saving	The quality of the saver is really good, I bought3of them and then one was given to me! I've been using it for over a week now and it's really saving money on my electric bill! It's very reliable and my in-laws love it!	Taobao Marketplace, a Chinese website for online shopping
Edison Technology Factory Store	King of Power Saving	Energy saver received, has been used, does have the effect, my home air conditioning does not use the energy saver3.6degrees of electricityan hour used the energy saver1.8 Indeed can be, and intend to buy two more	Taobao Marketplace, a Chinese website for online shopping
Edison Technology Factory Store	King of Power Saving	It is a recommended product! It works really well and the material is quite good! Worth recommending!	Taobao Marketplace, a Chinese website for online shopping
Edison Technology Factory Store	King of Power Saving	High-power economizer has received the goods, the logistics is very fast not dragging! Things I look at the workmanship is really quite good! Very satisfied	Taobao Marketplace, a Chinese website for online shopping
Edison Technology Factory Store	King of Power Saving	roommate recommended, said this thing is quite reliable, I a physics science students, I can not know? I took the circuit diagram to study, about a look is really so, I bought three directly sent home for parents to use, although the use of their money, but my filial piety can be clearly seen!	Taobao Marketplace, a Chinese website for online shopping
Edison Technology Factory Store	King of Power Saving	The saver is plugged in and shows that the red light is on and working in normal state, the red220should represent the voltage and the blue0.1,0.2is the current per second! It's pretty good.	Taobao Marketplace, a Chinese website for online shopping
Edison Technology Factory Store	King of Power Saving	roommate recommended, said this thing is quite reliable, I a physics science students, I can not know? I took the circuit diagram to study, about a look is really so, I bought three directly sent home for parents to use, although the use of their money, but my filial piety can be clearly seen!	Taobao Marketplace, a Chinese website for online shopping
Edison Technology Factory Store	King of Power Saving	Energy saver effect is really good, is also the country advocating energy saving and emission reduction, this green product is really good for everyone! Good quality I trust	Taobao Marketplace, a Chinese website for online shopping
Edison Technology Factory Store	King of Power Saving	I heard from a friend that this saver is very good, beforemore than200a month, plug in this saver a few dozen dollars a month, a month to earn back! I also come to a! After all, this year's weather does cost air conditioning! Ican't200 dollars	Taobao Marketplace, a Chinese website for online shopping
Edison Technology Factory Store	King of Power Saving	The product is great, pure aluminum look, pretty good quality, and covered by Pacific Insurance! This is the one I always recommend to my friends! I've been here many times! The price is not too high quite good	Taobao Marketplace, a Chinese website for online shopping
Edison Technology Factory Store	King of Power Saving	Really is a good product, this year the temperaturemore than40degrees, buy this really buy the right! How much more energy saving and environmental protection ah	Taobao Marketplace, a Chinese website for online shopping
Edison Technology Factory Store	King of Power Saving	The product is very good, there is almost no noise when you put it on, and you can hear the work when you listen carefully! Aluminum alloy material worthy of recognition	Taobao Marketplace, a Chinese website for online shopping
Edison Technology Factory Store	King of Power Saving	Summer essential, the temperature of41degreesthe past few days, hey hard to die these people, friends recommended this product reliable and stable, they used six months, bought three or four are this store, I let her send a link to buy a, say a word of conscience is quite good effect is also pretty good, I searched the search of Tmall sells more than a hundred, are a manufacturer! Give praise	Taobao Marketplace, a Chinese website for online shopping
Edison Technology Factory Store	King of Power Saving	Goods received, holding quite a weight, the quality looks really good, high-power a top three children, quite good!	Taobao Marketplace, a Chinese website for online shopping
Edison Technology Factory Store	King of Power Saving	The packaging is very good, the express delivery is strong, the seller is very reputable, the delivery speed is fast, the price is even more affordable than others.	Taobao Marketplace, a Chinese website for online shopping
Edison Technology Factory Store	King of Power Saving	Previously, my family's electricity bill is very high, since the use of this power saver, electricity costs down a lot of me, this is the second time to buy, I hope that on the basis of the original more on a little bit of summer air conditioning in our home is never off!	Taobao Marketplace, a Chinese website for online shopping
Edison Technology Factory Store	King of Power Saving	System default comments	Taobao Marketplace, a Chinese website for online shopping
Edison Technology Factory Store	King of Power Saving	The machine is not too big, not a waste of space, but the function is pretty awesome, very well made, I have purchased from him many times! I like it better.	Taobao Marketplace, a Chinese website for online shopping
Edison Technology Factory Store	King of Power Saving	Energy saver received, the appearance of aluminum alloy seems to be quite strong, I installed one at home, the effect has been good, this time is to help friends buy.	Taobao Marketplace, a Chinese website for online shopping
Edison Technology Factory Store	King of Power Saving	Received the goods, effective! Before my friend used, give me a recommendation, because my home is a mahjong room, so a said this product I immediately bought, before a month of electricity in the1000or so it, this is not to worry about, has been in use	Taobao Marketplace, a Chinese website for online shopping
Edison Technology Factory Store	King of Power Saving	Recently, the weather is very hot, the home air conditioning must be opened, although this year is difficult, but not too provincial, the electricity bill is a little more, but the neighbors recommended this power saver said their family last year have been used again, the effect is very good, a month since the family is also one hundred dollars, I decisively into the hands of my family a month have to be three	Taobao Marketplace, a Chinese website for online shopping
Edison Technology Factory Store	King of Power Saving	Friends recommended, specifically sent me the link I bought one! I heard them say it works well and the results are good, I'll try it too	Taobao Marketplace, a Chinese website for online shopping
Edison Technology Factory Store	King of Power Saving	A very practical product, the first two months to buy a son was broken, which can not be helped to buy a bar in his home, this cheap product is also reliable! Quite good, last time did not give a favorable comment this time to send a bar!	Taobao Marketplace, a Chinese website for online shopping
Edison Technology Factory Store	King of Power Saving	Goods received, the product is very good, or the manufacturers of the People's Insurance cooperation, feel more assured, has been tried, the effect is very much looking forward to, first give a favorable comment on it, logistics is really fast!	Taobao Marketplace, a Chinese website for online shopping
Edison Technology Factory Store	King of Power Saving	Just bought back and tested, I turned on all the appliances in the house, plugged in this saver for a full quarter of an hour, and it still feels OK! I've been testing it for the past few days, so I'll evaluate it further in two months!	Taobao Marketplace, a Chinese website for online shopping
Edison Technology Factory Store	King of Power Saving	The product was received and the logistics was fast! Much better than expected! Merchants are awesome!	Taobao Marketplace, a Chinese website for online shopping
Edison Technology Factory Store	King of Power Saving	Last year before the family used this product I, has been good, because the summer to the elderly used, their own search to buy another, this store cost-effective or genuine! Very good	Taobao Marketplace, a Chinese website for online shopping
Edison Technology Factory Store	King of Power Saving	The power savings are quite noticeable! Nice product!	Taobao Marketplace, a Chinese website for online shopping
Edison Technology Factory Store	King of Power Saving	The effect is very good! Everyone in the family says it's pretty good! Mom and dad especially like it, I am also very happy	Taobao Marketplace, a Chinese website for online shopping
Edison Technology Factory Store	King of Power Saving	Does it work?	Taobao Marketplace, a Chinese website for online shopping
Edison Technology Factory Store	King of Power Saving	Is it authentic?	Taobao Marketplace, a Chinese website for online shopping
Edison Technology Factory Store	King of Power Saving	Really save electricity? Please tell?	Taobao Marketplace, a Chinese website for online shopping
Edison Technology Factory Store	King of Power Saving	There are power savings? Or is it an IQ tax???	Taobao Marketplace, a Chinese website for online shopping

Edison Technology Factory Store	King of Power Saving	Does it work?	Taobao Marketplace, a Chinese website for online shopping
Edison Technology Factory Store	King of Power Saving	Genuine, great quality!	Taobao Marketplace, a Chinese website for online shopping
Edison Technology Factory Store	King of Power Saving	The effect is very good	Taobao Marketplace, a Chinese website for online shopping
Edison Technology Factory Store	King of Power Saving	The results are very noticewhen used for1month or more!	Taobao Marketplace, a Chinese website for online shopping
Edison Technology Factory Store	King of Power Saving	Those who have been using it for more than a month say it works great! The electricity bill is really less!	Taobao Marketplace, a Chinese website for online shopping
Edison Technology Factory Store	King of Power Saving	smart (phone, system, bomb etc)	Taobao Marketplace, a Chinese website for online shopping
Edison Technology Factory Store	Intelligent power saver	Power saving effect is very good, good workmanship material, very satisfied with a shopping!	Taobao Marketplace, a Chinese website for online shopping
Edison Technology Factory Store	Intelligent power saver	System default comments	Taobao Marketplace, a Chinese website for online shopping
Edison Technology Factory Store	Intelligent power saver	Nice, as described by the store, high sex-price ratio, good comments!!!	Taobao Marketplace, a Chinese website for online shopping
Edison Technology Factory Store	Intelligent power saver	Genuine, buy with confidence, the price is fair, satisfied!	Taobao Marketplace, a Chinese website for online shopping
Edison Technology Factory Store	Intelligent power saver	I bought two from him last year and have been using them ever since! It works pretty well! I've seen this saver getting pretty hot lately, so I came to get one! It's good to buy one more.	Taobao Marketplace, a Chinese website for online shopping
Edison Technology Factory Store	Intelligent power saver	The product works well and I started using it as soon as I received it! In the past few days, I feel that the electricity in the house has saved a lot! reliable	Taobao Marketplace, a Chinese website for online shopping
Edison Technology Factory Store	Intelligent power saver	Works pretty well! The stuff is small and pretty reliable! Been using it for a couple days now! Thanks to my buddy Wei for the recommendation!	Taobao Marketplace, a Chinese website for online shopping
Edison Technology Factory Store	Intelligent power saver	It's not a good idea to keep the air conditioner on all the time when it's so hot in the summer at home! I'm renting a house that was over a dollar at one time, better to spend a little money! Three is enough for a two-bedroom apartment!YYDS	Taobao Marketplace, a Chinese website for online shopping
Edison Technology Factory Store	Intelligent power saver	The product is effective. Very good! Trustworthy, second purchase! Very trustworthy!	Taobao Marketplace, a Chinese website for online shopping
Edison Technology Factory Store	Intelligent power saver	I've been using it for over a week and the results are still very noticeable! Very good, have repurchased3units! Express delivery! Looking forward to it! Let everyone in the family use it!	Taobao Marketplace, a Chinese website for online shopping
Edison Technology Factory Store	Intelligent power saver	System default comments	Taobao Marketplace, a Chinese website for online shopping
Edison Technology Factory Store	Intelligent power saver	It's a power saver, really good, and it doesn't take up much space when you plug it into your house! Satisfactory	Taobao Marketplace, a Chinese website for online shopping
Edison Technology Factory Store	Intelligent power saver	The electric bill for the house does get too much in the summer, big couple hundred a month! Hey! If I don't buy one of these, I'll feel like I'm losing millions.	Taobao Marketplace, a Chinese website for online shopping
Edison Technology Factory Store	Intelligent power saver	The product is very good, did nothold, directly buy three, in fact, my home appliances two is enough, just three air conditioning often open, the remaining one inserted kitchen refrigerator, but also quite good!	Taobao Marketplace, a Chinese website for online shopping
Edison Technology Factory Store	Intelligent power saver	System default comments	Taobao Marketplace, a Chinese website for online shopping
Edison Technology Factory Store	Intelligent power saver	System default comments	Taobao Marketplace, a Chinese website for online shopping
Edison Technology Factory Store	Intelligent power saver	This product was recommended by a friend, I feel quite cheap, buy a play who knows the effect is quite good! Really good! Bought three sent back home so that mom and dad also experience high-tech! The key is to save money not to save money to this is useless, I am very realistic!	Taobao Marketplace, a Chinese website for online shopping
Edison Technology Factory Store	Intelligent power saver	System default comments	Taobao Marketplace, a Chinese website for online shopping
Edison Technology Factory Store	Intelligent power saver	Saver received, good quality, good results, very satisfied, the price is not expensive!	Taobao Marketplace, a Chinese website for online shopping
Edison Technology Factory Store	Intelligent power saver	The product is very practical and cost-effective, this is a genuine product, have recommended!	Taobao Marketplace, a Chinese website for online shopping
Edison Technology Factory Store	Intelligent power saver	It's been over a week since I received it and it's pretty good stuff! It's easy to use, I just plug it into the plug board next to the TV at home! Feels like the effect is getting better every day Very cheerful! Haha, saving money is the hard way	Taobao Marketplace, a Chinese website for online shopping
Edison Technology Factory Store	Intelligent power saver	System default comments	Taobao Marketplace, a Chinese website for online shopping
Edison Technology Factory Store	Intelligent power saver	Home previously bought a large economizer used for several years, has been effective, this year the weather is hot, but also their own Taobao shopping brush to this product, I checked with my previous years to buy the large economizer a manufacturer, decisively bought three, arrived, quite good, I keep one,2to my mom sent back! It's great.	Taobao Marketplace, a Chinese website for online shopping
Edison Technology Factory Store	Intelligent power saver	System default comments	Taobao Marketplace, a Chinese website for online shopping
Edison Technology Factory Store	Intelligent power saver	Baby received, logistics is very fast, the description matches, more satisfied, praise,recommend~	Taobao Marketplace, a Chinese website for online shopping
Edison Technology Factory Store	Intelligent power saver	System default comments	Taobao Marketplace, a Chinese website for online shopping
Edison Technology Factory Store	Intelligent power saver	Early to see neighbors save a month of electricity quite a lot, that morning a piece to buy food asked to ask the original home installed a this, his family said that electrical appliances are not much this product is enough to use, I also buy a try, suitable and then buy a high-power will not, first try this	Taobao Marketplace, a Chinese website for online shopping
Edison Technology Factory Store	Intelligent power saver	System default comments	Taobao Marketplace, a Chinese website for online shopping

Edison Technology Factory Store	Intelligent power saver	Logistics is very fast, received, already looked at the product, very satisfied	Taobao Marketplace, a Chinese website for online shopping
Edison Technology Factory Store	Intelligent power saver	I have studied electrical appliances, power factor on power saving energy consumption Pullec useful, although there is a limit to reduce, but can save a little is a little, but also play a role in the protection of home appliances, stabilizing the electronic current, before I remember are very large a product, there are pointers, and now technological advances, are this small and exquisite type is quite good! Summer home three air conditioning a refrigerator and other appliances, with this review can save more than ten dollars a day! A month counted really quite a lot, quite good	Taobao Marketplace, a Chinese website for online shopping
Edison Technology Factory Store	Intelligent power saver	System default comments	Taobao Marketplace, a Chinese website for online shopping
Edison Technology Factory Store	Intelligent power saver	I got the baby. It's good.	Taobao Marketplace, a Chinese website for online shopping
Edison Technology Factory Store	Intelligent power saver	Good technology products, the boss said to buy two get one sex on a one-time buy two more, before buying a, has been using a good, and then brother-in-law came to my home by the way, I can not say, buy two more of their own it!	Taobao Marketplace, a Chinese website for online shopping
Edison Technology Factory Store	Intelligent power saver	I bought it with a skeptical attitude to try it out, and this trial has dispelled my doubts! Usually our home daily electricity consumption of 20 to 30 degrees, with the energy saver only15degreesper day! It's not bad.	Taobao Marketplace, a Chinese website for online shopping
Edison Technology Factory Store	Intelligent power saver	Since the installation of this product, the home's electricity consumption40%lessis there, my husband observed three months to summarize, my husband is a person who will live, trustworthy! The product is also good, this is the second time to buy for my parents and big sister to buy	Taobao Marketplace, a Chinese website for online shopping
Edison Technology Factory Store	Intelligent power saver	I received the appliance and have tried it out, I'll share a tip with you, try not to unplug this thing once it's plugged in and the closer it is to the main gate the better! My family has bought several, have experience in using	Taobao Marketplace, a Chinese website for online shopping
Edison Technology Factory Store	Intelligent power saver	Product quality is very good! Get back to plug in first to see the authenticity, I did not expect the effect is still quite obvious! Specialized scanning code authentication is genuine, write down I will rest assured to use!	Taobao Marketplace, a Chinese website for online shopping
Edison Technology Factory Store	Intelligent power saver	The effect of saving electricity is really good, but also a friend introduced to me, really reliable! The logistics is very fast and powerful	Taobao Marketplace, a Chinese website for online shopping
Edison Technology Factory Store	Intelligent power saver	Several friends around me have them in their homes, and I'm using them as well, and they still work very well.	Taobao Marketplace, a Chinese website for online shopping
Edison Technology Factory Store	Intelligent power saver	Are you sellers really saving power?	Taobao Marketplace, a Chinese website for online shopping
Xiao Cui Technology	a power saver (electronics)	The power saving effect is not obvious.	Taobao Marketplace, a Chinese website for online shopping
Xiao Cui Technology	a power saver (electronics)	The boss told me to buy the black$99 one and use it in my restaurant, I paid my electric bill yesterday, it's not bad, it does get partially less, the electric bill is not that high, it's a good thing, it's kind of satisfying	Taobao Marketplace, a Chinese website for online shopping
Xiao Cui Technology	a power saver (electronics)	The boss suggested buying two, the effect can be oh, usually every month is600yuan so, now446yuan, the effect is very good, the effect is very good, and then use two months to follow up the evaluation	Taobao Marketplace, a Chinese website for online shopping
Xiao Cui Technology	a power saver (electronics)	Try it for a month before commenting, I don't know whether it works or not	Taobao Marketplace, a Chinese website for online shopping
Xiao Cui Technology	a power saver (electronics)	Multi-buy	Taobao Marketplace, a Chinese website for online shopping
Xiao Cui Technology	a power saver (electronics)	Guys don't buy it, it doesn't work it doesn't save electricity, plugged in three of them don't work, it doesn't work at all spent$150tax, guys, pay attention it really doesn't work it really doesn't save electricity	Taobao Marketplace, a Chinese website for online shopping
Xiao Cui Technology	a power saver (electronics)	It doesn't work at all. Don't fall for it.	Taobao Marketplace, a Chinese website for online shopping
Xiao Cui Technology	a power saver (electronics)	This effect I am satisfied, the electricity bill finally came down, I this three-phase electricity use electricity is really too much, I factory a friend introduced me to buy, this month finally dry down the electricity bill, downmore than600yuan of electricity, a little bit unbelievable, never give other people good reviews, you must give your home good reviews	Taobao Marketplace, a Chinese website for online shopping
Xiao Cui Technology	a power saver (electronics)	I just started using it. I don't know what it's like.	Taobao Marketplace, a Chinese website for online shopping
Xiao Cui Technology	a power saver (electronics)	Works great, already paid the electric bill and it does save money on the electric bill	Taobao Marketplace, a Chinese website for online shopping
Xiao Cui Technology	a power saver (electronics)	My brother asked me to buy it, he used it himself and it worked, today I paid my electricity bill and it was much less than the month before, it's amazing!	Taobao Marketplace, a Chinese website for online shopping
Xiao Cui Technology	a power saver (electronics)	I bought this high power for the second time, my effect is very obvious, a month down, I can be quite a lot less, has been the second purchase, the	Taobao Marketplace, a Chinese website for online shopping
Xiao Cui Technology	a power saver (electronics)	Finally got my electric bill down. I've bought so many products and this one is the most noticeable.	Taobao Marketplace, a Chinese website for online shopping
Xiao Cui Technology	a power saver (electronics)	I haven't calculated the electric bill, I'll comment again after the next measurement.	Taobao Marketplace, a Chinese website for online shopping
Xiao Cui Technology	a power saver (electronics)	I bought it with the intention of trying it out, and the power saving does work!	Taobao Marketplace, a Chinese website for online shopping
Xiao Cui Technology	a power saver (electronics)	Boss recommended high power is not bad, I do not believe that this can save electricity in front, has paid the electricity bill twice before coming to the evaluation, this can really save electricity, I used to have beenabout300yuan, these two months with this high power, two times are almost180here, this time173yuan, served the effect of saving electricity is not bad	Taobao Marketplace, a Chinese website for online shopping
Xiao Cui Technology	a power saver (electronics)	Just started using it, haven't seen any effect yet	Taobao Marketplace, a Chinese website for online shopping
Xiao Cui Technology	a power saver (electronics)	I'll give you a good review first and look forward to the results.	Taobao Marketplace, a Chinese website for online shopping
Xiao Cui Technology	a power saver (electronics)	give it a try	Taobao Marketplace, a Chinese website for online shopping
Xiao Cui Technology	a power saver (electronics)	This black high power is really amazing, I usually500dollars, with this black high power99dollars of this, now it is353.53dollars, really cost-effective, have recommended to friends	Taobao Marketplace, a Chinese website for online shopping

Xiao Cui Technology	a power saver (electronics)	The product is very good, now that the air conditioning is on it doesn't seem to use as much power as it used to, this is okay haha	Taobao Marketplace, a Chinese website for online shopping
Xiao Cui Technology	a power saver (electronics)	Works great. Been using it for a week.	Taobao Marketplace, a Chinese website for online shopping
Xiao Cui Technology	a power saver (electronics)	Saving power received the logistics very quickly, friends recommended that with a good	Taobao Marketplace, a Chinese website for online shopping
Xiao Cui Technology	a power saver (electronics)	Received the goods, looking forward to the effect	Taobao Marketplace, a Chinese website for online shopping
Xiao Cui Technology	a power saver (electronics)	The product is very effective, my wife said paypal automatically pay the electricity bill, has been significantly less, the effect is very good	Taobao Marketplace, a Chinese website for online shopping
Xiao Cui Technology	a power saver (electronics)	This is not bad, I save a third of the electricity every month, this can be bought!	Taobao Marketplace, a Chinese website for online shopping
Xiao Cui Technology	a power saver (electronics)	I bought two at once, have paid the electricity bill twice, obviously using less power, electricity as usual, really good, effective!	Taobao Marketplace, a Chinese website for online shopping
Xiao Cui Technology	a power saver (electronics)	The product can be Oh, I am very satisfied, save me a lot of electricity	Taobao Marketplace, a Chinese website for online shopping
Xiao Cui Technology	a power saver (electronics)	I've paid my electricity bill a few times now, and since I bought this saver, I've seen a significant drop in power consumption, and the results are really great!	Taobao Marketplace, a Chinese website for online shopping
Xiao Cui Technology	a power saver (electronics)	Oh exempt, really good, the effect is still very good, just do not believe in the beginning, now believe it, you can save electricity, to give you a follow-up review.	Taobao Marketplace, a Chinese website for online shopping
Xiao Cui Technology	a power saver (electronics)	I'm using it with great results, this is good,	Taobao Marketplace, a Chinese website for online shopping
Xiao Cui Technology	a power saver (electronics)	Works great and really saves power	Taobao Marketplace, a Chinese website for online shopping
Xiao Cui Technology	a power saver (electronics)	I've tried many, and I'm happy with this one. It works well.	Taobao Marketplace, a Chinese website for online shopping
Xiao Cui Technology	a power saver (electronics)	I've bought this several times and it works well, I use it in my old home and new home	Taobao Marketplace, a Chinese website for online shopping
Xiao Cui Technology	a power saver (electronics)	This is pretty good, less money to spend on it, this is okay, it still works fine	Taobao Marketplace, a Chinese website for online shopping
Xiao Cui Technology	a power saver (electronics)	Already paid the electric bill, the overall down pretty good about38%savings, my girlfriends introduced me to buy the	Taobao Marketplace, a Chinese website for online shopping
Xiao Cui Technology	a power saver (electronics)	My husband bought it back and I was just talking about him when he bought it, now the power is down and it$139less than last month, so I'll give you a good review	Taobao Marketplace, a Chinese website for online shopping
Xiao Cui Technology	a power saver (electronics)	It works fine, send in I can save electricity every month	Taobao Marketplace, a Chinese website for online shopping
Xiao Cui Technology	a power saver (electronics)	My friend here to buy, gave me a, the effect is really good,eh, I can saveabout100dollarsthis month.	Taobao Marketplace, a Chinese website for online shopping
Xiao Cui Technology	a power saver (electronics)	It can save electricity, this one can, I'll recommend it to a friend for you	Taobao Marketplace, a Chinese website for online shopping
Xiao Cui Technology	a power saver (electronics)	I am half a year to pay, before so much, I use to save electrical appliances withmore than4months, this time the calculation can be about half less, because the amount of electricity I am more sensitive, all sure that this product has effect	Taobao Marketplace, a Chinese website for online shopping
Xiao Cui Technology	a power saver (electronics)	It works well, although not half of what I wanted to save, but it still counts for a lot of savings!	Taobao Marketplace, a Chinese website for online shopping
Xiao Cui Technology	a power saver (electronics)	I bought it to use for housing, my boyfriend said he had some money missing this month and said he wanted me to buy another one	Taobao Marketplace, a Chinese website for online shopping
Xiao Cui Technology	a power saver (electronics)	I bought three of them and have already paid my electric bill twice, this owner didn't lie to me, it really saves electricity	Taobao Marketplace, a Chinese website for online shopping
Xiao Cui Technology	a power saver (electronics)	There are a lot of big appliances at home, the air conditioner is very expensive in summer, I bought this in front of me, the effect on the air conditioner is very good, let me explain, I bought three in one breath, I use more electricity, a month downabout200dollarsless, very satisfied	Taobao Marketplace, a Chinese website for online shopping
Xiao Cui Technology	a power saver (electronics)	Already this old customer, also bought last year, power saving effect I am satisfied, plug in to save power, not plug in the power will go up, all the proof of the effect	Taobao Marketplace, a Chinese website for online shopping
Xiao Cui Technology	a power saver (electronics)	I've been using this for a long time now and this really works, it's good, kind of satisfactory	Taobao Marketplace, a Chinese website for online shopping
Xiao Cui Technology	a power saver (electronics)	didn't use the saver last month, but this month I could have gottenover$130less with the saver, and the results are obvious.	Taobao Marketplace, a Chinese website for online shopping
Xiao Cui Technology	a power saver (electronics)	I bought4energy savers, paid the electric bill today, lessmore than300yuan, anyway, not expensive, it's already very good, the boss said that more will be better, I've already ordered	Taobao Marketplace, a Chinese website for online shopping
Xiao Cui Technology	a power saver (electronics)	I'm a renter, usually it'sabout $400a month, this time I got two, I paid$276this month, it's pretty good	Taobao Marketplace, a Chinese website for online shopping
Xiao Cui Technology	a power saver (electronics)	My friend recommended to me, said the effect is very good, I bought two, this month less money, really can, just buy I do not believe, but has proved to have effect	Taobao Marketplace, a Chinese website for online shopping
Xiao Cui Technology	a power saver (electronics)	The third month, I'2800degreesa month, a breath of4save electrical appliances, to show you all, the first two months to savemore than400, this month to saveabout430dollars, this product is powerful	Taobao Marketplace, a Chinese website for online shopping
Xiao Cui Technology	a power saver (electronics)	I bought it to use for housing, my boyfriend said he had some money missing this month and said he wanted me to buy another one	Taobao Marketplace, a Chinese website for online shopping
Xiao Cui Technology	a power saver (electronics)	Second purchase, my husband said this gadget works pretty well, anyway, the electricity is not paid by me, he said it's good just can save money!	Taobao Marketplace, a Chinese website for online shopping

Xiao Cui Technology	a power saver (electronics)	This effect is good oh, has proved, really can, I basically every month can save electricity, and then buy two back home	Taobao Marketplace, a Chinese website for online shopping
Xiao Cui Technology	a power saver (electronics)	I've been using it for a long time, and it's working well. I think it's ideal.	Taobao Marketplace, a Chinese website for online shopping
Xiao Cui Technology	a power saver (electronics)	Buyer does not comment within15days	Taobao Marketplace, a Chinese website for online shopping
Xiao Cui Technology	a power saver (electronics)	I've been using it for so long, it works great, I'm very happy with it, it's not bad for air conditioning	Taobao Marketplace, a Chinese website for online shopping
Xiao Cui Technology	a power saver (electronics)	Buyer does not comment within15days	Taobao Marketplace, a Chinese website for online shopping
Xiao Cui Technology	a power saver (electronics)	Lying ghosts, no half-assed relationship and still the same as before	Taobao Marketplace, a Chinese website for online shopping
Xiao Cui Technology	a power saver (electronics)	Buyer does not comment within15days	Taobao Marketplace, a Chinese website for online shopping
Xiao Cui Technology	a power saver (electronics)	Not helpful at all, it's paid the IQ tax, I'm asking for a refund now, and if I don't get it, I'm going to file a complaint	Taobao Marketplace, a Chinese website for online shopping
Xiao Cui Technology	a power saver (electronics)	Buyer does not comment within15days	Taobao Marketplace, a Chinese website for online shopping
Xiao Cui Technology	a power saver (electronics)	increase ofmore than300 over the previous electricity bill. It's hard to believe that the meter readers are blind	Taobao Marketplace, a Chinese website for online shopping
Xiao Cui Technology	a power saver (electronics)	Buyer does not comment within15days	Taobao Marketplace, a Chinese website for online shopping
Xiao Cui Technology	a power saver (electronics)	Guys don't buy it, it doesn't work it doesn't save electricity, plugged in three of them don't work, it doesn't work at all spent$150tax, guys, pay attention it really doesn't work it really doesn't save electricity	Taobao Marketplace, a Chinese website for online shopping
Xiao Cui Technology	a power saver (electronics)	Buyer does not comment within15days	Taobao Marketplace, a Chinese website for online shopping
Xiao Cui Technology	a power saver (electronics)	an excellent	Taobao Marketplace, a Chinese website for online shopping
Xiao Cui Technology	a power saver (electronics)	Does it really work? Ask for the truth.	Taobao Marketplace, a Chinese website for online shopping
Xiao Cui Technology	a power saver (electronics)	Will there really be power savings?	Taobao Marketplace, a Chinese website for online shopping
Xiao Cui Technology	a power saver (electronics)	Is there any power saving effect	Taobao Marketplace, a Chinese website for online shopping
Xiao Cui Technology	a power saver (electronics)	If you've bought it, how is this power saver, does it really save electricity?	Taobao Marketplace, a Chinese website for online shopping
Xiao Cui Technology	a power saver (electronics)	IQ tax	Taobao Marketplace, a Chinese website for online shopping
Xiao Cui Technology	Intelligent power saver	Goods received good quality is genuine	Taobao Marketplace, a Chinese website for online shopping
Xiao Cui Technology	Intelligent power saver	Use it!	Taobao Marketplace, a Chinese website for online shopping
Xiao Cui Technology	Smart Saver	The boss suggested buying two, the effect can be oh, usually every month is600yuan so, now446yuan, the effect is very good, the effect is very good, and then use two months to follow up the evaluation	Taobao Marketplace, a Chinese website for online shopping
Xiao Cui Technology	Smart Saver	It's not bad, although it's not45%power saving rate that the owner said, I should beabout33%my own math, this product is okay	Taobao Marketplace, a Chinese website for online shopping
Xiao Cui Technology	Smart Saver	I didn't believe in it at first, but now I do. This product is okay, and the power saving effect is not bad.	Taobao Marketplace, a Chinese website for online shopping
Xiao Cui Technology	Smart Saver	My classmates asked me to buy it, my rental room uses a lot of electricity, after I bought this saver, I now pay the electricity bill does not have to be so much, anyway, you can reduce the amount of electricity, I'm still very satisfied with it, it's a good deal!	Taobao Marketplace, a Chinese website for online shopping
Xiao Cui Technology	Smart Saver	Bought two, I use them separately and have paid the electric bill twice now, the first time it was$176lessand this time it was$163less, personally I think it's ok.	Taobao Marketplace, a Chinese website for online shopping
Xiao Cui Technology	Smart Saver	The first time I bought a power saver, used for a period of time, wow, but also really effective, every day you can see that the power is less, I unplugged it if the power went up, this little thing is really magical, awesome!	Taobao Marketplace, a Chinese website for online shopping
Xiao Cui Technology	Smart Saver	Today I paid the electric bill, a few dozen dollars less, anyway, it is not expensive, it is already very good, the boss said to buy one more will be better, I have already placed an order	Taobao Marketplace, a Chinese website for online shopping
Xiao Cui Technology	Smart Saver	I used to buy in this home, the effect is very good, this time to buy two used in the new home, my new home electricity is relatively large, a month is about800yuan or so, I used two, and now the electricity bill down,more than590a month, it's really good, this home can be trusted	Taobao Marketplace, a Chinese website for online shopping
Xiao Cui Technology	Smart Saver	Boss this thing is okay, the power saving effect is not bad, after I use this thing, I have about32%lesspowerthis month, I use it again after a month, if I can still save32%, I come to give you a follow-up review.	Taobao Marketplace, a Chinese website for online shopping
Xiao Cui Technology	Smart Saver	I bought99dollars of high power, the first few months of electricity ismore than1280, did not use this product aremore than1200, this month I used, and now934yuan, you can compare, to buy99dollars this, this I personally think it is better, the effect is still satisfied	Taobao Marketplace, a Chinese website for online shopping
Xiao Cui Technology	Smart Saver	I'm on a smart meter and it still works pretty well, I've bought a couple of them and this one still works well	Taobao Marketplace, a Chinese website for online shopping
Xiao Cui Technology	Smart Saver	I bought the 3-phase saver,the $200+, and I usually have a lot of degrees. Since using this saver, I'veover470degreesless in a month,**, it's really possible to have a product like this, it's kind of empowering	Taobao Marketplace, a Chinese website for online shopping
Xiao Cui Technology	Smart Saver	I have been using a long time before I come to evaluate, I have paid2months, usually I am270dollarsa month, with the power saver is now basically every month to stay in theposition190dollars, the feeling of the effect is very good, I'm quite satisfied, my friend asked me to introduce to him, I also asked my [illegible]	Taobao Marketplace, a Chinese website for online shopping

Xiao Cui Technology	Smart Saver	I'm an electrician, was to try the psychological buy, did not expect the effect is not bad, this thing is not bad, now the science is really a generation to update, even I'm an electrician feel incredible, the effect is okay	Taobao Marketplace, a Chinese website for online shopping
Xiao Cui Technology	Smart Saver	I have been the third time to buy, these months with the power saver can be reduced by about33%so, it is very obvious, it is worth buying, the businessman is not bad, I have recommended to a few friends to buy, I hope that the next time you buy to give me a little discount, I'm always recommending friends to you	Taobao Marketplace, a Chinese website for online shopping
Xiao Cui Technology	Smart Saver	Has been used for a few months, since the use of power saver my power will come down, basically every month can save some money, this effect I think it is good, I use this meter, the home of the smart meter is also very effective, I asked the home of the father, pay the electricity bill is not as expensive as before, the old man is also very happy!	Taobao Marketplace, a Chinese website for online shopping
Xiao Cui Technology	Smart Saver	This in my hotel to use is not bad, the overall down is okay, to buy this to buy, truly recommended!	Taobao Marketplace, a Chinese website for online shopping
Xiao Cui Technology	Smart Saver	I bought800-2500degree one, I'm satisfied, it used toover $1900, I bought the black one and used it for a month, it was$1573 forthe month, wow Damn, it works fine, count it as very satisfying! I hope the store sells better and better, I'll use it for another month and come back to give you a follow up review	Taobao Marketplace, a Chinese website for online shopping
Xiao Cui Technology	Smart Saver	I'm a renter, usually it'sabout $400a month, this time I got two, I paid$276this month, it's pretty good	Taobao Marketplace, a Chinese website for online shopping
Xiao Cui Technology	Smart Saver	Bought several products, tried several products, this product has the effect, usuallymore than200, this time155, has been used for a few months, counting the awesome effect is what I want	Taobao Marketplace, a Chinese website for online shopping
Xiao Cui Technology	Smart Saver	I did not use the previous month to save appliances when the usual month is280dollars, I bought to save appliances, now a month of195dollars like this, the business did not cheat, you can buy	Taobao Marketplace, a Chinese website for online shopping
Xiao Cui Technology	Smart Saver	this is the chart	Taobao Marketplace, a Chinese website for online shopping
Xiao Cui Technology	Smart Saver	I rent an apartment, anyway, used to pay160+a monthelectricity, after using this thing, I pay72$, wow, can't believe it, but I'm very happy with it, and I'll continue to follow up on it later	Taobao Marketplace, a Chinese website for online shopping
Xiao Cui Technology	Smart Saver	My dad said he's happy that his electric bill was cut in half this month.	Taobao Marketplace, a Chinese website for online shopping
Xiao Cui Technology	Smart Saver	This works great, already paid the electric bill for a month and it's not too bad	Taobao Marketplace, a Chinese website for online shopping
Xiao Cui Technology	Smart Saver	I am half a year to pay, before so much, I use to save electrical appliances withmore than4months, this time the calculation can be about half less, because the amount of electricity I am more sensitive, all sure that this product has effect	Taobao Marketplace, a Chinese website for online shopping
Xiao Cui Technology	Smart Saver	This is good, do so many years of electricians, the first time I heard that this product can save electricity, buy back to try, not bad, not bad!	Taobao Marketplace, a Chinese website for online shopping
Xiao Cui Technology	Smart Saver	Our Guangdong side of the open air conditioning is very costly electricity, this effect on the air conditioning is not bad, but also really save electricity, try to buy the psychology, this can be	Taobao Marketplace, a Chinese website for online shopping
Xiao Cui Technology	Smart Saver	The boss told me to buy the black$99 one and use it in my restaurant, I paid my electric bill yesterday, it's not bad, it does get partially less, the electric bill is not that high, it's a good thing, it's kind of satisfying	Taobao Marketplace, a Chinese website for online shopping
Xiao Cui Technology	Smart Saver	System default comments	Taobao Marketplace, a Chinese website for online shopping
Xiao Cui Technology	Smart Saver	My friend recommended to me, said the effect is very good, I bought two, this month less money, really can, just buy I do not believe, but has proved to have effect	Taobao Marketplace, a Chinese website for online shopping
Xiao Cui Technology	Smart Saver	month before lastwas$198, after using the saver, this month$119,**, it really works, pretty good, want to buy two more back home, good	Taobao Marketplace, a Chinese website for online shopping
Xiao Cui Technology	Smart Saver	I've bought it for the third time, I didn't believe in it before, but now I do, it's very effective, I pay my electricity bill one day at a time, it's very obvious	Taobao Marketplace, a Chinese website for online shopping
Xiao Cui Technology	King of Power Saving	So far the effect is very good, I rent a house, the electricity bill has been reduced a little, quite satisfied, continue to come back to the feedback!	Taobao Marketplace, a Chinese website for online shopping
Xiao Cui Technology	King of Power Saving	The effect can be, friends introduced me to buy this product is not bad, quite satisfactory	Taobao Marketplace, a Chinese website for online shopping
Xiao Cui Technology	King of Power Saving	bought this one and it works fine, usually it's always close to$300 or so, this monthit's $181,**, it really saves electricity	Taobao Marketplace, a Chinese website for online shopping
Xiao Cui Technology	King of Power Saving	month before lastover$1100, but after using this$868left, which is not bad	Taobao Marketplace, a Chinese website for online shopping
Xiao Cui Technology	King of Power Saving	not good product	Taobao Marketplace, a Chinese website for online shopping
Xiao Cui Technology	King of Power Saving	It works well, I've bought it twice, my electricity bill has dropped, I think it's good.	Taobao Marketplace, a Chinese website for online shopping
Xiao Cui Technology	King of Power Saving	My husband asked me to buy it, it works well, and it really saves electricity, it's great!	Taobao Marketplace, a Chinese website for online shopping
Xiao Cui Technology	King of Power Saving	Effective, used for more than a month before commenting, can save electricity	Taobao Marketplace, a Chinese website for online shopping
Xiao Cui Technology	King of Power Saving	It's okay, I just don't know how much power it saves, let's use it and see.	Taobao Marketplace, a Chinese website for online shopping
Xiao Cui Technology	King of Power Saving	It's supposed to work, use it for 10 days or so and then pull it out! Do not dare to have it! My house has an elevator, the impact of the elevator can not start, change to another house without elevator house to try	Taobao Marketplace, a Chinese website for online shopping
Xiao Cui Technology	King of Power Saving	worthwhile	Taobao Marketplace, a Chinese website for online shopping
Xiao Cui Technology	King of Power Saving	One good review.	Taobao Marketplace, a Chinese website for online shopping
Xiao Cui Technology	King of Power Saving	Let's give it a good review for now,, use it for a while and then follow up!	Taobao Marketplace, a Chinese website for online shopping
Xiao Cui Technology	King of Power Saving	OK.	Taobao Marketplace, a Chinese website for online shopping

Xiao Cui Technology	King of Power Saving	I don't know how effective it is, use it first	Taobao Marketplace, a Chinese website for online shopping
Xiao Cui Technology	King of Power Saving	It feels different from the one I bought before, this one is a bit light and fluffy, the previous one was a bit weighty, I don't know if it works or not	Taobao Marketplace, a Chinese website for online shopping
Xiao Cui Technology	King of Power Saving	My friend recommended to me, said the effect is very good, I bought two, this month less money, really can, just buy I do not believe, but has proved to have effect	Taobao Marketplace, a Chinese website for online shopping
Xiao Cui Technology	King of Power Saving	I've been using it for a while and I'm very happy with it.	Taobao Marketplace, a Chinese website for online shopping
Xiao Cui Technology	King of Power Saving	System default comments	Taobao Marketplace, a Chinese website for online shopping
Xiao Cui Technology	King of Power Saving	Bought several products, tried several products, this product has the effect, usuallymore than200, this time155, has been used for a few months, counting the awesome effect is what I want	Taobao Marketplace, a Chinese website for online shopping
Xiao Cui Technology	King of Power Saving	favorable criticism	Taobao Marketplace, a Chinese website for online shopping
Xiao Cui Technology	King of Power Saving	My dad asked me to buy one, but after I paid the bill, I asked and he said it was a lot less this month, so he asked me to buy another one.	Taobao Marketplace, a Chinese website for online shopping
Xiao Cui Technology	King of Power Saving	It's a complete no-no. It's got a chip the size of a fingernail in it. You guys think it's gonna save power.	Taobao Marketplace, a Chinese website for online shopping
Xiao Cui Technology	King of Power Saving	For dormitory use Just one air conditioner Continuously record the power consumption of several days using and not using this device at the same time Tested to have the effect of power saving	Taobao Marketplace, a Chinese website for online shopping
Xiao Cui Technology	King of Power Saving	Just used it for a few days to try it out first no comment for now	Taobao Marketplace, a Chinese website for online shopping
Xiao Cui Technology	King of Power Saving	Said the effect is very good, every day can save some electricity, ready to buy another one, logistics is very fast, praise👍	Taobao Marketplace, a Chinese website for online shopping
Xiao Cui Technology	King of Power Saving	It's good. It works.	Taobao Marketplace, a Chinese website for online shopping
Xiao Cui Technology	King of Power Saving	I don't know yet.	Taobao Marketplace, a Chinese website for online shopping
Xiao Cui Technology	King of Power Saving	acceptable	Taobao Marketplace, a Chinese website for online shopping
Xiao Cui Technology	King of Power Saving	After using it for half a month it should work to save electricity! I won't know until I pay the bill at the end of the month.	Taobao Marketplace, a Chinese website for online shopping
Xiao Cui Technology	King of Power Saving	Just received the goods, do not know how the effect, after a period of time in the follow-up comment!	Taobao Marketplace, a Chinese website for online shopping
Xiao Cui Technology	King of Power Saving	The boss told me to buy the black$99 one and use it in my restaurant, I paid my electric bill yesterday, it's not bad, it does get partially less, the electric bill is not that high, it's a good thing, it's kind of satisfying	Taobao Marketplace, a Chinese website for online shopping
Xiao Cui Technology	King of Power Saving	I plugged in the refrigerator and air conditioning where better, the effect is quite OK, this is not a mistake to buy, satisfied with the results	Taobao Marketplace, a Chinese website for online shopping
Xiao Cui Technology	King of Power Saving	bought two of them, so far they work well, I've already paid my electric bill once, itabout $107less, I'll be back next month with a follow-up review.	Taobao Marketplace, a Chinese website for online shopping
Xiao Cui Technology	King of Power Saving	System default comments	Taobao Marketplace, a Chinese website for online shopping
Xiao Cui Technology	King of Power Saving	This is not bad, friends introduced, the electricity bill can really go down less, not bad	Taobao Marketplace, a Chinese website for online shopping
Xiao Cui Technology	King of Power Saving	Can say that this product is not bad, has been used for so long, power saving is quite good, this everyone can buy	Taobao Marketplace, a Chinese website for online shopping
Xiao Cui Technology	King of Power Saving	The effect is good, after using the electricity bill is quite a lot less, and then follow up feedback!	Taobao Marketplace, a Chinese website for online shopping
Xiao Cui Technology	King of Power Saving	Goods received! Scanning code is authentic, good value for money! Summer electricity consumption is high, purchased to save electricity costs, it is really practical! Logistics and customer service attitude is quite good, value for money!	Taobao Marketplace, a Chinese website for online shopping
Xiao Cui Technology	King of Power Saving	The saver is really good!11′follow up afteruse it for a while!	Taobao Marketplace, a Chinese website for online shopping
Xiao Cui Technology	King of Power Saving	My husband said he paid less for his electric bill this time and he said this is okay for saving electricity	Taobao Marketplace, a Chinese website for online shopping
Xiao Cui Technology	King of Power Saving	I don't feel so good.	Taobao Marketplace, a Chinese website for online shopping
Xiao Cui Technology	King of Power Saving	Effectiveness of subsequent advances	Taobao Marketplace, a Chinese website for online shopping
Xiao Cui Technology	King of Power Saving	I'll add more after I use it, but I'll give you a good review first.	Taobao Marketplace, a Chinese website for online shopping
Xiao Cui Technology	King of Power Saving	Alas not bad, for my air conditioning is okay, but also reduce quite a lot of electricity, good to use!	Taobao Marketplace, a Chinese website for online shopping
Xiao Cui Technology	King of Power Saving	I bought this one for the factory, it's not bad,over2,000degreesless this month, this thing is okay	Taobao Marketplace, a Chinese website for online shopping
Xiao Cui Technology	King of Power Saving	Good use, the old family dad said good use, the same open air conditioning, this month less quite a lot of good use, to give you a good review, the back to continue to follow up on the feedback	Taobao Marketplace, a Chinese website for online shopping
Xiao Cui Technology	King of Power Saving	I bought two black, milk tea store to pay the electricity bill, the effect is not bad, can save electricity, although not a lot, but this effect is I am satisfied, not bad	Taobao Marketplace, a Chinese website for online shopping

Xiao Cui Technology	King of Power Saving	I've heard it works. I'll try it.	Taobao Marketplace, a Chinese website for online shopping
Xiao Cui Technology	King of Power Saving	Just used a few days do not know how the effect of power saving, need to pay the electricity bill next month to know, looking forward to the use of results	Taobao Marketplace, a Chinese website for online shopping
Xiao Cui Technology	King of Power Saving	Expect power savings	Taobao Marketplace, a Chinese website for online shopping
Xiao Cui Technology	King of Power Saving	This is not bad, this month is so hot, I turn on the air conditioning every day, but calculated than the previous month to reduce some of the electricity bill, the effect of this thing is not bad, it works well, very satisfied with it.	Taobao Marketplace, a Chinese website for online shopping
Xiao Cui Technology	King of Power Saving	ve used it for a month to give feedback, so far the electricity bill is$102lessa month, I'll use it for a few more months and then come back with a follow-up review	Taobao Marketplace, a Chinese website for online shopping
Xiao Cui Technology	King of Power Saving	Used two months before the evaluation, the previous month also has the effect, this month the electricity bill also came down, seems to have the effect is good, usuallymore than700a month, now552yuan, feel pretty good, as if the effect on the air conditioning is quite good, I use the air conditioning test for a month, the feeling is not bad, so far it's very good, and after a while and then follow up on the	Taobao Marketplace, a Chinese website for online shopping
Xiao Cui Technology	King of Power Saving	Used how long, the effect is good, power saving effect is still quite obvious,	Taobao Marketplace, a Chinese website for online shopping
Xiao Cui Technology	King of Power Saving	I've only just started using it, I'll comment later.	Taobao Marketplace, a Chinese website for online shopping
Xiao Cui Technology	King of Power Saving	Substitute for some time before leveling	Taobao Marketplace, a Chinese website for online shopping
Xiao Cui Technology	King of Power Saving	Buy it and try it out first, use it for a month or so.	Taobao Marketplace, a Chinese website for online shopping
Xiao Cui Technology	King of Power Saving	Just started using it and don't know the results yet.	Taobao Marketplace, a Chinese website for online shopping
Xiao Cui Technology	King of Power Saving	Power saving pretty good good use, save a lot of electricity bill	Taobao Marketplace, a Chinese website for online shopping
Xiao Cui Technology	King of Power Saving	This effect I am satisfied, the electricity bill finally came down, I this three-phase electricity use electricity is really too much, I factory a friend introduced me to buy, this month finally dry down the electricity bill, downmore than600yuan of electricity, a little bit unbelievable, never give other people good reviews, you must give your home good reviews	Taobao Marketplace, a Chinese website for online shopping
Xiao Cui Technology	King of Power Saving	This black high-power is not bad, and ordered two, boss I give you real feedback to come, the effect is very good, this month's electricity bill obviously dropped part of the next time you buy remember to give me a discount, the next time you buy remember to give me a discount.	Taobao Marketplace, a Chinese website for online shopping
Xiao Cui Technology	King of Power Saving	Usuallymore than500degrees of electricitya month, now this329degrees,Julyis hotter, I use the air conditioning more, actually left329degrees, this model is good, I was to buy two, is not to buy the wrong, the product is good, the effect is really good, you have to give you a favorable review	Taobao Marketplace, a Chinese website for online shopping
Xiao Cui Technology	King of Power Saving	This works great, bought it a couple times	Taobao Marketplace, a Chinese website for online shopping
Xiao Cui Technology	King of Power Saving	Looks and feels great in red, will save on electric bill, will have to use it for a few months to find out.	Taobao Marketplace, a Chinese website for online shopping
Xiao Cui Technology	King of Power Saving	This high-power is not bad on, the first few months are1000degrees so, I casually brush in Taobao to this product, I bought back to try, I used a month after this month left 736degrees, scared me, I also called to the grid to confirm, really is736degrees, the effect is very good, and today bought a go back to the [illegible]	Taobao Marketplace, a Chinese website for online shopping
Xiao Cui Technology	King of Power Saving	After using it for so long, it's not bad, this little is ok, at least savemore than100per monthis the minimum,	Taobao Marketplace, a Chinese website for online shopping
Xiao Cui Technology	King of Power Saving	Just received and used for a while, only to know the effect, then evaluation	Taobao Marketplace, a Chinese website for online shopping
Xiao Cui Technology	King of Power Saving	Received, hope it works out well	Taobao Marketplace, a Chinese website for online shopping
Xiao Cui Technology	King of Power Saving	Let's give it a good review first! Don't know if it will be energy efficient yet, let's see and talk about it. Followed by in to review over.	Taobao Marketplace, a Chinese website for online shopping
Xiao Cui Technology	King of Power Saving	Electricity saves well. Know how much it costs a month	Taobao Marketplace, a Chinese website for online shopping
Xiao Cui Technology	King of Power Saving	Good seller, fast delivery, I am to help relatives buy, this power saver on electronic products to play a role in current stabilization, to protect the use of electronic products, but also to save electricity, the effect is quite obvious. It is worth buying.	Taobao Marketplace, a Chinese website for online shopping
Xiao Cui Technology	King of Power Saving	I bought three products, only left this one, this product can really save me electricity, the effect is up to my expectations, not bad!	Taobao Marketplace, a Chinese website for online shopping
Xiao Cui Technology	King of Power Saving	Satisfied, 5 stars	Taobao Marketplace, a Chinese website for online shopping
Xiao Cui Technology	King of Power Saving	My friend here to buy, gave me a, the effect is really good,eh, I can saveabout100dollarsthis month.	Taobao Marketplace, a Chinese website for online shopping
Xiao Cui Technology	King of Power Saving	I'm on a smart meter, and I save about30%a month, which I think is pretty good	Taobao Marketplace, a Chinese website for online shopping
Xiao Cui Technology	King of Power Saving	Since I bought this, the electricity bill can really be less, it's better, it's better	Taobao Marketplace, a Chinese website for online shopping
Xiao Cui Technology	King of Power Saving	I bought this high power for the second time, my effect is very obvious, a month down, I can be quite a lot less, has been the second purchase, the	Taobao Marketplace, a Chinese website for online shopping
Xiao Cui Technology	King of Power Saving	My brother-in-law asked me to buy, he said a month down the effect is very good, so he introduced me to buy, I have received, believe my brother-in-law's recommendation	Taobao Marketplace, a Chinese website for online shopping
Xiao Cui Technology	King of Power Saving	This high-power effect is really good, has bought another one back home, power-saving effect is also available	Taobao Marketplace, a Chinese website for online shopping
Xiao Cui Technology	King of Power Saving	My brother asked me to buy it, he used it himself and it worked, today I paid my electricity bill and it was much less than the month before, it's amazing!	Taobao Marketplace, a Chinese website for online shopping

Xiao Cui Technology	King of Power Saving	This works well, happy with the results	Taobao Marketplace, a Chinese website for online shopping
Xiao Cui Technology	King of Power Saving	It works well.	Taobao Marketplace, a Chinese website for online shopping
Xiao Cui Technology	King of Power Saving	It's pretty good. It works well.	Taobao Marketplace, a Chinese website for online shopping
Xiao Cui Technology	King of Power Saving	Finally got my electric bill down. I've bought so many products and this one is the most noticeable.	Taobao Marketplace, a Chinese website for online shopping
Xiao Cui Technology	King of Power Saving	The effect is very good, I have been running the air conditioning this month, the electricity bill is not much, this can be Oh, the effect is really good!	Taobao Marketplace, a Chinese website for online shopping
Xiao Cui Technology	King of Power Saving	two, already the second time to buy, although not too much, the calculation is about100degrees can be30degreesless, about this	Taobao Marketplace, a Chinese website for online shopping
Xiao Cui Technology	King of Power Saving	an excellent	Taobao Marketplace, a Chinese website for online shopping
Xiao Cui Technology	King of Power Saving	Second purchase, my husband said this gadget works pretty well, anyway, the electricity is not paid by me, he said it's good just can save money!	Taobao Marketplace, a Chinese website for online shopping
Xiao Cui Technology	King of Power Saving	I used for a long time again to review, this power saving effect is good, I save about 30% of power so, that is,100yuan can save about30yuan, honestly I, I came here to tell the truth, to avoid others stepping on the pit, the effect of this product is very good, you can buy	Taobao Marketplace, a Chinese website for online shopping
Xiao Cui Technology	King of Power Saving	It matches the description.	Taobao Marketplace, a Chinese website for online shopping
Xiao Cui Technology	King of Power Saving	Just received to start using, wait to use a period of time to follow up to see results	Taobao Marketplace, a Chinese website for online shopping
Xiao Cui Technology	King of Power Saving	This month's electricity bill has been paid, compared to the less quite a lot, not bad, although not a lot, first to my usual tomore than300yuan, and nowmore than210, this I am quite surprised, but also very satisfied with the electricity can be saved	Taobao Marketplace, a Chinese website for online shopping
Xiao Cui Technology	King of Power Saving	Without further ado, I ordered another one yesterday, I turned on the air conditioning, and it works well, this thing saves energy pretty well.	Taobao Marketplace, a Chinese website for online shopping
Xiao Cui Technology	King of Power Saving	This month's electricity bill has been paid, compared to the less quite a lot, not bad, although not a lot, first to my usual tomore than300yuan, and nowmore than210, this I am quite surprised, but also very satisfied with the electricity can be saved	Taobao Marketplace, a Chinese website for online shopping
Xiao Cui Technology	King of Power Saving	Boss recommended high power is not bad, I do not believe that this can save electricity in front, has paid the electricity bill twice before coming to the evaluation, this can really save electricity, I used to have beenabout300yuan, these two months with this high power, two times are almost180here, this time173yuan, served the effect of saving electricity is not bad	Taobao Marketplace, a Chinese website for online shopping
Xiao Cui Technology	King of Power Saving	Although it is not the40%power saving rateas claimed by the vendors, I calculate that28%-30%power saving rate is still available	Taobao Marketplace, a Chinese website for online shopping
Xiao Cui Technology	King of Power Saving	very happy	Taobao Marketplace, a Chinese website for online shopping
Xiao Cui Technology	King of Power Saving	Power Saving King is genuine, the summer came to the power more powerful, so buy a Power Saving King, we want to buy can rest assured that you can buy!	Taobao Marketplace, a Chinese website for online shopping
Xiao Cui Technology	King of Power Saving	I can comment again after some time	Taobao Marketplace, a Chinese website for online shopping
Xiao Cui Technology	King of Power Saving	Delete Xu's bad review	Taobao Marketplace, a Chinese website for online shopping
Xiao Cui Technology	King of Power Saving	(of an unmarried couple) be close	Taobao Marketplace, a Chinese website for online shopping
Xiao Cui Technology	King of Power Saving	I have bought a few times, I also recommended to my friends, all said that the effect, the product is not bad,the effectquite obvious	Taobao Marketplace, a Chinese website for online shopping
Xiao Cui Technology	King of Power Saving	This effect is good, good use only to give you a follow-up review, I bought a front back home, my father said that this month the effect is good, open the air conditioning electricity bill is also less, I rented a room is also good, this effect is very satisfied!	Taobao Marketplace, a Chinese website for online shopping
Xiao Cui Technology	King of Power Saving	Shit, it really works. Last month's electricity was$286, but after a month of using the power saver, I have$195left this month, which is not bad, so I'm counting on you now	Taobao Marketplace, a Chinese website for online shopping
Xiao Cui Technology	King of Power Saving	I've been using this since I've been using it, I'm now paying a lot less for my electricity bill, I have to give you a good review because I'm satisfied, give you a follow up review, it's really nice	Taobao Marketplace, a Chinese website for online shopping
Xiao Cui Technology	King of Power Saving	I run a processing plant. I got this one and it works pretty good.	Taobao Marketplace, a Chinese website for online shopping
Xiao Cui Technology	King of Power Saving	Today I paid the electric bill, a few dozen dollars less, anyway, it is not expensive, it is already very good, the boss said to buy one more will be better, I have already placed an order	Taobao Marketplace, a Chinese website for online shopping
Xiao Cui Technology	King of Power Saving	I've been paying for this for a long time, it really saves electricity, and the effect is quite good, I think it's quite satisfactory.	Taobao Marketplace, a Chinese website for online shopping
Xiao Cui Technology	King of Power Saving	I'm a renter, usually it'sabout $400a month, this time I got two, I paid$276this month, it's pretty good	Taobao Marketplace, a Chinese website for online shopping
Xiao Cui Technology	King of Power Saving	Wow, can Oh, the weather is hot open air conditioning effect is really good, this product is good, has been used for so long, moreok. power saving effect is not bad.	Taobao Marketplace, a Chinese website for online shopping
Xiao Cui Technology	King of Power Saving	I bought99dollars of high power, the first few months of electricity ismore than1280, did not use this product aremore than1200, this month I used, and now934yuan, you can compare, to buy99dollars this, this I personally think it is better, the effect is still satisfied	Taobao Marketplace, a Chinese website for online shopping
Xiao Cui Technology	King of Power Saving	This product is not bad Oh, I used so long, the effect is good Oh, I pull down the power saver electricity costs up, I plugged the power saver, power long less a lot, not bad!	Taobao Marketplace, a Chinese website for online shopping
Xiao Cui Technology	King of Power Saving	Second purchase, my husband said this gadget works pretty well, anyway, the electricity is not paid by me, he said it's good just can save money!	Taobao Marketplace, a Chinese website for online shopping
Xiao Cui Technology	King of Power Saving	This product works well and really saves a lot of power, it's really good	Taobao Marketplace, a Chinese website for online shopping

Xiao Cui Technology	King of Power Saving	Without further ado, the electric bill came down,$102less this month, personal satisfaction, it counts okay, give you a good review	Taobao Marketplace, a Chinese website for online shopping
Xiao Cui Technology	King of Power Saving	I paid my electric bill this time, it works well, I'm satisfied.	Taobao Marketplace, a Chinese website for online shopping
Xiao Cui Technology	King of Power Saving	Bought two of them, the effect is kind of okay, it's okay, it works well	Taobao Marketplace, a Chinese website for online shopping
Xiao Cui Technology	King of Power Saving	Without further ado, I've been using it for ages and it's better for the AC fridge,	Taobao Marketplace, a Chinese website for online shopping
Xiao Cui Technology	King of Power Saving	My dad asked me to buy one, but after I paid the bill, I asked and he said it was a lot less this month, so he asked me to buy another one.	Taobao Marketplace, a Chinese website for online shopping
Xiao Cui Technology	King of Power Saving	I've bought several, but this one has the best quality, it's amazing, it's about36% ofthe	Taobao Marketplace, a Chinese website for online shopping
Xiao Cui Technology	King of Power Saving	I bought a high-power, has been compared to two months, basically I am very satisfied, can save quite good, and then come back later to follow up on the evaluation, this model can be purchased	Taobao Marketplace, a Chinese website for online shopping
Xiao Cui Technology	King of Power Saving	This is a good product.	Taobao Marketplace, a Chinese website for online shopping
Xiao Cui Technology	King of Power Saving	I am half a year to pay, before so much, I use to save electrical appliances withmore than4months, this time the calculation can be about half less, because the amount of electricity I am more sensitive, all sure that this product has effect	Taobao Marketplace, a Chinese website for online shopping
Xiao Cui Technology	King of Power Saving	Wow, this works. This works. This works.	Taobao Marketplace, a Chinese website for online shopping
Xiao Cui Technology	King of Power Saving	I am opening a store, the use of electricity is relatively large, this is really effective, before not yet used, a month usuallymore than400yuan of electricity, after buying two power savers, this time only used195.75yuan, the effect is still quite satisfied	Taobao Marketplace, a Chinese website for online shopping
Xiao Cui Technology	King of Power Saving	I turn on the air conditioning effect is good, before turning on the air conditioning uses a lot of electricity, now with this energy-saving appliances, now turn on the air conditioning seems to be less electricity, the air conditioning does not consume so much electricity, now the technology is quite	Taobao Marketplace, a Chinese website for online shopping
Xiao Cui Technology	King of Power Saving	didn't use the saver last month, but this month I could have gottenover$130less with the saver, and the results are obvious.	Taobao Marketplace, a Chinese website for online shopping
Xiao Cui Technology	King of Power Saving	This thing works pretty well, I asked my friend to buy one too	Taobao Marketplace, a Chinese website for online shopping
Xiao Cui Technology	King of Power Saving	been proven to work, I saved about40%powerwhen I bought two, here's how I did it, here's a tip	Taobao Marketplace, a Chinese website for online shopping
Xiao Cui Technology	King of Power Saving	I introduced this to my coworkers, this works well, and my coworkers bought it too!	Taobao Marketplace, a Chinese website for online shopping
Xiao Cui Technology	King of Power Saving	My husband bought it back and I was just talking about him when he bought it, now the power is down and it$139less than last month, so I'll give you a good review	Taobao Marketplace, a Chinese website for online shopping
Xiao Cui Technology	King of Power Saving	This is really good, anyway, I have good results, paid the electricity bill is very obvious, I like this product	Taobao Marketplace, a Chinese website for online shopping
Xiao Cui Technology	King of Power Saving	My friend here to buy, gave me a, the effect is really good,eh, I can saveabout100dollarsthis month.	Taobao Marketplace, a Chinese website for online shopping
Xiao Cui Technology	King of Power Saving	Without further ado, this product power saving effect can be, count very satisfied, you can buy, this is good!	Taobao Marketplace, a Chinese website for online shopping
Xiao Cui Technology	King of Power Saving	I bought it to use for housing, my boyfriend said he had some money missing this month and said he wanted me to buy another one	Taobao Marketplace, a Chinese website for online shopping
Xiao Cui Technology	King of Power Saving	I've been trying it for a long time, it does work, I can get less power every month, it's really good, this product can be	Taobao Marketplace, a Chinese website for online shopping
Xiao Cui Technology	King of Power Saving	Already paid the electric bill, overall it's pretty good, my best friend introduced me to it	Taobao Marketplace, a Chinese website for online shopping
Xiao Cui Technology	King of Power Saving	I've had a lot less power since I bought this, and it's really working... nice... oh, this is good...	Taobao Marketplace, a Chinese website for online shopping
Xiao Cui Technology	King of Power Saving	There are a lot of big appliances at home, the air conditioner is very expensive in summer, I bought this in front of me, the effect on the air conditioner is very good, let me explain, I bought three in one breath, I use more electricity, a month downabout200dollarsless, very satisfied	Taobao Marketplace, a Chinese website for online shopping
Xiao Cui Technology	King of Power Saving	This model is not bad, really can this power-saving effect is good, give you a follow-up review, I hope the store is getting better and better	Taobao Marketplace, a Chinese website for online shopping
Xiao Cui Technology	King of Power Saving	I'm on a smart meter, and I save about30%a month, which I think is pretty good	Taobao Marketplace, a Chinese website for online shopping
Xiao Cui Technology	King of Power Saving	Since the purchase of two such high power, power down a lot, usuallyabout5,000yuan of electricity, the two times to pay the electricity bill shocked me, the remaining4,063yuan, there are really power-saving products, really can be, the effect of which I'm very satisfied, and would like to buy a few to my milk	Taobao Marketplace, a Chinese website for online shopping
Xiao Cui Technology	King of Power Saving	Since the purchase of two such high power, power down a lot, usuallyabout5,000yuan of electricity, the two times to pay the electricity bill shocked me, the remaining4,063yuan, there are really power-saving products, really can be, the effect of which I'm very satisfied, and would like to buy a few to my milk [illegible]	Taobao Marketplace, a Chinese website for online shopping
Xiao Cui Technology	King of Power Saving	Buyer does not comment within15days	Taobao Marketplace, a Chinese website for online shopping
Xiao Cui Technology	King of Power Saving	Not useful at all, used for a month, more power consumption, is a IQ tax, customer service attitude is not good, the problem is not given to solve, everyone careful to buy! It's just a scam.	Taobao Marketplace, a Chinese website for online shopping
Xiao Cui Technology	King of Power Saving	Buyer does not comment within15days	Taobao Marketplace, a Chinese website for online shopping
Xiao Cui Technology	King of Power Saving	and ineffective😤😤😤😤😤😤😤😤😤😤😤	Taobao Marketplace, a Chinese website for online shopping
Xiao Cui Technology	King of Power Saving	Buyer does not comment within15days	Taobao Marketplace, a Chinese website for online shopping

Xiao Cui Technology	King of Power Saving	Very good results, very satisfied	Taobao Marketplace, a Chinese website for online shopping
Xiao Cui Technology	King of Power Saving	Buyer does not comment within15days	Taobao Marketplace, a Chinese website for online shopping
Xiao Cui Technology	King of Power Saving	It's useless, don't fall for it, the more you use it the more electricity you get, it's too much of a scam, a scammer	Taobao Marketplace, a Chinese website for online shopping
Xiao Cui Technology	King of Power Saving	Buyer does not comment within15days	Taobao Marketplace, a Chinese website for online shopping
Xiao Cui Technology	King of Power Saving	I am very satisfied with the effect. I am an electrician, originally do not believe in this product, after using it, although it did not meet my expectations, but the effect is still very good, a month down to saveone-third of the electricity, now the technology is still possible	Taobao Marketplace, a Chinese website for online shopping
Xiao Cui Technology	King of Power Saving	Buyer does not comment within15days	Taobao Marketplace, a Chinese website for online shopping
Xiao Cui Technology	King of Power Saving	I've been using it for a month with no results at all, and the positive reviews are all blown out of proportion by cash coupons!	Taobao Marketplace, a Chinese website for online shopping
Xiao Cui Technology	King of Power Saving	Buyer does not comment within15days	Taobao Marketplace, a Chinese website for online shopping
Xiao Cui Technology	King of Power Saving	I bought this and now it works fine with the AC on, when I didn't use it itwas $600a month, now it's almost$500,00,$100less all.	Taobao Marketplace, a Chinese website for online shopping
Xiao Cui Technology	King of Power Saving	Buyer does not comment within15days	Taobao Marketplace, a Chinese website for online shopping
Xiao Cui Technology	King of Power Saving	Electricity bill direct policy1200deduction Is it saving flow or adding more electricity Electricity company sent, right?	Taobao Marketplace, a Chinese website for online shopping
Xiao Cui Technology	King of Power Saving	Buyer does not comment within15days	Taobao Marketplace, a Chinese website for online shopping
Xiao Cui Technology	King of Power Saving	It doesn't save electricity at all, and plugging this thing in seems to cost more electricity.	Taobao Marketplace, a Chinese website for online shopping
Xiao Cui Technology	King of Power Saving	Buyer does not comment within15days	Taobao Marketplace, a Chinese website for online shopping
Xiao Cui Technology	King of Power Saving	It's only been a couple days and it's broken.	Taobao Marketplace, a Chinese website for online shopping
Xiao Cui Technology	King of Power Saving	Buyer does not comment within15days	Taobao Marketplace, a Chinese website for online shopping
Xiao Cui Technology	King of Power Saving	Taobao more than ten years met the most disgusting business used a month did not see a little effect to find him to ask for a return At that time it wasAugust3 gave an address and then said that I tried a is a cheat thing does not reduce the I9.13to the return said that did not go through the consent of his sent to the past is not to give a refund copywriting half a year no matter how to use the package return package replacement.back of the people pay attention to⚠⚠⚠ avoid pit avoid pit avoid pit	Taobao Marketplace, a Chinese website for online shopping
Xiao Cui Technology	King of Power Saving	Buyer does not comment within15days	Taobao Marketplace, a Chinese website for online shopping
Xiao Cui Technology	King of Power Saving	Just a scam, no use at all, but also said that the useless can be returned, a month a little effect is not, and now do not give a refund! Just a scam, this store can still open down?	Taobao Marketplace, a Chinese website for online shopping
Xiao Cui Technology	King of Power Saving	Buyer does not comment within15days	Taobao Marketplace, a Chinese website for online shopping
Xiao Cui Technology	King of Power Saving	It's not power efficient at all. I'm returning it for a refund.	Taobao Marketplace, a Chinese website for online shopping
Xiao Cui Technology	King of Power Saving	Buyer does not comment within15days	Taobao Marketplace, a Chinese website for online shopping
Xiao Cui Technology	King of Power Saving	System default comments	Taobao Marketplace, a Chinese website for online shopping
Xiao Cui Technology	Love Home Intelligent Energy Saver	I just received it. I don't know if it's any good.	Taobao Marketplace, a Chinese website for online shopping
Xiao Cui Technology	Love Home Intelligent Energy Saver	I've been using this for a while, this is okay, acceptable, this is good	Taobao Marketplace, a Chinese website for online shopping
Xiao Cui Technology	Love Home Intelligent Energy Saver	I've tested it for20days and it works out to be aboutathirdlesspower, which is fine	Taobao Marketplace, a Chinese website for online shopping
Xiao Cui Technology	Love Home Intelligent Energy Saver	This is not a bad comparison for a while, it works pretty well	Taobao Marketplace, a Chinese website for online shopping
Xiao Cui Technology	Love Home Intelligent Energy Saver	I have bought a few times, I also recommended to my friends, all said that the effect, the product is not bad,the effectquite obvious	Taobao Marketplace, a Chinese website for online shopping
Xiao Cui Technology	Love Home Intelligent Energy Saver	I bought a high-power, the effect is not bad, really can, save almost one-third, continue to use, and then come back later to follow up!	Taobao Marketplace, a Chinese website for online shopping
Xiao Cui Technology	Love Home Intelligent Energy Saver	The electricity is paid for. It's working well.	Taobao Marketplace, a Chinese website for online shopping
Xiao Cui Technology	Love Home Intelligent Energy Saver	Since the purchase of two such high power, power down a lot, usuallyabout5,000yuan of electricity, the two times to pay the electricity bill shocked me, the remaining4,063yuan, but also really power-saving products, really can, the effect I am very satisfied, want to buy a few to my milk tea store to buy to	Taobao Marketplace, a Chinese website for online shopping
Xiao Cui Technology	Love Home Intelligent Energy Saver	I bought800-2500degree one, I'm satisfied, it used toover $1900, I bought the black one and used it for a month, it was$1573 forthe month, wow Damn, it works fine, count it as very satisfying! I hope the store sells better and better, I'll use it for another month and come back to give you a follow up review	Taobao Marketplace, a Chinese website for online shopping
Xiao Cui Technology	Love Home Intelligent Energy Saver	I deducted the electricity bill one day at a time, after using this can be less, the effect is kind of ideal	Taobao Marketplace, a Chinese website for online shopping

Xiao Cui Technology	Love Home Intelligent Energy Saver	This is better, it saves electricity	Taobao Marketplace, a Chinese website for online shopping
Xiao Cui Technology	Love Home Intelligent Energy Saver	Damn, it's really effective. Last month's electricity was$316, but after using the power saver for a month,$195left this month, which is not bad, so I'm counting on you now	Taobao Marketplace, a Chinese website for online shopping
Xiao Cui Technology	Love Home Intelligent Energy Saver	It's great. Good value for money.	Taobao Marketplace, a Chinese website for online shopping
Xiao Cui Technology	Love Home Intelligent Energy Saver	This can be oh, every month can be less, high power is not bad, real to give everyone advice, to buy buy this, this I am more satisfied	Taobao Marketplace, a Chinese website for online shopping
Xiao Cui Technology	Love Home Intelligent Energy Saver	System default comments	Taobao Marketplace, a Chinese website for online shopping
Xiao Cui Technology	Love Home Intelligent Energy Saver	Bought several products, tried several products, this product has the effect, usuallymore than280, this time155, has been used for a few months, counting the awesome effect is what I want	Taobao Marketplace, a Chinese website for online shopping
Xiao Cui Technology	Love Home Intelligent Energy Saver	Buyer does not comment within15days	Taobao Marketplace, a Chinese website for online shopping
Xiao Cui Technology	Love Home Intelligent Energy Saver	This is not bad, the effect is quite obvious, with the air conditioning effect is okay	Taobao Marketplace, a Chinese website for online shopping
Xiao Cui Technology	Love Home Intelligent Energy Saver	Buyer does not comment within15days	Taobao Marketplace, a Chinese website for online shopping
Xiao Cui Technology	Love Home Intelligent Energy Saver	This is nice. I've really saved on electricity this month.	Taobao Marketplace, a Chinese website for online shopping
Xiao Cui Technology	butler	not have	Taobao Marketplace, a Chinese website for online shopping
Enoch Grace Boutique	Copper Power Saving Specialist	Usually consume a lot of electricity, this side of the friend introduced to use a period of time, the feeling is very good, can save electricity. It's worth it!	Taobao Marketplace, a Chinese website for online shopping
Enoch Grace Boutique	Copper Power Saving Specialist	Very good is genuine,, logistics is very fast, recommended!	Taobao Marketplace, a Chinese website for online shopping
Enoch Grace Boutique	Copper Power Saving Specialist	Pro. Baby has received the appearance, the quality is very good, high price, energy saving is good, very satisfied, worth promoting	Taobao Marketplace, a Chinese website for online shopping
Enoch Grace Boutique	Copper Power Saving Specialist	Pro, the baby has received, appearance, quality is very good, cost-effective, energy-saving effect is good, very satisfied, know the promotion of the only product	Taobao Marketplace, a Chinese website for online shopping
Enoch Grace Boutique	Copper Power Saving Specialist	Received and looking forward to the results.	Taobao Marketplace, a Chinese website for online shopping
Enoch Grace Boutique	Copper Power Saving Specialist	Goods received awaiting verification.	Taobao Marketplace, a Chinese website for online shopping
Enoch Grace Boutique	Copper Power Saving Specialist	Just received, the effect of how not yet know, use and then evaluate	Taobao Marketplace, a Chinese website for online shopping
Enoch Grace Boutique	Copper Power Saving Specialist	Product quality is excellent, very good shopping experience	Taobao Marketplace, a Chinese website for online shopping
Enoch Grace Boutique	Copper Power Saving Specialist	Power-saving special, home power-saving magic weapon, the effect is surprisingly good	Taobao Marketplace, a Chinese website for online shopping
Enoch Grace Boutique	Copper Power Saving Specialist	I just received it. I'll comment after I use it.	Taobao Marketplace, a Chinese website for online shopping
Enoch Grace Boutique	Copper Power Saving Specialist	Packaging is very good. Product is formal. Documents are complete. Product certificate, manual, use, precautions are all there. Also bought insurance, good service attitude. Product quality is stable, used for a few days, power saving effect to be determined. But stabilize the voltage is very obvious, only3volts change. Appearance design is beautiful. Also sent the fuse, thoughtful. All5points praise.	Taobao Marketplace, a Chinese website for online shopping
Enoch Grace Boutique	Copper Power Saving Specialist	Haven't used it yet don't know the effect	Taobao Marketplace, a Chinese website for online shopping
Enoch Grace Boutique	Copper Power Saving Specialist	Received the baby, the same as described, the credit is real, after the installation of a significant power saving phenomenon, a pleasant shopping, highly recommended to buy this style.	Taobao Marketplace, a Chinese website for online shopping
Enoch Grace Boutique	Copper Power Saving Specialist	Saver appearance design is very nice, screen display voltage at a glance, received the goods on the use, looking forward to the effect.	Taobao Marketplace, a Chinese website for online shopping
Enoch Grace Boutique	Copper Power Saving Specialist	The item looks great, I'll comment after a month of passing it around, thanks.	Taobao Marketplace, a Chinese website for online shopping
Enoch Grace Boutique	Copper Power Saving Specialist	Exquisite, good quality, effective, practical and swift.	Taobao Marketplace, a Chinese website for online shopping
Enoch Grace Boutique	Copper Power Saving Specialist	Not bad.	Taobao Marketplace, a Chinese website for online shopping
Enoch Grace Boutique	Copper Power Saving Specialist	acceptable	Taobao Marketplace, a Chinese website for online shopping
Enoch Grace Boutique	Copper Power Saving Specialist	It's very good and works great, I'll buy it again next time!	Taobao Marketplace, a Chinese website for online shopping
Enoch Grace Boutique	Copper Power Saving Specialist	The collection quality is very good.	Taobao Marketplace, a Chinese website for online shopping
Enoch Grace Boutique	Copper Power Saving Specialist	Voltage display error	Taobao Marketplace, a Chinese website for online shopping
Enoch Grace Boutique	Copper Power Saving Specialist	Baby received, the appearance of the design is good, is in use, I hope to help me save a little electricity. Waiting for a period of time to come back to the review	Taobao Marketplace, a Chinese website for online shopping
Enoch Grace Boutique	Copper Power Saving Specialist	s ready,butI haven't used it yet.I don't know	Taobao Marketplace, a Chinese website for online shopping

Enoch Grace Boutique	Copper Power Saving Specialist	Really good quality, good power saving features.	Taobao Marketplace, a Chinese website for online shopping
Enoch Grace Boutique	Copper Power Saving Specialist	Nice, nice, bought it and used it, it works exceptionally well!	Taobao Marketplace, a Chinese website for online shopping
Enoch Grace Boutique	Copper Power Saving Specialist	Customer service attitude is very good, patience to answer questions, the product looks good, now first use the back to see the power savings less than when the time to comment!	Taobao Marketplace, a Chinese website for online shopping
Enoch Grace Boutique	Copper Power Saving Specialist	I've bought one before, used it for a couple years, changed where I live and bought another one, and the results are really good	Taobao Marketplace, a Chinese website for online shopping
Enoch Grace Boutique	Copper Power Saving Specialist	This is a great product to buy!	Taobao Marketplace, a Chinese website for online shopping
Enoch Grace Boutique	Copper Power Saving Specialist	Express delivery to the home very good quality is very good oh wait for the use of the follow-up evaluation	Taobao Marketplace, a Chinese website for online shopping
Enoch Grace Boutique	Copper Power Saving Specialist	The service was excellent.	Taobao Marketplace, a Chinese website for online shopping
Enoch Grace Boutique	Copper Power Saving Specialist	Smart Compensation Saver received, looks sturdy, love it. Am using it and will be buying it again next time.	Taobao Marketplace, a Chinese website for online shopping
Enoch Grace Boutique	Copper Power Saving Specialist	It's a good weight. I'll use it for a while and see what happens.	Taobao Marketplace, a Chinese website for online shopping
Enoch Grace Boutique	Copper Power Saving Specialist	Bought once good use this is the second box are exquisite worth buying	Taobao Marketplace, a Chinese website for online shopping
Enoch Grace Boutique	Copper Power Saving Specialist	Usedmore than2months to evaluate, power saving effect is really good, the first month is not obvious, thesecondis much better, looking forward to durability, the business is said to be2years of warranty	Taobao Marketplace, a Chinese website for online shopping
Enoch Grace Boutique	Copper Power Saving Specialist	Satisfied满满满满满满满满满满满，刚用期待效果	Taobao Marketplace, a Chinese website for online shopping
Enoch Grace Boutique	Copper Power Saving Specialist	Shipped quickly, easy to install, in use……Looking forward to saving power	Taobao Marketplace, a Chinese website for online shopping
Enoch Grace Boutique	Copper Power Saving Specialist	I just used it and look forward to using it to save power	Taobao Marketplace, a Chinese website for online shopping
Enoch Grace Boutique	Copper Power Saving Specialist	Very happy with the purchase, easy to use. Just buy it home and plug it into an outlet. Recommended to buy with confidence!	Taobao Marketplace, a Chinese website for online shopping
Enoch Grace Boutique	Copper Power Saving Specialist	Received the goods, the effect can not be seen until the use of a period of time.	Taobao Marketplace, a Chinese website for online shopping
Enoch Grace Boutique	Copper Power Saving Specialist	Good stuff at a good price works great fine workmanship	Taobao Marketplace, a Chinese website for online shopping
Enoch Grace Boutique	Copper Power Saving Specialist	The shipping was fast, the quality of the saver was excellent, and the customer was patient.	Taobao Marketplace, a Chinese website for online shopping
Enoch Grace Boutique	Copper Power Saving Specialist	Goods have been received, the appearance of good-looking, expect to save electricity, first give a good review!	Taobao Marketplace, a Chinese website for online shopping
Enoch Grace Boutique	Copper Power Saving Specialist	It breaks in half a year.	Taobao Marketplace, a Chinese website for online shopping
Enoch Grace Boutique	Copper Power Saving Specialist	Received on the plug and use Dan do not know how the effect, a month after the follow-up evaluation!	Taobao Marketplace, a Chinese website for online shopping
Enoch Grace Boutique	Copper Power Saving Specialist	Waiting for the effect	Taobao Marketplace, a Chinese website for online shopping
Enoch Grace Boutique	Copper Power Saving Specialist	Bold**	Taobao Marketplace, a Chinese website for online shopping
Enoch Grace Boutique	Copper Power Saving Specialist	Second time I have purchased this product and will continue to buy it if I need it in the future.	Taobao Marketplace, a Chinese website for online shopping
Enoch Grace Boutique	Copper Power Saving Specialist	Baby received, logistics is very fast, the description matches, more satisfied, praise, recommended!	Taobao Marketplace, a Chinese website for online shopping
Enoch Grace Boutique	Copper Power Saving Specialist	The workmanship is exquisite, the materials used are exquisite, to be used to really save electricity, then follow up the evaluation.	Taobao Marketplace, a Chinese website for online shopping
Enoch Grace Boutique	Copper Power Saving Specialist	A few months, did not see any effect, rather than saving electricity, it is better to spend money to buy his product money to buy electricity	Taobao Marketplace, a Chinese website for online shopping
Enoch Grace Boutique	Copper Power Saving Specialist	The merchandise is great.	Taobao Marketplace, a Chinese website for online shopping
Enoch Grace Boutique	Copper Power Saving Specialist	Already use it, after a month to know the effect, whether it saves electricity, looking forward to look forward to	Taobao Marketplace, a Chinese website for online shopping
Enoch Grace Boutique	Copper Power Saving Specialist	Very satisfied with the purchase Very easy to use Looking forward to energy saving and power saving After all, it is easier to save money than to earn money	Taobao Marketplace, a Chinese website for online shopping
Enoch Grace Boutique	Copper Power Saving Specialist	This product is really different from the one I bought before. I won't know how effective it is until I use it.	Taobao Marketplace, a Chinese website for online shopping
Enoch Grace Boutique	Copper Power Saving Specialist	I'll give it a good review for now and come back after I've used it!	Taobao Marketplace, a Chinese website for online shopping
Enoch Grace Boutique	Copper Power Saving Specialist	Very happy with the purchase, easy to use. Just buy it home and plug it into the socket. I recommend it to my coworkers!	Taobao Marketplace, a Chinese website for online shopping
Enoch Grace Boutique	Copper Power Saving Specialist	It's really quite good, it feels good to try it for the first time	Taobao Marketplace, a Chinese website for online shopping

Enoch Grace Boutique	Copper Power Saving Specialist	I can't see the results. I want to return it.	Taobao Marketplace, a Chinese website for online shopping
Enoch Grace Boutique	Copper Power Saving Specialist	Saving power received, unpacking has been plugged in the use, and so use a period of time in the follow-up.	Taobao Marketplace, a Chinese website for online shopping
Enoch Grace Boutique	Copper Power Saving Specialist	Saving50%is an exaggeration	Taobao Marketplace, a Chinese website for online shopping
Enoch Grace Boutique	Copper Power Saving Specialist	Received and installed, let's see how it works out	Taobao Marketplace, a Chinese website for online shopping
Enoch Grace Boutique	Copper Power Saving Specialist	It should feel pretty good!!!	Taobao Marketplace, a Chinese website for online shopping
Enoch Grace Boutique	Copper Power Saving Specialist	Very happy with it, love it very much!	Taobao Marketplace, a Chinese website for online shopping
Enoch Grace Boutique	Copper Power Saving Specialist	I've been using it for almost a month before I can comment, not an IQ tax. Before I didn't use it, it was 15, 16, even 20 dollars a day, after I used it it was 10, 9 dollars a day. There is a refrigerator in the house. The air conditioner is the most costly electricity, the rice cooker seller said it did not work. But this also saves a lot. Too much electricity per month can be used. Less estimated not much use.	Taobao Marketplace, a Chinese website for online shopping
Enoch Grace Boutique	Copper Power Saving Specialist	It really can. Not much to say.	Taobao Marketplace, a Chinese website for online shopping
Enoch Grace Boutique	Copper Power Saving Specialist	Logistics is very fast, received the goods to open looks very beautiful, good quality.	Taobao Marketplace, a Chinese website for online shopping
Enoch Grace Boutique	Copper Power Saving Specialist	Baby has received has been installed for a few days, the effect is still there. Later to follow up on the evaluation	Taobao Marketplace, a Chinese website for online shopping
Enoch Grace Boutique	Copper Power Saving Specialist	Power saving treasure received a month, today to give the evaluation of the business, Dad has been using this thing before, said it is very good use	Taobao Marketplace, a Chinese website for online shopping
Enoch Grace Boutique	Copper Power Saving Specialist	2together, received the goods for more than a month, only to evaluate, just now recommended friends link up	Taobao Marketplace, a Chinese website for online shopping
Enoch Grace Boutique	Copper Power Saving Specialist	May, $323electricity, June, $1,184for electricity. What's the point of saving electricity? You're paying taxes, aren't you	Taobao Marketplace, a Chinese website for online shopping
Enoch Grace Boutique	Copper Power Saving Specialist	dissatisfied	Taobao Marketplace, a Chinese website for online shopping
Enoch Grace Boutique	Copper Power Saving Specialist	Testing	Taobao Marketplace, a Chinese website for online shopping
Enoch Grace Boutique	Copper Power Saving Specialist	incompetent	Taobao Marketplace, a Chinese website for online shopping
Enoch Grace Boutique	Copper Power Saving Specialist	Other people introduced good use, with the attitude of trying to buy a, has been used in the store, the seller service is very good, after consulting the detailed answer, praise a, look forward to the use of effect	Taobao Marketplace, a Chinese website for online shopping
Enoch Grace Boutique	Copper Power Saving Specialist	I got it back and unpacked it, it looks great with the gold color casing, looking forward to using it afterward	Taobao Marketplace, a Chinese website for online shopping
Enoch Grace Boutique	Copper Power Saving Specialist	Goods received good quality energy-saving special	Taobao Marketplace, a Chinese website for online shopping
Enoch Grace Boutique	Copper Power Saving Specialist	Exquisite, ready to install near the meter, home air dryer, freezer, refrigerator, induction cooker is too much power consumption, buy this product should be good, two months after the follow-up evaluation.	Taobao Marketplace, a Chinese website for online shopping
Enoch Grace Boutique	Copper Power Saving Specialist	Second time I bought it, not bad, hope it works	Taobao Marketplace, a Chinese website for online shopping
Enoch Grace Boutique	Copper Power Saving Specialist	I used to buy a great power saver and now I'm buying another one	Taobao Marketplace, a Chinese website for online shopping
Enoch Grace Boutique	Copper Power Saving Specialist	Received, the appearance is relatively delicate, immediately put into use, look forward to the effect and then follow up!	Taobao Marketplace, a Chinese website for online shopping
Enoch Grace Boutique	Copper Power Saving Specialist	The saver is nice, and when plugged in, the induction cooktop seems to go faster. It should work, good review!	Taobao Marketplace, a Chinese website for online shopping
Enoch Grace Boutique	Copper Power Saving Specialist	Used it for a few days before I came to comment, frying is much faster. Will buy another one in a few days!	Taobao Marketplace, a Chinese website for online shopping
Enoch Grace Boutique	Copper Power Saving Specialist	1	Taobao Marketplace, a Chinese website for online shopping
Enoch Grace Boutique	Copper Power Saving Specialist	Used it for a few days and really saved a lot of electricity. It works well.	Taobao Marketplace, a Chinese website for online shopping
Enoch Grace Boutique	Copper Power Saving Specialist	Fine workmanship, pleasing to the eye on the outside, looking forward to the effect, tight packaging, fast logistics. Thank you for customer service patience and professional explanation.	Taobao Marketplace, a Chinese website for online shopping
Enoch Grace Boutique	Copper Power Saving Specialist	Just received the goods, the texture is good, try first, come back later	Taobao Marketplace, a Chinese website for online shopping
Enoch Grace Boutique	Copper Power Saving Specialist	Second purchase, nice and useful, energy saving little helper.	Taobao Marketplace, a Chinese website for online shopping
Enoch Grace Boutique	Copper Power Saving Specialist	Works pretty well for a while. Not bad.	Taobao Marketplace, a Chinese website for online shopping
Enoch Grace Boutique	Copper Power Saving Specialist	Okay, okay, okay, okay.	Taobao Marketplace, a Chinese website for online shopping
Enoch Grace Boutique	Copper Power Saving Specialist	To be honest, the quality of this workmanship and packaging is really better than the dozens of dollars I bought before, really a penny a penny, the effect is okay!	Taobao Marketplace, a Chinese website for online shopping
Enoch Grace Boutique	Copper Power Saving Specialist	Inadvertently found such a product, really only unexpected products, summer air conditioning high electricity costs, buy over to try, looked at a few, but also this is relatively good!	Taobao Marketplace, a Chinese website for online shopping

Enoch Grace Boutique	Copper Power Saving Specialist	Around friends have bought, recommended to me also bought anyway is not expensive, I did not expect to save electricity effect is quite good, this month's electricity bill than last month's electricity bill is more than20degreesless, surprised me, hurry up and also recommended to your friends around	Taobao Marketplace, a Chinese website for online shopping
Enoch Grace Boutique	Copper Power Saving Specialist	I received the goods and tried it, whether it saves electricity or not, wait and see	Taobao Marketplace, a Chinese website for online shopping
Enoch Grace Boutique	Copper Power Saving Specialist	I'll review it when I use it.	Taobao Marketplace, a Chinese website for online shopping
Enoch Grace Boutique	Copper Power Saving Specialist	Installation was easy, I mounted it directly on top of the exhaust and it didn't affect the appliance, good value for money, good	Taobao Marketplace, a Chinese website for online shopping
Enoch Grace Boutique	Copper Power Saving Specialist	The seller is very considerate	Taobao Marketplace, a Chinese website for online shopping
Enoch Grace Boutique	Copper Power Saving Specialist	I've been using it for a while and it doesn't save power at all, it uses more power. Never talk nonsense.	Taobao Marketplace, a Chinese website for online shopping
Enoch Grace Boutique	Copper Power Saving Specialist	Power saving experts have been listening to friends say Ting good use, every month can save not money it should be good, satisfied!	Taobao Marketplace, a Chinese website for online shopping
Enoch Grace Boutique	Copper Power Saving Specialist	Good review good review good review good review!	Taobao Marketplace, a Chinese website for online shopping
Enoch Grace Boutique	Copper Power Saving Specialist	Can show voltage Looking forward to using the results!	Taobao Marketplace, a Chinese website for online shopping
Enoch Grace Boutique	Copper Power Saving Specialist	In use and looking forward to the results.	Taobao Marketplace, a Chinese website for online shopping
Enoch Grace Boutique	Copper Power Saving Specialist	Very nice, looks noble	Taobao Marketplace, a Chinese website for online shopping
Enoch Grace Boutique	Copper Power Saving Specialist	Hope it works well!	Taobao Marketplace, a Chinese website for online shopping
Enoch Grace Boutique	Copper Power Saving Specialist	Merchants after-sales service attitude is very good, oh, during the fuse is broken, previously sent forget where to put, the merchants without saying a word on the fuse sent, for your service praise!	Taobao Marketplace, a Chinese website for online shopping
Enoch Grace Boutique	Copper Power Saving Specialist	Quality is very good, the seller shipped very quickly, very good, the voltage is very stable, cost-effective, worth buying!	Taobao Marketplace, a Chinese website for online shopping
Enoch Grace Boutique	Copper Power Saving Specialist	The price is very affordable, used for more than a month, no problems at all, the quality is very good, and the power consumption is also less than last month, praise!	Taobao Marketplace, a Chinese website for online shopping
Enoch Grace Boutique	Copper Power Saving Specialist	Saver received, stable voltage, very sturdy, works well, reasonable price, satisfaction	Taobao Marketplace, a Chinese website for online shopping
Enoch Grace Boutique	Copper Power Saving Specialist	Second purchase, logistics is fast, and power saving effective before buying a second time, will continue to pay attention!	Taobao Marketplace, a Chinese website for online shopping
Enoch Grace Boutique	Copper Power Saving Specialist	Logistics is fast, convenient and compact to use, five-star praise!	Taobao Marketplace, a Chinese website for online shopping
Enoch Grace Boutique	Copper Power Saving Specialist	Received the goods, the big brand is can, the quality is very good, trustworthy, recommended to you!	Taobao Marketplace, a Chinese website for online shopping
Enoch Grace Boutique	Copper Power Saving Specialist	Received at ！！！！！！！！！！	Taobao Marketplace, a Chinese website for online shopping
Enoch Grace Boutique	Copper Power Saving Specialist	It doesn't work, it doesn't save any money, and it's not based on science. Don't pay the IQ tax.	Taobao Marketplace, a Chinese website for online shopping
Enoch Grace Boutique	Copper Power Saving Specialist	The product is sturdy, the casing is metal, good quality, looking forward to good results!	Taobao Marketplace, a Chinese website for online shopping
Enoch Grace Boutique	Copper Power Saving Specialist	Looks classy, can tell if the voltage is high or low hope it works well	Taobao Marketplace, a Chinese website for online shopping
Enoch Grace Boutique	Copper Power Saving Specialist	I bought one3years ago and it works well, soon the summer is here and the air conditioner consumes more power, so I bought one more to strengthen the effect	Taobao Marketplace, a Chinese website for online shopping
Enoch Grace Boutique	Copper Power Saving Specialist	Baby received, looks very good in all aspects of craftsmanship, worth the purchase, the	Taobao Marketplace, a Chinese website for online shopping
Enoch Grace Boutique	Copper Power Saving Specialist	Baby received, I love it and satisfied!	Taobao Marketplace, a Chinese website for online shopping
Enoch Grace Boutique	Copper Power Saving Specialist	New intelligent high-power power saver received today has been used, good, you can rest assured that the purchase of	Taobao Marketplace, a Chinese website for online shopping
Enoch Grace Boutique	Copper Power Saving Specialist	Fine workmanship, the appearance of more than expected, plugged in and used one night, check the accounts, immediately see the effect, five-star praise, after a while to follow up!	Taobao Marketplace, a Chinese website for online shopping
Enoch Grace Boutique	Copper Power Saving Specialist	It's a real IQ tax.	Taobao Marketplace, a Chinese website for online shopping
Enoch Grace Boutique	Copper Power Saving Specialist	Fast shipping, energy saver home good, power saving effect is good, satisfied with the	Taobao Marketplace, a Chinese website for online shopping
Enoch Grace Boutique	Copper Power Saving Specialist	Friends introduced, said to use a very good, I also buy a try to see, received looks like the quality of workmanship is good, I look for the electrician to connect a socket installed on the main gate, so it should be more effective, after a month of follow-up!	Taobao Marketplace, a Chinese website for online shopping
Enoch Grace Boutique	Copper Power Saving Specialist	Baby received, the quality is particularly good, cost-effective, fast delivery, packaging is also very good, energy saving effect is also very good, very satisfied.	Taobao Marketplace, a Chinese website for online shopping
Enoch Grace Boutique	Copper Power Saving Specialist	Can be merchant description matches.	Taobao Marketplace, a Chinese website for online shopping
Enoch Grace Boutique	Copper Power Saving Specialist	an excellent	Taobao Marketplace, a Chinese website for online shopping

Enoch Grace Boutique	Copper Power Saving Specialist	I bought one last year and it worked well, summer is coming, I'll buy another one to install in the air-conditioned room	Taobao Marketplace, a Chinese website for online shopping
Enoch Grace Boutique	Copper Power Saving Specialist	Unintentionally see this thing, compared to a lot of home, only this evaluation is relatively good, received so far seems to be good, a little role, and then evaluate later	Taobao Marketplace, a Chinese website for online shopping
Enoch Grace Boutique	Copper Power Saving Specialist	Baby has been received and loved it. Will buy again. Will recommend to friends.	Taobao Marketplace, a Chinese website for online shopping
Enoch Grace Boutique	Copper Power Saving Specialist	I'll comment after I've used it for a while	Taobao Marketplace, a Chinese website for online shopping
Enoch Grace Boutique	Copper Power Saving Specialist	It's a scam, this thing has nothing to do with saving electricity, and the final conclusion:This thing is just used by scammers to cheat people!	Taobao Marketplace, a Chinese website for online shopping
Enoch Grace Boutique	Copper Power Saving Specialist	My Home Saver😌😌😌😌😌	Taobao Marketplace, a Chinese website for online shopping
Enoch Grace Boutique	Copper Power Saving Specialist	The logistics is super super fast! The quality is also very good, indeed value for money	Taobao Marketplace, a Chinese website for online shopping
Enoch Grace Boutique	Copper Power Saving Specialist	It looks good from the outside, but you have to use it for a few days to know how effective it is.	Taobao Marketplace, a Chinese website for online shopping
Enoch Grace Boutique	Copper Power Saving Specialist	long time ago I bought a, used a few years or good, the effect is quite satisfied, looking at the summer is here, the air conditioning is also more, and then buy a, this appearance quality, weight are much better than my previous, on the still don't know if it can be, to the summer to catch up with the next	Taobao Marketplace, a Chinese website for online shopping
Enoch Grace Boutique	Copper Power Saving Specialist	These savers work very well and are a great value for money.	Taobao Marketplace, a Chinese website for online shopping
Enoch Grace Boutique	Copper Power Saving Specialist	This saver works very well, is great value for money and is cheap!	Taobao Marketplace, a Chinese website for online shopping
Enoch Grace Boutique	Copper Power Saving Specialist	Received immediately used, and now also know how many degrees, before opening the home water heater, the living room lights will be darker, now is not the case, should have a certain stabilization of the voltage effect, follow-up follow-up evaluation	Taobao Marketplace, a Chinese website for online shopping
Enoch Grace Boutique	Copper Power Saving Specialist	very good	Taobao Marketplace, a Chinese website for online shopping
Enoch Grace Boutique	Copper Power Saving Specialist	This is really too😄,🎁packaging is very beautiful, baby workmanship is relatively fine, the business is particularly good ❤, there are problems are dealt with in a timely manner👍, it is indeed a quite satisfactory online shopping, will come back again😘	Taobao Marketplace, a Chinese website for online shopping
Enoch Grace Boutique	Copper Power Saving Specialist	The saver was well packaged and the power savings were good.	Taobao Marketplace, a Chinese website for online shopping
Enoch Grace Boutique	Copper Power Saving Specialist	an excellent	Taobao Marketplace, a Chinese website for online shopping
Enoch Grace Boutique	Copper Power Saving Specialist	The quality of is very good, very satisfied	Taobao Marketplace, a Chinese website for online shopping
Enoch Grace Boutique	Copper Power Saving Specialist	During the epidemic, the courier logistics is still quite fast, the next day received, in the customer service patience guide, quickly installed, let the electrician in the main switch connected to a socket plugged in, it is very convenient and safe, it is worth recommending!	Taobao Marketplace, a Chinese website for online shopping
Enoch Grace Boutique	Copper Power Saving Specialist	Beautifully packaged product certificates are authentic, very like, immediately used up foot power on it. Well, repurchase kudos👍	Taobao Marketplace, a Chinese website for online shopping
Enoch Grace Boutique	Copper Power Saving Specialist	First time buying this online, nice very nice	Taobao Marketplace, a Chinese website for online shopping
Enoch Grace Boutique	Copper Power Saving Specialist	Very nice. Much better than expected.	Taobao Marketplace, a Chinese website for online shopping
Enoch Grace Boutique	Copper Power Saving Specialist	At first I was worried about whether it would affect other electrical appliances in my home, and then used a few days later found that the worry is superfluous, or quite safe, recognize the brand is the most important, looking forward to the power-saving effect is great!	Taobao Marketplace, a Chinese website for online shopping
Enoch Grace Boutique	Copper Power Saving Specialist	The second one I bought, I haven't measured it exactly and precisely, but I see that the daily degrees seem to be somewhat useful. Anyway, I chose to believe it when I bought it and was more than satisfied with the great customer service.	Taobao Marketplace, a Chinese website for online shopping
Enoch Grace Boutique	Copper Power Saving Specialist	Cost-effective, does not take up space,more than100dollars to eat a meal is gone, this thing looks good quality, should be used for five or six years is not a problem, right, looking forward to saving electricity	Taobao Marketplace, a Chinese website for online shopping
Enoch Grace Boutique	Copper Power Saving Specialist	This is very easy to install, plug in the socket and it's ready, for people like me who know nothing about electrical appliances, it's really great, cost-effective and safe, the key is that it saves electricity, it's worth recommending!	Taobao Marketplace, a Chinese website for online shopping
Enoch Grace Boutique	Copper Power Saving Specialist	compared and saved some, about$60+less in2, which is great, when it works	Taobao Marketplace, a Chinese website for online shopping
Enoch Grace Boutique	Copper Power Saving Specialist	Received immediately used, found that you can display the voltage of the home, amazing, mainly very safe, small size, small children are not easy to touch, great, friends with the family said the effect is good, look forward to!	Taobao Marketplace, a Chinese website for online shopping
Enoch Grace Boutique	Copper Power Saving Specialist	Usually home justover100degrees, this winter will give me a burst meter, the electricity bill straight up, this winter is too cold, open the heating and air conditioning every day, and friends spit after the introduction of this power saver for me, the power saving effect is very obvious, it is worth recommending, I hope to help more friends.	Taobao Marketplace, a Chinese website for online shopping
Enoch Grace Boutique	Copper Power Saving Specialist	I'm very satisfied, I ordered another one for my parents in my hometown, it's not easy to make money these days, so if I can save a little bit, it's a little bit!	Taobao Marketplace, a Chinese website for online shopping
Enoch Grace Boutique	Copper Power Saving Specialist	Unintentionally found that there is such a product, specially bought to try, follow-up evaluation, the product looks quite good, design and packaging workmanship must be a lot of cost Unlike other products look is poor quality	Taobao Marketplace, a Chinese website for online shopping
Enoch Grace Boutique	Copper Power Saving Specialist	Home with how many degrees a day on the phone seems to have the effect is really obvious surprisingly less9degrees yesterday appliances are the same with the same time to see some of the said no effect may be different circuits and electrical appliances it product I am recommended	Taobao Marketplace, a Chinese website for online shopping
Enoch Grace Boutique	Copper Power Saving Specialist	Can Oh, look at the quality is good, look forward to power saving effect	Taobao Marketplace, a Chinese website for online shopping
Enoch Grace Boutique	Copper Power Saving Specialist	Used2days, there is a small meter at home, compared to the previous day almost18degrees, installed now12columns of13degreesa dayaccording to this day to save a5degrees or so, or very satisfied, is the hope that it can be maintained	Taobao Marketplace, a Chinese website for online shopping
Enoch Grace Boutique	Copper Power Saving Specialist	We found the courier.	Taobao Marketplace, a Chinese website for online shopping

Enoch Grace Boutique	Copper Power Saving Specialist	The product is really very good ah, the appearance of exquisite, and the same description of the manual, now just take delivery, do not know how the power-saving effect, if you want to buy, you can try to choose this	Taobao Marketplace, a Chinese website for online shopping
Enoch Grace Boutique	Copper Power Saving Specialist	It's good. It does work.	Taobao Marketplace, a Chinese website for online shopping
Enoch Grace Boutique	Copper Power Saving Specialist	Used for a few months and then evaluated, it does work, have repurchased for the second time. When you turn on the water heater, the number on the top goes down, and when you turn off the water heater, the number goes back up, so it's very obvious that it saves electricity!	Taobao Marketplace, a Chinese website for online shopping
Enoch Grace Boutique	Copper Power Saving Specialist	Baby received, can not wait to press on, looking forward to the effect, the appearance of the atmosphere on the top grade, fine workmanship, is genuine, looking forward to working together again!	Taobao Marketplace, a Chinese website for online shopping
Enoch Grace Boutique	Copper Power Saving Specialist	Second purchase, works great	Taobao Marketplace, a Chinese website for online shopping
Enoch Grace Boutique	Copper Power Saving Specialist	Baby received product quality appearance design is very good, but hopefully the effect is also very good.	Taobao Marketplace, a Chinese website for online shopping
Enoch Grace Boutique	Copper Power Saving Specialist	No effect at all, long-term red light is on instead of increasing the electricity bill	Taobao Marketplace, a Chinese website for online shopping
Enoch Grace Boutique	Copper Power Saving Specialist	In use, always220+ pretty stable, looking forward to power reduction.	Taobao Marketplace, a Chinese website for online shopping
Enoch Grace Boutique	Copper Power Saving Specialist	Good material, good quality, original genuine! Fine workmanship, clear display, just installed to show238volts, installed a few days, now showsabout228volts, the voltage should be smoothed down, effective, very practical, affordable, worth buying! Trustworthy!	Taobao Marketplace, a Chinese website for online shopping
Enoch Grace Boutique	Copper Power Saving Specialist	Baby received, look good workmanship. Looking forward to the power saving effect. Many friends are waiting to see the effect. Ready to start. Merchant shipping speed no comparison! Too fast!	Taobao Marketplace, a Chinese website for online shopping
Enoch Grace Boutique	Copper Power Saving Specialist	Received the goods, the goods are very good effect to a month or two to know	Taobao Marketplace, a Chinese website for online shopping
Enoch Grace Boutique	Copper Power Saving Specialist	Can't see the effect	Taobao Marketplace, a Chinese website for online shopping
Enoch Grace Boutique	Copper Power Saving Specialist	Baby received, customer service patience and thoughtful, something atmospheric, expect to save electricity, such as power-saving effect is good to evaluate again.	Taobao Marketplace, a Chinese website for online shopping
Enoch Grace Boutique	Copper Power Saving Specialist	Just arrived and installed to try, hope it works, things look good, customer service is also very patient.	Taobao Marketplace, a Chinese website for online shopping
Enoch Grace Boutique	Copper Power Saving Specialist	It doesn't work. I won't buy it again.	Taobao Marketplace, a Chinese website for online shopping
Enoch Grace Boutique	Copper Power Saving Specialist	an excellent	Taobao Marketplace, a Chinese website for online shopping
Enoch Grace Boutique	Copper Power Saving Specialist	a follow-up evaluation after the event	Taobao Marketplace, a Chinese website for online shopping
Enoch Grace Boutique	Copper Power Saving Specialist	have the National GridApp onmy phoneand have been looking at the power usage every day and have also carefully compared it to plugging this thing in$5to$7a dayin power usage and unplugging this thing$4to$6a dayin power usage, not only does it not save but it also consumes power! This thing has weight just a shell! Those who talk nonsense and say that the effect is very good, do you have a good conscience?	Taobao Marketplace, a Chinese website for online shopping
Enoch Grace Boutique	Copper Power Saving Specialist	The product has just arrived at home just used, do not know how the energy-saving situation, the appearance of the atmosphere looks like the quality can be. I hope to achieve energy-saving effect.	Taobao Marketplace, a Chinese website for online shopping
Enoch Grace Boutique	Copper Power Saving Specialist	No power savings at all, doubled my electric bill after a month of use, poor poor poor!	Taobao Marketplace, a Chinese website for online shopping
Enoch Grace Boutique	Copper Power Saving Specialist	Power Saving Expert received, plugged in and looking forward to saving power!	Taobao Marketplace, a Chinese website for online shopping
Enoch Grace Boutique	Copper Power Saving Specialist	Already installed and looking forward to the results!	Taobao Marketplace, a Chinese website for online shopping
Enoch Grace Boutique	Copper Power Saving Specialist	Received, first praise! Use it for a while and come back with a follow up review! Let's see how the real effect is.	Taobao Marketplace, a Chinese website for online shopping
Enoch Grace Boutique	Copper Power Saving Specialist	It's a rip off, don't buy it people! I used it until today,January12,2022, less than a month head to head, and the electric bill was$50expensive than it used to be, so spiteful that I decided to unplug it and not use it. Paid the IQ tax.	Taobao Marketplace, a Chinese website for online shopping
Enoch Grace Boutique	Copper Power Saving Specialist	Workmanship is good, the size is suitable, save power to be time to test, first to praise later follow-up.	Taobao Marketplace, a Chinese website for online shopping
Enoch Grace Boutique	Copper Power Saving Specialist	Try The seller is very good and very patient	Taobao Marketplace, a Chinese website for online shopping
Enoch Grace Boutique	Copper Power Saving Specialist	Really effective, so amazing ah, the electricity bill every day cell phone can be seen, I hope the effect will last, so far very satisfied with the	Taobao Marketplace, a Chinese website for online shopping
Enoch Grace Boutique	Copper Power Saving Specialist	The effect is very good, really too good to use, save a lot, very sorry indeed very save	Taobao Marketplace, a Chinese website for online shopping
Enoch Grace Boutique	Copper Power Saving Specialist	The goods have arrived Very satisfied with the logistics is also very fast	Taobao Marketplace, a Chinese website for online shopping
Enoch Grace Boutique	Copper Power Saving Specialist	The electric bill went up about ten dollars after a month or so of use	Taobao Marketplace, a Chinese website for online shopping
Enoch Grace Boutique	Copper Power Saving Specialist	It arrived and the merchant answered questions and was very reassuring! Good review!	Taobao Marketplace, a Chinese website for online shopping
Enoch Grace Boutique	Copper Power Saving Specialist	Intelligent compensation to achieve power saving effect, looking forward to the effect	Taobao Marketplace, a Chinese website for online shopping
Enoch Grace Boutique	Copper Power Saving Specialist	Well-packed, fast logistics, looks very atmospheric, wait for the power saving after use and then follow up.	Taobao Marketplace, a Chinese website for online shopping
Enoch Grace Boutique	Copper Power Saving Specialist	Baby received, the quality is very good, the seller has a very good service attitude, thank you for the seller's recommendation, next time also patronize!	Taobao Marketplace, a Chinese website for online shopping

Enoch Grace Boutique	Copper Power Saving Specialist	The product packaging is particularly good, the digital display is clear, the effect is in use, point a👍	Taobao Marketplace, a Chinese website for online shopping
Enoch Grace Boutique	Copper Power Saving Specialist	Repurchased many times, works great!	Taobao Marketplace, a Chinese website for online shopping
Enoch Grace Boutique	Copper Power Saving Specialist	Baby has been installed, easy to install, the seller also sent two protection tube. More looking forward to the effect of power saving, specific wait for a period of time after the use of a follow-up comment!	Taobao Marketplace, a Chinese website for online shopping
Enoch Grace Boutique	Copper Power Saving Specialist	Baby received the appearance of the beautiful atmosphere very much like	Taobao Marketplace, a Chinese website for online shopping
Enoch Grace Boutique	Copper Power Saving Specialist	Very nice because it's good quality.	Taobao Marketplace, a Chinese website for online shopping
Enoch Grace Boutique	Copper Power Saving Specialist	The quality is great and the results are noticeable. It does work. It's great.	Taobao Marketplace, a Chinese website for online shopping
Enoch Grace Boutique	Copper Power Saving Specialist	Baby received, try a week before coming to the evaluation, the quality is very good, er more power-saving	Taobao Marketplace, a Chinese website for online shopping
Enoch Grace Boutique	Copper Power Saving Specialist	Baby has been received, this is the second time I bought it and it works well. Give it to a friend.	Taobao Marketplace, a Chinese website for online shopping
Enoch Grace Boutique	Copper Power Saving Specialist	I've been using it for a month and it's working.	Taobao Marketplace, a Chinese website for online shopping
Enoch Grace Boutique	Copper Power Saving Specialist	I'm using it, looking forward to the results plus purchase	Taobao Marketplace, a Chinese website for online shopping
Enoch Grace Boutique	Copper Power Saving Specialist	Don't know how well it works wait a month	Taobao Marketplace, a Chinese website for online shopping
Enoch Grace Boutique	Copper Power Saving Specialist	No feeling bad	Taobao Marketplace, a Chinese website for online shopping
Enoch Grace Boutique	Copper Power Saving Specialist	Bought a long time, today on the flat price is worth buying the bezel, are aluminum alloy feel heavy inside a good new technology, this time seems to be a bit of power savings. I hope to keep saving	Taobao Marketplace, a Chinese website for online shopping
Enoch Grace Boutique	Copper Power Saving Specialist	What**energy saving,use a month after the electricity bill than the previous month also used more than 10 yuan,than the original also used nearly20yuan of money, electricity with the previous month is the same use,so remove the use of	Taobao Marketplace, a Chinese website for online shopping
Enoch Grace Boutique	Copper Power Saving Specialist	Goods to the home, although it is not used it first to the customer service attitude favorable, good to use and then follow up!	Taobao Marketplace, a Chinese website for online shopping
Enoch Grace Boutique	Copper Power Saving Specialist	Highly recommended by my dad, he said it's effective! I'll test it for a while and come back with a follow up review.	Taobao Marketplace, a Chinese website for online shopping
Enoch Grace Boutique	Copper Power Saving Specialist	Baby is very beautiful, easy to install and convenient, plug and play, customer service is very good, five stars!	Taobao Marketplace, a Chinese website for online shopping
Enoch Grace Boutique	Copper Power Saving Specialist	Baby received it, used for a period of time, very good, very practical, will continue to pay attention to drops	Taobao Marketplace, a Chinese website for online shopping
Enoch Grace Boutique	Copper Power Saving Specialist	Expect great results!	Taobao Marketplace, a Chinese website for online shopping
Enoch Grace Boutique	Copper Power Saving Specialist	I used it when I got home and look forward to the results!	Taobao Marketplace, a Chinese website for online shopping
Enoch Grace Boutique	Copper Power Saving Specialist	The saver finally arrived, the first time I checked the kitchen, just a pity that there is no unused socket, the crusher unplugged and pressed it, the saver screen shows238, I do not know if it is high or not?	Taobao Marketplace, a Chinese website for online shopping
Enoch Grace Boutique	Copper Power Saving Specialist	Logistics delivery is very fast, the seller's service attitude is very good, the product is consistent with the picture, very good shopping.	Taobao Marketplace, a Chinese website for online shopping
Enoch Grace Boutique	Copper Power Saving Specialist	Good installation, recommend family and friends to buy	Taobao Marketplace, a Chinese website for online shopping
Enoch Grace Boutique	Copper Power Saving Specialist	Received this brand new, authentic and great! Looking forward to the results. Bookmarked.	Taobao Marketplace, a Chinese website for online shopping
Enoch Grace Boutique	Copper Power Saving Specialist	I don't know if it's an IQ tax, use it for a few months and see, my family went searching for some reviews and they all told me to return it, I still want to try and see the results	Taobao Marketplace, a Chinese website for online shopping
Enoch Grace Boutique	Copper Power Saving Specialist	Looks good.	Taobao Marketplace, a Chinese website for online shopping
Enoch Grace Boutique	Copper Power Saving Specialist	The baby has been received and used right away, beautiful and generous. It should be very practical.	Taobao Marketplace, a Chinese website for online shopping
Enoch Grace Boutique	Copper Power Saving Specialist	Bao received the second purchase, it does save power, sell service attitude is good.	Taobao Marketplace, a Chinese website for online shopping
Enoch Grace Boutique	Copper Power Saving Specialist	I used it and thought it was great, the quality of the item was good and the shipping was pretty fast, all in all it was awesome!	Taobao Marketplace, a Chinese website for online shopping
Enoch Grace Boutique	Copper Power Saving Specialist	Not bad ah, looks very good big, South drifting ah, the first choice of rental ah, not by the landlord pit	Taobao Marketplace, a Chinese website for online shopping
Enoch Grace Boutique	Copper Power Saving Specialist	Multiple comparisons, finally chose this one, looking forward to the results, will follow up!	Taobao Marketplace, a Chinese website for online shopping
Enoch Grace Boutique	Copper Power Saving Specialist	Just used to be observed, the store is very enthusiastic	Taobao Marketplace, a Chinese website for online shopping
Enoch Grace Boutique	Copper Power Saving Specialist	Received the goods Feeling okay Looking forward to the effect	Taobao Marketplace, a Chinese website for online shopping
Enoch Grace Boutique	Copper Power Saving Specialist	The power savings are a little bit better, but not as great as they say.	Taobao Marketplace, a Chinese website for online shopping

Enoch Grace Boutique	Copper Power Saving Specialist	Received item, in use, will follow up!	Taobao Marketplace, a Chinese website for online shopping
Enoch Grace Boutique	Copper Power Saving Specialist	I'm using it and looking forward to really saving electricity.	Taobao Marketplace, a Chinese website for online shopping
Enoch Grace Boutique	Copper Power Saving Specialist	Good, good, very good, this is my second purchase here, used for two years, to save a few dozen dollars a month	Taobao Marketplace, a Chinese website for online shopping
Enoch Grace Boutique	Copper Power Saving Specialist	I've been busy with work lately and have been using it for a few days now, and the power has dwindled a bit. Baby is good, ready to get another one for the old family	Taobao Marketplace, a Chinese website for online shopping
Enoch Grace Boutique	Copper Power Saving Specialist	Have used it and look forward to the follow up.	Taobao Marketplace, a Chinese website for online shopping
Enoch Grace Boutique	Copper Power Saving Specialist	Used to come here to evaluate, not much to say, just2words, really can save	Taobao Marketplace, a Chinese website for online shopping
Enoch Grace Boutique	Copper Power Saving Specialist	Don't hesitate to buy wow! Doesn't feel cheap, very high end and exceptionally great!	Taobao Marketplace, a Chinese website for online shopping
Enoch Grace Boutique	Copper Power Saving Specialist	I've always bought from brick and mortar stores before, but this time I chose to buy online, not only is it authentic, but it's also affordable!😉works super well! It's also very aesthetically pleasing! Wall to wall recommendation! Will repurchase again if needed!	Taobao Marketplace, a Chinese website for online shopping
Enoch Grace Boutique	Copper Power Saving Specialist	Very good baby, looked at a lot of brands or feel at ease with this one, it is good, did not let me down, recommended to everyone.	Taobao Marketplace, a Chinese website for online shopping
Enoch Grace Boutique	Copper Power Saving Specialist	I'll tell you in a couple months!don't know yet if it's power saving or?If it's good, I'll buy it for the house next door!	Taobao Marketplace, a Chinese website for online shopping
Enoch Grace Boutique	Copper Power Saving Specialist	Non-satisfactory Good service Authentic	Taobao Marketplace, a Chinese website for online shopping
Enoch Grace Boutique	Copper Power Saving Specialist	Received the stuff, the quality is quite good, looking forward to the effect	Taobao Marketplace, a Chinese website for online shopping
Enoch Grace Boutique	Copper Power Saving Specialist	I feel it's more or less effective, I've reducedelectricity bill by more than200 dollarsthis month	Taobao Marketplace, a Chinese website for online shopping
Enoch Grace Boutique	Copper Power Saving Specialist	I received the goods, the old look is quite delicate. Try to use it, and then add comments if it's good ～.	Taobao Marketplace, a Chinese website for online shopping
Enoch Grace Boutique	Copper Power Saving Specialist	The power saver has been received very well, looking forward to the results in a month👍	Taobao Marketplace, a Chinese website for online shopping
Enoch Grace Boutique	Copper Power Saving Specialist	I have received the goods, I have purchased one before and felt good, I bought another one.	Taobao Marketplace, a Chinese website for online shopping
Enoch Grace Boutique	Copper Power Saving Specialist	It's very substantial when you pick it up and feels very high quality, I've already bought one before and it still felt really good, so I bought another one.	Taobao Marketplace, a Chinese website for online shopping
Enoch Grace Boutique	Copper Power Saving Specialist	Store attitude is very good,the middle of the exchange once,very cooperative. Now just use. Waiting for a month effect how to comment again	Taobao Marketplace, a Chinese website for online shopping
Enoch Grace Boutique	Copper Power Saving Specialist	Goods have been received tightly packed, logistics is very fast, the boss introduced a very clear and detailed, with a good and then go to evaluate.	Taobao Marketplace, a Chinese website for online shopping
Enoch Grace Boutique	Copper Power Saving Specialist	Logistics is very fast, the boss introduced a very detailed and clear, well-packed, and will patronize the store in the future!	Taobao Marketplace, a Chinese website for online shopping
Enoch Grace Boutique	Copper Power Saving Specialist	very good	Taobao Marketplace, a Chinese website for online shopping
Enoch Grace Boutique	Copper Power Saving Specialist	Received it a couple days ago and it looks good, happy with it	Taobao Marketplace, a Chinese website for online shopping
Enoch Grace Boutique	Copper Power Saving Specialist	Goods have been received, the appearance of the design is more gear, first try to use it, have the effect and then follow up.	Taobao Marketplace, a Chinese website for online shopping
Enoch Grace Boutique	Copper Power Saving Specialist	I do not know whether it is good or not, continue to pay attention to the quality of the look is OK and workmanship	Taobao Marketplace, a Chinese website for online shopping
Enoch Grace Boutique	Copper Power Saving Specialist	Effectiveness to be known after long term use. The material is good.	Taobao Marketplace, a Chinese website for online shopping
Enoch Grace Boutique	Copper Power Saving Specialist	Received it, very good quality, very happy with the workmanship. Use it for a while and then make an evaluation.	Taobao Marketplace, a Chinese website for online shopping
Enoch Grace Boutique	Copper Power Saving Specialist	favorable criticism	Taobao Marketplace, a Chinese website for online shopping
Enoch Grace Boutique	Copper Power Saving Specialist	Carefully compared to make sure it works, thanks to the seller, already repurchased a few units	Taobao Marketplace, a Chinese website for online shopping
Enoch Grace Boutique	Copper Power Saving Specialist	Baby received service attitude is very good	Taobao Marketplace, a Chinese website for online shopping
Enoch Grace Boutique	Copper Power Saving Specialist	Received the goods, the logistics is very fast first time to buy and use it to evaluate	Taobao Marketplace, a Chinese website for online shopping
Enoch Grace Boutique	Copper Power Saving Specialist	I don't know if I can save it, but I'll use it first.	Taobao Marketplace, a Chinese website for online shopping
Enoch Grace Boutique	Copper Power Saving Specialist	I received the baby and couldn't wait to plug it in and use it, it's superb.	Taobao Marketplace, a Chinese website for online shopping
Enoch Grace Boutique	Copper Power Saving Specialist	Received the saver took it home couldn't wait to use it! Gave it a 5 star review!	Taobao Marketplace, a Chinese website for online shopping
Enoch Grace Boutique	Copper Power Saving Specialist	Received the new smart high power saver! Took it home and couldn't wait to use it! Give five-star praise Oh!	Taobao Marketplace, a Chinese website for online shopping

Enoch Grace Boutique	Copper Power Saving Specialist	Very good quality👍	Taobao Marketplace, a Chinese website for online shopping
Enoch Grace Boutique	Copper Power Saving Specialist	I have received the baby and am using it now, so I'll come back with a follow-up review after a while.	Taobao Marketplace, a Chinese website for online shopping
Enoch Grace Boutique	Copper Power Saving Specialist	Just received the goods and plugged it in, friends introduced to buy it	Taobao Marketplace, a Chinese website for online shopping
Enoch Grace Boutique	Copper Power Saving Specialist	Visual inspection is okay, let's wait to pay the electricity bill after the follow-up review, I hope it's not paid IQ tax	Taobao Marketplace, a Chinese website for online shopping
Enoch Grace Boutique	Copper Power Saving Specialist	The stuff is good, I hope people hurry up and buy it	Taobao Marketplace, a Chinese website for online shopping
Enoch Grace Boutique	Copper Power Saving Specialist	Good quality, looking forward to the effect. Seller has great service and fast shipping.	Taobao Marketplace, a Chinese website for online shopping
Enoch Grace Boutique	Copper Power Saving Specialist	It doesn't feel like it's working, the meter is pretty much the same every day as it was before	Taobao Marketplace, a Chinese website for online shopping
Enoch Grace Boutique	Copper Power Saving Specialist	Energy saver appearance exquisite workmanship good, good quality, wait and see how the effect.	Taobao Marketplace, a Chinese website for online shopping
Enoch Grace Boutique	Copper Power Saving Specialist	Good review!	Taobao Marketplace, a Chinese website for online shopping
Enoch Grace Boutique	Copper Power Saving Specialist	It is authentic, trust the merchant, the baby has a great texture and the price is moderate, unlimited repurchase!	Taobao Marketplace, a Chinese website for online shopping
Enoch Grace Boutique	Copper Power Saving Specialist	Looking forward to the results after use	Taobao Marketplace, a Chinese website for online shopping
Enoch Grace Boutique	Copper Power Saving Specialist	It seems to really have some effect, I don't have much hope to save much, much more, it's good to be able to save, compare the electricity bill later and then look again	Taobao Marketplace, a Chinese website for online shopping
Enoch Grace Boutique	Copper Power Saving Specialist	Buy four or five and get free electricity?	Taobao Marketplace, a Chinese website for online shopping
Enoch Grace Boutique	Copper Power Saving Specialist	Is there an energy saving effect?	Taobao Marketplace, a Chinese website for online shopping
Enoch Grace Boutique	Copper Power Saving Specialist	Is there a perpetual motion machine in the world? Are there loads that don't consume electricity?	Taobao Marketplace, a Chinese website for online shopping
Enoch Grace Boutique	Copper Power Saving Specialist	I'd like to ask what diploma would buy this?	Taobao Marketplace, a Chinese website for online shopping
Enoch Grace Boutique	Copper Power Saving Specialist	Does it work? One for each floor?	Taobao Marketplace, a Chinese website for online shopping
Enoch Grace Boutique	Copper Power Saving Specialist	I think it's called a voltage regulator! How does this thing save electricity? It doesn't feel that amazing.	Taobao Marketplace, a Chinese website for online shopping
Enoch Grace Boutique	Copper Power Saving Specialist	Is this for 2-phase power?	Taobao Marketplace, a Chinese website for online shopping
Enoch Grace Boutique	Copper Power Saving Specialist	I'd like to askhow to look at thisif mine is235	Taobao Marketplace, a Chinese website for online shopping
Enoch Grace Boutique	Copper Power Saving Specialist	Has anyone measured with a multimeter what size capacitor is in there?	Taobao Marketplace, a Chinese website for online shopping
Enoch Grace Boutique	Copper Power Saving Specialist	Does it work? Does it save power?	Taobao Marketplace, a Chinese website for online shopping
Enoch Grace Boutique	Copper Power Saving Specialist	Is it good to install?	Taobao Marketplace, a Chinese website for online shopping
Enoch Grace Boutique	Copper Power Saving Specialist	Does this really work?	Taobao Marketplace, a Chinese website for online shopping
Enoch Grace Boutique	Copper Power Saving Specialist	Can I save electricity by installing a three-phase saver and having two-phase power go above it as well?	Taobao Marketplace, a Chinese website for online shopping
Enoch Grace Boutique	Copper Power Saving Specialist	It's actually a voltage stabilizer, right? Older generations of TV watchers had one in connected outside the TV set, is that correct please?	Taobao Marketplace, a Chinese website for online shopping
Enoch Grace Boutique	Copper Power Saving Specialist	Does it really save power, be honest, customer service don't bother!	Taobao Marketplace, a Chinese website for online shopping
Enoch Grace Boutique	Copper Power Saving Specialist	How does this work in practice?	Taobao Marketplace, a Chinese website for online shopping
Enoch Grace Boutique	Copper Power Saving Specialist	Save much power?	Taobao Marketplace, a Chinese website for online shopping
Enoch Grace Boutique	Copper Power Saving Specialist	Has it rung yet?	Taobao Marketplace, a Chinese website for online shopping
Enoch Grace Boutique	Copper Power Saving Specialist	Does it really save power?	Taobao Marketplace, a Chinese website for online shopping
Enoch Grace Boutique	Copper Power Saving Specialist	Does380vhome electric boiler work?	Taobao Marketplace, a Chinese website for online shopping
Enoch Grace Boutique	Copper Power Saving Specialist	Is this for 2-phase power?	Taobao Marketplace, a Chinese website for online shopping
Enoch Grace Boutique	Copper Power Saving Specialist	Does this save power?	Taobao Marketplace, a Chinese website for online shopping

Enoch Grace Boutique	Copper Power Saving Specialist	Let's be honest, does it really save power?	Taobao Marketplace, a Chinese website for online shopping
Enoch Grace Boutique	Copper Power Saving Specialist	Does it really save power?	Taobao Marketplace, a Chinese website for online shopping
Enoch Grace Boutique	Copper Power Saving Specialist	Does it save electricity? Really?	Taobao Marketplace, a Chinese website for online shopping
Enoch Grace Boutique	Copper Power Saving Specialist	Does a smart meter work?	Taobao Marketplace, a Chinese website for online shopping
Enoch Grace Boutique	Copper Power Saving Specialist	those of you whohave been using it2-3months Does it really save power?	Taobao Marketplace, a Chinese website for online shopping
Enoch Grace Boutique	Copper Power Saving Specialist	Wouldn't it be better to have one in each room?	Taobao Marketplace, a Chinese website for online shopping
Enoch Grace Boutique	Copper Power Saving Specialist	Does it work? Does it save power? Ask for a conscientious answer	Taobao Marketplace, a Chinese website for online shopping
Enoch Grace Boutique	Copper Power Saving Specialist	For all you real buyers out there, does this really save power?	Taobao Marketplace, a Chinese website for online shopping
Enoch Grace Boutique	Copper Power Saving Specialist	For those of you who have bought it, did you save on electricity?	Taobao Marketplace, a Chinese website for online shopping
Enoch Grace Boutique	Copper Power Saving Specialist	For those of you who have used it, does it really work? Please tell me the truth. Thank you.	Taobao Marketplace, a Chinese website for online shopping
Enoch Grace Boutique	Copper Power Saving Specialist	Did it work to burn out the circuits?	Taobao Marketplace, a Chinese website for online shopping
Enoch Grace Boutique	Copper Power Saving Specialist	Is there a national certification?	Taobao Marketplace, a Chinese website for online shopping
Enoch Grace Boutique	Copper Power Saving Specialist	Does it really work?	Taobao Marketplace, a Chinese website for online shopping
Enoch Grace Boutique	Copper Power Saving Specialist	How about this one? Does it really save electricity? How much is it good?	Taobao Marketplace, a Chinese website for online shopping
Enoch Grace Boutique	Copper Power Saving Specialist	Is this for 2-phase power?	Taobao Marketplace, a Chinese website for online shopping
Enoch Grace Boutique	Copper Power Saving Specialist	Can I ask what exactly will work to save electricity? Looking at my friends' answers above they seem to be ambiguous.	Taobao Marketplace, a Chinese website for online shopping
Enoch Grace Boutique	Copper Power Saving Specialist	Does pro really save power?	Taobao Marketplace, a Chinese website for online shopping
Enoch Grace Boutique	Copper Power Saving Specialist	Does it really save electricity?	Taobao Marketplace, a Chinese website for online shopping
Enoch Grace Boutique	Copper Power Saving Specialist	Just plug it into an outlet?	Taobao Marketplace, a Chinese website for online shopping
Enoch Grace Boutique	Copper Power Saving Specialist	Does it really work?	Taobao Marketplace, a Chinese website for online shopping
Enoch Grace Boutique	Copper Power Saving Specialist	Will plugging into the same outlet as the refrigerator and air conditioner cause unstable voltage to break the appliance? Please give me an honest answer.	Taobao Marketplace, a Chinese website for online shopping
Enoch Grace Boutique	Copper Power Saving Specialist	The Power Saver King that everyone uses, does it really save electricity?	Taobao Marketplace, a Chinese website for online shopping
Enoch Grace Boutique	Copper Power Saving Specialist	Let's be honest, does it really save power?	Taobao Marketplace, a Chinese website for online shopping
Enoch Grace Boutique	Copper Power Saving Specialist	Hello everyone! Is there any power saving with this saver?	Taobao Marketplace, a Chinese website for online shopping
Enoch Grace Boutique	Copper Power Saving Specialist	Does it affect IQ?	Taobao Marketplace, a Chinese website for online shopping
Enoch Grace Boutique	Copper Power Saving Specialist	Folks, does it really save electricity?	Taobao Marketplace, a Chinese website for online shopping
Enoch Grace Boutique	Copper Power Saving Specialist	Any disassembly pics yet?	Taobao Marketplace, a Chinese website for online shopping
Enoch Grace Boutique	Copper Power Saving Specialist	Are you sure it's a power saver?	Taobao Marketplace, a Chinese website for online shopping
Enoch Grace Boutique	Copper Power Saving Specialist	Do the ones that have used it really save power? I'm begging for the truth.	Taobao Marketplace, a Chinese website for online shopping
Enoch Grace Boutique	Copper Power Saving Specialist	With this power-saving device, do the electricians check it out?	Taobao Marketplace, a Chinese website for online shopping
Enoch Grace Boutique	Copper Power Saving Specialist	For all you real buyers out there, does this really save power?	Taobao Marketplace, a Chinese website for online shopping
Enoch Grace Boutique	Copper Power Saving Specialist	Is it a product that pays an IQ tax	Taobao Marketplace, a Chinese website for online shopping
Enoch Grace Boutique	Copper Power Saving Specialist	How does it work?	Taobao Marketplace, a Chinese website for online shopping
Enoch Grace Boutique	Copper Power Saving Specialist	Does this really save power?	Taobao Marketplace, a Chinese website for online shopping

Enoch Grace Boutique	Copper Power Saving Specialist	Is it an IQ tax?	Taobao Marketplace, a Chinese website for online shopping
Enoch Grace Boutique	Copper Power Saving Specialist	Does it really save power?	Taobao Marketplace, a Chinese website for online shopping
Enoch Grace Boutique	Copper Power Saving Specialist	Does it really save power?	Taobao Marketplace, a Chinese website for online shopping
Enoch Grace Boutique	Copper Power Saving Specialist	Does it really save power?	Taobao Marketplace, a Chinese website for online shopping
Enoch Grace Boutique	Copper Power Saving Specialist	Will it work if you put it on the main switch?	Taobao Marketplace, a Chinese website for online shopping
Enoch Grace Boutique	Copper Power Saving Specialist	Tell us if it works after 3 months of use, power saving or more power consuming, this isn't an appliance too, is it?	Taobao Marketplace, a Chinese website for online shopping
Enoch Grace Boutique	Copper Power Saving Specialist	What is the effect of3500winduction cooktop with this?	Taobao Marketplace, a Chinese website for online shopping
Enoch Grace Boutique	Copper Power Saving Specialist	Do you have a three-phase meter for that?	Taobao Marketplace, a Chinese website for online shopping
Enoch Grace Boutique	Copper Power Saving Specialist	Does it work for anyone who's used it?	Taobao Marketplace, a Chinese website for online shopping
Enoch Grace Boutique	Copper Power Saving Specialist	For those of you who have bought this power saver, does it really save power? Please tell me the truth.	Taobao Marketplace, a Chinese website for online shopping
Enoch Grace Boutique	Copper Power Saving Specialist	For those of you who have bought this power saver, does it really save power? Please tell me the truth.	Taobao Marketplace, a Chinese website for online shopping
Enoch Grace Boutique	Copper Power Saving Specialist	The display is a number that shows what	Taobao Marketplace, a Chinese website for online shopping
Enoch Grace Boutique	Copper Power Saving Specialist	Is it okay to have one energy efficient product for a home?	Taobao Marketplace, a Chinese website for online shopping
Enoch Grace Boutique	Copper Power Saving Specialist	Did people buy it and did it work? Is the effect good? Seller do not answer	Taobao Marketplace, a Chinese website for online shopping
Enoch Grace Boutique	Copper Power Saving Specialist	It's a real power saver.	Taobao Marketplace, a Chinese website for online shopping
Enoch Grace Boutique	Copper Power Saving Specialist	Has it rung yet?	Taobao Marketplace, a Chinese website for online shopping
Enoch Grace Boutique	Copper Power Saving Specialist	Is this product an appliance?	Taobao Marketplace, a Chinese website for online shopping
Enoch Grace Boutique	Copper Power Saving Specialist	Does it work?	Taobao Marketplace, a Chinese website for online shopping
Enoch Grace Boutique	Copper Power Saving Specialist	This......hasn'tresearched, do you want to try it? I'll tell you the results, and we'll buy four or five of them if we can.	Taobao Marketplace, a Chinese website for online shopping
Enoch Grace Boutique	Copper Power Saving Specialist	Doesn't feel like much use.	Taobao Marketplace, a Chinese website for online shopping
Enoch Grace Boutique	Copper Power Saving Specialist	I'm using it. I don't know yet.	Taobao Marketplace, a Chinese website for online shopping
Enoch Grace Boutique	Copper Power Saving Specialist	illiteracy	Taobao Marketplace, a Chinese website for online shopping
Enoch Grace Boutique	Copper Power Saving Specialist	It's better to connect it to the main switch line to be effective	Taobao Marketplace, a Chinese website for online shopping
Enoch Grace Boutique	Copper Power Saving Specialist	70-degreesavings from the same month last year	Taobao Marketplace, a Chinese website for online shopping
Enoch Grace Boutique	Copper Power Saving Specialist	It doesn't help, honestly. IQ tax.	Taobao Marketplace, a Chinese website for online shopping
Enoch Grace Boutique	Copper Power Saving Specialist	Feels like a power saver, and you can see the real-time voltage	Taobao Marketplace, a Chinese website for online shopping
Enoch Grace Boutique	Copper Power Saving Specialist	not great	Taobao Marketplace, a Chinese website for online shopping
Enoch Grace Boutique	Copper Power Saving Specialist	We have more than ten years of technical experience in making power savers. There are patents. The product is underwritten by PICC. Rest assured that the use of oh! Power saving rate of15-35%, why not fix a value. Because the degree of aging of the circuit of each home, the aging of appliances, the use of appliances are not the same. Energy saving effect is not the same. So there is a range! Just like the same model of air conditioner. New and old. Definitely the old one is more expensive. Because the old one has more wear and tear. So the savings will be more! There is also the brand and model of each appliance, power size, energy consumption and so on those factors are not the same. So there is a range of power	Taobao Marketplace, a Chinese website for online shopping
Enoch Grace Boutique	Copper Power Saving Specialist	Just plug it directly into the plug	Taobao Marketplace, a Chinese website for online shopping
Enoch Grace Boutique	Copper Power Saving Specialist	power saving rateis 15-35%, why not fix a value. Because the degree of aging of the circuit, the aging of the appliances, and the appliances used are different in each home. The energy saving effect is not the same. So there is a range! It's like the same model of air conditioner. New and old. Definitely the old one is more expensive. Because the old one has more losses. So the savings will be more! There is also the brand and model of electrical appliances, power size, energy consumption and so on those factors are not the same. So there is a range of power saving rate!	Taobao Marketplace, a Chinese website for online shopping
Enoch Grace Boutique	Copper Power Saving Specialist	Power saving and trustworthy	Taobao Marketplace, a Chinese website for online shopping
Enoch Grace Boutique	Copper Power Saving Specialist	good enough	Taobao Marketplace, a Chinese website for online shopping

Enoch Grace Boutique	Copper Power Saving Specialist	Home has been useful, control switch variable voltage float, can slightly let the table is not so fast, day by day, you say at once control how much electricity is not scientific! Hope it can help you!	Taobao Marketplace, a Chinese website for online shopping
Enoch Grace Boutique	Copper Power Saving Specialist	power saving rateis 15-35%, why not fix a value. Because the degree of aging of the circuit, the aging of the appliances, and the appliances used are different in each home. The energy saving effect is not the same. So there is a range! Just like the same model of air conditioner. New and old. Definitely the old one is more expensive. Because the old one has more wear and tear. So the savings will be more! There is also the brand and model of each appliance, power size, energy consumption and so on those factors are not the same. So there is a range of power saving rate! Not stealing electricity, not a mounted on the effect. The longer the use of time the better the effect. Electricity appliances, habits and so on almost the same case, can be used for a month or two. Comparison of previous electricity bills, it is best to compare the electricity bill of the same month in previous years, is also more accurate Oh.	Taobao Marketplace, a Chinese website for online shopping
Enoch Grace Boutique	Copper Power Saving Specialist	I'm not sure if it's working yet.	Taobao Marketplace, a Chinese website for online shopping
Enoch Grace Boutique	Copper Power Saving Specialist	It won't affect life haha. If you don't get close. You won't even hear a sound.	Taobao Marketplace, a Chinese website for online shopping
Enoch Grace Boutique	Copper Power Saving Specialist	Anyway, I'm saving more electricity than this time last year, and there's a reason I'm saving more than last month, because I didn't turn on the air conditioning in the cooler weather.	Taobao Marketplace, a Chinese website for online shopping
Enoch Grace Boutique	Copper Power Saving Specialist	Boiler. It doesn't work. - Oh.	Taobao Marketplace, a Chinese website for online shopping
Enoch Grace Boutique	Copper Power Saving Specialist	Yes pro.	Taobao Marketplace, a Chinese website for online shopping
Enoch Grace Boutique	Copper Power Saving Specialist	We have more than ten years of technical experience in making power savers. There are patents. The product is underwritten by PICC. Rest assured that the use of oh! Power saving rate of15-35%, why not fix a value. Because the degree of aging of the circuit of each home, the aging of appliances, the use of appliances are not the same. Energy saving effect is not the same. So there is a range! Just like the same model of air conditioner. New and old. Definitely the old one is more expensive. Because the old one has more wear and tear. So the savings will be more! There is also the brand and model of each appliance, power size, energy consumption and so on those factors are not the same. So there is a range of power	Taobao Marketplace, a Chinese website for online shopping
Enoch Grace Boutique	Copper Power Saving Specialist	We have more than ten years of technical experience in making power savers. There are patents. The product is underwritten by PICC. Rest assured that the use of oh! Power saving rate of15-35%, why not fix a value. Because the degree of aging of the circuit of each home, the aging of appliances, the use of appliances are not the same. Energy saving effect is not the same. So there is a range! Just like the same model of air conditioner. New and old. Definitely the old one is more expensive. Because the old one has more wear and tear. So the savings will be more! There is also the brand and model of each appliance, power size, energy consumption and so on those factors are not the same. So there is a range of power	Taobao Marketplace, a Chinese website for online shopping
Enoch Grace Boutique	Copper Power Saving Specialist	(usually used in the negative) have the possibility of	Taobao Marketplace, a Chinese website for online shopping
Enoch Grace Boutique	Copper Power Saving Specialist	I don't know yet. I haven't paid my electric bill yet.	Taobao Marketplace, a Chinese website for online shopping
Enoch Grace Boutique	Copper Power Saving Specialist	can	Taobao Marketplace, a Chinese website for online shopping
Enoch Grace Boutique	Copper Power Saving Specialist	Never noticed.	Taobao Marketplace, a Chinese website for online shopping
Enoch Grace Boutique	Copper Power Saving Specialist	Pro. Legitimate electricity savings are limited. It's not unlimited. Not the more you use, the better,	Taobao Marketplace, a Chinese website for online shopping
Enoch Grace Boutique	Copper Power Saving Specialist	power saving rateis 15-35%, why not fix a value. Because the degree of aging of the circuit, the aging of the appliances, and the appliances used are different in each home. The energy saving effect is not the same. So there is a range! Just like the same model of air conditioner. New and old. Definitely the old one is more expensive. Because the old one has more wear and tear. So the savings will be more! There is also the brand and model of each appliance, power size, energy consumption and so on those factors are not the same. So there is a range of power saving rate!	Taobao Marketplace, a Chinese website for online shopping
Enoch Grace Boutique	Copper Power Saving Specialist	Average. It's a little bit more efficient.	Taobao Marketplace, a Chinese website for online shopping
Enoch Grace Boutique	Copper Power Saving Specialist	It's okay to save.	Taobao Marketplace, a Chinese website for online shopping
Enoch Grace Boutique	Copper Power Saving Specialist	It works. It really does.	Taobao Marketplace, a Chinese website for online shopping
Enoch Grace Boutique	Copper Power Saving Specialist	Not yet.	Taobao Marketplace, a Chinese website for online shopping
Enoch Grace Boutique	Copper Power Saving Specialist	hasn't	Taobao Marketplace, a Chinese website for online shopping
Enoch Grace Boutique	Copper Power Saving Specialist	Applicable appliances Refrigerators, air conditioners, washing machines, freezers, sterilizers, copiers, printers, electric fans, hoods, vacuum cleaners, televisions, lamps with capacitors, and all electrical appliances with coils, motors, and electric……and any other inductive household appliances and equipment. Good effect. Electricity is directly converted into heat and other resistive appliances (induction cookers, electric water heaters, microwave ovens, ovens, electric kettles, irons, incandescent lamps), basically no effect! Power saving rate of15-35%, why not fix a value. Because the degree of aging of the circuit in each home, the aging of appliances, the use of appliances are not the same. Energy saving effect is also different.	Taobao Marketplace, a Chinese website for online shopping
Enoch Grace Boutique	Copper Power Saving Specialist	Applicable appliances Refrigerators, air conditioners, washing machines, freezers, sterilizers, copiers, printers, electric fans, hoods, vacuum cleaners, televisions, lamps with capacitors, and all electrical appliances with coils, motors, and electric……and any other inductive household appliances and equipment. Good effect. Electricity is directly converted into heat and other resistive appliances (induction cookers, electric water heaters, microwave ovens, ovens, electric kettles, irons, incandescent lamps), basically no effect! Power saving rate of15-35%, why not fix a value. Because the degree of aging of the circuit in each home, the aging of appliances, the use of appliances are not the same. Energy saving effect is also different. So there is a range! Just like the same model of air conditioner. New and old. Definitely the old one is more expensive. Because the old one has more wear and tear. So the savings will be more! There is also the brand and model of each appliance, power size, energy consumption and so on those factors are not the	Taobao Marketplace, a Chinese website for online shopping
Enoch Grace Boutique	Copper Power Saving Specialist	Yes pro.	Taobao Marketplace, a Chinese website for online shopping
Enoch Grace Boutique	Copper Power Saving Specialist	I don't know. I just used it.	Taobao Marketplace, a Chinese website for online shopping

Enoch Grace Boutique	Copper Power Saving Specialist	Still using it, let's see the power usage in a month	Taobao Marketplace, a Chinese website for online shopping
Enoch Grace Boutique	Copper Power Saving Specialist	It doesn't seem to be particularly effective.	Taobao Marketplace, a Chinese website for online shopping
Enoch Grace Boutique	Copper Power Saving Specialist	Any outlet under the same meter is fine, the whole house is effective. As long as it's not plugged into a remote corner outlet. Because of the remote corner socket. Usually at the end of the line, there are no appliances, the effect will be worse. It is recommended to plug in the outlet near the main switch. Or plug in the place where the appliances are concentrated. Or the outlet of the appliances that are often on (such as refrigerators, fish tanks). These places are better. Constant power on is fine!	Taobao Marketplace, a Chinese website for online shopping
Enoch Grace Boutique	Copper Power Saving Specialist	We have more than ten years of technical experience in making power savers. There are patents. The product is underwritten by PICC. Rest assured that the use of oh! Power saving rate of15-35%, why not fix a value. Because the degree of aging of the circuit of each home, the aging of appliances, the use of appliances are not the same. Energy saving effect is not the same. So there is a range! Just like the same model of air conditioner. New and old. Definitely the old one is more expensive. Because the old one has more wear and tear. So the savings will be more! There is also the brand and model of each appliance, power size, energy consumption and so on those factors are not the same. So there is a range of power	Taobao Marketplace, a Chinese website for online shopping
Enoch Grace Boutique	Copper Power Saving Specialist	It does not affect the longevity of wiring or appliances, and does not interfere with normal use.	Taobao Marketplace, a Chinese website for online shopping
Enoch Grace Boutique	Copper Power Saving Specialist	power saving rateis 15-35%, why not fix a value. Because the degree of aging of the circuit, the aging of the appliances, and the appliances used are different in each home. The energy saving effect is not the same. So there is a range! Just like the same model of air conditioner. New and old. Definitely the old one is more expensive. Because the old one has more wear and tear. So the savings will be more! There is also the brand and model of each appliance, power size, energy consumption and so on those factors are not the same. So there is a range of power saving rate!	Taobao Marketplace, a Chinese website for online shopping
Enoch Grace Boutique	Copper Power Saving Specialist	We have more than ten years of technical experience in making power savers. There are patents. The product is underwritten by PICC. Rest assured that the use of oh! Power saving rate of15-35%, why not fix a value. Because the degree of aging of the circuit of each home, the aging of appliances, the use of appliances are not the same. Energy saving effect is not the same. So there is a range! Just like the same model of air conditioner. New and old. Definitely the old one is more expensive. Because the old one has more wear and tear. So the savings will be more! There is also the brand and model of each appliance, power size, energy consumption and so on those factors are not the same. So there is a range of power	Taobao Marketplace, a Chinese website for online shopping
Enoch Grace Boutique	Copper Power Saving Specialist	It doesn't seem to feel like much:	Taobao Marketplace, a Chinese website for online shopping
Enoch Grace Boutique	Copper Power Saving Specialist	Yes, it will. The more you use it, the dumber it gets.	Taobao Marketplace, a Chinese website for online shopping
Enoch Grace Boutique	Copper Power Saving Specialist	power saving rateis 15-35%, why not fix a value. Because the degree of aging of the circuit, the aging of the appliances, and the appliances used are different in each home. The energy saving effect is not the same. So there is a range! Just like the same model of air conditioner. New and old. Definitely the old one is more expensive. Because the old one has more wear and tear. So the savings will be more! There is also the brand and model of each appliance, power size, energy consumption and so on those factors are not the same. So there is a range of power saving rate!	Taobao Marketplace, a Chinese website for online shopping
Enoch Grace Boutique	Copper Power Saving Specialist	hasn't	Taobao Marketplace, a Chinese website for online shopping
Enoch Grace Boutique	Copper Power Saving Specialist	inconclusive	Taobao Marketplace, a Chinese website for online shopping
Enoch Grace Boutique	Copper Power Saving Specialist	power saving rateis 15-35%, why not fix a value. Because the degree of aging of the circuit, the aging of the appliances, and the appliances used are different in each home. The energy saving effect is not the same. So there is a range! Just like the same model of air conditioner. New and old. Definitely the old one is more expensive. Because the old one has more wear and tear. So the savings will be more! There is also the brand and model of each appliance, power size, energy consumption and so on those factors are not the same. So there is a range of power saving rate!	Taobao Marketplace, a Chinese website for online shopping
Enoch Grace Boutique	Copper Power Saving Specialist	Who's going to check it at home?	Taobao Marketplace, a Chinese website for online shopping
Enoch Grace Boutique	Copper Power Saving Specialist	It's not working.	Taobao Marketplace, a Chinese website for online shopping
Enoch Grace Boutique	Copper Power Saving Specialist	Although I think it's an IQ tax, I still bought one and came back to dismantle it. I've been busy and haven't started it yet!	Taobao Marketplace, a Chinese website for online shopping
Enoch Grace Boutique	Copper Power Saving Specialist	Just plug it in, where it's a bit much where/near. It's actually a voltage regulator.	Taobao Marketplace, a Chinese website for online shopping
Enoch Grace Boutique	Copper Power Saving Specialist	It's supposed to save electricity, and I haven't done the math, it feels like it saves more than before	Taobao Marketplace, a Chinese website for online shopping
Enoch Grace Boutique	Copper Power Saving Specialist	Anyway, I don't see where my monthly electricity bill is less, it feels like it's more!	Taobao Marketplace, a Chinese website for online shopping
Enoch Grace Boutique	Copper Power Saving Specialist	It's no use.	Taobao Marketplace, a Chinese website for online shopping
Enoch Grace Boutique	Copper Power Saving Specialist	Applicable appliances Refrigerators, air conditioners, washing machines, freezers, sterilizers, copiers, printers, electric fans, hoods, vacuum cleaners, televisions, lamps with capacitors, and all electrical appliances with coils, motors, and electric……and any other inductive household appliances and equipment. Good effect. Electricity is directly converted into heat and other resistive appliances (induction cookers, electric water heaters, microwave ovens, ovens, electric kettles, irons, incandescent lamps), basically no effect! Power saving rate of15-35%, why not fix a value. Because the degree of aging of the circuit in each home, the aging of appliances, the use of appliances are not the same. Energy saving effect is also different.	Taobao Marketplace, a Chinese website for online shopping
Enoch Grace Boutique	Copper Power Saving Specialist	I've been using it for a month and it's still working, the electricity bill this monthis over170, with the air conditioner on	Taobao Marketplace, a Chinese website for online shopping
Enoch Grace Boutique	Copper Power Saving Specialist	Any outlet under the same meter is fine, the whole house is effective. As long as it's not plugged into a remote corner outlet. Because of the remote corner socket. Usually at the end of the line, there are no appliances, the effect will be worse. It is recommended to plug in the outlet near the main switch. Or plug in the place where the appliances are concentrated. Or the outlet of the appliances that are often on (such as refrigerators, fish tanks). These places are better. Continuously energized can be! You can also connect a three-pronged socket out of the main switch and plug it in oh (three-pronged socket ground wire do not have to connect, because the economizer only uses the fire wire and zero wire, made into a three-pronged plug is in order to plug more stable). Do not cut off the plug Oh, because if the plug is cut. Fear that the factory does not give warranty Oh. Our products are guaranteed2years. Productquality problems within2years is a new one.	Taobao Marketplace, a Chinese website for online shopping

Enoch Grace Boutique	Copper Power Saving Specialist	It's working.	Taobao Marketplace, a Chinese website for online shopping
Enoch Grace Boutique	Copper Power Saving Specialist	I'm using it, but I don't know how it works, and I feel like I can save some money!	Taobao Marketplace, a Chinese website for online shopping
Enoch Grace Boutique	Copper Power Saving Specialist	Hello. It is available in the store. You can also consult the online customer service.	Taobao Marketplace, a Chinese website for online shopping
Enoch Grace Boutique	Copper Power Saving Specialist	Haven't paid attention to it, bought it and plugged it into the line, haven't compared it, not quite sure if it will save power or not	Taobao Marketplace, a Chinese website for online shopping
Enoch Grace Boutique	Copper Power Saving Specialist	Applicable appliances Refrigerators, air conditioners, washing machines, freezers, sterilizers, copiers, printers, electric fans, hoods, vacuum cleaners, televisions, lamps with capacitors, and all electrical appliances with coils, motors, and electric......and any other inductive household appliances and equipment. Good effect. Electricity is directly converted into heat and other resistive appliances (induction cookers, electric water heaters, microwave ovens, ovens, electric kettles, irons, incandescent lamps), basically no effect! Power saving rate of15-35%, why not fix a value. Because the degree of aging of the circuit in each home, the aging of appliances, the use of appliances are not the same. Energy saving effect is also different. So there is a range! Just like the same model of air conditioner. New and old. Definitely the old one is more expensive. Because the old one has more wear and tear. So the savings will be more! There is also the brand and model of each appliance, power size, energy consumption and so on those factors are not the	Taobao Marketplace, a Chinese website for online shopping
Enoch Grace Boutique	Copper Power Saving Specialist	Applicable appliances Refrigerators, air conditioners, washing machines, freezers, sterilizers, copiers, printers, electric fans, hoods, vacuum cleaners, televisions, lamps with capacitors, and all electrical appliances with coils, motors, and electric......and any other inductive household appliances and equipment. Good effect. Electricity is directly converted into heat and other resistive appliances (induction cookers, electric water heaters, microwave ovens, ovens, electric kettles, irons, incandescent lamps), basically no effect! Power saving rate of15-35%, why not fix a value. Because the degree of aging of the circuit in each home, the aging of appliances, the use of appliances are not the same. Energy saving effect is also different. So there is a range! Just like the same model of air conditioner. New and old. Definitely the old one is more expensive. Because the old one has more wear and tear. So the savings will be more! There is also the brand and model of each appliance, power size, energy consumption and so on those factors are not the	Taobao Marketplace, a Chinese website for online shopping
Enoch Grace Boutique	Copper Power Saving Specialist	Real-time voltage	Taobao Marketplace, a Chinese website for online shopping
Enoch Grace Boutique	Copper Power Saving Specialist	You can consult the online customer service oh. According to the actual situation to determine.	Taobao Marketplace, a Chinese website for online shopping
Enoch Grace Boutique	Copper Power Saving Specialist	I haven't used it yet.	Taobao Marketplace, a Chinese website for online shopping
Enoch Grace Boutique	Copper Power Saving Specialist	It's not clear.	Taobao Marketplace, a Chinese website for online shopping
Enoch Grace Boutique	Copper Power Saving Specialist	No rattles. Can't even hear the sound without getting very close!	Taobao Marketplace, a Chinese website for online shopping
Enoch Grace Boutique	Copper Power Saving Specialist	It's no use.	Taobao Marketplace, a Chinese website for online shopping
Enoch Grace Boutique	Copper Power Saving Specialist	power saving rateis 15-35%, why not fix a value. Because the degree of aging of the circuit, the aging of the appliances, and the appliances used are different in each home. The energy saving effect is not the same. So there is a range! It's like the same model of air conditioner. New and old. Definitely the old one is more expensive. Because the old one has more losses. So the savings will be more! There is also the brand and model of electrical appliances, power size, energy consumption and so on those factors are not the same. So there is a range of power saving rate!	Taobao Marketplace, a Chinese website for online shopping
Enoch Grace Boutique	Blue Power Saving Specialist	Shipping was fast, stuff was well packaged, great price, I'm happy with and will repurchase	Taobao Marketplace, a Chinese website for online shopping
Enoch Grace Boutique	Blue Power Saving Specialist	favorable criticism	Taobao Marketplace, a Chinese website for online shopping
Enoch Grace Boutique	Blue Power Saving Specialist	Great value for money and effective, will buy again next time.	Taobao Marketplace, a Chinese website for online shopping
Enoch Grace Boutique	Blue Power Saving Specialist	The boss is very patient, looked at a lot of baby, and finally chose this one, sex price super high, suggest that the treasure can try this one	Taobao Marketplace, a Chinese website for online shopping
Enoch Grace Boutique	Blue Power Saving Specialist	It works very well and also saves power and protects the appliance,, the	Taobao Marketplace, a Chinese website for online shopping
Enoch Grace Boutique	Blue Power Saving Specialist	The thing received, a little heavy in the hand, whether it is power saving do not know yet, only wait for a period of time to know.	Taobao Marketplace, a Chinese website for online shopping
Enoch Grace Boutique	Blue Power Saving Specialist	Just got my shipment Not sure if it's any good yet	Taobao Marketplace, a Chinese website for online shopping
Enoch Grace Boutique	Blue Power Saving Specialist	Second purchase	Taobao Marketplace, a Chinese website for online shopping
Enoch Grace Boutique	Blue Power Saving Specialist	(of an unmarried couple) be close	Taobao Marketplace, a Chinese website for online shopping
Enoch Grace Boutique	Blue Power Saving Specialist	To be used after	Taobao Marketplace, a Chinese website for online shopping
Enoch Grace Boutique	Blue Power Saving Specialist	I haven't used it yet, but I heard my friend say that it can be used, and it costs a few hundred dollars a month for electricity. So I'm buying one to try it out.	Taobao Marketplace, a Chinese website for online shopping
Enoch Grace Boutique	Blue Power Saving Specialist	The stuff is great love it seller is very enthusiastic super praise	Taobao Marketplace, a Chinese website for online shopping
Enoch Grace Boutique	Blue Power Saving Specialist	Fast shipping, very satisfied	Taobao Marketplace, a Chinese website for online shopping
Enoch Grace Boutique	Blue Power Saving Specialist	The item is as described, the item was shipped quickly, in anticipation of good energy savings._x005f_x005f_x005f_x005f_x0001_ customer service with questions, OK, point of view.	Taobao Marketplace, a Chinese website for online shopping
Enoch Grace Boutique	Blue Power Saving Specialist	Very well packaged, works well	Taobao Marketplace, a Chinese website for online shopping

Enoch Grace Boutique	Blue Power Saving Specialist	started using it inMarchand have been using itover1month and now I'm back to rate it. electric bill came in, and it's more expensive than before when it wasn't even being used. Hundreds of dollars for a piece of junk to take home, buy with caution!!	Taobao Marketplace, a Chinese website for online shopping
Enoch Grace Boutique	Blue Power Saving Specialist	Home is to open the supermarket has a few refrigerators and a few freezers with electricity is very large, every month the electricity bill to pay the headache, a friend recommended to me this intelligent power saver, just started to use, the electricity bill to see seems to be less than a daymore than6dollars, the effect can be, follow-up evaluation	Taobao Marketplace, a Chinese website for online shopping
Enoch Grace Boutique	Blue Power Saving Specialist	Good quality and fast shipping from the store!	Taobao Marketplace, a Chinese website for online shopping
Enoch Grace Boutique	Blue Power Saving Specialist	It's a lie. It doesn't work.	Taobao Marketplace, a Chinese website for online shopping
Enoch Grace Boutique	Blue Power Saving Specialist	The goods are consistent with the description, the goods are shipped quickly, the goods are a little expensive, I believe that a penny a penny, I hope that the effect is obvious after use.	Taobao Marketplace, a Chinese website for online shopping
Enoch Grace Boutique	Blue Power Saving Specialist	Expect to save in use	Taobao Marketplace, a Chinese website for online shopping
Enoch Grace Boutique	Blue Power Saving Specialist	Before buying is also a big skepticism, see some bad reviews on the Internet, really try to know, used2months or so, the overall electricity bill dropped by nearly20%, very satisfied	Taobao Marketplace, a Chinese website for online shopping
Enoch Grace Boutique	Blue Power Saving Specialist	It doesn't work. It's just a lie.	Taobao Marketplace, a Chinese website for online shopping
Enoch Grace Boutique	Blue Power Saving Specialist	buy this I really do not hold any hope, overheard people talking about, use to try, the results of saving power not to mention, originally the home living room lamps often voltage instability motionless bad, this time with the lights actually do not flash, with a good, save electricity seems to determine less than30yuan, it seems that this thing is indeed still good	Taobao Marketplace, a Chinese website for online shopping
Enoch Grace Boutique	Blue Power Saving Specialist	Before listening to others say that this power saving effect can be, compared to the electricity bill is indeed effective, but also really good, highly recommended!	Taobao Marketplace, a Chinese website for online shopping
Enoch Grace Boutique	Blue Power Saving Specialist	Holding a try mentality, buy back to the home motor tried a few hours, it is indeed effective, ordinary air conditioning is also good, inverter class air conditioning time to be longer, the effect is a little worse, the overall is still very satisfactory	Taobao Marketplace, a Chinese website for online shopping
Enoch Grace Boutique	Blue Power Saving Specialist	How well does this thing work, the big guys are looking at the follow up reviews, right, so let's use it for now.	Taobao Marketplace, a Chinese website for online shopping
Enoch Grace Boutique	Blue Power Saving Specialist	First time buying this stuff, use it and see how it works, then come back with a follow up review	Taobao Marketplace, a Chinese website for online shopping
Enoch Grace Boutique	Blue Power Saving Specialist	It's really good, cost-effective and good quality, and the logistics are fast! It arrived in one day!	Taobao Marketplace, a Chinese website for online shopping
Enoch Grace Boutique	Blue Power Saving Specialist	Logistics is very fast, the product also looks very delicate, looking forward to energy saving effect is good!	Taobao Marketplace, a Chinese website for online shopping
Enoch Grace Boutique	Blue Power Saving Specialist	It's good quality.	Taobao Marketplace, a Chinese website for online shopping
Enoch Grace Boutique	Blue Power Saving Specialist	Second time I've bought it and it works well	Taobao Marketplace, a Chinese website for online shopping
Enoch Grace Boutique	Blue Power Saving Specialist	I'm using it right now. I'm waiting for the power savings.	Taobao Marketplace, a Chinese website for online shopping
Enoch Grace Boutique	Blue Power Saving Specialist	honest, it does have an effect, although the savings are not a lot, from1,000to the electricity bill tomore than700this month, the appliances are the same, the effect can be, it is worth recommending that you buy	Taobao Marketplace, a Chinese website for online shopping
Enoch Grace Boutique	Blue Power Saving Specialist	Inadvertently found this product, compared to a lot of home finally chose this, see the evaluation is good, I do not know how the effect, with1month after the follow-up, pre-sales service is very good, customer service is also very professional	Taobao Marketplace, a Chinese website for online shopping
Enoch Grace Boutique	Blue Power Saving Specialist	The goods are as described and are of very good quality. Value for money oh ！！！！ Very worth buying!!!	Taobao Marketplace, a Chinese website for online shopping
Enoch Grace Boutique	Blue Power Saving Specialist	Home children are still small,24hours are on the air conditioning, electricity costsmore than1,000a month, used a few days to compare, every day is reduced some, I hope the follow-up effect can be maintained	Taobao Marketplace, a Chinese website for online shopping
Enoch Grace Boutique	Blue Power Saving Specialist	Looking forward to the real effect, the follow-up real evaluation follow-up, so far to see the product and customer service is very professional	Taobao Marketplace, a Chinese website for online shopping
Enoch Grace Boutique	Blue Power Saving Specialist	Good quality, good comments	Taobao Marketplace, a Chinese website for online shopping
Enoch Grace Boutique	Blue Power Saving Specialist	It's good.	Taobao Marketplace, a Chinese website for online shopping
Enoch Grace Boutique	Blue Power Saving Specialist	I love the stuff and the quality is great, it's a great deal for the price!	Taobao Marketplace, a Chinese website for online shopping
Enoch Grace Boutique	Blue Power Saving Specialist	I've been using it for over a month and the results are really good.Over150lesselectricity billthan before	Taobao Marketplace, a Chinese website for online shopping
Enoch Grace Boutique	Blue Power Saving Specialist	Previously used What I did not expect is so many years, this store has been in, really good products My family on the refrigerator and air conditioning effect is available	Taobao Marketplace, a Chinese website for online shopping
Enoch Grace Boutique	Blue Power Saving Specialist	The logistics is very fast, the baby is very good, the shopkeeperNICE!	Taobao Marketplace, a Chinese website for online shopping
Enoch Grace Boutique	Blue Power Saving Specialist	Good review. The one I purchased does not come with a voltage value display, which is a slight disadvantage. Overall quality is very good. Exquisite workmanship. Nice.	Taobao Marketplace, a Chinese website for online shopping
Enoch Grace Boutique	Blue Power Saving Specialist	Intentionally open the air conditioning to compare a few days, did not use before30degrees up and downevery day After using now23degrees or so, the effect is still very obvious, depending on whether it can be a long-lasting effective	Taobao Marketplace, a Chinese website for online shopping
Enoch Grace Boutique	Blue Power Saving Specialist	Shipping was fast, stuff was well packaged, great price, I'm happy with👍and will repurchase	Taobao Marketplace, a Chinese website for online shopping
Enoch Grace Boutique	Blue Power Saving Specialist	Good or bad real use to know, see a lot of such and such a sound on the Internet, they really used it, anyway, my home effect is good, deliberately looking for an electrician to install the adapter cable in the position of the main switch	Taobao Marketplace, a Chinese website for online shopping

Enoch Grace Boutique	Blue Power Saving Specialist	Looking forward to good results……	Taobao Marketplace, a Chinese website for online shopping
Enoch Grace Boutique	Blue Power Saving Specialist	The quality of the stuff is very good, exactly the same as the seller's description, very satisfied!	Taobao Marketplace, a Chinese website for online shopping
Enoch Grace Boutique	Blue Power Saving Specialist	Previously bought other, publicity can save50%, the result is more electricity, I am a daily comparison, this time deliberately bought the most expensive this, the effect is about20%, very satisfied, especially on the air conditioning effect is very obvious, the old space at home with a long time, power consumption is very large with a significant improvement	Taobao Marketplace, a Chinese website for online shopping
Enoch Grace Boutique	Blue Power Saving Specialist	Great customer service and great price. Would recommend friends to repurchase.	Taobao Marketplace, a Chinese website for online shopping
Enoch Grace Boutique	Blue Power Saving Specialist	an excellent	Taobao Marketplace, a Chinese website for online shopping
Enoch Grace Boutique	Blue Power Saving Specialist	Baby East received has been used, indeed than I previously shot that section of the power saver to be good, fully functional with three-speed adjustment, very atmospheric, a look at the know very upscale, indeed value for money, worth having.	Taobao Marketplace, a Chinese website for online shopping
Enoch Grace Boutique	Blue Power Saving Specialist	Great. Looking forward to the results.	Taobao Marketplace, a Chinese website for online shopping
Enoch Grace Boutique	Blue Power Saving Specialist	Installed in August September electric bill was$15more	Taobao Marketplace, a Chinese website for online shopping
Enoch Grace Boutique	Blue Power Saving Specialist	ve been using it for more than 3,butelectricity bill is completely flat	Taobao Marketplace, a Chinese website for online shopping
Enoch Grace Boutique	Blue Power Saving Specialist	The stuff just arrived yesterday, started using it last night, guess it will take a while	Taobao Marketplace, a Chinese website for online shopping
Enoch Grace Boutique	Blue Power Saving Specialist	Through online communication, we know that the boss is a talented, handsome, good-looking, clear-eyed, and knowledgeable, literary and military, literary and military, rich in knowledge, talent. The products sold by the owner are also value for money, genuine and skillful. I will definitely buy back for a long time and will introduce my friends to buy, and I also hope that other users will trust and support the	Taobao Marketplace, a Chinese website for online shopping
Enoch Grace Boutique	Blue Power Saving Specialist	Not yet used, first praise, to be connected to the bus outlet effect will be good, and is a reasonable power saving, if the voltage at home is smooth, may not be able to save how much, but with better than not!	Taobao Marketplace, a Chinese website for online shopping
Enoch Grace Boutique	Blue Power Saving Specialist	Started testing the machine, the power saving feature is starting, hopefully it will be great, and hopefully after a month, I will be able to give the merchant a big shout out.	Taobao Marketplace, a Chinese website for online shopping
Enoch Grace Boutique	Blue Power Saving Specialist	It doesn't work.I've been using it for three,andthe bill is the same as before	Taobao Marketplace, a Chinese website for online shopping
Enoch Grace Boutique	Blue Power Saving Specialist	Try it first.	Taobao Marketplace, a Chinese website for online shopping
Enoch Grace Boutique	Blue Power Saving Specialist	Shipped quickly, has received the goods, the goods and the description is almost the same, the quality is not a problem, the effect is yet to be proved	Taobao Marketplace, a Chinese website for online shopping
Enoch Grace Boutique	Blue Power Saving Specialist	Power Saving King received is now starting to use, the quality of good workmanship to give a five-star!	Taobao Marketplace, a Chinese website for online shopping
Enoch Grace Boutique	Blue Power Saving Specialist	Received it, used it, and will see the results. I'll be back in a month to see the results and I'll comment again.	Taobao Marketplace, a Chinese website for online shopping
Enoch Grace Boutique	Blue Power Saving Specialist	The seller shipped in a timely manner, courier force, baby just got, look forward to the effect!	Taobao Marketplace, a Chinese website for online shopping
Enoch Grace Boutique	Blue Power Saving Specialist	Looks like good quality, using it to see the later results.	Taobao Marketplace, a Chinese website for online shopping
Enoch Grace Boutique	Blue Power Saving Specialist	I'll take it and see how it goes.	Taobao Marketplace, a Chinese website for online shopping
Enoch Grace Boutique	Blue Power Saving Specialist	Power saving effect is very obvious, save10% ofelectricity consumptionthan last month, recommendable	Taobao Marketplace, a Chinese website for online shopping
Enoch Grace Boutique	Blue Power Saving Specialist	Good product, second purchase	Taobao Marketplace, a Chinese website for online shopping
Enoch Grace Boutique	Blue Power Saving Specialist	It doesn't feel like it's working.	Taobao Marketplace, a Chinese website for online shopping
Enoch Grace Boutique	Blue Power Saving Specialist	Worth having, worth buying, worth paying attention to, worth trusting, a very good online shopping, I wish the store prosperous business!	Taobao Marketplace, a Chinese website for online shopping
Enoch Grace Boutique	Blue Power Saving Specialist	Tried it onMay21and now, day after day, it has no power saving feature at all, a waste of$298dollars. The store said to change the socket and continue to try, now it seems to have no power saving function at all. Do not recommend buying.	Taobao Marketplace, a Chinese website for online shopping
Enoch Grace Boutique	Blue Power Saving Specialist	The quality is pretty good, just as described	Taobao Marketplace, a Chinese website for online shopping
Enoch Grace Boutique	Blue Power Saving Specialist	Works well, saves power, doesn't take up much space, safe	Taobao Marketplace, a Chinese website for online shopping
Enoch Grace Boutique	Blue Power Saving Specialist	Super useful tool! Bought the utility and then evaluated it. Have been using the AC for a while now and the electric bill hasn't spiked like it did before! Really great product! Thanks!	Taobao Marketplace, a Chinese website for online shopping
Enoch Grace Boutique	Blue Power Saving Specialist	The color value is very high, the workmanship is also fine, hold in the hand also has a little weight, just started plugging in, do not know whether it will really save electricity, use a month after the follow-up!	Taobao Marketplace, a Chinese website for online shopping
Enoch Grace Boutique	Blue Power Saving Specialist	very happy	Taobao Marketplace, a Chinese website for online shopping
Enoch Grace Boutique	Blue Power Saving Specialist	Works great, very happy with it, saves power and electricity	Taobao Marketplace, a Chinese website for online shopping
Enoch Grace Boutique	Blue Power Saving Specialist	Very good, practical, worth having! My friend has used it practically and it does save electricity before I dared to get it! Looking forward to power saving effect?	Taobao Marketplace, a Chinese website for online shopping

Enoch Grace Boutique	Blue Power Saving Specialist	🆒It’s authentic, it’s the same as the ones you buy in the store, it’s easy and fast to buy online here, I’m assured of the quality, I’ll be back!!.^◡^.	Taobao Marketplace, a Chinese website for online shopping
Enoch Grace Boutique	Blue Power Saving Specialist	have seen this side of the hardware store sells, but it is very expensive, look at the brand themselves online to find a same, cheapermore than200, my home a month are close to2000degrees, with more than a month, these two months peak electricity, did not think that there is also lessthan300degrees, the effect is truly satisfied, truly recommended, a penny a penny	Taobao Marketplace, a Chinese website for online shopping
Enoch Grace Boutique	Blue Power Saving Specialist	Very good quality, fast logistics, very satisfied with a shopping	Taobao Marketplace, a Chinese website for online shopping
Enoch Grace Boutique	Blue Power Saving Specialist	pretty good	Taobao Marketplace, a Chinese website for online shopping
Enoch Grace Boutique	Blue Power Saving Specialist	Enoch2022intelligent energy saver, energy saving effect is very good, there are three gears can be adjusted, the home electric tiger🐯very useful, very satisfied with the quality of the product, the two days to use the product, the power dropped significantly, it seems to be able to save energy30%without a [illegible]	Taobao Marketplace, a Chinese website for online shopping
Enoch Grace Boutique	Blue Power Saving Specialist	Didn’t feel any power savings,506inJunethis year,530inJunelast year. and one less air conditioner this year	Taobao Marketplace, a Chinese website for online shopping
Enoch Grace Boutique	Blue Power Saving Specialist	The quality is good, and the warranty period, see this is what I bought, to choose to choose a big brand friends.	Taobao Marketplace, a Chinese website for online shopping
Enoch Grace Boutique	Blue Power Saving Specialist	Fooled and ineffective, advised not to buy!!!!	Taobao Marketplace, a Chinese website for online shopping
Enoch Grace Boutique	Blue Power Saving Specialist	I just received it I’ll come back with a review when I see the results	Taobao Marketplace, a Chinese website for online shopping
Enoch Grace Boutique	Blue Power Saving Specialist	The goods have been received, have not yet used, I heard others say it works well	Taobao Marketplace, a Chinese website for online shopping
Enoch Grace Boutique	Blue Power Saving Specialist	Quality is very good, and the seller described exactly the same, very satisfied,really like, completely exceeded expectations, the delivery speed is very fast	Taobao Marketplace, a Chinese website for online shopping
Enoch Grace Boutique	Blue Power Saving Specialist	Exquisite workmanship, the second one I bought, and I use it in my home supermarket!	Taobao Marketplace, a Chinese website for online shopping
Enoch Grace Boutique	Blue Power Saving Specialist	Before friends introduced, I was half-believing, used a period of time before coming to the evaluation, the day before yesterday to pay the electricity bill, it is indeed a lot less, I hope to be able to keep going	Taobao Marketplace, a Chinese website for online shopping
Enoch Grace Boutique	Blue Power Saving Specialist	Great value for money, very affordable, feels great to use, perfect.^◡^.	Taobao Marketplace, a Chinese website for online shopping
Enoch Grace Boutique	Blue Power Saving Specialist	Good	Taobao Marketplace, a Chinese website for online shopping
Enoch Grace Boutique	Blue Power Saving Specialist	It’s great, I’ll have to try it out before I add a review, customer service is on point.	Taobao Marketplace, a Chinese website for online shopping
Enoch Grace Boutique	Blue Power Saving Specialist	Friends have bought has been used for more than six months, said the power-saving effect is very good, recommended to me, I am open restaurant, try to use to see how the power-saving effect, then again praise recommended to you!	Taobao Marketplace, a Chinese website for online shopping
Enoch Grace Boutique	Blue Power Saving Specialist	But customer service is very satisfied! Had the patience to explain to me carefully the way to use, already installed, now follow up to see the electricity bill follow-up!	Taobao Marketplace, a Chinese website for online shopping
Enoch Grace Boutique	Blue Power Saving Specialist	Very good quality, good operation high power! Works very well!	Taobao Marketplace, a Chinese website for online shopping
Enoch Grace Boutique	Blue Power Saving Specialist	Last month leftabout20dollars, this month saved28, very grateful for the friend’s recommendation, I hope the power saving is more and more, recommended to everyone	Taobao Marketplace, a Chinese website for online shopping
Enoch Grace Boutique	Blue Power Saving Specialist	The quality is first class, the packaging is good, the logistics is fast, and the price is reasonable and affordable, I like it very much!	Taobao Marketplace, a Chinese website for online shopping
Enoch Grace Boutique	Blue Power Saving Specialist	Very good quality, very satisfied with the purchase. Customer service is very professional with questions and answers	Taobao Marketplace, a Chinese website for online shopping
Enoch Grace Boutique	Blue Power Saving Specialist	My friend kept saying that it was stealing electricity, don’t buy it, it’s illegal, so I didn’t listen, I did a lot of homework to research, and realized that it’s not like what he said, the principle is very important, if it’s illegal, then why is it still being sold normally, so I researched it by myself for a long time, and then I placed an order, and when I got it, I found out that it is indeed the same as I thought, it’s very good, it’s safe, reliable and I look forward to saving electricity!	Taobao Marketplace, a Chinese website for online shopping
Enoch Grace Boutique	Blue Power Saving Specialist	Things received, logistics is very fast, very satisfied	Taobao Marketplace, a Chinese website for online shopping
Enoch Grace Boutique	Blue Power Saving Specialist	The logistics arrived quickly	Taobao Marketplace, a Chinese website for online shopping
Enoch Grace Boutique	Blue Power Saving Specialist	Used for more than two months, power saving effect is very obvious, a very good shopping experience!	Taobao Marketplace, a Chinese website for online shopping
Enoch Grace Boutique	Blue Power Saving Specialist	Shipping was fast, arrived in two or three days, this color I love, cost-effective, verynice	Taobao Marketplace, a Chinese website for online shopping
Enoch Grace Boutique	Blue Power Saving Specialist	I have already purchased it for the second time, the power saving effect is good, and the product quality is safe, and the after-sales service attitude of the business is also very awesome.	Taobao Marketplace, a Chinese website for online shopping
Enoch Grace Boutique	Blue Power Saving Specialist	Packaging is very strong, well-protected; installation is also very simple does not require any technology, installed to keep the power on it; can also adjust the gear, set up a very humane use, the overall good	Taobao Marketplace, a Chinese website for online shopping
Enoch Grace Boutique	Blue Power Saving Specialist	The goods have been received, the object is consistent with the description, the delivery is also quite fast!	Taobao Marketplace, a Chinese website for online shopping
Enoch Grace Boutique	Blue Power Saving Specialist	Very satisfied with a shopping, customer service with questions and answers, very professional, reply quickly and efficiently!	Taobao Marketplace, a Chinese website for online shopping
Enoch Grace Boutique	Blue Power Saving Specialist	Super powerful oh, from the installation on a problem has not appeared, the performance is great, looking forward to future power saving effect ah!	Taobao Marketplace, a Chinese website for online shopping
Enoch Grace Boutique	Blue Power Saving Specialist	Very good quality and affordable price, shipping is strong, satisfied!	Taobao Marketplace, a Chinese website for online shopping

Enoch Grace Boutique	Blue Power Saving Specialist	favorable criticism	Taobao Marketplace, a Chinese website for online shopping
Enoch Grace Boutique	Blue Power Saving Specialist	The quality of the service is very good	Taobao Marketplace, a Chinese website for online shopping
Enoch Grace Boutique	Blue Power Saving Specialist	It's not much use. I haven't lost a dime in a month of use. Don't pay the IQ tax.	Taobao Marketplace, a Chinese website for online shopping
Enoch Grace Boutique	Blue Power Saving Specialist	Baby received the first time to open looks good, not yet installed, looking forward to good goods!	Taobao Marketplace, a Chinese website for online shopping
Enoch Grace Boutique	Blue Power Saving Specialist	Things received, good, logistics is also very fast five-star praise	Taobao Marketplace, a Chinese website for online shopping
Enoch Grace Boutique	Blue Power Saving Specialist	Merchants gave me a lot of fuse, unexpected surprise, originally do not believe, but customer service is very patient to explain the principle and the use of the appliance, it happens to be all in line with my home, so now come to recommend to you, absolutely good!	Taobao Marketplace, a Chinese website for online shopping
Enoch Grace Boutique	Blue Power Saving Specialist	Very good quality, good comments	Taobao Marketplace, a Chinese website for online shopping
Enoch Grace Boutique	Blue Power Saving Specialist	Business after-sales service attitude is very good, it is rare to meet such a good customer service, people beautiful heart, looking forward to the effect of power saving, and then follow up to recommend to everyone Oh.	Taobao Marketplace, a Chinese website for online shopping
Enoch Grace Boutique	Blue Power Saving Specialist	Just received, looks good quality, I directly inserted in the kitchen, induction cooker with more, the light will be dark, after the installation of the light brightness is normal, it should be more or less a little bit of the effect of it, after a month of follow-up!	Taobao Marketplace, a Chinese website for online shopping
Enoch Grace Boutique	Blue Power Saving Specialist	I first use to see, after all, with Poundland's price difference is a lot of times, I just use not a few days still do not know the specific effect and so late to come back to additional comments!	Taobao Marketplace, a Chinese website for online shopping
Enoch Grace Boutique	Blue Power Saving Specialist	Express is very fast, customer service attitude is also very good, specially praise, I asked before and after the first half of the month, customer service is really very patient, go home and immediately use the energy meter to try, the power is indeed reduced, the follow-up to see the cost of electricity to	Taobao Marketplace, a Chinese website for online shopping
Enoch Grace Boutique	Blue Power Saving Specialist	A minute a goods, the texture is very good, I like the blue color, can also be set to adjust the gear, very consistent with my home power consumption, and so I have the effect of power saving, then come back to the follow-up recommended to you, look forward to saving power 。。。。。。	Taobao Marketplace, a Chinese website for online shopping
Enoch Grace Boutique	Blue Power Saving Specialist	My friend's restaurant is to buy this, installed directly on the row, he said the effect is good, every month probably have to save a100yuan or so, a few months back to the capital, the longer you use the more power saving, so recommended to me, I just got has been used, and so on my power-saving effect of ha!	Taobao Marketplace, a Chinese website for online shopping
Enoch Grace Boutique	Blue Power Saving Specialist	The logistics is very fast, the package packaging is very strong looks good, try to see first, also heard friends said that good	Taobao Marketplace, a Chinese website for online shopping
Enoch Grace Boutique	Blue Power Saving Specialist	Summer has arrived, the air conditioning has been opened at night, Guangdong these days the weather is really hot, understand a long time, deliberately bought back to see the effect, compared to the air conditioning, is indeed a bit of an effect, a night less thanmore than5degrees, follow-up evaluation	Taobao Marketplace, a Chinese website for online shopping
Enoch Grace Boutique	Blue Power Saving Specialist	long time ago, I heard a friend say But they are the factory It is indeed a very good effect, but also in this buy, I also buy a try to see, epidemic situation at the moment, open the store is really not easy, set up the enhancement mode, I hope to save some of them, the quality seems to be very good	Taobao Marketplace, a Chinese website for online shopping
Enoch Grace Boutique	Blue Power Saving Specialist	The customer service staff is very good! Logistics is very fast! Value for money! Worth repurchasing! Customer service personnel attitude is very good! Logistics speed is very fast! Value for money! Worth repurchasing! Customer service personnel attitude is very good! Logistics speed is very fast! Value for money! Worth repurchasing! Customer service personnel attitude is very good! Logistics speed is very fast! Value for money! Worth repurchasing!	Taobao Marketplace, a Chinese website for online shopping
Enoch Grace Boutique	Blue Power Saving Specialist	The customer service staff is very good! Logistics is very fast! Value for money! Worth repurchasing! Customer service personnel attitude is very good! Logistics speed is very fast! Value for money! Worth repurchasing! Customer service personnel attitude is very good! Logistics speed is very fast! Value for money! Worth repurchasing! Customer service personnel attitude is very good! Logistics speed is very fast! Value for money! Worth repurchasing!	Taobao Marketplace, a Chinese website for online shopping
Enoch Grace Boutique	Blue Power Saving Specialist	Fine workmanship quality is good, good use is quite satisfactory	Taobao Marketplace, a Chinese website for online shopping
Enoch Grace Boutique	Blue Power Saving Specialist	At first, my side of the epidemic was returned, and then the business has free to send me back over, the service attitude is very awesome, next time if you need to continue to patronize.	Taobao Marketplace, a Chinese website for online shopping
Enoch Grace Boutique	Blue Power Saving Specialist	I'll add a positive review after I use it	Taobao Marketplace, a Chinese website for online shopping
Enoch Grace Boutique	Blue Power Saving Specialist	Previously went to Hong Kong to buy a, work, this time deliberately bought the most expensive, looks to be a lot higher than the previous, the home central air conditioning every day, compared to the effect is quite satisfactory, a day less20degrees or so, the overall is still more recommended this	Taobao Marketplace, a Chinese website for online shopping
Enoch Grace Boutique	Blue Power Saving Specialist	very good	Taobao Marketplace, a Chinese website for online shopping
Enoch Grace Boutique	Blue Power Saving Specialist	Saver received a good quality, fast shipping, seller service is also good, really satisfied!	Taobao Marketplace, a Chinese website for online shopping
Enoch Grace Boutique	Blue Power Saving Specialist	Received the goods, business service attitude is very good, logistics is also very strong, satisfied with the	Taobao Marketplace, a Chinese website for online shopping
Enoch Grace Boutique	Blue Power Saving Specialist	Although the price is slightly more expensive, it works much better than the other ones I bought last year, and you can also adjust the gear, and I heard from customer service that this one has the best power-saving effect, so I took the plunge; it's true that professionalism is in the hands of	Taobao Marketplace, a Chinese website for online shopping
Enoch Grace Boutique	Blue Power Saving Specialist	Good review ！！！！	Taobao Marketplace, a Chinese website for online shopping
Enoch Grace Boutique	Blue Power Saving Specialist	Good quality of goods, details, quality, workmanship are very good, shipping thief, express delivery, great~❀❀❀❀❀	Taobao Marketplace, a Chinese website for online shopping
Enoch Grace Boutique	Blue Power Saving Specialist	Logistics sent very quickly, SF brother handsome and kind, haha! The product is not broken at all, it is very good, looking forward to the effect of power saving Oh!	Taobao Marketplace, a Chinese website for online shopping
Enoch Grace Boutique	Blue Power Saving Specialist	It's Look at the online reviews really anything, mixed reviews. This evaluation is more real, or quite a lot of customers with good, I also intend to try, not to send it back, after these2days of observation, about14%savings, the results are still more than I expected, just see if you can use the lasting	Taobao Marketplace, a Chinese website for online shopping
Enoch Grace Boutique	Blue Power Saving Specialist	I've been using it for a while now, and the whole family has commented very favorably on it, so I recommend it.	Taobao Marketplace, a Chinese website for online shopping
Enoch Grace Boutique	Blue Power Saving Specialist	My friend used a few months after the power-saving effect is very good, recommended to me, my husband also said I pay IQ tax, do not give me to buy, and then I secretly bought and used, power-saving effect came out, he even changed his face and said that this thing is good, simple, convenient and safe; heh heh heh, the man words can not be trusted!	Taobao Marketplace, a Chinese website for online shopping

Enoch Grace Boutique	Blue Power Saving Specialist	over50bucksa month or so of use, still good, if you want to get one you can think about it, it's great	Taobao Marketplace, a Chinese website for online shopping
Enoch Grace Boutique	Blue Power Saving Specialist	Really like it, completely exceeded the expectations, the delivery speed is very fast, the packaging is very, tight, the logistics company has a very good service attitude, the delivery speed is very fast, very satisfied with a shopping!	Taobao Marketplace, a Chinese website for online shopping
Enoch Grace Boutique	Blue Power Saving Specialist	Saw that this baby can save electricity, with the mindset of trying. Received and installed to try, really save a lot, summer has it will not be afraid of air conditioning24hours open.	Taobao Marketplace, a Chinese website for online shopping
Enoch Grace Boutique	Blue Power Saving Specialist	Cost-effective is very good, power-saving effect is also better than my previous purchase ofmore than60yuan of that model, my family installed the meter, is indeed than not used to install the previous every day to save electricity, I have recommended around a few good friends to buy.	Taobao Marketplace, a Chinese website for online shopping
Enoch Grace Boutique	Blue Power Saving Specialist	Have never believed that there is a power saving this thing, and then overheard a friend talking about it, so I came back to search and with customer service to understand a lot, very patient to explain to me the principle of this and the product structure, compared to so many, or chose this one, it's great.	Taobao Marketplace, a Chinese website for online shopping
Enoch Grace Boutique	Blue Power Saving Specialist	's not heavy at all I bought it at a special promotion It Almost all of my friends are buying from this merchant I'm using it well It really works I recommend it now too	Taobao Marketplace, a Chinese website for online shopping
Enoch Grace Boutique	Blue Power Saving Specialist	Whether from the product packaging product shell plug quality and instructions certificate of conformity and so on details seem to kill any other product on the market, before also bought other, basically three no state, comparison test, the effect is real, probably has savedmore than160degrees, and then look at a month, good use will recommend to friends around the	Taobao Marketplace, a Chinese website for online shopping
Enoch Grace Boutique	Blue Power Saving Specialist	The quality is very good very good, power saving.	Taobao Marketplace, a Chinese website for online shopping
Enoch Grace Boutique	Blue Power Saving Specialist	Merchants at first said that you can save15%or so, I do not believe in my heart after a period of time to find that it really is a very good effect of power saving, integrity is very important, praise	Taobao Marketplace, a Chinese website for online shopping
Enoch Grace Boutique	Blue Power Saving Specialist	I love the blue casing, blue is my lucky color, surprisingly it saves power, love it, recommend it to you guys, my lucky blue!	Taobao Marketplace, a Chinese website for online shopping
Enoch Grace Boutique	Blue Power Saving Specialist	Previously bought168that is for the old home, the effect is very good. Then customer service recommended369this, is placed in my new home, because the new home appliances are more, two months, savingmore than30yuan of electricity, or good	Taobao Marketplace, a Chinese website for online shopping
Enoch Grace Boutique	Blue Power Saving Specialist	Very satisfied! 5 star reviews!	Taobao Marketplace, a Chinese website for online shopping
Enoch Grace Boutique	Blue Power Saving Specialist	Exquisite workmanship, multi-position adjustment, looks very high-end, pennies a penny, right, the effect of the comparison, or good, I hope to save all the time.	Taobao Marketplace, a Chinese website for online shopping
Enoch Grace Boutique	Blue Power Saving Specialist	Bought many times, the effect is really good, this time to move to a new home to install a high-power	Taobao Marketplace, a Chinese website for online shopping
Enoch Grace Boutique	Blue Power Saving Specialist	recommend highly	Taobao Marketplace, a Chinese website for online shopping
Enoch Grace Boutique	Blue Power Saving Specialist	This product is really good to use a month can really reduce hundreds of dollars in electricity bills to buy a few to friends and relatives!	Taobao Marketplace, a Chinese website for online shopping
Enoch Grace Boutique	Blue Power Saving Specialist	Previously bought a small power, use the effect is good, this time to replace this high power, looks good,3gears adjustable, hope the effect as always	Taobao Marketplace, a Chinese website for online shopping
Enoch Grace Boutique	Blue Power Saving Specialist	More than900degreesa month at home, the business said to set the standard mode can be, before the home to play hot pot sometimes trip, try today is normal, etc. will not flash should be the voltage stabilization, compared to the number of meters to save10degrees a day, very satisfied	Taobao Marketplace, a Chinese website for online shopping
Enoch Grace Boutique	Blue Power Saving Specialist	ve known about it for a long time Can't wait to receive it and follow the test method as told by the merchant The air conditioner at home uses a lot of electricity Measured it for one night It really saves About13% I'm quite satisfied with it Follow-up evaluation	Taobao Marketplace, a Chinese website for online shopping
Enoch Grace Boutique	Blue Power Saving Specialist	These two days compared, the effect is getting better and better, very satisfied, really did not think really magical, depending on the durability is not durable, the back also buy a few more units, hesitation can rest assured that the order, real customers do not spray.	Taobao Marketplace, a Chinese website for online shopping
Enoch Grace Boutique	Blue Power Saving Specialist	My two-story small flat building, electricity is quite a lot, sometimes more and sometimes less, my cousin recommended this can be adjusted to the level of energy-saving appliances to me, just used a few days found that the effect is good, the quality is very good and does not take up space, and force to	Taobao Marketplace, a Chinese website for online shopping
Enoch Grace Boutique	Blue Power Saving Specialist	Every day are compared to the phone on the software power consumption, really did not think the effect will be so obvious, reduced by a big half, a little shocked, real customers, highly recommended, asked the customer service to save so much normal, customer service said it is legal to save electricity, rest assured with different family power saving effect is different, I hope it will last!	Taobao Marketplace, a Chinese website for online shopping
Enoch Grace Boutique	Blue Power Saving Specialist	Product is great, just using it to see how it works any	Taobao Marketplace, a Chinese website for online shopping
Enoch Grace Boutique	Blue Power Saving Specialist	No effect at all, scamming all the time, not recommended, don't fall for it, people.	Taobao Marketplace, a Chinese website for online shopping
Enoch Grace Boutique	Blue Power Saving Specialist	Quite a lot of money, the quality is not good, no effect, don't be fooled!	Taobao Marketplace, a Chinese website for online shopping
Enoch Grace Boutique	Blue Power Saving Specialist	I think it's good, I just started using it, I'll come back in 2 months.	Taobao Marketplace, a Chinese website for online shopping
Enoch Grace Boutique	Blue Power Saving Specialist	Looks like a fine product, give it a good review! To be used after the effect to catch up with the review as the main. Customer service and SF Express is very satisfied.	Taobao Marketplace, a Chinese website for online shopping
Enoch Grace Boutique	Blue Power Saving Specialist	I don't know if it'll work.	Taobao Marketplace, a Chinese website for online shopping
Enoch Grace Boutique	Blue Power Saving Specialist	Have used it, took a chance on it and it actually worked and worked well.	Taobao Marketplace, a Chinese website for online shopping
Enoch Grace Boutique	Blue Power Saving Specialist	After a month of use, the electricity bill is relatively higher	Taobao Marketplace, a Chinese website for online shopping
Enoch Grace Boutique	Blue Power Saving Specialist	I've used it before, but now that the appliance has been added, it works well	Taobao Marketplace, a Chinese website for online shopping
Enoch Grace Boutique	Blue Power Saving Specialist	can	Taobao Marketplace, a Chinese website for online shopping
Enoch Grace Boutique	Blue Power Saving Specialist	After reading the certificate of compliance, the instruction manual, the insurance mark from PICC, all the paperwork shows that this machine has done a good job with the aura. Hope it works. Good review.	Taobao Marketplace, a Chinese website for online shopping

Enoch Grace Boutique	Blue Power Saving Specialist	It should work.	Taobao Marketplace, a Chinese website for online shopping
Enoch Grace Boutique	Blue Power Saving Specialist	Being used, can not see the effect, later additional evaluation	Taobao Marketplace, a Chinese website for online shopping
Enoch Grace Boutique	Blue Power Saving Specialist	Just received, installed under the seller's enthusiastic guidance, fast delivery and logistics. The three-stage design of the power saver is very humanized, and can be flexibly applied to different power consumption at different time points, one machine. Power-saving effect and warranty should be seen after a period of time to follow up.	Taobao Marketplace, a Chinese website for online shopping
Enoch Grace Boutique	Blue Power Saving Specialist	(of an unmarried couple) be close	Taobao Marketplace, a Chinese website for online shopping
Enoch Grace Boutique	Blue Power Saving Specialist	Shipping was really fast, received it the next day. The saver is well made and easy to use, and the customer service staff is careful and considerate in informing how to use it, which is great!	Taobao Marketplace, a Chinese website for online shopping
Enoch Grace Boutique	Blue Power Saving Specialist	I heard from a friend that it works well, so I'll try the results first.	Taobao Marketplace, a Chinese website for online shopping
Enoch Grace Boutique	Blue Power Saving Specialist	Saving appliances have been received, customer service attitude is very good, to send the use of pictures, according to the instructions have been installed to make asked, looking forward to good results, will buy back!	Taobao Marketplace, a Chinese website for online shopping
Enoch Grace Boutique	Blue Power Saving Specialist	Not seen to be effective	Taobao Marketplace, a Chinese website for online shopping
Enoch Grace Boutique	Blue Power Saving Specialist	Fast shipping, also already using it, machine looks good, looking forward to the results.	Taobao Marketplace, a Chinese website for online shopping
Enoch Grace Boutique	Blue Power Saving Specialist	Received it, looks good, looking forward to the results! If it's good, I'll recommend it to my friends and family.	Taobao Marketplace, a Chinese website for online shopping
Enoch Grace Boutique	Blue Power Saving Specialist	Baby received, exquisite workmanship, the seller's service is thoughtful, ask questions, five-star praise! Looking forward to the effect!	Taobao Marketplace, a Chinese website for online shopping
Enoch Grace Boutique	Blue Power Saving Specialist	It's good. It's good. It's good. It's good, good, good, good, good, good, good, good, good.	Taobao Marketplace, a Chinese website for online shopping
Enoch Grace Boutique	Blue Power Saving Specialist	(of an unmarried couple) be close	Taobao Marketplace, a Chinese website for online shopping
Enoch Grace Boutique	Blue Power Saving Specialist	It's installed. High-end atmosphere. Power-saving mode on.	Taobao Marketplace, a Chinese website for online shopping
Enoch Grace Boutique	Blue Power Saving Specialist	Received the item, looks exquisite, looking forward to the results.	Taobao Marketplace, a Chinese website for online shopping
Enoch Grace Boutique	Blue Power Saving Specialist	I'll comment when I've used it.	Taobao Marketplace, a Chinese website for online shopping
Enoch Grace Boutique	Blue Power Saving Specialist	Already installed, just started using it, looking forward to good results.	Taobao Marketplace, a Chinese website for online shopping
Enoch Grace Boutique	Blue Power Saving Specialist	It's not bad, I just plugged it in when it arrived and waited for a month to see if it saved me any money on my electricity bill.	Taobao Marketplace, a Chinese website for online shopping
Enoch Grace Boutique	Blue Power Saving Specialist	Waiting for the results	Taobao Marketplace, a Chinese website for online shopping
Enoch Grace Boutique	Blue Power Saving Specialist	Started using it, looking forward to the results	Taobao Marketplace, a Chinese website for online shopping
Enoch Grace Boutique	Blue Power Saving Specialist	an excellent	Taobao Marketplace, a Chinese website for online shopping
Enoch Grace Boutique	Blue Power Saving Specialist	Believe in science, believe in patents, and the end result is to expect to save power and money!	Taobao Marketplace, a Chinese website for online shopping
Enoch Grace Boutique	Blue Power Saving Specialist	First of all, to declare that I am buying a lot of units of the old customers, before there is no evaluation, during which I also saw some people questioned, I use down the effect has been very good, do not dare to say that a lot of savings,15%or so is there, this result can also ask for what?	Taobao Marketplace, a Chinese website for online shopping
Enoch Grace Boutique	Blue Power Saving Specialist	Good review to follow up later	Taobao Marketplace, a Chinese website for online shopping
Enoch Grace Boutique	Blue Power Saving Specialist	It didn't help at all and seemed to increase the power usage a bit.	Taobao Marketplace, a Chinese website for online shopping
Enoch Grace Boutique	Blue Power Saving Specialist	No effect at all, more than before.	Taobao Marketplace, a Chinese website for online shopping
Enoch Grace Boutique	Blue Power Saving Specialist	Summer air conditioning winter air conditioning, it is indeed very power-consuming, the family child is small, no way, all day are open, after using, save quite a lot, before aremore than800yuan a month, this month to see the electricity bill is682yuan, save quite a lot, very satisfied	Taobao Marketplace, a Chinese website for online shopping
Enoch Grace Boutique	Blue Power Saving Specialist	Received the power saver, bought the most expensive this, the packaging is very good, a little bit of weight, connect it to the main switch, from the surface feel good, my home power consumption ofabout400dollarsa month, looking forward to the effect,2months later to follow up!	Taobao Marketplace, a Chinese website for online shopping
Enoch Grace Boutique	Blue Power Saving Specialist	😉home villa a summer to open the central air conditioning is particularly power-consuming, the monthly electricity bill is basically in the1100-1300or so, while the usual electricity costs up to3hundred yuan, this time to Taobao to find a lot of home economizer, and ultimately chose this one because I saw that there is a formal inspection sheet, most of the users of the serious comments, and also is to communicate with the shopkeeper with a question-and-answer service attitude is very good! Immediately after receiving the goods began to install, plugged in and pressed to switch to normal mode, very	Taobao Marketplace, a Chinese website for online shopping
Enoch Grace Boutique	Blue Power Saving Specialist	My home ismore than380square feet of four-story villa, with four months, to speak a word of conscience this machine really does not have any effect, usually basic monthlyelectricity costsin350-400more or so, summer months that the big central air conditioning will generally be900-1200or so electricity costs, the last four months belong to the general month with this machine is still about350-400yuanper month, there is no effect at all! The last four months belong to the average month with this machine is still about 350-400 yuan per month, no effect at all. Quite disappointed, hehe!	Taobao Marketplace, a Chinese website for online shopping
Enoch Grace Boutique	Blue Power Saving Specialist	Things arrived, looks good, the product seems to be well-made, looking forward to a month after the energy-saving effect._x005f_x005F_x0001_ good service attitude	Taobao Marketplace, a Chinese website for online shopping
Enoch Grace Boutique	Blue Power Saving Specialist	I'll be back next time.	Taobao Marketplace, a Chinese website for online shopping

Enoch Grace Boutique	Blue Power Saving Specialist	Really good quality, the boss is very good. Looking forward to the effect, my home electricity bill used5000dollarsa year, it is a headache, see this immediately into the hands of the boss patiently explain, see the effect	Taobao Marketplace, a Chinese website for online shopping
Enoch Grace Boutique	Blue Power Saving Specialist	The quality of the stuff is very good, with the seller's description of the exact same, very satisfied,really like it, completely exceeded expectations, the delivery speed is very fast, the packaging is very careful, strict, the logistics company has a very good service attitude, the delivery speed is very fast, very satisfied with a shopping.	Taobao Marketplace, a Chinese website for online shopping
Enoch Grace Boutique	Blue Power Saving Specialist	The product looks well made and I look forward to the energy savings💪	Taobao Marketplace, a Chinese website for online shopping
Enoch Grace Boutique	Blue Power Saving Specialist	It doesn't work, and it's expensive.	Taobao Marketplace, a Chinese website for online shopping
Enoch Grace Boutique	Blue Power Saving Specialist	It looks good, I'll tell you after I use it!	Taobao Marketplace, a Chinese website for online shopping
Enoch Grace Boutique	Blue Power Saving Specialist	Can't see the point yet.	Taobao Marketplace, a Chinese website for online shopping
Enoch Grace Boutique	Blue Power Saving Specialist	Used before the evaluation, buyers said before buying much better, the results are really so, served, can indeed save power, great!	Taobao Marketplace, a Chinese website for online shopping
Enoch Grace Boutique	Blue Power Saving Specialist	Used a month before coming to evaluate, the effect is there, but not so magical, but my test is to save16%, I am personally very satisfied, after all, is not stealing electricity	Taobao Marketplace, a Chinese website for online shopping
Enoch Grace Boutique	Blue Power Saving Specialist	Confirmation of receipt line, did not try the effect, no evaluation	Taobao Marketplace, a Chinese website for online shopping
Enoch Grace Boutique	Blue Power Saving Specialist	I'm still trying it out.	Taobao Marketplace, a Chinese website for online shopping
Enoch Grace Boutique	Blue Power Saving Specialist	Received the goods to use to see, in the additional comments	Taobao Marketplace, a Chinese website for online shopping
Enoch Grace Boutique	Blue Power Saving Specialist	Power Saving King received, the boss has a very good service attitude, looking forward to the more fruitful to achieve the ideal, with the facts for the boss to publicize.	Taobao Marketplace, a Chinese website for online shopping
Enoch Grace Boutique	Blue Power Saving Specialist	I'll comment after I've used it and compared it.	Taobao Marketplace, a Chinese website for online shopping
Enoch Grace Boutique	Blue Power Saving Specialist	rare	Taobao Marketplace, a Chinese website for online shopping
Enoch Grace Boutique	Blue Power Saving Specialist	Pro, where do you have to plug it in to use it and does it have a power saving effect.	Taobao Marketplace, a Chinese website for online shopping
Enoch Grace Boutique	Blue Power Saving Specialist	Good name for an IQ blocker.	Taobao Marketplace, a Chinese website for online shopping
Enoch Grace Boutique	Blue Power Saving Specialist	How many of these would work bettera14Osquare foot house?	Taobao Marketplace, a Chinese website for online shopping
Enoch Grace Boutique	Blue Power Saving Specialist	Does it work, guys?	Taobao Marketplace, a Chinese website for online shopping
Enoch Grace Boutique	Blue Power Saving Specialist	I hope customers who have used it for a long time will tell me honestly whether it saves electricity or not. How much?	Taobao Marketplace, a Chinese website for online shopping
Enoch Grace Boutique	Blue Power Saving Specialist	Does the hair dryer work?	Taobao Marketplace, a Chinese website for online shopping
Enoch Grace Boutique	Blue Power Saving Specialist	If you buy a few more, you won't have to pay the electricity bill.	Taobao Marketplace, a Chinese website for online shopping
Enoch Grace Boutique	Blue Power Saving Specialist	Does it really save electricity?	Taobao Marketplace, a Chinese website for online shopping
Enoch Grace Boutique	Blue Power Saving Specialist	How many of these would work bettera14Osquare foot house?	Taobao Marketplace, a Chinese website for online shopping
Enoch Grace Boutique	Blue Power Saving Specialist	Cold storage380voltage three-phase electricity can be used	Taobao Marketplace, a Chinese website for online shopping
Enoch Grace Boutique	Blue Power Saving Specialist	Buyers, does this work?	Taobao Marketplace, a Chinese website for online shopping
Enoch Grace Boutique	Blue Power Saving Specialist	Is this stuff really effective if I've used it before? Is it legal?	Taobao Marketplace, a Chinese website for online shopping
Enoch Grace Boutique	Blue Power Saving Specialist	Do brothers and sisters who have bought it have any effect? Do not return to the merchants	Taobao Marketplace, a Chinese website for online shopping
Enoch Grace Boutique	Blue Power Saving Specialist	Why is it plugged into the socket? Can't it be connected directly to the main switch? All the appliances in the house go through the main switch.	Taobao Marketplace, a Chinese website for online shopping
Enoch Grace Boutique	Blue Power Saving Specialist	Hi, how long have you been using it and is it working?	Taobao Marketplace, a Chinese website for online shopping
Enoch Grace Boutique	Blue Power Saving Specialist	Does it work in a 3 phase oven if you've used a power saver? Does it save any electricity? Honestly.	Taobao Marketplace, a Chinese website for online shopping
Enoch Grace Boutique	Blue Power Saving Specialist	I hope those of you who have bought it can honestly answer the question of whether the price point is so high and whether it really works. Please, everyone.	Taobao Marketplace, a Chinese website for online shopping
Enoch Grace Boutique	Blue Power Saving Specialist	Is there a requirement as to where it should be installed, such as near the meter?	Taobao Marketplace, a Chinese website for online shopping
Enoch Grace Boutique	Blue Power Saving Specialist	Does this really work? It uses a lot of electricity in the summer	Taobao Marketplace, a Chinese website for online shopping
Enoch Grace Boutique	Blue Power Saving Specialist	Twelve thousand kilowatts a month for a convenience store. Does it work?	Taobao Marketplace, a Chinese website for online shopping

Enoch Grace Boutique	Blue Power Saving Specialist	usedbuyers whousingfor more than2months, and has there been a significant drop in electricity bills compared to the past	Taobao Marketplace, a Chinese website for online shopping
Enoch Grace Boutique	Blue Power Saving Specialist	Hi friends, please be honest does it really save electricity, looking forward to your answer.	Taobao Marketplace, a Chinese website for online shopping
Enoch Grace Boutique	Blue Power Saving Specialist	Can we really save electricity?	Taobao Marketplace, a Chinese website for online shopping
Enoch Grace Boutique	Blue Power Saving Specialist	I use it for a restaurant and it's over 4,000 degrees per month, will it work?	Taobao Marketplace, a Chinese website for online shopping
Enoch Grace Boutique	Blue Power Saving Specialist	Can I use 6,000 kilowatts of electricity? Three-phase.	Taobao Marketplace, a Chinese website for online shopping
Enoch Grace Boutique	Blue Power Saving Specialist	For those of you who have used it, tell me the truth, how did it work? Thank you.	Taobao Marketplace, a Chinese website for online shopping
Enoch Grace Boutique	Blue Power Saving Specialist	I need1,000kWh of electricityper month, how much can I save by buying him	Taobao Marketplace, a Chinese website for online shopping
Enoch Grace Boutique	Blue Power Saving Specialist	How many of these would work bettera14Osquare foot house?	Taobao Marketplace, a Chinese website for online shopping
Enoch Grace Boutique	Blue Power Saving Specialist	How many degrees can you save500degrees	Taobao Marketplace, a Chinese website for online shopping
Enoch Grace Boutique	Blue Power Saving Specialist	Anyone who has stuck with it? Does it save power?	Taobao Marketplace, a Chinese website for online shopping
Enoch Grace Boutique	Blue Power Saving Specialist	Does it really work?	Taobao Marketplace, a Chinese website for online shopping
Enoch Grace Boutique	Blue Power Saving Specialist	Does it work? Buyer's Answer.	Taobao Marketplace, a Chinese website for online shopping
Enoch Grace Boutique	Blue Power Saving Specialist	Is this saver safe for home use? Can it save electricity?	Taobao Marketplace, a Chinese website for online shopping
Enoch Grace Boutique	Blue Power Saving Specialist	Is that a capacitor in there?	Taobao Marketplace, a Chinese website for online shopping
Enoch Grace Boutique	Blue Power Saving Specialist	Does this power saver save electricity regardless of where it is plugged into the home, whether it is the appliances in the home that save more electricity or the appliances that are plugged in with it?	Taobao Marketplace, a Chinese website for online shopping
Enoch Grace Boutique	Blue Power Saving Specialist	How does it work for those who have used? Does it really save power How much does it save	Taobao Marketplace, a Chinese website for online shopping
Enoch Grace Boutique	Blue Power Saving Specialist	How to use	Taobao Marketplace, a Chinese website for online shopping
Enoch Grace Boutique	Blue Power Saving Specialist	The key is to save power or not	Taobao Marketplace, a Chinese website for online shopping
Enoch Grace Boutique	Blue Power Saving Specialist	How well does the air work with this, does the air power save electricity?	Taobao Marketplace, a Chinese website for online shopping
Enoch Grace Boutique	Blue Power Saving Specialist	Is this commercial video common to several?	Taobao Marketplace, a Chinese website for online shopping
Enoch Grace Boutique	Blue Power Saving Specialist	Does it have a stabilizing effect on voltage and current?	Taobao Marketplace, a Chinese website for online shopping
Enoch Grace Boutique	Blue Power Saving Specialist	Please, does it work?	Taobao Marketplace, a Chinese website for online shopping
Enoch Grace Boutique	Blue Power Saving Specialist	If you buy a few more, you won't have to pay the electricity bill.	Taobao Marketplace, a Chinese website for online shopping
Enoch Grace Boutique	Blue Power Saving Specialist	Does this saver work?	Taobao Marketplace, a Chinese website for online shopping
Enoch Grace Boutique	Blue Power Saving Specialist	Do you guys feel it's working?	Taobao Marketplace, a Chinese website for online shopping
Enoch Grace Boutique	Blue Power Saving Specialist	How did it work out?	Taobao Marketplace, a Chinese website for online shopping
Enoch Grace Boutique	Blue Power Saving Specialist	Can I use1900kWh of electricity up and downevery month?	Taobao Marketplace, a Chinese website for online shopping
Enoch Grace Boutique	Blue Power Saving Specialist	Is there a requirement as to where it should be installed, such as near the meter?	Taobao Marketplace, a Chinese website for online shopping
Enoch Grace Boutique	Blue Power Saving Specialist	Will the power company be fined if the electricity bill comes down less after this economizer is installed?	Taobao Marketplace, a Chinese website for online shopping
Enoch Grace Boutique	Blue Power Saving Specialist	The people who bought it don't know the law of conservation of energy.	Taobao Marketplace, a Chinese website for online shopping
Enoch Grace Boutique	Blue Power Saving Specialist	Does it really save power?	Taobao Marketplace, a Chinese website for online shopping
Enoch Grace Boutique	Blue Power Saving Specialist	Has anyone taken it apart?	Taobao Marketplace, a Chinese website for online shopping
Enoch Grace Boutique	Blue Power Saving Specialist	Does it really save power?	Taobao Marketplace, a Chinese website for online shopping
Enoch Grace Boutique	Blue Power Saving Specialist	Do you have to put a saver in every outlet? Wouldn't that be a lot of them?	Taobao Marketplace, a Chinese website for online shopping

Enoch Grace Boutique	Blue Power Saving Specialist	Does it work<br>Reply if it works	Taobao Marketplace, a Chinese website for online shopping
Enoch Grace Boutique	Blue Power Saving Specialist	IQ shield	Taobao Marketplace, a Chinese website for online shopping
Enoch Grace Boutique	Blue Power Saving Specialist	Why didn't you win the Nobel Prize?	Taobao Marketplace, a Chinese website for online shopping
Enoch Grace Boutique	Blue Power Saving Specialist	Is it true that you have to buy four for one meter for a building that is four stories high? It's the same meter.	Taobao Marketplace, a Chinese website for online shopping
Enoch Grace Boutique	Blue Power Saving Specialist	I'd say this needs to be plugged into the main switch, or where the power is highest, do you have one.	Taobao Marketplace, a Chinese website for online shopping
Enoch Grace Boutique	Blue Power Saving Specialist	There are no real results, folks.	Taobao Marketplace, a Chinese website for online shopping
Enoch Grace Boutique	Blue Power Saving Specialist	Power saving?	Taobao Marketplace, a Chinese website for online shopping
Enoch Grace Boutique	Blue Power Saving Specialist	Does it really save power?	Taobao Marketplace, a Chinese website for online shopping
Enoch Grace Boutique	Blue Power Saving Specialist	How many of these would work bettera14Osquare foot house?	Taobao Marketplace, a Chinese website for online shopping
Enoch Grace Boutique	Blue Power Saving Specialist	Which works better, this or a grain amplifier?	Taobao Marketplace, a Chinese website for online shopping
Enoch Grace Boutique	Blue Power Saving Specialist	Does it work in Malaysia?1350degreesa month	Taobao Marketplace, a Chinese website for online shopping
Enoch Grace Boutique	Blue Power Saving Specialist	May I ask the buyers pro, is this power saver good at saving electricity after use? Is there any power saving feature? Please tell me the truth!	Taobao Marketplace, a Chinese website for online shopping
Enoch Grace Boutique	Blue Power Saving Specialist	How effective?Twenty-fivepercent?	Taobao Marketplace, a Chinese website for online shopping
Enoch Grace Boutique	Blue Power Saving Specialist	Does this saver really work? How much can I save in a month?	Taobao Marketplace, a Chinese website for online shopping
Enoch Grace Boutique	Blue Power Saving Specialist	How does this power saving principle work with constant power?	Taobao Marketplace, a Chinese website for online shopping
Enoch Grace Boutique	Blue Power Saving Specialist	Can I buy a hundred meters and turn them upside down?	Taobao Marketplace, a Chinese website for online shopping
Enoch Grace Boutique	Blue Power Saving Specialist	Where should I install it? Or is it ok to install in the main switch of the entry electrical box?	Taobao Marketplace, a Chinese website for online shopping
Enoch Grace Boutique	Blue Power Saving Specialist	Does it work?	Taobao Marketplace, a Chinese website for online shopping
Enoch Grace Boutique	Blue Power Saving Specialist	Note to anyone who has bought one to see if it's a trifle?	Taobao Marketplace, a Chinese website for online shopping
Enoch Grace Boutique	Blue Power Saving Specialist	Just plug it into an outlet.	Taobao Marketplace, a Chinese website for online shopping
Enoch Grace Boutique	Blue Power Saving Specialist		Taobao Marketplace, a Chinese website for online shopping
Enoch Grace Boutique	Blue Power Saving Specialist	Usually one unit will do. Depending on the electricity consumption.369yuan this has three gears, the default mode100-500degrees, the standard mode500-1000degrees of electricity, enhanced mode1000-1800degrees of electricity.<br>According to the user's monthly electricity consumption adjusted to the appropriate level, the appropriate level of the best results. Continuously energized on the oh! Under the same meter, which socket can be, the whole house is effective.<br>As long as it is not inserted in the remote corner of the socket can be. Because the remote corner outlet. Generally at the end of the line, there are no appliances, the effect will be worse.<br>It is recommended to plug in the outlet near the main switch. Or plug in the place where the appliances are concentrated. Or the outlet of the appliances that are often on (such as refrigerators, fish tanks). These places are better. Constant power on is fine!	Taobao Marketplace, a Chinese website for online shopping
Enoch Grace Boutique	Blue Power Saving Specialist	power saving rateis 15-35%, why not fix a value. Because the degree of aging of the circuit, the aging of the appliances, and the appliances used are different in each home. The energy saving effect is not the same. So there is a range! Just like the same model of air conditioner. New and old. Definitely the old one is more expensive. Because the old one has more wear and tear. So the savings will be more! There is also the brand and model of each appliance, power size, energy consumption and so on those factors are not the same. So there is a range of power saving rate!	Taobao Marketplace, a Chinese website for online shopping
Enoch Grace Boutique	Blue Power Saving Specialist	Applicable appliances<br>Refrigerators, air conditioners, washing machines, freezers, sterilizers, copiers, printers, electric fans, hoods, vacuum cleaners, televisions, lamps with capacitors, and all electrical appliances with coils, motors, and electric……and any other inductive household appliances and equipment. Good effect.<br>Electricity is directly converted into heat and other resistive appliances (induction cookers, electric water heaters, microwave ovens, ovens, electric kettles, irons, incandescent lamps), basically no effect!<br>Power saving rate of15-35%, why not fix a value. Because the degree of aging of the circuit in each home, the aging of appliances, the use of appliances are not the same. Energy saving effect is also different. So there is a range! Just like the same model of air conditioner. New and old. Definitely the old one is more expensive. Because the old one has more wear and tear. So the savings will be more! There is also the brand and model of each appliance, power size, energy consumption and so on those factors are not the	Taobao Marketplace, a Chinese website for online shopping
Enoch Grace Boutique	Blue Power Saving Specialist	Motor motors, coils have an effect	Taobao Marketplace, a Chinese website for online shopping
Enoch Grace Boutique	Blue Power Saving Specialist	It's legal to save electricity, not steal it. It's not the more the merrier!	Taobao Marketplace, a Chinese website for online shopping
Enoch Grace Boutique	Blue Power Saving Specialist	power saving rateis 15-35%, why not fix a value. Because the degree of aging of the circuit, the aging of the appliances, and the appliances used are different in each home. The energy saving effect is not the same. So there is a range! Just like the same model of air conditioner. New and old. Definitely the old one is more expensive. Because the old one has more wear and tear. So the savings will be more! There is also the brand and model of each appliance, power size, energy consumption and so on those factors are not the same. So there is a range of power saving rate!	Taobao Marketplace, a Chinese website for online shopping

Enoch Grace Boutique	Blue Power Saving Specialist	Usually one unit will do. Depending on the electricity consumption.369yuan this has three gears, the default mode100-500degrees, the standard mode500-1000degrees of electricity, enhanced mode1000-1800degrees of electricity.<br>According to the user's monthly electricity consumption adjusted to the appropriate level, the appropriate level of the best results. Continuously energized on the oh! Under the same meter, which socket can be, the whole house is effective.<br>As long as it is not inserted in the remote corner of the socket can be. Because the remote corner outlet. Generally at the end of the line, there are no appliances, the effect will be worse.<br>It is recommended to plug in the outlet near the main switch. Or plug in the place where the appliances are concentrated. Or the outlet of the appliances that are often on (such as refrigerators, fish tanks). These places are better. Constant power on is fine!	Taobao Marketplace, a Chinese website for online shopping
Enoch Grace Boutique	Blue Power Saving Specialist	Pro. This is a single-phase economizer. To buy three-phase economizer can Oh. Details can consult online customer service oh	Taobao Marketplace, a Chinese website for online shopping
Enoch Grace Boutique	Blue Power Saving Specialist	It doesn't seem to be working.	Taobao Marketplace, a Chinese website for online shopping
Enoch Grace Boutique	Blue Power Saving Specialist	It doesn't work. Don't buy it.	Taobao Marketplace, a Chinese website for online shopping
Enoch Grace Boutique	Blue Power Saving Specialist	It doesn't feel obvious.	Taobao Marketplace, a Chinese website for online shopping
Enoch Grace Boutique	Blue Power Saving Specialist	Any outlet under the same meter is fine, the whole house is effective.<br>As long as it's not plugged into a remote corner outlet. Because of the remote corner socket. Usually at the end of the line, there are no appliances, the effect will be worse.<br>It is recommended to plug in the outlet near the main switch. Or plug in the place where the appliances are concentrated. Or the outlet of the appliances that are often on (such as refrigerators, fish tanks). These places are better. Continuously energized can be! You can also connect a three-pronged socket out of the main switch and plug it in oh (three-pronged socket ground wire do not have to connect, because the economizer only uses the fire wire and zero wire, made into a three-pronged plug is in order to plug more stable). Do not cut off the plug Oh, because if the plug is cut. Fear that the factory does not give	Taobao Marketplace, a Chinese website for online shopping
Enoch Grace Boutique	Blue Power Saving Specialist	Just started using Don't know the results yet	Taobao Marketplace, a Chinese website for online shopping
Enoch Grace Boutique	Blue Power Saving Specialist	Shouldn't save much.	Taobao Marketplace, a Chinese website for online shopping
Enoch Grace Boutique	Blue Power Saving Specialist	Applicable appliances<br>Refrigerators, air conditioners, washing machines, freezers, sterilizers, copiers, printers, electric fans, hoods, vacuum cleaners, televisions, lamps with capacitors, and all electrical appliances with coils, motors, and electric……and any other inductive household appliances and equipment. Good effect.<br>Electricity is directly converted into heat and other resistive appliances (induction cookers, electric water heaters, microwave ovens, ovens, electric kettles, irons, incandescent lamps), basically no effect!<br>Power saving rate of15-35%, why not fix a value. Because the degree of aging of the circuit in each home, the aging of appliances, the use of appliances are not the same. Energy saving effect is also different. So there is a range! Just like the same model of air conditioner. New and old. Definitely the old one is more expensive. Because the old one has more wear and tear. So the savings will be more! There is also the brand and model of each appliance, power size, energy consumption and so on those factors are not the	Taobao Marketplace, a Chinese website for online shopping
Enoch Grace Boutique	Blue Power Saving Specialist	Any outlet under the same meter is fine, the whole house is effective.<br>As long as it's not plugged into a remote corner outlet. Because of the remote corner socket. Usually at the end of the line, there are no appliances, the effect will be worse.<br>It is recommended to plug in the outlet near the main switch. Or plug in the place where the appliances are concentrated. Or the outlet of the appliances that are often on (such as refrigerators, fish tanks). These places are better. Constant power on is fine!	Taobao Marketplace, a Chinese website for online shopping
Enoch Grace Boutique	Blue Power Saving Specialist	We have more than ten years of technical experience in making power savers. There are patents.<br>The product is underwritten by PICC. Rest assured that the use of oh! Power saving rate of15-35%, why not fix a value. Because the degree of aging of the circuit of each home, the aging of appliances, the use of appliances are not the same. Energy saving effect is not the same. So there is a range! Just like the same model of air conditioner. New and old. Definitely the old one is more expensive. Because the old one has more wear and tear. So the savings will be more! There is also the brand and model of each appliance, power size, energy consumption and so on those factors are not the same. So there is a range of power	Taobao Marketplace, a Chinese website for online shopping
Enoch Grace Boutique	Blue Power Saving Specialist	It works. It's a hundred kilowatts or so.	Taobao Marketplace, a Chinese website for online shopping
Enoch Grace Boutique	Blue Power Saving Specialist	It's no fun!	Taobao Marketplace, a Chinese website for online shopping
Enoch Grace Boutique	Blue Power Saving Specialist	Just started using it. I don't know.	Taobao Marketplace, a Chinese website for online shopping
Enoch Grace Boutique	Blue Power Saving Specialist	I don't know, installed it and left it alone. It's not easy to test.	Taobao Marketplace, a Chinese website for online shopping
Enoch Grace Boutique	Blue Power Saving Specialist	I didn't feel my electric bill was less when I bought it.	Taobao Marketplace, a Chinese website for online shopping
Enoch Grace Boutique	Blue Power Saving Specialist	I don't know.	Taobao Marketplace, a Chinese website for online shopping
Enoch Grace Boutique	Blue Power Saving Specialist	You should be able to compare them next month when you pay your electric bill, it's been over a month since I used it	Taobao Marketplace, a Chinese website for online shopping
Enoch Grace Boutique	Blue Power Saving Specialist	$369this one has three gears, default mode100-500kWh, standard mode500-1000kWh, and enhanced mode1000-1800kWh.<br>According to the user's monthly electricity consumption adjusted to the appropriate level, the appropriate level of the best results. Continuously energized on the oh! Power saving rate15-35%, why not fix a value. Because the degree of aging of the circuit of each home, the aging of appliances, the use of appliances are not the same. Energy saving effect is not the same. So there is a range! Just like the same model of air conditioner. New and old. Definitely the old one is more expensive. Because the old one has more wear and tear. So the savings will be more! There is also the brand and model of each appliance,	Taobao Marketplace, a Chinese website for online shopping
Enoch Grace Boutique	Blue Power Saving Specialist	Usually one unit will do. Depending on the electricity consumption.369yuan this has three gears, the default mode100-500degrees, the standard mode500-1000degrees of electricity, enhanced mode1000-1800degrees of electricity.<br>According to the user's monthly electricity consumption adjusted to the appropriate level, the appropriate level of the best results. Continuously energized on the oh! Under the same meter, which socket can be, the whole house is effective.<br>As long as it is not inserted in the remote corner of the socket can be. Because the remote corner outlet. Generally at the end of the line, there are no appliances, the effect will be worse.<br>It is recommended to plug in the outlet near the main switch. Or plug in the place where the appliances are concentrated. Or the outlet of the appliances that are often on (such as refrigerators, fish tanks). These places are better. Constant power on is fine!	Taobao Marketplace, a Chinese website for online shopping

Enoch Grace Boutique	Blue Power Saving Specialist	Legitimate electricity savings. Space savings are limited, not unlimited. And how much you save depends entirely on the appliances and circuits used. Because not all appliances are effective. Electricity saving rate15-35%, why not fix a value. Because the degree of aging of the circuit, the aging of the appliances, the use of appliances are not the same in each home. The energy saving effect is not the same. So there is a range! Just like the same model of air conditioner. New and old. Definitely the old one is more expensive. Because the old one has more wear and tear. So the savings will be more! There is also the brand and model of each appliance, power size, energy consumption and so on those factors are	Taobao Marketplace, a Chinese website for online shopping
Enoch Grace Boutique	Blue Power Saving Specialist	I think it's okay.	Taobao Marketplace, a Chinese website for online shopping
Enoch Grace Boutique	Blue Power Saving Specialist	Useful.	Taobao Marketplace, a Chinese website for online shopping
Enoch Grace Boutique	Blue Power Saving Specialist	usefulness	Taobao Marketplace, a Chinese website for online shopping
Enoch Grace Boutique	Blue Power Saving Specialist	Power saving is so obvious oh! Every month my electric bill is nearly300dollars, installed the savera little more than200points, good stuff!	Taobao Marketplace, a Chinese website for online shopping
Enoch Grace Boutique	Blue Power Saving Specialist	There's more than just capacitors in there. There are also chips, circuit boards, electronic components!	Taobao Marketplace, a Chinese website for online shopping
Enoch Grace Boutique	Blue Power Saving Specialist	Any outlet under the same meter is fine, the whole house is effective.<br>As long as it's not plugged into a remote corner outlet. Because of the remote corner socket. Usually at the end of the line, there are no appliances, the effect will be worse.<br>It is recommended to plug in the outlet near the main switch. Or plug in the place where the appliances are concentrated. Or the outlet of the appliances that are often on (such as refrigerators, fish tanks). These places are better. Constant power on is fine!	Taobao Marketplace, a Chinese website for online shopping
Enoch Grace Boutique	Blue Power Saving Specialist	I don't know if it works.	Taobao Marketplace, a Chinese website for online shopping
Enoch Grace Boutique	Blue Power Saving Specialist	$369this one has three gears, default mode100-500kWh, standard mode500-1000kWh, and enhanced mode1000-1800kWh.<br>According to the user's monthly electricity consumption adjusted to the appropriate level, the appropriate level of the best results. Continuously energized on the oh! Under the same meter, which socket can be, the whole house is effective.<br>As long as it is not inserted in the remote corner of the socket can be. Because the remote corner outlet. Generally at the end of the line, there are no appliances, the effect will be worse.<br>It is recommended to plug in the outlet near the main switch. Or plug in the place where the appliances are concentrated. Or the outlet of the appliances that are often on (such as refrigerators, fish tanks).	Taobao Marketplace, a Chinese website for online shopping
Enoch Grace Boutique	Blue Power Saving Specialist	I don't see any results.	Taobao Marketplace, a Chinese website for online shopping
Enoch Grace Boutique	Blue Power Saving Specialist	The air will work.	Taobao Marketplace, a Chinese website for online shopping
Enoch Grace Boutique	Blue Power Saving Specialist	I don't know. I'm using it.	Taobao Marketplace, a Chinese website for online shopping
Enoch Grace Boutique	Blue Power Saving Specialist	There's a capacitor in there. It should stabilize the voltage and current.	Taobao Marketplace, a Chinese website for online shopping
Enoch Grace Boutique	Blue Power Saving Specialist	power saving rateis 15-35%, why not fix a value. Because the degree of aging of the circuit, the aging of the appliances, and the appliances used are different in each home. The energy saving effect is not the same. So there is a range! Just like the same model of air conditioner. New and old. Definitely the old one is more expensive. Because the old one has more wear and tear. So the savings will be more! There is also the brand and model of each appliance, power size, energy consumption and so on those factors are not the same. So there is a range of power saving rate! Legally save electricity. The savings are limited, not unlimited. And how much you save depends entirely on the appliances and circuits used. Because not all appliances are effective. Applicable appliances<br>Refrigerators, air conditioners, washing machines, freezers, sterilizers, photocopiers, printers, electric fans, hoods, vacuum cleaners, televisions, lamps with capacitors, and all appliances with coils, motors, and……and any other inductive household appliances and equipment. Good effect.<br>Electricity is directly converted into heat and other resistive appliances (induction cookers, electric	Taobao Marketplace, a Chinese website for online shopping
Enoch Grace Boutique	Blue Power Saving Specialist	It's legal to save electricity, not steal it. It's not the more the merrier!	Taobao Marketplace, a Chinese website for online shopping
Enoch Grace Boutique	Blue Power Saving Specialist	There's no change in the electricity bill.	Taobao Marketplace, a Chinese website for online shopping
Enoch Grace Boutique	Blue Power Saving Specialist	The effect is there depending on the home appliances, such as buying an air conditioner is inverter effect is small buy a fixed frequency effect is very obvious!	Taobao Marketplace, a Chinese website for online shopping
Enoch Grace Boutique	Blue Power Saving Specialist	power saving rateis 15-35%, why not fix a value. Because the degree of aging of the circuit, the aging of the appliances, and the appliances used are different in each home. The energy saving effect is not the same. So there is a range! Just like the same model of air conditioner. New and old. Definitely the old one is more expensive. Because the old one has more wear and tear. So the savings will be more! There is also the brand and model of each appliance, power size, energy consumption and so on those factors are not the same. So there is a range of power saving rate!	Taobao Marketplace, a Chinese website for online shopping
Enoch Grace Boutique	Blue Power Saving Specialist	It doesn't work. Don't buy it. I've been using it for a couple of months and it's not missing.	Taobao Marketplace, a Chinese website for online shopping
Enoch Grace Boutique	Blue Power Saving Specialist	Any outlet under the same meter is fine, the whole house is effective.<br>As long as it's not plugged into a remote corner outlet. Because of the remote corner socket. Usually at the end of the line, there are no appliances, the effect will be worse.<br>It is recommended to plug in the outlet near the main switch. Or plug in the place where the appliances are concentrated. Or the outlet of the appliances that are often on (such as refrigerators, fish tanks). These places are better. Constant power on is fine!	Taobao Marketplace, a Chinese website for online shopping
Enoch Grace Boutique	Blue Power Saving Specialist	It's not going to happen. It's legal to save electricity. It's not stealing electricity. Don't worry.	Taobao Marketplace, a Chinese website for online shopping
Enoch Grace Boutique	Blue Power Saving Specialist	It's working. It's smooth.	Taobao Marketplace, a Chinese website for online shopping
Enoch Grace Boutique	Blue Power Saving Specialist	On trial.	Taobao Marketplace, a Chinese website for online shopping
Enoch Grace Boutique	Blue Power Saving Specialist	I took it apart. There was a diamond inside.	Taobao Marketplace, a Chinese website for online shopping
Enoch Grace Boutique	Blue Power Saving Specialist	Actually, I'm not sure. I haven't done the math. I'll just buy one and plug it in.	Taobao Marketplace, a Chinese website for online shopping

Enoch Grace Boutique	Blue Power Saving Specialist	$369this one has three gears, default mode100-500kWh, standard mode500-1000kWh, and enhanced mode1000-1800kWh.<br>According to the user's monthly electricity consumption adjusted to the appropriate level, the appropriate level of the best results. Continuously energized on the oh! Under the same meter, which socket can be, the whole house is effective.<br>As long as it is not inserted in the remote corner of the socket can be. Because the remote corner outlet. Generally at the end of the line, there are no appliances, the effect will be worse.<br>It is recommended to plug in the outlet near the main switch. Or plug in the place where the appliances are concentrated. Or the outlet of the appliances that are often on (such as refrigerators, fish tanks).	Taobao Marketplace, a Chinese website for online shopping
Enoch Grace Boutique	Blue Power Saving Specialist	For example, air conditioners, refrigerators, washing machines, vacuum cleaners, hoods, fans, lamps with capacitors, computers, TVs, and all electrical appliances with coils and motors, all work well, as long as it is a perceptual electrical appliances work well.<br>If you are in a position to do so, you can also connect a billing meter, connect an outlet after the meter, and use a high-power air conditioner (big power obviously) to plug it in and open it10hours, and record how much electricity is used. Then plug in a power saver. Under the same conditions, the air conditioner is openfor 10hours, and record how much electricity is used. A comparison will know how much savings, this is the most accurate way to actually look at the meter. Saving is a little by little thing, and this is a legal power saving, not stealing electricity immediately see the effect, not a few days can see the obvious effect of oh.	Taobao Marketplace, a Chinese website for online shopping
Enoch Grace Boutique	Blue Power Saving Specialist	It doesn't work. Don't buy it if it's a scam.	Taobao Marketplace, a Chinese website for online shopping
Enoch Grace Boutique	Blue Power Saving Specialist	Ask the inventor. I don't see much of a savings.	Taobao Marketplace, a Chinese website for online shopping
Enoch Grace Boutique	Blue Power Saving Specialist	Power saving principle; automatic power factor compensation, simply put, part of the electrical appliances at home have a part to do with the useless power, part of the useful power, through the compensation device automatically adjust the difference between the useful and useless power.	Taobao Marketplace, a Chinese website for online shopping
Enoch Grace Boutique	Blue Power Saving Specialist	Any outlet under the same meter is fine, the whole house is effective.<br>As long as it's not plugged into a remote corner outlet. Because of the remote corner socket. Usually at the end of the line, there are no appliances, the effect will be worse.<br>It is recommended to plug in the outlet near the main switch. Or plug in the place where the appliances are concentrated. Or the outlet of the appliances that are often on (such as refrigerators, fish tanks). These places are better. Constant power on is fine!	Taobao Marketplace, a Chinese website for online shopping
Enoch Grace Boutique	Blue Power Saving Specialist	We have more than ten years of technical experience in making power savers. There are patents.<br>The product is underwritten by PICC. Rest assured that the use of oh!	Taobao Marketplace, a Chinese website for online shopping
Enoch Grace Boutique	Blue Power Saving Specialist	Seems to have a slight effect, but it is not very obvious, a month or more than six hundred, and not how to use electricity do not know where so much, this year, do not feel the cold, air conditioning, roasting fire did not open it!	Taobao Marketplace, a Chinese website for online shopping
Enoch Grace Boutique	Blue Power Saving Specialist	We have more than ten years of technical experience in making power savers. There are patents.<br>The product is underwritten by PICC. Rest assured that the use of oh! Power saving rate of15-35%, why not fix a value. Because the degree of aging of the circuit of each home, the aging of appliances, the use of appliances are not the same. Energy saving effect is not the same. So there is a range! Just like the same model of air conditioner. New and old. Definitely the old one is more expensive. Because the old one has more wear and tear. So the savings will be more! There is also the brand and model of each appliance, power size, energy consumption and so on those factors are not the same. So there is a range of power	Taobao Marketplace, a Chinese website for online shopping
Enoch Grace Boutique	Blue Power Saving Specialist	Usually one unit will do. Depending on the electricity consumption.369yuan this has three gears, the default mode100-500degrees, the standard mode500-1000degrees of electricity, enhanced mode1000-1800degrees of electricity.<br>According to the user's monthly electricity consumption adjusted to the appropriate level, the appropriate level of the best results. Continuously energized on the oh! Under the same meter, which socket can be, the whole house is effective.<br>As long as it is not inserted in the remote corner of the socket can be. Because the remote corner outlet. Generally at the end of the line, there are no appliances, the effect will be worse.<br>It is recommended to plug in the outlet near the main switch. Or plug in the place where the appliances are concentrated. Or the outlet of the appliances that are often on (such as refrigerators, fish tanks). These places are better. Constant power on is fine!	Taobao Marketplace, a Chinese website for online shopping
Enoch Grace Boutique	Blue Power Saving Specialist	Never used a grain amplifier.	Taobao Marketplace, a Chinese website for online shopping
Enoch Grace Boutique	Blue Power Saving Specialist	It doesn't seem to be working.	Taobao Marketplace, a Chinese website for online shopping
Enoch Grace Boutique	Blue Power Saving Specialist	power saving rateis 15-35%, why not fix a value. Because the degree of aging of the circuit, the aging of the appliances, and the appliances used are different in each home. The energy saving effect is not the same. So there is a range! Just like the same model of air conditioner. New and old. Definitely the old one is more expensive. Because the old one has more wear and tear. So the savings will be more! There is also the brand and model of each appliance, power size, energy consumption and so on those factors are not the same. So there is a range of power saving rate! Legally save electricity. The savings are limited, not unlimited. And how much you save depends entirely on the appliances and circuits used.<br>Because not all appliances are effective. Applicable appliances<br>Refrigerators, air conditioners, washing machines, freezers, sterilizers, photocopiers, printers, electric fans, hoods, vacuum cleaners, televisions, lamps with capacitors, and all appliances with coils, motors, and……and any other inductive household appliances and equipment. Good effect.<br>Electricity is directly converted into heat and other resistive appliances (induction cookers, electric	Taobao Marketplace, a Chinese website for online shopping
Enoch Grace Boutique	Blue Power Saving Specialist	power saving rateis 15-35%, why not fix a value. Because the degree of aging of the circuit, the aging of the appliances, and the appliances used are different in each home. The energy saving effect is not the same. So there is a range! Just like the same model of air conditioner. New and old. Definitely the old one is more expensive. Because the old one has more wear and tear. So the savings will be more! There is also the brand and model of each appliance, power size, energy consumption and so on those factors are not the same. So there is a range of power saving rate!	Taobao Marketplace, a Chinese website for online shopping
Enoch Grace Boutique	Blue Power Saving Specialist	We have more than ten years of technical experience in making power savers. There are patents.<br>The product is underwritten by PICC. Rest assured that the use of oh! Power saving rate of15-35%, why not fix a value. Because the degree of aging of the circuit of each home, the aging of appliances, the use of appliances are not the same. Energy saving effect is not the same. So there is a range! Just like the same model of air conditioner. New and old. Definitely the old one is more expensive. Because the old one has more wear and tear. So the savings will be more! There is also the brand and model of each appliance, power size, energy consumption and so on those factors are not the same. So there is a range of power	Taobao Marketplace, a Chinese website for online shopping
Enoch Grace Boutique	Blue Power Saving Specialist	Power saving experts intelligent power saver (scientific name: electric power reactive power in situ compensator) with safe electronic power saver original strong and weak mutual power saving principle and power saving practice, mutual restraint, so that the current is smooth and balanced, scientific practice has shown that the market application shows that the intelligent power saver with safe electronic power saver can stabilize the power supply voltage, filtering transient surges, smooth line currents, reduce the temperature of the line to improve the power factor, with power saving, Safety, protection to extend the service life of equipment and other multiple effects. The longer the use of time, the better the	Taobao Marketplace, a Chinese website for online shopping
Enoch Grace Boutique	Blue Power Saving Specialist	Can't. It's legal to save electricity not an illegal device.	Taobao Marketplace, a Chinese website for online shopping
Enoch Grace Boutique	Blue Power Saving Specialist	Yes. Close is best.	Taobao Marketplace, a Chinese website for online shopping

Enoch Grace Boutique	Blue Power Saving Specialist	Applicable appliances<br>Refrigerators, air conditioners, washing machines, freezers, sterilizers, copiers, printers, electric fans, hoods, vacuum cleaners, televisions, lamps with capacitors, and all electrical appliances with coils, motors, and electric……and any other inductive household appliances and equipment. Good effect. Electricity is directly converted into heat and other resistive appliances (induction cookers, electric water heaters, microwave ovens, ovens, electric kettles, irons, incandescent lamps), basically no effect! Power saving rate of15-35%, why not fix a value. Because the degree of aging of the circuit in each home, the aging of appliances, the use of appliances are not the same. Energy saving effect is also different. So there is a range! Just like the same model of air conditioner. New and old. Definitely the old one is more expensive. Because the old one has more wear and tear. So the savings will be more! There is also the brand and model of each appliance, power size, energy consumption and so on those factors are not the	Taobao Marketplace, a Chinese website for online shopping
Enoch Grace Boutique	Blue Power Saving Specialist	Personally, I think it's pretty good.	Taobao Marketplace, a Chinese website for online shopping
Enoch Grace Boutique	220V Commercial Power Saver	Some say it works, some say it doesn't… bought it to try.The appearance is consistent with the photo Good comments first	Taobao Marketplace, a Chinese website for online shopping
Enoch Grace Boutique	221V Commercial Power Saver	I don't know if it works, but I hope it's worth the price	Taobao Marketplace, a Chinese website for online shopping
Enoch Grace Boutique	222V Commercial Power Saver	How much less?	Taobao Marketplace, a Chinese website for online shopping
Enoch Grace Boutique	223V Commercial Power Saver	Can we save electricity? We need3000/4000kWha month, can we really save electricity?	Taobao Marketplace, a Chinese website for online shopping
Enoch Grace Boutique	224V Commercial Power Saver	Can I ask you guys if the saver works?	Taobao Marketplace, a Chinese website for online shopping
Enoch Grace Boutique	225V Commercial Power Saver	Does it really save electricity, have you bought it?	Taobao Marketplace, a Chinese website for online shopping
Enoch Grace Boutique	226V Commercial Power Saver	Does it work? Does it really save power?	Taobao Marketplace, a Chinese website for online shopping
Enoch Grace Boutique	227V Commercial Power Saver	I don't know, but myelectric billover6,000for two months inJuly and August	Taobao Marketplace, a Chinese website for online shopping
Enoch Grace Boutique	228V Commercial Power Saver	(usually used in the negative) have the possibility of	Taobao Marketplace, a Chinese website for online shopping
Enoch Grace Boutique	229V Commercial Power Saver	Applicable appliances<br>Refrigerators, air conditioners, washing machines, freezers, sterilizers, copiers, printers, electric fans, hoods, vacuum cleaners, televisions, lamps with capacitors, and all electrical appliances with coils, motors, and electric……and any other inductive household appliances and equipment. Good effect. Electricity is directly converted into heat and other resistive appliances (induction cookers, electric water heaters, microwave ovens, ovens, electric kettles, irons, incandescent lamps), basically no effect!	Taobao Marketplace, a Chinese website for online shopping
Enoch Grace Boutique	230V Commercial Power Saver	It's been installed for a month and it's a hundred dollars more than last month, so we'll see what happens when this month rolls around.	Taobao Marketplace, a Chinese website for online shopping
Enoch Grace Boutique	231V Commercial Power Saver	It is reasonable to say that it should save a little, you think of it this way, it is a start capacitor, high-power appliances start, your home meter at that time than Liu Xiang are running fast! It instantly gives electricity to reduce the impact on the meter! The impact of the small meter will run a little slower! But not as slow as Wu Da Lang.	Taobao Marketplace, a Chinese website for online shopping
ShangDian Technologies, Inc.	a power saver (electronics)	Looks more advanced than previous generations, very lightweight, easy to use, great!	Taobao Marketplace, a Chinese website for online shopping
ShangDian Technologies, Inc.	a power saver (electronics)	Clothing stores use, neighbors recommended to buy, said a month can savemore than70dollars, first plugged in to see the effect of the	Taobao Marketplace, a Chinese website for online shopping
ShangDian Technologies, Inc.	a power saver (electronics)	This really saves electricity, my house has been used for a while, the electricity bill is really less, recently the air conditioning is very costly, this month the electricity bill is really less, it's really good!	Taobao Marketplace, a Chinese website for online shopping
ShangDian Technologies, Inc.	a power saver (electronics)	This saver is really good, second purchase already.	Taobao Marketplace, a Chinese website for online shopping
ShangDian Technologies, Inc.	a power saver (electronics)	Power saving: good Price/performance ratio: good Exterior style: good looking	Taobao Marketplace, a Chinese website for online shopping
ShangDian Technologies, Inc.	a power saver (electronics)	Listening to people introduce the effect is good on the decisive to buy, the appearance of the packaging is better than expected. More high-power appliances at home, after receiving the goods directly plugged in and used!	Taobao Marketplace, a Chinese website for online shopping
ShangDian Technologies, Inc.	a power saver (electronics)	Looks more advanced than previous generations, very lightweight, easy to use, great!	Taobao Marketplace, a Chinese website for online shopping
ShangDian Technologies, Inc.	a power saver (electronics)	This really saves electricity, my family used for a while, the electricity bill is really less, summer air conditioning is very costly, this month the electricity bill is really less, it's really good!	Taobao Marketplace, a Chinese website for online shopping
ShangDian Technologies, Inc.	a power saver (electronics)	A very nice energy saver. Plugged it in the same day I bought it. It's a great value, well made, and the power savings are OK.	Taobao Marketplace, a Chinese website for online shopping
ShangDian Technologies, Inc.	a power saver (electronics)	Fake doesn't help a bit.	Taobao Marketplace, a Chinese website for online shopping
ShangDian Technologies, Inc.	a power saver (electronics)	Previously, I have been buying from other stores, all domestic plugs. Also need a converter, then see this store sales are very good, inadvertently consult, only to know that this store, the British plugs are also available, decisive order to buy, and then never need to use a converter!	Taobao Marketplace, a Chinese website for online shopping
ShangDian Technologies, Inc.	a power saver (electronics)	Second time I repurchased it, it works very well Value for money: it works better than the others and savesabout40degrees a month	Taobao Marketplace, a Chinese website for online shopping
ShangDian Technologies, Inc.	a power saver (electronics)	My brother asked me to buy it, he used it himself and it worked, today I paid my electricity bill and it was much less than the month before, it's amazing!	Taobao Marketplace, a Chinese website for online shopping
ShangDian Technologies, Inc.	a power saver (electronics)	Fine workmanship, the appearance is also good, hot days use too much electricity, bought this back to use, really save a lot of electricity, worth buying!	Taobao Marketplace, a Chinese website for online shopping
ShangDian Technologies, Inc.	a power saver (electronics)	Friends recommended to buy, said to save a lot of electricity, the value is also high, our dormitory before charging100electricity card, only5days, this time with8days. Indeed have effect!	Taobao Marketplace, a Chinese website for online shopping

ShangDian Technologies, Inc.	a power saver (electronics)	Used for a period of time to evaluate, the power-saving effect really did not let me down no longer need to worry about the power consumption of high-power appliances, the material is very good, the manual is simple and easy to understand there are indicators to see will be used, the quiet use of no noise is small and exquisite inserted where it does not get in the way of a very good product! Thanks to the seller, this time to friends also bought a few!	Taobao Marketplace, a Chinese website for online shopping
ShangDian Technologies, Inc.	a power saver (electronics)	Cost-effective, just received the goods today on the use of the home appliances are many, looking forward to a good effect, temporary so, with a period of time to say, the effect is good must follow up!	Taobao Marketplace, a Chinese website for online shopping
ShangDian Technologies, Inc.	a power saver (electronics)	I've been using it for a month or so, and I realize it's useless. It's like paying an IQ tax.	Taobao Marketplace, a Chinese website for online shopping
ShangDian Technologies, Inc.	a power saver (electronics)	Things received, very satisfied‼Really superb seller meticulous and conscientious, and the goods sent super fast, carefully packaged, trustworthy!	Taobao Marketplace, a Chinese website for online shopping
ShangDian Technologies, Inc.	a power saver (electronics)	Used for a period of time to evaluate, power-saving effect really did not let me down no longer need to worry about the power consumption of high-power appliances, the material is very good, the manual is simple and easy to understand there are lights to see will be used, the quiet use of no noise, small and exquisite inserted where it does not get in the way of a very good product! Thanks to the seller, this [illegible]	Taobao Marketplace, a Chinese website for online shopping
ShangDian Technologies, Inc.	a power saver (electronics)	Power saving effect: just started to use, do not know to, later in a chase level	Taobao Marketplace, a Chinese website for online shopping
ShangDian Technologies, Inc.	a power saver (electronics)	(of an unmarried couple) be close	Taobao Marketplace, a Chinese website for online shopping
ShangDian Technologies, Inc.	a power saver (electronics)	Recommended by the owner of the store next door, looked at his home does have to save electricity, decided to get. Indeed quite can, the voltage is much more stable, there is a reduction in electricity consumption.	Taobao Marketplace, a Chinese website for online shopping
ShangDian Technologies, Inc.	a power saver (electronics)	Not a bit of use, not a penny saved. Useless.	Taobao Marketplace, a Chinese website for online shopping
ShangDian Technologies, Inc.	a power saver (electronics)	I don't think it saves power.	Taobao Marketplace, a Chinese website for online shopping
ShangDian Technologies, Inc.	a power saver (electronics)	Power savings: very much looking forward to the power savings. Saving electricity is the best way to go in the summer. Thanks to the owner for the recommendation. Value for money: you get what you pay for Experience: it does save electricity throughout the day	Taobao Marketplace, a Chinese website for online shopping
ShangDian Technologies, Inc.	a power saver (electronics)	Appearance clean and beautiful, workmanship is very good, did not find any defects, personal feeling that the size is also quite suitable, used2, energy saving30%found that it is indeed quite good. Then use it and buy one for my sister's house!	Taobao Marketplace, a Chinese website for online shopping
ShangDian Technologies, Inc.	a power saver (electronics)	Refrigerator every start a computer black screen, separate row plug or so, online inquiries about the cause, and then buy a power saver back to try, but also really useful, can balance the current, occasionally once or twice will be black, than the previous start a black once a lot of improvement, the power saving effect of the follow-up and then see	Taobao Marketplace, a Chinese website for online shopping
ShangDian Technologies, Inc.	a power saver (electronics)	Simple and convenient, just take the plug board a plug, you can save electricity. A month can save dozens of dollars, a year down to save a lot, very good product cost-effective, customer service attitude is also very good, worth recommending!	Taobao Marketplace, a Chinese website for online shopping
ShangDian Technologies, Inc.	a power saver (electronics)	Energy-saving appliances are really good, the family has a three-horsepower air conditioning, last year, January down the electricity bill ofmore than1,000, this year, after the installation of energy-saving appliances can save50to100per month, really too cost-effective, super satisfied, must be five-star [illegible]	Taobao Marketplace, a Chinese website for online shopping
ShangDian Technologies, Inc.	a power saver (electronics)	Shipped quickly, very nice looking design and great material. Continue to follow up after use.	Taobao Marketplace, a Chinese website for online shopping
ShangDian Technologies, Inc.	a power saver (electronics)	Received on the direct use, usually too much electricity, buy a saving appliances to save some electricity, the effect is good, very satisfied!	Taobao Marketplace, a Chinese website for online shopping
ShangDian Technologies, Inc.	a power saver (electronics)	High-power super saver power saving effect is obvious, long service life, but also very safe, crazy power saving, a save in the end, factory direct, quality assurance, overload and over-voltage protection, elimination of harmonic advantages, reduce current	Taobao Marketplace, a Chinese website for online shopping
ShangDian Technologies, Inc.	a power saver (electronics)	For the real answer does it really save electricity thank you	Taobao Marketplace, a Chinese website for online shopping
ShangDian Technologies, Inc.	a power saver (electronics)	Does it work?	Taobao Marketplace, a Chinese website for online shopping
ShangDian Technologies, Inc.	a power saver (electronics)	It's not working.	Taobao Marketplace, a Chinese website for online shopping
ShangDian Technologies, Inc.	a power saver (electronics)	I just used it. I don't know yet.	Taobao Marketplace, a Chinese website for online shopping
ShangDian Technologies, Inc.	Intelligent power saver	The product is overall good, tried it for a few days and it feels ok. Customer service is good. Logistics is fast enough. First use a period of time to see if the power saving know. Then come back to the review.	Taobao Marketplace, a Chinese website for online shopping
ShangDian Technologies, Inc.	Intelligent power saver	Received immediately plugged in, or very save electricity, outside electricity is too expensive, save a little better.	Taobao Marketplace, a Chinese website for online shopping
ShangDian Technologies, Inc.	Intelligent power saver	Saving electricity and money is green and healthy, improving the efficiency of electricity use and reducing the loss of electricity. It's great!	Taobao Marketplace, a Chinese website for online shopping
ShangDian Technologies, Inc.	Intelligent power saver	Electricity bill at home ismore than200per month, with this power saver, you can save a fortune, especially convenient and simple	Taobao Marketplace, a Chinese website for online shopping
ShangDian Technologies, Inc.	Intelligent power saver	The air conditioner, refrigerator, TV, etc. at home is particularly power consumption, so I bought this power saver, just use it and do not know the results, but this product looks very formal, the packaging is particularly good, looking forward to power-saving effect.	Taobao Marketplace, a Chinese website for online shopping
ShangDian Technologies, Inc.	Intelligent power saver	Smooth voltage, scientific energy saving. Good value for money and energy saving! Good reviews!	Taobao Marketplace, a Chinese website for online shopping
ShangDian Technologies, Inc.	Intelligent power saver	Baby received, the courier is very powerful, well-packed, I can not wait to use, and so on three months of time, I will follow up and the actual usefulness of the product price is also very affordable, just look at the performance of the baby!	Taobao Marketplace, a Chinese website for online shopping
ShangDian Technologies, Inc.	Intelligent power saver	Plugged it in and everything looks good, we'll see what happens.	Taobao Marketplace, a Chinese website for online shopping
ShangDian Technologies, Inc.	Intelligent power saver	Products are really good I used two for the family, usually kitchen and bathroom water heaters with electricity is too expensive, because the water heater to open every day, the monthly electricity bill is too much, a few months down,electricity bill is less than half of the previous it, the effect is [illegible]	Taobao Marketplace, a Chinese website for online shopping
ShangDian Technologies, Inc.	Intelligent power saver	Energy saver received, not used before, I hope to realize the effect of power saving, logistics is very fast five-star praise!	Taobao Marketplace, a Chinese website for online shopping
ShangDian Technologies, Inc.	Intelligent power saver	Technology is productivity, saving a little is a little, quality assurance	Taobao Marketplace, a Chinese website for online shopping

ShangDian Technologies, Inc.	Intelligent power saver	Power-saving effect: really good Price-performance ratio: really good Appearance and style: quite hello Workmanship and material: quite good Experience of use: quite good.	Taobao Marketplace, a Chinese website for online shopping
ShangDian Technologies, Inc.	Intelligent power saver	Power-saving effect: received the goods on the plug but there is the effect of thinking of buying a plug on it Experience: obviously use less electricity	Taobao Marketplace, a Chinese website for online shopping
ShangDian Technologies, Inc.	Intelligent power saver	No point** $167 for electricitybefore I bought it,$168after I bought it	Taobao Marketplace, a Chinese website for online shopping
ShangDian Technologies, Inc.	Intelligent power saver	Meticulous workmanship and materials Easy to use, quick and simple Power saving effect is obvious Fast shipping and logistics.	Taobao Marketplace, a Chinese website for online shopping
ShangDian Technologies, Inc.	Intelligent power saver	After two days of testing. The effect is good!first day is about1/4, the second day is1/3, and is not"stealing electricity", is through the intelligent integrated circuit, automatically reduce the loss of electrical appliances to achieve the purpose of saving electricity! Recommended!	Taobao Marketplace, a Chinese website for online shopping
ShangDian Technologies, Inc.	Intelligent power saver	It feels good, worth buying, 5 stars now!	Taobao Marketplace, a Chinese website for online shopping
ShangDian Technologies, Inc.	Intelligent power saver	Very good quality, good results, very satisfied	Taobao Marketplace, a Chinese website for online shopping
ShangDian Technologies, Inc.	Intelligent power saver	I've been using it for about 20 days and it's not working at all, where does it save electricity? The electricity bill should be how much or how much a hair unchanged, directly is the IQ tax, I hope to help other people to buy with caution!	Taobao Marketplace, a Chinese website for online shopping
ShangDian Technologies, Inc.	Intelligent power saver	Saver has received, but real can save electricity, the quality is very good, I times I buy a few more, too good.	Taobao Marketplace, a Chinese website for online shopping
ShangDian Technologies, Inc.	Intelligent power saver	Don't hesitate to buy wow! Doesn't feel cheap, very high end and exceptionally great!	Taobao Marketplace, a Chinese website for online shopping
ShangDian Technologies, Inc.	Intelligent power saver	Still in the trial stage, looking forward to the results next month	Taobao Marketplace, a Chinese website for online shopping
ShangDian Technologies, Inc.	Intelligent power saver	It's been in use for a month and the electricity bill is about the same as last month, saving electricity is not working.	Taobao Marketplace, a Chinese website for online shopping
ShangDian Technologies, Inc.	Intelligent power saver	Just identify the receipt, look very beautiful, the results of large units of research is certainly excellent quality, the effect of trying to know, if you can I will recommend to friends in Hebei, good service, logistics is also very fast.	Taobao Marketplace, a Chinese website for online shopping
ShangDian Technologies, Inc.	Intelligent power saver	Easy to use, patient customer service, fast shipping, inexpensive, looking forward to power saving effect ! ! ! !	Taobao Marketplace, a Chinese website for online shopping
ShangDian Technologies, Inc.	Intelligent power saver	Thrifty received the goods, a friend recommended that the effect is very good, easy to use and plug it in! Good review!	Taobao Marketplace, a Chinese website for online shopping
ShangDian Technologies, Inc.	Intelligent power saver	In line with the description, the use of the effect to be verified. Logistics is very fast, timely delivery. First to give a favorable review!	Taobao Marketplace, a Chinese website for online shopping
ShangDian Technologies, Inc.	Intelligent power saver	Experience: not used, not recommended.	Taobao Marketplace, a Chinese website for online shopping
ShangDian Technologies, Inc.	Intelligent power saver	Received, the quality is very good, fine workmanship, very good, really save electricity	Taobao Marketplace, a Chinese website for online shopping
ShangDian Technologies, Inc.	Intelligent power saver	Power saving effect is not obvious	Taobao Marketplace, a Chinese website for online shopping
ShangDian Technologies, Inc.	Intelligent power saver	Technology is productivity, saving a little is a little, quality assurance	Taobao Marketplace, a Chinese website for online shopping
ShangDian Technologies, Inc.	Intelligent power saver	Value for money:, high Appearance style:(good looking, Experience of use:"recommendable	Taobao Marketplace, a Chinese website for online shopping
ShangDian Technologies, Inc.	Intelligent power saver	Choose this one from many merchants, the baby is of high quality, the price is favorable, cost-effective, and the customer service attitude is very good, the logistics is quite fast, give a favorable comment! There is a need, you can rest assured that the purchase.	Taobao Marketplace, a Chinese website for online shopping
ShangDian Technologies, Inc.	Intelligent power saver	Classmates recommended to buy, power saving effect is very satisfied, saving money, plug and play, very good, satisfied with all five praise!	Taobao Marketplace, a Chinese website for online shopping
ShangDian Technologies, Inc.	Intelligent power saver	I've been using it for almost half a month now and it does save me a lot of electricity, thanks!	Taobao Marketplace, a Chinese website for online shopping
ShangDian Technologies, Inc.	Intelligent power saver	Thought it was a real display, thought what the paper was to protect the display, but it turns out the display is just a piece of paper!	Taobao Marketplace, a Chinese website for online shopping
ShangDian Technologies, Inc.	Intelligent power saver	(of an unmarried couple) be close	Taobao Marketplace, a Chinese website for online shopping
ShangDian Technologies, Inc.	Intelligent power saver	Baby received, quite good, used, give five points, after three months in the follow-up Workmanship material: quite detailed, not for nothing	Taobao Marketplace, a Chinese website for online shopping
ShangDian Technologies, Inc.	Intelligent power saver	I've been using it for half a month, tested it and it doesn't work at all, buy with caution!	Taobao Marketplace, a Chinese website for online shopping
ShangDian Technologies, Inc.	Intelligent power saver	I don't know how it works yet, I won't know if it's working until I use it for a month.	Taobao Marketplace, a Chinese website for online shopping
ShangDian Technologies, Inc.	Intelligent power saver	On Trial	Taobao Marketplace, a Chinese website for online shopping
ShangDian Technologies, Inc.	Intelligent power saver	It's a scam. Don't buy it. It doesn't work.	Taobao Marketplace, a Chinese website for online shopping
ShangDian Technologies, Inc.	Intelligent power saver	Power savings: very good.	Taobao Marketplace, a Chinese website for online shopping
ShangDian Technologies, Inc.	Intelligent power saver	The appearance is a bit rough, use it first, don't know how effective it is	Taobao Marketplace, a Chinese website for online shopping
ShangDian Technologies, Inc.	Intelligent power saver	Looking forward to the results	Taobao Marketplace, a Chinese website for online shopping

ShangDian Technologies, Inc.	Intelligent power saver	Power saving effect: can have a certain effect, Cost-effective: high Appearance style: beautiful and generous Workmanship material: good Using experience: have a certain energy-saving effect.	Taobao Marketplace, a Chinese website for online shopping
ShangDian Technologies, Inc.	Intelligent power saver	unscientific product	Taobao Marketplace, a Chinese website for online shopping
ShangDian Technologies, Inc.	Intelligent power saver	this kind of	Taobao Marketplace, a Chinese website for online shopping
ShangDian Technologies, Inc.	Intelligent power saver	Bought two energy-saving appliances for the family, the kitchen and bathroom water heater is too expensive, the water heater to be turned on every day, the monthly electricity bill is a lot of use, with this, no longer afraid of the electricity bill is more than one month down the electricity bill can be less than half of the previous month it, the effect is particularly good!	Taobao Marketplace, a Chinese website for online shopping
ShangDian Technologies, Inc.	Intelligent power saver	heat is more costly I bought it and plugged it in when I The logistics was fast It's easy and convenient to plug it in directly	Taobao Marketplace, a Chinese website for online shopping
ShangDian Technologies, Inc.	Intelligent power saver	I've been using it for a month now and it really saves a lot of electricity, thanks to the merchant!	Taobao Marketplace, a Chinese website for online shopping
ShangDian Technologies, Inc.	Intelligent power saver	Repurchased, there are energy-saving effect, even if the energy saving10degrees per month electricity in the long term to calculate is also very substantial.	Taobao Marketplace, a Chinese website for online shopping
ShangDian Technologies, Inc.	Intelligent power saver	I'm using it and looking forward to the power saving effect!	Taobao Marketplace, a Chinese website for online shopping
ShangDian Technologies, Inc.	Intelligent power saver	I'm using it. I'm testing it.	Taobao Marketplace, a Chinese website for online shopping
ShangDian Technologies, Inc.	Intelligent power saver	Just received the goods, now have not felt out, wait to use a period of time and then comment on it, the product workmanship is quite beautiful, exquisite, the	Taobao Marketplace, a Chinese website for online shopping
ShangDian Technologies, Inc.	Intelligent power saver	My friend recommended it, and ll be back with a testimonial if I get the same results.	Taobao Marketplace, a Chinese website for online shopping
ShangDian Technologies, Inc.	Intelligent power saver	Received the baby, plugged it in and is using it, I don't know how effective it is, try it first.	Taobao Marketplace, a Chinese website for online shopping
ShangDian Technologies, Inc.	Intelligent power saver	I'll comment again after using it for a while to see how much difference in electricity costs.	Taobao Marketplace, a Chinese website for online shopping
ShangDian Technologies, Inc.	Intelligent power saver	It's useless. IQ tax.	Taobao Marketplace, a Chinese website for online shopping
ShangDian Technologies, Inc.	Intelligent power saver	I hope it works.	Taobao Marketplace, a Chinese website for online shopping
ShangDian Technologies, Inc.	Intelligent power saver	Definitely a great product, it's especially easy to use and plugs right into the plug board at home. In the past few days to turn on the air conditioning to see the meter turn a little slower than before. But the electricity consumption is a little less than before. This product is trustworthy, like	Taobao Marketplace, a Chinese website for online shopping
ShangDian Technologies, Inc.	Intelligent power saver	works!I don't know what makes sense, but it does save me money again on my electric bill, it'sNICE!	Taobao Marketplace, a Chinese website for online shopping
ShangDian Technologies, Inc.	Intelligent power saver	I'm using it now, I'll follow up if it works	Taobao Marketplace, a Chinese website for online shopping
ShangDian Technologies, Inc.	Intelligent power saver	Power saving effect: use it before evaluation, should be good	Taobao Marketplace, a Chinese website for online shopping
ShangDian Technologies, Inc.	Intelligent power saver	The actual test is effective. Small processing plant with this is still very suitable, basically every day to save a little bit, bashful, decided to buy a few more to the old family also installed😄	Taobao Marketplace, a Chinese website for online shopping
ShangDian Technologies, Inc.	Intelligent power saver	Saver is also a friend introduced to me to buy, she used a few after the effect is very good, let me also insist on using, now the landlord heart is too black, the meter adjusted very quickly, a month to hundreds of dollars of electricity. I haven't turned on the air conditioner much yet, and with this landlord I can't pay my electricity bill anymore!	Taobao Marketplace, a Chinese website for online shopping
ShangDian Technologies, Inc.	Intelligent power saver	It does save electricity, the thing works well, and the customer service attitude is also very good. At first I did not know what the principle, the businessman patiently explained, I downloaded the National Gridapp, after the installation of the daily power consumption can be seen, it does save electricity!	Taobao Marketplace, a Chinese website for online shopping
ShangDian Technologies, Inc.	Intelligent power saver	Blow not black, indeed have the effect of, the more you use the more you save, not much to say a month, save money plus a tank of gasoline is not a big problem, save money things, I think no one will refuse it, too on the head ah, we can rest assured that the purchase!	Taobao Marketplace, a Chinese website for online shopping
ShangDian Technologies, Inc.	Intelligent power saver	Works well and already has results, thanks to the merchant!	Taobao Marketplace, a Chinese website for online shopping
ShangDian Technologies, Inc.	Intelligent power saver	The new upgraded saver to try, I've been a long time user of the saver,ve been buying itsincethe8thgeneration, and I've bought it in almost every generation, now it's really getting better and better, and this time I also purchased6of themat once, and the effect is really nothing to speak of!	Taobao Marketplace, a Chinese website for online shopping
ShangDian Technologies, Inc.	Intelligent power saver	Intelligent power saver received, exquisite packaging, the use of power saving effect is very good, praise.	Taobao Marketplace, a Chinese website for online shopping
ShangDian Technologies, Inc.	Intelligent power saver	Power-saving effect: logistics is very fast the next day received, friends recommended that the effect is very good, easy to use and plug it up!	Taobao Marketplace, a Chinese website for online shopping
ShangDian Technologies, Inc.	Intelligent power saver	Purchased a power saver received on the put into use, rent a room with many electrical appliances, power consumption is very large, we have a piece of electricity or1.2dollars a time, with this power saver is a lot better, every day can save a few cents, very convenient and practical.	Taobao Marketplace, a Chinese website for online shopping
ShangDian Technologies, Inc.	Intelligent power saver	It's good.	Taobao Marketplace, a Chinese website for online shopping
ShangDian Technologies, Inc.	Intelligent power saver	The goods have been received, the packaging is intact, and the same as described, quite satisfied with the full five points praise!	Taobao Marketplace, a Chinese website for online shopping
ShangDian Technologies, Inc.	Intelligent power saver	Received it and plugged it in immediately. There is a Pacific Underwriting label, so feel free to use it. Home appliances are many, buy two more. Expect to achieve the effect of power saving. Logistics is very fast, the store service is also good, give five stars.	Taobao Marketplace, a Chinese website for online shopping
ShangDian Technologies, Inc.	Intelligent power saver	Intelligent power saver compact does not take up space, very good, very practical, received on the use, has been used for a few days, power-saving effect is good, only to use a period of time in order to know exactly how much electricity can be saved, the current effect is still good, ready to get another one	Taobao Marketplace, a Chinese website for online shopping
ShangDian Technologies, Inc.	Intelligent power saver	Affordable Good power saving Cost	Taobao Marketplace, a Chinese website for online shopping

ShangDian Technologies, Inc.	Intelligent power saver	Logistics is very fast Ao, the product received, received the product is not yet used and so on after use to evaluate	Taobao Marketplace, a Chinese website for online shopping
ShangDian Technologies, Inc.	Intelligent power saver	Previously bought a small, used can this time in the purchase of a high-power, multi-functional again in the price more to buy a put on the third floor feel good can save electricity is a good product[strong] to the merchant a good comment satisfied with	Taobao Marketplace, a Chinese website for online shopping
ShangDian Technologies, Inc.	Intelligent power saver	The product is well made, good quality, power saving and effective. And the price is affordable. Convinced that the effect will be more obvious as more time passes. Highly recommended and tips to connect it near commonly used high power appliances to enjoy better power saving effect.	Taobao Marketplace, a Chinese website for online shopping
ShangDian Technologies, Inc.	Intelligent power saver	Could this stuff be bad for the circuits?	Taobao Marketplace, a Chinese website for online shopping
ShangDian Technologies, Inc.	Intelligent power saver	Does it really save power?	Taobao Marketplace, a Chinese website for online shopping
ShangDian Technologies, Inc.	Intelligent power saver	For those of you who have used it, is this a real power saver?	Taobao Marketplace, a Chinese website for online shopping
ShangDian Technologies, Inc.	Intelligent power saver	Does it really save power for those who have used it? Or is it an IQ tax	Taobao Marketplace, a Chinese website for online shopping
ShangDian Technologies, Inc.	Intelligent power saver	If you use100kWh of electricityper day.How many kilowatt-hours can be saved per day.	Taobao Marketplace, a Chinese website for online shopping
ShangDian Technologies, Inc.	Intelligent power saver	A proper IQ tax.	Taobao Marketplace, a Chinese website for online shopping
ShangDian Technologies, Inc.	Intelligent power saver	If you've used it, does it save power?	Taobao Marketplace, a Chinese website for online shopping
ShangDian Technologies, Inc.	Intelligent power saver	For those who have used it, does this saver really work? Please tell me the truth.	Taobao Marketplace, a Chinese website for online shopping
ShangDian Technologies, Inc.	Intelligent power saver	If it really works, wouldn't it be more energy efficient to use one in each room?	Taobao Marketplace, a Chinese website for online shopping
ShangDian Technologies, Inc.	Intelligent power saver	Is it an IQ tax? Brothers.	Taobao Marketplace, a Chinese website for online shopping
ShangDian Technologies, Inc.	Intelligent power saver	For those of you who have used it, does this saver really save electricity?	Taobao Marketplace, a Chinese website for online shopping
ShangDian Technologies, Inc.	Intelligent power saver	Is it an IQ tax? Please answer if you have used it	Taobao Marketplace, a Chinese website for online shopping
ShangDian Technologies, Inc.	Intelligent power saver	How do you get around the law of conservation of energy?	Taobao Marketplace, a Chinese website for online shopping
ShangDian Technologies, Inc.	Intelligent power saver	Is it okay to buy just one at home? Is it okay to plug it in anywhere?	Taobao Marketplace, a Chinese website for online shopping
ShangDian Technologies, Inc.	Intelligent power saver	anyone believe in this stuffin2023/:085/:085	Taobao Marketplace, a Chinese website for online shopping
ShangDian Technologies, Inc.	Intelligent power saver	The light doesn't come on. Is it broken?	Taobao Marketplace, a Chinese website for online shopping
ShangDian Technologies, Inc.	Intelligent power saver	will not (act, happen etc)	Taobao Marketplace, a Chinese website for online shopping
ShangDian Technologies, Inc.	Intelligent power saver	It feels a little bit, but for30dollars it won't be bad, so it's quite useful	Taobao Marketplace, a Chinese website for online shopping
ShangDian Technologies, Inc.	Intelligent power saver	Figuring out what's in your heart doesn't matter so much if it is or isn't.	Taobao Marketplace, a Chinese website for online shopping
ShangDian Technologies, Inc.	Intelligent power saver	Not too noticeable, after all, it's only a 20 or 30 dollar gadget, I bought another one and am experimenting with it	Taobao Marketplace, a Chinese website for online shopping
ShangDian Technologies, Inc.	Intelligent power saver	It's a short period of time.	Taobao Marketplace, a Chinese website for online shopping
ShangDian Technologies, Inc.	Intelligent power saver	It feels like an IQtax/:Q	Taobao Marketplace, a Chinese website for online shopping
ShangDian Technologies, Inc.	Intelligent power saver	It saves electricity.	Taobao Marketplace, a Chinese website for online shopping
ShangDian Technologies, Inc.	Intelligent power saver	Saveabout$3a day	Taobao Marketplace, a Chinese website for online shopping
ShangDian Technologies, Inc.	Intelligent power saver	The Warlord ofLiangshan/:Q/:Q/:Q	Taobao Marketplace, a Chinese website for online shopping
ShangDian Technologies, Inc.	Intelligent power saver	Yes, to buy it is to fall for it.	Taobao Marketplace, a Chinese website for online shopping
ShangDian Technologies, Inc.	Intelligent power saver	It does save a lot of electricity. - It's good.	Taobao Marketplace, a Chinese website for online shopping
ShangDian Technologies, Inc.	Intelligent power saver	Save20%0	Taobao Marketplace, a Chinese website for online shopping
ShangDian Technologies, Inc.	Intelligent power saver	I don't know.	Taobao Marketplace, a Chinese website for online shopping
ShangDian Technologies, Inc.	Intelligent power saver	IQ tax. Don't buy it.	Taobao Marketplace, a Chinese website for online shopping
ShangDian Technologies, Inc.	Intelligent power saver	it or not,/:079	Taobao Marketplace, a Chinese website for online shopping

ShangDian Technologies, Inc.	Intelligent power saver	yea	Taobao Marketplace, a Chinese website for online shopping
ShangDian Technologies, Inc.	King of Power Saving	Has been plugged in a month, it does work, really save electricity, customer service attitude is very good to explain the very careful and professional! This month to savemore than70yuan electricity bill! I'm going to buy another one.	Taobao Marketplace, a Chinese website for online shopping
ShangDian Technologies, Inc.	King of Power Saving	Product workmanship is really nothing to speak of, look at the very high-grade, the use of the effect is very obvious, the electricity bill is40%less, summer electricity is too expensive, every time the electricity bill are meat pain, with this device is much more assured, every month to save money to eat a [illegible]	Taobao Marketplace, a Chinese website for online shopping
ShangDian Technologies, Inc.	King of Power Saving	Cost-effective: can be, appearance style: good , workmanship material: good, power-saving effect: to use for a period of time to see, the store promises120days does not achieve the effect can also be returned.👍	Taobao Marketplace, a Chinese website for online shopping
ShangDian Technologies, Inc.	King of Power Saving	Product workmanship is really nothing to speak of, look at the very high-grade, the use of the effect is very obvious, the electricity bill is40%less, summer electricity is too expensive, every time the electricity bill are meat pain, with this device is much more assured, every month to save money to eat a [illegible]	Taobao Marketplace, a Chinese website for online shopping
ShangDian Technologies, Inc.	King of Power Saving	Smart Saver received it. Packaging is very good. Much larger than the previous purchase, beautiful atmosphere looks very good. The effect of how to use a period of time and then comment. Merchants promise tobe refundable within90days! Thanks to the merchants!	Taobao Marketplace, a Chinese website for online shopping
ShangDian Technologies, Inc.	King of Power Saving	I'm not saving power right now. I'm just waiting two months to see.	Taobao Marketplace, a Chinese website for online shopping
ShangDian Technologies, Inc.	King of Power Saving	It does save electricity, the thing works well, and the customer service attitude is also very good. At first I did not know what principle, the businessman patiently explained, I downloaded the National Gridapp, after installation of the daily power consumption can be seen, it does save electricity	Taobao Marketplace, a Chinese website for online shopping
ShangDian Technologies, Inc.	King of Power Saving	Customer service was very professional in their instructions. Press to use on front end outlet at home! Current through the king of power saving. Work compensation effect is indeed very powerful, compared to previous years at this time to open the air conditioning a month are fast600degrees now the same with also smooth inmore than400degrees	Taobao Marketplace, a Chinese website for online shopping
ShangDian Technologies, Inc.	King of Power Saving	Indeed good, plugged in and used for half a month indeed less probably almost50degrees of electricity, the appearance of the open atmosphere high-end! And the manufacturer's warranty5years, try120days!	Taobao Marketplace, a Chinese website for online shopping
ShangDian Technologies, Inc.	King of Power Saving	Works great , I've purchased so many brands All of them made me return them because they really didn't work This brand I've been using regularly2months It really lets me see the results I like it I recommend It's the only one I've ever used that works the best.	Taobao Marketplace, a Chinese website for online shopping
ShangDian Technologies, Inc.	King of Power Saving	Experienced a month Indeed can save electricity,AugustthanJulylessthan60or so, very good Indeed authentic	Taobao Marketplace, a Chinese website for online shopping
ShangDian Technologies, Inc.	King of Power Saving	And back to buy it, my electricity bill is relatively high, before the customer service recommended that I buy2-3, I bought a try, the result is really effective quite obvious, hurry back to buy a, this is not yet received the goods, first to give a favorable comment, can save electricity really too happy! Thank	Taobao Marketplace, a Chinese website for online shopping
ShangDian Technologies, Inc.	King of Power Saving	Smart Saver received it. Packaging is very good. Much larger than the previous purchase, beautiful atmosphere looks very good. The effect of how to use a period of time and then comment. Merchants promise toreturn within120days! Thanks to the merchants!	Taobao Marketplace, a Chinese website for online shopping
ShangDian Technologies, Inc.	King of Power Saving	In-kind and the same as described, the quality is quite good, the seller's attitude is also good, ask questions and answer questions, the speed of delivery bar, it is worth buying!	Taobao Marketplace, a Chinese website for online shopping
ShangDian Technologies, Inc.	King of Power Saving	Second purchase, the old version was put in the old home. The new version has to be put in my new home. It's really effective and saves some money compared to usual.	Taobao Marketplace, a Chinese website for online shopping
ShangDian Technologies, Inc.	King of Power Saving	It works and saves electricity. Work room electricity machine more After using the same electricity consumption is indeed less60degrees of electricity Still very useful and cost-effective	Taobao Marketplace, a Chinese website for online shopping
ShangDian Technologies, Inc.	King of Power Saving	Very good quality with good results	Taobao Marketplace, a Chinese website for online shopping
ShangDian Technologies, Inc.	King of Power Saving	Appearance is very nice, the root of the same description, looks very good. Has been plugged in and used, intelligent display light can prompt the voltage, easy to use, value for money it!	Taobao Marketplace, a Chinese website for online shopping
ShangDian Technologies, Inc.	King of Power Saving	It is worth buying, happy to use, power saving and money saving, it is a necessary tool for home. Effective, safe and practical affordable.	Taobao Marketplace, a Chinese website for online shopping
ShangDian Technologies, Inc.	King of Power Saving	Quite satisfied, exquisite workmanship, the appearance of aluminum alloy design is very high-grade, there are anti-counterfeiting mark is genuine, received on the insertion of the use, I hope there will be good results	Taobao Marketplace, a Chinese website for online shopping
ShangDian Technologies, Inc.	King of Power Saving	This high-powered really good to use, I am also a friend introduced to him, his store used to save a lot of store, workmanship is also very good, the whole machine made of aluminum alloy, but also to send2, with real-time voltage display, up to this price	Taobao Marketplace, a Chinese website for online shopping
ShangDian Technologies, Inc.	King of Power Saving	Power saving effect:👍great Cost-effective: especially great Appearance style: beautiful and good Workmanship material: fine	Taobao Marketplace, a Chinese website for online shopping
ShangDian Technologies, Inc.	King of Power Saving	Baby received, logistics is very fast, thanks to the shopkeeper's very patient explanation, just tried to feel a little effect and also comes with air purification, in short, feel pretty good. First use a, late good will buy again.	Taobao Marketplace, a Chinese website for online shopping
ShangDian Technologies, Inc.	King of Power Saving	I didn't expect it to work so well, but after using it for a while I found that it does save a lot of money on electricity, which is very practical.	Taobao Marketplace, a Chinese website for online shopping
ShangDian Technologies, Inc.	King of Power Saving	Try it out for a while and see if it's an IQ tax before you say anything.☺ .	Taobao Marketplace, a Chinese website for online shopping
ShangDian Technologies, Inc.	King of Power Saving	I've been using it for a month and the power consumption hasn't changed at all. IQ tax.	Taobao Marketplace, a Chinese website for online shopping
ShangDian Technologies, Inc.	King of Power Saving	usuallymore than500degrees of electricitya month, now this329degrees,Augustis hotter, I use the air conditioning more, actually left329degrees, this model is good, I bought two, is not wrong to buy, the product is good, the effect is really good, you must give you a favorable review usuallymore than500degrees of electricitya month, now this329degrees,Julyis hotter, I use the air conditioning more, actually left329degree, this model is good, I bought two, is not to buy wrong, the product is good, the [illegible]	Taobao Marketplace, a Chinese website for online shopping
ShangDian Technologies, Inc.	King of Power Saving	So far I personally don't think there's been much of a reaction	Taobao Marketplace, a Chinese website for online shopping
ShangDian Technologies, Inc.	King of Power Saving	is an old customer of this store, bought3times, this power saving effect is really good, almost saveabout40%every month.Fine workmanship, beautiful, easy to use, is genuine, warranty5years is not worried about bad!	Taobao Marketplace, a Chinese website for online shopping
ShangDian Technologies, Inc.	King of Power Saving	This high-powered really good to use, I am also a friend introduced to him, his store used to save a lot of store, workmanship is also very good, the whole machine made of aluminum alloy, but also to send2, with real-time voltage display, up to this price	Taobao Marketplace, a Chinese website for online shopping
ShangDian Technologies, Inc.	King of Power Saving	There are a lot of big appliances at home, the air conditioner is very expensive in summer, I bought this in front of me, the effect on the air conditioner is very good, let me explain, I bought three in one breath, I use more electricity, a month downabout200dollarsless, very satisfied	Taobao Marketplace, a Chinese website for online shopping
ShangDian Technologies, Inc.	King of Power Saving	Home large recommended to buy a few more, summer home open two air conditioners, a refrigerator in addition to some appliances, a month to savemore than30percentof the electricity bill, there is no fine print, but also to protect household appliances, stabilize the current, the main plug in can be used	Taobao Marketplace, a Chinese website for online shopping

ShangDian Technologies, Inc.	King of Power Saving	Previously bought a small, used can this time in the purchase of a high-power, multi-functional again in the price more to buy a put on the third floor feel good can save electricity is a good product[strong] to the merchant a good comment satisfied with	Taobao Marketplace, a Chinese website for online shopping
ShangDian Technologies, Inc.	King of Power Saving	When the goods were received, I plugged it in and used it, it's very well made, the service is also very good, and some fuses were sent! Current looks much more stable!	Taobao Marketplace, a Chinese website for online shopping
ShangDian Technologies, Inc.	King of Power Saving	I've been using this product for a few months now and it's one of the best I've ever used, I've paid my electric bill a few times now and I'm probably saving a lot of money each month, it's a good product!	Taobao Marketplace, a Chinese website for online shopping
ShangDian Technologies, Inc.	King of Power Saving	The effect is particularly good,no longer afraid of the electricity bill more than one month down, the electricity bill than last month less than thirty percent or so, because the heat, the air conditioning to be open every day, every month the electricity bill is too much, after using this really let people too much worry about too cattle this power saver ! ! ! ! !	Taobao Marketplace, a Chinese website for online shopping
ShangDian Technologies, Inc.	King of Power Saving	Listening to the customer service end bought two separate plugged in, really toabout10days obviously see the effect, the home is a digital table, now every day can be less4-5degrees. Product5-year warranty also do not worry about after-sales problems, it is very good!	Taobao Marketplace, a Chinese website for online shopping
ShangDian Technologies, Inc.	King of Power Saving	Used for a period of time, the product is very stable, there is no sound, not debugging, just started a day to save2degrees, now basically every day isabout4-5degrees, you can always keep it oh, good product	Taobao Marketplace, a Chinese website for online shopping
ShangDian Technologies, Inc.	King of Power Saving	Good, are back to buy several times, this time to buy for a friend, his village installed charging pile, the effect is still obvious, ten days or so can see the effect, indeed less power consumption!	Taobao Marketplace, a Chinese website for online shopping
ShangDian Technologies, Inc.	King of Power Saving	This packaging is really too advanced, at first glance is a good thing, has been plugged into the power supply, good use will continue to buy back, ready to give parents and the store also buy a try!	Taobao Marketplace, a Chinese website for online shopping
ShangDian Technologies, Inc.	King of Power Saving	Simple and convenient, just that plug in, you can save electricity. A month can save dozens of dollars, a year down to save a lot of, very good product cost-effective, everyone can afford to buy and use!	Taobao Marketplace, a Chinese website for online shopping
ShangDian Technologies, Inc.	King of Power Saving	Saver really useful it, commercial electricity, electricity costs high daily electricity consumption is relatively large, the electricity bill can not eat, was just want to try, did not expect to use the monthly savings of a large amount of expenses it, happy!	Taobao Marketplace, a Chinese website for online shopping
ShangDian Technologies, Inc.	King of Power Saving	Received on the insertion of the meter down, currently more stable, home electricity is not tripped, I am an old house in the countryside! Home lines are a little messy At the same time open electrical appliances from time to time tripping, listening to the seller said the product has a voltage stabilization effect, decisive order. Currently experiencing2weeks indeed voltage is very stable there has been no tripping, the effect is very good, there is also a power-saving effect. Power-saving effect: very good Cost-effective: high Appearance style: good-looking Workmanship material: very good	Taobao Marketplace, a Chinese website for online shopping
ShangDian Technologies, Inc.	King of Power Saving	Every day are working 6-7 p.m. to go home, occasionally cooking, In the use of this energy saver before the monthly electricity bill ismore than150yuan or so, after the use of this energy saver is nowmore than60, up to80yuan, energy-saving appliances really have the effect of a good	Taobao Marketplace, a Chinese website for online shopping
ShangDian Technologies, Inc.	King of Power Saving	It is really unexpected that there is such a good thing, the moment you receive the product immediately used, the product is very formal, there are anti-counterfeiting labels There is also the purchase of insurance, my family use electricity with a lot, so ready to buy two more after the year!	Taobao Marketplace, a Chinese website for online shopping
ShangDian Technologies, Inc.	King of Power Saving	No noise, easy to use, logistics is fast, good service attitude, thank you seller sent two fuses said to spare. Electrician friend recommended to know that there is this thing, may have seen before thought it was a scam did not care, now believe it. Can be reasonable power saving pretty good!Very satisfied!	Taobao Marketplace, a Chinese website for online shopping
ShangDian Technologies, Inc.	King of Power Saving	pretty good	Taobao Marketplace, a Chinese website for online shopping
ShangDian Technologies, Inc.	King of Power Saving	Owns a restaurant Electricity costs over a thousand dollars a summer. This year, the business is not doing well, the electricity bill is still not less. A few days ago, the electrician came to repair the electric grill. He recommended this brand of power saver. Tried to buy three, a month to save about almost half of the electricity bill, really very satisfied, recommended to everyone!	Taobao Marketplace, a Chinese website for online shopping
ShangDian Technologies, Inc.	King of Power Saving	Saver received today, which is already bought the fourth, last year has bought a, after two with a significant effect, especially air conditioning, water heater, battery furnace most practical, effective, affordable, appearance and material is also okay! Really~worth having.	Taobao Marketplace, a Chinese website for online shopping
ShangDian Technologies, Inc.	King of Power Saving	Is it an IQ tax?	Taobao Marketplace, a Chinese website for online shopping
ShangDian Technologies, Inc.	King of Power Saving	Does it really save power? For those who have bought it	Taobao Marketplace, a Chinese website for online shopping
ShangDian Technologies, Inc.	King of Power Saving	yea	Taobao Marketplace, a Chinese website for online shopping
ShangDian Technologies, Inc.	King of Power Saving	I can't see it yet.	Taobao Marketplace, a Chinese website for online shopping
ShangDian Technologies, Inc.	Intelligent power saver	Fifty generations should be the latest model, the effect does seem to be a little stronger than the previous generations, the power saving rate is still in the test, see the effect of the next review, first give a positive review!	Taobao Marketplace, a Chinese website for online shopping
ShangDian Technologies, Inc.	Intelligent power saver	I thought it was an IQ tax, but it turns out it really saves money on your electricity bill, it's***rate, not bad, it's worth a good review	Taobao Marketplace, a Chinese website for online shopping
ShangDian Technologies, Inc.	Intelligent power saver	Can save a little bit of electricity, but there is no advertised55%, estimated atmore than40%, I do not know if it is the reason for inserting less, the overall is not bad, the seller said3months to achieve the best results, and then wait to see it	Taobao Marketplace, a Chinese website for online shopping
ShangDian Technologies, Inc.	Intelligent power saver	Installed in July during the peak of electricity consumption, to savemore than100a month, or a little effect, plugged into the main circuit can be, the closer to the meter, the better	Taobao Marketplace, a Chinese website for online shopping
ShangDian Technologies, Inc.	Intelligent power saver	Broken after a week of use, customer service has read and not returned… No shipping insurance. Whoever buys it regrets it Power saving effect: not seen User experience: very bad	Taobao Marketplace, a Chinese website for online shopping
ShangDian Technologies, Inc.	Intelligent power saver	Haven't paid the electric bill yet, not sure if it's working or not	Taobao Marketplace, a Chinese website for online shopping
ShangDian Technologies, Inc.	Intelligent power saver	Bad review,****, fake products	Taobao Marketplace, a Chinese website for online shopping
ShangDian Technologies, Inc.	Intelligent power saver	It's very good, it's really effective, and it saves electricity.	Taobao Marketplace, a Chinese website for online shopping
ShangDian Technologies, Inc.	Intelligent power saver	It doesn't help at all!	Taobao Marketplace, a Chinese website for online shopping
ShangDian Technologies, Inc.	Intelligent power saver	Workmanship material: very good very good indeed can save a lot of electricity very affordable	Taobao Marketplace, a Chinese website for online shopping
ShangDian Technologies, Inc.	Intelligent power saver	It didn't work, customer service was ignored, and looking at the National Grid electricity bill was more than enough	Taobao Marketplace, a Chinese website for online shopping

ShangDian Technologies, Inc.	Intelligent power saver	It's not an IQ tax. It'sa **tax**	Taobao Marketplace, a Chinese website for online shopping
ShangDian Technologies, Inc.	Intelligent power saver	I've paid the IQ tax again! Advise each residence not to be fooled Spirit before Baidu!	Taobao Marketplace, a Chinese website for online shopping
ShangDian Technologies, Inc.	Intelligent power saver	It does save electricity.	Taobao Marketplace, a Chinese website for online shopping
ShangDian Technologies, Inc.	Intelligent power saver	Great! Old family pig raising, use a lot of electricity, arranged for6, all installed, can savemore than500a month! The quality is also good, used for a period of time are not fault, is quite stable	Taobao Marketplace, a Chinese website for online shopping
ShangDian Technologies, Inc.	Intelligent power saver	Check the row of plugs can also be used, very convenient, do not have to plug into the wall, every month to pay the electricity bill is less, it's worth it, bought2power saver, but also sent a, a few days to Mom and Dad's house is also installed	Taobao Marketplace, a Chinese website for online shopping
ShangDian Technologies, Inc.	Intelligent power saver	Power-saving effect of the bar, this is the latest model of the 50th generation of power saver, than before the eight generation of twenty-four generations of the effect is too good, I used3months, just when the air conditioning with the most time to savemore than200dollarsper monthelectricity bill easily, [illegible]	Taobao Marketplace, a Chinese website for online shopping
ShangDian Technologies, Inc.	Intelligent power saver	It works.	Taobao Marketplace, a Chinese website for online shopping
ShangDian Technologies, Inc.	Intelligent power saver	Tried it, no power savings.	Taobao Marketplace, a Chinese website for online shopping
ShangDian Technologies, Inc.	Intelligent power saver	I'll comment after I use it and how it works when it's ten days old.	Taobao Marketplace, a Chinese website for online shopping
ShangDian Technologies, Inc.	Intelligent power saver	Bought it for a friend and have bought it multiple times with good reviews.	Taobao Marketplace, a Chinese website for online shopping
ShangDian Technologies, Inc.	Intelligent power saver	Speechless😒	Taobao Marketplace, a Chinese website for online shopping
ShangDian Technologies, Inc.	Intelligent power saver	Workmanship material: good value for money	Taobao Marketplace, a Chinese website for online shopping
ShangDian Technologies, Inc.	Intelligent power saver	It looks like it's not working.	Taobao Marketplace, a Chinese website for online shopping
ShangDian Technologies, Inc.	Intelligent power saver	charged$2a piece forpickup at the station	Taobao Marketplace, a Chinese website for online shopping
ShangDian Technologies, Inc.	Intelligent power saver	You won't know the power savings until you've used it for a few months	Taobao Marketplace, a Chinese website for online shopping
ShangDian Technologies, Inc.	Intelligent power saver	Just used it for a few days and haven't seen any results yet? I'll comment again in a month	Taobao Marketplace, a Chinese website for online shopping
ShangDian Technologies, Inc.	Intelligent power saver	It doesn't work.	Taobao Marketplace, a Chinese website for online shopping
ShangDian Technologies, Inc.	Intelligent power saver	Fast logistics, have not yet used, after use in the comments	Taobao Marketplace, a Chinese website for online shopping
ShangDian Technologies, Inc.	Intelligent power saver	Goods have been received, good quality, good results, worth buying.	Taobao Marketplace, a Chinese website for online shopping
ShangDian Technologies, Inc.	Intelligent power saver	It's supposed to effect energy conservation!	Taobao Marketplace, a Chinese website for online shopping
ShangDian Technologies, Inc.	Intelligent power saver	Power-saving effect: I don't know yet, use it first to see if it saves power. Appearance and style: good Workmanship and material: good Cost-effective: good	Taobao Marketplace, a Chinese website for online shopping
ShangDian Technologies, Inc.	Intelligent power saver	Things are broken! Customer service ignored	Taobao Marketplace, a Chinese website for online shopping
ShangDian Technologies, Inc.	Intelligent power saver	This time's was bought for a loved one to use, the evaluation in the above a single has been reviewed, praise.	Taobao Marketplace, a Chinese website for online shopping
ShangDian Technologies, Inc.	Intelligent power saver	Last year in curiosity to buy a, is also a few dollars, thought useful not good, useless lost is not a pity. Evaluation of some said that there are power saving, there are also said that the next power saving, but after their own calculations, the first two months is not so obvious, the third month has begun to obvious power saving, especially in June this year, the home of three air conditioning for a long time to open, they expect the electricity bill of the minimum five hundred on top of the results of only two hundred a few has not yet reached three hundred, absolutely satisfied, and then bought a few to the relatives of the family with. I can save on saving. Also, want to buy friends, do not look at the price to measure the good and the bad, probably all the same, cheap is okay, even if not lost is not	Taobao Marketplace, a Chinese website for online shopping
ShangDian Technologies, Inc.	Intelligent power saver	properly	Taobao Marketplace, a Chinese website for online shopping
ShangDian Technologies, Inc.	Intelligent power saver	You can only see the results after a month	Taobao Marketplace, a Chinese website for online shopping
ShangDian Technologies, Inc.	Intelligent power saver	Power-saving effect: currently in use it is not clear whether the power-saving effect can meet therequirements of the technical parameters of the productpower saving rate between15%and45%.	Taobao Marketplace, a Chinese website for online shopping
ShangDian Technologies, Inc.	Intelligent power saver	The power saving is good and genuine.	Taobao Marketplace, a Chinese website for online shopping
ShangDian Technologies, Inc.	Intelligent power saver	Good reviews do save some	Taobao Marketplace, a Chinese website for online shopping
ShangDian Technologies, Inc.	Intelligent power saver	Can't tell if it's good or bad now, I'll give it a favorable rating first, I'll use it for a while.	Taobao Marketplace, a Chinese website for online shopping
ShangDian Technologies, Inc.	Intelligent power saver	I just bought it to try the water, I don't know if it works. I'll use it for a while.	Taobao Marketplace, a Chinese website for online shopping
ShangDian Technologies, Inc.	Intelligent power saver	Good luck and good health and happiness and happiness and longevity!	Taobao Marketplace, a Chinese website for online shopping

ShangDian Technologies, Inc.	Intelligent power saver	have just used	Taobao Marketplace, a Chinese website for online shopping
ShangDian Technologies, Inc.	Intelligent power saver	Good luck, peace, joy, health and happiness	Taobao Marketplace, a Chinese website for online shopping
ShangDian Technologies, Inc.	Intelligent power saver	Buy two get one free , first praise Now not used, do not know what effect. I'm not sure what the effect is. I'll come back with a follow-up comment after I use it.	Taobao Marketplace, a Chinese website for online shopping
ShangDian Technologies, Inc.	Intelligent power saver	Buy two get one free , first praise Now not used, do not know what effect. I'm not sure what the effect is. I'll come back with a follow-up comment after I use it.	Taobao Marketplace, a Chinese website for online shopping
ShangDian Technologies, Inc.	Intelligent power saver	Good value for money worth buying	Taobao Marketplace, a Chinese website for online shopping
ShangDian Technologies, Inc.	Intelligent power saver	Power saving effect: to be evaluated after using it for a while.	Taobao Marketplace, a Chinese website for online shopping
ShangDian Technologies, Inc.	Intelligent power saver	It simply works!!! Saves big bucks a month!!!!	Taobao Marketplace, a Chinese website for online shopping
ShangDian Technologies, Inc.	Intelligent power saver	an excellent	Taobao Marketplace, a Chinese website for online shopping
ShangDian Technologies, Inc.	Intelligent power saver	Good packaging, fast logistics, product quality to be used and then comment.	Taobao Marketplace, a Chinese website for online shopping
ShangDian Technologies, Inc.	Intelligent power saver	I don't know yet.	Taobao Marketplace, a Chinese website for online shopping
ShangDian Technologies, Inc.	Intelligent power saver	acceptable	Taobao Marketplace, a Chinese website for online shopping
ShangDian Technologies, Inc.	Intelligent power saver	five-star review	Taobao Marketplace, a Chinese website for online shopping
ShangDian Technologies, Inc.	Intelligent power saver	useful and inexpensive	Taobao Marketplace, a Chinese website for online shopping
ShangDian Technologies, Inc.	Intelligent power saver	Nice baby, really knocks down the electric bill!	Taobao Marketplace, a Chinese website for online shopping
ShangDian Technologies, Inc.	Intelligent power saver	Great value for money and the power savings really pay off! It's great!	Taobao Marketplace, a Chinese website for online shopping
ShangDian Technologies, Inc.	Intelligent power saver	Power factor on energy saving and consumption reduction is really useful, although the reduction of energy consumption is limited, but can save a little is a little, but also to protect household appliances, stabilize the current and voltage, this is the main thing is easy to install, find a socket and plug it on the line. Very practical.	Taobao Marketplace, a Chinese website for online shopping
ShangDian Technologies, Inc.	Intelligent power saver	The 50th generation product has really been upgraded, almost10%more savings than the previous 8th and 16th generations, and the results are more noticeable, the point is that this generation doesn't get hot anymore, which is very satisfying, and I guess the life expectancy will be a lot longer as well.	Taobao Marketplace, a Chinese website for online shopping
ShangDian Technologies, Inc.	Intelligent power saver	It's good and inexpensive, save a little bit of results	Taobao Marketplace, a Chinese website for online shopping
ShangDian Technologies, Inc.	Intelligent power saver	Power saving effect: quite good Cost-effective: buy2send3very good Appearance style: beautiful Workmanship material: exquisite Using experience: plug and play, very convenient!	Taobao Marketplace, a Chinese website for online shopping
ShangDian Technologies, Inc.	Intelligent power saver	I've been using it for a while now, it does save electricity, satisfied	Taobao Marketplace, a Chinese website for online shopping
ShangDian Technologies, Inc.	Intelligent power saver	Very good product, solved my trouble, used more than twenty days, the effect of power saving is very good, has recommended to friends, every month for me to save money! Good good	Taobao Marketplace, a Chinese website for online shopping
ShangDian Technologies, Inc.	Intelligent power saver	latest models of the50generation, should be genuine, a variety of anti-counterfeiting mark and the Pacific insurance mark, the quality looks good, but also with the LCD display, the price is not expensive, my family uses less electricity, a month should be able to save dozens of dollars, summer air conditioning is estimated to be able to save more a month, has been very satisfied with the	Taobao Marketplace, a Chinese website for online shopping
ShangDian Technologies, Inc.	Intelligent power saver	I'll go! A month to save 300 electricity bill, too powerful, right? This little thing, our studio, the equipment is a little more, originally a month tomore than700dollars of electricity, with this period of time, this month to pay the electricity bill only paidmore than400, this effect is simply God!	Taobao Marketplace, a Chinese website for online shopping
ShangDian Technologies, Inc.	Intelligent power saver	Buy with the attitude of trying, I did not expect to be really useful, with40days, there is indeed a power saving, but the proportion of power saving is not too much, only35%or so, just asked the seller, said to be inserted into the main line road but also away from the meter a little closer to the place, you can reach50%, I change a place to plug in and try again for a month. Can save electricity, so first give a favorable comment, followed by a problem in the follow-up comment it	Taobao Marketplace, a Chinese website for online shopping
ShangDian Technologies, Inc.	Intelligent power saver	Power-saving effect: I am an electrician myself, this power saver is the real use of electronic waves automatically connected to track the decomposition of ionized electrons, in-situ compensation of reactive power, reduce effective power loss and waste, stabilize the voltage, to achieve energy saving and power saving, the overall effect is good, trustworthy, has purchased several, specially run to evaluate, will	Taobao Marketplace, a Chinese website for online shopping
ShangDian Technologies, Inc.	Intelligent power saver	Simply black science and technology, we opened the hotel, before a month of electricity costsmore than7,000, the installation ofninepower savers, this month's electricity costs only5,000more, the effect is too cattle it! Business is not good in these years, open source and cut back on expenditure, which saves a year down the food money is enough, really good!	Taobao Marketplace, a Chinese website for online shopping
ShangDian Technologies, Inc.	Intelligent power saver	Strongly recommend buying this fifty generation of power saver, the effect is surprisingly good, the first month the effect is general, to the second month directly less than200more than200dollars of electricity, really worth it, early to know that this high-tech how good ah, these years wasted a lot of money, especially the summer is here, a month of air conditioning fees to save a lot of ah! Like don't hesitate, hurry to order, directly buy2, but also send a fifty generation! Pro test effective!!!	Taobao Marketplace, a Chinese website for online shopping
ShangDian Technologies, Inc.	Intelligent power saver	The actual test is effective. Small processing plant with this is still very suitable, basically every day to save a little bit, bashful, decided to buy a few more to the old family also installed😄	Taobao Marketplace, a Chinese website for online shopping
ShangDian Technologies, Inc.	Intelligent power saver	Buyer does not comment within15days	Taobao Marketplace, a Chinese website for online shopping
ShangDian Technologies, Inc.	Intelligent power saver	It's not working at all.	Taobao Marketplace, a Chinese website for online shopping
ShangDian Technologies, Inc.	Intelligent power saver	Does it really save electricity? It's a disaster if you're sad	Taobao Marketplace, a Chinese website for online shopping

ShangDian Technologies, Inc.	Intelligent power saver	Does it have as many parts in it as a lighter?	Taobao Marketplace, a Chinese website for online shopping
ShangDian Technologies, Inc.	Intelligent power saver	Does this work?	Taobao Marketplace, a Chinese website for online shopping
ShangDian Technologies, Inc.	Intelligent power saver	Does it really work?	Taobao Marketplace, a Chinese website for online shopping
ShangDian Technologies, Inc.	Intelligent power saver	No power saving	Taobao Marketplace, a Chinese website for online shopping
ShangDian Technologies, Inc.	Intelligent power saver	You're right, it didn't really help at all, fooled myself, took a chance on it, bought it, took it apart and looked at it, and just threw it away.	Taobao Marketplace, a Chinese website for online shopping
ShangDian Technologies, Inc.	Intelligent power saver	Useful but more appliances in the house	Taobao Marketplace, a Chinese website for online shopping
ShangDian Technologies, Inc.	Intelligent power saver	Half a year less nearly six hundred degrees of electricity	Taobao Marketplace, a Chinese website for online shopping
ShangDian Technologies, Inc.	Power Saving Specialist	not have	Taobao Marketplace, a Chinese website for online shopping
BMI Digital Store	Gold Power Saving Specialist	Very well made, easy to use, looking forward to better power savings!	Taobao Marketplace, a Chinese website for online shopping
BMI Digital Store	Gold Power Saving Specialist	Same one I bought before.	Taobao Marketplace, a Chinese website for online shopping
BMI Digital Store	Gold Power Saving Specialist	Received and plugged in, plugged into a line with the refrigerator, with a period of time to evaluate again!	Taobao Marketplace, a Chinese website for online shopping
BMI Digital Store	Gold Power Saving Specialist	Good, already repurchased, the average household can save10-20dollarsa month on electricity bills	Taobao Marketplace, a Chinese website for online shopping
BMI Digital Store	Gold Power Saving Specialist	Tested. doesn't work	Taobao Marketplace, a Chinese website for online shopping
BMI Digital Store	Gold Power Saving Specialist	Used for a period of time is good, I hope to save electricity, good use will buy back!	Taobao Marketplace, a Chinese website for online shopping
BMI Digital Store	Gold Power Saving Specialist	Products work better	Taobao Marketplace, a Chinese website for online shopping
BMI Digital Store	Gold Power Saving Specialist	Received the courier directly open the plug, looking forward to a month how it is.	Taobao Marketplace, a Chinese website for online shopping
BMI Digital Store	Gold Power Saving Specialist	It looks okay, I just don't know if it works	Taobao Marketplace, a Chinese website for online shopping
BMI Digital Store	Gold Power Saving Specialist	I haven't used it for long, so how do I rate it?	Taobao Marketplace, a Chinese website for online shopping
BMI Digital Store	Gold Power Saving Specialist	It doesn't feel like it's doing much.	Taobao Marketplace, a Chinese website for online shopping
BMI Digital Store	Gold Power Saving Specialist	Baby received, and the same as in the picture, feel good, fast delivery	Taobao Marketplace, a Chinese website for online shopping
BMI Digital Store	Gold Power Saving Specialist	Just received the goods, someone else introduced seems to be good, after using the follow-up comment!	Taobao Marketplace, a Chinese website for online shopping
BMI Digital Store	Gold Power Saving Specialist	It's in use, looking forward to saving electricity, the material is very good	Taobao Marketplace, a Chinese website for online shopping
BMI Digital Store	Gold Power Saving Specialist	Baby bought back, beautifully packaged, beautiful products, product instructions in detail, but also the distribution of multi-grain safes, expect to make the meter turn a little slower, to save yourself some household, next month, after the electricity bill comes out and then follow up!	Taobao Marketplace, a Chinese website for online shopping
BMI Digital Store	Gold Power Saving Specialist	It was recommended by a friend, so far it feels okay, genuine product	Taobao Marketplace, a Chinese website for online shopping
BMI Digital Store	Gold Power Saving Specialist	Not bad, like it very much, it's worth buying	Taobao Marketplace, a Chinese website for online shopping
BMI Digital Store	Gold Power Saving Specialist	The product is well-packaged material is also good lightweight and convenient, reserved line is also enough, before the school teacher also introduced from a professional point of view, saving electricity to protect electrical appliances, is a good product	Taobao Marketplace, a Chinese website for online shopping
BMI Digital Store	Gold Power Saving Specialist	Haven't used it yet, will comment in a few days	Taobao Marketplace, a Chinese website for online shopping
BMI Digital Store	Gold Power Saving Specialist	Definitely one of the happiest shopping, the store has a great service and after-sales	Taobao Marketplace, a Chinese website for online shopping
BMI Digital Store	Gold Power Saving Specialist	The stuff feels okay, I haven't seen any heat after a few days of use, so let's see what's behind it	Taobao Marketplace, a Chinese website for online shopping
BMI Digital Store	Gold Power Saving Specialist	Got the stuff, hope it works.	Taobao Marketplace, a Chinese website for online shopping
BMI Digital Store	Gold Power Saving Specialist	The saver is pretty easy to use, let's use it first and see how it works, hopefully it will be as energy efficient as the seller said it would be!	Taobao Marketplace, a Chinese website for online shopping
BMI Digital Store	Gold Power Saving Specialist	Logistics fast quality, wait for the use of the evaluation again	Taobao Marketplace, a Chinese website for online shopping
BMI Digital Store	Gold Power Saving Specialist	It looks pretty good, let's see how much power it saves.	Taobao Marketplace, a Chinese website for online shopping
BMI Digital Store	Gold Power Saving Specialist	Yesterday received the baby, open feel good quality, the effect wait until after the trial and then follow up.	Taobao Marketplace, a Chinese website for online shopping

BMI Digital Store	Gold Power Saving Specialist	Logistics is fast, received, power saving, very satisfactory	Taobao Marketplace, a Chinese website for online shopping
BMI Digital Store	Gold Power Saving Specialist	This thing can not save electricity, and the more you use it, the more electricity, and will explode to scare*me, simply fall into his trap	Taobao Marketplace, a Chinese website for online shopping
BMI Digital Store	Gold Power Saving Specialist	The saver is easy to install, well made and practical, looking forward to good results.	Taobao Marketplace, a Chinese website for online shopping
BMI Digital Store	Gold Power Saving Specialist	Does it really work?	Taobao Marketplace, a Chinese website for online shopping
BMI Digital Store	Gold Power Saving Specialist	How does this work and does it feel like a power saver?	Taobao Marketplace, a Chinese website for online shopping
BMI Digital Store	Gold Power Saving Specialist	Does it have a shelf life? How long does it last?	Taobao Marketplace, a Chinese website for online shopping
BMI Digital Store	Gold Power Saving Specialist	Do digital power meters work?	Taobao Marketplace, a Chinese website for online shopping
BMI Digital Store	Gold Power Saving Specialist	Does it work?	Taobao Marketplace, a Chinese website for online shopping
BMI Digital Store	Gold Power Saving Specialist	So many amateurs are using it and none of them can tell a specific power saving figure? Can any of you test and compare to answer the question?	Taobao Marketplace, a Chinese website for online shopping
BMI Digital Store	Gold Power Saving Specialist	Does this save any electricity?	Taobao Marketplace, a Chinese website for online shopping
BMI Digital Store	Gold Power Saving Specialist	Does this power saver really work? Does it really save electricity?afford to buy now is what you can't afford to use?…….	Taobao Marketplace, a Chinese website for online shopping
BMI Digital Store	Gold Power Saving Specialist	Did you look closely? Does it really save electricity?	Taobao Marketplace, a Chinese website for online shopping
BMI Digital Store	Gold Power Saving Specialist	Does it have any effect on household appliances?	Taobao Marketplace, a Chinese website for online shopping
BMI Digital Store	Gold Power Saving Specialist	Does it save power?	Taobao Marketplace, a Chinese website for online shopping
BMI Digital Store	Gold Power Saving Specialist	Anyone who has used it? Does it save power or not?	Taobao Marketplace, a Chinese website for online shopping
BMI Digital Store	Gold Power Saving Specialist	May I ask you folks, is it true that this power saver will save?	Taobao Marketplace, a Chinese website for online shopping
BMI Digital Store	Gold Power Saving Specialist	Honey,does this stuff work?	Taobao Marketplace, a Chinese website for online shopping
BMI Digital Store	Gold Power Saving Specialist	Can you save electricity?	Taobao Marketplace, a Chinese website for online shopping
BMI Digital Store	Gold Power Saving Specialist	How's everyone doing with it?	Taobao Marketplace, a Chinese website for online shopping
BMI Digital Store	Gold Power Saving Specialist	Does it really save power? Is it safe?	Taobao Marketplace, a Chinese website for online shopping
BMI Digital Store	Gold Power Saving Specialist	How do I install it?	Taobao Marketplace, a Chinese website for online shopping
BMI Digital Store	Gold Power Saving Specialist	Can I ask if it really saves electricity?	Taobao Marketplace, a Chinese website for online shopping
BMI Digital Store	Gold Power Saving Specialist	Motor capacitance data for(12UF)which change two respectively(8UF) (14UF)which motor fast and slow?	Taobao Marketplace, a Chinese website for online shopping
BMI Digital Store	Gold Power Saving Specialist	Does it work? Is it safe?	Taobao Marketplace, a Chinese website for online shopping
BMI Digital Store	Gold Power Saving Specialist	Can anyone tell me if this power saving is really good? Do I need to buy more if I have a lot of appliances?	Taobao Marketplace, a Chinese website for online shopping
BMI Digital Store	Gold Power Saving Specialist	Pros: does this energy saver work? Thank you!	Taobao Marketplace, a Chinese website for online shopping
BMI Digital Store	Gold Power Saving Specialist	Who's been using it for a month and what are the results?	Taobao Marketplace, a Chinese website for online shopping
BMI Digital Store	Gold Power Saving Specialist	Aren't all energy saving meters nowadays?<br>Does it work?	Taobao Marketplace, a Chinese website for online shopping
BMI Digital Store	Gold Power Saving Specialist	Is the electricity bill really any less?	Taobao Marketplace, a Chinese website for online shopping
BMI Digital Store	Gold Power Saving Specialist	Will this affect the function of the electric shock protection switch	Taobao Marketplace, a Chinese website for online shopping
BMI Digital Store	Gold Power Saving Specialist	Does this thing really save power if you've used it?	Taobao Marketplace, a Chinese website for online shopping
BMI Digital Store	Gold Power Saving Specialist	Did it work? Thanks.	Taobao Marketplace, a Chinese website for online shopping
BMI Digital Store	Gold Power Saving Specialist	Does it save electricity?	Taobao Marketplace, a Chinese website for online shopping
BMI Digital Store	Gold Power Saving Specialist	Has anyone opened it to see what it looks like inside?	Taobao Marketplace, a Chinese website for online shopping

BMI Digital Store	Gold Power Saving Specialist	Does it really save power?	Taobao Marketplace, a Chinese website for online shopping
BMI Digital Store	Gold Power Saving Specialist	Does the triple power work?	Taobao Marketplace, a Chinese website for online shopping
BMI Digital Store	Gold Power Saving Specialist	Does it save electricity? How many kilowatts a month?	Taobao Marketplace, a Chinese website for online shopping
BMI Digital Store	Gold Power Saving Specialist	For those of you who have bought it, does this stuff really work? What is the principle.	Taobao Marketplace, a Chinese website for online shopping
BMI Digital Store	Gold Power Saving Specialist	How does it work?	Taobao Marketplace, a Chinese website for online shopping
BMI Digital Store	Gold Power Saving Specialist	Does it really save power? Thank you all.	Taobao Marketplace, a Chinese website for online shopping
BMI Digital Store	Gold Power Saving Specialist	Did you notice the meter flashing faster when the saver was plugged in?	Taobao Marketplace, a Chinese website for online shopping
BMI Digital Store	Gold Power Saving Specialist	Does it really save power?	Taobao Marketplace, a Chinese website for online shopping
BMI Digital Store	Gold Power Saving Specialist	How many kilowatt hours a day can you save?	Taobao Marketplace, a Chinese website for online shopping
BMI Digital Store	Gold Power Saving Specialist	Does it work? Is it appropriate to use a lot of electricity in the factory?	Taobao Marketplace, a Chinese website for online shopping
BMI Digital Store	Gold Power Saving Specialist	Hello, please, did you buy the saver and did it work?	Taobao Marketplace, a Chinese website for online shopping
BMI Digital Store	Gold Power Saving Specialist	About how much electricity is left when you use 100 degrees of electricity a month……	Taobao Marketplace, a Chinese website for online shopping
BMI Digital Store	Gold Power Saving Specialist	Power saving?	Taobao Marketplace, a Chinese website for online shopping
BMI Digital Store	Gold Power Saving Specialist	Does it work? How does it work?	Taobao Marketplace, a Chinese website for online shopping
BMI Digital Store	Gold Power Saving Specialist	How much electricity can you save in a month?	Taobao Marketplace, a Chinese website for online shopping
BMI Digital Store	Gold Power Saving Specialist	What do you think about it? Does it really save power?	Taobao Marketplace, a Chinese website for online shopping
BMI Digital Store	Gold Power Saving Specialist	Does this work for the newer machine meter reading? Will it save power?	Taobao Marketplace, a Chinese website for online shopping
BMI Digital Store	Gold Power Saving Specialist	How many kilowatt hours a day can you save?	Taobao Marketplace, a Chinese website for online shopping
BMI Digital Store	Gold Power Saving Specialist	What is the normal voltage?	Taobao Marketplace, a Chinese website for online shopping
BMI Digital Store	Gold Power Saving Specialist	Does it really save power please? Thanks for the answer!	Taobao Marketplace, a Chinese website for online shopping
BMI Digital Store	Gold Power Saving Specialist	What is the real difference betweenusing this to turn on the air conditioner10hours and not using it to air condition10hours?	Taobao Marketplace, a Chinese website for online shopping
BMI Digital Store	Gold Power Saving Specialist	Does this really work? Does it save power?	Taobao Marketplace, a Chinese website for online shopping
BMI Digital Store	Gold Power Saving Specialist	Does the air conditioner, refrigerator work?	Taobao Marketplace, a Chinese website for online shopping
BMI Digital Store	Gold Power Saving Specialist	Does anyone know the percentage of electricity that can be saved?	Taobao Marketplace, a Chinese website for online shopping
BMI Digital Store	Gold Power Saving Specialist	Are the circuits inside complicated?	Taobao Marketplace, a Chinese website for online shopping
BMI Digital Store	Gold Power Saving Specialist	Can I ask you guys if it really saves power?	Taobao Marketplace, a Chinese website for online shopping
BMI Digital Store	Gold Power Saving Specialist	For those of you who have sold it, does it really save power?	Taobao Marketplace, a Chinese website for online shopping
BMI Digital Store	Gold Power Saving Specialist	How does a power saver work? Know anything about it?	Taobao Marketplace, a Chinese website for online shopping
BMI Digital Store	Gold Power Saving Specialist	Is this really good?	Taobao Marketplace, a Chinese website for online shopping
BMI Digital Store	Gold Power Saving Specialist	I have4layers. Do I have to insert one in each layer	Taobao Marketplace, a Chinese website for online shopping
BMI Digital Store	Gold Power Saving Specialist	Does it really save power?	Taobao Marketplace, a Chinese website for online shopping
BMI Digital Store	Gold Power Saving Specialist	I would like to know if there are any that have been used for more than two months, and if so, how effective they are.	Taobao Marketplace, a Chinese website for online shopping
BMI Digital Store	Gold Power Saving Specialist	Purchased brothers, sisters, uncles and aunts of this energy-saving appliances in the end useful or not, about how much electricity can be saved!	Taobao Marketplace, a Chinese website for online shopping
BMI Digital Store	Gold Power Saving Specialist	Is it energy efficient? Is it safe?	Taobao Marketplace, a Chinese website for online shopping

BMI Digital Store	Gold Power Saving Specialist	How much electricity does this save?	Taobao Marketplace, a Chinese website for online shopping
BMI Digital Store	Gold Power Saving Specialist	Suppose the electricity bill for a month of normal use is three hundred dollars, one dollar a kilowatt hour. How much can you save in a month by using this	Taobao Marketplace, a Chinese website for online shopping
BMI Digital Store	Gold Power Saving Specialist	Does it save power or not? Can anyone answer this, thanks!	Taobao Marketplace, a Chinese website for online shopping
BMI Digital Store	Gold Power Saving Specialist	I have an electronic meter in my house and it's split into a spur for plugging in the air conditioner, does this save electricity?	Taobao Marketplace, a Chinese website for online shopping
BMI Digital Store	Gold Power Saving Specialist	Does it have a power saving effect? I hope all buyers will be honest!	Taobao Marketplace, a Chinese website for online shopping
BMI Digital Store	Gold Power Saving Specialist	Is it okay to buy one meter for many houses that share the same meter? Do you feel like you're saving electricity by using it?	Taobao Marketplace, a Chinese website for online shopping
BMI Digital Store	Gold Power Saving Specialist	Reactive power compensation? This stuff is only for three-phase power, right?	Taobao Marketplace, a Chinese website for online shopping
BMI Digital Store	Gold Power Saving Specialist	Does it really work?	Taobao Marketplace, a Chinese website for online shopping
BMI Digital Store	Gold Power Saving Specialist	By what principle do you save points?	Taobao Marketplace, a Chinese website for online shopping
BMI Digital Store	Gold Power Saving Specialist	Can't this thing really save power?	Taobao Marketplace, a Chinese website for online shopping
BMI Digital Store	Gold Power Saving Specialist	Does it really save electricity? How long does it usually take to have an effect and does it have air conditioning to save electricity bright and early! Please answer in good conscience! Thank you!	Taobao Marketplace, a Chinese website for online shopping
BMI Digital Store	Gold Power Saving Specialist	Friends Who bought it Does it really save electricity How much can you save in a month	Taobao Marketplace, a Chinese website for online shopping
BMI Digital Store	Gold Power Saving Specialist	Does it really work? Is it worth buying?	Taobao Marketplace, a Chinese website for online shopping
BMI Digital Store	Gold Power Saving Specialist	Does it work? Any noise?	Taobao Marketplace, a Chinese website for online shopping
BMI Digital Store	Gold Power Saving Specialist	Is there any power saving effect of this product?	Taobao Marketplace, a Chinese website for online shopping
BMI Digital Store	Gold Power Saving Specialist	Does it save power?	Taobao Marketplace, a Chinese website for online shopping
BMI Digital Store	Gold Power Saving Specialist	How much electricity does the best stuff save?	Taobao Marketplace, a Chinese website for online shopping
BMI Digital Store	Gold Power Saving Specialist	The landlord uses10Ahand read meter, I'm downover400kWhJanuary, 1.2a degree, how much can I save with this in January?	Taobao Marketplace, a Chinese website for online shopping
BMI Digital Store	Gold Power Saving Specialist	Is it genuine? Does it save power?	Taobao Marketplace, a Chinese website for online shopping
BMI Digital Store	Gold Power Saving Specialist	Are the power savings significant?	Taobao Marketplace, a Chinese website for online shopping
BMI Digital Store	Gold Power Saving Specialist	I would like to ask, does the product have differentiation requirements for mechanical meters and electronic meters?	Taobao Marketplace, a Chinese website for online shopping
BMI Digital Store	Gold Power Saving Specialist	I'm thinking of getting one, I wonder if it's power efficient?	Taobao Marketplace, a Chinese website for online shopping
BMI Digital Store	Gold Power Saving Specialist	Does it save power?	Taobao Marketplace, a Chinese website for online shopping
BMI Digital Store	Gold Power Saving Specialist	using itfor more than2months now how come the effect is not obvious often thenumber of237to243usually in240	Taobao Marketplace, a Chinese website for online shopping
BMI Digital Store	Gold Power Saving Specialist	Who is using itafter2months and how is it working?	Taobao Marketplace, a Chinese website for online shopping
BMI Digital Store	Gold Power Saving Specialist	Is this a capacitor?	Taobao Marketplace, a Chinese website for online shopping
BMI Digital Store	Gold Power Saving Specialist	Does it really save power? Please be honest.	Taobao Marketplace, a Chinese website for online shopping
BMI Digital Store	Gold Power Saving Specialist	Does it really save power?	Taobao Marketplace, a Chinese website for online shopping
BMI Digital Store	Gold Power Saving Specialist	I was wondering what size capacitors are in there?	Taobao Marketplace, a Chinese website for online shopping
BMI Digital Store	Gold Power Saving Specialist	What percent of electricity does this thing save?	Taobao Marketplace, a Chinese website for online shopping
BMI Digital Store	Gold Power Saving Specialist	Can you all tell me if this energy saver really achieves power savings?	Taobao Marketplace, a Chinese website for online shopping
BMI Digital Store	Gold Power Saving Specialist	Does this stuff work or not?	Taobao Marketplace, a Chinese website for online shopping
BMI Digital Store	Gold Power Saving Specialist	Saving appliances can really save electricity are not easy to earn money to say the conscience of words	Taobao Marketplace, a Chinese website for online shopping
BMI Digital Store	Gold Power Saving Specialist	Dear friends can you use two months have obvious results of your family ah	Taobao Marketplace, a Chinese website for online shopping

BMI Digital Store	Gold Power Saving Specialist	Can you really save on this stuff?	Taobao Marketplace, a Chinese website for online shopping
BMI Digital Store	Gold Power Saving Specialist	Does it really save electricity?	Taobao Marketplace, a Chinese website for online shopping
BMI Digital Store	Gold Power Saving Specialist	For those of you who have been using it for a long time, has it been a power saver?	Taobao Marketplace, a Chinese website for online shopping
BMI Digital Store	Gold Power Saving Specialist	Commercial electricity for opening a business is expensive, can this be saved?	Taobao Marketplace, a Chinese website for online shopping
BMI Digital Store	Gold Power Saving Specialist	Does this really save electricity? Does it work? What's the quality like?	Taobao Marketplace, a Chinese website for online shopping
BMI Digital Store	Gold Power Saving Specialist	Does it really save electricity? Does it work? This meter in my house is too fast, too.	Taobao Marketplace, a Chinese website for online shopping
BMI Digital Store	Gold Power Saving Specialist	Is it so amazing that plugging in any outlet can save electricity?	Taobao Marketplace, a Chinese website for online shopping
BMI Digital Store	Gold Power Saving Specialist	Does Nodebot work?	Taobao Marketplace, a Chinese website for online shopping
BMI Digital Store	Gold Power Saving Specialist	Can you use2or3a family?	Taobao Marketplace, a Chinese website for online shopping
BMI Digital Store	Gold Power Saving Specialist	Can I ask you folks if this saver you are using works?	Taobao Marketplace, a Chinese website for online shopping
BMI Digital Store	Gold Power Saving Specialist	Is it really possible to save electricity? I useabout100kWhper month, how much can I save? Thanks for the pro's help!	Taobao Marketplace, a Chinese website for online shopping
BMI Digital Store	Gold Power Saving Specialist	Does this slow down the meter?	Taobao Marketplace, a Chinese website for online shopping
BMI Digital Store	Gold Power Saving Specialist	Can you really save?	Taobao Marketplace, a Chinese website for online shopping
BMI Digital Store	Gold Power Saving Specialist	Does it really save power? I feel fakeit's all fII guessthere's no way for the user to test if he can save power anyway.	Taobao Marketplace, a Chinese website for online shopping
BMI Digital Store	Gold Power Saving Specialist	Does it work? Does it save electricity?	Taobao Marketplace, a Chinese website for online shopping
BMI Digital Store	Gold Power Saving Specialist	Can you please tell Meter Saver really saves electricity? How does it work?	Taobao Marketplace, a Chinese website for online shopping
BMI Digital Store	Gold Power Saving Specialist	It really saves power.	Taobao Marketplace, a Chinese website for online shopping
BMI Digital Store	Gold Power Saving Specialist	There's just one dedicated outlet for the air conditioner, and there's no extra outlet next to it, so where do I plug it in?	Taobao Marketplace, a Chinese website for online shopping
BMI Digital Store	Gold Power Saving Specialist	For those of you who have used it, may I ask? I can use300kWhper quarter, how much electricity can I save per quarter if I install this?	Taobao Marketplace, a Chinese website for online shopping
BMI Digital Store	Gold Power Saving Specialist	How much electricity can you save in an average month?	Taobao Marketplace, a Chinese website for online shopping
BMI Digital Store	Gold Power Saving Specialist	Does this thing work?	Taobao Marketplace, a Chinese website for online shopping
BMI Digital Store	Gold Power Saving Specialist	Does it really save power?	Taobao Marketplace, a Chinese website for online shopping
BMI Digital Store	Gold Power Saving Specialist	Does this go on the meter or on a plug in the house?	Taobao Marketplace, a Chinese website for online shopping
BMI Digital Store	Gold Power Saving Specialist	Does it really work! Friends?	Taobao Marketplace, a Chinese website for online shopping
BMI Digital Store	Gold Power Saving Specialist	How does this work friends. True or False	Taobao Marketplace, a Chinese website for online shopping
BMI Digital Store	Gold Power Saving Specialist	Does anyone really believe that?	Taobao Marketplace, a Chinese website for online shopping
BMI Digital Store	Gold Power Saving Specialist	Ask please can save electricity, right?	Taobao Marketplace, a Chinese website for online shopping
BMI Digital Store	Gold Power Saving Specialist	Does it really save electricity?Have any friends used it?	Taobao Marketplace, a Chinese website for online shopping
BMI Digital Store	Gold Power Saving Specialist	Hi, how does it feel when you use it and what percent of electricity can you save in a month? Thank you, sir.	Taobao Marketplace, a Chinese website for online shopping
BMI Digital Store	Gold Power Saving Specialist	Can you tell me how effective the power saving is? Thanks!	Taobao Marketplace, a Chinese website for online shopping
BMI Digital Store	Gold Power Saving Specialist	Have you used it for a long time? Does it feel useful.	Taobao Marketplace, a Chinese website for online shopping
BMI Digital Store	Gold Power Saving Specialist	We're always tripping here. Does it work?	Taobao Marketplace, a Chinese website for online shopping
BMI Digital Store	Gold Power Saving Specialist	Does it really save electricity? Please tell sincerely	Taobao Marketplace, a Chinese website for online shopping
BMI Digital Store	Gold Power Saving Specialist	Do home energy savers work, folks? Do they save electricity?	Taobao Marketplace, a Chinese website for online shopping

BMI Digital Store	Gold Power Saving Specialist	Hello everyone! Can you tell me if this saves electricity or not; roughly how much a month;	Taobao Marketplace, a Chinese website for online shopping
BMI Digital Store	Gold Power Saving Specialist	Hello friends! Does this Power Saver plug in, does the meter slow down or does the meter not go?	Taobao Marketplace, a Chinese website for online shopping
BMI Digital Store	Gold Power Saving Specialist	How does it work? Does it really work?	Taobao Marketplace, a Chinese website for online shopping
BMI Digital Store	Gold Power Saving Specialist	Wouldn't it be more energy efficient to plug in more than one?	Taobao Marketplace, a Chinese website for online shopping
BMI Digital Store	Gold Power Saving Specialist	In all conscience, really?	Taobao Marketplace, a Chinese website for online shopping
BMI Digital Store	Gold Power Saving Specialist	Already purchased brothers and sisters brothers and sisters uncles and aunts can really save some not? Is the good review a cashback brush? I'm not sure if I'm going to be able to do that. I don't know.	Taobao Marketplace, a Chinese website for online shopping
BMI Digital Store	Gold Power Saving Specialist	My houseover400kWha month. Isn't it necessary to use this. I don't have air conditioning. TV, washing machine, water heater, refrigerator.	Taobao Marketplace, a Chinese website for online shopping
BMI Digital Store	Gold Power Saving Specialist	Does it work?	Taobao Marketplace, a Chinese website for online shopping
BMI Digital Store	Gold Power Saving Specialist	Does it save electricity? How effective is it?	Taobao Marketplace, a Chinese website for online shopping
BMI Digital Store	Gold Power Saving Specialist	You can stick it anywhere, right?	Taobao Marketplace, a Chinese website for online shopping
BMI Digital Store	Gold Power Saving Specialist	Does this save power?	Taobao Marketplace, a Chinese website for online shopping
BMI Digital Store	Gold Power Saving Specialist	Excuse me folks, is it plugged into the first socket on the mains?	Taobao Marketplace, a Chinese website for online shopping
BMI Digital Store	Gold Power Saving Specialist	Home electricity use about one hundred and fifty to two hundred degrees a month, more than three hundred degrees of electricity in the summer, how to use it well	Taobao Marketplace, a Chinese website for online shopping
BMI Digital Store	Gold Power Saving Specialist	Hi all, does this thing really save power?	Taobao Marketplace, a Chinese website for online shopping
BMI Digital Store	Gold Power Saving Specialist	How does it work?	Taobao Marketplace, a Chinese website for online shopping
BMI Digital Store	Gold Power Saving Specialist	What's the best way to install it? Next to the meter?	Taobao Marketplace, a Chinese website for online shopping
BMI Digital Store	Gold Power Saving Specialist	Does it really save power?	Taobao Marketplace, a Chinese website for online shopping
BMI Digital Store	Gold Power Saving Specialist	Does it really save electricity?	Taobao Marketplace, a Chinese website for online shopping
BMI Digital Store	Gold Power Saving Specialist	Does anyone who has been using it for a few months save power?	Taobao Marketplace, a Chinese website for online shopping
BMI Digital Store	Gold Power Saving Specialist	Does it really save power?	Taobao Marketplace, a Chinese website for online shopping
BMI Digital Store	Gold Power Saving Specialist	Does it really save power?	Taobao Marketplace, a Chinese website for online shopping
BMI Digital Store	Gold Power Saving Specialist	Can any of you folks tell me the truth, does it work or not?Does it really save electricity? Please tell me the truth!	Taobao Marketplace, a Chinese website for online shopping
BMI Digital Store	Gold Power Saving Specialist	Is this a good pair of electronic watches?	Taobao Marketplace, a Chinese website for online shopping
BMI Digital Store	Gold Power Saving Specialist	How are you all feeling?	Taobao Marketplace, a Chinese website for online shopping
BMI Digital Store	Gold Power Saving Specialist	How does it work?	Taobao Marketplace, a Chinese website for online shopping
BMI Digital Store	Gold Power Saving Specialist	Does this work. Does it really save electricity?	Taobao Marketplace, a Chinese website for online shopping
BMI Digital Store	Gold Power Saving Specialist	Does this saver really work?	Taobao Marketplace, a Chinese website for online shopping
BMI Digital Store	Gold Power Saving Specialist	Does it save power?	Taobao Marketplace, a Chinese website for online shopping
BMI Digital Store	Gold Power Saving Specialist	How's it working out for you?	Taobao Marketplace, a Chinese website for online shopping
BMI Digital Store	Gold Power Saving Specialist	You guys bought it, what's the result? Does it really save electricity?	Taobao Marketplace, a Chinese website for online shopping
BMI Digital Store	Gold Power Saving Specialist	Dear Friends ~ If a familyhas 350kWh of electricitya month! How many to install!	Taobao Marketplace, a Chinese website for online shopping
BMI Digital Store	Gold Power Saving Specialist	Does this really work?	Taobao Marketplace, a Chinese website for online shopping
BMI Digital Store	Gold Power Saving Specialist	Does this power saver work?	Taobao Marketplace, a Chinese website for online shopping
BMI Digital Store	Gold Power Saving Specialist	Is the effect noticeable over time?	Taobao Marketplace, a Chinese website for online shopping

BMI Digital Store	Gold Power Saving Specialist	Really?	Taobao Marketplace, a Chinese website for online shopping
BMI Digital Store	Gold Power Saving Specialist	Is there a power saving effect?	Taobao Marketplace, a Chinese website for online shopping
BMI Digital Store	Gold Power Saving Specialist	How does it work? Does it work?	Taobao Marketplace, a Chinese website for online shopping
BMI Digital Store	Gold Power Saving Specialist	Will this thing explode?	Taobao Marketplace, a Chinese website for online shopping
BMI Digital Store	Gold Power Saving Specialist	Is it true that you can save electricity and how much can you save compared to last month?	Taobao Marketplace, a Chinese website for online shopping
BMI Digital Store	Gold Power Saving Specialist	Hello! Is it really possible to save electricity?	Taobao Marketplace, a Chinese website for online shopping
BMI Digital Store	Gold Power Saving Specialist	How many kWh can you save in a month	Taobao Marketplace, a Chinese website for online shopping
BMI Digital Store	Gold Power Saving Specialist	Can you save power?	Taobao Marketplace, a Chinese website for online shopping
BMI Digital Store	Gold Power Saving Specialist	Pros this thing you have used think you can use it, can not save electricity ah heh	Taobao Marketplace, a Chinese website for online shopping
BMI Digital Store	Gold Power Saving Specialist	Does anyone really have a power saving	Taobao Marketplace, a Chinese website for online shopping
BMI Digital Store	Gold Power Saving Specialist	Is it so amazing that plugging in any outlet can save electricity?	Taobao Marketplace, a Chinese website for online shopping
BMI Digital Store	Gold Power Saving Specialist	Does it work? Saves electricity.	Taobao Marketplace, a Chinese website for online shopping
BMI Digital Store	Gold Power Saving Specialist	Does it work? Does it work?	Taobao Marketplace, a Chinese website for online shopping
BMI Digital Store	Gold Power Saving Specialist	Does it work? Does it save electricity?	Taobao Marketplace, a Chinese website for online shopping
BMI Digital Store	Gold Power Saving Specialist	Will it work with low voltage at home?	Taobao Marketplace, a Chinese website for online shopping
BMI Digital Store	Gold Power Saving Specialist	Can this thing be plugged in anywhere?	Taobao Marketplace, a Chinese website for online shopping
BMI Digital Store	Gold Power Saving Specialist	It's disgusting. This thing doesn't do anything. It's a power hog.	Taobao Marketplace, a Chinese website for online shopping
BMI Digital Store	Gold Power Saving Specialist	Do the pro's really save electricity? I don't believe it.	Taobao Marketplace, a Chinese website for online shopping
BMI Digital Store	Gold Power Saving Specialist	Can you save electricity by putting it in a doorway?	Taobao Marketplace, a Chinese website for online shopping
BMI Digital Store	Gold Power Saving Specialist	Does it really save power? Does it have any effect?	Taobao Marketplace, a Chinese website for online shopping
BMI Digital Store	Gold Power Saving Specialist	Does it really work? Are there any returns that don't work?	Taobao Marketplace, a Chinese website for online shopping
BMI Digital Store	Gold Power Saving Specialist	What do you think of the Wen so-and-so bag reviews as below?	Taobao Marketplace, a Chinese website for online shopping
BMI Digital Store	Gold Power Saving Specialist	Does it really work?	Taobao Marketplace, a Chinese website for online shopping
BMI Digital Store	Gold Power Saving Specialist	For those who have used it, does it save power?	Taobao Marketplace, a Chinese website for online shopping
BMI Digital Store	Gold Power Saving Specialist	are4layersin total. Which floor is better to insert	Taobao Marketplace, a Chinese website for online shopping
BMI Digital Store	Gold Power Saving Specialist	Does it save power? What position do you plug it into?	Taobao Marketplace, a Chinese website for online shopping
BMI Digital Store	Gold Power Saving Specialist	Does it really save power?	Taobao Marketplace, a Chinese website for online shopping
BMI Digital Store	Gold Power Saving Specialist	anyone used itmore than2months with results?	Taobao Marketplace, a Chinese website for online shopping
BMI Digital Store	Gold Power Saving Specialist	Does this thing work?	Taobao Marketplace, a Chinese website for online shopping
BMI Digital Store	Gold Power Saving Specialist	Who has reliable evidence that it saves electricity	Taobao Marketplace, a Chinese website for online shopping
BMI Digital Store	Gold Power Saving Specialist	Does this save power?	Taobao Marketplace, a Chinese website for online shopping
BMI Digital Store	Gold Power Saving Specialist	Use a friend this power saving real power saving?	Taobao Marketplace, a Chinese website for online shopping
BMI Digital Store	Gold Power Saving Specialist	Does it really save electricity?	Taobao Marketplace, a Chinese website for online shopping
BMI Digital Store	Gold Power Saving Specialist	How does this thing work, I have a 3 phase 5 hp air conditioner at home, does it work?	Taobao Marketplace, a Chinese website for online shopping

BMI Digital Store	Gold Power Saving Specialist	Does it really work?	Taobao Marketplace, a Chinese website for online shopping
BMI Digital Store	Gold Power Saving Specialist	Folks, does it really save electricity?	Taobao Marketplace, a Chinese website for online shopping
BMI Digital Store	Gold Power Saving Specialist	For those who have already bought it, does it really save power?	Taobao Marketplace, a Chinese website for online shopping
BMI Digital Store	Gold Power Saving Specialist	Type in Baidu for energy saver and guess what happens?	Taobao Marketplace, a Chinese website for online shopping
BMI Digital Store	Gold Power Saving Specialist	Does it consume electricity by itself? How is the power saving effect?	Taobao Marketplace, a Chinese website for online shopping
BMI Digital Store	Gold Power Saving Specialist	Hello brothers and sisters. Can you please tell me if this product really saves power? Thanks and much appreciated.	Taobao Marketplace, a Chinese website for online shopping
BMI Digital Store	Gold Power Saving Specialist	Hello friends Can we really save electricity at home?	Taobao Marketplace, a Chinese website for online shopping
BMI Digital Store	Gold Power Saving Specialist	What are the power savings?	Taobao Marketplace, a Chinese website for online shopping
BMI Digital Store	Gold Power Saving Specialist	Will it save electricity, or not.	Taobao Marketplace, a Chinese website for online shopping
BMI Digital Store	Gold Power Saving Specialist	May I ask, this product, does it save electricity?	Taobao Marketplace, a Chinese website for online shopping
BMI Digital Store	Gold Power Saving Specialist	Is this power saving? Honest reply, thanks!	Taobao Marketplace, a Chinese website for online shopping
BMI Digital Store	Gold Power Saving Specialist	For those of you who have used it, does this thing really save power?	Taobao Marketplace, a Chinese website for online shopping
BMI Digital Store	Gold Power Saving Specialist	Does it really save electricity? What about conservation of energy? The principle should be reactive power compensation, right? Might as well just work on the meter	Taobao Marketplace, a Chinese website for online shopping
BMI Digital Store	Gold Power Saving Specialist	How is it working for all of you? Does it save power? Especially in the summer	Taobao Marketplace, a Chinese website for online shopping
BMI Digital Store	Gold Power Saving Specialist	Please, tell me how well it works?	Taobao Marketplace, a Chinese website for online shopping
BMI Digital Store	Gold Power Saving Specialist	Does this saver really save power? Answer if you're online. Thanks!	Taobao Marketplace, a Chinese website for online shopping
BMI Digital Store	Gold Power Saving Specialist	my housework on2phase power? Is it really the first electricity? Is it for home use?	Taobao Marketplace, a Chinese website for online shopping
BMI Digital Store	Gold Power Saving Specialist	I'm a restaurant. Can I use this?	Taobao Marketplace, a Chinese website for online shopping
BMI Digital Store	Gold Power Saving Specialist	I don't know if the landlord is using a substandard meter and the meter is a mechanical one old and costly, I don't know if the product will work, the	Taobao Marketplace, a Chinese website for online shopping
BMI Digital Store	Gold Power Saving Specialist	What's the principle of saving power?	Taobao Marketplace, a Chinese website for online shopping
BMI Digital Store	Gold Power Saving Specialist	Does it work, folks? Does it work? The electricity meter in our rented room is jumping so fast.	Taobao Marketplace, a Chinese website for online shopping
BMI Digital Store	Gold Power Saving Specialist	Does this really save power? Are there any side effects? Thanks!	Taobao Marketplace, a Chinese website for online shopping
BMI Digital Store	Gold Power Saving Specialist	Does it save electricity? How much can you save with air conditioning?	Taobao Marketplace, a Chinese website for online shopping
BMI Digital Store	Gold Power Saving Specialist	How long is the warranty on this one? Can you really name the power? Please be honest.	Taobao Marketplace, a Chinese website for online shopping
BMI Digital Store	Gold Power Saving Specialist	Will this affect the function of the home air switch?	Taobao Marketplace, a Chinese website for online shopping
BMI Digital Store	Gold Power Saving Specialist	There are a lot of people who say it doesn't work, some say it does, and those who say it does say where it's mounted.	Taobao Marketplace, a Chinese website for online shopping
BMI Digital Store	Gold Power Saving Specialist	How is the power saving effect? What's the quality like, any long time users give a big tell?	Taobao Marketplace, a Chinese website for online shopping
BMI Digital Store	Gold Power Saving Specialist	Does it really save power? There's not enough power to start.	Taobao Marketplace, a Chinese website for online shopping
BMI Digital Store	Gold Power Saving Specialist	Can you buy this and plug it into an outlet?	Taobao Marketplace, a Chinese website for online shopping
BMI Digital Store	Gold Power Saving Specialist	Can I ask if I save the fridge just plug it in there? Or do I plug it in anywhere that mentions saving electricity in the house?????	Taobao Marketplace, a Chinese website for online shopping
BMI Digital Store	Gold Power Saving Specialist	Can I use an induction cooker?	Taobao Marketplace, a Chinese website for online shopping
BMI Digital Store	Gold Power Saving Specialist	Power meter looks at0.7watts ofpower consumption, and the current felt by the electricity is20mA	Taobao Marketplace, a Chinese website for online shopping
BMI Digital Store	Gold Power Saving Specialist	work?	Taobao Marketplace, a Chinese website for online shopping
BMI Digital Store	Gold Power Saving Specialist	I would love to know what percent of electricity can be saved, to be precise, please reply	Taobao Marketplace, a Chinese website for online shopping

BMI Digital Store	Gold Power Saving Specialist	Can it be connected to the main indoor circuit breaker port?	Taobao Marketplace, a Chinese website for online shopping
BMI Digital Store	Gold Power Saving Specialist	Those who have a clear effectandsave electricity to check in,really work? Slippers.	Taobao Marketplace, a Chinese website for online shopping
BMI Digital Store	Gold Power Saving Specialist	Do you sell stolen appliances?	Taobao Marketplace, a Chinese website for online shopping
BMI Digital Store	Gold Power Saving Specialist	Does it work?	Taobao Marketplace, a Chinese website for online shopping
BMI Digital Store	Gold Power Saving Specialist	Does this really save power?	Taobao Marketplace, a Chinese website for online shopping
BMI Digital Store	Gold Power Saving Specialist	guys, has anyone used this thingfor more than2months and found that it uses significantly less power? The air conditioner is the only thing that consumes the most power in the summer.	Taobao Marketplace, a Chinese website for online shopping
BMI Digital Store	Gold Power Saving Specialist	Question for everyone, does it indeed save power? Thank you. - Thank you.	Taobao Marketplace, a Chinese website for online shopping
BMI Digital Store	Gold Power Saving Specialist	There's no way to turn the meter upside down.	Taobao Marketplace, a Chinese website for online shopping
BMI Digital Store	Gold Power Saving Specialist	Does it really save electricity? How much do you save in a month?	Taobao Marketplace, a Chinese website for online shopping
BMI Digital Store	Gold Power Saving Specialist	Has anyone tested a comparison of electricity usage using one appliance plus a meter specifically?	Taobao Marketplace, a Chinese website for online shopping
BMI Digital Store	Gold Power Saving Specialist	good,got it today andreally well	Taobao Marketplace, a Chinese website for online shopping
BMI Digital Store	Gold Power Saving Specialist	it work?	Taobao Marketplace, a Chinese website for online shopping
BMI Digital Store	Gold Power Saving Specialist	Does it work or not? Everyone says it doesn't.	Taobao Marketplace, a Chinese website for online shopping
BMI Digital Store	Gold Power Saving Specialist	usefulness	Taobao Marketplace, a Chinese website for online shopping
BMI Digital Store	Gold Power Saving Specialist	I don't know. I don't feel it.	Taobao Marketplace, a Chinese website for online shopping
BMI Digital Store	Gold Power Saving Specialist	One year warranty Works all the time	Taobao Marketplace, a Chinese website for online shopping
BMI Digital Store	Gold Power Saving Specialist	What to say. Tests felt ineffective. Not using it now.	Taobao Marketplace, a Chinese website for online shopping
BMI Digital Store	Gold Power Saving Specialist	not very effective	Taobao Marketplace, a Chinese website for online shopping
BMI Digital Store	Gold Power Saving Specialist	It's not as good as it says. I used an unknown phase.	Taobao Marketplace, a Chinese website for online shopping
BMI Digital Store	Gold Power Saving Specialist	The principle of reactive power compensation, home air conditioners, refrigerators, and other electrical appliances with motors and coils have good results.	Taobao Marketplace, a Chinese website for online shopping
BMI Digital Store	Gold Power Saving Specialist	There's nothing wrong with being wasteful.	Taobao Marketplace, a Chinese website for online shopping
BMI Digital Store	Gold Power Saving Specialist	Depends on how much you use in a day or a month！	Taobao Marketplace, a Chinese website for online shopping
BMI Digital Store	Gold Power Saving Specialist	I don't think so, I've been busy lately and haven't noticed, but I've been using it	Taobao Marketplace, a Chinese website for online shopping

BMI Digital Store	Gold Power Saving Specialist	I feel like would save me some money	Taobao Marketplace, a Chinese website for online shopping
BMI Digital Store	Gold Power Saving Specialist	I don't know. I just used it.	Taobao Marketplace, a Chinese website for online shopping
BMI Digital Store	Gold Power Saving Specialist	To be honest, I don't feel it's useful at all, and it's potentially power-hungry	Taobao Marketplace, a Chinese website for online shopping
BMI Digital Store	Gold Power Saving Specialist	I just bought it last month and I don't know yet	Taobao Marketplace, a Chinese website for online shopping
BMI Digital Store	Gold Power Saving Specialist	I don't see any nodes.	Taobao Marketplace, a Chinese website for online shopping
BMI Digital Store	Gold Power Saving Specialist	We don't know yet.	Taobao Marketplace, a Chinese website for online shopping
BMI Digital Store	Gold Power Saving Specialist	It's safe, there's no definitive answer as to whether or not it saves electricity	Taobao Marketplace, a Chinese website for online shopping
BMI Digital Store	Gold Power Saving Specialist	I plugged it directly into a five-hole outlet.	Taobao Marketplace, a Chinese website for online shopping
BMI Digital Store	Gold Power Saving Specialist	The effect is not obvious	Taobao Marketplace, a Chinese website for online shopping
BMI Digital Store	Gold Power Saving Specialist	I'm not sure about this one Haven't tried it	Taobao Marketplace, a Chinese website for online shopping
BMI Digital Store	Gold Power Saving Specialist	It works. It's safe.	Taobao Marketplace, a Chinese website for online shopping
BMI Digital Store	Gold Power Saving Specialist	It is not clear, the original is to open the empty electricity costs more, now do not open, is less, I do not know if it is useful or not	Taobao Marketplace, a Chinese website for online shopping
BMI Digital Store	Gold Power Saving Specialist	there are	Taobao Marketplace, a Chinese website for online shopping
BMI Digital Store	Gold Power Saving Specialist	I'm using it now, it hasn't been a month yet! I'll get back to you.	Taobao Marketplace, a Chinese website for online shopping
BMI Digital Store	Gold Power Saving Specialist	I'm not sure. I didn't ask the kids' grandparents! I'll find out when I pay the bill.	Taobao Marketplace, a Chinese website for online shopping
BMI Digital Store	Gold Power Saving Specialist	Yes, it will, saveabout30%, but don't let the bill collector know, it's not very effective for home use	Taobao Marketplace, a Chinese website for online shopping
BMI Digital Store	Gold Power Saving Specialist	It's fine. It's been fine for months.	Taobao Marketplace, a Chinese website for online shopping
BMI Digital Store	Gold Power Saving Specialist	works!I've saved almost 10 kilowatts this month.	Taobao Marketplace, a Chinese website for online shopping
BMI Digital Store	Gold Power Saving Specialist	Just used it ~ we'll see the results next month!	Taobao Marketplace, a Chinese website for online shopping
BMI Digital Store	Gold Power Saving Specialist	It's okay. It seems to save a little bit of electricity.	Taobao Marketplace, a Chinese website for online shopping
BMI Digital Store	Gold Power Saving Specialist	I haven't seen it I feel like it works I'd like to buy one in the	Taobao Marketplace, a Chinese website for online shopping
BMI Digital Store	Gold Power Saving Specialist	Power saving, very good very satisfied, thanks to the seller.	Taobao Marketplace, a Chinese website for online shopping
BMI Digital Store	Gold Power Saving Specialist	Not so sure about this one! Ask the boss.	Taobao Marketplace, a Chinese website for online shopping
BMI Digital Store	Gold Power Saving Specialist	No visible effect	Taobao Marketplace, a Chinese website for online shopping
BMI Digital Store	Gold Power Saving Specialist	not very useful	Taobao Marketplace, a Chinese website for online shopping
BMI Digital Store	Gold Power Saving Specialist	can	Taobao Marketplace, a Chinese website for online shopping
BMI Digital Store	Gold Power Saving Specialist	I don't know if it really saves power. Use it anyway.	Taobao Marketplace, a Chinese website for online shopping
BMI Digital Store	Gold Power Saving Specialist	I didn't notice.	Taobao Marketplace, a Chinese website for online shopping
BMI Digital Store	Gold Power Saving Specialist	You won't know until you've used it for a month.	Taobao Marketplace, a Chinese website for online shopping
BMI Digital Store	Gold Power Saving Specialist	Fake didn't save power	Taobao Marketplace, a Chinese website for online shopping
BMI Digital Store	Gold Power Saving Specialist	I've been using it for a month and just paid my electric bill and it feels like it's less than last month	Taobao Marketplace, a Chinese website for online shopping
BMI Digital Store	Gold Power Saving Specialist	Still on probation, not a good answer!	Taobao Marketplace, a Chinese website for online shopping
BMI Digital Store	Gold Power Saving Specialist	Doesn't seem like much of a savings.	Taobao Marketplace, a Chinese website for online shopping
BMI Digital Store	Gold Power Saving Specialist	Haven't paid the electric bill yet. Ha, ha.	Taobao Marketplace, a Chinese website for online shopping

BMI Digital Store	Gold Power Saving Specialist	nothing good be said about it	Taobao Marketplace, a Chinese website for online shopping
BMI Digital Store	Gold Power Saving Specialist	Didn't do the math. Should save some.	Taobao Marketplace, a Chinese website for online shopping
BMI Digital Store	Gold Power Saving Specialist	It's not working.	Taobao Marketplace, a Chinese website for online shopping
BMI Digital Store	Gold Power Saving Specialist	Guess it worked!	Taobao Marketplace, a Chinese website for online shopping
BMI Digital Store	Gold Power Saving Specialist	Dear, the general use of energy-saving appliances for a month, power saving is a little by little accumulation of oh! By the next month to pay the electricity bill will be able to see it!	Taobao Marketplace, a Chinese website for online shopping
BMI Digital Store	Gold Power Saving Specialist	Nowadays it's basically wide voltage220-240v	Taobao Marketplace, a Chinese website for online shopping
BMI Digital Store	Gold Power Saving Specialist	This month's electric bill is$12less than last time	Taobao Marketplace, a Chinese website for online shopping
BMI Digital Store	Gold Power Saving Specialist	I haven't looked hard enough.	Taobao Marketplace, a Chinese website for online shopping
BMI Digital Store	Gold Power Saving Specialist	I'm still watching! Void refund for what the seller says it looks like !!!! Think !!!!!	Taobao Marketplace, a Chinese website for online shopping
BMI Digital Store	Gold Power Saving Specialist	incompetent	Taobao Marketplace, a Chinese website for online shopping
BMI Digital Store	Gold Power Saving Specialist	You should be able to save 10 percent.	Taobao Marketplace, a Chinese website for online shopping
BMI Digital Store	Gold Power Saving Specialist	You can buy more appliances in your house, mainly those with motors, refrigerators washing machines range hoods and so on.	Taobao Marketplace, a Chinese website for online shopping
BMI Digital Store	Gold Power Saving Specialist	I don't think it's working.	Taobao Marketplace, a Chinese website for online shopping
BMI Digital Store	Gold Power Saving Specialist	I've been using it for over two years and it does work.	Taobao Marketplace, a Chinese website for online shopping
BMI Digital Store	Gold Power Saving Specialist	Not for a while. Wait for next month.	Taobao Marketplace, a Chinese website for online shopping
BMI Digital Store	Gold Power Saving Specialist	Not sure really, still in use	Taobao Marketplace, a Chinese website for online shopping
BMI Digital Store	Gold Power Saving Specialist	I don't know.	Taobao Marketplace, a Chinese website for online shopping
BMI Digital Store	Gold Power Saving Specialist	Just bought it less than a month ago, don't know	Taobao Marketplace, a Chinese website for online shopping
BMI Digital Store	Gold Power Saving Specialist	I just used it and don't know yet!	Taobao Marketplace, a Chinese website for online shopping
BMI Digital Store	Gold Power Saving Specialist	Already returned……used it for a month or so, or maybe it was too short of a time to see it	Taobao Marketplace, a Chinese website for online shopping
BMI Digital Store	Gold Power Saving Specialist	Safety can be, save or not you do not know now did not find the province	Taobao Marketplace, a Chinese website for online shopping
BMI Digital Store	Gold Power Saving Specialist	Last montharound370, this montharound140	Taobao Marketplace, a Chinese website for online shopping
BMI Digital Store	Gold Power Saving Specialist	senseless	Taobao Marketplace, a Chinese website for online shopping
BMI Digital Store	Gold Power Saving Specialist	Just used. We'll see at the end of the month.	Taobao Marketplace, a Chinese website for online shopping
BMI Digital Store	Gold Power Saving Specialist	No air conditioning, but there is an induction cooker, which is somewhat useful	Taobao Marketplace, a Chinese website for online shopping
BMI Digital Store	Gold Power Saving Specialist	I've been using it2months.	Taobao Marketplace, a Chinese website for online shopping
BMI Digital Store	Gold Power Saving Specialist	It's best to plug it into an outlet that uses a lot of electricity, and if the appliances are spread out you should still consider a few more for the effect to be noticeable	Taobao Marketplace, a Chinese website for online shopping
BMI Digital Store	Gold Power Saving Specialist	yea	Taobao Marketplace, a Chinese website for online shopping
BMI Digital Store	Gold Power Saving Specialist	I think so.	Taobao Marketplace, a Chinese website for online shopping
BMI Digital Store	Gold Power Saving Specialist	Stabilize the voltage, right? I don't get it.	Taobao Marketplace, a Chinese website for online shopping
BMI Digital Store	Gold Power Saving Specialist	(nonverbal grunt as interjection)	Taobao Marketplace, a Chinese website for online shopping
BMI Digital Store	Gold Power Saving Specialist	I don't seem to feel anything.	Taobao Marketplace, a Chinese website for online shopping
BMI Digital Store	Gold Power Saving Specialist	I didn't realize it was only half a month.It's time to pay the electric bill.	Taobao Marketplace, a Chinese website for online shopping
BMI Digital Store	Gold Power Saving Specialist	0	Taobao Marketplace, a Chinese website for online shopping

BMI Digital Store	Gold Power Saving Specialist	I don't know if it works without the noise, I don't think it works much, let's use it first.	Taobao Marketplace, a Chinese website for online shopping
BMI Digital Store	Gold Power Saving Specialist	There is some effect, not as good as he says, but a little bit of an effect of about10to15percent	Taobao Marketplace, a Chinese website for online shopping
BMI Digital Store	Gold Power Saving Specialist	Yeah, you can save a couple bucks a month.	Taobao Marketplace, a Chinese website for online shopping
BMI Digital Store	Gold Power Saving Specialist	Been using it for three months, no change for a month or two	Taobao Marketplace, a Chinese website for online shopping
BMI Digital Store	Gold Power Saving Specialist	There's no sense in using it or not using it.	Taobao Marketplace, a Chinese website for online shopping
BMI Digital Store	Gold Power Saving Specialist	In use, not sure yet	Taobao Marketplace, a Chinese website for online shopping
BMI Digital Store	Gold Power Saving Specialist	It doesn't feel particularly noticeable. I've been plugged in for a couple months now.	Taobao Marketplace, a Chinese website for online shopping
BMI Digital Store	Gold Power Saving Specialist	Power savings are a myth with this stuff. The state has never recognized this	Taobao Marketplace, a Chinese website for online shopping
BMI Digital Store	Gold Power Saving Specialist	Saveover20a month.	Taobao Marketplace, a Chinese website for online shopping
BMI Digital Store	Gold Power Saving Specialist	I don't know if I just used it, I don't know if it saves electricity	Taobao Marketplace, a Chinese website for online shopping
BMI Digital Store	Gold Power Saving Specialist	I don't know if it works yet.	Taobao Marketplace, a Chinese website for online shopping
BMI Digital Store	Gold Power Saving Specialist	It's also not very effective	Taobao Marketplace, a Chinese website for online shopping
BMI Digital Store	Gold Power Saving Specialist	I don't know if it's a capacitor or not, but it doesn't work as well as they say it saves power, and I don't feel like it works	Taobao Marketplace, a Chinese website for online shopping
BMI Digital Store	Gold Power Saving Specialist	It saves me%15degrees, this is temporary, look for a favorable review in two months.	Taobao Marketplace, a Chinese website for online shopping
BMI Digital Store	Gold Power Saving Specialist	Hmm?	Taobao Marketplace, a Chinese website for online shopping
BMI Digital Store	Gold Power Saving Specialist	I don't know about that.	Taobao Marketplace, a Chinese website for online shopping
BMI Digital Store	Gold Power Saving Specialist	ineffective	Taobao Marketplace, a Chinese website for online shopping
BMI Digital Store	Gold Power Saving Specialist	senseless	Taobao Marketplace, a Chinese website for online shopping
BMI Digital Store	Gold Power Saving Specialist	After using it for a while, I personally feel that it still works as a starting point!	Taobao Marketplace, a Chinese website for online shopping
BMI Digital Store	Gold Power Saving Specialist	Still in use	Taobao Marketplace, a Chinese website for online shopping
BMI Digital Store	Gold Power Saving Specialist	I don't feel it.	Taobao Marketplace, a Chinese website for online shopping
BMI Digital Store	Gold Power Saving Specialist	You can save2/10ths	Taobao Marketplace, a Chinese website for online shopping
BMI Digital Store	Gold Power Saving Specialist	I don't know yet. I'm using it.	Taobao Marketplace, a Chinese website for online shopping
BMI Digital Store	Gold Power Saving Specialist	inconspicuous	Taobao Marketplace, a Chinese website for online shopping
BMI Digital Store	Gold Power Saving Specialist	You can save some of it.	Taobao Marketplace, a Chinese website for online shopping
BMI Digital Store	Gold Power Saving Specialist	It feels okay.	Taobao Marketplace, a Chinese website for online shopping
BMI Digital Store	Gold Power Saving Specialist	Haven't paid the electric bill yet! I haven't measured it.	Taobao Marketplace, a Chinese website for online shopping
BMI Digital Store	Gold Power Saving Specialist	acceptable	Taobao Marketplace, a Chinese website for online shopping
BMI Digital Store	Gold Power Saving Specialist	I don't know yet.	Taobao Marketplace, a Chinese website for online shopping
BMI Digital Store	Gold Power Saving Specialist	Above200degrees can	Taobao Marketplace, a Chinese website for online shopping
BMI Digital Store	Gold Power Saving Specialist	It doesn't feel like anything.	Taobao Marketplace, a Chinese website for online shopping
BMI Digital Store	Gold Power Saving Specialist	no electricity saving	Taobao Marketplace, a Chinese website for online shopping
BMI Digital Store	Gold Power Saving Specialist	Saving appliances is concentrating electricity together to reduce the presence of voids, so naturally the meter is going to be slower.	Taobao Marketplace, a Chinese website for online shopping
BMI Digital Store	Gold Power Saving Specialist	Meters, okay?	Taobao Marketplace, a Chinese website for online shopping

BMI Digital Store	Gold Power Saving Specialist	Talk about a little experience. I originally had an old Power Saver King. It didn't make much sense at first. Then it became more noticeable as time went on. It's more obvious. We4to5people. Monthly electricity bill less than100yuan gas burner basically do not use. In addition to bathing. In addition to the heat of the day open air conditioning. My experience is really quite good. So I bought a better one.	Taobao Marketplace, a Chinese website for online shopping
BMI Digital Store	Gold Power Saving Specialist	should not	Taobao Marketplace, a Chinese website for online shopping
BMI Digital Store	Gold Power Saving Specialist	What?	Taobao Marketplace, a Chinese website for online shopping
BMI Digital Store	Gold Power Saving Specialist	I don't know, it's only been plugged in for a few days, I probably won't know until I pay my electric bill next month	Taobao Marketplace, a Chinese website for online shopping
BMI Digital Store	Gold Power Saving Specialist	I replaced it with one that has two outlets.	Taobao Marketplace, a Chinese website for online shopping
BMI Digital Store	Gold Power Saving Specialist	I've never tested them, I have2installed in my house, I'm not sure if they have compact fluorescent power, but I'm asking for self-compassion.	Taobao Marketplace, a Chinese website for online shopping
BMI Digital Store	Gold Power Saving Specialist	It feels like a savings, but I haven't bothered to do the math.	Taobao Marketplace, a Chinese website for online shopping
BMI Digital Store	Gold Power Saving Specialist	It helps a little, but not a lot.	Taobao Marketplace, a Chinese website for online shopping
BMI Digital Store	Gold Power Saving Specialist	No power saving, breaks down in a few days	Taobao Marketplace, a Chinese website for online shopping
BMI Digital Store	Gold Power Saving Specialist	On the home socket	Taobao Marketplace, a Chinese website for online shopping
BMI Digital Store	Gold Power Saving Specialist	It's used to stabilize the voltage. Saving power is the next best thing.	Taobao Marketplace, a Chinese website for online shopping
BMI Digital Store	Gold Power Saving Specialist	On Trial	Taobao Marketplace, a Chinese website for online shopping
BMI Digital Store	Gold Power Saving Specialist	It's a quarter less than the same month last year.	Taobao Marketplace, a Chinese website for online shopping
BMI Digital Store	Gold Power Saving Specialist	I don't get it either.	Taobao Marketplace, a Chinese website for online shopping
BMI Digital Store	Gold Power Saving Specialist	s not obvious.Maybe it doesn't work	Taobao Marketplace, a Chinese website for online shopping
BMI Digital Store	Gold Power Saving Specialist	I can't see it now.	Taobao Marketplace, a Chinese website for online shopping
BMI Digital Store	Gold Power Saving Specialist	Saving electricity is possible, but saving more is not possible	Taobao Marketplace, a Chinese website for online shopping
BMI Digital Store	Gold Power Saving Specialist	The voltage will be low when you use it. It's useful, isn't it?	Taobao Marketplace, a Chinese website for online shopping
BMI Digital Store	Gold Power Saving Specialist	It should have an effect.	Taobao Marketplace, a Chinese website for online shopping
BMI Digital Store	Gold Power Saving Specialist	It's not obvious. It should work.	Taobao Marketplace, a Chinese website for online shopping
BMI Digital Store	Gold Power Saving Specialist	A power-saving good good I want to buy a few units for friends to use can not be cheaper at a time in the order of5units can be based on how much money to me please reply	Taobao Marketplace, a Chinese website for online shopping
BMI Digital Store	Gold Power Saving Specialist	don't know yet. Illfind outat the end of this month	Taobao Marketplace, a Chinese website for online shopping
BMI Digital Store	Gold Power Saving Specialist	Turn slow it, I do not know if there is no province, buy for my mother-in-law they use, look at the company to buy	Taobao Marketplace, a Chinese website for online shopping
BMI Digital Store	Gold Power Saving Specialist	usefulness	Taobao Marketplace, a Chinese website for online shopping
BMI Digital Store	Gold Power Saving Specialist	No way.	Taobao Marketplace, a Chinese website for online shopping
BMI Digital Store	Gold Power Saving Specialist	The trial didn't turn out well!	Taobao Marketplace, a Chinese website for online shopping
BMI Digital Store	Gold Power Saving Specialist	I guess it's okay.	Taobao Marketplace, a Chinese website for online shopping
BMI Digital Store	Gold Power Saving Specialist	I think the meter's been tampered with.	Taobao Marketplace, a Chinese website for online shopping
BMI Digital Store	Gold Power Saving Specialist	I've been using it for two months now and it doesn't save any electricity. 10% more than before I've been fucking lied to. ! ! ! ! ! Whoever buys it loses money.	Taobao Marketplace, a Chinese website for online shopping
BMI Digital Store	Gold Power Saving Specialist	It's been less than two months since I put it in, so I'm not sure.	Taobao Marketplace, a Chinese website for online shopping
BMI Digital Store	Gold Power Saving Specialist	Plugging it in doesn't work. Try it	Taobao Marketplace, a Chinese website for online shopping
BMI Digital Store	Gold Power Saving Specialist	not very effective	Taobao Marketplace, a Chinese website for online shopping
BMI Digital Store	Gold Power Saving Specialist	Whichever socket will do.	Taobao Marketplace, a Chinese website for online shopping
BMI Digital Store	Gold Power Saving Specialist	I can't feel it right now. I'll have to watch it for a few months.	Taobao Marketplace, a Chinese website for online shopping

BMI Digital Store	Gold Power Saving Specialist	Power saving can be achieved by power saving compensation	Taobao Marketplace, a Chinese website for online shopping
BMI Digital Store	Gold Power Saving Specialist	Just plug it into the rechargeable battery	Taobao Marketplace, a Chinese website for online shopping
BMI Digital Store	Gold Power Saving Specialist	You don't need to deliberately install it next to the meter, just find an unused and infrequently used two-bit jack in your house and keep it plugged in all the time. It is important to note that it should be kept continuously energized as much as possible to ensure its reliable continuity.	Taobao Marketplace, a Chinese website for online shopping
BMI Digital Store	Gold Power Saving Specialist	How many degrees did you save?	Taobao Marketplace, a Chinese website for online shopping
BMI Digital Store	Gold Power Saving Specialist	I don't feel anything at the moment.	Taobao Marketplace, a Chinese website for online shopping
BMI Digital Store	Gold Power Saving Specialist	I've been using it for over two months and it's not working!	Taobao Marketplace, a Chinese website for online shopping
BMI Digital Store	Gold Power Saving Specialist	Sorry, I just started using it too. The effect is unknown……	Taobao Marketplace, a Chinese website for online shopping
BMI Digital Store	Gold Power Saving Specialist	Try it for a few days and you'll see.	Taobao Marketplace, a Chinese website for online shopping
BMI Digital Store	Gold Power Saving Specialist	I don't know It might work You'll have to try it yourself to find out Measured with a multimeter Changes when plugged in and unplugged	Taobao Marketplace, a Chinese website for online shopping
BMI Digital Store	Gold Power Saving Specialist	Didn't find anything wrong with it! Download Peanut Diary to buy a lot of Taobao internal coupons! It's a great deal! Invitation code756ZTRO	Taobao Marketplace, a Chinese website for online shopping
BMI Digital Store	Gold Power Saving Specialist	acceptable	Taobao Marketplace, a Chinese website for online shopping
BMI Digital Store	Gold Power Saving Specialist	Bad. True or not?	Taobao Marketplace, a Chinese website for online shopping
BMI Digital Store	Gold Power Saving Specialist	Really haven't broken it down, probably, but it's not as impressive as it's touted to be! It's just a good review.	Taobao Marketplace, a Chinese website for online shopping
BMI Digital Store	Gold Power Saving Specialist	It's not clear.	Taobao Marketplace, a Chinese website for online shopping
BMI Digital Store	Gold Power Saving Specialist	It doesn't save.	Taobao Marketplace, a Chinese website for online shopping
BMI Digital Store	Gold Power Saving Specialist	I haven't had it for a month yet I don't know how it works	Taobao Marketplace, a Chinese website for online shopping
BMI Digital Store	Gold Power Saving Specialist	It feels okay.	Taobao Marketplace, a Chinese website for online shopping
BMI Digital Store	Gold Power Saving Specialist	I bought it for a friend. I don't know if it's any good.	Taobao Marketplace, a Chinese website for online shopping
BMI Digital Store	Gold Power Saving Specialist	Useful, saves20electricity bill	Taobao Marketplace, a Chinese website for online shopping
BMI Digital Store	Gold Power Saving Specialist	(there are) some (who are...)	Taobao Marketplace, a Chinese website for online shopping
BMI Digital Store	Gold Power Saving Specialist	I can't see the power savings, but the quality seems to be up for replacing this one\.	Taobao Marketplace, a Chinese website for online shopping
BMI Digital Store	Gold Power Saving Specialist	I don't know.	Taobao Marketplace, a Chinese website for online shopping
BMI Digital Store	Gold Power Saving Specialist	hasn't	Taobao Marketplace, a Chinese website for online shopping
BMI Digital Store	Gold Power Saving Specialist	Didn't do a comparison test, huh?	Taobao Marketplace, a Chinese website for online shopping
BMI Digital Store	Gold Power Saving Specialist	There's no sign of an explosion.	Taobao Marketplace, a Chinese website for online shopping
BMI Digital Store	Gold Power Saving Specialist	I'm not sure if it saves power, but the voltage displayed is very stable.	Taobao Marketplace, a Chinese website for online shopping
BMI Digital Store	Gold Power Saving Specialist	No savings, I've been trying it for a few months and it's consuming more power than before	Taobao Marketplace, a Chinese website for online shopping
BMI Digital Store	Gold Power Saving Specialist	Save about twenty or thirty dollars.	Taobao Marketplace, a Chinese website for online shopping
BMI Digital Store	Gold Power Saving Specialist	I don't know. I feel it in my heart.	Taobao Marketplace, a Chinese website for online shopping
BMI Digital Store	Gold Power Saving Specialist	It's useless.	Taobao Marketplace, a Chinese website for online shopping
BMI Digital Store	Gold Power Saving Specialist	Save more than usual.	Taobao Marketplace, a Chinese website for online shopping
BMI Digital Store	Gold Power Saving Specialist	You can't be that dramatic.	Taobao Marketplace, a Chinese website for online shopping
BMI Digital Store	Gold Power Saving Specialist	I don't feel it either.	Taobao Marketplace, a Chinese website for online shopping
BMI Digital Store	Gold Power Saving Specialist	I don't know yet. I just used it for a few days.	Taobao Marketplace, a Chinese website for online shopping

BMI Digital Store	Gold Power Saving Specialist	Power saving has no effect!	Taobao Marketplace, a Chinese website for online shopping
BMI Digital Store	Gold Power Saving Specialist	The voltage in the house is stable and this has not happened for a while! I live in a commercial home where the voltage is all stable!	Taobao Marketplace, a Chinese website for online shopping
BMI Digital Store	Gold Power Saving Specialist	Plug it into the main line.	Taobao Marketplace, a Chinese website for online shopping
BMI Digital Store	Gold Power Saving Specialist	Testing	Taobao Marketplace, a Chinese website for online shopping
BMI Digital Store	Gold Power Saving Specialist	I don't know because the government gas prices went down	Taobao Marketplace, a Chinese website for online shopping
BMI Digital Store	Gold Power Saving Specialist	I don't know, does it save electricity, I know my home electric bill is over a thousand dollars a month	Taobao Marketplace, a Chinese website for online shopping
BMI Digital Store	Gold Power Saving Specialist	ineffective	Taobao Marketplace, a Chinese website for online shopping
BMI Digital Store	Gold Power Saving Specialist	' The family says it's working	Taobao Marketplace, a Chinese website for online shopping
BMI Digital Store	Gold Power Saving Specialist	It's good. The power savings are worth it.	Taobao Marketplace, a Chinese website for online shopping
BMI Digital Store	Gold Power Saving Specialist	We don't know yet.	Taobao Marketplace, a Chinese website for online shopping
BMI Digital Store	Gold Power Saving Specialist	should not	Taobao Marketplace, a Chinese website for online shopping
BMI Digital Store	Gold Power Saving Specialist	Any floor will do.	Taobao Marketplace, a Chinese website for online shopping
BMI Digital Store	Gold Power Saving Specialist	Power saving my ass. Balls don't work, they don't save any power at all. Get it while you can. I've had it for over a month. The cost of electricity is basically the same as usual. And possibly more.	Taobao Marketplace, a Chinese website for online shopping
BMI Digital Store	Gold Power Saving Specialist	I haven't felt it yet, but I think it's okay to read the description of the principle.	Taobao Marketplace, a Chinese website for online shopping
BMI Digital Store	Gold Power Saving Specialist	I don't know if it works or not, it's been plugged in. Haven't bothered to count it.	Taobao Marketplace, a Chinese website for online shopping
BMI Digital Store	Gold Power Saving Specialist	I don't know. I bought it and I went out of town to work.	Taobao Marketplace, a Chinese website for online shopping
BMI Digital Store	Gold Power Saving Specialist	acceptable	Taobao Marketplace, a Chinese website for online shopping
BMI Digital Store	Gold Power Saving Specialist	save electricity	Taobao Marketplace, a Chinese website for online shopping
BMI Digital Store	Gold Power Saving Specialist	Bought back and plugged in the door, power saving is not yet felt, may be long-term use can play a role in the effect it	Taobao Marketplace, a Chinese website for online shopping
BMI Digital Store	Gold Power Saving Specialist	Saves20-30%	Taobao Marketplace, a Chinese website for online shopping
BMI Digital Store	Gold Power Saving Specialist	Useful, my family bought one last year and another this year, the effect will come out after a long time of use	Taobao Marketplace, a Chinese website for online shopping
BMI Digital Store	Gold Power Saving Specialist	It's okay.	Taobao Marketplace, a Chinese website for online shopping
BMI Digital Store	Gold Power Saving Specialist	I've only been using it for a month, it's not very noticeable, so I'll use it again!	Taobao Marketplace, a Chinese website for online shopping
BMI Digital Store	Gold Power Saving Specialist	I just used it too. It's not bad.	Taobao Marketplace, a Chinese website for online shopping
BMI Digital Store	Gold Power Saving Specialist	(nonverbal grunt as interjection)	Taobao Marketplace, a Chinese website for online shopping
BMI Digital Store	Gold Power Saving Specialist	not bad	Taobao Marketplace, a Chinese website for online shopping
BMI Digital Store	Gold Power Saving Specialist	Appears to be less!	Taobao Marketplace, a Chinese website for online shopping
BMI Digital Store	Gold Power Saving Specialist	I bought, this product and haven't used it yet. Previously bought a small, soon burned out. I asked the merchant:product inserted into my home above the wire, whether the whole building are followed by power saving light ah? Merchants did not answer me......	Taobao Marketplace, a Chinese website for online shopping
BMI Digital Store	Gold Power Saving Specialist	How much electricity can be saved in a month	Taobao Marketplace, a Chinese website for online shopping
BMI Digital Store	Gold Power Saving Specialist	pointless	Taobao Marketplace, a Chinese website for online shopping
BMI Digital Store	Gold Power Saving Specialist	Ita bit of8percent savings	Taobao Marketplace, a Chinese website for online shopping
BMI Digital Store	Gold Power Saving Specialist	Yes, I've bought many of them for my friends	Taobao Marketplace, a Chinese website for online shopping
BMI Digital Store	Gold Power Saving Specialist	I've bought a few and they all say they work	Taobao Marketplace, a Chinese website for online shopping
BMI Digital Store	Gold Power Saving Specialist	It also just acts as a voltage stabilizer and still saves a little bit of	Taobao Marketplace, a Chinese website for online shopping

BMI Digital Store	Gold Power Saving Specialist	incompetent	Taobao Marketplace, a Chinese website for online shopping
BMI Digital Store	Gold Power Saving Specialist	You can't tell if it's a short period of time.	Taobao Marketplace, a Chinese website for online shopping
BMI Digital Store	Gold Power Saving Specialist	In use My friend bought it last year for New Year's Eve and said it worked So I'm getting one too	Taobao Marketplace, a Chinese website for online shopping
BMI Digital Store	Gold Power Saving Specialist	It will work, it will indeed save electricity, just plug it into any outlet in the house.	Taobao Marketplace, a Chinese website for online shopping
BMI Digital Store	Gold Power Saving Specialist	Of course you can use it. It'll save you some money on your electricity bill.	Taobao Marketplace, a Chinese website for online shopping
BMI Digital Store	Gold Power Saving Specialist	The reactive compensation's are useful in their own right, and it should be OK to think about them in terms of electrical power, otherwise the reactive compensation capacitors in the main distribution room aren't for nothing.	Taobao Marketplace, a Chinese website for online shopping
BMI Digital Store	Gold Power Saving Specialist	Go put up solar panels.	Taobao Marketplace, a Chinese website for online shopping
BMI Digital Store	Gold Power Saving Specialist	It's always tripping, it doesn't work. It's not costing me anything.	Taobao Marketplace, a Chinese website for online shopping
BMI Digital Store	Gold Power Saving Specialist	Now I'm watching it too. It doesn't save too much, justabout2degreesa day. I just used it2weeks I think! I've just been using it for 2 weeks!	Taobao Marketplace, a Chinese website for online shopping
BMI Digital Store	Gold Power Saving Specialist	I'm a restaurant, and my monthly electric bill isover600, and it used to beover700	Taobao Marketplace, a Chinese website for online shopping
BMI Digital Store	Gold Power Saving Specialist	It can be. That's a long process.	Taobao Marketplace, a Chinese website for online shopping
BMI Digital Store	Gold Power Saving Specialist	pointless	Taobao Marketplace, a Chinese website for online shopping
BMI Digital Store	Gold Power Saving Specialist	Plugged into a place that uses a lot of electricity, and it's been working fine for a couple of months.	Taobao Marketplace, a Chinese website for online shopping
BMI Digital Store	Gold Power Saving Specialist	I've been using it for several months now and there are significant power savings.	Taobao Marketplace, a Chinese website for online shopping
BMI Digital Store	Gold Power Saving Specialist	I don't know. It's a long shot.	Taobao Marketplace, a Chinese website for online shopping
BMI Digital Store	Gold Power Saving Specialist	Yes. Always in use.	Taobao Marketplace, a Chinese website for online shopping
BMI Digital Store	Gold Power Saving Specialist	Just plug it into any outlet in the house, but it doesn't seem to be working too well to me	Taobao Marketplace, a Chinese website for online shopping
BMI Digital Store	Gold Power Saving Specialist	It works. It's a little rusty on the inside.	Taobao Marketplace, a Chinese website for online shopping
BMI Digital Store	Gold Power Saving Specialist	I don't know yet.	Taobao Marketplace, a Chinese website for online shopping
BMI Digital Store	Gold Power Saving Specialist	It doesn't help at all. It's a lie.	Taobao Marketplace, a Chinese website for online shopping
BMI Digital Store	Gold Power Saving Specialist	You can only compare it to the previous month, there's no instrument to measure it accurately.	Taobao Marketplace, a Chinese website for online shopping
BMI Digital Store	Gold Power Saving Specialist	Any wiring that belongs to the house can be connected.	Taobao Marketplace, a Chinese website for online shopping
BMI Digital Store	Gold Power Saving Specialist	It's been three months. The electricity bill has gone up instead of down	Taobao Marketplace, a Chinese website for online shopping
BMI Digital Store	Gold Power Saving Specialist	hasn't	Taobao Marketplace, a Chinese website for online shopping
BMI Digital Store	Gold Power Saving Specialist	there are	Taobao Marketplace, a Chinese website for online shopping
BMI Digital Store	Gold Power Saving Specialist	general	Taobao Marketplace, a Chinese website for online shopping
BMI Digital Store	Gold Power Saving Specialist	It didn't work, instead it felt a little bit more. Don't buy it doesn't work	Taobao Marketplace, a Chinese website for online shopping
BMI Digital Store	Gold Power Saving Specialist	I can save my hotel two refrigerators electric fans air conditioning exhaust fans are on a month is only 300 yuan of electricity, right	Taobao Marketplace, a Chinese website for online shopping
BMI Digital Store	Gold Power Saving Specialist	Reversal is not available.	Taobao Marketplace, a Chinese website for online shopping
BMI Digital Store	Gold Power Saving Specialist	It's a little useful.	Taobao Marketplace, a Chinese website for online shopping
BMI Digital Store	Gold Power Saving Specialist	If you save 10 bucks a month, you'll be back on your feet in 7 or 8 months.	Taobao Marketplace, a Chinese website for online shopping
BMI Digital Store	Gold Power Saving Specialist	Let's try it.	Taobao Marketplace, a Chinese website for online shopping
BMI Digital Store	Gold Power Saving Specialist	But I don't know if it saves electricity.	Taobao Marketplace, a Chinese website for online shopping
BMI Digital Store	Gold Power Saving Specialist	It's useless.	Taobao Marketplace, a Chinese website for online shopping

BMI Digital Store	Blue Power Saving Specialist	favorable criticism	Taobao Marketplace, a Chinese website for online shopping
BMI Digital Store	Blue Power Saving Specialist	favorable criticism	Taobao Marketplace, a Chinese website for online shopping
BMI Digital Store	Blue Power Saving Specialist	This Power Saver is just as described by the seller and the voltage is stable. I'll comment again when I try it for two weeks, hopefully with good results!	Taobao Marketplace, a Chinese website for online shopping
BMI Digital Store	Blue Power Saving Specialist	This is the second time I bought it, it's really good, and the merchant is very responsible too	Taobao Marketplace, a Chinese website for online shopping
BMI Digital Store	Blue Power Saving Specialist	Does this save electricity?10degrees. How much does it save	Taobao Marketplace, a Chinese website for online shopping
BMI Digital Store	Blue Power Saving Specialist	What does this save power?	Taobao Marketplace, a Chinese website for online shopping
BMI Digital Store	Blue Power Saving Specialist	In using friends, does it really save power?	Taobao Marketplace, a Chinese website for online shopping
BMI Digital Store	Blue Power Saving Specialist	Does it really save electricity? Is it cost-effective?	Taobao Marketplace, a Chinese website for online shopping
BMI Digital Store	Blue Power Saving Specialist	Does this save power or does it save power instead?	Taobao Marketplace, a Chinese website for online shopping
BMI Digital Store	Blue Power Saving Specialist	Can you save electricity andhow much can you save100kWh? Is it a good deal?	Taobao Marketplace, a Chinese website for online shopping
BMI Digital Store	Blue Power Saving Specialist	For those of you who have bought it, does it really save power?	Taobao Marketplace, a Chinese website for online shopping
BMI Digital Store	Blue Power Saving Specialist	Does this really save power?	Taobao Marketplace, a Chinese website for online shopping
BMI Digital Store	Blue Power Saving Specialist	Can you please tell me, does this really save power?	Taobao Marketplace, a Chinese website for online shopping
BMI Digital Store	Blue Power Saving Specialist	What are the benefits of energy savers and how much electricity do they save	Taobao Marketplace, a Chinese website for online shopping
BMI Digital Store	Blue Power Saving Specialist	Can I ask the folks who have bought it if it really saves power?	Taobao Marketplace, a Chinese website for online shopping
BMI Digital Store	Blue Power Saving Specialist	Dear all Does this device really save power? Thank you all!	Taobao Marketplace, a Chinese website for online shopping
BMI Digital Store	Blue Power Saving Specialist	False. No power saving.	Taobao Marketplace, a Chinese website for online shopping
BMI Digital Store	Blue Power Saving Specialist	Ask if it really saves electricity	Taobao Marketplace, a Chinese website for online shopping
BMI Digital Store	Blue Power Saving Specialist	It should work.	Taobao Marketplace, a Chinese website for online shopping
BMI Digital Store	Blue Power Saving Specialist	I don't know what I'm doing to save electricity, but I'm paying less than I used to.	Taobao Marketplace, a Chinese website for online shopping
BMI Digital Store	Blue Power Saving Specialist	For example, air conditioners, refrigerators, washing machines, vacuum cleaners, hoods, fans, lamps with capacitors,, computers, televisions, and all electrical appliances with coils and motors, all work well, as long as the effect of sensible appliances are very good.	Taobao Marketplace, a Chinese website for online shopping
BMI Digital Store	Blue Power Saving Specialist	The principle of reactive power compensation, normal will certainly have the effect. Foreign countries have long been using such technology, because of the limitations of the domestic conditions, early are factories such as energy consumption is relatively large<br><br>enterprises will be in the distribution room to install such equipment (of course, the equipment to be much larger) the cost of ten thousand as a unit. With the improvement of technical level, power saving products gradually to ordinary families,<br><br>cost also fell to the acceptable range of the people, can be said to benefit the country and the people's good products;	Taobao Marketplace, a Chinese website for online shopping
BMI Digital Store	Blue Power Saving Specialist	The electric bill is less than it used to be, but I'm not sure if that has anything to do with it	Taobao Marketplace, a Chinese website for online shopping
BMI Digital Store	Blue Power Saving Specialist	It saves electricity.How much is100degrees? I don't know.	Taobao Marketplace, a Chinese website for online shopping
BMI Digital Store	Blue Power Saving Specialist	There are general power saving	Taobao Marketplace, a Chinese website for online shopping
BMI Digital Store	Blue Power Saving Specialist	I'm trying it out too.	Taobao Marketplace, a Chinese website for online shopping
BMI Digital Store	Blue Power Saving Specialist	OK	Taobao Marketplace, a Chinese website for online shopping
BMI Digital Store	Blue Power Saving Specialist	Just used it for a day, can't see it yet use it for a few days and then see	Taobao Marketplace, a Chinese website for online shopping
BMI Digital Store	Blue Power Saving Specialist	uncharted	Taobao Marketplace, a Chinese website for online shopping
BMI Digital Store	Blue Power Saving Specialist	Can't save power, as high as before	Taobao Marketplace, a Chinese website for online shopping

BMI Digital Store	Blue Power Saving Specialist	I'm still trying it out, but I hope it's as good as the description.	Taobao Marketplace, a Chinese website for online shopping
BMI Digital Store	Blue Power Saving Specialist	We are committed to60days trial invalid return, 2years time quality problems free of charge for new Oh, shipping insurance, pro can rest assured that the purchase Oh!<br><br>The principle of reactive power compensation, normal will certainly have the effect. Foreign countries have long been using such technology, because of the limitations of the domestic conditions, early are factories such as energy consumption is relatively large<br>enterprises will be in the distribution room to install such equipment (of course, the equipment to be much larger) the cost of the unit is also in the tens of thousands of units. With the improvement of the level of technology, power-saving products gradually to ordinary families,<br>cost also fell to the acceptable range of the people, can be said to benefit the country and the people's good products;<br><br>economizer effect test: after the meter connected to an outlet, with high-power air conditioning (big power obviously) plugged in to open up to10hours, record the number of degrees of electricity is how much.<br>Then plug the power saver up.same conditions under the air conditioning open10hours, record how much electricity. A comparison will know how much to save, this is the most accurate way to actually look at the meter degrees	Taobao Marketplace, a Chinese website for online shopping
BMI Digital Store	Deep Blue Power Saving Specialist	rare	Taobao Marketplace, a Chinese website for online shopping
BMI Digital Store	Deep Blue Power Saving Specialist	Very satisfied.	Taobao Marketplace, a Chinese website for online shopping
BMI Digital Store	Deep Blue Power Saving Specialist	Things look beautiful, the courier just arrived today isAugust21, the1sttime to use, hope he can save electricity, do not let me down. First give merchants a favorable review. Wait for a month or two before making comments. If it works well. Then definitely give the business a big positive feedback.I will comment2ndtime	Taobao Marketplace, a Chinese website for online shopping
BMI Digital Store	Deep Blue Power Saving Specialist	💌been wanting it for a long time, I decided to go for it, I feel great after using it, it's genuine and the delivery is great, ititsname👍	Taobao Marketplace, a Chinese website for online shopping
BMI Digital Store	Deep Blue Power Saving Specialist	It doesn't work.	Taobao Marketplace, a Chinese website for online shopping
BMI Digital Store	Deep Blue Power Saving Specialist	Baby looks high quality, my previous purchase didn't look as good as this one, hopefully it will help me save more on my electric bill.	Taobao Marketplace, a Chinese website for online shopping
BMI Digital Store	Deep Blue Power Saving Specialist	Good quality, use it for a few days and try it out	Taobao Marketplace, a Chinese website for online shopping
BMI Digital Store	Deep Blue Power Saving Specialist	Received the goods and immediately installed and debugged, expect the effect just😉If it's good will have to add additional comments	Taobao Marketplace, a Chinese website for online shopping
BMI Digital Store	Deep Blue Power Saving Specialist	few years ago I bought a, after moving do not know where to put, this time with this try to see, more than one gear, specially compared to the electricity bill degrees, the effect is not bad, a day to save10or so degrees	Taobao Marketplace, a Chinese website for online shopping
BMI Digital Store	Deep Blue Power Saving Specialist	Understand the later half a year, see the market mixed, this opened12years should be more reliable it, first use, the product3gears, looks quite advanced, follow-up as a matter of fact to assess the effect of	Taobao Marketplace, a Chinese website for online shopping
BMI Digital Store	Deep Blue Power Saving Specialist	Comparison of several before taking the picture, very satisfied, recommended!	Taobao Marketplace, a Chinese website for online shopping
BMI Digital Store	Deep Blue Power Saving Specialist	Detailed understanding, feel good, valuing the reputation of this family, received and tried a few days, it is indeed effective, so far more satisfactory	Taobao Marketplace, a Chinese website for online shopping
BMI Digital Store	Deep Blue Power Saving Specialist	Consulted a lot of home, and finally chose this one, valued also the store's reputation, can operate for12years, surely the product is good, with the next, compared to the previous daily degrees, the effect is very obvious, it is very unexpected	Taobao Marketplace, a Chinese website for online shopping
BMI Digital Store	Deep Blue Power Saving Specialist	ve been using it for a month now, and it really does save electricity,about $50less than our last month's electric bill, recommend	Taobao Marketplace, a Chinese website for online shopping
BMI Digital Store	Deep Blue Power Saving Specialist	Previously bought a, the effect is quite good, the summer came, air conditioning electricity costs a lot, deliberately bought this high-power,3gear adjustment, it does look high, just use seems to have an effect, because I can see the degree every day on the phone, save10degrees a day, the follow-up follow-up to see how much a month can save	Taobao Marketplace, a Chinese website for online shopping
BMI Digital Store	Deep Blue Power Saving Specialist	The second time I purchased, is to save electricity is very good, the quality of the product is also good, every time to buy with them, and discount logistics and fast.	Taobao Marketplace, a Chinese website for online shopping
BMI Digital Store	Deep Blue Power Saving Specialist	5years ago in his family bought a99yuan, used for many years the effect is really can, this time the home redecoration, electrical appliances also add a lot of summer is coming, air conditioning is very power-consuming, specially bought this flagship model, much larger than the previous one, and much heavier, the comparison, the effect is very satisfactory	Taobao Marketplace, a Chinese website for online shopping
BMI Digital Store	Deep Blue Power Saving Specialist	I've been using it for a month. The effect is great, all recommended to friends. Now I'd like to recommend it to you too!	Taobao Marketplace, a Chinese website for online shopping
BMI Digital Store	Deep Blue Power Saving Specialist	Customer service is very good, satisfied	Taobao Marketplace, a Chinese website for online shopping
BMI Digital Store	Deep Blue Power Saving Specialist	Adjustable gear, suitable for opening the restaurant I, off-peak season electricity costs are not the same, so we recommend to everyone to open the store can buy369this Oh, power saving effect is also very obvious	Taobao Marketplace, a Chinese website for online shopping
BMI Digital Store	Deep Blue Power Saving Specialist	Quality is very good, exquisite workmanship, is genuine, logistics is very fast, business service attitude is good.	Taobao Marketplace, a Chinese website for online shopping
BMI Digital Store	Deep Blue Power Saving Specialist	The quality is very good, good comments!	Taobao Marketplace, a Chinese website for online shopping
BMI Digital Store	Deep Blue Power Saving Specialist	Previously used a cheap model The effect is quite satisfactory This time the class new home air conditioning more with the boss to buy a powerful is indeed much larger than the previous3-speed adjustment looks good quality open the air conditioning to try power saving is very obvious Usually a night to15 [illegible] $11 satisfactory	Taobao Marketplace, a Chinese website for online shopping
BMI Digital Store	Deep Blue Power Saving Specialist	an excellent	Taobao Marketplace, a Chinese website for online shopping
BMI Digital Store	Deep Blue Power Saving Specialist	Saving power very good to use very good.	Taobao Marketplace, a Chinese website for online shopping
BMI Digital Store	Deep Blue Power Saving Specialist	The quality is very good, the first time I received the goods to open the first time to see, it is very good, very satisfied	Taobao Marketplace, a Chinese website for online shopping

BMI Digital Store	Deep Blue Power Saving Specialist	installing a billing meter in my house and observing it for a few weeks, it is true that is a power saver, which is much better than the one I bought before. I recommend it to you	Taobao Marketplace, a Chinese website for online shopping
BMI Digital Store	Deep Blue Power Saving Specialist	The item has been received, it's very nice, thick and substantial	Taobao Marketplace, a Chinese website for online shopping
BMI Digital Store	Deep Blue Power Saving Specialist	The store was right in recommending them, the quality is great, they are authentic and shipped from a large factory!~Very good shopping!	Taobao Marketplace, a Chinese website for online shopping
BMI Digital Store	Deep Blue Power Saving Specialist	Exquisite workmanship, manufacturer's heart, expect good use	Taobao Marketplace, a Chinese website for online shopping
BMI Digital Store	Deep Blue Power Saving Specialist	My housearound 51C electricityper dayand it doesn't hurt to use this	Taobao Marketplace, a Chinese website for online shopping
BMI Digital Store	Deep Blue Power Saving Specialist	Used a little bit, feel or have the effect, before also bought a few tens of dollars, are cheated, this I deliberately use the air conditioning test for a few days, so far probably save10or so points, follow-up evaluation	Taobao Marketplace, a Chinese website for online shopping
BMI Digital Store	Deep Blue Power Saving Specialist	Very good! If you need it, go ahead and place your order it's worth it!	Taobao Marketplace, a Chinese website for online shopping
BMI Digital Store	Deep Blue Power Saving Specialist	The saver works great, thanks to the seller, come back next time, special thanks to the seller!	Taobao Marketplace, a Chinese website for online shopping
BMI Digital Store	Deep Blue Power Saving Specialist	Good packaging, start to use, directly open enhancement to see, waiting for good news.	Taobao Marketplace, a Chinese website for online shopping
BMI Digital Store	Deep Blue Power Saving Specialist	What percentage can I save by using a lot of electricity in a commercial store? Approximately, please explain	Taobao Marketplace, a Chinese website for online shopping
BMI Digital Store	Deep Blue Power Saving Specialist	How many kilowatts can you save with one hundred degrees of electricity?	Taobao Marketplace, a Chinese website for online shopping
BMI Digital Store	Deep Blue Power Saving Specialist	What's the principle of this economizer?	Taobao Marketplace, a Chinese website for online shopping
BMI Digital Store	Deep Blue Power Saving Specialist	Is it complicated to install a power saver?	Taobao Marketplace, a Chinese website for online shopping
BMI Digital Store	Deep Blue Power Saving Specialist	Does it really save electricity? Store Do Not Disturb.	Taobao Marketplace, a Chinese website for online shopping
BMI Digital Store	Deep Blue Power Saving Specialist	Hello to all of you who have bought it as a purchase! Does this saver really save electricity? Thanks for the answer.	Taobao Marketplace, a Chinese website for online shopping
BMI Digital Store	Deep Blue Power Saving Specialist	May I ask if you used it and did it work? Does it really save power?	Taobao Marketplace, a Chinese website for online shopping
BMI Digital Store	Deep Blue Power Saving Specialist	Does it work?	Taobao Marketplace, a Chinese website for online shopping
BMI Digital Store	Deep Blue Power Saving Specialist	Is this made using quantum and graphene technology?	Taobao Marketplace, a Chinese website for online shopping
BMI Digital Store	Deep Blue Power Saving Specialist	Do heaters and water heaters work	Taobao Marketplace, a Chinese website for online shopping
BMI Digital Store	Deep Blue Power Saving Specialist	20kW electric heater with underfloor heatingon three phase powerwork?	Taobao Marketplace, a Chinese website for online shopping
BMI Digital Store	Deep Blue Power Saving Specialist	Can the meter be reversed after you buy a couple of them, is it possible to reverse the power to the power station?	Taobao Marketplace, a Chinese website for online shopping
BMI Digital Store	Deep Blue Power Saving Specialist	Does it really save electricity? How many kilowatts can be saved?	Taobao Marketplace, a Chinese website for online shopping
BMI Digital Store	Deep Blue Power Saving Specialist	Does saving electricity work?	Taobao Marketplace, a Chinese website for online shopping
BMI Digital Store	Deep Blue Power Saving Specialist	Question, does it really save electricity for those who have bought it? Is it true or not? Thank you!	Taobao Marketplace, a Chinese website for online shopping
BMI Digital Store	Deep Blue Power Saving Specialist	Baidu inputs a power saver, guess what happens	Taobao Marketplace, a Chinese website for online shopping
BMI Digital Store	Deep Blue Power Saving Specialist	Ask, please answer honestly, whether it saves electricity or not, thank you!	Taobao Marketplace, a Chinese website for online shopping
BMI Digital Store	Deep Blue Power Saving Specialist	Does it really save electricity for the folks who buy it?	Taobao Marketplace, a Chinese website for online shopping
BMI Digital Store	Deep Blue Power Saving Specialist	How do I install it?	Taobao Marketplace, a Chinese website for online shopping
BMI Digital Store	Deep Blue Power Saving Specialist	For those of you who have bought it, are there any power savings?	Taobao Marketplace, a Chinese website for online shopping
BMI Digital Store	Deep Blue Power Saving Specialist	Is this the kind built with quantum nanotechnology?	Taobao Marketplace, a Chinese website for online shopping
BMI Digital Store	Deep Blue Power Saving Specialist	Does it save electricity? How are brothers and sisters using it?	Taobao Marketplace, a Chinese website for online shopping
BMI Digital Store	Deep Blue Power Saving Specialist	Is it an IQ tax?	Taobao Marketplace, a Chinese website for online shopping
BMI Digital Store	Deep Blue Power Saving Specialist	My previous electric bill600to1100a month, after I used it it was500to800, not as much as thirty-five percent, fifteen to twenty percent is about right! Anyway, it's cost effective!	Taobao Marketplace, a Chinese website for online shopping
BMI Digital Store	Deep Blue Power Saving Specialist	Wono Buchi is here.	Taobao Marketplace, a Chinese website for online shopping

BMI Digital Store	Deep Blue Power Saving Specialist	Normal use of electricity actual effective electricity only 60% 40% of the ineffective equal to waste, the use of power-saving specialists<br><br>can be fully4% of the loss of electricity centralized reflux inside the economizer and will be the current loss in the internal formation of a complete reversal of through the firewire in the use of<br><br>and thus achieve the effect of power saving Oh,it is recommended that the pro-use of a two billing cycle and then compare the next, there are small surprises that you can not expect Oh;	Taobao Marketplace, a Chinese website for online shopping
BMI Digital Store	Deep Blue Power Saving Specialist	No installation required, just plug and play	Taobao Marketplace, a Chinese website for online shopping
BMI Digital Store	Deep Blue Power Saving Specialist	there are	Taobao Marketplace, a Chinese website for online shopping
BMI Digital Store	Deep Blue Power Saving Specialist	In the process of experiencing	Taobao Marketplace, a Chinese website for online shopping
BMI Digital Store	Deep Blue Power Saving Specialist	I haven't paid the electric bill for the new house.	Taobao Marketplace, a Chinese website for online shopping
BMI Digital Store	Deep Blue Power Saving Specialist	I haven't paid the electric bill for the new house.	Taobao Marketplace, a Chinese website for online shopping
BMI Digital Store	Deep Blue Power Saving Specialist	Well, it's synthesized using outer space technology.	Taobao Marketplace, a Chinese website for online shopping
BMI Digital Store	Deep Blue Power Saving Specialist	I was put in the store, only refrigerator air conditioning, save a hundred degrees a month	Taobao Marketplace, a Chinese website for online shopping
BMI Digital Store	Deep Blue Power Saving Specialist	I'll have to ask the store. Mine's regular electricity.	Taobao Marketplace, a Chinese website for online shopping
BMI Digital Store	Deep Blue Power Saving Specialist	I bought it and installed it, and it's been a long time now, I guess! It really doesn't do much.	Taobao Marketplace, a Chinese website for online shopping
BMI Digital Store	Deep Blue Power Saving Specialist	For example, air conditioners, refrigerators, washing machines, vacuum cleaners, hoods, fans, lamps with capacitors, computers, televisions, and all electrical appliances with coils and motors, are all effective, as long as the effect of perceptual appliances are very good.<br><br>Due to the use of electricity, electrical appliances of different types, specifications and the degree of newness, urban and rural power supply grid conditions are different reasons, will produce different energy-saving effect, the normal energy-saving appliances to choose the right words, the general power savings ofabout10-35%, each family is not quite the same, such as electrical appliances, the age of the family wiring, there will be a certain impact	Taobao Marketplace, a Chinese website for online shopping
BMI Digital Store	Deep Blue Power Saving Specialist	Returned	Taobao Marketplace, a Chinese website for online shopping
BMI Digital Store	Deep Blue Power Saving Specialist	It was only used for ten days last month, and we won't know this month's electric bill until next month.	Taobao Marketplace, a Chinese website for online shopping
BMI Digital Store	Deep Blue Power Saving Specialist	Power saving and voltage stabilizing effect for refrigerator, air conditioner and hood	Taobao Marketplace, a Chinese website for online shopping
BMI Digital Store	Deep Blue Power Saving Specialist	I haven't used it yet.	Taobao Marketplace, a Chinese website for online shopping
BMI Digital Store	Deep Blue Power Saving Specialist	For example, air conditioners, refrigerators, washing machines, vacuum cleaners, hoods, fans, lamps with capacitors,, computers, televisions, and all electrical appliances with coils and motors, all work well, as long as the effect of sensible appliances are very good.	Taobao Marketplace, a Chinese website for online shopping
BMI Digital Store	Deep Blue Power Saving Specialist	This kind of is plug and play Just plug in the home near the main switch on the idle socket can be. It is the whole house can play the effect of power saving oh!_x005f_x005f_x000b_ inserted in the vacant socket or power plug board can be, in general, inserted in the line of the larger power consumption or inserted in the power of electrical equipment near the socket. If the family has more electrical appliances or more branches, you can consider plugging the product in the whole line master switch closer	Taobao Marketplace, a Chinese website for online shopping
BMI Digital Store	Deep Blue Power Saving Specialist	Power Saver used to pay$180a month for electricitybut this month it's only$98	Taobao Marketplace, a Chinese website for online shopping
BMI Digital Store	Deep Blue Power Saving Specialist	It's not made of Earth material.	Taobao Marketplace, a Chinese website for online shopping
BMI Digital Store	Deep Blue Power Saving Specialist	I didn't do the math, but it should work.	Taobao Marketplace, a Chinese website for online shopping
BMI Digital Store	Deep Blue Power Saving Specialist	It's not working.	Taobao Marketplace, a Chinese website for online shopping
BMI Digital Store	Power saving voltage stabilizing compensator	not have	Taobao Marketplace, a Chinese website for online shopping
BMI Digital Store	Power saving voltage stabilizing compensator	Baby received, business attitude is very good. Waiting to use a period of time to evaluate.	Taobao Marketplace, a Chinese website for online shopping
BMI Digital Store	Power saving voltage stabilizing compensator	Let's use it.	Taobao Marketplace, a Chinese website for online shopping
BMI Digital Store	Power saving voltage stabilizing compensator	Received and satisfied.	Taobao Marketplace, a Chinese website for online shopping
BMI Digital Store	Power saving voltage stabilizing compensator	The company's electricity consumption ofmore than20,000 degreesa month, the electrician installed, the day of the comparison of a few hours, can indeed save some, about12%, so that a month counted quite a lot, very satisfied	Taobao Marketplace, a Chinese website for online shopping
BMI Digital Store	Power saving voltage stabilizing compensator	Fast shipping, good high power, long duration	Taobao Marketplace, a Chinese website for online shopping
BMI Digital Store	Power saving voltage stabilizing compensator	Hi, do power savers really save electricity?	Taobao Marketplace, a Chinese website for online shopping

BMI Digital Store	Power saving voltage stabilizing compensator	Does work?	Taobao Marketplace, a Chinese website for online shopping
BMI Digital Store	Power saving voltage stabilizing compensator	Has anyone used it for a couple months? Did it work?	Taobao Marketplace, a Chinese website for online shopping
BMI Digital Store	Power saving voltage stabilizing compensator	Who used it? Did it work?	Taobao Marketplace, a Chinese website for online shopping
BMI Digital Store	Power saving voltage stabilizing compensator	bogus	Taobao Marketplace, a Chinese website for online shopping
BMI Digital Store	Power saving voltage stabilizing compensator	How much of an effect has been achieved?	Taobao Marketplace, a Chinese website for online shopping
BMI Digital Store	Power saving voltage stabilizing compensator	How does this work?	Taobao Marketplace, a Chinese website for online shopping
BMI Digital Store	Power saving voltage stabilizing compensator	my house work with30+ kW of 3-phase, 4-wire power?	Taobao Marketplace, a Chinese website for online shopping
BMI Digital Store	Power saving voltage stabilizing compensator	What's the effect?	Taobao Marketplace, a Chinese website for online shopping
BMI Digital Store	Power saving voltage stabilizing compensator	Honey, is that true? My house uses 40,000 to 50,000 kilowatts a month, and he says it saves40% ? That's incredible.	Taobao Marketplace, a Chinese website for online shopping
BMI Digital Store	Power saving voltage stabilizing compensator	I have two-phase electricity and I use a lot of electricity every month, is this good?	Taobao Marketplace, a Chinese website for online shopping
BMI Digital Store	Power saving voltage stabilizing compensator	effectiveuse SaveElectronics ?How much can you save in a month Please answer	Taobao Marketplace, a Chinese website for online shopping
BMI Digital Store	Power saving voltage stabilizing compensator	Dear all, did the saver you bought work?	Taobao Marketplace, a Chinese website for online shopping
BMI Digital Store	Power saving voltage stabilizing compensator	Does it work. Does it really work?	Taobao Marketplace, a Chinese website for online shopping
BMI Digital Store	Power saving voltage stabilizing compensator	Ask if it works and if it really saves electricity.	Taobao Marketplace, a Chinese website for online shopping
BMI Digital Store	Power saving voltage stabilizing compensator	Just used a month the electricity bill is not less than last month is more than 3,000 wait in the use of two months to see June12to July12the electricity bill is14,000 the electricity bill July began to use12to August12more than17,000	Taobao Marketplace, a Chinese website for online shopping
BMI Digital Store	Power saving voltage stabilizing compensator	It's not working.	Taobao Marketplace, a Chinese website for online shopping
BMI Digital Store	Power saving voltage stabilizing compensator	Sixmonths	Taobao Marketplace, a Chinese website for online shopping
BMI Digital Store	Power saving voltage stabilizing compensator	I haven't yet, but the ones at home have worked	Taobao Marketplace, a Chinese website for online shopping
BMI Digital Store	Power saving voltage stabilizing compensator	truly	Taobao Marketplace, a Chinese website for online shopping
BMI Digital Store	Power saving voltage stabilizing compensator	I feel like it's the same with or without it.	Taobao Marketplace, a Chinese website for online shopping
BMI Digital Store	Power saving voltage stabilizing compensator	Normal use of electricity actual effective electricity only 60% 40% of the ineffective equal to waste, the use of power-saving specialists<br><br>can be fully4% of the loss of electricity centralized reflux inside the economizer and will be the current loss in the internal formation of a complete reversal of through the firewire in the use of<br><br>and thus achieve the effect of power saving Oh,it is recommended that the pro-use of a two billing cycle and then compare the next, there are small surprises that you can not expect Oh;	Taobao Marketplace, a Chinese website for online shopping
BMI Digital Store	Power saving voltage stabilizing compensator	It's not working.	Taobao Marketplace, a Chinese website for online shopping
BMI Digital Store	Power saving voltage stabilizing compensator	Just used it for a short time.	Taobao Marketplace, a Chinese website for online shopping
BMI Digital Store	Power saving voltage stabilizing compensator	I think it should save power.	Taobao Marketplace, a Chinese website for online shopping
BMI Digital Store	Power saving voltage stabilizing compensator	Dear, This is the three-phase power dedicated, two-phase power use more electricity, can use the enhanced version. Just ask the customer service.	Taobao Marketplace, a Chinese website for online shopping
BMI Digital Store	Power saving voltage stabilizing compensator	Not ideal, not feeling it!	Taobao Marketplace, a Chinese website for online shopping
BMI Digital Store	Power saving voltage stabilizing compensator	I've only been using it for two months and I don't feel any power savings.	Taobao Marketplace, a Chinese website for online shopping
BMI Digital Store	Power saving voltage stabilizing compensator	It's not time to read the meter yet, don't know yet? Monthly savings.	Taobao Marketplace, a Chinese website for online shopping
BMI Digital Store	Power saving voltage stabilizing compensator	I bought this is already the second, is for foreign customers order, good to make, not good to make the customer will not buy two of them	Taobao Marketplace, a Chinese website for online shopping
IXCHSQ Flagship Store	a power saver (electronics)	Shipping was fast, appearance is OK, it is not clear how to use the effect.	Taobao Marketplace, a Chinese website for online shopping
IXCHSQ Flagship Store	a power saver (electronics)	I just started using it, I don't know what it looks like, it's not bad, I'll come back and comment on it.	Taobao Marketplace, a Chinese website for online shopping

IXCHSQ Flagship Store	a power saver (electronics)	The quality is very good, the power saving effect is also very good, very satisfied with a shopping.	Taobao Marketplace, a Chinese website for online shopping
IXCHSQ Flagship Store	a power saver (electronics)	It does save electricity. After using it for a while, I've observed it. Almost every day can save at least half of the electricity or so, yesterday a night on the air conditioning, the water heater and take a shower, a night just use two degrees of electricity. It really can save electricity	Taobao Marketplace, a Chinese website for online shopping
IXCHSQ Flagship Store	Intelligent power saver	I don't know how well it works.	Taobao Marketplace, a Chinese website for online shopping
IXCHSQ Flagship Store	Intelligent power saver	(of an unmarried couple) be close	Taobao Marketplace, a Chinese website for online shopping
IXCHSQ Flagship Store	Intelligent power saver	Received, LCD display, thoughtful, every month power consumption within500degrees of electricity. Has been plugged in and used, observed a few days does have the effect, the courier is also very powerful less than three days to the goods. Thank you for the great support of merchants and logistics, has been recommended to relatives and friends. Will also buy back.	Taobao Marketplace, a Chinese website for online shopping
IXCHSQ Flagship Store	Intelligent power saver	Power saving effect: power saving effect is good, already repurchased the second one.	Taobao Marketplace, a Chinese website for online shopping
IXCHSQ Flagship Store	Intelligent power saver	Saving appliances are really useful it, commercial electricity, electricity costs high daily electricity consumption is relatively large, the electricity bill can not eat, was just to try, I did not expect to use the monthly savings of a large amount of expenses it, happy!	Taobao Marketplace, a Chinese website for online shopping
IXCHSQ Flagship Store	Intelligent power saver	Used half a month does save electricity, after plugging in to save me a lot of electricity, really and this baby hate each other ah, power-saving effect is really great, the appearance of the atmosphere is stylish, the use of the experience is perfect, does not take up space, the next time you need will buy again. Customer service is very enthusiastic, great!	Taobao Marketplace, a Chinese website for online shopping
IXCHSQ Flagship Store	Intelligent power saver	Baby received, well packaged, goods without damage. Appearance of workmanship and materials is okay. In fact, I personally do not believe that this thing can save electricity, unless it is through physical or mechanical interference with the normal operation of the meter, otherwise it is impossible. So many buyers say can save electricity, may be"three people into a tiger", I also buy one to comfort themselves. Looking forward to the miracle! The seller shipped quickly, the price is affordable, the courier service	Taobao Marketplace, a Chinese website for online shopping
IXCHSQ Flagship Store	Intelligent power saver	The seller shipped very quickly, the voltmeter's also very good sturdy, no damage. In-kind and the picture is exactly the same, the next time there is a need will come again, I wish the seller a prosperous business.	Taobao Marketplace, a Chinese website for online shopping
IXCHSQ Flagship Store	Intelligent power saver	The effect is very good, the electricity bill has dropped a lot worth repurchasing recommended! One month lessmore than160degrees Wasmore than600degrees Boss service attitude is very good Recommended friends to buy	Taobao Marketplace, a Chinese website for online shopping
IXCHSQ Flagship Store	Intelligent power saver	It's on trial.	Taobao Marketplace, a Chinese website for online shopping
IXCHSQ Flagship Store	Intelligent power saver	Power saving effect: good	Taobao Marketplace, a Chinese website for online shopping
IXCHSQ Flagship Store	Intelligent power saver	The effect is also too awesome it, usuallymore than800electricity billsper month, with three of this, each month down only to paymore than300dollars, really welfare products ！！！！	Taobao Marketplace, a Chinese website for online shopping
IXCHSQ Flagship Store	Intelligent power saver	Have not yet used, first give positive feedback, after use in the follow-up review	Taobao Marketplace, a Chinese website for online shopping
IXCHSQ Flagship Store	Intelligent power saver	Products are really good I used two for the family, usually kitchen and bathroom water heaters with electricity is too expensive, because the water heater to open every day, every month electricity is too much, no longer afraid of the electricity bill is too much, a few months down,electricity bill is less than half of it, the effect of the special good, very much like.	Taobao Marketplace, a Chinese website for online shopping
IXCHSQ Flagship Store	Intelligent power saver	The effect is really great! As soon as I received I immediately plugged it in, and now it has been used for many days, because my house does not have a warm day, every day to open the air conditioning, really hurt the electricity bill, after installing this, obviously found that the meter goes slower, has been	Taobao Marketplace, a Chinese website for online shopping
IXCHSQ Flagship Store	Intelligent power saver	Is it an IQ tax?	Taobao Marketplace, a Chinese website for online shopping
IXCHSQ Flagship Store	King of Power Saving	Very practical! Compared it to several others! Loved loved loved! Will buy from this family again next time!	Taobao Marketplace, a Chinese website for online shopping
IXCHSQ Flagship Store	King of Power Saving	The effect is particularly good, you can really save electricity, the meter jumps slowly, you can save several degrees of electricity a day it is worth recommending!	Taobao Marketplace, a Chinese website for online shopping
IXCHSQ Flagship Store	King of Power Saving	Logistics is very fast, the goods are very good worth buying, five-star praise, beautiful appearance, with a short time is very good, will recommend to friends, the	Taobao Marketplace, a Chinese website for online shopping
IXCHSQ Flagship Store	King of Power Saving	This power-saving products with a year before leveling it, now can not save electricity do not know, it is best to save electricity, so that later customers believe that this product, wait a year before leveling this section of the goods?	Taobao Marketplace, a Chinese website for online shopping
IXCHSQ Flagship Store	King of Power Saving	The effect is really good, after receiving the goods on the rush to try, originally do not believe this, the business said you can return, the results I used after this thing is really magical, just plugged in a split second on the meter does not jump, I turn on the high-power appliances, the meter jumped very slowly, a day down to the use of the2,3degrees of electricity, recommended that relatives and friends to [illegible]	Taobao Marketplace, a Chinese website for online shopping
IXCHSQ Flagship Store	King of Power Saving	The product can really, a month down the power saving is also very obvious less than ten degrees!	Taobao Marketplace, a Chinese website for online shopping
IXCHSQ Flagship Store	King of Power Saving	IQ tax again, hit from installing the saver, checked the comparative records from previous years and hey, it's higher every month😹	Taobao Marketplace, a Chinese website for online shopping
IXCHSQ Flagship Store	King of Power Saving	Don't fall for it, it came from pure pure IQ tax, threw it all away, not a lot of money, but the bad reviews have to be given on it.	Taobao Marketplace, a Chinese website for online shopping
IXCHSQ Flagship Store	King of Power Saving	Household power saver good quality, exquisite workmanship, power saving effect is obvious, protection of electrical appliances, harmonic inhibition, voltage stabilization, energy saving and power saving, intelligent display, can be down15%to45%of the electricityper month, power saving and money saving green, optimize the current and voltage, improve the efficiency of electricity, reduce the loss of	Taobao Marketplace, a Chinese website for online shopping
IXCHSQ Flagship Store	King of Power Saving	Saving appliances are exquisitely crafted, with texture, voltage, and leakage display, leakage can be alarmed, affordable, according to the principle, should be able to node electricity, looking forward to the miracle, first full5points praise.	Taobao Marketplace, a Chinese website for online shopping
IXCHSQ Flagship Store	King of Power Saving	Is it useful, please? Is it an IQ tax?	Taobao Marketplace, a Chinese website for online shopping
IXCHSQ Flagship Store	King of Power Saving	Are there any solid stats, really?	Taobao Marketplace, a Chinese website for online shopping
IXCHSQ Flagship Store	King of Power Saving	Nothing has changed.	Taobao Marketplace, a Chinese website for online shopping
IXCHSQ Flagship Store	King of Power Saving	Anyway, I used to have two of them in my house, and now that I have a bigger house and add another one, it feels like it's going to cost less in electricity, and I used to have those two for close to ten years now	Taobao Marketplace, a Chinese website for online shopping

Pinsent Technologies	King of Power Saving	The appearance of the economizer looks not bad quality, bought back in the use of data is more stable, whether the power saving after a while and then chase the evaluation	Taobao Marketplace, a Chinese website for online shopping
Pinsent Technologies	King of Power Saving	Not bad, it's cheap to pay for a month.	Taobao Marketplace, a Chinese website for online shopping
Pinsent Technologies	King of Power Saving	Power-saving effect: good, easy to install, I use the second one, every use to save dozens of degrees of electricity. Previously did not use this product when two or three hundred degrees per month, now more than a hundred degrees.	Taobao Marketplace, a Chinese website for online shopping
Pinsent Technologies	King of Power Saving	Power-saving effect: very good Cost-effective: very high Appearance style: simple Workmanship material: very good Experience: just feel with quite good, or friends sent the link introduced to say good use	Taobao Marketplace, a Chinese website for online shopping
Pinsent Technologies	King of Power Saving	Power saving effect: not bad	Taobao Marketplace, a Chinese website for online shopping
Pinsent Technologies	King of Power Saving	Power saving effect: very good power saver, already the second time to buy, the effect of the power saver is really obvious, thanks to the store, looking forward to the next purchase.	Taobao Marketplace, a Chinese website for online shopping
Pinsent Technologies	King of Power Saving	Power saving effect: power saving effect is great[illegible]Ness Cost effective: good Appearance style: luxury Exquisite Workmanship material: good Experience: has been the third time back to buy Very save point January save point me a lot of money Customer service sister is also very good service what she has to answer, quite comfortable	Taobao Marketplace, a Chinese website for online shopping
Pinsent Technologies	King of Power Saving	Buyer does not comment within15days	Taobao Marketplace, a Chinese website for online shopping
Pinsent Technologies	King of Power Saving	I've been using it for two months now and it feels like it uses the same amount of electricity. No effect! Waste of resources	Taobao Marketplace, a Chinese website for online shopping
Pinsent Technologies	King of Power Saving	Buyer does not comment within15days	Taobao Marketplace, a Chinese website for online shopping
Pinsent Technologies	King of Power Saving	The power saving effect is quite good, affordable and quality assurance. Workmanship is okay	Taobao Marketplace, a Chinese website for online shopping
Pinsent Technologies	King of Power Saving	Does it save power?	Taobao Marketplace, a Chinese website for online shopping
Pinsent Technologies	King of Power Saving	Does it work, folks?	Taobao Marketplace, a Chinese website for online shopping
Pinsent Technologies	King of Power Saving	Did it work?	Taobao Marketplace, a Chinese website for online shopping
Pinsent Technologies	King of Power Saving	What's the principle of this? Is it legal? What's the quality?	Taobao Marketplace, a Chinese website for online shopping
Pinsent Technologies	King of Power Saving	Does it really work? Thank you for the honest answer.	Taobao Marketplace, a Chinese website for online shopping
Pinsent Technologies	King of Power Saving	Does anyone who has used this thing really save power?	Taobao Marketplace, a Chinese website for online shopping
Pinsent Technologies	King of Power Saving	Does it help to raise your IQ?	Taobao Marketplace, a Chinese website for online shopping
Pinsent Technologies	King of Power Saving	Does this really save power? Looking for a real answer thanks!	Taobao Marketplace, a Chinese website for online shopping
Pinsent Technologies	King of Power Saving	Is there any god to buy one and disassemble it to see the inner structure?	Taobao Marketplace, a Chinese website for online shopping
Pinsent Technologies	King of Power Saving	Did it work, folks?	Taobao Marketplace, a Chinese website for online shopping
Pinsent Technologies	King of Power Saving	Does it really save power? Have you guys used it?	Taobao Marketplace, a Chinese website for online shopping
Pinsent Technologies	King of Power Saving	Is it true that the elderly in the house agree to turn on the air conditioner if you use this?	Taobao Marketplace, a Chinese website for online shopping
Pinsent Technologies	King of Power Saving	Is this just a voltmeter?	Taobao Marketplace, a Chinese website for online shopping
Pinsent Technologies	King of Power Saving	Can I ask the guys who have used it if it really works?	Taobao Marketplace, a Chinese website for online shopping
Pinsent Technologies	King of Power Saving	What's the principle of this? Thank you!	Taobao Marketplace, a Chinese website for online shopping
Pinsent Technologies	King of Power Saving	How many have actually paid the IQ tax?	Taobao Marketplace, a Chinese website for online shopping
Pinsent Technologies	King of Power Saving	Honestly, does using this save power, and how much a month?	Taobao Marketplace, a Chinese website for online shopping
Pinsent Technologies	King of Power Saving	Has anyone taken it out and looked at it?	Taobao Marketplace, a Chinese website for online shopping
Pinsent Technologies	King of Power Saving	Can buying this improve IQ? Children at home preparing for college entrance exams and midterm exams can let them buy a few more, the exams will be stable	Taobao Marketplace, a Chinese website for online shopping
Pinsent Technologies	King of Power Saving	Does this work for the pro's who have bought it?	Taobao Marketplace, a Chinese website for online shopping
Pinsent Technologies	King of Power Saving	For those of you who have bought it, does this power saver really save power?	Taobao Marketplace, a Chinese website for online shopping
Pinsent Technologies	King of Power Saving	Ask you guys if it really works or if it's a fake?	Taobao Marketplace, a Chinese website for online shopping
Pinsent Technologies	King of Power Saving	Note to anyone who has bought one to see if it's a trifle?	Taobao Marketplace, a Chinese website for online shopping

Pinsent Technologies	King of Power Saving	Will it trip the circuit breaker?	Taobao Marketplace, a Chinese website for online shopping
Pinsent Technologies	King of Power Saving	Did it work?	Taobao Marketplace, a Chinese website for online shopping
Pinsent Technologies	King of Power Saving	For buyers who have used it for a few months, how many kilowatts a month did it use before it was used? How many kilowatts a month after using it?	Taobao Marketplace, a Chinese website for online shopping
Pinsent Technologies	King of Power Saving	Can you take out the power company's evil smart meters?	Taobao Marketplace, a Chinese website for online shopping
Pinsent Technologies	King of Power Saving	Where does the saved electricity go?	Taobao Marketplace, a Chinese website for online shopping
Pinsent Technologies	King of Power Saving	Does it work? Please answer if you've used it for a few months	Taobao Marketplace, a Chinese website for online shopping
Pinsent Technologies	King of Power Saving	Does it work?	Taobao Marketplace, a Chinese website for online shopping
Pinsent Technologies	King of Power Saving	What is the principle of power saving	Taobao Marketplace, a Chinese website for online shopping
Pinsent Technologies	King of Power Saving	May I ask the pals who have used this product, does it really save power? Looking for a real answer. Thank you!	Taobao Marketplace, a Chinese website for online shopping
Pinsent Technologies	King of Power Saving	What's the principle?	Taobao Marketplace, a Chinese website for online shopping
Pinsent Technologies	King of Power Saving	What exactly is the principle by which this saves electricity?	Taobao Marketplace, a Chinese website for online shopping
Pinsent Technologies	King of Power Saving	Is it scientific to save electricity? Shouldn't how much power uses how much electricity correspond?	Taobao Marketplace, a Chinese website for online shopping
Pinsent Technologies	King of Power Saving	Does it work?	Taobao Marketplace, a Chinese website for online shopping
Pinsent Technologies	King of Power Saving	This Power Saver, is it really true? Can it save electricity?<br>Bosses	Taobao Marketplace, a Chinese website for online shopping
Pinsent Technologies	King of Power Saving	Is it useful to use a magnetic card meter now?	Taobao Marketplace, a Chinese website for online shopping
Pinsent Technologies	King of Power Saving	What is the principle behind the power saving of this stuff	Taobao Marketplace, a Chinese website for online shopping
Pinsent Technologies	King of Power Saving	Question for those who have used it, does the product really save power? How to install	Taobao Marketplace, a Chinese website for online shopping
Pinsent Technologies	King of Power Saving	The principle of power saving is to take up one jack and connect fewer appliances to save power, right?	Taobao Marketplace, a Chinese website for online shopping
Pinsent Technologies	King of Power Saving	To be honest with you all, does it really save electricity?	Taobao Marketplace, a Chinese website for online shopping
Pinsent Technologies	King of Power Saving	Can I use my house with three-phase electricity?	Taobao Marketplace, a Chinese website for online shopping
Pinsent Technologies	King of Power Saving	Is it true that the more you put in, the more power you save? One in each outlet.	Taobao Marketplace, a Chinese website for online shopping
Pinsent Technologies	King of Power Saving	it worked for a long time? How's the power saving? For real results Thank you	Taobao Marketplace, a Chinese website for online shopping
Pinsent Technologies	King of Power Saving	Can you save20%? For a month of combined use. If you have used it, please reply.	Taobao Marketplace, a Chinese website for online shopping
Pinsent Technologies	King of Power Saving	Please ask, how does it work is it worth buying? Does it really save electricity?	Taobao Marketplace, a Chinese website for online shopping
Pinsent Technologies	King of Power Saving	Does this thing save power anymore	Taobao Marketplace, a Chinese website for online shopping
Pinsent Technologies	King of Power Saving	Pro, where do you plug the saver in to use it and does it have a power saving effect.	Taobao Marketplace, a Chinese website for online shopping
Pinsent Technologies	King of Power Saving	What principle saves electricity?	Taobao Marketplace, a Chinese website for online shopping
Pinsent Technologies	King of Power Saving	Normal can, it is the reactive power compensation circuit, large shopping malls and enterprises are equipped with reactive capacitor compensation cabinet in the distribution room, the purpose is to compensate for reactive power to reduce losses.	Taobao Marketplace, a Chinese website for online shopping
Pinsent Technologies	King of Power Saving	I don't know yet.	Taobao Marketplace, a Chinese website for online shopping
Pinsent Technologies	King of Power Saving	It's my first time to buy it too I'll know if it works after this month.	Taobao Marketplace, a Chinese website for online shopping
Pinsent Technologies	King of Power Saving	It's true that it works, I've used it and it's obvious that my electricity bill is much less than before, and I've also had the air conditioner on for long periods of time, and my electricity bill is lower than it was before I didn't use it. It's legal.	Taobao Marketplace, a Chinese website for online shopping
Pinsent Technologies	King of Power Saving	I've been using it for a month and I don't know if it's saving any electricity.	Taobao Marketplace, a Chinese website for online shopping
Pinsent Technologies	King of Power Saving	Still in testing.	Taobao Marketplace, a Chinese website for online shopping
Pinsent Technologies	King of Power Saving	I haven't gotten my shipment yet. It's been two months.	Taobao Marketplace, a Chinese website for online shopping

Pinsent Technologies	King of Power Saving	I just bought it not long ago, I haven't experienced it yet, I'll let you know when I use one to see what it's like	Taobao Marketplace, a Chinese website for online shopping
Pinsent Technologies	King of Power Saving	No Why don't you try buying one	Taobao Marketplace, a Chinese website for online shopping
Pinsent Technologies	King of Power Saving	It's a little useful.	Taobao Marketplace, a Chinese website for online shopping
Pinsent Technologies	King of Power Saving	Not bad.	Taobao Marketplace, a Chinese website for online shopping
Pinsent Technologies	King of Power Saving	Maybe.	Taobao Marketplace, a Chinese website for online shopping
Pinsent Technologies	King of Power Saving	a power saver (electronics)	Taobao Marketplace, a Chinese website for online shopping
Pinsent Technologies	King of Power Saving	It seems like the electric bill is 20 or 30 dollars less these past two months.	Taobao Marketplace, a Chinese website for online shopping
Pinsent Technologies	King of Power Saving	let it be	Taobao Marketplace, a Chinese website for online shopping
Pinsent Technologies	King of Power Saving	Is really works, I used it and obviously my electricity bill is much less than before, and also run the air conditioning for a long time, the electricity bill is lower than before I didn't use it.	Taobao Marketplace, a Chinese website for online shopping
Pinsent Technologies	King of Power Saving	It's not working.	Taobao Marketplace, a Chinese website for online shopping
Pinsent Technologies	King of Power Saving	Yes, it's supposedly an IQ tax	Taobao Marketplace, a Chinese website for online shopping
Pinsent Technologies	King of Power Saving	Yes, buy it and put it on a hat for the kids to wear. That way they'll pass every test they take.	Taobao Marketplace, a Chinese website for online shopping
Pinsent Technologies	King of Power Saving	It doesn't seem to be working.	Taobao Marketplace, a Chinese website for online shopping
Pinsent Technologies	King of Power Saving	It doesn't help. The meter's jumping all over the place.	Taobao Marketplace, a Chinese website for online shopping
Pinsent Technologies	King of Power Saving	It doesn't seem to be working!	Taobao Marketplace, a Chinese website for online shopping
Pinsent Technologies	King of Power Saving	Can save, can't tell in a short time, takes a long time	Taobao Marketplace, a Chinese website for online shopping
Pinsent Technologies	King of Power Saving	will not (act, happen etc)	Taobao Marketplace, a Chinese website for online shopping
Pinsent Technologies	King of Power Saving	I've used it, and it feels like it's quite a power saver. Basically, it savesabout38%	Taobao Marketplace, a Chinese website for online shopping
Pinsent Technologies	King of Power Saving	It didn't work.	Taobao Marketplace, a Chinese website for online shopping
Pinsent Technologies	King of Power Saving	Don't be fooled. I threw mine away.	Taobao Marketplace, a Chinese website for online shopping
Pinsent Technologies	King of Power Saving	I don't know. It's true that the electricity bill is lower.	Taobao Marketplace, a Chinese website for online shopping
Pinsent Technologies	King of Power Saving	It works. I've been using it for years.	Taobao Marketplace, a Chinese website for online shopping
Pinsent Technologies	King of Power Saving	I don't know. I think so. He's a battery that recharges.	Taobao Marketplace, a Chinese website for online shopping
Pinsent Technologies	King of Power Saving	I don't think it's gonna help.	Taobao Marketplace, a Chinese website for online shopping
Pinsent Technologies	King of Power Saving	hasn't	Taobao Marketplace, a Chinese website for online shopping
Pinsent Technologies	King of Power Saving	Just used it for a few days. I don't know.	Taobao Marketplace, a Chinese website for online shopping
Pinsent Technologies	King of Power Saving	I don't think it's gonna help.	Taobao Marketplace, a Chinese website for online shopping
Pinsent Technologies	King of Power Saving	incompetent	Taobao Marketplace, a Chinese website for online shopping
Pinsent Technologies	King of Power Saving	short time. I haven't read the meter either. But the voltage has stabilized	Taobao Marketplace, a Chinese website for online shopping
Pinsent Technologies	King of Power Saving	I don't know.	Taobao Marketplace, a Chinese website for online shopping
Pinsent Technologies	King of Power Saving	I haven't used it for a long time and I don't know if it's working or not	Taobao Marketplace, a Chinese website for online shopping
Pinsent Technologies	King of Power Saving	IQ tax	Taobao Marketplace, a Chinese website for online shopping
Pinsent Technologies	King of Power Saving	I don't know yet, we'll talk about it later.	Taobao Marketplace, a Chinese website for online shopping
Pinsent Technologies	King of Power Saving	right	Taobao Marketplace, a Chinese website for online shopping

Pinsent Technologies	King of Power Saving	It's not working.	Taobao Marketplace, a Chinese website for online shopping
Pinsent Technologies	King of Power Saving	High voltage is fine.	Taobao Marketplace, a Chinese website for online shopping
Pinsent Technologies	King of Power Saving	Look at you, just buy it and see for yourself.	Taobao Marketplace, a Chinese website for online shopping
Pinsent Technologies	King of Power Saving	I don't seem to have much use for it, but I think it uses more electricity.	Taobao Marketplace, a Chinese website for online shopping
Pinsent Technologies	King of Power Saving	You're overthinking it. Just back off.	Taobao Marketplace, a Chinese website for online shopping
Pinsent Technologies	King of Power Saving	I use a lot of electricity here I used to pay over $400 for electricity without buying one Now it's over $240 Do you think you can save electricity Buy one like mine No need to buy another one	Taobao Marketplace, a Chinese website for online shopping
Pinsent Technologies	King of Power Saving	can	Taobao Marketplace, a Chinese website for online shopping
Pinsent Technologies	King of Power Saving	Any place that has a plug can be plugged in, but I don't know if it saves power, I didn't buy it personally	Taobao Marketplace, a Chinese website for online shopping
Pinsent Technologies	King of Power Saving	Maybe it's high tech, I'm not sure, but I bought it to try it out.	Taobao Marketplace, a Chinese website for online shopping
Intelligent power saver	Intelligent power saver	What the hell, it's been plugged in for a month, or two, and it hasn't saved me a dime on my electric bill, shit.	Taobao Marketplace, a Chinese website for online shopping
Intelligent power saver	Intelligent power saver	Experience: very, very good, very useful	Taobao Marketplace, a Chinese website for online shopping
Intelligent power saver	Intelligent power saver	System default comments	Taobao Marketplace, a Chinese website for online shopping
Intelligent power saver	Intelligent power saver	I don't see any slowing down, I feel like I'm getting faster, I'm dizzy.	Taobao Marketplace, a Chinese website for online shopping
Intelligent power saver	Intelligent power saver	Fast shipping, good business attitude, good product quality	Taobao Marketplace, a Chinese website for online shopping
Intelligent power saver	Intelligent power saver	Intelligent power saver received, do not yet know whether the power saving, first use it. Intelligent power saver received, do not know whether the power saving, first use it. Intelligent power saver received, do not know whether the power saving, first used. Intelligent energy-saving appliances received, do not know whether the power saving, first used.	Taobao Marketplace, a Chinese website for online shopping
Intelligent power saver	Intelligent power saver	Power saving effect: to be evaluated after use Price-performance ratio: to see the effect, to be used Appearance style: simple	Taobao Marketplace, a Chinese website for online shopping
Intelligent power saver	Intelligent power saver	It doesn't work. It doesn't work at all.	Taobao Marketplace, a Chinese website for online shopping
Intelligent power saver	Intelligent power saver	Sure enough, it's the IQ tax again, specially measured The electricity bill has not been reduced at all I also specially bought 2 Let my wife scolded Alas It's not easy to earn money Said that the Chinese people do not cheat the Chinese people it~	Taobao Marketplace, a Chinese website for online shopping
Intelligent power saver	Intelligent power saver	It doesn't help. Paying the IQ tax again	Taobao Marketplace, a Chinese website for online shopping
Intelligent power saver	Intelligent power saver	IQ tax. You guys rave about it to get your ten bucks back, right? I'll sacrifice.	Taobao Marketplace, a Chinese website for online shopping
Intelligent power saver	Intelligent power saver	Friends recommended that I buy this power saver, received the appearance of no damage immediately inserted into the power supply to work normally, the appearance of small and convenient, plugged in ignore, and does not interfere with the use of other electrical appliances in daily life, the shopkeeper's service attitude is very enthusiastic, I believe that the manufacturer's products, thank you	Taobao Marketplace, a Chinese website for online shopping
Intelligent power saver	Intelligent power saver	Things received, feel good, use a period of time, see if the effect is good, good and then buy a few more	Taobao Marketplace, a Chinese website for online shopping
Intelligent power saver	Intelligent power saver	This product is purely fraudulent products, not at all useful, said 100 within the refund, customer service**, a word back and no, everyone who then buy who is cheated, purely fraudulent products ！！！！！！	Taobao Marketplace, a Chinese website for online shopping
Intelligent power saver	Intelligent power saver	Power-saving effect: not clear Cost-effective: okay Appearance style: compact Workmanship material: okay Experience: simple operation, convenient	Taobao Marketplace, a Chinese website for online shopping
Intelligent power saver	Intelligent power saver	The effect is very good, deliberately used for a period of time to evaluate, the original electricity bill is very high, since the use of this power-saving device, the electricity bill to save a lot of too high, affordable, and save a large amount of electricity, compact, and very convenient, inserted in any	Taobao Marketplace, a Chinese website for online shopping
Intelligent power saver	Intelligent power saver	Before buying hesitated for a long time, asked the seller a lot of questions, the seller is very patient answer, the business said 100 days free trial, thinking of buying back to try. Used for a period of time, the effect is really quite good, is genuine, with a very good, really can save electricity, very good, will introduce friends to buy!	Taobao Marketplace, a Chinese website for online shopping
Intelligent power saver	Intelligent power saver	Saver quality is very good, the use of no sound, inserted where you can be very convenient, very satisfied, logistics is also very fast, every month with a lot of electricity, after using this save a lot of it, long time or can save a lot of it, has been repurchased for the second time ah, the family sent a mother's home to send one. Really like, customer service attitude is also quite good, value! In the future, there is a need to come back to buy	Taobao Marketplace, a Chinese website for online shopping
Intelligent power saver	Intelligent power saver	Mom there used a few days the effect is good, functional, the effect is good satisfaction!family said good, especially like Oh, this time finally bought the right, business service attitude is very good very good very👍	Taobao Marketplace, a Chinese website for online shopping
Intelligent power saver	Intelligent power saver	The long-awaited power saver finally received the goods, friends recommended, stable performance, good quality, with a very simple, unlike other still have to install, cost-effective really high, a month down should be able to save a lot of electricity, long-term or very cost-effective, value for money👍👍👍straight to thousands of households to rest assured that the use of the convenience of the use of it, customer service is also a good service satisfied!	Taobao Marketplace, a Chinese website for online shopping
Intelligent power saver	Intelligent power saver	Really too good to use, save electrical appliances are very formal, above the anti-counterfeiting labels, and genuine labels, good and inexpensive, according to your power consumption consulting merchants to use, the pro assured shot	Taobao Marketplace, a Chinese website for online shopping
Intelligent power saver	Intelligent power saver	With it and not it meter jump time is the same, I took it back at night and used it, the meter jumped the same time and did not use it jump time is the same, this thirty-some dollars is spent in vain, we used the cell phone's stopwatch for a long time to see if there is no effect, we still do not buy, no use!	Taobao Marketplace, a Chinese website for online shopping
Intelligent power saver	Intelligent power saver	We must not buy, inside is a small and small small electric street lamp, we must not be fooled, I was fooled, we must not be fooled, because I bought the inside is full of positive feedback, I also bought, fooled!	Taobao Marketplace, a Chinese website for online shopping

Intelligent power saver	Intelligent power saver	Workmanship material: can save electricity, this month can save a lot of money, thanks to the boss	Taobao Marketplace, a Chinese website for online shopping
Intelligent power saver	Intelligent power saver	Appearance and style: very beautiful, small and exquisite Workmanship material: plastic, very light can be carried	Taobao Marketplace, a Chinese website for online shopping
Intelligent power saver	Intelligent power saver	It's not a family that can use a pitchfork in the main house.	Taobao Marketplace, a Chinese website for online shopping
Intelligent power saver	Intelligent power saver	Does this work?	Taobao Marketplace, a Chinese website for online shopping
Intelligent power saver	Intelligent power saver	it work for those who have used it?Is it a safety hazard?	Taobao Marketplace, a Chinese website for online shopping
Intelligent power saver	Intelligent power saver	Is it better to buy one and be able to use outlets throughout the house or does it only come with one outlet? Trouble.	Taobao Marketplace, a Chinese website for online shopping
Intelligent power saver	Intelligent power saver	Does it really work?	Taobao Marketplace, a Chinese website for online shopping
Intelligent power saver	Intelligent power saver	It's an IQ tax, isn't it?	Taobao Marketplace, a Chinese website for online shopping
Intelligent power saver	Intelligent power saver	It's a handful.	Taobao Marketplace, a Chinese website for online shopping
Intelligent power saver	Intelligent power saver	Is it an IQ tax?	Taobao Marketplace, a Chinese website for online shopping
Intelligent power saver	Intelligent power saver	Harvesting the IQ tax again?	Taobao Marketplace, a Chinese website for online shopping
Intelligent power saver	Intelligent power saver	I can't feel it, I've been using it for a few months, but I don't feel any power saving.	Taobao Marketplace, a Chinese website for online shopping
Intelligent power saver	Intelligent power saver	Just used it10days or so, no obvious effect, I will know in January	Taobao Marketplace, a Chinese website for online shopping
Intelligent power saver	Intelligent power saver	It hasn't been a month yet, I don't know if it's working or not yet	Taobao Marketplace, a Chinese website for online shopping
Intelligent power saver	Intelligent power saver	This saver doesn't work at all. It's a waste of money.	Taobao Marketplace, a Chinese website for online shopping
Intelligent power saver	Intelligent power saver	It's useless. It's a waste of money.	Taobao Marketplace, a Chinese website for online shopping
Intelligent power saver	Intelligent power saver	I don't know yet.	Taobao Marketplace, a Chinese website for online shopping
Intelligent power saver	Intelligent power saver	IQ tax	Taobao Marketplace, a Chinese website for online shopping
Intelligent power saver	Intelligent power saver	don't know. I boughtsix.	Taobao Marketplace, a Chinese website for online shopping
Intelligent power saver	Intelligent power saver	yea	Taobao Marketplace, a Chinese website for online shopping
Intelligent power saver	a power saver (electronics)	Please do not buy, completely IQ tax,Julyelectricity business fully31days350dollars, plug this thing,August24days since359dollars, not only does not save electricity, and even cost electricity	Taobao Marketplace, a Chinese website for online shopping
Intelligent power saver	a power saver (electronics)	Plugged in a rental room, used it for a month before coming to evaluate, originally thought it would be less electricity bill, but this month's electricity bill is still higher than the previous months, Asked customer service, no one cares about it,Let me talk about it some other day.	Taobao Marketplace, a Chinese website for online shopping
Intelligent power saver	a power saver (electronics)	Used for a few days don't know the effect, customer service is average, you can watch the chat	Taobao Marketplace, a Chinese website for online shopping
Intelligent power saver	a power saver (electronics)	Just received in the afternoon, looked at the meter is indeed slower than before to see how much difference when paying the electricity bill. Power-saving effect: a table is to go slow Cost-effective: a good Appearance style:, beautiful Workmanship material: a fine	Taobao Marketplace, a Chinese website for online shopping
Intelligent power saver	a power saver (electronics)	Friends recommended, bought back and began to use, before every month need to pay three or four hundred electricity, I have to say that the effect is really very good, in a few days will continue to buy back a , this can be more energy-saving!	Taobao Marketplace, a Chinese website for online shopping
Intelligent power saver	a power saver (electronics)	Power savings: not useful at all, I'm returning it.	Taobao Marketplace, a Chinese website for online shopping
Intelligent power saver	a power saver (electronics)	Just started using it and how effective it is. Will comment again when I use it for a while.	Taobao Marketplace, a Chinese website for online shopping
Intelligent power saver	a power saver (electronics)	Good price, fast logistics, good service attitude, worthy of praise!	Taobao Marketplace, a Chinese website for online shopping
Intelligent power saver	a power saver (electronics)	Already used it and it feels good! Satisfied! Good review!	Taobao Marketplace, a Chinese website for online shopping
Intelligent power saver	a power saver (electronics)	This smart saver home model is quite good, and bought another one, have thone I boughtPoundlandfor16before, not only does it not save electricity but also burned my kettle, this saver can stabilize the voltage, save energy and reduce consumption, it's very good.	Taobao Marketplace, a Chinese website for online shopping
Intelligent power saver	a power saver (electronics)	Electricity swish, in the supermarket to see this power saver, the price tag to100hundred more, did not bother to buy, just in time to catch the recent activities, the price is quite affordable, buy back to install and use for a period of time, obviously feel that the electricity bill spent not so fast.	Taobao Marketplace, a Chinese website for online shopping
Intelligent power saver	a power saver (electronics)	Saving appliances are really too good to use, because the relatives of the family electricity is very expensive, after buying this device are said to be very effective, thanks to the patience of the seller's reception, and will recommend more relatives and friends!	Taobao Marketplace, a Chinese website for online shopping
Intelligent power saver	a power saver (electronics)	Energy saving appliances received today and used it, the cost-effectiveness, appearance and style is very good, exquisite workmanship! Energy-saving appliances as soon as received and started to enable, looks good quality!	Taobao Marketplace, a Chinese website for online shopping
Intelligent power saver	a power saver (electronics)	Received, looks pretty good, only a few days, the effect has not been compared out, next month the electricity bill out of the follow-up effect, the seller's attitude is very good, especially enthusiastic, I hope business is getting better and better!	Taobao Marketplace, a Chinese website for online shopping

Intelligent power saver	a power saver (electronics)	At the same time to buy three styles of power saver, this installed in the first floor of the home, the other two installed in the second floor of the home, to see which effect is better, but look at this power saver is very beautiful workmanship, first plugged into the socket to use for a period of time, to see the effect of how to come back to comment.	Taobao Marketplace, a Chinese website for online shopping
Intelligent power saver	a power saver (electronics)	Feel the effect is not bad, my side is living in a rental house. Originally thought that the smart meter can not be used, I did not think that the smart meter can also save electricity, consulting the store gave me a recommendation, decisive order to buy!	Taobao Marketplace, a Chinese website for online shopping
Intelligent power saver	a power saver (electronics)	The quality of this product is very good, excellent workmanship materials, it is worth recommending the purchase. Looking forward to everyone to see samples to buy. I score full marks.	Taobao Marketplace, a Chinese website for online shopping
Intelligent power saver	a power saver (electronics)	Power saving effect is very good, has shot the fourth, this time for the father-in-law's home, the old man is very like, energy saving, energy saving, compact and easy to use, protection of home appliances, voltage, current stabilization, very good use.	Taobao Marketplace, a Chinese website for online shopping
Intelligent power saver	a power saver (electronics)	Affordable and power saving, good quality	Taobao Marketplace, a Chinese website for online shopping
Intelligent power saver	a power saver (electronics)	there's nothing wrong with it	Taobao Marketplace, a Chinese website for online shopping
Intelligent power saver	a power saver (electronics)	This side is to live in an apartment, this side of everything is good is expensive electricity, this side to friends as guests, friends introduced me to buy this, just a few days is not too obvious, with the effect of the feeling is quite good!	Taobao Marketplace, a Chinese website for online shopping
Intelligent power saver	a power saver (electronics)	Now accommodation, usually consume more power, accidentally swiped this product, the appearance really attracted me, ordered one, as always, good quality, very like a product, very good!o(*￣︶￣*)o	Taobao Marketplace, a Chinese website for online shopping
Intelligent power saver	a power saver (electronics)	The electricity bill is a bit unbearable, see the neighbor's home installed this saver, the quality is very good, the appearance is very beautiful, get it plugged directly into the outlet	Taobao Marketplace, a Chinese website for online shopping
Intelligent power saver	a power saver (electronics)	Used more than one product, on this effect is the most stable, is indeed a penny a good, especially to the sales customer service point of praise, I would have liked to buy other models, said I do not use much electricity,more than80degreesa month, this is the right one, the gentleman to take the material	Taobao Marketplace, a Chinese website for online shopping
Intelligent power saver	a power saver (electronics)	This energy saver can save electricity is to save money plug it in the elderly will also use how convenient, the style is very like, really good! I want to buy2more	Taobao Marketplace, a Chinese website for online shopping
Intelligent power saver	a power saver (electronics)	Intelligent power saver use effect is very good, can effectively stabilize the voltage, reduce circuit losses to achieve the effect of energy saving. It is also inadvertently see a friend's home has to use, by his recommendation to buy a first use.	Taobao Marketplace, a Chinese website for online shopping
Intelligent power saver	a power saver (electronics)	Intelligent compensation economizer received, looks very sturdy, like it very much. It is being used, very satisfied with a shopping, very easy to use. Buy home and plug it into the socket. Recommended to buy with confidence!	Taobao Marketplace, a Chinese website for online shopping
Intelligent power saver	a power saver (electronics)	I am out to stay outside, this side of the meter do not know how to go very fast, just started not too obvious, the appearance is very delicate, looking forward to the effect	Taobao Marketplace, a Chinese website for online shopping
Intelligent power saver	a power saver (electronics)	System default comments	Taobao Marketplace, a Chinese website for online shopping
Intelligent power saver	a power saver (electronics)	Shipping is fast, the quality of the saver is very good, and the customer is patient. Just used it, looking forward to using it to save electricity, exquisite workmanship, and exquisite materials	Taobao Marketplace, a Chinese website for online shopping
Intelligent power saver	a power saver (electronics)	This side of the home due to the meter power consumption is relatively large, this side of the colleague introduced me to buy this with, he said that his family is also using this store to buy power-saving, I want to buy the link, looking forward to power-saving effect!	Taobao Marketplace, a Chinese website for online shopping
Intelligent power saver	a power saver (electronics)	I'm an electrician, was to try the psychological buy, did not expect the effect is not bad, this thing is not bad, now the science is really a generation to update, even I'm an electrician feel incredible, the effect is okay	Taobao Marketplace, a Chinese website for online shopping
Intelligent power saver	a power saver (electronics)	Logistics is very fast, my son immediately disassembled to play, was I beat up, haha, product quality is really good, was he fell after a little trace is not, praise!	Taobao Marketplace, a Chinese website for online shopping
Intelligent power saver	a power saver (electronics)	Energy saving effect is very good Use to save electricity and save money Can save electricity for the family, beautiful shape, exquisite workmanship worth buying.	Taobao Marketplace, a Chinese website for online shopping
Intelligent power saver	a power saver (electronics)	Goods received, intelligent power saver appearance design is good compact, good quality, read a lot of reviews say very save electricity, I immediately ordered to try, home appliances more effective will continue to buy back.	Taobao Marketplace, a Chinese website for online shopping
Intelligent power saver	a power saver (electronics)	Home appliances, electricity is very expensive, I hope that this device can save a little is a little, the quality of workmanship packaging, especially customer service attitude is really no words!	Taobao Marketplace, a Chinese website for online shopping
Intelligent power saver	a power saver (electronics)	Used for a week, everything is normal, and does not affect the line at home, and is very convenient, just plug in the socket can be, before a friend bought, the feedback is very good, I also asked him to buy me a, see after a month or two after the electricity bill.	Taobao Marketplace, a Chinese website for online shopping
Intelligent power saver	Intelligent Energy Saving Butler	It does save electricity, the thing works well, and the customer service attitude is also very good. At first I did not know what principle, the businessman patiently explained, I downloaded the National Gridapp, after the installation of the daily power consumption can be seen, it does save electricity	Taobao Marketplace, a Chinese website for online shopping
Intelligent power saver	Intelligent Energy Saving Butler	The owner of the fast delivery, as always, good to use, the family bought before the effect is quite good, in preparation for a few other houses are inserted	Taobao Marketplace, a Chinese website for online shopping
Intelligent power saver	Intelligent Energy Saving Butler	Received the goods look exquisite, just plugged in to use, the business said again to observe a hundred days, not much money to wait a hundred days, later must follow up the evaluation	Taobao Marketplace, a Chinese website for online shopping
Intelligent power saver	Intelligent Energy Saving Butler	System default comments	Taobao Marketplace, a Chinese website for online shopping
Intelligent power saver	Intelligent Energy Saving Butler	节电效果：这个节电器效果真的很好 性价比：价格优惠比其他滴性价比高很多呢 外观款式：外观设计非常好看，高档，大气 做工材质：材质采用真材实料，非常喜欢，结实耐用 使用体验：感觉给买家说滴一样，非常的不错👍👍👌	Taobao Marketplace, a Chinese website for online shopping
Intelligent power saver	Intelligent Energy Saving Butler	Just took delivery and plugged it in for a few days, whether it works or not, we'll know at the end of the month when we look at the electricity bill.	Taobao Marketplace, a Chinese website for online shopping
Intelligent power saver	Intelligent Energy Saving Butler	Logistics is particularly powerful, delivery speed is particularly fast, packaging quality is particularly good, installation is particularly convenient, customer service attitude is particularly good, explain particularly detailed, saving electricity effect is particularly Bashful, five-star praise!	Taobao Marketplace, a Chinese website for online shopping
Intelligent power saver	Intelligent Energy Saving Butler	Home water heater is too costly, friends recommended that good use, buy to try, quite good, quality first-class, packaging is also good, logistics is very fast, and the price is reasonable, very affordable, very like	Taobao Marketplace, a Chinese website for online shopping
Intelligent power saver	Intelligent Energy Saving Butler	The effect is really good, after receiving the goods on the rush to try, originally do not believe this, the business said you can return, the results I used after this thing is really magical, just plugged in a split second on the meter does not jump, I turn on the high-power appliances, the meter jumped very slowly, a day down to the use of the2,3degrees of electricity, recommended that relatives and friends to [illegible]	Taobao Marketplace, a Chinese website for online shopping
Intelligent power saver	Intelligent Energy Saving Butler	Baby received, the courier is very powerful, well-packed, I can not wait to use, and so on three months of time, I'll follow up and the actual usefulness of the product price is also very affordable, just look at the performance of the baby!	Taobao Marketplace, a Chinese website for online shopping

Intelligent power saver	Intelligent Energy Saving Butler	System default comments	Taobao Marketplace, a Chinese website for online shopping
Intelligent power saver	Intelligent Energy Saving Butler	After receiving, can not wait to open and use, the appearance of good-looking fashion, workmanship and materials are also very good, with a period of time to follow up!	Taobao Marketplace, a Chinese website for online shopping
Intelligent power saver	Intelligent Energy Saving Butler	Saver quality is very good, the use of no sound, inserted where you can be very convenient, very satisfied, logistics is also very fast, every month with a lot of electricity, after using this save a lot of it, long time or can save a lot of it, has been repurchased for the second time ah, the family sent a mother's home to send one. Really like, customer service attitude is also quite good, value! In the future, there is a need to come back to buy	Taobao Marketplace, a Chinese website for online shopping
Intelligent power saver	Intelligent Energy Saving Butler	Quality is very good, fine workmanship, friends introduced this artifact, genuinely good, power-saving effect is very obvious, love love love!	Taobao Marketplace, a Chinese website for online shopping
Intelligent power saver	Intelligent Energy Saving Butler	The power saver received, good quality, exquisite workmanship, power saving effect is obvious, protection of electrical appliances, the volume is not big, small and exquisite, the use of a few days no hot situation, the power saving effect is also very obvious, in a few days to come back to buy, give	Taobao Marketplace, a Chinese website for online shopping
Intelligent power saver	Intelligent Energy Saving Butler	The effect is really good, received the goods on the rush to try, originally do not believe that this, the business said that there is a warranty and dissatisfaction can be returned, used after the effect is really good, plugged in for a period of time to observe the meter, than before, a lot less electricity!	Taobao Marketplace, a Chinese website for online shopping
Intelligent power saver	Intelligent Energy Saving Butler	Saving power received, the appearance of the design is small and exquisite, the material is strong and easy to use simple, this time do not have to worry about the power bill, plugged in a very power-saving, and the price is also affordable very satisfied!	Taobao Marketplace, a Chinese website for online shopping
Intelligent power saver	Intelligent Energy Saving Butler	System default comments	Taobao Marketplace, a Chinese website for online shopping
Intelligent power saver	Intelligent Energy Saving Butler	Cost-effective is also very high, the quality is also very good, logistics is also fast ah! Small, simple, convenient, feel amazing, not much money can save money, very like that	Taobao Marketplace, a Chinese website for online shopping
Intelligent power saver	Intelligent Energy Saving Butler	Choose this one from many merchants, the baby is of high quality, the price is favorable, cost-effective, and the customer service attitude is very good, the logistics is quite fast, give a favorable comment! There is a need, you can rest assured that the purchase.	Taobao Marketplace, a Chinese website for online shopping
Intelligent power saver	Intelligent Energy Saving Butler	Brand new power saver just received! Power saving research unit product, well made and formal! There are detailed instructions! This product is simple and convenient, considering the more electrical equipment at home, buy to use to save electricity at home electricity bill!	Taobao Marketplace, a Chinese website for online shopping
Intelligent power saver	Intelligent Energy Saving Butler	Xat home really good to save ah !Received the goods can not wait to plug in , the meter I feel go slower ! Is it good to say that the power saving good obvious it!	Taobao Marketplace, a Chinese website for online shopping
Intelligent power saver	Intelligent Energy Saving Butler	System default comments	Taobao Marketplace, a Chinese website for online shopping
Intelligent power saver	Intelligent Energy Saving Butler	The thing is very beautifully made I do not know the exact effect If you look at the principle is right bought for the elderly at home before the old could not turn on the electrical appliances installed two for the family whether it is useful or not at least the old man willing to turn on the air conditioner	Taobao Marketplace, a Chinese website for online shopping
Intelligent power saver	Intelligent Energy Saving Butler	I am a half-yearly payment, before so much, this time the calculation can be about half less, because the electricity I am more sensitive, all sure that this product is effective	Taobao Marketplace, a Chinese website for online shopping
Intelligent power saver	Intelligent Energy Saving Butler	System default comments	Taobao Marketplace, a Chinese website for online shopping
Intelligent power saver	Intelligent Energy Saving Butler	Energy saver received, energy saving effect is good. The seller has a good service attitude, very patient to communicate the use of methods. Very good shopping experience, five stars!	Taobao Marketplace, a Chinese website for online shopping
Intelligent power saver	Intelligent Energy Saving Butler	Saving appliances are really useful, high electricity bills every day more power consumption, electricity bills can not eat, was just to try, I did not expect to use the monthly savings of a large amount of expenses, happy!	Taobao Marketplace, a Chinese website for online shopping
Intelligent power saver	Intelligent Energy Saving Butler	Does not take up space at all, such a small thing, really all the organs, after plugging it in, save a little effect is very obvious, which adopted the ten days and half a month to save a few dozen dollars, very good, it is worthwhile to get, in a few days and then back to buy!	Taobao Marketplace, a Chinese website for online shopping
Intelligent power saver	Intelligent Energy Saving Butler	The effect is also too good, right, praise, just arrived on the insertion of the use, compared to the previous meter, the degrees go much slower, can indeed play a power-saving effect, trustworthy recommended a product!	Taobao Marketplace, a Chinese website for online shopping
Intelligent power saver	Intelligent Energy Saving Butler	Does it really save power?	Taobao Marketplace, a Chinese website for online shopping
Intelligent power saver	Intelligent Energy Saving Butler	For those of you who bought one, did it really work?	Taobao Marketplace, a Chinese website for online shopping
Intelligent power saver	Intelligent Energy Saving Butler	It is the optimization of the circuit can save part of the electricity drop	Taobao Marketplace, a Chinese website for online shopping
Intelligent power saver	Intelligent Energy Saving Butler	Haven't used it long enough to see results	Taobao Marketplace, a Chinese website for online shopping
Intelligent power saver	King of Power Saving	IQ tax... Laughing my ass off... Doesn't help at all😂😂	Taobao Marketplace, a Chinese website for online shopping
Intelligent power saver	King of Power Saving	The effect is very good, deliberately used for a period of time to evaluate, the original electricity bill is very high, since the use of this power-saving device, the electricity bill to save a lot of scientific power-saving affordable, and save a large amount of electricity, something small, and very convenient, plugged into any outlet can be, it's really great to give the family to also arrange on the	Taobao Marketplace, a Chinese website for online shopping
Intelligent power saver	King of Power Saving	Is it really that good?	Taobao Marketplace, a Chinese website for online shopping
Intelligent power saver	King of Power Saving	Retard Tax Not helpful	Taobao Marketplace, a Chinese website for online shopping
Diyuan Electrical Appliances Flagship Store	Green Intelligent Power Saver	evaluation after use	Taobao Marketplace, a Chinese website for online shopping
Diyuan Electrical Appliances Flagship Store	Green Intelligent Power Saver	It looks too thin, I don't know how it is, let's use it for a month first and see how it goes	Taobao Marketplace, a Chinese website for online shopping
Diyuan Electrical Appliances Flagship Store	Green Intelligent Power Saver	The effect of this power saver is particularly great, the use of very simple, plug in the socket on theOK, too much power, electricity is also expensive, with this product directly on the electricity bill to save half, air conditioning, refrigerator and hot water heater are open every day, and has purchased a	Taobao Marketplace, a Chinese website for online shopping
Diyuan Electrical Appliances Flagship Store	Green Intelligent Power Saver	Dedicated to try for more than a month, mainly every day to use the same appliances under the experiment, with obvious results.Electricity costs fell by about half, really quite practical that save electricity and can save national resources good oh. The company also has the Pacific Ocean Insurance, safe and secure! The quality is very thick.	Taobao Marketplace, a Chinese website for online shopping

Diyuan Electrical Appliances Flagship Store	Green Intelligent Power Saver	I am a professional electrician, this power saver is really useful, the real use of electronic waves automatically connected to track the decomposition of ionized electrons, in-situ compensation, reduce the effective power loss and waste, stabilize the voltage to achieve the power connection, the overall is still quite good, it is worthwhile for everyone to buy.	Taobao Marketplace, a Chinese website for online shopping
Diyuan Electrical Appliances Flagship Store	Green Intelligent Power Saver	Energy-saving effect is very good, plugged in to see the meter is more obviously slow, the price is also more moderate, the appearance of the style is also very good-looking, workmanship is also very fine, use a few days down is very satisfied!!!!!	Taobao Marketplace, a Chinese website for online shopping
Diyuan Electrical Appliances Flagship Store	Green Intelligent Power Saver	3more, home and office are used, where more power is inserted in that neighborhood outlet, the effect is not bad, can save how much is how much	Taobao Marketplace, a Chinese website for online shopping
Diyuan Electrical Appliances Flagship Store	Green Intelligent Power Saver	It works great.	Taobao Marketplace, a Chinese website for online shopping
Diyuan Electrical Appliances Flagship Store	Green Intelligent Power Saver	There's no use at all.	Taobao Marketplace, a Chinese website for online shopping
Diyuan Electrical Appliances Flagship Store	Green Intelligent Power Saver	Power-saving effect: it is estimated that100yuan of electricity can savemore than40yuan Cost-effective: cost-effective, there is no cost, plug it in, a purchase of long-term benefit, to have been inserted into the home socket, it is very safe, recommended to buy	Taobao Marketplace, a Chinese website for online shopping
Diyuan Electrical Appliances Flagship Store	Green Intelligent Power Saver	Originally bought with the mindset of trying, but found that the effect seems to be still there, can be oh. Technology is awesome, feel the degree of electricity than before has decreased, very satisfactory	Taobao Marketplace, a Chinese website for online shopping
Diyuan Electrical Appliances Flagship Store	Green Intelligent Power Saver	Products are really good I used two for the family, usually kitchen and bathroom water heaters with electricity is too expensive, because the water heater to open every day, every month electricity is too much, no longer afraid of the electricity bill is too much, a few months down,electricity bill is less than half of it, the effect of the special good, very much like.	Taobao Marketplace, a Chinese website for online shopping
Diyuan Electrical Appliances Flagship Store	Green Intelligent Power Saver	How does it work?	Taobao Marketplace, a Chinese website for online shopping
Diyuan Electrical Appliances Flagship Store	Green Intelligent Power Saver	Is this punishable as fraud?	Taobao Marketplace, a Chinese website for online shopping
Diyuan Electrical Appliances Flagship Store	Green Intelligent Power Saver	No pie in the sky, proper IQ tax.	Taobao Marketplace, a Chinese website for online shopping
Diyuan Electrical Appliances Flagship Store	Green Intelligent Power Saver	Bad review,, fake	Taobao Marketplace, a Chinese website for online shopping
Diyuan Electrical Appliances Flagship Store	Green Intelligent Power Saver	/:O	Taobao Marketplace, a Chinese website for online shopping
Diyuan Electrical Appliances Flagship Store	Green Intelligent Power Saver	Haha😉	Taobao Marketplace, a Chinese website for online shopping
Diyuan Electrical Appliances Flagship Store	White Intelligent Power Saver	I don't know if it's working.	Taobao Marketplace, a Chinese website for online shopping
Diyuan Electrical Appliances Flagship Store	White Intelligent Power Saver	Goods received, all-powerful battery power saving, power, is the same as the introduction of the business, to give a favorable review	Taobao Marketplace, a Chinese website for online shopping
Diyuan Electrical Appliances Flagship Store	White Intelligent Power Saver	Things received, well-packed, the courier is also very fast, very good, really save electricity, praise!	Taobao Marketplace, a Chinese website for online shopping
Diyuan Electrical Appliances Flagship Store	White Intelligent Power Saver	Energy saver is also a friend introduced to me to buy, she used a few after the effect is very good, let me also insist on using, now the landlord is too**, the meter adjusted very quickly, a month to hundreds of dollars of electricity. I haven't turned on the air conditioner much yet, and with this the landlord can no longer pit my electricity bill!	Taobao Marketplace, a Chinese website for online shopping
Diyuan Electrical Appliances Flagship Store	White Intelligent Power Saver	Energy saver received, it is really high-tech too cow is very surprised, this thing can really save electricity. Before a monthmore than200electricity bill, now can save50dollarsa month, if the home power consumption; you can rest assured to get.	Taobao Marketplace, a Chinese website for online shopping
Diyuan Electrical Appliances Flagship Store	White Intelligent Power Saver	My family used two, the effect can be good, electrical appliances more power consumption is also large, and now a month of electrical appliances air conditioning has been open, each month can save several dozen dollars, has been using, very good!	Taobao Marketplace, a Chinese website for online shopping
Diyuan Electrical Appliances Flagship Store	White Intelligent Power Saver	I'll go! A month to save 300 electricity bill, too powerful, right? This little thing, our studio, the equipment is a little more, originally a month tomore than700dollars of electricity, with this period of time, this month to pay the electricity bill only paidmore than400, this effect is simply God!	Taobao Marketplace, a Chinese website for online shopping
Diyuan Electrical Appliances Flagship Store	White Intelligent Power Saver	This power saver is really a must-have for large families that consume electricity,2air conditioners athomenon-stop, with this power saver no longer worry about the electricity bill miso long	Taobao Marketplace, a Chinese website for online shopping
Diyuan Electrical Appliances Flagship Store	White Intelligent Power Saver	Great! Old family pig raising, use a lot of electricity, arranged for6, all installed, can savemore than500a month! The quality is also good, used for a period of time are not fault, is quite stable	Taobao Marketplace, a Chinese website for online shopping
Diyuan Electrical Appliances Flagship Store	White Intelligent Power Saver	The effect is very good, deliberately used for a period of time to evaluate the original electricity bill is very high, since the use of this power-saving device, electricity costs save a lot of cost-effective:too high, affordable, and save a large amount of electricity appearance style:small, and very convenient, inserted in any outlet.On the workmanship material:very good	Taobao Marketplace, a Chinese website for online shopping
Diyuan Electrical Appliances Flagship Store	White Intelligent Power Saver	I just used it. I don't know if it works.	Taobao Marketplace, a Chinese website for online shopping
Diyuan Electrical Appliances Flagship Store	White Intelligent Power Saver	two power savers for the family, the kitchen and bathroom water heater is too expensive_x005f_x005f_x0014_Ĉ, the water heater has to be turned on every day, every month the electricity bill isa lot ofuse, with this, no longer afraid of the electricity bill is too much, in January, down the electricity bill can be less than half of the previous month it □! H, the effect is	Taobao Marketplace, a Chinese website for online shopping
Diyuan Electrical Appliances Flagship Store	White Intelligent Power Saver	Bought two energy-saving appliances for the family, the kitchen and bathroom water heater is too expensive, the water heater to be turned on every day, the monthly electricity bill is a lot of use, with this, no longer afraid of the electricity bill is more than one month down the electricity bill can be less than half of the previous month it, the effect is particularly good!	Taobao Marketplace, a Chinese website for online shopping
Diyuan Electrical Appliances Flagship Store	White Intelligent Power Saver	Used for a period of time to look at the meter, check the price is indeed less, in the use of electricity plug on is slow, this instrument is really good, I also want to buy two, the bedroom and the living room are loaded with use experience, simple, convenient, fast!	Taobao Marketplace, a Chinese website for online shopping
Diyuan Electrical Appliances Flagship Store	White Intelligent Power Saver	The effect is really good, after receiving the goods will rush to try, originally do not believe that this, the business said that you can return, the results I used after this thing is really magical, plugged in does not jump, I turn on the high-power appliances, jumping is also very slow, a day down to the use of2,3degrees of electricity, recommended that relatives and friends purchase	Taobao Marketplace, a Chinese website for online shopping
Diyuan Electrical Appliances Flagship Store	White Intelligent Power Saver	Saving device received, really good, with the air conditioning tried, an hour without a saver with2.9degrees, with a saver an hour with1.1degrees, the beginning of not quite believe that this thing, the actual test to know that it is really useful, and intend to buy another.	Taobao Marketplace, a Chinese website for online shopping
Diyuan Electrical Appliances Flagship Store	White Intelligent Power Saver	fraudulent	Taobao Marketplace, a Chinese website for online shopping

Diyuan Electrical Appliances Flagship Store	White Intelligent Power Saver	The effect is very good, the electricity bill has dropped a lot worth repurchasing recommended! A month lessmore than160degrees originallymore than600degrees Boss service attitude is very good recommended friends to buy.	Taobao Marketplace, a Chinese website for online shopping
Diyuan Electrical Appliances Flagship Store	White Intelligent Power Saver	Second time I've bought this product and I do get by! Bought the plug in water heater there two months ago, and the recent electricity bill is indeed less, obviously, this summer weather is too hot to buy a plug in air conditioner and try again! Good product has to be used on the knife edge! Conscientious	Taobao Marketplace, a Chinese website for online shopping
Diyuan Electrical Appliances Flagship Store	White Intelligent Power Saver	The quality is first-class, the packaging is good, the logistics is fast, and the price is reasonable and very affordable	Taobao Marketplace, a Chinese website for online shopping
Diyuan Electrical Appliances Flagship Store	White Intelligent Power Saver	Friends with a good use recommended the purchase, down after the customer service attitude and timely, logistics is very fast, after the arrival of the plug to try, really good, like, can pay less for electricity later, want to save money friends can start to buy.	Taobao Marketplace, a Chinese website for online shopping
Diyuan Electrical Appliances Flagship Store	White Intelligent Power Saver	Turned on the air conditioner the last couple days and saw the meter turn a little slower than before. Electricity consumption is a little less than before. Stable performance , portable and convenient , the frequency of meter jumping has been significantly reduced!	Taobao Marketplace, a Chinese website for online shopping
Diyuan Electrical Appliances Flagship Store	White Intelligent Power Saver	Good cattle all this thing, bought4, received the goods on the rush to try, originally do not believe that this, the business said can be returned, the results I used after this thing is really magical, just plugged in a moment of the power meter does not jump, I put the high-power appliances are open, the meter jumped very slowly, a day down to3degrees of electricity, recommended relatives and friends to buy, really magical. Power saving effect: good Cost-effective: good Appearance style: good Workmanship material: [illegible]	Taobao Marketplace, a Chinese website for online shopping
Diyuan Electrical Appliances Flagship Store	White Intelligent Power Saver	Used a little, indeed can. Quite save electricity, the effect is not exaggerated, wait for a period of time to see how much can actually save, then certainly have to buy back, thanks to the seller at the time of the sale of the recommendation, to give a positive feedback!	Taobao Marketplace, a Chinese website for online shopping
Diyuan Electrical Appliances Flagship Store	White Intelligent Power Saver	(of an unmarried couple) be close	Taobao Marketplace, a Chinese website for online shopping
Diyuan Electrical Appliances Flagship Store	White Intelligent Power Saver	Power-saving effect: cow Price-performance ratio: good Appearance style: excellent	Taobao Marketplace, a Chinese website for online shopping
Diyuan Electrical Appliances Flagship Store	White Intelligent Power Saver	Small size, does not take up space, installed in the room really less electricity bill. About30degrees of electricitya day, work down indeed almost save10degrees of electricity The effect is really good, and scientific energy saving, very reassuring.	Taobao Marketplace, a Chinese website for online shopping
Diyuan Electrical Appliances Flagship Store	White Intelligent Power Saver	Products are really good I used two for the family, usually kitchen and bathroom water heaters with electricity is too expensive, because the water heater to open every day, every month electricity is too much, no longer afraid of the electricity bill is too much, a few months down,electricity bill is less than half of it, the effect of the special good, very much like.	Taobao Marketplace, a Chinese website for online shopping
Diyuan Electrical Appliances Flagship Store	White Intelligent Power Saver	I have studied electricity, I know the importance of power-saving factors on the circuit, this economizer can reduce reactive power, for complex circuits50%power saving rate is theoretically achievable, workmanship is also good, with a few years should not be a problem, it is worth recommending the	Taobao Marketplace, a Chinese website for online shopping
Diyuan Electrical Appliances Flagship Store	White Intelligent Power Saver	Does it really work, honestly?	Taobao Marketplace, a Chinese website for online shopping
Diyuan Electrical Appliances Flagship Store	White Intelligent Power Saver	Does this really work?	Taobao Marketplace, a Chinese website for online shopping
Diyuan Electrical Appliances Flagship Store	White Intelligent Power Saver	usefulness	Taobao Marketplace, a Chinese website for online shopping
Diyuan Electrical Appliances Flagship Store	White Intelligent Power Saver	It really works. I've saved over a hundred dollars in a month.	Taobao Marketplace, a Chinese website for online shopping
Diyuan Electrical Appliances Flagship Store	a power saver (electronics)	Haven't purchased online, but hopefully it will have the effect of saving energy on your electric bill	Taobao Marketplace, a Chinese website for online shopping
Diyuan Electrical Appliances Flagship Store	a power saver (electronics)	The power saver has been received for a few days, it is inserted in the living room, the living room has air conditioning, recently the weather is hot, the power saving effect is very obvious, calculated than the previous day to save about ten degrees of electricity, I have introduced it to my friends and relatives.	Taobao Marketplace, a Chinese website for online shopping
Diyuan Electrical Appliances Flagship Store	a power saver (electronics)	Good value for money, already using really power saving	Taobao Marketplace, a Chinese website for online shopping
Diyuan Electrical Appliances Flagship Store	a power saver (electronics)	Indeed very effective, because now are mobile paypal bill payment, before the normal electricity about half a month to charge100yuan electricity, summer air conditioning with a month300, we use this energy-saving products isabout28days100yuan electricity, is indeed a lot of savings, and to the old man to buy	Taobao Marketplace, a Chinese website for online shopping
Diyuan Electrical Appliances Flagship Store	a power saver (electronics)	Hello.How does the economizer work? How does it work? How many square feet to install one? Thank you for your help!	Taobao Marketplace, a Chinese website for online shopping
Diyuan Electrical Appliances Flagship Store	a power saver (electronics)	It's okay, plugged into an outlet, best to consult customer service.	Taobao Marketplace, a Chinese website for online shopping
Diyuan Electrical Appliances Flagship Store	King of Power Saving	Other people I do not know, anyway, I can save electricity, every month can bemore than100degreesless, I use more electricity, I bought two, oh together, the effect is very good, but also can make the air better, two birds with one stone	Taobao Marketplace, a Chinese website for online shopping
Diyuan Electrical Appliances Flagship Store	King of Power Saving	Saver received My wife compared it for a week said it does save Our house usually has a surprisingly large amount of electricity always feel like someone is stealing my appliances I don't know if it's strung together Used it mid-month to be able to pay less for electricity It's pretty good.	Taobao Marketplace, a Chinese website for online shopping
Diyuan Electrical Appliances Flagship Store	King of Power Saving	The effect is quite good, you can save. The seller promises no effect90days can be returned, buy also feel at ease	Taobao Marketplace, a Chinese website for online shopping
Lubao Boutique	a power saver (electronics)	Great material, good quality and arrived quickly!	JD.com
Lubao Boutique	a power saver (electronics)	Evaluate after you use it?	JD.com
Lubao Boutique	a power saver (electronics)	I'll comment when I use it to pay my electric bill	JD.com
Lubao Boutique	a power saver (electronics)	Just plugged it in for two days I don't know if it works or not The merchant said7days for a cycle After 7 days I'll follow up I hope it works[kudos]	JD.com
Lubao Boutique	a power saver (electronics)	Let's see how it goes.	JD.com
Lubao Boutique	a power saver (electronics)	can	JD.com
Lubao Boutique	a power saver (electronics)	received	JD.com
Lubao Boutique	a power saver (electronics)	. Received the item, logistics fast, worth waiting for the effect is good, can work, worthy of five-star praise! If the effect is good, or will buy back, I hope you, quality is guaranteed, we still believe that your products, can be as you say.	JD.com
Lubao Boutique	a power saver (electronics)	Likes and Likes and Likes and Likes and Likes and Likes and Likes and Likes and Likes and Likes and Likes and Likes and Likes and Likes.	JD.com
Lubao Boutique	a power saver (electronics)	Tiny, love it, good value. Hope the effect is also good. Merchant attitude is very good, logistics is also fast.	JD.com
Lubao Boutique	a power saver (electronics)	Order received, packaging is very good, logistics is very fast, the power saver received the goods on the use, do not know the power-saving effect, the first five-star praise, and so on with a period of time to see the effect of power saving in the additional evaluation!	JD.com
Lubao Boutique	a power saver (electronics)	I hope it saves power, try using it for a month first	JD.com
Lubao Boutique	a power saver (electronics)	I'll comment after using it.	JD.com
Lubao Boutique	a power saver (electronics)	Logistics is very fast, packaging is very good, the courier boy is very strong, two or three days to receive, really good.	JD.com
Lubao Boutique	a power saver (electronics)	First time buyer very small and not bulky hope it works	JD.com

Lubao Boutique	a power saver (electronics)	This user did not fill in a rating	JD.com
Lubao Boutique	a power saver (electronics)	This small power saving treasure plugged in a few days, power saving not yet know, the meter is very high can not see, very small, should have the effect of a month after the know la, small like does not take up	JD.com
Lubao Boutique	a power saver (electronics)	oh (interjection indicating that one has just learned sth)	JD.com
Lubao Boutique	a power saver (electronics)	Use it1month to know if it's good or bad.	JD.com
Lubao Boutique	a power saver (electronics)	This user feels the product is better	JD.com
Lubao Boutique	a power saver (electronics)	Affordable, fine workmanship, easy to use, compact design, the voltage is very stable, the product is beautiful, convenient and practical, good quality.	JD.com
Lubao Boutique	a power saver (electronics)	Saving appliances have been received, get home to use, saving appliances compact design, value is quite high, beautiful appearance, convenient and practical, I do not know how the effect of power saving, I hope there is an effect! First give merchants five-star praise it!	JD.com
Lubao Boutique	a power saver (electronics)	Genius invention! It's amazing.	JD.com
Lubao Boutique	a power saver (electronics)	This user did not fill in a rating	JD.com
Lubao Boutique	a power saver (electronics)	I'll see you after I use it to comment. It's just that the logistics is a bit slow	JD.com
Lubao Boutique	a power saver (electronics)	Received to use to see first good next time to return to know buy!	JD.com
Lubao Boutique	a power saver (electronics)	Trying it out now, not sure how it is yet?	JD.com
Lubao Boutique	a power saver (electronics)	Affordable, well made, compact design very much like it. The air conditioner can be used with	JD.com
Lubao Boutique	a power saver (electronics)	Small and refined, beautiful appearance, very satisfied with the purchase.	JD.com
Lubao Boutique	a power saver (electronics)	Just used it and don't know the effect	JD.com
Lubao Boutique	a power saver (electronics)	Value is quite high, looking forward to the results	JD.com
Lubao Boutique	a power saver (electronics)	I'm using it, I don't know the effect yet	JD.com
Lubao Boutique	a power saver (electronics)	Great quality, works well, will buy here from now on[big love]	JD.com
Lubao Boutique	a power saver (electronics)	Save electricity received, so like the color, good packaging, logistics is also very fast, customer service attitude.	JD.com
Lubao Boutique	a power saver (electronics)	Looking forward to the results	JD.com
Lubao Boutique	a power saver (electronics)	Logistics fast quality is good very satisfied with a shopping, beautiful design does not occupy space, power-saving small can do!	JD.com
Lubao Boutique	a power saver (electronics)	This user feels the product is better	JD.com
Lubao Boutique	a power saver (electronics)	Affordable, compact design, easy to use	JD.com
Lubao Boutique	a power saver (electronics)	Baby received, fast shipping logistics fast, received on the use of[love]plug-in, easy to use, recommended[cool]	JD.com
Lubao Boutique	a power saver (electronics)	Already using it. Hope it works.	JD.com
Lubao Boutique	a power saver (electronics)	New automatic economizer at the right price	JD.com
Lubao Boutique	a power saver (electronics)	an excellent	JD.com
Lubao Boutique	a power saver (electronics)	This user did not fill in a rating	JD.com
Lubao Boutique	a power saver (electronics)	Just received the goods, wait for the trial, if the effect is good, next time to buytwo	JD.com
Lubao Boutique	a power saver (electronics)	Received the goods, good quality pink is the color selected by the daughter, green light indicates energy-saving state, like the pro quick start Oh!	JD.com
Lubao Boutique	a power saver (electronics)	I'll comment after I've used it.	JD.com
Lubao Boutique	a power saver (electronics)	Miniature and compact design, I will comment after using it	JD.com
Lubao Boutique	a power saver (electronics)	Well made, affordable and great!	JD.com
Lubao Boutique	a power saver (electronics)	rare	JD.com
Lubao Boutique	a power saver (electronics)	Courier boy hard work, praise	JD.com
Lubao Boutique	a power saver (electronics)	Hahaha power-saving mode Use it and see Use it and follow up	JD.com
Lubao Boutique	a power saver (electronics)	It's good. Second time I've bought it.	JD.com
Lubao Boutique	a power saver (electronics)	The face is pretty good, so I hope the results are as good as that face!	JD.com
Lubao Boutique	a power saver (electronics)	Great product, guaranteed quality, recommended!	JD.com
Lubao Boutique	a power saver (electronics)	Looks premium, just got it natural can't tell the results yet	JD.com
Lubao Boutique	a power saver (electronics)	Things have been received, well-packed, good quality, seller service attitude is very good, cheap price, logistics is very fast, love it! Next time will buy back!	JD.com
Lubao Boutique	a power saver (electronics)	Thruster received, fine workmanship, looking good red and blue lights sometimes exchange Ming it is working!	JD.com
Lubao Boutique	a power saver (electronics)	It feels fine, next time another one	JD.com
Lubao Boutique	a power saver (electronics)	Received the goods do not know how effective?	JD.com
Lubao Boutique	a power saver (electronics)	Evaluate after a month.	JD.com
Lubao Boutique	a power saver (electronics)	Two or three times in a row.	JD.com
Lubao Boutique	a power saver (electronics)	Logistics is very fast, after using it to evaluate	JD.com
Lubao Boutique	a power saver (electronics)	The logistics is very fast packaging is good ah also very good-looking	JD.com
Lubao Boutique	a power saver (electronics)	Hello customer service, the goods received, good ah, thanks?	JD.com
Lubao Boutique	a power saver (electronics)	Power Saver is good planning to buy anotherone	JD.com
Lubao Boutique	a power saver (electronics)	Nice This saves appliances The last one I bought broke within a few days The merchant sent a replacement and it broke again This one is very good and convenient and the quality is also good Use it for a while and then review it again	JD.com
Lubao Boutique	a power saver (electronics)	Received it and used it, wait a few days to see if there is any effect	JD.com
Lubao Boutique	a power saver (electronics)	The goods have been received, use a period of time to see, in the evaluation.	JD.com
Lubao Boutique	a power saver (electronics)	It looks okay I don't know if it works or not	JD.com
Lubao Boutique	a power saver (electronics)	Purchased again, very good saver, saves so much electricity every month, it's amazing!	JD.com
Lubao Boutique	a power saver (electronics)	Good quality.	JD.com
Lubao Boutique	a power saver (electronics)	Saver received, small and beautiful, bought3, expect to subdue the speed of the meter, can it work? See the results[love] [love] [love] [love] [love]	JD.com
Lubao Boutique	a power saver (electronics)	I don't know how much power I can save, try using it	JD.com
Lubao Boutique	a power saver (electronics)	Received a year ago, used for a period of time, feel okay, the quality of the material is quite good, the face of the straight good, after a period of time to see the practicality of come back to the follow-up	JD.com
Lubao Boutique	a power saver (electronics)	Today the first seven three babies on the collection, the quality is good, beautiful, there are negative oxygen ions occurring, very good! Logistics is very fast! Praise praise!	JD.com
Lubao Boutique	a power saver (electronics)	Use the complaint time to reevaluate	JD.com
Lubao Boutique	a power saver (electronics)	Energy saver received, high quality and economical. Five points praise	JD.com
Lubao Boutique	a power saver (electronics)	It's nice, but you'll have to use it for a while to see if it works.	JD.com
Lubao Boutique	a power saver (electronics)	This user did not fill in a rating	JD.com
Lubao Boutique	a power saver (electronics)	I'm just starting to use it.	JD.com
Lubao Boutique	a power saver (electronics)	Okay👌	JD.com
Lubao Boutique	a power saver (electronics)	This energy-saving power king compact does not take up space, beautiful style and color is beautiful, I bought five this time, the family has air conditioning refrigerator, etc. with too much electricity, so I bought the king of power saving, started plugging in last night I hope to give me satisfied with the results, I believe that the businessman recommended this king of power saving is the old people's gospel.price, packaging is also very good, will recommend friends and family[Buy Buy Buy]	JD.com
Lubao Boutique	a power saver (electronics)	Goodreview~Goodreview~ [cool] [cool] [cool][cool	JD.com
Lubao Boutique	a power saver (electronics)	Good quality, worth buying	JD.com
Lubao Boutique	a power saver (electronics)	Things have been received, small and exquisite, can save electricity I do not know for the time being, a month after the follow-up evaluation	JD.com
Lubao Boutique	a power saver (electronics)	Just received it, looking forward to the results	JD.com
Lubao Boutique	a power saver (electronics)	I'm using it. I'm looking forward to the results.	JD.com
Lubao Boutique	a power saver (electronics)	pragmatic	JD.com

Lubao Boutique	a power saver (electronics)	Baby received, I bought before, but not this brand, my friend recommended this brand to me, should be the day wrong, small and exquisite, can save energy and electricity, this time it is for friends to buy.	JD.com
Lubao Boutique	a power saver (electronics)	The Power Saver is small, beautiful and effective, very happy with the purchase, stay tuned!	JD.com
Lubao Boutique	a power saver (electronics)	I have received the goods, fast shipping, with a good power-saving role, to be used in the future, and then add a follow-up review, the product is beautifully shaped This style is beautiful and colorful	JD.com
Lubao Boutique	a power saver (electronics)	This afternoon the king of power saving received the goods, a month to save a lot of power, good quality, very good, good customer service, packaging is very good, I give you!☆ ☆ ☆ ☆☆☆ ☆ stars praise	JD.com
Lubao Boutique	a power saver (electronics)	The goods have just arrived, use for a period of time to see, first give a fair evaluation of it	JD.com
Lubao Boutique	a power saver (electronics)	No noise, easy to use, good service attitude, beautiful, fine workmanship, affordable, good material, the voltage is very stable. Used a few days, I hope to have the effect	JD.com
Lubao Boutique	a power saver (electronics)	Already used, see the results continue to follow up	JD.com
Lubao Boutique	a power saver (electronics)	I use on this month can save electricity mode, I think this month can not will save how much electricity, and then see how much money can be saved💰electricity, I want to see if there is no effect anymore,[yay][envy][applaud][roses][roses]Electricity saver, save electric appliances received the goods, I see how much electricity to save electricity in a month, very good, good quality, fast logistics, customer service is very good, good packaging👌, good value, good attitude, I give a ☆☆ ★★★★★★☆	JD.com
Lubao Boutique	a power saver (electronics)	Power Saving King received, a month to save a lot of power, good quality, very good, fast logistics, customer service is very good, very cheap, good packaging. Good price and good attitude. Give ⑤ star	JD.com
Lubao Boutique	a power saver (electronics)	received	JD.com
Lubao Boutique	a power saver (electronics)	Good stuff[][][点赞][点赞]],packaging很好物流很快赞赞赞赞![Kudos][Kudos][Kudos]	JD.com
Lubao Boutique	a power saver (electronics)	Received, eye view is good, the effect of how to use to see the effect after the review.	JD.com
Lubao Boutique	a power saver (electronics)	I'll tell you when I've used it.	JD.com
Lubao Boutique	a power saver (electronics)	Received the goods, don't know the result yet	JD.com
Lubao Boutique	a power saver (electronics)	I don't know if it works, buy one and try	JD.com
Lubao Boutique	a power saver (electronics)	You can save65%energywith2of them, then you will see how much you can save, does it work.[yay][envy][applause][good][kudos][hugs][roses]	JD.com
Lubao Boutique	a power saver (electronics)	can	JD.com
Lubao Boutique	a power saver (electronics)	Logistics is very fast[smile]received the baby, hurry to try, I do not know if it is as they say really save electricity[smile]this month refrigerator air conditioning electricity let me just pay a hundred and five electricity bills, look at the results of the next month it is not the same as this month[grimace]look forward togood results[grimace]	JD.com
Lubao Boutique	a power saver (electronics)	[Big Love]	JD.com
Lubao Boutique	a power saver (electronics)	I'll follow up after I use it.	JD.com
Lubao Boutique	a power saver (electronics)	easy use	JD.com
Lubao Boutique	a power saver (electronics)	That was fast.	JD.com
Lubao Boutique	a power saver (electronics)	Express delivery is very fast, the saver received packaging intact. The method of use is simple and effective, it is worth everyone's trust to buy!	JD.com
Lubao Boutique	a power saver (electronics)	Easy to use, no noise, logistics is fast, the effect is not yet known.	JD.com
Lubao Boutique	a power saver (electronics)	It looks good, I'll follow up after I've used it!	JD.com
Lubao Boutique	a power saver (electronics)	I don't know. I'll use it first.	JD.com
Lubao Boutique	a power saver (electronics)	I hope it can save electricity, the weather is too hot, running the air conditioner uses a lot of electricity	JD.com
Lubao Boutique	a power saver (electronics)	It's okay It's good	JD.com
Lubao Boutique	a power saver (electronics)	Very good to use to save electrical appliances to use a good after the good to buy also recommend friends to buy also!	JD.com
Lubao Boutique	a power saver (electronics)	That's great. It does save electricity. It's nice!	JD.com
Lubao Boutique	a power saver (electronics)	No noise, easy to use, effective, fast logistics, good service attitude has been recommended to buy friends!	JD.com
Lubao Boutique	a power saver (electronics)	Bought ten units Used for20days Broken three (>д<) merchants replenished two over Said more can not afford to lose(ì_í.)	JD.com
Lubao Boutique	a power saver (electronics)	Logistics is very fast, have not used it yet, after a few days to comment on the quality and power saving issues!	JD.com
Lubao Boutique	a power saver (electronics)	Fast shipping Well packaged Genuine product Subtle and generous	JD.com
Lubao Boutique	a power saver (electronics)	The logistics is fast, the saver has arrived, I heard it will save electricity, I will start using it tonight, and I will come back in two months with a follow-up comment!	JD.com
Lubao Boutique	a power saver (electronics)	Energy saver received, with the seller said the same, convenient and practical, is genuine	JD.com
Lubao Boutique	a power saver (electronics)	Saving appliances arrived, to a week of use is quite good, customer service has a question and answer, good service attitude, logistics is very fast!	JD.com
Lubao Boutique	a power saver (electronics)	Easy to use, find a socket at home that is not used very much can be realized, black technology, genuine, good use will also buy back, praise!	JD.com
Lubao Boutique	a power saver (electronics)	No noise, easy to use compact size currently using two days in the test phase	JD.com
Lubao Boutique	a power saver (electronics)	Logistics is very fast, physical, easy to use, worth the experience of purchasing	JD.com
Lubao Boutique	a power saver (electronics)	Feel good, looking forward to the effect, genuine, wait for a period of time to see how much it can save, buy the second one	JD.com
Lubao Boutique	a power saver (electronics)	Saving appliances received, logistics is very fast, beautiful style, packaging is very good, I do not know how the effect, will always be watching, such as the effect of good will be back to buy.	JD.com
Lubao Boutique	a power saver (electronics)	Logistics is fast, the baby is good, the second time enough to buy useful[cool]	JD.com
Lubao Boutique	a power saver (electronics)	First time buyer, very small, not bulky, hope it works	JD.com
Lubao Boutique	a power saver (electronics)	I just started using it and I don't know if it works	JD.com
Lubao Boutique	a power saver (electronics)	Used to comment, courier service is very good, five-star praise!	JD.com
Lubao Boutique	a power saver (electronics)	No noise, easy to use, fast logistics, good packaging	JD.com
Lubao Boutique	a power saver (electronics)	No noise, easy to use, fast logistics. Just received the goods, the next second to plug it, the end of the month to see the effect. The design of the thing is very small, the first time to use this thing, I hope it works[smile]	JD.com
Lubao Boutique	a power saver (electronics)	Received used a few days, temporarily do not feel out, after a few days and then look, should be the effect is still good, like friends can order	JD.com
Lubao Boutique	a power saver (electronics)	The price is very beautiful, the stuff is still good and will continue to repurchase! All five points! Ness Ness ~ really like, completely exceeded expectations, shipping very fast, packaging very carefully, strict, fast shipping, very satisfied with a shopping feeling in kind better than the picture, the price and quality is proportional to the shopping is very satisfied, like the baby hurry up!	JD.com
Lubao Boutique	a power saver (electronics)	No noise, easy to use, fast logistics. Effective or not first use a month to see the effect, and then follow up	JD.com
Lubao Boutique	a power saver (electronics)	No noise, easy to use, feel high, depends on the later effect	JD.com
Lubao Boutique	a power saver (electronics)	Logistics is very fast, beautiful, affordable,, no noise, just received the goods today, first give a five-star praise, after a month and then see, if there is an effect, will follow up!	JD.com
Lubao Boutique	a power saver (electronics)	Baby received! Logistics is fast, the outer packaging is intact! Give positive feedback!	JD.com
Lubao Boutique	a power saver (electronics)	This is a great tool, finally the meter can be slowed down, no longer have to worry about electricity running fast!	JD.com
Lubao Boutique	a power saver (electronics)	Easy to use, effective, fast logistics, good attitude, good, genuine, beautiful	JD.com
Lubao Boutique	a power saver (electronics)	No noise, easy to use, fast logistics, beautiful, well packaged, good function, fine workmanship	JD.com
Lubao Boutique	a power saver (electronics)	It's authentic, inexpensive, fast delivery, and worth buying!	JD.com
Lubao Boutique	a power saver (electronics)	It is genuine! No noise, easy to use, effective, logistics is very fast, packaging is very good, good quality, good function, fine workmanship, affordable, good material, with convenient, the voltage is very stable	JD.com
Lubao Boutique	a power saver (electronics)	Very good quality, very energy efficient	JD.com
Lubao Boutique	a power saver (electronics)	No noise, logistics is fast, good use is genuine	JD.com
Lubao Boutique	a power saver (electronics)	Genuine, no noise, easy to use, effective, effective, logistics is fast, good attitude, good, beautiful, genuine, packaging is very good, good quality, good features	JD.com

Lubao Boutique	a power saver (electronics)	[big love] [big love] [big[love] [biglove]	JD.com
Lubao Boutique	a power saver (electronics)	Just received it today and hopefully it will save on electricity. Looking forward to it.	JD.com
Lubao Boutique	a power saver (electronics)	Packaging is very good no damage, this is a friend recommended to me! Said especially good use! I just used it and look forward to next month's results! Thank you for the store to launch such a good and save money! Great praise A	JD.com
Lubao Boutique	a power saver (electronics)	No noise, easy to use, beautiful. Use it for a while to see	JD.com
Lubao Boutique	a power saver (electronics)	Works great Saves a lot of electricity Oh	JD.com
Lubao Boutique	a power saver (electronics)	I've been using it since I got home and I'm looking forward to being useful	JD.com
Lubao Boutique	a power saver (electronics)	No noise,, easy to use, effective, fast logistics, good attitude, good use!	JD.com
Lubao Boutique	a power saver (electronics)	No noise, easy to use, effective, fast logistics	JD.com
Lubao Boutique	a power saver (electronics)	The goods have been received looks quite good small money exquisite	JD.com
Lubao Boutique	a power saver (electronics)	Whether it is power saving to be evaluated after using it for a while.	JD.com
Lubao Boutique	a power saver (electronics)	The baby received this automatic power saver power saving king power saving treasure god device intelligent regulator household power saving housekeeper, is very power saving,,, easy to use, logistics	JD.com
Lubao Boutique	a power saver (electronics)	This saver is compact and has a good value and saves power, love it, it's genuine.	JD.com
Lubao Boutique	a power saver (electronics)	Good packaging, fast delivery, small size, no noise, my friend recommended that I buy, she said she used for a period of time is very effective. Used a few days to evaluate, the effect is still quite obvious, inexpensive, worth buying!	JD.com
Lubao Boutique	a power saver (electronics)	Goods have been received, no noise, easy to use, expect good results	JD.com
Lubao Boutique	a power saver (electronics)	Goods received and so on after the use of good in the repurchase logistics in a timely manner, packaging is very good to use good to buy again.	JD.com
Lubao Boutique	a power saver (electronics)	No noise, fast logistics, genuine, well packaged	JD.com
Lubao Boutique	a power saver (electronics)	Let's try it and see how it works before we comment	JD.com
Lubao Boutique	a power saver (electronics)	Baby is very good, logistics is very fast, the effect of the use of after evaluation	JD.com
Lubao Boutique	a power saver (electronics)	I'll evaluate it after I use it.	JD.com
Lubao Boutique	a power saver (electronics)	The product has been received and is well made. Looking forward to using it!	JD.com
Lubao Boutique	a power saver (electronics)	Fine workmanship, affordable price, easy to use, no noise	JD.com
Lubao Boutique	a power saver (electronics)	I pressed it on, put it in the place that uses the most electricity, let's see how it is after two months, it's cheap!	JD.com
Lubao Boutique	a power saver (electronics)	Things received, logistics is very fast, customer service is very enthusiastic, used really good.	JD.com
Lubao Boutique	a power saver (electronics)	Phone okayFNFNCPLA weight loss weight loss on the home all those yearsCNCN CNCNKeen whisked Nishang you eat your sister registration um from where from where to eat chicken chicken pumping card airplane northern huachuang add the wrong skills to eat eat from where where Chen Nana put where northern huachuang stimulate stimulate stimulate you	JD.com
Lubao Boutique	a power saver (electronics)	I bought it for the second time and I feel good about it.	JD.com
Lubao Boutique	a power saver (electronics)	I plugged it in when I got back and I'm looking forward to the results.	JD.com
Lubao Boutique	a power saver (electronics)	It's very mini not temporary space, no noise.	JD.com
Lubao Boutique	a power saver (electronics)	The goods have been received compact and already used.looking forward to next month's results[snicker]	JD.com
Lubao Boutique	a power saver (electronics)	Easy to use, fast logistics, beautiful, well packaged, fine workmanship, affordable price	JD.com
Lubao Boutique	a power saver (electronics)	No sound,, easy to use, beautiful, very well packaged, good quality	JD.com
Lubao Boutique	a power saver (electronics)	This user feels the product is better	JD.com
Lubao Boutique	a power saver (electronics)	Received the goods, can not wait to use it, feel good, look forward to my review to see the effect of Yo!	JD.com
Lubao Boutique	a power saver (electronics)	Things received, immediately try it, do not know can see the power saving effect, use a period of time and then follow up!	JD.com
Lubao Boutique	a power saver (electronics)	The product can be good need to continue to buy again in the future	JD.com
Lubao Boutique	a power saver (electronics)	No noise, easy to use, effective is something I like an hope to be able to use the results, logistics is very fast	JD.com
Lubao Boutique	a power saver (electronics)	Power Saver was received easy to use and plugged in right away. Some small questions asked customer service, customer service attitude is very good. I hope to customer service said so good to use, and then after a period of time to comment, now give positive feedback, want to buy pro hurry to order it!	JD.com
Lubao Boutique	a power saver (electronics)	No noise, easy to use, logistics is fast, five stars!	JD.com
Lubao Boutique	a power saver (electronics)	No noise, easy to use, fast logistics, beautiful, well packaged	JD.com
Lubao Boutique	a power saver (electronics)	Intelligent power saving king received,,, easy to use, beautiful, genuine, well packaged, fine workmanship, no noise, electrical appliances are very good, give good comments.	JD.com
Lubao Boutique	a power saver (electronics)	Fast logistics, effective, easy to use, good attitude, good quality, affordable, good material, no noise	JD.com
Lubao Boutique	a power saver (electronics)	Easy to use, effective, beautiful, well packaged, well made, affordable	JD.com
Lubao Boutique	a power saver (electronics)	Energy saving appliance received, looks as described in the order, easy to use.	JD.com
Lubao Boutique	a power saver (electronics)	Let's see if there's any effect, then we'll add more	JD.com
Lubao Boutique	a power saver (electronics)	It works.	JD.com
Lubao Boutique	a power saver (electronics)	Easy to use, effective, fast logistics, good attitude, good, beautiful, genuine, well packaged	JD.com
Lubao Boutique	a power saver (electronics)	I don't know if it works.	JD.com
Lubao Boutique	a power saver (electronics)	Easy to use, beautiful, well packaged, very stable voltage	JD.com
Lubao Boutique	a power saver (electronics)	Easy to use, effective, fast logistics, good packaging, good quality, fine workmanship five stars!	JD.com
Lubao Boutique	a power saver (electronics)	Just plugged it in Feels pretty good The new model is just beautiful! Easy to use effect waiting for later	JD.com
Lubao Boutique	a power saver (electronics)	Easy to use, good attitude, fast logistics, beautiful, genuine, affordable, fine workmanship, the voltage is very stable, and so on after the use of the use of the results of the use of the flat	JD.com
Lubao Boutique	a power saver (electronics)	Saver received, logistics is very fast, packaging is very good, fine workmanship, affordable, no noise, additional comments after use.	JD.com
Lubao Boutique	a power saver (electronics)	Easy to use, great attitude, very well packaged	JD.com
Lubao Boutique	a power saver (electronics)	The appearance is super nice. If you want to take a shot, take a shot. Don't hesitate.	JD.com
Lubao Boutique	a power saver (electronics)	Logistics is fast, easy to use, beautiful, well packaged, beautiful, well made, no noise.	JD.com
Lubao Boutique	a power saver (electronics)	Effects need to be observed before commenting	JD.com
Lubao Boutique	a power saver (electronics)	The saver is plugged in, easy to use, looking forward to good results	JD.com
Lubao Boutique	a power saver (electronics)	Easy to use, fast logistics, good attitude	JD.com
Lubao Boutique	a power saver (electronics)	Easy to use, fast logistics, beautiful, well-packaged not yet used a while to the home to use	JD.com
Lubao Boutique	a power saver (electronics)	Easy to use, effective, fast logistics, good attitude, good use, good attitude, good packaging, good quality, affordable, no noise	JD.com
Lubao Boutique	a power saver (electronics)	The product received, now used, logistics is very fast, I wish you a prosperous business prosperity!	JD.com
Lubao Boutique	a power saver (electronics)	Easy to use, effective, fast logistics, good attitude	JD.com
Lubao Boutique	a power saver (electronics)	Logistics is very fast, good attitude, the goods have received, try to have the effect and then make a follow-up comment.	JD.com
Lubao Boutique	a power saver (electronics)	Easy to use, effective, fast logistics, good attitude	JD.com
Lubao Boutique	a power saver (electronics)	Logistics is very fast, received and used, the effect of how to need to look forward to.	JD.com
Lubao Boutique	a power saver (electronics)	The product received, small and exquisite, that is, plug to try. Expect as recommended by the merchant, powerful function, have obvious power-saving effect, thank you!	JD.com
Lubao Boutique	a power saver (electronics)	Easy to use,, fast logistics,, good attitude, trustworthy and purchase!	JD.com
Lubao Boutique	a power saver (electronics)	Automatic energy saver received, logistics fast, small, beautiful, easy to use, looking forward to the effect	JD.com
Lubao Boutique	a power saver (electronics)	The goods have been received, quite exquisite, expect good results	JD.com
Lubao Boutique	a power saver (electronics)	The product works well, have experimented with it, installed in place, the power saving effect will be obvious, trustworthy product!	JD.com
Lubao Boutique	a power saver (electronics)	This energy-saving electricity is also my first time to buy, friends they buy it is okay. Support the new technology products	JD.com
Lubao Boutique	a power saver (electronics)	Good quality, good use than the entity store cheaper, very satisfied	JD.com
Lubao Boutique	a power saver (electronics)	ve been using it for a while to follow up, it's a power saver than before, about17%or so	JD.com

Lubao Boutique	a power saver (electronics)	Baby is really good, I'm helping someone else to buy one, the last one I bought used really good, really save electricity, thanks to the manufacturer.	JD.com
Lubao Boutique	a power saver (electronics)	Hello, very very good, can next time in the purchase of a, beautiful, satisfied, I just want to connect to save money on electricity, the	JD.com
Lubao Boutique	a power saver (electronics)	works well , and the delivery is fast , If you want to buy it, hurry up and buy it	JD.com
Lubao Boutique	a power saver (electronics)	Logistics is very fast, the appearance is cool, the description is written in detail, it is the original genuine product, a one-time purchase of4units	JD.com
Lubao Boutique	a power saver (electronics)	This works very well, and it does save power, after using it.	JD.com
Lubao Boutique	a power saver (electronics)	Goods received, inexpensive, has been used, do not know can save electricity it	JD.com
Lubao Boutique	a power saver (electronics)	Very good baby will buy again!	JD.com
Lubao Boutique	a power saver (electronics)	It's very affordable, good value for money, kind of an affordable goodie, and hopefully saves electricity	JD.com
Lubao Boutique	a power saver (electronics)	Baby received very beautiful good value for money boss service attitude is very good like people quickly order ah	JD.com
Lubao Boutique	a power saver (electronics)	The product received, the quality is okay quickly came to buy.	JD.com
Lubao Boutique	a power saver (electronics)	Baby to arrive, looks very good, wait for the use of good in the follow-up comment.	JD.com
Lubao Boutique	a power saver (electronics)	The appearance is beautiful and delicate. Plugged in and ready to use. Will make a follow-up comment after verifying the effect.	JD.com
Lubao Boutique	a power saver (electronics)	Genuine really good, I bought three, just started to use when you can not feel, I use two or three months after the comparison, really good, everyone is worth enough to buy!	JD.com
Lubao Boutique	a power saver (electronics)	Received the stuff, have used it, later to see how the effect is	JD.com
Lubao Boutique	a power saver (electronics)	Saving electricity is very effective, save money on your monthly electricity bill, recommended to buy.	JD.com
Lubao Boutique	a power saver (electronics)	Product energy saving effect is obvious, recommended to buy.	JD.com
Lubao Boutique	a power saver (electronics)	Good quality, superb service, received it the next day!	JD.com
Lubao Boutique	a power saver (electronics)	Appearance and workmanship are very good, power saving effect to be observed.	JD.com
Lubao Boutique	a power saver (electronics)	Baby received, do not know if it is good, if good in the repurchase a	JD.com
Lubao Boutique	a power saver (electronics)	Intelligent power saving use for a period of time to have the effect to evaluate again	JD.com
Lubao Boutique	a power saver (electronics)	Received the goods immediately used, small size, does not take up space, hope to save electricity.	JD.com
Lubao Boutique	a power saver (electronics)	Workmanship is quite good, there is no odor, the effect can also be	JD.com
Lubao Boutique	a power saver (electronics)	The baby is very good shape beautiful just used to feel more comfortable in the heart of the business service is very good is genuine next month and then chase the product a very satisfactory cooperation to make some publicity so that we all save electricity packaging good logistics fast satisfied!	JD.com
Lubao Boutique	a power saver (electronics)	This power saving device can really save electricity it, used more than half a month to evaluate, really save money is very good will continue to use, recommended to friends, great!	JD.com
Lubao Boutique	a power saver (electronics)	It's convenient and really saves electricity.	JD.com
Lubao Boutique	a power saver (electronics)	Baby received, bought two at a time because the home power consumption, every day to save a little, day after day can be a lot of Oh!	JD.com
Lubao Boutique	a power saver (electronics)	Good workmanship, introduced by a neighbor, with detailed instructions. Plugging into any outlet in the house is fine	JD.com
Lubao Boutique	a power saver (electronics)	Look at the overall review is quite good, with the attitude of buying to try to buy, just used a few days, the feeling is good, and then use a month to comment on the specific effect	JD.com
Lubao Boutique	a power saver (electronics)	Home appliances are not much, but the power consumption is large, friends said to buy this power saving king works, easy to operate, plug and play, I hope to save some!	JD.com
Lubao Boutique	a power saver (electronics)	I didn't know there was such a thing as saving electricity A friend introduced it to me and it really works	JD.com
Lubao Boutique	a power saver (electronics)	Recommended by a circle of friends that it's good and power saving	JD.com
Lubao Boutique	a power saver (electronics)	My big brother bought it and said it's good to use it	JD.com
Lubao Boutique	a power saver (electronics)	Very happy with the shopping experience and fast shipping	JD.com
Lubao Boutique	a power saver (electronics)	Energy saver is good quality, high power, power saving, cost-effective	JD.com
Lubao Boutique	a power saver (electronics)	Home every month the electricity bill is particularly high, bought this miracle hope that the next month less, a month later to follow up evaluation	JD.com
Lubao Boutique	a power saver (electronics)	The results are noticeable and the electric bill has dropped significantly, love it so much.	JD.com
Lubao Boutique	a power saver (electronics)	I'll use it for a while and then comment Looks good haha	JD.com
Lubao Boutique	a power saver (electronics)	Bought several of these and they really save on electricity.	JD.com
Lubao Boutique	a power saver (electronics)	I used it, it really works well, if you need it, don't worry to buy it!	JD.com
Lubao Boutique	a power saver (electronics)	I received the stuff and the quality is good. The service of the courier is also good	JD.com
Lubao Boutique	a power saver (electronics)	Packaging carefully, very good, express box packaging, there is a layer of foam paper and a plastic bag, and the product's own box, the effect is not known for the time being, but look at the packaging is very good, there is a certificate of conformity, you can rest assured that the use of instructions are also very detailed, and intend to buy a few, a little bit not enough to use,800degrees of the proposed purchase of4, will be back to buy, can be used, no problems	JD.com
Lubao Boutique	a power saver (electronics)	The saver is very nice, good quality, good restoration with good use. Great! A month can save a lot of money, summer and winter is mainly the air conditioning is too expensive, with a power saver can rest	JD.com
Lubao Boutique	a power saver (electronics)	Cool days every day to open the heater is too costly, there is an energy-saving appliances beauty, power saving, cost-effective price, it is worth getting!	JD.com
Lubao Boutique	a power saver (electronics)	Effective and power-saving, as described, worth buying	JD.com
Lubao Boutique	a power saver (electronics)	Baby is really nice, looks good, got it back and used it, the	JD.com
Lubao Boutique	a power saver (electronics)	It has been installed and used, looks well made, hope it works.	JD.com
Lubao Boutique	a power saver (electronics)	Yesterday just received, tried last night, do not know if the effect is good, first try a few days, then see how the effect is	JD.com
Lubao Boutique	a power saver (electronics)	Baby received, to the home on the use, bought two, upstairs and downstairs each one.Neighbors have a said energy-saving.I hope to have the effect.Appearance of small and lovely.2after the best. Good reviews.	JD.com
Lubao Boutique	a power saver (electronics)	The baby was received and shipped quickly. Look also very delicate. Due to the large amount of electricity at a time to shoot two, save electricity with the use of the later to follow up!	JD.com
Lubao Boutique	a power saver (electronics)	Received the saver Looks great Waiting to use it for a month See how it works and then follow up	JD.com
Lubao Boutique	a power saver (electronics)	I received it and started using it right away to see how it works and buy another one my husband said.	JD.com
Lubao Boutique	a power saver (electronics)	Very good. Use it and see.	JD.com
Lubao Boutique	a power saver (electronics)	It's okay. It's good.	JD.com
Lubao Boutique	a power saver (electronics)	Have been trying to find a power saving king, the baby is very like or use, a plug to use, customer service attitude is very good, patiently explain the use, very straight to buy!	JD.com
Lubao Boutique	a power saver (electronics)	Works great, kudos for the quality for the money!	JD.com
Lubao Boutique	a power saver (electronics)	Overall evaluation is not bad, the quality can be. Effective function of this need time! Cost-effective mainly depends on the effect. use of experience is quite convenient drop appearance style square more regular workmanship material is quite strong drop, should be more durable! I hope it is useful, if it is useful, it will be sold again, not even if it is not used!	JD.com
Lubao Boutique	a power saver (electronics)	No damage to the packaging, fast logistics, fast delivery, good!	JD.com
Lubao Boutique	a power saver (electronics)	Easy to operate and clear instructions, expect power saving times!	JD.com
Lubao Boutique	a power saver (electronics)	Just received the goods, just started to try, feel still good, in the actual use and then continue to evaluate	JD.com
Lubao Boutique	a power saver (electronics)	Logistics is very fast, well-packed, received the goods good baby, immediately used on, looking forward to power-saving effect.	JD.com
Lubao Boutique	a power saver (electronics)	The first time I bought a power saver, I just started using it and it looks good, the power saving effect should help a lot, next time I will continue to buy it.	JD.com
Lubao Boutique	a power saver (electronics)	The quality is very good, beautiful, energy-saving and environmental protection, and the effect is good.	JD.com
Lubao Boutique	a power saver (electronics)	The quality of workmanship material is good, quite over the top, bright from the outside, from the introduction is the best choice for power saving at the moment! So bought two and hope to get effective	JD.com
Lubao Boutique	a power saver (electronics)	I've used something similar in the past at spell so and so, and it does have an effect on saving electricity, just that kind is too simple and not very safe. This time I bought this store, also for the safety and intuitive, digital display. Plugged in the computer host side, a very good feeling.	JD.com

Lubao Boutique	a power saver (electronics)	Easy to use and beautiful to look at!	JD.com
Lubao Boutique	a power saver (electronics)	Overall evaluation of the power saver is small and exquisite, beautiful appearance, carrying installation and use are very convenient, fine workmanship, there is indeed a power-saving effect, and can purify the air, I have bought three, this is to help a friend to buy, give five points.	JD.com
Lubao Boutique	a power saver (electronics)	Looking forward to saving electricity	JD.com
Lubao Boutique	a power saver (electronics)	Power Saving King this product is very practical, I first bought a, after a few days I bought three more.(National patented products, please rest assured that the purchase) do not seek to save electricity, but only to stabilize electricity, electricity consumption, install a few or peace of mind.	JD.com
Lubao Boutique	a power saver (electronics)	The product is practical, very satisfied, the price is not expensive, good but also practical, power saving and energy saving, very practical and satisfied with the shopping	JD.com
Lubao Boutique	a power saver (electronics)	The saver just arrived and tried it right away it should work great!	JD.com
Lubao Boutique	a power saver (electronics)	I see is fly long good really save electricity is genuine have want to buy see the advice of everyone to buy boss is very good!	JD.com
Lubao Boutique	a power saver (electronics)	Goods have been received, the goods are real, inexpensive and beautiful. Immediately inserted into the power supply, to wait a few days to observe the effect.	JD.com
Lubao Boutique	a power saver (electronics)	Overall good evaluation The effect function is very good Cost-effective	JD.com
Lubao Boutique	a power saver (electronics)	Received the power saver, the quality is excellent!	JD.com
Lubao Boutique	a power saver (electronics)	I received the item and started using it right away. Last month's electricity bill is very much this time with a month effect to try. I look forward to using it to save a little bit haha!	JD.com
Lubao Boutique	a power saver (electronics)	I've installed the power saver and will see if I can get a meter reading next month before I post a review!	JD.com
Lubao Boutique	a power saver (electronics)	A friend recommended it and said it would save a couple dozen bucks a month on the electric bill. So buy it to try. Looked at this thing is still pretty good.	JD.com
Lubao Boutique	a power saver (electronics)	Looks good, good quality, worth buying	JD.com
Lubao Boutique	a power saver (electronics)	Looks good , great quality and worth the money.	JD.com
Lubao Boutique	a power saver (electronics)	This product is not bad, second purchase	JD.com
Lubao Boutique	a power saver (electronics)	Overall evaluation received the goods, the first time to unpacking, feel the quality is still relatively good, and the description of the seller is still the same, very satisfied with the shipment speed is relatively fast, the logistics company has a very good attitude, the delivery speed is very fast, in general this time it is a very satisfied with the shopping, the	JD.com
Lubao Boutique	a power saver (electronics)	Effective, will come back later, wish the seller a prosperous business, wish the seller a big sale!	JD.com
Lubao Boutique	a power saver (electronics)	It should be okay to use it!	JD.com
Lubao Boutique	a power saver (electronics)	The goods arrived, still feel good, quite interesting, the first time to buy. Try to try, not bad	JD.com
Lubao Boutique	a power saver (electronics)	Baby received, it's beautiful and looks great. Looking forward to saving power.	JD.com
Lubao Boutique	a power saver (electronics)	Goods have been received, just plugged in and plugged in to obviously see the	JD.com
Lubao Boutique	a power saver (electronics)	It works great, it does save a lot of power, so if you need it, you can get it.	JD.com
Lubao Boutique	a power saver (electronics)	Great, great seller, great business, first time using it in decades.	JD.com
Lubao Boutique	a power saver (electronics)	The product has been received is using, like the merchant said, very good, next time to buy two more!	JD.com
Lubao Boutique	a power saver (electronics)	Good review, the product is well organized and has been used, I hope there is a good power saving effect	JD.com
Lubao Boutique	a power saver (electronics)	Good, logistics is fast, the seller's attitude is very good Oh!	JD.com
Lubao Boutique	a power saver (electronics)	I bought it and used it, it should be good, next we'll see what the electricity bill is this month	JD.com
Lubao Boutique	a power saver (electronics)	Received the goods looks good ready to start using look forward to saving power Oh!	JD.com
Lubao Boutique	a power saver (electronics)	Logistics is very fast, to the home to use a period of time, power saving does have the effect, and easy to use, small and exquisite, inexpensive and beautiful.	JD.com
Lubao Boutique	a power saver (electronics)	The voltage is very stable and effective.	JD.com
Lubao Boutique	a power saver (electronics)	Got the item, have bought several, one for both homes and one for the store, works great.	JD.com
Lubao Boutique	a power saver (electronics)	Power Saving Specialist has been received and is already being used, hopefully this month's electricity bill will go down	JD.com
Lubao Boutique	a power saver (electronics)	Nice saver, this is3rdpurchase, trustworthy product.	JD.com
Lubao Boutique	a power saver (electronics)	Electricity is less since I purchased this! Not bad not bad! Worth buying!	JD.com
Lubao Boutique	a power saver (electronics)	Baby is great, it's beautiful, reduces power consumption and doesn't take up much space	JD.com
Lubao Boutique	a power saver (electronics)	It's really a power saver, Ollie. If you like it, you can buy it.	JD.com
Lubao Boutique	a power saver (electronics)	Baby received, have had it for a while. It does save electricity, friends can buy with confidence.	JD.com
Lubao Boutique	a power saver (electronics)	Just received the item, used it as instructed and it looks just like the instructions. January after the follow-up evaluation.	JD.com
Lubao Boutique	a power saver (electronics)	This power-saving product is really good, have already taken three pieces of friends can feel free to buy.	JD.com
Lubao Boutique	a power saver (electronics)	I bought one for a classmate who used it and kept praising the Smart Saver as very good. She also said that she asked me to buy one for his two sisters.	JD.com
Lubao Boutique	a power saver (electronics)	I'm using it now, it's not expensive, I'll come back to review if it works!	JD.com
Lubao Boutique	a power saver (electronics)	This power saving expert is just great, if you like it, go ahead and order it, use it as soon as possible	JD.com
Lubao Boutique	a power saver (electronics)	I just opened it to see if I could save electricity for the following month! Wait and see! Look at the good effects of power saving. I'll buy it next time!	JD.com
Lubao Boutique	a power saver (electronics)	Things just received, the quality is okay, just see how the effect, energy-saving electricity can line it!	JD.com
Lubao Boutique	a power saver (electronics)	Expect the baby to last a long time in use, and anything that saves power is a good baby!	JD.com
Lubao Boutique	a power saver (electronics)	Baby arrived super fast, unpacked it and installed it immediately to see how it works.	JD.com
Lubao Boutique	a power saver (electronics)	I just started using it, I don't know if it's energy efficient or not	JD.com
Lubao Boutique	a power saver (electronics)	After the last few days, it's still working a bit.	JD.com
Lubao Boutique	a power saver (electronics)	I just received it and plugged it in, the merchant shipped it quickly, it should work well, thanks for the good intentions, thanks!	JD.com
Lubao Boutique	a power saver (electronics)	Goods have been received, logistics fast genuine goods, good use.	JD.com
Lubao Boutique	a power saver (electronics)	The logistics is fast, it really saves electricity, and I will patronize it again next time. Thank you boss!	JD.com
Lubao Boutique	a power saver (electronics)	Baby received, use to try, have effect continue to buy another one.	JD.com
Lubao Boutique	a power saver (electronics)	Use it as soon as you receive it it works well it's quick and easy and saves you money Recommended purchase	JD.com
Lubao Boutique	a power saver (electronics)	很好的^_^^_^效果杠杠的，买回家就试了一下^_^	JD.com
Lubao Boutique	a power saver (electronics)	Baby has been received, used, and is very satisfied! Place your order if you need it!	JD.com
Lubao Boutique	a power saver (electronics)	Baby has received looks good quality, now start to use if you can save electricity home to buy, then patronize the store. Service is good courier is also very good.	JD.com
Lubao Boutique	a power saver (electronics)	Good and beautiful, small size does not take up space, but also useful and very recommended to buy!	JD.com
Lubao Boutique	a power saver (electronics)	Product received, have bought similar energy saver before, this is very good oh!	JD.com
Lubao Boutique	a power saver (electronics)	Received the goods today, see a lot of comments above this product is good so buy a try, try to recommend a good friend also to buy!	JD.com
Lubao Boutique	a power saver (electronics)	Power saving officer is really good, I recommended this power saver to my classmates, my classmates have been used to praise the good, I helped my neighbor's sister to buy one, I myself also use, the node is really good, in the process of using both to protect the electrical appliances and to reduce the cost of living is trustworthy. After-sales service attitude is also very good, give them some praise.	JD.com
Lubao Boutique	a power saver (electronics)	I've been using it for a month now and I'm glad to hear it's working.	JD.com
Lubao Boutique	a power saver (electronics)	is said to be good to buy a try, it is very convenient to plug in the power on the line How much can be saved, use a period of time to report again	JD.com
Lubao Boutique	a power saver (electronics)	Feel pretty good, too much electricity at home, get one to try, next month good to continue to buy back, praise praise praise!	JD.com
Lubao Boutique	a power saver (electronics)	Very compact and lightweight stuff, just used it, will follow up after using it to see if it has any effect	JD.com
Lubao Boutique	a power saver (electronics)	The stuff works well, will introduce to the shower stuff works well, will introduce to the neighbors to buy in the future	JD.com
Lubao Boutique	a power saver (electronics)	Things are very received, the quality is very good, the price is also very favorable after the use of good, if you will continue to buy back	JD.com

Lubao Boutique	a power saver (electronics)	Just used it, looks good, will review later	JD.com
Lubao Boutique	a power saver (electronics)	Your goods are really good beauty off and good to use and science and save electricity cost and convenient and real and super save electricity king's ah really electricity king ah!	JD.com
Lubao Boutique	a power saver (electronics)	The quality feels good the effect is looking forward to, certainly not bad	JD.com
Lubao Boutique	a power saver (electronics)	Intelligent power saver is small in size and beautifully made,easy to use and simple to use,there are buy and gift,very afford,good value for money. I would recommend that you buy it.principle can save electricity	JD.com
Lubao Boutique	a power saver (electronics)	Back to try, against the meter, looked at the original did run a little slower than the original, continue to use to see!	JD.com
Lubao Boutique	a power saver (electronics)	I heard that this thing can save a lot of money on electricity so I bought it to try it out hopefully it will be as good as I think it will be thanks to the merchant. Looking forward to good results will comment again when it's good. Just used it.	JD.com
Lubao Boutique	a power saver (electronics)	This is a pretty good deal on this saver Used to buy them forover200 Bought two this time One upstairs One downstairs	JD.com
Lubao Boutique	a power saver (electronics)	The power saver has been received, looks good, easy to use, long term use, good reviews	JD.com
Lubao Boutique	a power saver (electronics)	Received a few days, look at the quality is quite good, the texture is very good, must come to a good comment!	JD.com
Lubao Boutique	a power saver (electronics)	Intelligent power saver, the king of power saving, easy to use, home electricity helper, too powerful!	JD.com
Lubao Boutique	a power saver (electronics)	My best friend said that she savedmore than20kilowattsa month onelectric billwith this daily saver at her houseI also bought several this time.	JD.com
Lubao Boutique	a power saver (electronics)	Previously bought and used very well, it does play an energy-saving and power-saving effect, electrical appliances are more again back to buy a, recommendable.	JD.com
Lubao Boutique	a power saver (electronics)	Will recommend friends to buy,good-looking,have the effect,back on the installation on, used a few days,anyway,the meter goes slow, the end of the month to see the effect,price cheap no problem	JD.com
Lubao Boutique	a power saver (electronics)	It can be a real power saver, my house uses a lot of electricity and it can save my houseabout 30+ electricitya month.	JD.com
Lubao Boutique	a power saver (electronics)	Friends of friends introduced to, I heard that it is very good, a year down can save a lot of electricity, you can buy a few more back to use!	JD.com
Lubao Boutique	a power saver (electronics)	Shipping was fast, already using it looking forward to some power savings.	JD.com
Lubao Boutique	a power saver (electronics)	Intelligent power saver is recommended by a friend to buy, said the family used quite good, two months did not save money, I bought this, looking forward to the effect of the	JD.com
Lubao Boutique	a power saver (electronics)	Friends of the family to use the first half of the year, said the effect before recommending me to buy, every day to use the same power consumption, last month there next month electricity bills really have to reduce, so I purchased. Thank you for recommending the good stuff, and can save a lot.	JD.com
Lubao Boutique	a power saver (electronics)	Every time you pay your electric bill, you realize that electricity is so expensive. This one is used as soon as I bought it, quick to help save electricity	JD.com
Lubao Boutique	a power saver (electronics)	Intelligent power saver fine workmanship quality is very good, the use of simple and convenient casually inserted in the home socket can be, looking forward to next month's electricity bill has been reduced,	JD.com
Lubao Boutique	a power saver (electronics)	Baby is still very good, can stabilize the voltage, with electricity can improve a little, need to plug in first two months the effect will be better!	JD.com
Lubao Boutique	a power saver (electronics)	Intelligent power saver received a few days, has begun to use, look forward to the effect, this intelligent power saver and put the authenticity of the two-dimensional code query, checked is genuine!	JD.com
Lubao Boutique	a power saver (electronics)	Received, very convenient does not take up space, quite like, saving more ready to buy a	JD.com
Lubao Boutique	a power saver (electronics)	Used this brand in the previous two years, quite good and effective, this time with my store bought3	JD.com
Lubao Boutique	a power saver (electronics)	Energy saver, inexpensive, nice and simple to install,nice'hope the home saves electricity	JD.com
Lubao Boutique	a power saver (electronics)	Feel pretty good, the family is now using electricity every month is really much, listening to friends say that this can save electricity, buy to try.	JD.com
Lubao Boutique	a power saver (electronics)	Intelligent power saver packaging is tightly received without damage, often use induction cooker cooking is very power consumption, friends recommended this power saver is very good.	JD.com
Lubao Boutique	a power saver (electronics)	I received it, I'm using it, I don't know the effect yet.	JD.com
Lubao Boutique	a power saver (electronics)	Received, very good, very like the compact does not take up space plug and play, it is worth buying!	JD.com
Lubao Boutique	a power saver (electronics)	It arrived super fast and the customer service was super nice and customer service was very patient! I can't believe how patiently I listened to my questions and look forward to the results.	JD.com
Lubao Boutique	a power saver (electronics)	Friends of the family to use the first half of the year, said there is an effect before recommending that I buy, every day to use the same power consumption, last month there next month the electricity bill is really reduced, so I also purchased. Thank you for recommending the good stuff, and can save a lot.	JD.com
Lubao Boutique	a power saver (electronics)	After plugging in the home voltage fluctuation is not as big as before, with in other stores to buy back the saver test box test, after plugging the saver up, the current is obviously less, great! It's a	JD.com
Lubao Boutique	a power saver (electronics)	Received two intelligent power saver,packaging is very good,inexpensive small East East,said as long as inserted into any one of the terminal board.On the same,I just, inserted in the desktop, after a period of time to know the effect,home appliances more than	JD.com
Lubao Boutique	a power saver (electronics)	Arrived quickly good quality has been in constant use	JD.com
Lubao Boutique	a power saver (electronics)	We bought one for our family and it works fine, we bought one for each of our relatives and it saves electricity	JD.com
Lubao Boutique	a power saver (electronics)	Really good, listen to the recommendation of friends over to buy, hope that in the future can save electricity and money very satisfied.	JD.com
Lubao Boutique	a power saver (electronics)	Received the baby immediately plugged in, simple operation, and very practical, worth having!	JD.com
Lubao Boutique	a power saver (electronics)	I was recommended by a friend to buy over, his family used two months said the effect is very good, I also buy to try, is currently in use, easy and quick installation!	JD.com
Lubao Boutique	a power saver (electronics)	Power saving device received, well packaged, arrived without damage, easy to use, affordable. Just started using it, looking forward to the effect.	JD.com
Lubao Boutique	a power saver (electronics)	It works super well! Also very aesthetically pleasing! Will repurchase again if needed!	JD.com
Lubao Boutique	a power saver (electronics)	I did not think that this thing can really save electricity it, has given the old family parents also bought, bought two boss sent a, thank you!	JD.com
Lubao Boutique	a power saver (electronics)	Received it, already plugged it in, ready to try to see if it can save power or not, if it's good, buy	JD.com
Lubao Boutique	a power saver (electronics)	Baby received. Already using it and looking forward to saving some money on my electric bill. Worth buying	JD.com
Lubao Boutique	a power saver (electronics)	Received and used, feel good, looking forward to the next month to save electricity, good to buy a few more	JD.com
Lubao Boutique	a power saver (electronics)	It's effective, it's a real power saver, I'll use this brand of saver from now on.	JD.com
Lubao Boutique	a power saver (electronics)	I've been using it for a while, and I've been calculating it every day, and it's working, and it's going to be more effective in a month.	JD.com
Lubao Boutique	a power saver (electronics)	It's already installed and it was easy to install, so I hope it will save electricity in the long run.	JD.com
Lubao Boutique	a power saver (electronics)	I pressed the kitchen I hope next month's electric bill will be less I feel It's good Haha I hope	JD.com
Lubao Boutique	a power saver (electronics)	Fine workmanship, thick material, power saving effect is very good. Cheap and affordable	JD.com
Lubao Boutique	a power saver (electronics)	Things received, friends recommended to buy overfirst used, useful to continue to buy back.	JD.com
Lubao Boutique	a power saver (electronics)	I heard that it can save electricity, so I bought it to try, nowadays the home is all electrical appliances, too much electricity, can save a little bit is a little bit haha	JD.com
Lubao Boutique	a power saver (electronics)	Received the goods immediately installed, looked pretty good, but also more compact and convenient , the price is also quite cheap	JD.com
Lubao Boutique	a power saver (electronics)	Energy saver has received,first try the effect of the two repurchase	JD.com
Lubao Boutique	a power saver (electronics)	I don't know until I use it, let's see the results later, the manufacturer said it takes three months to see the results.	JD.com
Lubao Boutique	a power saver (electronics)	The merchant shipped quickly, already used it, looking forward to saving power in the	JD.com
Lubao Boutique	a power saver (electronics)	Feels great! Better than the ones I bought before!	JD.com
Lubao Boutique	a power saver (electronics)	Logistics is very fast, the quality is very good, first use a month, good results and then come back to buy a	JD.com
Lubao Boutique	a power saver (electronics)	Power saving king received, baby is very good, logistics is very fast, the service is very thoughtful. Please feel free to buy.	JD.com
Lubao Boutique	a power saver (electronics)	It's not bad, I've been using it for a month now and my electric bill is a little less than last month.	JD.com
Lubao Boutique	a power saver (electronics)	Power Saving King, very good, just arrived home, very use our family, a month later to see the truth!	JD.com
Lubao Boutique	a power saver (electronics)	Good, received immediately plugged in to try a few days, obviously feel the effect Oh!	JD.com

Lubao Boutique	a power saver (electronics)	I just got here and tried it out We'll see after It looks great	JD.com
Lubao Boutique	a power saver (electronics)	Good, it does save electricity! If you need it, you can buy it with confidence, I bought another one	JD.com
Lubao Boutique	a power saver (electronics)	The quality is awesome! Voltage is stable! Power saving can be seen	JD.com
Lubao Boutique	a power saver (electronics)	Just received the baby today, wait for a month and then come back to review, look forward to good use!	JD.com
Lubao Boutique	a power saver (electronics)	Quality looks ok, power savings look to be seen later. Give five stars praise.	JD.com
Lubao Boutique	a power saver (electronics)	The quality is very good, two days to the goods an, with the same picture, behind the trial to see the effect of	JD.com
Lubao Boutique	a power saver (electronics)	Not bad, the price is also affordable, try can buy again!	JD.com
Lubao Boutique	a power saver (electronics)	Good Recommended by a friend Plugged it in when I got it back The first one has a small defect Customer service immediately replenished Customer service lady is very patient Will come back next time	JD.com
Lubao Boutique	a power saver (electronics)	The baby is good, consistent with the description, the store shipped quickly, praise! Just use, because my home meter is a digital meter, can not see the effect, wait for a period of time, look at the power consumption, only to know the effect.	JD.com
Lubao Boutique	a power saver (electronics)	Baby has been used, the effect is quite good, pro have the need to buy!	JD.com
Lubao Boutique	a power saver (electronics)	It does work, the difference between my electricity bill with the saver plugged in and without is several dollars a day, it's really a good thing to figure out that you're going to save hundreds of dollars a year on your electricity bill.	JD.com
Lubao Boutique	a power saver (electronics)	The description of the stuff matches, the logistics is in place, the service attitude is very good, give five stars, if necessary, will come back!	JD.com
Lubao Boutique	a power saver (electronics)	Received the item today and it was well packaged and of good quality I have already bought from this family	JD.com
Lubao Boutique	a power saver (electronics)	Power Saving King received, got it back and immediately used it. It looks pretty good. Use a month to see how much electricity can be saved!	JD.com
Lubao Boutique	a power saver (electronics)	The effect function must be able to use well, Cost-effective value Appearance style good-looking Workmanship material is very good	JD.com
Lubao Boutique	a power saver (electronics)	Feel pretty good, first use some time in the additional comments, the seller's service is very good!	JD.com
Lubao Boutique	a power saver (electronics)	Good quality, fast logistics, trustworthy and good product.	JD.com
Lubao Boutique	a power saver (electronics)	I just started using it for a month before I know if it's power saving or not	JD.com
Lubao Boutique	a power saver (electronics)	Received just tried, I do not know whether it can save electricity, to know after a period of time. Now give positive feedback, after seeing the effect before adding a follow-up evaluation.	JD.com
Lubao Boutique	a power saver (electronics)	Good quality, will buy again if needed	JD.com
Lubao Boutique	a power saver (electronics)	This is the kind of thing that is still verified by the future to know, and then add a follow-up review later. As for whether the description matches or not, I will give three stars first, and overall still give five stars for the time being.	JD.com
Lubao Boutique	a power saver (electronics)	Good use, save electricity cost, I'll introduce you to more buyers, I'll introduce you to Da Ying have two buyers	JD.com
Lubao Boutique	a power saver (electronics)	Appearancestyle'good quality, good service, received in three days	JD.com
Lubao Boutique	a power saver (electronics)	Cost-effective baby received just installed,,^,	JD.com
Lubao Boutique	a power saver (electronics)	My friend asked me to help him out and I brought two more for him.	JD.com
Lubao Boutique	a power saver (electronics)	Great quality Superb service Received in two days………	JD.com
Lubao Boutique	a power saver (electronics)	This technology is good, small size, big use, worth buying, can recommend friends to buy.	JD.com
Lubao Boutique	a power saver (electronics)	This power saving king is really good, received the day before yesterday, the day before yesterday night more than 8:00 pm plugged into yesterday night more than 9:00 pm only two degrees of electricity, and then I said to try not to unplug, more than 9:00 to 7:00 in the morning on the use of two and a half degrees, scares me to hurry up to plug in, it's really good!	JD.com
Lubao Boutique	a power saver (electronics)	Energy saver received, I'll install myself away from the meter nearest place, so that electricity can come out of the energy saver, you can automatically control the voltage, it's really quite good, I do not know if you can save electricity, first use two months to see the effect, look forward to will have good	JD.com
Lubao Boutique	a power saver (electronics)	I bought it after reading that people said it would work, and I hope it's as energy efficient as they say it is	JD.com
Lubao Boutique	a power saver (electronics)	The courier guy delivered the wrong item the first time, luckily he got it back for me, installed it almost a week ago and saved almost17%electricity	JD.com
Lubao Boutique	a power saver (electronics)	Saver received. The goods are regular, with Pacific insurance, there are instructions, you can rest assured that the use. Power-saving effect to be used one to two months after the follow-up evaluation.	JD.com
Lubao Boutique	a power saver (electronics)	Genuine, exquisite workmanship, easy to use, the effect is obvious, affordable, satisfactory, worth buying!	JD.com
Lubao Boutique	a power saver (electronics)	It does work, straight purchase, this brand is trustworthy, very good, used for a period of time, the point of view of a month significantly decreased.	JD.com
Lubao Boutique	a power saver (electronics)	Plugged into the socket, there is a small green light, always on, no sound at all, my friend also bought, he said it really saves electricity, the monthly electricity bill must be reduced	JD.com
Lubao Boutique	a power saver (electronics)	Good, fast logistics, product quality is very good, the effect of the use of a follow-up comment!	JD.com
Lubao Boutique	a power saver (electronics)	Exactly as described by the seller, very satisfied, really like, very fast shipping, packaging very carefully, very satisfied with a shopping. Follow-up with a follow-up review!	JD.com
Lubao Boutique	a power saver (electronics)	Received, well-packed, logistics, business service is attentive, energy-saving appliances fine workmanship, it is worth getting!	JD.com
Lubao Boutique	a power saver (electronics)	Not much of a reviewer, but this one is really great! The quality is very good, meticulous workmanship, the package is very tight, the courier service attitude is good, absolutely praise!	JD.com
Lubao Boutique	a power saver (electronics)	It is to see a friend's family used to buy. Have received the goods, looking forward to good results, the store's service is truly good.	JD.com
Lubao Boutique	a power saver (electronics)	.Because I have seen this product played on TV, I would like to buy one to try, so I will buy it and use it for a period of time to see the effect, if there is still, I will recommend it to my friends!	JD.com
Lubao Boutique	a power saver (electronics)	(Overall evaluation) baby received, to set on, look good, a month later, and then chase the evaluation of power-saving effect!	JD.com
Lubao Boutique	a power saver (electronics)	Plugged in does not take up space, beautiful and generous, affordable, power-saving performance is good, I installed a few units at home, there is a significant power-saving effect	JD.com
Lubao Boutique	a power saver (electronics)	Effective function is quite good Cost-effective is quite cheap Use experience is quite good Appearance style is quite nice Workmanship material is quite nice	JD.com
Lubao Boutique	a power saver (electronics)	Overall evaluation is very good Effective function is good Cost-effective Good using experience Good appearance and style Good workmanship and material	JD.com
Lubao Boutique	a power saver (electronics)	The store has a great service attitude! I received the product today and will follow up after using it!	JD.com
Lubao Boutique	a power saver (electronics)	The quality of the product is excellent and the customer is very thankful for the satisfaction!!!!	JD.com
Lubao Boutique	a power saver (electronics)	Baby received, first time to install, the product is beautifully made, customer service is very good! First five-star praise!	JD.com
Lubao Boutique	a power saver (electronics)	Baby arrived, very convenient, plugged into a good, this winter is not afraid of power consumption	JD.com
Lubao Boutique	a power saver (electronics)	Goods received, already used do not know how the effect, I hope to be able to meet the requirements!	JD.com
Lubao Boutique	a power saver (electronics)	The power saver received, as soon as I got back, I plugged it in and used it, looking forward to the power-saving effect!	JD.com
Lubao Boutique	a power saver (electronics)	I bought it back and plugged it in, observe it for a few days to see how the meter is doing, give it all 5 stars for now! To be followed up!	JD.com
Lubao Boutique	a power saver (electronics)	Very good, light and convenient, business customer service is also with the intimate, also good to talk to, must praise!	JD.com
Lubao Boutique	a power saver (electronics)	Power saving power saving super power saving, plugged in immediately the meter are turning slow, absolutely will save power!	JD.com
Lubao Boutique	a power saver (electronics)	Just received the goods on the use, others say good use and power saving I also buy a look, the home electricity bill is too high hope that really save electricity!	JD.com
Lubao Boutique	a power saver (electronics)	Power saving treasure has received, open a look, good refined, directly with, with good I want to buy a few, my home appliances, each socket is inserted into a, so that there is no fear of electricity.	JD.com
Lubao Boutique	a power saver (electronics)	Thruster received. Very good. Will patronize again next time.	JD.com
Lubao Boutique	a power saver (electronics)	It works really well, saved me a lot of money, good value for money, recommend buying!	JD.com
Lubao Boutique	a power saver (electronics)	Good stuff to share, how to save a few cigarettes a year!	JD.com
Lubao Boutique	a power saver (electronics)	The baby has been received! Install it and see! Hopefully it will save electricity next month! Thank you boss!	JD.com

Lubao Boutique	a power saver (electronics)	I'm using it. I saw my neighbor buying it and it worked well, so I bought it to try it out too.	JD.com
Lubao Boutique	a power saver (electronics)	Genuine Energy Saving Electricity Manager God Meter Air Conditioner Smart Save TV. Evaluated um	JD.com
Lubao Boutique	a power saver (electronics)	Kisses received the goods, thank you for allowing us to buy this product. Thank you.	JD.com
Lubao Boutique	a power saver (electronics)	The power saver received affordable,, well-packaged logistics very quickly and very satisfied with the shopping have the opportunity to come back to patronize, like the partners can try this product is really	JD.com
Novell Energy Saving Technology	Smart Saver	The store went out of business.	JD.com
energy-saving devices	King of Power Saving	The logistics is super fast, ordering late in the afternoon well, arriving in the afternoon of the next day, too about24hours to get the loan! Kudos to the merchant! Must give him five stars	JD.com
energy-saving devices	King of Power Saving	Good review!	JD.com
energy-saving devices	King of Power Saving	Item is great and gives5star reviews	JD.com
energy-saving devices	King of Power Saving	The product is very good and gives 5 stars	JD.com
energy-saving devices	High Power Super Saving	not have	JD.com
Ningde Science and Technology	Smart Saver	The store went out of business.	JD.com
Eight Catty Army Army (PRC armed forces)	a power saver (electronics)	This user feels the product is better	JD.com
Eight Catty Army Army (PRC armed forces)	a power saver (electronics)	I don't know if there is any power saving, but if it works, I will buy it again in the future.	JD.com
Eight Catty Army Army (PRC armed forces)	a power saver (electronics)	received	JD.com
Eight Catty Army Army (PRC armed forces)	a power saver (electronics)	an excellent	JD.com
Eight Catty Army Army (PRC armed forces)	a power saver (electronics)	incompetent	JD.com
Eight Catty Army Army (PRC armed forces)	a power saver (electronics)	The style is very new, the product is very small, beautiful design, affordable, easy to use, and the saver works well.	JD.com
Eight Catty Army Army (PRC armed forces)	a power saver (electronics)	I'm looking forward to helping me save on electricity for the fruit store	JD.com
Eight Catty Army Army (PRC armed forces)	a power saver (electronics)	Baby received, very good cheap beauty is a pleasant shopping, praise five stars!	JD.com
Eight Catty Army Army (PRC armed forces)	a power saver (electronics)	I got it. I don't know if it works.	JD.com
Eight Catty Army Army (PRC armed forces)	a power saver (electronics)	This user feels the product is better	JD.com
Eight Catty Army Army (PRC armed forces)	a power saver (electronics)	I'm coming. Okay	JD.com
Eight Catty Army Army (PRC armed forces)	a power saver (electronics)	This user feels the product is better	JD.com
Eight Catty Army Army (PRC armed forces)	a power saver (electronics)	This user feels the product is better	JD.com
Eight Catty Army Army (PRC armed forces)	a power saver (electronics)	This user feels the product is better	JD.com
Eight Catty Army Army (PRC armed forces)	a power saver (electronics)	Five stars, second purchase, good customer service, courier boy worked hard.	JD.com
Eight Catty Army Army (PRC armed forces)	a power saver (electronics)	Goods received, very good, thanks to the merchant!	JD.com
Eight Catty Army Army (PRC armed forces)	a power saver (electronics)	The style is new,[love it]hopefully power saving	JD.com
Eight Catty Army Army (PRC armed forces)	a power saver (electronics)	I don't know how it works yet.	JD.com
Eight Catty Army Army (PRC armed forces)	a power saver (electronics)	pretty good	JD.com
Eight Catty Army Army (PRC armed forces)	a power saver (electronics)	Commodities received, logistics speed is very good quality, simple style plug and play as to save power to be verified how much later there will be a follow-up.	JD.com
Eight Catty Army Army (PRC armed forces)	a power saver (electronics)	in use	JD.com
Eight Catty Army Army (PRC armed forces)	a power saver (electronics)	The style is very new, the product is very compact, the product is very compact, the product is very compact, the product is very compact, the product is very compact, the product is very compact, the power usage is high	JD.com
Eight Catty Army Army (PRC armed forces)	a power saver (electronics)	Have been using it for a few days now, hopefully it will save power.	JD.com
Eight Catty Army Army (PRC armed forces)	a power saver (electronics)	Very good👍👍, good quality👍👍👍	JD.com
Eight Catty Army Army (PRC armed forces)	a power saver (electronics)	This user feels the product is better	JD.com
Eight Catty Army Army (PRC armed forces)	a power saver (electronics)	It's really good. I'll give it a good review.	JD.com
Eight Catty Army Army (PRC armed forces)	a power saver (electronics)	This user feels the product is better	JD.com
Eight Catty Army Army (PRC armed forces)	a power saver (electronics)	Baby received, very good baby, worth buying, thanks to the business attentive service.	JD.com

Eight Catty Army Army (PRC armed forces)	a power saver (electronics)	Not good.[Chuckles]Same electricity	JD.com
Eight Catty Army Army (PRC armed forces)	a power saver (electronics)	This user feels the product is better	JD.com
Eight Catty Army Army (PRC armed forces)	a power saver (electronics)	Plug it in and use it for a while, observe to see if there is any significant power saving	JD.com
Eight Catty Army Army (PRC armed forces)	a power saver (electronics)	Not bad. I wonder if it's as good as it says.	JD.com
Eight Catty Army Army (PRC armed forces)	a power saver (electronics)	The product is compact and easy to use, we'll see how well it works!Looking forward to it!	JD.com
Eight Catty Army Army (PRC armed forces)	a power saver (electronics)	[big love] [big love] [big[love] [biglove]	JD.com
Eight Catty Army Army (PRC armed forces)	a power saver (electronics)	[Smiles] [Smiles]	JD.com
Eight Catty Army Army (PRC armed forces)	a power saver (electronics)	This user feels the product is better	JD.com
Eight Catty Army Army (PRC armed forces)	a power saver (electronics)	desirable	JD.com
Eight Catty Army Army (PRC armed forces)	a power saver (electronics)	The stuff has been received and it looks ok, it's good that it's working[smile]	JD.com
Eight Catty Army Army (PRC armed forces)	a power saver (electronics)	The quality is great and it feels like a perfect fit	JD.com
Eight Catty Army Army (PRC armed forces)	a power saver (electronics)	This user feels the product is better	JD.com
Eight Catty Army Army (PRC armed forces)	a power saver (electronics)	Started using it as soon as it arrived yesterday, looking forward to saving power	JD.com
Eight Catty Army Army (PRC armed forces)	a power saver (electronics)	The saver was received, well packaged and received in perfect condition, purch5this time, looking forward to next month's electricity bill	JD.com
Eight Catty Army Army (PRC armed forces)	a power saver (electronics)	Baby is very good, just arrived can not wait to use it, I like very much hope to achieve the desired effect of saving electricity and money, the future will also recommend to friends!	JD.com
Eight Catty Army Army (PRC armed forces)	a power saver (electronics)	Lifetime product warranty, the business is very thoughtful, I purchased5also gave me some freebies, and commitment to invalid full refund, now used for a period of time down, the feeling that the electricity bill is less, and so fully optimized after the belief that the effect will be even better, to the merchant five-star praise, and recommended	JD.com
Eight Catty Army Army (PRC armed forces)	a power saver (electronics)	This saver is not only good performance, but also high value, no matter the workmanship and materials are very good, this is the second time to buy, this time the purchase of8, to relatives and friends are used, everyone said I helped a lot	JD.com
Eight Catty Army Army (PRC armed forces)	a power saver (electronics)	Comparison has1month specially come over to evaluate, from the very beginning of a little less every day, the back of the stable, a day of the original20degrees to the current15degrees difference of a little bit, the effect is very good, the conscience of the product, to see some people say does not save electricity, I do not know how in the end to compare, recommended to rest assured that the purchase, the [illegible]	JD.com
Eight Catty Army Army (PRC armed forces)	a power saver (electronics)	two power savers for the family, the kitchen and bathroom water heater is too costly_x005f_x005f_x0014_, the water heater to be turned on every day, every month's electricity costs are a lot of use, with this, no longer afraid of the electricity bill is more than one month down to the electricity bill than the previous month less than half of it _x0014_,The effect is particularly good, very much like Will	JD.com
Eight Catty Army Army (PRC armed forces)	a power saver (electronics)	Received the goods can not wait to open and look at the power saver is very curious, above the authenticity of the logo, but also thoughtful purchase of insurance, business warranty5years, and the use of the manual, a very formal product	JD.com
Eight Catty Army Army (PRC armed forces)	a power saver (electronics)	Started to hold the attitude to try to buy, I did not expect this power saver is really useful, this month really save a lot of electricity bills!	JD.com
Eight Catty Army Army (PRC armed forces)	a power saver (electronics)	I did not think there are so magical things, before has been a waste of a lot of money, this time to buy2, plugged in after a smart display shows that the machine is working, good to use in recommending friends around, the shopkeeper service is also very good	JD.com
Eight Catty Army Army (PRC armed forces)	a power saver (electronics)	Saving appliances are also recommended by a friend, he bought5inone go last time, the electricity bill is really saved a lot, this time I also buy5	JD.com
Eight Catty Army Army (PRC armed forces)	a power saver (electronics)	It should work as we have been using the Saver and will repurchase if it's good.	JD.com
Eight Catty Army Army (PRC armed forces)	a power saver (electronics)	Smart saver is good, can save a lot every month, consulting customer service my family's electricity consumption, so purchased4, looking forward to the next month's electricity bill, the	JD.com
Eight Catty Army Army (PRC armed forces)	a power saver (electronics)	Friends used to recommend me over to buy, at first I do not believe this thing, hold the mind to try to buy3, the results are indeed unexpected, and will recommend friends to buy, now too many electrical appliances,1are several hundred of electricity, and now every day can save a dozen or so to the merchants [illegible]	JD.com
Eight Catty Army Army (PRC armed forces)	a power saver (electronics)	Baby received a few days of easy to use, only to see the effect, if the effect is very good, I will follow up, but also will introduce to others to buy!	JD.com
Eight Catty Army Army (PRC armed forces)	a power saver (electronics)	The saver is simply amazing, after plugging it in, it really saves a lot of electricity, really hate to meet with this saver.	JD.com
Eight Catty Army Army (PRC armed forces)	a power saver (electronics)	Just received and opened to check, packaging fine not rough, customer service can patiently answer questions, looking forward to the effect! Give the store5stars praise	JD.com
Eight Catty Army Army (PRC armed forces)	a power saver (electronics)	Relatives recommended, do not know that there is so good use of things, it is said that she last time to buy5,1month to savemore than100of the electricity bill, this time I also do not hesitate to buy5	JD.com
Eight Catty Army Army (PRC armed forces)	a power saver (electronics)	Thrifty received put into use, home appliances, power consumption is very large, we have a piece of electricity or1yuan1degree, with this thrifty is much better, every day can save a few cents, very convenient and practical, and energy-saving voltage is also stable, not bad	JD.com
Eight Catty Army Army (PRC armed forces)	a power saver (electronics)	The saver works well, plug it in and it works, where it consumes power plug it in, even I, a woman who doesn't do much, can use it successfully, it's very convenient and convenient!	JD.com
Eight Catty Army Army (PRC armed forces)	a power saver (electronics)	Good and inexpensive power saver, an investment for life, save a few dollars a day a year down can also save a lot of money, thanks to the store!	JD.com

Eight Catty Army Army (PRC armed forces)	a power saver (electronics)	Taobao recommended to me, or know that I use a lot of electricity, they recommended me this good stuff, so I did not hesitate to buy, looking forward to this month's electricity bill	JD.com
Eight Catty Army Army (PRC armed forces)	a power saver (electronics)	Workmanship is very good, the material is also a good chat, the shopkeeper service is also good, the purchase offivecomplimentary1to me, good if you will buy back	JD.com
Eight Catty Army Army (PRC armed forces)	a power saver (electronics)	Customer service introduced this is an energy saver, in situ compensation, it is also very convenient to use, where the power consumption plug where, but also can be moved at any time, do not need to be wired, is very suitable for us as a female user, to the merchant designers kudos	JD.com
Eight Catty Army Army (PRC armed forces)	a power saver (electronics)	Power-saving effect: energy-saving effect is better, very can save electricity, before the electricity bill is more, after using this product, the electricity bill made to save a lot, really too good to use!	JD.com
Eight Catty Army Army (PRC armed forces)	a power saver (electronics)	Saver has been used for a period of time, is still quite good, workmanship is also very good, the shopkeeper service is very good!	JD.com
Eight Catty Army Army (PRC armed forces)	a power saver (electronics)	The packaging is very delicate, the power saver is lightweight, I tried two places and the voltage is stable, I look forward to the power saving effect. Thank you for your help.	JD.com
Eight Catty Army Army (PRC armed forces)	a power saver (electronics)	The goods have been received, the appearance of the packaging exquisite……and easy to use, open store friends can buy this product! I tried it for a few days hoping to save myself some money on my electric bill! Next time will buy a few more	JD.com
Eight Catty Army Army (PRC armed forces)	a power saver (electronics)	Logistics is very fast, plugged in with a very dark night light, the instructions read and feel that it makes sense, I hope it can really save electricity, otherwise the home electricity bill is really quite high, especially in the winter, the first praise, and so on a month after the follow-up!	JD.com
Eight Catty Army Army (PRC armed forces)	a power saver (electronics)	Purchased5power saversat once, deliberately used for a period of time to comment on this model of power saver is really good to use, so far a total ofmore than70blocks of electricity saved, only ten days time, I believe that in the future will save more power	JD.com
Eight Catty Army Army (PRC armed forces)	a power saver (electronics)	Power-saving effect: power saver received, power saving effect is obvious, Cost-effective: cost-effective Appearance style: exquisite appearance, fine workmanship, open the air conditioning with two hours, feel very satisfied.	JD.com
Eight Catty Army Army (PRC armed forces)	a power saver (electronics)	Friends last time to buy2, the effect is good, every other month the electricity bill has dropped significantly, this time friends recommended that I come, the beginning did not know that there is something so good to use, this time to buy6, the family's self-build room is too much, it is really a one-time investment, lifelong benefit, the product of a lifetime warranty, the bad for free replacement of the new, the merchant to give the protection is very thoughtful, and the business is very	JD.com
Eight Catty Army Army (PRC armed forces)	a power saver (electronics)	first time to buy this energy saver, do not know that there is this thing, hold a try to buy the mentality, the business commitment to no effect full refund, after receiving the product there is a manual, it is very convenient, the saver is compact and does not take up space, my family's power consumption is very large, the purchase of6, the effect of the open after the chase comment	JD.com
Eight Catty Army Army (PRC armed forces)	a power saver (electronics)	Value for money: just received, then checked on the plug board, looked at the pulse meter, with the same appliances, with and without a saver. There is a real difference in the blinking frequency of the pulse meter light. With the saver plugged in, the meter pulse light blinks much slower. Not bad at.	JD.com
Eight Catty Army Army (PRC armed forces)	a power saver (electronics)	Very satisfied with a shopping, get back on the experience, the results do not know, should be effective, believe the business, I also see a lot of positive feedback before buying. Give five-star praise!	JD.com
Eight Catty Army Army (PRC armed forces)	a power saver (electronics)	Bought two, wait a day to bring to try, really good power saving, worth the commission there!	JD.com
Eight Catty Army Army (PRC armed forces)	a power saver (electronics)	The quality of this set is really, really great and the value is super high. When I received the goods are each individual box packaging, even the box is so good to see that I can not throw away, too much to like ~!	JD.com
Eight Catty Army Army (PRC armed forces)	a power saver (electronics)	It feels very promising, I consider that it should save electricity, the merchant promised very sure, it should be no problem	JD.com
Eight Catty Army Army (PRC armed forces)	a power saver (electronics)	Don't know the effect yet for now. I won't know the effect until I use it and then I will continue to evaluate it. But I have purchased it for the second time for my friend.	JD.com
Eight Catty Army Army (PRC armed forces)	a power saver (electronics)	The product is well crafted, insured, multi-functional, and has fake identification for peace of mind!	JD.com
Eight Catty Army Army (PRC armed forces)	a power saver (electronics)	It looks okay, I like it, and also send very gift next time in the repurchase.	JD.com
Eight Catty Army Army (PRC armed forces)	a power saver (electronics)	Very satisfied with a shopping, product quality is very good, protect the whole house to save electricity, all5stars	JD.com
Eight Catty Army Army (PRC armed forces)	a power saver (electronics)	Baby received, quite good plugged into the socket can logistics very quickly	JD.com
Eight Catty Army Army (PRC armed forces)	a power saver (electronics)	Goods have arrived, I used two days, really good ah, can save electricity, this hot days will not be afraid to open the air conditioning power consumption, worth to give merchants a big praise ah!	JD.com
Eight Catty Army Army (PRC armed forces)	a power saver (electronics)	Merchant service attitude is very good, the goods are consistent with the description of the merchant, will recommend to friends.	JD.com
Eight Catty Army Army (PRC armed forces)	a power saver (electronics)	As described, it is authentic, especially like.	JD.com
Eight Catty Army Army (PRC armed forces)	a power saver (electronics)	Affordable, economical product with beautiful design	JD.com
Eight Catty Army Army (PRC armed forces)	a power saver (electronics)	Power Saver works exceptionally well Good looking face Put it anywhere	JD.com
Eight Catty Army Army (PRC armed forces)	a power saver (electronics)	Electricity-saving appliances used, the meter seems to turn slower everyone can try to buy	JD.com
Eight Catty Army Army (PRC armed forces)	a power saver (electronics)	Good value for money, convenient and practical, logistics is also very fast.	JD.com
Eight Catty Army Army (PRC armed forces)	a power saver (electronics)	The goods have been received, the logistics is very fast, the service attitude is very good.	JD.com
Eight Catty Army Army (PRC armed forces)	a power saver (electronics)	Looking forward to it. Started using it today, hopefully it will be noticeable	JD.com
Eight Catty Army Army (PRC armed forces)	a power saver (electronics)	Product quality is very good, is genuine, service is very good logistics fast, worth buying!	JD.com
Eight Catty Army Army (PRC armed forces)	a power saver (electronics)	The product is affordable and effective, it is genuine, please feel free to buy.	JD.com

Eight Catty Army Army (PRC armed forces)	a power saver (electronics)	A very good energy saver! Much better quality than others, works great, worth buying!	JD.com
Eight Catty Army Army (PRC armed forces)	a power saver (electronics)	Very good, fast logistics, customer service is also very good, will introduce to friends in the future	JD.com
Eight Catty Army Army (PRC armed forces)	a power saver (electronics)	Nice and good, it's genuine, easy to use, not too expensive, if you need to buy this one in.	JD.com
Eight Catty Army Army (PRC armed forces)	a power saver (electronics)	Timely delivery, beautiful design,, affordable, the effect remains to be seen.	JD.com
Eight Catty Army Army (PRC armed forces)	a power saver (electronics)	Logistics is fast and cheap. The product is very good. To have to buy.	JD.com
Eight Catty Army Army (PRC armed forces)	a power saver (electronics)	It's authentic! The quality is great! Service is superb! Received it and couldn't wait to use it!	JD.com
Eight Catty Army Army (PRC armed forces)	a power saver (electronics)	I'm satisfied every time, and I'm even more satisfied with the baby I chose this time, and I'll buy again as always!	JD.com
Eight Catty Army Army (PRC armed forces)	a power saver (electronics)	Bought two, very small, immediately used, I hope it can really save power, look forward to the effect!	JD.com
Eight Catty Army Army (PRC armed forces)	a power saver (electronics)	Saving power received, looking forward to the effect, just started to use.not know if there is no effect yet, first use to see, have the effect and then follow up[smile]	JD.com
Eight Catty Army Army (PRC armed forces)	a power saver (electronics)	Shipping was fast, stuff was well packaged, great price, I'm happy with👍and will repurchase	JD.com
Eight Catty Army Army (PRC armed forces)	a power saver (electronics)	This user feels the product is better	JD.com
Eight Catty Army Army (PRC armed forces)	a power saver (electronics)	This user feels the product is better	JD.com
Eight Catty Army Army (PRC armed forces)	a power saver (electronics)	Used it, heard it recommended by a friend! Buy it and try[Big Love][Big Love]	JD.com
Eight Catty Army Army (PRC armed forces)	a power saver (electronics)	This user feels the product is better	JD.com
Eight Catty Army Army (PRC armed forces)	a power saver (electronics)	This user did not fill in a rating	JD.com
Eight Catty Army Army (PRC armed forces)	a power saver (electronics)	This user feels the product is better	JD.com
Eight Catty Army Army (PRC armed forces)	a power saver (electronics)	rare	JD.com
Eight Catty Army Army (PRC armed forces)	a power saver (electronics)	Not bad, just plug it in, convenient and practical	JD.com
Eight Catty Army Army (PRC armed forces)	a power saver (electronics)	Let's see if it works.	JD.com
Eight Catty Army Army (PRC armed forces)	a power saver (electronics)	The stuff is great really saves a lot of electricity! I love it.	JD.com
Eight Catty Army Army (PRC armed forces)	a power saver (electronics)	The meter won't slow down. Don't be fooled.	JD.com
Eight Catty Army Army (PRC armed forces)	a power saver (electronics)	This user feels the product is better	JD.com
Eight Catty Army Army (PRC armed forces)	a power saver (electronics)	Packaging is very good, the style of mini, the key is to save electricity, inexpensive and beautiful! Worth thousands of households to use and buy	JD.com
Eight Catty Army Army (PRC armed forces)	a power saver (electronics)	Good, good satisfaction<br>I didn't expect such a good product for the price	JD.com
Eight Catty Army Army (PRC armed forces)	a power saver (electronics)	Works exceptionally well.	JD.com
Eight Catty Army Army (PRC armed forces)	a power saver (electronics)	Looking forward to being useful	JD.com
Eight Catty Army Army (PRC armed forces)	a power saver (electronics)	[大爱]	JD.com
Eight Catty Army Army (PRC armed forces)	a power saver (electronics)	Goods received, logistics is very fast, beautiful style, but also can be used as a night light. Worth buying	JD.com
Eight Catty Army Army (PRC armed forces)	a power saver (electronics)	Great, really energy efficient, order and buy with confidence.	JD.com
Eight Catty Army Army (PRC armed forces)	a power saver (electronics)	Similar to a nightlight, the power saving mode is less than ideal.	JD.com
Eight Catty Army Army (PRC armed forces)	a power saver (electronics)	Fake ones don't save electricity anymore.	JD.com
Eight Catty Army Army (PRC armed forces)	a power saver (electronics)	Just received it. I think it's okay.	JD.com
Eight Catty Army Army (PRC armed forces)	a power saver (electronics)	This user feels the product is better	JD.com
Eight Catty Army Army (PRC armed forces)	a power saver (electronics)	have nothing to do	JD.com

Eight Catty Army Army (PRC armed forces)	a power saver (electronics)	I'll add more when I'm done.	JD.com
Eight Catty Army Army (PRC armed forces)	a power saver (electronics)	pretty good	JD.com
Eight Catty Army Army (PRC armed forces)	a power saver (electronics)	Very satisfied, very good, can	JD.com
Eight Catty Army Army (PRC armed forces)	a power saver (electronics)	Good. Very good.	JD.com
Eight Catty Army Army (PRC armed forces)	a power saver (electronics)	Received the goods, use to see how the effect! After that, I'll come back to evaluate.	JD.com
Eight Catty Army Army (PRC armed forces)	a power saver (electronics)	I've tried it for about ten days before commenting, and it can save one degree of electricity a day.	JD.com
Eight Catty Army Army (PRC armed forces)	a power saver (electronics)	The product received, I do not know if it is good, wait until you use it to evaluate, the	JD.com
Eight Catty Army Army (PRC armed forces)	a power saver (electronics)	[Big Love][Big Love]Save electricity, save money💰, buy it next time	JD.com
Eight Catty Army Army (PRC armed forces)	a power saver (electronics)	The merchandise is very good and really useful, the	JD.com
Eight Catty Army Army (PRC armed forces)	a power saver (electronics)	Still trying it out Not sure if it saves power or not	JD.com
Eight Catty Army Army (PRC armed forces)	a power saver (electronics)	Wish it had a power saving feature	JD.com
Eight Catty Army Army (PRC armed forces)	a power saver (electronics)	Shipped quickly, small and compact, just received it and opened it to look at it	JD.com
Eight Catty Army Army (PRC armed forces)	a power saver (electronics)	I've received the baby and I'll use it to see how it works.	JD.com
Eight Catty Army Army (PRC armed forces)	a power saver (electronics)	This user feels the product is better	JD.com
Eight Catty Army Army (PRC armed forces)	a power saver (electronics)	Still works well, looks and exquisite, very power-saving, logistics is also very fast.	JD.com
Eight Catty Army Army (PRC armed forces)	a power saver (electronics)	It's working fine.	JD.com
Eight Catty Army Army (PRC armed forces)	a power saver (electronics)	Got it. It's good quality.	JD.com
Eight Catty Army Army (PRC armed forces)	a power saver (electronics)	This user feels the product is better	JD.com
Eight Catty Army Army (PRC armed forces)	a power saver (electronics)	This user feels the product is better	JD.com
Eight Catty Army Army (PRC armed forces)	a power saver (electronics)	This user feels the product is better	JD.com
Eight Catty Army Army (PRC armed forces)	a power saver (electronics)	This user feels the product is better	JD.com
Eight Catty Army Army (PRC armed forces)	a power saver (electronics)	Satisfied with the look.	JD.com
Eight Catty Army Army (PRC armed forces)	a power saver (electronics)	This user feels the product is better	JD.com
Eight Catty Army Army (PRC armed forces)	a power saver (electronics)	Just received, quite good, courier boy service first-class	JD.com
Eight Catty Army Army (PRC armed forces)	a power saver (electronics)	This user feels the product is better	JD.com
Eight Catty Army Army (PRC armed forces)	a power saver (electronics)	Let's see if it's a real power saver before we judge it	JD.com
Eight Catty Army Army (PRC armed forces)	a power saver (electronics)	Merchants shipping fast, logistics is also fast. As to whether this product can save electricity, to use to see the effect before evaluation.	JD.com
Eight Catty Army Army (PRC armed forces)	a power saver (electronics)	This user feels the product is better	JD.com
Eight Catty Army Army (PRC armed forces)	a power saver (electronics)	This user feels the product is better	JD.com
Eight Catty Army Army (PRC armed forces)	a power saver (electronics)	It's not working.	JD.com
Eight Catty Army Army (PRC armed forces)	a power saver (electronics)	This user feels the product is better	JD.com
Eight Catty Army Army (PRC armed forces)	a power saver (electronics)	I gave it a good review just to see if it will work after using it.	JD.com
Eight Catty Army Army (PRC armed forces)	a power saver (electronics)	No comment for now, waiting to see the results	JD.com
Eight Catty Army Army (PRC armed forces)	a power saver (electronics)	I don't get it. Does it make a difference whether it works or not? Let's use it first.	JD.com

Eight Catty Army Army (PRC armed forces)	a power saver (electronics)	This user feels the product is better	JD.com
Eight Catty Army Army (PRC armed forces)	a power saver (electronics)	Save five dollars on electricity$15air conditioning never stops	JD.com
Eight Catty Army Army (PRC armed forces)	a power saver (electronics)	can	JD.com
Eight Catty Army Army (PRC armed forces)	a power saver (electronics)	This user feels the product is better	JD.com
Eight Catty Army Army (PRC armed forces)	a power saver (electronics)	This user feels the product is better	JD.com
Eight Catty Army Army (PRC armed forces)	a power saver (electronics)	I haven't used it yet, I'll comment afterward.	JD.com
Eight Catty Army Army (PRC armed forces)	a power saver (electronics)	What a****ing****inglie	JD.com
Eight Catty Army Army (PRC armed forces)	a power saver (electronics)	Great service and quality	JD.com
Eight Catty Army Army (PRC armed forces)	a power saver (electronics)	Good and worth buying, power saving and durable	JD.com
Eight Catty Army Army (PRC armed forces)	a power saver (electronics)	Still using it, don't know if it saves power yet	JD.com
Eight Catty Army Army (PRC armed forces)	a power saver (electronics)	This user feels the product is better	JD.com
Eight Catty Army Army (PRC armed forces)	a power saver (electronics)	Originally bought three are good, your home is the first time to buy the quality of how to wait to use to know.	JD.com
Eight Catty Army Army (PRC armed forces)	a power saver (electronics)	This user feels the product is better	JD.com
Eight Catty Army Army (PRC armed forces)	a power saver (electronics)	The saver has been received[kudos] [kudos][kudos[kudos[kudos[kudos] [kudos] [kudos] [kudos] [kudos] [kudos][kudos	JD.com
Eight Catty Army Army (PRC armed forces)	a power saver (electronics)	pretty good	JD.com
Eight Catty Army Army (PRC armed forces)	a power saver (electronics)	The product is very small, I don't know if it works or not, I'll use it first	JD.com
Eight Catty Army Army (PRC armed forces)	a power saver (electronics)	The power saving is not great, it's about the same as the original.	JD.com
Eight Catty Army Army (PRC armed forces)	a power saver (electronics)	This user feels the product is better	JD.com
Eight Catty Army Army (PRC armed forces)	a power saver (electronics)	This user feels the product is better	JD.com
Eight Catty Army Army (PRC armed forces)	a power saver (electronics)	This user feels the product is better	JD.com
Eight Catty Army Army (PRC armed forces)	a power saver (electronics)	This user feels the product is better	JD.com
Eight Catty Army Army (PRC armed forces)	a power saver (electronics)	I don't know if it's any good.	JD.com
Eight Catty Army Army (PRC armed forces)	a power saver (electronics)	This user feels the product is better	JD.com
Eight Catty Army Army (PRC armed forces)	a power saver (electronics)	This user did not fill in a rating	JD.com
Eight Catty Army Army (PRC armed forces)	a power saver (electronics)	It's good, I tried it and the voltage is much flatter, and I save a little bit every month.	JD.com
Eight Catty Army Army (PRC armed forces)	a power saver (electronics)	I don't know what the effect is yet, I'm using it	JD.com
Eight Catty Army Army (PRC armed forces)	a power saver (electronics)	This user feels the product is better	JD.com
Eight Catty Army Army (PRC armed forces)	a power saver (electronics)	I don't know.	JD.com
Eight Catty Army Army (PRC armed forces)	a power saver (electronics)	Great value<br>Great value for money<br>Worth the<br>👍👍👍👍👍	JD.com
Eight Catty Army Army (PRC armed forces)	a power saver (electronics)	Things received! The logistics is very fast, the quality is very good, very satisfied with a shopping that	JD.com
Eight Catty Army Army (PRC armed forces)	a power saver (electronics)	Power Saving King received, really save electricity, good price cheap	JD.com
Eight Catty Army Army (PRC armed forces)	a power saver (electronics)	Efficacy experience: test in<br>Current feeling: can<br>Function power: can<br>Appearance appearance: beautiful<br>Size: just right	JD.com
Eight Catty Army Army (PRC armed forces)	a power saver (electronics)	This user feels the product is better	JD.com

Eight Catty Army Army (PRC armed forces)	a power saver (electronics)	Very good, worth buying	JD.com
Eight Catty Army Army (PRC armed forces)	a power saver (electronics)	The logistics were fast, the packaging was good, and the saver was great.	JD.com
Eight Catty Army Army (PRC armed forces)	a power saver (electronics)	I don't know if it works.	JD.com
Eight Catty Army Army (PRC armed forces)	a power saver (electronics)	The good attitude of the CMT courier is so great.<br><br>Use it first and then comment.	JD.com
Eight Catty Army Army (PRC armed forces)	a power saver (electronics)	This user feels the product is better	JD.com
Eight Catty Army Army (PRC armed forces)	a power saver (electronics)	It's garbage. It's useless. It's an IQ tax. Garbage, garbage, garbage, garbage.	JD.com
Eight Catty Army Army (PRC armed forces)	a power saver (electronics)	This user feels the product is better	JD.com
Eight Catty Army Army (PRC armed forces)	a power saver (electronics)	Looking forward to the results	JD.com
Eight Catty Army Army (PRC armed forces)	a power saver (electronics)	Goods have been received, logistics is very fast, beautiful, with a period of time in the evaluation	JD.com
Eight Catty Army Army (PRC armed forces)	a power saver (electronics)	This user feels the product is better	JD.com
Eight Catty Army Army (PRC armed forces)	a power saver (electronics)	Goods received.	JD.com
Eight Catty Army Army (PRC armed forces)	a power saver (electronics)	Did a comparison and found this thing useless and a power hog.	JD.com
Eight Catty Army Army (PRC armed forces)	a power saver (electronics)	[big love][big love][big[love][biglove]	JD.com
Eight Catty Army Army (PRC armed forces)	a power saver (electronics)	I'll comment after I use it. Externally the workmanship is excellent. Great price.	JD.com
Eight Catty Army Army (PRC armed forces)	a power saver (electronics)	The goods have been received and used very satisfied. Courier service was good and fast. Kudos to you guys!	JD.com
Eight Catty Army Army (PRC armed forces)	a power saver (electronics)	Pretty compact, looking forward to the results	JD.com
Eight Catty Army Army (PRC armed forces)	a power saver (electronics)	[Smiles]	JD.com
Eight Catty Army Army (PRC armed forces)	a power saver (electronics)	Used to be at a flat rate	JD.com
Eight Catty Army Army (PRC armed forces)	a power saver (electronics)	We'll talk about it after we start using it.	JD.com
Eight Catty Army Army (PRC armed forces)	a power saver (electronics)	Product received immediately used, the home power consumption is too high, I hope that the power saver can really save power consumption, the effect is good also ready to buy2	JD.com
Eight Catty Army Army (PRC armed forces)	a power saver (electronics)	Save appliances received to buy two, see exquisite workmanship, have begun to use, everyone online reviews say the effect is good, should not be wrong!	JD.com
Eight Catty Army Army (PRC armed forces)	a power saver (electronics)	Shopping here is safe and convenient and fast, the courier boy has a very good attitude, this price is very powerful, very recommended, will continue to buy back[smile][smile][big love][big love][big love	JD.com
Eight Catty Army Army (PRC armed forces)	a power saver (electronics)	Received the item! Fully packaged and the saver is very powerful! Good reviews. For the country to advocate green low-carbon environmentally friendly life to dedicate a force! Thanks to the manufacturers of the invention! I hope that manufacturers produce more products that are favorable to the people!	JD.com
Eight Catty Army Army (PRC armed forces)	a power saver (electronics)	Product receipt flow quickly, the product is very compact, today began to plug in and use, after a month to see the effect.	JD.com
Eight Catty Army Army (PRC armed forces)	a power saver (electronics)	Intelligent economizer has arrived, logistics is fast, customer service attitude is very good	JD.com
Eight Catty Army Army (PRC armed forces)	a power saver (electronics)	quality goods Looking forward to the results	JD.com
Eight Catty Army Army (PRC armed forces)	a power saver (electronics)	Saving power baby received, consistent with the description, logistics is super fast, the seller has a good service attitude, praise!	JD.com
Eight Catty Army Army (PRC armed forces)	a power saver (electronics)	Goods received, easy to use, logistics is fast, good, good service attitude, to the business five-star praise.	JD.com
Eight Catty Army Army (PRC armed forces)	a power saver (electronics)	Fast shipping, looks good and perfect, very easy to use, the effect to be observed for a period of time to say.	JD.com
Eight Catty Army Army (PRC armed forces)	a power saver (electronics)	I've asked if it's effective to use one at home with a lot of power-consuming appliances. Thank you!	JD.com
Eight Catty Army Army (PRC armed forces)	a power saver (electronics)	Not bad hopefully it will save power, I'll comment after I use it thanks!	JD.com
Eight Catty Army Army (PRC armed forces)	a power saver (electronics)	Merchant service attitude is very good, logistics is also very fast, power saving to wait for a period of time to see it	JD.com
Eight Catty Army Army (PRC armed forces)	a power saver (electronics)	Hahahahaha good for you	JD.com
Eight Catty Army Army (PRC armed forces)	a power saver (electronics)	Things received, with can, good-looking, display normal, easy to use, with a period of time, only out of the effect, good in the follow-up comment |	JD.com

Eight Catty Army Army (PRC armed forces)	a power saver (electronics)	Things received, it is very simple to use, plugged in, very lightweight, received and plugged in, to see the follow-up effect, in the evaluation[smile]the same time with two	JD.com
Eight Catty Army Army (PRC armed forces)	a power saver (electronics)	This user feels the product is better	JD.com
Eight Catty Army Army (PRC armed forces)	a power saver (electronics)	quality goods Looking forward to the results	JD.com
Eight Catty Army Army (PRC armed forces)	a power saver (electronics)	I got it. It looks great. Thanks, boss.	JD.com
Eight Catty Army Army (PRC armed forces)	a power saver (electronics)	Received the goods, the appearance of no damage. This morning I used it, I don't know how effective it is. Regardless of the effect, will follow the review. Thank you for the business and logistics and courier boy!	JD.com
Eight Catty Army Army (PRC armed forces)	a power saver (electronics)	Cost-effective, will recommend friends to buy, good appearance, the appliance is very good, the appliance is very good, working properly, easy to use, no noise, effective, logistics is very fast, the appearance is very good, there is a role.	JD.com
Eight Catty Army Army (PRC armed forces)	a power saver (electronics)	After receiving the smart saver immediately plugged into the power supply, the indicator light is on, indicating normal operation. As for the effect, two months later to make a comparison, I believe the manufacturer's products, will make me satisfied.	JD.com
Eight Catty Army Army (PRC armed forces)	a power saver (electronics)	Express speed is getting faster and faster, product quality will be evaluated after use.	JD.com
Eight Catty Army Army (PRC armed forces)	a power saver (electronics)	The quality is very good, very satisfied with a shopping. Customer service has questions and answers, very professional really good, cost-effective and good quality, the logistics is also fast ah! Arrived in one day! Is genuine, trust merchants, baby texture is very good, the price is moderate, unlimited repurchase! Logistics and products are good, cost-effective, praise!	JD.com
Eight Catty Army Army (PRC armed forces)	a power saver (electronics)	Product receipt flow quickly, the product is very compact, today began to plug in and use, after a month to see the effect.	JD.com
Eight Catty Army Army (PRC armed forces)	a power saver (electronics)	Hello store, the power saver received, well-packed, logistics is very fast, something beautiful and generous, cuttings power, very beautiful, inexpensive, worth buying, after the use of follow-up, I gave all five-star praise.	JD.com
Eight Catty Army Army (PRC armed forces)	a power saver (electronics)	Have been installing it in the house for a few days. Looking forward to the power savings.[Cool]	JD.com
Eight Catty Army Army (PRC armed forces)	a power saver (electronics)	[Lots of love] [Lots of love] [Lof love] [Lotsof love]	JD.com
Eight Catty Army Army (PRC armed forces)	a power saver (electronics)	Got it last night. Used it right away	JD.com
Eight Catty Army Army (PRC armed forces)	a power saver (electronics)	Business: the power saver received, the appearance of the style can be, plug easy to use, give five-star praise, the effect of two months to see the use of follow-up, if the effect is good and then buy2units.	JD.com
Eight Catty Army Army (PRC armed forces)	a power saver (electronics)	Feels good to try	JD.com
Eight Catty Army Army (PRC armed forces)	a power saver (electronics)	EXPRESS RECEIPT: Very nice looking as good will buy again.	JD.com
Eight Catty Army Army (PRC armed forces)	a power saver (electronics)	The saver is very good and the owner is pretty nice too	JD.com
Eight Catty Army Army (PRC armed forces)	a power saver (electronics)	Just received the goods to plug into the electricity to see how good to make, I also want to buy a larger power, I do not know if there is a logistics very quickly? The style is very new, good service attitude. Wish merchants prosperous business, give five-star praise.	JD.com
Eight Catty Army Army (PRC armed forces)	a power saver (electronics)	Read a lot of reviews before deciding to buy this economizer, the appearance is very good. Wait a few days in the follow-up evaluation, I hope the goods are genuine, special praise for the courier boy, the day is very hot, the packaging is not damaged, hard work. Customer service also patiently answered a	JD.com
Eight Catty Army Army (PRC armed forces)	a power saver (electronics)	Have been installing it in the house for a few days. Looking forward to the power savings.	JD.com
Eight Catty Army Army (PRC armed forces)	a power saver (electronics)	Just received the goods to plug into the electricity to see how good to make, I also want to buy a larger power, I do not know if there is a logistics very quickly? The style is very new, good service attitude. Wish merchants prosperous business, give five-star praise.	JD.com
Eight Catty Army Army (PRC armed forces)	a power saver (electronics)	Product received immediately used, the home power consumption is too high, I hope that the power saver can really save power consumption, the effect is good also ready to buy2	JD.com
Eight Catty Army Army (PRC armed forces)	a power saver (electronics)	Hello store, online shopping new power saver received very quickly, compact and lightweight design, easy to operate, convenient and safe, for the summer power peak can highlight its energy-saving features, to be used to comment on its power-saving effect!	JD.com
Eight Catty Army Army (PRC armed forces)	a power saver (electronics)	Slows down and saves power well! Great customer service, fast shipping, and a conscientious and responsible courier. All give five stars!	JD.com
Eight Catty Army Army (PRC armed forces)	a power saver (electronics)	Received the item! Fully packaged and the saver is very powerful! Good reviews. For the country to advocate green low-carbon environmentally friendly life to dedicate a force! Thanks to the manufacturers of the invention! I hope that manufacturers produce more products that are favorable to the people!	JD.com
Eight Catty Army Army (PRC armed forces)	a power saver (electronics)	I have bought it before, plugged it into a power outlet at home and it works, very convenient and works well. Recommended to family and friends. Bought this time ready to give to friends.	JD.com
Eight Catty Army Army (PRC armed forces)	a power saver (electronics)	Cost-effective, will recommend friends to buy, good-looking, the appliance is very good, the appliance is very good, working properly, easy to use, no noise, effective, logistics is very fast	JD.com
Eight Catty Army Army (PRC armed forces)	a power saver (electronics)	This summer is too hot and use a lot of electricity, read a lot of reviews bought this saver, hope to save some electricity. Merchants ship quickly, logistics is also fast	JD.com
Eight Catty Army Army (PRC armed forces)	a power saver (electronics)	Very good looking, very light to use, match the description, match the description, as described, good service, the product is as described, weighty, power-saving effect is still okay	JD.com
Eight Catty Army Army (PRC armed forces)	a power saver (electronics)	Just received the goods to plug into the electricity to see how good to make, I also want to buy a larger power, I do not know if there is a logistics very quickly? The style is very new, business service	JD.com
Eight Catty Army Army (PRC armed forces)	a power saver (electronics)	Saving appliances received, the appearance of the style can be, the plug is easy to use, give five-star praise, the effect of two months to see the use of follow-up, if the effect is good and then buy2units.	JD.com
Eight Catty Army Army (PRC armed forces)	a power saver (electronics)	Have been installing it in the house for a few days. Looking forward to the power savings.[Cool]	JD.com
Eight Catty Army Army (PRC armed forces)	a power saver (electronics)	[Lots of love] [Lots of love] [Lof love] [Lotsof love]	JD.com

Eight Catty Army Army (PRC armed forces)	a power saver (electronics)	Just received the goods to plug into the electricity to see how good to make, I also want to buy a larger power, I do not know if there is a logistics very quickly? The style is very new, good service attitude. Wish merchants prosperous business, give five-star praise.	JD.com
Eight Catty Army Army (PRC armed forces)	a power saver (electronics)	I have bought it before, plugged it into a power outlet at home and it works, very convenient and works well. Recommended to family and friends. Bought this time ready to give to friends.	JD.com
Eight Catty Army Army (PRC armed forces)	a power saver (electronics)	Got it last night. Used it right away	JD.com
Eight Catty Army Army (PRC armed forces)	a power saver (electronics)	Read a lot of reviews before deciding to buy this economizer, the appearance is very good. Wait a few days in the follow-up evaluation, I hope the goods are genuine, special praise for the courier boy, the day is very hot, the packaging is not damaged, hard work. Customer service also patiently answered a	JD.com
Eight Catty Army Army (PRC armed forces)	a power saver (electronics)	Business: the power saver received, the appearance of the style can be, plug easy to use, give five-star praise, the effect of two months to see the use of follow-up, if the effect is good and then buy2units.	JD.com
Eight Catty Army Army (PRC armed forces)	a power saver (electronics)	Feels good to try	JD.com
Eight Catty Army Army (PRC armed forces)	a power saver (electronics)	EXPRESS RECEIPT: Very nice looking as good will buy again.	JD.com
Eight Catty Army Army (PRC armed forces)	a power saver (electronics)	The saver is very good and the owner is pretty nice too	JD.com
Duchene Home Dept.	In-situ compensated economizer	This user feels the product is better	JD.com
Duchene Home Dept.	In-situ compensated economizer	Received]see okay don't knowhow touseitfor a period of time is that he this manual are in English can't read it	JD.com
Duchene Home Dept.	In-situ compensated economizer	Goods have been received very well hope to work with you again next time.	JD.com
Duchene Home Dept.	In-situ compensated economizer	This user feels the product is better	JD.com
Duchene Home Dept.	In-situ compensated economizer	It doesn't feel like it's working. It's not saving any power.	JD.com
Duchene Home Dept.	In-situ compensated economizer	This user feels the product is better	JD.com
Duchene Home Dept.	In-situ compensated economizer	This user feels the product is better	JD.com
Duchene Home Dept.	In-situ compensated economizer	I feel duped. I can't understand all the English, not a single Chinese character.	JD.com
Duchene Home Dept.	In-situ compensated economizer	It's plugged in. Looking forward to the results.	JD.com
Duchene Home Dept.	In-situ compensated economizer	Instructions, can't read them,<br>Look at tomorrow, it'll save a few kWh.	JD.com
Duchene Home Dept.	In-situ compensated economizer	This user feels the product is better	JD.com
Duchene Home Dept.	In-situ compensated economizer	Well made, easy to use, beautiful baby	JD.com
Duchene Home Dept.	In-situ compensated economizer	The design is beautiful and durable.	JD.com
Duchene Home Dept.	In-situ compensated economizer	Save appliances received, logistics is very fast packaging is very good, and4, bought2for the neighbors, good value for money, very satisfied, really save electricity, praise!	JD.com
Duchene Home Dept.	In-situ compensated economizer	This user feels the product is better	JD.com
Duchene Home Dept.	In-situ compensated economizer	It really saves electricity, highly recommended	JD.com
Duchene Home Dept.	In-situ compensated economizer	It does save power.	JD.com
Duchene Home Dept.	In-situ compensated economizer	This user feels the product is better	JD.com
Duchene Home Dept.	In-situ compensated economizer	This power saver is good and as promised, it saves power and works well with no irritation Product performance is good I've bought it again for the second time!	JD.com
Duchene Home Dept.	In-situ compensated economizer	This user feels the product is better	JD.com
Duchene Home Dept.	In-situ compensated economizer	Really saves a lot of electricity, really good! I'm going to buy another one.	JD.com
Duchene Home Dept.	In-situ compensated economizer	Received the saver It's good and I'll keep buying it again	JD.com
Duchene Home Dept.	In-situ compensated economizer	Fine workmanship	JD.com
Duchene Home Dept.	In-situ compensated economizer	It's good. A friend wants to buy it.	JD.com
Duchene Home Dept.	In-situ compensated economizer	It's not working at all.	JD.com
Duchene Home Dept.	In-situ compensated economizer	The product is very effective, the business service attitude is very good, it is well worth buying, thank you boss!	JD.com
Duchene Home Dept.	In-situ compensated economizer	Logistics is super fast, looking forward to using the effect, and product quality!	JD.com
Duchene Home Dept.	In-situ compensated economizer	well, well, well.	JD.com
Duchene Home Dept.	In-situ compensated economizer	Things received logistics quite fast.	JD.com
Duchene Home Dept.	In-situ compensated economizer	This user did not fill in a rating	JD.com
Duchene Home Dept.	In-situ compensated economizer	I don't know if it's working yet.	JD.com
Duchene Home Dept.	In-situ compensated economizer	High power consumption, inconsistent with the facts, just use it, two degrees of electricity more overnight, the business said to use for half a month, every day risking two degrees of electricity more, this is half a month, or the same high power consumption	JD.com
Duchene Home Dept.	In-situ compensated economizer	This user feels the product is better	JD.com
Duchene Home Dept.	In-situ compensated economizer	This user feels the product is better	JD.com
Duchene Home Dept.	In-situ compensated economizer	This user did not fill in a rating	JD.com
Duchene Home Dept.	In-situ compensated economizer	I've already used it. I don't know how it works.	JD.com
Duchene Home Dept.	In-situ compensated economizer	This user feels the product is better	JD.com
Duchene Home Dept.	In-situ compensated economizer	This user feels the product is better	JD.com
Duchene Home Dept.	In-situ compensated economizer	This user feels the product is better	JD.com

Duchene Home Dept.	In-situ compensated economizer	A ghost of a scam. Don't buy it, people.	JD.com
Duchene Home Dept.	In-situ compensated economizer	This user feels the product is better	JD.com
Duchene Home Dept.	In-situ compensated economizer	This user feels the product is better	JD.com
Duchene Home Dept.	In-situ compensated economizer	This user feels the product is better	JD.com
Duchene Home Dept.	In-situ compensated economizer	I don't know if it works. I'll come back next time.	JD.com
Duchene Home Dept.	In-situ compensated economizer	courier boy is not helpful[fuming]not even give a phone call, randomly placed!	JD.com
Duchene Home Dept.	In-situ compensated economizer	I don't know if it will save power, I don't think it's possible with this thing, follow up	JD.com
Duchene Home Dept.	In-situ compensated economizer	This user feels the product is better	JD.com
Duchene Home Dept.	In-situ compensated economizer	I don't know if it works yet.	JD.com
Duchene Home Dept.	In-situ compensated economizer	an excellent	JD.com
Duchene Home Dept.	In-situ compensated economizer	It should work as we have been using the Saver and will repurchase if it's good.	JD.com
Duchene Home Dept.	In-situ compensated economizer	This user feels the product is better	JD.com
Duchene Home Dept.	In-situ compensated economizer	From the surface of the better workmanship, power saving or not need to be observed for a long time.	JD.com
Duchene Home Dept.	In-situ compensated economizer	This user feels the product is better	JD.com
Duchene Home Dept.	In-situ compensated economizer	This user feels the product is better	JD.com
Duchene Home Dept.	In-situ compensated economizer	It's okay.	JD.com
Duchene Home Dept.	In-situ compensated economizer	The best decision is really, you guys come over to buy this kind of is really not no fake, this is really come over to buy into you a note ah, to focus on me a person to buy very good, really decided to be [illegible]	JD.com
Duchene Home Dept.	In-situ compensated economizer	This user feels the product is better	JD.com
Duchene Home Dept.	In-situ compensated economizer	This user feels the product is better	JD.com
Duchene Home Dept.	In-situ compensated economizer	This user feels the product is better	JD.com
Duchene Home Dept.	In-situ compensated economizer	This user feels the product is better	JD.com
Duchene Home Dept.	In-situ compensated economizer	This user feels the product is better	JD.com
Duchene Home Dept.	In-situ compensated economizer	This user feels the product is better	JD.com
Duchene Home Dept.	In-situ compensated economizer	Very satisfied, logistics is also very fast, give 5 stars!	JD.com
Duchene Home Dept.	In-situ compensated economizer	First time to buy this saver, after receiving the goods to see the instructions, it is very convenient, the saver is small and does not take up space, my family uses a lot of electricity, after the effect, continue to buy back5! Kudos!	JD.com
Duchene Home Dept.	In-situ compensated economizer	This user did not fill in a rating	JD.com
Duchene Home Dept.	In-situ compensated economizer	Got it. Five stars.	JD.com
Duchene Home Dept.	In-situ compensated economizer	This user feels the product is better	JD.com
Duchene Home Dept.	In-situ compensated economizer	This user feels the product is better	JD.com
Duchene Home Dept.	In-situ compensated economizer	This user feels the product is better	JD.com
Duchene Home Dept.	In-situ compensated economizer	Rip off after a few days of use it doesn't save any power at all	JD.com
Duchene Home Dept.	In-situ compensated economizer	This user did not fill in a rating	JD.com
Duchene Home Dept.	In-situ compensated economizer	This user feels the product is better	JD.com
Duchene Home Dept.	In-situ compensated economizer	This user feels the product is better	JD.com
Duchene Home Dept.	In-situ compensated economizer	Two days no effect, business customer service said a month or two to optimize the good, I am looking at the table to use, expect effective	JD.com
Duchene Home Dept.	In-situ compensated economizer	This user feels the product is better	JD.com
Duchene Home Dept.	In-situ compensated economizer	We won't know until next month.	JD.com
Duchene Home Dept.	In-situ compensated economizer	evaluation after use	JD.com
Duchene Home Dept.	In-situ compensated economizer	Use it for a while and then comment	JD.com
Duchene Home Dept.	In-situ compensated economizer	[Smiles]It feels okay	JD.com
Duchene Home Dept.	In-situ compensated economizer	Express received, packaging complete very good, looks good,that good use, is really save electricity, worth buying.<br>Power-saving effect: good Cost-effective: very high Appearance style: simple Workmanship material: good Experience: buy two to send a, put the home every layer of a, hope that a month to see the effect, has been recommended to the family<br>Power-saving effect: can really save some good Cost-effective: cost-effective Appearance style: good-looking Workmanship material: good materials Experience: the effect is very good<br>This is already the second time to buy this kind of power saver, the quality is good, can effectively save the daily electricity, good use is not expensive, good, worth recommending Oh<br>Good, can play a role in the first inserted a, how much power savings can not be calculated, but inserted and not inserted into the meter word jumping fast or slow there is a difference between this store service is also very good, the same products sold very affordable, there are also prizes, the service to the right to recommend to everyone,<br>Relatives introduced, bought2, the appearance of good workmanship, the price is cheap, you can try. Simple to use. Use2effect hope better.<br><br>It's cold to turn on some electric heaters that meter is faster than an airplane. Used6.Sure enough there is an effect, has recommended relatives and friends to buy _<br>Power-saving effect: very obvious Cost-effectiveness: buy assured with happy Appearance style: beautiful and exquisite Workmanship material: real material Experience:1 *the most shallow and easy to understand the schematic diagram in history, but where literacy are clear 2 *cooking time is shortened by3minutes, the quality of the line power supply is significantly improved. Recommended for purchase to	JD.com

Duchene Home Dept.	In-situ compensated economizer	Power-saving effect: energy-saving effect is better, very can save electricity, before the electricity bill is more, after using this product, the electricity bill made to save a lot, really too good to use!	JD.com
Duchene Home Dept.	In-situ compensated economizer	Intelligent power saving system has been received20days. Energy saving effect is remarkable, cost-effective, I also alien beauty, fine workmanship, recently home cold, open the air conditioning every day before the monthly electricity bill inabout500yuan also used the intelligent power-saving system, after the monthly electricity bill can saveabout70yuan, recommended to everyone, really good to use	JD.com
Duchene Home Dept.	In-situ compensated economizer	Just received and opened to check, packaging fine not rough, customer service can patiently answer questions, looking forward to the effect! Give the store5stars praise	JD.com
Duchene Home Dept.	In-situ compensated economizer	Baby received a few days of easy to use, only to see the effect, if the effect is very good, I will follow up, but also will introduce to others to buy!	JD.com
Duchene Home Dept.	In-situ compensated economizer	The goods have been received, the appearance of the packaging exquisite……and easy to use, open store friends can buy this product! I tried it for a few days hoping to save myself some money on my electric bill! Next time will buy a few more	JD.com
Duchene Home Dept.	In-situ compensated economizer	Baby is very good, just arrived can not wait to use it, I like very much hope to achieve the desired effect of saving electricity and money, the future will also recommend to friends!	JD.com
Duchene Home Dept.	In-situ compensated economizer	The packaging is very delicate, the power saver is lightweight, I tried two places and the voltage is stable, I look forward to the power saving effect. Thank you for your help.	JD.com
Duchene Home Dept.	In-situ compensated economizer	Things received, try a day, the effect is good, so every month to save a little, down to the end of the year can save a lot of	JD.com
Duchene Home Dept.	In-situ compensated economizer	Merchants promise100days invalid full refund, no hesitation to buy6, try the next month's electricity bill in the end, equal to free use	JD.com
Duchene Home Dept.	In-situ compensated economizer	Friends recommended, do not yet know that there are so amazing things, the business gives100days of free use, decisively bought6	JD.com
Duchene Home Dept.	In-situ compensated economizer	Saving appliances are also recommended by a friend, he bought5inone go last time, the electricity bill is really saved a lot, this time I also buy5	JD.com
Duchene Home Dept.	In-situ compensated economizer	The saver works well, plug it in and it works, where it consumes power plug it in, even I, a woman who doesn't do much, can use it successfully, it's very convenient and convenient!	JD.com
Duchene Home Dept.	In-situ compensated economizer	The saver was received, well packaged and received in perfect condition, purch5this time, looking forward to next month's electricity bill	JD.com
Duchene Home Dept.	In-situ compensated economizer	Lifetime warranty, the business is very thoughtful, I purchased 5 I also sent me some gifts, and commitment to invalid full refund, now used for a period of time down, the feeling that the electricity bill is less, and so fully optimized after the belief that the effect will be even better, to the business of five-star praise, and recommended!	JD.com
Duchene Home Dept.	In-situ compensated economizer	Customer service introduced this is an energy saver, in situ compensation, it is also very convenient to use, where the power consumption plug where, but also can be moved at any time , do not need to be wired, it is very suitable for us as a female user, to the business designers kudos!	JD.com
Duchene Home Dept.	In-situ compensated economizer	Taobao recommended to me, or know that I use a lot of electricity, they recommended me this good thing, so I did not hesitate to buy, looking forward to this month's electricity bill!	JD.com
Duchene Home Dept.	In-situ compensated economizer	Received the goods can not wait to open and look at the power saver is very curious, above the authenticity of the logo, but also thoughtful purchase of insurance, business warranty of 5 years, and the use of the manual, a very formal product!	JD.com
Duchene Home Dept.	In-situ compensated economizer	Good and inexpensive power saver, an investment for life, save a few dollars a day a year down can also save a lot of money, thanks to the store!	JD.com
Duchene Home Dept.	In-situ compensated economizer	Purchased a total of 5 power saver, deliberately used for a period of time to comment, this section of the power saver is really good, and now a total of more than 70 blocks of electricity, only ten days of time, I believe that the future will save more power!	JD.com
Duchene Home Dept.	In-situ compensated economizer	Workmanship is very good, the material is also a good chat, the shopkeeper service is also good, the purchase of five complimentary 1 to me, good if you will buy back!	JD.com
Duchene Home Dept.	In-situ compensated economizer	This saver is not only good performance, but also high value, no matter the workmanship and materials are very good, this is the second time to buy, this time the purchase of 8, to relatives and friends are used, everyone said I helped a lot!	JD.com
Duchene Home Dept.	In-situ compensated economizer	Smart saver is good, can save a lot every month, consulting customer service my family's electricity consumption, so purchased 4, looking forward to the next month's electricity bill, the	JD.com
Duchene Home Dept.	In-situ compensated economizer	Started to hold the attitude to try to buy, I did not expect this power saver is really useful, this month really save a lot of electricity bills!	JD.com
Duchene Home Dept.	In-situ compensated economizer	My family's electricity consumption is particularly large, after consulting customer service, customer service carefully explained that I need to use 5 power saver to have the effect Oh, the beginning is also half-believe, and now used for a period of time down , indeed still have the effect, to the merchant	JD.com
Duchene Home Dept.	In-situ compensated economizer	Friends introduced me to buy, the store's energy-saving appliances genuine guarantee, plugged in really save a lot of electricity, my friends bought 5, I also do not hesitate to buy 5 this time!	JD.com
Duchene Home Dept.	In-situ compensated economizer	The saver is simply amazing, after plugging it in, it really saves a lot of electricity, really hate to meet with this saver.	JD.com
Duchene Home Dept.	In-situ compensated economizer	Saver has been used for a period of time, is still quite good, workmanship is also very good, the shopkeeper service is very good!	JD.com
Duchene Home Dept.	In-situ compensated economizer	Used for a period of time to evaluate, the power-saving effect really did not let me down no longer need to worry about the power consumption of high-power appliances, the material is very good, the manual is simple and easy to understand there are indicators will look at the use of quiet use of no noise small and exquisite inserted where it does not get in the way of a very good product!	JD.com
Duchene Home Dept.	In-situ compensated economizer	Baby is small, compact and well priced. It is the head quartet in the style and is perfect. Although the epidemic delayed a little time, but can understand. After using it for a few days, it feels very good, so don't worry about buying it, you'll be right!	JD.com
Duchene Home Dept.	In-situ compensated economizer	Baby received in good condition. Fast shipping, fine packaging, baby exquisite effect to be verified. Thank you for the integrity of the seller, thank you attentive customer service! Thank you dedicated courier! Five stars out of five!!! Wish you not forget the original intention, move forward, business is booming! Family happiness and well-being!!! Service: service attitude service quality is awesome!!!!	JD.com
Duchene Home Dept.	In-situ compensated economizer	Power saving effect: the power saver received, power saving effect is obvious, cost-effective: cost-effective very high Appearance style: exquisite appearance, fine workmanship, open the air conditioning with two hours, feel very satisfied.	JD.com
Duchene Home Dept.	In-situ compensated economizer	I did not think there are so magical things, before has been a waste of a lot of money, this time to buy 2, plugged in after a smart display shows that the machine is working, good to use in recommending friends around, the shopkeeper service is also very good!	JD.com
Duchene Home Dept.	In-situ compensated economizer	The first time to buy this energy saver, do not know that there is this thing, hold a try to buy the mentality, the business commitment to no effect full refund, after receiving the product there is a manual, it is very convenient, the saver is compact and does not take up space, my family's power consumption is very large, the purchase of 6, the effect of the open after the chase comment!	JD.com
Duchene Home Dept.	In-situ compensated economizer	Relatives recommended, do not know that there is so good use of things, it is said that she last time to buy 5, 1 month to save more than 100 of the electricity bill, this time I also do not hesitate to buy 5!	JD.com
Duchene Home Dept.	In-situ compensated economizer	Friends last time to buy 2, the effect is good, every other month the electricity bill has dropped significantly, this time friends recommended that I come, the beginning did not know that there is something so good to use, this time to buy 6, the family's self-build room is too much, it is really a one-time investment, lifelong benefit, the product of a lifetime warranty, the bad for free replacement of the new, the merchant to give the protection is very thoughtful, and the business is very good.	JD.com
Duchene Home Dept.	In-situ compensated economizer	Value for money: just received, then checked on the plug board, looked at the pulse meter, with the same appliances, with and without a saver. There is a real difference in the blinking frequency of the pulse meter light. With the saver plugged in, the meter pulse light blinks much slower. Not bad at all...	JD.com
Duchene Home Dept.	In-situ compensated economizer	Logistics is very fast, plugged in with a very dark night light, the instructions read and feel that it makes sense, I hope it can really save electricity, otherwise the home electricity bill is really quite high, especially in the winter, the first praise, and so on a month after the follow-up!	JD.com
Duchene Home Dept.	In-situ compensated economizer	Friends used to recommend that I come over to buy, at first I do not believe that this thing, with the intention of trying to buy 3, the results are indeed unexpected, and will recommend friends to buy, now too many electrical appliances, 1 month are several hundred of the electricity bill, and now every day can save a dozen or so to the merchant praise!	JD.com

Duchene Home Dept.	In-situ compensated economizer	Purposefully used for a period of time to evaluate, indeed useful, began to hear people say is the IQ tax, and now they do use and effect, fortunately they try, or else miss the good stuff, day after day can save a lot of business informed that the greater the power consumption needs to be the more energy-saving appliances to be more and more, this time to buy six!	JD.com
Duchene Home Dept.	In-situ compensated economizer	Seller service and can, very happy a shopping. Must give a praise, haha really affordable,,.	JD.com
Duchene Home Dept.	In-situ compensated economizer	I hope that if you want to buy can immediately start, baby received, recognize this.	JD.com
Duchene Home Dept.	In-situ compensated economizer	Logistics is very fast, the service attitude level can be, praise, is the first time to encounter such a heart of the seller!	JD.com
Duchene Home Dept.	In-situ compensated economizer	Friends are good. Service attitude hate line, so like, good value for money	JD.com
Duchene Home Dept.	In-situ compensated economizer	Fast logistics, love it and it's very generous	JD.com
Duchene Home Dept.	In-situ compensated economizer	I hope the more you do the better. Logistics fast praise, and the store described exactly the same, very can be shipped quickly service thoughtful, the	JD.com
Duchene Home Dept.	In-situ compensated economizer	Shipping was quick. Merchandise is also quite line. Love it so much, bought a lot of things are very can seller I will come back often, baby received really very good!	JD.com
Duchene Home Dept.	In-situ compensated economizer	Baby received quite like, baby received, the shopkeeper's service attitude really can be	JD.com
Duchene Home Dept.	In-situ compensated economizer	Anyone who likes it should hurry up and order it, they forgot, it's cheap	JD.com
Duchene Home Dept.	In-situ compensated economizer	It's good, in a word it's ok, I heard her say the quality is ok	JD.com
Duchene Home Dept.	In-situ compensated economizer	If you like the girl hurry up, so also, the stuff line. Trustworthy.	JD.com
Duchene Home Dept.	In-situ compensated economizer	All give can, I will be back, the quality is quite good price is very fair logistics very fast very good store, I hope the next new product will not let me down.	JD.com
Duchene Home Dept.	In-situ compensated economizer	Affordable, very fast very fast very fast thank you for the opportunity to work together again, overall it's worth buying for the price,,,	JD.com
Duchene Home Dept.	In-situ compensated economizer	Physical and store description of the same, logistics attitude is good, the key is the price is right quality looks pretty good	JD.com
Duchene Home Dept.	In-situ compensated economizer	And the store is very eager to respond to my questions, the quality can't be beat, praise be, the	JD.com
Duchene Home Dept.	In-situ compensated economizer	Just as soon as I received it, I couldn't wait to open it, praise the merchant, I love it This quality is very good ah, it is worth considering.	JD.com
Duchene Home Dept.	In-situ compensated economizer	Favorite the store, value for money service attitude is very good, praise,	JD.com
Duchene Home Dept.	In-situ compensated economizer	I heard her say the quality is okay, hahaha the seller is especially okay and affordable	JD.com
Duchene Home Dept.	In-situ compensated economizer	Express service is also very good, we can rest assured that the purchase, and will come back to buy!	JD.com
Duchene Home Dept.	In-situ compensated economizer	Logistics is very acceptable, affordable, good courier service	JD.com
Duchene Home Dept.	In-situ compensated economizer	I like it very much. Thank you boss, logistics can be fast ah, value for money, thank you!	JD.com
Duchene Home Dept.	In-situ compensated economizer	Thanks a lot. It fits. Love it.	JD.com
Duchene Home Dept.	In-situ compensated economizer	I received the goods the next day and the express delivery was fast. Will come back next time, very affordable	JD.com
Duchene Home Dept.	In-situ compensated economizer	Love it, kudos, kudos, and the shipping was very fast!	JD.com
Duchene Home Dept.	In-situ compensated economizer	Totally out of expectation, want to buy, love it!	JD.com
Duchene Home Dept.	In-situ compensated economizer	The seller shipped quickly, it is the kind I want Thank you very happy shopping, affordable Oh!	JD.com
Duchene Home Dept.	In-situ compensated economizer	Love it, very tightly packaged and affordable.	JD.com
Duchene Home Dept.	In-situ compensated economizer	Bought the baby, love it, can comment on it, can comment on it, can comment on it, can comment on it, can comment on it, can comment on it, can comment on it.	JD.com
Duchene Home Dept.	In-situ compensated economizer	The seller's service is also very commendable, it's ok, sorry. Only now come to evaluate	JD.com
Duchene Home Dept.	In-situ compensated economizer	The line is just as described in the photo, a pleasant purchase to review, but it's the first time I've laid my hands on it. Should say it's still affordable. Second time I've bought it.	JD.com
Duchene Home Dept.	In-situ compensated economizer	Said it's very ok, overall it's worth buying at this price, very good	JD.com
Duchene Home Dept.	In-situ compensated economizer	And the store is very enthusiastic to reply to my questions, the shopkeeper's service attitude is really OK, the shipment is very fast!	JD.com
Duchene Home Dept.	In-situ compensated economizer	Recommended purchase, awesome, great seller service!	JD.com
Duchene Home Dept.	In-situ compensated economizer	The line is quite comfortable logistics is also very fast, give a praise, has been an old customer	JD.com
Duchene Home Dept.	In-situ compensated economizer	My friend asked me to send her a link to buy this at this price. I bought it for a friend and it's a good price.	JD.com
Duchene Home Dept.	In-situ compensated economizer	Value for money service attitude is very good, shipping speed is quite fast praise, affordable. Big love, kudos, kudos, kudos.	JD.com
Duchene Home Dept.	In-situ compensated economizer	Originally bought with the heart to try, I wish the boss a prosperous business, was worried about the quality of	JD.com
Duchene Home Dept.	In-situ compensated economizer	I like this quality is very good ah, worth considering, customer service is very professional, the quality is okay	JD.com
Duchene Home Dept.	In-situ compensated economizer	It's great, I hope the next new product doesn't disappoint me.	JD.com
Duchene Home Dept.	In-situ compensated economizer	Worth buying, good seller service, very unexpected	JD.com
Duchene Home Dept.	In-situ compensated economizer	The stuff is fine, wrapped with care, quite fine	JD.com
Duchene Home Dept.	In-situ compensated economizer	Unintentionally came to you to buy the heart of the baby mood line baby received, very affordable, did not sign in time. Praise, praise.	JD.com
Duchene Home Dept.	In-situ compensated economizer	Looks good, much better than the one I bought at another place, very good!	JD.com
Duchene Home Dept.	In-situ compensated economizer	Cost-effective Oh very much like the future will continue to patronize, to buy a very can be a baby, something line	JD.com
Duchene Home Dept.	In-situ compensated economizer	Quality is fine, baby received, not the first time I've purchased. Fast shipping	JD.com
Duchene Home Dept.	In-situ compensated economizer	The quality is great, the price is affordable, it's the recipient who gets to the hand	JD.com
Duchene Home Dept.	In-situ compensated economizer	Things received very much like really level can be the seller to answer questions relentlessly meticulous and conscientious key is that things can be and goods sent quickly packaged carefully trustworthy, quite like, I use my personal experience to tell you all	JD.com
Duchene Home Dept.	In-situ compensated economizer	I hope the speed of delivery needs to be improved thank you, very ok seller definitely support, well well well well well well well	JD.com
Duchene Home Dept.	In-situ compensated economizer	Overall think it is okay, will come back, see can not hesitate to do so oh	JD.com

Duchene Home Dept.	In-situ compensated economizer	Just didn't have time to confirm receipt, the seller has a great service attitude, opened the package and looked at it right away	JD.com
Duchene Home Dept.	In-situ compensated economizer	Great value, just like the picture, and it's nice and really affordable!	JD.com
Duchene Home Dept.	In-situ compensated economizer	All in all great praise,, customer service attitude affordable, very patient store owner arrived in a timely manner, can be, never a TO!	JD.com
Duchene Home Dept.	In-situ compensated economizer	Can, line, packaging with care. Express delivery is also quite OK.	JD.com
Duchene Home Dept.	In-situ compensated economizer	I received the item the next day and love it, I will continue to patronize it. Seller service level also great!	JD.com
Duchene Home Dept.	In-situ compensated economizer	Support, love it, bought it for a coworker	JD.com
Duchene Home Dept.	In-situ compensated economizer	It can be, and it's cheap quality, quite like it.	JD.com
Duchene Home Dept.	In-situ compensated economizer	Very awesome, packed with care. Express delivery is also quite OK and fast. Next time will come again.	JD.com
Duchene Home Dept.	In-situ compensated economizer	Worth buying good value for money, like friends quickly get down to it, received in the morning, the baby received	JD.com
Duchene Home Dept.	In-situ compensated economizer	Can, have like the pro can decisively start, this price can buy such a good thing is quite cost-effective. Next time will continue to patronize. Overall quite good	JD.com
Duchene Home Dept.	In-situ compensated economizer	It's quite ok, genuinely ok. Kudos, kudos, affordable!	JD.com
Duchene Home Dept.	In-situ compensated economizer	I like a shopping, will often visit this store, and will come back to the store if needed!	JD.com
Duchene Home Dept.	In-situ compensated economizer	Meh, the delivery is all fine, and the seller's service is fine too	JD.com
Duchene Home Dept.	In-situ compensated economizer	Can I just say, I love it. It's good quality. It's worth considering. I like it.	JD.com
Duchene Home Dept.	In-situ compensated economizer	The price is very affordable, and the quality is quite acceptable praise bar, all very affordable, very praise, the	JD.com
Duchene Home Dept.	In-situ compensated economizer	I like it better. The logistics is also quite fast, very good and affordable	JD.com
Duchene Home Dept.	In-situ compensated economizer	It's quite ok, first time I bought from this store, I'll recognize this one.	JD.com
Duchene Home Dept.	In-situ compensated economizer	Really good value, the seller's service is also very commendable, unintentionally come to you this to buy the heart of the baby mood line baby received!	JD.com
Duchene Home Dept.	In-situ compensated economizer	Well worth the money, affordable and must be praised	JD.com
Duchene Home Dept.	In-situ compensated economizer	It's great, each one of them really liked it, and the stuff is very okay	JD.com
Duchene Home Dept.	In-situ compensated economizer	It's even better than expected. The price was fair and the shipping was pretty fast kudos, love it!	JD.com
Duchene Home Dept.	In-situ compensated economizer	A friend said to have bought here I look okay look so also buy to try, thank you, the goods arrived, much better than expected	JD.com
Duchene Home Dept.	In-situ compensated economizer	Thank you for your thoughtful service. Good luck with your business, a pleasant shopping, can comment, next time come back!	JD.com
Duchene Home Dept.	In-situ compensated economizer	I finally got what I wanted. Thanks to the seller, who has good service. The shipping speed was divine.	JD.com
Duchene Home Dept.	In-situ compensated economizer	It's okay, and I like it. Baby got it.	JD.com
Duchene Home Dept.	In-situ compensated economizer	Express very can seller packaged very can service attitude very can also quite can, each one is very like genuinely very like, is very like.	JD.com
Duchene Home Dept.	In-situ compensated economizer	Got it, love it, didn't wait a few days for nothing!	JD.com
Duchene Home Dept.	In-situ compensated economizer	Good value for money, baby received really very good, logistics is very can	JD.com
Duchene Home Dept.	In-situ compensated economizer	I really did not expect online shopping is still so interesting, logistics can be, the price is moderate. Much more cost-effective than the mall to buy	JD.com
Duchene Home Dept.	In-situ compensated economizer	Very fast very fast very fast thanks for the opportunity to work together again, this store reputation is quite good. Baby's are more like diamonds in general. Thank you so much and come back next time.	JD.com
Duchene Home Dept.	In-situ compensated economizer	Quality line, like the pro hurry up, overall feel like this shopping is very like.	JD.com
Duchene Home Dept.	In-situ compensated economizer	And the price is cheap quality is very good, quite comfortable, the look is also the same as the picture, quite like it!	JD.com
Duchene Home Dept.	In-situ compensated economizer	Go outside to buy are not this price, business attitude is very good, line. Service attitude is quite good.	JD.com
Duchene Home Dept.	In-situ compensated economizer	Love it, and it's ok, big ups, order soon if you like it.	JD.com
Duchene Home Dept.	In-situ compensated economizer	Very much like it, very much like it, can can can can can can can can	JD.com
Duchene Home Dept.	In-situ compensated economizer	Shipped quickly, will come back if needed, logistics is also fast!	JD.com
Duchene Home Dept.	In-situ compensated economizer	I tell you from my personal experience, it was a purchase I really enjoyed at a moderate price. Much better deal than buying at the mall	JD.com
Duchene Home Dept.	In-situ compensated economizer	Good, even asked me for a link to say also want to buy it, originally thought so cheap stuff	JD.com
Duchene Home Dept.	In-situ compensated economizer	Just got the courier can't wait to open the out of imagination, the baby is very like. Praise. Next time continue to patronize. Logistics is very fast, the price is very affordable	JD.com
Duchene Home Dept.	In-situ compensated economizer	Such a price can buy this quality is very good, buy a lot of things are very can seller I will often come, very recommendable!	JD.com
Duchene Home Dept.	In-situ compensated economizer	The price is still cheap, the store service is OK, and it's almost as good as what I'd buy outside of the store	JD.com
Duchene Home Dept.	In-situ compensated economizer	Logistics can be, next time again. Help you advertise, haha really affordable	JD.com
Duchene Home Dept.	In-situ compensated economizer	I love it, it's the first time I've come across such an attentive seller!	JD.com
Duchene Home Dept.	In-situ compensated economizer	Comparison has 1 month specially come over to evaluate, from the very beginning of a little less every day, the back of the stable, a day of the original 20 degrees to the current 15 degrees difference of a little bit, the effect is very good, the conscience of the product, to see some people say does not save electricity, I do not know how in the end to compare, recommended to rest assured that the purchase, the	JD.com
Duchene Home Dept.	In-situ compensated economizer	Effective, fast logistics, good quality, really save electricity, my home monthly electricity bill reduced by almost half! I have recommended my friends to buy and use, especially those who use a lot of electricity, the effect is more obvious. My home is a mahjong room, without the use of energy-saving inches of monthly electricity costs are about 500, with the use of energy-saving devices after the maximum of more than 200 yuan a month. The power saver is a real power saver! There is a power saver quality assurance genuine, friends can rest assured that the purchase and use, next time to come back to	JD.com
Duchene Home Dept.	In-situ compensated economizer	The actual test is effective! My family cooks by themselves every day, steam oven induction cooker almost a month to use more than 500 dollars of electricity, now after using this, more than 400 will be enough, although I do not understand what the reasoning, but it is true to save me money to save money on the electricity bill, it's very nice!	JD.com

Duchene Home Dept.	In-situ compensated economizer	two power savers for the family, the kitchen and bathroom water heater is too costly_x005f_x005f_x0014_, the water heater to be turned on every day, every month's electricity costs are a lot of use, with this, no longer afraid of the electricity bill is more than one month down to the electricity bill than the previous month less than half of it _x0014_,The effect is particularly good, very much like Will	JD.com
Duchene Home Dept.	In-situ compensated economizer	This power-saving appliance is very practical to use at home, the value is explosive, the store said it is the new 2023, upgraded appearance and power-saving chip, better effect and longer life, the	JD.com
Duchene Home Dept.	In-situ compensated economizer	Thrifty received put into use, home appliances, power consumption is very large, we have a piece of electricity or 1 yuan 1 degree, with this thrifty is much better, every day can save a few cents, very convenient and practical, and energy-saving voltage is also stable, not bad!	JD.com
Shangrui Electric	maximize energy savings (e.g. in gasoline and diesel fuel)	not be, or cease to exist	JD.com
Lai Xiao Xiao Boutique	smart butler	(of an unmarried couple) be close	JD.com
Lai Xiao Xiao Boutique	smart butler	Baby received, very satisfied worth buying.	JD.com
Lai Xiao Xiao Boutique	smart butler	Baby received, tried a few days a little power saving effect. And the price is cheap can get it.	JD.com
Lai Xiao Xiao Boutique	smart butler	[Kudos][Kudos]	JD.com
Lai Xiao Xiao Boutique	smart butler	This user feels the product is better	JD.com
Lai Xiao Xiao Boutique	smart butler	We'll see.	JD.com
Lai Xiao Xiao Boutique	smart butler	Shipping? was also awesome in a hurry!!!!	JD.com
Lai Xiao Xiao Boutique	smart butler	Express received, have begun to use, I hope to be able to save electricity effect	JD.com
Lai Xiao Xiao Boutique	smart butler	This user feels the product is better	JD.com
Lai Xiao Xiao Boutique	smart butler	This user feels the product is better	JD.com
Lai Xiao Xiao Boutique	smart butler	This item is well priced and economical, giving it a five star rating.	JD.com
Lai Xiao Xiao Boutique	smart butler	Baby received, thanks! I love it.	JD.com
Shuxin Lifestyle Store	In-situ compensated economizer	Next time. Help you advertise like a pro hurry up!	JD.com
Shuxin Lifestyle Store	In-situ compensated economizer	Hehehe, overall like it. Seller service is very can logistics very fast	JD.com
Shuxin Lifestyle Store	In-situ compensated economizer	Shipping was fast. The quality of the item was pretty good too. Love it.	JD.com
Shuxin Lifestyle Store	In-situ compensated economizer	Very happy with one purchase. Must give a shout out and will be back next time	JD.com
Shuxin Lifestyle Store	In-situ compensated economizer	Already started to use it, will review in a few days, the merchant is very responsible and has a very good attitude. Also very patient to explain, thanks to the merchant! Hope it really saves electricity	JD.com
Shuxin Lifestyle Store	In-situ compensated economizer	It's a power saver.	JD.com
Shuxin Lifestyle Store	In-situ compensated economizer	(of an unmarried couple) be close	JD.com
Shuxin Lifestyle Store	In-situ compensated economizer	This user finds merchants very good and gives it a 5 star rating	JD.com
Shuxin Lifestyle Store	In-situ compensated economizer	This user feels the product is better	JD.com
Shuxin Lifestyle Store	In-situ compensated economizer	This user feels the product is better	JD.com
Shuxin Lifestyle Store	In-situ compensated economizer	This works great thank you for all your hard work!	JD.com
Shuxin Lifestyle Store	In-situ compensated economizer	Very happy with one purchase. Must give a shout out and will be back next time	JD.com
Shuxin Lifestyle Store	In-situ compensated economizer	The picture is not the same as the item	JD.com
Shuxin Lifestyle Store	In-situ compensated economizer	Baby received, quite good like, logistics is very fast, the price is affordable!	JD.com
Shuxin Lifestyle Store	In-situ compensated economizer	Good service from the merchant and express delivery	JD.com
Shuxin Lifestyle Store	In-situ compensated economizer	You can't even buy it for this price outside. It's really good. I like it.	JD.com
Shuxin Lifestyle Store	In-situ compensated economizer	Very reputable seller the first time on the shipment, received awesome affordable, very much like like the pro can get down!	JD.com
Shuxin Lifestyle Store	In-situ compensated economizer	Shipping was fast. The quality of the item was pretty good too. Love it.	JD.com
Shuxin Lifestyle Store	In-situ compensated economizer	Cost-effective, coworkers see very like, seller service can be	JD.com
Shuxin Lifestyle Store	In-situ compensated economizer	After the shipment, I really have no words to say I love it, great value for money!	JD.com
Shuxin Lifestyle Store	In-situ compensated economizer	Hehehe, overall like it. Seller service is very can logistics very fast	JD.com
Shuxin Lifestyle Store	In-situ compensated economizer	(of an unmarried couple) be close	JD.com
Shuxin Lifestyle Store	In-situ compensated economizer	Next time. Help you advertise like a pro hurry up!	JD.com
Shuxin Lifestyle Store	Intelligent frequency conversion high power	not have	JD.com
Mustang Overseas Technology	King of Power Saving	Energy saving and environmental protection, good value for money, recommended to buy.	JD.com
Mustang Overseas Technology	King of Power Saving	This user feels the product is better	JD.com
Mustang Overseas Technology	King of Power Saving	Small square all-electricity saving	JD.com
Mustang Overseas Technology	King of Power Saving	I've repurchased it for the second time, it's really good	JD.com
Mustang Overseas Technology	King of Power Saving	It looks like it's pretty good. I hope it works.	JD.com
Mustang Overseas Technology	King of Power Saving	I've got the goods. - Thank you very much.	JD.com
Mustang Overseas Technology	King of Power Saving	I received it and used it right away, plugged it in and it immediately displayed numbers like 218, 219, 221, 225, 223 and the words power saving in progress and air purification. I assume the numbers are instantaneous voltage? I can plug it into any outlet I want. Easy to use, hope to see results in 1-2	JD.com
Mustang Overseas Technology	King of Power Saving	This user feels the product is better	JD.com
Mustang Overseas Technology	King of Power Saving	This user feels the product is better	JD.com

Mustang Overseas Technology	King of Power Saving	This user feels the product is better	JD.com
Mustang Overseas Technology	King of Power Saving	This user feels the product is better	JD.com
Mustang Overseas Technology	King of Power Saving	This is the second time I've purchased it and it works really well 👍, recommend it [smiles]	JD.com
Mustang Overseas Technology	King of Power Saving	The product isn't bad, it's aesthetically pleasing and works well	JD.com
Mustang Overseas Technology	King of Power Saving	Great, should work, already installed.	JD.com
Mustang Overseas Technology	King of Power Saving	Product quality is very good, logistics is very fast, really good power saving	JD.com
Mustang Overseas Technology	King of Power Saving	Aesthetically pleasing, product quality is guaranteed	JD.com
Mustang Overseas Technology	King of Power Saving	The product is not bad, it works very well, very good boss attitude is very good, good quality, next time will buy.	JD.com
Mustang Overseas Technology	King of Power Saving	I got it today, it was cheap, plugged right into the outlet and came with 2 extra safeties!	JD.com
Mustang Overseas Technology	King of Power Saving	This user feels the product is better	JD.com
Mustang Overseas Technology	King of Power Saving	The best gift ever given to thousands of families! Works great! Product quality is guaranteed! Trustworthy product!	JD.com
Mustang Overseas Technology	King of Power Saving	Express received quality is very good, next time to buy in this store!	JD.com
Mustang Overseas Technology	King of Power Saving	This user feels the product is better	JD.com
Mustang Overseas Technology	King of Power Saving	I just got it. I'll review it after a while.	JD.com
Mustang Overseas Technology	King of Power Saving	an excellent	JD.com
Mustang Overseas Technology	King of Power Saving	This product, beautiful and generous with a very convenient, and product statements one to the genuine article!	JD.com
Mustang Overseas Technology	King of Power Saving	Power Saving King received, especially like, I hope so to save me some, this product does not look bad, look forward to the effect, after a period of time to follow up the evaluation, [praise] [praise] [praise] [praise] [praise] [praise] [praise] [praise] [praise] [praise]	JD.com
Mustang Overseas Technology	King of Power Saving	The logistics is also fast and the Energy Saving King is exquisite. I hope it can help our businessmen who use a lot of electricity.	JD.com
Mustang Overseas Technology	King of Power Saving	How come I didn't get a refund?	JD.com
Mustang Overseas Technology	King of Power Saving	received	JD.com
Mustang Overseas Technology	King of Power Saving	This user feels the product is better	JD.com
Mustang Overseas Technology	King of Power Saving	Same as other people's goods: more expensive than other people's	JD.com
Mustang Overseas Technology	King of Power Saving	Good review Good review Good review Good review Good review	JD.com
Mustang Overseas Technology	King of Power Saving	It works well! Sends quickly too. Great product, feel trustworthy	JD.com
Mustang Overseas Technology	King of Power Saving	This user feels the product is better	JD.com
Mustang Overseas Technology	King of Power Saving	I'm using it now, I don't know if it's good or not, I'll come back and comment after I use it	JD.com
Mustang Overseas Technology	King of Power Saving	It doesn't look and feel like you guys are saying it works very well, it doesn't work very well.	JD.com
Mustang Overseas Technology	King of Power Saving	useless	JD.com
Mustang Overseas Technology	King of Power Saving	This product is very good, so hurry up and get it!	JD.com
Mustang Overseas Technology	King of Power Saving	Okay, okay. Thank you.	JD.com
Mustang Overseas Technology	King of Power Saving	With this product, I saved a lot of money on my electricity bill in a month. It is compact and beautiful.	JD.com
Mustang Overseas Technology	King of Power Saving	...	JD.com
Mustang Overseas Technology	King of Power Saving	Goods received to try the display is normal, I do not know how to use the power-saving effect, first give five-star praise.	JD.com
Mustang Overseas Technology	King of Power Saving	It's working great. I'm looking forward to a lower electric bill.	JD.com
Mustang Overseas Technology	King of Power Saving	The stuff is great, use it and recommend it to friends.	JD.com
Mustang Overseas Technology	King of Power Saving	This user feels the product is better	JD.com
Mustang Overseas Technology	King of Power Saving	Affordable great quality postal rush kudos!	JD.com
Mustang Overseas Technology	King of Power Saving	This user feels the product is better	JD.com
Mustang Overseas Technology	King of Power Saving	This user feels the product is better	JD.com
Mustang Overseas Technology	King of Power Saving	This user feels the product is better	JD.com
Mustang Overseas Technology	King of Power Saving	This user feels the product is better	JD.com
Mustang Overseas Technology	King of Power Saving	This user feels the product is better	JD.com
Mustang Overseas Technology	King of Power Saving	I bought this protection appliance to suppress harmonics, stabilize voltage, save energy and save electricity, this product is good to use [grimace] [grimace] [grimace] [grimace] [grimace] [grimace]	JD.com
Mustang Overseas Technology	King of Power Saving	Nice and cheap not too expensive love this kind of	JD.com
Mustang Overseas Technology	King of Power Saving	Product quality is good, the effect is very good, the price is affordable, all five stars praise!	JD.com
Mustang Overseas Technology	King of Power Saving	Pro, no savings at all. That sucks.	JD.com
Mustang Overseas Technology	King of Power Saving	This user feels the product is better	JD.com
Wangxin Electronic Digital	King of Power Saving	The store went out of business.	JD.com
Zichen Technology Digital	senior official in charge of electricity	not have	JD.com
Zichen Technology Digital	Whole house power saving	not have	JD.com
Zichen Technology Digital	Smart Saver	The product is good and it does save energy and electricity bills.	JD.com

Zichen Technology Digital	senior official in charge of electricity	not have	JD.com
Zichen Technology Digital	Power Saving Specialist	I'm just using it. I'm looking forward to the results.	JD.com
Zichen Technology Digital	Power Saving Specialist	Tried it, it really saves power, no longer need to save power, can not afford to turn on the air conditioning [love] [love] recommended to buy Oh!	JD.com
Zichen Technology Digital	Power Saving Specialist	Used more than ten days the effect is still good, logistics is also very fast, the product is also really save electricity	JD.com
Zichen Technology Digital	Power Saving Specialist	The product is as described, inexpensive, and the logistics are fast, giving it a five-star rating! This is another purchase!	JD.com
Qualcom Technology	Smart Saver	The store went out of business.	JD.com
Lao Si Daily Use Shop	King of Power Saving	The store went out of business.	jitterbug
Jinfeng Hongyuan Flagship Store	King of Power Saving	The store went out of business.	jitterbug
Lohan Commissary	King of Power Saving	The store went out of business.	jitterbug
Tian Tian Hao Gardening Shop	King of Power Saving	The store went out of business.	jitterbug
Longhua Creative Supply Chain	King of Power Saving	The store went out of business.	jitterbug
Lao Si Daily Use Shop	King of Power Saving	The store went out of business.	jitterbug
Quality U Choice Shop	King of Power Saving	The store went out of business.	jitterbug
spring and winter	King of Power Saving	The store went out of business.	jitterbug
Piovanle Daily-use Shop	King of Power Saving	The store went out of business.	jitterbug
Xuanjin Home Textile Specialized Shop	maximize energy savings (e.g. in gasoline and diesel fuel)	not have	Jingdong prefecture level city in Shandong
Xuanjin Home Textile	Ultrasonic power saver	not have	Jingdong prefecture level city in Shandong
Xuanjin Home Textile	Intelligent Power Saving Expert	not have	Jingdong prefecture level city in Shandong
Stowe Flagship Store	a power saver (electronics)	Absolutely good stuff, with particularly convenient and simple, directly into the home plug board on it. Now look at the meter does turn much slower than before, I now still open the air conditioning as usual to watch TV, and the original did not change at all, but the power really less. This product effect is	Jingdong prefecture level city in Shandong
Stowe Flagship Store	a power saver (electronics)	Saver received, the second time back to buy, before buying a installed in the old house effect is good, a month can really save a lot of electricity, the original night air conditioning power about ten degrees of electricity, now down to 4-5 degrees of electricity, and bought a put a part-time rental house, save a little salary, the quality of the material is still as good as ever, the use of a simple, randomly plugged into a socket on the line, it's very good, and is ready to buy two more to use!	Jingdong prefecture level city in Shandong
Stowe Flagship Store	a power saver (electronics)	Indeed very effective, because now are cell phone bill payment, summer air conditioning with a month of 300, we use this energy-saving products are less than 28 days or so ¥ 200 electricity is indeed a lot of savings, this time to buy two for the elderly, but also recommended that their relatives inside the home	Jingdong prefecture level city in Shandong
Stowe Flagship Store	a power saver (electronics)	I came to evaluate after using it, I feel the saver is very good, the meter runs slower, the power saving mode can be.	Jingdong prefecture level city in Shandong
Stowe Flagship Store	a power saver (electronics)	Value for money: just received, then checked on the plug board, looked at the pulse meter, with the same appliances, with and without a saver. There is a real difference in the blinking frequency of the pulse meter light. With the saver plugged in, the meter pulse light blinks much slower. It's really good...5 stars to the seller, also introduced to relatives to use!	Jingdong prefecture level city in Shandong
Stowe Flagship Store	a power saver (electronics)	Quite good, affordable price, fast logistics, recommended to buy!	Jingdong prefecture level city in Shandong
Stowe Flagship Store	a power saver (electronics)	Plug and play, stable voltage, the screen has a display when working, this winter home appliances, need more electricity, just buy to use, save electricity and save money, with instructions, not too big and does not take up space, the quality is very good, and the seller describes exactly the same!	Jingdong prefecture level city in Shandong
Stowe Flagship Store	a power saver (electronics)	I have received the Smart Power Saver. Perfectly packaged. Fast delivery. Boss customer service attitude is also very good. It's worth buying. I plugged it in and tried it and it really saves more power than the original. I will buy it again next time.	Jingdong prefecture level city in Shandong
Stowe Flagship Store	a power saver (electronics)	The original electricity bill is very high, since the use of this power saver, the electricity bill saved a lot, small, and very convenient, plugged into any outlet can be, affordable, and save a large amount of	Jingdong prefecture level city in Shandong
Stowe Flagship Store	a power saver (electronics)	Power-saving effect: power-saving effect is particularly obvious Cost-effective: cost-effective business Appearance and style: external small micro-work materials: fine workmanship Experience: use very good, non the first convenient	Jingdong prefecture level city in Shandong
Stowe Flagship Store	a power saver (electronics)	After using it for a few days, it feels quite good, it should be quite safe, and it also sends a plug, the merchant is good, wait for a period of time and then calculate whether it saves on the electricity bill and then add comments ha!	Jingdong prefecture level city in Shandong
Stowe Flagship Store	a power saver (electronics)	My friend recommended to me, said the effect is very good, I bought two, this month less money, really can, just buy I do not believe, but has proved to have effect	Jingdong prefecture level city in Shandong
Stowe Flagship Store	a power saver (electronics)	Just bought back, already used, these days to see the effect of it, and so have the effect will also come back to give positive feedback, the price is also super cheap!	Jingdong prefecture level city in Shandong
Stowe Flagship Store	a power saver (electronics)	It does work well, the store uses a lot of electricity, this time to buy back a few, each place that uses a lot of electricity to insert one. Summer is here, the electricity is too strong.	Jingdong prefecture level city in Shandong
Stowe Flagship Store	a power saver (electronics)	Power saving treasure received, as for the effect, according to the instructions written, a short period of time can not be seen, at least 2-3 months before the real effect, then follow up evaluation	Jingdong prefecture level city in Shandong
Stowe Flagship Store	a power saver (electronics)	The goods have just been received and have not been used yet looking forward to the results.	Jingdong prefecture level city in Shandong
Stowe Flagship Store	a power saver (electronics)	Good installation works great	Jingdong prefecture level city in Shandong
Stowe Flagship Store	a power saver (electronics)	The logistics is super super fast! The quality is also very good, indeed value for money	Jingdong prefecture level city in Shandong
Stowe Flagship Store	a power saver (electronics)	Has been used for a period of time before coming to comment on, really good, air conditioning a day open non-stop, pay the electricity bill when the heartache, with this, the home electricity is not at all worried about the bill out of contrast to save a lot of preparation in the hands of a few, each room An a	Jingdong prefecture level city in Shandong
Stowe Flagship Store	a power saver (electronics)	Saving device received, really good, with the air conditioning tried, an hour without a saver with 2.9 degrees, with a saver an hour with 1.1 degrees, the beginning of not quite believe that this thing, the actual test to know that it is really useful, and intend to buy another.	Jingdong prefecture level city in Shandong
Stowe Flagship Store	a power saver (electronics)	Power saving effect: this energy saver is still useful, day trial for three days, every day than not installed before the reduction of two dollars a day, every day to open the bedroom to open the air conditioning before fifteen dollars now thirteen dollars a day. Still a little use.	Jingdong prefecture level city in Shandong
Stowe Flagship Store	a power saver (electronics)	Originally bought for home use, used for two months, the electricity bill from more than 200 yuan per month, down to more than 140 yuan, there is indeed a power-saving effect, this time and sold two, the unit with. Merchant service attitude is really good.	Jingdong prefecture level city in Shandong
Stowe Flagship Store	a power saver (electronics)	Plug and play, stable voltage, the screen shows when working, this winter home appliances, need electricity in many places, just buy to use, save electricity and money, with instructions	Jingdong prefecture level city in Shandong
Stowe Flagship Store	a power saver (electronics)	Goods received, do not know if it is good. When I bought it, I saw that it was a Stowe brand or company's product. Received but not. I do not know what is the reason. Now I can only give this evaluation. I'm	Jingdong prefecture level city in Shandong
Stowe Flagship Store	a power saver (electronics)	The first time to buy online, but also look at the evaluation before buying, the quality is not bad, but also very cheap, the need for friends can rest assured to buy!	Jingdong prefecture level city in Shandong
Stowe Flagship Store	a power saver (electronics)	Great quality, bought it to my liking Awesome! Buy with confidence if you need it!	Jingdong prefecture level city in Shandong
Stowe Flagship Store	a power saver (electronics)	The quality is very good, completely exceeded the expectations, the delivery speed is very fast, the packaging is very careful and strict, very satisfied with a shopping	Jingdong prefecture level city in Shandong

Stowe Flagship Store	a power saver (electronics)	The effect is very good, the home uses less electricity is very energy efficient, cost-effective	Jingdong prefecture level city in Shandong
Stowe Flagship Store	a power saver (electronics)	Quality can't be beat, just like the store, especially like, and the price is favorable!	Jingdong prefecture level city in Shandong
Stowe Flagship Store	a power saver (electronics)	Baby received! Very unexpected, will be back next time ~ great seller, stay tuned!	Jingdong prefecture level city in Shandong
Stowe Flagship Store	a power saver (electronics)	Follow up review after a month is more real, waiting for it	Jingdong prefecture level city in Shandong
Stowe Flagship Store	a power saver (electronics)	It is the ideal product, good quality and fast logistics, worthy of praise!	Jingdong prefecture level city in Shandong
Stowe Flagship Store	a power saver (electronics)	The saver is great, the color is right, the installer is very professional	Jingdong prefecture level city in Shandong
Stowe Flagship Store	a power saver (electronics)	The effect of the power connection is particularly obvious, it is particularly easy to use, very satisfied, the	Jingdong prefecture level city in Shandong
Stowe Flagship Store	a power saver (electronics)	Goods are very energy-saving, used to be more than two hundred degrees, now there are only a hundred or so degrees	Jingdong prefecture level city in Shandong
Stowe Flagship Store	a power saver (electronics)	Good quality, fast shipping, good results, good value for money	Jingdong prefecture level city in Shandong
Stowe Flagship Store	a power saver (electronics)	Fast shipping, fine workmanship, very good, cost-effective	Jingdong prefecture level city in Shandong
Stowe Flagship Store	a power saver (electronics)	The meter is as described, the quality is quite good, energy-saving, worth buying oh~!	Jingdong prefecture level city in Shandong
Stowe Flagship Store	a power saver (electronics)	Great value for money, very affordable,, perfect . ^◡^.	Jingdong prefecture level city in Shandong
Stowe Flagship Store	a power saver (electronics)	Good!!! Worth recommending!	Jingdong prefecture level city in Shandong
Stowe Flagship Store	a power saver (electronics)	Great, fast logistics, great stuff, great value for money!	Jingdong prefecture level city in Shandong
Stowe Flagship Store	a power saver (electronics)	Fast logistics, very satisfied with the quality upon receipt	Jingdong prefecture level city in Shandong
Stowe Flagship Store	a power saver (electronics)	Great quality, plug and play, fast shipping!	Jingdong prefecture level city in Shandong
Stowe Flagship Store	a power saver (electronics)	Logistics received, quite satisfied	Jingdong prefecture level city in Shandong
Stowe Flagship Store	a power saver (electronics)	I don't know if it really works	Jingdong prefecture level city in Shandong
Stowe Flagship Store	a power saver (electronics)	It's bullshit. It's a plug light. Don't talk about power savings.	Jingdong prefecture level city in Shandong
Stowe Flagship Store	a power saver (electronics)	Fake and deceitful stuff that hasn't changed at all.	Jingdong prefecture level city in Shandong
Stowe Flagship Store	a power saver (electronics)	Power-saving effect: power-saving effect is particularly obvious Cost-effective: cost-effective business Appearance and style: external small micro-work materials: fine workmanship Experience: use very good, non the first convenient	Jingdong prefecture level city in Shandong
Stowe Flagship Store	a power saver (electronics)	After using it for a few days, it feels quite good, it should be quite safe, and it also sends a plug, the merchant is good, wait for a period of time and then calculate whether it saves on the electricity bill and then add comments ha!	Jingdong prefecture level city in Shandong
Stowe Flagship Store	a power saver (electronics)	My friend recommended to me, said the effect is very good, I bought two, this month less money, really can, just buy I do not believe, but has proved to have effect	Jingdong prefecture level city in Shandong
Stowe Flagship Store	a power saver (electronics)	Just bought back, already used, these days to see the effect of it, and so have the effect will also come back to give positive feedback, the price is also super cheap!	Jingdong prefecture level city in Shandong
Stowe Flagship Store	a power saver (electronics)	It does work well, the store uses a lot of electricity, this time to buy back a few, each place that uses a lot of electricity to insert one. Summer is here, the electricity is too strong.	Jingdong prefecture level city in Shandong
Stowe Flagship Store	a power saver (electronics)	Power saving treasure received, as for the effect, according to the instructions written, a short period of time can not be seen, at least 2-3 months before the real effect, then follow up evaluation	Jingdong prefecture level city in Shandong
Stowe Flagship Store	a power saver (electronics)	The goods have just been received and have not been used yet looking forward to the results.	Jingdong prefecture level city in Shandong
Stowe Flagship Store	a power saver (electronics)	Good installation works great	Jingdong prefecture level city in Shandong
Stowe Flagship Store	a power saver (electronics)	The logistics is super super fast! The quality is also very good, indeed value for money	Jingdong prefecture level city in Shandong
Stowe Flagship Store	a power saver (electronics)	Has been used for a period of time before coming to comment on, really good, air conditioning a day open non-stop, pay the electricity bill when the heartache, with this, the home electricity is not at all worried about the bill out of contrast to save a lot of preparation in the hands of a few, each room An a	Jingdong prefecture level city in Shandong
Stowe Flagship Store	a power saver (electronics)	Saving device received, really good, with the air conditioning tried, an hour without a saver with 2.9 degrees, with a saver an hour with 1.1 degrees, the beginning of not quite believe that this thing, the actual test to know that it is really useful, and intend to buy another.	Jingdong prefecture level city in Shandong
Stowe Flagship Store	a power saver (electronics)	Power saving effect: this energy saver is still useful, day trial for three days, every day than not installed before the reduction of two dollars a day, every day to open the bedroom to open the air conditioning before fifteen dollars now thirteen dollars a day. Still a little use.	Jingdong prefecture level city in Shandong
Stowe Flagship Store	a power saver (electronics)	Originally bought for home use, used for two months, the electricity bill from more than 200 yuan per month, down to more than 140 yuan, there is indeed a power-saving effect, this time and sold two, the unit with. Merchant service attitude is really good.	Jingdong prefecture level city in Shandong
Stowe Flagship Store	a power saver (electronics)	Plug and play, stable voltage, the screen shows when working, this winter home appliances, need electricity in many places, just buy to use, save electricity and money, with instructions	Jingdong prefecture level city in Shandong
Stowe Flagship Store	a power saver (electronics)	Goods received, do not know if it is good. When I bought it, I saw that it was a Stowe brand or company's product. Received but not. I do not know what is the reason. Now I can only give this evaluation. I'm	Jingdong prefecture level city in Shandong
Stowe Flagship Store	a power saver (electronics)	The first time to buy online, but also look at the evaluation before buying, the quality is not bad, but also very cheap, the need for friends can rest assured to buy!	Jingdong prefecture level city in Shandong
Stowe Flagship Store	a power saver (electronics)	🌈 Great quality, bought it to my liking 💕 Awesome! ✨ Buy with confidence if you need it!	Jingdong prefecture level city in Shandong
Stowe Flagship Store	a power saver (electronics)	The quality is very good, completely exceeded the expectations, the delivery speed is very fast, the packaging is very careful and strict, very satisfied with a shopping	Jingdong prefecture level city in Shandong
Stowe Flagship Store	a power saver (electronics)	The effect is very good, the home uses less electricity is very energy efficient, cost-effective	Jingdong prefecture level city in Shandong
Stowe Flagship Store	a power saver (electronics)	Quality can't be beat, just like the store, especially like, and the price is favorable!	Jingdong prefecture level city in Shandong
Stowe Flagship Store	a power saver (electronics)	Baby received! Very unexpected, will be back next time ~ great seller, stay tuned!	Jingdong prefecture level city in Shandong
Stowe Flagship Store	a power saver (electronics)	Follow up review after a month is more real, waiting for it	Jingdong prefecture level city in Shandong
Stowe Flagship Store	a power saver (electronics)	It is the ideal product, good quality and fast logistics, worthy of praise!	Jingdong prefecture level city in Shandong
Stowe Flagship Store	a power saver (electronics)	The saver is great, the color is right, the installer is very professional	Jingdong prefecture level city in Shandong
Stowe Flagship Store	a power saver (electronics)	The effect of the power connection is particularly obvious, it is particularly easy to use, very satisfied, the	Jingdong prefecture level city in Shandong
Stowe Flagship Store	a power saver (electronics)	Goods are very energy-saving, used to be more than two hundred degrees, now there are only a hundred or so degrees	Jingdong prefecture level city in Shandong
Stowe Flagship Store	a power saver (electronics)	Good quality, fast shipping, good results, good value for money	Jingdong prefecture level city in Shandong
Stowe Flagship Store	a power saver (electronics)	Fast shipping, fine workmanship, very good, cost-effective	Jingdong prefecture level city in Shandong

Stowe Flagship Store	a power saver (electronics)	The meter is as described, the quality is quite good, energy-saving, worth buying oh~!	Jingdong prefecture level city in Shandong
Stowe Flagship Store	a power saver (electronics)	Great value for money, very affordable,, perfect . ^◡^.	Jingdong prefecture level city in Shandong
Stowe Flagship Store	a power saver (electronics)	Good!!! Worth recommending!	Jingdong prefecture level city in Shandong
Stowe Flagship Store	a power saver (electronics)	Great, fast logistics, great stuff, great value for money!	Jingdong prefecture level city in Shandong
Stowe Flagship Store	a power saver (electronics)	Fast logistics, very satisfied with the quality upon receipt	Jingdong prefecture level city in Shandong
Stowe Flagship Store	a power saver (electronics)	Great quality, plug and play, fast shipping!	Jingdong prefecture level city in Shandong
Stowe Flagship Store	a power saver (electronics)	Logistics received, quite satisfied	Jingdong prefecture level city in Shandong
Stowe Flagship Store	a power saver (electronics)	I don't know if it really works	Jingdong prefecture level city in Shandong
Stowe Flagship Store	a power saver (electronics)	It's bullshit. It's a plug light. Don't talk about power savings.	Jingdong prefecture level city in Shandong
Stowe Flagship Store	a power saver (electronics)	Fake and deceitful stuff that hasn't changed at all.	Jingdong prefecture level city in Shandong
Stowe Flagship Store	a power saver (electronics)	Power-saving effect: power-saving effect is particularly obvious Cost-effective: cost-effective business Appearance and style: external small micro-work materials: fine workmanship Experience: use very good, non the first convenient	Jingdong prefecture level city in Shandong
Stowe Flagship Store	a power saver (electronics)	After using it for a few days, it feels quite good, it should be quite safe, and it also sends a plug, the merchant is good, wait for a period of time and then calculate whether it saves on the electricity bill and then add comments ha!	Jingdong prefecture level city in Shandong
Stowe Flagship Store	a power saver (electronics)	My friend recommended to me, said the effect is very good, I bought two, this month less money, really can, just buy I do not believe, but has proved to have effect	Jingdong prefecture level city in Shandong
Stowe Flagship Store	a power saver (electronics)	Just bought back, already used, these days to see the effect of it, and so have the effect will also come back to give positive feedback, the price is also super cheap!	Jingdong prefecture level city in Shandong
Stowe Flagship Store	a power saver (electronics)	It does work well, the store uses a lot of electricity, this time to buy back a few, each place that uses a lot of electricity to insert one. Summer is here, the electricity is too strong.	Jingdong prefecture level city in Shandong
Stowe Flagship Store	a power saver (electronics)	Power saving treasure received, as for the effect, according to the instructions written, a short period of time can not be seen, at least 2-3 months before the real effect, then follow up evaluation	Jingdong prefecture level city in Shandong
Stowe Flagship Store	a power saver (electronics)	The goods have just been received and have not been used yet looking forward to the results.	Jingdong prefecture level city in Shandong
Stowe Flagship Store	a power saver (electronics)	Good installation works great	Jingdong prefecture level city in Shandong
Stowe Flagship Store	a power saver (electronics)	The logistics is super super fast! The quality is also very good, indeed value for money	Jingdong prefecture level city in Shandong
Stowe Flagship Store	a power saver (electronics)	Has been used for a period of time before coming to comment on, really good, air conditioning a day open non-stop, pay the electricity bill when the heartache, with this, the home electricity is not at all worried about the bill out of contrast to save a lot of preparation in the hands of a few, each room An a	Jingdong prefecture level city in Shandong
Stowe Flagship Store	a power saver (electronics)	Saving device received, really good, with the air conditioning tried, an hour without a saver with 2.9 degrees, with a saver an hour with 1.1 degrees, the beginning of not quite believe that this thing, the actual test to know that it is really useful, and intend to buy another.	Jingdong prefecture level city in Shandong
Stowe Flagship Store	a power saver (electronics)	Power saving effect: this energy saver is still useful, day trial for three days, every day than not installed before the reduction of two dollars a day, every day to open the bedroom to open the air conditioning before fifteen dollars now thirteen dollars a day. Still a little use.	Jingdong prefecture level city in Shandong
Stowe Flagship Store	a power saver (electronics)	Originally bought for home use, used for two months, the electricity bill from more than 200 yuan per month, down to more than 140 yuan, there is indeed a power-saving effect, this time and sold two, the unit with. Merchant service attitude is really good.	Jingdong prefecture level city in Shandong
Stowe Flagship Store	a power saver (electronics)	Plug and play, stable voltage, the screen shows when working, this winter home appliances, need electricity in many places, just buy to use, save electricity and money, with instructions	Jingdong prefecture level city in Shandong
Stowe Flagship Store	a power saver (electronics)	Goods received, do not know if it is good. When I bought it, I saw that it was a Stowe brand or company's product. Received but not. I do not know what is the reason. Now I can only give this evaluation. I'm	Jingdong prefecture level city in Shandong
Stowe Flagship Store	a power saver (electronics)	The first time to buy online, but also look at the evaluation before buying, the quality is not bad, but also very cheap, the need for friends can rest assured to buy!	Jingdong prefecture level city in Shandong
Stowe Flagship Store	a power saver (electronics)	🌈 Great quality, bought it to my liking 💞 Awesome! ✨ Buy with confidence if you need it!	Jingdong prefecture level city in Shandong
Stowe Flagship Store	a power saver (electronics)	The quality is very good, completely exceeded the expectations, the delivery speed is very fast, the packaging is very careful and strict, very satisfied with a shopping	Jingdong prefecture level city in Shandong
Stowe Flagship Store	a power saver (electronics)	The effect is very good, the home uses less electricity is very energy efficient, cost-effective	Jingdong prefecture level city in Shandong
Stowe Flagship Store	a power saver (electronics)	Quality can't be beat, just like the store, especially like, and the price is favorable!	Jingdong prefecture level city in Shandong
Stowe Flagship Store	a power saver (electronics)	Baby received! Very unexpected, will be back next time ~ great seller, stay tuned!	Jingdong prefecture level city in Shandong
Stowe Flagship Store	a power saver (electronics)	Follow up review after a month is more real, waiting for it	Jingdong prefecture level city in Shandong
Stowe Flagship Store	a power saver (electronics)	It is the ideal product, good quality and fast logistics, worthy of praise!	Jingdong prefecture level city in Shandong
Stowe Flagship Store	a power saver (electronics)	The saver is great, the color is right, the installer is very professional	Jingdong prefecture level city in Shandong
Stowe Flagship Store	a power saver (electronics)	The effect of the power connection is particularly obvious, it is particularly easy to use, very satisfied, the	Jingdong prefecture level city in Shandong
Stowe Flagship Store	a power saver (electronics)	Goods are very energy-saving, used to be more than two hundred degrees, now there are only a hundred or so degrees	Jingdong prefecture level city in Shandong
Stowe Flagship Store	a power saver (electronics)	Good quality, fast shipping, good results, good value for money	Jingdong prefecture level city in Shandong
Stowe Flagship Store	a power saver (electronics)	Fast shipping, fine workmanship, very good, cost-effective	Jingdong prefecture level city in Shandong
Stowe Flagship Store	a power saver (electronics)	The meter is as described, the quality is quite good, energy-saving, worth buying oh~!	Jingdong prefecture level city in Shandong
Stowe Flagship Store	a power saver (electronics)	Great value for money, very affordable,, perfect . ^◡^.	Jingdong prefecture level city in Shandong
Stowe Flagship Store	a power saver (electronics)	Good!!! Worth recommending!	Jingdong prefecture level city in Shandong
Stowe Flagship Store	a power saver (electronics)	Great, fast logistics, great stuff, great value for money!	Jingdong prefecture level city in Shandong
Stowe Flagship Store	a power saver (electronics)	Fast logistics, very satisfied with the quality upon receipt	Jingdong prefecture level city in Shandong
Stowe Flagship Store	a power saver (electronics)	Great quality, plug and play, fast shipping!	Jingdong prefecture level city in Shandong
Stowe Flagship Store	a power saver (electronics)	Logistics received, quite satisfied	Jingdong prefecture level city in Shandong
Stowe Flagship Store	a power saver (electronics)	I don't know if it really works	Jingdong prefecture level city in Shandong
Stowe Flagship Store	a power saver (electronics)	It's bullshit. It's a plug light. Don't talk about power savings.	Jingdong prefecture level city in Shandong
Stowe Flagship Store	a power saver (electronics)	Fake and deceitful stuff that hasn't changed at all.	Jingdong prefecture level city in Shandong

Stowe Flagship Store	a power saver (electronics)	Power-saving effect: power-saving effect is particularly obvious Cost-effective: cost-effective business Appearance and style: external small micro-work materials: fine workmanship Experience: use very good, non the first convenient	Jingdong prefecture level city in Shandong
Stowe Flagship Store	a power saver (electronics)	After using it for a few days, it feels quite good, it should be quite safe, and it also sends a plug, the merchant is good, wait for a period of time and then calculate whether it saves on the electricity bill and then add comments ha!	Jingdong prefecture level city in Shandong
Stowe Flagship Store	a power saver (electronics)	My friend recommended to me, said the effect is very good, I bought two, this month less money, really can, just buy I do not believe, but has proved to have effect	Jingdong prefecture level city in Shandong
Stowe Flagship Store	a power saver (electronics)	Just bought back, already used, these days to see the effect of it, and so have the effect will also come back to give positive feedback, the price is also super cheap!	Jingdong prefecture level city in Shandong
Stowe Flagship Store	a power saver (electronics)	It does work well, the store uses a lot of electricity, this time to buy back a few, each place that uses a lot of electricity to insert one. Summer is here, the electricity is too strong.	Jingdong prefecture level city in Shandong
Stowe Flagship Store	a power saver (electronics)	Power saving treasure received, as for the effect, according to the instructions written, a short period of time can not be seen, at least 2-3 months before the real effect, then follow up evaluation	Jingdong prefecture level city in Shandong
Stowe Flagship Store	a power saver (electronics)	The goods have just been received and have not been used yet looking forward to the results.	Jingdong prefecture level city in Shandong
Stowe Flagship Store	a power saver (electronics)	Good installation works great	Jingdong prefecture level city in Shandong
Stowe Flagship Store	a power saver (electronics)	The logistics is super super fast! The quality is also very good, indeed value for money	Jingdong prefecture level city in Shandong
Stowe Flagship Store	a power saver (electronics)	Has been used for a period of time before coming to comment on, really good, air conditioning a day open non-stop, pay the electricity bill when the heartache, with this, the home electricity is not at all worried about the bill out of contrast to save a lot of preparation in the hands of a few, each room An a	Jingdong prefecture level city in Shandong
Stowe Flagship Store	a power saver (electronics)	Saving device received, really good, with the air conditioning tried, an hour without a saver with 2.9 degrees, with a saver an hour with 1.1 degrees, the beginning of not quite believe that this thing, the actual test to know that it is really useful, and intend to buy another.	Jingdong prefecture level city in Shandong
Stowe Flagship Store	a power saver (electronics)	Power saving effect: this energy saver is still useful, day trial for three days, every day than not installed before the reduction of two dollars a day, every day to open the bedroom to open the air conditioning before fifteen dollars now thirteen dollars a day. Still a little use.	Jingdong prefecture level city in Shandong
Stowe Flagship Store	a power saver (electronics)	Originally bought for home use, used for two months, the electricity bill from more than 200 yuan per month, down to more than 140 yuan, there is indeed a power-saving effect, this time and sold two, the unit with. Merchant service attitude is really good.	Jingdong prefecture level city in Shandong
Stowe Flagship Store	a power saver (electronics)	Plug and play, stable voltage, the screen shows when working, this winter home appliances, need electricity in many places, just buy to use, save electricity and money, with instructions	Jingdong prefecture level city in Shandong
Stowe Flagship Store	a power saver (electronics)	Goods received, do not know if it is good. When I bought it, I saw that it was a Stowe brand or company's product. Received but not. I do not know what is the reason. Now I can only give this evaluation. I'm	Jingdong prefecture level city in Shandong
Stowe Flagship Store	a power saver (electronics)	The first time to buy online, but also look at the evaluation before buying, the quality is not bad, but also very cheap, the need for friends can rest assured to buy!	Jingdong prefecture level city in Shandong
Stowe Flagship Store	a power saver (electronics)	🌈 Great quality, bought it to my liking 💗 Awesome! ✨ Buy with confidence if you need it!	Jingdong prefecture level city in Shandong
Stowe Flagship Store	a power saver (electronics)	The quality is very good, completely exceeded the expectations, the delivery speed is very fast, the packaging is very careful and strict, very satisfied with a shopping	Jingdong prefecture level city in Shandong
Stowe Flagship Store	a power saver (electronics)	The effect is very good, the home uses less electricity is very energy efficient, cost-effective	Jingdong prefecture level city in Shandong
Stowe Flagship Store	a power saver (electronics)	Quality can't be beat, just like the store, especially like, and the price is favorable!	Jingdong prefecture level city in Shandong
Stowe Flagship Store	a power saver (electronics)	Baby received! Very unexpected, will be back next time ~ great seller, stay tuned!	Jingdong prefecture level city in Shandong
Stowe Flagship Store	a power saver (electronics)	Follow up review after a month is more real, waiting for it	Jingdong prefecture level city in Shandong
Stowe Flagship Store	a power saver (electronics)	It is the ideal product, good quality and fast logistics, worthy of praise!	Jingdong prefecture level city in Shandong
Stowe Flagship Store	a power saver (electronics)	The saver is great, the color is right, the installer is very professional	Jingdong prefecture level city in Shandong
Stowe Flagship Store	a power saver (electronics)	The effect of the power connection is particularly obvious, it is particularly easy to use, very satisfied, the	Jingdong prefecture level city in Shandong
Stowe Flagship Store	a power saver (electronics)	Goods are very energy-saving, used to be more than two hundred degrees, now there are only a hundred or so degrees	Jingdong prefecture level city in Shandong
Stowe Flagship Store	a power saver (electronics)	Good quality, fast shipping, good results, good value for money	Jingdong prefecture level city in Shandong
Stowe Flagship Store	a power saver (electronics)	Fast shipping, fine workmanship, very good, cost-effective	Jingdong prefecture level city in Shandong
Stowe Flagship Store	a power saver (electronics)	The meter is as described, the quality is quite good, energy-saving, worth buying oh~!	Jingdong prefecture level city in Shandong
Stowe Flagship Store	a power saver (electronics)	Great value for money, very affordable,, perfect . ˆ◡ˆ.	Jingdong prefecture level city in Shandong
Stowe Flagship Store	a power saver (electronics)	Good!!! Worth recommending!	Jingdong prefecture level city in Shandong
Stowe Flagship Store	a power saver (electronics)	Great, fast logistics, great stuff, great value for money!	Jingdong prefecture level city in Shandong
Stowe Flagship Store	a power saver (electronics)	Fast logistics, very satisfied with the quality upon receipt	Jingdong prefecture level city in Shandong
Stowe Flagship Store	a power saver (electronics)	Great quality, plug and play, fast shipping!	Jingdong prefecture level city in Shandong
Stowe Flagship Store	a power saver (electronics)	Logistics received, quite satisfied	Jingdong prefecture level city in Shandong
Stowe Flagship Store	a power saver (electronics)	I don't know if it really works	Jingdong prefecture level city in Shandong
Stowe Flagship Store	a power saver (electronics)	It's bullshit. It's a plug light. Don't talk about power savings.	Jingdong prefecture level city in Shandong
Stowe Flagship Store	a power saver (electronics)	Fake and deceitful stuff that hasn't changed at all.	Jingdong prefecture level city in Shandong
Stowe Flagship Store	a power saver (electronics)	Power-saving effect: power-saving effect is particularly obvious Cost-effective: cost-effective business Appearance and style: external small micro-work materials: fine workmanship Experience: use very good, non the first convenient	Jingdong prefecture level city in Shandong
Stowe Flagship Store	a power saver (electronics)	After using it for a few days, it feels quite good, it should be quite safe, and it also sends a plug, the merchant is good, wait for a period of time and then calculate whether it saves on the electricity bill and then add comments ha!	Jingdong prefecture level city in Shandong
Stowe Flagship Store	a power saver (electronics)	My friend recommended to me, said the effect is very good, I bought two, this month less money, really can, just buy I do not believe, but has proved to have effect	Jingdong prefecture level city in Shandong
Stowe Flagship Store	a power saver (electronics)	Just bought back, already used, these days to see the effect of it, and so have the effect will also come back to give positive feedback, the price is also super cheap!	Jingdong prefecture level city in Shandong
Stowe Flagship Store	a power saver (electronics)	It does work well, the store uses a lot of electricity, this time to buy back a few, each place that uses a lot of electricity to insert one. Summer is here, the electricity is too strong.	Jingdong prefecture level city in Shandong
Stowe Flagship Store	a power saver (electronics)	Power saving treasure received, as for the effect, according to the instructions written, a short period of time can not be seen, at least 2-3 months before the real effect, then follow up evaluation	Jingdong prefecture level city in Shandong
Stowe Flagship Store	a power saver (electronics)	The goods have just been received and have not been used yet looking forward to the results.	Jingdong prefecture level city in Shandong
Stowe Flagship Store	a power saver (electronics)	Good installation works great	Jingdong prefecture level city in Shandong
Stowe Flagship Store	a power saver (electronics)	The logistics is super super fast! The quality is also very good, indeed value for money	Jingdong prefecture level city in Shandong

Stowe Flagship Store	a power saver (electronics)	Has been used for a period of time before coming to comment on, really good, air conditioning a day open non-stop, pay the electricity bill when the heartache, with this, the home electricity is not at all worried about the bill out of contrast to save a lot of preparation in the hands of a few, each room An a	Jingdong prefecture level city in Shandong
Stowe Flagship Store	a power saver (electronics)	Saving device received, really good, with the air conditioning tried, an hour without a saver with 2.9 degrees, with a saver an hour with 1.1 degrees, the beginning of not quite believe that this thing, the actual test to know that it is really useful, and intend to buy another.	Jingdong prefecture level city in Shandong
Stowe Flagship Store	a power saver (electronics)	Power saving effect: this energy saver is still useful, day trial for three days, every day than not installed before the reduction of two dollars a day, every day to open the bedroom to open the air conditioning before fifteen dollars now thirteen dollars a day. Still a little use.	Jingdong prefecture level city in Shandong
Stowe Flagship Store	a power saver (electronics)	Originally bought for home use, used for two months, the electricity bill from more than 200 yuan per month, down to more than 140 yuan, there is indeed a power-saving effect, this time and sold two, the unit with. Merchant service attitude is really good.	Jingdong prefecture level city in Shandong
Stowe Flagship Store	a power saver (electronics)	Plug and play, stable voltage, the screen shows when working, this winter home appliances, need electricity in many places, just buy to use, save electricity and money, with instructions	Jingdong prefecture level city in Shandong
Stowe Flagship Store	a power saver (electronics)	Goods received, do not know if it is good. When I bought it, I saw that it was a Stowe brand or company's product. Received but not. I do not know what is the reason. Now I can only give this evaluation. I'm	Jingdong prefecture level city in Shandong
Stowe Flagship Store	a power saver (electronics)	The first time to buy online, but also look at the evaluation before buying, the quality is not bad, but also very cheap, the need for friends can rest assured to buy!	Jingdong prefecture level city in Shandong
Stowe Flagship Store	a power saver (electronics)	Great quality, bought it to my liking Awesome! Buy with confidence if you need it!	Jingdong prefecture level city in Shandong
Stowe Flagship Store	a power saver (electronics)	The quality is very good, completely exceeded the expectations, the delivery speed is very fast, the packaging is very careful and strict, very satisfied with a shopping	Jingdong prefecture level city in Shandong
Stowe Flagship Store	a power saver (electronics)	The effect is very good, the home uses less electricity is very energy efficient, cost-effective	Jingdong prefecture level city in Shandong
Stowe Flagship Store	a power saver (electronics)	Quality can't be beat, just like the store, especially like, and the price is favorable!	Jingdong prefecture level city in Shandong
Stowe Flagship Store	a power saver (electronics)	Baby received! Very unexpected, will be back next time ~ great seller, stay tuned!	Jingdong prefecture level city in Shandong
Stowe Flagship Store	a power saver (electronics)	Follow up review after a month is more real, waiting for it	Jingdong prefecture level city in Shandong
Stowe Flagship Store	a power saver (electronics)	It is the ideal product, good quality and fast logistics, worthy of praise!	Jingdong prefecture level city in Shandong
Stowe Flagship Store	a power saver (electronics)	The saver is great, the color is right, the installer is very professional	Jingdong prefecture level city in Shandong
Stowe Flagship Store	a power saver (electronics)	The effect of the power connection is particularly obvious, it is particularly easy to use, very satisfied, the	Jingdong prefecture level city in Shandong
Stowe Flagship Store	a power saver (electronics)	Goods are very energy-saving, used to be more than two hundred degrees, now there are only a hundred or so degrees	Jingdong prefecture level city in Shandong
Stowe Flagship Store	a power saver (electronics)	Good quality, fast shipping, good results, good value for money	Jingdong prefecture level city in Shandong
Stowe Flagship Store	a power saver (electronics)	Fast shipping, fine workmanship, very good, cost-effective	Jingdong prefecture level city in Shandong
Stowe Flagship Store	a power saver (electronics)	The meter is as described, the quality is quite good, energy-saving, worth buying oh~!	Jingdong prefecture level city in Shandong
Stowe Flagship Store	a power saver (electronics)	Great value for money, very affordable,, perfect . ^◡^.	Jingdong prefecture level city in Shandong
Stowe Flagship Store	a power saver (electronics)	Good!!! Worth recommending!	Jingdong prefecture level city in Shandong
Stowe Flagship Store	a power saver (electronics)	Great, fast logistics, great stuff, great value for money!	Jingdong prefecture level city in Shandong
Stowe Flagship Store	a power saver (electronics)	Fast logistics, very satisfied with the quality upon receipt	Jingdong prefecture level city in Shandong
Stowe Flagship Store	a power saver (electronics)	Great quality, plug and play, fast shipping!	Jingdong prefecture level city in Shandong
Stowe Flagship Store	a power saver (electronics)	Logistics received, quite satisfied	Jingdong prefecture level city in Shandong
Stowe Flagship Store	a power saver (electronics)	I don't know if it really works	Jingdong prefecture level city in Shandong
Stowe Flagship Store	a power saver (electronics)	It's bullshit. It's a plug light. Don't talk about power savings.	Jingdong prefecture level city in Shandong
Stowe Flagship Store	a power saver (electronics)	Fake and deceitful stuff that hasn't changed at all.	Jingdong prefecture level city in Shandong
Stowe Flagship Store	a power saver (electronics)	Power-saving effect: power-saving effect is particularly obvious Cost-effective: cost-effective business Appearance and style: external small micro-work materials: fine workmanship Experience: use very good, non the first convenient	Jingdong prefecture level city in Shandong
Stowe Flagship Store	a power saver (electronics)	After using it for a few days, it feels quite good, it should be quite safe, and it also sends a plug, the merchant is good, wait for a period of time and then calculate whether it saves on the electricity bill and then add comments ha!	Jingdong prefecture level city in Shandong
Stowe Flagship Store	a power saver (electronics)	My friend recommended to me, said the effect is very good, I bought two, this month less money, really can, just buy I do not believe, but has proved to have effect	Jingdong prefecture level city in Shandong
Stowe Flagship Store	a power saver (electronics)	Just bought back, already used, these days to see the effect of it, and so have the effect will also come back to give positive feedback, the price is also super cheap!	Jingdong prefecture level city in Shandong
Stowe Flagship Store	a power saver (electronics)	It does work well, the store uses a lot of electricity, this time to buy back a few, each place that uses a lot of electricity to insert one. Summer is here, the electricity is too strong.	Jingdong prefecture level city in Shandong
Stowe Flagship Store	a power saver (electronics)	Power saving treasure received, as for the effect, according to the instructions written, a short period of time can not be seen, at least 2-3 months before the real effect, then follow up evaluation	Jingdong prefecture level city in Shandong
Stowe Flagship Store	a power saver (electronics)	The goods have just been received and have not been used yet looking forward to the results.	Jingdong prefecture level city in Shandong
Stowe Flagship Store	a power saver (electronics)	Good installation works great	Jingdong prefecture level city in Shandong
Stowe Flagship Store	a power saver (electronics)	The logistics is super super fast! The quality is also very good, indeed value for money	Jingdong prefecture level city in Shandong
Stowe Flagship Store	a power saver (electronics)	Has been used for a period of time before coming to comment on, really good, air conditioning a day open non-stop, pay the electricity bill when the heartache, with this, the home electricity is not at all worried about the bill out of contrast to save a lot of preparation in the hands of a few, each room An a	Jingdong prefecture level city in Shandong
Stowe Flagship Store	a power saver (electronics)	Saving device received, really good, with the air conditioning tried, an hour without a saver with 2.9 degrees, with a saver an hour with 1.1 degrees, the beginning of not quite believe that this thing, the actual test to know that it is really useful, and intend to buy another.	Jingdong prefecture level city in Shandong
Stowe Flagship Store	a power saver (electronics)	Power saving effect: this energy saver is still useful, day trial for three days, every day than not installed before the reduction of two dollars a day, every day to open the bedroom to open the air conditioning before fifteen dollars now thirteen dollars a day. Still a little use.	Jingdong prefecture level city in Shandong
Stowe Flagship Store	a power saver (electronics)	Originally bought for home use, used for two months, the electricity bill from more than 200 yuan per month, down to more than 140 yuan, there is indeed a power-saving effect, this time and sold two, the unit with. Merchant service attitude is really good.	Jingdong prefecture level city in Shandong
Stowe Flagship Store	a power saver (electronics)	Plug and play, stable voltage, the screen shows when working, this winter home appliances, need electricity in many places, just buy to use, save electricity and money, with instructions	Jingdong prefecture level city in Shandong
Stowe Flagship Store	a power saver (electronics)	Goods received, do not know if it is good. When I bought it, I saw that it was a Stowe brand or company's product. Received but not. I do not know what is the reason. Now I can only give this evaluation. I'm	Jingdong prefecture level city in Shandong
Stowe Flagship Store	a power saver (electronics)	The first time to buy online, but also look at the evaluation before buying, the quality is not bad, but also very cheap, the need for friends can rest assured to buy!	Jingdong prefecture level city in Shandong
Stowe Flagship Store	a power saver (electronics)	Great quality, bought it to my liking Awesome! Buy with confidence if you need it!	Jingdong prefecture level city in Shandong

Stowe Flagship Store	a power saver (electronics)	The quality is very good, completely exceeded the expectations, the delivery speed is very fast, the packaging is very careful and strict, very satisfied with a shopping	Jingdong prefecture level city in Shandong
Stowe Flagship Store	a power saver (electronics)	The effect is very good, the home uses less electricity is very energy efficient, cost-effective	Jingdong prefecture level city in Shandong
Stowe Flagship Store	a power saver (electronics)	Quality can't be beat, just like the store, especially like, and the price is favorable!	Jingdong prefecture level city in Shandong
Stowe Flagship Store	a power saver (electronics)	Baby received! Very unexpected, will be back next time ~ great seller, stay tuned!	Jingdong prefecture level city in Shandong
Stowe Flagship Store	a power saver (electronics)	Follow up review after a month is more real, waiting for it	Jingdong prefecture level city in Shandong
Stowe Flagship Store	a power saver (electronics)	It is the ideal product, good quality and fast logistics, worthy of praise!	Jingdong prefecture level city in Shandong
Stowe Flagship Store	a power saver (electronics)	The saver is great, the color is right, the installer is very professional	Jingdong prefecture level city in Shandong
Stowe Flagship Store	a power saver (electronics)	The effect of the power connection is particularly obvious, it is particularly easy to use, very satisfied, the	Jingdong prefecture level city in Shandong
Stowe Flagship Store	a power saver (electronics)	Goods are very energy-saving, used to be more than two hundred degrees, now there are only a hundred or so degrees	Jingdong prefecture level city in Shandong
Stowe Flagship Store	a power saver (electronics)	Fast shipping, fine workmanship, very good, cost-effective	Jingdong prefecture level city in Shandong
Stowe Flagship Store	a power saver (electronics)	Good quality, fast shipping, good results, good value for money	Jingdong prefecture level city in Shandong
Stowe Flagship Store	a power saver (electronics)	The meter is as described, the quality is quite good, energy-saving, worth buying oh~!	Jingdong prefecture level city in Shandong
Stowe Flagship Store	a power saver (electronics)	Great value for money, very affordable,, perfect . ˆ◡ˆ.	Jingdong prefecture level city in Shandong
Stowe Flagship Store	a power saver (electronics)	Good!!! Worth recommending!	Jingdong prefecture level city in Shandong
Stowe Flagship Store	a power saver (electronics)	Fast logistics, very satisfied with the quality upon receipt	Jingdong prefecture level city in Shandong
Stowe Flagship Store	a power saver (electronics)	Great, fast logistics, great stuff, great value for money!	Jingdong prefecture level city in Shandong
Stowe Flagship Store	a power saver (electronics)	Great quality, plug and play, fast shipping!	Jingdong prefecture level city in Shandong
Stowe Flagship Store	a power saver (electronics)	Logistics received, quite satisfied	Jingdong prefecture level city in Shandong
Stowe Flagship Store	a power saver (electronics)	It's bullshit. It's a plug light. Don't talk about power savings.	Jingdong prefecture level city in Shandong
Stowe Flagship Store	a power saver (electronics)	I don't know if it really works	Jingdong prefecture level city in Shandong
Stowe Flagship Store	a power saver (electronics)	Fake and deceitful stuff that hasn't changed at all.	Jingdong prefecture level city in Shandong
Stowe Flagship Store	a power saver (electronics)	Power-saving effect: power-saving effect is particularly obvious Cost-effective: cost-effective business Appearance and style: external small micro-work materials: fine workmanship Experience: use very good, non the first convenient	Jingdong prefecture level city in Shandong
Stowe Flagship Store	a power saver (electronics)	After using it for a few days, it feels quite good, it should be quite safe, and it also sends a plug, the merchant is good, wait for a period of time and then calculate whether it saves on the electricity bill and then add comments ha!	Jingdong prefecture level city in Shandong
Stowe Flagship Store	a power saver (electronics)	My friend recommended to me, said the effect is very good, I bought two, this month less money, really can, just buy I do not believe, but has proved to have effect	Jingdong prefecture level city in Shandong
Stowe Flagship Store	a power saver (electronics)	Just bought back, already used, these days to see the effect of it, and so have the effect will also come back to give positive feedback, the price is also super cheap!	Jingdong prefecture level city in Shandong
Stowe Flagship Store	a power saver (electronics)	It does work well, the store uses a lot of electricity, this time to buy back a few, each place that uses a lot of electricity to insert one. Summer is here, the electricity is too strong.	Jingdong prefecture level city in Shandong
Stowe Flagship Store	a power saver (electronics)	Power saving treasure received, as for the effect, according to the instructions written, a short period of time can not be seen, at least 2-3 months before the real effect, then follow up evaluation	Jingdong prefecture level city in Shandong
Stowe Flagship Store	a power saver (electronics)	The goods have just been received and have not been used yet looking forward to the results.	Jingdong prefecture level city in Shandong
Stowe Flagship Store	a power saver (electronics)	Good installation works great	Jingdong prefecture level city in Shandong
Stowe Flagship Store	a power saver (electronics)	The logistics is super super fast! The quality is also very good, indeed value for money	Jingdong prefecture level city in Shandong
Stowe Flagship Store	a power saver (electronics)	Has been used for a period of time before coming to comment on, really good, air conditioning a day open non-stop, pay the electricity bill when the heartache, with this, the home electricity is not at all worried about the bill out of contrast to save a lot of preparation in the hands of a few, each room An a	Jingdong prefecture level city in Shandong
Stowe Flagship Store	a power saver (electronics)	Saving device received, really good, with the air conditioning tried, an hour without a saver with 2.9 degrees, with a saver an hour with 1.1 degrees, the beginning of not quite believe that this thing, the actual test to know that it is really useful, and intend to buy another.	Jingdong prefecture level city in Shandong
Stowe Flagship Store	a power saver (electronics)	Power saving effect: this energy saver is still useful, day trial for three days, every day than not installed before the reduction of two dollars a day, every day to open the bedroom to open the air conditioning before fifteen dollars now thirteen dollars a day. Still a little use.	Jingdong prefecture level city in Shandong
Stowe Flagship Store	a power saver (electronics)	Originally bought for home use, used for two months, the electricity bill from more than 200 yuan per month, down to more than 140 yuan, there is indeed a power-saving effect, this time and sold two, the unit with. Merchant service attitude is really good.	Jingdong prefecture level city in Shandong
Stowe Flagship Store	a power saver (electronics)	Plug and play, stable voltage, the screen shows when working, this winter home appliances, need electricity in many places, just buy to use, save electricity and money, with instructions	Jingdong prefecture level city in Shandong
Stowe Flagship Store	a power saver (electronics)	Goods received, do not know if it is good. When I bought it, I saw that it was a Stowe brand or company's product. Received but not. I do not know what is the reason. Now I can only give this evaluation. I'm	Jingdong prefecture level city in Shandong
Stowe Flagship Store	a power saver (electronics)	The first time to buy online, but also look at the evaluation before buying, the quality is not bad, but also very cheap, the need for friends can rest assured to buy!	Jingdong prefecture level city in Shandong
Stowe Flagship Store	a power saver (electronics)	Great quality, bought it to my liking Awesome! Buy with confidence if you need it!	Jingdong prefecture level city in Shandong
Stowe Flagship Store	a power saver (electronics)	The quality is very good, completely exceeded the expectations, the delivery speed is very fast, the packaging is very careful and strict, very satisfied with a shopping	Jingdong prefecture level city in Shandong
Stowe Flagship Store	a power saver (electronics)	The effect is very good, the home uses less electricity is very energy efficient, cost-effective	Jingdong prefecture level city in Shandong
Stowe Flagship Store	a power saver (electronics)	Quality can't be beat, just like the store, especially like, and the price is favorable!	Jingdong prefecture level city in Shandong
Stowe Flagship Store	a power saver (electronics)	Baby received! Very unexpected, will be back next time ~ great seller, stay tuned!	Jingdong prefecture level city in Shandong
Stowe Flagship Store	a power saver (electronics)	Follow up review after a month is more real, waiting for it	Jingdong prefecture level city in Shandong
Stowe Flagship Store	a power saver (electronics)	It is the ideal product, good quality and fast logistics, worthy of praise!	Jingdong prefecture level city in Shandong
Stowe Flagship Store	a power saver (electronics)	The saver is great, the color is right, the installer is very professional	Jingdong prefecture level city in Shandong
Stowe Flagship Store	a power saver (electronics)	The effect of the power connection is particularly obvious, it is particularly easy to use, very satisfied, the	Jingdong prefecture level city in Shandong
Stowe Flagship Store	a power saver (electronics)	Goods are very energy-saving, used to be more than two hundred degrees, now there are only a hundred or so degrees	Jingdong prefecture level city in Shandong
Stowe Flagship Store	a power saver (electronics)	Good quality, fast shipping, good results, good value for money	Jingdong prefecture level city in Shandong

Stowe Flagship Store	a power saver (electronics)	Fast shipping, fine workmanship, very good, cost-effective	Jingdong prefecture level city in Shandong
Stowe Flagship Store	a power saver (electronics)	The meter is as described, the quality is quite good, energy-saving, worth buying oh~!	Jingdong prefecture level city in Shandong
Stowe Flagship Store	a power saver (electronics)	Great value for money, very affordable,, perfect . ^◡^.	Jingdong prefecture level city in Shandong
Stowe Flagship Store	a power saver (electronics)	Good!!! Worth recommending!	Jingdong prefecture level city in Shandong
Stowe Flagship Store	a power saver (electronics)	Great, fast logistics, great stuff, great value for money!	Jingdong prefecture level city in Shandong
Stowe Flagship Store	a power saver (electronics)	Fast logistics, very satisfied with the quality upon receipt	Jingdong prefecture level city in Shandong
Stowe Flagship Store	a power saver (electronics)	Great quality, plug and play, fast shipping!	Jingdong prefecture level city in Shandong
Stowe Flagship Store	a power saver (electronics)	Logistics received, quite satisfied	Jingdong prefecture level city in Shandong
Stowe Flagship Store	a power saver (electronics)	I don't know if it really works	Jingdong prefecture level city in Shandong
Stowe Flagship Store	a power saver (electronics)	It's bullshit. It's a plug light. Don't talk about power savings.	Jingdong prefecture level city in Shandong
Stowe Flagship Store	a power saver (electronics)	Fake and deceitful stuff that hasn't changed at all.	Jingdong prefecture level city in Shandong
Stowe Flagship Store	a power saver (electronics)	Power-saving effect: power-saving effect is particularly obvious Cost-effective: cost-effective business Appearance and style: external small micro-work materials: fine workmanship Experience: use very good, non the first convenient	Jingdong prefecture level city in Shandong
Stowe Flagship Store	a power saver (electronics)	After using it for a few days, it feels quite good, it should be quite safe, and it also sends a plug, the merchant is good, wait for a period of time and then calculate whether it saves on the electricity bill and then add comments ha!	Jingdong prefecture level city in Shandong
Stowe Flagship Store	a power saver (electronics)	My friend recommended to me, said the effect is very good, I bought two, this month less money, really can, just buy I do not believe, but has proved to have effect	Jingdong prefecture level city in Shandong
Stowe Flagship Store	a power saver (electronics)	Just bought back, already used, these days to see the effect of it, and so have the effect will also come back to give positive feedback, the price is also super cheap!	Jingdong prefecture level city in Shandong
Stowe Flagship Store	a power saver (electronics)	It does work well, the store uses a lot of electricity, this time to buy back a few, each place that uses a lot of electricity to insert one. Summer is here, the electricity is too strong.	Jingdong prefecture level city in Shandong
Stowe Flagship Store	a power saver (electronics)	Power saving treasure received, as for the effect, according to the instructions written, a short period of time can not be seen, at least 2-3 months before the real effect, then follow up evaluation	Jingdong prefecture level city in Shandong
Stowe Flagship Store	a power saver (electronics)	The goods have just been received and have not been used yet looking forward to the results.	Jingdong prefecture level city in Shandong
Stowe Flagship Store	a power saver (electronics)	Good installation works great	Jingdong prefecture level city in Shandong
Stowe Flagship Store	a power saver (electronics)	The logistics is super super fast! The quality is also very good, indeed value for money	Jingdong prefecture level city in Shandong
Stowe Flagship Store	a power saver (electronics)	Has been used for a period of time before coming to comment on, really good, air conditioning a day open non-stop, pay the electricity bill when the heartache, with this, the home electricity is not at all worried about the bill out of contrast to save a lot of preparation in the hands of a few, each room An a	Jingdong prefecture level city in Shandong
Stowe Flagship Store	a power saver (electronics)	Saving device received, really good, with the air conditioning tried, an hour without a saver with 2.9 degrees, with a saver an hour with 1.1 degrees, the beginning of not quite believe that this thing, the actual test to know that it is really useful, and intend to buy another.	Jingdong prefecture level city in Shandong
Stowe Flagship Store	a power saver (electronics)	Power saving effect: this energy saver is still useful, day trial for three days, every day than not installed before the reduction of two dollars a day, every day to open the bedroom to open the air conditioning before fifteen dollars now thirteen dollars a day. Still a little use.	Jingdong prefecture level city in Shandong
Stowe Flagship Store	a power saver (electronics)	Originally bought for home use, used for two months, the electricity bill from more than 200 yuan per month, down to more than 140 yuan, there is indeed a power-saving effect, this time and sold two, the unit with. Merchant service attitude is really good.	Jingdong prefecture level city in Shandong
Stowe Flagship Store	a power saver (electronics)	Plug and play, stable voltage, the screen shows when working, this winter home appliances, need electricity in many places, just buy to use, save electricity and money, with instructions	Jingdong prefecture level city in Shandong
Stowe Flagship Store	a power saver (electronics)	Goods received, do not know if it is good. When I bought it, I saw that it was a Stowe brand or company's product. Received but not. I do not know what is the reason. Now I can only give this evaluation. I'm	Jingdong prefecture level city in Shandong
Stowe Flagship Store	a power saver (electronics)	The first time to buy online, but also look at the evaluation before buying, the quality is not bad, but also very cheap, the need for friends can rest assured to buy!	Jingdong prefecture level city in Shandong
Stowe Flagship Store	a power saver (electronics)	🌈 Great quality, bought it to my liking 💕 Awesome! ✨ Buy with confidence if you need it!	Jingdong prefecture level city in Shandong
Stowe Flagship Store	a power saver (electronics)	The quality is very good, completely exceeded the expectations, the delivery speed is very fast, the packaging is very careful and strict, very satisfied with a shopping	Jingdong prefecture level city in Shandong
Stowe Flagship Store	a power saver (electronics)	The effect is very good, the home uses less electricity is very energy efficient, cost-effective	Jingdong prefecture level city in Shandong
Stowe Flagship Store	a power saver (electronics)	Quality can't be beat, just like the store, especially like, and the price is favorable!	Jingdong prefecture level city in Shandong
Stowe Flagship Store	a power saver (electronics)	Baby received! Very unexpected, will be back next time ~ great seller, stay tuned!	Jingdong prefecture level city in Shandong
Stowe Flagship Store	a power saver (electronics)	Follow up review after a month is more real, waiting for it	Jingdong prefecture level city in Shandong
Stowe Flagship Store	a power saver (electronics)	It is the ideal product, good quality and fast logistics, worthy of praise!	Jingdong prefecture level city in Shandong
Stowe Flagship Store	a power saver (electronics)	The saver is great, the color is right, the installer is very professional	Jingdong prefecture level city in Shandong
Stowe Flagship Store	a power saver (electronics)	The effect of the power connection is particularly obvious, it is particularly easy to use, very satisfied, the	Jingdong prefecture level city in Shandong
Stowe Flagship Store	a power saver (electronics)	Goods are very energy-saving, used to be more than two hundred degrees, now there are only a hundred or so degrees	Jingdong prefecture level city in Shandong
Stowe Flagship Store	a power saver (electronics)	Good quality, fast shipping, good results, good value for money	Jingdong prefecture level city in Shandong
Stowe Flagship Store	a power saver (electronics)	Fast shipping, fine workmanship, very good, cost-effective	Jingdong prefecture level city in Shandong
Stowe Flagship Store	a power saver (electronics)	The meter is as described, the quality is quite good, energy-saving, worth buying oh~!	Jingdong prefecture level city in Shandong
Stowe Flagship Store	a power saver (electronics)	Great value for money, very affordable,, perfect . ^◡^.	Jingdong prefecture level city in Shandong
Stowe Flagship Store	a power saver (electronics)	Good!!! Worth recommending!	Jingdong prefecture level city in Shandong
Stowe Flagship Store	a power saver (electronics)	Great, fast logistics, great stuff, great value for money!	Jingdong prefecture level city in Shandong
Stowe Flagship Store	a power saver (electronics)	Fast logistics, very satisfied with the quality upon receipt	Jingdong prefecture level city in Shandong
Stowe Flagship Store	a power saver (electronics)	Great quality, plug and play, fast shipping!	Jingdong prefecture level city in Shandong
Stowe Flagship Store	a power saver (electronics)	Logistics received, quite satisfied	Jingdong prefecture level city in Shandong
Stowe Flagship Store	a power saver (electronics)	I don't know if it really works	Jingdong prefecture level city in Shandong
Stowe Flagship Store	a power saver (electronics)	It's bullshit. It's a plug light. Don't talk about power savings.	Jingdong prefecture level city in Shandong

Stowe Flagship Store	a power saver (electronics)	Fake and deceitful stuff that hasn't changed at all.	Jingdong prefecture level city in Shandong
Stowe Flagship Store	a power saver (electronics)	Power-saving effect: power-saving effect is particularly obvious Cost-effective: cost-effective business Appearance and style: external small micro-work materials: fine workmanship Experience: use very good, non the first convenient	Jingdong prefecture level city in Shandong
Stowe Flagship Store	a power saver (electronics)	After using it for a few days, it feels quite good, it should be quite safe, and it also sends a plug, the merchant is good, wait for a period of time and then calculate whether it saves on the electricity bill and then add comments ha!	Jingdong prefecture level city in Shandong
Stowe Flagship Store	a power saver (electronics)	My friend recommended to me, said the effect is very good, I bought two, this month less money, really can, just buy I do not believe, but has proved to have effect	Jingdong prefecture level city in Shandong
Stowe Flagship Store	a power saver (electronics)	Just bought back, already used, these days to see the effect of it, and so have the effect will also come back to give positive feedback, the price is also super cheap!	Jingdong prefecture level city in Shandong
Stowe Flagship Store	a power saver (electronics)	It does work well, the store uses a lot of electricity, this time to buy back a few, each place that uses a lot of electricity to insert one. Summer is here, the electricity is too strong.	Jingdong prefecture level city in Shandong
Stowe Flagship Store	a power saver (electronics)	Power saving treasure received, as for the effect, according to the instructions written, a short period of time can not be seen, at least 2-3 months before the real effect, then follow up evaluation	Jingdong prefecture level city in Shandong
Stowe Flagship Store	a power saver (electronics)	The goods have just been received and have not been used yet looking forward to the results.	Jingdong prefecture level city in Shandong
Stowe Flagship Store	a power saver (electronics)	Good installation works great	Jingdong prefecture level city in Shandong
Stowe Flagship Store	a power saver (electronics)	The logistics is super super fast! The quality is also very good, indeed value for money	Jingdong prefecture level city in Shandong
Stowe Flagship Store	a power saver (electronics)	Has been used for a period of time before coming to comment on, really good, air conditioning a day open non-stop, pay the electricity bill when the heartache, with this, the home electricity is not at all worried about the bill out of contrast to save a lot of preparation in the hands of a few, each room An a	Jingdong prefecture level city in Shandong
Stowe Flagship Store	a power saver (electronics)	Saving device received, really good, with the air conditioning tried, an hour without a saver with 2.9 degrees, with a saver an hour with 1.1 degrees, the beginning of not quite believe that this thing, the actual test to know that it is really useful, and intend to buy another.	Jingdong prefecture level city in Shandong
Stowe Flagship Store	a power saver (electronics)	Power saving effect: this energy saver is still useful, day trial for three days, every day than not installed before the reduction of two dollars a day, every day to open the bedroom to open the air conditioning before fifteen dollars now thirteen dollars a day. Still a little use.	Jingdong prefecture level city in Shandong
Stowe Flagship Store	a power saver (electronics)	Originally bought for home use, used for two months, the electricity bill from more than 200 yuan per month, down to more than 140 yuan, there is indeed a power-saving effect, this time and sold two, the unit with. Merchant service attitude is really good.	Jingdong prefecture level city in Shandong
Stowe Flagship Store	a power saver (electronics)	Plug and play, stable voltage, the screen shows when working, this winter home appliances, need electricity in many places, just buy to use, save electricity and money, with instructions	Jingdong prefecture level city in Shandong
Stowe Flagship Store	a power saver (electronics)	Goods received, do not know if it is good. When I bought it, I saw that it was a Stowe brand or company's product. Received but not. I do not know what is the reason. Now I can only give this evaluation. I'm	Jingdong prefecture level city in Shandong
Stowe Flagship Store	a power saver (electronics)	The first time to buy online, but also look at the evaluation before buying, the quality is not bad, but also very cheap, the need for friends can rest assured to buy!	Jingdong prefecture level city in Shandong
Stowe Flagship Store	a power saver (electronics)	🌈 Great quality, bought it to my liking 💕 Awesome! ✨ Buy with confidence if you need it!	Jingdong prefecture level city in Shandong
Stowe Flagship Store	a power saver (electronics)	The quality is very good, completely exceeded the expectations, the delivery speed is very fast, the packaging is very careful and strict, very satisfied with a shopping	Jingdong prefecture level city in Shandong
Stowe Flagship Store	a power saver (electronics)	The effect is very good, the home uses less electricity is very energy efficient, cost-effective	Jingdong prefecture level city in Shandong
Stowe Flagship Store	a power saver (electronics)	Quality can't be beat, just like the store, especially like, and the price is favorable!	Jingdong prefecture level city in Shandong
Stowe Flagship Store	a power saver (electronics)	Baby received! Very unexpected, will be back next time ~ great seller, stay tuned!	Jingdong prefecture level city in Shandong
Stowe Flagship Store	a power saver (electronics)	Follow up review after a month is more real, waiting for it	Jingdong prefecture level city in Shandong
Stowe Flagship Store	a power saver (electronics)	It is the ideal product, good quality and fast logistics, worthy of praise!	Jingdong prefecture level city in Shandong
Stowe Flagship Store	a power saver (electronics)	The saver is great, the color is right, the installer is very professional	Jingdong prefecture level city in Shandong
Stowe Flagship Store	a power saver (electronics)	The effect of the power connection is particularly obvious, it is particularly easy to use, very satisfied, the	Jingdong prefecture level city in Shandong
Stowe Flagship Store	a power saver (electronics)	Goods are very energy-saving, used to be more than two hundred degrees, now there are only a hundred or so degrees	Jingdong prefecture level city in Shandong
Stowe Flagship Store	a power saver (electronics)	Good quality, fast shipping, good results, good value for money	Jingdong prefecture level city in Shandong
Stowe Flagship Store	a power saver (electronics)	Fast shipping, fine workmanship, very good, cost-effective	Jingdong prefecture level city in Shandong
Stowe Flagship Store	a power saver (electronics)	The meter is as described, the quality is quite good, energy-saving, worth buying oh~!	Jingdong prefecture level city in Shandong
Stowe Flagship Store	a power saver (electronics)	Great value for money, very affordable,, perfect . ˆ◡ˆ.	Jingdong prefecture level city in Shandong
Stowe Flagship Store	a power saver (electronics)	Good!!! Worth recommending!	Jingdong prefecture level city in Shandong
Stowe Flagship Store	a power saver (electronics)	Great, fast logistics, great stuff, great value for money!	Jingdong prefecture level city in Shandong
Stowe Flagship Store	a power saver (electronics)	Fast logistics, very satisfied with the quality upon receipt	Jingdong prefecture level city in Shandong
Stowe Flagship Store	a power saver (electronics)	Great quality, plug and play, fast shipping!	Jingdong prefecture level city in Shandong
Stowe Flagship Store	a power saver (electronics)	Logistics received, quite satisfied	Jingdong prefecture level city in Shandong
Stowe Flagship Store	a power saver (electronics)	I don't know if it really works	Jingdong prefecture level city in Shandong
Stowe Flagship Store	a power saver (electronics)	It's bullshit. It's a plug light. Don't talk about power savings.	Jingdong prefecture level city in Shandong
Stowe Flagship Store	a power saver (electronics)	Fake and deceitful stuff that hasn't changed at all.	Jingdong prefecture level city in Shandong
Stowe Flagship Store	a power saver (electronics)	Power-saving effect: power-saving effect is particularly obvious Cost-effective: cost-effective business Appearance and style: external small micro-work materials: fine workmanship Experience: use very good, non the first convenient	Jingdong prefecture level city in Shandong
Stowe Flagship Store	a power saver (electronics)	After using it for a few days, it feels quite good, it should be quite safe, and it also sends a plug, the merchant is good, wait for a period of time and then calculate whether it saves on the electricity bill and then add comments ha!	Jingdong prefecture level city in Shandong
Stowe Flagship Store	a power saver (electronics)	My friend recommended to me, said the effect is very good, I bought two, this month less money, really can, just buy I do not believe, but has proved to have effect	Jingdong prefecture level city in Shandong
Stowe Flagship Store	a power saver (electronics)	Just bought back, already used, these days to see the effect of it, and so have the effect will also come back to give positive feedback, the price is also super cheap!	Jingdong prefecture level city in Shandong
Stowe Flagship Store	a power saver (electronics)	It does work well, the store uses a lot of electricity, this time to buy back a few, each place that uses a lot of electricity to insert one. Summer is here, the electricity is too strong.	Jingdong prefecture level city in Shandong
Stowe Flagship Store	a power saver (electronics)	Power saving treasure received, as for the effect, according to the instructions written, a short period of time can not be seen, at least 2-3 months before the real effect, then follow up evaluation	Jingdong prefecture level city in Shandong
Stowe Flagship Store	a power saver (electronics)	The goods have just been received and have not been used yet looking forward to the results.	Jingdong prefecture level city in Shandong
Stowe Flagship Store	a power saver (electronics)	Good installation works great	Jingdong prefecture level city in Shandong

Stowe Flagship Store	a power saver (electronics)	The logistics is super super fast! The quality is also very good, indeed value for money	Jingdong prefecture level city in Shandong
Stowe Flagship Store	a power saver (electronics)	Has been used for a period of time before coming to comment on, really good, air conditioning a day open non-stop, pay the electricity bill when the heartache, with this, the home electricity is not at all worried about the bill out of contrast to save a lot of preparation in the hands of a few, each room An a	Jingdong prefecture level city in Shandong
Stowe Flagship Store	a power saver (electronics)	Saving device received, really good, with the air conditioning tried, an hour without a saver with 2.9 degrees, with a saver an hour with 1.1 degrees, the beginning of not quite believe that this thing, the actual test to know that it is really useful, and intend to buy another.	Jingdong prefecture level city in Shandong
Stowe Flagship Store	a power saver (electronics)	Power saving effect: this energy saver is still useful, day trial for three days, every day than not installed before the reduction of two dollars a day, every day to open the bedroom to open the air conditioning before fifteen dollars now thirteen dollars a day. Still a little use.	Jingdong prefecture level city in Shandong
Stowe Flagship Store	a power saver (electronics)	Originally bought for home use, used for two months, the electricity bill from more than 200 yuan per month, down to more than 140 yuan, there is indeed a power-saving effect, this time and sold two, the unit with. Merchant service attitude is really good.	Jingdong prefecture level city in Shandong
Stowe Flagship Store	a power saver (electronics)	Plug and play, stable voltage, the screen shows when working, this winter home appliances, need electricity in many places, just buy to use, save electricity and money, with instructions	Jingdong prefecture level city in Shandong
Stowe Flagship Store	a power saver (electronics)	Goods received, do not know if it is good. When I bought it, I saw that it was a Stowe brand or company's product. Received but not. I do not know what is the reason. Now I can only give this evaluation. I'm	Jingdong prefecture level city in Shandong
Stowe Flagship Store	a power saver (electronics)	The first time to buy online, but also look at the evaluation before buying, the quality is not bad, but also very cheap, the need for friends can rest assured to buy!	Jingdong prefecture level city in Shandong
Stowe Flagship Store	a power saver (electronics)	🌈 Great quality, bought it to my liking 💝 Awesome! ✨ Buy with confidence if you need it!	Jingdong prefecture level city in Shandong
Stowe Flagship Store	a power saver (electronics)	The quality is very good, completely exceeded the expectations, the delivery speed is very fast, the packaging is very careful and strict, very satisfied with a shopping	Jingdong prefecture level city in Shandong
Stowe Flagship Store	a power saver (electronics)	The effect is very good, the home uses less electricity is very energy efficient, cost-effective	Jingdong prefecture level city in Shandong
Stowe Flagship Store	a power saver (electronics)	Quality can't be beat, just like the store, especially like, and the price is favorable!	Jingdong prefecture level city in Shandong
Stowe Flagship Store	a power saver (electronics)	Baby received! Very unexpected, will be back next time ~ great seller, stay tuned!	Jingdong prefecture level city in Shandong
Stowe Flagship Store	a power saver (electronics)	Follow up review after a month is more real, waiting for it	Jingdong prefecture level city in Shandong
Stowe Flagship Store	a power saver (electronics)	It is the ideal product, good quality and fast logistics, worthy of praise!	Jingdong prefecture level city in Shandong
Stowe Flagship Store	a power saver (electronics)	The saver is great, the color is right, the installer is very professional	Jingdong prefecture level city in Shandong
Stowe Flagship Store	a power saver (electronics)	The effect of the power connection is particularly obvious, it is particularly easy to use, very satisfied, the	Jingdong prefecture level city in Shandong
Stowe Flagship Store	a power saver (electronics)	Goods are very energy-saving, used to be more than two hundred degrees, now there are only a hundred or so degrees	Jingdong prefecture level city in Shandong
Stowe Flagship Store	a power saver (electronics)	Good quality, fast shipping, good results, good value for money	Jingdong prefecture level city in Shandong
Stowe Flagship Store	a power saver (electronics)	Fast shipping, fine workmanship, very good, cost-effective	Jingdong prefecture level city in Shandong
Stowe Flagship Store	a power saver (electronics)	The meter is as described, the quality is quite good, energy-saving, worth buying oh~!	Jingdong prefecture level city in Shandong
Stowe Flagship Store	a power saver (electronics)	Great value for money, very affordable,, perfect . ˆ◡ˆ.	Jingdong prefecture level city in Shandong
Stowe Flagship Store	a power saver (electronics)	Good!!! Worth recommending!	Jingdong prefecture level city in Shandong
Stowe Flagship Store	a power saver (electronics)	Great, fast logistics, great stuff, great value for money!	Jingdong prefecture level city in Shandong
Stowe Flagship Store	a power saver (electronics)	Fast logistics, very satisfied with the quality upon receipt	Jingdong prefecture level city in Shandong
Stowe Flagship Store	a power saver (electronics)	Great quality, plug and play, fast shipping!	Jingdong prefecture level city in Shandong
Stowe Flagship Store	a power saver (electronics)	Logistics received, quite satisfied	Jingdong prefecture level city in Shandong
Stowe Flagship Store	a power saver (electronics)	I don't know if it really works	Jingdong prefecture level city in Shandong
Stowe Flagship Store	a power saver (electronics)	It's bullshit. It's a plug light. Don't talk about power savings.	Jingdong prefecture level city in Shandong
Stowe Flagship Store	a power saver (electronics)	Fake and deceitful stuff that hasn't changed at all.	Jingdong prefecture level city in Shandong
Stowe Flagship Store	a power saver (electronics)	Power-saving effect: power-saving effect is particularly obvious Cost-effective: cost-effective business Appearance and style: external small micro-work materials: fine workmanship Experience: use very good, non the first convenient	Jingdong prefecture level city in Shandong
Stowe Flagship Store	a power saver (electronics)	After using it for a few days, it feels quite good, it should be quite safe, and it also sends a plug, the merchant is good, wait for a period of time and then calculate whether it saves on the electricity bill and then add comments ha!	Jingdong prefecture level city in Shandong
Stowe Flagship Store	a power saver (electronics)	My friend recommended to me, said the effect is very good, I bought two, this month less money, really can, just buy I do not believe, but has proved to have effect	Jingdong prefecture level city in Shandong
Stowe Flagship Store	a power saver (electronics)	Just bought back, already used, these days to see the effect of it, and so have the effect will also come back to give positive feedback, the price is also super cheap!	Jingdong prefecture level city in Shandong
Stowe Flagship Store	a power saver (electronics)	It does work well, the store uses a lot of electricity, this time to buy back a few, each place that uses a lot of electricity to insert one. Summer is here, the electricity is too strong.	Jingdong prefecture level city in Shandong
Stowe Flagship Store	a power saver (electronics)	Power saving treasure received, as for the effect, according to the instructions written, a short period of time can not be seen, at least 2-3 months before the real effect, then follow up evaluation	Jingdong prefecture level city in Shandong
Stowe Flagship Store	a power saver (electronics)	The goods have just been received and have not been used yet looking forward to the results.	Jingdong prefecture level city in Shandong
Stowe Flagship Store	a power saver (electronics)	Good installation works great	Jingdong prefecture level city in Shandong
Stowe Flagship Store	a power saver (electronics)	The logistics is super super fast! The quality is also very good, indeed value for money	Jingdong prefecture level city in Shandong
Stowe Flagship Store	a power saver (electronics)	Has been used for a period of time before coming to comment on, really good, air conditioning a day open non-stop, pay the electricity bill when the heartache, with this, the home electricity is not at all worried about the bill out of contrast to save a lot of preparation in the hands of a few, each room An a	Jingdong prefecture level city in Shandong
Stowe Flagship Store	a power saver (electronics)	Saving device received, really good, with the air conditioning tried, an hour without a saver with 2.9 degrees, with a saver an hour with 1.1 degrees, the beginning of not quite believe that this thing, the actual test to know that it is really useful, and intend to buy another.	Jingdong prefecture level city in Shandong
Stowe Flagship Store	a power saver (electronics)	Power saving effect: this energy saver is still useful, day trial for three days, every day than not installed before the reduction of two dollars a day, every day to open the bedroom to open the air conditioning before fifteen dollars now thirteen dollars a day. Still a little use.	Jingdong prefecture level city in Shandong
Stowe Flagship Store	a power saver (electronics)	Originally bought for home use, used for two months, the electricity bill from more than 200 yuan per month, down to more than 140 yuan, there is indeed a power-saving effect, this time and sold two, the unit with. Merchant service attitude is really good.	Jingdong prefecture level city in Shandong
Stowe Flagship Store	a power saver (electronics)	Plug and play, stable voltage, the screen shows when working, this winter home appliances, need electricity in many places, just buy to use, save electricity and money, with instructions	Jingdong prefecture level city in Shandong
Stowe Flagship Store	a power saver (electronics)	Goods received, do not know if it is good. When I bought it, I saw that it was a Stowe brand or company's product. Received but not. I do not know what is the reason. Now I can only give this evaluation. I'm	Jingdong prefecture level city in Shandong
Stowe Flagship Store	a power saver (electronics)	The first time to buy online, but also look at the evaluation before buying, the quality is not bad, but also very cheap, the need for friends can rest assured to buy!	Jingdong prefecture level city in Shandong

Stowe Flagship Store	a power saver (electronics)	🌈 Great quality, bought it to my liking 💝 Awesome! ✨ Buy with confidence if you need it!	Jingdong prefecture level city in Shandong
Stowe Flagship Store	a power saver (electronics)	The quality is very good, completely exceeded the expectations, the delivery speed is very fast, the packaging is very careful and strict, very satisfied with a shopping	Jingdong prefecture level city in Shandong
Stowe Flagship Store	a power saver (electronics)	The effect is very good, the home uses less electricity is very energy efficient, cost-effective	Jingdong prefecture level city in Shandong
Stowe Flagship Store	a power saver (electronics)	Quality can't be beat, just like the store, especially like, and the price is favorable!	Jingdong prefecture level city in Shandong
Stowe Flagship Store	a power saver (electronics)	Baby received! Very unexpected, will be back next time ~ great seller, stay tuned!	Jingdong prefecture level city in Shandong
Stowe Flagship Store	a power saver (electronics)	Follow up review after a month is more real, waiting for it	Jingdong prefecture level city in Shandong
Stowe Flagship Store	a power saver (electronics)	It is the ideal product, good quality and fast logistics, worthy of praise!	Jingdong prefecture level city in Shandong
Stowe Flagship Store	a power saver (electronics)	The saver is great, the color is right, the installer is very professional	Jingdong prefecture level city in Shandong
Stowe Flagship Store	a power saver (electronics)	The effect of the power connection is particularly obvious, it is particularly easy to use, very satisfied, the	Jingdong prefecture level city in Shandong
Stowe Flagship Store	a power saver (electronics)	Goods are very energy-saving, used to be more than two hundred degrees, now there are only a hundred or so degrees	Jingdong prefecture level city in Shandong
Stowe Flagship Store	a power saver (electronics)	Good quality, fast shipping, good results, good value for money	Jingdong prefecture level city in Shandong
Stowe Flagship Store	a power saver (electronics)	Fast shipping, fine workmanship, very good, cost-effective	Jingdong prefecture level city in Shandong
Stowe Flagship Store	a power saver (electronics)	The meter is as described, the quality is quite good, energy-saving, worth buying oh~!	Jingdong prefecture level city in Shandong
Stowe Flagship Store	a power saver (electronics)	Great value for money, very affordable,, perfect . ^◡^.	Jingdong prefecture level city in Shandong
Stowe Flagship Store	a power saver (electronics)	Good!!! Worth recommending!	Jingdong prefecture level city in Shandong
Stowe Flagship Store	a power saver (electronics)	Great, fast logistics, great stuff, great value for money!	Jingdong prefecture level city in Shandong
Stowe Flagship Store	a power saver (electronics)	Fast logistics, very satisfied with the quality upon receipt	Jingdong prefecture level city in Shandong
Stowe Flagship Store	a power saver (electronics)	Great quality, plug and play, fast shipping!	Jingdong prefecture level city in Shandong
Stowe Flagship Store	a power saver (electronics)	Logistics received, quite satisfied	Jingdong prefecture level city in Shandong
Stowe Flagship Store	a power saver (electronics)	I don't know if it really works	Jingdong prefecture level city in Shandong
Stowe Flagship Store	a power saver (electronics)	It's bullshit. It's a plug light. Don't talk about power savings.	Jingdong prefecture level city in Shandong
Stowe Flagship Store	a power saver (electronics)	Fake and deceitful stuff that hasn't changed at all.	Jingdong prefecture level city in Shandong
Stowe Flagship Store	a power saver (electronics)	Power-saving effect: power-saving effect is particularly obvious Cost-effective: cost-effective business Appearance and style: external small micro-work materials: fine workmanship Experience: use very good, non the first convenient	Jingdong prefecture level city in Shandong
Stowe Flagship Store	a power saver (electronics)	After using it for a few days, it feels quite good, it should be quite safe, and it also sends a plug, the merchant is good, wait for a period of time and then calculate whether it saves on the electricity bill and then add comments ha!	Jingdong prefecture level city in Shandong
Stowe Flagship Store	a power saver (electronics)	My friend recommended to me, said the effect is very good, I bought two, this month less money, really can, just buy I do not believe, but has proved to have effect	Jingdong prefecture level city in Shandong
Stowe Flagship Store	a power saver (electronics)	Just bought back, already used, these days to see the effect of it, and so have the effect will also come back to give positive feedback, the price is also super cheap!	Jingdong prefecture level city in Shandong
Stowe Flagship Store	a power saver (electronics)	It does work well, the store uses a lot of electricity, this time to buy back a few, each place that uses a lot of electricity to insert one. Summer is here, the electricity is too strong.	Jingdong prefecture level city in Shandong
Stowe Flagship Store	a power saver (electronics)	Power saving treasure received, as for the effect, according to the instructions written, a short period of time can not be seen, at least 2-3 months before the real effect, then follow up evaluation	Jingdong prefecture level city in Shandong
Stowe Flagship Store	a power saver (electronics)	The goods have just been received and have not been used yet looking forward to the results.	Jingdong prefecture level city in Shandong
Stowe Flagship Store	a power saver (electronics)	Good installation works great	Jingdong prefecture level city in Shandong
Stowe Flagship Store	a power saver (electronics)	The logistics is super super fast! The quality is also very good, indeed value for money	Jingdong prefecture level city in Shandong
Stowe Flagship Store	a power saver (electronics)	Has been used for a period of time before coming to comment on, really good, air conditioning a day open non-stop, pay the electricity bill when the heartache, with this, the home electricity is not at all worried about the bill out of contrast to save a lot of preparation in the hands of a few, each room An a	Jingdong prefecture level city in Shandong
Stowe Flagship Store	a power saver (electronics)	Saving device received, really good, with the air conditioning tried, an hour without a saver with 2.9 degrees, with a saver an hour with 1.1 degrees, the beginning of not quite believe that this thing, the actual test to know that it is really useful, and intend to buy another.	Jingdong prefecture level city in Shandong
Stowe Flagship Store	a power saver (electronics)	Power saving effect: this energy saver is still useful, day trial for three days, every day than not installed before the reduction of two dollars a day, every day to open the bedroom to open the air conditioning before fifteen dollars now thirteen dollars a day. Still a little use.	Jingdong prefecture level city in Shandong
Stowe Flagship Store	a power saver (electronics)	Originally bought for home use, used for two months, the electricity bill from more than 200 yuan per month, down to more than 140 yuan, there is indeed a power-saving effect, this time and sold two, the unit with. Merchant service attitude is really good.	Jingdong prefecture level city in Shandong
Stowe Flagship Store	a power saver (electronics)	Plug and play, stable voltage, the screen shows when working, this winter home appliances, need electricity in many places, just buy to use, save electricity and money, with instructions	Jingdong prefecture level city in Shandong
Stowe Flagship Store	a power saver (electronics)	Goods received, do not know if it is good. When I bought it, I saw that it was a Stowe brand or company's product. Received but not. I do not know what is the reason. Now I can only give this evaluation. I'm	Jingdong prefecture level city in Shandong
Stowe Flagship Store	a power saver (electronics)	The first time to buy online, but also look at the evaluation before buying, the quality is not bad, but also very cheap, the need for friends can rest assured to buy!	Jingdong prefecture level city in Shandong
Stowe Flagship Store	a power saver (electronics)	🌈 Great quality, bought it to my liking 💝 Awesome! ✨ Buy with confidence if you need it!	Jingdong prefecture level city in Shandong
Stowe Flagship Store	a power saver (electronics)	The quality is very good, completely exceeded the expectations, the delivery speed is very fast, the packaging is very careful and strict, very satisfied with a shopping	Jingdong prefecture level city in Shandong
Stowe Flagship Store	a power saver (electronics)	The effect is very good, the home uses less electricity is very energy efficient, cost-effective	Jingdong prefecture level city in Shandong
Stowe Flagship Store	a power saver (electronics)	Quality can't be beat, just like the store, especially like, and the price is favorable!	Jingdong prefecture level city in Shandong
Stowe Flagship Store	a power saver (electronics)	Baby received! Very unexpected, will be back next time ~ great seller, stay tuned!	Jingdong prefecture level city in Shandong
Stowe Flagship Store	a power saver (electronics)	Follow up review after a month is more real, waiting for it	Jingdong prefecture level city in Shandong
Stowe Flagship Store	a power saver (electronics)	It is the ideal product, good quality and fast logistics, worthy of praise!	Jingdong prefecture level city in Shandong
Stowe Flagship Store	a power saver (electronics)	The saver is great, the color is right, the installer is very professional	Jingdong prefecture level city in Shandong
Stowe Flagship Store	a power saver (electronics)	The effect of the power connection is particularly obvious, it is particularly easy to use, very satisfied, the	Jingdong prefecture level city in Shandong
Stowe Flagship Store	a power saver (electronics)	Goods are very energy-saving, used to be more than two hundred degrees, now there are only a hundred or so degrees	Jingdong prefecture level city in Shandong

Stowe Flagship Store	a power saver (electronics)	Good quality, fast shipping, good results, good value for money	Jingdong prefecture level city in Shandong
Stowe Flagship Store	a power saver (electronics)	Fast shipping, fine workmanship, very good, cost-effective	Jingdong prefecture level city in Shandong
Stowe Flagship Store	a power saver (electronics)	The meter is as described, the quality is quite good, energy-saving, worth buying oh~!	Jingdong prefecture level city in Shandong
Stowe Flagship Store	a power saver (electronics)	Great value for money, very affordable,, perfect . ˆ◡ˆ.	Jingdong prefecture level city in Shandong
Stowe Flagship Store	a power saver (electronics)	Good!!! Worth recommending!	Jingdong prefecture level city in Shandong
Stowe Flagship Store	a power saver (electronics)	Great, fast logistics, great stuff, great value for money!	Jingdong prefecture level city in Shandong
Stowe Flagship Store	a power saver (electronics)	Fast logistics, very satisfied with the quality upon receipt	Jingdong prefecture level city in Shandong
Stowe Flagship Store	a power saver (electronics)	Great quality, plug and play, fast shipping!	Jingdong prefecture level city in Shandong
Stowe Flagship Store	a power saver (electronics)	Logistics received, quite satisfied	Jingdong prefecture level city in Shandong
Stowe Flagship Store	a power saver (electronics)	I don't know if it really works	Jingdong prefecture level city in Shandong
Stowe Flagship Store	a power saver (electronics)	It's bullshit. It's a plug light. Don't talk about power savings.	Jingdong prefecture level city in Shandong
Stowe Flagship Store	a power saver (electronics)	Fake and deceitful stuff that hasn't changed at all.	Jingdong prefecture level city in Shandong
Stowe Flagship Store	a power saver (electronics)	Power-saving effect: power-saving effect is particularly obvious Cost-effective: cost-effective business Appearance and style: external small micro-work materials: fine workmanship Experience: use very good, non the first convenient	Jingdong prefecture level city in Shandong
Stowe Flagship Store	a power saver (electronics)	After using it for a few days, it feels quite good, it should be quite safe, and it also sends a plug, the merchant is good, wait for a period of time and then calculate whether it saves on the electricity bill and then add comments ha!	Jingdong prefecture level city in Shandong
Stowe Flagship Store	a power saver (electronics)	My friend recommended to me, said the effect is very good, I bought two, this month less money, really can, just buy I do not believe, but has proved to have effect	Jingdong prefecture level city in Shandong
Stowe Flagship Store	a power saver (electronics)	Just bought back, already used, these days to see the effect of it, and so have the effect will also come back to give positive feedback, the price is also super cheap!	Jingdong prefecture level city in Shandong
Stowe Flagship Store	a power saver (electronics)	It does work well, the store uses a lot of electricity, this time to buy back a few, each place that uses a lot of electricity to insert one. Summer is here, the electricity is too strong.	Jingdong prefecture level city in Shandong
Stowe Flagship Store	a power saver (electronics)	Power saving treasure received, as for the effect, according to the instructions written, a short period of time can not be seen, at least 2-3 months before the real effect, then follow up evaluation	Jingdong prefecture level city in Shandong
Stowe Flagship Store	a power saver (electronics)	The goods have just been received and have not been used yet looking forward to the results.	Jingdong prefecture level city in Shandong
Stowe Flagship Store	a power saver (electronics)	Good installation works great	Jingdong prefecture level city in Shandong
Stowe Flagship Store	a power saver (electronics)	The logistics is super super fast! The quality is also very good, indeed value for money	Jingdong prefecture level city in Shandong
Stowe Flagship Store	a power saver (electronics)	Has been used for a period of time before coming to comment on, really good, air conditioning a day open non-stop, pay the electricity bill when the heartache, with this, the home electricity is not at all worried about the bill out of contrast to save a lot of preparation in the hands of a few, each room An a	Jingdong prefecture level city in Shandong
Stowe Flagship Store	a power saver (electronics)	Saving device received, really good, with the air conditioning tried, an hour without a saver with 2.9 degrees, with a saver an hour with 1.1 degrees, the beginning of not quite believe that this thing, the actual test to know that it is really useful, and intend to buy another.	Jingdong prefecture level city in Shandong
Stowe Flagship Store	a power saver (electronics)	Power saving effect: this energy saver is still useful, day trial for three days, every day than not installed before the reduction of two dollars a day, every day to open the bedroom to open the air conditioning before fifteen dollars now thirteen dollars a day. Still a little use.	Jingdong prefecture level city in Shandong
Stowe Flagship Store	a power saver (electronics)	Originally bought for home use, used for two months, the electricity bill from more than 200 yuan per month, down to more than 140 yuan, there is indeed a power-saving effect, this time and sold two, the unit with. Merchant service attitude is really good.	Jingdong prefecture level city in Shandong
Stowe Flagship Store	a power saver (electronics)	Plug and play, stable voltage, the screen shows when working, this winter home appliances, need electricity in many places, just buy to use, save electricity and money, with instructions	Jingdong prefecture level city in Shandong
Stowe Flagship Store	a power saver (electronics)	Goods received, do not know if it is good. When I bought it, I saw that it was a Stowe brand or company's product. Received but not. I do not know what is the reason. Now I can only give this evaluation. I'm	Jingdong prefecture level city in Shandong
Stowe Flagship Store	a power saver (electronics)	The first time to buy online, but also look at the evaluation before buying, the quality is not bad, but also very cheap, the need for friends can rest assured to buy!	Jingdong prefecture level city in Shandong
Stowe Flagship Store	a power saver (electronics)	Great quality, bought it to my liking Awesome! Buy with confidence if you need it!	Jingdong prefecture level city in Shandong
Stowe Flagship Store	a power saver (electronics)	The quality is very good, completely exceeded the expectations, the delivery speed is very fast, the packaging is very careful and strict, very satisfied with a shopping	Jingdong prefecture level city in Shandong
Stowe Flagship Store	a power saver (electronics)	The effect is very good, the home uses less electricity is very energy efficient, cost-effective	Jingdong prefecture level city in Shandong
Stowe Flagship Store	a power saver (electronics)	Quality can't be beat, just like the store, especially like, and the price is favorable!	Jingdong prefecture level city in Shandong
Stowe Flagship Store	a power saver (electronics)	Baby received! Very unexpected, will be back next time ~ great seller, stay tuned!	Jingdong prefecture level city in Shandong
Stowe Flagship Store	a power saver (electronics)	Follow up review after a month is more real, waiting for it	Jingdong prefecture level city in Shandong
Stowe Flagship Store	a power saver (electronics)	It is the ideal product, good quality and fast logistics, worthy of praise!	Jingdong prefecture level city in Shandong
Stowe Flagship Store	a power saver (electronics)	The saver is great, the color is right, the installer is very professional	Jingdong prefecture level city in Shandong
Stowe Flagship Store	a power saver (electronics)	The effect of the power connection is particularly obvious, it is particularly easy to use, very satisfied, the	Jingdong prefecture level city in Shandong
Stowe Flagship Store	a power saver (electronics)	Goods are very energy-saving, used to be more than two hundred degrees, now there are only a hundred or so degrees	Jingdong prefecture level city in Shandong
Stowe Flagship Store	a power saver (electronics)	Good quality, fast shipping, good results, good value for money	Jingdong prefecture level city in Shandong
Stowe Flagship Store	a power saver (electronics)	Fast shipping, fine workmanship, very good, cost-effective	Jingdong prefecture level city in Shandong
Stowe Flagship Store	a power saver (electronics)	The meter is as described, the quality is quite good, energy-saving, worth buying oh~!	Jingdong prefecture level city in Shandong
Stowe Flagship Store	a power saver (electronics)	Great value for money, very affordable,, perfect . ˆ◡ˆ.	Jingdong prefecture level city in Shandong
Stowe Flagship Store	a power saver (electronics)	Good!!! Worth recommending!	Jingdong prefecture level city in Shandong
Stowe Flagship Store	a power saver (electronics)	Great, fast logistics, great stuff, great value for money!	Jingdong prefecture level city in Shandong
Stowe Flagship Store	a power saver (electronics)	Fast logistics, very satisfied with the quality upon receipt	Jingdong prefecture level city in Shandong
Stowe Flagship Store	a power saver (electronics)	Great quality, plug and play, fast shipping!	Jingdong prefecture level city in Shandong
Stowe Flagship Store	a power saver (electronics)	Logistics received, quite satisfied	Jingdong prefecture level city in Shandong
Stowe Flagship Store	a power saver (electronics)	I don't know if it really works	Jingdong prefecture level city in Shandong

Stowe Flagship Store	a power saver (electronics)	It's bullshit. It's a plug light. Don't talk about power savings.	Jingdong prefecture level city in Shandong
Stowe Flagship Store	a power saver (electronics)	Fake and deceitful stuff that hasn't changed at all.	Jingdong prefecture level city in Shandong
Stowe Flagship Store	a power saver (electronics)	Power-saving effect: power-saving effect is particularly obvious Cost-effective: cost-effective business Appearance and style: external small micro-work materials: fine workmanship Experience: use very good, non the first convenient	Jingdong prefecture level city in Shandong
Stowe Flagship Store	a power saver (electronics)	After using it for a few days, it feels quite good, it should be quite safe, and it also sends a plug, the merchant is good, wait for a period of time and then calculate whether it saves on the electricity bill and then add comments ha!	Jingdong prefecture level city in Shandong
Stowe Flagship Store	a power saver (electronics)	My friend recommended to me, said the effect is very good, I bought two, this month less money, really can, just buy I do not believe, but has proved to have effect	Jingdong prefecture level city in Shandong
Stowe Flagship Store	a power saver (electronics)	Just bought back, already used, these days to see the effect of it, and so have the effect will also come back to give positive feedback, the price is also super cheap!	Jingdong prefecture level city in Shandong
Stowe Flagship Store	a power saver (electronics)	It does work well, the store uses a lot of electricity, this time to buy back a few, each place that uses a lot of electricity to insert one. Summer is here, the electricity is too strong.	Jingdong prefecture level city in Shandong
Stowe Flagship Store	a power saver (electronics)	Power saving treasure received, as for the effect, according to the instructions written, a short period of time can not be seen, at least 2-3 months before the real effect, then follow up evaluation	Jingdong prefecture level city in Shandong
Stowe Flagship Store	a power saver (electronics)	The goods have just been received and have not been used yet looking forward to the results.	Jingdong prefecture level city in Shandong
Stowe Flagship Store	a power saver (electronics)	Good installation works great	Jingdong prefecture level city in Shandong
Stowe Flagship Store	a power saver (electronics)	The logistics is super super fast! The quality is also very good, indeed value for money	Jingdong prefecture level city in Shandong
Stowe Flagship Store	a power saver (electronics)	Has been used for a period of time before coming to comment on, really good, air conditioning a day open non-stop, pay the electricity bill when the heartache, with this, the home electricity is not at all worried about the bill out of contrast to save a lot of preparation in the hands of a few, each room An a	Jingdong prefecture level city in Shandong
Stowe Flagship Store	a power saver (electronics)	Saving device received, really good, with the air conditioning tried, an hour without a saver with 2.9 degrees, with a saver an hour with 1.1 degrees, the beginning of not quite believe that this thing, the actual test to know that it is really useful, and intend to buy another.	Jingdong prefecture level city in Shandong
Stowe Flagship Store	a power saver (electronics)	Power saving effect: this energy saver is still useful, day trial for three days, every day than not installed before the reduction of two dollars a day, every day to open the bedroom to open the air conditioning before fifteen dollars now thirteen dollars a day. Still a little use.	Jingdong prefecture level city in Shandong
Stowe Flagship Store	a power saver (electronics)	Originally bought for home use, used for two months, the electricity bill from more than 200 yuan per month, down to more than 140 yuan, there is indeed a power-saving effect, this time and sold two, the unit with. Merchant service attitude is really good.	Jingdong prefecture level city in Shandong
Stowe Flagship Store	a power saver (electronics)	Plug and play, stable voltage, the screen shows when working, this winter home appliances, need electricity in many places, just buy to use, save electricity and money, with instructions	Jingdong prefecture level city in Shandong
Stowe Flagship Store	a power saver (electronics)	Goods received, do not know if it is good. When I bought it, I saw that it was a Stowe brand or company's product. Received but not. I do not know what is the reason. Now I can only give this evaluation. I'm	Jingdong prefecture level city in Shandong
Stowe Flagship Store	a power saver (electronics)	The first time to buy online, but also look at the evaluation before buying, the quality is not bad, but also very cheap, the need for friends can rest assured to buy!	Jingdong prefecture level city in Shandong
Stowe Flagship Store	a power saver (electronics)	🌈 Great quality, bought it to my liking 💞 Awesome! ✨ Buy with confidence if you need it!	Jingdong prefecture level city in Shandong
Stowe Flagship Store	a power saver (electronics)	The quality is very good, completely exceeded the expectations, the delivery speed is very fast, the packaging is very careful and strict, very satisfied with a shopping	Jingdong prefecture level city in Shandong
Stowe Flagship Store	a power saver (electronics)	The effect is very good, the home uses less electricity is very energy efficient, cost-effective	Jingdong prefecture level city in Shandong
Stowe Flagship Store	a power saver (electronics)	Quality can't be beat, just like the store, especially like, and the price is favorable!	Jingdong prefecture level city in Shandong
Stowe Flagship Store	a power saver (electronics)	Baby received! Very unexpected, will be back next time ~ great seller, stay tuned!	Jingdong prefecture level city in Shandong
Stowe Flagship Store	a power saver (electronics)	Follow up review after a month is more real, waiting for it	Jingdong prefecture level city in Shandong
Stowe Flagship Store	a power saver (electronics)	It is the ideal product, good quality and fast logistics, worthy of praise!	Jingdong prefecture level city in Shandong
Stowe Flagship Store	a power saver (electronics)	The saver is great, the color is right, the installer is very professional	Jingdong prefecture level city in Shandong
Stowe Flagship Store	a power saver (electronics)	The effect of the power connection is particularly obvious, it is particularly easy to use, very satisfied, the	Jingdong prefecture level city in Shandong
Stowe Flagship Store	a power saver (electronics)	Goods are very energy-saving, used to be more than two hundred degrees, now there are only a hundred or so degrees	Jingdong prefecture level city in Shandong
Stowe Flagship Store	a power saver (electronics)	Good quality, fast shipping, good results, good value for money	Jingdong prefecture level city in Shandong
Stowe Flagship Store	a power saver (electronics)	Fast shipping, fine workmanship, very good, cost-effective	Jingdong prefecture level city in Shandong
Stowe Flagship Store	a power saver (electronics)	The meter is as described, the quality is quite good, energy-saving, worth buying oh~!	Jingdong prefecture level city in Shandong
Stowe Flagship Store	a power saver (electronics)	Great value for money, very affordable,, perfect . ^◡^.	Jingdong prefecture level city in Shandong
Stowe Flagship Store	a power saver (electronics)	Good!!! Worth recommending!	Jingdong prefecture level city in Shandong
Stowe Flagship Store	a power saver (electronics)	Great, fast logistics, great stuff, great value for money!	Jingdong prefecture level city in Shandong
Stowe Flagship Store	a power saver (electronics)	Fast logistics, very satisfied with the quality upon receipt	Jingdong prefecture level city in Shandong
Stowe Flagship Store	a power saver (electronics)	Great quality, plug and play, fast shipping!	Jingdong prefecture level city in Shandong
Stowe Flagship Store	a power saver (electronics)	Logistics received, quite satisfied	Jingdong prefecture level city in Shandong
Stowe Flagship Store	a power saver (electronics)	I don't know if it really works	Jingdong prefecture level city in Shandong
Stowe Flagship Store	a power saver (electronics)	It's bullshit. It's a plug light. Don't talk about power savings.	Jingdong prefecture level city in Shandong
Stowe Flagship Store	a power saver (electronics)	Fake and deceitful stuff that hasn't changed at all.	Jingdong prefecture level city in Shandong
Stowe Flagship Store	a power saver (electronics)	Power-saving effect: power-saving effect is particularly obvious Cost-effective: cost-effective business Appearance and style: external small micro-work materials: fine workmanship Experience: use very good, non the first convenient	Jingdong prefecture level city in Shandong
Stowe Flagship Store	a power saver (electronics)	After using it for a few days, it feels quite good, it should be quite safe, and it also sends a plug, the merchant is good, wait for a period of time and then calculate whether it saves on the electricity bill and then add comments ha!	Jingdong prefecture level city in Shandong
Stowe Flagship Store	a power saver (electronics)	My friend recommended to me, said the effect is very good, I bought two, this month less money, really can, just buy I do not believe, but has proved to have effect	Jingdong prefecture level city in Shandong
Stowe Flagship Store	a power saver (electronics)	Just bought back, already used, these days to see the effect of it, and so have the effect will also come back to give positive feedback, the price is also super cheap!	Jingdong prefecture level city in Shandong
Stowe Flagship Store	a power saver (electronics)	It does work well, the store uses a lot of electricity, this time to buy back a few, each place that uses a lot of electricity to insert one. Summer is here, the electricity is too strong.	Jingdong prefecture level city in Shandong
Stowe Flagship Store	a power saver (electronics)	Power saving treasure received, as for the effect, according to the instructions written, a short period of time can not be seen, at least 2-3 months before the real effect, then follow up evaluation	Jingdong prefecture level city in Shandong
Stowe Flagship Store	a power saver (electronics)	The goods have just been received and have not been used yet looking forward to the results.	Jingdong prefecture level city in Shandong

Stowe Flagship Store	a power saver (electronics)	Good installation works great	Jingdong prefecture level city in Shandong
Stowe Flagship Store	a power saver (electronics)	The logistics is super super fast! The quality is also very good, indeed value for money	Jingdong prefecture level city in Shandong
Stowe Flagship Store	a power saver (electronics)	Has been used for a period of time before coming to comment on, really good, air conditioning a day open non-stop, pay the electricity bill when the heartache, with this, the home electricity is not at all worried about the bill out of contrast to save a lot of preparation in the hands of a few, each room An a	Jingdong prefecture level city in Shandong
Stowe Flagship Store	a power saver (electronics)	Saving device received, really good, with the air conditioning tried, an hour without a saver with 2.9 degrees, with a saver an hour with 1.1 degrees, the beginning of not quite believe that this thing, the actual test to know that it is really useful, and intend to buy another.	Jingdong prefecture level city in Shandong
Stowe Flagship Store	a power saver (electronics)	Power saving effect: this energy saver is still useful, day trial for three days, every day than not installed before the reduction of two dollars a day, every day to open the bedroom to open the air conditioning before fifteen dollars now thirteen dollars a day. Still a little use.	Jingdong prefecture level city in Shandong
Stowe Flagship Store	a power saver (electronics)	Originally bought for home use, used for two months, the electricity bill from more than 200 yuan per month, down to more than 140 yuan, there is indeed a power-saving effect, this time and sold two, the unit with. Merchant service attitude is really good.	Jingdong prefecture level city in Shandong
Stowe Flagship Store	a power saver (electronics)	Plug and play, stable voltage, the screen shows when working, this winter home appliances, need electricity in many places, just buy to use, save electricity and money, with instructions	Jingdong prefecture level city in Shandong
Stowe Flagship Store	a power saver (electronics)	The first time to buy online, but also look at the evaluation before buying, the quality is not bad, but also very cheap, the need for friends can rest assured to buy!	Jingdong prefecture level city in Shandong
Stowe Flagship Store	a power saver (electronics)	Goods received, do not know if it is good. When I bought it, I saw that it was a Stowe brand or company's product. Received but not. I do not know what is the reason. Now I can only give this evaluation. I'm	Jingdong prefecture level city in Shandong
Stowe Flagship Store	a power saver (electronics)	🌈 Great quality, bought it to my liking 💗 Awesome! ✨ Buy with confidence if you need it!	Jingdong prefecture level city in Shandong
Stowe Flagship Store	a power saver (electronics)	The quality is very good, completely exceeded the expectations, the delivery speed is very fast, the packaging is very careful and strict, very satisfied with a shopping	Jingdong prefecture level city in Shandong
Stowe Flagship Store	a power saver (electronics)	The effect is very good, the home uses less electricity is very energy efficient, cost-effective	Jingdong prefecture level city in Shandong
Stowe Flagship Store	a power saver (electronics)	Quality can't be beat, just like the store, especially like, and the price is favorable!	Jingdong prefecture level city in Shandong
Stowe Flagship Store	a power saver (electronics)	Baby received! Very unexpected, will be back next time ~ great seller, stay tuned!	Jingdong prefecture level city in Shandong
Stowe Flagship Store	a power saver (electronics)	It is the ideal product, good quality and fast logistics, worthy of praise!	Jingdong prefecture level city in Shandong
Stowe Flagship Store	a power saver (electronics)	Follow up review after a month is more real, waiting for it	Jingdong prefecture level city in Shandong
Stowe Flagship Store	a power saver (electronics)	The saver is great, the color is right, the installer is very professional	Jingdong prefecture level city in Shandong
Stowe Flagship Store	a power saver (electronics)	The effect of the power connection is particularly obvious, it is particularly easy to use, very satisfied, the	Jingdong prefecture level city in Shandong
Stowe Flagship Store	a power saver (electronics)	Goods are very energy-saving, used to be more than two hundred degrees, now there are only a hundred or so degrees	Jingdong prefecture level city in Shandong
Stowe Flagship Store	a power saver (electronics)	Good quality, fast shipping, good results, good value for money	Jingdong prefecture level city in Shandong
Stowe Flagship Store	a power saver (electronics)	Fast shipping, fine workmanship, very good, cost-effective	Jingdong prefecture level city in Shandong
Stowe Flagship Store	a power saver (electronics)	The meter is as described, the quality is quite good, energy-saving, worth buying oh~!	Jingdong prefecture level city in Shandong
Stowe Flagship Store	a power saver (electronics)	Great value for money, very affordable,, perfect . ^◡^.	Jingdong prefecture level city in Shandong
Stowe Flagship Store	a power saver (electronics)	Good!!! Worth recommending!	Jingdong prefecture level city in Shandong
Stowe Flagship Store	a power saver (electronics)	Great, fast logistics, great stuff, great value for money!	Jingdong prefecture level city in Shandong
Stowe Flagship Store	a power saver (electronics)	Fast logistics, very satisfied with the quality upon receipt	Jingdong prefecture level city in Shandong
Stowe Flagship Store	a power saver (electronics)	Great quality, plug and play, fast shipping!	Jingdong prefecture level city in Shandong
Stowe Flagship Store	a power saver (electronics)	Logistics received, quite satisfied	Jingdong prefecture level city in Shandong
Stowe Flagship Store	a power saver (electronics)	I don't know if it really works	Jingdong prefecture level city in Shandong
Stowe Flagship Store	a power saver (electronics)	It's bullshit. It's a plug light. Don't talk about power savings.	Jingdong prefecture level city in Shandong
Stowe Flagship Store	a power saver (electronics)	Fake and deceitful stuff that hasn't changed at all.	Jingdong prefecture level city in Shandong
Stowe Flagship Store	a power saver (electronics)	Power-saving effect: power-saving effect is particularly obvious Cost-effective: cost-effective business Appearance and style: external small micro-work materials: fine workmanship Experience: use very good, non the first convenient	Jingdong prefecture level city in Shandong
Stowe Flagship Store	a power saver (electronics)	After using it for a few days, it feels quite good, it should be quite safe, and it also sends a plug, the merchant is good, wait for a period of time and then calculate whether it saves on the electricity bill and then add comments ha!	Jingdong prefecture level city in Shandong
Stowe Flagship Store	a power saver (electronics)	My friend recommended to me, said the effect is very good, I bought two, this month less money, really can, just buy I do not believe, but has proved to have effect	Jingdong prefecture level city in Shandong
Stowe Flagship Store	a power saver (electronics)	Just bought back, already used, these days to see the effect of it, and so have the effect will also come back to give positive feedback, the price is also super cheap!	Jingdong prefecture level city in Shandong
Stowe Flagship Store	a power saver (electronics)	It does work well, the store uses a lot of electricity, this time to buy back a few, each place that uses a lot of electricity to insert one. Summer is here, the electricity is too strong.	Jingdong prefecture level city in Shandong
Stowe Flagship Store	a power saver (electronics)	Power saving treasure received, as for the effect, according to the instructions written, a short period of time can not be seen, at least 2-3 months before the real effect, then follow up evaluation	Jingdong prefecture level city in Shandong
Stowe Flagship Store	a power saver (electronics)	The goods have just been received and have not been used yet looking forward to the results.	Jingdong prefecture level city in Shandong
Stowe Flagship Store	a power saver (electronics)	Good installation works great	Jingdong prefecture level city in Shandong
Stowe Flagship Store	a power saver (electronics)	The logistics is super super fast! The quality is also very good, indeed value for money	Jingdong prefecture level city in Shandong
Stowe Flagship Store	a power saver (electronics)	Has been used for a period of time before coming to comment on, really good, air conditioning a day open non-stop, pay the electricity bill when the heartache, with this, the home electricity is not at all worried about the bill out of contrast to save a lot of preparation in the hands of a few, each room An a	Jingdong prefecture level city in Shandong
Stowe Flagship Store	a power saver (electronics)	Saving device received, really good, with the air conditioning tried, an hour without a saver with 2.9 degrees, with a saver an hour with 1.1 degrees, the beginning of not quite believe that this thing, the actual test to know that it is really useful, and intend to buy another.	Jingdong prefecture level city in Shandong
Stowe Flagship Store	a power saver (electronics)	Power saving effect: this energy saver is still useful, day trial for three days, every day than not installed before the reduction of two dollars a day, every day to open the bedroom to open the air conditioning before fifteen dollars now thirteen dollars a day. Still a little use.	Jingdong prefecture level city in Shandong
Stowe Flagship Store	a power saver (electronics)	Originally bought for home use, used for two months, the electricity bill from more than 200 yuan per month, down to more than 140 yuan, there is indeed a power-saving effect, this time and sold two, the unit with. Merchant service attitude is really good.	Jingdong prefecture level city in Shandong
Stowe Flagship Store	a power saver (electronics)	Plug and play, stable voltage, the screen shows when working, this winter home appliances, need electricity in many places, just buy to use, save electricity and money, with instructions	Jingdong prefecture level city in Shandong
Stowe Flagship Store	a power saver (electronics)	Goods received, do not know if it is good. When I bought it, I saw that it was a Stowe brand or company's product. Received but not. I do not know what is the reason. Now I can only give this evaluation. I'm	Jingdong prefecture level city in Shandong

Stowe Flagship Store	a power saver (electronics)	The first time to buy online, but also look at the evaluation before buying, the quality is not bad, but also very cheap, the need for friends can rest assured to buy!	Jingdong prefecture level city in Shandong
Stowe Flagship Store	a power saver (electronics)	🌈 Great quality, bought it to my liking 💛 Awesome! ✨ Buy with confidence if you need it!	Jingdong prefecture level city in Shandong
Stowe Flagship Store	a power saver (electronics)	The quality is very good, completely exceeded the expectations, the delivery speed is very fast, the packaging is very careful and strict, very satisfied with a shopping	Jingdong prefecture level city in Shandong
Stowe Flagship Store	a power saver (electronics)	The effect is very good, the home uses less electricity is very energy efficient, cost-effective	Jingdong prefecture level city in Shandong
Stowe Flagship Store	a power saver (electronics)	Quality can't be beat, just like the store, especially like, and the price is favorable!	Jingdong prefecture level city in Shandong
Stowe Flagship Store	a power saver (electronics)	Baby received! Very unexpected, will be back next time ~ great seller, stay tuned!	Jingdong prefecture level city in Shandong
Stowe Flagship Store	a power saver (electronics)	Follow up review after a month is more real, waiting for it	Jingdong prefecture level city in Shandong
Stowe Flagship Store	a power saver (electronics)	It is the ideal product, good quality and fast logistics, worthy of praise!	Jingdong prefecture level city in Shandong
Stowe Flagship Store	a power saver (electronics)	The saver is great, the color is right, the installer is very professional	Jingdong prefecture level city in Shandong
Stowe Flagship Store	a power saver (electronics)	The effect of the power connection is particularly obvious, it is particularly easy to use, very satisfied, the	Jingdong prefecture level city in Shandong
Stowe Flagship Store	a power saver (electronics)	Goods are very energy-saving, used to be more than two hundred degrees, now there are only a hundred or so degrees	Jingdong prefecture level city in Shandong
Stowe Flagship Store	a power saver (electronics)	Good quality, fast shipping, good results, good value for money	Jingdong prefecture level city in Shandong
Stowe Flagship Store	a power saver (electronics)	Fast shipping, fine workmanship, very good, cost-effective	Jingdong prefecture level city in Shandong
Stowe Flagship Store	a power saver (electronics)	The meter is as described, the quality is quite good, energy-saving, worth buying oh~!	Jingdong prefecture level city in Shandong
Stowe Flagship Store	a power saver (electronics)	Great value for money, very affordable,, perfect . ˆ◡ˆ.	Jingdong prefecture level city in Shandong
Stowe Flagship Store	a power saver (electronics)	Good!!! Worth recommending!	Jingdong prefecture level city in Shandong
Stowe Flagship Store	a power saver (electronics)	Great, fast logistics, great stuff, great value for money!	Jingdong prefecture level city in Shandong
Stowe Flagship Store	a power saver (electronics)	Fast logistics, very satisfied with the quality upon receipt	Jingdong prefecture level city in Shandong
Stowe Flagship Store	a power saver (electronics)	Great quality, plug and play, fast shipping!	Jingdong prefecture level city in Shandong
Stowe Flagship Store	a power saver (electronics)	Logistics received, quite satisfied	Jingdong prefecture level city in Shandong
Stowe Flagship Store	a power saver (electronics)	I don't know if it really works	Jingdong prefecture level city in Shandong
Stowe Flagship Store	a power saver (electronics)	It's bullshit. It's a plug light. Don't talk about power savings.	Jingdong prefecture level city in Shandong
Stowe Flagship Store	a power saver (electronics)	Fake and deceitful stuff that hasn't changed at all.	Jingdong prefecture level city in Shandong
Stowe Flagship Store	a power saver (electronics)	Power-saving effect: power-saving effect is particularly obvious Cost-effective: cost-effective business Appearance and style: external small micro-work materials: fine workmanship Experience: use very good, non the first convenient	Jingdong prefecture level city in Shandong
Stowe Flagship Store	a power saver (electronics)	After using it for a few days, it feels quite good, it should be quite safe, and it also sends a plug, the merchant is good, wait for a period of time and then calculate whether it saves on the electricity bill and then add comments ha!	Jingdong prefecture level city in Shandong
Stowe Flagship Store	a power saver (electronics)	My friend recommended to me, said the effect is very good, I bought two, this month less money, really can, just buy I do not believe, but has proved to have effect	Jingdong prefecture level city in Shandong
Stowe Flagship Store	a power saver (electronics)	Just bought back, already used, these days to see the effect of it, and so have the effect will also come back to give positive feedback, the price is also super cheap!	Jingdong prefecture level city in Shandong
Stowe Flagship Store	a power saver (electronics)	It does work well, the store uses a lot of electricity, this time to buy back a few, each place that uses a lot of electricity to insert one. Summer is here, the electricity is too strong.	Jingdong prefecture level city in Shandong
Stowe Flagship Store	a power saver (electronics)	Power saving treasure received, as for the effect, according to the instructions written, a short period of time can not be seen, at least 2-3 months before the real effect, then follow up evaluation	Jingdong prefecture level city in Shandong
Stowe Flagship Store	a power saver (electronics)	The goods have just been received and have not been used yet looking forward to the results.	Jingdong prefecture level city in Shandong
Stowe Flagship Store	a power saver (electronics)	Good installation works great	Jingdong prefecture level city in Shandong
Stowe Flagship Store	a power saver (electronics)	The logistics is super super fast! The quality is also very good, indeed value for money	Jingdong prefecture level city in Shandong
Stowe Flagship Store	a power saver (electronics)	Has been used for a period of time before coming to comment on, really good, air conditioning a day open non-stop, pay the electricity bill when the heartache, with this, the home electricity is not at all worried about the bill out of contrast to save a lot of preparation in the hands of a few, each room An a	Jingdong prefecture level city in Shandong
Stowe Flagship Store	a power saver (electronics)	Saving device received, really good, with the air conditioning tried, an hour without a saver with 2.9 degrees, with a saver an hour with 1.1 degrees, the beginning of not quite believe that this thing, the actual test to know that it is really useful, and intend to buy another.	Jingdong prefecture level city in Shandong
Stowe Flagship Store	a power saver (electronics)	Power saving effect: this energy saver is still useful, day trial for three days, every day than not installed before the reduction of two dollars a day, every day to open the bedroom to open the air conditioning before fifteen dollars now thirteen dollars a day. Still a little use.	Jingdong prefecture level city in Shandong
Stowe Flagship Store	a power saver (electronics)	Originally bought for home use, used for two months, the electricity bill from more than 200 yuan per month, down to more than 140 yuan, there is indeed a power-saving effect, this time and sold two, the unit with. Merchant service attitude is really good.	Jingdong prefecture level city in Shandong
Stowe Flagship Store	a power saver (electronics)	Plug and play, stable voltage, the screen shows when working, this winter home appliances, need electricity in many places, just buy to use, save electricity and money, with instructions	Jingdong prefecture level city in Shandong
Stowe Flagship Store	a power saver (electronics)	Goods received, do not know if it is good. When I bought it, I saw that it was a Stowe brand or company's product. Received but not. I do not know what is the reason. Now I can only give this evaluation. I'm	Jingdong prefecture level city in Shandong
Stowe Flagship Store	a power saver (electronics)	The first time to buy online, but also look at the evaluation before buying, the quality is not bad, but also very cheap, the need for friends can rest assured to buy!	Jingdong prefecture level city in Shandong
Stowe Flagship Store	a power saver (electronics)	🌈 Great quality, bought it to my liking 💛 Awesome! ✨ Buy with confidence if you need it!	Jingdong prefecture level city in Shandong
Stowe Flagship Store	a power saver (electronics)	The quality is very good, completely exceeded the expectations, the delivery speed is very fast, the packaging is very careful and strict, very satisfied with a shopping	Jingdong prefecture level city in Shandong
Stowe Flagship Store	a power saver (electronics)	The effect is very good, the home uses less electricity is very energy efficient, cost-effective	Jingdong prefecture level city in Shandong
Stowe Flagship Store	a power saver (electronics)	Quality can't be beat, just like the store, especially like, and the price is favorable!	Jingdong prefecture level city in Shandong
Stowe Flagship Store	a power saver (electronics)	Baby received! Very unexpected, will be back next time ~ great seller, stay tuned!	Jingdong prefecture level city in Shandong
Stowe Flagship Store	a power saver (electronics)	Follow up review after a month is more real, waiting for it	Jingdong prefecture level city in Shandong
Stowe Flagship Store	a power saver (electronics)	It is the ideal product, good quality and fast logistics, worthy of praise!	Jingdong prefecture level city in Shandong
Stowe Flagship Store	a power saver (electronics)	The saver is great, the color is right, the installer is very professional	Jingdong prefecture level city in Shandong
Stowe Flagship Store	a power saver (electronics)	The effect of the power connection is particularly obvious, it is particularly easy to use, very satisfied, the	Jingdong prefecture level city in Shandong

Stowe Flagship Store	a power saver (electronics)	Goods are very energy-saving, used to be more than two hundred degrees, now there are only a hundred or so degrees	Jingdong prefecture level city in Shandong
Stowe Flagship Store	a power saver (electronics)	Good quality, fast shipping, good results, good value for money	Jingdong prefecture level city in Shandong
Stowe Flagship Store	a power saver (electronics)	Fast shipping, fine workmanship, very good, cost-effective	Jingdong prefecture level city in Shandong
Stowe Flagship Store	a power saver (electronics)	The meter is as described, the quality is quite good, energy-saving, worth buying oh~!	Jingdong prefecture level city in Shandong
Stowe Flagship Store	a power saver (electronics)	Great value for money, very affordable,, perfect . ˆ◡ˆ.	Jingdong prefecture level city in Shandong
Stowe Flagship Store	a power saver (electronics)	Good!!! Worth recommending!	Jingdong prefecture level city in Shandong
Stowe Flagship Store	a power saver (electronics)	Great, fast logistics, great stuff, great value for money!	Jingdong prefecture level city in Shandong
Stowe Flagship Store	a power saver (electronics)	Fast logistics, very satisfied with the quality upon receipt	Jingdong prefecture level city in Shandong
Stowe Flagship Store	a power saver (electronics)	Great quality, plug and play, fast shipping!	Jingdong prefecture level city in Shandong
Stowe Flagship Store	a power saver (electronics)	Logistics received, quite satisfied	Jingdong prefecture level city in Shandong
Stowe Flagship Store	a power saver (electronics)	I don't know if it really works	Jingdong prefecture level city in Shandong
Stowe Flagship Store	a power saver (electronics)	It's bullshit. It's a plug light. Don't talk about power savings.	Jingdong prefecture level city in Shandong
Stowe Flagship Store	a power saver (electronics)	Fake and deceitful stuff that hasn't changed at all.	Jingdong prefecture level city in Shandong
Stowe Flagship Store	a power saver (electronics)	Power-saving effect: power-saving effect is particularly obvious Cost-effective: cost-effective business Appearance and style: external small micro-work materials: fine workmanship Experience: use very good, non the first convenient	Jingdong prefecture level city in Shandong
Stowe Flagship Store	a power saver (electronics)	After using it for a few days, it feels quite good, it should be quite safe, and it also sends a plug, the merchant is good, wait for a period of time and then calculate whether it saves on the electricity bill and then add comments ha!	Jingdong prefecture level city in Shandong
Stowe Flagship Store	a power saver (electronics)	My friend recommended to me, said the effect is very good, I bought two, this month less money, really can, just buy I do not believe, but has proved to have effect	Jingdong prefecture level city in Shandong
Stowe Flagship Store	a power saver (electronics)	Just bought back, already used, these days to see the effect of it, and so have the effect will also come back to give positive feedback, the price is also super cheap!	Jingdong prefecture level city in Shandong
Stowe Flagship Store	a power saver (electronics)	It does work well, the store uses a lot of electricity, this time to buy back a few, each place that uses a lot of electricity to insert one. Summer is here, the electricity is too strong.	Jingdong prefecture level city in Shandong
Stowe Flagship Store	a power saver (electronics)	Power saving treasure received, as for the effect, according to the instructions written, a short period of time can not be seen, at least 2-3 months before the real effect, then follow up evaluation	Jingdong prefecture level city in Shandong
Stowe Flagship Store	a power saver (electronics)	The goods have just been received and have not been used yet looking forward to the results.	Jingdong prefecture level city in Shandong
Stowe Flagship Store	a power saver (electronics)	Good installation works great	Jingdong prefecture level city in Shandong
Stowe Flagship Store	a power saver (electronics)	The logistics is super super fast! The quality is also very good, indeed value for money	Jingdong prefecture level city in Shandong
Stowe Flagship Store	a power saver (electronics)	Has been used for a period of time before coming to comment on, really good, air conditioning a day open non-stop, pay the electricity bill when the heartache, with this, the home electricity is not at all worried about the bill out of contrast to save a lot of preparation in the hands of a few, each room An a	Jingdong prefecture level city in Shandong
Stowe Flagship Store	a power saver (electronics)	Saving device received, really good, with the air conditioning tried, an hour without a saver with 2.9 degrees, with a saver an hour with 1.1 degrees, the beginning of not quite believe that this thing, the actual test to know that it is really useful, and intend to buy another.	Jingdong prefecture level city in Shandong
Stowe Flagship Store	a power saver (electronics)	Power saving effect: this energy saver is still useful, day trial for three days, every day than not installed before the reduction of two dollars a day, every day to open the bedroom to open the air conditioning before fifteen dollars now thirteen dollars a day. Still a little use.	Jingdong prefecture level city in Shandong
Stowe Flagship Store	a power saver (electronics)	Originally bought for home use, used for two months, the electricity bill from more than 200 yuan per month, down to more than 140 yuan, there is indeed a power-saving effect, this time and sold two, the unit with. Merchant service attitude is really good.	Jingdong prefecture level city in Shandong
Stowe Flagship Store	a power saver (electronics)	Plug and play, stable voltage, the screen shows when working, this winter home appliances, need electricity in many places, just buy to use, save electricity and money, with instructions	Jingdong prefecture level city in Shandong
Stowe Flagship Store	a power saver (electronics)	Goods received, do not know if it is good. When I bought it, I saw that it was a Stowe brand or company's product. Received but not. I do not know what is the reason. Now I can only give this evaluation. I'm	Jingdong prefecture level city in Shandong
Stowe Flagship Store	a power saver (electronics)	The first time to buy online, but also look at the evaluation before buying, the quality is not bad, but also very cheap, the need for friends can rest assured to buy!	Jingdong prefecture level city in Shandong
Stowe Flagship Store	a power saver (electronics)	Great quality, bought it to my liking Awesome! Buy with confidence if you need it!	Jingdong prefecture level city in Shandong
Stowe Flagship Store	a power saver (electronics)	The quality is very good, completely exceeded the expectations, the delivery speed is very fast, the packaging is very careful and strict, very satisfied with a shopping	Jingdong prefecture level city in Shandong
Stowe Flagship Store	a power saver (electronics)	The effect is very good, the home uses less electricity is very energy efficient, cost-effective	Jingdong prefecture level city in Shandong
Stowe Flagship Store	a power saver (electronics)	Quality can't be beat, just like the store, especially like, and the price is favorable!	Jingdong prefecture level city in Shandong
Stowe Flagship Store	a power saver (electronics)	Baby received! Very unexpected, will be back next time ~ great seller, stay tuned!	Jingdong prefecture level city in Shandong
Stowe Flagship Store	a power saver (electronics)	Follow up review after a month is more real, waiting for it	Jingdong prefecture level city in Shandong
Stowe Flagship Store	a power saver (electronics)	It is the ideal product, good quality and fast logistics, worthy of praise!	Jingdong prefecture level city in Shandong
Stowe Flagship Store	a power saver (electronics)	The saver is great, the color is right, the installer is very professional	Jingdong prefecture level city in Shandong
Stowe Flagship Store	a power saver (electronics)	The effect of the power connection is particularly obvious, it is particularly easy to use, very satisfied, the	Jingdong prefecture level city in Shandong
Stowe Flagship Store	a power saver (electronics)	Goods are very energy-saving, used to be more than two hundred degrees, now there are only a hundred or so degrees	Jingdong prefecture level city in Shandong
Stowe Flagship Store	a power saver (electronics)	Good quality, fast shipping, good results, good value for money	Jingdong prefecture level city in Shandong
Stowe Flagship Store	a power saver (electronics)	Fast shipping, fine workmanship, very good, cost-effective	Jingdong prefecture level city in Shandong
Stowe Flagship Store	a power saver (electronics)	The meter is as described, the quality is quite good, energy-saving, worth buying oh~!	Jingdong prefecture level city in Shandong
Stowe Flagship Store	a power saver (electronics)	Great value for money, very affordable,, perfect . ˆ◡ˆ.	Jingdong prefecture level city in Shandong
Stowe Flagship Store	a power saver (electronics)	Good!!! Worth recommending!	Jingdong prefecture level city in Shandong
Stowe Flagship Store	a power saver (electronics)	Great, fast logistics, great stuff, great value for money!	Jingdong prefecture level city in Shandong
Stowe Flagship Store	a power saver (electronics)	Fast logistics, very satisfied with the quality upon receipt	Jingdong prefecture level city in Shandong
Stowe Flagship Store	a power saver (electronics)	Great quality, plug and play, fast shipping!	Jingdong prefecture level city in Shandong
Stowe Flagship Store	a power saver (electronics)	Logistics received, quite satisfied	Jingdong prefecture level city in Shandong

Stowe Flagship Store	a power saver (electronics)	I don't know if it really works	Jingdong prefecture level city in Shandong
Stowe Flagship Store	a power saver (electronics)	It's bullshit. It's a plug light. Don't talk about power savings.	Jingdong prefecture level city in Shandong
Stowe Flagship Store	a power saver (electronics)	Fake and deceitful stuff that hasn't changed at all.	Jingdong prefecture level city in Shandong
Stowe Flagship Store	a power saver (electronics)	Power-saving effect: power-saving effect is particularly obvious Cost-effective: cost-effective business Appearance and style: external small micro-work materials: fine workmanship Experience: use very good, non the first convenient	Jingdong prefecture level city in Shandong
Stowe Flagship Store	a power saver (electronics)	After using it for a few days, it feels quite good, it should be quite safe, and it also sends a plug, the merchant is good, wait for a period of time and then calculate whether it saves on the electricity bill and then add comments ha!	Jingdong prefecture level city in Shandong
Stowe Flagship Store	a power saver (electronics)	My friend recommended to me, said the effect is very good, I bought two, this month less money, really can, just buy I do not believe, but has proved to have effect	Jingdong prefecture level city in Shandong
Stowe Flagship Store	a power saver (electronics)	Just bought back, already used, these days to see the effect of it, and so have the effect will also come back to give positive feedback, the price is also super cheap!	Jingdong prefecture level city in Shandong
Stowe Flagship Store	a power saver (electronics)	It does work well, the store uses a lot of electricity, this time to buy back a few, each place that uses a lot of electricity to insert one. Summer is here, the electricity is too strong.	Jingdong prefecture level city in Shandong
Stowe Flagship Store	a power saver (electronics)	Power saving treasure received, as for the effect, according to the instructions written, a short period of time can not be seen, at least 2-3 months before the real effect, then follow up evaluation	Jingdong prefecture level city in Shandong
Stowe Flagship Store	a power saver (electronics)	The goods have just been received and have not been used yet looking forward to the results.	Jingdong prefecture level city in Shandong
Stowe Flagship Store	a power saver (electronics)	Good installation works great	Jingdong prefecture level city in Shandong
Stowe Flagship Store	a power saver (electronics)	The logistics is super super fast! The quality is also very good, indeed value for money	Jingdong prefecture level city in Shandong
Stowe Flagship Store	a power saver (electronics)	Has been used for a period of time before coming to comment on, really good, air conditioning a day open non-stop, pay the electricity bill when the heartache, with this, the home electricity is not at all worried about the bill out of contrast to save a lot of preparation in the hands of a few, each room An a	Jingdong prefecture level city in Shandong
Stowe Flagship Store	a power saver (electronics)	Saving device received, really good, with the air conditioning tried, an hour without a saver with 2.9 degrees, with a saver an hour with 1.1 degrees, the beginning of not quite believe that this thing, the actual test to know that it is really useful, and intend to buy another.	Jingdong prefecture level city in Shandong
Stowe Flagship Store	a power saver (electronics)	Power saving effect: this energy saver is still useful, day trial for three days, every day than not installed before the reduction of two dollars a day, every day to open the bedroom to open the air conditioning before fifteen dollars now thirteen dollars a day. Still a little use.	Jingdong prefecture level city in Shandong
Stowe Flagship Store	a power saver (electronics)	Originally bought for home use, used for two months, the electricity bill from more than 200 yuan per month, down to more than 140 yuan, there is indeed a power-saving effect, this time and sold two, the unit with. Merchant service attitude is really good.	Jingdong prefecture level city in Shandong
Stowe Flagship Store	a power saver (electronics)	Plug and play, stable voltage, the screen shows when working, this winter home appliances, need electricity in many places, just buy to use, save electricity and money, with instructions	Jingdong prefecture level city in Shandong
Stowe Flagship Store	a power saver (electronics)	Goods received, do not know if it is good. When I bought it, I saw that it was a Stowe brand or company's product. Received but not. I do not know what is the reason. Now I can only give this evaluation. I'm	Jingdong prefecture level city in Shandong
Stowe Flagship Store	a power saver (electronics)	The first time to buy online, but also look at the evaluation before buying, the quality is not bad, but also very cheap, the need for friends can rest assured to buy!	Jingdong prefecture level city in Shandong
Stowe Flagship Store	a power saver (electronics)	Great quality, bought it to my liking Awesome! Buy with confidence if you need it!	Jingdong prefecture level city in Shandong
Stowe Flagship Store	a power saver (electronics)	The quality is very good, completely exceeded the expectations, the delivery speed is very fast, the packaging is very careful and strict, very satisfied with a shopping	Jingdong prefecture level city in Shandong
Stowe Flagship Store	a power saver (electronics)	The effect is very good, the home uses less electricity is very energy efficient, cost-effective	Jingdong prefecture level city in Shandong
Stowe Flagship Store	a power saver (electronics)	Quality can't be beat, just like the store, especially like, and the price is favorable!	Jingdong prefecture level city in Shandong
Stowe Flagship Store	a power saver (electronics)	Baby received! Very unexpected, will be back next time ~ great seller, stay tuned!	Jingdong prefecture level city in Shandong
Stowe Flagship Store	a power saver (electronics)	Follow up review after a month is more real, waiting for it	Jingdong prefecture level city in Shandong
Stowe Flagship Store	a power saver (electronics)	It is the ideal product, good quality and fast logistics, worthy of praise!	Jingdong prefecture level city in Shandong
Stowe Flagship Store	a power saver (electronics)	The saver is great, the color is right, the installer is very professional	Jingdong prefecture level city in Shandong
Stowe Flagship Store	a power saver (electronics)	The effect of the power connection is particularly obvious, it is particularly easy to use, very satisfied, the	Jingdong prefecture level city in Shandong
Stowe Flagship Store	a power saver (electronics)	Goods are very energy-saving, used to be more than two hundred degrees, now there are only a hundred or so degrees	Jingdong prefecture level city in Shandong
Stowe Flagship Store	a power saver (electronics)	Good quality, fast shipping, good results, good value for money	Jingdong prefecture level city in Shandong
Stowe Flagship Store	a power saver (electronics)	Fast shipping, fine workmanship, very good, cost-effective	Jingdong prefecture level city in Shandong
Stowe Flagship Store	a power saver (electronics)	The meter is as described, the quality is quite good, energy-saving, worth buying oh~!	Jingdong prefecture level city in Shandong
Stowe Flagship Store	a power saver (electronics)	Great value for money, very affordable,, perfect . ˆ◡ˆ.	Jingdong prefecture level city in Shandong
Stowe Flagship Store	a power saver (electronics)	Good!!! Worth recommending!	Jingdong prefecture level city in Shandong
Stowe Flagship Store	a power saver (electronics)	Great, fast logistics, great stuff, great value for money!	Jingdong prefecture level city in Shandong
Stowe Flagship Store	a power saver (electronics)	Fast logistics, very satisfied with the quality upon receipt	Jingdong prefecture level city in Shandong
Stowe Flagship Store	a power saver (electronics)	Great quality, plug and play, fast shipping!	Jingdong prefecture level city in Shandong
Stowe Flagship Store	a power saver (electronics)	Logistics received, quite satisfied	Jingdong prefecture level city in Shandong
Stowe Flagship Store	a power saver (electronics)	I don't know if it really works	Jingdong prefecture level city in Shandong
Stowe Flagship Store	a power saver (electronics)	It's bullshit. It's a plug light. Don't talk about power savings.	Jingdong prefecture level city in Shandong
Stowe Flagship Store	a power saver (electronics)	Fake and deceitful stuff that hasn't changed at all.	Jingdong prefecture level city in Shandong
Stowe Flagship Store	a power saver (electronics)	Power-saving effect: power-saving effect is particularly obvious Cost-effective: cost-effective business Appearance and style: external small micro-work materials: fine workmanship Experience: use very good, non the first convenient	Jingdong prefecture level city in Shandong
Stowe Flagship Store	a power saver (electronics)	After using it for a few days, it feels quite good, it should be quite safe, and it also sends a plug, the merchant is good, wait for a period of time and then calculate whether it saves on the electricity bill and then add comments ha!	Jingdong prefecture level city in Shandong
Stowe Flagship Store	a power saver (electronics)	My friend recommended to me, said the effect is very good, I bought two, this month less money, really can, just buy I do not believe, but has proved to have effect	Jingdong prefecture level city in Shandong
Stowe Flagship Store	a power saver (electronics)	Just bought back, already used, these days to see the effect of it, and so have the effect will also come back to give positive feedback, the price is also super cheap!	Jingdong prefecture level city in Shandong
Stowe Flagship Store	a power saver (electronics)	It does work well, the store uses a lot of electricity, this time to buy back a few, each place that uses a lot of electricity to insert one. Summer is here, the electricity is too strong.	Jingdong prefecture level city in Shandong
Stowe Flagship Store	a power saver (electronics)	Power saving treasure received, as for the effect, according to the instructions written, a short period of time can not be seen, at least 2-3 months before the real effect, then follow up evaluation	Jingdong prefecture level city in Shandong

Stowe Flagship Store	a power saver (electronics)	The goods have just been received and have not been used yet looking forward to the results.	Jingdong prefecture level city in Shandong
Stowe Flagship Store	a power saver (electronics)	Good installation works great	Jingdong prefecture level city in Shandong
Stowe Flagship Store	a power saver (electronics)	The logistics is super super fast! The quality is also very good, indeed value for money	Jingdong prefecture level city in Shandong
Stowe Flagship Store	a power saver (electronics)	Has been used for a period of time before coming to comment on, really good, air conditioning a day open non-stop, pay the electricity bill when the heartache, with this, the home electricity is not at all worried about the bill out of contrast to save a lot of preparation in the hands of a few, each room An a	Jingdong prefecture level city in Shandong
Stowe Flagship Store	a power saver (electronics)	Saving device received, really good, with the air conditioning tried, an hour without a saver with 2.9 degrees, with a saver an hour with 1.1 degrees, the beginning of not quite believe that this thing, the actual test to know that it is really useful, and intend to buy another.	Jingdong prefecture level city in Shandong
Stowe Flagship Store	a power saver (electronics)	Power saving effect: this energy saver is still useful, day trial for three days, every day than not installed before the reduction of two dollars a day, every day to open the bedroom to open the air conditioning before fifteen dollars now thirteen dollars a day. Still a little use.	Jingdong prefecture level city in Shandong
Stowe Flagship Store	a power saver (electronics)	Originally bought for home use, used for two months, the electricity bill from more than 200 yuan per month, down to more than 140 yuan, there is indeed a power-saving effect, this time and sold two, the unit with. Merchant service attitude is really good.	Jingdong prefecture level city in Shandong
Stowe Flagship Store	a power saver (electronics)	Plug and play, stable voltage, the screen shows when working, this winter home appliances, need electricity in many places, just buy to use, save electricity and money, with instructions	Jingdong prefecture level city in Shandong
Stowe Flagship Store	a power saver (electronics)	Goods received, do not know if it is good. When I bought it, I saw that it was a Stowe brand or company's product. Received but not. I do not know what is the reason. Now I can only give this evaluation. I'm	Jingdong prefecture level city in Shandong
Stowe Flagship Store	a power saver (electronics)	The first time to buy online, but also look at the evaluation before buying, the quality is not bad, but also very cheap, the need for friends can rest assured to buy!	Jingdong prefecture level city in Shandong
Stowe Flagship Store	a power saver (electronics)	🌈 Great quality, bought it to my liking 💞 Awesome! ✨ Buy with confidence if you need it!	Jingdong prefecture level city in Shandong
Stowe Flagship Store	a power saver (electronics)	The quality is very good, completely exceeded the expectations, the delivery speed is very fast, the packaging is very careful and strict, very satisfied with a shopping	Jingdong prefecture level city in Shandong
Stowe Flagship Store	a power saver (electronics)	The effect is very good, the home uses less electricity is very energy efficient, cost-effective	Jingdong prefecture level city in Shandong
Stowe Flagship Store	a power saver (electronics)	Quality can't be beat, just like the store, especially like, and the price is favorable!	Jingdong prefecture level city in Shandong
Stowe Flagship Store	a power saver (electronics)	Baby received! Very unexpected, will be back next time ~ great seller, stay tuned!	Jingdong prefecture level city in Shandong
Stowe Flagship Store	a power saver (electronics)	Follow up review after a month is more real, waiting for it	Jingdong prefecture level city in Shandong
Stowe Flagship Store	a power saver (electronics)	It is the ideal product, good quality and fast logistics, worthy of praise!	Jingdong prefecture level city in Shandong
Stowe Flagship Store	a power saver (electronics)	The saver is great, the color is right, the installer is very professional	Jingdong prefecture level city in Shandong
Stowe Flagship Store	a power saver (electronics)	The effect of the power connection is particularly obvious, it is particularly easy to use, very satisfied, the	Jingdong prefecture level city in Shandong
Stowe Flagship Store	a power saver (electronics)	Goods are very energy-saving, used to be more than two hundred degrees, now there are only a hundred or so degrees	Jingdong prefecture level city in Shandong
Stowe Flagship Store	a power saver (electronics)	Good quality, fast shipping, good results, good value for money	Jingdong prefecture level city in Shandong
Stowe Flagship Store	a power saver (electronics)	Fast shipping, fine workmanship, very good, cost-effective	Jingdong prefecture level city in Shandong
Stowe Flagship Store	a power saver (electronics)	The meter is as described, the quality is quite good, energy-saving, worth buying oh~!	Jingdong prefecture level city in Shandong
Stowe Flagship Store	a power saver (electronics)	Great value for money, very affordable,, perfect . ˆ◡ˆ.	Jingdong prefecture level city in Shandong
Stowe Flagship Store	a power saver (electronics)	Good!!! Worth recommending!	Jingdong prefecture level city in Shandong
Stowe Flagship Store	a power saver (electronics)	Great, fast logistics, great stuff, great value for money!	Jingdong prefecture level city in Shandong
Stowe Flagship Store	a power saver (electronics)	Fast logistics, very satisfied with the quality upon receipt	Jingdong prefecture level city in Shandong
Stowe Flagship Store	a power saver (electronics)	Great quality, plug and play, fast shipping!	Jingdong prefecture level city in Shandong
Stowe Flagship Store	a power saver (electronics)	Logistics received, quite satisfied	Jingdong prefecture level city in Shandong
Stowe Flagship Store	a power saver (electronics)	I don't know if it really works	Jingdong prefecture level city in Shandong
Stowe Flagship Store	a power saver (electronics)	It's bullshit. It's a plug light. Don't talk about power savings.	Jingdong prefecture level city in Shandong
Stowe Flagship Store	a power saver (electronics)	Fake and deceitful stuff that hasn't changed at all.	Jingdong prefecture level city in Shandong
Stowe Flagship Store	a power saver (electronics)	Power-saving effect: power-saving effect is particularly obvious Cost-effective: cost-effective business Appearance and style: external small micro-work materials: fine workmanship Experience: use very good, non the first convenient	Jingdong prefecture level city in Shandong
Stowe Flagship Store	a power saver (electronics)	After using it for a few days, it feels quite good, it should be quite safe, and it also sends a plug, the merchant is good, wait for a period of time and then calculate whether it saves on the electricity bill and then add comments ha!	Jingdong prefecture level city in Shandong
Stowe Flagship Store	a power saver (electronics)	My friend recommended to me, said the effect is very good, I bought two, this month less money, really can, just buy I do not believe, but has proved to have effect	Jingdong prefecture level city in Shandong
Stowe Flagship Store	a power saver (electronics)	Just bought back, already used, these days to see the effect of it, and so have the effect will also come back to give positive feedback, the price is also super cheap!	Jingdong prefecture level city in Shandong
Stowe Flagship Store	a power saver (electronics)	It does work well, the store uses a lot of electricity, this time to buy back a few, each place that uses a lot of electricity to insert one. Summer is here, the electricity is too strong.	Jingdong prefecture level city in Shandong
Stowe Flagship Store	a power saver (electronics)	Power saving treasure received, as for the effect, according to the instructions written, a short period of time can not be seen, at least 2-3 months before the real effect, then follow up evaluation	Jingdong prefecture level city in Shandong
Stowe Flagship Store	a power saver (electronics)	The goods have just been received and have not been used yet looking forward to the results.	Jingdong prefecture level city in Shandong
Stowe Flagship Store	a power saver (electronics)	Good installation works great	Jingdong prefecture level city in Shandong
Stowe Flagship Store	a power saver (electronics)	The logistics is super super fast! The quality is also very good, indeed value for money	Jingdong prefecture level city in Shandong
Stowe Flagship Store	a power saver (electronics)	Has been used for a period of time before coming to comment on, really good, air conditioning a day open non-stop, pay the electricity bill when the heartache, with this, the home electricity is not at all worried about the bill out of contrast to save a lot of preparation in the hands of a few, each room An a	Jingdong prefecture level city in Shandong
Stowe Flagship Store	a power saver (electronics)	Saving device received, really good, with the air conditioning tried, an hour without a saver with 2.9 degrees, with a saver an hour with 1.1 degrees, the beginning of not quite believe that this thing, the actual test to know that it is really useful, and intend to buy another.	Jingdong prefecture level city in Shandong
Stowe Flagship Store	a power saver (electronics)	Power saving effect: this energy saver is still useful, day trial for three days, every day than not installed before the reduction of two dollars a day, every day to open the bedroom to open the air conditioning before fifteen dollars now thirteen dollars a day. Still a little use.	Jingdong prefecture level city in Shandong
Stowe Flagship Store	a power saver (electronics)	Originally bought for home use, used for two months, the electricity bill from more than 200 yuan per month, down to more than 140 yuan, there is indeed a power-saving effect, this time and sold two, the unit with. Merchant service attitude is really good.	Jingdong prefecture level city in Shandong
Stowe Flagship Store	a power saver (electronics)	Plug and play, stable voltage, the screen shows when working, this winter home appliances, need electricity in many places, just buy to use, save electricity and money, with instructions	Jingdong prefecture level city in Shandong

Stowe Flagship Store	a power saver (electronics)	Goods received, do not know if it is good. When I bought it, I saw that it was a Stowe brand or company's product. Received but not. I do not know what is the reason. Now I can only give this evaluation. I'm	Jingdong prefecture level city in Shandong
Stowe Flagship Store	a power saver (electronics)	The first time to buy online, but also look at the evaluation before buying, the quality is not bad, but also very cheap, the need for friends can rest assured to buy!	Jingdong prefecture level city in Shandong
Stowe Flagship Store	a power saver (electronics)	Great quality, bought it to my liking Awesome! Buy with confidence if you need it!	Jingdong prefecture level city in Shandong
Stowe Flagship Store	a power saver (electronics)	The quality is very good, completely exceeded the expectations, the delivery speed is very fast, the packaging is very careful and strict, very satisfied with a shopping	Jingdong prefecture level city in Shandong
Stowe Flagship Store	a power saver (electronics)	The effect is very good, the home uses less electricity is very energy efficient, cost-effective	Jingdong prefecture level city in Shandong
Stowe Flagship Store	a power saver (electronics)	Quality can't be beat, just like the store, especially like, and the price is favorable!	Jingdong prefecture level city in Shandong
Stowe Flagship Store	a power saver (electronics)	Baby received! Very unexpected, will be back next time ~ great seller, stay tuned!	Jingdong prefecture level city in Shandong
Stowe Flagship Store	a power saver (electronics)	Follow up review after a month is more real, waiting for it	Jingdong prefecture level city in Shandong
Stowe Flagship Store	a power saver (electronics)	It is the ideal product, good quality and fast logistics, worthy of praise!	Jingdong prefecture level city in Shandong
Stowe Flagship Store	a power saver (electronics)	The saver is great, the color is right, the installer is very professional	Jingdong prefecture level city in Shandong
Stowe Flagship Store	a power saver (electronics)	The effect of the power connection is particularly obvious, it is particularly easy to use, very satisfied, the	Jingdong prefecture level city in Shandong
Stowe Flagship Store	a power saver (electronics)	Goods are very energy-saving, used to be more than two hundred degrees, now there are only a hundred or so degrees	Jingdong prefecture level city in Shandong
Stowe Flagship Store	a power saver (electronics)	Good quality, fast shipping, good results, good value for money	Jingdong prefecture level city in Shandong
Stowe Flagship Store	a power saver (electronics)	Fast shipping, fine workmanship, very good, cost-effective	Jingdong prefecture level city in Shandong
Stowe Flagship Store	a power saver (electronics)	The meter is as described, the quality is quite good, energy-saving, worth buying oh~!	Jingdong prefecture level city in Shandong
Stowe Flagship Store	a power saver (electronics)	Great value for money, very affordable,, perfect . ˆ◡ˆ.	Jingdong prefecture level city in Shandong
Stowe Flagship Store	a power saver (electronics)	Good!!! Worth recommending!	Jingdong prefecture level city in Shandong
Stowe Flagship Store	a power saver (electronics)	Great, fast logistics, great stuff, great value for money!	Jingdong prefecture level city in Shandong
Stowe Flagship Store	a power saver (electronics)	Fast logistics, very satisfied with the quality upon receipt	Jingdong prefecture level city in Shandong
Stowe Flagship Store	a power saver (electronics)	Great quality, plug and play, fast shipping!	Jingdong prefecture level city in Shandong
Stowe Flagship Store	a power saver (electronics)	Logistics received, quite satisfied	Jingdong prefecture level city in Shandong
Stowe Flagship Store	a power saver (electronics)	I don't know if it really works	Jingdong prefecture level city in Shandong
Stowe Flagship Store	a power saver (electronics)	It's bullshit. It's a plug light. Don't talk about power savings.	Jingdong prefecture level city in Shandong
Stowe Flagship Store	a power saver (electronics)	Fake and deceitful stuff that hasn't changed at all.	Jingdong prefecture level city in Shandong
Stowe Flagship Store	a power saver (electronics)	Power-saving effect: power-saving effect is particularly obvious Cost-effective: cost-effective business Appearance and style: external small micro-work materials: fine workmanship Experience: use very good, non the first convenient	Jingdong prefecture level city in Shandong
Stowe Flagship Store	a power saver (electronics)	After using it for a few days, it feels quite good, it should be quite safe, and it also sends a plug, the merchant is good, wait for a period of time and then calculate whether it saves on the electricity bill and then add comments ha!	Jingdong prefecture level city in Shandong
Stowe Flagship Store	a power saver (electronics)	My friend recommended to me, said the effect is very good, I bought two, this month less money, really can, just buy I do not believe, but has proved to have effect	Jingdong prefecture level city in Shandong
Stowe Flagship Store	a power saver (electronics)	Just bought back, already used, these days to see the effect of it, and so have the effect will also come back to give positive feedback, the price is also super cheap!	Jingdong prefecture level city in Shandong
Stowe Flagship Store	a power saver (electronics)	It does work well, the store uses a lot of electricity, this time to buy back a few, each place that uses a lot of electricity to insert one. Summer is here, the electricity is too strong.	Jingdong prefecture level city in Shandong
Stowe Flagship Store	a power saver (electronics)	Power saving treasure received, as for the effect, according to the instructions written, a short period of time can not be seen, at least 2-3 months before the real effect, then follow up evaluation	Jingdong prefecture level city in Shandong
Stowe Flagship Store	a power saver (electronics)	The goods have just been received and have not been used yet looking forward to the results.	Jingdong prefecture level city in Shandong
Stowe Flagship Store	a power saver (electronics)	Good installation works great	Jingdong prefecture level city in Shandong
Stowe Flagship Store	a power saver (electronics)	The logistics is super super fast! The quality is also very good, indeed value for money	Jingdong prefecture level city in Shandong
Stowe Flagship Store	a power saver (electronics)	Has been used for a period of time before coming to comment on, really good, air conditioning a day open non-stop, pay the electricity bill when the heartache, with this, the home electricity is not at all worried about the bill out of contrast to save a lot of preparation in the hands of a few, each room An a	Jingdong prefecture level city in Shandong
Stowe Flagship Store	a power saver (electronics)	Saving device received, really good, with the air conditioning tried, an hour without a saver with 2.9 degrees, with a saver an hour with 1.1 degrees, the beginning of not quite believe that this thing, the actual test to know that it is really useful, and intend to buy another.	Jingdong prefecture level city in Shandong
Stowe Flagship Store	a power saver (electronics)	Power saving effect: this energy saver is still useful, day trial for three days, every day than not installed before the reduction of two dollars a day, every day to open the bedroom to open the air conditioning before fifteen dollars now thirteen dollars a day. Still a little use.	Jingdong prefecture level city in Shandong
Stowe Flagship Store	a power saver (electronics)	Originally bought for home use, used for two months, the electricity bill from more than 200 yuan per month, down to more than 140 yuan, there is indeed a power-saving effect, this time and sold two, the unit with. Merchant service attitude is really good.	Jingdong prefecture level city in Shandong
Stowe Flagship Store	a power saver (electronics)	Plug and play, stable voltage, the screen shows when working, this winter home appliances, need electricity in many places, just buy to use, save electricity and money, with instructions	Jingdong prefecture level city in Shandong
Stowe Flagship Store	a power saver (electronics)	Goods received, do not know if it is good. When I bought it, I saw that it was a Stowe brand or company's product. Received but not. I do not know what is the reason. Now I can only give this evaluation. I'm	Jingdong prefecture level city in Shandong
Stowe Flagship Store	a power saver (electronics)	The first time to buy online, but also look at the evaluation before buying, the quality is not bad, but also very cheap, the need for friends can rest assured to buy!	Jingdong prefecture level city in Shandong
Stowe Flagship Store	a power saver (electronics)	Great quality, bought it to my liking Awesome! Buy with confidence if you need it!	Jingdong prefecture level city in Shandong
Stowe Flagship Store	a power saver (electronics)	The quality is very good, completely exceeded the expectations, the delivery speed is very fast, the packaging is very careful and strict, very satisfied with a shopping	Jingdong prefecture level city in Shandong
Stowe Flagship Store	a power saver (electronics)	The effect is very good, the home uses less electricity is very energy efficient, cost-effective	Jingdong prefecture level city in Shandong
Stowe Flagship Store	a power saver (electronics)	Quality can't be beat, just like the store, especially like, and the price is favorable!	Jingdong prefecture level city in Shandong
Stowe Flagship Store	a power saver (electronics)	Baby received! Very unexpected, will be back next time ~ great seller, stay tuned!	Jingdong prefecture level city in Shandong
Stowe Flagship Store	a power saver (electronics)	Follow up review after a month is more real, waiting for it	Jingdong prefecture level city in Shandong
Stowe Flagship Store	a power saver (electronics)	It is the ideal product, good quality and fast logistics, worthy of praise!	Jingdong prefecture level city in Shandong
Stowe Flagship Store	a power saver (electronics)	The saver is great, the color is right, the installer is very professional	Jingdong prefecture level city in Shandong

Stowe Flagship Store	a power saver (electronics)	The effect of the power connection is particularly obvious, it is particularly easy to use, very satisfied, the	Jingdong prefecture level city in Shandong
Stowe Flagship Store	a power saver (electronics)	Goods are very energy-saving, used to be more than two hundred degrees, now there are only a hundred or so degrees	Jingdong prefecture level city in Shandong
Stowe Flagship Store	a power saver (electronics)	Good quality, fast shipping, good results, good value for money	Jingdong prefecture level city in Shandong
Stowe Flagship Store	a power saver (electronics)	Fast shipping, fine workmanship, very good, cost-effective	Jingdong prefecture level city in Shandong
Stowe Flagship Store	a power saver (electronics)	The meter is as described, the quality is quite good, energy-saving, worth buying oh~!	Jingdong prefecture level city in Shandong
Stowe Flagship Store	a power saver (electronics)	Great value for money, very affordable,, perfect . ^◡^.	Jingdong prefecture level city in Shandong
Stowe Flagship Store	a power saver (electronics)	Good!!! Worth recommending!	Jingdong prefecture level city in Shandong
Stowe Flagship Store	a power saver (electronics)	Great, fast logistics, great stuff, great value for money!	Jingdong prefecture level city in Shandong
Stowe Flagship Store	a power saver (electronics)	Fast logistics, very satisfied with the quality upon receipt	Jingdong prefecture level city in Shandong
Stowe Flagship Store	a power saver (electronics)	Great quality, plug and play, fast shipping!	Jingdong prefecture level city in Shandong
Stowe Flagship Store	a power saver (electronics)	Logistics received, quite satisfied	Jingdong prefecture level city in Shandong
Stowe Flagship Store	a power saver (electronics)	I don't know if it really works	Jingdong prefecture level city in Shandong
Stowe Flagship Store	a power saver (electronics)	It's bullshit. It's a plug light. Don't talk about power savings.	Jingdong prefecture level city in Shandong
Stowe Flagship Store	a power saver (electronics)	Fake and deceitful stuff that hasn't changed at all.	Jingdong prefecture level city in Shandong
Stowe Flagship Store	a power saver (electronics)	Power-saving effect: power-saving effect is particularly obvious Cost-effective: cost-effective business Appearance and style: external small micro-work materials: fine workmanship Experience: use very good, non the first convenient	Jingdong prefecture level city in Shandong
Stowe Flagship Store	a power saver (electronics)	After using it for a few days, it feels quite good, it should be quite safe, and it also sends a plug, the merchant is good, wait for a period of time and then calculate whether it saves on the electricity bill and then add comments ha!	Jingdong prefecture level city in Shandong
Stowe Flagship Store	a power saver (electronics)	My friend recommended to me, said the effect is very good, I bought two, this month less money, really can, just buy I do not believe, but has proved to have effect	Jingdong prefecture level city in Shandong
Stowe Flagship Store	a power saver (electronics)	Just bought back, already used, these days to see the effect of it, and so have the effect will also come back to give positive feedback, the price is also super cheap!	Jingdong prefecture level city in Shandong
Stowe Flagship Store	a power saver (electronics)	It does work well, the store uses a lot of electricity, this time to buy back a few, each place that uses a lot of electricity to insert one. Summer is here, the electricity is too strong.	Jingdong prefecture level city in Shandong
Stowe Flagship Store	a power saver (electronics)	Power saving treasure received, as for the effect, according to the instructions written, a short period of time can not be seen, at least 2-3 months before the real effect, then follow up evaluation	Jingdong prefecture level city in Shandong
Stowe Flagship Store	a power saver (electronics)	The goods have just been received and have not been used yet looking forward to the results.	Jingdong prefecture level city in Shandong
Stowe Flagship Store	a power saver (electronics)	Good installation works great	Jingdong prefecture level city in Shandong
Stowe Flagship Store	a power saver (electronics)	The logistics is super super fast! The quality is also very good, indeed value for money	Jingdong prefecture level city in Shandong
Stowe Flagship Store	a power saver (electronics)	Has been used for a period of time before coming to comment on, really good, air conditioning a day open non-stop, pay the electricity bill when the heartache, with this, the home electricity is not at all worried about the bill out of contrast to save a lot of preparation in the hands of a few, each room An a	Jingdong prefecture level city in Shandong
Stowe Flagship Store	a power saver (electronics)	Saving device received, really good, with the air conditioning tried, an hour without a saver with 2.9 degrees, with a saver an hour with 1.1 degrees, the beginning of not quite believe that this thing, the actual test to know that it is really useful, and intend to buy another.	Jingdong prefecture level city in Shandong
Stowe Flagship Store	a power saver (electronics)	Power saving effect: this energy saver is still useful, day trial for three days, every day than not installed before the reduction of two dollars a day, every day to open the bedroom to open the air conditioning before fifteen dollars now thirteen dollars a day. Still a little use.	Jingdong prefecture level city in Shandong
Stowe Flagship Store	a power saver (electronics)	Originally bought for home use, used for two months, the electricity bill from more than 200 yuan per month, down to more than 140 yuan, there is indeed a power-saving effect, this time and sold two, the unit with. Merchant service attitude is really good.	Jingdong prefecture level city in Shandong
Stowe Flagship Store	a power saver (electronics)	Plug and play, stable voltage, the screen shows when working, this winter home appliances, need electricity in many places, just buy to use, save electricity and money, with instructions	Jingdong prefecture level city in Shandong
Stowe Flagship Store	a power saver (electronics)	Goods received, do not know if it is good. When I bought it, I saw that it was a Stowe brand or company's product. Received but not. I do not know what is the reason. Now I can only give this evaluation. I'm	Jingdong prefecture level city in Shandong
Stowe Flagship Store	a power saver (electronics)	The first time to buy online, but also look at the evaluation before buying, the quality is not bad, but also very cheap, the need for friends can rest assured to buy!	Jingdong prefecture level city in Shandong
Stowe Flagship Store	a power saver (electronics)	🌈 Great quality, bought it to my liking 💝 Awesome! ✨ Buy with confidence if you need it!	Jingdong prefecture level city in Shandong
Stowe Flagship Store	a power saver (electronics)	The quality is very good, completely exceeded the expectations, the delivery speed is very fast, the packaging is very careful and strict, very satisfied with a shopping	Jingdong prefecture level city in Shandong
Stowe Flagship Store	a power saver (electronics)	The effect is very good, the home uses less electricity is very energy efficient, cost-effective	Jingdong prefecture level city in Shandong
Stowe Flagship Store	a power saver (electronics)	Quality can't be beat, just like the store, especially like, and the price is favorable!	Jingdong prefecture level city in Shandong
Stowe Flagship Store	a power saver (electronics)	Baby received! Very unexpected, will be back next time ~ great seller, stay tuned!	Jingdong prefecture level city in Shandong
Stowe Flagship Store	a power saver (electronics)	Follow up review after a month is more real, waiting for it	Jingdong prefecture level city in Shandong
Stowe Flagship Store	a power saver (electronics)	It is the ideal product, good quality and fast logistics, worthy of praise!	Jingdong prefecture level city in Shandong
Stowe Flagship Store	a power saver (electronics)	The saver is great, the color is right, the installer is very professional	Jingdong prefecture level city in Shandong
Stowe Flagship Store	a power saver (electronics)	The effect of the power connection is particularly obvious, it is particularly easy to use, very satisfied, the	Jingdong prefecture level city in Shandong
Stowe Flagship Store	a power saver (electronics)	Goods are very energy-saving, used to be more than two hundred degrees, now there are only a hundred or so degrees	Jingdong prefecture level city in Shandong
Stowe Flagship Store	a power saver (electronics)	Good quality, fast shipping, good results, good value for money	Jingdong prefecture level city in Shandong
Stowe Flagship Store	a power saver (electronics)	Fast shipping, fine workmanship, very good, cost-effective	Jingdong prefecture level city in Shandong
Stowe Flagship Store	a power saver (electronics)	The meter is as described, the quality is quite good, energy-saving, worth buying oh~!	Jingdong prefecture level city in Shandong
Stowe Flagship Store	a power saver (electronics)	Great value for money, very affordable,, perfect . ^◡^.	Jingdong prefecture level city in Shandong
Stowe Flagship Store	a power saver (electronics)	Good!!! Worth recommending!	Jingdong prefecture level city in Shandong
Stowe Flagship Store	a power saver (electronics)	Great, fast logistics, great stuff, great value for money!	Jingdong prefecture level city in Shandong
Stowe Flagship Store	a power saver (electronics)	Fast logistics, very satisfied with the quality upon receipt	Jingdong prefecture level city in Shandong
Stowe Flagship Store	a power saver (electronics)	Great quality, plug and play, fast shipping!	Jingdong prefecture level city in Shandong

Stowe Flagship Store	a power saver (electronics)	Logistics received, quite satisfied	Jingdong prefecture level city in Shandong
Stowe Flagship Store	a power saver (electronics)	I don't know if it really works	Jingdong prefecture level city in Shandong
Stowe Flagship Store	a power saver (electronics)	It's bullshit. It's a plug light. Don't talk about power savings.	Jingdong prefecture level city in Shandong
Stowe Flagship Store	a power saver (electronics)	Fake and deceitful stuff that hasn't changed at all.	Jingdong prefecture level city in Shandong
Stowe Flagship Store	a power saver (electronics)	Power-saving effect: power-saving effect is particularly obvious Cost-effective: cost-effective business Appearance and style: external small micro-work materials: fine workmanship Experience: use very good, non the first convenient	Jingdong prefecture level city in Shandong
Stowe Flagship Store	a power saver (electronics)	After using it for a few days, it feels quite good, it should be quite safe, and it also sends a plug, the merchant is good, wait for a period of time and then calculate whether it saves on the electricity bill and then add comments ha!	Jingdong prefecture level city in Shandong
Stowe Flagship Store	a power saver (electronics)	My friend recommended to me, said the effect is very good, I bought two, this month less money, really can, just buy I do not believe, but has proved to have effect	Jingdong prefecture level city in Shandong
Stowe Flagship Store	a power saver (electronics)	Just bought back, already used, these days to see the effect of it, and so have the effect will also come back to give positive feedback, the price is also super cheap!	Jingdong prefecture level city in Shandong
Stowe Flagship Store	a power saver (electronics)	It does work well, the store uses a lot of electricity, this time to buy back a few, each place that uses a lot of electricity to insert one. Summer is here, the electricity is too strong.	Jingdong prefecture level city in Shandong
Stowe Flagship Store	a power saver (electronics)	Power saving treasure received, as for the effect, according to the instructions written, a short period of time can not be seen, at least 2-3 months before the real effect, then follow up evaluation	Jingdong prefecture level city in Shandong
Stowe Flagship Store	a power saver (electronics)	The goods have just been received and have not been used yet looking forward to the results.	Jingdong prefecture level city in Shandong
Stowe Flagship Store	a power saver (electronics)	Good installation works great	Jingdong prefecture level city in Shandong
Stowe Flagship Store	a power saver (electronics)	The logistics is super super fast! The quality is also very good, indeed value for money	Jingdong prefecture level city in Shandong
Stowe Flagship Store	a power saver (electronics)	Has been used for a period of time before coming to comment on, really good, air conditioning a day open non-stop, pay the electricity bill when the heartache, with this, the home electricity is not at all worried about the bill out of contrast to save a lot of preparation in the hands of a few, each room An a	Jingdong prefecture level city in Shandong
Stowe Flagship Store	a power saver (electronics)	Saving device received, really good, with the air conditioning tried, an hour without a saver with 2.9 degrees, with a saver an hour with 1.1 degrees, the beginning of not quite believe that this thing, the actual test to know that it is really useful, and intend to buy another.	Jingdong prefecture level city in Shandong
Stowe Flagship Store	a power saver (electronics)	Power saving effect: this energy saver is still useful, day trial for three days, every day than not installed before the reduction of two dollars a day, every day to open the bedroom to open the air conditioning before fifteen dollars now thirteen dollars a day. Still a little use.	Jingdong prefecture level city in Shandong
Stowe Flagship Store	a power saver (electronics)	Originally bought for home use, used for two months, the electricity bill from more than 200 yuan per month, down to more than 140 yuan, there is indeed a power-saving effect, this time and sold two, the unit with. Merchant service attitude is really good.	Jingdong prefecture level city in Shandong
Stowe Flagship Store	a power saver (electronics)	Plug and play, stable voltage, the screen shows when working, this winter home appliances, need electricity in many places, just buy to use, save electricity and money, with instructions	Jingdong prefecture level city in Shandong
Stowe Flagship Store	a power saver (electronics)	Goods received, do not know if it is good. When I bought it, I saw that it was a Stowe brand or company's product. Received but not. I do not know what is the reason. Now I can only give this evaluation. I'm	Jingdong prefecture level city in Shandong
Stowe Flagship Store	a power saver (electronics)	The first time to buy online, but also look at the evaluation before buying, the quality is not bad, but also very cheap, the need for friends can rest assured to buy!	Jingdong prefecture level city in Shandong
Stowe Flagship Store	a power saver (electronics)	Great quality, bought it to my liking Awesome! Buy with confidence if you need it!	Jingdong prefecture level city in Shandong
Stowe Flagship Store	a power saver (electronics)	The quality is very good, completely exceeded the expectations, the delivery speed is very fast, the packaging is very careful and strict, very satisfied with a shopping	Jingdong prefecture level city in Shandong
Stowe Flagship Store	a power saver (electronics)	The effect is very good, the home uses less electricity is very energy efficient, cost-effective	Jingdong prefecture level city in Shandong
Stowe Flagship Store	a power saver (electronics)	Quality can't be beat, just like the store, especially like, and the price is favorable!	Jingdong prefecture level city in Shandong
Stowe Flagship Store	a power saver (electronics)	Baby received! Very unexpected, will be back next time ~ great seller, stay tuned!	Jingdong prefecture level city in Shandong
Stowe Flagship Store	a power saver (electronics)	Follow up review after a month is more real, waiting for it	Jingdong prefecture level city in Shandong
Stowe Flagship Store	a power saver (electronics)	It is the ideal product, good quality and fast logistics, worthy of praise!	Jingdong prefecture level city in Shandong
Stowe Flagship Store	a power saver (electronics)	The saver is great, the color is right, the installer is very professional	Jingdong prefecture level city in Shandong
Stowe Flagship Store	a power saver (electronics)	The effect of the power connection is particularly obvious, it is particularly easy to use, very satisfied, the	Jingdong prefecture level city in Shandong
Stowe Flagship Store	a power saver (electronics)	Goods are very energy-saving, used to be more than two hundred degrees, now there are only a hundred or so degrees	Jingdong prefecture level city in Shandong
Stowe Flagship Store	a power saver (electronics)	Good quality, fast shipping, good results, good value for money	Jingdong prefecture level city in Shandong
Stowe Flagship Store	a power saver (electronics)	Fast shipping, fine workmanship, very good, cost-effective	Jingdong prefecture level city in Shandong
Stowe Flagship Store	a power saver (electronics)	The meter is as described, the quality is quite good, energy-saving, worth buying oh~!	Jingdong prefecture level city in Shandong
Stowe Flagship Store	a power saver (electronics)	Great value for money, very affordable,, perfect . ˆ◡ˆ.	Jingdong prefecture level city in Shandong
Stowe Flagship Store	a power saver (electronics)	Good!!! Worth recommending!	Jingdong prefecture level city in Shandong
Stowe Flagship Store	a power saver (electronics)	Great, fast logistics, great stuff, great value for money!	Jingdong prefecture level city in Shandong
Stowe Flagship Store	a power saver (electronics)	Fast logistics, very satisfied with the quality upon receipt	Jingdong prefecture level city in Shandong
Stowe Flagship Store	a power saver (electronics)	Great quality, plug and play, fast shipping!	Jingdong prefecture level city in Shandong
Stowe Flagship Store	a power saver (electronics)	Logistics received, quite satisfied	Jingdong prefecture level city in Shandong
Stowe Flagship Store	a power saver (electronics)	I don't know if it really works	Jingdong prefecture level city in Shandong
Stowe Flagship Store	a power saver (electronics)	It's bullshit. It's a plug light. Don't talk about power savings.	Jingdong prefecture level city in Shandong
Stowe Flagship Store	a power saver (electronics)	Fake and deceitful stuff that hasn't changed at all.	Jingdong prefecture level city in Shandong
Stowe Flagship Store	a power saver (electronics)	Power-saving effect: power-saving effect is particularly obvious Cost-effective: cost-effective business Appearance and style: external small micro-work materials: fine workmanship Experience: use very good, non the first convenient	Jingdong prefecture level city in Shandong
Stowe Flagship Store	a power saver (electronics)	After using it for a few days, it feels quite good, it should be quite safe, and it also sends a plug, the merchant is good, wait for a period of time and then calculate whether it saves on the electricity bill and then add comments ha!	Jingdong prefecture level city in Shandong
Stowe Flagship Store	a power saver (electronics)	My friend recommended to me, said the effect is very good, I bought two, this month less money, really can, just buy I do not believe, but has proved to have effect	Jingdong prefecture level city in Shandong
Stowe Flagship Store	a power saver (electronics)	Just bought back, already used, these days to see the effect of it, and so have the effect will also come back to give positive feedback, the price is also super cheap!	Jingdong prefecture level city in Shandong
Stowe Flagship Store	a power saver (electronics)	It does work well, the store uses a lot of electricity, this time to buy back a few, each place that uses a lot of electricity to insert one. Summer is here, the electricity is too strong.	Jingdong prefecture level city in Shandong

Stowe Flagship Store	a power saver (electronics)	Power saving treasure received, as for the effect, according to the instructions written, a short period of time can not be seen, at least 2-3 months before the real effect, then follow up evaluation	Jingdong prefecture level city in Shandong
Stowe Flagship Store	a power saver (electronics)	The goods have just been received and have not been used yet looking forward to the results.	Jingdong prefecture level city in Shandong
Stowe Flagship Store	a power saver (electronics)	Good installation works great	Jingdong prefecture level city in Shandong
Stowe Flagship Store	a power saver (electronics)	The logistics is super super fast! The quality is also very good, indeed value for money	Jingdong prefecture level city in Shandong
Stowe Flagship Store	a power saver (electronics)	Has been used for a period of time before coming to comment on, really good, air conditioning a day open non-stop, pay the electricity bill when the heartache, with this, the home electricity is not at all worried about the bill out of contrast to save a lot of preparation in the hands of a few, each room An a	Jingdong prefecture level city in Shandong
Stowe Flagship Store	a power saver (electronics)	Saving device received, really good, with the air conditioning tried, an hour without a saver with 2.9 degrees, with a saver an hour with 1.1 degrees, the beginning of not quite believe that this thing, the actual test to know that it is really useful, and intend to buy another.	Jingdong prefecture level city in Shandong
Stowe Flagship Store	a power saver (electronics)	Power saving effect: this energy saver is still useful, day trial for three days, every day than not installed before the reduction of two dollars a day, every day to open the bedroom to open the air conditioning before fifteen dollars now thirteen dollars a day. Still a little use.	Jingdong prefecture level city in Shandong
Stowe Flagship Store	a power saver (electronics)	Originally bought for home use, used for two months, the electricity bill from more than 200 yuan per month, down to more than 140 yuan, there is indeed a power-saving effect, this time and sold two, the unit with. Merchant service attitude is really good.	Jingdong prefecture level city in Shandong
Stowe Flagship Store	a power saver (electronics)	Plug and play, stable voltage, the screen shows when working, this winter home appliances, need electricity in many places, just buy to use, save electricity and money, with instructions	Jingdong prefecture level city in Shandong
Stowe Flagship Store	a power saver (electronics)	Goods received, do not know if it is good. When I bought it, I saw that it was a Stowe brand or company's product. Received but not. I do not know what is the reason. Now I can only give this evaluation. I'm	Jingdong prefecture level city in Shandong
Stowe Flagship Store	a power saver (electronics)	The first time to buy online, but also look at the evaluation before buying, the quality is not bad, but also very cheap, the need for friends can rest assured to buy!	Jingdong prefecture level city in Shandong
Stowe Flagship Store	a power saver (electronics)	🌈 Great quality, bought it to my liking 💞 Awesome! ✨ Buy with confidence if you need it!	Jingdong prefecture level city in Shandong
Stowe Flagship Store	a power saver (electronics)	The quality is very good, completely exceeded the expectations, the delivery speed is very fast, the packaging is very careful and strict, very satisfied with a shopping	Jingdong prefecture level city in Shandong
Stowe Flagship Store	a power saver (electronics)	The effect is very good, the home uses less electricity is very energy efficient, cost-effective	Jingdong prefecture level city in Shandong
Stowe Flagship Store	a power saver (electronics)	Quality can't be beat, just like the store, especially like, and the price is favorable!	Jingdong prefecture level city in Shandong
Stowe Flagship Store	a power saver (electronics)	Baby received! Very unexpected, will be back next time ~ great seller, stay tuned!	Jingdong prefecture level city in Shandong
Stowe Flagship Store	a power saver (electronics)	Follow up review after a month is more real, waiting for it	Jingdong prefecture level city in Shandong
Stowe Flagship Store	a power saver (electronics)	It is the ideal product, good quality and fast logistics, worthy of praise!	Jingdong prefecture level city in Shandong
Stowe Flagship Store	a power saver (electronics)	The saver is great, the color is right, the installer is very professional	Jingdong prefecture level city in Shandong
Stowe Flagship Store	a power saver (electronics)	The effect of the power connection is particularly obvious, it is particularly easy to use, very satisfied, the	Jingdong prefecture level city in Shandong
Stowe Flagship Store	a power saver (electronics)	Goods are very energy-saving, used to be more than two hundred degrees, now there are only a hundred or so degrees	Jingdong prefecture level city in Shandong
Stowe Flagship Store	a power saver (electronics)	Good quality, fast shipping, good results, good value for money	Jingdong prefecture level city in Shandong
Stowe Flagship Store	a power saver (electronics)	Fast shipping, fine workmanship, very good, cost-effective	Jingdong prefecture level city in Shandong
Stowe Flagship Store	a power saver (electronics)	The meter is as described, the quality is quite good, energy-saving, worth buying oh~!	Jingdong prefecture level city in Shandong
Stowe Flagship Store	a power saver (electronics)	Great value for money, very affordable,, perfect . ^◡^.	Jingdong prefecture level city in Shandong
Stowe Flagship Store	a power saver (electronics)	Good!!! Worth recommending!	Jingdong prefecture level city in Shandong
Stowe Flagship Store	a power saver (electronics)	Great, fast logistics, great stuff, great value for money!	Jingdong prefecture level city in Shandong
Stowe Flagship Store	a power saver (electronics)	Fast logistics, very satisfied with the quality upon receipt	Jingdong prefecture level city in Shandong
Stowe Flagship Store	a power saver (electronics)	Great quality, plug and play, fast shipping!	Jingdong prefecture level city in Shandong
Stowe Flagship Store	a power saver (electronics)	Logistics received, quite satisfied	Jingdong prefecture level city in Shandong
Stowe Flagship Store	a power saver (electronics)	I don't know if it really works	Jingdong prefecture level city in Shandong
Stowe Flagship Store	a power saver (electronics)	It's bullshit. It's a plug light. Don't talk about power savings.	Jingdong prefecture level city in Shandong
Stowe Flagship Store	a power saver (electronics)	Fake and deceitful stuff that hasn't changed at all.	Jingdong prefecture level city in Shandong
Stowe Flagship Store	a power saver (electronics)	Power-saving effect: power-saving effect is particularly obvious Cost-effective: cost-effective business Appearance and style: external small micro-work materials: fine workmanship Experience: use very good, non the first convenient	Jingdong prefecture level city in Shandong
Stowe Flagship Store	a power saver (electronics)	After using it for a few days, it feels quite good, it should be quite safe, and it also sends a plug, the merchant is good, wait for a period of time and then calculate whether it saves on the electricity bill and then add comments ha!	Jingdong prefecture level city in Shandong
Stowe Flagship Store	a power saver (electronics)	My friend recommended to me, said the effect is very good, I bought two, this month less money, really can, just buy I do not believe, but has proved to have effect	Jingdong prefecture level city in Shandong
Stowe Flagship Store	a power saver (electronics)	Just bought back, already used, these days to see the effect of it, and so have the effect will also come back to give positive feedback, the price is also super cheap!	Jingdong prefecture level city in Shandong
Stowe Flagship Store	a power saver (electronics)	It does work well, the store uses a lot of electricity, this time to buy back a few, each place that uses a lot of electricity to insert one. Summer is here, the electricity is too strong.	Jingdong prefecture level city in Shandong
Stowe Flagship Store	a power saver (electronics)	Power saving treasure received, as for the effect, according to the instructions written, a short period of time can not be seen, at least 2-3 months before the real effect, then follow up evaluation	Jingdong prefecture level city in Shandong
Stowe Flagship Store	a power saver (electronics)	The goods have just been received and have not been used yet looking forward to the results.	Jingdong prefecture level city in Shandong
Stowe Flagship Store	a power saver (electronics)	Good installation works great	Jingdong prefecture level city in Shandong
Stowe Flagship Store	a power saver (electronics)	The logistics is super super fast! The quality is also very good, indeed value for money	Jingdong prefecture level city in Shandong
Stowe Flagship Store	a power saver (electronics)	Has been used for a period of time before coming to comment on, really good, air conditioning a day open non-stop, pay the electricity bill when the heartache, with this, the home electricity is not at all worried about the bill out of contrast to save a lot of preparation in the hands of a few, each room An a	Jingdong prefecture level city in Shandong
Stowe Flagship Store	a power saver (electronics)	Saving device received, really good, with the air conditioning tried, an hour without a saver with 2.9 degrees, with a saver an hour with 1.1 degrees, the beginning of not quite believe that this thing, the actual test to know that it is really useful, and intend to buy another.	Jingdong prefecture level city in Shandong
Stowe Flagship Store	a power saver (electronics)	Power saving effect: this energy saver is still useful, day trial for three days, every day than not installed before the reduction of two dollars a day, every day to open the bedroom to open the air conditioning before fifteen dollars now thirteen dollars a day. Still a little use.	Jingdong prefecture level city in Shandong
Stowe Flagship Store	a power saver (electronics)	Originally bought for home use, used for two months, the electricity bill from more than 200 yuan per month, down to more than 140 yuan, there is indeed a power-saving effect, this time and sold two, the unit with. Merchant service attitude is really good.	Jingdong prefecture level city in Shandong

Stowe Flagship Store	a power saver (electronics)	Plug and play, stable voltage, the screen shows when working, this winter home appliances, need electricity in many places, just buy to use, save electricity and money, with instructions	Jingdong prefecture level city in Shandong
Stowe Flagship Store	a power saver (electronics)	Goods received, do not know if it is good. When I bought it, I saw that it was a Stowe brand or company's product. Received but not. I do not know what is the reason. Now I can only give this evaluation. I'm	Jingdong prefecture level city in Shandong
Stowe Flagship Store	a power saver (electronics)	The first time to buy online, but also look at the evaluation before buying, the quality is not bad, but also very cheap, the need for friends can rest assured to buy!	Jingdong prefecture level city in Shandong
Stowe Flagship Store	a power saver (electronics)	🌈 Great quality, bought it to my liking 💝 Awesome! ✨ Buy with confidence if you need it!	Jingdong prefecture level city in Shandong
Stowe Flagship Store	a power saver (electronics)	The quality is very good, completely exceeded the expectations, the delivery speed is very fast, the packaging is very careful and strict, very satisfied with a shopping	Jingdong prefecture level city in Shandong
Stowe Flagship Store	a power saver (electronics)	The effect is very good, the home uses less electricity is very energy efficient, cost-effective	Jingdong prefecture level city in Shandong
Stowe Flagship Store	a power saver (electronics)	Quality can't be beat, just like the store, especially like, and the price is favorable!	Jingdong prefecture level city in Shandong
Stowe Flagship Store	a power saver (electronics)	Baby received! Very unexpected, will be back next time ~ great seller, stay tuned!	Jingdong prefecture level city in Shandong
Stowe Flagship Store	a power saver (electronics)	Follow up review after a month is more real, waiting for it	Jingdong prefecture level city in Shandong
Stowe Flagship Store	a power saver (electronics)	It is the ideal product, good quality and fast logistics, worthy of praise!	Jingdong prefecture level city in Shandong
Stowe Flagship Store	a power saver (electronics)	The saver is great, the color is right, the installer is very professional	Jingdong prefecture level city in Shandong
Stowe Flagship Store	a power saver (electronics)	The effect of the power connection is particularly obvious, it is particularly easy to use, very satisfied, the	Jingdong prefecture level city in Shandong
Stowe Flagship Store	a power saver (electronics)	Good quality, fast shipping, good results, good value for money	Jingdong prefecture level city in Shandong
Stowe Flagship Store	a power saver (electronics)	Goods are very energy-saving, used to be more than two hundred degrees, now there are only a hundred or so degrees	Jingdong prefecture level city in Shandong
Stowe Flagship Store	a power saver (electronics)	Fast shipping, fine workmanship, very good, cost-effective	Jingdong prefecture level city in Shandong
Stowe Flagship Store	a power saver (electronics)	The meter is as described, the quality is quite good, energy-saving, worth buying oh~!	Jingdong prefecture level city in Shandong
Stowe Flagship Store	a power saver (electronics)	Great value for money, very affordable,, perfect . ˆ◡ˆ.	Jingdong prefecture level city in Shandong
Stowe Flagship Store	a power saver (electronics)	Good!!! Worth recommending!	Jingdong prefecture level city in Shandong
Stowe Flagship Store	a power saver (electronics)	Great, fast logistics, great stuff, great value for money!	Jingdong prefecture level city in Shandong
Stowe Flagship Store	a power saver (electronics)	Fast logistics, very satisfied with the quality upon receipt	Jingdong prefecture level city in Shandong
Stowe Flagship Store	a power saver (electronics)	Great quality, plug and play, fast shipping!	Jingdong prefecture level city in Shandong
Stowe Flagship Store	a power saver (electronics)	Logistics received, quite satisfied	Jingdong prefecture level city in Shandong
Stowe Flagship Store	a power saver (electronics)	I don't know if it really works	Jingdong prefecture level city in Shandong
Stowe Flagship Store	a power saver (electronics)	It's bullshit. It's a plug light. Don't talk about power savings.	Jingdong prefecture level city in Shandong
Stowe Flagship Store	a power saver (electronics)	Fake and deceitful stuff that hasn't changed at all.	Jingdong prefecture level city in Shandong
Stowe Flagship Store	a power saver (electronics)	Power-saving effect: power-saving effect is particularly obvious Cost-effective: cost-effective business Appearance and style: external small micro-work materials: fine workmanship Experience: use very good, non the first convenient	Jingdong prefecture level city in Shandong
Stowe Flagship Store	a power saver (electronics)	After using it for a few days, it feels quite good, it should be quite safe, and it also sends a plug, the merchant is good, wait for a period of time and then calculate whether it saves on the electricity bill and then add comments ha!	Jingdong prefecture level city in Shandong
Stowe Flagship Store	a power saver (electronics)	My friend recommended to me, said the effect is very good, I bought two, this month less money, really can, just buy I do not believe, but has proved to have effect	Jingdong prefecture level city in Shandong
Stowe Flagship Store	a power saver (electronics)	Just bought back, already used, these days to see the effect of it, and so have the effect will also come back to give positive feedback, the price is also super cheap!	Jingdong prefecture level city in Shandong
Stowe Flagship Store	a power saver (electronics)	It does work well, the store uses a lot of electricity, this time to buy back a few, each place that uses a lot of electricity to insert one. Summer is here, the electricity is too strong.	Jingdong prefecture level city in Shandong
Stowe Flagship Store	a power saver (electronics)	Power saving treasure received, as for the effect, according to the instructions written, a short period of time can not be seen, at least 2-3 months before the real effect, then follow up evaluation	Jingdong prefecture level city in Shandong
Stowe Flagship Store	a power saver (electronics)	The goods have just been received and have not been used yet looking forward to the results.	Jingdong prefecture level city in Shandong
Stowe Flagship Store	a power saver (electronics)	Good installation works great	Jingdong prefecture level city in Shandong
Stowe Flagship Store	a power saver (electronics)	The logistics is super super fast! The quality is also very good, indeed value for money	Jingdong prefecture level city in Shandong
Stowe Flagship Store	a power saver (electronics)	Has been used for a period of time before coming to comment on, really good, air conditioning a day open non-stop, pay the electricity bill when the heartache, with this, the home electricity is not at all worried about the bill out of contrast to save a lot of preparation in the hands of a few, each room An a	Jingdong prefecture level city in Shandong
Stowe Flagship Store	a power saver (electronics)	Saving device received, really good, with the air conditioning tried, an hour without a saver with 2.9 degrees, with a saver an hour with 1.1 degrees, the beginning of not quite believe that this thing, the actual test to know that it is really useful, and intend to buy another.	Jingdong prefecture level city in Shandong
Stowe Flagship Store	a power saver (electronics)	Power saving effect: this energy saver is still useful, day trial for three days, every day than not installed before the reduction of two dollars a day, every day to open the bedroom to open the air conditioning before fifteen dollars now thirteen dollars a day. Still a little use.	Jingdong prefecture level city in Shandong
Stowe Flagship Store	a power saver (electronics)	Originally bought for home use, used for two months, the electricity bill from more than 200 yuan per month, down to more than 140 yuan, there is indeed a power-saving effect, this time and sold two, the unit with. Merchant service attitude is really good.	Jingdong prefecture level city in Shandong
Stowe Flagship Store	a power saver (electronics)	Plug and play, stable voltage, the screen shows when working, this winter home appliances, need electricity in many places, just buy to use, save electricity and money, with instructions	Jingdong prefecture level city in Shandong
Stowe Flagship Store	a power saver (electronics)	Goods received, do not know if it is good. When I bought it, I saw that it was a Stowe brand or company's product. Received but not. I do not know what is the reason. Now I can only give this evaluation. I'm	Jingdong prefecture level city in Shandong
Stowe Flagship Store	a power saver (electronics)	The first time to buy online, but also look at the evaluation before buying, the quality is not bad, but also very cheap, the need for friends can rest assured to buy!	Jingdong prefecture level city in Shandong
Stowe Flagship Store	a power saver (electronics)	🌈 Great quality, bought it to my liking 💝 Awesome! ✨ Buy with confidence if you need it!	Jingdong prefecture level city in Shandong
Stowe Flagship Store	a power saver (electronics)	The quality is very good, completely exceeded the expectations, the delivery speed is very fast, the packaging is very careful and strict, very satisfied with a shopping	Jingdong prefecture level city in Shandong
Stowe Flagship Store	a power saver (electronics)	The effect is very good, the home uses less electricity is very energy efficient, cost-effective	Jingdong prefecture level city in Shandong
Stowe Flagship Store	a power saver (electronics)	Quality can't be beat, just like the store, especially like, and the price is favorable!	Jingdong prefecture level city in Shandong
Stowe Flagship Store	a power saver (electronics)	Baby received! Very unexpected, will be back next time ~ great seller, stay tuned!	Jingdong prefecture level city in Shandong
Stowe Flagship Store	a power saver (electronics)	Follow up review after a month is more real, waiting for it	Jingdong prefecture level city in Shandong
Stowe Flagship Store	a power saver (electronics)	It is the ideal product, good quality and fast logistics, worthy of praise!	Jingdong prefecture level city in Shandong

Stowe Flagship Store	a power saver (electronics)	The saver is great, the color is right, the installer is very professional	Jingdong prefecture level city in Shandong
Stowe Flagship Store	a power saver (electronics)	The effect of the power connection is particularly obvious, it is particularly easy to use, very satisfied, the	Jingdong prefecture level city in Shandong
Stowe Flagship Store	a power saver (electronics)	Goods are very energy-saving, used to be more than two hundred degrees, now there are only a hundred or so degrees	Jingdong prefecture level city in Shandong
Stowe Flagship Store	a power saver (electronics)	Good quality, fast shipping, good results, good value for money	Jingdong prefecture level city in Shandong
Stowe Flagship Store	a power saver (electronics)	Fast shipping, fine workmanship, very good, cost-effective	Jingdong prefecture level city in Shandong
Stowe Flagship Store	a power saver (electronics)	The meter is as described, the quality is quite good, energy-saving, worth buying oh~!	Jingdong prefecture level city in Shandong
Stowe Flagship Store	a power saver (electronics)	Great value for money, very affordable,, perfect . ^◡^.	Jingdong prefecture level city in Shandong
Stowe Flagship Store	a power saver (electronics)	Good!!! Worth recommending!	Jingdong prefecture level city in Shandong
Stowe Flagship Store	a power saver (electronics)	Great, fast logistics, great stuff, great value for money!	Jingdong prefecture level city in Shandong
Stowe Flagship Store	a power saver (electronics)	Fast logistics, very satisfied with the quality upon receipt	Jingdong prefecture level city in Shandong
Stowe Flagship Store	a power saver (electronics)	Great quality, plug and play, fast shipping!	Jingdong prefecture level city in Shandong
Stowe Flagship Store	a power saver (electronics)	Logistics received, quite satisfied	Jingdong prefecture level city in Shandong
Stowe Flagship Store	a power saver (electronics)	I don't know if it really works	Jingdong prefecture level city in Shandong
Stowe Flagship Store	a power saver (electronics)	It's bullshit. It's a plug light. Don't talk about power savings.	Jingdong prefecture level city in Shandong
Stowe Flagship Store	a power saver (electronics)	Fake and deceitful stuff that hasn't changed at all.	Jingdong prefecture level city in Shandong
Stowe Flagship Store	a power saver (electronics)	Power-saving effect: power-saving effect is particularly obvious Cost-effective: cost-effective business Appearance and style: external small micro-work materials: fine workmanship Experience: use very good, non the first convenient	Jingdong prefecture level city in Shandong
Stowe Flagship Store	a power saver (electronics)	After using it for a few days, it feels quite good, it should be quite safe, and it also sends a plug, the merchant is good, wait for a period of time and then calculate whether it saves on the electricity bill and then add comments ha!	Jingdong prefecture level city in Shandong
Stowe Flagship Store	a power saver (electronics)	My friend recommended to me, said the effect is very good, I bought two, this month less money, really can, just buy I do not believe, but has proved to have effect	Jingdong prefecture level city in Shandong
Stowe Flagship Store	a power saver (electronics)	Just bought back, already used, these days to see the effect of it, and so have the effect will also come back to give positive feedback, the price is also super cheap!	Jingdong prefecture level city in Shandong
Stowe Flagship Store	a power saver (electronics)	It does work well, the store uses a lot of electricity, this time to buy back a few, each place that uses a lot of electricity to insert one. Summer is here, the electricity is too strong.	Jingdong prefecture level city in Shandong
Stowe Flagship Store	a power saver (electronics)	Power saving treasure received, as for the effect, according to the instructions written, a short period of time can not be seen, at least 2-3 months before the real effect, then follow up evaluation	Jingdong prefecture level city in Shandong
Stowe Flagship Store	a power saver (electronics)	The goods have just been received and have not been used yet looking forward to the results.	Jingdong prefecture level city in Shandong
Stowe Flagship Store	a power saver (electronics)	Good installation works great	Jingdong prefecture level city in Shandong
Stowe Flagship Store	a power saver (electronics)	The logistics is super super fast! The quality is also very good, indeed value for money	Jingdong prefecture level city in Shandong
Stowe Flagship Store	a power saver (electronics)	Has been used for a period of time before coming to comment on, really good, air conditioning a day open non-stop, pay the electricity bill when the heartache, with this, the home electricity is not at all worried about the bill out of contrast to save a lot of preparation in the hands of a few, each room An a	Jingdong prefecture level city in Shandong
Stowe Flagship Store	a power saver (electronics)	Saving device received, really good, with the air conditioning tried, an hour without a saver with 2.9 degrees, with a saver an hour with 1.1 degrees, the beginning of not quite believe that this thing, the actual test to know that it is really useful, and intend to buy another.	Jingdong prefecture level city in Shandong
Stowe Flagship Store	a power saver (electronics)	Power saving effect: this energy saver is still useful, day trial for three days, every day than not installed before the reduction of two dollars a day, every day to open the bedroom to open the air conditioning before fifteen dollars now thirteen dollars a day. Still a little use.	Jingdong prefecture level city in Shandong
Stowe Flagship Store	a power saver (electronics)	Originally bought for home use, used for two months, the electricity bill from more than 200 yuan per month, down to more than 140 yuan, there is indeed a power-saving effect, this time and sold two, the unit with. Merchant service attitude is really good.	Jingdong prefecture level city in Shandong
Stowe Flagship Store	a power saver (electronics)	Plug and play, stable voltage, the screen shows when working, this winter home appliances, need electricity in many places, just buy to use, save electricity and money, with instructions	Jingdong prefecture level city in Shandong
Stowe Flagship Store	a power saver (electronics)	Goods received, do not know if it is good. When I bought it, I saw that it was a Stowe brand or company's product. Received but not. I do not know what is the reason. Now I can only give this evaluation. I'm	Jingdong prefecture level city in Shandong
Stowe Flagship Store	a power saver (electronics)	The first time to buy online, but also look at the evaluation before buying, the quality is not bad, but also very cheap, the need for friends can rest assured to buy!	Jingdong prefecture level city in Shandong
Stowe Flagship Store	a power saver (electronics)	Great quality, bought it to my liking Awesome! Buy with confidence if you need it!	Jingdong prefecture level city in Shandong
Stowe Flagship Store	a power saver (electronics)	The quality is very good, completely exceeded the expectations, the delivery speed is very fast, the packaging is very careful and strict, very satisfied with a shopping	Jingdong prefecture level city in Shandong
Stowe Flagship Store	a power saver (electronics)	The effect is very good, the home uses less electricity is very energy efficient, cost-effective	Jingdong prefecture level city in Shandong
Stowe Flagship Store	a power saver (electronics)	Quality can't be beat, just like the store, especially like, and the price is favorable!	Jingdong prefecture level city in Shandong
Stowe Flagship Store	a power saver (electronics)	Baby received! Very unexpected, will be back next time ~ great seller, stay tuned!	Jingdong prefecture level city in Shandong
Stowe Flagship Store	a power saver (electronics)	Follow up review after a month is more real, waiting for it	Jingdong prefecture level city in Shandong
Stowe Flagship Store	a power saver (electronics)	It is the ideal product, good quality and fast logistics, worthy of praise!	Jingdong prefecture level city in Shandong
Stowe Flagship Store	a power saver (electronics)	The saver is great, the color is right, the installer is very professional	Jingdong prefecture level city in Shandong
Stowe Flagship Store	a power saver (electronics)	The effect of the power connection is particularly obvious, it is particularly easy to use, very satisfied, the	Jingdong prefecture level city in Shandong
Stowe Flagship Store	a power saver (electronics)	Goods are very energy-saving, used to be more than two hundred degrees, now there are only a hundred or so degrees	Jingdong prefecture level city in Shandong
Stowe Flagship Store	a power saver (electronics)	Good quality, fast shipping, good results, good value for money	Jingdong prefecture level city in Shandong
Stowe Flagship Store	a power saver (electronics)	Fast shipping, fine workmanship, very good, cost-effective	Jingdong prefecture level city in Shandong
Stowe Flagship Store	a power saver (electronics)	The meter is as described, the quality is quite good, energy-saving, worth buying oh~!	Jingdong prefecture level city in Shandong
Stowe Flagship Store	a power saver (electronics)	Great value for money, very affordable,, perfect . ^◡^.	Jingdong prefecture level city in Shandong
Stowe Flagship Store	a power saver (electronics)	Good!!! Worth recommending!	Jingdong prefecture level city in Shandong
Stowe Flagship Store	a power saver (electronics)	Great, fast logistics, great stuff, great value for money!	Jingdong prefecture level city in Shandong
Stowe Flagship Store	a power saver (electronics)	Fast logistics, very satisfied with the quality upon receipt	Jingdong prefecture level city in Shandong

Stowe Flagship Store	a power saver (electronics)	Great quality, plug and play, fast shipping!	Jingdong prefecture level city in Shandong
Stowe Flagship Store	a power saver (electronics)	Logistics received, quite satisfied	Jingdong prefecture level city in Shandong
Stowe Flagship Store	a power saver (electronics)	I don't know if it really works	Jingdong prefecture level city in Shandong
Stowe Flagship Store	a power saver (electronics)	It's bullshit. It's a plug light. Don't talk about power savings.	Jingdong prefecture level city in Shandong
Stowe Flagship Store	a power saver (electronics)	Fake and deceitful stuff that hasn't changed at all.	Jingdong prefecture level city in Shandong
Stowe Flagship Store	a power saver (electronics)	Power-saving effect: power-saving effect is particularly obvious Cost-effective: cost-effective business Appearance and style: external small micro-work materials: fine workmanship Experience: use very good, non the first convenient	Jingdong prefecture level city in Shandong
Stowe Flagship Store	a power saver (electronics)	After using it for a few days, it feels quite good, it should be quite safe, and it also sends a plug, the merchant is good, wait for a period of time and then calculate whether it saves on the electricity bill and then add comments ha!	Jingdong prefecture level city in Shandong
Stowe Flagship Store	a power saver (electronics)	My friend recommended to me, said the effect is very good, I bought two, this month less money, really can, just buy I do not believe, but has proved to have effect	Jingdong prefecture level city in Shandong
Stowe Flagship Store	a power saver (electronics)	Just bought back, already used, these days to see the effect of it, and so have the effect will also come back to give positive feedback, the price is also super cheap!	Jingdong prefecture level city in Shandong
Stowe Flagship Store	a power saver (electronics)	It does work well, the store uses a lot of electricity, this time to buy back a few, each place that uses a lot of electricity to insert one. Summer is here, the electricity is too strong.	Jingdong prefecture level city in Shandong
Stowe Flagship Store	a power saver (electronics)	Power saving treasure received, as for the effect, according to the instructions written, a short period of time can not be seen, at least 2-3 months before the real effect, then follow up evaluation	Jingdong prefecture level city in Shandong
Stowe Flagship Store	a power saver (electronics)	The goods have just been received and have not been used yet looking forward to the results.	Jingdong prefecture level city in Shandong
Stowe Flagship Store	a power saver (electronics)	Good installation works great	Jingdong prefecture level city in Shandong
Stowe Flagship Store	a power saver (electronics)	The logistics is super super fast! The quality is also very good, indeed value for money	Jingdong prefecture level city in Shandong
Stowe Flagship Store	a power saver (electronics)	Has been used for a period of time before coming to comment on, really good, air conditioning a day open non-stop, pay the electricity bill when the heartache, with this, the home electricity is not at all worried about the bill out of contrast to save a lot of preparation in the hands of a few, each room An a	Jingdong prefecture level city in Shandong
Stowe Flagship Store	a power saver (electronics)	Saving device received, really good, with the air conditioning tried, an hour without a saver with 2.9 degrees, with a saver an hour with 1.1 degrees, the beginning of not quite believe that this thing, the actual test to know that it is really useful, and intend to buy another.	Jingdong prefecture level city in Shandong
Stowe Flagship Store	a power saver (electronics)	Power saving effect: this energy saver is still useful, day trial for three days, every day than not installed before the reduction of two dollars a day, every day to open the bedroom to open the air conditioning before fifteen dollars now thirteen dollars a day. Still a little use.	Jingdong prefecture level city in Shandong
Stowe Flagship Store	a power saver (electronics)	Originally bought for home use, used for two months, the electricity bill from more than 200 yuan per month, down to more than 140 yuan, there is indeed a power-saving effect, this time and sold two, the unit with. Merchant service attitude is really good.	Jingdong prefecture level city in Shandong
Stowe Flagship Store	a power saver (electronics)	Plug and play, stable voltage, the screen shows when working, this winter home appliances, need electricity in many places, just buy to use, save electricity and money, with instructions	Jingdong prefecture level city in Shandong
Stowe Flagship Store	a power saver (electronics)	Goods received, do not know if it is good. When I bought it, I saw that it was a Stowe brand or company's product. Received but not. I do not know what is the reason. Now I can only give this evaluation. I'm	Jingdong prefecture level city in Shandong
Stowe Flagship Store	a power saver (electronics)	The first time to buy online, but also look at the evaluation before buying, the quality is not bad, but also very cheap, the need for friends can rest assured to buy!	Jingdong prefecture level city in Shandong
Stowe Flagship Store	a power saver (electronics)	🌈 Great quality, bought it to my liking 💝 Awesome! ✨ Buy with confidence if you need it!	Jingdong prefecture level city in Shandong
Stowe Flagship Store	a power saver (electronics)	The quality is very good, completely exceeded the expectations, the delivery speed is very fast, the packaging is very careful and strict, very satisfied with a shopping	Jingdong prefecture level city in Shandong
Stowe Flagship Store	a power saver (electronics)	The effect is very good, the home uses less electricity is very energy efficient, cost-effective	Jingdong prefecture level city in Shandong
Stowe Flagship Store	a power saver (electronics)	Quality can't be beat, just like the store, especially like, and the price is favorable!	Jingdong prefecture level city in Shandong
Stowe Flagship Store	a power saver (electronics)	Baby received! Very unexpected, will be back next time ~ great seller, stay tuned!	Jingdong prefecture level city in Shandong
Stowe Flagship Store	a power saver (electronics)	Follow up review after a month is more real, waiting for it	Jingdong prefecture level city in Shandong
Stowe Flagship Store	a power saver (electronics)	It is the ideal product, good quality and fast logistics, worthy of praise!	Jingdong prefecture level city in Shandong
Stowe Flagship Store	a power saver (electronics)	The saver is great, the color is right, the installer is very professional	Jingdong prefecture level city in Shandong
Stowe Flagship Store	a power saver (electronics)	The effect of the power connection is particularly obvious, it is particularly easy to use, very satisfied, the	Jingdong prefecture level city in Shandong
Stowe Flagship Store	a power saver (electronics)	Goods are very energy-saving, used to be more than two hundred degrees, now there are only a hundred or so degrees	Jingdong prefecture level city in Shandong
Stowe Flagship Store	a power saver (electronics)	Good quality, fast shipping, good results, good value for money	Jingdong prefecture level city in Shandong
Stowe Flagship Store	a power saver (electronics)	Fast shipping, fine workmanship, very good, cost-effective	Jingdong prefecture level city in Shandong
Stowe Flagship Store	a power saver (electronics)	The meter is as described, the quality is quite good, energy-saving, worth buying oh~!	Jingdong prefecture level city in Shandong
Stowe Flagship Store	a power saver (electronics)	Great value for money, very affordable,, perfect . ^◡^.	Jingdong prefecture level city in Shandong
Stowe Flagship Store	a power saver (electronics)	Good!!! Worth recommending!	Jingdong prefecture level city in Shandong
Stowe Flagship Store	a power saver (electronics)	Great, fast logistics, great stuff, great value for money!	Jingdong prefecture level city in Shandong
Stowe Flagship Store	a power saver (electronics)	Fast logistics, very satisfied with the quality upon receipt	Jingdong prefecture level city in Shandong
Stowe Flagship Store	a power saver (electronics)	Great quality, plug and play, fast shipping!	Jingdong prefecture level city in Shandong
Stowe Flagship Store	a power saver (electronics)	Logistics received, quite satisfied	Jingdong prefecture level city in Shandong
Stowe Flagship Store	a power saver (electronics)	I don't know if it really works	Jingdong prefecture level city in Shandong
Stowe Flagship Store	a power saver (electronics)	It's bullshit. It's a plug light. Don't talk about power savings.	Jingdong prefecture level city in Shandong
Stowe Flagship Store	a power saver (electronics)	Fake and deceitful stuff that hasn't changed at all.	Jingdong prefecture level city in Shandong
Stowe Flagship Store	a power saver (electronics)	Power-saving effect: power-saving effect is particularly obvious Cost-effective: cost-effective business Appearance and style: external small micro-work materials: fine workmanship Experience: use very good, non the first convenient	Jingdong prefecture level city in Shandong
Stowe Flagship Store	a power saver (electronics)	After using it for a few days, it feels quite good, it should be quite safe, and it also sends a plug, the merchant is good, wait for a period of time and then calculate whether it saves on the electricity bill and then add comments ha!	Jingdong prefecture level city in Shandong
Stowe Flagship Store	a power saver (electronics)	My friend recommended to me, said the effect is very good, I bought two, this month less money, really can, just buy I do not believe, but has proved to have effect	Jingdong prefecture level city in Shandong
Stowe Flagship Store	a power saver (electronics)	Just bought back, already used, these days to see the effect of it, and so have the effect will also come back to give positive feedback, the price is also super cheap!	Jingdong prefecture level city in Shandong

Stowe Flagship Store	a power saver (electronics)	It does work well, the store uses a lot of electricity, this time to buy back a few, each place that uses a lot of electricity to insert one. Summer is here, the electricity is too strong.	Jingdong prefecture level city in Shandong
Stowe Flagship Store	a power saver (electronics)	Power saving treasure received, as for the effect, according to the instructions written, a short period of time can not be seen, at least 2-3 months before the real effect, then follow up evaluation	Jingdong prefecture level city in Shandong
Stowe Flagship Store	a power saver (electronics)	The goods have just been received and have not been used yet looking forward to the results.	Jingdong prefecture level city in Shandong
Stowe Flagship Store	a power saver (electronics)	Good installation works great	Jingdong prefecture level city in Shandong
Stowe Flagship Store	a power saver (electronics)	The logistics is super super fast! The quality is also very good, indeed value for money	Jingdong prefecture level city in Shandong
Stowe Flagship Store	a power saver (electronics)	Has been used for a period of time before coming to comment on, really good, air conditioning a day open non-stop, pay the electricity bill when the heartache, with this, the home electricity is not at all worried about the bill out of contrast to save a lot of preparation in the hands of a few, each room An a	Jingdong prefecture level city in Shandong
Stowe Flagship Store	a power saver (electronics)	Saving device received, really good, with the air conditioning tried, an hour without a saver with 2.9 degrees, with a saver an hour with 1.1 degrees, the beginning of not quite believe that this thing, the actual test to know that it is really useful, and intend to buy another.	Jingdong prefecture level city in Shandong
Stowe Flagship Store	a power saver (electronics)	Power saving effect: this energy saver is still useful, day trial for three days, every day than not installed before the reduction of two dollars a day, every day to open the bedroom to open the air conditioning before fifteen dollars now thirteen dollars a day. Still a little use.	Jingdong prefecture level city in Shandong
Stowe Flagship Store	a power saver (electronics)	Originally bought for home use, used for two months, the electricity bill from more than 200 yuan per month, down to more than 140 yuan, there is indeed a power-saving effect, this time and sold two, the unit with. Merchant service attitude is really good.	Jingdong prefecture level city in Shandong
Stowe Flagship Store	a power saver (electronics)	Plug and play, stable voltage, the screen shows when working, this winter home appliances, need electricity in many places, just buy to use, save electricity and money, with instructions	Jingdong prefecture level city in Shandong
Stowe Flagship Store	a power saver (electronics)	Goods received, do not know if it is good. When I bought it, I saw that it was a Stowe brand or company's product. Received but not. I do not know what is the reason. Now I can only give this evaluation. I'm	Jingdong prefecture level city in Shandong
Stowe Flagship Store	a power saver (electronics)	The first time to buy online, but also look at the evaluation before buying, the quality is not bad, but also very cheap, the need for friends can rest assured to buy!	Jingdong prefecture level city in Shandong
Stowe Flagship Store	a power saver (electronics)	🌈 Great quality, bought it to my liking 💛 Awesome! ✨ Buy with confidence if you need it!	Jingdong prefecture level city in Shandong
Stowe Flagship Store	a power saver (electronics)	The quality is very good, completely exceeded the expectations, the delivery speed is very fast, the packaging is very careful and strict, very satisfied with a shopping	Jingdong prefecture level city in Shandong
Stowe Flagship Store	a power saver (electronics)	The effect is very good, the home uses less electricity is very energy efficient, cost-effective	Jingdong prefecture level city in Shandong
Stowe Flagship Store	a power saver (electronics)	Quality can't be beat, just like the store, especially like, and the price is favorable!	Jingdong prefecture level city in Shandong
Stowe Flagship Store	a power saver (electronics)	Baby received! Very unexpected, will be back next time ~ great seller, stay tuned!	Jingdong prefecture level city in Shandong
Stowe Flagship Store	a power saver (electronics)	Follow up review after a month is more real, waiting for it	Jingdong prefecture level city in Shandong
Stowe Flagship Store	a power saver (electronics)	It is the ideal product, good quality and fast logistics, worthy of praise!	Jingdong prefecture level city in Shandong
Stowe Flagship Store	a power saver (electronics)	The saver is great, the color is right, the installer is very professional	Jingdong prefecture level city in Shandong
Stowe Flagship Store	a power saver (electronics)	The effect of the power connection is particularly obvious, it is particularly easy to use, very satisfied, the	Jingdong prefecture level city in Shandong
Stowe Flagship Store	a power saver (electronics)	Goods are very energy-saving, used to be more than two hundred degrees, now there are only a hundred or so degrees	Jingdong prefecture level city in Shandong
Stowe Flagship Store	a power saver (electronics)	Good quality, fast shipping, good results, good value for money	Jingdong prefecture level city in Shandong
Stowe Flagship Store	a power saver (electronics)	Fast shipping, fine workmanship, very good, cost-effective	Jingdong prefecture level city in Shandong
Stowe Flagship Store	a power saver (electronics)	The meter is as described, the quality is quite good, energy-saving, worth buying oh~!	Jingdong prefecture level city in Shandong
Stowe Flagship Store	a power saver (electronics)	Great value for money, very affordable,, perfect . ^◡^.	Jingdong prefecture level city in Shandong
Stowe Flagship Store	a power saver (electronics)	Good!!! Worth recommending!	Jingdong prefecture level city in Shandong
Stowe Flagship Store	a power saver (electronics)	Great, fast logistics, great stuff, great value for money!	Jingdong prefecture level city in Shandong
Stowe Flagship Store	a power saver (electronics)	Fast logistics, very satisfied with the quality upon receipt	Jingdong prefecture level city in Shandong
Stowe Flagship Store	a power saver (electronics)	Great quality, plug and play, fast shipping!	Jingdong prefecture level city in Shandong
Stowe Flagship Store	a power saver (electronics)	Logistics received, quite satisfied	Jingdong prefecture level city in Shandong
Stowe Flagship Store	a power saver (electronics)	I don't know if it really works	Jingdong prefecture level city in Shandong
Stowe Flagship Store	a power saver (electronics)	It's bullshit. It's a plug light. Don't talk about power savings.	Jingdong prefecture level city in Shandong
Stowe Flagship Store	a power saver (electronics)	Fake and deceitful stuff that hasn't changed at all.	Jingdong prefecture level city in Shandong
Stowe Flagship Store	a power saver (electronics)	Power-saving effect: power-saving effect is particularly obvious Cost-effective: cost-effective business Appearance and style: external small micro-work materials: fine workmanship Experience: use very good, non the first convenient	Jingdong prefecture level city in Shandong
Stowe Flagship Store	a power saver (electronics)	After using it for a few days, it feels quite good, it should be quite safe, and it also sends a plug, the merchant is good, wait for a period of time and then calculate whether it saves on the electricity bill and then add comments ha!	Jingdong prefecture level city in Shandong
Stowe Flagship Store	a power saver (electronics)	My friend recommended to me, said the effect is very good, I bought two, this month less money, really can, just buy I do not believe, but has proved to have effect	Jingdong prefecture level city in Shandong
Stowe Flagship Store	a power saver (electronics)	Just bought back, already used, these days to see the effect of it, and so have the effect will also come back to give positive feedback, the price is also super cheap!	Jingdong prefecture level city in Shandong
Stowe Flagship Store	a power saver (electronics)	It does work well, the store uses a lot of electricity, this time to buy back a few, each place that uses a lot of electricity to insert one. Summer is here, the electricity is too strong.	Jingdong prefecture level city in Shandong
Stowe Flagship Store	a power saver (electronics)	Power saving treasure received, as for the effect, according to the instructions written, a short period of time can not be seen, at least 2-3 months before the real effect, then follow up evaluation	Jingdong prefecture level city in Shandong
Stowe Flagship Store	a power saver (electronics)	The goods have just been received and have not been used yet looking forward to the results.	Jingdong prefecture level city in Shandong
Stowe Flagship Store	a power saver (electronics)	Good installation works great	Jingdong prefecture level city in Shandong
Stowe Flagship Store	a power saver (electronics)	The logistics is super super fast! The quality is also very good, indeed value for money	Jingdong prefecture level city in Shandong
Stowe Flagship Store	a power saver (electronics)	Has been used for a period of time before coming to comment on, really good, air conditioning a day open non-stop, pay the electricity bill when the heartache, with this, the home electricity is not at all worried about the bill out of contrast to save a lot of preparation in the hands of a few, each room An a	Jingdong prefecture level city in Shandong
Stowe Flagship Store	a power saver (electronics)	Saving device received, really good, with the air conditioning tried, an hour without a saver with 2.9 degrees, with a saver an hour with 1.1 degrees, the beginning of not quite believe that this thing, the actual test to know that it is really useful, and intend to buy another.	Jingdong prefecture level city in Shandong
Stowe Flagship Store	a power saver (electronics)	Power saving effect: this energy saver is still useful, day trial for three days, every day than not installed before the reduction of two dollars a day, every day to open the bedroom to open the air conditioning before fifteen dollars now thirteen dollars a day. Still a little use.	Jingdong prefecture level city in Shandong

Stowe Flagship Store	a power saver (electronics)	Originally bought for home use, used for two months, the electricity bill from more than 200 yuan per month, down to more than 140 yuan, there is indeed a power-saving effect, this time and sold two, the unit with. Merchant service attitude is really good.	Jingdong prefecture level city in Shandong
Stowe Flagship Store	a power saver (electronics)	Plug and play, stable voltage, the screen shows when working, this winter home appliances, need electricity in many places, just buy to use, save electricity and money, with instructions	Jingdong prefecture level city in Shandong
Stowe Flagship Store	a power saver (electronics)	Goods received, do not know if it is good. When I bought it, I saw that it was a Stowe brand or company's product. Received but not. I do not know what is the reason. Now I can only give this evaluation. I'm	Jingdong prefecture level city in Shandong
Stowe Flagship Store	a power saver (electronics)	The first time to buy online, but also look at the evaluation before buying, the quality is not bad, but also very cheap, the need for friends can rest assured to buy!	Jingdong prefecture level city in Shandong
Stowe Flagship Store	a power saver (electronics)	🌈 Great quality, bought it to my liking 💗 Awesome! ✨ Buy with confidence if you need it!	Jingdong prefecture level city in Shandong
Stowe Flagship Store	a power saver (electronics)	The quality is very good, completely exceeded the expectations, the delivery speed is very fast, the packaging is very careful and strict, very satisfied with a shopping	Jingdong prefecture level city in Shandong
Stowe Flagship Store	a power saver (electronics)	The effect is very good, the home uses less electricity is very energy efficient, cost-effective	Jingdong prefecture level city in Shandong
Stowe Flagship Store	a power saver (electronics)	Quality can't be beat, just like the store, especially like, and the price is favorable!	Jingdong prefecture level city in Shandong
Stowe Flagship Store	a power saver (electronics)	Baby received! Very unexpected, will be back next time ~ great seller, stay tuned!	Jingdong prefecture level city in Shandong
Stowe Flagship Store	a power saver (electronics)	Follow up review after a month is more real, waiting for it	Jingdong prefecture level city in Shandong
Stowe Flagship Store	a power saver (electronics)	It is the ideal product, good quality and fast logistics, worthy of praise!	Jingdong prefecture level city in Shandong
Stowe Flagship Store	a power saver (electronics)	The saver is great, the color is right, the installer is very professional	Jingdong prefecture level city in Shandong
Stowe Flagship Store	a power saver (electronics)	The effect of the power connection is particularly obvious, it is particularly easy to use, very satisfied, the	Jingdong prefecture level city in Shandong
Stowe Flagship Store	a power saver (electronics)	Goods are very energy-saving, used to be more than two hundred degrees, now there are only a hundred or so degrees	Jingdong prefecture level city in Shandong
Stowe Flagship Store	a power saver (electronics)	Good quality, fast shipping, good results, good value for money	Jingdong prefecture level city in Shandong
Stowe Flagship Store	a power saver (electronics)	Fast shipping, fine workmanship, very good, cost-effective	Jingdong prefecture level city in Shandong
Stowe Flagship Store	a power saver (electronics)	The meter is as described, the quality is quite good, energy-saving, worth buying oh~!	Jingdong prefecture level city in Shandong
Stowe Flagship Store	a power saver (electronics)	Great value for money, very affordable,, perfect . ˆ◡ˆ.	Jingdong prefecture level city in Shandong
Stowe Flagship Store	a power saver (electronics)	Good!!! Worth recommending!	Jingdong prefecture level city in Shandong
Stowe Flagship Store	a power saver (electronics)	Great, fast logistics, great stuff, great value for money!	Jingdong prefecture level city in Shandong
Stowe Flagship Store	a power saver (electronics)	Fast logistics, very satisfied with the quality upon receipt	Jingdong prefecture level city in Shandong
Stowe Flagship Store	a power saver (electronics)	Great quality, plug and play, fast shipping!	Jingdong prefecture level city in Shandong
Stowe Flagship Store	a power saver (electronics)	Logistics received, quite satisfied	Jingdong prefecture level city in Shandong
Stowe Flagship Store	a power saver (electronics)	I don't know if it really works	Jingdong prefecture level city in Shandong
Stowe Flagship Store	a power saver (electronics)	It's bullshit. It's a plug light. Don't talk about power savings.	Jingdong prefecture level city in Shandong
Stowe Flagship Store	a power saver (electronics)	Fake and deceitful stuff that hasn't changed at all.	Jingdong prefecture level city in Shandong
Stowe Flagship Store	a power saver (electronics)	Power-saving effect: power-saving effect is particularly obvious Cost-effective: cost-effective business Appearance and style: external small micro-work materials: fine workmanship Experience: use very good, non the first convenient	Jingdong prefecture level city in Shandong
Stowe Flagship Store	a power saver (electronics)	After using it for a few days, it feels quite good, it should be quite safe, and it also sends a plug, the merchant is good, wait for a period of time and then calculate whether it saves on the electricity bill and then add comments ha!	Jingdong prefecture level city in Shandong
Stowe Flagship Store	a power saver (electronics)	My friend recommended to me, said the effect is very good, I bought two, this month less money, really can, just buy I do not believe, but has proved to have effect	Jingdong prefecture level city in Shandong
Stowe Flagship Store	a power saver (electronics)	Just bought back, already used, these days to see the effect of it, and so have the effect will also come back to give positive feedback, the price is also super cheap!	Jingdong prefecture level city in Shandong
Stowe Flagship Store	a power saver (electronics)	It does work well, the store uses a lot of electricity, this time to buy back a few, each place that uses a lot of electricity to insert one. Summer is here, the electricity is too strong.	Jingdong prefecture level city in Shandong
Stowe Flagship Store	a power saver (electronics)	Power saving treasure received, as for the effect, according to the instructions written, a short period of time can not be seen, at least 2-3 months before the real effect, then follow up evaluation	Jingdong prefecture level city in Shandong
Stowe Flagship Store	a power saver (electronics)	The goods have just been received and have not been used yet looking forward to the results.	Jingdong prefecture level city in Shandong
Stowe Flagship Store	a power saver (electronics)	Good installation works great	Jingdong prefecture level city in Shandong
Stowe Flagship Store	a power saver (electronics)	The logistics is super super fast! The quality is also very good, indeed value for money	Jingdong prefecture level city in Shandong
Stowe Flagship Store	a power saver (electronics)	Has been used for a period of time before coming to comment on, really good, air conditioning a day open non-stop, pay the electricity bill when the heartache, with this, the home electricity is not at all worried about the bill out of contrast to save a lot of preparation in the hands of a few, each room An a	Jingdong prefecture level city in Shandong
Stowe Flagship Store	a power saver (electronics)	Saving device received, really good, with the air conditioning tried, an hour without a saver with 2.9 degrees, with a saver an hour with 1.1 degrees, the beginning of not quite believe that this thing, the actual test to know that it is really useful, and intend to buy another.	Jingdong prefecture level city in Shandong
Stowe Flagship Store	a power saver (electronics)	Power saving effect: this energy saver is still useful, day trial for three days, every day than not installed before the reduction of two dollars a day, every day to open the bedroom to open the air conditioning before fifteen dollars now thirteen dollars a day. Still a little use.	Jingdong prefecture level city in Shandong
Stowe Flagship Store	a power saver (electronics)	Originally bought for home use, used for two months, the electricity bill from more than 200 yuan per month, down to more than 140 yuan, there is indeed a power-saving effect, this time and sold two, the unit with. Merchant service attitude is really good.	Jingdong prefecture level city in Shandong
Stowe Flagship Store	a power saver (electronics)	Plug and play, stable voltage, the screen shows when working, this winter home appliances, need electricity in many places, just buy to use, save electricity and money, with instructions	Jingdong prefecture level city in Shandong
Stowe Flagship Store	a power saver (electronics)	Goods received, do not know if it is good. When I bought it, I saw that it was a Stowe brand or company's product. Received but not. I do not know what is the reason. Now I can only give this evaluation. I'm	Jingdong prefecture level city in Shandong
Stowe Flagship Store	a power saver (electronics)	The first time to buy online, but also look at the evaluation before buying, the quality is not bad, but also very cheap, the need for friends can rest assured to buy!	Jingdong prefecture level city in Shandong
Stowe Flagship Store	a power saver (electronics)	🌈 Great quality, bought it to my liking 💗 Awesome! ✨ Buy with confidence if you need it!	Jingdong prefecture level city in Shandong
Stowe Flagship Store	a power saver (electronics)	The quality is very good, completely exceeded the expectations, the delivery speed is very fast, the packaging is very careful and strict, very satisfied with a shopping	Jingdong prefecture level city in Shandong
Stowe Flagship Store	a power saver (electronics)	The effect is very good, the home uses less electricity is very energy efficient, cost-effective	Jingdong prefecture level city in Shandong
Stowe Flagship Store	a power saver (electronics)	Quality can't be beat, just like the store, especially like, and the price is favorable!	Jingdong prefecture level city in Shandong
Stowe Flagship Store	a power saver (electronics)	Baby received! Very unexpected, will be back next time ~ great seller, stay tuned!	Jingdong prefecture level city in Shandong
Stowe Flagship Store	a power saver (electronics)	Follow up review after a month is more real, waiting for it	Jingdong prefecture level city in Shandong

Stowe Flagship Store	a power saver (electronics)	It is the ideal product, good quality and fast logistics, worthy of praise!	Jingdong prefecture level city in Shandong
Stowe Flagship Store	a power saver (electronics)	The saver is great, the color is right, the installer is very professional	Jingdong prefecture level city in Shandong
Stowe Flagship Store	a power saver (electronics)	The effect of the power connection is particularly obvious, it is particularly easy to use, very satisfied, the	Jingdong prefecture level city in Shandong
Stowe Flagship Store	a power saver (electronics)	Goods are very energy-saving, used to be more than two hundred degrees, now there are only a hundred or so degrees	Jingdong prefecture level city in Shandong
Stowe Flagship Store	a power saver (electronics)	Good quality, fast shipping, good results, good value for money	Jingdong prefecture level city in Shandong
Stowe Flagship Store	a power saver (electronics)	Fast shipping, fine workmanship, very good, cost-effective	Jingdong prefecture level city in Shandong
Stowe Flagship Store	a power saver (electronics)	The meter is as described, the quality is quite good, energy-saving, worth buying oh~!	Jingdong prefecture level city in Shandong
Stowe Flagship Store	a power saver (electronics)	Great value for money, very affordable,, perfect . ^◡^.	Jingdong prefecture level city in Shandong
Stowe Flagship Store	a power saver (electronics)	Good!!! Worth recommending!	Jingdong prefecture level city in Shandong
Stowe Flagship Store	a power saver (electronics)	Great, fast logistics, great stuff, great value for money!	Jingdong prefecture level city in Shandong
Stowe Flagship Store	a power saver (electronics)	Fast logistics, very satisfied with the quality upon receipt	Jingdong prefecture level city in Shandong
Stowe Flagship Store	a power saver (electronics)	Great quality, plug and play, fast shipping!	Jingdong prefecture level city in Shandong
Stowe Flagship Store	a power saver (electronics)	Logistics received, quite satisfied	Jingdong prefecture level city in Shandong
Stowe Flagship Store	a power saver (electronics)	I don't know if it really works	Jingdong prefecture level city in Shandong
Stowe Flagship Store	a power saver (electronics)	It's bullshit. It's a plug light. Don't talk about power savings.	Jingdong prefecture level city in Shandong
Stowe Flagship Store	a power saver (electronics)	Fake and deceitful stuff that hasn't changed at all.	Jingdong prefecture level city in Shandong
Stowe Flagship Store	a power saver (electronics)	Power-saving effect: power-saving effect is particularly obvious Cost-effective: cost-effective business Appearance and style: external small micro-work materials: fine workmanship Experience: use very good, non the first convenient	Jingdong prefecture level city in Shandong
Stowe Flagship Store	a power saver (electronics)	After using it for a few days, it feels quite good, it should be quite safe, and it also sends a plug, the merchant is good, wait for a period of time and then calculate whether it saves on the electricity bill and then add comments ha!	Jingdong prefecture level city in Shandong
Stowe Flagship Store	a power saver (electronics)	My friend recommended to me, said the effect is very good, I bought two, this month less money, really can, just buy I do not believe, but has proved to have effect	Jingdong prefecture level city in Shandong
Stowe Flagship Store	a power saver (electronics)	Just bought back, already used, these days to see the effect of it, and so have the effect will also come back to give positive feedback, the price is also super cheap!	Jingdong prefecture level city in Shandong
Stowe Flagship Store	a power saver (electronics)	It does work well, the store uses a lot of electricity, this time to buy back a few, each place that uses a lot of electricity to insert one. Summer is here, the electricity is too strong.	Jingdong prefecture level city in Shandong
Stowe Flagship Store	a power saver (electronics)	Power saving treasure received, as for the effect, according to the instructions written, a short period of time can not be seen, at least 2-3 months before the real effect, then follow up evaluation	Jingdong prefecture level city in Shandong
Stowe Flagship Store	a power saver (electronics)	The goods have just been received and have not been used yet looking forward to the results.	Jingdong prefecture level city in Shandong
Stowe Flagship Store	a power saver (electronics)	Good installation works great	Jingdong prefecture level city in Shandong
Stowe Flagship Store	a power saver (electronics)	The logistics is super super fast! The quality is also very good, indeed value for money	Jingdong prefecture level city in Shandong
Stowe Flagship Store	a power saver (electronics)	Has been used for a period of time before coming to comment on, really good, air conditioning a day open non-stop, pay the electricity bill when the heartache, with this, the home electricity is not at all worried about the bill out of contrast to save a lot of preparation in the hands of a few, each room An a	Jingdong prefecture level city in Shandong
Stowe Flagship Store	a power saver (electronics)	Saving device received, really good, with the air conditioning tried, an hour without a saver with 2.9 degrees, with a saver an hour with 1.1 degrees, the beginning of not quite believe that this thing, the actual test to know that it is really useful, and intend to buy another.	Jingdong prefecture level city in Shandong
Stowe Flagship Store	a power saver (electronics)	Power saving effect: this energy saver is still useful, day trial for three days, every day than not installed before the reduction of two dollars a day, every day to open the bedroom to open the air conditioning before fifteen dollars now thirteen dollars a day. Still a little use.	Jingdong prefecture level city in Shandong
Stowe Flagship Store	a power saver (electronics)	Originally bought for home use, used for two months, the electricity bill from more than 200 yuan per month, down to more than 140 yuan, there is indeed a power-saving effect, this time and sold two, the unit with. Merchant service attitude is really good.	Jingdong prefecture level city in Shandong
Stowe Flagship Store	a power saver (electronics)	Plug and play, stable voltage, the screen shows when working, this winter home appliances, need electricity in many places, just buy to use, save electricity and money, with instructions	Jingdong prefecture level city in Shandong
Stowe Flagship Store	a power saver (electronics)	Goods received, do not know if it is good. When I bought it, I saw that it was a Stowe brand or company's product. Received but not. I do not know what is the reason. Now I can only give this evaluation. I'm	Jingdong prefecture level city in Shandong
Stowe Flagship Store	a power saver (electronics)	The first time to buy online, but also look at the evaluation before buying, the quality is not bad, but also very cheap, the need for friends can rest assured to buy!	Jingdong prefecture level city in Shandong
Stowe Flagship Store	a power saver (electronics)	🌈 Great quality, bought it to my liking 💞 Awesome! ✨ Buy with confidence if you need it!	Jingdong prefecture level city in Shandong
Stowe Flagship Store	a power saver (electronics)	The quality is very good, completely exceeded the expectations, the delivery speed is very fast, the packaging is very careful and strict, very satisfied with a shopping	Jingdong prefecture level city in Shandong
Stowe Flagship Store	a power saver (electronics)	The effect is very good, the home uses less electricity is very energy efficient, cost-effective	Jingdong prefecture level city in Shandong
Stowe Flagship Store	a power saver (electronics)	Quality can't be beat, just like the store, especially like, and the price is favorable!	Jingdong prefecture level city in Shandong
Stowe Flagship Store	a power saver (electronics)	Baby received! Very unexpected, will be back next time ~ great seller, stay tuned!	Jingdong prefecture level city in Shandong
Stowe Flagship Store	a power saver (electronics)	Follow up review after a month is more real, waiting for it	Jingdong prefecture level city in Shandong
Stowe Flagship Store	a power saver (electronics)	It is the ideal product, good quality and fast logistics, worthy of praise!	Jingdong prefecture level city in Shandong
Stowe Flagship Store	a power saver (electronics)	The saver is great, the color is right, the installer is very professional	Jingdong prefecture level city in Shandong
Stowe Flagship Store	a power saver (electronics)	The effect of the power connection is particularly obvious, it is particularly easy to use, very satisfied, the	Jingdong prefecture level city in Shandong
Stowe Flagship Store	a power saver (electronics)	Goods are very energy-saving, used to be more than two hundred degrees, now there are only a hundred or so degrees	Jingdong prefecture level city in Shandong
Stowe Flagship Store	a power saver (electronics)	Good quality, fast shipping, good results, good value for money	Jingdong prefecture level city in Shandong
Stowe Flagship Store	a power saver (electronics)	Fast shipping, fine workmanship, very good, cost-effective	Jingdong prefecture level city in Shandong
Stowe Flagship Store	a power saver (electronics)	The meter is as described, the quality is quite good, energy-saving, worth buying oh~!	Jingdong prefecture level city in Shandong
Stowe Flagship Store	a power saver (electronics)	Great value for money, very affordable,, perfect . ^◡^.	Jingdong prefecture level city in Shandong
Stowe Flagship Store	a power saver (electronics)	Good!!! Worth recommending!	Jingdong prefecture level city in Shandong
Stowe Flagship Store	a power saver (electronics)	Great, fast logistics, great stuff, great value for money!	Jingdong prefecture level city in Shandong

Stowe Flagship Store	a power saver (electronics)	Fast logistics, very satisfied with the quality upon receipt	Jingdong prefecture level city in Shandong
Stowe Flagship Store	a power saver (electronics)	Great quality, plug and play, fast shipping!	Jingdong prefecture level city in Shandong
Stowe Flagship Store	a power saver (electronics)	Logistics received, quite satisfied	Jingdong prefecture level city in Shandong
Stowe Flagship Store	a power saver (electronics)	I don't know if it really works	Jingdong prefecture level city in Shandong
Stowe Flagship Store	a power saver (electronics)	It's bullshit. It's a plug light. Don't talk about power savings.	Jingdong prefecture level city in Shandong
Stowe Flagship Store	a power saver (electronics)	Fake and deceitful stuff that hasn't changed at all.	Jingdong prefecture level city in Shandong
Stowe Flagship Store	a power saver (electronics)	Power-saving effect: power-saving effect is particularly obvious Cost-effective: cost-effective business Appearance and style: external small micro-work materials: fine workmanship Experience: use very good, non the first convenient	Jingdong prefecture level city in Shandong
Stowe Flagship Store	a power saver (electronics)	After using it for a few days, it feels quite good, it should be quite safe, and it also sends a plug, the merchant is good, wait for a period of time and then calculate whether it saves on the electricity bill and then add comments ha!	Jingdong prefecture level city in Shandong
Stowe Flagship Store	a power saver (electronics)	My friend recommended to me, said the effect is very good, I bought two, this month less money, really can, just buy I do not believe, but has proved to have effect	Jingdong prefecture level city in Shandong
Stowe Flagship Store	a power saver (electronics)	Just bought back, already used, these days to see the effect of it, and so have the effect will also come back to give positive feedback, the price is also super cheap!	Jingdong prefecture level city in Shandong
Stowe Flagship Store	a power saver (electronics)	It does work well, the store uses a lot of electricity, this time to buy back a few, each place that uses a lot of electricity to insert one. Summer is here, the electricity is too strong.	Jingdong prefecture level city in Shandong
Stowe Flagship Store	a power saver (electronics)	Power saving treasure received, as for the effect, according to the instructions written, a short period of time can not be seen, at least 2-3 months before the real effect, then follow up evaluation	Jingdong prefecture level city in Shandong
Stowe Flagship Store	a power saver (electronics)	The goods have just been received and have not been used yet looking forward to the results.	Jingdong prefecture level city in Shandong
Stowe Flagship Store	a power saver (electronics)	Good installation works great	Jingdong prefecture level city in Shandong
Stowe Flagship Store	a power saver (electronics)	The logistics is super super fast! The quality is also very good, indeed value for money	Jingdong prefecture level city in Shandong
Stowe Flagship Store	a power saver (electronics)	Has been used for a period of time before coming to comment on, really good, air conditioning a day open non-stop, pay the electricity bill when the heartache, with this, the home electricity is not at all worried about the bill out of contrast to save a lot of preparation in the hands of a few, each room An a	Jingdong prefecture level city in Shandong
Stowe Flagship Store	a power saver (electronics)	Saving device received, really good, with the air conditioning tried, an hour without a saver with 2.9 degrees, with a saver an hour with 1.1 degrees, the beginning of not quite believe that this thing, the actual test to know that it is really useful, and intend to buy another.	Jingdong prefecture level city in Shandong
Stowe Flagship Store	a power saver (electronics)	Power saving effect: this energy saver is still useful, day trial for three days, every day than not installed before the reduction of two dollars a day, every day to open the bedroom to open the air conditioning before fifteen dollars now thirteen dollars a day. Still a little use.	Jingdong prefecture level city in Shandong
Stowe Flagship Store	a power saver (electronics)	Originally bought for home use, used for two months, the electricity bill from more than 200 yuan per month, down to more than 140 yuan, there is indeed a power-saving effect, this time and sold two, the unit with. Merchant service attitude is really good.	Jingdong prefecture level city in Shandong
Stowe Flagship Store	a power saver (electronics)	Plug and play, stable voltage, the screen shows when working, this winter home appliances, need electricity in many places, just buy to use, save electricity and money, with instructions	Jingdong prefecture level city in Shandong
Stowe Flagship Store	a power saver (electronics)	Goods received, do not know if it is good. When I bought it, I saw that it was a Stowe brand or company's product. Received but not. I do not know what is the reason. Now I can only give this evaluation. I'm	Jingdong prefecture level city in Shandong
Stowe Flagship Store	a power saver (electronics)	The first time to buy online, but also look at the evaluation before buying, the quality is not bad, but also very cheap, the need for friends can rest assured to buy!	Jingdong prefecture level city in Shandong
Stowe Flagship Store	a power saver (electronics)	🌈 Great quality, bought it to my liking 💞 Awesome! ✨ Buy with confidence if you need it!	Jingdong prefecture level city in Shandong
Stowe Flagship Store	a power saver (electronics)	The quality is very good, completely exceeded the expectations, the delivery speed is very fast, the packaging is very careful and strict, very satisfied with a shopping	Jingdong prefecture level city in Shandong
Stowe Flagship Store	a power saver (electronics)	The effect is very good, the home uses less electricity is very energy efficient, cost-effective	Jingdong prefecture level city in Shandong
Stowe Flagship Store	a power saver (electronics)	Quality can't be beat, just like the store, especially like, and the price is favorable!	Jingdong prefecture level city in Shandong
Stowe Flagship Store	a power saver (electronics)	Baby received! Very unexpected, will be back next time ~ great seller, stay tuned!	Jingdong prefecture level city in Shandong
Stowe Flagship Store	a power saver (electronics)	Follow up review after a month is more real, waiting for it	Jingdong prefecture level city in Shandong
Stowe Flagship Store	a power saver (electronics)	It is the ideal product, good quality and fast logistics, worthy of praise!	Jingdong prefecture level city in Shandong
Stowe Flagship Store	a power saver (electronics)	The saver is great, the color is right, the installer is very professional	Jingdong prefecture level city in Shandong
Stowe Flagship Store	a power saver (electronics)	The effect of the power connection is particularly obvious, it is particularly easy to use, very satisfied, the	Jingdong prefecture level city in Shandong
Stowe Flagship Store	a power saver (electronics)	Goods are very energy-saving, used to be more than two hundred degrees, now there are only a hundred or so degrees	Jingdong prefecture level city in Shandong
Stowe Flagship Store	a power saver (electronics)	Good quality, fast shipping, good results, good value for money	Jingdong prefecture level city in Shandong
Stowe Flagship Store	a power saver (electronics)	Fast shipping, fine workmanship, very good, cost-effective	Jingdong prefecture level city in Shandong
Stowe Flagship Store	a power saver (electronics)	The meter is as described, the quality is quite good, energy-saving, worth buying oh~!	Jingdong prefecture level city in Shandong
Stowe Flagship Store	a power saver (electronics)	Great value for money, very affordable,, perfect . ˆ◡ˆ.	Jingdong prefecture level city in Shandong
Stowe Flagship Store	a power saver (electronics)	Good!!! Worth recommending!	Jingdong prefecture level city in Shandong
Stowe Flagship Store	a power saver (electronics)	Great, fast logistics, great stuff, great value for money!	Jingdong prefecture level city in Shandong
Stowe Flagship Store	a power saver (electronics)	Fast logistics, very satisfied with the quality upon receipt	Jingdong prefecture level city in Shandong
Stowe Flagship Store	a power saver (electronics)	Great quality, plug and play, fast shipping!	Jingdong prefecture level city in Shandong
Stowe Flagship Store	a power saver (electronics)	Logistics received, quite satisfied	Jingdong prefecture level city in Shandong
Stowe Flagship Store	a power saver (electronics)	I don't know if it really works	Jingdong prefecture level city in Shandong
Stowe Flagship Store	a power saver (electronics)	It's bullshit. It's a plug light. Don't talk about power savings.	Jingdong prefecture level city in Shandong
Stowe Flagship Store	a power saver (electronics)	Fake and deceitful stuff that hasn't changed at all.	Jingdong prefecture level city in Shandong
Stowe Flagship Store	a power saver (electronics)	Power-saving effect: power-saving effect is particularly obvious Cost-effective: cost-effective business Appearance and style: external small micro-work materials: fine workmanship Experience: use very good, non the first convenient	Jingdong prefecture level city in Shandong
Stowe Flagship Store	a power saver (electronics)	After using it for a few days, it feels quite good, it should be quite safe, and it also sends a plug, the merchant is good, wait for a period of time and then calculate whether it saves on the electricity bill and then add comments ha!	Jingdong prefecture level city in Shandong
Stowe Flagship Store	a power saver (electronics)	My friend recommended to me, said the effect is very good, I bought two, this month less money, really can, just buy I do not believe, but has proved to have effect	Jingdong prefecture level city in Shandong

Stowe Flagship Store	a power saver (electronics)	Just bought back, already used, these days to see the effect of it, and so have the effect will also come back to give positive feedback, the price is also super cheap!	Jingdong prefecture level city in Shandong
Stowe Flagship Store	a power saver (electronics)	It does work well, the store uses a lot of electricity, this time to buy back a few, each place that uses a lot of electricity to insert one. Summer is here, the electricity is too strong.	Jingdong prefecture level city in Shandong
Stowe Flagship Store	a power saver (electronics)	Power saving treasure received, as for the effect, according to the instructions written, a short period of time can not be seen, at least 2-3 months before the real effect, then follow up evaluation	Jingdong prefecture level city in Shandong
Stowe Flagship Store	a power saver (electronics)	The goods have just been received and have not been used yet looking forward to the results.	Jingdong prefecture level city in Shandong
Stowe Flagship Store	a power saver (electronics)	Good installation works great	Jingdong prefecture level city in Shandong
Stowe Flagship Store	a power saver (electronics)	The logistics is super super fast! The quality is also very good, indeed value for money	Jingdong prefecture level city in Shandong
Stowe Flagship Store	a power saver (electronics)	Has been used for a period of time before coming to comment on, really good, air conditioning a day open non-stop, pay the electricity bill when the heartache, with this, the home electricity is not at all worried about the bill out of contrast to save a lot of preparation in the hands of a few, each room An a	Jingdong prefecture level city in Shandong
Stowe Flagship Store	a power saver (electronics)	Saving device received, really good, with the air conditioning tried, an hour without a saver with 2.9 degrees, with a saver an hour with 1.1 degrees, the beginning of not quite believe that this thing, the actual test to know that it is really useful, and intend to buy another.	Jingdong prefecture level city in Shandong
Stowe Flagship Store	a power saver (electronics)	Power saving effect: this energy saver is still useful, day trial for three days, every day than not installed before the reduction of two dollars a day, every day to open the bedroom to open the air conditioning before fifteen dollars now thirteen dollars a day. Still a little use.	Jingdong prefecture level city in Shandong
Stowe Flagship Store	a power saver (electronics)	Originally bought for home use, used for two months, the electricity bill from more than 200 yuan per month, down to more than 140 yuan, there is indeed a power-saving effect, this time and sold two, the unit with. Merchant service attitude is really good.	Jingdong prefecture level city in Shandong
Stowe Flagship Store	a power saver (electronics)	Plug and play, stable voltage, the screen shows when working, this winter home appliances, need electricity in many places, just buy to use, save electricity and money, with instructions	Jingdong prefecture level city in Shandong
Stowe Flagship Store	a power saver (electronics)	Goods received, do not know if it is good. When I bought it, I saw that it was a Stowe brand or company's product. Received but not. I do not know what is the reason. Now I can only give this evaluation. I'm	Jingdong prefecture level city in Shandong
Stowe Flagship Store	a power saver (electronics)	The first time to buy online, but also look at the evaluation before buying, the quality is not bad, but also very cheap, the need for friends can rest assured to buy!	Jingdong prefecture level city in Shandong
Stowe Flagship Store	a power saver (electronics)	🌈 Great quality, bought it to my liking 💝 Awesome! ✨ Buy with confidence if you need it!	Jingdong prefecture level city in Shandong
Stowe Flagship Store	a power saver (electronics)	The quality is very good, completely exceeded the expectations, the delivery speed is very fast, the packaging is very careful and strict, very satisfied with a shopping	Jingdong prefecture level city in Shandong
Stowe Flagship Store	a power saver (electronics)	The effect is very good, the home uses less electricity is very energy efficient, cost-effective	Jingdong prefecture level city in Shandong
Stowe Flagship Store	a power saver (electronics)	Quality can't be beat, just like the store, especially like, and the price is favorable!	Jingdong prefecture level city in Shandong
Stowe Flagship Store	a power saver (electronics)	Baby received! Very unexpected, will be back next time ~ great seller, stay tuned!	Jingdong prefecture level city in Shandong
Stowe Flagship Store	a power saver (electronics)	Follow up review after a month is more real, waiting for it	Jingdong prefecture level city in Shandong
Stowe Flagship Store	a power saver (electronics)	It is the ideal product, good quality and fast logistics, worthy of praise!	Jingdong prefecture level city in Shandong
Stowe Flagship Store	a power saver (electronics)	The saver is great, the color is right, the installer is very professional	Jingdong prefecture level city in Shandong
Stowe Flagship Store	a power saver (electronics)	The effect of the power connection is particularly obvious, it is particularly easy to use, very satisfied, the	Jingdong prefecture level city in Shandong
Stowe Flagship Store	a power saver (electronics)	Goods are very energy-saving, used to be more than two hundred degrees, now there are only a hundred or so degrees	Jingdong prefecture level city in Shandong
Stowe Flagship Store	a power saver (electronics)	Good quality, fast shipping, good results, good value for money	Jingdong prefecture level city in Shandong
Stowe Flagship Store	a power saver (electronics)	Fast shipping, fine workmanship, very good, cost-effective	Jingdong prefecture level city in Shandong
Stowe Flagship Store	a power saver (electronics)	The meter is as described, the quality is quite good, energy-saving, worth buying oh~!	Jingdong prefecture level city in Shandong
Stowe Flagship Store	a power saver (electronics)	Great value for money, very affordable,, perfect . ˆ◡ˆ.	Jingdong prefecture level city in Shandong
Stowe Flagship Store	a power saver (electronics)	Good!!! Worth recommending!	Jingdong prefecture level city in Shandong
Stowe Flagship Store	a power saver (electronics)	Great, fast logistics, great stuff, great value for money!	Jingdong prefecture level city in Shandong
Stowe Flagship Store	a power saver (electronics)	Fast logistics, very satisfied with the quality upon receipt	Jingdong prefecture level city in Shandong
Stowe Flagship Store	a power saver (electronics)	Great quality, plug and play, fast shipping!	Jingdong prefecture level city in Shandong
Stowe Flagship Store	a power saver (electronics)	Logistics received, quite satisfied	Jingdong prefecture level city in Shandong
Stowe Flagship Store	a power saver (electronics)	I don't know if it really works	Jingdong prefecture level city in Shandong
Stowe Flagship Store	a power saver (electronics)	It's bullshit. It's a plug light. Don't talk about power savings.	Jingdong prefecture level city in Shandong
Stowe Flagship Store	a power saver (electronics)	Fake and deceitful stuff that hasn't changed at all.	Jingdong prefecture level city in Shandong
Stowe Flagship Store	a power saver (electronics)	Power-saving effect: power-saving effect is particularly obvious Cost-effective: cost-effective business Appearance and style: external small micro-work materials: fine workmanship Experience: use very good, non the first convenient	Jingdong prefecture level city in Shandong
Stowe Flagship Store	a power saver (electronics)	After using it for a few days, it feels quite good, it should be quite safe, and it also sends a plug, the merchant is good, wait for a period of time and then calculate whether it saves on the electricity bill and then add comments ha!	Jingdong prefecture level city in Shandong
Stowe Flagship Store	a power saver (electronics)	My friend recommended to me, said the effect is very good, I bought two, this month less money, really can, just buy I do not believe, but has proved to have effect	Jingdong prefecture level city in Shandong
Stowe Flagship Store	a power saver (electronics)	Just bought back, already used, these days to see the effect of it, and so have the effect will also come back to give positive feedback, the price is also super cheap!	Jingdong prefecture level city in Shandong
Stowe Flagship Store	a power saver (electronics)	It does work well, the store uses a lot of electricity, this time to buy back a few, each place that uses a lot of electricity to insert one. Summer is here, the electricity is too strong.	Jingdong prefecture level city in Shandong
Stowe Flagship Store	a power saver (electronics)	Power saving treasure received, as for the effect, according to the instructions written, a short period of time can not be seen, at least 2-3 months before the real effect, then follow up evaluation	Jingdong prefecture level city in Shandong
Stowe Flagship Store	a power saver (electronics)	The goods have just been received and have not been used yet looking forward to the results.	Jingdong prefecture level city in Shandong
Stowe Flagship Store	a power saver (electronics)	Good installation works great	Jingdong prefecture level city in Shandong
Stowe Flagship Store	a power saver (electronics)	The logistics is super super fast! The quality is also very good, indeed value for money	Jingdong prefecture level city in Shandong
Stowe Flagship Store	a power saver (electronics)	Has been used for a period of time before coming to comment on, really good, air conditioning a day open non-stop, pay the electricity bill when the heartache, with this, the home electricity is not at all worried about the bill out of contrast to save a lot of preparation in the hands of a few, each room An a	Jingdong prefecture level city in Shandong
Stowe Flagship Store	a power saver (electronics)	Saving device received, really good, with the air conditioning tried, an hour without a saver with 2.9 degrees, with a saver an hour with 1.1 degrees, the beginning of not quite believe that this thing, the actual test to know that it is really useful, and intend to buy another.	Jingdong prefecture level city in Shandong
Stowe Flagship Store	a power saver (electronics)	Power saving effect: this energy saver is still useful, day trial for three days, every day than not installed before the reduction of two dollars a day, every day to open the bedroom to open the air conditioning before fifteen dollars now thirteen dollars a day. Still a little use.	Jingdong prefecture level city in Shandong

Stowe Flagship Store	a power saver (electronics)	Originally bought for home use, used for two months, the electricity bill from more than 200 yuan per month, down to more than 140 yuan, there is indeed a power-saving effect, this time and sold two, the unit with. Merchant service attitude is really good.	Jingdong prefecture level city in Shandong
Stowe Flagship Store	a power saver (electronics)	Plug and play, stable voltage, the screen shows when working, this winter home appliances, need electricity in many places, just buy to use, save electricity and money, with instructions	Jingdong prefecture level city in Shandong
Stowe Flagship Store	a power saver (electronics)	Goods received, do not know if it is good. When I bought it, I saw that it was a Stowe brand or company's product. Received but not. I do not know what is the reason. Now I can only give this evaluation. I'm	Jingdong prefecture level city in Shandong
Stowe Flagship Store	a power saver (electronics)	The first time to buy online, but also look at the evaluation before buying, the quality is not bad, but also very cheap, the need for friends can rest assured to buy!	Jingdong prefecture level city in Shandong
Stowe Flagship Store	a power saver (electronics)	🌈 Great quality, bought it to my liking 💕 Awesome! ✨ Buy with confidence if you need it!	Jingdong prefecture level city in Shandong
Stowe Flagship Store	a power saver (electronics)	The quality is very good, completely exceeded the expectations, the delivery speed is very fast, the packaging is very careful and strict, very satisfied with a shopping	Jingdong prefecture level city in Shandong
Stowe Flagship Store	a power saver (electronics)	The effect is very good, the home uses less electricity is very energy efficient, cost-effective	Jingdong prefecture level city in Shandong
Stowe Flagship Store	a power saver (electronics)	Quality can't be beat, just like the store, especially like, and the price is favorable!	Jingdong prefecture level city in Shandong
Stowe Flagship Store	a power saver (electronics)	Baby received! Very unexpected, will be back next time ~ great seller, stay tuned!	Jingdong prefecture level city in Shandong
Stowe Flagship Store	a power saver (electronics)	Follow up review after a month is more real, waiting for it	Jingdong prefecture level city in Shandong
Stowe Flagship Store	a power saver (electronics)	It is the ideal product, good quality and fast logistics, worthy of praise!	Jingdong prefecture level city in Shandong
Stowe Flagship Store	a power saver (electronics)	The saver is great, the color is right, the installer is very professional	Jingdong prefecture level city in Shandong
Stowe Flagship Store	a power saver (electronics)	The effect of the power connection is particularly obvious, it is particularly easy to use, very satisfied, the	Jingdong prefecture level city in Shandong
Stowe Flagship Store	a power saver (electronics)	Goods are very energy-saving, used to be more than two hundred degrees, now there are only a hundred or so degrees	Jingdong prefecture level city in Shandong
Stowe Flagship Store	a power saver (electronics)	Good quality, fast shipping, good results, good value for money	Jingdong prefecture level city in Shandong
Stowe Flagship Store	a power saver (electronics)	Fast shipping, fine workmanship, very good, cost-effective	Jingdong prefecture level city in Shandong
Stowe Flagship Store	a power saver (electronics)	The meter is as described, the quality is quite good, energy-saving, worth buying oh~!	Jingdong prefecture level city in Shandong
Stowe Flagship Store	a power saver (electronics)	Great value for money, very affordable,, perfect . ˆ◡ˆ.	Jingdong prefecture level city in Shandong
Stowe Flagship Store	a power saver (electronics)	Good!!! Worth recommending!	Jingdong prefecture level city in Shandong
Stowe Flagship Store	a power saver (electronics)	Great, fast logistics, great stuff, great value for money!	Jingdong prefecture level city in Shandong
Stowe Flagship Store	a power saver (electronics)	Fast logistics, very satisfied with the quality upon receipt	Jingdong prefecture level city in Shandong
Stowe Flagship Store	a power saver (electronics)	Great quality, plug and play, fast shipping!	Jingdong prefecture level city in Shandong
Stowe Flagship Store	a power saver (electronics)	Logistics received, quite satisfied	Jingdong prefecture level city in Shandong
Stowe Flagship Store	a power saver (electronics)	I don't know if it really works	Jingdong prefecture level city in Shandong
Stowe Flagship Store	a power saver (electronics)	It's bullshit. It's a plug light. Don't talk about power savings.	Jingdong prefecture level city in Shandong
Stowe Flagship Store	a power saver (electronics)	Fake and deceitful stuff that hasn't changed at all.	Jingdong prefecture level city in Shandong
Stowe Flagship Store	a power saver (electronics)	Power-saving effect: power-saving effect is particularly obvious Cost-effective: cost-effective business Appearance and style: external small micro-work materials: fine workmanship Experience: use very good, non the first convenient	Jingdong prefecture level city in Shandong
Stowe Flagship Store	a power saver (electronics)	After using it for a few days, it feels quite good, it should be quite safe, and it also sends a plug, the merchant is good, wait for a period of time and then calculate whether it saves on the electricity bill and then add comments ha!	Jingdong prefecture level city in Shandong
Stowe Flagship Store	a power saver (electronics)	My friend recommended to me, said the effect is very good, I bought two, this month less money, really can, just buy I do not believe, but has proved to have effect	Jingdong prefecture level city in Shandong
Stowe Flagship Store	a power saver (electronics)	Just bought back, already used, these days to see the effect of it, and so have the effect will also come back to give positive feedback, the price is also super cheap!	Jingdong prefecture level city in Shandong
Stowe Flagship Store	a power saver (electronics)	It does work well, the store uses a lot of electricity, this time to buy back a few, each place that uses a lot of electricity to insert one. Summer is here, the electricity is too strong.	Jingdong prefecture level city in Shandong
Stowe Flagship Store	a power saver (electronics)	Power saving treasure received, as for the effect, according to the instructions written, a short period of time can not be seen, at least 2-3 months before the real effect, then follow up evaluation	Jingdong prefecture level city in Shandong
Stowe Flagship Store	a power saver (electronics)	The goods have just been received and have not been used yet looking forward to the results.	Jingdong prefecture level city in Shandong
Stowe Flagship Store	a power saver (electronics)	Good installation works great	Jingdong prefecture level city in Shandong
Stowe Flagship Store	a power saver (electronics)	The logistics is super super fast! The quality is also very good, indeed value for money	Jingdong prefecture level city in Shandong
Stowe Flagship Store	a power saver (electronics)	Has been used for a period of time before coming to comment on, really good, air conditioning a day open non-stop, pay the electricity bill when the heartache, with this, the home electricity is not at all worried about the bill out of contrast to save a lot of preparation in the hands of a few, each room An a	Jingdong prefecture level city in Shandong
Stowe Flagship Store	a power saver (electronics)	Saving device received, really good, with the air conditioning tried, an hour without a saver with 2.9 degrees, with a saver an hour with 1.1 degrees, the beginning of not quite believe that this thing, the actual test to know that it is really useful, and intend to buy another.	Jingdong prefecture level city in Shandong
Stowe Flagship Store	a power saver (electronics)	Power saving effect: this energy saver is still useful, day trial for three days, every day than not installed before the reduction of two dollars a day, every day to open the bedroom to open the air conditioning before fifteen dollars now thirteen dollars a day. Still a little use.	Jingdong prefecture level city in Shandong
Stowe Flagship Store	a power saver (electronics)	Originally bought for home use, used for two months, the electricity bill from more than 200 yuan per month, down to more than 140 yuan, there is indeed a power-saving effect, this time and sold two, the unit with. Merchant service attitude is really good.	Jingdong prefecture level city in Shandong
Stowe Flagship Store	a power saver (electronics)	Plug and play, stable voltage, the screen shows when working, this winter home appliances, need electricity in many places, just buy to use, save electricity and money, with instructions	Jingdong prefecture level city in Shandong
Stowe Flagship Store	a power saver (electronics)	Goods received, do not know if it is good. When I bought it, I saw that it was a Stowe brand or company's product. Received but not. I do not know what is the reason. Now I can only give this evaluation. I'm	Jingdong prefecture level city in Shandong
Stowe Flagship Store	a power saver (electronics)	The first time to buy online, but also look at the evaluation before buying, the quality is not bad, but also very cheap, the need for friends can rest assured to buy!	Jingdong prefecture level city in Shandong
Stowe Flagship Store	a power saver (electronics)	🌈 Great quality, bought it to my liking 💕 Awesome! ✨ Buy with confidence if you need it!	Jingdong prefecture level city in Shandong
Stowe Flagship Store	a power saver (electronics)	The quality is very good, completely exceeded the expectations, the delivery speed is very fast, the packaging is very careful and strict, very satisfied with a shopping	Jingdong prefecture level city in Shandong
Stowe Flagship Store	a power saver (electronics)	The effect is very good, the home uses less electricity is very energy efficient, cost-effective	Jingdong prefecture level city in Shandong
Stowe Flagship Store	a power saver (electronics)	Quality can't be beat, just like the store, especially like, and the price is favorable!	Jingdong prefecture level city in Shandong
Stowe Flagship Store	a power saver (electronics)	Baby received! Very unexpected, will be back next time ~ great seller, stay tuned!	Jingdong prefecture level city in Shandong
Stowe Flagship Store	a power saver (electronics)	Follow up review after a month is more real, waiting for it	Jingdong prefecture level city in Shandong

Stowe Flagship Store	a power saver (electronics)	It is the ideal product, good quality and fast logistics, worthy of praise!	Jingdong prefecture level city in Shandong
Stowe Flagship Store	a power saver (electronics)	The saver is great, the color is right, the installer is very professional	Jingdong prefecture level city in Shandong
Stowe Flagship Store	a power saver (electronics)	The effect of the power connection is particularly obvious, it is particularly easy to use, very satisfied, the	Jingdong prefecture level city in Shandong
Stowe Flagship Store	a power saver (electronics)	Goods are very energy-saving, used to be more than two hundred degrees, now there are only a hundred or so degrees	Jingdong prefecture level city in Shandong
Stowe Flagship Store	a power saver (electronics)	Good quality, fast shipping, good results, good value for money	Jingdong prefecture level city in Shandong
Stowe Flagship Store	a power saver (electronics)	Fast shipping, fine workmanship, very good, cost-effective	Jingdong prefecture level city in Shandong
Stowe Flagship Store	a power saver (electronics)	The meter is as described, the quality is quite good, energy-saving, worth buying oh~!	Jingdong prefecture level city in Shandong
Stowe Flagship Store	a power saver (electronics)	Great value for money, very affordable,, perfect . ˆ◡ˆ.	Jingdong prefecture level city in Shandong
Stowe Flagship Store	a power saver (electronics)	Good!!! Worth recommending!	Jingdong prefecture level city in Shandong
Stowe Flagship Store	a power saver (electronics)	Great, fast logistics, great stuff, great value for money!	Jingdong prefecture level city in Shandong
Stowe Flagship Store	a power saver (electronics)	Fast logistics, very satisfied with the quality upon receipt	Jingdong prefecture level city in Shandong
Stowe Flagship Store	a power saver (electronics)	Great quality, plug and play, fast shipping!	Jingdong prefecture level city in Shandong
Stowe Flagship Store	a power saver (electronics)	Logistics received, quite satisfied	Jingdong prefecture level city in Shandong
Stowe Flagship Store	a power saver (electronics)	I don't know if it really works	Jingdong prefecture level city in Shandong
Stowe Flagship Store	a power saver (electronics)	It's bullshit. It's a plug light. Don't talk about power savings.	Jingdong prefecture level city in Shandong
Stowe Flagship Store	a power saver (electronics)	Fake and deceitful stuff that hasn't changed at all.	Jingdong prefecture level city in Shandong
Stowe Flagship Store	a power saver (electronics)	Power-saving effect: power-saving effect is particularly obvious Cost-effective: cost-effective business Appearance and style: external small micro-work materials: fine workmanship Experience: use very good, non the first convenient	Jingdong prefecture level city in Shandong
Stowe Flagship Store	a power saver (electronics)	After using it for a few days, it feels quite good, it should be quite safe, and it also sends a plug, the merchant is good, wait for a period of time and then calculate whether it saves on the electricity bill and then add comments ha!	Jingdong prefecture level city in Shandong
Stowe Flagship Store	a power saver (electronics)	My friend recommended to me, said the effect is very good, I bought two, this month less money, really can, just buy I do not believe, but has proved to have effect	Jingdong prefecture level city in Shandong
Stowe Flagship Store	a power saver (electronics)	Just bought back, already used, these days to see the effect of it, and so have the effect will also come back to give positive feedback, the price is also super cheap!	Jingdong prefecture level city in Shandong
Stowe Flagship Store	a power saver (electronics)	It does work well, the store uses a lot of electricity, this time to buy back a few, each place that uses a lot of electricity to insert one. Summer is here, the electricity is too strong.	Jingdong prefecture level city in Shandong
Stowe Flagship Store	a power saver (electronics)	Power saving treasure received, as for the effect, according to the instructions written, a short period of time can not be seen, at least 2-3 months before the real effect, then follow up evaluation	Jingdong prefecture level city in Shandong
Stowe Flagship Store	a power saver (electronics)	The goods have just been received and have not been used yet looking forward to the results.	Jingdong prefecture level city in Shandong
Stowe Flagship Store	a power saver (electronics)	Good installation works great	Jingdong prefecture level city in Shandong
Stowe Flagship Store	a power saver (electronics)	The logistics is super super fast! The quality is also very good, indeed value for money	Jingdong prefecture level city in Shandong
Stowe Flagship Store	a power saver (electronics)	Has been used for a period of time before coming to comment on, really good, air conditioning a day open non-stop, pay the electricity bill when the heartache, with this, the home electricity is not at all worried about the bill out of contrast to save a lot of preparation in the hands of a few, each room An a	Jingdong prefecture level city in Shandong
Stowe Flagship Store	a power saver (electronics)	Saving device received, really good, with the air conditioning tried, an hour without a saver with 2.9 degrees, with a saver an hour with 1.1 degrees, the beginning of not quite believe that this thing, the actual test to know that it is really useful, and intend to buy another.	Jingdong prefecture level city in Shandong
Stowe Flagship Store	a power saver (electronics)	Power saving effect: this energy saver is still useful, day trial for three days, every day than not installed before the reduction of two dollars a day, every day to open the bedroom to open the air conditioning before fifteen dollars now thirteen dollars a day. Still a little use.	Jingdong prefecture level city in Shandong
Stowe Flagship Store	a power saver (electronics)	Originally bought for home use, used for two months, the electricity bill from more than 200 yuan per month, down to more than 140 yuan, there is indeed a power-saving effect, this time and sold two, the unit with. Merchant service attitude is really good.	Jingdong prefecture level city in Shandong
Stowe Flagship Store	a power saver (electronics)	Plug and play, stable voltage, the screen shows when working, this winter home appliances, need electricity in many places, just buy to use, save electricity and money, with instructions	Jingdong prefecture level city in Shandong
Stowe Flagship Store	a power saver (electronics)	Goods received, do not know if it is good. When I bought it, I saw that it was a Stowe brand or company's product. Received but not. I do not know what is the reason. Now I can only give this evaluation. I'm	Jingdong prefecture level city in Shandong
Stowe Flagship Store	a power saver (electronics)	The first time to buy online, but also look at the evaluation before buying, the quality is not bad, but also very cheap, the need for friends can rest assured to buy!	Jingdong prefecture level city in Shandong
Stowe Flagship Store	a power saver (electronics)	🌈 Great quality, bought it to my liking 💞 Awesome! ✨ Buy with confidence if you need it!	Jingdong prefecture level city in Shandong
Stowe Flagship Store	a power saver (electronics)	The quality is very good, completely exceeded the expectations, the delivery speed is very fast, the packaging is very careful and strict, very satisfied with a shopping	Jingdong prefecture level city in Shandong
Stowe Flagship Store	a power saver (electronics)	The effect is very good, the home uses less electricity is very energy efficient, cost-effective	Jingdong prefecture level city in Shandong
Stowe Flagship Store	a power saver (electronics)	Quality can't be beat, just like the store, especially like, and the price is favorable!	Jingdong prefecture level city in Shandong
Stowe Flagship Store	a power saver (electronics)	Baby received! Very unexpected, will be back next time ~ great seller, stay tuned!	Jingdong prefecture level city in Shandong
Stowe Flagship Store	a power saver (electronics)	Follow up review after a month is more real, waiting for it	Jingdong prefecture level city in Shandong
Stowe Flagship Store	a power saver (electronics)	It is the ideal product, good quality and fast logistics, worthy of praise!	Jingdong prefecture level city in Shandong
Stowe Flagship Store	a power saver (electronics)	The saver is great, the color is right, the installer is very professional	Jingdong prefecture level city in Shandong
Stowe Flagship Store	a power saver (electronics)	The effect of the power connection is particularly obvious, it is particularly easy to use, very satisfied, the	Jingdong prefecture level city in Shandong
Stowe Flagship Store	a power saver (electronics)	Goods are very energy-saving, used to be more than two hundred degrees, now there are only a hundred or so degrees	Jingdong prefecture level city in Shandong
Stowe Flagship Store	a power saver (electronics)	Good quality, fast shipping, good results, good value for money	Jingdong prefecture level city in Shandong
Stowe Flagship Store	a power saver (electronics)	Fast shipping, fine workmanship, very good, cost-effective	Jingdong prefecture level city in Shandong
Stowe Flagship Store	a power saver (electronics)	The meter is as described, the quality is quite good, energy-saving, worth buying oh~!	Jingdong prefecture level city in Shandong
Stowe Flagship Store	a power saver (electronics)	Great value for money, very affordable,, perfect . ˆ◡ˆ.	Jingdong prefecture level city in Shandong
Stowe Flagship Store	a power saver (electronics)	Good!!! Worth recommending!	Jingdong prefecture level city in Shandong
Stowe Flagship Store	a power saver (electronics)	Great, fast logistics, great stuff, great value for money!	Jingdong prefecture level city in Shandong

Stowe Flagship Store	a power saver (electronics)	Fast logistics, very satisfied with the quality upon receipt		Jingdong prefecture level city in Shandong
Stowe Flagship Store	a power saver (electronics)	Great quality, plug and play, fast shipping!		Jingdong prefecture level city in Shandong
Stowe Flagship Store	a power saver (electronics)	Logistics received, quite satisfied		Jingdong prefecture level city in Shandong
Stowe Flagship Store	a power saver (electronics)	I don't know if it really works		Jingdong prefecture level city in Shandong
Stowe Flagship Store	a power saver (electronics)	It's bullshit. It's a plug light. Don't talk about power savings.		Jingdong prefecture level city in Shandong
Stowe Flagship Store	a power saver (electronics)	Fake and deceitful stuff that hasn't changed at all.		Jingdong prefecture level city in Shandong
Stowe Flagship Store	a power saver (electronics)	Power-saving effect: power-saving effect is particularly obvious Cost-effective: cost-effective business Appearance and style: external small micro-work materials: fine workmanship Experience: use very good, non the first convenient		Jingdong prefecture level city in Shandong
Stowe Flagship Store	a power saver (electronics)	After using it for a few days, it feels quite good, it should be quite safe, and it also sends a plug, the merchant is good, wait for a period of time and then calculate whether it saves on the electricity bill and then add comments ha!		Jingdong prefecture level city in Shandong
Stowe Flagship Store	a power saver (electronics)	My friend recommended to me, said the effect is very good, I bought two, this month less money, really can, just buy I do not believe, but has proved to have effect		Jingdong prefecture level city in Shandong
Stowe Flagship Store	a power saver (electronics)	Just bought back, already used, these days to see the effect of it, and so have the effect will also come back to give positive feedback, the price is also super cheap!		Jingdong prefecture level city in Shandong
Stowe Flagship Store	a power saver (electronics)	It does work well, the store uses a lot of electricity, this time to buy back a few, each place that uses a lot of electricity to insert one. Summer is here, the electricity is too strong.		Jingdong prefecture level city in Shandong
Stowe Flagship Store	a power saver (electronics)	Power saving treasure received, as for the effect, according to the instructions written, a short period of time can not be seen, at least 2-3 months before the real effect, then follow up evaluation		Jingdong prefecture level city in Shandong
Stowe Flagship Store	a power saver (electronics)	The goods have just been received and have not been used yet looking forward to the results.		Jingdong prefecture level city in Shandong
Stowe Flagship Store	a power saver (electronics)	Good installation works great		Jingdong prefecture level city in Shandong
Stowe Flagship Store	a power saver (electronics)	The logistics is super super fast! The quality is also very good, indeed value for money		Jingdong prefecture level city in Shandong
Stowe Flagship Store	a power saver (electronics)	Has been used for a period of time before coming to comment on, really good, air conditioning a day open non-stop, pay the electricity bill when the heartache, with this, the home electricity is not at all worried about the bill out of contrast to save a lot of preparation in the hands of a few, each room An a		Jingdong prefecture level city in Shandong
Stowe Flagship Store	a power saver (electronics)	Saving device received, really good, with the air conditioning tried, an hour without a saver with 2.9 degrees, with a saver an hour with 1.1 degrees, the beginning of not quite believe that this thing, the actual test to know that it is really useful, and intend to buy another.		Jingdong prefecture level city in Shandong
Stowe Flagship Store	a power saver (electronics)	Power saving effect: this energy saver is still useful, day trial for three days, every day than not installed before the reduction of two dollars a day, every day to open the bedroom to open the air conditioning before fifteen dollars now thirteen dollars a day. Still a little use.		Jingdong prefecture level city in Shandong
Stowe Flagship Store	a power saver (electronics)	Originally bought for home use, used for two months, the electricity bill from more than 200 yuan per month, down to more than 140 yuan, there is indeed a power-saving effect, this time and sold two, the unit with. Merchant service attitude is really good.		Jingdong prefecture level city in Shandong
Stowe Flagship Store	a power saver (electronics)	Plug and play, stable voltage, the screen shows when working, this winter home appliances, need electricity in many places, just buy to use, save electricity and money, with instructions		Jingdong prefecture level city in Shandong
Stowe Flagship Store	a power saver (electronics)	Goods received, do not know if it is good. When I bought it, I saw that it was a Stowe brand or company's product. Received but not. I do not know what is the reason. Now I can only give this evaluation. I'm		Jingdong prefecture level city in Shandong
Stowe Flagship Store	a power saver (electronics)	The first time to buy online, but also look at the evaluation before buying, the quality is not bad, but also very cheap, the need for friends can rest assured to buy!		Jingdong prefecture level city in Shandong
Stowe Flagship Store	a power saver (electronics)	🌈 Great quality, bought it to my liking 💝 Awesome! ✨ Buy with confidence if you need it!		Jingdong prefecture level city in Shandong
Stowe Flagship Store	a power saver (electronics)	The quality is very good, completely exceeded the expectations, the delivery speed is very fast, the packaging is very careful and strict, very satisfied with a shopping		Jingdong prefecture level city in Shandong
Stowe Flagship Store	a power saver (electronics)	The effect is very good, the home uses less electricity is very energy efficient, cost-effective		Jingdong prefecture level city in Shandong
Stowe Flagship Store	a power saver (electronics)	Quality can't be beat, just like the store, especially like, and the price is favorable!		Jingdong prefecture level city in Shandong
Stowe Flagship Store	a power saver (electronics)	Baby received! Very unexpected, will be back next time ~ great seller, stay tuned!		Jingdong prefecture level city in Shandong
Stowe Flagship Store	a power saver (electronics)	Follow up review after a month is more real, waiting for it		Jingdong prefecture level city in Shandong
Stowe Flagship Store	a power saver (electronics)	It is the ideal product, good quality and fast logistics, worthy of praise!		Jingdong prefecture level city in Shandong
Stowe Flagship Store	a power saver (electronics)	The saver is great, the color is right, the installer is very professional		Jingdong prefecture level city in Shandong
Stowe Flagship Store	a power saver (electronics)	The effect of the power connection is particularly obvious, it is particularly easy to use, very satisfied, the		Jingdong prefecture level city in Shandong
Stowe Flagship Store	a power saver (electronics)	Goods are very energy-saving, used to be more than two hundred degrees, now there are only a hundred or so degrees		Jingdong prefecture level city in Shandong
Stowe Flagship Store	a power saver (electronics)	Good quality, fast shipping, good results, good value for money		Jingdong prefecture level city in Shandong
Stowe Flagship Store	a power saver (electronics)	Fast shipping, fine workmanship, very good, cost-effective		Jingdong prefecture level city in Shandong
Stowe Flagship Store	a power saver (electronics)	The meter is as described, the quality is quite good, energy-saving, worth buying oh~!		Jingdong prefecture level city in Shandong
Stowe Flagship Store	a power saver (electronics)	Great value for money, very affordable,, perfect . ^◡^.		Jingdong prefecture level city in Shandong
Stowe Flagship Store	a power saver (electronics)	Good!!! Worth recommending!		Jingdong prefecture level city in Shandong
Stowe Flagship Store	a power saver (electronics)	Great, fast logistics, great stuff, great value for money!		Jingdong prefecture level city in Shandong
Stowe Flagship Store	a power saver (electronics)	Fast logistics, very satisfied with the quality upon receipt		Jingdong prefecture level city in Shandong
Stowe Flagship Store	a power saver (electronics)	Great quality, plug and play, fast shipping!		Jingdong prefecture level city in Shandong
Stowe Flagship Store	a power saver (electronics)	Logistics received, quite satisfied		Jingdong prefecture level city in Shandong
Stowe Flagship Store	a power saver (electronics)	I don't know if it really works		Jingdong prefecture level city in Shandong
Stowe Flagship Store	a power saver (electronics)	It's bullshit. It's a plug light. Don't talk about power savings.		Jingdong prefecture level city in Shandong
Stowe Flagship Store	a power saver (electronics)	Fake and deceitful stuff that hasn't changed at all.		Jingdong prefecture level city in Shandong
Stowe Flagship Store	a power saver (electronics)	Power-saving effect: power-saving effect is particularly obvious Cost-effective: cost-effective business Appearance and style: external small micro-work materials: fine workmanship Experience: use very good, non the first convenient		Jingdong prefecture level city in Shandong
Stowe Flagship Store	a power saver (electronics)	After using it for a few days, it feels quite good, it should be quite safe, and it also sends a plug, the merchant is good, wait for a period of time and then calculate whether it saves on the electricity bill and then add comments ha!		Jingdong prefecture level city in Shandong
Stowe Flagship Store	a power saver (electronics)	My friend recommended to me, said the effect is very good, I bought two, this month less money, really can, just buy I do not believe, but has proved to have effect		Jingdong prefecture level city in Shandong

Stowe Flagship Store	a power saver (electronics)	Just bought back, already used, these days to see the effect of it, and so have the effect will also come back to give positive feedback, the price is also super cheap!	Jingdong prefecture level city in Shandong
Stowe Flagship Store	a power saver (electronics)	It does work well, the store uses a lot of electricity, this time to buy back a few, each place that uses a lot of electricity to insert one. Summer is here, the electricity is too strong.	Jingdong prefecture level city in Shandong
Stowe Flagship Store	a power saver (electronics)	Power saving treasure received, as for the effect, according to the instructions written, a short period of time can not be seen, at least 2-3 months before the real effect, then follow up evaluation	Jingdong prefecture level city in Shandong
Stowe Flagship Store	a power saver (electronics)	The goods have just been received and have not been used yet looking forward to the results.	Jingdong prefecture level city in Shandong
Stowe Flagship Store	a power saver (electronics)	Good installation works great	Jingdong prefecture level city in Shandong
Stowe Flagship Store	a power saver (electronics)	The logistics is super super fast! The quality is also very good, indeed value for money	Jingdong prefecture level city in Shandong
Stowe Flagship Store	a power saver (electronics)	Has been used for a period of time before coming to comment on, really good, air conditioning a day open non-stop, pay the electricity bill when the heartache, with this, the home electricity is not at all worried about the bill out of contrast to save a lot of preparation in the hands of a few, each room An a	Jingdong prefecture level city in Shandong
Stowe Flagship Store	a power saver (electronics)	Saving device received, really good, with the air conditioning tried, an hour without a saver with 2.9 degrees, with a saver an hour with 1.1 degrees, the beginning of not quite believe that this thing, the actual test to know that it is really useful, and intend to buy another.	Jingdong prefecture level city in Shandong
Stowe Flagship Store	a power saver (electronics)	Power saving effect: this energy saver is still useful, day trial for three days, every day than not installed before the reduction of two dollars a day, every day to open the bedroom to open the air conditioning before fifteen dollars now thirteen dollars a day. Still a little use.	Jingdong prefecture level city in Shandong
Stowe Flagship Store	a power saver (electronics)	Originally bought for home use, used for two months, the electricity bill from more than 200 yuan per month, down to more than 140 yuan, there is indeed a power-saving effect, this time and sold two, the unit with. Merchant service attitude is really good.	Jingdong prefecture level city in Shandong
Stowe Flagship Store	a power saver (electronics)	Plug and play, stable voltage, the screen shows when working, this winter home appliances, need electricity in many places, just buy to use, save electricity and money, with instructions	Jingdong prefecture level city in Shandong
Stowe Flagship Store	a power saver (electronics)	Goods received, do not know if it is good. When I bought it, I saw that it was a Stowe brand or company's product. Received but not. I do not know what is the reason. Now I can only give this evaluation. I'm	Jingdong prefecture level city in Shandong
Stowe Flagship Store	a power saver (electronics)	The first time to buy online, but also look at the evaluation before buying, the quality is not bad, but also very cheap, the need for friends can rest assured to buy!	Jingdong prefecture level city in Shandong
Stowe Flagship Store	a power saver (electronics)	🌈 Great quality, bought it to my liking 💞 Awesome! ✨ Buy with confidence if you need it!	Jingdong prefecture level city in Shandong
Stowe Flagship Store	a power saver (electronics)	The quality is very good, completely exceeded the expectations, the delivery speed is very fast, the packaging is very careful and strict, very satisfied with a shopping	Jingdong prefecture level city in Shandong
Stowe Flagship Store	a power saver (electronics)	The effect is very good, the home uses less electricity is very energy efficient, cost-effective	Jingdong prefecture level city in Shandong
Stowe Flagship Store	a power saver (electronics)	Quality can't be beat, just like the store, especially like, and the price is favorable!	Jingdong prefecture level city in Shandong
Stowe Flagship Store	a power saver (electronics)	Baby received! Very unexpected, will be back next time ~ great seller, stay tuned!	Jingdong prefecture level city in Shandong
Stowe Flagship Store	a power saver (electronics)	Follow up review after a month is more real, waiting for it	Jingdong prefecture level city in Shandong
Stowe Flagship Store	a power saver (electronics)	It is the ideal product, good quality and fast logistics, worthy of praise!	Jingdong prefecture level city in Shandong
Stowe Flagship Store	a power saver (electronics)	The saver is great, the color is right, the installer is very professional	Jingdong prefecture level city in Shandong
Stowe Flagship Store	a power saver (electronics)	The effect of the power connection is particularly obvious, it is particularly easy to use, very satisfied, the	Jingdong prefecture level city in Shandong
Stowe Flagship Store	a power saver (electronics)	Goods are very energy-saving, used to be more than two hundred degrees, now there are only a hundred or so degrees	Jingdong prefecture level city in Shandong
Stowe Flagship Store	a power saver (electronics)	Good quality, fast shipping, good results, good value for money	Jingdong prefecture level city in Shandong
Stowe Flagship Store	a power saver (electronics)	Fast shipping, fine workmanship, very good, cost-effective	Jingdong prefecture level city in Shandong
Stowe Flagship Store	a power saver (electronics)	The meter is as described, the quality is quite good, energy-saving, worth buying oh~!	Jingdong prefecture level city in Shandong
Stowe Flagship Store	a power saver (electronics)	Great value for money, very affordable,, perfect . ˆ◡ˆ.	Jingdong prefecture level city in Shandong
Stowe Flagship Store	a power saver (electronics)	Good!!! Worth recommending!	Jingdong prefecture level city in Shandong
Stowe Flagship Store	a power saver (electronics)	Great, fast logistics, great stuff, great value for money!	Jingdong prefecture level city in Shandong
Stowe Flagship Store	a power saver (electronics)	Fast logistics, very satisfied with the quality upon receipt	Jingdong prefecture level city in Shandong
Stowe Flagship Store	a power saver (electronics)	Great quality, plug and play, fast shipping!	Jingdong prefecture level city in Shandong
Stowe Flagship Store	a power saver (electronics)	Logistics received, quite satisfied	Jingdong prefecture level city in Shandong
Stowe Flagship Store	a power saver (electronics)	I don't know if it really works	Jingdong prefecture level city in Shandong
Stowe Flagship Store	a power saver (electronics)	It's bullshit. It's a plug light. Don't talk about power savings.	Jingdong prefecture level city in Shandong
Stowe Flagship Store	a power saver (electronics)	Fake and deceitful stuff that hasn't changed at all.	Jingdong prefecture level city in Shandong
Stowe Flagship Store	a power saver (electronics)	Power-saving effect: power-saving effect is particularly obvious Cost-effective: cost-effective business Appearance and style: external small micro-work materials: fine workmanship Experience: use very good, non the first convenient	Jingdong prefecture level city in Shandong
Stowe Flagship Store	a power saver (electronics)	After using it for a few days, it feels quite good, it should be quite safe, and it also sends a plug, the merchant is good, wait for a period of time and then calculate whether it saves on the electricity bill and then add comments ha!	Jingdong prefecture level city in Shandong
Stowe Flagship Store	a power saver (electronics)	My friend recommended to me, said the effect is very good, I bought two, this month less money, really can, just buy I do not believe, but has proved to have effect	Jingdong prefecture level city in Shandong
Stowe Flagship Store	a power saver (electronics)	Just bought back, already used, these days to see the effect of it, and so have the effect will also come back to give positive feedback, the price is also super cheap!	Jingdong prefecture level city in Shandong
Stowe Flagship Store	a power saver (electronics)	It does work well, the store uses a lot of electricity, this time to buy back a few, each place that uses a lot of electricity to insert one. Summer is here, the electricity is too strong.	Jingdong prefecture level city in Shandong
Stowe Flagship Store	a power saver (electronics)	Power saving treasure received, as for the effect, according to the instructions written, a short period of time can not be seen, at least 2-3 months before the real effect, then follow up evaluation	Jingdong prefecture level city in Shandong
Stowe Flagship Store	a power saver (electronics)	The goods have just been received and have not been used yet looking forward to the results.	Jingdong prefecture level city in Shandong
Stowe Flagship Store	a power saver (electronics)	Good installation works great	Jingdong prefecture level city in Shandong
Stowe Flagship Store	a power saver (electronics)	The logistics is super super fast! The quality is also very good, indeed value for money	Jingdong prefecture level city in Shandong
Stowe Flagship Store	a power saver (electronics)	Has been used for a period of time before coming to comment on, really good, air conditioning a day open non-stop, pay the electricity bill when the heartache, with this, the home electricity is not at all worried about the bill out of contrast to save a lot of preparation in the hands of a few, each room An a	Jingdong prefecture level city in Shandong
Stowe Flagship Store	a power saver (electronics)	Saving device received, really good, with the air conditioning tried, an hour without a saver with 2.9 degrees, with a saver an hour with 1.1 degrees, the beginning of not quite believe that this thing, the actual test to know that it is really useful, and intend to buy another.	Jingdong prefecture level city in Shandong
Stowe Flagship Store	a power saver (electronics)	Power saving effect: this energy saver is still useful, day trial for three days, every day than not installed before the reduction of two dollars a day, every day to open the bedroom to open the air conditioning before fifteen dollars now thirteen dollars a day. Still a little use.	Jingdong prefecture level city in Shandong

Stowe Flagship Store	a power saver (electronics)	Originally bought for home use, used for two months, the electricity bill from more than 200 yuan per month, down to more than 140 yuan, there is indeed a power-saving effect, this time and sold two, the unit with. Merchant service attitude is really good.	Jingdong prefecture level city in Shandong
Stowe Flagship Store	a power saver (electronics)	Plug and play, stable voltage, the screen shows when working, this winter home appliances, need electricity in many places, just buy to use, save electricity and money, with instructions	Jingdong prefecture level city in Shandong
Stowe Flagship Store	a power saver (electronics)	Goods received, do not know if it is good. When I bought it, I saw that it was a Stowe brand or company's product. Received but not. I do not know what is the reason. Now I can only give this evaluation. I'm	Jingdong prefecture level city in Shandong
Stowe Flagship Store	a power saver (electronics)	The first time to buy online, but also look at the evaluation before buying, the quality is not bad, but also very cheap, the need for friends can rest assured to buy!	Jingdong prefecture level city in Shandong
Stowe Flagship Store	a power saver (electronics)	Great quality, bought it to my liking Awesome! Buy with confidence if you need it!	Jingdong prefecture level city in Shandong
Stowe Flagship Store	a power saver (electronics)	The quality is very good, completely exceeded the expectations, the delivery speed is very fast, the packaging is very careful and strict, very satisfied with a shopping	Jingdong prefecture level city in Shandong
Stowe Flagship Store	a power saver (electronics)	The effect is very good, the home uses less electricity is very energy efficient, cost-effective	Jingdong prefecture level city in Shandong
Stowe Flagship Store	a power saver (electronics)	Quality can't be beat, just like the store, especially like, and the price is favorable!	Jingdong prefecture level city in Shandong
Stowe Flagship Store	a power saver (electronics)	Baby received! Very unexpected, will be back next time ~ great seller, stay tuned!	Jingdong prefecture level city in Shandong
Stowe Flagship Store	a power saver (electronics)	Follow up review after a month is more real, waiting for it	Jingdong prefecture level city in Shandong
Stowe Flagship Store	a power saver (electronics)	It is the ideal product, good quality and fast logistics, worthy of praise!	Jingdong prefecture level city in Shandong
Stowe Flagship Store	a power saver (electronics)	The saver is great, the color is right, the installer is very professional	Jingdong prefecture level city in Shandong
Stowe Flagship Store	a power saver (electronics)	The effect of the power connection is particularly obvious, it is particularly easy to use, very satisfied, the	Jingdong prefecture level city in Shandong
Stowe Flagship Store	a power saver (electronics)	Goods are very energy-saving, used to be more than two hundred degrees, now there are only a hundred or so degrees	Jingdong prefecture level city in Shandong
Stowe Flagship Store	a power saver (electronics)	Good quality, fast shipping, good results, good value for money	Jingdong prefecture level city in Shandong
Stowe Flagship Store	a power saver (electronics)	Fast shipping, fine workmanship, very good, cost-effective	Jingdong prefecture level city in Shandong
Stowe Flagship Store	a power saver (electronics)	The meter is as described, the quality is quite good, energy-saving, worth buying oh~!	Jingdong prefecture level city in Shandong
Stowe Flagship Store	a power saver (electronics)	Great value for money, very affordable,, perfect . ˆ◡ˆ.	Jingdong prefecture level city in Shandong
Stowe Flagship Store	a power saver (electronics)	Good!!! Worth recommending!	Jingdong prefecture level city in Shandong
Stowe Flagship Store	a power saver (electronics)	Great, fast logistics, great stuff, great value for money!	Jingdong prefecture level city in Shandong
Stowe Flagship Store	a power saver (electronics)	Fast logistics, very satisfied with the quality upon receipt	Jingdong prefecture level city in Shandong
Stowe Flagship Store	a power saver (electronics)	Great quality, plug and play, fast shipping!	Jingdong prefecture level city in Shandong
Stowe Flagship Store	a power saver (electronics)	Logistics received, quite satisfied	Jingdong prefecture level city in Shandong
Stowe Flagship Store	a power saver (electronics)	I don't know if it really works	Jingdong prefecture level city in Shandong
Stowe Flagship Store	a power saver (electronics)	It's bullshit. It's a plug light. Don't talk about power savings.	Jingdong prefecture level city in Shandong
Stowe Flagship Store	a power saver (electronics)	Fake and deceitful stuff that hasn't changed at all.	Jingdong prefecture level city in Shandong
Stowe Flagship Store	a power saver (electronics)	Power-saving effect: power-saving effect is particularly obvious Cost-effective: cost-effective business Appearance and style: external small micro-work materials: fine workmanship Experience: use very good, non the first convenient	Jingdong prefecture level city in Shandong
Stowe Flagship Store	a power saver (electronics)	After using it for a few days, it feels quite good, it should be quite safe, and it also sends a plug, the merchant is good, wait for a period of time and then calculate whether it saves on the electricity bill and then add comments ha!	Jingdong prefecture level city in Shandong
Stowe Flagship Store	a power saver (electronics)	My friend recommended to me, said the effect is very good, I bought two, this month less money, really can, just buy I do not believe, but has proved to have effect	Jingdong prefecture level city in Shandong
Stowe Flagship Store	a power saver (electronics)	Just bought back, already used, these days to see the effect of it, and so have the effect will also come back to give positive feedback, the price is also super cheap!	Jingdong prefecture level city in Shandong
Stowe Flagship Store	a power saver (electronics)	It does work well, the store uses a lot of electricity, this time to buy back a few, each place that uses a lot of electricity to insert one. Summer is here, the electricity is too strong.	Jingdong prefecture level city in Shandong
Stowe Flagship Store	a power saver (electronics)	Power saving treasure received, as for the effect, according to the instructions written, a short period of time can not be seen, at least 2-3 months before the real effect, then follow up evaluation	Jingdong prefecture level city in Shandong
Stowe Flagship Store	a power saver (electronics)	The goods have just been received and have not been used yet looking forward to the results.	Jingdong prefecture level city in Shandong
Stowe Flagship Store	a power saver (electronics)	Good installation works great	Jingdong prefecture level city in Shandong
Stowe Flagship Store	a power saver (electronics)	The logistics is super super fast! The quality is also very good, indeed value for money	Jingdong prefecture level city in Shandong
Stowe Flagship Store	a power saver (electronics)	Has been used for a period of time before coming to comment on, really good, air conditioning a day open non-stop, pay the electricity bill when the heartache, with this, the home electricity is not at all worried about the bill out of contrast to save a lot of preparation in the hands of a few, each room An a	Jingdong prefecture level city in Shandong
Stowe Flagship Store	a power saver (electronics)	Saving device received, really good, with the air conditioning tried, an hour without a saver with 2.9 degrees, with a saver an hour with 1.1 degrees, the beginning of not quite believe that this thing, the actual test to know that it is really useful, and intend to buy another.	Jingdong prefecture level city in Shandong
Stowe Flagship Store	a power saver (electronics)	Power saving effect: this energy saver is still useful, day trial for three days, every day than not installed before the reduction of two dollars a day, every day to open the bedroom to open the air conditioning before fifteen dollars now thirteen dollars a day. Still a little use.	Jingdong prefecture level city in Shandong
Stowe Flagship Store	a power saver (electronics)	Originally bought for home use, used for two months, the electricity bill from more than 200 yuan per month, down to more than 140 yuan, there is indeed a power-saving effect, this time and sold two, the unit with. Merchant service attitude is really good.	Jingdong prefecture level city in Shandong
Stowe Flagship Store	a power saver (electronics)	Plug and play, stable voltage, the screen shows when working, this winter home appliances, need electricity in many places, just buy to use, save electricity and money, with instructions	Jingdong prefecture level city in Shandong
Stowe Flagship Store	a power saver (electronics)	Goods received, do not know if it is good. When I bought it, I saw that it was a Stowe brand or company's product. Received but not. I do not know what is the reason. Now I can only give this evaluation. I'm	Jingdong prefecture level city in Shandong
Stowe Flagship Store	a power saver (electronics)	The first time to buy online, but also look at the evaluation before buying, the quality is not bad, but also very cheap, the need for friends can rest assured to buy!	Jingdong prefecture level city in Shandong
Stowe Flagship Store	a power saver (electronics)	Great quality, bought it to my liking Awesome! Buy with confidence if you need it!	Jingdong prefecture level city in Shandong
Stowe Flagship Store	a power saver (electronics)	The quality is very good, completely exceeded the expectations, the delivery speed is very fast, the packaging is very careful and strict, very satisfied with a shopping	Jingdong prefecture level city in Shandong
Stowe Flagship Store	a power saver (electronics)	The effect is very good, the home uses less electricity is very energy efficient, cost-effective	Jingdong prefecture level city in Shandong
Stowe Flagship Store	a power saver (electronics)	Quality can't be beat, just like the store, especially like, and the price is favorable!	Jingdong prefecture level city in Shandong
Stowe Flagship Store	a power saver (electronics)	Baby received! Very unexpected, will be back next time ~ great seller, stay tuned!	Jingdong prefecture level city in Shandong
Stowe Flagship Store	a power saver (electronics)	Follow up review after a month is more real, waiting for it	Jingdong prefecture level city in Shandong

Stowe Flagship Store	a power saver (electronics)	It is the ideal product, good quality and fast logistics, worthy of praise!	Jingdong prefecture level city in Shandong
Stowe Flagship Store	a power saver (electronics)	The saver is great, the color is right, the installer is very professional	Jingdong prefecture level city in Shandong
Stowe Flagship Store	a power saver (electronics)	The effect of the power connection is particularly obvious, it is particularly easy to use, very satisfied, the	Jingdong prefecture level city in Shandong
Stowe Flagship Store	a power saver (electronics)	Goods are very energy-saving, used to be more than two hundred degrees, now there are only a hundred or so degrees	Jingdong prefecture level city in Shandong
Stowe Flagship Store	a power saver (electronics)	Good quality, fast shipping, good results, good value for money	Jingdong prefecture level city in Shandong
Stowe Flagship Store	a power saver (electronics)	Fast shipping, fine workmanship, very good, cost-effective	Jingdong prefecture level city in Shandong
Stowe Flagship Store	a power saver (electronics)	The meter is as described, the quality is quite good, energy-saving, worth buying oh~!	Jingdong prefecture level city in Shandong
Stowe Flagship Store	a power saver (electronics)	Great value for money, very affordable,, perfect . ˆ◡ˆ.	Jingdong prefecture level city in Shandong
Stowe Flagship Store	a power saver (electronics)	Good!!! Worth recommending!	Jingdong prefecture level city in Shandong
Stowe Flagship Store	a power saver (electronics)	Great, fast logistics, great stuff, great value for money!	Jingdong prefecture level city in Shandong
Stowe Flagship Store	a power saver (electronics)	Fast logistics, very satisfied with the quality upon receipt	Jingdong prefecture level city in Shandong
Stowe Flagship Store	a power saver (electronics)	Great quality, plug and play, fast shipping!	Jingdong prefecture level city in Shandong
Stowe Flagship Store	a power saver (electronics)	Logistics received, quite satisfied	Jingdong prefecture level city in Shandong
Stowe Flagship Store	a power saver (electronics)	I don't know if it really works	Jingdong prefecture level city in Shandong
Stowe Flagship Store	a power saver (electronics)	It's bullshit. It's a plug light. Don't talk about power savings.	Jingdong prefecture level city in Shandong
Stowe Flagship Store	a power saver (electronics)	Fake and deceitful stuff that hasn't changed at all.	Jingdong prefecture level city in Shandong
Stowe Flagship Store	a power saver (electronics)	Power-saving effect: power-saving effect is particularly obvious Cost-effective: cost-effective business Appearance and style: external small micro-work materials: fine workmanship Experience: use very good, non the first convenient	Jingdong prefecture level city in Shandong
Stowe Flagship Store	a power saver (electronics)	After using it for a few days, it feels quite good, it should be quite safe, and it also sends a plug, the merchant is good, wait for a period of time and then calculate whether it saves on the electricity bill and then add comments ha!	Jingdong prefecture level city in Shandong
Stowe Flagship Store	a power saver (electronics)	My friend recommended to me, said the effect is very good, I bought two, this month less money, really can, just buy I do not believe, but has proved to have effect	Jingdong prefecture level city in Shandong
Stowe Flagship Store	a power saver (electronics)	Just bought back, already used, these days to see the effect of it, and so have the effect will also come back to give positive feedback, the price is also super cheap!	Jingdong prefecture level city in Shandong
Stowe Flagship Store	a power saver (electronics)	It does work well, the store uses a lot of electricity, this time to buy back a few, each place that uses a lot of electricity to insert one. Summer is here, the electricity is too strong.	Jingdong prefecture level city in Shandong
Stowe Flagship Store	a power saver (electronics)	Power saving treasure received, as for the effect, according to the instructions written, a short period of time can not be seen, at least 2-3 months before the real effect, then follow up evaluation	Jingdong prefecture level city in Shandong
Stowe Flagship Store	a power saver (electronics)	The goods have just been received and have not been used yet looking forward to the results.	Jingdong prefecture level city in Shandong
Stowe Flagship Store	a power saver (electronics)	Good installation works great	Jingdong prefecture level city in Shandong
Stowe Flagship Store	a power saver (electronics)	The logistics is super super fast! The quality is also very good, indeed value for money	Jingdong prefecture level city in Shandong
Stowe Flagship Store	a power saver (electronics)	Has been used for a period of time before coming to comment on, really good, air conditioning a day open non-stop, pay the electricity bill when the heartache, with this, the home electricity is not at all worried about the bill out of contrast to save a lot of preparation in the hands of a few, each room An a	Jingdong prefecture level city in Shandong
Stowe Flagship Store	a power saver (electronics)	Saving device received, really good, with the air conditioning tried, an hour without a saver with 2.9 degrees, with a saver an hour with 1.1 degrees, the beginning of not quite believe that this thing, the actual test to know that it is really useful, and intend to buy another.	Jingdong prefecture level city in Shandong
Stowe Flagship Store	a power saver (electronics)	Power saving effect: this energy saver is still useful, day trial for three days, every day than not installed before the reduction of two dollars a day, every day to open the bedroom to open the air conditioning before fifteen dollars now thirteen dollars a day. Still a little use.	Jingdong prefecture level city in Shandong
Stowe Flagship Store	a power saver (electronics)	Originally bought for home use, used for two months, the electricity bill from more than 200 yuan per month, down to more than 140 yuan, there is indeed a power-saving effect, this time and sold two, the unit with. Merchant service attitude is really good.	Jingdong prefecture level city in Shandong
Stowe Flagship Store	a power saver (electronics)	Plug and play, stable voltage, the screen shows when working, this winter home appliances, need electricity in many places, just buy to use, save electricity and money, with instructions	Jingdong prefecture level city in Shandong
Stowe Flagship Store	a power saver (electronics)	Goods received, do not know if it is good. When I bought it, I saw that it was a Stowe brand or company's product. Received but not. I do not know what is the reason. Now I can only give this evaluation. I'm	Jingdong prefecture level city in Shandong
Stowe Flagship Store	a power saver (electronics)	The first time to buy online, but also look at the evaluation before buying, the quality is not bad, but also very cheap, the need for friends can rest assured to buy!	Jingdong prefecture level city in Shandong
Stowe Flagship Store	a power saver (electronics)	🌈 Great quality, bought it to my liking 💝 Awesome! ✨ Buy with confidence if you need it!	Jingdong prefecture level city in Shandong
Stowe Flagship Store	a power saver (electronics)	The quality is very good, completely exceeded the expectations, the delivery speed is very fast, the packaging is very careful and strict, very satisfied with a shopping	Jingdong prefecture level city in Shandong
Stowe Flagship Store	a power saver (electronics)	The effect is very good, the home uses less electricity is very energy efficient, cost-effective	Jingdong prefecture level city in Shandong
Stowe Flagship Store	a power saver (electronics)	Quality can't be beat, just like the store, especially like, and the price is favorable!	Jingdong prefecture level city in Shandong
Stowe Flagship Store	a power saver (electronics)	Baby received! Very unexpected, will be back next time ~ great seller, stay tuned!	Jingdong prefecture level city in Shandong
Stowe Flagship Store	a power saver (electronics)	It is the ideal product, good quality and fast logistics, worthy of praise!	Jingdong prefecture level city in Shandong
Stowe Flagship Store	a power saver (electronics)	Follow up review after a month is more real, waiting for it	Jingdong prefecture level city in Shandong
Stowe Flagship Store	a power saver (electronics)	The saver is great, the color is right, the installer is very professional	Jingdong prefecture level city in Shandong
Stowe Flagship Store	a power saver (electronics)	The effect of the power connection is particularly obvious, it is particularly easy to use, very satisfied, the	Jingdong prefecture level city in Shandong
Stowe Flagship Store	a power saver (electronics)	Goods are very energy-saving, used to be more than two hundred degrees, now there are only a hundred or so degrees	Jingdong prefecture level city in Shandong
Stowe Flagship Store	a power saver (electronics)	Good quality, fast shipping, good results, good value for money	Jingdong prefecture level city in Shandong
Stowe Flagship Store	a power saver (electronics)	Fast shipping, fine workmanship, very good, cost-effective	Jingdong prefecture level city in Shandong
Stowe Flagship Store	a power saver (electronics)	The meter is as described, the quality is quite good, energy-saving, worth buying oh~!	Jingdong prefecture level city in Shandong
Stowe Flagship Store	a power saver (electronics)	Good!!! Worth recommending!	Jingdong prefecture level city in Shandong
Stowe Flagship Store	a power saver (electronics)	Great value for money, very affordable,, perfect . ˆ◡ˆ.	Jingdong prefecture level city in Shandong
Stowe Flagship Store	a power saver (electronics)	Great, fast logistics, great stuff, great value for money!	Jingdong prefecture level city in Shandong

Stowe Flagship Store	a power saver (electronics)	Fast logistics, very satisfied with the quality upon receipt	Jingdong prefecture level city in Shandong
Stowe Flagship Store	a power saver (electronics)	Great quality, plug and play, fast shipping!	Jingdong prefecture level city in Shandong
Stowe Flagship Store	a power saver (electronics)	Logistics received, quite satisfied	Jingdong prefecture level city in Shandong
Stowe Flagship Store	a power saver (electronics)	I don't know if it really works	Jingdong prefecture level city in Shandong
Stowe Flagship Store	a power saver (electronics)	It's bullshit. It's a plug light. Don't talk about power savings.	Jingdong prefecture level city in Shandong
Stowe Flagship Store	a power saver (electronics)	Fake and deceitful stuff that hasn't changed at all.	Jingdong prefecture level city in Shandong
Stowe Flagship Store	a power saver (electronics)	Power-saving effect: power-saving effect is particularly obvious Cost-effective: cost-effective business Appearance and style: external small micro-work materials: fine workmanship Experience: use very good, non the first convenient	Jingdong prefecture level city in Shandong
Stowe Flagship Store	a power saver (electronics)	After using it for a few days, it feels quite good, it should be quite safe, and it also sends a plug, the merchant is good, wait for a period of time and then calculate whether it saves on the electricity bill and then add comments ha!	Jingdong prefecture level city in Shandong
Stowe Flagship Store	a power saver (electronics)	My friend recommended to me, said the effect is very good, I bought two, this month less money, really can, just buy I do not believe, but has proved to have effect	Jingdong prefecture level city in Shandong
Stowe Flagship Store	a power saver (electronics)	Just bought back, already used, these days to see the effect of it, and so have the effect will also come back to give positive feedback, the price is also super cheap!	Jingdong prefecture level city in Shandong
Stowe Flagship Store	a power saver (electronics)	It does work well, the store uses a lot of electricity, this time to buy back a few, each place that uses a lot of electricity to insert one. Summer is here, the electricity is too strong.	Jingdong prefecture level city in Shandong
Stowe Flagship Store	a power saver (electronics)	Power saving treasure received, as for the effect, according to the instructions written, a short period of time can not be seen, at least 2-3 months before the real effect, then follow up evaluation	Jingdong prefecture level city in Shandong
Stowe Flagship Store	a power saver (electronics)	The goods have just been received and have not been used yet looking forward to the results.	Jingdong prefecture level city in Shandong
Stowe Flagship Store	a power saver (electronics)	Good installation works great	Jingdong prefecture level city in Shandong
Stowe Flagship Store	a power saver (electronics)	The logistics is super super fast! The quality is also very good, indeed value for money	Jingdong prefecture level city in Shandong
Stowe Flagship Store	a power saver (electronics)	Has been used for a period of time before coming to comment on, really good, air conditioning a day open non-stop, pay the electricity bill when the heartache, with this, the home electricity is not at all worried about the bill out of contrast to save a lot of preparation in the hands of a few, each room An a	Jingdong prefecture level city in Shandong
Stowe Flagship Store	a power saver (electronics)	Saving device received, really good, with the air conditioning tried, an hour without a saver with 2.9 degrees, with a saver an hour with 1.1 degrees, the beginning of not quite believe that this thing, the actual test to know that it is really useful, and intend to buy another.	Jingdong prefecture level city in Shandong
Stowe Flagship Store	a power saver (electronics)	Power saving effect: this energy saver is still useful, day trial for three days, every day than not installed before the reduction of two dollars a day, every day to open the bedroom to open the air conditioning before fifteen dollars now thirteen dollars a day. Still a little use.	Jingdong prefecture level city in Shandong
Stowe Flagship Store	a power saver (electronics)	Originally bought for home use, used for two months, the electricity bill from more than 200 yuan per month, down to more than 140 yuan, there is indeed a power-saving effect, this time and sold two, the unit with. Merchant service attitude is really good.	Jingdong prefecture level city in Shandong
Stowe Flagship Store	a power saver (electronics)	Plug and play, stable voltage, the screen shows when working, this winter home appliances, need electricity in many places, just buy to use, save electricity and money, with instructions	Jingdong prefecture level city in Shandong
Stowe Flagship Store	a power saver (electronics)	Goods received, do not know if it is good. When I bought it, I saw that it was a Stowe brand or company's product. Received but not. I do not know what is the reason. Now I can only give this evaluation. I'm	Jingdong prefecture level city in Shandong
Stowe Flagship Store	a power saver (electronics)	The first time to buy online, but also look at the evaluation before buying, the quality is not bad, but also very cheap, the need for friends can rest assured to buy!	Jingdong prefecture level city in Shandong
Stowe Flagship Store	a power saver (electronics)	Great quality, bought it to my liking Awesome! Buy with confidence if you need it!	Jingdong prefecture level city in Shandong
Stowe Flagship Store	a power saver (electronics)	The quality is very good, completely exceeded the expectations, the delivery speed is very fast, the packaging is very careful and strict, very satisfied with a shopping	Jingdong prefecture level city in Shandong
Stowe Flagship Store	a power saver (electronics)	The effect is very good, the home uses less electricity is very energy efficient, cost-effective	Jingdong prefecture level city in Shandong
Stowe Flagship Store	a power saver (electronics)	Quality can't be beat, just like the store, especially like, and the price is favorable!	Jingdong prefecture level city in Shandong
Stowe Flagship Store	a power saver (electronics)	Baby received! Very unexpected, will be back next time ~ great seller, stay tuned!	Jingdong prefecture level city in Shandong
Stowe Flagship Store	a power saver (electronics)	Follow up review after a month is more real, waiting for it	Jingdong prefecture level city in Shandong
Stowe Flagship Store	a power saver (electronics)	It is the ideal product, good quality and fast logistics, worthy of praise!	Jingdong prefecture level city in Shandong
Stowe Flagship Store	a power saver (electronics)	The saver is great, the color is right, the installer is very professional	Jingdong prefecture level city in Shandong
Stowe Flagship Store	a power saver (electronics)	The effect of the power connection is particularly obvious, it is particularly easy to use, very satisfied, the	Jingdong prefecture level city in Shandong
Stowe Flagship Store	a power saver (electronics)	Goods are very energy-saving, used to be more than two hundred degrees, now there are only a hundred or so degrees	Jingdong prefecture level city in Shandong
Stowe Flagship Store	a power saver (electronics)	Good quality, fast shipping, good results, good value for money	Jingdong prefecture level city in Shandong
Stowe Flagship Store	a power saver (electronics)	Fast shipping, fine workmanship, very good, cost-effective	Jingdong prefecture level city in Shandong
Stowe Flagship Store	a power saver (electronics)	The meter is as described, the quality is quite good, energy-saving, worth buying oh~!	Jingdong prefecture level city in Shandong
Stowe Flagship Store	a power saver (electronics)	Great value for money, very affordable,, perfect . ˆ◡ˆ.	Jingdong prefecture level city in Shandong
Stowe Flagship Store	a power saver (electronics)	Good!!! Worth recommending!	Jingdong prefecture level city in Shandong
Stowe Flagship Store	a power saver (electronics)	Great, fast logistics, great stuff, great value for money!	Jingdong prefecture level city in Shandong
Stowe Flagship Store	a power saver (electronics)	Fast logistics, very satisfied with the quality upon receipt	Jingdong prefecture level city in Shandong
Stowe Flagship Store	a power saver (electronics)	Great quality, plug and play, fast shipping!	Jingdong prefecture level city in Shandong
Stowe Flagship Store	a power saver (electronics)	Logistics received, quite satisfied	Jingdong prefecture level city in Shandong
Stowe Flagship Store	a power saver (electronics)	I don't know if it really works	Jingdong prefecture level city in Shandong
Stowe Flagship Store	a power saver (electronics)	It's bullshit. It's a plug light. Don't talk about power savings.	Jingdong prefecture level city in Shandong
Stowe Flagship Store	a power saver (electronics)	Fake and deceitful stuff that hasn't changed at all.	Jingdong prefecture level city in Shandong
Stowe Flagship Store	a power saver (electronics)	Power-saving effect: power-saving effect is particularly obvious Cost-effective: cost-effective business Appearance and style: external small micro-work materials: fine workmanship Experience: use very good, non the first convenient	Jingdong prefecture level city in Shandong
Stowe Flagship Store	a power saver (electronics)	After using it for a few days, it feels quite good, it should be quite safe, and it also sends a plug, the merchant is good, wait for a period of time and then calculate whether it saves on the electricity bill and then add comments ha!	Jingdong prefecture level city in Shandong
Stowe Flagship Store	a power saver (electronics)	My friend recommended to me, said the effect is very good, I bought two, this month less money, really can, just buy I do not believe, but has proved to have effect	Jingdong prefecture level city in Shandong

Stowe Flagship Store	a power saver (electronics)	Just bought back, already used, these days to see the effect of it, and so have the effect will also come back to give positive feedback, the price is also super cheap!	Jingdong prefecture level city in Shandong
Stowe Flagship Store	a power saver (electronics)	It does work well, the store uses a lot of electricity, this time to buy back a few, each place that uses a lot of electricity to insert one. Summer is here, the electricity is too strong.	Jingdong prefecture level city in Shandong
Stowe Flagship Store	a power saver (electronics)	Power saving treasure received, as for the effect, according to the instructions written, a short period of time can not be seen, at least 2-3 months before the real effect, then follow up evaluation	Jingdong prefecture level city in Shandong
Stowe Flagship Store	a power saver (electronics)	The goods have just been received and have not been used yet looking forward to the results.	Jingdong prefecture level city in Shandong
Stowe Flagship Store	a power saver (electronics)	Good installation works great	Jingdong prefecture level city in Shandong
Stowe Flagship Store	a power saver (electronics)	The logistics is super super fast! The quality is also very good, indeed value for money	Jingdong prefecture level city in Shandong
Stowe Flagship Store	a power saver (electronics)	Has been used for a period of time before coming to comment on, really good, air conditioning a day open non-stop, pay the electricity bill when the heartache, with this, the home electricity is not at all worried about the bill out of contrast to save a lot of preparation in the hands of a few, each room An a	Jingdong prefecture level city in Shandong
Stowe Flagship Store	a power saver (electronics)	Saving device received, really good, with the air conditioning tried, an hour without a saver with 2.9 degrees, with a saver an hour with 1.1 degrees, the beginning of not quite believe that this thing, the actual test to know that it is really useful, and intend to buy another.	Jingdong prefecture level city in Shandong
Stowe Flagship Store	a power saver (electronics)	Power saving effect: this energy saver is still useful, day trial for three days, every day than not installed before the reduction of two dollars a day, every day to open the bedroom to open the air conditioning before fifteen dollars now thirteen dollars a day. Still a little use.	Jingdong prefecture level city in Shandong
Stowe Flagship Store	a power saver (electronics)	Originally bought for home use, used for two months, the electricity bill from more than 200 yuan per month, down to more than 140 yuan, there is indeed a power-saving effect, this time and sold two, the unit with. Merchant service attitude is really good.	Jingdong prefecture level city in Shandong
Stowe Flagship Store	a power saver (electronics)	Plug and play, stable voltage, the screen shows when working, this winter home appliances, need electricity in many places, just buy to use, save electricity and money, with instructions	Jingdong prefecture level city in Shandong
Stowe Flagship Store	a power saver (electronics)	Goods received, do not know if it is good. When I bought it, I saw that it was a Stowe brand or company's product. Received but not. I do not know what is the reason. Now I can only give this evaluation. I'm	Jingdong prefecture level city in Shandong
Stowe Flagship Store	a power saver (electronics)	The first time to buy online, but also look at the evaluation before buying, the quality is not bad, but also very cheap, the need for friends can rest assured to buy!	Jingdong prefecture level city in Shandong
Stowe Flagship Store	a power saver (electronics)	🌈 Great quality, bought it to my liking 💝 Awesome! ✨ Buy with confidence if you need it!	Jingdong prefecture level city in Shandong
Stowe Flagship Store	a power saver (electronics)	The quality is very good, completely exceeded the expectations, the delivery speed is very fast, the packaging is very careful and strict, very satisfied with a shopping	Jingdong prefecture level city in Shandong
Stowe Flagship Store	a power saver (electronics)	The effect is very good, the home uses less electricity is very energy efficient, cost-effective	Jingdong prefecture level city in Shandong
Stowe Flagship Store	a power saver (electronics)	Quality can't be beat, just like the store, especially like, and the price is favorable!	Jingdong prefecture level city in Shandong
Stowe Flagship Store	a power saver (electronics)	Baby received! Very unexpected, will be back next time ~ great seller, stay tuned!	Jingdong prefecture level city in Shandong
Stowe Flagship Store	a power saver (electronics)	Follow up review after a month is more real, waiting for it	Jingdong prefecture level city in Shandong
Stowe Flagship Store	a power saver (electronics)	It is the ideal product, good quality and fast logistics, worthy of praise!	Jingdong prefecture level city in Shandong
Stowe Flagship Store	a power saver (electronics)	The saver is great, the color is right, the installer is very professional	Jingdong prefecture level city in Shandong
Stowe Flagship Store	a power saver (electronics)	The effect of the power connection is particularly obvious, it is particularly easy to use, very satisfied, the	Jingdong prefecture level city in Shandong
Stowe Flagship Store	a power saver (electronics)	Goods are very energy-saving, used to be more than two hundred degrees, now there are only a hundred or so degrees	Jingdong prefecture level city in Shandong
Stowe Flagship Store	a power saver (electronics)	Good quality, fast shipping, good results, good value for money	Jingdong prefecture level city in Shandong
Stowe Flagship Store	a power saver (electronics)	Fast shipping, fine workmanship, very good, cost-effective	Jingdong prefecture level city in Shandong
Stowe Flagship Store	a power saver (electronics)	The meter is as described, the quality is quite good, energy-saving, worth buying oh~!	Jingdong prefecture level city in Shandong
Stowe Flagship Store	a power saver (electronics)	Great value for money, very affordable,, perfect . ˆ◡ˆ.	Jingdong prefecture level city in Shandong
Stowe Flagship Store	a power saver (electronics)	Good!!! Worth recommending!	Jingdong prefecture level city in Shandong
Stowe Flagship Store	a power saver (electronics)	Great, fast logistics, great stuff, great value for money!	Jingdong prefecture level city in Shandong
Stowe Flagship Store	a power saver (electronics)	Fast logistics, very satisfied with the quality upon receipt	Jingdong prefecture level city in Shandong
Stowe Flagship Store	a power saver (electronics)	Great quality, plug and play, fast shipping!	Jingdong prefecture level city in Shandong
Stowe Flagship Store	a power saver (electronics)	Logistics received, quite satisfied	Jingdong prefecture level city in Shandong
Stowe Flagship Store	a power saver (electronics)	I don't know if it really works	Jingdong prefecture level city in Shandong
Stowe Flagship Store	a power saver (electronics)	It's bullshit. It's a plug light. Don't talk about power savings.	Jingdong prefecture level city in Shandong
Stowe Flagship Store	a power saver (electronics)	Fake and deceitful stuff that hasn't changed at all.	Jingdong prefecture level city in Shandong
Stowe Flagship Store	a power saver (electronics)	Power-saving effect: power-saving effect is particularly obvious Cost-effective: cost-effective business Appearance and style: external small micro-work materials: fine workmanship Experience: use very good, non the first convenient	Jingdong prefecture level city in Shandong
Stowe Flagship Store	a power saver (electronics)	After using it for a few days, it feels quite good, it should be quite safe, and it also sends a plug, the merchant is good, wait for a period of time and then calculate whether it saves on the electricity bill and then add comments ha!	Jingdong prefecture level city in Shandong
Stowe Flagship Store	a power saver (electronics)	My friend recommended to me, said the effect is very good, I bought two, this month less money, really can, just buy I do not believe, but has proved to have effect	Jingdong prefecture level city in Shandong
Stowe Flagship Store	a power saver (electronics)	Just bought back, already used, these days to see the effect of it, and so have the effect will also come back to give positive feedback, the price is also super cheap!	Jingdong prefecture level city in Shandong
Stowe Flagship Store	a power saver (electronics)	It does work well, the store uses a lot of electricity, this time to buy back a few, each place that uses a lot of electricity to insert one. Summer is here, the electricity is too strong.	Jingdong prefecture level city in Shandong
Stowe Flagship Store	a power saver (electronics)	Power saving treasure received, as for the effect, according to the instructions written, a short period of time can not be seen, at least 2-3 months before the real effect, then follow up evaluation	Jingdong prefecture level city in Shandong
Stowe Flagship Store	a power saver (electronics)	The goods have just been received and have not been used yet looking forward to the results.	Jingdong prefecture level city in Shandong
Stowe Flagship Store	a power saver (electronics)	Good installation works great	Jingdong prefecture level city in Shandong
Stowe Flagship Store	a power saver (electronics)	The logistics is super super fast! The quality is also very good, indeed value for money	Jingdong prefecture level city in Shandong
Stowe Flagship Store	a power saver (electronics)	Has been used for a period of time before coming to comment on, really good, air conditioning a day open non-stop, pay the electricity bill when the heartache, with this, the home electricity is not at all worried about the bill out of contrast to save a lot of preparation in the hands of a few, each room An a	Jingdong prefecture level city in Shandong
Stowe Flagship Store	a power saver (electronics)	Saving device received, really good, with the air conditioning tried, an hour without a saver with 2.9 degrees, with a saver an hour with 1.1 degrees, the beginning of not quite believe that this thing, the actual test to know that it is really useful, and intend to buy another.	Jingdong prefecture level city in Shandong
Stowe Flagship Store	a power saver (electronics)	Power saving effect: this energy saver is still useful, day trial for three days, every day than not installed before the reduction of two dollars a day, every day to open the bedroom to open the air conditioning before fifteen dollars now thirteen dollars a day. Still a little use.	Jingdong prefecture level city in Shandong

Stowe Flagship Store	a power saver (electronics)	Originally bought for home use, used for two months, the electricity bill from more than 200 yuan per month, down to more than 140 yuan, there is indeed a power-saving effect, this time and sold two, the unit with. Merchant service attitude is really good.	Jingdong prefecture level city in Shandong
Stowe Flagship Store	a power saver (electronics)	Plug and play, stable voltage, the screen shows when working, this winter home appliances, need electricity in many places, just buy to use, save electricity and money, with instructions	Jingdong prefecture level city in Shandong
Stowe Flagship Store	a power saver (electronics)	Goods received, do not know if it is good. When I bought it, I saw that it was a Stowe brand or company's product. Received but not. I do not know what is the reason. Now I can only give this evaluation. I'm	Jingdong prefecture level city in Shandong
Stowe Flagship Store	a power saver (electronics)	The first time to buy online, but also look at the evaluation before buying, the quality is not bad, but also very cheap, the need for friends can rest assured to buy!	Jingdong prefecture level city in Shandong
Stowe Flagship Store	a power saver (electronics)	🌈 Great quality, bought it to my liking 💝 Awesome! ✨ Buy with confidence if you need it!	Jingdong prefecture level city in Shandong
Stowe Flagship Store	a power saver (electronics)	The quality is very good, completely exceeded the expectations, the delivery speed is very fast, the packaging is very careful and strict, very satisfied with a shopping	Jingdong prefecture level city in Shandong
Stowe Flagship Store	a power saver (electronics)	The effect is very good, the home uses less electricity is very energy efficient, cost-effective	Jingdong prefecture level city in Shandong
Stowe Flagship Store	a power saver (electronics)	Quality can't be beat, just like the store, especially like, and the price is favorable!	Jingdong prefecture level city in Shandong
Stowe Flagship Store	a power saver (electronics)	Baby received! Very unexpected, will be back next time ~ great seller, stay tuned!	Jingdong prefecture level city in Shandong
Stowe Flagship Store	a power saver (electronics)	Follow up review after a month is more real, waiting for it	Jingdong prefecture level city in Shandong
Stowe Flagship Store	a power saver (electronics)	It is the ideal product, good quality and fast logistics, worthy of praise!	Jingdong prefecture level city in Shandong
Stowe Flagship Store	a power saver (electronics)	The saver is great, the color is right, the installer is very professional	Jingdong prefecture level city in Shandong
Stowe Flagship Store	a power saver (electronics)	The effect of the power connection is particularly obvious, it is particularly easy to use, very satisfied, the	Jingdong prefecture level city in Shandong
Stowe Flagship Store	a power saver (electronics)	Goods are very energy-saving, used to be more than two hundred degrees, now there are only a hundred or so degrees	Jingdong prefecture level city in Shandong
Stowe Flagship Store	a power saver (electronics)	Good quality, fast shipping, good results, good value for money	Jingdong prefecture level city in Shandong
Stowe Flagship Store	a power saver (electronics)	Fast shipping, fine workmanship, very good, cost-effective	Jingdong prefecture level city in Shandong
Stowe Flagship Store	a power saver (electronics)	The meter is as described, the quality is quite good, energy-saving, worth buying oh~!	Jingdong prefecture level city in Shandong
Stowe Flagship Store	a power saver (electronics)	Great value for money, very affordable,, perfect . ^◡^.	Jingdong prefecture level city in Shandong
Stowe Flagship Store	a power saver (electronics)	Good!!! Worth recommending!	Jingdong prefecture level city in Shandong
Stowe Flagship Store	a power saver (electronics)	Great, fast logistics, great stuff, great value for money!	Jingdong prefecture level city in Shandong
Stowe Flagship Store	a power saver (electronics)	Fast logistics, very satisfied with the quality upon receipt	Jingdong prefecture level city in Shandong
Stowe Flagship Store	a power saver (electronics)	Great quality, plug and play, fast shipping!	Jingdong prefecture level city in Shandong
Stowe Flagship Store	a power saver (electronics)	Logistics received, quite satisfied	Jingdong prefecture level city in Shandong
Stowe Flagship Store	a power saver (electronics)	I don't know if it really works	Jingdong prefecture level city in Shandong
Stowe Flagship Store	a power saver (electronics)	It's bullshit. It's a plug light. Don't talk about power savings.	Jingdong prefecture level city in Shandong
Stowe Flagship Store	a power saver (electronics)	Fake and deceitful stuff that hasn't changed at all.	Jingdong prefecture level city in Shandong
Stowe Flagship Store	a power saver (electronics)	Power-saving effect: power-saving effect is particularly obvious Cost-effective: cost-effective business Appearance and style: external small micro-work materials: fine workmanship Experience: use very good, non the first convenient	Jingdong prefecture level city in Shandong
Stowe Flagship Store	a power saver (electronics)	After using it for a few days, it feels quite good, it should be quite safe, and it also sends a plug, the merchant is good, wait for a period of time and then calculate whether it saves on the electricity bill and then add comments ha!	Jingdong prefecture level city in Shandong
Stowe Flagship Store	a power saver (electronics)	My friend recommended to me, said the effect is very good, I bought two, this month less money, really can, just buy I do not believe, but has proved to have effect	Jingdong prefecture level city in Shandong
Stowe Flagship Store	a power saver (electronics)	Just bought back, already used, these days to see the effect of it, and so have the effect will also come back to give positive feedback, the price is also super cheap!	Jingdong prefecture level city in Shandong
Stowe Flagship Store	a power saver (electronics)	It does work well, the store uses a lot of electricity, this time to buy back a few, each place that uses a lot of electricity to insert one. Summer is here, the electricity is too strong.	Jingdong prefecture level city in Shandong
Stowe Flagship Store	a power saver (electronics)	Power saving treasure received, as for the effect, according to the instructions written, a short period of time can not be seen, at least 2-3 months before the real effect, then follow up evaluation	Jingdong prefecture level city in Shandong
Stowe Flagship Store	a power saver (electronics)	The goods have just been received and have not been used yet looking forward to the results.	Jingdong prefecture level city in Shandong
Stowe Flagship Store	a power saver (electronics)	Good installation works great	Jingdong prefecture level city in Shandong
Stowe Flagship Store	a power saver (electronics)	The logistics is super super fast! The quality is also very good, indeed value for money	Jingdong prefecture level city in Shandong
Stowe Flagship Store	a power saver (electronics)	Has been used for a period of time before coming to comment on, really good, air conditioning a day open non-stop, pay the electricity bill when the heartache, with this, the home electricity is not at all worried about the bill out of contrast to save a lot of preparation in the hands of a few, each room An a	Jingdong prefecture level city in Shandong
Stowe Flagship Store	a power saver (electronics)	Saving device received, really good, with the air conditioning tried, an hour without a saver with 2.9 degrees, with a saver an hour with 1.1 degrees, the beginning of not quite believe that this thing, the actual test to know that it is really useful, and intend to buy another.	Jingdong prefecture level city in Shandong
Stowe Flagship Store	a power saver (electronics)	Power saving effect: this energy saver is still useful, day trial for three days, every day than not installed before the reduction of two dollars a day, every day to open the bedroom to open the air conditioning before fifteen dollars now thirteen dollars a day. Still a little use.	Jingdong prefecture level city in Shandong
Stowe Flagship Store	a power saver (electronics)	Originally bought for home use, used for two months, the electricity bill from more than 200 yuan per month, down to more than 140 yuan, there is indeed a power-saving effect, this time and sold two, the unit with. Merchant service attitude is really good.	Jingdong prefecture level city in Shandong
Stowe Flagship Store	a power saver (electronics)	Plug and play, stable voltage, the screen shows when working, this winter home appliances, need electricity in many places, just buy to use, save electricity and money, with instructions	Jingdong prefecture level city in Shandong
Stowe Flagship Store	a power saver (electronics)	Goods received, do not know if it is good. When I bought it, I saw that it was a Stowe brand or company's product. Received but not. I do not know what is the reason. Now I can only give this evaluation. I'm	Jingdong prefecture level city in Shandong
Stowe Flagship Store	a power saver (electronics)	The first time to buy online, but also look at the evaluation before buying, the quality is not bad, but also very cheap, the need for friends can rest assured to buy!	Jingdong prefecture level city in Shandong
Stowe Flagship Store	a power saver (electronics)	🌈 Great quality, bought it to my liking 💝 Awesome! ✨ Buy with confidence if you need it!	Jingdong prefecture level city in Shandong
Stowe Flagship Store	a power saver (electronics)	The quality is very good, completely exceeded the expectations, the delivery speed is very fast, the packaging is very careful and strict, very satisfied with a shopping	Jingdong prefecture level city in Shandong
Stowe Flagship Store	a power saver (electronics)	The effect is very good, the home uses less electricity is very energy efficient, cost-effective	Jingdong prefecture level city in Shandong
Stowe Flagship Store	a power saver (electronics)	Quality can't be beat, just like the store, especially like, and the price is favorable!	Jingdong prefecture level city in Shandong
Stowe Flagship Store	a power saver (electronics)	Baby received! Very unexpected, will be back next time ~ great seller, stay tuned!	Jingdong prefecture level city in Shandong
Stowe Flagship Store	a power saver (electronics)	Follow up review after a month is more real, waiting for it	Jingdong prefecture level city in Shandong

Stowe Flagship Store	a power saver (electronics)	It is the ideal product, good quality and fast logistics, worthy of praise!	Jingdong prefecture level city in Shandong
Stowe Flagship Store	a power saver (electronics)	The saver is great, the color is right, the installer is very professional	Jingdong prefecture level city in Shandong
Stowe Flagship Store	a power saver (electronics)	The effect of the power connection is particularly obvious, it is particularly easy to use, very satisfied, the	Jingdong prefecture level city in Shandong
Stowe Flagship Store	a power saver (electronics)	Goods are very energy-saving, used to be more than two hundred degrees, now there are only a hundred or so degrees	Jingdong prefecture level city in Shandong
Stowe Flagship Store	a power saver (electronics)	Good quality, fast shipping, good results, good value for money	Jingdong prefecture level city in Shandong
Stowe Flagship Store	a power saver (electronics)	Fast shipping, fine workmanship, very good, cost-effective	Jingdong prefecture level city in Shandong
Stowe Flagship Store	a power saver (electronics)	The meter is as described, the quality is quite good, energy-saving, worth buying oh~!	Jingdong prefecture level city in Shandong
Stowe Flagship Store	a power saver (electronics)	Great value for money, very affordable,, perfect . ˆ◡ˆ.	Jingdong prefecture level city in Shandong
Stowe Flagship Store	a power saver (electronics)	Good!!! Worth recommending!	Jingdong prefecture level city in Shandong
Stowe Flagship Store	a power saver (electronics)	Great, fast logistics, great stuff, great value for money!	Jingdong prefecture level city in Shandong
Stowe Flagship Store	a power saver (electronics)	Fast logistics, very satisfied with the quality upon receipt	Jingdong prefecture level city in Shandong
Stowe Flagship Store	a power saver (electronics)	Great quality, plug and play, fast shipping!	Jingdong prefecture level city in Shandong
Stowe Flagship Store	a power saver (electronics)	Logistics received, quite satisfied	Jingdong prefecture level city in Shandong
Stowe Flagship Store	a power saver (electronics)	I don't know if it really works	Jingdong prefecture level city in Shandong
Stowe Flagship Store	a power saver (electronics)	It's bullshit. It's a plug light. Don't talk about power savings.	Jingdong prefecture level city in Shandong
Stowe Flagship Store	a power saver (electronics)	Fake and deceitful stuff that hasn't changed at all.	Jingdong prefecture level city in Shandong
Stowe Flagship Store	a power saver (electronics)	Power-saving effect: power-saving effect is particularly obvious Cost-effective: cost-effective business Appearance and style: external small micro-work materials: fine workmanship Experience: use very good, non the first convenient	Jingdong prefecture level city in Shandong
Stowe Flagship Store	a power saver (electronics)	After using it for a few days, it feels quite good, it should be quite safe, and it also sends a plug, the merchant is good, wait for a period of time and then calculate whether it saves on the electricity bill and then add comments ha!	Jingdong prefecture level city in Shandong
Stowe Flagship Store	a power saver (electronics)	My friend recommended to me, said the effect is very good, I bought two, this month less money, really can, just buy I do not believe, but has proved to have effect	Jingdong prefecture level city in Shandong
Stowe Flagship Store	a power saver (electronics)	Just bought back, already used, these days to see the effect of it, and so have the effect will also come back to give positive feedback, the price is also super cheap!	Jingdong prefecture level city in Shandong
Stowe Flagship Store	a power saver (electronics)	It does work well, the store uses a lot of electricity, this time to buy back a few, each place that uses a lot of electricity to insert one. Summer is here, the electricity is too strong.	Jingdong prefecture level city in Shandong
Stowe Flagship Store	a power saver (electronics)	Power saving treasure received, as for the effect, according to the instructions written, a short period of time can not be seen, at least 2-3 months before the real effect, then follow up evaluation	Jingdong prefecture level city in Shandong
Stowe Flagship Store	a power saver (electronics)	The goods have just been received and have not been used yet looking forward to the results.	Jingdong prefecture level city in Shandong
Stowe Flagship Store	a power saver (electronics)	Good installation works great	Jingdong prefecture level city in Shandong
Stowe Flagship Store	a power saver (electronics)	The logistics is super super fast! The quality is also very good, indeed value for money	Jingdong prefecture level city in Shandong
Stowe Flagship Store	a power saver (electronics)	Has been used for a period of time before coming to comment on, really good, air conditioning a day open non-stop, pay the electricity bill when the heartache, with this, the home electricity is not at all worried about the bill out of contrast to save a lot of preparation in the hands of a few, each room An a	Jingdong prefecture level city in Shandong
Stowe Flagship Store	a power saver (electronics)	Saving device received, really good, with the air conditioning tried, an hour without a saver with 2.9 degrees, with a saver an hour with 1.1 degrees, the beginning of not quite believe that this thing, the actual test to know that it is really useful, and intend to buy another.	Jingdong prefecture level city in Shandong
Stowe Flagship Store	a power saver (electronics)	Power saving effect: this energy saver is still useful, day trial for three days, every day than not installed before the reduction of two dollars a day, every day to open the bedroom to open the air conditioning before fifteen dollars now thirteen dollars a day. Still a little use.	Jingdong prefecture level city in Shandong
Stowe Flagship Store	a power saver (electronics)	Originally bought for home use, used for two months, the electricity bill from more than 200 yuan per month, down to more than 140 yuan, there is indeed a power-saving effect, this time and sold two, the unit with. Merchant service attitude is really good.	Jingdong prefecture level city in Shandong
Stowe Flagship Store	a power saver (electronics)	Plug and play, stable voltage, the screen shows when working, this winter home appliances, need electricity in many places, just buy to use, save electricity and money, with instructions	Jingdong prefecture level city in Shandong
Stowe Flagship Store	a power saver (electronics)	Goods received, do not know if it is good. When I bought it, I saw that it was a Stowe brand or company's product. Received but not. I do not know what is the reason. Now I can only give this evaluation. I'm	Jingdong prefecture level city in Shandong
Stowe Flagship Store	a power saver (electronics)	The first time to buy online, but also look at the evaluation before buying, the quality is not bad, but also very cheap, the need for friends can rest assured to buy!	Jingdong prefecture level city in Shandong
Stowe Flagship Store	a power saver (electronics)	🌈 Great quality, bought it to my liking 💕 Awesome! ✨ Buy with confidence if you need it!	Jingdong prefecture level city in Shandong
Stowe Flagship Store	a power saver (electronics)	The quality is very good, completely exceeded the expectations, the delivery speed is very fast, the packaging is very careful and strict, very satisfied with a shopping	Jingdong prefecture level city in Shandong
Stowe Flagship Store	a power saver (electronics)	The effect is very good, the home uses less electricity is very energy efficient, cost-effective	Jingdong prefecture level city in Shandong
Stowe Flagship Store	a power saver (electronics)	Quality can't be beat, just like the store, especially like, and the price is favorable!	Jingdong prefecture level city in Shandong
Stowe Flagship Store	a power saver (electronics)	Baby received! Very unexpected, will be back next time ~ great seller, stay tuned!	Jingdong prefecture level city in Shandong
Stowe Flagship Store	a power saver (electronics)	Follow up review after a month is more real, waiting for it	Jingdong prefecture level city in Shandong
Stowe Flagship Store	a power saver (electronics)	It is the ideal product, good quality and fast logistics, worthy of praise!	Jingdong prefecture level city in Shandong
Stowe Flagship Store	a power saver (electronics)	The saver is great, the color is right, the installer is very professional	Jingdong prefecture level city in Shandong
Stowe Flagship Store	a power saver (electronics)	The effect of the power connection is particularly obvious, it is particularly easy to use, very satisfied, the	Jingdong prefecture level city in Shandong
Stowe Flagship Store	a power saver (electronics)	Goods are very energy-saving, used to be more than two hundred degrees, now there are only a hundred or so degrees	Jingdong prefecture level city in Shandong
Stowe Flagship Store	a power saver (electronics)	Good quality, fast shipping, good results, good value for money	Jingdong prefecture level city in Shandong
Stowe Flagship Store	a power saver (electronics)	Fast shipping, fine workmanship, very good, cost-effective	Jingdong prefecture level city in Shandong
Stowe Flagship Store	a power saver (electronics)	The meter is as described, the quality is quite good, energy-saving, worth buying oh~!	Jingdong prefecture level city in Shandong
Stowe Flagship Store	a power saver (electronics)	Great value for money, very affordable,, perfect . ˆ◡ˆ.	Jingdong prefecture level city in Shandong
Stowe Flagship Store	a power saver (electronics)	Good!!! Worth recommending!	Jingdong prefecture level city in Shandong
Stowe Flagship Store	a power saver (electronics)	Great, fast logistics, great stuff, great value for money!	Jingdong prefecture level city in Shandong

Stowe Flagship Store	a power saver (electronics)	Fast logistics, very satisfied with the quality upon receipt	Jingdong prefecture level city in Shandong
Stowe Flagship Store	a power saver (electronics)	Great quality, plug and play, fast shipping!	Jingdong prefecture level city in Shandong
Stowe Flagship Store	a power saver (electronics)	Logistics received, quite satisfied	Jingdong prefecture level city in Shandong
Stowe Flagship Store	a power saver (electronics)	I don't know if it really works	Jingdong prefecture level city in Shandong
Stowe Flagship Store	a power saver (electronics)	It's bullshit. It's a plug light. Don't talk about power savings.	Jingdong prefecture level city in Shandong
Stowe Flagship Store	a power saver (electronics)	Fake and deceitful stuff that hasn't changed at all.	Jingdong prefecture level city in Shandong
Stowe Flagship Store	a power saver (electronics)	Power-saving effect: power-saving effect is particularly obvious Cost-effective: cost-effective business Appearance and style: external small micro-work materials: fine workmanship Experience: use very good, non the first convenient	Jingdong prefecture level city in Shandong
Stowe Flagship Store	a power saver (electronics)	After using it for a few days, it feels quite good, it should be quite safe, and it also sends a plug, the merchant is good, wait for a period of time and then calculate whether it saves on the electricity bill and then add comments ha!	Jingdong prefecture level city in Shandong
Stowe Flagship Store	a power saver (electronics)	My friend recommended to me, said the effect is very good, I bought two, this month less money, really can, just buy I do not believe, but has proved to have effect	Jingdong prefecture level city in Shandong
Stowe Flagship Store	a power saver (electronics)	Just bought back, already used, these days to see the effect of it, and so have the effect will also come back to give positive feedback, the price is also super cheap!	Jingdong prefecture level city in Shandong
Stowe Flagship Store	a power saver (electronics)	It does work well, the store uses a lot of electricity, this time to buy back a few, each place that uses a lot of electricity to insert one. Summer is here, the electricity is too strong.	Jingdong prefecture level city in Shandong
Stowe Flagship Store	a power saver (electronics)	Power saving treasure received, as for the effect, according to the instructions written, a short period of time can not be seen, at least 2-3 months before the real effect, then follow up evaluation	Jingdong prefecture level city in Shandong
Stowe Flagship Store	a power saver (electronics)	The goods have just been received and have not been used yet looking forward to the results.	Jingdong prefecture level city in Shandong
Stowe Flagship Store	a power saver (electronics)	Good installation works great	Jingdong prefecture level city in Shandong
Stowe Flagship Store	a power saver (electronics)	The logistics is super super fast! The quality is also very good, indeed value for money	Jingdong prefecture level city in Shandong
Stowe Flagship Store	a power saver (electronics)	Has been used for a period of time before coming to comment on, really good, air conditioning a day open non-stop, pay the electricity bill when the heartache, with this, the home electricity is not at all worried about the bill out of contrast to save a lot of preparation in the hands of a few, each room An a	Jingdong prefecture level city in Shandong
Stowe Flagship Store	a power saver (electronics)	Saving device received, really good, with the air conditioning tried, an hour without a saver with 2.9 degrees, with a saver an hour with 1.1 degrees, the beginning of not quite believe that this thing, the actual test to know that it is really useful, and intend to buy another.	Jingdong prefecture level city in Shandong
Stowe Flagship Store	a power saver (electronics)	Power saving effect: this energy saver is still useful, day trial for three days, every day than not installed before the reduction of two dollars a day, every day to open the bedroom to open the air conditioning before fifteen dollars now thirteen dollars a day. Still a little use.	Jingdong prefecture level city in Shandong
Stowe Flagship Store	a power saver (electronics)	Originally bought for home use, used for two months, the electricity bill from more than 200 yuan per month, down to more than 140 yuan, there is indeed a power-saving effect, this time and sold two, the unit with. Merchant service attitude is really good.	Jingdong prefecture level city in Shandong
Stowe Flagship Store	a power saver (electronics)	Plug and play, stable voltage, the screen shows when working, this winter home appliances, need electricity in many places, just buy to use, save electricity and money, with instructions	Jingdong prefecture level city in Shandong
Stowe Flagship Store	a power saver (electronics)	Goods received, do not know if it is good. When I bought it, I saw that it was a Stowe brand or company's product. Received but not. I do not know what is the reason. Now I can only give this evaluation. I'm	Jingdong prefecture level city in Shandong
Stowe Flagship Store	a power saver (electronics)	The first time to buy online, but also look at the evaluation before buying, the quality is not bad, but also very cheap, the need for friends can rest assured to buy!	Jingdong prefecture level city in Shandong
Stowe Flagship Store	a power saver (electronics)	🌈 Great quality, bought it to my liking 💝 Awesome! ✨ Buy with confidence if you need it!	Jingdong prefecture level city in Shandong
Stowe Flagship Store	a power saver (electronics)	The quality is very good, completely exceeded the expectations, the delivery speed is very fast, the packaging is very careful and strict, very satisfied with a shopping	Jingdong prefecture level city in Shandong
Stowe Flagship Store	a power saver (electronics)	The effect is very good, the home uses less electricity is very energy efficient, cost-effective	Jingdong prefecture level city in Shandong
Stowe Flagship Store	a power saver (electronics)	Quality can't be beat, just like the store, especially like, and the price is favorable!	Jingdong prefecture level city in Shandong
Stowe Flagship Store	a power saver (electronics)	Baby received! Very unexpected, will be back next time ~ great seller, stay tuned!	Jingdong prefecture level city in Shandong
Stowe Flagship Store	a power saver (electronics)	Follow up review after a month is more real, waiting for it	Jingdong prefecture level city in Shandong
Stowe Flagship Store	a power saver (electronics)	It is the ideal product, good quality and fast logistics, worthy of praise!	Jingdong prefecture level city in Shandong
Stowe Flagship Store	a power saver (electronics)	The saver is great, the color is right, the installer is very professional	Jingdong prefecture level city in Shandong
Stowe Flagship Store	a power saver (electronics)	The effect of the power connection is particularly obvious, it is particularly easy to use, very satisfied, the	Jingdong prefecture level city in Shandong
Stowe Flagship Store	a power saver (electronics)	Goods are very energy-saving, used to be more than two hundred degrees, now there are only a hundred or so degrees	Jingdong prefecture level city in Shandong
Stowe Flagship Store	a power saver (electronics)	Good quality, fast shipping, good results, good value for money	Jingdong prefecture level city in Shandong
Stowe Flagship Store	a power saver (electronics)	Fast shipping, fine workmanship, very good, cost-effective	Jingdong prefecture level city in Shandong
Stowe Flagship Store	a power saver (electronics)	The meter is as described, the quality is quite good, energy-saving, worth buying oh~!	Jingdong prefecture level city in Shandong
Stowe Flagship Store	a power saver (electronics)	Great value for money, very affordable,, perfect . ˆ◡ˆ.	Jingdong prefecture level city in Shandong
Stowe Flagship Store	a power saver (electronics)	Good!!! Worth recommending!	Jingdong prefecture level city in Shandong
Stowe Flagship Store	a power saver (electronics)	Great, fast logistics, great stuff, great value for money!	Jingdong prefecture level city in Shandong
Stowe Flagship Store	a power saver (electronics)	Fast logistics, very satisfied with the quality upon receipt	Jingdong prefecture level city in Shandong
Stowe Flagship Store	a power saver (electronics)	Great quality, plug and play, fast shipping!	Jingdong prefecture level city in Shandong
Stowe Flagship Store	a power saver (electronics)	Logistics received, quite satisfied	Jingdong prefecture level city in Shandong
Stowe Flagship Store	a power saver (electronics)	I don't know if it really works	Jingdong prefecture level city in Shandong
Stowe Flagship Store	a power saver (electronics)	It's bullshit. It's a plug light. Don't talk about power savings.	Jingdong prefecture level city in Shandong
Stowe Flagship Store	a power saver (electronics)	Fake and deceitful stuff that hasn't changed at all.	Jingdong prefecture level city in Shandong
Stowe Flagship Store	a power saver (electronics)	Power-saving effect: power-saving effect is particularly obvious Cost-effective: cost-effective business Appearance and style: external small micro-work materials: fine workmanship Experience: use very good, non the first convenient	Jingdong prefecture level city in Shandong
Stowe Flagship Store	a power saver (electronics)	After using it for a few days, it feels quite good, it should be quite safe, and it also sends a plug, the merchant is good, wait for a period of time and then calculate whether it saves on the electricity bill and then add comments ha!	Jingdong prefecture level city in Shandong
Stowe Flagship Store	a power saver (electronics)	My friend recommended to me, said the effect is very good, I bought two, this month less money, really can, just buy I do not believe, but has proved to have effect	Jingdong prefecture level city in Shandong

Stowe Flagship Store	a power saver (electronics)	Just bought back, already used, these days to see the effect of it, and so have the effect will also come back to give positive feedback, the price is also super cheap!	Jingdong prefecture level city in Shandong
Stowe Flagship Store	a power saver (electronics)	It does work well, the store uses a lot of electricity, this time to buy back a few, each place that uses a lot of electricity to insert one. Summer is here, the electricity is too strong.	Jingdong prefecture level city in Shandong
Stowe Flagship Store	a power saver (electronics)	Power saving treasure received, as for the effect, according to the instructions written, a short period of time can not be seen, at least 2-3 months before the real effect, then follow up evaluation	Jingdong prefecture level city in Shandong
Stowe Flagship Store	a power saver (electronics)	The goods have just been received and have not been used yet looking forward to the results.	Jingdong prefecture level city in Shandong
Stowe Flagship Store	a power saver (electronics)	Good installation works great	Jingdong prefecture level city in Shandong
Stowe Flagship Store	a power saver (electronics)	The logistics is super super fast! The quality is also very good, indeed value for money	Jingdong prefecture level city in Shandong
Stowe Flagship Store	a power saver (electronics)	Has been used for a period of time before coming to comment on, really good, air conditioning a day open non-stop, pay the electricity bill when the heartache, with this, the home electricity is not at all worried about the bill out of contrast to save a lot of preparation in the hands of a few, each room An a	Jingdong prefecture level city in Shandong
Stowe Flagship Store	a power saver (electronics)	Saving device received, really good, with the air conditioning tried, an hour without a saver with 2.9 degrees, with a saver an hour with 1.1 degrees, the beginning of not quite believe that this thing, the actual test to know that it is really useful, and intend to buy another.	Jingdong prefecture level city in Shandong
Stowe Flagship Store	a power saver (electronics)	Power saving effect: this energy saver is still useful, day trial for three days, every day than not installed before the reduction of two dollars a day, every day to open the bedroom to open the air conditioning before fifteen dollars now thirteen dollars a day. Still a little use.	Jingdong prefecture level city in Shandong
Stowe Flagship Store	a power saver (electronics)	Originally bought for home use, used for two months, the electricity bill from more than 200 yuan per month, down to more than 140 yuan, there is indeed a power-saving effect, this time and sold two, the unit with. Merchant service attitude is really good.	Jingdong prefecture level city in Shandong
Stowe Flagship Store	a power saver (electronics)	Plug and play, stable voltage, the screen shows when working, this winter home appliances, need electricity in many places, just buy to use, save electricity and money, with instructions	Jingdong prefecture level city in Shandong
Stowe Flagship Store	a power saver (electronics)	Goods received, do not know if it is good. When I bought it, I saw that it was a Stowe brand or company's product. Received but not. I do not know what is the reason. Now I can only give this evaluation. I'm	Jingdong prefecture level city in Shandong
Stowe Flagship Store	a power saver (electronics)	The first time to buy online, but also look at the evaluation before buying, the quality is not bad, but also very cheap, the need for friends can rest assured to buy!	Jingdong prefecture level city in Shandong
Stowe Flagship Store	a power saver (electronics)	🌈 Great quality, bought it to my liking 💝 Awesome! ✨ Buy with confidence if you need it!	Jingdong prefecture level city in Shandong
Stowe Flagship Store	a power saver (electronics)	The quality is very good, completely exceeded the expectations, the delivery speed is very fast, the packaging is very careful and strict, very satisfied with a shopping	Jingdong prefecture level city in Shandong
Stowe Flagship Store	a power saver (electronics)	The effect is very good, the home uses less electricity is very energy efficient, cost-effective	Jingdong prefecture level city in Shandong
Stowe Flagship Store	a power saver (electronics)	Quality can't be beat, just like the store, especially like, and the price is favorable!	Jingdong prefecture level city in Shandong
Stowe Flagship Store	a power saver (electronics)	Baby received! Very unexpected, will be back next time ~ great seller, stay tuned!	Jingdong prefecture level city in Shandong
Stowe Flagship Store	a power saver (electronics)	Follow up review after a month is more real, waiting for it	Jingdong prefecture level city in Shandong
Stowe Flagship Store	a power saver (electronics)	It is the ideal product, good quality and fast logistics, worthy of praise!	Jingdong prefecture level city in Shandong
Stowe Flagship Store	a power saver (electronics)	The saver is great, the color is right, the installer is very professional	Jingdong prefecture level city in Shandong
Stowe Flagship Store	a power saver (electronics)	The effect of the power connection is particularly obvious, it is particularly easy to use, very satisfied, the	Jingdong prefecture level city in Shandong
Stowe Flagship Store	a power saver (electronics)	Goods are very energy-saving, used to be more than two hundred degrees, now there are only a hundred or so degrees	Jingdong prefecture level city in Shandong
Stowe Flagship Store	a power saver (electronics)	Good quality, fast shipping, good results, good value for money	Jingdong prefecture level city in Shandong
Stowe Flagship Store	a power saver (electronics)	Fast shipping, fine workmanship, very good, cost-effective	Jingdong prefecture level city in Shandong
Stowe Flagship Store	a power saver (electronics)	The meter is as described, the quality is quite good, energy-saving, worth buying oh~!	Jingdong prefecture level city in Shandong
Stowe Flagship Store	a power saver (electronics)	Great value for money, very affordable,, perfect . ˆ◡ˆ.	Jingdong prefecture level city in Shandong
Stowe Flagship Store	a power saver (electronics)	Good!!! Worth recommending!	Jingdong prefecture level city in Shandong
Stowe Flagship Store	a power saver (electronics)	Great, fast logistics, great stuff, great value for money!	Jingdong prefecture level city in Shandong
Stowe Flagship Store	a power saver (electronics)	Fast logistics, very satisfied with the quality upon receipt	Jingdong prefecture level city in Shandong
Stowe Flagship Store	a power saver (electronics)	Great quality, plug and play, fast shipping!	Jingdong prefecture level city in Shandong
Stowe Flagship Store	a power saver (electronics)	Logistics received, quite satisfied	Jingdong prefecture level city in Shandong
Stowe Flagship Store	a power saver (electronics)	I don't know if it really works	Jingdong prefecture level city in Shandong
Stowe Flagship Store	a power saver (electronics)	It's bullshit. It's a plug light. Don't talk about power savings.	Jingdong prefecture level city in Shandong
Stowe Flagship Store	a power saver (electronics)	Fake and deceitful stuff that hasn't changed at all.	Jingdong prefecture level city in Shandong
Stowe Flagship Store	a power saver (electronics)	Power-saving effect: power-saving effect is particularly obvious Cost-effective: cost-effective business Appearance and style: external small micro-work materials: fine workmanship Experience: use very good, non the first convenient	Jingdong prefecture level city in Shandong
Stowe Flagship Store	a power saver (electronics)	After using it for a few days, it feels quite good, it should be quite safe, and it also sends a plug, the merchant is good, wait for a period of time and then calculate whether it saves on the electricity bill and then add comments ha!	Jingdong prefecture level city in Shandong
Stowe Flagship Store	a power saver (electronics)	My friend recommended to me, said the effect is very good, I bought two, this month less money, really can, just buy I do not believe, but has proved to have effect	Jingdong prefecture level city in Shandong
Stowe Flagship Store	a power saver (electronics)	Just bought back, already used, these days to see the effect of it, and so have the effect will also come back to give positive feedback, the price is also super cheap!	Jingdong prefecture level city in Shandong
Stowe Flagship Store	a power saver (electronics)	It does work well, the store uses a lot of electricity, this time to buy back a few, each place that uses a lot of electricity to insert one. Summer is here, the electricity is too strong.	Jingdong prefecture level city in Shandong
Stowe Flagship Store	a power saver (electronics)	Power saving treasure received, as for the effect, according to the instructions written, a short period of time can not be seen, at least 2-3 months before the real effect, then follow up evaluation	Jingdong prefecture level city in Shandong
Stowe Flagship Store	a power saver (electronics)	The goods have just been received and have not been used yet looking forward to the results.	Jingdong prefecture level city in Shandong
Stowe Flagship Store	a power saver (electronics)	Good installation works great	Jingdong prefecture level city in Shandong
Stowe Flagship Store	a power saver (electronics)	The logistics is super super fast! The quality is also very good, indeed value for money	Jingdong prefecture level city in Shandong
Stowe Flagship Store	a power saver (electronics)	Has been used for a period of time before coming to comment on, really good, air conditioning a day open non-stop, pay the electricity bill when the heartache, with this, the home electricity is not at all worried about the bill out of contrast to save a lot of preparation in the hands of a few, each room An a	Jingdong prefecture level city in Shandong
Stowe Flagship Store	a power saver (electronics)	Saving device received, really good, with the air conditioning tried, an hour without a saver with 2.9 degrees, with a saver an hour with 1.1 degrees, the beginning of not quite believe that this thing, the actual test to know that it is really useful, and intend to buy another.	Jingdong prefecture level city in Shandong
Stowe Flagship Store	a power saver (electronics)	Power saving effect: this energy saver is still useful, day trial for three days, every day than not installed before the reduction of two dollars a day, every day to open the bedroom to open the air conditioning before fifteen dollars now thirteen dollars a day. Still a little use.	Jingdong prefecture level city in Shandong

Stowe Flagship Store	a power saver (electronics)	Originally bought for home use, used for two months, the electricity bill from more than 200 yuan per month, down to more than 140 yuan, there is indeed a power-saving effect, this time and sold two, the unit with. Merchant service attitude is really good.	Jingdong prefecture level city in Shandong
Stowe Flagship Store	a power saver (electronics)	Plug and play, stable voltage, the screen shows when working, this winter home appliances, need electricity in many places, just buy to use, save electricity and money, with instructions	Jingdong prefecture level city in Shandong
Stowe Flagship Store	a power saver (electronics)	Goods received, do not know if it is good. When I bought it, I saw that it was a Stowe brand or company's product. Received but not. I do not know what is the reason. Now I can only give this evaluation. I'm	Jingdong prefecture level city in Shandong
Stowe Flagship Store	a power saver (electronics)	The first time to buy online, but also look at the evaluation before buying, the quality is not bad, but also very cheap, the need for friends can rest assured to buy!	Jingdong prefecture level city in Shandong
Stowe Flagship Store	a power saver (electronics)	🌈 Great quality, bought it to my liking 💗 Awesome! ✨ Buy with confidence if you need it!	Jingdong prefecture level city in Shandong
Stowe Flagship Store	a power saver (electronics)	The quality is very good, completely exceeded the expectations, the delivery speed is very fast, the packaging is very careful and strict, very satisfied with a shopping	Jingdong prefecture level city in Shandong
Stowe Flagship Store	a power saver (electronics)	The effect is very good, the home uses less electricity is very energy efficient, cost-effective	Jingdong prefecture level city in Shandong
Stowe Flagship Store	a power saver (electronics)	Quality can't be beat, just like the store, especially like, and the price is favorable!	Jingdong prefecture level city in Shandong
Stowe Flagship Store	a power saver (electronics)	Baby received! Very unexpected, will be back next time ~ great seller, stay tuned!	Jingdong prefecture level city in Shandong
Stowe Flagship Store	a power saver (electronics)	Follow up review after a month is more real, waiting for it	Jingdong prefecture level city in Shandong
Stowe Flagship Store	a power saver (electronics)	It is the ideal product, good quality and fast logistics, worthy of praise!	Jingdong prefecture level city in Shandong
Stowe Flagship Store	a power saver (electronics)	The saver is great, the color is right, the installer is very professional	Jingdong prefecture level city in Shandong
Stowe Flagship Store	a power saver (electronics)	The effect of the power connection is particularly obvious, it is particularly easy to use, very satisfied, the	Jingdong prefecture level city in Shandong
Stowe Flagship Store	a power saver (electronics)	Goods are very energy-saving, used to be more than two hundred degrees, now there are only a hundred or so degrees	Jingdong prefecture level city in Shandong
Stowe Flagship Store	a power saver (electronics)	Good quality, fast shipping, good results, good value for money	Jingdong prefecture level city in Shandong
Stowe Flagship Store	a power saver (electronics)	Fast shipping, fine workmanship, very good, cost-effective	Jingdong prefecture level city in Shandong
Stowe Flagship Store	a power saver (electronics)	The meter is as described, the quality is quite good, energy-saving, worth buying oh~!	Jingdong prefecture level city in Shandong
Stowe Flagship Store	a power saver (electronics)	Great value for money, very affordable,, perfect . ˆ◡ˆ.	Jingdong prefecture level city in Shandong
Stowe Flagship Store	a power saver (electronics)	Good!!! Worth recommending!	Jingdong prefecture level city in Shandong
Stowe Flagship Store	a power saver (electronics)	Great, fast logistics, great stuff, great value for money!	Jingdong prefecture level city in Shandong
Stowe Flagship Store	a power saver (electronics)	Fast logistics, very satisfied with the quality upon receipt	Jingdong prefecture level city in Shandong
Stowe Flagship Store	a power saver (electronics)	Great quality, plug and play, fast shipping!	Jingdong prefecture level city in Shandong
Stowe Flagship Store	a power saver (electronics)	Logistics received, quite satisfied	Jingdong prefecture level city in Shandong
Stowe Flagship Store	a power saver (electronics)	I don't know if it really works	Jingdong prefecture level city in Shandong
Stowe Flagship Store	a power saver (electronics)	It's bullshit. It's a plug light. Don't talk about power savings.	Jingdong prefecture level city in Shandong
Stowe Flagship Store	a power saver (electronics)	Fake and deceitful stuff that hasn't changed at all.	Jingdong prefecture level city in Shandong
Stowe Flagship Store	a power saver (electronics)	Power-saving effect: power-saving effect is particularly obvious Cost-effective: cost-effective business Appearance and style: external small micro-work materials: fine workmanship Experience: use very good, non the first convenient	Jingdong prefecture level city in Shandong
Stowe Flagship Store	a power saver (electronics)	After using it for a few days, it feels quite good, it should be quite safe, and it also sends a plug, the merchant is good, wait for a period of time and then calculate whether it saves on the electricity bill and then add comments ha!	Jingdong prefecture level city in Shandong
Stowe Flagship Store	a power saver (electronics)	My friend recommended to me, said the effect is very good, I bought two, this month less money, really can, just buy I do not believe, but has proved to have effect	Jingdong prefecture level city in Shandong
Stowe Flagship Store	a power saver (electronics)	Just bought back, already used, these days to see the effect of it, and so have the effect will also come back to give positive feedback, the price is also super cheap!	Jingdong prefecture level city in Shandong
Stowe Flagship Store	a power saver (electronics)	It does work well, the store uses a lot of electricity, this time to buy back a few, each place that uses a lot of electricity to insert one. Summer is here, the electricity is too strong.	Jingdong prefecture level city in Shandong
Stowe Flagship Store	a power saver (electronics)	Power saving treasure received, as for the effect, according to the instructions written, a short period of time can not be seen, at least 2-3 months before the real effect, then follow up evaluation	Jingdong prefecture level city in Shandong
Stowe Flagship Store	a power saver (electronics)	The goods have just been received and have not been used yet looking forward to the results.	Jingdong prefecture level city in Shandong
Stowe Flagship Store	a power saver (electronics)	Good installation works great	Jingdong prefecture level city in Shandong
Stowe Flagship Store	a power saver (electronics)	The logistics is super super fast! The quality is also very good, indeed value for money	Jingdong prefecture level city in Shandong
Stowe Flagship Store	a power saver (electronics)	Has been used for a period of time before coming to comment on, really good, air conditioning a day open non-stop, pay the electricity bill when the heartache, with this, the home electricity is not at all worried about the bill out of contrast to save a lot of preparation in the hands of a few, each room An a	Jingdong prefecture level city in Shandong
Stowe Flagship Store	a power saver (electronics)	Saving device received, really good, with the air conditioning tried, an hour without a saver with 2.9 degrees, with a saver an hour with 1.1 degrees, the beginning of not quite believe that this thing, the actual test to know that it is really useful, and intend to buy another.	Jingdong prefecture level city in Shandong
Stowe Flagship Store	a power saver (electronics)	Power saving effect: this energy saver is still useful, day trial for three days, every day than not installed before the reduction of two dollars a day, every day to open the bedroom to open the air conditioning before fifteen dollars now thirteen dollars a day. Still a little use.	Jingdong prefecture level city in Shandong
Stowe Flagship Store	a power saver (electronics)	Originally bought for home use, used for two months, the electricity bill from more than 200 yuan per month, down to more than 140 yuan, there is indeed a power-saving effect, this time and sold two, the unit with. Merchant service attitude is really good.	Jingdong prefecture level city in Shandong
Stowe Flagship Store	a power saver (electronics)	Plug and play, stable voltage, the screen shows when working, this winter home appliances, need electricity in many places, just buy to use, save electricity and money, with instructions	Jingdong prefecture level city in Shandong
Stowe Flagship Store	a power saver (electronics)	Goods received, do not know if it is good. When I bought it, I saw that it was a Stowe brand or company's product. Received but not. I do not know what is the reason. Now I can only give this evaluation. I'm	Jingdong prefecture level city in Shandong
Stowe Flagship Store	a power saver (electronics)	The first time to buy online, but also look at the evaluation before buying, the quality is not bad, but also very cheap, the need for friends can rest assured to buy!	Jingdong prefecture level city in Shandong
Stowe Flagship Store	a power saver (electronics)	🌈 Great quality, bought it to my liking 💗 Awesome! ✨ Buy with confidence if you need it!	Jingdong prefecture level city in Shandong
Stowe Flagship Store	a power saver (electronics)	The quality is very good, completely exceeded the expectations, the delivery speed is very fast, the packaging is very careful and strict, very satisfied with a shopping	Jingdong prefecture level city in Shandong
Stowe Flagship Store	a power saver (electronics)	The effect is very good, the home uses less electricity is very energy efficient, cost-effective	Jingdong prefecture level city in Shandong
Stowe Flagship Store	a power saver (electronics)	Quality can't be beat, just like the store, especially like, and the price is favorable!	Jingdong prefecture level city in Shandong
Stowe Flagship Store	a power saver (electronics)	Baby received! Very unexpected, will be back next time ~ great seller, stay tuned!	Jingdong prefecture level city in Shandong
Stowe Flagship Store	a power saver (electronics)	Follow up review after a month is more real, waiting for it	Jingdong prefecture level city in Shandong

Stowe Flagship Store	a power saver (electronics)	It is the ideal product, good quality and fast logistics, worthy of praise!	Jingdong prefecture level city in Shandong
Stowe Flagship Store	a power saver (electronics)	The saver is great, the color is right, the installer is very professional	Jingdong prefecture level city in Shandong
Stowe Flagship Store	a power saver (electronics)	The effect of the power connection is particularly obvious, it is particularly easy to use, very satisfied, the	Jingdong prefecture level city in Shandong
Stowe Flagship Store	a power saver (electronics)	Goods are very energy-saving, used to be more than two hundred degrees, now there are only a hundred or so degrees	Jingdong prefecture level city in Shandong
Stowe Flagship Store	a power saver (electronics)	Good quality, fast shipping, good results, good value for money	Jingdong prefecture level city in Shandong
Stowe Flagship Store	a power saver (electronics)	Fast shipping, fine workmanship, very good, cost-effective	Jingdong prefecture level city in Shandong
Stowe Flagship Store	a power saver (electronics)	The meter is as described, the quality is quite good, energy-saving, worth buying oh~!	Jingdong prefecture level city in Shandong
Stowe Flagship Store	a power saver (electronics)	Great value for money, very affordable,, perfect . ˆ◡ˆ.	Jingdong prefecture level city in Shandong
Stowe Flagship Store	a power saver (electronics)	Good!!! Worth recommending!	Jingdong prefecture level city in Shandong
Stowe Flagship Store	a power saver (electronics)	Great, fast logistics, great stuff, great value for money!	Jingdong prefecture level city in Shandong
Stowe Flagship Store	a power saver (electronics)	Fast logistics, very satisfied with the quality upon receipt	Jingdong prefecture level city in Shandong
Stowe Flagship Store	a power saver (electronics)	Great quality, plug and play, fast shipping!	Jingdong prefecture level city in Shandong
Stowe Flagship Store	a power saver (electronics)	Logistics received, quite satisfied	Jingdong prefecture level city in Shandong
Stowe Flagship Store	a power saver (electronics)	I don't know if it really works	Jingdong prefecture level city in Shandong
Stowe Flagship Store	a power saver (electronics)	It's bullshit. It's a plug light. Don't talk about power savings.	Jingdong prefecture level city in Shandong
Stowe Flagship Store	a power saver (electronics)	Fake and deceitful stuff that hasn't changed at all.	Jingdong prefecture level city in Shandong
Stowe Flagship Store	a power saver (electronics)	Power-saving effect: power-saving effect is particularly obvious Cost-effective: cost-effective business Appearance and style: external small micro-work materials: fine workmanship Experience: use very good, non the first convenient	Jingdong prefecture level city in Shandong
Stowe Flagship Store	a power saver (electronics)	After using it for a few days, it feels quite good, it should be quite safe, and it also sends a plug, the merchant is good, wait for a period of time and then calculate whether it saves on the electricity bill and then add comments ha!	Jingdong prefecture level city in Shandong
Stowe Flagship Store	a power saver (electronics)	My friend recommended to me, said the effect is very good, I bought two, this month less money, really can, just buy I do not believe, but has proved to have effect	Jingdong prefecture level city in Shandong
Stowe Flagship Store	a power saver (electronics)	Just bought back, already used, these days to see the effect of it, and so have the effect will also come back to give positive feedback, the price is also super cheap!	Jingdong prefecture level city in Shandong
Stowe Flagship Store	a power saver (electronics)	It does work well, the store uses a lot of electricity, this time to buy back a few, each place that uses a lot of electricity to insert one. Summer is here, the electricity is too strong.	Jingdong prefecture level city in Shandong
Stowe Flagship Store	a power saver (electronics)	Power saving treasure received, as for the effect, according to the instructions written, a short period of time can not be seen, at least 2-3 months before the real effect, then follow up evaluation	Jingdong prefecture level city in Shandong
Stowe Flagship Store	a power saver (electronics)	The goods have just been received and have not been used yet looking forward to the results.	Jingdong prefecture level city in Shandong
Stowe Flagship Store	a power saver (electronics)	Good installation works great	Jingdong prefecture level city in Shandong
Stowe Flagship Store	a power saver (electronics)	The logistics is super super fast! The quality is also very good, indeed value for money	Jingdong prefecture level city in Shandong
Stowe Flagship Store	a power saver (electronics)	Has been used for a period of time before coming to comment on, really good, air conditioning a day open non-stop, pay the electricity bill when the heartache, with this, the home electricity is not at all worried about the bill out of contrast to save a lot of preparation in the hands of a few, each room An a	Jingdong prefecture level city in Shandong
Stowe Flagship Store	a power saver (electronics)	Saving device received, really good, with the air conditioning tried, an hour without a saver with 2.9 degrees, with a saver an hour with 1.1 degrees, the beginning of not quite believe that this thing, the actual test to know that it is really useful, and intend to buy another.	Jingdong prefecture level city in Shandong
Stowe Flagship Store	a power saver (electronics)	Power saving effect: this energy saver is still useful, day trial for three days, every day than not installed before the reduction of two dollars a day, every day to open the bedroom to open the air conditioning before fifteen dollars now thirteen dollars a day. Still a little use.	Jingdong prefecture level city in Shandong
Stowe Flagship Store	a power saver (electronics)	Originally bought for home use, used for two months, the electricity bill from more than 200 yuan per month, down to more than 140 yuan, there is indeed a power-saving effect, this time and sold two, the unit with. Merchant service attitude is really good.	Jingdong prefecture level city in Shandong
Stowe Flagship Store	a power saver (electronics)	Plug and play, stable voltage, the screen shows when working, this winter home appliances, need electricity in many places, just buy to use, save electricity and money, with instructions	Jingdong prefecture level city in Shandong
Stowe Flagship Store	a power saver (electronics)	Goods received, do not know if it is good. When I bought it, I saw that it was a Stowe brand or company's product. Received but not. I do not know what is the reason. Now I can only give this evaluation. I'm	Jingdong prefecture level city in Shandong
Stowe Flagship Store	a power saver (electronics)	The first time to buy online, but also look at the evaluation before buying, the quality is not bad, but also very cheap, the need for friends can rest assured to buy!	Jingdong prefecture level city in Shandong
Stowe Flagship Store	a power saver (electronics)	🌈 Great quality, bought it to my liking 💕 Awesome! ✨ Buy with confidence if you need it!	Jingdong prefecture level city in Shandong
Stowe Flagship Store	a power saver (electronics)	The quality is very good, completely exceeded the expectations, the delivery speed is very fast, the packaging is very careful and strict, very satisfied with a shopping	Jingdong prefecture level city in Shandong
Stowe Flagship Store	a power saver (electronics)	The effect is very good, the home uses less electricity is very energy efficient, cost-effective	Jingdong prefecture level city in Shandong
Stowe Flagship Store	a power saver (electronics)	Quality can't be beat, just like the store, especially like, and the price is favorable!	Jingdong prefecture level city in Shandong
Stowe Flagship Store	a power saver (electronics)	Baby received! Very unexpected, will be back next time ~ great seller, stay tuned!	Jingdong prefecture level city in Shandong
Stowe Flagship Store	a power saver (electronics)	Follow up review after a month is more real, waiting for it	Jingdong prefecture level city in Shandong
Stowe Flagship Store	a power saver (electronics)	It is the ideal product, good quality and fast logistics, worthy of praise!	Jingdong prefecture level city in Shandong
Stowe Flagship Store	a power saver (electronics)	The saver is great, the color is right, the installer is very professional	Jingdong prefecture level city in Shandong
Stowe Flagship Store	a power saver (electronics)	The effect of the power connection is particularly obvious, it is particularly easy to use, very satisfied, the	Jingdong prefecture level city in Shandong
Stowe Flagship Store	a power saver (electronics)	Goods are very energy-saving, used to be more than two hundred degrees, now there are only a hundred or so degrees	Jingdong prefecture level city in Shandong
Stowe Flagship Store	a power saver (electronics)	Good quality, fast shipping, good results, good value for money	Jingdong prefecture level city in Shandong
Stowe Flagship Store	a power saver (electronics)	Fast shipping, fine workmanship, very good, cost-effective	Jingdong prefecture level city in Shandong
Stowe Flagship Store	a power saver (electronics)	The meter is as described, the quality is quite good, energy-saving, worth buying oh~!	Jingdong prefecture level city in Shandong
Stowe Flagship Store	a power saver (electronics)	Great value for money, very affordable,, perfect . ˆ◡ˆ.	Jingdong prefecture level city in Shandong
Stowe Flagship Store	a power saver (electronics)	Good!!! Worth recommending!	Jingdong prefecture level city in Shandong
Stowe Flagship Store	a power saver (electronics)	Great, fast logistics, great stuff, great value for money!	Jingdong prefecture level city in Shandong

Stowe Flagship Store	a power saver (electronics)	Fast logistics, very satisfied with the quality upon receipt	Jingdong prefecture level city in Shandong
Stowe Flagship Store	a power saver (electronics)	Great quality, plug and play, fast shipping!	Jingdong prefecture level city in Shandong
Stowe Flagship Store	a power saver (electronics)	Logistics received, quite satisfied	Jingdong prefecture level city in Shandong
Stowe Flagship Store	a power saver (electronics)	I don't know if it really works	Jingdong prefecture level city in Shandong
Stowe Flagship Store	a power saver (electronics)	It's bullshit. It's a plug light. Don't talk about power savings.	Jingdong prefecture level city in Shandong
Stowe Flagship Store	a power saver (electronics)	Fake and deceitful stuff that hasn't changed at all.	Jingdong prefecture level city in Shandong
Stowe Flagship Store	a power saver (electronics)	Power-saving effect: power-saving effect is particularly obvious Cost-effective: cost-effective business Appearance and style: external small micro-work materials: fine workmanship Experience: use very good, non the first convenient	Jingdong prefecture level city in Shandong
Stowe Flagship Store	a power saver (electronics)	After using it for a few days, it feels quite good, it should be quite safe, and it also sends a plug, the merchant is good, wait for a period of time and then calculate whether it saves on the electricity bill and then add comments ha!	Jingdong prefecture level city in Shandong
Stowe Flagship Store	a power saver (electronics)	My friend recommended to me, said the effect is very good, I bought two, this month less money, really can, just buy I do not believe, but has proved to have effect	Jingdong prefecture level city in Shandong
Stowe Flagship Store	a power saver (electronics)	Just bought back, already used, these days to see the effect of it, and so have the effect will also come back to give positive feedback, the price is also super cheap!	Jingdong prefecture level city in Shandong
Stowe Flagship Store	a power saver (electronics)	It does work well, the store uses a lot of electricity, this time to buy back a few, each place that uses a lot of electricity to insert one. Summer is here, the electricity is too strong.	Jingdong prefecture level city in Shandong
Stowe Flagship Store	a power saver (electronics)	Power saving treasure received, as for the effect, according to the instructions written, a short period of time can not be seen, at least 2-3 months before the real effect, then follow up evaluation	Jingdong prefecture level city in Shandong
Stowe Flagship Store	a power saver (electronics)	The goods have just been received and have not been used yet looking forward to the results.	Jingdong prefecture level city in Shandong
Stowe Flagship Store	a power saver (electronics)	Good installation works great	Jingdong prefecture level city in Shandong
Stowe Flagship Store	a power saver (electronics)	The logistics is super super fast! The quality is also very good, indeed value for money	Jingdong prefecture level city in Shandong
Stowe Flagship Store	a power saver (electronics)	Has been used for a period of time before coming to comment on, really good, air conditioning a day open non-stop, pay the electricity bill when the heartache, with this, the home electricity is not at all worried about the bill out of contrast to save a lot of preparation in the hands of a few, each room An a	Jingdong prefecture level city in Shandong
Stowe Flagship Store	a power saver (electronics)	Saving device received, really good, with the air conditioning tried, an hour without a saver with 2.9 degrees, with a saver an hour with 1.1 degrees, the beginning of not quite believe that this thing, the actual test to know that it is really useful, and intend to buy another.	Jingdong prefecture level city in Shandong
Stowe Flagship Store	a power saver (electronics)	Power saving effect: this energy saver is still useful, day trial for three days, every day than not installed before the reduction of two dollars a day, every day to open the bedroom to open the air conditioning before fifteen dollars now thirteen dollars a day. Still a little use.	Jingdong prefecture level city in Shandong
Stowe Flagship Store	a power saver (electronics)	Originally bought for home use, used for two months, the electricity bill from more than 200 yuan per month, down to more than 140 yuan, there is indeed a power-saving effect, this time and sold two, the unit with. Merchant service attitude is really good.	Jingdong prefecture level city in Shandong
Stowe Flagship Store	a power saver (electronics)	Plug and play, stable voltage, the screen shows when working, this winter home appliances, need electricity in many places, just buy to use, save electricity and money, with instructions	Jingdong prefecture level city in Shandong
Stowe Flagship Store	a power saver (electronics)	Goods received, do not know if it is good. When I bought it, I saw that it was a Stowe brand or company's product. Received but not. I do not know what is the reason. Now I can only give this evaluation. I'm	Jingdong prefecture level city in Shandong
Stowe Flagship Store	a power saver (electronics)	The first time to buy online, but also look at the evaluation before buying, the quality is not bad, but also very cheap, the need for friends can rest assured to buy!	Jingdong prefecture level city in Shandong
Stowe Flagship Store	a power saver (electronics)	🌈 Great quality, bought it to my liking 💝 Awesome! ✨ Buy with confidence if you need it!	Jingdong prefecture level city in Shandong
Stowe Flagship Store	a power saver (electronics)	The quality is very good, completely exceeded the expectations, the delivery speed is very fast, the packaging is very careful and strict, very satisfied with a shopping	Jingdong prefecture level city in Shandong
Stowe Flagship Store	a power saver (electronics)	The effect is very good, the home uses less electricity is very energy efficient, cost-effective	Jingdong prefecture level city in Shandong
Stowe Flagship Store	a power saver (electronics)	Quality can't be beat, just like the store, especially like, and the price is favorable!	Jingdong prefecture level city in Shandong
Stowe Flagship Store	a power saver (electronics)	Baby received! Very unexpected, will be back next time ~ great seller, stay tuned!	Jingdong prefecture level city in Shandong
Stowe Flagship Store	a power saver (electronics)	Follow up review after a month is more real, waiting for it	Jingdong prefecture level city in Shandong
Stowe Flagship Store	a power saver (electronics)	It is the ideal product, good quality and fast logistics, worthy of praise!	Jingdong prefecture level city in Shandong
Stowe Flagship Store	a power saver (electronics)	The saver is great, the color is right, the installer is very professional	Jingdong prefecture level city in Shandong
Stowe Flagship Store	a power saver (electronics)	The effect of the power connection is particularly obvious, it is particularly easy to use, very satisfied, the	Jingdong prefecture level city in Shandong
Stowe Flagship Store	a power saver (electronics)	Goods are very energy-saving, used to be more than two hundred degrees, now there are only a hundred or so degrees	Jingdong prefecture level city in Shandong
Stowe Flagship Store	a power saver (electronics)	Good quality, fast shipping, good results, good value for money	Jingdong prefecture level city in Shandong
Stowe Flagship Store	a power saver (electronics)	Fast shipping, fine workmanship, very good, cost-effective	Jingdong prefecture level city in Shandong
Stowe Flagship Store	a power saver (electronics)	The meter is as described, the quality is quite good, energy-saving, worth buying oh~!	Jingdong prefecture level city in Shandong
Stowe Flagship Store	a power saver (electronics)	Great value for money, very affordable,, perfect . ˆ◡ˆ.	Jingdong prefecture level city in Shandong
Stowe Flagship Store	a power saver (electronics)	Good!!! Worth recommending!	Jingdong prefecture level city in Shandong
Stowe Flagship Store	a power saver (electronics)	Great, fast logistics, great stuff, great value for money!	Jingdong prefecture level city in Shandong
Stowe Flagship Store	a power saver (electronics)	Fast logistics, very satisfied with the quality upon receipt	Jingdong prefecture level city in Shandong
Stowe Flagship Store	a power saver (electronics)	Great quality, plug and play, fast shipping!	Jingdong prefecture level city in Shandong
Stowe Flagship Store	a power saver (electronics)	Logistics received, quite satisfied	Jingdong prefecture level city in Shandong
Stowe Flagship Store	a power saver (electronics)	I don't know if it really works	Jingdong prefecture level city in Shandong
Stowe Flagship Store	a power saver (electronics)	It's bullshit. It's a plug light. Don't talk about power savings.	Jingdong prefecture level city in Shandong
Stowe Flagship Store	a power saver (electronics)	Fake and deceitful stuff that hasn't changed at all.	Jingdong prefecture level city in Shandong
Stowe Flagship Store	a power saver (electronics)	Power-saving effect: power-saving effect is particularly obvious Cost-effective: cost-effective business Appearance and style: external small micro-work materials: fine workmanship Experience: use very good, non the first convenient	Jingdong prefecture level city in Shandong
Stowe Flagship Store	a power saver (electronics)	After using it for a few days, it feels quite good, it should be quite safe, and it also sends a plug, the merchant is good, wait for a period of time and then calculate whether it saves on the electricity bill and then add comments ha!	Jingdong prefecture level city in Shandong
Stowe Flagship Store	a power saver (electronics)	My friend recommended to me, said the effect is very good, I bought two, this month less money, really can, just buy I do not believe, but has proved to have effect	Jingdong prefecture level city in Shandong

Stowe Flagship Store	a power saver (electronics)	Just bought back, already used, these days to see the effect of it, and so have the effect will also come back to give positive feedback, the price is also super cheap!	Jingdong prefecture level city in Shandong
Stowe Flagship Store	a power saver (electronics)	It does work well, the store uses a lot of electricity, this time to buy back a few, each place that uses a lot of electricity to insert one. Summer is here, the electricity is too strong.	Jingdong prefecture level city in Shandong
Stowe Flagship Store	a power saver (electronics)	Power saving treasure received, as for the effect, according to the instructions written, a short period of time can not be seen, at least 2-3 months before the real effect, then follow up evaluation	Jingdong prefecture level city in Shandong
Stowe Flagship Store	a power saver (electronics)	The goods have just been received and have not been used yet looking forward to the results.	Jingdong prefecture level city in Shandong
Stowe Flagship Store	a power saver (electronics)	Good installation works great	Jingdong prefecture level city in Shandong
Stowe Flagship Store	a power saver (electronics)	The logistics is super super fast! The quality is also very good, indeed value for money	Jingdong prefecture level city in Shandong
Stowe Flagship Store	a power saver (electronics)	Has been used for a period of time before coming to comment on, really good, air conditioning a day open non-stop, pay the electricity bill when the heartache, with this, the home electricity is not at all worried about the bill out of contrast to save a lot of preparation in the hands of a few, each room An a	Jingdong prefecture level city in Shandong
Stowe Flagship Store	a power saver (electronics)	Saving device received, really good, with the air conditioning tried, an hour without a saver with 2.9 degrees, with a saver an hour with 1.1 degrees, the beginning of not quite believe that this thing, the actual test to know that it is really useful, and intend to buy another.	Jingdong prefecture level city in Shandong
Stowe Flagship Store	a power saver (electronics)	Power saving effect: this energy saver is still useful, day trial for three days, every day than not installed before the reduction of two dollars a day, every day to open the bedroom to open the air conditioning before fifteen dollars now thirteen dollars a day. Still a little use.	Jingdong prefecture level city in Shandong
Stowe Flagship Store	a power saver (electronics)	Originally bought for home use, used for two months, the electricity bill from more than 200 yuan per month, down to more than 140 yuan, there is indeed a power-saving effect, this time and sold two, the unit with. Merchant service attitude is really good.	Jingdong prefecture level city in Shandong
Stowe Flagship Store	a power saver (electronics)	Plug and play, stable voltage, the screen shows when working, this winter home appliances, need electricity in many places, just buy to use, save electricity and money, with instructions	Jingdong prefecture level city in Shandong
Stowe Flagship Store	a power saver (electronics)	Goods received, do not know if it is good. When I bought it, I saw that it was a Stowe brand or company's product. Received but not. I do not know what is the reason. Now I can only give this evaluation. I'm	Jingdong prefecture level city in Shandong
Stowe Flagship Store	a power saver (electronics)	The first time to buy online, but also look at the evaluation before buying, the quality is not bad, but also very cheap, the need for friends can rest assured to buy!	Jingdong prefecture level city in Shandong
Stowe Flagship Store	a power saver (electronics)	🌈 Great quality, bought it to my liking 💛 Awesome! ✨ Buy with confidence if you need it!	Jingdong prefecture level city in Shandong
Stowe Flagship Store	a power saver (electronics)	The quality is very good, completely exceeded the expectations, the delivery speed is very fast, the packaging is very careful and strict, very satisfied with a shopping	Jingdong prefecture level city in Shandong
Stowe Flagship Store	a power saver (electronics)	The effect is very good, the home uses less electricity is very energy efficient, cost-effective	Jingdong prefecture level city in Shandong
Stowe Flagship Store	a power saver (electronics)	Quality can't be beat, just like the store, especially like, and the price is favorable!	Jingdong prefecture level city in Shandong
Stowe Flagship Store	a power saver (electronics)	Baby received! Very unexpected, will be back next time ~ great seller, stay tuned!	Jingdong prefecture level city in Shandong
Stowe Flagship Store	a power saver (electronics)	Follow up review after a month is more real, waiting for it	Jingdong prefecture level city in Shandong
Stowe Flagship Store	a power saver (electronics)	It is the ideal product, good quality and fast logistics, worthy of praise!	Jingdong prefecture level city in Shandong
Stowe Flagship Store	a power saver (electronics)	The saver is great, the color is right, the installer is very professional	Jingdong prefecture level city in Shandong
Stowe Flagship Store	a power saver (electronics)	The effect of the power connection is particularly obvious, it is particularly easy to use, very satisfied, the	Jingdong prefecture level city in Shandong
Stowe Flagship Store	a power saver (electronics)	Goods are very energy-saving, used to be more than two hundred degrees, now there are only a hundred or so degrees	Jingdong prefecture level city in Shandong
Stowe Flagship Store	a power saver (electronics)	Good quality, fast shipping, good results, good value for money	Jingdong prefecture level city in Shandong
Stowe Flagship Store	a power saver (electronics)	Fast shipping, fine workmanship, very good, cost-effective	Jingdong prefecture level city in Shandong
Stowe Flagship Store	a power saver (electronics)	The meter is as described, the quality is quite good, energy-saving, worth buying oh~!	Jingdong prefecture level city in Shandong
Stowe Flagship Store	a power saver (electronics)	Great value for money, very affordable,, perfect . ˆ◡ˆ.	Jingdong prefecture level city in Shandong
Stowe Flagship Store	a power saver (electronics)	Good!!! Worth recommending!	Jingdong prefecture level city in Shandong
Stowe Flagship Store	a power saver (electronics)	Great, fast logistics, great stuff, great value for money!	Jingdong prefecture level city in Shandong
Stowe Flagship Store	a power saver (electronics)	Fast logistics, very satisfied with the quality upon receipt	Jingdong prefecture level city in Shandong
Stowe Flagship Store	a power saver (electronics)	Great quality, plug and play, fast shipping!	Jingdong prefecture level city in Shandong
Stowe Flagship Store	a power saver (electronics)	Logistics received, quite satisfied	Jingdong prefecture level city in Shandong
Stowe Flagship Store	a power saver (electronics)	I don't know if it really works	Jingdong prefecture level city in Shandong
Stowe Flagship Store	a power saver (electronics)	It's bullshit. It's a plug light. Don't talk about power savings.	Jingdong prefecture level city in Shandong
Stowe Flagship Store	a power saver (electronics)	Fake and deceitful stuff that hasn't changed at all.	Jingdong prefecture level city in Shandong
Stowe Flagship Store	a power saver (electronics)	Power-saving effect: power-saving effect is particularly obvious Cost-effective: cost-effective business Appearance and style: external small micro-work materials: fine workmanship Experience: use very good, non the first convenient	Jingdong prefecture level city in Shandong
Stowe Flagship Store	a power saver (electronics)	After using it for a few days, it feels quite good, it should be quite safe, and it also sends a plug, the merchant is good, wait for a period of time and then calculate whether it saves on the electricity bill and then add comments ha!	Jingdong prefecture level city in Shandong
Stowe Flagship Store	a power saver (electronics)	My friend recommended to me, said the effect is very good, I bought two, this month less money, really can, just buy I do not believe, but has proved to have effect	Jingdong prefecture level city in Shandong
Stowe Flagship Store	a power saver (electronics)	Just bought back, already used, these days to see the effect of it, and so have the effect will also come back to give positive feedback, the price is also super cheap!	Jingdong prefecture level city in Shandong
Stowe Flagship Store	a power saver (electronics)	It does work well, the store uses a lot of electricity, this time to buy back a few, each place that uses a lot of electricity to insert one. Summer is here, the electricity is too strong.	Jingdong prefecture level city in Shandong
Stowe Flagship Store	a power saver (electronics)	Power saving treasure received, as for the effect, according to the instructions written, a short period of time can not be seen, at least 2-3 months before the real effect, then follow up evaluation	Jingdong prefecture level city in Shandong
Stowe Flagship Store	a power saver (electronics)	The goods have just been received and have not been used yet looking forward to the results.	Jingdong prefecture level city in Shandong
Stowe Flagship Store	a power saver (electronics)	Good installation works great	Jingdong prefecture level city in Shandong
Stowe Flagship Store	a power saver (electronics)	The logistics is super super fast! The quality is also very good, indeed value for money	Jingdong prefecture level city in Shandong
Stowe Flagship Store	a power saver (electronics)	Has been used for a period of time before coming to comment on, really good, air conditioning a day open non-stop, pay the electricity bill when the heartache, with this, the home electricity is not at all worried about the bill out of contrast to save a lot of preparation in the hands of a few, each room An a	Jingdong prefecture level city in Shandong
Stowe Flagship Store	a power saver (electronics)	Saving device received, really good, with the air conditioning tried, an hour without a saver with 2.9 degrees, with a saver an hour with 1.1 degrees, the beginning of not quite believe that this thing, the actual test to know that it is really useful, and intend to buy another.	Jingdong prefecture level city in Shandong
Stowe Flagship Store	a power saver (electronics)	Power saving effect: this energy saver is still useful, day trial for three days, every day than not installed before the reduction of two dollars a day, every day to open the bedroom to open the air conditioning before fifteen dollars now thirteen dollars a day. Still a little use.	Jingdong prefecture level city in Shandong

Stowe Flagship Store	a power saver (electronics)	Originally bought for home use, used for two months, the electricity bill from more than 200 yuan per month, down to more than 140 yuan, there is indeed a power-saving effect, this time and sold two, the unit with. Merchant service attitude is really good.	Jingdong prefecture level city in Shandong
Stowe Flagship Store	a power saver (electronics)	Plug and play, stable voltage, the screen shows when working, this winter home appliances, need electricity in many places, just buy to use, save electricity and money, with instructions	Jingdong prefecture level city in Shandong
Stowe Flagship Store	a power saver (electronics)	Goods received, do not know if it is good. When I bought it, I saw that it was a Stowe brand or company's product. Received but not. I do not know what is the reason. Now I can only give this evaluation. I'm	Jingdong prefecture level city in Shandong
Stowe Flagship Store	a power saver (electronics)	The first time to buy online, but also look at the evaluation before buying, the quality is not bad, but also very cheap, the need for friends can rest assured to buy!	Jingdong prefecture level city in Shandong
Stowe Flagship Store	a power saver (electronics)	🌈 Great quality, bought it to my liking 💝 Awesome! ✨ Buy with confidence if you need it!	Jingdong prefecture level city in Shandong
Stowe Flagship Store	a power saver (electronics)	The quality is very good, completely exceeded the expectations, the delivery speed is very fast, the packaging is very careful and strict, very satisfied with a shopping	Jingdong prefecture level city in Shandong
Stowe Flagship Store	a power saver (electronics)	The effect is very good, the home uses less electricity is very energy efficient, cost-effective	Jingdong prefecture level city in Shandong
Stowe Flagship Store	a power saver (electronics)	Quality can't be beat, just like the store, especially like, and the price is favorable!	Jingdong prefecture level city in Shandong
Stowe Flagship Store	a power saver (electronics)	Baby received! Very unexpected, will be back next time ~ great seller, stay tuned!	Jingdong prefecture level city in Shandong
Stowe Flagship Store	a power saver (electronics)	Follow up review after a month is more real, waiting for it	Jingdong prefecture level city in Shandong
Stowe Flagship Store	a power saver (electronics)	It is the ideal product, good quality and fast logistics, worthy of praise!	Jingdong prefecture level city in Shandong
Stowe Flagship Store	a power saver (electronics)	The saver is great, the color is right, the installer is very professional	Jingdong prefecture level city in Shandong
Stowe Flagship Store	a power saver (electronics)	The effect of the power connection is particularly obvious, it is particularly easy to use, very satisfied, the	Jingdong prefecture level city in Shandong
Stowe Flagship Store	a power saver (electronics)	Goods are very energy-saving, used to be more than two hundred degrees, now there are only a hundred or so degrees	Jingdong prefecture level city in Shandong
Stowe Flagship Store	a power saver (electronics)	Good quality, fast shipping, good results, good value for money	Jingdong prefecture level city in Shandong
Stowe Flagship Store	a power saver (electronics)	Fast shipping, fine workmanship, very good, cost-effective	Jingdong prefecture level city in Shandong
Stowe Flagship Store	a power saver (electronics)	The meter is as described, the quality is quite good, energy-saving, worth buying oh~!	Jingdong prefecture level city in Shandong
Stowe Flagship Store	a power saver (electronics)	Great value for money, very affordable,, perfect . ˆ◡ˆ.	Jingdong prefecture level city in Shandong
Stowe Flagship Store	a power saver (electronics)	Good!!! Worth recommending!	Jingdong prefecture level city in Shandong
Stowe Flagship Store	a power saver (electronics)	Great, fast logistics, great stuff, great value for money!	Jingdong prefecture level city in Shandong
Stowe Flagship Store	a power saver (electronics)	Fast logistics, very satisfied with the quality upon receipt	Jingdong prefecture level city in Shandong
Stowe Flagship Store	a power saver (electronics)	Great quality, plug and play, fast shipping!	Jingdong prefecture level city in Shandong
Stowe Flagship Store	a power saver (electronics)	Logistics received, quite satisfied	Jingdong prefecture level city in Shandong
Stowe Flagship Store	a power saver (electronics)	I don't know if it really works	Jingdong prefecture level city in Shandong
Stowe Flagship Store	a power saver (electronics)	It's bullshit. It's a plug light. Don't talk about power savings.	Jingdong prefecture level city in Shandong
Stowe Flagship Store	a power saver (electronics)	Fake and deceitful stuff that hasn't changed at all.	Jingdong prefecture level city in Shandong
Stowe Flagship Store	a power saver (electronics)	Power-saving effect: power-saving effect is particularly obvious Cost-effective: cost-effective business Appearance and style: external small micro-work materials: fine workmanship Experience: use very good, non the first convenient	Jingdong prefecture level city in Shandong
Stowe Flagship Store	a power saver (electronics)	After using it for a few days, it feels quite good, it should be quite safe, and it also sends a plug, the merchant is good, wait for a period of time and then calculate whether it saves on the electricity bill and then add comments ha!	Jingdong prefecture level city in Shandong
Stowe Flagship Store	a power saver (electronics)	My friend recommended to me, said the effect is very good, I bought two, this month less money, really can, just buy I do not believe, but has proved to have effect	Jingdong prefecture level city in Shandong
Stowe Flagship Store	a power saver (electronics)	Just bought back, already used, these days to see the effect of it, and so have the effect will also come back to give positive feedback, the price is also super cheap!	Jingdong prefecture level city in Shandong
Stowe Flagship Store	a power saver (electronics)	It does work well, the store uses a lot of electricity, this time to buy back a few, each place that uses a lot of electricity to insert one. Summer is here, the electricity is too strong.	Jingdong prefecture level city in Shandong
Stowe Flagship Store	a power saver (electronics)	Power saving treasure received, as for the effect, according to the instructions written, a short period of time can not be seen, at least 2-3 months before the real effect, then follow up evaluation	Jingdong prefecture level city in Shandong
Stowe Flagship Store	a power saver (electronics)	The goods have just been received and have not been used yet looking forward to the results.	Jingdong prefecture level city in Shandong
Stowe Flagship Store	a power saver (electronics)	Good installation works great	Jingdong prefecture level city in Shandong
Stowe Flagship Store	a power saver (electronics)	The logistics is super super fast! The quality is also very good, indeed value for money	Jingdong prefecture level city in Shandong
Stowe Flagship Store	a power saver (electronics)	Has been used for a period of time before coming to comment on, really good, air conditioning a day open non-stop, pay the electricity bill when the heartache, with this, the home electricity is not at all worried about the bill out of contrast to save a lot of preparation in the hands of a few, each room An a	Jingdong prefecture level city in Shandong
Stowe Flagship Store	a power saver (electronics)	Saving device received, really good, with the air conditioning tried, an hour without a saver with 2.9 degrees, with a saver an hour with 1.1 degrees, the beginning of not quite believe that this thing, the actual test to know that it is really useful, and intend to buy another.	Jingdong prefecture level city in Shandong
Stowe Flagship Store	a power saver (electronics)	Power saving effect: this energy saver is still useful, day trial for three days, every day than not installed before the reduction of two dollars a day, every day to open the bedroom to open the air conditioning before fifteen dollars now thirteen dollars a day. Still a little use.	Jingdong prefecture level city in Shandong
Stowe Flagship Store	a power saver (electronics)	Originally bought for home use, used for two months, the electricity bill from more than 200 yuan per month, down to more than 140 yuan, there is indeed a power-saving effect, this time and sold two, the unit with. Merchant service attitude is really good.	Jingdong prefecture level city in Shandong
Stowe Flagship Store	a power saver (electronics)	Plug and play, stable voltage, the screen shows when working, this winter home appliances, need electricity in many places, just buy to use, save electricity and money, with instructions	Jingdong prefecture level city in Shandong
Stowe Flagship Store	a power saver (electronics)	Goods received, do not know if it is good. When I bought it, I saw that it was a Stowe brand or company's product. Received but not. I do not know what is the reason. Now I can only give this evaluation. I'm	Jingdong prefecture level city in Shandong
Stowe Flagship Store	a power saver (electronics)	The first time to buy online, but also look at the evaluation before buying, the quality is not bad, but also very cheap, the need for friends can rest assured to buy!	Jingdong prefecture level city in Shandong
Stowe Flagship Store	a power saver (electronics)	🌈 Great quality, bought it to my liking 💝 Awesome! ✨ Buy with confidence if you need it!	Jingdong prefecture level city in Shandong
Stowe Flagship Store	a power saver (electronics)	The quality is very good, completely exceeded the expectations, the delivery speed is very fast, the packaging is very careful and strict, very satisfied with a shopping	Jingdong prefecture level city in Shandong
Stowe Flagship Store	a power saver (electronics)	The effect is very good, the home uses less electricity is very energy efficient, cost-effective	Jingdong prefecture level city in Shandong
Stowe Flagship Store	a power saver (electronics)	Quality can't be beat, just like the store, especially like, and the price is favorable!	Jingdong prefecture level city in Shandong
Stowe Flagship Store	a power saver (electronics)	Baby received! Very unexpected, will be back next time ~ great seller, stay tuned!	Jingdong prefecture level city in Shandong
Stowe Flagship Store	a power saver (electronics)	Follow up review after a month is more real, waiting for it	Jingdong prefecture level city in Shandong

Stowe Flagship Store	a power saver (electronics)	It is the ideal product, good quality and fast logistics, worthy of praise!	Jingdong prefecture level city in Shandong
Stowe Flagship Store	a power saver (electronics)	The saver is great, the color is right, the installer is very professional	Jingdong prefecture level city in Shandong
Stowe Flagship Store	a power saver (electronics)	The effect of the power connection is particularly obvious, it is particularly easy to use, very satisfied, the	Jingdong prefecture level city in Shandong
Stowe Flagship Store	a power saver (electronics)	Goods are very energy-saving, used to be more than two hundred degrees, now there are only a hundred or so degrees	Jingdong prefecture level city in Shandong
Stowe Flagship Store	a power saver (electronics)	Good quality, fast shipping, good results, good value for money	Jingdong prefecture level city in Shandong
Stowe Flagship Store	a power saver (electronics)	Fast shipping, fine workmanship, very good, cost-effective	Jingdong prefecture level city in Shandong
Stowe Flagship Store	a power saver (electronics)	The meter is as described, the quality is quite good, energy-saving, worth buying oh~!	Jingdong prefecture level city in Shandong
Stowe Flagship Store	a power saver (electronics)	Great value for money, very affordable,, perfect . ^◡^.	Jingdong prefecture level city in Shandong
Stowe Flagship Store	a power saver (electronics)	Good!!! Worth recommending!	Jingdong prefecture level city in Shandong
Stowe Flagship Store	a power saver (electronics)	Great, fast logistics, great stuff, great value for money!	Jingdong prefecture level city in Shandong
Stowe Flagship Store	a power saver (electronics)	Fast logistics, very satisfied with the quality upon receipt	Jingdong prefecture level city in Shandong
Stowe Flagship Store	a power saver (electronics)	Great quality, plug and play, fast shipping!	Jingdong prefecture level city in Shandong
Stowe Flagship Store	a power saver (electronics)	Logistics received, quite satisfied	Jingdong prefecture level city in Shandong
Stowe Flagship Store	a power saver (electronics)	I don't know if it really works	Jingdong prefecture level city in Shandong
Stowe Flagship Store	a power saver (electronics)	It's bullshit. It's a plug light. Don't talk about power savings.	Jingdong prefecture level city in Shandong
Stowe Flagship Store	a power saver (electronics)	Fake and deceitful stuff that hasn't changed at all.	Jingdong prefecture level city in Shandong
Stowe Flagship Store	a power saver (electronics)	Power-saving effect: power-saving effect is particularly obvious Cost-effective: cost-effective business Appearance and style: external small micro-work materials: fine workmanship Experience: use very good, non the first convenient	Jingdong prefecture level city in Shandong
Stowe Flagship Store	a power saver (electronics)	After using it for a few days, it feels quite good, it should be quite safe, and it also sends a plug, the merchant is good, wait for a period of time and then calculate whether it saves on the electricity bill and then add comments ha!	Jingdong prefecture level city in Shandong
Stowe Flagship Store	a power saver (electronics)	My friend recommended to me, said the effect is very good, I bought two, this month less money, really can, just buy I do not believe, but has proved to have effect	Jingdong prefecture level city in Shandong
Stowe Flagship Store	a power saver (electronics)	Just bought back, already used, these days to see the effect of it, and so have the effect will also come back to give positive feedback, the price is also super cheap!	Jingdong prefecture level city in Shandong
Stowe Flagship Store	a power saver (electronics)	It does work well, the store uses a lot of electricity, this time to buy back a few, each place that uses a lot of electricity to insert one. Summer is here, the electricity is too strong.	Jingdong prefecture level city in Shandong
Stowe Flagship Store	a power saver (electronics)	Power saving treasure received, as for the effect, according to the instructions written, a short period of time can not be seen, at least 2-3 months before the real effect, then follow up evaluation	Jingdong prefecture level city in Shandong
Stowe Flagship Store	a power saver (electronics)	The goods have just been received and have not been used yet looking forward to the results.	Jingdong prefecture level city in Shandong
Stowe Flagship Store	a power saver (electronics)	Good installation works great	Jingdong prefecture level city in Shandong
Stowe Flagship Store	a power saver (electronics)	The logistics is super super fast! The quality is also very good, indeed value for money	Jingdong prefecture level city in Shandong
Stowe Flagship Store	a power saver (electronics)	Has been used for a period of time before coming to comment on, really good, air conditioning a day open non-stop, pay the electricity bill when the heartache, with this, the home electricity is not at all worried about the bill out of contrast to save a lot of preparation in the hands of a few, each room An a	Jingdong prefecture level city in Shandong
Stowe Flagship Store	a power saver (electronics)	Saving device received, really good, with the air conditioning tried, an hour without a saver with 2.9 degrees, with a saver an hour with 1.1 degrees, the beginning of not quite believe that this thing, the actual test to know that it is really useful, and intend to buy another.	Jingdong prefecture level city in Shandong
Stowe Flagship Store	a power saver (electronics)	Power saving effect: this energy saver is still useful, day trial for three days, every day than not installed before the reduction of two dollars a day, every day to open the bedroom to open the air conditioning before fifteen dollars now thirteen dollars a day. Still a little use.	Jingdong prefecture level city in Shandong
Stowe Flagship Store	a power saver (electronics)	Originally bought for home use, used for two months, the electricity bill from more than 200 yuan per month, down to more than 140 yuan, there is indeed a power-saving effect, this time and sold two, the unit with. Merchant service attitude is really good.	Jingdong prefecture level city in Shandong
Stowe Flagship Store	a power saver (electronics)	Plug and play, stable voltage, the screen shows when working, this winter home appliances, need electricity in many places, just buy to use, save electricity and money, with instructions	Jingdong prefecture level city in Shandong
Stowe Flagship Store	a power saver (electronics)	Goods received, do not know if it is good. When I bought it, I saw that it was a Stowe brand or company's product. Received but not. I do not know what is the reason. Now I can only give this evaluation. I'm	Jingdong prefecture level city in Shandong
Stowe Flagship Store	a power saver (electronics)	The first time to buy online, but also look at the evaluation before buying, the quality is not bad, but also very cheap, the need for friends can rest assured to buy!	Jingdong prefecture level city in Shandong
Stowe Flagship Store	a power saver (electronics)	🌈 Great quality, bought it to my liking 💝 Awesome! ✨ Buy with confidence if you need it!	Jingdong prefecture level city in Shandong
Stowe Flagship Store	a power saver (electronics)	The quality is very good, completely exceeded the expectations, the delivery speed is very fast, the packaging is very careful and strict, very satisfied with a shopping	Jingdong prefecture level city in Shandong
Stowe Flagship Store	a power saver (electronics)	The effect is very good, the home uses less electricity is very energy efficient, cost-effective	Jingdong prefecture level city in Shandong
Stowe Flagship Store	a power saver (electronics)	Quality can't be beat, just like the store, especially like, and the price is favorable!	Jingdong prefecture level city in Shandong
Stowe Flagship Store	a power saver (electronics)	Baby received! Very unexpected, will be back next time ~ great seller, stay tuned!	Jingdong prefecture level city in Shandong
Stowe Flagship Store	a power saver (electronics)	Follow up review after a month is more real, waiting for it	Jingdong prefecture level city in Shandong
Stowe Flagship Store	a power saver (electronics)	It is the ideal product, good quality and fast logistics, worthy of praise!	Jingdong prefecture level city in Shandong
Stowe Flagship Store	a power saver (electronics)	The saver is great, the color is right, the installer is very professional	Jingdong prefecture level city in Shandong
Stowe Flagship Store	a power saver (electronics)	The effect of the power connection is particularly obvious, it is particularly easy to use, very satisfied, the	Jingdong prefecture level city in Shandong
Stowe Flagship Store	a power saver (electronics)	Goods are very energy-saving, used to be more than two hundred degrees, now there are only a hundred or so degrees	Jingdong prefecture level city in Shandong
Stowe Flagship Store	a power saver (electronics)	Good quality, fast shipping, good results, good value for money	Jingdong prefecture level city in Shandong
Stowe Flagship Store	a power saver (electronics)	Fast shipping, fine workmanship, very good, cost-effective	Jingdong prefecture level city in Shandong
Stowe Flagship Store	a power saver (electronics)	The meter is as described, the quality is quite good, energy-saving, worth buying oh~!	Jingdong prefecture level city in Shandong
Stowe Flagship Store	a power saver (electronics)	Great value for money, very affordable,, perfect . ^◡^.	Jingdong prefecture level city in Shandong
Stowe Flagship Store	a power saver (electronics)	Good!!! Worth recommending!	Jingdong prefecture level city in Shandong
Stowe Flagship Store	a power saver (electronics)	Great, fast logistics, great stuff, great value for money!	Jingdong prefecture level city in Shandong

Stowe Flagship Store	a power saver (electronics)	Fast logistics, very satisfied with the quality upon receipt	Jingdong prefecture level city in Shandong
Stowe Flagship Store	a power saver (electronics)	Great quality, plug and play, fast shipping!	Jingdong prefecture level city in Shandong
Stowe Flagship Store	a power saver (electronics)	Logistics received, quite satisfied	Jingdong prefecture level city in Shandong
Stowe Flagship Store	a power saver (electronics)	I don't know if it really works	Jingdong prefecture level city in Shandong
Stowe Flagship Store	a power saver (electronics)	It's bullshit. It's a plug light. Don't talk about power savings.	Jingdong prefecture level city in Shandong
Stowe Flagship Store	a power saver (electronics)	Fake and deceitful stuff that hasn't changed at all.	Jingdong prefecture level city in Shandong
Stowe Flagship Store	a power saver (electronics)	Power-saving effect: power-saving effect is particularly obvious Cost-effective: cost-effective business Appearance and style: external small micro-work materials: fine workmanship Experience: use very good, non the first convenient	Jingdong prefecture level city in Shandong
Stowe Flagship Store	a power saver (electronics)	After using it for a few days, it feels quite good, it should be quite safe, and it also sends a plug, the merchant is good, wait for a period of time and then calculate whether it saves on the electricity bill and then add comments ha!	Jingdong prefecture level city in Shandong
Stowe Flagship Store	a power saver (electronics)	My friend recommended to me, said the effect is very good, I bought two, this month less money, really can, just buy I do not believe, but has proved to have effect	Jingdong prefecture level city in Shandong
Stowe Flagship Store	a power saver (electronics)	Just bought back, already used, these days to see the effect of it, and so have the effect will also come back to give positive feedback, the price is also super cheap!	Jingdong prefecture level city in Shandong
Stowe Flagship Store	a power saver (electronics)	It does work well, the store uses a lot of electricity, this time to buy back a few, each place that uses a lot of electricity to insert one. Summer is here, the electricity is too strong.	Jingdong prefecture level city in Shandong
Stowe Flagship Store	a power saver (electronics)	Power saving treasure received, as for the effect, according to the instructions written, a short period of time can not be seen, at least 2-3 months before the real effect, then follow up evaluation	Jingdong prefecture level city in Shandong
Stowe Flagship Store	a power saver (electronics)	The goods have just been received and have not been used yet looking forward to the results.	Jingdong prefecture level city in Shandong
Stowe Flagship Store	a power saver (electronics)	Good installation works great	Jingdong prefecture level city in Shandong
Stowe Flagship Store	a power saver (electronics)	The logistics is super super fast! The quality is also very good, indeed value for money	Jingdong prefecture level city in Shandong
Stowe Flagship Store	a power saver (electronics)	Has been used for a period of time before coming to comment on, really good, air conditioning a day open non-stop, pay the electricity bill when the heartache, with this, the home electricity is not at all worried about the bill out of contrast to save a lot of preparation in the hands of a few, each room An a	Jingdong prefecture level city in Shandong
Stowe Flagship Store	a power saver (electronics)	Saving device received, really good, with the air conditioning tried, an hour without a saver with 2.9 degrees, with a saver an hour with 1.1 degrees, the beginning of not quite believe that this thing, the actual test to know that it is really useful, and intend to buy another.	Jingdong prefecture level city in Shandong
Stowe Flagship Store	a power saver (electronics)	Power saving effect: this energy saver is still useful, day trial for three days, every day than not installed before the reduction of two dollars a day, every day to open the bedroom to open the air conditioning before fifteen dollars now thirteen dollars a day. Still a little use.	Jingdong prefecture level city in Shandong
Stowe Flagship Store	a power saver (electronics)	Originally bought for home use, used for two months, the electricity bill from more than 200 yuan per month, down to more than 140 yuan, there is indeed a power-saving effect, this time and sold two, the unit with. Merchant service attitude is really good.	Jingdong prefecture level city in Shandong
Stowe Flagship Store	a power saver (electronics)	Plug and play, stable voltage, the screen shows when working, this winter home appliances, need electricity in many places, just buy to use, save electricity and money, with instructions	Jingdong prefecture level city in Shandong
Stowe Flagship Store	a power saver (electronics)	Goods received, do not know if it is good. When I bought it, I saw that it was a Stowe brand or company's product. Received but not. I do not know what is the reason. Now I can only give this evaluation. I'm	Jingdong prefecture level city in Shandong
Stowe Flagship Store	a power saver (electronics)	The first time to buy online, but also look at the evaluation before buying, the quality is not bad, but also very cheap, the need for friends can rest assured to buy!	Jingdong prefecture level city in Shandong
Stowe Flagship Store	a power saver (electronics)	🌈 Great quality, bought it to my liking 💝 Awesome! ✨ Buy with confidence if you need it!	Jingdong prefecture level city in Shandong
Stowe Flagship Store	a power saver (electronics)	The quality is very good, completely exceeded the expectations, the delivery speed is very fast, the packaging is very careful and strict, very satisfied with a shopping	Jingdong prefecture level city in Shandong
Stowe Flagship Store	a power saver (electronics)	The effect is very good, the home uses less electricity is very energy efficient, cost-effective	Jingdong prefecture level city in Shandong
Stowe Flagship Store	a power saver (electronics)	Quality can't be beat, just like the store, especially like, and the price is favorable!	Jingdong prefecture level city in Shandong
Stowe Flagship Store	a power saver (electronics)	Baby received! Very unexpected, will be back next time ~ great seller, stay tuned!	Jingdong prefecture level city in Shandong
Stowe Flagship Store	a power saver (electronics)	Follow up review after a month is more real, waiting for it	Jingdong prefecture level city in Shandong
Stowe Flagship Store	a power saver (electronics)	It is the ideal product, good quality and fast logistics, worthy of praise!	Jingdong prefecture level city in Shandong
Stowe Flagship Store	a power saver (electronics)	The saver is great, the color is right, the installer is very professional	Jingdong prefecture level city in Shandong
Stowe Flagship Store	a power saver (electronics)	The effect of the power connection is particularly obvious, it is particularly easy to use, very satisfied, the	Jingdong prefecture level city in Shandong
Stowe Flagship Store	a power saver (electronics)	Goods are very energy-saving, used to be more than two hundred degrees, now there are only a hundred or so degrees	Jingdong prefecture level city in Shandong
Stowe Flagship Store	a power saver (electronics)	Good quality, fast shipping, good results, good value for money	Jingdong prefecture level city in Shandong
Stowe Flagship Store	a power saver (electronics)	Fast shipping, fine workmanship, very good, cost-effective	Jingdong prefecture level city in Shandong
Stowe Flagship Store	a power saver (electronics)	The meter is as described, the quality is quite good, energy-saving, worth buying oh~!	Jingdong prefecture level city in Shandong
Stowe Flagship Store	a power saver (electronics)	Great value for money, very affordable,, perfect . ˆ◡ˆ.	Jingdong prefecture level city in Shandong
Stowe Flagship Store	a power saver (electronics)	Good!!! Worth recommending!	Jingdong prefecture level city in Shandong
Stowe Flagship Store	a power saver (electronics)	Great, fast logistics, great stuff, great value for money!	Jingdong prefecture level city in Shandong
Stowe Flagship Store	a power saver (electronics)	Fast logistics, very satisfied with the quality upon receipt	Jingdong prefecture level city in Shandong
Stowe Flagship Store	a power saver (electronics)	Great quality, plug and play, fast shipping!	Jingdong prefecture level city in Shandong
Stowe Flagship Store	a power saver (electronics)	Logistics received, quite satisfied	Jingdong prefecture level city in Shandong
Stowe Flagship Store	a power saver (electronics)	I don't know if it really works	Jingdong prefecture level city in Shandong
Stowe Flagship Store	a power saver (electronics)	It's bullshit. It's a plug light. Don't talk about power savings.	Jingdong prefecture level city in Shandong
Stowe Flagship Store	a power saver (electronics)	Fake and deceitful stuff that hasn't changed at all.	Jingdong prefecture level city in Shandong
Stowe Flagship Store	a power saver (electronics)	Power-saving effect: power-saving effect is particularly obvious Cost-effective: cost-effective business Appearance and style: external small micro-work materials: fine workmanship Experience: use very good, non the first convenient	Jingdong prefecture level city in Shandong
Stowe Flagship Store	a power saver (electronics)	After using it for a few days, it feels quite good, it should be quite safe, and it also sends a plug, the merchant is good, wait for a period of time and then calculate whether it saves on the electricity bill and then add comments ha!	Jingdong prefecture level city in Shandong
Stowe Flagship Store	a power saver (electronics)	My friend recommended to me, said the effect is very good, I bought two, this month less money, really can, just buy I do not believe, but has proved to have effect	Jingdong prefecture level city in Shandong

Stowe Flagship Store	a power saver (electronics)	Just bought back, already used, these days to see the effect of it, and so have the effect will also come back to give positive feedback, the price is also super cheap!	Jingdong prefecture level city in Shandong
Stowe Flagship Store	a power saver (electronics)	It does work well, the store uses a lot of electricity, this time to buy back a few, each place that uses a lot of electricity to insert one. Summer is here, the electricity is too strong.	Jingdong prefecture level city in Shandong
Stowe Flagship Store	a power saver (electronics)	Power saving treasure received, as for the effect, according to the instructions written, a short period of time can not be seen, at least 2-3 months before the real effect, then follow up evaluation	Jingdong prefecture level city in Shandong
Stowe Flagship Store	a power saver (electronics)	The goods have just been received and have not been used yet looking forward to the results.	Jingdong prefecture level city in Shandong
Stowe Flagship Store	a power saver (electronics)	Good installation works great	Jingdong prefecture level city in Shandong
Stowe Flagship Store	a power saver (electronics)	The logistics is super super fast! The quality is also very good, indeed value for money	Jingdong prefecture level city in Shandong
Stowe Flagship Store	a power saver (electronics)	Has been used for a period of time before coming to comment on, really good, air conditioning a day open non-stop, pay the electricity bill when the heartache, with this, the home electricity is not at all worried about the bill out of contrast to save a lot of preparation in the hands of a few, each room An a	Jingdong prefecture level city in Shandong
Stowe Flagship Store	a power saver (electronics)	Saving device received, really good, with the air conditioning tried, an hour without a saver with 2.9 degrees, with a saver an hour with 1.1 degrees, the beginning of not quite believe that this thing, the actual test to know that it is really useful, and intend to buy another.	Jingdong prefecture level city in Shandong
Stowe Flagship Store	a power saver (electronics)	Power saving effect: this energy saver is still useful, day trial for three days, every day than not installed before the reduction of two dollars a day, every day to open the bedroom to open the air conditioning before fifteen dollars now thirteen dollars a day. Still a little use.	Jingdong prefecture level city in Shandong
Stowe Flagship Store	a power saver (electronics)	Originally bought for home use, used for two months, the electricity bill from more than 200 yuan per month, down to more than 140 yuan, there is indeed a power-saving effect, this time and sold two, the unit with. Merchant service attitude is really good.	Jingdong prefecture level city in Shandong
Stowe Flagship Store	a power saver (electronics)	Plug and play, stable voltage, the screen shows when working, this winter home appliances, need electricity in many places, just buy to use, save electricity and money, with instructions	Jingdong prefecture level city in Shandong
Stowe Flagship Store	a power saver (electronics)	Goods received, do not know if it is good. When I bought it, I saw that it was a Stowe brand or company's product. Received but not. I do not know what is the reason. Now I can only give this evaluation. I'm	Jingdong prefecture level city in Shandong
Stowe Flagship Store	a power saver (electronics)	The first time to buy online, but also look at the evaluation before buying, the quality is not bad, but also very cheap, the need for friends can rest assured to buy!	Jingdong prefecture level city in Shandong
Stowe Flagship Store	a power saver (electronics)	🌈 Great quality, bought it to my liking 💕 Awesome! ✨ Buy with confidence if you need it!	Jingdong prefecture level city in Shandong
Stowe Flagship Store	a power saver (electronics)	The quality is very good, completely exceeded the expectations, the delivery speed is very fast, the packaging is very careful and strict, very satisfied with a shopping	Jingdong prefecture level city in Shandong
Stowe Flagship Store	a power saver (electronics)	The effect is very good, the home uses less electricity is very energy efficient, cost-effective	Jingdong prefecture level city in Shandong
Stowe Flagship Store	a power saver (electronics)	Quality can't be beat, just like the store, especially like, and the price is favorable!	Jingdong prefecture level city in Shandong
Stowe Flagship Store	a power saver (electronics)	Baby received! Very unexpected, will be back next time ~ great seller, stay tuned!	Jingdong prefecture level city in Shandong
Stowe Flagship Store	a power saver (electronics)	Follow up review after a month is more real, waiting for it	Jingdong prefecture level city in Shandong
Stowe Flagship Store	a power saver (electronics)	It is the ideal product, good quality and fast logistics, worthy of praise!	Jingdong prefecture level city in Shandong
Stowe Flagship Store	a power saver (electronics)	The saver is great, the color is right, the installer is very professional	Jingdong prefecture level city in Shandong
Stowe Flagship Store	a power saver (electronics)	The effect of the power connection is particularly obvious, it is particularly easy to use, very satisfied, the	Jingdong prefecture level city in Shandong
Stowe Flagship Store	a power saver (electronics)	Goods are very energy-saving, used to be more than two hundred degrees, now there are only a hundred or so degrees	Jingdong prefecture level city in Shandong
Stowe Flagship Store	a power saver (electronics)	Good quality, fast shipping, good results, good value for money	Jingdong prefecture level city in Shandong
Stowe Flagship Store	a power saver (electronics)	Fast shipping, fine workmanship, very good, cost-effective	Jingdong prefecture level city in Shandong
Stowe Flagship Store	a power saver (electronics)	The meter is as described, the quality is quite good, energy-saving, worth buying oh~!	Jingdong prefecture level city in Shandong
Stowe Flagship Store	a power saver (electronics)	Great value for money, very affordable,, perfect . ^◡^.	Jingdong prefecture level city in Shandong
Stowe Flagship Store	a power saver (electronics)	Good!!! Worth recommending!	Jingdong prefecture level city in Shandong
Stowe Flagship Store	a power saver (electronics)	Great, fast logistics, great stuff, great value for money!	Jingdong prefecture level city in Shandong
Stowe Flagship Store	a power saver (electronics)	Fast logistics, very satisfied with the quality upon receipt	Jingdong prefecture level city in Shandong
Stowe Flagship Store	a power saver (electronics)	Great quality, plug and play, fast shipping!	Jingdong prefecture level city in Shandong
Stowe Flagship Store	a power saver (electronics)	Logistics received, quite satisfied	Jingdong prefecture level city in Shandong
Stowe Flagship Store	a power saver (electronics)	I don't know if it really works	Jingdong prefecture level city in Shandong
Stowe Flagship Store	a power saver (electronics)	It's bullshit. It's a plug light. Don't talk about power savings.	Jingdong prefecture level city in Shandong
Stowe Flagship Store	a power saver (electronics)	Fake and deceitful stuff that hasn't changed at all.	Jingdong prefecture level city in Shandong
Stowe Flagship Store	a power saver (electronics)	Power-saving effect: power-saving effect is particularly obvious Cost-effective: cost-effective business Appearance and style: external small micro-work materials: fine workmanship Experience: use very good, non the first convenient	Jingdong prefecture level city in Shandong
Stowe Flagship Store	a power saver (electronics)	After using it for a few days, it feels quite good, it should be quite safe, and it also sends a plug, the merchant is good, wait for a period of time and then calculate whether it saves on the electricity bill and then add comments ha!	Jingdong prefecture level city in Shandong
Stowe Flagship Store	a power saver (electronics)	My friend recommended to me, said the effect is very good, I bought two, this month less money, really can, just buy I do not believe, but has proved to have effect	Jingdong prefecture level city in Shandong
Stowe Flagship Store	a power saver (electronics)	Just bought back, already used, these days to see the effect of it, and so have the effect will also come back to give positive feedback, the price is also super cheap!	Jingdong prefecture level city in Shandong
Stowe Flagship Store	a power saver (electronics)	It does work well, the store uses a lot of electricity, this time to buy back a few, each place that uses a lot of electricity to insert one. Summer is here, the electricity is too strong.	Jingdong prefecture level city in Shandong
Stowe Flagship Store	a power saver (electronics)	Power saving treasure received, as for the effect, according to the instructions written, a short period of time can not be seen, at least 2-3 months before the real effect, then follow up evaluation	Jingdong prefecture level city in Shandong
Stowe Flagship Store	a power saver (electronics)	The goods have just been received and have not been used yet looking forward to the results.	Jingdong prefecture level city in Shandong
Stowe Flagship Store	a power saver (electronics)	Good installation works great	Jingdong prefecture level city in Shandong
Stowe Flagship Store	a power saver (electronics)	The logistics is super super fast! The quality is also very good, indeed value for money	Jingdong prefecture level city in Shandong
Stowe Flagship Store	a power saver (electronics)	Has been used for a period of time before coming to comment on, really good, air conditioning a day open non-stop, pay the electricity bill when the heartache, with this, the home electricity is not at all worried about the bill out of contrast to save a lot of preparation in the hands of a few, each room An a	Jingdong prefecture level city in Shandong
Stowe Flagship Store	a power saver (electronics)	Saving device received, really good, with the air conditioning tried, an hour without a saver with 2.9 degrees, with a saver an hour with 1.1 degrees, the beginning of not quite believe that this thing, the actual test to know that it is really useful, and intend to buy another.	Jingdong prefecture level city in Shandong
Stowe Flagship Store	a power saver (electronics)	Power saving effect: this energy saver is still useful, day trial for three days, every day than not installed before the reduction of two dollars a day, every day to open the bedroom to open the air conditioning before fifteen dollars now thirteen dollars a day. Still a little use.	Jingdong prefecture level city in Shandong

Stowe Flagship Store	a power saver (electronics)	Originally bought for home use, used for two months, the electricity bill from more than 200 yuan per month, down to more than 140 yuan, there is indeed a power-saving effect, this time and sold two, the unit with. Merchant service attitude is really good.	Jingdong prefecture level city in Shandong
Stowe Flagship Store	a power saver (electronics)	Plug and play, stable voltage, the screen shows when working, this winter home appliances, need electricity in many places, just buy to use, save electricity and money, with instructions	Jingdong prefecture level city in Shandong
Stowe Flagship Store	a power saver (electronics)	Goods received, do not know if it is good. When I bought it, I saw that it was a Stowe brand or company's product. Received but not. I do not know what is the reason. Now I can only give this evaluation. I'm	Jingdong prefecture level city in Shandong
Stowe Flagship Store	a power saver (electronics)	The first time to buy online, but also look at the evaluation before buying, the quality is not bad, but also very cheap, the need for friends can rest assured to buy!	Jingdong prefecture level city in Shandong
Stowe Flagship Store	a power saver (electronics)	🌈 Great quality, bought it to my liking 💝 Awesome! ✨ Buy with confidence if you need it!	Jingdong prefecture level city in Shandong
Stowe Flagship Store	a power saver (electronics)	The quality is very good, completely exceeded the expectations, the delivery speed is very fast, the packaging is very careful and strict, very satisfied with a shopping	Jingdong prefecture level city in Shandong
Stowe Flagship Store	a power saver (electronics)	The effect is very good, the home uses less electricity is very energy efficient, cost-effective	Jingdong prefecture level city in Shandong
Stowe Flagship Store	a power saver (electronics)	Quality can't be beat, just like the store, especially like, and the price is favorable!	Jingdong prefecture level city in Shandong
Stowe Flagship Store	a power saver (electronics)	Baby received! Very unexpected, will be back next time ~ great seller, stay tuned!	Jingdong prefecture level city in Shandong
Stowe Flagship Store	a power saver (electronics)	Follow up review after a month is more real, waiting for it	Jingdong prefecture level city in Shandong
Stowe Flagship Store	a power saver (electronics)	It is the ideal product, good quality and fast logistics, worthy of praise!	Jingdong prefecture level city in Shandong
Stowe Flagship Store	a power saver (electronics)	The saver is great, the color is right, the installer is very professional	Jingdong prefecture level city in Shandong
Stowe Flagship Store	a power saver (electronics)	The effect of the power connection is particularly obvious, it is particularly easy to use, very satisfied, the	Jingdong prefecture level city in Shandong
Stowe Flagship Store	a power saver (electronics)	Goods are very energy-saving, used to be more than two hundred degrees, now there are only a hundred or so degrees	Jingdong prefecture level city in Shandong
Stowe Flagship Store	a power saver (electronics)	Good quality, fast shipping, good results, good value for money	Jingdong prefecture level city in Shandong
Stowe Flagship Store	a power saver (electronics)	Fast shipping, fine workmanship, very good, cost-effective	Jingdong prefecture level city in Shandong
Stowe Flagship Store	a power saver (electronics)	The meter is as described, the quality is quite good, energy-saving, worth buying oh~!	Jingdong prefecture level city in Shandong
Stowe Flagship Store	a power saver (electronics)	Great value for money, very affordable,, perfect . ˆ◡ˆ.	Jingdong prefecture level city in Shandong
Stowe Flagship Store	a power saver (electronics)	Good!!! Worth recommending!	Jingdong prefecture level city in Shandong
Stowe Flagship Store	a power saver (electronics)	Great, fast logistics, great stuff, great value for money!	Jingdong prefecture level city in Shandong
Stowe Flagship Store	a power saver (electronics)	Fast logistics, very satisfied with the quality upon receipt	Jingdong prefecture level city in Shandong
Stowe Flagship Store	a power saver (electronics)	Great quality, plug and play, fast shipping!	Jingdong prefecture level city in Shandong
Stowe Flagship Store	a power saver (electronics)	Logistics received, quite satisfied	Jingdong prefecture level city in Shandong
Stowe Flagship Store	a power saver (electronics)	I don't know if it really works	Jingdong prefecture level city in Shandong
Stowe Flagship Store	a power saver (electronics)	It's bullshit. It's a plug light. Don't talk about power savings.	Jingdong prefecture level city in Shandong
Stowe Flagship Store	a power saver (electronics)	Fake and deceitful stuff that hasn't changed at all.	Jingdong prefecture level city in Shandong
Stowe Flagship Store	a power saver (electronics)	Power-saving effect: power-saving effect is particularly obvious Cost-effective: cost-effective business Appearance and style: external small micro-work materials: fine workmanship Experience: use very good, non the first convenient	Jingdong prefecture level city in Shandong
Stowe Flagship Store	a power saver (electronics)	After using it for a few days, it feels quite good, it should be quite safe, and it also sends a plug, the merchant is good, wait for a period of time and then calculate whether it saves on the electricity bill and then add comments ha!	Jingdong prefecture level city in Shandong
Stowe Flagship Store	a power saver (electronics)	My friend recommended to me, said the effect is very good, I bought two, this month less money, really can, just buy I do not believe, but has proved to have effect	Jingdong prefecture level city in Shandong
Stowe Flagship Store	a power saver (electronics)	Just bought back, already used, these days to see the effect of it, and so have the effect will also come back to give positive feedback, the price is also super cheap!	Jingdong prefecture level city in Shandong
Stowe Flagship Store	a power saver (electronics)	It does work well, the store uses a lot of electricity, this time to buy back a few, each place that uses a lot of electricity to insert one. Summer is here, the electricity is too strong.	Jingdong prefecture level city in Shandong
Stowe Flagship Store	a power saver (electronics)	Power saving treasure received, as for the effect, according to the instructions written, a short period of time can not be seen, at least 2-3 months before the real effect, then follow up evaluation	Jingdong prefecture level city in Shandong
Stowe Flagship Store	a power saver (electronics)	The goods have just been received and have not been used yet looking forward to the results.	Jingdong prefecture level city in Shandong
Stowe Flagship Store	a power saver (electronics)	Good installation works great	Jingdong prefecture level city in Shandong
Stowe Flagship Store	a power saver (electronics)	The logistics is super super fast! The quality is also very good, indeed value for money	Jingdong prefecture level city in Shandong
Stowe Flagship Store	a power saver (electronics)	Has been used for a period of time before coming to comment on, really good, air conditioning a day open non-stop, pay the electricity bill when the heartache, with this, the home electricity is not at all worried about the bill out of contrast to save a lot of preparation in the hands of a few, each room An a	Jingdong prefecture level city in Shandong
Stowe Flagship Store	a power saver (electronics)	Saving device received, really good, with the air conditioning tried, an hour without a saver with 2.9 degrees, with a saver an hour with 1.1 degrees, the beginning of not quite believe that this thing, the actual test to know that it is really useful, and intend to buy another.	Jingdong prefecture level city in Shandong
Stowe Flagship Store	a power saver (electronics)	Power saving effect: this energy saver is still useful, day trial for three days, every day than not installed before the reduction of two dollars a day, every day to open the bedroom to open the air conditioning before fifteen dollars now thirteen dollars a day. Still a little use.	Jingdong prefecture level city in Shandong
Stowe Flagship Store	a power saver (electronics)	Originally bought for home use, used for two months, the electricity bill from more than 200 yuan per month, down to more than 140 yuan, there is indeed a power-saving effect, this time and sold two, the unit with. Merchant service attitude is really good.	Jingdong prefecture level city in Shandong
Stowe Flagship Store	a power saver (electronics)	Plug and play, stable voltage, the screen shows when working, this winter home appliances, need electricity in many places, just buy to use, save electricity and money, with instructions	Jingdong prefecture level city in Shandong
Stowe Flagship Store	a power saver (electronics)	Goods received, do not know if it is good. When I bought it, I saw that it was a Stowe brand or company's product. Received but not. I do not know what is the reason. Now I can only give this evaluation. I'm	Jingdong prefecture level city in Shandong
Stowe Flagship Store	a power saver (electronics)	The first time to buy online, but also look at the evaluation before buying, the quality is not bad, but also very cheap, the need for friends can rest assured to buy!	Jingdong prefecture level city in Shandong
Stowe Flagship Store	a power saver (electronics)	🌈 Great quality, bought it to my liking 💝 Awesome! ✨ Buy with confidence if you need it!	Jingdong prefecture level city in Shandong
Stowe Flagship Store	a power saver (electronics)	The quality is very good, completely exceeded the expectations, the delivery speed is very fast, the packaging is very careful and strict, very satisfied with a shopping	Jingdong prefecture level city in Shandong
Stowe Flagship Store	a power saver (electronics)	The effect is very good, the home uses less electricity is very energy efficient, cost-effective	Jingdong prefecture level city in Shandong
Stowe Flagship Store	a power saver (electronics)	Quality can't be beat, just like the store, especially like, and the price is favorable!	Jingdong prefecture level city in Shandong
Stowe Flagship Store	a power saver (electronics)	Baby received! Very unexpected, will be back next time ~ great seller, stay tuned!	Jingdong prefecture level city in Shandong
Stowe Flagship Store	a power saver (electronics)	Follow up review after a month is more real, waiting for it	Jingdong prefecture level city in Shandong

Stowe Flagship Store	a power saver (electronics)	It is the ideal product, good quality and fast logistics, worthy of praise!	Jingdong prefecture level city in Shandong
Stowe Flagship Store	a power saver (electronics)	The saver is great, the color is right, the installer is very professional	Jingdong prefecture level city in Shandong
Stowe Flagship Store	a power saver (electronics)	The effect of the power connection is particularly obvious, it is particularly easy to use, very satisfied, the	Jingdong prefecture level city in Shandong
Stowe Flagship Store	a power saver (electronics)	Goods are very energy-saving, used to be more than two hundred degrees, now there are only a hundred or so degrees	Jingdong prefecture level city in Shandong
Stowe Flagship Store	a power saver (electronics)	Good quality, fast shipping, good results, good value for money	Jingdong prefecture level city in Shandong
Stowe Flagship Store	a power saver (electronics)	Fast shipping, fine workmanship, very good, cost-effective	Jingdong prefecture level city in Shandong
Stowe Flagship Store	a power saver (electronics)	The meter is as described, the quality is quite good, energy-saving, worth buying oh~!	Jingdong prefecture level city in Shandong
Stowe Flagship Store	a power saver (electronics)	Great value for money, very affordable,, perfect . ^◡^.	Jingdong prefecture level city in Shandong
Stowe Flagship Store	a power saver (electronics)	Good!!! Worth recommending!	Jingdong prefecture level city in Shandong
Stowe Flagship Store	a power saver (electronics)	Great, fast logistics, great stuff, great value for money!	Jingdong prefecture level city in Shandong
Stowe Flagship Store	a power saver (electronics)	Fast logistics, very satisfied with the quality upon receipt	Jingdong prefecture level city in Shandong
Stowe Flagship Store	a power saver (electronics)	Great quality, plug and play, fast shipping!	Jingdong prefecture level city in Shandong
Stowe Flagship Store	a power saver (electronics)	Logistics received, quite satisfied	Jingdong prefecture level city in Shandong
Stowe Flagship Store	a power saver (electronics)	I don't know if it really works	Jingdong prefecture level city in Shandong
Stowe Flagship Store	a power saver (electronics)	It's bullshit. It's a plug light. Don't talk about power savings.	Jingdong prefecture level city in Shandong
Stowe Flagship Store	a power saver (electronics)	Fake and deceitful stuff that hasn't changed at all.	Jingdong prefecture level city in Shandong
Stowe Flagship Store	a power saver (electronics)	Power-saving effect: power-saving effect is particularly obvious Cost-effective: cost-effective business Appearance and style: external small micro-work materials: fine workmanship Experience: use very good, non the first convenient	Jingdong prefecture level city in Shandong
Stowe Flagship Store	a power saver (electronics)	After using it for a few days, it feels quite good, it should be quite safe, and it also sends a plug, the merchant is good, wait for a period of time and then calculate whether it saves on the electricity bill and then add comments ha!	Jingdong prefecture level city in Shandong
Stowe Flagship Store	a power saver (electronics)	My friend recommended to me, said the effect is very good, I bought two, this month less money, really can, just buy I do not believe, but has proved to have effect	Jingdong prefecture level city in Shandong
Stowe Flagship Store	a power saver (electronics)	Just bought back, already used, these days to see the effect of it, and so have the effect will also come back to give positive feedback, the price is also super cheap!	Jingdong prefecture level city in Shandong
Stowe Flagship Store	a power saver (electronics)	It does work well, the store uses a lot of electricity, this time to buy back a few, each place that uses a lot of electricity to insert one. Summer is here, the electricity is too strong.	Jingdong prefecture level city in Shandong
Stowe Flagship Store	a power saver (electronics)	Power saving treasure received, as for the effect, according to the instructions written, a short period of time can not be seen, at least 2-3 months before the real effect, then follow up evaluation	Jingdong prefecture level city in Shandong
Stowe Flagship Store	a power saver (electronics)	The goods have just been received and have not been used yet looking forward to the results.	Jingdong prefecture level city in Shandong
Stowe Flagship Store	a power saver (electronics)	Good installation works great	Jingdong prefecture level city in Shandong
Stowe Flagship Store	a power saver (electronics)	The logistics is super super fast! The quality is also very good, indeed value for money	Jingdong prefecture level city in Shandong
Stowe Flagship Store	a power saver (electronics)	Has been used for a period of time before coming to comment on, really good, air conditioning a day open non-stop, pay the electricity bill when the heartache, with this, the home electricity is not at all worried about the bill out of contrast to save a lot of preparation in the hands of a few, each room An a	Jingdong prefecture level city in Shandong
Stowe Flagship Store	a power saver (electronics)	Saving device received, really good, with the air conditioning tried, an hour without a saver with 2.9 degrees, with a saver an hour with 1.1 degrees, the beginning of not quite believe that this thing, the actual test to know that it is really useful, and intend to buy another.	Jingdong prefecture level city in Shandong
Stowe Flagship Store	a power saver (electronics)	Power saving effect: this energy saver is still useful, day trial for three days, every day than not installed before the reduction of two dollars a day, every day to open the bedroom to open the air conditioning before fifteen dollars now thirteen dollars a day. Still a little use.	Jingdong prefecture level city in Shandong
Stowe Flagship Store	a power saver (electronics)	Originally bought for home use, used for two months, the electricity bill from more than 200 yuan per month, down to more than 140 yuan, there is indeed a power-saving effect, this time and sold two, the unit with. Merchant service attitude is really good.	Jingdong prefecture level city in Shandong
Stowe Flagship Store	a power saver (electronics)	Plug and play, stable voltage, the screen shows when working, this winter home appliances, need electricity in many places, just buy to use, save electricity and money, with instructions	Jingdong prefecture level city in Shandong
Stowe Flagship Store	a power saver (electronics)	Goods received, do not know if it is good. When I bought it, I saw that it was a Stowe brand or company's product. Received but not. I do not know what is the reason. Now I can only give this evaluation. I'm	Jingdong prefecture level city in Shandong
Stowe Flagship Store	a power saver (electronics)	The first time to buy online, but also look at the evaluation before buying, the quality is not bad, but also very cheap, the need for friends can rest assured to buy!	Jingdong prefecture level city in Shandong
Stowe Flagship Store	a power saver (electronics)	🌈 Great quality, bought it to my liking 💞 Awesome! ✨ Buy with confidence if you need it!	Jingdong prefecture level city in Shandong
Stowe Flagship Store	a power saver (electronics)	The quality is very good, completely exceeded the expectations, the delivery speed is very fast, the packaging is very careful and strict, very satisfied with a shopping	Jingdong prefecture level city in Shandong
Stowe Flagship Store	a power saver (electronics)	The effect is very good, the home uses less electricity is very energy efficient, cost-effective	Jingdong prefecture level city in Shandong
Stowe Flagship Store	a power saver (electronics)	Quality can't be beat, just like the store, especially like, and the price is favorable!	Jingdong prefecture level city in Shandong
Stowe Flagship Store	a power saver (electronics)	Baby received! Very unexpected, will be back next time ~ great seller, stay tuned!	Jingdong prefecture level city in Shandong
Stowe Flagship Store	a power saver (electronics)	Follow up review after a month is more real, waiting for it	Jingdong prefecture level city in Shandong
Stowe Flagship Store	a power saver (electronics)	It is the ideal product, good quality and fast logistics, worthy of praise!	Jingdong prefecture level city in Shandong
Stowe Flagship Store	a power saver (electronics)	The saver is great, the color is right, the installer is very professional	Jingdong prefecture level city in Shandong
Stowe Flagship Store	a power saver (electronics)	The effect of the power connection is particularly obvious, it is particularly easy to use, very satisfied, the	Jingdong prefecture level city in Shandong
Stowe Flagship Store	a power saver (electronics)	Goods are very energy-saving, used to be more than two hundred degrees, now there are only a hundred or so degrees	Jingdong prefecture level city in Shandong
Stowe Flagship Store	a power saver (electronics)	Good quality, fast shipping, good results, good value for money	Jingdong prefecture level city in Shandong
Stowe Flagship Store	a power saver (electronics)	Fast shipping, fine workmanship, very good, cost-effective	Jingdong prefecture level city in Shandong
Stowe Flagship Store	a power saver (electronics)	The meter is as described, the quality is quite good, energy-saving, worth buying oh~!	Jingdong prefecture level city in Shandong
Stowe Flagship Store	a power saver (electronics)	Great value for money, very affordable,, perfect . ^◡^.	Jingdong prefecture level city in Shandong
Stowe Flagship Store	a power saver (electronics)	Good!!! Worth recommending!	Jingdong prefecture level city in Shandong
Stowe Flagship Store	a power saver (electronics)	Great, fast logistics, great stuff, great value for money!	Jingdong prefecture level city in Shandong

Stowe Flagship Store	a power saver (electronics)	Fast logistics, very satisfied with the quality upon receipt	Jingdong prefecture level city in Shandong
Stowe Flagship Store	a power saver (electronics)	Great quality, plug and play, fast shipping!	Jingdong prefecture level city in Shandong
Stowe Flagship Store	a power saver (electronics)	Logistics received, quite satisfied	Jingdong prefecture level city in Shandong
Stowe Flagship Store	a power saver (electronics)	I don't know if it really works	Jingdong prefecture level city in Shandong
Stowe Flagship Store	a power saver (electronics)	It's bullshit. It's a plug light. Don't talk about power savings.	Jingdong prefecture level city in Shandong
Stowe Flagship Store	a power saver (electronics)	Fake and deceitful stuff that hasn't changed at all.	Jingdong prefecture level city in Shandong
Stowe Flagship Store	a power saver (electronics)	Power-saving effect: power-saving effect is particularly obvious Cost-effective: cost-effective business Appearance and style: external small micro-work materials: fine workmanship Experience: use very good, non the first convenient	Jingdong prefecture level city in Shandong
Stowe Flagship Store	a power saver (electronics)	After using it for a few days, it feels quite good, it should be quite safe, and it also sends a plug, the merchant is good, wait for a period of time and then calculate whether it saves on the electricity bill and then add comments ha!	Jingdong prefecture level city in Shandong
Stowe Flagship Store	a power saver (electronics)	My friend recommended to me, said the effect is very good, I bought two, this month less money, really can, just buy I do not believe, but has proved to have effect	Jingdong prefecture level city in Shandong
Stowe Flagship Store	a power saver (electronics)	Just bought back, already used, these days to see the effect of it, and so have the effect will also come back to give positive feedback, the price is also super cheap!	Jingdong prefecture level city in Shandong
Stowe Flagship Store	a power saver (electronics)	It does work well, the store uses a lot of electricity, this time to buy back a few, each place that uses a lot of electricity to insert one. Summer is here, the electricity is too strong.	Jingdong prefecture level city in Shandong
Stowe Flagship Store	a power saver (electronics)	Power saving treasure received, as for the effect, according to the instructions written, a short period of time can not be seen, at least 2-3 months before the real effect, then follow up evaluation	Jingdong prefecture level city in Shandong
Stowe Flagship Store	a power saver (electronics)	The goods have just been received and have not been used yet looking forward to the results.	Jingdong prefecture level city in Shandong
Stowe Flagship Store	a power saver (electronics)	Good installation works great	Jingdong prefecture level city in Shandong
Stowe Flagship Store	a power saver (electronics)	The logistics is super super fast! The quality is also very good, indeed value for money	Jingdong prefecture level city in Shandong
Stowe Flagship Store	a power saver (electronics)	Has been used for a period of time before coming to comment on, really good, air conditioning a day open non-stop, pay the electricity bill when the heartache, with this, the home electricity is not at all worried about the bill out of contrast to save a lot of preparation in the hands of a few, each room An a	Jingdong prefecture level city in Shandong
Stowe Flagship Store	a power saver (electronics)	Saving device received, really good, with the air conditioning tried, an hour without a saver with 2.9 degrees, with a saver an hour with 1.1 degrees, the beginning of not quite believe that this thing, the actual test to know that it is really useful, and intend to buy another.	Jingdong prefecture level city in Shandong
Stowe Flagship Store	a power saver (electronics)	Power saving effect: this energy saver is still useful, day trial for three days, every day than not installed before the reduction of two dollars a day, every day to open the bedroom to open the air conditioning before fifteen dollars now thirteen dollars a day. Still a little use.	Jingdong prefecture level city in Shandong
Stowe Flagship Store	a power saver (electronics)	Originally bought for home use, used for two months, the electricity bill from more than 200 yuan per month, down to more than 140 yuan, there is indeed a power-saving effect, this time and sold two, the unit with. Merchant service attitude is really good.	Jingdong prefecture level city in Shandong
Stowe Flagship Store	a power saver (electronics)	Plug and play, stable voltage, the screen shows when working, this winter home appliances, need electricity in many places, just buy to use, save electricity and money, with instructions	Jingdong prefecture level city in Shandong
Stowe Flagship Store	a power saver (electronics)	Goods received, do not know if it is good. When I bought it, I saw that it was a Stowe brand or company's product. Received but not. I do not know what is the reason. Now I can only give this evaluation. I'm	Jingdong prefecture level city in Shandong
Stowe Flagship Store	a power saver (electronics)	The first time to buy online, but also look at the evaluation before buying, the quality is not bad, but also very cheap, the need for friends can rest assured to buy!	Jingdong prefecture level city in Shandong
Stowe Flagship Store	a power saver (electronics)	🌈 Great quality, bought it to my liking 💞 Awesome! ✨ Buy with confidence if you need it!	Jingdong prefecture level city in Shandong
Stowe Flagship Store	a power saver (electronics)	The quality is very good, completely exceeded the expectations, the delivery speed is very fast, the packaging is very careful and strict, very satisfied with a shopping	Jingdong prefecture level city in Shandong
Stowe Flagship Store	a power saver (electronics)	The effect is very good, the home uses less electricity is very energy efficient, cost-effective	Jingdong prefecture level city in Shandong
Stowe Flagship Store	a power saver (electronics)	Quality can't be beat, just like the store, especially like, and the price is favorable!	Jingdong prefecture level city in Shandong
Stowe Flagship Store	a power saver (electronics)	Baby received! Very unexpected, will be back next time ~ great seller, stay tuned!	Jingdong prefecture level city in Shandong
Stowe Flagship Store	a power saver (electronics)	Follow up review after a month is more real, waiting for it	Jingdong prefecture level city in Shandong
Stowe Flagship Store	a power saver (electronics)	It is the ideal product, good quality and fast logistics, worthy of praise!	Jingdong prefecture level city in Shandong
Stowe Flagship Store	a power saver (electronics)	The saver is great, the color is right, the installer is very professional	Jingdong prefecture level city in Shandong
Stowe Flagship Store	a power saver (electronics)	The effect of the power connection is particularly obvious, it is particularly easy to use, very satisfied, the	Jingdong prefecture level city in Shandong
Stowe Flagship Store	a power saver (electronics)	Goods are very energy-saving, used to be more than two hundred degrees, now there are only a hundred or so degrees	Jingdong prefecture level city in Shandong
Stowe Flagship Store	a power saver (electronics)	Good quality, fast shipping, good results, good value for money	Jingdong prefecture level city in Shandong
Stowe Flagship Store	a power saver (electronics)	Fast shipping, fine workmanship, very good, cost-effective	Jingdong prefecture level city in Shandong
Stowe Flagship Store	a power saver (electronics)	The meter is as described, the quality is quite good, energy-saving, worth buying oh~!	Jingdong prefecture level city in Shandong
Stowe Flagship Store	a power saver (electronics)	Great value for money, very affordable,, perfect . ˆ◡ˆ.	Jingdong prefecture level city in Shandong
Stowe Flagship Store	a power saver (electronics)	Good!!! Worth recommending!	Jingdong prefecture level city in Shandong
Stowe Flagship Store	a power saver (electronics)	Great, fast logistics, great stuff, great value for money!	Jingdong prefecture level city in Shandong
Stowe Flagship Store	a power saver (electronics)	Fast logistics, very satisfied with the quality upon receipt	Jingdong prefecture level city in Shandong
Stowe Flagship Store	a power saver (electronics)	Great quality, plug and play, fast shipping!	Jingdong prefecture level city in Shandong
Stowe Flagship Store	a power saver (electronics)	Logistics received, quite satisfied	Jingdong prefecture level city in Shandong
Stowe Flagship Store	a power saver (electronics)	I don't know if it really works	Jingdong prefecture level city in Shandong
Stowe Flagship Store	a power saver (electronics)	It's bullshit. It's a plug light. Don't talk about power savings.	Jingdong prefecture level city in Shandong
Stowe Flagship Store	a power saver (electronics)	Fake and deceitful stuff that hasn't changed at all.	Jingdong prefecture level city in Shandong
Stowe Flagship Store	a power saver (electronics)	Power-saving effect: power-saving effect is particularly obvious Cost-effective: cost-effective business Appearance and style: external small micro-work materials: fine workmanship Experience: use very good, non the first convenient	Jingdong prefecture level city in Shandong
Stowe Flagship Store	a power saver (electronics)	After using it for a few days, it feels quite good, it should be quite safe, and it also sends a plug, the merchant is good, wait for a period of time and then calculate whether it saves on the electricity bill and then add comments ha!	Jingdong prefecture level city in Shandong
Stowe Flagship Store	a power saver (electronics)	My friend recommended to me, said the effect is very good, I bought two, this month less money, really can, just buy I do not believe, but has proved to have effect	Jingdong prefecture level city in Shandong

Stowe Flagship Store	a power saver (electronics)	Just bought back, already used, these days to see the effect of it, and so have the effect will also come back to give positive feedback, the price is also super cheap!	Jingdong prefecture level city in Shandong
Stowe Flagship Store	a power saver (electronics)	It does work well, the store uses a lot of electricity, this time to buy back a few, each place that uses a lot of electricity to insert one. Summer is here, the electricity is too strong.	Jingdong prefecture level city in Shandong
Stowe Flagship Store	a power saver (electronics)	Power saving treasure received, as for the effect, according to the instructions written, a short period of time can not be seen, at least 2-3 months before the real effect, then follow up evaluation	Jingdong prefecture level city in Shandong
Stowe Flagship Store	a power saver (electronics)	The goods have just been received and have not been used yet looking forward to the results.	Jingdong prefecture level city in Shandong
Stowe Flagship Store	a power saver (electronics)	Good installation works great	Jingdong prefecture level city in Shandong
Stowe Flagship Store	a power saver (electronics)	The logistics is super super fast! The quality is also very good, indeed value for money	Jingdong prefecture level city in Shandong
Stowe Flagship Store	a power saver (electronics)	Has been used for a period of time before coming to comment on, really good, air conditioning a day open non-stop, pay the electricity bill when the heartache, with this, the home electricity is not at all worried about the bill out of contrast to save a lot of preparation in the hands of a few, each room An a	Jingdong prefecture level city in Shandong
Stowe Flagship Store	a power saver (electronics)	Saving device received, really good, with the air conditioning tried, an hour without a saver with 2.9 degrees, with a saver an hour with 1.1 degrees, the beginning of not quite believe that this thing, the actual test to know that it is really useful, and intend to buy another.	Jingdong prefecture level city in Shandong
Stowe Flagship Store	a power saver (electronics)	Power saving effect: this energy saver is still useful, day trial for three days, every day than not installed before the reduction of two dollars a day, every day to open the bedroom to open the air conditioning before fifteen dollars now thirteen dollars a day. Still a little use.	Jingdong prefecture level city in Shandong
Stowe Flagship Store	a power saver (electronics)	Originally bought for home use, used for two months, the electricity bill from more than 200 yuan per month, down to more than 140 yuan, there is indeed a power-saving effect, this time and sold two, the unit with. Merchant service attitude is really good.	Jingdong prefecture level city in Shandong
Stowe Flagship Store	a power saver (electronics)	Plug and play, stable voltage, the screen shows when working, this winter home appliances, need electricity in many places, just buy to use, save electricity and money, with instructions	Jingdong prefecture level city in Shandong
Stowe Flagship Store	a power saver (electronics)	Goods received, do not know if it is good. When I bought it, I saw that it was a Stowe brand or company's product. Received but not. I do not know what is the reason. Now I can only give this evaluation. I'm	Jingdong prefecture level city in Shandong
Stowe Flagship Store	a power saver (electronics)	The first time to buy online, but also look at the evaluation before buying, the quality is not bad, but also very cheap, the need for friends can rest assured to buy!	Jingdong prefecture level city in Shandong
Stowe Flagship Store	a power saver (electronics)	🌈 Great quality, bought it to my liking 💞 Awesome! ✨ Buy with confidence if you need it!	Jingdong prefecture level city in Shandong
Stowe Flagship Store	a power saver (electronics)	The quality is very good, completely exceeded the expectations, the delivery speed is very fast, the packaging is very careful and strict, very satisfied with a shopping	Jingdong prefecture level city in Shandong
Stowe Flagship Store	a power saver (electronics)	The effect is very good, the home uses less electricity is very energy efficient, cost-effective	Jingdong prefecture level city in Shandong
Stowe Flagship Store	a power saver (electronics)	Quality can't be beat, just like the store, especially like, and the price is favorable!	Jingdong prefecture level city in Shandong
Stowe Flagship Store	a power saver (electronics)	Baby received! Very unexpected, will be back next time ~ great seller, stay tuned!	Jingdong prefecture level city in Shandong
Stowe Flagship Store	a power saver (electronics)	Follow up review after a month is more real, waiting for it	Jingdong prefecture level city in Shandong
Stowe Flagship Store	a power saver (electronics)	It is the ideal product, good quality and fast logistics, worthy of praise!	Jingdong prefecture level city in Shandong
Stowe Flagship Store	a power saver (electronics)	The saver is great, the color is right, the installer is very professional	Jingdong prefecture level city in Shandong
Stowe Flagship Store	a power saver (electronics)	The effect of the power connection is particularly obvious, it is particularly easy to use, very satisfied, the	Jingdong prefecture level city in Shandong
Stowe Flagship Store	a power saver (electronics)	Goods are very energy-saving, used to be more than two hundred degrees, now there are only a hundred or so degrees	Jingdong prefecture level city in Shandong
Stowe Flagship Store	a power saver (electronics)	Good quality, fast shipping, good results, good value for money	Jingdong prefecture level city in Shandong
Stowe Flagship Store	a power saver (electronics)	Fast shipping, fine workmanship, very good, cost-effective	Jingdong prefecture level city in Shandong
Stowe Flagship Store	a power saver (electronics)	The meter is as described, the quality is quite good, energy-saving, worth buying oh~!	Jingdong prefecture level city in Shandong
Stowe Flagship Store	a power saver (electronics)	Great value for money, very affordable,, perfect . ˆ◡ˆ.	Jingdong prefecture level city in Shandong
Stowe Flagship Store	a power saver (electronics)	Good!!! Worth recommending!	Jingdong prefecture level city in Shandong
Stowe Flagship Store	a power saver (electronics)	Great, fast logistics, great stuff, great value for money!	Jingdong prefecture level city in Shandong
Stowe Flagship Store	a power saver (electronics)	Fast logistics, very satisfied with the quality upon receipt	Jingdong prefecture level city in Shandong
Stowe Flagship Store	a power saver (electronics)	Great quality, plug and play, fast shipping!	Jingdong prefecture level city in Shandong
Stowe Flagship Store	a power saver (electronics)	Logistics received, quite satisfied	Jingdong prefecture level city in Shandong
Stowe Flagship Store	a power saver (electronics)	I don't know if it really works	Jingdong prefecture level city in Shandong
Stowe Flagship Store	a power saver (electronics)	It's bullshit. It's a plug light. Don't talk about power savings.	Jingdong prefecture level city in Shandong
Stowe Flagship Store	a power saver (electronics)	Fake and deceitful stuff that hasn't changed at all.	Jingdong prefecture level city in Shandong
Stowe Flagship Store	a power saver (electronics)	Power-saving effect: power-saving effect is particularly obvious Cost-effective: cost-effective business Appearance and style: external small micro-work materials: fine workmanship Experience: use very good, non the first convenient	Jingdong prefecture level city in Shandong
Stowe Flagship Store	a power saver (electronics)	After using it for a few days, it feels quite good, it should be quite safe, and it also sends a plug, the merchant is good, wait for a period of time and then calculate whether it saves on the electricity bill and then add comments ha!	Jingdong prefecture level city in Shandong
Stowe Flagship Store	a power saver (electronics)	My friend recommended to me, said the effect is very good, I bought two, this month less money, really can, just buy I do not believe, but has proved to have effect	Jingdong prefecture level city in Shandong
Stowe Flagship Store	a power saver (electronics)	Just bought back, already used, these days to see the effect of it, and so have the effect will also come back to give positive feedback, the price is also super cheap!	Jingdong prefecture level city in Shandong
Stowe Flagship Store	a power saver (electronics)	It does work well, the store uses a lot of electricity, this time to buy back a few, each place that uses a lot of electricity to insert one. Summer is here, the electricity is too strong.	Jingdong prefecture level city in Shandong
Stowe Flagship Store	a power saver (electronics)	Power saving treasure received, as for the effect, according to the instructions written, a short period of time can not be seen, at least 2-3 months before the real effect, then follow up evaluation	Jingdong prefecture level city in Shandong
Stowe Flagship Store	a power saver (electronics)	The goods have just been received and have not been used yet looking forward to the results.	Jingdong prefecture level city in Shandong
Stowe Flagship Store	a power saver (electronics)	Good installation works great	Jingdong prefecture level city in Shandong
Stowe Flagship Store	a power saver (electronics)	The logistics is super super fast! The quality is also very good, indeed value for money	Jingdong prefecture level city in Shandong
Stowe Flagship Store	a power saver (electronics)	Has been used for a period of time before coming to comment on, really good, air conditioning a day open non-stop, pay the electricity bill when the heartache, with this, the home electricity is not at all worried about the bill out of contrast to save a lot of preparation in the hands of a few, each room An a	Jingdong prefecture level city in Shandong
Stowe Flagship Store	a power saver (electronics)	Saving device received, really good, with the air conditioning tried, an hour without a saver with 2.9 degrees, with a saver an hour with 1.1 degrees, the beginning of not quite believe that this thing, the actual test to know that it is really useful, and intend to buy another.	Jingdong prefecture level city in Shandong
Stowe Flagship Store	a power saver (electronics)	Power saving effect: this energy saver is still useful, day trial for three days, every day than not installed before the reduction of two dollars a day, every day to open the bedroom to open the air conditioning before fifteen dollars now thirteen dollars a day. Still a little use.	Jingdong prefecture level city in Shandong

Stowe Flagship Store	a power saver (electronics)	Originally bought for home use, used for two months, the electricity bill from more than 200 yuan per month, down to more than 140 yuan, there is indeed a power-saving effect, this time and sold two, the unit with. Merchant service attitude is really good.	Jingdong prefecture level city in Shandong
Stowe Flagship Store	a power saver (electronics)	Plug and play, stable voltage, the screen shows when working, this winter home appliances, need electricity in many places, just buy to use, save electricity and money, with instructions	Jingdong prefecture level city in Shandong
Stowe Flagship Store	a power saver (electronics)	Goods received, do not know if it is good. When I bought it, I saw that it was a Stowe brand or company's product. Received but not. I do not know what is the reason. Now I can only give this evaluation. I'm	Jingdong prefecture level city in Shandong
Stowe Flagship Store	a power saver (electronics)	The first time to buy online, but also look at the evaluation before buying, the quality is not bad, but also very cheap, the need for friends can rest assured to buy!	Jingdong prefecture level city in Shandong
Stowe Flagship Store	a power saver (electronics)	Great quality, bought it to my liking Awesome! Buy with confidence if you need it!	Jingdong prefecture level city in Shandong
Stowe Flagship Store	a power saver (electronics)	The quality is very good, completely exceeded the expectations, the delivery speed is very fast, the packaging is very careful and strict, very satisfied with a shopping	Jingdong prefecture level city in Shandong
Stowe Flagship Store	a power saver (electronics)	The effect is very good, the home uses less electricity is very energy efficient, cost-effective	Jingdong prefecture level city in Shandong
Stowe Flagship Store	a power saver (electronics)	Quality can't be beat, just like the store, especially like, and the price is favorable!	Jingdong prefecture level city in Shandong
Stowe Flagship Store	a power saver (electronics)	Baby received! Very unexpected, will be back next time ~ great seller, stay tuned!	Jingdong prefecture level city in Shandong
Stowe Flagship Store	a power saver (electronics)	Follow up review after a month is more real, waiting for it	Jingdong prefecture level city in Shandong
Stowe Flagship Store	a power saver (electronics)	It is the ideal product, good quality and fast logistics, worthy of praise!	Jingdong prefecture level city in Shandong
Stowe Flagship Store	a power saver (electronics)	The saver is great, the color is right, the installer is very professional	Jingdong prefecture level city in Shandong
Stowe Flagship Store	a power saver (electronics)	The effect of the power connection is particularly obvious, it is particularly easy to use, very satisfied, the	Jingdong prefecture level city in Shandong
Stowe Flagship Store	a power saver (electronics)	Goods are very energy-saving, used to be more than two hundred degrees, now there are only a hundred or so degrees	Jingdong prefecture level city in Shandong
Stowe Flagship Store	a power saver (electronics)	Good quality, fast shipping, good results, good value for money	Jingdong prefecture level city in Shandong
Stowe Flagship Store	a power saver (electronics)	Fast shipping, fine workmanship, very good, cost-effective	Jingdong prefecture level city in Shandong
Stowe Flagship Store	a power saver (electronics)	The meter is as described, the quality is quite good, energy-saving, worth buying oh~!	Jingdong prefecture level city in Shandong
Stowe Flagship Store	a power saver (electronics)	Great value for money, very affordable,, perfect . ˆ◡ˆ.	Jingdong prefecture level city in Shandong
Stowe Flagship Store	a power saver (electronics)	Good!!! Worth recommending!	Jingdong prefecture level city in Shandong
Stowe Flagship Store	a power saver (electronics)	Great, fast logistics, great stuff, great value for money!	Jingdong prefecture level city in Shandong
Stowe Flagship Store	a power saver (electronics)	Fast logistics, very satisfied with the quality upon receipt	Jingdong prefecture level city in Shandong
Stowe Flagship Store	a power saver (electronics)	Great quality, plug and play, fast shipping!	Jingdong prefecture level city in Shandong
Stowe Flagship Store	a power saver (electronics)	Logistics received, quite satisfied	Jingdong prefecture level city in Shandong
Stowe Flagship Store	a power saver (electronics)	I don't know if it really works	Jingdong prefecture level city in Shandong
Stowe Flagship Store	a power saver (electronics)	It's bullshit. It's a plug light. Don't talk about power savings.	Jingdong prefecture level city in Shandong
Stowe Flagship Store	a power saver (electronics)	Fake and deceitful stuff that hasn't changed at all.	Jingdong prefecture level city in Shandong
Stowe Flagship Store	a power saver (electronics)	Power-saving effect: power-saving effect is particularly obvious Cost-effective: cost-effective business Appearance and style: external small micro-work materials: fine workmanship Experience: use very good, non the first convenient	Jingdong prefecture level city in Shandong
Stowe Flagship Store	a power saver (electronics)	After using it for a few days, it feels quite good, it should be quite safe, and it also sends a plug, the merchant is good, wait for a period of time and then calculate whether it saves on the electricity bill and then add comments ha!	Jingdong prefecture level city in Shandong
Stowe Flagship Store	a power saver (electronics)	My friend recommended to me, said the effect is very good, I bought two, this month less money, really can, just buy I do not believe, but has proved to have effect	Jingdong prefecture level city in Shandong
Stowe Flagship Store	a power saver (electronics)	Just bought back, already used, these days to see the effect of it, and so have the effect will also come back to give positive feedback, the price is also super cheap!	Jingdong prefecture level city in Shandong
Stowe Flagship Store	a power saver (electronics)	It does work well, the store uses a lot of electricity, this time to buy back a few, each place that uses a lot of electricity to insert one. Summer is here, the electricity is too strong.	Jingdong prefecture level city in Shandong
Stowe Flagship Store	a power saver (electronics)	Power saving treasure received, as for the effect, according to the instructions written, a short period of time can not be seen, at least 2-3 months before the real effect, then follow up evaluation	Jingdong prefecture level city in Shandong
Stowe Flagship Store	a power saver (electronics)	The goods have just been received and have not been used yet looking forward to the results.	Jingdong prefecture level city in Shandong
Stowe Flagship Store	a power saver (electronics)	Good installation works great	Jingdong prefecture level city in Shandong
Stowe Flagship Store	a power saver (electronics)	The logistics is super super fast! The quality is also very good, indeed value for money	Jingdong prefecture level city in Shandong
Stowe Flagship Store	a power saver (electronics)	Has been used for a period of time before coming to comment on, really good, air conditioning a day open non-stop, pay the electricity bill when the heartache, with this, the home electricity is not at all worried about the bill out of contrast to save a lot of preparation in the hands of a few, each room An a	Jingdong prefecture level city in Shandong
Stowe Flagship Store	a power saver (electronics)	Saving device received, really good, with the air conditioning tried, an hour without a saver with 2.9 degrees, with a saver an hour with 1.1 degrees, the beginning of not quite believe that this thing, the actual test to know that it is really useful, and intend to buy another.	Jingdong prefecture level city in Shandong
Stowe Flagship Store	a power saver (electronics)	Power saving effect: this energy saver is still useful, day trial for three days, every day than not installed before the reduction of two dollars a day, every day to open the bedroom to open the air conditioning before fifteen dollars now thirteen dollars a day. Still a little use.	Jingdong prefecture level city in Shandong
Stowe Flagship Store	a power saver (electronics)	Originally bought for home use, used for two months, the electricity bill from more than 200 yuan per month, down to more than 140 yuan, there is indeed a power-saving effect, this time and sold two, the unit with. Merchant service attitude is really good.	Jingdong prefecture level city in Shandong
Stowe Flagship Store	a power saver (electronics)	Plug and play, stable voltage, the screen shows when working, this winter home appliances, need electricity in many places, just buy to use, save electricity and money, with instructions	Jingdong prefecture level city in Shandong
Stowe Flagship Store	a power saver (electronics)	Goods received, do not know if it is good. When I bought it, I saw that it was a Stowe brand or company's product. Received but not. I do not know what is the reason. Now I can only give this evaluation. I'm	Jingdong prefecture level city in Shandong
Stowe Flagship Store	a power saver (electronics)	The first time to buy online, but also look at the evaluation before buying, the quality is not bad, but also very cheap, the need for friends can rest assured to buy!	Jingdong prefecture level city in Shandong
Stowe Flagship Store	a power saver (electronics)	Great quality, bought it to my liking Awesome! Buy with confidence if you need it!	Jingdong prefecture level city in Shandong
Stowe Flagship Store	a power saver (electronics)	The quality is very good, completely exceeded the expectations, the delivery speed is very fast, the packaging is very careful and strict, very satisfied with a shopping	Jingdong prefecture level city in Shandong
Stowe Flagship Store	a power saver (electronics)	The effect is very good, the home uses less electricity is very energy efficient, cost-effective	Jingdong prefecture level city in Shandong
Stowe Flagship Store	a power saver (electronics)	Quality can't be beat, just like the store, especially like, and the price is favorable!	Jingdong prefecture level city in Shandong
Stowe Flagship Store	a power saver (electronics)	Baby received! Very unexpected, will be back next time ~ great seller, stay tuned!	Jingdong prefecture level city in Shandong
Stowe Flagship Store	a power saver (electronics)	Follow up review after a month is more real, waiting for it	Jingdong prefecture level city in Shandong

Stowe Flagship Store	a power saver (electronics)	It is the ideal product, good quality and fast logistics, worthy of praise!	Jingdong prefecture level city in Shandong
Stowe Flagship Store	a power saver (electronics)	The saver is great, the color is right, the installer is very professional	Jingdong prefecture level city in Shandong
Stowe Flagship Store	a power saver (electronics)	The effect of the power connection is particularly obvious, it is particularly easy to use, very satisfied, the	Jingdong prefecture level city in Shandong
Stowe Flagship Store	a power saver (electronics)	Goods are very energy-saving, used to be more than two hundred degrees, now there are only a hundred or so degrees	Jingdong prefecture level city in Shandong
Stowe Flagship Store	a power saver (electronics)	Good quality, fast shipping, good results, good value for money	Jingdong prefecture level city in Shandong
Stowe Flagship Store	a power saver (electronics)	Fast shipping, fine workmanship, very good, cost-effective	Jingdong prefecture level city in Shandong
Stowe Flagship Store	a power saver (electronics)	The meter is as described, the quality is quite good, energy-saving, worth buying oh~!	Jingdong prefecture level city in Shandong
Stowe Flagship Store	a power saver (electronics)	Great value for money, very affordable,, perfect . ˆ◡ˆ.	Jingdong prefecture level city in Shandong
Stowe Flagship Store	a power saver (electronics)	Good!!! Worth recommending!	Jingdong prefecture level city in Shandong
Stowe Flagship Store	a power saver (electronics)	Great, fast logistics, great stuff, great value for money!	Jingdong prefecture level city in Shandong
Stowe Flagship Store	a power saver (electronics)	Fast logistics, very satisfied with the quality upon receipt	Jingdong prefecture level city in Shandong
Stowe Flagship Store	a power saver (electronics)	Great quality, plug and play, fast shipping!	Jingdong prefecture level city in Shandong
Stowe Flagship Store	a power saver (electronics)	Logistics received, quite satisfied	Jingdong prefecture level city in Shandong
Stowe Flagship Store	a power saver (electronics)	I don't know if it really works	Jingdong prefecture level city in Shandong
Stowe Flagship Store	a power saver (electronics)	It's bullshit. It's a plug light. Don't talk about power savings.	Jingdong prefecture level city in Shandong
Stowe Flagship Store	a power saver (electronics)	Fake and deceitful stuff that hasn't changed at all.	Jingdong prefecture level city in Shandong
Stowe Flagship Store	a power saver (electronics)	Power-saving effect: power-saving effect is particularly obvious Cost-effective: cost-effective business Appearance and style: external small micro-work materials: fine workmanship Experience: use very good, non the first convenient	Jingdong prefecture level city in Shandong
Stowe Flagship Store	a power saver (electronics)	After using it for a few days, it feels quite good, it should be quite safe, and it also sends a plug, the merchant is good, wait for a period of time and then calculate whether it saves on the electricity bill and then add comments ha!	Jingdong prefecture level city in Shandong
Stowe Flagship Store	a power saver (electronics)	My friend recommended to me, said the effect is very good, I bought two, this month less money, really can, just buy I do not believe, but has proved to have effect	Jingdong prefecture level city in Shandong
Stowe Flagship Store	a power saver (electronics)	Just bought back, already used, these days to see the effect of it, and so have the effect will also come back to give positive feedback, the price is also super cheap!	Jingdong prefecture level city in Shandong
Stowe Flagship Store	a power saver (electronics)	It does work well, the store uses a lot of electricity, this time to buy back a few, each place that uses a lot of electricity to insert one. Summer is here, the electricity is too strong.	Jingdong prefecture level city in Shandong
Stowe Flagship Store	a power saver (electronics)	Power saving treasure received, as for the effect, according to the instructions written, a short period of time can not be seen, at least 2-3 months before the real effect, then follow up evaluation	Jingdong prefecture level city in Shandong
Stowe Flagship Store	a power saver (electronics)	The goods have just been received and have not been used yet looking forward to the results.	Jingdong prefecture level city in Shandong
Stowe Flagship Store	a power saver (electronics)	Good installation works great	Jingdong prefecture level city in Shandong
Stowe Flagship Store	a power saver (electronics)	The logistics is super super fast! The quality is also very good, indeed value for money	Jingdong prefecture level city in Shandong
Stowe Flagship Store	a power saver (electronics)	Has been used for a period of time before coming to comment on, really good, air conditioning a day open non-stop, pay the electricity bill when the heartache, with this, the home electricity is not at all worried about the bill out of contrast to save a lot of preparation in the hands of a few, each room An a	Jingdong prefecture level city in Shandong
Stowe Flagship Store	a power saver (electronics)	Saving device received, really good, with the air conditioning tried, an hour without a saver with 2.9 degrees, with a saver an hour with 1.1 degrees, the beginning of not quite believe that this thing, the actual test to know that it is really useful, and intend to buy another.	Jingdong prefecture level city in Shandong
Stowe Flagship Store	a power saver (electronics)	Power saving effect: this energy saver is still useful, day trial for three days, every day than not installed before the reduction of two dollars a day, every day to open the bedroom to open the air conditioning before fifteen dollars now thirteen dollars a day. Still a little use.	Jingdong prefecture level city in Shandong
Stowe Flagship Store	a power saver (electronics)	Originally bought for home use, used for two months, the electricity bill from more than 200 yuan per month, down to more than 140 yuan, there is indeed a power-saving effect, this time and sold two, the unit with. Merchant service attitude is really good.	Jingdong prefecture level city in Shandong
Stowe Flagship Store	a power saver (electronics)	Plug and play, stable voltage, the screen shows when working, this winter home appliances, need electricity in many places, just buy to use, save electricity and money, with instructions	Jingdong prefecture level city in Shandong
Stowe Flagship Store	a power saver (electronics)	Goods received, do not know if it is good. When I bought it, I saw that it was a Stowe brand or company's product. Received but not. I do not know what is the reason. Now I can only give this evaluation. I'm	Jingdong prefecture level city in Shandong
Stowe Flagship Store	a power saver (electronics)	The first time to buy online, but also look at the evaluation before buying, the quality is not bad, but also very cheap, the need for friends can rest assured to buy!	Jingdong prefecture level city in Shandong
Stowe Flagship Store	a power saver (electronics)	Great quality, bought it to my liking Awesome! Buy with confidence if you need it!	Jingdong prefecture level city in Shandong
Stowe Flagship Store	a power saver (electronics)	The quality is very good, completely exceeded the expectations, the delivery speed is very fast, the packaging is very careful and strict, very satisfied with a shopping	Jingdong prefecture level city in Shandong
Stowe Flagship Store	a power saver (electronics)	The effect is very good, the home uses less electricity is very energy efficient, cost-effective	Jingdong prefecture level city in Shandong
Stowe Flagship Store	a power saver (electronics)	Quality can't be beat, just like the store, especially like, and the price is favorable!	Jingdong prefecture level city in Shandong
Stowe Flagship Store	a power saver (electronics)	Baby received! Very unexpected, will be back next time ~ great seller, stay tuned!	Jingdong prefecture level city in Shandong
Stowe Flagship Store	a power saver (electronics)	Follow up review after a month is more real, waiting for it	Jingdong prefecture level city in Shandong
Stowe Flagship Store	a power saver (electronics)	It is the ideal product, good quality and fast logistics, worthy of praise!	Jingdong prefecture level city in Shandong
Stowe Flagship Store	a power saver (electronics)	The saver is great, the color is right, the installer is very professional	Jingdong prefecture level city in Shandong
Stowe Flagship Store	a power saver (electronics)	The effect of the power connection is particularly obvious, it is particularly easy to use, very satisfied, the	Jingdong prefecture level city in Shandong
Stowe Flagship Store	a power saver (electronics)	Goods are very energy-saving, used to be more than two hundred degrees, now there are only a hundred or so degrees	Jingdong prefecture level city in Shandong
Stowe Flagship Store	a power saver (electronics)	Good quality, fast shipping, good results, good value for money	Jingdong prefecture level city in Shandong
Stowe Flagship Store	a power saver (electronics)	Fast shipping, fine workmanship, very good, cost-effective	Jingdong prefecture level city in Shandong
Stowe Flagship Store	a power saver (electronics)	The meter is as described, the quality is quite good, energy-saving, worth buying oh~!	Jingdong prefecture level city in Shandong
Stowe Flagship Store	a power saver (electronics)	Great value for money, very affordable,, perfect . ˆ◡ˆ.	Jingdong prefecture level city in Shandong
Stowe Flagship Store	a power saver (electronics)	Good!!! Worth recommending!	Jingdong prefecture level city in Shandong
Stowe Flagship Store	a power saver (electronics)	Great, fast logistics, great stuff, great value for money!	Jingdong prefecture level city in Shandong

Stowe Flagship Store	a power saver (electronics)	Fast logistics, very satisfied with the quality upon receipt	Jingdong prefecture level city in Shandong
Stowe Flagship Store	a power saver (electronics)	Great quality, plug and play, fast shipping!	Jingdong prefecture level city in Shandong
Stowe Flagship Store	a power saver (electronics)	Logistics received, quite satisfied	Jingdong prefecture level city in Shandong
Stowe Flagship Store	a power saver (electronics)	I don't know if it really works	Jingdong prefecture level city in Shandong
Stowe Flagship Store	a power saver (electronics)	It's bullshit. It's a plug light. Don't talk about power savings.	Jingdong prefecture level city in Shandong
Stowe Flagship Store	a power saver (electronics)	Fake and deceitful stuff that hasn't changed at all.	Jingdong prefecture level city in Shandong
Stowe Flagship Store	a power saver (electronics)	Power-saving effect: power-saving effect is particularly obvious Cost-effective: cost-effective business Appearance and style: external small micro-work materials: fine workmanship Experience: use very good, non the first convenient	Jingdong prefecture level city in Shandong
Stowe Flagship Store	a power saver (electronics)	After using it for a few days, it feels quite good, it should be quite safe, and it also sends a plug, the merchant is good, wait for a period of time and then calculate whether it saves on the electricity bill and then add comments ha!	Jingdong prefecture level city in Shandong
Stowe Flagship Store	a power saver (electronics)	My friend recommended to me, said the effect is very good, I bought two, this month less money, really can, just buy I do not believe, but has proved to have effect	Jingdong prefecture level city in Shandong
Stowe Flagship Store	a power saver (electronics)	Just bought back, already used, these days to see the effect of it, and so have the effect will also come back to give positive feedback, the price is also super cheap!	Jingdong prefecture level city in Shandong
Stowe Flagship Store	a power saver (electronics)	It does work well, the store uses a lot of electricity, this time to buy back a few, each place that uses a lot of electricity to insert one. Summer is here, the electricity is too strong.	Jingdong prefecture level city in Shandong
Stowe Flagship Store	a power saver (electronics)	Power saving treasure received, as for the effect, according to the instructions written, a short period of time can not be seen, at least 2-3 months before the real effect, then follow up evaluation	Jingdong prefecture level city in Shandong
Stowe Flagship Store	a power saver (electronics)	The goods have just been received and have not been used yet looking forward to the results.	Jingdong prefecture level city in Shandong
Stowe Flagship Store	a power saver (electronics)	Good installation works great	Jingdong prefecture level city in Shandong
Stowe Flagship Store	a power saver (electronics)	The logistics is super super fast! The quality is also very good, indeed value for money	Jingdong prefecture level city in Shandong
Stowe Flagship Store	a power saver (electronics)	Has been used for a period of time before coming to comment on, really good, air conditioning a day open non-stop, pay the electricity bill when the heartache, with this, the home electricity is not at all worried about the bill out of contrast to save a lot of preparation in the hands of a few, each room An a	Jingdong prefecture level city in Shandong
Stowe Flagship Store	a power saver (electronics)	Saving device received, really good, with the air conditioning tried, an hour without a saver with 2.9 degrees, with a saver an hour with 1.1 degrees, the beginning of not quite believe that this thing, the actual test to know that it is really useful, and intend to buy another.	Jingdong prefecture level city in Shandong
Stowe Flagship Store	a power saver (electronics)	Power saving effect: this energy saver is still useful, day trial for three days, every day than not installed before the reduction of two dollars a day, every day to open the bedroom to open the air conditioning before fifteen dollars now thirteen dollars a day. Still a little use.	Jingdong prefecture level city in Shandong
Stowe Flagship Store	a power saver (electronics)	Originally bought for home use, used for two months, the electricity bill from more than 200 yuan per month, down to more than 140 yuan, there is indeed a power-saving effect, this time and sold two, the unit with. Merchant service attitude is really good.	Jingdong prefecture level city in Shandong
Stowe Flagship Store	a power saver (electronics)	Plug and play, stable voltage, the screen shows when working, this winter home appliances, need electricity in many places, just buy to use, save electricity and money, with instructions	Jingdong prefecture level city in Shandong
Stowe Flagship Store	a power saver (electronics)	Goods received, do not know if it is good. When I bought it, I saw that it was a Stowe brand or company's product. Received but not. I do not know what is the reason. Now I can only give this evaluation. I'm	Jingdong prefecture level city in Shandong
Stowe Flagship Store	a power saver (electronics)	The first time to buy online, but also look at the evaluation before buying, the quality is not bad, but also very cheap, the need for friends can rest assured to buy!	Jingdong prefecture level city in Shandong
Stowe Flagship Store	a power saver (electronics)	🌈 Great quality, bought it to my liking 💞 Awesome! ✨ Buy with confidence if you need it!	Jingdong prefecture level city in Shandong
Stowe Flagship Store	a power saver (electronics)	The quality is very good, completely exceeded the expectations, the delivery speed is very fast, the packaging is very careful and strict, very satisfied with a shopping	Jingdong prefecture level city in Shandong
Stowe Flagship Store	a power saver (electronics)	The effect is very good, the home uses less electricity is very energy efficient, cost-effective	Jingdong prefecture level city in Shandong
Stowe Flagship Store	a power saver (electronics)	Quality can't be beat, just like the store, especially like, and the price is favorable!	Jingdong prefecture level city in Shandong
Stowe Flagship Store	a power saver (electronics)	Baby received! Very unexpected, will be back next time ~ great seller, stay tuned!	Jingdong prefecture level city in Shandong
Stowe Flagship Store	a power saver (electronics)	Follow up review after a month is more real, waiting for it	Jingdong prefecture level city in Shandong
Stowe Flagship Store	a power saver (electronics)	It is the ideal product, good quality and fast logistics, worthy of praise!	Jingdong prefecture level city in Shandong
Stowe Flagship Store	a power saver (electronics)	The saver is great, the color is right, the installer is very professional	Jingdong prefecture level city in Shandong
Stowe Flagship Store	a power saver (electronics)	The effect of the power connection is particularly obvious, it is particularly easy to use, very satisfied, the	Jingdong prefecture level city in Shandong
Stowe Flagship Store	a power saver (electronics)	Goods are very energy-saving, used to be more than two hundred degrees, now there are only a hundred or so degrees	Jingdong prefecture level city in Shandong
Stowe Flagship Store	a power saver (electronics)	Good quality, fast shipping, good results, good value for money	Jingdong prefecture level city in Shandong
Stowe Flagship Store	a power saver (electronics)	Fast shipping, fine workmanship, very good, cost-effective	Jingdong prefecture level city in Shandong
Stowe Flagship Store	a power saver (electronics)	The meter is as described, the quality is quite good, energy-saving, worth buying oh~!	Jingdong prefecture level city in Shandong
Stowe Flagship Store	a power saver (electronics)	Great value for money, very affordable,, perfect . ^◡^.	Jingdong prefecture level city in Shandong
Stowe Flagship Store	a power saver (electronics)	Good!!! Worth recommending!	Jingdong prefecture level city in Shandong
Stowe Flagship Store	a power saver (electronics)	Great, fast logistics, great stuff, great value for money!	Jingdong prefecture level city in Shandong
Stowe Flagship Store	a power saver (electronics)	Fast logistics, very satisfied with the quality upon receipt	Jingdong prefecture level city in Shandong
Stowe Flagship Store	a power saver (electronics)	Great quality, plug and play, fast shipping!	Jingdong prefecture level city in Shandong
Stowe Flagship Store	a power saver (electronics)	Logistics received, quite satisfied	Jingdong prefecture level city in Shandong
Stowe Flagship Store	a power saver (electronics)	I don't know if it really works	Jingdong prefecture level city in Shandong
Stowe Flagship Store	a power saver (electronics)	It's bullshit. It's a plug light. Don't talk about power savings.	Jingdong prefecture level city in Shandong
Stowe Flagship Store	a power saver (electronics)	Fake and deceitful stuff that hasn't changed at all.	Jingdong prefecture level city in Shandong
Stowe Flagship Store	a power saver (electronics)	Power-saving effect: power-saving effect is particularly obvious Cost-effective: cost-effective business Appearance and style: external small micro-work materials: fine workmanship Experience: use very good, non the first convenient	Jingdong prefecture level city in Shandong
Stowe Flagship Store	a power saver (electronics)	After using it for a few days, it feels quite good, it should be quite safe, and it also sends a plug, the merchant is good, wait for a period of time and then calculate whether it saves on the electricity bill and then add comments ha!	Jingdong prefecture level city in Shandong
Stowe Flagship Store	a power saver (electronics)	My friend recommended to me, said the effect is very good, I bought two, this month less money, really can, just buy I do not believe, but has proved to have effect	Jingdong prefecture level city in Shandong

Stowe Flagship Store	a power saver (electronics)	Just bought back, already used, these days to see the effect of it, and so have the effect will also come back to give positive feedback, the price is also super cheap!	Jingdong prefecture level city in Shandong
Stowe Flagship Store	a power saver (electronics)	It does work well, the store uses a lot of electricity, this time to buy back a few, each place that uses a lot of electricity to insert one. Summer is here, the electricity is too strong.	Jingdong prefecture level city in Shandong
Stowe Flagship Store	a power saver (electronics)	Power saving treasure received, as for the effect, according to the instructions written, a short period of time can not be seen, at least 2-3 months before the real effect, then follow up evaluation	Jingdong prefecture level city in Shandong
Stowe Flagship Store	a power saver (electronics)	The goods have just been received and have not been used yet looking forward to the results.	Jingdong prefecture level city in Shandong
Stowe Flagship Store	a power saver (electronics)	Good installation works great	Jingdong prefecture level city in Shandong
Stowe Flagship Store	a power saver (electronics)	The logistics is super super fast! The quality is also very good, indeed value for money	Jingdong prefecture level city in Shandong
Stowe Flagship Store	a power saver (electronics)	Has been used for a period of time before coming to comment on, really good, air conditioning a day open non-stop, pay the electricity bill when the heartache, with this, the home electricity is not at all worried about the bill out of contrast to save a lot of preparation in the hands of a few, each room An a	Jingdong prefecture level city in Shandong
Stowe Flagship Store	a power saver (electronics)	Saving device received, really good, with the air conditioning tried, an hour without a saver with 2.9 degrees, with a saver an hour with 1.1 degrees, the beginning of not quite believe that this thing, the actual test to know that it is really useful, and intend to buy another.	Jingdong prefecture level city in Shandong
Stowe Flagship Store	a power saver (electronics)	Power saving effect: this energy saver is still useful, day trial for three days, every day than not installed before the reduction of two dollars a day, every day to open the bedroom to open the air conditioning before fifteen dollars now thirteen dollars a day. Still a little use.	Jingdong prefecture level city in Shandong
Stowe Flagship Store	a power saver (electronics)	Originally bought for home use, used for two months, the electricity bill from more than 200 yuan per month, down to more than 140 yuan, there is indeed a power-saving effect, this time and sold two, the unit with. Merchant service attitude is really good.	Jingdong prefecture level city in Shandong
Stowe Flagship Store	a power saver (electronics)	Plug and play, stable voltage, the screen shows when working, this winter home appliances, need electricity in many places, just buy to use, save electricity and money, with instructions	Jingdong prefecture level city in Shandong
Stowe Flagship Store	a power saver (electronics)	Goods received, do not know if it is good. When I bought it, I saw that it was a Stowe brand or company's product. Received but not. I do not know what is the reason. Now I can only give this evaluation. I'm	Jingdong prefecture level city in Shandong
Stowe Flagship Store	a power saver (electronics)	The first time to buy online, but also look at the evaluation before buying, the quality is not bad, but also very cheap, the need for friends can rest assured to buy!	Jingdong prefecture level city in Shandong
Stowe Flagship Store	a power saver (electronics)	🌈 Great quality, bought it to my liking 💞 Awesome! ✨ Buy with confidence if you need it!	Jingdong prefecture level city in Shandong
Stowe Flagship Store	a power saver (electronics)	The quality is very good, completely exceeded the expectations, the delivery speed is very fast, the packaging is very careful and strict, very satisfied with a shopping	Jingdong prefecture level city in Shandong
Stowe Flagship Store	a power saver (electronics)	The effect is very good, the home uses less electricity is very energy efficient, cost-effective	Jingdong prefecture level city in Shandong
Stowe Flagship Store	a power saver (electronics)	Quality can't be beat, just like the store, especially like, and the price is favorable!	Jingdong prefecture level city in Shandong
Stowe Flagship Store	a power saver (electronics)	Baby received! Very unexpected, will be back next time ~ great seller, stay tuned!	Jingdong prefecture level city in Shandong
Stowe Flagship Store	a power saver (electronics)	Follow up review after a month is more real, waiting for it	Jingdong prefecture level city in Shandong
Stowe Flagship Store	a power saver (electronics)	It is the ideal product, good quality and fast logistics, worthy of praise!	Jingdong prefecture level city in Shandong
Stowe Flagship Store	a power saver (electronics)	The saver is great, the color is right, the installer is very professional	Jingdong prefecture level city in Shandong
Stowe Flagship Store	a power saver (electronics)	The effect of the power connection is particularly obvious, it is particularly easy to use, very satisfied, the	Jingdong prefecture level city in Shandong
Stowe Flagship Store	a power saver (electronics)	Goods are very energy-saving, used to be more than two hundred degrees, now there are only a hundred or so degrees	Jingdong prefecture level city in Shandong
Stowe Flagship Store	a power saver (electronics)	Good quality, fast shipping, good results, good value for money	Jingdong prefecture level city in Shandong
Stowe Flagship Store	a power saver (electronics)	Fast shipping, fine workmanship, very good, cost-effective	Jingdong prefecture level city in Shandong
Stowe Flagship Store	a power saver (electronics)	The meter is as described, the quality is quite good, energy-saving, worth buying oh~!	Jingdong prefecture level city in Shandong
Stowe Flagship Store	a power saver (electronics)	Great value for money, very affordable,, perfect . ˆ◡ˆ.	Jingdong prefecture level city in Shandong
Stowe Flagship Store	a power saver (electronics)	Good!!! Worth recommending!	Jingdong prefecture level city in Shandong
Stowe Flagship Store	a power saver (electronics)	Great, fast logistics, great stuff, great value for money!	Jingdong prefecture level city in Shandong
Stowe Flagship Store	a power saver (electronics)	Fast logistics, very satisfied with the quality upon receipt	Jingdong prefecture level city in Shandong
Stowe Flagship Store	a power saver (electronics)	Great quality, plug and play, fast shipping!	Jingdong prefecture level city in Shandong
Stowe Flagship Store	a power saver (electronics)	Logistics received, quite satisfied	Jingdong prefecture level city in Shandong
Stowe Flagship Store	a power saver (electronics)	I don't know if it really works	Jingdong prefecture level city in Shandong
Stowe Flagship Store	a power saver (electronics)	It's bullshit. It's a plug light. Don't talk about power savings.	Jingdong prefecture level city in Shandong
Stowe Flagship Store	a power saver (electronics)	Fake and deceitful stuff that hasn't changed at all.	Jingdong prefecture level city in Shandong
Stowe Flagship Store	a power saver (electronics)	Power-saving effect: power-saving effect is particularly obvious Cost-effective: cost-effective business Appearance and style: external small micro-work materials: fine workmanship Experience: use very good, non the first convenient	Jingdong prefecture level city in Shandong
Stowe Flagship Store	a power saver (electronics)	After using it for a few days, it feels quite good, it should be quite safe, and it also sends a plug, the merchant is good, wait for a period of time and then calculate whether it saves on the electricity bill and then add comments ha!	Jingdong prefecture level city in Shandong
Stowe Flagship Store	a power saver (electronics)	My friend recommended to me, said the effect is very good, I bought two, this month less money, really can, just buy I do not believe, but has proved to have effect	Jingdong prefecture level city in Shandong
Stowe Flagship Store	a power saver (electronics)	Just bought back, already used, these days to see the effect of it, and so have the effect will also come back to give positive feedback, the price is also super cheap!	Jingdong prefecture level city in Shandong
Stowe Flagship Store	a power saver (electronics)	It does work well, the store uses a lot of electricity, this time to buy back a few, each place that uses a lot of electricity to insert one. Summer is here, the electricity is too strong.	Jingdong prefecture level city in Shandong
Stowe Flagship Store	a power saver (electronics)	Power saving treasure received, as for the effect, according to the instructions written, a short period of time can not be seen, at least 2-3 months before the real effect, then follow up evaluation	Jingdong prefecture level city in Shandong
Stowe Flagship Store	a power saver (electronics)	The goods have just been received and have not been used yet looking forward to the results.	Jingdong prefecture level city in Shandong
Stowe Flagship Store	a power saver (electronics)	Good installation works great	Jingdong prefecture level city in Shandong
Stowe Flagship Store	a power saver (electronics)	The logistics is super super fast! The quality is also very good, indeed value for money	Jingdong prefecture level city in Shandong
Stowe Flagship Store	a power saver (electronics)	Has been used for a period of time before coming to comment on, really good, air conditioning a day open non-stop, pay the electricity bill when the heartache, with this, the home electricity is not at all worried about the bill out of contrast to save a lot of preparation in the hands of a few, each room An a	Jingdong prefecture level city in Shandong
Stowe Flagship Store	a power saver (electronics)	Saving device received, really good, with the air conditioning tried, an hour without a saver with 2.9 degrees, with a saver an hour with 1.1 degrees, the beginning of not quite believe that this thing, the actual test to know that it is really useful, and intend to buy another.	Jingdong prefecture level city in Shandong
Stowe Flagship Store	a power saver (electronics)	Power saving effect: this energy saver is still useful, day trial for three days, every day than not installed before the reduction of two dollars a day, every day to open the bedroom to open the air conditioning before fifteen dollars now thirteen dollars a day. Still a little use.	Jingdong prefecture level city in Shandong

Stowe Flagship Store	a power saver (electronics)	Originally bought for home use, used for two months, the electricity bill from more than 200 yuan per month, down to more than 140 yuan, there is indeed a power-saving effect, this time and sold two, the unit with. Merchant service attitude is really good.	Jingdong prefecture level city in Shandong
Stowe Flagship Store	a power saver (electronics)	Plug and play, stable voltage, the screen shows when working, this winter home appliances, need electricity in many places, just buy to use, save electricity and money, with instructions	Jingdong prefecture level city in Shandong
Stowe Flagship Store	a power saver (electronics)	Goods received, do not know if it is good. When I bought it, I saw that it was a Stowe brand or company's product. Received but not. I do not know what is the reason. Now I can only give this evaluation. I'm	Jingdong prefecture level city in Shandong
Stowe Flagship Store	a power saver (electronics)	The first time to buy online, but also look at the evaluation before buying, the quality is not bad, but also very cheap, the need for friends can rest assured to buy!	Jingdong prefecture level city in Shandong
Stowe Flagship Store	a power saver (electronics)	🌈 Great quality, bought it to my liking 💛 Awesome! ✨ Buy with confidence if you need it!	Jingdong prefecture level city in Shandong
Stowe Flagship Store	a power saver (electronics)	The quality is very good, completely exceeded the expectations, the delivery speed is very fast, the packaging is very careful and strict, very satisfied with a shopping	Jingdong prefecture level city in Shandong
Stowe Flagship Store	a power saver (electronics)	The effect is very good, the home uses less electricity is very energy efficient, cost-effective	Jingdong prefecture level city in Shandong
Stowe Flagship Store	a power saver (electronics)	Quality can't be beat, just like the store, especially like, and the price is favorable!	Jingdong prefecture level city in Shandong
Stowe Flagship Store	a power saver (electronics)	Baby received! Very unexpected, will be back next time ~ great seller, stay tuned!	Jingdong prefecture level city in Shandong
Stowe Flagship Store	a power saver (electronics)	Follow up review after a month is more real, waiting for it	Jingdong prefecture level city in Shandong
Stowe Flagship Store	a power saver (electronics)	It is the ideal product, good quality and fast logistics, worthy of praise!	Jingdong prefecture level city in Shandong
Stowe Flagship Store	a power saver (electronics)	The saver is great, the color is right, the installer is very professional	Jingdong prefecture level city in Shandong
Stowe Flagship Store	a power saver (electronics)	The effect of the power connection is particularly obvious, it is particularly easy to use, very satisfied, the	Jingdong prefecture level city in Shandong
Stowe Flagship Store	a power saver (electronics)	Goods are very energy-saving, used to be more than two hundred degrees, now there are only a hundred or so degrees	Jingdong prefecture level city in Shandong
Stowe Flagship Store	a power saver (electronics)	Good quality, fast shipping, good results, good value for money	Jingdong prefecture level city in Shandong
Stowe Flagship Store	a power saver (electronics)	Fast shipping, fine workmanship, very good, cost-effective	Jingdong prefecture level city in Shandong
Stowe Flagship Store	a power saver (electronics)	The meter is as described, the quality is quite good, energy-saving, worth buying oh~!	Jingdong prefecture level city in Shandong
Stowe Flagship Store	a power saver (electronics)	Great value for money, very affordable,, perfect . ˆ◡ˆ.	Jingdong prefecture level city in Shandong
Stowe Flagship Store	a power saver (electronics)	Good!!! Worth recommending!	Jingdong prefecture level city in Shandong
Stowe Flagship Store	a power saver (electronics)	Great, fast logistics, great stuff, great value for money!	Jingdong prefecture level city in Shandong
Stowe Flagship Store	a power saver (electronics)	Fast logistics, very satisfied with the quality upon receipt	Jingdong prefecture level city in Shandong
Stowe Flagship Store	a power saver (electronics)	Great quality, plug and play, fast shipping!	Jingdong prefecture level city in Shandong
Stowe Flagship Store	a power saver (electronics)	Logistics received, quite satisfied	Jingdong prefecture level city in Shandong
Stowe Flagship Store	a power saver (electronics)	I don't know if it really works	Jingdong prefecture level city in Shandong
Stowe Flagship Store	a power saver (electronics)	It's bullshit. It's a plug light. Don't talk about power savings.	Jingdong prefecture level city in Shandong
Stowe Flagship Store	a power saver (electronics)	Fake and deceitful stuff that hasn't changed at all.	Jingdong prefecture level city in Shandong
Stowe Flagship Store	a power saver (electronics)	Power-saving effect: power-saving effect is particularly obvious Cost-effective: cost-effective business Appearance and style: external small micro-work materials: fine workmanship Experience: use very good, non the first convenient	Jingdong prefecture level city in Shandong
Stowe Flagship Store	a power saver (electronics)	After using it for a few days, it feels quite good, it should be quite safe, and it also sends a plug, the merchant is good, wait for a period of time and then calculate whether it saves on the electricity bill and then add comments ha!	Jingdong prefecture level city in Shandong
Stowe Flagship Store	a power saver (electronics)	My friend recommended to me, said the effect is very good, I bought two, this month less money, really can, just buy I do not believe, but has proved to have effect	Jingdong prefecture level city in Shandong
Stowe Flagship Store	a power saver (electronics)	Just bought back, already used, these days to see the effect of it, and so have the effect will also come back to give positive feedback, the price is also super cheap!	Jingdong prefecture level city in Shandong
Stowe Flagship Store	a power saver (electronics)	It does work well, the store uses a lot of electricity, this time to buy back a few, each place that uses a lot of electricity to insert one. Summer is here, the electricity is too strong.	Jingdong prefecture level city in Shandong
Stowe Flagship Store	a power saver (electronics)	Power saving treasure received, as for the effect, according to the instructions written, a short period of time can not be seen, at least 2-3 months before the real effect, then follow up evaluation	Jingdong prefecture level city in Shandong
Stowe Flagship Store	a power saver (electronics)	The goods have just been received and have not been used yet looking forward to the results.	Jingdong prefecture level city in Shandong
Stowe Flagship Store	a power saver (electronics)	Good installation works great	Jingdong prefecture level city in Shandong
Stowe Flagship Store	a power saver (electronics)	The logistics is super super fast! The quality is also very good, indeed value for money	Jingdong prefecture level city in Shandong
Stowe Flagship Store	a power saver (electronics)	Has been used for a period of time before coming to comment on, really good, air conditioning a day open non-stop, pay the electricity bill when the heartache, with this, the home electricity is not at all worried about the bill out of contrast to save a lot of preparation in the hands of a few, each room An a	Jingdong prefecture level city in Shandong
Stowe Flagship Store	a power saver (electronics)	Saving device received, really good, with the air conditioning tried, an hour without a saver with 2.9 degrees, with a saver an hour with 1.1 degrees, the beginning of not quite believe that this thing, the actual test to know that it is really useful, and intend to buy another.	Jingdong prefecture level city in Shandong
Stowe Flagship Store	a power saver (electronics)	Power saving effect: this energy saver is still useful, day trial for three days, every day than not installed before the reduction of two dollars a day, every day to open the bedroom to open the air conditioning before fifteen dollars now thirteen dollars a day. Still a little use.	Jingdong prefecture level city in Shandong
Stowe Flagship Store	a power saver (electronics)	Originally bought for home use, used for two months, the electricity bill from more than 200 yuan per month, down to more than 140 yuan, there is indeed a power-saving effect, this time and sold two, the unit with. Merchant service attitude is really good.	Jingdong prefecture level city in Shandong
Stowe Flagship Store	a power saver (electronics)	Plug and play, stable voltage, the screen shows when working, this winter home appliances, need electricity in many places, just buy to use, save electricity and money, with instructions	Jingdong prefecture level city in Shandong
Stowe Flagship Store	a power saver (electronics)	Goods received, do not know if it is good. When I bought it, I saw that it was a Stowe brand or company's product. Received but not. I do not know what is the reason. Now I can only give this evaluation. I'm	Jingdong prefecture level city in Shandong
Stowe Flagship Store	a power saver (electronics)	The first time to buy online, but also look at the evaluation before buying, the quality is not bad, but also very cheap, the need for friends can rest assured to buy!	Jingdong prefecture level city in Shandong
Stowe Flagship Store	a power saver (electronics)	🌈 Great quality, bought it to my liking 💛 Awesome! ✨ Buy with confidence if you need it!	Jingdong prefecture level city in Shandong
Stowe Flagship Store	a power saver (electronics)	The quality is very good, completely exceeded the expectations, the delivery speed is very fast, the packaging is very careful and strict, very satisfied with a shopping	Jingdong prefecture level city in Shandong
Stowe Flagship Store	a power saver (electronics)	The effect is very good, the home uses less electricity is very energy efficient, cost-effective	Jingdong prefecture level city in Shandong
Stowe Flagship Store	a power saver (electronics)	Quality can't be beat, just like the store, especially like, and the price is favorable!	Jingdong prefecture level city in Shandong
Stowe Flagship Store	a power saver (electronics)	Baby received! Very unexpected, will be back next time ~ great seller, stay tuned!	Jingdong prefecture level city in Shandong
Stowe Flagship Store	a power saver (electronics)	Follow up review after a month is more real, waiting for it	Jingdong prefecture level city in Shandong

Stowe Flagship Store	a power saver (electronics)	It is the ideal product, good quality and fast logistics, worthy of praise!	Jingdong prefecture level city in Shandong
Stowe Flagship Store	a power saver (electronics)	The saver is great, the color is right, the installer is very professional	Jingdong prefecture level city in Shandong
Stowe Flagship Store	a power saver (electronics)	The effect of the power connection is particularly obvious, it is particularly easy to use, very satisfied, the	Jingdong prefecture level city in Shandong
Stowe Flagship Store	a power saver (electronics)	Goods are very energy-saving, used to be more than two hundred degrees, now there are only a hundred or so degrees	Jingdong prefecture level city in Shandong
Stowe Flagship Store	a power saver (electronics)	Good quality, fast shipping, good results, good value for money	Jingdong prefecture level city in Shandong
Stowe Flagship Store	a power saver (electronics)	Fast shipping, fine workmanship, very good, cost-effective	Jingdong prefecture level city in Shandong
Stowe Flagship Store	a power saver (electronics)	The meter is as described, the quality is quite good, energy-saving, worth buying oh~!	Jingdong prefecture level city in Shandong
Stowe Flagship Store	a power saver (electronics)	Great value for money, very affordable,, perfect . ˆ◡ˆ.	Jingdong prefecture level city in Shandong
Stowe Flagship Store	a power saver (electronics)	Good!!! Worth recommending!	Jingdong prefecture level city in Shandong
Stowe Flagship Store	a power saver (electronics)	Great, fast logistics, great stuff, great value for money!	Jingdong prefecture level city in Shandong
Stowe Flagship Store	a power saver (electronics)	Fast logistics, very satisfied with the quality upon receipt	Jingdong prefecture level city in Shandong
Stowe Flagship Store	a power saver (electronics)	Great quality, plug and play, fast shipping!	Jingdong prefecture level city in Shandong
Stowe Flagship Store	a power saver (electronics)	Logistics received, quite satisfied	Jingdong prefecture level city in Shandong
Stowe Flagship Store	a power saver (electronics)	I don't know if it really works	Jingdong prefecture level city in Shandong
Stowe Flagship Store	a power saver (electronics)	It's bullshit. It's a plug light. Don't talk about power savings.	Jingdong prefecture level city in Shandong
Stowe Flagship Store	a power saver (electronics)	Fake and deceitful stuff that hasn't changed at all.	Jingdong prefecture level city in Shandong
Stowe Flagship Store	a power saver (electronics)	Power-saving effect: power-saving effect is particularly obvious Cost-effective: cost-effective business Appearance and style: external small micro-work materials: fine workmanship Experience: use very good, non the first convenient	Jingdong prefecture level city in Shandong
Stowe Flagship Store	a power saver (electronics)	After using it for a few days, it feels quite good, it should be quite safe, and it also sends a plug, the merchant is good, wait for a period of time and then calculate whether it saves on the electricity bill and then add comments ha!	Jingdong prefecture level city in Shandong
Stowe Flagship Store	a power saver (electronics)	My friend recommended to me, said the effect is very good, I bought two, this month less money, really can, just buy I do not believe, but has proved to have effect	Jingdong prefecture level city in Shandong
Stowe Flagship Store	a power saver (electronics)	Just bought back, already used, these days to see the effect of it, and so have the effect will also come back to give positive feedback, the price is also super cheap!	Jingdong prefecture level city in Shandong
Stowe Flagship Store	a power saver (electronics)	It does work well, the store uses a lot of electricity, this time to buy back a few, each place that uses a lot of electricity to insert one. Summer is here, the electricity is too strong.	Jingdong prefecture level city in Shandong
Stowe Flagship Store	a power saver (electronics)	Power saving treasure received, as for the effect, according to the instructions written, a short period of time can not be seen, at least 2-3 months before the real effect, then follow up evaluation	Jingdong prefecture level city in Shandong
Stowe Flagship Store	a power saver (electronics)	The goods have just been received and have not been used yet looking forward to the results.	Jingdong prefecture level city in Shandong
Stowe Flagship Store	a power saver (electronics)	Good installation works great	Jingdong prefecture level city in Shandong
Stowe Flagship Store	a power saver (electronics)	The logistics is super super fast! The quality is also very good, indeed value for money	Jingdong prefecture level city in Shandong
Stowe Flagship Store	a power saver (electronics)	Has been used for a period of time before coming to comment on, really good, air conditioning a day open non-stop, pay the electricity bill when the heartache, with this, the home electricity is not at all worried about the bill out of contrast to save a lot of preparation in the hands of a few, each room An a	Jingdong prefecture level city in Shandong
Stowe Flagship Store	a power saver (electronics)	Saving device received, really good, with the air conditioning tried, an hour without a saver with 2.9 degrees, with a saver an hour with 1.1 degrees, the beginning of not quite believe that this thing, the actual test to know that it is really useful, and intend to buy another.	Jingdong prefecture level city in Shandong
Stowe Flagship Store	a power saver (electronics)	Power saving effect: this energy saver is still useful, day trial for three days, every day than not installed before the reduction of two dollars a day, every day to open the bedroom to open the air conditioning before fifteen dollars now thirteen dollars a day. Still a little use.	Jingdong prefecture level city in Shandong
Stowe Flagship Store	a power saver (electronics)	Originally bought for home use, used for two months, the electricity bill from more than 200 yuan per month, down to more than 140 yuan, there is indeed a power-saving effect, this time and sold two, the unit with. Merchant service attitude is really good.	Jingdong prefecture level city in Shandong
Stowe Flagship Store	a power saver (electronics)	Plug and play, stable voltage, the screen shows when working, this winter home appliances, need electricity in many places, just buy to use, save electricity and money, with instructions	Jingdong prefecture level city in Shandong
Stowe Flagship Store	a power saver (electronics)	Goods received, do not know if it is good. When I bought it, I saw that it was a Stowe brand or company's product. Received but not. I do not know what is the reason. Now I can only give this evaluation. I'm	Jingdong prefecture level city in Shandong
Stowe Flagship Store	a power saver (electronics)	The first time to buy online, but also look at the evaluation before buying, the quality is not bad, but also very cheap, the need for friends can rest assured to buy!	Jingdong prefecture level city in Shandong
Stowe Flagship Store	a power saver (electronics)	🌈 Great quality, bought it to my liking 💕 Awesome! ✨ Buy with confidence if you need it!	Jingdong prefecture level city in Shandong
Stowe Flagship Store	a power saver (electronics)	The quality is very good, completely exceeded the expectations, the delivery speed is very fast, the packaging is very careful and strict, very satisfied with a shopping	Jingdong prefecture level city in Shandong
Stowe Flagship Store	a power saver (electronics)	The effect is very good, the home uses less electricity is very energy efficient, cost-effective	Jingdong prefecture level city in Shandong
Stowe Flagship Store	a power saver (electronics)	Quality can't be beat, just like the store, especially like, and the price is favorable!	Jingdong prefecture level city in Shandong
Stowe Flagship Store	a power saver (electronics)	Baby received! Very unexpected, will be back next time ~ great seller, stay tuned!	Jingdong prefecture level city in Shandong
Stowe Flagship Store	a power saver (electronics)	Follow up review after a month is more real, waiting for it	Jingdong prefecture level city in Shandong
Stowe Flagship Store	a power saver (electronics)	It is the ideal product, good quality and fast logistics, worthy of praise!	Jingdong prefecture level city in Shandong
Stowe Flagship Store	a power saver (electronics)	The saver is great, the color is right, the installer is very professional	Jingdong prefecture level city in Shandong
Stowe Flagship Store	a power saver (electronics)	The effect of the power connection is particularly obvious, it is particularly easy to use, very satisfied, the	Jingdong prefecture level city in Shandong
Stowe Flagship Store	a power saver (electronics)	Goods are very energy-saving, used to be more than two hundred degrees, now there are only a hundred or so degrees	Jingdong prefecture level city in Shandong
Stowe Flagship Store	a power saver (electronics)	Good quality, fast shipping, good results, good value for money	Jingdong prefecture level city in Shandong
Stowe Flagship Store	a power saver (electronics)	Fast shipping, fine workmanship, very good, cost-effective	Jingdong prefecture level city in Shandong
Stowe Flagship Store	a power saver (electronics)	The meter is as described, the quality is quite good, energy-saving, worth buying oh~!	Jingdong prefecture level city in Shandong
Stowe Flagship Store	a power saver (electronics)	Great value for money, very affordable,, perfect . ˆ◡ˆ.	Jingdong prefecture level city in Shandong
Stowe Flagship Store	a power saver (electronics)	Good!!! Worth recommending!	Jingdong prefecture level city in Shandong
Stowe Flagship Store	a power saver (electronics)	Great, fast logistics, great stuff, great value for money!	Jingdong prefecture level city in Shandong

Stowe Flagship Store	a power saver (electronics)	Fast logistics, very satisfied with the quality upon receipt	Jingdong prefecture level city in Shandong
Stowe Flagship Store	a power saver (electronics)	Great quality, plug and play, fast shipping!	Jingdong prefecture level city in Shandong
Stowe Flagship Store	a power saver (electronics)	Logistics received, quite satisfied	Jingdong prefecture level city in Shandong
Stowe Flagship Store	a power saver (electronics)	I don't know if it really works	Jingdong prefecture level city in Shandong
Stowe Flagship Store	a power saver (electronics)	It's bullshit. It's a plug light. Don't talk about power savings.	Jingdong prefecture level city in Shandong
Stowe Flagship Store	a power saver (electronics)	Fake and deceitful stuff that hasn't changed at all.	Jingdong prefecture level city in Shandong
Stowe Flagship Store	a power saver (electronics)	Power-saving effect: power-saving effect is particularly obvious Cost-effective: cost-effective business Appearance and style: external small micro-work materials: fine workmanship Experience: use very good, non the first convenient	Jingdong prefecture level city in Shandong
Stowe Flagship Store	a power saver (electronics)	After using it for a few days, it feels quite good, it should be quite safe, and it also sends a plug, the merchant is good, wait for a period of time and then calculate whether it saves on the electricity bill and then add comments ha!	Jingdong prefecture level city in Shandong
Stowe Flagship Store	a power saver (electronics)	My friend recommended to me, said the effect is very good, I bought two, this month less money, really can, just buy I do not believe, but has proved to have effect	Jingdong prefecture level city in Shandong
Stowe Flagship Store	a power saver (electronics)	Just bought back, already used, these days to see the effect of it, and so have the effect will also come back to give positive feedback, the price is also super cheap!	Jingdong prefecture level city in Shandong
Stowe Flagship Store	a power saver (electronics)	It does work well, the store uses a lot of electricity, this time to buy back a few, each place that uses a lot of electricity to insert one. Summer is here, the electricity is too strong.	Jingdong prefecture level city in Shandong
Stowe Flagship Store	a power saver (electronics)	Power saving treasure received, as for the effect, according to the instructions written, a short period of time can not be seen, at least 2-3 months before the real effect, then follow up evaluation	Jingdong prefecture level city in Shandong
Stowe Flagship Store	a power saver (electronics)	The goods have just been received and have not been used yet looking forward to the results.	Jingdong prefecture level city in Shandong
Stowe Flagship Store	a power saver (electronics)	Good installation works great	Jingdong prefecture level city in Shandong
Stowe Flagship Store	a power saver (electronics)	The logistics is super super fast! The quality is also very good, indeed value for money	Jingdong prefecture level city in Shandong
Stowe Flagship Store	a power saver (electronics)	Has been used for a period of time before coming to comment on, really good, air conditioning a day open non-stop, pay the electricity bill when the heartache, with this, the home electricity is not at all worried about the bill out of contrast to save a lot of preparation in the hands of a few, each room An a	Jingdong prefecture level city in Shandong
Stowe Flagship Store	a power saver (electronics)	Saving device received, really good, with the air conditioning tried, an hour without a saver with 2.9 degrees, with a saver an hour with 1.1 degrees, the beginning of not quite believe that this thing, the actual test to know that it is really useful, and intend to buy another.	Jingdong prefecture level city in Shandong
Stowe Flagship Store	a power saver (electronics)	Power saving effect: this energy saver is still useful, day trial for three days, every day than not installed before the reduction of two dollars a day, every day to open the bedroom to open the air conditioning before fifteen dollars now thirteen dollars a day. Still a little use.	Jingdong prefecture level city in Shandong
Stowe Flagship Store	a power saver (electronics)	Originally bought for home use, used for two months, the electricity bill from more than 200 yuan per month, down to more than 140 yuan, there is indeed a power-saving effect, this time and sold two, the unit with. Merchant service attitude is really good.	Jingdong prefecture level city in Shandong
Stowe Flagship Store	a power saver (electronics)	Plug and play, stable voltage, the screen shows when working, this winter home appliances, need electricity in many places, just buy to use, save electricity and money, with instructions	Jingdong prefecture level city in Shandong
Stowe Flagship Store	a power saver (electronics)	Goods received, do not know if it is good. When I bought it, I saw that it was a Stowe brand or company's product. Received but not. I do not know what is the reason. Now I can only give this evaluation. I'm	Jingdong prefecture level city in Shandong
Stowe Flagship Store	a power saver (electronics)	The first time to buy online, but also look at the evaluation before buying, the quality is not bad, but also very cheap, the need for friends can rest assured to buy!	Jingdong prefecture level city in Shandong
Stowe Flagship Store	a power saver (electronics)	🌈 Great quality, bought it to my liking 💕 Awesome! ✨ Buy with confidence if you need it!	Jingdong prefecture level city in Shandong
Stowe Flagship Store	a power saver (electronics)	The quality is very good, completely exceeded the expectations, the delivery speed is very fast, the packaging is very careful and strict, very satisfied with a shopping	Jingdong prefecture level city in Shandong
Stowe Flagship Store	a power saver (electronics)	The effect is very good, the home uses less electricity is very energy efficient, cost-effective	Jingdong prefecture level city in Shandong
Stowe Flagship Store	a power saver (electronics)	Quality can't be beat, just like the store, especially like, and the price is favorable!	Jingdong prefecture level city in Shandong
Stowe Flagship Store	a power saver (electronics)	Baby received! Very unexpected, will be back next time ~ great seller, stay tuned!	Jingdong prefecture level city in Shandong
Stowe Flagship Store	a power saver (electronics)	Follow up review after a month is more real, waiting for it	Jingdong prefecture level city in Shandong
Stowe Flagship Store	a power saver (electronics)	It is the ideal product, good quality and fast logistics, worthy of praise!	Jingdong prefecture level city in Shandong
Stowe Flagship Store	a power saver (electronics)	The saver is great, the color is right, the installer is very professional	Jingdong prefecture level city in Shandong
Stowe Flagship Store	a power saver (electronics)	The effect of the power connection is particularly obvious, it is particularly easy to use, very satisfied, the	Jingdong prefecture level city in Shandong
Stowe Flagship Store	a power saver (electronics)	Goods are very energy-saving, used to be more than two hundred degrees, now there are only a hundred or so degrees	Jingdong prefecture level city in Shandong
Stowe Flagship Store	a power saver (electronics)	Good quality, fast shipping, good results, good value for money	Jingdong prefecture level city in Shandong
Stowe Flagship Store	a power saver (electronics)	Fast shipping, fine workmanship, very good, cost-effective	Jingdong prefecture level city in Shandong
Stowe Flagship Store	a power saver (electronics)	The meter is as described, the quality is quite good, energy-saving, worth buying oh~!	Jingdong prefecture level city in Shandong
Stowe Flagship Store	a power saver (electronics)	Great value for money, very affordable,, perfect . ˆ◡ˆ.	Jingdong prefecture level city in Shandong
Stowe Flagship Store	a power saver (electronics)	Good!!! Worth recommending!	Jingdong prefecture level city in Shandong
Stowe Flagship Store	a power saver (electronics)	Great, fast logistics, great stuff, great value for money!	Jingdong prefecture level city in Shandong
Stowe Flagship Store	a power saver (electronics)	Fast logistics, very satisfied with the quality upon receipt	Jingdong prefecture level city in Shandong
Stowe Flagship Store	a power saver (electronics)	Great quality, plug and play, fast shipping!	Jingdong prefecture level city in Shandong
Stowe Flagship Store	a power saver (electronics)	Logistics received, quite satisfied	Jingdong prefecture level city in Shandong
Stowe Flagship Store	a power saver (electronics)	I don't know if it really works	Jingdong prefecture level city in Shandong
Stowe Flagship Store	a power saver (electronics)	It's bullshit. It's a plug light. Don't talk about power savings.	Jingdong prefecture level city in Shandong
Stowe Flagship Store	a power saver (electronics)	Fake and deceitful stuff that hasn't changed at all.	Jingdong prefecture level city in Shandong
Stowe Flagship Store	a power saver (electronics)	Power-saving effect: power-saving effect is particularly obvious Cost-effective: cost-effective business Appearance and style: external small micro-work materials: fine workmanship Experience: use very good, non the first convenient	Jingdong prefecture level city in Shandong
Stowe Flagship Store	a power saver (electronics)	After using it for a few days, it feels quite good, it should be quite safe, and it also sends a plug, the merchant is good, wait for a period of time and then calculate whether it saves on the electricity bill and then add comments ha!	Jingdong prefecture level city in Shandong
Stowe Flagship Store	a power saver (electronics)	My friend recommended to me, said the effect is very good, I bought two, this month less money, really can, just buy I do not believe, but has proved to have effect	Jingdong prefecture level city in Shandong

Stowe Flagship Store	a power saver (electronics)	Just bought back, already used, these days to see the effect of it, and so have the effect will also come back to give positive feedback, the price is also super cheap!	Jingdong prefecture level city in Shandong
Stowe Flagship Store	a power saver (electronics)	It does work well, the store uses a lot of electricity, this time to buy back a few, each place that uses a lot of electricity to insert one. Summer is here, the electricity is too strong.	Jingdong prefecture level city in Shandong
Stowe Flagship Store	a power saver (electronics)	Power saving treasure received, as for the effect, according to the instructions written, a short period of time can not be seen, at least 2-3 months before the real effect, then follow up evaluation	Jingdong prefecture level city in Shandong
Stowe Flagship Store	a power saver (electronics)	The goods have just been received and have not been used yet looking forward to the results.	Jingdong prefecture level city in Shandong
Stowe Flagship Store	a power saver (electronics)	Good installation works great	Jingdong prefecture level city in Shandong
Stowe Flagship Store	a power saver (electronics)	The logistics is super super fast! The quality is also very good, indeed value for money	Jingdong prefecture level city in Shandong
Stowe Flagship Store	a power saver (electronics)	Has been used for a period of time before coming to comment on, really good, air conditioning a day open non-stop, pay the electricity bill when the heartache, with this, the home electricity is not at all worried about the bill out of contrast to save a lot of preparation in the hands of a few, each room An a	Jingdong prefecture level city in Shandong
Stowe Flagship Store	a power saver (electronics)	Saving device received, really good, with the air conditioning tried, an hour without a saver with 2.9 degrees, with a saver an hour with 1.1 degrees, the beginning of not quite believe that this thing, the actual test to know that it is really useful, and intend to buy another.	Jingdong prefecture level city in Shandong
Stowe Flagship Store	a power saver (electronics)	Power saving effect: this energy saver is still useful, day trial for three days, every day than not installed before the reduction of two dollars a day, every day to open the bedroom to open the air conditioning before fifteen dollars now thirteen dollars a day. Still a little use.	Jingdong prefecture level city in Shandong
Stowe Flagship Store	a power saver (electronics)	Originally bought for home use, used for two months, the electricity bill from more than 200 yuan per month, down to more than 140 yuan, there is indeed a power-saving effect, this time and sold two, the unit with. Merchant service attitude is really good.	Jingdong prefecture level city in Shandong
Stowe Flagship Store	a power saver (electronics)	Plug and play, stable voltage, the screen shows when working, this winter home appliances, need electricity in many places, just buy to use, save electricity and money, with instructions	Jingdong prefecture level city in Shandong
Stowe Flagship Store	a power saver (electronics)	Goods received, do not know if it is good. When I bought it, I saw that it was a Stowe brand or company's product. Received but not. I do not know what is the reason. Now I can only give this evaluation. I'm	Jingdong prefecture level city in Shandong
Stowe Flagship Store	a power saver (electronics)	The first time to buy online, but also look at the evaluation before buying, the quality is not bad, but also very cheap, the need for friends can rest assured to buy!	Jingdong prefecture level city in Shandong
Stowe Flagship Store	a power saver (electronics)	🌈 Great quality, bought it to my liking 💝 Awesome! ✨ Buy with confidence if you need it!	Jingdong prefecture level city in Shandong
Stowe Flagship Store	a power saver (electronics)	The quality is very good, completely exceeded the expectations, the delivery speed is very fast, the packaging is very careful and strict, very satisfied with a shopping	Jingdong prefecture level city in Shandong
Stowe Flagship Store	a power saver (electronics)	The effect is very good, the home uses less electricity is very energy efficient, cost-effective	Jingdong prefecture level city in Shandong
Stowe Flagship Store	a power saver (electronics)	Quality can't be beat, just like the store, especially like, and the price is favorable!	Jingdong prefecture level city in Shandong
Stowe Flagship Store	a power saver (electronics)	Baby received! Very unexpected, will be back next time ~ great seller, stay tuned!	Jingdong prefecture level city in Shandong
Stowe Flagship Store	a power saver (electronics)	Follow up review after a month is more real, waiting for it	Jingdong prefecture level city in Shandong
Stowe Flagship Store	a power saver (electronics)	It is the ideal product, good quality and fast logistics, worthy of praise!	Jingdong prefecture level city in Shandong
Stowe Flagship Store	a power saver (electronics)	The saver is great, the color is right, the installer is very professional	Jingdong prefecture level city in Shandong
Stowe Flagship Store	a power saver (electronics)	The effect of the power connection is particularly obvious, it is particularly easy to use, very satisfied, the	Jingdong prefecture level city in Shandong
Stowe Flagship Store	a power saver (electronics)	Goods are very energy-saving, used to be more than two hundred degrees, now there are only a hundred or so degrees	Jingdong prefecture level city in Shandong
Stowe Flagship Store	a power saver (electronics)	Good quality, fast shipping, good results, good value for money	Jingdong prefecture level city in Shandong
Stowe Flagship Store	a power saver (electronics)	Fast shipping, fine workmanship, very good, cost-effective	Jingdong prefecture level city in Shandong
Stowe Flagship Store	a power saver (electronics)	The meter is as described, the quality is quite good, energy-saving, worth buying oh~!	Jingdong prefecture level city in Shandong
Stowe Flagship Store	a power saver (electronics)	Great value for money, very affordable,, perfect . ˆ◡ˆ.	Jingdong prefecture level city in Shandong
Stowe Flagship Store	a power saver (electronics)	Good!!! Worth recommending!	Jingdong prefecture level city in Shandong
Stowe Flagship Store	a power saver (electronics)	Great, fast logistics, great stuff, great value for money!	Jingdong prefecture level city in Shandong
Stowe Flagship Store	a power saver (electronics)	Fast logistics, very satisfied with the quality upon receipt	Jingdong prefecture level city in Shandong
Stowe Flagship Store	a power saver (electronics)	Great quality, plug and play, fast shipping!	Jingdong prefecture level city in Shandong
Stowe Flagship Store	a power saver (electronics)	Logistics received, quite satisfied	Jingdong prefecture level city in Shandong
Stowe Flagship Store	a power saver (electronics)	I don't know if it really works	Jingdong prefecture level city in Shandong
Stowe Flagship Store	a power saver (electronics)	It's bullshit. It's a plug light. Don't talk about power savings.	Jingdong prefecture level city in Shandong
Stowe Flagship Store	a power saver (electronics)	Fake and deceitful stuff that hasn't changed at all.	Jingdong prefecture level city in Shandong
Stowe Flagship Store	a power saver (electronics)	Power-saving effect: power-saving effect is particularly obvious Cost-effective: cost-effective business Appearance and style: external small micro-work materials: fine workmanship Experience: use very good, non the first convenient	Jingdong prefecture level city in Shandong
Stowe Flagship Store	a power saver (electronics)	After using it for a few days, it feels quite good, it should be quite safe, and it also sends a plug, the merchant is good, wait for a period of time and then calculate whether it saves on the electricity bill and then add comments ha!	Jingdong prefecture level city in Shandong
Stowe Flagship Store	a power saver (electronics)	My friend recommended to me, said the effect is very good, I bought two, this month less money, really can, just buy I do not believe, but has proved to have effect	Jingdong prefecture level city in Shandong
Stowe Flagship Store	a power saver (electronics)	Just bought back, already used, these days to see the effect of it, and so have the effect will also come back to give positive feedback, the price is also super cheap!	Jingdong prefecture level city in Shandong
Stowe Flagship Store	a power saver (electronics)	It does work well, the store uses a lot of electricity, this time to buy back a few, each place that uses a lot of electricity to insert one. Summer is here, the electricity is too strong.	Jingdong prefecture level city in Shandong
Stowe Flagship Store	a power saver (electronics)	Power saving treasure received, as for the effect, according to the instructions written, a short period of time can not be seen, at least 2-3 months before the real effect, then follow up evaluation	Jingdong prefecture level city in Shandong
Stowe Flagship Store	a power saver (electronics)	The goods have just been received and have not been used yet looking forward to the results.	Jingdong prefecture level city in Shandong
Stowe Flagship Store	a power saver (electronics)	Good installation works great	Jingdong prefecture level city in Shandong
Stowe Flagship Store	a power saver (electronics)	The logistics is super super fast! The quality is also very good, indeed value for money	Jingdong prefecture level city in Shandong
Stowe Flagship Store	a power saver (electronics)	Has been used for a period of time before coming to comment on, really good, air conditioning a day open non-stop, pay the electricity bill when the heartache, with this, the home electricity is not at all worried about the bill out of contrast to save a lot of preparation in the hands of a few, each room An a	Jingdong prefecture level city in Shandong
Stowe Flagship Store	a power saver (electronics)	Saving device received, really good, with the air conditioning tried, an hour without a saver with 2.9 degrees, with a saver an hour with 1.1 degrees, the beginning of not quite believe that this thing, the actual test to know that it is really useful, and intend to buy another.	Jingdong prefecture level city in Shandong
Stowe Flagship Store	a power saver (electronics)	Power saving effect: this energy saver is still useful, day trial for three days, every day than not installed before the reduction of two dollars a day, every day to open the bedroom to open the air conditioning before fifteen dollars now thirteen dollars a day. Still a little use.	Jingdong prefecture level city in Shandong

Stowe Flagship Store	a power saver (electronics)	Originally bought for home use, used for two months, the electricity bill from more than 200 yuan per month, down to more than 140 yuan, there is indeed a power-saving effect, this time and sold two, the unit with. Merchant service attitude is really good.	Jingdong prefecture level city in Shandong
Stowe Flagship Store	a power saver (electronics)	Plug and play, stable voltage, the screen shows when working, this winter home appliances, need electricity in many places, just buy to use, save electricity and money, with instructions	Jingdong prefecture level city in Shandong
Stowe Flagship Store	a power saver (electronics)	Goods received, do not know if it is good. When I bought it, I saw that it was a Stowe brand or company's product. Received but not. I do not know what is the reason. Now I can only give this evaluation. I'm	Jingdong prefecture level city in Shandong
Stowe Flagship Store	a power saver (electronics)	The first time to buy online, but also look at the evaluation before buying, the quality is not bad, but also very cheap, the need for friends can rest assured to buy!	Jingdong prefecture level city in Shandong
Stowe Flagship Store	a power saver (electronics)	🌈 Great quality, bought it to my liking 💝 Awesome! ✨ Buy with confidence if you need it!	Jingdong prefecture level city in Shandong
Stowe Flagship Store	a power saver (electronics)	The quality is very good, completely exceeded the expectations, the delivery speed is very fast, the packaging is very careful and strict, very satisfied with a shopping	Jingdong prefecture level city in Shandong
Stowe Flagship Store	a power saver (electronics)	The effect is very good, the home uses less electricity is very energy efficient, cost-effective	Jingdong prefecture level city in Shandong
Stowe Flagship Store	a power saver (electronics)	Quality can't be beat, just like the store, especially like, and the price is favorable!	Jingdong prefecture level city in Shandong
Stowe Flagship Store	a power saver (electronics)	Baby received! Very unexpected, will be back next time ~ great seller, stay tuned!	Jingdong prefecture level city in Shandong
Stowe Flagship Store	a power saver (electronics)	Follow up review after a month is more real, waiting for it	Jingdong prefecture level city in Shandong
Stowe Flagship Store	a power saver (electronics)	It is the ideal product, good quality and fast logistics, worthy of praise!	Jingdong prefecture level city in Shandong
Stowe Flagship Store	a power saver (electronics)	The saver is great, the color is right, the installer is very professional	Jingdong prefecture level city in Shandong
Stowe Flagship Store	a power saver (electronics)	The effect of the power connection is particularly obvious, it is particularly easy to use, very satisfied, the	Jingdong prefecture level city in Shandong
Stowe Flagship Store	a power saver (electronics)	Goods are very energy-saving, used to be more than two hundred degrees, now there are only a hundred or so degrees	Jingdong prefecture level city in Shandong
Stowe Flagship Store	a power saver (electronics)	Good quality, fast shipping, good results, good value for money	Jingdong prefecture level city in Shandong
Stowe Flagship Store	a power saver (electronics)	Fast shipping, fine workmanship, very good, cost-effective	Jingdong prefecture level city in Shandong
Stowe Flagship Store	a power saver (electronics)	The meter is as described, the quality is quite good, energy-saving, worth buying oh~!	Jingdong prefecture level city in Shandong
Stowe Flagship Store	a power saver (electronics)	Great value for money, very affordable,, perfect . ˆ◡ˆ.	Jingdong prefecture level city in Shandong
Stowe Flagship Store	a power saver (electronics)	Good!!! Worth recommending!	Jingdong prefecture level city in Shandong
Stowe Flagship Store	a power saver (electronics)	Great, fast logistics, great stuff, great value for money!	Jingdong prefecture level city in Shandong
Stowe Flagship Store	a power saver (electronics)	Fast logistics, very satisfied with the quality upon receipt	Jingdong prefecture level city in Shandong
Stowe Flagship Store	a power saver (electronics)	Great quality, plug and play, fast shipping!	Jingdong prefecture level city in Shandong
Stowe Flagship Store	a power saver (electronics)	Logistics received, quite satisfied	Jingdong prefecture level city in Shandong
Stowe Flagship Store	a power saver (electronics)	I don't know if it really works	Jingdong prefecture level city in Shandong
Stowe Flagship Store	a power saver (electronics)	It's bullshit. It's a plug light. Don't talk about power savings.	Jingdong prefecture level city in Shandong
Stowe Flagship Store	a power saver (electronics)	Fake and deceitful stuff that hasn't changed at all.	Jingdong prefecture level city in Shandong
Stowe Flagship Store	a power saver (electronics)	Power-saving effect: power-saving effect is particularly obvious Cost-effective: cost-effective business Appearance and style: external small micro-work materials: fine workmanship Experience: use very good, non the first convenient	Jingdong prefecture level city in Shandong
Stowe Flagship Store	a power saver (electronics)	After using it for a few days, it feels quite good, it should be quite safe, and it also sends a plug, the merchant is good, wait for a period of time and then calculate whether it saves on the electricity bill and then add comments ha!	Jingdong prefecture level city in Shandong
Stowe Flagship Store	a power saver (electronics)	My friend recommended to me, said the effect is very good, I bought two, this month less money, really can, just buy I do not believe, but has proved to have effect	Jingdong prefecture level city in Shandong
Stowe Flagship Store	a power saver (electronics)	Just bought back, already used, these days to see the effect of it, and so have the effect will also come back to give positive feedback, the price is also super cheap!	Jingdong prefecture level city in Shandong
Stowe Flagship Store	a power saver (electronics)	It does work well, the store uses a lot of electricity, this time to buy back a few, each place that uses a lot of electricity to insert one. Summer is here, the electricity is too strong.	Jingdong prefecture level city in Shandong
Stowe Flagship Store	a power saver (electronics)	Power saving treasure received, as for the effect, according to the instructions written, a short period of time can not be seen, at least 2-3 months before the real effect, then follow up evaluation	Jingdong prefecture level city in Shandong
Stowe Flagship Store	a power saver (electronics)	The goods have just been received and have not been used yet looking forward to the results.	Jingdong prefecture level city in Shandong
Stowe Flagship Store	a power saver (electronics)	Good installation works great	Jingdong prefecture level city in Shandong
Stowe Flagship Store	a power saver (electronics)	The logistics is super super fast! The quality is also very good, indeed value for money	Jingdong prefecture level city in Shandong
Stowe Flagship Store	a power saver (electronics)	Has been used for a period of time before coming to comment on, really good, air conditioning a day open non-stop, pay the electricity bill when the heartache, with this, the home electricity is not at all worried about the bill out of contrast to save a lot of preparation in the hands of a few, each room An a	Jingdong prefecture level city in Shandong
Stowe Flagship Store	a power saver (electronics)	Saving device received, really good, with the air conditioning tried, an hour without a saver with 2.9 degrees, with a saver an hour with 1.1 degrees, the beginning of not quite believe that this thing, the actual test to know that it is really useful, and intend to buy another.	Jingdong prefecture level city in Shandong
Stowe Flagship Store	a power saver (electronics)	Power saving effect: this energy saver is still useful, day trial for three days, every day than not installed before the reduction of two dollars a day, every day to open the bedroom to open the air conditioning before fifteen dollars now thirteen dollars a day. Still a little use.	Jingdong prefecture level city in Shandong
Stowe Flagship Store	a power saver (electronics)	Originally bought for home use, used for two months, the electricity bill from more than 200 yuan per month, down to more than 140 yuan, there is indeed a power-saving effect, this time and sold two, the unit with. Merchant service attitude is really good.	Jingdong prefecture level city in Shandong
Stowe Flagship Store	a power saver (electronics)	Plug and play, stable voltage, the screen shows when working, this winter home appliances, need electricity in many places, just buy to use, save electricity and money, with instructions	Jingdong prefecture level city in Shandong
Stowe Flagship Store	a power saver (electronics)	Goods received, do not know if it is good. When I bought it, I saw that it was a Stowe brand or company's product. Received but not. I do not know what is the reason. Now I can only give this evaluation. I'm	Jingdong prefecture level city in Shandong
Stowe Flagship Store	a power saver (electronics)	The first time to buy online, but also look at the evaluation before buying, the quality is not bad, but also very cheap, the need for friends can rest assured to buy!	Jingdong prefecture level city in Shandong
Stowe Flagship Store	a power saver (electronics)	🌈 Great quality, bought it to my liking 💝 Awesome! ✨ Buy with confidence if you need it!	Jingdong prefecture level city in Shandong
Stowe Flagship Store	a power saver (electronics)	The quality is very good, completely exceeded the expectations, the delivery speed is very fast, the packaging is very careful and strict, very satisfied with a shopping	Jingdong prefecture level city in Shandong
Stowe Flagship Store	a power saver (electronics)	The effect is very good, the home uses less electricity is very energy efficient, cost-effective	Jingdong prefecture level city in Shandong
Stowe Flagship Store	a power saver (electronics)	Quality can't be beat, just like the store, especially like, and the price is favorable!	Jingdong prefecture level city in Shandong
Stowe Flagship Store	a power saver (electronics)	Baby received! Very unexpected, will be back next time ~ great seller, stay tuned!	Jingdong prefecture level city in Shandong
Stowe Flagship Store	a power saver (electronics)	Follow up review after a month is more real, waiting for it	Jingdong prefecture level city in Shandong

Stowe Flagship Store	a power saver (electronics)	It is the ideal product, good quality and fast logistics, worthy of praise!	Jingdong prefecture level city in Shandong
Stowe Flagship Store	a power saver (electronics)	The saver is great, the color is right, the installer is very professional	Jingdong prefecture level city in Shandong
Stowe Flagship Store	a power saver (electronics)	The effect of the power connection is particularly obvious, it is particularly easy to use, very satisfied, the	Jingdong prefecture level city in Shandong
Stowe Flagship Store	a power saver (electronics)	Goods are very energy-saving, used to be more than two hundred degrees, now there are only a hundred or so degrees	Jingdong prefecture level city in Shandong
Stowe Flagship Store	a power saver (electronics)	Good quality, fast shipping, good results, good value for money	Jingdong prefecture level city in Shandong
Stowe Flagship Store	a power saver (electronics)	Fast shipping, fine workmanship, very good, cost-effective	Jingdong prefecture level city in Shandong
Stowe Flagship Store	a power saver (electronics)	The meter is as described, the quality is quite good, energy-saving, worth buying oh~!	Jingdong prefecture level city in Shandong
Stowe Flagship Store	a power saver (electronics)	Great value for money, very affordable,, perfect . ^◡^.	Jingdong prefecture level city in Shandong
Stowe Flagship Store	a power saver (electronics)	Good!!! Worth recommending!	Jingdong prefecture level city in Shandong
Stowe Flagship Store	a power saver (electronics)	Great, fast logistics, great stuff, great value for money!	Jingdong prefecture level city in Shandong
Stowe Flagship Store	a power saver (electronics)	Fast logistics, very satisfied with the quality upon receipt	Jingdong prefecture level city in Shandong
Stowe Flagship Store	a power saver (electronics)	Great quality, plug and play, fast shipping!	Jingdong prefecture level city in Shandong
Stowe Flagship Store	a power saver (electronics)	Logistics received, quite satisfied	Jingdong prefecture level city in Shandong
Stowe Flagship Store	a power saver (electronics)	I don't know if it really works	Jingdong prefecture level city in Shandong
Stowe Flagship Store	a power saver (electronics)	It's bullshit. It's a plug light. Don't talk about power savings.	Jingdong prefecture level city in Shandong
Stowe Flagship Store	a power saver (electronics)	Fake and deceitful stuff that hasn't changed at all.	Jingdong prefecture level city in Shandong
Stowe Flagship Store	a power saver (electronics)	Power-saving effect: power-saving effect is particularly obvious Cost-effective: cost-effective business Appearance and style: external small micro-work materials: fine workmanship Experience: use very good, non the first convenient	Jingdong prefecture level city in Shandong
Stowe Flagship Store	a power saver (electronics)	After using it for a few days, it feels quite good, it should be quite safe, and it also sends a plug, the merchant is good, wait for a period of time and then calculate whether it saves on the electricity bill and then add comments ha!	Jingdong prefecture level city in Shandong
Stowe Flagship Store	a power saver (electronics)	My friend recommended to me, said the effect is very good, I bought two, this month less money, really can, just buy I do not believe, but has proved to have effect	Jingdong prefecture level city in Shandong
Stowe Flagship Store	a power saver (electronics)	Just bought back, already used, these days to see the effect of it, and so have the effect will also come back to give positive feedback, the price is also super cheap!	Jingdong prefecture level city in Shandong
Stowe Flagship Store	a power saver (electronics)	It does work well, the store uses a lot of electricity, this time to buy back a few, each place that uses a lot of electricity to insert one. Summer is here, the electricity is too strong.	Jingdong prefecture level city in Shandong
Stowe Flagship Store	a power saver (electronics)	Power saving treasure received, as for the effect, according to the instructions written, a short period of time can not be seen, at least 2-3 months before the real effect, then follow up evaluation	Jingdong prefecture level city in Shandong
Stowe Flagship Store	a power saver (electronics)	The goods have just been received and have not been used yet looking forward to the results.	Jingdong prefecture level city in Shandong
Stowe Flagship Store	a power saver (electronics)	Good installation works great	Jingdong prefecture level city in Shandong
Stowe Flagship Store	a power saver (electronics)	The logistics is super super fast! The quality is also very good, indeed value for money	Jingdong prefecture level city in Shandong
Stowe Flagship Store	a power saver (electronics)	Has been used for a period of time before coming to comment on, really good, air conditioning a day open non-stop, pay the electricity bill when the heartache, with this, the home electricity is not at all worried about the bill out of contrast to save a lot of preparation in the hands of a few, each room An a	Jingdong prefecture level city in Shandong
Stowe Flagship Store	a power saver (electronics)	Saving device received, really good, with the air conditioning tried, an hour without a saver with 2.9 degrees, with a saver an hour with 1.1 degrees, the beginning of not quite believe that this thing, the actual test to know that it is really useful, and intend to buy another.	Jingdong prefecture level city in Shandong
Stowe Flagship Store	a power saver (electronics)	Power saving effect: this energy saver is still useful, day trial for three days, every day than not installed before the reduction of two dollars a day, every day to open the bedroom to open the air conditioning before fifteen dollars now thirteen dollars a day. Still a little use.	Jingdong prefecture level city in Shandong
Stowe Flagship Store	a power saver (electronics)	Originally bought for home use, used for two months, the electricity bill from more than 200 yuan per month, down to more than 140 yuan, there is indeed a power-saving effect, this time and sold two, the unit with. Merchant service attitude is really good.	Jingdong prefecture level city in Shandong
Stowe Flagship Store	a power saver (electronics)	Plug and play, stable voltage, the screen shows when working, this winter home appliances, need electricity in many places, just buy to use, save electricity and money, with instructions	Jingdong prefecture level city in Shandong
Stowe Flagship Store	a power saver (electronics)	Goods received, do not know if it is good. When I bought it, I saw that it was a Stowe brand or company's product. Received but not. I do not know what is the reason. Now I can only give this evaluation. I'm	Jingdong prefecture level city in Shandong
Stowe Flagship Store	a power saver (electronics)	The first time to buy online, but also look at the evaluation before buying, the quality is not bad, but also very cheap, the need for friends can rest assured to buy!	Jingdong prefecture level city in Shandong
Stowe Flagship Store	a power saver (electronics)	🌈 Great quality, bought it to my liking 💝 Awesome! ✨ Buy with confidence if you need it!	Jingdong prefecture level city in Shandong
Stowe Flagship Store	a power saver (electronics)	The quality is very good, completely exceeded the expectations, the delivery speed is very fast, the packaging is very careful and strict, very satisfied with a shopping	Jingdong prefecture level city in Shandong
Stowe Flagship Store	a power saver (electronics)	The effect is very good, the home uses less electricity is very energy efficient, cost-effective	Jingdong prefecture level city in Shandong
Stowe Flagship Store	a power saver (electronics)	Quality can't be beat, just like the store, especially like, and the price is favorable!	Jingdong prefecture level city in Shandong
Stowe Flagship Store	a power saver (electronics)	Baby received! Very unexpected, will be back next time ~ great seller, stay tuned!	Jingdong prefecture level city in Shandong
Stowe Flagship Store	a power saver (electronics)	Follow up review after a month is more real, waiting for it	Jingdong prefecture level city in Shandong
Stowe Flagship Store	a power saver (electronics)	It is the ideal product, good quality and fast logistics, worthy of praise!	Jingdong prefecture level city in Shandong
Stowe Flagship Store	a power saver (electronics)	The saver is great, the color is right, the installer is very professional	Jingdong prefecture level city in Shandong
Stowe Flagship Store	a power saver (electronics)	The effect of the power connection is particularly obvious, it is particularly easy to use, very satisfied, the	Jingdong prefecture level city in Shandong
Stowe Flagship Store	a power saver (electronics)	Goods are very energy-saving, used to be more than two hundred degrees, now there are only a hundred or so degrees	Jingdong prefecture level city in Shandong
Stowe Flagship Store	a power saver (electronics)	Good quality, fast shipping, good results, good value for money	Jingdong prefecture level city in Shandong
Stowe Flagship Store	a power saver (electronics)	Fast shipping, fine workmanship, very good, cost-effective	Jingdong prefecture level city in Shandong
Stowe Flagship Store	a power saver (electronics)	The meter is as described, the quality is quite good, energy-saving, worth buying oh~!	Jingdong prefecture level city in Shandong
Stowe Flagship Store	a power saver (electronics)	Great value for money, very affordable,, perfect . ^◡^.	Jingdong prefecture level city in Shandong
Stowe Flagship Store	a power saver (electronics)	Good!!! Worth recommending!	Jingdong prefecture level city in Shandong
Stowe Flagship Store	a power saver (electronics)	Great, fast logistics, great stuff, great value for money!	Jingdong prefecture level city in Shandong

Stowe Flagship Store	a power saver (electronics)	Fast logistics, very satisfied with the quality upon receipt	Jingdong prefecture level city in Shandong
Stowe Flagship Store	a power saver (electronics)	Great quality, plug and play, fast shipping!	Jingdong prefecture level city in Shandong
Stowe Flagship Store	a power saver (electronics)	Logistics received, quite satisfied	Jingdong prefecture level city in Shandong
Stowe Flagship Store	a power saver (electronics)	I don't know if it really works	Jingdong prefecture level city in Shandong
Stowe Flagship Store	a power saver (electronics)	It's bullshit. It's a plug light. Don't talk about power savings.	Jingdong prefecture level city in Shandong
Stowe Flagship Store	a power saver (electronics)	Fake and deceitful stuff that hasn't changed at all.	Jingdong prefecture level city in Shandong
Stowe Flagship Store	a power saver (electronics)	Power-saving effect: power-saving effect is particularly obvious Cost-effective: cost-effective business Appearance and style: external small micro-work materials: fine workmanship Experience: use very good, non the first convenient	Jingdong prefecture level city in Shandong
Stowe Flagship Store	a power saver (electronics)	After using it for a few days, it feels quite good, it should be quite safe, and it also sends a plug, the merchant is good, wait for a period of time and then calculate whether it saves on the electricity bill and then add comments ha!	Jingdong prefecture level city in Shandong
Stowe Flagship Store	a power saver (electronics)	My friend recommended to me, said the effect is very good, I bought two, this month less money, really can, just buy I do not believe, but has proved to have effect	Jingdong prefecture level city in Shandong
Stowe Flagship Store	a power saver (electronics)	Just bought back, already used, these days to see the effect of it, and so have the effect will also come back to give positive feedback, the price is also super cheap!	Jingdong prefecture level city in Shandong
Stowe Flagship Store	a power saver (electronics)	It does work well, the store uses a lot of electricity, this time to buy back a few, each place that uses a lot of electricity to insert one. Summer is here, the electricity is too strong.	Jingdong prefecture level city in Shandong
Stowe Flagship Store	a power saver (electronics)	Power saving treasure received, as for the effect, according to the instructions written, a short period of time can not be seen, at least 2-3 months before the real effect, then follow up evaluation	Jingdong prefecture level city in Shandong
Stowe Flagship Store	a power saver (electronics)	The goods have just been received and have not been used yet looking forward to the results.	Jingdong prefecture level city in Shandong
Stowe Flagship Store	a power saver (electronics)	Good installation works great	Jingdong prefecture level city in Shandong
Stowe Flagship Store	a power saver (electronics)	The logistics is super super fast! The quality is also very good, indeed value for money	Jingdong prefecture level city in Shandong
Stowe Flagship Store	a power saver (electronics)	Has been used for a period of time before coming to comment on, really good, air conditioning a day open non-stop, pay the electricity bill when the heartache, with this, the home electricity is not at all worried about the bill out of contrast to save a lot of preparation in the hands of a few, each room An a	Jingdong prefecture level city in Shandong
Stowe Flagship Store	a power saver (electronics)	Saving device received, really good, with the air conditioning tried, an hour without a saver with 2.9 degrees, with a saver an hour with 1.1 degrees, the beginning of not quite believe that this thing, the actual test to know that it is really useful, and intend to buy another.	Jingdong prefecture level city in Shandong
Stowe Flagship Store	a power saver (electronics)	Power saving effect: this energy saver is still useful, day trial for three days, every day than not installed before the reduction of two dollars a day, every day to open the bedroom to open the air conditioning before fifteen dollars now thirteen dollars a day. Still a little use.	Jingdong prefecture level city in Shandong
Stowe Flagship Store	a power saver (electronics)	Originally bought for home use, used for two months, the electricity bill from more than 200 yuan per month, down to more than 140 yuan, there is indeed a power-saving effect, this time and sold two, the unit with. Merchant service attitude is really good.	Jingdong prefecture level city in Shandong
Stowe Flagship Store	a power saver (electronics)	Plug and play, stable voltage, the screen shows when working, this winter home appliances, need electricity in many places, just buy to use, save electricity and money, with instructions	Jingdong prefecture level city in Shandong
Stowe Flagship Store	a power saver (electronics)	Goods received, do not know if it is good. When I bought it, I saw that it was a Stowe brand or company's product. Received but not. I do not know what is the reason. Now I can only give this evaluation. I'm	Jingdong prefecture level city in Shandong
Stowe Flagship Store	a power saver (electronics)	The first time to buy online, but also look at the evaluation before buying, the quality is not bad, but also very cheap, the need for friends can rest assured to buy!	Jingdong prefecture level city in Shandong
Stowe Flagship Store	a power saver (electronics)	🌈 Great quality, bought it to my liking 💝 Awesome! ✨ Buy with confidence if you need it!	Jingdong prefecture level city in Shandong
Stowe Flagship Store	a power saver (electronics)	The quality is very good, completely exceeded the expectations, the delivery speed is very fast, the packaging is very careful and strict, very satisfied with a shopping	Jingdong prefecture level city in Shandong
Stowe Flagship Store	a power saver (electronics)	The effect is very good, the home uses less electricity is very energy efficient, cost-effective	Jingdong prefecture level city in Shandong
Stowe Flagship Store	a power saver (electronics)	Quality can't be beat, just like the store, especially like, and the price is favorable!	Jingdong prefecture level city in Shandong
Stowe Flagship Store	a power saver (electronics)	Baby received! Very unexpected, will be back next time ~ great seller, stay tuned!	Jingdong prefecture level city in Shandong
Stowe Flagship Store	a power saver (electronics)	Follow up review after a month is more real, waiting for it	Jingdong prefecture level city in Shandong
Stowe Flagship Store	a power saver (electronics)	It is the ideal product, good quality and fast logistics, worthy of praise!	Jingdong prefecture level city in Shandong
Stowe Flagship Store	a power saver (electronics)	The saver is great, the color is right, the installer is very professional	Jingdong prefecture level city in Shandong
Stowe Flagship Store	a power saver (electronics)	The effect of the power connection is particularly obvious, it is particularly easy to use, very satisfied, the	Jingdong prefecture level city in Shandong
Stowe Flagship Store	a power saver (electronics)	Goods are very energy-saving, used to be more than two hundred degrees, now there are only a hundred or so degrees	Jingdong prefecture level city in Shandong
Stowe Flagship Store	a power saver (electronics)	Good quality, fast shipping, good results, good value for money	Jingdong prefecture level city in Shandong
Stowe Flagship Store	a power saver (electronics)	Fast shipping, fine workmanship, very good, cost-effective	Jingdong prefecture level city in Shandong
Stowe Flagship Store	a power saver (electronics)	The meter is as described, the quality is quite good, energy-saving, worth buying oh~!	Jingdong prefecture level city in Shandong
Stowe Flagship Store	a power saver (electronics)	Great value for money, very affordable,, perfect . ˆ◡ˆ.	Jingdong prefecture level city in Shandong
Stowe Flagship Store	a power saver (electronics)	Good!!! Worth recommending!	Jingdong prefecture level city in Shandong
Stowe Flagship Store	a power saver (electronics)	Great, fast logistics, great stuff, great value for money!	Jingdong prefecture level city in Shandong
Stowe Flagship Store	a power saver (electronics)	Fast logistics, very satisfied with the quality upon receipt	Jingdong prefecture level city in Shandong
Stowe Flagship Store	a power saver (electronics)	Great quality, plug and play, fast shipping!	Jingdong prefecture level city in Shandong
Stowe Flagship Store	a power saver (electronics)	Logistics received, quite satisfied	Jingdong prefecture level city in Shandong
Stowe Flagship Store	a power saver (electronics)	I don't know if it really works	Jingdong prefecture level city in Shandong
Stowe Flagship Store	a power saver (electronics)	It's bullshit. It's a plug light. Don't talk about power savings.	Jingdong prefecture level city in Shandong
Stowe Flagship Store	a power saver (electronics)	Fake and deceitful stuff that hasn't changed at all.	Jingdong prefecture level city in Shandong
Stowe Flagship Store	a power saver (electronics)	Power-saving effect: power-saving effect is particularly obvious Cost-effective: cost-effective business Appearance and style: external small micro-work materials: fine workmanship Experience: use very good, non the first convenient	Jingdong prefecture level city in Shandong
Stowe Flagship Store	a power saver (electronics)	After using it for a few days, it feels quite good, it should be quite safe, and it also sends a plug, the merchant is good, wait for a period of time and then calculate whether it saves on the electricity bill and then add comments ha!	Jingdong prefecture level city in Shandong
Stowe Flagship Store	a power saver (electronics)	My friend recommended to me, said the effect is very good, I bought two, this month less money, really can, just buy I do not believe, but has proved to have effect	Jingdong prefecture level city in Shandong

Stowe Flagship Store	a power saver (electronics)	Just bought back, already used, these days to see the effect of it, and so have the effect will also come back to give positive feedback, the price is also super cheap!	Jingdong prefecture level city in Shandong
Stowe Flagship Store	a power saver (electronics)	It does work well, the store uses a lot of electricity, this time to buy back a few, each place that uses a lot of electricity to insert one. Summer is here, the electricity is too strong.	Jingdong prefecture level city in Shandong
Stowe Flagship Store	a power saver (electronics)	Power saving treasure received, as for the effect, according to the instructions written, a short period of time can not be seen, at least 2-3 months before the real effect, then follow up evaluation	Jingdong prefecture level city in Shandong
Stowe Flagship Store	a power saver (electronics)	The goods have just been received and have not been used yet looking forward to the results.	Jingdong prefecture level city in Shandong
Stowe Flagship Store	a power saver (electronics)	Good installation works great	Jingdong prefecture level city in Shandong
Stowe Flagship Store	a power saver (electronics)	The logistics is super super fast! The quality is also very good, indeed value for money	Jingdong prefecture level city in Shandong
Stowe Flagship Store	a power saver (electronics)	Has been used for a period of time before coming to comment on, really good, air conditioning a day open non-stop, pay the electricity bill when the heartache, with this, the home electricity is not at all worried about the bill out of contrast to save a lot of preparation in the hands of a few, each room An a	Jingdong prefecture level city in Shandong
Stowe Flagship Store	a power saver (electronics)	Saving device received, really good, with the air conditioning tried, an hour without a saver with 2.9 degrees, with a saver an hour with 1.1 degrees, the beginning of not quite believe that this thing, the actual test to know that it is really useful, and intend to buy another.	Jingdong prefecture level city in Shandong
Stowe Flagship Store	a power saver (electronics)	Power saving effect: this energy saver is still useful, day trial for three days, every day than not installed before the reduction of two dollars a day, every day to open the bedroom to open the air conditioning before fifteen dollars now thirteen dollars a day. Still a little use.	Jingdong prefecture level city in Shandong
Stowe Flagship Store	a power saver (electronics)	Originally bought for home use, used for two months, the electricity bill from more than 200 yuan per month, down to more than 140 yuan, there is indeed a power-saving effect, this time and sold two, the unit with. Merchant service attitude is really good.	Jingdong prefecture level city in Shandong
Stowe Flagship Store	a power saver (electronics)	Plug and play, stable voltage, the screen shows when working, this winter home appliances, need electricity in many places, just buy to use, save electricity and money, with instructions	Jingdong prefecture level city in Shandong
Stowe Flagship Store	a power saver (electronics)	Goods received, do not know if it is good. When I bought it, I saw that it was a Stowe brand or company's product. Received but not. I do not know what is the reason. Now I can only give this evaluation. I'm	Jingdong prefecture level city in Shandong
Stowe Flagship Store	a power saver (electronics)	The first time to buy online, but also look at the evaluation before buying, the quality is not bad, but also very cheap, the need for friends can rest assured to buy!	Jingdong prefecture level city in Shandong
Stowe Flagship Store	a power saver (electronics)	Great quality, bought it to my liking Awesome! Buy with confidence if you need it!	Jingdong prefecture level city in Shandong
Stowe Flagship Store	a power saver (electronics)	The quality is very good, completely exceeded the expectations, the delivery speed is very fast, the packaging is very careful and strict, very satisfied with a shopping	Jingdong prefecture level city in Shandong
Stowe Flagship Store	a power saver (electronics)	The effect is very good, the home uses less electricity is very energy efficient, cost-effective	Jingdong prefecture level city in Shandong
Stowe Flagship Store	a power saver (electronics)	Quality can't be beat, just like the store, especially like, and the price is favorable!	Jingdong prefecture level city in Shandong
Stowe Flagship Store	a power saver (electronics)	Baby received! Very unexpected, will be back next time ~ great seller, stay tuned!	Jingdong prefecture level city in Shandong
Stowe Flagship Store	a power saver (electronics)	Follow up review after a month is more real, waiting for it	Jingdong prefecture level city in Shandong
Stowe Flagship Store	a power saver (electronics)	It is the ideal product, good quality and fast logistics, worthy of praise!	Jingdong prefecture level city in Shandong
Stowe Flagship Store	a power saver (electronics)	The saver is great, the color is right, the installer is very professional	Jingdong prefecture level city in Shandong
Stowe Flagship Store	a power saver (electronics)	The effect of the power connection is particularly obvious, it is particularly easy to use, very satisfied, the	Jingdong prefecture level city in Shandong
Stowe Flagship Store	a power saver (electronics)	Goods are very energy-saving, used to be more than two hundred degrees, now there are only a hundred or so degrees	Jingdong prefecture level city in Shandong
Stowe Flagship Store	a power saver (electronics)	Good quality, fast shipping, good results, good value for money	Jingdong prefecture level city in Shandong
Stowe Flagship Store	a power saver (electronics)	Fast shipping, fine workmanship, very good, cost-effective	Jingdong prefecture level city in Shandong
Stowe Flagship Store	a power saver (electronics)	The meter is as described, the quality is quite good, energy-saving, worth buying oh~!	Jingdong prefecture level city in Shandong
Stowe Flagship Store	a power saver (electronics)	Great value for money, very affordable,, perfect . ˆ◡ˆ.	Jingdong prefecture level city in Shandong
Stowe Flagship Store	a power saver (electronics)	Good!!! Worth recommending!	Jingdong prefecture level city in Shandong
Stowe Flagship Store	a power saver (electronics)	Great, fast logistics, great stuff, great value for money!	Jingdong prefecture level city in Shandong
Stowe Flagship Store	a power saver (electronics)	Fast logistics, very satisfied with the quality upon receipt	Jingdong prefecture level city in Shandong
Stowe Flagship Store	a power saver (electronics)	Great quality, plug and play, fast shipping!	Jingdong prefecture level city in Shandong
Stowe Flagship Store	a power saver (electronics)	Logistics received, quite satisfied	Jingdong prefecture level city in Shandong
Stowe Flagship Store	a power saver (electronics)	I don't know if it really works	Jingdong prefecture level city in Shandong
Stowe Flagship Store	a power saver (electronics)	It's bullshit. It's a plug light. Don't talk about power savings.	Jingdong prefecture level city in Shandong
Stowe Flagship Store	a power saver (electronics)	Fake and deceitful stuff that hasn't changed at all.	Jingdong prefecture level city in Shandong
Stowe Flagship Store	a power saver (electronics)	Power-saving effect: power-saving effect is particularly obvious Cost-effective: cost-effective business Appearance and style: external small micro-work materials: fine workmanship Experience: use very good, non the first convenient	Jingdong prefecture level city in Shandong
Stowe Flagship Store	a power saver (electronics)	After using it for a few days, it feels quite good, it should be quite safe, and it also sends a plug, the merchant is good, wait for a period of time and then calculate whether it saves on the electricity bill and then add comments ha!	Jingdong prefecture level city in Shandong
Stowe Flagship Store	a power saver (electronics)	My friend recommended to me, said the effect is very good, I bought two, this month less money, really can, just buy I do not believe, but has proved to have effect	Jingdong prefecture level city in Shandong
Stowe Flagship Store	a power saver (electronics)	Just bought back, already used, these days to see the effect of it, and so have the effect will also come back to give positive feedback, the price is also super cheap!	Jingdong prefecture level city in Shandong
Stowe Flagship Store	a power saver (electronics)	It does work well, the store uses a lot of electricity, this time to buy back a few, each place that uses a lot of electricity to insert one. Summer is here, the electricity is too strong.	Jingdong prefecture level city in Shandong
Stowe Flagship Store	a power saver (electronics)	Power saving treasure received, as for the effect, according to the instructions written, a short period of time can not be seen, at least 2-3 months before the real effect, then follow up evaluation	Jingdong prefecture level city in Shandong
Stowe Flagship Store	a power saver (electronics)	The goods have just been received and have not been used yet looking forward to the results.	Jingdong prefecture level city in Shandong
Stowe Flagship Store	a power saver (electronics)	Good installation works great	Jingdong prefecture level city in Shandong
Stowe Flagship Store	a power saver (electronics)	The logistics is super super fast! The quality is also very good, indeed value for money	Jingdong prefecture level city in Shandong
Stowe Flagship Store	a power saver (electronics)	Has been used for a period of time before coming to comment on, really good, air conditioning a day open non-stop, pay the electricity bill when the heartache, with this, the home electricity is not at all worried about the bill out of contrast to save a lot of preparation in the hands of a few, each room An a	Jingdong prefecture level city in Shandong
Stowe Flagship Store	a power saver (electronics)	Saving device received, really good, with the air conditioning tried, an hour without a saver with 2.9 degrees, with a saver an hour with 1.1 degrees, the beginning of not quite believe that this thing, the actual test to know that it is really useful, and intend to buy another.	Jingdong prefecture level city in Shandong
Stowe Flagship Store	a power saver (electronics)	Power saving effect: this energy saver is still useful, day trial for three days, every day than not installed before the reduction of two dollars a day, every day to open the bedroom to open the air conditioning before fifteen dollars now thirteen dollars a day. Still a little use.	Jingdong prefecture level city in Shandong

Stowe Flagship Store	a power saver (electronics)	Originally bought for home use, used for two months, the electricity bill from more than 200 yuan per month, down to more than 140 yuan, there is indeed a power-saving effect, this time and sold two, the unit with. Merchant service attitude is really good.	Jingdong prefecture level city in Shandong
Stowe Flagship Store	a power saver (electronics)	Plug and play, stable voltage, the screen shows when working, this winter home appliances, need electricity in many places, just buy to use, save electricity and money, with instructions	Jingdong prefecture level city in Shandong
Stowe Flagship Store	a power saver (electronics)	Goods received, do not know if it is good. When I bought it, I saw that it was a Stowe brand or company's product. Received but not. I do not know what is the reason. Now I can only give this evaluation. I'm	Jingdong prefecture level city in Shandong
Stowe Flagship Store	a power saver (electronics)	The first time to buy online, but also look at the evaluation before buying, the quality is not bad, but also very cheap, the need for friends can rest assured to buy!	Jingdong prefecture level city in Shandong
Stowe Flagship Store	a power saver (electronics)	🌈 Great quality, bought it to my liking 💗 Awesome! ✨ Buy with confidence if you need it!	Jingdong prefecture level city in Shandong
Stowe Flagship Store	a power saver (electronics)	The quality is very good, completely exceeded the expectations, the delivery speed is very fast, the packaging is very careful and strict, very satisfied with a shopping	Jingdong prefecture level city in Shandong
Stowe Flagship Store	a power saver (electronics)	The effect is very good, the home uses less electricity is very energy efficient, cost-effective	Jingdong prefecture level city in Shandong
Stowe Flagship Store	a power saver (electronics)	Quality can't be beat, just like the store, especially like, and the price is favorable!	Jingdong prefecture level city in Shandong
Stowe Flagship Store	a power saver (electronics)	Baby received! Very unexpected, will be back next time ~ great seller, stay tuned!	Jingdong prefecture level city in Shandong
Stowe Flagship Store	a power saver (electronics)	Follow up review after a month is more real, waiting for it	Jingdong prefecture level city in Shandong
Stowe Flagship Store	a power saver (electronics)	It is the ideal product, good quality and fast logistics, worthy of praise!	Jingdong prefecture level city in Shandong
Stowe Flagship Store	a power saver (electronics)	The saver is great, the color is right, the installer is very professional	Jingdong prefecture level city in Shandong
Stowe Flagship Store	a power saver (electronics)	The effect of the power connection is particularly obvious, it is particularly easy to use, very satisfied, the	Jingdong prefecture level city in Shandong
Stowe Flagship Store	a power saver (electronics)	Goods are very energy-saving, used to be more than two hundred degrees, now there are only a hundred or so degrees	Jingdong prefecture level city in Shandong
Stowe Flagship Store	a power saver (electronics)	Good quality, fast shipping, good results, good value for money	Jingdong prefecture level city in Shandong
Stowe Flagship Store	a power saver (electronics)	Fast shipping, fine workmanship, very good, cost-effective	Jingdong prefecture level city in Shandong
Stowe Flagship Store	a power saver (electronics)	The meter is as described, the quality is quite good, energy-saving, worth buying oh~!	Jingdong prefecture level city in Shandong
Stowe Flagship Store	a power saver (electronics)	Great value for money, very affordable,, perfect . ^◡^.	Jingdong prefecture level city in Shandong
Stowe Flagship Store	a power saver (electronics)	Good!!! Worth recommending!	Jingdong prefecture level city in Shandong
Stowe Flagship Store	a power saver (electronics)	Great, fast logistics, great stuff, great value for money!	Jingdong prefecture level city in Shandong
Stowe Flagship Store	a power saver (electronics)	Fast logistics, very satisfied with the quality upon receipt	Jingdong prefecture level city in Shandong
Stowe Flagship Store	a power saver (electronics)	Great quality, plug and play, fast shipping!	Jingdong prefecture level city in Shandong
Stowe Flagship Store	a power saver (electronics)	Logistics received, quite satisfied	Jingdong prefecture level city in Shandong
Stowe Flagship Store	a power saver (electronics)	I don't know if it really works	Jingdong prefecture level city in Shandong
Stowe Flagship Store	a power saver (electronics)	It's bullshit. It's a plug light. Don't talk about power savings.	Jingdong prefecture level city in Shandong
Stowe Flagship Store	a power saver (electronics)	Fake and deceitful stuff that hasn't changed at all.	Jingdong prefecture level city in Shandong
Stowe Flagship Store	a power saver (electronics)	Power-saving effect: power-saving effect is particularly obvious Cost-effective: cost-effective business Appearance and style: external small micro-work materials: fine workmanship Experience: use very good, non the first convenient	Jingdong prefecture level city in Shandong
Stowe Flagship Store	a power saver (electronics)	After using it for a few days, it feels quite good, it should be quite safe, and it also sends a plug, the merchant is good, wait for a period of time and then calculate whether it saves on the electricity bill and then add comments ha!	Jingdong prefecture level city in Shandong
Stowe Flagship Store	a power saver (electronics)	My friend recommended to me, said the effect is very good, I bought two, this month less money, really can, just buy I do not believe, but has proved to have effect	Jingdong prefecture level city in Shandong
Stowe Flagship Store	a power saver (electronics)	Just bought back, already used, these days to see the effect of it, and so have the effect will also come back to give positive feedback, the price is also super cheap!	Jingdong prefecture level city in Shandong
Stowe Flagship Store	a power saver (electronics)	It does work well, the store uses a lot of electricity, this time to buy back a few, each place that uses a lot of electricity to insert one. Summer is here, the electricity is too strong.	Jingdong prefecture level city in Shandong
Stowe Flagship Store	a power saver (electronics)	Power saving treasure received, as for the effect, according to the instructions written, a short period of time can not be seen, at least 2-3 months before the real effect, then follow up evaluation	Jingdong prefecture level city in Shandong
Stowe Flagship Store	a power saver (electronics)	The goods have just been received and have not been used yet looking forward to the results.	Jingdong prefecture level city in Shandong
Stowe Flagship Store	a power saver (electronics)	Good installation works great	Jingdong prefecture level city in Shandong
Stowe Flagship Store	a power saver (electronics)	The logistics is super super fast! The quality is also very good, indeed value for money	Jingdong prefecture level city in Shandong
Stowe Flagship Store	a power saver (electronics)	Has been used for a period of time before coming to comment on, really good, air conditioning a day open non-stop, pay the electricity bill when the heartache, with this, the home electricity is not at all worried about the bill out of contrast to save a lot of preparation in the hands of a few, each room An a	Jingdong prefecture level city in Shandong
Stowe Flagship Store	a power saver (electronics)	Saving device received, really good, with the air conditioning tried, an hour without a saver with 2.9 degrees, with a saver an hour with 1.1 degrees, the beginning of not quite believe that this thing, the actual test to know that it is really useful, and intend to buy another.	Jingdong prefecture level city in Shandong
Stowe Flagship Store	a power saver (electronics)	Power saving effect: this energy saver is still useful, day trial for three days, every day than not installed before the reduction of two dollars a day, every day to open the bedroom to open the air conditioning before fifteen dollars now thirteen dollars a day. Still a little use.	Jingdong prefecture level city in Shandong
Stowe Flagship Store	a power saver (electronics)	Originally bought for home use, used for two months, the electricity bill from more than 200 yuan per month, down to more than 140 yuan, there is indeed a power-saving effect, this time and sold two, the unit with. Merchant service attitude is really good.	Jingdong prefecture level city in Shandong
Stowe Flagship Store	a power saver (electronics)	Plug and play, stable voltage, the screen shows when working, this winter home appliances, need electricity in many places, just buy to use, save electricity and money, with instructions	Jingdong prefecture level city in Shandong
Stowe Flagship Store	a power saver (electronics)	Goods received, do not know if it is good. When I bought it, I saw that it was a Stowe brand or company's product. Received but not. I do not know what is the reason. Now I can only give this evaluation. I'm	Jingdong prefecture level city in Shandong
Stowe Flagship Store	a power saver (electronics)	The first time to buy online, but also look at the evaluation before buying, the quality is not bad, but also very cheap, the need for friends can rest assured to buy!	Jingdong prefecture level city in Shandong
Stowe Flagship Store	a power saver (electronics)	🌈 Great quality, bought it to my liking 💗 Awesome! ✨ Buy with confidence if you need it!	Jingdong prefecture level city in Shandong
Stowe Flagship Store	a power saver (electronics)	The quality is very good, completely exceeded the expectations, the delivery speed is very fast, the packaging is very careful and strict, very satisfied with a shopping	Jingdong prefecture level city in Shandong
Stowe Flagship Store	a power saver (electronics)	The effect is very good, the home uses less electricity is very energy efficient, cost-effective	Jingdong prefecture level city in Shandong
Stowe Flagship Store	a power saver (electronics)	Quality can't be beat, just like the store, especially like, and the price is favorable!	Jingdong prefecture level city in Shandong
Stowe Flagship Store	a power saver (electronics)	Baby received! Very unexpected, will be back next time ~ great seller, stay tuned!	Jingdong prefecture level city in Shandong
Stowe Flagship Store	a power saver (electronics)	Follow up review after a month is more real, waiting for it	Jingdong prefecture level city in Shandong

Stowe Flagship Store	a power saver (electronics)	It is the ideal product, good quality and fast logistics, worthy of praise!	Jingdong prefecture level city in Shandong
Stowe Flagship Store	a power saver (electronics)	The saver is great, the color is right, the installer is very professional	Jingdong prefecture level city in Shandong
Stowe Flagship Store	a power saver (electronics)	The effect of the power connection is particularly obvious, it is particularly easy to use, very satisfied, the	Jingdong prefecture level city in Shandong
Stowe Flagship Store	a power saver (electronics)	Goods are very energy-saving, used to be more than two hundred degrees, now there are only a hundred or so degrees	Jingdong prefecture level city in Shandong
Stowe Flagship Store	a power saver (electronics)	Good quality, fast shipping, good results, good value for money	Jingdong prefecture level city in Shandong
Stowe Flagship Store	a power saver (electronics)	Fast shipping, fine workmanship, very good, cost-effective	Jingdong prefecture level city in Shandong
Stowe Flagship Store	a power saver (electronics)	The meter is as described, the quality is quite good, energy-saving, worth buying oh~!	Jingdong prefecture level city in Shandong
Stowe Flagship Store	a power saver (electronics)	Great value for money, very affordable,, perfect . ˆ◡ˆ.	Jingdong prefecture level city in Shandong
Stowe Flagship Store	a power saver (electronics)	Good!!! Worth recommending!	Jingdong prefecture level city in Shandong
Stowe Flagship Store	a power saver (electronics)	Great, fast logistics, great stuff, great value for money!	Jingdong prefecture level city in Shandong
Stowe Flagship Store	a power saver (electronics)	Fast logistics, very satisfied with the quality upon receipt	Jingdong prefecture level city in Shandong
Stowe Flagship Store	a power saver (electronics)	Great quality, plug and play, fast shipping!	Jingdong prefecture level city in Shandong
Stowe Flagship Store	a power saver (electronics)	Logistics received, quite satisfied	Jingdong prefecture level city in Shandong
Stowe Flagship Store	a power saver (electronics)	I don't know if it really works	Jingdong prefecture level city in Shandong
Stowe Flagship Store	a power saver (electronics)	It's bullshit. It's a plug light. Don't talk about power savings.	Jingdong prefecture level city in Shandong
Stowe Flagship Store	a power saver (electronics)	Fake and deceitful stuff that hasn't changed at all.	Jingdong prefecture level city in Shandong
Stowe Flagship Store	a power saver (electronics)	Power-saving effect: power-saving effect is particularly obvious Cost-effective: cost-effective business Appearance and style: external small micro-work materials: fine workmanship Experience: use very good, non the first convenient	Jingdong prefecture level city in Shandong
Stowe Flagship Store	a power saver (electronics)	After using it for a few days, it feels quite good, it should be quite safe, and it also sends a plug, the merchant is good, wait for a period of time and then calculate whether it saves on the electricity bill and then add comments ha!	Jingdong prefecture level city in Shandong
Stowe Flagship Store	a power saver (electronics)	My friend recommended to me, said the effect is very good, I bought two, this month less money, really can, just buy I do not believe, but has proved to have effect	Jingdong prefecture level city in Shandong
Stowe Flagship Store	a power saver (electronics)	Just bought back, already used, these days to see the effect of it, and so have the effect will also come back to give positive feedback, the price is also super cheap!	Jingdong prefecture level city in Shandong
Stowe Flagship Store	a power saver (electronics)	It does work well, the store uses a lot of electricity, this time to buy back a few, each place that uses a lot of electricity to insert one. Summer is here, the electricity is too strong.	Jingdong prefecture level city in Shandong
Stowe Flagship Store	a power saver (electronics)	Power saving treasure received, as for the effect, according to the instructions written, a short period of time can not be seen, at least 2-3 months before the real effect, then follow up evaluation	Jingdong prefecture level city in Shandong
Stowe Flagship Store	a power saver (electronics)	The goods have just been received and have not been used yet looking forward to the results.	Jingdong prefecture level city in Shandong
Stowe Flagship Store	a power saver (electronics)	Good installation works great	Jingdong prefecture level city in Shandong
Stowe Flagship Store	a power saver (electronics)	The logistics is super super fast! The quality is also very good, indeed value for money	Jingdong prefecture level city in Shandong
Stowe Flagship Store	a power saver (electronics)	Has been used for a period of time before coming to comment on, really good, air conditioning a day open non-stop, pay the electricity bill when the heartache, with this, the home electricity is not at all worried about the bill out of contrast to save a lot of preparation in the hands of a few, each room An a	Jingdong prefecture level city in Shandong
Stowe Flagship Store	a power saver (electronics)	Saving device received, really good, with the air conditioning tried, an hour without a saver with 2.9 degrees, with a saver an hour with 1.1 degrees, the beginning of not quite believe that this thing, the actual test to know that it is really useful, and intend to buy another.	Jingdong prefecture level city in Shandong
Stowe Flagship Store	a power saver (electronics)	Power saving effect: this energy saver is still useful, day trial for three days, every day than not installed before the reduction of two dollars a day, every day to open the bedroom to open the air conditioning before fifteen dollars now thirteen dollars a day. Still a little use.	Jingdong prefecture level city in Shandong
Stowe Flagship Store	a power saver (electronics)	Originally bought for home use, used for two months, the electricity bill from more than 200 yuan per month, down to more than 140 yuan, there is indeed a power-saving effect, this time and sold two, the unit with. Merchant service attitude is really good.	Jingdong prefecture level city in Shandong
Stowe Flagship Store	a power saver (electronics)	Plug and play, stable voltage, the screen shows when working, this winter home appliances, need electricity in many places, just buy to use, save electricity and money, with instructions	Jingdong prefecture level city in Shandong
Stowe Flagship Store	a power saver (electronics)	Goods received, do not know if it is good. When I bought it, I saw that it was a Stowe brand or company's product. Received but not. I do not know what is the reason. Now I can only give this evaluation. I'm	Jingdong prefecture level city in Shandong
Stowe Flagship Store	a power saver (electronics)	The first time to buy online, but also look at the evaluation before buying, the quality is not bad, but also very cheap, the need for friends can rest assured to buy!	Jingdong prefecture level city in Shandong
Stowe Flagship Store	a power saver (electronics)	Great quality, bought it to my liking Awesome! Buy with confidence if you need it!	Jingdong prefecture level city in Shandong
Stowe Flagship Store	a power saver (electronics)	The quality is very good, completely exceeded the expectations, the delivery speed is very fast, the packaging is very careful and strict, very satisfied with a shopping	Jingdong prefecture level city in Shandong
Stowe Flagship Store	a power saver (electronics)	The effect is very good, the home uses less electricity is very energy efficient, cost-effective	Jingdong prefecture level city in Shandong
Stowe Flagship Store	a power saver (electronics)	Quality can't be beat, just like the store, especially like, and the price is favorable!	Jingdong prefecture level city in Shandong
Stowe Flagship Store	a power saver (electronics)	Baby received! Very unexpected, will be back next time ~ great seller, stay tuned!	Jingdong prefecture level city in Shandong
Stowe Flagship Store	a power saver (electronics)	Follow up review after a month is more real, waiting for it	Jingdong prefecture level city in Shandong
Stowe Flagship Store	a power saver (electronics)	It is the ideal product, good quality and fast logistics, worthy of praise!	Jingdong prefecture level city in Shandong
Stowe Flagship Store	a power saver (electronics)	The saver is great, the color is right, the installer is very professional	Jingdong prefecture level city in Shandong
Stowe Flagship Store	a power saver (electronics)	The effect of the power connection is particularly obvious, it is particularly easy to use, very satisfied, the	Jingdong prefecture level city in Shandong
Stowe Flagship Store	a power saver (electronics)	Goods are very energy-saving, used to be more than two hundred degrees, now there are only a hundred or so degrees	Jingdong prefecture level city in Shandong
Stowe Flagship Store	a power saver (electronics)	Good quality, fast shipping, good results, good value for money	Jingdong prefecture level city in Shandong
Stowe Flagship Store	a power saver (electronics)	Fast shipping, fine workmanship, very good, cost-effective	Jingdong prefecture level city in Shandong
Stowe Flagship Store	a power saver (electronics)	The meter is as described, the quality is quite good, energy-saving, worth buying oh~!	Jingdong prefecture level city in Shandong
Stowe Flagship Store	a power saver (electronics)	Great value for money, very affordable,, perfect . ˆ◡ˆ.	Jingdong prefecture level city in Shandong
Stowe Flagship Store	a power saver (electronics)	Good!!! Worth recommending!	Jingdong prefecture level city in Shandong
Stowe Flagship Store	a power saver (electronics)	Great, fast logistics, great stuff, great value for money!	Jingdong prefecture level city in Shandong

Stowe Flagship Store	a power saver (electronics)	Fast logistics, very satisfied with the quality upon receipt	Jingdong prefecture level city in Shandong
Stowe Flagship Store	a power saver (electronics)	Great quality, plug and play, fast shipping!	Jingdong prefecture level city in Shandong
Stowe Flagship Store	a power saver (electronics)	Logistics received, quite satisfied	Jingdong prefecture level city in Shandong
Stowe Flagship Store	a power saver (electronics)	I don't know if it really works	Jingdong prefecture level city in Shandong
Stowe Flagship Store	a power saver (electronics)	It's bullshit. It's a plug light. Don't talk about power savings.	Jingdong prefecture level city in Shandong
Stowe Flagship Store	a power saver (electronics)	Fake and deceitful stuff that hasn't changed at all.	Jingdong prefecture level city in Shandong
Stowe Flagship Store	a power saver (electronics)	Power-saving effect: power-saving effect is particularly obvious Cost-effective: cost-effective business Appearance and style: external small micro-work materials: fine workmanship Experience: use very good, non the first convenient	Jingdong prefecture level city in Shandong
Stowe Flagship Store	a power saver (electronics)	After using it for a few days, it feels quite good, it should be quite safe, and it also sends a plug, the merchant is good, wait for a period of time and then calculate whether it saves on the electricity bill and then add comments ha!	Jingdong prefecture level city in Shandong
Stowe Flagship Store	a power saver (electronics)	My friend recommended to me, said the effect is very good, I bought two, this month less money, really can, just buy I do not believe, but has proved to have effect	Jingdong prefecture level city in Shandong
Stowe Flagship Store	a power saver (electronics)	Just bought back, already used, these days to see the effect of it, and so have the effect will also come back to give positive feedback, the price is also super cheap!	Jingdong prefecture level city in Shandong
Stowe Flagship Store	a power saver (electronics)	It does work well, the store uses a lot of electricity, this time to buy back a few, each place that uses a lot of electricity to insert one. Summer is here, the electricity is too strong.	Jingdong prefecture level city in Shandong
Stowe Flagship Store	a power saver (electronics)	Power saving treasure received, as for the effect, according to the instructions written, a short period of time can not be seen, at least 2-3 months before the real effect, then follow up evaluation	Jingdong prefecture level city in Shandong
Stowe Flagship Store	a power saver (electronics)	The goods have just been received and have not been used yet looking forward to the results.	Jingdong prefecture level city in Shandong
Stowe Flagship Store	a power saver (electronics)	Good installation works great	Jingdong prefecture level city in Shandong
Stowe Flagship Store	a power saver (electronics)	The logistics is super super fast! The quality is also very good, indeed value for money	Jingdong prefecture level city in Shandong
Stowe Flagship Store	a power saver (electronics)	Has been used for a period of time before coming to comment on, really good, air conditioning a day open non-stop, pay the electricity bill when the heartache, with this, the home electricity is not at all worried about the bill out of contrast to save a lot of preparation in the hands of a few, each room An a	Jingdong prefecture level city in Shandong
Stowe Flagship Store	a power saver (electronics)	Saving device received, really good, with the air conditioning tried, an hour without a saver with 2.9 degrees, with a saver an hour with 1.1 degrees, the beginning of not quite believe that this thing, the actual test to know that it is really useful, and intend to buy another.	Jingdong prefecture level city in Shandong
Stowe Flagship Store	a power saver (electronics)	Power saving effect: this energy saver is still useful, day trial for three days, every day than not installed before the reduction of two dollars a day, every day to open the bedroom to open the air conditioning before fifteen dollars now thirteen dollars a day. Still a little use.	Jingdong prefecture level city in Shandong
Stowe Flagship Store	a power saver (electronics)	Originally bought for home use, used for two months, the electricity bill from more than 200 yuan per month, down to more than 140 yuan, there is indeed a power-saving effect, this time and sold two, the unit with. Merchant service attitude is really good.	Jingdong prefecture level city in Shandong
Stowe Flagship Store	a power saver (electronics)	Plug and play, stable voltage, the screen shows when working, this winter home appliances, need electricity in many places, just buy to use, save electricity and money, with instructions	Jingdong prefecture level city in Shandong
Stowe Flagship Store	a power saver (electronics)	Goods received, do not know if it is good. When I bought it, I saw that it was a Stowe brand or company's product. Received but not. I do not know what is the reason. Now I can only give this evaluation. I'm	Jingdong prefecture level city in Shandong
Stowe Flagship Store	a power saver (electronics)	The first time to buy online, but also look at the evaluation before buying, the quality is not bad, but also very cheap, the need for friends can rest assured to buy!	Jingdong prefecture level city in Shandong
Stowe Flagship Store	a power saver (electronics)	Great quality, bought it to my liking Awesome! Buy with confidence if you need it!	Jingdong prefecture level city in Shandong
Stowe Flagship Store	a power saver (electronics)	The quality is very good, completely exceeded the expectations, the delivery speed is very fast, the packaging is very careful and strict, very satisfied with a shopping	Jingdong prefecture level city in Shandong
Stowe Flagship Store	a power saver (electronics)	The effect is very good, the home uses less electricity is very energy efficient, cost-effective	Jingdong prefecture level city in Shandong
Stowe Flagship Store	a power saver (electronics)	Quality can't be beat, just like the store, especially like, and the price is favorable!	Jingdong prefecture level city in Shandong
Stowe Flagship Store	a power saver (electronics)	Baby received! Very unexpected, will be back next time ~ great seller, stay tuned!	Jingdong prefecture level city in Shandong
Stowe Flagship Store	a power saver (electronics)	Follow up review after a month is more real, waiting for it	Jingdong prefecture level city in Shandong
Stowe Flagship Store	a power saver (electronics)	It is the ideal product, good quality and fast logistics, worthy of praise!	Jingdong prefecture level city in Shandong
Stowe Flagship Store	a power saver (electronics)	The saver is great, the color is right, the installer is very professional	Jingdong prefecture level city in Shandong
Stowe Flagship Store	a power saver (electronics)	The effect of the power connection is particularly obvious, it is particularly easy to use, very satisfied, the	Jingdong prefecture level city in Shandong
Stowe Flagship Store	a power saver (electronics)	Goods are very energy-saving, used to be more than two hundred degrees, now there are only a hundred or so degrees	Jingdong prefecture level city in Shandong
Stowe Flagship Store	a power saver (electronics)	Good quality, fast shipping, good results, good value for money	Jingdong prefecture level city in Shandong
Stowe Flagship Store	a power saver (electronics)	Fast shipping, fine workmanship, very good, cost-effective	Jingdong prefecture level city in Shandong
Stowe Flagship Store	a power saver (electronics)	The meter is as described, the quality is quite good, energy-saving, worth buying oh~!	Jingdong prefecture level city in Shandong
Stowe Flagship Store	a power saver (electronics)	Great value for money, very affordable,, perfect . ˆ◡ˆ.	Jingdong prefecture level city in Shandong
Stowe Flagship Store	a power saver (electronics)	Good!!! Worth recommending!	Jingdong prefecture level city in Shandong
Stowe Flagship Store	a power saver (electronics)	Great, fast logistics, great stuff, great value for money!	Jingdong prefecture level city in Shandong
Stowe Flagship Store	a power saver (electronics)	Fast logistics, very satisfied with the quality upon receipt	Jingdong prefecture level city in Shandong
Stowe Flagship Store	a power saver (electronics)	Great quality, plug and play, fast shipping!	Jingdong prefecture level city in Shandong
Stowe Flagship Store	a power saver (electronics)	Logistics received, quite satisfied	Jingdong prefecture level city in Shandong
Stowe Flagship Store	a power saver (electronics)	I don't know if it really works	Jingdong prefecture level city in Shandong
Stowe Flagship Store	a power saver (electronics)	It's bullshit. It's a plug light. Don't talk about power savings.	Jingdong prefecture level city in Shandong
Stowe Flagship Store	a power saver (electronics)	Fake and deceitful stuff that hasn't changed at all.	Jingdong prefecture level city in Shandong
Stowe Flagship Store	a power saver (electronics)	Power-saving effect: power-saving effect is particularly obvious Cost-effective: cost-effective business Appearance and style: external small micro-work materials: fine workmanship Experience: use very good, non the first convenient	Jingdong prefecture level city in Shandong
Stowe Flagship Store	a power saver (electronics)	After using it for a few days, it feels quite good, it should be quite safe, and it also sends a plug, the merchant is good, wait for a period of time and then calculate whether it saves on the electricity bill and then add comments ha!	Jingdong prefecture level city in Shandong
Stowe Flagship Store	a power saver (electronics)	My friend recommended to me, said the effect is very good, I bought two, this month less money, really can, just buy I do not believe, but has proved to have effect	Jingdong prefecture level city in Shandong

Stowe Flagship Store	a power saver (electronics)	Just bought back, already used, these days to see the effect of it, and so have the effect will also come back to give positive feedback, the price is also super cheap!	Jingdong prefecture level city in Shandong
Stowe Flagship Store	a power saver (electronics)	It does work well, the store uses a lot of electricity, this time to buy back a few, each place that uses a lot of electricity to insert one. Summer is here, the electricity is too strong.	Jingdong prefecture level city in Shandong
Stowe Flagship Store	a power saver (electronics)	Power saving treasure received, as for the effect, according to the instructions written, a short period of time can not be seen, at least 2-3 months before the real effect, then follow up evaluation	Jingdong prefecture level city in Shandong
Stowe Flagship Store	a power saver (electronics)	The goods have just been received and have not been used yet looking forward to the results.	Jingdong prefecture level city in Shandong
Stowe Flagship Store	a power saver (electronics)	Good installation works great	Jingdong prefecture level city in Shandong
Stowe Flagship Store	a power saver (electronics)	The logistics is super super fast! The quality is also very good, indeed value for money	Jingdong prefecture level city in Shandong
Stowe Flagship Store	a power saver (electronics)	Has been used for a period of time before coming to comment on, really good, air conditioning a day open non-stop, pay the electricity bill when the heartache, with this, the home electricity is not at all worried about the bill out of contrast to save a lot of preparation in the hands of a few, each room An a	Jingdong prefecture level city in Shandong
Stowe Flagship Store	a power saver (electronics)	Saving device received, really good, with the air conditioning tried, an hour without a saver with 2.9 degrees, with a saver an hour with 1.1 degrees, the beginning of not quite believe that this thing, the actual test to know that it is really useful, and intend to buy another.	Jingdong prefecture level city in Shandong
Stowe Flagship Store	a power saver (electronics)	Power saving effect: this energy saver is still useful, day trial for three days, every day than not installed before the reduction of two dollars a day, every day to open the bedroom to open the air conditioning before fifteen dollars now thirteen dollars a day. Still a little use.	Jingdong prefecture level city in Shandong
Stowe Flagship Store	a power saver (electronics)	Originally bought for home use, used for two months, the electricity bill from more than 200 yuan per month, down to more than 140 yuan, there is indeed a power-saving effect, this time and sold two, the unit with. Merchant service attitude is really good.	Jingdong prefecture level city in Shandong
Stowe Flagship Store	a power saver (electronics)	Plug and play, stable voltage, the screen shows when working, this winter home appliances, need electricity in many places, just buy to use, save electricity and money, with instructions	Jingdong prefecture level city in Shandong
Stowe Flagship Store	a power saver (electronics)	Goods received, do not know if it is good. When I bought it, I saw that it was a Stowe brand or company's product. Received but not. I do not know what is the reason. Now I can only give this evaluation. I'm	Jingdong prefecture level city in Shandong
Stowe Flagship Store	a power saver (electronics)	The first time to buy online, but also look at the evaluation before buying, the quality is not bad, but also very cheap, the need for friends can rest assured to buy!	Jingdong prefecture level city in Shandong
Stowe Flagship Store	a power saver (electronics)	Great quality, bought it to my liking Awesome! Buy with confidence if you need it!	Jingdong prefecture level city in Shandong
Stowe Flagship Store	a power saver (electronics)	The quality is very good, completely exceeded the expectations, the delivery speed is very fast, the packaging is very careful and strict, very satisfied with a shopping	Jingdong prefecture level city in Shandong
Stowe Flagship Store	a power saver (electronics)	The effect is very good, the home uses less electricity is very energy efficient, cost-effective	Jingdong prefecture level city in Shandong
Stowe Flagship Store	a power saver (electronics)	Quality can't be beat, just like the store, especially like, and the price is favorable!	Jingdong prefecture level city in Shandong
Stowe Flagship Store	a power saver (electronics)	Baby received! Very unexpected, will be back next time ~ great seller, stay tuned!	Jingdong prefecture level city in Shandong
Stowe Flagship Store	a power saver (electronics)	Follow up review after a month is more real, waiting for it	Jingdong prefecture level city in Shandong
Stowe Flagship Store	a power saver (electronics)	It is the ideal product, good quality and fast logistics, worthy of praise!	Jingdong prefecture level city in Shandong
Stowe Flagship Store	a power saver (electronics)	The saver is great, the color is right, the installer is very professional	Jingdong prefecture level city in Shandong
Stowe Flagship Store	a power saver (electronics)	The effect of the power connection is particularly obvious, it is particularly easy to use, very satisfied, the	Jingdong prefecture level city in Shandong
Stowe Flagship Store	a power saver (electronics)	Goods are very energy-saving, used to be more than two hundred degrees, now there are only a hundred or so degrees	Jingdong prefecture level city in Shandong
Stowe Flagship Store	a power saver (electronics)	Good quality, fast shipping, good results, good value for money	Jingdong prefecture level city in Shandong
Stowe Flagship Store	a power saver (electronics)	Fast shipping, fine workmanship, very good, cost-effective	Jingdong prefecture level city in Shandong
Stowe Flagship Store	a power saver (electronics)	The meter is as described, the quality is quite good, energy-saving, worth buying oh~!	Jingdong prefecture level city in Shandong
Stowe Flagship Store	a power saver (electronics)	Great value for money, very affordable,, perfect . ˆ◡ˆ.	Jingdong prefecture level city in Shandong
Stowe Flagship Store	a power saver (electronics)	Good!!! Worth recommending!	Jingdong prefecture level city in Shandong
Stowe Flagship Store	a power saver (electronics)	Great, fast logistics, great stuff, great value for money!	Jingdong prefecture level city in Shandong
Stowe Flagship Store	a power saver (electronics)	Fast logistics, very satisfied with the quality upon receipt	Jingdong prefecture level city in Shandong
Stowe Flagship Store	a power saver (electronics)	Great quality, plug and play, fast shipping!	Jingdong prefecture level city in Shandong
Stowe Flagship Store	a power saver (electronics)	Logistics received, quite satisfied	Jingdong prefecture level city in Shandong
Stowe Flagship Store	a power saver (electronics)	I don't know if it really works	Jingdong prefecture level city in Shandong
Stowe Flagship Store	a power saver (electronics)	It's bullshit. It's a plug light. Don't talk about power savings.	Jingdong prefecture level city in Shandong
Stowe Flagship Store	a power saver (electronics)	Fake and deceitful stuff that hasn't changed at all.	Jingdong prefecture level city in Shandong
Stowe Flagship Store	a power saver (electronics)	Power-saving effect: power-saving effect is particularly obvious Cost-effective: cost-effective business Appearance and style: external small micro-work materials: fine workmanship Experience: use very good, non the first convenient	Jingdong prefecture level city in Shandong
Stowe Flagship Store	a power saver (electronics)	After using it for a few days, it feels quite good, it should be quite safe, and it also sends a plug, the merchant is good, wait for a period of time and then calculate whether it saves on the electricity bill and then add comments ha!	Jingdong prefecture level city in Shandong
Stowe Flagship Store	a power saver (electronics)	My friend recommended to me, said the effect is very good, I bought two, this month less money, really can, just buy I do not believe, but has proved to have effect	Jingdong prefecture level city in Shandong
Stowe Flagship Store	a power saver (electronics)	Just bought back, already used, these days to see the effect of it, and so have the effect will also come back to give positive feedback, the price is also super cheap!	Jingdong prefecture level city in Shandong
Stowe Flagship Store	a power saver (electronics)	It does work well, the store uses a lot of electricity, this time to buy back a few, each place that uses a lot of electricity to insert one. Summer is here, the electricity is too strong.	Jingdong prefecture level city in Shandong
Stowe Flagship Store	a power saver (electronics)	Power saving treasure received, as for the effect, according to the instructions written, a short period of time can not be seen, at least 2-3 months before the real effect, then follow up evaluation	Jingdong prefecture level city in Shandong
Stowe Flagship Store	a power saver (electronics)	The goods have just been received and have not been used yet looking forward to the results.	Jingdong prefecture level city in Shandong
Stowe Flagship Store	a power saver (electronics)	Good installation works great	Jingdong prefecture level city in Shandong
Stowe Flagship Store	a power saver (electronics)	The logistics is super super fast! The quality is also very good, indeed value for money	Jingdong prefecture level city in Shandong
Stowe Flagship Store	a power saver (electronics)	Has been used for a period of time before coming to comment on, really good, air conditioning a day open non-stop, pay the electricity bill when the heartache, with this, the home electricity is not at all worried about the bill out of contrast to save a lot of preparation in the hands of a few, each room An a	Jingdong prefecture level city in Shandong
Stowe Flagship Store	a power saver (electronics)	Saving device received, really good, with the air conditioning tried, an hour without a saver with 2.9 degrees, with a saver an hour with 1.1 degrees, the beginning of not quite believe that this thing, the actual test to know that it is really useful, and intend to buy another.	Jingdong prefecture level city in Shandong
Stowe Flagship Store	a power saver (electronics)	Power saving effect: this energy saver is still useful, day trial for three days, every day than not installed before the reduction of two dollars a day, every day to open the bedroom to open the air conditioning before fifteen dollars now thirteen dollars a day. Still a little use.	Jingdong prefecture level city in Shandong

Stowe Flagship Store	a power saver (electronics)	Originally bought for home use, used for two months, the electricity bill from more than 200 yuan per month, down to more than 140 yuan, there is indeed a power-saving effect, this time and sold two, the unit with. Merchant service attitude is really good.	Jingdong prefecture level city in Shandong
Stowe Flagship Store	a power saver (electronics)	Plug and play, stable voltage, the screen shows when working, this winter home appliances, need electricity in many places, just buy to use, save electricity and money, with instructions	Jingdong prefecture level city in Shandong
Stowe Flagship Store	a power saver (electronics)	Goods received, do not know if it is good. When I bought it, I saw that it was a Stowe brand or company's product. Received but not. I do not know what is the reason. Now I can only give this evaluation. I'm	Jingdong prefecture level city in Shandong
Stowe Flagship Store	a power saver (electronics)	The first time to buy online, but also look at the evaluation before buying, the quality is not bad, but also very cheap, the need for friends can rest assured to buy!	Jingdong prefecture level city in Shandong
Stowe Flagship Store	a power saver (electronics)	🌈 Great quality, bought it to my liking 💗 Awesome! ✨ Buy with confidence if you need it!	Jingdong prefecture level city in Shandong
Stowe Flagship Store	a power saver (electronics)	The quality is very good, completely exceeded the expectations, the delivery speed is very fast, the packaging is very careful and strict, very satisfied with a shopping	Jingdong prefecture level city in Shandong
Stowe Flagship Store	a power saver (electronics)	The effect is very good, the home uses less electricity is very energy efficient, cost-effective	Jingdong prefecture level city in Shandong
Stowe Flagship Store	a power saver (electronics)	Quality can't be beat, just like the store, especially like, and the price is favorable!	Jingdong prefecture level city in Shandong
Stowe Flagship Store	a power saver (electronics)	Baby received! Very unexpected, will be back next time ~ great seller, stay tuned!	Jingdong prefecture level city in Shandong
Stowe Flagship Store	a power saver (electronics)	Follow up review after a month is more real, waiting for it	Jingdong prefecture level city in Shandong
Stowe Flagship Store	a power saver (electronics)	It is the ideal product, good quality and fast logistics, worthy of praise!	Jingdong prefecture level city in Shandong
Stowe Flagship Store	a power saver (electronics)	The saver is great, the color is right, the installer is very professional	Jingdong prefecture level city in Shandong
Stowe Flagship Store	a power saver (electronics)	The effect of the power connection is particularly obvious, it is particularly easy to use, very satisfied, the	Jingdong prefecture level city in Shandong
Stowe Flagship Store	a power saver (electronics)	Goods are very energy-saving, used to be more than two hundred degrees, now there are only a hundred or so degrees	Jingdong prefecture level city in Shandong
Stowe Flagship Store	a power saver (electronics)	Good quality, fast shipping, good results, good value for money	Jingdong prefecture level city in Shandong
Stowe Flagship Store	a power saver (electronics)	Fast shipping, fine workmanship, very good, cost-effective	Jingdong prefecture level city in Shandong
Stowe Flagship Store	a power saver (electronics)	The meter is as described, the quality is quite good, energy-saving, worth buying oh~!	Jingdong prefecture level city in Shandong
Stowe Flagship Store	a power saver (electronics)	Great value for money, very affordable,, perfect . ˆ◡ˆ.	Jingdong prefecture level city in Shandong
Stowe Flagship Store	a power saver (electronics)	Good!!! Worth recommending!	Jingdong prefecture level city in Shandong
Stowe Flagship Store	a power saver (electronics)	Great, fast logistics, great stuff, great value for money!	Jingdong prefecture level city in Shandong
Stowe Flagship Store	a power saver (electronics)	Fast logistics, very satisfied with the quality upon receipt	Jingdong prefecture level city in Shandong
Stowe Flagship Store	a power saver (electronics)	Great quality, plug and play, fast shipping!	Jingdong prefecture level city in Shandong
Stowe Flagship Store	a power saver (electronics)	Logistics received, quite satisfied	Jingdong prefecture level city in Shandong
Stowe Flagship Store	a power saver (electronics)	I don't know if it really works	Jingdong prefecture level city in Shandong
Stowe Flagship Store	a power saver (electronics)	It's bullshit. It's a plug light. Don't talk about power savings.	Jingdong prefecture level city in Shandong
Stowe Flagship Store	a power saver (electronics)	Fake and deceitful stuff that hasn't changed at all.	Jingdong prefecture level city in Shandong
Stowe Flagship Store	a power saver (electronics)	Power-saving effect: power-saving effect is particularly obvious Cost-effective: cost-effective business Appearance and style: external small micro-work materials: fine workmanship Experience: use very good, non the first convenient	Jingdong prefecture level city in Shandong
Stowe Flagship Store	a power saver (electronics)	After using it for a few days, it feels quite good, it should be quite safe, and it also sends a plug, the merchant is good, wait for a period of time and then calculate whether it saves on the electricity bill and then add comments ha!	Jingdong prefecture level city in Shandong
Stowe Flagship Store	a power saver (electronics)	My friend recommended to me, said the effect is very good, I bought two, this month less money, really can, just buy I do not believe, but has proved to have effect	Jingdong prefecture level city in Shandong
Stowe Flagship Store	a power saver (electronics)	Just bought back, already used, these days to see the effect of it, and so have the effect will also come back to give positive feedback, the price is also super cheap!	Jingdong prefecture level city in Shandong
Stowe Flagship Store	a power saver (electronics)	It does work well, the store uses a lot of electricity, this time to buy back a few, each place that uses a lot of electricity to insert one. Summer is here, the electricity is too strong.	Jingdong prefecture level city in Shandong
Stowe Flagship Store	a power saver (electronics)	Power saving treasure received, as for the effect, according to the instructions written, a short period of time can not be seen, at least 2-3 months before the real effect, then follow up evaluation	Jingdong prefecture level city in Shandong
Stowe Flagship Store	a power saver (electronics)	The goods have just been received and have not been used yet looking forward to the results.	Jingdong prefecture level city in Shandong
Stowe Flagship Store	a power saver (electronics)	Good installation works great	Jingdong prefecture level city in Shandong
Stowe Flagship Store	a power saver (electronics)	The logistics is super super fast! The quality is also very good, indeed value for money	Jingdong prefecture level city in Shandong
Stowe Flagship Store	a power saver (electronics)	Has been used for a period of time before coming to comment on, really good, air conditioning a day open non-stop, pay the electricity bill when the heartache, with this, the home electricity is not at all worried about the bill out of contrast to save a lot of preparation in the hands of a few, each room An a	Jingdong prefecture level city in Shandong
Stowe Flagship Store	a power saver (electronics)	Saving device received, really good, with the air conditioning tried, an hour without a saver with 2.9 degrees, with a saver an hour with 1.1 degrees, the beginning of not quite believe that this thing, the actual test to know that it is really useful, and intend to buy another.	Jingdong prefecture level city in Shandong
Stowe Flagship Store	a power saver (electronics)	Power saving effect: this energy saver is still useful, day trial for three days, every day than not installed before the reduction of two dollars a day, every day to open the bedroom to open the air conditioning before fifteen dollars now thirteen dollars a day. Still a little use.	Jingdong prefecture level city in Shandong
Stowe Flagship Store	a power saver (electronics)	Originally bought for home use, used for two months, the electricity bill from more than 200 yuan per month, down to more than 140 yuan, there is indeed a power-saving effect, this time and sold two, the unit with. Merchant service attitude is really good.	Jingdong prefecture level city in Shandong
Stowe Flagship Store	a power saver (electronics)	Plug and play, stable voltage, the screen shows when working, this winter home appliances, need electricity in many places, just buy to use, save electricity and money, with instructions	Jingdong prefecture level city in Shandong
Stowe Flagship Store	a power saver (electronics)	Goods received, do not know if it is good. When I bought it, I saw that it was a Stowe brand or company's product. Received but not. I do not know what is the reason. Now I can only give this evaluation. I'm	Jingdong prefecture level city in Shandong
Stowe Flagship Store	a power saver (electronics)	The first time to buy online, but also look at the evaluation before buying, the quality is not bad, but also very cheap, the need for friends can rest assured to buy!	Jingdong prefecture level city in Shandong
Stowe Flagship Store	a power saver (electronics)	🌈 Great quality, bought it to my liking 💗 Awesome! ✨ Buy with confidence if you need it!	Jingdong prefecture level city in Shandong
Stowe Flagship Store	a power saver (electronics)	The quality is very good, completely exceeded the expectations, the delivery speed is very fast, the packaging is very careful and strict, very satisfied with a shopping	Jingdong prefecture level city in Shandong
Stowe Flagship Store	a power saver (electronics)	The effect is very good, the home uses less electricity is very energy efficient, cost-effective	Jingdong prefecture level city in Shandong
Stowe Flagship Store	a power saver (electronics)	Quality can't be beat, just like the store, especially like, and the price is favorable!	Jingdong prefecture level city in Shandong
Stowe Flagship Store	a power saver (electronics)	Baby received! Very unexpected, will be back next time ~ great seller, stay tuned!	Jingdong prefecture level city in Shandong
Stowe Flagship Store	a power saver (electronics)	Follow up review after a month is more real, waiting for it	Jingdong prefecture level city in Shandong

Stowe Flagship Store	a power saver (electronics)	It is the ideal product, good quality and fast logistics, worthy of praise!	Jingdong prefecture level city in Shandong
Stowe Flagship Store	a power saver (electronics)	The saver is great, the color is right, the installer is very professional	Jingdong prefecture level city in Shandong
Stowe Flagship Store	a power saver (electronics)	The effect of the power connection is particularly obvious, it is particularly easy to use, very satisfied, the	Jingdong prefecture level city in Shandong
Stowe Flagship Store	a power saver (electronics)	Goods are very energy-saving, used to be more than two hundred degrees, now there are only a hundred or so degrees	Jingdong prefecture level city in Shandong
Stowe Flagship Store	a power saver (electronics)	Good quality, fast shipping, good results, good value for money	Jingdong prefecture level city in Shandong
Stowe Flagship Store	a power saver (electronics)	Fast shipping, fine workmanship, very good, cost-effective	Jingdong prefecture level city in Shandong
Stowe Flagship Store	a power saver (electronics)	The meter is as described, the quality is quite good, energy-saving, worth buying oh~!	Jingdong prefecture level city in Shandong
Stowe Flagship Store	a power saver (electronics)	Great value for money, very affordable,, perfect . ^◡^.	Jingdong prefecture level city in Shandong
Stowe Flagship Store	a power saver (electronics)	Good!!! Worth recommending!	Jingdong prefecture level city in Shandong
Stowe Flagship Store	a power saver (electronics)	Great, fast logistics, great stuff, great value for money!	Jingdong prefecture level city in Shandong
Stowe Flagship Store	a power saver (electronics)	Fast logistics, very satisfied with the quality upon receipt	Jingdong prefecture level city in Shandong
Stowe Flagship Store	a power saver (electronics)	Great quality, plug and play, fast shipping!	Jingdong prefecture level city in Shandong
Stowe Flagship Store	a power saver (electronics)	Logistics received, quite satisfied	Jingdong prefecture level city in Shandong
Stowe Flagship Store	a power saver (electronics)	I don't know if it really works	Jingdong prefecture level city in Shandong
Stowe Flagship Store	a power saver (electronics)	It's bullshit. It's a plug light. Don't talk about power savings.	Jingdong prefecture level city in Shandong
Stowe Flagship Store	a power saver (electronics)	Fake and deceitful stuff that hasn't changed at all.	Jingdong prefecture level city in Shandong
Stowe Flagship Store	a power saver (electronics)	Power-saving effect: power-saving effect is particularly obvious Cost-effective: cost-effective business Appearance and style: external small micro-work materials: fine workmanship Experience: use very good, non the first convenient	Jingdong prefecture level city in Shandong
Stowe Flagship Store	a power saver (electronics)	After using it for a few days, it feels quite good, it should be quite safe, and it also sends a plug, the merchant is good, wait for a period of time and then calculate whether it saves on the electricity bill and then add comments ha!	Jingdong prefecture level city in Shandong
Stowe Flagship Store	a power saver (electronics)	My friend recommended to me, said the effect is very good, I bought two, this month less money, really can, just buy I do not believe, but has proved to have effect	Jingdong prefecture level city in Shandong
Stowe Flagship Store	a power saver (electronics)	Just bought back, already used, these days to see the effect of it, and so have the effect will also come back to give positive feedback, the price is also super cheap!	Jingdong prefecture level city in Shandong
Stowe Flagship Store	a power saver (electronics)	It does work well, the store uses a lot of electricity, this time to buy back a few, each place that uses a lot of electricity to insert one. Summer is here, the electricity is too strong.	Jingdong prefecture level city in Shandong
Stowe Flagship Store	a power saver (electronics)	Power saving treasure received, as for the effect, according to the instructions written, a short period of time can not be seen, at least 2-3 months before the real effect, then follow up evaluation	Jingdong prefecture level city in Shandong
Stowe Flagship Store	a power saver (electronics)	The goods have just been received and have not been used yet looking forward to the results.	Jingdong prefecture level city in Shandong
Stowe Flagship Store	a power saver (electronics)	Good installation works great	Jingdong prefecture level city in Shandong
Stowe Flagship Store	a power saver (electronics)	The logistics is super super fast! The quality is also very good, indeed value for money	Jingdong prefecture level city in Shandong
Stowe Flagship Store	a power saver (electronics)	Has been used for a period of time before coming to comment on, really good, air conditioning a day open non-stop, pay the electricity bill when the heartache, with this, the home electricity is not at all worried about the bill out of contrast to save a lot of preparation in the hands of a few, each room An a	Jingdong prefecture level city in Shandong
Stowe Flagship Store	a power saver (electronics)	Saving device received, really good, with the air conditioning tried, an hour without a saver with 2.9 degrees, with a saver an hour with 1.1 degrees, the beginning of not quite believe that this thing, the actual test to know that it is really useful, and intend to buy another.	Jingdong prefecture level city in Shandong
Stowe Flagship Store	a power saver (electronics)	Power saving effect: this energy saver is still useful, day trial for three days, every day than not installed before the reduction of two dollars a day, every day to open the bedroom to open the air conditioning before fifteen dollars now thirteen dollars a day. Still a little use.	Jingdong prefecture level city in Shandong
Stowe Flagship Store	a power saver (electronics)	Originally bought for home use, used for two months, the electricity bill from more than 200 yuan per month, down to more than 140 yuan, there is indeed a power-saving effect, this time and sold two, the unit with. Merchant service attitude is really good.	Jingdong prefecture level city in Shandong
Stowe Flagship Store	a power saver (electronics)	Plug and play, stable voltage, the screen shows when working, this winter home appliances, need electricity in many places, just buy to use, save electricity and money, with instructions	Jingdong prefecture level city in Shandong
Stowe Flagship Store	a power saver (electronics)	Goods received, do not know if it is good. When I bought it, I saw that it was a Stowe brand or company's product. Received but not. I do not know what is the reason. Now I can only give this evaluation. I'm	Jingdong prefecture level city in Shandong
Stowe Flagship Store	a power saver (electronics)	The first time to buy online, but also look at the evaluation before buying, the quality is not bad, but also very cheap, the need for friends can rest assured to buy!	Jingdong prefecture level city in Shandong
Stowe Flagship Store	a power saver (electronics)	🌈 Great quality, bought it to my liking 💝 Awesome! ✨ Buy with confidence if you need it!	Jingdong prefecture level city in Shandong
Stowe Flagship Store	a power saver (electronics)	The quality is very good, completely exceeded the expectations, the delivery speed is very fast, the packaging is very careful and strict, very satisfied with a shopping	Jingdong prefecture level city in Shandong
Stowe Flagship Store	a power saver (electronics)	The effect is very good, the home uses less electricity is very energy efficient, cost-effective	Jingdong prefecture level city in Shandong
Stowe Flagship Store	a power saver (electronics)	Quality can't be beat, just like the store, especially like, and the price is favorable!	Jingdong prefecture level city in Shandong
Stowe Flagship Store	a power saver (electronics)	Baby received! Very unexpected, will be back next time ~ great seller, stay tuned!	Jingdong prefecture level city in Shandong
Stowe Flagship Store	a power saver (electronics)	Follow up review after a month is more real, waiting for it	Jingdong prefecture level city in Shandong
Stowe Flagship Store	a power saver (electronics)	It is the ideal product, good quality and fast logistics, worthy of praise!	Jingdong prefecture level city in Shandong
Stowe Flagship Store	a power saver (electronics)	The saver is great, the color is right, the installer is very professional	Jingdong prefecture level city in Shandong
Stowe Flagship Store	a power saver (electronics)	The effect of the power connection is particularly obvious, it is particularly easy to use, very satisfied, the	Jingdong prefecture level city in Shandong
Stowe Flagship Store	a power saver (electronics)	Goods are very energy-saving, used to be more than two hundred degrees, now there are only a hundred or so degrees	Jingdong prefecture level city in Shandong
Stowe Flagship Store	a power saver (electronics)	Good quality, fast shipping, good results, good value for money	Jingdong prefecture level city in Shandong
Stowe Flagship Store	a power saver (electronics)	Fast shipping, fine workmanship, very good, cost-effective	Jingdong prefecture level city in Shandong
Stowe Flagship Store	a power saver (electronics)	The meter is as described, the quality is quite good, energy-saving, worth buying oh~!	Jingdong prefecture level city in Shandong
Stowe Flagship Store	a power saver (electronics)	Great value for money, very affordable,, perfect . ^◡^.	Jingdong prefecture level city in Shandong
Stowe Flagship Store	a power saver (electronics)	Good!!! Worth recommending!	Jingdong prefecture level city in Shandong
Stowe Flagship Store	a power saver (electronics)	Great, fast logistics, great stuff, great value for money!	Jingdong prefecture level city in Shandong

Stowe Flagship Store	a power saver (electronics)	Fast logistics, very satisfied with the quality upon receipt	Jingdong prefecture level city in Shandong
Stowe Flagship Store	a power saver (electronics)	Great quality, plug and play, fast shipping!	Jingdong prefecture level city in Shandong
Stowe Flagship Store	a power saver (electronics)	Logistics received, quite satisfied	Jingdong prefecture level city in Shandong
Stowe Flagship Store	a power saver (electronics)	I don’t know if it really works	Jingdong prefecture level city in Shandong
Stowe Flagship Store	a power saver (electronics)	It’s bullshit. It’s a plug light. Don’t talk about power savings.	Jingdong prefecture level city in Shandong
Stowe Flagship Store	a power saver (electronics)	Fake and deceitful stuff that hasn’t changed at all.	Jingdong prefecture level city in Shandong
Stowe Flagship Store	a power saver (electronics)	Power-saving effect: power-saving effect is particularly obvious Cost-effective: cost-effective business Appearance and style: external small micro-work materials: fine workmanship Experience: use very good, non the first convenient	Jingdong prefecture level city in Shandong
Stowe Flagship Store	a power saver (electronics)	After using it for a few days, it feels quite good, it should be quite safe, and it also sends a plug, the merchant is good, wait for a period of time and then calculate whether it saves on the electricity bill and then add comments ha!	Jingdong prefecture level city in Shandong
Stowe Flagship Store	a power saver (electronics)	My friend recommended to me, said the effect is very good, I bought two, this month less money, really can, just buy I do not believe, but has proved to have effect	Jingdong prefecture level city in Shandong
Stowe Flagship Store	a power saver (electronics)	Just bought back, already used, these days to see the effect of it, and so have the effect will also come back to give positive feedback, the price is also super cheap!	Jingdong prefecture level city in Shandong
Stowe Flagship Store	a power saver (electronics)	It does work well, the store uses a lot of electricity, this time to buy back a few, each place that uses a lot of electricity to insert one. Summer is here, the electricity is too strong.	Jingdong prefecture level city in Shandong
Stowe Flagship Store	a power saver (electronics)	Power saving treasure received, as for the effect, according to the instructions written, a short period of time can not be seen, at least 2-3 months before the real effect, then follow up evaluation	Jingdong prefecture level city in Shandong
Stowe Flagship Store	a power saver (electronics)	The goods have just been received and have not been used yet looking forward to the results.	Jingdong prefecture level city in Shandong
Stowe Flagship Store	a power saver (electronics)	Good installation works great	Jingdong prefecture level city in Shandong
Stowe Flagship Store	a power saver (electronics)	The logistics is super super fast! The quality is also very good, indeed value for money	Jingdong prefecture level city in Shandong
Stowe Flagship Store	a power saver (electronics)	Has been used for a period of time before coming to comment on, really good, air conditioning a day open non-stop, pay the electricity bill when the heartache, with this, the home electricity is not at all worried about the bill out of contrast to save a lot of preparation in the hands of a few, each room An a	Jingdong prefecture level city in Shandong
Stowe Flagship Store	a power saver (electronics)	Saving device received, really good, with the air conditioning tried, an hour without a saver with 2.9 degrees, with a saver an hour with 1.1 degrees, the beginning of not quite believe that this thing, the actual test to know that it is really useful, and intend to buy another.	Jingdong prefecture level city in Shandong
Stowe Flagship Store	a power saver (electronics)	Power saving effect: this energy saver is still useful, day trial for three days, every day than not installed before the reduction of two dollars a day, every day to open the bedroom to open the air conditioning before fifteen dollars now thirteen dollars a day. Still a little use.	Jingdong prefecture level city in Shandong
Stowe Flagship Store	a power saver (electronics)	Originally bought for home use, used for two months, the electricity bill from more than 200 yuan per month, down to more than 140 yuan, there is indeed a power-saving effect, this time and sold two, the unit with. Merchant service attitude is really good.	Jingdong prefecture level city in Shandong
Stowe Flagship Store	a power saver (electronics)	Plug and play, stable voltage, the screen shows when working, this winter home appliances, need electricity in many places, just buy to use, save electricity and money, with instructions	Jingdong prefecture level city in Shandong
Stowe Flagship Store	a power saver (electronics)	Goods received, do not know if it is good. When I bought it, I saw that it was a Stowe brand or company’s product. Received but not. I do not know what is the reason. Now I can only give this evaluation. I’m	Jingdong prefecture level city in Shandong
Stowe Flagship Store	a power saver (electronics)	The first time to buy online, but also look at the evaluation before buying, the quality is not bad, but also very cheap, the need for friends can rest assured to buy!	Jingdong prefecture level city in Shandong
Stowe Flagship Store	a power saver (electronics)	🌈 Great quality, bought it to my liking 💕 Awesome! ✨ Buy with confidence if you need it!	Jingdong prefecture level city in Shandong
Stowe Flagship Store	a power saver (electronics)	The quality is very good, completely exceeded the expectations, the delivery speed is very fast, the packaging is very careful and strict, very satisfied with a shopping	Jingdong prefecture level city in Shandong
Stowe Flagship Store	a power saver (electronics)	The effect is very good, the home uses less electricity is very energy efficient, cost-effective	Jingdong prefecture level city in Shandong
Stowe Flagship Store	a power saver (electronics)	Quality can’t be beat, just like the store, especially like, and the price is favorable!	Jingdong prefecture level city in Shandong
Stowe Flagship Store	a power saver (electronics)	Baby received! Very unexpected, will be back next time ~ great seller, stay tuned!	Jingdong prefecture level city in Shandong
Stowe Flagship Store	a power saver (electronics)	Follow up review after a month is more real, waiting for it	Jingdong prefecture level city in Shandong
Stowe Flagship Store	a power saver (electronics)	It is the ideal product, good quality and fast logistics, worthy of praise!	Jingdong prefecture level city in Shandong
Stowe Flagship Store	a power saver (electronics)	The saver is great, the color is right, the installer is very professional	Jingdong prefecture level city in Shandong
Stowe Flagship Store	a power saver (electronics)	The effect of the power connection is particularly obvious, it is particularly easy to use, very satisfied, the	Jingdong prefecture level city in Shandong
Stowe Flagship Store	a power saver (electronics)	Goods are very energy-saving, used to be more than two hundred degrees, now there are only a hundred or so degrees	Jingdong prefecture level city in Shandong
Stowe Flagship Store	a power saver (electronics)	Good quality, fast shipping, good results, good value for money	Jingdong prefecture level city in Shandong
Stowe Flagship Store	a power saver (electronics)	Fast shipping, fine workmanship, very good, cost-effective	Jingdong prefecture level city in Shandong
Stowe Flagship Store	a power saver (electronics)	The meter is as described, the quality is quite good, energy-saving, worth buying oh~!	Jingdong prefecture level city in Shandong
Stowe Flagship Store	a power saver (electronics)	Great value for money, very affordable,, perfect . ˆ◡ˆ.	Jingdong prefecture level city in Shandong
Stowe Flagship Store	a power saver (electronics)	Good!!! Worth recommending!	Jingdong prefecture level city in Shandong
Stowe Flagship Store	a power saver (electronics)	Great, fast logistics, great stuff, great value for money!	Jingdong prefecture level city in Shandong
Stowe Flagship Store	a power saver (electronics)	Fast logistics, very satisfied with the quality upon receipt	Jingdong prefecture level city in Shandong
Stowe Flagship Store	a power saver (electronics)	Great quality, plug and play, fast shipping!	Jingdong prefecture level city in Shandong
Stowe Flagship Store	a power saver (electronics)	Logistics received, quite satisfied	Jingdong prefecture level city in Shandong
Stowe Flagship Store	a power saver (electronics)	I don’t know if it really works	Jingdong prefecture level city in Shandong
Stowe Flagship Store	a power saver (electronics)	It’s bullshit. It’s a plug light. Don’t talk about power savings.	Jingdong prefecture level city in Shandong
Stowe Flagship Store	a power saver (electronics)	Fake and deceitful stuff that hasn’t changed at all.	Jingdong prefecture level city in Shandong
Stowe Flagship Store	a power saver (electronics)	Power-saving effect: power-saving effect is particularly obvious Cost-effective: cost-effective business Appearance and style: external small micro-work materials: fine workmanship Experience: use very good, non the first convenient	Jingdong prefecture level city in Shandong
Stowe Flagship Store	a power saver (electronics)	After using it for a few days, it feels quite good, it should be quite safe, and it also sends a plug, the merchant is good, wait for a period of time and then calculate whether it saves on the electricity bill and then add comments ha!	Jingdong prefecture level city in Shandong
Stowe Flagship Store	a power saver (electronics)	My friend recommended to me, said the effect is very good, I bought two, this month less money, really can, just buy I do not believe, but has proved to have effect	Jingdong prefecture level city in Shandong

Stowe Flagship Store	a power saver (electronics)	Just bought back, already used, these days to see the effect of it, and so have the effect will also come back to give positive feedback, the price is also super cheap!	Jingdong prefecture level city in Shandong
Stowe Flagship Store	a power saver (electronics)	It does work well, the store uses a lot of electricity, this time to buy back a few, each place that uses a lot of electricity to insert one. Summer is here, the electricity is too strong.	Jingdong prefecture level city in Shandong
Stowe Flagship Store	a power saver (electronics)	Power saving treasure received, as for the effect, according to the instructions written, a short period of time can not be seen, at least 2-3 months before the real effect, then follow up evaluation	Jingdong prefecture level city in Shandong
Stowe Flagship Store	a power saver (electronics)	The goods have just been received and have not been used yet looking forward to the results.	Jingdong prefecture level city in Shandong
Stowe Flagship Store	a power saver (electronics)	Good installation works great	Jingdong prefecture level city in Shandong
Stowe Flagship Store	a power saver (electronics)	The logistics is super super fast! The quality is also very good, indeed value for money	Jingdong prefecture level city in Shandong
Stowe Flagship Store	a power saver (electronics)	Has been used for a period of time before coming to comment on, really good, air conditioning a day open non-stop, pay the electricity bill when the heartache, with this, the home electricity is not at all worried about the bill out of contrast to save a lot of preparation in the hands of a few, each room An a	Jingdong prefecture level city in Shandong
Stowe Flagship Store	a power saver (electronics)	Saving device received, really good, with the air conditioning tried, an hour without a saver with 2.9 degrees, with a saver an hour with 1.1 degrees, the beginning of not quite believe that this thing, the actual test to know that it is really useful, and intend to buy another.	Jingdong prefecture level city in Shandong
Stowe Flagship Store	a power saver (electronics)	Power saving effect: this energy saver is still useful, day trial for three days, every day than not installed before the reduction of two dollars a day, every day to open the bedroom to open the air conditioning before fifteen dollars now thirteen dollars a day. Still a little use.	Jingdong prefecture level city in Shandong
Stowe Flagship Store	a power saver (electronics)	Originally bought for home use, used for two months, the electricity bill from more than 200 yuan per month, down to more than 140 yuan, there is indeed a power-saving effect, this time and sold two, the unit with. Merchant service attitude is really good.	Jingdong prefecture level city in Shandong
Stowe Flagship Store	a power saver (electronics)	Plug and play, stable voltage, the screen shows when working, this winter home appliances, need electricity in many places, just buy to use, save electricity and money, with instructions	Jingdong prefecture level city in Shandong
Stowe Flagship Store	a power saver (electronics)	Goods received, do not know if it is good. When I bought it, I saw that it was a Stowe brand or company's product. Received but not. I do not know what is the reason. Now I can only give this evaluation. I'm	Jingdong prefecture level city in Shandong
Stowe Flagship Store	a power saver (electronics)	The first time to buy online, but also look at the evaluation before buying, the quality is not bad, but also very cheap, the need for friends can rest assured to buy!	Jingdong prefecture level city in Shandong
Stowe Flagship Store	a power saver (electronics)	🌈 Great quality, bought it to my liking 💞 Awesome! ✨ Buy with confidence if you need it!	Jingdong prefecture level city in Shandong
Stowe Flagship Store	a power saver (electronics)	The quality is very good, completely exceeded the expectations, the delivery speed is very fast, the packaging is very careful and strict, very satisfied with a shopping	Jingdong prefecture level city in Shandong
Stowe Flagship Store	a power saver (electronics)	The effect is very good, the home uses less electricity is very energy efficient, cost-effective	Jingdong prefecture level city in Shandong
Stowe Flagship Store	a power saver (electronics)	Quality can't be beat, just like the store, especially like, and the price is favorable!	Jingdong prefecture level city in Shandong
Stowe Flagship Store	a power saver (electronics)	Baby received! Very unexpected, will be back next time ~ great seller, stay tuned!	Jingdong prefecture level city in Shandong
Stowe Flagship Store	a power saver (electronics)	Follow up review after a month is more real, waiting for it	Jingdong prefecture level city in Shandong
Stowe Flagship Store	a power saver (electronics)	It is the ideal product, good quality and fast logistics, worthy of praise!	Jingdong prefecture level city in Shandong
Stowe Flagship Store	a power saver (electronics)	The saver is great, the color is right, the installer is very professional	Jingdong prefecture level city in Shandong
Stowe Flagship Store	a power saver (electronics)	The effect of the power connection is particularly obvious, it is particularly easy to use, very satisfied, the	Jingdong prefecture level city in Shandong
Stowe Flagship Store	a power saver (electronics)	Goods are very energy-saving, used to be more than two hundred degrees, now there are only a hundred or so degrees	Jingdong prefecture level city in Shandong
Stowe Flagship Store	a power saver (electronics)	Good quality, fast shipping, good results, good value for money	Jingdong prefecture level city in Shandong
Stowe Flagship Store	a power saver (electronics)	Fast shipping, fine workmanship, very good, cost-effective	Jingdong prefecture level city in Shandong
Stowe Flagship Store	a power saver (electronics)	The meter is as described, the quality is quite good, energy-saving, worth buying oh~!	Jingdong prefecture level city in Shandong
Stowe Flagship Store	a power saver (electronics)	Great value for money, very affordable,, perfect . ˆ◡ˆ.	Jingdong prefecture level city in Shandong
Stowe Flagship Store	a power saver (electronics)	Good!!! Worth recommending!	Jingdong prefecture level city in Shandong
Stowe Flagship Store	a power saver (electronics)	Great, fast logistics, great stuff, great value for money!	Jingdong prefecture level city in Shandong
Stowe Flagship Store	a power saver (electronics)	Fast logistics, very satisfied with the quality upon receipt	Jingdong prefecture level city in Shandong
Stowe Flagship Store	a power saver (electronics)	Great quality, plug and play, fast shipping!	Jingdong prefecture level city in Shandong
Stowe Flagship Store	a power saver (electronics)	Logistics received, quite satisfied	Jingdong prefecture level city in Shandong
Stowe Flagship Store	a power saver (electronics)	I don't know if it really works	Jingdong prefecture level city in Shandong
Stowe Flagship Store	a power saver (electronics)	It's bullshit. It's a plug light. Don't talk about power savings.	Jingdong prefecture level city in Shandong
Stowe Flagship Store	a power saver (electronics)	Fake and deceitful stuff that hasn't changed at all.	Jingdong prefecture level city in Shandong
Stowe Flagship Store	a power saver (electronics)	Power-saving effect: power-saving effect is particularly obvious Cost-effective: cost-effective business Appearance and style: external small micro-work materials: fine workmanship Experience: use very good, non the first convenient	Jingdong prefecture level city in Shandong
Stowe Flagship Store	a power saver (electronics)	After using it for a few days, it feels quite good, it should be quite safe, and it also sends a plug, the merchant is good, wait for a period of time and then calculate whether it saves on the electricity bill and then add comments ha!	Jingdong prefecture level city in Shandong
Stowe Flagship Store	a power saver (electronics)	My friend recommended to me, said the effect is very good, I bought two, this month less money, really can, just buy I do not believe, but has proved to have effect	Jingdong prefecture level city in Shandong
Stowe Flagship Store	a power saver (electronics)	Just bought back, already used, these days to see the effect of it, and so have the effect will also come back to give positive feedback, the price is also super cheap!	Jingdong prefecture level city in Shandong
Stowe Flagship Store	a power saver (electronics)	It does work well, the store uses a lot of electricity, this time to buy back a few, each place that uses a lot of electricity to insert one. Summer is here, the electricity is too strong.	Jingdong prefecture level city in Shandong
Stowe Flagship Store	a power saver (electronics)	Power saving treasure received, as for the effect, according to the instructions written, a short period of time can not be seen, at least 2-3 months before the real effect, then follow up evaluation	Jingdong prefecture level city in Shandong
Stowe Flagship Store	a power saver (electronics)	The goods have just been received and have not been used yet looking forward to the results.	Jingdong prefecture level city in Shandong
Stowe Flagship Store	a power saver (electronics)	Good installation works great	Jingdong prefecture level city in Shandong
Stowe Flagship Store	a power saver (electronics)	The logistics is super super fast! The quality is also very good, indeed value for money	Jingdong prefecture level city in Shandong
Stowe Flagship Store	a power saver (electronics)	Has been used for a period of time before coming to comment on, really good, air conditioning a day open non-stop, pay the electricity bill when the heartache, with this, the home electricity is not at all worried about the bill out of contrast to save a lot of preparation in the hands of a few, each room An a	Jingdong prefecture level city in Shandong
Stowe Flagship Store	a power saver (electronics)	Saving device received, really good, with the air conditioning tried, an hour without a saver with 2.9 degrees, with a saver an hour with 1.1 degrees, the beginning of not quite believe that this thing, the actual test to know that it is really useful, and intend to buy another.	Jingdong prefecture level city in Shandong
Stowe Flagship Store	a power saver (electronics)	Power saving effect: this energy saver is still useful, day trial for three days, every day than not installed before the reduction of two dollars a day, every day to open the bedroom to open the air conditioning before fifteen dollars now thirteen dollars a day. Still a little use.	Jingdong prefecture level city in Shandong

Stowe Flagship Store	a power saver (electronics)	Originally bought for home use, used for two months, the electricity bill from more than 200 yuan per month, down to more than 140 yuan, there is indeed a power-saving effect, this time and sold two, the unit with. Merchant service attitude is really good.	Jingdong prefecture level city in Shandong
Stowe Flagship Store	a power saver (electronics)	Plug and play, stable voltage, the screen shows when working, this winter home appliances, need electricity in many places, just buy to use, save electricity and money, with instructions	Jingdong prefecture level city in Shandong
Stowe Flagship Store	a power saver (electronics)	Goods received, do not know if it is good. When I bought it, I saw that it was a Stowe brand or company's product. Received but not. I do not know what is the reason. Now I can only give this evaluation. I'm	Jingdong prefecture level city in Shandong
Stowe Flagship Store	a power saver (electronics)	The first time to buy online, but also look at the evaluation before buying, the quality is not bad, but also very cheap, the need for friends can rest assured to buy!	Jingdong prefecture level city in Shandong
Stowe Flagship Store	a power saver (electronics)	🌈 Great quality, bought it to my liking 💗 Awesome! ✨ Buy with confidence if you need it!	Jingdong prefecture level city in Shandong
Stowe Flagship Store	a power saver (electronics)	The quality is very good, completely exceeded the expectations, the delivery speed is very fast, the packaging is very careful and strict, very satisfied with a shopping	Jingdong prefecture level city in Shandong
Stowe Flagship Store	a power saver (electronics)	The effect is very good, the home uses less electricity is very energy efficient, cost-effective	Jingdong prefecture level city in Shandong
Stowe Flagship Store	a power saver (electronics)	Quality can't be beat, just like the store, especially like, and the price is favorable!	Jingdong prefecture level city in Shandong
Stowe Flagship Store	a power saver (electronics)	Baby received! Very unexpected, will be back next time ~ great seller, stay tuned!	Jingdong prefecture level city in Shandong
Stowe Flagship Store	a power saver (electronics)	Follow up review after a month is more real, waiting for it	Jingdong prefecture level city in Shandong
Stowe Flagship Store	a power saver (electronics)	It is the ideal product, good quality and fast logistics, worthy of praise!	Jingdong prefecture level city in Shandong
Stowe Flagship Store	a power saver (electronics)	The saver is great, the color is right, the installer is very professional	Jingdong prefecture level city in Shandong
Stowe Flagship Store	a power saver (electronics)	The effect of the power connection is particularly obvious, it is particularly easy to use, very satisfied, the	Jingdong prefecture level city in Shandong
Stowe Flagship Store	a power saver (electronics)	Goods are very energy-saving, used to be more than two hundred degrees, now there are only a hundred or so degrees	Jingdong prefecture level city in Shandong
Stowe Flagship Store	a power saver (electronics)	Good quality, fast shipping, good results, good value for money	Jingdong prefecture level city in Shandong
Stowe Flagship Store	a power saver (electronics)	Fast shipping, fine workmanship, very good, cost-effective	Jingdong prefecture level city in Shandong
Stowe Flagship Store	a power saver (electronics)	The meter is as described, the quality is quite good, energy-saving, worth buying oh~!	Jingdong prefecture level city in Shandong
Stowe Flagship Store	a power saver (electronics)	Great value for money, very affordable,, perfect . ˆ◡ˆ.	Jingdong prefecture level city in Shandong
Stowe Flagship Store	a power saver (electronics)	Good!!! Worth recommending!	Jingdong prefecture level city in Shandong
Stowe Flagship Store	a power saver (electronics)	Great, fast logistics, great stuff, great value for money!	Jingdong prefecture level city in Shandong
Stowe Flagship Store	a power saver (electronics)	Fast logistics, very satisfied with the quality upon receipt	Jingdong prefecture level city in Shandong
Stowe Flagship Store	a power saver (electronics)	Great quality, plug and play, fast shipping!	Jingdong prefecture level city in Shandong
Stowe Flagship Store	a power saver (electronics)	Logistics received, quite satisfied	Jingdong prefecture level city in Shandong
Stowe Flagship Store	a power saver (electronics)	I don't know if it really works	Jingdong prefecture level city in Shandong
Stowe Flagship Store	a power saver (electronics)	It's bullshit. It's a plug light. Don't talk about power savings.	Jingdong prefecture level city in Shandong
Stowe Flagship Store	a power saver (electronics)	Fake and deceitful stuff that hasn't changed at all.	Jingdong prefecture level city in Shandong
Stowe Flagship Store	a power saver (electronics)	Power-saving effect: power-saving effect is particularly obvious Cost-effective: cost-effective business Appearance and style: external small micro-work materials: fine workmanship Experience: use very good, non the first convenient	Jingdong prefecture level city in Shandong
Stowe Flagship Store	a power saver (electronics)	After using it for a few days, it feels quite good, it should be quite safe, and it also sends a plug, the merchant is good, wait for a period of time and then calculate whether it saves on the electricity bill and then add comments ha!	Jingdong prefecture level city in Shandong
Stowe Flagship Store	a power saver (electronics)	My friend recommended to me, said the effect is very good, I bought two, this month less money, really can, just buy I do not believe, but has proved to have effect	Jingdong prefecture level city in Shandong
Stowe Flagship Store	a power saver (electronics)	Just bought back, already used, these days to see the effect of it, and so have the effect will also come back to give positive feedback, the price is also super cheap!	Jingdong prefecture level city in Shandong
Stowe Flagship Store	a power saver (electronics)	It does work well, the store uses a lot of electricity, this time to buy back a few, each place that uses a lot of electricity to insert one. Summer is here, the electricity is too strong.	Jingdong prefecture level city in Shandong
Stowe Flagship Store	a power saver (electronics)	Power saving treasure received, as for the effect, according to the instructions written, a short period of time can not be seen, at least 2-3 months before the real effect, then follow up evaluation	Jingdong prefecture level city in Shandong
Stowe Flagship Store	a power saver (electronics)	The goods have just been received and have not been used yet looking forward to the results.	Jingdong prefecture level city in Shandong
Stowe Flagship Store	a power saver (electronics)	Good installation works great	Jingdong prefecture level city in Shandong
Stowe Flagship Store	a power saver (electronics)	The logistics is super super fast! The quality is also very good, indeed value for money	Jingdong prefecture level city in Shandong
Stowe Flagship Store	a power saver (electronics)	Has been used for a period of time before coming to comment on, really good, air conditioning a day open non-stop, pay the electricity bill when the heartache, with this, the home electricity is not at all worried about the bill out of contrast to save a lot of preparation in the hands of a few, each room An a	Jingdong prefecture level city in Shandong
Stowe Flagship Store	a power saver (electronics)	Saving device received, really good, with the air conditioning tried, an hour without a saver with 2.9 degrees, with a saver an hour with 1.1 degrees, the beginning of not quite believe that this thing, the actual test to know that it is really useful, and intend to buy another.	Jingdong prefecture level city in Shandong
Stowe Flagship Store	a power saver (electronics)	Power saving effect: this energy saver is still useful, day trial for three days, every day than not installed before the reduction of two dollars a day, every day to open the bedroom to open the air conditioning before fifteen dollars now thirteen dollars a day. Still a little use.	Jingdong prefecture level city in Shandong
Stowe Flagship Store	a power saver (electronics)	Originally bought for home use, used for two months, the electricity bill from more than 200 yuan per month, down to more than 140 yuan, there is indeed a power-saving effect, this time and sold two, the unit with. Merchant service attitude is really good.	Jingdong prefecture level city in Shandong
Stowe Flagship Store	a power saver (electronics)	Plug and play, stable voltage, the screen shows when working, this winter home appliances, need electricity in many places, just buy to use, save electricity and money, with instructions	Jingdong prefecture level city in Shandong
Stowe Flagship Store	a power saver (electronics)	Goods received, do not know if it is good. When I bought it, I saw that it was a Stowe brand or company's product. Received but not. I do not know what is the reason. Now I can only give this evaluation. I'm	Jingdong prefecture level city in Shandong
Stowe Flagship Store	a power saver (electronics)	The first time to buy online, but also look at the evaluation before buying, the quality is not bad, but also very cheap, the need for friends can rest assured to buy!	Jingdong prefecture level city in Shandong
Stowe Flagship Store	a power saver (electronics)	🌈 Great quality, bought it to my liking 💗 Awesome! ✨ Buy with confidence if you need it!	Jingdong prefecture level city in Shandong
Stowe Flagship Store	a power saver (electronics)	The quality is very good, completely exceeded the expectations, the delivery speed is very fast, the packaging is very careful and strict, very satisfied with a shopping	Jingdong prefecture level city in Shandong
Stowe Flagship Store	a power saver (electronics)	The effect is very good, the home uses less electricity is very energy efficient, cost-effective	Jingdong prefecture level city in Shandong
Stowe Flagship Store	a power saver (electronics)	Quality can't be beat, just like the store, especially like, and the price is favorable!	Jingdong prefecture level city in Shandong
Stowe Flagship Store	a power saver (electronics)	Baby received! Very unexpected, will be back next time ~ great seller, stay tuned!	Jingdong prefecture level city in Shandong
Stowe Flagship Store	a power saver (electronics)	Follow up review after a month is more real, waiting for it	Jingdong prefecture level city in Shandong

Stowe Flagship Store	a power saver (electronics)	It is the ideal product, good quality and fast logistics, worthy of praise!	Jingdong prefecture level city in Shandong
Stowe Flagship Store	a power saver (electronics)	The saver is great, the color is right, the installer is very professional	Jingdong prefecture level city in Shandong
Stowe Flagship Store	a power saver (electronics)	The effect of the power connection is particularly obvious, it is particularly easy to use, very satisfied, the	Jingdong prefecture level city in Shandong
Stowe Flagship Store	a power saver (electronics)	Goods are very energy-saving, used to be more than two hundred degrees, now there are only a hundred or so degrees	Jingdong prefecture level city in Shandong
Stowe Flagship Store	a power saver (electronics)	Good quality, fast shipping, good results, good value for money	Jingdong prefecture level city in Shandong
Stowe Flagship Store	a power saver (electronics)	Fast shipping, fine workmanship, very good, cost-effective	Jingdong prefecture level city in Shandong
Stowe Flagship Store	a power saver (electronics)	The meter is as described, the quality is quite good, energy-saving, worth buying oh~!	Jingdong prefecture level city in Shandong
Stowe Flagship Store	a power saver (electronics)	Great value for money, very affordable,, perfect . ˆ◡ˆ.	Jingdong prefecture level city in Shandong
Stowe Flagship Store	a power saver (electronics)	Good!!! Worth recommending!	Jingdong prefecture level city in Shandong
Stowe Flagship Store	a power saver (electronics)	Great, fast logistics, great stuff, great value for money!	Jingdong prefecture level city in Shandong
Stowe Flagship Store	a power saver (electronics)	Fast logistics, very satisfied with the quality upon receipt	Jingdong prefecture level city in Shandong
Stowe Flagship Store	a power saver (electronics)	Great quality, plug and play, fast shipping!	Jingdong prefecture level city in Shandong
Stowe Flagship Store	a power saver (electronics)	Logistics received, quite satisfied	Jingdong prefecture level city in Shandong
Stowe Flagship Store	a power saver (electronics)	I don't know if it really works	Jingdong prefecture level city in Shandong
Stowe Flagship Store	a power saver (electronics)	It's bullshit. It's a plug light. Don't talk about power savings.	Jingdong prefecture level city in Shandong
Stowe Flagship Store	a power saver (electronics)	Fake and deceitful stuff that hasn't changed at all.	Jingdong prefecture level city in Shandong
Stowe Flagship Store	a power saver (electronics)	Power-saving effect: power-saving effect is particularly obvious Cost-effective: cost-effective business Appearance and style: external small micro-work materials: fine workmanship Experience: use very good, non the first convenient	Jingdong prefecture level city in Shandong
Stowe Flagship Store	a power saver (electronics)	After using it for a few days, it feels quite good, it should be quite safe, and it also sends a plug, the merchant is good, wait for a period of time and then calculate whether it saves on the electricity bill and then add comments ha!	Jingdong prefecture level city in Shandong
Stowe Flagship Store	a power saver (electronics)	My friend recommended to me, said the effect is very good, I bought two, this month less money, really can, just buy I do not believe, but has proved to have effect	Jingdong prefecture level city in Shandong
Stowe Flagship Store	a power saver (electronics)	Just bought back, already used, these days to see the effect of it, and so have the effect will also come back to give positive feedback, the price is also super cheap!	Jingdong prefecture level city in Shandong
Stowe Flagship Store	a power saver (electronics)	It does work well, the store uses a lot of electricity, this time to buy back a few, each place that uses a lot of electricity to insert one. Summer is here, the electricity is too strong.	Jingdong prefecture level city in Shandong
Stowe Flagship Store	a power saver (electronics)	Power saving treasure received, as for the effect, according to the instructions written, a short period of time can not be seen, at least 2-3 months before the real effect, then follow up evaluation	Jingdong prefecture level city in Shandong
Stowe Flagship Store	a power saver (electronics)	The goods have just been received and have not been used yet looking forward to the results.	Jingdong prefecture level city in Shandong
Stowe Flagship Store	a power saver (electronics)	Good installation works great	Jingdong prefecture level city in Shandong
Stowe Flagship Store	a power saver (electronics)	The logistics is super super fast! The quality is also very good, indeed value for money	Jingdong prefecture level city in Shandong
Stowe Flagship Store	a power saver (electronics)	Has been used for a period of time before coming to comment on, really good, air conditioning a day open non-stop, pay the electricity bill when the heartache, with this, the home electricity is not at all worried about the bill out of contrast to save a lot of preparation in the hands of a few, each room An a	Jingdong prefecture level city in Shandong
Stowe Flagship Store	a power saver (electronics)	Saving device received, really good, with the air conditioning tried, an hour without a saver with 2.9 degrees, with a saver an hour with 1.1 degrees, the beginning of not quite believe that this thing, the actual test to know that it is really useful, and intend to buy another.	Jingdong prefecture level city in Shandong
Stowe Flagship Store	a power saver (electronics)	Power saving effect: this energy saver is still useful, day trial for three days, every day than not installed before the reduction of two dollars a day, every day to open the bedroom to open the air conditioning before fifteen dollars now thirteen dollars a day. Still a little use.	Jingdong prefecture level city in Shandong
Stowe Flagship Store	a power saver (electronics)	Originally bought for home use, used for two months, the electricity bill from more than 200 yuan per month, down to more than 140 yuan, there is indeed a power-saving effect, this time and sold two, the unit with. Merchant service attitude is really good.	Jingdong prefecture level city in Shandong
Stowe Flagship Store	a power saver (electronics)	Plug and play, stable voltage, the screen shows when working, this winter home appliances, need electricity in many places, just buy to use, save electricity and money, with instructions	Jingdong prefecture level city in Shandong
Stowe Flagship Store	a power saver (electronics)	Goods received, do not know if it is good. When I bought it, I saw that it was a Stowe brand or company's product. Received but not. I do not know what is the reason. Now I can only give this evaluation. I'm	Jingdong prefecture level city in Shandong
Stowe Flagship Store	a power saver (electronics)	The first time to buy online, but also look at the evaluation before buying, the quality is not bad, but also very cheap, the need for friends can rest assured to buy!	Jingdong prefecture level city in Shandong
Stowe Flagship Store	a power saver (electronics)	🌈 Great quality, bought it to my liking 💝 Awesome! ✨ Buy with confidence if you need it!	Jingdong prefecture level city in Shandong
Stowe Flagship Store	a power saver (electronics)	The quality is very good, completely exceeded the expectations, the delivery speed is very fast, the packaging is very careful and strict, very satisfied with a shopping	Jingdong prefecture level city in Shandong
Stowe Flagship Store	a power saver (electronics)	The effect is very good, the home uses less electricity is very energy efficient, cost-effective	Jingdong prefecture level city in Shandong
Stowe Flagship Store	a power saver (electronics)	Quality can't be beat, just like the store, especially like, and the price is favorable!	Jingdong prefecture level city in Shandong
Stowe Flagship Store	a power saver (electronics)	Baby received! Very unexpected, will be back next time ~ great seller, stay tuned!	Jingdong prefecture level city in Shandong
Stowe Flagship Store	a power saver (electronics)	Follow up review after a month is more real, waiting for it	Jingdong prefecture level city in Shandong
Stowe Flagship Store	a power saver (electronics)	It is the ideal product, good quality and fast logistics, worthy of praise!	Jingdong prefecture level city in Shandong
Stowe Flagship Store	a power saver (electronics)	The saver is great, the color is right, the installer is very professional	Jingdong prefecture level city in Shandong
Stowe Flagship Store	a power saver (electronics)	The effect of the power connection is particularly obvious, it is particularly easy to use, very satisfied, the	Jingdong prefecture level city in Shandong
Stowe Flagship Store	a power saver (electronics)	Goods are very energy-saving, used to be more than two hundred degrees, now there are only a hundred or so degrees	Jingdong prefecture level city in Shandong
Stowe Flagship Store	a power saver (electronics)	Good quality, fast shipping, good results, good value for money	Jingdong prefecture level city in Shandong
Stowe Flagship Store	a power saver (electronics)	Fast shipping, fine workmanship, very good, cost-effective	Jingdong prefecture level city in Shandong
Stowe Flagship Store	a power saver (electronics)	The meter is as described, the quality is quite good, energy-saving, worth buying oh~!	Jingdong prefecture level city in Shandong
Stowe Flagship Store	a power saver (electronics)	Great value for money, very affordable,, perfect . ˆ◡ˆ.	Jingdong prefecture level city in Shandong
Stowe Flagship Store	a power saver (electronics)	Good!!! Worth recommending!	Jingdong prefecture level city in Shandong
Stowe Flagship Store	a power saver (electronics)	Great, fast logistics, great stuff, great value for money!	Jingdong prefecture level city in Shandong

Stowe Flagship Store	a power saver (electronics)	Fast logistics, very satisfied with the quality upon receipt	Jingdong prefecture level city in Shandong
Stowe Flagship Store	a power saver (electronics)	Great quality, plug and play, fast shipping!	Jingdong prefecture level city in Shandong
Stowe Flagship Store	a power saver (electronics)	Logistics received, quite satisfied	Jingdong prefecture level city in Shandong
Stowe Flagship Store	a power saver (electronics)	I don't know if it really works	Jingdong prefecture level city in Shandong
Stowe Flagship Store	a power saver (electronics)	It's bullshit. It's a plug light. Don't talk about power savings.	Jingdong prefecture level city in Shandong
Stowe Flagship Store	a power saver (electronics)	Fake and deceitful stuff that hasn't changed at all.	Jingdong prefecture level city in Shandong
Stowe Flagship Store	a power saver (electronics)	Power-saving effect: power-saving effect is particularly obvious Cost-effective: cost-effective business Appearance and style: external small micro-work materials: fine workmanship Experience: use very good, non the first convenient	Jingdong prefecture level city in Shandong
Stowe Flagship Store	a power saver (electronics)	After using it for a few days, it feels quite good, it should be quite safe, and it also sends a plug, the merchant is good, wait for a period of time and then calculate whether it saves on the electricity bill and then add comments ha!	Jingdong prefecture level city in Shandong
Stowe Flagship Store	a power saver (electronics)	My friend recommended to me, said the effect is very good, I bought two, this month less money, really can, just buy I do not believe, but has proved to have effect	Jingdong prefecture level city in Shandong
Stowe Flagship Store	a power saver (electronics)	Just bought back, already used, these days to see the effect of it, and so have the effect will also come back to give positive feedback, the price is also super cheap!	Jingdong prefecture level city in Shandong
Stowe Flagship Store	a power saver (electronics)	It does work well, the store uses a lot of electricity, this time to buy back a few, each place that uses a lot of electricity to insert one. Summer is here, the electricity is too strong.	Jingdong prefecture level city in Shandong
Stowe Flagship Store	a power saver (electronics)	Power saving treasure received, as for the effect, according to the instructions written, a short period of time can not be seen, at least 2-3 months before the real effect, then follow up evaluation	Jingdong prefecture level city in Shandong
Stowe Flagship Store	a power saver (electronics)	The goods have just been received and have not been used yet looking forward to the results.	Jingdong prefecture level city in Shandong
Stowe Flagship Store	a power saver (electronics)	Good installation works great	Jingdong prefecture level city in Shandong
Stowe Flagship Store	a power saver (electronics)	The logistics is super super fast! The quality is also very good, indeed value for money	Jingdong prefecture level city in Shandong
Stowe Flagship Store	a power saver (electronics)	Has been used for a period of time before coming to comment on, really good, air conditioning a day open non-stop, pay the electricity bill when the heartache, with this, the home electricity is not at all worried about the bill out of contrast to save a lot of preparation in the hands of a few, each room An a	Jingdong prefecture level city in Shandong
Stowe Flagship Store	a power saver (electronics)	Saving device received, really good, with the air conditioning tried, an hour without a saver with 2.9 degrees, with a saver an hour with 1.1 degrees, the beginning of not quite believe that this thing, the actual test to know that it is really useful, and intend to buy another.	Jingdong prefecture level city in Shandong
Stowe Flagship Store	a power saver (electronics)	Power saving effect: this energy saver is still useful, day trial for three days, every day than not installed before the reduction of two dollars a day, every day to open the bedroom to open the air conditioning before fifteen dollars now thirteen dollars a day. Still a little use.	Jingdong prefecture level city in Shandong
Stowe Flagship Store	a power saver (electronics)	Originally bought for home use, used for two months, the electricity bill from more than 200 yuan per month, down to more than 140 yuan, there is indeed a power-saving effect, this time and sold two, the unit with. Merchant service attitude is really good.	Jingdong prefecture level city in Shandong
Stowe Flagship Store	a power saver (electronics)	Plug and play, stable voltage, the screen shows when working, this winter home appliances, need electricity in many places, just buy to use, save electricity and money, with instructions	Jingdong prefecture level city in Shandong
Stowe Flagship Store	a power saver (electronics)	Goods received, do not know if it is good. When I bought it, I saw that it was a Stowe brand or company's product. Received but not. I do not know what is the reason. Now I can only give this evaluation. I'm	Jingdong prefecture level city in Shandong
Stowe Flagship Store	a power saver (electronics)	The first time to buy online, but also look at the evaluation before buying, the quality is not bad, but also very cheap, the need for friends can rest assured to buy!	Jingdong prefecture level city in Shandong
Stowe Flagship Store	a power saver (electronics)	🌈 Great quality, bought it to my liking 💝 Awesome! ✨ Buy with confidence if you need it!	Jingdong prefecture level city in Shandong
Stowe Flagship Store	a power saver (electronics)	The quality is very good, completely exceeded the expectations, the delivery speed is very fast, the packaging is very careful and strict, very satisfied with a shopping	Jingdong prefecture level city in Shandong
Stowe Flagship Store	a power saver (electronics)	The effect is very good, the home uses less electricity is very energy efficient, cost-effective	Jingdong prefecture level city in Shandong
Stowe Flagship Store	a power saver (electronics)	Quality can't be beat, just like the store, especially like, and the price is favorable!	Jingdong prefecture level city in Shandong
Stowe Flagship Store	a power saver (electronics)	Baby received! Very unexpected, will be back next time ~ great seller, stay tuned!	Jingdong prefecture level city in Shandong
Stowe Flagship Store	a power saver (electronics)	Follow up review after a month is more real, waiting for it	Jingdong prefecture level city in Shandong
Stowe Flagship Store	a power saver (electronics)	It is the ideal product, good quality and fast logistics, worthy of praise!	Jingdong prefecture level city in Shandong
Stowe Flagship Store	a power saver (electronics)	The saver is great, the color is right, the installer is very professional	Jingdong prefecture level city in Shandong
Stowe Flagship Store	a power saver (electronics)	The effect of the power connection is particularly obvious, it is particularly easy to use, very satisfied, the	Jingdong prefecture level city in Shandong
Stowe Flagship Store	a power saver (electronics)	Goods are very energy-saving, used to be more than two hundred degrees, now there are only a hundred or so degrees	Jingdong prefecture level city in Shandong
Stowe Flagship Store	a power saver (electronics)	Good quality, fast shipping, good results, good value for money	Jingdong prefecture level city in Shandong
Stowe Flagship Store	a power saver (electronics)	Fast shipping, fine workmanship, very good, cost-effective	Jingdong prefecture level city in Shandong
Stowe Flagship Store	a power saver (electronics)	The meter is as described, the quality is quite good, energy-saving, worth buying oh~!	Jingdong prefecture level city in Shandong
Stowe Flagship Store	a power saver (electronics)	Great value for money, very affordable,, perfect . ^◡^.	Jingdong prefecture level city in Shandong
Stowe Flagship Store	a power saver (electronics)	Good!!! Worth recommending!	Jingdong prefecture level city in Shandong
Stowe Flagship Store	a power saver (electronics)	Great, fast logistics, great stuff, great value for money!	Jingdong prefecture level city in Shandong
Stowe Flagship Store	a power saver (electronics)	Fast logistics, very satisfied with the quality upon receipt	Jingdong prefecture level city in Shandong
Stowe Flagship Store	a power saver (electronics)	Great quality, plug and play, fast shipping!	Jingdong prefecture level city in Shandong
Stowe Flagship Store	a power saver (electronics)	Logistics received, quite satisfied	Jingdong prefecture level city in Shandong
Stowe Flagship Store	a power saver (electronics)	I don't know if it really works	Jingdong prefecture level city in Shandong
Stowe Flagship Store	a power saver (electronics)	It's bullshit. It's a plug light. Don't talk about power savings.	Jingdong prefecture level city in Shandong
Stowe Flagship Store	a power saver (electronics)	Fake and deceitful stuff that hasn't changed at all.	Jingdong prefecture level city in Shandong
Stowe Flagship Store	a power saver (electronics)	Power-saving effect: power-saving effect is particularly obvious Cost-effective: cost-effective business Appearance and style: external small micro-work materials: fine workmanship Experience: use very good, non the first convenient	Jingdong prefecture level city in Shandong
Stowe Flagship Store	a power saver (electronics)	After using it for a few days, it feels quite good, it should be quite safe, and it also sends a plug, the merchant is good, wait for a period of time and then calculate whether it saves on the electricity bill and then add comments ha!	Jingdong prefecture level city in Shandong
Stowe Flagship Store	a power saver (electronics)	My friend recommended to me, said the effect is very good, I bought two, this month less money, really can, just buy I do not believe, but has proved to have effect	Jingdong prefecture level city in Shandong

Stowe Flagship Store	a power saver (electronics)	Just bought back, already used, these days to see the effect of it, and so have the effect will also come back to give positive feedback, the price is also super cheap!	Jingdong prefecture level city in Shandong
Stowe Flagship Store	a power saver (electronics)	It does work well, the store uses a lot of electricity, this time to buy back a few, each place that uses a lot of electricity to insert one. Summer is here, the electricity is too strong.	Jingdong prefecture level city in Shandong
Stowe Flagship Store	a power saver (electronics)	Power saving treasure received, as for the effect, according to the instructions written, a short period of time can not be seen, at least 2-3 months before the real effect, then follow up evaluation	Jingdong prefecture level city in Shandong
Stowe Flagship Store	a power saver (electronics)	The goods have just been received and have not been used yet looking forward to the results.	Jingdong prefecture level city in Shandong
Stowe Flagship Store	a power saver (electronics)	Good installation works great	Jingdong prefecture level city in Shandong
Stowe Flagship Store	a power saver (electronics)	The logistics is super super fast! The quality is also very good, indeed value for money	Jingdong prefecture level city in Shandong
Stowe Flagship Store	a power saver (electronics)	Has been used for a period of time before coming to comment on, really good, air conditioning a day open non-stop, pay the electricity bill when the heartache, with this, the home electricity is not at all worried about the bill out of contrast to save a lot of preparation in the hands of a few, each room An a	Jingdong prefecture level city in Shandong
Stowe Flagship Store	a power saver (electronics)	Saving device received, really good, with the air conditioning tried, an hour without a saver with 2.9 degrees, with a saver an hour with 1.1 degrees, the beginning of not quite believe that this thing, the actual test to know that it is really useful, and intend to buy another.	Jingdong prefecture level city in Shandong
Stowe Flagship Store	a power saver (electronics)	Power saving effect: this energy saver is still useful, day trial for three days, every day than not installed before the reduction of two dollars a day, every day to open the bedroom to open the air conditioning before fifteen dollars now thirteen dollars a day. Still a little use.	Jingdong prefecture level city in Shandong
Stowe Flagship Store	a power saver (electronics)	Originally bought for home use, used for two months, the electricity bill from more than 200 yuan per month, down to more than 140 yuan, there is indeed a power-saving effect, this time and sold two, the unit with. Merchant service attitude is really good.	Jingdong prefecture level city in Shandong
Stowe Flagship Store	a power saver (electronics)	Plug and play, stable voltage, the screen shows when working, this winter home appliances, need electricity in many places, just buy to use, save electricity and money, with instructions	Jingdong prefecture level city in Shandong
Stowe Flagship Store	a power saver (electronics)	Goods received, do not know if it is good. When I bought it, I saw that it was a Stowe brand or company's product. Received but not. I do not know what is the reason. Now I can only give this evaluation. I'm	Jingdong prefecture level city in Shandong
Stowe Flagship Store	a power saver (electronics)	The first time to buy online, but also look at the evaluation before buying, the quality is not bad, but also very cheap, the need for friends can rest assured to buy!	Jingdong prefecture level city in Shandong
Stowe Flagship Store	a power saver (electronics)	🌈 Great quality, bought it to my liking 💞 Awesome! ✨ Buy with confidence if you need it!	Jingdong prefecture level city in Shandong
Stowe Flagship Store	a power saver (electronics)	The quality is very good, completely exceeded the expectations, the delivery speed is very fast, the packaging is very careful and strict, very satisfied with a shopping	Jingdong prefecture level city in Shandong
Stowe Flagship Store	a power saver (electronics)	The effect is very good, the home uses less electricity is very energy efficient, cost-effective	Jingdong prefecture level city in Shandong
Stowe Flagship Store	a power saver (electronics)	Quality can't be beat, just like the store, especially like, and the price is favorable!	Jingdong prefecture level city in Shandong
Stowe Flagship Store	a power saver (electronics)	Baby received! Very unexpected, will be back next time ~ great seller, stay tuned!	Jingdong prefecture level city in Shandong
Stowe Flagship Store	a power saver (electronics)	Follow up review after a month is more real, waiting for it	Jingdong prefecture level city in Shandong
Stowe Flagship Store	a power saver (electronics)	It is the ideal product, good quality and fast logistics, worthy of praise!	Jingdong prefecture level city in Shandong
Stowe Flagship Store	a power saver (electronics)	The saver is great, the color is right, the installer is very professional	Jingdong prefecture level city in Shandong
Stowe Flagship Store	a power saver (electronics)	The effect of the power connection is particularly obvious, it is particularly easy to use, very satisfied, the	Jingdong prefecture level city in Shandong
Stowe Flagship Store	a power saver (electronics)	Goods are very energy-saving, used to be more than two hundred degrees, now there are only a hundred or so degrees	Jingdong prefecture level city in Shandong
Stowe Flagship Store	a power saver (electronics)	Good quality, fast shipping, good results, good value for money	Jingdong prefecture level city in Shandong
Stowe Flagship Store	a power saver (electronics)	Fast shipping, fine workmanship, very good, cost-effective	Jingdong prefecture level city in Shandong
Stowe Flagship Store	a power saver (electronics)	The meter is as described, the quality is quite good, energy-saving, worth buying oh~!	Jingdong prefecture level city in Shandong
Stowe Flagship Store	a power saver (electronics)	Great value for money, very affordable,, perfect . ˆ◡ˆ.	Jingdong prefecture level city in Shandong
Stowe Flagship Store	a power saver (electronics)	Good!!! Worth recommending!	Jingdong prefecture level city in Shandong
Stowe Flagship Store	a power saver (electronics)	Great, fast logistics, great stuff, great value for money!	Jingdong prefecture level city in Shandong
Stowe Flagship Store	a power saver (electronics)	Fast logistics, very satisfied with the quality upon receipt	Jingdong prefecture level city in Shandong
Stowe Flagship Store	a power saver (electronics)	Great quality, plug and play, fast shipping!	Jingdong prefecture level city in Shandong
Stowe Flagship Store	a power saver (electronics)	Logistics received, quite satisfied	Jingdong prefecture level city in Shandong
Stowe Flagship Store	a power saver (electronics)	I don't know if it really works	Jingdong prefecture level city in Shandong
Stowe Flagship Store	a power saver (electronics)	It's bullshit. It's a plug light. Don't talk about power savings.	Jingdong prefecture level city in Shandong
Stowe Flagship Store	a power saver (electronics)	Fake and deceitful stuff that hasn't changed at all.	Jingdong prefecture level city in Shandong
Stowe Flagship Store	a power saver (electronics)	Power-saving effect: power-saving effect is particularly obvious Cost-effective: cost-effective business Appearance and style: external small micro-work materials: fine workmanship Experience: use very good, non the first convenient	Jingdong prefecture level city in Shandong
Stowe Flagship Store	a power saver (electronics)	After using it for a few days, it feels quite good, it should be quite safe, and it also sends a plug, the merchant is good, wait for a period of time and then calculate whether it saves on the electricity bill and then add comments ha!	Jingdong prefecture level city in Shandong
Stowe Flagship Store	a power saver (electronics)	My friend recommended to me, said the effect is very good, I bought two, this month less money, really can, just buy I do not believe, but has proved to have effect	Jingdong prefecture level city in Shandong
Stowe Flagship Store	a power saver (electronics)	Just bought back, already used, these days to see the effect of it, and so have the effect will also come back to give positive feedback, the price is also super cheap!	Jingdong prefecture level city in Shandong
Stowe Flagship Store	a power saver (electronics)	It does work well, the store uses a lot of electricity, this time to buy back a few, each place that uses a lot of electricity to insert one. Summer is here, the electricity is too strong.	Jingdong prefecture level city in Shandong
Stowe Flagship Store	a power saver (electronics)	Power saving treasure received, as for the effect, according to the instructions written, a short period of time can not be seen, at least 2-3 months before the real effect, then follow up evaluation	Jingdong prefecture level city in Shandong
Stowe Flagship Store	a power saver (electronics)	The goods have just been received and have not been used yet looking forward to the results.	Jingdong prefecture level city in Shandong
Stowe Flagship Store	a power saver (electronics)	Good installation works great	Jingdong prefecture level city in Shandong
Stowe Flagship Store	a power saver (electronics)	The logistics is super super fast! The quality is also very good, indeed value for money	Jingdong prefecture level city in Shandong
Stowe Flagship Store	a power saver (electronics)	Has been used for a period of time before coming to comment on, really good, air conditioning a day open non-stop, pay the electricity bill when the heartache, with this, the home electricity is not at all worried about the bill out of contrast to save a lot of preparation in the hands of a few, each room An a	Jingdong prefecture level city in Shandong
Stowe Flagship Store	a power saver (electronics)	Saving device received, really good, with the air conditioning tried, an hour without a saver with 2.9 degrees, with a saver an hour with 1.1 degrees, the beginning of not quite believe that this thing, the actual test to know that it is really useful, and intend to buy another.	Jingdong prefecture level city in Shandong
Stowe Flagship Store	a power saver (electronics)	Power saving effect: this energy saver is still useful, day trial for three days, every day than not installed before the reduction of two dollars a day, every day to open the bedroom to open the air conditioning before fifteen dollars now thirteen dollars a day. Still a little use.	Jingdong prefecture level city in Shandong

Stowe Flagship Store	a power saver (electronics)	Originally bought for home use, used for two months, the electricity bill from more than 200 yuan per month, down to more than 140 yuan, there is indeed a power-saving effect, this time and sold two, the unit with. Merchant service attitude is really good.	Jingdong prefecture level city in Shandong
Stowe Flagship Store	a power saver (electronics)	Plug and play, stable voltage, the screen shows when working, this winter home appliances, need electricity in many places, just buy to use, save electricity and money, with instructions	Jingdong prefecture level city in Shandong
Stowe Flagship Store	a power saver (electronics)	Goods received, do not know if it is good. When I bought it, I saw that it was a Stowe brand or company's product. Received but not. I do not know what is the reason. Now I can only give this evaluation. I'm	Jingdong prefecture level city in Shandong
Stowe Flagship Store	a power saver (electronics)	The first time to buy online, but also look at the evaluation before buying, the quality is not bad, but also very cheap, the need for friends can rest assured to buy!	Jingdong prefecture level city in Shandong
Stowe Flagship Store	a power saver (electronics)	🌈 Great quality, bought it to my liking 💞 Awesome! ✨ Buy with confidence if you need it!	Jingdong prefecture level city in Shandong
Stowe Flagship Store	a power saver (electronics)	The quality is very good, completely exceeded the expectations, the delivery speed is very fast, the packaging is very careful and strict, very satisfied with a shopping	Jingdong prefecture level city in Shandong
Stowe Flagship Store	a power saver (electronics)	The effect is very good, the home uses less electricity is very energy efficient, cost-effective	Jingdong prefecture level city in Shandong
Stowe Flagship Store	a power saver (electronics)	Quality can't be beat, just like the store, especially like, and the price is favorable!	Jingdong prefecture level city in Shandong
Stowe Flagship Store	a power saver (electronics)	Baby received! Very unexpected, will be back next time ~ great seller, stay tuned!	Jingdong prefecture level city in Shandong
Stowe Flagship Store	a power saver (electronics)	Follow up review after a month is more real, waiting for it	Jingdong prefecture level city in Shandong
Stowe Flagship Store	a power saver (electronics)	It is the ideal product, good quality and fast logistics, worthy of praise!	Jingdong prefecture level city in Shandong
Stowe Flagship Store	a power saver (electronics)	The saver is great, the color is right, the installer is very professional	Jingdong prefecture level city in Shandong
Stowe Flagship Store	a power saver (electronics)	The effect of the power connection is particularly obvious, it is particularly easy to use, very satisfied, the	Jingdong prefecture level city in Shandong
Stowe Flagship Store	a power saver (electronics)	Goods are very energy-saving, used to be more than two hundred degrees, now there are only a hundred or so degrees	Jingdong prefecture level city in Shandong
Stowe Flagship Store	a power saver (electronics)	Good quality, fast shipping, good results, good value for money	Jingdong prefecture level city in Shandong
Stowe Flagship Store	a power saver (electronics)	Fast shipping, fine workmanship, very good, cost-effective	Jingdong prefecture level city in Shandong
Stowe Flagship Store	a power saver (electronics)	The meter is as described, the quality is quite good, energy-saving, worth buying oh~!	Jingdong prefecture level city in Shandong
Stowe Flagship Store	a power saver (electronics)	Great value for money, very affordable,, perfect . ˆ◡ˆ.	Jingdong prefecture level city in Shandong
Stowe Flagship Store	a power saver (electronics)	Good!!! Worth recommending!	Jingdong prefecture level city in Shandong
Stowe Flagship Store	a power saver (electronics)	Great, fast logistics, great stuff, great value for money!	Jingdong prefecture level city in Shandong
Stowe Flagship Store	a power saver (electronics)	Fast logistics, very satisfied with the quality upon receipt	Jingdong prefecture level city in Shandong
Stowe Flagship Store	a power saver (electronics)	Great quality, plug and play, fast shipping!	Jingdong prefecture level city in Shandong
Stowe Flagship Store	a power saver (electronics)	Logistics received, quite satisfied	Jingdong prefecture level city in Shandong
Stowe Flagship Store	a power saver (electronics)	I don't know if it really works	Jingdong prefecture level city in Shandong
Stowe Flagship Store	a power saver (electronics)	It's bullshit. It's a plug light. Don't talk about power savings.	Jingdong prefecture level city in Shandong
Stowe Flagship Store	a power saver (electronics)	Fake and deceitful stuff that hasn't changed at all.	Jingdong prefecture level city in Shandong
Stowe Flagship Store	a power saver (electronics)	Power-saving effect: power-saving effect is particularly obvious Cost-effective: cost-effective business Appearance and style: external small micro-work materials: fine workmanship Experience: use very good, non the first convenient	Jingdong prefecture level city in Shandong
Stowe Flagship Store	a power saver (electronics)	After using it for a few days, it feels quite good, it should be quite safe, and it also sends a plug, the merchant is good, wait for a period of time and then calculate whether it saves on the electricity bill and then add comments ha!	Jingdong prefecture level city in Shandong
Stowe Flagship Store	a power saver (electronics)	My friend recommended to me, said the effect is very good, I bought two, this month less money, really can, just buy I do not believe, but has proved to have effect	Jingdong prefecture level city in Shandong
Stowe Flagship Store	a power saver (electronics)	Just bought back, already used, these days to see the effect of it, and so have the effect will also come back to give positive feedback, the price is also super cheap!	Jingdong prefecture level city in Shandong
Stowe Flagship Store	a power saver (electronics)	It does work well, the store uses a lot of electricity, this time to buy back a few, each place that uses a lot of electricity to insert one. Summer is here, the electricity is too strong.	Jingdong prefecture level city in Shandong
Stowe Flagship Store	a power saver (electronics)	Power saving treasure received, as for the effect, according to the instructions written, a short period of time can not be seen, at least 2-3 months before the real effect, then follow up evaluation	Jingdong prefecture level city in Shandong
Stowe Flagship Store	a power saver (electronics)	The goods have just been received and have not been used yet looking forward to the results.	Jingdong prefecture level city in Shandong
Stowe Flagship Store	a power saver (electronics)	Good installation works great	Jingdong prefecture level city in Shandong
Stowe Flagship Store	a power saver (electronics)	The logistics is super super fast! The quality is also very good, indeed value for money	Jingdong prefecture level city in Shandong
Stowe Flagship Store	a power saver (electronics)	Has been used for a period of time before coming to comment on, really good, air conditioning a day open non-stop, pay the electricity bill when the heartache, with this, the home electricity is not at all worried about the bill out of contrast to save a lot of preparation in the hands of a few, each room An a	Jingdong prefecture level city in Shandong
Stowe Flagship Store	a power saver (electronics)	Saving device received, really good, with the air conditioning tried, an hour without a saver with 2.9 degrees, with a saver an hour with 1.1 degrees, the beginning of not quite believe that this thing, the actual test to know that it is really useful, and intend to buy another.	Jingdong prefecture level city in Shandong
Stowe Flagship Store	a power saver (electronics)	Power saving effect: this energy saver is still useful, day trial for three days, every day than not installed before the reduction of two dollars a day, every day to open the bedroom to open the air conditioning before fifteen dollars now thirteen dollars a day. Still a little use.	Jingdong prefecture level city in Shandong
Stowe Flagship Store	a power saver (electronics)	Originally bought for home use, used for two months, the electricity bill from more than 200 yuan per month, down to more than 140 yuan, there is indeed a power-saving effect, this time and sold two, the unit with. Merchant service attitude is really good.	Jingdong prefecture level city in Shandong
Stowe Flagship Store	a power saver (electronics)	Plug and play, stable voltage, the screen shows when working, this winter home appliances, need electricity in many places, just buy to use, save electricity and money, with instructions	Jingdong prefecture level city in Shandong
Stowe Flagship Store	a power saver (electronics)	Goods received, do not know if it is good. When I bought it, I saw that it was a Stowe brand or company's product. Received but not. I do not know what is the reason. Now I can only give this evaluation. I'm	Jingdong prefecture level city in Shandong
Stowe Flagship Store	a power saver (electronics)	The first time to buy online, but also look at the evaluation before buying, the quality is not bad, but also very cheap, the need for friends can rest assured to buy!	Jingdong prefecture level city in Shandong
Stowe Flagship Store	a power saver (electronics)	🌈 Great quality, bought it to my liking 💞 Awesome! ✨ Buy with confidence if you need it!	Jingdong prefecture level city in Shandong
Stowe Flagship Store	a power saver (electronics)	The quality is very good, completely exceeded the expectations, the delivery speed is very fast, the packaging is very careful and strict, very satisfied with a shopping	Jingdong prefecture level city in Shandong
Stowe Flagship Store	a power saver (electronics)	The effect is very good, the home uses less electricity is very energy efficient, cost-effective	Jingdong prefecture level city in Shandong
Stowe Flagship Store	a power saver (electronics)	Quality can't be beat, just like the store, especially like, and the price is favorable!	Jingdong prefecture level city in Shandong
Stowe Flagship Store	a power saver (electronics)	Baby received! Very unexpected, will be back next time ~ great seller, stay tuned!	Jingdong prefecture level city in Shandong
Stowe Flagship Store	a power saver (electronics)	Follow up review after a month is more real, waiting for it	Jingdong prefecture level city in Shandong

Stowe Flagship Store	a power saver (electronics)	It is the ideal product, good quality and fast logistics, worthy of praise!	Jingdong prefecture level city in Shandong
Stowe Flagship Store	a power saver (electronics)	The saver is great, the color is right, the installer is very professional	Jingdong prefecture level city in Shandong
Stowe Flagship Store	a power saver (electronics)	The effect of the power connection is particularly obvious, it is particularly easy to use, very satisfied, the	Jingdong prefecture level city in Shandong
Stowe Flagship Store	a power saver (electronics)	Goods are very energy-saving, used to be more than two hundred degrees, now there are only a hundred or so degrees	Jingdong prefecture level city in Shandong
Stowe Flagship Store	a power saver (electronics)	Good quality, fast shipping, good results, good value for money	Jingdong prefecture level city in Shandong
Stowe Flagship Store	a power saver (electronics)	Fast shipping, fine workmanship, very good, cost-effective	Jingdong prefecture level city in Shandong
Stowe Flagship Store	a power saver (electronics)	The meter is as described, the quality is quite good, energy-saving, worth buying oh~!	Jingdong prefecture level city in Shandong
Stowe Flagship Store	a power saver (electronics)	Great value for money, very affordable,, perfect . ˆ◡ˆ.	Jingdong prefecture level city in Shandong
Stowe Flagship Store	a power saver (electronics)	Good!!! Worth recommending!	Jingdong prefecture level city in Shandong
Stowe Flagship Store	a power saver (electronics)	Great, fast logistics, great stuff, great value for money!	Jingdong prefecture level city in Shandong
Stowe Flagship Store	a power saver (electronics)	Fast logistics, very satisfied with the quality upon receipt	Jingdong prefecture level city in Shandong
Stowe Flagship Store	a power saver (electronics)	Great quality, plug and play, fast shipping!	Jingdong prefecture level city in Shandong
Stowe Flagship Store	a power saver (electronics)	Logistics received, quite satisfied	Jingdong prefecture level city in Shandong
Stowe Flagship Store	a power saver (electronics)	I don't know if it really works	Jingdong prefecture level city in Shandong
Stowe Flagship Store	a power saver (electronics)	It's bullshit. It's a plug light. Don't talk about power savings.	Jingdong prefecture level city in Shandong
Stowe Flagship Store	a power saver (electronics)	Fake and deceitful stuff that hasn't changed at all.	Jingdong prefecture level city in Shandong
Stowe Flagship Store	a power saver (electronics)	Power-saving effect: power-saving effect is particularly obvious Cost-effective: cost-effective business Appearance and style: external small micro-work materials: fine workmanship Experience: use very good, non the first convenient	Jingdong prefecture level city in Shandong
Stowe Flagship Store	a power saver (electronics)	After using it for a few days, it feels quite good, it should be quite safe, and it also sends a plug, the merchant is good, wait for a period of time and then calculate whether it saves on the electricity bill and then add comments ha!	Jingdong prefecture level city in Shandong
Stowe Flagship Store	a power saver (electronics)	My friend recommended to me, said the effect is very good, I bought two, this month less money, really can, just buy I do not believe, but has proved to have effect	Jingdong prefecture level city in Shandong
Stowe Flagship Store	a power saver (electronics)	Just bought back, already used, these days to see the effect of it, and so have the effect will also come back to give positive feedback, the price is also super cheap!	Jingdong prefecture level city in Shandong
Stowe Flagship Store	a power saver (electronics)	It does work well, the store uses a lot of electricity, this time to buy back a few, each place that uses a lot of electricity to insert one. Summer is here, the electricity is too strong.	Jingdong prefecture level city in Shandong
Stowe Flagship Store	a power saver (electronics)	Power saving treasure received, as for the effect, according to the instructions written, a short period of time can not be seen, at least 2-3 months before the real effect, then follow up evaluation	Jingdong prefecture level city in Shandong
Stowe Flagship Store	a power saver (electronics)	The goods have just been received and have not been used yet looking forward to the results.	Jingdong prefecture level city in Shandong
Stowe Flagship Store	a power saver (electronics)	Good installation works great	Jingdong prefecture level city in Shandong
Stowe Flagship Store	a power saver (electronics)	The logistics is super super fast! The quality is also very good, indeed value for money	Jingdong prefecture level city in Shandong
Stowe Flagship Store	a power saver (electronics)	Has been used for a period of time before coming to comment on, really good, air conditioning a day open non-stop, pay the electricity bill when the heartache, with this, the home electricity is not at all worried about the bill out of contrast to save a lot of preparation in the hands of a few, each room An a	Jingdong prefecture level city in Shandong
Stowe Flagship Store	a power saver (electronics)	Saving device received, really good, with the air conditioning tried, an hour without a saver with 2.9 degrees, with a saver an hour with 1.1 degrees, the beginning of not quite believe that this thing, the actual test to know that it is really useful, and intend to buy another.	Jingdong prefecture level city in Shandong
Stowe Flagship Store	a power saver (electronics)	Power saving effect: this energy saver is still useful, day trial for three days, every day than not installed before the reduction of two dollars a day, every day to open the bedroom to open the air conditioning before fifteen dollars now thirteen dollars a day. Still a little use.	Jingdong prefecture level city in Shandong
Stowe Flagship Store	a power saver (electronics)	Originally bought for home use, used for two months, the electricity bill from more than 200 yuan per month, down to more than 140 yuan, there is indeed a power-saving effect, this time and sold two, the unit with. Merchant service attitude is really good.	Jingdong prefecture level city in Shandong
Stowe Flagship Store	a power saver (electronics)	Plug and play, stable voltage, the screen shows when working, this winter home appliances, need electricity in many places, just buy to use, save electricity and money, with instructions	Jingdong prefecture level city in Shandong
Stowe Flagship Store	a power saver (electronics)	Goods received, do not know if it is good. When I bought it, I saw that it was a Stowe brand or company's product. Received but not. I do not know what is the reason. Now I can only give this evaluation. I'm	Jingdong prefecture level city in Shandong
Stowe Flagship Store	a power saver (electronics)	The first time to buy online, but also look at the evaluation before buying, the quality is not bad, but also very cheap, the need for friends can rest assured to buy!	Jingdong prefecture level city in Shandong
Stowe Flagship Store	a power saver (electronics)	🌈 Great quality, bought it to my liking 💝 Awesome! ✨ Buy with confidence if you need it!	Jingdong prefecture level city in Shandong
Stowe Flagship Store	a power saver (electronics)	The quality is very good, completely exceeded the expectations, the delivery speed is very fast, the packaging is very careful and strict, very satisfied with a shopping	Jingdong prefecture level city in Shandong
Stowe Flagship Store	a power saver (electronics)	The effect is very good, the home uses less electricity is very energy efficient, cost-effective	Jingdong prefecture level city in Shandong
Stowe Flagship Store	a power saver (electronics)	Quality can't be beat, just like the store, especially like, and the price is favorable!	Jingdong prefecture level city in Shandong
Stowe Flagship Store	a power saver (electronics)	Baby received! Very unexpected, will be back next time ~ great seller, stay tuned!	Jingdong prefecture level city in Shandong
Stowe Flagship Store	a power saver (electronics)	Follow up review after a month is more real, waiting for it	Jingdong prefecture level city in Shandong
Stowe Flagship Store	a power saver (electronics)	It is the ideal product, good quality and fast logistics, worthy of praise!	Jingdong prefecture level city in Shandong
Stowe Flagship Store	a power saver (electronics)	The saver is great, the color is right, the installer is very professional	Jingdong prefecture level city in Shandong
Stowe Flagship Store	a power saver (electronics)	The effect of the power connection is particularly obvious, it is particularly easy to use, very satisfied, the	Jingdong prefecture level city in Shandong
Stowe Flagship Store	a power saver (electronics)	Goods are very energy-saving, used to be more than two hundred degrees, now there are only a hundred or so degrees	Jingdong prefecture level city in Shandong
Stowe Flagship Store	a power saver (electronics)	Good quality, fast shipping, good results, good value for money	Jingdong prefecture level city in Shandong
Stowe Flagship Store	a power saver (electronics)	Fast shipping, fine workmanship, very good, cost-effective	Jingdong prefecture level city in Shandong
Stowe Flagship Store	a power saver (electronics)	The meter is as described, the quality is quite good, energy-saving, worth buying oh~!	Jingdong prefecture level city in Shandong
Stowe Flagship Store	a power saver (electronics)	Great value for money, very affordable,, perfect . ˆ◡ˆ.	Jingdong prefecture level city in Shandong
Stowe Flagship Store	a power saver (electronics)	Good!!! Worth recommending!	Jingdong prefecture level city in Shandong
Stowe Flagship Store	a power saver (electronics)	Great, fast logistics, great stuff, great value for money!	Jingdong prefecture level city in Shandong

Stowe Flagship Store	a power saver (electronics)	Fast logistics, very satisfied with the quality upon receipt	Jingdong prefecture level city in Shandong
Stowe Flagship Store	a power saver (electronics)	Great quality, plug and play, fast shipping!	Jingdong prefecture level city in Shandong
Stowe Flagship Store	a power saver (electronics)	Logistics received, quite satisfied	Jingdong prefecture level city in Shandong
Stowe Flagship Store	a power saver (electronics)	I don't know if it really works	Jingdong prefecture level city in Shandong
Stowe Flagship Store	a power saver (electronics)	It's bullshit. It's a plug light. Don't talk about power savings.	Jingdong prefecture level city in Shandong
Stowe Flagship Store	a power saver (electronics)	Fake and deceitful stuff that hasn't changed at all.	Jingdong prefecture level city in Shandong
Stowe Flagship Store	a power saver (electronics)	Power-saving effect: power-saving effect is particularly obvious Cost-effective: cost-effective business Appearance and style: external small micro-work materials: fine workmanship Experience: use very good, non the first convenient	Jingdong prefecture level city in Shandong
Stowe Flagship Store	a power saver (electronics)	After using it for a few days, it feels quite good, it should be quite safe, and it also sends a plug, the merchant is good, wait for a period of time and then calculate whether it saves on the electricity bill and then add comments ha!	Jingdong prefecture level city in Shandong
Stowe Flagship Store	a power saver (electronics)	My friend recommended to me, said the effect is very good, I bought two, this month less money, really can, just buy I do not believe, but has proved to have effect	Jingdong prefecture level city in Shandong
Stowe Flagship Store	a power saver (electronics)	Just bought back, already used, these days to see the effect of it, and so have the effect will also come back to give positive feedback, the price is also super cheap!	Jingdong prefecture level city in Shandong
Stowe Flagship Store	a power saver (electronics)	It does work well, the store uses a lot of electricity, this time to buy back a few, each place that uses a lot of electricity to insert one. Summer is here, the electricity is too strong.	Jingdong prefecture level city in Shandong
Stowe Flagship Store	a power saver (electronics)	Power saving treasure received, as for the effect, according to the instructions written, a short period of time can not be seen, at least 2-3 months before the real effect, then follow up evaluation	Jingdong prefecture level city in Shandong
Stowe Flagship Store	a power saver (electronics)	The goods have just been received and have not been used yet looking forward to the results.	Jingdong prefecture level city in Shandong
Stowe Flagship Store	a power saver (electronics)	Good installation works great	Jingdong prefecture level city in Shandong
Stowe Flagship Store	a power saver (electronics)	The logistics is super super fast! The quality is also very good, indeed value for money	Jingdong prefecture level city in Shandong
Stowe Flagship Store	a power saver (electronics)	Has been used for a period of time before coming to comment on, really good, air conditioning a day open non-stop, pay the electricity bill when the heartache, with this, the home electricity is not at all worried about the bill out of contrast to save a lot of preparation in the hands of a few, each room An a	Jingdong prefecture level city in Shandong
Stowe Flagship Store	a power saver (electronics)	Saving device received, really good, with the air conditioning tried, an hour without a saver with 2.9 degrees, with a saver an hour with 1.1 degrees, the beginning of not quite believe that this thing, the actual test to know that it is really useful, and intend to buy another.	Jingdong prefecture level city in Shandong
Stowe Flagship Store	a power saver (electronics)	Power saving effect: this energy saver is still useful, day trial for three days, every day than not installed before the reduction of two dollars a day, every day to open the bedroom to open the air conditioning before fifteen dollars now thirteen dollars a day. Still a little use.	Jingdong prefecture level city in Shandong
Stowe Flagship Store	a power saver (electronics)	Originally bought for home use, used for two months, the electricity bill from more than 200 yuan per month, down to more than 140 yuan, there is indeed a power-saving effect, this time and sold two, the unit with. Merchant service attitude is really good.	Jingdong prefecture level city in Shandong
Stowe Flagship Store	a power saver (electronics)	Plug and play, stable voltage, the screen shows when working, this winter home appliances, need electricity in many places, just buy to use, save electricity and money, with instructions	Jingdong prefecture level city in Shandong
Stowe Flagship Store	a power saver (electronics)	Goods received, do not know if it is good. When I bought it, I saw that it was a Stowe brand or company's product. Received but not. I do not know what is the reason. Now I can only give this evaluation. I'm	Jingdong prefecture level city in Shandong
Stowe Flagship Store	a power saver (electronics)	The first time to buy online, but also look at the evaluation before buying, the quality is not bad, but also very cheap, the need for friends can rest assured to buy!	Jingdong prefecture level city in Shandong
Stowe Flagship Store	a power saver (electronics)	Great quality, bought it to my liking Awesome! Buy with confidence if you need it!	Jingdong prefecture level city in Shandong
Stowe Flagship Store	a power saver (electronics)	The quality is very good, completely exceeded the expectations, the delivery speed is very fast, the packaging is very careful and strict, very satisfied with a shopping	Jingdong prefecture level city in Shandong
Stowe Flagship Store	a power saver (electronics)	The effect is very good, the home uses less electricity is very energy efficient, cost-effective	Jingdong prefecture level city in Shandong
Stowe Flagship Store	a power saver (electronics)	Quality can't be beat, just like the store, especially like, and the price is favorable!	Jingdong prefecture level city in Shandong
Stowe Flagship Store	a power saver (electronics)	Baby received! Very unexpected, will be back next time ~ great seller, stay tuned!	Jingdong prefecture level city in Shandong
Stowe Flagship Store	a power saver (electronics)	Follow up review after a month is more real, waiting for it	Jingdong prefecture level city in Shandong
Stowe Flagship Store	a power saver (electronics)	It is the ideal product, good quality and fast logistics, worthy of praise!	Jingdong prefecture level city in Shandong
Stowe Flagship Store	a power saver (electronics)	The saver is great, the color is right, the installer is very professional	Jingdong prefecture level city in Shandong
Stowe Flagship Store	a power saver (electronics)	The effect of the power connection is particularly obvious, it is particularly easy to use, very satisfied, the	Jingdong prefecture level city in Shandong
Stowe Flagship Store	a power saver (electronics)	Goods are very energy-saving, used to be more than two hundred degrees, now there are only a hundred or so degrees	Jingdong prefecture level city in Shandong
Stowe Flagship Store	a power saver (electronics)	Good quality, fast shipping, good results, good value for money	Jingdong prefecture level city in Shandong
Stowe Flagship Store	a power saver (electronics)	Fast shipping, fine workmanship, very good, cost-effective	Jingdong prefecture level city in Shandong
Stowe Flagship Store	a power saver (electronics)	The meter is as described, the quality is quite good, energy-saving, worth buying oh~!	Jingdong prefecture level city in Shandong
Stowe Flagship Store	a power saver (electronics)	Great value for money, very affordable,, perfect . ˆ◡ˆ.	Jingdong prefecture level city in Shandong
Stowe Flagship Store	a power saver (electronics)	Good!!! Worth recommending!	Jingdong prefecture level city in Shandong
Stowe Flagship Store	a power saver (electronics)	Great, fast logistics, great stuff, great value for money!	Jingdong prefecture level city in Shandong
Stowe Flagship Store	a power saver (electronics)	Fast logistics, very satisfied with the quality upon receipt	Jingdong prefecture level city in Shandong
Stowe Flagship Store	a power saver (electronics)	Great quality, plug and play, fast shipping!	Jingdong prefecture level city in Shandong
Stowe Flagship Store	a power saver (electronics)	Logistics received, quite satisfied	Jingdong prefecture level city in Shandong
Stowe Flagship Store	a power saver (electronics)	I don't know if it really works	Jingdong prefecture level city in Shandong
Stowe Flagship Store	a power saver (electronics)	It's bullshit. It's a plug light. Don't talk about power savings.	Jingdong prefecture level city in Shandong
Stowe Flagship Store	a power saver (electronics)	Fake and deceitful stuff that hasn't changed at all.	Jingdong prefecture level city in Shandong
Stowe Flagship Store	King of Power Saving	I am a rural area, the home power consumption is too large, the population is relatively large, a day to use 5 air conditioners, a month of electricity costs are a thousand dollars, the customer service recommended that I use 3, 90 days within the package return, I just hold a try mentality, yesterday paid the electricity bill, I did not expect to really save more than 400 electricity, a month back to the capital, but also a warranty of 5 years, recommend that everyone buy!	Jingdong prefecture level city in Shandong
Stowe Flagship Store	King of Power Saving	The product is very good, very good, logistics is also very fast, before buying a, this time and bought two to the old family inside a, this side of the store and bought a, power-saving effect is also very good, like we open the store, summer ah, to open a few refrigerators, and air conditioning, quite suitable for the purchase of two of this installed inside the store!	Jingdong prefecture level city in Shandong

Stowe Flagship Store	King of Power Saving	Received on the insertion of the meter down, currently more stable, home electricity is not tripped, I am the old house in the countryside! Home lines are a bit chaotic while opening electrical appliances from time to time tripping, listening to the seller said the product has a stabilized voltage, decisive order. Currently experiencing 2 weeks indeed voltage is very stable without tripping, the effect is very good, there is also a power-saving effect. Power-saving effect: very good Cost-effective: high Appearance style: good-looking Workmanship material: very good	Jingdong prefecture level city in Shandong
Stowe Flagship Store	King of Power Saving	Power Saving King has been received, the quality is very good, used more than 10 days, found that it does feel less. And also can see the real-time voltage, stabilized a lot, good satisfaction!	Jingdong prefecture level city in Shandong
Stowe Flagship Store	King of Power Saving	Power saving effect: It works well. Calculated, than before not using this is reduced. Appearance and style: Exquisite and beautiful, high quality. The effect of using air conditioning in summer is more	Jingdong prefecture level city in Shandong
Stowe Flagship Store	King of Power Saving	Used for about a month, tried to really work, the electricity bill is less than 70 more, very suitable for the use of the workers now!	Jingdong prefecture level city in Shandong
Stowe Flagship Store	King of Power Saving	I am opening a milk tea store, this side of the feeling that the product is quite good, before not using it I dare not comment, with a month to compare, last month more than 780 degrees, this month is actually only 536 degrees, the effect is a lot better, after all, before the advances are too high, and now much better, feel the effect is very good!	Jingdong prefecture level city in Shandong
Stowe Flagship Store	King of Power Saving	I run a restaurant, and my electricity bill is over a thousand dollars a summer. This year's business is not doing well, and the electricity bill is still not small. A few days ago, the electrician came to repair the electric grill. Recommended this brand of power saver. Tried to buy three, a month to save about almost half of the electricity bill, truly very satisfied, recommended to everyone!	Jingdong prefecture level city in Shandong
Stowe Flagship Store	King of Power Saving	This high-power really good to use, I am also a friend introduced to his store with the use of a lot of electricity, workmanship is also very good, the whole machine made of aluminum alloy, but also send two fuses, with real-time voltage display affordable price!	Jingdong prefecture level city in Shandong
Stowe Flagship Store	King of Power Saving	At first used ten days did not see any effect, in accordance with the store said I used about thirty days to compare, after about thirty days or so, compared to the previous electricity bill does save, mainly air conditioning electricity, save about 50 more than 50 dollars, very satisfied!	Jingdong prefecture level city in Shandong
Stowe Flagship Store	King of Power Saving	This thing can really save electricity, I was introduced to buy by my friend, he used it very well, I used it and think it's also very good, it can really save electricity, save about half of the electricity every day, it's really good!	Jingdong prefecture level city in Shandong
Stowe Flagship Store	King of Power Saving	After using it for ten days or so, I checked the power consumption and it did drop, and customer service said it will save more electricity in the long run. The product is also insured, 5 years warranty do not have to worry about quality problems!	Jingdong prefecture level city in Shandong
Stowe Flagship Store	King of Power Saving	The product is good, before already bought a, this is the second, the effect is quite good, anyway, I see the daily electricity bill than before a lot less, this can be the choice!	Jingdong prefecture level city in Shandong
Stowe Flagship Store	King of Power Saving	Originally also very worried about things are not good, get the hand found completely over-anxious, five-star praise, the style is very generous, the quality is very good, the price is also very reasonable!	Jingdong prefecture level city in Shandong
Stowe Flagship Store	King of Power Saving	Quality is good, playing to the old family also buy a, repurchase, this quality is really good, recommended!	Jingdong prefecture level city in Shandong
Stowe Flagship Store	King of Power Saving	Thank you, the owner even sent two fuses.	Jingdong prefecture level city in Shandong
Stowe Flagship Store	King of Power Saving	This store has great stuff, relatively fast shipping and logistics	Jingdong prefecture level city in Shandong
Stowe Flagship Store	King of Power Saving	This product is based on the impossibility of saving electricity, I used 853 kWh of electricity in July, started using the product on August 1, and used 867 kWh of electricity by September 1, is this called	Jingdong prefecture level city in Shandong
Stowe Flagship Store	King of Power Saving	Power king received, to the home on the trial, when I plugged into the electric panel can not see the effect of power saving, the meter is still the same speed in the turn, I'll save the king of the manual to play look at the instructions, instructions to speak, have to have a day two days and half a month to save power obviously, then wait half a month to see the effect of power saving it! To half a month there are obvious power saving before continuing to chase Xu.	Jingdong prefecture level city in Shandong
Stowe Flagship Store	King of Power Saving	Received the goods, a look, did not see the manufacturer and production address, can only hold a try to see the mentality of the face	Jingdong prefecture level city in Shandong
Stowe Flagship Store	King of Power Saving	I've only been using it for a few days and I'm not sure if it's an IQ tax? Used to favorable reviews.	Jingdong prefecture level city in Shandong
Stowe Flagship Store	King of Power Saving	Appearance is okay, power saving next time to follow up the evaluation	Jingdong prefecture level city in Shandong
Stowe Flagship Store	King of Power Saving	Shorter time, but hopefully effective and power-saving, to be followed up in the future	Jingdong prefecture level city in Shandong
Stowe Flagship Store	King of Power Saving	Appearance is okay, I do not know if the power saving	Jingdong prefecture level city in Shandong
Stowe Flagship Store	King of Power Saving	Good service, fast logistics, Power Saving King is exquisite and beautiful, support the business, thank you!	Jingdong prefecture level city in Shandong
Stowe Flagship Store	King of Power Saving	Bought three at once to try first first.	Jingdong prefecture level city in Shandong
Stowe Flagship Store	King of Power Saving	The object is the same as the picture, the product is of high quality, the packaging is intact. High-end atmosphere, high power, power saving effect is obvious, average monthly power saving 50%, very satisfied.	Jingdong prefecture level city in Shandong
Stowe Flagship Store	King of Power Saving	Bought back and plugged in, really effective, before 16 degrees of electricity per day, now 8 degrees of electricity per day, open an air conditioner for 12 hours, decisive repurchase of a	Jingdong prefecture level city in Shandong
Stowe Flagship Store	King of Power Saving	The object is the same as the picture, the product is of high quality, and the package is intact. High-end atmosphere and high grade, high power	Jingdong prefecture level city in Shandong
Stowe Flagship Store	King of Power Saving	Goods received, I hope the store said can be energy-saving, first try a month, month after month to see the effect.	Jingdong prefecture level city in Shandong
Stowe Flagship Store	King of Power Saving	This is really good, the material and workmanship is particularly exquisite, the workmanship is really very good, I think I can recommend to my friends to buy	Jingdong prefecture level city in Shandong
Stowe Flagship Store	King of Power Saving	I have to praise, the quality is really good, and the packaging logistics are also good. Really like it, won't make you regret after getting it!	Jingdong prefecture level city in Shandong
Stowe Flagship Store	King of Power Saving	Reputable platform, efficient and professional service, buy with confidence and peace of mind!	Jingdong prefecture level city in Shandong
Stowe Flagship Store	King of Power Saving	Bought one on a trial basis to see if it works after 1-2 months.	Jingdong prefecture level city in Shandong
Stowe Flagship Store	King of Power Saving	Quality is very good, customer service attitude is very very praise, there are problems in a timely manner to solve the problem, the price is not expensive, super value, you can rest assured that the	Jingdong prefecture level city in Shandong
Stowe Flagship Store	King of Power Saving	With it no longer need to be afraid to use electricity, especially good, ready to buy a mom and dad that also	Jingdong prefecture level city in Shandong
Stowe Flagship Store	King of Power Saving	Baby received very like will return to buy the seller attitude is also very like will return to buy the seller attitude is also very like!	Jingdong prefecture level city in Shandong
Stowe Flagship Store	King of Power Saving	Jingdong Mall shopping convenience assured fast assured fast assured fast assured fast	Jingdong prefecture level city in Shandong
Stowe Flagship Store	King of Power Saving	Saving power received, the quality is very good, fine workmanship, especially good, much cheaper than the entity store, it is worth buying!	Jingdong prefecture level city in Shandong
Stowe Flagship Store	King of Power Saving	I did not expect the express delivery so quickly, very save point quality is super good, than the entity store is too cheap.	Jingdong prefecture level city in Shandong
Stowe Flagship Store	King of Power Saving	Have received the baby, very satisfied with a shopping, great worth buying!	Jingdong prefecture level city in Shandong
Stowe Flagship Store	King of Power Saving	This is really more powerful, very satisfied, more energy-saving, better, thank you boss.	Jingdong prefecture level city in Shandong
Stowe Flagship Store	King of Power Saving	Received has been used for two days, the air conditioning can boldly open it! Energy saving effect is really quite good	Jingdong prefecture level city in Shandong
Stowe Flagship Store	King of Power Saving	It is authentic, trust the merchant, the baby has a great texture and the price is moderate, unlimited repurchase!	Jingdong prefecture level city in Shandong
Stowe Flagship Store	King of Power Saving	Very good quality, fine workmanship, low power consumption, long warranty, fast shipping, economical and practical	Jingdong prefecture level city in Shandong
Stowe Flagship Store	King of Power Saving	It is genuine very good, the price is much cheaper, cost-effective, really special cost-effective, it is worth buying!	Jingdong prefecture level city in Shandong

Stowe Flagship Store	King of Power Saving	I've been using it for half a month now, it really saves power and the voltage is stable, it's very good	Jingdong prefecture level city in Shandong
Stowe Flagship Store	King of Power Saving	It's the ideal product ♡, good quality and fast logistics, worth the kudos!	Jingdong prefecture level city in Shandong
Stowe Flagship Store	King of Power Saving	The logistics is very fast, the baby is very good, the shopkeeper is NICE!	Jingdong prefecture level city in Shandong
Stowe Flagship Store	King of Power Saving	Good to use, good quality, save point durable	Jingdong prefecture level city in Shandong
Stowe Flagship Store	King of Power Saving	The quality is very good, customer service attitude is very, very awesome, there are problems in a timely manner to solve the problem	Jingdong prefecture level city in Shandong
Stowe Flagship Store	King of Power Saving	Quality is very good, the use of the effect is very good, more stable, quality assurance	Jingdong prefecture level city in Shandong
Stowe Flagship Store	King of Power Saving	Convenient, inexpensive, and see how it works out?	Jingdong prefecture level city in Shandong
Stowe Flagship Store	King of Power Saving	Very good quality, saves a lot of electricity, very good, worth buying	Jingdong prefecture level city in Shandong
Stowe Flagship Store	King of Power Saving	Power Saving King is easy to use, with the fruit of power saving to use easy to give!	Jingdong prefecture level city in Shandong
Stowe Flagship Store	King of Power Saving	This saver really works, good quality, safe and secure, very satisfied!	Jingdong prefecture level city in Shandong
Stowe Flagship Store	King of Power Saving	It works well, the voltage is very stable effect is good, the quality is also very good satisfaction	Jingdong prefecture level city in Shandong
Stowe Flagship Store	King of Power Saving	The logistics is super super fast! The quality is also very good, indeed value for money	Jingdong prefecture level city in Shandong
Stowe Flagship Store	King of Power Saving	I've only been using it for a month or so and it seems to be saving a lot of power	Jingdong prefecture level city in Shandong
Stowe Flagship Store	King of Power Saving	Very easy to operate, very good material, good value for money.	Jingdong prefecture level city in Shandong
Stowe Flagship Store	King of Power Saving	Aluminum alloy shell, durable, compact power-saving good effect	Jingdong prefecture level city in Shandong
Stowe Flagship Store	King of Power Saving	Nice device, easy to use, doesn't take up much space, recommendable!	Jingdong prefecture level city in Shandong
Stowe Flagship Store	King of Power Saving	Good quality, good price, fast logistics	Jingdong prefecture level city in Shandong
Stowe Flagship Store	King of Power Saving	Friends of the family in use recommended, the effect is good and recommendable!	Jingdong prefecture level city in Shandong
Stowe Flagship Store	King of Power Saving	Cheaper than brick and mortar stores worth buying is authentic	Jingdong prefecture level city in Shandong
Stowe Flagship Store	King of Power Saving	Merchants ship quickly, the price is cheaper than the physical store, good use	Jingdong prefecture level city in Shandong
Stowe Flagship Store	King of Power Saving	Very durable, affordable, fast shipping	Jingdong prefecture level city in Shandong
Stowe Flagship Store	King of Power Saving	Packaging tight quality is very good easy to use good results	Jingdong prefecture level city in Shandong
Stowe Flagship Store	King of Power Saving	Save the grid. It's in the whole house. It's saving money on the electric bill.	Jingdong prefecture level city in Shandong
Stowe Flagship Store	King of Power Saving	I used it today, it works well, I'm very satisfied	Jingdong prefecture level city in Shandong
Stowe Flagship Store	King of Power Saving	It really saves a lot of electricity after installation	Jingdong prefecture level city in Shandong
Stowe Flagship Store	King of Power Saving	Love it. Satisfied.	Jingdong prefecture level city in Shandong
Stowe Flagship Store	King of Power Saving	Doesn't feel like it's working.	Jingdong prefecture level city in Shandong
Stowe Flagship Store	King of Power Saving	I'll comment after using it.	Jingdong prefecture level city in Shandong
Stowe Flagship Store	King of Power Saving	I'm trying it out to see if it saves power, and I'll buy two more if it works well.	Jingdong prefecture level city in Shandong
Stowe Flagship Store	King of Power Saving	It is genuine very good, the price is much cheaper, cost-effective, really special cost-effective, it is worth buying!	Jingdong prefecture level city in Shandong
Stowe Flagship Store	King of Power Saving	This thing can really save electricity, I was introduced to buy by my friend, he used it very well, I used it and think it's also very good, it can really save electricity, save about half of the electricity every day, it's really good!	Jingdong prefecture level city in Shandong
Stowe Flagship Store	King of Power Saving	After using it for ten days or so, I checked the power consumption and it did drop, and customer service said it will save more electricity in the long run. The product is also insured, 5 years warranty do not have to worry about quality problems!	Jingdong prefecture level city in Shandong
Stowe Flagship Store	King of Power Saving	The product is good, before already bought a, this is the second, the effect is quite good, anyway, I see the daily electricity bill than before a lot less, this can be the choice!	Jingdong prefecture level city in Shandong
Stowe Flagship Store	King of Power Saving	Originally also very worried about things are not good, get the hand found completely over-anxious, five-star praise, the style is very generous, the quality is very good, the price is also very reasonable!	Jingdong prefecture level city in Shandong
Stowe Flagship Store	King of Power Saving	Quality is good, playing to the old family also buy a, repurchase, this quality is really good, recommended!	Jingdong prefecture level city in Shandong
Stowe Flagship Store	King of Power Saving	Thank you, the owner even sent two fuses.	Jingdong prefecture level city in Shandong
Stowe Flagship Store	King of Power Saving	This store has great stuff, relatively fast shipping and logistics	Jingdong prefecture level city in Shandong
Stowe Flagship Store	King of Power Saving	This product is based on the impossibility of saving electricity, I used 853 kWh of electricity in July, started using the product on August 1, and used 867 kWh of electricity by September 1, is this called	Jingdong prefecture level city in Shandong
Stowe Flagship Store	King of Power Saving	Power king received, to the home on the trial, when I plugged into the electric panel can not see the effect of power saving, the meter is still the same speed in the turn, I'll save the king of the manual to play look at the instructions, instructions to speak, have to have a day two days and half a month to save power obviously, then wait half a month to see the effect of power saving it! To half a month there are obvious power saving before continuing to chase Xu.	Jingdong prefecture level city in Shandong
Stowe Flagship Store	King of Power Saving	Received the goods, a look, did not see the manufacturer and production address, can only hold a try to see the mentality of the face	Jingdong prefecture level city in Shandong
Stowe Flagship Store	King of Power Saving	I've only been using it for a few days and I'm not sure if it's an IQ tax? Used to favorable reviews.	Jingdong prefecture level city in Shandong
Stowe Flagship Store	King of Power Saving	Appearance is okay, power saving next time to follow up the evaluation	Jingdong prefecture level city in Shandong
Stowe Flagship Store	King of Power Saving	Shorter time, but hopefully effective and power-saving, to be followed up in the future	Jingdong prefecture level city in Shandong
Stowe Flagship Store	King of Power Saving	Appearance is okay, I do not know if the power saving	Jingdong prefecture level city in Shandong
Stowe Flagship Store	King of Power Saving	Good service, fast logistics, Power Saving King is exquisite and beautiful, support the business, thank you!	Jingdong prefecture level city in Shandong
Stowe Flagship Store	King of Power Saving	Bought three at once to try first first.	Jingdong prefecture level city in Shandong
Stowe Flagship Store	King of Power Saving	The object is the same as the picture, the product is of high quality, the packaging is intact. High-end atmosphere, high power, power saving effect is obvious, average monthly power saving 50%, very satisfied.	Jingdong prefecture level city in Shandong
Stowe Flagship Store	King of Power Saving	Bought back and plugged in, really effective, before 16 degrees of electricity per day, now 8 degrees of electricity per day, open an air conditioner for 12 hours, decisive repurchase of a	Jingdong prefecture level city in Shandong
Stowe Flagship Store	King of Power Saving	The object is the same as the picture, the product is of high quality, and the package is intact. High-end atmosphere and high grade, high power	Jingdong prefecture level city in Shandong

Stowe Flagship Store	King of Power Saving	Goods received, I hope the store said can be energy-saving, first try a month, month after month to see the effect.	Jingdong prefecture level city in Shandong
Stowe Flagship Store	King of Power Saving	This is really good, the material and workmanship is particularly exquisite, the workmanship is really very good, I think I can recommend to my friends to buy	Jingdong prefecture level city in Shandong
Stowe Flagship Store	King of Power Saving	I have to praise, the quality is really good, and the packaging logistics are also good. Really like it, won't make you regret after getting it!	Jingdong prefecture level city in Shandong
Stowe Flagship Store	King of Power Saving	Reputable platform, efficient and professional service, buy with confidence and peace of mind!	Jingdong prefecture level city in Shandong
Stowe Flagship Store	King of Power Saving	Bought one on a trial basis to see if it works after 1-2 months.	Jingdong prefecture level city in Shandong
Stowe Flagship Store	King of Power Saving	Quality is very good, customer service attitude is very very praise, there are problems in a timely manner to solve the problem, the price is not expensive, super value, you can rest assured that the	Jingdong prefecture level city in Shandong
Stowe Flagship Store	King of Power Saving	With it no longer need to be afraid to use electricity, especially good, ready to buy a mom and dad that also	Jingdong prefecture level city in Shandong
Stowe Flagship Store	King of Power Saving	Baby received very like will return to buy the seller attitude is also very like will return to buy the seller attitude is also very like!	Jingdong prefecture level city in Shandong
Stowe Flagship Store	King of Power Saving	Jingdong Mall shopping convenience assured fast assured fast assured fast	Jingdong prefecture level city in Shandong
Stowe Flagship Store	King of Power Saving	Saving power received, the quality is very good, fine workmanship, especially good, much cheaper than the entity store, it is worth buying!	Jingdong prefecture level city in Shandong
Stowe Flagship Store	King of Power Saving	I did not expect the express delivery so quickly, very save point quality is super good, than the entity store is too cheap.	Jingdong prefecture level city in Shandong
Stowe Flagship Store	King of Power Saving	Have received the baby, very satisfied with a shopping, great worth buying!	Jingdong prefecture level city in Shandong
Stowe Flagship Store	King of Power Saving	This is really more powerful, very satisfied, more energy-saving, better, thank you boss.	Jingdong prefecture level city in Shandong
Stowe Flagship Store	King of Power Saving	Received has been used for two days, the air conditioning can boldly open it! Energy saving effect is really quite good	Jingdong prefecture level city in Shandong
Stowe Flagship Store	King of Power Saving	It is authentic, trust the merchant, the baby has a great texture and the price is moderate, unlimited repurchase!	Jingdong prefecture level city in Shandong
Stowe Flagship Store	King of Power Saving	It is genuine very good, the price is much cheaper, cost-effective, really special cost-effective, it is worth buying!	Jingdong prefecture level city in Shandong
Stowe Flagship Store	King of Power Saving	Very good quality, fine workmanship, low power consumption, long warranty, fast shipping, economical and practical	Jingdong prefecture level city in Shandong
Stowe Flagship Store	King of Power Saving	I've been using it for half a month now, it really saves power and the voltage is stable, it's very good	Jingdong prefecture level city in Shandong
Stowe Flagship Store	King of Power Saving	It's the ideal product 💝, good quality and fast logistics, worth the kudos!	Jingdong prefecture level city in Shandong
Stowe Flagship Store	King of Power Saving	The logistics is very fast, the baby is very good, the shopkeeper is NICE!	Jingdong prefecture level city in Shandong
Stowe Flagship Store	King of Power Saving	Good to use, good quality, save point durable	Jingdong prefecture level city in Shandong
Stowe Flagship Store	King of Power Saving	The quality is very good, customer service attitude is very, very awesome, there are problems in a timely manner to solve the problem	Jingdong prefecture level city in Shandong
Stowe Flagship Store	King of Power Saving	Quality is very good, the use of the effect is very good, more stable, quality assurance	Jingdong prefecture level city in Shandong
Stowe Flagship Store	King of Power Saving	Convenient, inexpensive, and see how it works out?	Jingdong prefecture level city in Shandong
Stowe Flagship Store	King of Power Saving	Very good quality, saves a lot of electricity, very good, worth buying	Jingdong prefecture level city in Shandong
Stowe Flagship Store	King of Power Saving	Power Saving King is easy to use, with the fruit of power saving to use easy to give!	Jingdong prefecture level city in Shandong
Stowe Flagship Store	King of Power Saving	This saver really works, good quality, safe and secure, very satisfied!	Jingdong prefecture level city in Shandong
Stowe Flagship Store	King of Power Saving	It works well, the voltage is very stable effect is good, the quality is also very good satisfaction	Jingdong prefecture level city in Shandong
Stowe Flagship Store	King of Power Saving	The logistics is super super fast! The quality is also very good, indeed value for money	Jingdong prefecture level city in Shandong
Stowe Flagship Store	King of Power Saving	I've only been using it for a month or so and it seems to be saving a lot of power	Jingdong prefecture level city in Shandong
Stowe Flagship Store	King of Power Saving	Aluminum alloy shell, durable, compact power-saving good effect	Jingdong prefecture level city in Shandong
Stowe Flagship Store	King of Power Saving	Very easy to operate, very good material, good value for money.	Jingdong prefecture level city in Shandong
Stowe Flagship Store	King of Power Saving	Nice device, easy to use, doesn't take up much space, recommendable!	Jingdong prefecture level city in Shandong
Stowe Flagship Store	King of Power Saving	Good quality, good price, fast logistics	Jingdong prefecture level city in Shandong
Stowe Flagship Store	King of Power Saving	Friends of the family in use recommended, the effect is good and recommendable!	Jingdong prefecture level city in Shandong
Stowe Flagship Store	King of Power Saving	Cheaper than brick and mortar stores worth buying is authentic	Jingdong prefecture level city in Shandong
Stowe Flagship Store	King of Power Saving	Merchants ship quickly, the price is cheaper than the physical store, good use	Jingdong prefecture level city in Shandong
Stowe Flagship Store	King of Power Saving	Very durable, affordable, fast shipping	Jingdong prefecture level city in Shandong
Stowe Flagship Store	King of Power Saving	Packaging tight quality is very good easy to use good results	Jingdong prefecture level city in Shandong
Stowe Flagship Store	King of Power Saving	Save the grid. It's in the whole house. It's saving money on the electric bill.	Jingdong prefecture level city in Shandong
Stowe Flagship Store	King of Power Saving	I used it today, it works well, I'm very satisfied	Jingdong prefecture level city in Shandong
Stowe Flagship Store	King of Power Saving	It really saves a lot of electricity after installation	Jingdong prefecture level city in Shandong
Stowe Flagship Store	King of Power Saving	Love it. Satisfied.	Jingdong prefecture level city in Shandong
Stowe Flagship Store	King of Power Saving	Doesn't feel like it's working.	Jingdong prefecture level city in Shandong
Stowe Flagship Store	King of Power Saving	I'm trying it out to see if it saves power, and I'll buy two more if it works well.	Jingdong prefecture level city in Shandong
Stowe Flagship Store	King of Power Saving	I'll comment after using it.	Jingdong prefecture level city in Shandong
Stowe Flagship Store	King of Power Saving	It is genuine very good, the price is much cheaper, cost-effective, really special cost-effective, it is worth buying!	Jingdong prefecture level city in Shandong
Stowe Flagship Store	King of Power Saving	This thing can really save electricity, I was introduced to buy by my friend, he used it very well, I used it and think it's also very good, it can really save electricity, save about half of the electricity every day, it's really good!	Jingdong prefecture level city in Shandong
Stowe Flagship Store	King of Power Saving	After using it for ten days or so, I checked the power consumption and it did drop, and customer service said it will save more electricity in the long run. The product is also insured, 5 years warranty do not have to worry about quality problems!	Jingdong prefecture level city in Shandong
Stowe Flagship Store	King of Power Saving	The product is good, before already bought a, this is the second, the effect is quite good, anyway, I see the daily electricity bill than before a lot less, this can be the choice!	Jingdong prefecture level city in Shandong

Stowe Flagship Store	King of Power Saving	Originally also very worried about things are not good, get the hand found completely over-anxious, five-star praise, the style is very generous, the quality is very good, the price is also very reasonable!	Jingdong prefecture level city in Shandong
Stowe Flagship Store	King of Power Saving	Quality is good, playing to the old family also buy a, repurchase, this quality is really good, recommended!	Jingdong prefecture level city in Shandong
Stowe Flagship Store	King of Power Saving	Thank you, the owner even sent two fuses.	Jingdong prefecture level city in Shandong
Stowe Flagship Store	King of Power Saving	This store has great stuff, relatively fast shipping and logistics	Jingdong prefecture level city in Shandong
Stowe Flagship Store	King of Power Saving	This product is based on the impossibility of saving electricity, I used 853 kWh of electricity in July, started using the product on August 1, and used 867 kWh of electricity by September 1, is this called	Jingdong prefecture level city in Shandong
Stowe Flagship Store	King of Power Saving	Power king received, to the home on the trial, when I plugged into the electric panel can not see the effect of power saving, the meter is still the same speed in the turn, I'll save the king of the manual to play look at the instructions, instructions to speak, have to have a day two days and half a month to save power obviously, then wait half a month to see the effect of power saving it! To half a month there are obvious power saving before continuing to chase Xu.	Jingdong prefecture level city in Shandong
Stowe Flagship Store	King of Power Saving	Received the goods, a look, did not see the manufacturer and production address, can only hold a try to see the mentality of the face	Jingdong prefecture level city in Shandong
Stowe Flagship Store	King of Power Saving	I've only been using it for a few days and I'm not sure if it's an IQ tax? Used to favorable reviews.	Jingdong prefecture level city in Shandong
Stowe Flagship Store	King of Power Saving	Appearance is okay, power saving next time to follow up the evaluation	Jingdong prefecture level city in Shandong
Stowe Flagship Store	King of Power Saving	Shorter time, but hopefully effective and power-saving, to be followed up in the future	Jingdong prefecture level city in Shandong
Stowe Flagship Store	King of Power Saving	Bought three at once to try first first.	Jingdong prefecture level city in Shandong
Stowe Flagship Store	King of Power Saving	The object is the same as the picture, the product is of high quality, the packaging is intact. High-end atmosphere, high power, power saving effect is obvious, average monthly power saving 50%, very satisfied.	Jingdong prefecture level city in Shandong
Stowe Flagship Store	King of Power Saving	Appearance is okay, I do not know if the power saving	Jingdong prefecture level city in Shandong
Stowe Flagship Store	King of Power Saving	Good service, fast logistics, Power Saving King is exquisite and beautiful, support the business, thank you!	Jingdong prefecture level city in Shandong
Stowe Flagship Store	King of Power Saving	Bought back and plugged in, really effective, before 16 degrees of electricity per day, now 8 degrees of electricity per day, open an air conditioner for 12 hours, decisive repurchase of a	Jingdong prefecture level city in Shandong
Stowe Flagship Store	King of Power Saving	The object is the same as the picture, the product is of high quality, and the package is intact. High-end atmosphere and high grade, high power	Jingdong prefecture level city in Shandong
Stowe Flagship Store	King of Power Saving	Goods received, I hope the store said can be energy-saving, first try a month, month after month to see the effect.	Jingdong prefecture level city in Shandong
Stowe Flagship Store	King of Power Saving	This is really good, the material and workmanship is particularly exquisite, the workmanship is really very good, I think I can recommend to my friends to buy	Jingdong prefecture level city in Shandong
Stowe Flagship Store	King of Power Saving	I have to praise, the quality is really good, and the packaging logistics are also good. Really like it, won't make you regret after getting it!	Jingdong prefecture level city in Shandong
Stowe Flagship Store	King of Power Saving	Reputable platform, efficient and professional service, buy with confidence and peace of mind!	Jingdong prefecture level city in Shandong
Stowe Flagship Store	King of Power Saving	Bought one on a trial basis to see if it works after 1-2 months.	Jingdong prefecture level city in Shandong
Stowe Flagship Store	King of Power Saving	Quality is very good, customer service attitude is very very praise, there are problems in a timely manner to solve the problem, the price is not expensive, super value, you can rest assured that the	Jingdong prefecture level city in Shandong
Stowe Flagship Store	King of Power Saving	With it no longer need to be afraid to use electricity, especially good, ready to buy a mom and dad that also	Jingdong prefecture level city in Shandong
Stowe Flagship Store	King of Power Saving	Baby received very like will return to buy the seller attitude is also very like will return to buy the seller attitude is also very like!	Jingdong prefecture level city in Shandong
Stowe Flagship Store	King of Power Saving	I did not expect the express delivery so quickly, very save point quality is super good, than the entity store is too cheap.	Jingdong prefecture level city in Shandong
Stowe Flagship Store	King of Power Saving	Saving power received, the quality is very good, fine workmanship, especially good, much cheaper than the entity store, it is worth buying!	Jingdong prefecture level city in Shandong
Stowe Flagship Store	King of Power Saving	Jingdong Mall shopping convenience assured fast assured fast assured fast assured fast	Jingdong prefecture level city in Shandong
Stowe Flagship Store	King of Power Saving	Have received the baby, very satisfied with a shopping, great worth buying!	Jingdong prefecture level city in Shandong
Stowe Flagship Store	King of Power Saving	This is really more powerful, very satisfied, more energy-saving, better, thank you boss.	Jingdong prefecture level city in Shandong
Stowe Flagship Store	King of Power Saving	Received has been used for two days, the air conditioning can boldly open it! Energy saving effect is really quite good	Jingdong prefecture level city in Shandong
Stowe Flagship Store	King of Power Saving	It is authentic, trust the merchant, the baby has a great texture and the price is moderate, unlimited repurchase!	Jingdong prefecture level city in Shandong
Stowe Flagship Store	King of Power Saving	It is genuine very good, the price is much cheaper, cost-effective, really special cost-effective, it is worth buying!	Jingdong prefecture level city in Shandong
Stowe Flagship Store	King of Power Saving	Very good quality, fine workmanship, low power consumption, long warranty, fast shipping, economical and practical	Jingdong prefecture level city in Shandong
Stowe Flagship Store	King of Power Saving	The logistics is very fast, the baby is very good, the shopkeeper is NICE!	Jingdong prefecture level city in Shandong
Stowe Flagship Store	King of Power Saving	It's the ideal product, good quality and fast logistics, worth the kudos!	Jingdong prefecture level city in Shandong
Stowe Flagship Store	King of Power Saving	I've been using it for half a month now, it really saves power and the voltage is stable, it's very good	Jingdong prefecture level city in Shandong
Stowe Flagship Store	King of Power Saving	Good to use, good quality, save point durable	Jingdong prefecture level city in Shandong
Stowe Flagship Store	King of Power Saving	The quality is very good, customer service attitude is very, very awesome, there are problems in a timely manner to solve the problem	Jingdong prefecture level city in Shandong
Stowe Flagship Store	King of Power Saving	Quality is very good, the use of the effect is very good, more stable, quality assurance	Jingdong prefecture level city in Shandong
Stowe Flagship Store	King of Power Saving	Convenient, inexpensive, and see how it works out?	Jingdong prefecture level city in Shandong
Stowe Flagship Store	King of Power Saving	Very good quality, saves a lot of electricity, very good, worth buying	Jingdong prefecture level city in Shandong
Stowe Flagship Store	King of Power Saving	Power Saving King is easy to use, with the fruit of power saving to use easy to give!	Jingdong prefecture level city in Shandong
Stowe Flagship Store	King of Power Saving	This saver really works, good quality, safe and secure, very satisfied!	Jingdong prefecture level city in Shandong
Stowe Flagship Store	King of Power Saving	It works well, the voltage is very stable effect is good, the quality is also very good satisfaction	Jingdong prefecture level city in Shandong
Stowe Flagship Store	King of Power Saving	The logistics is super super fast! The quality is also very good, indeed value for money	Jingdong prefecture level city in Shandong
Stowe Flagship Store	King of Power Saving	I've only been using it for a month or so and it seems to be saving a lot of power	Jingdong prefecture level city in Shandong
Stowe Flagship Store	King of Power Saving	Very easy to operate, very good material, good value for money.	Jingdong prefecture level city in Shandong
Stowe Flagship Store	King of Power Saving	Aluminum alloy shell, durable, compact power-saving good effect	Jingdong prefecture level city in Shandong
Stowe Flagship Store	King of Power Saving	Nice device, easy to use, doesn't take up much space, recommendable!	Jingdong prefecture level city in Shandong
Stowe Flagship Store	King of Power Saving	Good quality, good price, fast logistics	Jingdong prefecture level city in Shandong

Stowe Flagship Store	King of Power Saving	Friends of the family in use recommended, the effect is good and recommendable!	Jingdong prefecture level city in Shandong
Stowe Flagship Store	King of Power Saving	Cheaper than brick and mortar stores worth buying is authentic	Jingdong prefecture level city in Shandong
Stowe Flagship Store	King of Power Saving	Merchants ship quickly, the price is cheaper than the physical store, good use	Jingdong prefecture level city in Shandong
Stowe Flagship Store	King of Power Saving	Very durable, affordable, fast shipping	Jingdong prefecture level city in Shandong
Stowe Flagship Store	King of Power Saving	Packaging tight quality is very good easy to use good results	Jingdong prefecture level city in Shandong
Stowe Flagship Store	King of Power Saving	Save the grid. It's in the whole house. It's saving money on the electric bill.	Jingdong prefecture level city in Shandong
Stowe Flagship Store	King of Power Saving	I used it today, it works well, I'm very satisfied	Jingdong prefecture level city in Shandong
Stowe Flagship Store	King of Power Saving	It really saves a lot of electricity after installation	Jingdong prefecture level city in Shandong
Stowe Flagship Store	King of Power Saving	Love it. Satisfied.	Jingdong prefecture level city in Shandong
Stowe Flagship Store	King of Power Saving	Doesn't feel like it's working.	Jingdong prefecture level city in Shandong
Stowe Flagship Store	King of Power Saving	I'll comment after using it.	Jingdong prefecture level city in Shandong
Stowe Flagship Store	King of Power Saving	I'm trying it out to see if it saves power, and I'll buy two more if it works well.	Jingdong prefecture level city in Shandong
Stowe Flagship Store	King of Power Saving	It is genuine very good, the price is much cheaper, cost-effective, really special cost-effective, it is worth buying!	Jingdong prefecture level city in Shandong
Stowe Flagship Store	King of Power Saving	This thing can really save electricity, I was introduced to buy by my friend, he used it very well, I used it and think it's also very good, it can really save electricity, save about half of the electricity every day, it's really good!	Jingdong prefecture level city in Shandong
Stowe Flagship Store	King of Power Saving	After using it for ten days or so, I checked the power consumption and it did drop, and customer service said it will save more electricity in the long run. The product is also insured, 5 years warranty do not have to worry about quality problems!	Jingdong prefecture level city in Shandong
Stowe Flagship Store	King of Power Saving	The product is good, before already bought a, this is the second, the effect is quite good, anyway, I see the daily electricity bill than before a lot less, this can be the choice!	Jingdong prefecture level city in Shandong
Stowe Flagship Store	King of Power Saving	Originally also very worried about things are not good, get the hand found completely over-anxious, five-star praise, the style is very generous, the quality is very good, the price is also very reasonable!	Jingdong prefecture level city in Shandong
Stowe Flagship Store	King of Power Saving	Quality is good, playing to the old family also buy a, repurchase, this quality is really good, recommended!	Jingdong prefecture level city in Shandong
Stowe Flagship Store	King of Power Saving	Thank you, the owner even sent two fuses.	Jingdong prefecture level city in Shandong
Stowe Flagship Store	King of Power Saving	This store has great stuff, relatively fast shipping and logistics	Jingdong prefecture level city in Shandong
Stowe Flagship Store	King of Power Saving	This product is based on the impossibility of saving electricity, I used 853 kWh of electricity in July, started using the product on August 1, and used 867 kWh of electricity by September 1, is this called	Jingdong prefecture level city in Shandong
Stowe Flagship Store	King of Power Saving	Power king received, to the home on the trial, when I plugged into the electric panel can not see the effect of power saving, the meter is still the same speed in the turn, I'll save the king of the manual to play look at the instructions, instructions to speak, have to have a day two days and half a month to save power obviously, then wait half a month to see the effect of power saving it! To half a month there are obvious power saving before continuing to chase Xu.	Jingdong prefecture level city in Shandong
Stowe Flagship Store	King of Power Saving	Received the goods, a look, did not see the manufacturer and production address, can only hold a try to see the mentality of the face	Jingdong prefecture level city in Shandong
Stowe Flagship Store	King of Power Saving	I've only been using it for a few days and I'm not sure if it's an IQ tax? Used to favorable reviews.	Jingdong prefecture level city in Shandong
Stowe Flagship Store	King of Power Saving	Appearance is okay, power saving next time to follow up the evaluation	Jingdong prefecture level city in Shandong
Stowe Flagship Store	King of Power Saving	Shorter time, but hopefully effective and power-saving, to be followed up in the future	Jingdong prefecture level city in Shandong
Stowe Flagship Store	King of Power Saving	Appearance is okay, I do not know if the power saving	Jingdong prefecture level city in Shandong
Stowe Flagship Store	King of Power Saving	Good service, fast logistics, Power Saving King is exquisite and beautiful, support the business, thank you!	Jingdong prefecture level city in Shandong
Stowe Flagship Store	King of Power Saving	Bought three at once to try first first.	Jingdong prefecture level city in Shandong
Stowe Flagship Store	King of Power Saving	The object is the same as the picture, the product is of high quality, the packaging is intact. High-end atmosphere, high power, power saving effect is obvious, average monthly power saving 50%, very satisfied.	Jingdong prefecture level city in Shandong
Stowe Flagship Store	King of Power Saving	Bought back and plugged in, really effective, before 16 degrees of electricity per day, now 8 degrees of electricity per day, open an air conditioner for 12 hours, decisive repurchase of a	Jingdong prefecture level city in Shandong
Stowe Flagship Store	King of Power Saving	The object is the same as the picture, the product is of high quality, and the package is intact. High-end atmosphere and high grade, high power	Jingdong prefecture level city in Shandong
Stowe Flagship Store	King of Power Saving	Goods received, I hope the store said can be energy-saving, first try a month, month after month to see the effect.	Jingdong prefecture level city in Shandong
Stowe Flagship Store	King of Power Saving	This is really good, the material and workmanship is particularly exquisite, the workmanship is really very good, I think I can recommend to my friends to buy	Jingdong prefecture level city in Shandong
Stowe Flagship Store	King of Power Saving	I have to praise, the quality is really good, and the packaging logistics are also good. Really like it, won't make you regret after getting it!	Jingdong prefecture level city in Shandong
Stowe Flagship Store	King of Power Saving	Reputable platform, efficient and professional service, buy with confidence and peace of mind!	Jingdong prefecture level city in Shandong
Stowe Flagship Store	King of Power Saving	Bought one on a trial basis to see if it works after 1-2 months.	Jingdong prefecture level city in Shandong
Stowe Flagship Store	King of Power Saving	Quality is very good, customer service attitude is very very praise, there are problems in a timely manner to solve the problem, the price is not expensive, super value, you can rest assured that the	Jingdong prefecture level city in Shandong
Stowe Flagship Store	King of Power Saving	With it no longer need to be afraid to use electricity, especially good, ready to buy a mom and dad that also	Jingdong prefecture level city in Shandong
Stowe Flagship Store	King of Power Saving	Baby received very like will return to buy the seller attitude is also very like will return to buy the seller attitude is also very like!	Jingdong prefecture level city in Shandong
Stowe Flagship Store	King of Power Saving	Saving power received, the quality is very good, fine workmanship, especially good, much cheaper than the entity store, it is worth buying!	Jingdong prefecture level city in Shandong
Stowe Flagship Store	King of Power Saving	Jingdong Mall shopping convenience assured fast assured fast assured fast assured fast	Jingdong prefecture level city in Shandong
Stowe Flagship Store	King of Power Saving	I did not expect the express delivery so quickly, very save point quality is super good, than the entity store is too cheap.	Jingdong prefecture level city in Shandong
Stowe Flagship Store	King of Power Saving	Have received the baby, very satisfied with a shopping, great worth buying!	Jingdong prefecture level city in Shandong
Stowe Flagship Store	King of Power Saving	This is really more powerful, very satisfied, more energy-saving, better, thank you boss.	Jingdong prefecture level city in Shandong
Stowe Flagship Store	King of Power Saving	Received has been used for two days, the air conditioning can boldly open it! Energy saving effect is really quite good	Jingdong prefecture level city in Shandong
Stowe Flagship Store	King of Power Saving	It is authentic, trust the merchant, the baby has a great texture and the price is moderate, unlimited repurchase!	Jingdong prefecture level city in Shandong
Stowe Flagship Store	King of Power Saving	It is genuine very good, the price is much cheaper, cost-effective, really special cost-effective, it is worth buying!	Jingdong prefecture level city in Shandong
Stowe Flagship Store	King of Power Saving	Very good quality, fine workmanship, low power consumption, long warranty, fast shipping, economical and practical	Jingdong prefecture level city in Shandong

Stowe Flagship Store	King of Power Saving	I've been using it for half a month now, it really saves power and the voltage is stable, it's very good	Jingdong prefecture level city in Shandong
Stowe Flagship Store	King of Power Saving	The logistics is very fast, the baby is very good, the shopkeeper is NICE!	Jingdong prefecture level city in Shandong
Stowe Flagship Store	King of Power Saving	It's the ideal product 💖, good quality and fast logistics, worth the kudos!	Jingdong prefecture level city in Shandong
Stowe Flagship Store	King of Power Saving	Good to use, good quality, save point durable	Jingdong prefecture level city in Shandong
Stowe Flagship Store	King of Power Saving	The quality is very good, customer service attitude is very, very awesome, there are problems in a timely manner to solve the problem	Jingdong prefecture level city in Shandong
Stowe Flagship Store	King of Power Saving	Quality is very good, the use of the effect is very good, more stable, quality assurance	Jingdong prefecture level city in Shandong
Stowe Flagship Store	King of Power Saving	Convenient, inexpensive, and see how it works out?	Jingdong prefecture level city in Shandong
Stowe Flagship Store	King of Power Saving	Very good quality, saves a lot of electricity, very good, worth buying	Jingdong prefecture level city in Shandong
Stowe Flagship Store	King of Power Saving	Power Saving King is easy to use, with the fruit of power saving to use easy to give!	Jingdong prefecture level city in Shandong
Stowe Flagship Store	King of Power Saving	This saver really works, good quality, safe and secure, very satisfied!	Jingdong prefecture level city in Shandong
Stowe Flagship Store	King of Power Saving	It works well, the voltage is very stable effect is good, the quality is also very good satisfaction	Jingdong prefecture level city in Shandong
Stowe Flagship Store	King of Power Saving	The logistics is super super fast! The quality is also very good, indeed value for money	Jingdong prefecture level city in Shandong
Stowe Flagship Store	King of Power Saving	I've only been using it for a month or so and it seems to be saving a lot of power	Jingdong prefecture level city in Shandong
Stowe Flagship Store	King of Power Saving	Very easy to operate, very good material, good value for money.	Jingdong prefecture level city in Shandong
Stowe Flagship Store	King of Power Saving	Aluminum alloy shell, durable, compact power-saving good effect	Jingdong prefecture level city in Shandong
Stowe Flagship Store	King of Power Saving	Nice device, easy to use, doesn't take up much space, recommendable!	Jingdong prefecture level city in Shandong
Stowe Flagship Store	King of Power Saving	Good quality, good price, fast logistics	Jingdong prefecture level city in Shandong
Stowe Flagship Store	King of Power Saving	Friends of the family in use recommended, the effect is good and recommendable!	Jingdong prefecture level city in Shandong
Stowe Flagship Store	King of Power Saving	Cheaper than brick and mortar stores worth buying is authentic	Jingdong prefecture level city in Shandong
Stowe Flagship Store	King of Power Saving	Merchants ship quickly, the price is cheaper than the physical store, good use	Jingdong prefecture level city in Shandong
Stowe Flagship Store	King of Power Saving	Very durable, affordable, fast shipping	Jingdong prefecture level city in Shandong
Stowe Flagship Store	King of Power Saving	Packaging tight quality is very good easy to use good results	Jingdong prefecture level city in Shandong
Stowe Flagship Store	King of Power Saving	Save the grid. It's in the whole house. It's saving money on the electric bill.	Jingdong prefecture level city in Shandong
Stowe Flagship Store	King of Power Saving	I used it today, it works well, I'm very satisfied	Jingdong prefecture level city in Shandong
Stowe Flagship Store	King of Power Saving	It really saves a lot of electricity after installation	Jingdong prefecture level city in Shandong
Stowe Flagship Store	King of Power Saving	Love it. Satisfied.	Jingdong prefecture level city in Shandong
Stowe Flagship Store	King of Power Saving	Doesn't feel like it's working.	Jingdong prefecture level city in Shandong
Stowe Flagship Store	King of Power Saving	I'll comment after using it.	Jingdong prefecture level city in Shandong
Stowe Flagship Store	King of Power Saving	I'm trying it out to see if it saves power, and I'll buy two more if it works well.	Jingdong prefecture level city in Shandong
Stowe Flagship Store	King of Power Saving	It is genuine very good, the price is much cheaper, cost-effective, really special cost-effective, it is worth buying!	Jingdong prefecture level city in Shandong
Stowe Flagship Store	King of Power Saving	This thing can really save electricity, I was introduced to buy by my friend, he used it very well, I used it and think it's also very good, it can really save electricity, save about half of the electricity every day, it's really good!	Jingdong prefecture level city in Shandong
Stowe Flagship Store	King of Power Saving	After using it for ten days or so, I checked the power consumption and it did drop, and customer service said it will save more electricity in the long run. The product is also insured, 5 years warranty do not have to worry about quality problems!	Jingdong prefecture level city in Shandong
Stowe Flagship Store	King of Power Saving	The product is good, before already bought a, this is the second, the effect is quite good, anyway, I see the daily electricity bill than before a lot less, this can be the choice!	Jingdong prefecture level city in Shandong
Stowe Flagship Store	King of Power Saving	Originally also very worried about things are not good, get the hand found completely over-anxious, five-star praise, the style is very generous, the quality is very good, the price is also very reasonable!	Jingdong prefecture level city in Shandong
Stowe Flagship Store	King of Power Saving	Quality is good, playing to the old family also buy a, repurchase, this quality is really good, recommended!	Jingdong prefecture level city in Shandong
Stowe Flagship Store	King of Power Saving	Thank you, the owner even sent two fuses.	Jingdong prefecture level city in Shandong
Stowe Flagship Store	King of Power Saving	This store has great stuff, relatively fast shipping and logistics	Jingdong prefecture level city in Shandong
Stowe Flagship Store	King of Power Saving	This product is based on the impossibility of saving electricity, I used 853 kWh of electricity in July, started using the product on August 1, and used 867 kWh of electricity by September 1, is this called	Jingdong prefecture level city in Shandong
Stowe Flagship Store	King of Power Saving	Power king received, to the home on the trial, when I plugged into the electric panel can not see the effect of power saving, the meter is still the same speed in the turn, I'll save the king of the manual to play look at the instructions, instructions to speak, have to have a day two days and half a month to save power obviously, then wait half a month to see the effect of power saving it! To half a month there are obvious power saving before continuing to chase Xu.	Jingdong prefecture level city in Shandong
Stowe Flagship Store	King of Power Saving	Received the goods, a look, did not see the manufacturer and production address, can only hold a try to see the mentality of the face	Jingdong prefecture level city in Shandong
Stowe Flagship Store	King of Power Saving	I've only been using it for a few days and I'm not sure if it's an IQ tax? Used to favorable reviews.	Jingdong prefecture level city in Shandong
Stowe Flagship Store	King of Power Saving	Appearance is okay, power saving next time to follow up the evaluation	Jingdong prefecture level city in Shandong
Stowe Flagship Store	King of Power Saving	Shorter time, but hopefully effective and power-saving, to be followed up in the future	Jingdong prefecture level city in Shandong
Stowe Flagship Store	King of Power Saving	Appearance is okay, I do not know if the power saving	Jingdong prefecture level city in Shandong
Stowe Flagship Store	King of Power Saving	Good service, fast logistics, Power Saving King is exquisite and beautiful, support the business, thank you!	Jingdong prefecture level city in Shandong
Stowe Flagship Store	King of Power Saving	Bought three at once to try first first.	Jingdong prefecture level city in Shandong
Stowe Flagship Store	King of Power Saving	The object is the same as the picture, the product is of high quality, the packaging is intact. High-end atmosphere, high power, power saving effect is obvious, average monthly power saving 50%, very satisfied.	Jingdong prefecture level city in Shandong
Stowe Flagship Store	King of Power Saving	Bought back and plugged in, really effective, before 16 degrees of electricity per day, now 8 degrees of electricity per day, open an air conditioner for 12 hours, decisive repurchase of a	Jingdong prefecture level city in Shandong
Stowe Flagship Store	King of Power Saving	The object is the same as the picture, the product is of high quality, and the package is intact. High-end atmosphere and high grade, high power	Jingdong prefecture level city in Shandong

Stowe Flagship Store	King of Power Saving	Goods received, I hope the store said can be energy-saving, first try a month, month after month to see the effect.	Jingdong prefecture level city in Shandong
Stowe Flagship Store	King of Power Saving	This is really good, the material and workmanship is particularly exquisite, the workmanship is really very good, I think I can recommend to my friends to buy	Jingdong prefecture level city in Shandong
Stowe Flagship Store	King of Power Saving	I have to praise, the quality is really good, and the packaging logistics are also good. Really like it, won't make you regret after getting it!	Jingdong prefecture level city in Shandong
Stowe Flagship Store	King of Power Saving	Reputable platform, efficient and professional service, buy with confidence and peace of mind!	Jingdong prefecture level city in Shandong
Stowe Flagship Store	King of Power Saving	Bought one on a trial basis to see if it works after 1-2 months.	Jingdong prefecture level city in Shandong
Stowe Flagship Store	King of Power Saving	Quality is very good, customer service attitude is very very praise, there are problems in a timely manner to solve the problem, the price is not expensive, super value, you can rest assured that the	Jingdong prefecture level city in Shandong
Stowe Flagship Store	King of Power Saving	With it no longer need to be afraid to use electricity, especially good, ready to buy a mom and dad that also	Jingdong prefecture level city in Shandong
Stowe Flagship Store	King of Power Saving	Baby received very like will return to buy the seller attitude is also very like will return to buy the seller attitude is also very like!	Jingdong prefecture level city in Shandong
Stowe Flagship Store	King of Power Saving	Jingdong Mall shopping convenience assured fast assured fast assured fast assured fast	Jingdong prefecture level city in Shandong
Stowe Flagship Store	King of Power Saving	Saving power received, the quality is very good, fine workmanship, especially good, much cheaper than the entity store, it is worth buying!	Jingdong prefecture level city in Shandong
Stowe Flagship Store	King of Power Saving	I did not expect the express delivery so quickly, very save point quality is super good, than the entity store is too cheap.	Jingdong prefecture level city in Shandong
Stowe Flagship Store	King of Power Saving	Have received the baby, very satisfied with a shopping, great worth buying!	Jingdong prefecture level city in Shandong
Stowe Flagship Store	King of Power Saving	This is really more powerful, very satisfied, more energy-saving, better, thank you boss.	Jingdong prefecture level city in Shandong
Stowe Flagship Store	King of Power Saving	Received has been used for two days, the air conditioning can boldly open it! Energy saving effect is really quite good	Jingdong prefecture level city in Shandong
Stowe Flagship Store	King of Power Saving	It is authentic, trust the merchant, the baby has a great texture and the price is moderate, unlimited repurchase!	Jingdong prefecture level city in Shandong
Stowe Flagship Store	King of Power Saving	It is genuine very good, the price is much cheaper, cost-effective, really special cost-effective, it is worth buying!	Jingdong prefecture level city in Shandong
Stowe Flagship Store	King of Power Saving	Very good quality, fine workmanship, low power consumption, long warranty, fast shipping, economical and practical	Jingdong prefecture level city in Shandong
Stowe Flagship Store	King of Power Saving	I've been using it for half a month now, it really saves power and the voltage is stable, it's very good	Jingdong prefecture level city in Shandong
Stowe Flagship Store	King of Power Saving	It's the ideal product 💖, good quality and fast logistics, worth the kudos!	Jingdong prefecture level city in Shandong
Stowe Flagship Store	King of Power Saving	The logistics is very fast, the baby is very good, the shopkeeper is NICE!	Jingdong prefecture level city in Shandong
Stowe Flagship Store	King of Power Saving	Good to use, good quality, save point durable	Jingdong prefecture level city in Shandong
Stowe Flagship Store	King of Power Saving	The quality is very good, customer service attitude is very, very awesome, there are problems in a timely manner to solve the problem	Jingdong prefecture level city in Shandong
Stowe Flagship Store	King of Power Saving	Quality is very good, the use of the effect is very good, more stable, quality assurance	Jingdong prefecture level city in Shandong
Stowe Flagship Store	King of Power Saving	Convenient, inexpensive, and see how it works out?	Jingdong prefecture level city in Shandong
Stowe Flagship Store	King of Power Saving	Very good quality, saves a lot of electricity, very good, worth buying	Jingdong prefecture level city in Shandong
Stowe Flagship Store	King of Power Saving	Power Saving King is easy to use, with the fruit of power saving to use easy to give!	Jingdong prefecture level city in Shandong
Stowe Flagship Store	King of Power Saving	This saver really works, good quality, safe and secure, very satisfied!	Jingdong prefecture level city in Shandong
Stowe Flagship Store	King of Power Saving	It works well, the voltage is very stable effect is good, the quality is also very good satisfaction	Jingdong prefecture level city in Shandong
Stowe Flagship Store	King of Power Saving	The logistics is super super fast! The quality is also very good, indeed value for money	Jingdong prefecture level city in Shandong
Stowe Flagship Store	King of Power Saving	I've only been using it for a month or so and it seems to be saving a lot of power	Jingdong prefecture level city in Shandong
Stowe Flagship Store	King of Power Saving	Very easy to operate, very good material, good value for money.	Jingdong prefecture level city in Shandong
Stowe Flagship Store	King of Power Saving	Aluminum alloy shell, durable, compact power-saving good effect	Jingdong prefecture level city in Shandong
Stowe Flagship Store	King of Power Saving	Nice device, easy to use, doesn't take up much space, recommendable!	Jingdong prefecture level city in Shandong
Stowe Flagship Store	King of Power Saving	Good quality, good price, fast logistics	Jingdong prefecture level city in Shandong
Stowe Flagship Store	King of Power Saving	Friends of the family in use recommended, the effect is good and recommendable!	Jingdong prefecture level city in Shandong
Stowe Flagship Store	King of Power Saving	Cheaper than brick and mortar stores worth buying is authentic	Jingdong prefecture level city in Shandong
Stowe Flagship Store	King of Power Saving	Merchants ship quickly, the price is cheaper than the physical store, good use	Jingdong prefecture level city in Shandong
Stowe Flagship Store	King of Power Saving	Very durable, affordable, fast shipping	Jingdong prefecture level city in Shandong
Stowe Flagship Store	King of Power Saving	Packaging tight quality is very good easy to use good results	Jingdong prefecture level city in Shandong
Stowe Flagship Store	King of Power Saving	Save the grid. It's in the whole house. It's saving money on the electric bill.	Jingdong prefecture level city in Shandong
Stowe Flagship Store	King of Power Saving	I used it today, it works well, I'm very satisfied	Jingdong prefecture level city in Shandong
Stowe Flagship Store	King of Power Saving	It really saves a lot of electricity after installation	Jingdong prefecture level city in Shandong
Stowe Flagship Store	King of Power Saving	Love it. Satisfied.	Jingdong prefecture level city in Shandong
Stowe Flagship Store	King of Power Saving	Doesn't feel like it's working.	Jingdong prefecture level city in Shandong
Stowe Flagship Store	King of Power Saving	I'll comment after using it.	Jingdong prefecture level city in Shandong
Stowe Flagship Store	King of Power Saving	I'm trying it out to see if it saves power, and I'll buy two more if it works well.	Jingdong prefecture level city in Shandong
Stowe Flagship Store	King of Power Saving	It is genuine very good, the price is much cheaper, cost-effective, really special cost-effective, it is worth buying!	Jingdong prefecture level city in Shandong
Stowe Flagship Store	King of Power Saving	This thing can really save electricity, I was introduced to buy by my friend, he used it very well, I used it and think it's also very good, it can really save electricity, save about half of the electricity every day, it's really good!	Jingdong prefecture level city in Shandong
Stowe Flagship Store	King of Power Saving	After using it for ten days or so, I checked the power consumption and it did drop, and customer service said it will save more electricity in the long run. The product is also insured, 5 years warranty do not have to worry about quality problems!	Jingdong prefecture level city in Shandong
Stowe Flagship Store	King of Power Saving	The product is good, before already bought a, this is the second, the effect is quite good, anyway, I see the daily electricity bill than before a lot less, this can be the choice!	Jingdong prefecture level city in Shandong

Stowe Flagship Store	King of Power Saving	Originally also very worried about things are not good, get the hand found completely over-anxious, five-star praise, the style is very generous, the quality is very good, the price is also very reasonable!	Jingdong prefecture level city in Shandong
Stowe Flagship Store	King of Power Saving	Quality is good, playing to the old family also buy a, repurchase, this quality is really good, recommended!	Jingdong prefecture level city in Shandong
Stowe Flagship Store	King of Power Saving	Thank you, the owner even sent two fuses.	Jingdong prefecture level city in Shandong
Stowe Flagship Store	King of Power Saving	This store has great stuff, relatively fast shipping and logistics	Jingdong prefecture level city in Shandong
Stowe Flagship Store	King of Power Saving	This product is based on the impossibility of saving electricity, I used 853 kWh of electricity in July, started using the product on August 1, and used 867 kWh of electricity by September 1, is this called	Jingdong prefecture level city in Shandong
Stowe Flagship Store	King of Power Saving	Power king received, to the home on the trial, when I plugged into the electric panel can not see the effect of power saving, the meter is still the same speed in the turn, I'll save the king of the manual to play look at the instructions, instructions to speak, have to have a day two days and half a month to save power obviously, then wait half a month to see the effect of power saving it! To half a month there are obvious power saving before continuing to chase Xu.	Jingdong prefecture level city in Shandong
Stowe Flagship Store	King of Power Saving	Received the goods, a look, did not see the manufacturer and production address, can only hold a try to see the mentality of the face	Jingdong prefecture level city in Shandong
Stowe Flagship Store	King of Power Saving	I've only been using it for a few days and I'm not sure if it's an IQ tax? Used to favorable reviews.	Jingdong prefecture level city in Shandong
Stowe Flagship Store	King of Power Saving	Appearance is okay, power saving next time to follow up the evaluation	Jingdong prefecture level city in Shandong
Stowe Flagship Store	King of Power Saving	Shorter time, but hopefully effective and power-saving, to be followed up in the future	Jingdong prefecture level city in Shandong
Stowe Flagship Store	King of Power Saving	Appearance is okay, I do not know if the power saving	Jingdong prefecture level city in Shandong
Stowe Flagship Store	King of Power Saving	Good service, fast logistics, Power Saving King is exquisite and beautiful, support the business, thank you!	Jingdong prefecture level city in Shandong
Stowe Flagship Store	King of Power Saving	Bought three at once to try first first.	Jingdong prefecture level city in Shandong
Stowe Flagship Store	King of Power Saving	The object is the same as the picture, the product is of high quality, the packaging is intact. High-end atmosphere, high power, power saving effect is obvious, average monthly power saving 50%, very satisfied.	Jingdong prefecture level city in Shandong
Stowe Flagship Store	King of Power Saving	Bought back and plugged in, really effective, before 16 degrees of electricity per day, now 8 degrees of electricity per day, open an air conditioner for 12 hours, decisive repurchase of a	Jingdong prefecture level city in Shandong
Stowe Flagship Store	King of Power Saving	The object is the same as the picture, the product is of high quality, and the package is intact. High-end atmosphere and high grade, high power	Jingdong prefecture level city in Shandong
Stowe Flagship Store	King of Power Saving	Goods received, I hope the store said can be energy-saving, first try a month, month after month to see the effect.	Jingdong prefecture level city in Shandong
Stowe Flagship Store	King of Power Saving	This is really good, the material and workmanship is particularly exquisite, the workmanship is really very good, I think I can recommend to my friends to buy	Jingdong prefecture level city in Shandong
Stowe Flagship Store	King of Power Saving	I have to praise, the quality is really good, and the packaging logistics are also good. Really like it, won't make you regret after getting it!	Jingdong prefecture level city in Shandong
Stowe Flagship Store	King of Power Saving	Reputable platform, efficient and professional service, buy with confidence and peace of mind!	Jingdong prefecture level city in Shandong
Stowe Flagship Store	King of Power Saving	Bought one on a trial basis to see if it works after 1-2 months.	Jingdong prefecture level city in Shandong
Stowe Flagship Store	King of Power Saving	Quality is very good, customer service attitude is very very praise, there are problems in a timely manner to solve the problem, the price is not expensive, super value, you can rest assured that the	Jingdong prefecture level city in Shandong
Stowe Flagship Store	King of Power Saving	With it no longer need to be afraid to use electricity, especially good, ready to buy a mom and dad that also	Jingdong prefecture level city in Shandong
Stowe Flagship Store	King of Power Saving	Baby received very like will return to buy the seller attitude is also very like will return to buy the seller attitude is also very like!	Jingdong prefecture level city in Shandong
Stowe Flagship Store	King of Power Saving	Jingdong Mall shopping convenience assured fast assured fast assured fast assured fast	Jingdong prefecture level city in Shandong
Stowe Flagship Store	King of Power Saving	Saving power received, the quality is very good, fine workmanship, especially good, much cheaper than the entity store, it is worth buying!	Jingdong prefecture level city in Shandong
Stowe Flagship Store	King of Power Saving	I did not expect the express delivery so quickly, very save point quality is super good, than the entity store is too cheap.	Jingdong prefecture level city in Shandong
Stowe Flagship Store	King of Power Saving	Have received the baby, very satisfied with a shopping, great worth buying!	Jingdong prefecture level city in Shandong
Stowe Flagship Store	King of Power Saving	This is really more powerful, very satisfied, more energy-saving, better, thank you boss.	Jingdong prefecture level city in Shandong
Stowe Flagship Store	King of Power Saving	Received has been used for two days, the air conditioning can boldly open it! Energy saving effect is really quite good	Jingdong prefecture level city in Shandong
Stowe Flagship Store	King of Power Saving	It is authentic, trust the merchant, the baby has a great texture and the price is moderate, unlimited repurchase!	Jingdong prefecture level city in Shandong
Stowe Flagship Store	King of Power Saving	It is genuine very good, the price is much cheaper, cost-effective, really special cost-effective, it is worth buying!	Jingdong prefecture level city in Shandong
Stowe Flagship Store	King of Power Saving	Very good quality, fine workmanship, low power consumption, long warranty, fast shipping, economical and practical	Jingdong prefecture level city in Shandong
Stowe Flagship Store	King of Power Saving	I've been using it for half a month now, it really saves power and the voltage is stable, it's very good	Jingdong prefecture level city in Shandong
Stowe Flagship Store	King of Power Saving	It's the ideal product 💖, good quality and fast logistics, worth the kudos!	Jingdong prefecture level city in Shandong
Stowe Flagship Store	King of Power Saving	The logistics is very fast, the baby is very good, the shopkeeper is NICE!	Jingdong prefecture level city in Shandong
Stowe Flagship Store	King of Power Saving	Good to use, good quality, save point durable	Jingdong prefecture level city in Shandong
Stowe Flagship Store	King of Power Saving	The quality is very good, customer service attitude is very, very awesome, there are problems in a timely manner to solve the problem	Jingdong prefecture level city in Shandong
Stowe Flagship Store	King of Power Saving	Quality is very good, the use of the effect is very good, more stable, quality assurance	Jingdong prefecture level city in Shandong
Stowe Flagship Store	King of Power Saving	Convenient, inexpensive, and see how it works out?	Jingdong prefecture level city in Shandong
Stowe Flagship Store	King of Power Saving	Very good quality, saves a lot of electricity, very good, worth buying	Jingdong prefecture level city in Shandong
Stowe Flagship Store	King of Power Saving	Power Saving King is easy to use, with the fruit of power saving to use easy to give!	Jingdong prefecture level city in Shandong
Stowe Flagship Store	King of Power Saving	This saver really works, good quality, safe and secure, very satisfied!	Jingdong prefecture level city in Shandong
Stowe Flagship Store	King of Power Saving	It works well, the voltage is very stable effect is good, the quality is also very good satisfaction	Jingdong prefecture level city in Shandong
Stowe Flagship Store	King of Power Saving	The logistics is super super fast! The quality is also very good, indeed value for money	Jingdong prefecture level city in Shandong
Stowe Flagship Store	King of Power Saving	I've only been using it for a month or so and it seems to be saving a lot of power	Jingdong prefecture level city in Shandong
Stowe Flagship Store	King of Power Saving	Very easy to operate, very good material, good value for money.	Jingdong prefecture level city in Shandong
Stowe Flagship Store	King of Power Saving	Aluminum alloy shell, durable, compact power-saving good effect	Jingdong prefecture level city in Shandong
Stowe Flagship Store	King of Power Saving	Nice device, easy to use, doesn't take up much space, recommendable!	Jingdong prefecture level city in Shandong
Stowe Flagship Store	King of Power Saving	Good quality, good price, fast logistics	Jingdong prefecture level city in Shandong

Stowe Flagship Store	King of Power Saving	Friends of the family in use recommended, the effect is good and recommendable!	Jingdong prefecture level city in Shandong
Stowe Flagship Store	King of Power Saving	Cheaper than brick and mortar stores worth buying is authentic	Jingdong prefecture level city in Shandong
Stowe Flagship Store	King of Power Saving	Merchants ship quickly, the price is cheaper than the physical store, good use	Jingdong prefecture level city in Shandong
Stowe Flagship Store	King of Power Saving	Very durable, affordable, fast shipping	Jingdong prefecture level city in Shandong
Stowe Flagship Store	King of Power Saving	Packaging tight quality is very good easy to use good results	Jingdong prefecture level city in Shandong
Stowe Flagship Store	King of Power Saving	Save the grid. It's in the whole house. It's saving money on the electric bill.	Jingdong prefecture level city in Shandong
Stowe Flagship Store	King of Power Saving	I used it today, it works well, I'm very satisfied	Jingdong prefecture level city in Shandong
Stowe Flagship Store	King of Power Saving	It really saves a lot of electricity after installation	Jingdong prefecture level city in Shandong
Stowe Flagship Store	King of Power Saving	Love it. Satisfied.	Jingdong prefecture level city in Shandong
Stowe Flagship Store	King of Power Saving	Doesn't feel like it's working.	Jingdong prefecture level city in Shandong
Stowe Flagship Store	King of Power Saving	I'll comment after using it.	Jingdong prefecture level city in Shandong
Stowe Flagship Store	King of Power Saving	I'm trying it out to see if it saves power, and I'll buy two more if it works well.	Jingdong prefecture level city in Shandong
Stowe Flagship Store	King of Power Saving	It is genuine very good, the price is much cheaper, cost-effective, really special cost-effective, it is worth buying!	Jingdong prefecture level city in Shandong
Stowe Flagship Store	King of Power Saving	This thing can really save electricity, I was introduced to buy by my friend, he used it very well, I used it and think it's also very good, it can really save electricity, save about half of the electricity every day, it's really good!	Jingdong prefecture level city in Shandong
Stowe Flagship Store	King of Power Saving	After using it for ten days or so, I checked the power consumption and it did drop, and customer service said it will save more electricity in the long run. The product is also insured, 5 years warranty do not have to worry about quality problems!	Jingdong prefecture level city in Shandong
Stowe Flagship Store	King of Power Saving	The product is good, before already bought a, this is the second, the effect is quite good, anyway, I see the daily electricity bill than before a lot less, this can be the choice!	Jingdong prefecture level city in Shandong
Stowe Flagship Store	King of Power Saving	Originally also very worried about things are not good, get the hand found completely over-anxious, five-star praise, the style is very generous, the quality is very good, the price is also very reasonable!	Jingdong prefecture level city in Shandong
Stowe Flagship Store	King of Power Saving	Quality is good, playing to the old family also buy a, repurchase, this quality is really good, recommended!	Jingdong prefecture level city in Shandong
Stowe Flagship Store	King of Power Saving	Thank you, the owner even sent two fuses.	Jingdong prefecture level city in Shandong
Stowe Flagship Store	King of Power Saving	This store has great stuff, relatively fast shipping and logistics	Jingdong prefecture level city in Shandong
Stowe Flagship Store	King of Power Saving	This product is based on the impossibility of saving electricity, I used 853 kWh of electricity in July, started using the product on August 1, and used 867 kWh of electricity by September 1, is this called	Jingdong prefecture level city in Shandong
Stowe Flagship Store	King of Power Saving	Power king received, to the home on the trial, when I plugged into the electric panel can not see the effect of power saving, the meter is still the same speed in the turn, I'll save the king of the manual to play look at the instructions, instructions to speak, have to have a day two days and half a month to save power obviously, then wait half a month to see the effect of power saving it! To half a month there are obvious power saving before continuing to chase Xu.	Jingdong prefecture level city in Shandong
Stowe Flagship Store	King of Power Saving	Received the goods, a look, did not see the manufacturer and production address, can only hold a try to see the mentality of the face	Jingdong prefecture level city in Shandong
Stowe Flagship Store	King of Power Saving	I've only been using it for a few days and I'm not sure if it's an IQ tax? Used to favorable reviews.	Jingdong prefecture level city in Shandong
Stowe Flagship Store	King of Power Saving	Appearance is okay, power saving next time to follow up the evaluation	Jingdong prefecture level city in Shandong
Stowe Flagship Store	King of Power Saving	Shorter time, but hopefully effective and power-saving, to be followed up in the future	Jingdong prefecture level city in Shandong
Stowe Flagship Store	King of Power Saving	Appearance is okay, I do not know if the power saving	Jingdong prefecture level city in Shandong
Stowe Flagship Store	King of Power Saving	Good service, fast logistics, Power Saving King is exquisite and beautiful, support the business, thank you!	Jingdong prefecture level city in Shandong
Stowe Flagship Store	King of Power Saving	Bought three at once to try first first.	Jingdong prefecture level city in Shandong
Stowe Flagship Store	King of Power Saving	The object is the same as the picture, the product is of high quality, the packaging is intact. High-end atmosphere, high power, power saving effect is obvious, average monthly power saving 50%, very satisfied.	Jingdong prefecture level city in Shandong
Stowe Flagship Store	King of Power Saving	Bought back and plugged in, really effective, before 16 degrees of electricity per day, now 8 degrees of electricity per day, open an air conditioner for 12 hours, decisive repurchase of a	Jingdong prefecture level city in Shandong
Stowe Flagship Store	King of Power Saving	The object is the same as the picture, the product is of high quality, and the package is intact. High-end atmosphere and high grade, high power	Jingdong prefecture level city in Shandong
Stowe Flagship Store	King of Power Saving	Goods received, I hope the store said can be energy-saving, first try a month, month after month to see the effect.	Jingdong prefecture level city in Shandong
Stowe Flagship Store	King of Power Saving	This is really good, the material and workmanship is particularly exquisite, the workmanship is really very good, I think I can recommend to my friends to buy	Jingdong prefecture level city in Shandong
Stowe Flagship Store	King of Power Saving	I have to praise, the quality is really good, and the packaging logistics are also good. Really like it, won't make you regret after getting it!	Jingdong prefecture level city in Shandong
Stowe Flagship Store	King of Power Saving	Reputable platform, efficient and professional service, buy with confidence and peace of mind!	Jingdong prefecture level city in Shandong
Stowe Flagship Store	King of Power Saving	Bought one on a trial basis to see if it works after 1-2 months.	Jingdong prefecture level city in Shandong
Stowe Flagship Store	King of Power Saving	Quality is very good, customer service attitude is very very praise, there are problems in a timely manner to solve the problem, the price is not expensive, super value, you can rest assured that the	Jingdong prefecture level city in Shandong
Stowe Flagship Store	King of Power Saving	With it no longer need to be afraid to use electricity, especially good, ready to buy a mom and dad that also	Jingdong prefecture level city in Shandong
Stowe Flagship Store	King of Power Saving	Baby received very like will return to buy the seller attitude is also very like will return to buy the seller attitude is also very like!	Jingdong prefecture level city in Shandong
Stowe Flagship Store	King of Power Saving	Jingdong Mall shopping convenience assured fast assured fast assured fast assured fast	Jingdong prefecture level city in Shandong
Stowe Flagship Store	King of Power Saving	Saving power received, the quality is very good, fine workmanship, especially good, much cheaper than the entity store, it is worth buying!	Jingdong prefecture level city in Shandong
Stowe Flagship Store	King of Power Saving	I did not expect the express delivery so quickly, very save point quality is super good, than the entity store is too cheap.	Jingdong prefecture level city in Shandong
Stowe Flagship Store	King of Power Saving	Have received the baby, very satisfied with a shopping, great worth buying!	Jingdong prefecture level city in Shandong
Stowe Flagship Store	King of Power Saving	This is really more powerful, very satisfied, more energy-saving, better, thank you boss.	Jingdong prefecture level city in Shandong
Stowe Flagship Store	King of Power Saving	Received has been used for two days, the air conditioning can boldly open it! Energy saving effect is really quite good	Jingdong prefecture level city in Shandong
Stowe Flagship Store	King of Power Saving	It is authentic, trust the merchant, the baby has a great texture and the price is moderate, unlimited repurchase!	Jingdong prefecture level city in Shandong
Stowe Flagship Store	King of Power Saving	It is genuine very good, the price is much cheaper, cost-effective, really special cost-effective, it is worth buying!	Jingdong prefecture level city in Shandong
Stowe Flagship Store	King of Power Saving	Very good quality, fine workmanship, low power consumption, long warranty, fast shipping, economical and practical	Jingdong prefecture level city in Shandong

Stowe Flagship Store	King of Power Saving	I've been using it for half a month now, it really saves power and the voltage is stable, it's very good	Jingdong prefecture level city in Shandong
Stowe Flagship Store	King of Power Saving	It's the ideal product ❤, good quality and fast logistics, worth the kudos!	Jingdong prefecture level city in Shandong
Stowe Flagship Store	King of Power Saving	The logistics is very fast, the baby is very good, the shopkeeper is NICE!	Jingdong prefecture level city in Shandong
Stowe Flagship Store	King of Power Saving	Good to use, good quality, save point durable	Jingdong prefecture level city in Shandong
Stowe Flagship Store	King of Power Saving	The quality is very good, customer service attitude is very, very awesome, there are problems in a timely manner to solve the problem	Jingdong prefecture level city in Shandong
Stowe Flagship Store	King of Power Saving	Quality is very good, the use of the effect is very good, more stable, quality assurance	Jingdong prefecture level city in Shandong
Stowe Flagship Store	King of Power Saving	Convenient, inexpensive, and see how it works out?	Jingdong prefecture level city in Shandong
Stowe Flagship Store	King of Power Saving	Very good quality, saves a lot of electricity, very good, worth buying	Jingdong prefecture level city in Shandong
Stowe Flagship Store	King of Power Saving	Power Saving King is easy to use, with the fruit of power saving to use easy to give!	Jingdong prefecture level city in Shandong
Stowe Flagship Store	King of Power Saving	This saver really works, good quality, safe and secure, very satisfied!	Jingdong prefecture level city in Shandong
Stowe Flagship Store	King of Power Saving	It works well, the voltage is very stable effect is good, the quality is also very good satisfaction	Jingdong prefecture level city in Shandong
Stowe Flagship Store	King of Power Saving	The logistics is super super fast! The quality is also very good, indeed value for money	Jingdong prefecture level city in Shandong
Stowe Flagship Store	King of Power Saving	I've only been using it for a month or so and it seems to be saving a lot of power	Jingdong prefecture level city in Shandong
Stowe Flagship Store	King of Power Saving	Very easy to operate, very good material, good value for money.	Jingdong prefecture level city in Shandong
Stowe Flagship Store	King of Power Saving	Aluminum alloy shell, durable, compact power-saving good effect	Jingdong prefecture level city in Shandong
Stowe Flagship Store	King of Power Saving	Nice device, easy to use, doesn't take up much space, recommendable!	Jingdong prefecture level city in Shandong
Stowe Flagship Store	King of Power Saving	Good quality, good price, fast logistics	Jingdong prefecture level city in Shandong
Stowe Flagship Store	King of Power Saving	Friends of the family in use recommended, the effect is good and recommendable!	Jingdong prefecture level city in Shandong
Stowe Flagship Store	King of Power Saving	Cheaper than brick and mortar stores worth buying is authentic	Jingdong prefecture level city in Shandong
Stowe Flagship Store	King of Power Saving	Merchants ship quickly, the price is cheaper than the physical store, good use	Jingdong prefecture level city in Shandong
Stowe Flagship Store	King of Power Saving	Very durable, affordable, fast shipping	Jingdong prefecture level city in Shandong
Stowe Flagship Store	King of Power Saving	Packaging tight quality is very good easy to use good results	Jingdong prefecture level city in Shandong
Stowe Flagship Store	King of Power Saving	Save the grid. It's in the whole house. It's saving money on the electric bill.	Jingdong prefecture level city in Shandong
Stowe Flagship Store	King of Power Saving	I used it today, it works well, I'm very satisfied	Jingdong prefecture level city in Shandong
Stowe Flagship Store	King of Power Saving	It really saves a lot of electricity after installation	Jingdong prefecture level city in Shandong
Stowe Flagship Store	King of Power Saving	Love it. Satisfied.	Jingdong prefecture level city in Shandong
Stowe Flagship Store	King of Power Saving	Doesn't feel like it's working.	Jingdong prefecture level city in Shandong
Stowe Flagship Store	King of Power Saving	I'll comment after using it.	Jingdong prefecture level city in Shandong
Stowe Flagship Store	King of Power Saving	I'm trying it out to see if it saves power, and I'll buy two more if it works well.	Jingdong prefecture level city in Shandong
Stowe Flagship Store	King of Power Saving	It is genuine very good, the price is much cheaper, cost-effective, really special cost-effective, it is worth buying!	Jingdong prefecture level city in Shandong
Stowe Flagship Store	King of Power Saving	This thing can really save electricity, I was introduced to buy by my friend, he used it very well, I used it and think it's also very good, it can really save electricity, save about half of the electricity every day, it's really good!	Jingdong prefecture level city in Shandong
Stowe Flagship Store	King of Power Saving	After using it for ten days or so, I checked the power consumption and it did drop, and customer service said it will save more electricity in the long run. The product is also insured, 5 years warranty do not have to worry about quality problems!	Jingdong prefecture level city in Shandong
Stowe Flagship Store	King of Power Saving	The product is good, before already bought a, this is the second, the effect is quite good, anyway, I see the daily electricity bill than before a lot less, this can be the choice!	Jingdong prefecture level city in Shandong
Stowe Flagship Store	King of Power Saving	Originally also very worried about things are not good, get the hand found completely over-anxious, five-star praise, the style is very generous, the quality is very good, the price is also very reasonable!	Jingdong prefecture level city in Shandong
Stowe Flagship Store	King of Power Saving	Quality is good, playing to the old family also buy a, repurchase, this quality is really good, recommended!	Jingdong prefecture level city in Shandong
Stowe Flagship Store	King of Power Saving	Thank you, the owner even sent two fuses.	Jingdong prefecture level city in Shandong
Stowe Flagship Store	King of Power Saving	This store has great stuff, relatively fast shipping and logistics	Jingdong prefecture level city in Shandong
Stowe Flagship Store	King of Power Saving	This product is based on the impossibility of saving electricity, I used 853 kWh of electricity in July, started using the product on August 1, and used 867 kWh of electricity by September 1, is this called	Jingdong prefecture level city in Shandong
Stowe Flagship Store	King of Power Saving	Power king received, to the home on the trial, when I plugged into the electric panel can not see the effect of power saving, the meter is still the same speed in the turn, I'll save the king of the manual to play look at the instructions, instructions to speak, have to have a day two days and half a month to save power obviously, then wait half a month to see the effect of power saving it! To half a month there are obvious power saving before continuing to chase Xu.	Jingdong prefecture level city in Shandong
Stowe Flagship Store	King of Power Saving	Received the goods, a look, did not see the manufacturer and production address, can only hold a try to see the mentality of the face	Jingdong prefecture level city in Shandong
Stowe Flagship Store	King of Power Saving	I've only been using it for a few days and I'm not sure if it's an IQ tax? Used to favorable reviews.	Jingdong prefecture level city in Shandong
Stowe Flagship Store	King of Power Saving	Appearance is okay, power saving next time to follow up the evaluation	Jingdong prefecture level city in Shandong
Stowe Flagship Store	King of Power Saving	Shorter time, but hopefully effective and power-saving, to be followed up in the future	Jingdong prefecture level city in Shandong
Stowe Flagship Store	King of Power Saving	Appearance is okay, I do not know if the power saving	Jingdong prefecture level city in Shandong
Stowe Flagship Store	King of Power Saving	Good service, fast logistics, Power Saving King is exquisite and beautiful, support the business, thank you!	Jingdong prefecture level city in Shandong
Stowe Flagship Store	King of Power Saving	Bought three at once to try first first.	Jingdong prefecture level city in Shandong
Stowe Flagship Store	King of Power Saving	The object is the same as the picture, the product is of high quality, the packaging is intact. High-end atmosphere, high power, power saving effect is obvious, average monthly power saving 50%, very satisfied.	Jingdong prefecture level city in Shandong
Stowe Flagship Store	King of Power Saving	Bought back and plugged in, really effective, before 16 degrees of electricity per day, now 8 degrees of electricity per day, open an air conditioner for 12 hours, decisive repurchase of a	Jingdong prefecture level city in Shandong
Stowe Flagship Store	King of Power Saving	The object is the same as the picture, the product is of high quality, and the package is intact. High-end atmosphere and high grade, high power	Jingdong prefecture level city in Shandong

Stowe Flagship Store	King of Power Saving	Goods received, I hope the store said can be energy-saving, first try a month, month after month to see the effect.	Jingdong prefecture level city in Shandong
Stowe Flagship Store	King of Power Saving	This is really good, the material and workmanship is particularly exquisite, the workmanship is really very good, I think I can recommend to my friends to buy	Jingdong prefecture level city in Shandong
Stowe Flagship Store	King of Power Saving	I have to praise, the quality is really good, and the packaging logistics are also good. Really like it, won't make you regret after getting it!	Jingdong prefecture level city in Shandong
Stowe Flagship Store	King of Power Saving	Reputable platform, efficient and professional service, buy with confidence and peace of mind!	Jingdong prefecture level city in Shandong
Stowe Flagship Store	King of Power Saving	Bought one on a trial basis to see if it works after 1-2 months.	Jingdong prefecture level city in Shandong
Stowe Flagship Store	King of Power Saving	Quality is very good, customer service attitude is very very praise, there are problems in a timely manner to solve the problem, the price is not expensive, super value, you can rest assured that the	Jingdong prefecture level city in Shandong
Stowe Flagship Store	King of Power Saving	With it no longer need to be afraid to use electricity, especially good, ready to buy a mom and dad that also	Jingdong prefecture level city in Shandong
Stowe Flagship Store	King of Power Saving	Baby received very like will return to buy the seller attitude is also very like will return to buy the seller attitude is also very like!	Jingdong prefecture level city in Shandong
Stowe Flagship Store	King of Power Saving	Jingdong Mall shopping convenience assured fast assured fast assured fast assured fast	Jingdong prefecture level city in Shandong
Stowe Flagship Store	King of Power Saving	Saving power received, the quality is very good, fine workmanship, especially good, much cheaper than the entity store, it is worth buying!	Jingdong prefecture level city in Shandong
Stowe Flagship Store	King of Power Saving	I did not expect the express delivery so quickly, very save point quality is super good, than the entity store is too cheap.	Jingdong prefecture level city in Shandong
Stowe Flagship Store	King of Power Saving	Have received the baby, very satisfied with a shopping, great worth buying!	Jingdong prefecture level city in Shandong
Stowe Flagship Store	King of Power Saving	This is really more powerful, very satisfied, more energy-saving, better, thank you boss.	Jingdong prefecture level city in Shandong
Stowe Flagship Store	King of Power Saving	Received has been used for two days, the air conditioning can boldly open it! Energy saving effect is really quite good	Jingdong prefecture level city in Shandong
Stowe Flagship Store	King of Power Saving	It is authentic, trust the merchant, the baby has a great texture and the price is moderate, unlimited repurchase!	Jingdong prefecture level city in Shandong
Stowe Flagship Store	King of Power Saving	It is genuine very good, the price is much cheaper, cost-effective, really special cost-effective, it is worth buying!	Jingdong prefecture level city in Shandong
Stowe Flagship Store	King of Power Saving	Very good quality, fine workmanship, low power consumption, long warranty, fast shipping, economical and practical	Jingdong prefecture level city in Shandong
Stowe Flagship Store	King of Power Saving	I've been using it for half a month now, it really saves power and the voltage is stable, it's very good	Jingdong prefecture level city in Shandong
Stowe Flagship Store	King of Power Saving	It's the ideal product 💖, good quality and fast logistics, worth the kudos!	Jingdong prefecture level city in Shandong
Stowe Flagship Store	King of Power Saving	The logistics is very fast, the baby is very good, the shopkeeper is NICE!	Jingdong prefecture level city in Shandong
Stowe Flagship Store	King of Power Saving	Good to use, good quality, save point durable	Jingdong prefecture level city in Shandong
Stowe Flagship Store	King of Power Saving	The quality is very good, customer service attitude is very, very awesome, there are problems in a timely manner to solve the problem	Jingdong prefecture level city in Shandong
Stowe Flagship Store	King of Power Saving	Quality is very good, the use of the effect is very good, more stable, quality assurance	Jingdong prefecture level city in Shandong
Stowe Flagship Store	King of Power Saving	Convenient, inexpensive, and see how it works out?	Jingdong prefecture level city in Shandong
Stowe Flagship Store	King of Power Saving	Very good quality, saves a lot of electricity, very good, worth buying	Jingdong prefecture level city in Shandong
Stowe Flagship Store	King of Power Saving	Power Saving King is easy to use, with the fruit of power saving to use easy to give!	Jingdong prefecture level city in Shandong
Stowe Flagship Store	King of Power Saving	This saver really works, good quality, safe and secure, very satisfied!	Jingdong prefecture level city in Shandong
Stowe Flagship Store	King of Power Saving	It works well, the voltage is very stable effect is good, the quality is also very good satisfaction	Jingdong prefecture level city in Shandong
Stowe Flagship Store	King of Power Saving	The logistics is super super fast! The quality is also very good, indeed value for money	Jingdong prefecture level city in Shandong
Stowe Flagship Store	King of Power Saving	I've only been using it for a month or so and it seems to be saving a lot of power	Jingdong prefecture level city in Shandong
Stowe Flagship Store	King of Power Saving	Very easy to operate, very good material, good value for money.	Jingdong prefecture level city in Shandong
Stowe Flagship Store	King of Power Saving	Aluminum alloy shell, durable, compact power-saving good effect	Jingdong prefecture level city in Shandong
Stowe Flagship Store	King of Power Saving	Nice device, easy to use, doesn't take up much space, recommendable!	Jingdong prefecture level city in Shandong
Stowe Flagship Store	King of Power Saving	Good quality, good price, fast logistics	Jingdong prefecture level city in Shandong
Stowe Flagship Store	King of Power Saving	Friends of the family in use recommended, the effect is good and recommendable!	Jingdong prefecture level city in Shandong
Stowe Flagship Store	King of Power Saving	Cheaper than brick and mortar stores worth buying is authentic	Jingdong prefecture level city in Shandong
Stowe Flagship Store	King of Power Saving	Merchants ship quickly, the price is cheaper than the physical store, good use	Jingdong prefecture level city in Shandong
Stowe Flagship Store	King of Power Saving	Very durable, affordable, fast shipping	Jingdong prefecture level city in Shandong
Stowe Flagship Store	King of Power Saving	Packaging tight quality is very good easy to use good results	Jingdong prefecture level city in Shandong
Stowe Flagship Store	King of Power Saving	Save the grid. It's in the whole house. It's saving money on the electric bill.	Jingdong prefecture level city in Shandong
Stowe Flagship Store	King of Power Saving	I used it today, it works well, I'm very satisfied	Jingdong prefecture level city in Shandong
Stowe Flagship Store	King of Power Saving	It really saves a lot of electricity after installation	Jingdong prefecture level city in Shandong
Stowe Flagship Store	King of Power Saving	Love it. Satisfied.	Jingdong prefecture level city in Shandong
Stowe Flagship Store	King of Power Saving	Doesn't feel like it's working.	Jingdong prefecture level city in Shandong
Stowe Flagship Store	King of Power Saving	I'll comment after using it.	Jingdong prefecture level city in Shandong
Stowe Flagship Store	King of Power Saving	I'm trying it out to see if it saves power, and I'll buy two more if it works well.	Jingdong prefecture level city in Shandong
Stowe Flagship Store	King of Power Saving	It is genuine very good, the price is much cheaper, cost-effective, really special cost-effective, it is worth buying!	Jingdong prefecture level city in Shandong
Stowe Flagship Store	King of Power Saving	This thing can really save electricity, I was introduced to buy by my friend, he used it very well, I used it and think it's also very good, it can really save electricity, save about half of the electricity every day, it's really good!	Jingdong prefecture level city in Shandong
Stowe Flagship Store	King of Power Saving	After using it for ten days or so, I checked the power consumption and it did drop, and customer service said it will save more electricity in the long run. The product is also insured, 5 years warranty do not have to worry about quality problems!	Jingdong prefecture level city in Shandong
Stowe Flagship Store	King of Power Saving	The product is good, before already bought a, this is the second, the effect is quite good, anyway, I see the daily electricity bill than before a lot less, this can be the choice!	Jingdong prefecture level city in Shandong

Stowe Flagship Store	King of Power Saving	Originally also very worried about things are not good, get the hand found completely over-anxious, five-star praise, the style is very generous, the quality is very good, the price is also very reasonable!	Jingdong prefecture level city in Shandong
Stowe Flagship Store	King of Power Saving	Quality is good, playing to the old family also buy a, repurchase, this quality is really good, recommended!	Jingdong prefecture level city in Shandong
Stowe Flagship Store	King of Power Saving	Thank you, the owner even sent two fuses.	Jingdong prefecture level city in Shandong
Stowe Flagship Store	King of Power Saving	This store has great stuff, relatively fast shipping and logistics	Jingdong prefecture level city in Shandong
Stowe Flagship Store	King of Power Saving	This product is based on the impossibility of saving electricity, I used 853 kWh of electricity in July, started using the product on August 1, and used 867 kWh of electricity by September 1, is this called	Jingdong prefecture level city in Shandong
Stowe Flagship Store	King of Power Saving	Power king received, to the home on the trial, when I plugged into the electric panel can not see the effect of power saving, the meter is still the same speed in the turn, I'll save the king of the manual to play look at the instructions, instructions to speak, have to have a day two days and half a month to save power obviously, then wait half a month to see the effect of power saving it! To half a month there are obvious power saving before continuing to chase Xu.	Jingdong prefecture level city in Shandong
Stowe Flagship Store	King of Power Saving	Received the goods, a look, did not see the manufacturer and production address, can only hold a try to see the mentality of the face	Jingdong prefecture level city in Shandong
Stowe Flagship Store	King of Power Saving	I've only been using it for a few days and I'm not sure if it's an IQ tax? Used to favorable reviews.	Jingdong prefecture level city in Shandong
Stowe Flagship Store	King of Power Saving	Appearance is okay, power saving next time to follow up the evaluation	Jingdong prefecture level city in Shandong
Stowe Flagship Store	King of Power Saving	Shorter time, but hopefully effective and power-saving, to be followed up in the future	Jingdong prefecture level city in Shandong
Stowe Flagship Store	King of Power Saving	Appearance is okay, I do not know if the power saving	Jingdong prefecture level city in Shandong
Stowe Flagship Store	King of Power Saving	Good service, fast logistics, Power Saving King is exquisite and beautiful, support the business, thank you!	Jingdong prefecture level city in Shandong
Stowe Flagship Store	King of Power Saving	Bought three at once to try first first.	Jingdong prefecture level city in Shandong
Stowe Flagship Store	King of Power Saving	The object is the same as the picture, the product is of high quality, the packaging is intact. High-end atmosphere, high power, power saving effect is obvious, average monthly power saving 50%, very satisfied.	Jingdong prefecture level city in Shandong
Stowe Flagship Store	King of Power Saving	Bought back and plugged in, really effective, before 16 degrees of electricity per day, now 8 degrees of electricity per day, open an air conditioner for 12 hours, decisive repurchase of a	Jingdong prefecture level city in Shandong
Stowe Flagship Store	King of Power Saving	The object is the same as the picture, the product is of high quality, and the package is intact. High-end atmosphere and high grade, high power	Jingdong prefecture level city in Shandong
Stowe Flagship Store	King of Power Saving	Goods received, I hope the store said can be energy-saving, first try a month, month after month to see the effect.	Jingdong prefecture level city in Shandong
Stowe Flagship Store	King of Power Saving	This is really good, the material and workmanship is particularly exquisite, the workmanship is really very good, I think I can recommend to my friends to buy	Jingdong prefecture level city in Shandong
Stowe Flagship Store	King of Power Saving	I have to praise, the quality is really good, and the packaging logistics are also good. Really like it, won't make you regret after getting it!	Jingdong prefecture level city in Shandong
Stowe Flagship Store	King of Power Saving	Reputable platform, efficient and professional service, buy with confidence and peace of mind!	Jingdong prefecture level city in Shandong
Stowe Flagship Store	King of Power Saving	Bought one on a trial basis to see if it works after 1-2 months.	Jingdong prefecture level city in Shandong
Stowe Flagship Store	King of Power Saving	Quality is very good, customer service attitude is very very praise, there are problems in a timely manner to solve the problem, the price is not expensive, super value, you can rest assured that the	Jingdong prefecture level city in Shandong
Stowe Flagship Store	King of Power Saving	With it no longer need to be afraid to use electricity, especially good, ready to buy a mom and dad that also	Jingdong prefecture level city in Shandong
Stowe Flagship Store	King of Power Saving	Baby received very like will return to buy the seller attitude is also very like will return to buy the seller attitude is also very like!	Jingdong prefecture level city in Shandong
Stowe Flagship Store	King of Power Saving	Jingdong Mall shopping convenience assured fast assured fast assured fast assured fast	Jingdong prefecture level city in Shandong
Stowe Flagship Store	King of Power Saving	Saving power received, the quality is very good, fine workmanship, especially good, much cheaper than the entity store, it is worth buying!	Jingdong prefecture level city in Shandong
Stowe Flagship Store	King of Power Saving	I did not expect the express delivery so quickly, very save point quality is super good, than the entity store is too cheap.	Jingdong prefecture level city in Shandong
Stowe Flagship Store	King of Power Saving	Have received the baby, very satisfied with a shopping, great worth buying!	Jingdong prefecture level city in Shandong
Stowe Flagship Store	King of Power Saving	This is really more powerful, very satisfied, more energy-saving, better, thank you boss.	Jingdong prefecture level city in Shandong
Stowe Flagship Store	King of Power Saving	Received has been used for two days, the air conditioning can boldly open it! Energy saving effect is really quite good	Jingdong prefecture level city in Shandong
Stowe Flagship Store	King of Power Saving	It is authentic, trust the merchant, the baby has a great texture and the price is moderate, unlimited repurchase!	Jingdong prefecture level city in Shandong
Stowe Flagship Store	King of Power Saving	It is genuine very good, the price is much cheaper, cost-effective, really special cost-effective, it is worth buying!	Jingdong prefecture level city in Shandong
Stowe Flagship Store	King of Power Saving	Very good quality, fine workmanship, low power consumption, long warranty, fast shipping, economical and practical	Jingdong prefecture level city in Shandong
Stowe Flagship Store	King of Power Saving	I've been using it for half a month now, it really saves power and the voltage is stable, it's very good	Jingdong prefecture level city in Shandong
Stowe Flagship Store	King of Power Saving	It's the ideal product 💝, good quality and fast logistics, worth the kudos!	Jingdong prefecture level city in Shandong
Stowe Flagship Store	King of Power Saving	The logistics is very fast, the baby is very good, the shopkeeper is NICE!	Jingdong prefecture level city in Shandong
Stowe Flagship Store	King of Power Saving	Good to use, good quality, save point durable	Jingdong prefecture level city in Shandong
Stowe Flagship Store	King of Power Saving	The quality is very good, customer service attitude is very, very awesome, there are problems in a timely manner to solve the problem	Jingdong prefecture level city in Shandong
Stowe Flagship Store	King of Power Saving	Quality is very good, the use of the effect is very good, more stable, quality assurance	Jingdong prefecture level city in Shandong
Stowe Flagship Store	King of Power Saving	Convenient, inexpensive, and see how it works out?	Jingdong prefecture level city in Shandong
Stowe Flagship Store	King of Power Saving	Very good quality, saves a lot of electricity, very good, worth buying	Jingdong prefecture level city in Shandong
Stowe Flagship Store	King of Power Saving	Power Saving King is easy to use, with the fruit of power saving to use easy to give!	Jingdong prefecture level city in Shandong
Stowe Flagship Store	King of Power Saving	It works well, the voltage is very stable effect is good, the quality is also very good satisfaction	Jingdong prefecture level city in Shandong
Stowe Flagship Store	King of Power Saving	This saver really works, good quality, safe and secure, very satisfied!	Jingdong prefecture level city in Shandong
Stowe Flagship Store	King of Power Saving	The logistics is super super fast! The quality is also very good, indeed value for money	Jingdong prefecture level city in Shandong
Stowe Flagship Store	King of Power Saving	I've only been using it for a month or so and it seems to be saving a lot of power	Jingdong prefecture level city in Shandong
Stowe Flagship Store	King of Power Saving	Very easy to operate, very good material, good value for money.	Jingdong prefecture level city in Shandong
Stowe Flagship Store	King of Power Saving	Aluminum alloy shell, durable, compact power-saving good effect	Jingdong prefecture level city in Shandong
Stowe Flagship Store	King of Power Saving	Nice device, easy to use, doesn't take up much space, recommendable!	Jingdong prefecture level city in Shandong
Stowe Flagship Store	King of Power Saving	Good quality, good price, fast logistics	Jingdong prefecture level city in Shandong

Stowe Flagship Store	King of Power Saving	Friends of the family in use recommended, the effect is good and recommendable!	Jingdong prefecture level city in Shandong
Stowe Flagship Store	King of Power Saving	Cheaper than brick and mortar stores worth buying is authentic	Jingdong prefecture level city in Shandong
Stowe Flagship Store	King of Power Saving	Merchants ship quickly, the price is cheaper than the physical store, good use	Jingdong prefecture level city in Shandong
Stowe Flagship Store	King of Power Saving	Very durable, affordable, fast shipping	Jingdong prefecture level city in Shandong
Stowe Flagship Store	King of Power Saving	Packaging tight quality is very good easy to use good results	Jingdong prefecture level city in Shandong
Stowe Flagship Store	King of Power Saving	Save the grid. It's in the whole house. It's saving money on the electric bill.	Jingdong prefecture level city in Shandong
Stowe Flagship Store	King of Power Saving	I used it today, it works well, I'm very satisfied	Jingdong prefecture level city in Shandong
Stowe Flagship Store	King of Power Saving	It really saves a lot of electricity after installation	Jingdong prefecture level city in Shandong
Stowe Flagship Store	King of Power Saving	Love it. Satisfied.	Jingdong prefecture level city in Shandong
Stowe Flagship Store	King of Power Saving	Doesn't feel like it's working.	Jingdong prefecture level city in Shandong
Stowe Flagship Store	King of Power Saving	I'll comment after using it.	Jingdong prefecture level city in Shandong
Stowe Flagship Store	King of Power Saving	I'm trying it out to see if it saves power, and I'll buy two more if it works well.	Jingdong prefecture level city in Shandong
Stowe Flagship Store	King of Power Saving	It is genuine very good, the price is much cheaper, cost-effective, really special cost-effective, it is worth buying!	Jingdong prefecture level city in Shandong
Stowe Flagship Store	King of Power Saving	This thing can really save electricity, I was introduced to buy by my friend, he used it very well, I used it and think it's also very good, it can really save electricity, save about half of the electricity every day, it's really good!	Jingdong prefecture level city in Shandong
Stowe Flagship Store	King of Power Saving	After using it for ten days or so, I checked the power consumption and it did drop, and customer service said it will save more electricity in the long run. The product is also insured, 5 years warranty do not have to worry about quality problems!	Jingdong prefecture level city in Shandong
Stowe Flagship Store	King of Power Saving	The product is good, before already bought a, this is the second, the effect is quite good, anyway, I see the daily electricity bill than before a lot less, this can be the choice!	Jingdong prefecture level city in Shandong
Stowe Flagship Store	King of Power Saving	Originally also very worried about things are not good, get the hand found completely over-anxious, five-star praise, the style is very generous, the quality is very good, the price is also very reasonable!	Jingdong prefecture level city in Shandong
Stowe Flagship Store	King of Power Saving	Quality is good, playing to the old family also buy a, repurchase, this quality is really good, recommended!	Jingdong prefecture level city in Shandong
Stowe Flagship Store	King of Power Saving	Thank you, the owner even sent two fuses.	Jingdong prefecture level city in Shandong
Stowe Flagship Store	King of Power Saving	This store has great stuff, relatively fast shipping and logistics	Jingdong prefecture level city in Shandong
Stowe Flagship Store	King of Power Saving	This product is based on the impossibility of saving electricity, I used 853 kWh of electricity in July, started using the product on August 1, and used 867 kWh of electricity by September 1, is this called	Jingdong prefecture level city in Shandong
Stowe Flagship Store	King of Power Saving	Power king received, to the home on the trial, when I plugged into the electric panel can not see the effect of power saving, the meter is still the same speed in the turn, I'll save the king of the manual to play look at the instructions, instructions to speak, have to have a day two days and half a month to save power obviously, then wait half a month to see the effect of power saving it! To half a month there are obvious power saving before continuing to chase Xu.	Jingdong prefecture level city in Shandong
Stowe Flagship Store	King of Power Saving	Received the goods, a look, did not see the manufacturer and production address, can only hold a try to see the mentality of the face	Jingdong prefecture level city in Shandong
Stowe Flagship Store	King of Power Saving	I've only been using it for a few days and I'm not sure if it's an IQ tax? Used to favorable reviews.	Jingdong prefecture level city in Shandong
Stowe Flagship Store	King of Power Saving	Appearance is okay, power saving next time to follow up the evaluation	Jingdong prefecture level city in Shandong
Stowe Flagship Store	King of Power Saving	Shorter time, but hopefully effective and power-saving, to be followed up in the future	Jingdong prefecture level city in Shandong
Stowe Flagship Store	King of Power Saving	Appearance is okay, I do not know if the power saving	Jingdong prefecture level city in Shandong
Stowe Flagship Store	King of Power Saving	Good service, fast logistics, Power Saving King is exquisite and beautiful, support the business, thank you!	Jingdong prefecture level city in Shandong
Stowe Flagship Store	King of Power Saving	Bought three at once to try first first.	Jingdong prefecture level city in Shandong
Stowe Flagship Store	King of Power Saving	The object is the same as the picture, the product is of high quality, the packaging is intact. High-end atmosphere, high power, power saving effect is obvious, average monthly power saving 50%, very satisfied.	Jingdong prefecture level city in Shandong
Stowe Flagship Store	King of Power Saving	Bought back and plugged in, really effective, before 16 degrees of electricity per day, now 8 degrees of electricity per day, open an air conditioner for 12 hours, decisive repurchase of a	Jingdong prefecture level city in Shandong
Stowe Flagship Store	King of Power Saving	The object is the same as the picture, the product is of high quality, and the package is intact. High-end atmosphere and high grade, high power	Jingdong prefecture level city in Shandong
Stowe Flagship Store	King of Power Saving	Goods received, I hope the store said can be energy-saving, first try a month, month after month to see the effect.	Jingdong prefecture level city in Shandong
Stowe Flagship Store	King of Power Saving	This is really good, the material and workmanship is particularly exquisite, the workmanship is really very good, I think I can recommend to my friends to buy	Jingdong prefecture level city in Shandong
Stowe Flagship Store	King of Power Saving	I have to praise, the quality is really good, and the packaging logistics are also good. Really like it, won't make you regret after getting it!	Jingdong prefecture level city in Shandong
Stowe Flagship Store	King of Power Saving	Reputable platform, efficient and professional service, buy with confidence and peace of mind!	Jingdong prefecture level city in Shandong
Stowe Flagship Store	King of Power Saving	Bought one on a trial basis to see if it works after 1-2 months.	Jingdong prefecture level city in Shandong
Stowe Flagship Store	King of Power Saving	Quality is very good, customer service attitude is very very praise, there are problems in a timely manner to solve the problem, the price is not expensive, super value, you can rest assured that the	Jingdong prefecture level city in Shandong
Stowe Flagship Store	King of Power Saving	With it no longer need to be afraid to use electricity, especially good, ready to buy a mom and dad that also	Jingdong prefecture level city in Shandong
Stowe Flagship Store	King of Power Saving	Baby received very like will return to buy the seller attitude is also very like will return to buy the seller attitude is also very like!	Jingdong prefecture level city in Shandong
Stowe Flagship Store	King of Power Saving	Jingdong Mall shopping convenience assured fast assured fast assured fast assured fast	Jingdong prefecture level city in Shandong
Stowe Flagship Store	King of Power Saving	Saving power received, the quality is very good, fine workmanship, especially good, much cheaper than the entity store, it is worth buying!	Jingdong prefecture level city in Shandong
Stowe Flagship Store	King of Power Saving	I did not expect the express delivery so quickly, very save point quality is super good, than the entity store is too cheap.	Jingdong prefecture level city in Shandong
Stowe Flagship Store	King of Power Saving	Have received the baby, very satisfied with a shopping, great worth buying!	Jingdong prefecture level city in Shandong
Stowe Flagship Store	King of Power Saving	This is really more powerful, very satisfied, more energy-saving, better, thank you boss.	Jingdong prefecture level city in Shandong
Stowe Flagship Store	King of Power Saving	Received has been used for two days, the air conditioning can boldly open it! Energy saving effect is really quite good	Jingdong prefecture level city in Shandong
Stowe Flagship Store	King of Power Saving	It is authentic, trust the merchant, the baby has a great texture and the price is moderate, unlimited repurchase!	Jingdong prefecture level city in Shandong
Stowe Flagship Store	King of Power Saving	It is genuine very good, the price is much cheaper, cost-effective, really special cost-effective, it is worth buying!	Jingdong prefecture level city in Shandong
Stowe Flagship Store	King of Power Saving	Very good quality, fine workmanship, low power consumption, long warranty, fast shipping, economical and practical	Jingdong prefecture level city in Shandong

Stowe Flagship Store	King of Power Saving	I've been using it for half a month now, it really saves power and the voltage is stable, it's very good	Jingdong prefecture level city in Shandong
Stowe Flagship Store	King of Power Saving	It's the ideal product 💝, good quality and fast logistics, worth the kudos!	Jingdong prefecture level city in Shandong
Stowe Flagship Store	King of Power Saving	The logistics is very fast, the baby is very good, the shopkeeper is NICE!	Jingdong prefecture level city in Shandong
Stowe Flagship Store	King of Power Saving	Good to use, good quality, save point durable	Jingdong prefecture level city in Shandong
Stowe Flagship Store	King of Power Saving	The quality is very good, customer service attitude is very, very awesome, there are problems in a timely manner to solve the problem	Jingdong prefecture level city in Shandong
Stowe Flagship Store	King of Power Saving	Quality is very good, the use of the effect is very good, more stable, quality assurance	Jingdong prefecture level city in Shandong
Stowe Flagship Store	King of Power Saving	Very good quality, saves a lot of electricity, very good, worth buying	Jingdong prefecture level city in Shandong
Stowe Flagship Store	King of Power Saving	Convenient, inexpensive, and see how it works out?	Jingdong prefecture level city in Shandong
Stowe Flagship Store	King of Power Saving	Power Saving King is easy to use, with the fruit of power saving to use easy to give!	Jingdong prefecture level city in Shandong
Stowe Flagship Store	King of Power Saving	This saver really works, good quality, safe and secure, very satisfied!	Jingdong prefecture level city in Shandong
Stowe Flagship Store	King of Power Saving	It works well, the voltage is very stable effect is good, the quality is also very good satisfaction	Jingdong prefecture level city in Shandong
Stowe Flagship Store	King of Power Saving	The logistics is super super fast! The quality is also very good, indeed value for money	Jingdong prefecture level city in Shandong
Stowe Flagship Store	King of Power Saving	I've only been using it for a month or so and it seems to be saving a lot of power	Jingdong prefecture level city in Shandong
Stowe Flagship Store	King of Power Saving	Very easy to operate, very good material, good value for money.	Jingdong prefecture level city in Shandong
Stowe Flagship Store	King of Power Saving	Aluminum alloy shell, durable, compact power-saving good effect	Jingdong prefecture level city in Shandong
Stowe Flagship Store	King of Power Saving	Nice device, easy to use, doesn't take up much space, recommendable!	Jingdong prefecture level city in Shandong
Stowe Flagship Store	King of Power Saving	Good quality, good price, fast logistics	Jingdong prefecture level city in Shandong
Stowe Flagship Store	King of Power Saving	Friends of the family in use recommended, the effect is good and recommendable!	Jingdong prefecture level city in Shandong
Stowe Flagship Store	King of Power Saving	Cheaper than brick and mortar stores worth buying is authentic	Jingdong prefecture level city in Shandong
Stowe Flagship Store	King of Power Saving	Merchants ship quickly, the price is cheaper than the physical store, good use	Jingdong prefecture level city in Shandong
Stowe Flagship Store	King of Power Saving	Very durable, affordable, fast shipping	Jingdong prefecture level city in Shandong
Stowe Flagship Store	King of Power Saving	Packaging tight quality is very good easy to use good results	Jingdong prefecture level city in Shandong
Stowe Flagship Store	King of Power Saving	Save the grid. It's in the whole house. It's saving money on the electric bill.	Jingdong prefecture level city in Shandong
Stowe Flagship Store	King of Power Saving	I used it today, it works well, I'm very satisfied	Jingdong prefecture level city in Shandong
Stowe Flagship Store	King of Power Saving	It really saves a lot of electricity after installation	Jingdong prefecture level city in Shandong
Stowe Flagship Store	King of Power Saving	Love it. Satisfied.	Jingdong prefecture level city in Shandong
Stowe Flagship Store	King of Power Saving	Doesn't feel like it's working.	Jingdong prefecture level city in Shandong
Stowe Flagship Store	King of Power Saving	I'll comment after using it.	Jingdong prefecture level city in Shandong
Stowe Flagship Store	King of Power Saving	I'm trying it out to see if it saves power, and I'll buy two more if it works well.	Jingdong prefecture level city in Shandong
Stowe Flagship Store	King of Power Saving	It is genuine very good, the price is much cheaper, cost-effective, really special cost-effective, it is worth buying!	Jingdong prefecture level city in Shandong
Stowe Flagship Store	King of Power Saving	This thing can really save electricity, I was introduced to buy by my friend, he used it very well, I used it and think it's also very good, it can really save electricity, save about half of the electricity every day, it's really good!	Jingdong prefecture level city in Shandong
Stowe Flagship Store	King of Power Saving	After using it for ten days or so, I checked the power consumption and it did drop, and customer service said it will save more electricity in the long run. The product is also insured, 5 years warranty do not have to worry about quality problems!	Jingdong prefecture level city in Shandong
Stowe Flagship Store	King of Power Saving	The product is good, before already bought a, this is the second, the effect is quite good, anyway, I see the daily electricity bill than before a lot less, this can be the choice!	Jingdong prefecture level city in Shandong
Stowe Flagship Store	King of Power Saving	Originally also very worried about things are not good, get the hand found completely over-anxious, five-star praise, the style is very generous, the quality is very good, the price is also very reasonable!	Jingdong prefecture level city in Shandong
Stowe Flagship Store	King of Power Saving	Quality is good, playing to the old family also buy a, repurchase, this quality is really good, recommended!	Jingdong prefecture level city in Shandong
Stowe Flagship Store	King of Power Saving	Thank you, the owner even sent two fuses.	Jingdong prefecture level city in Shandong
Stowe Flagship Store	King of Power Saving	This store has great stuff, relatively fast shipping and logistics	Jingdong prefecture level city in Shandong
Stowe Flagship Store	King of Power Saving	This product is based on the impossibility of saving electricity, I used 853 kWh of electricity in July, started using the product on August 1, and used 867 kWh of electricity by September 1, is this called	Jingdong prefecture level city in Shandong
Stowe Flagship Store	King of Power Saving	Power king received, to the home on the trial, when I plugged into the electric panel can not see the effect of power saving, the meter is still the same speed in the turn, I'll save the king of the manual to play look at the instructions, instructions to speak, have to have a day two days and half a month to save power obviously, then wait half a month to see the effect of power saving it! To half a month there are obvious power saving before continuing to chase Xu.	Jingdong prefecture level city in Shandong
Stowe Flagship Store	King of Power Saving	Received the goods, a look, did not see the manufacturer and production address, can only hold a try to see the mentality of the face	Jingdong prefecture level city in Shandong
Stowe Flagship Store	King of Power Saving	I've only been using it for a few days and I'm not sure if it's an IQ tax? Used to favorable reviews.	Jingdong prefecture level city in Shandong
Stowe Flagship Store	King of Power Saving	Appearance is okay, power saving next time to follow up the evaluation	Jingdong prefecture level city in Shandong
Stowe Flagship Store	King of Power Saving	Shorter time, but hopefully effective and power-saving, to be followed up in the future	Jingdong prefecture level city in Shandong
Stowe Flagship Store	King of Power Saving	Appearance is okay, I do not know if the power saving	Jingdong prefecture level city in Shandong
Stowe Flagship Store	King of Power Saving	Good service, fast logistics, Power Saving King is exquisite and beautiful, support the business, thank you!	Jingdong prefecture level city in Shandong
Stowe Flagship Store	King of Power Saving	Bought three at once to try first first.	Jingdong prefecture level city in Shandong
Stowe Flagship Store	King of Power Saving	The object is the same as the picture, the product is of high quality, the packaging is intact. High-end atmosphere, high power, power saving effect is obvious, average monthly power saving 50%, very satisfied.	Jingdong prefecture level city in Shandong
Stowe Flagship Store	King of Power Saving	Bought back and plugged in, really effective, before 16 degrees of electricity per day, now 8 degrees of electricity per day, open an air conditioner for 12 hours, decisive repurchase of a	Jingdong prefecture level city in Shandong
Stowe Flagship Store	King of Power Saving	The object is the same as the picture, the product is of high quality, and the package is intact. High-end atmosphere and high grade, high power	Jingdong prefecture level city in Shandong

Stowe Flagship Store	King of Power Saving	Goods received, I hope the store said can be energy-saving, first try a month, month after month to see the effect.	Jingdong prefecture level city in Shandong
Stowe Flagship Store	King of Power Saving	This is really good, the material and workmanship is particularly exquisite, the workmanship is really very good, I think I can recommend to my friends to buy	Jingdong prefecture level city in Shandong
Stowe Flagship Store	King of Power Saving	I have to praise, the quality is really good, and the packaging logistics are also good. Really like it, won't make you regret after getting it!	Jingdong prefecture level city in Shandong
Stowe Flagship Store	King of Power Saving	Reputable platform, efficient and professional service, buy with confidence and peace of mind!	Jingdong prefecture level city in Shandong
Stowe Flagship Store	King of Power Saving	Bought one on a trial basis to see if it works after 1-2 months.	Jingdong prefecture level city in Shandong
Stowe Flagship Store	King of Power Saving	Quality is very good, customer service attitude is very very praise, there are problems in a timely manner to solve the problem, the price is not expensive, super value, you can rest assured that the	Jingdong prefecture level city in Shandong
Stowe Flagship Store	King of Power Saving	With it no longer need to be afraid to use electricity, especially good, ready to buy a mom and dad that also	Jingdong prefecture level city in Shandong
Stowe Flagship Store	King of Power Saving	Baby received very like will return to buy the seller attitude is also very like will return to buy the seller attitude is also very like!	Jingdong prefecture level city in Shandong
Stowe Flagship Store	King of Power Saving	Jingdong Mall shopping convenience assured fast assured fast assured fast assured fast	Jingdong prefecture level city in Shandong
Stowe Flagship Store	King of Power Saving	Saving power received, the quality is very good, fine workmanship, especially good, much cheaper than the entity store, it is worth buying!	Jingdong prefecture level city in Shandong
Stowe Flagship Store	King of Power Saving	I did not expect the express delivery so quickly, very save point quality is super good, than the entity store is too cheap.	Jingdong prefecture level city in Shandong
Stowe Flagship Store	King of Power Saving	Have received the baby, very satisfied with a shopping, great worth buying!	Jingdong prefecture level city in Shandong
Stowe Flagship Store	King of Power Saving	This is really more powerful, very satisfied, more energy-saving, better, thank you boss.	Jingdong prefecture level city in Shandong
Stowe Flagship Store	King of Power Saving	Received has been used for two days, the air conditioning can boldly open it! Energy saving effect is really quite good	Jingdong prefecture level city in Shandong
Stowe Flagship Store	King of Power Saving	It is authentic, trust the merchant, the baby has a great texture and the price is moderate, unlimited repurchase!	Jingdong prefecture level city in Shandong
Stowe Flagship Store	King of Power Saving	It is genuine very good, the price is much cheaper, cost-effective, really special cost-effective, it is worth buying!	Jingdong prefecture level city in Shandong
Stowe Flagship Store	King of Power Saving	Very good quality, fine workmanship, low power consumption, long warranty, fast shipping, economical and practical	Jingdong prefecture level city in Shandong
Stowe Flagship Store	King of Power Saving	I've been using it for half a month now, it really saves power and the voltage is stable, it's very good	Jingdong prefecture level city in Shandong
Stowe Flagship Store	King of Power Saving	It's the ideal product 💝, good quality and fast logistics, worth the kudos!	Jingdong prefecture level city in Shandong
Stowe Flagship Store	King of Power Saving	The logistics is very fast, the baby is very good, the shopkeeper is NICE!	Jingdong prefecture level city in Shandong
Stowe Flagship Store	King of Power Saving	Good to use, good quality, save point durable	Jingdong prefecture level city in Shandong
Stowe Flagship Store	King of Power Saving	The quality is very good, customer service attitude is very, very awesome, there are problems in a timely manner to solve the problem	Jingdong prefecture level city in Shandong
Stowe Flagship Store	King of Power Saving	Quality is very good, the use of the effect is very good, more stable, quality assurance	Jingdong prefecture level city in Shandong
Stowe Flagship Store	King of Power Saving	Convenient, inexpensive, and see how it works out?	Jingdong prefecture level city in Shandong
Stowe Flagship Store	King of Power Saving	Very good quality, saves a lot of electricity, very good, worth buying	Jingdong prefecture level city in Shandong
Stowe Flagship Store	King of Power Saving	Power Saving King is easy to use, with the fruit of power saving to use easy to give!	Jingdong prefecture level city in Shandong
Stowe Flagship Store	King of Power Saving	This saver really works, good quality, safe and secure, very satisfied!	Jingdong prefecture level city in Shandong
Stowe Flagship Store	King of Power Saving	It works well, the voltage is very stable effect is good, the quality is also very good satisfaction	Jingdong prefecture level city in Shandong
Stowe Flagship Store	King of Power Saving	The logistics is super super fast! The quality is also very good, indeed value for money	Jingdong prefecture level city in Shandong
Stowe Flagship Store	King of Power Saving	I've only been using it for a month or so and it seems to be saving a lot of power	Jingdong prefecture level city in Shandong
Stowe Flagship Store	King of Power Saving	Very easy to operate, very good material, good value for money.	Jingdong prefecture level city in Shandong
Stowe Flagship Store	King of Power Saving	Aluminum alloy shell, durable, compact power-saving good effect	Jingdong prefecture level city in Shandong
Stowe Flagship Store	King of Power Saving	Nice device, easy to use, doesn't take up much space, recommendable!	Jingdong prefecture level city in Shandong
Stowe Flagship Store	King of Power Saving	Good quality, good price, fast logistics	Jingdong prefecture level city in Shandong
Stowe Flagship Store	King of Power Saving	Friends of the family in use recommended, the effect is good and recommendable!	Jingdong prefecture level city in Shandong
Stowe Flagship Store	King of Power Saving	Cheaper than brick and mortar stores worth buying is authentic	Jingdong prefecture level city in Shandong
Stowe Flagship Store	King of Power Saving	Merchants ship quickly, the price is cheaper than the physical store, good use	Jingdong prefecture level city in Shandong
Stowe Flagship Store	King of Power Saving	Very durable, affordable, fast shipping	Jingdong prefecture level city in Shandong
Stowe Flagship Store	King of Power Saving	Packaging tight quality is very good easy to use good results	Jingdong prefecture level city in Shandong
Stowe Flagship Store	King of Power Saving	Save the grid. It's in the whole house. It's saving money on the electric bill.	Jingdong prefecture level city in Shandong
Stowe Flagship Store	King of Power Saving	I used it today, it works well, I'm very satisfied	Jingdong prefecture level city in Shandong
Stowe Flagship Store	King of Power Saving	It really saves a lot of electricity after installation	Jingdong prefecture level city in Shandong
Stowe Flagship Store	King of Power Saving	Love it. Satisfied.	Jingdong prefecture level city in Shandong
Stowe Flagship Store	King of Power Saving	Doesn't feel like it's working.	Jingdong prefecture level city in Shandong
Stowe Flagship Store	King of Power Saving	I'll comment after using it.	Jingdong prefecture level city in Shandong
Stowe Flagship Store	King of Power Saving	I'm trying it out to see if it saves power, and I'll buy two more if it works well.	Jingdong prefecture level city in Shandong
Stowe Flagship Store	King of Power Saving	It is genuine very good, the price is much cheaper, cost-effective, really special cost-effective, it is worth buying!	Jingdong prefecture level city in Shandong
Stowe Flagship Store	King of Power Saving	This thing can really save electricity, I was introduced to buy by my friend, he used it very well, I used it and think it's also very good, it can really save electricity, save about half of the electricity every day, it's really good!	Jingdong prefecture level city in Shandong
Stowe Flagship Store	King of Power Saving	After using it for ten days or so, I checked the power consumption and it did drop, and customer service said it will save more electricity in the long run. The product is also insured, 5 years warranty do not have to worry about quality problems!	Jingdong prefecture level city in Shandong
Stowe Flagship Store	King of Power Saving	The product is good, before already bought a, this is the second, the effect is quite good, anyway, I see the daily electricity bill than before a lot less, this can be the choice!	Jingdong prefecture level city in Shandong

Stowe Flagship Store	King of Power Saving	Originally also very worried about things are not good, get the hand found completely over-anxious, five-star praise, the style is very generous, the quality is very good, the price is also very reasonable!	Jingdong prefecture level city in Shandong
Stowe Flagship Store	King of Power Saving	Quality is good, playing to the old family also buy a, repurchase, this quality is really good, recommended!	Jingdong prefecture level city in Shandong
Stowe Flagship Store	King of Power Saving	Thank you, the owner even sent two fuses.	Jingdong prefecture level city in Shandong
Stowe Flagship Store	King of Power Saving	This store has great stuff, relatively fast shipping and logistics	Jingdong prefecture level city in Shandong
Stowe Flagship Store	King of Power Saving	This product is based on the impossibility of saving electricity, I used 853 kWh of electricity in July, started using the product on August 1, and used 867 kWh of electricity by September 1, is this called	Jingdong prefecture level city in Shandong
Stowe Flagship Store	King of Power Saving	Power king received, to the home on the trial, when I plugged into the electric panel can not see the effect of power saving, the meter is still the same speed in the turn, I'll save the king of the manual to play look at the instructions, instructions to speak, have to have a day two days and half a month to save power obviously, then wait half a month to see the effect of power saving it! To half a month there are obvious power saving before continuing to chase Xu.	Jingdong prefecture level city in Shandong
Stowe Flagship Store	King of Power Saving	Received the goods, a look, did not see the manufacturer and production address, can only hold a try to see the mentality of the face	Jingdong prefecture level city in Shandong
Stowe Flagship Store	King of Power Saving	I've only been using it for a few days and I'm not sure if it's an IQ tax? Used to favorable reviews.	Jingdong prefecture level city in Shandong
Stowe Flagship Store	King of Power Saving	Appearance is okay, power saving next time to follow up the evaluation	Jingdong prefecture level city in Shandong
Stowe Flagship Store	King of Power Saving	Shorter time, but hopefully effective and power-saving, to be followed up in the future	Jingdong prefecture level city in Shandong
Stowe Flagship Store	King of Power Saving	Appearance is okay, I do not know if the power saving	Jingdong prefecture level city in Shandong
Stowe Flagship Store	King of Power Saving	Good service, fast logistics, Power Saving King is exquisite and beautiful, support the business, thank you!	Jingdong prefecture level city in Shandong
Stowe Flagship Store	King of Power Saving	Bought three at once to try first first.	Jingdong prefecture level city in Shandong
Stowe Flagship Store	King of Power Saving	The object is the same as the picture, the product is of high quality, the packaging is intact. High-end atmosphere, high power, power saving effect is obvious, average monthly power saving 50%, very satisfied.	Jingdong prefecture level city in Shandong
Stowe Flagship Store	King of Power Saving	Bought back and plugged in, really effective, before 16 degrees of electricity per day, now 8 degrees of electricity per day, open an air conditioner for 12 hours, decisive repurchase of a	Jingdong prefecture level city in Shandong
Stowe Flagship Store	King of Power Saving	The object is the same as the picture, the product is of high quality, and the package is intact. High-end atmosphere and high grade, high power	Jingdong prefecture level city in Shandong
Stowe Flagship Store	King of Power Saving	Goods received, I hope the store said can be energy-saving, first try a month, month after month to see the effect.	Jingdong prefecture level city in Shandong
Stowe Flagship Store	King of Power Saving	This is really good, the material and workmanship is particularly exquisite, the workmanship is really very good, I think I can recommend to my friends to buy	Jingdong prefecture level city in Shandong
Stowe Flagship Store	King of Power Saving	I have to praise, the quality is really good, and the packaging logistics are also good. Really like it, won't make you regret after getting it!	Jingdong prefecture level city in Shandong
Stowe Flagship Store	King of Power Saving	Reputable platform, efficient and professional service, buy with confidence and peace of mind!	Jingdong prefecture level city in Shandong
Stowe Flagship Store	King of Power Saving	Bought one on a trial basis to see if it works after 1-2 months.	Jingdong prefecture level city in Shandong
Stowe Flagship Store	King of Power Saving	Quality is very good, customer service attitude is very very praise, there are problems in a timely manner to solve the problem, the price is not expensive, super value, you can rest assured that the	Jingdong prefecture level city in Shandong
Stowe Flagship Store	King of Power Saving	With it no longer need to be afraid to use electricity, especially good, ready to buy a mom and dad that also	Jingdong prefecture level city in Shandong
Stowe Flagship Store	King of Power Saving	Baby received very like will return to buy the seller attitude is also very like will return to buy the seller attitude is also very like!	Jingdong prefecture level city in Shandong
Stowe Flagship Store	King of Power Saving	Jingdong Mall shopping convenience assured fast assured fast assured fast assured fast	Jingdong prefecture level city in Shandong
Stowe Flagship Store	King of Power Saving	Saving power received, the quality is very good, fine workmanship, especially good, much cheaper than the entity store, it is worth buying!	Jingdong prefecture level city in Shandong
Stowe Flagship Store	King of Power Saving	I did not expect the express delivery so quickly, very save point quality is super good, than the entity store is too cheap.	Jingdong prefecture level city in Shandong
Stowe Flagship Store	King of Power Saving	Have received the baby, very satisfied with a shopping, great worth buying!	Jingdong prefecture level city in Shandong
Stowe Flagship Store	King of Power Saving	This is really more powerful, very satisfied, more energy-saving, better, thank you boss.	Jingdong prefecture level city in Shandong
Stowe Flagship Store	King of Power Saving	Received has been used for two days, the air conditioning can boldly open it! Energy saving effect is really quite good	Jingdong prefecture level city in Shandong
Stowe Flagship Store	King of Power Saving	It is authentic, trust the merchant, the baby has a great texture and the price is moderate, unlimited repurchase!	Jingdong prefecture level city in Shandong
Stowe Flagship Store	King of Power Saving	It is genuine very good, the price is much cheaper, cost-effective, really special cost-effective, it is worth buying!	Jingdong prefecture level city in Shandong
Stowe Flagship Store	King of Power Saving	Very good quality, fine workmanship, low power consumption, long warranty, fast shipping, economical and practical	Jingdong prefecture level city in Shandong
Stowe Flagship Store	King of Power Saving	I've been using it for half a month now, it really saves power and the voltage is stable, it's very good	Jingdong prefecture level city in Shandong
Stowe Flagship Store	King of Power Saving	It's the ideal product , good quality and fast logistics, worth the kudos!	Jingdong prefecture level city in Shandong
Stowe Flagship Store	King of Power Saving	The logistics is very fast, the baby is very good, the shopkeeper is NICE!	Jingdong prefecture level city in Shandong
Stowe Flagship Store	King of Power Saving	Good to use, good quality, save point durable	Jingdong prefecture level city in Shandong
Stowe Flagship Store	King of Power Saving	The quality is very good, customer service attitude is very, very awesome, there are problems in a timely manner to solve the problem	Jingdong prefecture level city in Shandong
Stowe Flagship Store	King of Power Saving	Quality is very good, the use of the effect is very good, more stable, quality assurance	Jingdong prefecture level city in Shandong
Stowe Flagship Store	King of Power Saving	Convenient, inexpensive, and see how it works out?	Jingdong prefecture level city in Shandong
Stowe Flagship Store	King of Power Saving	Very good quality, saves a lot of electricity, very good, worth buying	Jingdong prefecture level city in Shandong
Stowe Flagship Store	King of Power Saving	Power Saving King is easy to use, with the fruit of power saving to use easy to give!	Jingdong prefecture level city in Shandong
Stowe Flagship Store	King of Power Saving	This saver really works, good quality, safe and secure, very satisfied!	Jingdong prefecture level city in Shandong
Stowe Flagship Store	King of Power Saving	It works well, the voltage is very stable effect is good, the quality is also very good satisfaction	Jingdong prefecture level city in Shandong
Stowe Flagship Store	King of Power Saving	The logistics is super super fast! The quality is also very good, indeed value for money	Jingdong prefecture level city in Shandong
Stowe Flagship Store	King of Power Saving	I've only been using it for a month or so and it seems to be saving a lot of power	Jingdong prefecture level city in Shandong
Stowe Flagship Store	King of Power Saving	Very easy to operate, very good material, good value for money.	Jingdong prefecture level city in Shandong
Stowe Flagship Store	King of Power Saving	Aluminum alloy shell, durable, compact power-saving good effect	Jingdong prefecture level city in Shandong
Stowe Flagship Store	King of Power Saving	Nice device, easy to use, doesn't take up much space, recommendable!	Jingdong prefecture level city in Shandong
Stowe Flagship Store	King of Power Saving	Good quality, good price, fast logistics	Jingdong prefecture level city in Shandong

Stowe Flagship Store	King of Power Saving	Friends of the family in use recommended, the effect is good and recommendable!	Jingdong prefecture level city in Shandong
Stowe Flagship Store	King of Power Saving	Cheaper than brick and mortar stores worth buying is authentic	Jingdong prefecture level city in Shandong
Stowe Flagship Store	King of Power Saving	Merchants ship quickly, the price is cheaper than the physical store, good use	Jingdong prefecture level city in Shandong
Stowe Flagship Store	King of Power Saving	Very durable, affordable, fast shipping	Jingdong prefecture level city in Shandong
Stowe Flagship Store	King of Power Saving	Packaging tight quality is very good easy to use good results	Jingdong prefecture level city in Shandong
Stowe Flagship Store	King of Power Saving	Save the grid. It's in the whole house. It's saving money on the electric bill.	Jingdong prefecture level city in Shandong
Stowe Flagship Store	King of Power Saving	I used it today, it works well, I'm very satisfied	Jingdong prefecture level city in Shandong
Stowe Flagship Store	King of Power Saving	It really saves a lot of electricity after installation	Jingdong prefecture level city in Shandong
Stowe Flagship Store	King of Power Saving	Love it. Satisfied.	Jingdong prefecture level city in Shandong
Stowe Flagship Store	King of Power Saving	Doesn't feel like it's working.	Jingdong prefecture level city in Shandong
Stowe Flagship Store	King of Power Saving	I'll comment after using it.	Jingdong prefecture level city in Shandong
Stowe Flagship Store	King of Power Saving	I'm trying it out to see if it saves power, and I'll buy two more if it works well.	Jingdong prefecture level city in Shandong
Stowe Flagship Store	King of Power Saving	It is genuine very good, the price is much cheaper, cost-effective, really special cost-effective, it is worth buying!	Jingdong prefecture level city in Shandong
Stowe Flagship Store	King of Power Saving	This thing can really save electricity, I was introduced to buy by my friend, he used it very well, I used it and think it's also very good, it can really save electricity, save about half of the electricity every day, it's really good!	Jingdong prefecture level city in Shandong
Stowe Flagship Store	King of Power Saving	After using it for ten days or so, I checked the power consumption and it did drop, and customer service said it will save more electricity in the long run. The product is also insured, 5 years warranty do not have to worry about quality problems!	Jingdong prefecture level city in Shandong
Stowe Flagship Store	King of Power Saving	The product is good, before already bought a, this is the second, the effect is quite good, anyway, I see the daily electricity bill than before a lot less, this can be the choice!	Jingdong prefecture level city in Shandong
Stowe Flagship Store	King of Power Saving	Originally also very worried about things are not good, get the hand found completely over-anxious, five-star praise, the style is very generous, the quality is very good, the price is also very reasonable!	Jingdong prefecture level city in Shandong
Stowe Flagship Store	King of Power Saving	Quality is good, playing to the old family also buy a, repurchase, this quality is really good, recommended!	Jingdong prefecture level city in Shandong
Stowe Flagship Store	King of Power Saving	Thank you, the owner even sent two fuses.	Jingdong prefecture level city in Shandong
Stowe Flagship Store	King of Power Saving	This store has great stuff, relatively fast shipping and logistics	Jingdong prefecture level city in Shandong
Stowe Flagship Store	King of Power Saving	This product is based on the impossibility of saving electricity, I used 853 kWh of electricity in July, started using the product on August 1, and used 867 kWh of electricity by September 1, is this called	Jingdong prefecture level city in Shandong
Stowe Flagship Store	King of Power Saving	Power king received, to the home on the trial, when I plugged into the electric panel can not see the effect of power saving, the meter is still the same speed in the turn, I'll save the king of the manual to play look at the instructions, instructions to speak, have to have a day two days and half a month to save power obviously, then wait half a month to see the effect of power saving it! To half a month there are obvious power saving before continuing to chase Xu.	Jingdong prefecture level city in Shandong
Stowe Flagship Store	King of Power Saving	Received the goods, a look, did not see the manufacturer and production address, can only hold a try to see the mentality of the face	Jingdong prefecture level city in Shandong
Stowe Flagship Store	King of Power Saving	I've only been using it for a few days and I'm not sure if it's an IQ tax? Used to favorable reviews.	Jingdong prefecture level city in Shandong
Stowe Flagship Store	King of Power Saving	Appearance is okay, power saving next time to follow up the evaluation	Jingdong prefecture level city in Shandong
Stowe Flagship Store	King of Power Saving	Shorter time, but hopefully effective and power-saving, to be followed up in the future	Jingdong prefecture level city in Shandong
Stowe Flagship Store	King of Power Saving	Appearance is okay, I do not know if the power saving	Jingdong prefecture level city in Shandong
Stowe Flagship Store	King of Power Saving	Good service, fast logistics, Power Saving King is exquisite and beautiful, support the business, thank you!	Jingdong prefecture level city in Shandong
Stowe Flagship Store	King of Power Saving	Bought three at once to try first first.	Jingdong prefecture level city in Shandong
Stowe Flagship Store	King of Power Saving	The object is the same as the picture, the product is of high quality, the packaging is intact. High-end atmosphere, high power, power saving effect is obvious, average monthly power saving 50%, very satisfied.	Jingdong prefecture level city in Shandong
Stowe Flagship Store	King of Power Saving	Bought back and plugged in, really effective, before 16 degrees of electricity per day, now 8 degrees of electricity per day, open an air conditioner for 12 hours, decisive repurchase of a	Jingdong prefecture level city in Shandong
Stowe Flagship Store	King of Power Saving	The object is the same as the picture, the product is of high quality, and the package is intact. High-end atmosphere and high grade, high power	Jingdong prefecture level city in Shandong
Stowe Flagship Store	King of Power Saving	Goods received, I hope the store said can be energy-saving, first try a month, month after month to see the effect.	Jingdong prefecture level city in Shandong
Stowe Flagship Store	King of Power Saving	This is really good, the material and workmanship is particularly exquisite, the workmanship is really very good, I think I can recommend to my friends to buy	Jingdong prefecture level city in Shandong
Stowe Flagship Store	King of Power Saving	I have to praise, the quality is really good, and the packaging logistics are also good. Really like it, won't make you regret after getting it!	Jingdong prefecture level city in Shandong
Stowe Flagship Store	King of Power Saving	Reputable platform, efficient and professional service, buy with confidence and peace of mind!	Jingdong prefecture level city in Shandong
Stowe Flagship Store	King of Power Saving	Bought one on a trial basis to see if it works after 1-2 months.	Jingdong prefecture level city in Shandong
Stowe Flagship Store	King of Power Saving	Quality is very good, customer service attitude is very very praise, there are problems in a timely manner to solve the problem, the price is not expensive, super value, you can rest assured that the	Jingdong prefecture level city in Shandong
Stowe Flagship Store	King of Power Saving	With it no longer need to be afraid to use electricity, especially good, ready to buy a mom and dad that also	Jingdong prefecture level city in Shandong
Stowe Flagship Store	King of Power Saving	Baby received very like will return to buy the seller attitude is also very like will return to buy the seller attitude is also very like!	Jingdong prefecture level city in Shandong
Stowe Flagship Store	King of Power Saving	Jingdong Mall shopping convenience assured fast assured fast assured fast assured fast	Jingdong prefecture level city in Shandong
Stowe Flagship Store	King of Power Saving	Saving power received, the quality is very good, fine workmanship, especially good, much cheaper than the entity store, it is worth buying!	Jingdong prefecture level city in Shandong
Stowe Flagship Store	King of Power Saving	I did not expect the express delivery so quickly, very save point quality is super good, than the entity store is too cheap.	Jingdong prefecture level city in Shandong
Stowe Flagship Store	King of Power Saving	Have received the baby, very satisfied with a shopping, great worth buying!	Jingdong prefecture level city in Shandong
Stowe Flagship Store	King of Power Saving	This is really more powerful, very satisfied, more energy-saving, better, thank you boss.	Jingdong prefecture level city in Shandong
Stowe Flagship Store	King of Power Saving	Received has been used for two days, the air conditioning can boldly open it! Energy saving effect is really quite good	Jingdong prefecture level city in Shandong
Stowe Flagship Store	King of Power Saving	It is authentic, trust the merchant, the baby has a great texture and the price is moderate, unlimited repurchase!	Jingdong prefecture level city in Shandong
Stowe Flagship Store	King of Power Saving	It is genuine very good, the price is much cheaper, cost-effective, really special cost-effective, it is worth buying!	Jingdong prefecture level city in Shandong
Stowe Flagship Store	King of Power Saving	Very good quality, fine workmanship, low power consumption, long warranty, fast shipping, economical and practical	Jingdong prefecture level city in Shandong

Stowe Flagship Store	King of Power Saving	I've been using it for half a month now, it really saves power and the voltage is stable, it's very good	Jingdong prefecture level city in Shandong
Stowe Flagship Store	King of Power Saving	It's the ideal product 💝, good quality and fast logistics, worth the kudos!	Jingdong prefecture level city in Shandong
Stowe Flagship Store	King of Power Saving	The logistics is very fast, the baby is very good, the shopkeeper is NICE!	Jingdong prefecture level city in Shandong
Stowe Flagship Store	King of Power Saving	Good to use, good quality, save point durable	Jingdong prefecture level city in Shandong
Stowe Flagship Store	King of Power Saving	The quality is very good, customer service attitude is very, very awesome, there are problems in a timely manner to solve the problem	Jingdong prefecture level city in Shandong
Stowe Flagship Store	King of Power Saving	Quality is very good, the use of the effect is very good, more stable, quality assurance	Jingdong prefecture level city in Shandong
Stowe Flagship Store	King of Power Saving	Convenient, inexpensive, and see how it works out?	Jingdong prefecture level city in Shandong
Stowe Flagship Store	King of Power Saving	Very good quality, saves a lot of electricity, very good, worth buying	Jingdong prefecture level city in Shandong
Stowe Flagship Store	King of Power Saving	Power Saving King is easy to use, with the fruit of power saving to use easy to give!	Jingdong prefecture level city in Shandong
Stowe Flagship Store	King of Power Saving	This saver really works, good quality, safe and secure, very satisfied!	Jingdong prefecture level city in Shandong
Stowe Flagship Store	King of Power Saving	It works well, the voltage is very stable effect is good, the quality is also very good satisfaction	Jingdong prefecture level city in Shandong
Stowe Flagship Store	King of Power Saving	The logistics is super super fast! The quality is also very good, indeed value for money	Jingdong prefecture level city in Shandong
Stowe Flagship Store	King of Power Saving	I've only been using it for a month or so and it seems to be saving a lot of power	Jingdong prefecture level city in Shandong
Stowe Flagship Store	King of Power Saving	Very easy to operate, very good material, good value for money.	Jingdong prefecture level city in Shandong
Stowe Flagship Store	King of Power Saving	Aluminum alloy shell, durable, compact power-saving good effect	Jingdong prefecture level city in Shandong
Stowe Flagship Store	King of Power Saving	Nice device, easy to use, doesn't take up much space, recommendable!	Jingdong prefecture level city in Shandong
Stowe Flagship Store	King of Power Saving	Good quality, good price, fast logistics	Jingdong prefecture level city in Shandong
Stowe Flagship Store	King of Power Saving	Friends of the family in use recommended, the effect is good and recommendable!	Jingdong prefecture level city in Shandong
Stowe Flagship Store	King of Power Saving	Cheaper than brick and mortar stores worth buying is authentic	Jingdong prefecture level city in Shandong
Stowe Flagship Store	King of Power Saving	Merchants ship quickly, the price is cheaper than the physical store, good use	Jingdong prefecture level city in Shandong
Stowe Flagship Store	King of Power Saving	Very durable, affordable, fast shipping	Jingdong prefecture level city in Shandong
Stowe Flagship Store	King of Power Saving	Packaging tight quality is very good easy to use good results	Jingdong prefecture level city in Shandong
Stowe Flagship Store	King of Power Saving	Save the grid. It's in the whole house. It's saving money on the electric bill.	Jingdong prefecture level city in Shandong
Stowe Flagship Store	King of Power Saving	I used it today, it works well, I'm very satisfied	Jingdong prefecture level city in Shandong
Stowe Flagship Store	King of Power Saving	It really saves a lot of electricity after installation	Jingdong prefecture level city in Shandong
Stowe Flagship Store	King of Power Saving	Love it. Satisfied.	Jingdong prefecture level city in Shandong
Stowe Flagship Store	King of Power Saving	Doesn't feel like it's working.	Jingdong prefecture level city in Shandong
Stowe Flagship Store	King of Power Saving	I'll comment after using it.	Jingdong prefecture level city in Shandong
Stowe Flagship Store	King of Power Saving	I'm trying it out to see if it saves power, and I'll buy two more if it works well.	Jingdong prefecture level city in Shandong
Stowe Flagship Store	King of Power Saving	It is genuine very good, the price is much cheaper, cost-effective, really special cost-effective, it is worth buying!	Jingdong prefecture level city in Shandong
Stowe Flagship Store	King of Power Saving	This thing can really save electricity, I was introduced to buy by my friend, he used it very well, I used it and think it's also very good, it can really save electricity, save about half of the electricity every day, it's really good!	Jingdong prefecture level city in Shandong
Stowe Flagship Store	King of Power Saving	After using it for ten days or so, I checked the power consumption and it did drop, and customer service said it will save more electricity in the long run. The product is also insured, 5 years warranty do not have to worry about quality problems!	Jingdong prefecture level city in Shandong
Stowe Flagship Store	King of Power Saving	The product is good, before already bought a, this is the second, the effect is quite good, anyway, I see the daily electricity bill than before a lot less, this can be the choice!	Jingdong prefecture level city in Shandong
Stowe Flagship Store	King of Power Saving	Originally also very worried about things are not good, get the hand found completely over-anxious, five-star praise, the style is very generous, the quality is very good, the price is also very reasonable!	Jingdong prefecture level city in Shandong
Stowe Flagship Store	King of Power Saving	Quality is good, playing to the old family also buy a, repurchase, this quality is really good, recommended!	Jingdong prefecture level city in Shandong
Stowe Flagship Store	King of Power Saving	Thank you, the owner even sent two fuses.	Jingdong prefecture level city in Shandong
Stowe Flagship Store	King of Power Saving	This store has great stuff, relatively fast shipping and logistics	Jingdong prefecture level city in Shandong
Stowe Flagship Store	King of Power Saving	This product is based on the impossibility of saving electricity, I used 853 kWh of electricity in July, started using the product on August 1, and used 867 kWh of electricity by September 1, is this called	Jingdong prefecture level city in Shandong
Stowe Flagship Store	King of Power Saving	Power king received, to the home on the trial, when I plugged into the electric panel can not see the effect of power saving, the meter is still the same speed in the turn, I'll save the king of the manual to play look at the instructions, instructions to speak, have to have a day two days and half a month to save power obviously, then wait half a month to see the effect of power saving it! To half a month there are obvious power saving before continuing to chase Xu.	Jingdong prefecture level city in Shandong
Stowe Flagship Store	King of Power Saving	Received the goods, a look, did not see the manufacturer and production address, can only hold a try to see the mentality of the face	Jingdong prefecture level city in Shandong
Stowe Flagship Store	King of Power Saving	I've only been using it for a few days and I'm not sure if it's an IQ tax? Used to favorable reviews.	Jingdong prefecture level city in Shandong
Stowe Flagship Store	King of Power Saving	Appearance is okay, power saving next time to follow up the evaluation	Jingdong prefecture level city in Shandong
Stowe Flagship Store	King of Power Saving	Shorter time, but hopefully effective and power-saving, to be followed up in the future	Jingdong prefecture level city in Shandong
Stowe Flagship Store	King of Power Saving	Appearance is okay, I do not know if the power saving	Jingdong prefecture level city in Shandong
Stowe Flagship Store	King of Power Saving	Good service, fast logistics, Power Saving King is exquisite and beautiful, support the business, thank you!	Jingdong prefecture level city in Shandong
Stowe Flagship Store	King of Power Saving	Bought three at once to try first first.	Jingdong prefecture level city in Shandong
Stowe Flagship Store	King of Power Saving	The object is the same as the picture, the product is of high quality, the packaging is intact. High-end atmosphere, high power, power saving effect is obvious, average monthly power saving 50%, very satisfied.	Jingdong prefecture level city in Shandong
Stowe Flagship Store	King of Power Saving	Bought back and plugged in, really effective, before 16 degrees of electricity per day, now 8 degrees of electricity per day, open an air conditioner for 12 hours, decisive repurchase of a	Jingdong prefecture level city in Shandong
Stowe Flagship Store	King of Power Saving	The object is the same as the picture, the product is of high quality, and the package is intact. High-end atmosphere and high grade, high power	Jingdong prefecture level city in Shandong

Stowe Flagship Store	King of Power Saving	Goods received, I hope the store said can be energy-saving, first try a month, month after month to see the effect.	Jingdong prefecture level city in Shandong
Stowe Flagship Store	King of Power Saving	This is really good, the material and workmanship is particularly exquisite, the workmanship is really very good, I think I can recommend to my friends to buy	Jingdong prefecture level city in Shandong
Stowe Flagship Store	King of Power Saving	I have to praise, the quality is really good, and the packaging logistics are also good. Really like it, won't make you regret after getting it!	Jingdong prefecture level city in Shandong
Stowe Flagship Store	King of Power Saving	Reputable platform, efficient and professional service, buy with confidence and peace of mind!	Jingdong prefecture level city in Shandong
Stowe Flagship Store	King of Power Saving	Bought one on a trial basis to see if it works after 1-2 months.	Jingdong prefecture level city in Shandong
Stowe Flagship Store	King of Power Saving	Quality is very good, customer service attitude is very very praise, there are problems in a timely manner to solve the problem, the price is not expensive, super value, you can rest assured that the	Jingdong prefecture level city in Shandong
Stowe Flagship Store	King of Power Saving	With it no longer need to be afraid to use electricity, especially good, ready to buy a mom and dad that also	Jingdong prefecture level city in Shandong
Stowe Flagship Store	King of Power Saving	Baby received very like will return to buy the seller attitude is also very like will return to buy the seller attitude is also very like!	Jingdong prefecture level city in Shandong
Stowe Flagship Store	King of Power Saving	Jingdong Mall shopping convenience assured fast assured fast assured fast assured fast	Jingdong prefecture level city in Shandong
Stowe Flagship Store	King of Power Saving	Saving power received, the quality is very good, fine workmanship, especially good, much cheaper than the entity store, it is worth buying!	Jingdong prefecture level city in Shandong
Stowe Flagship Store	King of Power Saving	I did not expect the express delivery so quickly, very save point quality is super good, than the entity store is too cheap.	Jingdong prefecture level city in Shandong
Stowe Flagship Store	King of Power Saving	Have received the baby, very satisfied with a shopping, great worth buying!	Jingdong prefecture level city in Shandong
Stowe Flagship Store	King of Power Saving	This is really more powerful, very satisfied, more energy-saving, better, thank you boss.	Jingdong prefecture level city in Shandong
Stowe Flagship Store	King of Power Saving	Received has been used for two days, the air conditioning can boldly open it! Energy saving effect is really quite good	Jingdong prefecture level city in Shandong
Stowe Flagship Store	King of Power Saving	It is authentic, trust the merchant, the baby has a great texture and the price is moderate, unlimited repurchase!	Jingdong prefecture level city in Shandong
Stowe Flagship Store	King of Power Saving	It is genuine very good, the price is much cheaper, cost-effective, really special cost-effective, it is worth buying!	Jingdong prefecture level city in Shandong
Stowe Flagship Store	King of Power Saving	Very good quality, fine workmanship, low power consumption, long warranty, fast shipping, economical and practical	Jingdong prefecture level city in Shandong
Stowe Flagship Store	King of Power Saving	I've been using it for half a month now, it really saves power and the voltage is stable, it's very good	Jingdong prefecture level city in Shandong
Stowe Flagship Store	King of Power Saving	It's the ideal product 💖, good quality and fast logistics, worth the kudos!	Jingdong prefecture level city in Shandong
Stowe Flagship Store	King of Power Saving	The logistics is very fast, the baby is very good, the shopkeeper is NICE!	Jingdong prefecture level city in Shandong
Stowe Flagship Store	King of Power Saving	Good to use, good quality, save point durable	Jingdong prefecture level city in Shandong
Stowe Flagship Store	King of Power Saving	The quality is very good, customer service attitude is very, very awesome, there are problems in a timely manner to solve the problem	Jingdong prefecture level city in Shandong
Stowe Flagship Store	King of Power Saving	Quality is very good, the use of the effect is very good, more stable, quality assurance	Jingdong prefecture level city in Shandong
Stowe Flagship Store	King of Power Saving	Convenient, inexpensive, and see how it works out?	Jingdong prefecture level city in Shandong
Stowe Flagship Store	King of Power Saving	Very good quality, saves a lot of electricity, very good, worth buying	Jingdong prefecture level city in Shandong
Stowe Flagship Store	King of Power Saving	Power Saving King is easy to use, with the fruit of power saving to use easy to give!	Jingdong prefecture level city in Shandong
Stowe Flagship Store	King of Power Saving	This saver really works, good quality, safe and secure, very satisfied!	Jingdong prefecture level city in Shandong
Stowe Flagship Store	King of Power Saving	It works well, the voltage is very stable effect is good, the quality is also very good satisfaction	Jingdong prefecture level city in Shandong
Stowe Flagship Store	King of Power Saving	The logistics is super super fast! The quality is also very good, indeed value for money	Jingdong prefecture level city in Shandong
Stowe Flagship Store	King of Power Saving	I've only been using it for a month or so and it seems to be saving a lot of power	Jingdong prefecture level city in Shandong
Stowe Flagship Store	King of Power Saving	Very easy to operate, very good material, good value for money.	Jingdong prefecture level city in Shandong
Stowe Flagship Store	King of Power Saving	Aluminum alloy shell, durable, compact power-saving good effect	Jingdong prefecture level city in Shandong
Stowe Flagship Store	King of Power Saving	Nice device, easy to use, doesn't take up much space, recommendable!	Jingdong prefecture level city in Shandong
Stowe Flagship Store	King of Power Saving	Good quality, good price, fast logistics	Jingdong prefecture level city in Shandong
Stowe Flagship Store	King of Power Saving	Friends of the family in use recommended, the effect is good and recommendable!	Jingdong prefecture level city in Shandong
Stowe Flagship Store	King of Power Saving	Cheaper than brick and mortar stores worth buying is authentic	Jingdong prefecture level city in Shandong
Stowe Flagship Store	King of Power Saving	Merchants ship quickly, the price is cheaper than the physical store, good use	Jingdong prefecture level city in Shandong
Stowe Flagship Store	King of Power Saving	Very durable, affordable, fast shipping	Jingdong prefecture level city in Shandong
Stowe Flagship Store	King of Power Saving	Packaging tight quality is very good easy to use good results	Jingdong prefecture level city in Shandong
Stowe Flagship Store	King of Power Saving	Save the grid. It's in the whole house. It's saving money on the electric bill.	Jingdong prefecture level city in Shandong
Stowe Flagship Store	King of Power Saving	I used it today, it works well, I'm very satisfied	Jingdong prefecture level city in Shandong
Stowe Flagship Store	King of Power Saving	It really saves a lot of electricity after installation	Jingdong prefecture level city in Shandong
Stowe Flagship Store	King of Power Saving	Love it. Satisfied.	Jingdong prefecture level city in Shandong
Stowe Flagship Store	King of Power Saving	Doesn't feel like it's working.	Jingdong prefecture level city in Shandong
Stowe Flagship Store	King of Power Saving	I'll comment after using it.	Jingdong prefecture level city in Shandong
Stowe Flagship Store	King of Power Saving	I'm trying it out to see if it saves power, and I'll buy two more if it works well.	Jingdong prefecture level city in Shandong
Stowe Flagship Store	King of Power Saving	It is genuine very good, the price is much cheaper, cost-effective, really special cost-effective, it is worth buying!	Jingdong prefecture level city in Shandong
Stowe Flagship Store	King of Power Saving	This thing can really save electricity, I was introduced to buy by my friend, he used it very well, I used it and think it's also very good, it can really save electricity, save about half of the electricity every day, it's really good!	Jingdong prefecture level city in Shandong
Stowe Flagship Store	King of Power Saving	After using it for ten days or so, I checked the power consumption and it did drop, and customer service said it will save more electricity in the long run. The product is also insured, 5 years warranty do not have to worry about quality problems!	Jingdong prefecture level city in Shandong
Stowe Flagship Store	King of Power Saving	The product is good, before already bought a, this is the second, the effect is quite good, anyway, I see the daily electricity bill than before a lot less, this can be the choice!	Jingdong prefecture level city in Shandong

Stowe Flagship Store	King of Power Saving	Originally also very worried about things are not good, get the hand found completely over-anxious, five-star praise, the style is very generous, the quality is very good, the price is also very reasonable!	Jingdong prefecture level city in Shandong
Stowe Flagship Store	King of Power Saving	Quality is good, playing to the old family also buy a, repurchase, this quality is really good, recommended!	Jingdong prefecture level city in Shandong
Stowe Flagship Store	King of Power Saving	Thank you, the owner even sent two fuses.	Jingdong prefecture level city in Shandong
Stowe Flagship Store	King of Power Saving	This store has great stuff, relatively fast shipping and logistics	Jingdong prefecture level city in Shandong
Stowe Flagship Store	King of Power Saving	This product is based on the impossibility of saving electricity, I used 853 kWh of electricity in July, started using the product on August 1, and used 867 kWh of electricity by September 1, is this called	Jingdong prefecture level city in Shandong
Stowe Flagship Store	King of Power Saving	Power king received, to the home on the trial, when I plugged into the electric panel can not see the effect of power saving, the meter is still the same speed in the turn, I'll save the king of the manual to play look at the instructions, instructions to speak, have to have a day two days and half a month to save power obviously, then wait half a month to see the effect of power saving it! To half a month there are obvious power saving before continuing to chase Xu.	Jingdong prefecture level city in Shandong
Stowe Flagship Store	King of Power Saving	Received the goods, a look, did not see the manufacturer and production address, can only hold a try to see the mentality of the face	Jingdong prefecture level city in Shandong
Stowe Flagship Store	King of Power Saving	I've only been using it for a few days and I'm not sure if it's an IQ tax? Used to favorable reviews.	Jingdong prefecture level city in Shandong
Stowe Flagship Store	King of Power Saving	Appearance is okay, power saving next time to follow up the evaluation	Jingdong prefecture level city in Shandong
Stowe Flagship Store	King of Power Saving	Shorter time, but hopefully effective and power-saving, to be followed up in the future	Jingdong prefecture level city in Shandong
Stowe Flagship Store	King of Power Saving	Appearance is okay, I do not know if the power saving	Jingdong prefecture level city in Shandong
Stowe Flagship Store	King of Power Saving	Good service, fast logistics, Power Saving King is exquisite and beautiful, support the business, thank you!	Jingdong prefecture level city in Shandong
Stowe Flagship Store	King of Power Saving	Bought three at once to try first first.	Jingdong prefecture level city in Shandong
Stowe Flagship Store	King of Power Saving	The object is the same as the picture, the product is of high quality, the packaging is intact. High-end atmosphere, high power, power saving effect is obvious, average monthly power saving 50%, very satisfied.	Jingdong prefecture level city in Shandong
Stowe Flagship Store	King of Power Saving	Bought back and plugged in, really effective, before 16 degrees of electricity per day, now 8 degrees of electricity per day, open an air conditioner for 12 hours, decisive repurchase of a	Jingdong prefecture level city in Shandong
Stowe Flagship Store	King of Power Saving	The object is the same as the picture, the product is of high quality, and the package is intact. High-end atmosphere and high grade, high power	Jingdong prefecture level city in Shandong
Stowe Flagship Store	King of Power Saving	Goods received, I hope the store said can be energy-saving, first try a month, month after month to see the effect.	Jingdong prefecture level city in Shandong
Stowe Flagship Store	King of Power Saving	This is really good, the material and workmanship is particularly exquisite, the workmanship is really very good, I think I can recommend to my friends to buy	Jingdong prefecture level city in Shandong
Stowe Flagship Store	King of Power Saving	I have to praise, the quality is really good, and the packaging logistics are also good. Really like it, won't make you regret after getting it!	Jingdong prefecture level city in Shandong
Stowe Flagship Store	King of Power Saving	Reputable platform, efficient and professional service, buy with confidence and peace of mind!	Jingdong prefecture level city in Shandong
Stowe Flagship Store	King of Power Saving	Bought one on a trial basis to see if it works after 1-2 months.	Jingdong prefecture level city in Shandong
Stowe Flagship Store	King of Power Saving	Quality is very good, customer service attitude is very very praise, there are problems in a timely manner to solve the problem, the price is not expensive, super value, you can rest assured that the	Jingdong prefecture level city in Shandong
Stowe Flagship Store	King of Power Saving	With it no longer need to be afraid to use electricity, especially good, ready to buy a mom and dad that also	Jingdong prefecture level city in Shandong
Stowe Flagship Store	King of Power Saving	Baby received very like will return to buy the seller attitude is also very like will return to buy the seller attitude is also very like!	Jingdong prefecture level city in Shandong
Stowe Flagship Store	King of Power Saving	Jingdong Mall shopping convenience assured fast assured fast assured fast assured fast	Jingdong prefecture level city in Shandong
Stowe Flagship Store	King of Power Saving	Saving power received, the quality is very good, fine workmanship, especially good, much cheaper than the entity store, it is worth buying!	Jingdong prefecture level city in Shandong
Stowe Flagship Store	King of Power Saving	I did not expect the express delivery so quickly, very save point quality is super good, than the entity store is too cheap.	Jingdong prefecture level city in Shandong
Stowe Flagship Store	King of Power Saving	Have received the baby, very satisfied with a shopping, great worth buying!	Jingdong prefecture level city in Shandong
Stowe Flagship Store	King of Power Saving	This is really more powerful, very satisfied, more energy-saving, better, thank you boss.	Jingdong prefecture level city in Shandong
Stowe Flagship Store	King of Power Saving	Received has been used for two days, the air conditioning can boldly open it! Energy saving effect is really quite good	Jingdong prefecture level city in Shandong
Stowe Flagship Store	King of Power Saving	It is authentic, trust the merchant, the baby has a great texture and the price is moderate, unlimited repurchase!	Jingdong prefecture level city in Shandong
Stowe Flagship Store	King of Power Saving	It is genuine very good, the price is much cheaper, cost-effective, really special cost-effective, it is worth buying!	Jingdong prefecture level city in Shandong
Stowe Flagship Store	King of Power Saving	Very good quality, fine workmanship, low power consumption, long warranty, fast shipping, economical and practical	Jingdong prefecture level city in Shandong
Stowe Flagship Store	King of Power Saving	I've been using it for half a month now, it really saves power and the voltage is stable, it's very good	Jingdong prefecture level city in Shandong
Stowe Flagship Store	King of Power Saving	It's the ideal product , good quality and fast logistics, worth the kudos!	Jingdong prefecture level city in Shandong
Stowe Flagship Store	King of Power Saving	The logistics is very fast, the baby is very good, the shopkeeper is NICE!	Jingdong prefecture level city in Shandong
Stowe Flagship Store	King of Power Saving	Good to use, good quality, save point durable	Jingdong prefecture level city in Shandong
Stowe Flagship Store	King of Power Saving	The quality is very good, customer service attitude is very, very awesome, there are problems in a timely manner to solve the problem	Jingdong prefecture level city in Shandong
Stowe Flagship Store	King of Power Saving	Quality is very good, the use of the effect is very good, more stable, quality assurance	Jingdong prefecture level city in Shandong
Stowe Flagship Store	King of Power Saving	Convenient, inexpensive, and see how it works out?	Jingdong prefecture level city in Shandong
Stowe Flagship Store	King of Power Saving	Very good quality, saves a lot of electricity, very good, worth buying	Jingdong prefecture level city in Shandong
Stowe Flagship Store	King of Power Saving	Power Saving King is easy to use, with the fruit of power saving to use easy to give!	Jingdong prefecture level city in Shandong
Stowe Flagship Store	King of Power Saving	This saver really works, good quality, safe and secure, very satisfied!	Jingdong prefecture level city in Shandong
Stowe Flagship Store	King of Power Saving	It works well, the voltage is very stable effect is good, the quality is also very good satisfaction	Jingdong prefecture level city in Shandong
Stowe Flagship Store	King of Power Saving	The logistics is super super fast! The quality is also very good, indeed value for money	Jingdong prefecture level city in Shandong
Stowe Flagship Store	King of Power Saving	I've only been using it for a month or so and it seems to be saving a lot of power	Jingdong prefecture level city in Shandong
Stowe Flagship Store	King of Power Saving	Very easy to operate, very good material, good value for money.	Jingdong prefecture level city in Shandong
Stowe Flagship Store	King of Power Saving	Aluminum alloy shell, durable, compact power-saving good effect	Jingdong prefecture level city in Shandong
Stowe Flagship Store	King of Power Saving	Nice device, easy to use, doesn't take up much space, recommendable!	Jingdong prefecture level city in Shandong
Stowe Flagship Store	King of Power Saving	Good quality, good price, fast logistics	Jingdong prefecture level city in Shandong

Stowe Flagship Store	King of Power Saving	Friends of the family in use recommended, the effect is good and recommendable!	Jingdong prefecture level city in Shandong
Stowe Flagship Store	King of Power Saving	Cheaper than brick and mortar stores worth buying is authentic	Jingdong prefecture level city in Shandong
Stowe Flagship Store	King of Power Saving	Merchants ship quickly, the price is cheaper than the physical store, good use	Jingdong prefecture level city in Shandong
Stowe Flagship Store	King of Power Saving	Very durable, affordable, fast shipping	Jingdong prefecture level city in Shandong
Stowe Flagship Store	King of Power Saving	Packaging tight quality is very good easy to use good results	Jingdong prefecture level city in Shandong
Stowe Flagship Store	King of Power Saving	Save the grid. It's in the whole house. It's saving money on the electric bill.	Jingdong prefecture level city in Shandong
Stowe Flagship Store	King of Power Saving	I used it today, it works well, I'm very satisfied	Jingdong prefecture level city in Shandong
Stowe Flagship Store	King of Power Saving	It really saves a lot of electricity after installation	Jingdong prefecture level city in Shandong
Stowe Flagship Store	King of Power Saving	Love it. Satisfied.	Jingdong prefecture level city in Shandong
Stowe Flagship Store	King of Power Saving	Doesn't feel like it's working.	Jingdong prefecture level city in Shandong
Stowe Flagship Store	King of Power Saving	I'll comment after using it.	Jingdong prefecture level city in Shandong
Stowe Flagship Store	King of Power Saving	I'm trying it out to see if it saves power, and I'll buy two more if it works well.	Jingdong prefecture level city in Shandong
Stowe Flagship Store	King of Power Saving	It is genuine very good, the price is much cheaper, cost-effective, really special cost-effective, it is worth buying!	Jingdong prefecture level city in Shandong
Stowe Flagship Store	King of Power Saving	This thing can really save electricity, I was introduced to buy by my friend, he used it very well, I used it and think it's also very good, it can really save electricity, save about half of the electricity every day, it's really good!	Jingdong prefecture level city in Shandong
Stowe Flagship Store	King of Power Saving	After using it for ten days or so, I checked the power consumption and it did drop, and customer service said it will save more electricity in the long run. The product is also insured, 5 years warranty do not have to worry about quality problems!	Jingdong prefecture level city in Shandong
Stowe Flagship Store	King of Power Saving	The product is good, before already bought a, this is the second, the effect is quite good, anyway, I see the daily electricity bill than before a lot less, this can be the choice!	Jingdong prefecture level city in Shandong
Stowe Flagship Store	King of Power Saving	Originally also very worried about things are not good, get the hand found completely over-anxious, five-star praise, the style is very generous, the quality is very good, the price is also very reasonable!	Jingdong prefecture level city in Shandong
Stowe Flagship Store	King of Power Saving	Quality is good, playing to the old family also buy a, repurchase, this quality is really good, recommended!	Jingdong prefecture level city in Shandong
Stowe Flagship Store	King of Power Saving	Thank you, the owner even sent two fuses.	Jingdong prefecture level city in Shandong
Stowe Flagship Store	King of Power Saving	This store has great stuff, relatively fast shipping and logistics	Jingdong prefecture level city in Shandong
Stowe Flagship Store	King of Power Saving	This product is based on the impossibility of saving electricity, I used 853 kWh of electricity in July, started using the product on August 1, and used 867 kWh of electricity by September 1, is this called	Jingdong prefecture level city in Shandong
Stowe Flagship Store	King of Power Saving	Power king received, to the home on the trial, when I plugged into the electric panel can not see the effect of power saving, the meter is still the same speed in the turn, I'll save the king of the manual to play look at the instructions, instructions to speak, have to have a day two days and half a month to save power obviously, then wait half a month to see the effect of power saving it! To half a month there are obvious power saving before continuing to chase Xu.	Jingdong prefecture level city in Shandong
Stowe Flagship Store	King of Power Saving	Received the goods, a look, did not see the manufacturer and production address, can only hold a try to see the mentality of the face	Jingdong prefecture level city in Shandong
Stowe Flagship Store	King of Power Saving	I've only been using it for a few days and I'm not sure if it's an IQ tax? Used to favorable reviews.	Jingdong prefecture level city in Shandong
Stowe Flagship Store	King of Power Saving	Appearance is okay, power saving next time to follow up the evaluation	Jingdong prefecture level city in Shandong
Stowe Flagship Store	King of Power Saving	Shorter time, but hopefully effective and power-saving, to be followed up in the future	Jingdong prefecture level city in Shandong
Stowe Flagship Store	King of Power Saving	Appearance is okay, I do not know if the power saving	Jingdong prefecture level city in Shandong
Stowe Flagship Store	King of Power Saving	Good service, fast logistics, Power Saving King is exquisite and beautiful, support the business, thank you!	Jingdong prefecture level city in Shandong
Stowe Flagship Store	King of Power Saving	Bought three at once to try first first.	Jingdong prefecture level city in Shandong
Stowe Flagship Store	King of Power Saving	The object is the same as the picture, the product is of high quality, the packaging is intact. High-end atmosphere, high power, power saving effect is obvious, average monthly power saving 50%, very satisfied.	Jingdong prefecture level city in Shandong
Stowe Flagship Store	King of Power Saving	Bought back and plugged in, really effective, before 16 degrees of electricity per day, now 8 degrees of electricity per day, open an air conditioner for 12 hours, decisive repurchase of a	Jingdong prefecture level city in Shandong
Stowe Flagship Store	King of Power Saving	The object is the same as the picture, the product is of high quality, and the package is intact. High-end atmosphere and high grade, high power	Jingdong prefecture level city in Shandong
Stowe Flagship Store	King of Power Saving	Goods received, I hope the store said can be energy-saving, first try a month, month after month to see the effect.	Jingdong prefecture level city in Shandong
Stowe Flagship Store	King of Power Saving	This is really good, the material and workmanship is particularly exquisite, the workmanship is really very good, I think I can recommend to my friends to buy	Jingdong prefecture level city in Shandong
Stowe Flagship Store	King of Power Saving	I have to praise, the quality is really good, and the packaging logistics are also good. Really like it, won't make you regret after getting it!	Jingdong prefecture level city in Shandong
Stowe Flagship Store	King of Power Saving	Reputable platform, efficient and professional service, buy with confidence and peace of mind!	Jingdong prefecture level city in Shandong
Stowe Flagship Store	King of Power Saving	Bought one on a trial basis to see if it works after 1-2 months.	Jingdong prefecture level city in Shandong
Stowe Flagship Store	King of Power Saving	Quality is very good, customer service attitude is very very praise, there are problems in a timely manner to solve the problem, the price is not expensive, super value, you can rest assured that the	Jingdong prefecture level city in Shandong
Stowe Flagship Store	King of Power Saving	With it no longer need to be afraid to use electricity, especially good, ready to buy a mom and dad that also	Jingdong prefecture level city in Shandong
Stowe Flagship Store	King of Power Saving	Baby received very like will return to buy the seller attitude is also very like will return to buy the seller attitude is also very like!	Jingdong prefecture level city in Shandong
Stowe Flagship Store	King of Power Saving	Jingdong Mall shopping convenience assured fast assured fast assured fast assured fast	Jingdong prefecture level city in Shandong
Stowe Flagship Store	King of Power Saving	Saving power received, the quality is very good, fine workmanship, especially good, much cheaper than the entity store, it is worth buying!	Jingdong prefecture level city in Shandong
Stowe Flagship Store	King of Power Saving	I did not expect the express delivery so quickly, very save point quality is super good, than the entity store is too cheap.	Jingdong prefecture level city in Shandong
Stowe Flagship Store	King of Power Saving	Have received the baby, very satisfied with a shopping, great worth buying!	Jingdong prefecture level city in Shandong
Stowe Flagship Store	King of Power Saving	This is really more powerful, very satisfied, more energy-saving, better, thank you boss.	Jingdong prefecture level city in Shandong
Stowe Flagship Store	King of Power Saving	Received has been used for two days, the air conditioning can boldly open it! Energy saving effect is really quite good	Jingdong prefecture level city in Shandong
Stowe Flagship Store	King of Power Saving	It is authentic, trust the merchant, the baby has a great texture and the price is moderate, unlimited repurchase!	Jingdong prefecture level city in Shandong
Stowe Flagship Store	King of Power Saving	It is genuine very good, the price is much cheaper, cost-effective, really special cost-effective, it is worth buying!	Jingdong prefecture level city in Shandong
Stowe Flagship Store	King of Power Saving	Very good quality, fine workmanship, low power consumption, long warranty, fast shipping, economical and practical	Jingdong prefecture level city in Shandong

Stowe Flagship Store	King of Power Saving	I've been using it for half a month now, it really saves power and the voltage is stable, it's very good	Jingdong prefecture level city in Shandong
Stowe Flagship Store	King of Power Saving	It's the ideal product 💝, good quality and fast logistics, worth the kudos!	Jingdong prefecture level city in Shandong
Stowe Flagship Store	King of Power Saving	The logistics is very fast, the baby is very good, the shopkeeper is NICE!	Jingdong prefecture level city in Shandong
Stowe Flagship Store	King of Power Saving	Good to use, good quality, save point durable	Jingdong prefecture level city in Shandong
Stowe Flagship Store	King of Power Saving	The quality is very good, customer service attitude is very, very awesome, there are problems in a timely manner to solve the problem	Jingdong prefecture level city in Shandong
Stowe Flagship Store	King of Power Saving	Quality is very good, the use of the effect is very good, more stable, quality assurance	Jingdong prefecture level city in Shandong
Stowe Flagship Store	King of Power Saving	Convenient, inexpensive, and see how it works out?	Jingdong prefecture level city in Shandong
Stowe Flagship Store	King of Power Saving	Very good quality, saves a lot of electricity, very good, worth buying	Jingdong prefecture level city in Shandong
Stowe Flagship Store	King of Power Saving	Power Saving King is easy to use, with the fruit of power saving to use easy to give!	Jingdong prefecture level city in Shandong
Stowe Flagship Store	King of Power Saving	This saver really works, good quality, safe and secure, very satisfied!	Jingdong prefecture level city in Shandong
Stowe Flagship Store	King of Power Saving	It works well, the voltage is very stable effect is good, the quality is also very good satisfaction	Jingdong prefecture level city in Shandong
Stowe Flagship Store	King of Power Saving	The logistics is super super fast! The quality is also very good, indeed value for money	Jingdong prefecture level city in Shandong
Stowe Flagship Store	King of Power Saving	I've only been using it for a month or so and it seems to be saving a lot of power	Jingdong prefecture level city in Shandong
Stowe Flagship Store	King of Power Saving	Very easy to operate, very good material, good value for money.	Jingdong prefecture level city in Shandong
Stowe Flagship Store	King of Power Saving	Aluminum alloy shell, durable, compact power-saving good effect	Jingdong prefecture level city in Shandong
Stowe Flagship Store	King of Power Saving	Nice device, easy to use, doesn't take up much space, recommendable!	Jingdong prefecture level city in Shandong
Stowe Flagship Store	King of Power Saving	Good quality, good price, fast logistics	Jingdong prefecture level city in Shandong
Stowe Flagship Store	King of Power Saving	Friends of the family in use recommended, the effect is good and recommendable!	Jingdong prefecture level city in Shandong
Stowe Flagship Store	King of Power Saving	Cheaper than brick and mortar stores worth buying is authentic	Jingdong prefecture level city in Shandong
Stowe Flagship Store	King of Power Saving	Merchants ship quickly, the price is cheaper than the physical store, good use	Jingdong prefecture level city in Shandong
Stowe Flagship Store	King of Power Saving	Very durable, affordable, fast shipping	Jingdong prefecture level city in Shandong
Stowe Flagship Store	King of Power Saving	Packaging tight quality is very good easy to use good results	Jingdong prefecture level city in Shandong
Stowe Flagship Store	King of Power Saving	Save the grid. It's in the whole house. It's saving money on the electric bill.	Jingdong prefecture level city in Shandong
Stowe Flagship Store	King of Power Saving	I used it today, it works well, I'm very satisfied	Jingdong prefecture level city in Shandong
Stowe Flagship Store	King of Power Saving	It really saves a lot of electricity after installation	Jingdong prefecture level city in Shandong
Stowe Flagship Store	King of Power Saving	Love it. Satisfied.	Jingdong prefecture level city in Shandong
Stowe Flagship Store	King of Power Saving	Doesn't feel like it's working.	Jingdong prefecture level city in Shandong
Stowe Flagship Store	King of Power Saving	I'll comment after using it.	Jingdong prefecture level city in Shandong
Stowe Flagship Store	King of Power Saving	I'm trying it out to see if it saves power, and I'll buy two more if it works well.	Jingdong prefecture level city in Shandong
Stowe Flagship Store	King of Power Saving	It is genuine very good, the price is much cheaper, cost-effective, really special cost-effective, it is worth buying!	Jingdong prefecture level city in Shandong
Stowe Flagship Store	King of Power Saving	This thing can really save electricity, I was introduced to buy by my friend, he used it very well, I used it and think it's also very good, it can really save electricity, save about half of the electricity every day, it's really good!	Jingdong prefecture level city in Shandong
Stowe Flagship Store	King of Power Saving	After using it for ten days or so, I checked the power consumption and it did drop, and customer service said it will save more electricity in the long run. The product is also insured, 5 years warranty do not have to worry about quality problems!	Jingdong prefecture level city in Shandong
Stowe Flagship Store	King of Power Saving	The product is good, before already bought a, this is the second, the effect is quite good, anyway, I see the daily electricity bill than before a lot less, this can be the choice!	Jingdong prefecture level city in Shandong
Stowe Flagship Store	King of Power Saving	Originally also very worried about things are not good, get the hand found completely over-anxious, five-star praise, the style is very generous, the quality is very good, the price is also very reasonable!	Jingdong prefecture level city in Shandong
Stowe Flagship Store	King of Power Saving	Quality is good, playing to the old family also buy a, repurchase, this quality is really good, recommended!	Jingdong prefecture level city in Shandong
Stowe Flagship Store	King of Power Saving	Thank you, the owner even sent two fuses.	Jingdong prefecture level city in Shandong
Stowe Flagship Store	King of Power Saving	This store has great stuff, relatively fast shipping and logistics	Jingdong prefecture level city in Shandong
Stowe Flagship Store	King of Power Saving	This product is based on the impossibility of saving electricity, I used 853 kWh of electricity in July, started using the product on August 1, and used 867 kWh of electricity by September 1, is this called	Jingdong prefecture level city in Shandong
Stowe Flagship Store	King of Power Saving	Power king received, to the home on the trial, when I plugged into the electric panel can not see the effect of power saving, the meter is still the same speed in the turn, I'll save the king of the manual to play look at the instructions, instructions to speak, have to have a day two days and half a month to save power obviously, then wait half a month to see the effect of power saving it! To half a month there are obvious power saving before continuing to chase Xu.	Jingdong prefecture level city in Shandong
Stowe Flagship Store	King of Power Saving	Received the goods, a look, did not see the manufacturer and production address, can only hold a try to see the mentality of the face	Jingdong prefecture level city in Shandong
Stowe Flagship Store	King of Power Saving	I've only been using it for a few days and I'm not sure if it's an IQ tax? Used to favorable reviews.	Jingdong prefecture level city in Shandong
Stowe Flagship Store	King of Power Saving	Appearance is okay, power saving next time to follow up the evaluation	Jingdong prefecture level city in Shandong
Stowe Flagship Store	King of Power Saving	Shorter time, but hopefully effective and power-saving, to be followed up in the future	Jingdong prefecture level city in Shandong
Stowe Flagship Store	King of Power Saving	Appearance is okay, I do not know if the power saving	Jingdong prefecture level city in Shandong
Stowe Flagship Store	King of Power Saving	Good service, fast logistics, Power Saving King is exquisite and beautiful, support the business, thank you!	Jingdong prefecture level city in Shandong
Stowe Flagship Store	King of Power Saving	Bought three at once to try first first.	Jingdong prefecture level city in Shandong
Stowe Flagship Store	King of Power Saving	The object is the same as the picture, the product is of high quality, the packaging is intact. High-end atmosphere, high power, power saving effect is obvious, average monthly power saving 50%, very satisfied.	Jingdong prefecture level city in Shandong
Stowe Flagship Store	King of Power Saving	Bought back and plugged in, really effective, before 16 degrees of electricity per day, now 8 degrees of electricity per day, open an air conditioner for 12 hours, decisive repurchase of a	Jingdong prefecture level city in Shandong
Stowe Flagship Store	King of Power Saving	The object is the same as the picture, the product is of high quality, and the package is intact. High-end atmosphere and high grade, high power	Jingdong prefecture level city in Shandong

Stowe Flagship Store	King of Power Saving	Goods received, I hope the store said can be energy-saving, first try a month, month after month to see the effect.	Jingdong prefecture level city in Shandong
Stowe Flagship Store	King of Power Saving	This is really good, the material and workmanship is particularly exquisite, the workmanship is really very good, I think I can recommend to my friends to buy	Jingdong prefecture level city in Shandong
Stowe Flagship Store	King of Power Saving	I have to praise, the quality is really good, and the packaging logistics are also good. Really like it, won't make you regret after getting it!	Jingdong prefecture level city in Shandong
Stowe Flagship Store	King of Power Saving	Reputable platform, efficient and professional service, buy with confidence and peace of mind!	Jingdong prefecture level city in Shandong
Stowe Flagship Store	King of Power Saving	Bought one on a trial basis to see if it works after 1-2 months.	Jingdong prefecture level city in Shandong
Stowe Flagship Store	King of Power Saving	Quality is very good, customer service attitude is very very praise, there are problems in a timely manner to solve the problem, the price is not expensive, super value, you can rest assured that the	Jingdong prefecture level city in Shandong
Stowe Flagship Store	King of Power Saving	With it no longer need to be afraid to use electricity, especially good, ready to buy a mom and dad that also	Jingdong prefecture level city in Shandong
Stowe Flagship Store	King of Power Saving	Baby received very like will return to buy the seller attitude is also very like will return to buy the seller attitude is also very like!	Jingdong prefecture level city in Shandong
Stowe Flagship Store	King of Power Saving	Jingdong Mall shopping convenience assured fast assured fast assured fast assured fast	Jingdong prefecture level city in Shandong
Stowe Flagship Store	King of Power Saving	Saving power received, the quality is very good, fine workmanship, especially good, much cheaper than the entity store, it is worth buying!	Jingdong prefecture level city in Shandong
Stowe Flagship Store	King of Power Saving	I did not expect the express delivery so quickly, very save point quality is super good, than the entity store is too cheap.	Jingdong prefecture level city in Shandong
Stowe Flagship Store	King of Power Saving	Have received the baby, very satisfied with a shopping, great worth buying!	Jingdong prefecture level city in Shandong
Stowe Flagship Store	King of Power Saving	This is really more powerful, very satisfied, more energy-saving, better, thank you boss.	Jingdong prefecture level city in Shandong
Stowe Flagship Store	King of Power Saving	Received has been used for two days, the air conditioning can boldly open it! Energy saving effect is really quite good	Jingdong prefecture level city in Shandong
Stowe Flagship Store	King of Power Saving	It is authentic, trust the merchant, the baby has a great texture and the price is moderate, unlimited repurchase!	Jingdong prefecture level city in Shandong
Stowe Flagship Store	King of Power Saving	It is genuine very good, the price is much cheaper, cost-effective, really special cost-effective, it is worth buying!	Jingdong prefecture level city in Shandong
Stowe Flagship Store	King of Power Saving	Very good quality, fine workmanship, low power consumption, long warranty, fast shipping, economical and practical	Jingdong prefecture level city in Shandong
Stowe Flagship Store	King of Power Saving	I've been using it for half a month now, it really saves power and the voltage is stable, it's very good	Jingdong prefecture level city in Shandong
Stowe Flagship Store	King of Power Saving	It's the ideal product 💖, good quality and fast logistics, worth the kudos!	Jingdong prefecture level city in Shandong
Stowe Flagship Store	King of Power Saving	The logistics is very fast, the baby is very good, the shopkeeper is NICE!	Jingdong prefecture level city in Shandong
Stowe Flagship Store	King of Power Saving	Good to use, good quality, save point durable	Jingdong prefecture level city in Shandong
Stowe Flagship Store	King of Power Saving	The quality is very good, customer service attitude is very, very awesome, there are problems in a timely manner to solve the problem	Jingdong prefecture level city in Shandong
Stowe Flagship Store	King of Power Saving	Quality is very good, the use of the effect is very good, more stable, quality assurance	Jingdong prefecture level city in Shandong
Stowe Flagship Store	King of Power Saving	Convenient, inexpensive, and see how it works out?	Jingdong prefecture level city in Shandong
Stowe Flagship Store	King of Power Saving	Very good quality, saves a lot of electricity, very good, worth buying	Jingdong prefecture level city in Shandong
Stowe Flagship Store	King of Power Saving	Power Saving King is easy to use, with the fruit of power saving to use easy to give!	Jingdong prefecture level city in Shandong
Stowe Flagship Store	King of Power Saving	This saver really works, good quality, safe and secure, very satisfied!	Jingdong prefecture level city in Shandong
Stowe Flagship Store	King of Power Saving	It works well, the voltage is very stable effect is good, the quality is also very good satisfaction	Jingdong prefecture level city in Shandong
Stowe Flagship Store	King of Power Saving	The logistics is super super fast! The quality is also very good, indeed value for money	Jingdong prefecture level city in Shandong
Stowe Flagship Store	King of Power Saving	I've only been using it for a month or so and it seems to be saving a lot of power	Jingdong prefecture level city in Shandong
Stowe Flagship Store	King of Power Saving	Very easy to operate, very good material, good value for money.	Jingdong prefecture level city in Shandong
Stowe Flagship Store	King of Power Saving	Aluminum alloy shell, durable, compact power-saving good effect	Jingdong prefecture level city in Shandong
Stowe Flagship Store	King of Power Saving	Nice device, easy to use, doesn't take up much space, recommendable!	Jingdong prefecture level city in Shandong
Stowe Flagship Store	King of Power Saving	Good quality, good price, fast logistics	Jingdong prefecture level city in Shandong
Stowe Flagship Store	King of Power Saving	Friends of the family in use recommended, the effect is good and recommendable!	Jingdong prefecture level city in Shandong
Stowe Flagship Store	King of Power Saving	Cheaper than brick and mortar stores worth buying is authentic	Jingdong prefecture level city in Shandong
Stowe Flagship Store	King of Power Saving	Merchants ship quickly, the price is cheaper than the physical store, good use	Jingdong prefecture level city in Shandong
Stowe Flagship Store	King of Power Saving	Very durable, affordable, fast shipping	Jingdong prefecture level city in Shandong
Stowe Flagship Store	King of Power Saving	Packaging tight quality is very good easy to use good results	Jingdong prefecture level city in Shandong
Stowe Flagship Store	King of Power Saving	Save the grid. It's in the whole house. It's saving money on the electric bill.	Jingdong prefecture level city in Shandong
Stowe Flagship Store	King of Power Saving	I used it today, it works well, I'm very satisfied	Jingdong prefecture level city in Shandong
Stowe Flagship Store	King of Power Saving	It really saves a lot of electricity after installation	Jingdong prefecture level city in Shandong
Stowe Flagship Store	King of Power Saving	Love it. Satisfied.	Jingdong prefecture level city in Shandong
Stowe Flagship Store	King of Power Saving	Doesn't feel like it's working.	Jingdong prefecture level city in Shandong
Stowe Flagship Store	King of Power Saving	I'll comment after using it.	Jingdong prefecture level city in Shandong
Stowe Flagship Store	King of Power Saving	I'm trying it out to see if it saves power, and I'll buy two more if it works well.	Jingdong prefecture level city in Shandong
Stowe Flagship Store	King of Power Saving	It is genuine very good, the price is much cheaper, cost-effective, really special cost-effective, it is worth buying!	Jingdong prefecture level city in Shandong
Stowe Flagship Store	King of Power Saving	This thing can really save electricity, I was introduced to buy by my friend, he used it very well, I used it and think it's also very good, it can really save electricity, save about half of the electricity every day, it's really good!	Jingdong prefecture level city in Shandong
Stowe Flagship Store	King of Power Saving	After using it for ten days or so, I checked the power consumption and it did drop, and customer service said it will save more electricity in the long run. The product is also insured, 5 years warranty do not have to worry about quality problems!	Jingdong prefecture level city in Shandong
Stowe Flagship Store	King of Power Saving	The product is good, before already bought a, this is the second, the effect is quite good, anyway, I see the daily electricity bill than before a lot less, this can be the choice!	Jingdong prefecture level city in Shandong

Stowe Flagship Store	King of Power Saving	Originally also very worried about things are not good, get the hand found completely over-anxious, five-star praise, the style is very generous, the quality is very good, the price is also very reasonable!	Jingdong prefecture level city in Shandong
Stowe Flagship Store	King of Power Saving	Quality is good, playing to the old family also buy a, repurchase, this quality is really good, recommended!	Jingdong prefecture level city in Shandong
Stowe Flagship Store	King of Power Saving	Thank you, the owner even sent two fuses.	Jingdong prefecture level city in Shandong
Stowe Flagship Store	King of Power Saving	This store has great stuff, relatively fast shipping and logistics	Jingdong prefecture level city in Shandong
Stowe Flagship Store	King of Power Saving	This product is based on the impossibility of saving electricity, I used 853 kWh of electricity in July, started using the product on August 1, and used 867 kWh of electricity by September 1, is this called	Jingdong prefecture level city in Shandong
Stowe Flagship Store	King of Power Saving	Power king received, to the home on the trial, when I plugged into the electric panel can not see the effect of power saving, the meter is still the same speed in the turn, I'll save the king of the manual to play look at the instructions, instructions to speak, have to have a day two days and half a month to save power obviously, then wait half a month to see the effect of power saving it! To half a month there are obvious power saving before continuing to chase Xu.	Jingdong prefecture level city in Shandong
Stowe Flagship Store	King of Power Saving	Received the goods, a look, did not see the manufacturer and production address, can only hold a try to see the mentality of the face	Jingdong prefecture level city in Shandong
Stowe Flagship Store	King of Power Saving	I've only been using it for a few days and I'm not sure if it's an IQ tax? Used to favorable reviews.	Jingdong prefecture level city in Shandong
Stowe Flagship Store	King of Power Saving	Appearance is okay, power saving next time to follow up the evaluation	Jingdong prefecture level city in Shandong
Stowe Flagship Store	King of Power Saving	Shorter time, but hopefully effective and power-saving, to be followed up in the future	Jingdong prefecture level city in Shandong
Stowe Flagship Store	King of Power Saving	Appearance is okay, I do not know if the power saving	Jingdong prefecture level city in Shandong
Stowe Flagship Store	King of Power Saving	Good service, fast logistics, Power Saving King is exquisite and beautiful, support the business, thank you!	Jingdong prefecture level city in Shandong
Stowe Flagship Store	King of Power Saving	Bought three at once to try first first.	Jingdong prefecture level city in Shandong
Stowe Flagship Store	King of Power Saving	The object is the same as the picture, the product is of high quality, the packaging is intact. High-end atmosphere, high power, power saving effect is obvious, average monthly power saving 50%, very satisfied.	Jingdong prefecture level city in Shandong
Stowe Flagship Store	King of Power Saving	Bought back and plugged in, really effective, before 16 degrees of electricity per day, now 8 degrees of electricity per day, open an air conditioner for 12 hours, decisive repurchase of a	Jingdong prefecture level city in Shandong
Stowe Flagship Store	King of Power Saving	The object is the same as the picture, the product is of high quality, and the package is intact. High-end atmosphere and high grade, high power	Jingdong prefecture level city in Shandong
Stowe Flagship Store	King of Power Saving	Goods received, I hope the store said can be energy-saving, first try a month, month after month to see the effect.	Jingdong prefecture level city in Shandong
Stowe Flagship Store	King of Power Saving	This is really good, the material and workmanship is particularly exquisite, the workmanship is really very good, I think I can recommend to my friends to buy	Jingdong prefecture level city in Shandong
Stowe Flagship Store	King of Power Saving	I have to praise, the quality is really good, and the packaging logistics are also good. Really like it, won't make you regret after getting it!	Jingdong prefecture level city in Shandong
Stowe Flagship Store	King of Power Saving	Reputable platform, efficient and professional service, buy with confidence and peace of mind!	Jingdong prefecture level city in Shandong
Stowe Flagship Store	King of Power Saving	Bought one on a trial basis to see if it works after 1-2 months.	Jingdong prefecture level city in Shandong
Stowe Flagship Store	King of Power Saving	Quality is very good, customer service attitude is very very praise, there are problems in a timely manner to solve the problem, the price is not expensive, super value, you can rest assured that the	Jingdong prefecture level city in Shandong
Stowe Flagship Store	King of Power Saving	With it no longer need to be afraid to use electricity, especially good, ready to buy a mom and dad that also	Jingdong prefecture level city in Shandong
Stowe Flagship Store	King of Power Saving	Baby received very like will return to buy the seller attitude is also very like will return to buy the seller attitude is also very like!	Jingdong prefecture level city in Shandong
Stowe Flagship Store	King of Power Saving	Jingdong Mall shopping convenience assured fast assured fast assured fast assured fast	Jingdong prefecture level city in Shandong
Stowe Flagship Store	King of Power Saving	Saving power received, the quality is very good, fine workmanship, especially good, much cheaper than the entity store, it is worth buying!	Jingdong prefecture level city in Shandong
Stowe Flagship Store	King of Power Saving	I did not expect the express delivery so quickly, very save point quality is super good, than the entity store is too cheap.	Jingdong prefecture level city in Shandong
Stowe Flagship Store	King of Power Saving	Have received the baby, very satisfied with a shopping, great worth buying!	Jingdong prefecture level city in Shandong
Stowe Flagship Store	King of Power Saving	This is really more powerful, very satisfied, more energy-saving, better, thank you boss.	Jingdong prefecture level city in Shandong
Stowe Flagship Store	King of Power Saving	Received has been used for two days, the air conditioning can boldly open it! Energy saving effect is really quite good	Jingdong prefecture level city in Shandong
Stowe Flagship Store	King of Power Saving	It is authentic, trust the merchant, the baby has a great texture and the price is moderate, unlimited repurchase!	Jingdong prefecture level city in Shandong
Stowe Flagship Store	King of Power Saving	It is genuine very good, the price is much cheaper, cost-effective, really special cost-effective, it is worth buying!	Jingdong prefecture level city in Shandong
Stowe Flagship Store	King of Power Saving	Very good quality, fine workmanship, low power consumption, long warranty, fast shipping, economical and practical	Jingdong prefecture level city in Shandong
Stowe Flagship Store	King of Power Saving	I've been using it for half a month now, it really saves power and the voltage is stable, it's very good	Jingdong prefecture level city in Shandong
Stowe Flagship Store	King of Power Saving	It's the ideal product, good quality and fast logistics, worth the kudos!	Jingdong prefecture level city in Shandong
Stowe Flagship Store	King of Power Saving	The logistics is very fast, the baby is very good, the shopkeeper is NICE!	Jingdong prefecture level city in Shandong
Stowe Flagship Store	King of Power Saving	Good to use, good quality, save point durable	Jingdong prefecture level city in Shandong
Stowe Flagship Store	King of Power Saving	The quality is very good, customer service attitude is very, very awesome, there are problems in a timely manner to solve the problem	Jingdong prefecture level city in Shandong
Stowe Flagship Store	King of Power Saving	Quality is very good, the use of the effect is very good, more stable, quality assurance	Jingdong prefecture level city in Shandong
Stowe Flagship Store	King of Power Saving	Convenient, inexpensive, and see how it works out?	Jingdong prefecture level city in Shandong
Stowe Flagship Store	King of Power Saving	Very good quality, saves a lot of electricity, very good, worth buying	Jingdong prefecture level city in Shandong
Stowe Flagship Store	King of Power Saving	Power Saving King is easy to use, with the fruit of power saving to use easy to give!	Jingdong prefecture level city in Shandong
Stowe Flagship Store	King of Power Saving	This saver really works, good quality, safe and secure, very satisfied!	Jingdong prefecture level city in Shandong
Stowe Flagship Store	King of Power Saving	It works well, the voltage is very stable effect is good, the quality is also very good satisfaction	Jingdong prefecture level city in Shandong
Stowe Flagship Store	King of Power Saving	The logistics is super super fast! The quality is also very good, indeed value for money	Jingdong prefecture level city in Shandong
Stowe Flagship Store	King of Power Saving	I've only been using it for a month or so and it seems to be saving a lot of power	Jingdong prefecture level city in Shandong
Stowe Flagship Store	King of Power Saving	Very easy to operate, very good material, good value for money.	Jingdong prefecture level city in Shandong
Stowe Flagship Store	King of Power Saving	Aluminum alloy shell, durable, compact power-saving good effect	Jingdong prefecture level city in Shandong
Stowe Flagship Store	King of Power Saving	Nice device, easy to use, doesn't take up much space, recommendable!	Jingdong prefecture level city in Shandong
Stowe Flagship Store	King of Power Saving	Good quality, good price, fast logistics	Jingdong prefecture level city in Shandong

Stowe Flagship Store	King of Power Saving	Friends of the family in use recommended, the effect is good and recommendable!	Jingdong prefecture level city in Shandong
Stowe Flagship Store	King of Power Saving	Cheaper than brick and mortar stores worth buying is authentic	Jingdong prefecture level city in Shandong
Stowe Flagship Store	King of Power Saving	Merchants ship quickly, the price is cheaper than the physical store, good use	Jingdong prefecture level city in Shandong
Stowe Flagship Store	King of Power Saving	Very durable, affordable, fast shipping	Jingdong prefecture level city in Shandong
Stowe Flagship Store	King of Power Saving	Packaging tight quality is very good easy to use good results	Jingdong prefecture level city in Shandong
Stowe Flagship Store	King of Power Saving	Save the grid. It's in the whole house. It's saving money on the electric bill.	Jingdong prefecture level city in Shandong
Stowe Flagship Store	King of Power Saving	I used it today, it works well, I'm very satisfied	Jingdong prefecture level city in Shandong
Stowe Flagship Store	King of Power Saving	It really saves a lot of electricity after installation	Jingdong prefecture level city in Shandong
Stowe Flagship Store	King of Power Saving	Love it. Satisfied.	Jingdong prefecture level city in Shandong
Stowe Flagship Store	King of Power Saving	Doesn't feel like it's working.	Jingdong prefecture level city in Shandong
Stowe Flagship Store	King of Power Saving	I'll comment after using it.	Jingdong prefecture level city in Shandong
Stowe Flagship Store	King of Power Saving	I'm trying it out to see if it saves power, and I'll buy two more if it works well.	Jingdong prefecture level city in Shandong
Stowe Flagship Store	King of Power Saving	It is genuine very good, the price is much cheaper, cost-effective, really special cost-effective, it is worth buying!	Jingdong prefecture level city in Shandong
Stowe Flagship Store	King of Power Saving	This thing can really save electricity, I was introduced to buy by my friend, he used it very well, I used it and think it's also very good, it can really save electricity, save about half of the electricity every day, it's really good!	Jingdong prefecture level city in Shandong
Stowe Flagship Store	King of Power Saving	After using it for ten days or so, I checked the power consumption and it did drop, and customer service said it will save more electricity in the long run. The product is also insured, 5 years warranty do not have to worry about quality problems!	Jingdong prefecture level city in Shandong
Stowe Flagship Store	King of Power Saving	The product is good, before already bought a, this is the second, the effect is quite good, anyway, I see the daily electricity bill than before a lot less, this can be the choice!	Jingdong prefecture level city in Shandong
Stowe Flagship Store	King of Power Saving	Originally also very worried about things are not good, get the hand found completely over-anxious, five-star praise, the style is very generous, the quality is very good, the price is also very reasonable!	Jingdong prefecture level city in Shandong
Stowe Flagship Store	King of Power Saving	Quality is good, playing to the old family also buy a, repurchase, this quality is really good, recommended!	Jingdong prefecture level city in Shandong
Stowe Flagship Store	King of Power Saving	Thank you, the owner even sent two fuses.	Jingdong prefecture level city in Shandong
Stowe Flagship Store	King of Power Saving	This store has great stuff, relatively fast shipping and logistics	Jingdong prefecture level city in Shandong
Stowe Flagship Store	King of Power Saving	This product is based on the impossibility of saving electricity, I used 853 kWh of electricity in July, started using the product on August 1, and used 867 kWh of electricity by September 1, is this called	Jingdong prefecture level city in Shandong
Stowe Flagship Store	King of Power Saving	Power king received, to the home on the trial, when I plugged into the electric panel can not see the effect of power saving, the meter is still the same speed in the turn, I'll save the king of the manual to play look at the instructions, instructions to speak, have to have a day two days and half a month to save power obviously, then wait half a month to see the effect of power saving it! To half a month there are obvious power saving before continuing to chase Xu.	Jingdong prefecture level city in Shandong
Stowe Flagship Store	King of Power Saving	Received the goods, a look, did not see the manufacturer and production address, can only hold a try to see the mentality of the face	Jingdong prefecture level city in Shandong
Stowe Flagship Store	King of Power Saving	I've only been using it for a few days and I'm not sure if it's an IQ tax? Used to favorable reviews.	Jingdong prefecture level city in Shandong
Stowe Flagship Store	King of Power Saving	Appearance is okay, power saving next time to follow up the evaluation	Jingdong prefecture level city in Shandong
Stowe Flagship Store	King of Power Saving	Shorter time, but hopefully effective and power-saving, to be followed up in the future	Jingdong prefecture level city in Shandong
Stowe Flagship Store	King of Power Saving	Appearance is okay, I do not know if the power saving	Jingdong prefecture level city in Shandong
Stowe Flagship Store	King of Power Saving	Good service, fast logistics, Power Saving King is exquisite and beautiful, support the business, thank you!	Jingdong prefecture level city in Shandong
Stowe Flagship Store	King of Power Saving	Bought three at once to try first first.	Jingdong prefecture level city in Shandong
Stowe Flagship Store	King of Power Saving	The object is the same as the picture, the product is of high quality, the packaging is intact. High-end atmosphere, high power, power saving effect is obvious, average monthly power saving 50%, very satisfied.	Jingdong prefecture level city in Shandong
Stowe Flagship Store	King of Power Saving	Bought back and plugged in, really effective, before 16 degrees of electricity per day, now 8 degrees of electricity per day, open an air conditioner for 12 hours, decisive repurchase of a	Jingdong prefecture level city in Shandong
Stowe Flagship Store	King of Power Saving	The object is the same as the picture, the product is of high quality, and the package is intact. High-end atmosphere and high grade, high power	Jingdong prefecture level city in Shandong
Stowe Flagship Store	King of Power Saving	Goods received, I hope the store said can be energy-saving, first try a month, month after month to see the effect.	Jingdong prefecture level city in Shandong
Stowe Flagship Store	King of Power Saving	This is really good, the material and workmanship is particularly exquisite, the workmanship is really very good, I think I can recommend to my friends to buy	Jingdong prefecture level city in Shandong
Stowe Flagship Store	King of Power Saving	I have to praise, the quality is really good, and the packaging logistics are also good. Really like it, won't make you regret after getting it!	Jingdong prefecture level city in Shandong
Stowe Flagship Store	King of Power Saving	Reputable platform, efficient and professional service, buy with confidence and peace of mind!	Jingdong prefecture level city in Shandong
Stowe Flagship Store	King of Power Saving	Bought one on a trial basis to see if it works after 1-2 months.	Jingdong prefecture level city in Shandong
Stowe Flagship Store	King of Power Saving	Quality is very good, customer service attitude is very very praise, there are problems in a timely manner to solve the problem, the price is not expensive, super value, you can rest assured that the	Jingdong prefecture level city in Shandong
Stowe Flagship Store	King of Power Saving	With it no longer need to be afraid to use electricity, especially good, ready to buy a mom and dad that also	Jingdong prefecture level city in Shandong
Stowe Flagship Store	King of Power Saving	Baby received very like will return to buy the seller attitude is also very like will return to buy the seller attitude is also very like!	Jingdong prefecture level city in Shandong
Stowe Flagship Store	King of Power Saving	Jingdong Mall shopping convenience assured fast assured fast assured fast assured fast	Jingdong prefecture level city in Shandong
Stowe Flagship Store	King of Power Saving	Saving power received, the quality is very good, fine workmanship, especially good, much cheaper than the entity store, it is worth buying!	Jingdong prefecture level city in Shandong
Stowe Flagship Store	King of Power Saving	I did not expect the express delivery so quickly, very save point quality is super good, than the entity store is too cheap.	Jingdong prefecture level city in Shandong
Stowe Flagship Store	King of Power Saving	Have received the baby, very satisfied with a shopping, great worth buying!	Jingdong prefecture level city in Shandong
Stowe Flagship Store	King of Power Saving	This is really more powerful, very satisfied, more energy-saving, better, thank you boss.	Jingdong prefecture level city in Shandong
Stowe Flagship Store	King of Power Saving	Received has been used for two days, the air conditioning can boldly open it! Energy saving effect is really quite good	Jingdong prefecture level city in Shandong
Stowe Flagship Store	King of Power Saving	It is authentic, trust the merchant, the baby has a great texture and the price is moderate, unlimited repurchase!	Jingdong prefecture level city in Shandong
Stowe Flagship Store	King of Power Saving	It is genuine very good, the price is much cheaper, cost-effective, really special cost-effective, it is worth buying!	Jingdong prefecture level city in Shandong
Stowe Flagship Store	King of Power Saving	Very good quality, fine workmanship, low power consumption, long warranty, fast shipping, economical and practical	Jingdong prefecture level city in Shandong

Stowe Flagship Store	King of Power Saving	I've been using it for half a month now, it really saves power and the voltage is stable, it's very good	Jingdong prefecture level city in Shandong
Stowe Flagship Store	King of Power Saving	It's the ideal product ♡, good quality and fast logistics, worth the kudos!	Jingdong prefecture level city in Shandong
Stowe Flagship Store	King of Power Saving	The logistics is very fast, the baby is very good, the shopkeeper is NICE!	Jingdong prefecture level city in Shandong
Stowe Flagship Store	King of Power Saving	Good to use, good quality, save point durable	Jingdong prefecture level city in Shandong
Stowe Flagship Store	King of Power Saving	The quality is very good, customer service attitude is very, very awesome, there are problems in a timely manner to solve the problem	Jingdong prefecture level city in Shandong
Stowe Flagship Store	King of Power Saving	Quality is very good, the use of the effect is very good, more stable, quality assurance	Jingdong prefecture level city in Shandong
Stowe Flagship Store	King of Power Saving	Convenient, inexpensive, and see how it works out?	Jingdong prefecture level city in Shandong
Stowe Flagship Store	King of Power Saving	Very good quality, saves a lot of electricity, very good, worth buying	Jingdong prefecture level city in Shandong
Stowe Flagship Store	King of Power Saving	Power Saving King is easy to use, with the fruit of power saving to use easy to give!	Jingdong prefecture level city in Shandong
Stowe Flagship Store	King of Power Saving	This saver really works, good quality, safe and secure, very satisfied!	Jingdong prefecture level city in Shandong
Stowe Flagship Store	King of Power Saving	It works well, the voltage is very stable effect is good, the quality is also very good satisfaction	Jingdong prefecture level city in Shandong
Stowe Flagship Store	King of Power Saving	The logistics is super super fast! The quality is also very good, indeed value for money	Jingdong prefecture level city in Shandong
Stowe Flagship Store	King of Power Saving	I've only been using it for a month or so and it seems to be saving a lot of power	Jingdong prefecture level city in Shandong
Stowe Flagship Store	King of Power Saving	Very easy to operate, very good material, good value for money.	Jingdong prefecture level city in Shandong
Stowe Flagship Store	King of Power Saving	Aluminum alloy shell, durable, compact power-saving good effect	Jingdong prefecture level city in Shandong
Stowe Flagship Store	King of Power Saving	Nice device, easy to use, doesn't take up much space, recommendable!	Jingdong prefecture level city in Shandong
Stowe Flagship Store	King of Power Saving	Good quality, good price, fast logistics	Jingdong prefecture level city in Shandong
Stowe Flagship Store	King of Power Saving	Friends of the family in use recommended, the effect is good and recommendable!	Jingdong prefecture level city in Shandong
Stowe Flagship Store	King of Power Saving	Cheaper than brick and mortar stores worth buying is authentic	Jingdong prefecture level city in Shandong
Stowe Flagship Store	King of Power Saving	Merchants ship quickly, the price is cheaper than the physical store, good use	Jingdong prefecture level city in Shandong
Stowe Flagship Store	King of Power Saving	Very durable, affordable, fast shipping	Jingdong prefecture level city in Shandong
Stowe Flagship Store	King of Power Saving	Packaging tight quality is very good easy to use good results	Jingdong prefecture level city in Shandong
Stowe Flagship Store	King of Power Saving	Save the grid. It's in the whole house. It's saving money on the electric bill.	Jingdong prefecture level city in Shandong
Stowe Flagship Store	King of Power Saving	I used it today, it works well, I'm very satisfied	Jingdong prefecture level city in Shandong
Stowe Flagship Store	King of Power Saving	It really saves a lot of electricity after installation	Jingdong prefecture level city in Shandong
Stowe Flagship Store	King of Power Saving	Love it. Satisfied.	Jingdong prefecture level city in Shandong
Stowe Flagship Store	King of Power Saving	Doesn't feel like it's working.	Jingdong prefecture level city in Shandong
Stowe Flagship Store	King of Power Saving	I'll comment after using it.	Jingdong prefecture level city in Shandong
Stowe Flagship Store	King of Power Saving	I'm trying it out to see if it saves power, and I'll buy two more if it works well.	Jingdong prefecture level city in Shandong
Stowe Flagship Store	King of Power Saving	It is genuine very good, the price is much cheaper, cost-effective, really special cost-effective, it is worth buying!	Jingdong prefecture level city in Shandong
Stowe Flagship Store	King of Power Saving	This thing can really save electricity, I was introduced to buy by my friend, he used it very well, I used it and think it's also very good, it can really save electricity, save about half of the electricity every day, it's really good!	Jingdong prefecture level city in Shandong
Stowe Flagship Store	King of Power Saving	After using it for ten days or so, I checked the power consumption and it did drop, and customer service said it will save more electricity in the long run. The product is also insured, 5 years warranty do not have to worry about quality problems!	Jingdong prefecture level city in Shandong
Stowe Flagship Store	King of Power Saving	The product is good, before already bought a, this is the second, the effect is quite good, anyway, I see the daily electricity bill than before a lot less, this can be the choice!	Jingdong prefecture level city in Shandong
Stowe Flagship Store	King of Power Saving	Originally also very worried about things are not good, get the hand found completely over-anxious, five-star praise, the style is very generous, the quality is very good, the price is also very reasonable!	Jingdong prefecture level city in Shandong
Stowe Flagship Store	King of Power Saving	Quality is good, playing to the old family also buy a, repurchase, this quality is really good, recommended!	Jingdong prefecture level city in Shandong
Stowe Flagship Store	King of Power Saving	Thank you, the owner even sent two fuses.	Jingdong prefecture level city in Shandong
Stowe Flagship Store	King of Power Saving	This store has great stuff, relatively fast shipping and logistics	Jingdong prefecture level city in Shandong
Stowe Flagship Store	King of Power Saving	This product is based on the impossibility of saving electricity, I used 853 kWh of electricity in July,<br>started using the product on August 1, and used 867 kWh of electricity by September 1, is this called	Jingdong prefecture level city in Shandong
Stowe Flagship Store	King of Power Saving	Power king received, to the home on the trial, when I plugged into the electric panel can not see the effect of power saving, the meter is still the same speed in the turn, I'll save the king of the manual to play look at the instructions, instructions to speak, have to have a day two days and half a month to save power obviously, then wait half a month to see the effect of power saving it! To half a month there are obvious power saving before continuing to chase Xu.	Jingdong prefecture level city in Shandong
Stowe Flagship Store	King of Power Saving	Received the goods, a look, did not see the manufacturer and production address, can only hold a try to see the mentality of the face	Jingdong prefecture level city in Shandong
Stowe Flagship Store	King of Power Saving	I've only been using it for a few days and I'm not sure if it's an IQ tax? Used to favorable reviews.	Jingdong prefecture level city in Shandong
Stowe Flagship Store	King of Power Saving	Appearance is okay, power saving next time to follow up the evaluation	Jingdong prefecture level city in Shandong
Stowe Flagship Store	King of Power Saving	Shorter time, but hopefully effective and power-saving, to be followed up in the future	Jingdong prefecture level city in Shandong
Stowe Flagship Store	King of Power Saving	Appearance is okay, I do not know if the power saving	Jingdong prefecture level city in Shandong
Stowe Flagship Store	King of Power Saving	Good service, fast logistics, Power Saving King is exquisite and beautiful, support the business, thank you!	Jingdong prefecture level city in Shandong
Stowe Flagship Store	King of Power Saving	Bought three at once to try first first.	Jingdong prefecture level city in Shandong
Stowe Flagship Store	King of Power Saving	The object is the same as the picture, the product is of high quality, the packaging is intact. High-end atmosphere, high power, power saving effect is obvious, average monthly power saving 50%, very satisfied.	Jingdong prefecture level city in Shandong
Stowe Flagship Store	King of Power Saving	Bought back and plugged in, really effective, before 16 degrees of electricity per day, now 8 degrees of<br>electricity per day, open an air conditioner for 12 hours, decisive repurchase of a	Jingdong prefecture level city in Shandong
Stowe Flagship Store	King of Power Saving	The object is the same as the picture, the product is of high quality, and the package is intact. High-<br>end atmosphere and high grade, high power	Jingdong prefecture level city in Shandong

Stowe Flagship Store	King of Power Saving	Goods received, I hope the store said can be energy-saving, first try a month, month after month to see the effect.	Jingdong prefecture level city in Shandong
Stowe Flagship Store	King of Power Saving	This is really good, the material and workmanship is particularly exquisite, the workmanship is really very good, I think I can recommend to my friends to buy	Jingdong prefecture level city in Shandong
Stowe Flagship Store	King of Power Saving	I have to praise, the quality is really good, and the packaging logistics are also good. Really like it, won't make you regret after getting it!	Jingdong prefecture level city in Shandong
Stowe Flagship Store	King of Power Saving	Reputable platform, efficient and professional service, buy with confidence and peace of mind!	Jingdong prefecture level city in Shandong
Stowe Flagship Store	King of Power Saving	Bought one on a trial basis to see if it works after 1-2 months.	Jingdong prefecture level city in Shandong
Stowe Flagship Store	King of Power Saving	Quality is very good, customer service attitude is very very praise, there are problems in a timely manner to solve the problem, the price is not expensive, super value, you can rest assured that the	Jingdong prefecture level city in Shandong
Stowe Flagship Store	King of Power Saving	With it no longer need to be afraid to use electricity, especially good, ready to buy a mom and dad that also	Jingdong prefecture level city in Shandong
Stowe Flagship Store	King of Power Saving	Baby received very like will return to buy the seller attitude is also very like will return to buy the seller attitude is also very like!	Jingdong prefecture level city in Shandong
Stowe Flagship Store	King of Power Saving	Jingdong Mall shopping convenience assured fast assured fast assured fast assured fast	Jingdong prefecture level city in Shandong
Stowe Flagship Store	King of Power Saving	Saving power received, the quality is very good, fine workmanship, especially good, much cheaper than the entity store, it is worth buying!	Jingdong prefecture level city in Shandong
Stowe Flagship Store	King of Power Saving	I did not expect the express delivery so quickly, very save point quality is super good, than the entity store is too cheap.	Jingdong prefecture level city in Shandong
Stowe Flagship Store	King of Power Saving	Have received the baby, very satisfied with a shopping, great worth buying!	Jingdong prefecture level city in Shandong
Stowe Flagship Store	King of Power Saving	This is really more powerful, very satisfied, more energy-saving, better, thank you boss.	Jingdong prefecture level city in Shandong
Stowe Flagship Store	King of Power Saving	Received has been used for two days, the air conditioning can boldly open it! Energy saving effect is really quite good	Jingdong prefecture level city in Shandong
Stowe Flagship Store	King of Power Saving	It is authentic, trust the merchant, the baby has a great texture and the price is moderate, unlimited repurchase!	Jingdong prefecture level city in Shandong
Stowe Flagship Store	King of Power Saving	It is genuine very good, the price is much cheaper, cost-effective, really special cost-effective, it is worth buying!	Jingdong prefecture level city in Shandong
Stowe Flagship Store	King of Power Saving	Very good quality, fine workmanship, low power consumption, long warranty, fast shipping, economical and practical	Jingdong prefecture level city in Shandong
Stowe Flagship Store	King of Power Saving	I've been using it for half a month now, it really saves power and the voltage is stable, it's very good	Jingdong prefecture level city in Shandong
Stowe Flagship Store	King of Power Saving	It's the ideal product 💝, good quality and fast logistics, worth the kudos!	Jingdong prefecture level city in Shandong
Stowe Flagship Store	King of Power Saving	The logistics is very fast, the baby is very good, the shopkeeper is NICE!	Jingdong prefecture level city in Shandong
Stowe Flagship Store	King of Power Saving	Good to use, good quality, save point durable	Jingdong prefecture level city in Shandong
Stowe Flagship Store	King of Power Saving	The quality is very good, customer service attitude is very, very awesome, there are problems in a timely manner to solve the problem	Jingdong prefecture level city in Shandong
Stowe Flagship Store	King of Power Saving	Quality is very good, the use of the effect is very good, more stable, quality assurance	Jingdong prefecture level city in Shandong
Stowe Flagship Store	King of Power Saving	Convenient, inexpensive, and see how it works out?	Jingdong prefecture level city in Shandong
Stowe Flagship Store	King of Power Saving	Very good quality, saves a lot of electricity, very good, worth buying	Jingdong prefecture level city in Shandong
Stowe Flagship Store	King of Power Saving	Power Saving King is easy to use, with the fruit of power saving to use easy to give!	Jingdong prefecture level city in Shandong
Stowe Flagship Store	King of Power Saving	This saver really works, good quality, safe and secure, very satisfied!	Jingdong prefecture level city in Shandong
Stowe Flagship Store	King of Power Saving	It works well, the voltage is very stable effect is good, the quality is also very good satisfaction	Jingdong prefecture level city in Shandong
Stowe Flagship Store	King of Power Saving	The logistics is super super fast! The quality is also very good, indeed value for money	Jingdong prefecture level city in Shandong
Stowe Flagship Store	King of Power Saving	I've only been using it for a month or so and it seems to be saving a lot of power	Jingdong prefecture level city in Shandong
Stowe Flagship Store	King of Power Saving	Very easy to operate, very good material, good value for money.	Jingdong prefecture level city in Shandong
Stowe Flagship Store	King of Power Saving	Aluminum alloy shell, durable, compact power-saving good effect	Jingdong prefecture level city in Shandong
Stowe Flagship Store	King of Power Saving	Nice device, easy to use, doesn't take up much space, recommendable!	Jingdong prefecture level city in Shandong
Stowe Flagship Store	King of Power Saving	Good quality, good price, fast logistics	Jingdong prefecture level city in Shandong
Stowe Flagship Store	King of Power Saving	Friends of the family in use recommended, the effect is good and recommendable!	Jingdong prefecture level city in Shandong
Stowe Flagship Store	King of Power Saving	Cheaper than brick and mortar stores worth buying is authentic	Jingdong prefecture level city in Shandong
Stowe Flagship Store	King of Power Saving	Merchants ship quickly, the price is cheaper than the physical store, good use	Jingdong prefecture level city in Shandong
Stowe Flagship Store	King of Power Saving	Very durable, affordable, fast shipping	Jingdong prefecture level city in Shandong
Stowe Flagship Store	King of Power Saving	Packaging tight quality is very good easy to use good results	Jingdong prefecture level city in Shandong
Stowe Flagship Store	King of Power Saving	Save the grid. It's in the whole house. It's saving money on the electric bill.	Jingdong prefecture level city in Shandong
Stowe Flagship Store	King of Power Saving	I used it today, it works well, I'm very satisfied	Jingdong prefecture level city in Shandong
Stowe Flagship Store	King of Power Saving	It really saves a lot of electricity after installation	Jingdong prefecture level city in Shandong
Stowe Flagship Store	King of Power Saving	Love it. Satisfied.	Jingdong prefecture level city in Shandong
Stowe Flagship Store	King of Power Saving	Doesn't feel like it's working.	Jingdong prefecture level city in Shandong
Stowe Flagship Store	King of Power Saving	I'll comment after using it.	Jingdong prefecture level city in Shandong
Stowe Flagship Store	King of Power Saving	I'm trying it out to see if it saves power, and I'll buy two more if it works well.	Jingdong prefecture level city in Shandong
Stowe Flagship Store	King of Power Saving	It is genuine very good, the price is much cheaper, cost-effective, really special cost-effective, it is worth buying!	Jingdong prefecture level city in Shandong
Stowe Flagship Store	King of Power Saving	This thing can really save electricity, I was introduced to buy by my friend, he used it very well, I used it and think it's also very good, it can really save electricity, save about half of the electricity every day, it's really good!	Jingdong prefecture level city in Shandong
Stowe Flagship Store	King of Power Saving	After using it for ten days or so, I checked the power consumption and it did drop, and customer service said it will save more electricity in the long run. The product is also insured, 5 years warranty do not have to worry about quality problems!	Jingdong prefecture level city in Shandong
Stowe Flagship Store	King of Power Saving	The product is good, before already bought a, this is the second, the effect is quite good, anyway, I see the daily electricity bill than before a lot less, this can be the choice!	Jingdong prefecture level city in Shandong

Stowe Flagship Store	King of Power Saving	Originally also very worried about things are not good, get the hand found completely over-anxious, five-star praise, the style is very generous, the quality is very good, the price is also very reasonable!	Jingdong prefecture level city in Shandong
Stowe Flagship Store	King of Power Saving	Quality is good, playing to the old family also buy a, repurchase, this quality is really good, recommended!	Jingdong prefecture level city in Shandong
Stowe Flagship Store	King of Power Saving	Thank you, the owner even sent two fuses.	Jingdong prefecture level city in Shandong
Stowe Flagship Store	King of Power Saving	This store has great stuff, relatively fast shipping and logistics	Jingdong prefecture level city in Shandong
Stowe Flagship Store	King of Power Saving	This product is based on the impossibility of saving electricity, I used 853 kWh of electricity in July, started using the product on August 1, and used 867 kWh of electricity by September 1, is this called	Jingdong prefecture level city in Shandong
Stowe Flagship Store	King of Power Saving	Power king received, to the home on the trial, when I plugged into the electric panel can not see the effect of power saving, the meter is still the same speed in the turn, I'll save the king of the manual to play look at the instructions, instructions to speak, have to have a day two days and half a month to save power obviously, then wait half a month to see the effect of power saving it! To half a month there are obvious power saving before continuing to chase Xu.	Jingdong prefecture level city in Shandong
Stowe Flagship Store	King of Power Saving	Received the goods, a look, did not see the manufacturer and production address, can only hold a try to see the mentality of the face	Jingdong prefecture level city in Shandong
Stowe Flagship Store	King of Power Saving	I've only been using it for a few days and I'm not sure if it's an IQ tax? Used to favorable reviews.	Jingdong prefecture level city in Shandong
Stowe Flagship Store	King of Power Saving	Appearance is okay, power saving next time to follow up the evaluation	Jingdong prefecture level city in Shandong
Stowe Flagship Store	King of Power Saving	Shorter time, but hopefully effective and power-saving, to be followed up in the future	Jingdong prefecture level city in Shandong
Stowe Flagship Store	King of Power Saving	Appearance is okay, I do not know if the power saving	Jingdong prefecture level city in Shandong
Stowe Flagship Store	King of Power Saving	Good service, fast logistics, Power Saving King is exquisite and beautiful, support the business, thank you!	Jingdong prefecture level city in Shandong
Stowe Flagship Store	King of Power Saving	Bought three at once to try first first.	Jingdong prefecture level city in Shandong
Stowe Flagship Store	King of Power Saving	The object is the same as the picture, the product is of high quality, the packaging is intact. High-end atmosphere, high power, power saving effect is obvious, average monthly power saving 50%, very satisfied.	Jingdong prefecture level city in Shandong
Stowe Flagship Store	King of Power Saving	Bought back and plugged in, really effective, before 16 degrees of electricity per day, now 8 degrees of electricity per day, open an air conditioner for 12 hours, decisive repurchase of a	Jingdong prefecture level city in Shandong
Stowe Flagship Store	King of Power Saving	The object is the same as the picture, the product is of high quality, and the package is intact. High-end atmosphere and high grade, high power	Jingdong prefecture level city in Shandong
Stowe Flagship Store	King of Power Saving	Goods received, I hope the store said can be energy-saving, first try a month, month after month to see the effect.	Jingdong prefecture level city in Shandong
Stowe Flagship Store	King of Power Saving	This is really good, the material and workmanship is particularly exquisite, the workmanship is really very good, I think I can recommend to my friends to buy	Jingdong prefecture level city in Shandong
Stowe Flagship Store	King of Power Saving	I have to praise, the quality is really good, and the packaging logistics are also good. Really like it, won't make you regret after getting it!	Jingdong prefecture level city in Shandong
Stowe Flagship Store	King of Power Saving	Reputable platform, efficient and professional service, buy with confidence and peace of mind!	Jingdong prefecture level city in Shandong
Stowe Flagship Store	King of Power Saving	Bought one on a trial basis to see if it works after 1-2 months.	Jingdong prefecture level city in Shandong
Stowe Flagship Store	King of Power Saving	Quality is very good, customer service attitude is very very praise, there are problems in a timely manner to solve the problem, the price is not expensive, super value, you can rest assured that the	Jingdong prefecture level city in Shandong
Stowe Flagship Store	King of Power Saving	With it no longer need to be afraid to use electricity, especially good, ready to buy a mom and dad that also	Jingdong prefecture level city in Shandong
Stowe Flagship Store	King of Power Saving	Baby received very like will return to buy the seller attitude is also very like will return to buy the seller attitude is also very like!	Jingdong prefecture level city in Shandong
Stowe Flagship Store	King of Power Saving	Jingdong Mall shopping convenience assured fast assured fast assured fast assured fast	Jingdong prefecture level city in Shandong
Stowe Flagship Store	King of Power Saving	Saving power received, the quality is very good, fine workmanship, especially good, much cheaper than the entity store, it is worth buying!	Jingdong prefecture level city in Shandong
Stowe Flagship Store	King of Power Saving	I did not expect the express delivery so quickly, very save point quality is super good, than the entity store is too cheap.	Jingdong prefecture level city in Shandong
Stowe Flagship Store	King of Power Saving	Have received the baby, very satisfied with a shopping, great worth buying!	Jingdong prefecture level city in Shandong
Stowe Flagship Store	King of Power Saving	This is really more powerful, very satisfied, more energy-saving, better, thank you boss.	Jingdong prefecture level city in Shandong
Stowe Flagship Store	King of Power Saving	Received has been used for two days, the air conditioning can boldly open it! Energy saving effect is really quite good	Jingdong prefecture level city in Shandong
Stowe Flagship Store	King of Power Saving	It is authentic, trust the merchant, the baby has a great texture and the price is moderate, unlimited repurchase!	Jingdong prefecture level city in Shandong
Stowe Flagship Store	King of Power Saving	It is genuine very good, the price is much cheaper, cost-effective, really special cost-effective, it is worth buying!	Jingdong prefecture level city in Shandong
Stowe Flagship Store	King of Power Saving	Very good quality, fine workmanship, low power consumption, long warranty, fast shipping, economical and practical	Jingdong prefecture level city in Shandong
Stowe Flagship Store	King of Power Saving	I've been using it for half a month now, it really saves power and the voltage is stable, it's very good	Jingdong prefecture level city in Shandong
Stowe Flagship Store	King of Power Saving	It's the ideal product, good quality and fast logistics, worth the kudos!	Jingdong prefecture level city in Shandong
Stowe Flagship Store	King of Power Saving	The logistics is very fast, the baby is very good, the shopkeeper is NICE!	Jingdong prefecture level city in Shandong
Stowe Flagship Store	King of Power Saving	Good to use, good quality, save point durable	Jingdong prefecture level city in Shandong
Stowe Flagship Store	King of Power Saving	The quality is very good, customer service attitude is very, very awesome, there are problems in a timely manner to solve the problem	Jingdong prefecture level city in Shandong
Stowe Flagship Store	King of Power Saving	Quality is very good, the use of the effect is very good, more stable, quality assurance	Jingdong prefecture level city in Shandong
Stowe Flagship Store	King of Power Saving	Convenient, inexpensive, and see how it works out?	Jingdong prefecture level city in Shandong
Stowe Flagship Store	King of Power Saving	Very good quality, saves a lot of electricity, very good, worth buying	Jingdong prefecture level city in Shandong
Stowe Flagship Store	King of Power Saving	Power Saving King is easy to use, with the fruit of power saving to use easy to give!	Jingdong prefecture level city in Shandong
Stowe Flagship Store	King of Power Saving	This saver really works, good quality, safe and secure, very satisfied!	Jingdong prefecture level city in Shandong
Stowe Flagship Store	King of Power Saving	It works well, the voltage is very stable effect is good, the quality is also very good satisfaction	Jingdong prefecture level city in Shandong
Stowe Flagship Store	King of Power Saving	The logistics is super super fast! The quality is also very good, indeed value for money	Jingdong prefecture level city in Shandong
Stowe Flagship Store	King of Power Saving	I've only been using it for a month or so and it seems to be saving a lot of power	Jingdong prefecture level city in Shandong
Stowe Flagship Store	King of Power Saving	Very easy to operate, very good material, good value for money.	Jingdong prefecture level city in Shandong
Stowe Flagship Store	King of Power Saving	Aluminum alloy shell, durable, compact power-saving good effect	Jingdong prefecture level city in Shandong
Stowe Flagship Store	King of Power Saving	Nice device, easy to use, doesn't take up much space, recommendable!	Jingdong prefecture level city in Shandong
Stowe Flagship Store	King of Power Saving	Good quality, good price, fast logistics	Jingdong prefecture level city in Shandong

Stowe Flagship Store	King of Power Saving	Friends of the family in use recommended, the effect is good and recommendable!	Jingdong prefecture level city in Shandong
Stowe Flagship Store	King of Power Saving	Cheaper than brick and mortar stores worth buying is authentic	Jingdong prefecture level city in Shandong
Stowe Flagship Store	King of Power Saving	Merchants ship quickly, the price is cheaper than the physical store, good use	Jingdong prefecture level city in Shandong
Stowe Flagship Store	King of Power Saving	Very durable, affordable, fast shipping	Jingdong prefecture level city in Shandong
Stowe Flagship Store	King of Power Saving	Packaging tight quality is very good easy to use good results	Jingdong prefecture level city in Shandong
Stowe Flagship Store	King of Power Saving	Save the grid. It's in the whole house. It's saving money on the electric bill.	Jingdong prefecture level city in Shandong
Stowe Flagship Store	King of Power Saving	I used it today, it works well, I'm very satisfied	Jingdong prefecture level city in Shandong
Stowe Flagship Store	King of Power Saving	It really saves a lot of electricity after installation	Jingdong prefecture level city in Shandong
Stowe Flagship Store	King of Power Saving	Love it. Satisfied.	Jingdong prefecture level city in Shandong
Stowe Flagship Store	King of Power Saving	Doesn't feel like it's working.	Jingdong prefecture level city in Shandong
Stowe Flagship Store	King of Power Saving	I'll comment after using it.	Jingdong prefecture level city in Shandong
Stowe Flagship Store	King of Power Saving	I'm trying it out to see if it saves power, and I'll buy two more if it works well.	Jingdong prefecture level city in Shandong
Stowe Flagship Store	King of Power Saving	It is genuine very good, the price is much cheaper, cost-effective, really special cost-effective, it is worth buying!	Jingdong prefecture level city in Shandong
Stowe Flagship Store	King of Power Saving	This thing can really save electricity, I was introduced to buy by my friend, he used it very well, I used it and think it's also very good, it can really save electricity, save about half of the electricity every day, it's really good!	Jingdong prefecture level city in Shandong
Stowe Flagship Store	King of Power Saving	After using it for ten days or so, I checked the power consumption and it did drop, and customer service said it will save more electricity in the long run. The product is also insured, 5 years warranty do not have to worry about quality problems!	Jingdong prefecture level city in Shandong
Stowe Flagship Store	King of Power Saving	The product is good, before already bought a, this is the second, the effect is quite good, anyway, I see the daily electricity bill than before a lot less, this can be the choice!	Jingdong prefecture level city in Shandong
Stowe Flagship Store	King of Power Saving	Originally also very worried about things are not good, get the hand found completely over-anxious, five-star praise, the style is very generous, the quality is very good, the price is also very reasonable!	Jingdong prefecture level city in Shandong
Stowe Flagship Store	King of Power Saving	Quality is good, playing to the old family also buy a, repurchase, this quality is really good, recommended!	Jingdong prefecture level city in Shandong
Stowe Flagship Store	King of Power Saving	Thank you, the owner even sent two fuses.	Jingdong prefecture level city in Shandong
Stowe Flagship Store	King of Power Saving	This store has great stuff, relatively fast shipping and logistics	Jingdong prefecture level city in Shandong
Stowe Flagship Store	King of Power Saving	This product is based on the impossibility of saving electricity, I used 853 kWh of electricity in July, started using the product on August 1, and used 867 kWh of electricity by September 1, is this called	Jingdong prefecture level city in Shandong
Stowe Flagship Store	King of Power Saving	Power king received, to the home on the trial, when I plugged into the electric panel can not see the effect of power saving, the meter is still the same speed in the turn, I'll save the king of the manual to play look at the instructions, instructions to speak, have to have a day two days and half a month to save power obviously, then wait half a month to see the effect of power saving it! To half a month there are obvious power saving before continuing to chase Xu.	Jingdong prefecture level city in Shandong
Stowe Flagship Store	King of Power Saving	Received the goods, a look, did not see the manufacturer and production address, can only hold a try to see the mentality of the face	Jingdong prefecture level city in Shandong
Stowe Flagship Store	King of Power Saving	I've only been using it for a few days and I'm not sure if it's an IQ tax? Used to favorable reviews.	Jingdong prefecture level city in Shandong
Stowe Flagship Store	King of Power Saving	Appearance is okay, power saving next time to follow up the evaluation	Jingdong prefecture level city in Shandong
Stowe Flagship Store	King of Power Saving	Shorter time, but hopefully effective and power-saving, to be followed up in the future	Jingdong prefecture level city in Shandong
Stowe Flagship Store	King of Power Saving	Appearance is okay, I do not know if the power saving	Jingdong prefecture level city in Shandong
Stowe Flagship Store	King of Power Saving	Good service, fast logistics, Power Saving King is exquisite and beautiful, support the business, thank you!	Jingdong prefecture level city in Shandong
Stowe Flagship Store	King of Power Saving	Bought three at once to try first first.	Jingdong prefecture level city in Shandong
Stowe Flagship Store	King of Power Saving	The object is the same as the picture, the product is of high quality, the packaging is intact. High-end atmosphere, high power, power saving effect is obvious, average monthly power saving 50%, very satisfied.	Jingdong prefecture level city in Shandong
Stowe Flagship Store	King of Power Saving	Bought back and plugged in, really effective, before 16 degrees of electricity per day, now 8 degrees of electricity per day, open an air conditioner for 12 hours, decisive repurchase of a	Jingdong prefecture level city in Shandong
Stowe Flagship Store	King of Power Saving	The object is the same as the picture, the product is of high quality, and the package is intact. High-end atmosphere and high grade, high power	Jingdong prefecture level city in Shandong
Stowe Flagship Store	King of Power Saving	Goods received, I hope the store said can be energy-saving, first try a month, month after month to see the effect.	Jingdong prefecture level city in Shandong
Stowe Flagship Store	King of Power Saving	This is really good, the material and workmanship is particularly exquisite, the workmanship is really very good, I think I can recommend to my friends to buy	Jingdong prefecture level city in Shandong
Stowe Flagship Store	King of Power Saving	I have to praise, the quality is really good, and the packaging logistics are also good. Really like it, won't make you regret after getting it!	Jingdong prefecture level city in Shandong
Stowe Flagship Store	King of Power Saving	Reputable platform, efficient and professional service, buy with confidence and peace of mind!	Jingdong prefecture level city in Shandong
Stowe Flagship Store	King of Power Saving	Bought one on a trial basis to see if it works after 1-2 months.	Jingdong prefecture level city in Shandong
Stowe Flagship Store	King of Power Saving	Quality is very good, customer service attitude is very very praise, there are problems in a timely manner to solve the problem, the price is not expensive, super value, you can rest assured that the	Jingdong prefecture level city in Shandong
Stowe Flagship Store	King of Power Saving	With it no longer need to be afraid to use electricity, especially good, ready to buy a mom and dad that also	Jingdong prefecture level city in Shandong
Stowe Flagship Store	King of Power Saving	Baby received very like will return to buy the seller attitude is also very like will return to buy the seller attitude is also very like!	Jingdong prefecture level city in Shandong
Stowe Flagship Store	King of Power Saving	Jingdong Mall shopping convenience assured fast assured fast assured fast assured fast	Jingdong prefecture level city in Shandong
Stowe Flagship Store	King of Power Saving	Saving power received, the quality is very good, fine workmanship, especially good, much cheaper than the entity store, it is worth buying!	Jingdong prefecture level city in Shandong
Stowe Flagship Store	King of Power Saving	I did not expect the express delivery so quickly, very save point quality is super good, than the entity store is too cheap.	Jingdong prefecture level city in Shandong
Stowe Flagship Store	King of Power Saving	Have received the baby, very satisfied with a shopping, great worth buying!	Jingdong prefecture level city in Shandong
Stowe Flagship Store	King of Power Saving	This is really more powerful, very satisfied, more energy-saving, better, thank you boss.	Jingdong prefecture level city in Shandong
Stowe Flagship Store	King of Power Saving	Received has been used for two days, the air conditioning can boldly open it! Energy saving effect is really quite good	Jingdong prefecture level city in Shandong
Stowe Flagship Store	King of Power Saving	It is authentic, trust the merchant, the baby has a great texture and the price is moderate, unlimited repurchase!	Jingdong prefecture level city in Shandong
Stowe Flagship Store	King of Power Saving	It is genuine very good, the price is much cheaper, cost-effective, really special cost-effective, it is worth buying!	Jingdong prefecture level city in Shandong
Stowe Flagship Store	King of Power Saving	Very good quality, fine workmanship, low power consumption, long warranty, fast shipping, economical and practical	Jingdong prefecture level city in Shandong

Stowe Flagship Store	King of Power Saving	I've been using it for half a month now, it really saves power and the voltage is stable, it's very good	Jingdong prefecture level city in Shandong
Stowe Flagship Store	King of Power Saving	It's the ideal product ♡, good quality and fast logistics, worth the kudos!	Jingdong prefecture level city in Shandong
Stowe Flagship Store	King of Power Saving	The logistics is very fast, the baby is very good, the shopkeeper is NICE!	Jingdong prefecture level city in Shandong
Stowe Flagship Store	King of Power Saving	Good to use, good quality, save point durable	Jingdong prefecture level city in Shandong
Stowe Flagship Store	King of Power Saving	The quality is very good, customer service attitude is very, very awesome, there are problems in a timely manner to solve the problem	Jingdong prefecture level city in Shandong
Stowe Flagship Store	King of Power Saving	Quality is very good, the use of the effect is very good, more stable, quality assurance	Jingdong prefecture level city in Shandong
Stowe Flagship Store	King of Power Saving	Convenient, inexpensive, and see how it works out?	Jingdong prefecture level city in Shandong
Stowe Flagship Store	King of Power Saving	Very good quality, saves a lot of electricity, very good, worth buying	Jingdong prefecture level city in Shandong
Stowe Flagship Store	King of Power Saving	Power Saving King is easy to use, with the fruit of power saving to use easy to give!	Jingdong prefecture level city in Shandong
Stowe Flagship Store	King of Power Saving	This saver really works, good quality, safe and secure, very satisfied!	Jingdong prefecture level city in Shandong
Stowe Flagship Store	King of Power Saving	It works well, the voltage is very stable effect is good, the quality is also very good satisfaction	Jingdong prefecture level city in Shandong
Stowe Flagship Store	King of Power Saving	The logistics is super super fast! The quality is also very good, indeed value for money	Jingdong prefecture level city in Shandong
Stowe Flagship Store	King of Power Saving	I've only been using it for a month or so and it seems to be saving a lot of power	Jingdong prefecture level city in Shandong
Stowe Flagship Store	King of Power Saving	Very easy to operate, very good material, good value for money.	Jingdong prefecture level city in Shandong
Stowe Flagship Store	King of Power Saving	Aluminum alloy shell, durable, compact power-saving good effect	Jingdong prefecture level city in Shandong
Stowe Flagship Store	King of Power Saving	Nice device, easy to use, doesn't take up much space, recommendable!	Jingdong prefecture level city in Shandong
Stowe Flagship Store	King of Power Saving	Good quality, good price, fast logistics	Jingdong prefecture level city in Shandong
Stowe Flagship Store	King of Power Saving	Friends of the family in use recommended, the effect is good and recommendable!	Jingdong prefecture level city in Shandong
Stowe Flagship Store	King of Power Saving	Cheaper than brick and mortar stores worth buying is authentic	Jingdong prefecture level city in Shandong
Stowe Flagship Store	King of Power Saving	Merchants ship quickly, the price is cheaper than the physical store, good use	Jingdong prefecture level city in Shandong
Stowe Flagship Store	King of Power Saving	Very durable, affordable, fast shipping	Jingdong prefecture level city in Shandong
Stowe Flagship Store	King of Power Saving	Packaging tight quality is very good easy to use good results	Jingdong prefecture level city in Shandong
Stowe Flagship Store	King of Power Saving	Save the grid. It's in the whole house. It's saving money on the electric bill.	Jingdong prefecture level city in Shandong
Stowe Flagship Store	King of Power Saving	I used it today, it works well, I'm very satisfied	Jingdong prefecture level city in Shandong
Stowe Flagship Store	King of Power Saving	It really saves a lot of electricity after installation	Jingdong prefecture level city in Shandong
Stowe Flagship Store	King of Power Saving	Love it. Satisfied.	Jingdong prefecture level city in Shandong
Stowe Flagship Store	King of Power Saving	Doesn't feel like it's working.	Jingdong prefecture level city in Shandong
Stowe Flagship Store	King of Power Saving	I'll comment after using it.	Jingdong prefecture level city in Shandong
Stowe Flagship Store	King of Power Saving	I'm trying it out to see if it saves power, and I'll buy two more if it works well.	Jingdong prefecture level city in Shandong
Stowe Flagship Store	King of Power Saving	It is genuine very good, the price is much cheaper, cost-effective, really special cost-effective, it is worth buying!	Jingdong prefecture level city in Shandong
Stowe Flagship Store	King of Power Saving	This thing can really save electricity, I was introduced to buy by my friend, he used it very well, I used it and think it's also very good, it can really save electricity, save about half of the electricity every day, it's really good!	Jingdong prefecture level city in Shandong
Stowe Flagship Store	King of Power Saving	After using it for ten days or so, I checked the power consumption and it did drop, and customer service said it will save more electricity in the long run. The product is also insured, 5 years warranty do not have to worry about quality problems!	Jingdong prefecture level city in Shandong
Stowe Flagship Store	King of Power Saving	The product is good, before already bought a, this is the second, the effect is quite good, anyway, I see the daily electricity bill than before a lot less, this can be the choice!	Jingdong prefecture level city in Shandong
Stowe Flagship Store	King of Power Saving	Originally also very worried about things are not good, get the hand found completely over-anxious, five-star praise, the style is very generous, the quality is very good, the price is also very reasonable!	Jingdong prefecture level city in Shandong
Stowe Flagship Store	King of Power Saving	Quality is good, playing to the old family also buy a, repurchase, this quality is really good, recommended!	Jingdong prefecture level city in Shandong
Stowe Flagship Store	King of Power Saving	Thank you, the owner even sent two fuses.	Jingdong prefecture level city in Shandong
Stowe Flagship Store	King of Power Saving	This store has great stuff, relatively fast shipping and logistics	Jingdong prefecture level city in Shandong
Stowe Flagship Store	King of Power Saving	This product is based on the impossibility of saving electricity, I used 853 kWh of electricity in July, started using the product on August 1, and used 867 kWh of electricity by September 1, is this called	Jingdong prefecture level city in Shandong
Stowe Flagship Store	King of Power Saving	Power king received, to the home on the trial, when I plugged into the electric panel can not see the effect of power saving, the meter is still the same speed in the turn, I'll save the king of the manual to play look at the instructions, instructions to speak, have to have a day two days and half a month to save power obviously, then wait half a month to see the effect of power saving it! To half a month there are obvious power saving before continuing to chase Xu.	Jingdong prefecture level city in Shandong
Stowe Flagship Store	King of Power Saving	Received the goods, a look, did not see the manufacturer and production address, can only hold a try to see the mentality of the face	Jingdong prefecture level city in Shandong
Stowe Flagship Store	King of Power Saving	I've only been using it for a few days and I'm not sure if it's an IQ tax? Used to favorable reviews.	Jingdong prefecture level city in Shandong
Stowe Flagship Store	King of Power Saving	Appearance is okay, power saving next time to follow up the evaluation	Jingdong prefecture level city in Shandong
Stowe Flagship Store	King of Power Saving	Shorter time, but hopefully effective and power-saving, to be followed up in the future	Jingdong prefecture level city in Shandong
Stowe Flagship Store	King of Power Saving	Appearance is okay, I do not know if the power saving	Jingdong prefecture level city in Shandong
Stowe Flagship Store	King of Power Saving	Good service, fast logistics, Power Saving King is exquisite and beautiful, support the business, thank you!	Jingdong prefecture level city in Shandong
Stowe Flagship Store	King of Power Saving	Bought three at once to try first first.	Jingdong prefecture level city in Shandong
Stowe Flagship Store	King of Power Saving	The object is the same as the picture, the product is of high quality, the packaging is intact. High-end atmosphere, high power, power saving effect is obvious, average monthly power saving 50%, very satisfied.	Jingdong prefecture level city in Shandong
Stowe Flagship Store	King of Power Saving	Bought back and plugged in, really effective, before 16 degrees of electricity per day, now 8 degrees of electricity per day, open an air conditioner for 12 hours, decisive repurchase of a	Jingdong prefecture level city in Shandong
Stowe Flagship Store	King of Power Saving	The object is the same as the picture, the product is of high quality, and the package is intact. High-end atmosphere and high grade, high power	Jingdong prefecture level city in Shandong

Stowe Flagship Store	King of Power Saving	Goods received, I hope the store said can be energy-saving, first try a month, month after month to see the effect.	Jingdong prefecture level city in Shandong
Stowe Flagship Store	King of Power Saving	This is really good, the material and workmanship is particularly exquisite, the workmanship is really very good, I think I can recommend to my friends to buy	Jingdong prefecture level city in Shandong
Stowe Flagship Store	King of Power Saving	I have to praise, the quality is really good, and the packaging logistics are also good. Really like it, won't make you regret after getting it!	Jingdong prefecture level city in Shandong
Stowe Flagship Store	King of Power Saving	Reputable platform, efficient and professional service, buy with confidence and peace of mind!	Jingdong prefecture level city in Shandong
Stowe Flagship Store	King of Power Saving	Bought one on a trial basis to see if it works after 1-2 months.	Jingdong prefecture level city in Shandong
Stowe Flagship Store	King of Power Saving	Quality is very good, customer service attitude is very very praise, there are problems in a timely manner to solve the problem, the price is not expensive, super value, you can rest assured that the	Jingdong prefecture level city in Shandong
Stowe Flagship Store	King of Power Saving	With it no longer need to be afraid to use electricity, especially good, ready to buy a mom and dad that also	Jingdong prefecture level city in Shandong
Stowe Flagship Store	King of Power Saving	Baby received very like will return to buy the seller attitude is also very like will return to buy the seller attitude is also very like!	Jingdong prefecture level city in Shandong
Stowe Flagship Store	King of Power Saving	Jingdong Mall shopping convenience assured fast assured fast assured fast assured fast	Jingdong prefecture level city in Shandong
Stowe Flagship Store	King of Power Saving	Saving power received, the quality is very good, fine workmanship, especially good, much cheaper than the entity store, it is worth buying!	Jingdong prefecture level city in Shandong
Stowe Flagship Store	King of Power Saving	I did not expect the express delivery so quickly, very save point quality is super good, than the entity store is too cheap.	Jingdong prefecture level city in Shandong
Stowe Flagship Store	King of Power Saving	Have received the baby, very satisfied with a shopping, great worth buying!	Jingdong prefecture level city in Shandong
Stowe Flagship Store	King of Power Saving	This is really more powerful, very satisfied, more energy-saving, better, thank you boss.	Jingdong prefecture level city in Shandong
Stowe Flagship Store	King of Power Saving	Received has been used for two days, the air conditioning can boldly open it! Energy saving effect is really quite good	Jingdong prefecture level city in Shandong
Stowe Flagship Store	King of Power Saving	It is authentic, trust the merchant, the baby has a great texture and the price is moderate, unlimited repurchase!	Jingdong prefecture level city in Shandong
Stowe Flagship Store	King of Power Saving	It is genuine very good, the price is much cheaper, cost-effective, really special cost-effective, it is worth buying!	Jingdong prefecture level city in Shandong
Stowe Flagship Store	King of Power Saving	Very good quality, fine workmanship, low power consumption, long warranty, fast shipping, economical and practical	Jingdong prefecture level city in Shandong
Stowe Flagship Store	King of Power Saving	I've been using it for half a month now, it really saves power and the voltage is stable, it's very good	Jingdong prefecture level city in Shandong
Stowe Flagship Store	King of Power Saving	It's the ideal product 💝, good quality and fast logistics, worth the kudos!	Jingdong prefecture level city in Shandong
Stowe Flagship Store	King of Power Saving	The logistics is very fast, the baby is very good, the shopkeeper is NICE!	Jingdong prefecture level city in Shandong
Stowe Flagship Store	King of Power Saving	Good to use, good quality, save point durable	Jingdong prefecture level city in Shandong
Stowe Flagship Store	King of Power Saving	The quality is very good, customer service attitude is very, very awesome, there are problems in a timely manner to solve the problem	Jingdong prefecture level city in Shandong
Stowe Flagship Store	King of Power Saving	Quality is very good, the use of the effect is very good, more stable, quality assurance	Jingdong prefecture level city in Shandong
Stowe Flagship Store	King of Power Saving	Convenient, inexpensive, and see how it works out?	Jingdong prefecture level city in Shandong
Stowe Flagship Store	King of Power Saving	Very good quality, saves a lot of electricity, very good, worth buying	Jingdong prefecture level city in Shandong
Stowe Flagship Store	King of Power Saving	Power Saving King is easy to use, with the fruit of power saving to use easy to give!	Jingdong prefecture level city in Shandong
Stowe Flagship Store	King of Power Saving	This saver really works, good quality, safe and secure, very satisfied!	Jingdong prefecture level city in Shandong
Stowe Flagship Store	King of Power Saving	It works well, the voltage is very stable effect is good, the quality is also very good satisfaction	Jingdong prefecture level city in Shandong
Stowe Flagship Store	King of Power Saving	The logistics is super super fast! The quality is also very good, indeed value for money	Jingdong prefecture level city in Shandong
Stowe Flagship Store	King of Power Saving	I've only been using it for a month or so and it seems to be saving a lot of power	Jingdong prefecture level city in Shandong
Stowe Flagship Store	King of Power Saving	Very easy to operate, very good material, good value for money.	Jingdong prefecture level city in Shandong
Stowe Flagship Store	King of Power Saving	Aluminum alloy shell, durable, compact power-saving good effect	Jingdong prefecture level city in Shandong
Stowe Flagship Store	King of Power Saving	Nice device, easy to use, doesn't take up much space, recommendable!	Jingdong prefecture level city in Shandong
Stowe Flagship Store	King of Power Saving	Good quality, good price, fast logistics	Jingdong prefecture level city in Shandong
Stowe Flagship Store	King of Power Saving	Friends of the family in use recommended, the effect is good and recommendable!	Jingdong prefecture level city in Shandong
Stowe Flagship Store	King of Power Saving	Cheaper than brick and mortar stores worth buying is authentic	Jingdong prefecture level city in Shandong
Stowe Flagship Store	King of Power Saving	Merchants ship quickly, the price is cheaper than the physical store, good use	Jingdong prefecture level city in Shandong
Stowe Flagship Store	King of Power Saving	Very durable, affordable, fast shipping	Jingdong prefecture level city in Shandong
Stowe Flagship Store	King of Power Saving	Packaging tight quality is very good easy to use good results	Jingdong prefecture level city in Shandong
Stowe Flagship Store	King of Power Saving	Save the grid. It's in the whole house. It's saving money on the electric bill.	Jingdong prefecture level city in Shandong
Stowe Flagship Store	King of Power Saving	I used it today, it works well, I'm very satisfied	Jingdong prefecture level city in Shandong
Stowe Flagship Store	King of Power Saving	It really saves a lot of electricity after installation	Jingdong prefecture level city in Shandong
Stowe Flagship Store	King of Power Saving	Love it. Satisfied.	Jingdong prefecture level city in Shandong
Stowe Flagship Store	King of Power Saving	Doesn't feel like it's working.	Jingdong prefecture level city in Shandong
Stowe Flagship Store	King of Power Saving	I'll comment after using it.	Jingdong prefecture level city in Shandong
Stowe Flagship Store	King of Power Saving	I'm trying it out to see if it saves power, and I'll buy two more if it works well.	Jingdong prefecture level city in Shandong
Stowe Flagship Store	King of Power Saving	It is genuine very good, the price is much cheaper, cost-effective, really special cost-effective, it is worth buying!	Jingdong prefecture level city in Shandong
Stowe Flagship Store	King of Power Saving	This thing can really save electricity, I was introduced to buy by my friend, he used it very well, I used it and think it's also very good, it can really save electricity, save about half of the electricity every day, it's really good!	Jingdong prefecture level city in Shandong
Stowe Flagship Store	King of Power Saving	After using it for ten days or so, I checked the power consumption and it did drop, and customer service said it will save more electricity in the long run. The product is also insured, 5 years warranty do not have to worry about quality problems!	Jingdong prefecture level city in Shandong
Stowe Flagship Store	King of Power Saving	The product is good, before already bought a, this is the second, the effect is quite good, anyway, I see the daily electricity bill than before a lot less, this can be the choice!	Jingdong prefecture level city in Shandong

Stowe Flagship Store	King of Power Saving	Originally also very worried about things are not good, get the hand found completely over-anxious, five-star praise, the style is very generous, the quality is very good, the price is also very reasonable!	Jingdong prefecture level city in Shandong
Stowe Flagship Store	King of Power Saving	Quality is good, playing to the old family also buy a, repurchase, this quality is really good, recommended!	Jingdong prefecture level city in Shandong
Stowe Flagship Store	King of Power Saving	Thank you, the owner even sent two fuses.	Jingdong prefecture level city in Shandong
Stowe Flagship Store	King of Power Saving	This store has great stuff, relatively fast shipping and logistics	Jingdong prefecture level city in Shandong
Stowe Flagship Store	King of Power Saving	This product is based on the impossibility of saving electricity, I used 853 kWh of electricity in July, started using the product on August 1, and used 867 kWh of electricity by September 1, is this called	Jingdong prefecture level city in Shandong
Stowe Flagship Store	King of Power Saving	Power king received, to the home on the trial, when I plugged into the electric panel can not see the effect of power saving, the meter is still the same speed in the turn, I'll save the king of the manual to play look at the instructions, instructions to speak, have to have a day two days and half a month to save power obviously, then wait half a month to see the effect of power saving it! To half a month there are obvious power saving before continuing to chase Xu.	Jingdong prefecture level city in Shandong
Stowe Flagship Store	King of Power Saving	Received the goods, a look, did not see the manufacturer and production address, can only hold a try to see the mentality of the face	Jingdong prefecture level city in Shandong
Stowe Flagship Store	King of Power Saving	I've only been using it for a few days and I'm not sure if it's an IQ tax? Used to favorable reviews.	Jingdong prefecture level city in Shandong
Stowe Flagship Store	King of Power Saving	Appearance is okay, power saving next time to follow up the evaluation	Jingdong prefecture level city in Shandong
Stowe Flagship Store	King of Power Saving	Shorter time, but hopefully effective and power-saving, to be followed up in the future	Jingdong prefecture level city in Shandong
Stowe Flagship Store	King of Power Saving	Appearance is okay, I do not know if the power saving	Jingdong prefecture level city in Shandong
Stowe Flagship Store	King of Power Saving	Good service, fast logistics, Power Saving King is exquisite and beautiful, support the business, thank you!	Jingdong prefecture level city in Shandong
Stowe Flagship Store	King of Power Saving	Bought three at once to try first first.	Jingdong prefecture level city in Shandong
Stowe Flagship Store	King of Power Saving	The object is the same as the picture, the product is of high quality, the packaging is intact. High-end atmosphere, high power, power saving effect is obvious, average monthly power saving 50%, very satisfied.	Jingdong prefecture level city in Shandong
Stowe Flagship Store	King of Power Saving	Bought back and plugged in, really effective, before 16 degrees of electricity per day, now 8 degrees of electricity per day, open an air conditioner for 12 hours, decisive repurchase of a	Jingdong prefecture level city in Shandong
Stowe Flagship Store	King of Power Saving	The object is the same as the picture, the product is of high quality, and the package is intact. High-end atmosphere and high grade, high power	Jingdong prefecture level city in Shandong
Stowe Flagship Store	King of Power Saving	Goods received, I hope the store said can be energy-saving, first try a month, month after month to see the effect.	Jingdong prefecture level city in Shandong
Stowe Flagship Store	King of Power Saving	This is really good, the material and workmanship is particularly exquisite, the workmanship is really very good, I think I can recommend to my friends to buy	Jingdong prefecture level city in Shandong
Stowe Flagship Store	King of Power Saving	I have to praise, the quality is really good, and the packaging logistics are also good. Really like it, won't make you regret after getting it!	Jingdong prefecture level city in Shandong
Stowe Flagship Store	King of Power Saving	Reputable platform, efficient and professional service, buy with confidence and peace of mind!	Jingdong prefecture level city in Shandong
Stowe Flagship Store	King of Power Saving	Bought one on a trial basis to see if it works after 1-2 months.	Jingdong prefecture level city in Shandong
Stowe Flagship Store	King of Power Saving	Quality is very good, customer service attitude is very very praise, there are problems in a timely manner to solve the problem, the price is not expensive, super value, you can rest assured that the	Jingdong prefecture level city in Shandong
Stowe Flagship Store	King of Power Saving	With it no longer need to be afraid to use electricity, especially good, ready to buy a mom and dad that also	Jingdong prefecture level city in Shandong
Stowe Flagship Store	King of Power Saving	Baby received very like will return to buy the seller attitude is also very like will return to buy the seller attitude is also very like!	Jingdong prefecture level city in Shandong
Stowe Flagship Store	King of Power Saving	Jingdong Mall shopping convenience assured fast assured fast assured fast assured fast	Jingdong prefecture level city in Shandong
Stowe Flagship Store	King of Power Saving	Saving power received, the quality is very good, fine workmanship, especially good, much cheaper than the entity store, it is worth buying!	Jingdong prefecture level city in Shandong
Stowe Flagship Store	King of Power Saving	I did not expect the express delivery so quickly, very save point quality is super good, than the entity store is too cheap.	Jingdong prefecture level city in Shandong
Stowe Flagship Store	King of Power Saving	Have received the baby, very satisfied with a shopping, great worth buying!	Jingdong prefecture level city in Shandong
Stowe Flagship Store	King of Power Saving	This is really more powerful, very satisfied, more energy-saving, better, thank you boss.	Jingdong prefecture level city in Shandong
Stowe Flagship Store	King of Power Saving	Received has been used for two days, the air conditioning can boldly open it! Energy saving effect is really quite good	Jingdong prefecture level city in Shandong
Stowe Flagship Store	King of Power Saving	It is authentic, trust the merchant, the baby has a great texture and the price is moderate, unlimited repurchase!	Jingdong prefecture level city in Shandong
Stowe Flagship Store	King of Power Saving	It is genuine very good, the price is much cheaper, cost-effective, really special cost-effective, it is worth buying!	Jingdong prefecture level city in Shandong
Stowe Flagship Store	King of Power Saving	Very good quality, fine workmanship, low power consumption, long warranty, fast shipping, economical and practical	Jingdong prefecture level city in Shandong
Stowe Flagship Store	King of Power Saving	I've been using it for half a month now, it really saves power and the voltage is stable, it's very good	Jingdong prefecture level city in Shandong
Stowe Flagship Store	King of Power Saving	It's the ideal product 💞, good quality and fast logistics, worth the kudos!	Jingdong prefecture level city in Shandong
Stowe Flagship Store	King of Power Saving	The logistics is very fast, the baby is very good, the shopkeeper is NICE!	Jingdong prefecture level city in Shandong
Stowe Flagship Store	King of Power Saving	Good to use, good quality, save point durable	Jingdong prefecture level city in Shandong
Stowe Flagship Store	King of Power Saving	The quality is very good, customer service attitude is very, very awesome, there are problems in a timely manner to solve the problem	Jingdong prefecture level city in Shandong
Stowe Flagship Store	King of Power Saving	Quality is very good, the use of the effect is very good, more stable, quality assurance	Jingdong prefecture level city in Shandong
Stowe Flagship Store	King of Power Saving	Convenient, inexpensive, and see how it works out?	Jingdong prefecture level city in Shandong
Stowe Flagship Store	King of Power Saving	Very good quality, saves a lot of electricity, very good, worth buying	Jingdong prefecture level city in Shandong
Stowe Flagship Store	King of Power Saving	Power Saving King is easy to use, with the fruit of power saving to use easy to give!	Jingdong prefecture level city in Shandong
Stowe Flagship Store	King of Power Saving	This saver really works, good quality, safe and secure, very satisfied!	Jingdong prefecture level city in Shandong
Stowe Flagship Store	King of Power Saving	It works well, the voltage is very stable effect is good, the quality is also very good satisfaction	Jingdong prefecture level city in Shandong
Stowe Flagship Store	King of Power Saving	The logistics is super super fast! The quality is also very good, indeed value for money	Jingdong prefecture level city in Shandong
Stowe Flagship Store	King of Power Saving	I've only been using it for a month or so and it seems to be saving a lot of power	Jingdong prefecture level city in Shandong
Stowe Flagship Store	King of Power Saving	Very easy to operate, very good material, good value for money.	Jingdong prefecture level city in Shandong
Stowe Flagship Store	King of Power Saving	Aluminum alloy shell, durable, compact power-saving good effect	Jingdong prefecture level city in Shandong
Stowe Flagship Store	King of Power Saving	Nice device, easy to use, doesn't take up much space, recommendable!	Jingdong prefecture level city in Shandong
Stowe Flagship Store	King of Power Saving	Good quality, good price, fast logistics	Jingdong prefecture level city in Shandong

Stowe Flagship Store	King of Power Saving	Friends of the family in use recommended, the effect is good and recommendable!	Jingdong prefecture level city in Shandong
Stowe Flagship Store	King of Power Saving	Cheaper than brick and mortar stores worth buying is authentic	Jingdong prefecture level city in Shandong
Stowe Flagship Store	King of Power Saving	Merchants ship quickly, the price is cheaper than the physical store, good use	Jingdong prefecture level city in Shandong
Stowe Flagship Store	King of Power Saving	Very durable, affordable, fast shipping	Jingdong prefecture level city in Shandong
Stowe Flagship Store	King of Power Saving	Packaging tight quality is very good easy to use good results	Jingdong prefecture level city in Shandong
Stowe Flagship Store	King of Power Saving	Save the grid. It's in the whole house. It's saving money on the electric bill.	Jingdong prefecture level city in Shandong
Stowe Flagship Store	King of Power Saving	I used it today, it works well, I'm very satisfied	Jingdong prefecture level city in Shandong
Stowe Flagship Store	King of Power Saving	It really saves a lot of electricity after installation	Jingdong prefecture level city in Shandong
Stowe Flagship Store	King of Power Saving	Love it. Satisfied.	Jingdong prefecture level city in Shandong
Stowe Flagship Store	King of Power Saving	Doesn't feel like it's working.	Jingdong prefecture level city in Shandong
Stowe Flagship Store	King of Power Saving	I'll comment after using it.	Jingdong prefecture level city in Shandong
Stowe Flagship Store	King of Power Saving	I'm trying it out to see if it saves power, and I'll buy two more if it works well.	Jingdong prefecture level city in Shandong
Stowe Flagship Store	King of Power Saving	It is genuine very good, the price is much cheaper, cost-effective, really special cost-effective, it is worth buying!	Jingdong prefecture level city in Shandong
Stowe Flagship Store	King of Power Saving	This thing can really save electricity, I was introduced to buy by my friend, he used it very well, I used it and think it's also very good, it can really save electricity, save about half of the electricity every day, it's really good!	Jingdong prefecture level city in Shandong
Stowe Flagship Store	King of Power Saving	After using it for ten days or so, I checked the power consumption and it did drop, and customer service said it will save more electricity in the long run. The product is also insured, 5 years warranty do not have to worry about quality problems!	Jingdong prefecture level city in Shandong
Stowe Flagship Store	King of Power Saving	The product is good, before already bought a, this is the second, the effect is quite good, anyway, I see the daily electricity bill than before a lot less, this can be the choice!	Jingdong prefecture level city in Shandong
Stowe Flagship Store	King of Power Saving	Originally also very worried about things are not good, get the hand found completely over-anxious, five-star praise, the style is very generous, the quality is very good, the price is also very reasonable!	Jingdong prefecture level city in Shandong
Stowe Flagship Store	King of Power Saving	Quality is good, playing to the old family also buy a, repurchase, this quality is really good, recommended!	Jingdong prefecture level city in Shandong
Stowe Flagship Store	King of Power Saving	Thank you, the owner even sent two fuses.	Jingdong prefecture level city in Shandong
Stowe Flagship Store	King of Power Saving	This store has great stuff, relatively fast shipping and logistics	Jingdong prefecture level city in Shandong
Stowe Flagship Store	King of Power Saving	This product is based on the impossibility of saving electricity, I used 853 kWh of electricity in July, started using the product on August 1, and used 867 kWh of electricity by September 1, is this called	Jingdong prefecture level city in Shandong
Stowe Flagship Store	King of Power Saving	Power king received, to the home on the trial, when I plugged into the electric panel can not see the effect of power saving, the meter is still the same speed in the turn, I'll save the king of the manual to play look at the instructions, instructions to speak, have to have a day two days and half a month to save power obviously, then wait half a month to see the effect of power saving it! To half a month there are obvious power saving before continuing to chase Xu.	Jingdong prefecture level city in Shandong
Stowe Flagship Store	King of Power Saving	Received the goods, a look, did not see the manufacturer and production address, can only hold a try to see the mentality of the face	Jingdong prefecture level city in Shandong
Stowe Flagship Store	King of Power Saving	I've only been using it for a few days and I'm not sure if it's an IQ tax? Used to favorable reviews.	Jingdong prefecture level city in Shandong
Stowe Flagship Store	King of Power Saving	Appearance is okay, power saving next time to follow up the evaluation	Jingdong prefecture level city in Shandong
Stowe Flagship Store	King of Power Saving	Shorter time, but hopefully effective and power-saving, to be followed up in the future	Jingdong prefecture level city in Shandong
Stowe Flagship Store	King of Power Saving	Appearance is okay, I do not know if the power saving	Jingdong prefecture level city in Shandong
Stowe Flagship Store	King of Power Saving	Good service, fast logistics, Power Saving King is exquisite and beautiful, support the business, thank you!	Jingdong prefecture level city in Shandong
Stowe Flagship Store	King of Power Saving	Bought three at once to try first first.	Jingdong prefecture level city in Shandong
Stowe Flagship Store	King of Power Saving	The object is the same as the picture, the product is of high quality, the packaging is intact. High-end atmosphere, high power, power saving effect is obvious, average monthly power saving 50%, very satisfied.	Jingdong prefecture level city in Shandong
Stowe Flagship Store	King of Power Saving	Bought back and plugged in, really effective, before 16 degrees of electricity per day, now 8 degrees of electricity per day, open an air conditioner for 12 hours, decisive repurchase of a	Jingdong prefecture level city in Shandong
Stowe Flagship Store	King of Power Saving	The object is the same as the picture, the product is of high quality, and the package is intact. High-end atmosphere and high grade, high power	Jingdong prefecture level city in Shandong
Stowe Flagship Store	King of Power Saving	Goods received, I hope the store said can be energy-saving, first try a month, month after month to see the effect.	Jingdong prefecture level city in Shandong
Stowe Flagship Store	King of Power Saving	This is really good, the material and workmanship is particularly exquisite, the workmanship is really very good, I think I can recommend to my friends to buy	Jingdong prefecture level city in Shandong
Stowe Flagship Store	King of Power Saving	I have to praise, the quality is really good, and the packaging logistics are also good. Really like it, won't make you regret after getting it!	Jingdong prefecture level city in Shandong
Stowe Flagship Store	King of Power Saving	Reputable platform, efficient and professional service, buy with confidence and peace of mind!	Jingdong prefecture level city in Shandong
Stowe Flagship Store	King of Power Saving	Bought one on a trial basis to see if it works after 1-2 months.	Jingdong prefecture level city in Shandong
Stowe Flagship Store	King of Power Saving	Quality is very good, customer service attitude is very very praise, there are problems in a timely manner to solve the problem, the price is not expensive, super value, you can rest assured that the	Jingdong prefecture level city in Shandong
Stowe Flagship Store	King of Power Saving	With it no longer need to be afraid to use electricity, especially good, ready to buy a mom and dad that also	Jingdong prefecture level city in Shandong
Stowe Flagship Store	King of Power Saving	Baby received very like will return to buy the seller attitude is also very like will return to buy the seller attitude is also very like!	Jingdong prefecture level city in Shandong
Stowe Flagship Store	King of Power Saving	Jingdong Mall shopping convenience assured fast assured fast assured fast assured fast	Jingdong prefecture level city in Shandong
Stowe Flagship Store	King of Power Saving	Saving power received, the quality is very good, fine workmanship, especially good, much cheaper than the entity store, it is worth buying!	Jingdong prefecture level city in Shandong
Stowe Flagship Store	King of Power Saving	I did not expect the express delivery so quickly, very save point quality is super good, than the entity store is too cheap.	Jingdong prefecture level city in Shandong
Stowe Flagship Store	King of Power Saving	Have received the baby, very satisfied with a shopping, great worth buying!	Jingdong prefecture level city in Shandong
Stowe Flagship Store	King of Power Saving	This is really more powerful, very satisfied, more energy-saving, better, thank you boss.	Jingdong prefecture level city in Shandong
Stowe Flagship Store	King of Power Saving	Received has been used for two days, the air conditioning can boldly open it! Energy saving effect is really quite good	Jingdong prefecture level city in Shandong
Stowe Flagship Store	King of Power Saving	It is authentic, trust the merchant, the baby has a great texture and the price is moderate, unlimited repurchase!	Jingdong prefecture level city in Shandong
Stowe Flagship Store	King of Power Saving	It is genuine very good, the price is much cheaper, cost-effective, really special cost-effective, it is worth buying!	Jingdong prefecture level city in Shandong
Stowe Flagship Store	King of Power Saving	Very good quality, fine workmanship, low power consumption, long warranty, fast shipping, economical and practical	Jingdong prefecture level city in Shandong

Stowe Flagship Store	King of Power Saving	I've been using it for half a month now, it really saves power and the voltage is stable, it's very good	Jingdong prefecture level city in Shandong
Stowe Flagship Store	King of Power Saving	It's the ideal product 💖, good quality and fast logistics, worth the kudos!	Jingdong prefecture level city in Shandong
Stowe Flagship Store	King of Power Saving	The logistics is very fast, the baby is very good, the shopkeeper is NICE!	Jingdong prefecture level city in Shandong
Stowe Flagship Store	King of Power Saving	Good to use, good quality, save point durable	Jingdong prefecture level city in Shandong
Stowe Flagship Store	King of Power Saving	The quality is very good, customer service attitude is very, very awesome, there are problems in a timely manner to solve the problem	Jingdong prefecture level city in Shandong
Stowe Flagship Store	King of Power Saving	Quality is very good, the use of the effect is very good, more stable, quality assurance	Jingdong prefecture level city in Shandong
Stowe Flagship Store	King of Power Saving	Convenient, inexpensive, and see how it works out?	Jingdong prefecture level city in Shandong
Stowe Flagship Store	King of Power Saving	Very good quality, saves a lot of electricity, very good, worth buying	Jingdong prefecture level city in Shandong
Stowe Flagship Store	King of Power Saving	Power Saving King is easy to use, with the fruit of power saving to use easy to give!	Jingdong prefecture level city in Shandong
Stowe Flagship Store	King of Power Saving	This saver really works, good quality, safe and secure, very satisfied!	Jingdong prefecture level city in Shandong
Stowe Flagship Store	King of Power Saving	It works well, the voltage is very stable effect is good, the quality is also very good satisfaction	Jingdong prefecture level city in Shandong
Stowe Flagship Store	King of Power Saving	The logistics is super super fast! The quality is also very good, indeed value for money	Jingdong prefecture level city in Shandong
Stowe Flagship Store	King of Power Saving	I've only been using it for a month or so and it seems to be saving a lot of power	Jingdong prefecture level city in Shandong
Stowe Flagship Store	King of Power Saving	Very easy to operate, very good material, good value for money.	Jingdong prefecture level city in Shandong
Stowe Flagship Store	King of Power Saving	Aluminum alloy shell, durable, compact power-saving good effect	Jingdong prefecture level city in Shandong
Stowe Flagship Store	King of Power Saving	Nice device, easy to use, doesn't take up much space, recommendable!	Jingdong prefecture level city in Shandong
Stowe Flagship Store	King of Power Saving	Good quality, good price, fast logistics	Jingdong prefecture level city in Shandong
Stowe Flagship Store	King of Power Saving	Friends of the family in use recommended, the effect is good and recommendable!	Jingdong prefecture level city in Shandong
Stowe Flagship Store	King of Power Saving	Cheaper than brick and mortar stores worth buying is authentic	Jingdong prefecture level city in Shandong
Stowe Flagship Store	King of Power Saving	Merchants ship quickly, the price is cheaper than the physical store, good use	Jingdong prefecture level city in Shandong
Stowe Flagship Store	King of Power Saving	Very durable, affordable, fast shipping	Jingdong prefecture level city in Shandong
Stowe Flagship Store	King of Power Saving	Packaging tight quality is very good easy to use good results	Jingdong prefecture level city in Shandong
Stowe Flagship Store	King of Power Saving	Save the grid. It's in the whole house. It's saving money on the electric bill.	Jingdong prefecture level city in Shandong
Stowe Flagship Store	King of Power Saving	I used it today, it works well, I'm very satisfied	Jingdong prefecture level city in Shandong
Stowe Flagship Store	King of Power Saving	It really saves a lot of electricity after installation	Jingdong prefecture level city in Shandong
Stowe Flagship Store	King of Power Saving	Love it. Satisfied.	Jingdong prefecture level city in Shandong
Stowe Flagship Store	King of Power Saving	Doesn't feel like it's working.	Jingdong prefecture level city in Shandong
Stowe Flagship Store	King of Power Saving	I'll comment after using it.	Jingdong prefecture level city in Shandong
Stowe Flagship Store	King of Power Saving	I'm trying it out to see if it saves power, and I'll buy two more if it works well.	Jingdong prefecture level city in Shandong
Stowe Flagship Store	King of Power Saving	It is genuine very good, the price is much cheaper, cost-effective, really special cost-effective, it is worth buying!	Jingdong prefecture level city in Shandong
Stowe Flagship Store	King of Power Saving	This thing can really save electricity, I was introduced to buy by my friend, he used it very well, I used it and think it's also very good, it can really save electricity, save about half of the electricity every day, it's really good!	Jingdong prefecture level city in Shandong
Stowe Flagship Store	King of Power Saving	After using it for ten days or so, I checked the power consumption and it did drop, and customer service said it will save more electricity in the long run. The product is also insured, 5 years warranty do not have to worry about quality problems!	Jingdong prefecture level city in Shandong
Stowe Flagship Store	King of Power Saving	The product is good, before already bought a, this is the second, the effect is quite good, anyway, I see the daily electricity bill than before a lot less, this can be the choice!	Jingdong prefecture level city in Shandong
Stowe Flagship Store	King of Power Saving	Originally also very worried about things are not good, get the hand found completely over-anxious, five-star praise, the style is very generous, the quality is very good, the price is also very reasonable!	Jingdong prefecture level city in Shandong
Stowe Flagship Store	King of Power Saving	Quality is good, playing to the old family also buy a, repurchase, this quality is really good, recommended!	Jingdong prefecture level city in Shandong
Stowe Flagship Store	King of Power Saving	Thank you, the owner even sent two fuses.	Jingdong prefecture level city in Shandong
Stowe Flagship Store	King of Power Saving	This store has great stuff, relatively fast shipping and logistics	Jingdong prefecture level city in Shandong
Stowe Flagship Store	King of Power Saving	This product is based on the impossibility of saving electricity, I used 853 kWh of electricity in July, started using the product on August 1, and used 867 kWh of electricity by September 1, is this called	Jingdong prefecture level city in Shandong
Stowe Flagship Store	King of Power Saving	Power king received, to the home on the trial, when I plugged into the electric panel can not see the effect of power saving, the meter is still the same speed in the turn, I'll save the king of the manual to play look at the instructions, instructions to speak, have to have a day two days and half a month to save power obviously, then wait half a month to see the effect of power saving it! To half a month there are obvious power saving before continuing to chase Xu.	Jingdong prefecture level city in Shandong
Stowe Flagship Store	King of Power Saving	Received the goods, a look, did not see the manufacturer and production address, can only hold a try to see the mentality of the face	Jingdong prefecture level city in Shandong
Stowe Flagship Store	King of Power Saving	I've only been using it for a few days and I'm not sure if it's an IQ tax? Used to favorable reviews.	Jingdong prefecture level city in Shandong
Stowe Flagship Store	King of Power Saving	Appearance is okay, power saving next time to follow up the evaluation	Jingdong prefecture level city in Shandong
Stowe Flagship Store	King of Power Saving	Shorter time, but hopefully effective and power-saving, to be followed up in the future	Jingdong prefecture level city in Shandong
Stowe Flagship Store	King of Power Saving	Appearance is okay, I do not know if the power saving	Jingdong prefecture level city in Shandong
Stowe Flagship Store	King of Power Saving	Good service, fast logistics, Power Saving King is exquisite and beautiful, support the business, thank you!	Jingdong prefecture level city in Shandong
Stowe Flagship Store	King of Power Saving	Bought three at once to try first first.	Jingdong prefecture level city in Shandong
Stowe Flagship Store	King of Power Saving	The object is the same as the picture, the product is of high quality, the packaging is intact. High-end atmosphere, high power, power saving effect is obvious, average monthly power saving 50%, very satisfied.	Jingdong prefecture level city in Shandong
Stowe Flagship Store	King of Power Saving	Bought back and plugged in, really effective, before 16 degrees of electricity per day, now 8 degrees of electricity per day, open an air conditioner for 12 hours, decisive repurchase of a	Jingdong prefecture level city in Shandong
Stowe Flagship Store	King of Power Saving	The object is the same as the picture, the product is of high quality, and the package is intact. High-end atmosphere and high grade, high power	Jingdong prefecture level city in Shandong

Stowe Flagship Store	King of Power Saving	Goods received, I hope the store said can be energy-saving, first try a month, month after month to see the effect.	Jingdong prefecture level city in Shandong
Stowe Flagship Store	King of Power Saving	This is really good, the material and workmanship is particularly exquisite, the workmanship is really very good, I think I can recommend to my friends to buy	Jingdong prefecture level city in Shandong
Stowe Flagship Store	King of Power Saving	I have to praise, the quality is really good, and the packaging logistics are also good. Really like it, won't make you regret after getting it!	Jingdong prefecture level city in Shandong
Stowe Flagship Store	King of Power Saving	Reputable platform, efficient and professional service, buy with confidence and peace of mind!	Jingdong prefecture level city in Shandong
Stowe Flagship Store	King of Power Saving	Bought one on a trial basis to see if it works after 1-2 months.	Jingdong prefecture level city in Shandong
Stowe Flagship Store	King of Power Saving	Quality is very good, customer service attitude is very very praise, there are problems in a timely manner to solve the problem, the price is not expensive, super value, you can rest assured that the	Jingdong prefecture level city in Shandong
Stowe Flagship Store	King of Power Saving	With it no longer need to be afraid to use electricity, especially good, ready to buy a mom and dad that also	Jingdong prefecture level city in Shandong
Stowe Flagship Store	King of Power Saving	Baby received very like will return to buy the seller attitude is also very like will return to buy the seller attitude is also very like!	Jingdong prefecture level city in Shandong
Stowe Flagship Store	King of Power Saving	Jingdong Mall shopping convenience assured fast assured fast assured fast assured fast	Jingdong prefecture level city in Shandong
Stowe Flagship Store	King of Power Saving	Saving power received, the quality is very good, fine workmanship, especially good, much cheaper than the entity store, it is worth buying!	Jingdong prefecture level city in Shandong
Stowe Flagship Store	King of Power Saving	I did not expect the express delivery so quickly, very save point quality is super good, than the entity store is too cheap.	Jingdong prefecture level city in Shandong
Stowe Flagship Store	King of Power Saving	Have received the baby, very satisfied with a shopping, great worth buying!	Jingdong prefecture level city in Shandong
Stowe Flagship Store	King of Power Saving	This is really more powerful, very satisfied, more energy-saving, better, thank you boss.	Jingdong prefecture level city in Shandong
Stowe Flagship Store	King of Power Saving	Received has been used for two days, the air conditioning can boldly open it! Energy saving effect is really quite good	Jingdong prefecture level city in Shandong
Stowe Flagship Store	King of Power Saving	It is authentic, trust the merchant, the baby has a great texture and the price is moderate, unlimited repurchase!	Jingdong prefecture level city in Shandong
Stowe Flagship Store	King of Power Saving	It is genuine very good, the price is much cheaper, cost-effective, really special cost-effective, it is worth buying!	Jingdong prefecture level city in Shandong
Stowe Flagship Store	King of Power Saving	Very good quality, fine workmanship, low power consumption, long warranty, fast shipping, economical and practical	Jingdong prefecture level city in Shandong
Stowe Flagship Store	King of Power Saving	I've been using it for half a month now, it really saves power and the voltage is stable, it's very good	Jingdong prefecture level city in Shandong
Stowe Flagship Store	King of Power Saving	It's the ideal product 💖, good quality and fast logistics, worth the kudos!	Jingdong prefecture level city in Shandong
Stowe Flagship Store	King of Power Saving	The logistics is very fast, the baby is very good, the shopkeeper is NICE!	Jingdong prefecture level city in Shandong
Stowe Flagship Store	King of Power Saving	Good to use, good quality, save point durable	Jingdong prefecture level city in Shandong
Stowe Flagship Store	King of Power Saving	The quality is very good, customer service attitude is very, very awesome, there are problems in a timely manner to solve the problem	Jingdong prefecture level city in Shandong
Stowe Flagship Store	King of Power Saving	Quality is very good, the use of the effect is very good, more stable, quality assurance	Jingdong prefecture level city in Shandong
Stowe Flagship Store	King of Power Saving	Convenient, inexpensive, and see how it works out?	Jingdong prefecture level city in Shandong
Stowe Flagship Store	King of Power Saving	Very good quality, saves a lot of electricity, very good, worth buying	Jingdong prefecture level city in Shandong
Stowe Flagship Store	King of Power Saving	Power Saving King is easy to use, with the fruit of power saving to use easy to give!	Jingdong prefecture level city in Shandong
Stowe Flagship Store	King of Power Saving	This saver really works, good quality, safe and secure, very satisfied!	Jingdong prefecture level city in Shandong
Stowe Flagship Store	King of Power Saving	It works well, the voltage is very stable effect is good, the quality is also very good satisfaction	Jingdong prefecture level city in Shandong
Stowe Flagship Store	King of Power Saving	The logistics is super super fast! The quality is also very good, indeed value for money	Jingdong prefecture level city in Shandong
Stowe Flagship Store	King of Power Saving	I've only been using it for a month or so and it seems to be saving a lot of power	Jingdong prefecture level city in Shandong
Stowe Flagship Store	King of Power Saving	Very easy to operate, very good material, good value for money.	Jingdong prefecture level city in Shandong
Stowe Flagship Store	King of Power Saving	Aluminum alloy shell, durable, compact power-saving good effect	Jingdong prefecture level city in Shandong
Stowe Flagship Store	King of Power Saving	Nice device, easy to use, doesn't take up much space, recommendable!	Jingdong prefecture level city in Shandong
Stowe Flagship Store	King of Power Saving	Good quality, good price, fast logistics	Jingdong prefecture level city in Shandong
Stowe Flagship Store	King of Power Saving	Friends of the family in use recommended, the effect is good and recommendable!	Jingdong prefecture level city in Shandong
Stowe Flagship Store	King of Power Saving	Cheaper than brick and mortar stores worth buying is authentic	Jingdong prefecture level city in Shandong
Stowe Flagship Store	King of Power Saving	Merchants ship quickly, the price is cheaper than the physical store, good use	Jingdong prefecture level city in Shandong
Stowe Flagship Store	King of Power Saving	Very durable, affordable, fast shipping	Jingdong prefecture level city in Shandong
Stowe Flagship Store	King of Power Saving	Packaging tight quality is very good easy to use good results	Jingdong prefecture level city in Shandong
Stowe Flagship Store	King of Power Saving	Save the grid. It's in the whole house. It's saving money on the electric bill.	Jingdong prefecture level city in Shandong
Stowe Flagship Store	King of Power Saving	I used it today, it works well, I'm very satisfied	Jingdong prefecture level city in Shandong
Stowe Flagship Store	King of Power Saving	It really saves a lot of electricity after installation	Jingdong prefecture level city in Shandong
Stowe Flagship Store	King of Power Saving	Love it. Satisfied.	Jingdong prefecture level city in Shandong
Stowe Flagship Store	King of Power Saving	Doesn't feel like it's working.	Jingdong prefecture level city in Shandong
Stowe Flagship Store	King of Power Saving	I'll comment after using it.	Jingdong prefecture level city in Shandong
Stowe Flagship Store	King of Power Saving	I'm trying it out to see if it saves power, and I'll buy two more if it works well.	Jingdong prefecture level city in Shandong
Stowe Flagship Store	King of Power Saving	It is genuine very good, the price is much cheaper, cost-effective, really special cost-effective, it is worth buying!	Jingdong prefecture level city in Shandong
Stowe Flagship Store	King of Power Saving	This thing can really save electricity, I was introduced to buy by my friend, he used it very well, I used it and think it's also very good, it can really save electricity, save about half of the electricity every day, it's really good!	Jingdong prefecture level city in Shandong
Stowe Flagship Store	King of Power Saving	After using it for ten days or so, I checked the power consumption and it did drop, and customer service said it will save more electricity in the long run. The product is also insured, 5 years warranty do not have to worry about quality problems!	Jingdong prefecture level city in Shandong
Stowe Flagship Store	King of Power Saving	The product is good, before already bought a, this is the second, the effect is quite good, anyway, I see the daily electricity bill than before a lot less, this can be the choice!	Jingdong prefecture level city in Shandong

Stowe Flagship Store	King of Power Saving	Originally also very worried about things are not good, get the hand found completely over-anxious, five-star praise, the style is very generous, the quality is very good, the price is also very reasonable!	Jingdong prefecture level city in Shandong
Stowe Flagship Store	King of Power Saving	Quality is good, playing to the old family also buy a, repurchase, this quality is really good, recommended!	Jingdong prefecture level city in Shandong
Stowe Flagship Store	King of Power Saving	Thank you, the owner even sent two fuses.	Jingdong prefecture level city in Shandong
Stowe Flagship Store	King of Power Saving	This store has great stuff, relatively fast shipping and logistics	Jingdong prefecture level city in Shandong
Stowe Flagship Store	King of Power Saving	This product is based on the impossibility of saving electricity, I used 853 kWh of electricity in July, started using the product on August 1, and used 867 kWh of electricity by September 1, is this called	Jingdong prefecture level city in Shandong
Stowe Flagship Store	King of Power Saving	Power king received, to the home on the trial, when I plugged into the electric panel can not see the effect of power saving, the meter is still the same speed in the turn, I'll save the king of the manual to play look at the instructions, instructions to speak, have to have a day two days and half a month to save power obviously, then wait half a month to see the effect of power saving it! To half a month there are obvious power saving before continuing to chase Xu.	Jingdong prefecture level city in Shandong
Stowe Flagship Store	King of Power Saving	Received the goods, a look, did not see the manufacturer and production address, can only hold a try to see the mentality of the face	Jingdong prefecture level city in Shandong
Stowe Flagship Store	King of Power Saving	I've only been using it for a few days and I'm not sure if it's an IQ tax? Used to favorable reviews.	Jingdong prefecture level city in Shandong
Stowe Flagship Store	King of Power Saving	Appearance is okay, power saving next time to follow up the evaluation	Jingdong prefecture level city in Shandong
Stowe Flagship Store	King of Power Saving	Shorter time, but hopefully effective and power-saving, to be followed up in the future	Jingdong prefecture level city in Shandong
Stowe Flagship Store	King of Power Saving	Appearance is okay, I do not know if the power saving	Jingdong prefecture level city in Shandong
Stowe Flagship Store	King of Power Saving	Good service, fast logistics, Power Saving King is exquisite and beautiful, support the business, thank you!	Jingdong prefecture level city in Shandong
Stowe Flagship Store	King of Power Saving	Bought three at once to try first first.	Jingdong prefecture level city in Shandong
Stowe Flagship Store	King of Power Saving	The object is the same as the picture, the product is of high quality, the packaging is intact. High-end atmosphere, high power, power saving effect is obvious, average monthly power saving 50%, very satisfied.	Jingdong prefecture level city in Shandong
Stowe Flagship Store	King of Power Saving	Bought back and plugged in, really effective, before 16 degrees of electricity per day, now 8 degrees of electricity per day, open an air conditioner for 12 hours, decisive repurchase of a	Jingdong prefecture level city in Shandong
Stowe Flagship Store	King of Power Saving	The object is the same as the picture, the product is of high quality, and the package is intact. High-end atmosphere and high grade, high power	Jingdong prefecture level city in Shandong
Stowe Flagship Store	King of Power Saving	Goods received, I hope the store said can be energy-saving, first try a month, month after month to see the effect.	Jingdong prefecture level city in Shandong
Stowe Flagship Store	King of Power Saving	This is really good, the material and workmanship is particularly exquisite, the workmanship is really very good, I think I can recommend to my friends to buy	Jingdong prefecture level city in Shandong
Stowe Flagship Store	King of Power Saving	I have to praise, the quality is really good, and the packaging logistics are also good. Really like it, won't make you regret after getting it!	Jingdong prefecture level city in Shandong
Stowe Flagship Store	King of Power Saving	Reputable platform, efficient and professional service, buy with confidence and peace of mind!	Jingdong prefecture level city in Shandong
Stowe Flagship Store	King of Power Saving	Bought one on a trial basis to see if it works after 1-2 months.	Jingdong prefecture level city in Shandong
Stowe Flagship Store	King of Power Saving	Quality is very good, customer service attitude is very very praise, there are problems in a timely manner to solve the problem, the price is not expensive, super value, you can rest assured that the	Jingdong prefecture level city in Shandong
Stowe Flagship Store	King of Power Saving	With it no longer need to be afraid to use electricity, especially good, ready to buy a mom and dad that also	Jingdong prefecture level city in Shandong
Stowe Flagship Store	King of Power Saving	Baby received very like will return to buy the seller attitude is also very like will return to buy the seller attitude is also very like!	Jingdong prefecture level city in Shandong
Stowe Flagship Store	King of Power Saving	Jingdong Mall shopping convenience assured fast assured fast assured fast assured fast	Jingdong prefecture level city in Shandong
Stowe Flagship Store	King of Power Saving	Saving power received, the quality is very good, fine workmanship, especially good, much cheaper than the entity store, it is worth buying!	Jingdong prefecture level city in Shandong
Stowe Flagship Store	King of Power Saving	I did not expect the express delivery so quickly, very save point quality is super good, than the entity store is too cheap.	Jingdong prefecture level city in Shandong
Stowe Flagship Store	King of Power Saving	Have received the baby, very satisfied with a shopping, great worth buying!	Jingdong prefecture level city in Shandong
Stowe Flagship Store	King of Power Saving	This is really more powerful, very satisfied, more energy-saving, better, thank you boss.	Jingdong prefecture level city in Shandong
Stowe Flagship Store	King of Power Saving	Received has been used for two days, the air conditioning can boldly open it! Energy saving effect is really quite good	Jingdong prefecture level city in Shandong
Stowe Flagship Store	King of Power Saving	It is authentic, trust the merchant, the baby has a great texture and the price is moderate, unlimited repurchase!	Jingdong prefecture level city in Shandong
Stowe Flagship Store	King of Power Saving	It is genuine very good, the price is much cheaper, cost-effective, really special cost-effective, it is worth buying!	Jingdong prefecture level city in Shandong
Stowe Flagship Store	King of Power Saving	Very good quality, fine workmanship, low power consumption, long warranty, fast shipping, economical and practical	Jingdong prefecture level city in Shandong
Stowe Flagship Store	King of Power Saving	I've been using it for half a month now, it really saves power and the voltage is stable, it's very good	Jingdong prefecture level city in Shandong
Stowe Flagship Store	King of Power Saving	It's the ideal product 💞, good quality and fast logistics, worth the kudos!	Jingdong prefecture level city in Shandong
Stowe Flagship Store	King of Power Saving	The logistics is very fast, the baby is very good, the shopkeeper is NICE!	Jingdong prefecture level city in Shandong
Stowe Flagship Store	King of Power Saving	Good to use, good quality, save point durable	Jingdong prefecture level city in Shandong
Stowe Flagship Store	King of Power Saving	The quality is very good, customer service attitude is very, very awesome, there are problems in a timely manner to solve the problem	Jingdong prefecture level city in Shandong
Stowe Flagship Store	King of Power Saving	Quality is very good, the use of the effect is very good, more stable, quality assurance	Jingdong prefecture level city in Shandong
Stowe Flagship Store	King of Power Saving	Convenient, inexpensive, and see how it works out?	Jingdong prefecture level city in Shandong
Stowe Flagship Store	King of Power Saving	Very good quality, saves a lot of electricity, very good, worth buying	Jingdong prefecture level city in Shandong
Stowe Flagship Store	King of Power Saving	Power Saving King is easy to use, with the fruit of power saving to use easy to give!	Jingdong prefecture level city in Shandong
Stowe Flagship Store	King of Power Saving	This saver really works, good quality, safe and secure, very satisfied!	Jingdong prefecture level city in Shandong
Stowe Flagship Store	King of Power Saving	It works well, the voltage is very stable effect is good, the quality is also very good satisfaction	Jingdong prefecture level city in Shandong
Stowe Flagship Store	King of Power Saving	The logistics is super super fast! The quality is also very good, indeed value for money	Jingdong prefecture level city in Shandong
Stowe Flagship Store	King of Power Saving	I've only been using it for a month or so and it seems to be saving a lot of power	Jingdong prefecture level city in Shandong
Stowe Flagship Store	King of Power Saving	Very easy to operate, very good material, good value for money.	Jingdong prefecture level city in Shandong
Stowe Flagship Store	King of Power Saving	Aluminum alloy shell, durable, compact power-saving good effect	Jingdong prefecture level city in Shandong
Stowe Flagship Store	King of Power Saving	Nice device, easy to use, doesn't take up much space, recommendable!	Jingdong prefecture level city in Shandong
Stowe Flagship Store	King of Power Saving	Good quality, good price, fast logistics	Jingdong prefecture level city in Shandong

Stowe Flagship Store	King of Power Saving	Friends of the family in use recommended, the effect is good and recommendable!	Jingdong prefecture level city in Shandong
Stowe Flagship Store	King of Power Saving	Cheaper than brick and mortar stores worth buying is authentic	Jingdong prefecture level city in Shandong
Stowe Flagship Store	King of Power Saving	Merchants ship quickly, the price is cheaper than the physical store, good use	Jingdong prefecture level city in Shandong
Stowe Flagship Store	King of Power Saving	Very durable, affordable, fast shipping	Jingdong prefecture level city in Shandong
Stowe Flagship Store	King of Power Saving	Packaging tight quality is very good easy to use good results	Jingdong prefecture level city in Shandong
Stowe Flagship Store	King of Power Saving	Save the grid. It's in the whole house. It's saving money on the electric bill.	Jingdong prefecture level city in Shandong
Stowe Flagship Store	King of Power Saving	I used it today, it works well, I'm very satisfied	Jingdong prefecture level city in Shandong
Stowe Flagship Store	King of Power Saving	It really saves a lot of electricity after installation	Jingdong prefecture level city in Shandong
Stowe Flagship Store	King of Power Saving	Love it. Satisfied.	Jingdong prefecture level city in Shandong
Stowe Flagship Store	King of Power Saving	Doesn't feel like it's working.	Jingdong prefecture level city in Shandong
Stowe Flagship Store	King of Power Saving	I'll comment after using it.	Jingdong prefecture level city in Shandong
Stowe Flagship Store	King of Power Saving	I'm trying it out to see if it saves power, and I'll buy two more if it works well.	Jingdong prefecture level city in Shandong
Stowe Flagship Store	King of Power Saving	It is genuine very good, the price is much cheaper, cost-effective, really special cost-effective, it is worth buying!	Jingdong prefecture level city in Shandong
Stowe Flagship Store	King of Power Saving	This thing can really save electricity, I was introduced to buy by my friend, he used it very well, I used it and think it's also very good, it can really save electricity, save about half of the electricity every day, it's really good!	Jingdong prefecture level city in Shandong
Stowe Flagship Store	King of Power Saving	After using it for ten days or so, I checked the power consumption and it did drop, and customer service said it will save more electricity in the long run. The product is also insured, 5 years warranty do not have to worry about quality problems!	Jingdong prefecture level city in Shandong
Stowe Flagship Store	King of Power Saving	The product is good, before already bought a, this is the second, the effect is quite good, anyway, I see the daily electricity bill than before a lot less, this can be the choice!	Jingdong prefecture level city in Shandong
Stowe Flagship Store	King of Power Saving	Originally also very worried about things are not good, get the hand found completely over-anxious, five-star praise, the style is very generous, the quality is very good, the price is also very reasonable!	Jingdong prefecture level city in Shandong
Stowe Flagship Store	King of Power Saving	Quality is good, playing to the old family also buy a, repurchase, this quality is really good, recommended!	Jingdong prefecture level city in Shandong
Stowe Flagship Store	King of Power Saving	Thank you, the owner even sent two fuses.	Jingdong prefecture level city in Shandong
Stowe Flagship Store	King of Power Saving	This store has great stuff, relatively fast shipping and logistics	Jingdong prefecture level city in Shandong
Stowe Flagship Store	King of Power Saving	This product is based on the impossibility of saving electricity, I used 853 kWh of electricity in July, started using the product on August 1, and used 867 kWh of electricity by September 1, is this called	Jingdong prefecture level city in Shandong
Stowe Flagship Store	King of Power Saving	Power king received, to the home on the trial, when I plugged into the electric panel can not see the effect of power saving, the meter is still the same speed in the turn, I'll save the king of the manual to play look at the instructions, instructions to speak, have to have a day two days and half a month to save power obviously, then wait half a month to see the effect of power saving it! To half a month there are obvious power saving before continuing to chase Xu.	Jingdong prefecture level city in Shandong
Stowe Flagship Store	King of Power Saving	Received the goods, a look, did not see the manufacturer and production address, can only hold a try to see the mentality of the face	Jingdong prefecture level city in Shandong
Stowe Flagship Store	King of Power Saving	I've only been using it for a few days and I'm not sure if it's an IQ tax? Used to favorable reviews.	Jingdong prefecture level city in Shandong
Stowe Flagship Store	King of Power Saving	Appearance is okay, power saving next time to follow up the evaluation	Jingdong prefecture level city in Shandong
Stowe Flagship Store	King of Power Saving	Shorter time, but hopefully effective and power-saving, to be followed up in the future	Jingdong prefecture level city in Shandong
Stowe Flagship Store	King of Power Saving	Appearance is okay, I do not know if the power saving	Jingdong prefecture level city in Shandong
Stowe Flagship Store	King of Power Saving	Good service, fast logistics, Power Saving King is exquisite and beautiful, support the business, thank you!	Jingdong prefecture level city in Shandong
Stowe Flagship Store	King of Power Saving	Bought three at once to try first first.	Jingdong prefecture level city in Shandong
Stowe Flagship Store	King of Power Saving	The object is the same as the picture, the product is of high quality, the packaging is intact. High-end atmosphere, high power, power saving effect is obvious, average monthly power saving 50%, very satisfied.	Jingdong prefecture level city in Shandong
Stowe Flagship Store	King of Power Saving	Bought back and plugged in, really effective, before 16 degrees of electricity per day, now 8 degrees of electricity per day, open an air conditioner for 12 hours, decisive repurchase of a	Jingdong prefecture level city in Shandong
Stowe Flagship Store	King of Power Saving	The object is the same as the picture, the product is of high quality, and the package is intact. High-end atmosphere and high grade, high power	Jingdong prefecture level city in Shandong
Stowe Flagship Store	King of Power Saving	Goods received, I hope the store said can be energy-saving, first try a month, month after month to see the effect.	Jingdong prefecture level city in Shandong
Stowe Flagship Store	King of Power Saving	This is really good, the material and workmanship is particularly exquisite, the workmanship is really very good, I think I can recommend to my friends to buy	Jingdong prefecture level city in Shandong
Stowe Flagship Store	King of Power Saving	I have to praise, the quality is really good, and the packaging logistics are also good. Really like it, won't make you regret after getting it!	Jingdong prefecture level city in Shandong
Stowe Flagship Store	King of Power Saving	Reputable platform, efficient and professional service, buy with confidence and peace of mind!	Jingdong prefecture level city in Shandong
Stowe Flagship Store	King of Power Saving	Bought one on a trial basis to see if it works after 1-2 months.	Jingdong prefecture level city in Shandong
Stowe Flagship Store	King of Power Saving	Quality is very good, customer service attitude is very very praise, there are problems in a timely manner to solve the problem, the price is not expensive, super value, you can rest assured that the	Jingdong prefecture level city in Shandong
Stowe Flagship Store	King of Power Saving	With it no longer need to be afraid to use electricity, especially good, ready to buy a mom and dad that also	Jingdong prefecture level city in Shandong
Stowe Flagship Store	King of Power Saving	Baby received very like will return to buy the seller attitude is also very like will return to buy the seller attitude is also very like!	Jingdong prefecture level city in Shandong
Stowe Flagship Store	King of Power Saving	Jingdong Mall shopping convenience assured fast assured fast assured fast assured fast	Jingdong prefecture level city in Shandong
Stowe Flagship Store	King of Power Saving	Saving power received, the quality is very good, fine workmanship, especially good, much cheaper than the entity store, it is worth buying!	Jingdong prefecture level city in Shandong
Stowe Flagship Store	King of Power Saving	I did not expect the express delivery so quickly, very save point quality is super good, than the entity store is too cheap.	Jingdong prefecture level city in Shandong
Stowe Flagship Store	King of Power Saving	Have received the baby, very satisfied with a shopping, great worth buying!	Jingdong prefecture level city in Shandong
Stowe Flagship Store	King of Power Saving	This is really more powerful, very satisfied, more energy-saving, better, thank you boss.	Jingdong prefecture level city in Shandong
Stowe Flagship Store	King of Power Saving	Received has been used for two days, the air conditioning can boldly open it! Energy saving effect is really quite good	Jingdong prefecture level city in Shandong
Stowe Flagship Store	King of Power Saving	It is authentic, trust the merchant, the baby has a great texture and the price is moderate, unlimited repurchase!	Jingdong prefecture level city in Shandong
Stowe Flagship Store	King of Power Saving	It is genuine very good, the price is much cheaper, cost-effective, really special cost-effective, it is worth buying!	Jingdong prefecture level city in Shandong
Stowe Flagship Store	King of Power Saving	Very good quality, fine workmanship, low power consumption, long warranty, fast shipping, economical and practical	Jingdong prefecture level city in Shandong

Stowe Flagship Store	King of Power Saving	I've been using it for half a month now, it really saves power and the voltage is stable, it's very good	Jingdong prefecture level city in Shandong
Stowe Flagship Store	King of Power Saving	It's the ideal product 💞, good quality and fast logistics, worth the kudos!	Jingdong prefecture level city in Shandong
Stowe Flagship Store	King of Power Saving	The logistics is very fast, the baby is very good, the shopkeeper is NICE!	Jingdong prefecture level city in Shandong
Stowe Flagship Store	King of Power Saving	Good to use, good quality, save point durable	Jingdong prefecture level city in Shandong
Stowe Flagship Store	King of Power Saving	The quality is very good, customer service attitude is very, very awesome, there are problems in a timely manner to solve the problem	Jingdong prefecture level city in Shandong
Stowe Flagship Store	King of Power Saving	Quality is very good, the use of the effect is very good, more stable, quality assurance	Jingdong prefecture level city in Shandong
Stowe Flagship Store	King of Power Saving	Convenient, inexpensive, and see how it works out?	Jingdong prefecture level city in Shandong
Stowe Flagship Store	King of Power Saving	Very good quality, saves a lot of electricity, very good, worth buying	Jingdong prefecture level city in Shandong
Stowe Flagship Store	King of Power Saving	Power Saving King is easy to use, with the fruit of power saving to use easy to give!	Jingdong prefecture level city in Shandong
Stowe Flagship Store	King of Power Saving	This saver really works, good quality, safe and secure, very satisfied!	Jingdong prefecture level city in Shandong
Stowe Flagship Store	King of Power Saving	It works well, the voltage is very stable effect is good, the quality is also very good satisfaction	Jingdong prefecture level city in Shandong
Stowe Flagship Store	King of Power Saving	The logistics is super super fast! The quality is also very good, indeed value for money	Jingdong prefecture level city in Shandong
Stowe Flagship Store	King of Power Saving	I've only been using it for a month or so and it seems to be saving a lot of power	Jingdong prefecture level city in Shandong
Stowe Flagship Store	King of Power Saving	Very easy to operate, very good material, good value for money.	Jingdong prefecture level city in Shandong
Stowe Flagship Store	King of Power Saving	Aluminum alloy shell, durable, compact power-saving good effect	Jingdong prefecture level city in Shandong
Stowe Flagship Store	King of Power Saving	Nice device, easy to use, doesn't take up much space, recommendable!	Jingdong prefecture level city in Shandong
Stowe Flagship Store	King of Power Saving	Good quality, good price, fast logistics	Jingdong prefecture level city in Shandong
Stowe Flagship Store	King of Power Saving	Friends of the family in use recommended, the effect is good and recommendable!	Jingdong prefecture level city in Shandong
Stowe Flagship Store	King of Power Saving	Cheaper than brick and mortar stores worth buying is authentic	Jingdong prefecture level city in Shandong
Stowe Flagship Store	King of Power Saving	Merchants ship quickly, the price is cheaper than the physical store, good use	Jingdong prefecture level city in Shandong
Stowe Flagship Store	King of Power Saving	Very durable, affordable, fast shipping	Jingdong prefecture level city in Shandong
Stowe Flagship Store	King of Power Saving	Packaging tight quality is very good easy to use good results	Jingdong prefecture level city in Shandong
Stowe Flagship Store	King of Power Saving	Save the grid. It's in the whole house. It's saving money on the electric bill.	Jingdong prefecture level city in Shandong
Stowe Flagship Store	King of Power Saving	I used it today, it works well, I'm very satisfied	Jingdong prefecture level city in Shandong
Stowe Flagship Store	King of Power Saving	It really saves a lot of electricity after installation	Jingdong prefecture level city in Shandong
Stowe Flagship Store	King of Power Saving	Love it. Satisfied.	Jingdong prefecture level city in Shandong
Stowe Flagship Store	King of Power Saving	Doesn't feel like it's working.	Jingdong prefecture level city in Shandong
Stowe Flagship Store	King of Power Saving	I'll comment after using it.	Jingdong prefecture level city in Shandong
Stowe Flagship Store	King of Power Saving	I'm trying it out to see if it saves power, and I'll buy two more if it works well.	Jingdong prefecture level city in Shandong
Stowe Flagship Store	King of Power Saving	It is genuine very good, the price is much cheaper, cost-effective, really special cost-effective, it is worth buying!	Jingdong prefecture level city in Shandong
Stowe Flagship Store	King of Power Saving	This thing can really save electricity, I was introduced to buy by my friend, he used it very well, I used it and think it's also very good, it can really save electricity, save about half of the electricity every day, it's really good!	Jingdong prefecture level city in Shandong
Stowe Flagship Store	King of Power Saving	After using it for ten days or so, I checked the power consumption and it did drop, and customer service said it will save more electricity in the long run. The product is also insured, 5 years warranty do not have to worry about quality problems!	Jingdong prefecture level city in Shandong
Stowe Flagship Store	King of Power Saving	The product is good, before already bought a, this is the second, the effect is quite good, anyway, I see the daily electricity bill than before a lot less, this can be the choice!	Jingdong prefecture level city in Shandong
Stowe Flagship Store	King of Power Saving	Originally also very worried about things are not good, get the hand found completely over-anxious, five-star praise, the style is very generous, the quality is very good, the price is also very reasonable!	Jingdong prefecture level city in Shandong
Stowe Flagship Store	King of Power Saving	Quality is good, playing to the old family also buy a, repurchase, this quality is really good, recommended!	Jingdong prefecture level city in Shandong
Stowe Flagship Store	King of Power Saving	Thank you, the owner even sent two fuses.	Jingdong prefecture level city in Shandong
Stowe Flagship Store	King of Power Saving	This store has great stuff, relatively fast shipping and logistics	Jingdong prefecture level city in Shandong
Stowe Flagship Store	King of Power Saving	This product is based on the impossibility of saving electricity, I used 853 kWh of electricity in July, started using the product on August 1, and used 867 kWh of electricity by September 1, is this called	Jingdong prefecture level city in Shandong
Stowe Flagship Store	King of Power Saving	Power king received, to the home on the trial, when I plugged into the electric panel can not see the effect of power saving, the meter is still the same speed in the turn, I'll save the king of the manual to play look at the instructions, instructions to speak, have to have a day two days and half a month to save power obviously, then wait half a month to see the effect of power saving it! To half a month there are obvious power saving before continuing to chase Xu.	Jingdong prefecture level city in Shandong
Stowe Flagship Store	King of Power Saving	Received the goods, a look, did not see the manufacturer and production address, can only hold a try to see the mentality of the face	Jingdong prefecture level city in Shandong
Stowe Flagship Store	King of Power Saving	I've only been using it for a few days and I'm not sure if it's an IQ tax? Used to favorable reviews.	Jingdong prefecture level city in Shandong
Stowe Flagship Store	King of Power Saving	Appearance is okay, power saving next time to follow up the evaluation	Jingdong prefecture level city in Shandong
Stowe Flagship Store	King of Power Saving	Shorter time, but hopefully effective and power-saving, to be followed up in the future	Jingdong prefecture level city in Shandong
Stowe Flagship Store	King of Power Saving	Appearance is okay, I do not know if the power saving	Jingdong prefecture level city in Shandong
Stowe Flagship Store	King of Power Saving	Good service, fast logistics, Power Saving King is exquisite and beautiful, support the business, thank you!	Jingdong prefecture level city in Shandong
Stowe Flagship Store	King of Power Saving	Bought three at once to try first first.	Jingdong prefecture level city in Shandong
Stowe Flagship Store	King of Power Saving	The object is the same as the picture, the product is of high quality, the packaging is intact. High-end atmosphere, high power, power saving effect is obvious, average monthly power saving 50%, very satisfied.	Jingdong prefecture level city in Shandong
Stowe Flagship Store	King of Power Saving	Bought back and plugged in, really effective, before 16 degrees of electricity per day, now 8 degrees of electricity per day, open an air conditioner for 12 hours, decisive repurchase of a	Jingdong prefecture level city in Shandong
Stowe Flagship Store	King of Power Saving	The object is the same as the picture, the product is of high quality, and the package is intact. High-end atmosphere and high grade, high power	Jingdong prefecture level city in Shandong

Stowe Flagship Store	King of Power Saving	Goods received, I hope the store said can be energy-saving, first try a month, month after month to see the effect.	Jingdong prefecture level city in Shandong
Stowe Flagship Store	King of Power Saving	This is really good, the material and workmanship is particularly exquisite, the workmanship is really very good, I think I can recommend to my friends to buy	Jingdong prefecture level city in Shandong
Stowe Flagship Store	King of Power Saving	I have to praise, the quality is really good, and the packaging logistics are also good. Really like it, won't make you regret after getting it!	Jingdong prefecture level city in Shandong
Stowe Flagship Store	King of Power Saving	Reputable platform, efficient and professional service, buy with confidence and peace of mind!	Jingdong prefecture level city in Shandong
Stowe Flagship Store	King of Power Saving	Bought one on a trial basis to see if it works after 1-2 months.	Jingdong prefecture level city in Shandong
Stowe Flagship Store	King of Power Saving	Quality is very good, customer service attitude is very very praise, there are problems in a timely manner to solve the problem, the price is not expensive, super value, you can rest assured that the	Jingdong prefecture level city in Shandong
Stowe Flagship Store	King of Power Saving	With it no longer need to be afraid to use electricity, especially good, ready to buy a mom and dad that also	Jingdong prefecture level city in Shandong
Stowe Flagship Store	King of Power Saving	Baby received very like will return to buy the seller attitude is also very like will return to buy the seller attitude is also very like!	Jingdong prefecture level city in Shandong
Stowe Flagship Store	King of Power Saving	Jingdong Mall shopping convenience assured fast assured fast assured fast assured fast	Jingdong prefecture level city in Shandong
Stowe Flagship Store	King of Power Saving	Saving power received, the quality is very good, fine workmanship, especially good, much cheaper than the entity store, it is worth buying!	Jingdong prefecture level city in Shandong
Stowe Flagship Store	King of Power Saving	I did not expect the express delivery so quickly, very save point quality is super good, than the entity store is too cheap.	Jingdong prefecture level city in Shandong
Stowe Flagship Store	King of Power Saving	Have received the baby, very satisfied with a shopping, great worth buying!	Jingdong prefecture level city in Shandong
Stowe Flagship Store	King of Power Saving	This is really more powerful, very satisfied, more energy-saving, better, thank you boss.	Jingdong prefecture level city in Shandong
Stowe Flagship Store	King of Power Saving	Received has been used for two days, the air conditioning can boldly open it! Energy saving effect is really quite good	Jingdong prefecture level city in Shandong
Stowe Flagship Store	King of Power Saving	It is authentic, trust the merchant, the baby has a great texture and the price is moderate, unlimited repurchase!	Jingdong prefecture level city in Shandong
Stowe Flagship Store	King of Power Saving	It is genuine very good, the price is much cheaper, cost-effective, really special cost-effective, it is worth buying!	Jingdong prefecture level city in Shandong
Stowe Flagship Store	King of Power Saving	Very good quality, fine workmanship, low power consumption, long warranty, fast shipping, economical and practical	Jingdong prefecture level city in Shandong
Stowe Flagship Store	King of Power Saving	I've been using it for half a month now, it really saves power and the voltage is stable, it's very good	Jingdong prefecture level city in Shandong
Stowe Flagship Store	King of Power Saving	It's the ideal product 💝, good quality and fast logistics, worth the kudos!	Jingdong prefecture level city in Shandong
Stowe Flagship Store	King of Power Saving	The logistics is very fast, the baby is very good, the shopkeeper is NICE!	Jingdong prefecture level city in Shandong
Stowe Flagship Store	King of Power Saving	Good to use, good quality, save point durable	Jingdong prefecture level city in Shandong
Stowe Flagship Store	King of Power Saving	The quality is very good, customer service attitude is very, very awesome, there are problems in a timely manner to solve the problem	Jingdong prefecture level city in Shandong
Stowe Flagship Store	King of Power Saving	Quality is very good, the use of the effect is very good, more stable, quality assurance	Jingdong prefecture level city in Shandong
Stowe Flagship Store	King of Power Saving	Convenient, inexpensive, and see how it works out?	Jingdong prefecture level city in Shandong
Stowe Flagship Store	King of Power Saving	Very good quality, saves a lot of electricity, very good, worth buying	Jingdong prefecture level city in Shandong
Stowe Flagship Store	King of Power Saving	Power Saving King is easy to use, with the fruit of power saving to use easy to give!	Jingdong prefecture level city in Shandong
Stowe Flagship Store	King of Power Saving	This saver really works, good quality, safe and secure, very satisfied!	Jingdong prefecture level city in Shandong
Stowe Flagship Store	King of Power Saving	It works well, the voltage is very stable effect is good, the quality is also very good satisfaction	Jingdong prefecture level city in Shandong
Stowe Flagship Store	King of Power Saving	The logistics is super super fast! The quality is also very good, indeed value for money	Jingdong prefecture level city in Shandong
Stowe Flagship Store	King of Power Saving	I've only been using it for a month or so and it seems to be saving a lot of power	Jingdong prefecture level city in Shandong
Stowe Flagship Store	King of Power Saving	Very easy to operate, very good material, good value for money.	Jingdong prefecture level city in Shandong
Stowe Flagship Store	King of Power Saving	Aluminum alloy shell, durable, compact power-saving good effect	Jingdong prefecture level city in Shandong
Stowe Flagship Store	King of Power Saving	Nice device, easy to use, doesn't take up much space, recommendable!	Jingdong prefecture level city in Shandong
Stowe Flagship Store	King of Power Saving	Good quality, good price, fast logistics	Jingdong prefecture level city in Shandong
Stowe Flagship Store	King of Power Saving	Friends of the family in use recommended, the effect is good and recommendable!	Jingdong prefecture level city in Shandong
Stowe Flagship Store	King of Power Saving	Cheaper than brick and mortar stores worth buying is authentic	Jingdong prefecture level city in Shandong
Stowe Flagship Store	King of Power Saving	Merchants ship quickly, the price is cheaper than the physical store, good use	Jingdong prefecture level city in Shandong
Stowe Flagship Store	King of Power Saving	Very durable, affordable, fast shipping	Jingdong prefecture level city in Shandong
Stowe Flagship Store	King of Power Saving	Packaging tight quality is very good easy to use good results	Jingdong prefecture level city in Shandong
Stowe Flagship Store	King of Power Saving	Save the grid. It's in the whole house. It's saving money on the electric bill.	Jingdong prefecture level city in Shandong
Stowe Flagship Store	King of Power Saving	I used it today, it works well, I'm very satisfied	Jingdong prefecture level city in Shandong
Stowe Flagship Store	King of Power Saving	It really saves a lot of electricity after installation	Jingdong prefecture level city in Shandong
Stowe Flagship Store	King of Power Saving	Love it. Satisfied.	Jingdong prefecture level city in Shandong
Stowe Flagship Store	King of Power Saving	Doesn't feel like it's working.	Jingdong prefecture level city in Shandong
Stowe Flagship Store	King of Power Saving	I'll comment after using it.	Jingdong prefecture level city in Shandong
Stowe Flagship Store	King of Power Saving	I'm trying it out to see if it saves power, and I'll buy two more if it works well.	Jingdong prefecture level city in Shandong
Stowe Flagship Store	King of Power Saving	It is genuine very good, the price is much cheaper, cost-effective, really special cost-effective, it is worth buying!	Jingdong prefecture level city in Shandong
Stowe Flagship Store	King of Power Saving	This thing can really save electricity, I was introduced to buy by my friend, he used it very well, I used it and think it's also very good, it can really save electricity, save about half of the electricity every day, it's really good!	Jingdong prefecture level city in Shandong
Stowe Flagship Store	King of Power Saving	After using it for ten days or so, I checked the power consumption and it did drop, and customer service said it will save more electricity in the long run. The product is also insured, 5 years warranty do not have to worry about quality problems!	Jingdong prefecture level city in Shandong
Stowe Flagship Store	King of Power Saving	The product is good, before already bought a, this is the second, the effect is quite good, anyway, I see the daily electricity bill than before a lot less, this can be the choice!	Jingdong prefecture level city in Shandong

Stowe Flagship Store	King of Power Saving	Originally also very worried about things are not good, get the hand found completely over-anxious, five-star praise, the style is very generous, the quality is very good, the price is also very reasonable!	Jingdong prefecture level city in Shandong
Stowe Flagship Store	King of Power Saving	Quality is good, playing to the old family also buy a, repurchase, this quality is really good, recommended!	Jingdong prefecture level city in Shandong
Stowe Flagship Store	King of Power Saving	Thank you, the owner even sent two fuses.	Jingdong prefecture level city in Shandong
Stowe Flagship Store	King of Power Saving	This store has great stuff, relatively fast shipping and logistics	Jingdong prefecture level city in Shandong
Stowe Flagship Store	King of Power Saving	This product is based on the impossibility of saving electricity, I used 853 kWh of electricity in July, started using the product on August 1, and used 867 kWh of electricity by September 1, is this called	Jingdong prefecture level city in Shandong
Stowe Flagship Store	King of Power Saving	Power king received, to the home on the trial, when I plugged into the electric panel can not see the effect of power saving, the meter is still the same speed in the turn, I'll save the king of the manual to play look at the instructions, instructions to speak, have to have a day two days and half a month to save power obviously, then wait half a month to see the effect of power saving it! To half a month there are obvious power saving before continuing to chase Xu.	Jingdong prefecture level city in Shandong
Stowe Flagship Store	King of Power Saving	Received the goods, a look, did not see the manufacturer and production address, can only hold a try to see the mentality of the face	Jingdong prefecture level city in Shandong
Stowe Flagship Store	King of Power Saving	I've only been using it for a few days and I'm not sure if it's an IQ tax? Used to favorable reviews.	Jingdong prefecture level city in Shandong
Stowe Flagship Store	King of Power Saving	Appearance is okay, power saving next time to follow up the evaluation	Jingdong prefecture level city in Shandong
Stowe Flagship Store	King of Power Saving	Shorter time, but hopefully effective and power-saving, to be followed up in the future	Jingdong prefecture level city in Shandong
Stowe Flagship Store	King of Power Saving	Appearance is okay, I do not know if the power saving	Jingdong prefecture level city in Shandong
Stowe Flagship Store	King of Power Saving	Good service, fast logistics, Power Saving King is exquisite and beautiful, support the business, thank you!	Jingdong prefecture level city in Shandong
Stowe Flagship Store	King of Power Saving	Bought three at once to try first first.	Jingdong prefecture level city in Shandong
Stowe Flagship Store	King of Power Saving	The object is the same as the picture, the product is of high quality, the packaging is intact. High-end atmosphere, high power, power saving effect is obvious, average monthly power saving 50%, very satisfied.	Jingdong prefecture level city in Shandong
Stowe Flagship Store	King of Power Saving	Bought back and plugged in, really effective, before 16 degrees of electricity per day, now 8 degrees of electricity per day, open an air conditioner for 12 hours, decisive repurchase of a	Jingdong prefecture level city in Shandong
Stowe Flagship Store	King of Power Saving	The object is the same as the picture, the product is of high quality, and the package is intact. High-end atmosphere and high grade, high power	Jingdong prefecture level city in Shandong
Stowe Flagship Store	King of Power Saving	Goods received, I hope the store said can be energy-saving, first try a month, month after month to see the effect.	Jingdong prefecture level city in Shandong
Stowe Flagship Store	King of Power Saving	This is really good, the material and workmanship is particularly exquisite, the workmanship is really very good, I think I can recommend to my friends to buy	Jingdong prefecture level city in Shandong
Stowe Flagship Store	King of Power Saving	I have to praise, the quality is really good, and the packaging logistics are also good. Really like it, won't make you regret after getting it!	Jingdong prefecture level city in Shandong
Stowe Flagship Store	King of Power Saving	Reputable platform, efficient and professional service, buy with confidence and peace of mind!	Jingdong prefecture level city in Shandong
Stowe Flagship Store	King of Power Saving	Bought one on a trial basis to see if it works after 1-2 months.	Jingdong prefecture level city in Shandong
Stowe Flagship Store	King of Power Saving	Quality is very good, customer service attitude is very very praise, there are problems in a timely manner to solve the problem, the price is not expensive, super value, you can rest assured that the	Jingdong prefecture level city in Shandong
Stowe Flagship Store	King of Power Saving	With it no longer need to be afraid to use electricity, especially good, ready to buy a mom and dad that also	Jingdong prefecture level city in Shandong
Stowe Flagship Store	King of Power Saving	Baby received very like will return to buy the seller attitude is also very like will return to buy the seller attitude is also very like!	Jingdong prefecture level city in Shandong
Stowe Flagship Store	King of Power Saving	Jingdong Mall shopping convenience assured fast assured fast assured fast assured fast	Jingdong prefecture level city in Shandong
Stowe Flagship Store	King of Power Saving	Saving power received, the quality is very good, fine workmanship, especially good, much cheaper than the entity store, it is worth buying!	Jingdong prefecture level city in Shandong
Stowe Flagship Store	King of Power Saving	I did not expect the express delivery so quickly, very save point quality is super good, than the entity store is too cheap.	Jingdong prefecture level city in Shandong
Stowe Flagship Store	King of Power Saving	Have received the baby, very satisfied with a shopping, great worth buying!	Jingdong prefecture level city in Shandong
Stowe Flagship Store	King of Power Saving	This is really more powerful, very satisfied, more energy-saving, better, thank you boss.	Jingdong prefecture level city in Shandong
Stowe Flagship Store	King of Power Saving	Received has been used for two days, the air conditioning can boldly open it! Energy saving effect is really quite good	Jingdong prefecture level city in Shandong
Stowe Flagship Store	King of Power Saving	It is authentic, trust the merchant, the baby has a great texture and the price is moderate, unlimited repurchase!	Jingdong prefecture level city in Shandong
Stowe Flagship Store	King of Power Saving	It is genuine very good, the price is much cheaper, cost-effective, really special cost-effective, it is worth buying!	Jingdong prefecture level city in Shandong
Stowe Flagship Store	King of Power Saving	Very good quality, fine workmanship, low power consumption, long warranty, fast shipping, economical and practical	Jingdong prefecture level city in Shandong
Stowe Flagship Store	King of Power Saving	I've been using it for half a month now, it really saves power and the voltage is stable, it's very good	Jingdong prefecture level city in Shandong
Stowe Flagship Store	King of Power Saving	It's the ideal product, good quality and fast logistics, worth the kudos!	Jingdong prefecture level city in Shandong
Stowe Flagship Store	King of Power Saving	The logistics is very fast, the baby is very good, the shopkeeper is NICE!	Jingdong prefecture level city in Shandong
Stowe Flagship Store	King of Power Saving	Good to use, good quality, save point durable	Jingdong prefecture level city in Shandong
Stowe Flagship Store	King of Power Saving	The quality is very good, customer service attitude is very, very awesome, there are problems in a timely manner to solve the problem	Jingdong prefecture level city in Shandong
Stowe Flagship Store	King of Power Saving	Quality is very good, the use of the effect is very good, more stable, quality assurance	Jingdong prefecture level city in Shandong
Stowe Flagship Store	King of Power Saving	Convenient, inexpensive, and see how it works out?	Jingdong prefecture level city in Shandong
Stowe Flagship Store	King of Power Saving	Very good quality, saves a lot of electricity, very good, worth buying	Jingdong prefecture level city in Shandong
Stowe Flagship Store	King of Power Saving	Power Saving King is easy to use, with the fruit of power saving to use easy to give!	Jingdong prefecture level city in Shandong
Stowe Flagship Store	King of Power Saving	This saver really works, good quality, safe and secure, very satisfied!	Jingdong prefecture level city in Shandong
Stowe Flagship Store	King of Power Saving	It works well, the voltage is very stable effect is good, the quality is also very good satisfaction	Jingdong prefecture level city in Shandong
Stowe Flagship Store	King of Power Saving	The logistics is super super fast! The quality is also very good, indeed value for money	Jingdong prefecture level city in Shandong
Stowe Flagship Store	King of Power Saving	I've only been using it for a month or so and it seems to be saving a lot of power	Jingdong prefecture level city in Shandong
Stowe Flagship Store	King of Power Saving	Very easy to operate, very good material, good value for money.	Jingdong prefecture level city in Shandong
Stowe Flagship Store	King of Power Saving	Aluminum alloy shell, durable, compact power-saving good effect	Jingdong prefecture level city in Shandong
Stowe Flagship Store	King of Power Saving	Nice device, easy to use, doesn't take up much space, recommendable!	Jingdong prefecture level city in Shandong
Stowe Flagship Store	King of Power Saving	Good quality, good price, fast logistics	Jingdong prefecture level city in Shandong

Stowe Flagship Store	King of Power Saving	Friends of the family in use recommended, the effect is good and recommendable!	Jingdong prefecture level city in Shandong
Stowe Flagship Store	King of Power Saving	Cheaper than brick and mortar stores worth buying is authentic	Jingdong prefecture level city in Shandong
Stowe Flagship Store	King of Power Saving	Merchants ship quickly, the price is cheaper than the physical store, good use	Jingdong prefecture level city in Shandong
Stowe Flagship Store	King of Power Saving	Very durable, affordable, fast shipping	Jingdong prefecture level city in Shandong
Stowe Flagship Store	King of Power Saving	Packaging tight quality is very good easy to use good results	Jingdong prefecture level city in Shandong
Stowe Flagship Store	King of Power Saving	Save the grid. It's in the whole house. It's saving money on the electric bill.	Jingdong prefecture level city in Shandong
Stowe Flagship Store	King of Power Saving	I used it today, it works well, I'm very satisfied	Jingdong prefecture level city in Shandong
Stowe Flagship Store	King of Power Saving	It really saves a lot of electricity after installation	Jingdong prefecture level city in Shandong
Stowe Flagship Store	King of Power Saving	Love it. Satisfied.	Jingdong prefecture level city in Shandong
Stowe Flagship Store	King of Power Saving	Doesn't feel like it's working.	Jingdong prefecture level city in Shandong
Stowe Flagship Store	King of Power Saving	I'll comment after using it.	Jingdong prefecture level city in Shandong
Stowe Flagship Store	King of Power Saving	I'm trying it out to see if it saves power, and I'll buy two more if it works well.	Jingdong prefecture level city in Shandong
Stowe Flagship Store	King of Power Saving	It is genuine very good, the price is much cheaper, cost-effective, really special cost-effective, it is worth buying!	Jingdong prefecture level city in Shandong
Stowe Flagship Store	King of Power Saving	This thing can really save electricity, I was introduced to buy by my friend, he used it very well, I used it and think it's also very good, it can really save electricity, save about half of the electricity every day, it's really good!	Jingdong prefecture level city in Shandong
Stowe Flagship Store	King of Power Saving	After using it for ten days or so, I checked the power consumption and it did drop, and customer service said it will save more electricity in the long run. The product is also insured, 5 years warranty do not have to worry about quality problems!	Jingdong prefecture level city in Shandong
Stowe Flagship Store	King of Power Saving	The product is good, before already bought a, this is the second, the effect is quite good, anyway, I see the daily electricity bill than before a lot less, this can be the choice!	Jingdong prefecture level city in Shandong
Stowe Flagship Store	King of Power Saving	Originally also very worried about things are not good, get the hand found completely over-anxious, five-star praise, the style is very generous, the quality is very good, the price is also very reasonable!	Jingdong prefecture level city in Shandong
Stowe Flagship Store	King of Power Saving	Quality is good, playing to the old family also buy a, repurchase, this quality is really good, recommended!	Jingdong prefecture level city in Shandong
Stowe Flagship Store	King of Power Saving	Thank you, the owner even sent two fuses.	Jingdong prefecture level city in Shandong
Stowe Flagship Store	King of Power Saving	This store has great stuff, relatively fast shipping and logistics	Jingdong prefecture level city in Shandong
Stowe Flagship Store	King of Power Saving	This product is based on the impossibility of saving electricity, I used 853 kWh of electricity in July, started using the product on August 1, and used 867 kWh of electricity by September 1, is this called	Jingdong prefecture level city in Shandong
Stowe Flagship Store	King of Power Saving	Power king received, to the home on the trial, when I plugged into the electric panel can not see the effect of power saving, the meter is still the same speed in the turn, I'll save the king of the manual to play look at the instructions, instructions to speak, have to have a day two days and half a month to save power obviously, then wait half a month to see the effect of power saving it! To half a month there are obvious power saving before continuing to chase Xu.	Jingdong prefecture level city in Shandong
Stowe Flagship Store	King of Power Saving	Received the goods, a look, did not see the manufacturer and production address, can only hold a try to see the mentality of the face	Jingdong prefecture level city in Shandong
Stowe Flagship Store	King of Power Saving	I've only been using it for a few days and I'm not sure if it's an IQ tax? Used to favorable reviews.	Jingdong prefecture level city in Shandong
Stowe Flagship Store	King of Power Saving	Appearance is okay, power saving next time to follow up the evaluation	Jingdong prefecture level city in Shandong
Stowe Flagship Store	King of Power Saving	Shorter time, but hopefully effective and power-saving, to be followed up in the future	Jingdong prefecture level city in Shandong
Stowe Flagship Store	King of Power Saving	Appearance is okay, I do not know if the power saving	Jingdong prefecture level city in Shandong
Stowe Flagship Store	King of Power Saving	Good service, fast logistics, Power Saving King is exquisite and beautiful, support the business, thank you!	Jingdong prefecture level city in Shandong
Stowe Flagship Store	King of Power Saving	Bought three at once to try first first.	Jingdong prefecture level city in Shandong
Stowe Flagship Store	King of Power Saving	The object is the same as the picture, the product is of high quality, the packaging is intact. High-end atmosphere, high power, power saving effect is obvious, average monthly power saving 50%, very satisfied.	Jingdong prefecture level city in Shandong
Stowe Flagship Store	King of Power Saving	Bought back and plugged in, really effective, before 16 degrees of electricity per day, now 8 degrees of electricity per day, open an air conditioner for 12 hours, decisive repurchase of a	Jingdong prefecture level city in Shandong
Stowe Flagship Store	King of Power Saving	The object is the same as the picture, the product is of high quality, and the package is intact. High-end atmosphere and high grade, high power	Jingdong prefecture level city in Shandong
Stowe Flagship Store	King of Power Saving	Goods received, I hope the store said can be energy-saving, first try a month, month after month to see the effect.	Jingdong prefecture level city in Shandong
Stowe Flagship Store	King of Power Saving	This is really good, the material and workmanship is particularly exquisite, the workmanship is really very good, I think I can recommend to my friends to buy	Jingdong prefecture level city in Shandong
Stowe Flagship Store	King of Power Saving	I have to praise, the quality is really good, and the packaging logistics are also good. Really like it, won't make you regret after getting it!	Jingdong prefecture level city in Shandong
Stowe Flagship Store	King of Power Saving	Reputable platform, efficient and professional service, buy with confidence and peace of mind!	Jingdong prefecture level city in Shandong
Stowe Flagship Store	King of Power Saving	Bought one on a trial basis to see if it works after 1-2 months.	Jingdong prefecture level city in Shandong
Stowe Flagship Store	King of Power Saving	Quality is very good, customer service attitude is very very praise, there are problems in a timely manner to solve the problem, the price is not expensive, super value, you can rest assured that the	Jingdong prefecture level city in Shandong
Stowe Flagship Store	King of Power Saving	With it no longer need to be afraid to use electricity, especially good, ready to buy a mom and dad that also	Jingdong prefecture level city in Shandong
Stowe Flagship Store	King of Power Saving	Baby received very like will return to buy the seller attitude is also very like will return to buy the seller attitude is also very like!	Jingdong prefecture level city in Shandong
Stowe Flagship Store	King of Power Saving	Jingdong Mall shopping convenience assured fast assured fast assured fast assured fast	Jingdong prefecture level city in Shandong
Stowe Flagship Store	King of Power Saving	Saving power received, the quality is very good, fine workmanship, especially good, much cheaper than the entity store, it is worth buying!	Jingdong prefecture level city in Shandong
Stowe Flagship Store	King of Power Saving	I did not expect the express delivery so quickly, very save point quality is super good, than the entity store is too cheap.	Jingdong prefecture level city in Shandong
Stowe Flagship Store	King of Power Saving	Have received the baby, very satisfied with a shopping, great worth buying!	Jingdong prefecture level city in Shandong
Stowe Flagship Store	King of Power Saving	This is really more powerful, very satisfied, more energy-saving, better, thank you boss.	Jingdong prefecture level city in Shandong
Stowe Flagship Store	King of Power Saving	Received has been used for two days, the air conditioning can boldly open it! Energy saving effect is really quite good	Jingdong prefecture level city in Shandong
Stowe Flagship Store	King of Power Saving	It is authentic, trust the merchant, the baby has a great texture and the price is moderate, unlimited repurchase!	Jingdong prefecture level city in Shandong
Stowe Flagship Store	King of Power Saving	It is genuine very good, the price is much cheaper, cost-effective, really special cost-effective, it is worth buying!	Jingdong prefecture level city in Shandong
Stowe Flagship Store	King of Power Saving	Very good quality, fine workmanship, low power consumption, long warranty, fast shipping, economical and practical	Jingdong prefecture level city in Shandong

Stowe Flagship Store	King of Power Saving	I've been using it for half a month now, it really saves power and the voltage is stable, it's very good	Jingdong prefecture level city in Shandong
Stowe Flagship Store	King of Power Saving	It's the ideal product 💝, good quality and fast logistics, worth the kudos!	Jingdong prefecture level city in Shandong
Stowe Flagship Store	King of Power Saving	The logistics is very fast, the baby is very good, the shopkeeper is NICE!	Jingdong prefecture level city in Shandong
Stowe Flagship Store	King of Power Saving	Good to use, good quality, save point durable	Jingdong prefecture level city in Shandong
Stowe Flagship Store	King of Power Saving	The quality is very good, customer service attitude is very, very awesome, there are problems in a timely manner to solve the problem	Jingdong prefecture level city in Shandong
Stowe Flagship Store	King of Power Saving	Quality is very good, the use of the effect is very good, more stable, quality assurance	Jingdong prefecture level city in Shandong
Stowe Flagship Store	King of Power Saving	Convenient, inexpensive, and see how it works out?	Jingdong prefecture level city in Shandong
Stowe Flagship Store	King of Power Saving	Very good quality, saves a lot of electricity, very good, worth buying	Jingdong prefecture level city in Shandong
Stowe Flagship Store	King of Power Saving	Power Saving King is easy to use, with the fruit of power saving to use easy to give!	Jingdong prefecture level city in Shandong
Stowe Flagship Store	King of Power Saving	This saver really works, good quality, safe and secure, very satisfied!	Jingdong prefecture level city in Shandong
Stowe Flagship Store	King of Power Saving	It works well, the voltage is very stable effect is good, the quality is also very good satisfaction	Jingdong prefecture level city in Shandong
Stowe Flagship Store	King of Power Saving	The logistics is super super fast! The quality is also very good, indeed value for money	Jingdong prefecture level city in Shandong
Stowe Flagship Store	King of Power Saving	I've only been using it for a month or so and it seems to be saving a lot of power	Jingdong prefecture level city in Shandong
Stowe Flagship Store	King of Power Saving	Very easy to operate, very good material, good value for money.	Jingdong prefecture level city in Shandong
Stowe Flagship Store	King of Power Saving	Aluminum alloy shell, durable, compact power-saving good effect	Jingdong prefecture level city in Shandong
Stowe Flagship Store	King of Power Saving	Nice device, easy to use, doesn't take up much space, recommendable!	Jingdong prefecture level city in Shandong
Stowe Flagship Store	King of Power Saving	Good quality, good price, fast logistics	Jingdong prefecture level city in Shandong
Stowe Flagship Store	King of Power Saving	Friends of the family in use recommended, the effect is good and recommendable!	Jingdong prefecture level city in Shandong
Stowe Flagship Store	King of Power Saving	Cheaper than brick and mortar stores worth buying is authentic	Jingdong prefecture level city in Shandong
Stowe Flagship Store	King of Power Saving	Merchants ship quickly, the price is cheaper than the physical store, good use	Jingdong prefecture level city in Shandong
Stowe Flagship Store	King of Power Saving	Very durable, affordable, fast shipping	Jingdong prefecture level city in Shandong
Stowe Flagship Store	King of Power Saving	Packaging tight quality is very good easy to use good results	Jingdong prefecture level city in Shandong
Stowe Flagship Store	King of Power Saving	Save the grid. It's in the whole house. It's saving money on the electric bill.	Jingdong prefecture level city in Shandong
Stowe Flagship Store	King of Power Saving	I used it today, it works well, I'm very satisfied	Jingdong prefecture level city in Shandong
Stowe Flagship Store	King of Power Saving	It really saves a lot of electricity after installation	Jingdong prefecture level city in Shandong
Stowe Flagship Store	King of Power Saving	Love it. Satisfied.	Jingdong prefecture level city in Shandong
Stowe Flagship Store	King of Power Saving	Doesn't feel like it's working.	Jingdong prefecture level city in Shandong
Stowe Flagship Store	King of Power Saving	I'll comment after using it.	Jingdong prefecture level city in Shandong
Stowe Flagship Store	King of Power Saving	I'm trying it out to see if it saves power, and I'll buy two more if it works well.	Jingdong prefecture level city in Shandong
Stowe Flagship Store	King of Power Saving	It is genuine very good, the price is much cheaper, cost-effective, really special cost-effective, it is worth buying!	Jingdong prefecture level city in Shandong
Stowe Flagship Store	King of Power Saving	This thing can really save electricity, I was introduced to buy by my friend, he used it very well, I used it and think it's also very good, it can really save electricity, save about half of the electricity every day, it's really good!	Jingdong prefecture level city in Shandong
Stowe Flagship Store	King of Power Saving	After using it for ten days or so, I checked the power consumption and it did drop, and customer service said it will save more electricity in the long run. The product is also insured, 5 years warranty do not have to worry about quality problems!	Jingdong prefecture level city in Shandong
Stowe Flagship Store	King of Power Saving	The product is good, before already bought a, this is the second, the effect is quite good, anyway, I see the daily electricity bill than before a lot less, this can be the choice!	Jingdong prefecture level city in Shandong
Stowe Flagship Store	King of Power Saving	Originally also very worried about things are not good, get the hand found completely over-anxious, five-star praise, the style is very generous, the quality is very good, the price is also very reasonable!	Jingdong prefecture level city in Shandong
Stowe Flagship Store	King of Power Saving	Quality is good, playing to the old family also buy a, repurchase, this quality is really good, recommended!	Jingdong prefecture level city in Shandong
Stowe Flagship Store	King of Power Saving	Thank you, the owner even sent two fuses.	Jingdong prefecture level city in Shandong
Stowe Flagship Store	King of Power Saving	This store has great stuff, relatively fast shipping and logistics	Jingdong prefecture level city in Shandong
Stowe Flagship Store	King of Power Saving	This product is based on the impossibility of saving electricity, I used 853 kWh of electricity in July, started using the product on August 1, and used 867 kWh of electricity by September 1, is this called	Jingdong prefecture level city in Shandong
Stowe Flagship Store	King of Power Saving	Power king received, to the home on the trial, when I plugged into the electric panel can not see the effect of power saving, the meter is still the same speed in the turn, I'll save the king of the manual to play look at the instructions, instructions to speak, have to have a day two days and half a month to save power obviously, then wait half a month to see the effect of power saving it! To half a month there are obvious power saving before continuing to chase Xu.	Jingdong prefecture level city in Shandong
Stowe Flagship Store	King of Power Saving	Received the goods, a look, did not see the manufacturer and production address, can only hold a try to see the mentality of the face	Jingdong prefecture level city in Shandong
Stowe Flagship Store	King of Power Saving	I've only been using it for a few days and I'm not sure if it's an IQ tax? Used to favorable reviews.	Jingdong prefecture level city in Shandong
Stowe Flagship Store	King of Power Saving	Appearance is okay, power saving next time to follow up the evaluation	Jingdong prefecture level city in Shandong
Stowe Flagship Store	King of Power Saving	Shorter time, but hopefully effective and power-saving, to be followed up in the future	Jingdong prefecture level city in Shandong
Stowe Flagship Store	King of Power Saving	Appearance is okay, I do not know if the power saving	Jingdong prefecture level city in Shandong
Stowe Flagship Store	King of Power Saving	Good service, fast logistics, Power Saving King is exquisite and beautiful, support the business, thank you!	Jingdong prefecture level city in Shandong
Stowe Flagship Store	King of Power Saving	Bought three at once to try first first.	Jingdong prefecture level city in Shandong
Stowe Flagship Store	King of Power Saving	The object is the same as the picture, the product is of high quality, the packaging is intact. High-end atmosphere, high power, power saving effect is obvious, average monthly power saving 50%, very satisfied.	Jingdong prefecture level city in Shandong
Stowe Flagship Store	King of Power Saving	Bought back and plugged in, really effective, before 16 degrees of electricity per day, now 8 degrees of electricity per day, open an air conditioner for 12 hours, decisive repurchase of a	Jingdong prefecture level city in Shandong
Stowe Flagship Store	King of Power Saving	The object is the same as the picture, the product is of high quality, and the package is intact. High-end atmosphere and high grade, high power	Jingdong prefecture level city in Shandong

Stowe Flagship Store	King of Power Saving	Goods received, I hope the store said can be energy-saving, first try a month, month after month to see the effect.	Jingdong prefecture level city in Shandong
Stowe Flagship Store	King of Power Saving	This is really good, the material and workmanship is particularly exquisite, the workmanship is really very good, I think I can recommend to my friends to buy	Jingdong prefecture level city in Shandong
Stowe Flagship Store	King of Power Saving	I have to praise, the quality is really good, and the packaging logistics are also good. Really like it, won't make you regret after getting it!	Jingdong prefecture level city in Shandong
Stowe Flagship Store	King of Power Saving	Reputable platform, efficient and professional service, buy with confidence and peace of mind!	Jingdong prefecture level city in Shandong
Stowe Flagship Store	King of Power Saving	Bought one on a trial basis to see if it works after 1-2 months.	Jingdong prefecture level city in Shandong
Stowe Flagship Store	King of Power Saving	Quality is very good, customer service attitude is very very praise, there are problems in a timely manner to solve the problem, the price is not expensive, super value, you can rest assured that the	Jingdong prefecture level city in Shandong
Stowe Flagship Store	King of Power Saving	With it no longer need to be afraid to use electricity, especially good, ready to buy a mom and dad that also	Jingdong prefecture level city in Shandong
Stowe Flagship Store	King of Power Saving	Baby received very like will return to buy the seller attitude is also very like will return to buy the seller attitude is also very like!	Jingdong prefecture level city in Shandong
Stowe Flagship Store	King of Power Saving	Jingdong Mall shopping convenience assured fast assured fast assured fast assured fast	Jingdong prefecture level city in Shandong
Stowe Flagship Store	King of Power Saving	Saving power received, the quality is very good, fine workmanship, especially good, much cheaper than the entity store, it is worth buying!	Jingdong prefecture level city in Shandong
Stowe Flagship Store	King of Power Saving	I did not expect the express delivery so quickly, very save point quality is super good, than the entity store is too cheap.	Jingdong prefecture level city in Shandong
Stowe Flagship Store	King of Power Saving	Have received the baby, very satisfied with a shopping, great worth buying!	Jingdong prefecture level city in Shandong
Stowe Flagship Store	King of Power Saving	This is really more powerful, very satisfied, more energy-saving, better, thank you boss.	Jingdong prefecture level city in Shandong
Stowe Flagship Store	King of Power Saving	Received has been used for two days, the air conditioning can boldly open it! Energy saving effect is really quite good	Jingdong prefecture level city in Shandong
Stowe Flagship Store	King of Power Saving	It is authentic, trust the merchant, the baby has a great texture and the price is moderate, unlimited repurchase!	Jingdong prefecture level city in Shandong
Stowe Flagship Store	King of Power Saving	It is genuine very good, the price is much cheaper, cost-effective, really special cost-effective, it is worth buying!	Jingdong prefecture level city in Shandong
Stowe Flagship Store	King of Power Saving	Very good quality, fine workmanship, low power consumption, long warranty, fast shipping, economical and practical	Jingdong prefecture level city in Shandong
Stowe Flagship Store	King of Power Saving	I've been using it for half a month now, it really saves power and the voltage is stable, it's very good	Jingdong prefecture level city in Shandong
Stowe Flagship Store	King of Power Saving	It's the ideal product, good quality and fast logistics, worth the kudos!	Jingdong prefecture level city in Shandong
Stowe Flagship Store	King of Power Saving	The logistics is very fast, the baby is very good, the shopkeeper is NICE!	Jingdong prefecture level city in Shandong
Stowe Flagship Store	King of Power Saving	Good to use, good quality, save point durable	Jingdong prefecture level city in Shandong
Stowe Flagship Store	King of Power Saving	The quality is very good, customer service attitude is very, very awesome, there are problems in a timely manner to solve the problem	Jingdong prefecture level city in Shandong
Stowe Flagship Store	King of Power Saving	Quality is very good, the use of the effect is very good, more stable, quality assurance	Jingdong prefecture level city in Shandong
Stowe Flagship Store	King of Power Saving	Convenient, inexpensive, and see how it works out?	Jingdong prefecture level city in Shandong
Stowe Flagship Store	King of Power Saving	Very good quality, saves a lot of electricity, very good, worth buying	Jingdong prefecture level city in Shandong
Stowe Flagship Store	King of Power Saving	Power Saving King is easy to use, with the fruit of power saving to use easy to give!	Jingdong prefecture level city in Shandong
Stowe Flagship Store	King of Power Saving	This saver really works, good quality, safe and secure, very satisfied!	Jingdong prefecture level city in Shandong
Stowe Flagship Store	King of Power Saving	It works well, the voltage is very stable effect is good, the quality is also very good satisfaction	Jingdong prefecture level city in Shandong
Stowe Flagship Store	King of Power Saving	The logistics is super super fast! The quality is also very good, indeed value for money	Jingdong prefecture level city in Shandong
Stowe Flagship Store	King of Power Saving	I've only been using it for a month or so and it seems to be saving a lot of power	Jingdong prefecture level city in Shandong
Stowe Flagship Store	King of Power Saving	Very easy to operate, very good material, good value for money.	Jingdong prefecture level city in Shandong
Stowe Flagship Store	King of Power Saving	Aluminum alloy shell, durable, compact power-saving good effect	Jingdong prefecture level city in Shandong
Stowe Flagship Store	King of Power Saving	Nice device, easy to use, doesn't take up much space, recommendable!	Jingdong prefecture level city in Shandong
Stowe Flagship Store	King of Power Saving	Good quality, good price, fast logistics	Jingdong prefecture level city in Shandong
Stowe Flagship Store	King of Power Saving	Friends of the family in use recommended, the effect is good and recommendable!	Jingdong prefecture level city in Shandong
Stowe Flagship Store	King of Power Saving	Cheaper than brick and mortar stores worth buying is authentic	Jingdong prefecture level city in Shandong
Stowe Flagship Store	King of Power Saving	Merchants ship quickly, the price is cheaper than the physical store, good use	Jingdong prefecture level city in Shandong
Stowe Flagship Store	King of Power Saving	Very durable, affordable, fast shipping	Jingdong prefecture level city in Shandong
Stowe Flagship Store	King of Power Saving	Packaging tight quality is very good easy to use good results	Jingdong prefecture level city in Shandong
Stowe Flagship Store	King of Power Saving	Save the grid. It's in the whole house. It's saving money on the electric bill.	Jingdong prefecture level city in Shandong
Stowe Flagship Store	King of Power Saving	I used it today, it works well, I'm very satisfied	Jingdong prefecture level city in Shandong
Stowe Flagship Store	King of Power Saving	It really saves a lot of electricity after installation	Jingdong prefecture level city in Shandong
Stowe Flagship Store	King of Power Saving	Love it. Satisfied.	Jingdong prefecture level city in Shandong
Stowe Flagship Store	King of Power Saving	Doesn't feel like it's working.	Jingdong prefecture level city in Shandong
Stowe Flagship Store	King of Power Saving	I'll comment after using it.	Jingdong prefecture level city in Shandong
Stowe Flagship Store	King of Power Saving	I'm trying it out to see if it saves power, and I'll buy two more if it works well.	Jingdong prefecture level city in Shandong
Stowe Flagship Store	King of Power Saving	It is genuine very good, the price is much cheaper, cost-effective, really special cost-effective, it is worth buying!	Jingdong prefecture level city in Shandong
Stowe Flagship Store	King of Power Saving	This thing can really save electricity, I was introduced to buy by my friend, he used it very well, I used it and think it's also very good, it can really save electricity, save about half of the electricity every day, it's really good!	Jingdong prefecture level city in Shandong
Stowe Flagship Store	King of Power Saving	After using it for ten days or so, I checked the power consumption and it did drop, and customer service said it will save more electricity in the long run. The product is also insured, 5 years warranty do not have to worry about quality problems!	Jingdong prefecture level city in Shandong
Stowe Flagship Store	King of Power Saving	The product is good, before already bought a, this is the second, the effect is quite good, anyway, I see the daily electricity bill than before a lot less, this can be the choice!	Jingdong prefecture level city in Shandong

Stowe Flagship Store	King of Power Saving	Originally also very worried about things are not good, get the hand found completely over-anxious, five-star praise, the style is very generous, the quality is very good, the price is also very reasonable!	Jingdong prefecture level city in Shandong
Stowe Flagship Store	King of Power Saving	Quality is good, playing to the old family also buy a, repurchase, this quality is really good, recommended!	Jingdong prefecture level city in Shandong
Stowe Flagship Store	King of Power Saving	Thank you, the owner even sent two fuses.	Jingdong prefecture level city in Shandong
Stowe Flagship Store	King of Power Saving	This store has great stuff, relatively fast shipping and logistics	Jingdong prefecture level city in Shandong
Stowe Flagship Store	King of Power Saving	This product is based on the impossibility of saving electricity, I used 853 kWh of electricity in July, started using the product on August 1, and used 867 kWh of electricity by September 1, is this called	Jingdong prefecture level city in Shandong
Stowe Flagship Store	King of Power Saving	Power king received, to the home on the trial, when I plugged into the electric panel can not see the effect of power saving, the meter is still the same speed in the turn, I'll save the king of the manual to play look at the instructions, instructions to speak, have to have a day two days and half a month to save power obviously, then wait half a month to see the effect of power saving it! To half a month there are obvious power saving before continuing to chase Xu.	Jingdong prefecture level city in Shandong
Stowe Flagship Store	King of Power Saving	Received the goods, a look, did not see the manufacturer and production address, can only hold a try to see the mentality of the face	Jingdong prefecture level city in Shandong
Stowe Flagship Store	King of Power Saving	I've only been using it for a few days and I'm not sure if it's an IQ tax? Used to favorable reviews.	Jingdong prefecture level city in Shandong
Stowe Flagship Store	King of Power Saving	Appearance is okay, power saving next time to follow up the evaluation	Jingdong prefecture level city in Shandong
Stowe Flagship Store	King of Power Saving	Shorter time, but hopefully effective and power-saving, to be followed up in the future	Jingdong prefecture level city in Shandong
Stowe Flagship Store	King of Power Saving	Appearance is okay, I do not know if the power saving	Jingdong prefecture level city in Shandong
Stowe Flagship Store	King of Power Saving	Good service, fast logistics, Power Saving King is exquisite and beautiful, support the business, thank you!	Jingdong prefecture level city in Shandong
Stowe Flagship Store	King of Power Saving	Bought three at once to try first first.	Jingdong prefecture level city in Shandong
Stowe Flagship Store	King of Power Saving	The object is the same as the picture, the product is of high quality, the packaging is intact. High-end atmosphere, high power, power saving effect is obvious, average monthly power saving 50%, very satisfied.	Jingdong prefecture level city in Shandong
Stowe Flagship Store	King of Power Saving	Bought back and plugged in, really effective, before 16 degrees of electricity per day, now 8 degrees of electricity per day, open an air conditioner for 12 hours, decisive repurchase of a	Jingdong prefecture level city in Shandong
Stowe Flagship Store	King of Power Saving	The object is the same as the picture, the product is of high quality, and the package is intact. High-end atmosphere and high grade, high power	Jingdong prefecture level city in Shandong
Stowe Flagship Store	King of Power Saving	Goods received, I hope the store said can be energy-saving, first try a month, month after month to see the effect.	Jingdong prefecture level city in Shandong
Stowe Flagship Store	King of Power Saving	This is really good, the material and workmanship is particularly exquisite, the workmanship is really very good, I think I can recommend to my friends to buy	Jingdong prefecture level city in Shandong
Stowe Flagship Store	King of Power Saving	I have to praise, the quality is really good, and the packaging logistics are also good. Really like it, won't make you regret after getting it!	Jingdong prefecture level city in Shandong
Stowe Flagship Store	King of Power Saving	Reputable platform, efficient and professional service, buy with confidence and peace of mind!	Jingdong prefecture level city in Shandong
Stowe Flagship Store	King of Power Saving	Bought one on a trial basis to see if it works after 1-2 months.	Jingdong prefecture level city in Shandong
Stowe Flagship Store	King of Power Saving	Quality is very good, customer service attitude is very very praise, there are problems in a timely manner to solve the problem, the price is not expensive, super value, you can rest assured that the	Jingdong prefecture level city in Shandong
Stowe Flagship Store	King of Power Saving	With it no longer need to be afraid to use electricity, especially good, ready to buy a mom and dad that also	Jingdong prefecture level city in Shandong
Stowe Flagship Store	King of Power Saving	Baby received very like will return to buy the seller attitude is also very like will return to buy the seller attitude is also very like!	Jingdong prefecture level city in Shandong
Stowe Flagship Store	King of Power Saving	Jingdong Mall shopping convenience assured fast assured fast assured fast assured fast	Jingdong prefecture level city in Shandong
Stowe Flagship Store	King of Power Saving	Saving power received, the quality is very good, fine workmanship, especially good, much cheaper than the entity store, it is worth buying!	Jingdong prefecture level city in Shandong
Stowe Flagship Store	King of Power Saving	I did not expect the express delivery so quickly, very save point quality is super good, than the entity store is too cheap.	Jingdong prefecture level city in Shandong
Stowe Flagship Store	King of Power Saving	Have received the baby, very satisfied with a shopping, great worth buying!	Jingdong prefecture level city in Shandong
Stowe Flagship Store	King of Power Saving	This is really more powerful, very satisfied, more energy-saving, better, thank you boss.	Jingdong prefecture level city in Shandong
Stowe Flagship Store	King of Power Saving	Received has been used for two days, the air conditioning can boldly open it! Energy saving effect is really quite good	Jingdong prefecture level city in Shandong
Stowe Flagship Store	King of Power Saving	It is authentic, trust the merchant, the baby has a great texture and the price is moderate, unlimited repurchase!	Jingdong prefecture level city in Shandong
Stowe Flagship Store	King of Power Saving	It is genuine very good, the price is much cheaper, cost-effective, really special cost-effective, it is worth buying!	Jingdong prefecture level city in Shandong
Stowe Flagship Store	King of Power Saving	Very good quality, fine workmanship, low power consumption, long warranty, fast shipping, economical and practical	Jingdong prefecture level city in Shandong
Stowe Flagship Store	King of Power Saving	I've been using it for half a month now, it really saves power and the voltage is stable, it's very good	Jingdong prefecture level city in Shandong
Stowe Flagship Store	King of Power Saving	It's the ideal product, good quality and fast logistics, worth the kudos!	Jingdong prefecture level city in Shandong
Stowe Flagship Store	King of Power Saving	The logistics is very fast, the baby is very good, the shopkeeper is NICE!	Jingdong prefecture level city in Shandong
Stowe Flagship Store	King of Power Saving	Good to use, good quality, save point durable	Jingdong prefecture level city in Shandong
Stowe Flagship Store	King of Power Saving	The quality is very good, customer service attitude is very, very awesome, there are problems in a timely manner to solve the problem	Jingdong prefecture level city in Shandong
Stowe Flagship Store	King of Power Saving	Quality is very good, the use of the effect is very good, more stable, quality assurance	Jingdong prefecture level city in Shandong
Stowe Flagship Store	King of Power Saving	Convenient, inexpensive, and see how it works out?	Jingdong prefecture level city in Shandong
Stowe Flagship Store	King of Power Saving	Very good quality, saves a lot of electricity, very good, worth buying	Jingdong prefecture level city in Shandong
Stowe Flagship Store	King of Power Saving	Power Saving King is easy to use, with the fruit of power saving to use easy to give!	Jingdong prefecture level city in Shandong
Stowe Flagship Store	King of Power Saving	This saver really works, good quality, safe and secure, very satisfied!	Jingdong prefecture level city in Shandong
Stowe Flagship Store	King of Power Saving	It works well, the voltage is very stable effect is good, the quality is also very good satisfaction	Jingdong prefecture level city in Shandong
Stowe Flagship Store	King of Power Saving	The logistics is super super fast! The quality is also very good, indeed value for money	Jingdong prefecture level city in Shandong
Stowe Flagship Store	King of Power Saving	I've only been using it for a month or so and it seems to be saving a lot of power	Jingdong prefecture level city in Shandong
Stowe Flagship Store	King of Power Saving	Very easy to operate, very good material, good value for money.	Jingdong prefecture level city in Shandong
Stowe Flagship Store	King of Power Saving	Aluminum alloy shell, durable, compact power-saving good effect	Jingdong prefecture level city in Shandong
Stowe Flagship Store	King of Power Saving	Nice device, easy to use, doesn't take up much space, recommendable!	Jingdong prefecture level city in Shandong
Stowe Flagship Store	King of Power Saving	Good quality, good price, fast logistics	Jingdong prefecture level city in Shandong

Stowe Flagship Store	King of Power Saving	Friends of the family in use recommended, the effect is good and recommendable!	Jingdong prefecture level city in Shandong
Stowe Flagship Store	King of Power Saving	Cheaper than brick and mortar stores worth buying is authentic	Jingdong prefecture level city in Shandong
Stowe Flagship Store	King of Power Saving	Merchants ship quickly, the price is cheaper than the physical store, good use	Jingdong prefecture level city in Shandong
Stowe Flagship Store	King of Power Saving	Very durable, affordable, fast shipping	Jingdong prefecture level city in Shandong
Stowe Flagship Store	King of Power Saving	Packaging tight quality is very good easy to use good results	Jingdong prefecture level city in Shandong
Stowe Flagship Store	King of Power Saving	Save the grid. It's in the whole house. It's saving money on the electric bill.	Jingdong prefecture level city in Shandong
Stowe Flagship Store	King of Power Saving	I used it today, it works well, I'm very satisfied	Jingdong prefecture level city in Shandong
Stowe Flagship Store	King of Power Saving	It really saves a lot of electricity after installation	Jingdong prefecture level city in Shandong
Stowe Flagship Store	King of Power Saving	Love it. Satisfied.	Jingdong prefecture level city in Shandong
Stowe Flagship Store	King of Power Saving	Doesn't feel like it's working.	Jingdong prefecture level city in Shandong
Stowe Flagship Store	King of Power Saving	I'll comment after using it.	Jingdong prefecture level city in Shandong
Stowe Flagship Store	King of Power Saving	I'm trying it out to see if it saves power, and I'll buy two more if it works well.	Jingdong prefecture level city in Shandong
Stowe Flagship Store	King of Power Saving	It is genuine very good, the price is much cheaper, cost-effective, really special cost-effective, it is worth buying!	Jingdong prefecture level city in Shandong
Stowe Flagship Store	King of Power Saving	This thing can really save electricity, I was introduced to buy by my friend, he used it very well, I used it and think it's also very good, it can really save electricity, save about half of the electricity every day, it's really good!	Jingdong prefecture level city in Shandong
Stowe Flagship Store	King of Power Saving	After using it for ten days or so, I checked the power consumption and it did drop, and customer service said it will save more electricity in the long run. The product is also insured, 5 years warranty do not have to worry about quality problems!	Jingdong prefecture level city in Shandong
Stowe Flagship Store	King of Power Saving	The product is good, before already bought a, this is the second, the effect is quite good, anyway, I see the daily electricity bill than before a lot less, this can be the choice!	Jingdong prefecture level city in Shandong
Stowe Flagship Store	King of Power Saving	Originally also very worried about things are not good, get the hand found completely over-anxious, five-star praise, the style is very generous, the quality is very good, the price is also very reasonable!	Jingdong prefecture level city in Shandong
Stowe Flagship Store	King of Power Saving	Quality is good, playing to the old family also buy a, repurchase, this quality is really good, recommended!	Jingdong prefecture level city in Shandong
Stowe Flagship Store	King of Power Saving	Thank you, the owner even sent two fuses.	Jingdong prefecture level city in Shandong
Stowe Flagship Store	King of Power Saving	This store has great stuff, relatively fast shipping and logistics	Jingdong prefecture level city in Shandong
Stowe Flagship Store	King of Power Saving	This product is based on the impossibility of saving electricity, I used 853 kWh of electricity in July, started using the product on August 1, and used 867 kWh of electricity by September 1, is this called	Jingdong prefecture level city in Shandong
Stowe Flagship Store	King of Power Saving	Power king received, to the home on the trial, when I plugged into the electric panel can not see the effect of power saving, the meter is still the same speed in the turn, I'll save the king of the manual to play look at the instructions, instructions to speak, have to have a day two days and half a month to save power obviously, then wait half a month to see the effect of power saving it! To half a month there are obvious power saving before continuing to chase Xu.	Jingdong prefecture level city in Shandong
Stowe Flagship Store	King of Power Saving	Received the goods, a look, did not see the manufacturer and production address, can only hold a try to see the mentality of the face	Jingdong prefecture level city in Shandong
Stowe Flagship Store	King of Power Saving	I've only been using it for a few days and I'm not sure if it's an IQ tax? Used to favorable reviews.	Jingdong prefecture level city in Shandong
Stowe Flagship Store	King of Power Saving	Appearance is okay, power saving next time to follow up the evaluation	Jingdong prefecture level city in Shandong
Stowe Flagship Store	King of Power Saving	Shorter time, but hopefully effective and power-saving, to be followed up in the future	Jingdong prefecture level city in Shandong
Stowe Flagship Store	King of Power Saving	Appearance is okay, I do not know if the power saving	Jingdong prefecture level city in Shandong
Stowe Flagship Store	King of Power Saving	Good service, fast logistics, Power Saving King is exquisite and beautiful, support the business, thank you!	Jingdong prefecture level city in Shandong
Stowe Flagship Store	King of Power Saving	Bought three at once to try first first.	Jingdong prefecture level city in Shandong
Stowe Flagship Store	King of Power Saving	The object is the same as the picture, the product is of high quality, the packaging is intact. High-end atmosphere, high power, power saving effect is obvious, average monthly power saving 50%, very satisfied.	Jingdong prefecture level city in Shandong
Stowe Flagship Store	King of Power Saving	Bought back and plugged in, really effective, before 16 degrees of electricity per day, now 8 degrees of electricity per day, open an air conditioner for 12 hours, decisive repurchase of a	Jingdong prefecture level city in Shandong
Stowe Flagship Store	King of Power Saving	The object is the same as the picture, the product is of high quality, and the package is intact. High-end atmosphere and high grade, high power	Jingdong prefecture level city in Shandong
Stowe Flagship Store	King of Power Saving	Goods received, I hope the store said can be energy-saving, first try a month, month after month to see the effect.	Jingdong prefecture level city in Shandong
Stowe Flagship Store	King of Power Saving	This is really good, the material and workmanship is particularly exquisite, the workmanship is really very good, I think I can recommend to my friends to buy	Jingdong prefecture level city in Shandong
Stowe Flagship Store	King of Power Saving	I have to praise, the quality is really good, and the packaging logistics are also good. Really like it, won't make you regret after getting it!	Jingdong prefecture level city in Shandong
Stowe Flagship Store	King of Power Saving	Reputable platform, efficient and professional service, buy with confidence and peace of mind!	Jingdong prefecture level city in Shandong
Stowe Flagship Store	King of Power Saving	Bought one on a trial basis to see if it works after 1-2 months.	Jingdong prefecture level city in Shandong
Stowe Flagship Store	King of Power Saving	Quality is very good, customer service attitude is very very praise, there are problems in a timely manner to solve the problem, the price is not expensive, super value, you can rest assured that the	Jingdong prefecture level city in Shandong
Stowe Flagship Store	King of Power Saving	With it no longer need to be afraid to use electricity, especially good, ready to buy a mom and dad that also	Jingdong prefecture level city in Shandong
Stowe Flagship Store	King of Power Saving	Baby received very like will return to buy the seller attitude is also very like will return to buy the seller attitude is also very like!	Jingdong prefecture level city in Shandong
Stowe Flagship Store	King of Power Saving	Jingdong Mall shopping convenience assured fast assured fast assured fast assured fast	Jingdong prefecture level city in Shandong
Stowe Flagship Store	King of Power Saving	Saving power received, the quality is very good, fine workmanship, especially good, much cheaper than the entity store, it is worth buying!	Jingdong prefecture level city in Shandong
Stowe Flagship Store	King of Power Saving	I did not expect the express delivery so quickly, very save point quality is super good, than the entity store is too cheap.	Jingdong prefecture level city in Shandong
Stowe Flagship Store	King of Power Saving	Have received the baby, very satisfied with a shopping, great worth buying!	Jingdong prefecture level city in Shandong
Stowe Flagship Store	King of Power Saving	This is really more powerful, very satisfied, more energy-saving, better, thank you boss.	Jingdong prefecture level city in Shandong
Stowe Flagship Store	King of Power Saving	Received has been used for two days, the air conditioning can boldly open it! Energy saving effect is really quite good	Jingdong prefecture level city in Shandong
Stowe Flagship Store	King of Power Saving	It is authentic, trust the merchant, the baby has a great texture and the price is moderate, unlimited repurchase!	Jingdong prefecture level city in Shandong
Stowe Flagship Store	King of Power Saving	It is genuine very good, the price is much cheaper, cost-effective, really special cost-effective, it is worth buying!	Jingdong prefecture level city in Shandong
Stowe Flagship Store	King of Power Saving	Very good quality, fine workmanship, low power consumption, long warranty, fast shipping, economical and practical	Jingdong prefecture level city in Shandong

Stowe Flagship Store	King of Power Saving	I've been using it for half a month now, it really saves power and the voltage is stable, it's very good	Jingdong prefecture level city in Shandong
Stowe Flagship Store	King of Power Saving	It's the ideal product 💝, good quality and fast logistics, worth the kudos!	Jingdong prefecture level city in Shandong
Stowe Flagship Store	King of Power Saving	The logistics is very fast, the baby is very good, the shopkeeper is NICE!	Jingdong prefecture level city in Shandong
Stowe Flagship Store	King of Power Saving	Good to use, good quality, save point durable	Jingdong prefecture level city in Shandong
Stowe Flagship Store	King of Power Saving	The quality is very good, customer service attitude is very, very awesome, there are problems in a timely manner to solve the problem	Jingdong prefecture level city in Shandong
Stowe Flagship Store	King of Power Saving	Quality is very good, the use of the effect is very good, more stable, quality assurance	Jingdong prefecture level city in Shandong
Stowe Flagship Store	King of Power Saving	Convenient, inexpensive, and see how it works out?	Jingdong prefecture level city in Shandong
Stowe Flagship Store	King of Power Saving	Very good quality, saves a lot of electricity, very good, worth buying	Jingdong prefecture level city in Shandong
Stowe Flagship Store	King of Power Saving	Power Saving King is easy to use, with the fruit of power saving to use easy to give!	Jingdong prefecture level city in Shandong
Stowe Flagship Store	King of Power Saving	This saver really works, good quality, safe and secure, very satisfied!	Jingdong prefecture level city in Shandong
Stowe Flagship Store	King of Power Saving	It works well, the voltage is very stable effect is good, the quality is also very good satisfaction	Jingdong prefecture level city in Shandong
Stowe Flagship Store	King of Power Saving	The logistics is super super fast! The quality is also very good, indeed value for money	Jingdong prefecture level city in Shandong
Stowe Flagship Store	King of Power Saving	I've only been using it for a month or so and it seems to be saving a lot of power	Jingdong prefecture level city in Shandong
Stowe Flagship Store	King of Power Saving	Very easy to operate, very good material, good value for money.	Jingdong prefecture level city in Shandong
Stowe Flagship Store	King of Power Saving	Aluminum alloy shell, durable, compact power-saving good effect	Jingdong prefecture level city in Shandong
Stowe Flagship Store	King of Power Saving	Nice device, easy to use, doesn't take up much space, recommendable!	Jingdong prefecture level city in Shandong
Stowe Flagship Store	King of Power Saving	Good quality, good price, fast logistics	Jingdong prefecture level city in Shandong
Stowe Flagship Store	King of Power Saving	Friends of the family in use recommended, the effect is good and recommendable!	Jingdong prefecture level city in Shandong
Stowe Flagship Store	King of Power Saving	Cheaper than brick and mortar stores worth buying is authentic	Jingdong prefecture level city in Shandong
Stowe Flagship Store	King of Power Saving	Merchants ship quickly, the price is cheaper than the physical store, good use	Jingdong prefecture level city in Shandong
Stowe Flagship Store	King of Power Saving	Very durable, affordable, fast shipping	Jingdong prefecture level city in Shandong
Stowe Flagship Store	King of Power Saving	Packaging tight quality is very good easy to use good results	Jingdong prefecture level city in Shandong
Stowe Flagship Store	King of Power Saving	Save the grid. It's in the whole house. It's saving money on the electric bill.	Jingdong prefecture level city in Shandong
Stowe Flagship Store	King of Power Saving	I used it today, it works well, I'm very satisfied	Jingdong prefecture level city in Shandong
Stowe Flagship Store	King of Power Saving	It really saves a lot of electricity after installation	Jingdong prefecture level city in Shandong
Stowe Flagship Store	King of Power Saving	Love it. Satisfied.	Jingdong prefecture level city in Shandong
Stowe Flagship Store	King of Power Saving	Doesn't feel like it's working.	Jingdong prefecture level city in Shandong
Stowe Flagship Store	King of Power Saving	I'll comment after using it.	Jingdong prefecture level city in Shandong
Stowe Flagship Store	King of Power Saving	I'm trying it out to see if it saves power, and I'll buy two more if it works well.	Jingdong prefecture level city in Shandong
Stowe Flagship Store	King of Power Saving	It is genuine very good, the price is much cheaper, cost-effective, really special cost-effective, it is worth buying!	Jingdong prefecture level city in Shandong
Stowe Flagship Store	King of Power Saving	This thing can really save electricity, I was introduced to buy by my friend, he used it very well, I used it and think it's also very good, it can really save electricity, save about half of the electricity every day, it's really good!	Jingdong prefecture level city in Shandong
Stowe Flagship Store	King of Power Saving	After using it for ten days or so, I checked the power consumption and it did drop, and customer service said it will save more electricity in the long run. The product is also insured, 5 years warranty do not have to worry about quality problems!	Jingdong prefecture level city in Shandong
Stowe Flagship Store	King of Power Saving	The product is good, before already bought a, this is the second, the effect is quite good, anyway, I see the daily electricity bill than before a lot less, this can be the choice!	Jingdong prefecture level city in Shandong
Stowe Flagship Store	King of Power Saving	Originally also very worried about things are not good, get the hand found completely over-anxious, five-star praise, the style is very generous, the quality is very good, the price is also very reasonable!	Jingdong prefecture level city in Shandong
Stowe Flagship Store	King of Power Saving	Quality is good, playing to the old family also buy a, repurchase, this quality is really good, recommended!	Jingdong prefecture level city in Shandong
Stowe Flagship Store	King of Power Saving	Thank you, the owner even sent two fuses.	Jingdong prefecture level city in Shandong
Stowe Flagship Store	King of Power Saving	This store has great stuff, relatively fast shipping and logistics	Jingdong prefecture level city in Shandong
Stowe Flagship Store	King of Power Saving	This product is based on the impossibility of saving electricity, I used 853 kWh of electricity in July, started using the product on August 1, and used 867 kWh of electricity by September 1, is this called	Jingdong prefecture level city in Shandong
Stowe Flagship Store	King of Power Saving	Power king received, to the home on the trial, when I plugged into the electric panel can not see the effect of power saving, the meter is still the same speed in the turn, I'll save the king of the manual to play look at the instructions, instructions to speak, have to have a day two days and half a month to save power obviously, then wait half a month to see the effect of power saving it! To half a month there are obvious power saving before continuing to chase Xu.	Jingdong prefecture level city in Shandong
Stowe Flagship Store	King of Power Saving	Received the goods, a look, did not see the manufacturer and production address, can only hold a try to see the mentality of the face	Jingdong prefecture level city in Shandong
Stowe Flagship Store	King of Power Saving	I've only been using it for a few days and I'm not sure if it's an IQ tax? Used to favorable reviews.	Jingdong prefecture level city in Shandong
Stowe Flagship Store	King of Power Saving	Appearance is okay, power saving next time to follow up the evaluation	Jingdong prefecture level city in Shandong
Stowe Flagship Store	King of Power Saving	Shorter time, but hopefully effective and power-saving, to be followed up in the future	Jingdong prefecture level city in Shandong
Stowe Flagship Store	King of Power Saving	Appearance is okay, I do not know if the power saving	Jingdong prefecture level city in Shandong
Stowe Flagship Store	King of Power Saving	Good service, fast logistics, Power Saving King is exquisite and beautiful, support the business, thank you!	Jingdong prefecture level city in Shandong
Stowe Flagship Store	King of Power Saving	Bought three at once to try first first.	Jingdong prefecture level city in Shandong
Stowe Flagship Store	King of Power Saving	The object is the same as the picture, the product is of high quality, the packaging is intact. High-end atmosphere, high power, power saving effect is obvious, average monthly power saving 50%, very satisfied.	Jingdong prefecture level city in Shandong
Stowe Flagship Store	King of Power Saving	Bought back and plugged in, really effective, before 16 degrees of electricity per day, now 8 degrees of electricity per day, open an air conditioner for 12 hours, decisive repurchase of a	Jingdong prefecture level city in Shandong
Stowe Flagship Store	King of Power Saving	The object is the same as the picture, the product is of high quality, and the package is intact. High-end atmosphere and high grade, high power	Jingdong prefecture level city in Shandong

Stowe Flagship Store	King of Power Saving	Goods received, I hope the store said can be energy-saving, first try a month, month after month to see the effect.	Jingdong prefecture level city in Shandong
Stowe Flagship Store	King of Power Saving	This is really good, the material and workmanship is particularly exquisite, the workmanship is really very good, I think I can recommend to my friends to buy	Jingdong prefecture level city in Shandong
Stowe Flagship Store	King of Power Saving	I have to praise, the quality is really good, and the packaging logistics are also good. Really like it, won't make you regret after getting it!	Jingdong prefecture level city in Shandong
Stowe Flagship Store	King of Power Saving	Reputable platform, efficient and professional service, buy with confidence and peace of mind!	Jingdong prefecture level city in Shandong
Stowe Flagship Store	King of Power Saving	Bought one on a trial basis to see if it works after 1-2 months.	Jingdong prefecture level city in Shandong
Stowe Flagship Store	King of Power Saving	Quality is very good, customer service attitude is very very praise, there are problems in a timely manner to solve the problem, the price is not expensive, super value, you can rest assured that the	Jingdong prefecture level city in Shandong
Stowe Flagship Store	King of Power Saving	With it no longer need to be afraid to use electricity, especially good, ready to buy a mom and dad that also	Jingdong prefecture level city in Shandong
Stowe Flagship Store	King of Power Saving	Baby received very like will return to buy the seller attitude is also very like will return to buy the seller attitude is also very like!	Jingdong prefecture level city in Shandong
Stowe Flagship Store	King of Power Saving	Jingdong Mall shopping convenience assured fast assured fast assured fast assured fast	Jingdong prefecture level city in Shandong
Stowe Flagship Store	King of Power Saving	Saving power received, the quality is very good, fine workmanship, especially good, much cheaper than the entity store, it is worth buying!	Jingdong prefecture level city in Shandong
Stowe Flagship Store	King of Power Saving	I did not expect the express delivery so quickly, very save point quality is super good, than the entity store is too cheap.	Jingdong prefecture level city in Shandong
Stowe Flagship Store	King of Power Saving	Have received the baby, very satisfied with a shopping, great worth buying!	Jingdong prefecture level city in Shandong
Stowe Flagship Store	King of Power Saving	This is really more powerful, very satisfied, more energy-saving, better, thank you boss.	Jingdong prefecture level city in Shandong
Stowe Flagship Store	King of Power Saving	Received has been used for two days, the air conditioning can boldly open it! Energy saving effect is really quite good	Jingdong prefecture level city in Shandong
Stowe Flagship Store	King of Power Saving	It is authentic, trust the merchant, the baby has a great texture and the price is moderate, unlimited repurchase!	Jingdong prefecture level city in Shandong
Stowe Flagship Store	King of Power Saving	It is genuine very good, the price is much cheaper, cost-effective, really special cost-effective, it is worth buying!	Jingdong prefecture level city in Shandong
Stowe Flagship Store	King of Power Saving	Very good quality, fine workmanship, low power consumption, long warranty, fast shipping, economical and practical	Jingdong prefecture level city in Shandong
Stowe Flagship Store	King of Power Saving	I've been using it for half a month now, it really saves power and the voltage is stable, it's very good	Jingdong prefecture level city in Shandong
Stowe Flagship Store	King of Power Saving	It's the ideal product , good quality and fast logistics, worth the kudos!	Jingdong prefecture level city in Shandong
Stowe Flagship Store	King of Power Saving	The logistics is very fast, the baby is very good, the shopkeeper is NICE!	Jingdong prefecture level city in Shandong
Stowe Flagship Store	King of Power Saving	Good to use, good quality, save point durable	Jingdong prefecture level city in Shandong
Stowe Flagship Store	King of Power Saving	The quality is very good, customer service attitude is very, very awesome, there are problems in a timely manner to solve the problem	Jingdong prefecture level city in Shandong
Stowe Flagship Store	King of Power Saving	Quality is very good, the use of the effect is very good, more stable, quality assurance	Jingdong prefecture level city in Shandong
Stowe Flagship Store	King of Power Saving	Convenient, inexpensive, and see how it works out?	Jingdong prefecture level city in Shandong
Stowe Flagship Store	King of Power Saving	Very good quality, saves a lot of electricity, very good, worth buying	Jingdong prefecture level city in Shandong
Stowe Flagship Store	King of Power Saving	Power Saving King is easy to use, with the fruit of power saving to use easy to give!	Jingdong prefecture level city in Shandong
Stowe Flagship Store	King of Power Saving	This saver really works, good quality, safe and secure, very satisfied!	Jingdong prefecture level city in Shandong
Stowe Flagship Store	King of Power Saving	It works well, the voltage is very stable effect is good, the quality is also very good satisfaction	Jingdong prefecture level city in Shandong
Stowe Flagship Store	King of Power Saving	The logistics is super super fast! The quality is also very good, indeed value for money	Jingdong prefecture level city in Shandong
Stowe Flagship Store	King of Power Saving	I've only been using it for a month or so and it seems to be saving a lot of power	Jingdong prefecture level city in Shandong
Stowe Flagship Store	King of Power Saving	Very easy to operate, very good material, good value for money.	Jingdong prefecture level city in Shandong
Stowe Flagship Store	King of Power Saving	Aluminum alloy shell, durable, compact power-saving good effect	Jingdong prefecture level city in Shandong
Stowe Flagship Store	King of Power Saving	Nice device, easy to use, doesn't take up much space, recommendable!	Jingdong prefecture level city in Shandong
Stowe Flagship Store	King of Power Saving	Good quality, good price, fast logistics	Jingdong prefecture level city in Shandong
Stowe Flagship Store	King of Power Saving	Friends of the family in use recommended, the effect is good and recommendable!	Jingdong prefecture level city in Shandong
Stowe Flagship Store	King of Power Saving	Cheaper than brick and mortar stores worth buying is authentic	Jingdong prefecture level city in Shandong
Stowe Flagship Store	King of Power Saving	Merchants ship quickly, the price is cheaper than the physical store, good use	Jingdong prefecture level city in Shandong
Stowe Flagship Store	King of Power Saving	Very durable, affordable, fast shipping	Jingdong prefecture level city in Shandong
Stowe Flagship Store	King of Power Saving	Packaging tight quality is very good easy to use good results	Jingdong prefecture level city in Shandong
Stowe Flagship Store	King of Power Saving	Save the grid. It's in the whole house. It's saving money on the electric bill.	Jingdong prefecture level city in Shandong
Stowe Flagship Store	King of Power Saving	I used it today, it works well, I'm very satisfied	Jingdong prefecture level city in Shandong
Stowe Flagship Store	King of Power Saving	It really saves a lot of electricity after installation	Jingdong prefecture level city in Shandong
Stowe Flagship Store	King of Power Saving	Love it. Satisfied.	Jingdong prefecture level city in Shandong
Stowe Flagship Store	King of Power Saving	Doesn't feel like it's working.	Jingdong prefecture level city in Shandong
Stowe Flagship Store	King of Power Saving	I'll comment after using it.	Jingdong prefecture level city in Shandong
Stowe Flagship Store	King of Power Saving	I'm trying it out to see if it saves power, and I'll buy two more if it works well.	Jingdong prefecture level city in Shandong
Stowe Flagship Store	King of Power Saving	It is genuine very good, the price is much cheaper, cost-effective, really special cost-effective, it is worth buying!	Jingdong prefecture level city in Shandong
Stowe Flagship Store	King of Power Saving	This thing can really save electricity, I was introduced to buy by my friend, he used it very well, I used it and think it's also very good, it can really save electricity, save about half of the electricity every day, it's really good!	Jingdong prefecture level city in Shandong
Stowe Flagship Store	King of Power Saving	After using it for ten days or so, I checked the power consumption and it did drop, and customer service said it will save more electricity in the long run. The product is also insured, 5 years warranty do not have to worry about quality problems!	Jingdong prefecture level city in Shandong
Stowe Flagship Store	King of Power Saving	The product is good, before already bought a, this is the second, the effect is quite good, anyway, I see the daily electricity bill than before a lot less, this can be the choice!	Jingdong prefecture level city in Shandong

Stowe Flagship Store	King of Power Saving	Originally also very worried about things are not good, get the hand found completely over-anxious, five-star praise, the style is very generous, the quality is very good, the price is also very reasonable!	Jingdong prefecture level city in Shandong
Stowe Flagship Store	King of Power Saving	Quality is good, playing to the old family also buy a, repurchase, this quality is really good, recommended!	Jingdong prefecture level city in Shandong
Stowe Flagship Store	King of Power Saving	Thank you, the owner even sent two fuses.	Jingdong prefecture level city in Shandong
Stowe Flagship Store	King of Power Saving	This store has great stuff, relatively fast shipping and logistics	Jingdong prefecture level city in Shandong
Stowe Flagship Store	King of Power Saving	This product is based on the impossibility of saving electricity, I used 853 kWh of electricity in July, started using the product on August 1, and used 867 kWh of electricity by September 1, is this called	Jingdong prefecture level city in Shandong
Stowe Flagship Store	King of Power Saving	Power king received, to the home on the trial, when I plugged into the electric panel can not see the effect of power saving, the meter is still the same speed in the turn, I'll save the king of the manual to play look at the instructions, instructions to speak, have to have a day two days and half a month to save power obviously, then wait half a month to see the effect of power saving it! To half a month there are obvious power saving before continuing to chase Xu.	Jingdong prefecture level city in Shandong
Stowe Flagship Store	King of Power Saving	Received the goods, a look, did not see the manufacturer and production address, can only hold a try to see the mentality of the face	Jingdong prefecture level city in Shandong
Stowe Flagship Store	King of Power Saving	I've only been using it for a few days and I'm not sure if it's an IQ tax? Used to favorable reviews.	Jingdong prefecture level city in Shandong
Stowe Flagship Store	King of Power Saving	Appearance is okay, power saving next time to follow up the evaluation	Jingdong prefecture level city in Shandong
Stowe Flagship Store	King of Power Saving	Shorter time, but hopefully effective and power-saving, to be followed up in the future	Jingdong prefecture level city in Shandong
Stowe Flagship Store	King of Power Saving	Appearance is okay, I do not know if the power saving	Jingdong prefecture level city in Shandong
Stowe Flagship Store	King of Power Saving	Good service, fast logistics, Power Saving King is exquisite and beautiful, support the business, thank you!	Jingdong prefecture level city in Shandong
Stowe Flagship Store	King of Power Saving	Bought three at once to try first first.	Jingdong prefecture level city in Shandong
Stowe Flagship Store	King of Power Saving	The object is the same as the picture, the product is of high quality, the packaging is intact. High-end atmosphere, high power, power saving effect is obvious, average monthly power saving 50%, very satisfied.	Jingdong prefecture level city in Shandong
Stowe Flagship Store	King of Power Saving	Bought back and plugged in, really effective, before 16 degrees of electricity per day, now 8 degrees of electricity per day, open an air conditioner for 12 hours, decisive repurchase of a	Jingdong prefecture level city in Shandong
Stowe Flagship Store	King of Power Saving	The object is the same as the picture, the product is of high quality, and the package is intact. High-end atmosphere and high grade, high power	Jingdong prefecture level city in Shandong
Stowe Flagship Store	King of Power Saving	Goods received, I hope the store said can be energy-saving, first try a month, month after month to see the effect.	Jingdong prefecture level city in Shandong
Stowe Flagship Store	King of Power Saving	This is really good, the material and workmanship is particularly exquisite, the workmanship is really very good, I think I can recommend to my friends to buy	Jingdong prefecture level city in Shandong
Stowe Flagship Store	King of Power Saving	I have to praise, the quality is really good, and the packaging logistics are also good. Really like it, won't make you regret after getting it!	Jingdong prefecture level city in Shandong
Stowe Flagship Store	King of Power Saving	Reputable platform, efficient and professional service, buy with confidence and peace of mind!	Jingdong prefecture level city in Shandong
Stowe Flagship Store	King of Power Saving	Bought one on a trial basis to see if it works after 1-2 months.	Jingdong prefecture level city in Shandong
Stowe Flagship Store	King of Power Saving	Quality is very good, customer service attitude is very very praise, there are problems in a timely manner to solve the problem, the price is not expensive, super value, you can rest assured that the	Jingdong prefecture level city in Shandong
Stowe Flagship Store	King of Power Saving	With it no longer need to be afraid to use electricity, especially good, ready to buy a mom and dad that also	Jingdong prefecture level city in Shandong
Stowe Flagship Store	King of Power Saving	Baby received very like will return to buy the seller attitude is also very like will return to buy the seller attitude is also very like!	Jingdong prefecture level city in Shandong
Stowe Flagship Store	King of Power Saving	Jingdong Mall shopping convenience assured fast assured fast assured fast assured fast	Jingdong prefecture level city in Shandong
Stowe Flagship Store	King of Power Saving	Saving power received, the quality is very good, fine workmanship, especially good, much cheaper than the entity store, it is worth buying!	Jingdong prefecture level city in Shandong
Stowe Flagship Store	King of Power Saving	I did not expect the express delivery so quickly, very save point quality is super good, than the entity store is too cheap.	Jingdong prefecture level city in Shandong
Stowe Flagship Store	King of Power Saving	Have received the baby, very satisfied with a shopping, great worth buying!	Jingdong prefecture level city in Shandong
Stowe Flagship Store	King of Power Saving	This is really more powerful, very satisfied, more energy-saving, better, thank you boss.	Jingdong prefecture level city in Shandong
Stowe Flagship Store	King of Power Saving	Received has been used for two days, the air conditioning can boldly open it! Energy saving effect is really quite good	Jingdong prefecture level city in Shandong
Stowe Flagship Store	King of Power Saving	It is authentic, trust the merchant, the baby has a great texture and the price is moderate, unlimited repurchase!	Jingdong prefecture level city in Shandong
Stowe Flagship Store	King of Power Saving	It is genuine very good, the price is much cheaper, cost-effective, really special cost-effective, it is worth buying!	Jingdong prefecture level city in Shandong
Stowe Flagship Store	King of Power Saving	Very good quality, fine workmanship, low power consumption, long warranty, fast shipping, economical and practical	Jingdong prefecture level city in Shandong
Stowe Flagship Store	King of Power Saving	I've been using it for half a month now, it really saves power and the voltage is stable, it's very good	Jingdong prefecture level city in Shandong
Stowe Flagship Store	King of Power Saving	It's the ideal product 💝, good quality and fast logistics, worth the kudos!	Jingdong prefecture level city in Shandong
Stowe Flagship Store	King of Power Saving	The logistics is very fast, the baby is very good, the shopkeeper is NICE!	Jingdong prefecture level city in Shandong
Stowe Flagship Store	King of Power Saving	Good to use, good quality, save point durable	Jingdong prefecture level city in Shandong
Stowe Flagship Store	King of Power Saving	The quality is very good, customer service attitude is very, very awesome, there are problems in a timely manner to solve the problem	Jingdong prefecture level city in Shandong
Stowe Flagship Store	King of Power Saving	Quality is very good, the use of the effect is very good, more stable, quality assurance	Jingdong prefecture level city in Shandong
Stowe Flagship Store	King of Power Saving	Convenient, inexpensive, and see how it works out?	Jingdong prefecture level city in Shandong
Stowe Flagship Store	King of Power Saving	Very good quality, saves a lot of electricity, very good, worth buying	Jingdong prefecture level city in Shandong
Stowe Flagship Store	King of Power Saving	Power Saving King is easy to use, with the fruit of power saving to use easy to give!	Jingdong prefecture level city in Shandong
Stowe Flagship Store	King of Power Saving	This saver really works, good quality, safe and secure, very satisfied!	Jingdong prefecture level city in Shandong
Stowe Flagship Store	King of Power Saving	It works well, the voltage is very stable effect is good, the quality is also very good satisfaction	Jingdong prefecture level city in Shandong
Stowe Flagship Store	King of Power Saving	The logistics is super super fast! The quality is also very good, indeed value for money	Jingdong prefecture level city in Shandong
Stowe Flagship Store	King of Power Saving	I've only been using it for a month or so and it seems to be saving a lot of power	Jingdong prefecture level city in Shandong
Stowe Flagship Store	King of Power Saving	Very easy to operate, very good material, good value for money.	Jingdong prefecture level city in Shandong
Stowe Flagship Store	King of Power Saving	Aluminum alloy shell, durable, compact power-saving good effect	Jingdong prefecture level city in Shandong
Stowe Flagship Store	King of Power Saving	Nice device, easy to use, doesn't take up much space, recommendable!	Jingdong prefecture level city in Shandong
Stowe Flagship Store	King of Power Saving	Good quality, good price, fast logistics	Jingdong prefecture level city in Shandong

Stowe Flagship Store	King of Power Saving	Friends of the family in use recommended, the effect is good and recommendable!	Jingdong prefecture level city in Shandong
Stowe Flagship Store	King of Power Saving	Cheaper than brick and mortar stores worth buying is authentic	Jingdong prefecture level city in Shandong
Stowe Flagship Store	King of Power Saving	Merchants ship quickly, the price is cheaper than the physical store, good use	Jingdong prefecture level city in Shandong
Stowe Flagship Store	King of Power Saving	Very durable, affordable, fast shipping	Jingdong prefecture level city in Shandong
Stowe Flagship Store	King of Power Saving	Packaging tight quality is very good easy to use good results	Jingdong prefecture level city in Shandong
Stowe Flagship Store	King of Power Saving	Save the grid. It's in the whole house. It's saving money on the electric bill.	Jingdong prefecture level city in Shandong
Stowe Flagship Store	King of Power Saving	I used it today, it works well, I'm very satisfied	Jingdong prefecture level city in Shandong
Stowe Flagship Store	King of Power Saving	It really saves a lot of electricity after installation	Jingdong prefecture level city in Shandong
Stowe Flagship Store	King of Power Saving	Love it. Satisfied.	Jingdong prefecture level city in Shandong
Stowe Flagship Store	King of Power Saving	Doesn't feel like it's working.	Jingdong prefecture level city in Shandong
Stowe Flagship Store	King of Power Saving	I'll comment after using it.	Jingdong prefecture level city in Shandong
Stowe Flagship Store	King of Power Saving	I'm trying it out to see if it saves power, and I'll buy two more if it works well.	Jingdong prefecture level city in Shandong
Stowe Flagship Store	King of Power Saving	It is genuine very good, the price is much cheaper, cost-effective, really special cost-effective, it is worth buying!	Jingdong prefecture level city in Shandong
Stowe Flagship Store	King of Power Saving	This thing can really save electricity, I was introduced to buy by my friend, he used it very well, I used it and think it's also very good, it can really save electricity, save about half of the electricity every day, it's really good!	Jingdong prefecture level city in Shandong
Stowe Flagship Store	King of Power Saving	After using it for ten days or so, I checked the power consumption and it did drop, and customer service said it will save more electricity in the long run. The product is also insured, 5 years warranty do not have to worry about quality problems!	Jingdong prefecture level city in Shandong
Stowe Flagship Store	King of Power Saving	The product is good, before already bought a, this is the second, the effect is quite good, anyway, I see the daily electricity bill than before a lot less, this can be the choice!	Jingdong prefecture level city in Shandong
Stowe Flagship Store	King of Power Saving	Originally also very worried about things are not good, get the hand found completely over-anxious, five-star praise, the style is very generous, the quality is very good, the price is also very reasonable!	Jingdong prefecture level city in Shandong
Stowe Flagship Store	King of Power Saving	Quality is good, playing to the old family also buy a, repurchase, this quality is really good, recommended!	Jingdong prefecture level city in Shandong
Stowe Flagship Store	King of Power Saving	Thank you, the owner even sent two fuses.	Jingdong prefecture level city in Shandong
Stowe Flagship Store	King of Power Saving	This store has great stuff, relatively fast shipping and logistics	Jingdong prefecture level city in Shandong
Stowe Flagship Store	King of Power Saving	This product is based on the impossibility of saving electricity, I used 853 kWh of electricity in July, started using the product on August 1, and used 867 kWh of electricity by September 1, is this called	Jingdong prefecture level city in Shandong
Stowe Flagship Store	King of Power Saving	Power king received, to the home on the trial, when I plugged into the electric panel can not see the effect of power saving, the meter is still the same speed in the turn, I'll save the king of the manual to play look at the instructions, instructions to speak, have to have a day two days and half a month to save power obviously, then wait half a month to see the effect of power saving it! To half a month there are obvious power saving before continuing to chase Xu.	Jingdong prefecture level city in Shandong
Stowe Flagship Store	King of Power Saving	Received the goods, a look, did not see the manufacturer and production address, can only hold a try to see the mentality of the face	Jingdong prefecture level city in Shandong
Stowe Flagship Store	King of Power Saving	I've only been using it for a few days and I'm not sure if it's an IQ tax? Used to favorable reviews.	Jingdong prefecture level city in Shandong
Stowe Flagship Store	King of Power Saving	Appearance is okay, power saving next time to follow up the evaluation	Jingdong prefecture level city in Shandong
Stowe Flagship Store	King of Power Saving	Shorter time, but hopefully effective and power-saving, to be followed up in the future	Jingdong prefecture level city in Shandong
Stowe Flagship Store	King of Power Saving	Appearance is okay, I do not know if the power saving	Jingdong prefecture level city in Shandong
Stowe Flagship Store	King of Power Saving	Good service, fast logistics, Power Saving King is exquisite and beautiful, support the business, thank you!	Jingdong prefecture level city in Shandong
Stowe Flagship Store	King of Power Saving	Bought three at once to try first first.	Jingdong prefecture level city in Shandong
Stowe Flagship Store	King of Power Saving	The object is the same as the picture, the product is of high quality, the packaging is intact. High-end atmosphere, high power, power saving effect is obvious, average monthly power saving 50%, very satisfied.	Jingdong prefecture level city in Shandong
Stowe Flagship Store	King of Power Saving	Bought back and plugged in, really effective, before 16 degrees of electricity per day, now 8 degrees of electricity per day, open an air conditioner for 12 hours, decisive repurchase of a	Jingdong prefecture level city in Shandong
Stowe Flagship Store	King of Power Saving	The object is the same as the picture, the product is of high quality, and the package is intact. High-end atmosphere and high grade, high power	Jingdong prefecture level city in Shandong
Stowe Flagship Store	King of Power Saving	Goods received, I hope the store said can be energy-saving, first try a month, month after month to see the effect.	Jingdong prefecture level city in Shandong
Stowe Flagship Store	King of Power Saving	This is really good, the material and workmanship is particularly exquisite, the workmanship is really very good, I think I can recommend to my friends to buy	Jingdong prefecture level city in Shandong
Stowe Flagship Store	King of Power Saving	I have to praise, the quality is really good, and the packaging logistics are also good. Really like it, won't make you regret after getting it!	Jingdong prefecture level city in Shandong
Stowe Flagship Store	King of Power Saving	Reputable platform, efficient and professional service, buy with confidence and peace of mind!	Jingdong prefecture level city in Shandong
Stowe Flagship Store	King of Power Saving	Bought one on a trial basis to see if it works after 1-2 months.	Jingdong prefecture level city in Shandong
Stowe Flagship Store	King of Power Saving	Quality is very good, customer service attitude is very very praise, there are problems in a timely manner to solve the problem, the price is not expensive, super value, you can rest assured that the	Jingdong prefecture level city in Shandong
Stowe Flagship Store	King of Power Saving	With it no longer need to be afraid to use electricity, especially good, ready to buy a mom and dad that also	Jingdong prefecture level city in Shandong
Stowe Flagship Store	King of Power Saving	Baby received very like will return to buy the seller attitude is also very like will return to buy the seller attitude is also very like!	Jingdong prefecture level city in Shandong
Stowe Flagship Store	King of Power Saving	Jingdong Mall shopping convenience assured fast assured fast assured fast assured fast	Jingdong prefecture level city in Shandong
Stowe Flagship Store	King of Power Saving	Saving power received, the quality is very good, fine workmanship, especially good, much cheaper than the entity store, it is worth buying!	Jingdong prefecture level city in Shandong
Stowe Flagship Store	King of Power Saving	I did not expect the express delivery so quickly, very save point quality is super good, than the entity store is too cheap.	Jingdong prefecture level city in Shandong
Stowe Flagship Store	King of Power Saving	Have received the baby, very satisfied with a shopping, great worth buying!	Jingdong prefecture level city in Shandong
Stowe Flagship Store	King of Power Saving	This is really more powerful, very satisfied, more energy-saving, better, thank you boss.	Jingdong prefecture level city in Shandong
Stowe Flagship Store	King of Power Saving	Received has been used for two days, the air conditioning can boldly open it! Energy saving effect is really quite good	Jingdong prefecture level city in Shandong
Stowe Flagship Store	King of Power Saving	It is authentic, trust the merchant, the baby has a great texture and the price is moderate, unlimited repurchase!	Jingdong prefecture level city in Shandong
Stowe Flagship Store	King of Power Saving	It is genuine very good, the price is much cheaper, cost-effective, really special cost-effective, it is worth buying!	Jingdong prefecture level city in Shandong
Stowe Flagship Store	King of Power Saving	Very good quality, fine workmanship, low power consumption, long warranty, fast shipping, economical and practical	Jingdong prefecture level city in Shandong

Stowe Flagship Store	King of Power Saving	I've been using it for half a month now, it really saves power and the voltage is stable, it's very good	Jingdong prefecture level city in Shandong
Stowe Flagship Store	King of Power Saving	It's the ideal product ♡, good quality and fast logistics, worth the kudos!	Jingdong prefecture level city in Shandong
Stowe Flagship Store	King of Power Saving	The logistics is very fast, the baby is very good, the shopkeeper is NICE!	Jingdong prefecture level city in Shandong
Stowe Flagship Store	King of Power Saving	Good to use, good quality, save point durable	Jingdong prefecture level city in Shandong
Stowe Flagship Store	King of Power Saving	The quality is very good, customer service attitude is very, very awesome, there are problems in a timely manner to solve the problem	Jingdong prefecture level city in Shandong
Stowe Flagship Store	King of Power Saving	Quality is very good, the use of the effect is very good, more stable, quality assurance	Jingdong prefecture level city in Shandong
Stowe Flagship Store	King of Power Saving	Convenient, inexpensive, and see how it works out?	Jingdong prefecture level city in Shandong
Stowe Flagship Store	King of Power Saving	Very good quality, saves a lot of electricity, very good, worth buying	Jingdong prefecture level city in Shandong
Stowe Flagship Store	King of Power Saving	Power Saving King is easy to use, with the fruit of power saving to use easy to give!	Jingdong prefecture level city in Shandong
Stowe Flagship Store	King of Power Saving	This saver really works, good quality, safe and secure, very satisfied!	Jingdong prefecture level city in Shandong
Stowe Flagship Store	King of Power Saving	It works well, the voltage is very stable effect is good, the quality is also very good satisfaction	Jingdong prefecture level city in Shandong
Stowe Flagship Store	King of Power Saving	The logistics is super super fast! The quality is also very good, indeed value for money	Jingdong prefecture level city in Shandong
Stowe Flagship Store	King of Power Saving	I've only been using it for a month or so and it seems to be saving a lot of power	Jingdong prefecture level city in Shandong
Stowe Flagship Store	King of Power Saving	Very easy to operate, very good material, good value for money.	Jingdong prefecture level city in Shandong
Stowe Flagship Store	King of Power Saving	Aluminum alloy shell, durable, compact power-saving good effect	Jingdong prefecture level city in Shandong
Stowe Flagship Store	King of Power Saving	Nice device, easy to use, doesn't take up much space, recommendable!	Jingdong prefecture level city in Shandong
Stowe Flagship Store	King of Power Saving	Good quality, good price, fast logistics	Jingdong prefecture level city in Shandong
Stowe Flagship Store	King of Power Saving	Friends of the family in use recommended, the effect is good and recommendable!	Jingdong prefecture level city in Shandong
Stowe Flagship Store	King of Power Saving	Cheaper than brick and mortar stores worth buying is authentic	Jingdong prefecture level city in Shandong
Stowe Flagship Store	King of Power Saving	Merchants ship quickly, the price is cheaper than the physical store, good use	Jingdong prefecture level city in Shandong
Stowe Flagship Store	King of Power Saving	Very durable, affordable, fast shipping	Jingdong prefecture level city in Shandong
Stowe Flagship Store	King of Power Saving	Packaging tight quality is very good easy to use good results	Jingdong prefecture level city in Shandong
Stowe Flagship Store	King of Power Saving	Save the grid. It's in the whole house. It's saving money on the electric bill.	Jingdong prefecture level city in Shandong
Stowe Flagship Store	King of Power Saving	I used it today, it works well, I'm very satisfied	Jingdong prefecture level city in Shandong
Stowe Flagship Store	King of Power Saving	It really saves a lot of electricity after installation	Jingdong prefecture level city in Shandong
Stowe Flagship Store	King of Power Saving	Love it. Satisfied.	Jingdong prefecture level city in Shandong
Stowe Flagship Store	King of Power Saving	Doesn't feel like it's working.	Jingdong prefecture level city in Shandong
Stowe Flagship Store	King of Power Saving	I'll comment after using it.	Jingdong prefecture level city in Shandong
Stowe Flagship Store	King of Power Saving	I'm trying it out to see if it saves power, and I'll buy two more if it works well.	Jingdong prefecture level city in Shandong
Stowe Flagship Store	King of Power Saving	It is genuine very good, the price is much cheaper, cost-effective, really special cost-effective, it is worth buying!	Jingdong prefecture level city in Shandong
Stowe Flagship Store	King of Power Saving	This thing can really save electricity, I was introduced to buy by my friend, he used it very well, I used it and think it's also very good, it can really save electricity, save about half of the electricity every day, it's really good!	Jingdong prefecture level city in Shandong
Stowe Flagship Store	King of Power Saving	After using it for ten days or so, I checked the power consumption and it did drop, and customer service said it will save more electricity in the long run. The product is also insured, 5 years warranty do not have to worry about quality problems!	Jingdong prefecture level city in Shandong
Stowe Flagship Store	King of Power Saving	The product is good, before already bought a, this is the second, the effect is quite good, anyway, I see the daily electricity bill than before a lot less, this can be the choice!	Jingdong prefecture level city in Shandong
Stowe Flagship Store	King of Power Saving	Originally also very worried about things are not good, get the hand found completely over-anxious, five-star praise, the style is very generous, the quality is very good, the price is also very reasonable!	Jingdong prefecture level city in Shandong
Stowe Flagship Store	King of Power Saving	Quality is good, playing to the old family also buy a, repurchase, this quality is really good, recommended!	Jingdong prefecture level city in Shandong
Stowe Flagship Store	King of Power Saving	Thank you, the owner even sent two fuses.	Jingdong prefecture level city in Shandong
Stowe Flagship Store	King of Power Saving	This store has great stuff, relatively fast shipping and logistics	Jingdong prefecture level city in Shandong
Stowe Flagship Store	King of Power Saving	This product is based on the impossibility of saving electricity, I used 853 kWh of electricity in July, started using the product on August 1, and used 867 kWh of electricity by September 1, is this called	Jingdong prefecture level city in Shandong
Stowe Flagship Store	King of Power Saving	Power king received, to the home on the trial, when I plugged into the electric panel can not see the effect of power saving, the meter is still the same speed in the turn, I'll save the king of the manual to play look at the instructions, instructions to speak, have to have a day two days and half a month to save power obviously, then wait half a month to see the effect of power saving it! To half a month there are obvious power saving before continuing to chase Xu.	Jingdong prefecture level city in Shandong
Stowe Flagship Store	King of Power Saving	Received the goods, a look, did not see the manufacturer and production address, can only hold a try to see the mentality of the face	Jingdong prefecture level city in Shandong
Stowe Flagship Store	King of Power Saving	I've only been using it for a few days and I'm not sure if it's an IQ tax? Used to favorable reviews.	Jingdong prefecture level city in Shandong
Stowe Flagship Store	King of Power Saving	Appearance is okay, power saving next time to follow up the evaluation	Jingdong prefecture level city in Shandong
Stowe Flagship Store	King of Power Saving	Shorter time, but hopefully effective and power-saving, to be followed up in the future	Jingdong prefecture level city in Shandong
Stowe Flagship Store	King of Power Saving	Appearance is okay, I do not know if the power saving	Jingdong prefecture level city in Shandong
Stowe Flagship Store	King of Power Saving	Good service, fast logistics, Power Saving King is exquisite and beautiful, support the business, thank you!	Jingdong prefecture level city in Shandong
Stowe Flagship Store	King of Power Saving	Bought three at once to try first first.	Jingdong prefecture level city in Shandong
Stowe Flagship Store	King of Power Saving	The object is the same as the picture, the product is of high quality, the packaging is intact. High-end atmosphere, high power, power saving effect is obvious, average monthly power saving 50%, very satisfied.	Jingdong prefecture level city in Shandong
Stowe Flagship Store	King of Power Saving	Bought back and plugged in, really effective, before 16 degrees of electricity per day, now 8 degrees of electricity per day, open an air conditioner for 12 hours, decisive repurchase of a	Jingdong prefecture level city in Shandong
Stowe Flagship Store	King of Power Saving	The object is the same as the picture, the product is of high quality, and the package is intact. High-end atmosphere and high grade, high power	Jingdong prefecture level city in Shandong

Stowe Flagship Store	King of Power Saving	Goods received, I hope the store said can be energy-saving, first try a month, month after month to see the effect.	Jingdong prefecture level city in Shandong
Stowe Flagship Store	King of Power Saving	This is really good, the material and workmanship is particularly exquisite, the workmanship is really very good, I think I can recommend to my friends to buy	Jingdong prefecture level city in Shandong
Stowe Flagship Store	King of Power Saving	I have to praise, the quality is really good, and the packaging logistics are also good. Really like it, won't make you regret after getting it!	Jingdong prefecture level city in Shandong
Stowe Flagship Store	King of Power Saving	Reputable platform, efficient and professional service, buy with confidence and peace of mind!	Jingdong prefecture level city in Shandong
Stowe Flagship Store	King of Power Saving	Bought one on a trial basis to see if it works after 1-2 months.	Jingdong prefecture level city in Shandong
Stowe Flagship Store	King of Power Saving	Quality is very good, customer service attitude is very very praise, there are problems in a timely manner to solve the problem, the price is not expensive, super value, you can rest assured that the	Jingdong prefecture level city in Shandong
Stowe Flagship Store	King of Power Saving	With it no longer need to be afraid to use electricity, especially good, ready to buy a mom and dad that also	Jingdong prefecture level city in Shandong
Stowe Flagship Store	King of Power Saving	Baby received very like will return to buy the seller attitude is also very like will return to buy the seller attitude is also very like!	Jingdong prefecture level city in Shandong
Stowe Flagship Store	King of Power Saving	Jingdong Mall shopping convenience assured fast assured fast assured fast assured fast	Jingdong prefecture level city in Shandong
Stowe Flagship Store	King of Power Saving	Saving power received, the quality is very good, fine workmanship, especially good, much cheaper than the entity store, it is worth buying!	Jingdong prefecture level city in Shandong
Stowe Flagship Store	King of Power Saving	I did not expect the express delivery so quickly, very save point quality is super good, than the entity store is too cheap.	Jingdong prefecture level city in Shandong
Stowe Flagship Store	King of Power Saving	Have received the baby, very satisfied with a shopping, great worth buying!	Jingdong prefecture level city in Shandong
Stowe Flagship Store	King of Power Saving	This is really more powerful, very satisfied, more energy-saving, better, thank you boss.	Jingdong prefecture level city in Shandong
Stowe Flagship Store	King of Power Saving	Received has been used for two days, the air conditioning can boldly open it! Energy saving effect is really quite good	Jingdong prefecture level city in Shandong
Stowe Flagship Store	King of Power Saving	It is authentic, trust the merchant, the baby has a great texture and the price is moderate, unlimited repurchase!	Jingdong prefecture level city in Shandong
Stowe Flagship Store	King of Power Saving	It is genuine very good, the price is much cheaper, cost-effective, really special cost-effective, it is worth buying!	Jingdong prefecture level city in Shandong
Stowe Flagship Store	King of Power Saving	Very good quality, fine workmanship, low power consumption, long warranty, fast shipping, economical and practical	Jingdong prefecture level city in Shandong
Stowe Flagship Store	King of Power Saving	I've been using it for half a month now, it really saves power and the voltage is stable, it's very good	Jingdong prefecture level city in Shandong
Stowe Flagship Store	King of Power Saving	It's the ideal product 💞, good quality and fast logistics, worth the kudos!	Jingdong prefecture level city in Shandong
Stowe Flagship Store	King of Power Saving	The logistics is very fast, the baby is very good, the shopkeeper is NICE!	Jingdong prefecture level city in Shandong
Stowe Flagship Store	King of Power Saving	Good to use, good quality, save point durable	Jingdong prefecture level city in Shandong
Stowe Flagship Store	King of Power Saving	The quality is very good, customer service attitude is very, very awesome, there are problems in a timely manner to solve the problem	Jingdong prefecture level city in Shandong
Stowe Flagship Store	King of Power Saving	Quality is very good, the use of the effect is very good, more stable, quality assurance	Jingdong prefecture level city in Shandong
Stowe Flagship Store	King of Power Saving	Convenient, inexpensive, and see how it works out?	Jingdong prefecture level city in Shandong
Stowe Flagship Store	King of Power Saving	Very good quality, saves a lot of electricity, very good, worth buying	Jingdong prefecture level city in Shandong
Stowe Flagship Store	King of Power Saving	Power Saving King is easy to use, with the fruit of power saving to use easy to give!	Jingdong prefecture level city in Shandong
Stowe Flagship Store	King of Power Saving	This saver really works, good quality, safe and secure, very satisfied!	Jingdong prefecture level city in Shandong
Stowe Flagship Store	King of Power Saving	It works well, the voltage is very stable effect is good, the quality is also very good satisfaction	Jingdong prefecture level city in Shandong
Stowe Flagship Store	King of Power Saving	The logistics is super super fast! The quality is also very good, indeed value for money	Jingdong prefecture level city in Shandong
Stowe Flagship Store	King of Power Saving	I've only been using it for a month or so and it seems to be saving a lot of power	Jingdong prefecture level city in Shandong
Stowe Flagship Store	King of Power Saving	Very easy to operate, very good material, good value for money.	Jingdong prefecture level city in Shandong
Stowe Flagship Store	King of Power Saving	Aluminum alloy shell, durable, compact power-saving good effect	Jingdong prefecture level city in Shandong
Stowe Flagship Store	King of Power Saving	Nice device, easy to use, doesn't take up much space, recommendable!	Jingdong prefecture level city in Shandong
Stowe Flagship Store	King of Power Saving	Good quality, good price, fast logistics	Jingdong prefecture level city in Shandong
Stowe Flagship Store	King of Power Saving	Friends of the family in use recommended, the effect is good and recommendable!	Jingdong prefecture level city in Shandong
Stowe Flagship Store	King of Power Saving	Cheaper than brick and mortar stores worth buying is authentic	Jingdong prefecture level city in Shandong
Stowe Flagship Store	King of Power Saving	Merchants ship quickly, the price is cheaper than the physical store, good use	Jingdong prefecture level city in Shandong
Stowe Flagship Store	King of Power Saving	Very durable, affordable, fast shipping	Jingdong prefecture level city in Shandong
Stowe Flagship Store	King of Power Saving	Packaging tight quality is very good easy to use good results	Jingdong prefecture level city in Shandong
Stowe Flagship Store	King of Power Saving	Save the grid. It's in the whole house. It's saving money on the electric bill.	Jingdong prefecture level city in Shandong
Stowe Flagship Store	King of Power Saving	I used it today, it works well, I'm very satisfied	Jingdong prefecture level city in Shandong
Stowe Flagship Store	King of Power Saving	It really saves a lot of electricity after installation	Jingdong prefecture level city in Shandong
Stowe Flagship Store	King of Power Saving	Love it. Satisfied.	Jingdong prefecture level city in Shandong
Stowe Flagship Store	King of Power Saving	Doesn't feel like it's working.	Jingdong prefecture level city in Shandong
Stowe Flagship Store	King of Power Saving	I'll comment after using it.	Jingdong prefecture level city in Shandong
Stowe Flagship Store	King of Power Saving	I'm trying it out to see if it saves power, and I'll buy two more if it works well.	Jingdong prefecture level city in Shandong
Stowe Flagship Store	King of Power Saving	It is genuine very good, the price is much cheaper, cost-effective, really special cost-effective, it is worth buying!	Jingdong prefecture level city in Shandong
Stowe Flagship Store	King of Power Saving	This thing can really save electricity, I was introduced to buy by my friend, he used it very well, I used it and think it's also very good, it can really save electricity, save about half of the electricity every day, it's really good!	Jingdong prefecture level city in Shandong
Stowe Flagship Store	King of Power Saving	After using it for ten days or so, I checked the power consumption and it did drop, and customer service said it will save more electricity in the long run. The product is also insured, 5 years warranty do not have to worry about quality problems!	Jingdong prefecture level city in Shandong
Stowe Flagship Store	King of Power Saving	The product is good, before already bought a, this is the second, the effect is quite good, anyway, I see the daily electricity bill than before a lot less, this can be the choice!	Jingdong prefecture level city in Shandong

Stowe Flagship Store	King of Power Saving	Originally also very worried about things are not good, get the hand found completely over-anxious, five-star praise, the style is very generous, the quality is very good, the price is also very reasonable!	Jingdong prefecture level city in Shandong
Stowe Flagship Store	King of Power Saving	Quality is good, playing to the old family also buy a, repurchase, this quality is really good, recommended!	Jingdong prefecture level city in Shandong
Stowe Flagship Store	King of Power Saving	Thank you, the owner even sent two fuses.	Jingdong prefecture level city in Shandong
Stowe Flagship Store	King of Power Saving	This store has great stuff, relatively fast shipping and logistics	Jingdong prefecture level city in Shandong
Stowe Flagship Store	King of Power Saving	This product is based on the impossibility of saving electricity, I used 853 kWh of electricity in July, started using the product on August 1, and used 867 kWh of electricity by September 1, is this called	Jingdong prefecture level city in Shandong
Stowe Flagship Store	King of Power Saving	Power king received, to the home on the trial, when I plugged into the electric panel can not see the effect of power saving, the meter is still the same speed in the turn, I'll save the king of the manual to play look at the instructions, instructions to speak, have to have a day two days and half a month to save power obviously, then wait half a month to see the effect of power saving it! To half a month there are obvious power saving before continuing to chase Xu.	Jingdong prefecture level city in Shandong
Stowe Flagship Store	King of Power Saving	Received the goods, a look, did not see the manufacturer and production address, can only hold a try to see the mentality of the face	Jingdong prefecture level city in Shandong
Stowe Flagship Store	King of Power Saving	I've only been using it for a few days and I'm not sure if it's an IQ tax? Used to favorable reviews.	Jingdong prefecture level city in Shandong
Stowe Flagship Store	King of Power Saving	Appearance is okay, power saving next time to follow up the evaluation	Jingdong prefecture level city in Shandong
Stowe Flagship Store	King of Power Saving	Shorter time, but hopefully effective and power-saving, to be followed up in the future	Jingdong prefecture level city in Shandong
Stowe Flagship Store	King of Power Saving	Appearance is okay, I do not know if the power saving	Jingdong prefecture level city in Shandong
Stowe Flagship Store	King of Power Saving	Good service, fast logistics, Power Saving King is exquisite and beautiful, support the business, thank you!	Jingdong prefecture level city in Shandong
Stowe Flagship Store	King of Power Saving	Bought three at once to try first first.	Jingdong prefecture level city in Shandong
Stowe Flagship Store	King of Power Saving	The object is the same as the picture, the product is of high quality, the packaging is intact. High-end atmosphere, high power, power saving effect is obvious, average monthly power saving 50%, very satisfied.	Jingdong prefecture level city in Shandong
Stowe Flagship Store	King of Power Saving	Bought back and plugged in, really effective, before 16 degrees of electricity per day, now 8 degrees of electricity per day, open an air conditioner for 12 hours, decisive repurchase of a	Jingdong prefecture level city in Shandong
Stowe Flagship Store	King of Power Saving	The object is the same as the picture, the product is of high quality, and the package is intact. High-end atmosphere and high grade, high power	Jingdong prefecture level city in Shandong
Stowe Flagship Store	King of Power Saving	Goods received, I hope the store said can be energy-saving, first try a month, month after month to see the effect.	Jingdong prefecture level city in Shandong
Stowe Flagship Store	King of Power Saving	This is really good, the material and workmanship is particularly exquisite, the workmanship is really very good, I think I can recommend to my friends to buy	Jingdong prefecture level city in Shandong
Stowe Flagship Store	King of Power Saving	I have to praise, the quality is really good, and the packaging logistics are also good. Really like it, won't make you regret after getting it!	Jingdong prefecture level city in Shandong
Stowe Flagship Store	King of Power Saving	Reputable platform, efficient and professional service, buy with confidence and peace of mind!	Jingdong prefecture level city in Shandong
Stowe Flagship Store	King of Power Saving	Bought one on a trial basis to see if it works after 1-2 months.	Jingdong prefecture level city in Shandong
Stowe Flagship Store	King of Power Saving	Quality is very good, customer service attitude is very very praise, there are problems in a timely manner to solve the problem, the price is not expensive, super value, you can rest assured that the	Jingdong prefecture level city in Shandong
Stowe Flagship Store	King of Power Saving	With it no longer need to be afraid to use electricity, especially good, ready to buy a mom and dad that also	Jingdong prefecture level city in Shandong
Stowe Flagship Store	King of Power Saving	Baby received very like will return to buy the seller attitude is also very like will return to buy the seller attitude is also very like!	Jingdong prefecture level city in Shandong
Stowe Flagship Store	King of Power Saving	Jingdong Mall shopping convenience assured fast assured fast assured fast assured fast	Jingdong prefecture level city in Shandong
Stowe Flagship Store	King of Power Saving	Saving power received, the quality is very good, fine workmanship, especially good, much cheaper than the entity store, it is worth buying!	Jingdong prefecture level city in Shandong
Stowe Flagship Store	King of Power Saving	I did not expect the express delivery so quickly, very save point quality is super good, than the entity store is too cheap.	Jingdong prefecture level city in Shandong
Stowe Flagship Store	King of Power Saving	Have received the baby, very satisfied with a shopping, great worth buying!	Jingdong prefecture level city in Shandong
Stowe Flagship Store	King of Power Saving	This is really more powerful, very satisfied, more energy-saving, better, thank you boss.	Jingdong prefecture level city in Shandong
Stowe Flagship Store	King of Power Saving	Received has been used for two days, the air conditioning can boldly open it! Energy saving effect is really quite good	Jingdong prefecture level city in Shandong
Stowe Flagship Store	King of Power Saving	It is authentic, trust the merchant, the baby has a great texture and the price is moderate, unlimited repurchase!	Jingdong prefecture level city in Shandong
Stowe Flagship Store	King of Power Saving	It is genuine very good, the price is much cheaper, cost-effective, really special cost-effective, it is worth buying!	Jingdong prefecture level city in Shandong
Stowe Flagship Store	King of Power Saving	Very good quality, fine workmanship, low power consumption, long warranty, fast shipping, economical and practical	Jingdong prefecture level city in Shandong
Stowe Flagship Store	King of Power Saving	I've been using it for half a month now, it really saves power and the voltage is stable, it's very good	Jingdong prefecture level city in Shandong
Stowe Flagship Store	King of Power Saving	It's the ideal product 💖, good quality and fast logistics, worth the kudos!	Jingdong prefecture level city in Shandong
Stowe Flagship Store	King of Power Saving	The logistics is very fast, the baby is very good, the shopkeeper is NICE!	Jingdong prefecture level city in Shandong
Stowe Flagship Store	King of Power Saving	Good to use, good quality, save point durable	Jingdong prefecture level city in Shandong
Stowe Flagship Store	King of Power Saving	The quality is very good, customer service attitude is very, very awesome, there are problems in a timely manner to solve the problem	Jingdong prefecture level city in Shandong
Stowe Flagship Store	King of Power Saving	Quality is very good, the use of the effect is very good, more stable, quality assurance	Jingdong prefecture level city in Shandong
Stowe Flagship Store	King of Power Saving	Convenient, inexpensive, and see how it works out?	Jingdong prefecture level city in Shandong
Stowe Flagship Store	King of Power Saving	Very good quality, saves a lot of electricity, very good, worth buying	Jingdong prefecture level city in Shandong
Stowe Flagship Store	King of Power Saving	Power Saving King is easy to use, with the fruit of power saving to use easy to give!	Jingdong prefecture level city in Shandong
Stowe Flagship Store	King of Power Saving	This saver really works, good quality, safe and secure, very satisfied!	Jingdong prefecture level city in Shandong
Stowe Flagship Store	King of Power Saving	It works well, the voltage is very stable effect is good, the quality is also very good satisfaction	Jingdong prefecture level city in Shandong
Stowe Flagship Store	King of Power Saving	The logistics is super super fast! The quality is also very good, indeed value for money	Jingdong prefecture level city in Shandong
Stowe Flagship Store	King of Power Saving	I've only been using it for a month or so and it seems to be saving a lot of power	Jingdong prefecture level city in Shandong
Stowe Flagship Store	King of Power Saving	Very easy to operate, very good material, good value for money.	Jingdong prefecture level city in Shandong
Stowe Flagship Store	King of Power Saving	Aluminum alloy shell, durable, compact power-saving good effect	Jingdong prefecture level city in Shandong
Stowe Flagship Store	King of Power Saving	Nice device, easy to use, doesn't take up much space, recommendable!	Jingdong prefecture level city in Shandong
Stowe Flagship Store	King of Power Saving	Good quality, good price, fast logistics	Jingdong prefecture level city in Shandong

Stowe Flagship Store	King of Power Saving	Friends of the family in use recommended, the effect is good and recommendable!	Jingdong prefecture level city in Shandong
Stowe Flagship Store	King of Power Saving	Cheaper than brick and mortar stores worth buying is authentic	Jingdong prefecture level city in Shandong
Stowe Flagship Store	King of Power Saving	Merchants ship quickly, the price is cheaper than the physical store, good use	Jingdong prefecture level city in Shandong
Stowe Flagship Store	King of Power Saving	Very durable, affordable, fast shipping	Jingdong prefecture level city in Shandong
Stowe Flagship Store	King of Power Saving	Packaging tight quality is very good easy to use good results	Jingdong prefecture level city in Shandong
Stowe Flagship Store	King of Power Saving	Save the grid. It's in the whole house. It's saving money on the electric bill.	Jingdong prefecture level city in Shandong
Stowe Flagship Store	King of Power Saving	I used it today, it works well, I'm very satisfied	Jingdong prefecture level city in Shandong
Stowe Flagship Store	King of Power Saving	It really saves a lot of electricity after installation	Jingdong prefecture level city in Shandong
Stowe Flagship Store	King of Power Saving	Love it. Satisfied.	Jingdong prefecture level city in Shandong
Stowe Flagship Store	King of Power Saving	Doesn't feel like it's working.	Jingdong prefecture level city in Shandong
Stowe Flagship Store	King of Power Saving	I'll comment after using it.	Jingdong prefecture level city in Shandong
Stowe Flagship Store	King of Power Saving	I'm trying it out to see if it saves power, and I'll buy two more if it works well.	Jingdong prefecture level city in Shandong
Stowe Flagship Store	King of Power Saving	It is genuine very good, the price is much cheaper, cost-effective, really special cost-effective, it is worth buying!	Jingdong prefecture level city in Shandong
Stowe Flagship Store	King of Power Saving	This thing can really save electricity, I was introduced to buy by my friend, he used it very well, I used it and think it's also very good, it can really save electricity, save about half of the electricity every day, it's really good!	Jingdong prefecture level city in Shandong
Stowe Flagship Store	King of Power Saving	After using it for ten days or so, I checked the power consumption and it did drop, and customer service said it will save more electricity in the long run. The product is also insured, 5 years warranty do not have to worry about quality problems!	Jingdong prefecture level city in Shandong
Stowe Flagship Store	King of Power Saving	The product is good, before already bought a, this is the second, the effect is quite good, anyway, I see the daily electricity bill than before a lot less, this can be the choice!	Jingdong prefecture level city in Shandong
Stowe Flagship Store	King of Power Saving	Originally also very worried about things are not good, get the hand found completely over-anxious, five-star praise, the style is very generous, the quality is very good, the price is also very reasonable!	Jingdong prefecture level city in Shandong
Stowe Flagship Store	King of Power Saving	Quality is good, playing to the old family also buy a, repurchase, this quality is really good, recommended!	Jingdong prefecture level city in Shandong
Stowe Flagship Store	King of Power Saving	Thank you, the owner even sent two fuses.	Jingdong prefecture level city in Shandong
Stowe Flagship Store	King of Power Saving	This store has great stuff, relatively fast shipping and logistics	Jingdong prefecture level city in Shandong
Stowe Flagship Store	King of Power Saving	This product is based on the impossibility of saving electricity, I used 853 kWh of electricity in July, started using the product on August 1, and used 867 kWh of electricity by September 1, is this called	Jingdong prefecture level city in Shandong
Stowe Flagship Store	King of Power Saving	Power king received, to the home on the trial, when I plugged into the electric panel can not see the effect of power saving, the meter is still the same speed in the turn, I'll save the king of the manual to play look at the instructions, instructions to speak, have to have a day two days and half a month to save power obviously, then wait half a month to see the effect of power saving it! To half a month there are obvious power saving before continuing to chase Xu.	Jingdong prefecture level city in Shandong
Stowe Flagship Store	King of Power Saving	Received the goods, a look, did not see the manufacturer and production address, can only hold a try to see the mentality of the face	Jingdong prefecture level city in Shandong
Stowe Flagship Store	King of Power Saving	I've only been using it for a few days and I'm not sure if it's an IQ tax? Used to favorable reviews.	Jingdong prefecture level city in Shandong
Stowe Flagship Store	King of Power Saving	Appearance is okay, power saving next time to follow up the evaluation	Jingdong prefecture level city in Shandong
Stowe Flagship Store	King of Power Saving	Shorter time, but hopefully effective and power-saving, to be followed up in the future	Jingdong prefecture level city in Shandong
Stowe Flagship Store	King of Power Saving	Appearance is okay, I do not know if the power saving	Jingdong prefecture level city in Shandong
Stowe Flagship Store	King of Power Saving	Good service, fast logistics, Power Saving King is exquisite and beautiful, support the business, thank you!	Jingdong prefecture level city in Shandong
Stowe Flagship Store	King of Power Saving	Bought three at once to try first first.	Jingdong prefecture level city in Shandong
Stowe Flagship Store	King of Power Saving	The object is the same as the picture, the product is of high quality, the packaging is intact. High-end atmosphere, high power, power saving effect is obvious, average monthly power saving 50%, very satisfied.	Jingdong prefecture level city in Shandong
Stowe Flagship Store	King of Power Saving	Bought back and plugged in, really effective, before 16 degrees of electricity per day, now 8 degrees of electricity per day, open an air conditioner for 12 hours, decisive repurchase of a	Jingdong prefecture level city in Shandong
Stowe Flagship Store	King of Power Saving	The object is the same as the picture, the product is of high quality, and the package is intact. High-end atmosphere and high grade, high power	Jingdong prefecture level city in Shandong
Stowe Flagship Store	King of Power Saving	Goods received, I hope the store said can be energy-saving, first try a month, month after month to see the effect.	Jingdong prefecture level city in Shandong
Stowe Flagship Store	King of Power Saving	This is really good, the material and workmanship is particularly exquisite, the workmanship is really very good, I think I can recommend to my friends to buy	Jingdong prefecture level city in Shandong
Stowe Flagship Store	King of Power Saving	I have to praise, the quality is really good, and the packaging logistics are also good. Really like it, won't make you regret after getting it!	Jingdong prefecture level city in Shandong
Stowe Flagship Store	King of Power Saving	Reputable platform, efficient and professional service, buy with confidence and peace of mind!	Jingdong prefecture level city in Shandong
Stowe Flagship Store	King of Power Saving	Bought one on a trial basis to see if it works after 1-2 months.	Jingdong prefecture level city in Shandong
Stowe Flagship Store	King of Power Saving	Quality is very good, customer service attitude is very very praise, there are problems in a timely manner to solve the problem, the price is not expensive, super value, you can rest assured that the	Jingdong prefecture level city in Shandong
Stowe Flagship Store	King of Power Saving	With it no longer need to be afraid to use electricity, especially good, ready to buy a mom and dad that also	Jingdong prefecture level city in Shandong
Stowe Flagship Store	King of Power Saving	Baby received very like will return to buy the seller attitude is also very like will return to buy the seller attitude is also very like!	Jingdong prefecture level city in Shandong
Stowe Flagship Store	King of Power Saving	Jingdong Mall shopping convenience assured fast assured fast assured fast assured fast	Jingdong prefecture level city in Shandong
Stowe Flagship Store	King of Power Saving	Saving power received, the quality is very good, fine workmanship, especially good, much cheaper than the entity store, it is worth buying!	Jingdong prefecture level city in Shandong
Stowe Flagship Store	King of Power Saving	I did not expect the express delivery so quickly, very save point quality is super good, than the entity store is too cheap.	Jingdong prefecture level city in Shandong
Stowe Flagship Store	King of Power Saving	Have received the baby, very satisfied with a shopping, great worth buying!	Jingdong prefecture level city in Shandong
Stowe Flagship Store	King of Power Saving	This is really more powerful, very satisfied, more energy-saving, better, thank you boss.	Jingdong prefecture level city in Shandong
Stowe Flagship Store	King of Power Saving	Received has been used for two days, the air conditioning can boldly open it! Energy saving effect is really quite good	Jingdong prefecture level city in Shandong
Stowe Flagship Store	King of Power Saving	It is authentic, trust the merchant, the baby has a great texture and the price is moderate, unlimited repurchase!	Jingdong prefecture level city in Shandong
Stowe Flagship Store	King of Power Saving	It is genuine very good, the price is much cheaper, cost-effective, really special cost-effective, it is worth buying!	Jingdong prefecture level city in Shandong
Stowe Flagship Store	King of Power Saving	Very good quality, fine workmanship, low power consumption, long warranty, fast shipping, economical and practical	Jingdong prefecture level city in Shandong

Stowe Flagship Store	King of Power Saving	I've been using it for half a month now, it really saves power and the voltage is stable, it's very good	Jingdong prefecture level city in Shandong
Stowe Flagship Store	King of Power Saving	It's the ideal product ♡, good quality and fast logistics, worth the kudos!	Jingdong prefecture level city in Shandong
Stowe Flagship Store	King of Power Saving	The logistics is very fast, the baby is very good, the shopkeeper is NICE!	Jingdong prefecture level city in Shandong
Stowe Flagship Store	King of Power Saving	Good to use, good quality, save point durable	Jingdong prefecture level city in Shandong
Stowe Flagship Store	King of Power Saving	The quality is very good, customer service attitude is very, very awesome, there are problems in a timely manner to solve the problem	Jingdong prefecture level city in Shandong
Stowe Flagship Store	King of Power Saving	Quality is very good, the use of the effect is very good, more stable, quality assurance	Jingdong prefecture level city in Shandong
Stowe Flagship Store	King of Power Saving	Convenient, inexpensive, and see how it works out?	Jingdong prefecture level city in Shandong
Stowe Flagship Store	King of Power Saving	Very good quality, saves a lot of electricity, very good, worth buying	Jingdong prefecture level city in Shandong
Stowe Flagship Store	King of Power Saving	Power Saving King is easy to use, with the fruit of power saving to use easy to give!	Jingdong prefecture level city in Shandong
Stowe Flagship Store	King of Power Saving	This saver really works, good quality, safe and secure, very satisfied!	Jingdong prefecture level city in Shandong
Stowe Flagship Store	King of Power Saving	It works well, the voltage is very stable effect is good, the quality is also very good satisfaction	Jingdong prefecture level city in Shandong
Stowe Flagship Store	King of Power Saving	The logistics is super super fast! The quality is also very good, indeed value for money	Jingdong prefecture level city in Shandong
Stowe Flagship Store	King of Power Saving	I've only been using it for a month or so and it seems to be saving a lot of power	Jingdong prefecture level city in Shandong
Stowe Flagship Store	King of Power Saving	Very easy to operate, very good material, good value for money.	Jingdong prefecture level city in Shandong
Stowe Flagship Store	King of Power Saving	Aluminum alloy shell, durable, compact power-saving good effect	Jingdong prefecture level city in Shandong
Stowe Flagship Store	King of Power Saving	Nice device, easy to use, doesn't take up much space, recommendable!	Jingdong prefecture level city in Shandong
Stowe Flagship Store	King of Power Saving	Good quality, good price, fast logistics	Jingdong prefecture level city in Shandong
Stowe Flagship Store	King of Power Saving	Friends of the family in use recommended, the effect is good and recommendable!	Jingdong prefecture level city in Shandong
Stowe Flagship Store	King of Power Saving	Cheaper than brick and mortar stores worth buying is authentic	Jingdong prefecture level city in Shandong
Stowe Flagship Store	King of Power Saving	Merchants ship quickly, the price is cheaper than the physical store, good use	Jingdong prefecture level city in Shandong
Stowe Flagship Store	King of Power Saving	Very durable, affordable, fast shipping	Jingdong prefecture level city in Shandong
Stowe Flagship Store	King of Power Saving	Packaging tight quality is very good easy to use good results	Jingdong prefecture level city in Shandong
Stowe Flagship Store	King of Power Saving	Save the grid. It's in the whole house. It's saving money on the electric bill.	Jingdong prefecture level city in Shandong
Stowe Flagship Store	King of Power Saving	I used it today, it works well, I'm very satisfied	Jingdong prefecture level city in Shandong
Stowe Flagship Store	King of Power Saving	It really saves a lot of electricity after installation	Jingdong prefecture level city in Shandong
Stowe Flagship Store	King of Power Saving	Love it. Satisfied.	Jingdong prefecture level city in Shandong
Stowe Flagship Store	King of Power Saving	Doesn't feel like it's working.	Jingdong prefecture level city in Shandong
Stowe Flagship Store	King of Power Saving	I'll comment after using it.	Jingdong prefecture level city in Shandong
Stowe Flagship Store	King of Power Saving	I'm trying it out to see if it saves power, and I'll buy two more if it works well.	Jingdong prefecture level city in Shandong
Stowe Flagship Store	King of Power Saving	It is genuine very good, the price is much cheaper, cost-effective, really special cost-effective, it is worth buying!	Jingdong prefecture level city in Shandong
Stowe Flagship Store	King of Power Saving	This thing can really save electricity, I was introduced to buy by my friend, he used it very well, I used it and think it's also very good, it can really save electricity, save about half of the electricity every day, it's really good!	Jingdong prefecture level city in Shandong
Stowe Flagship Store	King of Power Saving	After using it for ten days or so, I checked the power consumption and it did drop, and customer service said it will save more electricity in the long run. The product is also insured, 5 years warranty do not have to worry about quality problems!	Jingdong prefecture level city in Shandong
Stowe Flagship Store	King of Power Saving	The product is good, before already bought a, this is the second, the effect is quite good, anyway, I see the daily electricity bill than before a lot less, this can be the choice!	Jingdong prefecture level city in Shandong
Stowe Flagship Store	King of Power Saving	Originally also very worried about things are not good, get the hand found completely over-anxious, five-star praise, the style is very generous, the quality is very good, the price is also very reasonable!	Jingdong prefecture level city in Shandong
Stowe Flagship Store	King of Power Saving	Quality is good, playing to the old family also buy a, repurchase, this quality is really good, recommended!	Jingdong prefecture level city in Shandong
Stowe Flagship Store	King of Power Saving	Thank you, the owner even sent two fuses.	Jingdong prefecture level city in Shandong
Stowe Flagship Store	King of Power Saving	This store has great stuff, relatively fast shipping and logistics	Jingdong prefecture level city in Shandong
Stowe Flagship Store	King of Power Saving	This product is based on the impossibility of saving electricity, I used 853 kWh of electricity in July, started using the product on August 1, and used 867 kWh of electricity by September 1, is this called	Jingdong prefecture level city in Shandong
Stowe Flagship Store	King of Power Saving	Power king received, to the home on the trial, when I plugged into the electric panel can not see the effect of power saving, the meter is still the same speed in the turn, I'll save the king of the manual to play look at the instructions, instructions to speak, have to have a day two days and half a month to save power obviously, then wait half a month to see the effect of power saving it! To half a month there are obvious power saving before continuing to chase Xu.	Jingdong prefecture level city in Shandong
Stowe Flagship Store	King of Power Saving	Received the goods, a look, did not see the manufacturer and production address, can only hold a try to see the mentality of the face	Jingdong prefecture level city in Shandong
Stowe Flagship Store	King of Power Saving	I've only been using it for a few days and I'm not sure if it's an IQ tax? Used to favorable reviews.	Jingdong prefecture level city in Shandong
Stowe Flagship Store	King of Power Saving	Appearance is okay, power saving next time to follow up the evaluation	Jingdong prefecture level city in Shandong
Stowe Flagship Store	King of Power Saving	Shorter time, but hopefully effective and power-saving, to be followed up in the future	Jingdong prefecture level city in Shandong
Stowe Flagship Store	King of Power Saving	Appearance is okay, I do not know if the power saving	Jingdong prefecture level city in Shandong
Stowe Flagship Store	King of Power Saving	Good service, fast logistics, Power Saving King is exquisite and beautiful, support the business, thank you!	Jingdong prefecture level city in Shandong
Stowe Flagship Store	King of Power Saving	Bought three at once to try first first.	Jingdong prefecture level city in Shandong
Stowe Flagship Store	King of Power Saving	The object is the same as the picture, the product is of high quality, the packaging is intact. High-end atmosphere, high power, power saving effect is obvious, average monthly power saving 50%, very satisfied.	Jingdong prefecture level city in Shandong
Stowe Flagship Store	King of Power Saving	Bought back and plugged in, really effective, before 16 degrees of electricity per day, now 8 degrees of electricity per day, open an air conditioner for 12 hours, decisive repurchase of a	Jingdong prefecture level city in Shandong
Stowe Flagship Store	King of Power Saving	The object is the same as the picture, the product is of high quality, and the package is intact. High-end atmosphere and high grade, high power	Jingdong prefecture level city in Shandong

Stowe Flagship Store	King of Power Saving	Goods received, I hope the store said can be energy-saving, first try a month, month after month to see the effect.	Jingdong prefecture level city in Shandong
Stowe Flagship Store	King of Power Saving	This is really good, the material and workmanship is particularly exquisite, the workmanship is really very good, I think I can recommend to my friends to buy	Jingdong prefecture level city in Shandong
Stowe Flagship Store	King of Power Saving	I have to praise, the quality is really good, and the packaging logistics are also good. Really like it, won't make you regret after getting it!	Jingdong prefecture level city in Shandong
Stowe Flagship Store	King of Power Saving	Reputable platform, efficient and professional service, buy with confidence and peace of mind!	Jingdong prefecture level city in Shandong
Stowe Flagship Store	King of Power Saving	Bought one on a trial basis to see if it works after 1-2 months.	Jingdong prefecture level city in Shandong
Stowe Flagship Store	King of Power Saving	Quality is very good, customer service attitude is very very praise, there are problems in a timely manner to solve the problem, the price is not expensive, super value, you can rest assured that the	Jingdong prefecture level city in Shandong
Stowe Flagship Store	King of Power Saving	With it no longer need to be afraid to use electricity, especially good, ready to buy a mom and dad that also	Jingdong prefecture level city in Shandong
Stowe Flagship Store	King of Power Saving	Baby received very like will return to buy the seller attitude is also very like will return to buy the seller attitude is also very like!	Jingdong prefecture level city in Shandong
Stowe Flagship Store	King of Power Saving	Jingdong Mall shopping convenience assured fast assured fast assured fast assured fast	Jingdong prefecture level city in Shandong
Stowe Flagship Store	King of Power Saving	Saving power received, the quality is very good, fine workmanship, especially good, much cheaper than the entity store, it is worth buying!	Jingdong prefecture level city in Shandong
Stowe Flagship Store	King of Power Saving	I did not expect the express delivery so quickly, very save point quality is super good, than the entity store is too cheap.	Jingdong prefecture level city in Shandong
Stowe Flagship Store	King of Power Saving	Have received the baby, very satisfied with a shopping, great worth buying!	Jingdong prefecture level city in Shandong
Stowe Flagship Store	King of Power Saving	This is really more powerful, very satisfied, more energy-saving, better, thank you boss.	Jingdong prefecture level city in Shandong
Stowe Flagship Store	King of Power Saving	Received has been used for two days, the air conditioning can boldly open it! Energy saving effect is really quite good	Jingdong prefecture level city in Shandong
Stowe Flagship Store	King of Power Saving	It is authentic, trust the merchant, the baby has a great texture and the price is moderate, unlimited repurchase!	Jingdong prefecture level city in Shandong
Stowe Flagship Store	King of Power Saving	It is genuine very good, the price is much cheaper, cost-effective, really special cost-effective, it is worth buying!	Jingdong prefecture level city in Shandong
Stowe Flagship Store	King of Power Saving	Very good quality, fine workmanship, low power consumption, long warranty, fast shipping, economical and practical	Jingdong prefecture level city in Shandong
Stowe Flagship Store	King of Power Saving	I've been using it for half a month now, it really saves power and the voltage is stable, it's very good	Jingdong prefecture level city in Shandong
Stowe Flagship Store	King of Power Saving	It's the ideal product 💖, good quality and fast logistics, worth the kudos!	Jingdong prefecture level city in Shandong
Stowe Flagship Store	King of Power Saving	The logistics is very fast, the baby is very good, the shopkeeper is NICE!	Jingdong prefecture level city in Shandong
Stowe Flagship Store	King of Power Saving	Good to use, good quality, save point durable	Jingdong prefecture level city in Shandong
Stowe Flagship Store	King of Power Saving	The quality is very good, customer service attitude is very, very awesome, there are problems in a timely manner to solve the problem	Jingdong prefecture level city in Shandong
Stowe Flagship Store	King of Power Saving	Quality is very good, the use of the effect is very good, more stable, quality assurance	Jingdong prefecture level city in Shandong
Stowe Flagship Store	King of Power Saving	Convenient, inexpensive, and see how it works out?	Jingdong prefecture level city in Shandong
Stowe Flagship Store	King of Power Saving	Very good quality, saves a lot of electricity, very good, worth buying	Jingdong prefecture level city in Shandong
Stowe Flagship Store	King of Power Saving	Power Saving King is easy to use, with the fruit of power saving to use easy to give!	Jingdong prefecture level city in Shandong
Stowe Flagship Store	King of Power Saving	This saver really works, good quality, safe and secure, very satisfied!	Jingdong prefecture level city in Shandong
Stowe Flagship Store	King of Power Saving	It works well, the voltage is very stable effect is good, the quality is also very good satisfaction	Jingdong prefecture level city in Shandong
Stowe Flagship Store	King of Power Saving	The logistics is super super fast! The quality is also very good, indeed value for money	Jingdong prefecture level city in Shandong
Stowe Flagship Store	King of Power Saving	I've only been using it for a month or so and it seems to be saving a lot of power	Jingdong prefecture level city in Shandong
Stowe Flagship Store	King of Power Saving	Very easy to operate, very good material, good value for money.	Jingdong prefecture level city in Shandong
Stowe Flagship Store	King of Power Saving	Aluminum alloy shell, durable, compact power-saving good effect	Jingdong prefecture level city in Shandong
Stowe Flagship Store	King of Power Saving	Nice device, easy to use, doesn't take up much space, recommendable!	Jingdong prefecture level city in Shandong
Stowe Flagship Store	King of Power Saving	Good quality, good price, fast logistics	Jingdong prefecture level city in Shandong
Stowe Flagship Store	King of Power Saving	Friends of the family in use recommended, the effect is good and recommendable!	Jingdong prefecture level city in Shandong
Stowe Flagship Store	King of Power Saving	Cheaper than brick and mortar stores worth buying is authentic	Jingdong prefecture level city in Shandong
Stowe Flagship Store	King of Power Saving	Merchants ship quickly, the price is cheaper than the physical store, good use	Jingdong prefecture level city in Shandong
Stowe Flagship Store	King of Power Saving	Very durable, affordable, fast shipping	Jingdong prefecture level city in Shandong
Stowe Flagship Store	King of Power Saving	Packaging tight quality is very good easy to use good results	Jingdong prefecture level city in Shandong
Stowe Flagship Store	King of Power Saving	Save the grid. It's in the whole house. It's saving money on the electric bill.	Jingdong prefecture level city in Shandong
Stowe Flagship Store	King of Power Saving	I used it today, it works well, I'm very satisfied	Jingdong prefecture level city in Shandong
Stowe Flagship Store	King of Power Saving	It really saves a lot of electricity after installation	Jingdong prefecture level city in Shandong
Stowe Flagship Store	King of Power Saving	Love it. Satisfied.	Jingdong prefecture level city in Shandong
Stowe Flagship Store	King of Power Saving	Doesn't feel like it's working.	Jingdong prefecture level city in Shandong
Stowe Flagship Store	King of Power Saving	I'll comment after using it.	Jingdong prefecture level city in Shandong
Stowe Flagship Store	King of Power Saving	I'm trying it out to see if it saves power, and I'll buy two more if it works well.	Jingdong prefecture level city in Shandong
Stowe Flagship Store	King of Power Saving	It is genuine very good, the price is much cheaper, cost-effective, really special cost-effective, it is worth buying!	Jingdong prefecture level city in Shandong
Stowe Flagship Store	King of Power Saving	This thing can really save electricity, I was introduced to buy by my friend, he used it very well, I used it and think it's also very good, it can really save electricity, save about half of the electricity every day, it's really good!	Jingdong prefecture level city in Shandong
Stowe Flagship Store	King of Power Saving	After using it for ten days or so, I checked the power consumption and it did drop, and customer service said it will save more electricity in the long run. The product is also insured, 5 years warranty do not have to worry about quality problems!	Jingdong prefecture level city in Shandong
Stowe Flagship Store	King of Power Saving	The product is good, before already bought a, this is the second, the effect is quite good, anyway, I see the daily electricity bill than before a lot less, this can be the choice!	Jingdong prefecture level city in Shandong

Stowe Flagship Store	King of Power Saving	Originally also very worried about things are not good, get the hand found completely over-anxious, five-star praise, the style is very generous, the quality is very good, the price is also very reasonable!	Jingdong prefecture level city in Shandong
Stowe Flagship Store	King of Power Saving	Quality is good, playing to the old family also buy a, repurchase, this quality is really good, recommended!	Jingdong prefecture level city in Shandong
Stowe Flagship Store	King of Power Saving	Thank you, the owner even sent two fuses.	Jingdong prefecture level city in Shandong
Stowe Flagship Store	King of Power Saving	This store has great stuff, relatively fast shipping and logistics	Jingdong prefecture level city in Shandong
Stowe Flagship Store	King of Power Saving	This product is based on the impossibility of saving electricity, I used 853 kWh of electricity in July, started using the product on August 1, and used 867 kWh of electricity by September 1, is this called	Jingdong prefecture level city in Shandong
Stowe Flagship Store	King of Power Saving	Power king received, to the home on the trial, when I plugged into the electric panel can not see the effect of power saving, the meter is still the same speed in the turn, I'll save the king of the manual to play look at the instructions, instructions to speak, have to have a day two days and half a month to save power obviously, then wait half a month to see the effect of power saving it! To half a month there are obvious power saving before continuing to chase Xu.	Jingdong prefecture level city in Shandong
Stowe Flagship Store	King of Power Saving	Received the goods, a look, did not see the manufacturer and production address, can only hold a try to see the mentality of the face	Jingdong prefecture level city in Shandong
Stowe Flagship Store	King of Power Saving	I've only been using it for a few days and I'm not sure if it's an IQ tax? Used to favorable reviews.	Jingdong prefecture level city in Shandong
Stowe Flagship Store	King of Power Saving	Appearance is okay, power saving next time to follow up the evaluation	Jingdong prefecture level city in Shandong
Stowe Flagship Store	King of Power Saving	Shorter time, but hopefully effective and power-saving, to be followed up in the future	Jingdong prefecture level city in Shandong
Stowe Flagship Store	King of Power Saving	Appearance is okay, I do not know if the power saving	Jingdong prefecture level city in Shandong
Stowe Flagship Store	King of Power Saving	Good service, fast logistics, Power Saving King is exquisite and beautiful, support the business, thank you!	Jingdong prefecture level city in Shandong
Stowe Flagship Store	King of Power Saving	Bought three at once to try first first.	Jingdong prefecture level city in Shandong
Stowe Flagship Store	King of Power Saving	The object is the same as the picture, the product is of high quality, the packaging is intact. High-end atmosphere, high power, power saving effect is obvious, average monthly power saving 50%, very satisfied.	Jingdong prefecture level city in Shandong
Stowe Flagship Store	King of Power Saving	Bought back and plugged in, really effective, before 16 degrees of electricity per day, now 8 degrees of electricity per day, open an air conditioner for 12 hours, decisive repurchase of a	Jingdong prefecture level city in Shandong
Stowe Flagship Store	King of Power Saving	The object is the same as the picture, the product is of high quality, and the package is intact. High-end atmosphere and high grade, high power	Jingdong prefecture level city in Shandong
Stowe Flagship Store	King of Power Saving	Goods received, I hope the store said can be energy-saving, first try a month, month after month to see the effect.	Jingdong prefecture level city in Shandong
Stowe Flagship Store	King of Power Saving	This is really good, the material and workmanship is particularly exquisite, the workmanship is really very good, I think I can recommend to my friends to buy	Jingdong prefecture level city in Shandong
Stowe Flagship Store	King of Power Saving	I have to praise, the quality is really good, and the packaging logistics are also good. Really like it, won't make you regret after getting it!	Jingdong prefecture level city in Shandong
Stowe Flagship Store	King of Power Saving	Reputable platform, efficient and professional service, buy with confidence and peace of mind!	Jingdong prefecture level city in Shandong
Stowe Flagship Store	King of Power Saving	Bought one on a trial basis to see if it works after 1-2 months.	Jingdong prefecture level city in Shandong
Stowe Flagship Store	King of Power Saving	Quality is very good, customer service attitude is very very praise, there are problems in a timely manner to solve the problem, the price is not expensive, super value, you can rest assured that the	Jingdong prefecture level city in Shandong
Stowe Flagship Store	King of Power Saving	With it no longer need to be afraid to use electricity, especially good, ready to buy a mom and dad that also	Jingdong prefecture level city in Shandong
Stowe Flagship Store	King of Power Saving	Baby received very like will return to buy the seller attitude is also very like will return to buy the seller attitude is also very like!	Jingdong prefecture level city in Shandong
Stowe Flagship Store	King of Power Saving	Jingdong Mall shopping convenience assured fast assured fast assured fast assured fast	Jingdong prefecture level city in Shandong
Stowe Flagship Store	King of Power Saving	Saving power received, the quality is very good, fine workmanship, especially good, much cheaper than the entity store, it is worth buying!	Jingdong prefecture level city in Shandong
Stowe Flagship Store	King of Power Saving	I did not expect the express delivery so quickly, very save point quality is super good, than the entity store is too cheap.	Jingdong prefecture level city in Shandong
Stowe Flagship Store	King of Power Saving	Have received the baby, very satisfied with a shopping, great worth buying!	Jingdong prefecture level city in Shandong
Stowe Flagship Store	King of Power Saving	This is really more powerful, very satisfied, more energy-saving, better, thank you boss.	Jingdong prefecture level city in Shandong
Stowe Flagship Store	King of Power Saving	Received has been used for two days, the air conditioning can boldly open it! Energy saving effect is really quite good	Jingdong prefecture level city in Shandong
Stowe Flagship Store	King of Power Saving	It is authentic, trust the merchant, the baby has a great texture and the price is moderate, unlimited repurchase!	Jingdong prefecture level city in Shandong
Stowe Flagship Store	King of Power Saving	It is genuine very good, the price is much cheaper, cost-effective, really special cost-effective, it is worth buying!	Jingdong prefecture level city in Shandong
Stowe Flagship Store	King of Power Saving	Very good quality, fine workmanship, low power consumption, long warranty, fast shipping, economical and practical	Jingdong prefecture level city in Shandong
Stowe Flagship Store	King of Power Saving	I've been using it for half a month now, it really saves power and the voltage is stable, it's very good	Jingdong prefecture level city in Shandong
Stowe Flagship Store	King of Power Saving	It's the ideal product 💝, good quality and fast logistics, worth the kudos!	Jingdong prefecture level city in Shandong
Stowe Flagship Store	King of Power Saving	The logistics is very fast, the baby is very good, the shopkeeper is NICE!	Jingdong prefecture level city in Shandong
Stowe Flagship Store	King of Power Saving	Good to use, good quality, save point durable	Jingdong prefecture level city in Shandong
Stowe Flagship Store	King of Power Saving	The quality is very good, customer service attitude is very, very awesome, there are problems in a timely manner to solve the problem	Jingdong prefecture level city in Shandong
Stowe Flagship Store	King of Power Saving	Quality is very good, the use of the effect is very good, more stable, quality assurance	Jingdong prefecture level city in Shandong
Stowe Flagship Store	King of Power Saving	Convenient, inexpensive, and see how it works out?	Jingdong prefecture level city in Shandong
Stowe Flagship Store	King of Power Saving	Very good quality, saves a lot of electricity, very good, worth buying	Jingdong prefecture level city in Shandong
Stowe Flagship Store	King of Power Saving	Power Saving King is easy to use, with the fruit of power saving to use easy to give!	Jingdong prefecture level city in Shandong
Stowe Flagship Store	King of Power Saving	This saver really works, good quality, safe and secure, very satisfied!	Jingdong prefecture level city in Shandong
Stowe Flagship Store	King of Power Saving	It works well, the voltage is very stable effect is good, the quality is also very good satisfaction	Jingdong prefecture level city in Shandong
Stowe Flagship Store	King of Power Saving	The logistics is super super fast! The quality is also very good, indeed value for money	Jingdong prefecture level city in Shandong
Stowe Flagship Store	King of Power Saving	I've only been using it for a month or so and it seems to be saving a lot of power	Jingdong prefecture level city in Shandong
Stowe Flagship Store	King of Power Saving	Very easy to operate, very good material, good value for money.	Jingdong prefecture level city in Shandong
Stowe Flagship Store	King of Power Saving	Aluminum alloy shell, durable, compact power-saving good effect	Jingdong prefecture level city in Shandong
Stowe Flagship Store	King of Power Saving	Nice device, easy to use, doesn't take up much space, recommendable!	Jingdong prefecture level city in Shandong
Stowe Flagship Store	King of Power Saving	Good quality, good price, fast logistics	Jingdong prefecture level city in Shandong

Stowe Flagship Store	King of Power Saving	Friends of the family in use recommended, the effect is good and recommendable!	Jingdong prefecture level city in Shandong
Stowe Flagship Store	King of Power Saving	Cheaper than brick and mortar stores worth buying is authentic	Jingdong prefecture level city in Shandong
Stowe Flagship Store	King of Power Saving	Merchants ship quickly, the price is cheaper than the physical store, good use	Jingdong prefecture level city in Shandong
Stowe Flagship Store	King of Power Saving	Very durable, affordable, fast shipping	Jingdong prefecture level city in Shandong
Stowe Flagship Store	King of Power Saving	Packaging tight quality is very good easy to use good results	Jingdong prefecture level city in Shandong
Stowe Flagship Store	King of Power Saving	Save the grid. It's in the whole house. It's saving money on the electric bill.	Jingdong prefecture level city in Shandong
Stowe Flagship Store	King of Power Saving	I used it today, it works well, I'm very satisfied	Jingdong prefecture level city in Shandong
Stowe Flagship Store	King of Power Saving	It really saves a lot of electricity after installation	Jingdong prefecture level city in Shandong
Stowe Flagship Store	King of Power Saving	Love it. Satisfied.	Jingdong prefecture level city in Shandong
Stowe Flagship Store	King of Power Saving	Doesn't feel like it's working.	Jingdong prefecture level city in Shandong
Stowe Flagship Store	King of Power Saving	I'll comment after using it.	Jingdong prefecture level city in Shandong
Stowe Flagship Store	King of Power Saving	I'm trying it out to see if it saves power, and I'll buy two more if it works well.	Jingdong prefecture level city in Shandong
Stowe Flagship Store	King of Power Saving	It is genuine very good, the price is much cheaper, cost-effective, really special cost-effective, it is worth buying!	Jingdong prefecture level city in Shandong
Stowe Flagship Store	King of Power Saving	This thing can really save electricity, I was introduced to buy by my friend, he used it very well, I used it and think it's also very good, it can really save electricity, save about half of the electricity every day, it's really good!	Jingdong prefecture level city in Shandong
Stowe Flagship Store	King of Power Saving	After using it for ten days or so, I checked the power consumption and it did drop, and customer service said it will save more electricity in the long run. The product is also insured, 5 years warranty do not have to worry about quality problems!	Jingdong prefecture level city in Shandong
Stowe Flagship Store	King of Power Saving	The product is good, before already bought a, this is the second, the effect is quite good, anyway, I see the daily electricity bill than before a lot less, this can be the choice!	Jingdong prefecture level city in Shandong
Stowe Flagship Store	King of Power Saving	Originally also very worried about things are not good, get the hand found completely over-anxious, five-star praise, the style is very generous, the quality is very good, the price is also very reasonable!	Jingdong prefecture level city in Shandong
Stowe Flagship Store	King of Power Saving	Quality is good, playing to the old family also buy a, repurchase, this quality is really good, recommended!	Jingdong prefecture level city in Shandong
Stowe Flagship Store	King of Power Saving	Thank you, the owner even sent two fuses.	Jingdong prefecture level city in Shandong
Stowe Flagship Store	King of Power Saving	This store has great stuff, relatively fast shipping and logistics	Jingdong prefecture level city in Shandong
Stowe Flagship Store	King of Power Saving	This product is based on the impossibility of saving electricity, I used 853 kWh of electricity in July, started using the product on August 1, and used 867 kWh of electricity by September 1, is this called	Jingdong prefecture level city in Shandong
Stowe Flagship Store	King of Power Saving	Power king received, to the home on the trial, when I plugged into the electric panel can not see the effect of power saving, the meter is still the same speed in the turn, I'll save the king of the manual to play look at the instructions, instructions to speak, have to have a day two days and half a month to save power obviously, then wait half a month to see the effect of power saving it! To half a month there are obvious power saving before continuing to chase Xu.	Jingdong prefecture level city in Shandong
Stowe Flagship Store	King of Power Saving	Received the goods, a look, did not see the manufacturer and production address, can only hold a try to see the mentality of the face	Jingdong prefecture level city in Shandong
Stowe Flagship Store	King of Power Saving	I've only been using it for a few days and I'm not sure if it's an IQ tax? Used to favorable reviews.	Jingdong prefecture level city in Shandong
Stowe Flagship Store	King of Power Saving	Appearance is okay, power saving next time to follow up the evaluation	Jingdong prefecture level city in Shandong
Stowe Flagship Store	King of Power Saving	Shorter time, but hopefully effective and power-saving, to be followed up in the future	Jingdong prefecture level city in Shandong
Stowe Flagship Store	King of Power Saving	Appearance is okay, I do not know if the power saving	Jingdong prefecture level city in Shandong
Stowe Flagship Store	King of Power Saving	Good service, fast logistics, Power Saving King is exquisite and beautiful, support the business, thank you!	Jingdong prefecture level city in Shandong
Stowe Flagship Store	King of Power Saving	Bought three at once to try first first.	Jingdong prefecture level city in Shandong
Stowe Flagship Store	King of Power Saving	The object is the same as the picture, the product is of high quality, the packaging is intact. High-end atmosphere, high power, power saving effect is obvious, average monthly power saving 50%, very satisfied.	Jingdong prefecture level city in Shandong
Stowe Flagship Store	King of Power Saving	Bought back and plugged in, really effective, before 16 degrees of electricity per day, now 8 degrees of electricity per day, open an air conditioner for 12 hours, decisive repurchase of a	Jingdong prefecture level city in Shandong
Stowe Flagship Store	King of Power Saving	The object is the same as the picture, the product is of high quality, and the package is intact. High-end atmosphere and high grade, high power	Jingdong prefecture level city in Shandong
Stowe Flagship Store	King of Power Saving	Goods received, I hope the store said can be energy-saving, first try a month, month after month to see the effect.	Jingdong prefecture level city in Shandong
Stowe Flagship Store	King of Power Saving	This is really good, the material and workmanship is particularly exquisite, the workmanship is really very good, I think I can recommend to my friends to buy	Jingdong prefecture level city in Shandong
Stowe Flagship Store	King of Power Saving	I have to praise, the quality is really good, and the packaging logistics are also good. Really like it, won't make you regret after getting it!	Jingdong prefecture level city in Shandong
Stowe Flagship Store	King of Power Saving	Reputable platform, efficient and professional service, buy with confidence and peace of mind!	Jingdong prefecture level city in Shandong
Stowe Flagship Store	King of Power Saving	Bought one on a trial basis to see if it works after 1-2 months.	Jingdong prefecture level city in Shandong
Stowe Flagship Store	King of Power Saving	Quality is very good, customer service attitude is very very praise, there are problems in a timely manner to solve the problem, the price is not expensive, super value, you can rest assured that the	Jingdong prefecture level city in Shandong
Stowe Flagship Store	King of Power Saving	With it no longer need to be afraid to use electricity, especially good, ready to buy a mom and dad that also	Jingdong prefecture level city in Shandong
Stowe Flagship Store	King of Power Saving	Baby received very like will return to buy the seller attitude is also very like will return to buy the seller attitude is also very like!	Jingdong prefecture level city in Shandong
Stowe Flagship Store	King of Power Saving	Jingdong Mall shopping convenience assured fast assured fast assured fast assured fast	Jingdong prefecture level city in Shandong
Stowe Flagship Store	King of Power Saving	Saving power received, the quality is very good, fine workmanship, especially good, much cheaper than the entity store, it is worth buying!	Jingdong prefecture level city in Shandong
Stowe Flagship Store	King of Power Saving	I did not expect the express delivery so quickly, very save point quality is super good, than the entity store is too cheap.	Jingdong prefecture level city in Shandong
Stowe Flagship Store	King of Power Saving	Have received the baby, very satisfied with a shopping, great worth buying!	Jingdong prefecture level city in Shandong
Stowe Flagship Store	King of Power Saving	This is really more powerful, very satisfied, more energy-saving, better, thank you boss.	Jingdong prefecture level city in Shandong
Stowe Flagship Store	King of Power Saving	Received has been used for two days, the air conditioning can boldly open it! Energy saving effect is really quite good	Jingdong prefecture level city in Shandong
Stowe Flagship Store	King of Power Saving	It is authentic, trust the merchant, the baby has a great texture and the price is moderate, unlimited repurchase!	Jingdong prefecture level city in Shandong
Stowe Flagship Store	King of Power Saving	It is genuine very good, the price is much cheaper, cost-effective, really special cost-effective, it is worth buying!	Jingdong prefecture level city in Shandong
Stowe Flagship Store	King of Power Saving	Very good quality, fine workmanship, low power consumption, long warranty, fast shipping, economical and practical	Jingdong prefecture level city in Shandong

Stowe Flagship Store	King of Power Saving	I've been using it for half a month now, it really saves power and the voltage is stable, it's very good	Jingdong prefecture level city in Shandong
Stowe Flagship Store	King of Power Saving	It's the ideal product ♡, good quality and fast logistics, worth the kudos!	Jingdong prefecture level city in Shandong
Stowe Flagship Store	King of Power Saving	The logistics is very fast, the baby is very good, the shopkeeper is NICE!	Jingdong prefecture level city in Shandong
Stowe Flagship Store	King of Power Saving	Good to use, good quality, save point durable	Jingdong prefecture level city in Shandong
Stowe Flagship Store	King of Power Saving	The quality is very good, customer service attitude is very, very awesome, there are problems in a timely manner to solve the problem	Jingdong prefecture level city in Shandong
Stowe Flagship Store	King of Power Saving	Quality is very good, the use of the effect is very good, more stable, quality assurance	Jingdong prefecture level city in Shandong
Stowe Flagship Store	King of Power Saving	Convenient, inexpensive, and see how it works out?	Jingdong prefecture level city in Shandong
Stowe Flagship Store	King of Power Saving	Very good quality, saves a lot of electricity, very good, worth buying	Jingdong prefecture level city in Shandong
Stowe Flagship Store	King of Power Saving	Power Saving King is easy to use, with the fruit of power saving to use easy to give!	Jingdong prefecture level city in Shandong
Stowe Flagship Store	King of Power Saving	This saver really works, good quality, safe and secure, very satisfied!	Jingdong prefecture level city in Shandong
Stowe Flagship Store	King of Power Saving	It works well, the voltage is very stable effect is good, the quality is also very good satisfaction	Jingdong prefecture level city in Shandong
Stowe Flagship Store	King of Power Saving	The logistics is super super fast! The quality is also very good, indeed value for money	Jingdong prefecture level city in Shandong
Stowe Flagship Store	King of Power Saving	I've only been using it for a month or so and it seems to be saving a lot of power	Jingdong prefecture level city in Shandong
Stowe Flagship Store	King of Power Saving	Very easy to operate, very good material, good value for money.	Jingdong prefecture level city in Shandong
Stowe Flagship Store	King of Power Saving	Aluminum alloy shell, durable, compact power-saving good effect	Jingdong prefecture level city in Shandong
Stowe Flagship Store	King of Power Saving	Nice device, easy to use, doesn't take up much space, recommendable!	Jingdong prefecture level city in Shandong
Stowe Flagship Store	King of Power Saving	Good quality, good price, fast logistics	Jingdong prefecture level city in Shandong
Stowe Flagship Store	King of Power Saving	Friends of the family in use recommended, the effect is good and recommendable!	Jingdong prefecture level city in Shandong
Stowe Flagship Store	King of Power Saving	Cheaper than brick and mortar stores worth buying is authentic	Jingdong prefecture level city in Shandong
Stowe Flagship Store	King of Power Saving	Merchants ship quickly, the price is cheaper than the physical store, good use	Jingdong prefecture level city in Shandong
Stowe Flagship Store	King of Power Saving	Very durable, affordable, fast shipping	Jingdong prefecture level city in Shandong
Stowe Flagship Store	King of Power Saving	Packaging tight quality is very good easy to use good results	Jingdong prefecture level city in Shandong
Stowe Flagship Store	King of Power Saving	Save the grid. It's in the whole house. It's saving money on the electric bill.	Jingdong prefecture level city in Shandong
Stowe Flagship Store	King of Power Saving	I used it today, it works well, I'm very satisfied	Jingdong prefecture level city in Shandong
Stowe Flagship Store	King of Power Saving	It really saves a lot of electricity after installation	Jingdong prefecture level city in Shandong
Stowe Flagship Store	King of Power Saving	Love it. Satisfied.	Jingdong prefecture level city in Shandong
Stowe Flagship Store	King of Power Saving	Doesn't feel like it's working.	Jingdong prefecture level city in Shandong
Stowe Flagship Store	King of Power Saving	I'll comment after using it.	Jingdong prefecture level city in Shandong
Stowe Flagship Store	King of Power Saving	I'm trying it out to see if it saves power, and I'll buy two more if it works well.	Jingdong prefecture level city in Shandong
Stowe Flagship Store	King of Power Saving	It is genuine very good, the price is much cheaper, cost-effective, really special cost-effective, it is worth buying!	Jingdong prefecture level city in Shandong
Stowe Flagship Store	King of Power Saving	This thing can really save electricity, I was introduced to buy by my friend, he used it very well, I used it and think it's also very good, it can really save electricity, save about half of the electricity every day, it's really good!	Jingdong prefecture level city in Shandong
Stowe Flagship Store	King of Power Saving	After using it for ten days or so, I checked the power consumption and it did drop, and customer service said it will save more electricity in the long run. The product is also insured, 5 years warranty do not have to worry about quality problems!	Jingdong prefecture level city in Shandong
Stowe Flagship Store	King of Power Saving	The product is good, before already bought a, this is the second, the effect is quite good, anyway, I see the daily electricity bill than before a lot less, this can be the choice!	Jingdong prefecture level city in Shandong
Stowe Flagship Store	King of Power Saving	Originally also very worried about things are not good, get the hand found completely over-anxious, five-star praise, the style is very generous, the quality is very good, the price is also very reasonable!	Jingdong prefecture level city in Shandong
Stowe Flagship Store	King of Power Saving	Quality is good, playing to the old family also buy a, repurchase, this quality is really good, recommended!	Jingdong prefecture level city in Shandong
Stowe Flagship Store	King of Power Saving	Thank you, the owner even sent two fuses.	Jingdong prefecture level city in Shandong
Stowe Flagship Store	King of Power Saving	This store has great stuff, relatively fast shipping and logistics	Jingdong prefecture level city in Shandong
Stowe Flagship Store	King of Power Saving	This product is based on the impossibility of saving electricity, I used 853 kWh of electricity in July, started using the product on August 1, and used 867 kWh of electricity by September 1, is this called	Jingdong prefecture level city in Shandong
Stowe Flagship Store	King of Power Saving	Power king received, to the home on the trial, when I plugged into the electric panel can not see the effect of power saving, the meter is still the same speed in the turn, I'll save the king of the manual to play look at the instructions, instructions to speak, have to have a day two days and half a month to save power obviously, then wait half a month to see the effect of power saving it! To half a month there are obvious power saving before continuing to chase Xu.	Jingdong prefecture level city in Shandong
Stowe Flagship Store	King of Power Saving	Received the goods, a look, did not see the manufacturer and production address, can only hold a try to see the mentality of the face	Jingdong prefecture level city in Shandong
Stowe Flagship Store	King of Power Saving	I've only been using it for a few days and I'm not sure if it's an IQ tax? Used to favorable reviews.	Jingdong prefecture level city in Shandong
Stowe Flagship Store	King of Power Saving	Appearance is okay, power saving next time to follow up the evaluation	Jingdong prefecture level city in Shandong
Stowe Flagship Store	King of Power Saving	Shorter time, but hopefully effective and power-saving, to be followed up in the future	Jingdong prefecture level city in Shandong
Stowe Flagship Store	King of Power Saving	Appearance is okay, I do not know if the power saving	Jingdong prefecture level city in Shandong
Stowe Flagship Store	King of Power Saving	Good service, fast logistics, Power Saving King is exquisite and beautiful, support the business, thank you!	Jingdong prefecture level city in Shandong
Stowe Flagship Store	King of Power Saving	Bought three at once to try first first.	Jingdong prefecture level city in Shandong
Stowe Flagship Store	King of Power Saving	The object is the same as the picture, the product is of high quality, the packaging is intact. High-end atmosphere, high power, power saving effect is obvious, average monthly power saving 50%, very satisfied.	Jingdong prefecture level city in Shandong
Stowe Flagship Store	King of Power Saving	Bought back and plugged in, really effective, before 16 degrees of electricity per day, now 8 degrees of electricity per day, open an air conditioner for 12 hours, decisive repurchase of a	Jingdong prefecture level city in Shandong
Stowe Flagship Store	King of Power Saving	The object is the same as the picture, the product is of high quality, and the package is intact. High-end atmosphere and high grade, high power	Jingdong prefecture level city in Shandong

Stowe Flagship Store	King of Power Saving	Goods received, I hope the store said can be energy-saving, first try a month, month after month to see the effect.	Jingdong prefecture level city in Shandong
Stowe Flagship Store	King of Power Saving	This is really good, the material and workmanship is particularly exquisite, the workmanship is really very good, I think I can recommend to my friends to buy	Jingdong prefecture level city in Shandong
Stowe Flagship Store	King of Power Saving	I have to praise, the quality is really good, and the packaging logistics are also good. Really like it, won't make you regret after getting it!	Jingdong prefecture level city in Shandong
Stowe Flagship Store	King of Power Saving	Reputable platform, efficient and professional service, buy with confidence and peace of mind!	Jingdong prefecture level city in Shandong
Stowe Flagship Store	King of Power Saving	Bought one on a trial basis to see if it works after 1-2 months.	Jingdong prefecture level city in Shandong
Stowe Flagship Store	King of Power Saving	Quality is very good, customer service attitude is very very praise, there are problems in a timely manner to solve the problem, the price is not expensive, super value, you can rest assured that the	Jingdong prefecture level city in Shandong
Stowe Flagship Store	King of Power Saving	With it no longer need to be afraid to use electricity, especially good, ready to buy a mom and dad that also	Jingdong prefecture level city in Shandong
Stowe Flagship Store	King of Power Saving	Baby received very like will return to buy the seller attitude is also very like will return to buy the seller attitude is also very like!	Jingdong prefecture level city in Shandong
Stowe Flagship Store	King of Power Saving	Jingdong Mall shopping convenience assured fast assured fast assured fast assured fast	Jingdong prefecture level city in Shandong
Stowe Flagship Store	King of Power Saving	Saving power received, the quality is very good, fine workmanship, especially good, much cheaper than the entity store, it is worth buying!	Jingdong prefecture level city in Shandong
Stowe Flagship Store	King of Power Saving	I did not expect the express delivery so quickly, very save point quality is super good, than the entity store is too cheap.	Jingdong prefecture level city in Shandong
Stowe Flagship Store	King of Power Saving	Have received the baby, very satisfied with a shopping, great worth buying!	Jingdong prefecture level city in Shandong
Stowe Flagship Store	King of Power Saving	This is really more powerful, very satisfied, more energy-saving, better, thank you boss.	Jingdong prefecture level city in Shandong
Stowe Flagship Store	King of Power Saving	Received has been used for two days, the air conditioning can boldly open it! Energy saving effect is really quite good	Jingdong prefecture level city in Shandong
Stowe Flagship Store	King of Power Saving	It is authentic, trust the merchant, the baby has a great texture and the price is moderate, unlimited repurchase!	Jingdong prefecture level city in Shandong
Stowe Flagship Store	King of Power Saving	It is genuine very good, the price is much cheaper, cost-effective, really special cost-effective, it is worth buying!	Jingdong prefecture level city in Shandong
Stowe Flagship Store	King of Power Saving	Very good quality, fine workmanship, low power consumption, long warranty, fast shipping, economical and practical	Jingdong prefecture level city in Shandong
Stowe Flagship Store	King of Power Saving	I've been using it for half a month now, it really saves power and the voltage is stable, it's very good	Jingdong prefecture level city in Shandong
Stowe Flagship Store	King of Power Saving	It's the ideal product 💝, good quality and fast logistics, worth the kudos!	Jingdong prefecture level city in Shandong
Stowe Flagship Store	King of Power Saving	The logistics is very fast, the baby is very good, the shopkeeper is NICE!	Jingdong prefecture level city in Shandong
Stowe Flagship Store	King of Power Saving	Good to use, good quality, save point durable	Jingdong prefecture level city in Shandong
Stowe Flagship Store	King of Power Saving	The quality is very good, customer service attitude is very, very awesome, there are problems in a timely manner to solve the problem	Jingdong prefecture level city in Shandong
Stowe Flagship Store	King of Power Saving	Quality is very good, the use of the effect is very good, more stable, quality assurance	Jingdong prefecture level city in Shandong
Stowe Flagship Store	King of Power Saving	Convenient, inexpensive, and see how it works out?	Jingdong prefecture level city in Shandong
Stowe Flagship Store	King of Power Saving	Very good quality, saves a lot of electricity, very good, worth buying	Jingdong prefecture level city in Shandong
Stowe Flagship Store	King of Power Saving	Power Saving King is easy to use, with the fruit of power saving to use easy to give!	Jingdong prefecture level city in Shandong
Stowe Flagship Store	King of Power Saving	This saver really works, good quality, safe and secure, very satisfied!	Jingdong prefecture level city in Shandong
Stowe Flagship Store	King of Power Saving	It works well, the voltage is very stable effect is good, the quality is also very good satisfaction	Jingdong prefecture level city in Shandong
Stowe Flagship Store	King of Power Saving	The logistics is super super fast! The quality is also very good, indeed value for money	Jingdong prefecture level city in Shandong
Stowe Flagship Store	King of Power Saving	I've only been using it for a month or so and it seems to be saving a lot of power	Jingdong prefecture level city in Shandong
Stowe Flagship Store	King of Power Saving	Very easy to operate, very good material, good value for money.	Jingdong prefecture level city in Shandong
Stowe Flagship Store	King of Power Saving	Aluminum alloy shell, durable, compact power-saving good effect	Jingdong prefecture level city in Shandong
Stowe Flagship Store	King of Power Saving	Nice device, easy to use, doesn't take up much space, recommendable!	Jingdong prefecture level city in Shandong
Stowe Flagship Store	King of Power Saving	Good quality, good price, fast logistics	Jingdong prefecture level city in Shandong
Stowe Flagship Store	King of Power Saving	Friends of the family in use recommended, the effect is good and recommendable!	Jingdong prefecture level city in Shandong
Stowe Flagship Store	King of Power Saving	Cheaper than brick and mortar stores worth buying is authentic	Jingdong prefecture level city in Shandong
Stowe Flagship Store	King of Power Saving	Merchants ship quickly, the price is cheaper than the physical store, good use	Jingdong prefecture level city in Shandong
Stowe Flagship Store	King of Power Saving	Very durable, affordable, fast shipping	Jingdong prefecture level city in Shandong
Stowe Flagship Store	King of Power Saving	Packaging tight quality is very good easy to use good results	Jingdong prefecture level city in Shandong
Stowe Flagship Store	King of Power Saving	Save the grid. It's in the whole house. It's saving money on the electric bill.	Jingdong prefecture level city in Shandong
Stowe Flagship Store	King of Power Saving	I used it today, it works well, I'm very satisfied	Jingdong prefecture level city in Shandong
Stowe Flagship Store	King of Power Saving	It really saves a lot of electricity after installation	Jingdong prefecture level city in Shandong
Stowe Flagship Store	King of Power Saving	Love it. Satisfied.	Jingdong prefecture level city in Shandong
Stowe Flagship Store	King of Power Saving	Doesn't feel like it's working.	Jingdong prefecture level city in Shandong
Stowe Flagship Store	King of Power Saving	I'll comment after using it.	Jingdong prefecture level city in Shandong
Stowe Flagship Store	King of Power Saving	I'm trying it out to see if it saves power, and I'll buy two more if it works well.	Jingdong prefecture level city in Shandong
Stowe Flagship Store	King of Power Saving	It is genuine very good, the price is much cheaper, cost-effective, really special cost-effective, it is worth buying!	Jingdong prefecture level city in Shandong
Stowe Flagship Store	King of Power Saving	This thing can really save electricity, I was introduced to buy by my friend, he used it very well, I used it and think it's also very good, it can really save electricity, save about half of the electricity every day, it's really good!	Jingdong prefecture level city in Shandong
Stowe Flagship Store	King of Power Saving	After using it for ten days or so, I checked the power consumption and it did drop, and customer service said it will save more electricity in the long run. The product is also insured, 5 years warranty do not have to worry about quality problems!	Jingdong prefecture level city in Shandong
Stowe Flagship Store	King of Power Saving	The product is good, before already bought a, this is the second, the effect is quite good, anyway, I see the daily electricity bill than before a lot less, this can be the choice!	Jingdong prefecture level city in Shandong

Stowe Flagship Store	King of Power Saving	Originally also very worried about things are not good, get the hand found completely over-anxious, five-star praise, the style is very generous, the quality is very good, the price is also very reasonable!	Jingdong prefecture level city in Shandong
Stowe Flagship Store	King of Power Saving	Quality is good, playing to the old family also buy a, repurchase, this quality is really good, recommended!	Jingdong prefecture level city in Shandong
Stowe Flagship Store	King of Power Saving	Thank you, the owner even sent two fuses.	Jingdong prefecture level city in Shandong
Stowe Flagship Store	King of Power Saving	This store has great stuff, relatively fast shipping and logistics	Jingdong prefecture level city in Shandong
Stowe Flagship Store	King of Power Saving	This product is based on the impossibility of saving electricity, I used 853 kWh of electricity in July, started using the product on August 1, and used 867 kWh of electricity by September 1, is this called	Jingdong prefecture level city in Shandong
Stowe Flagship Store	King of Power Saving	Power king received, to the home on the trial, when I plugged into the electric panel can not see the effect of power saving, the meter is still the same speed in the turn, I'll save the king of the manual to play look at the instructions, instructions to speak, have to have a day two days and half a month to save power obviously, then wait half a month to see the effect of power saving it! To half a month there are obvious power saving before continuing to chase Xu.	Jingdong prefecture level city in Shandong
Stowe Flagship Store	King of Power Saving	Received the goods, a look, did not see the manufacturer and production address, can only hold a try to see the mentality of the face	Jingdong prefecture level city in Shandong
Stowe Flagship Store	King of Power Saving	I've only been using it for a few days and I'm not sure if it's an IQ tax? Used to favorable reviews.	Jingdong prefecture level city in Shandong
Stowe Flagship Store	King of Power Saving	Appearance is okay, power saving next time to follow up the evaluation	Jingdong prefecture level city in Shandong
Stowe Flagship Store	King of Power Saving	Shorter time, but hopefully effective and power-saving, to be followed up in the future	Jingdong prefecture level city in Shandong
Stowe Flagship Store	King of Power Saving	Appearance is okay, I do not know if the power saving	Jingdong prefecture level city in Shandong
Stowe Flagship Store	King of Power Saving	Bought three at once to try first first.	Jingdong prefecture level city in Shandong
Stowe Flagship Store	King of Power Saving	Bought back and plugged in, really effective, before 16 degrees of electricity per day, now 8 degrees of electricity per day, open an air conditioner for 12 hours, decisive repurchase of a	Jingdong prefecture level city in Shandong
Stowe Flagship Store	King of Power Saving	The object is the same as the picture, the product is of high quality, the packaging is intact. High-end atmosphere, high power, power saving effect is obvious, average monthly power saving 50%, very satisfied.	Jingdong prefecture level city in Shandong
Stowe Flagship Store	King of Power Saving	The object is the same as the picture, the product is of high quality, and the package is intact. High-end atmosphere and high grade, high power	Jingdong prefecture level city in Shandong
Stowe Flagship Store	King of Power Saving	Good service, fast logistics, Power Saving King is exquisite and beautiful, support the business, thank you!	Jingdong prefecture level city in Shandong
Stowe Flagship Store	King of Power Saving	Goods received, I hope the store said can be energy-saving, first try a month, month after month to see the effect.	Jingdong prefecture level city in Shandong
Stowe Flagship Store	King of Power Saving	This is really good, the material and workmanship is particularly exquisite, the workmanship is really very good, I think I can recommend to my friends to buy	Jingdong prefecture level city in Shandong
Stowe Flagship Store	King of Power Saving	I have to praise, the quality is really good, and the packaging logistics are also good. Really like it, won't make you regret after getting it!	Jingdong prefecture level city in Shandong
Stowe Flagship Store	King of Power Saving	Reputable platform, efficient and professional service, buy with confidence and peace of mind!	Jingdong prefecture level city in Shandong
Stowe Flagship Store	King of Power Saving	Bought one on a trial basis to see if it works after 1-2 months.	Jingdong prefecture level city in Shandong
Stowe Flagship Store	King of Power Saving	Quality is very good, customer service attitude is very very praise, there are problems in a timely manner to solve the problem, the price is not expensive, super value, you can rest assured that the	Jingdong prefecture level city in Shandong
Stowe Flagship Store	King of Power Saving	With it no longer need to be afraid to use electricity, especially good, ready to buy a mom and dad that also	Jingdong prefecture level city in Shandong
Stowe Flagship Store	King of Power Saving	Baby received very like will return to buy the seller attitude is also very like will return to buy the seller attitude is also very like!	Jingdong prefecture level city in Shandong
Stowe Flagship Store	King of Power Saving	Jingdong Mall shopping convenience assured fast assured fast assured fast	Jingdong prefecture level city in Shandong
Stowe Flagship Store	King of Power Saving	Saving power received, the quality is very good, fine workmanship, especially good, much cheaper than the entity store, it is worth buying!	Jingdong prefecture level city in Shandong
Stowe Flagship Store	King of Power Saving	I did not expect the express delivery so quickly, very save point quality is super good, than the entity store is too cheap.	Jingdong prefecture level city in Shandong
Stowe Flagship Store	King of Power Saving	Have received the baby, very satisfied with a shopping, great worth buying!	Jingdong prefecture level city in Shandong
Stowe Flagship Store	King of Power Saving	This is really more powerful, very satisfied, more energy-saving, better, thank you boss.	Jingdong prefecture level city in Shandong
Stowe Flagship Store	King of Power Saving	Received has been used for two days, the air conditioning can boldly open it! Energy saving effect is really quite good	Jingdong prefecture level city in Shandong
Stowe Flagship Store	King of Power Saving	It is authentic, trust the merchant, the baby has a great texture and the price is moderate, unlimited repurchase!	Jingdong prefecture level city in Shandong
Stowe Flagship Store	King of Power Saving	It is genuine very good, the price is much cheaper, cost-effective, really special cost-effective, it is worth buying!	Jingdong prefecture level city in Shandong
Stowe Flagship Store	King of Power Saving	Very good quality, fine workmanship, low power consumption, long warranty, fast shipping, economical and practical	Jingdong prefecture level city in Shandong
Stowe Flagship Store	King of Power Saving	I've been using it for half a month now, it really saves power and the voltage is stable, it's very good	Jingdong prefecture level city in Shandong
Stowe Flagship Store	King of Power Saving	It's the ideal product 💝, good quality and fast logistics, worth the kudos!	Jingdong prefecture level city in Shandong
Stowe Flagship Store	King of Power Saving	The logistics is very fast, the baby is very good, the shopkeeper is NICE!	Jingdong prefecture level city in Shandong
Stowe Flagship Store	King of Power Saving	Good to use, good quality, save point durable	Jingdong prefecture level city in Shandong
Stowe Flagship Store	King of Power Saving	The quality is very good, customer service attitude is very, very awesome, there are problems in a timely manner to solve the problem	Jingdong prefecture level city in Shandong
Stowe Flagship Store	King of Power Saving	Quality is very good, the use of the effect is very good, more stable, quality assurance	Jingdong prefecture level city in Shandong
Stowe Flagship Store	King of Power Saving	Convenient, inexpensive, and see how it works out?	Jingdong prefecture level city in Shandong
Stowe Flagship Store	King of Power Saving	Very good quality, saves a lot of electricity, very good, worth buying	Jingdong prefecture level city in Shandong
Stowe Flagship Store	King of Power Saving	Power Saving King is easy to use, with the fruit of power saving to use easy to give!	Jingdong prefecture level city in Shandong
Stowe Flagship Store	King of Power Saving	This saver really works, good quality, safe and secure, very satisfied!	Jingdong prefecture level city in Shandong
Stowe Flagship Store	King of Power Saving	It works well, the voltage is very stable effect is good, the quality is also very good satisfaction	Jingdong prefecture level city in Shandong
Stowe Flagship Store	King of Power Saving	The logistics is super super fast! The quality is also very good, indeed value for money	Jingdong prefecture level city in Shandong
Stowe Flagship Store	King of Power Saving	I've only been using it for a month or so and it seems to be saving a lot of power	Jingdong prefecture level city in Shandong
Stowe Flagship Store	King of Power Saving	Very easy to operate, very good material, good value for money.	Jingdong prefecture level city in Shandong
Stowe Flagship Store	King of Power Saving	Aluminum alloy shell, durable, compact power-saving good effect	Jingdong prefecture level city in Shandong
Stowe Flagship Store	King of Power Saving	Nice device, easy to use, doesn't take up much space, recommendable!	Jingdong prefecture level city in Shandong
Stowe Flagship Store	King of Power Saving	Good quality, good price, fast logistics	Jingdong prefecture level city in Shandong

Stowe Flagship Store	King of Power Saving	Friends of the family in use recommended, the effect is good and recommendable!	Jingdong prefecture level city in Shandong
Stowe Flagship Store	King of Power Saving	Cheaper than brick and mortar stores worth buying is authentic	Jingdong prefecture level city in Shandong
Stowe Flagship Store	King of Power Saving	Merchants ship quickly, the price is cheaper than the physical store, good use	Jingdong prefecture level city in Shandong
Stowe Flagship Store	King of Power Saving	Very durable, affordable, fast shipping	Jingdong prefecture level city in Shandong
Stowe Flagship Store	King of Power Saving	Packaging tight quality is very good easy to use good results	Jingdong prefecture level city in Shandong
Stowe Flagship Store	King of Power Saving	Save the grid. It's in the whole house. It's saving money on the electric bill.	Jingdong prefecture level city in Shandong
Stowe Flagship Store	King of Power Saving	I used it today, it works well, I'm very satisfied	Jingdong prefecture level city in Shandong
Stowe Flagship Store	King of Power Saving	It really saves a lot of electricity after installation	Jingdong prefecture level city in Shandong
Stowe Flagship Store	King of Power Saving	Love it. Satisfied.	Jingdong prefecture level city in Shandong
Stowe Flagship Store	King of Power Saving	Doesn't feel like it's working.	Jingdong prefecture level city in Shandong
Stowe Flagship Store	King of Power Saving	I'll comment after using it.	Jingdong prefecture level city in Shandong
Stowe Flagship Store	King of Power Saving	I'm trying it out to see if it saves power, and I'll buy two more if it works well.	Jingdong prefecture level city in Shandong
Stowe Flagship Store	King of Power Saving	It is genuine very good, the price is much cheaper, cost-effective, really special cost-effective, it is worth buying!	Jingdong prefecture level city in Shandong
Stowe Flagship Store	King of Power Saving	This thing can really save electricity, I was introduced to buy by my friend, he used it very well, I used it and think it's also very good, it can really save electricity, save about half of the electricity every day, it's really good!	Jingdong prefecture level city in Shandong
Stowe Flagship Store	King of Power Saving	After using it for ten days or so, I checked the power consumption and it did drop, and customer service said it will save more electricity in the long run. The product is also insured, 5 years warranty do not have to worry about quality problems!	Jingdong prefecture level city in Shandong
Stowe Flagship Store	King of Power Saving	The product is good, before already bought a, this is the second, the effect is quite good, anyway, I see the daily electricity bill than before a lot less, this can be the choice!	Jingdong prefecture level city in Shandong
Stowe Flagship Store	King of Power Saving	Originally also very worried about things are not good, get the hand found completely over-anxious, five-star praise, the style is very generous, the quality is very good, the price is also very reasonable!	Jingdong prefecture level city in Shandong
Stowe Flagship Store	King of Power Saving	Quality is good, playing to the old family also buy a, repurchase, this quality is really good, recommended!	Jingdong prefecture level city in Shandong
Stowe Flagship Store	King of Power Saving	Thank you, the owner even sent two fuses.	Jingdong prefecture level city in Shandong
Stowe Flagship Store	King of Power Saving	This store has great stuff, relatively fast shipping and logistics	Jingdong prefecture level city in Shandong
Stowe Flagship Store	King of Power Saving	This product is based on the impossibility of saving electricity, I used 853 kWh of electricity in July, started using the product on August 1, and used 867 kWh of electricity by September 1, is this called	Jingdong prefecture level city in Shandong
Stowe Flagship Store	King of Power Saving	Power king received, to the home on the trial, when I plugged into the electric panel can not see the effect of power saving, the meter is still the same speed in the turn, I'll save the king of the manual to play look at the instructions, instructions to speak, have to have a day two days and half a month to save power obviously, then wait half a month to see the effect of power saving it! To half a month there are obvious power saving before continuing to chase Xu.	Jingdong prefecture level city in Shandong
Stowe Flagship Store	King of Power Saving	Received the goods, a look, did not see the manufacturer and production address, can only hold a try to see the mentality of the face	Jingdong prefecture level city in Shandong
Stowe Flagship Store	King of Power Saving	I've only been using it for a few days and I'm not sure if it's an IQ tax? Used to favorable reviews.	Jingdong prefecture level city in Shandong
Stowe Flagship Store	King of Power Saving	Appearance is okay, power saving next time to follow up the evaluation	Jingdong prefecture level city in Shandong
Stowe Flagship Store	King of Power Saving	Shorter time, but hopefully effective and power-saving, to be followed up in the future	Jingdong prefecture level city in Shandong
Stowe Flagship Store	King of Power Saving	Appearance is okay, I do not know if the power saving	Jingdong prefecture level city in Shandong
Stowe Flagship Store	King of Power Saving	Good service, fast logistics, Power Saving King is exquisite and beautiful, support the business, thank you!	Jingdong prefecture level city in Shandong
Stowe Flagship Store	King of Power Saving	Bought three at once to try first first.	Jingdong prefecture level city in Shandong
Stowe Flagship Store	King of Power Saving	The object is the same as the picture, the product is of high quality, the packaging is intact. High-end atmosphere, high power, power saving effect is obvious, average monthly power saving 50%, very satisfied.	Jingdong prefecture level city in Shandong
Stowe Flagship Store	King of Power Saving	Bought back and plugged in, really effective, before 16 degrees of electricity per day, now 8 degrees of electricity per day, open an air conditioner for 12 hours, decisive repurchase of a	Jingdong prefecture level city in Shandong
Stowe Flagship Store	King of Power Saving	The object is the same as the picture, the product is of high quality, and the package is intact. High-end atmosphere and high grade, high power	Jingdong prefecture level city in Shandong
Stowe Flagship Store	King of Power Saving	Goods received, I hope the store said can be energy-saving, first try a month, month after month to see the effect.	Jingdong prefecture level city in Shandong
Stowe Flagship Store	King of Power Saving	This is really good, the material and workmanship is particularly exquisite, the workmanship is really very good, I think I can recommend to my friends to buy	Jingdong prefecture level city in Shandong
Stowe Flagship Store	King of Power Saving	I have to praise, the quality is really good, and the packaging logistics are also good. Really like it, won't make you regret after getting it!	Jingdong prefecture level city in Shandong
Stowe Flagship Store	King of Power Saving	Reputable platform, efficient and professional service, buy with confidence and peace of mind!	Jingdong prefecture level city in Shandong
Stowe Flagship Store	King of Power Saving	Bought one on a trial basis to see if it works after 1-2 months.	Jingdong prefecture level city in Shandong
Stowe Flagship Store	King of Power Saving	Quality is very good, customer service attitude is very very praise, there are problems in a timely manner to solve the problem, the price is not expensive, super value, you can rest assured that the	Jingdong prefecture level city in Shandong
Stowe Flagship Store	King of Power Saving	With it no longer need to be afraid to use electricity, especially good, ready to buy a mom and dad that also	Jingdong prefecture level city in Shandong
Stowe Flagship Store	King of Power Saving	Baby received very like will return to buy the seller attitude is also very like will return to buy the seller attitude is also very like!	Jingdong prefecture level city in Shandong
Stowe Flagship Store	King of Power Saving	Jingdong Mall shopping convenience assured fast assured fast assured fast assured fast	Jingdong prefecture level city in Shandong
Stowe Flagship Store	King of Power Saving	Saving power received, the quality is very good, fine workmanship, especially good, much cheaper than the entity store, it is worth buying!	Jingdong prefecture level city in Shandong
Stowe Flagship Store	King of Power Saving	I did not expect the express delivery so quickly, very save point quality is super good, than the entity store is too cheap.	Jingdong prefecture level city in Shandong
Stowe Flagship Store	King of Power Saving	Have received the baby, very satisfied with a shopping, great worth buying!	Jingdong prefecture level city in Shandong
Stowe Flagship Store	King of Power Saving	This is really more powerful, very satisfied, more energy-saving, better, thank you boss.	Jingdong prefecture level city in Shandong
Stowe Flagship Store	King of Power Saving	Received has been used for two days, the air conditioning can boldly open it! Energy saving effect is really quite good	Jingdong prefecture level city in Shandong
Stowe Flagship Store	King of Power Saving	It is authentic, trust the merchant, the baby has a great texture and the price is moderate, unlimited repurchase!	Jingdong prefecture level city in Shandong
Stowe Flagship Store	King of Power Saving	It is genuine very good, the price is much cheaper, cost-effective, really special cost-effective, it is worth buying!	Jingdong prefecture level city in Shandong
Stowe Flagship Store	King of Power Saving	Very good quality, fine workmanship, low power consumption, long warranty, fast shipping, economical and practical	Jingdong prefecture level city in Shandong

Stowe Flagship Store	King of Power Saving	I've been using it for half a month now, it really saves power and the voltage is stable, it's very good	Jingdong prefecture level city in Shandong
Stowe Flagship Store	King of Power Saving	It's the ideal product 💖, good quality and fast logistics, worth the kudos!	Jingdong prefecture level city in Shandong
Stowe Flagship Store	King of Power Saving	The logistics is very fast, the baby is very good, the shopkeeper is NICE!	Jingdong prefecture level city in Shandong
Stowe Flagship Store	King of Power Saving	Good to use, good quality, save point durable	Jingdong prefecture level city in Shandong
Stowe Flagship Store	King of Power Saving	The quality is very good, customer service attitude is very, very awesome, there are problems in a timely manner to solve the problem	Jingdong prefecture level city in Shandong
Stowe Flagship Store	King of Power Saving	Quality is very good, the use of the effect is very good, more stable, quality assurance	Jingdong prefecture level city in Shandong
Stowe Flagship Store	King of Power Saving	Convenient, inexpensive, and see how it works out?	Jingdong prefecture level city in Shandong
Stowe Flagship Store	King of Power Saving	Very good quality, saves a lot of electricity, very good, worth buying	Jingdong prefecture level city in Shandong
Stowe Flagship Store	King of Power Saving	Power Saving King is easy to use, with the fruit of power saving to use easy to give!	Jingdong prefecture level city in Shandong
Stowe Flagship Store	King of Power Saving	This saver really works, good quality, safe and secure, very satisfied!	Jingdong prefecture level city in Shandong
Stowe Flagship Store	King of Power Saving	It works well, the voltage is very stable effect is good, the quality is also very good satisfaction	Jingdong prefecture level city in Shandong
Stowe Flagship Store	King of Power Saving	The logistics is super super fast! The quality is also very good, indeed value for money	Jingdong prefecture level city in Shandong
Stowe Flagship Store	King of Power Saving	I've only been using it for a month or so and it seems to be saving a lot of power	Jingdong prefecture level city in Shandong
Stowe Flagship Store	King of Power Saving	Very easy to operate, very good material, good value for money.	Jingdong prefecture level city in Shandong
Stowe Flagship Store	King of Power Saving	Aluminum alloy shell, durable, compact power-saving good effect	Jingdong prefecture level city in Shandong
Stowe Flagship Store	King of Power Saving	Nice device, easy to use, doesn't take up much space, recommendable!	Jingdong prefecture level city in Shandong
Stowe Flagship Store	King of Power Saving	Good quality, good price, fast logistics	Jingdong prefecture level city in Shandong
Stowe Flagship Store	King of Power Saving	Friends of the family in use recommended, the effect is good and recommendable!	Jingdong prefecture level city in Shandong
Stowe Flagship Store	King of Power Saving	Cheaper than brick and mortar stores worth buying is authentic	Jingdong prefecture level city in Shandong
Stowe Flagship Store	King of Power Saving	Merchants ship quickly, the price is cheaper than the physical store, good use	Jingdong prefecture level city in Shandong
Stowe Flagship Store	King of Power Saving	Very durable, affordable, fast shipping	Jingdong prefecture level city in Shandong
Stowe Flagship Store	King of Power Saving	Packaging tight quality is very good easy to use good results	Jingdong prefecture level city in Shandong
Stowe Flagship Store	King of Power Saving	Save the grid. It's in the whole house. It's saving money on the electric bill.	Jingdong prefecture level city in Shandong
Stowe Flagship Store	King of Power Saving	I used it today, it works well, I'm very satisfied	Jingdong prefecture level city in Shandong
Stowe Flagship Store	King of Power Saving	It really saves a lot of electricity after installation	Jingdong prefecture level city in Shandong
Stowe Flagship Store	King of Power Saving	Love it. Satisfied.	Jingdong prefecture level city in Shandong
Stowe Flagship Store	King of Power Saving	Doesn't feel like it's working.	Jingdong prefecture level city in Shandong
Stowe Flagship Store	King of Power Saving	I'll comment after using it.	Jingdong prefecture level city in Shandong
Stowe Flagship Store	King of Power Saving	I'm trying it out to see if it saves power, and I'll buy two more if it works well.	Jingdong prefecture level city in Shandong
Stowe Flagship Store	King of Power Saving	It is genuine very good, the price is much cheaper, cost-effective, really special cost-effective, it is worth buying!	Jingdong prefecture level city in Shandong
Stowe Flagship Store	King of Power Saving	This thing can really save electricity, I was introduced to buy by my friend, he used it very well, I used it and think it's also very good, it can really save electricity, save about half of the electricity every day, it's really good!	Jingdong prefecture level city in Shandong
Stowe Flagship Store	King of Power Saving	After using it for ten days or so, I checked the power consumption and it did drop, and customer service said it will save more electricity in the long run. The product is also insured, 5 years warranty do not have to worry about quality problems!	Jingdong prefecture level city in Shandong
Stowe Flagship Store	King of Power Saving	The product is good, before already bought a, this is the second, the effect is quite good, anyway, I see the daily electricity bill than before a lot less, this can be the choice!	Jingdong prefecture level city in Shandong
Stowe Flagship Store	King of Power Saving	Originally also very worried about things are not good, get the hand found completely over-anxious, five-star praise, the style is very generous, the quality is very good, the price is also very reasonable!	Jingdong prefecture level city in Shandong
Stowe Flagship Store	King of Power Saving	Quality is good, playing to the old family also buy a, repurchase, this quality is really good, recommended!	Jingdong prefecture level city in Shandong
Stowe Flagship Store	King of Power Saving	Thank you, the owner even sent two fuses.	Jingdong prefecture level city in Shandong
Stowe Flagship Store	King of Power Saving	This store has great stuff, relatively fast shipping and logistics	Jingdong prefecture level city in Shandong
Stowe Flagship Store	King of Power Saving	This product is based on the impossibility of saving electricity, I used 853 kWh of electricity in July, started using the product on August 1, and used 867 kWh of electricity by September 1, is this called	Jingdong prefecture level city in Shandong
Stowe Flagship Store	King of Power Saving	Power king received, to the home on the trial, when I plugged into the electric panel can not see the effect of power saving, the meter is still the same speed in the turn, I'll save the king of the manual to play look at the instructions, instructions to speak, have to have a day two days and half a month to save power obviously, then wait half a month to see the effect of power saving it! To half a month there are obvious power saving before continuing to chase Xu.	Jingdong prefecture level city in Shandong
Stowe Flagship Store	King of Power Saving	Received the goods, a look, did not see the manufacturer and production address, can only hold a try to see the mentality of the face	Jingdong prefecture level city in Shandong
Stowe Flagship Store	King of Power Saving	I've only been using it for a few days and I'm not sure if it's an IQ tax? Used to favorable reviews.	Jingdong prefecture level city in Shandong
Stowe Flagship Store	King of Power Saving	Appearance is okay, power saving next time to follow up the evaluation	Jingdong prefecture level city in Shandong
Stowe Flagship Store	King of Power Saving	Shorter time, but hopefully effective and power-saving, to be followed up in the future	Jingdong prefecture level city in Shandong
Stowe Flagship Store	King of Power Saving	Appearance is okay, I do not know if the power saving	Jingdong prefecture level city in Shandong
Stowe Flagship Store	King of Power Saving	Good service, fast logistics, Power Saving King is exquisite and beautiful, support the business, thank you!	Jingdong prefecture level city in Shandong
Stowe Flagship Store	King of Power Saving	Bought three at once to try first first.	Jingdong prefecture level city in Shandong
Stowe Flagship Store	King of Power Saving	The object is the same as the picture, the product is of high quality, the packaging is intact. High-end atmosphere, high power, power saving effect is obvious, average monthly power saving 50%, very satisfied.	Jingdong prefecture level city in Shandong
Stowe Flagship Store	King of Power Saving	Bought back and plugged in, really effective, before 16 degrees of electricity per day, now 8 degrees of electricity per day, open an air conditioner for 12 hours, decisive repurchase of a	Jingdong prefecture level city in Shandong
Stowe Flagship Store	King of Power Saving	The object is the same as the picture, the product is of high quality, and the package is intact. High-end atmosphere and high grade, high power	Jingdong prefecture level city in Shandong

Stowe Flagship Store	King of Power Saving	Goods received, I hope the store said can be energy-saving, first try a month, month after month to see the effect.	Jingdong prefecture level city in Shandong
Stowe Flagship Store	King of Power Saving	This is really good, the material and workmanship is particularly exquisite, the workmanship is really very good, I think I can recommend to my friends to buy	Jingdong prefecture level city in Shandong
Stowe Flagship Store	King of Power Saving	I have to praise, the quality is really good, and the packaging logistics are also good. Really like it, won't make you regret after getting it!	Jingdong prefecture level city in Shandong
Stowe Flagship Store	King of Power Saving	Reputable platform, efficient and professional service, buy with confidence and peace of mind!	Jingdong prefecture level city in Shandong
Stowe Flagship Store	King of Power Saving	Bought one on a trial basis to see if it works after 1-2 months.	Jingdong prefecture level city in Shandong
Stowe Flagship Store	King of Power Saving	Quality is very good, customer service attitude is very very praise, there are problems in a timely manner to solve the problem, the price is not expensive, super value, you can rest assured that the	Jingdong prefecture level city in Shandong
Stowe Flagship Store	King of Power Saving	With it no longer need to be afraid to use electricity, especially good, ready to buy a mom and dad that also	Jingdong prefecture level city in Shandong
Stowe Flagship Store	King of Power Saving	Baby received very like will return to buy the seller attitude is also very like will return to buy the seller attitude is also very like!	Jingdong prefecture level city in Shandong
Stowe Flagship Store	King of Power Saving	Jingdong Mall shopping convenience assured fast assured fast assured fast assured fast	Jingdong prefecture level city in Shandong
Stowe Flagship Store	King of Power Saving	Saving power received, the quality is very good, fine workmanship, especially good, much cheaper than the entity store, it is worth buying!	Jingdong prefecture level city in Shandong
Stowe Flagship Store	King of Power Saving	I did not expect the express delivery so quickly, very save point quality is super good, than the entity store is too cheap.	Jingdong prefecture level city in Shandong
Stowe Flagship Store	King of Power Saving	Have received the baby, very satisfied with a shopping, great worth buying!	Jingdong prefecture level city in Shandong
Stowe Flagship Store	King of Power Saving	This is really more powerful, very satisfied, more energy-saving, better, thank you boss.	Jingdong prefecture level city in Shandong
Stowe Flagship Store	King of Power Saving	Received has been used for two days, the air conditioning can boldly open it! Energy saving effect is really quite good	Jingdong prefecture level city in Shandong
Stowe Flagship Store	King of Power Saving	It is authentic, trust the merchant, the baby has a great texture and the price is moderate, unlimited repurchase!	Jingdong prefecture level city in Shandong
Stowe Flagship Store	King of Power Saving	It is genuine very good, the price is much cheaper, cost-effective, really special cost-effective, it is worth buying!	Jingdong prefecture level city in Shandong
Stowe Flagship Store	King of Power Saving	Very good quality, fine workmanship, low power consumption, long warranty, fast shipping, economical and practical	Jingdong prefecture level city in Shandong
Stowe Flagship Store	King of Power Saving	I've been using it for half a month now, it really saves power and the voltage is stable, it's very good	Jingdong prefecture level city in Shandong
Stowe Flagship Store	King of Power Saving	It's the ideal product 💕, good quality and fast logistics, worth the kudos!	Jingdong prefecture level city in Shandong
Stowe Flagship Store	King of Power Saving	The logistics is very fast, the baby is very good, the shopkeeper is NICE!	Jingdong prefecture level city in Shandong
Stowe Flagship Store	King of Power Saving	Good to use, good quality, save point durable	Jingdong prefecture level city in Shandong
Stowe Flagship Store	King of Power Saving	The quality is very good, customer service attitude is very, very awesome, there are problems in a timely manner to solve the problem	Jingdong prefecture level city in Shandong
Stowe Flagship Store	King of Power Saving	Quality is very good, the use of the effect is very good, more stable, quality assurance	Jingdong prefecture level city in Shandong
Stowe Flagship Store	King of Power Saving	Convenient, inexpensive, and see how it works out?	Jingdong prefecture level city in Shandong
Stowe Flagship Store	King of Power Saving	Very good quality, saves a lot of electricity, very good, worth buying	Jingdong prefecture level city in Shandong
Stowe Flagship Store	King of Power Saving	Power Saving King is easy to use, with the fruit of power saving to use easy to give!	Jingdong prefecture level city in Shandong
Stowe Flagship Store	King of Power Saving	This saver really works, good quality, safe and secure, very satisfied!	Jingdong prefecture level city in Shandong
Stowe Flagship Store	King of Power Saving	It works well, the voltage is very stable effect is good, the quality is also very good satisfaction	Jingdong prefecture level city in Shandong
Stowe Flagship Store	King of Power Saving	The logistics is super super fast! The quality is also very good, indeed value for money	Jingdong prefecture level city in Shandong
Stowe Flagship Store	King of Power Saving	I've only been using it for a month or so and it seems to be saving a lot of power	Jingdong prefecture level city in Shandong
Stowe Flagship Store	King of Power Saving	Very easy to operate, very good material, good value for money.	Jingdong prefecture level city in Shandong
Stowe Flagship Store	King of Power Saving	Aluminum alloy shell, durable, compact power-saving good effect	Jingdong prefecture level city in Shandong
Stowe Flagship Store	King of Power Saving	Nice device, easy to use, doesn't take up much space, recommendable!	Jingdong prefecture level city in Shandong
Stowe Flagship Store	King of Power Saving	Good quality, good price, fast logistics	Jingdong prefecture level city in Shandong
Stowe Flagship Store	King of Power Saving	Friends of the family in use recommended, the effect is good and recommendable!	Jingdong prefecture level city in Shandong
Stowe Flagship Store	King of Power Saving	Cheaper than brick and mortar stores worth buying is authentic	Jingdong prefecture level city in Shandong
Stowe Flagship Store	King of Power Saving	Merchants ship quickly, the price is cheaper than the physical store, good use	Jingdong prefecture level city in Shandong
Stowe Flagship Store	King of Power Saving	Very durable, affordable, fast shipping	Jingdong prefecture level city in Shandong
Stowe Flagship Store	King of Power Saving	Packaging tight quality is very good easy to use good results	Jingdong prefecture level city in Shandong
Stowe Flagship Store	King of Power Saving	Save the grid. It's in the whole house. It's saving money on the electric bill.	Jingdong prefecture level city in Shandong
Stowe Flagship Store	King of Power Saving	I used it today, it works well, I'm very satisfied	Jingdong prefecture level city in Shandong
Stowe Flagship Store	King of Power Saving	It really saves a lot of electricity after installation	Jingdong prefecture level city in Shandong
Stowe Flagship Store	King of Power Saving	Love it. Satisfied.	Jingdong prefecture level city in Shandong
Stowe Flagship Store	King of Power Saving	Doesn't feel like it's working.	Jingdong prefecture level city in Shandong
Stowe Flagship Store	King of Power Saving	I'll comment after using it.	Jingdong prefecture level city in Shandong
Stowe Flagship Store	King of Power Saving	I'm trying it out to see if it saves power, and I'll buy two more if it works well.	Jingdong prefecture level city in Shandong
Stowe Flagship Store	King of Power Saving	It is genuine very good, the price is much cheaper, cost-effective, really special cost-effective, it is worth buying!	Jingdong prefecture level city in Shandong
Stowe Flagship Store	King of Power Saving	This thing can really save electricity, I was introduced to buy by my friend, he used it very well, I used it and think it's also very good, it can really save electricity, save about half of the electricity every day, it's really good!	Jingdong prefecture level city in Shandong
Stowe Flagship Store	King of Power Saving	After using it for ten days or so, I checked the power consumption and it did drop, and customer service said it will save more electricity in the long run. The product is also insured, 5 years warranty do not have to worry about quality problems!	Jingdong prefecture level city in Shandong
Stowe Flagship Store	King of Power Saving	The product is good, before already bought a, this is the second, the effect is quite good, anyway, I see the daily electricity bill than before a lot less, this can be the choice!	Jingdong prefecture level city in Shandong

Stowe Flagship Store	King of Power Saving	Originally also very worried about things are not good, get the hand found completely over-anxious, five-star praise, the style is very generous, the quality is very good, the price is also very reasonable!	Jingdong prefecture level city in Shandong
Stowe Flagship Store	King of Power Saving	Quality is good, playing to the old family also buy a, repurchase, this quality is really good, recommended!	Jingdong prefecture level city in Shandong
Stowe Flagship Store	King of Power Saving	Thank you, the owner even sent two fuses.	Jingdong prefecture level city in Shandong
Stowe Flagship Store	King of Power Saving	This store has great stuff, relatively fast shipping and logistics	Jingdong prefecture level city in Shandong
Stowe Flagship Store	King of Power Saving	This product is based on the impossibility of saving electricity, I used 853 kWh of electricity in July, started using the product on August 1, and used 867 kWh of electricity by September 1, is this called	Jingdong prefecture level city in Shandong
Stowe Flagship Store	King of Power Saving	Power king received, to the home on the trial, when I plugged into the electric panel can not see the effect of power saving, the meter is still the same speed in the turn, I'll save the king of the manual to play look at the instructions, instructions to speak, have to have a day two days and half a month to save power obviously, then wait half a month to see the effect of power saving it! To half a month there are obvious power saving before continuing to chase Xu.	Jingdong prefecture level city in Shandong
Stowe Flagship Store	King of Power Saving	Received the goods, a look, did not see the manufacturer and production address, can only hold a try to see the mentality of the face	Jingdong prefecture level city in Shandong
Stowe Flagship Store	King of Power Saving	I've only been using it for a few days and I'm not sure if it's an IQ tax? Used to favorable reviews.	Jingdong prefecture level city in Shandong
Stowe Flagship Store	King of Power Saving	Appearance is okay, power saving next time to follow up the evaluation	Jingdong prefecture level city in Shandong
Stowe Flagship Store	King of Power Saving	Shorter time, but hopefully effective and power-saving, to be followed up in the future	Jingdong prefecture level city in Shandong
Stowe Flagship Store	King of Power Saving	Appearance is okay, I do not know if the power saving	Jingdong prefecture level city in Shandong
Stowe Flagship Store	King of Power Saving	Good service, fast logistics, Power Saving King is exquisite and beautiful, support the business, thank you!	Jingdong prefecture level city in Shandong
Stowe Flagship Store	King of Power Saving	Bought three at once to try first first.	Jingdong prefecture level city in Shandong
Stowe Flagship Store	King of Power Saving	The object is the same as the picture, the product is of high quality, the packaging is intact. High-end atmosphere, high power, power saving effect is obvious, average monthly power saving 50%, very satisfied.	Jingdong prefecture level city in Shandong
Stowe Flagship Store	King of Power Saving	Bought back and plugged in, really effective, before 16 degrees of electricity per day, now 8 degrees of electricity per day, open an air conditioner for 12 hours, decisive repurchase of a	Jingdong prefecture level city in Shandong
Stowe Flagship Store	King of Power Saving	The object is the same as the picture, the product is of high quality, and the package is intact. High-end atmosphere and high grade, high power	Jingdong prefecture level city in Shandong
Stowe Flagship Store	King of Power Saving	Goods received, I hope the store said can be energy-saving, first try a month, month after month to see the effect.	Jingdong prefecture level city in Shandong
Stowe Flagship Store	King of Power Saving	This is really good, the material and workmanship is particularly exquisite, the workmanship is really very good, I think I can recommend to my friends to buy	Jingdong prefecture level city in Shandong
Stowe Flagship Store	King of Power Saving	I have to praise, the quality is really good, and the packaging logistics are also good. Really like it, won't make you regret after getting it!	Jingdong prefecture level city in Shandong
Stowe Flagship Store	King of Power Saving	Reputable platform, efficient and professional service, buy with confidence and peace of mind!	Jingdong prefecture level city in Shandong
Stowe Flagship Store	King of Power Saving	Bought one on a trial basis to see if it works after 1-2 months.	Jingdong prefecture level city in Shandong
Stowe Flagship Store	King of Power Saving	Quality is very good, customer service attitude is very very praise, there are problems in a timely manner to solve the problem, the price is not expensive, super value, you can rest assured that the	Jingdong prefecture level city in Shandong
Stowe Flagship Store	King of Power Saving	With it no longer need to be afraid to use electricity, especially good, ready to buy a mom and dad that also	Jingdong prefecture level city in Shandong
Stowe Flagship Store	King of Power Saving	Baby received very like will return to buy the seller attitude is also very like will return to buy the seller attitude is also very like!	Jingdong prefecture level city in Shandong
Stowe Flagship Store	King of Power Saving	Jingdong Mall shopping convenience assured fast assured fast assured fast assured fast	Jingdong prefecture level city in Shandong
Stowe Flagship Store	King of Power Saving	Saving power received, the quality is very good, fine workmanship, especially good, much cheaper than the entity store, it is worth buying!	Jingdong prefecture level city in Shandong
Stowe Flagship Store	King of Power Saving	I did not expect the express delivery so quickly, very save point quality is super good, than the entity store is too cheap.	Jingdong prefecture level city in Shandong
Stowe Flagship Store	King of Power Saving	Have received the baby, very satisfied with a shopping, great worth buying!	Jingdong prefecture level city in Shandong
Stowe Flagship Store	King of Power Saving	This is really more powerful, very satisfied, more energy-saving, better, thank you boss.	Jingdong prefecture level city in Shandong
Stowe Flagship Store	King of Power Saving	Received has been used for two days, the air conditioning can boldly open it! Energy saving effect is really quite good	Jingdong prefecture level city in Shandong
Stowe Flagship Store	King of Power Saving	It is authentic, trust the merchant, the baby has a great texture and the price is moderate, unlimited repurchase!	Jingdong prefecture level city in Shandong
Stowe Flagship Store	King of Power Saving	It is genuine very good, the price is much cheaper, cost-effective, really special cost-effective, it is worth buying!	Jingdong prefecture level city in Shandong
Stowe Flagship Store	King of Power Saving	Very good quality, fine workmanship, low power consumption, long warranty, fast shipping, economical and practical	Jingdong prefecture level city in Shandong
Stowe Flagship Store	King of Power Saving	I've been using it for half a month now, it really saves power and the voltage is stable, it's very good	Jingdong prefecture level city in Shandong
Stowe Flagship Store	King of Power Saving	It's the ideal product , good quality and fast logistics, worth the kudos!	Jingdong prefecture level city in Shandong
Stowe Flagship Store	King of Power Saving	The logistics is very fast, the baby is very good, the shopkeeper is NICE!	Jingdong prefecture level city in Shandong
Stowe Flagship Store	King of Power Saving	Good to use, good quality, save point durable	Jingdong prefecture level city in Shandong
Stowe Flagship Store	King of Power Saving	The quality is very good, customer service attitude is very, very awesome, there are problems in a timely manner to solve the problem	Jingdong prefecture level city in Shandong
Stowe Flagship Store	King of Power Saving	Quality is very good, the use of the effect is very good, more stable, quality assurance	Jingdong prefecture level city in Shandong
Stowe Flagship Store	King of Power Saving	Convenient, inexpensive, and see how it works out?	Jingdong prefecture level city in Shandong
Stowe Flagship Store	King of Power Saving	Very good quality, saves a lot of electricity, very good, worth buying	Jingdong prefecture level city in Shandong
Stowe Flagship Store	King of Power Saving	Power Saving King is easy to use, with the fruit of power saving to use easy to give!	Jingdong prefecture level city in Shandong
Stowe Flagship Store	King of Power Saving	This saver really works, good quality, safe and secure, very satisfied!	Jingdong prefecture level city in Shandong
Stowe Flagship Store	King of Power Saving	It works well, the voltage is very stable effect is good, the quality is also very good satisfaction	Jingdong prefecture level city in Shandong
Stowe Flagship Store	King of Power Saving	The logistics is super super fast! The quality is also very good, indeed value for money	Jingdong prefecture level city in Shandong
Stowe Flagship Store	King of Power Saving	I've only been using it for a month or so and it seems to be saving a lot of power	Jingdong prefecture level city in Shandong
Stowe Flagship Store	King of Power Saving	Very easy to operate, very good material, good value for money.	Jingdong prefecture level city in Shandong
Stowe Flagship Store	King of Power Saving	Aluminum alloy shell, durable, compact power-saving good effect	Jingdong prefecture level city in Shandong
Stowe Flagship Store	King of Power Saving	Nice device, easy to use, doesn't take up much space, recommendable!	Jingdong prefecture level city in Shandong
Stowe Flagship Store	King of Power Saving	Good quality, good price, fast logistics	Jingdong prefecture level city in Shandong

Stowe Flagship Store	King of Power Saving	Friends of the family in use recommended, the effect is good and recommendable!	Jingdong prefecture level city in Shandong
Stowe Flagship Store	King of Power Saving	Cheaper than brick and mortar stores worth buying is authentic	Jingdong prefecture level city in Shandong
Stowe Flagship Store	King of Power Saving	Merchants ship quickly, the price is cheaper than the physical store, good use	Jingdong prefecture level city in Shandong
Stowe Flagship Store	King of Power Saving	Very durable, affordable, fast shipping	Jingdong prefecture level city in Shandong
Stowe Flagship Store	King of Power Saving	Packaging tight quality is very good easy to use good results	Jingdong prefecture level city in Shandong
Stowe Flagship Store	King of Power Saving	Save the grid. It's in the whole house. It's saving money on the electric bill.	Jingdong prefecture level city in Shandong
Stowe Flagship Store	King of Power Saving	I used it today, it works well, I'm very satisfied	Jingdong prefecture level city in Shandong
Stowe Flagship Store	King of Power Saving	It really saves a lot of electricity after installation	Jingdong prefecture level city in Shandong
Stowe Flagship Store	King of Power Saving	Love it. Satisfied.	Jingdong prefecture level city in Shandong
Stowe Flagship Store	King of Power Saving	Doesn't feel like it's working.	Jingdong prefecture level city in Shandong
Stowe Flagship Store	King of Power Saving	I'll comment after using it.	Jingdong prefecture level city in Shandong
Stowe Flagship Store	King of Power Saving	I'm trying it out to see if it saves power, and I'll buy two more if it works well.	Jingdong prefecture level city in Shandong
Stowe Flagship Store	King of Power Saving	It is genuine very good, the price is much cheaper, cost-effective, really special cost-effective, it is worth buying!	Jingdong prefecture level city in Shandong
Stowe Flagship Store	King of Power Saving	This thing can really save electricity, I was introduced to buy by my friend, he used it very well, I used it and think it's also very good, it can really save electricity, save about half of the electricity every day, it's really good!	Jingdong prefecture level city in Shandong
Stowe Flagship Store	King of Power Saving	After using it for ten days or so, I checked the power consumption and it did drop, and customer service said it will save more electricity in the long run. The product is also insured, 5 years warranty do not have to worry about quality problems!	Jingdong prefecture level city in Shandong
Stowe Flagship Store	King of Power Saving	The product is good, before already bought a, this is the second, the effect is quite good, anyway, I see the daily electricity bill than before a lot less, this can be the choice!	Jingdong prefecture level city in Shandong
Stowe Flagship Store	King of Power Saving	Originally also very worried about things are not good, get the hand found completely over-anxious, five-star praise, the style is very generous, the quality is very good, the price is also very reasonable!	Jingdong prefecture level city in Shandong
Stowe Flagship Store	King of Power Saving	Quality is good, playing to the old family also buy a, repurchase, this quality is really good, recommended!	Jingdong prefecture level city in Shandong
Stowe Flagship Store	King of Power Saving	Thank you, the owner even sent two fuses.	Jingdong prefecture level city in Shandong
Stowe Flagship Store	King of Power Saving	This store has great stuff, relatively fast shipping and logistics	Jingdong prefecture level city in Shandong
Stowe Flagship Store	King of Power Saving	This product is based on the impossibility of saving electricity, I used 853 kWh of electricity in July, started using the product on August 1, and used 867 kWh of electricity by September 1, is this called	Jingdong prefecture level city in Shandong
Stowe Flagship Store	King of Power Saving	Power king received, to the home on the trial, when I plugged into the electric panel can not see the effect of power saving, the meter is still the same speed in the turn, I'll save the king of the manual to play look at the instructions, instructions to speak, have to have a day two days and half a month to save power obviously, then wait half a month to see the effect of power saving it! To half a month there are obvious power saving before continuing to chase Xu.	Jingdong prefecture level city in Shandong
Stowe Flagship Store	King of Power Saving	Received the goods, a look, did not see the manufacturer and production address, can only hold a try to see the mentality of the face	Jingdong prefecture level city in Shandong
Stowe Flagship Store	King of Power Saving	I've only been using it for a few days and I'm not sure if it's an IQ tax? Used to favorable reviews.	Jingdong prefecture level city in Shandong
Stowe Flagship Store	King of Power Saving	Appearance is okay, power saving next time to follow up the evaluation	Jingdong prefecture level city in Shandong
Stowe Flagship Store	King of Power Saving	Shorter time, but hopefully effective and power-saving, to be followed up in the future	Jingdong prefecture level city in Shandong
Stowe Flagship Store	King of Power Saving	Appearance is okay, I do not know if the power saving	Jingdong prefecture level city in Shandong
Stowe Flagship Store	King of Power Saving	Good service, fast logistics, Power Saving King is exquisite and beautiful, support the business, thank you!	Jingdong prefecture level city in Shandong
Stowe Flagship Store	King of Power Saving	Bought three at once to try first first.	Jingdong prefecture level city in Shandong
Stowe Flagship Store	King of Power Saving	The object is the same as the picture, the product is of high quality, the packaging is intact. High-end atmosphere, high power, power saving effect is obvious, average monthly power saving 50%, very satisfied.	Jingdong prefecture level city in Shandong
Stowe Flagship Store	King of Power Saving	Bought back and plugged in, really effective, before 16 degrees of electricity per day, now 8 degrees of electricity per day, open an air conditioner for 12 hours, decisive repurchase of a	Jingdong prefecture level city in Shandong
Stowe Flagship Store	King of Power Saving	The object is the same as the picture, the product is of high quality, and the package is intact. High-end atmosphere and high grade, high power	Jingdong prefecture level city in Shandong
Stowe Flagship Store	King of Power Saving	Goods received, I hope the store said can be energy-saving, first try a month, month after month to see the effect.	Jingdong prefecture level city in Shandong
Stowe Flagship Store	King of Power Saving	This is really good, the material and workmanship is particularly exquisite, the workmanship is really very good, I think I can recommend to my friends to buy	Jingdong prefecture level city in Shandong
Stowe Flagship Store	King of Power Saving	I have to praise, the quality is really good, and the packaging logistics are also good. Really like it, won't make you regret after getting it!	Jingdong prefecture level city in Shandong
Stowe Flagship Store	King of Power Saving	Reputable platform, efficient and professional service, buy with confidence and peace of mind!	Jingdong prefecture level city in Shandong
Stowe Flagship Store	King of Power Saving	Bought one on a trial basis to see if it works after 1-2 months.	Jingdong prefecture level city in Shandong
Stowe Flagship Store	King of Power Saving	Quality is very good, customer service attitude is very very praise, there are problems in a timely manner to solve the problem, the price is not expensive, super value, you can rest assured that the	Jingdong prefecture level city in Shandong
Stowe Flagship Store	King of Power Saving	With it no longer need to be afraid to use electricity, especially good, ready to buy a mom and dad that also	Jingdong prefecture level city in Shandong
Stowe Flagship Store	King of Power Saving	Baby received very like will return to buy the seller attitude is also very like will return to buy the seller attitude is also very like!	Jingdong prefecture level city in Shandong
Stowe Flagship Store	King of Power Saving	Jingdong Mall shopping convenience assured fast assured fast assured fast assured fast	Jingdong prefecture level city in Shandong
Stowe Flagship Store	King of Power Saving	Saving power received, the quality is very good, fine workmanship, especially good, much cheaper than the entity store, it is worth buying!	Jingdong prefecture level city in Shandong
Stowe Flagship Store	King of Power Saving	I did not expect the express delivery so quickly, very save point quality is super good, than the entity store is too cheap.	Jingdong prefecture level city in Shandong
Stowe Flagship Store	King of Power Saving	Have received the baby, very satisfied with a shopping, great worth buying!	Jingdong prefecture level city in Shandong
Stowe Flagship Store	King of Power Saving	This is really more powerful, very satisfied, more energy-saving, better, thank you boss.	Jingdong prefecture level city in Shandong
Stowe Flagship Store	King of Power Saving	Received has been used for two days, the air conditioning can boldly open it! Energy saving effect is really quite good	Jingdong prefecture level city in Shandong
Stowe Flagship Store	King of Power Saving	It is authentic, trust the merchant, the baby has a great texture and the price is moderate, unlimited repurchase!	Jingdong prefecture level city in Shandong
Stowe Flagship Store	King of Power Saving	It is genuine very good, the price is much cheaper, cost-effective, really special cost-effective, it is worth buying!	Jingdong prefecture level city in Shandong
Stowe Flagship Store	King of Power Saving	Very good quality, fine workmanship, low power consumption, long warranty, fast shipping, economical and practical	Jingdong prefecture level city in Shandong

Stowe Flagship Store	King of Power Saving	I've been using it for half a month now, it really saves power and the voltage is stable, it's very good	Jingdong prefecture level city in Shandong
Stowe Flagship Store	King of Power Saving	It's the ideal product 💗, good quality and fast logistics, worth the kudos!	Jingdong prefecture level city in Shandong
Stowe Flagship Store	King of Power Saving	The logistics is very fast, the baby is very good, the shopkeeper is NICE!	Jingdong prefecture level city in Shandong
Stowe Flagship Store	King of Power Saving	Good to use, good quality, save point durable	Jingdong prefecture level city in Shandong
Stowe Flagship Store	King of Power Saving	The quality is very good, customer service attitude is very, very awesome, there are problems in a timely manner to solve the problem	Jingdong prefecture level city in Shandong
Stowe Flagship Store	King of Power Saving	Quality is very good, the use of the effect is very good, more stable, quality assurance	Jingdong prefecture level city in Shandong
Stowe Flagship Store	King of Power Saving	Convenient, inexpensive, and see how it works out?	Jingdong prefecture level city in Shandong
Stowe Flagship Store	King of Power Saving	Very good quality, saves a lot of electricity, very good, worth buying	Jingdong prefecture level city in Shandong
Stowe Flagship Store	King of Power Saving	Power Saving King is easy to use, with the fruit of power saving to use easy to give!	Jingdong prefecture level city in Shandong
Stowe Flagship Store	King of Power Saving	This saver really works, good quality, safe and secure, very satisfied!	Jingdong prefecture level city in Shandong
Stowe Flagship Store	King of Power Saving	It works well, the voltage is very stable effect is good, the quality is also very good satisfaction	Jingdong prefecture level city in Shandong
Stowe Flagship Store	King of Power Saving	The logistics is super super fast! The quality is also very good, indeed value for money	Jingdong prefecture level city in Shandong
Stowe Flagship Store	King of Power Saving	I've only been using it for a month or so and it seems to be saving a lot of power	Jingdong prefecture level city in Shandong
Stowe Flagship Store	King of Power Saving	Very easy to operate, very good material, good value for money.	Jingdong prefecture level city in Shandong
Stowe Flagship Store	King of Power Saving	Aluminum alloy shell, durable, compact power-saving good effect	Jingdong prefecture level city in Shandong
Stowe Flagship Store	King of Power Saving	Nice device, easy to use, doesn't take up much space, recommendable!	Jingdong prefecture level city in Shandong
Stowe Flagship Store	King of Power Saving	Good quality, good price, fast logistics	Jingdong prefecture level city in Shandong
Stowe Flagship Store	King of Power Saving	Friends of the family in use recommended, the effect is good and recommendable!	Jingdong prefecture level city in Shandong
Stowe Flagship Store	King of Power Saving	Cheaper than brick and mortar stores worth buying is authentic	Jingdong prefecture level city in Shandong
Stowe Flagship Store	King of Power Saving	Merchants ship quickly, the price is cheaper than the physical store, good use	Jingdong prefecture level city in Shandong
Stowe Flagship Store	King of Power Saving	Very durable, affordable, fast shipping	Jingdong prefecture level city in Shandong
Stowe Flagship Store	King of Power Saving	Packaging tight quality is very good easy to use good results	Jingdong prefecture level city in Shandong
Stowe Flagship Store	King of Power Saving	Save the grid. It's in the whole house. It's saving money on the electric bill.	Jingdong prefecture level city in Shandong
Stowe Flagship Store	King of Power Saving	I used it today, it works well, I'm very satisfied	Jingdong prefecture level city in Shandong
Stowe Flagship Store	King of Power Saving	It really saves a lot of electricity after installation	Jingdong prefecture level city in Shandong
Stowe Flagship Store	King of Power Saving	Love it. Satisfied.	Jingdong prefecture level city in Shandong
Stowe Flagship Store	King of Power Saving	Doesn't feel like it's working.	Jingdong prefecture level city in Shandong
Stowe Flagship Store	King of Power Saving	I'll comment after using it.	Jingdong prefecture level city in Shandong
Stowe Flagship Store	King of Power Saving	I'm trying it out to see if it saves power, and I'll buy two more if it works well.	Jingdong prefecture level city in Shandong
Stowe Flagship Store	King of Power Saving	It is genuine very good, the price is much cheaper, cost-effective, really special cost-effective, it is worth buying!	Jingdong prefecture level city in Shandong
Stowe Flagship Store	King of Power Saving	This thing can really save electricity, I was introduced to buy by my friend, he used it very well, I used it and think it's also very good, it can really save electricity, save about half of the electricity every day, it's really good!	Jingdong prefecture level city in Shandong
Stowe Flagship Store	King of Power Saving	After using it for ten days or so, I checked the power consumption and it did drop, and customer service said it will save more electricity in the long run. The product is also insured, 5 years warranty do not have to worry about quality problems!	Jingdong prefecture level city in Shandong
Stowe Flagship Store	King of Power Saving	The product is good, before already bought a, this is the second, the effect is quite good, anyway, I see the daily electricity bill than before a lot less, this can be the choice!	Jingdong prefecture level city in Shandong
Stowe Flagship Store	King of Power Saving	Originally also very worried about things are not good, get the hand found completely over-anxious, five-star praise, the style is very generous, the quality is very good, the price is also very reasonable!	Jingdong prefecture level city in Shandong
Stowe Flagship Store	King of Power Saving	Quality is good, playing to the old family also buy a, repurchase, this quality is really good, recommended!	Jingdong prefecture level city in Shandong
Stowe Flagship Store	King of Power Saving	Thank you, the owner even sent two fuses.	Jingdong prefecture level city in Shandong
Stowe Flagship Store	King of Power Saving	This store has great stuff, relatively fast shipping and logistics	Jingdong prefecture level city in Shandong
Stowe Flagship Store	King of Power Saving	This product is based on the impossibility of saving electricity, I used 853 kWh of electricity in July, started using the product on August 1, and used 867 kWh of electricity by September 1, is this called	Jingdong prefecture level city in Shandong
Stowe Flagship Store	King of Power Saving	Power king received, to the home on the trial, when I plugged into the electric panel can not see the effect of power saving, the meter is still the same speed in the turn, I'll save the king of the manual to play look at the instructions, instructions to speak, have to have a day two days and half a month to save power obviously, then wait half a month to see the effect of power saving it! To half a month there are obvious power saving before continuing to chase Xu.	Jingdong prefecture level city in Shandong
Stowe Flagship Store	King of Power Saving	Received the goods, a look, did not see the manufacturer and production address, can only hold a try to see the mentality of the face	Jingdong prefecture level city in Shandong
Stowe Flagship Store	King of Power Saving	I've only been using it for a few days and I'm not sure if it's an IQ tax? Used to favorable reviews.	Jingdong prefecture level city in Shandong
Stowe Flagship Store	King of Power Saving	Appearance is okay, power saving next time to follow up the evaluation	Jingdong prefecture level city in Shandong
Stowe Flagship Store	King of Power Saving	Shorter time, but hopefully effective and power-saving, to be followed up in the future	Jingdong prefecture level city in Shandong
Stowe Flagship Store	King of Power Saving	Appearance is okay, I do not know if the power saving	Jingdong prefecture level city in Shandong
Stowe Flagship Store	King of Power Saving	Good service, fast logistics, Power Saving King is exquisite and beautiful, support the business, thank you!	Jingdong prefecture level city in Shandong
Stowe Flagship Store	King of Power Saving	Bought three at once to try first first.	Jingdong prefecture level city in Shandong
Stowe Flagship Store	King of Power Saving	The object is the same as the picture, the product is of high quality, the packaging is intact. High-end atmosphere, high power, power saving effect is obvious, average monthly power saving 50%, very satisfied.	Jingdong prefecture level city in Shandong
Stowe Flagship Store	King of Power Saving	Bought back and plugged in, really effective, before 16 degrees of electricity per day, now 8 degrees of electricity per day, open an air conditioner for 12 hours, decisive repurchase of a	Jingdong prefecture level city in Shandong
Stowe Flagship Store	King of Power Saving	The object is the same as the picture, the product is of high quality, and the package is intact. High-end atmosphere and high grade, high power	Jingdong prefecture level city in Shandong

Stowe Flagship Store	King of Power Saving	Goods received, I hope the store said can be energy-saving, first try a month, month after month to see the effect.	Jingdong prefecture level city in Shandong
Stowe Flagship Store	King of Power Saving	This is really good, the material and workmanship is particularly exquisite, the workmanship is really very good, I think I can recommend to my friends to buy	Jingdong prefecture level city in Shandong
Stowe Flagship Store	King of Power Saving	I have to praise, the quality is really good, and the packaging logistics are also good. Really like it, won't make you regret after getting it!	Jingdong prefecture level city in Shandong
Stowe Flagship Store	King of Power Saving	Reputable platform, efficient and professional service, buy with confidence and peace of mind!	Jingdong prefecture level city in Shandong
Stowe Flagship Store	King of Power Saving	Bought one on a trial basis to see if it works after 1-2 months.	Jingdong prefecture level city in Shandong
Stowe Flagship Store	King of Power Saving	Quality is very good, customer service attitude is very very praise, there are problems in a timely manner to solve the problem, the price is not expensive, super value, you can rest assured that the	Jingdong prefecture level city in Shandong
Stowe Flagship Store	King of Power Saving	With it no longer need to be afraid to use electricity, especially good, ready to buy a mom and dad that also	Jingdong prefecture level city in Shandong
Stowe Flagship Store	King of Power Saving	Baby received very like will return to buy the seller attitude is also very like will return to buy the seller attitude is also very like!	Jingdong prefecture level city in Shandong
Stowe Flagship Store	King of Power Saving	Jingdong Mall shopping convenience assured fast assured fast assured fast assured fast	Jingdong prefecture level city in Shandong
Stowe Flagship Store	King of Power Saving	Saving power received, the quality is very good, fine workmanship, especially good, much cheaper than the entity store, it is worth buying!	Jingdong prefecture level city in Shandong
Stowe Flagship Store	King of Power Saving	I did not expect the express delivery so quickly, very save point quality is super good, than the entity store is too cheap.	Jingdong prefecture level city in Shandong
Stowe Flagship Store	King of Power Saving	Have received the baby, very satisfied with a shopping, great worth buying!	Jingdong prefecture level city in Shandong
Stowe Flagship Store	King of Power Saving	This is really more powerful, very satisfied, more energy-saving, better, thank you boss.	Jingdong prefecture level city in Shandong
Stowe Flagship Store	King of Power Saving	Received has been used for two days, the air conditioning can boldly open it! Energy saving effect is really quite good	Jingdong prefecture level city in Shandong
Stowe Flagship Store	King of Power Saving	It is authentic, trust the merchant, the baby has a great texture and the price is moderate, unlimited repurchase!	Jingdong prefecture level city in Shandong
Stowe Flagship Store	King of Power Saving	It is genuine very good, the price is much cheaper, cost-effective, really special cost-effective, it is worth buying!	Jingdong prefecture level city in Shandong
Stowe Flagship Store	King of Power Saving	Very good quality, fine workmanship, low power consumption, long warranty, fast shipping, economical and practical	Jingdong prefecture level city in Shandong
Stowe Flagship Store	King of Power Saving	I've been using it for half a month now, it really saves power and the voltage is stable, it's very good	Jingdong prefecture level city in Shandong
Stowe Flagship Store	King of Power Saving	It's the ideal product, good quality and fast logistics, worth the kudos!	Jingdong prefecture level city in Shandong
Stowe Flagship Store	King of Power Saving	The logistics is very fast, the baby is very good, the shopkeeper is NICE!	Jingdong prefecture level city in Shandong
Stowe Flagship Store	King of Power Saving	Good to use, good quality, save point durable	Jingdong prefecture level city in Shandong
Stowe Flagship Store	King of Power Saving	The quality is very good, customer service attitude is very, very awesome, there are problems in a timely manner to solve the problem	Jingdong prefecture level city in Shandong
Stowe Flagship Store	King of Power Saving	Quality is very good, the use of the effect is very good, more stable, quality assurance	Jingdong prefecture level city in Shandong
Stowe Flagship Store	King of Power Saving	Convenient, inexpensive, and see how it works out?	Jingdong prefecture level city in Shandong
Stowe Flagship Store	King of Power Saving	Very good quality, saves a lot of electricity, very good, worth buying	Jingdong prefecture level city in Shandong
Stowe Flagship Store	King of Power Saving	Power Saving King is easy to use, with the fruit of power saving to use easy to give!	Jingdong prefecture level city in Shandong
Stowe Flagship Store	King of Power Saving	This saver really works, good quality, safe and secure, very satisfied!	Jingdong prefecture level city in Shandong
Stowe Flagship Store	King of Power Saving	It works well, the voltage is very stable effect is good, the quality is also very good satisfaction	Jingdong prefecture level city in Shandong
Stowe Flagship Store	King of Power Saving	The logistics is super super fast! The quality is also very good, indeed value for money	Jingdong prefecture level city in Shandong
Stowe Flagship Store	King of Power Saving	I've only been using it for a month or so and it seems to be saving a lot of power	Jingdong prefecture level city in Shandong
Stowe Flagship Store	King of Power Saving	Very easy to operate, very good material, good value for money.	Jingdong prefecture level city in Shandong
Stowe Flagship Store	King of Power Saving	Aluminum alloy shell, durable, compact power-saving good effect	Jingdong prefecture level city in Shandong
Stowe Flagship Store	King of Power Saving	Nice device, easy to use, doesn't take up much space, recommendable!	Jingdong prefecture level city in Shandong
Stowe Flagship Store	King of Power Saving	Good quality, good price, fast logistics	Jingdong prefecture level city in Shandong
Stowe Flagship Store	King of Power Saving	Friends of the family in use recommended, the effect is good and recommendable!	Jingdong prefecture level city in Shandong
Stowe Flagship Store	King of Power Saving	Cheaper than brick and mortar stores worth buying is authentic	Jingdong prefecture level city in Shandong
Stowe Flagship Store	King of Power Saving	Merchants ship quickly, the price is cheaper than the physical store, good use	Jingdong prefecture level city in Shandong
Stowe Flagship Store	King of Power Saving	Very durable, affordable, fast shipping	Jingdong prefecture level city in Shandong
Stowe Flagship Store	King of Power Saving	Packaging tight quality is very good easy to use good results	Jingdong prefecture level city in Shandong
Stowe Flagship Store	King of Power Saving	Save the grid. It's in the whole house. It's saving money on the electric bill.	Jingdong prefecture level city in Shandong
Stowe Flagship Store	King of Power Saving	I used it today, it works well, I'm very satisfied	Jingdong prefecture level city in Shandong
Stowe Flagship Store	King of Power Saving	It really saves a lot of electricity after installation	Jingdong prefecture level city in Shandong
Stowe Flagship Store	King of Power Saving	Love it. Satisfied.	Jingdong prefecture level city in Shandong
Stowe Flagship Store	King of Power Saving	Doesn't feel like it's working.	Jingdong prefecture level city in Shandong
Stowe Flagship Store	King of Power Saving	I'll comment after using it.	Jingdong prefecture level city in Shandong
Stowe Flagship Store	King of Power Saving	I'm trying it out to see if it saves power, and I'll buy two more if it works well.	Jingdong prefecture level city in Shandong
Stowe Flagship Store	King of Power Saving	It is genuine very good, the price is much cheaper, cost-effective, really special cost-effective, it is worth buying!	Jingdong prefecture level city in Shandong
Stowe Flagship Store	King of Power Saving	This thing can really save electricity, I was introduced to buy by my friend, he used it very well, I used it and think it's also very good, it can really save electricity, save about half of the electricity every day, it's really good!	Jingdong prefecture level city in Shandong
Stowe Flagship Store	King of Power Saving	After using it for ten days or so, I checked the power consumption and it did drop, and customer service said it will save more electricity in the long run. The product is also insured, 5 years warranty do not have to worry about quality problems!	Jingdong prefecture level city in Shandong
Stowe Flagship Store	King of Power Saving	The product is good, before already bought a, this is the second, the effect is quite good, anyway, I see the daily electricity bill than before a lot less, this can be the choice!	Jingdong prefecture level city in Shandong

Stowe Flagship Store	King of Power Saving	Originally also very worried about things are not good, get the hand found completely over-anxious, five-star praise, the style is very generous, the quality is very good, the price is also very reasonable!	Jingdong prefecture level city in Shandong
Stowe Flagship Store	King of Power Saving	Quality is good, playing to the old family also buy a, repurchase, this quality is really good, recommended!	Jingdong prefecture level city in Shandong
Stowe Flagship Store	King of Power Saving	Thank you, the owner even sent two fuses.	Jingdong prefecture level city in Shandong
Stowe Flagship Store	King of Power Saving	This store has great stuff, relatively fast shipping and logistics	Jingdong prefecture level city in Shandong
Stowe Flagship Store	King of Power Saving	This product is based on the impossibility of saving electricity, I used 853 kWh of electricity in July, started using the product on August 1, and used 867 kWh of electricity by September 1, is this called	Jingdong prefecture level city in Shandong
Stowe Flagship Store	King of Power Saving	Power king received, to the home on the trial, when I plugged into the electric panel can not see the effect of power saving, the meter is still the same speed in the turn, I'll save the king of the manual to play look at the instructions, instructions to speak, have to have a day two days and half a month to save power obviously, then wait half a month to see the effect of power saving it! To half a month there are obvious power saving before continuing to chase Xu.	Jingdong prefecture level city in Shandong
Stowe Flagship Store	King of Power Saving	Received the goods, a look, did not see the manufacturer and production address, can only hold a try to see the mentality of the face	Jingdong prefecture level city in Shandong
Stowe Flagship Store	King of Power Saving	I've only been using it for a few days and I'm not sure if it's an IQ tax? Used to favorable reviews.	Jingdong prefecture level city in Shandong
Stowe Flagship Store	King of Power Saving	Appearance is okay, power saving next time to follow up the evaluation	Jingdong prefecture level city in Shandong
Stowe Flagship Store	King of Power Saving	Shorter time, but hopefully effective and power-saving, to be followed up in the future	Jingdong prefecture level city in Shandong
Stowe Flagship Store	King of Power Saving	Appearance is okay, I do not know if the power saving	Jingdong prefecture level city in Shandong
Stowe Flagship Store	King of Power Saving	Good service, fast logistics, Power Saving King is exquisite and beautiful, support the business, thank you!	Jingdong prefecture level city in Shandong
Stowe Flagship Store	King of Power Saving	Bought three at once to try first first.	Jingdong prefecture level city in Shandong
Stowe Flagship Store	King of Power Saving	The object is the same as the picture, the product is of high quality, the packaging is intact. High-end atmosphere, high power, power saving effect is obvious, average monthly power saving 50%, very satisfied.	Jingdong prefecture level city in Shandong
Stowe Flagship Store	King of Power Saving	Bought back and plugged in, really effective, before 16 degrees of electricity per day, now 8 degrees of electricity per day, open an air conditioner for 12 hours, decisive repurchase of a	Jingdong prefecture level city in Shandong
Stowe Flagship Store	King of Power Saving	The object is the same as the picture, the product is of high quality, and the package is intact. High-end atmosphere and high grade, high power	Jingdong prefecture level city in Shandong
Stowe Flagship Store	King of Power Saving	Goods received, I hope the store said can be energy-saving, first try a month, month after month to see the effect.	Jingdong prefecture level city in Shandong
Stowe Flagship Store	King of Power Saving	This is really good, the material and workmanship is particularly exquisite, the workmanship is really very good, I think I can recommend to my friends to buy	Jingdong prefecture level city in Shandong
Stowe Flagship Store	King of Power Saving	I have to praise, the quality is really good, and the packaging logistics are also good. Really like it, won't make you regret after getting it!	Jingdong prefecture level city in Shandong
Stowe Flagship Store	King of Power Saving	Reputable platform, efficient and professional service, buy with confidence and peace of mind!	Jingdong prefecture level city in Shandong
Stowe Flagship Store	King of Power Saving	Bought one on a trial basis to see if it works after 1-2 months.	Jingdong prefecture level city in Shandong
Stowe Flagship Store	King of Power Saving	Quality is very good, customer service attitude is very very praise, there are problems in a timely manner to solve the problem, the price is not expensive, super value, you can rest assured that the	Jingdong prefecture level city in Shandong
Stowe Flagship Store	King of Power Saving	With it no longer need to be afraid to use electricity, especially good, ready to buy a mom and dad that also	Jingdong prefecture level city in Shandong
Stowe Flagship Store	King of Power Saving	Baby received very like will return to buy the seller attitude is also very like will return to buy the seller attitude is also very like!	Jingdong prefecture level city in Shandong
Stowe Flagship Store	King of Power Saving	Jingdong Mall shopping convenience assured fast assured fast assured fast assured fast	Jingdong prefecture level city in Shandong
Stowe Flagship Store	King of Power Saving	Saving power received, the quality is very good, fine workmanship, especially good, much cheaper than the entity store, it is worth buying!	Jingdong prefecture level city in Shandong
Stowe Flagship Store	King of Power Saving	I did not expect the express delivery so quickly, very save point quality is super good, than the entity store is too cheap.	Jingdong prefecture level city in Shandong
Stowe Flagship Store	King of Power Saving	Have received the baby, very satisfied with a shopping, great worth buying!	Jingdong prefecture level city in Shandong
Stowe Flagship Store	King of Power Saving	This is really more powerful, very satisfied, more energy-saving, better, thank you boss.	Jingdong prefecture level city in Shandong
Stowe Flagship Store	King of Power Saving	Received has been used for two days, the air conditioning can boldly open it! Energy saving effect is really quite good	Jingdong prefecture level city in Shandong
Stowe Flagship Store	King of Power Saving	It is authentic, trust the merchant, the baby has a great texture and the price is moderate, unlimited repurchase!	Jingdong prefecture level city in Shandong
Stowe Flagship Store	King of Power Saving	It is genuine very good, the price is much cheaper, cost-effective, really special cost-effective, it is worth buying!	Jingdong prefecture level city in Shandong
Stowe Flagship Store	King of Power Saving	Very good quality, fine workmanship, low power consumption, long warranty, fast shipping, economical and practical	Jingdong prefecture level city in Shandong
Stowe Flagship Store	King of Power Saving	I've been using it for half a month now, it really saves power and the voltage is stable, it's very good	Jingdong prefecture level city in Shandong
Stowe Flagship Store	King of Power Saving	It's the ideal product, good quality and fast logistics, worth the kudos!	Jingdong prefecture level city in Shandong
Stowe Flagship Store	King of Power Saving	The logistics is very fast, the baby is very good, the shopkeeper is NICE!	Jingdong prefecture level city in Shandong
Stowe Flagship Store	King of Power Saving	Good to use, good quality, save point durable	Jingdong prefecture level city in Shandong
Stowe Flagship Store	King of Power Saving	The quality is very good, customer service attitude is very, very awesome, there are problems in a timely manner to solve the problem	Jingdong prefecture level city in Shandong
Stowe Flagship Store	King of Power Saving	Quality is very good, the use of the effect is very good, more stable, quality assurance	Jingdong prefecture level city in Shandong
Stowe Flagship Store	King of Power Saving	Convenient, inexpensive, and see how it works out?	Jingdong prefecture level city in Shandong
Stowe Flagship Store	King of Power Saving	Very good quality, saves a lot of electricity, very good, worth buying	Jingdong prefecture level city in Shandong
Stowe Flagship Store	King of Power Saving	Power Saving King is easy to use, with the fruit of power saving to use easy to give!	Jingdong prefecture level city in Shandong
Stowe Flagship Store	King of Power Saving	This saver really works, good quality, safe and secure, very satisfied!	Jingdong prefecture level city in Shandong
Stowe Flagship Store	King of Power Saving	It works well, the voltage is very stable effect is good, the quality is also very good satisfaction	Jingdong prefecture level city in Shandong
Stowe Flagship Store	King of Power Saving	The logistics is super super fast! The quality is also very good, indeed value for money	Jingdong prefecture level city in Shandong
Stowe Flagship Store	King of Power Saving	I've only been using it for a month or so and it seems to be saving a lot of power	Jingdong prefecture level city in Shandong
Stowe Flagship Store	King of Power Saving	Very easy to operate, very good material, good value for money.	Jingdong prefecture level city in Shandong
Stowe Flagship Store	King of Power Saving	Aluminum alloy shell, durable, compact power-saving good effect	Jingdong prefecture level city in Shandong
Stowe Flagship Store	King of Power Saving	Nice device, easy to use, doesn't take up much space, recommendable!	Jingdong prefecture level city in Shandong
Stowe Flagship Store	King of Power Saving	Good quality, good price, fast logistics	Jingdong prefecture level city in Shandong

Stowe Flagship Store	King of Power Saving	Friends of the family in use recommended, the effect is good and recommendable!	Jingdong prefecture level city in Shandong
Stowe Flagship Store	King of Power Saving	Cheaper than brick and mortar stores worth buying is authentic	Jingdong prefecture level city in Shandong
Stowe Flagship Store	King of Power Saving	Merchants ship quickly, the price is cheaper than the physical store, good use	Jingdong prefecture level city in Shandong
Stowe Flagship Store	King of Power Saving	Very durable, affordable, fast shipping	Jingdong prefecture level city in Shandong
Stowe Flagship Store	King of Power Saving	Packaging tight quality is very good easy to use good results	Jingdong prefecture level city in Shandong
Stowe Flagship Store	King of Power Saving	Save the grid. It's in the whole house. It's saving money on the electric bill.	Jingdong prefecture level city in Shandong
Stowe Flagship Store	King of Power Saving	I used it today, it works well, I'm very satisfied	Jingdong prefecture level city in Shandong
Stowe Flagship Store	King of Power Saving	It really saves a lot of electricity after installation	Jingdong prefecture level city in Shandong
Stowe Flagship Store	King of Power Saving	Love it. Satisfied.	Jingdong prefecture level city in Shandong
Stowe Flagship Store	King of Power Saving	Doesn't feel like it's working.	Jingdong prefecture level city in Shandong
Stowe Flagship Store	King of Power Saving	I'll comment after using it.	Jingdong prefecture level city in Shandong
Stowe Flagship Store	King of Power Saving	I'm trying it out to see if it saves power, and I'll buy two more if it works well.	Jingdong prefecture level city in Shandong
Stowe Flagship Store	King of Power Saving	It is genuine very good, the price is much cheaper, cost-effective, really special cost-effective, it is worth buying!	Jingdong prefecture level city in Shandong
Stowe Flagship Store	King of Power Saving	This thing can really save electricity, I was introduced to buy by my friend, he used it very well, I used it and think it's also very good, it can really save electricity, save about half of the electricity every day, it's really good!	Jingdong prefecture level city in Shandong
Stowe Flagship Store	King of Power Saving	After using it for ten days or so, I checked the power consumption and it did drop, and customer service said it will save more electricity in the long run. The product is also insured, 5 years warranty do not have to worry about quality problems!	Jingdong prefecture level city in Shandong
Stowe Flagship Store	King of Power Saving	The product is good, before already bought a, this is the second, the effect is quite good, anyway, I see the daily electricity bill than before a lot less, this can be the choice!	Jingdong prefecture level city in Shandong
Stowe Flagship Store	King of Power Saving	Originally also very worried about things are not good, get the hand found completely over-anxious, five-star praise, the style is very generous, the quality is very good, the price is also very reasonable!	Jingdong prefecture level city in Shandong
Stowe Flagship Store	King of Power Saving	Quality is good, playing to the old family also buy a, repurchase, this quality is really good, recommended!	Jingdong prefecture level city in Shandong
Stowe Flagship Store	King of Power Saving	Thank you, the owner even sent two fuses.	Jingdong prefecture level city in Shandong
Stowe Flagship Store	King of Power Saving	This store has great stuff, relatively fast shipping and logistics	Jingdong prefecture level city in Shandong
Stowe Flagship Store	King of Power Saving	This product is based on the impossibility of saving electricity, I used 853 kWh of electricity in July, started using the product on August 1, and used 867 kWh of electricity by September 1, is this called	Jingdong prefecture level city in Shandong
Stowe Flagship Store	King of Power Saving	Power king received, to the home on the trial, when I plugged into the electric panel can not see the effect of power saving, the meter is still the same speed in the turn, I'll save the king of the manual to play look at the instructions, instructions to speak, have to have a day two days and half a month to save power obviously, then wait half a month to see the effect of power saving it! To half a month there are obvious power saving before continuing to chase Xu.	Jingdong prefecture level city in Shandong
Stowe Flagship Store	King of Power Saving	Received the goods, a look, did not see the manufacturer and production address, can only hold a try to see the mentality of the face	Jingdong prefecture level city in Shandong
Stowe Flagship Store	King of Power Saving	I've only been using it for a few days and I'm not sure if it's an IQ tax? Used to favorable reviews.	Jingdong prefecture level city in Shandong
Stowe Flagship Store	King of Power Saving	Appearance is okay, power saving next time to follow up the evaluation	Jingdong prefecture level city in Shandong
Stowe Flagship Store	King of Power Saving	Shorter time, but hopefully effective and power-saving, to be followed up in the future	Jingdong prefecture level city in Shandong
Stowe Flagship Store	King of Power Saving	Appearance is okay, I do not know if the power saving	Jingdong prefecture level city in Shandong
Stowe Flagship Store	King of Power Saving	Good service, fast logistics, Power Saving King is exquisite and beautiful, support the business, thank you!	Jingdong prefecture level city in Shandong
Stowe Flagship Store	King of Power Saving	Bought three at once to try first first.	Jingdong prefecture level city in Shandong
Stowe Flagship Store	King of Power Saving	The object is the same as the picture, the product is of high quality, the packaging is intact. High-end atmosphere, high power, power saving effect is obvious, average monthly power saving 50%, very satisfied.	Jingdong prefecture level city in Shandong
Stowe Flagship Store	King of Power Saving	Bought back and plugged in, really effective, before 16 degrees of electricity per day, now 8 degrees of electricity per day, open an air conditioner for 12 hours, decisive repurchase of a	Jingdong prefecture level city in Shandong
Stowe Flagship Store	King of Power Saving	The object is the same as the picture, the product is of high quality, and the package is intact. High-end atmosphere and high grade, high power	Jingdong prefecture level city in Shandong
Stowe Flagship Store	King of Power Saving	Goods received, I hope the store said can be energy-saving, first try a month, month after month to see the effect.	Jingdong prefecture level city in Shandong
Stowe Flagship Store	King of Power Saving	This is really good, the material and workmanship is particularly exquisite, the workmanship is really very good, I think I can recommend to my friends to buy	Jingdong prefecture level city in Shandong
Stowe Flagship Store	King of Power Saving	I have to praise, the quality is really good, and the packaging logistics are also good. Really like it, won't make you regret after getting it!	Jingdong prefecture level city in Shandong
Stowe Flagship Store	King of Power Saving	Reputable platform, efficient and professional service, buy with confidence and peace of mind!	Jingdong prefecture level city in Shandong
Stowe Flagship Store	King of Power Saving	Bought one on a trial basis to see if it works after 1-2 months.	Jingdong prefecture level city in Shandong
Stowe Flagship Store	King of Power Saving	Quality is very good, customer service attitude is very very praise, there are problems in a timely manner to solve the problem, the price is not expensive, super value, you can rest assured that the	Jingdong prefecture level city in Shandong
Stowe Flagship Store	King of Power Saving	With it no longer need to be afraid to use electricity, especially good, ready to buy a mom and dad that also	Jingdong prefecture level city in Shandong
Stowe Flagship Store	King of Power Saving	Baby received very like will return to buy the seller attitude is also very like will return to buy the seller attitude is also very like!	Jingdong prefecture level city in Shandong
Stowe Flagship Store	King of Power Saving	Jingdong Mall shopping convenience assured fast assured fast assured fast assured fast	Jingdong prefecture level city in Shandong
Stowe Flagship Store	King of Power Saving	Saving power received, the quality is very good, fine workmanship, especially good, much cheaper than the entity store, it is worth buying!	Jingdong prefecture level city in Shandong
Stowe Flagship Store	King of Power Saving	I did not expect the express delivery so quickly, very save point quality is super good, than the entity store is too cheap.	Jingdong prefecture level city in Shandong
Stowe Flagship Store	King of Power Saving	Have received the baby, very satisfied with a shopping, great worth buying!	Jingdong prefecture level city in Shandong
Stowe Flagship Store	King of Power Saving	This is really more powerful, very satisfied, more energy-saving, better, thank you boss.	Jingdong prefecture level city in Shandong
Stowe Flagship Store	King of Power Saving	Received has been used for two days, the air conditioning can boldly open it! Energy saving effect is really quite good	Jingdong prefecture level city in Shandong
Stowe Flagship Store	King of Power Saving	It is authentic, trust the merchant, the baby has a great texture and the price is moderate, unlimited repurchase!	Jingdong prefecture level city in Shandong
Stowe Flagship Store	King of Power Saving	It is genuine very good, the price is much cheaper, cost-effective, really special cost-effective, it is worth buying!	Jingdong prefecture level city in Shandong
Stowe Flagship Store	King of Power Saving	Very good quality, fine workmanship, low power consumption, long warranty, fast shipping, economical and practical	Jingdong prefecture level city in Shandong

Stowe Flagship Store	King of Power Saving	I've been using it for half a month now, it really saves power and the voltage is stable, it's very good	Jingdong prefecture level city in Shandong
Stowe Flagship Store	King of Power Saving	It's the ideal product 💝, good quality and fast logistics, worth the kudos!	Jingdong prefecture level city in Shandong
Stowe Flagship Store	King of Power Saving	The logistics is very fast, the baby is very good, the shopkeeper is NICE!	Jingdong prefecture level city in Shandong
Stowe Flagship Store	King of Power Saving	Good to use, good quality, save point durable	Jingdong prefecture level city in Shandong
Stowe Flagship Store	King of Power Saving	The quality is very good, customer service attitude is very, very awesome, there are problems in a timely manner to solve the problem	Jingdong prefecture level city in Shandong
Stowe Flagship Store	King of Power Saving	Quality is very good, the use of the effect is very good, more stable, quality assurance	Jingdong prefecture level city in Shandong
Stowe Flagship Store	King of Power Saving	Convenient, inexpensive, and see how it works out?	Jingdong prefecture level city in Shandong
Stowe Flagship Store	King of Power Saving	Very good quality, saves a lot of electricity, very good, worth buying	Jingdong prefecture level city in Shandong
Stowe Flagship Store	King of Power Saving	Power Saving King is easy to use, with the fruit of power saving to use easy to give!	Jingdong prefecture level city in Shandong
Stowe Flagship Store	King of Power Saving	This saver really works, good quality, safe and secure, very satisfied!	Jingdong prefecture level city in Shandong
Stowe Flagship Store	King of Power Saving	It works well, the voltage is very stable effect is good, the quality is also very good satisfaction	Jingdong prefecture level city in Shandong
Stowe Flagship Store	King of Power Saving	The logistics is super super fast! The quality is also very good, indeed value for money	Jingdong prefecture level city in Shandong
Stowe Flagship Store	King of Power Saving	I've only been using it for a month or so and it seems to be saving a lot of power	Jingdong prefecture level city in Shandong
Stowe Flagship Store	King of Power Saving	Very easy to operate, very good material, good value for money.	Jingdong prefecture level city in Shandong
Stowe Flagship Store	King of Power Saving	Aluminum alloy shell, durable, compact power-saving good effect	Jingdong prefecture level city in Shandong
Stowe Flagship Store	King of Power Saving	Nice device, easy to use, doesn't take up much space, recommendable!	Jingdong prefecture level city in Shandong
Stowe Flagship Store	King of Power Saving	Good quality, good price, fast logistics	Jingdong prefecture level city in Shandong
Stowe Flagship Store	King of Power Saving	Friends of the family in use recommended, the effect is good and recommendable!	Jingdong prefecture level city in Shandong
Stowe Flagship Store	King of Power Saving	Cheaper than brick and mortar stores worth buying is authentic	Jingdong prefecture level city in Shandong
Stowe Flagship Store	King of Power Saving	Merchants ship quickly, the price is cheaper than the physical store, good use	Jingdong prefecture level city in Shandong
Stowe Flagship Store	King of Power Saving	Very durable, affordable, fast shipping	Jingdong prefecture level city in Shandong
Stowe Flagship Store	King of Power Saving	Packaging tight quality is very good easy to use good results	Jingdong prefecture level city in Shandong
Stowe Flagship Store	King of Power Saving	Save the grid. It's in the whole house. It's saving money on the electric bill.	Jingdong prefecture level city in Shandong
Stowe Flagship Store	King of Power Saving	I used it today, it works well, I'm very satisfied	Jingdong prefecture level city in Shandong
Stowe Flagship Store	King of Power Saving	It really saves a lot of electricity after installation	Jingdong prefecture level city in Shandong
Stowe Flagship Store	King of Power Saving	Love it. Satisfied.	Jingdong prefecture level city in Shandong
Stowe Flagship Store	King of Power Saving	Doesn't feel like it's working.	Jingdong prefecture level city in Shandong
Stowe Flagship Store	King of Power Saving	I'll comment after using it.	Jingdong prefecture level city in Shandong
Stowe Flagship Store	King of Power Saving	I'm trying it out to see if it saves power, and I'll buy two more if it works well.	Jingdong prefecture level city in Shandong
Stowe Flagship Store	King of Power Saving	It is genuine very good, the price is much cheaper, cost-effective, really special cost-effective, it is worth buying!	Jingdong prefecture level city in Shandong
Stowe Flagship Store	King of Power Saving	This thing can really save electricity, I was introduced to buy by my friend, he used it very well, I used it and think it's also very good, it can really save electricity, save about half of the electricity every day, it's really good!	Jingdong prefecture level city in Shandong
Stowe Flagship Store	King of Power Saving	After using it for ten days or so, I checked the power consumption and it did drop, and customer service said it will save more electricity in the long run. The product is also insured, 5 years warranty do not have to worry about quality problems!	Jingdong prefecture level city in Shandong
Stowe Flagship Store	King of Power Saving	The product is good, before already bought a, this is the second, the effect is quite good, anyway, I see the daily electricity bill than before a lot less, this can be the choice!	Jingdong prefecture level city in Shandong
Stowe Flagship Store	King of Power Saving	Originally also very worried about things are not good, get the hand found completely over-anxious, five-star praise, the style is very generous, the quality is very good, the price is also very reasonable!	Jingdong prefecture level city in Shandong
Stowe Flagship Store	King of Power Saving	Quality is good, playing to the old family also buy a, repurchase, this quality is really good, recommended!	Jingdong prefecture level city in Shandong
Stowe Flagship Store	King of Power Saving	Thank you, the owner even sent two fuses.	Jingdong prefecture level city in Shandong
Stowe Flagship Store	King of Power Saving	This store has great stuff, relatively fast shipping and logistics	Jingdong prefecture level city in Shandong
Stowe Flagship Store	King of Power Saving	This product is based on the impossibility of saving electricity, I used 853 kWh of electricity in July, started using the product on August 1, and used 867 kWh of electricity by September 1, is this called	Jingdong prefecture level city in Shandong
Stowe Flagship Store	King of Power Saving	Power king received, to the home on the trial, when I plugged into the electric panel can not see the effect of power saving, the meter is still the same speed in the turn, I'll save the king of the manual to play look at the instructions, instructions to speak, have to have a day two days and half a month to save power obviously, then wait half a month to see the effect of power saving it! To half a month there are obvious power saving before continuing to chase Xu.	Jingdong prefecture level city in Shandong
Stowe Flagship Store	King of Power Saving	Received the goods, a look, did not see the manufacturer and production address, can only hold a try to see the mentality of the face	Jingdong prefecture level city in Shandong
Stowe Flagship Store	King of Power Saving	I've only been using it for a few days and I'm not sure if it's an IQ tax? Used to favorable reviews.	Jingdong prefecture level city in Shandong
Stowe Flagship Store	King of Power Saving	Appearance is okay, power saving next time to follow up the evaluation	Jingdong prefecture level city in Shandong
Stowe Flagship Store	King of Power Saving	Shorter time, but hopefully effective and power-saving, to be followed up in the future	Jingdong prefecture level city in Shandong
Stowe Flagship Store	King of Power Saving	Appearance is okay, I do not know if the power saving	Jingdong prefecture level city in Shandong
Stowe Flagship Store	King of Power Saving	Good service, fast logistics, Power Saving King is exquisite and beautiful, support the business, thank you!	Jingdong prefecture level city in Shandong
Stowe Flagship Store	King of Power Saving	Bought three at once to try first first.	Jingdong prefecture level city in Shandong
Stowe Flagship Store	King of Power Saving	The object is the same as the picture, the product is of high quality, the packaging is intact. High-end atmosphere, high power, power saving effect is obvious, average monthly power saving 50%, very satisfied.	Jingdong prefecture level city in Shandong
Stowe Flagship Store	King of Power Saving	Bought back and plugged in, really effective, before 16 degrees of electricity per day, now 8 degrees of electricity per day, open an air conditioner for 12 hours, decisive repurchase of a	Jingdong prefecture level city in Shandong
Stowe Flagship Store	King of Power Saving	The object is the same as the picture, the product is of high quality, and the package is intact. High-end atmosphere and high grade, high power	Jingdong prefecture level city in Shandong

Stowe Flagship Store	King of Power Saving	Goods received, I hope the store said can be energy-saving, first try a month, month after month to see the effect.	Jingdong prefecture level city in Shandong
Stowe Flagship Store	King of Power Saving	This is really good, the material and workmanship is particularly exquisite, the workmanship is really very good, I think I can recommend to my friends to buy	Jingdong prefecture level city in Shandong
Stowe Flagship Store	King of Power Saving	I have to praise, the quality is really good, and the packaging logistics are also good. Really like it, won't make you regret after getting it!	Jingdong prefecture level city in Shandong
Stowe Flagship Store	King of Power Saving	Reputable platform, efficient and professional service, buy with confidence and peace of mind!	Jingdong prefecture level city in Shandong
Stowe Flagship Store	King of Power Saving	Bought one on a trial basis to see if it works after 1-2 months.	Jingdong prefecture level city in Shandong
Stowe Flagship Store	King of Power Saving	Quality is very good, customer service attitude is very very praise, there are problems in a timely manner to solve the problem, the price is not expensive, super value, you can rest assured that the	Jingdong prefecture level city in Shandong
Stowe Flagship Store	King of Power Saving	With it no longer need to be afraid to use electricity, especially good, ready to buy a mom and dad that also	Jingdong prefecture level city in Shandong
Stowe Flagship Store	King of Power Saving	Baby received very like will return to buy the seller attitude is also very like will return to buy the seller attitude is also very like!	Jingdong prefecture level city in Shandong
Stowe Flagship Store	King of Power Saving	Jingdong Mall shopping convenience assured fast assured fast assured fast assured fast	Jingdong prefecture level city in Shandong
Stowe Flagship Store	King of Power Saving	Saving power received, the quality is very good, fine workmanship, especially good, much cheaper than the entity store, it is worth buying!	Jingdong prefecture level city in Shandong
Stowe Flagship Store	King of Power Saving	I did not expect the express delivery so quickly, very save point quality is super good, than the entity store is too cheap.	Jingdong prefecture level city in Shandong
Stowe Flagship Store	King of Power Saving	Have received the baby, very satisfied with a shopping, great worth buying!	Jingdong prefecture level city in Shandong
Stowe Flagship Store	King of Power Saving	This is really more powerful, very satisfied, more energy-saving, better, thank you boss.	Jingdong prefecture level city in Shandong
Stowe Flagship Store	King of Power Saving	Received has been used for two days, the air conditioning can boldly open it! Energy saving effect is really quite good	Jingdong prefecture level city in Shandong
Stowe Flagship Store	King of Power Saving	It is authentic, trust the merchant, the baby has a great texture and the price is moderate, unlimited repurchase!	Jingdong prefecture level city in Shandong
Stowe Flagship Store	King of Power Saving	It is genuine very good, the price is much cheaper, cost-effective, really special cost-effective, it is worth buying!	Jingdong prefecture level city in Shandong
Stowe Flagship Store	King of Power Saving	Very good quality, fine workmanship, low power consumption, long warranty, fast shipping, economical and practical	Jingdong prefecture level city in Shandong
Stowe Flagship Store	King of Power Saving	I've been using it for half a month now, it really saves power and the voltage is stable, it's very good	Jingdong prefecture level city in Shandong
Stowe Flagship Store	King of Power Saving	It's the ideal product 💝, good quality and fast logistics, worth the kudos!	Jingdong prefecture level city in Shandong
Stowe Flagship Store	King of Power Saving	The logistics is very fast, the baby is very good, the shopkeeper is NICE!	Jingdong prefecture level city in Shandong
Stowe Flagship Store	King of Power Saving	Good to use, good quality, save point durable	Jingdong prefecture level city in Shandong
Stowe Flagship Store	King of Power Saving	The quality is very good, customer service attitude is very, very awesome, there are problems in a timely manner to solve the problem	Jingdong prefecture level city in Shandong
Stowe Flagship Store	King of Power Saving	Quality is very good, the use of the effect is very good, more stable, quality assurance	Jingdong prefecture level city in Shandong
Stowe Flagship Store	King of Power Saving	Convenient, inexpensive, and see how it works out?	Jingdong prefecture level city in Shandong
Stowe Flagship Store	King of Power Saving	Very good quality, saves a lot of electricity, very good, worth buying	Jingdong prefecture level city in Shandong
Stowe Flagship Store	King of Power Saving	Power Saving King is easy to use, with the fruit of power saving to use easy to give!	Jingdong prefecture level city in Shandong
Stowe Flagship Store	King of Power Saving	This saver really works, good quality, safe and secure, very satisfied!	Jingdong prefecture level city in Shandong
Stowe Flagship Store	King of Power Saving	It works well, the voltage is very stable effect is good, the quality is also very good satisfaction	Jingdong prefecture level city in Shandong
Stowe Flagship Store	King of Power Saving	The logistics is super super fast! The quality is also very good, indeed value for money	Jingdong prefecture level city in Shandong
Stowe Flagship Store	King of Power Saving	I've only been using it for a month or so and it seems to be saving a lot of power	Jingdong prefecture level city in Shandong
Stowe Flagship Store	King of Power Saving	Very easy to operate, very good material, good value for money.	Jingdong prefecture level city in Shandong
Stowe Flagship Store	King of Power Saving	Aluminum alloy shell, durable, compact power-saving good effect	Jingdong prefecture level city in Shandong
Stowe Flagship Store	King of Power Saving	Nice device, easy to use, doesn't take up much space, recommendable!	Jingdong prefecture level city in Shandong
Stowe Flagship Store	King of Power Saving	Good quality, good price, fast logistics	Jingdong prefecture level city in Shandong
Stowe Flagship Store	King of Power Saving	Friends of the family in use recommended, the effect is good and recommendable!	Jingdong prefecture level city in Shandong
Stowe Flagship Store	King of Power Saving	Cheaper than brick and mortar stores worth buying is authentic	Jingdong prefecture level city in Shandong
Stowe Flagship Store	King of Power Saving	Merchants ship quickly, the price is cheaper than the physical store, good use	Jingdong prefecture level city in Shandong
Stowe Flagship Store	King of Power Saving	Very durable, affordable, fast shipping	Jingdong prefecture level city in Shandong
Stowe Flagship Store	King of Power Saving	Packaging tight quality is very good easy to use good results	Jingdong prefecture level city in Shandong
Stowe Flagship Store	King of Power Saving	Save the grid. It's in the whole house. It's saving money on the electric bill.	Jingdong prefecture level city in Shandong
Stowe Flagship Store	King of Power Saving	I used it today, it works well, I'm very satisfied	Jingdong prefecture level city in Shandong
Stowe Flagship Store	King of Power Saving	It really saves a lot of electricity after installation	Jingdong prefecture level city in Shandong
Stowe Flagship Store	King of Power Saving	Love it. Satisfied.	Jingdong prefecture level city in Shandong
Stowe Flagship Store	King of Power Saving	Doesn't feel like it's working.	Jingdong prefecture level city in Shandong
Stowe Flagship Store	King of Power Saving	I'll comment after using it.	Jingdong prefecture level city in Shandong
Stowe Flagship Store	King of Power Saving	I'm trying it out to see if it saves power, and I'll buy two more if it works well.	Jingdong prefecture level city in Shandong
Stowe Flagship Store	King of Power Saving	It is genuine very good, the price is much cheaper, cost-effective, really special cost-effective, it is worth buying!	Jingdong prefecture level city in Shandong
Stowe Flagship Store	King of Power Saving	This thing can really save electricity, I was introduced to buy by my friend, he used it very well, I used it and think it's also very good, it can really save electricity, save about half of the electricity every day, it's really good!	Jingdong prefecture level city in Shandong
Stowe Flagship Store	King of Power Saving	After using it for ten days or so, I checked the power consumption and it did drop, and customer service said it will save more electricity in the long run. The product is also insured, 5 years warranty do not have to worry about quality problems!	Jingdong prefecture level city in Shandong
Stowe Flagship Store	King of Power Saving	The product is good, before already bought a, this is the second, the effect is quite good, anyway, I see the daily electricity bill than before a lot less, this can be the choice!	Jingdong prefecture level city in Shandong

Stowe Flagship Store	King of Power Saving	Originally also very worried about things are not good, get the hand found completely over-anxious, five-star praise, the style is very generous, the quality is very good, the price is also very reasonable!	Jingdong prefecture level city in Shandong
Stowe Flagship Store	King of Power Saving	Quality is good, playing to the old family also buy a, repurchase, this quality is really good, recommended!	Jingdong prefecture level city in Shandong
Stowe Flagship Store	King of Power Saving	Thank you, the owner even sent two fuses.	Jingdong prefecture level city in Shandong
Stowe Flagship Store	King of Power Saving	This store has great stuff, relatively fast shipping and logistics	Jingdong prefecture level city in Shandong
Stowe Flagship Store	King of Power Saving	This product is based on the impossibility of saving electricity, I used 853 kWh of electricity in July, started using the product on August 1, and used 867 kWh of electricity by September 1, is this called	Jingdong prefecture level city in Shandong
Stowe Flagship Store	King of Power Saving	Power king received, to the home on the trial, when I plugged into the electric panel can not see the effect of power saving, the meter is still the same speed in the turn, I'll save the king of the manual to play look at the instructions, instructions to speak, have to have a day two days and half a month to save power obviously, then wait half a month to see the effect of power saving it! To half a month there are obvious power saving before continuing to chase Xu.	Jingdong prefecture level city in Shandong
Stowe Flagship Store	King of Power Saving	Received the goods, a look, did not see the manufacturer and production address, can only hold a try to see the mentality of the face	Jingdong prefecture level city in Shandong
Stowe Flagship Store	King of Power Saving	I've only been using it for a few days and I'm not sure if it's an IQ tax? Used to favorable reviews.	Jingdong prefecture level city in Shandong
Stowe Flagship Store	King of Power Saving	Appearance is okay, power saving next time to follow up the evaluation	Jingdong prefecture level city in Shandong
Stowe Flagship Store	King of Power Saving	Shorter time, but hopefully effective and power-saving, to be followed up in the future	Jingdong prefecture level city in Shandong
Stowe Flagship Store	King of Power Saving	Appearance is okay, I do not know if the power saving	Jingdong prefecture level city in Shandong
Stowe Flagship Store	King of Power Saving	Good service, fast logistics, Power Saving King is exquisite and beautiful, support the business, thank you!	Jingdong prefecture level city in Shandong
Stowe Flagship Store	King of Power Saving	Bought three at once to try first first.	Jingdong prefecture level city in Shandong
Stowe Flagship Store	King of Power Saving	The object is the same as the picture, the product is of high quality, the packaging is intact. High-end atmosphere, high power, power saving effect is obvious, average monthly power saving 50%, very satisfied.	Jingdong prefecture level city in Shandong
Stowe Flagship Store	King of Power Saving	Bought back and plugged in, really effective, before 16 degrees of electricity per day, now 8 degrees of electricity per day, open an air conditioner for 12 hours, decisive repurchase of a	Jingdong prefecture level city in Shandong
Stowe Flagship Store	King of Power Saving	The object is the same as the picture, the product is of high quality, and the package is intact. High-end atmosphere and high grade, high power	Jingdong prefecture level city in Shandong
Stowe Flagship Store	King of Power Saving	Goods received, I hope the store said can be energy-saving, first try a month, month after month to see the effect.	Jingdong prefecture level city in Shandong
Stowe Flagship Store	King of Power Saving	This is really good, the material and workmanship is particularly exquisite, the workmanship is really very good, I think I can recommend to my friends to buy	Jingdong prefecture level city in Shandong
Stowe Flagship Store	King of Power Saving	I have to praise, the quality is really good, and the packaging logistics are also good. Really like it, won't make you regret after getting it!	Jingdong prefecture level city in Shandong
Stowe Flagship Store	King of Power Saving	Reputable platform, efficient and professional service, buy with confidence and peace of mind!	Jingdong prefecture level city in Shandong
Stowe Flagship Store	King of Power Saving	Bought one on a trial basis to see if it works after 1-2 months.	Jingdong prefecture level city in Shandong
Stowe Flagship Store	King of Power Saving	Quality is very good, customer service attitude is very very praise, there are problems in a timely manner to solve the problem, the price is not expensive, super value, you can rest assured that the	Jingdong prefecture level city in Shandong
Stowe Flagship Store	King of Power Saving	With it no longer need to be afraid to use electricity, especially good, ready to buy a mom and dad that also	Jingdong prefecture level city in Shandong
Stowe Flagship Store	King of Power Saving	Baby received very like will return to buy the seller attitude is also very like will return to buy the seller attitude is also very like!	Jingdong prefecture level city in Shandong
Stowe Flagship Store	King of Power Saving	Jingdong Mall shopping convenience assured fast assured fast assured fast assured fast	Jingdong prefecture level city in Shandong
Stowe Flagship Store	King of Power Saving	Saving power received, the quality is very good, fine workmanship, especially good, much cheaper than the entity store, it is worth buying!	Jingdong prefecture level city in Shandong
Stowe Flagship Store	King of Power Saving	I did not expect the express delivery so quickly, very save point quality is super good, than the entity store is too cheap.	Jingdong prefecture level city in Shandong
Stowe Flagship Store	King of Power Saving	Have received the baby, very satisfied with a shopping, great worth buying!	Jingdong prefecture level city in Shandong
Stowe Flagship Store	King of Power Saving	This is really more powerful, very satisfied, more energy-saving, better, thank you boss.	Jingdong prefecture level city in Shandong
Stowe Flagship Store	King of Power Saving	Received has been used for two days, the air conditioning can boldly open it! Energy saving effect is really quite good	Jingdong prefecture level city in Shandong
Stowe Flagship Store	King of Power Saving	It is authentic, trust the merchant, the baby has a great texture and the price is moderate, unlimited repurchase!	Jingdong prefecture level city in Shandong
Stowe Flagship Store	King of Power Saving	It is genuine very good, the price is much cheaper, cost-effective, really special cost-effective, it is worth buying!	Jingdong prefecture level city in Shandong
Stowe Flagship Store	King of Power Saving	Very good quality, fine workmanship, low power consumption, long warranty, fast shipping, economical and practical	Jingdong prefecture level city in Shandong
Stowe Flagship Store	King of Power Saving	I've been using it for half a month now, it really saves power and the voltage is stable, it's very good	Jingdong prefecture level city in Shandong
Stowe Flagship Store	King of Power Saving	It's the ideal product, good quality and fast logistics, worth the kudos!	Jingdong prefecture level city in Shandong
Stowe Flagship Store	King of Power Saving	The logistics is very fast, the baby is very good, the shopkeeper is NICE!	Jingdong prefecture level city in Shandong
Stowe Flagship Store	King of Power Saving	Good to use, good quality, save point durable	Jingdong prefecture level city in Shandong
Stowe Flagship Store	King of Power Saving	The quality is very good, customer service attitude is very, very awesome, there are problems in a timely manner to solve the problem	Jingdong prefecture level city in Shandong
Stowe Flagship Store	King of Power Saving	Quality is very good, the use of the effect is very good, more stable, quality assurance	Jingdong prefecture level city in Shandong
Stowe Flagship Store	King of Power Saving	Convenient, inexpensive, and see how it works out?	Jingdong prefecture level city in Shandong
Stowe Flagship Store	King of Power Saving	Very good quality, saves a lot of electricity, very good, worth buying	Jingdong prefecture level city in Shandong
Stowe Flagship Store	King of Power Saving	Power Saving King is easy to use, with the fruit of power saving to use easy to give!	Jingdong prefecture level city in Shandong
Stowe Flagship Store	King of Power Saving	This saver really works, good quality, safe and secure, very satisfied!	Jingdong prefecture level city in Shandong
Stowe Flagship Store	King of Power Saving	It works well, the voltage is very stable effect is good, the quality is also very good satisfaction	Jingdong prefecture level city in Shandong
Stowe Flagship Store	King of Power Saving	The logistics is super super fast! The quality is also very good, indeed value for money	Jingdong prefecture level city in Shandong
Stowe Flagship Store	King of Power Saving	I've only been using it for a month or so and it seems to be saving a lot of power	Jingdong prefecture level city in Shandong
Stowe Flagship Store	King of Power Saving	Very easy to operate, very good material, good value for money.	Jingdong prefecture level city in Shandong
Stowe Flagship Store	King of Power Saving	Aluminum alloy shell, durable, compact power-saving good effect	Jingdong prefecture level city in Shandong
Stowe Flagship Store	King of Power Saving	Nice device, easy to use, doesn't take up much space, recommendable!	Jingdong prefecture level city in Shandong
Stowe Flagship Store	King of Power Saving	Good quality, good price, fast logistics	Jingdong prefecture level city in Shandong

Stowe Flagship Store	King of Power Saving	Friends of the family in use recommended, the effect is good and recommendable!	Jingdong prefecture level city in Shandong
Stowe Flagship Store	King of Power Saving	Cheaper than brick and mortar stores worth buying is authentic	Jingdong prefecture level city in Shandong
Stowe Flagship Store	King of Power Saving	Merchants ship quickly, the price is cheaper than the physical store, good use	Jingdong prefecture level city in Shandong
Stowe Flagship Store	King of Power Saving	Very durable, affordable, fast shipping	Jingdong prefecture level city in Shandong
Stowe Flagship Store	King of Power Saving	Packaging tight quality is very good easy to use good results	Jingdong prefecture level city in Shandong
Stowe Flagship Store	King of Power Saving	Save the grid. It's in the whole house. It's saving money on the electric bill.	Jingdong prefecture level city in Shandong
Stowe Flagship Store	King of Power Saving	I used it today, it works well, I'm very satisfied	Jingdong prefecture level city in Shandong
Stowe Flagship Store	King of Power Saving	It really saves a lot of electricity after installation	Jingdong prefecture level city in Shandong
Stowe Flagship Store	King of Power Saving	Love it. Satisfied.	Jingdong prefecture level city in Shandong
Stowe Flagship Store	King of Power Saving	Doesn't feel like it's working.	Jingdong prefecture level city in Shandong
Stowe Flagship Store	King of Power Saving	I'll comment after using it.	Jingdong prefecture level city in Shandong
Stowe Flagship Store	King of Power Saving	I'm trying it out to see if it saves power, and I'll buy two more if it works well.	Jingdong prefecture level city in Shandong
Stowe Flagship Store	King of Power Saving	It is genuine very good, the price is much cheaper, cost-effective, really special cost-effective, it is worth buying!	Jingdong prefecture level city in Shandong
Stowe Flagship Store	King of Power Saving	This thing can really save electricity, I was introduced to buy by my friend, he used it very well, I used it and think it's also very good, it can really save electricity, save about half of the electricity every day, it's really good!	Jingdong prefecture level city in Shandong
Stowe Flagship Store	King of Power Saving	After using it for ten days or so, I checked the power consumption and it did drop, and customer service said it will save more electricity in the long run. The product is also insured, 5 years warranty do not have to worry about quality problems!	Jingdong prefecture level city in Shandong
Stowe Flagship Store	King of Power Saving	The product is good, before already bought a, this is the second, the effect is quite good, anyway, I see the daily electricity bill than before a lot less, this can be the choice!	Jingdong prefecture level city in Shandong
Stowe Flagship Store	King of Power Saving	Originally also very worried about things are not good, get the hand found completely over-anxious, five-star praise, the style is very generous, the quality is very good, the price is also very reasonable!	Jingdong prefecture level city in Shandong
Stowe Flagship Store	King of Power Saving	Quality is good, playing to the old family also buy a, repurchase, this quality is really good, recommended!	Jingdong prefecture level city in Shandong
Stowe Flagship Store	King of Power Saving	Thank you, the owner even sent two fuses.	Jingdong prefecture level city in Shandong
Stowe Flagship Store	King of Power Saving	This store has great stuff, relatively fast shipping and logistics	Jingdong prefecture level city in Shandong
Stowe Flagship Store	King of Power Saving	This product is based on the impossibility of saving electricity, I used 853 kWh of electricity in July, started using the product on August 1, and used 867 kWh of electricity by September 1, is this called	Jingdong prefecture level city in Shandong
Stowe Flagship Store	King of Power Saving	Power king received, to the home on the trial, when I plugged into the electric panel can not see the effect of power saving, the meter is still the same speed in the turn, I'll save the king of the manual to play look at the instructions, instructions to speak, have to have a day two days and half a month to save power obviously, then wait half a month to see the effect of power saving it! To half a month there are obvious power saving before continuing to chase Xu.	Jingdong prefecture level city in Shandong
Stowe Flagship Store	King of Power Saving	Received the goods, a look, did not see the manufacturer and production address, can only hold a try to see the mentality of the face	Jingdong prefecture level city in Shandong
Stowe Flagship Store	King of Power Saving	I've only been using it for a few days and I'm not sure if it's an IQ tax? Used to favorable reviews.	Jingdong prefecture level city in Shandong
Stowe Flagship Store	King of Power Saving	Appearance is okay, power saving next time to follow up the evaluation	Jingdong prefecture level city in Shandong
Stowe Flagship Store	King of Power Saving	Shorter time, but hopefully effective and power-saving, to be followed up in the future	Jingdong prefecture level city in Shandong
Stowe Flagship Store	King of Power Saving	Appearance is okay, I do not know if the power saving	Jingdong prefecture level city in Shandong
Stowe Flagship Store	King of Power Saving	Good service, fast logistics, Power Saving King is exquisite and beautiful, support the business, thank you!	Jingdong prefecture level city in Shandong
Stowe Flagship Store	King of Power Saving	Bought three at once to try first first.	Jingdong prefecture level city in Shandong
Stowe Flagship Store	King of Power Saving	The object is the same as the picture, the product is of high quality, the packaging is intact. High-end atmosphere, high power, power saving effect is obvious, average monthly power saving 50%, very satisfied.	Jingdong prefecture level city in Shandong
Stowe Flagship Store	King of Power Saving	Bought back and plugged in, really effective, before 16 degrees of electricity per day, now 8 degrees of electricity per day, open an air conditioner for 12 hours, decisive repurchase of a	Jingdong prefecture level city in Shandong
Stowe Flagship Store	King of Power Saving	The object is the same as the picture, the product is of high quality, and the package is intact. High-end atmosphere and high grade, high power	Jingdong prefecture level city in Shandong
Stowe Flagship Store	King of Power Saving	Goods received, I hope the store said can be energy-saving, first try a month, month after month to see the effect.	Jingdong prefecture level city in Shandong
Stowe Flagship Store	King of Power Saving	This is really good, the material and workmanship is particularly exquisite, the workmanship is really very good, I think I can recommend to my friends to buy	Jingdong prefecture level city in Shandong
Stowe Flagship Store	King of Power Saving	I have to praise, the quality is really good, and the packaging logistics are also good. Really like it, won't make you regret after getting it!	Jingdong prefecture level city in Shandong
Stowe Flagship Store	King of Power Saving	Reputable platform, efficient and professional service, buy with confidence and peace of mind!	Jingdong prefecture level city in Shandong
Stowe Flagship Store	King of Power Saving	Bought one on a trial basis to see if it works after 1-2 months.	Jingdong prefecture level city in Shandong
Stowe Flagship Store	King of Power Saving	Quality is very good, customer service attitude is very very praise, there are problems in a timely manner to solve the problem, the price is not expensive, super value, you can rest assured that the	Jingdong prefecture level city in Shandong
Stowe Flagship Store	King of Power Saving	With it no longer need to be afraid to use electricity, especially good, ready to buy a mom and dad that also	Jingdong prefecture level city in Shandong
Stowe Flagship Store	King of Power Saving	Baby received very like will return to buy the seller attitude is also very like will return to buy the seller attitude is also very like!	Jingdong prefecture level city in Shandong
Stowe Flagship Store	King of Power Saving	Jingdong Mall shopping convenience assured fast assured fast assured fast assured fast	Jingdong prefecture level city in Shandong
Stowe Flagship Store	King of Power Saving	Saving power received, the quality is very good, fine workmanship, especially good, much cheaper than the entity store, it is worth buying!	Jingdong prefecture level city in Shandong
Stowe Flagship Store	King of Power Saving	I did not expect the express delivery so quickly, very save point quality is super good, than the entity store is too cheap.	Jingdong prefecture level city in Shandong
Stowe Flagship Store	King of Power Saving	Have received the baby, very satisfied with a shopping, great worth buying!	Jingdong prefecture level city in Shandong
Stowe Flagship Store	King of Power Saving	This is really more powerful, very satisfied, more energy-saving, better, thank you boss.	Jingdong prefecture level city in Shandong
Stowe Flagship Store	King of Power Saving	Received has been used for two days, the air conditioning can boldly open it! Energy saving effect is really quite good	Jingdong prefecture level city in Shandong
Stowe Flagship Store	King of Power Saving	It is authentic, trust the merchant, the baby has a great texture and the price is moderate, unlimited repurchase!	Jingdong prefecture level city in Shandong
Stowe Flagship Store	King of Power Saving	It is genuine very good, the price is much cheaper, cost-effective, really special cost-effective, it is worth buying!	Jingdong prefecture level city in Shandong
Stowe Flagship Store	King of Power Saving	Very good quality, fine workmanship, low power consumption, long warranty, fast shipping, economical and practical	Jingdong prefecture level city in Shandong

Stowe Flagship Store	King of Power Saving	I've been using it for half a month now, it really saves power and the voltage is stable, it's very good	Jingdong prefecture level city in Shandong
Stowe Flagship Store	King of Power Saving	It's the ideal product 💝, good quality and fast logistics, worth the kudos!	Jingdong prefecture level city in Shandong
Stowe Flagship Store	King of Power Saving	The logistics is very fast, the baby is very good, the shopkeeper is NICE!	Jingdong prefecture level city in Shandong
Stowe Flagship Store	King of Power Saving	Good to use, good quality, save point durable	Jingdong prefecture level city in Shandong
Stowe Flagship Store	King of Power Saving	The quality is very good, customer service attitude is very, very awesome, there are problems in a timely manner to solve the problem	Jingdong prefecture level city in Shandong
Stowe Flagship Store	King of Power Saving	Quality is very good, the use of the effect is very good, more stable, quality assurance	Jingdong prefecture level city in Shandong
Stowe Flagship Store	King of Power Saving	Convenient, inexpensive, and see how it works out?	Jingdong prefecture level city in Shandong
Stowe Flagship Store	King of Power Saving	Very good quality, saves a lot of electricity, very good, worth buying	Jingdong prefecture level city in Shandong
Stowe Flagship Store	King of Power Saving	Power Saving King is easy to use, with the fruit of power saving to use easy to give!	Jingdong prefecture level city in Shandong
Stowe Flagship Store	King of Power Saving	This saver really works, good quality, safe and secure, very satisfied!	Jingdong prefecture level city in Shandong
Stowe Flagship Store	King of Power Saving	It works well, the voltage is very stable effect is good, the quality is also very good satisfaction	Jingdong prefecture level city in Shandong
Stowe Flagship Store	King of Power Saving	The logistics is super super fast! The quality is also very good, indeed value for money	Jingdong prefecture level city in Shandong
Stowe Flagship Store	King of Power Saving	I've only been using it for a month or so and it seems to be saving a lot of power	Jingdong prefecture level city in Shandong
Stowe Flagship Store	King of Power Saving	Very easy to operate, very good material, good value for money.	Jingdong prefecture level city in Shandong
Stowe Flagship Store	King of Power Saving	Aluminum alloy shell, durable, compact power-saving good effect	Jingdong prefecture level city in Shandong
Stowe Flagship Store	King of Power Saving	Nice device, easy to use, doesn't take up much space, recommendable!	Jingdong prefecture level city in Shandong
Stowe Flagship Store	King of Power Saving	Good quality, good price, fast logistics	Jingdong prefecture level city in Shandong
Stowe Flagship Store	King of Power Saving	Friends of the family in use recommended, the effect is good and recommendable!	Jingdong prefecture level city in Shandong
Stowe Flagship Store	King of Power Saving	Cheaper than brick and mortar stores worth buying is authentic	Jingdong prefecture level city in Shandong
Stowe Flagship Store	King of Power Saving	Merchants ship quickly, the price is cheaper than the physical store, good use	Jingdong prefecture level city in Shandong
Stowe Flagship Store	King of Power Saving	Very durable, affordable, fast shipping	Jingdong prefecture level city in Shandong
Stowe Flagship Store	King of Power Saving	Packaging tight quality is very good easy to use good results	Jingdong prefecture level city in Shandong
Stowe Flagship Store	King of Power Saving	Save the grid. It's in the whole house. It's saving money on the electric bill.	Jingdong prefecture level city in Shandong
Stowe Flagship Store	King of Power Saving	I used it today, it works well, I'm very satisfied	Jingdong prefecture level city in Shandong
Stowe Flagship Store	King of Power Saving	It really saves a lot of electricity after installation	Jingdong prefecture level city in Shandong
Stowe Flagship Store	King of Power Saving	Love it. Satisfied.	Jingdong prefecture level city in Shandong
Stowe Flagship Store	King of Power Saving	Doesn't feel like it's working.	Jingdong prefecture level city in Shandong
Stowe Flagship Store	King of Power Saving	I'll comment after using it.	Jingdong prefecture level city in Shandong
Stowe Flagship Store	King of Power Saving	I'm trying it out to see if it saves power, and I'll buy two more if it works well.	Jingdong prefecture level city in Shandong
Stowe Flagship Store	King of Power Saving	It is genuine very good, the price is much cheaper, cost-effective, really special cost-effective, it is worth buying!	Jingdong prefecture level city in Shandong
Stowe Flagship Store	King of Power Saving	This thing can really save electricity, I was introduced to buy by my friend, he used it very well, I used it and think it's also very good, it can really save electricity, save about half of the electricity every day, it's really good!	Jingdong prefecture level city in Shandong
Stowe Flagship Store	King of Power Saving	After using it for ten days or so, I checked the power consumption and it did drop, and customer service said it will save more electricity in the long run. The product is also insured, 5 years warranty do not have to worry about quality problems!	Jingdong prefecture level city in Shandong
Stowe Flagship Store	King of Power Saving	The product is good, before already bought a, this is the second, the effect is quite good, anyway, I see the daily electricity bill than before a lot less, this can be the choice!	Jingdong prefecture level city in Shandong
Stowe Flagship Store	King of Power Saving	Originally also very worried about things are not good, get the hand found completely over-anxious, five-star praise, the style is very generous, the quality is very good, the price is also very reasonable!	Jingdong prefecture level city in Shandong
Stowe Flagship Store	King of Power Saving	Quality is good, playing to the old family also buy a, repurchase, this quality is really good, recommended!	Jingdong prefecture level city in Shandong
Stowe Flagship Store	King of Power Saving	Thank you, the owner even sent two fuses.	Jingdong prefecture level city in Shandong
Stowe Flagship Store	King of Power Saving	This store has great stuff, relatively fast shipping and logistics	Jingdong prefecture level city in Shandong
Stowe Flagship Store	King of Power Saving	This product is based on the impossibility of saving electricity, I used 853 kWh of electricity in July, started using the product on August 1, and used 867 kWh of electricity by September 1, is this called	Jingdong prefecture level city in Shandong
Stowe Flagship Store	King of Power Saving	Power king received, to the home on the trial, when I plugged into the electric panel can not see the effect of power saving, the meter is still the same speed in the turn, I'll save the king of the manual to play look at the instructions, instructions to speak, have to have a day two days and half a month to save power obviously, then wait half a month to see the effect of power saving it! To half a month there are obvious power saving before continuing to chase Xu.	Jingdong prefecture level city in Shandong
Stowe Flagship Store	King of Power Saving	Received the goods, a look, did not see the manufacturer and production address, can only hold a try to see the mentality of the face	Jingdong prefecture level city in Shandong
Stowe Flagship Store	King of Power Saving	I've only been using it for a few days and I'm not sure if it's an IQ tax? Used to favorable reviews.	Jingdong prefecture level city in Shandong
Stowe Flagship Store	King of Power Saving	Appearance is okay, power saving next time to follow up the evaluation	Jingdong prefecture level city in Shandong
Stowe Flagship Store	King of Power Saving	Shorter time, but hopefully effective and power-saving, to be followed up in the future	Jingdong prefecture level city in Shandong
Stowe Flagship Store	King of Power Saving	Appearance is okay, I do not know if the power saving	Jingdong prefecture level city in Shandong
Stowe Flagship Store	King of Power Saving	Good service, fast logistics, Power Saving King is exquisite and beautiful, support the business, thank you!	Jingdong prefecture level city in Shandong
Stowe Flagship Store	King of Power Saving	Bought three at once to try first first.	Jingdong prefecture level city in Shandong
Stowe Flagship Store	King of Power Saving	The object is the same as the picture, the product is of high quality, the packaging is intact. High-end atmosphere, high power, power saving effect is obvious, average monthly power saving 50%, very satisfied.	Jingdong prefecture level city in Shandong
Stowe Flagship Store	King of Power Saving	Bought back and plugged in, really effective, before 16 degrees of electricity per day, now 8 degrees of electricity per day, open an air conditioner for 12 hours, decisive repurchase of a	Jingdong prefecture level city in Shandong
Stowe Flagship Store	King of Power Saving	The object is the same as the picture, the product is of high quality, and the package is intact. High-end atmosphere and high grade, high power	Jingdong prefecture level city in Shandong

Stowe Flagship Store	King of Power Saving	Goods received, I hope the store said can be energy-saving, first try a month, month after month to see the effect.	Jingdong prefecture level city in Shandong
Stowe Flagship Store	King of Power Saving	This is really good, the material and workmanship is particularly exquisite, the workmanship is really very good, I think I can recommend to my friends to buy	Jingdong prefecture level city in Shandong
Stowe Flagship Store	King of Power Saving	I have to praise, the quality is really good, and the packaging logistics are also good. Really like it, won't make you regret after getting it!	Jingdong prefecture level city in Shandong
Stowe Flagship Store	King of Power Saving	Reputable platform, efficient and professional service, buy with confidence and peace of mind!	Jingdong prefecture level city in Shandong
Stowe Flagship Store	King of Power Saving	Bought one on a trial basis to see if it works after 1-2 months.	Jingdong prefecture level city in Shandong
Stowe Flagship Store	King of Power Saving	Quality is very good, customer service attitude is very very praise, there are problems in a timely manner to solve the problem, the price is not expensive, super value, you can rest assured that the	Jingdong prefecture level city in Shandong
Stowe Flagship Store	King of Power Saving	With it no longer need to be afraid to use electricity, especially good, ready to buy a mom and dad that also	Jingdong prefecture level city in Shandong
Stowe Flagship Store	King of Power Saving	Baby received very like will return to buy the seller attitude is also very like will return to buy the seller attitude is also very like!	Jingdong prefecture level city in Shandong
Stowe Flagship Store	King of Power Saving	Jingdong Mall shopping convenience assured fast assured fast assured fast assured fast	Jingdong prefecture level city in Shandong
Stowe Flagship Store	King of Power Saving	Saving power received, the quality is very good, fine workmanship, especially good, much cheaper than the entity store, it is worth buying!	Jingdong prefecture level city in Shandong
Stowe Flagship Store	King of Power Saving	I did not expect the express delivery so quickly, very save point quality is super good, than the entity store is too cheap.	Jingdong prefecture level city in Shandong
Stowe Flagship Store	King of Power Saving	Have received the baby, very satisfied with a shopping, great worth buying!	Jingdong prefecture level city in Shandong
Stowe Flagship Store	King of Power Saving	This is really more powerful, very satisfied, more energy-saving, better, thank you boss.	Jingdong prefecture level city in Shandong
Stowe Flagship Store	King of Power Saving	Received has been used for two days, the air conditioning can boldly open it! Energy saving effect is really quite good	Jingdong prefecture level city in Shandong
Stowe Flagship Store	King of Power Saving	It is authentic, trust the merchant, the baby has a great texture and the price is moderate, unlimited repurchase!	Jingdong prefecture level city in Shandong
Stowe Flagship Store	King of Power Saving	It is genuine very good, the price is much cheaper, cost-effective, really special cost-effective, it is worth buying!	Jingdong prefecture level city in Shandong
Stowe Flagship Store	King of Power Saving	Very good quality, fine workmanship, low power consumption, long warranty, fast shipping, economical and practical	Jingdong prefecture level city in Shandong
Stowe Flagship Store	King of Power Saving	I've been using it for half a month now, it really saves power and the voltage is stable, it's very good	Jingdong prefecture level city in Shandong
Stowe Flagship Store	King of Power Saving	It's the ideal product 💝, good quality and fast logistics, worth the kudos!	Jingdong prefecture level city in Shandong
Stowe Flagship Store	King of Power Saving	The logistics is very fast, the baby is very good, the shopkeeper is NICE!	Jingdong prefecture level city in Shandong
Stowe Flagship Store	King of Power Saving	Good to use, good quality, save point durable	Jingdong prefecture level city in Shandong
Stowe Flagship Store	King of Power Saving	The quality is very good, customer service attitude is very, very awesome, there are problems in a timely manner to solve the problem	Jingdong prefecture level city in Shandong
Stowe Flagship Store	King of Power Saving	Quality is very good, the use of the effect is very good, more stable, quality assurance	Jingdong prefecture level city in Shandong
Stowe Flagship Store	King of Power Saving	Convenient, inexpensive, and see how it works out?	Jingdong prefecture level city in Shandong
Stowe Flagship Store	King of Power Saving	Very good quality, saves a lot of electricity, very good, worth buying	Jingdong prefecture level city in Shandong
Stowe Flagship Store	King of Power Saving	Power Saving King is easy to use, with the fruit of power saving to use easy to give!	Jingdong prefecture level city in Shandong
Stowe Flagship Store	King of Power Saving	This saver really works, good quality, safe and secure, very satisfied!	Jingdong prefecture level city in Shandong
Stowe Flagship Store	King of Power Saving	It works well, the voltage is very stable effect is good, the quality is also very good satisfaction	Jingdong prefecture level city in Shandong
Stowe Flagship Store	King of Power Saving	The logistics is super super fast! The quality is also very good, indeed value for money	Jingdong prefecture level city in Shandong
Stowe Flagship Store	King of Power Saving	I've only been using it for a month or so and it seems to be saving a lot of power	Jingdong prefecture level city in Shandong
Stowe Flagship Store	King of Power Saving	Very easy to operate, very good material, good value for money.	Jingdong prefecture level city in Shandong
Stowe Flagship Store	King of Power Saving	Aluminum alloy shell, durable, compact power-saving good effect	Jingdong prefecture level city in Shandong
Stowe Flagship Store	King of Power Saving	Nice device, easy to use, doesn't take up much space, recommendable!	Jingdong prefecture level city in Shandong
Stowe Flagship Store	King of Power Saving	Good quality, good price, fast logistics	Jingdong prefecture level city in Shandong
Stowe Flagship Store	King of Power Saving	Friends of the family in use recommended, the effect is good and recommendable!	Jingdong prefecture level city in Shandong
Stowe Flagship Store	King of Power Saving	Cheaper than brick and mortar stores worth buying is authentic	Jingdong prefecture level city in Shandong
Stowe Flagship Store	King of Power Saving	Merchants ship quickly, the price is cheaper than the physical store, good use	Jingdong prefecture level city in Shandong
Stowe Flagship Store	King of Power Saving	Very durable, affordable, fast shipping	Jingdong prefecture level city in Shandong
Stowe Flagship Store	King of Power Saving	Packaging tight quality is very good easy to use good results	Jingdong prefecture level city in Shandong
Stowe Flagship Store	King of Power Saving	Save the grid. It's in the whole house. It's saving money on the electric bill.	Jingdong prefecture level city in Shandong
Stowe Flagship Store	King of Power Saving	I used it today, it works well, I'm very satisfied	Jingdong prefecture level city in Shandong
Stowe Flagship Store	King of Power Saving	It really saves a lot of electricity after installation	Jingdong prefecture level city in Shandong
Stowe Flagship Store	King of Power Saving	Love it. Satisfied.	Jingdong prefecture level city in Shandong
Stowe Flagship Store	King of Power Saving	Doesn't feel like it's working.	Jingdong prefecture level city in Shandong
Stowe Flagship Store	King of Power Saving	I'll comment after using it.	Jingdong prefecture level city in Shandong
Stowe Flagship Store	King of Power Saving	I'm trying it out to see if it saves power, and I'll buy two more if it works well.	Jingdong prefecture level city in Shandong
Stowe Flagship Store	King of Power Saving	It is genuine very good, the price is much cheaper, cost-effective, really special cost-effective, it is worth buying!	Jingdong prefecture level city in Shandong
Stowe Flagship Store	King of Power Saving	This thing can really save electricity, I was introduced to buy by my friend, he used it very well, I used it and think it's also very good, it can really save electricity, save about half of the electricity every day, it's really good!	Jingdong prefecture level city in Shandong
Stowe Flagship Store	King of Power Saving	After using it for ten days or so, I checked the power consumption and it did drop, and customer service said it will save more electricity in the long run. The product is also insured, 5 years warranty do not have to worry about quality problems!	Jingdong prefecture level city in Shandong
Stowe Flagship Store	King of Power Saving	The product is good, before already bought a, this is the second, the effect is quite good, anyway, I see the daily electricity bill than before a lot less, this can be the choice!	Jingdong prefecture level city in Shandong

Stowe Flagship Store	King of Power Saving	Originally also very worried about things are not good, get the hand found completely over-anxious, five-star praise, the style is very generous, the quality is very good, the price is also very reasonable!	Jingdong prefecture level city in Shandong
Stowe Flagship Store	King of Power Saving	Quality is good, playing to the old family also buy a, repurchase, this quality is really good, recommended!	Jingdong prefecture level city in Shandong
Stowe Flagship Store	King of Power Saving	Thank you, the owner even sent two fuses.	Jingdong prefecture level city in Shandong
Stowe Flagship Store	King of Power Saving	This store has great stuff, relatively fast shipping and logistics	Jingdong prefecture level city in Shandong
Stowe Flagship Store	King of Power Saving	This product is based on the impossibility of saving electricity, I used 853 kWh of electricity in July, started using the product on August 1, and used 867 kWh of electricity by September 1, is this called	Jingdong prefecture level city in Shandong
Stowe Flagship Store	King of Power Saving	Power king received, to the home on the trial, when I plugged into the electric panel can not see the effect of power saving, the meter is still the same speed in the turn, I'll save the king of the manual to play look at the instructions, instructions to speak, have to have a day two days and half a month to save power obviously, then wait half a month to see the effect of power saving it! To half a month there are obvious power saving before continuing to chase Xu.	Jingdong prefecture level city in Shandong
Stowe Flagship Store	King of Power Saving	Received the goods, a look, did not see the manufacturer and production address, can only hold a try to see the mentality of the face	Jingdong prefecture level city in Shandong
Stowe Flagship Store	King of Power Saving	I've only been using it for a few days and I'm not sure if it's an IQ tax? Used to favorable reviews.	Jingdong prefecture level city in Shandong
Stowe Flagship Store	King of Power Saving	Appearance is okay, power saving next time to follow up the evaluation	Jingdong prefecture level city in Shandong
Stowe Flagship Store	King of Power Saving	Shorter time, but hopefully effective and power-saving, to be followed up in the future	Jingdong prefecture level city in Shandong
Stowe Flagship Store	King of Power Saving	Appearance is okay, I do not know if the power saving	Jingdong prefecture level city in Shandong
Stowe Flagship Store	King of Power Saving	Good service, fast logistics, Power Saving King is exquisite and beautiful, support the business, thank you!	Jingdong prefecture level city in Shandong
Stowe Flagship Store	King of Power Saving	Bought three at once to try first first.	Jingdong prefecture level city in Shandong
Stowe Flagship Store	King of Power Saving	The object is the same as the picture, the product is of high quality, the packaging is intact. High-end atmosphere, high power, power saving effect is obvious, average monthly power saving 50%, very satisfied.	Jingdong prefecture level city in Shandong
Stowe Flagship Store	King of Power Saving	Bought back and plugged in, really effective, before 16 degrees of electricity per day, now 8 degrees of electricity per day, open an air conditioner for 12 hours, decisive repurchase of a	Jingdong prefecture level city in Shandong
Stowe Flagship Store	King of Power Saving	The object is the same as the picture, the product is of high quality, and the package is intact. High-end atmosphere and high grade, high power	Jingdong prefecture level city in Shandong
Stowe Flagship Store	King of Power Saving	Goods received, I hope the store said can be energy-saving, first try a month, month after month to see the effect.	Jingdong prefecture level city in Shandong
Stowe Flagship Store	King of Power Saving	This is really good, the material and workmanship is particularly exquisite, the workmanship is really very good, I think I can recommend to my friends to buy	Jingdong prefecture level city in Shandong
Stowe Flagship Store	King of Power Saving	I have to praise, the quality is really good, and the packaging logistics are also good. Really like it, won't make you regret after getting it!	Jingdong prefecture level city in Shandong
Stowe Flagship Store	King of Power Saving	Reputable platform, efficient and professional service, buy with confidence and peace of mind!	Jingdong prefecture level city in Shandong
Stowe Flagship Store	King of Power Saving	Bought one on a trial basis to see if it works after 1-2 months.	Jingdong prefecture level city in Shandong
Stowe Flagship Store	King of Power Saving	Quality is very good, customer service attitude is very very praise, there are problems in a timely manner to solve the problem, the price is not expensive, super value, you can rest assured that the	Jingdong prefecture level city in Shandong
Stowe Flagship Store	King of Power Saving	With it no longer need to be afraid to use electricity, especially good, ready to buy a mom and dad that also	Jingdong prefecture level city in Shandong
Stowe Flagship Store	King of Power Saving	Baby received very like will return to buy the seller attitude is also very like will return to buy the seller attitude is also very like!	Jingdong prefecture level city in Shandong
Stowe Flagship Store	King of Power Saving	Jingdong Mall shopping convenience assured fast assured fast assured fast assured fast	Jingdong prefecture level city in Shandong
Stowe Flagship Store	King of Power Saving	Saving power received, the quality is very good, fine workmanship, especially good, much cheaper than the entity store, it is worth buying!	Jingdong prefecture level city in Shandong
Stowe Flagship Store	King of Power Saving	I did not expect the express delivery so quickly, very save point quality is super good, than the entity store is too cheap.	Jingdong prefecture level city in Shandong
Stowe Flagship Store	King of Power Saving	Have received the baby, very satisfied with a shopping, great worth buying!	Jingdong prefecture level city in Shandong
Stowe Flagship Store	King of Power Saving	This is really more powerful, very satisfied, more energy-saving, better, thank you boss.	Jingdong prefecture level city in Shandong
Stowe Flagship Store	King of Power Saving	Received has been used for two days, the air conditioning can boldly open it! Energy saving effect is really quite good	Jingdong prefecture level city in Shandong
Stowe Flagship Store	King of Power Saving	It is authentic, trust the merchant, the baby has a great texture and the price is moderate, unlimited repurchase!	Jingdong prefecture level city in Shandong
Stowe Flagship Store	King of Power Saving	It is genuine very good, the price is much cheaper, cost-effective, really special cost-effective, it is worth buying!	Jingdong prefecture level city in Shandong
Stowe Flagship Store	King of Power Saving	Very good quality, fine workmanship, low power consumption, long warranty, fast shipping, economical and practical	Jingdong prefecture level city in Shandong
Stowe Flagship Store	King of Power Saving	I've been using it for half a month now, it really saves power and the voltage is stable, it's very good	Jingdong prefecture level city in Shandong
Stowe Flagship Store	King of Power Saving	It's the ideal product , good quality and fast logistics, worth the kudos!	Jingdong prefecture level city in Shandong
Stowe Flagship Store	King of Power Saving	The logistics is very fast, the baby is very good, the shopkeeper is NICE!	Jingdong prefecture level city in Shandong
Stowe Flagship Store	King of Power Saving	Good to use, good quality, save point durable	Jingdong prefecture level city in Shandong
Stowe Flagship Store	King of Power Saving	The quality is very good, customer service attitude is very, very awesome, there are problems in a timely manner to solve the problem	Jingdong prefecture level city in Shandong
Stowe Flagship Store	King of Power Saving	Quality is very good, the use of the effect is very good, more stable, quality assurance	Jingdong prefecture level city in Shandong
Stowe Flagship Store	King of Power Saving	Convenient, inexpensive, and see how it works out?	Jingdong prefecture level city in Shandong
Stowe Flagship Store	King of Power Saving	Very good quality, saves a lot of electricity, very good, worth buying	Jingdong prefecture level city in Shandong
Stowe Flagship Store	King of Power Saving	Power Saving King is easy to use, with the fruit of power saving to use easy to give!	Jingdong prefecture level city in Shandong
Stowe Flagship Store	King of Power Saving	This saver really works, good quality, safe and secure, very satisfied!	Jingdong prefecture level city in Shandong
Stowe Flagship Store	King of Power Saving	It works well, the voltage is very stable effect is good, the quality is also very good satisfaction	Jingdong prefecture level city in Shandong
Stowe Flagship Store	King of Power Saving	The logistics is super super fast! The quality is also very good, indeed value for money	Jingdong prefecture level city in Shandong
Stowe Flagship Store	King of Power Saving	I've only been using it for a month or so and it seems to be saving a lot of power	Jingdong prefecture level city in Shandong
Stowe Flagship Store	King of Power Saving	Very easy to operate, very good material, good value for money.	Jingdong prefecture level city in Shandong
Stowe Flagship Store	King of Power Saving	Aluminum alloy shell, durable, compact power-saving good effect	Jingdong prefecture level city in Shandong
Stowe Flagship Store	King of Power Saving	Nice device, easy to use, doesn't take up much space, recommendable!	Jingdong prefecture level city in Shandong
Stowe Flagship Store	King of Power Saving	Good quality, good price, fast logistics	Jingdong prefecture level city in Shandong

Stowe Flagship Store	King of Power Saving	Friends of the family in use recommended, the effect is good and recommendable!	Jingdong prefecture level city in Shandong
Stowe Flagship Store	King of Power Saving	Cheaper than brick and mortar stores worth buying is authentic	Jingdong prefecture level city in Shandong
Stowe Flagship Store	King of Power Saving	Merchants ship quickly, the price is cheaper than the physical store, good use	Jingdong prefecture level city in Shandong
Stowe Flagship Store	King of Power Saving	Very durable, affordable, fast shipping	Jingdong prefecture level city in Shandong
Stowe Flagship Store	King of Power Saving	Packaging tight quality is very good easy to use good results	Jingdong prefecture level city in Shandong
Stowe Flagship Store	King of Power Saving	Save the grid. It's in the whole house. It's saving money on the electric bill.	Jingdong prefecture level city in Shandong
Stowe Flagship Store	King of Power Saving	I used it today, it works well, I'm very satisfied	Jingdong prefecture level city in Shandong
Stowe Flagship Store	King of Power Saving	It really saves a lot of electricity after installation	Jingdong prefecture level city in Shandong
Stowe Flagship Store	King of Power Saving	Love it. Satisfied.	Jingdong prefecture level city in Shandong
Stowe Flagship Store	King of Power Saving	Doesn't feel like it's working.	Jingdong prefecture level city in Shandong
Stowe Flagship Store	King of Power Saving	I'll comment after using it.	Jingdong prefecture level city in Shandong
Stowe Flagship Store	King of Power Saving	I'm trying it out to see if it saves power, and I'll buy two more if it works well.	Jingdong prefecture level city in Shandong
Stowe Flagship Store	King of Power Saving	It is genuine very good, the price is much cheaper, cost-effective, really special cost-effective, it is worth buying!	Jingdong prefecture level city in Shandong
Stowe Flagship Store	King of Power Saving	This thing can really save electricity, I was introduced to buy by my friend, he used it very well, I used it and think it's also very good, it can really save electricity, save about half of the electricity every day, it's really good!	Jingdong prefecture level city in Shandong
Stowe Flagship Store	King of Power Saving	After using it for ten days or so, I checked the power consumption and it did drop, and customer service said it will save more electricity in the long run. The product is also insured, 5 years warranty do not have to worry about quality problems!	Jingdong prefecture level city in Shandong
Stowe Flagship Store	King of Power Saving	The product is good, before already bought a, this is the second, the effect is quite good, anyway, I see the daily electricity bill than before a lot less, this can be the choice!	Jingdong prefecture level city in Shandong
Stowe Flagship Store	King of Power Saving	Originally also very worried about things are not good, get the hand found completely over-anxious, five-star praise, the style is very generous, the quality is very good, the price is also very reasonable!	Jingdong prefecture level city in Shandong
Stowe Flagship Store	King of Power Saving	Quality is good, playing to the old family also buy a, repurchase, this quality is really good, recommended!	Jingdong prefecture level city in Shandong
Stowe Flagship Store	King of Power Saving	Thank you, the owner even sent two fuses.	Jingdong prefecture level city in Shandong
Stowe Flagship Store	King of Power Saving	This store has great stuff, relatively fast shipping and logistics	Jingdong prefecture level city in Shandong
Stowe Flagship Store	King of Power Saving	This product is based on the impossibility of saving electricity, I used 853 kWh of electricity in July, started using the product on August 1, and used 867 kWh of electricity by September 1, is this called	Jingdong prefecture level city in Shandong
Stowe Flagship Store	King of Power Saving	Power king received, to the home on the trial, when I plugged into the electric panel can not see the effect of power saving, the meter is still the same speed in the turn, I'll save the king of the manual to play look at the instructions, instructions to speak, have to have a day two days and half a month to save power obviously, then wait half a month to see the effect of power saving it! To half a month there are obvious power saving before continuing to chase Xu.	Jingdong prefecture level city in Shandong
Stowe Flagship Store	King of Power Saving	Received the goods, a look, did not see the manufacturer and production address, can only hold a try to see the mentality of the face	Jingdong prefecture level city in Shandong
Stowe Flagship Store	King of Power Saving	I've only been using it for a few days and I'm not sure if it's an IQ tax? Used to favorable reviews.	Jingdong prefecture level city in Shandong
Stowe Flagship Store	King of Power Saving	Appearance is okay, power saving next time to follow up the evaluation	Jingdong prefecture level city in Shandong
Stowe Flagship Store	King of Power Saving	Shorter time, but hopefully effective and power-saving, to be followed up in the future	Jingdong prefecture level city in Shandong
Stowe Flagship Store	King of Power Saving	Appearance is okay, I do not know if the power saving	Jingdong prefecture level city in Shandong
Stowe Flagship Store	King of Power Saving	Good service, fast logistics, Power Saving King is exquisite and beautiful, support the business, thank you!	Jingdong prefecture level city in Shandong
Stowe Flagship Store	King of Power Saving	Bought three at once to try first first.	Jingdong prefecture level city in Shandong
Stowe Flagship Store	King of Power Saving	The object is the same as the picture, the product is of high quality, the packaging is intact. High-end atmosphere, high power, power saving effect is obvious, average monthly power saving 50%, very satisfied.	Jingdong prefecture level city in Shandong
Stowe Flagship Store	King of Power Saving	Bought back and plugged in, really effective, before 16 degrees of electricity per day, now 8 degrees of electricity per day, open an air conditioner for 12 hours, decisive repurchase of a	Jingdong prefecture level city in Shandong
Stowe Flagship Store	King of Power Saving	The object is the same as the picture, the product is of high quality, and the package is intact. High-end atmosphere and high grade, high power	Jingdong prefecture level city in Shandong
Stowe Flagship Store	King of Power Saving	Goods received, I hope the store said can be energy-saving, first try a month, month after month to see the effect.	Jingdong prefecture level city in Shandong
Stowe Flagship Store	King of Power Saving	This is really good, the material and workmanship is particularly exquisite, the workmanship is really very good, I think I can recommend to my friends to buy	Jingdong prefecture level city in Shandong
Stowe Flagship Store	King of Power Saving	I have to praise, the quality is really good, and the packaging logistics are also good. Really like it, won't make you regret after getting it!	Jingdong prefecture level city in Shandong
Stowe Flagship Store	King of Power Saving	Reputable platform, efficient and professional service, buy with confidence and peace of mind!	Jingdong prefecture level city in Shandong
Stowe Flagship Store	King of Power Saving	Bought one on a trial basis to see if it works after 1-2 months.	Jingdong prefecture level city in Shandong
Stowe Flagship Store	King of Power Saving	Quality is very good, customer service attitude is very very praise, there are problems in a timely manner to solve the problem, the price is not expensive, super value, you can rest assured that the	Jingdong prefecture level city in Shandong
Stowe Flagship Store	King of Power Saving	With it no longer need to be afraid to use electricity, especially good, ready to buy a mom and dad that also	Jingdong prefecture level city in Shandong
Stowe Flagship Store	King of Power Saving	Baby received very like will return to buy the seller attitude is also very like will return to buy the seller attitude is also very like!	Jingdong prefecture level city in Shandong
Stowe Flagship Store	King of Power Saving	Jingdong Mall shopping convenience assured fast assured fast assured fast assured fast	Jingdong prefecture level city in Shandong
Stowe Flagship Store	King of Power Saving	Saving power received, the quality is very good, fine workmanship, especially good, much cheaper than the entity store, it is worth buying!	Jingdong prefecture level city in Shandong
Stowe Flagship Store	King of Power Saving	I did not expect the express delivery so quickly, very save point quality is super good, than the entity store is too cheap.	Jingdong prefecture level city in Shandong
Stowe Flagship Store	King of Power Saving	Have received the baby, very satisfied with a shopping, great worth buying!	Jingdong prefecture level city in Shandong
Stowe Flagship Store	King of Power Saving	This is really more powerful, very satisfied, more energy-saving, better, thank you boss.	Jingdong prefecture level city in Shandong
Stowe Flagship Store	King of Power Saving	Received has been used for two days, the air conditioning can boldly open it! Energy saving effect is really quite good	Jingdong prefecture level city in Shandong
Stowe Flagship Store	King of Power Saving	It is authentic, trust the merchant, the baby has a great texture and the price is moderate, unlimited repurchase!	Jingdong prefecture level city in Shandong
Stowe Flagship Store	King of Power Saving	It is genuine very good, the price is much cheaper, cost-effective, really special cost-effective, it is worth buying!	Jingdong prefecture level city in Shandong
Stowe Flagship Store	King of Power Saving	Very good quality, fine workmanship, low power consumption, long warranty, fast shipping, economical and practical	Jingdong prefecture level city in Shandong

Stowe Flagship Store	King of Power Saving	I've been using it for half a month now, it really saves power and the voltage is stable, it's very good	Jingdong prefecture level city in Shandong
Stowe Flagship Store	King of Power Saving	It's the ideal product 💝, good quality and fast logistics, worth the kudos!	Jingdong prefecture level city in Shandong
Stowe Flagship Store	King of Power Saving	The logistics is very fast, the baby is very good, the shopkeeper is NICE!	Jingdong prefecture level city in Shandong
Stowe Flagship Store	King of Power Saving	Good to use, good quality, save point durable	Jingdong prefecture level city in Shandong
Stowe Flagship Store	King of Power Saving	The quality is very good, customer service attitude is very, very awesome, there are problems in a timely manner to solve the problem	Jingdong prefecture level city in Shandong
Stowe Flagship Store	King of Power Saving	Quality is very good, the use of the effect is very good, more stable, quality assurance	Jingdong prefecture level city in Shandong
Stowe Flagship Store	King of Power Saving	Convenient, inexpensive, and see how it works out?	Jingdong prefecture level city in Shandong
Stowe Flagship Store	King of Power Saving	Very good quality, saves a lot of electricity, very good, worth buying	Jingdong prefecture level city in Shandong
Stowe Flagship Store	King of Power Saving	Power Saving King is easy to use, with the fruit of power saving to use easy to give!	Jingdong prefecture level city in Shandong
Stowe Flagship Store	King of Power Saving	This saver really works, good quality, safe and secure, very satisfied!	Jingdong prefecture level city in Shandong
Stowe Flagship Store	King of Power Saving	It works well, the voltage is very stable effect is good, the quality is also very good satisfaction	Jingdong prefecture level city in Shandong
Stowe Flagship Store	King of Power Saving	The logistics is super super fast! The quality is also very good, indeed value for money	Jingdong prefecture level city in Shandong
Stowe Flagship Store	King of Power Saving	I've only been using it for a month or so and it seems to be saving a lot of power	Jingdong prefecture level city in Shandong
Stowe Flagship Store	King of Power Saving	Very easy to operate, very good material, good value for money.	Jingdong prefecture level city in Shandong
Stowe Flagship Store	King of Power Saving	Aluminum alloy shell, durable, compact power-saving good effect	Jingdong prefecture level city in Shandong
Stowe Flagship Store	King of Power Saving	Nice device, easy to use, doesn't take up much space, recommendable!	Jingdong prefecture level city in Shandong
Stowe Flagship Store	King of Power Saving	Good quality, good price, fast logistics	Jingdong prefecture level city in Shandong
Stowe Flagship Store	King of Power Saving	Friends of the family in use recommended, the effect is good and recommendable!	Jingdong prefecture level city in Shandong
Stowe Flagship Store	King of Power Saving	Cheaper than brick and mortar stores worth buying is authentic	Jingdong prefecture level city in Shandong
Stowe Flagship Store	King of Power Saving	Merchants ship quickly, the price is cheaper than the physical store, good use	Jingdong prefecture level city in Shandong
Stowe Flagship Store	King of Power Saving	Very durable, affordable, fast shipping	Jingdong prefecture level city in Shandong
Stowe Flagship Store	King of Power Saving	Packaging tight quality is very good easy to use good results	Jingdong prefecture level city in Shandong
Stowe Flagship Store	King of Power Saving	Save the grid. It's in the whole house. It's saving money on the electric bill.	Jingdong prefecture level city in Shandong
Stowe Flagship Store	King of Power Saving	I used it today, it works well, I'm very satisfied	Jingdong prefecture level city in Shandong
Stowe Flagship Store	King of Power Saving	It really saves a lot of electricity after installation	Jingdong prefecture level city in Shandong
Stowe Flagship Store	King of Power Saving	Love it. Satisfied.	Jingdong prefecture level city in Shandong
Stowe Flagship Store	King of Power Saving	Doesn't feel like it's working.	Jingdong prefecture level city in Shandong
Stowe Flagship Store	King of Power Saving	I'll comment after using it.	Jingdong prefecture level city in Shandong
Stowe Flagship Store	King of Power Saving	I'm trying it out to see if it saves power, and I'll buy two more if it works well.	Jingdong prefecture level city in Shandong
Stowe Flagship Store	King of Power Saving	It is genuine very good, the price is much cheaper, cost-effective, really special cost-effective, it is worth buying!	Jingdong prefecture level city in Shandong
Stowe Flagship Store	King of Power Saving	This thing can really save electricity, I was introduced to buy by my friend, he used it very well, I used it and think it's also very good, it can really save electricity, save about half of the electricity every day, it's really good!	Jingdong prefecture level city in Shandong
Stowe Flagship Store	King of Power Saving	After using it for ten days or so, I checked the power consumption and it did drop, and customer service said it will save more electricity in the long run. The product is also insured, 5 years warranty do not have to worry about quality problems!	Jingdong prefecture level city in Shandong
Stowe Flagship Store	King of Power Saving	The product is good, before already bought a, this is the second, the effect is quite good, anyway, I see the daily electricity bill than before a lot less, this can be the choice!	Jingdong prefecture level city in Shandong
Stowe Flagship Store	King of Power Saving	Originally also very worried about things are not good, get the hand found completely over-anxious, five-star praise, the style is very generous, the quality is very good, the price is also very reasonable!	Jingdong prefecture level city in Shandong
Stowe Flagship Store	King of Power Saving	Quality is good, playing to the old family also buy a, repurchase, this quality is really good, recommended!	Jingdong prefecture level city in Shandong
Stowe Flagship Store	King of Power Saving	Thank you, the owner even sent two fuses.	Jingdong prefecture level city in Shandong
Stowe Flagship Store	King of Power Saving	This store has great stuff, relatively fast shipping and logistics	Jingdong prefecture level city in Shandong
Stowe Flagship Store	King of Power Saving	This product is based on the impossibility of saving electricity, I used 853 kWh of electricity in July, started using the product on August 1, and used 867 kWh of electricity by September 1, is this called	Jingdong prefecture level city in Shandong
Stowe Flagship Store	King of Power Saving	Power king received, to the home on the trial, when I plugged into the electric panel can not see the effect of power saving, the meter is still the same speed in the turn, I'll save the king of the manual to play look at the instructions, instructions to speak, have to have a day two days and half a month to save power obviously, then wait half a month to see the effect of power saving it! To half a month there are obvious power saving before continuing to chase Xu.	Jingdong prefecture level city in Shandong
Stowe Flagship Store	King of Power Saving	Received the goods, a look, did not see the manufacturer and production address, can only hold a try to see the mentality of the face	Jingdong prefecture level city in Shandong
Stowe Flagship Store	King of Power Saving	I've only been using it for a few days and I'm not sure if it's an IQ tax? Used to favorable reviews.	Jingdong prefecture level city in Shandong
Stowe Flagship Store	King of Power Saving	Appearance is okay, power saving next time to follow up the evaluation	Jingdong prefecture level city in Shandong
Stowe Flagship Store	King of Power Saving	Shorter time, but hopefully effective and power-saving, to be followed up in the future	Jingdong prefecture level city in Shandong
Stowe Flagship Store	King of Power Saving	Appearance is okay, I do not know if the power saving	Jingdong prefecture level city in Shandong
Stowe Flagship Store	King of Power Saving	Good service, fast logistics, Power Saving King is exquisite and beautiful, support the business, thank you!	Jingdong prefecture level city in Shandong
Stowe Flagship Store	King of Power Saving	Bought three at once to try first first.	Jingdong prefecture level city in Shandong
Stowe Flagship Store	King of Power Saving	The object is the same as the picture, the product is of high quality, the packaging is intact. High-end atmosphere, high power, power saving effect is obvious, average monthly power saving 50%, very satisfied.	Jingdong prefecture level city in Shandong
Stowe Flagship Store	King of Power Saving	Bought back and plugged in, really effective, before 16 degrees of electricity per day, now 8 degrees of electricity per day, open an air conditioner for 12 hours, decisive repurchase of a	Jingdong prefecture level city in Shandong
Stowe Flagship Store	King of Power Saving	The object is the same as the picture, the product is of high quality, and the package is intact. High-end atmosphere and high grade, high power	Jingdong prefecture level city in Shandong

Stowe Flagship Store	King of Power Saving	Goods received, I hope the store said can be energy-saving, first try a month, month after month to see the effect.	Jingdong prefecture level city in Shandong
Stowe Flagship Store	King of Power Saving	This is really good, the material and workmanship is particularly exquisite, the workmanship is really very good, I think I can recommend to my friends to buy	Jingdong prefecture level city in Shandong
Stowe Flagship Store	King of Power Saving	I have to praise, the quality is really good, and the packaging logistics are also good. Really like it, won't make you regret after getting it!	Jingdong prefecture level city in Shandong
Stowe Flagship Store	King of Power Saving	Reputable platform, efficient and professional service, buy with confidence and peace of mind!	Jingdong prefecture level city in Shandong
Stowe Flagship Store	King of Power Saving	Bought one on a trial basis to see if it works after 1-2 months.	Jingdong prefecture level city in Shandong
Stowe Flagship Store	King of Power Saving	Quality is very good, customer service attitude is very very praise, there are problems in a timely manner to solve the problem, the price is not expensive, super value, you can rest assured that the	Jingdong prefecture level city in Shandong
Stowe Flagship Store	King of Power Saving	With it no longer need to be afraid to use electricity, especially good, ready to buy a mom and dad that also	Jingdong prefecture level city in Shandong
Stowe Flagship Store	King of Power Saving	Baby received very like will return to buy the seller attitude is also very like will return to buy the seller attitude is also very like!	Jingdong prefecture level city in Shandong
Stowe Flagship Store	King of Power Saving	Jingdong Mall shopping convenience assured fast assured fast assured fast assured fast	Jingdong prefecture level city in Shandong
Stowe Flagship Store	King of Power Saving	Saving power received, the quality is very good, fine workmanship, especially good, much cheaper than the entity store, it is worth buying!	Jingdong prefecture level city in Shandong
Stowe Flagship Store	King of Power Saving	I did not expect the express delivery so quickly, very save point quality is super good, than the entity store is too cheap.	Jingdong prefecture level city in Shandong
Stowe Flagship Store	King of Power Saving	Have received the baby, very satisfied with a shopping, great worth buying!	Jingdong prefecture level city in Shandong
Stowe Flagship Store	King of Power Saving	This is really more powerful, very satisfied, more energy-saving, better, thank you boss.	Jingdong prefecture level city in Shandong
Stowe Flagship Store	King of Power Saving	Received has been used for two days, the air conditioning can boldly open it! Energy saving effect is really quite good	Jingdong prefecture level city in Shandong
Stowe Flagship Store	King of Power Saving	It is authentic, trust the merchant, the baby has a great texture and the price is moderate, unlimited repurchase!	Jingdong prefecture level city in Shandong
Stowe Flagship Store	King of Power Saving	It is genuine very good, the price is much cheaper, cost-effective, really special cost-effective, it is worth buying!	Jingdong prefecture level city in Shandong
Stowe Flagship Store	King of Power Saving	Very good quality, fine workmanship, low power consumption, long warranty, fast shipping, economical and practical	Jingdong prefecture level city in Shandong
Stowe Flagship Store	King of Power Saving	I've been using it for half a month now, it really saves power and the voltage is stable, it's very good	Jingdong prefecture level city in Shandong
Stowe Flagship Store	King of Power Saving	It's the ideal product 💖, good quality and fast logistics, worth the kudos!	Jingdong prefecture level city in Shandong
Stowe Flagship Store	King of Power Saving	The logistics is very fast, the baby is very good, the shopkeeper is NICE!	Jingdong prefecture level city in Shandong
Stowe Flagship Store	King of Power Saving	Good to use, good quality, save point durable	Jingdong prefecture level city in Shandong
Stowe Flagship Store	King of Power Saving	The quality is very good, customer service attitude is very, very awesome, there are problems in a timely manner to solve the problem	Jingdong prefecture level city in Shandong
Stowe Flagship Store	King of Power Saving	Quality is very good, the use of the effect is very good, more stable, quality assurance	Jingdong prefecture level city in Shandong
Stowe Flagship Store	King of Power Saving	Convenient, inexpensive, and see how it works out?	Jingdong prefecture level city in Shandong
Stowe Flagship Store	King of Power Saving	Very good quality, saves a lot of electricity, very good, worth buying	Jingdong prefecture level city in Shandong
Stowe Flagship Store	King of Power Saving	Power Saving King is easy to use, with the fruit of power saving to use easy to give!	Jingdong prefecture level city in Shandong
Stowe Flagship Store	King of Power Saving	This saver really works, good quality, safe and secure, very satisfied!	Jingdong prefecture level city in Shandong
Stowe Flagship Store	King of Power Saving	It works well, the voltage is very stable effect is good, the quality is also very good satisfaction	Jingdong prefecture level city in Shandong
Stowe Flagship Store	King of Power Saving	The logistics is super super fast! The quality is also very good, indeed value for money	Jingdong prefecture level city in Shandong
Stowe Flagship Store	King of Power Saving	I've only been using it for a month or so and it seems to be saving a lot of power	Jingdong prefecture level city in Shandong
Stowe Flagship Store	King of Power Saving	Very easy to operate, very good material, good value for money.	Jingdong prefecture level city in Shandong
Stowe Flagship Store	King of Power Saving	Aluminum alloy shell, durable, compact power-saving good effect	Jingdong prefecture level city in Shandong
Stowe Flagship Store	King of Power Saving	Nice device, easy to use, doesn't take up much space, recommendable!	Jingdong prefecture level city in Shandong
Stowe Flagship Store	King of Power Saving	Good quality, good price, fast logistics	Jingdong prefecture level city in Shandong
Stowe Flagship Store	King of Power Saving	Friends of the family in use recommended, the effect is good and recommendable!	Jingdong prefecture level city in Shandong
Stowe Flagship Store	King of Power Saving	Cheaper than brick and mortar stores worth buying is authentic	Jingdong prefecture level city in Shandong
Stowe Flagship Store	King of Power Saving	Merchants ship quickly, the price is cheaper than the physical store, good use	Jingdong prefecture level city in Shandong
Stowe Flagship Store	King of Power Saving	Very durable, affordable, fast shipping	Jingdong prefecture level city in Shandong
Stowe Flagship Store	King of Power Saving	Packaging tight quality is very good easy to use good results	Jingdong prefecture level city in Shandong
Stowe Flagship Store	King of Power Saving	Save the grid. It's in the whole house. It's saving money on the electric bill.	Jingdong prefecture level city in Shandong
Stowe Flagship Store	King of Power Saving	I used it today, it works well, I'm very satisfied	Jingdong prefecture level city in Shandong
Stowe Flagship Store	King of Power Saving	It really saves a lot of electricity after installation	Jingdong prefecture level city in Shandong
Stowe Flagship Store	King of Power Saving	Love it. Satisfied.	Jingdong prefecture level city in Shandong
Stowe Flagship Store	King of Power Saving	Doesn't feel like it's working.	Jingdong prefecture level city in Shandong
Stowe Flagship Store	King of Power Saving	I'll comment after using it.	Jingdong prefecture level city in Shandong
Stowe Flagship Store	King of Power Saving	I'm trying it out to see if it saves power, and I'll buy two more if it works well.	Jingdong prefecture level city in Shandong
Stowe Flagship Store	King of Power Saving	It is genuine very good, the price is much cheaper, cost-effective, really special cost-effective, it is worth buying!	Jingdong prefecture level city in Shandong
Stowe Flagship Store	King of Power Saving	This thing can really save electricity, I was introduced to buy by my friend, he used it very well, I used it and think it's also very good, it can really save electricity, save about half of the electricity every day, it's really good!	Jingdong prefecture level city in Shandong
Stowe Flagship Store	King of Power Saving	After using it for ten days or so, I checked the power consumption and it did drop, and customer service said it will save more electricity in the long run. The product is also insured, 5 years warranty do not have to worry about quality problems!	Jingdong prefecture level city in Shandong
Stowe Flagship Store	King of Power Saving	The product is good, before already bought a, this is the second, the effect is quite good, anyway, I see the daily electricity bill than before a lot less, this can be the choice!	Jingdong prefecture level city in Shandong

Stowe Flagship Store	King of Power Saving	Originally also very worried about things are not good, get the hand found completely over-anxious, five-star praise, the style is very generous, the quality is very good, the price is also very reasonable!	Jingdong prefecture level city in Shandong
Stowe Flagship Store	King of Power Saving	Quality is good, playing to the old family also buy a, repurchase, this quality is really good, recommended!	Jingdong prefecture level city in Shandong
Stowe Flagship Store	King of Power Saving	Thank you, the owner even sent two fuses.	Jingdong prefecture level city in Shandong
Stowe Flagship Store	King of Power Saving	This store has great stuff, relatively fast shipping and logistics	Jingdong prefecture level city in Shandong
Stowe Flagship Store	King of Power Saving	This product is based on the impossibility of saving electricity, I used 853 kWh of electricity in July, started using the product on August 1, and used 867 kWh of electricity by September 1, is this called	Jingdong prefecture level city in Shandong
Stowe Flagship Store	King of Power Saving	Power king received, to the home on the trial, when I plugged into the electric panel can not see the effect of power saving, the meter is still the same speed in the turn, I'll save the king of the manual to play look at the instructions, instructions to speak, have to have a day two days and half a month to save power obviously, then wait half a month to see the effect of power saving it! To half a month there are obvious power saving before continuing to chase Xu.	Jingdong prefecture level city in Shandong
Stowe Flagship Store	King of Power Saving	Received the goods, a look, did not see the manufacturer and production address, can only hold a try to see the mentality of the face	Jingdong prefecture level city in Shandong
Stowe Flagship Store	King of Power Saving	I've only been using it for a few days and I'm not sure if it's an IQ tax? Used to favorable reviews.	Jingdong prefecture level city in Shandong
Stowe Flagship Store	King of Power Saving	Appearance is okay, power saving next time to follow up the evaluation	Jingdong prefecture level city in Shandong
Stowe Flagship Store	King of Power Saving	Shorter time, but hopefully effective and power-saving, to be followed up in the future	Jingdong prefecture level city in Shandong
Stowe Flagship Store	King of Power Saving	Appearance is okay, I do not know if the power saving	Jingdong prefecture level city in Shandong
Stowe Flagship Store	King of Power Saving	Good service, fast logistics, Power Saving King is exquisite and beautiful, support the business, thank you!	Jingdong prefecture level city in Shandong
Stowe Flagship Store	King of Power Saving	Bought three at once to try first first.	Jingdong prefecture level city in Shandong
Stowe Flagship Store	King of Power Saving	The object is the same as the picture, the product is of high quality, the packaging is intact. High-end atmosphere, high power, power saving effect is obvious, average monthly power saving 50%, very satisfied.	Jingdong prefecture level city in Shandong
Stowe Flagship Store	King of Power Saving	Bought back and plugged in, really effective, before 16 degrees of electricity per day, now 8 degrees of electricity per day, open an air conditioner for 12 hours, decisive repurchase of a	Jingdong prefecture level city in Shandong
Stowe Flagship Store	King of Power Saving	The object is the same as the picture, the product is of high quality, and the package is intact. High-end atmosphere and high grade, high power	Jingdong prefecture level city in Shandong
Stowe Flagship Store	King of Power Saving	Goods received, I hope the store said can be energy-saving, first try a month, month after month to see the effect.	Jingdong prefecture level city in Shandong
Stowe Flagship Store	King of Power Saving	This is really good, the material and workmanship is particularly exquisite, the workmanship is really very good, I think I can recommend to my friends to buy	Jingdong prefecture level city in Shandong
Stowe Flagship Store	King of Power Saving	I have to praise, the quality is really good, and the packaging logistics are also good. Really like it, won't make you regret after getting it!	Jingdong prefecture level city in Shandong
Stowe Flagship Store	King of Power Saving	Reputable platform, efficient and professional service, buy with confidence and peace of mind!	Jingdong prefecture level city in Shandong
Stowe Flagship Store	King of Power Saving	Bought one on a trial basis to see if it works after 1-2 months.	Jingdong prefecture level city in Shandong
Stowe Flagship Store	King of Power Saving	Quality is very good, customer service attitude is very very praise, there are problems in a timely manner to solve the problem, the price is not expensive, super value, you can rest assured that the	Jingdong prefecture level city in Shandong
Stowe Flagship Store	King of Power Saving	With it no longer need to be afraid to use electricity, especially good, ready to buy a mom and dad that also	Jingdong prefecture level city in Shandong
Stowe Flagship Store	King of Power Saving	Baby received very like will return to buy the seller attitude is also very like will return to buy the seller attitude is also very like!	Jingdong prefecture level city in Shandong
Stowe Flagship Store	King of Power Saving	Jingdong Mall shopping convenience assured fast assured fast assured fast assured fast	Jingdong prefecture level city in Shandong
Stowe Flagship Store	King of Power Saving	Saving power received, the quality is very good, fine workmanship, especially good, much cheaper than the entity store, it is worth buying!	Jingdong prefecture level city in Shandong
Stowe Flagship Store	King of Power Saving	I did not expect the express delivery so quickly, very save point quality is super good, than the entity store is too cheap.	Jingdong prefecture level city in Shandong
Stowe Flagship Store	King of Power Saving	Have received the baby, very satisfied with a shopping, great worth buying!	Jingdong prefecture level city in Shandong
Stowe Flagship Store	King of Power Saving	This is really more powerful, very satisfied, more energy-saving, better, thank you boss.	Jingdong prefecture level city in Shandong
Stowe Flagship Store	King of Power Saving	Received has been used for two days, the air conditioning can boldly open it! Energy saving effect is really quite good	Jingdong prefecture level city in Shandong
Stowe Flagship Store	King of Power Saving	It is authentic, trust the merchant, the baby has a great texture and the price is moderate, unlimited repurchase!	Jingdong prefecture level city in Shandong
Stowe Flagship Store	King of Power Saving	It is genuine very good, the price is much cheaper, cost-effective, really special cost-effective, it is worth buying!	Jingdong prefecture level city in Shandong
Stowe Flagship Store	King of Power Saving	Very good quality, fine workmanship, low power consumption, long warranty, fast shipping, economical and practical	Jingdong prefecture level city in Shandong
Stowe Flagship Store	King of Power Saving	I've been using it for half a month now, it really saves power and the voltage is stable, it's very good	Jingdong prefecture level city in Shandong
Stowe Flagship Store	King of Power Saving	It's the ideal product 💝, good quality and fast logistics, worth the kudos!	Jingdong prefecture level city in Shandong
Stowe Flagship Store	King of Power Saving	The logistics is very fast, the baby is very good, the shopkeeper is NICE!	Jingdong prefecture level city in Shandong
Stowe Flagship Store	King of Power Saving	Good to use, good quality, save point durable	Jingdong prefecture level city in Shandong
Stowe Flagship Store	King of Power Saving	The quality is very good, customer service attitude is very, very awesome, there are problems in a timely manner to solve the problem	Jingdong prefecture level city in Shandong
Stowe Flagship Store	King of Power Saving	Quality is very good, the use of the effect is very good, more stable, quality assurance	Jingdong prefecture level city in Shandong
Stowe Flagship Store	King of Power Saving	Convenient, inexpensive, and see how it works out?	Jingdong prefecture level city in Shandong
Stowe Flagship Store	King of Power Saving	Very good quality, saves a lot of electricity, very good, worth buying	Jingdong prefecture level city in Shandong
Stowe Flagship Store	King of Power Saving	Power Saving King is easy to use, with the fruit of power saving to use easy to give!	Jingdong prefecture level city in Shandong
Stowe Flagship Store	King of Power Saving	This saver really works, good quality, safe and secure, very satisfied!	Jingdong prefecture level city in Shandong
Stowe Flagship Store	King of Power Saving	It works well, the voltage is very stable effect is good, the quality is also very good satisfaction	Jingdong prefecture level city in Shandong
Stowe Flagship Store	King of Power Saving	The logistics is super super fast! The quality is also very good, indeed value for money	Jingdong prefecture level city in Shandong
Stowe Flagship Store	King of Power Saving	I've only been using it for a month or so and it seems to be saving a lot of power	Jingdong prefecture level city in Shandong
Stowe Flagship Store	King of Power Saving	Very easy to operate, very good material, good value for money.	Jingdong prefecture level city in Shandong
Stowe Flagship Store	King of Power Saving	Aluminum alloy shell, durable, compact power-saving good effect	Jingdong prefecture level city in Shandong
Stowe Flagship Store	King of Power Saving	Nice device, easy to use, doesn't take up much space, recommendable!	Jingdong prefecture level city in Shandong
Stowe Flagship Store	King of Power Saving	Good quality, good price, fast logistics	Jingdong prefecture level city in Shandong

Stowe Flagship Store	King of Power Saving	Friends of the family in use recommended, the effect is good and recommendable!	Jingdong prefecture level city in Shandong
Stowe Flagship Store	King of Power Saving	Cheaper than brick and mortar stores worth buying is authentic	Jingdong prefecture level city in Shandong
Stowe Flagship Store	King of Power Saving	Merchants ship quickly, the price is cheaper than the physical store, good use	Jingdong prefecture level city in Shandong
Stowe Flagship Store	King of Power Saving	Very durable, affordable, fast shipping	Jingdong prefecture level city in Shandong
Stowe Flagship Store	King of Power Saving	Packaging tight quality is very good easy to use good results	Jingdong prefecture level city in Shandong
Stowe Flagship Store	King of Power Saving	Save the grid. It's in the whole house. It's saving money on the electric bill.	Jingdong prefecture level city in Shandong
Stowe Flagship Store	King of Power Saving	I used it today, it works well, I'm very satisfied	Jingdong prefecture level city in Shandong
Stowe Flagship Store	King of Power Saving	It really saves a lot of electricity after installation	Jingdong prefecture level city in Shandong
Stowe Flagship Store	King of Power Saving	Love it. Satisfied.	Jingdong prefecture level city in Shandong
Stowe Flagship Store	King of Power Saving	Doesn't feel like it's working.	Jingdong prefecture level city in Shandong
Stowe Flagship Store	King of Power Saving	I'll comment after using it.	Jingdong prefecture level city in Shandong
Stowe Flagship Store	King of Power Saving	I'm trying it out to see if it saves power, and I'll buy two more if it works well.	Jingdong prefecture level city in Shandong
Stowe Flagship Store	King of Power Saving	It is genuine very good, the price is much cheaper, cost-effective, really special cost-effective, it is worth buying!	Jingdong prefecture level city in Shandong
Stowe Flagship Store	King of Power Saving	This thing can really save electricity, I was introduced to buy by my friend, he used it very well, I used it and think it's also very good, it can really save electricity, save about half of the electricity every day, it's really good!	Jingdong prefecture level city in Shandong
Stowe Flagship Store	King of Power Saving	After using it for ten days or so, I checked the power consumption and it did drop, and customer service said it will save more electricity in the long run. The product is also insured, 5 years warranty do not have to worry about quality problems!	Jingdong prefecture level city in Shandong
Stowe Flagship Store	King of Power Saving	The product is good, before already bought a, this is the second, the effect is quite good, anyway, I see the daily electricity bill than before a lot less, this can be the choice!	Jingdong prefecture level city in Shandong
Stowe Flagship Store	King of Power Saving	Originally also very worried about things are not good, get the hand found completely over-anxious, five-star praise, the style is very generous, the quality is very good, the price is also very reasonable!	Jingdong prefecture level city in Shandong
Stowe Flagship Store	King of Power Saving	Quality is good, playing to the old family also buy a, repurchase, this quality is really good, recommended!	Jingdong prefecture level city in Shandong
Stowe Flagship Store	King of Power Saving	Thank you, the owner even sent two fuses.	Jingdong prefecture level city in Shandong
Stowe Flagship Store	King of Power Saving	This store has great stuff, relatively fast shipping and logistics	Jingdong prefecture level city in Shandong
Stowe Flagship Store	King of Power Saving	This product is based on the impossibility of saving electricity, I used 853 kWh of electricity in July, started using the product on August 1, and used 867 kWh of electricity by September 1, is this called	Jingdong prefecture level city in Shandong
Stowe Flagship Store	King of Power Saving	Power king received, to the home on the trial, when I plugged into the electric panel can not see the effect of power saving, the meter is still the same speed in the turn, I'll save the king of the manual to play look at the instructions, instructions to speak, have to have a day two days and half a month to save power obviously, then wait half a month to see the effect of power saving it! To half a month there are obvious power saving before continuing to chase Xu.	Jingdong prefecture level city in Shandong
Stowe Flagship Store	King of Power Saving	Received the goods, a look, did not see the manufacturer and production address, can only hold a try to see the mentality of the face	Jingdong prefecture level city in Shandong
Stowe Flagship Store	King of Power Saving	I've only been using it for a few days and I'm not sure if it's an IQ tax? Used to favorable reviews.	Jingdong prefecture level city in Shandong
Stowe Flagship Store	King of Power Saving	Appearance is okay, power saving next time to follow up the evaluation	Jingdong prefecture level city in Shandong
Stowe Flagship Store	King of Power Saving	Shorter time, but hopefully effective and power-saving, to be followed up in the future	Jingdong prefecture level city in Shandong
Stowe Flagship Store	King of Power Saving	Appearance is okay, I do not know if the power saving	Jingdong prefecture level city in Shandong
Stowe Flagship Store	King of Power Saving	Good service, fast logistics, Power Saving King is exquisite and beautiful, support the business, thank you!	Jingdong prefecture level city in Shandong
Stowe Flagship Store	King of Power Saving	Bought three at once to try first first.	Jingdong prefecture level city in Shandong
Stowe Flagship Store	King of Power Saving	The object is the same as the picture, the product is of high quality, the packaging is intact. High-end atmosphere, high power, power saving effect is obvious, average monthly power saving 50%, very satisfied.	Jingdong prefecture level city in Shandong
Stowe Flagship Store	King of Power Saving	Bought back and plugged in, really effective, before 16 degrees of electricity per day, now 8 degrees of electricity per day, open an air conditioner for 12 hours, decisive repurchase of a	Jingdong prefecture level city in Shandong
Stowe Flagship Store	King of Power Saving	The object is the same as the picture, the product is of high quality, and the package is intact. High-end atmosphere and high grade, high power	Jingdong prefecture level city in Shandong
Stowe Flagship Store	King of Power Saving	Goods received, I hope the store said can be energy-saving, first try a month, month after month to see the effect.	Jingdong prefecture level city in Shandong
Stowe Flagship Store	King of Power Saving	This is really good, the material and workmanship is particularly exquisite, the workmanship is really very good, I think I can recommend to my friends to buy	Jingdong prefecture level city in Shandong
Stowe Flagship Store	King of Power Saving	I have to praise, the quality is really good, and the packaging logistics are also good. Really like it, won't make you regret after getting it!	Jingdong prefecture level city in Shandong
Stowe Flagship Store	King of Power Saving	Reputable platform, efficient and professional service, buy with confidence and peace of mind!	Jingdong prefecture level city in Shandong
Stowe Flagship Store	King of Power Saving	Bought one on a trial basis to see if it works after 1-2 months.	Jingdong prefecture level city in Shandong
Stowe Flagship Store	King of Power Saving	Quality is very good, customer service attitude is very very praise, there are problems in a timely manner to solve the problem, the price is not expensive, super value, you can rest assured that the	Jingdong prefecture level city in Shandong
Stowe Flagship Store	King of Power Saving	With it no longer need to be afraid to use electricity, especially good, ready to buy a mom and dad that also	Jingdong prefecture level city in Shandong
Stowe Flagship Store	King of Power Saving	Baby received very like will return to buy the seller attitude is also very like will return to buy the seller attitude is also very like!	Jingdong prefecture level city in Shandong
Stowe Flagship Store	King of Power Saving	Jingdong Mall shopping convenience assured fast assured fast assured fast assured fast	Jingdong prefecture level city in Shandong
Stowe Flagship Store	King of Power Saving	Saving power received, the quality is very good, fine workmanship, especially good, much cheaper than the entity store, it is worth buying!	Jingdong prefecture level city in Shandong
Stowe Flagship Store	King of Power Saving	I did not expect the express delivery so quickly, very save point quality is super good, than the entity store is too cheap.	Jingdong prefecture level city in Shandong
Stowe Flagship Store	King of Power Saving	Have received the baby, very satisfied with a shopping, great worth buying!	Jingdong prefecture level city in Shandong
Stowe Flagship Store	King of Power Saving	This is really more powerful, very satisfied, more energy-saving, better, thank you boss.	Jingdong prefecture level city in Shandong
Stowe Flagship Store	King of Power Saving	Received has been used for two days, the air conditioning can boldly open it! Energy saving effect is really quite good	Jingdong prefecture level city in Shandong
Stowe Flagship Store	King of Power Saving	It is authentic, trust the merchant, the baby has a great texture and the price is moderate, unlimited repurchase!	Jingdong prefecture level city in Shandong
Stowe Flagship Store	King of Power Saving	It is genuine very good, the price is much cheaper, cost-effective, really special cost-effective, it is worth buying!	Jingdong prefecture level city in Shandong
Stowe Flagship Store	King of Power Saving	Very good quality, fine workmanship, low power consumption, long warranty, fast shipping, economical and practical	Jingdong prefecture level city in Shandong

Stowe Flagship Store	King of Power Saving	I've been using it for half a month now, it really saves power and the voltage is stable, it's very good	Jingdong prefecture level city in Shandong
Stowe Flagship Store	King of Power Saving	It's the ideal product 💝, good quality and fast logistics, worth the kudos!	Jingdong prefecture level city in Shandong
Stowe Flagship Store	King of Power Saving	The logistics is very fast, the baby is very good, the shopkeeper is NICE!	Jingdong prefecture level city in Shandong
Stowe Flagship Store	King of Power Saving	Good to use, good quality, save point durable	Jingdong prefecture level city in Shandong
Stowe Flagship Store	King of Power Saving	The quality is very good, customer service attitude is very, very awesome, there are problems in a timely manner to solve the problem	Jingdong prefecture level city in Shandong
Stowe Flagship Store	King of Power Saving	Quality is very good, the use of the effect is very good, more stable, quality assurance	Jingdong prefecture level city in Shandong
Stowe Flagship Store	King of Power Saving	Convenient, inexpensive, and see how it works out?	Jingdong prefecture level city in Shandong
Stowe Flagship Store	King of Power Saving	Very good quality, saves a lot of electricity, very good, worth buying	Jingdong prefecture level city in Shandong
Stowe Flagship Store	King of Power Saving	Power Saving King is easy to use, with the fruit of power saving to use easy to give!	Jingdong prefecture level city in Shandong
Stowe Flagship Store	King of Power Saving	This saver really works, good quality, safe and secure, very satisfied!	Jingdong prefecture level city in Shandong
Stowe Flagship Store	King of Power Saving	It works well, the voltage is very stable effect is good, the quality is also very good satisfaction	Jingdong prefecture level city in Shandong
Stowe Flagship Store	King of Power Saving	The logistics is super super fast! The quality is also very good, indeed value for money	Jingdong prefecture level city in Shandong
Stowe Flagship Store	King of Power Saving	I've only been using it for a month or so and it seems to be saving a lot of power	Jingdong prefecture level city in Shandong
Stowe Flagship Store	King of Power Saving	Very easy to operate, very good material, good value for money.	Jingdong prefecture level city in Shandong
Stowe Flagship Store	King of Power Saving	Aluminum alloy shell, durable, compact power-saving good effect	Jingdong prefecture level city in Shandong
Stowe Flagship Store	King of Power Saving	Nice device, easy to use, doesn't take up much space, recommendable!	Jingdong prefecture level city in Shandong
Stowe Flagship Store	King of Power Saving	Good quality, good price, fast logistics	Jingdong prefecture level city in Shandong
Stowe Flagship Store	King of Power Saving	Friends of the family in use recommended, the effect is good and recommendable!	Jingdong prefecture level city in Shandong
Stowe Flagship Store	King of Power Saving	Cheaper than brick and mortar stores worth buying is authentic	Jingdong prefecture level city in Shandong
Stowe Flagship Store	King of Power Saving	Merchants ship quickly, the price is cheaper than the physical store, good use	Jingdong prefecture level city in Shandong
Stowe Flagship Store	King of Power Saving	Very durable, affordable, fast shipping	Jingdong prefecture level city in Shandong
Stowe Flagship Store	King of Power Saving	Packaging tight quality is very good easy to use good results	Jingdong prefecture level city in Shandong
Stowe Flagship Store	King of Power Saving	Save the grid. It's in the whole house. It's saving money on the electric bill.	Jingdong prefecture level city in Shandong
Stowe Flagship Store	King of Power Saving	I used it today, it works well, I'm very satisfied	Jingdong prefecture level city in Shandong
Stowe Flagship Store	King of Power Saving	It really saves a lot of electricity after installation	Jingdong prefecture level city in Shandong
Stowe Flagship Store	King of Power Saving	Love it. Satisfied.	Jingdong prefecture level city in Shandong
Stowe Flagship Store	King of Power Saving	Doesn't feel like it's working.	Jingdong prefecture level city in Shandong
Stowe Flagship Store	King of Power Saving	I'll comment after using it.	Jingdong prefecture level city in Shandong
Stowe Flagship Store	King of Power Saving	I'm trying it out to see if it saves power, and I'll buy two more if it works well.	Jingdong prefecture level city in Shandong
Stowe Flagship Store	King of Power Saving	It is genuine very good, the price is much cheaper, cost-effective, really special cost-effective, it is worth buying!	Jingdong prefecture level city in Shandong
Stowe Flagship Store	King of Power Saving	This thing can really save electricity, I was introduced to buy by my friend, he used it very well, I used it and think it's also very good, it can really save electricity, save about half of the electricity every day, it's really good!	Jingdong prefecture level city in Shandong
Stowe Flagship Store	King of Power Saving	After using it for ten days or so, I checked the power consumption and it did drop, and customer service said it will save more electricity in the long run. The product is also insured, 5 years warranty do not have to worry about quality problems!	Jingdong prefecture level city in Shandong
Stowe Flagship Store	King of Power Saving	The product is good, before already bought a, this is the second, the effect is quite good, anyway, I see the daily electricity bill than before a lot less, this can be the choice!	Jingdong prefecture level city in Shandong
Stowe Flagship Store	King of Power Saving	Originally also very worried about things are not good, get the hand found completely over-anxious, five-star praise, the style is very generous, the quality is very good, the price is also very reasonable!	Jingdong prefecture level city in Shandong
Stowe Flagship Store	King of Power Saving	Quality is good, playing to the old family also buy a, repurchase, this quality is really good, recommended!	Jingdong prefecture level city in Shandong
Stowe Flagship Store	King of Power Saving	Thank you, the owner even sent two fuses.	Jingdong prefecture level city in Shandong
Stowe Flagship Store	King of Power Saving	This store has great stuff, relatively fast shipping and logistics	Jingdong prefecture level city in Shandong
Stowe Flagship Store	King of Power Saving	This product is based on the impossibility of saving electricity, I used 853 kWh of electricity in July, started using the product on August 1, and used 867 kWh of electricity by September 1, is this called	Jingdong prefecture level city in Shandong
Stowe Flagship Store	King of Power Saving	Power king received, to the home on the trial, when I plugged into the electric panel can not see the effect of power saving, the meter is still the same speed in the turn, I'll save the king of the manual to play look at the instructions, instructions to speak, have to have a day two days and half a month to save power obviously, then wait half a month to see the effect of power saving it! To half a month there are obvious power saving before continuing to chase Xu.	Jingdong prefecture level city in Shandong
Stowe Flagship Store	King of Power Saving	Received the goods, a look, did not see the manufacturer and production address, can only hold a try to see the mentality of the face	Jingdong prefecture level city in Shandong
Stowe Flagship Store	King of Power Saving	I've only been using it for a few days and I'm not sure if it's an IQ tax? Used to favorable reviews.	Jingdong prefecture level city in Shandong
Stowe Flagship Store	King of Power Saving	Appearance is okay, power saving next time to follow up the evaluation	Jingdong prefecture level city in Shandong
Stowe Flagship Store	King of Power Saving	Shorter time, but hopefully effective and power-saving, to be followed up in the future	Jingdong prefecture level city in Shandong
Stowe Flagship Store	King of Power Saving	Appearance is okay, I do not know if the power saving	Jingdong prefecture level city in Shandong
Stowe Flagship Store	King of Power Saving	Good service, fast logistics, Power Saving King is exquisite and beautiful, support the business, thank you!	Jingdong prefecture level city in Shandong
Stowe Flagship Store	King of Power Saving	Bought three at once to try first first.	Jingdong prefecture level city in Shandong
Stowe Flagship Store	King of Power Saving	The object is the same as the picture, the product is of high quality, the packaging is intact. High-end atmosphere, high power, power saving effect is obvious, average monthly power saving 50%, very satisfied.	Jingdong prefecture level city in Shandong
Stowe Flagship Store	King of Power Saving	Bought back and plugged in, really effective, before 16 degrees of electricity per day, now 8 degrees of electricity per day, open an air conditioner for 12 hours, decisive repurchase of a	Jingdong prefecture level city in Shandong
Stowe Flagship Store	King of Power Saving	The object is the same as the picture, the product is of high quality, and the package is intact. High-end atmosphere and high grade, high power	Jingdong prefecture level city in Shandong

Stowe Flagship Store	King of Power Saving	Goods received, I hope the store said can be energy-saving, first try a month, month after month to see the effect.	Jingdong prefecture level city in Shandong
Stowe Flagship Store	King of Power Saving	This is really good, the material and workmanship is particularly exquisite, the workmanship is really very good, I think I can recommend to my friends to buy	Jingdong prefecture level city in Shandong
Stowe Flagship Store	King of Power Saving	I have to praise, the quality is really good, and the packaging logistics are also good. Really like it, won't make you regret after getting it!	Jingdong prefecture level city in Shandong
Stowe Flagship Store	King of Power Saving	Reputable platform, efficient and professional service, buy with confidence and peace of mind!	Jingdong prefecture level city in Shandong
Stowe Flagship Store	King of Power Saving	Bought one on a trial basis to see if it works after 1-2 months.	Jingdong prefecture level city in Shandong
Stowe Flagship Store	King of Power Saving	Quality is very good, customer service attitude is very very praise, there are problems in a timely manner to solve the problem, the price is not expensive, super value, you can rest assured that the	Jingdong prefecture level city in Shandong
Stowe Flagship Store	King of Power Saving	With it no longer need to be afraid to use electricity, especially good, ready to buy a mom and dad that also	Jingdong prefecture level city in Shandong
Stowe Flagship Store	King of Power Saving	Baby received very like will return to buy the seller attitude is also very like will return to buy the seller attitude is also very like!	Jingdong prefecture level city in Shandong
Stowe Flagship Store	King of Power Saving	Jingdong Mall shopping convenience assured fast assured fast assured fast assured fast	Jingdong prefecture level city in Shandong
Stowe Flagship Store	King of Power Saving	Saving power received, the quality is very good, fine workmanship, especially good, much cheaper than the entity store, it is worth buying!	Jingdong prefecture level city in Shandong
Stowe Flagship Store	King of Power Saving	I did not expect the express delivery so quickly, very save point quality is super good, than the entity store is too cheap.	Jingdong prefecture level city in Shandong
Stowe Flagship Store	King of Power Saving	Have received the baby, very satisfied with a shopping, great worth buying!	Jingdong prefecture level city in Shandong
Stowe Flagship Store	King of Power Saving	This is really more powerful, very satisfied, more energy-saving, better, thank you boss.	Jingdong prefecture level city in Shandong
Stowe Flagship Store	King of Power Saving	Received has been used for two days, the air conditioning can boldly open it! Energy saving effect is really quite good	Jingdong prefecture level city in Shandong
Stowe Flagship Store	King of Power Saving	It is authentic, trust the merchant, the baby has a great texture and the price is moderate, unlimited repurchase!	Jingdong prefecture level city in Shandong
Stowe Flagship Store	King of Power Saving	It is genuine very good, the price is much cheaper, cost-effective, really special cost-effective, it is worth buying!	Jingdong prefecture level city in Shandong
Stowe Flagship Store	King of Power Saving	Very good quality, fine workmanship, low power consumption, long warranty, fast shipping, economical and practical	Jingdong prefecture level city in Shandong
Stowe Flagship Store	King of Power Saving	I've been using it for half a month now, it really saves power and the voltage is stable, it's very good	Jingdong prefecture level city in Shandong
Stowe Flagship Store	King of Power Saving	It's the ideal product ♡, good quality and fast logistics, worth the kudos!	Jingdong prefecture level city in Shandong
Stowe Flagship Store	King of Power Saving	The logistics is very fast, the baby is very good, the shopkeeper is NICE!	Jingdong prefecture level city in Shandong
Stowe Flagship Store	King of Power Saving	Good to use, good quality, save point durable	Jingdong prefecture level city in Shandong
Stowe Flagship Store	King of Power Saving	The quality is very good, customer service attitude is very, very awesome, there are problems in a timely manner to solve the problem	Jingdong prefecture level city in Shandong
Stowe Flagship Store	King of Power Saving	Quality is very good, the use of the effect is very good, more stable, quality assurance	Jingdong prefecture level city in Shandong
Stowe Flagship Store	King of Power Saving	Convenient, inexpensive, and see how it works out?	Jingdong prefecture level city in Shandong
Stowe Flagship Store	King of Power Saving	Very good quality, saves a lot of electricity, very good, worth buying	Jingdong prefecture level city in Shandong
Stowe Flagship Store	King of Power Saving	Power Saving King is easy to use, with the fruit of power saving to use easy to give!	Jingdong prefecture level city in Shandong
Stowe Flagship Store	King of Power Saving	This saver really works, good quality, safe and secure, very satisfied!	Jingdong prefecture level city in Shandong
Stowe Flagship Store	King of Power Saving	It works well, the voltage is very stable effect is good, the quality is also very good satisfaction	Jingdong prefecture level city in Shandong
Stowe Flagship Store	King of Power Saving	The logistics is super super fast! The quality is also very good, indeed value for money	Jingdong prefecture level city in Shandong
Stowe Flagship Store	King of Power Saving	I've only been using it for a month or so and it seems to be saving a lot of power	Jingdong prefecture level city in Shandong
Stowe Flagship Store	King of Power Saving	Very easy to operate, very good material, good value for money.	Jingdong prefecture level city in Shandong
Stowe Flagship Store	King of Power Saving	Aluminum alloy shell, durable, compact power-saving good effect	Jingdong prefecture level city in Shandong
Stowe Flagship Store	King of Power Saving	Nice device, easy to use, doesn't take up much space, recommendable!	Jingdong prefecture level city in Shandong
Stowe Flagship Store	King of Power Saving	Good quality, good price, fast logistics	Jingdong prefecture level city in Shandong
Stowe Flagship Store	King of Power Saving	Friends of the family in use recommended, the effect is good and recommendable!	Jingdong prefecture level city in Shandong
Stowe Flagship Store	King of Power Saving	Cheaper than brick and mortar stores worth buying is authentic	Jingdong prefecture level city in Shandong
Stowe Flagship Store	King of Power Saving	Merchants ship quickly, the price is cheaper than the physical store, good use	Jingdong prefecture level city in Shandong
Stowe Flagship Store	King of Power Saving	Very durable, affordable, fast shipping	Jingdong prefecture level city in Shandong
Stowe Flagship Store	King of Power Saving	Packaging tight quality is very good easy to use good results	Jingdong prefecture level city in Shandong
Stowe Flagship Store	King of Power Saving	Save the grid. It's in the whole house. It's saving money on the electric bill.	Jingdong prefecture level city in Shandong
Stowe Flagship Store	King of Power Saving	I used it today, it works well, I'm very satisfied	Jingdong prefecture level city in Shandong
Stowe Flagship Store	King of Power Saving	It really saves a lot of electricity after installation	Jingdong prefecture level city in Shandong
Stowe Flagship Store	King of Power Saving	Love it. Satisfied.	Jingdong prefecture level city in Shandong
Stowe Flagship Store	King of Power Saving	Doesn't feel like it's working.	Jingdong prefecture level city in Shandong
Stowe Flagship Store	King of Power Saving	I'll comment after using it.	Jingdong prefecture level city in Shandong
Stowe Flagship Store	King of Power Saving	I'm trying it out to see if it saves power, and I'll buy two more if it works well.	Jingdong prefecture level city in Shandong
Stowe Flagship Store	King of Power Saving	It is genuine very good, the price is much cheaper, cost-effective, really special cost-effective, it is worth buying!	Jingdong prefecture level city in Shandong
Stowe Flagship Store	King of Power Saving	This thing can really save electricity, I was introduced to buy by my friend, he used it very well, I used it and think it's also very good, it can really save electricity, save about half of the electricity every day, it's really good!	Jingdong prefecture level city in Shandong
Stowe Flagship Store	King of Power Saving	After using it for ten days or so, I checked the power consumption and it did drop, and customer service said it will save more electricity in the long run. The product is also insured, 5 years warranty do not have to worry about quality problems!	Jingdong prefecture level city in Shandong
Stowe Flagship Store	King of Power Saving	The product is good, before already bought a, this is the second, the effect is quite good, anyway, I see the daily electricity bill than before a lot less, this can be the choice!	Jingdong prefecture level city in Shandong

Stowe Flagship Store	King of Power Saving	Originally also very worried about things are not good, get the hand found completely over-anxious, five-star praise, the style is very generous, the quality is very good, the price is also very reasonable!	Jingdong prefecture level city in Shandong
Stowe Flagship Store	King of Power Saving	Quality is good, playing to the old family also buy a, repurchase, this quality is really good, recommended!	Jingdong prefecture level city in Shandong
Stowe Flagship Store	King of Power Saving	Thank you, the owner even sent two fuses.	Jingdong prefecture level city in Shandong
Stowe Flagship Store	King of Power Saving	This store has great stuff, relatively fast shipping and logistics	Jingdong prefecture level city in Shandong
Stowe Flagship Store	King of Power Saving	This product is based on the impossibility of saving electricity, I used 853 kWh of electricity in July, started using the product on August 1, and used 867 kWh of electricity by September 1, is this called	Jingdong prefecture level city in Shandong
Stowe Flagship Store	King of Power Saving	Power king received, to the home on the trial, when I plugged into the electric panel can not see the effect of power saving, the meter is still the same speed in the turn, I'll save the king of the manual to play look at the instructions, instructions to speak, have to have a day two days and half a month to save power obviously, then wait half a month to see the effect of power saving it! To half a month there are obvious power saving before continuing to chase Xu.	Jingdong prefecture level city in Shandong
Stowe Flagship Store	King of Power Saving	Received the goods, a look, did not see the manufacturer and production address, can only hold a try to see the mentality of the face	Jingdong prefecture level city in Shandong
Stowe Flagship Store	King of Power Saving	I've only been using it for a few days and I'm not sure if it's an IQ tax? Used to favorable reviews.	Jingdong prefecture level city in Shandong
Stowe Flagship Store	King of Power Saving	Appearance is okay, power saving next time to follow up the evaluation	Jingdong prefecture level city in Shandong
Stowe Flagship Store	King of Power Saving	Shorter time, but hopefully effective and power-saving, to be followed up in the future	Jingdong prefecture level city in Shandong
Stowe Flagship Store	King of Power Saving	Appearance is okay, I do not know if the power saving	Jingdong prefecture level city in Shandong
Stowe Flagship Store	King of Power Saving	Good service, fast logistics, Power Saving King is exquisite and beautiful, support the business, thank you!	Jingdong prefecture level city in Shandong
Stowe Flagship Store	King of Power Saving	Bought three at once to try first first.	Jingdong prefecture level city in Shandong
Stowe Flagship Store	King of Power Saving	The object is the same as the picture, the product is of high quality, the packaging is intact. High-end atmosphere, high power, power saving effect is obvious, average monthly power saving 50%, very satisfied.	Jingdong prefecture level city in Shandong
Stowe Flagship Store	King of Power Saving	Bought back and plugged in, really effective, before 16 degrees of electricity per day, now 8 degrees of electricity per day, open an air conditioner for 12 hours, decisive repurchase of a	Jingdong prefecture level city in Shandong
Stowe Flagship Store	King of Power Saving	The object is the same as the picture, the product is of high quality, and the package is intact. High-end atmosphere and high grade, high power	Jingdong prefecture level city in Shandong
Stowe Flagship Store	King of Power Saving	Goods received, I hope the store said can be energy-saving, first try a month, month after month to see the effect.	Jingdong prefecture level city in Shandong
Stowe Flagship Store	King of Power Saving	This is really good, the material and workmanship is particularly exquisite, the workmanship is really very good, I think I can recommend to my friends to buy	Jingdong prefecture level city in Shandong
Stowe Flagship Store	King of Power Saving	I have to praise, the quality is really good, and the packaging logistics are also good. Really like it, won't make you regret after getting it!	Jingdong prefecture level city in Shandong
Stowe Flagship Store	King of Power Saving	Reputable platform, efficient and professional service, buy with confidence and peace of mind!	Jingdong prefecture level city in Shandong
Stowe Flagship Store	King of Power Saving	Bought one on a trial basis to see if it works after 1-2 months.	Jingdong prefecture level city in Shandong
Stowe Flagship Store	King of Power Saving	Quality is very good, customer service attitude is very very praise, there are problems in a timely manner to solve the problem, the price is not expensive, super value, you can rest assured that the	Jingdong prefecture level city in Shandong
Stowe Flagship Store	King of Power Saving	With it no longer need to be afraid to use electricity, especially good, ready to buy a mom and dad that also	Jingdong prefecture level city in Shandong
Stowe Flagship Store	King of Power Saving	Baby received very like will return to buy the seller attitude is also very like will return to buy the seller attitude is also very like!	Jingdong prefecture level city in Shandong
Stowe Flagship Store	King of Power Saving	Jingdong Mall shopping convenience assured fast assured fast assured fast assured fast	Jingdong prefecture level city in Shandong
Stowe Flagship Store	King of Power Saving	Saving power received, the quality is very good, fine workmanship, especially good, much cheaper than the entity store, it is worth buying!	Jingdong prefecture level city in Shandong
Stowe Flagship Store	King of Power Saving	I did not expect the express delivery so quickly, very save point quality is super good, than the entity store is too cheap.	Jingdong prefecture level city in Shandong
Stowe Flagship Store	King of Power Saving	Have received the baby, very satisfied with a shopping, great worth buying!	Jingdong prefecture level city in Shandong
Stowe Flagship Store	King of Power Saving	This is really more powerful, very satisfied, more energy-saving, better, thank you boss.	Jingdong prefecture level city in Shandong
Stowe Flagship Store	King of Power Saving	Received has been used for two days, the air conditioning can boldly open it! Energy saving effect is really quite good	Jingdong prefecture level city in Shandong
Stowe Flagship Store	King of Power Saving	It is authentic, trust the merchant, the baby has a great texture and the price is moderate, unlimited repurchase!	Jingdong prefecture level city in Shandong
Stowe Flagship Store	King of Power Saving	It is genuine very good, the price is much cheaper, cost-effective, really special cost-effective, it is worth buying!	Jingdong prefecture level city in Shandong
Stowe Flagship Store	King of Power Saving	Very good quality, fine workmanship, low power consumption, long warranty, fast shipping, economical and practical	Jingdong prefecture level city in Shandong
Stowe Flagship Store	King of Power Saving	I've been using it for half a month now, it really saves power and the voltage is stable, it's very good	Jingdong prefecture level city in Shandong
Stowe Flagship Store	King of Power Saving	It's the ideal product 💝, good quality and fast logistics, worth the kudos!	Jingdong prefecture level city in Shandong
Stowe Flagship Store	King of Power Saving	The logistics is very fast, the baby is very good, the shopkeeper is NICE!	Jingdong prefecture level city in Shandong
Stowe Flagship Store	King of Power Saving	Good to use, good quality, save point durable	Jingdong prefecture level city in Shandong
Stowe Flagship Store	King of Power Saving	The quality is very good, customer service attitude is very, very awesome, there are problems in a timely manner to solve the problem	Jingdong prefecture level city in Shandong
Stowe Flagship Store	King of Power Saving	Quality is very good, the use of the effect is very good, more stable, quality assurance	Jingdong prefecture level city in Shandong
Stowe Flagship Store	King of Power Saving	Convenient, inexpensive, and see how it works out?	Jingdong prefecture level city in Shandong
Stowe Flagship Store	King of Power Saving	Very good quality, saves a lot of electricity, very good, worth buying	Jingdong prefecture level city in Shandong
Stowe Flagship Store	King of Power Saving	Power Saving King is easy to use, with the fruit of power saving to use easy to give!	Jingdong prefecture level city in Shandong
Stowe Flagship Store	King of Power Saving	This saver really works, good quality, safe and secure, very satisfied!	Jingdong prefecture level city in Shandong
Stowe Flagship Store	King of Power Saving	It works well, the voltage is very stable effect is good, the quality is also very good satisfaction	Jingdong prefecture level city in Shandong
Stowe Flagship Store	King of Power Saving	The logistics is super super fast! The quality is also very good, indeed value for money	Jingdong prefecture level city in Shandong
Stowe Flagship Store	King of Power Saving	I've only been using it for a month or so and it seems to be saving a lot of power	Jingdong prefecture level city in Shandong
Stowe Flagship Store	King of Power Saving	Very easy to operate, very good material, good value for money.	Jingdong prefecture level city in Shandong
Stowe Flagship Store	King of Power Saving	Aluminum alloy shell, durable, compact power-saving good effect	Jingdong prefecture level city in Shandong
Stowe Flagship Store	King of Power Saving	Nice device, easy to use, doesn't take up much space, recommendable!	Jingdong prefecture level city in Shandong
Stowe Flagship Store	King of Power Saving	Good quality, good price, fast logistics	Jingdong prefecture level city in Shandong

Stowe Flagship Store	King of Power Saving	Friends of the family in use recommended, the effect is good and recommendable!	Jingdong prefecture level city in Shandong
Stowe Flagship Store	King of Power Saving	Cheaper than brick and mortar stores worth buying is authentic	Jingdong prefecture level city in Shandong
Stowe Flagship Store	King of Power Saving	Merchants ship quickly, the price is cheaper than the physical store, good use	Jingdong prefecture level city in Shandong
Stowe Flagship Store	King of Power Saving	Very durable, affordable, fast shipping	Jingdong prefecture level city in Shandong
Stowe Flagship Store	King of Power Saving	Packaging tight quality is very good easy to use good results	Jingdong prefecture level city in Shandong
Stowe Flagship Store	King of Power Saving	Save the grid. It's in the whole house. It's saving money on the electric bill.	Jingdong prefecture level city in Shandong
Stowe Flagship Store	King of Power Saving	I used it today, it works well, I'm very satisfied	Jingdong prefecture level city in Shandong
Stowe Flagship Store	King of Power Saving	It really saves a lot of electricity after installation	Jingdong prefecture level city in Shandong
Stowe Flagship Store	King of Power Saving	Love it. Satisfied.	Jingdong prefecture level city in Shandong
Stowe Flagship Store	King of Power Saving	Doesn't feel like it's working.	Jingdong prefecture level city in Shandong
Stowe Flagship Store	King of Power Saving	I'll comment after using it.	Jingdong prefecture level city in Shandong
Stowe Flagship Store	King of Power Saving	I'm trying it out to see if it saves power, and I'll buy two more if it works well.	Jingdong prefecture level city in Shandong
Stowe Flagship Store	King of Power Saving	It is genuine very good, the price is much cheaper, cost-effective, really special cost-effective, it is worth buying!	Jingdong prefecture level city in Shandong
Stowe Flagship Store	King of Power Saving	This thing can really save electricity, I was introduced to buy by my friend, he used it very well, I used it and think it's also very good, it can really save electricity, save about half of the electricity every day, it's really good!	Jingdong prefecture level city in Shandong
Stowe Flagship Store	King of Power Saving	After using it for ten days or so, I checked the power consumption and it did drop, and customer service said it will save more electricity in the long run. The product is also insured, 5 years warranty do not have to worry about quality problems!	Jingdong prefecture level city in Shandong
Stowe Flagship Store	King of Power Saving	The product is good, before already bought a, this is the second, the effect is quite good, anyway, I see the daily electricity bill than before a lot less, this can be the choice!	Jingdong prefecture level city in Shandong
Stowe Flagship Store	King of Power Saving	Originally also very worried about things are not good, get the hand found completely over-anxious, five-star praise, the style is very generous, the quality is very good, the price is also very reasonable!	Jingdong prefecture level city in Shandong
Stowe Flagship Store	King of Power Saving	Quality is good, playing to the old family also buy a, repurchase, this quality is really good, recommended!	Jingdong prefecture level city in Shandong
Stowe Flagship Store	King of Power Saving	Thank you, the owner even sent two fuses.	Jingdong prefecture level city in Shandong
Stowe Flagship Store	King of Power Saving	This store has great stuff, relatively fast shipping and logistics	Jingdong prefecture level city in Shandong
Stowe Flagship Store	King of Power Saving	This product is based on the impossibility of saving electricity, I used 853 kWh of electricity in July, started using the product on August 1, and used 867 kWh of electricity by September 1, is this called	Jingdong prefecture level city in Shandong
Stowe Flagship Store	King of Power Saving	Power king received, to the home on the trial, when I plugged into the electric panel can not see the effect of power saving, the meter is still the same speed in the turn, I'll save the king of the manual to play look at the instructions, instructions to speak, have to have a day two days and half a month to save power obviously, then wait half a month to see the effect of power saving it! To half a month there are obvious power saving before continuing to chase Xu.	Jingdong prefecture level city in Shandong
Stowe Flagship Store	King of Power Saving	Received the goods, a look, did not see the manufacturer and production address, can only hold a try to see the mentality of the face	Jingdong prefecture level city in Shandong
Stowe Flagship Store	King of Power Saving	I've only been using it for a few days and I'm not sure if it's an IQ tax? Used to favorable reviews.	Jingdong prefecture level city in Shandong
Stowe Flagship Store	King of Power Saving	Appearance is okay, power saving next time to follow up the evaluation	Jingdong prefecture level city in Shandong
Stowe Flagship Store	King of Power Saving	Shorter time, but hopefully effective and power-saving, to be followed up in the future	Jingdong prefecture level city in Shandong
Stowe Flagship Store	King of Power Saving	Appearance is okay, I do not know if the power saving	Jingdong prefecture level city in Shandong
Stowe Flagship Store	King of Power Saving	Good service, fast logistics, Power Saving King is exquisite and beautiful, support the business, thank you!	Jingdong prefecture level city in Shandong
Stowe Flagship Store	King of Power Saving	Bought three at once to try first first.	Jingdong prefecture level city in Shandong
Stowe Flagship Store	King of Power Saving	The object is the same as the picture, the product is of high quality, the packaging is intact. High-end atmosphere, high power, power saving effect is obvious, average monthly power saving 50%, very satisfied.	Jingdong prefecture level city in Shandong
Stowe Flagship Store	King of Power Saving	Bought back and plugged in, really effective, before 16 degrees of electricity per day, now 8 degrees of electricity per day, open an air conditioner for 12 hours, decisive repurchase of a	Jingdong prefecture level city in Shandong
Stowe Flagship Store	King of Power Saving	The object is the same as the picture, the product is of high quality, and the package is intact. High-end atmosphere and high grade, high power	Jingdong prefecture level city in Shandong
Stowe Flagship Store	King of Power Saving	Goods received, I hope the store said can be energy-saving, first try a month, month after month to see the effect.	Jingdong prefecture level city in Shandong
Stowe Flagship Store	King of Power Saving	This is really good, the material and workmanship is particularly exquisite, the workmanship is really very good, I think I can recommend to my friends to buy	Jingdong prefecture level city in Shandong
Stowe Flagship Store	King of Power Saving	I have to praise, the quality is really good, and the packaging logistics are also good. Really like it, won't make you regret after getting it!	Jingdong prefecture level city in Shandong
Stowe Flagship Store	King of Power Saving	Reputable platform, efficient and professional service, buy with confidence and peace of mind!	Jingdong prefecture level city in Shandong
Stowe Flagship Store	King of Power Saving	Bought one on a trial basis to see if it works after 1-2 months.	Jingdong prefecture level city in Shandong
Stowe Flagship Store	King of Power Saving	Quality is very good, customer service attitude is very very praise, there are problems in a timely manner to solve the problem, the price is not expensive, super value, you can rest assured that the	Jingdong prefecture level city in Shandong
Stowe Flagship Store	King of Power Saving	With it no longer need to be afraid to use electricity, especially good, ready to buy a mom and dad that also	Jingdong prefecture level city in Shandong
Stowe Flagship Store	King of Power Saving	Baby received very like will return to buy the seller attitude is also very like will return to buy the seller attitude is also very like!	Jingdong prefecture level city in Shandong
Stowe Flagship Store	King of Power Saving	Jingdong Mall shopping convenience assured fast assured fast assured fast assured fast	Jingdong prefecture level city in Shandong
Stowe Flagship Store	King of Power Saving	Saving power received, the quality is very good, fine workmanship, especially good, much cheaper than the entity store, it is worth buying!	Jingdong prefecture level city in Shandong
Stowe Flagship Store	King of Power Saving	I did not expect the express delivery so quickly, very save point quality is super good, than the entity store is too cheap.	Jingdong prefecture level city in Shandong
Stowe Flagship Store	King of Power Saving	Have received the baby, very satisfied with a shopping, great worth buying!	Jingdong prefecture level city in Shandong
Stowe Flagship Store	King of Power Saving	This is really more powerful, very satisfied, more energy-saving, better, thank you boss.	Jingdong prefecture level city in Shandong
Stowe Flagship Store	King of Power Saving	Received has been used for two days, the air conditioning can boldly open it! Energy saving effect is really quite good	Jingdong prefecture level city in Shandong
Stowe Flagship Store	King of Power Saving	It is authentic, trust the merchant, the baby has a great texture and the price is moderate, unlimited repurchase!	Jingdong prefecture level city in Shandong
Stowe Flagship Store	King of Power Saving	It is genuine very good, the price is much cheaper, cost-effective, really special cost-effective, it is worth buying!	Jingdong prefecture level city in Shandong
Stowe Flagship Store	King of Power Saving	Very good quality, fine workmanship, low power consumption, long warranty, fast shipping, economical and practical	Jingdong prefecture level city in Shandong

Stowe Flagship Store	King of Power Saving	I've been using it for half a month now, it really saves power and the voltage is stable, it's very good	Jingdong prefecture level city in Shandong
Stowe Flagship Store	King of Power Saving	It's the ideal product 💝, good quality and fast logistics, worth the kudos!	Jingdong prefecture level city in Shandong
Stowe Flagship Store	King of Power Saving	The logistics is very fast, the baby is very good, the shopkeeper is NICE!	Jingdong prefecture level city in Shandong
Stowe Flagship Store	King of Power Saving	Good to use, good quality, save point durable	Jingdong prefecture level city in Shandong
Stowe Flagship Store	King of Power Saving	The quality is very good, customer service attitude is very, very awesome, there are problems in a timely manner to solve the problem	Jingdong prefecture level city in Shandong
Stowe Flagship Store	King of Power Saving	Quality is very good, the use of the effect is very good, more stable, quality assurance	Jingdong prefecture level city in Shandong
Stowe Flagship Store	King of Power Saving	Convenient, inexpensive, and see how it works out?	Jingdong prefecture level city in Shandong
Stowe Flagship Store	King of Power Saving	Very good quality, saves a lot of electricity, very good, worth buying	Jingdong prefecture level city in Shandong
Stowe Flagship Store	King of Power Saving	Power Saving King is easy to use, with the fruit of power saving to use easy to give!	Jingdong prefecture level city in Shandong
Stowe Flagship Store	King of Power Saving	This saver really works, good quality, safe and secure, very satisfied!	Jingdong prefecture level city in Shandong
Stowe Flagship Store	King of Power Saving	It works well, the voltage is very stable effect is good, the quality is also very good satisfaction	Jingdong prefecture level city in Shandong
Stowe Flagship Store	King of Power Saving	The logistics is super super fast! The quality is also very good, indeed value for money	Jingdong prefecture level city in Shandong
Stowe Flagship Store	King of Power Saving	I've only been using it for a month or so and it seems to be saving a lot of power	Jingdong prefecture level city in Shandong
Stowe Flagship Store	King of Power Saving	Very easy to operate, very good material, good value for money.	Jingdong prefecture level city in Shandong
Stowe Flagship Store	King of Power Saving	Aluminum alloy shell, durable, compact power-saving good effect	Jingdong prefecture level city in Shandong
Stowe Flagship Store	King of Power Saving	Nice device, easy to use, doesn't take up much space, recommendable!	Jingdong prefecture level city in Shandong
Stowe Flagship Store	King of Power Saving	Good quality, good price, fast logistics	Jingdong prefecture level city in Shandong
Stowe Flagship Store	King of Power Saving	Friends of the family in use recommended, the effect is good and recommendable!	Jingdong prefecture level city in Shandong
Stowe Flagship Store	King of Power Saving	Cheaper than brick and mortar stores worth buying is authentic	Jingdong prefecture level city in Shandong
Stowe Flagship Store	King of Power Saving	Merchants ship quickly, the price is cheaper than the physical store, good use	Jingdong prefecture level city in Shandong
Stowe Flagship Store	King of Power Saving	Very durable, affordable, fast shipping	Jingdong prefecture level city in Shandong
Stowe Flagship Store	King of Power Saving	Packaging tight quality is very good easy to use good results	Jingdong prefecture level city in Shandong
Stowe Flagship Store	King of Power Saving	Save the grid. It's in the whole house. It's saving money on the electric bill.	Jingdong prefecture level city in Shandong
Stowe Flagship Store	King of Power Saving	I used it today, it works well, I'm very satisfied	Jingdong prefecture level city in Shandong
Stowe Flagship Store	King of Power Saving	It really saves a lot of electricity after installation	Jingdong prefecture level city in Shandong
Stowe Flagship Store	King of Power Saving	Love it. Satisfied.	Jingdong prefecture level city in Shandong
Stowe Flagship Store	King of Power Saving	Doesn't feel like it's working.	Jingdong prefecture level city in Shandong
Stowe Flagship Store	King of Power Saving	I'll comment after using it.	Jingdong prefecture level city in Shandong
Stowe Flagship Store	King of Power Saving	I'm trying it out to see if it saves power, and I'll buy two more if it works well.	Jingdong prefecture level city in Shandong
Stowe Flagship Store	King of Power Saving	It is genuine very good, the price is much cheaper, cost-effective, really special cost-effective, it is worth buying!	Jingdong prefecture level city in Shandong
Stowe Flagship Store	King of Power Saving	This thing can really save electricity, I was introduced to buy by my friend, he used it very well, I used it and think it's also very good, it can really save electricity, save about half of the electricity every day, it's really good!	Jingdong prefecture level city in Shandong
Stowe Flagship Store	King of Power Saving	After using it for ten days or so, I checked the power consumption and it did drop, and customer service said it will save more electricity in the long run. The product is also insured, 5 years warranty do not have to worry about quality problems!	Jingdong prefecture level city in Shandong
Stowe Flagship Store	King of Power Saving	The product is good, before already bought a, this is the second, the effect is quite good, anyway, I see the daily electricity bill than before a lot less, this can be the choice!	Jingdong prefecture level city in Shandong
Stowe Flagship Store	King of Power Saving	Originally also very worried about things are not good, get the hand found completely over-anxious, five-star praise, the style is very generous, the quality is very good, the price is also very reasonable!	Jingdong prefecture level city in Shandong
Stowe Flagship Store	King of Power Saving	Quality is good, playing to the old family also buy a, repurchase, this quality is really good, recommended!	Jingdong prefecture level city in Shandong
Stowe Flagship Store	King of Power Saving	Thank you, the owner even sent two fuses.	Jingdong prefecture level city in Shandong
Stowe Flagship Store	King of Power Saving	This store has great stuff, relatively fast shipping and logistics	Jingdong prefecture level city in Shandong
Stowe Flagship Store	King of Power Saving	This product is based on the impossibility of saving electricity, I used 853 kWh of electricity in July, started using the product on August 1, and used 867 kWh of electricity by September 1, is this called	Jingdong prefecture level city in Shandong
Stowe Flagship Store	King of Power Saving	Power king received, to the home on the trial, when I plugged into the electric panel can not see the effect of power saving, the meter is still the same speed in the turn, I'll save the king of the manual to play look at the instructions, instructions to speak, have to have a day two days and half a month to save power obviously, then wait half a month to see the effect of power saving it! To half a month there are obvious power saving before continuing to chase Xu.	Jingdong prefecture level city in Shandong
Stowe Flagship Store	King of Power Saving	Received the goods, a look, did not see the manufacturer and production address, can only hold a try to see the mentality of the face	Jingdong prefecture level city in Shandong
Stowe Flagship Store	King of Power Saving	I've only been using it for a few days and I'm not sure if it's an IQ tax? Used to favorable reviews.	Jingdong prefecture level city in Shandong
Stowe Flagship Store	King of Power Saving	Appearance is okay, power saving next time to follow up the evaluation	Jingdong prefecture level city in Shandong
Stowe Flagship Store	King of Power Saving	Shorter time, but hopefully effective and power-saving, to be followed up in the future	Jingdong prefecture level city in Shandong
Stowe Flagship Store	King of Power Saving	Appearance is okay, I do not know if the power saving	Jingdong prefecture level city in Shandong
Stowe Flagship Store	King of Power Saving	Good service, fast logistics, Power Saving King is exquisite and beautiful, support the business, thank you!	Jingdong prefecture level city in Shandong
Stowe Flagship Store	King of Power Saving	Bought three at once to try first first.	Jingdong prefecture level city in Shandong
Stowe Flagship Store	King of Power Saving	The object is the same as the picture, the product is of high quality, the packaging is intact. High-end atmosphere, high power, power saving effect is obvious, average monthly power saving 50%, very satisfied.	Jingdong prefecture level city in Shandong
Stowe Flagship Store	King of Power Saving	Bought back and plugged in, really effective, before 16 degrees of electricity per day, now 8 degrees of electricity per day, open an air conditioner for 12 hours, decisive repurchase of a	Jingdong prefecture level city in Shandong
Stowe Flagship Store	King of Power Saving	The object is the same as the picture, the product is of high quality, and the package is intact. High-end atmosphere and high grade, high power	Jingdong prefecture level city in Shandong

Stowe Flagship Store	King of Power Saving	Goods received, I hope the store said can be energy-saving, first try a month, month after month to see the effect.	Jingdong prefecture level city in Shandong
Stowe Flagship Store	King of Power Saving	This is really good, the material and workmanship is particularly exquisite, the workmanship is really very good, I think I can recommend to my friends to buy	Jingdong prefecture level city in Shandong
Stowe Flagship Store	King of Power Saving	I have to praise, the quality is really good, and the packaging logistics are also good. Really like it, won't make you regret after getting it!	Jingdong prefecture level city in Shandong
Stowe Flagship Store	King of Power Saving	Reputable platform, efficient and professional service, buy with confidence and peace of mind!	Jingdong prefecture level city in Shandong
Stowe Flagship Store	King of Power Saving	Bought one on a trial basis to see if it works after 1-2 months.	Jingdong prefecture level city in Shandong
Stowe Flagship Store	King of Power Saving	Quality is very good, customer service attitude is very very praise, there are problems in a timely manner to solve the problem, the price is not expensive, super value, you can rest assured that the	Jingdong prefecture level city in Shandong
Stowe Flagship Store	King of Power Saving	With it no longer need to be afraid to use electricity, especially good, ready to buy a mom and dad that also	Jingdong prefecture level city in Shandong
Stowe Flagship Store	King of Power Saving	Baby received very like will return to buy the seller attitude is also very like will return to buy the seller attitude is also very like!	Jingdong prefecture level city in Shandong
Stowe Flagship Store	King of Power Saving	Jingdong Mall shopping convenience assured fast assured fast assured fast assured fast	Jingdong prefecture level city in Shandong
Stowe Flagship Store	King of Power Saving	Saving power received, the quality is very good, fine workmanship, especially good, much cheaper than the entity store, it is worth buying!	Jingdong prefecture level city in Shandong
Stowe Flagship Store	King of Power Saving	I did not expect the express delivery so quickly, very save point quality is super good, than the entity store is too cheap.	Jingdong prefecture level city in Shandong
Stowe Flagship Store	King of Power Saving	Have received the baby, very satisfied with a shopping, great worth buying!	Jingdong prefecture level city in Shandong
Stowe Flagship Store	King of Power Saving	This is really more powerful, very satisfied, more energy-saving, better, thank you boss.	Jingdong prefecture level city in Shandong
Stowe Flagship Store	King of Power Saving	Received has been used for two days, the air conditioning can boldly open it! Energy saving effect is really quite good	Jingdong prefecture level city in Shandong
Stowe Flagship Store	King of Power Saving	It is authentic, trust the merchant, the baby has a great texture and the price is moderate, unlimited repurchase!	Jingdong prefecture level city in Shandong
Stowe Flagship Store	King of Power Saving	It is genuine very good, the price is much cheaper, cost-effective, really special cost-effective, it is worth buying!	Jingdong prefecture level city in Shandong
Stowe Flagship Store	King of Power Saving	Very good quality, fine workmanship, low power consumption, long warranty, fast shipping, economical and practical	Jingdong prefecture level city in Shandong
Stowe Flagship Store	King of Power Saving	I've been using it for half a month now, it really saves power and the voltage is stable, it's very good	Jingdong prefecture level city in Shandong
Stowe Flagship Store	King of Power Saving	It's the ideal product 💝, good quality and fast logistics, worth the kudos!	Jingdong prefecture level city in Shandong
Stowe Flagship Store	King of Power Saving	The logistics is very fast, the baby is very good, the shopkeeper is NICE!	Jingdong prefecture level city in Shandong
Stowe Flagship Store	King of Power Saving	Good to use, good quality, save point durable	Jingdong prefecture level city in Shandong
Stowe Flagship Store	King of Power Saving	The quality is very good, customer service attitude is very, very awesome, there are problems in a timely manner to solve the problem	Jingdong prefecture level city in Shandong
Stowe Flagship Store	King of Power Saving	Quality is very good, the use of the effect is very good, more stable, quality assurance	Jingdong prefecture level city in Shandong
Stowe Flagship Store	King of Power Saving	Convenient, inexpensive, and see how it works out?	Jingdong prefecture level city in Shandong
Stowe Flagship Store	King of Power Saving	Very good quality, saves a lot of electricity, very good, worth buying	Jingdong prefecture level city in Shandong
Stowe Flagship Store	King of Power Saving	Power Saving King is easy to use, with the fruit of power saving to use easy to give!	Jingdong prefecture level city in Shandong
Stowe Flagship Store	King of Power Saving	This saver really works, good quality, safe and secure, very satisfied!	Jingdong prefecture level city in Shandong
Stowe Flagship Store	King of Power Saving	It works well, the voltage is very stable effect is good, the quality is also very good satisfaction	Jingdong prefecture level city in Shandong
Stowe Flagship Store	King of Power Saving	The logistics is super super fast! The quality is also very good, indeed value for money	Jingdong prefecture level city in Shandong
Stowe Flagship Store	King of Power Saving	I've only been using it for a month or so and it seems to be saving a lot of power	Jingdong prefecture level city in Shandong
Stowe Flagship Store	King of Power Saving	Very easy to operate, very good material, good value for money.	Jingdong prefecture level city in Shandong
Stowe Flagship Store	King of Power Saving	Aluminum alloy shell, durable, compact power-saving good effect	Jingdong prefecture level city in Shandong
Stowe Flagship Store	King of Power Saving	Nice device, easy to use, doesn't take up much space, recommendable!	Jingdong prefecture level city in Shandong
Stowe Flagship Store	King of Power Saving	Good quality, good price, fast logistics	Jingdong prefecture level city in Shandong
Stowe Flagship Store	King of Power Saving	Friends of the family in use recommended, the effect is good and recommendable!	Jingdong prefecture level city in Shandong
Stowe Flagship Store	King of Power Saving	Cheaper than brick and mortar stores worth buying is authentic	Jingdong prefecture level city in Shandong
Stowe Flagship Store	King of Power Saving	Merchants ship quickly, the price is cheaper than the physical store, good use	Jingdong prefecture level city in Shandong
Stowe Flagship Store	King of Power Saving	Very durable, affordable, fast shipping	Jingdong prefecture level city in Shandong
Stowe Flagship Store	King of Power Saving	Packaging tight quality is very good easy to use good results	Jingdong prefecture level city in Shandong
Stowe Flagship Store	King of Power Saving	Save the grid. It's in the whole house. It's saving money on the electric bill.	Jingdong prefecture level city in Shandong
Stowe Flagship Store	King of Power Saving	I used it today, it works well, I'm very satisfied	Jingdong prefecture level city in Shandong
Stowe Flagship Store	King of Power Saving	It really saves a lot of electricity after installation	Jingdong prefecture level city in Shandong
Stowe Flagship Store	King of Power Saving	Love it. Satisfied.	Jingdong prefecture level city in Shandong
Stowe Flagship Store	King of Power Saving	Doesn't feel like it's working.	Jingdong prefecture level city in Shandong
Stowe Flagship Store	King of Power Saving	I'll comment after using it.	Jingdong prefecture level city in Shandong
Stowe Flagship Store	King of Power Saving	I'm trying it out to see if it saves power, and I'll buy two more if it works well.	Jingdong prefecture level city in Shandong
Stowe Flagship Store	King of Power Saving	It is genuine very good, the price is much cheaper, cost-effective, really special cost-effective, it is worth buying!	Jingdong prefecture level city in Shandong
Stowe Flagship Store	King of Power Saving	This thing can really save electricity, I was introduced to buy by my friend, he used it very well, I used it and think it's also very good, it can really save electricity, save about half of the electricity every day, it's really good!	Jingdong prefecture level city in Shandong
Stowe Flagship Store	King of Power Saving	After using it for ten days or so, I checked the power consumption and it did drop, and customer service said it will save more electricity in the long run. The product is also insured, 5 years warranty do not have to worry about quality problems!	Jingdong prefecture level city in Shandong
Stowe Flagship Store	King of Power Saving	The product is good, before already bought a, this is the second, the effect is quite good, anyway, I see the daily electricity bill than before a lot less, this can be the choice!	Jingdong prefecture level city in Shandong

Stowe Flagship Store	King of Power Saving	Originally also very worried about things are not good, get the hand found completely over-anxious, five-star praise, the style is very generous, the quality is very good, the price is also very reasonable!	Jingdong prefecture level city in Shandong
Stowe Flagship Store	King of Power Saving	Quality is good, playing to the old family also buy a, repurchase, this quality is really good, recommended!	Jingdong prefecture level city in Shandong
Stowe Flagship Store	King of Power Saving	Thank you, the owner even sent two fuses.	Jingdong prefecture level city in Shandong
Stowe Flagship Store	King of Power Saving	This store has great stuff, relatively fast shipping and logistics	Jingdong prefecture level city in Shandong
Stowe Flagship Store	King of Power Saving	This product is based on the impossibility of saving electricity, I used 853 kWh of electricity in July, started using the product on August 1, and used 867 kWh of electricity by September 1, is this called	Jingdong prefecture level city in Shandong
Stowe Flagship Store	King of Power Saving	Power king received, to the home on the trial, when I plugged into the electric panel can not see the effect of power saving, the meter is still the same speed in the turn, I'll save the king of the manual to play look at the instructions, instructions to speak, have to have a day two days and half a month to save power obviously, then wait half a month to see the effect of power saving it! To half a month there are obvious power saving before continuing to chase Xu.	Jingdong prefecture level city in Shandong
Stowe Flagship Store	King of Power Saving	Received the goods, a look, did not see the manufacturer and production address, can only hold a try to see the mentality of the face	Jingdong prefecture level city in Shandong
Stowe Flagship Store	King of Power Saving	I've only been using it for a few days and I'm not sure if it's an IQ tax? Used to favorable reviews.	Jingdong prefecture level city in Shandong
Stowe Flagship Store	King of Power Saving	Appearance is okay, power saving next time to follow up the evaluation	Jingdong prefecture level city in Shandong
Stowe Flagship Store	King of Power Saving	Shorter time, but hopefully effective and power-saving, to be followed up in the future	Jingdong prefecture level city in Shandong
Stowe Flagship Store	King of Power Saving	Appearance is okay, I do not know if the power saving	Jingdong prefecture level city in Shandong
Stowe Flagship Store	King of Power Saving	Good service, fast logistics, Power Saving King is exquisite and beautiful, support the business, thank you!	Jingdong prefecture level city in Shandong
Stowe Flagship Store	King of Power Saving	Bought three at once to try first first.	Jingdong prefecture level city in Shandong
Stowe Flagship Store	King of Power Saving	The object is the same as the picture, the product is of high quality, the packaging is intact. High-end atmosphere, high power, power saving effect is obvious, average monthly power saving 50%, very satisfied.	Jingdong prefecture level city in Shandong
Stowe Flagship Store	King of Power Saving	Bought back and plugged in, really effective, before 16 degrees of electricity per day, now 8 degrees of electricity per day, open an air conditioner for 12 hours, decisive repurchase of a	Jingdong prefecture level city in Shandong
Stowe Flagship Store	King of Power Saving	The object is the same as the picture, the product is of high quality, and the package is intact. High-end atmosphere and high grade, high power	Jingdong prefecture level city in Shandong
Stowe Flagship Store	King of Power Saving	Goods received, I hope the store said can be energy-saving, first try a month, month after month to see the effect.	Jingdong prefecture level city in Shandong
Stowe Flagship Store	King of Power Saving	This is really good, the material and workmanship is particularly exquisite, the workmanship is really very good, I think I can recommend to my friends to buy	Jingdong prefecture level city in Shandong
Stowe Flagship Store	King of Power Saving	I have to praise, the quality is really good, and the packaging logistics are also good. Really like it, won't make you regret after getting it!	Jingdong prefecture level city in Shandong
Stowe Flagship Store	King of Power Saving	Reputable platform, efficient and professional service, buy with confidence and peace of mind!	Jingdong prefecture level city in Shandong
Stowe Flagship Store	King of Power Saving	Bought one on a trial basis to see if it works after 1-2 months.	Jingdong prefecture level city in Shandong
Stowe Flagship Store	King of Power Saving	Quality is very good, customer service attitude is very very praise, there are problems in a timely manner to solve the problem, the price is not expensive, super value, you can rest assured that the	Jingdong prefecture level city in Shandong
Stowe Flagship Store	King of Power Saving	With it no longer need to be afraid to use electricity, especially good, ready to buy a mom and dad that also	Jingdong prefecture level city in Shandong
Stowe Flagship Store	King of Power Saving	Baby received very like will return to buy the seller attitude is also very like will return to buy the seller attitude is also very like!	Jingdong prefecture level city in Shandong
Stowe Flagship Store	King of Power Saving	Jingdong Mall shopping convenience assured fast assured fast assured fast assured fast	Jingdong prefecture level city in Shandong
Stowe Flagship Store	King of Power Saving	Saving power received, the quality is very good, fine workmanship, especially good, much cheaper than the entity store, it is worth buying!	Jingdong prefecture level city in Shandong
Stowe Flagship Store	King of Power Saving	I did not expect the express delivery so quickly, very save point quality is super good, than the entity store is too cheap.	Jingdong prefecture level city in Shandong
Stowe Flagship Store	King of Power Saving	Have received the baby, very satisfied with a shopping, great worth buying!	Jingdong prefecture level city in Shandong
Stowe Flagship Store	King of Power Saving	This is really more powerful, very satisfied, more energy-saving, better, thank you boss.	Jingdong prefecture level city in Shandong
Stowe Flagship Store	King of Power Saving	Received has been used for two days, the air conditioning can boldly open it! Energy saving effect is really quite good	Jingdong prefecture level city in Shandong
Stowe Flagship Store	King of Power Saving	It is authentic, trust the merchant, the baby has a great texture and the price is moderate, unlimited repurchase!	Jingdong prefecture level city in Shandong
Stowe Flagship Store	King of Power Saving	It is genuine very good, the price is much cheaper, cost-effective, really special cost-effective, it is worth buying!	Jingdong prefecture level city in Shandong
Stowe Flagship Store	King of Power Saving	Very good quality, fine workmanship, low power consumption, long warranty, fast shipping, economical and practical	Jingdong prefecture level city in Shandong
Stowe Flagship Store	King of Power Saving	I've been using it for half a month now, it really saves power and the voltage is stable, it's very good	Jingdong prefecture level city in Shandong
Stowe Flagship Store	King of Power Saving	It's the ideal product 💝, good quality and fast logistics, worth the kudos!	Jingdong prefecture level city in Shandong
Stowe Flagship Store	King of Power Saving	The logistics is very fast, the baby is very good, the shopkeeper is NICE!	Jingdong prefecture level city in Shandong
Stowe Flagship Store	King of Power Saving	Good to use, good quality, save point durable	Jingdong prefecture level city in Shandong
Stowe Flagship Store	King of Power Saving	The quality is very good, customer service attitude is very, very awesome, there are problems in a timely manner to solve the problem	Jingdong prefecture level city in Shandong
Stowe Flagship Store	King of Power Saving	Quality is very good, the use of the effect is very good, more stable, quality assurance	Jingdong prefecture level city in Shandong
Stowe Flagship Store	King of Power Saving	Convenient, inexpensive, and see how it works out?	Jingdong prefecture level city in Shandong
Stowe Flagship Store	King of Power Saving	Very good quality, saves a lot of electricity, very good, worth buying	Jingdong prefecture level city in Shandong
Stowe Flagship Store	King of Power Saving	Power Saving King is easy to use, with the fruit of power saving to use easy to give!	Jingdong prefecture level city in Shandong
Stowe Flagship Store	King of Power Saving	This saver really works, good quality, safe and secure, very satisfied!	Jingdong prefecture level city in Shandong
Stowe Flagship Store	King of Power Saving	It works well, the voltage is very stable effect is good, the quality is also very good satisfaction	Jingdong prefecture level city in Shandong
Stowe Flagship Store	King of Power Saving	The logistics is super super fast! The quality is also very good, indeed value for money	Jingdong prefecture level city in Shandong
Stowe Flagship Store	King of Power Saving	I've only been using it for a month or so and it seems to be saving a lot of power	Jingdong prefecture level city in Shandong
Stowe Flagship Store	King of Power Saving	Very easy to operate, very good material, good value for money.	Jingdong prefecture level city in Shandong
Stowe Flagship Store	King of Power Saving	Aluminum alloy shell, durable, compact power-saving good effect	Jingdong prefecture level city in Shandong
Stowe Flagship Store	King of Power Saving	Nice device, easy to use, doesn't take up much space, recommendable!	Jingdong prefecture level city in Shandong
Stowe Flagship Store	King of Power Saving	Good quality, good price, fast logistics	Jingdong prefecture level city in Shandong

Stowe Flagship Store	King of Power Saving	Friends of the family in use recommended, the effect is good and recommendable!	Jingdong prefecture level city in Shandong
Stowe Flagship Store	King of Power Saving	Cheaper than brick and mortar stores worth buying is authentic	Jingdong prefecture level city in Shandong
Stowe Flagship Store	King of Power Saving	Merchants ship quickly, the price is cheaper than the physical store, good use	Jingdong prefecture level city in Shandong
Stowe Flagship Store	King of Power Saving	Very durable, affordable, fast shipping	Jingdong prefecture level city in Shandong
Stowe Flagship Store	King of Power Saving	Packaging tight quality is very good easy to use good results	Jingdong prefecture level city in Shandong
Stowe Flagship Store	King of Power Saving	Save the grid. It's in the whole house. It's saving money on the electric bill.	Jingdong prefecture level city in Shandong
Stowe Flagship Store	King of Power Saving	I used it today, it works well, I'm very satisfied	Jingdong prefecture level city in Shandong
Stowe Flagship Store	King of Power Saving	It really saves a lot of electricity after installation	Jingdong prefecture level city in Shandong
Stowe Flagship Store	King of Power Saving	Love it. Satisfied.	Jingdong prefecture level city in Shandong
Stowe Flagship Store	King of Power Saving	Doesn't feel like it's working.	Jingdong prefecture level city in Shandong
Stowe Flagship Store	King of Power Saving	I'll comment after using it.	Jingdong prefecture level city in Shandong
Stowe Flagship Store	King of Power Saving	I'm trying it out to see if it saves power, and I'll buy two more if it works well.	Jingdong prefecture level city in Shandong
Stowe Flagship Store	King of Power Saving	It is genuine very good, the price is much cheaper, cost-effective, really special cost-effective, it is worth buying!	Jingdong prefecture level city in Shandong
Stowe Flagship Store	King of Power Saving	This thing can really save electricity, I was introduced to buy by my friend, he used it very well, I used it and think it's also very good, it can really save electricity, save about half of the electricity every day, it's really good!	Jingdong prefecture level city in Shandong
Stowe Flagship Store	King of Power Saving	After using it for ten days or so, I checked the power consumption and it did drop, and customer service said it will save more electricity in the long run. The product is also insured, 5 years warranty do not have to worry about quality problems!	Jingdong prefecture level city in Shandong
Stowe Flagship Store	King of Power Saving	The product is good, before already bought a, this is the second, the effect is quite good, anyway, I see the daily electricity bill than before a lot less, this can be the choice!	Jingdong prefecture level city in Shandong
Stowe Flagship Store	King of Power Saving	Originally also very worried about things are not good, get the hand found completely over-anxious, five-star praise, the style is very generous, the quality is very good, the price is also very reasonable!	Jingdong prefecture level city in Shandong
Stowe Flagship Store	King of Power Saving	Quality is good, playing to the old family also buy a, repurchase, this quality is really good, recommended!	Jingdong prefecture level city in Shandong
Stowe Flagship Store	King of Power Saving	Thank you, the owner even sent two fuses.	Jingdong prefecture level city in Shandong
Stowe Flagship Store	King of Power Saving	This store has great stuff, relatively fast shipping and logistics	Jingdong prefecture level city in Shandong
Stowe Flagship Store	King of Power Saving	This product is based on the impossibility of saving electricity, I used 853 kWh of electricity in July, started using the product on August 1, and used 867 kWh of electricity by September 1, is this called	Jingdong prefecture level city in Shandong
Stowe Flagship Store	King of Power Saving	Power king received, to the home on the trial, when I plugged into the electric panel can not see the effect of power saving, the meter is still the same speed in the turn, I'll save the king of the manual to play look at the instructions, instructions to speak, have to have a day two days and half a month to save power obviously, then wait half a month to see the effect of power saving it! To half a month there are obvious power saving before continuing to chase Xu.	Jingdong prefecture level city in Shandong
Stowe Flagship Store	King of Power Saving	Received the goods, a look, did not see the manufacturer and production address, can only hold a try to see the mentality of the face	Jingdong prefecture level city in Shandong
Stowe Flagship Store	King of Power Saving	I've only been using it for a few days and I'm not sure if it's an IQ tax? Used to favorable reviews.	Jingdong prefecture level city in Shandong
Stowe Flagship Store	King of Power Saving	Appearance is okay, power saving next time to follow up the evaluation	Jingdong prefecture level city in Shandong
Stowe Flagship Store	King of Power Saving	Shorter time, but hopefully effective and power-saving, to be followed up in the future	Jingdong prefecture level city in Shandong
Stowe Flagship Store	King of Power Saving	Appearance is okay, I do not know if the power saving	Jingdong prefecture level city in Shandong
Stowe Flagship Store	King of Power Saving	Good service, fast logistics, Power Saving King is exquisite and beautiful, support the business, thank you!	Jingdong prefecture level city in Shandong
Stowe Flagship Store	King of Power Saving	Bought three at once to try first first.	Jingdong prefecture level city in Shandong
Stowe Flagship Store	King of Power Saving	The object is the same as the picture, the product is of high quality, the packaging is intact. High-end atmosphere, high power, power saving effect is obvious, average monthly power saving 50%, very satisfied.	Jingdong prefecture level city in Shandong
Stowe Flagship Store	King of Power Saving	Bought back and plugged in, really effective, before 16 degrees of electricity per day, now 8 degrees of electricity per day, open an air conditioner for 12 hours, decisive repurchase of a	Jingdong prefecture level city in Shandong
Stowe Flagship Store	King of Power Saving	The object is the same as the picture, the product is of high quality, and the package is intact. High-end atmosphere and high grade, high power	Jingdong prefecture level city in Shandong
Stowe Flagship Store	King of Power Saving	Goods received, I hope the store said can be energy-saving, first try a month, month after month to see the effect.	Jingdong prefecture level city in Shandong
Stowe Flagship Store	King of Power Saving	This is really good, the material and workmanship is particularly exquisite, the workmanship is really very good, I think I can recommend to my friends to buy	Jingdong prefecture level city in Shandong
Stowe Flagship Store	King of Power Saving	I have to praise, the quality is really good, and the packaging logistics are also good. Really like it, won't make you regret after getting it!	Jingdong prefecture level city in Shandong
Stowe Flagship Store	King of Power Saving	Reputable platform, efficient and professional service, buy with confidence and peace of mind!	Jingdong prefecture level city in Shandong
Stowe Flagship Store	King of Power Saving	Bought one on a trial basis to see if it works after 1-2 months.	Jingdong prefecture level city in Shandong
Stowe Flagship Store	King of Power Saving	Quality is very good, customer service attitude is very very praise, there are problems in a timely manner to solve the problem, the price is not expensive, super value, you can rest assured that the	Jingdong prefecture level city in Shandong
Stowe Flagship Store	King of Power Saving	With it no longer need to be afraid to use electricity, especially good, ready to buy a mom and dad that also	Jingdong prefecture level city in Shandong
Stowe Flagship Store	King of Power Saving	Baby received very like will return to buy the seller attitude is also very like will return to buy the seller attitude is also very like!	Jingdong prefecture level city in Shandong
Stowe Flagship Store	King of Power Saving	Jingdong Mall shopping convenience assured fast assured fast assured fast assured fast	Jingdong prefecture level city in Shandong
Stowe Flagship Store	King of Power Saving	Saving power received, the quality is very good, fine workmanship, especially good, much cheaper than the entity store, it is worth buying!	Jingdong prefecture level city in Shandong
Stowe Flagship Store	King of Power Saving	I did not expect the express delivery so quickly, very save point quality is super good, than the entity store is too cheap.	Jingdong prefecture level city in Shandong
Stowe Flagship Store	King of Power Saving	Have received the baby, very satisfied with a shopping, great worth buying!	Jingdong prefecture level city in Shandong
Stowe Flagship Store	King of Power Saving	This is really more powerful, very satisfied, more energy-saving, better, thank you boss.	Jingdong prefecture level city in Shandong
Stowe Flagship Store	King of Power Saving	Received has been used for two days, the air conditioning can boldly open it! Energy saving effect is really quite good	Jingdong prefecture level city in Shandong
Stowe Flagship Store	King of Power Saving	It is authentic, trust the merchant, the baby has a great texture and the price is moderate, unlimited repurchase!	Jingdong prefecture level city in Shandong
Stowe Flagship Store	King of Power Saving	It is genuine very good, the price is much cheaper, cost-effective, really special cost-effective, it is worth buying!	Jingdong prefecture level city in Shandong
Stowe Flagship Store	King of Power Saving	Very good quality, fine workmanship, low power consumption, long warranty, fast shipping, economical and practical	Jingdong prefecture level city in Shandong

Stowe Flagship Store	King of Power Saving	I've been using it for half a month now, it really saves power and the voltage is stable, it's very good	Jingdong prefecture level city in Shandong
Stowe Flagship Store	King of Power Saving	It's the ideal product ♡, good quality and fast logistics, worth the kudos!	Jingdong prefecture level city in Shandong
Stowe Flagship Store	King of Power Saving	The logistics is very fast, the baby is very good, the shopkeeper is NICE!	Jingdong prefecture level city in Shandong
Stowe Flagship Store	King of Power Saving	Good to use, good quality, save point durable	Jingdong prefecture level city in Shandong
Stowe Flagship Store	King of Power Saving	The quality is very good, customer service attitude is very, very awesome, there are problems in a timely manner to solve the problem	Jingdong prefecture level city in Shandong
Stowe Flagship Store	King of Power Saving	Quality is very good, the use of the effect is very good, more stable, quality assurance	Jingdong prefecture level city in Shandong
Stowe Flagship Store	King of Power Saving	Convenient, inexpensive, and see how it works out?	Jingdong prefecture level city in Shandong
Stowe Flagship Store	King of Power Saving	Very good quality, saves a lot of electricity, very good, worth buying	Jingdong prefecture level city in Shandong
Stowe Flagship Store	King of Power Saving	Power Saving King is easy to use, with the fruit of power saving to use easy to give!	Jingdong prefecture level city in Shandong
Stowe Flagship Store	King of Power Saving	This saver really works, good quality, safe and secure, very satisfied!	Jingdong prefecture level city in Shandong
Stowe Flagship Store	King of Power Saving	It works well, the voltage is very stable effect is good, the quality is also very good satisfaction	Jingdong prefecture level city in Shandong
Stowe Flagship Store	King of Power Saving	The logistics is super super fast! The quality is also very good, indeed value for money	Jingdong prefecture level city in Shandong
Stowe Flagship Store	King of Power Saving	I've only been using it for a month or so and it seems to be saving a lot of power	Jingdong prefecture level city in Shandong
Stowe Flagship Store	King of Power Saving	Very easy to operate, very good material, good value for money.	Jingdong prefecture level city in Shandong
Stowe Flagship Store	King of Power Saving	Aluminum alloy shell, durable, compact power-saving good effect	Jingdong prefecture level city in Shandong
Stowe Flagship Store	King of Power Saving	Nice device, easy to use, doesn't take up much space, recommendable!	Jingdong prefecture level city in Shandong
Stowe Flagship Store	King of Power Saving	Good quality, good price, fast logistics	Jingdong prefecture level city in Shandong
Stowe Flagship Store	King of Power Saving	Friends of the family in use recommended, the effect is good and recommendable!	Jingdong prefecture level city in Shandong
Stowe Flagship Store	King of Power Saving	Cheaper than brick and mortar stores worth buying is authentic	Jingdong prefecture level city in Shandong
Stowe Flagship Store	King of Power Saving	Merchants ship quickly, the price is cheaper than the physical store, good use	Jingdong prefecture level city in Shandong
Stowe Flagship Store	King of Power Saving	Very durable, affordable, fast shipping	Jingdong prefecture level city in Shandong
Stowe Flagship Store	King of Power Saving	Packaging tight quality is very good easy to use good results	Jingdong prefecture level city in Shandong
Stowe Flagship Store	King of Power Saving	Save the grid. It's in the whole house. It's saving money on the electric bill.	Jingdong prefecture level city in Shandong
Stowe Flagship Store	King of Power Saving	I used it today, it works well, I'm very satisfied	Jingdong prefecture level city in Shandong
Stowe Flagship Store	King of Power Saving	It really saves a lot of electricity after installation	Jingdong prefecture level city in Shandong
Stowe Flagship Store	King of Power Saving	Love it. Satisfied.	Jingdong prefecture level city in Shandong
Stowe Flagship Store	King of Power Saving	Doesn't feel like it's working.	Jingdong prefecture level city in Shandong
Stowe Flagship Store	King of Power Saving	I'll comment after using it.	Jingdong prefecture level city in Shandong
Stowe Flagship Store	King of Power Saving	I'm trying it out to see if it saves power, and I'll buy two more if it works well.	Jingdong prefecture level city in Shandong
Stowe Flagship Store	King of Power Saving	It is genuine very good, the price is much cheaper, cost-effective, really special cost-effective, it is worth buying!	Jingdong prefecture level city in Shandong
Stowe Flagship Store	King of Power Saving	This thing can really save electricity, I was introduced to buy by my friend, he used it very well, I used it and think it's also very good, it can really save electricity, save about half of the electricity every day, it's really good!	Jingdong prefecture level city in Shandong
Stowe Flagship Store	King of Power Saving	After using it for ten days or so, I checked the power consumption and it did drop, and customer service said it will save more electricity in the long run. The product is also insured, 5 years warranty do not have to worry about quality problems!	Jingdong prefecture level city in Shandong
Stowe Flagship Store	King of Power Saving	The product is good, before already bought a, this is the second, the effect is quite good, anyway, I see the daily electricity bill than before a lot less, this can be the choice!	Jingdong prefecture level city in Shandong
Stowe Flagship Store	King of Power Saving	Originally also very worried about things are not good, get the hand found completely over-anxious, five-star praise, the style is very generous, the quality is very good, the price is also very reasonable!	Jingdong prefecture level city in Shandong
Stowe Flagship Store	King of Power Saving	Quality is good, playing to the old family also buy a, repurchase, this quality is really good, recommended!	Jingdong prefecture level city in Shandong
Stowe Flagship Store	King of Power Saving	Thank you, the owner even sent two fuses.	Jingdong prefecture level city in Shandong
Stowe Flagship Store	King of Power Saving	This store has great stuff, relatively fast shipping and logistics	Jingdong prefecture level city in Shandong
Stowe Flagship Store	King of Power Saving	This product is based on the impossibility of saving electricity, I used 853 kWh of electricity in July, started using the product on August 1, and used 867 kWh of electricity by September 1, is this called	Jingdong prefecture level city in Shandong
Stowe Flagship Store	King of Power Saving	Power king received, to the home on the trial, when I plugged into the electric panel can not see the effect of power saving, the meter is still the same speed in the turn, I'll save the king of the manual to play look at the instructions, instructions to speak, have to have a day two days and half a month to save power obviously, then wait half a month to see the effect of power saving it! To half a month there are obvious power saving before continuing to chase Xu.	Jingdong prefecture level city in Shandong
Stowe Flagship Store	King of Power Saving	Received the goods, a look, did not see the manufacturer and production address, can only hold a try to see the mentality of the face	Jingdong prefecture level city in Shandong
Stowe Flagship Store	King of Power Saving	I've only been using it for a few days and I'm not sure if it's an IQ tax? Used to favorable reviews.	Jingdong prefecture level city in Shandong
Stowe Flagship Store	King of Power Saving	Appearance is okay, power saving next time to follow up the evaluation	Jingdong prefecture level city in Shandong
Stowe Flagship Store	King of Power Saving	Shorter time, but hopefully effective and power-saving, to be followed up in the future	Jingdong prefecture level city in Shandong
Stowe Flagship Store	King of Power Saving	Appearance is okay, I do not know if the power saving	Jingdong prefecture level city in Shandong
Stowe Flagship Store	King of Power Saving	Good service, fast logistics, Power Saving King is exquisite and beautiful, support the business, thank you!	Jingdong prefecture level city in Shandong
Stowe Flagship Store	King of Power Saving	Bought three at once to try first first.	Jingdong prefecture level city in Shandong
Stowe Flagship Store	King of Power Saving	The object is the same as the picture, the product is of high quality, the packaging is intact. High-end atmosphere, high power, power saving effect is obvious, average monthly power saving 50%, very satisfied.	Jingdong prefecture level city in Shandong
Stowe Flagship Store	King of Power Saving	Bought back and plugged in, really effective, before 16 degrees of electricity per day, now 8 degrees of electricity per day, open an air conditioner for 12 hours, decisive repurchase of a	Jingdong prefecture level city in Shandong
Stowe Flagship Store	King of Power Saving	The object is the same as the picture, the product is of high quality, and the package is intact. High-end atmosphere and high grade, high power	Jingdong prefecture level city in Shandong

Stowe Flagship Store	King of Power Saving	Goods received, I hope the store said can be energy-saving, first try a month, month after month to see the effect.	Jingdong prefecture level city in Shandong
Stowe Flagship Store	King of Power Saving	This is really good, the material and workmanship is particularly exquisite, the workmanship is really very good, I think I can recommend to my friends to buy	Jingdong prefecture level city in Shandong
Stowe Flagship Store	King of Power Saving	I have to praise, the quality is really good, and the packaging logistics are also good. Really like it, won't make you regret after getting it!	Jingdong prefecture level city in Shandong
Stowe Flagship Store	King of Power Saving	Reputable platform, efficient and professional service, buy with confidence and peace of mind!	Jingdong prefecture level city in Shandong
Stowe Flagship Store	King of Power Saving	Bought one on a trial basis to see if it works after 1-2 months.	Jingdong prefecture level city in Shandong
Stowe Flagship Store	King of Power Saving	Quality is very good, customer service attitude is very very praise, there are problems in a timely manner to solve the problem, the price is not expensive, super value, you can rest assured that the	Jingdong prefecture level city in Shandong
Stowe Flagship Store	King of Power Saving	With it no longer need to be afraid to use electricity, especially good, ready to buy a mom and dad that also	Jingdong prefecture level city in Shandong
Stowe Flagship Store	King of Power Saving	Baby received very like will return to buy the seller attitude is also very like will return to buy the seller attitude is also very like!	Jingdong prefecture level city in Shandong
Stowe Flagship Store	King of Power Saving	Jingdong Mall shopping convenience assured fast assured fast assured fast assured fast	Jingdong prefecture level city in Shandong
Stowe Flagship Store	King of Power Saving	Saving power received, the quality is very good, fine workmanship, especially good, much cheaper than the entity store, it is worth buying!	Jingdong prefecture level city in Shandong
Stowe Flagship Store	King of Power Saving	I did not expect the express delivery so quickly, very save point quality is super good, than the entity store is too cheap.	Jingdong prefecture level city in Shandong
Stowe Flagship Store	King of Power Saving	Have received the baby, very satisfied with a shopping, great worth buying!	Jingdong prefecture level city in Shandong
Stowe Flagship Store	King of Power Saving	This is really more powerful, very satisfied, more energy-saving, better, thank you boss.	Jingdong prefecture level city in Shandong
Stowe Flagship Store	King of Power Saving	Received has been used for two days, the air conditioning can boldly open it! Energy saving effect is really quite good	Jingdong prefecture level city in Shandong
Stowe Flagship Store	King of Power Saving	It is authentic, trust the merchant, the baby has a great texture and the price is moderate, unlimited repurchase!	Jingdong prefecture level city in Shandong
Stowe Flagship Store	King of Power Saving	It is genuine very good, the price is much cheaper, cost-effective, really special cost-effective, it is worth buying!	Jingdong prefecture level city in Shandong
Stowe Flagship Store	King of Power Saving	Very good quality, fine workmanship, low power consumption, long warranty, fast shipping, economical and practical	Jingdong prefecture level city in Shandong
Stowe Flagship Store	King of Power Saving	I've been using it for half a month now, it really saves power and the voltage is stable, it's very good	Jingdong prefecture level city in Shandong
Stowe Flagship Store	King of Power Saving	It's the ideal product ♡, good quality and fast logistics, worth the kudos!	Jingdong prefecture level city in Shandong
Stowe Flagship Store	King of Power Saving	The logistics is very fast, the baby is very good, the shopkeeper is NICE!	Jingdong prefecture level city in Shandong
Stowe Flagship Store	King of Power Saving	Good to use, good quality, save point durable	Jingdong prefecture level city in Shandong
Stowe Flagship Store	King of Power Saving	The quality is very good, customer service attitude is very, very awesome, there are problems in a timely manner to solve the problem	Jingdong prefecture level city in Shandong
Stowe Flagship Store	King of Power Saving	Quality is very good, the use of the effect is very good, more stable, quality assurance	Jingdong prefecture level city in Shandong
Stowe Flagship Store	King of Power Saving	Convenient, inexpensive, and see how it works out?	Jingdong prefecture level city in Shandong
Stowe Flagship Store	King of Power Saving	Very good quality, saves a lot of electricity, very good, worth buying	Jingdong prefecture level city in Shandong
Stowe Flagship Store	King of Power Saving	Power Saving King is easy to use, with the fruit of power saving to use easy to give!	Jingdong prefecture level city in Shandong
Stowe Flagship Store	King of Power Saving	This saver really works, good quality, safe and secure, very satisfied!	Jingdong prefecture level city in Shandong
Stowe Flagship Store	King of Power Saving	It works well, the voltage is very stable effect is good, the quality is also very good satisfaction	Jingdong prefecture level city in Shandong
Stowe Flagship Store	King of Power Saving	The logistics is super super fast! The quality is also very good, indeed value for money	Jingdong prefecture level city in Shandong
Stowe Flagship Store	King of Power Saving	I've only been using it for a month or so and it seems to be saving a lot of power	Jingdong prefecture level city in Shandong
Stowe Flagship Store	King of Power Saving	Very easy to operate, very good material, good value for money.	Jingdong prefecture level city in Shandong
Stowe Flagship Store	King of Power Saving	Aluminum alloy shell, durable, compact power-saving good effect	Jingdong prefecture level city in Shandong
Stowe Flagship Store	King of Power Saving	Nice device, easy to use, doesn't take up much space, recommendable!	Jingdong prefecture level city in Shandong
Stowe Flagship Store	King of Power Saving	Good quality, good price, fast logistics	Jingdong prefecture level city in Shandong
Stowe Flagship Store	King of Power Saving	Friends of the family in use recommended, the effect is good and recommendable!	Jingdong prefecture level city in Shandong
Stowe Flagship Store	King of Power Saving	Cheaper than brick and mortar stores worth buying is authentic	Jingdong prefecture level city in Shandong
Stowe Flagship Store	King of Power Saving	Merchants ship quickly, the price is cheaper than the physical store, good use	Jingdong prefecture level city in Shandong
Stowe Flagship Store	King of Power Saving	Very durable, affordable, fast shipping	Jingdong prefecture level city in Shandong
Stowe Flagship Store	King of Power Saving	Packaging tight quality is very good easy to use good results	Jingdong prefecture level city in Shandong
Stowe Flagship Store	King of Power Saving	Save the grid. It's in the whole house. It's saving money on the electric bill.	Jingdong prefecture level city in Shandong
Stowe Flagship Store	King of Power Saving	I used it today, it works well, I'm very satisfied	Jingdong prefecture level city in Shandong
Stowe Flagship Store	King of Power Saving	It really saves a lot of electricity after installation	Jingdong prefecture level city in Shandong
Stowe Flagship Store	King of Power Saving	Love it. Satisfied.	Jingdong prefecture level city in Shandong
Stowe Flagship Store	King of Power Saving	Doesn't feel like it's working.	Jingdong prefecture level city in Shandong
Stowe Flagship Store	King of Power Saving	I'll comment after using it.	Jingdong prefecture level city in Shandong
Stowe Flagship Store	King of Power Saving	I'm trying it out to see if it saves power, and I'll buy two more if it works well.	Jingdong prefecture level city in Shandong
Stowe Flagship Store	King of Power Saving	It is genuine very good, the price is much cheaper, cost-effective, really special cost-effective, it is worth buying!	Jingdong prefecture level city in Shandong
Stowe Flagship Store	King of Power Saving	This thing can really save electricity, I was introduced to buy by my friend, he used it very well, I used it and think it's also very good, it can really save electricity, save about half of the electricity every day, it's really good!	Jingdong prefecture level city in Shandong
Stowe Flagship Store	King of Power Saving	After using it for ten days or so, I checked the power consumption and it did drop, and customer service said it will save more electricity in the long run. The product is also insured, 5 years warranty do not have to worry about quality problems!	Jingdong prefecture level city in Shandong
Stowe Flagship Store	King of Power Saving	The product is good, before already bought a, this is the second, the effect is quite good, anyway, I see the daily electricity bill than before a lot less, this can be the choice!	Jingdong prefecture level city in Shandong

Stowe Flagship Store	King of Power Saving	Originally also very worried about things are not good, get the hand found completely over-anxious, five-star praise, the style is very generous, the quality is very good, the price is also very reasonable!	Jingdong prefecture level city in Shandong
Stowe Flagship Store	King of Power Saving	Quality is good, playing to the old family also buy a, repurchase, this quality is really good, recommended!	Jingdong prefecture level city in Shandong
Stowe Flagship Store	King of Power Saving	Thank you, the owner even sent two fuses.	Jingdong prefecture level city in Shandong
Stowe Flagship Store	King of Power Saving	This store has great stuff, relatively fast shipping and logistics	Jingdong prefecture level city in Shandong
Stowe Flagship Store	King of Power Saving	This product is based on the impossibility of saving electricity, I used 853 kWh of electricity in July, started using the product on August 1, and used 867 kWh of electricity by September 1, is this called	Jingdong prefecture level city in Shandong
Stowe Flagship Store	King of Power Saving	Power king received, to the home on the trial, when I plugged into the electric panel can not see the effect of power saving, the meter is still the same speed in the turn, I'll save the king of the manual to play look at the instructions, instructions to speak, have to have a day two days and half a month to save power obviously, then wait half a month to see the effect of power saving it! To half a month there are obvious power saving before continuing to chase Xu.	Jingdong prefecture level city in Shandong
Stowe Flagship Store	King of Power Saving	Received the goods, a look, did not see the manufacturer and production address, can only hold a try to see the mentality of the face	Jingdong prefecture level city in Shandong
Stowe Flagship Store	King of Power Saving	I've only been using it for a few days and I'm not sure if it's an IQ tax? Used to favorable reviews.	Jingdong prefecture level city in Shandong
Stowe Flagship Store	King of Power Saving	Appearance is okay, power saving next time to follow up the evaluation	Jingdong prefecture level city in Shandong
Stowe Flagship Store	King of Power Saving	Shorter time, but hopefully effective and power-saving, to be followed up in the future	Jingdong prefecture level city in Shandong
Stowe Flagship Store	King of Power Saving	Appearance is okay, I do not know if the power saving	Jingdong prefecture level city in Shandong
Stowe Flagship Store	King of Power Saving	Good service, fast logistics, Power Saving King is exquisite and beautiful, support the business, thank you!	Jingdong prefecture level city in Shandong
Stowe Flagship Store	King of Power Saving	Bought three at once to try first first.	Jingdong prefecture level city in Shandong
Stowe Flagship Store	King of Power Saving	The object is the same as the picture, the product is of high quality, the packaging is intact. High-end atmosphere, high power, power saving effect is obvious, average monthly power saving 50%, very satisfied.	Jingdong prefecture level city in Shandong
Stowe Flagship Store	King of Power Saving	Bought back and plugged in, really effective, before 16 degrees of electricity per day, now 8 degrees of electricity per day, open an air conditioner for 12 hours, decisive repurchase of a	Jingdong prefecture level city in Shandong
Stowe Flagship Store	King of Power Saving	The object is the same as the picture, the product is of high quality, and the package is intact. High-end atmosphere and high grade, high power	Jingdong prefecture level city in Shandong
Stowe Flagship Store	King of Power Saving	Goods received, I hope the store said can be energy-saving, first try a month, month after month to see the effect.	Jingdong prefecture level city in Shandong
Stowe Flagship Store	King of Power Saving	This is really good, the material and workmanship is particularly exquisite, the workmanship is really very good, I think I can recommend to my friends to buy	Jingdong prefecture level city in Shandong
Stowe Flagship Store	King of Power Saving	I have to praise, the quality is really good, and the packaging logistics are also good. Really like it, won't make you regret after getting it!	Jingdong prefecture level city in Shandong
Stowe Flagship Store	King of Power Saving	Reputable platform, efficient and professional service, buy with confidence and peace of mind!	Jingdong prefecture level city in Shandong
Stowe Flagship Store	King of Power Saving	Bought one on a trial basis to see if it works after 1-2 months.	Jingdong prefecture level city in Shandong
Stowe Flagship Store	King of Power Saving	Quality is very good, customer service attitude is very very praise, there are problems in a timely manner to solve the problem, the price is not expensive, super value, you can rest assured that the	Jingdong prefecture level city in Shandong
Stowe Flagship Store	King of Power Saving	With it no longer need to be afraid to use electricity, especially good, ready to buy a mom and dad that also	Jingdong prefecture level city in Shandong
Stowe Flagship Store	King of Power Saving	Baby received very like will return to buy the seller attitude is also very like will return to buy the seller attitude is also very like!	Jingdong prefecture level city in Shandong
Stowe Flagship Store	King of Power Saving	Jingdong Mall shopping convenience assured fast assured fast assured fast assured fast	Jingdong prefecture level city in Shandong
Stowe Flagship Store	King of Power Saving	Saving power received, the quality is very good, fine workmanship, especially good, much cheaper than the entity store, it is worth buying!	Jingdong prefecture level city in Shandong
Stowe Flagship Store	King of Power Saving	I did not expect the express delivery so quickly, very save point quality is super good, than the entity store is too cheap.	Jingdong prefecture level city in Shandong
Stowe Flagship Store	King of Power Saving	Have received the baby, very satisfied with a shopping, great worth buying!	Jingdong prefecture level city in Shandong
Stowe Flagship Store	King of Power Saving	This is really more powerful, very satisfied, more energy-saving, better, thank you boss.	Jingdong prefecture level city in Shandong
Stowe Flagship Store	King of Power Saving	Received has been used for two days, the air conditioning can boldly open it! Energy saving effect is really quite good	Jingdong prefecture level city in Shandong
Stowe Flagship Store	King of Power Saving	It is authentic, trust the merchant, the baby has a great texture and the price is moderate, unlimited repurchase!	Jingdong prefecture level city in Shandong
Stowe Flagship Store	King of Power Saving	It is genuine very good, the price is much cheaper, cost-effective, really special cost-effective, it is worth buying!	Jingdong prefecture level city in Shandong
Stowe Flagship Store	King of Power Saving	Very good quality, fine workmanship, low power consumption, long warranty, fast shipping, economical and practical	Jingdong prefecture level city in Shandong
Stowe Flagship Store	King of Power Saving	I've been using it for half a month now, it really saves power and the voltage is stable, it's very good	Jingdong prefecture level city in Shandong
Stowe Flagship Store	King of Power Saving	It's the ideal product 💝, good quality and fast logistics, worth the kudos!	Jingdong prefecture level city in Shandong
Stowe Flagship Store	King of Power Saving	The logistics is very fast, the baby is very good, the shopkeeper is NICE!	Jingdong prefecture level city in Shandong
Stowe Flagship Store	King of Power Saving	Good to use, good quality, save point durable	Jingdong prefecture level city in Shandong
Stowe Flagship Store	King of Power Saving	The quality is very good, customer service attitude is very, very awesome, there are problems in a timely manner to solve the problem	Jingdong prefecture level city in Shandong
Stowe Flagship Store	King of Power Saving	Quality is very good, the use of the effect is very good, more stable, quality assurance	Jingdong prefecture level city in Shandong
Stowe Flagship Store	King of Power Saving	Convenient, inexpensive, and see how it works out?	Jingdong prefecture level city in Shandong
Stowe Flagship Store	King of Power Saving	Very good quality, saves a lot of electricity, very good, worth buying	Jingdong prefecture level city in Shandong
Stowe Flagship Store	King of Power Saving	Power Saving King is easy to use, with the fruit of power saving to use easy to give!	Jingdong prefecture level city in Shandong
Stowe Flagship Store	King of Power Saving	This saver really works, good quality, safe and secure, very satisfied!	Jingdong prefecture level city in Shandong
Stowe Flagship Store	King of Power Saving	It works well, the voltage is very stable effect is good, the quality is also very good satisfaction	Jingdong prefecture level city in Shandong
Stowe Flagship Store	King of Power Saving	The logistics is super super fast! The quality is also very good, indeed value for money	Jingdong prefecture level city in Shandong
Stowe Flagship Store	King of Power Saving	I've only been using it for a month or so and it seems to be saving a lot of power	Jingdong prefecture level city in Shandong
Stowe Flagship Store	King of Power Saving	Very easy to operate, very good material, good value for money.	Jingdong prefecture level city in Shandong
Stowe Flagship Store	King of Power Saving	Aluminum alloy shell, durable, compact power-saving good effect	Jingdong prefecture level city in Shandong
Stowe Flagship Store	King of Power Saving	Nice device, easy to use, doesn't take up much space, recommendable!	Jingdong prefecture level city in Shandong
Stowe Flagship Store	King of Power Saving	Good quality, good price, fast logistics	Jingdong prefecture level city in Shandong

Stowe Flagship Store	King of Power Saving	Friends of the family in use recommended, the effect is good and recommendable!	Jingdong prefecture level city in Shandong
Stowe Flagship Store	King of Power Saving	Cheaper than brick and mortar stores worth buying is authentic	Jingdong prefecture level city in Shandong
Stowe Flagship Store	King of Power Saving	Merchants ship quickly, the price is cheaper than the physical store, good use	Jingdong prefecture level city in Shandong
Stowe Flagship Store	King of Power Saving	Very durable, affordable, fast shipping	Jingdong prefecture level city in Shandong
Stowe Flagship Store	King of Power Saving	Packaging tight quality is very good easy to use good results	Jingdong prefecture level city in Shandong
Stowe Flagship Store	King of Power Saving	Save the grid. It's in the whole house. It's saving money on the electric bill.	Jingdong prefecture level city in Shandong
Stowe Flagship Store	King of Power Saving	I used it today, it works well, I'm very satisfied	Jingdong prefecture level city in Shandong
Stowe Flagship Store	King of Power Saving	It really saves a lot of electricity after installation	Jingdong prefecture level city in Shandong
Stowe Flagship Store	King of Power Saving	Love it. Satisfied.	Jingdong prefecture level city in Shandong
Stowe Flagship Store	King of Power Saving	Doesn't feel like it's working.	Jingdong prefecture level city in Shandong
Stowe Flagship Store	King of Power Saving	I'll comment after using it.	Jingdong prefecture level city in Shandong
Stowe Flagship Store	King of Power Saving	I'm trying it out to see if it saves power, and I'll buy two more if it works well.	Jingdong prefecture level city in Shandong
Stowe Flagship Store	King of Power Saving	It is genuine very good, the price is much cheaper, cost-effective, really special cost-effective, it is worth buying!	Jingdong prefecture level city in Shandong
Stowe Flagship Store	King of Power Saving	This thing can really save electricity, I was introduced to buy by my friend, he used it very well, I used it and think it's also very good, it can really save electricity, save about half of the electricity every day, it's really good!	Jingdong prefecture level city in Shandong
Stowe Flagship Store	King of Power Saving	After using it for ten days or so, I checked the power consumption and it did drop, and customer service said it will save more electricity in the long run. The product is also insured, 5 years warranty do not have to worry about quality problems!	Jingdong prefecture level city in Shandong
Stowe Flagship Store	King of Power Saving	The product is good, before already bought a, this is the second, the effect is quite good, anyway, I see the daily electricity bill than before a lot less, this can be the choice!	Jingdong prefecture level city in Shandong
Stowe Flagship Store	King of Power Saving	Originally also very worried about things are not good, get the hand found completely over-anxious, five-star praise, the style is very generous, the quality is very good, the price is also very reasonable!	Jingdong prefecture level city in Shandong
Stowe Flagship Store	King of Power Saving	Quality is good, playing to the old family also buy a, repurchase, this quality is really good, recommended!	Jingdong prefecture level city in Shandong
Stowe Flagship Store	King of Power Saving	Thank you, the owner even sent two fuses.	Jingdong prefecture level city in Shandong
Stowe Flagship Store	King of Power Saving	This store has great stuff, relatively fast shipping and logistics	Jingdong prefecture level city in Shandong
Stowe Flagship Store	King of Power Saving	This product is based on the impossibility of saving electricity, I used 853 kWh of electricity in July, started using the product on August 1, and used 867 kWh of electricity by September 1, is this called	Jingdong prefecture level city in Shandong
Stowe Flagship Store	King of Power Saving	Power king received, to the home on the trial, when I plugged into the electric panel can not see the effect of power saving, the meter is still the same speed in the turn, I'll save the king of the manual to play look at the instructions, instructions to speak, have to have a day two days and half a month to save power obviously, then wait half a month to see the effect of power saving it! To half a month there are obvious power saving before continuing to chase Xu.	Jingdong prefecture level city in Shandong
Stowe Flagship Store	King of Power Saving	Received the goods, a look, did not see the manufacturer and production address, can only hold a try to see the mentality of the face	Jingdong prefecture level city in Shandong
Stowe Flagship Store	King of Power Saving	I've only been using it for a few days and I'm not sure if it's an IQ tax? Used to favorable reviews.	Jingdong prefecture level city in Shandong
Stowe Flagship Store	King of Power Saving	Appearance is okay, power saving next time to follow up the evaluation	Jingdong prefecture level city in Shandong
Stowe Flagship Store	King of Power Saving	Shorter time, but hopefully effective and power-saving, to be followed up in the future	Jingdong prefecture level city in Shandong
Stowe Flagship Store	King of Power Saving	Appearance is okay, I do not know if the power saving	Jingdong prefecture level city in Shandong
Stowe Flagship Store	King of Power Saving	Good service, fast logistics, Power Saving King is exquisite and beautiful, support the business, thank you!	Jingdong prefecture level city in Shandong
Stowe Flagship Store	King of Power Saving	Bought three at once to try first first.	Jingdong prefecture level city in Shandong
Stowe Flagship Store	King of Power Saving	The object is the same as the picture, the product is of high quality, the packaging is intact. High-end atmosphere, high power, power saving effect is obvious, average monthly power saving 50%, very satisfied.	Jingdong prefecture level city in Shandong
Stowe Flagship Store	King of Power Saving	Bought back and plugged in, really effective, before 16 degrees of electricity per day, now 8 degrees of electricity per day, open an air conditioner for 12 hours, decisive repurchase of a	Jingdong prefecture level city in Shandong
Stowe Flagship Store	King of Power Saving	The object is the same as the picture, the product is of high quality, and the package is intact. High-end atmosphere and high grade, high power	Jingdong prefecture level city in Shandong
Stowe Flagship Store	King of Power Saving	Goods received, I hope the store said can be energy-saving, first try a month, month after month to see the effect.	Jingdong prefecture level city in Shandong
Stowe Flagship Store	King of Power Saving	This is really good, the material and workmanship is particularly exquisite, the workmanship is really very good, I think I can recommend to my friends to buy	Jingdong prefecture level city in Shandong
Stowe Flagship Store	King of Power Saving	I have to praise, the quality is really good, and the packaging logistics are also good. Really like it, won't make you regret after getting it!	Jingdong prefecture level city in Shandong
Stowe Flagship Store	King of Power Saving	Reputable platform, efficient and professional service, buy with confidence and peace of mind!	Jingdong prefecture level city in Shandong
Stowe Flagship Store	King of Power Saving	Bought one on a trial basis to see if it works after 1-2 months.	Jingdong prefecture level city in Shandong
Stowe Flagship Store	King of Power Saving	Quality is very good, customer service attitude is very very praise, there are problems in a timely manner to solve the problem, the price is not expensive, super value, you can rest assured that the	Jingdong prefecture level city in Shandong
Stowe Flagship Store	King of Power Saving	With it no longer need to be afraid to use electricity, especially good, ready to buy a mom and dad that also	Jingdong prefecture level city in Shandong
Stowe Flagship Store	King of Power Saving	Baby received very like will return to buy the seller attitude is also very like will return to buy the seller attitude is also very like!	Jingdong prefecture level city in Shandong
Stowe Flagship Store	King of Power Saving	Jingdong Mall shopping convenience assured fast assured fast assured fast assured fast	Jingdong prefecture level city in Shandong
Stowe Flagship Store	King of Power Saving	Saving power received, the quality is very good, fine workmanship, especially good, much cheaper than the entity store, it is worth buying!	Jingdong prefecture level city in Shandong
Stowe Flagship Store	King of Power Saving	I did not expect the express delivery so quickly, very save point quality is super good, than the entity store is too cheap.	Jingdong prefecture level city in Shandong
Stowe Flagship Store	King of Power Saving	Have received the baby, very satisfied with a shopping, great worth buying!	Jingdong prefecture level city in Shandong
Stowe Flagship Store	King of Power Saving	This is really more powerful, very satisfied, more energy-saving, better, thank you boss.	Jingdong prefecture level city in Shandong
Stowe Flagship Store	King of Power Saving	Received has been used for two days, the air conditioning can boldly open it! Energy saving effect is really quite good	Jingdong prefecture level city in Shandong
Stowe Flagship Store	King of Power Saving	It is authentic, trust the merchant, the baby has a great texture and the price is moderate, unlimited repurchase!	Jingdong prefecture level city in Shandong
Stowe Flagship Store	King of Power Saving	It is genuine very good, the price is much cheaper, cost-effective, really special cost-effective, it is worth buying!	Jingdong prefecture level city in Shandong
Stowe Flagship Store	King of Power Saving	Very good quality, fine workmanship, low power consumption, long warranty, fast shipping, economical and practical	Jingdong prefecture level city in Shandong

Stowe Flagship Store	King of Power Saving	I've been using it for half a month now, it really saves power and the voltage is stable, it's very good	Jingdong prefecture level city in Shandong
Stowe Flagship Store	King of Power Saving	It's the ideal product 💝, good quality and fast logistics, worth the kudos!	Jingdong prefecture level city in Shandong
Stowe Flagship Store	King of Power Saving	The logistics is very fast, the baby is very good, the shopkeeper is NICE!	Jingdong prefecture level city in Shandong
Stowe Flagship Store	King of Power Saving	Good to use, good quality, save point durable	Jingdong prefecture level city in Shandong
Stowe Flagship Store	King of Power Saving	The quality is very good, customer service attitude is very, very awesome, there are problems in a timely manner to solve the problem	Jingdong prefecture level city in Shandong
Stowe Flagship Store	King of Power Saving	Quality is very good, the use of the effect is very good, more stable, quality assurance	Jingdong prefecture level city in Shandong
Stowe Flagship Store	King of Power Saving	Convenient, inexpensive, and see how it works out?	Jingdong prefecture level city in Shandong
Stowe Flagship Store	King of Power Saving	Very good quality, saves a lot of electricity, very good, worth buying	Jingdong prefecture level city in Shandong
Stowe Flagship Store	King of Power Saving	Power Saving King is easy to use, with the fruit of power saving to use easy to give!	Jingdong prefecture level city in Shandong
Stowe Flagship Store	King of Power Saving	This saver really works, good quality, safe and secure, very satisfied!	Jingdong prefecture level city in Shandong
Stowe Flagship Store	King of Power Saving	It works well, the voltage is very stable effect is good, the quality is also very good satisfaction	Jingdong prefecture level city in Shandong
Stowe Flagship Store	King of Power Saving	The logistics is super super fast! The quality is also very good, indeed value for money	Jingdong prefecture level city in Shandong
Stowe Flagship Store	King of Power Saving	I've only been using it for a month or so and it seems to be saving a lot of power	Jingdong prefecture level city in Shandong
Stowe Flagship Store	King of Power Saving	Very easy to operate, very good material, good value for money.	Jingdong prefecture level city in Shandong
Stowe Flagship Store	King of Power Saving	Aluminum alloy shell, durable, compact power-saving good effect	Jingdong prefecture level city in Shandong
Stowe Flagship Store	King of Power Saving	Nice device, easy to use, doesn't take up much space, recommendable!	Jingdong prefecture level city in Shandong
Stowe Flagship Store	King of Power Saving	Good quality, good price, fast logistics	Jingdong prefecture level city in Shandong
Stowe Flagship Store	King of Power Saving	Friends of the family in use recommended, the effect is good and recommendable!	Jingdong prefecture level city in Shandong
Stowe Flagship Store	King of Power Saving	Cheaper than brick and mortar stores worth buying is authentic	Jingdong prefecture level city in Shandong
Stowe Flagship Store	King of Power Saving	Merchants ship quickly, the price is cheaper than the physical store, good use	Jingdong prefecture level city in Shandong
Stowe Flagship Store	King of Power Saving	Very durable, affordable, fast shipping	Jingdong prefecture level city in Shandong
Stowe Flagship Store	King of Power Saving	Packaging tight quality is very good easy to use good results	Jingdong prefecture level city in Shandong
Stowe Flagship Store	King of Power Saving	Save the grid. It's in the whole house. It's saving money on the electric bill.	Jingdong prefecture level city in Shandong
Stowe Flagship Store	King of Power Saving	I used it today, it works well, I'm very satisfied	Jingdong prefecture level city in Shandong
Stowe Flagship Store	King of Power Saving	It really saves a lot of electricity after installation	Jingdong prefecture level city in Shandong
Stowe Flagship Store	King of Power Saving	Love it. Satisfied.	Jingdong prefecture level city in Shandong
Stowe Flagship Store	King of Power Saving	Doesn't feel like it's working.	Jingdong prefecture level city in Shandong
Stowe Flagship Store	King of Power Saving	I'll comment after using it.	Jingdong prefecture level city in Shandong
Stowe Flagship Store	King of Power Saving	I'm trying it out to see if it saves power, and I'll buy two more if it works well.	Jingdong prefecture level city in Shandong
Stowe Flagship Store	King of Power Saving	It is genuine very good, the price is much cheaper, cost-effective, really special cost-effective, it is worth buying!	Jingdong prefecture level city in Shandong
Stowe Flagship Store	King of Power Saving	This thing can really save electricity, I was introduced to buy by my friend, he used it very well, I used it and think it's also very good, it can really save electricity, save about half of the electricity every day, it's really good!	Jingdong prefecture level city in Shandong
Stowe Flagship Store	King of Power Saving	After using it for ten days or so, I checked the power consumption and it did drop, and customer service said it will save more electricity in the long run. The product is also insured, 5 years warranty do not have to worry about quality problems!	Jingdong prefecture level city in Shandong
Stowe Flagship Store	King of Power Saving	The product is good, before already bought a, this is the second, the effect is quite good, anyway, I see the daily electricity bill than before a lot less, this can be the choice!	Jingdong prefecture level city in Shandong
Stowe Flagship Store	King of Power Saving	Originally also very worried about things are not good, get the hand found completely over-anxious, five-star praise, the style is very generous, the quality is very good, the price is also very reasonable!	Jingdong prefecture level city in Shandong
Stowe Flagship Store	King of Power Saving	Quality is good, playing to the old family also buy a, repurchase, this quality is really good, recommended!	Jingdong prefecture level city in Shandong
Stowe Flagship Store	King of Power Saving	Thank you, the owner even sent two fuses.	Jingdong prefecture level city in Shandong
Stowe Flagship Store	King of Power Saving	This store has great stuff, relatively fast shipping and logistics	Jingdong prefecture level city in Shandong
Stowe Flagship Store	King of Power Saving	This product is based on the impossibility of saving electricity, I used 853 kWh of electricity in July, started using the product on August 1, and used 867 kWh of electricity by September 1, is this called	Jingdong prefecture level city in Shandong
Stowe Flagship Store	King of Power Saving	Power king received, to the home on the trial, when I plugged into the electric panel can not see the effect of power saving, the meter is still the same speed in the turn, I'll save the king of the manual to play look at the instructions, instructions to speak, have to have a day two days and half a month to save power obviously, then wait half a month to see the effect of power saving it! To half a month there are obvious power saving before continuing to chase Xu.	Jingdong prefecture level city in Shandong
Stowe Flagship Store	King of Power Saving	Received the goods, a look, did not see the manufacturer and production address, can only hold a try to see the mentality of the face	Jingdong prefecture level city in Shandong
Stowe Flagship Store	King of Power Saving	I've only been using it for a few days and I'm not sure if it's an IQ tax? Used to favorable reviews.	Jingdong prefecture level city in Shandong
Stowe Flagship Store	King of Power Saving	Appearance is okay, power saving next time to follow up the evaluation	Jingdong prefecture level city in Shandong
Stowe Flagship Store	King of Power Saving	Shorter time, but hopefully effective and power-saving, to be followed up in the future	Jingdong prefecture level city in Shandong
Stowe Flagship Store	King of Power Saving	Appearance is okay, I do not know if the power saving	Jingdong prefecture level city in Shandong
Stowe Flagship Store	King of Power Saving	Good service, fast logistics, Power Saving King is exquisite and beautiful, support the business, thank you!	Jingdong prefecture level city in Shandong
Stowe Flagship Store	King of Power Saving	Bought three at once to try first first.	Jingdong prefecture level city in Shandong
Stowe Flagship Store	King of Power Saving	The object is the same as the picture, the product is of high quality, the packaging is intact. High-end atmosphere, high power, power saving effect is obvious, average monthly power saving 50%, very satisfied.	Jingdong prefecture level city in Shandong
Stowe Flagship Store	King of Power Saving	Bought back and plugged in, really effective, before 16 degrees of electricity per day, now 8 degrees of electricity per day, open an air conditioner for 12 hours, decisive repurchase of a	Jingdong prefecture level city in Shandong
Stowe Flagship Store	King of Power Saving	The object is the same as the picture, the product is of high quality, and the package is intact. High-end atmosphere and high grade, high power	Jingdong prefecture level city in Shandong

Stowe Flagship Store	King of Power Saving	Goods received, I hope the store said can be energy-saving, first try a month, month after month to see the effect.	Jingdong prefecture level city in Shandong
Stowe Flagship Store	King of Power Saving	This is really good, the material and workmanship is particularly exquisite, the workmanship is really very good, I think I can recommend to my friends to buy	Jingdong prefecture level city in Shandong
Stowe Flagship Store	King of Power Saving	I have to praise, the quality is really good, and the packaging logistics are also good. Really like it, won't make you regret after getting it!	Jingdong prefecture level city in Shandong
Stowe Flagship Store	King of Power Saving	Reputable platform, efficient and professional service, buy with confidence and peace of mind!	Jingdong prefecture level city in Shandong
Stowe Flagship Store	King of Power Saving	Bought one on a trial basis to see if it works after 1-2 months.	Jingdong prefecture level city in Shandong
Stowe Flagship Store	King of Power Saving	Quality is very good, customer service attitude is very very praise, there are problems in a timely manner to solve the problem, the price is not expensive, super value, you can rest assured that the	Jingdong prefecture level city in Shandong
Stowe Flagship Store	King of Power Saving	With it no longer need to be afraid to use electricity, especially good, ready to buy a mom and dad that also	Jingdong prefecture level city in Shandong
Stowe Flagship Store	King of Power Saving	Baby received very like will return to buy the seller attitude is also very like will return to buy the seller attitude is also very like!	Jingdong prefecture level city in Shandong
Stowe Flagship Store	King of Power Saving	Jingdong Mall shopping convenience assured fast assured fast assured fast assured fast	Jingdong prefecture level city in Shandong
Stowe Flagship Store	King of Power Saving	Saving power received, the quality is very good, fine workmanship, especially good, much cheaper than the entity store, it is worth buying!	Jingdong prefecture level city in Shandong
Stowe Flagship Store	King of Power Saving	I did not expect the express delivery so quickly, very save point quality is super good, than the entity store is too cheap.	Jingdong prefecture level city in Shandong
Stowe Flagship Store	King of Power Saving	Have received the baby, very satisfied with a shopping, great worth buying!	Jingdong prefecture level city in Shandong
Stowe Flagship Store	King of Power Saving	This is really more powerful, very satisfied, more energy-saving, better, thank you boss.	Jingdong prefecture level city in Shandong
Stowe Flagship Store	King of Power Saving	Received has been used for two days, the air conditioning can boldly open it! Energy saving effect is really quite good	Jingdong prefecture level city in Shandong
Stowe Flagship Store	King of Power Saving	It is authentic, trust the merchant, the baby has a great texture and the price is moderate, unlimited repurchase!	Jingdong prefecture level city in Shandong
Stowe Flagship Store	King of Power Saving	It is genuine very good, the price is much cheaper, cost-effective, really special cost-effective, it is worth buying!	Jingdong prefecture level city in Shandong
Stowe Flagship Store	King of Power Saving	Very good quality, fine workmanship, low power consumption, long warranty, fast shipping, economical and practical	Jingdong prefecture level city in Shandong
Stowe Flagship Store	King of Power Saving	I've been using it for half a month now, it really saves power and the voltage is stable, it's very good	Jingdong prefecture level city in Shandong
Stowe Flagship Store	King of Power Saving	It's the ideal product 💝, good quality and fast logistics, worth the kudos!	Jingdong prefecture level city in Shandong
Stowe Flagship Store	King of Power Saving	The logistics is very fast, the baby is very good, the shopkeeper is NICE!	Jingdong prefecture level city in Shandong
Stowe Flagship Store	King of Power Saving	Good to use, good quality, save point durable	Jingdong prefecture level city in Shandong
Stowe Flagship Store	King of Power Saving	The quality is very good, customer service attitude is very, very awesome, there are problems in a timely manner to solve the problem	Jingdong prefecture level city in Shandong
Stowe Flagship Store	King of Power Saving	Quality is very good, the use of the effect is very good, more stable, quality assurance	Jingdong prefecture level city in Shandong
Stowe Flagship Store	King of Power Saving	Convenient, inexpensive, and see how it works out?	Jingdong prefecture level city in Shandong
Stowe Flagship Store	King of Power Saving	Very good quality, saves a lot of electricity, very good, worth buying	Jingdong prefecture level city in Shandong
Stowe Flagship Store	King of Power Saving	Power Saving King is easy to use, with the fruit of power saving to use easy to give!	Jingdong prefecture level city in Shandong
Stowe Flagship Store	King of Power Saving	This saver really works, good quality, safe and secure, very satisfied!	Jingdong prefecture level city in Shandong
Stowe Flagship Store	King of Power Saving	It works well, the voltage is very stable effect is good, the quality is also very good satisfaction	Jingdong prefecture level city in Shandong
Stowe Flagship Store	King of Power Saving	The logistics is super super fast! The quality is also very good, indeed value for money	Jingdong prefecture level city in Shandong
Stowe Flagship Store	King of Power Saving	I've only been using it for a month or so and it seems to be saving a lot of power	Jingdong prefecture level city in Shandong
Stowe Flagship Store	King of Power Saving	Very easy to operate, very good material, good value for money.	Jingdong prefecture level city in Shandong
Stowe Flagship Store	King of Power Saving	Aluminum alloy shell, durable, compact power-saving good effect	Jingdong prefecture level city in Shandong
Stowe Flagship Store	King of Power Saving	Nice device, easy to use, doesn't take up much space, recommendable!	Jingdong prefecture level city in Shandong
Stowe Flagship Store	King of Power Saving	Good quality, good price, fast logistics	Jingdong prefecture level city in Shandong
Stowe Flagship Store	King of Power Saving	Friends of the family in use recommended, the effect is good and recommendable!	Jingdong prefecture level city in Shandong
Stowe Flagship Store	King of Power Saving	Cheaper than brick and mortar stores worth buying is authentic	Jingdong prefecture level city in Shandong
Stowe Flagship Store	King of Power Saving	Merchants ship quickly, the price is cheaper than the physical store, good use	Jingdong prefecture level city in Shandong
Stowe Flagship Store	King of Power Saving	Very durable, affordable, fast shipping	Jingdong prefecture level city in Shandong
Stowe Flagship Store	King of Power Saving	Packaging tight quality is very good easy to use good results	Jingdong prefecture level city in Shandong
Stowe Flagship Store	King of Power Saving	Save the grid. It's in the whole house. It's saving money on the electric bill.	Jingdong prefecture level city in Shandong
Stowe Flagship Store	King of Power Saving	I used it today, it works well, I'm very satisfied	Jingdong prefecture level city in Shandong
Stowe Flagship Store	King of Power Saving	It really saves a lot of electricity after installation	Jingdong prefecture level city in Shandong
Stowe Flagship Store	King of Power Saving	Love it. Satisfied.	Jingdong prefecture level city in Shandong
Stowe Flagship Store	King of Power Saving	Doesn't feel like it's working.	Jingdong prefecture level city in Shandong
Stowe Flagship Store	King of Power Saving	I'll comment after using it.	Jingdong prefecture level city in Shandong
Stowe Flagship Store	King of Power Saving	I'm trying it out to see if it saves power, and I'll buy two more if it works well.	Jingdong prefecture level city in Shandong
Stowe Flagship Store	King of Power Saving	It is genuine very good, the price is much cheaper, cost-effective, really special cost-effective, it is worth buying!	Jingdong prefecture level city in Shandong
Stowe Flagship Store	King of Power Saving	This thing can really save electricity, I was introduced to buy by my friend, he used it very well, I used it and think it's also very good, it can really save electricity, save about half of the electricity every day, it's really good!	Jingdong prefecture level city in Shandong
Stowe Flagship Store	King of Power Saving	After using it for ten days or so, I checked the power consumption and it did drop, and customer service said it will save more electricity in the long run. The product is also insured, 5 years warranty do not have to worry about quality problems!	Jingdong prefecture level city in Shandong
Stowe Flagship Store	King of Power Saving	The product is good, before already bought a, this is the second, the effect is quite good, anyway, I see the daily electricity bill than before a lot less, this can be the choice!	Jingdong prefecture level city in Shandong

Stowe Flagship Store	King of Power Saving	Originally also very worried about things are not good, get the hand found completely over-anxious, five-star praise, the style is very generous, the quality is very good, the price is also very reasonable!	Jingdong prefecture level city in Shandong
Stowe Flagship Store	King of Power Saving	Quality is good, playing to the old family also buy a, repurchase, this quality is really good, recommended!	Jingdong prefecture level city in Shandong
Stowe Flagship Store	King of Power Saving	Thank you, the owner even sent two fuses.	Jingdong prefecture level city in Shandong
Stowe Flagship Store	King of Power Saving	This store has great stuff, relatively fast shipping and logistics	Jingdong prefecture level city in Shandong
Stowe Flagship Store	King of Power Saving	This product is based on the impossibility of saving electricity, I used 853 kWh of electricity in July, started using the product on August 1, and used 867 kWh of electricity by September 1, is this called	Jingdong prefecture level city in Shandong
Stowe Flagship Store	King of Power Saving	Power king received, to the home on the trial, when I plugged into the electric panel can not see the effect of power saving, the meter is still the same speed in the turn, I'll save the king of the manual to play look at the instructions, instructions to speak, have to have a day two days and half a month to save power obviously, then wait half a month to see the effect of power saving it! To half a month there are obvious power saving before continuing to chase Xu.	Jingdong prefecture level city in Shandong
Stowe Flagship Store	King of Power Saving	Received the goods, a look, did not see the manufacturer and production address, can only hold a try to see the mentality of the face	Jingdong prefecture level city in Shandong
Stowe Flagship Store	King of Power Saving	I've only been using it for a few days and I'm not sure if it's an IQ tax? Used to favorable reviews.	Jingdong prefecture level city in Shandong
Stowe Flagship Store	King of Power Saving	Appearance is okay, power saving next time to follow up the evaluation	Jingdong prefecture level city in Shandong
Stowe Flagship Store	King of Power Saving	Shorter time, but hopefully effective and power-saving, to be followed up in the future	Jingdong prefecture level city in Shandong
Stowe Flagship Store	King of Power Saving	Appearance is okay, I do not know if the power saving	Jingdong prefecture level city in Shandong
Stowe Flagship Store	King of Power Saving	Good service, fast logistics, Power Saving King is exquisite and beautiful, support the business, thank you!	Jingdong prefecture level city in Shandong
Stowe Flagship Store	King of Power Saving	Bought three at once to try first first.	Jingdong prefecture level city in Shandong
Stowe Flagship Store	King of Power Saving	The object is the same as the picture, the product is of high quality, the packaging is intact. High-end atmosphere, high power, power saving effect is obvious, average monthly power saving 50%, very satisfied.	Jingdong prefecture level city in Shandong
Stowe Flagship Store	King of Power Saving	Bought back and plugged in, really effective, before 16 degrees of electricity per day, now 8 degrees of electricity per day, open an air conditioner for 12 hours, decisive repurchase of a	Jingdong prefecture level city in Shandong
Stowe Flagship Store	King of Power Saving	The object is the same as the picture, the product is of high quality, and the package is intact. High-end atmosphere and high grade, high power	Jingdong prefecture level city in Shandong
Stowe Flagship Store	King of Power Saving	Goods received, I hope the store said can be energy-saving, first try a month, month after month to see the effect.	Jingdong prefecture level city in Shandong
Stowe Flagship Store	King of Power Saving	This is really good, the material and workmanship is particularly exquisite, the workmanship is really very good, I think I can recommend to my friends to buy	Jingdong prefecture level city in Shandong
Stowe Flagship Store	King of Power Saving	I have to praise, the quality is really good, and the packaging logistics are also good. Really like it, won't make you regret after getting it!	Jingdong prefecture level city in Shandong
Stowe Flagship Store	King of Power Saving	Reputable platform, efficient and professional service, buy with confidence and peace of mind!	Jingdong prefecture level city in Shandong
Stowe Flagship Store	King of Power Saving	Bought one on a trial basis to see if it works after 1-2 months.	Jingdong prefecture level city in Shandong
Stowe Flagship Store	King of Power Saving	Quality is very good, customer service attitude is very very praise, there are problems in a timely manner to solve the problem, the price is not expensive, super value, you can rest assured that the	Jingdong prefecture level city in Shandong
Stowe Flagship Store	King of Power Saving	With it no longer need to be afraid to use electricity, especially good, ready to buy a mom and dad that also	Jingdong prefecture level city in Shandong
Stowe Flagship Store	King of Power Saving	Baby received very like will return to buy the seller attitude is also very like will return to buy the seller attitude is also very like!	Jingdong prefecture level city in Shandong
Stowe Flagship Store	King of Power Saving	Jingdong Mall shopping convenience assured fast assured fast assured fast assured fast	Jingdong prefecture level city in Shandong
Stowe Flagship Store	King of Power Saving	Saving power received, the quality is very good, fine workmanship, especially good, much cheaper than the entity store, it is worth buying!	Jingdong prefecture level city in Shandong
Stowe Flagship Store	King of Power Saving	I did not expect the express delivery so quickly, very save point quality is super good, than the entity store is too cheap.	Jingdong prefecture level city in Shandong
Stowe Flagship Store	King of Power Saving	Have received the baby, very satisfied with a shopping, great worth buying!	Jingdong prefecture level city in Shandong
Stowe Flagship Store	King of Power Saving	This is really more powerful, very satisfied, more energy-saving, better, thank you boss.	Jingdong prefecture level city in Shandong
Stowe Flagship Store	King of Power Saving	Received has been used for two days, the air conditioning can boldly open it! Energy saving effect is really quite good	Jingdong prefecture level city in Shandong
Stowe Flagship Store	King of Power Saving	It is authentic, trust the merchant, the baby has a great texture and the price is moderate, unlimited repurchase!	Jingdong prefecture level city in Shandong
Stowe Flagship Store	King of Power Saving	It is genuine very good, the price is much cheaper, cost-effective, really special cost-effective, it is worth buying!	Jingdong prefecture level city in Shandong
Stowe Flagship Store	King of Power Saving	Very good quality, fine workmanship, low power consumption, long warranty, fast shipping, economical and practical	Jingdong prefecture level city in Shandong
Stowe Flagship Store	King of Power Saving	I've been using it for half a month now, it really saves power and the voltage is stable, it's very good	Jingdong prefecture level city in Shandong
Stowe Flagship Store	King of Power Saving	It's the ideal product 💞, good quality and fast logistics, worth the kudos!	Jingdong prefecture level city in Shandong
Stowe Flagship Store	King of Power Saving	The logistics is very fast, the baby is very good, the shopkeeper is NICE!	Jingdong prefecture level city in Shandong
Stowe Flagship Store	King of Power Saving	Good to use, good quality, save point durable	Jingdong prefecture level city in Shandong
Stowe Flagship Store	King of Power Saving	The quality is very good, customer service attitude is very, very awesome, there are problems in a timely manner to solve the problem	Jingdong prefecture level city in Shandong
Stowe Flagship Store	King of Power Saving	Quality is very good, the use of the effect is very good, more stable, quality assurance	Jingdong prefecture level city in Shandong
Stowe Flagship Store	King of Power Saving	Convenient, inexpensive, and see how it works out?	Jingdong prefecture level city in Shandong
Stowe Flagship Store	King of Power Saving	Very good quality, saves a lot of electricity, very good, worth buying	Jingdong prefecture level city in Shandong
Stowe Flagship Store	King of Power Saving	Power Saving King is easy to use, with the fruit of power saving to use easy to give!	Jingdong prefecture level city in Shandong
Stowe Flagship Store	King of Power Saving	This saver really works, good quality, safe and secure, very satisfied!	Jingdong prefecture level city in Shandong
Stowe Flagship Store	King of Power Saving	It works well, the voltage is very stable effect is good, the quality is also very good satisfaction	Jingdong prefecture level city in Shandong
Stowe Flagship Store	King of Power Saving	The logistics is super super fast! The quality is also very good, indeed value for money	Jingdong prefecture level city in Shandong
Stowe Flagship Store	King of Power Saving	I've only been using it for a month or so and it seems to be saving a lot of power	Jingdong prefecture level city in Shandong
Stowe Flagship Store	King of Power Saving	Very easy to operate, very good material, good value for money.	Jingdong prefecture level city in Shandong
Stowe Flagship Store	King of Power Saving	Aluminum alloy shell, durable, compact power-saving good effect	Jingdong prefecture level city in Shandong
Stowe Flagship Store	King of Power Saving	Nice device, easy to use, doesn't take up much space, recommendable!	Jingdong prefecture level city in Shandong
Stowe Flagship Store	King of Power Saving	Good quality, good price, fast logistics	Jingdong prefecture level city in Shandong

Stowe Flagship Store	King of Power Saving	Friends of the family in use recommended, the effect is good and recommendable!	Jingdong prefecture level city in Shandong
Stowe Flagship Store	King of Power Saving	Cheaper than brick and mortar stores worth buying is authentic	Jingdong prefecture level city in Shandong
Stowe Flagship Store	King of Power Saving	Merchants ship quickly, the price is cheaper than the physical store, good use	Jingdong prefecture level city in Shandong
Stowe Flagship Store	King of Power Saving	Very durable, affordable, fast shipping	Jingdong prefecture level city in Shandong
Stowe Flagship Store	King of Power Saving	Packaging tight quality is very good easy to use good results	Jingdong prefecture level city in Shandong
Stowe Flagship Store	King of Power Saving	Save the grid. It's in the whole house. It's saving money on the electric bill.	Jingdong prefecture level city in Shandong
Stowe Flagship Store	King of Power Saving	I used it today, it works well, I'm very satisfied	Jingdong prefecture level city in Shandong
Stowe Flagship Store	King of Power Saving	It really saves a lot of electricity after installation	Jingdong prefecture level city in Shandong
Stowe Flagship Store	King of Power Saving	Love it. Satisfied.	Jingdong prefecture level city in Shandong
Stowe Flagship Store	King of Power Saving	Doesn't feel like it's working.	Jingdong prefecture level city in Shandong
Stowe Flagship Store	King of Power Saving	I'll comment after using it.	Jingdong prefecture level city in Shandong
Stowe Flagship Store	King of Power Saving	I'm trying it out to see if it saves power, and I'll buy two more if it works well.	Jingdong prefecture level city in Shandong
Stowe Flagship Store	King of Power Saving	It is genuine very good, the price is much cheaper, cost-effective, really special cost-effective, it is worth buying!	Jingdong prefecture level city in Shandong
Stowe Flagship Store	King of Power Saving	This thing can really save electricity, I was introduced to buy by my friend, he used it very well, I used it and think it's also very good, it can really save electricity, save about half of the electricity every day, it's really good!	Jingdong prefecture level city in Shandong
Stowe Flagship Store	King of Power Saving	After using it for ten days or so, I checked the power consumption and it did drop, and customer service said it will save more electricity in the long run. The product is also insured, 5 years warranty do not have to worry about quality problems!	Jingdong prefecture level city in Shandong
Stowe Flagship Store	King of Power Saving	The product is good, before already bought a, this is the second, the effect is quite good, anyway, I see the daily electricity bill than before a lot less, this can be the choice!	Jingdong prefecture level city in Shandong
Stowe Flagship Store	King of Power Saving	Originally also very worried about things are not good, get the hand found completely over-anxious, five-star praise, the style is very generous, the quality is very good, the price is also very reasonable!	Jingdong prefecture level city in Shandong
Stowe Flagship Store	King of Power Saving	Quality is good, playing to the old family also buy a, repurchase, this quality is really good, recommended!	Jingdong prefecture level city in Shandong
Stowe Flagship Store	King of Power Saving	Thank you, the owner even sent two fuses.	Jingdong prefecture level city in Shandong
Stowe Flagship Store	King of Power Saving	This store has great stuff, relatively fast shipping and logistics	Jingdong prefecture level city in Shandong
Stowe Flagship Store	King of Power Saving	This product is based on the impossibility of saving electricity, I used 853 kWh of electricity in July, started using the product on August 1, and used 867 kWh of electricity by September 1, is this called	Jingdong prefecture level city in Shandong
Stowe Flagship Store	King of Power Saving	Power king received, to the home on the trial, when I plugged into the electric panel can not see the effect of power saving, the meter is still the same speed in the turn, I'll save the king of the manual to play look at the instructions, instructions to speak, have to have a day two days and half a month to save power obviously, then wait half a month to see the effect of power saving it! To half a month there are obvious power saving before continuing to chase Xu.	Jingdong prefecture level city in Shandong
Stowe Flagship Store	King of Power Saving	Received the goods, a look, did not see the manufacturer and production address, can only hold a try to see the mentality of the face	Jingdong prefecture level city in Shandong
Stowe Flagship Store	King of Power Saving	I've only been using it for a few days and I'm not sure if it's an IQ tax? Used to favorable reviews.	Jingdong prefecture level city in Shandong
Stowe Flagship Store	King of Power Saving	Appearance is okay, power saving next time to follow up the evaluation	Jingdong prefecture level city in Shandong
Stowe Flagship Store	King of Power Saving	Shorter time, but hopefully effective and power-saving, to be followed up in the future	Jingdong prefecture level city in Shandong
Stowe Flagship Store	King of Power Saving	Appearance is okay, I do not know if the power saving	Jingdong prefecture level city in Shandong
Stowe Flagship Store	King of Power Saving	Good service, fast logistics, Power Saving King is exquisite and beautiful, support the business, thank you!	Jingdong prefecture level city in Shandong
Stowe Flagship Store	King of Power Saving	Bought three at once to try first first.	Jingdong prefecture level city in Shandong
Stowe Flagship Store	King of Power Saving	The object is the same as the picture, the product is of high quality, the packaging is intact. High-end atmosphere, high power, power saving effect is obvious, average monthly power saving 50%, very satisfied.	Jingdong prefecture level city in Shandong
Stowe Flagship Store	King of Power Saving	Bought back and plugged in, really effective, before 16 degrees of electricity per day, now 8 degrees of electricity per day, open an air conditioner for 12 hours, decisive repurchase of a	Jingdong prefecture level city in Shandong
Stowe Flagship Store	King of Power Saving	The object is the same as the picture, the product is of high quality, and the package is intact. High-end atmosphere and high grade, high power	Jingdong prefecture level city in Shandong
Stowe Flagship Store	King of Power Saving	Goods received, I hope the store said can be energy-saving, first try a month, month after month to see the effect.	Jingdong prefecture level city in Shandong
Stowe Flagship Store	King of Power Saving	This is really good, the material and workmanship is particularly exquisite, the workmanship is really very good, I think I can recommend to my friends to buy	Jingdong prefecture level city in Shandong
Stowe Flagship Store	King of Power Saving	I have to praise, the quality is really good, and the packaging logistics are also good. Really like it, won't make you regret after getting it!	Jingdong prefecture level city in Shandong
Stowe Flagship Store	King of Power Saving	Reputable platform, efficient and professional service, buy with confidence and peace of mind!	Jingdong prefecture level city in Shandong
Stowe Flagship Store	King of Power Saving	Bought one on a trial basis to see if it works after 1-2 months.	Jingdong prefecture level city in Shandong
Stowe Flagship Store	King of Power Saving	Quality is very good, customer service attitude is very very praise, there are problems in a timely manner to solve the problem, the price is not expensive, super value, you can rest assured that the	Jingdong prefecture level city in Shandong
Stowe Flagship Store	King of Power Saving	With it no longer need to be afraid to use electricity, especially good, ready to buy a mom and dad that also	Jingdong prefecture level city in Shandong
Stowe Flagship Store	King of Power Saving	Baby received very like will return to buy the seller attitude is also very like will return to buy the seller attitude is also very like!	Jingdong prefecture level city in Shandong
Stowe Flagship Store	King of Power Saving	Jingdong Mall shopping convenience assured fast assured fast assured fast assured fast	Jingdong prefecture level city in Shandong
Stowe Flagship Store	King of Power Saving	Saving power received, the quality is very good, fine workmanship, especially good, much cheaper than the entity store, it is worth buying!	Jingdong prefecture level city in Shandong
Stowe Flagship Store	King of Power Saving	I did not expect the express delivery so quickly, very save point quality is super good, than the entity store is too cheap.	Jingdong prefecture level city in Shandong
Stowe Flagship Store	King of Power Saving	Have received the baby, very satisfied with a shopping, great worth buying!	Jingdong prefecture level city in Shandong
Stowe Flagship Store	King of Power Saving	This is really more powerful, very satisfied, more energy-saving, better, thank you boss.	Jingdong prefecture level city in Shandong
Stowe Flagship Store	King of Power Saving	Received has been used for two days, the air conditioning can boldly open it! Energy saving effect is really quite good	Jingdong prefecture level city in Shandong
Stowe Flagship Store	King of Power Saving	It is authentic, trust the merchant, the baby has a great texture and the price is moderate, unlimited repurchase!	Jingdong prefecture level city in Shandong
Stowe Flagship Store	King of Power Saving	It is genuine very good, the price is much cheaper, cost-effective, really special cost-effective, it is worth buying!	Jingdong prefecture level city in Shandong
Stowe Flagship Store	King of Power Saving	Very good quality, fine workmanship, low power consumption, long warranty, fast shipping, economical and practical	Jingdong prefecture level city in Shandong

Stowe Flagship Store	King of Power Saving	I've been using it for half a month now, it really saves power and the voltage is stable, it's very good	Jingdong prefecture level city in Shandong
Stowe Flagship Store	King of Power Saving	It's the ideal product ❤, good quality and fast logistics, worth the kudos!	Jingdong prefecture level city in Shandong
Stowe Flagship Store	King of Power Saving	The logistics is very fast, the baby is very good, the shopkeeper is NICE!	Jingdong prefecture level city in Shandong
Stowe Flagship Store	King of Power Saving	Good to use, good quality, save point durable	Jingdong prefecture level city in Shandong
Stowe Flagship Store	King of Power Saving	The quality is very good, customer service attitude is very, very awesome, there are problems in a timely manner to solve the problem	Jingdong prefecture level city in Shandong
Stowe Flagship Store	King of Power Saving	Quality is very good, the use of the effect is very good, more stable, quality assurance	Jingdong prefecture level city in Shandong
Stowe Flagship Store	King of Power Saving	Convenient, inexpensive, and see how it works out?	Jingdong prefecture level city in Shandong
Stowe Flagship Store	King of Power Saving	Very good quality, saves a lot of electricity, very good, worth buying	Jingdong prefecture level city in Shandong
Stowe Flagship Store	King of Power Saving	Power Saving King is easy to use, with the fruit of power saving to use easy to give!	Jingdong prefecture level city in Shandong
Stowe Flagship Store	King of Power Saving	This saver really works, good quality, safe and secure, very satisfied!	Jingdong prefecture level city in Shandong
Stowe Flagship Store	King of Power Saving	It works well, the voltage is very stable effect is good, the quality is also very good satisfaction	Jingdong prefecture level city in Shandong
Stowe Flagship Store	King of Power Saving	The logistics is super super fast! The quality is also very good, indeed value for money	Jingdong prefecture level city in Shandong
Stowe Flagship Store	King of Power Saving	I've only been using it for a month or so and it seems to be saving a lot of power	Jingdong prefecture level city in Shandong
Stowe Flagship Store	King of Power Saving	Very easy to operate, very good material, good value for money.	Jingdong prefecture level city in Shandong
Stowe Flagship Store	King of Power Saving	Aluminum alloy shell, durable, compact power-saving good effect	Jingdong prefecture level city in Shandong
Stowe Flagship Store	King of Power Saving	Nice device, easy to use, doesn't take up much space, recommendable!	Jingdong prefecture level city in Shandong
Stowe Flagship Store	King of Power Saving	Good quality, good price, fast logistics	Jingdong prefecture level city in Shandong
Stowe Flagship Store	King of Power Saving	Friends of the family in use recommended, the effect is good and recommendable!	Jingdong prefecture level city in Shandong
Stowe Flagship Store	King of Power Saving	Cheaper than brick and mortar stores worth buying is authentic	Jingdong prefecture level city in Shandong
Stowe Flagship Store	King of Power Saving	Merchants ship quickly, the price is cheaper than the physical store, good use	Jingdong prefecture level city in Shandong
Stowe Flagship Store	King of Power Saving	Very durable, affordable, fast shipping	Jingdong prefecture level city in Shandong
Stowe Flagship Store	King of Power Saving	Packaging tight quality is very good easy to use good results	Jingdong prefecture level city in Shandong
Stowe Flagship Store	King of Power Saving	Save the grid. It's in the whole house. It's saving money on the electric bill.	Jingdong prefecture level city in Shandong
Stowe Flagship Store	King of Power Saving	I used it today, it works well, I'm very satisfied	Jingdong prefecture level city in Shandong
Stowe Flagship Store	King of Power Saving	It really saves a lot of electricity after installation	Jingdong prefecture level city in Shandong
Stowe Flagship Store	King of Power Saving	Love it. Satisfied.	Jingdong prefecture level city in Shandong
Stowe Flagship Store	King of Power Saving	Doesn't feel like it's working.	Jingdong prefecture level city in Shandong
Stowe Flagship Store	King of Power Saving	I'll comment after using it.	Jingdong prefecture level city in Shandong
Stowe Flagship Store	King of Power Saving	I'm trying it out to see if it saves power, and I'll buy two more if it works well.	Jingdong prefecture level city in Shandong
Stowe Flagship Store	King of Power Saving	It is genuine very good, the price is much cheaper, cost-effective, really special cost-effective, it is worth buying!	Jingdong prefecture level city in Shandong
Stowe Flagship Store	King of Power Saving	This thing can really save electricity, I was introduced to buy by my friend, he used it very well, I used it and think it's also very good, it can really save electricity, save about half of the electricity every day, it's really good!	Jingdong prefecture level city in Shandong
Stowe Flagship Store	King of Power Saving	After using it for ten days or so, I checked the power consumption and it did drop, and customer service said it will save more electricity in the long run. The product is also insured, 5 years warranty do not have to worry about quality problems!	Jingdong prefecture level city in Shandong
Stowe Flagship Store	King of Power Saving	The product is good, before already bought a, this is the second, the effect is quite good, anyway, I see the daily electricity bill than before a lot less, this can be the choice!	Jingdong prefecture level city in Shandong
Stowe Flagship Store	King of Power Saving	Originally also very worried about things are not good, get the hand found completely over-anxious, five-star praise, the style is very generous, the quality is very good, the price is also very reasonable!	Jingdong prefecture level city in Shandong
Stowe Flagship Store	King of Power Saving	Quality is good, playing to the old family also buy a, repurchase, this quality is really good, recommended!	Jingdong prefecture level city in Shandong
Stowe Flagship Store	King of Power Saving	Thank you, the owner even sent two fuses.	Jingdong prefecture level city in Shandong
Stowe Flagship Store	King of Power Saving	This store has great stuff, relatively fast shipping and logistics	Jingdong prefecture level city in Shandong
Stowe Flagship Store	King of Power Saving	This product is based on the impossibility of saving electricity, I used 853 kWh of electricity in July, started using the product on August 1, and used 867 kWh of electricity by September 1, is this called	Jingdong prefecture level city in Shandong
Stowe Flagship Store	King of Power Saving	Power king received, to the home on the trial, when I plugged into the electric panel can not see the effect of power saving, the meter is still the same speed in the turn, I'll save the king of the manual to play look at the instructions, instructions to speak, have to have a day two days and half a month to save power obviously, then wait half a month to see the effect of power saving it! To half a month there are obvious power saving before continuing to chase Xu.	Jingdong prefecture level city in Shandong
Stowe Flagship Store	King of Power Saving	Received the goods, a look, did not see the manufacturer and production address, can only hold a try to see the mentality of the face	Jingdong prefecture level city in Shandong
Stowe Flagship Store	King of Power Saving	I've only been using it for a few days and I'm not sure if it's an IQ tax? Used to favorable reviews.	Jingdong prefecture level city in Shandong
Stowe Flagship Store	King of Power Saving	Appearance is okay, power saving next time to follow up the evaluation	Jingdong prefecture level city in Shandong
Stowe Flagship Store	King of Power Saving	Shorter time, but hopefully effective and power-saving, to be followed up in the future	Jingdong prefecture level city in Shandong
Stowe Flagship Store	King of Power Saving	Appearance is okay, I do not know if the power saving	Jingdong prefecture level city in Shandong
Stowe Flagship Store	King of Power Saving	Good service, fast logistics, Power Saving King is exquisite and beautiful, support the business, thank you!	Jingdong prefecture level city in Shandong
Stowe Flagship Store	King of Power Saving	Bought three at once to try first first.	Jingdong prefecture level city in Shandong
Stowe Flagship Store	King of Power Saving	The object is the same as the picture, the product is of high quality, the packaging is intact. High-end atmosphere, high power, power saving effect is obvious, average monthly power saving 50%, very satisfied.	Jingdong prefecture level city in Shandong
Stowe Flagship Store	King of Power Saving	Bought back and plugged in, really effective, before 16 degrees of electricity per day, now 8 degrees of electricity per day, open an air conditioner for 12 hours, decisive repurchase of a	Jingdong prefecture level city in Shandong
Stowe Flagship Store	King of Power Saving	The object is the same as the picture, the product is of high quality, and the package is intact. High-end atmosphere and high grade, high power	Jingdong prefecture level city in Shandong

Stowe Flagship Store	King of Power Saving	Goods received, I hope the store said can be energy-saving, first try a month, month after month to see the effect.	Jingdong prefecture level city in Shandong
Stowe Flagship Store	King of Power Saving	This is really good, the material and workmanship is particularly exquisite, the workmanship is really very good, I think I can recommend to my friends to buy	Jingdong prefecture level city in Shandong
Stowe Flagship Store	King of Power Saving	I have to praise, the quality is really good, and the packaging logistics are also good. Really like it, won't make you regret after getting it!	Jingdong prefecture level city in Shandong
Stowe Flagship Store	King of Power Saving	Reputable platform, efficient and professional service, buy with confidence and peace of mind!	Jingdong prefecture level city in Shandong
Stowe Flagship Store	King of Power Saving	Bought one on a trial basis to see if it works after 1-2 months.	Jingdong prefecture level city in Shandong
Stowe Flagship Store	King of Power Saving	Quality is very good, customer service attitude is very very praise, there are problems in a timely manner to solve the problem, the price is not expensive, super value, you can rest assured that the	Jingdong prefecture level city in Shandong
Stowe Flagship Store	King of Power Saving	With it no longer need to be afraid to use electricity, especially good, ready to buy a mom and dad that also	Jingdong prefecture level city in Shandong
Stowe Flagship Store	King of Power Saving	Baby received very like will return to buy the seller attitude is also very like will return to buy the seller attitude is also very like!	Jingdong prefecture level city in Shandong
Stowe Flagship Store	King of Power Saving	Jingdong Mall shopping convenience assured fast assured fast assured fast assured fast	Jingdong prefecture level city in Shandong
Stowe Flagship Store	King of Power Saving	Saving power received, the quality is very good, fine workmanship, especially good, much cheaper than the entity store, it is worth buying!	Jingdong prefecture level city in Shandong
Stowe Flagship Store	King of Power Saving	I did not expect the express delivery so quickly, very save point quality is super good, than the entity store is too cheap.	Jingdong prefecture level city in Shandong
Stowe Flagship Store	King of Power Saving	Have received the baby, very satisfied with a shopping, great worth buying!	Jingdong prefecture level city in Shandong
Stowe Flagship Store	King of Power Saving	This is really more powerful, very satisfied, more energy-saving, better, thank you boss.	Jingdong prefecture level city in Shandong
Stowe Flagship Store	King of Power Saving	Received has been used for two days, the air conditioning can boldly open it! Energy saving effect is really quite good	Jingdong prefecture level city in Shandong
Stowe Flagship Store	King of Power Saving	It is authentic, trust the merchant, the baby has a great texture and the price is moderate, unlimited repurchase!	Jingdong prefecture level city in Shandong
Stowe Flagship Store	King of Power Saving	It is genuine very good, the price is much cheaper, cost-effective, really special cost-effective, it is worth buying!	Jingdong prefecture level city in Shandong
Stowe Flagship Store	King of Power Saving	Very good quality, fine workmanship, low power consumption, long warranty, fast shipping, economical and practical	Jingdong prefecture level city in Shandong
Stowe Flagship Store	King of Power Saving	I've been using it for half a month now, it really saves power and the voltage is stable, it's very good	Jingdong prefecture level city in Shandong
Stowe Flagship Store	King of Power Saving	It's the ideal product 💞, good quality and fast logistics, worth the kudos!	Jingdong prefecture level city in Shandong
Stowe Flagship Store	King of Power Saving	The logistics is very fast, the baby is very good, the shopkeeper is NICE!	Jingdong prefecture level city in Shandong
Stowe Flagship Store	King of Power Saving	Good to use, good quality, save point durable	Jingdong prefecture level city in Shandong
Stowe Flagship Store	King of Power Saving	The quality is very good, customer service attitude is very, very awesome, there are problems in a timely manner to solve the problem	Jingdong prefecture level city in Shandong
Stowe Flagship Store	King of Power Saving	Quality is very good, the use of the effect is very good, more stable, quality assurance	Jingdong prefecture level city in Shandong
Stowe Flagship Store	King of Power Saving	Convenient, inexpensive, and see how it works out?	Jingdong prefecture level city in Shandong
Stowe Flagship Store	King of Power Saving	Very good quality, saves a lot of electricity, very good, worth buying	Jingdong prefecture level city in Shandong
Stowe Flagship Store	King of Power Saving	Power Saving King is easy to use, with the fruit of power saving to use easy to give!	Jingdong prefecture level city in Shandong
Stowe Flagship Store	King of Power Saving	This saver really works, good quality, safe and secure, very satisfied!	Jingdong prefecture level city in Shandong
Stowe Flagship Store	King of Power Saving	It works well, the voltage is very stable effect is good, the quality is also very good satisfaction	Jingdong prefecture level city in Shandong
Stowe Flagship Store	King of Power Saving	The logistics is super super fast! The quality is also very good, indeed value for money	Jingdong prefecture level city in Shandong
Stowe Flagship Store	King of Power Saving	I've only been using it for a month or so and it seems to be saving a lot of power	Jingdong prefecture level city in Shandong
Stowe Flagship Store	King of Power Saving	Very easy to operate, very good material, good value for money.	Jingdong prefecture level city in Shandong
Stowe Flagship Store	King of Power Saving	Aluminum alloy shell, durable, compact power-saving good effect	Jingdong prefecture level city in Shandong
Stowe Flagship Store	King of Power Saving	Nice device, easy to use, doesn't take up much space, recommendable!	Jingdong prefecture level city in Shandong
Stowe Flagship Store	King of Power Saving	Good quality, good price, fast logistics	Jingdong prefecture level city in Shandong
Stowe Flagship Store	King of Power Saving	Friends of the family in use recommended, the effect is good and recommendable!	Jingdong prefecture level city in Shandong
Stowe Flagship Store	King of Power Saving	Cheaper than brick and mortar stores worth buying is authentic	Jingdong prefecture level city in Shandong
Stowe Flagship Store	King of Power Saving	Merchants ship quickly, the price is cheaper than the physical store, good use	Jingdong prefecture level city in Shandong
Stowe Flagship Store	King of Power Saving	Very durable, affordable, fast shipping	Jingdong prefecture level city in Shandong
Stowe Flagship Store	King of Power Saving	Packaging tight quality is very good easy to use good results	Jingdong prefecture level city in Shandong
Stowe Flagship Store	King of Power Saving	Save the grid. It's in the whole house. It's saving money on the electric bill.	Jingdong prefecture level city in Shandong
Stowe Flagship Store	King of Power Saving	I used it today, it works well, I'm very satisfied	Jingdong prefecture level city in Shandong
Stowe Flagship Store	King of Power Saving	It really saves a lot of electricity after installation	Jingdong prefecture level city in Shandong
Stowe Flagship Store	King of Power Saving	Love it. Satisfied.	Jingdong prefecture level city in Shandong
Stowe Flagship Store	King of Power Saving	Doesn't feel like it's working.	Jingdong prefecture level city in Shandong
Stowe Flagship Store	King of Power Saving	I'll comment after using it.	Jingdong prefecture level city in Shandong
Stowe Flagship Store	King of Power Saving	I'm trying it out to see if it saves power, and I'll buy two more if it works well.	Jingdong prefecture level city in Shandong
Stowe Flagship Store	King of Power Saving	It is genuine very good, the price is much cheaper, cost-effective, really special cost-effective, it is worth buying!	Jingdong prefecture level city in Shandong
Stowe Flagship Store	King of Power Saving	This thing can really save electricity, I was introduced to buy by my friend, he used it very well, I used it and think it's also very good, it can really save electricity, save about half of the electricity every day, it's really good!	Jingdong prefecture level city in Shandong
Stowe Flagship Store	King of Power Saving	After using it for ten days or so, I checked the power consumption and it did drop, and customer service said it will save more electricity in the long run. The product is also insured, 5 years warranty do not have to worry about quality problems!	Jingdong prefecture level city in Shandong
Stowe Flagship Store	King of Power Saving	The product is good, before already bought a, this is the second, the effect is quite good, anyway, I see the daily electricity bill than before a lot less, this can be the choice!	Jingdong prefecture level city in Shandong

Stowe Flagship Store	King of Power Saving	Originally also very worried about things are not good, get the hand found completely over-anxious, five-star praise, the style is very generous, the quality is very good, the price is also very reasonable!	Jingdong prefecture level city in Shandong
Stowe Flagship Store	King of Power Saving	Quality is good, playing to the old family also buy a, repurchase, this quality is really good, recommended!	Jingdong prefecture level city in Shandong
Stowe Flagship Store	King of Power Saving	Thank you, the owner even sent two fuses.	Jingdong prefecture level city in Shandong
Stowe Flagship Store	King of Power Saving	This store has great stuff, relatively fast shipping and logistics	Jingdong prefecture level city in Shandong
Stowe Flagship Store	King of Power Saving	This product is based on the impossibility of saving electricity, I used 853 kWh of electricity in July, started using the product on August 1, and used 867 kWh of electricity by September 1, is this called	Jingdong prefecture level city in Shandong
Stowe Flagship Store	King of Power Saving	Power king received, to the home on the trial, when I plugged into the electric panel can not see the effect of power saving, the meter is still the same speed in the turn, I'll save the king of the manual to play look at the instructions, instructions to speak, have to have a day two days and half a month to save power obviously, then wait half a month to see the effect of power saving it! To half a month there are obvious power saving before continuing to chase Xu.	Jingdong prefecture level city in Shandong
Stowe Flagship Store	King of Power Saving	Received the goods, a look, did not see the manufacturer and production address, can only hold a try to see the mentality of the face	Jingdong prefecture level city in Shandong
Stowe Flagship Store	King of Power Saving	I've only been using it for a few days and I'm not sure if it's an IQ tax? Used to favorable reviews.	Jingdong prefecture level city in Shandong
Stowe Flagship Store	King of Power Saving	Appearance is okay, power saving next time to follow up the evaluation	Jingdong prefecture level city in Shandong
Stowe Flagship Store	King of Power Saving	Shorter time, but hopefully effective and power-saving, to be followed up in the future	Jingdong prefecture level city in Shandong
Stowe Flagship Store	King of Power Saving	Appearance is okay, I do not know if the power saving	Jingdong prefecture level city in Shandong
Stowe Flagship Store	King of Power Saving	Good service, fast logistics, Power Saving King is exquisite and beautiful, support the business, thank you!	Jingdong prefecture level city in Shandong
Stowe Flagship Store	King of Power Saving	Bought three at once to try first first.	Jingdong prefecture level city in Shandong
Stowe Flagship Store	King of Power Saving	The object is the same as the picture, the product is of high quality, the packaging is intact. High-end atmosphere, high power, power saving effect is obvious, average monthly power saving 50%, very satisfied.	Jingdong prefecture level city in Shandong
Stowe Flagship Store	King of Power Saving	Bought back and plugged in, really effective, before 16 degrees of electricity per day, now 8 degrees of electricity per day, open an air conditioner for 12 hours, decisive repurchase of a	Jingdong prefecture level city in Shandong
Stowe Flagship Store	King of Power Saving	The object is the same as the picture, the product is of high quality, and the package is intact. High-end atmosphere and high grade, high power	Jingdong prefecture level city in Shandong
Stowe Flagship Store	King of Power Saving	Goods received, I hope the store said can be energy-saving, first try a month, month after month to see the effect.	Jingdong prefecture level city in Shandong
Stowe Flagship Store	King of Power Saving	This is really good, the material and workmanship is particularly exquisite, the workmanship is really very good, I think I can recommend to my friends to buy	Jingdong prefecture level city in Shandong
Stowe Flagship Store	King of Power Saving	I have to praise, the quality is really good, and the packaging logistics are also good. Really like it, won't make you regret after getting it!	Jingdong prefecture level city in Shandong
Stowe Flagship Store	King of Power Saving	Reputable platform, efficient and professional service, buy with confidence and peace of mind!	Jingdong prefecture level city in Shandong
Stowe Flagship Store	King of Power Saving	Bought one on a trial basis to see if it works after 1-2 months.	Jingdong prefecture level city in Shandong
Stowe Flagship Store	King of Power Saving	Quality is very good, customer service attitude is very very praise, there are problems in a timely manner to solve the problem, the price is not expensive, super value, you can rest assured that the	Jingdong prefecture level city in Shandong
Stowe Flagship Store	King of Power Saving	With it no longer need to be afraid to use electricity, especially good, ready to buy a mom and dad that also	Jingdong prefecture level city in Shandong
Stowe Flagship Store	King of Power Saving	Baby received very like will return to buy the seller attitude is also very like will return to buy the seller attitude is also very like!	Jingdong prefecture level city in Shandong
Stowe Flagship Store	King of Power Saving	Jingdong Mall shopping convenience assured fast assured fast assured fast assured fast	Jingdong prefecture level city in Shandong
Stowe Flagship Store	King of Power Saving	Saving power received, the quality is very good, fine workmanship, especially good, much cheaper than the entity store, it is worth buying!	Jingdong prefecture level city in Shandong
Stowe Flagship Store	King of Power Saving	I did not expect the express delivery so quickly, very save point quality is super good, than the entity store is too cheap.	Jingdong prefecture level city in Shandong
Stowe Flagship Store	King of Power Saving	Have received the baby, very satisfied with a shopping, great worth buying!	Jingdong prefecture level city in Shandong
Stowe Flagship Store	King of Power Saving	This is really more powerful, very satisfied, more energy-saving, better, thank you boss.	Jingdong prefecture level city in Shandong
Stowe Flagship Store	King of Power Saving	Received has been used for two days, the air conditioning can boldly open it! Energy saving effect is really quite good	Jingdong prefecture level city in Shandong
Stowe Flagship Store	King of Power Saving	It is authentic, trust the merchant, the baby has a great texture and the price is moderate, unlimited repurchase!	Jingdong prefecture level city in Shandong
Stowe Flagship Store	King of Power Saving	It is genuine very good, the price is much cheaper, cost-effective, really special cost-effective, it is worth buying!	Jingdong prefecture level city in Shandong
Stowe Flagship Store	King of Power Saving	Very good quality, fine workmanship, low power consumption, long warranty, fast shipping, economical and practical	Jingdong prefecture level city in Shandong
Stowe Flagship Store	King of Power Saving	I've been using it for half a month now, it really saves power and the voltage is stable, it's very good	Jingdong prefecture level city in Shandong
Stowe Flagship Store	King of Power Saving	It's the ideal product, good quality and fast logistics, worth the kudos!	Jingdong prefecture level city in Shandong
Stowe Flagship Store	King of Power Saving	The logistics is very fast, the baby is very good, the shopkeeper is NICE!	Jingdong prefecture level city in Shandong
Stowe Flagship Store	King of Power Saving	Good to use, good quality, save point durable	Jingdong prefecture level city in Shandong
Stowe Flagship Store	King of Power Saving	The quality is very good, customer service attitude is very, very awesome, there are problems in a timely manner to solve the problem	Jingdong prefecture level city in Shandong
Stowe Flagship Store	King of Power Saving	Quality is very good, the use of the effect is very good, more stable, quality assurance	Jingdong prefecture level city in Shandong
Stowe Flagship Store	King of Power Saving	Convenient, inexpensive, and see how it works out?	Jingdong prefecture level city in Shandong
Stowe Flagship Store	King of Power Saving	Very good quality, saves a lot of electricity, very good, worth buying	Jingdong prefecture level city in Shandong
Stowe Flagship Store	King of Power Saving	Power Saving King is easy to use, with the fruit of power saving to use easy to give!	Jingdong prefecture level city in Shandong
Stowe Flagship Store	King of Power Saving	This saver really works, good quality, safe and secure, very satisfied!	Jingdong prefecture level city in Shandong
Stowe Flagship Store	King of Power Saving	It works well, the voltage is very stable effect is good, the quality is also very good satisfaction	Jingdong prefecture level city in Shandong
Stowe Flagship Store	King of Power Saving	The logistics is super super fast! The quality is also very good, indeed value for money	Jingdong prefecture level city in Shandong
Stowe Flagship Store	King of Power Saving	I've only been using it for a month or so and it seems to be saving a lot of power	Jingdong prefecture level city in Shandong
Stowe Flagship Store	King of Power Saving	Very easy to operate, very good material, good value for money.	Jingdong prefecture level city in Shandong
Stowe Flagship Store	King of Power Saving	Aluminum alloy shell, durable, compact power-saving good effect	Jingdong prefecture level city in Shandong
Stowe Flagship Store	King of Power Saving	Nice device, easy to use, doesn't take up much space, recommendable!	Jingdong prefecture level city in Shandong
Stowe Flagship Store	King of Power Saving	Good quality, good price, fast logistics	Jingdong prefecture level city in Shandong

Stowe Flagship Store	King of Power Saving	Friends of the family in use recommended, the effect is good and recommendable!	Jingdong prefecture level city in Shandong
Stowe Flagship Store	King of Power Saving	Cheaper than brick and mortar stores worth buying is authentic	Jingdong prefecture level city in Shandong
Stowe Flagship Store	King of Power Saving	Merchants ship quickly, the price is cheaper than the physical store, good use	Jingdong prefecture level city in Shandong
Stowe Flagship Store	King of Power Saving	Very durable, affordable, fast shipping	Jingdong prefecture level city in Shandong
Stowe Flagship Store	King of Power Saving	Packaging tight quality is very good easy to use good results	Jingdong prefecture level city in Shandong
Stowe Flagship Store	King of Power Saving	Save the grid. It's in the whole house. It's saving money on the electric bill.	Jingdong prefecture level city in Shandong
Stowe Flagship Store	King of Power Saving	I used it today, it works well, I'm very satisfied	Jingdong prefecture level city in Shandong
Stowe Flagship Store	King of Power Saving	It really saves a lot of electricity after installation	Jingdong prefecture level city in Shandong
Stowe Flagship Store	King of Power Saving	Love it. Satisfied.	Jingdong prefecture level city in Shandong
Stowe Flagship Store	King of Power Saving	Doesn't feel like it's working.	Jingdong prefecture level city in Shandong
Stowe Flagship Store	King of Power Saving	I'll comment after using it.	Jingdong prefecture level city in Shandong
Stowe Flagship Store	King of Power Saving	I'm trying it out to see if it saves power, and I'll buy two more if it works well.	Jingdong prefecture level city in Shandong
Stowe Flagship Store	King of Power Saving	It is genuine very good, the price is much cheaper, cost-effective, really special cost-effective, it is worth buying!	Jingdong prefecture level city in Shandong
Stowe Flagship Store	King of Power Saving	This thing can really save electricity, I was introduced to buy by my friend, he used it very well, I used it and think it's also very good, it can really save electricity, save about half of the electricity every day, it's really good!	Jingdong prefecture level city in Shandong
Stowe Flagship Store	King of Power Saving	After using it for ten days or so, I checked the power consumption and it did drop, and customer service said it will save more electricity in the long run. The product is also insured, 5 years warranty do not have to worry about quality problems!	Jingdong prefecture level city in Shandong
Stowe Flagship Store	King of Power Saving	The product is good, before already bought a, this is the second, the effect is quite good, anyway, I see the daily electricity bill than before a lot less, this can be the choice!	Jingdong prefecture level city in Shandong
Stowe Flagship Store	King of Power Saving	Originally also very worried about things are not good, get the hand found completely over-anxious, five-star praise, the style is very generous, the quality is very good, the price is also very reasonable!	Jingdong prefecture level city in Shandong
Stowe Flagship Store	King of Power Saving	Quality is good, playing to the old family also buy a, repurchase, this quality is really good, recommended!	Jingdong prefecture level city in Shandong
Stowe Flagship Store	King of Power Saving	Thank you, the owner even sent two fuses.	Jingdong prefecture level city in Shandong
Stowe Flagship Store	King of Power Saving	This store has great stuff, relatively fast shipping and logistics	Jingdong prefecture level city in Shandong
Stowe Flagship Store	King of Power Saving	This product is based on the impossibility of saving electricity, I used 853 kWh of electricity in July, started using the product on August 1, and used 867 kWh of electricity by September 1, is this called	Jingdong prefecture level city in Shandong
Stowe Flagship Store	King of Power Saving	Power king received, to the home on the trial, when I plugged into the electric panel can not see the effect of power saving, the meter is still the same speed in the turn, I'll save the king of the manual to play look at the instructions, instructions to speak, have to have a day two days and half a month to save power obviously, then wait half a month to see the effect of power saving it! To half a month there are obvious power saving before continuing to chase Xu.	Jingdong prefecture level city in Shandong
Stowe Flagship Store	King of Power Saving	Received the goods, a look, did not see the manufacturer and production address, can only hold a try to see the mentality of the face	Jingdong prefecture level city in Shandong
Stowe Flagship Store	King of Power Saving	I've only been using it for a few days and I'm not sure if it's an IQ tax? Used to favorable reviews.	Jingdong prefecture level city in Shandong
Stowe Flagship Store	King of Power Saving	Appearance is okay, power saving next time to follow up the evaluation	Jingdong prefecture level city in Shandong
Stowe Flagship Store	King of Power Saving	Shorter time, but hopefully effective and power-saving, to be followed up in the future	Jingdong prefecture level city in Shandong
Stowe Flagship Store	King of Power Saving	Appearance is okay, I do not know if the power saving	Jingdong prefecture level city in Shandong
Stowe Flagship Store	King of Power Saving	Good service, fast logistics, Power Saving King is exquisite and beautiful, support the business, thank you!	Jingdong prefecture level city in Shandong
Stowe Flagship Store	King of Power Saving	Bought three at once to try first first.	Jingdong prefecture level city in Shandong
Stowe Flagship Store	King of Power Saving	The object is the same as the picture, the product is of high quality, the packaging is intact. High-end atmosphere, high power, power saving effect is obvious, average monthly power saving 50%, very satisfied.	Jingdong prefecture level city in Shandong
Stowe Flagship Store	King of Power Saving	Bought back and plugged in, really effective, before 16 degrees of electricity per day, now 8 degrees of electricity per day, open an air conditioner for 12 hours, decisive repurchase of a	Jingdong prefecture level city in Shandong
Stowe Flagship Store	King of Power Saving	The object is the same as the picture, the product is of high quality, and the package is intact. High-end atmosphere and high grade, high power	Jingdong prefecture level city in Shandong
Stowe Flagship Store	King of Power Saving	Goods received, I hope the store said can be energy-saving, first try a month, month after month to see the effect.	Jingdong prefecture level city in Shandong
Stowe Flagship Store	King of Power Saving	This is really good, the material and workmanship is particularly exquisite, the workmanship is really very good, I think I can recommend to my friends to buy	Jingdong prefecture level city in Shandong
Stowe Flagship Store	King of Power Saving	I have to praise, the quality is really good, and the packaging logistics are also good. Really like it, won't make you regret after getting it!	Jingdong prefecture level city in Shandong
Stowe Flagship Store	King of Power Saving	Reputable platform, efficient and professional service, buy with confidence and peace of mind!	Jingdong prefecture level city in Shandong
Stowe Flagship Store	King of Power Saving	Bought one on a trial basis to see if it works after 1-2 months.	Jingdong prefecture level city in Shandong
Stowe Flagship Store	King of Power Saving	Quality is very good, customer service attitude is very very praise, there are problems in a timely manner to solve the problem, the price is not expensive, super value, you can rest assured that the	Jingdong prefecture level city in Shandong
Stowe Flagship Store	King of Power Saving	With it no longer need to be afraid to use electricity, especially good, ready to buy a mom and dad that also	Jingdong prefecture level city in Shandong
Stowe Flagship Store	King of Power Saving	Baby received very like will return to buy the seller attitude is also very like will return to buy the seller attitude is also very like!	Jingdong prefecture level city in Shandong
Stowe Flagship Store	King of Power Saving	Jingdong Mall shopping convenience assured fast assured fast assured fast assured fast	Jingdong prefecture level city in Shandong
Stowe Flagship Store	King of Power Saving	Saving power received, the quality is very good, fine workmanship, especially good, much cheaper than the entity store, it is worth buying!	Jingdong prefecture level city in Shandong
Stowe Flagship Store	King of Power Saving	I did not expect the express delivery so quickly, very save point quality is super good, than the entity store is too cheap.	Jingdong prefecture level city in Shandong
Stowe Flagship Store	King of Power Saving	Have received the baby, very satisfied with a shopping, great worth buying!	Jingdong prefecture level city in Shandong
Stowe Flagship Store	King of Power Saving	This is really more powerful, very satisfied, more energy-saving, better, thank you boss.	Jingdong prefecture level city in Shandong
Stowe Flagship Store	King of Power Saving	Received has been used for two days, the air conditioning can boldly open it! Energy saving effect is really quite good	Jingdong prefecture level city in Shandong
Stowe Flagship Store	King of Power Saving	It is authentic, trust the merchant, the baby has a great texture and the price is moderate, unlimited repurchase!	Jingdong prefecture level city in Shandong
Stowe Flagship Store	King of Power Saving	It is genuine very good, the price is much cheaper, cost-effective, really special cost-effective, it is worth buying!	Jingdong prefecture level city in Shandong
Stowe Flagship Store	King of Power Saving	Very good quality, fine workmanship, low power consumption, long warranty, fast shipping, economical and practical	Jingdong prefecture level city in Shandong

Stowe Flagship Store	King of Power Saving	I've been using it for half a month now, it really saves power and the voltage is stable, it's very good	Jingdong prefecture level city in Shandong
Stowe Flagship Store	King of Power Saving	It's the ideal product 💝, good quality and fast logistics, worth the kudos!	Jingdong prefecture level city in Shandong
Stowe Flagship Store	King of Power Saving	The logistics is very fast, the baby is very good, the shopkeeper is NICE!	Jingdong prefecture level city in Shandong
Stowe Flagship Store	King of Power Saving	Good to use, good quality, save point durable	Jingdong prefecture level city in Shandong
Stowe Flagship Store	King of Power Saving	The quality is very good, customer service attitude is very, very awesome, there are problems in a timely manner to solve the problem	Jingdong prefecture level city in Shandong
Stowe Flagship Store	King of Power Saving	Quality is very good, the use of the effect is very good, more stable, quality assurance	Jingdong prefecture level city in Shandong
Stowe Flagship Store	King of Power Saving	Convenient, inexpensive, and see how it works out?	Jingdong prefecture level city in Shandong
Stowe Flagship Store	King of Power Saving	Very good quality, saves a lot of electricity, very good, worth buying	Jingdong prefecture level city in Shandong
Stowe Flagship Store	King of Power Saving	Power Saving King is easy to use, with the fruit of power saving to use easy to give!	Jingdong prefecture level city in Shandong
Stowe Flagship Store	King of Power Saving	This saver really works, good quality, safe and secure, very satisfied!	Jingdong prefecture level city in Shandong
Stowe Flagship Store	King of Power Saving	It works well, the voltage is very stable effect is good, the quality is also very good satisfaction	Jingdong prefecture level city in Shandong
Stowe Flagship Store	King of Power Saving	The logistics is super super fast! The quality is also very good, indeed value for money	Jingdong prefecture level city in Shandong
Stowe Flagship Store	King of Power Saving	I've only been using it for a month or so and it seems to be saving a lot of power	Jingdong prefecture level city in Shandong
Stowe Flagship Store	King of Power Saving	Very easy to operate, very good material, good value for money.	Jingdong prefecture level city in Shandong
Stowe Flagship Store	King of Power Saving	Aluminum alloy shell, durable, compact power-saving good effect	Jingdong prefecture level city in Shandong
Stowe Flagship Store	King of Power Saving	Nice device, easy to use, doesn't take up much space, recommendable!	Jingdong prefecture level city in Shandong
Stowe Flagship Store	King of Power Saving	Good quality, good price, fast logistics	Jingdong prefecture level city in Shandong
Stowe Flagship Store	King of Power Saving	Friends of the family in use recommended, the effect is good and recommendable!	Jingdong prefecture level city in Shandong
Stowe Flagship Store	King of Power Saving	Cheaper than brick and mortar stores worth buying is authentic	Jingdong prefecture level city in Shandong
Stowe Flagship Store	King of Power Saving	Merchants ship quickly, the price is cheaper than the physical store, good use	Jingdong prefecture level city in Shandong
Stowe Flagship Store	King of Power Saving	Very durable, affordable, fast shipping	Jingdong prefecture level city in Shandong
Stowe Flagship Store	King of Power Saving	Packaging tight quality is very good easy to use good results	Jingdong prefecture level city in Shandong
Stowe Flagship Store	King of Power Saving	Save the grid. It's in the whole house. It's saving money on the electric bill.	Jingdong prefecture level city in Shandong
Stowe Flagship Store	King of Power Saving	I used it today, it works well, I'm very satisfied	Jingdong prefecture level city in Shandong
Stowe Flagship Store	King of Power Saving	It really saves a lot of electricity after installation	Jingdong prefecture level city in Shandong
Stowe Flagship Store	King of Power Saving	Love it. Satisfied.	Jingdong prefecture level city in Shandong
Stowe Flagship Store	King of Power Saving	Doesn't feel like it's working.	Jingdong prefecture level city in Shandong
Stowe Flagship Store	King of Power Saving	I'll comment after using it.	Jingdong prefecture level city in Shandong
Stowe Flagship Store	King of Power Saving	I'm trying it out to see if it saves power, and I'll buy two more if it works well.	Jingdong prefecture level city in Shandong
Stowe Flagship Store	King of Power Saving	It is genuine very good, the price is much cheaper, cost-effective, really special cost-effective, it is worth buying!	Jingdong prefecture level city in Shandong
Stowe Flagship Store	King of Power Saving	This thing can really save electricity, I was introduced to buy by my friend, he used it very well, I used it and think it's also very good, it can really save electricity, save about half of the electricity every day, it's really good!	Jingdong prefecture level city in Shandong
Stowe Flagship Store	King of Power Saving	After using it for ten days or so, I checked the power consumption and it did drop, and customer service said it will save more electricity in the long run. The product is also insured, 5 years warranty do not have to worry about quality problems!	Jingdong prefecture level city in Shandong
Stowe Flagship Store	King of Power Saving	The product is good, before already bought a, this is the second, the effect is quite good, anyway, I see the daily electricity bill than before a lot less, this can be the choice!	Jingdong prefecture level city in Shandong
Stowe Flagship Store	King of Power Saving	Originally also very worried about things are not good, get the hand found completely over-anxious, five-star praise, the style is very generous, the quality is very good, the price is also very reasonable!	Jingdong prefecture level city in Shandong
Stowe Flagship Store	King of Power Saving	Quality is good, playing to the old family also buy a, repurchase, this quality is really good, recommended!	Jingdong prefecture level city in Shandong
Stowe Flagship Store	King of Power Saving	Thank you, the owner even sent two fuses.	Jingdong prefecture level city in Shandong
Stowe Flagship Store	King of Power Saving	This store has great stuff, relatively fast shipping and logistics	Jingdong prefecture level city in Shandong
Stowe Flagship Store	King of Power Saving	This product is based on the impossibility of saving electricity, I used 853 kWh of electricity in July, started using the product on August 1, and used 867 kWh of electricity by September 1, is this called	Jingdong prefecture level city in Shandong
Stowe Flagship Store	King of Power Saving	Power king received, to the home on the trial, when I plugged into the electric panel can not see the effect of power saving, the meter is still the same speed in the turn, I'll save the king of the manual to play look at the instructions, instructions to speak, have to have a day two days and half a month to save power obviously, then wait half a month to see the effect of power saving it! To half a month there are obvious power saving before continuing to chase Xu.	Jingdong prefecture level city in Shandong
Stowe Flagship Store	King of Power Saving	Received the goods, a look, did not see the manufacturer and production address, can only hold a try to see the mentality of the face	Jingdong prefecture level city in Shandong
Stowe Flagship Store	King of Power Saving	I've only been using it for a few days and I'm not sure if it's an IQ tax? Used to favorable reviews.	Jingdong prefecture level city in Shandong
Stowe Flagship Store	King of Power Saving	Appearance is okay, power saving next time to follow up the evaluation	Jingdong prefecture level city in Shandong
Stowe Flagship Store	King of Power Saving	Shorter time, but hopefully effective and power-saving, to be followed up in the future	Jingdong prefecture level city in Shandong
Stowe Flagship Store	King of Power Saving	Appearance is okay, I do not know if the power saving	Jingdong prefecture level city in Shandong
Stowe Flagship Store	King of Power Saving	Good service, fast logistics, Power Saving King is exquisite and beautiful, support the business, thank you!	Jingdong prefecture level city in Shandong
Stowe Flagship Store	King of Power Saving	Bought three at once to try first first.	Jingdong prefecture level city in Shandong
Stowe Flagship Store	King of Power Saving	The object is the same as the picture, the product is of high quality, the packaging is intact. High-end atmosphere, high power, power saving effect is obvious, average monthly power saving 50%, very satisfied.	Jingdong prefecture level city in Shandong
Stowe Flagship Store	King of Power Saving	Bought back and plugged in, really effective, before 16 degrees of electricity per day, now 8 degrees of electricity per day, open an air conditioner for 12 hours, decisive repurchase of a	Jingdong prefecture level city in Shandong
Stowe Flagship Store	King of Power Saving	The object is the same as the picture, the product is of high quality, and the package is intact. High-end atmosphere and high grade, high power	Jingdong prefecture level city in Shandong

Stowe Flagship Store	King of Power Saving	Goods received, I hope the store said can be energy-saving, first try a month, month after month to see the effect.	Jingdong prefecture level city in Shandong
Stowe Flagship Store	King of Power Saving	This is really good, the material and workmanship is particularly exquisite, the workmanship is really very good, I think I can recommend to my friends to buy	Jingdong prefecture level city in Shandong
Stowe Flagship Store	King of Power Saving	I have to praise, the quality is really good, and the packaging logistics are also good. Really like it, won't make you regret after getting it!	Jingdong prefecture level city in Shandong
Stowe Flagship Store	King of Power Saving	Reputable platform, efficient and professional service, buy with confidence and peace of mind!	Jingdong prefecture level city in Shandong
Stowe Flagship Store	King of Power Saving	Bought one on a trial basis to see if it works after 1-2 months.	Jingdong prefecture level city in Shandong
Stowe Flagship Store	King of Power Saving	Quality is very good, customer service attitude is very very praise, there are problems in a timely manner to solve the problem, the price is not expensive, super value, you can rest assured that the	Jingdong prefecture level city in Shandong
Stowe Flagship Store	King of Power Saving	With it no longer need to be afraid to use electricity, especially good, ready to buy a mom and dad that also	Jingdong prefecture level city in Shandong
Stowe Flagship Store	King of Power Saving	Baby received very like will return to buy the seller attitude is also very like will return to buy the seller attitude is also very like!	Jingdong prefecture level city in Shandong
Stowe Flagship Store	King of Power Saving	Jingdong Mall shopping convenience assured fast assured fast assured fast assured fast	Jingdong prefecture level city in Shandong
Stowe Flagship Store	King of Power Saving	Saving power received, the quality is very good, fine workmanship, especially good, much cheaper than the entity store, it is worth buying!	Jingdong prefecture level city in Shandong
Stowe Flagship Store	King of Power Saving	I did not expect the express delivery so quickly, very save point quality is super good, than the entity store is too cheap.	Jingdong prefecture level city in Shandong
Stowe Flagship Store	King of Power Saving	Have received the baby, very satisfied with a shopping, great worth buying!	Jingdong prefecture level city in Shandong
Stowe Flagship Store	King of Power Saving	This is really more powerful, very satisfied, more energy-saving, better, thank you boss.	Jingdong prefecture level city in Shandong
Stowe Flagship Store	King of Power Saving	Received has been used for two days, the air conditioning can boldly open it! Energy saving effect is really quite good	Jingdong prefecture level city in Shandong
Stowe Flagship Store	King of Power Saving	It is authentic, trust the merchant, the baby has a great texture and the price is moderate, unlimited repurchase!	Jingdong prefecture level city in Shandong
Stowe Flagship Store	King of Power Saving	It is genuine very good, the price is much cheaper, cost-effective, really special cost-effective, it is worth buying!	Jingdong prefecture level city in Shandong
Stowe Flagship Store	King of Power Saving	Very good quality, fine workmanship, low power consumption, long warranty, fast shipping, economical and practical	Jingdong prefecture level city in Shandong
Stowe Flagship Store	King of Power Saving	I've been using it for half a month now, it really saves power and the voltage is stable, it's very good	Jingdong prefecture level city in Shandong
Stowe Flagship Store	King of Power Saving	It's the ideal product 💝, good quality and fast logistics, worth the kudos!	Jingdong prefecture level city in Shandong
Stowe Flagship Store	King of Power Saving	The logistics is very fast, the baby is very good, the shopkeeper is NICE!	Jingdong prefecture level city in Shandong
Stowe Flagship Store	King of Power Saving	Good to use, good quality, save point durable	Jingdong prefecture level city in Shandong
Stowe Flagship Store	King of Power Saving	The quality is very good, customer service attitude is very, very awesome, there are problems in a timely manner to solve the problem	Jingdong prefecture level city in Shandong
Stowe Flagship Store	King of Power Saving	Quality is very good, the use of the effect is very good, more stable, quality assurance	Jingdong prefecture level city in Shandong
Stowe Flagship Store	King of Power Saving	Convenient, inexpensive, and see how it works out?	Jingdong prefecture level city in Shandong
Stowe Flagship Store	King of Power Saving	Very good quality, saves a lot of electricity, very good, worth buying	Jingdong prefecture level city in Shandong
Stowe Flagship Store	King of Power Saving	Power Saving King is easy to use, with the fruit of power saving to use easy to give!	Jingdong prefecture level city in Shandong
Stowe Flagship Store	King of Power Saving	This saver really works, good quality, safe and secure, very satisfied!	Jingdong prefecture level city in Shandong
Stowe Flagship Store	King of Power Saving	The logistics is super super fast! The quality is also very good, indeed value for money	Jingdong prefecture level city in Shandong
Stowe Flagship Store	King of Power Saving	It works well, the voltage is very stable effect is good, the quality is also very good satisfaction	Jingdong prefecture level city in Shandong
Stowe Flagship Store	King of Power Saving	I've only been using it for a month or so and it seems to be saving a lot of power	Jingdong prefecture level city in Shandong
Stowe Flagship Store	King of Power Saving	Very easy to operate, very good material, good value for money.	Jingdong prefecture level city in Shandong
Stowe Flagship Store	King of Power Saving	Aluminum alloy shell, durable, compact power-saving good effect	Jingdong prefecture level city in Shandong
Stowe Flagship Store	King of Power Saving	Nice device, easy to use, doesn't take up much space, recommendable!	Jingdong prefecture level city in Shandong
Stowe Flagship Store	King of Power Saving	Good quality, good price, fast logistics	Jingdong prefecture level city in Shandong
Stowe Flagship Store	King of Power Saving	Friends of the family in use recommended, the effect is good and recommendable!	Jingdong prefecture level city in Shandong
Stowe Flagship Store	King of Power Saving	Cheaper than brick and mortar stores worth buying is authentic	Jingdong prefecture level city in Shandong
Stowe Flagship Store	King of Power Saving	Merchants ship quickly, the price is cheaper than the physical store, good use	Jingdong prefecture level city in Shandong
Stowe Flagship Store	King of Power Saving	Very durable, affordable, fast shipping	Jingdong prefecture level city in Shandong
Stowe Flagship Store	King of Power Saving	Packaging tight quality is very good easy to use good results	Jingdong prefecture level city in Shandong
Stowe Flagship Store	King of Power Saving	Save the grid. It's in the whole house. It's saving money on the electric bill.	Jingdong prefecture level city in Shandong
Stowe Flagship Store	King of Power Saving	I used it today, it works well, I'm very satisfied	Jingdong prefecture level city in Shandong
Stowe Flagship Store	King of Power Saving	It really saves a lot of electricity after installation	Jingdong prefecture level city in Shandong
Stowe Flagship Store	King of Power Saving	Love it. Satisfied.	Jingdong prefecture level city in Shandong
Stowe Flagship Store	King of Power Saving	Doesn't feel like it's working.	Jingdong prefecture level city in Shandong
Stowe Flagship Store	King of Power Saving	I'll comment after using it.	Jingdong prefecture level city in Shandong
Stowe Flagship Store	King of Power Saving	I'm trying it out to see if it saves power, and I'll buy two more if it works well.	Jingdong prefecture level city in Shandong
Stowe Flagship Store	King of Power Saving	It is genuine very good, the price is much cheaper, cost-effective, really special cost-effective, it is worth buying!	Jingdong prefecture level city in Shandong
Stowe Flagship Store	King of Power Saving	This thing can really save electricity, I was introduced to buy by my friend, he used it very well, I used it and think it's also very good, it can really save electricity, save about half of the electricity every day, it's really good!	Jingdong prefecture level city in Shandong
Stowe Flagship Store	King of Power Saving	After using it for ten days or so, I checked the power consumption and it did drop, and customer service said it will save more electricity in the long run. The product is also insured, 5 years warranty do not have to worry about quality problems!	Jingdong prefecture level city in Shandong
Stowe Flagship Store	King of Power Saving	The product is good, before already bought a, this is the second, the effect is quite good, anyway, I see the daily electricity bill than before a lot less, this can be the choice!	Jingdong prefecture level city in Shandong

Stowe Flagship Store	King of Power Saving	Originally also very worried about things are not good, get the hand found completely over-anxious, five-star praise, the style is very generous, the quality is very good, the price is also very reasonable!	Jingdong prefecture level city in Shandong
Stowe Flagship Store	King of Power Saving	Quality is good, playing to the old family also buy a, repurchase, this quality is really good, recommended!	Jingdong prefecture level city in Shandong
Stowe Flagship Store	King of Power Saving	Thank you, the owner even sent two fuses.	Jingdong prefecture level city in Shandong
Stowe Flagship Store	King of Power Saving	This store has great stuff, relatively fast shipping and logistics	Jingdong prefecture level city in Shandong
Stowe Flagship Store	King of Power Saving	This product is based on the impossibility of saving electricity, I used 853 kWh of electricity in July, started using the product on August 1, and used 867 kWh of electricity by September 1, is this called	Jingdong prefecture level city in Shandong
Stowe Flagship Store	King of Power Saving	Power king received, to the home on the trial, when I plugged into the electric panel can not see the effect of power saving, the meter is still the same speed in the turn, I'll save the king of the manual to play look at the instructions, instructions to speak, have to have a day two days and half a month to save power obviously, then wait half a month to see the effect of power saving it! To half a month there are obvious power saving before continuing to chase Xu.	Jingdong prefecture level city in Shandong
Stowe Flagship Store	King of Power Saving	Received the goods, a look, did not see the manufacturer and production address, can only hold a try to see the mentality of the face	Jingdong prefecture level city in Shandong
Stowe Flagship Store	King of Power Saving	I've only been using it for a few days and I'm not sure if it's an IQ tax? Used to favorable reviews.	Jingdong prefecture level city in Shandong
Stowe Flagship Store	King of Power Saving	Appearance is okay, power saving next time to follow up the evaluation	Jingdong prefecture level city in Shandong
Stowe Flagship Store	King of Power Saving	Shorter time, but hopefully effective and power-saving, to be followed up in the future	Jingdong prefecture level city in Shandong
Stowe Flagship Store	King of Power Saving	Appearance is okay, I do not know if the power saving	Jingdong prefecture level city in Shandong
Stowe Flagship Store	King of Power Saving	Good service, fast logistics, Power Saving King is exquisite and beautiful, support the business, thank you!	Jingdong prefecture level city in Shandong
Stowe Flagship Store	King of Power Saving	Bought three at once to try first first.	Jingdong prefecture level city in Shandong
Stowe Flagship Store	King of Power Saving	The object is the same as the picture, the product is of high quality, the packaging is intact. High-end atmosphere, high power, power saving effect is obvious, average monthly power saving 50%, very satisfied.	Jingdong prefecture level city in Shandong
Stowe Flagship Store	King of Power Saving	Bought back and plugged in, really effective, before 16 degrees of electricity per day, now 8 degrees of electricity per day, open an air conditioner for 12 hours, decisive repurchase of a	Jingdong prefecture level city in Shandong
Stowe Flagship Store	King of Power Saving	The object is the same as the picture, the product is of high quality, and the package is intact. High-end atmosphere and high grade, high power	Jingdong prefecture level city in Shandong
Stowe Flagship Store	King of Power Saving	Goods received, I hope the store said can be energy-saving, first try a month, month after month to see the effect.	Jingdong prefecture level city in Shandong
Stowe Flagship Store	King of Power Saving	This is really good, the material and workmanship is particularly exquisite, the workmanship is really very good, I think I can recommend to my friends to buy	Jingdong prefecture level city in Shandong
Stowe Flagship Store	King of Power Saving	I have to praise, the quality is really good, and the packaging logistics are also good. Really like it, won't make you regret after getting it!	Jingdong prefecture level city in Shandong
Stowe Flagship Store	King of Power Saving	Reputable platform, efficient and professional service, buy with confidence and peace of mind!	Jingdong prefecture level city in Shandong
Stowe Flagship Store	King of Power Saving	Bought one on a trial basis to see if it works after 1-2 months.	Jingdong prefecture level city in Shandong
Stowe Flagship Store	King of Power Saving	Quality is very good, customer service attitude is very very praise, there are problems in a timely manner to solve the problem, the price is not expensive, super value, you can rest assured that the	Jingdong prefecture level city in Shandong
Stowe Flagship Store	King of Power Saving	With it no longer need to be afraid to use electricity, especially good, ready to buy a mom and dad that also	Jingdong prefecture level city in Shandong
Stowe Flagship Store	King of Power Saving	Baby received very like will return to buy the seller attitude is also very like will return to buy the seller attitude is also very like!	Jingdong prefecture level city in Shandong
Stowe Flagship Store	King of Power Saving	Jingdong Mall shopping convenience assured fast assured fast assured fast assured fast	Jingdong prefecture level city in Shandong
Stowe Flagship Store	King of Power Saving	Saving power received, the quality is very good, fine workmanship, especially good, much cheaper than the entity store, it is worth buying!	Jingdong prefecture level city in Shandong
Stowe Flagship Store	King of Power Saving	I did not expect the express delivery so quickly, very save point quality is super good, than the entity store is too cheap.	Jingdong prefecture level city in Shandong
Stowe Flagship Store	King of Power Saving	Have received the baby, very satisfied with a shopping, great worth buying!	Jingdong prefecture level city in Shandong
Stowe Flagship Store	King of Power Saving	This is really more powerful, very satisfied, more energy-saving, better, thank you boss.	Jingdong prefecture level city in Shandong
Stowe Flagship Store	King of Power Saving	Received has been used for two days, the air conditioning can boldly open it! Energy saving effect is really quite good	Jingdong prefecture level city in Shandong
Stowe Flagship Store	King of Power Saving	It is authentic, trust the merchant, the baby has a great texture and the price is moderate, unlimited repurchase!	Jingdong prefecture level city in Shandong
Stowe Flagship Store	King of Power Saving	It is genuine very good, the price is much cheaper, cost-effective, really special cost-effective, it is worth buying!	Jingdong prefecture level city in Shandong
Stowe Flagship Store	King of Power Saving	Very good quality, fine workmanship, low power consumption, long warranty, fast shipping, economical and practical	Jingdong prefecture level city in Shandong
Stowe Flagship Store	King of Power Saving	I've been using it for half a month now, it really saves power and the voltage is stable, it's very good	Jingdong prefecture level city in Shandong
Stowe Flagship Store	King of Power Saving	It's the ideal product 💝, good quality and fast logistics, worth the kudos!	Jingdong prefecture level city in Shandong
Stowe Flagship Store	King of Power Saving	The logistics is very fast, the baby is very good, the shopkeeper is NICE!	Jingdong prefecture level city in Shandong
Stowe Flagship Store	King of Power Saving	Good to use, good quality, save point durable	Jingdong prefecture level city in Shandong
Stowe Flagship Store	King of Power Saving	The quality is very good, customer service attitude is very, very awesome, there are problems in a timely manner to solve the problem	Jingdong prefecture level city in Shandong
Stowe Flagship Store	King of Power Saving	Quality is very good, the use of the effect is very good, more stable, quality assurance	Jingdong prefecture level city in Shandong
Stowe Flagship Store	King of Power Saving	Convenient, inexpensive, and see how it works out?	Jingdong prefecture level city in Shandong
Stowe Flagship Store	King of Power Saving	Very good quality, saves a lot of electricity, very good, worth buying	Jingdong prefecture level city in Shandong
Stowe Flagship Store	King of Power Saving	Power Saving King is easy to use, with the fruit of power saving to use easy to give!	Jingdong prefecture level city in Shandong
Stowe Flagship Store	King of Power Saving	This saver really works, good quality, safe and secure, very satisfied!	Jingdong prefecture level city in Shandong
Stowe Flagship Store	King of Power Saving	It works well, the voltage is very stable effect is good, the quality is also very good satisfaction	Jingdong prefecture level city in Shandong
Stowe Flagship Store	King of Power Saving	The logistics is super super fast! The quality is also very good, indeed value for money	Jingdong prefecture level city in Shandong
Stowe Flagship Store	King of Power Saving	I've only been using it for a month or so and it seems to be saving a lot of power	Jingdong prefecture level city in Shandong
Stowe Flagship Store	King of Power Saving	Very easy to operate, very good material, good value for money.	Jingdong prefecture level city in Shandong
Stowe Flagship Store	King of Power Saving	Aluminum alloy shell, durable, compact power-saving good effect	Jingdong prefecture level city in Shandong
Stowe Flagship Store	King of Power Saving	Nice device, easy to use, doesn't take up much space, recommendable!	Jingdong prefecture level city in Shandong
Stowe Flagship Store	King of Power Saving	Good quality, good price, fast logistics	Jingdong prefecture level city in Shandong

Stowe Flagship Store	King of Power Saving	Friends of the family in use recommended, the effect is good and recommendable!	Jingdong prefecture level city in Shandong
Stowe Flagship Store	King of Power Saving	Cheaper than brick and mortar stores worth buying is authentic	Jingdong prefecture level city in Shandong
Stowe Flagship Store	King of Power Saving	Merchants ship quickly, the price is cheaper than the physical store, good use	Jingdong prefecture level city in Shandong
Stowe Flagship Store	King of Power Saving	Very durable, affordable, fast shipping	Jingdong prefecture level city in Shandong
Stowe Flagship Store	King of Power Saving	Packaging tight quality is very good easy to use good results	Jingdong prefecture level city in Shandong
Stowe Flagship Store	King of Power Saving	Save the grid. It's in the whole house. It's saving money on the electric bill.	Jingdong prefecture level city in Shandong
Stowe Flagship Store	King of Power Saving	I used it today, it works well, I'm very satisfied	Jingdong prefecture level city in Shandong
Stowe Flagship Store	King of Power Saving	It really saves a lot of electricity after installation	Jingdong prefecture level city in Shandong
Stowe Flagship Store	King of Power Saving	Love it. Satisfied.	Jingdong prefecture level city in Shandong
Stowe Flagship Store	King of Power Saving	Doesn't feel like it's working.	Jingdong prefecture level city in Shandong
Stowe Flagship Store	King of Power Saving	I'll comment after using it.	Jingdong prefecture level city in Shandong
Stowe Flagship Store	King of Power Saving	I'm trying it out to see if it saves power, and I'll buy two more if it works well.	Jingdong prefecture level city in Shandong
Stowe Flagship Store	King of Power Saving	It is genuine very good, the price is much cheaper, cost-effective, really special cost-effective, it is worth buying!	Jingdong prefecture level city in Shandong
Stowe Flagship Store	King of Power Saving	This thing can really save electricity, I was introduced to buy by my friend, he used it very well, I used it and think it's also very good, it can really save electricity, save about half of the electricity every day, it's really good!	Jingdong prefecture level city in Shandong
Stowe Flagship Store	King of Power Saving	After using it for ten days or so, I checked the power consumption and it did drop, and customer service said it will save more electricity in the long run. The product is also insured, 5 years warranty do not have to worry about quality problems!	Jingdong prefecture level city in Shandong
Stowe Flagship Store	King of Power Saving	The product is good, before already bought a, this is the second, the effect is quite good, anyway, I see the daily electricity bill than before a lot less, this can be the choice!	Jingdong prefecture level city in Shandong
Stowe Flagship Store	King of Power Saving	Originally also very worried about things are not good, get the hand found completely over-anxious, five-star praise, the style is very generous, the quality is very good, the price is also very reasonable!	Jingdong prefecture level city in Shandong
Stowe Flagship Store	King of Power Saving	Quality is good, playing to the old family also buy a, repurchase, this quality is really good, recommended!	Jingdong prefecture level city in Shandong
Stowe Flagship Store	King of Power Saving	Thank you, the owner even sent two fuses.	Jingdong prefecture level city in Shandong
Stowe Flagship Store	King of Power Saving	This store has great stuff, relatively fast shipping and logistics	Jingdong prefecture level city in Shandong
Stowe Flagship Store	King of Power Saving	This product is based on the impossibility of saving electricity, I used 853 kWh of electricity in July, started using the product on August 1, and used 867 kWh of electricity by September 1, is this called	Jingdong prefecture level city in Shandong
Stowe Flagship Store	King of Power Saving	Power king received, to the home on the trial, when I plugged into the electric panel can not see the effect of power saving, the meter is still the same speed in the turn, I'll save the king of the manual to play look at the instructions, instructions to speak, have to have a day two days and half a month to save power obviously, then wait half a month to see the effect of power saving it! To half a month there are obvious power saving before continuing to chase Xu.	Jingdong prefecture level city in Shandong
Stowe Flagship Store	King of Power Saving	Received the goods, a look, did not see the manufacturer and production address, can only hold a try to see the mentality of the face	Jingdong prefecture level city in Shandong
Stowe Flagship Store	King of Power Saving	I've only been using it for a few days and I'm not sure if it's an IQ tax? Used to favorable reviews.	Jingdong prefecture level city in Shandong
Stowe Flagship Store	King of Power Saving	Appearance is okay, power saving next time to follow up the evaluation	Jingdong prefecture level city in Shandong
Stowe Flagship Store	King of Power Saving	Shorter time, but hopefully effective and power-saving, to be followed up in the future	Jingdong prefecture level city in Shandong
Stowe Flagship Store	King of Power Saving	Appearance is okay, I do not know if the power saving	Jingdong prefecture level city in Shandong
Stowe Flagship Store	King of Power Saving	Good service, fast logistics, Power Saving King is exquisite and beautiful, support the business, thank you!	Jingdong prefecture level city in Shandong
Stowe Flagship Store	King of Power Saving	Bought three at once to try first first.	Jingdong prefecture level city in Shandong
Stowe Flagship Store	King of Power Saving	The object is the same as the picture, the product is of high quality, the packaging is intact. High-end atmosphere, high power, power saving effect is obvious, average monthly power saving 50%, very satisfied.	Jingdong prefecture level city in Shandong
Stowe Flagship Store	King of Power Saving	Bought back and plugged in, really effective, before 16 degrees of electricity per day, now 8 degrees of electricity per day, open an air conditioner for 12 hours, decisive repurchase of a	Jingdong prefecture level city in Shandong
Stowe Flagship Store	King of Power Saving	The object is the same as the picture, the product is of high quality, and the package is intact. High-end atmosphere and high grade, high power	Jingdong prefecture level city in Shandong
Stowe Flagship Store	King of Power Saving	Goods received, I hope the store said can be energy-saving, first try a month, month after month to see the effect.	Jingdong prefecture level city in Shandong
Stowe Flagship Store	King of Power Saving	This is really good, the material and workmanship is particularly exquisite, the workmanship is really very good, I think I can recommend to my friends to buy	Jingdong prefecture level city in Shandong
Stowe Flagship Store	King of Power Saving	I have to praise, the quality is really good, and the packaging logistics are also good. Really like it, won't make you regret after getting it!	Jingdong prefecture level city in Shandong
Stowe Flagship Store	King of Power Saving	Reputable platform, efficient and professional service, buy with confidence and peace of mind!	Jingdong prefecture level city in Shandong
Stowe Flagship Store	King of Power Saving	Bought one on a trial basis to see if it works after 1-2 months.	Jingdong prefecture level city in Shandong
Stowe Flagship Store	King of Power Saving	Quality is very good, customer service attitude is very very praise, there are problems in a timely manner to solve the problem, the price is not expensive, super value, you can rest assured that the	Jingdong prefecture level city in Shandong
Stowe Flagship Store	King of Power Saving	With it no longer need to be afraid to use electricity, especially good, ready to buy a mom and dad that also	Jingdong prefecture level city in Shandong
Stowe Flagship Store	King of Power Saving	Baby received very like will return to buy the seller attitude is also very like will return to buy the seller attitude is also very like!	Jingdong prefecture level city in Shandong
Stowe Flagship Store	King of Power Saving	Jingdong Mall shopping convenience assured fast assured fast assured fast assured fast	Jingdong prefecture level city in Shandong
Stowe Flagship Store	King of Power Saving	Saving power received, the quality is very good, fine workmanship, especially good, much cheaper than the entity store, it is worth buying!	Jingdong prefecture level city in Shandong
Stowe Flagship Store	King of Power Saving	I did not expect the express delivery so quickly, very save point quality is super good, than the entity store is too cheap.	Jingdong prefecture level city in Shandong
Stowe Flagship Store	King of Power Saving	Have received the baby, very satisfied with a shopping, great worth buying!	Jingdong prefecture level city in Shandong
Stowe Flagship Store	King of Power Saving	This is really more powerful, very satisfied, more energy-saving, better, thank you boss.	Jingdong prefecture level city in Shandong
Stowe Flagship Store	King of Power Saving	Received has been used for two days, the air conditioning can boldly open it! Energy saving effect is really quite good	Jingdong prefecture level city in Shandong
Stowe Flagship Store	King of Power Saving	It is authentic, trust the merchant, the baby has a great texture and the price is moderate, unlimited repurchase!	Jingdong prefecture level city in Shandong
Stowe Flagship Store	King of Power Saving	It is genuine very good, the price is much cheaper, cost-effective, really special cost-effective, it is worth buying!	Jingdong prefecture level city in Shandong
Stowe Flagship Store	King of Power Saving	Very good quality, fine workmanship, low power consumption, long warranty, fast shipping, economical and practical	Jingdong prefecture level city in Shandong

Stowe Flagship Store	King of Power Saving	I've been using it for half a month now, it really saves power and the voltage is stable, it's very good	Jingdong prefecture level city in Shandong
Stowe Flagship Store	King of Power Saving	It's the ideal product ♡, good quality and fast logistics, worth the kudos!	Jingdong prefecture level city in Shandong
Stowe Flagship Store	King of Power Saving	The logistics is very fast, the baby is very good, the shopkeeper is NICE!	Jingdong prefecture level city in Shandong
Stowe Flagship Store	King of Power Saving	Good to use, good quality, save point durable	Jingdong prefecture level city in Shandong
Stowe Flagship Store	King of Power Saving	The quality is very good, customer service attitude is very, very awesome, there are problems in a timely manner to solve the problem	Jingdong prefecture level city in Shandong
Stowe Flagship Store	King of Power Saving	Quality is very good, the use of the effect is very good, more stable, quality assurance	Jingdong prefecture level city in Shandong
Stowe Flagship Store	King of Power Saving	Convenient, inexpensive, and see how it works out?	Jingdong prefecture level city in Shandong
Stowe Flagship Store	King of Power Saving	Very good quality, saves a lot of electricity, very good, worth buying	Jingdong prefecture level city in Shandong
Stowe Flagship Store	King of Power Saving	Power Saving King is easy to use, with the fruit of power saving to use easy to give!	Jingdong prefecture level city in Shandong
Stowe Flagship Store	King of Power Saving	This saver really works, good quality, safe and secure, very satisfied!	Jingdong prefecture level city in Shandong
Stowe Flagship Store	King of Power Saving	It works well, the voltage is very stable effect is good, the quality is also very good satisfaction	Jingdong prefecture level city in Shandong
Stowe Flagship Store	King of Power Saving	The logistics is super super fast! The quality is also very good, indeed value for money	Jingdong prefecture level city in Shandong
Stowe Flagship Store	King of Power Saving	I've only been using it for a month or so and it seems to be saving a lot of power	Jingdong prefecture level city in Shandong
Stowe Flagship Store	King of Power Saving	Very easy to operate, very good material, good value for money.	Jingdong prefecture level city in Shandong
Stowe Flagship Store	King of Power Saving	Aluminum alloy shell, durable, compact power-saving good effect	Jingdong prefecture level city in Shandong
Stowe Flagship Store	King of Power Saving	Nice device, easy to use, doesn't take up much space, recommendable!	Jingdong prefecture level city in Shandong
Stowe Flagship Store	King of Power Saving	Good quality, good price, fast logistics	Jingdong prefecture level city in Shandong
Stowe Flagship Store	King of Power Saving	Friends of the family in use recommended, the effect is good and recommendable!	Jingdong prefecture level city in Shandong
Stowe Flagship Store	King of Power Saving	Cheaper than brick and mortar stores worth buying is authentic	Jingdong prefecture level city in Shandong
Stowe Flagship Store	King of Power Saving	Merchants ship quickly, the price is cheaper than the physical store, good use	Jingdong prefecture level city in Shandong
Stowe Flagship Store	King of Power Saving	Very durable, affordable, fast shipping	Jingdong prefecture level city in Shandong
Stowe Flagship Store	King of Power Saving	Packaging tight quality is very good easy to use good results	Jingdong prefecture level city in Shandong
Stowe Flagship Store	King of Power Saving	Save the grid. It's in the whole house. It's saving money on the electric bill.	Jingdong prefecture level city in Shandong
Stowe Flagship Store	King of Power Saving	I used it today, it works well, I'm very satisfied	Jingdong prefecture level city in Shandong
Stowe Flagship Store	King of Power Saving	It really saves a lot of electricity after installation	Jingdong prefecture level city in Shandong
Stowe Flagship Store	King of Power Saving	Love it. Satisfied.	Jingdong prefecture level city in Shandong
Stowe Flagship Store	King of Power Saving	Doesn't feel like it's working.	Jingdong prefecture level city in Shandong
Stowe Flagship Store	King of Power Saving	I'll comment after using it.	Jingdong prefecture level city in Shandong
Stowe Flagship Store	King of Power Saving	I'm trying it out to see if it saves power, and I'll buy two more if it works well.	Jingdong prefecture level city in Shandong
Stowe Flagship Store	King of Power Saving	It is genuine very good, the price is much cheaper, cost-effective, really special cost-effective, it is worth buying!	Jingdong prefecture level city in Shandong
Stowe Flagship Store	King of Power Saving	This thing can really save electricity, I was introduced to buy by my friend, he used it very well, I used it and think it's also very good, it can really save electricity, save about half of the electricity every day, it's really good!	Jingdong prefecture level city in Shandong
Stowe Flagship Store	King of Power Saving	After using it for ten days or so, I checked the power consumption and it did drop, and customer service said it will save more electricity in the long run. The product is also insured, 5 years warranty do not have to worry about quality problems!	Jingdong prefecture level city in Shandong
Stowe Flagship Store	King of Power Saving	The product is good, before already bought a, this is the second, the effect is quite good, anyway, I see the daily electricity bill than before a lot less, this can be the choice!	Jingdong prefecture level city in Shandong
Stowe Flagship Store	King of Power Saving	Originally also very worried about things are not good, get the hand found completely over-anxious, five-star praise, the style is very generous, the quality is very good, the price is also very reasonable!	Jingdong prefecture level city in Shandong
Stowe Flagship Store	King of Power Saving	Quality is good, playing to the old family also buy a, repurchase, this quality is really good, recommended!	Jingdong prefecture level city in Shandong
Stowe Flagship Store	King of Power Saving	Thank you, the owner even sent two fuses.	Jingdong prefecture level city in Shandong
Stowe Flagship Store	King of Power Saving	This store has great stuff, relatively fast shipping and logistics	Jingdong prefecture level city in Shandong
Stowe Flagship Store	King of Power Saving	This product is based on the impossibility of saving electricity, I used 853 kWh of electricity in July, started using the product on August 1, and used 867 kWh of electricity by September 1, is this called	Jingdong prefecture level city in Shandong
Stowe Flagship Store	King of Power Saving	Power king received, to the home on the trial, when I plugged into the electric panel can not see the effect of power saving, the meter is still the same speed in the turn, I'll save the king of the manual to play look at the instructions, instructions to speak, have to have a day two days and half a month to save power obviously, then wait half a month to see the effect of power saving it! To half a month there are obvious power saving before continuing to chase Xu.	Jingdong prefecture level city in Shandong
Stowe Flagship Store	King of Power Saving	Received the goods, a look, did not see the manufacturer and production address, can only hold a try to see the mentality of the face	Jingdong prefecture level city in Shandong
Stowe Flagship Store	King of Power Saving	I've only been using it for a few days and I'm not sure if it's an IQ tax? Used to favorable reviews.	Jingdong prefecture level city in Shandong
Stowe Flagship Store	King of Power Saving	Appearance is okay, power saving next time to follow up the evaluation	Jingdong prefecture level city in Shandong
Stowe Flagship Store	King of Power Saving	Shorter time, but hopefully effective and power-saving, to be followed up in the future	Jingdong prefecture level city in Shandong
Stowe Flagship Store	King of Power Saving	Appearance is okay, I do not know if the power saving	Jingdong prefecture level city in Shandong
Stowe Flagship Store	King of Power Saving	Good service, fast logistics, Power Saving King is exquisite and beautiful, support the business, thank you!	Jingdong prefecture level city in Shandong
Stowe Flagship Store	King of Power Saving	Bought three at once to try first first.	Jingdong prefecture level city in Shandong
Stowe Flagship Store	King of Power Saving	The object is the same as the picture, the product is of high quality, the packaging is intact. High-end atmosphere, high power, power saving effect is obvious, average monthly power saving 50%, very satisfied.	Jingdong prefecture level city in Shandong
Stowe Flagship Store	King of Power Saving	Bought back and plugged in, really effective, before 16 degrees of electricity per day, now 8 degrees of electricity per day, open an air conditioner for 12 hours, decisive repurchase of a	Jingdong prefecture level city in Shandong
Stowe Flagship Store	King of Power Saving	The object is the same as the picture, the product is of high quality, and the package is intact. High-end atmosphere and high grade, high power	Jingdong prefecture level city in Shandong

Stowe Flagship Store	King of Power Saving	Goods received, I hope the store said can be energy-saving, first try a month, month after month to see the effect.	Jingdong prefecture level city in Shandong
Stowe Flagship Store	King of Power Saving	This is really good, the material and workmanship is particularly exquisite, the workmanship is really very good, I think I can recommend to my friends to buy	Jingdong prefecture level city in Shandong
Stowe Flagship Store	King of Power Saving	I have to praise, the quality is really good, and the packaging logistics are also good. Really like it, won't make you regret after getting it!	Jingdong prefecture level city in Shandong
Stowe Flagship Store	King of Power Saving	Reputable platform, efficient and professional service, buy with confidence and peace of mind!	Jingdong prefecture level city in Shandong
Stowe Flagship Store	King of Power Saving	Bought one on a trial basis to see if it works after 1-2 months.	Jingdong prefecture level city in Shandong
Stowe Flagship Store	King of Power Saving	Quality is very good, customer service attitude is very very praise, there are problems in a timely manner to solve the problem, the price is not expensive, super value, you can rest assured that the	Jingdong prefecture level city in Shandong
Stowe Flagship Store	King of Power Saving	With it no longer need to be afraid to use electricity, especially good, ready to buy a mom and dad that also	Jingdong prefecture level city in Shandong
Stowe Flagship Store	King of Power Saving	Baby received very like will return to buy the seller attitude is also very like will return to buy the seller attitude is also very like!	Jingdong prefecture level city in Shandong
Stowe Flagship Store	King of Power Saving	Jingdong Mall shopping convenience assured fast assured fast assured fast assured fast	Jingdong prefecture level city in Shandong
Stowe Flagship Store	King of Power Saving	Saving power received, the quality is very good, fine workmanship, especially good, much cheaper than the entity store, it is worth buying!	Jingdong prefecture level city in Shandong
Stowe Flagship Store	King of Power Saving	I did not expect the express delivery so quickly, very save point quality is super good, than the entity store is too cheap.	Jingdong prefecture level city in Shandong
Stowe Flagship Store	King of Power Saving	Have received the baby, very satisfied with a shopping, great worth buying!	Jingdong prefecture level city in Shandong
Stowe Flagship Store	King of Power Saving	This is really more powerful, very satisfied, more energy-saving, better, thank you boss.	Jingdong prefecture level city in Shandong
Stowe Flagship Store	King of Power Saving	Received has been used for two days, the air conditioning can boldly open it! Energy saving effect is really quite good	Jingdong prefecture level city in Shandong
Stowe Flagship Store	King of Power Saving	It is authentic, trust the merchant, the baby has a great texture and the price is moderate, unlimited repurchase!	Jingdong prefecture level city in Shandong
Stowe Flagship Store	King of Power Saving	It is genuine very good, the price is much cheaper, cost-effective, really special cost-effective, it is worth buying!	Jingdong prefecture level city in Shandong
Stowe Flagship Store	King of Power Saving	Very good quality, fine workmanship, low power consumption, long warranty, fast shipping, economical and practical	Jingdong prefecture level city in Shandong
Stowe Flagship Store	King of Power Saving	I've been using it for half a month now, it really saves power and the voltage is stable, it's very good	Jingdong prefecture level city in Shandong
Stowe Flagship Store	King of Power Saving	It's the ideal product 💝, good quality and fast logistics, worth the kudos!	Jingdong prefecture level city in Shandong
Stowe Flagship Store	King of Power Saving	The logistics is very fast, the baby is very good, the shopkeeper is NICE!	Jingdong prefecture level city in Shandong
Stowe Flagship Store	King of Power Saving	Good to use, good quality, save point durable	Jingdong prefecture level city in Shandong
Stowe Flagship Store	King of Power Saving	The quality is very good, customer service attitude is very, very awesome, there are problems in a timely manner to solve the problem	Jingdong prefecture level city in Shandong
Stowe Flagship Store	King of Power Saving	Quality is very good, the use of the effect is very good, more stable, quality assurance	Jingdong prefecture level city in Shandong
Stowe Flagship Store	King of Power Saving	Convenient, inexpensive, and see how it works out?	Jingdong prefecture level city in Shandong
Stowe Flagship Store	King of Power Saving	Very good quality, saves a lot of electricity, very good, worth buying	Jingdong prefecture level city in Shandong
Stowe Flagship Store	King of Power Saving	Power Saving King is easy to use, with the fruit of power saving to use easy to give!	Jingdong prefecture level city in Shandong
Stowe Flagship Store	King of Power Saving	This saver really works, good quality, safe and secure, very satisfied!	Jingdong prefecture level city in Shandong
Stowe Flagship Store	King of Power Saving	It works well, the voltage is very stable effect is good, the quality is also very good satisfaction	Jingdong prefecture level city in Shandong
Stowe Flagship Store	King of Power Saving	The logistics is super super fast! The quality is also very good, indeed value for money	Jingdong prefecture level city in Shandong
Stowe Flagship Store	King of Power Saving	I've only been using it for a month or so and it seems to be saving a lot of power	Jingdong prefecture level city in Shandong
Stowe Flagship Store	King of Power Saving	Very easy to operate, very good material, good value for money.	Jingdong prefecture level city in Shandong
Stowe Flagship Store	King of Power Saving	Aluminum alloy shell, durable, compact power-saving good effect	Jingdong prefecture level city in Shandong
Stowe Flagship Store	King of Power Saving	Nice device, easy to use, doesn't take up much space, recommendable!	Jingdong prefecture level city in Shandong
Stowe Flagship Store	King of Power Saving	Good quality, good price, fast logistics	Jingdong prefecture level city in Shandong
Stowe Flagship Store	King of Power Saving	Friends of the family in use recommended, the effect is good and recommendable!	Jingdong prefecture level city in Shandong
Stowe Flagship Store	King of Power Saving	Cheaper than brick and mortar stores worth buying is authentic	Jingdong prefecture level city in Shandong
Stowe Flagship Store	King of Power Saving	Merchants ship quickly, the price is cheaper than the physical store, good use	Jingdong prefecture level city in Shandong
Stowe Flagship Store	King of Power Saving	Very durable, affordable, fast shipping	Jingdong prefecture level city in Shandong
Stowe Flagship Store	King of Power Saving	Packaging tight quality is very good easy to use good results	Jingdong prefecture level city in Shandong
Stowe Flagship Store	King of Power Saving	Save the grid. It's in the whole house. It's saving money on the electric bill.	Jingdong prefecture level city in Shandong
Stowe Flagship Store	King of Power Saving	I used it today, it works well, I'm very satisfied	Jingdong prefecture level city in Shandong
Stowe Flagship Store	King of Power Saving	It really saves a lot of electricity after installation	Jingdong prefecture level city in Shandong
Stowe Flagship Store	King of Power Saving	Love it. Satisfied.	Jingdong prefecture level city in Shandong
Stowe Flagship Store	King of Power Saving	Doesn't feel like it's working.	Jingdong prefecture level city in Shandong
Stowe Flagship Store	King of Power Saving	I'll comment after using it.	Jingdong prefecture level city in Shandong
Stowe Flagship Store	King of Power Saving	I'm trying it out to see if it saves power, and I'll buy two more if it works well.	Jingdong prefecture level city in Shandong
Stowe Flagship Store	King of Power Saving	It is genuine very good, the price is much cheaper, cost-effective, really special cost-effective, it is worth buying!	Jingdong prefecture level city in Shandong
Stowe Flagship Store	King of Power Saving	This thing can really save electricity, I was introduced to buy by my friend, he used it very well, I used it and think it's also very good, it can really save electricity, save about half of the electricity every day, it's really good!	Jingdong prefecture level city in Shandong
Stowe Flagship Store	King of Power Saving	After using it for ten days or so, I checked the power consumption and it did drop, and customer service said it will save more electricity in the long run. The product is also insured, 5 years warranty do not have to worry about quality problems!	Jingdong prefecture level city in Shandong
Stowe Flagship Store	King of Power Saving	The product is good, before already bought a, this is the second, the effect is quite good, anyway, I see the daily electricity bill than before a lot less, this can be the choice!	Jingdong prefecture level city in Shandong

Stowe Flagship Store	King of Power Saving	Originally also very worried about things are not good, get the hand found completely over-anxious, five-star praise, the style is very generous, the quality is very good, the price is also very reasonable!	Jingdong prefecture level city in Shandong
Stowe Flagship Store	King of Power Saving	Quality is good, playing to the old family also buy a, repurchase, this quality is really good, recommended!	Jingdong prefecture level city in Shandong
Stowe Flagship Store	King of Power Saving	Thank you, the owner even sent two fuses.	Jingdong prefecture level city in Shandong
Stowe Flagship Store	King of Power Saving	This store has great stuff, relatively fast shipping and logistics	Jingdong prefecture level city in Shandong
Stowe Flagship Store	King of Power Saving	This product is based on the impossibility of saving electricity, I used 853 kWh of electricity in July, started using the product on August 1, and used 867 kWh of electricity by September 1, is this called	Jingdong prefecture level city in Shandong
Stowe Flagship Store	King of Power Saving	Power king received, to the home on the trial, when I plugged into the electric panel can not see the effect of power saving, the meter is still the same speed in the turn, I'll save the king of the manual to play look at the instructions, instructions to speak, have to have a day two days and half a month to save power obviously, then wait half a month to see the effect of power saving it! To half a month there are obvious power saving before continuing to chase Xu.	Jingdong prefecture level city in Shandong
Stowe Flagship Store	King of Power Saving	Received the goods, a look, did not see the manufacturer and production address, can only hold a try to see the mentality of the face	Jingdong prefecture level city in Shandong
Stowe Flagship Store	King of Power Saving	I've only been using it for a few days and I'm not sure if it's an IQ tax? Used to favorable reviews.	Jingdong prefecture level city in Shandong
Stowe Flagship Store	King of Power Saving	Appearance is okay, power saving next time to follow up the evaluation	Jingdong prefecture level city in Shandong
Stowe Flagship Store	King of Power Saving	Shorter time, but hopefully effective and power-saving, to be followed up in the future	Jingdong prefecture level city in Shandong
Stowe Flagship Store	King of Power Saving	Appearance is okay, I do not know if the power saving	Jingdong prefecture level city in Shandong
Stowe Flagship Store	King of Power Saving	Good service, fast logistics, Power Saving King is exquisite and beautiful, support the business, thank you!	Jingdong prefecture level city in Shandong
Stowe Flagship Store	King of Power Saving	Bought three at once to try first first.	Jingdong prefecture level city in Shandong
Stowe Flagship Store	King of Power Saving	The object is the same as the picture, the product is of high quality, the packaging is intact. High-end atmosphere, high power, power saving effect is obvious, average monthly power saving 50%, very satisfied.	Jingdong prefecture level city in Shandong
Stowe Flagship Store	King of Power Saving	Bought back and plugged in, really effective, before 16 degrees of electricity per day, now 8 degrees of electricity per day, open an air conditioner for 12 hours, decisive repurchase of a	Jingdong prefecture level city in Shandong
Stowe Flagship Store	King of Power Saving	The object is the same as the picture, the product is of high quality, and the package is intact. High-end atmosphere and high grade, high power	Jingdong prefecture level city in Shandong
Stowe Flagship Store	King of Power Saving	Goods received, I hope the store said can be energy-saving, first try a month, month after month to see the effect.	Jingdong prefecture level city in Shandong
Stowe Flagship Store	King of Power Saving	This is really good, the material and workmanship is particularly exquisite, the workmanship is really very good, I think I can recommend to my friends to buy	Jingdong prefecture level city in Shandong
Stowe Flagship Store	King of Power Saving	I have to praise, the quality is really good, and the packaging logistics are also good. Really like it, won't make you regret after getting it!	Jingdong prefecture level city in Shandong
Stowe Flagship Store	King of Power Saving	Reputable platform, efficient and professional service, buy with confidence and peace of mind!	Jingdong prefecture level city in Shandong
Stowe Flagship Store	King of Power Saving	Bought one on a trial basis to see if it works after 1-2 months.	Jingdong prefecture level city in Shandong
Stowe Flagship Store	King of Power Saving	Quality is very good, customer service attitude is very very praise, there are problems in a timely manner to solve the problem, the price is not expensive, super value, you can rest assured that the	Jingdong prefecture level city in Shandong
Stowe Flagship Store	King of Power Saving	With it no longer need to be afraid to use electricity, especially good, ready to buy a mom and dad that also	Jingdong prefecture level city in Shandong
Stowe Flagship Store	King of Power Saving	Baby received very like will return to buy the seller attitude is also very like will return to buy the seller attitude is also very like!	Jingdong prefecture level city in Shandong
Stowe Flagship Store	King of Power Saving	Jingdong Mall shopping convenience assured fast assured fast assured fast assured fast	Jingdong prefecture level city in Shandong
Stowe Flagship Store	King of Power Saving	Saving power received, the quality is very good, fine workmanship, especially good, much cheaper than the entity store, it is worth buying!	Jingdong prefecture level city in Shandong
Stowe Flagship Store	King of Power Saving	I did not expect the express delivery so quickly, very save point quality is super good, than the entity store is too cheap.	Jingdong prefecture level city in Shandong
Stowe Flagship Store	King of Power Saving	Have received the baby, very satisfied with a shopping, great worth buying!	Jingdong prefecture level city in Shandong
Stowe Flagship Store	King of Power Saving	This is really more powerful, very satisfied, more energy-saving, better, thank you boss.	Jingdong prefecture level city in Shandong
Stowe Flagship Store	King of Power Saving	Received has been used for two days, the air conditioning can boldly open it! Energy saving effect is really quite good	Jingdong prefecture level city in Shandong
Stowe Flagship Store	King of Power Saving	It is authentic, trust the merchant, the baby has a great texture and the price is moderate, unlimited repurchase!	Jingdong prefecture level city in Shandong
Stowe Flagship Store	King of Power Saving	It is genuine very good, the price is much cheaper, cost-effective, really special cost-effective, it is worth buying!	Jingdong prefecture level city in Shandong
Stowe Flagship Store	King of Power Saving	Very good quality, fine workmanship, low power consumption, long warranty, fast shipping, economical and practical	Jingdong prefecture level city in Shandong
Stowe Flagship Store	King of Power Saving	I've been using it for half a month now, it really saves power and the voltage is stable, it's very good	Jingdong prefecture level city in Shandong
Stowe Flagship Store	King of Power Saving	It's the ideal product, good quality and fast logistics, worth the kudos!	Jingdong prefecture level city in Shandong
Stowe Flagship Store	King of Power Saving	The logistics is very fast, the baby is very good, the shopkeeper is NICE!	Jingdong prefecture level city in Shandong
Stowe Flagship Store	King of Power Saving	Good to use, good quality, save point durable	Jingdong prefecture level city in Shandong
Stowe Flagship Store	King of Power Saving	The quality is very good, customer service attitude is very, very awesome, there are problems in a timely manner to solve the problem	Jingdong prefecture level city in Shandong
Stowe Flagship Store	King of Power Saving	Quality is very good, the use of the effect is very good, more stable, quality assurance	Jingdong prefecture level city in Shandong
Stowe Flagship Store	King of Power Saving	Convenient, inexpensive, and see how it works out?	Jingdong prefecture level city in Shandong
Stowe Flagship Store	King of Power Saving	Very good quality, saves a lot of electricity, very good, worth buying	Jingdong prefecture level city in Shandong
Stowe Flagship Store	King of Power Saving	Power Saving King is easy to use, with the fruit of power saving to use easy to give!	Jingdong prefecture level city in Shandong
Stowe Flagship Store	King of Power Saving	This saver really works, good quality, safe and secure, very satisfied!	Jingdong prefecture level city in Shandong
Stowe Flagship Store	King of Power Saving	It works well, the voltage is very stable effect is good, the quality is also very good satisfaction	Jingdong prefecture level city in Shandong
Stowe Flagship Store	King of Power Saving	The logistics is super super fast! The quality is also very good, indeed value for money	Jingdong prefecture level city in Shandong
Stowe Flagship Store	King of Power Saving	I've only been using it for a month or so and it seems to be saving a lot of power	Jingdong prefecture level city in Shandong
Stowe Flagship Store	King of Power Saving	Very easy to operate, very good material, good value for money.	Jingdong prefecture level city in Shandong
Stowe Flagship Store	King of Power Saving	Aluminum alloy shell, durable, compact power-saving good effect	Jingdong prefecture level city in Shandong
Stowe Flagship Store	King of Power Saving	Nice device, easy to use, doesn't take up much space, recommendable!	Jingdong prefecture level city in Shandong
Stowe Flagship Store	King of Power Saving	Good quality, good price, fast logistics	Jingdong prefecture level city in Shandong

Stowe Flagship Store	King of Power Saving	Friends of the family in use recommended, the effect is good and recommendable!	Jingdong prefecture level city in Shandong
Stowe Flagship Store	King of Power Saving	Cheaper than brick and mortar stores worth buying is authentic	Jingdong prefecture level city in Shandong
Stowe Flagship Store	King of Power Saving	Merchants ship quickly, the price is cheaper than the physical store, good use	Jingdong prefecture level city in Shandong
Stowe Flagship Store	King of Power Saving	Very durable, affordable, fast shipping	Jingdong prefecture level city in Shandong
Stowe Flagship Store	King of Power Saving	Packaging tight quality is very good easy to use good results	Jingdong prefecture level city in Shandong
Stowe Flagship Store	King of Power Saving	Save the grid. It's in the whole house. It's saving money on the electric bill.	Jingdong prefecture level city in Shandong
Stowe Flagship Store	King of Power Saving	I used it today, it works well, I'm very satisfied	Jingdong prefecture level city in Shandong
Stowe Flagship Store	King of Power Saving	It really saves a lot of electricity after installation	Jingdong prefecture level city in Shandong
Stowe Flagship Store	King of Power Saving	Love it. Satisfied.	Jingdong prefecture level city in Shandong
Stowe Flagship Store	King of Power Saving	Doesn't feel like it's working.	Jingdong prefecture level city in Shandong
Stowe Flagship Store	King of Power Saving	I'll comment after using it.	Jingdong prefecture level city in Shandong
Stowe Flagship Store	King of Power Saving	I'm trying it out to see if it saves power, and I'll buy two more if it works well.	Jingdong prefecture level city in Shandong
Stowe Flagship Store	King of Power Saving	It is genuine very good, the price is much cheaper, cost-effective, really special cost-effective, it is worth buying!	Jingdong prefecture level city in Shandong
Stowe Flagship Store	King of Power Saving	This thing can really save electricity, I was introduced to buy by my friend, he used it very well, I used it and think it's also very good, it can really save electricity, save about half of the electricity every day, it's really good!	Jingdong prefecture level city in Shandong
Stowe Flagship Store	King of Power Saving	After using it for ten days or so, I checked the power consumption and it did drop, and customer service said it will save more electricity in the long run. The product is also insured, 5 years warranty do not have to worry about quality problems!	Jingdong prefecture level city in Shandong
Stowe Flagship Store	King of Power Saving	The product is good, before already bought a, this is the second, the effect is quite good, anyway, I see the daily electricity bill than before a lot less, this can be the choice!	Jingdong prefecture level city in Shandong
Stowe Flagship Store	King of Power Saving	Originally also very worried about things are not good, get the hand found completely over-anxious, five-star praise, the style is very generous, the quality is very good, the price is also very reasonable!	Jingdong prefecture level city in Shandong
Stowe Flagship Store	King of Power Saving	Quality is good, playing to the old family also buy a, repurchase, this quality is really good, recommended!	Jingdong prefecture level city in Shandong
Stowe Flagship Store	King of Power Saving	Thank you, the owner even sent two fuses.	Jingdong prefecture level city in Shandong
Stowe Flagship Store	King of Power Saving	This store has great stuff, relatively fast shipping and logistics	Jingdong prefecture level city in Shandong
Stowe Flagship Store	King of Power Saving	This product is based on the impossibility of saving electricity, I used 853 kWh of electricity in July, started using the product on August 1, and used 867 kWh of electricity by September 1, is this called	Jingdong prefecture level city in Shandong
Stowe Flagship Store	King of Power Saving	Power king received, to the home on the trial, when I plugged into the electric panel can not see the effect of power saving, the meter is still the same speed in the turn, I'll save the king of the manual to play look at the instructions, instructions to speak, have to have a day two days and half a month to save power obviously, then wait half a month to see the effect of power saving it! To half a month there are obvious power saving before continuing to chase Xu.	Jingdong prefecture level city in Shandong
Stowe Flagship Store	King of Power Saving	Received the goods, a look, did not see the manufacturer and production address, can only hold a try to see the mentality of the face	Jingdong prefecture level city in Shandong
Stowe Flagship Store	King of Power Saving	I've only been using it for a few days and I'm not sure if it's an IQ tax? Used to favorable reviews.	Jingdong prefecture level city in Shandong
Stowe Flagship Store	King of Power Saving	Appearance is okay, power saving next time to follow up the evaluation	Jingdong prefecture level city in Shandong
Stowe Flagship Store	King of Power Saving	Shorter time, but hopefully effective and power-saving, to be followed up in the future	Jingdong prefecture level city in Shandong
Stowe Flagship Store	King of Power Saving	Appearance is okay, I do not know if the power saving	Jingdong prefecture level city in Shandong
Stowe Flagship Store	King of Power Saving	Good service, fast logistics, Power Saving King is exquisite and beautiful, support the business, thank you!	Jingdong prefecture level city in Shandong
Stowe Flagship Store	King of Power Saving	Bought three at once to try first first.	Jingdong prefecture level city in Shandong
Stowe Flagship Store	King of Power Saving	The object is the same as the picture, the product is of high quality, the packaging is intact. High-end atmosphere, high power, power saving effect is obvious, average monthly power saving 50%, very satisfied.	Jingdong prefecture level city in Shandong
Stowe Flagship Store	King of Power Saving	Bought back and plugged in, really effective, before 16 degrees of electricity per day, now 8 degrees of electricity per day, open an air conditioner for 12 hours, decisive repurchase of a	Jingdong prefecture level city in Shandong
Stowe Flagship Store	King of Power Saving	The object is the same as the picture, the product is of high quality, and the package is intact. High-end atmosphere and high grade, high power	Jingdong prefecture level city in Shandong
Stowe Flagship Store	King of Power Saving	Goods received, I hope the store said can be energy-saving, first try a month, month after month to see the effect.	Jingdong prefecture level city in Shandong
Stowe Flagship Store	King of Power Saving	This is really good, the material and workmanship is particularly exquisite, the workmanship is really very good, I think I can recommend to my friends to buy	Jingdong prefecture level city in Shandong
Stowe Flagship Store	King of Power Saving	I have to praise, the quality is really good, and the packaging logistics are also good. Really like it, won't make you regret after getting it!	Jingdong prefecture level city in Shandong
Stowe Flagship Store	King of Power Saving	Reputable platform, efficient and professional service, buy with confidence and peace of mind!	Jingdong prefecture level city in Shandong
Stowe Flagship Store	King of Power Saving	Bought one on a trial basis to see if it works after 1-2 months.	Jingdong prefecture level city in Shandong
Stowe Flagship Store	King of Power Saving	Quality is very good, customer service attitude is very very praise, there are problems in a timely manner to solve the problem, the price is not expensive, super value, you can rest assured that the	Jingdong prefecture level city in Shandong
Stowe Flagship Store	King of Power Saving	With it no longer need to be afraid to use electricity, especially good, ready to buy a mom and dad that also	Jingdong prefecture level city in Shandong
Stowe Flagship Store	King of Power Saving	Baby received very like will return to buy the seller attitude is also very like will return to buy the seller attitude is also very like!	Jingdong prefecture level city in Shandong
Stowe Flagship Store	King of Power Saving	Jingdong Mall shopping convenience assured fast assured fast assured fast assured fast	Jingdong prefecture level city in Shandong
Stowe Flagship Store	King of Power Saving	Saving power received, the quality is very good, fine workmanship, especially good, much cheaper than the entity store, it is worth buying!	Jingdong prefecture level city in Shandong
Stowe Flagship Store	King of Power Saving	I did not expect the express delivery so quickly, very save point quality is super good, than the entity store is too cheap.	Jingdong prefecture level city in Shandong
Stowe Flagship Store	King of Power Saving	Have received the baby, very satisfied with a shopping, great worth buying!	Jingdong prefecture level city in Shandong
Stowe Flagship Store	King of Power Saving	This is really more powerful, very satisfied, more energy-saving, better, thank you boss.	Jingdong prefecture level city in Shandong
Stowe Flagship Store	King of Power Saving	Received has been used for two days, the air conditioning can boldly open it! Energy saving effect is really quite good	Jingdong prefecture level city in Shandong
Stowe Flagship Store	King of Power Saving	It is authentic, trust the merchant, the baby has a great texture and the price is moderate, unlimited repurchase!	Jingdong prefecture level city in Shandong
Stowe Flagship Store	King of Power Saving	It is genuine very good, the price is much cheaper, cost-effective, really special cost-effective, it is worth buying!	Jingdong prefecture level city in Shandong
Stowe Flagship Store	King of Power Saving	Very good quality, fine workmanship, low power consumption, long warranty, fast shipping, economical and practical	Jingdong prefecture level city in Shandong

Stowe Flagship Store	King of Power Saving	I've been using it for half a month now, it really saves power and the voltage is stable, it's very good	Jingdong prefecture level city in Shandong
Stowe Flagship Store	King of Power Saving	It's the ideal product 💝, good quality and fast logistics, worth the kudos!	Jingdong prefecture level city in Shandong
Stowe Flagship Store	King of Power Saving	The logistics is very fast, the baby is very good, the shopkeeper is NICE!	Jingdong prefecture level city in Shandong
Stowe Flagship Store	King of Power Saving	Good to use, good quality, save point durable	Jingdong prefecture level city in Shandong
Stowe Flagship Store	King of Power Saving	The quality is very good, customer service attitude is very, very awesome, there are problems in a timely manner to solve the problem	Jingdong prefecture level city in Shandong
Stowe Flagship Store	King of Power Saving	Quality is very good, the use of the effect is very good, more stable, quality assurance	Jingdong prefecture level city in Shandong
Stowe Flagship Store	King of Power Saving	Convenient, inexpensive, and see how it works out?	Jingdong prefecture level city in Shandong
Stowe Flagship Store	King of Power Saving	Very good quality, saves a lot of electricity, very good, worth buying	Jingdong prefecture level city in Shandong
Stowe Flagship Store	King of Power Saving	Power Saving King is easy to use, with the fruit of power saving to use easy to give!	Jingdong prefecture level city in Shandong
Stowe Flagship Store	King of Power Saving	This saver really works, good quality, safe and secure, very satisfied!	Jingdong prefecture level city in Shandong
Stowe Flagship Store	King of Power Saving	It works well, the voltage is very stable effect is good, the quality is also very good satisfaction	Jingdong prefecture level city in Shandong
Stowe Flagship Store	King of Power Saving	The logistics is super super fast! The quality is also very good, indeed value for money	Jingdong prefecture level city in Shandong
Stowe Flagship Store	King of Power Saving	I've only been using it for a month or so and it seems to be saving a lot of power	Jingdong prefecture level city in Shandong
Stowe Flagship Store	King of Power Saving	Very easy to operate, very good material, good value for money.	Jingdong prefecture level city in Shandong
Stowe Flagship Store	King of Power Saving	Aluminum alloy shell, durable, compact power-saving good effect	Jingdong prefecture level city in Shandong
Stowe Flagship Store	King of Power Saving	Nice device, easy to use, doesn't take up much space, recommendable!	Jingdong prefecture level city in Shandong
Stowe Flagship Store	King of Power Saving	Good quality, good price, fast logistics	Jingdong prefecture level city in Shandong
Stowe Flagship Store	King of Power Saving	Friends of the family in use recommended, the effect is good and recommendable!	Jingdong prefecture level city in Shandong
Stowe Flagship Store	King of Power Saving	Cheaper than brick and mortar stores worth buying is authentic	Jingdong prefecture level city in Shandong
Stowe Flagship Store	King of Power Saving	Merchants ship quickly, the price is cheaper than the physical store, good use	Jingdong prefecture level city in Shandong
Stowe Flagship Store	King of Power Saving	Very durable, affordable, fast shipping	Jingdong prefecture level city in Shandong
Stowe Flagship Store	King of Power Saving	Packaging tight quality is very good easy to use good results	Jingdong prefecture level city in Shandong
Stowe Flagship Store	King of Power Saving	Save the grid. It's in the whole house. It's saving money on the electric bill.	Jingdong prefecture level city in Shandong
Stowe Flagship Store	King of Power Saving	I used it today, it works well, I'm very satisfied	Jingdong prefecture level city in Shandong
Stowe Flagship Store	King of Power Saving	It really saves a lot of electricity after installation	Jingdong prefecture level city in Shandong
Stowe Flagship Store	King of Power Saving	Love it. Satisfied.	Jingdong prefecture level city in Shandong
Stowe Flagship Store	King of Power Saving	Doesn't feel like it's working.	Jingdong prefecture level city in Shandong
Stowe Flagship Store	King of Power Saving	I'll comment after using it.	Jingdong prefecture level city in Shandong
Stowe Flagship Store	King of Power Saving	I'm trying it out to see if it saves power, and I'll buy two more if it works well.	Jingdong prefecture level city in Shandong
Stowe Flagship Store	King of Power Saving	It is genuine very good, the price is much cheaper, cost-effective, really special cost-effective, it is worth buying!	Jingdong prefecture level city in Shandong
Stowe Flagship Store	King of Power Saving	This thing can really save electricity, I was introduced to buy by my friend, he used it very well, I used it and think it's also very good, it can really save electricity, save about half of the electricity every day, it's really good!	Jingdong prefecture level city in Shandong
Stowe Flagship Store	King of Power Saving	After using it for ten days or so, I checked the power consumption and it did drop, and customer service said it will save more electricity in the long run. The product is also insured, 5 years warranty do not have to worry about quality problems!	Jingdong prefecture level city in Shandong
Stowe Flagship Store	King of Power Saving	The product is good, before already bought a, this is the second, the effect is quite good, anyway, I see the daily electricity bill than before a lot less, this can be the choice!	Jingdong prefecture level city in Shandong
Stowe Flagship Store	King of Power Saving	Originally also very worried about things are not good, get the hand found completely over-anxious, five-star praise, the style is very generous, the quality is very good, the price is also very reasonable!	Jingdong prefecture level city in Shandong
Stowe Flagship Store	King of Power Saving	Quality is good, playing to the old family also buy a, repurchase, this quality is really good, recommended!	Jingdong prefecture level city in Shandong
Stowe Flagship Store	King of Power Saving	Thank you, the owner even sent two fuses.	Jingdong prefecture level city in Shandong
Stowe Flagship Store	King of Power Saving	This store has great stuff, relatively fast shipping and logistics	Jingdong prefecture level city in Shandong
Stowe Flagship Store	King of Power Saving	This product is based on the impossibility of saving electricity, I used 853 kWh of electricity in July, started using the product on August 1, and used 867 kWh of electricity by September 1, is this called	Jingdong prefecture level city in Shandong
Stowe Flagship Store	King of Power Saving	Power king received, to the home on the trial, when I plugged into the electric panel can not see the effect of power saving, the meter is still the same speed in the turn, I'll save the king of the manual to play look at the instructions, instructions to speak, have to have a day two days and half a month to save power obviously, then wait half a month to see the effect of power saving it! To half a month there are obvious power saving before continuing to chase Xu.	Jingdong prefecture level city in Shandong
Stowe Flagship Store	King of Power Saving	Received the goods, a look, did not see the manufacturer and production address, can only hold a try to see the mentality of the face	Jingdong prefecture level city in Shandong
Stowe Flagship Store	King of Power Saving	I've only been using it for a few days and I'm not sure if it's an IQ tax? Used to favorable reviews.	Jingdong prefecture level city in Shandong
Stowe Flagship Store	King of Power Saving	Appearance is okay, power saving next time to follow up the evaluation	Jingdong prefecture level city in Shandong
Stowe Flagship Store	King of Power Saving	Shorter time, but hopefully effective and power-saving, to be followed up in the future	Jingdong prefecture level city in Shandong
Stowe Flagship Store	King of Power Saving	Appearance is okay, I do not know if the power saving	Jingdong prefecture level city in Shandong
Stowe Flagship Store	King of Power Saving	Good service, fast logistics, Power Saving King is exquisite and beautiful, support the business, thank you!	Jingdong prefecture level city in Shandong
Stowe Flagship Store	King of Power Saving	Bought three at once to try first first.	Jingdong prefecture level city in Shandong
Stowe Flagship Store	King of Power Saving	The object is the same as the picture, the product is of high quality, the packaging is intact. High-end atmosphere, high power, power saving effect is obvious, average monthly power saving 50%, very satisfied.	Jingdong prefecture level city in Shandong
Stowe Flagship Store	King of Power Saving	Bought back and plugged in, really effective, before 16 degrees of electricity per day, now 8 degrees of electricity per day, open an air conditioner for 12 hours, decisive repurchase of a	Jingdong prefecture level city in Shandong
Stowe Flagship Store	King of Power Saving	The object is the same as the picture, the product is of high quality, and the package is intact. High-end atmosphere and high grade, high power	Jingdong prefecture level city in Shandong

Stowe Flagship Store	King of Power Saving	Goods received, I hope the store said can be energy-saving, first try a month, month after month to see the effect.	Jingdong prefecture level city in Shandong
Stowe Flagship Store	King of Power Saving	This is really good, the material and workmanship is particularly exquisite, the workmanship is really very good, I think I can recommend to my friends to buy	Jingdong prefecture level city in Shandong
Stowe Flagship Store	King of Power Saving	I have to praise, the quality is really good, and the packaging logistics are also good. Really like it, won't make you regret after getting it!	Jingdong prefecture level city in Shandong
Stowe Flagship Store	King of Power Saving	Reputable platform, efficient and professional service, buy with confidence and peace of mind!	Jingdong prefecture level city in Shandong
Stowe Flagship Store	King of Power Saving	Bought one on a trial basis to see if it works after 1-2 months.	Jingdong prefecture level city in Shandong
Stowe Flagship Store	King of Power Saving	Quality is very good, customer service attitude is very very praise, there are problems in a timely manner to solve the problem, the price is not expensive, super value, you can rest assured that the	Jingdong prefecture level city in Shandong
Stowe Flagship Store	King of Power Saving	With it no longer need to be afraid to use electricity, especially good, ready to buy a mom and dad that also	Jingdong prefecture level city in Shandong
Stowe Flagship Store	King of Power Saving	Baby received very like will return to buy the seller attitude is also very like will return to buy the seller attitude is also very like!	Jingdong prefecture level city in Shandong
Stowe Flagship Store	King of Power Saving	Jingdong Mall shopping convenience assured fast assured fast assured fast assured fast	Jingdong prefecture level city in Shandong
Stowe Flagship Store	King of Power Saving	Saving power received, the quality is very good, fine workmanship, especially good, much cheaper than the entity store, it is worth buying!	Jingdong prefecture level city in Shandong
Stowe Flagship Store	King of Power Saving	I did not expect the express delivery so quickly, very save point quality is super good, than the entity store is too cheap.	Jingdong prefecture level city in Shandong
Stowe Flagship Store	King of Power Saving	Have received the baby, very satisfied with a shopping, great worth buying!	Jingdong prefecture level city in Shandong
Stowe Flagship Store	King of Power Saving	This is really more powerful, very satisfied, more energy-saving, better, thank you boss.	Jingdong prefecture level city in Shandong
Stowe Flagship Store	King of Power Saving	Received has been used for two days, the air conditioning can boldly open it! Energy saving effect is really quite good	Jingdong prefecture level city in Shandong
Stowe Flagship Store	King of Power Saving	It is authentic, trust the merchant, the baby has a great texture and the price is moderate, unlimited repurchase!	Jingdong prefecture level city in Shandong
Stowe Flagship Store	King of Power Saving	It is genuine very good, the price is much cheaper, cost-effective, really special cost-effective, it is worth buying!	Jingdong prefecture level city in Shandong
Stowe Flagship Store	King of Power Saving	Very good quality, fine workmanship, low power consumption, long warranty, fast shipping, economical and practical	Jingdong prefecture level city in Shandong
Stowe Flagship Store	King of Power Saving	I've been using it for half a month now, it really saves power and the voltage is stable, it's very good	Jingdong prefecture level city in Shandong
Stowe Flagship Store	King of Power Saving	It's the ideal product 💝, good quality and fast logistics, worth the kudos!	Jingdong prefecture level city in Shandong
Stowe Flagship Store	King of Power Saving	The logistics is very fast, the baby is very good, the shopkeeper is NICE!	Jingdong prefecture level city in Shandong
Stowe Flagship Store	King of Power Saving	Good to use, good quality, save point durable	Jingdong prefecture level city in Shandong
Stowe Flagship Store	King of Power Saving	The quality is very good, customer service attitude is very, very awesome, there are problems in a timely manner to solve the problem	Jingdong prefecture level city in Shandong
Stowe Flagship Store	King of Power Saving	Quality is very good, the use of the effect is very good, more stable, quality assurance	Jingdong prefecture level city in Shandong
Stowe Flagship Store	King of Power Saving	Convenient, inexpensive, and see how it works out?	Jingdong prefecture level city in Shandong
Stowe Flagship Store	King of Power Saving	Very good quality, saves a lot of electricity, very good, worth buying	Jingdong prefecture level city in Shandong
Stowe Flagship Store	King of Power Saving	Power Saving King is easy to use, with the fruit of power saving to use easy to give!	Jingdong prefecture level city in Shandong
Stowe Flagship Store	King of Power Saving	This saver really works, good quality, safe and secure, very satisfied!	Jingdong prefecture level city in Shandong
Stowe Flagship Store	King of Power Saving	It works well, the voltage is very stable effect is good, the quality is also very good satisfaction	Jingdong prefecture level city in Shandong
Stowe Flagship Store	King of Power Saving	The logistics is super super fast! The quality is also very good, indeed value for money	Jingdong prefecture level city in Shandong
Stowe Flagship Store	King of Power Saving	I've only been using it for a month or so and it seems to be saving a lot of power	Jingdong prefecture level city in Shandong
Stowe Flagship Store	King of Power Saving	Very easy to operate, very good material, good value for money.	Jingdong prefecture level city in Shandong
Stowe Flagship Store	King of Power Saving	Aluminum alloy shell, durable, compact power-saving good effect	Jingdong prefecture level city in Shandong
Stowe Flagship Store	King of Power Saving	Nice device, easy to use, doesn't take up much space, recommendable!	Jingdong prefecture level city in Shandong
Stowe Flagship Store	King of Power Saving	Good quality, good price, fast logistics	Jingdong prefecture level city in Shandong
Stowe Flagship Store	King of Power Saving	Friends of the family in use recommended, the effect is good and recommendable!	Jingdong prefecture level city in Shandong
Stowe Flagship Store	King of Power Saving	Cheaper than brick and mortar stores worth buying is authentic	Jingdong prefecture level city in Shandong
Stowe Flagship Store	King of Power Saving	Merchants ship quickly, the price is cheaper than the physical store, good use	Jingdong prefecture level city in Shandong
Stowe Flagship Store	King of Power Saving	Very durable, affordable, fast shipping	Jingdong prefecture level city in Shandong
Stowe Flagship Store	King of Power Saving	Packaging tight quality is very good easy to use good results	Jingdong prefecture level city in Shandong
Stowe Flagship Store	King of Power Saving	Save the grid. It's in the whole house. It's saving money on the electric bill.	Jingdong prefecture level city in Shandong
Stowe Flagship Store	King of Power Saving	I used it today, it works well, I'm very satisfied	Jingdong prefecture level city in Shandong
Stowe Flagship Store	King of Power Saving	It really saves a lot of electricity after installation	Jingdong prefecture level city in Shandong
Stowe Flagship Store	King of Power Saving	Love it. Satisfied.	Jingdong prefecture level city in Shandong
Stowe Flagship Store	King of Power Saving	Doesn't feel like it's working.	Jingdong prefecture level city in Shandong
Stowe Flagship Store	King of Power Saving	I'll comment after using it.	Jingdong prefecture level city in Shandong
Stowe Flagship Store	King of Power Saving	I'm trying it out to see if it saves power, and I'll buy two more if it works well.	Jingdong prefecture level city in Shandong
Stowe Flagship Store	King of Power Saving	It is genuine very good, the price is much cheaper, cost-effective, really special cost-effective, it is worth buying!	Jingdong prefecture level city in Shandong
Stowe Flagship Store	King of Power Saving	This thing can really save electricity, I was introduced to buy by my friend, he used it very well, I used it and think it's also very good, it can really save electricity, save about half of the electricity every day, it's really good!	Jingdong prefecture level city in Shandong
Stowe Flagship Store	King of Power Saving	After using it for ten days or so, I checked the power consumption and it did drop, and customer service said it will save more electricity in the long run. The product is also insured, 5 years warranty do not have to worry about quality problems!	Jingdong prefecture level city in Shandong
Stowe Flagship Store	King of Power Saving	The product is good, before already bought a, this is the second, the effect is quite good, anyway, I see the daily electricity bill than before a lot less, this can be the choice!	Jingdong prefecture level city in Shandong

Stowe Flagship Store	King of Power Saving	Originally also very worried about things are not good, get the hand found completely over-anxious, five-star praise, the style is very generous, the quality is very good, the price is also very reasonable!	Jingdong prefecture level city in Shandong
Stowe Flagship Store	King of Power Saving	Quality is good, playing to the old family also buy a, repurchase, this quality is really good, recommended!	Jingdong prefecture level city in Shandong
Stowe Flagship Store	King of Power Saving	Thank you, the owner even sent two fuses.	Jingdong prefecture level city in Shandong
Stowe Flagship Store	King of Power Saving	This store has great stuff, relatively fast shipping and logistics	Jingdong prefecture level city in Shandong
Stowe Flagship Store	King of Power Saving	This product is based on the impossibility of saving electricity, I used 853 kWh of electricity in July, started using the product on August 1, and used 867 kWh of electricity by September 1, is this called	Jingdong prefecture level city in Shandong
Stowe Flagship Store	King of Power Saving	Power king received, to the home on the trial, when I plugged into the electric panel can not see the effect of power saving, the meter is still the same speed in the turn, I'll save the king of the manual to play look at the instructions, instructions to speak, have to have a day two days and half a month to save power obviously, then wait half a month to see the effect of power saving it! To half a month there are obvious power saving before continuing to chase Xu.	Jingdong prefecture level city in Shandong
Stowe Flagship Store	King of Power Saving	Received the goods, a look, did not see the manufacturer and production address, can only hold a try to see the mentality of the face	Jingdong prefecture level city in Shandong
Stowe Flagship Store	King of Power Saving	I've only been using it for a few days and I'm not sure if it's an IQ tax? Used to favorable reviews.	Jingdong prefecture level city in Shandong
Stowe Flagship Store	King of Power Saving	Appearance is okay, power saving next time to follow up the evaluation	Jingdong prefecture level city in Shandong
Stowe Flagship Store	King of Power Saving	Shorter time, but hopefully effective and power-saving, to be followed up in the future	Jingdong prefecture level city in Shandong
Stowe Flagship Store	King of Power Saving	Appearance is okay, I do not know if the power saving	Jingdong prefecture level city in Shandong
Stowe Flagship Store	King of Power Saving	Good service, fast logistics, Power Saving King is exquisite and beautiful, support the business, thank you!	Jingdong prefecture level city in Shandong
Stowe Flagship Store	King of Power Saving	Bought three at once to try first first.	Jingdong prefecture level city in Shandong
Stowe Flagship Store	King of Power Saving	The object is the same as the picture, the product is of high quality, the packaging is intact. High-end atmosphere, high power, power saving effect is obvious, average monthly power saving 50%, very satisfied.	Jingdong prefecture level city in Shandong
Stowe Flagship Store	King of Power Saving	Bought back and plugged in, really effective, before 16 degrees of electricity per day, now 8 degrees of electricity per day, open an air conditioner for 12 hours, decisive repurchase of a	Jingdong prefecture level city in Shandong
Stowe Flagship Store	King of Power Saving	The object is the same as the picture, the product is of high quality, and the package is intact. High-end atmosphere and high grade, high power	Jingdong prefecture level city in Shandong
Stowe Flagship Store	King of Power Saving	Goods received, I hope the store said can be energy-saving, first try a month, month after month to see the effect.	Jingdong prefecture level city in Shandong
Stowe Flagship Store	King of Power Saving	This is really good, the material and workmanship is particularly exquisite, the workmanship is really very good, I think I can recommend to my friends to buy	Jingdong prefecture level city in Shandong
Stowe Flagship Store	King of Power Saving	I have to praise, the quality is really good, and the packaging logistics are also good. Really like it, won't make you regret after getting it!	Jingdong prefecture level city in Shandong
Stowe Flagship Store	King of Power Saving	Reputable platform, efficient and professional service, buy with confidence and peace of mind!	Jingdong prefecture level city in Shandong
Stowe Flagship Store	King of Power Saving	Bought one on a trial basis to see if it works after 1-2 months.	Jingdong prefecture level city in Shandong
Stowe Flagship Store	King of Power Saving	Quality is very good, customer service attitude is very very praise, there are problems in a timely manner to solve the problem, the price is not expensive, super value, you can rest assured that the	Jingdong prefecture level city in Shandong
Stowe Flagship Store	King of Power Saving	With it no longer need to be afraid to use electricity, especially good, ready to buy a mom and dad that also	Jingdong prefecture level city in Shandong
Stowe Flagship Store	King of Power Saving	Baby received very like will return to buy the seller attitude is also very like will return to buy the seller attitude is also very like!	Jingdong prefecture level city in Shandong
Stowe Flagship Store	King of Power Saving	Jingdong Mall shopping convenience assured fast assured fast assured fast assured fast	Jingdong prefecture level city in Shandong
Stowe Flagship Store	King of Power Saving	Saving power received, the quality is very good, fine workmanship, especially good, much cheaper than the entity store, it is worth buying!	Jingdong prefecture level city in Shandong
Stowe Flagship Store	King of Power Saving	I did not expect the express delivery so quickly, very save point quality is super good, than the entity store is too cheap.	Jingdong prefecture level city in Shandong
Stowe Flagship Store	King of Power Saving	Have received the baby, very satisfied with a shopping, great worth buying!	Jingdong prefecture level city in Shandong
Stowe Flagship Store	King of Power Saving	This is really more powerful, very satisfied, more energy-saving, better, thank you boss.	Jingdong prefecture level city in Shandong
Stowe Flagship Store	King of Power Saving	Received has been used for two days, the air conditioning can boldly open it! Energy saving effect is really quite good	Jingdong prefecture level city in Shandong
Stowe Flagship Store	King of Power Saving	It is authentic, trust the merchant, the baby has a great texture and the price is moderate, unlimited repurchase!	Jingdong prefecture level city in Shandong
Stowe Flagship Store	King of Power Saving	It is genuine very good, the price is much cheaper, cost-effective, really special cost-effective, it is worth buying!	Jingdong prefecture level city in Shandong
Stowe Flagship Store	King of Power Saving	Very good quality, fine workmanship, low power consumption, long warranty, fast shipping, economical and practical	Jingdong prefecture level city in Shandong
Stowe Flagship Store	King of Power Saving	I've been using it for half a month now, it really saves power and the voltage is stable, it's very good	Jingdong prefecture level city in Shandong
Stowe Flagship Store	King of Power Saving	It's the ideal product 💝, good quality and fast logistics, worth the kudos!	Jingdong prefecture level city in Shandong
Stowe Flagship Store	King of Power Saving	The logistics is very fast, the baby is very good, the shopkeeper is NICE!	Jingdong prefecture level city in Shandong
Stowe Flagship Store	King of Power Saving	Good to use, good quality, save point durable	Jingdong prefecture level city in Shandong
Stowe Flagship Store	King of Power Saving	The quality is very good, customer service attitude is very, very awesome, there are problems in a timely manner to solve the problem	Jingdong prefecture level city in Shandong
Stowe Flagship Store	King of Power Saving	Quality is very good, the use of the effect is very good, more stable, quality assurance	Jingdong prefecture level city in Shandong
Stowe Flagship Store	King of Power Saving	Convenient, inexpensive, and see how it works out?	Jingdong prefecture level city in Shandong
Stowe Flagship Store	King of Power Saving	Very good quality, saves a lot of electricity, very good, worth buying	Jingdong prefecture level city in Shandong
Stowe Flagship Store	King of Power Saving	Power Saving King is easy to use, with the fruit of power saving to use easy to give!	Jingdong prefecture level city in Shandong
Stowe Flagship Store	King of Power Saving	This saver really works, good quality, safe and secure, very satisfied!	Jingdong prefecture level city in Shandong
Stowe Flagship Store	King of Power Saving	It works well, the voltage is very stable effect is good, the quality is also very good satisfaction	Jingdong prefecture level city in Shandong
Stowe Flagship Store	King of Power Saving	The logistics is super super fast! The quality is also very good, indeed value for money	Jingdong prefecture level city in Shandong
Stowe Flagship Store	King of Power Saving	I've only been using it for a month or so and it seems to be saving a lot of power	Jingdong prefecture level city in Shandong
Stowe Flagship Store	King of Power Saving	Very easy to operate, very good material, good value for money.	Jingdong prefecture level city in Shandong
Stowe Flagship Store	King of Power Saving	Aluminum alloy shell, durable, compact power-saving good effect	Jingdong prefecture level city in Shandong
Stowe Flagship Store	King of Power Saving	Nice device, easy to use, doesn't take up much space, recommendable!	Jingdong prefecture level city in Shandong
Stowe Flagship Store	King of Power Saving	Good quality, good price, fast logistics	Jingdong prefecture level city in Shandong

Stowe Flagship Store	King of Power Saving	Friends of the family in use recommended, the effect is good and recommendable!	Jingdong prefecture level city in Shandong
Stowe Flagship Store	King of Power Saving	Cheaper than brick and mortar stores worth buying is authentic	Jingdong prefecture level city in Shandong
Stowe Flagship Store	King of Power Saving	Merchants ship quickly, the price is cheaper than the physical store, good use	Jingdong prefecture level city in Shandong
Stowe Flagship Store	King of Power Saving	Very durable, affordable, fast shipping	Jingdong prefecture level city in Shandong
Stowe Flagship Store	King of Power Saving	Packaging tight quality is very good easy to use good results	Jingdong prefecture level city in Shandong
Stowe Flagship Store	King of Power Saving	Save the grid. It's in the whole house. It's saving money on the electric bill.	Jingdong prefecture level city in Shandong
Stowe Flagship Store	King of Power Saving	I used it today, it works well, I'm very satisfied	Jingdong prefecture level city in Shandong
Stowe Flagship Store	King of Power Saving	It really saves a lot of electricity after installation	Jingdong prefecture level city in Shandong
Stowe Flagship Store	King of Power Saving	Love it. Satisfied.	Jingdong prefecture level city in Shandong
Stowe Flagship Store	King of Power Saving	Doesn't feel like it's working.	Jingdong prefecture level city in Shandong
Stowe Flagship Store	King of Power Saving	I'll comment after using it.	Jingdong prefecture level city in Shandong
Stowe Flagship Store	King of Power Saving	I'm trying it out to see if it saves power, and I'll buy two more if it works well.	Jingdong prefecture level city in Shandong
Stowe Flagship Store	King of Power Saving	It is genuine very good, the price is much cheaper, cost-effective, really special cost-effective, it is worth buying!	Jingdong prefecture level city in Shandong
Stowe Flagship Store	King of Power Saving	This thing can really save electricity, I was introduced to buy by my friend, he used it very well, I used it and think it's also very good, it can really save electricity, save about half of the electricity every day, it's really good!	Jingdong prefecture level city in Shandong
Stowe Flagship Store	King of Power Saving	After using it for ten days or so, I checked the power consumption and it did drop, and customer service said it will save more electricity in the long run. The product is also insured, 5 years warranty do not have to worry about quality problems!	Jingdong prefecture level city in Shandong
Stowe Flagship Store	King of Power Saving	The product is good, before already bought a, this is the second, the effect is quite good, anyway, I see the daily electricity bill than before a lot less, this can be the choice!	Jingdong prefecture level city in Shandong
Stowe Flagship Store	King of Power Saving	Originally also very worried about things are not good, get the hand found completely over-anxious, five-star praise, the style is very generous, the quality is very good, the price is also very reasonable!	Jingdong prefecture level city in Shandong
Stowe Flagship Store	King of Power Saving	Quality is good, playing to the old family also buy a, repurchase, this quality is really good, recommended!	Jingdong prefecture level city in Shandong
Stowe Flagship Store	King of Power Saving	Thank you, the owner even sent two fuses.	Jingdong prefecture level city in Shandong
Stowe Flagship Store	King of Power Saving	This store has great stuff, relatively fast shipping and logistics	Jingdong prefecture level city in Shandong
Stowe Flagship Store	King of Power Saving	This product is based on the impossibility of saving electricity, I used 853 kWh of electricity in July, started using the product on August 1, and used 867 kWh of electricity by September 1, is this called	Jingdong prefecture level city in Shandong
Stowe Flagship Store	King of Power Saving	Power king received, to the home on the trial, when I plugged into the electric panel can not see the effect of power saving, the meter is still the same speed in the turn, I'll save the king of the manual to play look at the instructions, instructions to speak, have to have a day two days and half a month to save power obviously, then wait half a month to see the effect of power saving it! To half a month there are obvious power saving before continuing to chase Xu.	Jingdong prefecture level city in Shandong
Stowe Flagship Store	King of Power Saving	Received the goods, a look, did not see the manufacturer and production address, can only hold a try to see the mentality of the face	Jingdong prefecture level city in Shandong
Stowe Flagship Store	King of Power Saving	I've only been using it for a few days and I'm not sure if it's an IQ tax? Used to favorable reviews.	Jingdong prefecture level city in Shandong
Stowe Flagship Store	King of Power Saving	Appearance is okay, power saving next time to follow up the evaluation	Jingdong prefecture level city in Shandong
Stowe Flagship Store	King of Power Saving	Shorter time, but hopefully effective and power-saving, to be followed up in the future	Jingdong prefecture level city in Shandong
Stowe Flagship Store	King of Power Saving	Appearance is okay, I do not know if the power saving	Jingdong prefecture level city in Shandong
Stowe Flagship Store	King of Power Saving	Good service, fast logistics, Power Saving King is exquisite and beautiful, support the business, thank you!	Jingdong prefecture level city in Shandong
Stowe Flagship Store	King of Power Saving	Bought three at once to try first first.	Jingdong prefecture level city in Shandong
Stowe Flagship Store	King of Power Saving	The object is the same as the picture, the product is of high quality, the packaging is intact. High-end atmosphere, high power, power saving effect is obvious, average monthly power saving 50%, very satisfied.	Jingdong prefecture level city in Shandong
Stowe Flagship Store	King of Power Saving	Bought back and plugged in, really effective, before 16 degrees of electricity per day, now 8 degrees of electricity per day, open an air conditioner for 12 hours, decisive repurchase of a	Jingdong prefecture level city in Shandong
Stowe Flagship Store	King of Power Saving	The object is the same as the picture, the product is of high quality, and the package is intact. High-end atmosphere and high grade, high power	Jingdong prefecture level city in Shandong
Stowe Flagship Store	King of Power Saving	Goods received, I hope the store said can be energy-saving, first try a month, month after month to see the effect.	Jingdong prefecture level city in Shandong
Stowe Flagship Store	King of Power Saving	This is really good, the material and workmanship is particularly exquisite, the workmanship is really very good, I think I can recommend to my friends to buy	Jingdong prefecture level city in Shandong
Stowe Flagship Store	King of Power Saving	I have to praise, the quality is really good, and the packaging logistics are also good. Really like it, won't make you regret after getting it!	Jingdong prefecture level city in Shandong
Stowe Flagship Store	King of Power Saving	Reputable platform, efficient and professional service, buy with confidence and peace of mind!	Jingdong prefecture level city in Shandong
Stowe Flagship Store	King of Power Saving	Bought one on a trial basis to see if it works after 1-2 months.	Jingdong prefecture level city in Shandong
Stowe Flagship Store	King of Power Saving	Quality is very good, customer service attitude is very very praise, there are problems in a timely manner to solve the problem, the price is not expensive, super value, you can rest assured that the	Jingdong prefecture level city in Shandong
Stowe Flagship Store	King of Power Saving	With it no longer need to be afraid to use electricity, especially good, ready to buy a mom and dad that also	Jingdong prefecture level city in Shandong
Stowe Flagship Store	King of Power Saving	Baby received very like will return to buy the seller attitude is also very like will return to buy the seller attitude is also very like!	Jingdong prefecture level city in Shandong
Stowe Flagship Store	King of Power Saving	Jingdong Mall shopping convenience assured fast assured fast assured fast assured fast	Jingdong prefecture level city in Shandong
Stowe Flagship Store	King of Power Saving	Saving power received, the quality is very good, fine workmanship, especially good, much cheaper than the entity store, it is worth buying!	Jingdong prefecture level city in Shandong
Stowe Flagship Store	King of Power Saving	I did not expect the express delivery so quickly, very save point quality is super good, than the entity store is too cheap.	Jingdong prefecture level city in Shandong
Stowe Flagship Store	King of Power Saving	Have received the baby, very satisfied with a shopping, great worth buying!	Jingdong prefecture level city in Shandong
Stowe Flagship Store	King of Power Saving	This is really more powerful, very satisfied, more energy-saving, better, thank you boss.	Jingdong prefecture level city in Shandong
Stowe Flagship Store	King of Power Saving	Received has been used for two days, the air conditioning can boldly open it! Energy saving effect is really quite good	Jingdong prefecture level city in Shandong
Stowe Flagship Store	King of Power Saving	It is authentic, trust the merchant, the baby has a great texture and the price is moderate, unlimited repurchase!	Jingdong prefecture level city in Shandong
Stowe Flagship Store	King of Power Saving	It is genuine very good, the price is much cheaper, cost-effective, really special cost-effective, it is worth buying!	Jingdong prefecture level city in Shandong
Stowe Flagship Store	King of Power Saving	Very good quality, fine workmanship, low power consumption, long warranty, fast shipping, economical and practical	Jingdong prefecture level city in Shandong

Stowe Flagship Store	King of Power Saving	I've been using it for half a month now, it really saves power and the voltage is stable, it's very good	Jingdong prefecture level city in Shandong
Stowe Flagship Store	King of Power Saving	It's the ideal product 💝, good quality and fast logistics, worth the kudos!	Jingdong prefecture level city in Shandong
Stowe Flagship Store	King of Power Saving	The logistics is very fast, the baby is very good, the shopkeeper is NICE!	Jingdong prefecture level city in Shandong
Stowe Flagship Store	King of Power Saving	Good to use, good quality, save point durable	Jingdong prefecture level city in Shandong
Stowe Flagship Store	King of Power Saving	The quality is very good, customer service attitude is very, very awesome, there are problems in a timely manner to solve the problem	Jingdong prefecture level city in Shandong
Stowe Flagship Store	King of Power Saving	Quality is very good, the use of the effect is very good, more stable, quality assurance	Jingdong prefecture level city in Shandong
Stowe Flagship Store	King of Power Saving	Convenient, inexpensive, and see how it works out?	Jingdong prefecture level city in Shandong
Stowe Flagship Store	King of Power Saving	Very good quality, saves a lot of electricity, very good, worth buying	Jingdong prefecture level city in Shandong
Stowe Flagship Store	King of Power Saving	Power Saving King is easy to use, with the fruit of power saving to use easy to give!	Jingdong prefecture level city in Shandong
Stowe Flagship Store	King of Power Saving	This saver really works, good quality, safe and secure, very satisfied!	Jingdong prefecture level city in Shandong
Stowe Flagship Store	King of Power Saving	It works well, the voltage is very stable effect is good, the quality is also very good satisfaction	Jingdong prefecture level city in Shandong
Stowe Flagship Store	King of Power Saving	The logistics is super super fast! The quality is also very good, indeed value for money	Jingdong prefecture level city in Shandong
Stowe Flagship Store	King of Power Saving	I've only been using it for a month or so and it seems to be saving a lot of power	Jingdong prefecture level city in Shandong
Stowe Flagship Store	King of Power Saving	Very easy to operate, very good material, good value for money.	Jingdong prefecture level city in Shandong
Stowe Flagship Store	King of Power Saving	Aluminum alloy shell, durable, compact power-saving good effect	Jingdong prefecture level city in Shandong
Stowe Flagship Store	King of Power Saving	Nice device, easy to use, doesn't take up much space, recommendable!	Jingdong prefecture level city in Shandong
Stowe Flagship Store	King of Power Saving	Good quality, good price, fast logistics	Jingdong prefecture level city in Shandong
Stowe Flagship Store	King of Power Saving	Friends of the family in use recommended, the effect is good and recommendable!	Jingdong prefecture level city in Shandong
Stowe Flagship Store	King of Power Saving	Cheaper than brick and mortar stores worth buying is authentic	Jingdong prefecture level city in Shandong
Stowe Flagship Store	King of Power Saving	Merchants ship quickly, the price is cheaper than the physical store, good use	Jingdong prefecture level city in Shandong
Stowe Flagship Store	King of Power Saving	Very durable, affordable, fast shipping	Jingdong prefecture level city in Shandong
Stowe Flagship Store	King of Power Saving	Packaging tight quality is very good easy to use good results	Jingdong prefecture level city in Shandong
Stowe Flagship Store	King of Power Saving	Save the grid. It's in the whole house. It's saving money on the electric bill.	Jingdong prefecture level city in Shandong
Stowe Flagship Store	King of Power Saving	I used it today, it works well, I'm very satisfied	Jingdong prefecture level city in Shandong
Stowe Flagship Store	King of Power Saving	It really saves a lot of electricity after installation	Jingdong prefecture level city in Shandong
Stowe Flagship Store	King of Power Saving	Love it. Satisfied.	Jingdong prefecture level city in Shandong
Stowe Flagship Store	King of Power Saving	Doesn't feel like it's working.	Jingdong prefecture level city in Shandong
Stowe Flagship Store	King of Power Saving	I'll comment after using it.	Jingdong prefecture level city in Shandong
Stowe Flagship Store	King of Power Saving	I'm trying it out to see if it saves power, and I'll buy two more if it works well.	Jingdong prefecture level city in Shandong
Stowe Flagship Store	King of Power Saving	It is genuine very good, the price is much cheaper, cost-effective, really special cost-effective, it is worth buying!	Jingdong prefecture level city in Shandong
Stowe Flagship Store	King of Power Saving	This thing can really save electricity, I was introduced to buy by my friend, he used it very well, I used it and think it's also very good, it can really save electricity, save about half of the electricity every day, it's really good!	Jingdong prefecture level city in Shandong
Stowe Flagship Store	King of Power Saving	After using it for ten days or so, I checked the power consumption and it did drop, and customer service said it will save more electricity in the long run. The product is also insured, 5 years warranty do not have to worry about quality problems!	Jingdong prefecture level city in Shandong
Stowe Flagship Store	King of Power Saving	The product is good, before already bought a, this is the second, the effect is quite good, anyway, I see the daily electricity bill than before a lot less, this can be the choice!	Jingdong prefecture level city in Shandong
Stowe Flagship Store	King of Power Saving	Originally also very worried about things are not good, get the hand found completely over-anxious, five-star praise, the style is very generous, the quality is very good, the price is also very reasonable!	Jingdong prefecture level city in Shandong
Stowe Flagship Store	King of Power Saving	Quality is good, playing to the old family also buy a, repurchase, this quality is really good, recommended!	Jingdong prefecture level city in Shandong
Stowe Flagship Store	King of Power Saving	Thank you, the owner even sent two fuses.	Jingdong prefecture level city in Shandong
Stowe Flagship Store	King of Power Saving	This store has great stuff, relatively fast shipping and logistics	Jingdong prefecture level city in Shandong
Stowe Flagship Store	King of Power Saving	This product is based on the impossibility of saving electricity, I used 853 kWh of electricity in July, started using the product on August 1, and used 867 kWh of electricity by September 1, is this called	Jingdong prefecture level city in Shandong
Stowe Flagship Store	King of Power Saving	Power king received, to the home on the trial, when I plugged into the electric panel can not see the effect of power saving, the meter is still the same speed in the turn, I'll save the king of the manual to play look at the instructions, instructions to speak, have to have a day two days and half a month to save power obviously, then wait half a month to see the effect of power saving it! To half a month there are obvious power saving before continuing to chase Xu.	Jingdong prefecture level city in Shandong
Stowe Flagship Store	King of Power Saving	Received the goods, a look, did not see the manufacturer and production address, can only hold a try to see the mentality of the face	Jingdong prefecture level city in Shandong
Stowe Flagship Store	King of Power Saving	I've only been using it for a few days and I'm not sure if it's an IQ tax? Used to favorable reviews.	Jingdong prefecture level city in Shandong
Stowe Flagship Store	King of Power Saving	Appearance is okay, power saving next time to follow up the evaluation	Jingdong prefecture level city in Shandong
Stowe Flagship Store	King of Power Saving	Shorter time, but hopefully effective and power-saving, to be followed up in the future	Jingdong prefecture level city in Shandong
Stowe Flagship Store	King of Power Saving	Appearance is okay, I do not know if the power saving	Jingdong prefecture level city in Shandong
Stowe Flagship Store	King of Power Saving	Good service, fast logistics, Power Saving King is exquisite and beautiful, support the business, thank you!	Jingdong prefecture level city in Shandong
Stowe Flagship Store	King of Power Saving	Bought three at once to try first first.	Jingdong prefecture level city in Shandong
Stowe Flagship Store	King of Power Saving	The object is the same as the picture, the product is of high quality, the packaging is intact. High-end atmosphere, high power, power saving effect is obvious, average monthly power saving 50%, very satisfied.	Jingdong prefecture level city in Shandong
Stowe Flagship Store	King of Power Saving	Bought back and plugged in, really effective, before 16 degrees of electricity per day, now 8 degrees of electricity per day, open an air conditioner for 12 hours, decisive repurchase of a	Jingdong prefecture level city in Shandong
Stowe Flagship Store	King of Power Saving	The object is the same as the picture, the product is of high quality, and the package is intact. High-end atmosphere and high grade, high power	Jingdong prefecture level city in Shandong

Stowe Flagship Store	King of Power Saving	Goods received, I hope the store said can be energy-saving, first try a month, month after month to see the effect.	Jingdong prefecture level city in Shandong
Stowe Flagship Store	King of Power Saving	This is really good, the material and workmanship is particularly exquisite, the workmanship is really very good, I think I can recommend to my friends to buy	Jingdong prefecture level city in Shandong
Stowe Flagship Store	King of Power Saving	I have to praise, the quality is really good, and the packaging logistics are also good. Really like it, won't make you regret after getting it!	Jingdong prefecture level city in Shandong
Stowe Flagship Store	King of Power Saving	Reputable platform, efficient and professional service, buy with confidence and peace of mind!	Jingdong prefecture level city in Shandong
Stowe Flagship Store	King of Power Saving	Bought one on a trial basis to see if it works after 1-2 months.	Jingdong prefecture level city in Shandong
Stowe Flagship Store	King of Power Saving	Quality is very good, customer service attitude is very very praise, there are problems in a timely manner to solve the problem, the price is not expensive, super value, you can rest assured that the	Jingdong prefecture level city in Shandong
Stowe Flagship Store	King of Power Saving	With it no longer need to be afraid to use electricity, especially good, ready to buy a mom and dad that also	Jingdong prefecture level city in Shandong
Stowe Flagship Store	King of Power Saving	Baby received very like will return to buy the seller attitude is also very like will return to buy the seller attitude is also very like!	Jingdong prefecture level city in Shandong
Stowe Flagship Store	King of Power Saving	Jingdong Mall shopping convenience assured fast assured fast assured fast assured fast	Jingdong prefecture level city in Shandong
Stowe Flagship Store	King of Power Saving	Saving power received, the quality is very good, fine workmanship, especially good, much cheaper than the entity store, it is worth buying!	Jingdong prefecture level city in Shandong
Stowe Flagship Store	King of Power Saving	I did not expect the express delivery so quickly, very save point quality is super good, than the entity store is too cheap.	Jingdong prefecture level city in Shandong
Stowe Flagship Store	King of Power Saving	Have received the baby, very satisfied with a shopping, great worth buying!	Jingdong prefecture level city in Shandong
Stowe Flagship Store	King of Power Saving	This is really more powerful, very satisfied, more energy-saving, better, thank you boss.	Jingdong prefecture level city in Shandong
Stowe Flagship Store	King of Power Saving	Received has been used for two days, the air conditioning can boldly open it! Energy saving effect is really quite good	Jingdong prefecture level city in Shandong
Stowe Flagship Store	King of Power Saving	It is authentic, trust the merchant, the baby has a great texture and the price is moderate, unlimited repurchase!	Jingdong prefecture level city in Shandong
Stowe Flagship Store	King of Power Saving	It is genuine very good, the price is much cheaper, cost-effective, really special cost-effective, it is worth buying!	Jingdong prefecture level city in Shandong
Stowe Flagship Store	King of Power Saving	Very good quality, fine workmanship, low power consumption, long warranty, fast shipping, economical and practical	Jingdong prefecture level city in Shandong
Stowe Flagship Store	King of Power Saving	I've been using it for half a month now, it really saves power and the voltage is stable, it's very good	Jingdong prefecture level city in Shandong
Stowe Flagship Store	King of Power Saving	It's the ideal product 💝, good quality and fast logistics, worth the kudos!	Jingdong prefecture level city in Shandong
Stowe Flagship Store	King of Power Saving	The logistics is very fast, the baby is very good, the shopkeeper is NICE!	Jingdong prefecture level city in Shandong
Stowe Flagship Store	King of Power Saving	Good to use, good quality, save point durable	Jingdong prefecture level city in Shandong
Stowe Flagship Store	King of Power Saving	The quality is very good, customer service attitude is very, very awesome, there are problems in a timely manner to solve the problem	Jingdong prefecture level city in Shandong
Stowe Flagship Store	King of Power Saving	Quality is very good, the use of the effect is very good, more stable, quality assurance	Jingdong prefecture level city in Shandong
Stowe Flagship Store	King of Power Saving	Convenient, inexpensive, and see how it works out?	Jingdong prefecture level city in Shandong
Stowe Flagship Store	King of Power Saving	Very good quality, saves a lot of electricity, very good, worth buying	Jingdong prefecture level city in Shandong
Stowe Flagship Store	King of Power Saving	Power Saving King is easy to use, with the fruit of power saving to use easy to give!	Jingdong prefecture level city in Shandong
Stowe Flagship Store	King of Power Saving	This saver really works, good quality, safe and secure, very satisfied!	Jingdong prefecture level city in Shandong
Stowe Flagship Store	King of Power Saving	It works well, the voltage is very stable effect is good, the quality is also very good satisfaction	Jingdong prefecture level city in Shandong
Stowe Flagship Store	King of Power Saving	The logistics is super super fast! The quality is also very good, indeed value for money	Jingdong prefecture level city in Shandong
Stowe Flagship Store	King of Power Saving	I've only been using it for a month or so and it seems to be saving a lot of power	Jingdong prefecture level city in Shandong
Stowe Flagship Store	King of Power Saving	Very easy to operate, very good material, good value for money.	Jingdong prefecture level city in Shandong
Stowe Flagship Store	King of Power Saving	Aluminum alloy shell, durable, compact power-saving good effect	Jingdong prefecture level city in Shandong
Stowe Flagship Store	King of Power Saving	Nice device, easy to use, doesn't take up much space, recommendable!	Jingdong prefecture level city in Shandong
Stowe Flagship Store	King of Power Saving	Good quality, good price, fast logistics	Jingdong prefecture level city in Shandong
Stowe Flagship Store	King of Power Saving	Friends of the family in use recommended, the effect is good and recommendable!	Jingdong prefecture level city in Shandong
Stowe Flagship Store	King of Power Saving	Cheaper than brick and mortar stores worth buying is authentic	Jingdong prefecture level city in Shandong
Stowe Flagship Store	King of Power Saving	Merchants ship quickly, the price is cheaper than the physical store, good use	Jingdong prefecture level city in Shandong
Stowe Flagship Store	King of Power Saving	Very durable, affordable, fast shipping	Jingdong prefecture level city in Shandong
Stowe Flagship Store	King of Power Saving	Packaging tight quality is very good easy to use good results	Jingdong prefecture level city in Shandong
Stowe Flagship Store	King of Power Saving	Save the grid. It's in the whole house. It's saving money on the electric bill.	Jingdong prefecture level city in Shandong
Stowe Flagship Store	King of Power Saving	I used it today, it works well, I'm very satisfied	Jingdong prefecture level city in Shandong
Stowe Flagship Store	King of Power Saving	It really saves a lot of electricity after installation	Jingdong prefecture level city in Shandong
Stowe Flagship Store	King of Power Saving	Love it. Satisfied.	Jingdong prefecture level city in Shandong
Stowe Flagship Store	King of Power Saving	Doesn't feel like it's working.	Jingdong prefecture level city in Shandong
Stowe Flagship Store	King of Power Saving	I'll comment after using it.	Jingdong prefecture level city in Shandong
Stowe Flagship Store	King of Power Saving	I'm trying it out to see if it saves power, and I'll buy two more if it works well.	Jingdong prefecture level city in Shandong
Stowe Flagship Store	King of Power Saving	It is genuine very good, the price is much cheaper, cost-effective, really special cost-effective, it is worth buying!	Jingdong prefecture level city in Shandong
Stowe Flagship Store	King of Power Saving	This thing can really save electricity, I was introduced to buy by my friend, he used it very well, I used it and think it's also very good, it can really save electricity, save about half of the electricity every day, it's really good!	Jingdong prefecture level city in Shandong
Stowe Flagship Store	King of Power Saving	After using it for ten days or so, I checked the power consumption and it did drop, and customer service said it will save more electricity in the long run. The product is also insured, 5 years warranty do not have to worry about quality problems!	Jingdong prefecture level city in Shandong
Stowe Flagship Store	King of Power Saving	The product is good, before already bought a, this is the second, the effect is quite good, anyway, I see the daily electricity bill than before a lot less, this can be the choice!	Jingdong prefecture level city in Shandong

Stowe Flagship Store	King of Power Saving	Originally also very worried about things are not good, get the hand found completely over-anxious, five-star praise, the style is very generous, the quality is very good, the price is also very reasonable!	Jingdong prefecture level city in Shandong
Stowe Flagship Store	King of Power Saving	Quality is good, playing to the old family also buy a, repurchase, this quality is really good, recommended!	Jingdong prefecture level city in Shandong
Stowe Flagship Store	King of Power Saving	Thank you, the owner even sent two fuses.	Jingdong prefecture level city in Shandong
Stowe Flagship Store	King of Power Saving	This store has great stuff, relatively fast shipping and logistics	Jingdong prefecture level city in Shandong
Stowe Flagship Store	King of Power Saving	This product is based on the impossibility of saving electricity, I used 853 kWh of electricity in July, started using the product on August 1, and used 867 kWh of electricity by September 1, is this called	Jingdong prefecture level city in Shandong
Stowe Flagship Store	King of Power Saving	Power king received, to the home on the trial, when I plugged into the electric panel can not see the effect of power saving, the meter is still the same speed in the turn, I'll save the king of the manual to play look at the instructions, instructions to speak, have to have a day two days and half a month to save power obviously, then wait half a month to see the effect of power saving it! To half a month there are obvious power saving before continuing to chase Xu.	Jingdong prefecture level city in Shandong
Stowe Flagship Store	King of Power Saving	Received the goods, a look, did not see the manufacturer and production address, can only hold a try to see the mentality of the face	Jingdong prefecture level city in Shandong
Stowe Flagship Store	King of Power Saving	I've only been using it for a few days and I'm not sure if it's an IQ tax? Used to favorable reviews.	Jingdong prefecture level city in Shandong
Stowe Flagship Store	King of Power Saving	Appearance is okay, power saving next time to follow up the evaluation	Jingdong prefecture level city in Shandong
Stowe Flagship Store	King of Power Saving	Shorter time, but hopefully effective and power-saving, to be followed up in the future	Jingdong prefecture level city in Shandong
Stowe Flagship Store	King of Power Saving	Appearance is okay, I do not know if the power saving	Jingdong prefecture level city in Shandong
Stowe Flagship Store	King of Power Saving	Good service, fast logistics, Power Saving King is exquisite and beautiful, support the business, thank you!	Jingdong prefecture level city in Shandong
Stowe Flagship Store	King of Power Saving	Bought three at once to try first first.	Jingdong prefecture level city in Shandong
Stowe Flagship Store	King of Power Saving	The object is the same as the picture, the product is of high quality, the packaging is intact. High-end atmosphere, high power, power saving effect is obvious, average monthly power saving 50%, very satisfied.	Jingdong prefecture level city in Shandong
Stowe Flagship Store	King of Power Saving	Bought back and plugged in, really effective, before 16 degrees of electricity per day, now 8 degrees of electricity per day, open an air conditioner for 12 hours, decisive repurchase of a	Jingdong prefecture level city in Shandong
Stowe Flagship Store	King of Power Saving	The object is the same as the picture, the product is of high quality, and the package is intact. High-end atmosphere and high grade, high power	Jingdong prefecture level city in Shandong
Stowe Flagship Store	King of Power Saving	Goods received, I hope the store said can be energy-saving, first try a month, month after month to see the effect.	Jingdong prefecture level city in Shandong
Stowe Flagship Store	King of Power Saving	This is really good, the material and workmanship is particularly exquisite, the workmanship is really very good, I think I can recommend to my friends to buy	Jingdong prefecture level city in Shandong
Stowe Flagship Store	King of Power Saving	I have to praise, the quality is really good, and the packaging logistics are also good. Really like it, won't make you regret after getting it!	Jingdong prefecture level city in Shandong
Stowe Flagship Store	King of Power Saving	Reputable platform, efficient and professional service, buy with confidence and peace of mind!	Jingdong prefecture level city in Shandong
Stowe Flagship Store	King of Power Saving	Bought one on a trial basis to see if it works after 1-2 months.	Jingdong prefecture level city in Shandong
Stowe Flagship Store	King of Power Saving	Quality is very good, customer service attitude is very very praise, there are problems in a timely manner to solve the problem, the price is not expensive, super value, you can rest assured that the	Jingdong prefecture level city in Shandong
Stowe Flagship Store	King of Power Saving	With it no longer need to be afraid to use electricity, especially good, ready to buy a mom and dad that also	Jingdong prefecture level city in Shandong
Stowe Flagship Store	King of Power Saving	Baby received very like will return to buy the seller attitude is also very like will return to buy the seller attitude is also very like!	Jingdong prefecture level city in Shandong
Stowe Flagship Store	King of Power Saving	Jingdong Mall shopping convenience assured fast assured fast assured fast assured fast	Jingdong prefecture level city in Shandong
Stowe Flagship Store	King of Power Saving	Saving power received, the quality is very good, fine workmanship, especially good, much cheaper than the entity store, it is worth buying!	Jingdong prefecture level city in Shandong
Stowe Flagship Store	King of Power Saving	I did not expect the express delivery so quickly, very save point quality is super good, than the entity store is too cheap.	Jingdong prefecture level city in Shandong
Stowe Flagship Store	King of Power Saving	Have received the baby, very satisfied with a shopping, great worth buying!	Jingdong prefecture level city in Shandong
Stowe Flagship Store	King of Power Saving	This is really more powerful, very satisfied, more energy-saving, better, thank you boss.	Jingdong prefecture level city in Shandong
Stowe Flagship Store	King of Power Saving	Received has been used for two days, the air conditioning can boldly open it! Energy saving effect is really quite good	Jingdong prefecture level city in Shandong
Stowe Flagship Store	King of Power Saving	It is authentic, trust the merchant, the baby has a great texture and the price is moderate, unlimited repurchase!	Jingdong prefecture level city in Shandong
Stowe Flagship Store	King of Power Saving	It is genuine very good, the price is much cheaper, cost-effective, really special cost-effective, it is worth buying!	Jingdong prefecture level city in Shandong
Stowe Flagship Store	King of Power Saving	Very good quality, fine workmanship, low power consumption, long warranty, fast shipping, economical and practical	Jingdong prefecture level city in Shandong
Stowe Flagship Store	King of Power Saving	I've been using it for half a month now, it really saves power and the voltage is stable, it's very good	Jingdong prefecture level city in Shandong
Stowe Flagship Store	King of Power Saving	It's the ideal product 💝, good quality and fast logistics, worth the kudos!	Jingdong prefecture level city in Shandong
Stowe Flagship Store	King of Power Saving	The logistics is very fast, the baby is very good, the shopkeeper is NICE!	Jingdong prefecture level city in Shandong
Stowe Flagship Store	King of Power Saving	Good to use, good quality, save point durable	Jingdong prefecture level city in Shandong
Stowe Flagship Store	King of Power Saving	The quality is very good, customer service attitude is very, very awesome, there are problems in a timely manner to solve the problem	Jingdong prefecture level city in Shandong
Stowe Flagship Store	King of Power Saving	Quality is very good, the use of the effect is very good, more stable, quality assurance	Jingdong prefecture level city in Shandong
Stowe Flagship Store	King of Power Saving	Convenient, inexpensive, and see how it works out?	Jingdong prefecture level city in Shandong
Stowe Flagship Store	King of Power Saving	Very good quality, saves a lot of electricity, very good, worth buying	Jingdong prefecture level city in Shandong
Stowe Flagship Store	King of Power Saving	Power Saving King is easy to use, with the fruit of power saving to use easy to give!	Jingdong prefecture level city in Shandong
Stowe Flagship Store	King of Power Saving	This saver really works, good quality, safe and secure, very satisfied!	Jingdong prefecture level city in Shandong
Stowe Flagship Store	King of Power Saving	It works well, the voltage is very stable effect is good, the quality is also very good satisfaction	Jingdong prefecture level city in Shandong
Stowe Flagship Store	King of Power Saving	The logistics is super super fast! The quality is also very good, indeed value for money	Jingdong prefecture level city in Shandong
Stowe Flagship Store	King of Power Saving	I've only been using it for a month or so and it seems to be saving a lot of power	Jingdong prefecture level city in Shandong
Stowe Flagship Store	King of Power Saving	Very easy to operate, very good material, good value for money.	Jingdong prefecture level city in Shandong
Stowe Flagship Store	King of Power Saving	Aluminum alloy shell, durable, compact power-saving good effect	Jingdong prefecture level city in Shandong
Stowe Flagship Store	King of Power Saving	Nice device, easy to use, doesn't take up much space, recommendable!	Jingdong prefecture level city in Shandong
Stowe Flagship Store	King of Power Saving	Good quality, good price, fast logistics	Jingdong prefecture level city in Shandong

Stowe Flagship Store	King of Power Saving	Friends of the family in use recommended, the effect is good and recommendable!	Jingdong prefecture level city in Shandong
Stowe Flagship Store	King of Power Saving	Cheaper than brick and mortar stores worth buying is authentic	Jingdong prefecture level city in Shandong
Stowe Flagship Store	King of Power Saving	Merchants ship quickly, the price is cheaper than the physical store, good use	Jingdong prefecture level city in Shandong
Stowe Flagship Store	King of Power Saving	Very durable, affordable, fast shipping	Jingdong prefecture level city in Shandong
Stowe Flagship Store	King of Power Saving	Packaging tight quality is very good easy to use good results	Jingdong prefecture level city in Shandong
Stowe Flagship Store	King of Power Saving	Save the grid. It's in the whole house. It's saving money on the electric bill.	Jingdong prefecture level city in Shandong
Stowe Flagship Store	King of Power Saving	I used it today, it works well, I'm very satisfied	Jingdong prefecture level city in Shandong
Stowe Flagship Store	King of Power Saving	It really saves a lot of electricity after installation	Jingdong prefecture level city in Shandong
Stowe Flagship Store	King of Power Saving	Love it. Satisfied.	Jingdong prefecture level city in Shandong
Stowe Flagship Store	King of Power Saving	Doesn't feel like it's working.	Jingdong prefecture level city in Shandong
Stowe Flagship Store	King of Power Saving	I'll comment after using it.	Jingdong prefecture level city in Shandong
Stowe Flagship Store	King of Power Saving	I'm trying it out to see if it saves power, and I'll buy two more if it works well.	Jingdong prefecture level city in Shandong
Stowe Flagship Store	King of Power Saving	It is genuine very good, the price is much cheaper, cost-effective, really special cost-effective, it is worth buying!	Jingdong prefecture level city in Shandong
Stowe Flagship Store	King of Power Saving	This thing can really save electricity, I was introduced to buy by my friend, he used it very well, I used it and think it's also very good, it can really save electricity, save about half of the electricity every day, it's really good!	Jingdong prefecture level city in Shandong
Stowe Flagship Store	King of Power Saving	After using it for ten days or so, I checked the power consumption and it did drop, and customer service said it will save more electricity in the long run. The product is also insured, 5 years warranty do not have to worry about quality problems!	Jingdong prefecture level city in Shandong
Stowe Flagship Store	King of Power Saving	The product is good, before already bought a, this is the second, the effect is quite good, anyway, I see the daily electricity bill than before a lot less, this can be the choice!	Jingdong prefecture level city in Shandong
Stowe Flagship Store	King of Power Saving	Originally also very worried about things are not good, get the hand found completely over-anxious, five-star praise, the style is very generous, the quality is very good, the price is also very reasonable!	Jingdong prefecture level city in Shandong
Stowe Flagship Store	King of Power Saving	Quality is good, playing to the old family also buy a, repurchase, this quality is really good, recommended!	Jingdong prefecture level city in Shandong
Stowe Flagship Store	King of Power Saving	Thank you, the owner even sent two fuses.	Jingdong prefecture level city in Shandong
Stowe Flagship Store	King of Power Saving	This store has great stuff, relatively fast shipping and logistics	Jingdong prefecture level city in Shandong
Stowe Flagship Store	King of Power Saving	This product is based on the impossibility of saving electricity, I used 853 kWh of electricity in July, started using the product on August 1, and used 867 kWh of electricity by September 1, is this called	Jingdong prefecture level city in Shandong
Stowe Flagship Store	King of Power Saving	Power king received, to the home on the trial, when I plugged into the electric panel can not see the effect of power saving, the meter is still the same speed in the turn, I'll save the king of the manual to play look at the instructions, instructions to speak, have to have a day two days and half a month to save power obviously, then wait half a month to see the effect of power saving it! To half a month there are obvious power saving before continuing to chase Xu.	Jingdong prefecture level city in Shandong
Stowe Flagship Store	King of Power Saving	Received the goods, a look, did not see the manufacturer and production address, can only hold a try to see the mentality of the face	Jingdong prefecture level city in Shandong
Stowe Flagship Store	King of Power Saving	I've only been using it for a few days and I'm not sure if it's an IQ tax? Used to favorable reviews.	Jingdong prefecture level city in Shandong
Stowe Flagship Store	King of Power Saving	Appearance is okay, power saving next time to follow up the evaluation	Jingdong prefecture level city in Shandong
Stowe Flagship Store	King of Power Saving	Shorter time, but hopefully effective and power-saving, to be followed up in the future	Jingdong prefecture level city in Shandong
Stowe Flagship Store	King of Power Saving	Appearance is okay, I do not know if the power saving	Jingdong prefecture level city in Shandong
Stowe Flagship Store	King of Power Saving	Good service, fast logistics, Power Saving King is exquisite and beautiful, support the business, thank you!	Jingdong prefecture level city in Shandong
Stowe Flagship Store	King of Power Saving	Bought three at once to try first first.	Jingdong prefecture level city in Shandong
Stowe Flagship Store	King of Power Saving	The object is the same as the picture, the product is of high quality, the packaging is intact. High-end atmosphere, high power, power saving effect is obvious, average monthly power saving 50%, very satisfied.	Jingdong prefecture level city in Shandong
Stowe Flagship Store	King of Power Saving	Bought back and plugged in, really effective, before 16 degrees of electricity per day, now 8 degrees of electricity per day, open an air conditioner for 12 hours, decisive repurchase of a	Jingdong prefecture level city in Shandong
Stowe Flagship Store	King of Power Saving	The object is the same as the picture, the product is of high quality, and the package is intact. High-end atmosphere and high grade, high power	Jingdong prefecture level city in Shandong
Stowe Flagship Store	King of Power Saving	Goods received, I hope the store said can be energy-saving, first try a month, month after month to see the effect.	Jingdong prefecture level city in Shandong
Stowe Flagship Store	King of Power Saving	This is really good, the material and workmanship is particularly exquisite, the workmanship is really very good, I think I can recommend to my friends to buy	Jingdong prefecture level city in Shandong
Stowe Flagship Store	King of Power Saving	I have to praise, the quality is really good, and the packaging logistics are also good. Really like it, won't make you regret after getting it!	Jingdong prefecture level city in Shandong
Stowe Flagship Store	King of Power Saving	Reputable platform, efficient and professional service, buy with confidence and peace of mind!	Jingdong prefecture level city in Shandong
Stowe Flagship Store	King of Power Saving	Bought one on a trial basis to see if it works after 1-2 months.	Jingdong prefecture level city in Shandong
Stowe Flagship Store	King of Power Saving	Quality is very good, customer service attitude is very very praise, there are problems in a timely manner to solve the problem, the price is not expensive, super value, you can rest assured that the	Jingdong prefecture level city in Shandong
Stowe Flagship Store	King of Power Saving	With it no longer need to be afraid to use electricity, especially good, ready to buy a mom and dad that also	Jingdong prefecture level city in Shandong
Stowe Flagship Store	King of Power Saving	Baby received very like will return to buy the seller attitude is also very like will return to buy the seller attitude is also very like!	Jingdong prefecture level city in Shandong
Stowe Flagship Store	King of Power Saving	Jingdong Mall shopping convenience assured fast assured fast assured fast assured fast	Jingdong prefecture level city in Shandong
Stowe Flagship Store	King of Power Saving	Saving power received, the quality is very good, fine workmanship, especially good, much cheaper than the entity store, it is worth buying!	Jingdong prefecture level city in Shandong
Stowe Flagship Store	King of Power Saving	I did not expect the express delivery so quickly, very save point quality is super good, than the entity store is too cheap.	Jingdong prefecture level city in Shandong
Stowe Flagship Store	King of Power Saving	Have received the baby, very satisfied with a shopping, great worth buying!	Jingdong prefecture level city in Shandong
Stowe Flagship Store	King of Power Saving	This is really more powerful, very satisfied, more energy-saving, better, thank you boss.	Jingdong prefecture level city in Shandong
Stowe Flagship Store	King of Power Saving	Received has been used for two days, the air conditioning can boldly open it! Energy saving effect is really quite good	Jingdong prefecture level city in Shandong
Stowe Flagship Store	King of Power Saving	It is authentic, trust the merchant, the baby has a great texture and the price is moderate, unlimited repurchase!	Jingdong prefecture level city in Shandong
Stowe Flagship Store	King of Power Saving	It is genuine very good, the price is much cheaper, cost-effective, really special cost-effective, it is worth buying!	Jingdong prefecture level city in Shandong
Stowe Flagship Store	King of Power Saving	Very good quality, fine workmanship, low power consumption, long warranty, fast shipping, economical and practical	Jingdong prefecture level city in Shandong

Stowe Flagship Store	King of Power Saving	I've been using it for half a month now, it really saves power and the voltage is stable, it's very good	Jingdong prefecture level city in Shandong
Stowe Flagship Store	King of Power Saving	It's the ideal product 💝, good quality and fast logistics, worth the kudos!	Jingdong prefecture level city in Shandong
Stowe Flagship Store	King of Power Saving	The logistics is very fast, the baby is very good, the shopkeeper is NICE!	Jingdong prefecture level city in Shandong
Stowe Flagship Store	King of Power Saving	Good to use, good quality, save point durable	Jingdong prefecture level city in Shandong
Stowe Flagship Store	King of Power Saving	The quality is very good, customer service attitude is very, very awesome, there are problems in a timely manner to solve the problem	Jingdong prefecture level city in Shandong
Stowe Flagship Store	King of Power Saving	Quality is very good, the use of the effect is very good, more stable, quality assurance	Jingdong prefecture level city in Shandong
Stowe Flagship Store	King of Power Saving	Convenient, inexpensive, and see how it works out?	Jingdong prefecture level city in Shandong
Stowe Flagship Store	King of Power Saving	Very good quality, saves a lot of electricity, very good, worth buying	Jingdong prefecture level city in Shandong
Stowe Flagship Store	King of Power Saving	Power Saving King is easy to use, with the fruit of power saving to use easy to give!	Jingdong prefecture level city in Shandong
Stowe Flagship Store	King of Power Saving	This saver really works, good quality, safe and secure, very satisfied!	Jingdong prefecture level city in Shandong
Stowe Flagship Store	King of Power Saving	It works well, the voltage is very stable effect is good, the quality is also very good satisfaction	Jingdong prefecture level city in Shandong
Stowe Flagship Store	King of Power Saving	The logistics is super super fast! The quality is also very good, indeed value for money	Jingdong prefecture level city in Shandong
Stowe Flagship Store	King of Power Saving	I've only been using it for a month or so and it seems to be saving a lot of power	Jingdong prefecture level city in Shandong
Stowe Flagship Store	King of Power Saving	Very easy to operate, very good material, good value for money.	Jingdong prefecture level city in Shandong
Stowe Flagship Store	King of Power Saving	Aluminum alloy shell, durable, compact power-saving good effect	Jingdong prefecture level city in Shandong
Stowe Flagship Store	King of Power Saving	Nice device, easy to use, doesn't take up much space, recommendable!	Jingdong prefecture level city in Shandong
Stowe Flagship Store	King of Power Saving	Good quality, good price, fast logistics	Jingdong prefecture level city in Shandong
Stowe Flagship Store	King of Power Saving	Friends of the family in use recommended, the effect is good and recommendable!	Jingdong prefecture level city in Shandong
Stowe Flagship Store	King of Power Saving	Cheaper than brick and mortar stores worth buying is authentic	Jingdong prefecture level city in Shandong
Stowe Flagship Store	King of Power Saving	Merchants ship quickly, the price is cheaper than the physical store, good use	Jingdong prefecture level city in Shandong
Stowe Flagship Store	King of Power Saving	Very durable, affordable, fast shipping	Jingdong prefecture level city in Shandong
Stowe Flagship Store	King of Power Saving	Packaging tight quality is very good easy to use good results	Jingdong prefecture level city in Shandong
Stowe Flagship Store	King of Power Saving	Save the grid. It's in the whole house. It's saving money on the electric bill.	Jingdong prefecture level city in Shandong
Stowe Flagship Store	King of Power Saving	I used it today, it works well, I'm very satisfied	Jingdong prefecture level city in Shandong
Stowe Flagship Store	King of Power Saving	It really saves a lot of electricity after installation	Jingdong prefecture level city in Shandong
Stowe Flagship Store	King of Power Saving	Love it. Satisfied.	Jingdong prefecture level city in Shandong
Stowe Flagship Store	King of Power Saving	Doesn't feel like it's working.	Jingdong prefecture level city in Shandong
Stowe Flagship Store	King of Power Saving	I'll comment after using it.	Jingdong prefecture level city in Shandong
Stowe Flagship Store	King of Power Saving	I'm trying it out to see if it saves power, and I'll buy two more if it works well.	Jingdong prefecture level city in Shandong
Stowe Flagship Store	King of Power Saving	It is genuine very good, the price is much cheaper, cost-effective, really special cost-effective, it is worth buying!	Jingdong prefecture level city in Shandong
Stowe Flagship Store	King of Power Saving	This thing can really save electricity, I was introduced to buy by my friend, he used it very well, I used it and think it's also very good, it can really save electricity, save about half of the electricity every day, it's really good!	Jingdong prefecture level city in Shandong
Stowe Flagship Store	King of Power Saving	After using it for ten days or so, I checked the power consumption and it did drop, and customer service said it will save more electricity in the long run. The product is also insured, 5 years warranty do not have to worry about quality problems!	Jingdong prefecture level city in Shandong
Stowe Flagship Store	King of Power Saving	The product is good, before already bought a, this is the second, the effect is quite good, anyway, I see the daily electricity bill than before a lot less, this can be the choice!	Jingdong prefecture level city in Shandong
Stowe Flagship Store	King of Power Saving	Originally also very worried about things are not good, get the hand found completely over-anxious, five-star praise, the style is very generous, the quality is very good, the price is also very reasonable!	Jingdong prefecture level city in Shandong
Stowe Flagship Store	King of Power Saving	Quality is good, playing to the old family also buy a, repurchase, this quality is really good, recommended!	Jingdong prefecture level city in Shandong
Stowe Flagship Store	King of Power Saving	Thank you, the owner even sent two fuses.	Jingdong prefecture level city in Shandong
Stowe Flagship Store	King of Power Saving	This store has great stuff, relatively fast shipping and logistics	Jingdong prefecture level city in Shandong
Stowe Flagship Store	King of Power Saving	This product is based on the impossibility of saving electricity, I used 853 kWh of electricity in July, started using the product on August 1, and used 867 kWh of electricity by September 1, is this called	Jingdong prefecture level city in Shandong
Stowe Flagship Store	King of Power Saving	Power king received, to the home on the trial, when I plugged into the electric panel can not see the effect of power saving, the meter is still the same speed in the turn, I'll save the king of the manual to play look at the instructions, instructions to speak, have to have a day two days and half a month to save power obviously, then wait half a month to see the effect of power saving it! To half a month there are obvious power saving before continuing to chase Xu.	Jingdong prefecture level city in Shandong
Stowe Flagship Store	King of Power Saving	Received the goods, a look, did not see the manufacturer and production address, can only hold a try to see the mentality of the face	Jingdong prefecture level city in Shandong
Stowe Flagship Store	King of Power Saving	I've only been using it for a few days and I'm not sure if it's an IQ tax? Used to favorable reviews.	Jingdong prefecture level city in Shandong
Stowe Flagship Store	King of Power Saving	Appearance is okay, power saving next time to follow up the evaluation	Jingdong prefecture level city in Shandong
Stowe Flagship Store	King of Power Saving	Shorter time, but hopefully effective and power-saving, to be followed up in the future	Jingdong prefecture level city in Shandong
Stowe Flagship Store	King of Power Saving	Appearance is okay, I do not know if the power saving	Jingdong prefecture level city in Shandong
Stowe Flagship Store	King of Power Saving	Good service, fast logistics, Power Saving King is exquisite and beautiful, support the business, thank you!	Jingdong prefecture level city in Shandong
Stowe Flagship Store	King of Power Saving	Bought three at once to try first first.	Jingdong prefecture level city in Shandong
Stowe Flagship Store	King of Power Saving	The object is the same as the picture, the product is of high quality, the packaging is intact. High-end atmosphere, high power, power saving effect is obvious, average monthly power saving 50%, very satisfied.	Jingdong prefecture level city in Shandong
Stowe Flagship Store	King of Power Saving	Bought back and plugged in, really effective, before 16 degrees of electricity per day, now 8 degrees of electricity per day, open an air conditioner for 12 hours, decisive repurchase of a	Jingdong prefecture level city in Shandong
Stowe Flagship Store	King of Power Saving	The object is the same as the picture, the product is of high quality, and the package is intact. High-end atmosphere and high grade, high power	Jingdong prefecture level city in Shandong

Stowe Flagship Store	King of Power Saving	Goods received, I hope the store said can be energy-saving, first try a month, month after month to see the effect.	Jingdong prefecture level city in Shandong
Stowe Flagship Store	King of Power Saving	This is really good, the material and workmanship is particularly exquisite, the workmanship is really very good, I think I can recommend to my friends to buy	Jingdong prefecture level city in Shandong
Stowe Flagship Store	King of Power Saving	I have to praise, the quality is really good, and the packaging logistics are also good. Really like it, won't make you regret after getting it!	Jingdong prefecture level city in Shandong
Stowe Flagship Store	King of Power Saving	Reputable platform, efficient and professional service, buy with confidence and peace of mind!	Jingdong prefecture level city in Shandong
Stowe Flagship Store	King of Power Saving	Bought one on a trial basis to see if it works after 1-2 months.	Jingdong prefecture level city in Shandong
Stowe Flagship Store	King of Power Saving	Quality is very good, customer service attitude is very very praise, there are problems in a timely manner to solve the problem, the price is not expensive, super value, you can rest assured that the	Jingdong prefecture level city in Shandong
Stowe Flagship Store	King of Power Saving	With it no longer need to be afraid to use electricity, especially good, ready to buy a mom and dad that also	Jingdong prefecture level city in Shandong
Stowe Flagship Store	King of Power Saving	Baby received very like will return to buy the seller attitude is also very like will return to buy the seller attitude is also very like!	Jingdong prefecture level city in Shandong
Stowe Flagship Store	King of Power Saving	Jingdong Mall shopping convenience assured fast assured fast assured fast assured fast	Jingdong prefecture level city in Shandong
Stowe Flagship Store	King of Power Saving	Saving power received, the quality is very good, fine workmanship, especially good, much cheaper than the entity store, it is worth buying!	Jingdong prefecture level city in Shandong
Stowe Flagship Store	King of Power Saving	I did not expect the express delivery so quickly, very save point quality is super good, than the entity store is too cheap.	Jingdong prefecture level city in Shandong
Stowe Flagship Store	King of Power Saving	Have received the baby, very satisfied with a shopping, great worth buying!	Jingdong prefecture level city in Shandong
Stowe Flagship Store	King of Power Saving	This is really more powerful, very satisfied, more energy-saving, better, thank you boss.	Jingdong prefecture level city in Shandong
Stowe Flagship Store	King of Power Saving	Received has been used for two days, the air conditioning can boldly open it! Energy saving effect is really quite good	Jingdong prefecture level city in Shandong
Stowe Flagship Store	King of Power Saving	It is authentic, trust the merchant, the baby has a great texture and the price is moderate, unlimited repurchase!	Jingdong prefecture level city in Shandong
Stowe Flagship Store	King of Power Saving	It is genuine very good, the price is much cheaper, cost-effective, really special cost-effective, it is worth buying!	Jingdong prefecture level city in Shandong
Stowe Flagship Store	King of Power Saving	Very good quality, fine workmanship, low power consumption, long warranty, fast shipping, economical and practical	Jingdong prefecture level city in Shandong
Stowe Flagship Store	King of Power Saving	I've been using it for half a month now, it really saves power and the voltage is stable, it's very good	Jingdong prefecture level city in Shandong
Stowe Flagship Store	King of Power Saving	It's the ideal product 💖, good quality and fast logistics, worth the kudos!	Jingdong prefecture level city in Shandong
Stowe Flagship Store	King of Power Saving	The logistics is very fast, the baby is very good, the shopkeeper is NICE!	Jingdong prefecture level city in Shandong
Stowe Flagship Store	King of Power Saving	Good to use, good quality, save point durable	Jingdong prefecture level city in Shandong
Stowe Flagship Store	King of Power Saving	The quality is very good, customer service attitude is very, very awesome, there are problems in a timely manner to solve the problem	Jingdong prefecture level city in Shandong
Stowe Flagship Store	King of Power Saving	Quality is very good, the use of the effect is very good, more stable, quality assurance	Jingdong prefecture level city in Shandong
Stowe Flagship Store	King of Power Saving	Convenient, inexpensive, and see how it works out?	Jingdong prefecture level city in Shandong
Stowe Flagship Store	King of Power Saving	Very good quality, saves a lot of electricity, very good, worth buying	Jingdong prefecture level city in Shandong
Stowe Flagship Store	King of Power Saving	Power Saving King is easy to use, with the fruit of power saving to use easy to give!	Jingdong prefecture level city in Shandong
Stowe Flagship Store	King of Power Saving	This saver really works, good quality, safe and secure, very satisfied!	Jingdong prefecture level city in Shandong
Stowe Flagship Store	King of Power Saving	It works well, the voltage is very stable effect is good, the quality is also very good satisfaction	Jingdong prefecture level city in Shandong
Stowe Flagship Store	King of Power Saving	The logistics is super super fast! The quality is also very good, indeed value for money	Jingdong prefecture level city in Shandong
Stowe Flagship Store	King of Power Saving	I've only been using it for a month or so and it seems to be saving a lot of power	Jingdong prefecture level city in Shandong
Stowe Flagship Store	King of Power Saving	Very easy to operate, very good material, good value for money.	Jingdong prefecture level city in Shandong
Stowe Flagship Store	King of Power Saving	Aluminum alloy shell, durable, compact power-saving good effect	Jingdong prefecture level city in Shandong
Stowe Flagship Store	King of Power Saving	Nice device, easy to use, doesn't take up much space, recommendable!	Jingdong prefecture level city in Shandong
Stowe Flagship Store	King of Power Saving	Good quality, good price, fast logistics	Jingdong prefecture level city in Shandong
Stowe Flagship Store	King of Power Saving	Friends of the family in use recommended, the effect is good and recommendable!	Jingdong prefecture level city in Shandong
Stowe Flagship Store	King of Power Saving	Cheaper than brick and mortar stores worth buying is authentic	Jingdong prefecture level city in Shandong
Stowe Flagship Store	King of Power Saving	Merchants ship quickly, the price is cheaper than the physical store, good use	Jingdong prefecture level city in Shandong
Stowe Flagship Store	King of Power Saving	Very durable, affordable, fast shipping	Jingdong prefecture level city in Shandong
Stowe Flagship Store	King of Power Saving	Packaging tight quality is very good easy to use good results	Jingdong prefecture level city in Shandong
Stowe Flagship Store	King of Power Saving	Save the grid. It's in the whole house. It's saving money on the electric bill.	Jingdong prefecture level city in Shandong
Stowe Flagship Store	King of Power Saving	I used it today, it works well, I'm very satisfied	Jingdong prefecture level city in Shandong
Stowe Flagship Store	King of Power Saving	It really saves a lot of electricity after installation	Jingdong prefecture level city in Shandong
Stowe Flagship Store	King of Power Saving	Love it. Satisfied.	Jingdong prefecture level city in Shandong
Stowe Flagship Store	King of Power Saving	Doesn't feel like it's working.	Jingdong prefecture level city in Shandong
Stowe Flagship Store	King of Power Saving	I'll comment after using it.	Jingdong prefecture level city in Shandong
Stowe Flagship Store	King of Power Saving	I'm trying it out to see if it saves power, and I'll buy two more if it works well.	Jingdong prefecture level city in Shandong
Stowe Flagship Store	King of Power Saving	It is genuine very good, the price is much cheaper, cost-effective, really special cost-effective, it is worth buying!	Jingdong prefecture level city in Shandong
Stowe Flagship Store	King of Power Saving	This thing can really save electricity, I was introduced to buy by my friend, he used it very well, I used it and think it's also very good, it can really save electricity, save about half of the electricity every day, it's really good!	Jingdong prefecture level city in Shandong
Stowe Flagship Store	King of Power Saving	After using it for ten days or so, I checked the power consumption and it did drop, and customer service said it will save more electricity in the long run. The product is also insured, 5 years warranty do not have to worry about quality problems!	Jingdong prefecture level city in Shandong
Stowe Flagship Store	King of Power Saving	The product is good, before already bought a, this is the second, the effect is quite good, anyway, I see the daily electricity bill than before a lot less, this can be the choice!	Jingdong prefecture level city in Shandong

Stowe Flagship Store	King of Power Saving	Originally also very worried about things are not good, get the hand found completely over-anxious, five-star praise, the style is very generous, the quality is very good, the price is also very reasonable!	Jingdong prefecture level city in Shandong
Stowe Flagship Store	King of Power Saving	Quality is good, playing to the old family also buy a, repurchase, this quality is really good, recommended!	Jingdong prefecture level city in Shandong
Stowe Flagship Store	King of Power Saving	Thank you, the owner even sent two fuses.	Jingdong prefecture level city in Shandong
Stowe Flagship Store	King of Power Saving	This store has great stuff, relatively fast shipping and logistics	Jingdong prefecture level city in Shandong
Stowe Flagship Store	King of Power Saving	This product is based on the impossibility of saving electricity, I used 853 kWh of electricity in July, started using the product on August 1, and used 867 kWh of electricity by September 1, is this called	Jingdong prefecture level city in Shandong
Stowe Flagship Store	King of Power Saving	Power king received, to the home on the trial, when I plugged into the electric panel can not see the effect of power saving, the meter is still the same speed in the turn, I'll save the king of the manual to play look at the instructions, instructions to speak, have to have a day two days and half a month to save power obviously, then wait half a month to see the effect of power saving it! To half a month there are obvious power saving before continuing to chase Xu.	Jingdong prefecture level city in Shandong
Stowe Flagship Store	King of Power Saving	Received the goods, a look, did not see the manufacturer and production address, can only hold a try to see the mentality of the face	Jingdong prefecture level city in Shandong
Stowe Flagship Store	King of Power Saving	I've only been using it for a few days and I'm not sure if it's an IQ tax? Used to favorable reviews.	Jingdong prefecture level city in Shandong
Stowe Flagship Store	King of Power Saving	Appearance is okay, power saving next time to follow up the evaluation	Jingdong prefecture level city in Shandong
Stowe Flagship Store	King of Power Saving	Shorter time, but hopefully effective and power-saving, to be followed up in the future	Jingdong prefecture level city in Shandong
Stowe Flagship Store	King of Power Saving	Appearance is okay, I do not know if the power saving	Jingdong prefecture level city in Shandong
Stowe Flagship Store	King of Power Saving	Good service, fast logistics, Power Saving King is exquisite and beautiful, support the business, thank you!	Jingdong prefecture level city in Shandong
Stowe Flagship Store	King of Power Saving	Bought three at once to try first first.	Jingdong prefecture level city in Shandong
Stowe Flagship Store	King of Power Saving	The object is the same as the picture, the product is of high quality, the packaging is intact. High-end atmosphere, high power, power saving effect is obvious, average monthly power saving 50%, very satisfied.	Jingdong prefecture level city in Shandong
Stowe Flagship Store	King of Power Saving	Bought back and plugged in, really effective, before 16 degrees of electricity per day, now 8 degrees of electricity per day, open an air conditioner for 12 hours, decisive repurchase of a	Jingdong prefecture level city in Shandong
Stowe Flagship Store	King of Power Saving	The object is the same as the picture, the product is of high quality, and the package is intact. High-end atmosphere and high grade, high power	Jingdong prefecture level city in Shandong
Stowe Flagship Store	King of Power Saving	Goods received, I hope the store said can be energy-saving, first try a month, month after month to see the effect.	Jingdong prefecture level city in Shandong
Stowe Flagship Store	King of Power Saving	This is really good, the material and workmanship is particularly exquisite, the workmanship is really very good, I think I can recommend to my friends to buy	Jingdong prefecture level city in Shandong
Stowe Flagship Store	King of Power Saving	I have to praise, the quality is really good, and the packaging logistics are also good. Really like it, won't make you regret after getting it!	Jingdong prefecture level city in Shandong
Stowe Flagship Store	King of Power Saving	Reputable platform, efficient and professional service, buy with confidence and peace of mind!	Jingdong prefecture level city in Shandong
Stowe Flagship Store	King of Power Saving	Bought one on a trial basis to see if it works after 1-2 months.	Jingdong prefecture level city in Shandong
Stowe Flagship Store	King of Power Saving	Quality is very good, customer service attitude is very very praise, there are problems in a timely manner to solve the problem, the price is not expensive, super value, you can rest assured that the	Jingdong prefecture level city in Shandong
Stowe Flagship Store	King of Power Saving	With it no longer need to be afraid to use electricity, especially good, ready to buy a mom and dad that also	Jingdong prefecture level city in Shandong
Stowe Flagship Store	King of Power Saving	Baby received very like will return to buy the seller attitude is also very like will return to buy the seller attitude is also very like!	Jingdong prefecture level city in Shandong
Stowe Flagship Store	King of Power Saving	Jingdong Mall shopping convenience assured fast assured fast assured fast assured fast	Jingdong prefecture level city in Shandong
Stowe Flagship Store	King of Power Saving	Saving power received, the quality is very good, fine workmanship, especially good, much cheaper than the entity store, it is worth buying!	Jingdong prefecture level city in Shandong
Stowe Flagship Store	King of Power Saving	I did not expect the express delivery so quickly, very save point quality is super good, than the entity store is too cheap.	Jingdong prefecture level city in Shandong
Stowe Flagship Store	King of Power Saving	Have received the baby, very satisfied with a shopping, great worth buying!	Jingdong prefecture level city in Shandong
Stowe Flagship Store	King of Power Saving	This is really more powerful, very satisfied, more energy-saving, better, thank you boss.	Jingdong prefecture level city in Shandong
Stowe Flagship Store	King of Power Saving	Received has been used for two days, the air conditioning can boldly open it! Energy saving effect is really quite good	Jingdong prefecture level city in Shandong
Stowe Flagship Store	King of Power Saving	It is authentic, trust the merchant, the baby has a great texture and the price is moderate, unlimited repurchase!	Jingdong prefecture level city in Shandong
Stowe Flagship Store	King of Power Saving	It is genuine very good, the price is much cheaper, cost-effective, really special cost-effective, it is worth buying!	Jingdong prefecture level city in Shandong
Stowe Flagship Store	King of Power Saving	Very good quality, fine workmanship, low power consumption, long warranty, fast shipping, economical and practical	Jingdong prefecture level city in Shandong
Stowe Flagship Store	King of Power Saving	I've been using it for half a month now, it really saves power and the voltage is stable, it's very good	Jingdong prefecture level city in Shandong
Stowe Flagship Store	King of Power Saving	It's the ideal product 💞, good quality and fast logistics, worth the kudos!	Jingdong prefecture level city in Shandong
Stowe Flagship Store	King of Power Saving	The logistics is very fast, the baby is very good, the shopkeeper is NICE!	Jingdong prefecture level city in Shandong
Stowe Flagship Store	King of Power Saving	Good to use, good quality, save point durable	Jingdong prefecture level city in Shandong
Stowe Flagship Store	King of Power Saving	The quality is very good, customer service attitude is very, very awesome, there are problems in a timely manner to solve the problem	Jingdong prefecture level city in Shandong
Stowe Flagship Store	King of Power Saving	Quality is very good, the use of the effect is very good, more stable, quality assurance	Jingdong prefecture level city in Shandong
Stowe Flagship Store	King of Power Saving	Convenient, inexpensive, and see how it works out?	Jingdong prefecture level city in Shandong
Stowe Flagship Store	King of Power Saving	Very good quality, saves a lot of electricity, very good, worth buying	Jingdong prefecture level city in Shandong
Stowe Flagship Store	King of Power Saving	Power Saving King is easy to use, with the fruit of power saving to use easy to give!	Jingdong prefecture level city in Shandong
Stowe Flagship Store	King of Power Saving	This saver really works, good quality, safe and secure, very satisfied!	Jingdong prefecture level city in Shandong
Stowe Flagship Store	King of Power Saving	It works well, the voltage is very stable effect is good, the quality is also very good satisfaction	Jingdong prefecture level city in Shandong
Stowe Flagship Store	King of Power Saving	The logistics is super super fast! The quality is also very good, indeed value for money	Jingdong prefecture level city in Shandong
Stowe Flagship Store	King of Power Saving	I've only been using it for a month or so and it seems to be saving a lot of power	Jingdong prefecture level city in Shandong
Stowe Flagship Store	King of Power Saving	Very easy to operate, very good material, good value for money.	Jingdong prefecture level city in Shandong
Stowe Flagship Store	King of Power Saving	Aluminum alloy shell, durable, compact power-saving good effect	Jingdong prefecture level city in Shandong
Stowe Flagship Store	King of Power Saving	Nice device, easy to use, doesn't take up much space, recommendable!	Jingdong prefecture level city in Shandong
Stowe Flagship Store	King of Power Saving	Good quality, good price, fast logistics	Jingdong prefecture level city in Shandong

Stowe Flagship Store	King of Power Saving	Friends of the family in use recommended, the effect is good and recommendable!	Jingdong prefecture level city in Shandong
Stowe Flagship Store	King of Power Saving	Cheaper than brick and mortar stores worth buying is authentic	Jingdong prefecture level city in Shandong
Stowe Flagship Store	King of Power Saving	Merchants ship quickly, the price is cheaper than the physical store, good use	Jingdong prefecture level city in Shandong
Stowe Flagship Store	King of Power Saving	Very durable, affordable, fast shipping	Jingdong prefecture level city in Shandong
Stowe Flagship Store	King of Power Saving	Packaging tight quality is very good easy to use good results	Jingdong prefecture level city in Shandong
Stowe Flagship Store	King of Power Saving	Save the grid. It's in the whole house. It's saving money on the electric bill.	Jingdong prefecture level city in Shandong
Stowe Flagship Store	King of Power Saving	I used it today, it works well, I'm very satisfied	Jingdong prefecture level city in Shandong
Stowe Flagship Store	King of Power Saving	It really saves a lot of electricity after installation	Jingdong prefecture level city in Shandong
Stowe Flagship Store	King of Power Saving	Love it. Satisfied.	Jingdong prefecture level city in Shandong
Stowe Flagship Store	King of Power Saving	Doesn't feel like it's working.	Jingdong prefecture level city in Shandong
Stowe Flagship Store	King of Power Saving	I'll comment after using it.	Jingdong prefecture level city in Shandong
Stowe Flagship Store	King of Power Saving	I'm trying it out to see if it saves power, and I'll buy two more if it works well.	Jingdong prefecture level city in Shandong
Stowe Flagship Store	King of Power Saving	It is genuine very good, the price is much cheaper, cost-effective, really special cost-effective, it is worth buying!	Jingdong prefecture level city in Shandong
Stowe Flagship Store	King of Power Saving	This thing can really save electricity, I was introduced to buy by my friend, he used it very well, I used it and think it's also very good, it can really save electricity, save about half of the electricity every day, it's really good!	Jingdong prefecture level city in Shandong
Stowe Flagship Store	King of Power Saving	After using it for ten days or so, I checked the power consumption and it did drop, and customer service said it will save more electricity in the long run. The product is also insured, 5 years warranty do not have to worry about quality problems!	Jingdong prefecture level city in Shandong
Stowe Flagship Store	King of Power Saving	The product is good, before already bought a, this is the second, the effect is quite good, anyway, I see the daily electricity bill than before a lot less, this can be the choice!	Jingdong prefecture level city in Shandong
Stowe Flagship Store	King of Power Saving	Originally also very worried about things are not good, get the hand found completely over-anxious, five-star praise, the style is very generous, the quality is very good, the price is also very reasonable!	Jingdong prefecture level city in Shandong
Stowe Flagship Store	King of Power Saving	Quality is good, playing to the old family also buy a, repurchase, this quality is really good, recommended!	Jingdong prefecture level city in Shandong
Stowe Flagship Store	King of Power Saving	Thank you, the owner even sent two fuses.	Jingdong prefecture level city in Shandong
Stowe Flagship Store	King of Power Saving	This store has great stuff, relatively fast shipping and logistics	Jingdong prefecture level city in Shandong
Stowe Flagship Store	King of Power Saving	This product is based on the impossibility of saving electricity, I used 853 kWh of electricity in July, started using the product on August 1, and used 867 kWh of electricity by September 1, is this called	Jingdong prefecture level city in Shandong
Stowe Flagship Store	King of Power Saving	Power king received, to the home on the trial, when I plugged into the electric panel can not see the effect of power saving, the meter is still the same speed in the turn, I'll save the king of the manual to play look at the instructions, instructions to speak, have to have a day two days and half a month to save power obviously, then wait half a month to see the effect of power saving it! To half a month there are obvious power saving before continuing to chase Xu.	Jingdong prefecture level city in Shandong
Stowe Flagship Store	King of Power Saving	Received the goods, a look, did not see the manufacturer and production address, can only hold a try to see the mentality of the face	Jingdong prefecture level city in Shandong
Stowe Flagship Store	King of Power Saving	I've only been using it for a few days and I'm not sure if it's an IQ tax? Used to favorable reviews.	Jingdong prefecture level city in Shandong
Stowe Flagship Store	King of Power Saving	Appearance is okay, power saving next time to follow up the evaluation	Jingdong prefecture level city in Shandong
Stowe Flagship Store	King of Power Saving	Shorter time, but hopefully effective and power-saving, to be followed up in the future	Jingdong prefecture level city in Shandong
Stowe Flagship Store	King of Power Saving	Appearance is okay, I do not know if the power saving	Jingdong prefecture level city in Shandong
Stowe Flagship Store	King of Power Saving	Good service, fast logistics, Power Saving King is exquisite and beautiful, support the business, thank you!	Jingdong prefecture level city in Shandong
Stowe Flagship Store	King of Power Saving	Bought three at once to try first first.	Jingdong prefecture level city in Shandong
Stowe Flagship Store	King of Power Saving	The object is the same as the picture, the product is of high quality, the packaging is intact. High-end atmosphere, high power, power saving effect is obvious, average monthly power saving 50%, very satisfied.	Jingdong prefecture level city in Shandong
Stowe Flagship Store	King of Power Saving	Bought back and plugged in, really effective, before 16 degrees of electricity per day, now 8 degrees of electricity per day, open an air conditioner for 12 hours, decisive repurchase of a	Jingdong prefecture level city in Shandong
Stowe Flagship Store	King of Power Saving	The object is the same as the picture, the product is of high quality, and the package is intact. High-end atmosphere and high grade, high power	Jingdong prefecture level city in Shandong
Stowe Flagship Store	King of Power Saving	Goods received, I hope the store said can be energy-saving, first try a month, month after month to see the effect.	Jingdong prefecture level city in Shandong
Stowe Flagship Store	King of Power Saving	This is really good, the material and workmanship is particularly exquisite, the workmanship is really very good, I think I can recommend to my friends to buy	Jingdong prefecture level city in Shandong
Stowe Flagship Store	King of Power Saving	I have to praise, the quality is really good, and the packaging logistics are also good. Really like it, won't make you regret after getting it!	Jingdong prefecture level city in Shandong
Stowe Flagship Store	King of Power Saving	Reputable platform, efficient and professional service, buy with confidence and peace of mind!	Jingdong prefecture level city in Shandong
Stowe Flagship Store	King of Power Saving	Bought one on a trial basis to see if it works after 1-2 months.	Jingdong prefecture level city in Shandong
Stowe Flagship Store	King of Power Saving	Quality is very good, customer service attitude is very very praise, there are problems in a timely manner to solve the problem, the price is not expensive, super value, you can rest assured that the	Jingdong prefecture level city in Shandong
Stowe Flagship Store	King of Power Saving	With it no longer need to be afraid to use electricity, especially good, ready to buy a mom and dad that also	Jingdong prefecture level city in Shandong
Stowe Flagship Store	King of Power Saving	Baby received very like will return to buy the seller attitude is also very like will return to buy the seller attitude is also very like!	Jingdong prefecture level city in Shandong
Stowe Flagship Store	King of Power Saving	Jingdong Mall shopping convenience assured fast assured fast assured fast assured fast	Jingdong prefecture level city in Shandong
Stowe Flagship Store	King of Power Saving	Saving power received, the quality is very good, fine workmanship, especially good, much cheaper than the entity store, it is worth buying!	Jingdong prefecture level city in Shandong
Stowe Flagship Store	King of Power Saving	I did not expect the express delivery so quickly, very save point quality is super good, than the entity store is too cheap.	Jingdong prefecture level city in Shandong
Stowe Flagship Store	King of Power Saving	Have received the baby, very satisfied with a shopping, great worth buying!	Jingdong prefecture level city in Shandong
Stowe Flagship Store	King of Power Saving	This is really more powerful, very satisfied, more energy-saving, better, thank you boss.	Jingdong prefecture level city in Shandong
Stowe Flagship Store	King of Power Saving	Received has been used for two days, the air conditioning can boldly open it! Energy saving effect is really quite good	Jingdong prefecture level city in Shandong
Stowe Flagship Store	King of Power Saving	It is authentic, trust the merchant, the baby has a great texture and the price is moderate, unlimited repurchase!	Jingdong prefecture level city in Shandong
Stowe Flagship Store	King of Power Saving	It is genuine very good, the price is much cheaper, cost-effective, really special cost-effective, it is worth buying!	Jingdong prefecture level city in Shandong
Stowe Flagship Store	King of Power Saving	Very good quality, fine workmanship, low power consumption, long warranty, fast shipping, economical and practical	Jingdong prefecture level city in Shandong

Stowe Flagship Store	King of Power Saving	I've been using it for half a month now, it really saves power and the voltage is stable, it's very good	Jingdong prefecture level city in Shandong
Stowe Flagship Store	King of Power Saving	It's the ideal product 💝, good quality and fast logistics, worth the kudos!	Jingdong prefecture level city in Shandong
Stowe Flagship Store	King of Power Saving	The logistics is very fast, the baby is very good, the shopkeeper is NICE!	Jingdong prefecture level city in Shandong
Stowe Flagship Store	King of Power Saving	Good to use, good quality, save point durable	Jingdong prefecture level city in Shandong
Stowe Flagship Store	King of Power Saving	The quality is very good, customer service attitude is very, very awesome, there are problems in a timely manner to solve the problem	Jingdong prefecture level city in Shandong
Stowe Flagship Store	King of Power Saving	Quality is very good, the use of the effect is very good, more stable, quality assurance	Jingdong prefecture level city in Shandong
Stowe Flagship Store	King of Power Saving	Convenient, inexpensive, and see how it works out?	Jingdong prefecture level city in Shandong
Stowe Flagship Store	King of Power Saving	Very good quality, saves a lot of electricity, very good, worth buying	Jingdong prefecture level city in Shandong
Stowe Flagship Store	King of Power Saving	Power Saving King is easy to use, with the fruit of power saving to use easy to give!	Jingdong prefecture level city in Shandong
Stowe Flagship Store	King of Power Saving	This saver really works, good quality, safe and secure, very satisfied!	Jingdong prefecture level city in Shandong
Stowe Flagship Store	King of Power Saving	It works well, the voltage is very stable effect is good, the quality is also very good satisfaction	Jingdong prefecture level city in Shandong
Stowe Flagship Store	King of Power Saving	The logistics is super super fast! The quality is also very good, indeed value for money	Jingdong prefecture level city in Shandong
Stowe Flagship Store	King of Power Saving	I've only been using it for a month or so and it seems to be saving a lot of power	Jingdong prefecture level city in Shandong
Stowe Flagship Store	King of Power Saving	Very easy to operate, very good material, good value for money.	Jingdong prefecture level city in Shandong
Stowe Flagship Store	King of Power Saving	Aluminum alloy shell, durable, compact power-saving good effect	Jingdong prefecture level city in Shandong
Stowe Flagship Store	King of Power Saving	Nice device, easy to use, doesn't take up much space, recommendable!	Jingdong prefecture level city in Shandong
Stowe Flagship Store	King of Power Saving	Good quality, good price, fast logistics	Jingdong prefecture level city in Shandong
Stowe Flagship Store	King of Power Saving	Friends of the family in use recommended, the effect is good and recommendable!	Jingdong prefecture level city in Shandong
Stowe Flagship Store	King of Power Saving	Cheaper than brick and mortar stores worth buying is authentic	Jingdong prefecture level city in Shandong
Stowe Flagship Store	King of Power Saving	Merchants ship quickly, the price is cheaper than the physical store, good use	Jingdong prefecture level city in Shandong
Stowe Flagship Store	King of Power Saving	Very durable, affordable, fast shipping	Jingdong prefecture level city in Shandong
Stowe Flagship Store	King of Power Saving	Packaging tight quality is very good easy to use good results	Jingdong prefecture level city in Shandong
Stowe Flagship Store	King of Power Saving	Save the grid. It's in the whole house. It's saving money on the electric bill.	Jingdong prefecture level city in Shandong
Stowe Flagship Store	King of Power Saving	I used it today, it works well, I'm very satisfied	Jingdong prefecture level city in Shandong
Stowe Flagship Store	King of Power Saving	It really saves a lot of electricity after installation	Jingdong prefecture level city in Shandong
Stowe Flagship Store	King of Power Saving	Love it. Satisfied.	Jingdong prefecture level city in Shandong
Stowe Flagship Store	King of Power Saving	Doesn't feel like it's working.	Jingdong prefecture level city in Shandong
Stowe Flagship Store	King of Power Saving	I'll comment after using it.	Jingdong prefecture level city in Shandong
Stowe Flagship Store	King of Power Saving	I'm trying it out to see if it saves power, and I'll buy two more if it works well.	Jingdong prefecture level city in Shandong
Stowe Flagship Store	King of Power Saving	It is genuine very good, the price is much cheaper, cost-effective, really special cost-effective, it is worth buying!	Jingdong prefecture level city in Shandong
Stowe Flagship Store	King of Power Saving	This thing can really save electricity, I was introduced to buy by my friend, he used it very well, I used it and think it's also very good, it can really save electricity, save about half of the electricity every day, it's really good!	Jingdong prefecture level city in Shandong
Stowe Flagship Store	King of Power Saving	After using it for ten days or so, I checked the power consumption and it did drop, and customer service said it will save more electricity in the long run. The product is also insured, 5 years warranty do not have to worry about quality problems!	Jingdong prefecture level city in Shandong
Stowe Flagship Store	King of Power Saving	The product is good, before already bought a, this is the second, the effect is quite good, anyway, I see the daily electricity bill than before a lot less, this can be the choice!	Jingdong prefecture level city in Shandong
Stowe Flagship Store	King of Power Saving	Originally also very worried about things are not good, get the hand found completely over-anxious, five-star praise, the style is very generous, the quality is very good, the price is also very reasonable!	Jingdong prefecture level city in Shandong
Stowe Flagship Store	King of Power Saving	Quality is good, playing to the old family also buy a, repurchase, this quality is really good, recommended!	Jingdong prefecture level city in Shandong
Stowe Flagship Store	King of Power Saving	Thank you, the owner even sent two fuses.	Jingdong prefecture level city in Shandong
Stowe Flagship Store	King of Power Saving	This store has great stuff, relatively fast shipping and logistics	Jingdong prefecture level city in Shandong
Stowe Flagship Store	King of Power Saving	This product is based on the impossibility of saving electricity, I used 853 kWh of electricity in July, started using the product on August 1, and used 867 kWh of electricity by September 1, is this called	Jingdong prefecture level city in Shandong
Stowe Flagship Store	King of Power Saving	Power king received, to the home on the trial, when I plugged into the electric panel can not see the effect of power saving, the meter is still the same speed in the turn, I'll save the king of the manual to play look at the instructions, instructions to speak, have to have a day two days and half a month to save power obviously, then wait half a month to see the effect of power saving it! To half a month there are obvious power saving before continuing to chase Xu.	Jingdong prefecture level city in Shandong
Stowe Flagship Store	King of Power Saving	Received the goods, a look, did not see the manufacturer and production address, can only hold a try to see the mentality of the face	Jingdong prefecture level city in Shandong
Stowe Flagship Store	King of Power Saving	I've only been using it for a few days and I'm not sure if it's an IQ tax? Used to favorable reviews.	Jingdong prefecture level city in Shandong
Stowe Flagship Store	King of Power Saving	Appearance is okay, power saving next time to follow up the evaluation	Jingdong prefecture level city in Shandong
Stowe Flagship Store	King of Power Saving	Shorter time, but hopefully effective and power-saving, to be followed up in the future	Jingdong prefecture level city in Shandong
Stowe Flagship Store	King of Power Saving	Appearance is okay, I do not know if the power saving	Jingdong prefecture level city in Shandong
Stowe Flagship Store	King of Power Saving	Good service, fast logistics, Power Saving King is exquisite and beautiful, support the business, thank you!	Jingdong prefecture level city in Shandong
Stowe Flagship Store	King of Power Saving	Bought three at once to try first first.	Jingdong prefecture level city in Shandong
Stowe Flagship Store	King of Power Saving	The object is the same as the picture, the product is of high quality, the packaging is intact. High-end atmosphere, high power, power saving effect is obvious, average monthly power saving 50%, very satisfied.	Jingdong prefecture level city in Shandong
Stowe Flagship Store	King of Power Saving	Bought back and plugged in, really effective, before 16 degrees of electricity per day, now 8 degrees of electricity per day, open an air conditioner for 12 hours, decisive repurchase of a	Jingdong prefecture level city in Shandong
Stowe Flagship Store	King of Power Saving	The object is the same as the picture, the product is of high quality, and the package is intact. High-end atmosphere and high grade, high power	Jingdong prefecture level city in Shandong

Stowe Flagship Store	King of Power Saving	Goods received, I hope the store said can be energy-saving, first try a month, month after month to see the effect.	Jingdong prefecture level city in Shandong
Stowe Flagship Store	King of Power Saving	This is really good, the material and workmanship is particularly exquisite, the workmanship is really very good, I think I can recommend to my friends to buy	Jingdong prefecture level city in Shandong
Stowe Flagship Store	King of Power Saving	I have to praise, the quality is really good, and the packaging logistics are also good. Really like it, won't make you regret after getting it!	Jingdong prefecture level city in Shandong
Stowe Flagship Store	King of Power Saving	Reputable platform, efficient and professional service, buy with confidence and peace of mind!	Jingdong prefecture level city in Shandong
Stowe Flagship Store	King of Power Saving	Bought one on a trial basis to see if it works after 1-2 months.	Jingdong prefecture level city in Shandong
Stowe Flagship Store	King of Power Saving	Quality is very good, customer service attitude is very very praise, there are problems in a timely manner to solve the problem, the price is not expensive, super value, you can rest assured that the	Jingdong prefecture level city in Shandong
Stowe Flagship Store	King of Power Saving	With it no longer need to be afraid to use electricity, especially good, ready to buy a mom and dad that also	Jingdong prefecture level city in Shandong
Stowe Flagship Store	King of Power Saving	Baby received very like will return to buy the seller attitude is also very like will return to buy the seller attitude is also very like!	Jingdong prefecture level city in Shandong
Stowe Flagship Store	King of Power Saving	Jingdong Mall shopping convenience assured fast assured fast assured fast assured fast	Jingdong prefecture level city in Shandong
Stowe Flagship Store	King of Power Saving	Saving power received, the quality is very good, fine workmanship, especially good, much cheaper than the entity store, it is worth buying!	Jingdong prefecture level city in Shandong
Stowe Flagship Store	King of Power Saving	I did not expect the express delivery so quickly, very save point quality is super good, than the entity store is too cheap.	Jingdong prefecture level city in Shandong
Stowe Flagship Store	King of Power Saving	Have received the baby, very satisfied with a shopping, great worth buying!	Jingdong prefecture level city in Shandong
Stowe Flagship Store	King of Power Saving	This is really more powerful, very satisfied, more energy-saving, better, thank you boss.	Jingdong prefecture level city in Shandong
Stowe Flagship Store	King of Power Saving	Received has been used for two days, the air conditioning can boldly open it! Energy saving effect is really quite good	Jingdong prefecture level city in Shandong
Stowe Flagship Store	King of Power Saving	It is authentic, trust the merchant, the baby has a great texture and the price is moderate, unlimited repurchase!	Jingdong prefecture level city in Shandong
Stowe Flagship Store	King of Power Saving	It is genuine very good, the price is much cheaper, cost-effective, really special cost-effective, it is worth buying!	Jingdong prefecture level city in Shandong
Stowe Flagship Store	King of Power Saving	Very good quality, fine workmanship, low power consumption, long warranty, fast shipping, economical and practical	Jingdong prefecture level city in Shandong
Stowe Flagship Store	King of Power Saving	I've been using it for half a month now, it really saves power and the voltage is stable, it's very good	Jingdong prefecture level city in Shandong
Stowe Flagship Store	King of Power Saving	It's the ideal product 💖, good quality and fast logistics, worth the kudos!	Jingdong prefecture level city in Shandong
Stowe Flagship Store	King of Power Saving	The logistics is very fast, the baby is very good, the shopkeeper is NICE!	Jingdong prefecture level city in Shandong
Stowe Flagship Store	King of Power Saving	Good to use, good quality, save point durable	Jingdong prefecture level city in Shandong
Stowe Flagship Store	King of Power Saving	The quality is very good, customer service attitude is very, very awesome, there are problems in a timely manner to solve the problem	Jingdong prefecture level city in Shandong
Stowe Flagship Store	King of Power Saving	Quality is very good, the use of the effect is very good, more stable, quality assurance	Jingdong prefecture level city in Shandong
Stowe Flagship Store	King of Power Saving	Convenient, inexpensive, and see how it works out?	Jingdong prefecture level city in Shandong
Stowe Flagship Store	King of Power Saving	Very good quality, saves a lot of electricity, very good, worth buying	Jingdong prefecture level city in Shandong
Stowe Flagship Store	King of Power Saving	Power Saving King is easy to use, with the fruit of power saving to use easy to give!	Jingdong prefecture level city in Shandong
Stowe Flagship Store	King of Power Saving	This saver really works, good quality, safe and secure, very satisfied!	Jingdong prefecture level city in Shandong
Stowe Flagship Store	King of Power Saving	It works well, the voltage is very stable effect is good, the quality is also very good satisfaction	Jingdong prefecture level city in Shandong
Stowe Flagship Store	King of Power Saving	The logistics is super super fast! The quality is also very good, indeed value for money	Jingdong prefecture level city in Shandong
Stowe Flagship Store	King of Power Saving	I've only been using it for a month or so and it seems to be saving a lot of power	Jingdong prefecture level city in Shandong
Stowe Flagship Store	King of Power Saving	Very easy to operate, very good material, good value for money.	Jingdong prefecture level city in Shandong
Stowe Flagship Store	King of Power Saving	Aluminum alloy shell, durable, compact power-saving good effect	Jingdong prefecture level city in Shandong
Stowe Flagship Store	King of Power Saving	Nice device, easy to use, doesn't take up much space, recommendable!	Jingdong prefecture level city in Shandong
Stowe Flagship Store	King of Power Saving	Good quality, good price, fast logistics	Jingdong prefecture level city in Shandong
Stowe Flagship Store	King of Power Saving	Friends of the family in use recommended, the effect is good and recommendable!	Jingdong prefecture level city in Shandong
Stowe Flagship Store	King of Power Saving	Cheaper than brick and mortar stores worth buying is authentic	Jingdong prefecture level city in Shandong
Stowe Flagship Store	King of Power Saving	Merchants ship quickly, the price is cheaper than the physical store, good use	Jingdong prefecture level city in Shandong
Stowe Flagship Store	King of Power Saving	Very durable, affordable, fast shipping	Jingdong prefecture level city in Shandong
Stowe Flagship Store	King of Power Saving	Packaging tight quality is very good easy to use good results	Jingdong prefecture level city in Shandong
Stowe Flagship Store	King of Power Saving	Save the grid. It's in the whole house. It's saving money on the electric bill.	Jingdong prefecture level city in Shandong
Stowe Flagship Store	King of Power Saving	I used it today, it works well, I'm very satisfied	Jingdong prefecture level city in Shandong
Stowe Flagship Store	King of Power Saving	It really saves a lot of electricity after installation	Jingdong prefecture level city in Shandong
Stowe Flagship Store	King of Power Saving	Love it. Satisfied.	Jingdong prefecture level city in Shandong
Stowe Flagship Store	King of Power Saving	Doesn't feel like it's working.	Jingdong prefecture level city in Shandong
Stowe Flagship Store	King of Power Saving	I'll comment after using it.	Jingdong prefecture level city in Shandong
Stowe Flagship Store	King of Power Saving	I'm trying it out to see if it saves power, and I'll buy two more if it works well.	Jingdong prefecture level city in Shandong
Stowe Flagship Store	King of Power Saving	It is genuine very good, the price is much cheaper, cost-effective, really special cost-effective, it is worth buying!	Jingdong prefecture level city in Shandong
Stowe Flagship Store	King of Power Saving	This thing can really save electricity, I was introduced to buy by my friend, he used it very well, I used it and think it's also very good, it can really save electricity, save about half of the electricity every day, it's really good!	Jingdong prefecture level city in Shandong
Stowe Flagship Store	King of Power Saving	After using it for ten days or so, I checked the power consumption and it did drop, and customer service said it will save more electricity in the long run. The product is also insured, 5 years warranty do not have to worry about quality problems!	Jingdong prefecture level city in Shandong
Stowe Flagship Store	King of Power Saving	The product is good, before already bought a, this is the second, the effect is quite good, anyway, I see the daily electricity bill than before a lot less, this can be the choice!	Jingdong prefecture level city in Shandong

Stowe Flagship Store	King of Power Saving	Originally also very worried about things are not good, get the hand found completely over-anxious, five-star praise, the style is very generous, the quality is very good, the price is also very reasonable!	Jingdong prefecture level city in Shandong
Stowe Flagship Store	King of Power Saving	Quality is good, playing to the old family also buy a, repurchase, this quality is really good, recommended!	Jingdong prefecture level city in Shandong
Stowe Flagship Store	King of Power Saving	Thank you, the owner even sent two fuses.	Jingdong prefecture level city in Shandong
Stowe Flagship Store	King of Power Saving	This store has great stuff, relatively fast shipping and logistics	Jingdong prefecture level city in Shandong
Stowe Flagship Store	King of Power Saving	This product is based on the impossibility of saving electricity, I used 853 kWh of electricity in July, started using the product on August 1, and used 867 kWh of electricity by September 1, is this called	Jingdong prefecture level city in Shandong
Stowe Flagship Store	King of Power Saving	Power king received, to the home on the trial, when I plugged into the electric panel can not see the effect of power saving, the meter is still the same speed in the turn, I'll save the king of the manual to play look at the instructions, instructions to speak, have to have a day two days and half a month to save power obviously, then wait half a month to see the effect of power saving it! To half a month there are obvious power saving before continuing to chase Xu.	Jingdong prefecture level city in Shandong
Stowe Flagship Store	King of Power Saving	Received the goods, a look, did not see the manufacturer and production address, can only hold a try to see the mentality of the face	Jingdong prefecture level city in Shandong
Stowe Flagship Store	King of Power Saving	I've only been using it for a few days and I'm not sure if it's an IQ tax? Used to favorable reviews.	Jingdong prefecture level city in Shandong
Stowe Flagship Store	King of Power Saving	Appearance is okay, power saving next time to follow up the evaluation	Jingdong prefecture level city in Shandong
Stowe Flagship Store	King of Power Saving	Shorter time, but hopefully effective and power-saving, to be followed up in the future	Jingdong prefecture level city in Shandong
Stowe Flagship Store	King of Power Saving	Appearance is okay, I do not know if the power saving	Jingdong prefecture level city in Shandong
Stowe Flagship Store	King of Power Saving	Good service, fast logistics, Power Saving King is exquisite and beautiful, support the business, thank you!	Jingdong prefecture level city in Shandong
Stowe Flagship Store	King of Power Saving	Bought three at once to try first first.	Jingdong prefecture level city in Shandong
Stowe Flagship Store	King of Power Saving	The object is the same as the picture, the product is of high quality, the packaging is intact. High-end atmosphere, high power, power saving effect is obvious, average monthly power saving 50%, very satisfied.	Jingdong prefecture level city in Shandong
Stowe Flagship Store	King of Power Saving	Bought back and plugged in, really effective, before 16 degrees of electricity per day, now 8 degrees of electricity per day, open an air conditioner for 12 hours, decisive repurchase of a	Jingdong prefecture level city in Shandong
Stowe Flagship Store	King of Power Saving	The object is the same as the picture, the product is of high quality, and the package is intact. High-end atmosphere and high grade, high power	Jingdong prefecture level city in Shandong
Stowe Flagship Store	King of Power Saving	Goods received, I hope the store said can be energy-saving, first try a month, month after month to see the effect.	Jingdong prefecture level city in Shandong
Stowe Flagship Store	King of Power Saving	This is really good, the material and workmanship is particularly exquisite, the workmanship is really very good, I think I can recommend to my friends to buy	Jingdong prefecture level city in Shandong
Stowe Flagship Store	King of Power Saving	I have to praise, the quality is really good, and the packaging logistics are also good. Really like it, won't make you regret after getting it!	Jingdong prefecture level city in Shandong
Stowe Flagship Store	King of Power Saving	Reputable platform, efficient and professional service, buy with confidence and peace of mind!	Jingdong prefecture level city in Shandong
Stowe Flagship Store	King of Power Saving	Bought one on a trial basis to see if it works after 1-2 months.	Jingdong prefecture level city in Shandong
Stowe Flagship Store	King of Power Saving	Quality is very good, customer service attitude is very very praise, there are problems in a timely manner to solve the problem, the price is not expensive, super value, you can rest assured that the	Jingdong prefecture level city in Shandong
Stowe Flagship Store	King of Power Saving	With it no longer need to be afraid to use electricity, especially good, ready to buy a mom and dad that also	Jingdong prefecture level city in Shandong
Stowe Flagship Store	King of Power Saving	Baby received very like will return to buy the seller attitude is also very like will return to buy the seller attitude is also very like!	Jingdong prefecture level city in Shandong
Stowe Flagship Store	King of Power Saving	Jingdong Mall shopping convenience assured fast assured fast assured fast assured fast	Jingdong prefecture level city in Shandong
Stowe Flagship Store	King of Power Saving	Saving power received, the quality is very good, fine workmanship, especially good, much cheaper than the entity store, it is worth buying!	Jingdong prefecture level city in Shandong
Stowe Flagship Store	King of Power Saving	I did not expect the express delivery so quickly, very save point quality is super good, than the entity store is too cheap.	Jingdong prefecture level city in Shandong
Stowe Flagship Store	King of Power Saving	Have received the baby, very satisfied with a shopping, great worth buying!	Jingdong prefecture level city in Shandong
Stowe Flagship Store	King of Power Saving	This is really more powerful, very satisfied, more energy-saving, better, thank you boss.	Jingdong prefecture level city in Shandong
Stowe Flagship Store	King of Power Saving	Received has been used for two days, the air conditioning can boldly open it! Energy saving effect is really quite good	Jingdong prefecture level city in Shandong
Stowe Flagship Store	King of Power Saving	It is authentic, trust the merchant, the baby has a great texture and the price is moderate, unlimited repurchase!	Jingdong prefecture level city in Shandong
Stowe Flagship Store	King of Power Saving	It is genuine very good, the price is much cheaper, cost-effective, really special cost-effective, it is worth buying!	Jingdong prefecture level city in Shandong
Stowe Flagship Store	King of Power Saving	Very good quality, fine workmanship, low power consumption, long warranty, fast shipping, economical and practical	Jingdong prefecture level city in Shandong
Stowe Flagship Store	King of Power Saving	I've been using it for half a month now, it really saves power and the voltage is stable, it's very good	Jingdong prefecture level city in Shandong
Stowe Flagship Store	King of Power Saving	It's the ideal product 💞, good quality and fast logistics, worth the kudos!	Jingdong prefecture level city in Shandong
Stowe Flagship Store	King of Power Saving	The logistics is very fast, the baby is very good, the shopkeeper is NICE!	Jingdong prefecture level city in Shandong
Stowe Flagship Store	King of Power Saving	Good to use, good quality, save point durable	Jingdong prefecture level city in Shandong
Stowe Flagship Store	King of Power Saving	The quality is very good, customer service attitude is very, very awesome, there are problems in a timely manner to solve the problem	Jingdong prefecture level city in Shandong
Stowe Flagship Store	King of Power Saving	Quality is very good, the use of the effect is very good, more stable, quality assurance	Jingdong prefecture level city in Shandong
Stowe Flagship Store	King of Power Saving	Convenient, inexpensive, and see how it works out?	Jingdong prefecture level city in Shandong
Stowe Flagship Store	King of Power Saving	Very good quality, saves a lot of electricity, very good, worth buying	Jingdong prefecture level city in Shandong
Stowe Flagship Store	King of Power Saving	Power Saving King is easy to use, with the fruit of power saving to use easy to give!	Jingdong prefecture level city in Shandong
Stowe Flagship Store	King of Power Saving	This saver really works, good quality, safe and secure, very satisfied!	Jingdong prefecture level city in Shandong
Stowe Flagship Store	King of Power Saving	It works well, the voltage is very stable effect is good, the quality is also very good satisfaction	Jingdong prefecture level city in Shandong
Stowe Flagship Store	King of Power Saving	The logistics is super super fast! The quality is also very good, indeed value for money	Jingdong prefecture level city in Shandong
Stowe Flagship Store	King of Power Saving	I've only been using it for a month or so and it seems to be saving a lot of power	Jingdong prefecture level city in Shandong
Stowe Flagship Store	King of Power Saving	Very easy to operate, very good material, good value for money.	Jingdong prefecture level city in Shandong
Stowe Flagship Store	King of Power Saving	Aluminum alloy shell, durable, compact power-saving good effect	Jingdong prefecture level city in Shandong
Stowe Flagship Store	King of Power Saving	Nice device, easy to use, doesn't take up much space, recommendable!	Jingdong prefecture level city in Shandong
Stowe Flagship Store	King of Power Saving	Good quality, good price, fast logistics	Jingdong prefecture level city in Shandong

Stowe Flagship Store	King of Power Saving	Friends of the family in use recommended, the effect is good and recommendable!	Jingdong prefecture level city in Shandong
Stowe Flagship Store	King of Power Saving	Cheaper than brick and mortar stores worth buying is authentic	Jingdong prefecture level city in Shandong
Stowe Flagship Store	King of Power Saving	Merchants ship quickly, the price is cheaper than the physical store, good use	Jingdong prefecture level city in Shandong
Stowe Flagship Store	King of Power Saving	Very durable, affordable, fast shipping	Jingdong prefecture level city in Shandong
Stowe Flagship Store	King of Power Saving	Packaging tight quality is very good easy to use good results	Jingdong prefecture level city in Shandong
Stowe Flagship Store	King of Power Saving	Save the grid. It's in the whole house. It's saving money on the electric bill.	Jingdong prefecture level city in Shandong
Stowe Flagship Store	King of Power Saving	I used it today, it works well, I'm very satisfied	Jingdong prefecture level city in Shandong
Stowe Flagship Store	King of Power Saving	It really saves a lot of electricity after installation	Jingdong prefecture level city in Shandong
Stowe Flagship Store	King of Power Saving	Love it. Satisfied.	Jingdong prefecture level city in Shandong
Stowe Flagship Store	King of Power Saving	Doesn't feel like it's working.	Jingdong prefecture level city in Shandong
Stowe Flagship Store	King of Power Saving	I'll comment after using it.	Jingdong prefecture level city in Shandong
Stowe Flagship Store	King of Power Saving	I'm trying it out to see if it saves power, and I'll buy two more if it works well.	Jingdong prefecture level city in Shandong
Stowe Flagship Store	King of Power Saving	It is genuine very good, the price is much cheaper, cost-effective, really special cost-effective, it is worth buying!	Jingdong prefecture level city in Shandong
Stowe Flagship Store	King of Power Saving	This thing can really save electricity, I was introduced to buy by my friend, he used it very well, I used it and think it's also very good, it can really save electricity, save about half of the electricity every day, it's really good!	Jingdong prefecture level city in Shandong
Stowe Flagship Store	King of Power Saving	After using it for ten days or so, I checked the power consumption and it did drop, and customer service said it will save more electricity in the long run. The product is also insured, 5 years warranty do not have to worry about quality problems!	Jingdong prefecture level city in Shandong
Stowe Flagship Store	King of Power Saving	The product is good, before already bought a, this is the second, the effect is quite good, anyway, I see the daily electricity bill than before a lot less, this can be the choice!	Jingdong prefecture level city in Shandong
Stowe Flagship Store	King of Power Saving	Originally also very worried about things are not good, get the hand found completely over-anxious, five-star praise, the style is very generous, the quality is very good, the price is also very reasonable!	Jingdong prefecture level city in Shandong
Stowe Flagship Store	King of Power Saving	Quality is good, playing to the old family also buy a, repurchase, this quality is really good, recommended!	Jingdong prefecture level city in Shandong
Stowe Flagship Store	King of Power Saving	Thank you, the owner even sent two fuses.	Jingdong prefecture level city in Shandong
Stowe Flagship Store	King of Power Saving	This store has great stuff, relatively fast shipping and logistics	Jingdong prefecture level city in Shandong
Stowe Flagship Store	King of Power Saving	This product is based on the impossibility of saving electricity, I used 853 kWh of electricity in July, started using the product on August 1, and used 867 kWh of electricity by September 1, is this called	Jingdong prefecture level city in Shandong
Stowe Flagship Store	King of Power Saving	Power king received, to the home on the trial, when I plugged into the electric panel can not see the effect of power saving, the meter is still the same speed in the turn, I'll save the king of the manual to play look at the instructions, instructions to speak, have to have a day two days and half a month to save power obviously, then wait half a month to see the effect of power saving it! To half a month there are obvious power saving before continuing to chase Xu.	Jingdong prefecture level city in Shandong
Stowe Flagship Store	King of Power Saving	Received the goods, a look, did not see the manufacturer and production address, can only hold a try to see the mentality of the face	Jingdong prefecture level city in Shandong
Stowe Flagship Store	King of Power Saving	I've only been using it for a few days and I'm not sure if it's an IQ tax? Used to favorable reviews.	Jingdong prefecture level city in Shandong
Stowe Flagship Store	King of Power Saving	Appearance is okay, power saving next time to follow up the evaluation	Jingdong prefecture level city in Shandong
Stowe Flagship Store	King of Power Saving	Shorter time, but hopefully effective and power-saving, to be followed up in the future	Jingdong prefecture level city in Shandong
Stowe Flagship Store	King of Power Saving	Appearance is okay, I do not know if the power saving	Jingdong prefecture level city in Shandong
Stowe Flagship Store	King of Power Saving	Good service, fast logistics, Power Saving King is exquisite and beautiful, support the business, thank you!	Jingdong prefecture level city in Shandong
Stowe Flagship Store	King of Power Saving	Bought three at once to try first first.	Jingdong prefecture level city in Shandong
Stowe Flagship Store	King of Power Saving	The object is the same as the picture, the product is of high quality, the packaging is intact. High-end atmosphere, high power, power saving effect is obvious, average monthly power saving 50%, very satisfied.	Jingdong prefecture level city in Shandong
Stowe Flagship Store	King of Power Saving	Bought back and plugged in, really effective, before 16 degrees of electricity per day, now 8 degrees of electricity per day, open an air conditioner for 12 hours, decisive repurchase of a	Jingdong prefecture level city in Shandong
Stowe Flagship Store	King of Power Saving	The object is the same as the picture, the product is of high quality, and the package is intact. High-end atmosphere and high grade, high power	Jingdong prefecture level city in Shandong
Stowe Flagship Store	King of Power Saving	Goods received, I hope the store said can be energy-saving, first try a month, month after month to see the effect.	Jingdong prefecture level city in Shandong
Stowe Flagship Store	King of Power Saving	This is really good, the material and workmanship is particularly exquisite, the workmanship is really very good, I think I can recommend to my friends to buy	Jingdong prefecture level city in Shandong
Stowe Flagship Store	King of Power Saving	I have to praise, the quality is really good, and the packaging logistics are also good. Really like it, won't make you regret after getting it!	Jingdong prefecture level city in Shandong
Stowe Flagship Store	King of Power Saving	Reputable platform, efficient and professional service, buy with confidence and peace of mind!	Jingdong prefecture level city in Shandong
Stowe Flagship Store	King of Power Saving	Bought one on a trial basis to see if it works after 1-2 months.	Jingdong prefecture level city in Shandong
Stowe Flagship Store	King of Power Saving	Quality is very good, customer service attitude is very very praise, there are problems in a timely manner to solve the problem, the price is not expensive, super value, you can rest assured that the	Jingdong prefecture level city in Shandong
Stowe Flagship Store	King of Power Saving	With it no longer need to be afraid to use electricity, especially good, ready to buy a mom and dad that also	Jingdong prefecture level city in Shandong
Stowe Flagship Store	King of Power Saving	Baby received very like will return to buy the seller attitude is also very like will return to buy the seller attitude is also very like!	Jingdong prefecture level city in Shandong
Stowe Flagship Store	King of Power Saving	Jingdong Mall shopping convenience assured fast assured fast assured fast assured fast	Jingdong prefecture level city in Shandong
Stowe Flagship Store	King of Power Saving	Saving power received, the quality is very good, fine workmanship, especially good, much cheaper than the entity store, it is worth buying!	Jingdong prefecture level city in Shandong
Stowe Flagship Store	King of Power Saving	I did not expect the express delivery so quickly, very save point quality is super good, than the entity store is too cheap.	Jingdong prefecture level city in Shandong
Stowe Flagship Store	King of Power Saving	Have received the baby, very satisfied with a shopping, great worth buying!	Jingdong prefecture level city in Shandong
Stowe Flagship Store	King of Power Saving	This is really more powerful, very satisfied, more energy-saving, better, thank you boss.	Jingdong prefecture level city in Shandong
Stowe Flagship Store	King of Power Saving	Received has been used for two days, the air conditioning can boldly open it! Energy saving effect is really quite good	Jingdong prefecture level city in Shandong
Stowe Flagship Store	King of Power Saving	It is authentic, trust the merchant, the baby has a great texture and the price is moderate, unlimited repurchase!	Jingdong prefecture level city in Shandong
Stowe Flagship Store	King of Power Saving	It is genuine very good, the price is much cheaper, cost-effective, really special cost-effective, it is worth buying!	Jingdong prefecture level city in Shandong
Stowe Flagship Store	King of Power Saving	Very good quality, fine workmanship, low power consumption, long warranty, fast shipping, economical and practical	Jingdong prefecture level city in Shandong

Stowe Flagship Store	King of Power Saving	I've been using it for half a month now, it really saves power and the voltage is stable, it's very good	Jingdong prefecture level city in Shandong
Stowe Flagship Store	King of Power Saving	It's the ideal product 💗, good quality and fast logistics, worth the kudos!	Jingdong prefecture level city in Shandong
Stowe Flagship Store	King of Power Saving	The logistics is very fast, the baby is very good, the shopkeeper is NICE!	Jingdong prefecture level city in Shandong
Stowe Flagship Store	King of Power Saving	Good to use, good quality, save point durable	Jingdong prefecture level city in Shandong
Stowe Flagship Store	King of Power Saving	The quality is very good, customer service attitude is very, very awesome, there are problems in a timely manner to solve the problem	Jingdong prefecture level city in Shandong
Stowe Flagship Store	King of Power Saving	Quality is very good, the use of the effect is very good, more stable, quality assurance	Jingdong prefecture level city in Shandong
Stowe Flagship Store	King of Power Saving	Convenient, inexpensive, and see how it works out?	Jingdong prefecture level city in Shandong
Stowe Flagship Store	King of Power Saving	Very good quality, saves a lot of electricity, very good, worth buying	Jingdong prefecture level city in Shandong
Stowe Flagship Store	King of Power Saving	Power Saving King is easy to use, with the fruit of power saving to use easy to give!	Jingdong prefecture level city in Shandong
Stowe Flagship Store	King of Power Saving	This saver really works, good quality, safe and secure, very satisfied!	Jingdong prefecture level city in Shandong
Stowe Flagship Store	King of Power Saving	It works well, the voltage is very stable effect is good, the quality is also very good satisfaction	Jingdong prefecture level city in Shandong
Stowe Flagship Store	King of Power Saving	The logistics is super super fast! The quality is also very good, indeed value for money	Jingdong prefecture level city in Shandong
Stowe Flagship Store	King of Power Saving	I've only been using it for a month or so and it seems to be saving a lot of power	Jingdong prefecture level city in Shandong
Stowe Flagship Store	King of Power Saving	Very easy to operate, very good material, good value for money.	Jingdong prefecture level city in Shandong
Stowe Flagship Store	King of Power Saving	Aluminum alloy shell, durable, compact power-saving good effect	Jingdong prefecture level city in Shandong
Stowe Flagship Store	King of Power Saving	Nice device, easy to use, doesn't take up much space, recommendable!	Jingdong prefecture level city in Shandong
Stowe Flagship Store	King of Power Saving	Good quality, good price, fast logistics	Jingdong prefecture level city in Shandong
Stowe Flagship Store	King of Power Saving	Friends of the family in use recommended, the effect is good and recommendable!	Jingdong prefecture level city in Shandong
Stowe Flagship Store	King of Power Saving	Cheaper than brick and mortar stores worth buying is authentic	Jingdong prefecture level city in Shandong
Stowe Flagship Store	King of Power Saving	Merchants ship quickly, the price is cheaper than the physical store, good use	Jingdong prefecture level city in Shandong
Stowe Flagship Store	King of Power Saving	Very durable, affordable, fast shipping	Jingdong prefecture level city in Shandong
Stowe Flagship Store	King of Power Saving	Packaging tight quality is very good easy to use good results	Jingdong prefecture level city in Shandong
Stowe Flagship Store	King of Power Saving	Save the grid. It's in the whole house. It's saving money on the electric bill.	Jingdong prefecture level city in Shandong
Stowe Flagship Store	King of Power Saving	I used it today, it works well, I'm very satisfied	Jingdong prefecture level city in Shandong
Stowe Flagship Store	King of Power Saving	It really saves a lot of electricity after installation	Jingdong prefecture level city in Shandong
Stowe Flagship Store	King of Power Saving	Love it. Satisfied.	Jingdong prefecture level city in Shandong
Stowe Flagship Store	King of Power Saving	Doesn't feel like it's working.	Jingdong prefecture level city in Shandong
Stowe Flagship Store	King of Power Saving	I'll comment after using it.	Jingdong prefecture level city in Shandong
Stowe Flagship Store	King of Power Saving	I'm trying it out to see if it saves power, and I'll buy two more if it works well.	Jingdong prefecture level city in Shandong
Stowe Flagship Store	King of Power Saving	It is genuine very good, the price is much cheaper, cost-effective, really special cost-effective, it is worth buying!	Jingdong prefecture level city in Shandong
Stowe Flagship Store	King of Power Saving	This thing can really save electricity, I was introduced to buy by my friend, he used it very well, I used it and think it's also very good, it can really save electricity, save about half of the electricity every day, it's really good!	Jingdong prefecture level city in Shandong
Stowe Flagship Store	King of Power Saving	After using it for ten days or so, I checked the power consumption and it did drop, and customer service said it will save more electricity in the long run. The product is also insured, 5 years warranty do not have to worry about quality problems!	Jingdong prefecture level city in Shandong
Stowe Flagship Store	King of Power Saving	The product is good, before already bought a, this is the second, the effect is quite good, anyway, I see the daily electricity bill than before a lot less, this can be the choice!	Jingdong prefecture level city in Shandong
Stowe Flagship Store	King of Power Saving	Originally also very worried about things are not good, get the hand found completely over-anxious, five-star praise, the style is very generous, the quality is very good, the price is also very reasonable!	Jingdong prefecture level city in Shandong
Stowe Flagship Store	King of Power Saving	Quality is good, playing to the old family also buy a, repurchase, this quality is really good, recommended!	Jingdong prefecture level city in Shandong
Stowe Flagship Store	King of Power Saving	Thank you, the owner even sent two fuses.	Jingdong prefecture level city in Shandong
Stowe Flagship Store	King of Power Saving	This store has great stuff, relatively fast shipping and logistics	Jingdong prefecture level city in Shandong
Stowe Flagship Store	King of Power Saving	This product is based on the impossibility of saving electricity, I used 853 kWh of electricity in July, started using the product on August 1, and used 867 kWh of electricity by September 1, is this called	Jingdong prefecture level city in Shandong
Stowe Flagship Store	King of Power Saving	Power king received, to the home on the trial, when I plugged into the electric panel can not see the effect of power saving, the meter is still the same speed in the turn, I'll save the king of the manual to play look at the instructions, instructions to speak, have to have a day two days and half a month to save power obviously, then wait half a month to see the effect of power saving it! To half a month there are obvious power saving before continuing to chase Xu.	Jingdong prefecture level city in Shandong
Stowe Flagship Store	King of Power Saving	Received the goods, a look, did not see the manufacturer and production address, can only hold a try to see the mentality of the face	Jingdong prefecture level city in Shandong
Stowe Flagship Store	King of Power Saving	I've only been using it for a few days and I'm not sure if it's an IQ tax? Used to favorable reviews.	Jingdong prefecture level city in Shandong
Stowe Flagship Store	King of Power Saving	Appearance is okay, power saving next time to follow up the evaluation	Jingdong prefecture level city in Shandong
Stowe Flagship Store	King of Power Saving	Shorter time, but hopefully effective and power-saving, to be followed up in the future	Jingdong prefecture level city in Shandong
Stowe Flagship Store	King of Power Saving	Appearance is okay, I do not know if the power saving	Jingdong prefecture level city in Shandong
Stowe Flagship Store	King of Power Saving	Good service, fast logistics, Power Saving King is exquisite and beautiful, support the business, thank you!	Jingdong prefecture level city in Shandong
Stowe Flagship Store	King of Power Saving	Bought three at once to try first first.	Jingdong prefecture level city in Shandong
Stowe Flagship Store	King of Power Saving	The object is the same as the picture, the product is of high quality, the packaging is intact. High-end atmosphere, high power, power saving effect is obvious, average monthly power saving 50%, very satisfied.	Jingdong prefecture level city in Shandong
Stowe Flagship Store	King of Power Saving	Bought back and plugged in, really effective, before 16 degrees of electricity per day, now 8 degrees of electricity per day, open an air conditioner for 12 hours, decisive repurchase of a	Jingdong prefecture level city in Shandong
Stowe Flagship Store	King of Power Saving	The object is the same as the picture, the product is of high quality, and the package is intact. High-end atmosphere and high grade, high power	Jingdong prefecture level city in Shandong

Stowe Flagship Store	King of Power Saving	Goods received, I hope the store said can be energy-saving, first try a month, month after month to see the effect.	Jingdong prefecture level city in Shandong
Stowe Flagship Store	King of Power Saving	This is really good, the material and workmanship is particularly exquisite, the workmanship is really very good, I think I can recommend to my friends to buy	Jingdong prefecture level city in Shandong
Stowe Flagship Store	King of Power Saving	I have to praise, the quality is really good, and the packaging logistics are also good. Really like it, won't make you regret after getting it!	Jingdong prefecture level city in Shandong
Stowe Flagship Store	King of Power Saving	Reputable platform, efficient and professional service, buy with confidence and peace of mind!	Jingdong prefecture level city in Shandong
Stowe Flagship Store	King of Power Saving	Bought one on a trial basis to see if it works after 1-2 months.	Jingdong prefecture level city in Shandong
Stowe Flagship Store	King of Power Saving	Quality is very good, customer service attitude is very very praise, there are problems in a timely manner to solve the problem, the price is not expensive, super value, you can rest assured that the	Jingdong prefecture level city in Shandong
Stowe Flagship Store	King of Power Saving	With it no longer need to be afraid to use electricity, especially good, ready to buy a mom and dad that also	Jingdong prefecture level city in Shandong
Stowe Flagship Store	King of Power Saving	Baby received very like will return to buy the seller attitude is also very like will return to buy the seller attitude is also very like!	Jingdong prefecture level city in Shandong
Stowe Flagship Store	King of Power Saving	Jingdong Mall shopping convenience assured fast assured fast assured fast assured fast	Jingdong prefecture level city in Shandong
Stowe Flagship Store	King of Power Saving	Saving power received, the quality is very good, fine workmanship, especially good, much cheaper than the entity store, it is worth buying!	Jingdong prefecture level city in Shandong
Stowe Flagship Store	King of Power Saving	I did not expect the express delivery so quickly, very save point quality is super good, than the entity store is too cheap.	Jingdong prefecture level city in Shandong
Stowe Flagship Store	King of Power Saving	Have received the baby, very satisfied with a shopping, great worth buying!	Jingdong prefecture level city in Shandong
Stowe Flagship Store	King of Power Saving	This is really more powerful, very satisfied, more energy-saving, better, thank you boss.	Jingdong prefecture level city in Shandong
Stowe Flagship Store	King of Power Saving	Received has been used for two days, the air conditioning can boldly open it! Energy saving effect is really quite good	Jingdong prefecture level city in Shandong
Stowe Flagship Store	King of Power Saving	It is authentic, trust the merchant, the baby has a great texture and the price is moderate, unlimited repurchase!	Jingdong prefecture level city in Shandong
Stowe Flagship Store	King of Power Saving	It is genuine very good, the price is much cheaper, cost-effective, really special cost-effective, it is worth buying!	Jingdong prefecture level city in Shandong
Stowe Flagship Store	King of Power Saving	Very good quality, fine workmanship, low power consumption, long warranty, fast shipping, economical and practical	Jingdong prefecture level city in Shandong
Stowe Flagship Store	King of Power Saving	I've been using it for half a month now, it really saves power and the voltage is stable, it's very good	Jingdong prefecture level city in Shandong
Stowe Flagship Store	King of Power Saving	It's the ideal product 💝, good quality and fast logistics, worth the kudos!	Jingdong prefecture level city in Shandong
Stowe Flagship Store	King of Power Saving	The logistics is very fast, the baby is very good, the shopkeeper is NICE!	Jingdong prefecture level city in Shandong
Stowe Flagship Store	King of Power Saving	Good to use, good quality, save point durable	Jingdong prefecture level city in Shandong
Stowe Flagship Store	King of Power Saving	The quality is very good, customer service attitude is very, very awesome, there are problems in a timely manner to solve the problem	Jingdong prefecture level city in Shandong
Stowe Flagship Store	King of Power Saving	Quality is very good, the use of the effect is very good, more stable, quality assurance	Jingdong prefecture level city in Shandong
Stowe Flagship Store	King of Power Saving	Convenient, inexpensive, and see how it works out?	Jingdong prefecture level city in Shandong
Stowe Flagship Store	King of Power Saving	Very good quality, saves a lot of electricity, very good, worth buying	Jingdong prefecture level city in Shandong
Stowe Flagship Store	King of Power Saving	Power Saving King is easy to use, with the fruit of power saving to use easy to give!	Jingdong prefecture level city in Shandong
Stowe Flagship Store	King of Power Saving	This saver really works, good quality, safe and secure, very satisfied!	Jingdong prefecture level city in Shandong
Stowe Flagship Store	King of Power Saving	It works well, the voltage is very stable effect is good, the quality is also very good satisfaction	Jingdong prefecture level city in Shandong
Stowe Flagship Store	King of Power Saving	The logistics is super super fast! The quality is also very good, indeed value for money	Jingdong prefecture level city in Shandong
Stowe Flagship Store	King of Power Saving	I've only been using it for a month or so and it seems to be saving a lot of power	Jingdong prefecture level city in Shandong
Stowe Flagship Store	King of Power Saving	Very easy to operate, very good material, good value for money.	Jingdong prefecture level city in Shandong
Stowe Flagship Store	King of Power Saving	Aluminum alloy shell, durable, compact power-saving good effect	Jingdong prefecture level city in Shandong
Stowe Flagship Store	King of Power Saving	Nice device, easy to use, doesn't take up much space, recommendable!	Jingdong prefecture level city in Shandong
Stowe Flagship Store	King of Power Saving	Good quality, good price, fast logistics	Jingdong prefecture level city in Shandong
Stowe Flagship Store	King of Power Saving	Friends of the family in use recommended, the effect is good and recommendable!	Jingdong prefecture level city in Shandong
Stowe Flagship Store	King of Power Saving	Cheaper than brick and mortar stores worth buying is authentic	Jingdong prefecture level city in Shandong
Stowe Flagship Store	King of Power Saving	Merchants ship quickly, the price is cheaper than the physical store, good use	Jingdong prefecture level city in Shandong
Stowe Flagship Store	King of Power Saving	Very durable, affordable, fast shipping	Jingdong prefecture level city in Shandong
Stowe Flagship Store	King of Power Saving	Packaging tight quality is very good easy to use good results	Jingdong prefecture level city in Shandong
Stowe Flagship Store	King of Power Saving	Save the grid. It's in the whole house. It's saving money on the electric bill.	Jingdong prefecture level city in Shandong
Stowe Flagship Store	King of Power Saving	I used it today, it works well, I'm very satisfied	Jingdong prefecture level city in Shandong
Stowe Flagship Store	King of Power Saving	It really saves a lot of electricity after installation	Jingdong prefecture level city in Shandong
Stowe Flagship Store	King of Power Saving	Love it. Satisfied.	Jingdong prefecture level city in Shandong
Stowe Flagship Store	King of Power Saving	Doesn't feel like it's working.	Jingdong prefecture level city in Shandong
Stowe Flagship Store	King of Power Saving	I'll comment after using it.	Jingdong prefecture level city in Shandong
Stowe Flagship Store	King of Power Saving	I'm trying it out to see if it saves power, and I'll buy two more if it works well.	Jingdong prefecture level city in Shandong
Stowe Flagship Store	King of Power Saving	It is genuine very good, the price is much cheaper, cost-effective, really special cost-effective, it is worth buying!	Jingdong prefecture level city in Shandong
Stowe Flagship Store	King of Power Saving	This thing can really save electricity, I was introduced to buy by my friend, he used it very well, I used it and think it's also very good, it can really save electricity, save about half of the electricity every day, it's really good!	Jingdong prefecture level city in Shandong
Stowe Flagship Store	King of Power Saving	After using it for ten days or so, I checked the power consumption and it did drop, and customer service said it will save more electricity in the long run. The product is also insured, 5 years warranty do not have to worry about quality problems!	Jingdong prefecture level city in Shandong
Stowe Flagship Store	King of Power Saving	The product is good, before already bought a, this is the second, the effect is quite good, anyway, I see the daily electricity bill than before a lot less, this can be the choice!	Jingdong prefecture level city in Shandong

Stowe Flagship Store	King of Power Saving	Originally also very worried about things are not good, get the hand found completely over-anxious, five-star praise, the style is very generous, the quality is very good, the price is also very reasonable!	Jingdong prefecture level city in Shandong
Stowe Flagship Store	King of Power Saving	Quality is good, playing to the old family also buy a, repurchase, this quality is really good, recommended!	Jingdong prefecture level city in Shandong
Stowe Flagship Store	King of Power Saving	Thank you, the owner even sent two fuses.	Jingdong prefecture level city in Shandong
Stowe Flagship Store	King of Power Saving	This store has great stuff, relatively fast shipping and logistics	Jingdong prefecture level city in Shandong
Stowe Flagship Store	King of Power Saving	This product is based on the impossibility of saving electricity, I used 853 kWh of electricity in July, started using the product on August 1, and used 867 kWh of electricity by September 1, is this called	Jingdong prefecture level city in Shandong
Stowe Flagship Store	King of Power Saving	Power king received, to the home on the trial, when I plugged into the electric panel can not see the effect of power saving, the meter is still the same speed in the turn, I'll save the king of the manual to play look at the instructions, instructions to speak, have to have a day two days and half a month to save power obviously, then wait half a month to see the effect of power saving it! To half a month there are obvious power saving before continuing to chase Xu.	Jingdong prefecture level city in Shandong
Stowe Flagship Store	King of Power Saving	Received the goods, a look, did not see the manufacturer and production address, can only hold a try to see the mentality of the face	Jingdong prefecture level city in Shandong
Stowe Flagship Store	King of Power Saving	I've only been using it for a few days and I'm not sure if it's an IQ tax? Used to favorable reviews.	Jingdong prefecture level city in Shandong
Stowe Flagship Store	King of Power Saving	Appearance is okay, power saving next time to follow up the evaluation	Jingdong prefecture level city in Shandong
Stowe Flagship Store	King of Power Saving	Shorter time, but hopefully effective and power-saving, to be followed up in the future	Jingdong prefecture level city in Shandong
Stowe Flagship Store	King of Power Saving	Appearance is okay, I do not know if the power saving	Jingdong prefecture level city in Shandong
Stowe Flagship Store	King of Power Saving	Good service, fast logistics, Power Saving King is exquisite and beautiful, support the business, thank you!	Jingdong prefecture level city in Shandong
Stowe Flagship Store	King of Power Saving	Bought three at once to try first first.	Jingdong prefecture level city in Shandong
Stowe Flagship Store	King of Power Saving	The object is the same as the picture, the product is of high quality, the packaging is intact. High-end atmosphere, high power, power saving effect is obvious, average monthly power saving 50%, very satisfied.	Jingdong prefecture level city in Shandong
Stowe Flagship Store	King of Power Saving	Bought back and plugged in, really effective, before 16 degrees of electricity per day, now 8 degrees of electricity per day, open an air conditioner for 12 hours, decisive repurchase of a	Jingdong prefecture level city in Shandong
Stowe Flagship Store	King of Power Saving	The object is the same as the picture, the product is of high quality, and the package is intact. High-end atmosphere and high grade, high power	Jingdong prefecture level city in Shandong
Stowe Flagship Store	King of Power Saving	Goods received, I hope the store said can be energy-saving, first try a month, month after month to see the effect.	Jingdong prefecture level city in Shandong
Stowe Flagship Store	King of Power Saving	This is really good, the material and workmanship is particularly exquisite, the workmanship is really very good, I think I can recommend to my friends to buy	Jingdong prefecture level city in Shandong
Stowe Flagship Store	King of Power Saving	I have to praise, the quality is really good, and the packaging logistics are also good. Really like it, won't make you regret after getting it!	Jingdong prefecture level city in Shandong
Stowe Flagship Store	King of Power Saving	Reputable platform, efficient and professional service, buy with confidence and peace of mind!	Jingdong prefecture level city in Shandong
Stowe Flagship Store	King of Power Saving	Bought one on a trial basis to see if it works after 1-2 months.	Jingdong prefecture level city in Shandong
Stowe Flagship Store	King of Power Saving	Quality is very good, customer service attitude is very very praise, there are problems in a timely manner to solve the problem, the price is not expensive, super value, you can rest assured that the	Jingdong prefecture level city in Shandong
Stowe Flagship Store	King of Power Saving	With it no longer need to be afraid to use electricity, especially good, ready to buy a mom and dad that also	Jingdong prefecture level city in Shandong
Stowe Flagship Store	King of Power Saving	Baby received very like will return to buy the seller attitude is also very like will return to buy the seller attitude is also very like!	Jingdong prefecture level city in Shandong
Stowe Flagship Store	King of Power Saving	Jingdong Mall shopping convenience assured fast assured fast assured fast assured fast	Jingdong prefecture level city in Shandong
Stowe Flagship Store	King of Power Saving	Saving power received, the quality is very good, fine workmanship, especially good, much cheaper than the entity store, it is worth buying!	Jingdong prefecture level city in Shandong
Stowe Flagship Store	King of Power Saving	I did not expect the express delivery so quickly, very save point quality is super good, than the entity store is too cheap.	Jingdong prefecture level city in Shandong
Stowe Flagship Store	King of Power Saving	Have received the baby, very satisfied with a shopping, great worth buying!	Jingdong prefecture level city in Shandong
Stowe Flagship Store	King of Power Saving	This is really more powerful, very satisfied, more energy-saving, better, thank you boss.	Jingdong prefecture level city in Shandong
Stowe Flagship Store	King of Power Saving	Received has been used for two days, the air conditioning can boldly open it! Energy saving effect is really quite good	Jingdong prefecture level city in Shandong
Stowe Flagship Store	King of Power Saving	It is authentic, trust the merchant, the baby has a great texture and the price is moderate, unlimited repurchase!	Jingdong prefecture level city in Shandong
Stowe Flagship Store	King of Power Saving	It is genuine very good, the price is much cheaper, cost-effective, really special cost-effective, it is worth buying!	Jingdong prefecture level city in Shandong
Stowe Flagship Store	King of Power Saving	Very good quality, fine workmanship, low power consumption, long warranty, fast shipping, economical and practical	Jingdong prefecture level city in Shandong
Stowe Flagship Store	King of Power Saving	I've been using it for half a month now, it really saves power and the voltage is stable, it's very good	Jingdong prefecture level city in Shandong
Stowe Flagship Store	King of Power Saving	It's the ideal product 💝, good quality and fast logistics, worth the kudos!	Jingdong prefecture level city in Shandong
Stowe Flagship Store	King of Power Saving	The logistics is very fast, the baby is very good, the shopkeeper is NICE!	Jingdong prefecture level city in Shandong
Stowe Flagship Store	King of Power Saving	Good to use, good quality, save point durable	Jingdong prefecture level city in Shandong
Stowe Flagship Store	King of Power Saving	The quality is very good, customer service attitude is very, very awesome, there are problems in a timely manner to solve the problem	Jingdong prefecture level city in Shandong
Stowe Flagship Store	King of Power Saving	Quality is very good, the use of the effect is very good, more stable, quality assurance	Jingdong prefecture level city in Shandong
Stowe Flagship Store	King of Power Saving	Convenient, inexpensive, and see how it works out?	Jingdong prefecture level city in Shandong
Stowe Flagship Store	King of Power Saving	Very good quality, saves a lot of electricity, very good, worth buying	Jingdong prefecture level city in Shandong
Stowe Flagship Store	King of Power Saving	Power Saving King is easy to use, with the fruit of power saving to use easy to give!	Jingdong prefecture level city in Shandong
Stowe Flagship Store	King of Power Saving	This saver really works, good quality, safe and secure, very satisfied!	Jingdong prefecture level city in Shandong
Stowe Flagship Store	King of Power Saving	It works well, the voltage is very stable effect is good, the quality is also very good satisfaction	Jingdong prefecture level city in Shandong
Stowe Flagship Store	King of Power Saving	The logistics is super super fast! The quality is also very good, indeed value for money	Jingdong prefecture level city in Shandong
Stowe Flagship Store	King of Power Saving	I've only been using it for a month or so and it seems to be saving a lot of power	Jingdong prefecture level city in Shandong
Stowe Flagship Store	King of Power Saving	Very easy to operate, very good material, good value for money.	Jingdong prefecture level city in Shandong
Stowe Flagship Store	King of Power Saving	Aluminum alloy shell, durable, compact power-saving good effect	Jingdong prefecture level city in Shandong
Stowe Flagship Store	King of Power Saving	Nice device, easy to use, doesn't take up much space, recommendable!	Jingdong prefecture level city in Shandong
Stowe Flagship Store	King of Power Saving	Good quality, good price, fast logistics	Jingdong prefecture level city in Shandong

Stowe Flagship Store	King of Power Saving	Friends of the family in use recommended, the effect is good and recommendable!	Jingdong prefecture level city in Shandong
Stowe Flagship Store	King of Power Saving	Cheaper than brick and mortar stores worth buying is authentic	Jingdong prefecture level city in Shandong
Stowe Flagship Store	King of Power Saving	Merchants ship quickly, the price is cheaper than the physical store, good use	Jingdong prefecture level city in Shandong
Stowe Flagship Store	King of Power Saving	Very durable, affordable, fast shipping	Jingdong prefecture level city in Shandong
Stowe Flagship Store	King of Power Saving	Packaging tight quality is very good easy to use good results	Jingdong prefecture level city in Shandong
Stowe Flagship Store	King of Power Saving	Save the grid. It's in the whole house. It's saving money on the electric bill.	Jingdong prefecture level city in Shandong
Stowe Flagship Store	King of Power Saving	I used it today, it works well, I'm very satisfied	Jingdong prefecture level city in Shandong
Stowe Flagship Store	King of Power Saving	It really saves a lot of electricity after installation	Jingdong prefecture level city in Shandong
Stowe Flagship Store	King of Power Saving	Love it. Satisfied.	Jingdong prefecture level city in Shandong
Stowe Flagship Store	King of Power Saving	Doesn't feel like it's working.	Jingdong prefecture level city in Shandong
Stowe Flagship Store	King of Power Saving	I'll comment after using it.	Jingdong prefecture level city in Shandong
Stowe Flagship Store	King of Power Saving	I'm trying it out to see if it saves power, and I'll buy two more if it works well.	Jingdong prefecture level city in Shandong
Stowe Flagship Store	King of Power Saving	It is genuine very good, the price is much cheaper, cost-effective, really special cost-effective, it is worth buying!	Jingdong prefecture level city in Shandong
Stowe Flagship Store	King of Power Saving	This thing can really save electricity, I was introduced to buy by my friend, he used it very well, I used it and think it's also very good, it can really save electricity, save about half of the electricity every day, it's really good!	Jingdong prefecture level city in Shandong
Stowe Flagship Store	King of Power Saving	After using it for ten days or so, I checked the power consumption and it did drop, and customer service said it will save more electricity in the long run. The product is also insured, 5 years warranty do not have to worry about quality problems!	Jingdong prefecture level city in Shandong
Stowe Flagship Store	King of Power Saving	The product is good, before already bought a, this is the second, the effect is quite good, anyway, I see the daily electricity bill than before a lot less, this can be the choice!	Jingdong prefecture level city in Shandong
Stowe Flagship Store	King of Power Saving	Originally also very worried about things are not good, get the hand found completely over-anxious, five-star praise, the style is very generous, the quality is very good, the price is also very reasonable!	Jingdong prefecture level city in Shandong
Stowe Flagship Store	King of Power Saving	Quality is good, playing to the old family also buy a, repurchase, this quality is really good, recommended!	Jingdong prefecture level city in Shandong
Stowe Flagship Store	King of Power Saving	Thank you, the owner even sent two fuses.	Jingdong prefecture level city in Shandong
Stowe Flagship Store	King of Power Saving	This store has great stuff, relatively fast shipping and logistics	Jingdong prefecture level city in Shandong
Stowe Flagship Store	King of Power Saving	This product is based on the impossibility of saving electricity, I used 853 kWh of electricity in July, started using the product on August 1, and used 867 kWh of electricity by September 1, is this called	Jingdong prefecture level city in Shandong
Stowe Flagship Store	King of Power Saving	Power king received, to the home on the trial, when I plugged into the electric panel can not see the effect of power saving, the meter is still the same speed in the turn, I'll save the king of the manual to play look at the instructions, instructions to speak, have to have a day two days and half a month to save power obviously, then wait half a month to see the effect of power saving it! To half a month there are obvious power saving before continuing to chase Xu.	Jingdong prefecture level city in Shandong
Stowe Flagship Store	King of Power Saving	Received the goods, a look, did not see the manufacturer and production address, can only hold a try to see the mentality of the face	Jingdong prefecture level city in Shandong
Stowe Flagship Store	King of Power Saving	I've only been using it for a few days and I'm not sure if it's an IQ tax? Used to favorable reviews.	Jingdong prefecture level city in Shandong
Stowe Flagship Store	King of Power Saving	Appearance is okay, power saving next time to follow up the evaluation	Jingdong prefecture level city in Shandong
Stowe Flagship Store	King of Power Saving	Shorter time, but hopefully effective and power-saving, to be followed up in the future	Jingdong prefecture level city in Shandong
Stowe Flagship Store	King of Power Saving	Appearance is okay, I do not know if the power saving	Jingdong prefecture level city in Shandong
Stowe Flagship Store	King of Power Saving	Good service, fast logistics, Power Saving King is exquisite and beautiful, support the business, thank you!	Jingdong prefecture level city in Shandong
Stowe Flagship Store	King of Power Saving	Bought three at once to try first first.	Jingdong prefecture level city in Shandong
Stowe Flagship Store	King of Power Saving	The object is the same as the picture, the product is of high quality, the packaging is intact. High-end atmosphere, high power, power saving effect is obvious, average monthly power saving 50%, very satisfied.	Jingdong prefecture level city in Shandong
Stowe Flagship Store	King of Power Saving	Bought back and plugged in, really effective, before 16 degrees of electricity per day, now 8 degrees of electricity per day, open an air conditioner for 12 hours, decisive repurchase of a	Jingdong prefecture level city in Shandong
Stowe Flagship Store	King of Power Saving	The object is the same as the picture, the product is of high quality, and the package is intact. High-end atmosphere and high grade, high power	Jingdong prefecture level city in Shandong
Stowe Flagship Store	King of Power Saving	Goods received, I hope the store said can be energy-saving, first try a month, month after month to see the effect.	Jingdong prefecture level city in Shandong
Stowe Flagship Store	King of Power Saving	This is really good, the material and workmanship is particularly exquisite, the workmanship is really very good, I think I can recommend to my friends to buy	Jingdong prefecture level city in Shandong
Stowe Flagship Store	King of Power Saving	I have to praise, the quality is really good, and the packaging logistics are also good. Really like it, won't make you regret after getting it!	Jingdong prefecture level city in Shandong
Stowe Flagship Store	King of Power Saving	Reputable platform, efficient and professional service, buy with confidence and peace of mind!	Jingdong prefecture level city in Shandong
Stowe Flagship Store	King of Power Saving	Bought one on a trial basis to see if it works after 1-2 months.	Jingdong prefecture level city in Shandong
Stowe Flagship Store	King of Power Saving	Quality is very good, customer service attitude is very very praise, there are problems in a timely manner to solve the problem, the price is not expensive, super value, you can rest assured that the	Jingdong prefecture level city in Shandong
Stowe Flagship Store	King of Power Saving	With it no longer need to be afraid to use electricity, especially good, ready to buy a mom and dad that also	Jingdong prefecture level city in Shandong
Stowe Flagship Store	King of Power Saving	Baby received very like will return to buy the seller attitude is also very like will return to buy the seller attitude is also very like!	Jingdong prefecture level city in Shandong
Stowe Flagship Store	King of Power Saving	Jingdong Mall shopping convenience assured fast assured fast assured fast assured fast	Jingdong prefecture level city in Shandong
Stowe Flagship Store	King of Power Saving	Saving power received, the quality is very good, fine workmanship, especially good, much cheaper than the entity store, it is worth buying!	Jingdong prefecture level city in Shandong
Stowe Flagship Store	King of Power Saving	I did not expect the express delivery so quickly, very save point quality is super good, than the entity store is too cheap.	Jingdong prefecture level city in Shandong
Stowe Flagship Store	King of Power Saving	Have received the baby, very satisfied with a shopping, great worth buying!	Jingdong prefecture level city in Shandong
Stowe Flagship Store	King of Power Saving	This is really more powerful, very satisfied, more energy-saving, better, thank you boss.	Jingdong prefecture level city in Shandong
Stowe Flagship Store	King of Power Saving	Received has been used for two days, the air conditioning can boldly open it! Energy saving effect is really quite good	Jingdong prefecture level city in Shandong
Stowe Flagship Store	King of Power Saving	It is authentic, trust the merchant, the baby has a great texture and the price is moderate, unlimited repurchase!	Jingdong prefecture level city in Shandong
Stowe Flagship Store	King of Power Saving	It is genuine very good, the price is much cheaper, cost-effective, really special cost-effective, it is worth buying!	Jingdong prefecture level city in Shandong
Stowe Flagship Store	King of Power Saving	Very good quality, fine workmanship, low power consumption, long warranty, fast shipping, economical and practical	Jingdong prefecture level city in Shandong

Stowe Flagship Store	King of Power Saving	I've been using it for half a month now, it really saves power and the voltage is stable, it's very good	Jingdong prefecture level city in Shandong
Stowe Flagship Store	King of Power Saving	It's the ideal product 💝, good quality and fast logistics, worth the kudos!	Jingdong prefecture level city in Shandong
Stowe Flagship Store	King of Power Saving	The logistics is very fast, the baby is very good, the shopkeeper is NICE!	Jingdong prefecture level city in Shandong
Stowe Flagship Store	King of Power Saving	Good to use, good quality, save point durable	Jingdong prefecture level city in Shandong
Stowe Flagship Store	King of Power Saving	The quality is very good, customer service attitude is very, very awesome, there are problems in a timely manner to solve the problem	Jingdong prefecture level city in Shandong
Stowe Flagship Store	King of Power Saving	Quality is very good, the use of the effect is very good, more stable, quality assurance	Jingdong prefecture level city in Shandong
Stowe Flagship Store	King of Power Saving	Convenient, inexpensive, and see how it works out?	Jingdong prefecture level city in Shandong
Stowe Flagship Store	King of Power Saving	Very good quality, saves a lot of electricity, very good, worth buying	Jingdong prefecture level city in Shandong
Stowe Flagship Store	King of Power Saving	Power Saving King is easy to use, with the fruit of power saving to use easy to give!	Jingdong prefecture level city in Shandong
Stowe Flagship Store	King of Power Saving	This saver really works, good quality, safe and secure, very satisfied!	Jingdong prefecture level city in Shandong
Stowe Flagship Store	King of Power Saving	It works well, the voltage is very stable effect is good, the quality is also very good satisfaction	Jingdong prefecture level city in Shandong
Stowe Flagship Store	King of Power Saving	The logistics is super super fast! The quality is also very good, indeed value for money	Jingdong prefecture level city in Shandong
Stowe Flagship Store	King of Power Saving	I've only been using it for a month or so and it seems to be saving a lot of power	Jingdong prefecture level city in Shandong
Stowe Flagship Store	King of Power Saving	Very easy to operate, very good material, good value for money.	Jingdong prefecture level city in Shandong
Stowe Flagship Store	King of Power Saving	Aluminum alloy shell, durable, compact power-saving good effect	Jingdong prefecture level city in Shandong
Stowe Flagship Store	King of Power Saving	Nice device, easy to use, doesn't take up much space, recommendable!	Jingdong prefecture level city in Shandong
Stowe Flagship Store	King of Power Saving	Good quality, good price, fast logistics	Jingdong prefecture level city in Shandong
Stowe Flagship Store	King of Power Saving	Friends of the family in use recommended, the effect is good and recommendable!	Jingdong prefecture level city in Shandong
Stowe Flagship Store	King of Power Saving	Cheaper than brick and mortar stores worth buying is authentic	Jingdong prefecture level city in Shandong
Stowe Flagship Store	King of Power Saving	Merchants ship quickly, the price is cheaper than the physical store, good use	Jingdong prefecture level city in Shandong
Stowe Flagship Store	King of Power Saving	Very durable, affordable, fast shipping	Jingdong prefecture level city in Shandong
Stowe Flagship Store	King of Power Saving	Packaging tight quality is very good easy to use good results	Jingdong prefecture level city in Shandong
Stowe Flagship Store	King of Power Saving	Save the grid. It's in the whole house. It's saving money on the electric bill.	Jingdong prefecture level city in Shandong
Stowe Flagship Store	King of Power Saving	I used it today, it works well, I'm very satisfied	Jingdong prefecture level city in Shandong
Stowe Flagship Store	King of Power Saving	It really saves a lot of electricity after installation	Jingdong prefecture level city in Shandong
Stowe Flagship Store	King of Power Saving	Love it. Satisfied.	Jingdong prefecture level city in Shandong
Stowe Flagship Store	King of Power Saving	Doesn't feel like it's working.	Jingdong prefecture level city in Shandong
Stowe Flagship Store	King of Power Saving	I'll comment after using it.	Jingdong prefecture level city in Shandong
Stowe Flagship Store	King of Power Saving	I'm trying it out to see if it saves power, and I'll buy two more if it works well.	Jingdong prefecture level city in Shandong
Stowe Flagship Store	King of Power Saving	It is genuine very good, the price is much cheaper, cost-effective, really special cost-effective, it is worth buying!	Jingdong prefecture level city in Shandong
Stowe Flagship Store	King of Power Saving	This thing can really save electricity, I was introduced to buy by my friend, he used it very well, I used it and think it's also very good, it can really save electricity, save about half of the electricity every day, it's really good!	Jingdong prefecture level city in Shandong
Stowe Flagship Store	King of Power Saving	After using it for ten days or so, I checked the power consumption and it did drop, and customer service said it will save more electricity in the long run. The product is also insured, 5 years warranty do not have to worry about quality problems!	Jingdong prefecture level city in Shandong
Stowe Flagship Store	King of Power Saving	The product is good, before already bought a, this is the second, the effect is quite good, anyway, I see the daily electricity bill than before a lot less, this can be the choice!	Jingdong prefecture level city in Shandong
Stowe Flagship Store	King of Power Saving	Originally also very worried about things are not good, get the hand found completely over-anxious, five-star praise, the style is very generous, the quality is very good, the price is also very reasonable!	Jingdong prefecture level city in Shandong
Stowe Flagship Store	King of Power Saving	Quality is good, playing to the old family also buy a, repurchase, this quality is really good, recommended!	Jingdong prefecture level city in Shandong
Stowe Flagship Store	King of Power Saving	Thank you, the owner even sent two fuses.	Jingdong prefecture level city in Shandong
Stowe Flagship Store	King of Power Saving	This store has great stuff, relatively fast shipping and logistics	Jingdong prefecture level city in Shandong
Stowe Flagship Store	King of Power Saving	This product is based on the impossibility of saving electricity, I used 853 kWh of electricity in July, started using the product on August 1, and used 867 kWh of electricity by September 1, is this called	Jingdong prefecture level city in Shandong
Stowe Flagship Store	King of Power Saving	Power king received, to the home on the trial, when I plugged into the electric panel can not see the effect of power saving, the meter is still the same speed in the turn, I'll save the king of the manual to play look at the instructions, instructions to speak, have to have a day two days and half a month to save power obviously, then wait half a month to see the effect of power saving it! To half a month there are obvious power saving before continuing to chase Xu.	Jingdong prefecture level city in Shandong
Stowe Flagship Store	King of Power Saving	Received the goods, a look, did not see the manufacturer and production address, can only hold a try to see the mentality of the face	Jingdong prefecture level city in Shandong
Stowe Flagship Store	King of Power Saving	I've only been using it for a few days and I'm not sure if it's an IQ tax? Used to favorable reviews.	Jingdong prefecture level city in Shandong
Stowe Flagship Store	King of Power Saving	Appearance is okay, power saving next time to follow up the evaluation	Jingdong prefecture level city in Shandong
Stowe Flagship Store	King of Power Saving	Shorter time, but hopefully effective and power-saving, to be followed up in the future	Jingdong prefecture level city in Shandong
Stowe Flagship Store	King of Power Saving	Appearance is okay, I do not know if the power saving	Jingdong prefecture level city in Shandong
Stowe Flagship Store	King of Power Saving	Good service, fast logistics, Power Saving King is exquisite and beautiful, support the business, thank you!	Jingdong prefecture level city in Shandong
Stowe Flagship Store	King of Power Saving	Bought three at once to try first first.	Jingdong prefecture level city in Shandong
Stowe Flagship Store	King of Power Saving	The object is the same as the picture, the product is of high quality, the packaging is intact. High-end atmosphere, high power, power saving effect is obvious, average monthly power saving 50%, very satisfied.	Jingdong prefecture level city in Shandong
Stowe Flagship Store	King of Power Saving	Bought back and plugged in, really effective, before 16 degrees of electricity per day, now 8 degrees of electricity per day, open an air conditioner for 12 hours, decisive repurchase of a	Jingdong prefecture level city in Shandong
Stowe Flagship Store	King of Power Saving	The object is the same as the picture, the product is of high quality, and the package is intact. High-end atmosphere and high grade, high power	Jingdong prefecture level city in Shandong

Stowe Flagship Store	King of Power Saving	Goods received, I hope the store said can be energy-saving, first try a month, month after month to see the effect.	Jingdong prefecture level city in Shandong
Stowe Flagship Store	King of Power Saving	This is really good, the material and workmanship is particularly exquisite, the workmanship is really very good, I think I can recommend to my friends to buy	Jingdong prefecture level city in Shandong
Stowe Flagship Store	King of Power Saving	I have to praise, the quality is really good, and the packaging logistics are also good. Really like it, won't make you regret after getting it!	Jingdong prefecture level city in Shandong
Stowe Flagship Store	King of Power Saving	Reputable platform, efficient and professional service, buy with confidence and peace of mind!	Jingdong prefecture level city in Shandong
Stowe Flagship Store	King of Power Saving	Bought one on a trial basis to see if it works after 1-2 months.	Jingdong prefecture level city in Shandong
Stowe Flagship Store	King of Power Saving	Quality is very good, customer service attitude is very very praise, there are problems in a timely manner to solve the problem, the price is not expensive, super value, you can rest assured that the	Jingdong prefecture level city in Shandong
Stowe Flagship Store	King of Power Saving	With it no longer need to be afraid to use electricity, especially good, ready to buy a mom and dad that also	Jingdong prefecture level city in Shandong
Stowe Flagship Store	King of Power Saving	Baby received very like will return to buy the seller attitude is also very like will return to buy the seller attitude is also very like!	Jingdong prefecture level city in Shandong
Stowe Flagship Store	King of Power Saving	Jingdong Mall shopping convenience assured fast assured fast assured fast assured fast	Jingdong prefecture level city in Shandong
Stowe Flagship Store	King of Power Saving	Saving power received, the quality is very good, fine workmanship, especially good, much cheaper than the entity store, it is worth buying!	Jingdong prefecture level city in Shandong
Stowe Flagship Store	King of Power Saving	I did not expect the express delivery so quickly, very save point quality is super good, than the entity store is too cheap.	Jingdong prefecture level city in Shandong
Stowe Flagship Store	King of Power Saving	Have received the baby, very satisfied with a shopping, great worth buying!	Jingdong prefecture level city in Shandong
Stowe Flagship Store	King of Power Saving	This is really more powerful, very satisfied, more energy-saving, better, thank you boss.	Jingdong prefecture level city in Shandong
Stowe Flagship Store	King of Power Saving	Received has been used for two days, the air conditioning can boldly open it! Energy saving effect is really quite good	Jingdong prefecture level city in Shandong
Stowe Flagship Store	King of Power Saving	It is authentic, trust the merchant, the baby has a great texture and the price is moderate, unlimited repurchase!	Jingdong prefecture level city in Shandong
Stowe Flagship Store	King of Power Saving	It is genuine very good, the price is much cheaper, cost-effective, really special cost-effective, it is worth buying!	Jingdong prefecture level city in Shandong
Stowe Flagship Store	King of Power Saving	Very good quality, fine workmanship, low power consumption, long warranty, fast shipping, economical and practical	Jingdong prefecture level city in Shandong
Stowe Flagship Store	King of Power Saving	I've been using it for half a month now, it really saves power and the voltage is stable, it's very good	Jingdong prefecture level city in Shandong
Stowe Flagship Store	King of Power Saving	It's the ideal product 💝, good quality and fast logistics, worth the kudos!	Jingdong prefecture level city in Shandong
Stowe Flagship Store	King of Power Saving	The logistics is very fast, the baby is very good, the shopkeeper is NICE!	Jingdong prefecture level city in Shandong
Stowe Flagship Store	King of Power Saving	Good to use, good quality, save point durable	Jingdong prefecture level city in Shandong
Stowe Flagship Store	King of Power Saving	The quality is very good, customer service attitude is very, very awesome, there are problems in a timely manner to solve the problem	Jingdong prefecture level city in Shandong
Stowe Flagship Store	King of Power Saving	Quality is very good, the use of the effect is very good, more stable, quality assurance	Jingdong prefecture level city in Shandong
Stowe Flagship Store	King of Power Saving	Convenient, inexpensive, and see how it works out?	Jingdong prefecture level city in Shandong
Stowe Flagship Store	King of Power Saving	Very good quality, saves a lot of electricity, very good, worth buying	Jingdong prefecture level city in Shandong
Stowe Flagship Store	King of Power Saving	Power Saving King is easy to use, with the fruit of power saving to use easy to give!	Jingdong prefecture level city in Shandong
Stowe Flagship Store	King of Power Saving	This saver really works, good quality, safe and secure, very satisfied!	Jingdong prefecture level city in Shandong
Stowe Flagship Store	King of Power Saving	It works well, the voltage is very stable effect is good, the quality is also very good satisfaction	Jingdong prefecture level city in Shandong
Stowe Flagship Store	King of Power Saving	The logistics is super super fast! The quality is also very good, indeed value for money	Jingdong prefecture level city in Shandong
Stowe Flagship Store	King of Power Saving	I've only been using it for a month or so and it seems to be saving a lot of power	Jingdong prefecture level city in Shandong
Stowe Flagship Store	King of Power Saving	Very easy to operate, very good material, good value for money.	Jingdong prefecture level city in Shandong
Stowe Flagship Store	King of Power Saving	Aluminum alloy shell, durable, compact power-saving good effect	Jingdong prefecture level city in Shandong
Stowe Flagship Store	King of Power Saving	Nice device, easy to use, doesn't take up much space, recommendable!	Jingdong prefecture level city in Shandong
Stowe Flagship Store	King of Power Saving	Good quality, good price, fast logistics	Jingdong prefecture level city in Shandong
Stowe Flagship Store	King of Power Saving	Friends of the family in use recommended, the effect is good and recommendable!	Jingdong prefecture level city in Shandong
Stowe Flagship Store	King of Power Saving	Cheaper than brick and mortar stores worth buying is authentic	Jingdong prefecture level city in Shandong
Stowe Flagship Store	King of Power Saving	Merchants ship quickly, the price is cheaper than the physical store, good use	Jingdong prefecture level city in Shandong
Stowe Flagship Store	King of Power Saving	Very durable, affordable, fast shipping	Jingdong prefecture level city in Shandong
Stowe Flagship Store	King of Power Saving	Packaging tight quality is very good easy to use good results	Jingdong prefecture level city in Shandong
Stowe Flagship Store	King of Power Saving	Save the grid. It's in the whole house. It's saving money on the electric bill.	Jingdong prefecture level city in Shandong
Stowe Flagship Store	King of Power Saving	I used it today, it works well, I'm very satisfied	Jingdong prefecture level city in Shandong
Stowe Flagship Store	King of Power Saving	It really saves a lot of electricity after installation	Jingdong prefecture level city in Shandong
Stowe Flagship Store	King of Power Saving	Love it. Satisfied.	Jingdong prefecture level city in Shandong
Stowe Flagship Store	King of Power Saving	Doesn't feel like it's working.	Jingdong prefecture level city in Shandong
Stowe Flagship Store	King of Power Saving	I'll comment after using it.	Jingdong prefecture level city in Shandong
Stowe Flagship Store	King of Power Saving	I'm trying it out to see if it saves power, and I'll buy two more if it works well.	Jingdong prefecture level city in Shandong
Stowe Flagship Store	King of Power Saving	It is genuine very good, the price is much cheaper, cost-effective, really special cost-effective, it is worth buying!	Jingdong prefecture level city in Shandong
Stowe Flagship Store	King of Power Saving	This thing can really save electricity, I was introduced to buy by my friend, he used it very well, I used it and think it's also very good, it can really save electricity, save about half of the electricity every day, it's really good!	Jingdong prefecture level city in Shandong
Stowe Flagship Store	King of Power Saving	After using it for ten days or so, I checked the power consumption and it did drop, and customer service said it will save more electricity in the long run. The product is also insured, 5 years warranty do not have to worry about quality problems!	Jingdong prefecture level city in Shandong
Stowe Flagship Store	King of Power Saving	The product is good, before already bought a, this is the second, the effect is quite good, anyway, I see the daily electricity bill than before a lot less, this can be the choice!	Jingdong prefecture level city in Shandong

Stowe Flagship Store	King of Power Saving	Originally also very worried about things are not good, get the hand found completely over-anxious, five-star praise, the style is very generous, the quality is very good, the price is also very reasonable!	Jingdong prefecture level city in Shandong
Stowe Flagship Store	King of Power Saving	Quality is good, playing to the old family also buy a, repurchase, this quality is really good, recommended!	Jingdong prefecture level city in Shandong
Stowe Flagship Store	King of Power Saving	Thank you, the owner even sent two fuses.	Jingdong prefecture level city in Shandong
Stowe Flagship Store	King of Power Saving	This store has great stuff, relatively fast shipping and logistics	Jingdong prefecture level city in Shandong
Stowe Flagship Store	King of Power Saving	This product is based on the impossibility of saving electricity, I used 853 kWh of electricity in July, started using the product on August 1, and used 867 kWh of electricity by September 1, is this called	Jingdong prefecture level city in Shandong
Stowe Flagship Store	King of Power Saving	Power king received, to the home on the trial, when I plugged into the electric panel can not see the effect of power saving, the meter is still the same speed in the turn, I'll save the king of the manual to play look at the instructions, instructions to speak, have to have a day two days and half a month to save power obviously, then wait half a month to see the effect of power saving it! To half a month there are obvious power saving before continuing to chase Xu.	Jingdong prefecture level city in Shandong
Stowe Flagship Store	King of Power Saving	Received the goods, a look, did not see the manufacturer and production address, can only hold a try to see the mentality of the face	Jingdong prefecture level city in Shandong
Stowe Flagship Store	King of Power Saving	I've only been using it for a few days and I'm not sure if it's an IQ tax? Used to favorable reviews.	Jingdong prefecture level city in Shandong
Stowe Flagship Store	King of Power Saving	Appearance is okay, power saving next time to follow up the evaluation	Jingdong prefecture level city in Shandong
Stowe Flagship Store	King of Power Saving	Shorter time, but hopefully effective and power-saving, to be followed up in the future	Jingdong prefecture level city in Shandong
Stowe Flagship Store	King of Power Saving	Appearance is okay, I do not know if the power saving	Jingdong prefecture level city in Shandong
Stowe Flagship Store	King of Power Saving	Good service, fast logistics, Power Saving King is exquisite and beautiful, support the business, thank you!	Jingdong prefecture level city in Shandong
Stowe Flagship Store	King of Power Saving	Bought three at once to try first first.	Jingdong prefecture level city in Shandong
Stowe Flagship Store	King of Power Saving	The object is the same as the picture, the product is of high quality, the packaging is intact. High-end atmosphere, high power, power saving effect is obvious, average monthly power saving 50%, very satisfied.	Jingdong prefecture level city in Shandong
Stowe Flagship Store	King of Power Saving	Bought back and plugged in, really effective, before 16 degrees of electricity per day, now 8 degrees of electricity per day, open an air conditioner for 12 hours, decisive repurchase of a	Jingdong prefecture level city in Shandong
Stowe Flagship Store	King of Power Saving	The object is the same as the picture, the product is of high quality, and the package is intact. High-end atmosphere and high grade, high power	Jingdong prefecture level city in Shandong
Stowe Flagship Store	King of Power Saving	Goods received, I hope the store said can be energy-saving, first try a month, month after month to see the effect.	Jingdong prefecture level city in Shandong
Stowe Flagship Store	King of Power Saving	This is really good, the material and workmanship is particularly exquisite, the workmanship is really very good, I think I can recommend to my friends to buy	Jingdong prefecture level city in Shandong
Stowe Flagship Store	King of Power Saving	I have to praise, the quality is really good, and the packaging logistics are also good. Really like it, won't make you regret after getting it!	Jingdong prefecture level city in Shandong
Stowe Flagship Store	King of Power Saving	Reputable platform, efficient and professional service, buy with confidence and peace of mind!	Jingdong prefecture level city in Shandong
Stowe Flagship Store	King of Power Saving	Bought one on a trial basis to see if it works after 1-2 months.	Jingdong prefecture level city in Shandong
Stowe Flagship Store	King of Power Saving	Quality is very good, customer service attitude is very very praise, there are problems in a timely manner to solve the problem, the price is not expensive, super value, you can rest assured that the	Jingdong prefecture level city in Shandong
Stowe Flagship Store	King of Power Saving	With it no longer need to be afraid to use electricity, especially good, ready to buy a mom and dad that also	Jingdong prefecture level city in Shandong
Stowe Flagship Store	King of Power Saving	Baby received very like will return to buy the seller attitude is also very like will return to buy the seller attitude is also very like!	Jingdong prefecture level city in Shandong
Stowe Flagship Store	King of Power Saving	Jingdong Mall shopping convenience assured fast assured fast assured fast assured fast	Jingdong prefecture level city in Shandong
Stowe Flagship Store	King of Power Saving	Saving power received, the quality is very good, fine workmanship, especially good, much cheaper than the entity store, it is worth buying!	Jingdong prefecture level city in Shandong
Stowe Flagship Store	King of Power Saving	I did not expect the express delivery so quickly, very save point quality is super good, than the entity store is too cheap.	Jingdong prefecture level city in Shandong
Stowe Flagship Store	King of Power Saving	Have received the baby, very satisfied with a shopping, great worth buying!	Jingdong prefecture level city in Shandong
Stowe Flagship Store	King of Power Saving	This is really more powerful, very satisfied, more energy-saving, better, thank you boss.	Jingdong prefecture level city in Shandong
Stowe Flagship Store	King of Power Saving	Received has been used for two days, the air conditioning can boldly open it! Energy saving effect is really quite good	Jingdong prefecture level city in Shandong
Stowe Flagship Store	King of Power Saving	It is authentic, trust the merchant, the baby has a great texture and the price is moderate, unlimited repurchase!	Jingdong prefecture level city in Shandong
Stowe Flagship Store	King of Power Saving	It is genuine very good, the price is much cheaper, cost-effective, really special cost-effective, it is worth buying!	Jingdong prefecture level city in Shandong
Stowe Flagship Store	King of Power Saving	Very good quality, fine workmanship, low power consumption, long warranty, fast shipping, economical and practical	Jingdong prefecture level city in Shandong
Stowe Flagship Store	King of Power Saving	I've been using it for half a month now, it really saves power and the voltage is stable, it's very good	Jingdong prefecture level city in Shandong
Stowe Flagship Store	King of Power Saving	It's the ideal product , good quality and fast logistics, worth the kudos!	Jingdong prefecture level city in Shandong
Stowe Flagship Store	King of Power Saving	The logistics is very fast, the baby is very good, the shopkeeper is NICE!	Jingdong prefecture level city in Shandong
Stowe Flagship Store	King of Power Saving	Good to use, good quality, save point durable	Jingdong prefecture level city in Shandong
Stowe Flagship Store	King of Power Saving	The quality is very good, customer service attitude is very, very awesome, there are problems in a timely manner to solve the problem	Jingdong prefecture level city in Shandong
Stowe Flagship Store	King of Power Saving	Quality is very good, the use of the effect is very good, more stable, quality assurance	Jingdong prefecture level city in Shandong
Stowe Flagship Store	King of Power Saving	Convenient, inexpensive, and see how it works out?	Jingdong prefecture level city in Shandong
Stowe Flagship Store	King of Power Saving	Very good quality, saves a lot of electricity, very good, worth buying	Jingdong prefecture level city in Shandong
Stowe Flagship Store	King of Power Saving	Power Saving King is easy to use, with the fruit of power saving to use easy to give!	Jingdong prefecture level city in Shandong
Stowe Flagship Store	King of Power Saving	This saver really works, good quality, safe and secure, very satisfied!	Jingdong prefecture level city in Shandong
Stowe Flagship Store	King of Power Saving	It works well, the voltage is very stable effect is good, the quality is also very good satisfaction	Jingdong prefecture level city in Shandong
Stowe Flagship Store	King of Power Saving	The logistics is super super fast! The quality is also very good, indeed value for money	Jingdong prefecture level city in Shandong
Stowe Flagship Store	King of Power Saving	I've only been using it for a month or so and it seems to be saving a lot of power	Jingdong prefecture level city in Shandong
Stowe Flagship Store	King of Power Saving	Very easy to operate, very good material, good value for money.	Jingdong prefecture level city in Shandong
Stowe Flagship Store	King of Power Saving	Aluminum alloy shell, durable, compact power-saving good effect	Jingdong prefecture level city in Shandong
Stowe Flagship Store	King of Power Saving	Nice device, easy to use, doesn't take up much space, recommendable!	Jingdong prefecture level city in Shandong
Stowe Flagship Store	King of Power Saving	Good quality, good price, fast logistics	Jingdong prefecture level city in Shandong

Stowe Flagship Store	King of Power Saving	Friends of the family in use recommended, the effect is good and recommendable!	Jingdong prefecture level city in Shandong
Stowe Flagship Store	King of Power Saving	Cheaper than brick and mortar stores worth buying is authentic	Jingdong prefecture level city in Shandong
Stowe Flagship Store	King of Power Saving	Merchants ship quickly, the price is cheaper than the physical store, good use	Jingdong prefecture level city in Shandong
Stowe Flagship Store	King of Power Saving	Very durable, affordable, fast shipping	Jingdong prefecture level city in Shandong
Stowe Flagship Store	King of Power Saving	Packaging tight quality is very good easy to use good results	Jingdong prefecture level city in Shandong
Stowe Flagship Store	King of Power Saving	Save the grid. It's in the whole house. It's saving money on the electric bill.	Jingdong prefecture level city in Shandong
Stowe Flagship Store	King of Power Saving	I used it today, it works well, I'm very satisfied	Jingdong prefecture level city in Shandong
Stowe Flagship Store	King of Power Saving	It really saves a lot of electricity after installation	Jingdong prefecture level city in Shandong
Stowe Flagship Store	King of Power Saving	Love it. Satisfied.	Jingdong prefecture level city in Shandong
Stowe Flagship Store	King of Power Saving	Doesn't feel like it's working.	Jingdong prefecture level city in Shandong
Stowe Flagship Store	King of Power Saving	I'll comment after using it.	Jingdong prefecture level city in Shandong
Stowe Flagship Store	King of Power Saving	I'm trying it out to see if it saves power, and I'll buy two more if it works well.	Jingdong prefecture level city in Shandong
Stowe Flagship Store	King of Power Saving	It is genuine very good, the price is much cheaper, cost-effective, really special cost-effective, it is worth buying!	Jingdong prefecture level city in Shandong
Jinfeng Hongyuan Flagship Store	Electricity savings1	Just received the goods on the use, watching the current has been declining, and so used in January to see the electricity bill in the evaluation, Jingdong logistics is fast, well-packed, to the business, logistics point praise praise praise praise	Jingdong prefecture level city in Shandong
Jinfeng Hongyuan Flagship Store	Electricity savings1	Shipping is too fast, the packaging is very good, the quality is very good, shopping in Jingdong is assured, good customer service, after-sales protection.	Jingdong prefecture level city in Shandong
Jinfeng Hongyuan Flagship Store	Electricity savings1	The product is superbly made and already in use, 有 insurance certification should produce results!	Jingdong prefecture level city in Shandong
Jinfeng Hongyuan Flagship Store	Electricity savings1	Good product, since the purchase of this product, the home electricity bill is less, less than last month's electricity bill ah, good product!	Jingdong prefecture level city in Shandong
Jinfeng Hongyuan Flagship Store	Electricity savings1	The goods have been received, showing voltage fluctuations, the effect will have to wait for a follow-up review!	Jingdong prefecture level city in Shandong
Jinfeng Hongyuan Flagship Store	Electricity savings1	The top of KYO's has always been my most trusted!!!!	Jingdong prefecture level city in Shandong
Jinfeng Hongyuan Flagship Store	Electricity savings1	I believe that Jingdong recommended Jinfeng Hongyuan power saver, small and exquisite, this is the second time to buy. Believe all the goods of Jingdong Mall, the quality should be no problem.	Jingdong prefecture level city in Shandong
Jinfeng Hongyuan Flagship Store	Electricity savings1	I don't know if it's a power saver. It's a couple bucks. I hope it works.	Jingdong prefecture level city in Shandong
Jinfeng Hongyuan Flagship Store	Electricity savings1	I don't know what to say about this stuff. I don't know if it works or not, it's kind of a psychological purchase. Dormitory use to see the meter can not be so good-looking. You have to wait until the time to deduct the utility bill to know.	Jingdong prefecture level city in Shandong
Jinfeng Hongyuan Flagship Store	Electricity savings1	Energy savers don't work / they're all a bunch of hoaxes.	Jingdong prefecture level city in Shandong
Jinfeng Hongyuan Flagship Store	Electricity savings1	Received the goods, used for more than a week, it's okay, sure a little effect	Jingdong prefecture level city in Shandong
Jinfeng Hongyuan Flagship Store	Electricity savings1	Well made and compact! Looking forward to the effect!	Jingdong prefecture level city in Shandong
Jinfeng Hongyuan Flagship Store	Electricity savings1	It has arrived and we'll see how it works.	Jingdong prefecture level city in Shandong
Jinfeng Hongyuan Flagship Store	Electricity savings1	Good. Good. It's worth it.	Jingdong prefecture level city in Shandong
Jinfeng Hongyuan Flagship Store	Electricity savings1	How come there's no indicator light?	Jingdong prefecture level city in Shandong
Jinfeng Hongyuan Flagship Store	Electricity savings1	The machine that uses electricity works quite well. This saves	Jingdong prefecture level city in Shandong
Jinfeng Hongyuan Flagship Store	Electricity savings1	It's good for home use and feels quite energy efficient	Jingdong prefecture level city in Shandong
Jinfeng Hongyuan Flagship Store	Electricity savings1	I regret buying this product, after using it for two days it doesn't work at all, still so many kilowatts per day just like the original.	Jingdong prefecture level city in Shandong
Jinfeng Hongyuan Flagship Store	Electricity savings1	Works very well and has some scientific merit.	Jingdong prefecture level city in Shandong
Jinfeng Hongyuan Flagship Store	Electricity savings1	Logistics is also very fast this good power saving I use two months before commenting!	Jingdong prefecture level city in Shandong
Jinfeng Hongyuan Flagship Store	Electricity savings1	Repeat customer. Very good.	Jingdong prefecture level city in Shandong
Jinfeng Hongyuan Flagship Store	Electricity savings1	It's good stuff. I've been using it for a month now and it's a real power saver.	Jingdong prefecture level city in Shandong
Jinfeng Hongyuan Flagship Store	Electricity savings1	Jingdong platform to buy things, has been very assured, quality assurance. Jingdong Express is as good as ever, complete packaging, delivery, service attitude to go! The family has always wanted to buy an energy-saving and power-saving equipment, selected half a day to choose this one, the value is very good, used for a period of time to follow up, to see whether it can achieve the real purpose of power saving, I	Jingdong prefecture level city in Shandong
Jinfeng Hongyuan Flagship Store	Electricity savings1	Works great, inexpensive and will be back next time.	Jingdong prefecture level city in Shandong
Jinfeng Hongyuan Flagship Store	Electricity savings1	Baby received, looks good, wait for a month to see how it works?	Jingdong prefecture level city in Shandong
Jinfeng Hongyuan Flagship Store	Electricity savings1	This product is not bad quite good well customer service said quite power saving that look at the customer service to see if the accuracy of the look at all aspects of the color is also quite beautiful	Jingdong prefecture level city in Shandong
Jinfeng Hongyuan Flagship Store	Electricity savings1	Just got back and tried it feels pretty good, just don't know how the quality is, give a good comment first	Jingdong prefecture level city in Shandong
Jinfeng Hongyuan Flagship Store	Electricity savings1	Received the product, trying it out, hope it helps!	Jingdong prefecture level city in Shandong
Jinfeng Hongyuan Flagship Store	Electricity savings1	Works great, saves electricity a dollar a day and a night	Jingdong prefecture level city in Shandong
Jinfeng Hongyuan Flagship Store	Electricity savings1	Baby received, look at the workmanship is still very fine, is genuine. I hope that this summer to open the air conditioning electricity bill is no longer high scary!	Jingdong prefecture level city in Shandong
Jinfeng Hongyuan Flagship Store	Electricity savings1	It's here. I don't know how it's going to work, but I'm going to use it.	Jingdong prefecture level city in Shandong
Jinfeng Hongyuan Flagship Store	Electricity savings1	Power saver received, Jingdong logistics first-class, business customer service attitude is good, one by one to introduce the product performance, back on the horse plugged in, it is very simple, there is a voltage indicator, to be a few months to see how the effect of power-saving to come back to a good follow-up, but I believe that Jinfenghong products.	Jingdong prefecture level city in Shandong
Jinfeng Hongyuan Flagship Store	Electricity savings1	Took the third one...the super power-saving kind!	Jingdong prefecture level city in Shandong
Jinfeng Hongyuan Flagship Store	Electricity savings1	First time buyer, customer service is very polite and courteous, conscientious business, support! Baby has received 🎈, super like, high value and practical, super like! ❥❥❥❥❥ Very good quality, very satisfied with a shopping. Customer service has a question and answer, very professional finally received the goods, very, very much like, exquisite and beautiful workmanship, has been praised by many people, really too happy, is already a loyal fan of his family, will continue to buy buy buy buy!	Jingdong prefecture level city in Shandong

Jinfeng Hongyuan Flagship Store	Electricity savings1	That's good. That's good. That's really good.	Jingdong prefecture level city in Shandong
Jinfeng Hongyuan Flagship Store	Electricity savings1	Saver received, good-looking design, voltage at a glance, and the merchant description, save some about 12% or so	Jingdong prefecture level city in Shandong
Jinfeng Hongyuan Flagship Store	Electricity savings1	Intelligent power saver is small and does not take up space, very good, very practical, received and used, packaging is tight, logistics is also very fast, satisfied with a shopping!	Jingdong prefecture level city in Shandong
Jinfeng Hongyuan Flagship Store	Electricity savings1	The stuff is good! Well made, compact, alloy case, shows voltage! Very like! Kudos!	Jingdong prefecture level city in Shandong
Jinfeng Hongyuan Flagship Store	Electricity savings1	I just purchased this power saver from Jingdong, inserted in the socket shows the number 206, a moment and 207 back and forth display, I do not know what it means, my home voltage should be normal, because it is the owner of the community's own power plant to put, first use for a period of time in the positive feedback it, first use in the look at it!	Jingdong prefecture level city in Shandong
Jinfeng Hongyuan Flagship Store	Electricity savings1	Very delicate and compact, there is a convenient intelligent display prompts the voltage, you can know the voltage at a glance, there is also very easy to use, a plug can be used, you can save electricity	Jingdong prefecture level city in Shandong
Jinfeng Hongyuan Flagship Store	Electricity savings1	Logistics is fast, ask customer service said can save electricity, but only use two months to see the effect, use two months in the evaluation.	Jingdong prefecture level city in Shandong
Jinfeng Hongyuan Flagship Store	Electricity savings1	The quality is very good.	Jingdong prefecture level city in Shandong
Jinfeng Hongyuan Flagship Store	Electricity savings1	Smart Saver has been received and will be evaluated after use	Jingdong prefecture level city in Shandong
Jinfeng Hongyuan Flagship Store	Electricity savings1	Home electricity is too waste, a month more than 300 dollars of electricity, and then the family heard that this can save a lot of electricity, let me order to buy a, anyway, will not, buy on the buy, thought it has to be a pretty big box it, delivery to the home to see a very small, plug-and-play, you can really save electricity, it's worth it!	Jingdong prefecture level city in Shandong
Jinfeng Hongyuan Flagship Store	Electricity savings1	Smart Saver received. Unpacking the box is exquisite. The material workmanship is very good. Plugged in and tried the space. Did not open the air conditioning voltage display 206 air conditioning open up the air conditioning display for 194. whether to save electricity to a month later to pay the electricity bill will know. Logistics is also very fast service is also in place.	Jingdong prefecture level city in Shandong
Jinfeng Hongyuan Flagship Store	Electricity savings1	The product feels pretty good and I hope it works, as the store owner said it would. Works!!!	Jingdong prefecture level city in Shandong
Jinfeng Hongyuan Flagship Store	Electricity savings1	Baby received, physical very quickly, just plugged in hope that it can be as everyone said those power saving, with two months after additional comments, first give a five-star praise.	Jingdong prefecture level city in Shandong
Jinfeng Hongyuan Flagship Store	Electricity savings1	Good, very good, very good, you deserve it, whoever uses it knows, the stuff works great, will buy it again in the future.	Jingdong prefecture level city in Shandong
Jinfeng Hongyuan Flagship Store	Electricity savings1	Don't hesitate to buy wow! No cheap feeling, very high-end, especially great! It is genuine, and the same as the entity store, the price is also favorable, decisive turn powder 💗 catch up to do activities when you buy, the price of a penny for a penny ♡, from the outside looking very beautiful 💗, the material workmanship is good 💗, feel that the purchase is worth it, and the logistics is super	Jingdong prefecture level city in Shandong
Jinfeng Hongyuan Flagship Store	Electricity savings1	Logistics is fast! Very good quality! Very beautiful! Next time to patronize!	Jingdong prefecture level city in Shandong
Jinfeng Hongyuan Flagship Store	Electricity savings1	Really like it, completely exceeded expectations, the shipping speed is very fast, the packaging is very careful and strict, the delivery speed is very fast, very satisfied with a shopping Baby received, and the description is the same, love it!	Jingdong prefecture level city in Shandong
Jinfeng Hongyuan Flagship Store	Electricity savings1	Very good, if you need to order!	Jingdong prefecture level city in Shandong
Jinfeng Hongyuan Flagship Store	Electricity savings1	So far, plugged in the voltage is more stable, as for the effect of how to quietly wait until the payment of the electricity bill to compare, the appearance of the commodity design is very good, the real thing!	Jingdong prefecture level city in Shandong
Jinfeng Hongyuan Flagship Store	Electricity savings1	📩 I've been wanting it for a long time, I decided to go for it, I feel great after using it, it's genuine and the delivery is great, it lives up to its name👍	Jingdong prefecture level city in Shandong
Jinfeng Hongyuan Flagship Store	Electricity savings1	Looking forward to it!!! I don't know if it's working or not, let's look forward to it!!!! How much is saved，，，，，，，，， Saving a degree is a degree with long term use！！！！！！！	Jingdong prefecture level city in Shandong
Jinfeng Hongyuan Flagship Store	Electricity savings1	After using it for a while, I am seriously and responsibly saying that this saver is really very power efficient. Two rooms with the air conditioner on at night for one night, plus watching TV, charging cell phones, lights, etc. etc. only used one degree of electricity. So I bought several more and it's really	Jingdong prefecture level city in Shandong
Jinfeng Hongyuan Flagship Store	Electricity savings1	It arrived quickly, and the box is solid and delicate. Baby shell is thick aluminum and by multi-layer coating. The inner core is heavy, and the power cord and plug are genuine products from a large manufacturer. It is the best power saver I have found after choosing all of them!	Jingdong prefecture level city in Shandong
Jinfeng Hongyuan Flagship Store	Electricity savings1	The saver was received, genuine guarantee, good quality...	Jingdong prefecture level city in Shandong
Jinfeng Hongyuan Flagship Store	Electricity savings1	Logistics was fast! The product is easy to use!	Jingdong prefecture level city in Shandong
Jinfeng Hongyuan Flagship Store	Electricity savings1	Look appearance is okay, use a period of time to know how it is. Jingdong Mall, trustworthy! Home appliances, cell phones and computers are almost 100% bought by Jingdong. Ordered the night before yesterday, received yesterday. Jingdong logistics, the fastest in the network!	Jingdong prefecture level city in Shandong
Jinfeng Hongyuan Flagship Store	Electricity savings1	Very good, perfect packaging, home energy saver to determine the effect of power saving and also play a role in stabilizing the voltage Very good products	Jingdong prefecture level city in Shandong
Jinfeng Hongyuan Flagship Store	Electricity savings1	Smart Saver received, looks very exquisite. The method of use is simple, and it has been used. The later effect, with time to test.	Jingdong prefecture level city in Shandong
Jinfeng Hongyuan Flagship Store	Electricity savings1	Both the material and workmanship exceeded expectations, great value, and looks great on oh. The seller also shipped much earlier than expected, will come back. Buy it, you will not regret it! Full five points. Both the material and workmanship exceeded expectations, great value, wear it also looks good Oh. Seller delivery is also much earlier than expected, will come again. Buy it, will not regret it! Full	Jingdong prefecture level city in Shandong
Jinfeng Hongyuan Flagship Store	Electricity savings1	The appearance is clean and beautiful, the workmanship is very good, I didn't find any defects, I personally feel that the size is also quite suitable, I've used it for 2 months, I didn't find out exactly how energy efficient it is. Try using it again!	Jingdong prefecture level city in Shandong
Jinfeng Hongyuan Flagship Store	Electricity savings1	Power saving device received, product certification, accessories, etc. complete, very good workmanship, very practical power-saving effect is also very obvious.	Jingdong prefecture level city in Shandong
Jinfeng Hongyuan Flagship Store	Electricity savings1	Looked at a lot of think buy this one, see a lot of reviews say it works, a month with save nearly twenty degrees of electricity, there are sixty days of return guarantee, with a good and then come back to the	Jingdong prefecture level city in Shandong
Jinfeng Hongyuan Flagship Store	Electricity savings1	I received the power saving device, and I'm using it. I hope I can get the desired effect, and I'm trying to force it to work quickly.	Jingdong prefecture level city in Shandong
Jinfeng Hongyuan Flagship Store	Electricity savings1	Voltage is stable, already trying it out and waiting for the results to happen, saving electricity will follow up next month!	Jingdong prefecture level city in Shandong
Jinfeng Hongyuan Flagship Store	Electricity savings1	Jingdong Mall self-supporting goods of excellent quality, logistics is also very fast, worth recommending 👏👏👏👏 excellent!	Jingdong prefecture level city in Shandong
Jinfeng Hongyuan Flagship Store	Electricity savings1	Power saving has been received, the appearance of the beautiful, I have already been the second time to buy the use of down the effect is very good!	Jingdong prefecture level city in Shandong
Jinfeng Hongyuan Flagship Store	Electricity savings1	Energy saver received very happy, the color is also very good looking forward to a very good effect can save electricity	Jingdong prefecture level city in Shandong
Jinfeng Hongyuan Flagship Store	Electricity savings1	It's small and easy to plug in and use. It displays the voltage value when plugged in and	Jingdong prefecture level city in Shandong
Jinfeng Hongyuan Flagship Store	Electricity savings1	This saver really works well, because I installed one at home about seven or eight years ago, when there is no voltage display, now there is a voltage display, the appearance also looks much better, the electricity bill is also saved, the stability of the home appliances is also good, the best of both	Jingdong prefecture level city in Shandong
Jinfeng Hongyuan Flagship Store	Electricity savings1	Ordered yesterday, arrived today in use. Shipping was fast. The effect is looking forward to.	Jingdong prefecture level city in Shandong
Jinfeng Hongyuan Flagship Store	Electricity savings1	This thing, I don't know what the principle is. Why it can save electricity. Wait for a month to use it first, see if it will save electricity bill, then come back to evaluate. There is one thing that does not fit the principle ah. The law of conservation of energy.	Jingdong prefecture level city in Shandong
Jinfeng Hongyuan Flagship Store	Electricity savings1	Home mom listen to others say this can save electricity, specially let buy a, later a very large, go back to see a particularly small, directly plugged into the electricity can be, no need to install, the price is also very cheap, the first time with this product, do not know if it is not good to use, temporarily try for a period of time, if it is good to use, recommend that you buy this one, the	Jingdong prefecture level city in Shandong

Jinfeng Hongyuan Flagship Store	Electricity savings1	I've been using it for a few days, and I think the workmanship and materials are pretty good, so we'll have to look at the next bill for the real energy savings, and then we'll add more comments.	Jingdong prefecture level city in Shandong
Jinfeng Hongyuan Flagship Store	Electricity savings1	Energy saver is well made, energy saving and power saving, and anti-counterfeiting code, it is a regular commodity!	Jingdong prefecture level city in Shandong
Jinfeng Hongyuan Flagship Store	Electricity savings1	Jinfeng Hongyuan, buy back home, as a mouse, haha. First of all, normal electricity for 1 month, the power did not increase, the electricity bill feels smoother, year-on-year electricity costs in the low, whether it is very energy-saving still have to be used for a long time to know. All 5 stars ☆	Jingdong prefecture level city in Shandong
Jinfeng Hongyuan Flagship Store	Electricity savings1	The box is well packaged and beautiful! I hope to save electricity with it haha!	Jingdong prefecture level city in Shandong
Jinfeng Hongyuan Flagship Store	Electricity savings1	Ordered last night, arrived this morning, first try the effect before making comments	Jingdong prefecture level city in Shandong
Jinfeng Hongyuan Flagship Store	Electricity savings1	Looks good. Don't know the effect yet.	Jingdong prefecture level city in Shandong
Jinfeng Hongyuan Flagship Store	Electricity savings1	Jinfeng Hongyuan intelligent power saver has exquisite workmanship and modern fashionable design style. It is a good home economizer . I believe that the goods purchased from Jingdong will not disappoint our	Jingdong prefecture level city in Shandong
Jinfeng Hongyuan Flagship Store	Electricity savings1	Inadvertently flipped to this power saver, never heard of, feel very novel, the price is not expensive, so buy to try, see a month after the electricity bill will be less, recently the home air-conditioning open a lot of power consumption is very large.	Jingdong prefecture level city in Shandong
Jinfeng Hongyuan Flagship Store	Electricity savings1	The quality of the goods is very good workmanship, but also sent five fuse	Jingdong prefecture level city in Shandong
Jinfeng Hongyuan Flagship Store	Electricity savings1	I'm trying it out. I don't know if it'll work.	Jingdong prefecture level city in Shandong
Jinfeng Hongyuan Flagship Store	Electricity savings1	There is no effect yet do not know, is to look at the official sale, buy to try it! Use according to the instructions, plugged in, used for a period of time, anyway, do not know how, after a few months to see it, to see if the average month than before will be less electricity.	Jingdong prefecture level city in Shandong
Jinfeng Hongyuan Flagship Store	Electricity savings1	Received the goods, the first time to unpacking, the quality is very good, consistent with the description of the seller, completely exceeded my expectations, the packaging is very careful, strict, in general this time is very satisfied with a shopping, thanks to the seller.	Jingdong prefecture level city in Shandong
Jinfeng Hongyuan Flagship Store	Electricity savings1	The energy saver was received and used for a few days with good results.	Jingdong prefecture level city in Shandong
Jinfeng Hongyuan Flagship Store	Electricity savings1	Previously I have paid attention to this product, but I think that the monthly electricity consumption at home is not large, there is no need to save electricity savings. After a second thought, energy saving is the responsibility of each of our citizens, the winter is coming, this year's winter is cold, heating and all electric appliances, can save is to save, the price is not expensive, so buy to try. I analyze the principle of power saving, should be able to save electricity, but the larger the power saving effect is about good. To be tried after a month or two to see the effect and then evaluate. Jingdong trustworthy.	Jingdong prefecture level city in Shandong
Jinfeng Hongyuan Flagship Store	Electricity savings1	It just arrived and I can't wait to use it, and the customer service is great! There is also voltage showing up which is nice! I don't know if it can save a bit I won't know until I've used it for a month! When the time comes to come back to the follow-up review!	Jingdong prefecture level city in Shandong
Jinfeng Hongyuan Flagship Store	Electricity savings1	Checked out the reviews from numerous users. The shipment is still very large, so I decided to buy it anyway to use it and see the effect after a month. Jingdong Mall here to buy safe, convenient and fast, this price is very powerful 💪, very recommended.	Jingdong prefecture level city in Shandong
Jinfeng Hongyuan Flagship Store	Electricity savings1	Purchased two at the same time to use together. The other one works fine, the other has problems with the numbers not sure if the machine is faulty. Power saving effect can not be observed. Feel the effect is not very obvious like?	Jingdong prefecture level city in Shandong
Jinfeng Hongyuan Flagship Store	Electricity savings1	Looking forward to it!!! I don't know if it's working or not, let's look forward to it!!!! How much is saved , , , , , , , , , Saving a degree is a degree with long term use ! ! ! ! ! ! !	Jingdong prefecture level city in Shandong
Jinfeng Hongyuan Flagship Store	Electricity savings1	Received, directly on the use of the special convenient	Jingdong prefecture level city in Shandong
Jinfeng Hongyuan Flagship Store	Electricity savings1	Goods received a few days well-packed, the same day opened and plugged in to try to be normal is not sure what the power-saving effect. After a month with a follow-up evaluation.	Jingdong prefecture level city in Shandong
Jinfeng Hongyuan Flagship Store	Electricity savings1	I bought it and used it, I don't know how it works yet, we'll see later. Use the effect of it	Jingdong prefecture level city in Shandong
Jinfeng Hongyuan Flagship Store	Electricity savings1	Photographed 2, the old family also put a, with a small January, there should be some effect, with a long period of time to comment, with a long time to see the obvious effect, hard courier brother.	Jingdong prefecture level city in Shandong
Jinfeng Hongyuan Flagship Store	Electricity savings1	Experience: I am an electrician, this is the real use of electronic waves automatically connected to track the decomposition of ionized electrons, in situ compensation of reactive power, reduce effective power loss and waste, stabilize the voltage to achieve energy saving and power saving, the overall effect	Jingdong prefecture level city in Shandong
Jinfeng Hongyuan Flagship Store	Electricity savings1	Great looking and practical! Easy to use and save! Saves 15% in a month of comparison	Jingdong prefecture level city in Shandong
Jinfeng Hongyuan Flagship Store	Electricity savings1	Product quality is very good! Packaging is very nice, like a cell phone box, thick and well protected! Service is also great! Logistics is very fast! Thank you for your business! Very honest!	Jingdong prefecture level city in Shandong
Jinfeng Hongyuan Flagship Store	Electricity savings1	In several products inside the choice of this purchase for a period of time, but also need to use a few months to compare last year's electricity bill before you can analyze the savings in electricity, to the summer peak electricity consumption should be more obvious.	Jingdong prefecture level city in Shandong
Jinfeng Hongyuan Flagship Store	Electricity savings1	The goods have long been received, after more than a month to comment, the home electricity bill is indeed reduced, worth buying!	Jingdong prefecture level city in Shandong
Jinfeng Hongyuan Flagship Store	Electricity savings1	Baby received, the logistics is strong, the quality is reliable, it is worth having and recommending!	Jingdong prefecture level city in Shandong
Jinfeng Hongyuan Flagship Store	Electricity savings1	Using a conservation appliance, you can achieve a 10-15% savings in electricity usage.	Jingdong prefecture level city in Shandong
Jinfeng Hongyuan Flagship Store	Electricity savings1	Quality is very good, metal shell, touch is very good, very recommended Oh ~, the subsequent effect of continuous follow-up	Jingdong prefecture level city in Shandong
Jinfeng Hongyuan Flagship Store	Electricity savings1	The product is beautiful, practical, worth buying. The product is beautiful, practical, good quality, fast logistics product is beautiful, practical, beautiful and elegant, very classy, worth buying!	Jingdong prefecture level city in Shandong
Jinfeng Hongyuan Flagship Store	Electricity savings1	Just received the item and have already used it. Didn't know much about these products before. I hope it is as effective and useful as the product advertises.	Jingdong prefecture level city in Shandong
Jinfeng Hongyuan Flagship Store	Electricity savings1	A month two people used more than five hundred degrees of electricity, in a hurry and then add a power saver, I hope to have an effect, Jingdong shopping is very cool, logistics is very fast especially good service attitude delivery, this hot day is not easy, thank you!	Jingdong prefecture level city in Shandong
Jinfeng Hongyuan Flagship Store	Electricity savings1	Just received the baby, first plugged in to try to use a month and then additional evaluation it	Jingdong prefecture level city in Shandong
Jinfeng Hongyuan Flagship Store	Electricity savings1	Goods fast, the power saver has been received, a friend recommended to buy that can save electricity. Exquisite and compact is very convenient, plug and play. The effect is good, the price is affordable, the next need will patronize again!	Jingdong prefecture level city in Shandong
Jinfeng Hongyuan Flagship Store	Electricity savings1	Packaging is better than expected, sent a few fuse, power saving effect in the end do not know, try a period of time to say!	Jingdong prefecture level city in Shandong
Jinfeng Hongyuan Flagship Store	Electricity savings1	Shipped quickly, immediately installed, read countless reviews were not sure if this thing is effective, was going to buy three at once, ultimately decided to first use a try the effect, if the power ring significantly reduced, then will buy a few more!	Jingdong prefecture level city in Shandong
Jinfeng Hongyuan Flagship Store	Electricity savings1	Used for a period of time to evaluate, not to a month still do not know whether it can save electricity, the voltage has a display!	Jingdong prefecture level city in Shandong
Jinfeng Hongyuan Flagship Store	Electricity savings1	I don't know if it saves electricity or not, but I'm using it anyway, so I hope it saves a little bit, electricity is too expensive	Jingdong prefecture level city in Shandong
Jinfeng Hongyuan Flagship Store	Electricity savings1	The power saver has been received and I am very happy with it! Just waiting to see how the power saving works!	Jingdong prefecture level city in Shandong
Jinfeng Hongyuan Flagship Store	Electricity savings1	Saving power has been received, logistics is quite fast, the quality is not bad, small and a little weight, has been used to look forward to the effect of	Jingdong prefecture level city in Shandong
Jinfeng Hongyuan Flagship Store	Electricity savings1	The logistics is very fast, the effect is obvious and trustworthy.	Jingdong prefecture level city in Shandong
Jinfeng Hongyuan Flagship Store	Electricity savings1	Good quality, good quality, beautiful, beautiful, beautiful, beautiful, beautiful, very worth buying, worth buying, worth buying, worth buying, worth customizing!	Jingdong prefecture level city in Shandong

Jinfeng Hongyuan Flagship Store	Electricity savings1	I've used it for now, I don't know if it saves electricity	Jingdong prefecture level city in Shandong
Jinfeng Hongyuan Flagship Store	Electricity savings1	Bought two at a time, logistics is very fast, the product is beautifully packaged, after two months to see the results	Jingdong prefecture level city in Shandong
Jinfeng Hongyuan Flagship Store	Electricity savings1	The workmanship is really fine, the packaging is also good, the regular factory production ~ use a break in the evaluation of the effectiveness of the	Jingdong prefecture level city in Shandong
Jinfeng Hongyuan Flagship Store	Electricity savings1	Sometimes 219. Sometimes 220 in this case	Jingdong prefecture level city in Shandong
Jinfeng Hongyuan Flagship Store	Electricity savings1	It is the ideal product, good quality, logistics is also very fast, worthy of praise!	Jingdong prefecture level city in Shandong
Jinfeng Hongyuan Flagship Store	Electricity savings1	Store service is very good, the courier received the next day. Product packaging is very good, just use the quality to be tested.	Jingdong prefecture level city in Shandong
Jinfeng Hongyuan Flagship Store	Electricity savings1	I've been using it for almost a month now and I feel like it's working.	Jingdong prefecture level city in Shandong
Jinfeng Hongyuan Flagship Store	Electricity savings1	Very satisfied, very restful, very pretty, very beautiful, very much enjoyed! Well worth it!	Jingdong prefecture level city in Shandong
Jinfeng Hongyuan Flagship Store	Electricity savings1	Baby especially like, the value of high-quality good, a very good shopping experience, attentive service, fast logistics, distribution is also very thoughtful, attitude is also very good, value for money ah, decisive praise! Quality is quite good, logistics is also very fast, praise!	Jingdong prefecture level city in Shandong
Jinfeng Hongyuan Flagship Store	Electricity savings1	The shape of the item is basically the same as ordered, the effect is to be examined.	Jingdong prefecture level city in Shandong
Jinfeng Hongyuan Flagship Store	Electricity savings1	Appearance style: good-looking workmanship material: very good, a small one, very delicate Experience: still in use, in the process of using the shopkeeper is very nice to have a careful explanation Use for a period of time in the look at the results	Jingdong prefecture level city in Shandong
Jinfeng Hongyuan Flagship Store	Electricity savings1	As described, compact and well packaged	Jingdong prefecture level city in Shandong
Jinfeng Hongyuan Flagship Store	Electricity savings1	Fine workmanship. Test in progress. Let's see how it works.	Jingdong prefecture level city in Shandong
Jinfeng Hongyuan Flagship Store	Electricity savings1	The goods have been received, I'm very satisfied and love it	Jingdong prefecture level city in Shandong
Jinfeng Hongyuan Flagship Store	Electricity savings1	Safety! Whether you can save electricity, you can roughly feel it in 2 months. The principle is simple. Before the electrical appliances and circuits inductive more, now the electrical appliances integrated, the home is not a lot of inductive circuits, but used certainly save no problem! Wiring according to the requirements of the manual is good, if it is not convenient, see the socket plug.	Jingdong prefecture level city in Shandong
Jinfeng Hongyuan Flagship Store	Electricity savings1	Great product, looking forward to how much power I can save, first time buyer!	Jingdong prefecture level city in Shandong
Jinfeng Hongyuan Flagship Store	Electricity savings1	The product is exquisite, beautifully packaged, and the customer service is excellent. Just waiting to see how the power saving effect is, looking forward to it!	Jingdong prefecture level city in Shandong
Jinfeng Hongyuan Flagship Store	Electricity savings1	Express very quickly very satisfied with the first use to see how the effect to know, if you can save 10 percent even if acceptable, if you can not meet this requirement is obviously not much effect. Obviously.	Jingdong prefecture level city in Shandong
Jinfeng Hongyuan Flagship Store	Electricity savings1	Very delicate and compact, there is a very convenient intelligent display prompts the voltage, you can know the voltage at a glance, there is very easy to use, a plug can be used, I hope to save money on	Jingdong prefecture level city in Shandong
Jinfeng Hongyuan Flagship Store	Electricity savings1	First time buyer is it really save after use	Jingdong prefecture level city in Shandong
Jinfeng Hongyuan Flagship Store	Electricity savings1	Purchased again and will be for a long time yet.	Jingdong prefecture level city in Shandong
Jinfeng Hongyuan Flagship Store	Electricity savings1	Bought it to stock up, came back and plugged it in, hope it works	Jingdong prefecture level city in Shandong
Jinfeng Hongyuan Flagship Store	Electricity savings1	Baby received the logistics very fast very like very satisfied praise a	Jingdong prefecture level city in Shandong
Jinfeng Hongyuan Flagship Store	Electricity savings1	Beginning the process of using, easy to evaluate more later.	Jingdong prefecture level city in Shandong
Jinfeng Hongyuan Flagship Store	Electricity savings1	The goods have been received, just do not know how effective	Jingdong prefecture level city in Shandong
Jinfeng Hongyuan Flagship Store	Electricity savings1	It feels okay. I wish there were more events.	Jingdong prefecture level city in Shandong
Jinfeng Hongyuan Flagship Store	Electricity savings1	Things have been received, just plugged in, I do not know if it works, wait a few days to have the effect of additional	Jingdong prefecture level city in Shandong
Jinfeng Hongyuan Flagship Store	Electricity savings1	The effect of this power saver is really good, the house more, a few air conditioners are open every day, not used before a month to say less than seven hundred eight hundred, used after three months only nine	Jingdong prefecture level city in Shandong
Jinfeng Hongyuan Flagship Store	Electricity savings1	Cheap, good and easy to use, recommend it.	Jingdong prefecture level city in Shandong
Jinfeng Hongyuan Flagship Store	Electricity savings1	Bought this device, to the home for a long time, wait to finish a month to see how, good can recommend friends to buy, we use the electricity every month scary, a month of electricity costs more than a thousand, can not afford to hurt ah! I hope this power saving can save me some money, the beginning of the shoot order did not pay attention to look at the address, sent elsewhere to go, that side called to find out, hurry to communicate with customer service, customer service is very careful to help me get it done, call to that side to help me turn around, thank you!	Jingdong prefecture level city in Shandong
Jinfeng Hongyuan Flagship Store	Electricity savings1	I don't know the effect if it, use it first to see.	Jingdong prefecture level city in Shandong
Jinfeng Hongyuan Flagship Store	Electricity savings1	Looks good. I won't know if it saves power until I use it.	Jingdong prefecture level city in Shandong
Jinfeng Hongyuan Flagship Store	Electricity savings1	Goods received, shipping speed is very fast, yesterday afternoon more than two o'clock shot, just express delivery, but also sent a few insurance, very good business, the display of the number do not understand what is the meaning of the display said voltage, do not understand this, try a month to see how, good and	Jingdong prefecture level city in Shandong
Jinfeng Hongyuan Flagship Store	Electricity savings1	What ** play also, save electricity more and more save more and more, save chicken hair electricity, are cheating, this is a night of air conditioning with electricity, a night with 11 electricity word, this is	Jingdong prefecture level city in Shandong
Jinfeng Hongyuan Flagship Store	Electricity savings1	Received the product, hope it works!	Jingdong prefecture level city in Shandong
Jinfeng Hongyuan Flagship Store	Electricity savings1	Bought home and used very convenient, easy to operate, quite good	Jingdong prefecture level city in Shandong
Jinfeng Hongyuan Flagship Store	Electricity savings1	It's really good, cost-effective and good quality, and the logistics are fast! It arrived in one day! Really good, cost-effective and good quality, logistics is fast ah! It arrived in one day!	Jingdong prefecture level city in Shandong
Jinfeng Hongyuan Flagship Store	Electricity savings1	Works well, stabilizes voltage and saves power	Jingdong prefecture level city in Shandong
Jinfeng Hongyuan Flagship Store	Electricity savings1	I'll use it right away and look forward to good results	Jingdong prefecture level city in Shandong
Jinfeng Hongyuan Flagship Store	Electricity savings1	Recommended by a friend, said it works well, so I bought one, the effect to be tested	Jingdong prefecture level city in Shandong
Jinfeng Hongyuan Flagship Store	Electricity savings1	I didn't know it would save electricity.	Jingdong prefecture level city in Shandong
Jinfeng Hongyuan Flagship Store	Electricity savings1	I've been using it for a week, I don't know what's going on yet I hope it works well and saves power I'll follow up after a month of use	Jingdong prefecture level city in Shandong
Jinfeng Hongyuan Flagship Store	Electricity savings1	Very well packaged, looks great, don't know how it works	Jingdong prefecture level city in Shandong
Jinfeng Hongyuan Flagship Store	Electricity savings1	Genuine, good quality, really saves energy.	Jingdong prefecture level city in Shandong
Jinfeng Hongyuan Flagship Store	Electricity savings1	I was worried that the stuff is not good, but when I got it, I realized that I was completely over-anxious, 5 stars!	Jingdong prefecture level city in Shandong
Jinfeng Hongyuan Flagship Store	Electricity savings1	Very good and satisfied trustworthy	Jingdong prefecture level city in Shandong
Jinfeng Hongyuan Flagship Store	Electricity savings1	I don't know if it saves power	Jingdong prefecture level city in Shandong

Jinfeng Hongyuan Flagship Store	Electricity savings1	The quality of the product is very good, beautiful and generous applicable fast delivery speed good service attitude	Jingdong prefecture level city in Shandong
Jinfeng Hongyuan Flagship Store	Electricity savings1	Let's use it for a while and then we'll follow up	Jingdong prefecture level city in Shandong
Jinfeng Hongyuan Flagship Store	Electricity savings1	I've only been using it for a month, I don't know if there's any change yet, I'll come back with a follow-up comment after checking the meter!	Jingdong prefecture level city in Shandong
Jinfeng Hongyuan Flagship Store	Electricity savings1	I don't know if it works, I read the instructions and online reviews, I hope I won't be disappointed, if I use it, I will buy it again. Jingdong Express, fast and guaranteed service.	Jingdong prefecture level city in Shandong
Jinfeng Hongyuan Flagship Store	Electricity savings1	It feels like it saves quite a bit huh?	Jingdong prefecture level city in Shandong
Jinfeng Hongyuan Flagship Store	Electricity savings1	I've been using it for a while now, and it still seems to work and have a power saving effect	Jingdong prefecture level city in Shandong
Jinfeng Hongyuan Flagship Store	Electricity savings1	Looks good. It says 235. The 222 bar voltage is shown as high. I don't know if that's right. We'll see.	Jingdong prefecture level city in Shandong
Jinfeng Hongyuan Flagship Store	Electricity savings1	Baby is really good, do really careful, worth to have, fast delivery!	Jingdong prefecture level city in Shandong
Jinfeng Hongyuan Flagship Store	Electricity savings1	Quite delicate and compact, simple and convenient to use. Things are still very good, bought two at a time. Very satisfied with an online purchase.	Jingdong prefecture level city in Shandong
Jinfeng Hongyuan Flagship Store	Electricity savings1	Feel very good, the use of unclear, ask customer service, timely explanation, big brand, trustworthy	Jingdong prefecture level city in Shandong
Jinfeng Hongyuan Flagship Store	Electricity savings1	Thrifty received a few days did not make a comment is to see how the quality of the product now feel the quality of good appearance is also powerful seller service is very good to buy the product is also attached to the 5 fuse thought very thoughtful!	Jingdong prefecture level city in Shandong
Jinfeng Hongyuan Flagship Store	Electricity savings1	Big brand, with a very assured, the quality is very good, in time for the event to buy very cost-effective, customer service is really very good Oh ~!	Jingdong prefecture level city in Shandong
Jinfeng Hongyuan Flagship Store	Electricity savings1	I ordered yesterday and received it this morning, I heard my friend say that it can save electricity, I don't know if it works, I'll come back to make a comment after using it.	Jingdong prefecture level city in Shandong
Jinfeng Hongyuan Flagship Store	Electricity savings1	I've been using it for over a month and it doesn't seem to be working!	Jingdong prefecture level city in Shandong
Jinfeng Hongyuan Flagship Store	Electricity savings1	Used it, the family has always been in the habit of using energy-saving devices, this is a new one. Digital display voltage is very good, can see that the voltage is more stable.	Jingdong prefecture level city in Shandong
Jinfeng Hongyuan Flagship Store	Electricity savings1	I haven't used it for more than a month, but it looks good. It's easy to plug into an outlet and it works.	Jingdong prefecture level city in Shandong
Jinfeng Hongyuan Flagship Store	Electricity savings1	Good Oh...appearance is very beautiful...just plugged in with...see the effect of it...with a period of time in the additional evaluation...	Jingdong prefecture level city in Shandong
Jinfeng Hongyuan Flagship Store	Electricity savings1	I've received the goods, whether it's good or not, I'll know after I use it.	Jingdong prefecture level city in Shandong
Jinfeng Hongyuan Flagship Store	Electricity savings1	Very good, logistics is fast, the stuff is very powerful and cost-effective! Can feel the meter slowing down	Jingdong prefecture level city in Shandong
Jinfeng Hongyuan Flagship Store	Electricity savings1	Logistics is very fast, get it immediately after use, easy to operate, plug in the power supply can be used, just do not know to the use of the effect of how.	Jingdong prefecture level city in Shandong
Jinfeng Hongyuan Flagship Store	Electricity savings1	Quality is very over the top, quality is guaranteed, quite a good choice, a lot of friends to see me use all want to buy, I recommend it!	Jingdong prefecture level city in Shandong
Jinfeng Hongyuan Flagship Store	Electricity savings1	The appearance is clean and beautiful, the workmanship is very good, did not find any flaws, I personally feel that the size is also quite suitable, looking for a long time to choose this one, big love! The price is very beautiful, things are still good, will continue to buy back! All five points! Ness Ness ~ logistics speed is really no words, is a word: fast. Commodities from the outer packaging is very high-grade, really a penny a penny, expensive has the reason for expensive	Jingdong prefecture level city in Shandong
Jinfeng Hongyuan Flagship Store	Electricity savings1	The logistics is very fast, the texture is very good, genuine, I like it very much.	Jingdong prefecture level city in Shandong
Jinfeng Hongyuan Flagship Store	Electricity savings1	It's been a few days since I put it on, I can't see any results now, waiting for a month to say, steady steady steady.	Jingdong prefecture level city in Shandong
Jinfeng Hongyuan Flagship Store	Electricity savings1	I've been using it for a while now and it should be fine, so I'm buying it for the second time.	Jingdong prefecture level city in Shandong
Jinfeng Hongyuan Flagship Store	Electricity savings1	Power Saving King has been received, looks good, quite delicate, looking forward to using it to save electricity	Jingdong prefecture level city in Shandong
Jinfeng Hongyuan Flagship Store	Electricity savings1	My mom said the home uses a little too much electricity, so I bought a Power Saver to try it out, I don't know how effective it is. Expect it to be a good thing!	Jingdong prefecture level city in Shandong
Jinfeng Hongyuan Flagship Store	Electricity savings1	Thank you Jingdong, logistics is very fast order to receipt only one day. Believe in Jingdong. Favorable comments	Jingdong prefecture level city in Shandong
Jinfeng Hongyuan Flagship Store	Electricity savings1	Saved some money on the electric bill compared to last month, watch this month again, no problems so far	Jingdong prefecture level city in Shandong
Jinfeng Hongyuan Flagship Store	Electricity savings1	Energy saver exquisite workmanship, beautiful style, more features, just bought, power saving effect to be used for a period of time, then evaluate!	Jingdong prefecture level city in Shandong
Jinfeng Hongyuan Flagship Store	Electricity savings1	This thing does save power is more compact to hang on the wall.	Jingdong prefecture level city in Shandong
Jinfeng Hongyuan Flagship Store	Electricity savings1	I've been buying it for a while now, I haven't actually measured it, but it still feels like it's effective	Jingdong prefecture level city in Shandong
Jinfeng Hongyuan Flagship Store	Electricity savings1	I've already used it since I bought it, I don't know how effective it is yet, I'll comment on it later.	Jingdong prefecture level city in Shandong
Jinfeng Hongyuan Flagship Store	Electricity savings1	Baby received, careful appearance, logistics is very fast, worth buying!	Jingdong prefecture level city in Shandong
Jinfeng Hongyuan Flagship Store	Electricity savings1	It's great, it's just not the same in hand, I bought it at that other cheaper online one for about the same price as this one. But then oops, doesn't work at all.	Jingdong prefecture level city in Shandong
Jinfeng Hongyuan Flagship Store	Electricity savings1	The product is exquisite, but you have to use it for a month to know how it works. I'll comment again after a month.	Jingdong prefecture level city in Shandong
Jinfeng Hongyuan Flagship Store	Electricity savings1	I've been using it for a while now, and it doesn't feel like it's working, and it doesn't save any power.	Jingdong prefecture level city in Shandong
Jinfeng Hongyuan Flagship Store	Electricity savings1	Affordable price, fast delivery, Jingdong self-supporting guarantee	Jingdong prefecture level city in Shandong
Jinfeng Hongyuan Flagship Store	Electricity savings1	Packaging is very good, no damage, good quality, fine workmanship Packaging is very good, no damage, good quality, fine workmanship Packaging is very good, no damage, good quality, fine workmanship Packaging is very good, no damage, good quality, fine workmanship	Jingdong prefecture level city in Shandong
Jinfeng Hongyuan Flagship Store	Electricity savings1	Big brand, with a very assured, the quality is very good, in time for the event to buy very cost-effective, customer service is really very good Oh ~!	Jingdong prefecture level city in Shandong
Jinfeng Hongyuan Flagship Store	Electricity savings1	Used a few days, the specific effect is not yet clear, looking forward to the effect as described by the merchant!	Jingdong prefecture level city in Shandong
Jinfeng Hongyuan Flagship Store	Electricity savings1	I just used it and don't know how it works, I'll follow up after using it!	Jingdong prefecture level city in Shandong
Jinfeng Hongyuan Flagship Store	Electricity savings1	Well, it's been installed and now I'm waiting to see the results, BOE shopping, hopefully I won't be disappointed!	Jingdong prefecture level city in Shandong
Jinfeng Hongyuan Flagship Store	Electricity savings1	Goods received, plugged in the voltage from 229 to 222, do not know whether to save electricity, that depends on the situation.	Jingdong prefecture level city in Shandong
Jinfeng Hongyuan Flagship Store	Electricity savings1	This is the second time I have bought the product and I feel it really saves electricity! Use it again for a while and then evaluate!	Jingdong prefecture level city in Shandong
Jinfeng Hongyuan Flagship Store	Electricity savings1	Overall, it's really good, and the customer service is great, you'll get a message back almost immediately when you speak, it's great value for money, I really recommend it!	Jingdong prefecture level city in Shandong
Jinfeng Hongyuan Flagship Store	Electricity savings1	Have purchased many times, a must have for the family, affordable purchase price, fast delivery, beautifully packaged!	Jingdong prefecture level city in Shandong
Jinfeng Hongyuan Flagship Store	Electricity savings1	I bought it after a friend introduced it, he said it is effective and can save electricity. I bought it to try, try a period of time and then follow up!	Jingdong prefecture level city in Shandong
Jinfeng Hongyuan Flagship Store	Electricity savings1	Received it, trying it out tonight, and bought one earlier.	Jingdong prefecture level city in Shandong

Jinfeng Hongyuan Flagship Store	Electricity savings1	It will take a month or two to see if it saves power or not.	Jingdong prefecture level city in Shandong
Jinfeng Hongyuan Flagship Store	Electricity savings1	Looked good in the instructions! Bought one and pressed it on! Use it and see! If it works! I'll buy some more!	Jingdong prefecture level city in Shandong
Jinfeng Hongyuan Flagship Store	Electricity savings1	Goods have been received packaging can also things are not bad appearance, with a use to know, now evaluation is nonsense.	Jingdong prefecture level city in Shandong
Jinfeng Hongyuan Flagship Store	Electricity savings1	Logistics is very fast, packaging is very good, the second unit, as always, looking forward to the effect of	Jingdong prefecture level city in Shandong
Jinfeng Hongyuan Flagship Store	Electricity savings1	I received the goods today, the quality of goods is very good, I am very satisfied ah.	Jingdong prefecture level city in Shandong
Jinfeng Hongyuan Flagship Store	Electricity savings1	Buy one and try it, I wonder if it has a power saving effect?	Jingdong prefecture level city in Shandong
Jinfeng Hongyuan Flagship Store	Electricity savings1	🔍 The item was received, well packaged, logistics is very fast, customer service attitude is very positive, there are questions answered in a timely manner! Overall very satisfied!	Jingdong prefecture level city in Shandong
Jinfeng Hongyuan Flagship Store	Electricity savings1	Baby packaging is very good, purchase submission goods quickly delivered to the home, use and then follow up.	Jingdong prefecture level city in Shandong
Jinfeng Hongyuan Flagship Store	Electricity savings1	My friend asked me to buy an extra one so he could try it too.	Jingdong prefecture level city in Shandong
Jinfeng Hongyuan Flagship Store	Electricity savings1	I've just put it on, I won't know the result until a month later if it will really save power.	Jingdong prefecture level city in Shandong
Jinfeng Hongyuan Flagship Store	Electricity savings1	It's good quality. Hopefully it'll help save power.	Jingdong prefecture level city in Shandong
Jinfeng Hongyuan Flagship Store	Electricity savings1	The appearance is clean and beautiful, the workmanship is very good, I did not find any flaws, I personally feel that the size is also quite suitable, I have been looking for a long time to choose this	Jingdong prefecture level city in Shandong
Jinfeng Hongyuan Flagship Store	Electricity savings1	Logistics is fast! Baby looks great! The price is fair! Wish the store business is prosperous!	Jingdong prefecture level city in Shandong
Jinfeng Hongyuan Flagship Store	Electricity savings1	Jingdong express fast, good, have used, sit and wait to save money...	Jingdong prefecture level city in Shandong
Jinfeng Hongyuan Flagship Store	Electricity savings1	In use, it didn't work as expected and there was nothing noticeable in terms of power savings.	Jingdong prefecture level city in Shandong
Jinfeng Hongyuan Flagship Store	Electricity savings1	Just started using it and looking forward to the results.	Jingdong prefecture level city in Shandong
Jinfeng Hongyuan Flagship Store	Electricity savings1	The quality is very good, the metal shell is very good, is genuine, energy saving and power saving, logistics is very fast, there is a need to buy o, recommend everyone, very satisfied with an online	Jingdong prefecture level city in Shandong
Jinfeng Hongyuan Flagship Store	Electricity savings1	Received, I do not know if the product is good, used to see if it is good to come back to buy!	Jingdong prefecture level city in Shandong
Jinfeng Hongyuan Flagship Store	Electricity savings1	Jinfeng source of intelligent power saving treasure also used less than three months, the last thunder may be burned, the home of the electric send gate is not on, only the old man at home, no way only their own in the field and run back to find out what is the problem with the circuit, the quality is still not very good, thunder is easy to burn!	Jingdong prefecture level city in Shandong
Jinfeng Hongyuan Flagship Store	Electricity savings1	Saving power just started to use, I do not know if there is no effect, but read a lot of reviews are said to be very good so I bought, baby All aspects are good There are spare 2 fuses, late good and then follow	Jingdong prefecture level city in Shandong
Jinfeng Hongyuan Flagship Store	Electricity savings1	Use it for a few days and see, the delivery is quite fast	Jingdong prefecture level city in Shandong
Jinfeng Hongyuan Flagship Store	Electricity savings1	Small, well made and easy to use.	Jingdong prefecture level city in Shandong
Jinfeng Hongyuan Flagship Store	Electricity savings1	Beautifully packaged, it is genuine. Whether it is power saving, use it to evaluate.	Jingdong prefecture level city in Shandong
Jinfeng Hongyuan Flagship Store	Electricity savings1	Power saver. Used two of them and they do save quite a bit of power.	Jingdong prefecture level city in Shandong
Jinfeng Hongyuan Flagship Store	Electricity savings1	Workmanship is not bad, small and does not take up space, is also do not know whether the power saving, buy a to try!	Jingdong prefecture level city in Shandong
Jinfeng Hongyuan Flagship Store	Electricity savings1	The logistics is very fast, the courier attitude is very good, worth buying	Jingdong prefecture level city in Shandong
Jinfeng Hongyuan Flagship Store	Electricity savings1	The effect is not known until it is used.	Jingdong prefecture level city in Shandong
Jinfeng Hongyuan Flagship Store	Electricity savings1	Just arrived plugged in won't know for a month	Jingdong prefecture level city in Shandong
Jinfeng Hongyuan Flagship Store	Electricity savings1	This is really good to use the pull to realize the savings ah think again for my sister's family to buy a	Jingdong prefecture level city in Shandong
Jinfeng Hongyuan Flagship Store	Electricity savings1	Lies, a month with a few hundred electricity are no savings, family use	Jingdong prefecture level city in Shandong
Jinfeng Hongyuan Flagship Store	Electricity savings1	Express very quickly, look at the fine workmanship also sent a few fuse, look forward to can really save electricity	Jingdong prefecture level city in Shandong
Jinfeng Hongyuan Flagship Store	Electricity savings1	I didn't realize it was so small.	Jingdong prefecture level city in Shandong
Jinfeng Hongyuan Flagship Store	Electricity savings1	Logistics courier is very fast, the courier service is very good	Jingdong prefecture level city in Shandong
Jinfeng Hongyuan Flagship Store	Electricity savings1	Used for a period of time the effect is quite good indeed than the previous power saving	Jingdong prefecture level city in Shandong
Jinfeng Hongyuan Flagship Store	Electricity savings1	Logistics is super fast, the product is in use, see the effect...	Jingdong prefecture level city in Shandong
Jinfeng Hongyuan Flagship Store	Electricity savings1	Baby got it, tried it out and it didn't work at all. How can I use the tips to save power?	Jingdong prefecture level city in Shandong
Jinfeng Hongyuan Flagship Store	Electricity savings1	Power Saving King received, immediately used has been very stable without any sound good, good service, fast logistics inexpensive and beautiful worth repurchasing!	Jingdong prefecture level city in Shandong
Jinfeng Hongyuan Flagship Store	Electricity savings1	Logistics courier is very fast, the courier service is good	Jingdong prefecture level city in Shandong
Jinfeng Hongyuan Flagship Store	Electricity savings1	Good to recommend, the first month of air conditioning only 1 week, the electricity bill has reduced about 100 yuan, may be to reduce the use of air conditioning is also related to the other or as usual electricity, estimated that the longer you use the more you can save electricity.	Jingdong prefecture level city in Shandong
Jinfeng Hongyuan Flagship Store	Electricity savings1	It doesn't work. I've been using it for 20 days and it doesn't work.	Jingdong prefecture level city in Shandong
Jinfeng Hongyuan Flagship Store	Electricity savings1	Fast shipping, good quality, affordable price, very satisfied with a shopping.	Jingdong prefecture level city in Shandong
Jinfeng Hongyuan Flagship Store	Electricity savings1	Try it for a month to see if it saves money.	Jingdong prefecture level city in Shandong
Jinfeng Hongyuan Flagship Store	Electricity savings1	It's in use. I'll comment again after a while.	Jingdong prefecture level city in Shandong
Jinfeng Hongyuan Flagship Store	Electricity savings1	Goods have received look good 👍👍👍👍, use a period of time and then look at the	Jingdong prefecture level city in Shandong
Jinfeng Hongyuan Flagship Store	Electricity savings1	No, it doesn't work. It's useless. It's garbage.	Jingdong prefecture level city in Shandong
Jinfeng Hongyuan Flagship Store	Electricity savings1	I've been using it for almost 2 months now and I don't feel any reduction in my electricity bill.	Jingdong prefecture level city in Shandong
Jinfeng Hongyuan Flagship Store	Electricity savings1	I've received the goods, the logistics is really fast, ordered yesterday, arrived today.	Jingdong prefecture level city in Shandong
Jinfeng Hongyuan Flagship Store	Electricity savings1	And the backup insurance.	Jingdong prefecture level city in Shandong
Jinfeng Hongyuan Flagship Store	Electricity savings1	The appearance of this product is good, easy to use and install, the effect of how there is no use to wait for time to verify that the voltage displayed is different from the voltage measured by other meters, the gap is not too large, the follow-up to add the effect.	Jingdong prefecture level city in Shandong

Jinfeng Hongyuan Flagship Store	Electricity savings1	Baby received it 👌, really great ah! 🌈The quality can't be beat, very satisfied! 🆗Happy happy happy 😃	Jingdong prefecture level city in Shandong
Jinfeng Hongyuan Flagship Store	Electricity savings1	I'll follow up with a review after using it for a while, hopefully it won't bring me back a disappointment!	Jingdong prefecture level city in Shandong
Jinfeng Hongyuan Flagship Store	Electricity savings1	In use and looking forward to results! Good review to send	Jingdong prefecture level city in Shandong
Jinfeng Hongyuan Flagship Store	Electricity savings1	Intelligent power saver received, the volume is not big, small and exquisite, quite good, Jingdong Express is also quite fast, the	Jingdong prefecture level city in Shandong
Jinfeng Hongyuan Flagship Store	Electricity savings1	I don't know how to use it, keep using it	Jingdong prefecture level city in Shandong
Jinfeng Hongyuan Flagship Store	Electricity savings1	Very good for saving electricity	Jingdong prefecture level city in Shandong
Jinfeng Hongyuan Flagship Store	Electricity savings1	Seriously, did not use, feel also more power consumption, the peak is bought back to use the first time, the next day unplugged, the power consumption is back to more than ten degrees (the frequency of power use in the past few days is pretty much the same), feel the IQ tax paid 😂	Jingdong prefecture level city in Shandong
Jinfeng Hongyuan Flagship Store	Electricity savings1	I haven't seen any effect on the power saving, but the voltage stabilization looks good.	Jingdong prefecture level city in Shandong
Jinfeng Hongyuan Flagship Store	Electricity savings1	It's very good, I'll come back to it after using it for a while	Jingdong prefecture level city in Shandong
Jinfeng Hongyuan Flagship Store	Electricity savings1	Already used it and see the results.	Jingdong prefecture level city in Shandong
Jinfeng Hongyuan Flagship Store	Electricity savings1	Fast shipping, beautiful packaging, attentive service, the use of the effect, to be followed up after the evaluation.	Jingdong prefecture level city in Shandong
Jinfeng Hongyuan Flagship Store	Electricity savings1	The process of using this product is more than satisfactory	Jingdong prefecture level city in Shandong
Jinfeng Hongyuan Flagship Store	Electricity savings1	Very good East East cost-effective! Every time so buy more assured, and the logistics is also very fast. It is also very good to use, so I will often come to buy in the future, and will also introduce to	Jingdong prefecture level city in Shandong
Jinfeng Hongyuan Flagship Store	Electricity savings1	But still understand how effective it is!	Jingdong prefecture level city in Shandong
Jinfeng Hongyuan Flagship Store	Electricity savings1	Fast shipping, great logistics, great stuff, great price.	Jingdong prefecture level city in Shandong
Jinfeng Hongyuan Flagship Store	Electricity savings1	I'll review it in a month and give it 5 stars if it's good	Jingdong prefecture level city in Shandong
Jinfeng Hongyuan Flagship Store	Electricity savings1	I've saved a lot of electricity in the past month, so I'm glad to hear that.	Jingdong prefecture level city in Shandong
Jinfeng Hongyuan Flagship Store	Electricity savings1	Baby just received and installed! Looks genuinely good, workmanship feels fine! Looking forward to the effect! Said to see the effect in two months! Waiting for if really have the effect must come to follow up! The boss is absolutely responsible! Asked and answered! Just for the boss this attitude must also	Jingdong prefecture level city in Shandong
Jinfeng Hongyuan Flagship Store	Electricity savings1	This baby is very delicate, I'll comment after I've used it	Jingdong prefecture level city in Shandong
Jinfeng Hongyuan Flagship Store	Electricity savings1	It's been over two months. It's not working. It's just like it was.	Jingdong prefecture level city in Shandong
Jinfeng Hongyuan Flagship Store	Electricity savings1	Fast shipping and logistics, let's see if it works.	Jingdong prefecture level city in Shandong
Jinfeng Hongyuan Flagship Store	Electricity savings1	Just received the goods, try it to know if it can really save electricity	Jingdong prefecture level city in Shandong
Jinfeng Hongyuan Flagship Store	Electricity savings1	Not bad. Not bad. Not bad.	Jingdong prefecture level city in Shandong
Jinfeng Hongyuan Flagship Store	Electricity savings1	Not sure if it works. I'll comment again if it works.	Jingdong prefecture level city in Shandong
Jinfeng Hongyuan Flagship Store	Electricity savings1	Safe and energy-saving helpers to use!	Jingdong prefecture level city in Shandong
Jinfeng Hongyuan Flagship Store	Electricity savings1	Good, first time buyer. Used good can	Jingdong prefecture level city in Shandong
Jinfeng Hongyuan Flagship Store	Electricity savings1	It feels okay, I guess! I won't know until I use it for a while!	Jingdong prefecture level city in Shandong
Jinfeng Hongyuan Flagship Store	Electricity savings1	The saver matches the description and has been used, waiting for a follow up review after two months.	Jingdong prefecture level city in Shandong
Jinfeng Hongyuan Flagship Store	Electricity savings1	Received, the appearance of the business as described, the effect of how to use a period of time to evaluate.	Jingdong prefecture level city in Shandong
Jinfeng Hongyuan Flagship Store	Electricity savings1	Still using it don't know how effective it is, I'll comment after three months	Jingdong prefecture level city in Shandong
Jinfeng Hongyuan Flagship Store	Electricity savings1	The stuff is very good, the packaging is also very good logistics is also very fast	Jingdong prefecture level city in Shandong
Jinfeng Hongyuan Flagship Store	Electricity savings1	I'll follow up with a review when I use it, hopefully I won't be disappointed	Jingdong prefecture level city in Shandong
Jinfeng Hongyuan Flagship Store	Electricity savings1	Cancel Houjiaying Logistics.	Jingdong prefecture level city in Shandong
Jinfeng Hongyuan Flagship Store	Electricity savings1	It's very good, it's worth buying.	Jingdong prefecture level city in Shandong
Jinfeng Hongyuan Flagship Store	Electricity savings1	My friend bought it to save electricity and referred me to buy it! I'll buy it and try it out! I will know how much electricity I can save next month, looking forward to it! 4-500 dollars a month electricity bill is horrible! I hope it can really help me save some electricity!	Jingdong prefecture level city in Shandong
Jinfeng Hongyuan Flagship Store	Electricity savings1	Just received it and installed it.	Jingdong prefecture level city in Shandong
Jinfeng Hongyuan Flagship Store	Electricity savings1	Very nice. This one is my favorite.	Jingdong prefecture level city in Shandong
Jinfeng Hongyuan Flagship Store	Electricity savings1	Good value for money worth buying very good	Jingdong prefecture level city in Shandong
Jinfeng Hongyuan Flagship Store	Electricity savings1	Very energy-saving, small and exquisite, does not take up space, very satisfied, complete packaging, no damage, customer service explanation is also very serious	Jingdong prefecture level city in Shandong
Jinfeng Hongyuan Flagship Store	Electricity savings1	Received immediately used, really effective, do not understand electricity, but looked at the meter every day, recommended that you can use, especially this weather every day to open the air conditioning, with him not afraid of the electricity bill.	Jingdong prefecture level city in Shandong
Jinfeng Hongyuan Flagship Store	Electricity savings1	Nice, good value for money, nice stuff.	Jingdong prefecture level city in Shandong
Jinfeng Hongyuan Flagship Store	Electricity savings1	The packaging of the product is very good, the logistics of the product is very fast, and the quality of the product will be evaluated after use!	Jingdong prefecture level city in Shandong
Jinfeng Hongyuan Flagship Store	Electricity savings1	Express is very, well very fast, give a compliment 👍	Jingdong prefecture level city in Shandong
Jinfeng Hongyuan Flagship Store	Electricity savings1	Packaging is very good, fast delivery, used a night and a day did not feel the power saving. Use a month to see if it saves power.	Jingdong prefecture level city in Shandong
Jinfeng Hongyuan Flagship Store	Electricity savings1	Seems to be a bit of a power saver.	Jingdong prefecture level city in Shandong
Jinfeng Hongyuan Flagship Store	Electricity savings1	Bought this for someone else and it came over so fast, ###!	Jingdong prefecture level city in Shandong
Jinfeng Hongyuan Flagship Store	Electricity savings1	Very delicate, just received a period of time to use in the follow-up evaluation	Jingdong prefecture level city in Shandong
Jinfeng Hongyuan Flagship Store	Electricity savings1	It's been installed for a while now, not sure if it's working or not	Jingdong prefecture level city in Shandong
Jinfeng Hongyuan Flagship Store	Electricity savings1	The item looks okay, I just don't know if it works or not.	Jingdong prefecture level city in Shandong

Jinfeng Hongyuan Flagship Store	Electricity savings1	The goods have been received, but there is a screw missing	Jingdong prefecture level city in Shandong
Jinfeng Hongyuan Flagship Store	Electricity savings1	I didn't believe it before, but after receiving the goods today and trying it out, it really works!	Jingdong prefecture level city in Shandong
Jinfeng Hongyuan Flagship Store	Electricity savings1	Received, this thing will not know whether it is the effect of saving electricity, no matter how to say that there should always be a little use it, otherwise why develop it I think there is still the value of	Jingdong prefecture level city in Shandong
Jinfeng Hongyuan Flagship Store	Electricity savings1	Another one just arrived.	Jingdong prefecture level city in Shandong
Jinfeng Hongyuan Flagship Store	Electricity savings1	I don't feel anything, ordinary farmers can't go to TEDA, just use it first, it's safe.	Jingdong prefecture level city in Shandong
Jinfeng Hongyuan Flagship Store	Electricity savings1	Just purchased and will follow up after using it for a while	Jingdong prefecture level city in Shandong
Jinfeng Hongyuan Flagship Store	Electricity savings1	I'm trying it out now. I'll comment after I use it.	Jingdong prefecture level city in Shandong
Jinfeng Hongyuan Flagship Store	Electricity savings1	Received the item, in use, hope it works.	Jingdong prefecture level city in Shandong
Jinfeng Hongyuan Flagship Store	Electricity savings1	It works well. Good review.	Jingdong prefecture level city in Shandong
Jinfeng Hongyuan Flagship Store	Electricity savings1	All a scam, no effect at all, I bought too many fakes in Poundland before, I did not think that Jingdong is also the same	Jingdong prefecture level city in Shandong
Jinfeng Hongyuan Flagship Store	Electricity savings1	I used the saver as soon as I received it, it's in good shape and still working!	Jingdong prefecture level city in Shandong
Jinfeng Hongyuan Flagship Store	Electricity savings1	It works. I don't know what to do with the picture, but it works.	Jingdong prefecture level city in Shandong
Jinfeng Hongyuan Flagship Store	Electricity savings1	A very good shopping experience, attentive service, fast logistics, delivery is also very thoughtful, attitude is also very good, value for money ah, decisive praise!	Jingdong prefecture level city in Shandong
Jinfeng Hongyuan Flagship Store	Electricity savings1	The product is consistent with the presentation picture.	Jingdong prefecture level city in Shandong
Jinfeng Hongyuan Flagship Store	Electricity savings1	Baby received, use a period of time to evaluate, wish the boss business prosperity!	Jingdong prefecture level city in Shandong
Jinfeng Hongyuan Flagship Store	Electricity savings1	The stuff is better! Recommended!	Jingdong prefecture level city in Shandong
Jinfeng Hongyuan Flagship Store	Electricity savings1	I don't know if it works or not, I'll use it for a while before I comment on it.	Jingdong prefecture level city in Shandong
Jinfeng Hongyuan Flagship Store	Electricity savings1	Cost-effective, I like it, next time will come back Baby received, very surprised, the quality is very good, very satisfied with a shopping. I will continue to buy back, the store is trustworthy	Jingdong prefecture level city in Shandong
Jinfeng Hongyuan Flagship Store	Electricity savings1	Plugged it in and used it for a few days, not sure if it saves power yet	Jingdong prefecture level city in Shandong
Jinfeng Hongyuan Flagship Store	Electricity savings1	I'm trying it out and will follow up if it seems to be working!	Jingdong prefecture level city in Shandong
Jinfeng Hongyuan Flagship Store	Electricity savings1	Works pretty well, big kudos 👍👍👍👍👍👍👍👍👍👍👍	Jingdong prefecture level city in Shandong
Jinfeng Hongyuan Flagship Store	Electricity savings1	Just put it on, we'll see how it looks in a month.	Jingdong prefecture level city in Shandong
Jinfeng Hongyuan Flagship Store	Electricity savings1	The baby is very nice and I've already recommended buying it for all my friends! The main thing is that it not only saves electricity, I was more concerned about the fact that it can adjust the current, usually wiring problems. Now I found that it is really good, it is the effect I want!	Jingdong prefecture level city in Shandong
Jinfeng Hongyuan Flagship Store	Electricity savings1	Baby arrived, looking forward to his results, hopefully it will be good	Jingdong prefecture level city in Shandong
Jinfeng Hongyuan Flagship Store	Electricity savings1	Things have been received, look at the workmanship and packaging are good, I do not know how the power-saving effect? Use a period of time to see the effect	Jingdong prefecture level city in Shandong
Jinfeng Hongyuan Flagship Store	Electricity savings1	The price is right, the shape is beautiful and does not take up space, use it for a while to see the effect	Jingdong prefecture level city in Shandong
Jinfeng Hongyuan Flagship Store	Electricity savings1	Can't save electricity, it doesn't work!	Jingdong prefecture level city in Shandong
Jinfeng Hongyuan Flagship Store	Electricity savings1	It's pretty convenient, just plug it in and it works. There's no noise, plug it in and leave it alone, it's very hassle free! Good workmanship and value for money.	Jingdong prefecture level city in Shandong
Jinfeng Hongyuan Flagship Store	Electricity savings1	Just received the goods have been installed, try two months later to comment!	Jingdong prefecture level city in Shandong
Jinfeng Hongyuan Flagship Store	Electricity savings1	I received the stuff, I haven't used it yet, and overall it's fine.	Jingdong prefecture level city in Shandong
Jinfeng Hongyuan Flagship Store	Electricity savings1	Couldn't use a return at all.	Jingdong prefecture level city in Shandong
Jinfeng Hongyuan Flagship Store	Electricity savings1	Second purchase.	Jingdong prefecture level city in Shandong
Jinfeng Hongyuan Flagship Store	Electricity savings1	Very good quality, high tech and practical.	Jingdong prefecture level city in Shandong
Jinfeng Hongyuan Flagship Store	Electricity savings1	Very good, very great! Very satisfied, thanks to the store!	Jingdong prefecture level city in Shandong
Jinfeng Hongyuan Flagship Store	Electricity savings1	Just used it, don't know if it's good or not, hope it works well	Jingdong prefecture level city in Shandong
Jinfeng Hongyuan Flagship Store	Electricity savings1	Saving power has been received, the product is good, good like.	Jingdong prefecture level city in Shandong
Jinfeng Hongyuan Flagship Store	Electricity savings1	Received the goods and used it for two months before evaluation	Jingdong prefecture level city in Shandong
Jinfeng Hongyuan Flagship Store	Electricity savings1	It looks a little small, I do not know how effective, first use to see the effect, there is a small adjustable button, I do not know this is to adjust to that position to work well	Jingdong prefecture level city in Shandong
Jinfeng Hongyuan Flagship Store	Electricity savings1	Simple, just plug it in, there are spare fuses, can't say how well it works, no comparison	Jingdong prefecture level city in Shandong
Jinfeng Hongyuan Flagship Store	Electricity savings1	It's been a few days since I used it, and the power's gone up.	Jingdong prefecture level city in Shandong
Jinfeng Hongyuan Flagship Store	Electricity savings1	The owner was very nice and sent a spare fuse.	Jingdong prefecture level city in Shandong
Jinfeng Hongyuan Flagship Store	Electricity savings1	It's authentic and it's not expensive and it's great to use, I use it all the time and it's great 🎉✨ just don't know if it really saves any points	Jingdong prefecture level city in Shandong
Jinfeng Hongyuan Flagship Store	Electricity savings1	The information is good, it is the child to buy, the child read it is also quite happy, I hope to be able to match the child's preparation for the midterm examination! Because of the epidemic, it is not too fast, but I also give full credit to the express delivery!	Jingdong prefecture level city in Shandong
Jinfeng Hongyuan Flagship Store	Electricity savings1	This power saver comes to the logistics very quickly, the business attitude is also very good, ** very comfortable, this brand is trustworthy, and buy in this store than in the physical store to buy cheaper,	Jingdong prefecture level city in Shandong
Jinfeng Hongyuan Flagship Store	Electricity savings1	It's very well made and the materials used are okay. This is just a few days old, so I don't know how it will work, only next month.	Jingdong prefecture level city in Shandong
Jinfeng Hongyuan Flagship Store	Electricity savings1	Already used on, have not tested the effect, wait for a couple of months to add comments.	Jingdong prefecture level city in Shandong
Jinfeng Hongyuan Flagship Store	Electricity savings1	Baby received, packaging is very thoughtful, already in use, should soon see the effect.	Jingdong prefecture level city in Shandong
Jinfeng Hongyuan Flagship Store	Electricity savings1	Just received in the office to try, to the home installed, the voltage are different, and do not know if there is no effect. Use a period of time and then follow up.	Jingdong prefecture level city in Shandong
Jinfeng Hongyuan Flagship Store	Electricity savings1	Just received the goods, is trying, looking forward to the effect, first give a favorable comment, two months later, then additional evaluation, please be patient.	Jingdong prefecture level city in Shandong
Jinfeng Hongyuan Flagship Store	Electricity savings1	The item has just been received, well packaged, results to be verified.	Jingdong prefecture level city in Shandong

Jinfeng Hongyuan Flagship Store	Electricity savings1	Packaging logistics are very good, the next look at how the effect, before I see others use the effect is good, as I bought this can also have good results.	Jingdong prefecture level city in Shandong
Jinfeng Hongyuan Flagship Store	Electricity savings1	I don't know if it's a real power saver or a fake power saver, but I've used it since I bought it, so I'll give you a good review.	Jingdong prefecture level city in Shandong
Jinfeng Hongyuan Flagship Store	Electricity savings1	Good quality, genuine goods, much better than imagined before purchase, next time will come back to care!	Jingdong prefecture level city in Shandong
Jinfeng Hongyuan Flagship Store	Electricity savings1	Logistics is very fast good use again	Jingdong prefecture level city in Shandong
Jinfeng Hongyuan Flagship Store	Electricity savings1	Received the goods. Let's see if it saves power in a month and then we'll follow up!	Jingdong prefecture level city in Shandong
Jinfeng Hongyuan Flagship Store	Electricity savings1	I bought it yesterday and it arrived today, I bought two of them, I hope they work well, I'll come back after a while to give a follow-up review	Jingdong prefecture level city in Shandong
Jinfeng Hongyuan Flagship Store	Electricity savings1	The home air conditioner consumes a lot of electricity, so I bought it and used it in the hope that there is A	Jingdong prefecture level city in Shandong
Jinfeng Hongyuan Flagship Store	Electricity savings1	The product is excellent and very current.	Jingdong prefecture level city in Shandong
Jinfeng Hongyuan Flagship Store	Electricity savings1	With the description, it is the most practical and good products that households can need, and it is an ideal shopping, the courier is also fast, and the service attitude is enthusiastic!	Jingdong prefecture level city in Shandong
Jinfeng Hongyuan Flagship Store	Electricity savings1	The Power Saver is easy to use, works well, is stable, and plugs into the top of the outlet	Jingdong prefecture level city in Shandong
Jinfeng Hongyuan Flagship Store	Electricity savings1	Overall still can, excellent craftsmanship, power saving do not know for the time being, after the use of the evaluation	Jingdong prefecture level city in Shandong
Jinfeng Hongyuan Flagship Store	Electricity savings1	Baby received on the plug, but also with four fuses, look at the quality is okay, I do not know whether it is useful, use and then follow up.	Jingdong prefecture level city in Shandong
Jinfeng Hongyuan Flagship Store	Electricity savings1	I'll evaluate it after a while, I don't know if it's good or not yet, and if it can save electricity	Jingdong prefecture level city in Shandong
Jinfeng Hongyuan Flagship Store	Electricity savings1	Jingdong self-operated purchase more assured delivery fast quality assurance	Jingdong prefecture level city in Shandong
Jinfeng Hongyuan Flagship Store	Electricity savings1	Shipping was fast, stuff was well packaged, great price, I'm happy with it 👍 and will repurchase!	Jingdong prefecture level city in Shandong
Jinfeng Hongyuan Flagship Store	Electricity savings1	The quality of the product is very good and I will definitely come back again next time.	Jingdong prefecture level city in Shandong
Jinfeng Hongyuan Flagship Store	Electricity savings1	I'll follow up with a review after I use it.	Jingdong prefecture level city in Shandong
Jinfeng Hongyuan Flagship Store	Electricity savings1	Hello, the goods are perfectly packaged, save power for a period of time to evaluate again!	Jingdong prefecture level city in Shandong
Jinfeng Hongyuan Flagship Store	Electricity savings1	Not bad, pretty good, good value for money, pretty good	Jingdong prefecture level city in Shandong
Jinfeng Hongyuan Flagship Store	Electricity savings1	The packaging is very good, received immediately used, looking forward to the effect in.	Jingdong prefecture level city in Shandong
Jinfeng Hongyuan Flagship Store	Electricity savings1	See how much power you save at the end of the month	Jingdong prefecture level city in Shandong
Jinfeng Hongyuan Flagship Store	Electricity savings1	Easy to use, affordable, beautiful color	Jingdong prefecture level city in Shandong
Jinfeng Hongyuan Flagship Store	Electricity savings1	It's taking too long to put together an order. It could be an epidemic.	Jingdong prefecture level city in Shandong
Jinfeng Hongyuan Flagship Store	Electricity savings1	I don't feel much after using it, no change in my electric bill 。。。。。。。	Jingdong prefecture level city in Shandong
Jinfeng Hongyuan Flagship Store	Electricity savings1	Logistics is very fast, the quality of the economizer is good, good quality and cheap, it is worth buying, I hope there is a power saving effect!	Jingdong prefecture level city in Shandong
Jinfeng Hongyuan Flagship Store	Electricity savings1	Feeling okay, in a couple of months to try to rely on it before making a comment. Let's give it a five-star review first!	Jingdong prefecture level city in Shandong
Jinfeng Hongyuan Flagship Store	Electricity savings1	Power saving device has been hesitating before in the end to buy or not to buy, after consideration, consideration, in consideration, or decided to buy a try. Things received can not wait to open a look, and the seller described the same, I hope to use 2 months to have the effect, two months to follow up on	Jingdong prefecture level city in Shandong
Jinfeng Hongyuan Flagship Store	Electricity savings1	Thank you Jingdong, logistics is very fast order to receipt only one day. Believe in Jingdong. Favorable comments	Jingdong prefecture level city in Shandong
Jinfeng Hongyuan Flagship Store	Electricity savings1	I used one to use it, but it didn't work very well, what to do	Jingdong prefecture level city in Shandong
Jinfeng Hongyuan Flagship Store	Electricity savings1	Quality and packaging are very good, fast shipping, good service, buy with confidence	Jingdong prefecture level city in Shandong
Jinfeng Hongyuan Flagship Store	Electricity savings1	Use a period of time to evaluate, the quality is very good, the price is affordable, logistics is also very fast	Jingdong prefecture level city in Shandong
Jinfeng Hongyuan Flagship Store	Electricity savings1	Goods received, the day of the order tomorrow delivery real God speed, delivery force, the product to be used after a period of time and then low comment!	Jingdong prefecture level city in Shandong
Jinfeng Hongyuan Flagship Store	Electricity savings1	I don't know, how effective it is, let's talk about it when the time comes, whether it saves power or not, how much, unknown.	Jingdong prefecture level city in Shandong
Jinfeng Hongyuan Flagship Store	Electricity savings1	It does save some electricity, and I don't want to use something that isn't legal! Used nearly ~ electricity last month, probably not much more this month with the AC on!	Jingdong prefecture level city in Shandong
Jinfeng Hongyuan Flagship Store	Electricity savings1	Well-packed, excellent quality, service satisfaction good value for money	Jingdong prefecture level city in Shandong
Jinfeng Hongyuan Flagship Store	Electricity savings1	Express very quickly, the goods received last night, today used a day of goods can not heat, looking forward to whether it can save electricity, with a month and then comment, is used well will buy again.	Jingdong prefecture level city in Shandong
Jinfeng Hongyuan Flagship Store	Electricity savings1	It's pretty good, hopefully it'll be a real power saver.	Jingdong prefecture level city in Shandong
Jinfeng Hongyuan Flagship Store	Electricity savings1	The goods received in use, can not see the effect of energy saving, after a month to know!	Jingdong prefecture level city in Shandong
Jinfeng Hongyuan Flagship Store	Electricity savings1	Received the goods, looking forward to better surprise, with the effect of the follow-up comment!	Jingdong prefecture level city in Shandong
Jinfeng Hongyuan Flagship Store	Electricity savings1	It's not bad, it's still in use and should save some	Jingdong prefecture level city in Shandong
Jinfeng Hongyuan Flagship Store	Electricity savings1	Logistics is very fast, easy to install, plugged in on it,, the effect of the use of the later to evaluate!	Jingdong prefecture level city in Shandong
Jinfeng Hongyuan Flagship Store	Electricity savings1	The stuff is recommendable to buy very good.	Jingdong prefecture level city in Shandong
Jinfeng Hongyuan Flagship Store	Electricity savings1	Voltmeter measurements of voltage are way off, aren't they?	Jingdong prefecture level city in Shandong
Jinfeng Hongyuan Flagship Store	Electricity savings1	Install it to the socket immediately after purchase and try to see if it can save electricity after one month.	Jingdong prefecture level city in Shandong
Jinfeng Hongyuan Flagship Store	Electricity savings1	I've been using it for almost a month now, and I won't know if it saves electricity until I pay the bill.	Jingdong prefecture level city in Shandong
Jinfeng Hongyuan Flagship Store	Electricity savings1	I bought one in the past and it was fine, so I'm buying it back.	Jingdong prefecture level city in Shandong
Jinfeng Hongyuan Flagship Store	Electricity savings1	Service is very good, this thing is small and exquisite to use, just waiting to use the effect of a period of time	Jingdong prefecture level city in Shandong
Jinfeng Hongyuan Flagship Store	Electricity savings1	Not sure how well it works yet	Jingdong prefecture level city in Shandong
Jinfeng Hongyuan Flagship Store	Electricity savings1	Kyodo goods habitually good reviews	Jingdong prefecture level city in Shandong
Jinfeng Hongyuan Flagship Store	Electricity savings1	Appearance is OK, whether it is power saving remains to be seen.	Jingdong prefecture level city in Shandong
Jinfeng Hongyuan Flagship Store	Electricity savings1	I bought it and used it, I just don't know if it really saves power	Jingdong prefecture level city in Shandong

Jinfeng Hongyuan Flagship Store	Electricity savings1	The saver is well made and of good quality, I will evaluate it after using it for a while.	Jingdong prefecture level city in Shandong
Jinfeng Hongyuan Flagship Store	Electricity savings1	Just received in use, use for a period of time to see if there is power saving	Jingdong prefecture level city in Shandong
Jinfeng Hongyuan Flagship Store	Electricity savings1	Used 3 days before commenting, it really saves electricity, I look at the "power calendar" after using one less degree of electricity per day, so I think it's worth it to buy this, I will introduce my friends	Jingdong prefecture level city in Shandong
Jinfeng Hongyuan Flagship Store	Electricity savings1	Reliable quality, very well packaged and matches the real thing.	Jingdong prefecture level city in Shandong
Jinfeng Hongyuan Flagship Store	Electricity savings1	Logistics is still quite fast, ha in the trial	Jingdong prefecture level city in Shandong
Jinfeng Hongyuan Flagship Store	Electricity savings1	Consultant service attitude is very good, the courier is also very powerful, the first day of the order the next day to arrive. Saver received used and then add comments!	Jingdong prefecture level city in Shandong
Jinfeng Hongyuan Flagship Store	Electricity savings1	I just got home to try it out and see how it goes.	Jingdong prefecture level city in Shandong
Jinfeng Hongyuan Flagship Store	Electricity savings1	Well made and weighted instrument. Kudos! Put it into use immediately, don't know if it saves power, will evaluate later!	Jingdong prefecture level city in Shandong
Jinfeng Hongyuan Flagship Store	Electricity savings1	I've just put it in place. I'll give you a review in ten days.	Jingdong prefecture level city in Shandong
Jinfeng Hongyuan Flagship Store	Electricity savings1	It looks very delicate, not bad. Well, I don't know if it can save power or not, I have to try it for a while and see. If it can really save electricity. Uh, if it works well. Then additional comments. Anyway, thanks for the logistics, thanks to Jingdong. Well, it deserves some praise.	Jingdong prefecture level city in Shandong
Jinfeng Hongyuan Flagship Store	Electricity savings1	Bought a month, the feeling is that there is a certain effect, a month to save a lot of money	Jingdong prefecture level city in Shandong
Jinfeng Hongyuan Flagship Store	Electricity savings1	Goods received, the physical and the same as the picture, logistics is also very fast, the service attitude is very good, it is worth buying!	Jingdong prefecture level city in Shandong
Jinfeng Hongyuan Flagship Store	Electricity savings1	I've used it, I don't know whether it saves electricity or not, I'll talk about it after a while.	Jingdong prefecture level city in Shandong
Jinfeng Hongyuan Flagship Store	Electricity savings1	August only began to use, October to know, have no power-saving effect, to the in to evaluate!	Jingdong prefecture level city in Shandong
Jinfeng Hongyuan Flagship Store	Electricity savings1	Goods received: I do not know to the factory products used to save electricity!	Jingdong prefecture level city in Shandong
Jinfeng Hongyuan Flagship Store	Electricity savings1	Don't know if it works, bought it anyway.	Jingdong prefecture level city in Shandong
Jinfeng Hongyuan Flagship Store	Electricity savings1	Still testing, will follow up next month to evaluate how much electricity is saved	Jingdong prefecture level city in Shandong
Jinfeng Hongyuan Flagship Store	Electricity savings1	I'm still testing it, I'll comment after I use it.	Jingdong prefecture level city in Shandong
Jinfeng Hongyuan Flagship Store	Electricity savings1	Fast service, praiseworthy. Good item pre-style, first praise, the use of the effect to be evaluated after use.	Jingdong prefecture level city in Shandong
Jinfeng Hongyuan Flagship Store	Electricity savings1	The power saving effect is more obvious through verified use.	Jingdong prefecture level city in Shandong
Jinfeng Hongyuan Flagship Store	Electricity savings1	Logistics is very fast yesterday order today arrived. The product was immediately used, and after a month to see its effect.	Jingdong prefecture level city in Shandong
Jinfeng Hongyuan Flagship Store	Electricity savings1	Power saving is not yet known, as far as the TV and audio are concerned, I feel that there is a significant quality improvement, and I recommend using a good product!	Jingdong prefecture level city in Shandong
Jinfeng Hongyuan Flagship Store	Electricity savings1	Good power savings, tens of dollars a month saved	Jingdong prefecture level city in Shandong
Jinfeng Hongyuan Flagship Store	Electricity savings1	Already took two, great value! You can save 30% on your electric bill in a month. A summer must-have!	Jingdong prefecture level city in Shandong
Jinfeng Hongyuan Flagship Store	Electricity savings1	Received the item, plugged it into a home outlet and it showed 230 volts (not that high with a multimeter). Let's use it first!	Jingdong prefecture level city in Shandong
Jinfeng Hongyuan Flagship Store	Electricity savings1	Expecting results in the experiment	Jingdong prefecture level city in Shandong
Jinfeng Hongyuan Flagship Store	Electricity savings1	I don't know if it really saves power.	Jingdong prefecture level city in Shandong
Jinfeng Hongyuan Flagship Store	Electricity savings1	It's very good and the efficacy is very visible.	Jingdong prefecture level city in Shandong
Jinfeng Hongyuan Flagship Store	Electricity savings1	It's good and worth buying with confidence	Jingdong prefecture level city in Shandong
Jinfeng Hongyuan Flagship Store	Electricity savings1	The goods have been received, just do not know if it is effective	Jingdong prefecture level city in Shandong
Jinfeng Hongyuan Flagship Store	Electricity savings1	It's already in use, but based on some of the descriptions, I suspect that to use it, you need to have one in series on each line, which seems to work for all appliances, that's my guess, but I feel as though the principle of the thing seems to require it.	Jingdong prefecture level city in Shandong
Jinfeng Hongyuan Flagship Store	Electricity savings1	Fast delivery, already in use, looks good!	Jingdong prefecture level city in Shandong
Jinfeng Hongyuan Flagship Store	Electricity savings1	Hoping to save 30% of power as presented in the graphic	Jingdong prefecture level city in Shandong
Jinfeng Hongyuan Flagship Store	Electricity savings1	Things look good, power saving do not know now, with a month after the continued evaluation	Jingdong prefecture level city in Shandong
Jinfeng Hongyuan Flagship Store	Electricity savings1	Already used it but not sure if it will top out!	Jingdong prefecture level city in Shandong
Jinfeng Hongyuan Flagship Store	Electricity savings1	Looking forward to using the results, the product quality looks okay.	Jingdong prefecture level city in Shandong
Jinfeng Hongyuan Flagship Store	Electricity savings1	It's somewhat effective and saves money more or less 💰	Jingdong prefecture level city in Shandong
Jinfeng Hongyuan Flagship Store	Electricity savings1	Got it, it's great, looking forward to saving power.	Jingdong prefecture level city in Shandong
Jinfeng Hongyuan Flagship Store	Electricity savings1	I don't know if it's working yet, I'll follow up when I get there	Jingdong prefecture level city in Shandong
Jinfeng Hongyuan Flagship Store	Electricity savings1	The product received, good workmanship, fast logistics, good inner packaging, the outer bag is damaged, but fortunately did not hurt the product, retrieved and inserted according to the instructions, looking forward to the effect is good, with a period of time to evaluate, I believe that the Jingdong.	Jingdong prefecture level city in Shandong
Jinfeng Hongyuan Flagship Store	Electricity savings1	Since it is a new product, it is said to work, so if it works well for a month, then I will give you a good review.	Jingdong prefecture level city in Shandong
Jinfeng Hongyuan Flagship Store	Electricity savings1	The quality of the product is very good, very well made, well packaged, easy to use and very convenient!	Jingdong prefecture level city in Shandong
Jinfeng Hongyuan Flagship Store	Electricity savings1	Good price for the item, good functionality	Jingdong prefecture level city in Shandong
Jinfeng Hongyuan Flagship Store	Electricity savings1	Received the first time to use it, the stuff is very good, specifics wait for a month to come back to feedback to see the effect.	Jingdong prefecture level city in Shandong
Jinfeng Hongyuan Flagship Store	Electricity savings1	Just received, the courier is too bad, not notified directly to put the Feng Nest courier cabinet	Jingdong prefecture level city in Shandong
Jinfeng Hongyuan Flagship Store	Electricity savings1	Baby received, the second time to buy, plugged in and used do not know if there is no effect. Baby received, the second time to buy, plugged in and used do not know if there is no effect. Baby received, the second time to buy, plugged in and used do not know if there is no effect.	Jingdong prefecture level city in Shandong
Jinfeng Hongyuan Flagship Store	Electricity savings1	It's okay if it saves power. It's not a bad product.	Jingdong prefecture level city in Shandong
Jinfeng Hongyuan Flagship Store	Electricity savings1	Received, very good, meticulous packaging, open a look is genuine, meticulous workmanship, the manual is very detailed, has been used, there is no effect on the only look at the next month's electricity bill, when I can I buy a few more to send people!	Jingdong prefecture level city in Shandong
Jinfeng Hongyuan Flagship Store	Electricity savings1	The quality is very good very delicate express delivery is too fast	Jingdong prefecture level city in Shandong

Jinfeng Hongyuan Flagship Store	Electricity savings1	I won't know until a month after I start using it today.	Jingdong prefecture level city in Shandong
Jinfeng Hongyuan Flagship Store	Electricity savings1	It doesn't work at all, the customer service attitude is very bad, use it or not!	Jingdong prefecture level city in Shandong
Jinfeng Hongyuan Flagship Store	Electricity savings1	Temporarily try, do not know the effect, Jingdong logistics delivery very quickly	Jingdong prefecture level city in Shandong
Jinfeng Hongyuan Flagship Store	Electricity savings1	It doesn't help. It's comforting.	Jingdong prefecture level city in Shandong
Jinfeng Hongyuan Flagship Store	Electricity savings1	It's great, use it before you say anything.	Jingdong prefecture level city in Shandong
Jinfeng Hongyuan Flagship Store	Electricity savings1	Received, already used, looking forward to a good a effect!	Jingdong prefecture level city in Shandong
Jinfeng Hongyuan Flagship Store	Electricity savings1	I don't know if it works yet, I won't know until I use it for a few months.	Jingdong prefecture level city in Shandong
Jinfeng Hongyuan Flagship Store	Electricity savings1	It takes a long time to get there. There's no change now.	Jingdong prefecture level city in Shandong
Jinfeng Hongyuan Flagship Store	Electricity savings1	Jingdong's delivery never disappoints, or so fast, and the stuff is great, next time you need to come back.	Jingdong prefecture level city in Shandong
Jinfeng Hongyuan Flagship Store	Electricity savings1	At first it was ok today, after using it for a while it didn't work anymore	Jingdong prefecture level city in Shandong
Jinfeng Hongyuan Flagship Store	Electricity savings1	Just received the goods, do not know the effect of using a few days before there may be effect	Jingdong prefecture level city in Shandong
Jinfeng Hongyuan Flagship Store	Electricity savings1	The logistics was fast, I received it the next day, I've purchased it for the second time and have been using it all along	Jingdong prefecture level city in Shandong
Jinfeng Hongyuan Flagship Store	Electricity savings1	Inexpensive, fast delivery, very pleased!	Jingdong prefecture level city in Shandong
Jinfeng Hongyuan Flagship Store	Electricity savings1	Received the goods immediately plugged directly into the use of power saving yet to be good news!	Jingdong prefecture level city in Shandong
Jinfeng Hongyuan Flagship Store	Electricity savings1	The service attitude is very good, and the Jingdong self-owned products can be trusted.	Jingdong prefecture level city in Shandong
Jinfeng Hongyuan Flagship Store	Electricity savings1	The product is good, I do not know if it can save play a power saving effect	Jingdong prefecture level city in Shandong
Jinfeng Hongyuan Flagship Store	Electricity savings1	We won't know how effective it is until we use it for a while, so I'll give you a good review now.	Jingdong prefecture level city in Shandong
Jinfeng Hongyuan Flagship Store	Electricity savings1	The quality is pretty good, just started using it, hopefully it will deliver results.	Jingdong prefecture level city in Shandong
Jinfeng Hongyuan Flagship Store	Electricity savings1	I've plugged it in first, and will follow up after I've used it to see how it works.	Jingdong prefecture level city in Shandong
Jinfeng Hongyuan Flagship Store	Electricity savings1	Things have arrived, the quality is good, the effect is also equivalent to can ah	Jingdong prefecture level city in Shandong
Jinfeng Hongyuan Flagship Store	Electricity savings1	It's not worth it to brag about power savings.	Jingdong prefecture level city in Shandong
Jinfeng Hongyuan Flagship Store	Electricity savings1	I don't know if it's an IQ tax, but I'll use it for now.	Jingdong prefecture level city in Shandong
Jinfeng Hongyuan Flagship Store	Electricity savings1	I don't feel the power saving yet, I can't know until I use it for 2 months.	Jingdong prefecture level city in Shandong
Jinfeng Hongyuan Flagship Store	Electricity savings1	There's nothing wrong with this at the moment.	Jingdong prefecture level city in Shandong
Jinfeng Hongyuan Flagship Store	Electricity savings1	It's authentic and worth buying	Jingdong prefecture level city in Shandong
Jinfeng Hongyuan Flagship Store	Electricity savings1	It's good. We'll see how it goes.	Jingdong prefecture level city in Shandong
Jinfeng Hongyuan Flagship Store	Electricity savings1	I don't know if it's working or not in the middle of using it, we'll see when the month is over	Jingdong prefecture level city in Shandong
Jinfeng Hongyuan Flagship Store	Electricity savings1	I'm not going to buy anything from Jingdong anymore, so I'll give it a good review	Jingdong prefecture level city in Shandong
Jinfeng Hongyuan Flagship Store	Electricity savings1	I'm not going to buy anything from Jingdong anymore, so I'll give it a good review	Jingdong prefecture level city in Shandong
Jinfeng Hongyuan Flagship Store	Electricity savings1	Just received the item and will see how it works in a month.	Jingdong prefecture level city in Shandong
Jinfeng Hongyuan Flagship Store	Electricity savings1	I don't know if it saves electricity, but it's plugged into an outlet every day anyway	Jingdong prefecture level city in Shandong
Jinfeng Hongyuan Flagship Store	Electricity savings1	I don't know if it works yet.	Jingdong prefecture level city in Shandong
Jinfeng Hongyuan Flagship Store	Electricity savings1	Plugged it in when I got back, waiting for a month to see the results	Jingdong prefecture level city in Shandong
Jinfeng Hongyuan Flagship Store	Electricity savings1	Effects remain to be seen, but it looks good quality and runs fine	Jingdong prefecture level city in Shandong
Jinfeng Hongyuan Flagship Store	Electricity savings1	The product received logistics very quickly, has not yet been used to test! After use in the evaluation!	Jingdong prefecture level city in Shandong
Jinfeng Hongyuan Flagship Store	Electricity savings1	The power saver is in use, and I don't know how much electricity has been saved after using it for more than a month, and the display voltmeter is very good.	Jingdong prefecture level city in Shandong
Jinfeng Hongyuan Flagship Store	Electricity savings1	It's so good, isn't it? You're a real Shanghainese, aren't you?	Jingdong prefecture level city in Shandong
Jinfeng Hongyuan Flagship Store	Electricity savings1	Great quality great great 👍👍👍👍	Jingdong prefecture level city in Shandong
Jinfeng Hongyuan Flagship Store	Electricity savings1	I'm using it, I don't know if it's working yet.	Jingdong prefecture level city in Shandong
Jinfeng Hongyuan Flagship Store	Electricity savings1	Just used it for more than 10 days and haven't seen any results yet	Jingdong prefecture level city in Shandong
Jinfeng Hongyuan Flagship Store	Electricity savings1	The product arrived home feeling like it should work	Jingdong prefecture level city in Shandong
Jinfeng Hongyuan Flagship Store	Electricity savings1	Just received the goods, use it and comment back	Jingdong prefecture level city in Shandong
Jinfeng Hongyuan Flagship Store	Electricity savings1	Try it for a month and then evaluate	Jingdong prefecture level city in Shandong
Jinfeng Hongyuan Flagship Store	Electricity savings1	I don't know if it saves power yet, but I'll comment again after using it for a while.	Jingdong prefecture level city in Shandong
Jinfeng Hongyuan Flagship Store	Electricity savings1	The product is better than I thought, the quality and appearance are very good, the voltage is stable, and I'm looking forward to a good power saving effect.	Jingdong prefecture level city in Shandong
Jinfeng Hongyuan Flagship Store	Electricity savings1	Received and scanned the QR code to check that it is genuine, have plugged it in, can it save power, looking forward to the advertised effect. Customary praise, 5 points!	Jingdong prefecture level city in Shandong
Jinfeng Hongyuan Flagship Store	Electricity savings1	The goods have been received and have not yet been used. Do not know how the effect.	Jingdong prefecture level city in Shandong
Jinfeng Hongyuan Flagship Store	Electricity savings1	Hopefully, you get what you pay for, and you get what you pay for!	Jingdong prefecture level city in Shandong
Jinfeng Hongyuan Flagship Store	Electricity savings1	Just put it on and it feels like it's turning as fast as a meter! Looking forward to the results!	Jingdong prefecture level city in Shandong
Jinfeng Hongyuan Flagship Store	Electricity savings1	Overall look on the product quality is good, I do not know is not the effect of power saving, wait to see and then follow up!	Jingdong prefecture level city in Shandong
Jinfeng Hongyuan Flagship Store	Electricity savings1	The logistics courier is very fast, the courier service attitude is very good	Jingdong prefecture level city in Shandong

Jinfeng Hongyuan Flagship Store	Electricity savings1	The product has been received, it works well worth buying	Jingdong prefecture level city in Shandong
Jinfeng Hongyuan Flagship Store	Electricity savings1	How did it not work? Is it purely an IQ tax?	Jingdong prefecture level city in Shandong
Jinfeng Hongyuan Flagship Store	Electricity savings1	It looks good, I don't know how well it works.	Jingdong prefecture level city in Shandong
Jinfeng Hongyuan Flagship Store	Electricity savings1	It does seem to be a bit of a power saver, I just don't know if the more expensive one is more power efficient?	Jingdong prefecture level city in Shandong
Jinfeng Hongyuan Flagship Store	Electricity savings1	Is genuine very good, the price is much cheaper, cost-effective, really special paddle just to do activities, the price is much cheaper, cost-effective, really special paddle counting, packaging is also very high-end atmospheric class, it is worth buying!	Jingdong prefecture level city in Shandong
Jinfeng Hongyuan Flagship Store	Electricity savings1	I received it yesterday and used it right away, I hope it works, I'll wait for next month to come back to the conclusion, I'm looking forward to it. Five hearts praise!	Jingdong prefecture level city in Shandong
Jinfeng Hongyuan Flagship Store	Electricity savings1	Looks good, packed properly, received it today, am using it, will see results in a month!	Jingdong prefecture level city in Shandong
Jinfeng Hongyuan Flagship Store	Electricity savings1	Regular product Jingdong self-support trustworthy. There is an effect Come back to the review. 😍😍😍😍	Jingdong prefecture level city in Shandong
Jinfeng Hongyuan Flagship Store	Electricity savings1	I haven't used it yet, hopefully it will work as a power saver, and will comment again if it works well.	Jingdong prefecture level city in Shandong
Jinfeng Hongyuan Flagship Store	Electricity savings1	Shipping was fast, logistics was fast, service was good, and I look forward to good results from the item!	Jingdong prefecture level city in Shandong
Jinfeng Hongyuan Flagship Store	Electricity savings1	This product is this compensator, it has to be at the main switch to be useful, haven't compared the final result, the	Jingdong prefecture level city in Shandong
Jinfeng Hongyuan Flagship Store	Electricity savings1	Packaging is perfect . I've already started using it, so I'm looking forward to the results.	Jingdong prefecture level city in Shandong
Jinfeng Hongyuan Flagship Store	Electricity savings1	I just got it back. I'll comment after I use it.	Jingdong prefecture level city in Shandong
Jinfeng Hongyuan Flagship Store	Electricity savings1	Big brand, with a very assured, very good quality, catch the event to buy very cost-effective to take advantage of the offer, hurry to get, it is genuine, the packaging is also very good, no breakage, the quality is not to speak of, great 👍 logistics super super fast! Quality is also very good, indeed value	Jingdong prefecture level city in Shandong
Jinfeng Hongyuan Flagship Store	Electricity savings1	The quality is pretty good. The price is also quite affordable.	Jingdong prefecture level city in Shandong
Jinfeng Hongyuan Flagship Store	Electricity savings1	I did not expect the courier so fast to arrive yesterday just ordered, the head is a little smaller than I thought, well, the picture looks bigger. The appearance still looks good, as for the practicality and effectiveness of the use of the only know.	Jingdong prefecture level city in Shandong
Jinfeng Hongyuan Flagship Store	Electricity savings1	I don't know. Looking forward to it.	Jingdong prefecture level city in Shandong
Jinfeng Hongyuan Flagship Store	Electricity savings1	Baby received, very delicate, very nice, like. Just don't know if it can really save electricity. Baby received, very delicate, very good-looking, like. Just don't know if it can really save electricity.	Jingdong prefecture level city in Shandong
Jinfeng Hongyuan Flagship Store	Electricity savings1	I'll use it for a month and then comment on the quality of the item	Jingdong prefecture level city in Shandong
Jinfeng Hongyuan Flagship Store	Electricity savings1	It feels ok, use it for a while and see how it works before adding more comments.	Jingdong prefecture level city in Shandong
Jinfeng Hongyuan Flagship Store	Electricity savings1	In use, see results in a month.	Jingdong prefecture level city in Shandong
Jinfeng Hongyuan Flagship Store	Electricity savings1	Jingdong's logistics and quality are very good, the service attitude is very good, the logistics is very fast	Jingdong prefecture level city in Shandong
Jinfeng Hongyuan Flagship Store	Electricity savings1	Intelligent power saver received, well-packed logistics is also quite fast, easy to use, looking forward to good results!	Jingdong prefecture level city in Shandong
Jinfeng Hongyuan Flagship Store	Electricity savings1	The quality is unknown at this point, I won't know until I use it.	Jingdong prefecture level city in Shandong
Jinfeng Hongyuan Flagship Store	Electricity savings1	It's a good product. It really saves power and doesn't take up much space.	Jingdong prefecture level city in Shandong
Jinfeng Hongyuan Flagship Store	Electricity savings1	Goods have been received, packaging is very good, good to see the effect of future use in the follow-up.	Jingdong prefecture level city in Shandong
Jinfeng Hongyuan Flagship Store	Electricity savings1	The product is in perfect shape and the light weight is worth it!	Jingdong prefecture level city in Shandong
Jinfeng Hongyuan Flagship Store	Electricity savings1	Just got home and plugged it in. It's good.	Jingdong prefecture level city in Shandong
Jinfeng Hongyuan Flagship Store	Electricity savings1	The logistics are quick, just to see the results.	Jingdong prefecture level city in Shandong
Jinfeng Hongyuan Flagship Store	Electricity savings1	Short time can not feel here to leave a message, to be followed up later!	Jingdong prefecture level city in Shandong
Jinfeng Hongyuan Flagship Store	Electricity savings1	This user thinks the product is good	Jingdong prefecture level city in Shandong
Jinfeng Hongyuan Flagship Store	Electricity savings1	Received the baby, to be used to see the effect of it!	Jingdong prefecture level city in Shandong
Jinfeng Hongyuan Flagship Store	Electricity savings1	You have to wait a month or two to see how it works.	Jingdong prefecture level city in Shandong
Jinfeng Hongyuan Flagship Store	Electricity savings1	Not bad, good, like it, don't know if it's good, try it first.	Jingdong prefecture level city in Shandong
Jinfeng Hongyuan Flagship Store	Electricity savings1	The goods are good value for money and the logistics is very fast and good service!	Jingdong prefecture level city in Shandong
Jinfeng Hongyuan Flagship Store	Electricity savings1	I don't know how well it works, we'll see in a month	Jingdong prefecture level city in Shandong
Jinfeng Hongyuan Flagship Store	Electricity savings1	The product is fine, but r saving power or not I won't know until I use it for two months. The display and lights take electricity too?	Jingdong prefecture level city in Shandong
Jinfeng Hongyuan Flagship Store	Electricity savings1	I've used it. I don't know how it works yet.	Jingdong prefecture level city in Shandong
Jinfeng Hongyuan Flagship Store	Electricity savings1	I can't say until I compare them.	Jingdong prefecture level city in Shandong
Jinfeng Hongyuan Flagship Store	Electricity savings1	It's ok in the process of using 。。。。	Jingdong prefecture level city in Shandong
Jinfeng Hongyuan Flagship Store	Electricity savings1	Jinfeng Hongyuan Intelligent Power Saver Household Electricity Meter Electricity Saving Appliance High Power Enhanced Power Saving King Energy Saving Treasure Butler Control Non Stealing Electricity Reversing Not Going Interfering Slow Turner Artifact King Gold Yellow. Haven't tested it accurately yet, really need to wait a few months to test it before I have a say.	Jingdong prefecture level city in Shandong
Jinfeng Hongyuan Flagship Store	Electricity savings1	Don't know how effective it is yet? I hope it works well and I'll come back next time	Jingdong prefecture level city in Shandong
Jinfeng Hongyuan Flagship Store	Electricity savings1	Saving device has been received, material workmanship are very good ah! Have not yet begun to use. First observe the daily electricity consumption and cost of the home meter, and then plug in the power saver to use, so as to compare the use of the effect is good!	Jingdong prefecture level city in Shandong
Jinfeng Hongyuan Flagship Store	Electricity savings1	Merchants shipping extremely fast, Jingdong logistics safe and fast, I believe that the product can play a safe use of electricity, the role of scientific power saving, first praise, the effect of how to be used for a period of time and then the pursuit of bubble!	Jingdong prefecture level city in Shandong
Jinfeng Hongyuan Flagship Store	Electricity savings1	I just bought it. I don't know yet.	Jingdong prefecture level city in Shandong
Jinfeng Hongyuan Flagship Store	Electricity savings1	It is genuine, good quality, and it is very reassuring to use. The logistics is very fast, and the business service attitude is on point.	Jingdong prefecture level city in Shandong
Jinfeng Hongyuan Flagship Store	Electricity savings1	The quality is fine, I don't know how effective it is.	Jingdong prefecture level city in Shandong
Jinfeng Hongyuan Flagship Store	Electricity savings1	The stuff is back and I expect it will save the electricity	Jingdong prefecture level city in Shandong

Jinfeng Hongyuan Flagship Store	Electricity savings1	I'll give it 4 stars first and see what happens in a couple of months and then follow up	Jingdong prefecture level city in Shandong
Jinfeng Hongyuan Flagship Store	Electricity savings1	It's not bad. It's in the mind.	Jingdong prefecture level city in Shandong
Jinfeng Hongyuan Flagship Store	Electricity savings1	Received the goods have not yet started to use do not know whether it is good	Jingdong prefecture level city in Shandong
Jinfeng Hongyuan Flagship Store	Electricity savings1	This user thinks the product is good	Jingdong prefecture level city in Shandong
Jinfeng Hongyuan Flagship Store	Electricity savings1	Hello! The saver was received, thank you! Not yet used, but from the packaging looks very good quality, exquisite workmanship. Logistics is quite fast, if it is really very power saving, I will come back to	Jingdong prefecture level city in Shandong
Jinfeng Hongyuan Flagship Store	Electricity savings1	I've been using it for a few days now, I'll comment again after a month or two of use	Jingdong prefecture level city in Shandong
Jinfeng Hongyuan Flagship Store	Electricity savings1	I don't know. Bought it for my father at a good price.	Jingdong prefecture level city in Shandong
Jinfeng Hongyuan Flagship Store	Electricity savings1	Plugged it in the same day I bought it and the voltage showed 223, 225 looking forward to the results	Jingdong prefecture level city in Shandong
Jinfeng Hongyuan Flagship Store	Electricity savings1	Still in use, the biggest role is to stabilize the current, reduce losses, product quality on the look is very good, with this feeling quite good, see colleagues are using, power saving effect should be good	Jingdong prefecture level city in Shandong
Jinfeng Hongyuan Flagship Store	Electricity savings1	How to receive goods but no invoice and warranty more than it ...... quality is the premise of reputation!	Jingdong prefecture level city in Shandong
Jinfeng Hongyuan Flagship Store	Electricity savings1	Received but not used Continued evaluation	Jingdong prefecture level city in Shandong
Jinfeng Hongyuan Flagship Store	Electricity savings1	I just received the product, I don't know if it works well yet	Jingdong prefecture level city in Shandong
Jinfeng Hongyuan Flagship Store	Electricity savings1	Received it and used it, not sure it really saves electricity use it and then evaluate it, first give a 5 star review!!!!	Jingdong prefecture level city in Shandong
Jinfeng Hongyuan Flagship Store	Electricity savings1	It's good to use, it's very good to use, and it smells OK too	Jingdong prefecture level city in Shandong
Jinfeng Hongyuan Flagship Store	Electricity savings1	Packaging is very good, feel the product is not bad, how much power saving with the only know	Jingdong prefecture level city in Shandong
Jinfeng Hongyuan Flagship Store	Electricity savings1	Additional comments to be made after use	Jingdong prefecture level city in Shandong
Jinfeng Hongyuan Flagship Store	Electricity savings1	The product has been installed, try a few months and then comment!	Jingdong prefecture level city in Shandong
Jinfeng Hongyuan Flagship Store	Electricity savings1	I've bought it, let's use it for now, I'll comment after a month	Jingdong prefecture level city in Shandong
Jinfeng Hongyuan Flagship Store	Electricity savings1	Try it out and then follow up	Jingdong prefecture level city in Shandong
Jinfeng Hongyuan Flagship Store	Electricity savings1	It's good in theory, it doesn't feel good in practice.	Jingdong prefecture level city in Shandong
Jinfeng Hongyuan Flagship Store	Electricity savings1	I haven't tried it yet. We'll see.	Jingdong prefecture level city in Shandong
Jinfeng Hongyuan Flagship Store	Electricity savings1	Great item, second purchase!	Jingdong prefecture level city in Shandong
Jinfeng Hongyuan Flagship Store	Electricity savings1	Goods received now use it, if it does save power, plus reevaluate.	Jingdong prefecture level city in Shandong
Jinfeng Hongyuan Flagship Store	Electricity savings1	The stuff is light and small, the stuff is good, the courier service is good	Jingdong prefecture level city in Shandong
Jinfeng Hongyuan Flagship Store	Electricity savings1	I haven't used it yet. I'm looking forward to it.	Jingdong prefecture level city in Shandong
Jinfeng Hongyuan Flagship Store	Electricity savings1	Express received, in use do not know if there is no power saving	Jingdong prefecture level city in Shandong
Jinfeng Hongyuan Flagship Store	Electricity savings1	The quality of the product is quite good! The power saving mode is still getting used to it, can't say yet, how much power it saves.	Jingdong prefecture level city in Shandong
Jinfeng Hongyuan Flagship Store	Electricity savings1	The goods have been received, just started to use, now I do not know how the quality, after a period of time to say.	Jingdong prefecture level city in Shandong
Jinfeng Hongyuan Flagship Store	Electricity savings1	I received the stuff, used it for a few days to evaluate, the stuff is very good and good, worth buying.	Jingdong prefecture level city in Shandong
Jinfeng Hongyuan Flagship Store	Electricity savings1	I've been using it for a while, but I don't know if it's good or if it saves electricity! What the heck, let's give it a good review first. And for the record, I'm not helping him**.	Jingdong prefecture level city in Shandong
Jinfeng Hongyuan Flagship Store	Electricity savings1	Haven't used it yet, looks good, try it for 60 days, might be good, great review!	Jingdong prefecture level city in Shandong
Jinfeng Hongyuan Flagship Store	Electricity savings1	Baby received, logistics is very fast. Ordered last night, received this morning. Received the goods immediately plugged in the power supply, save power to the first of next month to know.	Jingdong prefecture level city in Shandong
Jinfeng Hongyuan Flagship Store	Electricity savings1	First, I'll give you a good review and use it.	Jingdong prefecture level city in Shandong
Jinfeng Hongyuan Flagship Store	Electricity savings1	Just arrived to follow up with a genuine review	Jingdong prefecture level city in Shandong
Jinfeng Hongyuan Flagship Store	Electricity savings1	Not bad pretty fast, good quality.	Jingdong prefecture level city in Shandong
Jinfeng Hongyuan Flagship Store	Electricity savings1	Recommended, use it and hopefully it will save some electricity	Jingdong prefecture level city in Shandong
Jinfeng Hongyuan Flagship Store	Electricity savings1	The stuff looks ok, I don't know the effect if it.	Jingdong prefecture level city in Shandong
Jinfeng Hongyuan Flagship Store	Electricity savings1	The stuff looks OK, the stuff looks OK.	Jingdong prefecture level city in Shandong
Jinfeng Hongyuan Flagship Store	Electricity savings1	The item has been received, after using it in a follow-up review. Satisfied with the purchase.	Jingdong prefecture level city in Shandong
Jinfeng Hongyuan Flagship Store	Electricity savings1	Has been normal plug-in use, at present do not know how to see, use down to see and then evaluation	Jingdong prefecture level city in Shandong
Jinfeng Hongyuan Flagship Store	Electricity savings1	Commodities make noise.	Jingdong prefecture level city in Shandong
Jinfeng Hongyuan Flagship Store	Electricity savings1	After using it for a few days and observing it, the saving is about ten percent	Jingdong prefecture level city in Shandong
Jinfeng Hongyuan Flagship Store	Electricity savings1	The goods received, the quality looks quite OK. First use it, the effect is not good to say.	Jingdong prefecture level city in Shandong
Jinfeng Hongyuan Flagship Store	Electricity savings1	In use, follow up next month	Jingdong prefecture level city in Shandong
Jinfeng Hongyuan Flagship Store	Electricity savings1	I just received the goods and have not yet used it to comment after using it	Jingdong prefecture level city in Shandong
Jinfeng Hongyuan Flagship Store	Electricity savings1	So far I don't know if it really saves electricity yet! Spinning waiting a couple months to find out! Good reviews first!	Jingdong prefecture level city in Shandong
Jinfeng Hongyuan Flagship Store	Electricity savings1	Small and exquisite, looking forward to the effect, to be evaluated 6666666666666	Jingdong prefecture level city in Shandong
Jinfeng Hongyuan Flagship Store	Electricity savings1	I just used it, I don't know if it's effective, I'll comment again after a month	Jingdong prefecture level city in Shandong
Jinfeng Hongyuan Flagship Store	Electricity savings1	I just used it, I don't know if it's good or not, I'll comment later.	Jingdong prefecture level city in Shandong
Jinfeng Hongyuan Flagship Store	Electricity savings1	Baby received, Baobei quite good, use and then come back to comment!	Jingdong prefecture level city in Shandong
Jinfeng Hongyuan Flagship Store	Electricity savings1	Good quality power saving is fake	Jingdong prefecture level city in Shandong

Jinfeng Hongyuan Flagship Store	Electricity savings1	The packaging of the goods is atmospheric, the quality is exquisite, and I look forward to saving electricity.	Jingdong prefecture level city in Shandong
Jinfeng Hongyuan Flagship Store	Electricity savings1	The packaging is great and the quality is good!	Jingdong prefecture level city in Shandong
Jinfeng Hongyuan Flagship Store	Electricity savings1	I don't see any effect.	Jingdong prefecture level city in Shandong
Jinfeng Hongyuan Flagship Store	Electricity savings1	Already used, but did not see obvious results	Jingdong prefecture level city in Shandong
Jinfeng Hongyuan Flagship Store	Electricity savings1	It's good. I'm just using it. I don't know what it's like.	Jingdong prefecture level city in Shandong
Jinfeng Hongyuan Flagship Store	Electricity savings1	I just used it. I'll comment later.	Jingdong prefecture level city in Shandong
Jinfeng Hongyuan Flagship Store	Electricity savings1	I installed it when it arrived, try it for a month and then come back for a follow-up review	Jingdong prefecture level city in Shandong
Jinfeng Hongyuan Flagship Store	Electricity savings1	Goods arrived a few days fastidious does not call to pick up the goods, the quality is average	Jingdong prefecture level city in Shandong
Jinfeng Hongyuan Flagship Store	Electricity savings1	This user thinks the product is good	Jingdong prefecture level city in Shandong
Jinfeng Hongyuan Flagship Store	Electricity savings1	Roger that.	Jingdong prefecture level city in Shandong
Jinfeng Hongyuan Flagship Store	Electricity savings1	Second purchase was ok.	Jingdong prefecture level city in Shandong
Jinfeng Hongyuan Flagship Store	Electricity savings1	The merchant is very reputable and the product is good value for money. Logistics is fast!	Jingdong prefecture level city in Shandong
Jinfeng Hongyuan Flagship Store	Electricity savings1	The quality of the baby is very good, I have always trusted Kyodo	Jingdong prefecture level city in Shandong
Jinfeng Hongyuan Flagship Store	Electricity savings1	We'll see how it goes when we get it.	Jingdong prefecture level city in Shandong
Jinfeng Hongyuan Flagship Store	Electricity savings1	I just used it. I don't know if it works.	Jingdong prefecture level city in Shandong
Jinfeng Hongyuan Flagship Store	Electricity savings1	Good quality, used for a month to save about 20%, power saving effect is good	Jingdong prefecture level city in Shandong
Jinfeng Hongyuan Flagship Store	Electricity savings1	I'll give you a good review before I use it.	Jingdong prefecture level city in Shandong
Jinfeng Hongyuan Flagship Store	Electricity savings1	Received, satisfied with the follow-up evaluation	Jingdong prefecture level city in Shandong
Jinfeng Hongyuan Flagship Store	Electricity savings1	Immediate results, saving electricity and money	Jingdong prefecture level city in Shandong
Jinfeng Hongyuan Flagship Store	Electricity savings1	Measured to save a little	Jingdong prefecture level city in Shandong
Jinfeng Hongyuan Flagship Store	Electricity savings1	This user thinks the product is good	Jingdong prefecture level city in Shandong
Jinfeng Hongyuan Flagship Store	Electricity savings1	This user thinks the product is good	Jingdong prefecture level city in Shandong
Jinfeng Hongyuan Flagship Store	Electricity savings1	Product quality and logistics are very good, worth buying	Jingdong prefecture level city in Shandong
Jinfeng Hongyuan Flagship Store	Electricity savings1	Appearance looks ok! Express delivery was fast! Service is still relatively enthusiastic.	Jingdong prefecture level city in Shandong
Jinfeng Hongyuan Flagship Store	Electricity savings1	It doesn't feel like it's working.	Jingdong prefecture level city in Shandong
Jinfeng Hongyuan Flagship Store	Electricity savings1	Well made and looks good. Used it with other appliances and felt as if it had some effect.	Jingdong prefecture level city in Shandong
Jinfeng Hongyuan Flagship Store	Electricity savings1	Haven't used it yet, will follow up later	Jingdong prefecture level city in Shandong
Jinfeng Hongyuan Flagship Store	Electricity savings1	Things received, used a few days the plug is a little hot unplugged did not work	Jingdong prefecture level city in Shandong
Jinfeng Hongyuan Flagship Store	Electricity savings1	Look at the meter again in a month.	Jingdong prefecture level city in Shandong
Jinfeng Hongyuan Flagship Store	Electricity savings1	This one didn't work at all.	Jingdong prefecture level city in Shandong
Jinfeng Hongyuan Flagship Store	Electricity savings1	It's small. I've seen friends with much bigger ones.	Jingdong prefecture level city in Shandong
Jinfeng Hongyuan Flagship Store	Electricity savings1	The item was received and the quality is good, it will be nice to see how the power saving rate is down the road, the item is great.	Jingdong prefecture level city in Shandong
Jinfeng Hongyuan Flagship Store	Electricity savings1	Cheap and affordable quality is quite good, logistics is also very fast, praise! , baby good 😉, cost-effective very high, big brand trustworthy 👍	Jingdong prefecture level city in Shandong
Jinfeng Hongyuan Flagship Store	Electricity savings1	Used it for a while it really saves electricity ⚡ thanks 🈶 such a good product, compact and practical, every family should buy one ✨	Jingdong prefecture level city in Shandong
Jinfeng Hongyuan Flagship Store	Electricity savings1	Very satisfied with the purchase Next time I will come back	Jingdong prefecture level city in Shandong
Jinfeng Hongyuan Flagship Store	Electricity savings1	Logistics is fast. Good quality, used one before and it does save power	Jingdong prefecture level city in Shandong
Jinfeng Hongyuan Flagship Store	Electricity savings1	I don't know if it works.	Jingdong prefecture level city in Shandong
Jinfeng Hongyuan Flagship Store	Electricity savings1	Good goods, very solid, good.	Jingdong prefecture level city in Shandong
Jinfeng Hongyuan Flagship Store	Electricity savings1	This user thinks the product is good	Jingdong prefecture level city in Shandong
Jinfeng Hongyuan Flagship Store	Electricity savings1	I've been using it for half a month, my electricity bill is much lower, and the quality is very good, so don't worry about buying it!	Jingdong prefecture level city in Shandong
Jinfeng Hongyuan Flagship Store	Electricity savings1	I received the goods today and used it right away, looking forward to the effect	Jingdong prefecture level city in Shandong
Jinfeng Hongyuan Flagship Store	Electricity savings1	The merchandise is great. I am going to recommend it to my friends! Will buy again next time.	Jingdong prefecture level city in Shandong
Jinfeng Hongyuan Flagship Store	Electricity savings1	Let's give it a good review first, it's working fine so far, and I'll follow up with a review,...	Jingdong prefecture level city in Shandong
Jinfeng Hongyuan Flagship Store	Electricity savings1	Product quality is very good, looking forward to using the results	Jingdong prefecture level city in Shandong
Jinfeng Hongyuan Flagship Store	Electricity savings1	I just can't detect it. - Save it.	Jingdong prefecture level city in Shandong
Jinfeng Hongyuan Flagship Store	Electricity savings1	It arrived and is being used, so we'll see how it works!	Jingdong prefecture level city in Shandong
Jinfeng Hongyuan Flagship Store	Electricity savings1	Received yesterday thanks! This energy saver is available . Safe! But in the end how much energy can be saved must be used for a period of time to know, if the real energy saving next time to buy again.	Jingdong prefecture level city in Shandong
Jinfeng Hongyuan Flagship Store	Electricity savings1	Just used it, don't know if it saves power	Jingdong prefecture level city in Shandong
Jinfeng Hongyuan Flagship Store	Electricity savings1	I'll rate it first, is it a tease wait two months and see? It's been four days and it's not noticeable!	Jingdong prefecture level city in Shandong
Jinfeng Hongyuan Flagship Store	Electricity savings1	Workmanship is quite good, just started to use, the effect of the follow-up evaluation.	Jingdong prefecture level city in Shandong
Jinfeng Hongyuan Flagship Store	Electricity savings1	Received the goods used for a few days before evaluation, the effect is quite good	Jingdong prefecture level city in Shandong

Jinfeng Hongyuan Flagship Store	Electricity savings1	Try it for a month and then evaluate	Jingdong prefecture level city in Shandong
Jinfeng Hongyuan Flagship Store	Electricity savings1	Just received the goods, the packaging is very good, the appearance of the economizer feels good, I do not know how the effect of power saving, with a month or two and then follow up!	Jingdong prefecture level city in Shandong
Jinfeng Hongyuan Flagship Store	Electricity savings1	It's quite small and delicate, I don't know if it's useful yet.	Jingdong prefecture level city in Shandong
Jinfeng Hongyuan Flagship Store	Electricity savings1	Just received it, use it for a few days before commenting	Jingdong prefecture level city in Shandong
Jinfeng Hongyuan Flagship Store	Electricity savings1	Does this thing work! When it arrived, the button for the safety was loose. I'm a little afraid of fire!	Jingdong prefecture level city in Shandong
Jinfeng Hongyuan Flagship Store	Electricity savings1	I received it. I don't know how it works.	Jingdong prefecture level city in Shandong
Jinfeng Hongyuan Flagship Store	Electricity savings1	This user thinks the product is good	Jingdong prefecture level city in Shandong
Jinfeng Hongyuan Flagship Store	Electricity savings1	Customer service was great! Small i problem was taken care of in a timely manner! Effectiveness to be tested by time! No rush!!!	Jingdong prefecture level city in Shandong
Jinfeng Hongyuan Flagship Store	Electricity savings1	I put it on right after I received it and it looks compact. I believe the high technology should save electricity.	Jingdong prefecture level city in Shandong
Jinfeng Hongyuan Flagship Store	Electricity savings1	Logistics courier very quickly, the first day under the order the next day to receive, off pieces whether power saving is the most important thing, things are exquisite workmanship, a month later in the follow-up evaluation whether power saving	Jingdong prefecture level city in Shandong
Jinfeng Hongyuan Flagship Store	Electricity savings1	The delivery is pretty fast, I'm using it, have to have it for a while to see results, looking forward to it.	Jingdong prefecture level city in Shandong
Jinfeng Hongyuan Flagship Store	Electricity savings1	See pretty good just started to use a month later to give you a real evaluation, should be useful.	Jingdong prefecture level city in Shandong
Jinfeng Hongyuan Flagship Store	Electricity savings1	And then comment.	Jingdong prefecture level city in Shandong
Jinfeng Hongyuan Flagship Store	Electricity savings1	Received the goods, wait for the test to evaluate	Jingdong prefecture level city in Shandong
Jinfeng Hongyuan Flagship Store	Electricity savings1	This user thinks the product is good	Jingdong prefecture level city in Shandong
Jinfeng Hongyuan Flagship Store	Electricity savings1	I'm already using it, I just don't know how to judge the power savings. I won't know until I use it for a season.	Jingdong prefecture level city in Shandong
Jinfeng Hongyuan Flagship Store	Electricity savings1	Works. Says it works.	Jingdong prefecture level city in Shandong
Jinfeng Hongyuan Flagship Store	Electricity savings1	Very good. Satisfied.	Jingdong prefecture level city in Shandong
Jinfeng Hongyuan Flagship Store	Electricity savings1	This user thinks the product is good	Jingdong prefecture level city in Shandong
Jinfeng Hongyuan Flagship Store	Electricity savings1	It's working. Obviously, it's less expensive.	Jingdong prefecture level city in Shandong
Jinfeng Hongyuan Flagship Store	Electricity savings1	The second one is up, the first one still works! It's reactive power compensation! This is well understood if you want a professional!	Jingdong prefecture level city in Shandong
Jinfeng Hongyuan Flagship Store	Electricity savings1	It feels as if there are some nodes, but they need to be tested and observed over time, thanks.	Jingdong prefecture level city in Shandong
Jinfeng Hongyuan Flagship Store	Electricity savings1	I've received the goods, the energy saving effect will be evaluated after using it for a period of time, and all the services are very good, praise!	Jingdong prefecture level city in Shandong
Jinfeng Hongyuan Flagship Store	Electricity savings1	I don't know how well it works yet, I bought it and plugged it in, so we'll see if it saves some power or not!	Jingdong prefecture level city in Shandong
Jinfeng Hongyuan Flagship Store	Electricity savings1	Baby a bunch of knife quality is very good, previously bought a quality is not very good. It really works and has results.	Jingdong prefecture level city in Shandong
Jinfeng Hongyuan Flagship Store	Electricity savings1	Things have arrived, it looks good, just started to use, as to whether or not there is a business said the power-saving effect, but also to observe the effect, now can not evaluate	Jingdong prefecture level city in Shandong
Jinfeng Hongyuan Flagship Store	Electricity savings1	I haven't used it yet. I'll comment afterward.	Jingdong prefecture level city in Shandong
Jinfeng Hongyuan Flagship Store	Electricity savings1	I don't know if it's any good.	Jingdong prefecture level city in Shandong
Jinfeng Hongyuan Flagship Store	Electricity savings1	I don't know how well it works.	Jingdong prefecture level city in Shandong
Jinfeng Hongyuan Flagship Store	Electricity savings1	The baby has been received! Uh-huh! Good, quite exquisite, small and exquisite I thought it was big. Dare to tightly plugged in the electricity, just wait for the effect later!	Jingdong prefecture level city in Shandong
Jinfeng Hongyuan Flagship Store	Electricity savings1	The packaging is sturdy, I tried it right after I received it, so I'm looking forward to the results.	Jingdong prefecture level city in Shandong
Jinfeng Hongyuan Flagship Store	Electricity savings1	Received the item and installed it! Don't know if it works well! Will follow up with a review later! I hope it works!	Jingdong prefecture level city in Shandong
Jinfeng Hongyuan Flagship Store	Electricity savings1	Things used on the effect of waiting to use a little to say, but things are very good. Logistics is very fast, business service is very good.	Jingdong prefecture level city in Shandong
Jinfeng Hongyuan Flagship Store	Electricity savings1	This user thinks the product is good	Jingdong prefecture level city in Shandong
Jinfeng Hongyuan Flagship Store	Electricity savings1	I'm really using it. It feels good.	Jingdong prefecture level city in Shandong
Jinfeng Hongyuan Flagship Store	Electricity savings1	Good, Jingdong logistics is very fast, intelligent energy-saving appliances	Jingdong prefecture level city in Shandong
Jinfeng Hongyuan Flagship Store	Electricity savings1	It's not bad. It really saves energy.	Jingdong prefecture level city in Shandong
Jinfeng Hongyuan Flagship Store	Electricity savings1	The logistics is fast, praise	Jingdong prefecture level city in Shandong
Jinfeng Hongyuan Flagship Store	Electricity savings1	This user thinks the product is good	Jingdong prefecture level city in Shandong
Jinfeng Hongyuan Flagship Store	Electricity savings1	The first time I placed two, and today I placed another one, a friend introduced it, said it is particularly power-saving	Jingdong prefecture level city in Shandong
Jinfeng Hongyuan Flagship Store	Electricity savings1	I don't know how effective it is. Let's leave it at home for a while and see.	Jingdong prefecture level city in Shandong
Jinfeng Hongyuan Flagship Store	Electricity savings1	Dear customer service! I would like to ask you how to understand the digital display (increase/decrease jump) on the top of the saver? Thank you for your help.	Jingdong prefecture level city in Shandong
Jinfeng Hongyuan Flagship Store	Electricity savings1	This user thinks the product is good	Jingdong prefecture level city in Shandong
Jinfeng Hongyuan Flagship Store	Electricity savings1	The goods have been received, packaging exquisite, is using, later to see how the effect?	Jingdong prefecture level city in Shandong
Jinfeng Hongyuan Flagship Store	Electricity savings1	The saver received, the boss shipped very quickly, yesterday shot today arrived!	Jingdong prefecture level city in Shandong
Jinfeng Hongyuan Flagship Store	Electricity savings1	Beautiful appearance, well packaged. Recording data to see if I can lower my electric bill a bit.	Jingdong prefecture level city in Shandong
Jinfeng Hongyuan Flagship Store	Electricity savings1	Things received, the packaging is quite good, the effect of the use and then come back to evaluate.	Jingdong prefecture level city in Shandong
Jinfeng Hongyuan Flagship Store	Electricity savings1	Easy to use, just plug it in. But there is a current sound.	Jingdong prefecture level city in Shandong
Jinfeng Hongyuan Flagship Store	Electricity savings1	Fast logistics, easy to use and labor-saving	Jingdong prefecture level city in Shandong
Jinfeng Hongyuan Flagship Store	Electricity savings1	Received, thank you!	Jingdong prefecture level city in Shandong
Jinfeng Hongyuan Flagship Store	Electricity savings1	Second time I've bought it. It's good.	Jingdong prefecture level city in Shandong

Jinfeng Hongyuan Flagship Store	Electricity savings1	I've only been using it for a short time, so I'm looking forward to the results.	Jingdong prefecture level city in Shandong
Jinfeng Hongyuan Flagship Store	Electricity savings1	Good stuff, good stuff, good stuff good stuff.	Jingdong prefecture level city in Shandong
Jinfeng Hongyuan Flagship Store	Electricity savings1	Buy your stuff is not always good I have been using it for almost two months now, it does not save electricity at all the electricity bill is about ten dollars a day this is still called the king of power	Jingdong prefecture level city in Shandong
Jinfeng Hongyuan Flagship Store	Electricity savings1	Express arrived, the quality is very good, worth the purchase.	Jingdong prefecture level city in Shandong
Jinfeng Hongyuan Flagship Store	Electricity savings1	Energy saving has been received, now the voltage is more stable, energy-saving effect to see a few days and then chase flat, the service attitude is very good, the courier is relatively fast, I am satisfied.	Jingdong prefecture level city in Shandong
Jinfeng Hongyuan Flagship Store	Electricity savings1	Stuff installed, numbers are smaller than when installed, hopefully it works	Jingdong prefecture level city in Shandong
Jinfeng Hongyuan Flagship Store	Electricity savings1	Good appearance, fine workmanship, just bought, use up the feeling of power saving effect is good, worth buying!	Jingdong prefecture level city in Shandong
Jinfeng Hongyuan Flagship Store	Electricity savings1	Economical and practical 👍 Always use it	Jingdong prefecture level city in Shandong
Jinfeng Hongyuan Flagship Store	Electricity savings1	Just used it, don't know the effect yet, will come back to follow up after the effect. Hope it works well(▽)	Jingdong prefecture level city in Shandong
Jinfeng Hongyuan Flagship Store	Electricity savings1	The goods have been received, use it to see in the evaluation.	Jingdong prefecture level city in Shandong
Jinfeng Hongyuan Flagship Store	Electricity savings1	Doesn't it save electricity? The boil kettle still goes faster when you press the meter.	Jingdong prefecture level city in Shandong
Jinfeng Hongyuan Flagship Store	Electricity savings1	Already used. Will patronize again in the future	Jingdong prefecture level city in Shandong
Jinfeng Hongyuan Flagship Store	Electricity savings1	Works great, just plug it in, results to be checked, satisfied.	Jingdong prefecture level city in Shandong
Jinfeng Hongyuan Flagship Store	Electricity savings1	Second purchase. Works well 😍😍😍😍	Jingdong prefecture level city in Shandong
Jinfeng Hongyuan Flagship Store	Electricity savings1	Prompt shipment, very satisfied	Jingdong prefecture level city in Shandong
Jinfeng Hongyuan Flagship Store	Electricity savings1	It's been less than a month, let's see how it works, hopefully it's power efficient	Jingdong prefecture level city in Shandong
Jinfeng Hongyuan Flagship Store	Electricity savings1	Use a month in say.	Jingdong prefecture level city in Shandong
Jinfeng Hongyuan Flagship Store	Electricity savings1	I've saved a lot of electricity since I've been using it.	Jingdong prefecture level city in Shandong
Jinfeng Hongyuan Flagship Store	Electricity savings1	It's fast, looks and feels good on the outside, try it out and evaluate.	Jingdong prefecture level city in Shandong
Jinfeng Hongyuan Flagship Store	Electricity savings1	I haven't used it. I just used it. I don't know if it's any good.	Jingdong prefecture level city in Shandong
Jinfeng Hongyuan Flagship Store	Electricity savings1	Quality can be, power saving now can not see just use, after a while in the look at the power saving effect	Jingdong prefecture level city in Shandong
Jinfeng Hongyuan Flagship Store	Electricity savings1	The logistics is very fast, and it seems to have some effect	Jingdong prefecture level city in Shandong
Jinfeng Hongyuan Flagship Store	Electricity savings1	Good, but the effects remain to be seen	Jingdong prefecture level city in Shandong
Jinfeng Hongyuan Flagship Store	Electricity savings1	Baby is basically the same as the description, shipping quickly, worth a buy Good price, logistics is strong, worth repurchasing!	Jingdong prefecture level city in Shandong
Jinfeng Hongyuan Flagship Store	Electricity savings1	I bought it for my dad, I don't know much about it, if he thinks it's good then it must be good 👍	Jingdong prefecture level city in Shandong
Jinfeng Hongyuan Flagship Store	Electricity savings1	Goods received. It's working fine. Display voltage. After a period of time to see the effect in the addition to the level of theory	Jingdong prefecture level city in Shandong
Jinfeng Hongyuan Flagship Store	Electricity savings1	The stuff is pretty good, I just don't know how well it saves power.	Jingdong prefecture level city in Shandong
Jinfeng Hongyuan Flagship Store	Electricity savings1	Logistics is a little too fast 👍	Jingdong prefecture level city in Shandong
Jinfeng Hongyuan Flagship Store	Electricity savings1	Received the goods used for a period of time before coming, good, beautiful appearance, really stable flow	Jingdong prefecture level city in Shandong
Jinfeng Hongyuan Flagship Store	Electricity savings1	Things received, look at the packaging is very good, not yet, to the power saving in the follow-up evaluation	Jingdong prefecture level city in Shandong
Jinfeng Hongyuan Flagship Store	Electricity savings1	Baby is very good! Use a few days before to evaluate, I personally test really effective.	Jingdong prefecture level city in Shandong
Jinfeng Hongyuan Flagship Store	Electricity savings1	I received the goods are in use, I do not know how effective? Can it save energy? Let's see.	Jingdong prefecture level city in Shandong
Jinfeng Hongyuan Flagship Store	Electricity savings1	It's really good and has some power saving effect.	Jingdong prefecture level city in Shandong
Jinfeng Hongyuan Flagship Store	Electricity savings1	It's good quality, well made, and I hope it'll save on electricity over time.	Jingdong prefecture level city in Shandong
Jinfeng Hongyuan Flagship Store	Electricity savings1	I just used it, I don't know how it works yet, but I'll give it a good review first.	Jingdong prefecture level city in Shandong
Jinfeng Hongyuan Flagship Store	Electricity savings1	Second purchase, the delivery guy was fast and called ahead to deliver.	Jingdong prefecture level city in Shandong
Jinfeng Hongyuan Flagship Store	Electricity savings1	Goods have been received, but have not yet used, used to see how the effect, in the evaluation of fertilizer	Jingdong prefecture level city in Shandong
Jinfeng Hongyuan Flagship Store	Electricity savings1	The delivery is very fast. It's the same in kind. It's working.	Jingdong prefecture level city in Shandong
Jinfeng Hongyuan Flagship Store	Electricity savings1	Fast logistics, good quality, more satisfied	Jingdong prefecture level city in Shandong
Jinfeng Hongyuan Flagship Store	Electricity savings1	I've been here for almost a month now and there is a very noticeable difference than it is worth buying.	Jingdong prefecture level city in Shandong
Jinfeng Hongyuan Flagship Store	Electricity savings1	Fast shipping, received the next day, plug and play, but do not know how effective to not break down!	Jingdong prefecture level city in Shandong
Jinfeng Hongyuan Flagship Store	Electricity savings1	The logistics are great I've been using it for a while before commenting on it This saves electricity	Jingdong prefecture level city in Shandong
Jinfeng Hongyuan Flagship Store	Electricity savings1	Wait to use it for a while to know if it's energy efficient	Jingdong prefecture level city in Shandong
Jinfeng Hongyuan Flagship Store	Electricity savings1	Just received it, haven't used it yet don't know if it's any good.	Jingdong prefecture level city in Shandong
Jinfeng Hongyuan Flagship Store	Electricity savings1	It really doesn't work, and the electric bill is still the same amount every month.	Jingdong prefecture level city in Shandong
Jinfeng Hongyuan Flagship Store	Electricity savings1	It's well packaged and looks fine	Jingdong prefecture level city in Shandong
Jinfeng Hongyuan Flagship Store	Electricity savings1	Very good. Will patronize again if needed.	Jingdong prefecture level city in Shandong
Jinfeng Hongyuan Flagship Store	Electricity savings1	I have received the product and installed it, try it for a while and see how it works.	Jingdong prefecture level city in Shandong
Jinfeng Hongyuan Flagship Store	Electricity savings1	I've been using it for a month now...I don't feel like there's any noticeable power savings...last month it was $130 this month it's $123...let's see if I can use it for another month	Jingdong prefecture level city in Shandong
Jinfeng Hongyuan Flagship Store	Electricity savings1	Just received it, yet to try it and see if it works.	Jingdong prefecture level city in Shandong
Jinfeng Hongyuan Flagship Store	Electricity savings1	It's easy to use and has a power saving credit!	Jingdong prefecture level city in Shandong

Jinfeng Hongyuan Flagship Store	Electricity savings1	It's very good. I've already bought two of them.	Jingdong prefecture level city in Shandong
Jinfeng Hongyuan Flagship Store	Electricity savings1	It's very well made, packaged meticulously, and I hope it works!	Jingdong prefecture level city in Shandong
Jinfeng Hongyuan Flagship Store	Electricity savings1	Goods have been received have not yet used it, used to comment again	Jingdong prefecture level city in Shandong
Jinfeng Hongyuan Flagship Store	Electricity savings1	Looking forward to good results	Jingdong prefecture level city in Shandong
Jinfeng Hongyuan Flagship Store	Electricity savings1	Not sure if it saves power or not. Still trying it out.	Jingdong prefecture level city in Shandong
Jinfeng Hongyuan Flagship Store	Electricity savings1	It also really saves on electricity! Superb!	Jingdong prefecture level city in Shandong
Jinfeng Hongyuan Flagship Store	Electricity savings1	Didn't work at all, want to return it	Jingdong prefecture level city in Shandong
Jinfeng Hongyuan Flagship Store	Electricity savings1	Uh-huh! It's working, I've been using it for a month and it saves 20% of electricity compared to before.	Jingdong prefecture level city in Shandong
Jinfeng Hongyuan Flagship Store	Electricity savings1	Should be a little bit of a power saver, I wonder how long it will take to save back the money I spent on this one	Jingdong prefecture level city in Shandong
Jinfeng Hongyuan Flagship Store	Electricity savings1	Exquisite appearance and stable voltage	Jingdong prefecture level city in Shandong
Jinfeng Hongyuan Flagship Store	Electricity savings1	After using it for a few days it feels like it saves electricity.	Jingdong prefecture level city in Shandong
Jinfeng Hongyuan Flagship Store	Electricity savings1	Second purchase and its pending good results.	Jingdong prefecture level city in Shandong
Jinfeng Hongyuan Flagship Store	Electricity savings1	The appearance of the workmanship is exquisite and meticulous, beautiful appearance, just to see if there is no effect!	Jingdong prefecture level city in Shandong
Jinfeng Hongyuan Flagship Store	Electricity savings1	Let's see what happens in a month.	Jingdong prefecture level city in Shandong
Jinfeng Hongyuan Flagship Store	Electricity savings1	Trial, now short time not yet obvious effect	Jingdong prefecture level city in Shandong
Jinfeng Hongyuan Flagship Store	Electricity savings1	I'm using it now, don't know how well it works, it looks great!	Jingdong prefecture level city in Shandong
Jinfeng Hongyuan Flagship Store	Electricity savings1	There is no role! Now it's true that I don't know!	Jingdong prefecture level city in Shandong
Jinfeng Hongyuan Flagship Store	Electricity savings1	Purchased for the second time, power saving not sure if it's ok? Let's talk about it after using it	Jingdong prefecture level city in Shandong
Jinfeng Hongyuan Flagship Store	Electricity savings1	I just received it, I have to try it for a month to know how it works.	Jingdong prefecture level city in Shandong
Jinfeng Hongyuan Flagship Store	Electricity savings1	I just used it for an hour, and the meter's still spinning so fast it's not much use.	Jingdong prefecture level city in Shandong
Jinfeng Hongyuan Flagship Store	Electricity savings1	Not bad, logistics is fast	Jingdong prefecture level city in Shandong
Jinfeng Hongyuan Flagship Store	Electricity savings1	I don't know if it works. I'll find out next month when I pay the bill. It's easy. Just plug it in.	Jingdong prefecture level city in Shandong
Jinfeng Hongyuan Flagship Store	Electricity savings1	Logistics is particularly fast, received the goods are genuine, looking forward to power saving effect	Jingdong prefecture level city in Shandong
Jinfeng Hongyuan Flagship Store	Electricity savings1	First time buying this product like this one from this family use it and then evaluate.	Jingdong prefecture level city in Shandong
Jinfeng Hongyuan Flagship Store	Electricity savings1	Lightning arrived, the product is very good, before all do not know there is such a baby, in a friend's home has seen, and then bought to try, has been used for a period of time before coming to the evaluation, the work is very stable, so far looked very good, will introduce friends to buy!	Jingdong prefecture level city in Shandong
Jinfeng Hongyuan Flagship Store	Electricity savings1	I was a bit skeptical when I heard that I could save money on my electricity bill by installing this, I don't know if the power saving effect is really what it is said to be. But since I bought it, I just have to put it on and try it out. I hope it lives up to its name and saves me the money I spent on the device	Jingdong prefecture level city in Shandong
Jinfeng Hongyuan Flagship Store	Electricity savings1	The quality of the stuff is okay, but I'm not sure if it works, so I'll try using it first.	Jingdong prefecture level city in Shandong
Jinfeng Hongyuan Flagship Store	Electricity savings1	It should work. The current is much more stable.	Jingdong prefecture level city in Shandong
Jinfeng Hongyuan Flagship Store	Electricity savings1	The store has a great service attitude	Jingdong prefecture level city in Shandong
Jinfeng Hongyuan Flagship Store	Electricity savings1	Let's wait until we've used it for a month.	Jingdong prefecture level city in Shandong
Jinfeng Hongyuan Flagship Store	Electricity savings1	I'm using it and I don't know if it's a power saver or not	Jingdong prefecture level city in Shandong
Jinfeng Hongyuan Flagship Store	Electricity savings1	Original and authentic guarantee quality is good now.	Jingdong prefecture level city in Shandong
Jinfeng Hongyuan Flagship Store	Electricity savings1	Great, very happy with it, will buy a couple more next time	Jingdong prefecture level city in Shandong
Jinfeng Hongyuan Flagship Store	Electricity savings1	I just used it, I don't know if it works yet, I'll come back after using it...	Jingdong prefecture level city in Shandong
Jinfeng Hongyuan Flagship Store	Electricity savings1	It's still being tested.	Jingdong prefecture level city in Shandong
Jinfeng Hongyuan Flagship Store	Electricity savings1	I've been using it for a month and it's not working.	Jingdong prefecture level city in Shandong
Jinfeng Hongyuan Flagship Store	Electricity savings1	It's basically nothing!	Jingdong prefecture level city in Shandong
Jinfeng Hongyuan Flagship Store	Electricity savings1	It's not bad, it kind of works, support it.	Jingdong prefecture level city in Shandong
Jinfeng Hongyuan Flagship Store	Electricity savings1	I just received it last night, let's try it first to see how it works, it looks good!	Jingdong prefecture level city in Shandong
Jinfeng Hongyuan Flagship Store	Electricity savings1	The current is very stable, fine workmanship, small and exquisite, very satisfied	Jingdong prefecture level city in Shandong
Jinfeng Hongyuan Flagship Store	Electricity savings1	Not bad, exactly how much electricity can be saved; still have to follow up the evaluation	Jingdong prefecture level city in Shandong
Jinfeng Hongyuan Flagship Store	Electricity savings1	Logistics is also very fast, next time there is an opportunity to introduce friends to buy!	Jingdong prefecture level city in Shandong
Jinfeng Hongyuan Flagship Store	Electricity savings1	Voltage display and actual do not pay	Jingdong prefecture level city in Shandong
Jinfeng Hongyuan Flagship Store	Electricity savings1	Second purchase, still saves a little on the electric bill	Jingdong prefecture level city in Shandong
Jinfeng Hongyuan Flagship Store	Electricity savings1	Order received, currently trying it out, thanks a lot.	Jingdong prefecture level city in Shandong
Jinfeng Hongyuan Flagship Store	Electricity savings1	I haven't used it for a long time yet, I don't know if the effect is good or not, looking forward to it	Jingdong prefecture level city in Shandong
Jinfeng Hongyuan Flagship Store	Electricity savings1	Good, working, I just don't know if it will save electricity... 5 stars for now.	Jingdong prefecture level city in Shandong
Jinfeng Hongyuan Flagship Store	Electricity savings1	Great product, just as the owner said it would be.	Jingdong prefecture level city in Shandong
Jinfeng Hongyuan Flagship Store	Electricity savings1	Shipping was fast, I don't know if it works well or not, I have to use it for a while first.	Jingdong prefecture level city in Shandong
Jinfeng Hongyuan Flagship Store	Electricity savings1	I don't feel that it saves much electricity, but I'll give it a try. It's been more than a month now, and the electricity bill is still a bit high.	Jingdong prefecture level city in Shandong

Jinfeng Hongyuan Flagship Store	Electricity savings1	Got it. Just used it.	Jingdong prefecture level city in Shandong
Jinfeng Hongyuan Flagship Store	Electricity savings1	Use it for a while and then comment.	Jingdong prefecture level city in Shandong
Jinfeng Hongyuan Flagship Store	Electricity savings1	Used it, see how it works with it and then comment. The quality is very good!	Jingdong prefecture level city in Shandong
Jinfeng Hongyuan Flagship Store	Electricity savings1	I'll comment again after a while.	Jingdong prefecture level city in Shandong
Jinfeng Hongyuan Flagship Store	Electricity savings1	It's easy to operate, affordable and works well, love it.	Jingdong prefecture level city in Shandong
Jinfeng Hongyuan Flagship Store	Electricity savings1	It's good. You can try it. It's cheap.	Jingdong prefecture level city in Shandong
Jinfeng Hongyuan Flagship Store	Electricity savings1	It's in use, so we'll see how it works after a while!	Jingdong prefecture level city in Shandong
Jinfeng Hongyuan Flagship Store	Electricity savings1	Service is pretty good, not sure about the quality yet.	Jingdong prefecture level city in Shandong
Jinfeng Hongyuan Flagship Store	Electricity savings1	Just used it and will see the results in a month 😃	Jingdong prefecture level city in Shandong
Jinfeng Hongyuan Flagship Store	Electricity savings1	Saving electricity is expensive and good	Jingdong prefecture level city in Shandong
Jinfeng Hongyuan Flagship Store	Electricity savings1	Pretty good, 5 stars! Will buy again next time seller service is very good ! ! ! ! Good quality! Will recommend to friends!	Jingdong prefecture level city in Shandong
Jinfeng Hongyuan Flagship Store	Electricity savings1	In use, with results naturally have follow-up reviews!	Jingdong prefecture level city in Shandong
Jinfeng Hongyuan Flagship Store	Electricity savings1	I'm using it now, I'll come back with results, it'll work.	Jingdong prefecture level city in Shandong
Jinfeng Hongyuan Flagship Store	Electricity savings1	Good, nice, fast shipping, cheap, worth having!	Jingdong prefecture level city in Shandong
Jinfeng Hongyuan Flagship Store	Electricity savings1	Logistics is fast.	Jingdong prefecture level city in Shandong
Jinfeng Hongyuan Flagship Store	Electricity savings1	I have just started using the product and will be reviewing it later.	Jingdong prefecture level city in Shandong
Jinfeng Hongyuan Flagship Store	Electricity savings1	I'll comment after I use it.	Jingdong prefecture level city in Shandong
Jinfeng Hongyuan Flagship Store	Electricity savings1	I don't know if it's working or not, though I'll try plugging it in.	Jingdong prefecture level city in Shandong
Jinfeng Hongyuan Flagship Store	Electricity savings1	It really saves electricity and contributes to energy savings in disguise	Jingdong prefecture level city in Shandong
Jinfeng Hongyuan Flagship Store	Electricity savings1	It feels good in the experience.	Jingdong prefecture level city in Shandong
Jinfeng Hongyuan Flagship Store	Electricity savings1	The product is better than I thought it would be, the quality looks great, the voltage is stable, and I'm looking forward to some great power savings!	Jingdong prefecture level city in Shandong
Jinfeng Hongyuan Flagship Store	Electricity savings1	I'm using it now looking forward to saving some points,, I'm feeding back	Jingdong prefecture level city in Shandong
Jinfeng Hongyuan Flagship Store	Electricity savings1	Displayed voltage below 200.	Jingdong prefecture level city in Shandong
Jinfeng Hongyuan Flagship Store	Electricity savings1	I haven't seen any great results, but it should last about a hundred or eighty years.	Jingdong prefecture level city in Shandong
Jinfeng Hongyuan Flagship Store	Electricity savings1	I don't know if it works or not, but I keep it plugged in anyway, so I hope it works!	Jingdong prefecture level city in Shandong
Jinfeng Hongyuan Flagship Store	Electricity savings1	Reasonable price, good quality, fast delivery, great service, already a repurchase.	Jingdong prefecture level city in Shandong
Jinfeng Hongyuan Flagship Store	Electricity savings1	Second purchase, looking forward to the results	Jingdong prefecture level city in Shandong
Jinfeng Hongyuan Flagship Store	Electricity savings1	The quality of this product feels like it should work in a use test! Just how many kilowatts of electricity must be wasted per day with the red light and digital display on for long periods of time?	Jingdong prefecture level city in Shandong
Jinfeng Hongyuan Flagship Store	Electricity savings1	It looks good, we'll see if we use it.	Jingdong prefecture level city in Shandong
Jinfeng Hongyuan Flagship Store	Electricity savings1	Great stuff, second purchase!	Jingdong prefecture level city in Shandong
Jinfeng Hongyuan Flagship Store	Electricity savings1	It is indeed very practical and saves electricity at home	Jingdong prefecture level city in Shandong
Jinfeng Hongyuan Flagship Store	Electricity savings1	Goods received, the physical and the same as the picture, logistics is also very fast!	Jingdong prefecture level city in Shandong
Jinfeng Hongyuan Flagship Store	Electricity savings1	I'll use it for a while and see how it goes. I'll buy it again if it's good.	Jingdong prefecture level city in Shandong
Jinfeng Hongyuan Flagship Store	Electricity savings1	In use, voltage monitoring, the effect of how to wait for next month's electricity consumption confirmation	Jingdong prefecture level city in Shandong
Jinfeng Hongyuan Flagship Store	Electricity savings1	It's still pretty good.	Jingdong prefecture level city in Shandong
Jinfeng Hongyuan Flagship Store	Electricity savings1	It's been plugged in for a month and there hasn't been a big change yet, since it usually uses very little electricity	Jingdong prefecture level city in Shandong
Jinfeng Hongyuan Flagship Store	Electricity savings1	Let's wait for a month to see if it works	Jingdong prefecture level city in Shandong
Jinfeng Hongyuan Flagship Store	Electricity savings1	Good, good, good, good, good, good, good, good, good, good, good, good, good, good, good.	Jingdong prefecture level city in Shandong
Jinfeng Hongyuan Flagship Store	Electricity savings1	The product received the trial a few days, feel good, praise!	Jingdong prefecture level city in Shandong
Jinfeng Hongyuan Flagship Store	Electricity savings1	After six or seven hours of use, 250 volts won't save you any power.	Jingdong prefecture level city in Shandong
Jinfeng Hongyuan Flagship Store	Electricity savings1	I haven't used it yet, I wonder if it saves electricity?	Jingdong prefecture level city in Shandong
Jinfeng Hongyuan Flagship Store	Electricity savings1	Baby value for money, love it, next time will come back is genuine, trust the business, baby texture is very good, the price is moderate, unlimited repurchase!	Jingdong prefecture level city in Shandong
Jinfeng Hongyuan Flagship Store	Electricity savings1	Power saving effect is obvious! Raising tropical fish daily power consumption, after using the daily power savings of more than 3 degrees, currently about 8 degrees / day!	Jingdong prefecture level city in Shandong
Jinfeng Hongyuan Flagship Store	Electricity savings1	Just used it, unknown results.	Jingdong prefecture level city in Shandong
Jinfeng Hongyuan Flagship Store	Electricity savings1	The goods received, well, look forward to the effect. Logistics is very fast, thanks to the platform, thanks to Jingdong. I hope it can save electricity.	Jingdong prefecture level city in Shandong
Jinfeng Hongyuan Flagship Store	Electricity savings1	Not sure 有 effect?	Jingdong prefecture level city in Shandong
Jinfeng Hongyuan Flagship Store	Electricity savings1	I'd say it's a 5% savings.	Jingdong prefecture level city in Shandong
Jinfeng Hongyuan Flagship Store	Electricity savings1	I received it and am using it, I don't know how effective it is, I'll evaluate it afterwards	Jingdong prefecture level city in Shandong
Jinfeng Hongyuan Flagship Store	Electricity savings1	Baby received not yet used, first give praise	Jingdong prefecture level city in Shandong
Jinfeng Hongyuan Flagship Store	Electricity savings1	Looks good, I'll evaluate it later	Jingdong prefecture level city in Shandong
Jinfeng Hongyuan Flagship Store	Electricity savings1	The goods have been received in use, the overall feeling is not bad!	Jingdong prefecture level city in Shandong

Jinfeng Hongyuan Flagship Store	Electricity savings1	It's working. This is the second one. This stuff has to be used over a long period of time to be effective. And it only works on inductive loads, i.e. appliances containing electric motors. Use it.	Jingdong prefecture level city in Shandong
Jinfeng Hongyuan Flagship Store	Electricity savings1	Use it for a while and see how it works.	Jingdong prefecture level city in Shandong
Jinfeng Hongyuan Flagship Store	Electricity savings1	Good courier service and the item is very nice and practical.	Jingdong prefecture level city in Shandong
Jinfeng Hongyuan Flagship Store	Electricity savings1	It doesn't do anything and it's a waste of electricity.	Jingdong prefecture level city in Shandong
Jinfeng Hongyuan Flagship Store	Electricity savings1	I haven't used it yet. It should be good.	Jingdong prefecture level city in Shandong
Jinfeng Hongyuan Flagship Store	Electricity savings1	That's good. OK, that's good.	Jingdong prefecture level city in Shandong
Jinfeng Hongyuan Flagship Store	Electricity savings1	It's a rip off, doesn't work, doesn't save electricity, does the job to well, and I've wasted a couple dozen dollars again.	Jingdong prefecture level city in Shandong
Jinfeng Hongyuan Flagship Store	Electricity savings1	Use it first, the effect is not as good as the road, then we will comment again	Jingdong prefecture level city in Shandong
Jinfeng Hongyuan Flagship Store	Electricity savings1	The price is affordable, the logistics is strong, just use four or five days, the effect will be added later!	Jingdong prefecture level city in Shandong
Jinfeng Hongyuan Flagship Store	Electricity savings1	It's easy to use and works fine.	Jingdong prefecture level city in Shandong
Jinfeng Hongyuan Flagship Store	Electricity savings1	It doesn't feel like it's working.	Jingdong prefecture level city in Shandong
Jinfeng Hongyuan Flagship Store	Electricity savings1	I'm using it. We'll see what happens in a couple months.	Jingdong prefecture level city in Shandong
Jinfeng Hongyuan Flagship Store	Electricity savings1	I just got it back to try it out and I'll comment later	Jingdong prefecture level city in Shandong
Jinfeng Hongyuan Flagship Store	Electricity savings1	Less than two months. Can't tell.	Jingdong prefecture level city in Shandong
Jinfeng Hongyuan Flagship Store	Electricity savings1	No power saving effect	Jingdong prefecture level city in Shandong
Jinfeng Hongyuan Flagship Store	Electricity savings1	Express Express Very quickly not only to the power saver can not save electricity, with two months, look at the evaluation put.	Jingdong prefecture level city in Shandong
Jinfeng Hongyuan Flagship Store	Electricity savings1	Genuine products are packaged well!	Jingdong prefecture level city in Shandong
Jinfeng Hongyuan Flagship Store	Electricity savings1	I've been using it for a while, but I don't know if it saves electricity. I hope it works.	Jingdong prefecture level city in Shandong
Jinfeng Hongyuan Flagship Store	Electricity savings1	Good service, fast logistics, the effect is to be used to know	Jingdong prefecture level city in Shandong
Jinfeng Hongyuan Flagship Store	Electricity savings1	Just used it, not sure about the power saving effect	Jingdong prefecture level city in Shandong
Jinfeng Hongyuan Flagship Store	Electricity savings1	The stuff is ok, save power or not, will comment after a month.	Jingdong prefecture level city in Shandong
Jinfeng Hongyuan Flagship Store	Electricity savings1	Used it for a month to save %25, normal is a little over 100, it's great! I want to buy one in.	Jingdong prefecture level city in Shandong
Jinfeng Hongyuan Flagship Store	Electricity savings1	I'm using it and looking forward to the results	Jingdong prefecture level city in Shandong
Jinfeng Hongyuan Flagship Store	Electricity savings1	The quality is very good and the workmanship is exquisite. The use effect is also very good.	Jingdong prefecture level city in Shandong
Jinfeng Hongyuan Flagship Store	Electricity savings1	Merchant service attitude is very good, logistics speed is super fast, online evaluation is very good, the first time to use, a month to know the specific effect, the effect is good, and then additional	Jingdong prefecture level city in Shandong
Jinfeng Hongyuan Flagship Store	Electricity savings1	Works great, solves the problem of high power usage in the house	Jingdong prefecture level city in Shandong
Jinfeng Hongyuan Flagship Store	Electricity savings1	To receive! Very delicate! It's not clear how effective it is, I won't know until I use it!!!!	Jingdong prefecture level city in Shandong
Jinfeng Hongyuan Flagship Store	Electricity savings1	I've just been using it for a while and I don't feel like it's too noticeable.	Jingdong prefecture level city in Shandong
Jinfeng Hongyuan Flagship Store	Electricity savings1	Already used, no visible effect for the time being	Jingdong prefecture level city in Shandong
Jinfeng Hongyuan Flagship Store	Electricity savings1	I can't feel the effect.	Jingdong prefecture level city in Shandong
Jinfeng Hongyuan Flagship Store	Electricity savings1	Use it for a while and see the results	Jingdong prefecture level city in Shandong
Jinfeng Hongyuan Flagship Store	Electricity savings1	The price is more reasonable, the power-saving effect is to be verified, the delivery is fast, and the courier service is good.	Jingdong prefecture level city in Shandong
Jinfeng Hongyuan Flagship Store	Electricity savings1	Quality is very good, consistent with the seller's description, very satisfied, very like, very fast shipping, packaging is very careful and strict, the logistics company has a very good service attitude, fast delivery, very satisfied with a shopping, very satisfied. Recommended to buy, cost-effective, the service attitude of the business is very enthusiastic, providing such high quality goods.	Jingdong prefecture level city in Shandong
Jinfeng Hongyuan Flagship Store	Electricity savings1	I don't know.	Jingdong prefecture level city in Shandong
Jinfeng Hongyuan Flagship Store	Electricity savings1	I didn't get the delivery.	Jingdong prefecture level city in Shandong
Jinfeng Hongyuan Flagship Store	Electricity savings1	It works, it does have power savings, better than expected, will repurchase.	Jingdong prefecture level city in Shandong
Jinfeng Hongyuan Flagship Store	Electricity savings1	That's where the five fuses are.	Jingdong prefecture level city in Shandong
Jinfeng Hongyuan Flagship Store	Electricity savings1	Don't buy it, it doesn't work and you can't return it.	Jingdong prefecture level city in Shandong
Jinfeng Hongyuan Flagship Store	Electricity savings1	I hope it works. Wait a month.	Jingdong prefecture level city in Shandong
Jinfeng Hongyuan Flagship Store	Electricity savings1	Nothing**, buy with caution	Jingdong prefecture level city in Shandong
Jinfeng Hongyuan Flagship Store	Electricity savings1	I'm using it and waiting for the results	Jingdong prefecture level city in Shandong
Jinfeng Hongyuan Flagship Store	Electricity savings1	Saver quality is very good, has always felt that the home power consumption is very large, this is the company's electrician recommended, said he bought this home is really able to save a little electricity, I also came to buy, the effect if it is good, then buy, a floor a...	Jingdong prefecture level city in Shandong
Jinfeng Hongyuan Flagship Store	Electricity savings1	Good product, satisfied with purchase	Jingdong prefecture level city in Shandong
Jinfeng Hongyuan Flagship Store	Electricity savings1	Occupying the time has not yet found the power saving, occupying not evaluation	Jingdong prefecture level city in Shandong
Jinfeng Hongyuan Flagship Store	Electricity savings1	Haven't tested the results yet. To be followed up with comments!	Jingdong prefecture level city in Shandong
Jinfeng Hongyuan Flagship Store	Electricity savings1	After experiencing it for a month, I don't seem to know how to save it, and I don't pay attention to it anyway	Jingdong prefecture level city in Shandong
Jinfeng Hongyuan Flagship Store	Electricity savings1	It's useless. It's a waste of electricity.	Jingdong prefecture level city in Shandong
Jinfeng Hongyuan Flagship Store	Electricity savings1	I don't know if it's a power saver. It's a couple bucks. I hope it works.	Jingdong prefecture level city in Shandong
Jinfeng Hongyuan Flagship Store	Electricity savings1	I don't know what to say about this stuff. I don't know if it works or not, it's kind of a psychological purchase. Dormitory use to see the meter can not be not so good-looking. You have to wait until the time to deduct the utility bill to know.	Jingdong prefecture level city in Shandong

Jinfeng Hongyuan Flagship Store	Electricity savings1	Energy savers don't work / they're all a bunch of hoaxes.	Jingdong prefecture level city in Shandong
Jinfeng Hongyuan Flagship Store	Electricity savings1	Received the goods, used for more than a week, it's okay, sure a little effect	Jingdong prefecture level city in Shandong
Jinfeng Hongyuan Flagship Store	Electricity savings1	Well made and compact! Looking forward to the effect!	Jingdong prefecture level city in Shandong
Jinfeng Hongyuan Flagship Store	Electricity savings1	It has arrived and we'll see how it works.	Jingdong prefecture level city in Shandong
Jinfeng Hongyuan Flagship Store	Electricity savings1	Good. Good. It's worth it.	Jingdong prefecture level city in Shandong
Jinfeng Hongyuan Flagship Store	Electricity savings1	How come there's no indicator light?	Jingdong prefecture level city in Shandong
Jinfeng Hongyuan Flagship Store	Electricity savings1	The machine that uses electricity works quite well. This saves	Jingdong prefecture level city in Shandong
Jinfeng Hongyuan Flagship Store	Electricity savings1	It's good for home use and feels quite energy efficient	Jingdong prefecture level city in Shandong
Jinfeng Hongyuan Flagship Store	Electricity savings1	I regret buying this product, after using it for two days it doesn't work at all, still so many kilowatts per day just like the original.	Jingdong prefecture level city in Shandong
Jinfeng Hongyuan Flagship Store	Electricity savings1	Works very well and has some scientific merit.	Jingdong prefecture level city in Shandong
Jinfeng Hongyuan Flagship Store	Electricity savings1	Logistics is also very fast this good power saving I use two months before commenting!	Jingdong prefecture level city in Shandong
Jinfeng Hongyuan Flagship Store	Electricity savings1	Repeat customer. Very good.	Jingdong prefecture level city in Shandong
Jinfeng Hongyuan Flagship Store	Electricity savings1	It's good stuff. I've been using it for a month now and it's a real power saver.	Jingdong prefecture level city in Shandong
Jinfeng Hongyuan Flagship Store	Electricity savings1	Jingdong platform to buy things, has been very assured, quality assurance. Jingdong Express is as good as ever, complete packaging, delivery, service attitude to go! The family has always wanted to buy an energy-saving and power-saving equipment, selected half a day to choose this one, the value is very good, used for a period of time to follow up, to see whether it can achieve the real purpose of power saving, I	Jingdong prefecture level city in Shandong
Jinfeng Hongyuan Flagship Store	Electricity savings1	Works great, inexpensive and will be back next time.	Jingdong prefecture level city in Shandong
Jinfeng Hongyuan Flagship Store	Electricity savings1	Baby received, looks good, wait for a month to see how it works?	Jingdong prefecture level city in Shandong
Jinfeng Hongyuan Flagship Store	Electricity savings1	This product is not bad quite good well customer service said quite power saving that look at the customer service to see if the accuracy of the look at all aspects of the color is also quite beautiful	Jingdong prefecture level city in Shandong
Jinfeng Hongyuan Flagship Store	Electricity savings1	Just got back and tried it feels pretty good, just don't know how the quality is, give a good comment first	Jingdong prefecture level city in Shandong
Jinfeng Hongyuan Flagship Store	Electricity savings1	Received the product, trying it out, hope it helps!	Jingdong prefecture level city in Shandong
Jinfeng Hongyuan Flagship Store	Electricity savings1	Works great, saves electricity a dollar a day and a night	Jingdong prefecture level city in Shandong
Jinfeng Hongyuan Flagship Store	Electricity savings1	Baby received, look at the workmanship is still very fine, is genuine. I hope that this summer to open the air conditioning electricity bill is no longer high scary!	Jingdong prefecture level city in Shandong
Jinfeng Hongyuan Flagship Store	Electricity savings1	It's here. I don't know how it's going to work, but I'm going to use it.	Jingdong prefecture level city in Shandong
Jinfeng Hongyuan Flagship Store	Electricity savings1	Power saver received, Jingdong logistics first-class, business customer service attitude is good, one by one to introduce the product performance, back on the horse plugged in, it is very simple, there is a voltage indicator, to be a few months to see how the effect of power-saving to come back to a good follow-up, but I believe that Jinfenghong products.	Jingdong prefecture level city in Shandong
Jinfeng Hongyuan Flagship Store	Electricity savings1	Took the third one...the super power-saving kind!	Jingdong prefecture level city in Shandong
Jinfeng Hongyuan Flagship Store	Electricity savings1	First time buyer, customer service is very polite and courteous, conscientious business, support! Baby has received 🎈, super like, high value and practical, super like! ❥❥❥❥❥ Very good quality, very satisfied with a shopping. Customer service has a question and answer, very professional finally received the goods, very, very much like, exquisite and beautiful workmanship, has been praised by many people, really too happy, is already a loyal fan of his family, will continue to buy buy buy buy!	Jingdong prefecture level city in Shandong
Jinfeng Hongyuan Flagship Store	Electricity savings1	That's good. That's good. That's really good.	Jingdong prefecture level city in Shandong
Jinfeng Hongyuan Flagship Store	Electricity savings1	Saver received, good-looking design, voltage at a glance, and the merchant description, save some about 12% or so	Jingdong prefecture level city in Shandong
Jinfeng Hongyuan Flagship Store	Electricity savings1	Intelligent power saver is small and does not take up space, very good, very practical, received and used, packaging is tight, logistics is also very fast, satisfied with a shopping!	Jingdong prefecture level city in Shandong
Jinfeng Hongyuan Flagship Store	Electricity savings1	The stuff is good! Well made, compact, alloy case, shows voltage! Very like! Kudos!	Jingdong prefecture level city in Shandong
Jinfeng Hongyuan Flagship Store	Electricity savings1	I just purchased this power saver from Jingdong, inserted in the socket shows the number 206, a moment and 207 back and forth display, I do not know what it means, my home voltage should be normal, because it is the owner of the community's own power plant to put, first use for a period of time in the positive feedback it, first use in the look at it!	Jingdong prefecture level city in Shandong
Jinfeng Hongyuan Flagship Store	Electricity savings1	Very delicate and compact, there is a convenient intelligent display prompts the voltage, you can know the voltage at a glance, there is also very easy to use, a plug can be used, you can save electricity	Jingdong prefecture level city in Shandong
Jinfeng Hongyuan Flagship Store	Electricity savings1	Logistics is fast, ask customer service said can save electricity, but only use two months to see the effect, use two months in the evaluation.	Jingdong prefecture level city in Shandong
Jinfeng Hongyuan Flagship Store	Electricity savings1	The quality is very good.	Jingdong prefecture level city in Shandong
Jinfeng Hongyuan Flagship Store	Electricity savings1	Smart Saver has been received and will be evaluated after use	Jingdong prefecture level city in Shandong
Jinfeng Hongyuan Flagship Store	Electricity savings1	Home electricity is too waste, a month more than 300 dollars of electricity, and then the family heard that this can save a lot of electricity, let me order to buy a, anyway, will not, buy on the buy, thought it has to be a pretty big box it, delivery to the home to see a very small, plug-and-play, you can really save electricity, it's worth it!	Jingdong prefecture level city in Shandong
Jinfeng Hongyuan Flagship Store	Electricity savings1	Smart Saver received. Unpacking the box is exquisite. The material workmanship is very good. Plugged in and tried the space. Did not open the air conditioning voltage display 206 air conditioning open up the air conditioning display for 194. whether to save electricity to a month later to pay the electricity bill will know. Logistics is also very fast service is also in place.	Jingdong prefecture level city in Shandong
Jinfeng Hongyuan Flagship Store	Electricity savings1	The product feels pretty good and I hope it works, as the store owner said it would. Works!!!	Jingdong prefecture level city in Shandong
Jinfeng Hongyuan Flagship Store	Electricity savings1	Baby received, physical very quickly, just plugged in hope that it can be as everyone said those power saving, with two months after additional comments, first give a five-star praise.	Jingdong prefecture level city in Shandong
Jinfeng Hongyuan Flagship Store	Electricity savings1	Good, very good, very good, you deserve it, whoever uses it knows, the stuff works great, will buy it again in the future.	Jingdong prefecture level city in Shandong
Jinfeng Hongyuan Flagship Store	Electricity savings1	Don't hesitate to buy wow! No cheap feeling, very high-end, especially great! It is genuine, and the same as the entity store, the price is also favorable, decisive turn powder 💗 catch up to do activities when you buy, the price of a penny for a penny ♡, from the outside looking very beautiful 💗, the material workmanship is good 💗, feel that the purchase is worth it, and the logistics is super	Jingdong prefecture level city in Shandong
Jinfeng Hongyuan Flagship Store	Electricity savings1	Logistics is fast! Very good quality! Very beautiful! Next time to patronize!	Jingdong prefecture level city in Shandong
Jinfeng Hongyuan Flagship Store	Electricity savings1	Really like it, completely exceeded expectations, the shipping speed is very fast, the packaging is very careful and strict, the delivery speed is very fast, very satisfied with a shopping Baby received, and the description is the same, love it!	Jingdong prefecture level city in Shandong
Jinfeng Hongyuan Flagship Store	Electricity savings1	Very good, if you need to order!	Jingdong prefecture level city in Shandong

Jinfeng Hongyuan Flagship Store	Electricity savings1	So far, plugged in the voltage is more stable, as for the effect of how to quietly wait until the payment of the electricity bill to compare, the appearance of the commodity design is very good, the real thing!	Jingdong prefecture level city in Shandong
Jinfeng Hongyuan Flagship Store	Electricity savings1	📩 I've been wanting it for a long time, I decided to go for it, I feel great after using it, it's genuine and the delivery is great, it lives up to its name👍	Jingdong prefecture level city in Shandong
Jinfeng Hongyuan Flagship Store	Electricity savings1	Looking forward to it!!! I don't know if it's working or not, let's look forward to it!!!! How much is saved ，，，，，，，，， Saving a degree is a degree with long term use ！！！！！！！	Jingdong prefecture level city in Shandong
Jinfeng Hongyuan Flagship Store	Electricity savings1	After using it for a while, I am seriously and responsibly saying that this saver is really very power efficient. Two rooms with the air conditioner on at night for one night, plus watching TV, charging cell phones, lights, etc. etc. only used one degree of electricity. So I bought several more and it's really	Jingdong prefecture level city in Shandong
Jinfeng Hongyuan Flagship Store	Electricity savings1	It arrived quickly, and the box is solid and delicate. Baby shell is thick aluminum and by multi-layer coating. The inner core is heavy, and the power cord and plug are genuine products from a large manufacturer. It is the best power saver I have found after choosing all of them!	Jingdong prefecture level city in Shandong
Jinfeng Hongyuan Flagship Store	Electricity savings1	The saver was received, genuine guarantee, good quality...	Jingdong prefecture level city in Shandong
Jinfeng Hongyuan Flagship Store	Electricity savings1	Logistics was fast! The product is easy to use!	Jingdong prefecture level city in Shandong
Jinfeng Hongyuan Flagship Store	Electricity savings1	Look appearance is okay, use a period of time to know how it is. Jingdong Mall, trustworthy! Home appliances, cell phones and computers are almost 100% bought by Jingdong. Ordered the night before yesterday, received yesterday. Jingdong logistics, the fastest in the network!	Jingdong prefecture level city in Shandong
Jinfeng Hongyuan Flagship Store	Electricity savings1	Very good, perfect packaging, home energy saver to determine the effect of power saving and also play a role in stabilizing the voltage Very good products	Jingdong prefecture level city in Shandong
Jinfeng Hongyuan Flagship Store	Electricity savings1	Smart Saver received, looks very exquisite. The method of use is simple, and it has been used. The later effect, with time to test.	Jingdong prefecture level city in Shandong
Jinfeng Hongyuan Flagship Store	Electricity savings1	Both the material and workmanship exceeded expectations, great value, and looks great on oh. The seller also shipped much earlier than expected, will come back. Buy it, you will not regret it! Full five points. Both the material and workmanship exceeded expectations, great value, wear it also looks good Oh. Seller delivery is also much earlier than expected, will come again. Buy it, will not regret it! Full	Jingdong prefecture level city in Shandong
Jinfeng Hongyuan Flagship Store	Electricity savings1	The appearance is clean and beautiful, the workmanship is very good, I didn't find any defects, I personally feel that the size is also quite suitable, I've used it for 2 months, I didn't find out exactly how energy efficient it is. Try using it again!	Jingdong prefecture level city in Shandong
Jinfeng Hongyuan Flagship Store	Electricity savings1	Power saving device received, product certification, accessories, etc. complete, very good workmanship, very practical power-saving effect is also very obvious.	Jingdong prefecture level city in Shandong
Jinfeng Hongyuan Flagship Store	Electricity savings1	Looked at a lot of think buy this one, see a lot of reviews say it works, a month with save nearly twenty degrees of electricity, there are sixty days of return guarantee, with a good and then come back to the	Jingdong prefecture level city in Shandong
Jinfeng Hongyuan Flagship Store	Electricity savings1	I received the power saving device, and I'm using it. I hope I can get the desired effect, and I'm trying to force it to work quickly.	Jingdong prefecture level city in Shandong
Jinfeng Hongyuan Flagship Store	Electricity savings1	Voltage is stable, already trying it out and waiting for the results to happen, saving electricity will follow up next month!	Jingdong prefecture level city in Shandong
Jinfeng Hongyuan Flagship Store	Electricity savings1	Jingdong Mall self-supporting goods of excellent quality, logistics is also very fast, worth recommending 👏👏👏👏 excellent!	Jingdong prefecture level city in Shandong
Jinfeng Hongyuan Flagship Store	Electricity savings1	Power saving has been received, the appearance of the beautiful, I have already been the second time to buy the use of down the effect is very good!	Jingdong prefecture level city in Shandong
Jinfeng Hongyuan Flagship Store	Electricity savings1	Energy saver received very happy, the color is also very good looking forward to a very good effect can save electricity	Jingdong prefecture level city in Shandong
Jinfeng Hongyuan Flagship Store	Electricity savings1	It's small and easy to plug in and use. It displays the voltage value when plugged in and	Jingdong prefecture level city in Shandong
Jinfeng Hongyuan Flagship Store	Electricity savings1	This saver really works well, because I installed one at home about seven or eight years ago, when there is no voltage display, now there is a voltage display, the appearance also looks much better, the electricity bill is also saved, the stability of the home appliances is also good, the best of both	Jingdong prefecture level city in Shandong
Jinfeng Hongyuan Flagship Store	Electricity savings1	Ordered yesterday, arrived today in use. Shipping was fast. The effect is looking forward to.	Jingdong prefecture level city in Shandong
Jinfeng Hongyuan Flagship Store	Electricity savings1	This thing, I don't know what the principle is. Why it can save electricity. Wait for a month to use it first, see if it will save electricity bill, then come back to evaluate. There is one thing that does not fit the principle ah. The law of conservation of energy.	Jingdong prefecture level city in Shandong
Jinfeng Hongyuan Flagship Store	Electricity savings1	Home mom listen to others say this can save electricity, specially let buy a, later a very large, go back to see a particularly small, directly plugged into the electricity can be, no need to install, the price is also very cheap, the first time with this product, do not know if it is not good to use, temporarily try for a period of time, if it is good to use, recommend that you buy this one, the	Jingdong prefecture level city in Shandong
Jinfeng Hongyuan Flagship Store	Electricity savings1	I've been using it for a few days, and I think the workmanship and materials are pretty good, so we'll have to look at the next bill for the real energy savings, and then we'll add more comments.	Jingdong prefecture level city in Shandong
Jinfeng Hongyuan Flagship Store	Electricity savings1	Energy saver is well made, energy saving and power saving, and anti-counterfeiting code, it is a regular commodity!	Jingdong prefecture level city in Shandong
Jinfeng Hongyuan Flagship Store	Electricity savings1	Jinfeng Hongyuan, buy back home, as a mouse, haha. First of all, normal electricity for 1 month, the power did not increase, the electricity bill feels smoother, year-on-year electricity costs in the low, whether it is very energy-saving still have to be used for a long time to know. All 5 stars ☆	Jingdong prefecture level city in Shandong
Jinfeng Hongyuan Flagship Store	Electricity savings1	The box is well packaged and beautiful! I hope to save electricity with it haha!	Jingdong prefecture level city in Shandong
Jinfeng Hongyuan Flagship Store	Electricity savings1	Ordered last night, arrived this morning, first try the effect before making comments	Jingdong prefecture level city in Shandong
Jinfeng Hongyuan Flagship Store	Electricity savings1	Looks good. Don't know the effect yet.	Jingdong prefecture level city in Shandong
Jinfeng Hongyuan Flagship Store	Electricity savings1	Jinfeng Hongyuan intelligent power saver has exquisite workmanship and modern fashionable design style. It is a good home economizer . I believe that the goods purchased from Jingdong will not disappoint our	Jingdong prefecture level city in Shandong
Jinfeng Hongyuan Flagship Store	Electricity savings1	Inadvertently flipped to this power saver, never heard of, feel very novel, the price is not expensive, so buy to try, see a month after the electricity bill will be less, recently the home air-conditioning open a lot of power consumption is very large.	Jingdong prefecture level city in Shandong
Jinfeng Hongyuan Flagship Store	Electricity savings1	The quality of the goods is very good workmanship, but also sent five fuse	Jingdong prefecture level city in Shandong
Jinfeng Hongyuan Flagship Store	Electricity savings1	I'm trying it out. I don't know if it'll work.	Jingdong prefecture level city in Shandong
Jinfeng Hongyuan Flagship Store	Electricity savings1	There is no effect yet do not know, is to look at the official sale, buy to try it! Use according to the instructions, plugged in, used for a period of time, anyway, do not know how, after a few months to see it, to see if the average month than before will be less electricity.	Jingdong prefecture level city in Shandong
Jinfeng Hongyuan Flagship Store	Electricity savings1	Received the goods, the first time to unpacking, the quality is very good, consistent with the description of the seller, completely exceeded my expectations, the packaging is very careful, strict, in general this time is very satisfied with a shopping, thanks to the seller.	Jingdong prefecture level city in Shandong
Jinfeng Hongyuan Flagship Store	Electricity savings1	The energy saver was received and used for a few days with good results.	Jingdong prefecture level city in Shandong
Jinfeng Hongyuan Flagship Store	Electricity savings1	Previously I have paid attention to this product, but I think that the monthly electricity consumption at home is not large, there is no need to save electricity savings. After a second thought, energy saving is the responsibility of each of our citizens, the winter is coming, this year's winter is cold, heating and all electric appliances, can save is to save, the price is not expensive, so buy to try. I analyze the principle of power saving, should be able to save electricity, but the larger the power saving effect is about good. To be tried after a month or two to see the effect and then evaluate. Jingdong trustworthy.	Jingdong prefecture level city in Shandong
Jinfeng Hongyuan Flagship Store	Electricity savings1	It just arrived and I can't wait to use it, and the customer service is great! There is also voltage showing up which is nice! I don't know if it can save a bit I won't know until I've used it for a month! When the time comes to come back to the follow-up review!	Jingdong prefecture level city in Shandong
Jinfeng Hongyuan Flagship Store	Electricity savings1	Checked out the reviews from numerous users. The shipment is still very large, so I decided to buy it anyway to use it and see the effect after a month. Jingdong Mall here to buy safe, convenient and fast, this price is very powerful 💪, very recommended.	Jingdong prefecture level city in Shandong
Jinfeng Hongyuan Flagship Store	Electricity savings1	Purchased two at the same time to use together. The other one works fine, the other has problems with the numbers not sure if the machine is faulty. Power saving effect can not be observed. Feel the effect is not very obvious like?	Jingdong prefecture level city in Shandong

Jinfeng Hongyuan Flagship Store	Electricity savings1	Looking forward to it!!! I don't know if it's working or not, let's look forward to it!!!! How much is saved , , , , , , , , , Saving a degree is a degree with long term use ! ! ! ! ! ! !	Jingdong prefecture level city in Shandong
Jinfeng Hongyuan Flagship Store	Electricity savings1	Received, directly on the use of the special convenient	Jingdong prefecture level city in Shandong
Jinfeng Hongyuan Flagship Store	Electricity savings1	Goods received a few days well-packed, the same day opened and plugged in to try to be normal is not sure what the power-saving effect. After a month with a follow-up evaluation.	Jingdong prefecture level city in Shandong
Jinfeng Hongyuan Flagship Store	Electricity savings1	I bought it and used it, I don't know how it works yet, we'll see later. Use the effect of it	Jingdong prefecture level city in Shandong
Jinfeng Hongyuan Flagship Store	Electricity savings1	Photographed 2, the old family also put a, with a small January, there should be some effect, with a long period of time to comment, with a long time to see the obvious effect, hard courier brother.	Jingdong prefecture level city in Shandong
Jinfeng Hongyuan Flagship Store	Electricity savings1	Experience: I am an electrician, this is the real use of electronic waves automatically connected to track the decomposition of ionized electrons, in situ compensation of reactive power, reduce effective power loss and waste, stabilize the voltage to achieve energy saving and power saving, the overall effect	Jingdong prefecture level city in Shandong
Jinfeng Hongyuan Flagship Store	Electricity savings1	Great looking and practical! Easy to use and save! Saves 15% in a month of comparison	Jingdong prefecture level city in Shandong
Jinfeng Hongyuan Flagship Store	Electricity savings1	Product quality is very good! Packaging is very nice, like a cell phone box, thick and well protected! Service is also great! Logistics is very fast! Thank you for your business! Very honest!	Jingdong prefecture level city in Shandong
Jinfeng Hongyuan Flagship Store	Electricity savings1	In several products inside the choice of this purchase for a period of time, but also need to use a few months to compare last year's electricity bill before you can analyze the savings in electricity, to the summer peak electricity consumption should be more obvious.	Jingdong prefecture level city in Shandong
Jinfeng Hongyuan Flagship Store	Electricity savings1	The goods have long been received, after more than a month to comment, the home electricity bill is indeed reduced, worth buying!	Jingdong prefecture level city in Shandong
Jinfeng Hongyuan Flagship Store	Electricity savings1	Baby received, the logistics is strong, the quality is reliable, it is worth having and recommending!	Jingdong prefecture level city in Shandong
Jinfeng Hongyuan Flagship Store	Electricity savings1	Using a conservation appliance, you can achieve a 10-15% savings in electricity usage.	Jingdong prefecture level city in Shandong
Jinfeng Hongyuan Flagship Store	Electricity savings1	Quality is very good, metal shell, touch is very good, very recommended Oh ~, the subsequent effect of continuous follow-up	Jingdong prefecture level city in Shandong
Jinfeng Hongyuan Flagship Store	Electricity savings1	The product is beautiful, practical, worth buying. The product is beautiful, practical, good quality, fast logistics product is beautiful, practical, beautiful and elegant, very classy, worth buying!	Jingdong prefecture level city in Shandong
Jinfeng Hongyuan Flagship Store	Electricity savings1	Just received the item and have already used it. Didn't know much about these products before. I hope it is as effective and useful as the product advertises.	Jingdong prefecture level city in Shandong
Jinfeng Hongyuan Flagship Store	Electricity savings1	A month two people used more than five hundred degrees of electricity, in a hurry and then add a power saver, I hope to have an effect, Jingdong shopping is very cool, logistics is very fast especially good service attitude delivery, this hot day is not easy, thank you!	Jingdong prefecture level city in Shandong
Jinfeng Hongyuan Flagship Store	Electricity savings1	Just received the baby, first plugged in to try to use a month and then additional evaluation it	Jingdong prefecture level city in Shandong
Jinfeng Hongyuan Flagship Store	Electricity savings1	Goods fast, the power saver has been received, a friend recommended to buy that can save electricity. Exquisite and compact is very convenient, plug and play. The effect is good, the price is affordable, the next need will patronize again!	Jingdong prefecture level city in Shandong
Jinfeng Hongyuan Flagship Store	Electricity savings1	Packaging is better than expected, sent a few fuse, power saving effect in the end do not know, try a period of time to say!	Jingdong prefecture level city in Shandong
Jinfeng Hongyuan Flagship Store	Electricity savings1	Shipped quickly, immediately installed, read countless reviews were not sure if this thing is effective, was going to buy three at once, ultimately decided to first use a try the effect, if the power ring significantly reduced, then will buy a few more!	Jingdong prefecture level city in Shandong
Jinfeng Hongyuan Flagship Store	Electricity savings1	Used for a period of time to evaluate, not to a month still do not know whether it can save electricity, the voltage has a display!	Jingdong prefecture level city in Shandong
Jinfeng Hongyuan Flagship Store	Electricity savings1	I don't know if it saves electricity or not, but I'm using it anyway, so I hope it saves a little bit, electricity is too expensive	Jingdong prefecture level city in Shandong
Jinfeng Hongyuan Flagship Store	Electricity savings1	The power saver has been received and I am very happy with it! Just waiting to see how the power saving works!	Jingdong prefecture level city in Shandong
Jinfeng Hongyuan Flagship Store	Electricity savings1	Saving power has been received, logistics is quite fast, the quality is not bad, small and a little weight, has been used to look forward to the effect of	Jingdong prefecture level city in Shandong
Jinfeng Hongyuan Flagship Store	Electricity savings1	The logistics is very fast, the effect is obvious and trustworthy.	Jingdong prefecture level city in Shandong
Jinfeng Hongyuan Flagship Store	Electricity savings1	Good quality, good quality, beautiful, beautiful, beautiful, beautiful, beautiful, very worth buying, worth buying, worth buying, worth buying, worth customizing!	Jingdong prefecture level city in Shandong
Jinfeng Hongyuan Flagship Store	Electricity savings1	I've used it for now, I don't know if it saves electricity	Jingdong prefecture level city in Shandong
Jinfeng Hongyuan Flagship Store	Electricity savings1	Bought two at a time, logistics is very fast, the product is beautifully packaged, after two months to see the results	Jingdong prefecture level city in Shandong
Jinfeng Hongyuan Flagship Store	Electricity savings1	The workmanship is really fine, the packaging is also good, the regular factory production ~ use a break in the evaluation of the effectiveness of the	Jingdong prefecture level city in Shandong
Jinfeng Hongyuan Flagship Store	Electricity savings1	Sometimes 219. Sometimes 220 in this case	Jingdong prefecture level city in Shandong
Jinfeng Hongyuan Flagship Store	Electricity savings1	It is the ideal product, good quality, logistics is also very fast, worthy of praise!	Jingdong prefecture level city in Shandong
Jinfeng Hongyuan Flagship Store	Electricity savings1	Store service is very good, the courier received the next day. Product packaging is very good, just use the quality to be tested.	Jingdong prefecture level city in Shandong
Jinfeng Hongyuan Flagship Store	Electricity savings1	I've been using it for almost a month now and I feel like it's working.	Jingdong prefecture level city in Shandong
Jinfeng Hongyuan Flagship Store	Electricity savings1	Very satisfied, very restful, very pretty, very beautiful, very much enjoyed! Well worth it!	Jingdong prefecture level city in Shandong
Jinfeng Hongyuan Flagship Store	Electricity savings1	Baby especially like, the value of high-quality good, a very good shopping experience, attentive service, fast logistics, distribution is also very thoughtful, attitude is also very good, value for money ah, decisive praise! Quality is quite good, logistics is also very fast, praise!	Jingdong prefecture level city in Shandong
Jinfeng Hongyuan Flagship Store	Electricity savings1	The shape of the item is basically the same as ordered, the effect is to be examined.	Jingdong prefecture level city in Shandong
Jinfeng Hongyuan Flagship Store	Electricity savings1	Appearance style: good-looking workmanship material: very good, a small one, very delicate Experience: still in use, in the process of using the shopkeeper is very nice to have a careful explanation Use for a period of time in the look at the results	Jingdong prefecture level city in Shandong
Jinfeng Hongyuan Flagship Store	Electricity savings1	As described, compact and well packaged	Jingdong prefecture level city in Shandong
Jinfeng Hongyuan Flagship Store	Electricity savings1	Fine workmanship. Test in progress. Let's see how it works.	Jingdong prefecture level city in Shandong
Jinfeng Hongyuan Flagship Store	Electricity savings1	The goods have been received, I'm very satisfied and love it	Jingdong prefecture level city in Shandong
Jinfeng Hongyuan Flagship Store	Electricity savings1	Safety! Whether you can save electricity, you can roughly feel it in 2 months. The principle is simple. Before the electrical appliances and circuits inductive more, now the electrical appliances integrated, the home is not a lot of inductive circuits, but used certainly save no problem! Wiring according to the requirements of the manual is good, if it is not convenient, see the socket plug.	Jingdong prefecture level city in Shandong
Jinfeng Hongyuan Flagship Store	Electricity savings1	Great product, looking forward to how much power I can save, first time buyer!	Jingdong prefecture level city in Shandong
Jinfeng Hongyuan Flagship Store	Electricity savings1	The product is exquisite, beautifully packaged, and the customer service is excellent. Just waiting to see how the power saving effect is, looking forward to it!	Jingdong prefecture level city in Shandong
Jinfeng Hongyuan Flagship Store	Electricity savings1	Express very quickly very satisfied with the first use to see how the effect to know, if you can save 10 percent even if acceptable, if you can not meet this requirement is obviously not much effect. Obviously.	Jingdong prefecture level city in Shandong
Jinfeng Hongyuan Flagship Store	Electricity savings1	Very delicate and compact, there is a very convenient intelligent display prompts the voltage, you can know the voltage at a glance, there is very easy to use, a plug can be used, I hope to save money on	Jingdong prefecture level city in Shandong
Jinfeng Hongyuan Flagship Store	Electricity savings1	First time buyer is it really save after use	Jingdong prefecture level city in Shandong
Jinfeng Hongyuan Flagship Store	Electricity savings1	Purchased again and will be for a long time yet.	Jingdong prefecture level city in Shandong

Jinfeng Hongyuan Flagship Store	Electricity savings1	Bought it to stock up, came back and plugged it in, hope it works	Jingdong prefecture level city in Shandong
Jinfeng Hongyuan Flagship Store	Electricity savings1	Baby received the logistics very fast very like very satisfied praise a	Jingdong prefecture level city in Shandong
Jinfeng Hongyuan Flagship Store	Electricity savings1	Beginning the process of using, easy to evaluate more later.	Jingdong prefecture level city in Shandong
Jinfeng Hongyuan Flagship Store	Electricity savings1	The goods have been received, just do not know how effective	Jingdong prefecture level city in Shandong
Jinfeng Hongyuan Flagship Store	Electricity savings1	It feels okay. I wish there were more events.	Jingdong prefecture level city in Shandong
Jinfeng Hongyuan Flagship Store	Electricity savings1	Things have been received, just plugged in, I do not know if it works, wait a few days to have the effect of additional	Jingdong prefecture level city in Shandong
Jinfeng Hongyuan Flagship Store	Electricity savings1	The effect of this power saver is really good, the house more, a few air conditioners are open every day, not used before a month to say less than seven hundred eight hundred, used after three months only nine	Jingdong prefecture level city in Shandong
Jinfeng Hongyuan Flagship Store	Electricity savings1	Cheap, good and easy to use, recommend it.	Jingdong prefecture level city in Shandong
Jinfeng Hongyuan Flagship Store	Electricity savings1	Bought this device, to the home for a long time, wait to finish a month to see how, good can recommend friends to buy, we use the electricity every month scary, a month of electricity costs more than a thousand, can not afford to hurt ah! I hope this power saving can save me some money, the beginning of the shoot order did not pay attention to look at the address, sent elsewhere to go, that side called to find out, hurry to communicate with customer service, customer service is very careful to help me get it done, call to that side to help me turn around, thank you!	Jingdong prefecture level city in Shandong
Jinfeng Hongyuan Flagship Store	Electricity savings1	I don't know the effect if it, use it first to see.	Jingdong prefecture level city in Shandong
Jinfeng Hongyuan Flagship Store	Electricity savings1	Looks good. I won't know if it saves power until I use it.	Jingdong prefecture level city in Shandong
Jinfeng Hongyuan Flagship Store	Electricity savings1	Goods received, shipping speed is very fast, yesterday afternoon more than two o'clock shot, just express delivery, but also sent a few insurance, very good business, the display of the number do not understand what is the meaning of the display said voltage, do not understand this, try a month to see how, good and	Jingdong prefecture level city in Shandong
Jinfeng Hongyuan Flagship Store	Electricity savings1	What ** play also, save electricity more and more save more and more, save chicken hair electricity, are cheating, this is a night of air conditioning with electricity, a night with 11 electricity word, this is	Jingdong prefecture level city in Shandong
Jinfeng Hongyuan Flagship Store	Electricity savings1	Received the product, hope it works!	Jingdong prefecture level city in Shandong
Jinfeng Hongyuan Flagship Store	Electricity savings1	Bought home and used very convenient, easy to operate, quite good	Jingdong prefecture level city in Shandong
Jinfeng Hongyuan Flagship Store	Electricity savings1	It's really good, cost-effective and good quality, and the logistics are fast! It arrived in one day! Really good, cost-effective and good quality, logistics is fast ah! It arrived in one day!	Jingdong prefecture level city in Shandong
Jinfeng Hongyuan Flagship Store	Electricity savings1	Works well, stabilizes voltage and saves power	Jingdong prefecture level city in Shandong
Jinfeng Hongyuan Flagship Store	Electricity savings1	I'll use it right away and look forward to good results	Jingdong prefecture level city in Shandong
Jinfeng Hongyuan Flagship Store	Electricity savings1	Recommended by a friend, said it works well, so I bought one, the effect to be tested	Jingdong prefecture level city in Shandong
Jinfeng Hongyuan Flagship Store	Electricity savings1	I didn't know it would save electricity.	Jingdong prefecture level city in Shandong
Jinfeng Hongyuan Flagship Store	Electricity savings1	I've been using it for a week, I don't know what's going on yet I hope it works well and saves power I'll follow up after a month of use	Jingdong prefecture level city in Shandong
Jinfeng Hongyuan Flagship Store	Electricity savings1	Very well packaged, looks great, don't know how it works	Jingdong prefecture level city in Shandong
Jinfeng Hongyuan Flagship Store	Electricity savings1	Genuine, good quality, really saves energy.	Jingdong prefecture level city in Shandong
Jinfeng Hongyuan Flagship Store	Electricity savings1	I was worried that the stuff is not good, but when I got it, I realized that I was completely over-anxious, 5 stars!	Jingdong prefecture level city in Shandong
Jinfeng Hongyuan Flagship Store	Electricity savings1	Very good and satisfied trustworthy	Jingdong prefecture level city in Shandong
Jinfeng Hongyuan Flagship Store	Electricity savings1	I don't know if it saves power	Jingdong prefecture level city in Shandong
Jinfeng Hongyuan Flagship Store	Electricity savings1	The quality of the product is very good, beautiful and generous applicable fast delivery speed good service attitude	Jingdong prefecture level city in Shandong
Jinfeng Hongyuan Flagship Store	Electricity savings1	Let's use it for a while and then we'll follow up	Jingdong prefecture level city in Shandong
Jinfeng Hongyuan Flagship Store	Electricity savings1	I've only been using it for a month, I don't know if there's any change yet, I'll come back with a follow-up comment after checking the meter!	Jingdong prefecture level city in Shandong
Jinfeng Hongyuan Flagship Store	Electricity savings1	I don't know if it works, I read the instructions and online reviews, I hope I won't be disappointed, if I use it, I will buy it again. Jingdong Express, fast and guaranteed service.	Jingdong prefecture level city in Shandong
Jinfeng Hongyuan Flagship Store	Electricity savings1	It feels like it saves quite a bit huh?	Jingdong prefecture level city in Shandong
Jinfeng Hongyuan Flagship Store	Electricity savings1	I've been using it for a while now, and it still seems to work and have a power saving effect	Jingdong prefecture level city in Shandong
Jinfeng Hongyuan Flagship Store	Electricity savings1	Looks good. It says 235. The 222 bar voltage is shown as high. I don't know if that's right. We'll see.	Jingdong prefecture level city in Shandong
Jinfeng Hongyuan Flagship Store	Electricity savings1	Baby is really good, do really careful, worth to have, fast delivery!	Jingdong prefecture level city in Shandong
Jinfeng Hongyuan Flagship Store	Electricity savings1	Quite delicate and compact, simple and convenient to use. Things are still very good, bought two at a time. Very satisfied with an online purchase.	Jingdong prefecture level city in Shandong
Jinfeng Hongyuan Flagship Store	Electricity savings1	Feel very good, the use of unclear, ask customer service, timely explanation, big brand, trustworthy	Jingdong prefecture level city in Shandong
Jinfeng Hongyuan Flagship Store	Electricity savings1	Thrifty received a few days did not make a comment is to see how the quality of the product now feel the quality of good appearance is also powerful seller service is very good to buy the product is also attached to the 5 fuse thought very thoughtful!	Jingdong prefecture level city in Shandong
Jinfeng Hongyuan Flagship Store	Electricity savings1	Big brand, with a very assured, the quality is very good, in time for the event to buy very cost-effective, customer service is really very good Oh ~!	Jingdong prefecture level city in Shandong
Jinfeng Hongyuan Flagship Store	Electricity savings1	I ordered yesterday and received it this morning, I heard my friend say that it can save electricity, I don't know if it works, I'll come back to make a comment after using it.	Jingdong prefecture level city in Shandong
Jinfeng Hongyuan Flagship Store	Electricity savings1	I've been using it for over a month and it doesn't seem to be working!	Jingdong prefecture level city in Shandong
Jinfeng Hongyuan Flagship Store	Electricity savings1	Used it, the family has always been in the habit of using energy-saving devices, this is a new one. Digital display voltage is very good, can see that the voltage is more stable.	Jingdong prefecture level city in Shandong
Jinfeng Hongyuan Flagship Store	Electricity savings1	I haven't used it for more than a month, but it looks good. It's easy to plug into an outlet and it works.	Jingdong prefecture level city in Shandong
Jinfeng Hongyuan Flagship Store	Electricity savings1	Good Oh...appearance is very beautiful...just plugged in with...see the effect of it...with a period of time in the additional evaluation...	Jingdong prefecture level city in Shandong
Jinfeng Hongyuan Flagship Store	Electricity savings1	I've received the goods, whether it's good or not, I'll know after I use it.	Jingdong prefecture level city in Shandong
Jinfeng Hongyuan Flagship Store	Electricity savings1	Very good, logistics is fast, the stuff is very powerful and cost-effective! Can feel the meter slowing down	Jingdong prefecture level city in Shandong
Jinfeng Hongyuan Flagship Store	Electricity savings1	Logistics is very fast, get it immediately after use, easy to operate, plug in the power supply can be used, just do not know to the use of the effect of how.	Jingdong prefecture level city in Shandong
Jinfeng Hongyuan Flagship Store	Electricity savings1	Quality is very over the top, quality is guaranteed, quite a good choice, a lot of friends to see me use all want to buy, I recommend it!	Jingdong prefecture level city in Shandong

Jinfeng Hongyuan Flagship Store	Electricity savings1	The appearance is clean and beautiful, the workmanship is very good, did not find any flaws, I personally feel that the size is also quite suitable, looking for a long time to choose this one, big love! The price is very beautiful, things are still good, will continue to buy back! All five points! Ness Ness ~ logistics speed is really no words, is a word: fast. Commodities from the outer packaging is very high-grade, really a penny a penny, expensive has the reason for expensive	Jingdong prefecture level city in Shandong
Jinfeng Hongyuan Flagship Store	Electricity savings1	The logistics is very fast, the texture is very good, genuine, I like it very much.	Jingdong prefecture level city in Shandong
Jinfeng Hongyuan Flagship Store	Electricity savings1	It's been a few days since I put it on, I can't see any results now, waiting for a month to say, steady steady steady.	Jingdong prefecture level city in Shandong
Jinfeng Hongyuan Flagship Store	Electricity savings1	I've been using it for a while now and it should be fine, so I'm buying it for the second time.	Jingdong prefecture level city in Shandong
Jinfeng Hongyuan Flagship Store	Electricity savings1	Power Saving King has been received, looks good, quite delicate, looking forward to using it to save electricity	Jingdong prefecture level city in Shandong
Jinfeng Hongyuan Flagship Store	Electricity savings1	My mom said the home uses a little too much electricity, so I bought a Power Saver to try it out, I don't know how effective it is. Expect it to be a good thing!	Jingdong prefecture level city in Shandong
Jinfeng Hongyuan Flagship Store	Electricity savings1	Thank you Jingdong, logistics is very fast order to receipt only one day. Believe in Jingdong. Favorable comments	Jingdong prefecture level city in Shandong
Jinfeng Hongyuan Flagship Store	Electricity savings1	Saved some money on the electric bill compared to last month, watch this month again, no problems so far	Jingdong prefecture level city in Shandong
Jinfeng Hongyuan Flagship Store	Electricity savings1	Energy saver exquisite workmanship, beautiful style, more features, just bought, power saving effect to be used for a period of time, then evaluate!	Jingdong prefecture level city in Shandong
Jinfeng Hongyuan Flagship Store	Electricity savings1	This thing does save power is more compact to hang on the wall.	Jingdong prefecture level city in Shandong
Jinfeng Hongyuan Flagship Store	Electricity savings1	I've been buying it for a while now, I haven't actually measured it, but it still feels like it's effective	Jingdong prefecture level city in Shandong
Jinfeng Hongyuan Flagship Store	Electricity savings1	I've already used it since I bought it, I don't know how effective it is yet, I'll comment on it later.	Jingdong prefecture level city in Shandong
Jinfeng Hongyuan Flagship Store	Electricity savings1	Baby received, careful appearance, logistics is very fast, worth buying!	Jingdong prefecture level city in Shandong
Jinfeng Hongyuan Flagship Store	Electricity savings1	It's great, it's just not the same in hand, I bought it at that other cheaper online one for about the same price as this one. But then oops, doesn't work at all.	Jingdong prefecture level city in Shandong
Jinfeng Hongyuan Flagship Store	Electricity savings1	The product is exquisite, but you have to use it for a month to know how it works. I'll comment again after a month.	Jingdong prefecture level city in Shandong
Jinfeng Hongyuan Flagship Store	Electricity savings1	I've been using it for a while now, and it doesn't feel like it's working, and it doesn't save any power.	Jingdong prefecture level city in Shandong
Jinfeng Hongyuan Flagship Store	Electricity savings1	Affordable price, fast delivery, Jingdong self-supporting guarantee	Jingdong prefecture level city in Shandong
Jinfeng Hongyuan Flagship Store	Electricity savings1	Packaging is very good, no damage, good quality, fine workmanship Packaging is very good, no damage, good quality, fine workmanship Packaging is very good, no damage, good quality, fine workmanship Packaging is very good, no damage, good quality, fine workmanship	Jingdong prefecture level city in Shandong
Jinfeng Hongyuan Flagship Store	Electricity savings1	Big brand, with a very assured, the quality is very good, in time for the event to buy very cost-effective, customer service is really very good Oh ~!	Jingdong prefecture level city in Shandong
Jinfeng Hongyuan Flagship Store	Electricity savings1	Used a few days, the specific effect is not yet clear, looking forward to the effect as described by the merchant!	Jingdong prefecture level city in Shandong
Jinfeng Hongyuan Flagship Store	Electricity savings1	I just used it and don't know how it works, I'll follow up after using it!	Jingdong prefecture level city in Shandong
Jinfeng Hongyuan Flagship Store	Electricity savings1	Well, it's been installed and now I'm waiting to see the results, BOE shopping, hopefully I won't be disappointed!	Jingdong prefecture level city in Shandong
Jinfeng Hongyuan Flagship Store	Electricity savings1	Goods received, plugged in the voltage from 229 to 222, do not know whether to save electricity, that depends on the situation.	Jingdong prefecture level city in Shandong
Jinfeng Hongyuan Flagship Store	Electricity savings1	This is the second time I have bought the product and I feel it really saves electricity! Use it again for a while and then evaluate!	Jingdong prefecture level city in Shandong
Jinfeng Hongyuan Flagship Store	Electricity savings1	Overall, it's really good, and the customer service is great, you'll get a message back almost immediately when you speak, it's great value for money, I really recommend it!	Jingdong prefecture level city in Shandong
Jinfeng Hongyuan Flagship Store	Electricity savings1	✨ Have purchased many times, a must have for the family, affordable purchase price, fast delivery, 🎁 beautifully packaged!	Jingdong prefecture level city in Shandong
Jinfeng Hongyuan Flagship Store	Electricity savings1	I bought it after a friend introduced it, he said it is effective and can save electricity. I bought it to try, try a period of time and then follow up!	Jingdong prefecture level city in Shandong
Jinfeng Hongyuan Flagship Store	Electricity savings1	Received it, trying it out tonight, and bought one earlier.	Jingdong prefecture level city in Shandong
Jinfeng Hongyuan Flagship Store	Electricity savings1	It will take a month or two to see if it saves power or not.	Jingdong prefecture level city in Shandong
Jinfeng Hongyuan Flagship Store	Electricity savings1	Looked good in the instructions! Bought one and pressed it on! Use it and see! If it works! I'll buy some more!	Jingdong prefecture level city in Shandong
Jinfeng Hongyuan Flagship Store	Electricity savings1	Goods have been received packaging can also things are not bad appearance, with a use to know, now evaluation is nonsense.	Jingdong prefecture level city in Shandong
Jinfeng Hongyuan Flagship Store	Electricity savings1	Logistics is very fast, packaging is very good, the second unit, as always, looking forward to the effect of	Jingdong prefecture level city in Shandong
Jinfeng Hongyuan Flagship Store	Electricity savings1	I received the goods today, the quality of goods is very good, I am very satisfied ah.	Jingdong prefecture level city in Shandong
Jinfeng Hongyuan Flagship Store	Electricity savings1	Buy one and try it, I wonder if it has a power saving effect?	Jingdong prefecture level city in Shandong
Jinfeng Hongyuan Flagship Store	Electricity savings1	🎈 The item was received, well packaged, logistics is very fast, customer service attitude is very positive, there are questions answered in a timely manner! Overall very satisfied!	Jingdong prefecture level city in Shandong
Jinfeng Hongyuan Flagship Store	Electricity savings1	Baby packaging is very good, purchase submission goods quickly delivered to the home, use and then follow up.	Jingdong prefecture level city in Shandong
Jinfeng Hongyuan Flagship Store	Electricity savings1	My friend asked me to buy an extra one so he could try it too.	Jingdong prefecture level city in Shandong
Jinfeng Hongyuan Flagship Store	Electricity savings1	I've just put it on, I won't know the result until a month later if it will really save power.	Jingdong prefecture level city in Shandong
Jinfeng Hongyuan Flagship Store	Electricity savings1	It's good quality. Hopefully it'll help save power.	Jingdong prefecture level city in Shandong
Jinfeng Hongyuan Flagship Store	Electricity savings1	The appearance is clean and beautiful, the workmanship is very good, I did not find any flaws, I personally feel that the size is also quite suitable, I have been looking for a long time to choose this	Jingdong prefecture level city in Shandong
Jinfeng Hongyuan Flagship Store	Electricity savings1	Logistics is fast! Baby looks great! The price is fair! Wish the store business is prosperous!	Jingdong prefecture level city in Shandong
Jinfeng Hongyuan Flagship Store	Electricity savings1	Jingdong express fast, good, have used, sit and wait to save money...	Jingdong prefecture level city in Shandong
Jinfeng Hongyuan Flagship Store	Electricity savings1	In use, it didn't work as expected and there was nothing noticeable in terms of power savings.	Jingdong prefecture level city in Shandong
Jinfeng Hongyuan Flagship Store	Electricity savings1	Just started using it and looking forward to the results.	Jingdong prefecture level city in Shandong
Jinfeng Hongyuan Flagship Store	Electricity savings1	The quality is very good, the metal shell is very good, is genuine, energy saving and power saving, logistics is very fast, there is a need to buy o, recommend everyone, very satisfied with an online	Jingdong prefecture level city in Shandong
Jinfeng Hongyuan Flagship Store	Electricity savings1	Received, I do not know if the product is good, used to see if it is good to come back to buy!	Jingdong prefecture level city in Shandong
Jinfeng Hongyuan Flagship Store	Electricity savings1	Jinfeng source of intelligent power saving treasure also used less than three months, the last thunder may be burned, the home of the electric send gate is not on, only the old man at home, no way only their own in the field and run back to find out what is the problem with the circuit, the quality is still not very good, thunder is easy to burn!	Jingdong prefecture level city in Shandong
Jinfeng Hongyuan Flagship Store	Electricity savings1	Saving power just started to use, I do not know if there is no effect, but read a lot of reviews are said to be very good so I bought, baby All aspects are good There are spare 2 fuses, late good and then follow	Jingdong prefecture level city in Shandong

Jinfeng Hongyuan Flagship Store	Electricity savings1	Use it for a few days and see, the delivery is quite fast	Jingdong prefecture level city in Shandong
Jinfeng Hongyuan Flagship Store	Electricity savings1	Small, well made and easy to use.	Jingdong prefecture level city in Shandong
Jinfeng Hongyuan Flagship Store	Electricity savings1	Beautifully packaged, it is genuine. Whether it is power saving, use it to evaluate.	Jingdong prefecture level city in Shandong
Jinfeng Hongyuan Flagship Store	Electricity savings1	Power saver. Used two of them and they do save quite a bit of power.	Jingdong prefecture level city in Shandong
Jinfeng Hongyuan Flagship Store	Electricity savings1	Workmanship is not bad, small and does not take up space, is also do not know whether the power saving, buy a to try!	Jingdong prefecture level city in Shandong
Jinfeng Hongyuan Flagship Store	Electricity savings1	The logistics is very fast, the courier attitude is very good, worth buying	Jingdong prefecture level city in Shandong
Jinfeng Hongyuan Flagship Store	Electricity savings1	The effect is not known until it is used.	Jingdong prefecture level city in Shandong
Jinfeng Hongyuan Flagship Store	Electricity savings1	Just arrived plugged in won't know for a month	Jingdong prefecture level city in Shandong
Jinfeng Hongyuan Flagship Store	Electricity savings1	This is really good to use the pull to realize the savings ah think again for my sister's family to buy a	Jingdong prefecture level city in Shandong
Jinfeng Hongyuan Flagship Store	Electricity savings1	Lies, a month with a few hundred electricity are no savings, family use	Jingdong prefecture level city in Shandong
Jinfeng Hongyuan Flagship Store	Electricity savings1	Express very quickly, look at the fine workmanship also sent a few fuse, look forward to can really save electricity	Jingdong prefecture level city in Shandong
Jinfeng Hongyuan Flagship Store	Electricity savings1	I didn't realize it was so small.	Jingdong prefecture level city in Shandong
Jinfeng Hongyuan Flagship Store	Electricity savings1	Logistics courier is very fast, the courier service is very good	Jingdong prefecture level city in Shandong
Jinfeng Hongyuan Flagship Store	Electricity savings1	Used for a period of time the effect is quite good indeed than the previous power saving	Jingdong prefecture level city in Shandong
Jinfeng Hongyuan Flagship Store	Electricity savings1	Logistics is super fast, the product is in use, see the effect...	Jingdong prefecture level city in Shandong
Jinfeng Hongyuan Flagship Store	Electricity savings1	Baby got it, tried it out and it didn't work at all. How can I use the tips to save power?	Jingdong prefecture level city in Shandong
Jinfeng Hongyuan Flagship Store	Electricity savings1	Power Saving King received, immediately used has been very stable without any sound good, good service, fast logistics inexpensive and beautiful worth repurchasing!	Jingdong prefecture level city in Shandong
Jinfeng Hongyuan Flagship Store	Electricity savings1	Logistics courier is very fast, the courier service is good	Jingdong prefecture level city in Shandong
Jinfeng Hongyuan Flagship Store	Electricity savings1	Good to recommend, the first month of air conditioning only 1 week, the electricity bill has reduced about 100 yuan, may be to reduce the use of air conditioning is also related to the other or as usual electricity, estimated that the longer you use the more you can save electricity.	Jingdong prefecture level city in Shandong
Jinfeng Hongyuan Flagship Store	Electricity savings1	It doesn't work. I've been using it for 20 days and it doesn't work.	Jingdong prefecture level city in Shandong
Jinfeng Hongyuan Flagship Store	Electricity savings1	Fast shipping, good quality, affordable price, very satisfied with a shopping.	Jingdong prefecture level city in Shandong
Jinfeng Hongyuan Flagship Store	Electricity savings1	Try it for a month to see if it saves money.	Jingdong prefecture level city in Shandong
Jinfeng Hongyuan Flagship Store	Electricity savings1	It's in use. I'll comment again after a while.	Jingdong prefecture level city in Shandong
Jinfeng Hongyuan Flagship Store	Electricity savings1	Goods have received look good 👍👍👍👍, use a period of time and then look at the	Jingdong prefecture level city in Shandong
Jinfeng Hongyuan Flagship Store	Electricity savings1	No, it doesn't work. It's useless. It's garbage.	Jingdong prefecture level city in Shandong
Jinfeng Hongyuan Flagship Store	Electricity savings1	I've been using it for almost 2 months now and I don't feel any reduction in my electricity bill.	Jingdong prefecture level city in Shandong
Jinfeng Hongyuan Flagship Store	Electricity savings1	I've received the goods, the logistics is really fast, ordered yesterday, arrived today.	Jingdong prefecture level city in Shandong
Jinfeng Hongyuan Flagship Store	Electricity savings1	And the backup insurance.	Jingdong prefecture level city in Shandong
Jinfeng Hongyuan Flagship Store	Electricity savings1	The appearance of this product is good, easy to use and install, the effect of how there is no use to wait for time to verify that the voltage displayed is different from the voltage measured by other meters, the gap is not too large, the follow-up to add the effect.	Jingdong prefecture level city in Shandong
Jinfeng Hongyuan Flagship Store	Electricity savings1	Baby received it 👌, really great ah! 🌈The quality can't be beat, very satisfied! 🆗Happy happy happy 😃	Jingdong prefecture level city in Shandong
Jinfeng Hongyuan Flagship Store	Electricity savings1	I'll follow up with a review after using it for a while, hopefully it won't bring me back a disappointment!	Jingdong prefecture level city in Shandong
Jinfeng Hongyuan Flagship Store	Electricity savings1	In use and looking forward to results! Good review to send	Jingdong prefecture level city in Shandong
Jinfeng Hongyuan Flagship Store	Electricity savings1	Intelligent power saver received, the volume is not big, small and exquisite, quite good, Jingdong Express is also quite fast, the	Jingdong prefecture level city in Shandong
Jinfeng Hongyuan Flagship Store	Electricity savings1	I don't know how to use it, keep using it	Jingdong prefecture level city in Shandong
Jinfeng Hongyuan Flagship Store	Electricity savings1	Very good for saving electricity	Jingdong prefecture level city in Shandong
Jinfeng Hongyuan Flagship Store	Electricity savings1	Seriously, did not use, feel also more power consumption, the peak is bought back to use the first time, the next day unplugged, the power consumption is back to more than ten degrees (the frequency of power use in the past few days is pretty much the same), feel the IQ tax paid 😂	Jingdong prefecture level city in Shandong
Jinfeng Hongyuan Flagship Store	Electricity savings1	I haven't seen any effect on the power saving, but the voltage stabilization looks good.	Jingdong prefecture level city in Shandong
Jinfeng Hongyuan Flagship Store	Electricity savings1	It's very good, I'll come back to it after using it for a while	Jingdong prefecture level city in Shandong
Jinfeng Hongyuan Flagship Store	Electricity savings1	Already used it and see the results.	Jingdong prefecture level city in Shandong
Jinfeng Hongyuan Flagship Store	Electricity savings1	Fast shipping, beautiful packaging, attentive service, the use of the effect, to be followed up after the evaluation.	Jingdong prefecture level city in Shandong
Jinfeng Hongyuan Flagship Store	Electricity savings1	The process of using this product is more than satisfactory	Jingdong prefecture level city in Shandong
Jinfeng Hongyuan Flagship Store	Electricity savings1	Very good East East cost-effective! Every time so buy more assured, and the logistics is also very fast. It is also very good to use, so I will often come to buy in the future, and will also introduce to	Jingdong prefecture level city in Shandong
Jinfeng Hongyuan Flagship Store	Electricity savings1	But still understand how effective it is!	Jingdong prefecture level city in Shandong
Jinfeng Hongyuan Flagship Store	Electricity savings1	Fast shipping, great logistics, great stuff, great price.	Jingdong prefecture level city in Shandong
Jinfeng Hongyuan Flagship Store	Electricity savings1	I'll review it in a month and give it 5 stars if it's good	Jingdong prefecture level city in Shandong
Jinfeng Hongyuan Flagship Store	Electricity savings1	I've saved a lot of electricity in the past month, so I'm glad to hear that.	Jingdong prefecture level city in Shandong
Jinfeng Hongyuan Flagship Store	Electricity savings1	Baby just received and installed! Looks genuinely good, workmanship feels fine! Looking forward to the effect! Said to see the effect in two months! Waiting for if really have the effect must come to follow up! The boss is absolutely responsible! Asked and answered! Just for the boss this attitude must also	Jingdong prefecture level city in Shandong
Jinfeng Hongyuan Flagship Store	Electricity savings1	This baby is very delicate, I'll comment after I've used it	Jingdong prefecture level city in Shandong
Jinfeng Hongyuan Flagship Store	Electricity savings1	It's been over two months. It's not working. It's just like it was.	Jingdong prefecture level city in Shandong

Jinfeng Hongyuan Flagship Store	Electricity savings1	Fast shipping and logistics, let's see if it works.	Jingdong prefecture level city in Shandong
Jinfeng Hongyuan Flagship Store	Electricity savings1	Just received the goods, try it to know if it can really save electricity	Jingdong prefecture level city in Shandong
Jinfeng Hongyuan Flagship Store	Electricity savings1	Not bad. Not bad. Not bad.	Jingdong prefecture level city in Shandong
Jinfeng Hongyuan Flagship Store	Electricity savings1	Not sure if it works. I'll comment again if it works.	Jingdong prefecture level city in Shandong
Jinfeng Hongyuan Flagship Store	Electricity savings1	Safe and energy-saving helpers to use!	Jingdong prefecture level city in Shandong
Jinfeng Hongyuan Flagship Store	Electricity savings1	Good, first time buyer. Used good can	Jingdong prefecture level city in Shandong
Jinfeng Hongyuan Flagship Store	Electricity savings1	It feels okay, I guess! I won't know until I use it for a while!	Jingdong prefecture level city in Shandong
Jinfeng Hongyuan Flagship Store	Electricity savings1	The saver matches the description and has been used, waiting for a follow up review after two months.	Jingdong prefecture level city in Shandong
Jinfeng Hongyuan Flagship Store	Electricity savings1	Received, the appearance of the business as described, the effect of how to use a period of time to evaluate.	Jingdong prefecture level city in Shandong
Jinfeng Hongyuan Flagship Store	Electricity savings1	Still using it don't know how effective it is, I'll comment after three months	Jingdong prefecture level city in Shandong
Jinfeng Hongyuan Flagship Store	Electricity savings1	The stuff is very good, the packaging is also very good logistics is also very fast	Jingdong prefecture level city in Shandong
Jinfeng Hongyuan Flagship Store	Electricity savings1	I'll follow up with a review when I use it, hopefully I won't be disappointed	Jingdong prefecture level city in Shandong
Jinfeng Hongyuan Flagship Store	Electricity savings1	Cancel Houjiaying Logistics.	Jingdong prefecture level city in Shandong
Jinfeng Hongyuan Flagship Store	Electricity savings1	It's very good, it's worth buying.	Jingdong prefecture level city in Shandong
Jinfeng Hongyuan Flagship Store	Electricity savings1	My friend bought it to save electricity and referred me to buy it! I'll buy it and try it out! I will know how much electricity I can save next month, looking forward to it! 4-500 dollars a month electricity bill is horrible! I hope it can really help me save some electricity!	Jingdong prefecture level city in Shandong
Jinfeng Hongyuan Flagship Store	Electricity savings1	Just received it and installed it.	Jingdong prefecture level city in Shandong
Jinfeng Hongyuan Flagship Store	Electricity savings1	Very nice. This one is my favorite.	Jingdong prefecture level city in Shandong
Jinfeng Hongyuan Flagship Store	Electricity savings1	Good value for money worth buying very good	Jingdong prefecture level city in Shandong
Jinfeng Hongyuan Flagship Store	Electricity savings1	Very energy-saving, small and exquisite, does not take up space, very satisfied, complete packaging, no damage, customer service explanation is also very serious	Jingdong prefecture level city in Shandong
Jinfeng Hongyuan Flagship Store	Electricity savings1	Received immediately used, really effective, do not understand electricity, but looked at the meter every day, recommended that you can use, especially this weather every day to open the air conditioning, with him not afraid of the electricity bill.	Jingdong prefecture level city in Shandong
Jinfeng Hongyuan Flagship Store	Electricity savings1	Nice, good value for money, nice stuff.	Jingdong prefecture level city in Shandong
Jinfeng Hongyuan Flagship Store	Electricity savings1	The packaging of the product is very good, the logistics of the product is very fast, and the quality of the product will be evaluated after use!	Jingdong prefecture level city in Shandong
Jinfeng Hongyuan Flagship Store	Electricity savings1	Express is very, well very fast, give a compliment 👍	Jingdong prefecture level city in Shandong
Jinfeng Hongyuan Flagship Store	Electricity savings1	Packaging is very good, fast delivery, used a night and a day did not feel the power saving. Use a month to see if it saves power.	Jingdong prefecture level city in Shandong
Jinfeng Hongyuan Flagship Store	Electricity savings1	Seems to be a bit of a power saver.	Jingdong prefecture level city in Shandong
Jinfeng Hongyuan Flagship Store	Electricity savings1	Bought this for someone else and it came over so fast, ###!	Jingdong prefecture level city in Shandong
Jinfeng Hongyuan Flagship Store	Electricity savings1	Very delicate, just received a period of time to use in the follow-up evaluation	Jingdong prefecture level city in Shandong
Jinfeng Hongyuan Flagship Store	Electricity savings1	It's been installed for a while now, not sure if it's working or not	Jingdong prefecture level city in Shandong
Jinfeng Hongyuan Flagship Store	Electricity savings1	The item looks okay, I just don't know if it works or not.	Jingdong prefecture level city in Shandong
Jinfeng Hongyuan Flagship Store	Electricity savings1	The goods have been received, but there is a screw missing	Jingdong prefecture level city in Shandong
Jinfeng Hongyuan Flagship Store	Electricity savings1	I didn't believe it before, but after receiving the goods today and trying it out, it really works!	Jingdong prefecture level city in Shandong
Jinfeng Hongyuan Flagship Store	Electricity savings1	Received, this thing will not know whether it is the effect of saving electricity, no matter how to say that there should always be a little use it, otherwise why develop it I think there is still the value of	Jingdong prefecture level city in Shandong
Jinfeng Hongyuan Flagship Store	Electricity savings1	Another one just arrived.	Jingdong prefecture level city in Shandong
Jinfeng Hongyuan Flagship Store	Electricity savings1	I don't feel anything, ordinary farmers can't go to TEDA, just use it first, it's safe.	Jingdong prefecture level city in Shandong
Jinfeng Hongyuan Flagship Store	Electricity savings1	Just purchased and will follow up after using it for a while	Jingdong prefecture level city in Shandong
Jinfeng Hongyuan Flagship Store	Electricity savings1	I'm trying it out now. I'll comment after I use it.	Jingdong prefecture level city in Shandong
Jinfeng Hongyuan Flagship Store	Electricity savings1	Received the item, in use, hope it works.	Jingdong prefecture level city in Shandong
Jinfeng Hongyuan Flagship Store	Electricity savings1	It works well. Good review.	Jingdong prefecture level city in Shandong
Jinfeng Hongyuan Flagship Store	Electricity savings1	All a scam, no effect at all, I bought too many fakes in Poundland before, I did not think that Jingdong is also the same	Jingdong prefecture level city in Shandong
Jinfeng Hongyuan Flagship Store	Electricity savings1	I used the saver as soon as I received it, it's in good shape and still working!	Jingdong prefecture level city in Shandong
Jinfeng Hongyuan Flagship Store	Electricity savings1	It works. I don't know what to do with the picture, but it works.	Jingdong prefecture level city in Shandong
Jinfeng Hongyuan Flagship Store	Electricity savings1	A very good shopping experience, attentive service, fast logistics, delivery is also very thoughtful, attitude is also very good, value for money ah, decisive praise!	Jingdong prefecture level city in Shandong
Jinfeng Hongyuan Flagship Store	Electricity savings1	The product is consistent with the presentation picture.	Jingdong prefecture level city in Shandong
Jinfeng Hongyuan Flagship Store	Electricity savings1	Baby received, use a period of time to evaluate, wish the boss business prosperity!	Jingdong prefecture level city in Shandong
Jinfeng Hongyuan Flagship Store	Electricity savings1	The stuff is better! Recommended!	Jingdong prefecture level city in Shandong
Jinfeng Hongyuan Flagship Store	Electricity savings1	I don't know if it works or not, I'll use it for a while before I comment on it.	Jingdong prefecture level city in Shandong
Jinfeng Hongyuan Flagship Store	Electricity savings1	Cost-effective, I like it, next time will come back Baby received, very surprised, the quality is very good, very satisfied with a shopping. I will continue to buy back, the store is trustworthy	Jingdong prefecture level city in Shandong
Jinfeng Hongyuan Flagship Store	Electricity savings1	Plugged it in and used it for a few days, not sure if it saves power yet	Jingdong prefecture level city in Shandong
Jinfeng Hongyuan Flagship Store	Electricity savings1	I'm trying it out and will follow up if it seems to be working!	Jingdong prefecture level city in Shandong
Jinfeng Hongyuan Flagship Store	Electricity savings1	Works pretty well, big kudos 👍👍👍👍👍👍👍👍👍👍👍	Jingdong prefecture level city in Shandong

Jinfeng Hongyuan Flagship Store	Electricity savings1	Just put it on, we'll see how it looks in a month.	Jingdong prefecture level city in Shandong
Jinfeng Hongyuan Flagship Store	Electricity savings1	The baby is very nice and I've already recommended buying it for all my friends! The main thing is that it not only saves electricity, I was more concerned about the fact that it can adjust the current, usually wiring problems. Now I found that it is really good, it is the effect I want!	Jingdong prefecture level city in Shandong
Jinfeng Hongyuan Flagship Store	Electricity savings1	Baby arrived, looking forward to his results, hopefully it will be good	Jingdong prefecture level city in Shandong
Jinfeng Hongyuan Flagship Store	Electricity savings1	Things have been received, look at the workmanship and packaging are good, I do not know how the power-saving effect? Use a period of time to see the effect	Jingdong prefecture level city in Shandong
Jinfeng Hongyuan Flagship Store	Electricity savings1	The price is right, the shape is beautiful and does not take up space, use it for a while to see the effect	Jingdong prefecture level city in Shandong
Jinfeng Hongyuan Flagship Store	Electricity savings1	Can't save electricity, it doesn't work!	Jingdong prefecture level city in Shandong
Jinfeng Hongyuan Flagship Store	Electricity savings1	It's pretty convenient, just plug it in and it works. There's no noise, plug it in and leave it alone, it's very hassle free! Good workmanship and value for money.	Jingdong prefecture level city in Shandong
Jinfeng Hongyuan Flagship Store	Electricity savings1	Just received the goods have been installed, try two months later to comment!	Jingdong prefecture level city in Shandong
Jinfeng Hongyuan Flagship Store	Electricity savings1	I received the stuff, I haven't used it yet, and overall it's fine.	Jingdong prefecture level city in Shandong
Jinfeng Hongyuan Flagship Store	Electricity savings1	Couldn't use a return at all.	Jingdong prefecture level city in Shandong
Jinfeng Hongyuan Flagship Store	Electricity savings1	Second purchase.	Jingdong prefecture level city in Shandong
Jinfeng Hongyuan Flagship Store	Electricity savings1	Very good quality, high tech and practical.	Jingdong prefecture level city in Shandong
Jinfeng Hongyuan Flagship Store	Electricity savings1	Very good, very great! Very satisfied, thanks to the store!	Jingdong prefecture level city in Shandong
Jinfeng Hongyuan Flagship Store	Electricity savings1	Just used it, don't know if it's good or not, hope it works well	Jingdong prefecture level city in Shandong
Jinfeng Hongyuan Flagship Store	Electricity savings1	Saving power has been received, the product is good, good like.	Jingdong prefecture level city in Shandong
Jinfeng Hongyuan Flagship Store	Electricity savings1	Received the goods and used it for two months before evaluation	Jingdong prefecture level city in Shandong
Jinfeng Hongyuan Flagship Store	Electricity savings1	It looks a little small, I do not know how effective, first use to see the effect, there is a small adjustable button, I do not know this is to adjust to that position to work well	Jingdong prefecture level city in Shandong
Jinfeng Hongyuan Flagship Store	Electricity savings1	Simple, just plug it in, there are spare fuses, can't say how well it works, no comparison	Jingdong prefecture level city in Shandong
Jinfeng Hongyuan Flagship Store	Electricity savings1	It's been a few days since I used it, and the power's gone up.	Jingdong prefecture level city in Shandong
Jinfeng Hongyuan Flagship Store	Electricity savings1	The owner was very nice and sent a spare fuse.	Jingdong prefecture level city in Shandong
Jinfeng Hongyuan Flagship Store	Electricity savings1	It's authentic and it's not expensive and it's great to use, I use it all the time and it's great 🎉✨ just don't know if it really saves any points	Jingdong prefecture level city in Shandong
Jinfeng Hongyuan Flagship Store	Electricity savings1	The information is good, it is the child to buy, the child read it is also quite happy, I hope to be able to match the child's preparation for the midterm examination! Because of the epidemic, it is not too fast, but I also give full credit to the express delivery!	Jingdong prefecture level city in Shandong
Jinfeng Hongyuan Flagship Store	Electricity savings1	This power saver comes to the logistics very quickly, the business attitude is also very good, ** very comfortable, this brand is trustworthy, and buy in this store than in the physical store to buy cheaper,	Jingdong prefecture level city in Shandong
Jinfeng Hongyuan Flagship Store	Electricity savings1	It's very well made and the materials used are okay. This is just a few days old, so I don't know how it will work, only next month.	Jingdong prefecture level city in Shandong
Jinfeng Hongyuan Flagship Store	Electricity savings1	Already used on, have not tested the effect, wait for a couple of months to add comments.	Jingdong prefecture level city in Shandong
Jinfeng Hongyuan Flagship Store	Electricity savings1	Baby received, packaging is very thoughtful, already in use, should soon see the effect.	Jingdong prefecture level city in Shandong
Jinfeng Hongyuan Flagship Store	Electricity savings1	Just received in the office to try, to the home installed, the voltage are different, and do not know if there is no effect. Use a period of time and then follow up.	Jingdong prefecture level city in Shandong
Jinfeng Hongyuan Flagship Store	Electricity savings1	Just received the goods, is trying, looking forward to the effect, first give a favorable comment, two months later, then additional evaluation, please be patient.	Jingdong prefecture level city in Shandong
Jinfeng Hongyuan Flagship Store	Electricity savings1	The item has just been received, well packaged, results to be verified.	Jingdong prefecture level city in Shandong
Jinfeng Hongyuan Flagship Store	Electricity savings1	Packaging logistics are very good, the next look at how the effect, before I see others use the effect is good, as I bought this can also have good results.	Jingdong prefecture level city in Shandong
Jinfeng Hongyuan Flagship Store	Electricity savings1	I don't know if it's a real power saver or a fake power saver, but I've used it since I bought it, so I'll give you a good review.	Jingdong prefecture level city in Shandong
Jinfeng Hongyuan Flagship Store	Electricity savings1	Good quality, genuine goods, much better than imagined before purchase, next time will come back to care!	Jingdong prefecture level city in Shandong
Jinfeng Hongyuan Flagship Store	Electricity savings1	Logistics is very fast good use again	Jingdong prefecture level city in Shandong
Jinfeng Hongyuan Flagship Store	Electricity savings1	Received the goods. Let's see if it saves power in a month and then we'll follow up!	Jingdong prefecture level city in Shandong
Jinfeng Hongyuan Flagship Store	Electricity savings1	I bought it yesterday and it arrived today, I bought two of them, I hope they work well, I'll come back after a while to give a follow-up review	Jingdong prefecture level city in Shandong
Jinfeng Hongyuan Flagship Store	Electricity savings1	The home air conditioner consumes a lot of electricity, so I bought it and used it in the hope that there is A	Jingdong prefecture level city in Shandong
Jinfeng Hongyuan Flagship Store	Electricity savings1	The product is excellent and very current.	Jingdong prefecture level city in Shandong
Jinfeng Hongyuan Flagship Store	Electricity savings1	With the description, it is the most practical and good products that households can need, and it is an ideal shopping, the courier is also fast, and the service attitude is enthusiastic!	Jingdong prefecture level city in Shandong
Jinfeng Hongyuan Flagship Store	Electricity savings1	The Power Saver is easy to use, works well, is stable, and plugs into the top of the outlet	Jingdong prefecture level city in Shandong
Jinfeng Hongyuan Flagship Store	Electricity savings1	Overall still can, excellent craftsmanship, power saving do not know for the time being, after the use of the evaluation	Jingdong prefecture level city in Shandong
Jinfeng Hongyuan Flagship Store	Electricity savings1	Baby received on the plug, but also with four fuses, look at the quality is okay, I do not know whether it is useful, use and then follow up.	Jingdong prefecture level city in Shandong
Jinfeng Hongyuan Flagship Store	Electricity savings1	I'll evaluate it after a while, I don't know if it's good or not yet, and if it can save electricity	Jingdong prefecture level city in Shandong
Jinfeng Hongyuan Flagship Store	Electricity savings1	Jingdong self-operated purchase more assured delivery fast quality assurance	Jingdong prefecture level city in Shandong
Jinfeng Hongyuan Flagship Store	Electricity savings1	Shipping was fast, stuff was well packaged, great price, I'm happy with it 👍 and will repurchase!	Jingdong prefecture level city in Shandong
Jinfeng Hongyuan Flagship Store	Electricity savings1	The quality of the product is very good and I will definitely come back again next time.	Jingdong prefecture level city in Shandong
Jinfeng Hongyuan Flagship Store	Electricity savings1	I'll follow up with a review after I use it.	Jingdong prefecture level city in Shandong
Jinfeng Hongyuan Flagship Store	Electricity savings1	Hello, the goods are perfectly packaged, save power for a period of time to evaluate again!	Jingdong prefecture level city in Shandong
Jinfeng Hongyuan Flagship Store	Electricity savings1	Not bad, pretty good, good value for money, pretty good	Jingdong prefecture level city in Shandong
Jinfeng Hongyuan Flagship Store	Electricity savings1	The packaging is very good, received immediately used, looking forward to the effect in.	Jingdong prefecture level city in Shandong
Jinfeng Hongyuan Flagship Store	Electricity savings1	See how much power you save at the end of the month	Jingdong prefecture level city in Shandong

Jinfeng Hongyuan Flagship Store	Electricity savings1	Easy to use, affordable, beautiful color	Jingdong prefecture level city in Shandong
Jinfeng Hongyuan Flagship Store	Electricity savings1	It's taking too long to put together an order. It could be an epidemic.	Jingdong prefecture level city in Shandong
Jinfeng Hongyuan Flagship Store	Electricity savings1	I don't feel much after using it, no change in my electric bill 。。。。。。。	Jingdong prefecture level city in Shandong
Jinfeng Hongyuan Flagship Store	Electricity savings1	Logistics is very fast, the quality of the economizer is good, good quality and cheap, it is worth buying, I hope there is a power saving effect!	Jingdong prefecture level city in Shandong
Jinfeng Hongyuan Flagship Store	Electricity savings1	Feeling okay, in a couple of months to try to rely on it before making a comment. Let's give it a five-star review first!	Jingdong prefecture level city in Shandong
Jinfeng Hongyuan Flagship Store	Electricity savings1	Power saving device has been hesitating before in the end to buy or not to buy, after consideration, consideration, in consideration, or decided to buy a try. Things received can not wait to open a look, and the seller described the same, I hope to use 2 months to have the effect, two months to follow up on	Jingdong prefecture level city in Shandong
Jinfeng Hongyuan Flagship Store	Electricity savings1	Thank you Jingdong, logistics is very fast order to receipt only one day. Believe in Jingdong. Favorable comments	Jingdong prefecture level city in Shandong
Jinfeng Hongyuan Flagship Store	Electricity savings1	I used one to use it, but it didn't work very well, what to do	Jingdong prefecture level city in Shandong
Jinfeng Hongyuan Flagship Store	Electricity savings1	Quality and packaging are very good, fast shipping, good service, buy with confidence	Jingdong prefecture level city in Shandong
Jinfeng Hongyuan Flagship Store	Electricity savings1	Use a period of time to evaluate, the quality is very good, the price is affordable, logistics is also very fast	Jingdong prefecture level city in Shandong
Jinfeng Hongyuan Flagship Store	Electricity savings1	Goods received, the day of the order tomorrow delivery real God speed, delivery force, the product to be used after a period of time and then low comment!	Jingdong prefecture level city in Shandong
Jinfeng Hongyuan Flagship Store	Electricity savings1	I don't know, how effective it is, let's talk about it when the time comes, whether it saves power or not, how much, unknown.	Jingdong prefecture level city in Shandong
Jinfeng Hongyuan Flagship Store	Electricity savings1	It does save some electricity, and I don't want to use something that isn't legal! Used nearly ~ electricity last month, probably not much more this month with the AC on!	Jingdong prefecture level city in Shandong
Jinfeng Hongyuan Flagship Store	Electricity savings1	Well-packed, excellent quality, service satisfaction good value for money	Jingdong prefecture level city in Shandong
Jinfeng Hongyuan Flagship Store	Electricity savings1	Express very quickly, the goods received last night, today used a day of goods can not heat, looking forward to whether it can save electricity, with a month and then comment, is used well will buy again.	Jingdong prefecture level city in Shandong
Jinfeng Hongyuan Flagship Store	Electricity savings1	It's pretty good, hopefully it'll be a real power saver.	Jingdong prefecture level city in Shandong
Jinfeng Hongyuan Flagship Store	Electricity savings1	The goods received in use, can not see the effect of energy saving, after a month to know!	Jingdong prefecture level city in Shandong
Jinfeng Hongyuan Flagship Store	Electricity savings1	Received the goods, looking forward to better surprise, with the effect of the follow-up comment!	Jingdong prefecture level city in Shandong
Jinfeng Hongyuan Flagship Store	Electricity savings1	It's not bad, it's still in use and should save some	Jingdong prefecture level city in Shandong
Jinfeng Hongyuan Flagship Store	Electricity savings1	Logistics is very fast, easy to install, plugged in on it,, the effect of the use of the later to evaluate!	Jingdong prefecture level city in Shandong
Jinfeng Hongyuan Flagship Store	Electricity savings1	The stuff is recommendable to buy very good.	Jingdong prefecture level city in Shandong
Jinfeng Hongyuan Flagship Store	Electricity savings1	Voltmeter measurements of voltage are way off, aren't they?	Jingdong prefecture level city in Shandong
Jinfeng Hongyuan Flagship Store	Electricity savings1	Install it to the socket immediately after purchase and try to see if it can save electricity after one month.	Jingdong prefecture level city in Shandong
Jinfeng Hongyuan Flagship Store	Electricity savings1	I've been using it for almost a month now, and I won't know if it saves electricity until I pay the bill.	Jingdong prefecture level city in Shandong
Jinfeng Hongyuan Flagship Store	Electricity savings1	I bought one in the past and it was fine, so I'm buying it back.	Jingdong prefecture level city in Shandong
Jinfeng Hongyuan Flagship Store	Electricity savings1	Service is very good, this thing is small and exquisite to use, just waiting to use the effect of a period of time	Jingdong prefecture level city in Shandong
Jinfeng Hongyuan Flagship Store	Electricity savings1	Not sure how well it works yet	Jingdong prefecture level city in Shandong
Jinfeng Hongyuan Flagship Store	Electricity savings1	Kyodo goods habitually good reviews	Jingdong prefecture level city in Shandong
Jinfeng Hongyuan Flagship Store	Electricity savings1	Appearance is OK, whether it is power saving remains to be seen.	Jingdong prefecture level city in Shandong
Jinfeng Hongyuan Flagship Store	Electricity savings1	I bought it and used it, I just don't know if it really saves power	Jingdong prefecture level city in Shandong
Jinfeng Hongyuan Flagship Store	Electricity savings1	The saver is well made and of good quality, I will evaluate it after using it for a while.	Jingdong prefecture level city in Shandong
Jinfeng Hongyuan Flagship Store	Electricity savings1	Just received in use, use for a period of time to see if there is power saving	Jingdong prefecture level city in Shandong
Jinfeng Hongyuan Flagship Store	Electricity savings1	Used 3 days before commenting, it really saves electricity, I look at the "power calendar" after using one less degree of electricity per day, so I think it's worth it to buy this, I will introduce my friends	Jingdong prefecture level city in Shandong
Jinfeng Hongyuan Flagship Store	Electricity savings1	Reliable quality, very well packaged and matches the real thing.	Jingdong prefecture level city in Shandong
Jinfeng Hongyuan Flagship Store	Electricity savings1	Logistics is still quite fast, ha in the trial	Jingdong prefecture level city in Shandong
Jinfeng Hongyuan Flagship Store	Electricity savings1	Consultant service attitude is very good, the courier is also very powerful, the first day of the order the next day to arrive. Saver received used and then add comments!	Jingdong prefecture level city in Shandong
Jinfeng Hongyuan Flagship Store	Electricity savings1	I just got home to try it out and see how it goes.	Jingdong prefecture level city in Shandong
Jinfeng Hongyuan Flagship Store	Electricity savings1	Well made and weighted instrument. Kudos! Put it into use immediately, don't know if it saves power, will evaluate later!	Jingdong prefecture level city in Shandong
Jinfeng Hongyuan Flagship Store	Electricity savings1	I've just put it in place. I'll give you a review in ten days.	Jingdong prefecture level city in Shandong
Jinfeng Hongyuan Flagship Store	Electricity savings1	It looks very delicate, not bad. Well, I don't know if it can save power or not, I have to try it for a while and see. If it can really save electricity. Uh, if it works well. Then additional comments. Anyway, thanks for the logistics, thanks to Jingdong. Well, it deserves some praise.	Jingdong prefecture level city in Shandong
Jinfeng Hongyuan Flagship Store	Electricity savings1	Bought a month, the feeling is that there is a certain effect, a month to save a lot of money	Jingdong prefecture level city in Shandong
Jinfeng Hongyuan Flagship Store	Electricity savings1	Goods received, the physical and the same as the picture, logistics is also very fast, the service attitude is very good, it is worth buying!	Jingdong prefecture level city in Shandong
Jinfeng Hongyuan Flagship Store	Electricity savings1	I've used it, I don't know whether it saves electricity or not, I'll talk about it after a while.	Jingdong prefecture level city in Shandong
Jinfeng Hongyuan Flagship Store	Electricity savings1	August only began to use, October to know, have no power-saving effect, to the in to evaluate!	Jingdong prefecture level city in Shandong
Jinfeng Hongyuan Flagship Store	Electricity savings1	Goods received: I do not know to the factory products used to save electricity!	Jingdong prefecture level city in Shandong
Jinfeng Hongyuan Flagship Store	Electricity savings1	Don't know if it works, bought it anyway.	Jingdong prefecture level city in Shandong
Jinfeng Hongyuan Flagship Store	Electricity savings1	Still testing, will follow up next month to evaluate how much electricity is saved	Jingdong prefecture level city in Shandong
Jinfeng Hongyuan Flagship Store	Electricity savings1	I'm still testing it, I'll comment after I use it.	Jingdong prefecture level city in Shandong
Jinfeng Hongyuan Flagship Store	Electricity savings1	Fast service, praiseworthy. Good item pre-style, first praise, the use of the effect to be evaluated after use.	Jingdong prefecture level city in Shandong
Jinfeng Hongyuan Flagship Store	Electricity savings1	The power saving effect is more obvious through verified use.	Jingdong prefecture level city in Shandong

Jinfeng Hongyuan Flagship Store	Electricity savings1	Logistics is very fast yesterday order today arrived. The product was immediately used, and after a month to see its effect.	Jingdong prefecture level city in Shandong
Jinfeng Hongyuan Flagship Store	Electricity savings1	Power saving is not yet known, as far as the TV and audio are concerned, I feel that there is a significant quality improvement, and I recommend using a good product!	Jingdong prefecture level city in Shandong
Jinfeng Hongyuan Flagship Store	Electricity savings1	Good power savings, tens of dollars a month saved	Jingdong prefecture level city in Shandong
Jinfeng Hongyuan Flagship Store	Electricity savings1	Already took two, great value! You can save 30% on your electric bill in a month. A summer must-have!	Jingdong prefecture level city in Shandong
Jinfeng Hongyuan Flagship Store	Electricity savings1	Received the item, plugged it into a home outlet and it showed 230 volts (not that high with a multimeter). Let's use it first!	Jingdong prefecture level city in Shandong
Jinfeng Hongyuan Flagship Store	Electricity savings1	Expecting results in the experiment	Jingdong prefecture level city in Shandong
Jinfeng Hongyuan Flagship Store	Electricity savings1	I don't know if it really saves power.	Jingdong prefecture level city in Shandong
Jinfeng Hongyuan Flagship Store	Electricity savings1	It's very good and the efficacy is very visible.	Jingdong prefecture level city in Shandong
Jinfeng Hongyuan Flagship Store	Electricity savings1	It's good and worth buying with confidence	Jingdong prefecture level city in Shandong
Jinfeng Hongyuan Flagship Store	Electricity savings1	The goods have been received, just do not know if it is effective	Jingdong prefecture level city in Shandong
Jinfeng Hongyuan Flagship Store	Electricity savings1	It's already in use, but based on some of the descriptions, I suspect that to use it, you need to have one in series on each line, which seems to work for all appliances, that's my guess, but I feel as though the principle of the thing seems to require it.	Jingdong prefecture level city in Shandong
Jinfeng Hongyuan Flagship Store	Electricity savings1	Fast delivery, already in use, looks good!	Jingdong prefecture level city in Shandong
Jinfeng Hongyuan Flagship Store	Electricity savings1	Hoping to save 30% of power as presented in the graphic	Jingdong prefecture level city in Shandong
Jinfeng Hongyuan Flagship Store	Electricity savings1	Things look good, power saving do not know now, with a month after the continued evaluation	Jingdong prefecture level city in Shandong
Jinfeng Hongyuan Flagship Store	Electricity savings1	Already used it but not sure if it will top out!	Jingdong prefecture level city in Shandong
Jinfeng Hongyuan Flagship Store	Electricity savings1	Looking forward to using the results, the product quality looks okay.	Jingdong prefecture level city in Shandong
Jinfeng Hongyuan Flagship Store	Electricity savings1	It's somewhat effective and saves money more or less 💰	Jingdong prefecture level city in Shandong
Jinfeng Hongyuan Flagship Store	Electricity savings1	Got it, it's great, looking forward to saving power.	Jingdong prefecture level city in Shandong
Jinfeng Hongyuan Flagship Store	Electricity savings1	I don't know if it's working yet, I'll follow up when I get there	Jingdong prefecture level city in Shandong
Jinfeng Hongyuan Flagship Store	Electricity savings1	The product received, good workmanship, fast logistics, good inner packaging, the outer bag is damaged, but fortunately did not hurt the product, retrieved and inserted according to the instructions, looking forward to the effect is good, with a period of time to evaluate, I believe that the Jingdong.	Jingdong prefecture level city in Shandong
Jinfeng Hongyuan Flagship Store	Electricity savings1	Since it is a new product, it is said to work, so if it works well for a month, then I will give you a good review.	Jingdong prefecture level city in Shandong
Jinfeng Hongyuan Flagship Store	Electricity savings1	The quality of the product is very good, very well made, well packaged, easy to use and very convenient!	Jingdong prefecture level city in Shandong
Jinfeng Hongyuan Flagship Store	Electricity savings1	Good price for the item, good functionality	Jingdong prefecture level city in Shandong
Jinfeng Hongyuan Flagship Store	Electricity savings1	Received the first time to use it, the stuff is very good, specifics wait for a month to come back to feedback to see the effect.	Jingdong prefecture level city in Shandong
Jinfeng Hongyuan Flagship Store	Electricity savings1	Just received, the courier is too bad, not notified directly to put the Feng Nest courier cabinet	Jingdong prefecture level city in Shandong
Jinfeng Hongyuan Flagship Store	Electricity savings1	Baby received, the second time to buy, plugged in and used do not know if there is no effect. Baby received, the second time to buy, plugged in and used do not know if there is no effect. Baby received, the second time to buy, plugged in and used do not know if there is no effect.	Jingdong prefecture level city in Shandong
Jinfeng Hongyuan Flagship Store	Electricity savings1	It's okay if it saves power. It's not a bad product.	Jingdong prefecture level city in Shandong
Jinfeng Hongyuan Flagship Store	Electricity savings1	Received, very good, meticulous packaging, open a look is genuine, meticulous workmanship, the manual is very detailed, has been used, there is no effect on the only look at the next month's electricity bill, when I can I buy a few more to send people!	Jingdong prefecture level city in Shandong
Jinfeng Hongyuan Flagship Store	Electricity savings1	The quality is very good very delicate express delivery is too fast	Jingdong prefecture level city in Shandong
Jinfeng Hongyuan Flagship Store	Electricity savings1	I won't know until a month after I start using it today.	Jingdong prefecture level city in Shandong
Jinfeng Hongyuan Flagship Store	Electricity savings1	It doesn't work at all, the customer service attitude is very bad, use it or not!	Jingdong prefecture level city in Shandong
Jinfeng Hongyuan Flagship Store	Electricity savings1	Temporarily try, do not know the effect, Jingdong logistics delivery very quickly	Jingdong prefecture level city in Shandong
Jinfeng Hongyuan Flagship Store	Electricity savings1	It doesn't help. It's comforting.	Jingdong prefecture level city in Shandong
Jinfeng Hongyuan Flagship Store	Electricity savings1	It's great, use it before you say anything.	Jingdong prefecture level city in Shandong
Jinfeng Hongyuan Flagship Store	Electricity savings1	Received, already used, looking forward to a good a effect!	Jingdong prefecture level city in Shandong
Jinfeng Hongyuan Flagship Store	Electricity savings1	I don't know if it works yet, I won't know until I use it for a few months.	Jingdong prefecture level city in Shandong
Jinfeng Hongyuan Flagship Store	Electricity savings1	It takes a long time to get there. There's no change now.	Jingdong prefecture level city in Shandong
Jinfeng Hongyuan Flagship Store	Electricity savings1	Jingdong's delivery never disappoints, or so fast, and the stuff is great, next time you need to come back.	Jingdong prefecture level city in Shandong
Jinfeng Hongyuan Flagship Store	Electricity savings1	At first it was ok today, after using it for a while it didn't work anymore	Jingdong prefecture level city in Shandong
Jinfeng Hongyuan Flagship Store	Electricity savings1	Just received the goods, do not know the effect of using a few days before there may be effect	Jingdong prefecture level city in Shandong
Jinfeng Hongyuan Flagship Store	Electricity savings1	The logistics was fast, I received it the next day, I've purchased it for the second time and have been using it all along	Jingdong prefecture level city in Shandong
Jinfeng Hongyuan Flagship Store	Electricity savings1	Inexpensive, fast delivery, very pleased!	Jingdong prefecture level city in Shandong
Jinfeng Hongyuan Flagship Store	Electricity savings1	Received the goods immediately plugged directly into the use of power saving yet to be good news!	Jingdong prefecture level city in Shandong
Jinfeng Hongyuan Flagship Store	Electricity savings1	The service attitude is very good, and the Jingdong self-owned products can be trusted.	Jingdong prefecture level city in Shandong
Jinfeng Hongyuan Flagship Store	Electricity savings1	The product is good, I do not know if it can save play a power saving effect	Jingdong prefecture level city in Shandong
Jinfeng Hongyuan Flagship Store	Electricity savings1	We won't know how effective it is until we use it for a while, so I'll give you a good review now.	Jingdong prefecture level city in Shandong
Jinfeng Hongyuan Flagship Store	Electricity savings1	The quality is pretty good, just started using it, hopefully it will deliver results.	Jingdong prefecture level city in Shandong
Jinfeng Hongyuan Flagship Store	Electricity savings1	I've plugged it in first, and will follow up after I've used it to see how it works.	Jingdong prefecture level city in Shandong
Jinfeng Hongyuan Flagship Store	Electricity savings1	Things have arrived, the quality is good, the effect is also equivalent to can ah	Jingdong prefecture level city in Shandong

Jinfeng Hongyuan Flagship Store	Electricity savings1	It's not worth it to brag about power savings.	Jingdong prefecture level city in Shandong
Jinfeng Hongyuan Flagship Store	Electricity savings1	I don't know if it's an IQ tax, but I'll use it for now.	Jingdong prefecture level city in Shandong
Jinfeng Hongyuan Flagship Store	Electricity savings1	I don't feel the power saving yet, I can't know until I use it for 2 months.	Jingdong prefecture level city in Shandong
Jinfeng Hongyuan Flagship Store	Electricity savings1	There's nothing wrong with this at the moment.	Jingdong prefecture level city in Shandong
Jinfeng Hongyuan Flagship Store	Electricity savings1	It's authentic and worth buying	Jingdong prefecture level city in Shandong
Jinfeng Hongyuan Flagship Store	Electricity savings1	It's good. We'll see how it goes.	Jingdong prefecture level city in Shandong
Jinfeng Hongyuan Flagship Store	Electricity savings1	I don't know if it's working or not in the middle of using it, we'll see when the month is over	Jingdong prefecture level city in Shandong
Jinfeng Hongyuan Flagship Store	Electricity savings1	I'm not going to buy anything from Jingdong anymore, so I'll give it a good review	Jingdong prefecture level city in Shandong
Jinfeng Hongyuan Flagship Store	Electricity savings1	I'm not going to buy anything from Jingdong anymore, so I'll give it a good review	Jingdong prefecture level city in Shandong
Jinfeng Hongyuan Flagship Store	Electricity savings1	Just received the item and will see how it works in a month.	Jingdong prefecture level city in Shandong
Jinfeng Hongyuan Flagship Store	Electricity savings1	I don't know if it saves electricity, but it's plugged into an outlet every day anyway	Jingdong prefecture level city in Shandong
Jinfeng Hongyuan Flagship Store	Electricity savings1	I don't know if it works yet.	Jingdong prefecture level city in Shandong
Jinfeng Hongyuan Flagship Store	Electricity savings1	Plugged it in when I got back, waiting for a month to see the results	Jingdong prefecture level city in Shandong
Jinfeng Hongyuan Flagship Store	Electricity savings1	Effects remain to be seen, but it looks good quality and runs fine	Jingdong prefecture level city in Shandong
Jinfeng Hongyuan Flagship Store	Electricity savings1	The product received logistics very quickly, has not yet been used to test! After use in the evaluation!	Jingdong prefecture level city in Shandong
Jinfeng Hongyuan Flagship Store	Electricity savings1	The power saver is in use, and I don't know how much electricity has been saved after using it for more than a month, and the display voltmeter is very good.	Jingdong prefecture level city in Shandong
Jinfeng Hongyuan Flagship Store	Electricity savings1	It's so good, isn't it? You're a real Shanghainese, aren't you?	Jingdong prefecture level city in Shandong
Jinfeng Hongyuan Flagship Store	Electricity savings1	Great quality great great great 👍👍👍👍	Jingdong prefecture level city in Shandong
Jinfeng Hongyuan Flagship Store	Electricity savings1	I'm using it, I don't know if it's working yet.	Jingdong prefecture level city in Shandong
Jinfeng Hongyuan Flagship Store	Electricity savings1	Just used it for more than 10 days and haven't seen any results yet	Jingdong prefecture level city in Shandong
Jinfeng Hongyuan Flagship Store	Electricity savings1	The product arrived home feeling like it should work	Jingdong prefecture level city in Shandong
Jinfeng Hongyuan Flagship Store	Electricity savings1	Just received the goods, use it and comment back	Jingdong prefecture level city in Shandong
Jinfeng Hongyuan Flagship Store	Electricity savings1	Try it for a month and then evaluate	Jingdong prefecture level city in Shandong
Jinfeng Hongyuan Flagship Store	Electricity savings1	I don't know if it saves power yet, but I'll comment again after using it for a while.	Jingdong prefecture level city in Shandong
Jinfeng Hongyuan Flagship Store	Electricity savings1	The product is better than I thought, the quality and appearance are very good, the voltage is stable, and I'm looking forward to a good power saving effect.	Jingdong prefecture level city in Shandong
Jinfeng Hongyuan Flagship Store	Electricity savings1	Received and scanned the QR code to check that it is genuine, have plugged it in, can it save power, looking forward to the advertised effect. Customary praise, 5 points!	Jingdong prefecture level city in Shandong
Jinfeng Hongyuan Flagship Store	Electricity savings1	The goods have been received and have not yet been used. Do not know how the effect.	Jingdong prefecture level city in Shandong
Jinfeng Hongyuan Flagship Store	Electricity savings1	Hopefully, you get what you pay for, and you get what you pay for!	Jingdong prefecture level city in Shandong
Jinfeng Hongyuan Flagship Store	Electricity savings1	Just put it on and it feels like it's turning as fast as a meter! Looking forward to the results!	Jingdong prefecture level city in Shandong
Jinfeng Hongyuan Flagship Store	Electricity savings1	Overall look on the product quality is good, I do not know is not the effect of power saving, wait to see and then follow up!	Jingdong prefecture level city in Shandong
Jinfeng Hongyuan Flagship Store	Electricity savings1	The logistics courier is very fast, the courier service attitude is very good	Jingdong prefecture level city in Shandong
Jinfeng Hongyuan Flagship Store	Electricity savings1	The product has been received, it works well worth buying	Jingdong prefecture level city in Shandong
Jinfeng Hongyuan Flagship Store	Electricity savings1	How did it not work? Is it purely an IQ tax?	Jingdong prefecture level city in Shandong
Jinfeng Hongyuan Flagship Store	Electricity savings1	It looks good, I don't know how well it works.	Jingdong prefecture level city in Shandong
Jinfeng Hongyuan Flagship Store	Electricity savings1	It does seem to be a bit of a power saver, I just don't know if the more expensive one is more power efficient?	Jingdong prefecture level city in Shandong
Jinfeng Hongyuan Flagship Store	Electricity savings1	Is genuine very good, the price is much cheaper, cost-effective, really special paddle just to do activities, the price is much cheaper, cost-effective, really special paddle counting, packaging is also very high-end atmospheric class, it is worth buying!	Jingdong prefecture level city in Shandong
Jinfeng Hongyuan Flagship Store	Electricity savings1	I received it yesterday and used it right away, I hope it works, I'll wait for next month to come back to the conclusion, I'm looking forward to it. Five hearts praise!	Jingdong prefecture level city in Shandong
Jinfeng Hongyuan Flagship Store	Electricity savings1	Looks good, packed properly, received it today, am using it, will see results in a month!	Jingdong prefecture level city in Shandong
Jinfeng Hongyuan Flagship Store	Electricity savings1	Regular product Jingdong self-support trustworthy. There is an effect Come back to the review. 😍😍😍😍	Jingdong prefecture level city in Shandong
Jinfeng Hongyuan Flagship Store	Electricity savings1	I haven't used it yet, hopefully it will work as a power saver, and will comment again if it works well.	Jingdong prefecture level city in Shandong
Jinfeng Hongyuan Flagship Store	Electricity savings1	Shipping was fast, logistics was fast, service was good, and I look forward to good results from the item!	Jingdong prefecture level city in Shandong
Jinfeng Hongyuan Flagship Store	Electricity savings1	This product is this compensator, it has to be at the main switch to be useful, haven't compared the final result, the	Jingdong prefecture level city in Shandong
Jinfeng Hongyuan Flagship Store	Electricity savings1	Packaging is perfect . I've already started using it, so I'm looking forward to the results.	Jingdong prefecture level city in Shandong
Jinfeng Hongyuan Flagship Store	Electricity savings1	I just got it back. I'll comment after I use it.	Jingdong prefecture level city in Shandong
Jinfeng Hongyuan Flagship Store	Electricity savings1	Big brand, with a very assured, very good quality, catch the event to buy very cost-effective to take advantage of the offer, hurry to get, it is genuine, the packaging is also very good, no breakage, the quality is not to speak of, great 👍 logistics super super fast! Quality is also very good, indeed value	Jingdong prefecture level city in Shandong
Jinfeng Hongyuan Flagship Store	Electricity savings1	The quality is pretty good. The price is also quite affordable.	Jingdong prefecture level city in Shandong
Jinfeng Hongyuan Flagship Store	Electricity savings1	I did not expect the courier so fast to arrive yesterday just ordered, the head is a little smaller than I thought, well, the picture looks bigger. The appearance still looks good, as for the practicality and effectiveness of the use of the only know.	Jingdong prefecture level city in Shandong
Jinfeng Hongyuan Flagship Store	Electricity savings1	I don't know. Looking forward to it.	Jingdong prefecture level city in Shandong
Jinfeng Hongyuan Flagship Store	Electricity savings1	Baby received, very delicate, very nice, like. Just don't know if it can really save electricity. Baby received, very delicate, very good-looking, like. Just don't know if it can really save electricity.	Jingdong prefecture level city in Shandong
Jinfeng Hongyuan Flagship Store	Electricity savings1	I'll use it for a month and then comment on the quality of the item	Jingdong prefecture level city in Shandong

Jinfeng Hongyuan Flagship Store	Electricity savings1	It feels ok, use it for a while and see how it works before adding more comments.	Jingdong prefecture level city in Shandong
Jinfeng Hongyuan Flagship Store	Electricity savings1	In use, see results in a month.	Jingdong prefecture level city in Shandong
Jinfeng Hongyuan Flagship Store	Electricity savings1	Jingdong's logistics and quality are very good, the service attitude is very good, the logistics is very fast	Jingdong prefecture level city in Shandong
Jinfeng Hongyuan Flagship Store	Electricity savings1	Intelligent power saver received, well-packed logistics is also quite fast, easy to use, looking forward to good results!	Jingdong prefecture level city in Shandong
Jinfeng Hongyuan Flagship Store	Electricity savings1	The quality is unknown at this point, I won't know until I use it.	Jingdong prefecture level city in Shandong
Jinfeng Hongyuan Flagship Store	Electricity savings1	It's a good product. It really saves power and doesn't take up much space.	Jingdong prefecture level city in Shandong
Jinfeng Hongyuan Flagship Store	Electricity savings1	Goods have been received, packaging is very good, good to see the effect of future use in the follow-up.	Jingdong prefecture level city in Shandong
Jinfeng Hongyuan Flagship Store	Electricity savings1	The product is in perfect shape and the light weight is worth it!	Jingdong prefecture level city in Shandong
Jinfeng Hongyuan Flagship Store	Electricity savings1	Just got home and plugged it in. It's good.	Jingdong prefecture level city in Shandong
Jinfeng Hongyuan Flagship Store	Electricity savings1	The logistics are quick, just to see the results.	Jingdong prefecture level city in Shandong
Jinfeng Hongyuan Flagship Store	Electricity savings1	Short time can not feel here to leave a message, to be followed up later!	Jingdong prefecture level city in Shandong
Jinfeng Hongyuan Flagship Store	Electricity savings1	This user thinks the product is good	Jingdong prefecture level city in Shandong
Jinfeng Hongyuan Flagship Store	Electricity savings1	Received the baby, to be used to see the effect of it!	Jingdong prefecture level city in Shandong
Jinfeng Hongyuan Flagship Store	Electricity savings1	You have to wait a month or two to see how it works.	Jingdong prefecture level city in Shandong
Jinfeng Hongyuan Flagship Store	Electricity savings1	Not bad, good, like it, don't know if it's good, try it first.	Jingdong prefecture level city in Shandong
Jinfeng Hongyuan Flagship Store	Electricity savings1	The goods are good value for money and the logistics is very fast and good service!	Jingdong prefecture level city in Shandong
Jinfeng Hongyuan Flagship Store	Electricity savings1	I don't know how well it works, we'll see in a month	Jingdong prefecture level city in Shandong
Jinfeng Hongyuan Flagship Store	Electricity savings1	The product is fine, but r saving power or not I won't know until I use it for two months. The display and lights take electricity too?	Jingdong prefecture level city in Shandong
Jinfeng Hongyuan Flagship Store	Electricity savings1	I've used it. I don't know how it works yet.	Jingdong prefecture level city in Shandong
Jinfeng Hongyuan Flagship Store	Electricity savings1	I can't say until I compare them.	Jingdong prefecture level city in Shandong
Jinfeng Hongyuan Flagship Store	Electricity savings1	It's ok in the process of using 。。。。	Jingdong prefecture level city in Shandong
Jinfeng Hongyuan Flagship Store	Electricity savings1	Jinfeng Hongyuan Intelligent Power Saver Household Electricity Meter Electricity Saving Appliance High Power Enhanced Power Saving King Energy Saving Treasure Butler Control Non Stealing Electricity Reversing Not Going Interfering Slow Turner Artifact King Gold Yellow. Haven't tested it accurately yet, really need to wait a few months to test it before I have a say.	Jingdong prefecture level city in Shandong
Jinfeng Hongyuan Flagship Store	Electricity savings1	Don't know how effective it is yet? I hope it works well and I'll come back next time	Jingdong prefecture level city in Shandong
Jinfeng Hongyuan Flagship Store	Electricity savings1	Saving device has been received, material workmanship are very good ah! Have not yet begun to use. First observe the daily electricity consumption and cost of the home meter, and then plug in the power saver to use, so as to compare the use of the effect is good!	Jingdong prefecture level city in Shandong
Jinfeng Hongyuan Flagship Store	Electricity savings1	Merchants shipping extremely fast, Jingdong logistics safe and fast, I believe that the product can play a safe use of electricity, the role of scientific power saving, first praise, the effect of how to be used for a period of time and then the pursuit of bubble!	Jingdong prefecture level city in Shandong
Jinfeng Hongyuan Flagship Store	Electricity savings1	I just bought it. I don't know yet.	Jingdong prefecture level city in Shandong
Jinfeng Hongyuan Flagship Store	Electricity savings1	It is genuine, good quality, and it is very reassuring to use. The logistics is very fast, and the business service attitude is on point.	Jingdong prefecture level city in Shandong
Jinfeng Hongyuan Flagship Store	Electricity savings1	The quality is fine, I don't know how effective it is.	Jingdong prefecture level city in Shandong
Jinfeng Hongyuan Flagship Store	Electricity savings1	The stuff is back and I expect it will save the electricity	Jingdong prefecture level city in Shandong
Jinfeng Hongyuan Flagship Store	Electricity savings1	I'll give it 4 stars first and see what happens in a couple of months and then follow up	Jingdong prefecture level city in Shandong
Jinfeng Hongyuan Flagship Store	Electricity savings1	It's not bad. It's in the mind.	Jingdong prefecture level city in Shandong
Jinfeng Hongyuan Flagship Store	Electricity savings1	Received the goods have not yet started to use do not know whether it is good	Jingdong prefecture level city in Shandong
Jinfeng Hongyuan Flagship Store	Electricity savings1	This user thinks the product is good	Jingdong prefecture level city in Shandong
Jinfeng Hongyuan Flagship Store	Electricity savings1	Hello! The saver was received, thank you! Not yet used, but from the packaging looks very good quality, exquisite workmanship. Logistics is quite fast, if it is really very power saving, I will come back to	Jingdong prefecture level city in Shandong
Jinfeng Hongyuan Flagship Store	Electricity savings1	I've been using it for a few days now, I'll comment again after a month or two of use	Jingdong prefecture level city in Shandong
Jinfeng Hongyuan Flagship Store	Electricity savings1	I don't know. Bought it for my father at a good price.	Jingdong prefecture level city in Shandong
Jinfeng Hongyuan Flagship Store	Electricity savings1	Plugged it in the same day I bought it and the voltage showed 223, 225 looking forward to the results	Jingdong prefecture level city in Shandong
Jinfeng Hongyuan Flagship Store	Electricity savings1	Still in use, the biggest role is to stabilize the current, reduce losses, product quality on the look is very good, with this feeling quite good, see colleagues are using, power saving effect should be good	Jingdong prefecture level city in Shandong
Jinfeng Hongyuan Flagship Store	Electricity savings1	How to receive goods but no invoice and warranty more than it ...... quality is the premise of reputation!	Jingdong prefecture level city in Shandong
Jinfeng Hongyuan Flagship Store	Electricity savings1	Received but not used Continued evaluation	Jingdong prefecture level city in Shandong
Jinfeng Hongyuan Flagship Store	Electricity savings1	I just received the product, I don't know if it works well yet	Jingdong prefecture level city in Shandong
Jinfeng Hongyuan Flagship Store	Electricity savings1	Received it and used it, not sure it really saves electricity use it and then evaluate it, first give a 5 star review!!!!	Jingdong prefecture level city in Shandong
Jinfeng Hongyuan Flagship Store	Electricity savings1	It's good to use, it's very good to use, and it smells OK too	Jingdong prefecture level city in Shandong
Jinfeng Hongyuan Flagship Store	Electricity savings1	Packaging is very good, feel the product is not bad, how much power saving with the only know	Jingdong prefecture level city in Shandong
Jinfeng Hongyuan Flagship Store	Electricity savings1	Additional comments to be made after use	Jingdong prefecture level city in Shandong
Jinfeng Hongyuan Flagship Store	Electricity savings1	The product has been installed, try a few months and then comment!	Jingdong prefecture level city in Shandong
Jinfeng Hongyuan Flagship Store	Electricity savings1	I've bought it, let's use it for now, I'll comment after a month	Jingdong prefecture level city in Shandong
Jinfeng Hongyuan Flagship Store	Electricity savings1	Try it out and then follow up	Jingdong prefecture level city in Shandong
Jinfeng Hongyuan Flagship Store	Electricity savings1	It's good in theory, it doesn't feel good in practice.	Jingdong prefecture level city in Shandong

Jinfeng Hongyuan Flagship Store	Electricity savings1	I haven't tried it yet. We'll see.	Jingdong prefecture level city in Shandong
Jinfeng Hongyuan Flagship Store	Electricity savings1	Great item, second purchase!	Jingdong prefecture level city in Shandong
Jinfeng Hongyuan Flagship Store	Electricity savings1	Goods received now use it, if it does save power, plus reevaluate.	Jingdong prefecture level city in Shandong
Jinfeng Hongyuan Flagship Store	Electricity savings1	The stuff is light and small, the stuff is good, the courier service is good	Jingdong prefecture level city in Shandong
Jinfeng Hongyuan Flagship Store	Electricity savings1	I haven't used it yet. I'm looking forward to it.	Jingdong prefecture level city in Shandong
Jinfeng Hongyuan Flagship Store	Electricity savings1	Express received, in use do not know if there is no power saving	Jingdong prefecture level city in Shandong
Jinfeng Hongyuan Flagship Store	Electricity savings1	The quality of the product is quite good! The power saving mode is still getting used to it, can't say yet, how much power it saves.	Jingdong prefecture level city in Shandong
Jinfeng Hongyuan Flagship Store	Electricity savings1	The goods have been received, just started to use, now I do not know how the quality, after a period of time to say.	Jingdong prefecture level city in Shandong
Jinfeng Hongyuan Flagship Store	Electricity savings1	I received the stuff, used it for a few days to evaluate, the stuff is very good and good, worth buying.	Jingdong prefecture level city in Shandong
Jinfeng Hongyuan Flagship Store	Electricity savings1	I've been using it for a while, but I don't know if it's good or if it saves electricity! What the heck, let's give it a good review first. And for the record, I'm not helping him**.	Jingdong prefecture level city in Shandong
Jinfeng Hongyuan Flagship Store	Electricity savings1	Haven't used it yet, looks good, try it for 60 days, might be good, great review!	Jingdong prefecture level city in Shandong
Jinfeng Hongyuan Flagship Store	Electricity savings1	Baby received, logistics is very fast. Ordered last night, received this morning. Received the goods immediately plugged in the power supply, save power to the first of next month to know.	Jingdong prefecture level city in Shandong
Jinfeng Hongyuan Flagship Store	Electricity savings1	First, I'll give you a good review and use it.	Jingdong prefecture level city in Shandong
Jinfeng Hongyuan Flagship Store	Electricity savings1	Just arrived to follow up with a genuine review	Jingdong prefecture level city in Shandong
Jinfeng Hongyuan Flagship Store	Electricity savings1	Not bad pretty fast, good quality.	Jingdong prefecture level city in Shandong
Jinfeng Hongyuan Flagship Store	Electricity savings1	Recommended, use it and hopefully it will save some electricity	Jingdong prefecture level city in Shandong
Jinfeng Hongyuan Flagship Store	Electricity savings1	The stuff looks ok, I don't know the effect if it.	Jingdong prefecture level city in Shandong
Jinfeng Hongyuan Flagship Store	Electricity savings1	The stuff looks OK, the stuff looks OK.	Jingdong prefecture level city in Shandong
Jinfeng Hongyuan Flagship Store	Electricity savings1	The item has been received, after using it in a follow-up review. Satisfied with the purchase.	Jingdong prefecture level city in Shandong
Jinfeng Hongyuan Flagship Store	Electricity savings1	Has been normal plug-in use, at present do not know how to see, use down to see and then evaluation	Jingdong prefecture level city in Shandong
Jinfeng Hongyuan Flagship Store	Electricity savings1	Commodities make noise.	Jingdong prefecture level city in Shandong
Jinfeng Hongyuan Flagship Store	Electricity savings1	After using it for a few days and observing it, the saving is about ten percent	Jingdong prefecture level city in Shandong
Jinfeng Hongyuan Flagship Store	Electricity savings1	The goods received, the quality looks quite OK. First use it, the effect is not good to say.	Jingdong prefecture level city in Shandong
Jinfeng Hongyuan Flagship Store	Electricity savings1	In use, follow up next month	Jingdong prefecture level city in Shandong
Jinfeng Hongyuan Flagship Store	Electricity savings1	I just received the goods and have not yet used it to comment after using it	Jingdong prefecture level city in Shandong
Jinfeng Hongyuan Flagship Store	Electricity savings1	So far I don't know if it really saves electricity yet! Spinning waiting a couple months to find out! Good reviews first!	Jingdong prefecture level city in Shandong
Jinfeng Hongyuan Flagship Store	Electricity savings1	Small and exquisite, looking forward to the effect, to be evaluated 6666666666666	Jingdong prefecture level city in Shandong
Jinfeng Hongyuan Flagship Store	Electricity savings1	I just used it, I don't know if it's effective, I'll comment again after a month	Jingdong prefecture level city in Shandong
Jinfeng Hongyuan Flagship Store	Electricity savings1	I just used it, I don't know if it's good or not, I'll comment later.	Jingdong prefecture level city in Shandong
Jinfeng Hongyuan Flagship Store	Electricity savings1	Baby received, Baobei quite good, use and then come back to comment!	Jingdong prefecture level city in Shandong
Jinfeng Hongyuan Flagship Store	Electricity savings1	Good quality power saving is fake	Jingdong prefecture level city in Shandong
Jinfeng Hongyuan Flagship Store	Electricity savings1	The packaging of the goods is atmospheric, the quality is exquisite, and I look forward to saving electricity.	Jingdong prefecture level city in Shandong
Jinfeng Hongyuan Flagship Store	Electricity savings1	The packaging is great and the quality is good!	Jingdong prefecture level city in Shandong
Jinfeng Hongyuan Flagship Store	Electricity savings1	I don't see any effect.	Jingdong prefecture level city in Shandong
Jinfeng Hongyuan Flagship Store	Electricity savings1	Already used, but did not see obvious results	Jingdong prefecture level city in Shandong
Jinfeng Hongyuan Flagship Store	Electricity savings1	It's good. I'm just using it. I don't know what it's like.	Jingdong prefecture level city in Shandong
Jinfeng Hongyuan Flagship Store	Electricity savings1	I just used it. I'll comment later.	Jingdong prefecture level city in Shandong
Jinfeng Hongyuan Flagship Store	Electricity savings1	I installed it when it arrived, try it for a month and then come back for a follow-up review	Jingdong prefecture level city in Shandong
Jinfeng Hongyuan Flagship Store	Electricity savings1	Goods arrived a few days fastidious does not call to pick up the goods, the quality is average	Jingdong prefecture level city in Shandong
Jinfeng Hongyuan Flagship Store	Electricity savings1	This user thinks the product is good	Jingdong prefecture level city in Shandong
Jinfeng Hongyuan Flagship Store	Electricity savings1	Roger that.	Jingdong prefecture level city in Shandong
Jinfeng Hongyuan Flagship Store	Electricity savings1	Second purchase was ok.	Jingdong prefecture level city in Shandong
Jinfeng Hongyuan Flagship Store	Electricity savings1	The merchant is very reputable and the product is good value for money. Logistics is fast!	Jingdong prefecture level city in Shandong
Jinfeng Hongyuan Flagship Store	Electricity savings1	The quality of the baby is very good, I have always trusted Kyodo	Jingdong prefecture level city in Shandong
Jinfeng Hongyuan Flagship Store	Electricity savings1	We'll see how it goes when we get it.	Jingdong prefecture level city in Shandong
Jinfeng Hongyuan Flagship Store	Electricity savings1	I just used it. I don't know if it works.	Jingdong prefecture level city in Shandong
Jinfeng Hongyuan Flagship Store	Electricity savings1	Good quality, used for a month to save about 20%, power saving effect is good	Jingdong prefecture level city in Shandong
Jinfeng Hongyuan Flagship Store	Electricity savings1	I'll give you a good review before I use it.	Jingdong prefecture level city in Shandong
Jinfeng Hongyuan Flagship Store	Electricity savings1	Received, satisfied with the follow-up evaluation	Jingdong prefecture level city in Shandong
Jinfeng Hongyuan Flagship Store	Electricity savings1	Immediate results, saving electricity and money	Jingdong prefecture level city in Shandong
Jinfeng Hongyuan Flagship Store	Electricity savings1	Measured to save a little	Jingdong prefecture level city in Shandong

Jinfeng Hongyuan Flagship Store	Electricity savings1	This user thinks the product is good	Jingdong prefecture level city in Shandong
Jinfeng Hongyuan Flagship Store	Electricity savings1	This user thinks the product is good	Jingdong prefecture level city in Shandong
Jinfeng Hongyuan Flagship Store	Electricity savings1	Product quality and logistics are very good, worth buying	Jingdong prefecture level city in Shandong
Jinfeng Hongyuan Flagship Store	Electricity savings1	Appearance looks ok! Express delivery was fast! Service is still relatively enthusiastic.	Jingdong prefecture level city in Shandong
Jinfeng Hongyuan Flagship Store	Electricity savings1	It doesn't feel like it's working.	Jingdong prefecture level city in Shandong
Jinfeng Hongyuan Flagship Store	Electricity savings1	Well made and looks good. Used it with other appliances and felt as if it had some effect.	Jingdong prefecture level city in Shandong
Jinfeng Hongyuan Flagship Store	Electricity savings1	Haven't used it yet, will follow up later	Jingdong prefecture level city in Shandong
Jinfeng Hongyuan Flagship Store	Electricity savings1	Things received, used a few days the plug is a little hot unplugged did not work	Jingdong prefecture level city in Shandong
Jinfeng Hongyuan Flagship Store	Electricity savings1	Look at the meter again in a month.	Jingdong prefecture level city in Shandong
Jinfeng Hongyuan Flagship Store	Electricity savings1	This one didn't work at all.	Jingdong prefecture level city in Shandong
Jinfeng Hongyuan Flagship Store	Electricity savings1	It's small. I've seen friends with much bigger ones.	Jingdong prefecture level city in Shandong
Jinfeng Hongyuan Flagship Store	Electricity savings1	The item was received and the quality is good, it will be nice to see how the power saving rate is down the road, the item is great.	Jingdong prefecture level city in Shandong
Jinfeng Hongyuan Flagship Store	Electricity savings1	Cheap and affordable quality is quite good, logistics is also very fast, praise! , baby good 😉, cost-effective very high, big brand trustworthy 👍	Jingdong prefecture level city in Shandong
Jinfeng Hongyuan Flagship Store	Electricity savings1	Used it for a while it really saves electricity ⚡ thanks 🈶 such a good product, compact and practical, every family should buy one ✨	Jingdong prefecture level city in Shandong
Jinfeng Hongyuan Flagship Store	Electricity savings1	Very satisfied with the purchase Next time I will come back	Jingdong prefecture level city in Shandong
Jinfeng Hongyuan Flagship Store	Electricity savings1	Logistics is fast. Good quality, used one before and it does save power	Jingdong prefecture level city in Shandong
Jinfeng Hongyuan Flagship Store	Electricity savings1	I don't know if it works.	Jingdong prefecture level city in Shandong
Jinfeng Hongyuan Flagship Store	Electricity savings1	Good goods, very solid, good.	Jingdong prefecture level city in Shandong
Jinfeng Hongyuan Flagship Store	Electricity savings1	This user thinks the product is good	Jingdong prefecture level city in Shandong
Jinfeng Hongyuan Flagship Store	Electricity savings1	I've been using it for half a month, my electricity bill is much lower, and the quality is very good, so don't worry about buying it!	Jingdong prefecture level city in Shandong
Jinfeng Hongyuan Flagship Store	Electricity savings1	I received the goods today and used it right away, looking forward to the effect	Jingdong prefecture level city in Shandong
Jinfeng Hongyuan Flagship Store	Electricity savings1	The merchandise is great. I am going to recommend it to my friends! Will buy again next time.	Jingdong prefecture level city in Shandong
Jinfeng Hongyuan Flagship Store	Electricity savings1	Let's give it a good review first, it's working fine so far, and I'll follow up with a review,...	Jingdong prefecture level city in Shandong
Jinfeng Hongyuan Flagship Store	Electricity savings1	Product quality is very good, looking forward to using the results	Jingdong prefecture level city in Shandong
Jinfeng Hongyuan Flagship Store	Electricity savings1	I just can't detect it. - Save it.	Jingdong prefecture level city in Shandong
Jinfeng Hongyuan Flagship Store	Electricity savings1	It arrived and is being used, so we'll see how it works!	Jingdong prefecture level city in Shandong
Jinfeng Hongyuan Flagship Store	Electricity savings1	Received yesterday thanks! This energy saver is available . Safe! But in the end how much energy can be saved must be used for a period of time to know, if the real energy saving next time to buy again.	Jingdong prefecture level city in Shandong
Jinfeng Hongyuan Flagship Store	Electricity savings1	Just used it, don't know if it saves power	Jingdong prefecture level city in Shandong
Jinfeng Hongyuan Flagship Store	Electricity savings1	I'll rate it first, is it a tease wait two months and see? It's been four days and it's not noticeable!	Jingdong prefecture level city in Shandong
Jinfeng Hongyuan Flagship Store	Electricity savings1	Workmanship is quite good, just started to use, the effect of the follow-up evaluation.	Jingdong prefecture level city in Shandong
Jinfeng Hongyuan Flagship Store	Electricity savings1	Received the goods used for a few days before evaluation, the effect is quite good	Jingdong prefecture level city in Shandong
Jinfeng Hongyuan Flagship Store	Electricity savings1	Try it for a month and then evaluate	Jingdong prefecture level city in Shandong
Jinfeng Hongyuan Flagship Store	Electricity savings1	Just received the goods, the packaging is very good, the appearance of the economizer feels good, I do not know how the effect of power saving, with a month or two and then follow up!	Jingdong prefecture level city in Shandong
Jinfeng Hongyuan Flagship Store	Electricity savings1	It's quite small and delicate, I don't know if it's useful yet.	Jingdong prefecture level city in Shandong
Jinfeng Hongyuan Flagship Store	Electricity savings1	Just received it, use it for a few days before commenting	Jingdong prefecture level city in Shandong
Jinfeng Hongyuan Flagship Store	Electricity savings1	Does this thing work! When it arrived, the button for the safety was loose. I'm a little afraid of fire!	Jingdong prefecture level city in Shandong
Jinfeng Hongyuan Flagship Store	Electricity savings1	I received it. I don't know how it works.	Jingdong prefecture level city in Shandong
Jinfeng Hongyuan Flagship Store	Electricity savings1	This user thinks the product is good	Jingdong prefecture level city in Shandong
Jinfeng Hongyuan Flagship Store	Electricity savings1	Customer service was great! Small i problem was taken care of in a timely manner! Effectiveness to be tested by time! No rush!!!	Jingdong prefecture level city in Shandong
Jinfeng Hongyuan Flagship Store	Electricity savings1	I put it on right after I received it and it looks compact. I believe the high technology should save electricity.	Jingdong prefecture level city in Shandong
Jinfeng Hongyuan Flagship Store	Electricity savings1	Logistics courier very quickly, the first day under the order the next day to receive, off pieces whether power saving is the most important thing, things are exquisite workmanship, a month later in the follow-up evaluation whether power saving	Jingdong prefecture level city in Shandong
Jinfeng Hongyuan Flagship Store	Electricity savings1	The delivery is pretty fast, I'm using it, have to have it for a while to see results, looking forward to it.	Jingdong prefecture level city in Shandong
Jinfeng Hongyuan Flagship Store	Electricity savings1	See pretty good just started to use a month later to give you a real evaluation, should be useful.	Jingdong prefecture level city in Shandong
Jinfeng Hongyuan Flagship Store	Electricity savings1	And then comment.	Jingdong prefecture level city in Shandong
Jinfeng Hongyuan Flagship Store	Electricity savings1	Received the goods, wait for the test to evaluate	Jingdong prefecture level city in Shandong
Jinfeng Hongyuan Flagship Store	Electricity savings1	This user thinks the product is good	Jingdong prefecture level city in Shandong
Jinfeng Hongyuan Flagship Store	Electricity savings1	I'm already using it, I just don't know how to judge the power savings. I won't know until I use it for a season.	Jingdong prefecture level city in Shandong
Jinfeng Hongyuan Flagship Store	Electricity savings1	Works. Says it works.	Jingdong prefecture level city in Shandong
Jinfeng Hongyuan Flagship Store	Electricity savings1	Very good. Satisfied.	Jingdong prefecture level city in Shandong
Jinfeng Hongyuan Flagship Store	Electricity savings1	This user thinks the product is good	Jingdong prefecture level city in Shandong
Jinfeng Hongyuan Flagship Store	Electricity savings1	It's working. Obviously, it's less expensive.	Jingdong prefecture level city in Shandong

Jinfeng Hongyuan Flagship Store	Electricity savings1	The second one is up, the first one still works! It's reactive power compensation! This is well understood if you want a professional!	Jingdong prefecture level city in Shandong
Jinfeng Hongyuan Flagship Store	Electricity savings1	It feels as if there are some nodes, but they need to be tested and observed over time, thanks.	Jingdong prefecture level city in Shandong
Jinfeng Hongyuan Flagship Store	Electricity savings1	I've received the goods, the energy saving effect will be evaluated after using it for a period of time, and all the services are very good, praise!	Jingdong prefecture level city in Shandong
Jinfeng Hongyuan Flagship Store	Electricity savings1	I don't know how well it works yet, I bought it and plugged it in, so we'll see if it saves some power or not!	Jingdong prefecture level city in Shandong
Jinfeng Hongyuan Flagship Store	Electricity savings1	Baby a bunch of knife quality is very good, previously bought a quality is not very good. It really works and has results.	Jingdong prefecture level city in Shandong
Jinfeng Hongyuan Flagship Store	Electricity savings1	Things have arrived, it looks good, just started to use, as to whether or not there is a business said the power-saving effect, but also to observe the effect, now can not evaluate	Jingdong prefecture level city in Shandong
Jinfeng Hongyuan Flagship Store	Electricity savings1	I haven't used it yet. I'll comment afterward.	Jingdong prefecture level city in Shandong
Jinfeng Hongyuan Flagship Store	Electricity savings1	I don't know if it's any good.	Jingdong prefecture level city in Shandong
Jinfeng Hongyuan Flagship Store	Electricity savings1	I don't know how well it works.	Jingdong prefecture level city in Shandong
Jinfeng Hongyuan Flagship Store	Electricity savings1	The baby has been received! Uh-huh! Good, quite exquisite, small and exquisite I thought it was big. Dare to tightly plugged in the electricity, just wait for the effect later!	Jingdong prefecture level city in Shandong
Jinfeng Hongyuan Flagship Store	Electricity savings1	The packaging is sturdy, I tried it right after I received it, so I'm looking forward to the results.	Jingdong prefecture level city in Shandong
Jinfeng Hongyuan Flagship Store	Electricity savings1	Received the item and installed it! Don't know if it works well! Will follow up with a review later! I hope it works!	Jingdong prefecture level city in Shandong
Jinfeng Hongyuan Flagship Store	Electricity savings1	Things used on the effect of waiting to use a little to say, but things are very good. Logistics is very fast, business service is very good.	Jingdong prefecture level city in Shandong
Jinfeng Hongyuan Flagship Store	Electricity savings1	This user thinks the product is good	Jingdong prefecture level city in Shandong
Jinfeng Hongyuan Flagship Store	Electricity savings1	I'm really using it. It feels good.	Jingdong prefecture level city in Shandong
Jinfeng Hongyuan Flagship Store	Electricity savings1	Good, Jingdong logistics is very fast, intelligent energy-saving appliances	Jingdong prefecture level city in Shandong
Jinfeng Hongyuan Flagship Store	Electricity savings1	It's not bad. It really saves energy.	Jingdong prefecture level city in Shandong
Jinfeng Hongyuan Flagship Store	Electricity savings1	The logistics is fast, praise	Jingdong prefecture level city in Shandong
Jinfeng Hongyuan Flagship Store	Electricity savings1	This user thinks the product is good	Jingdong prefecture level city in Shandong
Jinfeng Hongyuan Flagship Store	Electricity savings1	The first time I placed two, and today I placed another one, a friend introduced it, said it is particularly power-saving	Jingdong prefecture level city in Shandong
Jinfeng Hongyuan Flagship Store	Electricity savings1	I don't know how effective it is. Let's leave it at home for a while and see.	Jingdong prefecture level city in Shandong
Jinfeng Hongyuan Flagship Store	Electricity savings1	Dear customer service! I would like to ask you how to understand the digital display (increase/decrease jump) on the top of the saver? Thank you for your help.	Jingdong prefecture level city in Shandong
Jinfeng Hongyuan Flagship Store	Electricity savings1	This user thinks the product is good	Jingdong prefecture level city in Shandong
Jinfeng Hongyuan Flagship Store	Electricity savings1	The goods have been received, packaging exquisite, is using, later to see how the effect?	Jingdong prefecture level city in Shandong
Jinfeng Hongyuan Flagship Store	Electricity savings1	The saver received, the boss shipped very quickly, yesterday shot today arrived!	Jingdong prefecture level city in Shandong
Jinfeng Hongyuan Flagship Store	Electricity savings1	Beautiful appearance, well packaged. Recording data to see if I can lower my electric bill a bit.	Jingdong prefecture level city in Shandong
Jinfeng Hongyuan Flagship Store	Electricity savings1	Things received, the packaging is quite good, the effect of the use and then come back to evaluate.	Jingdong prefecture level city in Shandong
Jinfeng Hongyuan Flagship Store	Electricity savings1	Easy to use, just plug it in. But there is a current sound.	Jingdong prefecture level city in Shandong
Jinfeng Hongyuan Flagship Store	Electricity savings1	Fast logistics, easy to use and labor-saving	Jingdong prefecture level city in Shandong
Jinfeng Hongyuan Flagship Store	Electricity savings1	Received, thank you!	Jingdong prefecture level city in Shandong
Jinfeng Hongyuan Flagship Store	Electricity savings1	Second time I've bought it. It's good.	Jingdong prefecture level city in Shandong
Jinfeng Hongyuan Flagship Store	Electricity savings1	I've only been using it for a short time, so I'm looking forward to the results.	Jingdong prefecture level city in Shandong
Jinfeng Hongyuan Flagship Store	Electricity savings1	Good stuff, good stuff, good stuff good stuff.	Jingdong prefecture level city in Shandong
Jinfeng Hongyuan Flagship Store	Electricity savings1	Buy your stuff is not always good I have been using it for almost two months now, it does not save electricity at all the electricity bill is about ten dollars a day this is still called the king of power	Jingdong prefecture level city in Shandong
Jinfeng Hongyuan Flagship Store	Electricity savings1	Express arrived, the quality is very good, worth the purchase.	Jingdong prefecture level city in Shandong
Jinfeng Hongyuan Flagship Store	Electricity savings1	Energy saving has been received, now the voltage is more stable, energy-saving effect to see a few days and then chase flat, the service attitude is very good, the courier is relatively fast, I am satisfied.	Jingdong prefecture level city in Shandong
Jinfeng Hongyuan Flagship Store	Electricity savings1	Stuff installed, numbers are smaller than when installed, hopefully it works	Jingdong prefecture level city in Shandong
Jinfeng Hongyuan Flagship Store	Electricity savings1	Good appearance, fine workmanship, just bought, use up the feeling of power saving effect is good, worth buying!	Jingdong prefecture level city in Shandong
Jinfeng Hongyuan Flagship Store	Electricity savings1	Economical and practical 👍 Always use it	Jingdong prefecture level city in Shandong
Jinfeng Hongyuan Flagship Store	Electricity savings1	Just used it, don't know the effect yet, will come back to follow up after the effect. Hope it works well(▽)	Jingdong prefecture level city in Shandong
Jinfeng Hongyuan Flagship Store	Electricity savings1	The goods have been received, use it to see in the evaluation.	Jingdong prefecture level city in Shandong
Jinfeng Hongyuan Flagship Store	Electricity savings1	Doesn't it save electricity? The boil kettle still goes faster when you press the meter.	Jingdong prefecture level city in Shandong
Jinfeng Hongyuan Flagship Store	Electricity savings1	Already used. Will patronize again in the future	Jingdong prefecture level city in Shandong
Jinfeng Hongyuan Flagship Store	Electricity savings1	Works great, just plug it in, results to be checked, satisfied.	Jingdong prefecture level city in Shandong
Jinfeng Hongyuan Flagship Store	Electricity savings1	Second purchase. Works well 😍😍😍😍	Jingdong prefecture level city in Shandong
Jinfeng Hongyuan Flagship Store	Electricity savings1	Prompt shipment, very satisfied	Jingdong prefecture level city in Shandong
Jinfeng Hongyuan Flagship Store	Electricity savings1	It's been less than a month, let's see how it works, hopefully it's power efficient	Jingdong prefecture level city in Shandong
Jinfeng Hongyuan Flagship Store	Electricity savings1	Use a month in say.	Jingdong prefecture level city in Shandong
Jinfeng Hongyuan Flagship Store	Electricity savings1	I've saved a lot of electricity since I've been using it.	Jingdong prefecture level city in Shandong
Jinfeng Hongyuan Flagship Store	Electricity savings1	It's fast, looks and feels good on the outside, try it out and evaluate.	Jingdong prefecture level city in Shandong
Jinfeng Hongyuan Flagship Store	Electricity savings1	I haven't used it. I just used it. I don't know if it's any good.	Jingdong prefecture level city in Shandong

Jinfeng Hongyuan Flagship Store	Electricity savings1	Quality can be, power saving now can not see just use, after a while in the look at the power saving effect	Jingdong prefecture level city in Shandong
Jinfeng Hongyuan Flagship Store	Electricity savings1	The logistics is very fast, and it seems to have some effect	Jingdong prefecture level city in Shandong
Jinfeng Hongyuan Flagship Store	Electricity savings1	Good, but the effects remain to be seen	Jingdong prefecture level city in Shandong
Jinfeng Hongyuan Flagship Store	Electricity savings1	Baby is basically the same as the description, shipping quickly, worth a buy Good price, logistics is strong, worth repurchasing!	Jingdong prefecture level city in Shandong
Jinfeng Hongyuan Flagship Store	Electricity savings1	I bought it for my dad, I don't know much about it, if he thinks it's good then it must be good 👍	Jingdong prefecture level city in Shandong
Jinfeng Hongyuan Flagship Store	Electricity savings1	Goods received. It's working fine. Display voltage. After a period of time to see the effect in the addition to the level of theory	Jingdong prefecture level city in Shandong
Jinfeng Hongyuan Flagship Store	Electricity savings1	The stuff is pretty good, I just don't know how well it saves power.	Jingdong prefecture level city in Shandong
Jinfeng Hongyuan Flagship Store	Electricity savings1	Logistics is a little too fast 👍	Jingdong prefecture level city in Shandong
Jinfeng Hongyuan Flagship Store	Electricity savings1	Received the goods used for a period of time before coming, good, beautiful appearance, really stable flow	Jingdong prefecture level city in Shandong
Jinfeng Hongyuan Flagship Store	Electricity savings1	Things received, look at the packaging is very good, not yet, to the power saving in the follow-up evaluation	Jingdong prefecture level city in Shandong
Jinfeng Hongyuan Flagship Store	Electricity savings1	Baby is very good! Use a few days before to evaluate, I personally test really effective.	Jingdong prefecture level city in Shandong
Jinfeng Hongyuan Flagship Store	Electricity savings1	I received the goods are in use, I do not know how effective? Can it save energy? Let's see.	Jingdong prefecture level city in Shandong
Jinfeng Hongyuan Flagship Store	Electricity savings1	It's really good and has some power saving effect.	Jingdong prefecture level city in Shandong
Jinfeng Hongyuan Flagship Store	Electricity savings1	It's good quality, well made, and I hope it'll save on electricity over time.	Jingdong prefecture level city in Shandong
Jinfeng Hongyuan Flagship Store	Electricity savings1	I just used it, I don't know how it works yet, but I'll give it a good review first.	Jingdong prefecture level city in Shandong
Jinfeng Hongyuan Flagship Store	Electricity savings1	Second purchase, the delivery guy was fast and called ahead to deliver.	Jingdong prefecture level city in Shandong
Jinfeng Hongyuan Flagship Store	Electricity savings1	Goods have been received, but have not yet used, used to see how the effect, in the evaluation of fertilizer	Jingdong prefecture level city in Shandong
Jinfeng Hongyuan Flagship Store	Electricity savings1	The delivery is very fast. It's the same in kind. It's working.	Jingdong prefecture level city in Shandong
Jinfeng Hongyuan Flagship Store	Electricity savings1	Fast logistics, good quality, more satisfied	Jingdong prefecture level city in Shandong
Jinfeng Hongyuan Flagship Store	Electricity savings1	I've been here for almost a month now and there is a very noticeable difference than it is worth buying.	Jingdong prefecture level city in Shandong
Jinfeng Hongyuan Flagship Store	Electricity savings1	Fast shipping, received the next day, plug and play, but do not know how effective to not break down!	Jingdong prefecture level city in Shandong
Jinfeng Hongyuan Flagship Store	Electricity savings1	The logistics are great I've been using it for a while before commenting on it This saves electricity	Jingdong prefecture level city in Shandong
Jinfeng Hongyuan Flagship Store	Electricity savings1	Wait to use it for a while to know if it's energy efficient	Jingdong prefecture level city in Shandong
Jinfeng Hongyuan Flagship Store	Electricity savings1	Just received it, haven't used it yet don't know if it's any good.	Jingdong prefecture level city in Shandong
Jinfeng Hongyuan Flagship Store	Electricity savings1	It really doesn't work, and the electric bill is still the same amount every month.	Jingdong prefecture level city in Shandong
Jinfeng Hongyuan Flagship Store	Electricity savings1	It's well packaged and looks fine	Jingdong prefecture level city in Shandong
Jinfeng Hongyuan Flagship Store	Electricity savings1	Very good. Will patronize again if needed.	Jingdong prefecture level city in Shandong
Jinfeng Hongyuan Flagship Store	Electricity savings1	I have received the product and installed it, try it for a while and see how it works.	Jingdong prefecture level city in Shandong
Jinfeng Hongyuan Flagship Store	Electricity savings1	I've been using it for a month now...I don't feel like there's any noticeable power savings...last month it was $130 this month it's $123...let's see if I can use it for another month	Jingdong prefecture level city in Shandong
Jinfeng Hongyuan Flagship Store	Electricity savings1	Just received it, yet to try it and see if it works.	Jingdong prefecture level city in Shandong
Jinfeng Hongyuan Flagship Store	Electricity savings1	It's easy to use and has a power saving credit!	Jingdong prefecture level city in Shandong
Jinfeng Hongyuan Flagship Store	Electricity savings1	It's very good. I've already bought two of them.	Jingdong prefecture level city in Shandong
Jinfeng Hongyuan Flagship Store	Electricity savings1	It's very well made, packaged meticulously, and I hope it works!	Jingdong prefecture level city in Shandong
Jinfeng Hongyuan Flagship Store	Electricity savings1	Goods have been received have not yet used it, used to comment again	Jingdong prefecture level city in Shandong
Jinfeng Hongyuan Flagship Store	Electricity savings1	Looking forward to good results	Jingdong prefecture level city in Shandong
Jinfeng Hongyuan Flagship Store	Electricity savings1	Not sure if it saves power or not. Still trying it out.	Jingdong prefecture level city in Shandong
Jinfeng Hongyuan Flagship Store	Electricity savings1	It also really saves on electricity! Superb!	Jingdong prefecture level city in Shandong
Jinfeng Hongyuan Flagship Store	Electricity savings1	Didn't work at all, want to return it	Jingdong prefecture level city in Shandong
Jinfeng Hongyuan Flagship Store	Electricity savings1	Uh-huh! It's working, I've been using it for a month and it saves 20% of electricity compared to before.	Jingdong prefecture level city in Shandong
Jinfeng Hongyuan Flagship Store	Electricity savings1	Should be a little bit of a power saver, I wonder how long it will take to save back the money I spent on this one	Jingdong prefecture level city in Shandong
Jinfeng Hongyuan Flagship Store	Electricity savings1	Exquisite appearance and stable voltage	Jingdong prefecture level city in Shandong
Jinfeng Hongyuan Flagship Store	Electricity savings1	After using it for a few days it feels like it saves electricity.	Jingdong prefecture level city in Shandong
Jinfeng Hongyuan Flagship Store	Electricity savings1	Second purchase and its pending good results.	Jingdong prefecture level city in Shandong
Jinfeng Hongyuan Flagship Store	Electricity savings1	The appearance of the workmanship is exquisite and meticulous, beautiful appearance, just to see if there is no effect!	Jingdong prefecture level city in Shandong
Jinfeng Hongyuan Flagship Store	Electricity savings1	Let's see what happens in a month.	Jingdong prefecture level city in Shandong
Jinfeng Hongyuan Flagship Store	Electricity savings1	Trial, now short time not yet obvious effect	Jingdong prefecture level city in Shandong
Jinfeng Hongyuan Flagship Store	Electricity savings1	I'm using it now, don't know how well it works, it looks great!	Jingdong prefecture level city in Shandong
Jinfeng Hongyuan Flagship Store	Electricity savings1	There is no role! Now it's true that I don't know!	Jingdong prefecture level city in Shandong
Jinfeng Hongyuan Flagship Store	Electricity savings1	Purchased for the second time, power saving not sure if it's ok? Let's talk about it after using it	Jingdong prefecture level city in Shandong
Jinfeng Hongyuan Flagship Store	Electricity savings1	I just received it, I have to try it for a month to know how it works.	Jingdong prefecture level city in Shandong
Jinfeng Hongyuan Flagship Store	Electricity savings1	I just used it for an hour, and the meter's still spinning so fast it's not much use.	Jingdong prefecture level city in Shandong

Jinfeng Hongyuan Flagship Store	Electricity savings1	Not bad, logistics is fast	Jingdong prefecture level city in Shandong
Jinfeng Hongyuan Flagship Store	Electricity savings1	I don't know if it works. I'll find out next month when I pay the bill. It's easy. Just plug it in.	Jingdong prefecture level city in Shandong
Jinfeng Hongyuan Flagship Store	Electricity savings1	Logistics is particularly fast, received the goods are genuine, looking forward to power saving effect	Jingdong prefecture level city in Shandong
Jinfeng Hongyuan Flagship Store	Electricity savings1	First time buying this product like this one from this family use it and then evaluate.	Jingdong prefecture level city in Shandong
Jinfeng Hongyuan Flagship Store	Electricity savings1	Lightning arrived, the product is very good, before all do not know there is such a baby, in a friend's home has seen, and then bought to try, has been used for a period of time before coming to the evaluation, the work is very stable, so far looked very good, will introduce friends to buy!	Jingdong prefecture level city in Shandong
Jinfeng Hongyuan Flagship Store	Electricity savings1	I was a bit skeptical when I heard that I could save money on my electricity bill by installing this, I don't know if the power saving effect is really what it is said to be. But since I bought it, I just have to put it on and try it out. I hope it lives up to its name and saves me the money I spent on the device	Jingdong prefecture level city in Shandong
Jinfeng Hongyuan Flagship Store	Electricity savings1	The quality of the stuff is okay, but I'm not sure if it works, so I'll try using it first.	Jingdong prefecture level city in Shandong
Jinfeng Hongyuan Flagship Store	Electricity savings1	It should work. The current is much more stable.	Jingdong prefecture level city in Shandong
Jinfeng Hongyuan Flagship Store	Electricity savings1	The store has a great service attitude	Jingdong prefecture level city in Shandong
Jinfeng Hongyuan Flagship Store	Electricity savings1	Let's wait until we've used it for a month.	Jingdong prefecture level city in Shandong
Jinfeng Hongyuan Flagship Store	Electricity savings1	I'm using it and I don't know if it's a power saver or not	Jingdong prefecture level city in Shandong
Jinfeng Hongyuan Flagship Store	Electricity savings1	Original and authentic guarantee quality is good now.	Jingdong prefecture level city in Shandong
Jinfeng Hongyuan Flagship Store	Electricity savings1	Great, very happy with it, will buy a couple more next time	Jingdong prefecture level city in Shandong
Jinfeng Hongyuan Flagship Store	Electricity savings1	I just used it, I don't know if it works yet, I'll come back after using it...	Jingdong prefecture level city in Shandong
Jinfeng Hongyuan Flagship Store	Electricity savings1	It's still being tested.	Jingdong prefecture level city in Shandong
Jinfeng Hongyuan Flagship Store	Electricity savings1	I've been using it for a month and it's not working.	Jingdong prefecture level city in Shandong
Jinfeng Hongyuan Flagship Store	Electricity savings1	It's basically nothing!	Jingdong prefecture level city in Shandong
Jinfeng Hongyuan Flagship Store	Electricity savings1	It's not bad, it kind of works, support it.	Jingdong prefecture level city in Shandong
Jinfeng Hongyuan Flagship Store	Electricity savings1	I just received it last night, let's try it first to see how it works, it looks good!	Jingdong prefecture level city in Shandong
Jinfeng Hongyuan Flagship Store	Electricity savings1	The current is very stable, fine workmanship, small and exquisite, very satisfied	Jingdong prefecture level city in Shandong
Jinfeng Hongyuan Flagship Store	Electricity savings1	Not bad, exactly how much electricity can be saved; still have to follow up the evaluation	Jingdong prefecture level city in Shandong
Jinfeng Hongyuan Flagship Store	Electricity savings1	Logistics is also very fast, next time there is an opportunity to introduce friends to buy!	Jingdong prefecture level city in Shandong
Jinfeng Hongyuan Flagship Store	Electricity savings1	Voltage display and actual do not pay	Jingdong prefecture level city in Shandong
Jinfeng Hongyuan Flagship Store	Electricity savings1	Second purchase, still saves a little on the electric bill	Jingdong prefecture level city in Shandong
Jinfeng Hongyuan Flagship Store	Electricity savings1	Order received, currently trying it out, thanks a lot.	Jingdong prefecture level city in Shandong
Jinfeng Hongyuan Flagship Store	Electricity savings1	I haven't used it for a long time yet, I don't know if the effect is good or not, looking forward to it	Jingdong prefecture level city in Shandong
Jinfeng Hongyuan Flagship Store	Electricity savings1	Good, working, I just don't know if it will save electricity... 5 stars for now.	Jingdong prefecture level city in Shandong
Jinfeng Hongyuan Flagship Store	Electricity savings1	Great product, just as the owner said it would be.	Jingdong prefecture level city in Shandong
Jinfeng Hongyuan Flagship Store	Electricity savings1	Shipping was fast, I don't know if it works well or not, I have to use it for a while first.	Jingdong prefecture level city in Shandong
Jinfeng Hongyuan Flagship Store	Electricity savings1	I don't feel that it saves much electricity, but I'll give it a try. It's been more than a month now, and the electricity bill is still a bit high.	Jingdong prefecture level city in Shandong
Jinfeng Hongyuan Flagship Store	Electricity savings1	Got it. Just used it.	Jingdong prefecture level city in Shandong
Jinfeng Hongyuan Flagship Store	Electricity savings1	Use it for a while and then comment.	Jingdong prefecture level city in Shandong
Jinfeng Hongyuan Flagship Store	Electricity savings1	Used it, see how it works with it and then comment. The quality is very good!	Jingdong prefecture level city in Shandong
Jinfeng Hongyuan Flagship Store	Electricity savings1	I'll comment again after a while.	Jingdong prefecture level city in Shandong
Jinfeng Hongyuan Flagship Store	Electricity savings1	It's easy to operate, affordable and works well, love it.	Jingdong prefecture level city in Shandong
Jinfeng Hongyuan Flagship Store	Electricity savings1	It's good. You can try it. It's cheap.	Jingdong prefecture level city in Shandong
Jinfeng Hongyuan Flagship Store	Electricity savings1	It's in use, so we'll see how it works after a while!	Jingdong prefecture level city in Shandong
Jinfeng Hongyuan Flagship Store	Electricity savings1	Service is pretty good, not sure about the quality yet.	Jingdong prefecture level city in Shandong
Jinfeng Hongyuan Flagship Store	Electricity savings1	Just used it and will see the results in a month 😀	Jingdong prefecture level city in Shandong
Jinfeng Hongyuan Flagship Store	Electricity savings1	Saving electricity is expensive and good	Jingdong prefecture level city in Shandong
Jinfeng Hongyuan Flagship Store	Electricity savings1	Pretty good, 5 stars! Will buy again next time seller service is very good !!!! Good quality! Will recommend to friends!	Jingdong prefecture level city in Shandong
Jinfeng Hongyuan Flagship Store	Electricity savings1	In use, with results naturally have follow-up reviews!	Jingdong prefecture level city in Shandong
Jinfeng Hongyuan Flagship Store	Electricity savings1	I'm using it now, I'll come back with results, it'll work.	Jingdong prefecture level city in Shandong
Jinfeng Hongyuan Flagship Store	Electricity savings1	Good, nice, fast shipping, cheap, worth having!	Jingdong prefecture level city in Shandong
Jinfeng Hongyuan Flagship Store	Electricity savings1	Logistics is fast.	Jingdong prefecture level city in Shandong
Jinfeng Hongyuan Flagship Store	Electricity savings1	I have just started using the product and will be reviewing it later.	Jingdong prefecture level city in Shandong
Jinfeng Hongyuan Flagship Store	Electricity savings1	I'll comment after I use it.	Jingdong prefecture level city in Shandong
Jinfeng Hongyuan Flagship Store	Electricity savings1	I don't know if it's working or not, though I'll try plugging it in.	Jingdong prefecture level city in Shandong
Jinfeng Hongyuan Flagship Store	Electricity savings1	It really saves electricity and contributes to energy savings in disguise	Jingdong prefecture level city in Shandong
Jinfeng Hongyuan Flagship Store	Electricity savings1	It feels good in the experience.	Jingdong prefecture level city in Shandong

Jinfeng Hongyuan Flagship Store	Electricity savings1	The product is better than I thought it would be, the quality looks great, the voltage is stable, and I'm looking forward to some great power savings!	Jingdong prefecture level city in Shandong
Jinfeng Hongyuan Flagship Store	Electricity savings1	I'm using it now looking forward to saving some points,, I'm feeding back	Jingdong prefecture level city in Shandong
Jinfeng Hongyuan Flagship Store	Electricity savings1	Displayed voltage below 200.	Jingdong prefecture level city in Shandong
Jinfeng Hongyuan Flagship Store	Electricity savings1	I haven't seen any great results, but it should last about a hundred or eighty years.	Jingdong prefecture level city in Shandong
Jinfeng Hongyuan Flagship Store	Electricity savings1	I don't know if it works or not, but I keep it plugged in anyway, so I hope it works!	Jingdong prefecture level city in Shandong
Jinfeng Hongyuan Flagship Store	Electricity savings1	Reasonable price, good quality, fast delivery, great service, already a repurchase.	Jingdong prefecture level city in Shandong
Jinfeng Hongyuan Flagship Store	Electricity savings1	Second purchase, looking forward to the results	Jingdong prefecture level city in Shandong
Jinfeng Hongyuan Flagship Store	Electricity savings1	The quality of this product feels like it should work in a use test! Just how many kilowatts of electricity must be wasted per day with the red light and digital display on for long periods of time?	Jingdong prefecture level city in Shandong
Jinfeng Hongyuan Flagship Store	Electricity savings1	It looks good, we'll see if we use it.	Jingdong prefecture level city in Shandong
Jinfeng Hongyuan Flagship Store	Electricity savings1	Great stuff, second purchase!	Jingdong prefecture level city in Shandong
Jinfeng Hongyuan Flagship Store	Electricity savings1	It is indeed very practical and saves electricity at home	Jingdong prefecture level city in Shandong
Jinfeng Hongyuan Flagship Store	Electricity savings1	Goods received, the physical and the same as the picture, logistics is also very fast!	Jingdong prefecture level city in Shandong
Jinfeng Hongyuan Flagship Store	Electricity savings1	I'll use it for a while and see how it goes. I'll buy it again if it's good.	Jingdong prefecture level city in Shandong
Jinfeng Hongyuan Flagship Store	Electricity savings1	In use, voltage monitoring, the effect of how to wait for next month's electricity consumption confirmation	Jingdong prefecture level city in Shandong
Jinfeng Hongyuan Flagship Store	Electricity savings1	It's still pretty good.	Jingdong prefecture level city in Shandong
Jinfeng Hongyuan Flagship Store	Electricity savings1	It's been plugged in for a month and there hasn't been a big change yet, since it usually uses very little electricity	Jingdong prefecture level city in Shandong
Jinfeng Hongyuan Flagship Store	Electricity savings1	Let's wait for a month to see if it works	Jingdong prefecture level city in Shandong
Jinfeng Hongyuan Flagship Store	Electricity savings1	Good, good, good, good, good, good, good, good, good, good, good, good, good, good, good.	Jingdong prefecture level city in Shandong
Jinfeng Hongyuan Flagship Store	Electricity savings1	The product received the trial a few days, feel good, praise!	Jingdong prefecture level city in Shandong
Jinfeng Hongyuan Flagship Store	Electricity savings1	After six or seven hours of use, 250 volts won't save you any power.	Jingdong prefecture level city in Shandong
Jinfeng Hongyuan Flagship Store	Electricity savings1	I haven't used it yet, I wonder if it saves electricity?	Jingdong prefecture level city in Shandong
Jinfeng Hongyuan Flagship Store	Electricity savings1	Baby value for money, love it, next time will come back is genuine, trust the business, baby texture is very good, the price is moderate, unlimited repurchase!	Jingdong prefecture level city in Shandong
Jinfeng Hongyuan Flagship Store	Electricity savings1	Power saving effect is obvious! Raising tropical fish daily power consumption, after using the daily power savings of more than 3 degrees, currently about 8 degrees / day!	Jingdong prefecture level city in Shandong
Jinfeng Hongyuan Flagship Store	Electricity savings1	Just used it, unknown results.	Jingdong prefecture level city in Shandong
Jinfeng Hongyuan Flagship Store	Electricity savings1	The goods received, well, look forward to the effect. Logistics is very fast, thanks to the platform, thanks to Jingdong. I hope it can save electricity.	Jingdong prefecture level city in Shandong
Jinfeng Hongyuan Flagship Store	Electricity savings1	Not sure 有 effect?	Jingdong prefecture level city in Shandong
Jinfeng Hongyuan Flagship Store	Electricity savings1	I'd say it's a 5% savings.	Jingdong prefecture level city in Shandong
Jinfeng Hongyuan Flagship Store	Electricity savings1	I received it and am using it, I don't know how effective it is, I'll evaluate it afterwards	Jingdong prefecture level city in Shandong
Jinfeng Hongyuan Flagship Store	Electricity savings1	Baby received not yet used, first give praise	Jingdong prefecture level city in Shandong
Jinfeng Hongyuan Flagship Store	Electricity savings1	Looks good, I'll evaluate it later	Jingdong prefecture level city in Shandong
Jinfeng Hongyuan Flagship Store	Electricity savings1	The goods have been received in use, the overall feeling is not bad!	Jingdong prefecture level city in Shandong
Jinfeng Hongyuan Flagship Store	Electricity savings1	It's working. This is the second one. This stuff has to be used over a long period of time to be effective. And it only works on inductive loads, i.e. appliances containing electric motors. Use it.	Jingdong prefecture level city in Shandong
Jinfeng Hongyuan Flagship Store	Electricity savings1	Use it for a while and see how it works.	Jingdong prefecture level city in Shandong
Jinfeng Hongyuan Flagship Store	Electricity savings1	Good courier service and the item is very nice and practical.	Jingdong prefecture level city in Shandong
Jinfeng Hongyuan Flagship Store	Electricity savings1	It doesn't do anything and it's a waste of electricity.	Jingdong prefecture level city in Shandong
Jinfeng Hongyuan Flagship Store	Electricity savings1	I haven't used it yet. It should be good.	Jingdong prefecture level city in Shandong
Jinfeng Hongyuan Flagship Store	Electricity savings1	That's good. OK, that's good.	Jingdong prefecture level city in Shandong
Jinfeng Hongyuan Flagship Store	Electricity savings1	It's a rip off, doesn't work, doesn't save electricity, does the job to well, and I've wasted a couple dozen dollars again.	Jingdong prefecture level city in Shandong
Jinfeng Hongyuan Flagship Store	Electricity savings1	Use it first, the effect is not as good as the road, then we will comment again	Jingdong prefecture level city in Shandong
Jinfeng Hongyuan Flagship Store	Electricity savings1	The price is affordable, the logistics is strong, just use four or five days, the effect will be added later!	Jingdong prefecture level city in Shandong
Jinfeng Hongyuan Flagship Store	Electricity savings1	It's easy to use and works fine.	Jingdong prefecture level city in Shandong
Jinfeng Hongyuan Flagship Store	Electricity savings1	It doesn't feel like it's working.	Jingdong prefecture level city in Shandong
Jinfeng Hongyuan Flagship Store	Electricity savings1	I'm using it. We'll see what happens in a couple months.	Jingdong prefecture level city in Shandong
Jinfeng Hongyuan Flagship Store	Electricity savings1	I just got it back to try it out and I'll comment later	Jingdong prefecture level city in Shandong
Jinfeng Hongyuan Flagship Store	Electricity savings1	Less than two months. Can't tell.	Jingdong prefecture level city in Shandong
Jinfeng Hongyuan Flagship Store	Electricity savings1	No power saving effect	Jingdong prefecture level city in Shandong
Jinfeng Hongyuan Flagship Store	Electricity savings1	Express Express Very quickly not only to the power saver can not save electricity, with two months, look at the evaluation put.	Jingdong prefecture level city in Shandong
Jinfeng Hongyuan Flagship Store	Electricity savings1	Genuine products are packaged well!	Jingdong prefecture level city in Shandong
Jinfeng Hongyuan Flagship Store	Electricity savings1	I've been using it for a while, but I don't know if it saves electricity. I hope it works.	Jingdong prefecture level city in Shandong
Jinfeng Hongyuan Flagship Store	Electricity savings1	Good service, fast logistics, the effect is to be used to know	Jingdong prefecture level city in Shandong
Jinfeng Hongyuan Flagship Store	Electricity savings1	Just used it, not sure about the power saving effect	Jingdong prefecture level city in Shandong

Jinfeng Hongyuan Flagship Store	Electricity savings1	The stuff is ok, save power or not, will comment after a month.	Jingdong prefecture level city in Shandong
Jinfeng Hongyuan Flagship Store	Electricity savings1	Used it for a month to save %25, normal is a little over 100, it's great! I want to buy one in.	Jingdong prefecture level city in Shandong
Jinfeng Hongyuan Flagship Store	Electricity savings1	I'm using it and looking forward to the results	Jingdong prefecture level city in Shandong
Jinfeng Hongyuan Flagship Store	Electricity savings1	The quality is very good and the workmanship is exquisite. The use effect is also very good.	Jingdong prefecture level city in Shandong
Jinfeng Hongyuan Flagship Store	Electricity savings1	Merchant service attitude is very good, logistics speed is super fast, online evaluation is very good, the first time to use, a month to know the specific effect, the effect is good, and then additional	Jingdong prefecture level city in Shandong
Jinfeng Hongyuan Flagship Store	Electricity savings1	Works great, solves the problem of high power usage in the house	Jingdong prefecture level city in Shandong
Jinfeng Hongyuan Flagship Store	Electricity savings1	To receive! Very delicate! It's not clear how effective it is, I won't know until I use it!!!!	Jingdong prefecture level city in Shandong
Jinfeng Hongyuan Flagship Store	Electricity savings1	I've just been using it for a while and I don't feel like it's too noticeable.	Jingdong prefecture level city in Shandong
Jinfeng Hongyuan Flagship Store	Electricity savings1	Already used, no visible effect for the time being	Jingdong prefecture level city in Shandong
Jinfeng Hongyuan Flagship Store	Electricity savings1	I can't feel the effect.	Jingdong prefecture level city in Shandong
Jinfeng Hongyuan Flagship Store	Electricity savings1	Use it for a while and see the results	Jingdong prefecture level city in Shandong
Jinfeng Hongyuan Flagship Store	Electricity savings1	The price is more reasonable, the power-saving effect is to be verified, the delivery is fast, and the courier service is good.	Jingdong prefecture level city in Shandong
Jinfeng Hongyuan Flagship Store	Electricity savings1	Quality is very good, consistent with the seller's description, very satisfied, very like, very fast shipping, packaging is very careful and strict, the logistics company has a very good service attitude, fast delivery, very satisfied with a shopping, very satisfied. Recommended to buy, cost-effective, the service attitude of the business is very enthusiastic, providing such high quality goods.	Jingdong prefecture level city in Shandong
Jinfeng Hongyuan Flagship Store	Electricity savings1	I don't know.	Jingdong prefecture level city in Shandong
Jinfeng Hongyuan Flagship Store	Electricity savings1	I didn't get the delivery.	Jingdong prefecture level city in Shandong
Jinfeng Hongyuan Flagship Store	Electricity savings1	It works, it does have power savings, better than expected, will repurchase.	Jingdong prefecture level city in Shandong
Jinfeng Hongyuan Flagship Store	Electricity savings1	That's where the five fuses are.	Jingdong prefecture level city in Shandong
Jinfeng Hongyuan Flagship Store	Electricity savings1	Don't buy it, it doesn't work and you can't return it.	Jingdong prefecture level city in Shandong
Jinfeng Hongyuan Flagship Store	Electricity savings1	I hope it works. Wait a month.	Jingdong prefecture level city in Shandong
Jinfeng Hongyuan Flagship Store	Electricity savings1	Nothing**, buy with caution	Jingdong prefecture level city in Shandong
Jinfeng Hongyuan Flagship Store	Electricity savings1	I'm using it and waiting for the results	Jingdong prefecture level city in Shandong
Jinfeng Hongyuan Flagship Store	Electricity savings1	Saver quality is very good, has always felt that the home power consumption is very large, this is the company's electrician recommended, said he bought this home is really able to save a little electricity, I also came to buy, the effect if it is good, then buy, a floor a...	Jingdong prefecture level city in Shandong
Jinfeng Hongyuan Flagship Store	Electricity savings1	Good product, satisfied with purchase	Jingdong prefecture level city in Shandong
Jinfeng Hongyuan Flagship Store	Electricity savings1	Occupying the time has not yet found the power saving, occupying not evaluation	Jingdong prefecture level city in Shandong
Jinfeng Hongyuan Flagship Store	Electricity savings1	Haven't tested the results yet. To be followed up with comments!	Jingdong prefecture level city in Shandong
Jinfeng Hongyuan Flagship Store	Electricity savings1	After experiencing it for a month, I don't seem to know how to save it, and I don't pay attention to it anyway	Jingdong prefecture level city in Shandong
Jinfeng Hongyuan Flagship Store	Electricity savings1	It's useless. It's a waste of electricity.	Jingdong prefecture level city in Shandong
Jinfeng Hongyuan Flagship Store	Electricity savings1	I don't know if it's a power saver. It's a couple bucks. I hope it works.	Jingdong prefecture level city in Shandong
Jinfeng Hongyuan Flagship Store	Electricity savings1	I don't know what to say about this stuff. I don't know if it works or not, it's kind of a psychological purchase. Dormitory use to see the meter can not be not so good-looking. You have to wait until the time to deduct the utility bill to know.	Jingdong prefecture level city in Shandong
Jinfeng Hongyuan Flagship Store	Electricity savings1	Energy savers don't work / they're all a bunch of hoaxes.	Jingdong prefecture level city in Shandong
Jinfeng Hongyuan Flagship Store	Electricity savings1	Received the goods, used for more than a week, it's okay, sure a little effect	Jingdong prefecture level city in Shandong
Jinfeng Hongyuan Flagship Store	Electricity savings1	Well made and compact! Looking forward to the effect!	Jingdong prefecture level city in Shandong
Jinfeng Hongyuan Flagship Store	Electricity savings1	It has arrived and we'll see how it works.	Jingdong prefecture level city in Shandong
Jinfeng Hongyuan Flagship Store	Electricity savings1	Good. Good. It's worth it.	Jingdong prefecture level city in Shandong
Jinfeng Hongyuan Flagship Store	Electricity savings1	How come there's no indicator light?	Jingdong prefecture level city in Shandong
Jinfeng Hongyuan Flagship Store	Electricity savings1	The machine that uses electricity works quite well. This saves	Jingdong prefecture level city in Shandong
Jinfeng Hongyuan Flagship Store	Electricity savings1	It's good for home use and feels quite energy efficient	Jingdong prefecture level city in Shandong
Jinfeng Hongyuan Flagship Store	Electricity savings1	I regret buying this product, after using it for two days it doesn't work at all, still so many kilowatts per day just like the original.	Jingdong prefecture level city in Shandong
Jinfeng Hongyuan Flagship Store	Electricity savings1	Works very well and has some scientific merit.	Jingdong prefecture level city in Shandong
Jinfeng Hongyuan Flagship Store	Electricity savings1	Logistics is also very fast this good power saving I use two months before commenting!	Jingdong prefecture level city in Shandong
Jinfeng Hongyuan Flagship Store	Electricity savings1	Repeat customer. Very good.	Jingdong prefecture level city in Shandong
Jinfeng Hongyuan Flagship Store	Electricity savings1	It's good stuff. I've been using it for a month now and it's a real power saver.	Jingdong prefecture level city in Shandong
Jinfeng Hongyuan Flagship Store	Electricity savings1	Jingdong platform to buy things, has been very assured, quality assurance. Jingdong Express is as good as ever, complete packaging, delivery, service attitude to go! The family has always wanted to buy an energy-saving and power-saving equipment, selected half a day to choose this one, the value is very good, used for a period of time to follow up, to see whether it can achieve the real purpose of power saving, I	Jingdong prefecture level city in Shandong
Jinfeng Hongyuan Flagship Store	Electricity savings1	Works great, inexpensive and will be back next time.	Jingdong prefecture level city in Shandong
Jinfeng Hongyuan Flagship Store	Electricity savings1	Baby received, looks good, wait for a month to see how it works?	Jingdong prefecture level city in Shandong
Jinfeng Hongyuan Flagship Store	Electricity savings1	This product is not bad quite good well customer service said quite power saving that look at the customer service to see if the accuracy of the look at all aspects of the color is also quite beautiful	Jingdong prefecture level city in Shandong
Jinfeng Hongyuan Flagship Store	Electricity savings1	Just got back and tried it feels pretty good, just don't know how the quality is, give a good comment first	Jingdong prefecture level city in Shandong
Jinfeng Hongyuan Flagship Store	Electricity savings1	Received the product, trying it out, hope it helps!	Jingdong prefecture level city in Shandong

Jinfeng Hongyuan Flagship Store	Electricity savings1	Works great, saves electricity a dollar a day and a night	Jingdong prefecture level city in Shandong
Jinfeng Hongyuan Flagship Store	Electricity savings1	Baby received, look at the workmanship is still very fine, is genuine. I hope that this summer to open the air conditioning electricity bill is no longer high scary!	Jingdong prefecture level city in Shandong
Jinfeng Hongyuan Flagship Store	Electricity savings1	It's here. I don't know how it's going to work, but I'm going to use it.	Jingdong prefecture level city in Shandong
Jinfeng Hongyuan Flagship Store	Electricity savings1	Power saver received, Jingdong logistics first-class, business customer service attitude is good, one by one to introduce the product performance, back on the horse plugged in, it is very simple, there is a voltage indicator, to be a few months to see how the effect of power-saving to come back to a good follow-up, but I believe that Jinfenghong products.	Jingdong prefecture level city in Shandong
Jinfeng Hongyuan Flagship Store	Electricity savings1	Took the third one...the super power-saving kind!	Jingdong prefecture level city in Shandong
Jinfeng Hongyuan Flagship Store	Electricity savings1	First time buyer, customer service is very polite and courteous, conscientious business, support! Baby has received 🔍, super like, high value and practical, super like! ❥❥❥❥❥ Very good quality, very satisfied with a shopping. Customer service has a question and answer, very professional finally received the goods, very, very much like, exquisite and beautiful workmanship, has been praised by many people, really too happy, is already a loyal fan of his family, will continue to buy buy buy buy!	Jingdong prefecture level city in Shandong
Jinfeng Hongyuan Flagship Store	Electricity savings1	That's good. That's good. That's really good.	Jingdong prefecture level city in Shandong
Jinfeng Hongyuan Flagship Store	Electricity savings1	Saver received, good-looking design, voltage at a glance, and the merchant description, save some about 12% or so	Jingdong prefecture level city in Shandong
Jinfeng Hongyuan Flagship Store	Electricity savings1	Intelligent power saver is small and does not take up space, very good, very practical, received and used, packaging is tight, logistics is also very fast, satisfied with a shopping!	Jingdong prefecture level city in Shandong
Jinfeng Hongyuan Flagship Store	Electricity savings1	The stuff is good! Well made, compact, alloy case, shows voltage! Very like! Kudos!	Jingdong prefecture level city in Shandong
Jinfeng Hongyuan Flagship Store	Electricity savings1	I just purchased this power saver from Jingdong, inserted in the socket shows the number 206, a moment and 207 back and forth display, I do not know what it means, my home voltage should be normal, because it is the owner of the community's own power plant to put, first use for a period of time in the positive feedback it, first use in the look at it!	Jingdong prefecture level city in Shandong
Jinfeng Hongyuan Flagship Store	Electricity savings1	Very delicate and compact, there is a convenient intelligent display prompts the voltage, you can know the voltage at a glance, there is also very easy to use, a plug can be used, you can save electricity	Jingdong prefecture level city in Shandong
Jinfeng Hongyuan Flagship Store	Electricity savings1	Logistics is fast, ask customer service said can save electricity, but only use two months to see the effect, use two months in the evaluation.	Jingdong prefecture level city in Shandong
Jinfeng Hongyuan Flagship Store	Electricity savings1	The quality is very good.	Jingdong prefecture level city in Shandong
Jinfeng Hongyuan Flagship Store	Electricity savings1	Smart Saver has been received and will be evaluated after use	Jingdong prefecture level city in Shandong
Jinfeng Hongyuan Flagship Store	Electricity savings1	Home electricity is too waste, a month more than 300 dollars of electricity, and then the family heard that this can save a lot of electricity, let me order to buy a, anyway, will not, buy on the buy, thought it has to be a pretty big box it, delivery to the home to see a very small, plug-and-play, you can really save electricity, it's worth it!	Jingdong prefecture level city in Shandong
Jinfeng Hongyuan Flagship Store	Electricity savings1	Smart Saver received. Unpacking the box is exquisite. The material workmanship is very good. Plugged in and tried the space. Did not open the air conditioning voltage display 206 air conditioning open up the air conditioning display for 194. whether to save electricity to a month later to pay the electricity bill will know. Logistics is also very fast service is also in place.	Jingdong prefecture level city in Shandong
Jinfeng Hongyuan Flagship Store	Electricity savings1	The product feels pretty good and I hope it works, as the store owner said it would. Works!!!	Jingdong prefecture level city in Shandong
Jinfeng Hongyuan Flagship Store	Electricity savings1	Baby received, physical very quickly, just plugged in hope that it can be as everyone said those power saving, with two months after additional comments, first give a five-star praise.	Jingdong prefecture level city in Shandong
Jinfeng Hongyuan Flagship Store	Electricity savings1	Good, very good, very good, you deserve it, whoever uses it knows, the stuff works great, will buy it again in the future.	Jingdong prefecture level city in Shandong
Jinfeng Hongyuan Flagship Store	Electricity savings1	Don't hesitate to buy wow! No cheap feeling, very high-end, especially great! It is genuine, and the same as the entity store, the price is also favorable, decisive turn powder 💗 catch up to do activities when you buy, the price of a penny for a penny ♡, from the outside looking very beautiful ♡, the material workmanship is good 💗, feel that the purchase is worth it, and the logistics is super	Jingdong prefecture level city in Shandong
Jinfeng Hongyuan Flagship Store	Electricity savings1	Logistics is fast! Very good quality! Very beautiful! Next time to patronize!	Jingdong prefecture level city in Shandong
Jinfeng Hongyuan Flagship Store	Electricity savings1	Really like it, completely exceeded expectations, the shipping speed is very fast, the packaging is very careful and strict, the delivery speed is very fast, very satisfied with a shopping Baby received, and the description is the same, love it!	Jingdong prefecture level city in Shandong
Jinfeng Hongyuan Flagship Store	Electricity savings1	Very good, if you need to order!	Jingdong prefecture level city in Shandong
Jinfeng Hongyuan Flagship Store	Electricity savings1	So far, plugged in the voltage is more stable, as for the effect of how to quietly wait until the payment of the electricity bill to compare, the appearance of the commodity design is very good, the real thing!	Jingdong prefecture level city in Shandong
Jinfeng Hongyuan Flagship Store	Electricity savings1	📩 I've been wanting it for a long time, I decided to go for it, I feel great after using it, it's genuine and the delivery is great, it lives up to its name👍	Jingdong prefecture level city in Shandong
Jinfeng Hongyuan Flagship Store	Electricity savings1	Looking forward to it!!! I don't know if it's working or not, let's look forward to it!!!! How much is saved , , , , , , , , , Saving a degree is a degree with long term use ! ! ! ! ! ! !	Jingdong prefecture level city in Shandong
Jinfeng Hongyuan Flagship Store	Electricity savings1	After using it for a while, I am seriously and responsibly saying that this saver is really very power efficient. Two rooms with the air conditioner on at night for one night, plus watching TV, charging cell phones, lights, etc. etc. only used one degree of electricity. So I bought several more and it's really	Jingdong prefecture level city in Shandong
Jinfeng Hongyuan Flagship Store	Electricity savings1	It arrived quickly, and the box is solid and delicate. Baby shell is thick aluminum and by multi-layer coating. The inner core is heavy, and the power cord and plug are genuine products from a large manufacturer. It is the best power saver I have found after choosing all of them!	Jingdong prefecture level city in Shandong
Jinfeng Hongyuan Flagship Store	Electricity savings1	The saver was received, genuine guarantee, good quality...	Jingdong prefecture level city in Shandong
Jinfeng Hongyuan Flagship Store	Electricity savings1	Logistics was fast! The product is easy to use!	Jingdong prefecture level city in Shandong
Jinfeng Hongyuan Flagship Store	Electricity savings1	Look appearance is okay, use a period of time to know how it is. Jingdong Mall, trustworthy! Home appliances, cell phones and computers are almost 100% bought by Jingdong. Ordered the night before yesterday, received yesterday. Jingdong logistics, the fastest in the network!	Jingdong prefecture level city in Shandong
Jinfeng Hongyuan Flagship Store	Electricity savings1	Very good, perfect packaging, home energy saver to determine the effect of power saving and also play a role in stabilizing the voltage Very good products	Jingdong prefecture level city in Shandong
Jinfeng Hongyuan Flagship Store	Electricity savings1	Smart Saver received, looks very exquisite. The method of use is simple, and it has been used. The later effect, with time to test.	Jingdong prefecture level city in Shandong
Jinfeng Hongyuan Flagship Store	Electricity savings1	Both the material and workmanship exceeded expectations, great value, and looks great on oh. The seller also shipped much earlier than expected, will come back. Buy it, you will not regret it! Full five points. Both the material and workmanship exceeded expectations, great value, wear it also looks good Oh. Seller delivery is also much earlier than expected, will come again. Buy it, will not regret it! Full	Jingdong prefecture level city in Shandong
Jinfeng Hongyuan Flagship Store	Electricity savings1	The appearance is clean and beautiful, the workmanship is very good, I didn't find any defects, I personally feel that the size is also quite suitable, I've used it for 2 months, I didn't find out exactly how energy efficient it is. Try using it again!	Jingdong prefecture level city in Shandong
Jinfeng Hongyuan Flagship Store	Electricity savings1	Power saving device received, product certification, accessories, etc. complete, very good workmanship, very practical power-saving effect is also very obvious.	Jingdong prefecture level city in Shandong
Jinfeng Hongyuan Flagship Store	Electricity savings1	Looked at a lot of think buy this one, see a lot of reviews say it works, a month with save nearly twenty degrees of electricity, there are sixty days of return guarantee, with a good and then come back to the	Jingdong prefecture level city in Shandong
Jinfeng Hongyuan Flagship Store	Electricity savings1	I received the power saving device, and I'm using it. I hope I can get the desired effect, and I'm trying to force it to work quickly.	Jingdong prefecture level city in Shandong
Jinfeng Hongyuan Flagship Store	Electricity savings1	Voltage is stable, already trying it out and waiting for the results to happen, saving electricity will follow up next month!	Jingdong prefecture level city in Shandong
Jinfeng Hongyuan Flagship Store	Electricity savings1	Jingdong Mall self-supporting goods of excellent quality, logistics is also very fast, worth recommending 👏👏👏👏 excellent!	Jingdong prefecture level city in Shandong

Jinfeng Hongyuan Flagship Store	Electricity savings1	Power saving has been received, the appearance of the beautiful, I have already been the second time to buy the use of down the effect is very good!	Jingdong prefecture level city in Shandong
Jinfeng Hongyuan Flagship Store	Electricity savings1	Energy saver received very happy, the color is also very good looking forward to a very good effect can save electricity	Jingdong prefecture level city in Shandong
Jinfeng Hongyuan Flagship Store	Electricity savings1	It's small and easy to plug in and use. It displays the voltage value when plugged in and	Jingdong prefecture level city in Shandong
Jinfeng Hongyuan Flagship Store	Electricity savings1	This saver really works well, because I installed one at home about seven or eight years ago, when there is no voltage display, now there is a voltage display, the appearance also looks much better, the electricity bill is also saved, the stability of the home appliances is also good, the best of both	Jingdong prefecture level city in Shandong
Jinfeng Hongyuan Flagship Store	Electricity savings1	Ordered yesterday, arrived today in use. Shipping was fast. The effect is looking forward to.	Jingdong prefecture level city in Shandong
Jinfeng Hongyuan Flagship Store	Electricity savings1	This thing, I don't know what the principle is. Why it can save electricity. Wait for a month to use it first, see if it will save electricity bill, then come back to evaluate. There is one thing that does not fit the principle ah. The law of conservation of energy.	Jingdong prefecture level city in Shandong
Jinfeng Hongyuan Flagship Store	Electricity savings1	Home mom listen to others say this can save electricity, specially let buy a, later a very large, go back to see a particularly small, directly plugged into the electricity can be, no need to install, the price is also very cheap, the first time with this product, do not know if it is not good to use, temporarily try for a period of time, if it is good to use, recommend that you buy this one, the	Jingdong prefecture level city in Shandong
Jinfeng Hongyuan Flagship Store	Electricity savings1	I've been using it for a few days, and I think the workmanship and materials are pretty good, so we'll have to look at the next bill for the real energy savings, and then we'll add more comments.	Jingdong prefecture level city in Shandong
Jinfeng Hongyuan Flagship Store	Electricity savings1	Energy saver is well made, energy saving and power saving, and anti-counterfeiting code, it is a regular commodity!	Jingdong prefecture level city in Shandong
Jinfeng Hongyuan Flagship Store	Electricity savings1	Jinfeng Hongyuan, buy back home, as a mouse, haha. First of all, normal electricity for 1 month, the power did not increase, the electricity bill feels smoother, year-on-year electricity costs in the low, whether it is very energy-saving still have to be used for a long time to know. All 5 stars ☆	Jingdong prefecture level city in Shandong
Jinfeng Hongyuan Flagship Store	Electricity savings1	The box is well packaged and beautiful! I hope to save electricity with it haha!	Jingdong prefecture level city in Shandong
Jinfeng Hongyuan Flagship Store	Electricity savings1	Ordered last night, arrived this morning, first try the effect before making comments	Jingdong prefecture level city in Shandong
Jinfeng Hongyuan Flagship Store	Electricity savings1	Looks good. Don't know the effect yet.	Jingdong prefecture level city in Shandong
Jinfeng Hongyuan Flagship Store	Electricity savings1	Jinfeng Hongyuan intelligent power saver has exquisite workmanship and modern fashionable design style. It is a good home economizer . I believe that the goods purchased from Jingdong will not disappoint our	Jingdong prefecture level city in Shandong
Jinfeng Hongyuan Flagship Store	Electricity savings1	Inadvertently flipped to this power saver, never heard of, feel very novel, the price is not expensive, so buy to try, see a month after the electricity bill will be less, recently the home air-conditioning open a lot of power consumption is very large.	Jingdong prefecture level city in Shandong
Jinfeng Hongyuan Flagship Store	Electricity savings1	The quality of the goods is very good workmanship, but also sent five fuse	Jingdong prefecture level city in Shandong
Jinfeng Hongyuan Flagship Store	Electricity savings1	I'm trying it out. I don't know if it'll work.	Jingdong prefecture level city in Shandong
Jinfeng Hongyuan Flagship Store	Electricity savings1	There is no effect yet do not know, is to look at the official sale, buy to try it! Use according to the instructions, plugged in, used for a period of time, anyway, do not know how, after a few months to see it, to see if the average month than before will be less electricity.	Jingdong prefecture level city in Shandong
Jinfeng Hongyuan Flagship Store	Electricity savings1	Received the goods, the first time to unpacking, the quality is very good, consistent with the description of the seller, completely exceeded my expectations, the packaging is very careful, strict, in general this time is very satisfied with a shopping, thanks to the seller.	Jingdong prefecture level city in Shandong
Jinfeng Hongyuan Flagship Store	Electricity savings1	The energy saver was received and used for a few days with good results.	Jingdong prefecture level city in Shandong
Jinfeng Hongyuan Flagship Store	Electricity savings1	Previously I have paid attention to this product, but I think that the monthly electricity consumption at home is not large, there is no need to save electricity savings. After a second thought, energy saving is the responsibility of each of our citizens, the winter is coming, this year's winter is cold, heating and all electric appliances, can save is to save, the price is not expensive, so buy to try. I analyze the principle of power saving, should be able to save electricity, but the larger the power saving effect is about good. To be tried after a month or two to see the effect and then evaluate. Jingdong trustworthy.	Jingdong prefecture level city in Shandong
Jinfeng Hongyuan Flagship Store	Electricity savings1	It just arrived and I can't wait to use it, and the customer service is great! There is also voltage showing up which is nice! I don't know if it can save a bit I won't know until I've used it for a month! When the time comes to come back to the follow-up review!	Jingdong prefecture level city in Shandong
Jinfeng Hongyuan Flagship Store	Electricity savings1	Checked out the reviews from numerous users. The shipment is still very large, so I decided to buy it anyway to use it and see the effect after a month. Jingdong Mall here to buy safe, convenient and fast, this price is very powerful 💪, very recommended.	Jingdong prefecture level city in Shandong
Jinfeng Hongyuan Flagship Store	Electricity savings1	Purchased two at the same time to use together. The other one works fine, the other has problems with the numbers not sure if the machine is faulty. Power saving effect can not be observed. Feel the effect is not very obvious like?	Jingdong prefecture level city in Shandong
Jinfeng Hongyuan Flagship Store	Electricity savings1	Looking forward to it!!! I don't know if it's working or not, let's look forward to it!!!! How much is saved , , , , , , , , , Saving a degree is a degree with long term use ! ! ! ! ! ! !	Jingdong prefecture level city in Shandong
Jinfeng Hongyuan Flagship Store	Electricity savings1	Received, directly on the use of the special convenient	Jingdong prefecture level city in Shandong
Jinfeng Hongyuan Flagship Store	Electricity savings1	Goods received a few days well-packed, the same day opened and plugged in to try to be normal is not sure what the power-saving effect. After a month with a follow-up evaluation.	Jingdong prefecture level city in Shandong
Jinfeng Hongyuan Flagship Store	Electricity savings1	I bought it and used it, I don't know how it works yet, we'll see later. Use the effect of it	Jingdong prefecture level city in Shandong
Jinfeng Hongyuan Flagship Store	Electricity savings1	Photographed 2, the old family also put a, with a small January, there should be some effect, with a long period of time to comment, with a long time to see the obvious effect, hard courier brother.	Jingdong prefecture level city in Shandong
Jinfeng Hongyuan Flagship Store	Electricity savings1	Experience: I am an electrician, this is the real use of electronic waves automatically connected to track the decomposition of ionized electrons, in situ compensation of reactive power, reduce effective power loss and waste, stabilize the voltage to achieve energy saving and power saving, the overall effect	Jingdong prefecture level city in Shandong
Jinfeng Hongyuan Flagship Store	Electricity savings1	Great looking and practical! Easy to use and save! Saves 15% in a month of comparison	Jingdong prefecture level city in Shandong
Jinfeng Hongyuan Flagship Store	Electricity savings1	Product quality is very good! Packaging is very nice, like a cell phone box, thick and well protected! Service is also great! Logistics is very fast! Thank you for your business! Very honest!	Jingdong prefecture level city in Shandong
Jinfeng Hongyuan Flagship Store	Electricity savings1	In several products inside the choice of this purchase for a period of time, but also need to use a few months to compare last year's electricity bill before you can analyze the savings in electricity, to the summer peak electricity consumption should be more obvious.	Jingdong prefecture level city in Shandong
Jinfeng Hongyuan Flagship Store	Electricity savings1	The goods have long been received, after more than a month to comment, the home electricity bill is indeed reduced, worth buying!	Jingdong prefecture level city in Shandong
Jinfeng Hongyuan Flagship Store	Electricity savings1	Baby received, the logistics is strong, the quality is reliable, it is worth having and recommending!	Jingdong prefecture level city in Shandong
Jinfeng Hongyuan Flagship Store	Electricity savings1	Using a conservation appliance, you can achieve a 10-15% savings in electricity usage.	Jingdong prefecture level city in Shandong
Jinfeng Hongyuan Flagship Store	Electricity savings1	Quality is very good, metal shell, touch is very good, very recommended Oh ~, the subsequent effect of continuous follow-up	Jingdong prefecture level city in Shandong
Jinfeng Hongyuan Flagship Store	Electricity savings1	The product is beautiful, practical, worth buying. The product is beautiful, practical, good quality, fast logistics product is beautiful, practical, beautiful and elegant, very classy, worth buying!	Jingdong prefecture level city in Shandong
Jinfeng Hongyuan Flagship Store	Electricity savings1	Just received the item and have already used it. Didn't know much about these products before. I hope it is as effective and useful as the product advertises.	Jingdong prefecture level city in Shandong
Jinfeng Hongyuan Flagship Store	Electricity savings1	A month two people used more than five hundred degrees of electricity, in a hurry and then add a power saver, I hope to have an effect, Jingdong shopping is very cool, logistics is very fast especially good service attitude delivery, this hot day is not easy, thank you!	Jingdong prefecture level city in Shandong
Jinfeng Hongyuan Flagship Store	Electricity savings1	Just received the baby, first plugged in to try to use a month and then additional evaluation it	Jingdong prefecture level city in Shandong
Jinfeng Hongyuan Flagship Store	Electricity savings1	Goods fast, the power saver has been received, a friend recommended to buy that can save electricity. Exquisite and compact is very convenient, plug and play. The effect is good, the price is affordable, the next need will patronize again!	Jingdong prefecture level city in Shandong

Jinfeng Hongyuan Flagship Store	Electricity savings1	Packaging is better than expected, sent a few fuse, power saving effect in the end do not know, try a period of time to say!	Jingdong prefecture level city in Shandong
Jinfeng Hongyuan Flagship Store	Electricity savings1	Shipped quickly, immediately installed, read countless reviews were not sure if this thing is effective, was going to buy three at once, ultimately decided to first use a try the effect, if the power ring significantly reduced, then will buy a few more!	Jingdong prefecture level city in Shandong
Jinfeng Hongyuan Flagship Store	Electricity savings1	Used for a period of time to evaluate, not to a month still do not know whether it can save electricity, the voltage has a display!	Jingdong prefecture level city in Shandong
Jinfeng Hongyuan Flagship Store	Electricity savings1	I don't know if it saves electricity or not, but I'm using it anyway, so I hope it saves a little bit, electricity is too expensive	Jingdong prefecture level city in Shandong
Jinfeng Hongyuan Flagship Store	Electricity savings1	The power saver has been received and I am very happy with it! Just waiting to see how the power saving works!	Jingdong prefecture level city in Shandong
Jinfeng Hongyuan Flagship Store	Electricity savings1	Saving power has been received, logistics is quite fast, the quality is not bad, small and a little weight, has been used to look forward to the effect of	Jingdong prefecture level city in Shandong
Jinfeng Hongyuan Flagship Store	Electricity savings1	The logistics is very fast, the effect is obvious and trustworthy.	Jingdong prefecture level city in Shandong
Jinfeng Hongyuan Flagship Store	Electricity savings1	Good quality, good quality, beautiful, beautiful, beautiful, beautiful, beautiful, very worth buying, worth buying, worth buying, worth buying, worth customizing!	Jingdong prefecture level city in Shandong
Jinfeng Hongyuan Flagship Store	Electricity savings1	I've used it for now, I don't know if it saves electricity	Jingdong prefecture level city in Shandong
Jinfeng Hongyuan Flagship Store	Electricity savings1	Bought two at a time, logistics is very fast, the product is beautifully packaged, after two months to see the results	Jingdong prefecture level city in Shandong
Jinfeng Hongyuan Flagship Store	Electricity savings1	The workmanship is really fine, the packaging is also good, the regular factory production ~ use a break in the evaluation of the effectiveness of the	Jingdong prefecture level city in Shandong
Jinfeng Hongyuan Flagship Store	Electricity savings1	Sometimes 219. Sometimes 220 in this case	Jingdong prefecture level city in Shandong
Jinfeng Hongyuan Flagship Store	Electricity savings1	It is the ideal product, good quality, logistics is also very fast, worthy of praise!	Jingdong prefecture level city in Shandong
Jinfeng Hongyuan Flagship Store	Electricity savings1	Store service is very good, the courier received the next day. Product packaging is very good, just use the quality to be tested.	Jingdong prefecture level city in Shandong
Jinfeng Hongyuan Flagship Store	Electricity savings1	I've been using it for almost a month now and I feel like it's working.	Jingdong prefecture level city in Shandong
Jinfeng Hongyuan Flagship Store	Electricity savings1	Very satisfied, very restful, very pretty, very beautiful, very much enjoyed! Well worth it!	Jingdong prefecture level city in Shandong
Jinfeng Hongyuan Flagship Store	Electricity savings1	Baby especially like, the value of high-quality good, a very good shopping experience, attentive service, fast logistics, distribution is also very thoughtful, attitude is also very good, value for money ah, decisive praise! Quality is quite good, logistics is also very fast, praise!	Jingdong prefecture level city in Shandong
Jinfeng Hongyuan Flagship Store	Electricity savings1	The shape of the item is basically the same as ordered, the effect is to be examined.	Jingdong prefecture level city in Shandong
Jinfeng Hongyuan Flagship Store	Electricity savings1	Appearance style: good-looking workmanship material: very good, a small one, very delicate Experience: still in use, in the process of using the shopkeeper is very nice to have a careful explanation Use for a period of time in the look at the results	Jingdong prefecture level city in Shandong
Jinfeng Hongyuan Flagship Store	Electricity savings1	As described, compact and well packaged	Jingdong prefecture level city in Shandong
Jinfeng Hongyuan Flagship Store	Electricity savings1	Fine workmanship. Test in progress. Let's see how it works.	Jingdong prefecture level city in Shandong
Jinfeng Hongyuan Flagship Store	Electricity savings1	The goods have been received, I'm very satisfied and love it	Jingdong prefecture level city in Shandong
Jinfeng Hongyuan Flagship Store	Electricity savings1	Safety! Whether you can save electricity, you can roughly feel it in 2 months. The principle is simple. Before the electrical appliances and circuits inductive more, now the electrical appliances integrated, the home is not a lot of inductive circuits, but used certainly save no problem! Wiring according to the requirements of the manual is good, if it is not convenient, see the socket plug.	Jingdong prefecture level city in Shandong
Jinfeng Hongyuan Flagship Store	Electricity savings1	Great product, looking forward to how much power I can save, first time buyer!	Jingdong prefecture level city in Shandong
Jinfeng Hongyuan Flagship Store	Electricity savings1	The product is exquisite, beautifully packaged, and the customer service is excellent. Just waiting to see how the power saving effect is, looking forward to it!	Jingdong prefecture level city in Shandong
Jinfeng Hongyuan Flagship Store	Electricity savings1	Express very quickly very satisfied with the first use to see how the effect to know, if you can save 10 percent even if acceptable, if you can not meet this requirement is obviously not much effect. Obviously.	Jingdong prefecture level city in Shandong
Jinfeng Hongyuan Flagship Store	Electricity savings1	Very delicate and compact, there is a very convenient intelligent display prompts the voltage, you can know the voltage at a glance, there is very easy to use, a plug can be used, I hope to save money on	Jingdong prefecture level city in Shandong
Jinfeng Hongyuan Flagship Store	Electricity savings1	First time buyer is it really save after use	Jingdong prefecture level city in Shandong
Jinfeng Hongyuan Flagship Store	Electricity savings1	Purchased again and will be for a long time yet.	Jingdong prefecture level city in Shandong
Jinfeng Hongyuan Flagship Store	Electricity savings1	Bought it to stock up, came back and plugged it in, hope it works	Jingdong prefecture level city in Shandong
Jinfeng Hongyuan Flagship Store	Electricity savings1	Baby received the logistics very fast very like very satisfied praise a	Jingdong prefecture level city in Shandong
Jinfeng Hongyuan Flagship Store	Electricity savings1	Beginning the process of using, easy to evaluate more later.	Jingdong prefecture level city in Shandong
Jinfeng Hongyuan Flagship Store	Electricity savings1	The goods have been received, just do not know how effective	Jingdong prefecture level city in Shandong
Jinfeng Hongyuan Flagship Store	Electricity savings1	It feels okay. I wish there were more events.	Jingdong prefecture level city in Shandong
Jinfeng Hongyuan Flagship Store	Electricity savings1	Things have been received, just plugged in, I do not know if it works, wait a few days to have the effect of additional	Jingdong prefecture level city in Shandong
Jinfeng Hongyuan Flagship Store	Electricity savings1	The effect of this power saver is really good, the house more, a few air conditioners are open every day, not used before a month to say less than seven hundred eight hundred, used after three months only nine	Jingdong prefecture level city in Shandong
Jinfeng Hongyuan Flagship Store	Electricity savings1	Cheap, good and easy to use, recommend it.	Jingdong prefecture level city in Shandong
Jinfeng Hongyuan Flagship Store	Electricity savings1	Bought this device, to the home for a long time, wait to finish a month to see how, good can recommend friends to buy, we use the electricity every month scary, a month of electricity costs more than a thousand, can not afford to hurt ah! I hope this power saving can save me some money, the beginning of the shoot order did not pay attention to look at the address, sent elsewhere to go, that side called to find out, hurry to communicate with customer service, customer service is very careful to help me get it done, call to that side to help me turn around, thank you!	Jingdong prefecture level city in Shandong
Jinfeng Hongyuan Flagship Store	Electricity savings1	I don't know the effect if it, use it first to see.	Jingdong prefecture level city in Shandong
Jinfeng Hongyuan Flagship Store	Electricity savings1	Looks good. I won't know if it saves power until I use it.	Jingdong prefecture level city in Shandong
Jinfeng Hongyuan Flagship Store	Electricity savings1	Goods received, shipping speed is very fast, yesterday afternoon more than two o'clock shot, just express delivery, but also sent a few insurance, very good business, the display of the number do not understand what is the meaning of the display said voltage, do not understand this, try a month to see how, good and	Jingdong prefecture level city in Shandong
Jinfeng Hongyuan Flagship Store	Electricity savings1	What ** play also, save electricity more and more save more and more, save chicken hair electricity, are cheating, this is a night of air conditioning with electricity, a night with 11 electricity word, this is	Jingdong prefecture level city in Shandong
Jinfeng Hongyuan Flagship Store	Electricity savings1	Received the product, hope it works!	Jingdong prefecture level city in Shandong
Jinfeng Hongyuan Flagship Store	Electricity savings1	Bought home and used very convenient, easy to operate, quite good	Jingdong prefecture level city in Shandong
Jinfeng Hongyuan Flagship Store	Electricity savings1	It's really good, cost-effective and good quality, and the logistics are fast! It arrived in one day! Really good, cost-effective and good quality, logistics is fast ah! It arrived in one day!	Jingdong prefecture level city in Shandong
Jinfeng Hongyuan Flagship Store	Electricity savings1	Works well, stabilizes voltage and saves power	Jingdong prefecture level city in Shandong

Jinfeng Hongyuan Flagship Store	Electricity savings1	I'll use it right away and look forward to good results	Jingdong prefecture level city in Shandong
Jinfeng Hongyuan Flagship Store	Electricity savings1	Recommended by a friend, said it works well, so I bought one, the effect to be tested	Jingdong prefecture level city in Shandong
Jinfeng Hongyuan Flagship Store	Electricity savings1	I didn't know it would save electricity.	Jingdong prefecture level city in Shandong
Jinfeng Hongyuan Flagship Store	Electricity savings1	I've been using it for a week, I don't know what's going on yet I hope it works well and saves power I'll follow up after a month of use	Jingdong prefecture level city in Shandong
Jinfeng Hongyuan Flagship Store	Electricity savings1	Very well packaged, looks great, don't know how it works	Jingdong prefecture level city in Shandong
Jinfeng Hongyuan Flagship Store	Electricity savings1	Genuine, good quality, really saves energy.	Jingdong prefecture level city in Shandong
Jinfeng Hongyuan Flagship Store	Electricity savings1	I was worried that the stuff is not good, but when I got it, I realized that I was completely over-anxious, 5 stars!	Jingdong prefecture level city in Shandong
Jinfeng Hongyuan Flagship Store	Electricity savings1	Very good and satisfied trustworthy	Jingdong prefecture level city in Shandong
Jinfeng Hongyuan Flagship Store	Electricity savings1	I don't know if it saves power	Jingdong prefecture level city in Shandong
Jinfeng Hongyuan Flagship Store	Electricity savings1	The quality of the product is very good, beautiful and generous applicable fast delivery speed good service attitude	Jingdong prefecture level city in Shandong
Jinfeng Hongyuan Flagship Store	Electricity savings1	Let's use it for a while and then we'll follow up	Jingdong prefecture level city in Shandong
Jinfeng Hongyuan Flagship Store	Electricity savings1	I've only been using it for a month, I don't know if there's any change yet, I'll come back with a follow-up comment after checking the meter!	Jingdong prefecture level city in Shandong
Jinfeng Hongyuan Flagship Store	Electricity savings1	I don't know if it works, I read the instructions and online reviews, I hope I won't be disappointed, if I use it, I will buy it again. Jingdong Express, fast and guaranteed service.	Jingdong prefecture level city in Shandong
Jinfeng Hongyuan Flagship Store	Electricity savings1	It feels like it saves quite a bit huh?	Jingdong prefecture level city in Shandong
Jinfeng Hongyuan Flagship Store	Electricity savings1	I've been using it for a while now, and it still seems to work and have a power saving effect	Jingdong prefecture level city in Shandong
Jinfeng Hongyuan Flagship Store	Electricity savings1	Looks good. It says 235. The 222 bar voltage is shown as high. I don't know if that's right. We'll see.	Jingdong prefecture level city in Shandong
Jinfeng Hongyuan Flagship Store	Electricity savings1	Baby is really good, do really careful, worth to have, fast delivery!	Jingdong prefecture level city in Shandong
Jinfeng Hongyuan Flagship Store	Electricity savings1	Quite delicate and compact, simple and convenient to use. Things are still very good, bought two at a time. Very satisfied with an online purchase.	Jingdong prefecture level city in Shandong
Jinfeng Hongyuan Flagship Store	Electricity savings1	Feel very good, the use of unclear, ask customer service, timely explanation, big brand, trustworthy	Jingdong prefecture level city in Shandong
Jinfeng Hongyuan Flagship Store	Electricity savings1	Thrifty received a few days did not make a comment is to see how the quality of the product now feel the quality of good appearance is also powerful seller service is very good to buy the product is also attached to the 5 fuse thought very thoughtful!	Jingdong prefecture level city in Shandong
Jinfeng Hongyuan Flagship Store	Electricity savings1	Big brand, with a very assured, the quality is very good, in time for the event to buy very cost-effective, customer service is really very good Oh ~!	Jingdong prefecture level city in Shandong
Jinfeng Hongyuan Flagship Store	Electricity savings1	I ordered yesterday and received it this morning, I heard my friend say that it can save electricity, I don't know if it works, I'll come back to make a comment after using it.	Jingdong prefecture level city in Shandong
Jinfeng Hongyuan Flagship Store	Electricity savings1	I've been using it for over a month and it doesn't seem to be working!	Jingdong prefecture level city in Shandong
Jinfeng Hongyuan Flagship Store	Electricity savings1	Used it, the family has always been in the habit of using energy-saving devices, this is a new one. Digital display voltage is very good, can see that the voltage is more stable.	Jingdong prefecture level city in Shandong
Jinfeng Hongyuan Flagship Store	Electricity savings1	I haven't used it for more than a month, but it looks good. It's easy to plug into an outlet and it works.	Jingdong prefecture level city in Shandong
Jinfeng Hongyuan Flagship Store	Electricity savings1	Good Oh...appearance is very beautiful...just plugged in with...see the effect of it...with a period of time in the additional evaluation...	Jingdong prefecture level city in Shandong
Jinfeng Hongyuan Flagship Store	Electricity savings1	I've received the goods, whether it's good or not, I'll know after I use it.	Jingdong prefecture level city in Shandong
Jinfeng Hongyuan Flagship Store	Electricity savings1	Very good, logistics is fast, the stuff is very powerful and cost-effective! Can feel the meter slowing down	Jingdong prefecture level city in Shandong
Jinfeng Hongyuan Flagship Store	Electricity savings1	Logistics is very fast, get it immediately after use, easy to operate, plug in the power supply can be used, just do not know to the use of the effect of how.	Jingdong prefecture level city in Shandong
Jinfeng Hongyuan Flagship Store	Electricity savings1	Quality is very over the top, quality is guaranteed, quite a good choice, a lot of friends to see me use all want to buy, I recommend it!	Jingdong prefecture level city in Shandong
Jinfeng Hongyuan Flagship Store	Electricity savings1	The appearance is clean and beautiful, the workmanship is very good, did not find any flaws, I personally feel that the size is also quite suitable, looking for a long time to choose this one, big love! The price is very beautiful, things are still good, will continue to buy back! All five points! Ness Ness ~ logistics speed is really no words, is a word: fast. Commodities from the outer packaging is very high-grade, really a penny a penny, expensive has the reason for expensive	Jingdong prefecture level city in Shandong
Jinfeng Hongyuan Flagship Store	Electricity savings1	The logistics is very fast, the texture is very good, genuine, I like it very much.	Jingdong prefecture level city in Shandong
Jinfeng Hongyuan Flagship Store	Electricity savings1	It's been a few days since I put it on, I can't see any results now, waiting for a month to say, steady steady steady.	Jingdong prefecture level city in Shandong
Jinfeng Hongyuan Flagship Store	Electricity savings1	I've been using it for a while now and it should be fine, so I'm buying it for the second time.	Jingdong prefecture level city in Shandong
Jinfeng Hongyuan Flagship Store	Electricity savings1	Power Saving King has been received, looks good, quite delicate, looking forward to using it to save electricity	Jingdong prefecture level city in Shandong
Jinfeng Hongyuan Flagship Store	Electricity savings1	My mom said the home uses a little too much electricity, so I bought a Power Saver to try it out, I don't know how effective it is. Expect it to be a good thing!	Jingdong prefecture level city in Shandong
Jinfeng Hongyuan Flagship Store	Electricity savings1	Thank you Jingdong, logistics is very fast order to receipt only one day. Believe in Jingdong. Favorable comments	Jingdong prefecture level city in Shandong
Jinfeng Hongyuan Flagship Store	Electricity savings1	Saved some money on the electric bill compared to last month, watch this month again, no problems so far	Jingdong prefecture level city in Shandong
Jinfeng Hongyuan Flagship Store	Electricity savings1	Energy saver exquisite workmanship, beautiful style, more features, just bought, power saving effect to be used for a period of time, then evaluate!	Jingdong prefecture level city in Shandong
Jinfeng Hongyuan Flagship Store	Electricity savings1	This thing does save power is more compact to hang on the wall.	Jingdong prefecture level city in Shandong
Jinfeng Hongyuan Flagship Store	Electricity savings1	I've been buying it for a while now, I haven't actually measured it, but it still feels like it's effective	Jingdong prefecture level city in Shandong
Jinfeng Hongyuan Flagship Store	Electricity savings1	I've already used it since I bought it, I don't know how effective it is yet, I'll comment on it later.	Jingdong prefecture level city in Shandong
Jinfeng Hongyuan Flagship Store	Electricity savings1	Baby received, careful appearance, logistics is very fast, worth buying!	Jingdong prefecture level city in Shandong
Jinfeng Hongyuan Flagship Store	Electricity savings1	It's great, it's just not the same in hand, I bought it at that other cheaper online one for about the same price as this one. But then oops, doesn't work at all.	Jingdong prefecture level city in Shandong
Jinfeng Hongyuan Flagship Store	Electricity savings1	The product is exquisite, but you have to use it for a month to know how it works. I'll comment again after a month.	Jingdong prefecture level city in Shandong
Jinfeng Hongyuan Flagship Store	Electricity savings1	I've been using it for a while now, and it doesn't feel like it's working, and it doesn't save any power.	Jingdong prefecture level city in Shandong
Jinfeng Hongyuan Flagship Store	Electricity savings1	Affordable price, fast delivery, Jingdong self-supporting guarantee	Jingdong prefecture level city in Shandong
Jinfeng Hongyuan Flagship Store	Electricity savings1	Packaging is very good, no damage, good quality, fine workmanship Packaging is very good, no damage, good quality, fine workmanship Packaging is very good, no damage, good quality, fine workmanship Packaging is very good, no damage, good quality, fine workmanship	Jingdong prefecture level city in Shandong
Jinfeng Hongyuan Flagship Store	Electricity savings1	Big brand, with a very assured, the quality is very good, in time for the event to buy very cost-effective, customer service is really very good Oh ~!	Jingdong prefecture level city in Shandong

Jinfeng Hongyuan Flagship Store	Electricity savings1	Used a few days, the specific effect is not yet clear, looking forward to the effect as described by the merchant!	Jingdong prefecture level city in Shandong
Jinfeng Hongyuan Flagship Store	Electricity savings1	I just used it and don't know how it works, I'll follow up after using it!	Jingdong prefecture level city in Shandong
Jinfeng Hongyuan Flagship Store	Electricity savings1	Well, it's been installed and now I'm waiting to see the results, BOE shopping, hopefully I won't be disappointed!	Jingdong prefecture level city in Shandong
Jinfeng Hongyuan Flagship Store	Electricity savings1	Goods received, plugged in the voltage from 229 to 222, do not know whether to save electricity, that depends on the situation.	Jingdong prefecture level city in Shandong
Jinfeng Hongyuan Flagship Store	Electricity savings1	This is the second time I have bought the product and I feel it really saves electricity! Use it again for a while and then evaluate!	Jingdong prefecture level city in Shandong
Jinfeng Hongyuan Flagship Store	Electricity savings1	Overall, it's really good, and the customer service is great, you'll get a message back almost immediately when you speak, it's great value for money, I really recommend it!	Jingdong prefecture level city in Shandong
Jinfeng Hongyuan Flagship Store	Electricity savings1	✨ Have purchased many times, a must have for the family, affordable purchase price, fast delivery, 🎁 beautifully packaged!	Jingdong prefecture level city in Shandong
Jinfeng Hongyuan Flagship Store	Electricity savings1	I bought it after a friend introduced it, he said it is effective and can save electricity. I bought it to try, try a period of time and then follow up!	Jingdong prefecture level city in Shandong
Jinfeng Hongyuan Flagship Store	Electricity savings1	Received it, trying it out tonight, and bought one earlier.	Jingdong prefecture level city in Shandong
Jinfeng Hongyuan Flagship Store	Electricity savings1	It will take a month or two to see if it saves power or not.	Jingdong prefecture level city in Shandong
Jinfeng Hongyuan Flagship Store	Electricity savings1	Looked good in the instructions! Bought one and pressed it on! Use it and see! If it works! I'll buy some more!	Jingdong prefecture level city in Shandong
Jinfeng Hongyuan Flagship Store	Electricity savings1	Goods have been received packaging can also things are not bad appearance, with a use to know, now evaluation is nonsense.	Jingdong prefecture level city in Shandong
Jinfeng Hongyuan Flagship Store	Electricity savings1	Logistics is very fast, packaging is very good, the second unit, as always, looking forward to the effect of	Jingdong prefecture level city in Shandong
Jinfeng Hongyuan Flagship Store	Electricity savings1	I received the goods today, the quality of goods is very good, I am very satisfied ah.	Jingdong prefecture level city in Shandong
Jinfeng Hongyuan Flagship Store	Electricity savings1	Buy one and try it, I wonder if it has a power saving effect?	Jingdong prefecture level city in Shandong
Jinfeng Hongyuan Flagship Store	Electricity savings1	🔍 The item was received, well packaged, logistics is very fast, customer service attitude is very positive, there are questions answered in a timely manner! Overall very satisfied!	Jingdong prefecture level city in Shandong
Jinfeng Hongyuan Flagship Store	Electricity savings1	Baby packaging is very good, purchase submission goods quickly delivered to the home, use and then follow up.	Jingdong prefecture level city in Shandong
Jinfeng Hongyuan Flagship Store	Electricity savings1	My friend asked me to buy an extra one so he could try it too.	Jingdong prefecture level city in Shandong
Jinfeng Hongyuan Flagship Store	Electricity savings1	I've just put it on, I won't know the result until a month later if it will really save power.	Jingdong prefecture level city in Shandong
Jinfeng Hongyuan Flagship Store	Electricity savings1	It's good quality. Hopefully it'll help save power.	Jingdong prefecture level city in Shandong
Jinfeng Hongyuan Flagship Store	Electricity savings1	The appearance is clean and beautiful, the workmanship is very good, I did not find any flaws, I personally feel that the size is also quite suitable, I have been looking for a long time to choose this	Jingdong prefecture level city in Shandong
Jinfeng Hongyuan Flagship Store	Electricity savings1	Logistics is fast! Baby looks great! The price is fair! Wish the store business is prosperous!	Jingdong prefecture level city in Shandong
Jinfeng Hongyuan Flagship Store	Electricity savings1	Jingdong express fast, good, have used, sit and wait to save money...	Jingdong prefecture level city in Shandong
Jinfeng Hongyuan Flagship Store	Electricity savings1	In use, it didn't work as expected and there was nothing noticeable in terms of power savings.	Jingdong prefecture level city in Shandong
Jinfeng Hongyuan Flagship Store	Electricity savings1	Just started using it and looking forward to the results.	Jingdong prefecture level city in Shandong
Jinfeng Hongyuan Flagship Store	Electricity savings1	The quality is very good, the metal shell is very good, is genuine, energy saving and power saving, logistics is very fast, there is a need to buy o, recommend everyone, very satisfied with an online	Jingdong prefecture level city in Shandong
Jinfeng Hongyuan Flagship Store	Electricity savings1	Received, I do not know if the product is good, used to see if it is good to come back to buy!	Jingdong prefecture level city in Shandong
Jinfeng Hongyuan Flagship Store	Electricity savings1	Jinfeng source of intelligent power saving treasure also used less than three months, the last thunder may be burned, the home of the electric send gate is not on, only the old man at home, no way only their own in the field and run back to find out what is the problem with the circuit, the quality is still not very good, thunder is easy to burn!	Jingdong prefecture level city in Shandong
Jinfeng Hongyuan Flagship Store	Electricity savings1	Saving power just started to use, I do not know if there is no effect, but read a lot of reviews are said to be very good so I bought, baby All aspects are good There are spare 2 fuses, late good and then follow	Jingdong prefecture level city in Shandong
Jinfeng Hongyuan Flagship Store	Electricity savings1	Use it for a few days and see, the delivery is quite fast	Jingdong prefecture level city in Shandong
Jinfeng Hongyuan Flagship Store	Electricity savings1	Small, well made and easy to use.	Jingdong prefecture level city in Shandong
Jinfeng Hongyuan Flagship Store	Electricity savings1	Beautifully packaged, it is genuine. Whether it is power saving, use it to evaluate.	Jingdong prefecture level city in Shandong
Jinfeng Hongyuan Flagship Store	Electricity savings1	Power saver. Used two of them and they do save quite a bit of power.	Jingdong prefecture level city in Shandong
Jinfeng Hongyuan Flagship Store	Electricity savings1	Workmanship is not bad, small and does not take up space, is also do not know whether the power saving, buy a to try!	Jingdong prefecture level city in Shandong
Jinfeng Hongyuan Flagship Store	Electricity savings1	The logistics is very fast, the courier attitude is very good, worth buying	Jingdong prefecture level city in Shandong
Jinfeng Hongyuan Flagship Store	Electricity savings1	The effect is not known until it is used.	Jingdong prefecture level city in Shandong
Jinfeng Hongyuan Flagship Store	Electricity savings1	Just arrived plugged in won't know for a month	Jingdong prefecture level city in Shandong
Jinfeng Hongyuan Flagship Store	Electricity savings1	This is really good to use the pull to realize the savings ah think again for my sister's family to buy a	Jingdong prefecture level city in Shandong
Jinfeng Hongyuan Flagship Store	Electricity savings1	Lies, a month with a few hundred electricity are no savings, family use	Jingdong prefecture level city in Shandong
Jinfeng Hongyuan Flagship Store	Electricity savings1	Express very quickly, look at the fine workmanship also sent a few fuse, look forward to can really save electricity	Jingdong prefecture level city in Shandong
Jinfeng Hongyuan Flagship Store	Electricity savings1	I didn't realize it was so small.	Jingdong prefecture level city in Shandong
Jinfeng Hongyuan Flagship Store	Electricity savings1	Logistics courier is very fast, the courier service is very good	Jingdong prefecture level city in Shandong
Jinfeng Hongyuan Flagship Store	Electricity savings1	Used for a period of time the effect is quite good indeed than the previous power saving	Jingdong prefecture level city in Shandong
Jinfeng Hongyuan Flagship Store	Electricity savings1	Logistics is super fast, the product is in use, see the effect...	Jingdong prefecture level city in Shandong
Jinfeng Hongyuan Flagship Store	Electricity savings1	Baby got it, tried it out and it didn't work at all. How can I use the tips to save power?	Jingdong prefecture level city in Shandong
Jinfeng Hongyuan Flagship Store	Electricity savings1	Power Saving King received, immediately used has been very stable without any sound good, good service, fast logistics inexpensive and beautiful worth repurchasing!	Jingdong prefecture level city in Shandong
Jinfeng Hongyuan Flagship Store	Electricity savings1	Logistics courier is very fast, the courier service is good	Jingdong prefecture level city in Shandong
Jinfeng Hongyuan Flagship Store	Electricity savings1	Good to recommend, the first month of air conditioning only 1 week, the electricity bill has reduced about 100 yuan, may be to reduce the use of air conditioning is also related to the other or as usual electricity, estimated that the longer you use the more you can save electricity.	Jingdong prefecture level city in Shandong
Jinfeng Hongyuan Flagship Store	Electricity savings1	It doesn't work. I've been using it for 20 days and it doesn't work.	Jingdong prefecture level city in Shandong
Jinfeng Hongyuan Flagship Store	Electricity savings1	Fast shipping, good quality, affordable price, very satisfied with a shopping.	Jingdong prefecture level city in Shandong

Jinfeng Hongyuan Flagship Store	Electricity savings1	Try it for a month to see if it saves money.	Jingdong prefecture level city in Shandong
Jinfeng Hongyuan Flagship Store	Electricity savings1	It's in use. I'll comment again after a while.	Jingdong prefecture level city in Shandong
Jinfeng Hongyuan Flagship Store	Electricity savings1	Goods have received look good 👍👍👍👍, use a period of time and then look at the	Jingdong prefecture level city in Shandong
Jinfeng Hongyuan Flagship Store	Electricity savings1	No, it doesn't work. It's useless. It's garbage.	Jingdong prefecture level city in Shandong
Jinfeng Hongyuan Flagship Store	Electricity savings1	I've been using it for almost 2 months now and I don't feel any reduction in my electricity bill.	Jingdong prefecture level city in Shandong
Jinfeng Hongyuan Flagship Store	Electricity savings1	I've received the goods, the logistics is really fast, ordered yesterday, arrived today.	Jingdong prefecture level city in Shandong
Jinfeng Hongyuan Flagship Store	Electricity savings1	And the backup insurance.	Jingdong prefecture level city in Shandong
Jinfeng Hongyuan Flagship Store	Electricity savings1	The appearance of this product is good, easy to use and install, the effect of how there is no use to wait for time to verify that the voltage displayed is different from the voltage measured by other meters, the gap is not too large, the follow-up to add the effect.	Jingdong prefecture level city in Shandong
Jinfeng Hongyuan Flagship Store	Electricity savings1	Baby received it 👌, really great ah! 🌈The quality can't be beat, very satisfied! 🆗Happy happy happy 😉	Jingdong prefecture level city in Shandong
Jinfeng Hongyuan Flagship Store	Electricity savings1	I'll follow up with a review after using it for a while, hopefully it won't bring me back a disappointment!	Jingdong prefecture level city in Shandong
Jinfeng Hongyuan Flagship Store	Electricity savings1	In use and looking forward to results! Good review to send	Jingdong prefecture level city in Shandong
Jinfeng Hongyuan Flagship Store	Electricity savings1	Intelligent power saver received, the volume is not big, small and exquisite, quite good, Jingdong Express is also quite fast, the	Jingdong prefecture level city in Shandong
Jinfeng Hongyuan Flagship Store	Electricity savings1	I don't know how to use it, keep using it	Jingdong prefecture level city in Shandong
Jinfeng Hongyuan Flagship Store	Electricity savings1	Very good for saving electricity	Jingdong prefecture level city in Shandong
Jinfeng Hongyuan Flagship Store	Electricity savings1	Seriously, did not use, feel also more power consumption, the peak is bought back to use the first time, the next day unplugged, the power consumption is back to more than ten degrees (the frequency of power use in the past few days is pretty much the same), feel the IQ tax paid 😭	Jingdong prefecture level city in Shandong
Jinfeng Hongyuan Flagship Store	Electricity savings1	I haven't seen any effect on the power saving, but the voltage stabilization looks good.	Jingdong prefecture level city in Shandong
Jinfeng Hongyuan Flagship Store	Electricity savings1	It's very good, I'll come back to it after using it for a while	Jingdong prefecture level city in Shandong
Jinfeng Hongyuan Flagship Store	Electricity savings1	Already used it and see the results.	Jingdong prefecture level city in Shandong
Jinfeng Hongyuan Flagship Store	Electricity savings1	Fast shipping, beautiful packaging, attentive service, the use of the effect, to be followed up after the evaluation.	Jingdong prefecture level city in Shandong
Jinfeng Hongyuan Flagship Store	Electricity savings1	The process of using this product is more than satisfactory	Jingdong prefecture level city in Shandong
Jinfeng Hongyuan Flagship Store	Electricity savings1	Very good East East cost-effective! Every time so buy more assured, and the logistics is also very fast. It is also very good to use, so I will often come to buy in the future, and will also introduce to	Jingdong prefecture level city in Shandong
Jinfeng Hongyuan Flagship Store	Electricity savings1	But still understand how effective it is!	Jingdong prefecture level city in Shandong
Jinfeng Hongyuan Flagship Store	Electricity savings1	Fast shipping, great logistics, great stuff, great price.	Jingdong prefecture level city in Shandong
Jinfeng Hongyuan Flagship Store	Electricity savings1	I'll review it in a month and give it 5 stars if it's good	Jingdong prefecture level city in Shandong
Jinfeng Hongyuan Flagship Store	Electricity savings1	I've saved a lot of electricity in the past month, so I'm glad to hear that.	Jingdong prefecture level city in Shandong
Jinfeng Hongyuan Flagship Store	Electricity savings1	Baby just received and installed! Looks genuinely good, workmanship feels fine! Looking forward to the effect! Said to see the effect in two months! Waiting for if really have the effect must come to follow up! The boss is absolutely responsible! Asked and answered! Just for the boss this attitude must also	Jingdong prefecture level city in Shandong
Jinfeng Hongyuan Flagship Store	Electricity savings1	This baby is very delicate, I'll comment after I've used it	Jingdong prefecture level city in Shandong
Jinfeng Hongyuan Flagship Store	Electricity savings1	It's been over two months. It's not working. It's just like it was.	Jingdong prefecture level city in Shandong
Jinfeng Hongyuan Flagship Store	Electricity savings1	Fast shipping and logistics, let's see if it works.	Jingdong prefecture level city in Shandong
Jinfeng Hongyuan Flagship Store	Electricity savings1	Just received the goods, try it to know if it can really save electricity	Jingdong prefecture level city in Shandong
Jinfeng Hongyuan Flagship Store	Electricity savings1	Not bad. Not bad. Not bad.	Jingdong prefecture level city in Shandong
Jinfeng Hongyuan Flagship Store	Electricity savings1	Not sure if it works. I'll comment again if it works.	Jingdong prefecture level city in Shandong
Jinfeng Hongyuan Flagship Store	Electricity savings1	Safe and energy-saving helpers to use!	Jingdong prefecture level city in Shandong
Jinfeng Hongyuan Flagship Store	Electricity savings1	Good, first time buyer. Used good can	Jingdong prefecture level city in Shandong
Jinfeng Hongyuan Flagship Store	Electricity savings1	It feels okay, I guess! I won't know until I use it for a while!	Jingdong prefecture level city in Shandong
Jinfeng Hongyuan Flagship Store	Electricity savings1	The saver matches the description and has been used, waiting for a follow up review after two months.	Jingdong prefecture level city in Shandong
Jinfeng Hongyuan Flagship Store	Electricity savings1	Received, the appearance of the business as described, the effect of how to use a period of time to evaluate.	Jingdong prefecture level city in Shandong
Jinfeng Hongyuan Flagship Store	Electricity savings1	Still using it don't know how effective it is, I'll comment after three months	Jingdong prefecture level city in Shandong
Jinfeng Hongyuan Flagship Store	Electricity savings1	The stuff is very good, the packaging is also very good logistics is also very fast	Jingdong prefecture level city in Shandong
Jinfeng Hongyuan Flagship Store	Electricity savings1	I'll follow up with a review when I use it, hopefully I won't be disappointed	Jingdong prefecture level city in Shandong
Jinfeng Hongyuan Flagship Store	Electricity savings1	Cancel Houjiaying Logistics.	Jingdong prefecture level city in Shandong
Jinfeng Hongyuan Flagship Store	Electricity savings1	It's very good, it's worth buying.	Jingdong prefecture level city in Shandong
Jinfeng Hongyuan Flagship Store	Electricity savings1	My friend bought it to save electricity and referred me to buy it! I'll buy it and try it out! I will know how much electricity I can save next month, looking forward to it! 4-500 dollars a month electricity bill is horrible! I hope it can really help me save some electricity!	Jingdong prefecture level city in Shandong
Jinfeng Hongyuan Flagship Store	Electricity savings1	Just received it and installed it.	Jingdong prefecture level city in Shandong
Jinfeng Hongyuan Flagship Store	Electricity savings1	Very nice. This one is my favorite.	Jingdong prefecture level city in Shandong
Jinfeng Hongyuan Flagship Store	Electricity savings1	Good value for money worth buying very good	Jingdong prefecture level city in Shandong
Jinfeng Hongyuan Flagship Store	Electricity savings1	Very energy-saving, small and exquisite, does not take up space, very satisfied, complete packaging, no damage, customer service explanation is also very serious	Jingdong prefecture level city in Shandong
Jinfeng Hongyuan Flagship Store	Electricity savings1	Received immediately used, really effective, do not understand electricity, but looked at the meter every day, recommended that you can use, especially this weather every day to open the air conditioning, with him not afraid of the electricity bill.	Jingdong prefecture level city in Shandong
Jinfeng Hongyuan Flagship Store	Electricity savings1	Nice, good value for money, nice stuff.	Jingdong prefecture level city in Shandong

Jinfeng Hongyuan Flagship Store	Electricity savings1	The packaging of the product is very good, the logistics of the product is very fast, and the quality of the product will be evaluated after use!	Jingdong prefecture level city in Shandong
Jinfeng Hongyuan Flagship Store	Electricity savings1	Express is very, well very fast, give a compliment 👍	Jingdong prefecture level city in Shandong
Jinfeng Hongyuan Flagship Store	Electricity savings1	Packaging is very good, fast delivery, used a night and a day did not feel the power saving. Use a month to see if it saves power.	Jingdong prefecture level city in Shandong
Jinfeng Hongyuan Flagship Store	Electricity savings1	Seems to be a bit of a power saver.	Jingdong prefecture level city in Shandong
Jinfeng Hongyuan Flagship Store	Electricity savings1	Bought this for someone else and it came over so fast, ###!	Jingdong prefecture level city in Shandong
Jinfeng Hongyuan Flagship Store	Electricity savings1	Very delicate, just received a period of time to use in the follow-up evaluation	Jingdong prefecture level city in Shandong
Jinfeng Hongyuan Flagship Store	Electricity savings1	It's been installed for a while now, not sure if it's working or not	Jingdong prefecture level city in Shandong
Jinfeng Hongyuan Flagship Store	Electricity savings1	The item looks okay, I just don't know if it works or not.	Jingdong prefecture level city in Shandong
Jinfeng Hongyuan Flagship Store	Electricity savings1	The goods have been received, but there is a screw missing	Jingdong prefecture level city in Shandong
Jinfeng Hongyuan Flagship Store	Electricity savings1	I didn't believe it before, but after receiving the goods today and trying it out, it really works!	Jingdong prefecture level city in Shandong
Jinfeng Hongyuan Flagship Store	Electricity savings1	Received, this thing will not know whether it is the effect of saving electricity, no matter how to say that there should always be a little use it, otherwise why develop it I think there is still the value of	Jingdong prefecture level city in Shandong
Jinfeng Hongyuan Flagship Store	Electricity savings1	Another one just arrived.	Jingdong prefecture level city in Shandong
Jinfeng Hongyuan Flagship Store	Electricity savings1	I don't feel anything, ordinary farmers can't go to TEDA, just use it first, it's safe.	Jingdong prefecture level city in Shandong
Jinfeng Hongyuan Flagship Store	Electricity savings1	Just purchased and will follow up after using it for a while	Jingdong prefecture level city in Shandong
Jinfeng Hongyuan Flagship Store	Electricity savings1	I'm trying it out now. I'll comment after I use it.	Jingdong prefecture level city in Shandong
Jinfeng Hongyuan Flagship Store	Electricity savings1	Received the item, in use, hope it works.	Jingdong prefecture level city in Shandong
Jinfeng Hongyuan Flagship Store	Electricity savings1	It works well. Good review.	Jingdong prefecture level city in Shandong
Jinfeng Hongyuan Flagship Store	Electricity savings1	All a scam, no effect at all, I bought too many fakes in Poundland before, I did not think that Jingdong is also the same	Jingdong prefecture level city in Shandong
Jinfeng Hongyuan Flagship Store	Electricity savings1	I used the saver as soon as I received it, it's in good shape and still working!	Jingdong prefecture level city in Shandong
Jinfeng Hongyuan Flagship Store	Electricity savings1	It works. I don't know what to do with the picture, but it works.	Jingdong prefecture level city in Shandong
Jinfeng Hongyuan Flagship Store	Electricity savings1	A very good shopping experience, attentive service, fast logistics, delivery is also very thoughtful, attitude is also very good, value for money ah, decisive praise!	Jingdong prefecture level city in Shandong
Jinfeng Hongyuan Flagship Store	Electricity savings1	The product is consistent with the presentation picture.	Jingdong prefecture level city in Shandong
Jinfeng Hongyuan Flagship Store	Electricity savings1	Baby received, use a period of time to evaluate, wish the boss business prosperity!	Jingdong prefecture level city in Shandong
Jinfeng Hongyuan Flagship Store	Electricity savings1	The stuff is better! Recommended!	Jingdong prefecture level city in Shandong
Jinfeng Hongyuan Flagship Store	Electricity savings1	I don't know if it works or not, I'll use it for a while before I comment on it.	Jingdong prefecture level city in Shandong
Jinfeng Hongyuan Flagship Store	Electricity savings1	Cost-effective, I like it, next time will come back Baby received, very surprised, the quality is very good, very satisfied with a shopping. I will continue to buy back, the store is trustworthy	Jingdong prefecture level city in Shandong
Jinfeng Hongyuan Flagship Store	Electricity savings1	Plugged it in and used it for a few days, not sure if it saves power yet	Jingdong prefecture level city in Shandong
Jinfeng Hongyuan Flagship Store	Electricity savings1	I'm trying it out and will follow up if it seems to be working!	Jingdong prefecture level city in Shandong
Jinfeng Hongyuan Flagship Store	Electricity savings1	Works pretty well, big kudos 👍👍👍👍👍👍👍👍👍👍👍	Jingdong prefecture level city in Shandong
Jinfeng Hongyuan Flagship Store	Electricity savings1	Just put it on, we'll see how it looks in a month.	Jingdong prefecture level city in Shandong
Jinfeng Hongyuan Flagship Store	Electricity savings1	The baby is very nice and I've already recommended buying it for all my friends! The main thing is that it not only saves electricity, I was more concerned about the fact that it can adjust the current, usually wiring problems. Now I found that it is really good, it is the effect I want!	Jingdong prefecture level city in Shandong
Jinfeng Hongyuan Flagship Store	Electricity savings1	Baby arrived, looking forward to his results, hopefully it will be good	Jingdong prefecture level city in Shandong
Jinfeng Hongyuan Flagship Store	Electricity savings1	Things have been received, look at the workmanship and packaging are good, I do not know how the power-saving effect? Use a period of time to see the effect	Jingdong prefecture level city in Shandong
Jinfeng Hongyuan Flagship Store	Electricity savings1	The price is right, the shape is beautiful and does not take up space, use it for a while to see the effect	Jingdong prefecture level city in Shandong
Jinfeng Hongyuan Flagship Store	Electricity savings1	Can't save electricity, it doesn't work!	Jingdong prefecture level city in Shandong
Jinfeng Hongyuan Flagship Store	Electricity savings1	It's pretty convenient, just plug it in and it works. There's no noise, plug it in and leave it alone, it's very hassle free! Good workmanship and value for money.	Jingdong prefecture level city in Shandong
Jinfeng Hongyuan Flagship Store	Electricity savings1	Just received the goods have been installed, try two months later to comment!	Jingdong prefecture level city in Shandong
Jinfeng Hongyuan Flagship Store	Electricity savings1	I received the stuff, I haven't used it yet, and overall it's fine.	Jingdong prefecture level city in Shandong
Jinfeng Hongyuan Flagship Store	Electricity savings1	Couldn't use a return at all.	Jingdong prefecture level city in Shandong
Jinfeng Hongyuan Flagship Store	Electricity savings1	Second purchase.	Jingdong prefecture level city in Shandong
Jinfeng Hongyuan Flagship Store	Electricity savings1	Very good quality, high tech and practical.	Jingdong prefecture level city in Shandong
Jinfeng Hongyuan Flagship Store	Electricity savings1	Very good, very great! Very satisfied, thanks to the store!	Jingdong prefecture level city in Shandong
Jinfeng Hongyuan Flagship Store	Electricity savings1	Just used it, don't know if it's good or not, hope it works well	Jingdong prefecture level city in Shandong
Jinfeng Hongyuan Flagship Store	Electricity savings1	Saving power has been received, the product is good, good like.	Jingdong prefecture level city in Shandong
Jinfeng Hongyuan Flagship Store	Electricity savings1	Received the goods and used it for two months before evaluation	Jingdong prefecture level city in Shandong
Jinfeng Hongyuan Flagship Store	Electricity savings1	It looks a little small, I do not know how effective, first use to see the effect, there is a small adjustable button, I do not know this is to adjust to that position to work well	Jingdong prefecture level city in Shandong
Jinfeng Hongyuan Flagship Store	Electricity savings1	Simple, just plug it in, there are spare fuses, can't say how well it works, no comparison	Jingdong prefecture level city in Shandong
Jinfeng Hongyuan Flagship Store	Electricity savings1	It's been a few days since I used it, and the power's gone up.	Jingdong prefecture level city in Shandong
Jinfeng Hongyuan Flagship Store	Electricity savings1	The owner was very nice and sent a spare fuse.	Jingdong prefecture level city in Shandong
Jinfeng Hongyuan Flagship Store	Electricity savings1	It's authentic and it's not expensive and it's great to use, I use it all the time and it's great 🎉✨ just don't know if it really saves any points	Jingdong prefecture level city in Shandong

Jinfeng Hongyuan Flagship Store	Electricity savings1	The information is good, it is the child to buy, the child read it is also quite happy, I hope to be able to match the child's preparation for the midterm examination! Because of the epidemic, it is not too fast, but I also give full credit to the express delivery!	Jingdong prefecture level city in Shandong
Jinfeng Hongyuan Flagship Store	Electricity savings1	This power saver comes to the logistics very quickly, the business attitude is also very good, ** very comfortable, this brand is trustworthy, and buy in this store than in the physical store to buy cheaper,	Jingdong prefecture level city in Shandong
Jinfeng Hongyuan Flagship Store	Electricity savings1	It's very well made and the materials used are okay. This is just a few days old, so I don't know how it will work, only next month.	Jingdong prefecture level city in Shandong
Jinfeng Hongyuan Flagship Store	Electricity savings1	Already used on, have not tested the effect, wait for a couple of months to add comments.	Jingdong prefecture level city in Shandong
Jinfeng Hongyuan Flagship Store	Electricity savings1	Baby received, packaging is very thoughtful, already in use, should soon see the effect.	Jingdong prefecture level city in Shandong
Jinfeng Hongyuan Flagship Store	Electricity savings1	Just received in the office to try, to the home installed, the voltage are different, and do not know if there is no effect. Use a period of time and then follow up.	Jingdong prefecture level city in Shandong
Jinfeng Hongyuan Flagship Store	Electricity savings1	Just received the goods, is trying, looking forward to the effect, first give a favorable comment, two months later, then additional evaluation, please be patient.	Jingdong prefecture level city in Shandong
Jinfeng Hongyuan Flagship Store	Electricity savings1	The item has just been received, well packaged, results to be verified.	Jingdong prefecture level city in Shandong
Jinfeng Hongyuan Flagship Store	Electricity savings1	Packaging logistics are very good, the next look at how the effect, before I see others use the effect is good, as I bought this can also have good results.	Jingdong prefecture level city in Shandong
Jinfeng Hongyuan Flagship Store	Electricity savings1	I don't know if it's a real power saver or a fake power saver, but I've used it since I bought it, so I'll give you a good review.	Jingdong prefecture level city in Shandong
Jinfeng Hongyuan Flagship Store	Electricity savings1	Good quality, genuine goods, much better than imagined before purchase, next time will come back to care!	Jingdong prefecture level city in Shandong
Jinfeng Hongyuan Flagship Store	Electricity savings1	Logistics is very fast good use again	Jingdong prefecture level city in Shandong
Jinfeng Hongyuan Flagship Store	Electricity savings1	Received the goods. Let's see if it saves power in a month and then we'll follow up!	Jingdong prefecture level city in Shandong
Jinfeng Hongyuan Flagship Store	Electricity savings1	I bought it yesterday and it arrived today, I bought two of them, I hope they work well, I'll come back after a while to give a follow-up review	Jingdong prefecture level city in Shandong
Jinfeng Hongyuan Flagship Store	Electricity savings1	The home air conditioner consumes a lot of electricity, so I bought it and used it in the hope that there is A	Jingdong prefecture level city in Shandong
Jinfeng Hongyuan Flagship Store	Electricity savings1	The product is excellent and very current.	Jingdong prefecture level city in Shandong
Jinfeng Hongyuan Flagship Store	Electricity savings1	With the description, it is the most practical and good products that households can need, and it is an ideal shopping, the courier is also fast, and the service attitude is enthusiastic!	Jingdong prefecture level city in Shandong
Jinfeng Hongyuan Flagship Store	Electricity savings1	The Power Saver is easy to use, works well, is stable, and plugs into the top of the outlet	Jingdong prefecture level city in Shandong
Jinfeng Hongyuan Flagship Store	Electricity savings1	Overall still can, excellent craftsmanship, power saving do not know for the time being, after the use of the evaluation	Jingdong prefecture level city in Shandong
Jinfeng Hongyuan Flagship Store	Electricity savings1	Baby received on the plug, but also with four fuses, look at the quality is okay, I do not know whether it is useful, use and then follow up.	Jingdong prefecture level city in Shandong
Jinfeng Hongyuan Flagship Store	Electricity savings1	I'll evaluate it after a while, I don't know if it's good or not yet, and if it can save electricity	Jingdong prefecture level city in Shandong
Jinfeng Hongyuan Flagship Store	Electricity savings1	Jingdong self-operated purchase more assured delivery fast quality assurance	Jingdong prefecture level city in Shandong
Jinfeng Hongyuan Flagship Store	Electricity savings1	Shipping was fast, stuff was well packaged, great price, I'm happy with it 👍 and will repurchase!	Jingdong prefecture level city in Shandong
Jinfeng Hongyuan Flagship Store	Electricity savings1	The quality of the product is very good and I will definitely come back again next time.	Jingdong prefecture level city in Shandong
Jinfeng Hongyuan Flagship Store	Electricity savings1	I'll follow up with a review after I use it.	Jingdong prefecture level city in Shandong
Jinfeng Hongyuan Flagship Store	Electricity savings1	Hello, the goods are perfectly packaged, save power for a period of time to evaluate again!	Jingdong prefecture level city in Shandong
Jinfeng Hongyuan Flagship Store	Electricity savings1	Not bad, pretty good, good value for money, pretty good	Jingdong prefecture level city in Shandong
Jinfeng Hongyuan Flagship Store	Electricity savings1	The packaging is very good, received immediately used, looking forward to the effect in.	Jingdong prefecture level city in Shandong
Jinfeng Hongyuan Flagship Store	Electricity savings1	See how much power you save at the end of the month	Jingdong prefecture level city in Shandong
Jinfeng Hongyuan Flagship Store	Electricity savings1	Easy to use, affordable, beautiful color	Jingdong prefecture level city in Shandong
Jinfeng Hongyuan Flagship Store	Electricity savings1	It's taking too long to put together an order. It could be an epidemic.	Jingdong prefecture level city in Shandong
Jinfeng Hongyuan Flagship Store	Electricity savings1	I don't feel much after using it, no change in my electric bill 。。。。。。。	Jingdong prefecture level city in Shandong
Jinfeng Hongyuan Flagship Store	Electricity savings1	Logistics is very fast, the quality of the economizer is good, good quality and cheap, it is worth buying, I hope there is a power saving effect!	Jingdong prefecture level city in Shandong
Jinfeng Hongyuan Flagship Store	Electricity savings1	Feeling okay, in a couple of months to try to rely on it before making a comment. Let's give it a five-star review first!	Jingdong prefecture level city in Shandong
Jinfeng Hongyuan Flagship Store	Electricity savings1	Power saving device has been hesitating before in the end to buy or not to buy, after consideration, consideration, in consideration, or decided to buy a try. Things received can not wait to open a look, and the seller described the same, I hope to use 2 months to have the effect, two months to follow up on	Jingdong prefecture level city in Shandong
Jinfeng Hongyuan Flagship Store	Electricity savings1	Thank you Jingdong, logistics is very fast order to receipt only one day. Believe in Jingdong. Favorable comments	Jingdong prefecture level city in Shandong
Jinfeng Hongyuan Flagship Store	Electricity savings1	I used one to use it, but it didn't work very well, what to do	Jingdong prefecture level city in Shandong
Jinfeng Hongyuan Flagship Store	Electricity savings1	Quality and packaging are very good, fast shipping, good service, buy with confidence	Jingdong prefecture level city in Shandong
Jinfeng Hongyuan Flagship Store	Electricity savings1	Use a period of time to evaluate, the quality is very good, the price is affordable, logistics is also very fast	Jingdong prefecture level city in Shandong
Jinfeng Hongyuan Flagship Store	Electricity savings1	Goods received, the day of the order tomorrow delivery real God speed, delivery force, the product to be used after a period of time and then low comment!	Jingdong prefecture level city in Shandong
Jinfeng Hongyuan Flagship Store	Electricity savings1	I don't know, how effective it is, let's talk about it when the time comes, whether it saves power or not, how much, unknown.	Jingdong prefecture level city in Shandong
Jinfeng Hongyuan Flagship Store	Electricity savings1	It does save some electricity, and I don't want to use something that isn't legal! Used nearly ~ electricity last month, probably not much more this month with the AC on!	Jingdong prefecture level city in Shandong
Jinfeng Hongyuan Flagship Store	Electricity savings1	Well-packed, excellent quality, service satisfaction good value for money	Jingdong prefecture level city in Shandong
Jinfeng Hongyuan Flagship Store	Electricity savings1	Express very quickly, the goods received last night, today used a day of goods can not heat, looking forward to whether it can save electricity, with a month and then comment, is used well will buy again.	Jingdong prefecture level city in Shandong
Jinfeng Hongyuan Flagship Store	Electricity savings1	It's pretty good, hopefully it'll be a real power saver.	Jingdong prefecture level city in Shandong
Jinfeng Hongyuan Flagship Store	Electricity savings1	The goods received in use, can not see the effect of energy saving, after a month to know!	Jingdong prefecture level city in Shandong
Jinfeng Hongyuan Flagship Store	Electricity savings1	Received the goods, looking forward to better surprise, with the effect of the follow-up comment!	Jingdong prefecture level city in Shandong
Jinfeng Hongyuan Flagship Store	Electricity savings1	It's not bad, it's still in use and should save some	Jingdong prefecture level city in Shandong
Jinfeng Hongyuan Flagship Store	Electricity savings1	Logistics is very fast, easy to install, plugged in on it,, the effect of the use of the later to evaluate!	Jingdong prefecture level city in Shandong
Jinfeng Hongyuan Flagship Store	Electricity savings1	The stuff is recommendable to buy very good.	Jingdong prefecture level city in Shandong

Jinfeng Hongyuan Flagship Store	Electricity savings1	Voltmeter measurements of voltage are way off, aren't they?	Jingdong prefecture level city in Shandong
Jinfeng Hongyuan Flagship Store	Electricity savings1	Install it to the socket immediately after purchase and try to see if it can save electricity after one month.	Jingdong prefecture level city in Shandong
Jinfeng Hongyuan Flagship Store	Electricity savings1	I've been using it for almost a month now, and I won't know if it saves electricity until I pay the bill.	Jingdong prefecture level city in Shandong
Jinfeng Hongyuan Flagship Store	Electricity savings1	I bought one in the past and it was fine, so I'm buying it back.	Jingdong prefecture level city in Shandong
Jinfeng Hongyuan Flagship Store	Electricity savings1	Service is very good, this thing is small and exquisite to use, just waiting to use the effect of a period of time	Jingdong prefecture level city in Shandong
Jinfeng Hongyuan Flagship Store	Electricity savings1	Not sure how well it works yet	Jingdong prefecture level city in Shandong
Jinfeng Hongyuan Flagship Store	Electricity savings1	Kyodo goods habitually good reviews	Jingdong prefecture level city in Shandong
Jinfeng Hongyuan Flagship Store	Electricity savings1	Appearance is OK, whether it is power saving remains to be seen.	Jingdong prefecture level city in Shandong
Jinfeng Hongyuan Flagship Store	Electricity savings1	I bought it and used it, I just don't know if it really saves power	Jingdong prefecture level city in Shandong
Jinfeng Hongyuan Flagship Store	Electricity savings1	The saver is well made and of good quality, I will evaluate it after using it for a while.	Jingdong prefecture level city in Shandong
Jinfeng Hongyuan Flagship Store	Electricity savings1	Just received in use, use for a period of time to see if there is power saving	Jingdong prefecture level city in Shandong
Jinfeng Hongyuan Flagship Store	Electricity savings1	Used 3 days before commenting, it really saves electricity, I look at the "power calendar" after using one less degree of electricity per day, so I think it's worth it to buy this, I will introduce my friends	Jingdong prefecture level city in Shandong
Jinfeng Hongyuan Flagship Store	Electricity savings1	Reliable quality, very well packaged and matches the real thing.	Jingdong prefecture level city in Shandong
Jinfeng Hongyuan Flagship Store	Electricity savings1	Logistics is still quite fast, ha in the trial	Jingdong prefecture level city in Shandong
Jinfeng Hongyuan Flagship Store	Electricity savings1	Consultant service attitude is very good, the courier is also very powerful, the first day of the order the next day to arrive. Saver received used and then add comments!	Jingdong prefecture level city in Shandong
Jinfeng Hongyuan Flagship Store	Electricity savings1	I just got home to try it out and see how it goes.	Jingdong prefecture level city in Shandong
Jinfeng Hongyuan Flagship Store	Electricity savings1	Well made and weighted instrument. Kudos! Put it into use immediately, don't know if it saves power, will evaluate later!	Jingdong prefecture level city in Shandong
Jinfeng Hongyuan Flagship Store	Electricity savings1	I've just put it in place. I'll give you a review in ten days.	Jingdong prefecture level city in Shandong
Jinfeng Hongyuan Flagship Store	Electricity savings1	It looks very delicate, not bad. Well, I don't know if it can save power or not, I have to try it for a while and see. If it can really save electricity. Uh, if it works well. Then additional comments. Anyway, thanks for the logistics, thanks to Jingdong. Well, it deserves some praise.	Jingdong prefecture level city in Shandong
Jinfeng Hongyuan Flagship Store	Electricity savings1	Bought a month, the feeling is that there is a certain effect, a month to save a lot of money	Jingdong prefecture level city in Shandong
Jinfeng Hongyuan Flagship Store	Electricity savings1	Goods received, the physical and the same as the picture, logistics is also very fast, the service attitude is very good, it is worth buying!	Jingdong prefecture level city in Shandong
Jinfeng Hongyuan Flagship Store	Electricity savings1	I've used it, I don't know whether it saves electricity or not, I'll talk about it after a while.	Jingdong prefecture level city in Shandong
Jinfeng Hongyuan Flagship Store	Electricity savings1	August only began to use, October to know, have no power-saving effect, to the in to evaluate!	Jingdong prefecture level city in Shandong
Jinfeng Hongyuan Flagship Store	Electricity savings1	Goods received: I do not know to the factory products used to save electricity!	Jingdong prefecture level city in Shandong
Jinfeng Hongyuan Flagship Store	Electricity savings1	Don't know if it works, bought it anyway.	Jingdong prefecture level city in Shandong
Jinfeng Hongyuan Flagship Store	Electricity savings1	Still testing, will follow up next month to evaluate how much electricity is saved	Jingdong prefecture level city in Shandong
Jinfeng Hongyuan Flagship Store	Electricity savings1	I'm still testing it, I'll comment after I use it.	Jingdong prefecture level city in Shandong
Jinfeng Hongyuan Flagship Store	Electricity savings1	Fast service, praiseworthy. Good item pre-style, first praise, the use of the effect to be evaluated after use.	Jingdong prefecture level city in Shandong
Jinfeng Hongyuan Flagship Store	Electricity savings1	The power saving effect is more obvious through verified use.	Jingdong prefecture level city in Shandong
Jinfeng Hongyuan Flagship Store	Electricity savings1	Logistics is very fast yesterday order today arrived. The product was immediately used, and after a month to see its effect.	Jingdong prefecture level city in Shandong
Jinfeng Hongyuan Flagship Store	Electricity savings1	Power saving is not yet known, as far as the TV and audio are concerned, I feel that there is a significant quality improvement, and I recommend using a good product!	Jingdong prefecture level city in Shandong
Jinfeng Hongyuan Flagship Store	Electricity savings1	Good power savings, tens of dollars a month saved	Jingdong prefecture level city in Shandong
Jinfeng Hongyuan Flagship Store	Electricity savings1	Already took two, great value! You can save 30% on your electric bill in a month. A summer must-have!	Jingdong prefecture level city in Shandong
Jinfeng Hongyuan Flagship Store	Electricity savings1	Received the item, plugged it into a home outlet and it showed 230 volts (not that high with a multimeter). Let's use it first!	Jingdong prefecture level city in Shandong
Jinfeng Hongyuan Flagship Store	Electricity savings1	Expecting results in the experiment	Jingdong prefecture level city in Shandong
Jinfeng Hongyuan Flagship Store	Electricity savings1	I don't know if it really saves power.	Jingdong prefecture level city in Shandong
Jinfeng Hongyuan Flagship Store	Electricity savings1	It's very good and the efficacy is very visible.	Jingdong prefecture level city in Shandong
Jinfeng Hongyuan Flagship Store	Electricity savings1	It's good and worth buying with confidence	Jingdong prefecture level city in Shandong
Jinfeng Hongyuan Flagship Store	Electricity savings1	The goods have been received, just do not know if it is effective	Jingdong prefecture level city in Shandong
Jinfeng Hongyuan Flagship Store	Electricity savings1	It's already in use, but based on some of the descriptions, I suspect that to use it, you need to have one in series on each line, which seems to work for all appliances, that's my guess, but I feel as though the principle of the thing seems to require it.	Jingdong prefecture level city in Shandong
Jinfeng Hongyuan Flagship Store	Electricity savings1	Fast delivery, already in use, looks good!	Jingdong prefecture level city in Shandong
Jinfeng Hongyuan Flagship Store	Electricity savings1	Hoping to save 30% of power as presented in the graphic	Jingdong prefecture level city in Shandong
Jinfeng Hongyuan Flagship Store	Electricity savings1	Things look good, power saving do not know now, with a month after the continued evaluation	Jingdong prefecture level city in Shandong
Jinfeng Hongyuan Flagship Store	Electricity savings1	Already used it but not sure if it will top out!	Jingdong prefecture level city in Shandong
Jinfeng Hongyuan Flagship Store	Electricity savings1	Looking forward to using the results, the product quality looks okay.	Jingdong prefecture level city in Shandong
Jinfeng Hongyuan Flagship Store	Electricity savings1	It's somewhat effective and saves money more or less 💰	Jingdong prefecture level city in Shandong
Jinfeng Hongyuan Flagship Store	Electricity savings1	Got it, it's great, looking forward to saving power.	Jingdong prefecture level city in Shandong
Jinfeng Hongyuan Flagship Store	Electricity savings1	I don't know if it's working yet, I'll follow up when I get there	Jingdong prefecture level city in Shandong
Jinfeng Hongyuan Flagship Store	Electricity savings1	The product received, good workmanship, fast logistics, good inner packaging, the outer bag is damaged, but fortunately did not hurt the product, retrieved and inserted according to the instructions, looking forward to the effect is good, with a period of time to evaluate, I believe that the Jingdong.	Jingdong prefecture level city in Shandong
Jinfeng Hongyuan Flagship Store	Electricity savings1	Since it is a new product, it is said to work, so if it works well for a month, then I will give you a good review.	Jingdong prefecture level city in Shandong

Jinfeng Hongyuan Flagship Store	Electricity savings1	The quality of the product is very good, very well made, well packaged, easy to use and very convenient!	Jingdong prefecture level city in Shandong
Jinfeng Hongyuan Flagship Store	Electricity savings1	Good price for the item, good functionality	Jingdong prefecture level city in Shandong
Jinfeng Hongyuan Flagship Store	Electricity savings1	Received the first time to use it, the stuff is very good, specifics wait for a month to come back to feedback to see the effect.	Jingdong prefecture level city in Shandong
Jinfeng Hongyuan Flagship Store	Electricity savings1	Just received, the courier is too bad, not notified directly to put the Feng Nest courier cabinet	Jingdong prefecture level city in Shandong
Jinfeng Hongyuan Flagship Store	Electricity savings1	Baby received, the second time to buy, plugged in and used do not know if there is no effect. Baby received, the second time to buy, plugged in and used do not know if there is no effect. Baby received, the second time to buy, plugged in and used do not know if there is no effect.	Jingdong prefecture level city in Shandong
Jinfeng Hongyuan Flagship Store	Electricity savings1	It's okay if it saves power. It's not a bad product.	Jingdong prefecture level city in Shandong
Jinfeng Hongyuan Flagship Store	Electricity savings1	Received, very good, meticulous packaging, open a look is genuine, meticulous workmanship, the manual is very detailed, has been used, there is no effect on the only look at the next month's electricity bill, when I can I buy a few more to send people!	Jingdong prefecture level city in Shandong
Jinfeng Hongyuan Flagship Store	Electricity savings1	The quality is very good very delicate express delivery is too fast	Jingdong prefecture level city in Shandong
Jinfeng Hongyuan Flagship Store	Electricity savings1	I won't know until a month after I start using it today.	Jingdong prefecture level city in Shandong
Jinfeng Hongyuan Flagship Store	Electricity savings1	It doesn't work at all, the customer service attitude is very bad, use it or not!	Jingdong prefecture level city in Shandong
Jinfeng Hongyuan Flagship Store	Electricity savings1	Temporarily try, do not know the effect, Jingdong logistics delivery very quickly	Jingdong prefecture level city in Shandong
Jinfeng Hongyuan Flagship Store	Electricity savings1	It doesn't help. It's comforting.	Jingdong prefecture level city in Shandong
Jinfeng Hongyuan Flagship Store	Electricity savings1	It's great, use it before you say anything.	Jingdong prefecture level city in Shandong
Jinfeng Hongyuan Flagship Store	Electricity savings1	Received, already used, looking forward to a good a effect!	Jingdong prefecture level city in Shandong
Jinfeng Hongyuan Flagship Store	Electricity savings1	I don't know if it works yet, I won't know until I use it for a few months.	Jingdong prefecture level city in Shandong
Jinfeng Hongyuan Flagship Store	Electricity savings1	It takes a long time to get there. There's no change now.	Jingdong prefecture level city in Shandong
Jinfeng Hongyuan Flagship Store	Electricity savings1	Jingdong's delivery never disappoints, or so fast, and the stuff is great, next time you need to come back.	Jingdong prefecture level city in Shandong
Jinfeng Hongyuan Flagship Store	Electricity savings1	At first it was ok today, after using it for a while it didn't work anymore	Jingdong prefecture level city in Shandong
Jinfeng Hongyuan Flagship Store	Electricity savings1	Just received the goods, do not know the effect of using a few days before there may be effect	Jingdong prefecture level city in Shandong
Jinfeng Hongyuan Flagship Store	Electricity savings1	The logistics was fast, I received it the next day, I've purchased it for the second time and have been using it all along	Jingdong prefecture level city in Shandong
Jinfeng Hongyuan Flagship Store	Electricity savings1	Inexpensive, fast delivery, very pleased!	Jingdong prefecture level city in Shandong
Jinfeng Hongyuan Flagship Store	Electricity savings1	Received the goods immediately plugged directly into the use of power saving yet to be good news!	Jingdong prefecture level city in Shandong
Jinfeng Hongyuan Flagship Store	Electricity savings1	The service attitude is very good, and the Jingdong self-owned products can be trusted.	Jingdong prefecture level city in Shandong
Jinfeng Hongyuan Flagship Store	Electricity savings1	The product is good, I do not know if it can save play a power saving effect	Jingdong prefecture level city in Shandong
Jinfeng Hongyuan Flagship Store	Electricity savings1	We won't know how effective it is until we use it for a while, so I'll give you a good review now.	Jingdong prefecture level city in Shandong
Jinfeng Hongyuan Flagship Store	Electricity savings1	The quality is pretty good, just started using it, hopefully it will deliver results.	Jingdong prefecture level city in Shandong
Jinfeng Hongyuan Flagship Store	Electricity savings1	I've plugged it in first, and will follow up after I've used it to see how it works.	Jingdong prefecture level city in Shandong
Jinfeng Hongyuan Flagship Store	Electricity savings1	Things have arrived, the quality is good, the effect is also equivalent to can ah	Jingdong prefecture level city in Shandong
Jinfeng Hongyuan Flagship Store	Electricity savings1	It's not worth it to brag about power savings.	Jingdong prefecture level city in Shandong
Jinfeng Hongyuan Flagship Store	Electricity savings1	I don't know if it's an IQ tax, but I'll use it for now.	Jingdong prefecture level city in Shandong
Jinfeng Hongyuan Flagship Store	Electricity savings1	I don't feel the power saving yet, I can't know until I use it for 2 months.	Jingdong prefecture level city in Shandong
Jinfeng Hongyuan Flagship Store	Electricity savings1	There's nothing wrong with this at the moment.	Jingdong prefecture level city in Shandong
Jinfeng Hongyuan Flagship Store	Electricity savings1	It's authentic and worth buying	Jingdong prefecture level city in Shandong
Jinfeng Hongyuan Flagship Store	Electricity savings1	It's good. We'll see how it goes.	Jingdong prefecture level city in Shandong
Jinfeng Hongyuan Flagship Store	Electricity savings1	I don't know if it's working or not in the middle of using it, we'll see when the month is over	Jingdong prefecture level city in Shandong
Jinfeng Hongyuan Flagship Store	Electricity savings1	I'm not going to buy anything from Jingdong anymore, so I'll give it a good review	Jingdong prefecture level city in Shandong
Jinfeng Hongyuan Flagship Store	Electricity savings1	I'm not going to buy anything from Jingdong anymore, so I'll give it a good review	Jingdong prefecture level city in Shandong
Jinfeng Hongyuan Flagship Store	Electricity savings1	Just received the item and will see how it works in a month.	Jingdong prefecture level city in Shandong
Jinfeng Hongyuan Flagship Store	Electricity savings1	I don't know if it saves electricity, but it's plugged into an outlet every day anyway	Jingdong prefecture level city in Shandong
Jinfeng Hongyuan Flagship Store	Electricity savings1	I don't know if it works yet.	Jingdong prefecture level city in Shandong
Jinfeng Hongyuan Flagship Store	Electricity savings1	Plugged it in when I got back, waiting for a month to see the results	Jingdong prefecture level city in Shandong
Jinfeng Hongyuan Flagship Store	Electricity savings1	Effects remain to be seen, but it looks good quality and runs fine	Jingdong prefecture level city in Shandong
Jinfeng Hongyuan Flagship Store	Electricity savings1	The product received logistics very quickly, has not yet been used to test! After use in the evaluation!	Jingdong prefecture level city in Shandong
Jinfeng Hongyuan Flagship Store	Electricity savings1	The power saver is in use, and I don't know how much electricity has been saved after using it for more than a month, and the display voltmeter is very good.	Jingdong prefecture level city in Shandong
Jinfeng Hongyuan Flagship Store	Electricity savings1	It's so good, isn't it? You're a real Shanghainese, aren't you?	Jingdong prefecture level city in Shandong
Jinfeng Hongyuan Flagship Store	Electricity savings1	Great quality great great great 👍👍👍👍	Jingdong prefecture level city in Shandong
Jinfeng Hongyuan Flagship Store	Electricity savings1	I'm using it, I don't know if it's working yet.	Jingdong prefecture level city in Shandong
Jinfeng Hongyuan Flagship Store	Electricity savings1	Just used it for more than 10 days and haven't seen any results yet	Jingdong prefecture level city in Shandong
Jinfeng Hongyuan Flagship Store	Electricity savings1	The product arrived home feeling like it should work	Jingdong prefecture level city in Shandong
Jinfeng Hongyuan Flagship Store	Electricity savings1	Just received the goods, use it and comment back	Jingdong prefecture level city in Shandong

Jinfeng Hongyuan Flagship Store	Electricity savings1	Try it for a month and then evaluate	Jingdong prefecture level city in Shandong
Jinfeng Hongyuan Flagship Store	Electricity savings1	I don't know if it saves power yet, but I'll comment again after using it for a while.	Jingdong prefecture level city in Shandong
Jinfeng Hongyuan Flagship Store	Electricity savings1	The product is better than I thought, the quality and appearance are very good, the voltage is stable, and I'm looking forward to a good power saving effect.	Jingdong prefecture level city in Shandong
Jinfeng Hongyuan Flagship Store	Electricity savings1	Received and scanned the QR code to check that it is genuine, have plugged it in, can it save power, looking forward to the advertised effect. Customary praise, 5 points!	Jingdong prefecture level city in Shandong
Jinfeng Hongyuan Flagship Store	Electricity savings1	The goods have been received and have not yet been used. Do not know how the effect.	Jingdong prefecture level city in Shandong
Jinfeng Hongyuan Flagship Store	Electricity savings1	Hopefully, you get what you pay for, and you get what you pay for!	Jingdong prefecture level city in Shandong
Jinfeng Hongyuan Flagship Store	Electricity savings1	Just put it on and it feels like it's turning as fast as a meter! Looking forward to the results!	Jingdong prefecture level city in Shandong
Jinfeng Hongyuan Flagship Store	Electricity savings1	Overall look on the product quality is good, I do not know is not the effect of power saving, wait to see and then follow up!	Jingdong prefecture level city in Shandong
Jinfeng Hongyuan Flagship Store	Electricity savings1	The logistics courier is very fast, the courier service attitude is very good	Jingdong prefecture level city in Shandong
Jinfeng Hongyuan Flagship Store	Electricity savings1	The product has been received, it works well worth buying	Jingdong prefecture level city in Shandong
Jinfeng Hongyuan Flagship Store	Electricity savings1	How did it not work? Is it purely an IQ tax?	Jingdong prefecture level city in Shandong
Jinfeng Hongyuan Flagship Store	Electricity savings1	It looks good, I don't know how well it works.	Jingdong prefecture level city in Shandong
Jinfeng Hongyuan Flagship Store	Electricity savings1	It does seem to be a bit of a power saver, I just don't know if the more expensive one is more power efficient?	Jingdong prefecture level city in Shandong
Jinfeng Hongyuan Flagship Store	Electricity savings1	Is genuine very good, the price is much cheaper, cost-effective, really special paddle just to do activities, the price is much cheaper, cost-effective, really special paddle counting, packaging is also very high-end atmospheric class, it is worth buying!	Jingdong prefecture level city in Shandong
Jinfeng Hongyuan Flagship Store	Electricity savings1	I received it yesterday and used it right away, I hope it works, I'll wait for next month to come back to the conclusion, I'm looking forward to it. Five hearts praise!	Jingdong prefecture level city in Shandong
Jinfeng Hongyuan Flagship Store	Electricity savings1	Looks good, packed properly, received it today, am using it, will see results in a month!	Jingdong prefecture level city in Shandong
Jinfeng Hongyuan Flagship Store	Electricity savings1	Regular product Jingdong self-support trustworthy. There is an effect Come back to the review. 😍😍😍😍	Jingdong prefecture level city in Shandong
Jinfeng Hongyuan Flagship Store	Electricity savings1	I haven't used it yet, hopefully it will work as a power saver, and will comment again if it works well.	Jingdong prefecture level city in Shandong
Jinfeng Hongyuan Flagship Store	Electricity savings1	Shipping was fast, logistics was fast, service was good, and I look forward to good results from the item!	Jingdong prefecture level city in Shandong
Jinfeng Hongyuan Flagship Store	Electricity savings1	This product is this compensator, it has to be at the main switch to be useful, haven't compared the final result, the	Jingdong prefecture level city in Shandong
Jinfeng Hongyuan Flagship Store	Electricity savings1	Packaging is perfect . I've already started using it, so I'm looking forward to the results.	Jingdong prefecture level city in Shandong
Jinfeng Hongyuan Flagship Store	Electricity savings1	I just got it back. I'll comment after I use it.	Jingdong prefecture level city in Shandong
Jinfeng Hongyuan Flagship Store	Electricity savings1	Big brand, with a very assured, very good quality, catch the event to buy very cost-effective to take advantage of the offer, hurry to get, it is genuine, the packaging is also very good, no breakage, the quality is not to speak of, great 👍 logistics super super fast! Quality is also very good, indeed value	Jingdong prefecture level city in Shandong
Jinfeng Hongyuan Flagship Store	Electricity savings1	The quality is pretty good. The price is also quite affordable.	Jingdong prefecture level city in Shandong
Jinfeng Hongyuan Flagship Store	Electricity savings1	I did not expect the courier so fast to arrive yesterday just ordered, the head is a little smaller than I thought, well, the picture looks bigger. The appearance still looks good, as for the practicality and effectiveness of the use of the only know.	Jingdong prefecture level city in Shandong
Jinfeng Hongyuan Flagship Store	Electricity savings1	I don't know. Looking forward to it.	Jingdong prefecture level city in Shandong
Jinfeng Hongyuan Flagship Store	Electricity savings1	Baby received, very delicate, very nice, like. Just don't know if it can really save electricity. Baby received, very delicate, very good-looking, like. Just don't know if it can really save electricity.	Jingdong prefecture level city in Shandong
Jinfeng Hongyuan Flagship Store	Electricity savings1	I'll use it for a month and then comment on the quality of the item	Jingdong prefecture level city in Shandong
Jinfeng Hongyuan Flagship Store	Electricity savings1	It feels ok, use it for a while and see how it works before adding more comments.	Jingdong prefecture level city in Shandong
Jinfeng Hongyuan Flagship Store	Electricity savings1	In use, see results in a month.	Jingdong prefecture level city in Shandong
Jinfeng Hongyuan Flagship Store	Electricity savings1	Jingdong's logistics and quality are very good, the service attitude is very good, the logistics is very fast	Jingdong prefecture level city in Shandong
Jinfeng Hongyuan Flagship Store	Electricity savings1	Intelligent power saver received, well-packed logistics is also quite fast, easy to use, looking forward to good results!	Jingdong prefecture level city in Shandong
Jinfeng Hongyuan Flagship Store	Electricity savings1	The quality is unknown at this point, I won't know until I use it.	Jingdong prefecture level city in Shandong
Jinfeng Hongyuan Flagship Store	Electricity savings1	It's a good product. It really saves power and doesn't take up much space.	Jingdong prefecture level city in Shandong
Jinfeng Hongyuan Flagship Store	Electricity savings1	Goods have been received, packaging is very good, good to see the effect of future use in the follow-up.	Jingdong prefecture level city in Shandong
Jinfeng Hongyuan Flagship Store	Electricity savings1	The product is in perfect shape and the light weight is worth it!	Jingdong prefecture level city in Shandong
Jinfeng Hongyuan Flagship Store	Electricity savings1	Just got home and plugged it in. It's good.	Jingdong prefecture level city in Shandong
Jinfeng Hongyuan Flagship Store	Electricity savings1	The logistics are quick, just to see the results.	Jingdong prefecture level city in Shandong
Jinfeng Hongyuan Flagship Store	Electricity savings1	Short time can not feel here to leave a message, to be followed up later!	Jingdong prefecture level city in Shandong
Jinfeng Hongyuan Flagship Store	Electricity savings1	This user thinks the product is good	Jingdong prefecture level city in Shandong
Jinfeng Hongyuan Flagship Store	Electricity savings1	Received the baby, to be used to see the effect of it!	Jingdong prefecture level city in Shandong
Jinfeng Hongyuan Flagship Store	Electricity savings1	You have to wait a month or two to see how it works.	Jingdong prefecture level city in Shandong
Jinfeng Hongyuan Flagship Store	Electricity savings1	Not bad, good, like it, don't know if it's good, try it first.	Jingdong prefecture level city in Shandong
Jinfeng Hongyuan Flagship Store	Electricity savings1	The goods are good value for money and the logistics is very fast and good service!	Jingdong prefecture level city in Shandong
Jinfeng Hongyuan Flagship Store	Electricity savings1	I don't know how well it works, we'll see in a month	Jingdong prefecture level city in Shandong
Jinfeng Hongyuan Flagship Store	Electricity savings1	The product is fine, but r saving power or not I won't know until I use it for two months. The display and lights take electricity too?	Jingdong prefecture level city in Shandong
Jinfeng Hongyuan Flagship Store	Electricity savings1	I've used it. I don't know how it works yet.	Jingdong prefecture level city in Shandong
Jinfeng Hongyuan Flagship Store	Electricity savings1	I can't say until I compare them.	Jingdong prefecture level city in Shandong
Jinfeng Hongyuan Flagship Store	Electricity savings1	It's ok in the process of using 。。。。	Jingdong prefecture level city in Shandong

Jinfeng Hongyuan Flagship Store	Electricity savings1	Jinfeng Hongyuan Intelligent Power Saver Household Electricity Meter Electricity Saving Appliance High Power Enhanced Power Saving King Energy Saving Treasure Butler Control Non Stealing Electricity Reversing Not Going Interfering Slow Turner Artifact King Gold Yellow. Haven't tested it accurately yet, really need to wait a few months to test it before I have a say.	Jingdong prefecture level city in Shandong
Jinfeng Hongyuan Flagship Store	Electricity savings1	Don't know how effective it is yet? I hope it works well and I'll come back next time	Jingdong prefecture level city in Shandong
Jinfeng Hongyuan Flagship Store	Electricity savings1	Saving device has been received, material workmanship are very good ah! Have not yet begun to use. First observe the daily electricity consumption and cost of the home meter, and then plug in the power saver to use, so as to compare the use of the effect is good!	Jingdong prefecture level city in Shandong
Jinfeng Hongyuan Flagship Store	Electricity savings1	Merchants shipping extremely fast, Jingdong logistics safe and fast, I believe that the product can play a safe use of electricity, the role of scientific power saving, first praise, the effect of how to be used for a period of time and then the pursuit of bubble!	Jingdong prefecture level city in Shandong
Jinfeng Hongyuan Flagship Store	Electricity savings1	I just bought it. I don't know yet.	Jingdong prefecture level city in Shandong
Jinfeng Hongyuan Flagship Store	Electricity savings1	It is genuine, good quality, and it is very reassuring to use. The logistics is very fast, and the business service attitude is on point.	Jingdong prefecture level city in Shandong
Jinfeng Hongyuan Flagship Store	Electricity savings1	The quality is fine, I don't know how effective it is.	Jingdong prefecture level city in Shandong
Jinfeng Hongyuan Flagship Store	Electricity savings1	The stuff is back and I expect it will save the electricity	Jingdong prefecture level city in Shandong
Jinfeng Hongyuan Flagship Store	Electricity savings1	I'll give it 4 stars first and see what happens in a couple of months and then follow up	Jingdong prefecture level city in Shandong
Jinfeng Hongyuan Flagship Store	Electricity savings1	It's not bad. It's in the mind.	Jingdong prefecture level city in Shandong
Jinfeng Hongyuan Flagship Store	Electricity savings1	Received the goods have not yet started to use do not know whether it is good	Jingdong prefecture level city in Shandong
Jinfeng Hongyuan Flagship Store	Electricity savings1	This user thinks the product is good	Jingdong prefecture level city in Shandong
Jinfeng Hongyuan Flagship Store	Electricity savings1	Hello! The saver was received, thank you! Not yet used, but from the packaging looks very good quality, exquisite workmanship. Logistics is quite fast, if it is really very power saving, I will come back to	Jingdong prefecture level city in Shandong
Jinfeng Hongyuan Flagship Store	Electricity savings1	I've been using it for a few days now, I'll comment again after a month or two of use	Jingdong prefecture level city in Shandong
Jinfeng Hongyuan Flagship Store	Electricity savings1	I don't know. Bought it for my father at a good price.	Jingdong prefecture level city in Shandong
Jinfeng Hongyuan Flagship Store	Electricity savings1	Plugged it in the same day I bought it and the voltage showed 223, 225 looking forward to the results	Jingdong prefecture level city in Shandong
Jinfeng Hongyuan Flagship Store	Electricity savings1	Still in use, the biggest role is to stabilize the current, reduce losses, product quality on the look is very good, with this feeling quite good, see colleagues are using, power saving effect should be good	Jingdong prefecture level city in Shandong
Jinfeng Hongyuan Flagship Store	Electricity savings1	How to receive goods but no invoice and warranty more than it ...... quality is the premise of reputation!	Jingdong prefecture level city in Shandong
Jinfeng Hongyuan Flagship Store	Electricity savings1	Received but not used Continued evaluation	Jingdong prefecture level city in Shandong
Jinfeng Hongyuan Flagship Store	Electricity savings1	I just received the product, I don't know if it works well yet	Jingdong prefecture level city in Shandong
Jinfeng Hongyuan Flagship Store	Electricity savings1	Received it and used it, not sure it really saves electricity use it and then evaluate it, first give a 5 star review!!!!	Jingdong prefecture level city in Shandong
Jinfeng Hongyuan Flagship Store	Electricity savings1	It's good to use, it's very good to use, and it smells OK too	Jingdong prefecture level city in Shandong
Jinfeng Hongyuan Flagship Store	Electricity savings1	Packaging is very good, feel the product is not bad, how much power saving with the only know	Jingdong prefecture level city in Shandong
Jinfeng Hongyuan Flagship Store	Electricity savings1	Additional comments to be made after use	Jingdong prefecture level city in Shandong
Jinfeng Hongyuan Flagship Store	Electricity savings1	The product has been installed, try a few months and then comment!	Jingdong prefecture level city in Shandong
Jinfeng Hongyuan Flagship Store	Electricity savings1	I've bought it, let's use it for now, I'll comment after a month	Jingdong prefecture level city in Shandong
Jinfeng Hongyuan Flagship Store	Electricity savings1	Try it out and then follow up	Jingdong prefecture level city in Shandong
Jinfeng Hongyuan Flagship Store	Electricity savings1	It's good in theory, it doesn't feel good in practice.	Jingdong prefecture level city in Shandong
Jinfeng Hongyuan Flagship Store	Electricity savings1	I haven't tried it yet. We'll see.	Jingdong prefecture level city in Shandong
Jinfeng Hongyuan Flagship Store	Electricity savings1	Great item, second purchase!	Jingdong prefecture level city in Shandong
Jinfeng Hongyuan Flagship Store	Electricity savings1	Goods received now use it, if it does save power, plus reevaluate.	Jingdong prefecture level city in Shandong
Jinfeng Hongyuan Flagship Store	Electricity savings1	The stuff is light and small, the stuff is good, the courier service is good	Jingdong prefecture level city in Shandong
Jinfeng Hongyuan Flagship Store	Electricity savings1	I haven't used it yet. I'm looking forward to it.	Jingdong prefecture level city in Shandong
Jinfeng Hongyuan Flagship Store	Electricity savings1	Express received, in use do not know if there is no power saving	Jingdong prefecture level city in Shandong
Jinfeng Hongyuan Flagship Store	Electricity savings1	The quality of the product is quite good! The power saving mode is still getting used to it, can't say yet, how much power it saves.	Jingdong prefecture level city in Shandong
Jinfeng Hongyuan Flagship Store	Electricity savings1	The goods have been received, just started to use, now I do not know how the quality, after a period of time to say.	Jingdong prefecture level city in Shandong
Jinfeng Hongyuan Flagship Store	Electricity savings1	I received the stuff, used it for a few days to evaluate, the stuff is very good and good, worth buying.	Jingdong prefecture level city in Shandong
Jinfeng Hongyuan Flagship Store	Electricity savings1	I've been using it for a while, but I don't know if it's good or if it saves electricity! What the heck, let's give it a good review first. And for the record, I'm not helping him**.	Jingdong prefecture level city in Shandong
Jinfeng Hongyuan Flagship Store	Electricity savings1	Haven't used it yet, looks good, try it for 60 days, might be good, great review!	Jingdong prefecture level city in Shandong
Jinfeng Hongyuan Flagship Store	Electricity savings1	Baby received, logistics is very fast. Ordered last night, received this morning. Received the goods immediately plugged in the power supply, save power to the first of next month to know.	Jingdong prefecture level city in Shandong
Jinfeng Hongyuan Flagship Store	Electricity savings1	First, I'll give you a good review and use it.	Jingdong prefecture level city in Shandong
Jinfeng Hongyuan Flagship Store	Electricity savings1	Just arrived to follow up with a genuine review	Jingdong prefecture level city in Shandong
Jinfeng Hongyuan Flagship Store	Electricity savings1	Not bad pretty fast, good quality.	Jingdong prefecture level city in Shandong
Jinfeng Hongyuan Flagship Store	Electricity savings1	Recommended, use it and hopefully it will save some electricity	Jingdong prefecture level city in Shandong
Jinfeng Hongyuan Flagship Store	Electricity savings1	The stuff looks ok, I don't know the effect if it.	Jingdong prefecture level city in Shandong
Jinfeng Hongyuan Flagship Store	Electricity savings1	The stuff looks OK, the stuff looks OK.	Jingdong prefecture level city in Shandong
Jinfeng Hongyuan Flagship Store	Electricity savings1	The item has been received, after using it in a follow-up review. Satisfied with the purchase.	Jingdong prefecture level city in Shandong
Jinfeng Hongyuan Flagship Store	Electricity savings1	Has been normal plug-in use, at present do not know how to see, use down to see and then evaluation	Jingdong prefecture level city in Shandong
Jinfeng Hongyuan Flagship Store	Electricity savings1	Commodities make noise.	Jingdong prefecture level city in Shandong

Jinfeng Hongyuan Flagship Store	Electricity savings1	After using it for a few days and observing it, the saving is about ten percent	Jingdong prefecture level city in Shandong
Jinfeng Hongyuan Flagship Store	Electricity savings1	The goods received, the quality looks quite OK. First use it, the effect is not good to say.	Jingdong prefecture level city in Shandong
Jinfeng Hongyuan Flagship Store	Electricity savings1	In use, follow up next month	Jingdong prefecture level city in Shandong
Jinfeng Hongyuan Flagship Store	Electricity savings1	I just received the goods and have not yet used it to comment after using it	Jingdong prefecture level city in Shandong
Jinfeng Hongyuan Flagship Store	Electricity savings1	So far I don't know if it really saves electricity yet! Spinning waiting a couple months to find out! Good reviews first!	Jingdong prefecture level city in Shandong
Jinfeng Hongyuan Flagship Store	Electricity savings1	Small and exquisite, looking forward to the effect, to be evaluated 6666666666666	Jingdong prefecture level city in Shandong
Jinfeng Hongyuan Flagship Store	Electricity savings1	I just used it, I don't know if it's effective, I'll comment again after a month	Jingdong prefecture level city in Shandong
Jinfeng Hongyuan Flagship Store	Electricity savings1	I just used it, I don't know if it's good or not, I'll comment later.	Jingdong prefecture level city in Shandong
Jinfeng Hongyuan Flagship Store	Electricity savings1	Baby received, Baobei quite good, use and then come back to comment!	Jingdong prefecture level city in Shandong
Jinfeng Hongyuan Flagship Store	Electricity savings1	Good quality power saving is fake	Jingdong prefecture level city in Shandong
Jinfeng Hongyuan Flagship Store	Electricity savings1	The packaging of the goods is atmospheric, the quality is exquisite, and I look forward to saving electricity.	Jingdong prefecture level city in Shandong
Jinfeng Hongyuan Flagship Store	Electricity savings1	The packaging is great and the quality is good!	Jingdong prefecture level city in Shandong
Jinfeng Hongyuan Flagship Store	Electricity savings1	I don't see any effect.	Jingdong prefecture level city in Shandong
Jinfeng Hongyuan Flagship Store	Electricity savings1	Already used, but did not see obvious results	Jingdong prefecture level city in Shandong
Jinfeng Hongyuan Flagship Store	Electricity savings1	It's good. I'm just using it. I don't know what it's like.	Jingdong prefecture level city in Shandong
Jinfeng Hongyuan Flagship Store	Electricity savings1	I just used it. I'll comment later.	Jingdong prefecture level city in Shandong
Jinfeng Hongyuan Flagship Store	Electricity savings1	I installed it when it arrived, try it for a month and then come back for a follow-up review	Jingdong prefecture level city in Shandong
Jinfeng Hongyuan Flagship Store	Electricity savings1	Goods arrived a few days fastidious does not call to pick up the goods, the quality is average	Jingdong prefecture level city in Shandong
Jinfeng Hongyuan Flagship Store	Electricity savings1	This user thinks the product is good	Jingdong prefecture level city in Shandong
Jinfeng Hongyuan Flagship Store	Electricity savings1	Roger that.	Jingdong prefecture level city in Shandong
Jinfeng Hongyuan Flagship Store	Electricity savings1	Second purchase was ok.	Jingdong prefecture level city in Shandong
Jinfeng Hongyuan Flagship Store	Electricity savings1	The merchant is very reputable and the product is good value for money. Logistics is fast!	Jingdong prefecture level city in Shandong
Jinfeng Hongyuan Flagship Store	Electricity savings1	The quality of the baby is very good, I have always trusted Kyodo	Jingdong prefecture level city in Shandong
Jinfeng Hongyuan Flagship Store	Electricity savings1	We'll see how it goes when we get it.	Jingdong prefecture level city in Shandong
Jinfeng Hongyuan Flagship Store	Electricity savings1	I just used it. I don't know if it works.	Jingdong prefecture level city in Shandong
Jinfeng Hongyuan Flagship Store	Electricity savings1	Good quality, used for a month to save about 20%, power saving effect is good	Jingdong prefecture level city in Shandong
Jinfeng Hongyuan Flagship Store	Electricity savings1	I'll give you a good review before I use it.	Jingdong prefecture level city in Shandong
Jinfeng Hongyuan Flagship Store	Electricity savings1	Received, satisfied with the follow-up evaluation	Jingdong prefecture level city in Shandong
Jinfeng Hongyuan Flagship Store	Electricity savings1	Immediate results, saving electricity and money	Jingdong prefecture level city in Shandong
Jinfeng Hongyuan Flagship Store	Electricity savings1	Measured to save a little	Jingdong prefecture level city in Shandong
Jinfeng Hongyuan Flagship Store	Electricity savings1	This user thinks the product is good	Jingdong prefecture level city in Shandong
Jinfeng Hongyuan Flagship Store	Electricity savings1	This user thinks the product is good	Jingdong prefecture level city in Shandong
Jinfeng Hongyuan Flagship Store	Electricity savings1	Product quality and logistics are very good, worth buying	Jingdong prefecture level city in Shandong
Jinfeng Hongyuan Flagship Store	Electricity savings1	Appearance looks ok! Express delivery was fast! Service is still relatively enthusiastic.	Jingdong prefecture level city in Shandong
Jinfeng Hongyuan Flagship Store	Electricity savings1	It doesn't feel like it's working.	Jingdong prefecture level city in Shandong
Jinfeng Hongyuan Flagship Store	Electricity savings1	Well made and looks good. Used it with other appliances and felt as if it had some effect.	Jingdong prefecture level city in Shandong
Jinfeng Hongyuan Flagship Store	Electricity savings1	Haven't used it yet, will follow up later	Jingdong prefecture level city in Shandong
Jinfeng Hongyuan Flagship Store	Electricity savings1	Things received, used a few days the plug is a little hot unplugged did not work	Jingdong prefecture level city in Shandong
Jinfeng Hongyuan Flagship Store	Electricity savings1	Look at the meter again in a month.	Jingdong prefecture level city in Shandong
Jinfeng Hongyuan Flagship Store	Electricity savings1	This one didn't work at all.	Jingdong prefecture level city in Shandong
Jinfeng Hongyuan Flagship Store	Electricity savings1	It's small. I've seen friends with much bigger ones.	Jingdong prefecture level city in Shandong
Jinfeng Hongyuan Flagship Store	Electricity savings1	The item was received and the quality is good, it will be nice to see how the power saving rate is down the road, the item is great.	Jingdong prefecture level city in Shandong
Jinfeng Hongyuan Flagship Store	Electricity savings1	Cheap and affordable quality is quite good, logistics is also very fast, praise! , baby good 😊, cost-effective very high, big brand trustworthy 👍	Jingdong prefecture level city in Shandong
Jinfeng Hongyuan Flagship Store	Electricity savings1	Used it for a while it really saves electricity ⚡ thanks 🈶 such a good product, compact and practical, every family should buy one ✨	Jingdong prefecture level city in Shandong
Jinfeng Hongyuan Flagship Store	Electricity savings1	Very satisfied with the purchase Next time I will come back	Jingdong prefecture level city in Shandong
Jinfeng Hongyuan Flagship Store	Electricity savings1	Logistics is fast. Good quality, used one before and it does save power	Jingdong prefecture level city in Shandong
Jinfeng Hongyuan Flagship Store	Electricity savings1	I don't know if it works.	Jingdong prefecture level city in Shandong
Jinfeng Hongyuan Flagship Store	Electricity savings1	Good goods, very solid, good.	Jingdong prefecture level city in Shandong
Jinfeng Hongyuan Flagship Store	Electricity savings1	This user thinks the product is good	Jingdong prefecture level city in Shandong
Jinfeng Hongyuan Flagship Store	Electricity savings1	I've been using it for half a month, my electricity bill is much lower, and the quality is very good, so don't worry about buying it!	Jingdong prefecture level city in Shandong
Jinfeng Hongyuan Flagship Store	Electricity savings1	I received the goods today and used it right away, looking forward to the effect	Jingdong prefecture level city in Shandong

Jinfeng Hongyuan Flagship Store	Electricity savings1	The merchandise is great. I am going to recommend it to my friends! Will buy again next time.	Jingdong prefecture level city in Shandong
Jinfeng Hongyuan Flagship Store	Electricity savings1	Let's give it a good review first, it's working fine so far, and I'll follow up with a review,...	Jingdong prefecture level city in Shandong
Jinfeng Hongyuan Flagship Store	Electricity savings1	Product quality is very good, looking forward to using the results	Jingdong prefecture level city in Shandong
Jinfeng Hongyuan Flagship Store	Electricity savings1	I just can't detect it. - Save it.	Jingdong prefecture level city in Shandong
Jinfeng Hongyuan Flagship Store	Electricity savings1	It arrived and is being used, so we'll see how it works!	Jingdong prefecture level city in Shandong
Jinfeng Hongyuan Flagship Store	Electricity savings1	Received yesterday thanks! This energy saver is available . Safe! But in the end how much energy can be saved must be used for a period of time to know, if the real energy saving next time to buy again.	Jingdong prefecture level city in Shandong
Jinfeng Hongyuan Flagship Store	Electricity savings1	Just used it, don't know if it saves power	Jingdong prefecture level city in Shandong
Jinfeng Hongyuan Flagship Store	Electricity savings1	I'll rate it first, is it a tease wait two months and see? It's been four days and it's not noticeable!	Jingdong prefecture level city in Shandong
Jinfeng Hongyuan Flagship Store	Electricity savings1	Workmanship is quite good, just started to use, the effect of the follow-up evaluation.	Jingdong prefecture level city in Shandong
Jinfeng Hongyuan Flagship Store	Electricity savings1	Received the goods used for a few days before evaluation, the effect is quite good	Jingdong prefecture level city in Shandong
Jinfeng Hongyuan Flagship Store	Electricity savings1	Try it for a month and then evaluate	Jingdong prefecture level city in Shandong
Jinfeng Hongyuan Flagship Store	Electricity savings1	Just received the goods, the packaging is very good, the appearance of the economizer feels good, I do not know how the effect of power saving, with a month or two and then follow up!	Jingdong prefecture level city in Shandong
Jinfeng Hongyuan Flagship Store	Electricity savings1	It's quite small and delicate, I don't know if it's useful yet.	Jingdong prefecture level city in Shandong
Jinfeng Hongyuan Flagship Store	Electricity savings1	Just received it, use it for a few days before commenting	Jingdong prefecture level city in Shandong
Jinfeng Hongyuan Flagship Store	Electricity savings1	Does this thing work! When it arrived, the button for the safety was loose. I'm a little afraid of fire!	Jingdong prefecture level city in Shandong
Jinfeng Hongyuan Flagship Store	Electricity savings1	I received it. I don't know how it works.	Jingdong prefecture level city in Shandong
Jinfeng Hongyuan Flagship Store	Electricity savings1	This user thinks the product is good	Jingdong prefecture level city in Shandong
Jinfeng Hongyuan Flagship Store	Electricity savings1	Customer service was great! Small i problem was taken care of in a timely manner! Effectiveness to be tested by time! No rush!!!	Jingdong prefecture level city in Shandong
Jinfeng Hongyuan Flagship Store	Electricity savings1	I put it on right after I received it and it looks compact. I believe the high technology should save electricity.	Jingdong prefecture level city in Shandong
Jinfeng Hongyuan Flagship Store	Electricity savings1	Logistics courier very quickly, the first day under the order the next day to receive, off pieces whether power saving is the most important thing, things are exquisite workmanship, a month later in the follow-up evaluation whether power saving	Jingdong prefecture level city in Shandong
Jinfeng Hongyuan Flagship Store	Electricity savings1	The delivery is pretty fast, I'm using it, have to have it for a while to see results, looking forward to it.	Jingdong prefecture level city in Shandong
Jinfeng Hongyuan Flagship Store	Electricity savings1	See pretty good just started to use a month later to give you a real evaluation, should be useful.	Jingdong prefecture level city in Shandong
Jinfeng Hongyuan Flagship Store	Electricity savings1	And then comment.	Jingdong prefecture level city in Shandong
Jinfeng Hongyuan Flagship Store	Electricity savings1	Received the goods, wait for the test to evaluate	Jingdong prefecture level city in Shandong
Jinfeng Hongyuan Flagship Store	Electricity savings1	This user thinks the product is good	Jingdong prefecture level city in Shandong
Jinfeng Hongyuan Flagship Store	Electricity savings1	I'm already using it, I just don't know how to judge the power savings. I won't know until I use it for a season.	Jingdong prefecture level city in Shandong
Jinfeng Hongyuan Flagship Store	Electricity savings1	Works. Says it works.	Jingdong prefecture level city in Shandong
Jinfeng Hongyuan Flagship Store	Electricity savings1	Very good. Satisfied.	Jingdong prefecture level city in Shandong
Jinfeng Hongyuan Flagship Store	Electricity savings1	This user thinks the product is good	Jingdong prefecture level city in Shandong
Jinfeng Hongyuan Flagship Store	Electricity savings1	It's working. Obviously, it's less expensive.	Jingdong prefecture level city in Shandong
Jinfeng Hongyuan Flagship Store	Electricity savings1	The second one is up, the first one still works! It's reactive power compensation! This is well understood if you want a professional!	Jingdong prefecture level city in Shandong
Jinfeng Hongyuan Flagship Store	Electricity savings1	It feels as if there are some nodes, but they need to be tested and observed over time, thanks.	Jingdong prefecture level city in Shandong
Jinfeng Hongyuan Flagship Store	Electricity savings1	I've received the goods, the energy saving effect will be evaluated after using it for a period of time, and all the services are very good, praise!	Jingdong prefecture level city in Shandong
Jinfeng Hongyuan Flagship Store	Electricity savings1	I don't know how well it works yet, I bought it and plugged it in, so we'll see if it saves some power or not!	Jingdong prefecture level city in Shandong
Jinfeng Hongyuan Flagship Store	Electricity savings1	Baby a bunch of knife quality is very good, previously bought a quality is not very good. It really works and has results.	Jingdong prefecture level city in Shandong
Jinfeng Hongyuan Flagship Store	Electricity savings1	Things have arrived, it looks good, just started to use, as to whether or not there is a business said the power-saving effect, but also to observe the effect, now can not evaluate	Jingdong prefecture level city in Shandong
Jinfeng Hongyuan Flagship Store	Electricity savings1	I haven't used it yet. I'll comment afterward.	Jingdong prefecture level city in Shandong
Jinfeng Hongyuan Flagship Store	Electricity savings1	I don't know if it's any good.	Jingdong prefecture level city in Shandong
Jinfeng Hongyuan Flagship Store	Electricity savings1	I don't know how well it works.	Jingdong prefecture level city in Shandong
Jinfeng Hongyuan Flagship Store	Electricity savings1	The baby has been received! Uh-huh! Good, quite exquisite, small and exquisite I thought it was big. Dare to tightly plugged in the electricity, just wait for the effect later!	Jingdong prefecture level city in Shandong
Jinfeng Hongyuan Flagship Store	Electricity savings1	The packaging is sturdy, I tried it right after I received it, so I'm looking forward to the results.	Jingdong prefecture level city in Shandong
Jinfeng Hongyuan Flagship Store	Electricity savings1	Received the item and installed it! Don't know if it works well! Will follow up with a review later! I hope it works!	Jingdong prefecture level city in Shandong
Jinfeng Hongyuan Flagship Store	Electricity savings1	Things used on the effect of waiting to use a little to say, but things are very good. Logistics is very fast, business service is very good.	Jingdong prefecture level city in Shandong
Jinfeng Hongyuan Flagship Store	Electricity savings1	This user thinks the product is good	Jingdong prefecture level city in Shandong
Jinfeng Hongyuan Flagship Store	Electricity savings1	I'm really using it. It feels good.	Jingdong prefecture level city in Shandong
Jinfeng Hongyuan Flagship Store	Electricity savings1	Good, Jingdong logistics is very fast, intelligent energy-saving appliances	Jingdong prefecture level city in Shandong
Jinfeng Hongyuan Flagship Store	Electricity savings1	It's not bad. It really saves energy.	Jingdong prefecture level city in Shandong
Jinfeng Hongyuan Flagship Store	Electricity savings1	The logistics is fast, praise	Jingdong prefecture level city in Shandong
Jinfeng Hongyuan Flagship Store	Electricity savings1	This user thinks the product is good	Jingdong prefecture level city in Shandong
Jinfeng Hongyuan Flagship Store	Electricity savings1	The first time I placed two, and today I placed another one, a friend introduced it, said it is particularly power-saving	Jingdong prefecture level city in Shandong
Jinfeng Hongyuan Flagship Store	Electricity savings1	I don't know how effective it is. Let's leave it at home for a while and see.	Jingdong prefecture level city in Shandong

Jinfeng Hongyuan Flagship Store	Electricity savings1	Dear customer service! I would like to ask you how to understand the digital display (increase/decrease jump) on the top of the saver? Thank you for your help.	Jingdong prefecture level city in Shandong
Jinfeng Hongyuan Flagship Store	Electricity savings1	This user thinks the product is good	Jingdong prefecture level city in Shandong
Jinfeng Hongyuan Flagship Store	Electricity savings1	The goods have been received, packaging exquisite, is using, later to see how the effect?	Jingdong prefecture level city in Shandong
Jinfeng Hongyuan Flagship Store	Electricity savings1	The saver received, the boss shipped very quickly, yesterday shot today arrived!	Jingdong prefecture level city in Shandong
Jinfeng Hongyuan Flagship Store	Electricity savings1	Beautiful appearance, well packaged. Recording data to see if I can lower my electric bill a bit.	Jingdong prefecture level city in Shandong
Jinfeng Hongyuan Flagship Store	Electricity savings1	Things received, the packaging is quite good, the effect of the use and then come back to evaluate.	Jingdong prefecture level city in Shandong
Jinfeng Hongyuan Flagship Store	Electricity savings1	Easy to use, just plug it in. But there is a current sound.	Jingdong prefecture level city in Shandong
Jinfeng Hongyuan Flagship Store	Electricity savings1	Fast logistics, easy to use and labor-saving	Jingdong prefecture level city in Shandong
Jinfeng Hongyuan Flagship Store	Electricity savings1	Received, thank you!	Jingdong prefecture level city in Shandong
Jinfeng Hongyuan Flagship Store	Electricity savings1	Second time I've bought it. It's good.	Jingdong prefecture level city in Shandong
Jinfeng Hongyuan Flagship Store	Electricity savings1	I've only been using it for a short time, so I'm looking forward to the results.	Jingdong prefecture level city in Shandong
Jinfeng Hongyuan Flagship Store	Electricity savings1	Good stuff, good stuff, good stuff good stuff.	Jingdong prefecture level city in Shandong
Jinfeng Hongyuan Flagship Store	Electricity savings1	Buy your stuff is not always good I have been using it for almost two months now, it does not save electricity at all the electricity bill is about ten dollars a day this is still called the king of power	Jingdong prefecture level city in Shandong
Jinfeng Hongyuan Flagship Store	Electricity savings1	Express arrived, the quality is very good, worth the purchase.	Jingdong prefecture level city in Shandong
Jinfeng Hongyuan Flagship Store	Electricity savings1	Energy saving has been received, now the voltage is more stable, energy-saving effect to see a few days and then chase flat, the service attitude is very good, the courier is relatively fast, I am satisfied.	Jingdong prefecture level city in Shandong
Jinfeng Hongyuan Flagship Store	Electricity savings1	Stuff installed, numbers are smaller than when installed, hopefully it works	Jingdong prefecture level city in Shandong
Jinfeng Hongyuan Flagship Store	Electricity savings1	Good appearance, fine workmanship, just bought, use up the feeling of power saving effect is good, worth buying!	Jingdong prefecture level city in Shandong
Jinfeng Hongyuan Flagship Store	Electricity savings1	Economical and practical 👍 Always use it	Jingdong prefecture level city in Shandong
Jinfeng Hongyuan Flagship Store	Electricity savings1	Just used it, don't know the effect yet, will come back to follow up after the effect. Hope it works well(▽)	Jingdong prefecture level city in Shandong
Jinfeng Hongyuan Flagship Store	Electricity savings1	The goods have been received, use it to see in the evaluation.	Jingdong prefecture level city in Shandong
Jinfeng Hongyuan Flagship Store	Electricity savings1	Doesn't it save electricity? The boil kettle still goes faster when you press the meter.	Jingdong prefecture level city in Shandong
Jinfeng Hongyuan Flagship Store	Electricity savings1	Already used. Will patronize again in the future	Jingdong prefecture level city in Shandong
Jinfeng Hongyuan Flagship Store	Electricity savings1	Works great, just plug it in, results to be checked, satisfied.	Jingdong prefecture level city in Shandong
Jinfeng Hongyuan Flagship Store	Electricity savings1	Second purchase. Works well 😍😍😍😍	Jingdong prefecture level city in Shandong
Jinfeng Hongyuan Flagship Store	Electricity savings1	Prompt shipment, very satisfied	Jingdong prefecture level city in Shandong
Jinfeng Hongyuan Flagship Store	Electricity savings1	It's been less than a month, let's see how it works, hopefully it's power efficient	Jingdong prefecture level city in Shandong
Jinfeng Hongyuan Flagship Store	Electricity savings1	Use a month in say.	Jingdong prefecture level city in Shandong
Jinfeng Hongyuan Flagship Store	Electricity savings1	I've saved a lot of electricity since I've been using it.	Jingdong prefecture level city in Shandong
Jinfeng Hongyuan Flagship Store	Electricity savings1	It's fast, looks and feels good on the outside, try it out and evaluate.	Jingdong prefecture level city in Shandong
Jinfeng Hongyuan Flagship Store	Electricity savings1	I haven't used it. I just used it. I don't know if it's any good.	Jingdong prefecture level city in Shandong
Jinfeng Hongyuan Flagship Store	Electricity savings1	Quality can be, power saving now can not see just use, after a while in the look at the power saving effect	Jingdong prefecture level city in Shandong
Jinfeng Hongyuan Flagship Store	Electricity savings1	The logistics is very fast, and it seems to have some effect	Jingdong prefecture level city in Shandong
Jinfeng Hongyuan Flagship Store	Electricity savings1	Good, but the effects remain to be seen	Jingdong prefecture level city in Shandong
Jinfeng Hongyuan Flagship Store	Electricity savings1	Baby is basically the same as the description, shipping quickly, worth a buy Good price, logistics is strong, worth repurchasing!	Jingdong prefecture level city in Shandong
Jinfeng Hongyuan Flagship Store	Electricity savings1	I bought it for my dad, I don't know much about it, if he thinks it's good then it must be good 👍	Jingdong prefecture level city in Shandong
Jinfeng Hongyuan Flagship Store	Electricity savings1	Goods received. It's working fine. Display voltage. After a period of time to see the effect in the addition to the level of theory	Jingdong prefecture level city in Shandong
Jinfeng Hongyuan Flagship Store	Electricity savings1	The stuff is pretty good, I just don't know how well it saves power.	Jingdong prefecture level city in Shandong
Jinfeng Hongyuan Flagship Store	Electricity savings1	Logistics is a little too fast 👍	Jingdong prefecture level city in Shandong
Jinfeng Hongyuan Flagship Store	Electricity savings1	Received the goods used for a period of time before coming, good, beautiful appearance, really stable flow	Jingdong prefecture level city in Shandong
Jinfeng Hongyuan Flagship Store	Electricity savings1	Things received, look at the packaging is very good, not yet, to the power saving in the follow-up evaluation	Jingdong prefecture level city in Shandong
Jinfeng Hongyuan Flagship Store	Electricity savings1	Baby is very good! Use a few days before to evaluate, I personally test really effective.	Jingdong prefecture level city in Shandong
Jinfeng Hongyuan Flagship Store	Electricity savings1	I received the goods are in use, I do not know how effective? Can it save energy? Let's see.	Jingdong prefecture level city in Shandong
Jinfeng Hongyuan Flagship Store	Electricity savings1	It's really good and has some power saving effect.	Jingdong prefecture level city in Shandong
Jinfeng Hongyuan Flagship Store	Electricity savings1	It's good quality, well made, and I hope it'll save on electricity over time.	Jingdong prefecture level city in Shandong
Jinfeng Hongyuan Flagship Store	Electricity savings1	I just used it, I don't know how it works yet, but I'll give it a good review first.	Jingdong prefecture level city in Shandong
Jinfeng Hongyuan Flagship Store	Electricity savings1	Second purchase, the delivery guy was fast and called ahead to deliver.	Jingdong prefecture level city in Shandong
Jinfeng Hongyuan Flagship Store	Electricity savings1	Goods have been received, but have not yet used, used to see how the effect, in the evaluation of fertilizer	Jingdong prefecture level city in Shandong
Jinfeng Hongyuan Flagship Store	Electricity savings1	The delivery is very fast. It's the same in kind. It's working.	Jingdong prefecture level city in Shandong
Jinfeng Hongyuan Flagship Store	Electricity savings1	Fast logistics, good quality, more satisfied	Jingdong prefecture level city in Shandong
Jinfeng Hongyuan Flagship Store	Electricity savings1	I've been here for almost a month now and there is a very noticeable difference than it is worth buying.	Jingdong prefecture level city in Shandong
Jinfeng Hongyuan Flagship Store	Electricity savings1	Fast shipping, received the next day, plug and play, but do not know how effective to not break down!	Jingdong prefecture level city in Shandong

Jinfeng Hongyuan Flagship Store	Electricity savings1	The logistics are great I've been using it for a while before commenting on it This saves electricity	Jingdong prefecture level city in Shandong
Jinfeng Hongyuan Flagship Store	Electricity savings1	Wait to use it for a while to know if it's energy efficient	Jingdong prefecture level city in Shandong
Jinfeng Hongyuan Flagship Store	Electricity savings1	Just received it, haven't used it yet don't know if it's any good.	Jingdong prefecture level city in Shandong
Jinfeng Hongyuan Flagship Store	Electricity savings1	It really doesn't work, and the electric bill is still the same amount every month.	Jingdong prefecture level city in Shandong
Jinfeng Hongyuan Flagship Store	Electricity savings1	It's well packaged and looks fine	Jingdong prefecture level city in Shandong
Jinfeng Hongyuan Flagship Store	Electricity savings1	Very good. Will patronize again if needed.	Jingdong prefecture level city in Shandong
Jinfeng Hongyuan Flagship Store	Electricity savings1	I have received the product and installed it, try it for a while and see how it works.	Jingdong prefecture level city in Shandong
Jinfeng Hongyuan Flagship Store	Electricity savings1	I've been using it for a month now...I don't feel like there's any noticeable power savings...last month it was $130 this month it's $123...let's see if I can use it for another month	Jingdong prefecture level city in Shandong
Jinfeng Hongyuan Flagship Store	Electricity savings1	Just received it, yet to try it and see if it works.	Jingdong prefecture level city in Shandong
Jinfeng Hongyuan Flagship Store	Electricity savings1	It's easy to use and has a power saving credit!	Jingdong prefecture level city in Shandong
Jinfeng Hongyuan Flagship Store	Electricity savings1	It's very good. I've already bought two of them.	Jingdong prefecture level city in Shandong
Jinfeng Hongyuan Flagship Store	Electricity savings1	It's very well made, packaged meticulously, and I hope it works!	Jingdong prefecture level city in Shandong
Jinfeng Hongyuan Flagship Store	Electricity savings1	Goods have been received have not yet used it, used to comment again	Jingdong prefecture level city in Shandong
Jinfeng Hongyuan Flagship Store	Electricity savings1	Looking forward to good results	Jingdong prefecture level city in Shandong
Jinfeng Hongyuan Flagship Store	Electricity savings1	Not sure if it saves power or not. Still trying it out.	Jingdong prefecture level city in Shandong
Jinfeng Hongyuan Flagship Store	Electricity savings1	It also really saves on electricity! Superb!	Jingdong prefecture level city in Shandong
Jinfeng Hongyuan Flagship Store	Electricity savings1	Didn't work at all, want to return it	Jingdong prefecture level city in Shandong
Jinfeng Hongyuan Flagship Store	Electricity savings1	Uh-huh! It's working, I've been using it for a month and it saves 20% of electricity compared to before.	Jingdong prefecture level city in Shandong
Jinfeng Hongyuan Flagship Store	Electricity savings1	Should be a little bit of a power saver, I wonder how long it will take to save back the money I spent on this one	Jingdong prefecture level city in Shandong
Jinfeng Hongyuan Flagship Store	Electricity savings1	Exquisite appearance and stable voltage	Jingdong prefecture level city in Shandong
Jinfeng Hongyuan Flagship Store	Electricity savings1	After using it for a few days it feels like it saves electricity.	Jingdong prefecture level city in Shandong
Jinfeng Hongyuan Flagship Store	Electricity savings1	Second purchase and its pending good results.	Jingdong prefecture level city in Shandong
Jinfeng Hongyuan Flagship Store	Electricity savings1	The appearance of the workmanship is exquisite and meticulous, beautiful appearance, just to see if there is no effect!	Jingdong prefecture level city in Shandong
Jinfeng Hongyuan Flagship Store	Electricity savings1	Let's see what happens in a month.	Jingdong prefecture level city in Shandong
Jinfeng Hongyuan Flagship Store	Electricity savings1	Trial, now short time not yet obvious effect	Jingdong prefecture level city in Shandong
Jinfeng Hongyuan Flagship Store	Electricity savings1	I'm using it now, don't know how well it works, it looks great!	Jingdong prefecture level city in Shandong
Jinfeng Hongyuan Flagship Store	Electricity savings1	There is no role! Now it's true that I don't know!	Jingdong prefecture level city in Shandong
Jinfeng Hongyuan Flagship Store	Electricity savings1	Purchased for the second time, power saving not sure if it's ok? Let's talk about it after using it	Jingdong prefecture level city in Shandong
Jinfeng Hongyuan Flagship Store	Electricity savings1	I just received it, I have to try it for a month to know how it works.	Jingdong prefecture level city in Shandong
Jinfeng Hongyuan Flagship Store	Electricity savings1	I just used it for an hour, and the meter's still spinning so fast it's not much use.	Jingdong prefecture level city in Shandong
Jinfeng Hongyuan Flagship Store	Electricity savings1	Not bad, logistics is fast	Jingdong prefecture level city in Shandong
Jinfeng Hongyuan Flagship Store	Electricity savings1	I don't know if it works. I'll find out next month when I pay the bill. It's easy. Just plug it in.	Jingdong prefecture level city in Shandong
Jinfeng Hongyuan Flagship Store	Electricity savings1	Logistics is particularly fast, received the goods are genuine, looking forward to power saving effect	Jingdong prefecture level city in Shandong
Jinfeng Hongyuan Flagship Store	Electricity savings1	First time buying this product like this one from this family use it and then evaluate.	Jingdong prefecture level city in Shandong
Jinfeng Hongyuan Flagship Store	Electricity savings1	Lightning arrived, the product is very good, before all do not know there is such a baby, in a friend's home has seen, and then bought to try, has been used for a period of time before coming to the evaluation, the work is very stable, so far looked very good, will introduce friends to buy!	Jingdong prefecture level city in Shandong
Jinfeng Hongyuan Flagship Store	Electricity savings1	I was a bit skeptical when I heard that I could save money on my electricity bill by installing this, I don't know if the power saving effect is really what it is said to be. But since I bought it, I just have to put it on and try it out. I hope it lives up to its name and saves me the money I spent on the device	Jingdong prefecture level city in Shandong
Jinfeng Hongyuan Flagship Store	Electricity savings1	The quality of the stuff is okay, but I'm not sure if it works, so I'll try using it first.	Jingdong prefecture level city in Shandong
Jinfeng Hongyuan Flagship Store	Electricity savings1	It should work. The current is much more stable.	Jingdong prefecture level city in Shandong
Jinfeng Hongyuan Flagship Store	Electricity savings1	The store has a great service attitude	Jingdong prefecture level city in Shandong
Jinfeng Hongyuan Flagship Store	Electricity savings1	Let's wait until we've used it for a month.	Jingdong prefecture level city in Shandong
Jinfeng Hongyuan Flagship Store	Electricity savings1	I'm using it and I don't know if it's a power saver or not	Jingdong prefecture level city in Shandong
Jinfeng Hongyuan Flagship Store	Electricity savings1	Original and authentic guarantee quality is good now.	Jingdong prefecture level city in Shandong
Jinfeng Hongyuan Flagship Store	Electricity savings1	Great, very happy with it, will buy a couple more next time	Jingdong prefecture level city in Shandong
Jinfeng Hongyuan Flagship Store	Electricity savings1	I just used it, I don't know if it works yet, I'll come back after using it...	Jingdong prefecture level city in Shandong
Jinfeng Hongyuan Flagship Store	Electricity savings1	It's still being tested.	Jingdong prefecture level city in Shandong
Jinfeng Hongyuan Flagship Store	Electricity savings1	I've been using it for a month and it's not working.	Jingdong prefecture level city in Shandong
Jinfeng Hongyuan Flagship Store	Electricity savings1	It's basically nothing!	Jingdong prefecture level city in Shandong
Jinfeng Hongyuan Flagship Store	Electricity savings1	It's not bad, it kind of works, support it.	Jingdong prefecture level city in Shandong
Jinfeng Hongyuan Flagship Store	Electricity savings1	I just received it last night, let's try it first to see how it works, it looks good!	Jingdong prefecture level city in Shandong
Jinfeng Hongyuan Flagship Store	Electricity savings1	The current is very stable, fine workmanship, small and exquisite, very satisfied	Jingdong prefecture level city in Shandong

Jinfeng Hongyuan Flagship Store	Electricity savings1	Not bad, exactly how much electricity can be saved; still have to follow up the evaluation	Jingdong prefecture level city in Shandong
Jinfeng Hongyuan Flagship Store	Electricity savings1	Logistics is also very fast, next time there is an opportunity to introduce friends to buy!	Jingdong prefecture level city in Shandong
Jinfeng Hongyuan Flagship Store	Electricity savings1	Voltage display and actual do not pay	Jingdong prefecture level city in Shandong
Jinfeng Hongyuan Flagship Store	Electricity savings1	Second purchase, still saves a little on the electric bill	Jingdong prefecture level city in Shandong
Jinfeng Hongyuan Flagship Store	Electricity savings1	Order received, currently trying it out, thanks a lot.	Jingdong prefecture level city in Shandong
Jinfeng Hongyuan Flagship Store	Electricity savings1	I haven't used it for a long time yet, I don't know if the effect is good or not, looking forward to it	Jingdong prefecture level city in Shandong
Jinfeng Hongyuan Flagship Store	Electricity savings1	Good, working, I just don't know if it will save electricity... 5 stars for now.	Jingdong prefecture level city in Shandong
Jinfeng Hongyuan Flagship Store	Electricity savings1	Great product, just as the owner said it would be.	Jingdong prefecture level city in Shandong
Jinfeng Hongyuan Flagship Store	Electricity savings1	Shipping was fast, I don't know if it works well or not, I have to use it for a while first.	Jingdong prefecture level city in Shandong
Jinfeng Hongyuan Flagship Store	Electricity savings1	I don't feel that it saves much electricity, but I'll give it a try. It's been more than a month now, and the electricity bill is still a bit high.	Jingdong prefecture level city in Shandong
Jinfeng Hongyuan Flagship Store	Electricity savings1	Got it. Just used it.	Jingdong prefecture level city in Shandong
Jinfeng Hongyuan Flagship Store	Electricity savings1	Use it for a while and then comment.	Jingdong prefecture level city in Shandong
Jinfeng Hongyuan Flagship Store	Electricity savings1	Used it, see how it works with it and then comment. The quality is very good!	Jingdong prefecture level city in Shandong
Jinfeng Hongyuan Flagship Store	Electricity savings1	I'll comment again after a while.	Jingdong prefecture level city in Shandong
Jinfeng Hongyuan Flagship Store	Electricity savings1	It's easy to operate, affordable and works well, love it.	Jingdong prefecture level city in Shandong
Jinfeng Hongyuan Flagship Store	Electricity savings1	It's good. You can try it. It's cheap.	Jingdong prefecture level city in Shandong
Jinfeng Hongyuan Flagship Store	Electricity savings1	It's in use, so we'll see how it works after a while!	Jingdong prefecture level city in Shandong
Jinfeng Hongyuan Flagship Store	Electricity savings1	Service is pretty good, not sure about the quality yet.	Jingdong prefecture level city in Shandong
Jinfeng Hongyuan Flagship Store	Electricity savings1	Just used it and will see the results in a month ☺	Jingdong prefecture level city in Shandong
Jinfeng Hongyuan Flagship Store	Electricity savings1	Saving electricity is expensive and good	Jingdong prefecture level city in Shandong
Jinfeng Hongyuan Flagship Store	Electricity savings1	Pretty good, 5 stars! Will buy again next time seller service is very good ！！！！ Good quality! Will recommend to friends!	Jingdong prefecture level city in Shandong
Jinfeng Hongyuan Flagship Store	Electricity savings1	In use, with results naturally have follow-up reviews!	Jingdong prefecture level city in Shandong
Jinfeng Hongyuan Flagship Store	Electricity savings1	I'm using it now, I'll come back with results, it'll work.	Jingdong prefecture level city in Shandong
Jinfeng Hongyuan Flagship Store	Electricity savings1	Good, nice, fast shipping, cheap, worth having!	Jingdong prefecture level city in Shandong
Jinfeng Hongyuan Flagship Store	Electricity savings1	Logistics is fast.	Jingdong prefecture level city in Shandong
Jinfeng Hongyuan Flagship Store	Electricity savings1	I have just started using the product and will be reviewing it later.	Jingdong prefecture level city in Shandong
Jinfeng Hongyuan Flagship Store	Electricity savings1	I'll comment after I use it.	Jingdong prefecture level city in Shandong
Jinfeng Hongyuan Flagship Store	Electricity savings1	I don't know if it's working or not, though I'll try plugging it in.	Jingdong prefecture level city in Shandong
Jinfeng Hongyuan Flagship Store	Electricity savings1	It really saves electricity and contributes to energy savings in disguise	Jingdong prefecture level city in Shandong
Jinfeng Hongyuan Flagship Store	Electricity savings1	It feels good in the experience.	Jingdong prefecture level city in Shandong
Jinfeng Hongyuan Flagship Store	Electricity savings1	The product is better than I thought it would be, the quality looks great, the voltage is stable, and I'm looking forward to some great power savings!	Jingdong prefecture level city in Shandong
Jinfeng Hongyuan Flagship Store	Electricity savings1	I'm using it now looking forward to saving some points,, I'm feeding back	Jingdong prefecture level city in Shandong
Jinfeng Hongyuan Flagship Store	Electricity savings1	Displayed voltage below 200.	Jingdong prefecture level city in Shandong
Jinfeng Hongyuan Flagship Store	Electricity savings1	I haven't seen any great results, but it should last about a hundred or eighty years.	Jingdong prefecture level city in Shandong
Jinfeng Hongyuan Flagship Store	Electricity savings1	I don't know if it works or not, but I keep it plugged in anyway, so I hope it works!	Jingdong prefecture level city in Shandong
Jinfeng Hongyuan Flagship Store	Electricity savings1	Reasonable price, good quality, fast delivery, great service, already a repurchase.	Jingdong prefecture level city in Shandong
Jinfeng Hongyuan Flagship Store	Electricity savings1	Second purchase, looking forward to the results	Jingdong prefecture level city in Shandong
Jinfeng Hongyuan Flagship Store	Electricity savings1	The quality of this product feels like it should work in a use test! Just how many kilowatts of electricity must be wasted per day with the red light and digital display on for long periods of time?	Jingdong prefecture level city in Shandong
Jinfeng Hongyuan Flagship Store	Electricity savings1	It looks good, we'll see if we use it.	Jingdong prefecture level city in Shandong
Jinfeng Hongyuan Flagship Store	Electricity savings1	Great stuff, second purchase!	Jingdong prefecture level city in Shandong
Jinfeng Hongyuan Flagship Store	Electricity savings1	It is indeed very practical and saves electricity at home	Jingdong prefecture level city in Shandong
Jinfeng Hongyuan Flagship Store	Electricity savings1	Goods received, the physical and the same as the picture, logistics is also very fast!	Jingdong prefecture level city in Shandong
Jinfeng Hongyuan Flagship Store	Electricity savings1	I'll use it for a while and see how it goes. I'll buy it again if it's good.	Jingdong prefecture level city in Shandong
Jinfeng Hongyuan Flagship Store	Electricity savings1	In use, voltage monitoring, the effect of how to wait for next month's electricity consumption confirmation	Jingdong prefecture level city in Shandong
Jinfeng Hongyuan Flagship Store	Electricity savings1	It's still pretty good.	Jingdong prefecture level city in Shandong
Jinfeng Hongyuan Flagship Store	Electricity savings1	It's been plugged in for a month and there hasn't been a big change yet, since it usually uses very little electricity	Jingdong prefecture level city in Shandong
Jinfeng Hongyuan Flagship Store	Electricity savings1	Let's wait for a month to see if it works	Jingdong prefecture level city in Shandong
Jinfeng Hongyuan Flagship Store	Electricity savings1	Good, good, good, good, good, good, good, good, good, good, good, good, good, good, good.	Jingdong prefecture level city in Shandong
Jinfeng Hongyuan Flagship Store	Electricity savings1	The product received the trial a few days, feel good, praise!	Jingdong prefecture level city in Shandong
Jinfeng Hongyuan Flagship Store	Electricity savings1	After six or seven hours of use, 250 volts won't save you any power.	Jingdong prefecture level city in Shandong
Jinfeng Hongyuan Flagship Store	Electricity savings1	I haven't used it yet, I wonder if it saves electricity?	Jingdong prefecture level city in Shandong

Jinfeng Hongyuan Flagship Store	Electricity savings1	Baby value for money, love it, next time will come back is genuine, trust the business, baby texture is very good, the price is moderate, unlimited repurchase!	Jingdong prefecture level city in Shandong
Jinfeng Hongyuan Flagship Store	Electricity savings1	Power saving effect is obvious! Raising tropical fish daily power consumption, after using the daily power savings of more than 3 degrees, currently about 8 degrees / day!	Jingdong prefecture level city in Shandong
Jinfeng Hongyuan Flagship Store	Electricity savings1	Just used it, unknown results.	Jingdong prefecture level city in Shandong
Jinfeng Hongyuan Flagship Store	Electricity savings1	The goods received, well, look forward to the effect. Logistics is very fast, thanks to the platform, thanks to Jingdong. I hope it can save electricity.	Jingdong prefecture level city in Shandong
Jinfeng Hongyuan Flagship Store	Electricity savings1	Not sure 有 effect?	Jingdong prefecture level city in Shandong
Jinfeng Hongyuan Flagship Store	Electricity savings1	I'd say it's a 5% savings.	Jingdong prefecture level city in Shandong
Jinfeng Hongyuan Flagship Store	Electricity savings1	I received it and am using it, I don't know how effective it is, I'll evaluate it afterwards	Jingdong prefecture level city in Shandong
Jinfeng Hongyuan Flagship Store	Electricity savings1	Baby received not yet used, first give praise	Jingdong prefecture level city in Shandong
Jinfeng Hongyuan Flagship Store	Electricity savings1	Looks good, I'll evaluate it later	Jingdong prefecture level city in Shandong
Jinfeng Hongyuan Flagship Store	Electricity savings1	The goods have been received in use, the overall feeling is not bad!	Jingdong prefecture level city in Shandong
Jinfeng Hongyuan Flagship Store	Electricity savings1	It's working. This is the second one. This stuff has to be used over a long period of time to be effective. And it only works on inductive loads, i.e. appliances containing electric motors. Use it.	Jingdong prefecture level city in Shandong
Jinfeng Hongyuan Flagship Store	Electricity savings1	Use it for a while and see how it works.	Jingdong prefecture level city in Shandong
Jinfeng Hongyuan Flagship Store	Electricity savings1	Good courier service and the item is very nice and practical.	Jingdong prefecture level city in Shandong
Jinfeng Hongyuan Flagship Store	Electricity savings1	It doesn't do anything and it's a waste of electricity.	Jingdong prefecture level city in Shandong
Jinfeng Hongyuan Flagship Store	Electricity savings1	I haven't used it yet. It should be good.	Jingdong prefecture level city in Shandong
Jinfeng Hongyuan Flagship Store	Electricity savings1	That's good. OK, that's good.	Jingdong prefecture level city in Shandong
Jinfeng Hongyuan Flagship Store	Electricity savings1	It's a rip off, doesn't work, doesn't save electricity, does the job to well, and I've wasted a couple dozen dollars again.	Jingdong prefecture level city in Shandong
Jinfeng Hongyuan Flagship Store	Electricity savings1	Use it first, the effect is not as good as the road, then we will comment again	Jingdong prefecture level city in Shandong
Jinfeng Hongyuan Flagship Store	Electricity savings1	The price is affordable, the logistics is strong, just use four or five days, the effect will be added later!	Jingdong prefecture level city in Shandong
Jinfeng Hongyuan Flagship Store	Electricity savings1	It's easy to use and works fine.	Jingdong prefecture level city in Shandong
Jinfeng Hongyuan Flagship Store	Electricity savings1	It doesn't feel like it's working.	Jingdong prefecture level city in Shandong
Jinfeng Hongyuan Flagship Store	Electricity savings1	I'm using it. We'll see what happens in a couple months.	Jingdong prefecture level city in Shandong
Jinfeng Hongyuan Flagship Store	Electricity savings1	I just got it back to try it out and I'll comment later	Jingdong prefecture level city in Shandong
Jinfeng Hongyuan Flagship Store	Electricity savings1	Less than two months. Can't tell.	Jingdong prefecture level city in Shandong
Jinfeng Hongyuan Flagship Store	Electricity savings1	No power saving effect	Jingdong prefecture level city in Shandong
Jinfeng Hongyuan Flagship Store	Electricity savings1	Express Express Very quickly not only to the power saver can not save electricity, with two months, look at the evaluation put.	Jingdong prefecture level city in Shandong
Jinfeng Hongyuan Flagship Store	Electricity savings1	Genuine products are packaged well!	Jingdong prefecture level city in Shandong
Jinfeng Hongyuan Flagship Store	Electricity savings1	I've been using it for a while, but I don't know if it saves electricity. I hope it works.	Jingdong prefecture level city in Shandong
Jinfeng Hongyuan Flagship Store	Electricity savings1	Good service, fast logistics, the effect is to be used to know	Jingdong prefecture level city in Shandong
Jinfeng Hongyuan Flagship Store	Electricity savings1	Just used it, not sure about the power saving effect	Jingdong prefecture level city in Shandong
Jinfeng Hongyuan Flagship Store	Electricity savings1	The stuff is ok, save power or not, will comment after a month.	Jingdong prefecture level city in Shandong
Jinfeng Hongyuan Flagship Store	Electricity savings1	Used it for a month to save %25, normal is a little over 100, it's great! I want to buy one in.	Jingdong prefecture level city in Shandong
Jinfeng Hongyuan Flagship Store	Electricity savings1	I'm using it and looking forward to the results	Jingdong prefecture level city in Shandong
Jinfeng Hongyuan Flagship Store	Electricity savings1	The quality is very good and the workmanship is exquisite. The use effect is also very good.	Jingdong prefecture level city in Shandong
Jinfeng Hongyuan Flagship Store	Electricity savings1	Merchant service attitude is very good, logistics speed is super fast, online evaluation is very good, the first time to use, a month to know the specific effect, the effect is good, and then additional	Jingdong prefecture level city in Shandong
Jinfeng Hongyuan Flagship Store	Electricity savings1	Works great, solves the problem of high power usage in the house	Jingdong prefecture level city in Shandong
Jinfeng Hongyuan Flagship Store	Electricity savings1	To receive! Very delicate! It's not clear how effective it is, I won't know until I use it!!!!	Jingdong prefecture level city in Shandong
Jinfeng Hongyuan Flagship Store	Electricity savings1	I've just been using it for a while and I don't feel like it's too noticeable.	Jingdong prefecture level city in Shandong
Jinfeng Hongyuan Flagship Store	Electricity savings1	Already used, no visible effect for the time being	Jingdong prefecture level city in Shandong
Jinfeng Hongyuan Flagship Store	Electricity savings1	I can't feel the effect.	Jingdong prefecture level city in Shandong
Jinfeng Hongyuan Flagship Store	Electricity savings1	Use it for a while and see the results	Jingdong prefecture level city in Shandong
Jinfeng Hongyuan Flagship Store	Electricity savings1	The price is more reasonable, the power-saving effect is to be verified, the delivery is fast, and the courier service is good.	Jingdong prefecture level city in Shandong
Jinfeng Hongyuan Flagship Store	Electricity savings1	Quality is very good, consistent with the seller's description, very satisfied, very like, very fast shipping, packaging is very careful and strict, the logistics company has a very good service attitude, fast delivery, very satisfied with a shopping, very satisfied. Recommended to buy, cost-effective, the service attitude of the business is very enthusiastic, providing such high quality goods.	Jingdong prefecture level city in Shandong
Jinfeng Hongyuan Flagship Store	Electricity savings1	I don't know.	Jingdong prefecture level city in Shandong
Jinfeng Hongyuan Flagship Store	Electricity savings1	I didn't get the delivery.	Jingdong prefecture level city in Shandong
Jinfeng Hongyuan Flagship Store	Electricity savings1	It works, it does have power savings, better than expected, will repurchase.	Jingdong prefecture level city in Shandong
Jinfeng Hongyuan Flagship Store	Electricity savings1	That's where the five fuses are.	Jingdong prefecture level city in Shandong
Jinfeng Hongyuan Flagship Store	Electricity savings1	Don't buy it, it doesn't work and you can't return it.	Jingdong prefecture level city in Shandong
Jinfeng Hongyuan Flagship Store	Electricity savings1	I hope it works. Wait a month.	Jingdong prefecture level city in Shandong
Jinfeng Hongyuan Flagship Store	Electricity savings1	Nothing**, buy with caution	Jingdong prefecture level city in Shandong

Jinfeng Hongyuan Flagship Store	Electricity savings1	I'm using it and waiting for the results	Jingdong prefecture level city in Shandong
Jinfeng Hongyuan Flagship Store	Electricity savings1	Saver quality is very good, has always felt that the home power consumption is very large, this is the company's electrician recommended, said he bought this home is really able to save a little electricity, I also came to buy, the effect if it is good, then buy, a floor a...	Jingdong prefecture level city in Shandong
Jinfeng Hongyuan Flagship Store	Electricity savings1	Good product, satisfied with purchase	Jingdong prefecture level city in Shandong
Jinfeng Hongyuan Flagship Store	Electricity savings1	Occupying the time has not yet found the power saving, occupying not evaluation	Jingdong prefecture level city in Shandong
Jinfeng Hongyuan Flagship Store	Electricity savings1	Haven't tested the results yet. To be followed up with comments!	Jingdong prefecture level city in Shandong
Jinfeng Hongyuan Flagship Store	Electricity savings1	After experiencing it for a month, I don't seem to know how to save it, and I don't pay attention to it anyway	Jingdong prefecture level city in Shandong
Jinfeng Hongyuan Flagship Store	Electricity savings1	It's useless. It's a waste of electricity.	Jingdong prefecture level city in Shandong
Jinfeng Hongyuan Flagship Store	Electricity savings1	I don't know if it's a power saver. It's a couple bucks. I hope it works.	Jingdong prefecture level city in Shandong
Jinfeng Hongyuan Flagship Store	Electricity savings1	I don't know what to say about this stuff. I don't know if it works or not, it's kind of a psychological purchase. Dormitory use to see the meter can not be not so good-looking. You have to wait until the time to deduct the utility bill to know.	Jingdong prefecture level city in Shandong
Jinfeng Hongyuan Flagship Store	Electricity savings1	Energy savers don't work / they're all a bunch of hoaxes.	Jingdong prefecture level city in Shandong
Jinfeng Hongyuan Flagship Store	Electricity savings1	Received the goods, used for more than a week, it's okay, sure a little effect	Jingdong prefecture level city in Shandong
Jinfeng Hongyuan Flagship Store	Electricity savings1	Well made and compact! Looking forward to the effect!	Jingdong prefecture level city in Shandong
Jinfeng Hongyuan Flagship Store	Electricity savings1	It has arrived and we'll see how it works.	Jingdong prefecture level city in Shandong
Jinfeng Hongyuan Flagship Store	Electricity savings1	Good. Good. It's worth it.	Jingdong prefecture level city in Shandong
Jinfeng Hongyuan Flagship Store	Electricity savings1	How come there's no indicator light?	Jingdong prefecture level city in Shandong
Jinfeng Hongyuan Flagship Store	Electricity savings1	The machine that uses electricity works quite well. This saves	Jingdong prefecture level city in Shandong
Jinfeng Hongyuan Flagship Store	Electricity savings1	It's good for home use and feels quite energy efficient	Jingdong prefecture level city in Shandong
Jinfeng Hongyuan Flagship Store	Electricity savings1	I regret buying this product, after using it for two days it doesn't work at all, still so many kilowatts per day just like the original.	Jingdong prefecture level city in Shandong
Jinfeng Hongyuan Flagship Store	Electricity savings1	Works very well and has some scientific merit.	Jingdong prefecture level city in Shandong
Jinfeng Hongyuan Flagship Store	Electricity savings1	Logistics is also very fast this good power saving I use two months before commenting!	Jingdong prefecture level city in Shandong
Jinfeng Hongyuan Flagship Store	Electricity savings1	Repeat customer. Very good.	Jingdong prefecture level city in Shandong
Jinfeng Hongyuan Flagship Store	Electricity savings1	It's good stuff. I've been using it for a month now and it's a real power saver.	Jingdong prefecture level city in Shandong
Jinfeng Hongyuan Flagship Store	Electricity savings1	Jingdong platform to buy things, has been very assured, quality assurance. Jingdong Express is as good as ever, complete packaging, delivery, service attitude to go! The family has always wanted to buy an energy-saving and power-saving equipment, selected half a day to choose this one, the value is very good, used for a period of time to follow up, to see whether it can achieve the real purpose of power saving, I	Jingdong prefecture level city in Shandong
Jinfeng Hongyuan Flagship Store	Electricity savings1	Works great, inexpensive and will be back next time.	Jingdong prefecture level city in Shandong
Jinfeng Hongyuan Flagship Store	Electricity savings1	Baby received, looks good, wait for a month to see how it works?	Jingdong prefecture level city in Shandong
Jinfeng Hongyuan Flagship Store	Electricity savings1	This product is not bad quite good well customer service said quite power saving that look at the customer service to see if the accuracy of the look at all aspects of the color is also quite beautiful	Jingdong prefecture level city in Shandong
Jinfeng Hongyuan Flagship Store	Electricity savings1	Just got back and tried it feels pretty good, just don't know how the quality is, give a good comment first	Jingdong prefecture level city in Shandong
Jinfeng Hongyuan Flagship Store	Electricity savings1	Received the product, trying it out, hope it helps!	Jingdong prefecture level city in Shandong
Jinfeng Hongyuan Flagship Store	Electricity savings1	Works great, saves electricity a dollar a day and a night	Jingdong prefecture level city in Shandong
Jinfeng Hongyuan Flagship Store	Electricity savings1	Baby received, look at the workmanship is still very fine, is genuine. I hope that this summer to open the air conditioning electricity bill is no longer high scary!	Jingdong prefecture level city in Shandong
Jinfeng Hongyuan Flagship Store	Electricity savings1	It's here. I don't know how it's going to work, but I'm going to use it.	Jingdong prefecture level city in Shandong
Jinfeng Hongyuan Flagship Store	Electricity savings1	Power saver received, Jingdong logistics first-class, business customer service attitude is good, one by one to introduce the product performance, back on the horse plugged in, it is very simple, there is a voltage indicator, to be a few months to see how the effect of power-saving to come back to a good follow-up, but I believe that Jinfenghong products.	Jingdong prefecture level city in Shandong
Jinfeng Hongyuan Flagship Store	Electricity savings1	Took the third one...the super power-saving kind!	Jingdong prefecture level city in Shandong
Jinfeng Hongyuan Flagship Store	Electricity savings1	First time buyer, customer service is very polite and courteous, conscientious business, support! Baby has received 🎈, super like, high value and practical, super like! ❥❥❥❥❥ Very good quality, very satisfied with a shopping. Customer service has a question and answer, very professional finally received the goods, very, very much like, exquisite and beautiful workmanship, has been praised by many people, really too happy, is already a loyal fan of his family, will continue to buy buy buy buy!	Jingdong prefecture level city in Shandong
Jinfeng Hongyuan Flagship Store	Electricity savings1	That's good. That's good. That's really good.	Jingdong prefecture level city in Shandong
Jinfeng Hongyuan Flagship Store	Electricity savings1	Saver received, good-looking design, voltage at a glance, and the merchant description, save some about 12% or so	Jingdong prefecture level city in Shandong
Jinfeng Hongyuan Flagship Store	Electricity savings1	Intelligent power saver is small and does not take up space, very good, very practical, received and used, packaging is tight, logistics is also very fast, satisfied with a shopping!	Jingdong prefecture level city in Shandong
Jinfeng Hongyuan Flagship Store	Electricity savings1	The stuff is good! Well made, compact, alloy case, shows voltage! Very like! Kudos!	Jingdong prefecture level city in Shandong
Jinfeng Hongyuan Flagship Store	Electricity savings1	I just purchased this power saver from Jingdong, inserted in the socket shows the number 206, a moment and 207 back and forth display, I do not know what it means, my home voltage should be normal, because it is the owner of the community's own power plant to put, first use for a period of time in the positive feedback it, first use in the look at it!	Jingdong prefecture level city in Shandong
Jinfeng Hongyuan Flagship Store	Electricity savings1	Very delicate and compact, there is a convenient intelligent display prompts the voltage, you can know the voltage at a glance, there is also very easy to use, a plug can be used, you can save electricity	Jingdong prefecture level city in Shandong
Jinfeng Hongyuan Flagship Store	Electricity savings1	Logistics is fast, ask customer service said can save electricity, but only use two months to see the effect, use two months in the evaluation.	Jingdong prefecture level city in Shandong
Jinfeng Hongyuan Flagship Store	Electricity savings1	The quality is very good.	Jingdong prefecture level city in Shandong
Jinfeng Hongyuan Flagship Store	Electricity savings1	Smart Saver has been received and will be evaluated after use	Jingdong prefecture level city in Shandong
Jinfeng Hongyuan Flagship Store	Electricity savings1	Home electricity is too waste, a month more than 300 dollars of electricity, and then the family heard that this can save a lot of electricity, let me order to buy a, anyway, will not, buy on the buy, thought it has to be a pretty big box it, delivery to the home to see a very small, plug-and-play, you can really save electricity, it's worth it!	Jingdong prefecture level city in Shandong

Jinfeng Hongyuan Flagship Store	Electricity savings1	Smart Saver received. Unpacking the box is exquisite. The material workmanship is very good. Plugged in and tried the space. Did not open the air conditioning voltage display 206 air conditioning open up the air conditioning display for 194. whether to save electricity to a month later to pay the electricity bill will know. Logistics is also very fast service is also in place.	Jingdong prefecture level city in Shandong
Jinfeng Hongyuan Flagship Store	Electricity savings1	The product feels pretty good and I hope it works, as the store owner said it would. Works!!!	Jingdong prefecture level city in Shandong
Jinfeng Hongyuan Flagship Store	Electricity savings1	Baby received, physical very quickly, just plugged in hope that it can be as everyone said those power saving, with two months after additional comments, first give a five-star praise.	Jingdong prefecture level city in Shandong
Jinfeng Hongyuan Flagship Store	Electricity savings1	Good, very good, very good, you deserve it, whoever uses it knows, the stuff works great, will buy it again in the future.	Jingdong prefecture level city in Shandong
Jinfeng Hongyuan Flagship Store	Electricity savings1	Don't hesitate to buy wow! No cheap feeling, very high-end, especially great! It is genuine, and the same as the entity store, the price is also favorable, decisive turn powder 💗 catch up to do activities when you buy, the price of a penny for a penny 🤍, from the outside looking very beautiful 🤍, the material workmanship is good 💗, feel that the purchase is worth it, and the logistics is super	Jingdong prefecture level city in Shandong
Jinfeng Hongyuan Flagship Store	Electricity savings1	Logistics is fast! Very good quality! Very beautiful! Next time to patronize!	Jingdong prefecture level city in Shandong
Jinfeng Hongyuan Flagship Store	Electricity savings1	Really like it, completely exceeded expectations, the shipping speed is very fast, the packaging is very careful and strict, the delivery speed is very fast, very satisfied with a shopping Baby received, and the description is the same, love it!	Jingdong prefecture level city in Shandong
Jinfeng Hongyuan Flagship Store	Electricity savings1	Very good, if you need to order!	Jingdong prefecture level city in Shandong
Jinfeng Hongyuan Flagship Store	Electricity savings1	So far, plugged in the voltage is more stable, as for the effect of how to quietly wait until the payment of the electricity bill to compare, the appearance of the commodity design is very good, the real thing!	Jingdong prefecture level city in Shandong
Jinfeng Hongyuan Flagship Store	Electricity savings1	📩 I've been wanting it for a long time, I decided to go for it, I feel great after using it, it's genuine and the delivery is great, it lives up to its name👍	Jingdong prefecture level city in Shandong
Jinfeng Hongyuan Flagship Store	Electricity savings1	Looking forward to it!!! I don't know if it's working or not, let's look forward to it!!!! How much is saved ，，，，，，，，， Saving a degree is a degree with long term use ！！！！！！！	Jingdong prefecture level city in Shandong
Jinfeng Hongyuan Flagship Store	Electricity savings1	After using it for a while, I am seriously and responsibly saying that this saver is really very power efficient. Two rooms with the air conditioner on at night for one night, plus watching TV, charging cell phones, lights, etc. etc. only used one degree of electricity. So I bought several more and it's really	Jingdong prefecture level city in Shandong
Jinfeng Hongyuan Flagship Store	Electricity savings1	It arrived quickly, and the box is solid and delicate. Baby shell is thick aluminum and by multi-layer coating. The inner core is heavy, and the power cord and plug are genuine products from a large manufacturer. It is the best power saver I have found after choosing all of them!	Jingdong prefecture level city in Shandong
Jinfeng Hongyuan Flagship Store	Electricity savings1	The saver was received, genuine guarantee, good quality...	Jingdong prefecture level city in Shandong
Jinfeng Hongyuan Flagship Store	Electricity savings1	Logistics was fast! The product is easy to use!	Jingdong prefecture level city in Shandong
Jinfeng Hongyuan Flagship Store	Electricity savings1	Look appearance is okay, use a period of time to know how it is. Jingdong Mall, trustworthy! Home appliances, cell phones and computers are almost 100% bought by Jingdong. Ordered the night before yesterday, received yesterday. Jingdong logistics, the fastest in the network!	Jingdong prefecture level city in Shandong
Jinfeng Hongyuan Flagship Store	Electricity savings1	Very good, perfect packaging, home energy saver to determine the effect of power saving and also play a role in stabilizing the voltage Very good products	Jingdong prefecture level city in Shandong
Jinfeng Hongyuan Flagship Store	Electricity savings1	Smart Saver received, looks very exquisite. The method of use is simple, and it has been used. The later effect, with time to test.	Jingdong prefecture level city in Shandong
Jinfeng Hongyuan Flagship Store	Electricity savings1	Both the material and workmanship exceeded expectations, great value, and looks great on oh. The seller also shipped much earlier than expected, will come back. Buy it, you will not regret it! Full five points. Both the material and workmanship exceeded expectations, great value, wear it also looks good Oh. Seller delivery is also much earlier than expected, will come again. Buy it, will not regret it! Full	Jingdong prefecture level city in Shandong
Jinfeng Hongyuan Flagship Store	Electricity savings1	The appearance is clean and beautiful, the workmanship is very good, I didn't find any defects, I personally feel that the size is also quite suitable, I've used it for 2 months, I didn't find out exactly how energy efficient it is. Try using it again!	Jingdong prefecture level city in Shandong
Jinfeng Hongyuan Flagship Store	Electricity savings1	Power saving device received, product certification, accessories, etc. complete, very good workmanship, very practical power-saving effect is also very obvious.	Jingdong prefecture level city in Shandong
Jinfeng Hongyuan Flagship Store	Electricity savings1	Looked at a lot of think buy this one, see a lot of reviews say it works, a month with save nearly twenty degrees of electricity, there are sixty days of return guarantee, with a good and then come back to the	Jingdong prefecture level city in Shandong
Jinfeng Hongyuan Flagship Store	Electricity savings1	I received the power saving device, and I'm using it. I hope I can get the desired effect, and I'm trying to force it to work quickly.	Jingdong prefecture level city in Shandong
Jinfeng Hongyuan Flagship Store	Electricity savings1	Voltage is stable, already trying it out and waiting for the results to happen, saving electricity will follow up next month!	Jingdong prefecture level city in Shandong
Jinfeng Hongyuan Flagship Store	Electricity savings1	Jingdong Mall self-supporting goods of excellent quality, logistics is also very fast, worth recommending 👏👏👏👏 excellent!	Jingdong prefecture level city in Shandong
Jinfeng Hongyuan Flagship Store	Electricity savings1	Power saving has been received, the appearance of the beautiful, I have already been the second time to buy the use of down the effect is very good!	Jingdong prefecture level city in Shandong
Jinfeng Hongyuan Flagship Store	Electricity savings1	Energy saver received very happy, the color is also very good looking forward to a very good effect can save electricity	Jingdong prefecture level city in Shandong
Jinfeng Hongyuan Flagship Store	Electricity savings1	It's small and easy to plug in and use. It displays the voltage value when plugged in and	Jingdong prefecture level city in Shandong
Jinfeng Hongyuan Flagship Store	Electricity savings1	This saver really works well, because I installed one at home about seven or eight years ago, when there is no voltage display, now there is a voltage display, the appearance also looks much better, the electricity bill is also saved, the stability of the home appliances is also good, the best of both	Jingdong prefecture level city in Shandong
Jinfeng Hongyuan Flagship Store	Electricity savings1	Ordered yesterday, arrived today in use. Shipping was fast. The effect is looking forward to.	Jingdong prefecture level city in Shandong
Jinfeng Hongyuan Flagship Store	Electricity savings1	This thing, I don't know what the principle is. Why it can save electricity. Wait for a month to use it first, see if it will save electricity bill, then come back to evaluate. There is one thing that does not fit the principle ah. The law of conservation of energy.	Jingdong prefecture level city in Shandong
Jinfeng Hongyuan Flagship Store	Electricity savings1	Home mom listen to others say this can save electricity, specially let buy a, later a very large, go back to see a particularly small, directly plugged into the electricity can be, no need to install, the price is also very cheap, the first time with this product, do not know if it is not good to use, temporarily try for a period of time, if it is good to use, recommend that you buy this one, the	Jingdong prefecture level city in Shandong
Jinfeng Hongyuan Flagship Store	Electricity savings1	I've been using it for a few days, and I think the workmanship and materials are pretty good, so we'll have to look at the next bill for the real energy savings, and then we'll add more comments.	Jingdong prefecture level city in Shandong
Jinfeng Hongyuan Flagship Store	Electricity savings1	Energy saver is well made, energy saving and power saving, and anti-counterfeiting code, it is a regular commodity!	Jingdong prefecture level city in Shandong
Jinfeng Hongyuan Flagship Store	Electricity savings1	Jinfeng Hongyuan, buy back home, as a mouse, haha. First of all, normal electricity for 1 month, the power did not increase, the electricity bill feels smoother, year-on-year electricity costs in the low, whether it is very energy-saving still have to be used for a long time to know. All 5 stars ☆	Jingdong prefecture level city in Shandong
Jinfeng Hongyuan Flagship Store	Electricity savings1	The box is well packaged and beautiful! I hope to save electricity with it haha!	Jingdong prefecture level city in Shandong
Jinfeng Hongyuan Flagship Store	Electricity savings1	Ordered last night, arrived this morning, first try the effect before making comments	Jingdong prefecture level city in Shandong
Jinfeng Hongyuan Flagship Store	Electricity savings1	Looks good. Don't know the effect yet.	Jingdong prefecture level city in Shandong
Jinfeng Hongyuan Flagship Store	Electricity savings1	Jinfeng Hongyuan intelligent power saver has exquisite workmanship and modern fashionable design style. It is a good home economizer . I believe that the goods purchased from Jingdong will not disappoint our	Jingdong prefecture level city in Shandong
Jinfeng Hongyuan Flagship Store	Electricity savings1	Inadvertently flipped to this power saver, never heard of, feel very novel, the price is not expensive, so buy to try, see a month after the electricity bill will be less, recently the home air-conditioning open a lot of power consumption is very large.	Jingdong prefecture level city in Shandong
Jinfeng Hongyuan Flagship Store	Electricity savings1	The quality of the goods is very good workmanship, but also sent five fuse	Jingdong prefecture level city in Shandong
Jinfeng Hongyuan Flagship Store	Electricity savings1	I'm trying it out. I don't know if it'll work.	Jingdong prefecture level city in Shandong

Jinfeng Hongyuan Flagship Store	Electricity savings1	There is no effect yet do not know, is to look at the official sale, buy to try it! Use according to the instructions, plugged in, used for a period of time, anyway, do not know how, after a few months to see it, to see if the average month than before will be less electricity.	Jingdong prefecture level city in Shandong
Jinfeng Hongyuan Flagship Store	Electricity savings1	Received the goods, the first time to unpacking, the quality is very good, consistent with the description of the seller, completely exceeded my expectations, the packaging is very careful, strict, in general this time is very satisfied with a shopping, thanks to the seller.	Jingdong prefecture level city in Shandong
Jinfeng Hongyuan Flagship Store	Electricity savings1	The energy saver was received and used for a few days with good results.	Jingdong prefecture level city in Shandong
Jinfeng Hongyuan Flagship Store	Electricity savings1	Previously I have paid attention to this product, but I think that the monthly electricity consumption at home is not large, there is no need to save electricity savings. After a second thought, energy saving is the responsibility of each of our citizens, the winter is coming, this year's winter is cold, heating and all electric appliances, can save is to save, the price is not expensive, so buy to try. I analyze the principle of power saving, should be able to save electricity, but the larger the power saving effect is about good. To be tried after a month or two to see the effect and then evaluate. Jingdong trustworthy.	Jingdong prefecture level city in Shandong
Jinfeng Hongyuan Flagship Store	Electricity savings1	It just arrived and I can't wait to use it, and the customer service is great! There is also voltage showing up which is nice! I don't know if it can save a bit I won't know until I've used it for a month! When the time comes to come back to the follow-up review!	Jingdong prefecture level city in Shandong
Jinfeng Hongyuan Flagship Store	Electricity savings1	Checked out the reviews from numerous users. The shipment is still very large, so I decided to buy it anyway to use it and see the effect after a month. Jingdong Mall here to buy safe, convenient and fast, this price is very powerful 💪, very recommended.	Jingdong prefecture level city in Shandong
Jinfeng Hongyuan Flagship Store	Electricity savings1	Purchased two at the same time to use together. The other one works fine, the other has problems with the numbers not sure if the machine is faulty. Power saving effect can not be observed. Feel the effect is not very obvious like?	Jingdong prefecture level city in Shandong
Jinfeng Hongyuan Flagship Store	Electricity savings1	Looking forward to it!!! I don't know if it's working or not, let's look forward to it!!!! How much is saved ，，，，，，，，， Saving a degree is a degree with long term use ！！！！！！！	Jingdong prefecture level city in Shandong
Jinfeng Hongyuan Flagship Store	Electricity savings1	Received, directly on the use of the special convenient	Jingdong prefecture level city in Shandong
Jinfeng Hongyuan Flagship Store	Electricity savings1	Goods received a few days well-packed, the same day opened and plugged in to try to be normal is not sure what the power-saving effect. After a month with a follow-up evaluation.	Jingdong prefecture level city in Shandong
Jinfeng Hongyuan Flagship Store	Electricity savings1	I bought it and used it, I don't know how it works yet, we'll see later. Use the effect of it	Jingdong prefecture level city in Shandong
Jinfeng Hongyuan Flagship Store	Electricity savings1	Photographed 2, the old family also put a, with a small January, there should be some effect, with a long period of time to comment, with a long time to see the obvious effect, hard courier brother.	Jingdong prefecture level city in Shandong
Jinfeng Hongyuan Flagship Store	Electricity savings1	Experience: I am an electrician, this is the real use of electronic waves automatically connected to track the decomposition of ionized electrons, in situ compensation of reactive power, reduce effective power loss and waste, stabilize the voltage to achieve energy saving and power saving, the overall effect	Jingdong prefecture level city in Shandong
Jinfeng Hongyuan Flagship Store	Electricity savings1	Great looking and practical! Easy to use and save! Saves 15% in a month of comparison	Jingdong prefecture level city in Shandong
Jinfeng Hongyuan Flagship Store	Electricity savings1	Product quality is very good! Packaging is very nice, like a cell phone box, thick and well protected! Service is also great! Logistics is very fast! Thank you for your business! Very honest!	Jingdong prefecture level city in Shandong
Jinfeng Hongyuan Flagship Store	Electricity savings1	In several products inside the choice of this purchase for a period of time, but also need to use a few months to compare last year's electricity bill before you can analyze the savings in electricity, to the summer peak electricity consumption should be more obvious.	Jingdong prefecture level city in Shandong
Jinfeng Hongyuan Flagship Store	Electricity savings1	The goods have long been received, after more than a month to comment, the home electricity bill is indeed reduced, worth buying!	Jingdong prefecture level city in Shandong
Jinfeng Hongyuan Flagship Store	Electricity savings1	Baby received, the logistics is strong, the quality is reliable, it is worth having and recommending!	Jingdong prefecture level city in Shandong
Jinfeng Hongyuan Flagship Store	Electricity savings1	Using a conservation appliance, you can achieve a 10-15% savings in electricity usage.	Jingdong prefecture level city in Shandong
Jinfeng Hongyuan Flagship Store	Electricity savings1	Quality is very good, metal shell, touch is very good, very recommended Oh ~, the subsequent effect of continuous follow-up	Jingdong prefecture level city in Shandong
Jinfeng Hongyuan Flagship Store	Electricity savings1	The product is beautiful, practical, worth buying. The product is beautiful, practical, good quality, fast logistics product is beautiful, practical, beautiful and elegant, very classy, worth buying!	Jingdong prefecture level city in Shandong
Jinfeng Hongyuan Flagship Store	Electricity savings1	Just received the item and have already used it. Didn't know much about these products before. I hope it is as effective and useful as the product advertises.	Jingdong prefecture level city in Shandong
Jinfeng Hongyuan Flagship Store	Electricity savings1	A month two people used more than five hundred degrees of electricity, in a hurry and then add a power saver, I hope to have an effect, Jingdong shopping is very cool, logistics is very fast especially good service attitude delivery, this hot day is not easy, thank you!	Jingdong prefecture level city in Shandong
Jinfeng Hongyuan Flagship Store	Electricity savings1	Just received the baby, first plugged in to try to use a month and then additional evaluation it	Jingdong prefecture level city in Shandong
Jinfeng Hongyuan Flagship Store	Electricity savings1	Goods fast, the power saver has been received, a friend recommended to buy that can save electricity. Exquisite and compact is very convenient, plug and play. The effect is good, the price is affordable, the next need will patronize again!	Jingdong prefecture level city in Shandong
Jinfeng Hongyuan Flagship Store	Electricity savings1	Packaging is better than expected, sent a few fuse, power saving effect in the end do not know, try a period of time to say!	Jingdong prefecture level city in Shandong
Jinfeng Hongyuan Flagship Store	Electricity savings1	Shipped quickly, immediately installed, read countless reviews were not sure if this thing is effective, was going to buy three at once, ultimately decided to first use a try the effect, if the power ring significantly reduced, then will buy a few more!	Jingdong prefecture level city in Shandong
Jinfeng Hongyuan Flagship Store	Electricity savings1	Used for a period of time to evaluate, not to a month still do not know whether it can save electricity, the voltage has a display!	Jingdong prefecture level city in Shandong
Jinfeng Hongyuan Flagship Store	Electricity savings1	I don't know if it saves electricity or not, but I'm using it anyway, so I hope it saves a little bit, electricity is too expensive	Jingdong prefecture level city in Shandong
Jinfeng Hongyuan Flagship Store	Electricity savings1	The power saver has been received and I am very happy with it! Just waiting to see how the power saving works!	Jingdong prefecture level city in Shandong
Jinfeng Hongyuan Flagship Store	Electricity savings1	Saving power has been received, logistics is quite fast, the quality is not bad, small and a little weight, has been used to look forward to the effect of	Jingdong prefecture level city in Shandong
Jinfeng Hongyuan Flagship Store	Electricity savings1	The logistics is very fast, the effect is obvious and trustworthy.	Jingdong prefecture level city in Shandong
Jinfeng Hongyuan Flagship Store	Electricity savings1	Good quality, good quality, beautiful, beautiful, beautiful, beautiful, beautiful, very worth buying, worth buying, worth buying, worth buying, worth customizing!	Jingdong prefecture level city in Shandong
Jinfeng Hongyuan Flagship Store	Electricity savings1	I've used it for now, I don't know if it saves electricity	Jingdong prefecture level city in Shandong
Jinfeng Hongyuan Flagship Store	Electricity savings1	Bought two at a time, logistics is very fast, the product is beautifully packaged, after two months to see the results	Jingdong prefecture level city in Shandong
Jinfeng Hongyuan Flagship Store	Electricity savings1	The workmanship is really fine, the packaging is also good, the regular factory production ~ use a break in the evaluation of the effectiveness of the	Jingdong prefecture level city in Shandong
Jinfeng Hongyuan Flagship Store	Electricity savings1	Sometimes 219. Sometimes 220 in this case	Jingdong prefecture level city in Shandong
Jinfeng Hongyuan Flagship Store	Electricity savings1	It is the ideal product, good quality, logistics is also very fast, worthy of praise!	Jingdong prefecture level city in Shandong
Jinfeng Hongyuan Flagship Store	Electricity savings1	Store service is very good, the courier received the next day. Product packaging is very good, just use the quality to be tested.	Jingdong prefecture level city in Shandong
Jinfeng Hongyuan Flagship Store	Electricity savings1	I've been using it for almost a month now and I feel like it's working.	Jingdong prefecture level city in Shandong
Jinfeng Hongyuan Flagship Store	Electricity savings1	Very satisfied, very restful, very pretty, very beautiful, very much enjoyed! Well worth it!	Jingdong prefecture level city in Shandong
Jinfeng Hongyuan Flagship Store	Electricity savings1	Baby especially like, the value of high-quality good, a very good shopping experience, attentive service, fast logistics, distribution is also very thoughtful, attitude is also very good, value for money ah, decisive praise! Quality is quite good, logistics is also very fast, praise!	Jingdong prefecture level city in Shandong
Jinfeng Hongyuan Flagship Store	Electricity savings1	The shape of the item is basically the same as ordered, the effect is to be examined.	Jingdong prefecture level city in Shandong

Jinfeng Hongyuan Flagship Store	Electricity savings1	Appearance style: good-looking workmanship material: very good, a small one, very delicate Experience: still in use, in the process of using the shopkeeper is very nice to have a careful explanation Use for a period of time in the look at the results	Jingdong prefecture level city in Shandong
Jinfeng Hongyuan Flagship Store	Electricity savings1	As described, compact and well packaged	Jingdong prefecture level city in Shandong
Jinfeng Hongyuan Flagship Store	Electricity savings1	Fine workmanship. Test in progress. Let's see how it works.	Jingdong prefecture level city in Shandong
Jinfeng Hongyuan Flagship Store	Electricity savings1	The goods have been received, I'm very satisfied and love it	Jingdong prefecture level city in Shandong
Jinfeng Hongyuan Flagship Store	Electricity savings1	Safety! Whether you can save electricity, you can roughly feel it in 2 months. The principle is simple. Before the electrical appliances and circuits inductive more, now the electrical appliances integrated, the home is not a lot of inductive circuits, but used certainly save no problem! Wiring according to the requirements of the manual is good, if it is not convenient, see the socket plug.	Jingdong prefecture level city in Shandong
Jinfeng Hongyuan Flagship Store	Electricity savings1	Great product, looking forward to how much power I can save, first time buyer!	Jingdong prefecture level city in Shandong
Jinfeng Hongyuan Flagship Store	Electricity savings1	The product is exquisite, beautifully packaged, and the customer service is excellent. Just waiting to see how the power saving effect is, looking forward to it!	Jingdong prefecture level city in Shandong
Jinfeng Hongyuan Flagship Store	Electricity savings1	Express very quickly very satisfied with the first use to see how the effect to know, if you can save 10 percent even if acceptable, if you can not meet this requirement is obviously not much effect. Obviously.	Jingdong prefecture level city in Shandong
Jinfeng Hongyuan Flagship Store	Electricity savings1	Very delicate and compact, there is a very convenient intelligent display prompts the voltage, you can know the voltage at a glance, there is very easy to use, a plug can be used, I hope to save money on	Jingdong prefecture level city in Shandong
Jinfeng Hongyuan Flagship Store	Electricity savings1	First time buyer is it really save after use	Jingdong prefecture level city in Shandong
Jinfeng Hongyuan Flagship Store	Electricity savings1	Purchased again and will be for a long time yet.	Jingdong prefecture level city in Shandong
Jinfeng Hongyuan Flagship Store	Electricity savings1	Bought it to stock up, came back and plugged it in, hope it works	Jingdong prefecture level city in Shandong
Jinfeng Hongyuan Flagship Store	Electricity savings1	Baby received the logistics very fast very like very satisfied praise a	Jingdong prefecture level city in Shandong
Jinfeng Hongyuan Flagship Store	Electricity savings1	Beginning the process of using, easy to evaluate more later.	Jingdong prefecture level city in Shandong
Jinfeng Hongyuan Flagship Store	Electricity savings1	The goods have been received, just do not know how effective	Jingdong prefecture level city in Shandong
Jinfeng Hongyuan Flagship Store	Electricity savings1	It feels okay. I wish there were more events.	Jingdong prefecture level city in Shandong
Jinfeng Hongyuan Flagship Store	Electricity savings1	Things have been received, just plugged in, I do not know if it works, wait a few days to have the effect of additional	Jingdong prefecture level city in Shandong
Jinfeng Hongyuan Flagship Store	Electricity savings1	The effect of this power saver is really good, the house more, a few air conditioners are open every day, not used before a month to say less than seven hundred eight hundred, used after three months only nine	Jingdong prefecture level city in Shandong
Jinfeng Hongyuan Flagship Store	Electricity savings1	Cheap, good and easy to use, recommend it.	Jingdong prefecture level city in Shandong
Jinfeng Hongyuan Flagship Store	Electricity savings1	Bought this device, to the home for a long time, wait to finish a month to see how, good can recommend friends to buy, we use the electricity every month scary, a month of electricity costs more than a thousand, can not afford to hurt ah! I hope this power saving can save me some money, the beginning of the shoot order did not pay attention to look at the address, sent elsewhere to go, that side called to find out, hurry to communicate with customer service, customer service is very careful to help me get it done, call to that side to help me turn around, thank you!	Jingdong prefecture level city in Shandong
Jinfeng Hongyuan Flagship Store	Electricity savings1	I don't know the effect if it, use it first to see.	Jingdong prefecture level city in Shandong
Jinfeng Hongyuan Flagship Store	Electricity savings1	Looks good. I won't know if it saves power until I use it.	Jingdong prefecture level city in Shandong
Jinfeng Hongyuan Flagship Store	Electricity savings1	Goods received, shipping speed is very fast, yesterday afternoon more than two o'clock shot, just express delivery, but also sent a few insurance, very good business, the display of the number do not understand what is the meaning of the display said voltage, do not understand this, try a month to see how, good and	Jingdong prefecture level city in Shandong
Jinfeng Hongyuan Flagship Store	Electricity savings1	What ** play also, save electricity more and more save more and more, save chicken hair electricity, are cheating, this is a night of air conditioning with electricity, a night with 11 electricity word, this is	Jingdong prefecture level city in Shandong
Jinfeng Hongyuan Flagship Store	Electricity savings1	Received the product, hope it works!	Jingdong prefecture level city in Shandong
Jinfeng Hongyuan Flagship Store	Electricity savings1	Bought home and used very convenient, easy to operate, quite good	Jingdong prefecture level city in Shandong
Jinfeng Hongyuan Flagship Store	Electricity savings1	It's really good, cost-effective and good quality, and the logistics are fast! It arrived in one day! Really good, cost-effective and good quality, logistics is fast ah! It arrived in one day!	Jingdong prefecture level city in Shandong
Jinfeng Hongyuan Flagship Store	Electricity savings1	Works well, stabilizes voltage and saves power	Jingdong prefecture level city in Shandong
Jinfeng Hongyuan Flagship Store	Electricity savings1	I'll use it right away and look forward to good results	Jingdong prefecture level city in Shandong
Jinfeng Hongyuan Flagship Store	Electricity savings1	Recommended by a friend, said it works well, so I bought one, the effect to be tested	Jingdong prefecture level city in Shandong
Jinfeng Hongyuan Flagship Store	Electricity savings1	I didn't know it would save electricity.	Jingdong prefecture level city in Shandong
Jinfeng Hongyuan Flagship Store	Electricity savings1	I've been using it for a week, I don't know what's going on yet I hope it works well and saves power I'll follow up after a month of use	Jingdong prefecture level city in Shandong
Jinfeng Hongyuan Flagship Store	Electricity savings1	Very well packaged, looks great, don't know how it works	Jingdong prefecture level city in Shandong
Jinfeng Hongyuan Flagship Store	Electricity savings1	Genuine, good quality, really saves energy.	Jingdong prefecture level city in Shandong
Jinfeng Hongyuan Flagship Store	Electricity savings1	I was worried that the stuff is not good, but when I got it, I realized that I was completely over-anxious, 5 stars!	Jingdong prefecture level city in Shandong
Jinfeng Hongyuan Flagship Store	Electricity savings1	Very good and satisfied trustworthy	Jingdong prefecture level city in Shandong
Jinfeng Hongyuan Flagship Store	Electricity savings1	I don't know if it saves power	Jingdong prefecture level city in Shandong
Jinfeng Hongyuan Flagship Store	Electricity savings1	The quality of the product is very good, beautiful and generous applicable fast delivery speed good service attitude	Jingdong prefecture level city in Shandong
Jinfeng Hongyuan Flagship Store	Electricity savings1	Let's use it for a while and then we'll follow up	Jingdong prefecture level city in Shandong
Jinfeng Hongyuan Flagship Store	Electricity savings1	I've only been using it for a month, I don't know if there's any change yet, I'll come back with a follow-up comment after checking the meter!	Jingdong prefecture level city in Shandong
Jinfeng Hongyuan Flagship Store	Electricity savings1	I don't know if it works, I read the instructions and online reviews, I hope I won't be disappointed, if I use it, I will buy it again. Jingdong Express, fast and guaranteed service.	Jingdong prefecture level city in Shandong
Jinfeng Hongyuan Flagship Store	Electricity savings1	It feels like it saves quite a bit huh?	Jingdong prefecture level city in Shandong
Jinfeng Hongyuan Flagship Store	Electricity savings1	I've been using it for a while now, and it still seems to work and have a power saving effect	Jingdong prefecture level city in Shandong
Jinfeng Hongyuan Flagship Store	Electricity savings1	Looks good. It says 235. The 222 bar voltage is shown as high. I don't know if that's right. We'll see.	Jingdong prefecture level city in Shandong
Jinfeng Hongyuan Flagship Store	Electricity savings1	Baby is really good, do really careful, worth to have, fast delivery!	Jingdong prefecture level city in Shandong
Jinfeng Hongyuan Flagship Store	Electricity savings1	Quite delicate and compact, simple and convenient to use. Things are still very good, bought two at a time. Very satisfied with an online purchase.	Jingdong prefecture level city in Shandong
Jinfeng Hongyuan Flagship Store	Electricity savings1	Feel very good, the use of unclear, ask customer service, timely explanation, big brand, trustworthy	Jingdong prefecture level city in Shandong

Jinfeng Hongyuan Flagship Store	Electricity savings1	Thrifty received a few days did not make a comment is to see how the quality of the product now feel the quality of good appearance is also powerful seller service is very good to buy the product is also attached to the 5 fuse thought very thoughtful!	Jingdong prefecture level city in Shandong
Jinfeng Hongyuan Flagship Store	Electricity savings1	Big brand, with a very assured, the quality is very good, in time for the event to buy very cost-effective, customer service is really very good Oh ~!	Jingdong prefecture level city in Shandong
Jinfeng Hongyuan Flagship Store	Electricity savings1	I ordered yesterday and received it this morning, I heard my friend say that it can save electricity, I don't know if it works, I'll come back to make a comment after using it.	Jingdong prefecture level city in Shandong
Jinfeng Hongyuan Flagship Store	Electricity savings1	I've been using it for over a month and it doesn't seem to be working!	Jingdong prefecture level city in Shandong
Jinfeng Hongyuan Flagship Store	Electricity savings1	Used it, the family has always been in the habit of using energy-saving devices, this is a new one. Digital display voltage is very good, can see that the voltage is more stable.	Jingdong prefecture level city in Shandong
Jinfeng Hongyuan Flagship Store	Electricity savings1	I haven't used it for more than a month, but it looks good. It's easy to plug into an outlet and it works.	Jingdong prefecture level city in Shandong
Jinfeng Hongyuan Flagship Store	Electricity savings1	Good Oh...appearance is very beautiful...just plugged in with...see the effect of it...with a period of time in the additional evaluation...	Jingdong prefecture level city in Shandong
Jinfeng Hongyuan Flagship Store	Electricity savings1	I've received the goods, whether it's good or not, I'll know after I use it.	Jingdong prefecture level city in Shandong
Jinfeng Hongyuan Flagship Store	Electricity savings1	Very good, logistics is fast, the stuff is very powerful and cost-effective! Can feel the meter slowing down	Jingdong prefecture level city in Shandong
Jinfeng Hongyuan Flagship Store	Electricity savings1	Logistics is very fast, get it immediately after use, easy to operate, plug in the power supply can be used, just do not know to the use of the effect of how.	Jingdong prefecture level city in Shandong
Jinfeng Hongyuan Flagship Store	Electricity savings1	Quality is very over the top, quality is guaranteed, quite a good choice, a lot of friends to see me use all want to buy, I recommend it!	Jingdong prefecture level city in Shandong
Jinfeng Hongyuan Flagship Store	Electricity savings1	The appearance is clean and beautiful, the workmanship is very good, did not find any flaws, I personally feel that the size is also quite suitable, looking for a long time to choose this one, big love! The price is very beautiful, things are still good, will continue to buy back! All five points! Ness Ness ~ logistics speed is really no words, is a word: fast. Commodities from the outer packaging is very high-grade, really a penny a penny, expensive has the reason for expensive	Jingdong prefecture level city in Shandong
Jinfeng Hongyuan Flagship Store	Electricity savings1	The logistics is very fast, the texture is very good, genuine, I like it very much.	Jingdong prefecture level city in Shandong
Jinfeng Hongyuan Flagship Store	Electricity savings1	It's been a few days since I put it on, I can't see any results now, waiting for a month to say, steady steady steady.	Jingdong prefecture level city in Shandong
Jinfeng Hongyuan Flagship Store	Electricity savings1	I've been using it for a while now and it should be fine, so I'm buying it for the second time.	Jingdong prefecture level city in Shandong
Jinfeng Hongyuan Flagship Store	Electricity savings1	Power Saving King has been received, looks good, quite delicate, looking forward to using it to save electricity	Jingdong prefecture level city in Shandong
Jinfeng Hongyuan Flagship Store	Electricity savings1	My mom said the home uses a little too much electricity, so I bought a Power Saver to try it out, I don't know how effective it is. Expect it to be a good thing!	Jingdong prefecture level city in Shandong
Jinfeng Hongyuan Flagship Store	Electricity savings1	Thank you Jingdong, logistics is very fast order to receipt only one day. Believe in Jingdong. Favorable comments	Jingdong prefecture level city in Shandong
Jinfeng Hongyuan Flagship Store	Electricity savings1	Saved some money on the electric bill compared to last month, watch this month again, no problems so far	Jingdong prefecture level city in Shandong
Jinfeng Hongyuan Flagship Store	Electricity savings1	Energy saver exquisite workmanship, beautiful style, more features, just bought, power saving effect to be used for a period of time, then evaluate!	Jingdong prefecture level city in Shandong
Jinfeng Hongyuan Flagship Store	Electricity savings1	This thing does save power is more compact to hang on the wall.	Jingdong prefecture level city in Shandong
Jinfeng Hongyuan Flagship Store	Electricity savings1	I've been buying it for a while now, I haven't actually measured it, but it still feels like it's effective	Jingdong prefecture level city in Shandong
Jinfeng Hongyuan Flagship Store	Electricity savings1	I've already used it since I bought it, I don't know how effective it is yet, I'll comment on it later.	Jingdong prefecture level city in Shandong
Jinfeng Hongyuan Flagship Store	Electricity savings1	Baby received, careful appearance, logistics is very fast, worth buying!	Jingdong prefecture level city in Shandong
Jinfeng Hongyuan Flagship Store	Electricity savings1	It's great, it's just not the same in hand, I bought it at that other cheaper online one for about the same price as this one. But then oops, doesn't work at all.	Jingdong prefecture level city in Shandong
Jinfeng Hongyuan Flagship Store	Electricity savings1	The product is exquisite, but you have to use it for a month to know how it works. I'll comment again after a month.	Jingdong prefecture level city in Shandong
Jinfeng Hongyuan Flagship Store	Electricity savings1	I've been using it for a while now, and it doesn't feel like it's working, and it doesn't save any power.	Jingdong prefecture level city in Shandong
Jinfeng Hongyuan Flagship Store	Electricity savings1	Affordable price, fast delivery, Jingdong self-supporting guarantee	Jingdong prefecture level city in Shandong
Jinfeng Hongyuan Flagship Store	Electricity savings1	Packaging is very good, no damage, good quality, fine workmanship Packaging is very good, no damage, good quality, fine workmanship Packaging is very good, no damage, good quality, fine workmanship Packaging is very good, no damage, good quality, fine workmanship	Jingdong prefecture level city in Shandong
Jinfeng Hongyuan Flagship Store	Electricity savings1	Big brand, with a very assured, the quality is very good, in time for the event to buy very cost-effective, customer service is really very good Oh ~!	Jingdong prefecture level city in Shandong
Jinfeng Hongyuan Flagship Store	Electricity savings1	Used a few days, the specific effect is not yet clear, looking forward to the effect as described by the merchant!	Jingdong prefecture level city in Shandong
Jinfeng Hongyuan Flagship Store	Electricity savings1	I just used it and don't know how it works, I'll follow up after using it!	Jingdong prefecture level city in Shandong
Jinfeng Hongyuan Flagship Store	Electricity savings1	Well, it's been installed and now I'm waiting to see the results, BOE shopping, hopefully I won't be disappointed!	Jingdong prefecture level city in Shandong
Jinfeng Hongyuan Flagship Store	Electricity savings1	Goods received, plugged in the voltage from 229 to 222, do not know whether to save electricity, that depends on the situation.	Jingdong prefecture level city in Shandong
Jinfeng Hongyuan Flagship Store	Electricity savings1	This is the second time I have bought the product and I feel it really saves electricity! Use it again for a while and then evaluate!	Jingdong prefecture level city in Shandong
Jinfeng Hongyuan Flagship Store	Electricity savings1	Overall, it's really good, and the customer service is great, you'll get a message back almost immediately when you speak, it's great value for money, I really recommend it!	Jingdong prefecture level city in Shandong
Jinfeng Hongyuan Flagship Store	Electricity savings1	Have purchased many times, a must have for the family, affordable purchase price, fast delivery, beautifully packaged!	Jingdong prefecture level city in Shandong
Jinfeng Hongyuan Flagship Store	Electricity savings1	I bought it after a friend introduced it, he said it is effective and can save electricity. I bought it to try, try a period of time and then follow up!	Jingdong prefecture level city in Shandong
Jinfeng Hongyuan Flagship Store	Electricity savings1	Received it, trying it out tonight, and bought one earlier.	Jingdong prefecture level city in Shandong
Jinfeng Hongyuan Flagship Store	Electricity savings1	It will take a month or two to see if it saves power or not.	Jingdong prefecture level city in Shandong
Jinfeng Hongyuan Flagship Store	Electricity savings1	Looked good in the instructions! Bought one and pressed it on! Use it and see! If it works! I'll buy some more!	Jingdong prefecture level city in Shandong
Jinfeng Hongyuan Flagship Store	Electricity savings1	Goods have been received packaging can also things are not bad appearance, with a use to know, now evaluation is nonsense.	Jingdong prefecture level city in Shandong
Jinfeng Hongyuan Flagship Store	Electricity savings1	Logistics is very fast, packaging is very good, the second unit, as always, looking forward to the effect of	Jingdong prefecture level city in Shandong
Jinfeng Hongyuan Flagship Store	Electricity savings1	I received the goods today, the quality of goods is very good, I am very satisfied ah.	Jingdong prefecture level city in Shandong
Jinfeng Hongyuan Flagship Store	Electricity savings1	Buy one and try it, I wonder if it has a power saving effect?	Jingdong prefecture level city in Shandong
Jinfeng Hongyuan Flagship Store	Electricity savings1	The item was received, well packaged, logistics is very fast, customer service attitude is very positive, there are questions answered in a timely manner! Overall very satisfied!	Jingdong prefecture level city in Shandong
Jinfeng Hongyuan Flagship Store	Electricity savings1	Baby packaging is very good, purchase submission goods quickly delivered to the home, use and then follow up.	Jingdong prefecture level city in Shandong
Jinfeng Hongyuan Flagship Store	Electricity savings1	My friend asked me to buy an extra one so he could try it too.	Jingdong prefecture level city in Shandong
Jinfeng Hongyuan Flagship Store	Electricity savings1	I've just put it on, I won't know the result until a month later if it will really save power.	Jingdong prefecture level city in Shandong

Jinfeng Hongyuan Flagship Store	Electricity savings1	It's good quality. Hopefully it'll help save power.	Jingdong prefecture level city in Shandong
Jinfeng Hongyuan Flagship Store	Electricity savings1	The appearance is clean and beautiful, the workmanship is very good, I did not find any flaws, I personally feel that the size is also quite suitable, I have been looking for a long time to choose this	Jingdong prefecture level city in Shandong
Jinfeng Hongyuan Flagship Store	Electricity savings1	Logistics is fast! Baby looks great! The price is fair! Wish the store business is prosperous!	Jingdong prefecture level city in Shandong
Jinfeng Hongyuan Flagship Store	Electricity savings1	Jingdong express fast, good, have used, sit and wait to save money...	Jingdong prefecture level city in Shandong
Jinfeng Hongyuan Flagship Store	Electricity savings1	In use, it didn't work as expected and there was nothing noticeable in terms of power savings.	Jingdong prefecture level city in Shandong
Jinfeng Hongyuan Flagship Store	Electricity savings1	Just started using it and looking forward to the results.	Jingdong prefecture level city in Shandong
Jinfeng Hongyuan Flagship Store	Electricity savings1	The quality is very good, the metal shell is very good, is genuine, energy saving and power saving, logistics is very fast, there is a need to buy o, recommend everyone, very satisfied with an online	Jingdong prefecture level city in Shandong
Jinfeng Hongyuan Flagship Store	Electricity savings1	Received, I do not know if the product is good, used to see if it is good to come back to buy!	Jingdong prefecture level city in Shandong
Jinfeng Hongyuan Flagship Store	Electricity savings1	Jinfeng source of intelligent power saving treasure also used less than three months, the last thunder may be burned, the home of the electric send gate is not on, only the old man at home, no way only their own in the field and run back to find out what is the problem with the circuit, the quality is still not very good, thunder is easy to burn!	Jingdong prefecture level city in Shandong
Jinfeng Hongyuan Flagship Store	Electricity savings1	Saving power just started to use, I do not know if there is no effect, but read a lot of reviews are said to be very good so I bought, baby All aspects are good There are spare 2 fuses, late good and then follow	Jingdong prefecture level city in Shandong
Jinfeng Hongyuan Flagship Store	Electricity savings1	Use it for a few days and see, the delivery is quite fast	Jingdong prefecture level city in Shandong
Jinfeng Hongyuan Flagship Store	Electricity savings1	Small, well made and easy to use.	Jingdong prefecture level city in Shandong
Jinfeng Hongyuan Flagship Store	Electricity savings1	Beautifully packaged, it is genuine. Whether it is power saving, use it to evaluate.	Jingdong prefecture level city in Shandong
Jinfeng Hongyuan Flagship Store	Electricity savings1	Power saver. Used two of them and they do save quite a bit of power.	Jingdong prefecture level city in Shandong
Jinfeng Hongyuan Flagship Store	Electricity savings1	Workmanship is not bad, small and does not take up space, is also do not know whether the power saving, buy a to try!	Jingdong prefecture level city in Shandong
Jinfeng Hongyuan Flagship Store	Electricity savings1	The logistics is very fast, the courier attitude is very good, worth buying	Jingdong prefecture level city in Shandong
Jinfeng Hongyuan Flagship Store	Electricity savings1	The effect is not known until it is used.	Jingdong prefecture level city in Shandong
Jinfeng Hongyuan Flagship Store	Electricity savings1	Just arrived plugged in won't know for a month	Jingdong prefecture level city in Shandong
Jinfeng Hongyuan Flagship Store	Electricity savings1	This is really good to use the pull to realize the savings ah think again for my sister's family to buy a	Jingdong prefecture level city in Shandong
Jinfeng Hongyuan Flagship Store	Electricity savings1	Lies, a month with a few hundred electricity are no savings, family use	Jingdong prefecture level city in Shandong
Jinfeng Hongyuan Flagship Store	Electricity savings1	Express very quickly, look at the fine workmanship also sent a few fuse, look forward to can really save electricity	Jingdong prefecture level city in Shandong
Jinfeng Hongyuan Flagship Store	Electricity savings1	I didn't realize it was so small.	Jingdong prefecture level city in Shandong
Jinfeng Hongyuan Flagship Store	Electricity savings1	Logistics courier is very fast, the courier service is very good	Jingdong prefecture level city in Shandong
Jinfeng Hongyuan Flagship Store	Electricity savings1	Used for a period of time the effect is quite good indeed than the previous power saving	Jingdong prefecture level city in Shandong
Jinfeng Hongyuan Flagship Store	Electricity savings1	Logistics is super fast, the product is in use, see the effect...	Jingdong prefecture level city in Shandong
Jinfeng Hongyuan Flagship Store	Electricity savings1	Baby got it, tried it out and it didn't work at all. How can I use the tips to save power?	Jingdong prefecture level city in Shandong
Jinfeng Hongyuan Flagship Store	Electricity savings1	Power Saving King received, immediately used has been very stable without any sound good, good service, fast logistics inexpensive and beautiful worth repurchasing!	Jingdong prefecture level city in Shandong
Jinfeng Hongyuan Flagship Store	Electricity savings1	Logistics courier is very fast, the courier service is good	Jingdong prefecture level city in Shandong
Jinfeng Hongyuan Flagship Store	Electricity savings1	Good to recommend, the first month of air conditioning only 1 week, the electricity bill has reduced about 100 yuan, may be to reduce the use of air conditioning is also related to the other or as usual electricity, estimated that the longer you use the more you can save electricity.	Jingdong prefecture level city in Shandong
Jinfeng Hongyuan Flagship Store	Electricity savings1	It doesn't work. I've been using it for 20 days and it doesn't work.	Jingdong prefecture level city in Shandong
Jinfeng Hongyuan Flagship Store	Electricity savings1	Fast shipping, good quality, affordable price, very satisfied with a shopping.	Jingdong prefecture level city in Shandong
Jinfeng Hongyuan Flagship Store	Electricity savings1	Try it for a month to see if it saves money.	Jingdong prefecture level city in Shandong
Jinfeng Hongyuan Flagship Store	Electricity savings1	It's in use. I'll comment again after a while.	Jingdong prefecture level city in Shandong
Jinfeng Hongyuan Flagship Store	Electricity savings1	Goods have received look good 👍👍👍👍, use a period of time and then look at the	Jingdong prefecture level city in Shandong
Jinfeng Hongyuan Flagship Store	Electricity savings1	No, it doesn't work. It's useless. It's garbage.	Jingdong prefecture level city in Shandong
Jinfeng Hongyuan Flagship Store	Electricity savings1	I've been using it for almost 2 months now and I don't feel any reduction in my electricity bill.	Jingdong prefecture level city in Shandong
Jinfeng Hongyuan Flagship Store	Electricity savings1	I've received the goods, the logistics is really fast, ordered yesterday, arrived today.	Jingdong prefecture level city in Shandong
Jinfeng Hongyuan Flagship Store	Electricity savings1	And the backup insurance.	Jingdong prefecture level city in Shandong
Jinfeng Hongyuan Flagship Store	Electricity savings1	The appearance of this product is good, easy to use and install, the effect of how there is no use to wait for time to verify that the voltage displayed is different from the voltage measured by other meters, the gap is not too large, the follow-up to add the effect.	Jingdong prefecture level city in Shandong
Jinfeng Hongyuan Flagship Store	Electricity savings1	Baby received it 👌, really great ah! 🌈The quality can't be beat, very satisfied! 🆗Happy happy happy 😃	Jingdong prefecture level city in Shandong
Jinfeng Hongyuan Flagship Store	Electricity savings1	I'll follow up with a review after using it for a while, hopefully it won't bring me back a disappointment!	Jingdong prefecture level city in Shandong
Jinfeng Hongyuan Flagship Store	Electricity savings1	In use and looking forward to results! Good review to send	Jingdong prefecture level city in Shandong
Jinfeng Hongyuan Flagship Store	Electricity savings1	Intelligent power saver received, the volume is not big, small and exquisite, quite good, Jingdong Express is also quite fast, the	Jingdong prefecture level city in Shandong
Jinfeng Hongyuan Flagship Store	Electricity savings1	I don't know how to use it, keep using it	Jingdong prefecture level city in Shandong
Jinfeng Hongyuan Flagship Store	Electricity savings1	Very good for saving electricity	Jingdong prefecture level city in Shandong
Jinfeng Hongyuan Flagship Store	Electricity savings1	Seriously, did not use, feel also more power consumption, the peak is bought back to use the first time, the next day unplugged, the power consumption is back to more than ten degrees (the frequency of power use in the past few days is pretty much the same), feel the IQ tax paid 😂	Jingdong prefecture level city in Shandong
Jinfeng Hongyuan Flagship Store	Electricity savings1	I haven't seen any effect on the power saving, but the voltage stabilization looks good.	Jingdong prefecture level city in Shandong
Jinfeng Hongyuan Flagship Store	Electricity savings1	It's very good, I'll come back to it after using it for a while	Jingdong prefecture level city in Shandong
Jinfeng Hongyuan Flagship Store	Electricity savings1	Already used it and see the results.	Jingdong prefecture level city in Shandong

Jinfeng Hongyuan Flagship Store	Electricity savings1	Fast shipping, beautiful packaging, attentive service, the use of the effect, to be followed up after the evaluation.	Jingdong prefecture level city in Shandong
Jinfeng Hongyuan Flagship Store	Electricity savings1	The process of using this product is more than satisfactory	Jingdong prefecture level city in Shandong
Jinfeng Hongyuan Flagship Store	Electricity savings1	Very good East East cost-effective! Every time so buy more assured, and the logistics is also very fast. It is also very good to use, so I will often come to buy in the future, and will also introduce to	Jingdong prefecture level city in Shandong
Jinfeng Hongyuan Flagship Store	Electricity savings1	But still understand how effective it is!	Jingdong prefecture level city in Shandong
Jinfeng Hongyuan Flagship Store	Electricity savings1	Fast shipping, great logistics, great stuff, great price.	Jingdong prefecture level city in Shandong
Jinfeng Hongyuan Flagship Store	Electricity savings1	I'll review it in a month and give it 5 stars if it's good	Jingdong prefecture level city in Shandong
Jinfeng Hongyuan Flagship Store	Electricity savings1	I've saved a lot of electricity in the past month, so I'm glad to hear that.	Jingdong prefecture level city in Shandong
Jinfeng Hongyuan Flagship Store	Electricity savings1	Baby just received and installed! Looks genuinely good, workmanship feels fine! Looking forward to the effect! Said to see the effect in two months! Waiting for if really have the effect must come to follow up! The boss is absolutely responsible! Asked and answered! Just for the boss this attitude must also	Jingdong prefecture level city in Shandong
Jinfeng Hongyuan Flagship Store	Electricity savings1	This baby is very delicate, I'll comment after I've used it	Jingdong prefecture level city in Shandong
Jinfeng Hongyuan Flagship Store	Electricity savings1	It's been over two months. It's not working. It's just like it was.	Jingdong prefecture level city in Shandong
Jinfeng Hongyuan Flagship Store	Electricity savings1	Fast shipping and logistics, let's see if it works.	Jingdong prefecture level city in Shandong
Jinfeng Hongyuan Flagship Store	Electricity savings1	Just received the goods, try it to know if it can really save electricity	Jingdong prefecture level city in Shandong
Jinfeng Hongyuan Flagship Store	Electricity savings1	Not bad. Not bad. Not bad.	Jingdong prefecture level city in Shandong
Jinfeng Hongyuan Flagship Store	Electricity savings1	Not sure if it works. I'll comment again if it works.	Jingdong prefecture level city in Shandong
Jinfeng Hongyuan Flagship Store	Electricity savings1	Safe and energy-saving helpers to use!	Jingdong prefecture level city in Shandong
Jinfeng Hongyuan Flagship Store	Electricity savings1	Good, first time buyer. Used good can	Jingdong prefecture level city in Shandong
Jinfeng Hongyuan Flagship Store	Electricity savings1	It feels okay, I guess! I won't know until I use it for a while!	Jingdong prefecture level city in Shandong
Jinfeng Hongyuan Flagship Store	Electricity savings1	The saver matches the description and has been used, waiting for a follow up review after two months.	Jingdong prefecture level city in Shandong
Jinfeng Hongyuan Flagship Store	Electricity savings1	Received, the appearance of the business as described, the effect of how to use a period of time to evaluate.	Jingdong prefecture level city in Shandong
Jinfeng Hongyuan Flagship Store	Electricity savings1	Still using it don't know how effective it is, I'll comment after three months	Jingdong prefecture level city in Shandong
Jinfeng Hongyuan Flagship Store	Electricity savings1	The stuff is very good, the packaging is also very good logistics is also very fast	Jingdong prefecture level city in Shandong
Jinfeng Hongyuan Flagship Store	Electricity savings1	I'll follow up with a review when I use it, hopefully I won't be disappointed	Jingdong prefecture level city in Shandong
Jinfeng Hongyuan Flagship Store	Electricity savings1	Cancel Houjiaying Logistics.	Jingdong prefecture level city in Shandong
Jinfeng Hongyuan Flagship Store	Electricity savings1	It's very good, it's worth buying.	Jingdong prefecture level city in Shandong
Jinfeng Hongyuan Flagship Store	Electricity savings1	My friend bought it to save electricity and referred me to buy it! I'll buy it and try it out! I will know how much electricity I can save next month, looking forward to it! 4-500 dollars a month electricity bill is horrible! I hope it can really help me save some electricity!	Jingdong prefecture level city in Shandong
Jinfeng Hongyuan Flagship Store	Electricity savings1	Just received it and installed it.	Jingdong prefecture level city in Shandong
Jinfeng Hongyuan Flagship Store	Electricity savings1	Very nice. This one is my favorite.	Jingdong prefecture level city in Shandong
Jinfeng Hongyuan Flagship Store	Electricity savings1	Good value for money worth buying very good	Jingdong prefecture level city in Shandong
Jinfeng Hongyuan Flagship Store	Electricity savings1	Very energy-saving, small and exquisite, does not take up space, very satisfied, complete packaging, no damage, customer service explanation is also very serious	Jingdong prefecture level city in Shandong
Jinfeng Hongyuan Flagship Store	Electricity savings1	Received immediately used, really effective, do not understand electricity, but looked at the meter every day, recommended that you can use, especially this weather every day to open the air conditioning, with him not afraid of the electricity bill.	Jingdong prefecture level city in Shandong
Jinfeng Hongyuan Flagship Store	Electricity savings1	Nice, good value for money, nice stuff.	Jingdong prefecture level city in Shandong
Jinfeng Hongyuan Flagship Store	Electricity savings1	The packaging of the product is very good, the logistics of the product is very fast, and the quality of the product will be evaluated after use!	Jingdong prefecture level city in Shandong
Jinfeng Hongyuan Flagship Store	Electricity savings1	Express is very, well very fast, give a compliment 👍	Jingdong prefecture level city in Shandong
Jinfeng Hongyuan Flagship Store	Electricity savings1	Packaging is very good, fast delivery, used a night and a day did not feel the power saving. Use a month to see if it saves power.	Jingdong prefecture level city in Shandong
Jinfeng Hongyuan Flagship Store	Electricity savings1	Seems to be a bit of a power saver.	Jingdong prefecture level city in Shandong
Jinfeng Hongyuan Flagship Store	Electricity savings1	Bought this for someone else and it came over so fast, ###!	Jingdong prefecture level city in Shandong
Jinfeng Hongyuan Flagship Store	Electricity savings1	Very delicate, just received a period of time to use in the follow-up evaluation	Jingdong prefecture level city in Shandong
Jinfeng Hongyuan Flagship Store	Electricity savings1	It's been installed for a while now, not sure if it's working or not	Jingdong prefecture level city in Shandong
Jinfeng Hongyuan Flagship Store	Electricity savings1	The item looks okay, I just don't know if it works or not.	Jingdong prefecture level city in Shandong
Jinfeng Hongyuan Flagship Store	Electricity savings1	The goods have been received, but there is a screw missing	Jingdong prefecture level city in Shandong
Jinfeng Hongyuan Flagship Store	Electricity savings1	I didn't believe it before, but after receiving the goods today and trying it out, it really works!	Jingdong prefecture level city in Shandong
Jinfeng Hongyuan Flagship Store	Electricity savings1	Received, this thing will not know whether it is the effect of saving electricity, no matter how to say that there should always be a little use it, otherwise why develop it I think there is still the value of	Jingdong prefecture level city in Shandong
Jinfeng Hongyuan Flagship Store	Electricity savings1	Another one just arrived.	Jingdong prefecture level city in Shandong
Jinfeng Hongyuan Flagship Store	Electricity savings1	I don't feel anything, ordinary farmers can't go to TEDA, just use it first, it's safe.	Jingdong prefecture level city in Shandong
Jinfeng Hongyuan Flagship Store	Electricity savings1	Just purchased and will follow up after using it for a while	Jingdong prefecture level city in Shandong
Jinfeng Hongyuan Flagship Store	Electricity savings1	I'm trying it out now. I'll comment after I use it.	Jingdong prefecture level city in Shandong
Jinfeng Hongyuan Flagship Store	Electricity savings1	Received the item, in use, hope it works.	Jingdong prefecture level city in Shandong
Jinfeng Hongyuan Flagship Store	Electricity savings1	It works well. Good review.	Jingdong prefecture level city in Shandong
Jinfeng Hongyuan Flagship Store	Electricity savings1	All a scam, no effect at all, I bought too many fakes in Poundland before, I did not think that Jingdong is also the same	Jingdong prefecture level city in Shandong
Jinfeng Hongyuan Flagship Store	Electricity savings1	I used the saver as soon as I received it, it's in good shape and still working!	Jingdong prefecture level city in Shandong

Jinfeng Hongyuan Flagship Store	Electricity savings1	It works. I don't know what to do with the picture, but it works.	Jingdong prefecture level city in Shandong
Jinfeng Hongyuan Flagship Store	Electricity savings1	A very good shopping experience, attentive service, fast logistics, delivery is also very thoughtful, attitude is also very good, value for money ah, decisive praise!	Jingdong prefecture level city in Shandong
Jinfeng Hongyuan Flagship Store	Electricity savings1	The product is consistent with the presentation picture.	Jingdong prefecture level city in Shandong
Jinfeng Hongyuan Flagship Store	Electricity savings1	Baby received, use a period of time to evaluate, wish the boss business prosperity!	Jingdong prefecture level city in Shandong
Jinfeng Hongyuan Flagship Store	Electricity savings1	The stuff is better! Recommended!	Jingdong prefecture level city in Shandong
Jinfeng Hongyuan Flagship Store	Electricity savings1	I don't know if it works or not, I'll use it for a while before I comment on it.	Jingdong prefecture level city in Shandong
Jinfeng Hongyuan Flagship Store	Electricity savings1	Cost-effective, I like it, next time will come back Baby received, very surprised, the quality is very good, very satisfied with a shopping, I will continue to buy back, the store is trustworthy	Jingdong prefecture level city in Shandong
Jinfeng Hongyuan Flagship Store	Electricity savings1	Plugged it in and used it for a few days, not sure if it saves power yet	Jingdong prefecture level city in Shandong
Jinfeng Hongyuan Flagship Store	Electricity savings1	I'm trying it out and will follow up if it seems to be working!	Jingdong prefecture level city in Shandong
Jinfeng Hongyuan Flagship Store	Electricity savings1	Works pretty well, big kudos 👍👍👍👍👍👍👍👍👍👍👍👍	Jingdong prefecture level city in Shandong
Jinfeng Hongyuan Flagship Store	Electricity savings1	Just put it on, we'll see how it looks in a month.	Jingdong prefecture level city in Shandong
Jinfeng Hongyuan Flagship Store	Electricity savings1	The baby is very nice and I've already recommended buying it for all my friends! The main thing is that it not only saves electricity, I was more concerned about the fact that it can adjust the current, usually wiring problems. Now I found that it is really good, it is the effect I want!	Jingdong prefecture level city in Shandong
Jinfeng Hongyuan Flagship Store	Electricity savings1	Baby arrived, looking forward to his results, hopefully it will be good	Jingdong prefecture level city in Shandong
Jinfeng Hongyuan Flagship Store	Electricity savings1	Things have been received, look at the workmanship and packaging are good, I do not know how the power-saving effect? Use a period of time to see the effect	Jingdong prefecture level city in Shandong
Jinfeng Hongyuan Flagship Store	Electricity savings1	The price is right, the shape is beautiful and does not take up space, use it for a while to see the effect	Jingdong prefecture level city in Shandong
Jinfeng Hongyuan Flagship Store	Electricity savings1	Can't save electricity, it doesn't work!	Jingdong prefecture level city in Shandong
Jinfeng Hongyuan Flagship Store	Electricity savings1	It's pretty convenient, just plug it in and it works. There's no noise, plug it in and leave it alone, it's very hassle free! Good workmanship and value for money.	Jingdong prefecture level city in Shandong
Jinfeng Hongyuan Flagship Store	Electricity savings1	Just received the goods have been installed, try two months later to comment!	Jingdong prefecture level city in Shandong
Jinfeng Hongyuan Flagship Store	Electricity savings1	I received the stuff, I haven't used it yet, and overall it's fine.	Jingdong prefecture level city in Shandong
Jinfeng Hongyuan Flagship Store	Electricity savings1	Couldn't use a return at all.	Jingdong prefecture level city in Shandong
Jinfeng Hongyuan Flagship Store	Electricity savings1	Second purchase.	Jingdong prefecture level city in Shandong
Jinfeng Hongyuan Flagship Store	Electricity savings1	Very good quality, high tech and practical.	Jingdong prefecture level city in Shandong
Jinfeng Hongyuan Flagship Store	Electricity savings1	Very good, very great! Very satisfied, thanks to the store!	Jingdong prefecture level city in Shandong
Jinfeng Hongyuan Flagship Store	Electricity savings1	Just used it, don't know if it's good or not, hope it works well	Jingdong prefecture level city in Shandong
Jinfeng Hongyuan Flagship Store	Electricity savings1	Saving power has been received, the product is good, good like.	Jingdong prefecture level city in Shandong
Jinfeng Hongyuan Flagship Store	Electricity savings1	Received the goods and used it for two months before evaluation	Jingdong prefecture level city in Shandong
Jinfeng Hongyuan Flagship Store	Electricity savings1	It looks a little small, I do not know how effective, first use to see the effect, there is a small adjustable button, I do not know this is to adjust to that position to work well	Jingdong prefecture level city in Shandong
Jinfeng Hongyuan Flagship Store	Electricity savings1	Simple, just plug it in, there are spare fuses, can't say how well it works, no comparison	Jingdong prefecture level city in Shandong
Jinfeng Hongyuan Flagship Store	Electricity savings1	It's been a few days since I used it, and the power's gone up.	Jingdong prefecture level city in Shandong
Jinfeng Hongyuan Flagship Store	Electricity savings1	The owner was very nice and sent a spare fuse.	Jingdong prefecture level city in Shandong
Jinfeng Hongyuan Flagship Store	Electricity savings1	It's authentic and it's not expensive and it's great to use, I use it all the time and it's great 🎉✨ just don't know if it really saves any points	Jingdong prefecture level city in Shandong
Jinfeng Hongyuan Flagship Store	Electricity savings1	The information is good, it is the child to buy, the child read it is also quite happy, I hope to be able to match the child's preparation for the midterm examination! Because of the epidemic, it is not too fast, but I also give full credit to the express delivery!	Jingdong prefecture level city in Shandong
Jinfeng Hongyuan Flagship Store	Electricity savings1	This power saver comes to the logistics very quickly, the business attitude is also very good, ** very comfortable, this brand is trustworthy, and buy in this store than in the physical store to buy cheaper,	Jingdong prefecture level city in Shandong
Jinfeng Hongyuan Flagship Store	Electricity savings1	It's very well made and the materials used are okay. This is just a few days old, so I don't know how it will work, only next month.	Jingdong prefecture level city in Shandong
Jinfeng Hongyuan Flagship Store	Electricity savings1	Already used on, have not tested the effect, wait for a couple of months to add comments.	Jingdong prefecture level city in Shandong
Jinfeng Hongyuan Flagship Store	Electricity savings1	Baby received, packaging is very thoughtful, already in use, should soon see the effect.	Jingdong prefecture level city in Shandong
Jinfeng Hongyuan Flagship Store	Electricity savings1	Just received in the office to try, to the home installed, the voltage are different, and do not know if there is no effect. Use a period of time and then follow up.	Jingdong prefecture level city in Shandong
Jinfeng Hongyuan Flagship Store	Electricity savings1	Just received the goods, is trying, looking forward to the effect, first give a favorable comment, two months later, then additional evaluation, please be patient.	Jingdong prefecture level city in Shandong
Jinfeng Hongyuan Flagship Store	Electricity savings1	The item has just been received, well packaged, results to be verified.	Jingdong prefecture level city in Shandong
Jinfeng Hongyuan Flagship Store	Electricity savings1	Packaging logistics are very good, the next look at how the effect, before I see others use the effect is good, as I bought this can also have good results.	Jingdong prefecture level city in Shandong
Jinfeng Hongyuan Flagship Store	Electricity savings1	I don't know if it's a real power saver or a fake power saver, but I've used it since I bought it, so I'll give you a good review.	Jingdong prefecture level city in Shandong
Jinfeng Hongyuan Flagship Store	Electricity savings1	Good quality, genuine goods, much better than imagined before purchase, next time will come back to care!	Jingdong prefecture level city in Shandong
Jinfeng Hongyuan Flagship Store	Electricity savings1	Logistics is very fast good use again	Jingdong prefecture level city in Shandong
Jinfeng Hongyuan Flagship Store	Electricity savings1	Received the goods. Let's see if it saves power in a month and then we'll follow up!	Jingdong prefecture level city in Shandong
Jinfeng Hongyuan Flagship Store	Electricity savings1	I bought it yesterday and it arrived today, I bought two of them, I hope they work well, I'll come back after a while to give a follow-up review	Jingdong prefecture level city in Shandong
Jinfeng Hongyuan Flagship Store	Electricity savings1	The home air conditioner consumes a lot of electricity, so I bought it and used it in the hope that there is A	Jingdong prefecture level city in Shandong
Jinfeng Hongyuan Flagship Store	Electricity savings1	The product is excellent and very current.	Jingdong prefecture level city in Shandong
Jinfeng Hongyuan Flagship Store	Electricity savings1	With the description, it is the most practical and good products that households can need, and it is an ideal shopping, the courier is also fast, and the service attitude is enthusiastic!	Jingdong prefecture level city in Shandong
Jinfeng Hongyuan Flagship Store	Electricity savings1	The Power Saver is easy to use, works well, is stable, and plugs into the top of the outlet	Jingdong prefecture level city in Shandong
Jinfeng Hongyuan Flagship Store	Electricity savings1	Overall still can, excellent craftsmanship, power saving do not know for the time being, after the use of the evaluation	Jingdong prefecture level city in Shandong

Jinfeng Hongyuan Flagship Store	Electricity savings1	Baby received on the plug, but also with four fuses, look at the quality is okay, I do not know whether it is useful, use and then follow up.	Jingdong prefecture level city in Shandong
Jinfeng Hongyuan Flagship Store	Electricity savings1	I'll evaluate it after a while, I don't know if it's good or not yet, and if it can save electricity	Jingdong prefecture level city in Shandong
Jinfeng Hongyuan Flagship Store	Electricity savings1	Jingdong self-operated purchase more assured delivery fast quality assurance	Jingdong prefecture level city in Shandong
Jinfeng Hongyuan Flagship Store	Electricity savings1	Shipping was fast, stuff was well packaged, great price, I'm happy with it 👍 and will repurchase!	Jingdong prefecture level city in Shandong
Jinfeng Hongyuan Flagship Store	Electricity savings1	The quality of the product is very good and I will definitely come back again next time.	Jingdong prefecture level city in Shandong
Jinfeng Hongyuan Flagship Store	Electricity savings1	I'll follow up with a review after I use it.	Jingdong prefecture level city in Shandong
Jinfeng Hongyuan Flagship Store	Electricity savings1	Hello, the goods are perfectly packaged, save power for a period of time to evaluate again!	Jingdong prefecture level city in Shandong
Jinfeng Hongyuan Flagship Store	Electricity savings1	Not bad, pretty good, good value for money, pretty good	Jingdong prefecture level city in Shandong
Jinfeng Hongyuan Flagship Store	Electricity savings1	The packaging is very good, received immediately used, looking forward to the effect in.	Jingdong prefecture level city in Shandong
Jinfeng Hongyuan Flagship Store	Electricity savings1	See how much power you save at the end of the month	Jingdong prefecture level city in Shandong
Jinfeng Hongyuan Flagship Store	Electricity savings1	Easy to use, affordable, beautiful color	Jingdong prefecture level city in Shandong
Jinfeng Hongyuan Flagship Store	Electricity savings1	It's taking too long to put together an order. It could be an epidemic.	Jingdong prefecture level city in Shandong
Jinfeng Hongyuan Flagship Store	Electricity savings1	I don't feel much after using it, no change in my electric bill 。。。。。。。	Jingdong prefecture level city in Shandong
Jinfeng Hongyuan Flagship Store	Electricity savings1	Logistics is very fast, the quality of the economizer is good, good quality and cheap, it is worth buying, I hope there is a power saving effect!	Jingdong prefecture level city in Shandong
Jinfeng Hongyuan Flagship Store	Electricity savings1	Feeling okay, in a couple of months to try to rely on it before making a comment. Let's give it a five-star review first!	Jingdong prefecture level city in Shandong
Jinfeng Hongyuan Flagship Store	Electricity savings1	Power saving device has been hesitating before in the end to buy or not to buy, after consideration, consideration, in consideration, or decided to buy a try. Things received can not wait to open a look, and the seller described the same, I hope to use 2 months to have the effect, two months to follow up on	Jingdong prefecture level city in Shandong
Jinfeng Hongyuan Flagship Store	Electricity savings1	Thank you Jingdong, logistics is very fast order to receipt only one day. Believe in Jingdong. Favorable comments	Jingdong prefecture level city in Shandong
Jinfeng Hongyuan Flagship Store	Electricity savings1	I used one to use it, but it didn't work very well, what to do	Jingdong prefecture level city in Shandong
Jinfeng Hongyuan Flagship Store	Electricity savings1	Quality and packaging are very good, fast shipping, good service, buy with confidence	Jingdong prefecture level city in Shandong
Jinfeng Hongyuan Flagship Store	Electricity savings1	Use a period of time to evaluate, the quality is very good, the price is affordable, logistics is also very fast	Jingdong prefecture level city in Shandong
Jinfeng Hongyuan Flagship Store	Electricity savings1	Goods received, the day of the order tomorrow delivery real God speed, delivery force, the product to be used after a period of time and then low comment!	Jingdong prefecture level city in Shandong
Jinfeng Hongyuan Flagship Store	Electricity savings1	I don't know, how effective it is, let's talk about it when the time comes, whether it saves power or not, how much, unknown.	Jingdong prefecture level city in Shandong
Jinfeng Hongyuan Flagship Store	Electricity savings1	It does save some electricity, and I don't want to use something that isn't legal! Used nearly ~ electricity last month, probably not much more this month with the AC on!	Jingdong prefecture level city in Shandong
Jinfeng Hongyuan Flagship Store	Electricity savings1	Well-packed, excellent quality, service satisfaction good value for money	Jingdong prefecture level city in Shandong
Jinfeng Hongyuan Flagship Store	Electricity savings1	Express very quickly, the goods received last night, today used a day of goods can not heat, looking forward to whether it can save electricity, with a month and then comment, is used well will buy again.	Jingdong prefecture level city in Shandong
Jinfeng Hongyuan Flagship Store	Electricity savings1	It's pretty good, hopefully it'll be a real power saver.	Jingdong prefecture level city in Shandong
Jinfeng Hongyuan Flagship Store	Electricity savings1	The goods received in use, can not see the effect of energy saving, after a month to know!	Jingdong prefecture level city in Shandong
Jinfeng Hongyuan Flagship Store	Electricity savings1	Received the goods, looking forward to better surprise, with the effect of the follow-up comment!	Jingdong prefecture level city in Shandong
Jinfeng Hongyuan Flagship Store	Electricity savings1	It's not bad, it's still in use and should save some	Jingdong prefecture level city in Shandong
Jinfeng Hongyuan Flagship Store	Electricity savings1	Logistics is very fast, easy to install, plugged in on it,, the effect of the use of the later to evaluate!	Jingdong prefecture level city in Shandong
Jinfeng Hongyuan Flagship Store	Electricity savings1	The stuff is recommendable to buy very good.	Jingdong prefecture level city in Shandong
Jinfeng Hongyuan Flagship Store	Electricity savings1	Voltmeter measurements of voltage are way off, aren't they?	Jingdong prefecture level city in Shandong
Jinfeng Hongyuan Flagship Store	Electricity savings1	Install it to the socket immediately after purchase and try to see if it can save electricity after one month.	Jingdong prefecture level city in Shandong
Jinfeng Hongyuan Flagship Store	Electricity savings1	I've been using it for almost a month now, and I won't know if it saves electricity until I pay the bill.	Jingdong prefecture level city in Shandong
Jinfeng Hongyuan Flagship Store	Electricity savings1	I bought one in the past and it was fine, so I'm buying it back.	Jingdong prefecture level city in Shandong
Jinfeng Hongyuan Flagship Store	Electricity savings1	Service is very good, this thing is small and exquisite to use, just waiting to use the effect of a period of time	Jingdong prefecture level city in Shandong
Jinfeng Hongyuan Flagship Store	Electricity savings1	Not sure how well it works yet	Jingdong prefecture level city in Shandong
Jinfeng Hongyuan Flagship Store	Electricity savings1	Kyodo goods habitually good reviews	Jingdong prefecture level city in Shandong
Jinfeng Hongyuan Flagship Store	Electricity savings1	Appearance is OK, whether it is power saving remains to be seen.	Jingdong prefecture level city in Shandong
Jinfeng Hongyuan Flagship Store	Electricity savings1	I bought it and used it, I just don't know if it really saves power	Jingdong prefecture level city in Shandong
Jinfeng Hongyuan Flagship Store	Electricity savings1	The saver is well made and of good quality, I will evaluate it after using it for a while.	Jingdong prefecture level city in Shandong
Jinfeng Hongyuan Flagship Store	Electricity savings1	Just received in use, use for a period of time to see if there is power saving	Jingdong prefecture level city in Shandong
Jinfeng Hongyuan Flagship Store	Electricity savings1	Used 3 days before commenting, it really saves electricity, I look at the "power calendar" after using one less degree of electricity per day, so I think it's worth it to buy this, I will introduce my friends	Jingdong prefecture level city in Shandong
Jinfeng Hongyuan Flagship Store	Electricity savings1	Reliable quality, very well packaged and matches the real thing.	Jingdong prefecture level city in Shandong
Jinfeng Hongyuan Flagship Store	Electricity savings1	Logistics is still quite fast, ha in the trial	Jingdong prefecture level city in Shandong
Jinfeng Hongyuan Flagship Store	Electricity savings1	Consultant service attitude is very good, the courier is also very powerful, the first day of the order the next day to arrive. Saver received used and then add comments!	Jingdong prefecture level city in Shandong
Jinfeng Hongyuan Flagship Store	Electricity savings1	I just got home to try it out and see how it goes.	Jingdong prefecture level city in Shandong
Jinfeng Hongyuan Flagship Store	Electricity savings1	Well made and weighted instrument. Kudos! Put it into use immediately, don't know if it saves power, will evaluate later!	Jingdong prefecture level city in Shandong
Jinfeng Hongyuan Flagship Store	Electricity savings1	I've just put it in place. I'll give you a review in ten days.	Jingdong prefecture level city in Shandong
Jinfeng Hongyuan Flagship Store	Electricity savings1	It looks very delicate, not bad. Well, I don't know if it can save power or not, I have to try it for a while and see. If it can really save electricity. Uh, if it works well. Then additional comments. Anyway, thanks for the logistics, thanks to Jingdong. Well, it deserves some praise.	Jingdong prefecture level city in Shandong

Jinfeng Hongyuan Flagship Store	Electricity savings1	Bought a month, the feeling is that there is a certain effect, a month to save a lot of money	Jingdong prefecture level city in Shandong
Jinfeng Hongyuan Flagship Store	Electricity savings1	Goods received, the physical and the same as the picture, logistics is also very fast, the service attitude is very good, it is worth buying!	Jingdong prefecture level city in Shandong
Jinfeng Hongyuan Flagship Store	Electricity savings1	I've used it, I don't know whether it saves electricity or not, I'll talk about it after a while.	Jingdong prefecture level city in Shandong
Jinfeng Hongyuan Flagship Store	Electricity savings1	August only began to use, October to know, have no power-saving effect, to the in to evaluate!	Jingdong prefecture level city in Shandong
Jinfeng Hongyuan Flagship Store	Electricity savings1	Goods received: I do not know to the factory products used to save electricity!	Jingdong prefecture level city in Shandong
Jinfeng Hongyuan Flagship Store	Electricity savings1	Don't know if it works, bought it anyway.	Jingdong prefecture level city in Shandong
Jinfeng Hongyuan Flagship Store	Electricity savings1	Still testing, will follow up next month to evaluate how much electricity is saved	Jingdong prefecture level city in Shandong
Jinfeng Hongyuan Flagship Store	Electricity savings1	I'm still testing it, I'll comment after I use it.	Jingdong prefecture level city in Shandong
Jinfeng Hongyuan Flagship Store	Electricity savings1	Fast service, praiseworthy. Good item pre-style, first praise, the use of the effect to be evaluated after use.	Jingdong prefecture level city in Shandong
Jinfeng Hongyuan Flagship Store	Electricity savings1	The power saving effect is more obvious through verified use.	Jingdong prefecture level city in Shandong
Jinfeng Hongyuan Flagship Store	Electricity savings1	Logistics is very fast yesterday order today arrived. The product was immediately used, and after a month to see its effect.	Jingdong prefecture level city in Shandong
Jinfeng Hongyuan Flagship Store	Electricity savings1	Power saving is not yet known, as far as the TV and audio are concerned, I feel that there is a significant quality improvement, and I recommend using a good product!	Jingdong prefecture level city in Shandong
Jinfeng Hongyuan Flagship Store	Electricity savings1	Good power savings, tens of dollars a month saved	Jingdong prefecture level city in Shandong
Jinfeng Hongyuan Flagship Store	Electricity savings1	Already took two, great value! You can save 30% on your electric bill in a month. A summer must-have!	Jingdong prefecture level city in Shandong
Jinfeng Hongyuan Flagship Store	Electricity savings1	Received the item, plugged it into a home outlet and it showed 230 volts (not that high with a multimeter). Let's use it first!	Jingdong prefecture level city in Shandong
Jinfeng Hongyuan Flagship Store	Electricity savings1	Expecting results in the experiment	Jingdong prefecture level city in Shandong
Jinfeng Hongyuan Flagship Store	Electricity savings1	I don't know if it really saves power.	Jingdong prefecture level city in Shandong
Jinfeng Hongyuan Flagship Store	Electricity savings1	It's very good and the efficacy is very visible.	Jingdong prefecture level city in Shandong
Jinfeng Hongyuan Flagship Store	Electricity savings1	It's good and worth buying with confidence	Jingdong prefecture level city in Shandong
Jinfeng Hongyuan Flagship Store	Electricity savings1	The goods have been received, just do not know if it is effective	Jingdong prefecture level city in Shandong
Jinfeng Hongyuan Flagship Store	Electricity savings1	It's already in use, but based on some of the descriptions, I suspect that to use it, you need to have one in series on each line, which seems to work for all appliances, that's my guess, but I feel as though the principle of the thing seems to require it.	Jingdong prefecture level city in Shandong
Jinfeng Hongyuan Flagship Store	Electricity savings1	Fast delivery, already in use, looks good!	Jingdong prefecture level city in Shandong
Jinfeng Hongyuan Flagship Store	Electricity savings1	Hoping to save 30% of power as presented in the graphic	Jingdong prefecture level city in Shandong
Jinfeng Hongyuan Flagship Store	Electricity savings1	Things look good, power saving do not know now, with a month after the continued evaluation	Jingdong prefecture level city in Shandong
Jinfeng Hongyuan Flagship Store	Electricity savings1	Already used it but not sure if it will top out!	Jingdong prefecture level city in Shandong
Jinfeng Hongyuan Flagship Store	Electricity savings1	Looking forward to using the results, the product quality looks okay.	Jingdong prefecture level city in Shandong
Jinfeng Hongyuan Flagship Store	Electricity savings1	It's somewhat effective and saves money more or less 💰	Jingdong prefecture level city in Shandong
Jinfeng Hongyuan Flagship Store	Electricity savings1	Got it, it's great, looking forward to saving power.	Jingdong prefecture level city in Shandong
Jinfeng Hongyuan Flagship Store	Electricity savings1	I don't know if it's working yet, I'll follow up when I get there	Jingdong prefecture level city in Shandong
Jinfeng Hongyuan Flagship Store	Electricity savings1	The product received, good workmanship, fast logistics, good inner packaging, the outer bag is damaged, but fortunately did not hurt the product, retrieved and inserted according to the instructions, looking forward to the effect is good, with a period of time to evaluate, I believe that the Jingdong.	Jingdong prefecture level city in Shandong
Jinfeng Hongyuan Flagship Store	Electricity savings1	Since it is a new product, it is said to work, so if it works well for a month, then I will give you a good review.	Jingdong prefecture level city in Shandong
Jinfeng Hongyuan Flagship Store	Electricity savings1	The quality of the product is very good, very well made, well packaged, easy to use and very convenient!	Jingdong prefecture level city in Shandong
Jinfeng Hongyuan Flagship Store	Electricity savings1	Good price for the item, good functionality	Jingdong prefecture level city in Shandong
Jinfeng Hongyuan Flagship Store	Electricity savings1	Received the first time to use it, the stuff is very good, specifics wait for a month to come back to feedback to see the effect.	Jingdong prefecture level city in Shandong
Jinfeng Hongyuan Flagship Store	Electricity savings1	Just received, the courier is too bad, not notified directly to put the Feng Nest courier cabinet	Jingdong prefecture level city in Shandong
Jinfeng Hongyuan Flagship Store	Electricity savings1	Baby received, the second time to buy, plugged in and used do not know if there is no effect. Baby received, the second time to buy, plugged in and used do not know if there is no effect. Baby received, the second time to buy, plugged in and used do not know if there is no effect.	Jingdong prefecture level city in Shandong
Jinfeng Hongyuan Flagship Store	Electricity savings1	It's okay if it saves power. It's not a bad product.	Jingdong prefecture level city in Shandong
Jinfeng Hongyuan Flagship Store	Electricity savings1	Received, very good, meticulous packaging, open a look is genuine, meticulous workmanship, the manual is very detailed, has been used, there is no effect on the only look at the next month's electricity bill, when I can I buy a few more to send people!	Jingdong prefecture level city in Shandong
Jinfeng Hongyuan Flagship Store	Electricity savings1	The quality is very good very delicate express delivery is too fast	Jingdong prefecture level city in Shandong
Jinfeng Hongyuan Flagship Store	Electricity savings1	I won't know until a month after I start using it today.	Jingdong prefecture level city in Shandong
Jinfeng Hongyuan Flagship Store	Electricity savings1	It doesn't work at all, the customer service attitude is very bad, use it or not!	Jingdong prefecture level city in Shandong
Jinfeng Hongyuan Flagship Store	Electricity savings1	Temporarily try, do not know the effect, Jingdong logistics delivery very quickly	Jingdong prefecture level city in Shandong
Jinfeng Hongyuan Flagship Store	Electricity savings1	It doesn't help. It's comforting.	Jingdong prefecture level city in Shandong
Jinfeng Hongyuan Flagship Store	Electricity savings1	It's great, use it before you say anything.	Jingdong prefecture level city in Shandong
Jinfeng Hongyuan Flagship Store	Electricity savings1	Received, already used, looking forward to a good a effect!	Jingdong prefecture level city in Shandong
Jinfeng Hongyuan Flagship Store	Electricity savings1	I don't know if it works yet, I won't know until I use it for a few months.	Jingdong prefecture level city in Shandong
Jinfeng Hongyuan Flagship Store	Electricity savings1	It takes a long time to get there. There's no change now.	Jingdong prefecture level city in Shandong
Jinfeng Hongyuan Flagship Store	Electricity savings1	Jingdong's delivery never disappoints, or so fast, and the stuff is great, next time you need to come back.	Jingdong prefecture level city in Shandong
Jinfeng Hongyuan Flagship Store	Electricity savings1	At first it was ok today, after using it for a while it didn't work anymore	Jingdong prefecture level city in Shandong

Jinfeng Hongyuan Flagship Store	Electricity savings1	Just received the goods, do not know the effect of using a few days before there may be effect	Jingdong prefecture level city in Shandong
Jinfeng Hongyuan Flagship Store	Electricity savings1	The logistics was fast, I received it the next day, I've purchased it for the second time and have been using it all along	Jingdong prefecture level city in Shandong
Jinfeng Hongyuan Flagship Store	Electricity savings1	Inexpensive, fast delivery, very pleased!	Jingdong prefecture level city in Shandong
Jinfeng Hongyuan Flagship Store	Electricity savings1	Received the goods immediately plugged directly into the use of power saving yet to be good news!	Jingdong prefecture level city in Shandong
Jinfeng Hongyuan Flagship Store	Electricity savings1	The service attitude is very good, and the Jingdong self-owned products can be trusted.	Jingdong prefecture level city in Shandong
Jinfeng Hongyuan Flagship Store	Electricity savings1	The product is good, I do not know if it can save play a power saving effect	Jingdong prefecture level city in Shandong
Jinfeng Hongyuan Flagship Store	Electricity savings1	We won't know how effective it is until we use it for a while, so I'll give you a good review now.	Jingdong prefecture level city in Shandong
Jinfeng Hongyuan Flagship Store	Electricity savings1	The quality is pretty good, just started using it, hopefully it will deliver results.	Jingdong prefecture level city in Shandong
Jinfeng Hongyuan Flagship Store	Electricity savings1	I've plugged it in first, and will follow up after I've used it to see how it works.	Jingdong prefecture level city in Shandong
Jinfeng Hongyuan Flagship Store	Electricity savings1	Things have arrived, the quality is good, the effect is also equivalent to can ah	Jingdong prefecture level city in Shandong
Jinfeng Hongyuan Flagship Store	Electricity savings1	It's not worth it to brag about power savings.	Jingdong prefecture level city in Shandong
Jinfeng Hongyuan Flagship Store	Electricity savings1	I don't know if it's an IQ tax, but I'll use it for now.	Jingdong prefecture level city in Shandong
Jinfeng Hongyuan Flagship Store	Electricity savings1	I don't feel the power saving yet, I can't know until I use it for 2 months.	Jingdong prefecture level city in Shandong
Jinfeng Hongyuan Flagship Store	Electricity savings1	There's nothing wrong with this at the moment.	Jingdong prefecture level city in Shandong
Jinfeng Hongyuan Flagship Store	Electricity savings1	It's authentic and worth buying	Jingdong prefecture level city in Shandong
Jinfeng Hongyuan Flagship Store	Electricity savings1	It's good. We'll see how it goes.	Jingdong prefecture level city in Shandong
Jinfeng Hongyuan Flagship Store	Electricity savings1	I don't know if it's working or not in the middle of using it, we'll see when the month is over	Jingdong prefecture level city in Shandong
Jinfeng Hongyuan Flagship Store	Electricity savings1	I'm not going to buy anything from Jingdong anymore, so I'll give it a good review	Jingdong prefecture level city in Shandong
Jinfeng Hongyuan Flagship Store	Electricity savings1	I'm not going to buy anything from Jingdong anymore, so I'll give it a good review	Jingdong prefecture level city in Shandong
Jinfeng Hongyuan Flagship Store	Electricity savings1	Just received the item and will see how it works in a month.	Jingdong prefecture level city in Shandong
Jinfeng Hongyuan Flagship Store	Electricity savings1	I don't know if it saves electricity, but it's plugged into an outlet every day anyway	Jingdong prefecture level city in Shandong
Jinfeng Hongyuan Flagship Store	Electricity savings1	I don't know if it works yet.	Jingdong prefecture level city in Shandong
Jinfeng Hongyuan Flagship Store	Electricity savings1	Plugged it in when I got back, waiting for a month to see the results	Jingdong prefecture level city in Shandong
Jinfeng Hongyuan Flagship Store	Electricity savings1	Effects remain to be seen, but it looks good quality and runs fine	Jingdong prefecture level city in Shandong
Jinfeng Hongyuan Flagship Store	Electricity savings1	The product received logistics very quickly, has not yet been used to test! After use in the evaluation!	Jingdong prefecture level city in Shandong
Jinfeng Hongyuan Flagship Store	Electricity savings1	The power saver is in use, and I don't know how much electricity has been saved after using it for more than a month, and the display voltmeter is very good.	Jingdong prefecture level city in Shandong
Jinfeng Hongyuan Flagship Store	Electricity savings1	It's so good, isn't it? You're a real Shanghainese, aren't you?	Jingdong prefecture level city in Shandong
Jinfeng Hongyuan Flagship Store	Electricity savings1	Great quality great great great 👍👍👍👍	Jingdong prefecture level city in Shandong
Jinfeng Hongyuan Flagship Store	Electricity savings1	I'm using it, I don't know if it's working yet.	Jingdong prefecture level city in Shandong
Jinfeng Hongyuan Flagship Store	Electricity savings1	Just used it for more than 10 days and haven't seen any results yet	Jingdong prefecture level city in Shandong
Jinfeng Hongyuan Flagship Store	Electricity savings1	The product arrived home feeling like it should work	Jingdong prefecture level city in Shandong
Jinfeng Hongyuan Flagship Store	Electricity savings1	Just received the goods, use it and comment back	Jingdong prefecture level city in Shandong
Jinfeng Hongyuan Flagship Store	Electricity savings1	Try it for a month and then evaluate	Jingdong prefecture level city in Shandong
Jinfeng Hongyuan Flagship Store	Electricity savings1	I don't know if it saves power yet, but I'll comment again after using it for a while.	Jingdong prefecture level city in Shandong
Jinfeng Hongyuan Flagship Store	Electricity savings1	The product is better than I thought, the quality and appearance are very good, the voltage is stable, and I'm looking forward to a good power saving effect.	Jingdong prefecture level city in Shandong
Jinfeng Hongyuan Flagship Store	Electricity savings1	Received and scanned the QR code to check that it is genuine, have plugged it in, can it save power, looking forward to the advertised effect. Customary praise, 5 points!	Jingdong prefecture level city in Shandong
Jinfeng Hongyuan Flagship Store	Electricity savings1	The goods have been received and have not yet been used. Do not know how the effect.	Jingdong prefecture level city in Shandong
Jinfeng Hongyuan Flagship Store	Electricity savings1	Hopefully, you get what you pay for, and you get what you pay for!	Jingdong prefecture level city in Shandong
Jinfeng Hongyuan Flagship Store	Electricity savings1	Just put it on and it feels like it's turning as fast as a meter! Looking forward to the results!	Jingdong prefecture level city in Shandong
Jinfeng Hongyuan Flagship Store	Electricity savings1	Overall look on the product quality is good, I do not know is not the effect of power saving, wait to see and then follow up!	Jingdong prefecture level city in Shandong
Jinfeng Hongyuan Flagship Store	Electricity savings1	The logistics courier is very fast, the courier service attitude is very good	Jingdong prefecture level city in Shandong
Jinfeng Hongyuan Flagship Store	Electricity savings1	The product has been received, it works well worth buying	Jingdong prefecture level city in Shandong
Jinfeng Hongyuan Flagship Store	Electricity savings1	How did it not work? Is it purely an IQ tax?	Jingdong prefecture level city in Shandong
Jinfeng Hongyuan Flagship Store	Electricity savings1	It looks good, I don't know how well it works.	Jingdong prefecture level city in Shandong
Jinfeng Hongyuan Flagship Store	Electricity savings1	It does seem to be a bit of a power saver, I just don't know if the more expensive one is more power efficient?	Jingdong prefecture level city in Shandong
Jinfeng Hongyuan Flagship Store	Electricity savings1	Is genuine very good, the price is much cheaper, cost-effective, really special paddle just to do activities, the price is much cheaper, cost-effective, really special paddle counting, packaging is also very high-end atmospheric class, it is worth buying!	Jingdong prefecture level city in Shandong
Jinfeng Hongyuan Flagship Store	Electricity savings1	I received it yesterday and used it right away, I hope it works, I'll wait for next month to come back to the conclusion, I'm looking forward to it. Five hearts praise!	Jingdong prefecture level city in Shandong
Jinfeng Hongyuan Flagship Store	Electricity savings1	Looks good, packed properly, received it today, am using it, will see results in a month!	Jingdong prefecture level city in Shandong
Jinfeng Hongyuan Flagship Store	Electricity savings1	Regular product Jingdong self-support trustworthy. There is an effect Come back to the review. 😍😍😍😍	Jingdong prefecture level city in Shandong
Jinfeng Hongyuan Flagship Store	Electricity savings1	I haven't used it yet, hopefully it will work as a power saver, and will comment again if it works well.	Jingdong prefecture level city in Shandong
Jinfeng Hongyuan Flagship Store	Electricity savings1	Shipping was fast, logistics was fast, service was good, and I look forward to good results from the item!	Jingdong prefecture level city in Shandong

Jinfeng Hongyuan Flagship Store	Electricity savings1	This product is this compensator, it has to be at the main switch to be useful, haven't compared the final result, the	Jingdong prefecture level city in Shandong
Jinfeng Hongyuan Flagship Store	Electricity savings1	Packaging is perfect . I've already started using it, so I'm looking forward to the results.	Jingdong prefecture level city in Shandong
Jinfeng Hongyuan Flagship Store	Electricity savings1	I just got it back. I'll comment after I use it.	Jingdong prefecture level city in Shandong
Jinfeng Hongyuan Flagship Store	Electricity savings1	Big brand, with a very assured, very good quality, catch the event to buy very cost-effective to take advantage of the offer, hurry to get, it is genuine, the packaging is also very good, no breakage, the quality is not to speak of, great 👍 logistics super super fast! Quality is also very good, indeed value	Jingdong prefecture level city in Shandong
Jinfeng Hongyuan Flagship Store	Electricity savings1	The quality is pretty good. The price is also quite affordable.	Jingdong prefecture level city in Shandong
Jinfeng Hongyuan Flagship Store	Electricity savings1	I did not expect the courier so fast to arrive yesterday just ordered, the head is a little smaller than I thought, well, the picture looks bigger. The appearance still looks good, as for the practicality and effectiveness of the use of the only know.	Jingdong prefecture level city in Shandong
Jinfeng Hongyuan Flagship Store	Electricity savings1	I don't know. Looking forward to it.	Jingdong prefecture level city in Shandong
Jinfeng Hongyuan Flagship Store	Electricity savings1	Baby received, very delicate, very nice, like. Just don't know if it can really save electricity. Baby received, very delicate, very good-looking, like. Just don't know if it can really save electricity.	Jingdong prefecture level city in Shandong
Jinfeng Hongyuan Flagship Store	Electricity savings1	I'll use it for a month and then comment on the quality of the item	Jingdong prefecture level city in Shandong
Jinfeng Hongyuan Flagship Store	Electricity savings1	It feels ok, use it for a while and see how it works before adding more comments.	Jingdong prefecture level city in Shandong
Jinfeng Hongyuan Flagship Store	Electricity savings1	In use, see results in a month.	Jingdong prefecture level city in Shandong
Jinfeng Hongyuan Flagship Store	Electricity savings1	Jingdong's logistics and quality are very good, the service attitude is very good, the logistics is very fast	Jingdong prefecture level city in Shandong
Jinfeng Hongyuan Flagship Store	Electricity savings1	Intelligent power saver received, well-packed logistics is also quite fast, easy to use, looking forward to good results!	Jingdong prefecture level city in Shandong
Jinfeng Hongyuan Flagship Store	Electricity savings1	The quality is unknown at this point, I won't know until I use it.	Jingdong prefecture level city in Shandong
Jinfeng Hongyuan Flagship Store	Electricity savings1	It's a good product. It really saves power and doesn't take up much space.	Jingdong prefecture level city in Shandong
Jinfeng Hongyuan Flagship Store	Electricity savings1	Goods have been received, packaging is very good, good to see the effect of future use in the follow-up.	Jingdong prefecture level city in Shandong
Jinfeng Hongyuan Flagship Store	Electricity savings1	The product is in perfect shape and the light weight is worth it!	Jingdong prefecture level city in Shandong
Jinfeng Hongyuan Flagship Store	Electricity savings1	Just got home and plugged it in. It's good.	Jingdong prefecture level city in Shandong
Jinfeng Hongyuan Flagship Store	Electricity savings1	The logistics are quick, just to see the results.	Jingdong prefecture level city in Shandong
Jinfeng Hongyuan Flagship Store	Electricity savings1	Short time can not feel here to leave a message, to be followed up later!	Jingdong prefecture level city in Shandong
Jinfeng Hongyuan Flagship Store	Electricity savings1	This user thinks the product is good	Jingdong prefecture level city in Shandong
Jinfeng Hongyuan Flagship Store	Electricity savings1	Received the baby, to be used to see the effect of it!	Jingdong prefecture level city in Shandong
Jinfeng Hongyuan Flagship Store	Electricity savings1	You have to wait a month or two to see how it works.	Jingdong prefecture level city in Shandong
Jinfeng Hongyuan Flagship Store	Electricity savings1	Not bad, good, like it, don't know if it's good, try it first.	Jingdong prefecture level city in Shandong
Jinfeng Hongyuan Flagship Store	Electricity savings1	The goods are good value for money and the logistics is very fast and good service!	Jingdong prefecture level city in Shandong
Jinfeng Hongyuan Flagship Store	Electricity savings1	I don't know how well it works, we'll see in a month	Jingdong prefecture level city in Shandong
Jinfeng Hongyuan Flagship Store	Electricity savings1	The product is fine, but r saving power or not I won't know until I use it for two months. The display and lights take electricity too?	Jingdong prefecture level city in Shandong
Jinfeng Hongyuan Flagship Store	Electricity savings1	I've used it. I don't know how it works yet.	Jingdong prefecture level city in Shandong
Jinfeng Hongyuan Flagship Store	Electricity savings1	I can't say until I compare them.	Jingdong prefecture level city in Shandong
Jinfeng Hongyuan Flagship Store	Electricity savings1	It's ok in the process of using 。。。。	Jingdong prefecture level city in Shandong
Jinfeng Hongyuan Flagship Store	Electricity savings1	Jinfeng Hongyuan Intelligent Power Saver Household Electricity Meter Electricity Saving Appliance High Power Enhanced Power Saving King Energy Saving Treasure Butler Control Non Stealing Electricity Reversing Not Going Interfering Slow Turner Artifact King Gold Yellow. Haven't tested it accurately yet, really need to wait a few months to test it before I have a say.	Jingdong prefecture level city in Shandong
Jinfeng Hongyuan Flagship Store	Electricity savings1	Don't know how effective it is yet? I hope it works well and I'll come back next time	Jingdong prefecture level city in Shandong
Jinfeng Hongyuan Flagship Store	Electricity savings1	Saving device has been received, material workmanship are very good ah! Have not yet begun to use. First observe the daily electricity consumption and cost of the home meter, and then plug in the power saver to use, so as to compare the use of the effect is good!	Jingdong prefecture level city in Shandong
Jinfeng Hongyuan Flagship Store	Electricity savings1	Merchants shipping extremely fast, Jingdong logistics safe and fast, I believe that the product can play a safe use of electricity, the role of scientific power saving, first praise, the effect of how to be used for a period of time and then the pursuit of bubble!	Jingdong prefecture level city in Shandong
Jinfeng Hongyuan Flagship Store	Electricity savings1	I just bought it. I don't know yet.	Jingdong prefecture level city in Shandong
Jinfeng Hongyuan Flagship Store	Electricity savings1	It is genuine, good quality, and it is very reassuring to use. The logistics is very fast, and the business service attitude is on point.	Jingdong prefecture level city in Shandong
Jinfeng Hongyuan Flagship Store	Electricity savings1	The quality is fine, I don't know how effective it is.	Jingdong prefecture level city in Shandong
Jinfeng Hongyuan Flagship Store	Electricity savings1	The stuff is back and I expect it will save the electricity	Jingdong prefecture level city in Shandong
Jinfeng Hongyuan Flagship Store	Electricity savings1	I'll give it 4 stars first and see what happens in a couple of months and then follow up	Jingdong prefecture level city in Shandong
Jinfeng Hongyuan Flagship Store	Electricity savings1	It's not bad. It's in the mind.	Jingdong prefecture level city in Shandong
Jinfeng Hongyuan Flagship Store	Electricity savings1	Received the goods have not yet started to use do not know whether it is good	Jingdong prefecture level city in Shandong
Jinfeng Hongyuan Flagship Store	Electricity savings1	This user thinks the product is good	Jingdong prefecture level city in Shandong
Jinfeng Hongyuan Flagship Store	Electricity savings1	Hello! The saver was received, thank you! Not yet used, but from the packaging looks very good quality, exquisite workmanship. Logistics is quite fast, if it is really very power saving, I will come back to	Jingdong prefecture level city in Shandong
Jinfeng Hongyuan Flagship Store	Electricity savings1	I've been using it for a few days now, I'll comment again after a month or two of use	Jingdong prefecture level city in Shandong
Jinfeng Hongyuan Flagship Store	Electricity savings1	I don't know. Bought it for my father at a good price.	Jingdong prefecture level city in Shandong
Jinfeng Hongyuan Flagship Store	Electricity savings1	Plugged it in the same day I bought it and the voltage showed 223, 225 looking forward to the results	Jingdong prefecture level city in Shandong
Jinfeng Hongyuan Flagship Store	Electricity savings1	Still in use, the biggest role is to stabilize the current, reduce losses, product quality on the look is very good, with this feeling quite good, see colleagues are using, power saving effect should be good	Jingdong prefecture level city in Shandong
Jinfeng Hongyuan Flagship Store	Electricity savings1	How to receive goods but no invoice and warranty more than it ...... quality is the premise of reputation!	Jingdong prefecture level city in Shandong

Jinfeng Hongyuan Flagship Store	Electricity savings1	Received but not used Continued evaluation	Jingdong prefecture level city in Shandong
Jinfeng Hongyuan Flagship Store	Electricity savings1	I just received the product, I don't know if it works well yet	Jingdong prefecture level city in Shandong
Jinfeng Hongyuan Flagship Store	Electricity savings1	Received it and used it, not sure it really saves electricity use it and then evaluate it, first give a 5 star review!!!!	Jingdong prefecture level city in Shandong
Jinfeng Hongyuan Flagship Store	Electricity savings1	It's good to use, it's very good to use, and it smells OK too	Jingdong prefecture level city in Shandong
Jinfeng Hongyuan Flagship Store	Electricity savings1	Packaging is very good, feel the product is not bad, how much power saving with the only know	Jingdong prefecture level city in Shandong
Jinfeng Hongyuan Flagship Store	Electricity savings1	Additional comments to be made after use	Jingdong prefecture level city in Shandong
Jinfeng Hongyuan Flagship Store	Electricity savings1	The product has been installed, try a few months and then comment!	Jingdong prefecture level city in Shandong
Jinfeng Hongyuan Flagship Store	Electricity savings1	I've bought it, let's use it for now, I'll comment after a month	Jingdong prefecture level city in Shandong
Jinfeng Hongyuan Flagship Store	Electricity savings1	Try it out and then follow up	Jingdong prefecture level city in Shandong
Jinfeng Hongyuan Flagship Store	Electricity savings1	It's good in theory, it doesn't feel good in practice.	Jingdong prefecture level city in Shandong
Jinfeng Hongyuan Flagship Store	Electricity savings1	I haven't tried it yet. We'll see.	Jingdong prefecture level city in Shandong
Jinfeng Hongyuan Flagship Store	Electricity savings1	Great item, second purchase!	Jingdong prefecture level city in Shandong
Jinfeng Hongyuan Flagship Store	Electricity savings1	Goods received now use it, if it does save power, plus reevaluate.	Jingdong prefecture level city in Shandong
Jinfeng Hongyuan Flagship Store	Electricity savings1	The stuff is light and small, the stuff is good, the courier service is good	Jingdong prefecture level city in Shandong
Jinfeng Hongyuan Flagship Store	Electricity savings1	I haven't used it yet. I'm looking forward to it.	Jingdong prefecture level city in Shandong
Jinfeng Hongyuan Flagship Store	Electricity savings1	Express received, in use do not know if there is no power saving	Jingdong prefecture level city in Shandong
Jinfeng Hongyuan Flagship Store	Electricity savings1	The quality of the product is quite good! The power saving mode is still getting used to it, can't say yet, how much power it saves.	Jingdong prefecture level city in Shandong
Jinfeng Hongyuan Flagship Store	Electricity savings1	The goods have been received, just started to use, now I do not know how the quality, after a period of time to say.	Jingdong prefecture level city in Shandong
Jinfeng Hongyuan Flagship Store	Electricity savings1	I received the stuff, used it for a few days to evaluate, the stuff is very good and good, worth buying.	Jingdong prefecture level city in Shandong
Jinfeng Hongyuan Flagship Store	Electricity savings1	I've been using it for a while, but I don't know if it's good or if it saves electricity! What the heck, let's give it a good review first. And for the record, I'm not helping him**.	Jingdong prefecture level city in Shandong
Jinfeng Hongyuan Flagship Store	Electricity savings1	Haven't used it yet, looks good, try it for 60 days, might be good, great review!	Jingdong prefecture level city in Shandong
Jinfeng Hongyuan Flagship Store	Electricity savings1	Baby received, logistics is very fast. Ordered last night, received this morning. Received the goods immediately plugged in the power supply, save power to the first of next month to know.	Jingdong prefecture level city in Shandong
Jinfeng Hongyuan Flagship Store	Electricity savings1	First, I'll give you a good review and use it.	Jingdong prefecture level city in Shandong
Jinfeng Hongyuan Flagship Store	Electricity savings1	Just arrived to follow up with a genuine review	Jingdong prefecture level city in Shandong
Jinfeng Hongyuan Flagship Store	Electricity savings1	Not bad pretty fast, good quality.	Jingdong prefecture level city in Shandong
Jinfeng Hongyuan Flagship Store	Electricity savings1	Recommended, use it and hopefully it will save some electricity	Jingdong prefecture level city in Shandong
Jinfeng Hongyuan Flagship Store	Electricity savings1	The stuff looks ok, I don't know the effect if it.	Jingdong prefecture level city in Shandong
Jinfeng Hongyuan Flagship Store	Electricity savings1	The stuff looks OK, the stuff looks OK.	Jingdong prefecture level city in Shandong
Jinfeng Hongyuan Flagship Store	Electricity savings1	The item has been received, after using it in a follow-up review. Satisfied with the purchase.	Jingdong prefecture level city in Shandong
Jinfeng Hongyuan Flagship Store	Electricity savings1	Has been normal plug-in use, at present do not know how to see, use down to see and then evaluation	Jingdong prefecture level city in Shandong
Jinfeng Hongyuan Flagship Store	Electricity savings1	Commodities make noise.	Jingdong prefecture level city in Shandong
Jinfeng Hongyuan Flagship Store	Electricity savings1	After using it for a few days and observing it, the saving is about ten percent	Jingdong prefecture level city in Shandong
Jinfeng Hongyuan Flagship Store	Electricity savings1	The goods received, the quality looks quite OK. First use it, the effect is not good to say.	Jingdong prefecture level city in Shandong
Jinfeng Hongyuan Flagship Store	Electricity savings1	In use, follow up next month	Jingdong prefecture level city in Shandong
Jinfeng Hongyuan Flagship Store	Electricity savings1	I just received the goods and have not yet used it to comment after using it	Jingdong prefecture level city in Shandong
Jinfeng Hongyuan Flagship Store	Electricity savings1	So far I don't know if it really saves electricity yet! Spinning waiting a couple months to find out! Good reviews first!	Jingdong prefecture level city in Shandong
Jinfeng Hongyuan Flagship Store	Electricity savings1	Small and exquisite, looking forward to the effect, to be evaluated 6666666666666	Jingdong prefecture level city in Shandong
Jinfeng Hongyuan Flagship Store	Electricity savings1	I just used it, I don't know if it's effective, I'll comment again after a month	Jingdong prefecture level city in Shandong
Jinfeng Hongyuan Flagship Store	Electricity savings1	I just used it, I don't know if it's good or not, I'll comment later.	Jingdong prefecture level city in Shandong
Jinfeng Hongyuan Flagship Store	Electricity savings1	Baby received, Baobei quite good, use and then come back to comment!	Jingdong prefecture level city in Shandong
Jinfeng Hongyuan Flagship Store	Electricity savings1	Good quality power saving is fake	Jingdong prefecture level city in Shandong
Jinfeng Hongyuan Flagship Store	Electricity savings1	The packaging of the goods is atmospheric, the quality is exquisite, and I look forward to saving electricity.	Jingdong prefecture level city in Shandong
Jinfeng Hongyuan Flagship Store	Electricity savings1	The packaging is great and the quality is good!	Jingdong prefecture level city in Shandong
Jinfeng Hongyuan Flagship Store	Electricity savings1	I don't see any effect.	Jingdong prefecture level city in Shandong
Jinfeng Hongyuan Flagship Store	Electricity savings1	Already used, but did not see obvious results	Jingdong prefecture level city in Shandong
Jinfeng Hongyuan Flagship Store	Electricity savings1	It's good. I'm just using it. I don't know what it's like.	Jingdong prefecture level city in Shandong
Jinfeng Hongyuan Flagship Store	Electricity savings1	I just used it. I'll comment later.	Jingdong prefecture level city in Shandong
Jinfeng Hongyuan Flagship Store	Electricity savings1	I installed it when it arrived, try it for a month and then come back for a follow-up review	Jingdong prefecture level city in Shandong
Jinfeng Hongyuan Flagship Store	Electricity savings1	Goods arrived a few days fastidious does not call to pick up the goods, the quality is average	Jingdong prefecture level city in Shandong
Jinfeng Hongyuan Flagship Store	Electricity savings1	This user thinks the product is good	Jingdong prefecture level city in Shandong
Jinfeng Hongyuan Flagship Store	Electricity savings1	Roger that.	Jingdong prefecture level city in Shandong

Jinfeng Hongyuan Flagship Store	Electricity savings1	Second purchase was ok.	Jingdong prefecture level city in Shandong
Jinfeng Hongyuan Flagship Store	Electricity savings1	The merchant is very reputable and the product is good value for money. Logistics is fast!	Jingdong prefecture level city in Shandong
Jinfeng Hongyuan Flagship Store	Electricity savings1	The quality of the baby is very good, I have always trusted Kyodo	Jingdong prefecture level city in Shandong
Jinfeng Hongyuan Flagship Store	Electricity savings1	We'll see how it goes when we get it.	Jingdong prefecture level city in Shandong
Jinfeng Hongyuan Flagship Store	Electricity savings1	I just used it. I don't know if it works.	Jingdong prefecture level city in Shandong
Jinfeng Hongyuan Flagship Store	Electricity savings1	Good quality, used for a month to save about 20%, power saving effect is good	Jingdong prefecture level city in Shandong
Jinfeng Hongyuan Flagship Store	Electricity savings1	I'll give you a good review before I use it.	Jingdong prefecture level city in Shandong
Jinfeng Hongyuan Flagship Store	Electricity savings1	Received, satisfied with the follow-up evaluation	Jingdong prefecture level city in Shandong
Jinfeng Hongyuan Flagship Store	Electricity savings1	Immediate results, saving electricity and money	Jingdong prefecture level city in Shandong
Jinfeng Hongyuan Flagship Store	Electricity savings1	Measured to save a little	Jingdong prefecture level city in Shandong
Jinfeng Hongyuan Flagship Store	Electricity savings1	This user thinks the product is good	Jingdong prefecture level city in Shandong
Jinfeng Hongyuan Flagship Store	Electricity savings1	This user thinks the product is good	Jingdong prefecture level city in Shandong
Jinfeng Hongyuan Flagship Store	Electricity savings1	Product quality and logistics are very good, worth buying	Jingdong prefecture level city in Shandong
Jinfeng Hongyuan Flagship Store	Electricity savings1	Appearance looks ok! Express delivery was fast! Service is still relatively enthusiastic.	Jingdong prefecture level city in Shandong
Jinfeng Hongyuan Flagship Store	Electricity savings1	It doesn't feel like it's working.	Jingdong prefecture level city in Shandong
Jinfeng Hongyuan Flagship Store	Electricity savings1	Well made and looks good. Used it with other appliances and felt as if it had some effect.	Jingdong prefecture level city in Shandong
Jinfeng Hongyuan Flagship Store	Electricity savings1	Haven't used it yet, will follow up later	Jingdong prefecture level city in Shandong
Jinfeng Hongyuan Flagship Store	Electricity savings1	Things received, used a few days the plug is a little hot unplugged did not work	Jingdong prefecture level city in Shandong
Jinfeng Hongyuan Flagship Store	Electricity savings1	Look at the meter again in a month.	Jingdong prefecture level city in Shandong
Jinfeng Hongyuan Flagship Store	Electricity savings1	This one didn't work at all.	Jingdong prefecture level city in Shandong
Jinfeng Hongyuan Flagship Store	Electricity savings1	It's small. I've seen friends with much bigger ones.	Jingdong prefecture level city in Shandong
Jinfeng Hongyuan Flagship Store	Electricity savings1	The item was received and the quality is good, it will be nice to see how the power saving rate is down the road, the item is great.	Jingdong prefecture level city in Shandong
Jinfeng Hongyuan Flagship Store	Electricity savings1	Cheap and affordable quality is quite good, logistics is also very fast, praise! , baby good 😊, cost-effective very high, big brand trustworthy 👍	Jingdong prefecture level city in Shandong
Jinfeng Hongyuan Flagship Store	Electricity savings1	Used it for a while it really saves electricity ⚡ thanks 🈶 such a good product, compact and practical, every family should buy one ✨	Jingdong prefecture level city in Shandong
Jinfeng Hongyuan Flagship Store	Electricity savings1	Very satisfied with the purchase Next time I will come back	Jingdong prefecture level city in Shandong
Jinfeng Hongyuan Flagship Store	Electricity savings1	Logistics is fast. Good quality, used one before and it does save power	Jingdong prefecture level city in Shandong
Jinfeng Hongyuan Flagship Store	Electricity savings1	I don't know if it works.	Jingdong prefecture level city in Shandong
Jinfeng Hongyuan Flagship Store	Electricity savings1	Good goods, very solid, good.	Jingdong prefecture level city in Shandong
Jinfeng Hongyuan Flagship Store	Electricity savings1	This user thinks the product is good	Jingdong prefecture level city in Shandong
Jinfeng Hongyuan Flagship Store	Electricity savings1	I've been using it for half a month, my electricity bill is much lower, and the quality is very good, so don't worry about buying it!	Jingdong prefecture level city in Shandong
Jinfeng Hongyuan Flagship Store	Electricity savings1	I received the goods today and used it right away, looking forward to the effect	Jingdong prefecture level city in Shandong
Jinfeng Hongyuan Flagship Store	Electricity savings1	The merchandise is great. I am going to recommend it to my friends! Will buy again next time.	Jingdong prefecture level city in Shandong
Jinfeng Hongyuan Flagship Store	Electricity savings1	Let's give it a good review first, it's working fine so far, and I'll follow up with a review,...	Jingdong prefecture level city in Shandong
Jinfeng Hongyuan Flagship Store	Electricity savings1	Product quality is very good, looking forward to using the results	Jingdong prefecture level city in Shandong
Jinfeng Hongyuan Flagship Store	Electricity savings1	I just can't detect it. - Save it.	Jingdong prefecture level city in Shandong
Jinfeng Hongyuan Flagship Store	Electricity savings1	It arrived and is being used, so we'll see how it works!	Jingdong prefecture level city in Shandong
Jinfeng Hongyuan Flagship Store	Electricity savings1	Received yesterday thanks! This energy saver is available . Safe! But in the end how much energy can be saved must be used for a period of time to know, if the real energy saving next time to buy again.	Jingdong prefecture level city in Shandong
Jinfeng Hongyuan Flagship Store	Electricity savings1	Just used it, don't know if it saves power	Jingdong prefecture level city in Shandong
Jinfeng Hongyuan Flagship Store	Electricity savings1	I'll rate it first, is it a tease wait two months and see? It's been four days and it's not noticeable!	Jingdong prefecture level city in Shandong
Jinfeng Hongyuan Flagship Store	Electricity savings1	Workmanship is quite good, just started to use, the effect of the follow-up evaluation.	Jingdong prefecture level city in Shandong
Jinfeng Hongyuan Flagship Store	Electricity savings1	Received the goods used for a few days before evaluation, the effect is quite good	Jingdong prefecture level city in Shandong
Jinfeng Hongyuan Flagship Store	Electricity savings1	Try it for a month and then evaluate	Jingdong prefecture level city in Shandong
Jinfeng Hongyuan Flagship Store	Electricity savings1	Just received the goods, the packaging is very good, the appearance of the economizer feels good, I do not know how the effect of power saving, with a month or two and then follow up!	Jingdong prefecture level city in Shandong
Jinfeng Hongyuan Flagship Store	Electricity savings1	It's quite small and delicate, I don't know if it's useful yet.	Jingdong prefecture level city in Shandong
Jinfeng Hongyuan Flagship Store	Electricity savings1	Just received it, use it for a few days before commenting	Jingdong prefecture level city in Shandong
Jinfeng Hongyuan Flagship Store	Electricity savings1	Does this thing work! When it arrived, the button for the safety was loose. I'm a little afraid of fire!	Jingdong prefecture level city in Shandong
Jinfeng Hongyuan Flagship Store	Electricity savings1	I received it. I don't know how it works.	Jingdong prefecture level city in Shandong
Jinfeng Hongyuan Flagship Store	Electricity savings1	This user thinks the product is good	Jingdong prefecture level city in Shandong
Jinfeng Hongyuan Flagship Store	Electricity savings1	Customer service was great! Small i problem was taken care of in a timely manner! Effectiveness to be tested by time! No rush!!!	Jingdong prefecture level city in Shandong
Jinfeng Hongyuan Flagship Store	Electricity savings1	I put it on right after I received it and it looks compact. I believe the high technology should save electricity.	Jingdong prefecture level city in Shandong
Jinfeng Hongyuan Flagship Store	Electricity savings1	Logistics courier very quickly, the first day under the order the next day to receive, off pieces whether power saving is the most important thing, things are exquisite workmanship, a month later in the follow-up evaluation whether power saving	Jingdong prefecture level city in Shandong

Jinfeng Hongyuan Flagship Store	Electricity savings1	The delivery is pretty fast, I'm using it, have to have it for a while to see results, looking forward to it.	Jingdong prefecture level city in Shandong
Jinfeng Hongyuan Flagship Store	Electricity savings1	See pretty good just started to use a month later to give you a real evaluation, should be useful.	Jingdong prefecture level city in Shandong
Jinfeng Hongyuan Flagship Store	Electricity savings1	And then comment.	Jingdong prefecture level city in Shandong
Jinfeng Hongyuan Flagship Store	Electricity savings1	Received the goods, wait for the test to evaluate	Jingdong prefecture level city in Shandong
Jinfeng Hongyuan Flagship Store	Electricity savings1	This user thinks the product is good	Jingdong prefecture level city in Shandong
Jinfeng Hongyuan Flagship Store	Electricity savings1	I'm already using it, I just don't know how to judge the power savings. I won't know until I use it for a season.	Jingdong prefecture level city in Shandong
Jinfeng Hongyuan Flagship Store	Electricity savings1	Works. Says it works.	Jingdong prefecture level city in Shandong
Jinfeng Hongyuan Flagship Store	Electricity savings1	Very good. Satisfied.	Jingdong prefecture level city in Shandong
Jinfeng Hongyuan Flagship Store	Electricity savings1	This user thinks the product is good	Jingdong prefecture level city in Shandong
Jinfeng Hongyuan Flagship Store	Electricity savings1	It's working. Obviously, it's less expensive.	Jingdong prefecture level city in Shandong
Jinfeng Hongyuan Flagship Store	Electricity savings1	The second one is up, the first one still works! It's reactive power compensation! This is well understood if you want a professional!	Jingdong prefecture level city in Shandong
Jinfeng Hongyuan Flagship Store	Electricity savings1	It feels as if there are some nodes, but they need to be tested and observed over time, thanks.	Jingdong prefecture level city in Shandong
Jinfeng Hongyuan Flagship Store	Electricity savings1	I've received the goods, the energy saving effect will be evaluated after using it for a period of time, and all the services are very good, praise!	Jingdong prefecture level city in Shandong
Jinfeng Hongyuan Flagship Store	Electricity savings1	I don't know how well it works yet, I bought it and plugged it in, so we'll see if it saves some power or not!	Jingdong prefecture level city in Shandong
Jinfeng Hongyuan Flagship Store	Electricity savings1	Baby a bunch of knife quality is very good, previously bought a quality is not very good. It really works and has results.	Jingdong prefecture level city in Shandong
Jinfeng Hongyuan Flagship Store	Electricity savings1	Things have arrived, it looks good, just started to use, as to whether or not there is a business said the power-saving effect, but also to observe the effect, now can not evaluate	Jingdong prefecture level city in Shandong
Jinfeng Hongyuan Flagship Store	Electricity savings1	I haven't used it yet. I'll comment afterward.	Jingdong prefecture level city in Shandong
Jinfeng Hongyuan Flagship Store	Electricity savings1	I don't know if it's any good.	Jingdong prefecture level city in Shandong
Jinfeng Hongyuan Flagship Store	Electricity savings1	I don't know how well it works.	Jingdong prefecture level city in Shandong
Jinfeng Hongyuan Flagship Store	Electricity savings1	The baby has been received! Uh-huh! Good, quite exquisite, small and exquisite I thought it was big. Dare to tightly plugged in the electricity, just wait for the effect later!	Jingdong prefecture level city in Shandong
Jinfeng Hongyuan Flagship Store	Electricity savings1	The packaging is sturdy, I tried it right after I received it, so I'm looking forward to the results.	Jingdong prefecture level city in Shandong
Jinfeng Hongyuan Flagship Store	Electricity savings1	Received the item and installed it! Don't know if it works well! Will follow up with a review later! I hope it works!	Jingdong prefecture level city in Shandong
Jinfeng Hongyuan Flagship Store	Electricity savings1	Things used on the effect of waiting to use a little to say, but things are very good. Logistics is very fast, business service is very good.	Jingdong prefecture level city in Shandong
Jinfeng Hongyuan Flagship Store	Electricity savings1	This user thinks the product is good	Jingdong prefecture level city in Shandong
Jinfeng Hongyuan Flagship Store	Electricity savings1	I'm really using it. It feels good.	Jingdong prefecture level city in Shandong
Jinfeng Hongyuan Flagship Store	Electricity savings1	Good, Jingdong logistics is very fast, intelligent energy-saving appliances	Jingdong prefecture level city in Shandong
Jinfeng Hongyuan Flagship Store	Electricity savings1	It's not bad. It really saves energy.	Jingdong prefecture level city in Shandong
Jinfeng Hongyuan Flagship Store	Electricity savings1	The logistics is fast, praise	Jingdong prefecture level city in Shandong
Jinfeng Hongyuan Flagship Store	Electricity savings1	This user thinks the product is good	Jingdong prefecture level city in Shandong
Jinfeng Hongyuan Flagship Store	Electricity savings1	The first time I placed two, and today I placed another one, a friend introduced it, said it is particularly power-saving	Jingdong prefecture level city in Shandong
Jinfeng Hongyuan Flagship Store	Electricity savings1	I don't know how effective it is. Let's leave it at home for a while and see.	Jingdong prefecture level city in Shandong
Jinfeng Hongyuan Flagship Store	Electricity savings1	Dear customer service! I would like to ask you how to understand the digital display (increase/decrease jump) on the top of the saver? Thank you for your help.	Jingdong prefecture level city in Shandong
Jinfeng Hongyuan Flagship Store	Electricity savings1	This user thinks the product is good	Jingdong prefecture level city in Shandong
Jinfeng Hongyuan Flagship Store	Electricity savings1	The goods have been received, packaging exquisite, is using, later to see how the effect?	Jingdong prefecture level city in Shandong
Jinfeng Hongyuan Flagship Store	Electricity savings1	The saver received, the boss shipped very quickly, yesterday shot today arrived!	Jingdong prefecture level city in Shandong
Jinfeng Hongyuan Flagship Store	Electricity savings1	Beautiful appearance, well packaged. Recording data to see if I can lower my electric bill a bit.	Jingdong prefecture level city in Shandong
Jinfeng Hongyuan Flagship Store	Electricity savings1	Things received, the packaging is quite good, the effect of the use and then come back to evaluate.	Jingdong prefecture level city in Shandong
Jinfeng Hongyuan Flagship Store	Electricity savings1	Easy to use, just plug it in. But there is a current sound.	Jingdong prefecture level city in Shandong
Jinfeng Hongyuan Flagship Store	Electricity savings1	Fast logistics, easy to use and labor-saving	Jingdong prefecture level city in Shandong
Jinfeng Hongyuan Flagship Store	Electricity savings1	Received, thank you!	Jingdong prefecture level city in Shandong
Jinfeng Hongyuan Flagship Store	Electricity savings1	Second time I've bought it. It's good.	Jingdong prefecture level city in Shandong
Jinfeng Hongyuan Flagship Store	Electricity savings1	I've only been using it for a short time, so I'm looking forward to the results.	Jingdong prefecture level city in Shandong
Jinfeng Hongyuan Flagship Store	Electricity savings1	Good stuff, good stuff, good stuff good stuff.	Jingdong prefecture level city in Shandong
Jinfeng Hongyuan Flagship Store	Electricity savings1	Buy your stuff is not always good I have been using it for almost two months now, it does not save electricity at all the electricity bill is about ten dollars a day this is still called the king of power	Jingdong prefecture level city in Shandong
Jinfeng Hongyuan Flagship Store	Electricity savings1	Express arrived, the quality is very good, worth the purchase.	Jingdong prefecture level city in Shandong
Jinfeng Hongyuan Flagship Store	Electricity savings1	Energy saving has been received, now the voltage is more stable, energy-saving effect to see a few days and then chase flat, the service attitude is very good, the courier is relatively fast, I am satisfied.	Jingdong prefecture level city in Shandong
Jinfeng Hongyuan Flagship Store	Electricity savings1	Stuff installed, numbers are smaller than when installed, hopefully it works	Jingdong prefecture level city in Shandong
Jinfeng Hongyuan Flagship Store	Electricity savings1	Good appearance, fine workmanship, just bought, use up the feeling of power saving effect is good, worth buying!	Jingdong prefecture level city in Shandong
Jinfeng Hongyuan Flagship Store	Electricity savings1	Economical and practical 👍 Always use it	Jingdong prefecture level city in Shandong
Jinfeng Hongyuan Flagship Store	Electricity savings1	Just used it, don't know the effect yet, will come back to follow up after the effect. Hope it works well(▽)	Jingdong prefecture level city in Shandong
Jinfeng Hongyuan Flagship Store	Electricity savings1	The goods have been received, use it to see in the evaluation.	Jingdong prefecture level city in Shandong

Jinfeng Hongyuan Flagship Store	Electricity savings1	Doesn't it save electricity? The boil kettle still goes faster when you press the meter.	Jingdong prefecture level city in Shandong
Jinfeng Hongyuan Flagship Store	Electricity savings1	Already used. Will patronize again in the future	Jingdong prefecture level city in Shandong
Jinfeng Hongyuan Flagship Store	Electricity savings1	Works great, just plug it in, results to be checked, satisfied.	Jingdong prefecture level city in Shandong
Jinfeng Hongyuan Flagship Store	Electricity savings1	Second purchase. Works well 😍😍😍😍	Jingdong prefecture level city in Shandong
Jinfeng Hongyuan Flagship Store	Electricity savings1	Prompt shipment, very satisfied	Jingdong prefecture level city in Shandong
Jinfeng Hongyuan Flagship Store	Electricity savings1	It's been less than a month, let's see how it works, hopefully it's power efficient	Jingdong prefecture level city in Shandong
Jinfeng Hongyuan Flagship Store	Electricity savings1	Use a month in say.	Jingdong prefecture level city in Shandong
Jinfeng Hongyuan Flagship Store	Electricity savings1	I've saved a lot of electricity since I've been using it.	Jingdong prefecture level city in Shandong
Jinfeng Hongyuan Flagship Store	Electricity savings1	It's fast, looks and feels good on the outside, try it out and evaluate.	Jingdong prefecture level city in Shandong
Jinfeng Hongyuan Flagship Store	Electricity savings1	I haven't used it. I just used it. I don't know if it's any good.	Jingdong prefecture level city in Shandong
Jinfeng Hongyuan Flagship Store	Electricity savings1	Quality can be, power saving now can not see just use, after a while in the look at the power saving effect	Jingdong prefecture level city in Shandong
Jinfeng Hongyuan Flagship Store	Electricity savings1	The logistics is very fast, and it seems to have some effect	Jingdong prefecture level city in Shandong
Jinfeng Hongyuan Flagship Store	Electricity savings1	Good, but the effects remain to be seen	Jingdong prefecture level city in Shandong
Jinfeng Hongyuan Flagship Store	Electricity savings1	Baby is basically the same as the description, shipping quickly, worth a buy Good price, logistics is strong, worth repurchasing!	Jingdong prefecture level city in Shandong
Jinfeng Hongyuan Flagship Store	Electricity savings1	I bought it for my dad, I don't know much about it, if he thinks it's good then it must be good 👍	Jingdong prefecture level city in Shandong
Jinfeng Hongyuan Flagship Store	Electricity savings1	Goods received. It's working fine. Display voltage. After a period of time to see the effect in the addition to the level of theory	Jingdong prefecture level city in Shandong
Jinfeng Hongyuan Flagship Store	Electricity savings1	The stuff is pretty good, I just don't know how well it saves power.	Jingdong prefecture level city in Shandong
Jinfeng Hongyuan Flagship Store	Electricity savings1	Logistics is a little too fast 👍	Jingdong prefecture level city in Shandong
Jinfeng Hongyuan Flagship Store	Electricity savings1	Received the goods used for a period of time before coming, good, beautiful appearance, really stable flow	Jingdong prefecture level city in Shandong
Jinfeng Hongyuan Flagship Store	Electricity savings1	Things received, look at the packaging is very good, not yet, to the power saving in the follow-up evaluation	Jingdong prefecture level city in Shandong
Jinfeng Hongyuan Flagship Store	Electricity savings1	Baby is very good! Use a few days before to evaluate, I personally test really effective.	Jingdong prefecture level city in Shandong
Jinfeng Hongyuan Flagship Store	Electricity savings1	I received the goods are in use, I do not know how effective? Can it save energy? Let's see.	Jingdong prefecture level city in Shandong
Jinfeng Hongyuan Flagship Store	Electricity savings1	It's really good and has some power saving effect.	Jingdong prefecture level city in Shandong
Jinfeng Hongyuan Flagship Store	Electricity savings1	It's good quality, well made, and I hope it'll save on electricity over time.	Jingdong prefecture level city in Shandong
Jinfeng Hongyuan Flagship Store	Electricity savings1	I just used it, I don't know how it works yet, but I'll give it a good review first.	Jingdong prefecture level city in Shandong
Jinfeng Hongyuan Flagship Store	Electricity savings1	Second purchase, the delivery guy was fast and called ahead to deliver.	Jingdong prefecture level city in Shandong
Jinfeng Hongyuan Flagship Store	Electricity savings1	Goods have been received, but have not yet used, used to see how the effect, in the evaluation of fertilizer	Jingdong prefecture level city in Shandong
Jinfeng Hongyuan Flagship Store	Electricity savings1	The delivery is very fast. It's the same in kind. It's working.	Jingdong prefecture level city in Shandong
Jinfeng Hongyuan Flagship Store	Electricity savings1	Fast logistics, good quality, more satisfied	Jingdong prefecture level city in Shandong
Jinfeng Hongyuan Flagship Store	Electricity savings1	I've been here for almost a month now and there is a very noticeable difference than it is worth buying.	Jingdong prefecture level city in Shandong
Jinfeng Hongyuan Flagship Store	Electricity savings1	Fast shipping, received the next day, plug and play, but do not know how effective to not break down!	Jingdong prefecture level city in Shandong
Jinfeng Hongyuan Flagship Store	Electricity savings1	The logistics are great I've been using it for a while before commenting on it This saves electricity	Jingdong prefecture level city in Shandong
Jinfeng Hongyuan Flagship Store	Electricity savings1	Wait to use it for a while to know if it's energy efficient	Jingdong prefecture level city in Shandong
Jinfeng Hongyuan Flagship Store	Electricity savings1	Just received it, haven't used it yet don't know if it's any good.	Jingdong prefecture level city in Shandong
Jinfeng Hongyuan Flagship Store	Electricity savings1	It really doesn't work, and the electric bill is still the same amount every month.	Jingdong prefecture level city in Shandong
Jinfeng Hongyuan Flagship Store	Electricity savings1	It's well packaged and looks fine	Jingdong prefecture level city in Shandong
Jinfeng Hongyuan Flagship Store	Electricity savings1	Very good. Will patronize again if needed.	Jingdong prefecture level city in Shandong
Jinfeng Hongyuan Flagship Store	Electricity savings1	I have received the product and installed it, try it for a while and see how it works.	Jingdong prefecture level city in Shandong
Jinfeng Hongyuan Flagship Store	Electricity savings1	I've been using it for a month now...I don't feel like there's any noticeable power savings...last month it was $130 this month it's $123...let's see if I can use it for another month	Jingdong prefecture level city in Shandong
Jinfeng Hongyuan Flagship Store	Electricity savings1	Just received it, yet to try it and see if it works.	Jingdong prefecture level city in Shandong
Jinfeng Hongyuan Flagship Store	Electricity savings1	It's easy to use and has a power saving credit!	Jingdong prefecture level city in Shandong
Jinfeng Hongyuan Flagship Store	Electricity savings1	It's very good. I've already bought two of them.	Jingdong prefecture level city in Shandong
Jinfeng Hongyuan Flagship Store	Electricity savings1	It's very well made, packaged meticulously, and I hope it works!	Jingdong prefecture level city in Shandong
Jinfeng Hongyuan Flagship Store	Electricity savings1	Goods have been received have not yet used it, used to comment again	Jingdong prefecture level city in Shandong
Jinfeng Hongyuan Flagship Store	Electricity savings1	Looking forward to good results	Jingdong prefecture level city in Shandong
Jinfeng Hongyuan Flagship Store	Electricity savings1	Not sure if it saves power or not. Still trying it out.	Jingdong prefecture level city in Shandong
Jinfeng Hongyuan Flagship Store	Electricity savings1	It also really saves on electricity! Superb!	Jingdong prefecture level city in Shandong
Jinfeng Hongyuan Flagship Store	Electricity savings1	Didn't work at all, want to return it	Jingdong prefecture level city in Shandong
Jinfeng Hongyuan Flagship Store	Electricity savings1	Uh-huh! It's working, I've been using it for a month and it saves 20% of electricity compared to before.	Jingdong prefecture level city in Shandong
Jinfeng Hongyuan Flagship Store	Electricity savings1	Should be a little bit of a power saver, I wonder how long it will take to save back the money I spent on this one	Jingdong prefecture level city in Shandong
Jinfeng Hongyuan Flagship Store	Electricity savings1	Exquisite appearance and stable voltage	Jingdong prefecture level city in Shandong

Jinfeng Hongyuan Flagship Store	Electricity savings1	After using it for a few days it feels like it saves electricity.	Jingdong prefecture level city in Shandong
Jinfeng Hongyuan Flagship Store	Electricity savings1	Second purchase and its pending good results.	Jingdong prefecture level city in Shandong
Jinfeng Hongyuan Flagship Store	Electricity savings1	The appearance of the workmanship is exquisite and meticulous, beautiful appearance, just to see if there is no effect!	Jingdong prefecture level city in Shandong
Jinfeng Hongyuan Flagship Store	Electricity savings1	Let's see what happens in a month.	Jingdong prefecture level city in Shandong
Jinfeng Hongyuan Flagship Store	Electricity savings1	Trial, now short time not yet obvious effect	Jingdong prefecture level city in Shandong
Jinfeng Hongyuan Flagship Store	Electricity savings1	I'm using it now, don't know how well it works, it looks great!	Jingdong prefecture level city in Shandong
Jinfeng Hongyuan Flagship Store	Electricity savings1	There is no role! Now it's true that I don't know!	Jingdong prefecture level city in Shandong
Jinfeng Hongyuan Flagship Store	Electricity savings1	Purchased for the second time, power saving not sure if it's ok? Let's talk about it after using it	Jingdong prefecture level city in Shandong
Jinfeng Hongyuan Flagship Store	Electricity savings1	I just received it, I have to try it for a month to know how it works.	Jingdong prefecture level city in Shandong
Jinfeng Hongyuan Flagship Store	Electricity savings1	I just used it for an hour, and the meter's still spinning so fast it's not much use.	Jingdong prefecture level city in Shandong
Jinfeng Hongyuan Flagship Store	Electricity savings1	Not bad, logistics is fast	Jingdong prefecture level city in Shandong
Jinfeng Hongyuan Flagship Store	Electricity savings1	I don't know if it works. I'll find out next month when I pay the bill. It's easy. Just plug it in.	Jingdong prefecture level city in Shandong
Jinfeng Hongyuan Flagship Store	Electricity savings1	Logistics is particularly fast, received the goods are genuine, looking forward to power saving effect	Jingdong prefecture level city in Shandong
Jinfeng Hongyuan Flagship Store	Electricity savings1	First time buying this product like this one from this family use it and then evaluate.	Jingdong prefecture level city in Shandong
Jinfeng Hongyuan Flagship Store	Electricity savings1	Lightning arrived, the product is very good, before all do not know there is such a baby, in a friend's home has seen, and then bought to try, has been used for a period of time before coming to the evaluation, the work is very stable, so far looked very good, will introduce friends to buy!	Jingdong prefecture level city in Shandong
Jinfeng Hongyuan Flagship Store	Electricity savings1	I was a bit skeptical when I heard that I could save money on my electricity bill by installing this, I don't know if the power saving effect is really what it is said to be. But since I bought it, I just have to put it on and try it out. I hope it lives up to its name and saves me the money I spent on the device	Jingdong prefecture level city in Shandong
Jinfeng Hongyuan Flagship Store	Electricity savings1	The quality of the stuff is okay, but I'm not sure if it works, so I'll try using it first.	Jingdong prefecture level city in Shandong
Jinfeng Hongyuan Flagship Store	Electricity savings1	It should work. The current is much more stable.	Jingdong prefecture level city in Shandong
Jinfeng Hongyuan Flagship Store	Electricity savings1	The store has a great service attitude	Jingdong prefecture level city in Shandong
Jinfeng Hongyuan Flagship Store	Electricity savings1	Let's wait until we've used it for a month.	Jingdong prefecture level city in Shandong
Jinfeng Hongyuan Flagship Store	Electricity savings1	I'm using it and I don't know if it's a power saver or not	Jingdong prefecture level city in Shandong
Jinfeng Hongyuan Flagship Store	Electricity savings1	Original and authentic guarantee quality is good now.	Jingdong prefecture level city in Shandong
Jinfeng Hongyuan Flagship Store	Electricity savings1	Great, very happy with it, will buy a couple more next time	Jingdong prefecture level city in Shandong
Jinfeng Hongyuan Flagship Store	Electricity savings1	I just used it, I don't know if it works yet, I'll come back after using it...	Jingdong prefecture level city in Shandong
Jinfeng Hongyuan Flagship Store	Electricity savings1	It's still being tested.	Jingdong prefecture level city in Shandong
Jinfeng Hongyuan Flagship Store	Electricity savings1	I've been using it for a month and it's not working.	Jingdong prefecture level city in Shandong
Jinfeng Hongyuan Flagship Store	Electricity savings1	It's basically nothing!	Jingdong prefecture level city in Shandong
Jinfeng Hongyuan Flagship Store	Electricity savings1	It's not bad, it kind of works, support it.	Jingdong prefecture level city in Shandong
Jinfeng Hongyuan Flagship Store	Electricity savings1	I just received it last night, let's try it first to see how it works, it looks good!	Jingdong prefecture level city in Shandong
Jinfeng Hongyuan Flagship Store	Electricity savings1	The current is very stable, fine workmanship, small and exquisite, very satisfied	Jingdong prefecture level city in Shandong
Jinfeng Hongyuan Flagship Store	Electricity savings1	Not bad, exactly how much electricity can be saved; still have to follow up the evaluation	Jingdong prefecture level city in Shandong
Jinfeng Hongyuan Flagship Store	Electricity savings1	Logistics is also very fast, next time there is an opportunity to introduce friends to buy!	Jingdong prefecture level city in Shandong
Jinfeng Hongyuan Flagship Store	Electricity savings1	Voltage display and actual do not pay	Jingdong prefecture level city in Shandong
Jinfeng Hongyuan Flagship Store	Electricity savings1	Second purchase, still saves a little on the electric bill	Jingdong prefecture level city in Shandong
Jinfeng Hongyuan Flagship Store	Electricity savings1	Order received, currently trying it out, thanks a lot.	Jingdong prefecture level city in Shandong
Jinfeng Hongyuan Flagship Store	Electricity savings1	I haven't used it for a long time yet, I don't know if the effect is good or not, looking forward to it	Jingdong prefecture level city in Shandong
Jinfeng Hongyuan Flagship Store	Electricity savings1	Good, working, I just don't know if it will save electricity... 5 stars for now.	Jingdong prefecture level city in Shandong
Jinfeng Hongyuan Flagship Store	Electricity savings1	Great product, just as the owner said it would be.	Jingdong prefecture level city in Shandong
Jinfeng Hongyuan Flagship Store	Electricity savings1	Shipping was fast, I don't know if it works well or not, I have to use it for a while first.	Jingdong prefecture level city in Shandong
Jinfeng Hongyuan Flagship Store	Electricity savings1	I don't feel that it saves much electricity, but I'll give it a try. It's been more than a month now, and the electricity bill is still a bit high.	Jingdong prefecture level city in Shandong
Jinfeng Hongyuan Flagship Store	Electricity savings1	Got it. Just used it.	Jingdong prefecture level city in Shandong
Jinfeng Hongyuan Flagship Store	Electricity savings1	Use it for a while and then comment.	Jingdong prefecture level city in Shandong
Jinfeng Hongyuan Flagship Store	Electricity savings1	Used it, see how it works with it and then comment. The quality is very good!	Jingdong prefecture level city in Shandong
Jinfeng Hongyuan Flagship Store	Electricity savings1	I'll comment again after a while.	Jingdong prefecture level city in Shandong
Jinfeng Hongyuan Flagship Store	Electricity savings1	It's easy to operate, affordable and works well, love it.	Jingdong prefecture level city in Shandong
Jinfeng Hongyuan Flagship Store	Electricity savings1	It's good. You can try it. It's cheap.	Jingdong prefecture level city in Shandong
Jinfeng Hongyuan Flagship Store	Electricity savings1	It's in use, so we'll see how it works after a while!	Jingdong prefecture level city in Shandong
Jinfeng Hongyuan Flagship Store	Electricity savings1	Service is pretty good, not sure about the quality yet.	Jingdong prefecture level city in Shandong
Jinfeng Hongyuan Flagship Store	Electricity savings1	Just used it and will see the results in a month 😀	Jingdong prefecture level city in Shandong
Jinfeng Hongyuan Flagship Store	Electricity savings1	Saving electricity is expensive and good	Jingdong prefecture level city in Shandong

Jinfeng Hongyuan Flagship Store	Electricity savings1	Pretty good, 5 stars! Will buy again next time seller service is very good !!!! Good quality! Will recommend to friends!	Jingdong prefecture level city in Shandong
Jinfeng Hongyuan Flagship Store	Electricity savings1	In use, with results naturally have follow-up reviews!	Jingdong prefecture level city in Shandong
Jinfeng Hongyuan Flagship Store	Electricity savings1	I'm using it now, I'll come back with results, it'll work.	Jingdong prefecture level city in Shandong
Jinfeng Hongyuan Flagship Store	Electricity savings1	Good, nice, fast shipping, cheap, worth having!	Jingdong prefecture level city in Shandong
Jinfeng Hongyuan Flagship Store	Electricity savings1	Logistics is fast.	Jingdong prefecture level city in Shandong
Jinfeng Hongyuan Flagship Store	Electricity savings1	I have just started using the product and will be reviewing it later.	Jingdong prefecture level city in Shandong
Jinfeng Hongyuan Flagship Store	Electricity savings1	I'll comment after I use it.	Jingdong prefecture level city in Shandong
Jinfeng Hongyuan Flagship Store	Electricity savings1	I don't know if it's working or not, though I'll try plugging it in.	Jingdong prefecture level city in Shandong
Jinfeng Hongyuan Flagship Store	Electricity savings1	It really saves electricity and contributes to energy savings in disguise	Jingdong prefecture level city in Shandong
Jinfeng Hongyuan Flagship Store	Electricity savings1	It feels good in the experience.	Jingdong prefecture level city in Shandong
Jinfeng Hongyuan Flagship Store	Electricity savings1	The product is better than I thought it would be, the quality looks great, the voltage is stable, and I'm looking forward to some great power savings!	Jingdong prefecture level city in Shandong
Jinfeng Hongyuan Flagship Store	Electricity savings1	I'm using it now looking forward to saving some points,, I'm feeding back	Jingdong prefecture level city in Shandong
Jinfeng Hongyuan Flagship Store	Electricity savings1	Displayed voltage below 200.	Jingdong prefecture level city in Shandong
Jinfeng Hongyuan Flagship Store	Electricity savings1	I haven't seen any great results, but it should last about a hundred or eighty years.	Jingdong prefecture level city in Shandong
Jinfeng Hongyuan Flagship Store	Electricity savings1	I don't know if it works or not, but I keep it plugged in anyway, so I hope it works!	Jingdong prefecture level city in Shandong
Jinfeng Hongyuan Flagship Store	Electricity savings1	Reasonable price, good quality, fast delivery, great service, already a repurchase.	Jingdong prefecture level city in Shandong
Jinfeng Hongyuan Flagship Store	Electricity savings1	Second purchase, looking forward to the results	Jingdong prefecture level city in Shandong
Jinfeng Hongyuan Flagship Store	Electricity savings1	The quality of this product feels like it should work in a use test! Just how many kilowatts of electricity must be wasted per day with the red light and digital display on for long periods of time?	Jingdong prefecture level city in Shandong
Jinfeng Hongyuan Flagship Store	Electricity savings1	It looks good, we'll see if we use it.	Jingdong prefecture level city in Shandong
Jinfeng Hongyuan Flagship Store	Electricity savings1	Great stuff, second purchase!	Jingdong prefecture level city in Shandong
Jinfeng Hongyuan Flagship Store	Electricity savings1	It is indeed very practical and saves electricity at home	Jingdong prefecture level city in Shandong
Jinfeng Hongyuan Flagship Store	Electricity savings1	Goods received, the physical and the same as the picture, logistics is also very fast!	Jingdong prefecture level city in Shandong
Jinfeng Hongyuan Flagship Store	Electricity savings1	I'll use it for a while and see how it goes. I'll buy it again if it's good.	Jingdong prefecture level city in Shandong
Jinfeng Hongyuan Flagship Store	Electricity savings1	In use, voltage monitoring, the effect of how to wait for next month's electricity consumption confirmation	Jingdong prefecture level city in Shandong
Jinfeng Hongyuan Flagship Store	Electricity savings1	It's still pretty good.	Jingdong prefecture level city in Shandong
Jinfeng Hongyuan Flagship Store	Electricity savings1	It's been plugged in for a month and there hasn't been a big change yet, since it usually uses very little electricity	Jingdong prefecture level city in Shandong
Jinfeng Hongyuan Flagship Store	Electricity savings1	Let's wait for a month to see if it works	Jingdong prefecture level city in Shandong
Jinfeng Hongyuan Flagship Store	Electricity savings1	Good, good, good, good, good, good, good, good, good, good, good, good, good, good, good.	Jingdong prefecture level city in Shandong
Jinfeng Hongyuan Flagship Store	Electricity savings1	The product received the trial a few days, feel good, praise!	Jingdong prefecture level city in Shandong
Jinfeng Hongyuan Flagship Store	Electricity savings1	After six or seven hours of use, 250 volts won't save you any power.	Jingdong prefecture level city in Shandong
Jinfeng Hongyuan Flagship Store	Electricity savings1	I haven't used it yet, I wonder if it saves electricity?	Jingdong prefecture level city in Shandong
Jinfeng Hongyuan Flagship Store	Electricity savings1	Baby value for money, love it, next time will come back is genuine, trust the business, baby texture is very good, the price is moderate, unlimited repurchase!	Jingdong prefecture level city in Shandong
Jinfeng Hongyuan Flagship Store	Electricity savings1	Power saving effect is obvious! Raising tropical fish daily power consumption, after using the daily power savings of more than 3 degrees, currently about 8 degrees / day!	Jingdong prefecture level city in Shandong
Jinfeng Hongyuan Flagship Store	Electricity savings1	Just used it, unknown results.	Jingdong prefecture level city in Shandong
Jinfeng Hongyuan Flagship Store	Electricity savings1	The goods received, well, look forward to the effect. Logistics is very fast, thanks to the platform, thanks to Jingdong. I hope it can save electricity.	Jingdong prefecture level city in Shandong
Jinfeng Hongyuan Flagship Store	Electricity savings1	Not sure 有 effect?	Jingdong prefecture level city in Shandong
Jinfeng Hongyuan Flagship Store	Electricity savings1	I'd say it's a 5% savings.	Jingdong prefecture level city in Shandong
Jinfeng Hongyuan Flagship Store	Electricity savings1	I received it and am using it, I don't know how effective it is, I'll evaluate it afterwards	Jingdong prefecture level city in Shandong
Jinfeng Hongyuan Flagship Store	Electricity savings1	Baby received not yet used, first give praise	Jingdong prefecture level city in Shandong
Jinfeng Hongyuan Flagship Store	Electricity savings1	Looks good, I'll evaluate it later	Jingdong prefecture level city in Shandong
Jinfeng Hongyuan Flagship Store	Electricity savings1	The goods have been received in use, the overall feeling is not bad!	Jingdong prefecture level city in Shandong
Jinfeng Hongyuan Flagship Store	Electricity savings1	It's working. This is the second one. This stuff has to be used over a long period of time to be effective. And it only works on inductive loads, i.e. appliances containing electric motors. Use it.	Jingdong prefecture level city in Shandong
Jinfeng Hongyuan Flagship Store	Electricity savings1	Use it for a while and see how it works.	Jingdong prefecture level city in Shandong
Jinfeng Hongyuan Flagship Store	Electricity savings1	Good courier service and the item is very nice and practical.	Jingdong prefecture level city in Shandong
Jinfeng Hongyuan Flagship Store	Electricity savings1	It doesn't do anything and it's a waste of electricity.	Jingdong prefecture level city in Shandong
Jinfeng Hongyuan Flagship Store	Electricity savings1	I haven't used it yet. It should be good.	Jingdong prefecture level city in Shandong
Jinfeng Hongyuan Flagship Store	Electricity savings1	That's good. OK, that's good.	Jingdong prefecture level city in Shandong
Jinfeng Hongyuan Flagship Store	Electricity savings1	It's a rip off, doesn't work, doesn't save electricity, does the job to well, and I've wasted a couple dozen dollars again.	Jingdong prefecture level city in Shandong
Jinfeng Hongyuan Flagship Store	Electricity savings1	Use it first, the effect is not as good as the road, then we will comment again	Jingdong prefecture level city in Shandong
Jinfeng Hongyuan Flagship Store	Electricity savings1	The price is affordable, the logistics is strong, just use four or five days, the effect will be added later!	Jingdong prefecture level city in Shandong
Jinfeng Hongyuan Flagship Store	Electricity savings1	It's easy to use and works fine.	Jingdong prefecture level city in Shandong

Jinfeng Hongyuan Flagship Store	Electricity savings1	It doesn't feel like it's working.	Jingdong prefecture level city in Shandong
Jinfeng Hongyuan Flagship Store	Electricity savings1	I'm using it. We'll see what happens in a couple months.	Jingdong prefecture level city in Shandong
Jinfeng Hongyuan Flagship Store	Electricity savings1	I just got it back to try it out and I'll comment later	Jingdong prefecture level city in Shandong
Jinfeng Hongyuan Flagship Store	Electricity savings1	Less than two months. Can't tell.	Jingdong prefecture level city in Shandong
Jinfeng Hongyuan Flagship Store	Electricity savings1	No power saving effect	Jingdong prefecture level city in Shandong
Jinfeng Hongyuan Flagship Store	Electricity savings1	Express Express Very quickly not only to the power saver can not save electricity, with two months, look at the evaluation put.	Jingdong prefecture level city in Shandong
Jinfeng Hongyuan Flagship Store	Electricity savings1	Genuine products are packaged well!	Jingdong prefecture level city in Shandong
Jinfeng Hongyuan Flagship Store	Electricity savings1	I've been using it for a while, but I don't know if it saves electricity. I hope it works.	Jingdong prefecture level city in Shandong
Jinfeng Hongyuan Flagship Store	Electricity savings1	Good service, fast logistics, the effect is to be used to know	Jingdong prefecture level city in Shandong
Jinfeng Hongyuan Flagship Store	Electricity savings1	Just used it, not sure about the power saving effect	Jingdong prefecture level city in Shandong
Jinfeng Hongyuan Flagship Store	Electricity savings1	The stuff is ok, save power or not, will comment after a month.	Jingdong prefecture level city in Shandong
Jinfeng Hongyuan Flagship Store	Electricity savings1	Used it for a month to save %25, normal is a little over 100, it's great! I want to buy one in.	Jingdong prefecture level city in Shandong
Jinfeng Hongyuan Flagship Store	Electricity savings1	I'm using it and looking forward to the results	Jingdong prefecture level city in Shandong
Jinfeng Hongyuan Flagship Store	Electricity savings1	The quality is very good and the workmanship is exquisite. The use effect is also very good.	Jingdong prefecture level city in Shandong
Jinfeng Hongyuan Flagship Store	Electricity savings1	Merchant service attitude is very good, logistics speed is super fast, online evaluation is very good, the first time to use, a month to know the specific effect, the effect is good, and then additional	Jingdong prefecture level city in Shandong
Jinfeng Hongyuan Flagship Store	Electricity savings1	Works great, solves the problem of high power usage in the house	Jingdong prefecture level city in Shandong
Jinfeng Hongyuan Flagship Store	Electricity savings1	To receive! Very delicate! It's not clear how effective it is, I won't know until I use it!!!!	Jingdong prefecture level city in Shandong
Jinfeng Hongyuan Flagship Store	Electricity savings1	I've just been using it for a while and I don't feel like it's too noticeable.	Jingdong prefecture level city in Shandong
Jinfeng Hongyuan Flagship Store	Electricity savings1	Already used, no visible effect for the time being	Jingdong prefecture level city in Shandong
Jinfeng Hongyuan Flagship Store	Electricity savings1	I can't feel the effect.	Jingdong prefecture level city in Shandong
Jinfeng Hongyuan Flagship Store	Electricity savings1	Use it for a while and see the results	Jingdong prefecture level city in Shandong
Jinfeng Hongyuan Flagship Store	Electricity savings1	The price is more reasonable, the power-saving effect is to be verified, the delivery is fast, and the courier service is good.	Jingdong prefecture level city in Shandong
Jinfeng Hongyuan Flagship Store	Electricity savings1	Quality is very good, consistent with the seller's description, very satisfied, very like, very fast shipping, packaging is very careful and strict, the logistics company has a very good service attitude, fast delivery, very satisfied with a shopping, very satisfied. Recommended to buy, cost-effective, the service attitude of the business is very enthusiastic, providing such high quality goods.	Jingdong prefecture level city in Shandong
Jinfeng Hongyuan Flagship Store	Electricity savings1	I don't know.	Jingdong prefecture level city in Shandong
Jinfeng Hongyuan Flagship Store	Electricity savings1	I didn't get the delivery.	Jingdong prefecture level city in Shandong
Jinfeng Hongyuan Flagship Store	Electricity savings1	It works, it does have power savings, better than expected, will repurchase.	Jingdong prefecture level city in Shandong
Jinfeng Hongyuan Flagship Store	Electricity savings1	That's where the five fuses are.	Jingdong prefecture level city in Shandong
Jinfeng Hongyuan Flagship Store	Electricity savings1	Don't buy it, it doesn't work and you can't return it.	Jingdong prefecture level city in Shandong
Jinfeng Hongyuan Flagship Store	Electricity savings1	I hope it works. Wait a month.	Jingdong prefecture level city in Shandong
Jinfeng Hongyuan Flagship Store	Electricity savings1	Nothing**, buy with caution	Jingdong prefecture level city in Shandong
Jinfeng Hongyuan Flagship Store	Electricity savings1	I'm using it and waiting for the results	Jingdong prefecture level city in Shandong
Jinfeng Hongyuan Flagship Store	Electricity savings1	Saver quality is very good, has always felt that the home power consumption is very large, this is the company's electrician recommended, said he bought this home is really able to save a little electricity, I also came to buy, the effect if it is good, then buy, a floor a...	Jingdong prefecture level city in Shandong
Jinfeng Hongyuan Flagship Store	Electricity savings1	Good product, satisfied with purchase	Jingdong prefecture level city in Shandong
Jinfeng Hongyuan Flagship Store	Electricity savings1	Occupying the time has not yet found the power saving, occupying not evaluation	Jingdong prefecture level city in Shandong
Jinfeng Hongyuan Flagship Store	Electricity savings1	Haven't tested the results yet. To be followed up with comments!	Jingdong prefecture level city in Shandong
Jinfeng Hongyuan Flagship Store	Electricity savings1	After experiencing it for a month, I don't seem to know how to save it, and I don't pay attention to it anyway	Jingdong prefecture level city in Shandong
Jinfeng Hongyuan Flagship Store	Electricity savings1	It's useless. It's a waste of electricity.	Jingdong prefecture level city in Shandong
Jinfeng Hongyuan Flagship Store	Electricity savings1	I don't know if it's a power saver. It's a couple bucks. I hope it works.	Jingdong prefecture level city in Shandong
Jinfeng Hongyuan Flagship Store	Electricity savings1	I don't know what to say about this stuff. I don't know if it works or not, it's kind of a psychological purchase. Dormitory use to see the meter can not be not so good-looking. You have to wait until the time to deduct the utility bill to know.	Jingdong prefecture level city in Shandong
Jinfeng Hongyuan Flagship Store	Electricity savings1	Energy savers don't work / they're all a bunch of hoaxes.	Jingdong prefecture level city in Shandong
Jinfeng Hongyuan Flagship Store	Electricity savings1	Received the goods, used for more than a week, it's okay, sure a little effect	Jingdong prefecture level city in Shandong
Jinfeng Hongyuan Flagship Store	Electricity savings1	Well made and compact! Looking forward to the effect!	Jingdong prefecture level city in Shandong
Jinfeng Hongyuan Flagship Store	Electricity savings1	It has arrived and we'll see how it works.	Jingdong prefecture level city in Shandong
Jinfeng Hongyuan Flagship Store	Electricity savings1	Good. Good. It's worth it.	Jingdong prefecture level city in Shandong
Jinfeng Hongyuan Flagship Store	Electricity savings1	How come there's no indicator light?	Jingdong prefecture level city in Shandong
Jinfeng Hongyuan Flagship Store	Electricity savings1	The machine that uses electricity works quite well. This saves	Jingdong prefecture level city in Shandong
Jinfeng Hongyuan Flagship Store	Electricity savings1	It's good for home use and feels quite energy efficient	Jingdong prefecture level city in Shandong
Jinfeng Hongyuan Flagship Store	Electricity savings1	I regret buying this product, after using it for two days it doesn't work at all, still so many kilowatts per day just like the original.	Jingdong prefecture level city in Shandong
Jinfeng Hongyuan Flagship Store	Electricity savings1	Works very well and has some scientific merit.	Jingdong prefecture level city in Shandong

Jinfeng Hongyuan Flagship Store	Electricity savings1	Logistics is also very fast this good power saving I use two months before commenting!	Jingdong prefecture level city in Shandong
Jinfeng Hongyuan Flagship Store	Electricity savings1	Repeat customer. Very good.	Jingdong prefecture level city in Shandong
Jinfeng Hongyuan Flagship Store	Electricity savings1	It's good stuff. I've been using it for a month now and it's a real power saver.	Jingdong prefecture level city in Shandong
Jinfeng Hongyuan Flagship Store	Electricity savings1	Jingdong platform to buy things, has been very assured, quality assurance. Jingdong Express is as good as ever, complete packaging, delivery, service attitude to go! The family has always wanted to buy an energy-saving and power-saving equipment, selected half a day to choose this one, the value is very good, used for a period of time to follow up, to see whether it can achieve the real purpose of power saving, I	Jingdong prefecture level city in Shandong
Jinfeng Hongyuan Flagship Store	Electricity savings1	Works great, inexpensive and will be back next time.	Jingdong prefecture level city in Shandong
Jinfeng Hongyuan Flagship Store	Electricity savings1	Baby received, looks good, wait for a month to see how it works?	Jingdong prefecture level city in Shandong
Jinfeng Hongyuan Flagship Store	Electricity savings1	This product is not bad quite good well customer service said quite power saving that look at the customer service to see if the accuracy of the look at all aspects of the color is also quite beautiful	Jingdong prefecture level city in Shandong
Jinfeng Hongyuan Flagship Store	Electricity savings1	Just got back and tried it feels pretty good, just don't know how the quality is, give a good comment first	Jingdong prefecture level city in Shandong
Jinfeng Hongyuan Flagship Store	Electricity savings1	Received the product, trying it out, hope it helps!	Jingdong prefecture level city in Shandong
Jinfeng Hongyuan Flagship Store	Electricity savings1	Works great, saves electricity a dollar a day and a night	Jingdong prefecture level city in Shandong
Jinfeng Hongyuan Flagship Store	Electricity savings1	Baby received, look at the workmanship is still very fine, is genuine. I hope that this summer to open the air conditioning electricity bill is no longer high scary!	Jingdong prefecture level city in Shandong
Jinfeng Hongyuan Flagship Store	Electricity savings1	It's here. I don't know how it's going to work, but I'm going to use it.	Jingdong prefecture level city in Shandong
Jinfeng Hongyuan Flagship Store	Electricity savings1	Power saver received, Jingdong logistics first-class, business customer service attitude is good, one by one to introduce the product performance, back on the horse plugged in, it is very simple, there is a voltage indicator, to be a few months to see how the effect of power-saving to come back to a good follow-up, but I believe that Jinfenghong products.	Jingdong prefecture level city in Shandong
Jinfeng Hongyuan Flagship Store	Electricity savings1	Took the third one...the super power-saving kind!	Jingdong prefecture level city in Shandong
Jinfeng Hongyuan Flagship Store	Electricity savings1	First time buyer, customer service is very polite and courteous, conscientious business, support! Baby has received 🔍, super like, high value and practical, super like! ➧➧➧➧➧ Very good quality, very satisfied with a shopping. Customer service has a question and answer, very professional finally received the goods, very, very much like, exquisite and beautiful workmanship, has been praised by many people, really too happy, is already a loyal fan of his family, will continue to buy buy buy buy!	Jingdong prefecture level city in Shandong
Jinfeng Hongyuan Flagship Store	Electricity savings1	That's good. That's good. That's really good.	Jingdong prefecture level city in Shandong
Jinfeng Hongyuan Flagship Store	Electricity savings1	Saver received, good-looking design, voltage at a glance, and the merchant description, save some about 12% or so	Jingdong prefecture level city in Shandong
Jinfeng Hongyuan Flagship Store	Electricity savings1	Intelligent power saver is small and does not take up space, very good, very practical, received and used, packaging is tight, logistics is also very fast, satisfied with a shopping!	Jingdong prefecture level city in Shandong
Jinfeng Hongyuan Flagship Store	Electricity savings1	The stuff is good! Well made, compact, alloy case, shows voltage! Very like! Kudos!	Jingdong prefecture level city in Shandong
Jinfeng Hongyuan Flagship Store	Electricity savings1	I just purchased this power saver from Jingdong, inserted in the socket shows the number 206, a moment and 207 back and forth display, I do not know what it means, my home voltage should be normal, because it is the owner of the community's own power plant to put, first use for a period of time in the positive feedback it, first use in the look at it!	Jingdong prefecture level city in Shandong
Jinfeng Hongyuan Flagship Store	Electricity savings1	Very delicate and compact, there is a convenient intelligent display prompts the voltage, you can know the voltage at a glance, there is also very easy to use, a plug can be used, you can save electricity	Jingdong prefecture level city in Shandong
Jinfeng Hongyuan Flagship Store	Electricity savings1	Logistics is fast, ask customer service said can save electricity, but only use two months to see the effect, use two months in the evaluation.	Jingdong prefecture level city in Shandong
Jinfeng Hongyuan Flagship Store	Electricity savings1	The quality is very good.	Jingdong prefecture level city in Shandong
Jinfeng Hongyuan Flagship Store	Electricity savings1	Smart Saver has been received and will be evaluated after use	Jingdong prefecture level city in Shandong
Jinfeng Hongyuan Flagship Store	Electricity savings1	Home electricity is too waste, a month more than 300 dollars of electricity, and then the family heard that this can save a lot of electricity, let me order to buy a, anyway, will not, buy on the buy, thought it has to be a pretty big box it, delivery to the home to see a very small, plug-and-play, you can really save electricity, it's worth it!	Jingdong prefecture level city in Shandong
Jinfeng Hongyuan Flagship Store	Electricity savings1	Smart Saver received. Unpacking the box is exquisite. The material workmanship is very good. Plugged in and tried the space. Did not open the air conditioning voltage display 206 air conditioning open up the air conditioning display for 194. whether to save electricity to a month later to pay the electricity bill will know. Logistics is also very fast service is also in place.	Jingdong prefecture level city in Shandong
Jinfeng Hongyuan Flagship Store	Electricity savings1	The product feels pretty good and I hope it works, as the store owner said it would. Works!!!	Jingdong prefecture level city in Shandong
Jinfeng Hongyuan Flagship Store	Electricity savings1	Baby received, physical very quickly, just plugged in hope that it can be as everyone said those power saving, with two months after additional comments, first give a five-star praise.	Jingdong prefecture level city in Shandong
Jinfeng Hongyuan Flagship Store	Electricity savings1	Good, very good, very good, you deserve it, whoever uses it knows, the stuff works great, will buy it again in the future.	Jingdong prefecture level city in Shandong
Jinfeng Hongyuan Flagship Store	Electricity savings1	Don't hesitate to buy wow! No cheap feeling, very high-end, especially great! It is genuine, and the same as the entity store, the price is also favorable, decisive turn powder ♡ catch up to do activities when you buy, the price of a penny for a penny ♡, from the outside looking very beautiful ♡, the material workmanship is good ♡, feel that the purchase is worth it, and the logistics is super	Jingdong prefecture level city in Shandong
Jinfeng Hongyuan Flagship Store	Electricity savings1	Logistics is fast! Very good quality! Very beautiful! Next time to patronize!	Jingdong prefecture level city in Shandong
Jinfeng Hongyuan Flagship Store	Electricity savings1	Really like it, completely exceeded expectations, the shipping speed is very fast, the packaging is very careful and strict, the delivery speed is very fast, very satisfied with a shopping Baby received, and the description is the same, love it!	Jingdong prefecture level city in Shandong
Jinfeng Hongyuan Flagship Store	Electricity savings1	Very good, if you need to order!	Jingdong prefecture level city in Shandong
Jinfeng Hongyuan Flagship Store	Electricity savings1	So far, plugged in the voltage is more stable, as for the effect of how to quietly wait until the payment of the electricity bill to compare, the appearance of the commodity design is very good, the real thing!	Jingdong prefecture level city in Shandong
Jinfeng Hongyuan Flagship Store	Electricity savings1	💌 I've been wanting it for a long time, I decided to go for it, I feel great after using it, it's genuine and the delivery is great, it lives up to its name👍	Jingdong prefecture level city in Shandong
Jinfeng Hongyuan Flagship Store	Electricity savings1	Looking forward to it!!! I don't know if it's working or not, let's look forward to it!!!! How much is saved , , , , , , , , , Saving a degree is a degree with long term use ! ! ! ! ! ! !	Jingdong prefecture level city in Shandong
Jinfeng Hongyuan Flagship Store	Electricity savings1	After using it for a while, I am seriously and responsibly saying that this saver is really very power efficient. Two rooms with the air conditioner on at night for one night, plus watching TV, charging cell phones, lights, etc. etc. only used one degree of electricity. So I bought several more and it's really	Jingdong prefecture level city in Shandong
Jinfeng Hongyuan Flagship Store	Electricity savings1	It arrived quickly, and the box is solid and delicate. Baby shell is thick aluminum and by multi-layer coating. The inner core is heavy, and the power cord and plug are genuine products from a large manufacturer. It is the best power saver I have found after choosing all of them!	Jingdong prefecture level city in Shandong
Jinfeng Hongyuan Flagship Store	Electricity savings1	The saver was received, genuine guarantee, good quality...	Jingdong prefecture level city in Shandong
Jinfeng Hongyuan Flagship Store	Electricity savings1	Logistics was fast! The product is easy to use!	Jingdong prefecture level city in Shandong
Jinfeng Hongyuan Flagship Store	Electricity savings1	Look appearance is okay, use a period of time to know how it is. Jingdong Mall, trustworthy! Home appliances, cell phones and computers are almost 100% bought by Jingdong. Ordered the night before yesterday, received yesterday. Jingdong logistics, the fastest in the network!	Jingdong prefecture level city in Shandong
Jinfeng Hongyuan Flagship Store	Electricity savings1	Very good, perfect packaging, home energy saver to determine the effect of power saving and also play a role in stabilizing the voltage Very good products	Jingdong prefecture level city in Shandong

Jinfeng Hongyuan Flagship Store	Electricity savings1	Smart Saver received, looks very exquisite. The method of use is simple, and it has been used. The later effect, with time to test.	Jingdong prefecture level city in Shandong
Jinfeng Hongyuan Flagship Store	Electricity savings1	Both the material and workmanship exceeded expectations, great value, and looks great on oh. The seller also shipped much earlier than expected, will come back. Buy it, you will not regret it! Full five points. Both the material and workmanship exceeded expectations, great value, wear it also looks good Oh. Seller delivery is also much earlier than expected, will come again. Buy it, will not regret it! Full	Jingdong prefecture level city in Shandong
Jinfeng Hongyuan Flagship Store	Electricity savings1	The appearance is clean and beautiful, the workmanship is very good, I didn't find any defects, I personally feel that the size is also quite suitable, I've used it for 2 months, I didn't find out exactly how energy efficient it is. Try using it again!	Jingdong prefecture level city in Shandong
Jinfeng Hongyuan Flagship Store	Electricity savings1	Power saving device received, product certification, accessories, etc. complete, very good workmanship, very practical power-saving effect is also very obvious.	Jingdong prefecture level city in Shandong
Jinfeng Hongyuan Flagship Store	Electricity savings1	Looked at a lot of think buy this one, see a lot of reviews say it works, a month with save nearly twenty degrees of electricity, there are sixty days of return guarantee, with a good and then come back to the	Jingdong prefecture level city in Shandong
Jinfeng Hongyuan Flagship Store	Electricity savings1	I received the power saving device, and I'm using it. I hope I can get the desired effect, and I'm trying to force it to work quickly.	Jingdong prefecture level city in Shandong
Jinfeng Hongyuan Flagship Store	Electricity savings1	Voltage is stable, already trying it out and waiting for the results to happen, saving electricity will follow up next month!	Jingdong prefecture level city in Shandong
Jinfeng Hongyuan Flagship Store	Electricity savings1	Jingdong Mall self-supporting goods of excellent quality, logistics is also very fast, worth recommending 👏👏👏👏 excellent!	Jingdong prefecture level city in Shandong
Jinfeng Hongyuan Flagship Store	Electricity savings1	Power saving has been received, the appearance of the beautiful, I have already been the second time to buy the use of down the effect is very good!	Jingdong prefecture level city in Shandong
Jinfeng Hongyuan Flagship Store	Electricity savings1	Energy saver received very happy, the color is also very good looking forward to a very good effect can save electricity	Jingdong prefecture level city in Shandong
Jinfeng Hongyuan Flagship Store	Electricity savings1	It's small and easy to plug in and use. It displays the voltage value when plugged in and	Jingdong prefecture level city in Shandong
Jinfeng Hongyuan Flagship Store	Electricity savings1	This saver really works well, because I installed one at home about seven or eight years ago, when there is no voltage display, now there is a voltage display, the appearance also looks much better, the electricity bill is also saved, the stability of the home appliances is also good, the best of both	Jingdong prefecture level city in Shandong
Jinfeng Hongyuan Flagship Store	Electricity savings1	Ordered yesterday, arrived today in use. Shipping was fast. The effect is looking forward to.	Jingdong prefecture level city in Shandong
Jinfeng Hongyuan Flagship Store	Electricity savings1	This thing, I don't know what the principle is. Why it can save electricity. Wait for a month to use it first, see if it will save electricity bill, then come back to evaluate. There is one thing that does not fit the principle ah. The law of conservation of energy.	Jingdong prefecture level city in Shandong
Jinfeng Hongyuan Flagship Store	Electricity savings1	Home mom listen to others say this can save electricity, specially let buy a, later a very large, go back to see a particularly small, directly plugged into the electricity can be, no need to install, the price is also very cheap, the first time with this product, do not know if it is not good to use, temporarily try for a period of time, if it is good to use, recommend that you buy this one, the	Jingdong prefecture level city in Shandong
Jinfeng Hongyuan Flagship Store	Electricity savings1	I've been using it for a few days, and I think the workmanship and materials are pretty good, so we'll have to look at the next bill for the real energy savings, and then we'll add more comments.	Jingdong prefecture level city in Shandong
Jinfeng Hongyuan Flagship Store	Electricity savings1	Energy saver is well made, energy saving and power saving, and anti-counterfeiting code, it is a regular commodity!	Jingdong prefecture level city in Shandong
Jinfeng Hongyuan Flagship Store	Electricity savings1	Jinfeng Hongyuan, buy back home, as a mouse, haha. First of all, normal electricity for 1 month, the power did not increase, the electricity bill feels smoother, year-on-year electricity costs in the low, whether it is very energy-saving still have to be used for a long time to know. All 5 stars ☆	Jingdong prefecture level city in Shandong
Jinfeng Hongyuan Flagship Store	Electricity savings1	The box is well packaged and beautiful! I hope to save electricity with it haha!	Jingdong prefecture level city in Shandong
Jinfeng Hongyuan Flagship Store	Electricity savings1	Ordered last night, arrived this morning, first try the effect before making comments	Jingdong prefecture level city in Shandong
Jinfeng Hongyuan Flagship Store	Electricity savings1	Looks good. Don't know the effect yet.	Jingdong prefecture level city in Shandong
Jinfeng Hongyuan Flagship Store	Electricity savings1	Jinfeng Hongyuan intelligent power saver has exquisite workmanship and modern fashionable design style. It is a good home economizer . I believe that the goods purchased from Jingdong will not disappoint our	Jingdong prefecture level city in Shandong
Jinfeng Hongyuan Flagship Store	Electricity savings1	Inadvertently flipped to this power saver, never heard of, feel very novel, the price is not expensive, so buy to try, see a month after the electricity bill will be less, recently the home air-conditioning open a lot of power consumption is very large.	Jingdong prefecture level city in Shandong
Jinfeng Hongyuan Flagship Store	Electricity savings1	The quality of the goods is very good workmanship, but also sent five fuse	Jingdong prefecture level city in Shandong
Jinfeng Hongyuan Flagship Store	Electricity savings1	I'm trying it out. I don't know if it'll work.	Jingdong prefecture level city in Shandong
Jinfeng Hongyuan Flagship Store	Electricity savings1	There is no effect yet do not know, is to look at the official sale, buy to try it! Use according to the instructions, plugged in, used for a period of time, anyway, do not know how, after a few months to see it, to see if the average month than before will be less electricity.	Jingdong prefecture level city in Shandong
Jinfeng Hongyuan Flagship Store	Electricity savings1	Received the goods, the first time to unpacking, the quality is very good, consistent with the description of the seller, completely exceeded my expectations, the packaging is very careful, strict, in general this time is very satisfied with a shopping, thanks to the seller.	Jingdong prefecture level city in Shandong
Jinfeng Hongyuan Flagship Store	Electricity savings1	The energy saver was received and used for a few days with good results.	Jingdong prefecture level city in Shandong
Jinfeng Hongyuan Flagship Store	Electricity savings1	Previously I have paid attention to this product, but I think that the monthly electricity consumption at home is not large, there is no need to save electricity savings. After a second thought, energy saving is the responsibility of each of our citizens, the winter is coming, this year's winter is cold, heating and all electric appliances, can save is to save, the price is not expensive, so buy to try. I analyze the principle of power saving, should be able to save electricity, but the larger the power saving effect is about good. To be tried after a month or two to see the effect and then evaluate. Jingdong trustworthy.	Jingdong prefecture level city in Shandong
Jinfeng Hongyuan Flagship Store	Electricity savings1	It just arrived and I can't wait to use it, and the customer service is great! There is also voltage showing up which is nice! I don't know if it can save a bit I won't know until I've used it for a month! When the time comes to come back to the follow-up review!	Jingdong prefecture level city in Shandong
Jinfeng Hongyuan Flagship Store	Electricity savings1	Checked out the reviews from numerous users. The shipment is still very large, so I decided to buy it anyway to use it and see the effect after a month. Jingdong Mall here to buy safe, convenient and fast, this price is very powerful 💪, very recommended.	Jingdong prefecture level city in Shandong
Jinfeng Hongyuan Flagship Store	Electricity savings1	Purchased two at the same time to use together. The other one works fine, the other has problems with the numbers not sure if the machine is faulty. Power saving effect can not be observed. Feel the effect is not very obvious like?	Jingdong prefecture level city in Shandong
Jinfeng Hongyuan Flagship Store	Electricity savings1	Looking forward to it!!! I don't know if it's working or not, let's look forward to it!!!! How much is saved , , , , , , , , , Saving a degree is a degree with long term use ! ! ! ! ! ! !	Jingdong prefecture level city in Shandong
Jinfeng Hongyuan Flagship Store	Electricity savings1	Received, directly on the use of the special convenient	Jingdong prefecture level city in Shandong
Jinfeng Hongyuan Flagship Store	Electricity savings1	Goods received a few days well-packed, the same day opened and plugged in to try to be normal is not sure what the power-saving effect. After a month with a follow-up evaluation.	Jingdong prefecture level city in Shandong
Jinfeng Hongyuan Flagship Store	Electricity savings1	I bought it and used it, I don't know how it works yet, we'll see later. Use the effect of it	Jingdong prefecture level city in Shandong
Jinfeng Hongyuan Flagship Store	Electricity savings1	Photographed 2, the old family also put a, with a small January, there should be some effect, with a long period of time to comment, with a long time to see the obvious effect, hard courier brother.	Jingdong prefecture level city in Shandong
Jinfeng Hongyuan Flagship Store	Electricity savings1	Experience: I am an electrician, this is the real use of electronic waves automatically connected to track the decomposition of ionized electrons, in situ compensation of reactive power, reduce effective power loss and waste, stabilize the voltage to achieve energy saving and power saving, the overall effect	Jingdong prefecture level city in Shandong
Jinfeng Hongyuan Flagship Store	Electricity savings1	Great looking and practical! Easy to use and save! Saves 15% in a month of comparison	Jingdong prefecture level city in Shandong
Jinfeng Hongyuan Flagship Store	Electricity savings1	Product quality is very good! Packaging is very nice, like a cell phone box, thick and well protected! Service is also great! Logistics is very fast! Thank you for your business! Very honest!	Jingdong prefecture level city in Shandong
Jinfeng Hongyuan Flagship Store	Electricity savings1	In several products inside the choice of this purchase for a period of time, but also need to use a few months to compare last year's electricity bill before you can analyze the savings in electricity, to the summer peak electricity consumption should be more obvious.	Jingdong prefecture level city in Shandong

Jinfeng Hongyuan Flagship Store	Electricity savings1	The goods have long been received, after more than a month to comment, the home electricity bill is indeed reduced, worth buying!	Jingdong prefecture level city in Shandong
Jinfeng Hongyuan Flagship Store	Electricity savings1	Baby received, the logistics is strong, the quality is reliable, it is worth having and recommending!	Jingdong prefecture level city in Shandong
Jinfeng Hongyuan Flagship Store	Electricity savings1	Using a conservation appliance, you can achieve a 10-15% savings in electricity usage.	Jingdong prefecture level city in Shandong
Jinfeng Hongyuan Flagship Store	Electricity savings1	Quality is very good, metal shell, touch is very good, very recommended Oh ~, the subsequent effect of continuous follow-up	Jingdong prefecture level city in Shandong
Jinfeng Hongyuan Flagship Store	Electricity savings1	The product is beautiful, practical, worth buying. The product is beautiful, practical, good quality, fast logistics product is beautiful, practical, beautiful and elegant, very classy, worth buying!	Jingdong prefecture level city in Shandong
Jinfeng Hongyuan Flagship Store	Electricity savings1	Just received the item and have already used it. Didn't know much about these products before. I hope it is as effective and useful as the product advertises.	Jingdong prefecture level city in Shandong
Jinfeng Hongyuan Flagship Store	Electricity savings1	A month two people used more than five hundred degrees of electricity, in a hurry and then add a power saver, I hope to have an effect, Jingdong shopping is very cool, logistics is very fast especially good service attitude delivery, this hot day is not easy, thank you!	Jingdong prefecture level city in Shandong
Jinfeng Hongyuan Flagship Store	Electricity savings1	Just received the baby, first plugged in to try to use a month and then additional evaluation it	Jingdong prefecture level city in Shandong
Jinfeng Hongyuan Flagship Store	Electricity savings1	Goods fast, the power saver has been received, a friend recommended to buy that can save electricity. Exquisite and compact is very convenient, plug and play. The effect is good, the price is affordable, the next need will patronize again!	Jingdong prefecture level city in Shandong
Jinfeng Hongyuan Flagship Store	Electricity savings1	Packaging is better than expected, sent a few fuse, power saving effect in the end do not know, try a period of time to say!	Jingdong prefecture level city in Shandong
Jinfeng Hongyuan Flagship Store	Electricity savings1	Shipped quickly, immediately installed, read countless reviews were not sure if this thing is effective, was going to buy three at once, ultimately decided to first use a try the effect, if the power ring significantly reduced, then will buy a few more!	Jingdong prefecture level city in Shandong
Jinfeng Hongyuan Flagship Store	Electricity savings1	Used for a period of time to evaluate, not to a month still do not know whether it can save electricity, the voltage has a display!	Jingdong prefecture level city in Shandong
Jinfeng Hongyuan Flagship Store	Electricity savings1	I don't know if it saves electricity or not, but I'm using it anyway, so I hope it saves a little bit, electricity is too expensive	Jingdong prefecture level city in Shandong
Jinfeng Hongyuan Flagship Store	Electricity savings1	The power saver has been received and I am very happy with it! Just waiting to see how the power saving works!	Jingdong prefecture level city in Shandong
Jinfeng Hongyuan Flagship Store	Electricity savings1	Saving power has been received, logistics is quite fast, the quality is not bad, small and a little weight, has been used to look forward to the effect of	Jingdong prefecture level city in Shandong
Jinfeng Hongyuan Flagship Store	Electricity savings1	The logistics is very fast, the effect is obvious and trustworthy.	Jingdong prefecture level city in Shandong
Jinfeng Hongyuan Flagship Store	Electricity savings1	Good quality, good quality, beautiful, beautiful, beautiful, beautiful, beautiful, very worth buying, worth buying, worth buying, worth buying, worth customizing!	Jingdong prefecture level city in Shandong
Jinfeng Hongyuan Flagship Store	Electricity savings1	I've used it for now, I don't know if it saves electricity	Jingdong prefecture level city in Shandong
Jinfeng Hongyuan Flagship Store	Electricity savings1	Bought two at a time, logistics is very fast, the product is beautifully packaged, after two months to see the results	Jingdong prefecture level city in Shandong
Jinfeng Hongyuan Flagship Store	Electricity savings1	The workmanship is really fine, the packaging is also good, the regular factory production ~ use a break in the evaluation of the effectiveness of the	Jingdong prefecture level city in Shandong
Jinfeng Hongyuan Flagship Store	Electricity savings1	Sometimes 219. Sometimes 220 in this case	Jingdong prefecture level city in Shandong
Jinfeng Hongyuan Flagship Store	Electricity savings1	It is the ideal product, good quality, logistics is also very fast, worthy of praise!	Jingdong prefecture level city in Shandong
Jinfeng Hongyuan Flagship Store	Electricity savings1	Store service is very good, the courier received the next day. Product packaging is very good, just use the quality to be tested.	Jingdong prefecture level city in Shandong
Jinfeng Hongyuan Flagship Store	Electricity savings1	I've been using it for almost a month now and I feel like it's working.	Jingdong prefecture level city in Shandong
Jinfeng Hongyuan Flagship Store	Electricity savings1	Very satisfied, very restful, very pretty, very beautiful, very much enjoyed! Well worth it!	Jingdong prefecture level city in Shandong
Jinfeng Hongyuan Flagship Store	Electricity savings1	Baby especially like, the value of high-quality good, a very good shopping experience, attentive service, fast logistics, distribution is also very thoughtful, attitude is also very good, value for money ah, decisive praise! Quality is quite good, logistics is also very fast, praise!	Jingdong prefecture level city in Shandong
Jinfeng Hongyuan Flagship Store	Electricity savings1	The shape of the item is basically the same as ordered, the effect is to be examined.	Jingdong prefecture level city in Shandong
Jinfeng Hongyuan Flagship Store	Electricity savings1	Appearance style: good-looking workmanship material: very good, a small one, very delicate Experience: still in use, in the process of using the shopkeeper is very nice to have a careful explanation Use for a period of time in the look at the results	Jingdong prefecture level city in Shandong
Jinfeng Hongyuan Flagship Store	Electricity savings1	As described, compact and well packaged	Jingdong prefecture level city in Shandong
Jinfeng Hongyuan Flagship Store	Electricity savings1	Fine workmanship. Test in progress. Let's see how it works.	Jingdong prefecture level city in Shandong
Jinfeng Hongyuan Flagship Store	Electricity savings1	The goods have been received, I'm very satisfied and love it	Jingdong prefecture level city in Shandong
Jinfeng Hongyuan Flagship Store	Electricity savings1	Safety! Whether you can save electricity, you can roughly feel it in 2 months. The principle is simple. Before the electrical appliances and circuits inductive more, now the electrical appliances integrated, the home is not a lot of inductive circuits, but used certainly save no problem! Wiring according to the requirements of the manual is good, if it is not convenient, see the socket plug.	Jingdong prefecture level city in Shandong
Jinfeng Hongyuan Flagship Store	Electricity savings1	Great product, looking forward to how much power I can save, first time buyer!	Jingdong prefecture level city in Shandong
Jinfeng Hongyuan Flagship Store	Electricity savings1	The product is exquisite, beautifully packaged, and the customer service is excellent. Just waiting to see how the power saving effect is, looking forward to it!	Jingdong prefecture level city in Shandong
Jinfeng Hongyuan Flagship Store	Electricity savings1	Express very quickly very satisfied with the first use to see how the effect to know, if you can save 10 percent even if acceptable, if you can not meet this requirement is obviously not much effect. Obviously.	Jingdong prefecture level city in Shandong
Jinfeng Hongyuan Flagship Store	Electricity savings1	Very delicate and compact, there is a very convenient intelligent display prompts the voltage, you can know the voltage at a glance, there is very easy to use, a plug can be used, I hope to save money on	Jingdong prefecture level city in Shandong
Jinfeng Hongyuan Flagship Store	Electricity savings1	First time buyer is it really save after use	Jingdong prefecture level city in Shandong
Jinfeng Hongyuan Flagship Store	Electricity savings1	Purchased again and will be for a long time yet.	Jingdong prefecture level city in Shandong
Jinfeng Hongyuan Flagship Store	Electricity savings1	Bought it to stock up, came back and plugged it in, hope it works	Jingdong prefecture level city in Shandong
Jinfeng Hongyuan Flagship Store	Electricity savings1	Baby received the logistics very fast very like very satisfied praise a	Jingdong prefecture level city in Shandong
Jinfeng Hongyuan Flagship Store	Electricity savings1	Beginning the process of using, easy to evaluate more later.	Jingdong prefecture level city in Shandong
Jinfeng Hongyuan Flagship Store	Electricity savings1	The goods have been received, just do not know how effective	Jingdong prefecture level city in Shandong
Jinfeng Hongyuan Flagship Store	Electricity savings1	It feels okay. I wish there were more events.	Jingdong prefecture level city in Shandong
Jinfeng Hongyuan Flagship Store	Electricity savings1	Things have been received, just plugged in, I do not know if it works, wait a few days to have the effect of additional	Jingdong prefecture level city in Shandong
Jinfeng Hongyuan Flagship Store	Electricity savings1	The effect of this power saver is really good, the house more, a few air conditioners are open every day, not used before a month to say less than seven hundred eight hundred, used after three months only nine	Jingdong prefecture level city in Shandong
Jinfeng Hongyuan Flagship Store	Electricity savings1	Cheap, good and easy to use, recommend it.	Jingdong prefecture level city in Shandong

Jinfeng Hongyuan Flagship Store	Electricity savings1	Bought this device, to the home for a long time, wait to finish a month to see how, good can recommend friends to buy, we use the electricity every month scary, a month of electricity costs more than a thousand, can not afford to hurt ah! I hope this power saving can save me some money, the beginning of the shoot order did not pay attention to look at the address, sent elsewhere to go, that side called to find out, hurry to communicate with customer service, customer service is very careful to help me get it done, call to that side to help me turn around, thank you!	Jingdong prefecture level city in Shandong
Jinfeng Hongyuan Flagship Store	Electricity savings1	I don't know the effect if it, use it first to see.	Jingdong prefecture level city in Shandong
Jinfeng Hongyuan Flagship Store	Electricity savings1	Looks good. I won't know if it saves power until I use it.	Jingdong prefecture level city in Shandong
Jinfeng Hongyuan Flagship Store	Electricity savings1	Goods received, shipping speed is very fast, yesterday afternoon more than two o'clock shot, just express delivery, but also sent a few insurance, very good business, the display of the number do not understand what is the meaning of the display said voltage, do not understand this, try a month to see how, good and	Jingdong prefecture level city in Shandong
Jinfeng Hongyuan Flagship Store	Electricity savings1	What ** play also, save electricity more and more save more and more, save chicken hair electricity, are cheating, this is a night of air conditioning with electricity, a night with 11 electricity word, this is	Jingdong prefecture level city in Shandong
Jinfeng Hongyuan Flagship Store	Electricity savings1	Received the product, hope it works!	Jingdong prefecture level city in Shandong
Jinfeng Hongyuan Flagship Store	Electricity savings1	Bought home and used very convenient, easy to operate, quite good	Jingdong prefecture level city in Shandong
Jinfeng Hongyuan Flagship Store	Electricity savings1	It's really good, cost-effective and good quality, and the logistics are fast! It arrived in one day! Really good, cost-effective and good quality, logistics is fast ah! It arrived in one day!	Jingdong prefecture level city in Shandong
Jinfeng Hongyuan Flagship Store	Electricity savings1	Works well, stabilizes voltage and saves power	Jingdong prefecture level city in Shandong
Jinfeng Hongyuan Flagship Store	Electricity savings1	I'll use it right away and look forward to good results	Jingdong prefecture level city in Shandong
Jinfeng Hongyuan Flagship Store	Electricity savings1	Recommended by a friend, said it works well, so I bought one, the effect to be tested	Jingdong prefecture level city in Shandong
Jinfeng Hongyuan Flagship Store	Electricity savings1	I didn't know it would save electricity.	Jingdong prefecture level city in Shandong
Jinfeng Hongyuan Flagship Store	Electricity savings1	I've been using it for a week, I don't know what's going on yet I hope it works well and saves power I'll follow up after a month of use	Jingdong prefecture level city in Shandong
Jinfeng Hongyuan Flagship Store	Electricity savings1	Very well packaged, looks great, don't know how it works	Jingdong prefecture level city in Shandong
Jinfeng Hongyuan Flagship Store	Electricity savings1	Genuine, good quality, really saves energy.	Jingdong prefecture level city in Shandong
Jinfeng Hongyuan Flagship Store	Electricity savings1	I was worried that the stuff is not good, but when I got it, I realized that I was completely over-anxious, 5 stars!	Jingdong prefecture level city in Shandong
Jinfeng Hongyuan Flagship Store	Electricity savings1	Very good and satisfied trustworthy	Jingdong prefecture level city in Shandong
Jinfeng Hongyuan Flagship Store	Electricity savings1	I don't know if it saves power	Jingdong prefecture level city in Shandong
Jinfeng Hongyuan Flagship Store	Electricity savings1	The quality of the product is very good, beautiful and generous applicable fast delivery speed good service attitude	Jingdong prefecture level city in Shandong
Jinfeng Hongyuan Flagship Store	Electricity savings1	Let's use it for a while and then we'll follow up	Jingdong prefecture level city in Shandong
Jinfeng Hongyuan Flagship Store	Electricity savings1	I've only been using it for a month, I don't know if there's any change yet, I'll come back with a follow-up comment after checking the meter!	Jingdong prefecture level city in Shandong
Jinfeng Hongyuan Flagship Store	Electricity savings1	I don't know if it works, I read the instructions and online reviews, I hope I won't be disappointed, if I use it, I will buy it again. Jingdong Express, fast and guaranteed service.	Jingdong prefecture level city in Shandong
Jinfeng Hongyuan Flagship Store	Electricity savings1	It feels like it saves quite a bit huh?	Jingdong prefecture level city in Shandong
Jinfeng Hongyuan Flagship Store	Electricity savings1	I've been using it for a while now, and it still seems to work and have a power saving effect	Jingdong prefecture level city in Shandong
Jinfeng Hongyuan Flagship Store	Electricity savings1	Looks good. It says 235. The 222 bar voltage is shown as high. I don't know if that's right. We'll see.	Jingdong prefecture level city in Shandong
Jinfeng Hongyuan Flagship Store	Electricity savings1	Baby is really good, do really careful, worth to have, fast delivery!	Jingdong prefecture level city in Shandong
Jinfeng Hongyuan Flagship Store	Electricity savings1	Quite delicate and compact, simple and convenient to use. Things are still very good, bought two at a time. Very satisfied with an online purchase.	Jingdong prefecture level city in Shandong
Jinfeng Hongyuan Flagship Store	Electricity savings1	Feel very good, the use of unclear, ask customer service, timely explanation, big brand, trustworthy	Jingdong prefecture level city in Shandong
Jinfeng Hongyuan Flagship Store	Electricity savings1	Thrifty received a few days did not make a comment is to see how the quality of the product now feel the quality of good appearance is also powerful seller service is very good to buy the product is also attached to the 5 fuse thought very thoughtful!	Jingdong prefecture level city in Shandong
Jinfeng Hongyuan Flagship Store	Electricity savings1	Big brand, with a very assured, the quality is very good, in time for the event to buy very cost-effective, customer service is really very good Oh ~!	Jingdong prefecture level city in Shandong
Jinfeng Hongyuan Flagship Store	Electricity savings1	I ordered yesterday and received it this morning, I heard my friend say that it can save electricity, I don't know if it works, I'll come back to make a comment after using it.	Jingdong prefecture level city in Shandong
Jinfeng Hongyuan Flagship Store	Electricity savings1	I've been using it for over a month and it doesn't seem to be working!	Jingdong prefecture level city in Shandong
Jinfeng Hongyuan Flagship Store	Electricity savings1	Used it, the family has always been in the habit of using energy-saving devices, this is a new one. Digital display voltage is very good, can see that the voltage is more stable.	Jingdong prefecture level city in Shandong
Jinfeng Hongyuan Flagship Store	Electricity savings1	I haven't used it for more than a month, but it looks good. It's easy to plug into an outlet and it works.	Jingdong prefecture level city in Shandong
Jinfeng Hongyuan Flagship Store	Electricity savings1	Good Oh...appearance is very beautiful...just plugged in with...see the effect of it...with a period of time in the additional evaluation...	Jingdong prefecture level city in Shandong
Jinfeng Hongyuan Flagship Store	Electricity savings1	I've received the goods, whether it's good or not, I'll know after I use it.	Jingdong prefecture level city in Shandong
Jinfeng Hongyuan Flagship Store	Electricity savings1	Very good, logistics is fast, the stuff is very powerful and cost-effective! Can feel the meter slowing down	Jingdong prefecture level city in Shandong
Jinfeng Hongyuan Flagship Store	Electricity savings1	Logistics is very fast, get it immediately after use, easy to operate, plug in the power supply can be used, just do not know to the use of the effect of how.	Jingdong prefecture level city in Shandong
Jinfeng Hongyuan Flagship Store	Electricity savings1	Quality is very over the top, quality is guaranteed, quite a good choice, a lot of friends to see me use all want to buy, I recommend it!	Jingdong prefecture level city in Shandong
Jinfeng Hongyuan Flagship Store	Electricity savings1	The appearance is clean and beautiful, the workmanship is very good, did not find any flaws, I personally feel that the size is also quite suitable, looking for a long time to choose this one, big love! The price is very beautiful, things are still good, will continue to buy back! All five points! Ness Ness ~ logistics speed is really no words, is a word: fast. Commodities from the outer packaging is very high-grade, really a penny a penny, expensive has the reason for expensive	Jingdong prefecture level city in Shandong
Jinfeng Hongyuan Flagship Store	Electricity savings1	The logistics is very fast, the texture is very good, genuine, I like it very much.	Jingdong prefecture level city in Shandong
Jinfeng Hongyuan Flagship Store	Electricity savings1	It's been a few days since I put it on, I can't see any results now, waiting for a month to say, steady steady steady.	Jingdong prefecture level city in Shandong
Jinfeng Hongyuan Flagship Store	Electricity savings1	I've been using it for a while now and it should be fine, so I'm buying it for the second time.	Jingdong prefecture level city in Shandong
Jinfeng Hongyuan Flagship Store	Electricity savings1	Power Saving King has been received, looks good, quite delicate, looking forward to using it to save electricity	Jingdong prefecture level city in Shandong
Jinfeng Hongyuan Flagship Store	Electricity savings1	My mom said the home uses a little too much electricity, so I bought a Power Saver to try it out, I don't know how effective it is. Expect it to be a good thing!	Jingdong prefecture level city in Shandong
Jinfeng Hongyuan Flagship Store	Electricity savings1	Thank you Jingdong, logistics is very fast order to receipt only one day. Believe in Jingdong. Favorable comments	Jingdong prefecture level city in Shandong
Jinfeng Hongyuan Flagship Store	Electricity savings1	Saved some money on the electric bill compared to last month, watch this month again, no problems so far	Jingdong prefecture level city in Shandong

Jinfeng Hongyuan Flagship Store	Electricity savings1	Energy saver exquisite workmanship, beautiful style, more features, just bought, power saving effect to be used for a period of time, then evaluate!	Jingdong prefecture level city in Shandong
Jinfeng Hongyuan Flagship Store	Electricity savings1	This thing does save power is more compact to hang on the wall.	Jingdong prefecture level city in Shandong
Jinfeng Hongyuan Flagship Store	Electricity savings1	I've been buying it for a while now, I haven't actually measured it, but it still feels like it's effective	Jingdong prefecture level city in Shandong
Jinfeng Hongyuan Flagship Store	Electricity savings1	I've already used it since I bought it, I don't know how effective it is yet, I'll comment on it later.	Jingdong prefecture level city in Shandong
Jinfeng Hongyuan Flagship Store	Electricity savings1	Baby received, careful appearance, logistics is very fast, worth buying!	Jingdong prefecture level city in Shandong
Jinfeng Hongyuan Flagship Store	Electricity savings1	It's great, it's just not the same in hand, I bought it at that other cheaper online one for about the same price as this one. But then oops, doesn't work at all.	Jingdong prefecture level city in Shandong
Jinfeng Hongyuan Flagship Store	Electricity savings1	The product is exquisite, but you have to use it for a month to know how it works. I'll comment again after a month.	Jingdong prefecture level city in Shandong
Jinfeng Hongyuan Flagship Store	Electricity savings1	I've been using it for a while now, and it doesn't feel like it's working, and it doesn't save any power.	Jingdong prefecture level city in Shandong
Jinfeng Hongyuan Flagship Store	Electricity savings1	Affordable price, fast delivery, Jingdong self-supporting guarantee	Jingdong prefecture level city in Shandong
Jinfeng Hongyuan Flagship Store	Electricity savings1	Packaging is very good, no damage, good quality, fine workmanship Packaging is very good, no damage, good quality, fine workmanship Packaging is very good, no damage, good quality, fine workmanship Packaging is very good, no damage, good quality, fine workmanship	Jingdong prefecture level city in Shandong
Jinfeng Hongyuan Flagship Store	Electricity savings1	Big brand, with a very assured, the quality is very good, in time for the event to buy very cost-effective, customer service is really very good Oh ~!	Jingdong prefecture level city in Shandong
Jinfeng Hongyuan Flagship Store	Electricity savings1	Used a few days, the specific effect is not yet clear, looking forward to the effect as described by the merchant!	Jingdong prefecture level city in Shandong
Jinfeng Hongyuan Flagship Store	Electricity savings1	I just used it and don't know how it works, I'll follow up after using it!	Jingdong prefecture level city in Shandong
Jinfeng Hongyuan Flagship Store	Electricity savings1	Well, it's been installed and now I'm waiting to see the results, BOE shopping, hopefully I won't be disappointed!	Jingdong prefecture level city in Shandong
Jinfeng Hongyuan Flagship Store	Electricity savings1	Goods received, plugged in the voltage from 229 to 222, do not know whether to save electricity, that depends on the situation.	Jingdong prefecture level city in Shandong
Jinfeng Hongyuan Flagship Store	Electricity savings1	This is the second time I have bought the product and I feel it really saves electricity! Use it again for a while and then evaluate!	Jingdong prefecture level city in Shandong
Jinfeng Hongyuan Flagship Store	Electricity savings1	Overall, it's really good, and the customer service is great, you'll get a message back almost immediately when you speak, it's great value for money, I really recommend it!	Jingdong prefecture level city in Shandong
Jinfeng Hongyuan Flagship Store	Electricity savings1	Have purchased many times, a must have for the family, affordable purchase price, fast delivery, beautifully packaged!	Jingdong prefecture level city in Shandong
Jinfeng Hongyuan Flagship Store	Electricity savings1	I bought it after a friend introduced it, he said it is effective and can save electricity. I bought it to try, try a period of time and then follow up!	Jingdong prefecture level city in Shandong
Jinfeng Hongyuan Flagship Store	Electricity savings1	Received it, trying it out tonight, and bought one earlier.	Jingdong prefecture level city in Shandong
Jinfeng Hongyuan Flagship Store	Electricity savings1	It will take a month or two to see if it saves power or not.	Jingdong prefecture level city in Shandong
Jinfeng Hongyuan Flagship Store	Electricity savings1	Looked good in the instructions! Bought one and pressed it on! Use it and see! If it works! I'll buy some more!	Jingdong prefecture level city in Shandong
Jinfeng Hongyuan Flagship Store	Electricity savings1	Goods have been received packaging can also things are not bad appearance, with a use to know, now evaluation is nonsense.	Jingdong prefecture level city in Shandong
Jinfeng Hongyuan Flagship Store	Electricity savings1	Logistics is very fast, packaging is very good, the second unit, as always, looking forward to the effect of	Jingdong prefecture level city in Shandong
Jinfeng Hongyuan Flagship Store	Electricity savings1	I received the goods today, the quality of goods is very good, I am very satisfied ah.	Jingdong prefecture level city in Shandong
Jinfeng Hongyuan Flagship Store	Electricity savings1	Buy one and try it, I wonder if it has a power saving effect?	Jingdong prefecture level city in Shandong
Jinfeng Hongyuan Flagship Store	Electricity savings1	The item was received, well packaged, logistics is very fast, customer service attitude is very positive, there are questions answered in a timely manner! Overall very satisfied!	Jingdong prefecture level city in Shandong
Jinfeng Hongyuan Flagship Store	Electricity savings1	Baby packaging is very good, purchase submission goods quickly delivered to the home, use and then follow up.	Jingdong prefecture level city in Shandong
Jinfeng Hongyuan Flagship Store	Electricity savings1	My friend asked me to buy an extra one so he could try it too.	Jingdong prefecture level city in Shandong
Jinfeng Hongyuan Flagship Store	Electricity savings1	I've just put it on, I won't know the result until a month later if it will really save power.	Jingdong prefecture level city in Shandong
Jinfeng Hongyuan Flagship Store	Electricity savings1	It's good quality. Hopefully it'll help save power.	Jingdong prefecture level city in Shandong
Jinfeng Hongyuan Flagship Store	Electricity savings1	The appearance is clean and beautiful, the workmanship is very good, I did not find any flaws, I personally feel that the size is also quite suitable, I have been looking for a long time to choose this	Jingdong prefecture level city in Shandong
Jinfeng Hongyuan Flagship Store	Electricity savings1	Logistics is fast! Baby looks great! The price is fair! Wish the store business is prosperous!	Jingdong prefecture level city in Shandong
Jinfeng Hongyuan Flagship Store	Electricity savings1	Jingdong express fast, good, have used, sit and wait to save money...	Jingdong prefecture level city in Shandong
Jinfeng Hongyuan Flagship Store	Electricity savings1	In use, it didn't work as expected and there was nothing noticeable in terms of power savings.	Jingdong prefecture level city in Shandong
Jinfeng Hongyuan Flagship Store	Electricity savings1	Just started using it and looking forward to the results.	Jingdong prefecture level city in Shandong
Jinfeng Hongyuan Flagship Store	Electricity savings1	The quality is very good, the metal shell is very good, is genuine, energy saving and power saving, logistics is very fast, there is a need to buy o, recommend everyone, very satisfied with an online	Jingdong prefecture level city in Shandong
Jinfeng Hongyuan Flagship Store	Electricity savings1	Received, I do not know if the product is good, used to see if it is good to come back to buy!	Jingdong prefecture level city in Shandong
Jinfeng Hongyuan Flagship Store	Electricity savings1	Jinfeng source of intelligent power saving treasure also used less than three months, the last thunder may be burned, the home of the electric send gate is not on, only the old man at home, no way only their own in the field and run back to find out what is the problem with the circuit, the quality is still not very good, thunder is easy to burn!	Jingdong prefecture level city in Shandong
Jinfeng Hongyuan Flagship Store	Electricity savings1	Saving power just started to use, I do not know if there is no effect, but read a lot of reviews are said to be very good so I bought, baby All aspects are good There are spare 2 fuses, late good and then follow	Jingdong prefecture level city in Shandong
Jinfeng Hongyuan Flagship Store	Electricity savings1	Use it for a few days and see, the delivery is quite fast	Jingdong prefecture level city in Shandong
Jinfeng Hongyuan Flagship Store	Electricity savings1	Small, well made and easy to use.	Jingdong prefecture level city in Shandong
Jinfeng Hongyuan Flagship Store	Electricity savings1	Beautifully packaged, it is genuine. Whether it is power saving, use it to evaluate.	Jingdong prefecture level city in Shandong
Jinfeng Hongyuan Flagship Store	Electricity savings1	Power saver. Used two of them and they do save quite a bit of power.	Jingdong prefecture level city in Shandong
Jinfeng Hongyuan Flagship Store	Electricity savings1	Workmanship is not bad, small and does not take up space, is also do not know whether the power saving, buy a to try!	Jingdong prefecture level city in Shandong
Jinfeng Hongyuan Flagship Store	Electricity savings1	The logistics is very fast, the courier attitude is very good, worth buying	Jingdong prefecture level city in Shandong
Jinfeng Hongyuan Flagship Store	Electricity savings1	The effect is not known until it is used.	Jingdong prefecture level city in Shandong
Jinfeng Hongyuan Flagship Store	Electricity savings1	Just arrived plugged in won't know for a month	Jingdong prefecture level city in Shandong
Jinfeng Hongyuan Flagship Store	Electricity savings1	This is really good to use the pull to realize the savings ah think again for my sister's family to buy a	Jingdong prefecture level city in Shandong
Jinfeng Hongyuan Flagship Store	Electricity savings1	Lies, a month with a few hundred electricity are no savings, family use	Jingdong prefecture level city in Shandong

Jinfeng Hongyuan Flagship Store	Electricity savings1	Express very quickly, look at the fine workmanship also sent a few fuse, look forward to can really save electricity	Jingdong prefecture level city in Shandong
Jinfeng Hongyuan Flagship Store	Electricity savings1	I didn't realize it was so small.	Jingdong prefecture level city in Shandong
Jinfeng Hongyuan Flagship Store	Electricity savings1	Logistics courier is very fast, the courier service is very good	Jingdong prefecture level city in Shandong
Jinfeng Hongyuan Flagship Store	Electricity savings1	Used for a period of time the effect is quite good indeed than the previous power saving	Jingdong prefecture level city in Shandong
Jinfeng Hongyuan Flagship Store	Electricity savings1	Logistics is super fast, the product is in use, see the effect...	Jingdong prefecture level city in Shandong
Jinfeng Hongyuan Flagship Store	Electricity savings1	Baby got it, tried it out and it didn't work at all. How can I use the tips to save power?	Jingdong prefecture level city in Shandong
Jinfeng Hongyuan Flagship Store	Electricity savings1	Power Saving King received, immediately used has been very stable without any sound good, good service, fast logistics inexpensive and beautiful worth repurchasing!	Jingdong prefecture level city in Shandong
Jinfeng Hongyuan Flagship Store	Electricity savings1	Logistics courier is very fast, the courier service is good	Jingdong prefecture level city in Shandong
Jinfeng Hongyuan Flagship Store	Electricity savings1	Good to recommend, the first month of air conditioning only 1 week, the electricity bill has reduced about 100 yuan, may be to reduce the use of air conditioning is also related to the other or as usual electricity, estimated that the longer you use the more you can save electricity.	Jingdong prefecture level city in Shandong
Jinfeng Hongyuan Flagship Store	Electricity savings1	It doesn't work. I've been using it for 20 days and it doesn't work.	Jingdong prefecture level city in Shandong
Jinfeng Hongyuan Flagship Store	Electricity savings1	Fast shipping, good quality, affordable price, very satisfied with a shopping.	Jingdong prefecture level city in Shandong
Jinfeng Hongyuan Flagship Store	Electricity savings1	Try it for a month to see if it saves money.	Jingdong prefecture level city in Shandong
Jinfeng Hongyuan Flagship Store	Electricity savings1	It's in use. I'll comment again after a while.	Jingdong prefecture level city in Shandong
Jinfeng Hongyuan Flagship Store	Electricity savings1	Goods have received look good 👍👍👍👍, use a period of time and then look at the	Jingdong prefecture level city in Shandong
Jinfeng Hongyuan Flagship Store	Electricity savings1	No, it doesn't work. It's useless. It's garbage.	Jingdong prefecture level city in Shandong
Jinfeng Hongyuan Flagship Store	Electricity savings1	I've been using it for almost 2 months now and I don't feel any reduction in my electricity bill.	Jingdong prefecture level city in Shandong
Jinfeng Hongyuan Flagship Store	Electricity savings1	I've received the goods, the logistics is really fast, ordered yesterday, arrived today.	Jingdong prefecture level city in Shandong
Jinfeng Hongyuan Flagship Store	Electricity savings1	And the backup insurance.	Jingdong prefecture level city in Shandong
Jinfeng Hongyuan Flagship Store	Electricity savings1	The appearance of this product is good, easy to use and install, the effect of how there is no use to wait for time to verify that the voltage displayed is different from the voltage measured by other meters, the gap is not too large, the follow-up to add the effect.	Jingdong prefecture level city in Shandong
Jinfeng Hongyuan Flagship Store	Electricity savings1	Baby received it 👌, really great ah! 🌈The quality can't be beat, very satisfied! 🆗Happy happy happy 😊	Jingdong prefecture level city in Shandong
Jinfeng Hongyuan Flagship Store	Electricity savings1	I'll follow up with a review after using it for a while, hopefully it won't bring me back a disappointment!	Jingdong prefecture level city in Shandong
Jinfeng Hongyuan Flagship Store	Electricity savings1	In use and looking forward to results! Good review to send	Jingdong prefecture level city in Shandong
Jinfeng Hongyuan Flagship Store	Electricity savings1	Intelligent power saver received, the volume is not big, small and exquisite, quite good, Jingdong Express is also quite fast, the	Jingdong prefecture level city in Shandong
Jinfeng Hongyuan Flagship Store	Electricity savings1	I don't know how to use it, keep using it	Jingdong prefecture level city in Shandong
Jinfeng Hongyuan Flagship Store	Electricity savings1	Very good for saving electricity	Jingdong prefecture level city in Shandong
Jinfeng Hongyuan Flagship Store	Electricity savings1	Seriously, did not use, feel also more power consumption, the peak is bought back to use the first time, the next day unplugged, the power consumption is back to more than ten degrees (the frequency of power use in the past few days is pretty much the same), feel the IQ tax paid 😂	Jingdong prefecture level city in Shandong
Jinfeng Hongyuan Flagship Store	Electricity savings1	I haven't seen any effect on the power saving, but the voltage stabilization looks good.	Jingdong prefecture level city in Shandong
Jinfeng Hongyuan Flagship Store	Electricity savings1	It's very good, I'll come back to it after using it for a while	Jingdong prefecture level city in Shandong
Jinfeng Hongyuan Flagship Store	Electricity savings1	Already used it and see the results.	Jingdong prefecture level city in Shandong
Jinfeng Hongyuan Flagship Store	Electricity savings1	Fast shipping, beautiful packaging, attentive service, the use of the effect, to be followed up after the evaluation.	Jingdong prefecture level city in Shandong
Jinfeng Hongyuan Flagship Store	Electricity savings1	The process of using this product is more than satisfactory	Jingdong prefecture level city in Shandong
Jinfeng Hongyuan Flagship Store	Electricity savings1	Very good East East cost-effective! Every time so buy more assured, and the logistics is also very fast. It is also very good to use, so I will often come to buy in the future, and will also introduce to	Jingdong prefecture level city in Shandong
Jinfeng Hongyuan Flagship Store	Electricity savings1	But still understand how effective it is!	Jingdong prefecture level city in Shandong
Jinfeng Hongyuan Flagship Store	Electricity savings1	Fast shipping, great logistics, great stuff, great price.	Jingdong prefecture level city in Shandong
Jinfeng Hongyuan Flagship Store	Electricity savings1	I'll review it in a month and give it 5 stars if it's good	Jingdong prefecture level city in Shandong
Jinfeng Hongyuan Flagship Store	Electricity savings1	I've saved a lot of electricity in the past month, so I'm glad to hear that.	Jingdong prefecture level city in Shandong
Jinfeng Hongyuan Flagship Store	Electricity savings1	Baby just received and installed! Looks genuinely good, workmanship feels fine! Looking forward to the effect! Said to see the effect in two months! Waiting for if really have the effect must come to follow up! The boss is absolutely responsible! Asked and answered! Just for the boss this attitude must also	Jingdong prefecture level city in Shandong
Jinfeng Hongyuan Flagship Store	Electricity savings1	This baby is very delicate, I'll comment after I've used it	Jingdong prefecture level city in Shandong
Jinfeng Hongyuan Flagship Store	Electricity savings1	It's been over two months. It's not working. It's just like it was.	Jingdong prefecture level city in Shandong
Jinfeng Hongyuan Flagship Store	Electricity savings1	Fast shipping and logistics, let's see if it works.	Jingdong prefecture level city in Shandong
Jinfeng Hongyuan Flagship Store	Electricity savings1	Just received the goods, try it to know if it can really save electricity	Jingdong prefecture level city in Shandong
Jinfeng Hongyuan Flagship Store	Electricity savings1	Not bad. Not bad. Not bad.	Jingdong prefecture level city in Shandong
Jinfeng Hongyuan Flagship Store	Electricity savings1	Not sure if it works. I'll comment again if it works.	Jingdong prefecture level city in Shandong
Jinfeng Hongyuan Flagship Store	Electricity savings1	Safe and energy-saving helpers to use!	Jingdong prefecture level city in Shandong
Jinfeng Hongyuan Flagship Store	Electricity savings1	Good, first time buyer. Used good can	Jingdong prefecture level city in Shandong
Jinfeng Hongyuan Flagship Store	Electricity savings1	It feels okay, I guess! I won't know until I use it for a while!	Jingdong prefecture level city in Shandong
Jinfeng Hongyuan Flagship Store	Electricity savings1	The saver matches the description and has been used, waiting for a follow up review after two months.	Jingdong prefecture level city in Shandong
Jinfeng Hongyuan Flagship Store	Electricity savings1	Received, the appearance of the business as described, the effect of how to use a period of time to evaluate.	Jingdong prefecture level city in Shandong
Jinfeng Hongyuan Flagship Store	Electricity savings1	Still using it don't know how effective it is, I'll comment after three months	Jingdong prefecture level city in Shandong

Jinfeng Hongyuan Flagship Store	Electricity savings1	The stuff is very good, the packaging is also very good logistics is also very fast	Jingdong prefecture level city in Shandong
Jinfeng Hongyuan Flagship Store	Electricity savings1	I'll follow up with a review when I use it, hopefully I won't be disappointed	Jingdong prefecture level city in Shandong
Jinfeng Hongyuan Flagship Store	Electricity savings1	Cancel Houjiaying Logistics.	Jingdong prefecture level city in Shandong
Jinfeng Hongyuan Flagship Store	Electricity savings1	It's very good, it's worth buying.	Jingdong prefecture level city in Shandong
Jinfeng Hongyuan Flagship Store	Electricity savings1	My friend bought it to save electricity and referred me to buy it! I'll buy it and try it out! I will know how much electricity I can save next month, looking forward to it! 4-500 dollars a month electricity bill is horrible! I hope it can really help me save some electricity!	Jingdong prefecture level city in Shandong
Jinfeng Hongyuan Flagship Store	Electricity savings1	Just received it and installed it.	Jingdong prefecture level city in Shandong
Jinfeng Hongyuan Flagship Store	Electricity savings1	Very nice. This one is my favorite.	Jingdong prefecture level city in Shandong
Jinfeng Hongyuan Flagship Store	Electricity savings1	Good value for money worth buying very good	Jingdong prefecture level city in Shandong
Jinfeng Hongyuan Flagship Store	Electricity savings1	Very energy-saving, small and exquisite, does not take up space, very satisfied, complete packaging, no damage, customer service explanation is also very serious	Jingdong prefecture level city in Shandong
Jinfeng Hongyuan Flagship Store	Electricity savings1	Received immediately used, really effective, do not understand electricity, but looked at the meter every day, recommended that you can use, especially this weather every day to open the air conditioning, with him not afraid of the electricity bill.	Jingdong prefecture level city in Shandong
Jinfeng Hongyuan Flagship Store	Electricity savings1	Nice, good value for money, nice stuff.	Jingdong prefecture level city in Shandong
Jinfeng Hongyuan Flagship Store	Electricity savings1	The packaging of the product is very good, the logistics of the product is very fast, and the quality of the product will be evaluated after use!	Jingdong prefecture level city in Shandong
Jinfeng Hongyuan Flagship Store	Electricity savings1	Express is very, well very fast, give a compliment 👍	Jingdong prefecture level city in Shandong
Jinfeng Hongyuan Flagship Store	Electricity savings1	Packaging is very good, fast delivery, used a night and a day did not feel the power saving. Use a month to see if it saves power.	Jingdong prefecture level city in Shandong
Jinfeng Hongyuan Flagship Store	Electricity savings1	Seems to be a bit of a power saver.	Jingdong prefecture level city in Shandong
Jinfeng Hongyuan Flagship Store	Electricity savings1	Bought this for someone else and it came over so fast, ###!	Jingdong prefecture level city in Shandong
Jinfeng Hongyuan Flagship Store	Electricity savings1	Very delicate, just received a period of time to use in the follow-up evaluation	Jingdong prefecture level city in Shandong
Jinfeng Hongyuan Flagship Store	Electricity savings1	It's been installed for a while now, not sure if it's working or not	Jingdong prefecture level city in Shandong
Jinfeng Hongyuan Flagship Store	Electricity savings1	The item looks okay, I just don't know if it works or not.	Jingdong prefecture level city in Shandong
Jinfeng Hongyuan Flagship Store	Electricity savings1	The goods have been received, but there is a screw missing	Jingdong prefecture level city in Shandong
Jinfeng Hongyuan Flagship Store	Electricity savings1	I didn't believe it before, but after receiving the goods today and trying it out, it really works!	Jingdong prefecture level city in Shandong
Jinfeng Hongyuan Flagship Store	Electricity savings1	Received, this thing will not know whether it is the effect of saving electricity, no matter how to say that there should always be a little use it, otherwise why develop it I think there is still the value of	Jingdong prefecture level city in Shandong
Jinfeng Hongyuan Flagship Store	Electricity savings1	Another one just arrived.	Jingdong prefecture level city in Shandong
Jinfeng Hongyuan Flagship Store	Electricity savings1	I don't feel anything, ordinary farmers can't go to TEDA, just use it first, it's safe.	Jingdong prefecture level city in Shandong
Jinfeng Hongyuan Flagship Store	Electricity savings1	Just purchased and will follow up after using it for a while	Jingdong prefecture level city in Shandong
Jinfeng Hongyuan Flagship Store	Electricity savings1	I'm trying it out now. I'll comment after I use it.	Jingdong prefecture level city in Shandong
Jinfeng Hongyuan Flagship Store	Electricity savings1	Received the item, in use, hope it works.	Jingdong prefecture level city in Shandong
Jinfeng Hongyuan Flagship Store	Electricity savings1	It works well. Good review.	Jingdong prefecture level city in Shandong
Jinfeng Hongyuan Flagship Store	Electricity savings1	All a scam, no effect at all, I bought too many fakes in Poundland before, I did not think that Jingdong is also the same	Jingdong prefecture level city in Shandong
Jinfeng Hongyuan Flagship Store	Electricity savings1	I used the saver as soon as I received it, it's in good shape and still working!	Jingdong prefecture level city in Shandong
Jinfeng Hongyuan Flagship Store	Electricity savings1	It works. I don't know what to do with the picture, but it works.	Jingdong prefecture level city in Shandong
Jinfeng Hongyuan Flagship Store	Electricity savings1	A very good shopping experience, attentive service, fast logistics, delivery is also very thoughtful, attitude is also very good, value for money ah, decisive praise!	Jingdong prefecture level city in Shandong
Jinfeng Hongyuan Flagship Store	Electricity savings1	The product is consistent with the presentation picture.	Jingdong prefecture level city in Shandong
Jinfeng Hongyuan Flagship Store	Electricity savings1	Baby received, use a period of time to evaluate, wish the boss business prosperity!	Jingdong prefecture level city in Shandong
Jinfeng Hongyuan Flagship Store	Electricity savings1	The stuff is better! Recommended!	Jingdong prefecture level city in Shandong
Jinfeng Hongyuan Flagship Store	Electricity savings1	I don't know if it works or not, I'll use it for a while before I comment on it.	Jingdong prefecture level city in Shandong
Jinfeng Hongyuan Flagship Store	Electricity savings1	Cost-effective, I like it, next time will come back Baby received, very surprised, the quality is very good, very satisfied with a shopping. I will continue to buy back, the store is trustworthy	Jingdong prefecture level city in Shandong
Jinfeng Hongyuan Flagship Store	Electricity savings1	Plugged it in and used it for a few days, not sure if it saves power yet	Jingdong prefecture level city in Shandong
Jinfeng Hongyuan Flagship Store	Electricity savings1	I'm trying it out and will follow up if it seems to be working!	Jingdong prefecture level city in Shandong
Jinfeng Hongyuan Flagship Store	Electricity savings1	Works pretty well, big kudos 👍👍👍👍👍👍👍👍👍👍👍	Jingdong prefecture level city in Shandong
Jinfeng Hongyuan Flagship Store	Electricity savings1	Just put it on, we'll see how it looks in a month.	Jingdong prefecture level city in Shandong
Jinfeng Hongyuan Flagship Store	Electricity savings1	The baby is very nice and I've already recommended buying it for all my friends! The main thing is that it not only saves electricity, I was more concerned about the fact that it can adjust the current, usually wiring problems. Now I found that it is really good, it is the effect I want!	Jingdong prefecture level city in Shandong
Jinfeng Hongyuan Flagship Store	Electricity savings1	Baby arrived, looking forward to his results, hopefully it will be good	Jingdong prefecture level city in Shandong
Jinfeng Hongyuan Flagship Store	Electricity savings1	Things have been received, look at the workmanship and packaging are good, I do not know how the power-saving effect? Use a period of time to see the effect	Jingdong prefecture level city in Shandong
Jinfeng Hongyuan Flagship Store	Electricity savings1	The price is right, the shape is beautiful and does not take up space, use it for a while to see the effect	Jingdong prefecture level city in Shandong
Jinfeng Hongyuan Flagship Store	Electricity savings1	Can't save electricity, it doesn't work!	Jingdong prefecture level city in Shandong
Jinfeng Hongyuan Flagship Store	Electricity savings1	It's pretty convenient, just plug it in and it works. There's no noise, plug it in and leave it alone, it's very hassle free! Good workmanship and value for money.	Jingdong prefecture level city in Shandong
Jinfeng Hongyuan Flagship Store	Electricity savings1	Just received the goods have been installed, try two months later to comment!	Jingdong prefecture level city in Shandong
Jinfeng Hongyuan Flagship Store	Electricity savings1	I received the stuff, I haven't used it yet, and overall it's fine.	Jingdong prefecture level city in Shandong
Jinfeng Hongyuan Flagship Store	Electricity savings1	Couldn't use a return at all.	Jingdong prefecture level city in Shandong

Jinfeng Hongyuan Flagship Store	Electricity savings1	Second purchase.	Jingdong prefecture level city in Shandong
Jinfeng Hongyuan Flagship Store	Electricity savings1	Very good quality, high tech and practical.	Jingdong prefecture level city in Shandong
Jinfeng Hongyuan Flagship Store	Electricity savings1	Very good, very great! Very satisfied, thanks to the store!	Jingdong prefecture level city in Shandong
Jinfeng Hongyuan Flagship Store	Electricity savings1	Just used it, don't know if it's good or not, hope it works well	Jingdong prefecture level city in Shandong
Jinfeng Hongyuan Flagship Store	Electricity savings1	Saving power has been received, the product is good, good like.	Jingdong prefecture level city in Shandong
Jinfeng Hongyuan Flagship Store	Electricity savings1	Received the goods and used it for two months before evaluation	Jingdong prefecture level city in Shandong
Jinfeng Hongyuan Flagship Store	Electricity savings1	It looks a little small, I do not know how effective, first use to see the effect, there is a small adjustable button, I do not know this is to adjust to that position to work well	Jingdong prefecture level city in Shandong
Jinfeng Hongyuan Flagship Store	Electricity savings1	Simple, just plug it in, there are spare fuses, can't say how well it works, no comparison	Jingdong prefecture level city in Shandong
Jinfeng Hongyuan Flagship Store	Electricity savings1	It's been a few days since I used it, and the power's gone up.	Jingdong prefecture level city in Shandong
Jinfeng Hongyuan Flagship Store	Electricity savings1	The owner was very nice and sent a spare fuse.	Jingdong prefecture level city in Shandong
Jinfeng Hongyuan Flagship Store	Electricity savings1	It's authentic and it's not expensive and it's great to use, I use it all the time and it's great just don't know if it really saves any points	Jingdong prefecture level city in Shandong
Jinfeng Hongyuan Flagship Store	Electricity savings1	The information is good, it is the child to buy, the child read it is also quite happy, I hope to be able to match the child's preparation for the midterm examination! Because of the epidemic, it is not too fast, but I also give full credit to the express delivery!	Jingdong prefecture level city in Shandong
Jinfeng Hongyuan Flagship Store	Electricity savings1	This power saver comes to the logistics very quickly, the business attitude is also very good, ** very comfortable, this brand is trustworthy, and buy in this store than in the physical store to buy cheaper,	Jingdong prefecture level city in Shandong
Jinfeng Hongyuan Flagship Store	Electricity savings1	It's very well made and the materials used are okay. This is just a few days old, so I don't know how it will work, only next month.	Jingdong prefecture level city in Shandong
Jinfeng Hongyuan Flagship Store	Electricity savings1	Already used on, have not tested the effect, wait for a couple of months to add comments.	Jingdong prefecture level city in Shandong
Jinfeng Hongyuan Flagship Store	Electricity savings1	Baby received, packaging is very thoughtful, already in use, should soon see the effect.	Jingdong prefecture level city in Shandong
Jinfeng Hongyuan Flagship Store	Electricity savings1	Just received in the office to try, to the home installed, the voltage are different, and do not know if there is no effect. Use a period of time and then follow up.	Jingdong prefecture level city in Shandong
Jinfeng Hongyuan Flagship Store	Electricity savings1	Just received the goods, is trying, looking forward to the effect, first give a favorable comment, two months later, then additional evaluation, please be patient.	Jingdong prefecture level city in Shandong
Jinfeng Hongyuan Flagship Store	Electricity savings1	The item has just been received, well packaged, results to be verified.	Jingdong prefecture level city in Shandong
Jinfeng Hongyuan Flagship Store	Electricity savings1	Packaging logistics are very good, the next look at how the effect, before I see others use the effect is good, as I bought this can also have good results.	Jingdong prefecture level city in Shandong
Jinfeng Hongyuan Flagship Store	Electricity savings1	I don't know if it's a real power saver or a fake power saver, but I've used it since I bought it, so I'll give you a good review.	Jingdong prefecture level city in Shandong
Jinfeng Hongyuan Flagship Store	Electricity savings1	Good quality, genuine goods, much better than imagined before purchase, next time will come back to care!	Jingdong prefecture level city in Shandong
Jinfeng Hongyuan Flagship Store	Electricity savings1	Logistics is very fast good use again	Jingdong prefecture level city in Shandong
Jinfeng Hongyuan Flagship Store	Electricity savings1	Received the goods. Let's see if it saves power in a month and then we'll follow up!	Jingdong prefecture level city in Shandong
Jinfeng Hongyuan Flagship Store	Electricity savings1	I bought it yesterday and it arrived today, I bought two of them, I hope they work well, I'll come back after a while to give a follow-up review	Jingdong prefecture level city in Shandong
Jinfeng Hongyuan Flagship Store	Electricity savings1	The home air conditioner consumes a lot of electricity, so I bought it and used it in the hope that there is A	Jingdong prefecture level city in Shandong
Jinfeng Hongyuan Flagship Store	Electricity savings1	The product is excellent and very current.	Jingdong prefecture level city in Shandong
Jinfeng Hongyuan Flagship Store	Electricity savings1	With the description, it is the most practical and good products that households can need, and it is an ideal shopping, the courier is also fast, and the service attitude is enthusiastic!	Jingdong prefecture level city in Shandong
Jinfeng Hongyuan Flagship Store	Electricity savings1	The Power Saver is easy to use, works well, is stable, and plugs into the top of the outlet	Jingdong prefecture level city in Shandong
Jinfeng Hongyuan Flagship Store	Electricity savings1	Overall still can, excellent craftsmanship, power saving do not know for the time being, after the use of the evaluation	Jingdong prefecture level city in Shandong
Jinfeng Hongyuan Flagship Store	Electricity savings1	Baby received on the plug, but also with four fuses, look at the quality is okay, I do not know whether it is useful, use and then follow up.	Jingdong prefecture level city in Shandong
Jinfeng Hongyuan Flagship Store	Electricity savings1	I'll evaluate it after a while, I don't know if it's good or not yet, and if it can save electricity	Jingdong prefecture level city in Shandong
Jinfeng Hongyuan Flagship Store	Electricity savings1	Jingdong self-operated purchase more assured delivery fast quality assurance	Jingdong prefecture level city in Shandong
Jinfeng Hongyuan Flagship Store	Electricity savings1	Shipping was fast, stuff was well packaged, great price, I'm happy with it and will repurchase!	Jingdong prefecture level city in Shandong
Jinfeng Hongyuan Flagship Store	Electricity savings1	The quality of the product is very good and I will definitely come back again next time.	Jingdong prefecture level city in Shandong
Jinfeng Hongyuan Flagship Store	Electricity savings1	I'll follow up with a review after I use it.	Jingdong prefecture level city in Shandong
Jinfeng Hongyuan Flagship Store	Electricity savings1	Hello, the goods are perfectly packaged, save power for a period of time to evaluate again!	Jingdong prefecture level city in Shandong
Jinfeng Hongyuan Flagship Store	Electricity savings1	Not bad, pretty good, good value for money, pretty good	Jingdong prefecture level city in Shandong
Jinfeng Hongyuan Flagship Store	Electricity savings1	The packaging is very good, received immediately used, looking forward to the effect in.	Jingdong prefecture level city in Shandong
Jinfeng Hongyuan Flagship Store	Electricity savings1	See how much power you save at the end of the month	Jingdong prefecture level city in Shandong
Jinfeng Hongyuan Flagship Store	Electricity savings1	Easy to use, affordable, beautiful color	Jingdong prefecture level city in Shandong
Jinfeng Hongyuan Flagship Store	Electricity savings1	It's taking too long to put together an order. It could be an epidemic.	Jingdong prefecture level city in Shandong
Jinfeng Hongyuan Flagship Store	Electricity savings1	I don't feel much after using it, no change in my electric bill 。。。。。。。	Jingdong prefecture level city in Shandong
Jinfeng Hongyuan Flagship Store	Electricity savings1	Logistics is very fast, the quality of the economizer is good, good quality and cheap, it is worth buying, I hope there is a power saving effect!	Jingdong prefecture level city in Shandong
Jinfeng Hongyuan Flagship Store	Electricity savings1	Feeling okay, in a couple of months to try to rely on it before making a comment. Let's give it a five-star review first!	Jingdong prefecture level city in Shandong
Jinfeng Hongyuan Flagship Store	Electricity savings1	Power saving device has been hesitating before in the end to buy or not to buy, after consideration, consideration, in consideration, or decided to buy a try. Things received can not wait to open a look, and the seller described the same, I hope to use 2 months to have the effect, two months to follow up on	Jingdong prefecture level city in Shandong
Jinfeng Hongyuan Flagship Store	Electricity savings1	Thank you Jingdong, logistics is very fast order to receipt only one day. Believe in Jingdong. Favorable comments	Jingdong prefecture level city in Shandong
Jinfeng Hongyuan Flagship Store	Electricity savings1	I used one to use it, but it didn't work very well, what to do	Jingdong prefecture level city in Shandong
Jinfeng Hongyuan Flagship Store	Electricity savings1	Quality and packaging are very good, fast shipping, good service, buy with confidence	Jingdong prefecture level city in Shandong
Jinfeng Hongyuan Flagship Store	Electricity savings1	Use a period of time to evaluate, the quality is very good, the price is affordable, logistics is also very fast	Jingdong prefecture level city in Shandong

Jinfeng Hongyuan Flagship Store	Electricity savings1	Goods received, the day of the order tomorrow delivery real God speed, delivery force, the product to be used after a period of time and then low comment!	Jingdong prefecture level city in Shandong
Jinfeng Hongyuan Flagship Store	Electricity savings1	I don't know, how effective it is, let's talk about it when the time comes, whether it saves power or not, how much, unknown.	Jingdong prefecture level city in Shandong
Jinfeng Hongyuan Flagship Store	Electricity savings1	It does save some electricity, and I don't want to use something that isn't legal! Used nearly ~ electricity last month, probably not much more this month with the AC on!	Jingdong prefecture level city in Shandong
Jinfeng Hongyuan Flagship Store	Electricity savings1	Well-packed, excellent quality, service satisfaction good value for money	Jingdong prefecture level city in Shandong
Jinfeng Hongyuan Flagship Store	Electricity savings1	Express very quickly, the goods received last night, today used a day of goods can not heat, looking forward to whether it can save electricity, with a month and then comment, is used well will buy again.	Jingdong prefecture level city in Shandong
Jinfeng Hongyuan Flagship Store	Electricity savings1	It's pretty good, hopefully it'll be a real power saver.	Jingdong prefecture level city in Shandong
Jinfeng Hongyuan Flagship Store	Electricity savings1	The goods received in use, can not see the effect of energy saving, after a month to know!	Jingdong prefecture level city in Shandong
Jinfeng Hongyuan Flagship Store	Electricity savings1	Received the goods, looking forward to better surprise, with the effect of the follow-up comment!	Jingdong prefecture level city in Shandong
Jinfeng Hongyuan Flagship Store	Electricity savings1	It's not bad, it's still in use and should save some	Jingdong prefecture level city in Shandong
Jinfeng Hongyuan Flagship Store	Electricity savings1	Logistics is very fast, easy to install, plugged in on it,, the effect of the use of the later to evaluate!	Jingdong prefecture level city in Shandong
Jinfeng Hongyuan Flagship Store	Electricity savings1	The stuff is recommendable to buy very good.	Jingdong prefecture level city in Shandong
Jinfeng Hongyuan Flagship Store	Electricity savings1	Voltmeter measurements of voltage are way off, aren't they?	Jingdong prefecture level city in Shandong
Jinfeng Hongyuan Flagship Store	Electricity savings1	Install it to the socket immediately after purchase and try to see if it can save electricity after one month.	Jingdong prefecture level city in Shandong
Jinfeng Hongyuan Flagship Store	Electricity savings1	I've been using it for almost a month now, and I won't know if it saves electricity until I pay the bill.	Jingdong prefecture level city in Shandong
Jinfeng Hongyuan Flagship Store	Electricity savings1	I bought one in the past and it was fine, so I'm buying it back.	Jingdong prefecture level city in Shandong
Jinfeng Hongyuan Flagship Store	Electricity savings1	Service is very good, this thing is small and exquisite to use, just waiting to use the effect of a period of time	Jingdong prefecture level city in Shandong
Jinfeng Hongyuan Flagship Store	Electricity savings1	Not sure how well it works yet	Jingdong prefecture level city in Shandong
Jinfeng Hongyuan Flagship Store	Electricity savings1	Kyodo goods habitually good reviews	Jingdong prefecture level city in Shandong
Jinfeng Hongyuan Flagship Store	Electricity savings1	Appearance is OK, whether it is power saving remains to be seen.	Jingdong prefecture level city in Shandong
Jinfeng Hongyuan Flagship Store	Electricity savings1	I bought it and used it, I just don't know if it really saves power	Jingdong prefecture level city in Shandong
Jinfeng Hongyuan Flagship Store	Electricity savings1	The saver is well made and of good quality, I will evaluate it after using it for a while.	Jingdong prefecture level city in Shandong
Jinfeng Hongyuan Flagship Store	Electricity savings1	Just received in use, use for a period of time to see if there is power saving	Jingdong prefecture level city in Shandong
Jinfeng Hongyuan Flagship Store	Electricity savings1	Used 3 days before commenting, it really saves electricity, I look at the "power calendar" after using one less degree of electricity per day, so I think it's worth it to buy this, I will introduce my friends	Jingdong prefecture level city in Shandong
Jinfeng Hongyuan Flagship Store	Electricity savings1	Reliable quality, very well packaged and matches the real thing.	Jingdong prefecture level city in Shandong
Jinfeng Hongyuan Flagship Store	Electricity savings1	Logistics is still quite fast, ha in the trial	Jingdong prefecture level city in Shandong
Jinfeng Hongyuan Flagship Store	Electricity savings1	Consultant service attitude is very good, the courier is also very powerful, the first day of the order the next day to arrive. Saver received used and then add comments!	Jingdong prefecture level city in Shandong
Jinfeng Hongyuan Flagship Store	Electricity savings1	I just got home to try it out and see how it goes.	Jingdong prefecture level city in Shandong
Jinfeng Hongyuan Flagship Store	Electricity savings1	Well made and weighted instrument. Kudos! Put it into use immediately, don't know if it saves power, will evaluate later!	Jingdong prefecture level city in Shandong
Jinfeng Hongyuan Flagship Store	Electricity savings1	I've just put it in place. I'll give you a review in ten days.	Jingdong prefecture level city in Shandong
Jinfeng Hongyuan Flagship Store	Electricity savings1	It looks very delicate, not bad. Well, I don't know if it can save power or not, I have to try it for a while and see. If it can really save electricity. Uh, if it works well. Then additional comments. Anyway, thanks for the logistics, thanks to Jingdong. Well, it deserves some praise.	Jingdong prefecture level city in Shandong
Jinfeng Hongyuan Flagship Store	Electricity savings1	Bought a month, the feeling is that there is a certain effect, a month to save a lot of money	Jingdong prefecture level city in Shandong
Jinfeng Hongyuan Flagship Store	Electricity savings1	Goods received, the physical and the same as the picture, logistics is also very fast, the service attitude is very good, it is worth buying!	Jingdong prefecture level city in Shandong
Jinfeng Hongyuan Flagship Store	Electricity savings1	I've used it, I don't know whether it saves electricity or not, I'll talk about it after a while.	Jingdong prefecture level city in Shandong
Jinfeng Hongyuan Flagship Store	Electricity savings1	August only began to use, October to know, have no power-saving effect, to the in to evaluate!	Jingdong prefecture level city in Shandong
Jinfeng Hongyuan Flagship Store	Electricity savings1	Goods received: I do not know to the factory products used to save electricity!	Jingdong prefecture level city in Shandong
Jinfeng Hongyuan Flagship Store	Electricity savings1	Don't know if it works, bought it anyway.	Jingdong prefecture level city in Shandong
Jinfeng Hongyuan Flagship Store	Electricity savings1	Still testing, will follow up next month to evaluate how much electricity is saved	Jingdong prefecture level city in Shandong
Jinfeng Hongyuan Flagship Store	Electricity savings1	I'm still testing it, I'll comment after I use it.	Jingdong prefecture level city in Shandong
Jinfeng Hongyuan Flagship Store	Electricity savings1	Fast service, praiseworthy. Good item pre-style, first praise, the use of the effect to be evaluated after use.	Jingdong prefecture level city in Shandong
Jinfeng Hongyuan Flagship Store	Electricity savings1	The power saving effect is more obvious through verified use.	Jingdong prefecture level city in Shandong
Jinfeng Hongyuan Flagship Store	Electricity savings1	Logistics is very fast yesterday order today arrived. The product was immediately used, and after a month to see its effect.	Jingdong prefecture level city in Shandong
Jinfeng Hongyuan Flagship Store	Electricity savings1	Power saving is not yet known, as far as the TV and audio are concerned, I feel that there is a significant quality improvement, and I recommend using a good product!	Jingdong prefecture level city in Shandong
Jinfeng Hongyuan Flagship Store	Electricity savings1	Good power savings, tens of dollars a month saved	Jingdong prefecture level city in Shandong
Jinfeng Hongyuan Flagship Store	Electricity savings1	Already took two, great value! You can save 30% on your electric bill in a month. A summer must-have!	Jingdong prefecture level city in Shandong
Jinfeng Hongyuan Flagship Store	Electricity savings1	Received the item, plugged it into a home outlet and it showed 230 volts (not that high with a multimeter). Let's use it first!	Jingdong prefecture level city in Shandong
Jinfeng Hongyuan Flagship Store	Electricity savings1	Expecting results in the experiment	Jingdong prefecture level city in Shandong
Jinfeng Hongyuan Flagship Store	Electricity savings1	I don't know if it really saves power.	Jingdong prefecture level city in Shandong
Jinfeng Hongyuan Flagship Store	Electricity savings1	It's very good and the efficacy is very visible.	Jingdong prefecture level city in Shandong
Jinfeng Hongyuan Flagship Store	Electricity savings1	It's good and worth buying with confidence	Jingdong prefecture level city in Shandong
Jinfeng Hongyuan Flagship Store	Electricity savings1	The goods have been received, just do not know if it is effective	Jingdong prefecture level city in Shandong

Jinfeng Hongyuan Flagship Store	Electricity savings1	It's already in use, but based on some of the descriptions, I suspect that to use it, you need to have one in series on each line, which seems to work for all appliances, that's my guess, but I feel as though the principle of the thing seems to require it.	Jingdong prefecture level city in Shandong
Jinfeng Hongyuan Flagship Store	Electricity savings1	Fast delivery, already in use, looks good!	Jingdong prefecture level city in Shandong
Jinfeng Hongyuan Flagship Store	Electricity savings1	Hoping to save 30% of power as presented in the graphic	Jingdong prefecture level city in Shandong
Jinfeng Hongyuan Flagship Store	Electricity savings1	Things look good, power saving do not know now, with a month after the continued evaluation	Jingdong prefecture level city in Shandong
Jinfeng Hongyuan Flagship Store	Electricity savings1	Already used it but not sure if it will top out!	Jingdong prefecture level city in Shandong
Jinfeng Hongyuan Flagship Store	Electricity savings1	Looking forward to using the results, the product quality looks okay.	Jingdong prefecture level city in Shandong
Jinfeng Hongyuan Flagship Store	Electricity savings1	It's somewhat effective and saves money more or less 💰	Jingdong prefecture level city in Shandong
Jinfeng Hongyuan Flagship Store	Electricity savings1	Got it, it's great, looking forward to saving power.	Jingdong prefecture level city in Shandong
Jinfeng Hongyuan Flagship Store	Electricity savings1	I don't know if it's working yet, I'll follow up when I get there	Jingdong prefecture level city in Shandong
Jinfeng Hongyuan Flagship Store	Electricity savings1	The product received, good workmanship, fast logistics, good inner packaging, the outer bag is damaged, but fortunately did not hurt the product, retrieved and inserted according to the instructions, looking forward to the effect is good, with a period of time to evaluate, I believe that the Jingdong.	Jingdong prefecture level city in Shandong
Jinfeng Hongyuan Flagship Store	Electricity savings1	Since it is a new product, it is said to work, so if it works well for a month, then I will give you a good review.	Jingdong prefecture level city in Shandong
Jinfeng Hongyuan Flagship Store	Electricity savings1	The quality of the product is very good, very well made, well packaged, easy to use and very convenient!	Jingdong prefecture level city in Shandong
Jinfeng Hongyuan Flagship Store	Electricity savings1	Good price for the item, good functionality	Jingdong prefecture level city in Shandong
Jinfeng Hongyuan Flagship Store	Electricity savings1	Received the first time to use it, the stuff is very good, specifics wait for a month to come back to feedback to see the effect.	Jingdong prefecture level city in Shandong
Jinfeng Hongyuan Flagship Store	Electricity savings1	Just received, the courier is too bad, not notified directly to put the Feng Nest courier cabinet	Jingdong prefecture level city in Shandong
Jinfeng Hongyuan Flagship Store	Electricity savings1	Baby received, the second time to buy, plugged in and used do not know if there is no effect. Baby received, the second time to buy, plugged in and used do not know if there is no effect. Baby received, the second time to buy, plugged in and used do not know if there is no effect.	Jingdong prefecture level city in Shandong
Jinfeng Hongyuan Flagship Store	Electricity savings1	It's okay if it saves power. It's not a bad product.	Jingdong prefecture level city in Shandong
Jinfeng Hongyuan Flagship Store	Electricity savings1	Received, very good, meticulous packaging, open a look is genuine, meticulous workmanship, the manual is very detailed, has been used, there is no effect on the only look at the next month's electricity bill, when I can I buy a few more to send people!	Jingdong prefecture level city in Shandong
Jinfeng Hongyuan Flagship Store	Electricity savings1	The quality is very good very delicate express delivery is too fast	Jingdong prefecture level city in Shandong
Jinfeng Hongyuan Flagship Store	Electricity savings1	I won't know until a month after I start using it today.	Jingdong prefecture level city in Shandong
Jinfeng Hongyuan Flagship Store	Electricity savings1	It doesn't work at all, the customer service attitude is very bad, use it or not!	Jingdong prefecture level city in Shandong
Jinfeng Hongyuan Flagship Store	Electricity savings1	Temporarily try, do not know the effect, Jingdong logistics delivery very quickly	Jingdong prefecture level city in Shandong
Jinfeng Hongyuan Flagship Store	Electricity savings1	It doesn't help. It's comforting.	Jingdong prefecture level city in Shandong
Jinfeng Hongyuan Flagship Store	Electricity savings1	It's great, use it before you say anything.	Jingdong prefecture level city in Shandong
Jinfeng Hongyuan Flagship Store	Electricity savings1	Received, already used, looking forward to a good a effect!	Jingdong prefecture level city in Shandong
Jinfeng Hongyuan Flagship Store	Electricity savings1	I don't know if it works yet, I won't know until I use it for a few months.	Jingdong prefecture level city in Shandong
Jinfeng Hongyuan Flagship Store	Electricity savings1	It takes a long time to get there. There's no change now.	Jingdong prefecture level city in Shandong
Jinfeng Hongyuan Flagship Store	Electricity savings1	Jingdong's delivery never disappoints, or so fast, and the stuff is great, next time you need to come back.	Jingdong prefecture level city in Shandong
Jinfeng Hongyuan Flagship Store	Electricity savings1	At first it was ok today, after using it for a while it didn't work anymore	Jingdong prefecture level city in Shandong
Jinfeng Hongyuan Flagship Store	Electricity savings1	Just received the goods, do not know the effect of using a few days before there may be effect	Jingdong prefecture level city in Shandong
Jinfeng Hongyuan Flagship Store	Electricity savings1	The logistics was fast, I received it the next day, I've purchased it for the second time and have been using it all along	Jingdong prefecture level city in Shandong
Jinfeng Hongyuan Flagship Store	Electricity savings1	Inexpensive, fast delivery, very pleased!	Jingdong prefecture level city in Shandong
Jinfeng Hongyuan Flagship Store	Electricity savings1	Received the goods immediately plugged directly into the use of power saving yet to be good news!	Jingdong prefecture level city in Shandong
Jinfeng Hongyuan Flagship Store	Electricity savings1	The service attitude is very good, and the Jingdong self-owned products can be trusted.	Jingdong prefecture level city in Shandong
Jinfeng Hongyuan Flagship Store	Electricity savings1	The product is good, I do not know if it can save play a power saving effect	Jingdong prefecture level city in Shandong
Jinfeng Hongyuan Flagship Store	Electricity savings1	We won't know how effective it is until we use it for a while, so I'll give you a good review now.	Jingdong prefecture level city in Shandong
Jinfeng Hongyuan Flagship Store	Electricity savings1	The quality is pretty good, just started using it, hopefully it will deliver results.	Jingdong prefecture level city in Shandong
Jinfeng Hongyuan Flagship Store	Electricity savings1	I've plugged it in first, and will follow up after I've used it to see how it works.	Jingdong prefecture level city in Shandong
Jinfeng Hongyuan Flagship Store	Electricity savings1	Things have arrived, the quality is good, the effect is also equivalent to can ah	Jingdong prefecture level city in Shandong
Jinfeng Hongyuan Flagship Store	Electricity savings1	It's not worth it to brag about power savings.	Jingdong prefecture level city in Shandong
Jinfeng Hongyuan Flagship Store	Electricity savings1	I don't know if it's an IQ tax, but I'll use it for now.	Jingdong prefecture level city in Shandong
Jinfeng Hongyuan Flagship Store	Electricity savings1	I don't feel the power saving yet, I can't know until I use it for 2 months.	Jingdong prefecture level city in Shandong
Jinfeng Hongyuan Flagship Store	Electricity savings1	There's nothing wrong with this at the moment.	Jingdong prefecture level city in Shandong
Jinfeng Hongyuan Flagship Store	Electricity savings1	It's authentic and worth buying	Jingdong prefecture level city in Shandong
Jinfeng Hongyuan Flagship Store	Electricity savings1	It's good. We'll see how it goes.	Jingdong prefecture level city in Shandong
Jinfeng Hongyuan Flagship Store	Electricity savings1	I don't know if it's working or not in the middle of using it, we'll see when the month is over	Jingdong prefecture level city in Shandong
Jinfeng Hongyuan Flagship Store	Electricity savings1	I'm not going to buy anything from Jingdong anymore, so I'll give it a good review	Jingdong prefecture level city in Shandong
Jinfeng Hongyuan Flagship Store	Electricity savings1	I'm not going to buy anything from Jingdong anymore, so I'll give it a good review	Jingdong prefecture level city in Shandong
Jinfeng Hongyuan Flagship Store	Electricity savings1	Just received the item and will see how it works in a month.	Jingdong prefecture level city in Shandong

Jinfeng Hongyuan Flagship Store	Electricity savings1	I don't know if it saves electricity, but it's plugged into an outlet every day anyway	Jingdong prefecture level city in Shandong
Jinfeng Hongyuan Flagship Store	Electricity savings1	I don't know if it works yet.	Jingdong prefecture level city in Shandong
Jinfeng Hongyuan Flagship Store	Electricity savings1	Plugged it in when I got back, waiting for a month to see the results	Jingdong prefecture level city in Shandong
Jinfeng Hongyuan Flagship Store	Electricity savings1	Effects remain to be seen, but it looks good quality and runs fine	Jingdong prefecture level city in Shandong
Jinfeng Hongyuan Flagship Store	Electricity savings1	The product received logistics very quickly, has not yet been used to test! After use in the evaluation!	Jingdong prefecture level city in Shandong
Jinfeng Hongyuan Flagship Store	Electricity savings1	The power saver is in use, and I don't know how much electricity has been saved after using it for more than a month, and the display voltmeter is very good.	Jingdong prefecture level city in Shandong
Jinfeng Hongyuan Flagship Store	Electricity savings1	It's so good, isn't it? You're a real Shanghainese, aren't you?	Jingdong prefecture level city in Shandong
Jinfeng Hongyuan Flagship Store	Electricity savings1	Great quality great great great 👍👍👍👍	Jingdong prefecture level city in Shandong
Jinfeng Hongyuan Flagship Store	Electricity savings1	I'm using it, I don't know if it's working yet.	Jingdong prefecture level city in Shandong
Jinfeng Hongyuan Flagship Store	Electricity savings1	Just used it for more than 10 days and haven't seen any results yet	Jingdong prefecture level city in Shandong
Jinfeng Hongyuan Flagship Store	Electricity savings1	The product arrived home feeling like it should work	Jingdong prefecture level city in Shandong
Jinfeng Hongyuan Flagship Store	Electricity savings1	Just received the goods, use it and comment back	Jingdong prefecture level city in Shandong
Jinfeng Hongyuan Flagship Store	Electricity savings1	Try it for a month and then evaluate	Jingdong prefecture level city in Shandong
Jinfeng Hongyuan Flagship Store	Electricity savings1	I don't know if it saves power yet, but I'll comment again after using it for a while.	Jingdong prefecture level city in Shandong
Jinfeng Hongyuan Flagship Store	Electricity savings1	The product is better than I thought, the quality and appearance are very good, the voltage is stable, and I'm looking forward to a good power saving effect.	Jingdong prefecture level city in Shandong
Jinfeng Hongyuan Flagship Store	Electricity savings1	Received and scanned the QR code to check that it is genuine, have plugged it in, can it save power, looking forward to the advertised effect. Customary praise, 5 points!	Jingdong prefecture level city in Shandong
Jinfeng Hongyuan Flagship Store	Electricity savings1	The goods have been received and have not yet been used. Do not know how the effect.	Jingdong prefecture level city in Shandong
Jinfeng Hongyuan Flagship Store	Electricity savings1	Hopefully, you get what you pay for, and you get what you pay for!	Jingdong prefecture level city in Shandong
Jinfeng Hongyuan Flagship Store	Electricity savings1	Just put it on and it feels like it's turning as fast as a meter! Looking forward to the results!	Jingdong prefecture level city in Shandong
Jinfeng Hongyuan Flagship Store	Electricity savings1	Overall look on the product quality is good, I do not know is not the effect of power saving, wait to see and then follow up!	Jingdong prefecture level city in Shandong
Jinfeng Hongyuan Flagship Store	Electricity savings1	The logistics courier is very fast, the courier service attitude is very good	Jingdong prefecture level city in Shandong
Jinfeng Hongyuan Flagship Store	Electricity savings1	The product has been received, it works well worth buying	Jingdong prefecture level city in Shandong
Jinfeng Hongyuan Flagship Store	Electricity savings1	How did it not work? Is it purely an IQ tax?	Jingdong prefecture level city in Shandong
Jinfeng Hongyuan Flagship Store	Electricity savings1	It looks good, I don't know how well it works.	Jingdong prefecture level city in Shandong
Jinfeng Hongyuan Flagship Store	Electricity savings1	It does seem to be a bit of a power saver, I just don't know if the more expensive one is more power efficient?	Jingdong prefecture level city in Shandong
Jinfeng Hongyuan Flagship Store	Electricity savings1	Is genuine very good, the price is much cheaper, cost-effective, really special paddle just to do activities, the price is much cheaper, cost-effective, really special paddle counting, packaging is also very high-end atmospheric class, it is worth buying!	Jingdong prefecture level city in Shandong
Jinfeng Hongyuan Flagship Store	Electricity savings1	I received it yesterday and used it right away, I hope it works, I'll wait for next month to come back to the conclusion, I'm looking forward to it. Five hearts praise!	Jingdong prefecture level city in Shandong
Jinfeng Hongyuan Flagship Store	Electricity savings1	Looks good, packed properly, received it today, am using it, will see results in a month!	Jingdong prefecture level city in Shandong
Jinfeng Hongyuan Flagship Store	Electricity savings1	Regular product Jingdong self-support trustworthy. There is an effect Come back to the review. 😍😍😍😍	Jingdong prefecture level city in Shandong
Jinfeng Hongyuan Flagship Store	Electricity savings1	I haven't used it yet, hopefully it will work as a power saver, and will comment again if it works well.	Jingdong prefecture level city in Shandong
Jinfeng Hongyuan Flagship Store	Electricity savings1	Shipping was fast, logistics was fast, service was good, and I look forward to good results from the item!	Jingdong prefecture level city in Shandong
Jinfeng Hongyuan Flagship Store	Electricity savings1	This product is this compensator, it has to be at the main switch to be useful, haven't compared the final result, the	Jingdong prefecture level city in Shandong
Jinfeng Hongyuan Flagship Store	Electricity savings1	Packaging is perfect . I've already started using it, so I'm looking forward to the results.	Jingdong prefecture level city in Shandong
Jinfeng Hongyuan Flagship Store	Electricity savings1	I just got it back. I'll comment after I use it.	Jingdong prefecture level city in Shandong
Jinfeng Hongyuan Flagship Store	Electricity savings1	Big brand, with a very assured, very good quality, catch the event to buy very cost-effective to take advantage of the offer, hurry to get, it is genuine, the packaging is also very good, no breakage, the quality is not to speak of, great 👍 logistics super super fast! Quality is also very good, indeed value	Jingdong prefecture level city in Shandong
Jinfeng Hongyuan Flagship Store	Electricity savings1	The quality is pretty good. The price is also quite affordable.	Jingdong prefecture level city in Shandong
Jinfeng Hongyuan Flagship Store	Electricity savings1	I did not expect the courier so fast to arrive yesterday just ordered, the head is a little smaller than I thought, well, the picture looks bigger. The appearance still looks good, as for the practicality and effectiveness of the use of the only know.	Jingdong prefecture level city in Shandong
Jinfeng Hongyuan Flagship Store	Electricity savings1	I don't know. Looking forward to it.	Jingdong prefecture level city in Shandong
Jinfeng Hongyuan Flagship Store	Electricity savings1	Baby received, very delicate, very nice, like. Just don't know if it can really save electricity. Baby received, very delicate, very good-looking, like. Just don't know if it can really save electricity.	Jingdong prefecture level city in Shandong
Jinfeng Hongyuan Flagship Store	Electricity savings1	I'll use it for a month and then comment on the quality of the item	Jingdong prefecture level city in Shandong
Jinfeng Hongyuan Flagship Store	Electricity savings1	It feels ok, use it for a while and see how it works before adding more comments.	Jingdong prefecture level city in Shandong
Jinfeng Hongyuan Flagship Store	Electricity savings1	In use, see results in a month.	Jingdong prefecture level city in Shandong
Jinfeng Hongyuan Flagship Store	Electricity savings1	Jingdong's logistics and quality are very good, the service attitude is very good, the logistics is very fast	Jingdong prefecture level city in Shandong
Jinfeng Hongyuan Flagship Store	Electricity savings1	Intelligent power saver received, well-packed logistics is also quite fast, easy to use, looking forward to good results!	Jingdong prefecture level city in Shandong
Jinfeng Hongyuan Flagship Store	Electricity savings1	The quality is unknown at this point, I won't know until I use it.	Jingdong prefecture level city in Shandong
Jinfeng Hongyuan Flagship Store	Electricity savings1	It's a good product. It really saves power and doesn't take up much space.	Jingdong prefecture level city in Shandong
Jinfeng Hongyuan Flagship Store	Electricity savings1	Goods have been received, packaging is very good, good to see the effect of future use in the follow-up.	Jingdong prefecture level city in Shandong
Jinfeng Hongyuan Flagship Store	Electricity savings1	The product is in perfect shape and the light weight is worth it!	Jingdong prefecture level city in Shandong
Jinfeng Hongyuan Flagship Store	Electricity savings1	Just got home and plugged it in. It's good.	Jingdong prefecture level city in Shandong
Jinfeng Hongyuan Flagship Store	Electricity savings1	The logistics are quick, just to see the results.	Jingdong prefecture level city in Shandong

Jinfeng Hongyuan Flagship Store	Electricity savings1	Short time can not feel here to leave a message, to be followed up later!	Jingdong prefecture level city in Shandong
Jinfeng Hongyuan Flagship Store	Electricity savings1	This user thinks the product is good	Jingdong prefecture level city in Shandong
Jinfeng Hongyuan Flagship Store	Electricity savings1	Received the baby, to be used to see the effect of it!	Jingdong prefecture level city in Shandong
Jinfeng Hongyuan Flagship Store	Electricity savings1	You have to wait a month or two to see how it works.	Jingdong prefecture level city in Shandong
Jinfeng Hongyuan Flagship Store	Electricity savings1	Not bad, good, like it, don't know if it's good, try it first.	Jingdong prefecture level city in Shandong
Jinfeng Hongyuan Flagship Store	Electricity savings1	The goods are good value for money and the logistics is very fast and good service!	Jingdong prefecture level city in Shandong
Jinfeng Hongyuan Flagship Store	Electricity savings1	I don't know how well it works, we'll see in a month	Jingdong prefecture level city in Shandong
Jinfeng Hongyuan Flagship Store	Electricity savings1	The product is fine, but r saving power or not I won't know until I use it for two months. The display and lights take electricity too?	Jingdong prefecture level city in Shandong
Jinfeng Hongyuan Flagship Store	Electricity savings1	I've used it. I don't know how it works yet.	Jingdong prefecture level city in Shandong
Jinfeng Hongyuan Flagship Store	Electricity savings1	I can't say until I compare them.	Jingdong prefecture level city in Shandong
Jinfeng Hongyuan Flagship Store	Electricity savings1	It's ok in the process of using 。。。。	Jingdong prefecture level city in Shandong
Jinfeng Hongyuan Flagship Store	Electricity savings1	Jinfeng Hongyuan Intelligent Power Saver Household Electricity Meter Electricity Saving Appliance High Power Enhanced Power Saving King Energy Saving Treasure Butler Control Non Stealing Electricity Reversing Not Going Interfering Slow Turner Artifact King Gold Yellow. Haven't tested it accurately yet, really need to wait a few months to test it before I have a say.	Jingdong prefecture level city in Shandong
Jinfeng Hongyuan Flagship Store	Electricity savings1	Don't know how effective it is yet? I hope it works well and I'll come back next time	Jingdong prefecture level city in Shandong
Jinfeng Hongyuan Flagship Store	Electricity savings1	Saving device has been received, material workmanship are very good ah! Have not yet begun to use. First observe the daily electricity consumption and cost of the home meter, and then plug in the power saver to use, so as to compare the use of the effect is good!	Jingdong prefecture level city in Shandong
Jinfeng Hongyuan Flagship Store	Electricity savings1	Merchants shipping extremely fast, Jingdong logistics safe and fast, I believe that the product can play a safe use of electricity, the role of scientific power saving, first praise, the effect of how to be used for a period of time and then the pursuit of bubble!	Jingdong prefecture level city in Shandong
Jinfeng Hongyuan Flagship Store	Electricity savings1	I just bought it. I don't know yet.	Jingdong prefecture level city in Shandong
Jinfeng Hongyuan Flagship Store	Electricity savings1	It is genuine, good quality, and it is very reassuring to use. The logistics is very fast, and the business service attitude is on point.	Jingdong prefecture level city in Shandong
Jinfeng Hongyuan Flagship Store	Electricity savings1	The quality is fine, I don't know how effective it is.	Jingdong prefecture level city in Shandong
Jinfeng Hongyuan Flagship Store	Electricity savings1	The stuff is back and I expect it will save the electricity	Jingdong prefecture level city in Shandong
Jinfeng Hongyuan Flagship Store	Electricity savings1	I'll give it 4 stars first and see what happens in a couple of months and then follow up	Jingdong prefecture level city in Shandong
Jinfeng Hongyuan Flagship Store	Electricity savings1	It's not bad. It's in the mind.	Jingdong prefecture level city in Shandong
Jinfeng Hongyuan Flagship Store	Electricity savings1	Received the goods have not yet started to use do not know whether it is good	Jingdong prefecture level city in Shandong
Jinfeng Hongyuan Flagship Store	Electricity savings1	This user thinks the product is good	Jingdong prefecture level city in Shandong
Jinfeng Hongyuan Flagship Store	Electricity savings1	Hello! The saver was received, thank you! Not yet used, but from the packaging looks very good quality, exquisite workmanship. Logistics is quite fast, if it is really very power saving, I will come back to	Jingdong prefecture level city in Shandong
Jinfeng Hongyuan Flagship Store	Electricity savings1	I've been using it for a few days now, I'll comment again after a month or two of use	Jingdong prefecture level city in Shandong
Jinfeng Hongyuan Flagship Store	Electricity savings1	I don't know. Bought it for my father at a good price.	Jingdong prefecture level city in Shandong
Jinfeng Hongyuan Flagship Store	Electricity savings1	Plugged it in the same day I bought it and the voltage showed 223, 225 looking forward to the results	Jingdong prefecture level city in Shandong
Jinfeng Hongyuan Flagship Store	Electricity savings1	Still in use, the biggest role is to stabilize the current, reduce losses, product quality on the look is very good, with this feeling quite good, see colleagues are using, power saving effect should be good	Jingdong prefecture level city in Shandong
Jinfeng Hongyuan Flagship Store	Electricity savings1	How to receive goods but no invoice and warranty more than it ...... quality is the premise of reputation!	Jingdong prefecture level city in Shandong
Jinfeng Hongyuan Flagship Store	Electricity savings1	Received but not used Continued evaluation	Jingdong prefecture level city in Shandong
Jinfeng Hongyuan Flagship Store	Electricity savings1	I just received the product, I don't know if it works well yet	Jingdong prefecture level city in Shandong
Jinfeng Hongyuan Flagship Store	Electricity savings1	Received it and used it, not sure it really saves electricity use it and then evaluate it, first give a 5 star review!!!!	Jingdong prefecture level city in Shandong
Jinfeng Hongyuan Flagship Store	Electricity savings1	It's good to use, it's very good to use, and it smells OK too	Jingdong prefecture level city in Shandong
Jinfeng Hongyuan Flagship Store	Electricity savings1	Packaging is very good, feel the product is not bad, how much power saving with the only know	Jingdong prefecture level city in Shandong
Jinfeng Hongyuan Flagship Store	Electricity savings1	Additional comments to be made after use	Jingdong prefecture level city in Shandong
Jinfeng Hongyuan Flagship Store	Electricity savings1	The product has been installed, try a few months and then comment!	Jingdong prefecture level city in Shandong
Jinfeng Hongyuan Flagship Store	Electricity savings1	I've bought it, let's use it for now, I'll comment after a month	Jingdong prefecture level city in Shandong
Jinfeng Hongyuan Flagship Store	Electricity savings1	Try it out and then follow up	Jingdong prefecture level city in Shandong
Jinfeng Hongyuan Flagship Store	Electricity savings1	It's good in theory, it doesn't feel good in practice.	Jingdong prefecture level city in Shandong
Jinfeng Hongyuan Flagship Store	Electricity savings1	I haven't tried it yet. We'll see.	Jingdong prefecture level city in Shandong
Jinfeng Hongyuan Flagship Store	Electricity savings1	Great item, second purchase!	Jingdong prefecture level city in Shandong
Jinfeng Hongyuan Flagship Store	Electricity savings1	Goods received now use it, if it does save power, plus reevaluate.	Jingdong prefecture level city in Shandong
Jinfeng Hongyuan Flagship Store	Electricity savings1	The stuff is light and small, the stuff is good, the courier service is good	Jingdong prefecture level city in Shandong
Jinfeng Hongyuan Flagship Store	Electricity savings1	I haven't used it yet. I'm looking forward to it.	Jingdong prefecture level city in Shandong
Jinfeng Hongyuan Flagship Store	Electricity savings1	Express received, in use do not know if there is no power saving	Jingdong prefecture level city in Shandong
Jinfeng Hongyuan Flagship Store	Electricity savings1	The quality of the product is quite good! The power saving mode is still getting used to it, can't say yet, how much power it saves.	Jingdong prefecture level city in Shandong
Jinfeng Hongyuan Flagship Store	Electricity savings1	The goods have been received, just started to use, now I do not know how the quality, after a period of time to say.	Jingdong prefecture level city in Shandong
Jinfeng Hongyuan Flagship Store	Electricity savings1	I received the stuff, used it for a few days to evaluate, the stuff is very good and good, worth buying.	Jingdong prefecture level city in Shandong
Jinfeng Hongyuan Flagship Store	Electricity savings1	I've been using it for a while, but I don't know if it's good or if it saves electricity! What the heck, let's give it a good review first. And for the record, I'm not helping him**.	Jingdong prefecture level city in Shandong

Jinfeng Hongyuan Flagship Store	Electricity savings1	Haven't used it yet, looks good, try it for 60 days, might be good, great review!	Jingdong prefecture level city in Shandong
Jinfeng Hongyuan Flagship Store	Electricity savings1	Baby received, logistics is very fast. Ordered last night, received this morning. Received the goods immediately plugged in the power supply, save power to the first of next month to know.	Jingdong prefecture level city in Shandong
Jinfeng Hongyuan Flagship Store	Electricity savings1	First, I'll give you a good review and use it.	Jingdong prefecture level city in Shandong
Jinfeng Hongyuan Flagship Store	Electricity savings1	Just arrived to follow up with a genuine review	Jingdong prefecture level city in Shandong
Jinfeng Hongyuan Flagship Store	Electricity savings1	Not bad pretty fast, good quality.	Jingdong prefecture level city in Shandong
Jinfeng Hongyuan Flagship Store	Electricity savings1	Recommended, use it and hopefully it will save some electricity	Jingdong prefecture level city in Shandong
Jinfeng Hongyuan Flagship Store	Electricity savings1	The stuff looks ok, I don't know the effect if it.	Jingdong prefecture level city in Shandong
Jinfeng Hongyuan Flagship Store	Electricity savings1	The stuff looks OK, the stuff looks OK.	Jingdong prefecture level city in Shandong
Jinfeng Hongyuan Flagship Store	Electricity savings1	The item has been received, after using it in a follow-up review. Satisfied with the purchase.	Jingdong prefecture level city in Shandong
Jinfeng Hongyuan Flagship Store	Electricity savings1	Has been normal plug-in use, at present do not know how to see, use down to see and then evaluation	Jingdong prefecture level city in Shandong
Jinfeng Hongyuan Flagship Store	Electricity savings1	Commodities make noise.	Jingdong prefecture level city in Shandong
Jinfeng Hongyuan Flagship Store	Electricity savings1	After using it for a few days and observing it, the saving is about ten percent	Jingdong prefecture level city in Shandong
Jinfeng Hongyuan Flagship Store	Electricity savings1	The goods received, the quality looks quite OK. First use it, the effect is not good to say.	Jingdong prefecture level city in Shandong
Jinfeng Hongyuan Flagship Store	Electricity savings1	In use, follow up next month	Jingdong prefecture level city in Shandong
Jinfeng Hongyuan Flagship Store	Electricity savings1	I just received the goods and have not yet used it to comment after using it	Jingdong prefecture level city in Shandong
Jinfeng Hongyuan Flagship Store	Electricity savings1	So far I don't know if it really saves electricity yet! Spinning waiting a couple months to find out! Good reviews first!	Jingdong prefecture level city in Shandong
Jinfeng Hongyuan Flagship Store	Electricity savings1	Small and exquisite, looking forward to the effect, to be evaluated 6666666666666	Jingdong prefecture level city in Shandong
Jinfeng Hongyuan Flagship Store	Electricity savings1	I just used it, I don't know if it's effective, I'll comment again after a month	Jingdong prefecture level city in Shandong
Jinfeng Hongyuan Flagship Store	Electricity savings1	I just used it, I don't know if it's good or not, I'll comment later.	Jingdong prefecture level city in Shandong
Jinfeng Hongyuan Flagship Store	Electricity savings1	Baby received, Baobei quite good, use and then come back to comment!	Jingdong prefecture level city in Shandong
Jinfeng Hongyuan Flagship Store	Electricity savings1	Good quality power saving is fake	Jingdong prefecture level city in Shandong
Jinfeng Hongyuan Flagship Store	Electricity savings1	The packaging of the goods is atmospheric, the quality is exquisite, and I look forward to saving electricity.	Jingdong prefecture level city in Shandong
Jinfeng Hongyuan Flagship Store	Electricity savings1	The packaging is great and the quality is good!	Jingdong prefecture level city in Shandong
Jinfeng Hongyuan Flagship Store	Electricity savings1	I don't see any effect.	Jingdong prefecture level city in Shandong
Jinfeng Hongyuan Flagship Store	Electricity savings1	Already used, but did not see obvious results	Jingdong prefecture level city in Shandong
Jinfeng Hongyuan Flagship Store	Electricity savings1	It's good. I'm just using it. I don't know what it's like.	Jingdong prefecture level city in Shandong
Jinfeng Hongyuan Flagship Store	Electricity savings1	I just used it. I'll comment later.	Jingdong prefecture level city in Shandong
Jinfeng Hongyuan Flagship Store	Electricity savings1	I installed it when it arrived, try it for a month and then come back for a follow-up review	Jingdong prefecture level city in Shandong
Jinfeng Hongyuan Flagship Store	Electricity savings1	Goods arrived a few days fastidious does not call to pick up the goods, the quality is average	Jingdong prefecture level city in Shandong
Jinfeng Hongyuan Flagship Store	Electricity savings1	This user thinks the product is good	Jingdong prefecture level city in Shandong
Jinfeng Hongyuan Flagship Store	Electricity savings1	Roger that.	Jingdong prefecture level city in Shandong
Jinfeng Hongyuan Flagship Store	Electricity savings1	Second purchase was ok.	Jingdong prefecture level city in Shandong
Jinfeng Hongyuan Flagship Store	Electricity savings1	The merchant is very reputable and the product is good value for money. Logistics is fast!	Jingdong prefecture level city in Shandong
Jinfeng Hongyuan Flagship Store	Electricity savings1	The quality of the baby is very good, I have always trusted Kyodo	Jingdong prefecture level city in Shandong
Jinfeng Hongyuan Flagship Store	Electricity savings1	We'll see how it goes when we get it.	Jingdong prefecture level city in Shandong
Jinfeng Hongyuan Flagship Store	Electricity savings1	I just used it. I don't know if it works.	Jingdong prefecture level city in Shandong
Jinfeng Hongyuan Flagship Store	Electricity savings1	Good quality, used for a month to save about 20%, power saving effect is good	Jingdong prefecture level city in Shandong
Jinfeng Hongyuan Flagship Store	Electricity savings1	I'll give you a good review before I use it.	Jingdong prefecture level city in Shandong
Jinfeng Hongyuan Flagship Store	Electricity savings1	Received, satisfied with the follow-up evaluation	Jingdong prefecture level city in Shandong
Jinfeng Hongyuan Flagship Store	Electricity savings1	Immediate results, saving electricity and money	Jingdong prefecture level city in Shandong
Jinfeng Hongyuan Flagship Store	Electricity savings1	Measured to save a little	Jingdong prefecture level city in Shandong
Jinfeng Hongyuan Flagship Store	Electricity savings1	This user thinks the product is good	Jingdong prefecture level city in Shandong
Jinfeng Hongyuan Flagship Store	Electricity savings1	This user thinks the product is good	Jingdong prefecture level city in Shandong
Jinfeng Hongyuan Flagship Store	Electricity savings1	Product quality and logistics are very good, worth buying	Jingdong prefecture level city in Shandong
Jinfeng Hongyuan Flagship Store	Electricity savings1	Appearance looks ok! Express delivery was fast! Service is still relatively enthusiastic.	Jingdong prefecture level city in Shandong
Jinfeng Hongyuan Flagship Store	Electricity savings1	It doesn't feel like it's working.	Jingdong prefecture level city in Shandong
Jinfeng Hongyuan Flagship Store	Electricity savings1	Well made and looks good. Used it with other appliances and felt as if it had some effect.	Jingdong prefecture level city in Shandong
Jinfeng Hongyuan Flagship Store	Electricity savings1	Haven't used it yet, will follow up later	Jingdong prefecture level city in Shandong
Jinfeng Hongyuan Flagship Store	Electricity savings1	Things received, used a few days the plug is a little hot unplugged did not work	Jingdong prefecture level city in Shandong
Jinfeng Hongyuan Flagship Store	Electricity savings1	Look at the meter again in a month.	Jingdong prefecture level city in Shandong
Jinfeng Hongyuan Flagship Store	Electricity savings1	This one didn't work at all.	Jingdong prefecture level city in Shandong

Jinfeng Hongyuan Flagship Store	Electricity savings1	It's small. I've seen friends with much bigger ones.	Jingdong prefecture level city in Shandong
Jinfeng Hongyuan Flagship Store	Electricity savings1	The item was received and the quality is good, it will be nice to see how the power saving rate is down the road, the item is great.	Jingdong prefecture level city in Shandong
Jinfeng Hongyuan Flagship Store	Electricity savings1	Cheap and affordable quality is quite good, logistics is also very fast, praise! , baby good 😊, cost-effective very high, big brand trustworthy 👍	Jingdong prefecture level city in Shandong
Jinfeng Hongyuan Flagship Store	Electricity savings1	Used it for a while it really saves electricity ⚡ thanks 🈶 such a good product, compact and practical, every family should buy one ✨	Jingdong prefecture level city in Shandong
Jinfeng Hongyuan Flagship Store	Electricity savings1	Very satisfied with the purchase Next time I will come back	Jingdong prefecture level city in Shandong
Jinfeng Hongyuan Flagship Store	Electricity savings1	Logistics is fast. Good quality, used one before and it does save power	Jingdong prefecture level city in Shandong
Jinfeng Hongyuan Flagship Store	Electricity savings1	I don't know if it works.	Jingdong prefecture level city in Shandong
Jinfeng Hongyuan Flagship Store	Electricity savings1	Good goods, very solid, good.	Jingdong prefecture level city in Shandong
Jinfeng Hongyuan Flagship Store	Electricity savings1	This user thinks the product is good	Jingdong prefecture level city in Shandong
Jinfeng Hongyuan Flagship Store	Electricity savings1	I've been using it for half a month, my electricity bill is much lower, and the quality is very good, so don't worry about buying it!	Jingdong prefecture level city in Shandong
Jinfeng Hongyuan Flagship Store	Electricity savings1	I received the goods today and used it right away, looking forward to the effect	Jingdong prefecture level city in Shandong
Jinfeng Hongyuan Flagship Store	Electricity savings1	The merchandise is great. I am going to recommend it to my friends! Will buy again next time.	Jingdong prefecture level city in Shandong
Jinfeng Hongyuan Flagship Store	Electricity savings1	Let's give it a good review first, it's working fine so far, and I'll follow up with a review,...	Jingdong prefecture level city in Shandong
Jinfeng Hongyuan Flagship Store	Electricity savings1	Product quality is very good, looking forward to using the results	Jingdong prefecture level city in Shandong
Jinfeng Hongyuan Flagship Store	Electricity savings1	I just can't detect it. - Save it.	Jingdong prefecture level city in Shandong
Jinfeng Hongyuan Flagship Store	Electricity savings1	It arrived and is being used, so we'll see how it works!	Jingdong prefecture level city in Shandong
Jinfeng Hongyuan Flagship Store	Electricity savings1	Received yesterday thanks! This energy saver is available . Safe! But in the end how much energy can be saved must be used for a period of time to know, if the real energy saving next time to buy again.	Jingdong prefecture level city in Shandong
Jinfeng Hongyuan Flagship Store	Electricity savings1	Just used it, don't know if it saves power	Jingdong prefecture level city in Shandong
Jinfeng Hongyuan Flagship Store	Electricity savings1	I'll rate it first, is it a tease wait two months and see? It's been four days and it's not noticeable!	Jingdong prefecture level city in Shandong
Jinfeng Hongyuan Flagship Store	Electricity savings1	Workmanship is quite good, just started to use, the effect of the follow-up evaluation.	Jingdong prefecture level city in Shandong
Jinfeng Hongyuan Flagship Store	Electricity savings1	Received the goods used for a few days before evaluation, the effect is quite good	Jingdong prefecture level city in Shandong
Jinfeng Hongyuan Flagship Store	Electricity savings1	Try it for a month and then evaluate	Jingdong prefecture level city in Shandong
Jinfeng Hongyuan Flagship Store	Electricity savings1	Just received the goods, the packaging is very good, the appearance of the economizer feels good, I do not know how the effect of power saving, with a month or two and then follow up!	Jingdong prefecture level city in Shandong
Jinfeng Hongyuan Flagship Store	Electricity savings1	It's quite small and delicate, I don't know if it's useful yet.	Jingdong prefecture level city in Shandong
Jinfeng Hongyuan Flagship Store	Electricity savings1	Just received it, use it for a few days before commenting	Jingdong prefecture level city in Shandong
Jinfeng Hongyuan Flagship Store	Electricity savings1	Does this thing work! When it arrived, the button for the safety was loose. I'm a little afraid of fire!	Jingdong prefecture level city in Shandong
Jinfeng Hongyuan Flagship Store	Electricity savings1	I received it. I don't know how it works.	Jingdong prefecture level city in Shandong
Jinfeng Hongyuan Flagship Store	Electricity savings1	This user thinks the product is good	Jingdong prefecture level city in Shandong
Jinfeng Hongyuan Flagship Store	Electricity savings1	Customer service was great! Small i problem was taken care of in a timely manner! Effectiveness to be tested by time! No rush!!!	Jingdong prefecture level city in Shandong
Jinfeng Hongyuan Flagship Store	Electricity savings1	I put it on right after I received it and it looks compact. I believe the high technology should save electricity.	Jingdong prefecture level city in Shandong
Jinfeng Hongyuan Flagship Store	Electricity savings1	Logistics courier very quickly, the first day under the order the next day to receive, off pieces whether power saving is the most important thing, things are exquisite workmanship, a month later in the follow-up evaluation whether power saving	Jingdong prefecture level city in Shandong
Jinfeng Hongyuan Flagship Store	Electricity savings1	The delivery is pretty fast, I'm using it, have to have it for a while to see results, looking forward to it.	Jingdong prefecture level city in Shandong
Jinfeng Hongyuan Flagship Store	Electricity savings1	See pretty good just started to use a month later to give you a real evaluation, should be useful.	Jingdong prefecture level city in Shandong
Jinfeng Hongyuan Flagship Store	Electricity savings1	And then comment.	Jingdong prefecture level city in Shandong
Jinfeng Hongyuan Flagship Store	Electricity savings1	Received the goods, wait for the test to evaluate	Jingdong prefecture level city in Shandong
Jinfeng Hongyuan Flagship Store	Electricity savings1	This user thinks the product is good	Jingdong prefecture level city in Shandong
Jinfeng Hongyuan Flagship Store	Electricity savings1	I'm already using it, I just don't know how to judge the power savings. I won't know until I use it for a season.	Jingdong prefecture level city in Shandong
Jinfeng Hongyuan Flagship Store	Electricity savings1	Works. Says it works.	Jingdong prefecture level city in Shandong
Jinfeng Hongyuan Flagship Store	Electricity savings1	Very good. Satisfied.	Jingdong prefecture level city in Shandong
Jinfeng Hongyuan Flagship Store	Electricity savings1	This user thinks the product is good	Jingdong prefecture level city in Shandong
Jinfeng Hongyuan Flagship Store	Electricity savings1	It's working. Obviously, it's less expensive.	Jingdong prefecture level city in Shandong
Jinfeng Hongyuan Flagship Store	Electricity savings1	The second one is up, the first one still works! It's reactive power compensation! This is well understood if you want a professional!	Jingdong prefecture level city in Shandong
Jinfeng Hongyuan Flagship Store	Electricity savings1	It feels as if there are some nodes, but they need to be tested and observed over time, thanks.	Jingdong prefecture level city in Shandong
Jinfeng Hongyuan Flagship Store	Electricity savings1	I've received the goods, the energy saving effect will be evaluated after using it for a period of time, and all the services are very good, praise!	Jingdong prefecture level city in Shandong
Jinfeng Hongyuan Flagship Store	Electricity savings1	I don't know how well it works yet, I bought it and plugged it in, so we'll see if it saves some power or not!	Jingdong prefecture level city in Shandong
Jinfeng Hongyuan Flagship Store	Electricity savings1	Baby a bunch of knife quality is very good, previously bought a quality is not very good. It really works and has results.	Jingdong prefecture level city in Shandong
Jinfeng Hongyuan Flagship Store	Electricity savings1	Things have arrived, it looks good, just started to use, as to whether or not there is a business said the power-saving effect, but also to observe the effect, now can not evaluate	Jingdong prefecture level city in Shandong
Jinfeng Hongyuan Flagship Store	Electricity savings1	I haven't used it yet. I'll comment afterward.	Jingdong prefecture level city in Shandong
Jinfeng Hongyuan Flagship Store	Electricity savings1	I don't know if it's any good.	Jingdong prefecture level city in Shandong
Jinfeng Hongyuan Flagship Store	Electricity savings1	I don't know how well it works.	Jingdong prefecture level city in Shandong
Jinfeng Hongyuan Flagship Store	Electricity savings1	The baby has been received! Uh-huh! Good, quite exquisite, small and exquisite I thought it was big. Dare to tightly plugged in the electricity, just wait for the effect later!	Jingdong prefecture level city in Shandong

Jinfeng Hongyuan Flagship Store	Electricity savings1	The packaging is sturdy, I tried it right after I received it, so I'm looking forward to the results.	Jingdong prefecture level city in Shandong
Jinfeng Hongyuan Flagship Store	Electricity savings1	Received the item and installed it! Don't know if it works well! Will follow up with a review later! I hope it works!	Jingdong prefecture level city in Shandong
Jinfeng Hongyuan Flagship Store	Electricity savings1	Things used on the effect of waiting to use a little to say, but things are very good. Logistics is very fast, business service is very good.	Jingdong prefecture level city in Shandong
Jinfeng Hongyuan Flagship Store	Electricity savings1	This user thinks the product is good	Jingdong prefecture level city in Shandong
Jinfeng Hongyuan Flagship Store	Electricity savings1	I'm really using it. It feels good.	Jingdong prefecture level city in Shandong
Jinfeng Hongyuan Flagship Store	Electricity savings1	Good, Jingdong logistics is very fast, intelligent energy-saving appliances	Jingdong prefecture level city in Shandong
Jinfeng Hongyuan Flagship Store	Electricity savings1	It's not bad. It really saves energy.	Jingdong prefecture level city in Shandong
Jinfeng Hongyuan Flagship Store	Electricity savings1	The logistics is fast, praise	Jingdong prefecture level city in Shandong
Jinfeng Hongyuan Flagship Store	Electricity savings1	This user thinks the product is good	Jingdong prefecture level city in Shandong
Jinfeng Hongyuan Flagship Store	Electricity savings1	The first time I placed two, and today I placed another one, a friend introduced it, said it is particularly power-saving	Jingdong prefecture level city in Shandong
Jinfeng Hongyuan Flagship Store	Electricity savings1	I don't know how effective it is. Let's leave it at home for a while and see.	Jingdong prefecture level city in Shandong
Jinfeng Hongyuan Flagship Store	Electricity savings1	Dear customer service! I would like to ask you how to understand the digital display (increase/decrease jump) on the top of the saver? Thank you for your help.	Jingdong prefecture level city in Shandong
Jinfeng Hongyuan Flagship Store	Electricity savings1	This user thinks the product is good	Jingdong prefecture level city in Shandong
Jinfeng Hongyuan Flagship Store	Electricity savings1	The goods have been received, packaging exquisite, is using, later to see how the effect?	Jingdong prefecture level city in Shandong
Jinfeng Hongyuan Flagship Store	Electricity savings1	The saver received, the boss shipped very quickly, yesterday shot today arrived!	Jingdong prefecture level city in Shandong
Jinfeng Hongyuan Flagship Store	Electricity savings1	Beautiful appearance, well packaged. Recording data to see if I can lower my electric bill a bit.	Jingdong prefecture level city in Shandong
Jinfeng Hongyuan Flagship Store	Electricity savings1	Things received, the packaging is quite good, the effect of the use and then come back to evaluate.	Jingdong prefecture level city in Shandong
Jinfeng Hongyuan Flagship Store	Electricity savings1	Easy to use, just plug it in. But there is a current sound.	Jingdong prefecture level city in Shandong
Jinfeng Hongyuan Flagship Store	Electricity savings1	Fast logistics, easy to use and labor-saving	Jingdong prefecture level city in Shandong
Jinfeng Hongyuan Flagship Store	Electricity savings1	Received, thank you!	Jingdong prefecture level city in Shandong
Jinfeng Hongyuan Flagship Store	Electricity savings1	Second time I've bought it. It's good.	Jingdong prefecture level city in Shandong
Jinfeng Hongyuan Flagship Store	Electricity savings1	I've only been using it for a short time, so I'm looking forward to the results.	Jingdong prefecture level city in Shandong
Jinfeng Hongyuan Flagship Store	Electricity savings1	Good stuff, good stuff, good stuff good stuff.	Jingdong prefecture level city in Shandong
Jinfeng Hongyuan Flagship Store	Electricity savings1	Buy your stuff is not always good I have been using it for almost two months now, it does not save electricity at all the electricity bill is about ten dollars a day this is still called the king of power	Jingdong prefecture level city in Shandong
Jinfeng Hongyuan Flagship Store	Electricity savings1	Express arrived, the quality is very good, worth the purchase.	Jingdong prefecture level city in Shandong
Jinfeng Hongyuan Flagship Store	Electricity savings1	Energy saving has been received, now the voltage is more stable, energy-saving effect to see a few days and then chase flat, the service attitude is very good, the courier is relatively fast, I am satisfied.	Jingdong prefecture level city in Shandong
Jinfeng Hongyuan Flagship Store	Electricity savings1	Stuff installed, numbers are smaller than when installed, hopefully it works	Jingdong prefecture level city in Shandong
Jinfeng Hongyuan Flagship Store	Electricity savings1	Good appearance, fine workmanship, just bought, use up the feeling of power saving effect is good, worth buying!	Jingdong prefecture level city in Shandong
Jinfeng Hongyuan Flagship Store	Electricity savings1	Economical and practical 👍 Always use it	Jingdong prefecture level city in Shandong
Jinfeng Hongyuan Flagship Store	Electricity savings1	Just used it, don't know the effect yet, will come back to follow up after the effect. Hope it works well(▽)	Jingdong prefecture level city in Shandong
Jinfeng Hongyuan Flagship Store	Electricity savings1	The goods have been received, use it to see in the evaluation.	Jingdong prefecture level city in Shandong
Jinfeng Hongyuan Flagship Store	Electricity savings1	Doesn't it save electricity? The boil kettle still goes faster when you press the meter.	Jingdong prefecture level city in Shandong
Jinfeng Hongyuan Flagship Store	Electricity savings1	Already used. Will patronize again in the future	Jingdong prefecture level city in Shandong
Jinfeng Hongyuan Flagship Store	Electricity savings1	Works great, just plug it in, results to be checked, satisfied.	Jingdong prefecture level city in Shandong
Jinfeng Hongyuan Flagship Store	Electricity savings1	Second purchase. Works well 😍😍😍😍	Jingdong prefecture level city in Shandong
Jinfeng Hongyuan Flagship Store	Electricity savings1	Prompt shipment, very satisfied	Jingdong prefecture level city in Shandong
Jinfeng Hongyuan Flagship Store	Electricity savings1	It's been less than a month, let's see how it works, hopefully it's power efficient	Jingdong prefecture level city in Shandong
Jinfeng Hongyuan Flagship Store	Electricity savings1	Use a month in say.	Jingdong prefecture level city in Shandong
Jinfeng Hongyuan Flagship Store	Electricity savings1	I've saved a lot of electricity since I've been using it.	Jingdong prefecture level city in Shandong
Jinfeng Hongyuan Flagship Store	Electricity savings1	It's fast, looks and feels good on the outside, try it out and evaluate.	Jingdong prefecture level city in Shandong
Jinfeng Hongyuan Flagship Store	Electricity savings1	I haven't used it. I just used it. I don't know if it's any good.	Jingdong prefecture level city in Shandong
Jinfeng Hongyuan Flagship Store	Electricity savings1	Quality can be, power saving now can not see just use, after a while in the look at the power saving effect	Jingdong prefecture level city in Shandong
Jinfeng Hongyuan Flagship Store	Electricity savings1	The logistics is very fast, and it seems to have some effect	Jingdong prefecture level city in Shandong
Jinfeng Hongyuan Flagship Store	Electricity savings1	Good, but the effects remain to be seen	Jingdong prefecture level city in Shandong
Jinfeng Hongyuan Flagship Store	Electricity savings1	Baby is basically the same as the description, shipping quickly, worth a buy Good price, logistics is strong, worth repurchasing!	Jingdong prefecture level city in Shandong
Jinfeng Hongyuan Flagship Store	Electricity savings1	I bought it for my dad, I don't know much about it, if he thinks it's good then it must be good 👍	Jingdong prefecture level city in Shandong
Jinfeng Hongyuan Flagship Store	Electricity savings1	Goods received. It's working fine. Display voltage. After a period of time to see the effect in the addition to the level of theory	Jingdong prefecture level city in Shandong
Jinfeng Hongyuan Flagship Store	Electricity savings1	The stuff is pretty good, I just don't know how well it saves power.	Jingdong prefecture level city in Shandong
Jinfeng Hongyuan Flagship Store	Electricity savings1	Logistics is a little too fast 👍	Jingdong prefecture level city in Shandong
Jinfeng Hongyuan Flagship Store	Electricity savings1	Received the goods used for a period of time before coming, good, beautiful appearance, really stable flow	Jingdong prefecture level city in Shandong
Jinfeng Hongyuan Flagship Store	Electricity savings1	Things received, look at the packaging is very good, not yet, to the power saving in the follow-up evaluation	Jingdong prefecture level city in Shandong

Jinfeng Hongyuan Flagship Store	Electricity savings1	Baby is very good! Use a few days before to evaluate, I personally test really effective.	Jingdong prefecture level city in Shandong
Jinfeng Hongyuan Flagship Store	Electricity savings1	I received the goods are in use, I do not know how effective? Can it save energy? Let's see.	Jingdong prefecture level city in Shandong
Jinfeng Hongyuan Flagship Store	Electricity savings1	It's really good and has some power saving effect.	Jingdong prefecture level city in Shandong
Jinfeng Hongyuan Flagship Store	Electricity savings1	It's good quality, well made, and I hope it'll save on electricity over time.	Jingdong prefecture level city in Shandong
Jinfeng Hongyuan Flagship Store	Electricity savings1	I just used it, I don't know how it works yet, but I'll give it a good review first.	Jingdong prefecture level city in Shandong
Jinfeng Hongyuan Flagship Store	Electricity savings1	Second purchase, the delivery guy was fast and called ahead to deliver.	Jingdong prefecture level city in Shandong
Jinfeng Hongyuan Flagship Store	Electricity savings1	Goods have been received, but have not yet used, used to see how the effect, in the evaluation of fertilizer	Jingdong prefecture level city in Shandong
Jinfeng Hongyuan Flagship Store	Electricity savings1	The delivery is very fast. It's the same in kind. It's working.	Jingdong prefecture level city in Shandong
Jinfeng Hongyuan Flagship Store	Electricity savings1	Fast logistics, good quality, more satisfied	Jingdong prefecture level city in Shandong
Jinfeng Hongyuan Flagship Store	Electricity savings1	I've been here for almost a month now and there is a very noticeable difference than it is worth buying.	Jingdong prefecture level city in Shandong
Jinfeng Hongyuan Flagship Store	Electricity savings1	Fast shipping, received the next day, plug and play, but do not know how effective to not break down!	Jingdong prefecture level city in Shandong
Jinfeng Hongyuan Flagship Store	Electricity savings1	The logistics are great I've been using it for a while before commenting on it This saves electricity	Jingdong prefecture level city in Shandong
Jinfeng Hongyuan Flagship Store	Electricity savings1	Wait to use it for a while to know if it's energy efficient	Jingdong prefecture level city in Shandong
Jinfeng Hongyuan Flagship Store	Electricity savings1	Just received it, haven't used it yet don't know if it's any good.	Jingdong prefecture level city in Shandong
Jinfeng Hongyuan Flagship Store	Electricity savings1	It really doesn't work, and the electric bill is still the same amount every month.	Jingdong prefecture level city in Shandong
Jinfeng Hongyuan Flagship Store	Electricity savings1	It's well packaged and looks fine	Jingdong prefecture level city in Shandong
Jinfeng Hongyuan Flagship Store	Electricity savings1	Very good. Will patronize again if needed.	Jingdong prefecture level city in Shandong
Jinfeng Hongyuan Flagship Store	Electricity savings1	I have received the product and installed it, try it for a while and see how it works.	Jingdong prefecture level city in Shandong
Jinfeng Hongyuan Flagship Store	Electricity savings1	I've been using it for a month now...I don't feel like there's any noticeable power savings...last month it was $130 this month it's $123...let's see if I can use it for another month	Jingdong prefecture level city in Shandong
Jinfeng Hongyuan Flagship Store	Electricity savings1	Just received it, yet to try it and see if it works.	Jingdong prefecture level city in Shandong
Jinfeng Hongyuan Flagship Store	Electricity savings1	It's easy to use and has a power saving credit!	Jingdong prefecture level city in Shandong
Jinfeng Hongyuan Flagship Store	Electricity savings1	It's very good. I've already bought two of them.	Jingdong prefecture level city in Shandong
Jinfeng Hongyuan Flagship Store	Electricity savings1	It's very well made, packaged meticulously, and I hope it works!	Jingdong prefecture level city in Shandong
Jinfeng Hongyuan Flagship Store	Electricity savings1	Goods have been received have not yet used it, used to comment again	Jingdong prefecture level city in Shandong
Jinfeng Hongyuan Flagship Store	Electricity savings1	Looking forward to good results	Jingdong prefecture level city in Shandong
Jinfeng Hongyuan Flagship Store	Electricity savings1	Not sure if it saves power or not. Still trying it out.	Jingdong prefecture level city in Shandong
Jinfeng Hongyuan Flagship Store	Electricity savings1	It also really saves on electricity! Superb!	Jingdong prefecture level city in Shandong
Jinfeng Hongyuan Flagship Store	Electricity savings1	Didn't work at all, want to return it	Jingdong prefecture level city in Shandong
Jinfeng Hongyuan Flagship Store	Electricity savings1	Uh-huh! It's working, I've been using it for a month and it saves 20% of electricity compared to before.	Jingdong prefecture level city in Shandong
Jinfeng Hongyuan Flagship Store	Electricity savings1	Should be a little bit of a power saver, I wonder how long it will take to save back the money I spent on this one	Jingdong prefecture level city in Shandong
Jinfeng Hongyuan Flagship Store	Electricity savings1	Exquisite appearance and stable voltage	Jingdong prefecture level city in Shandong
Jinfeng Hongyuan Flagship Store	Electricity savings1	After using it for a few days it feels like it saves electricity.	Jingdong prefecture level city in Shandong
Jinfeng Hongyuan Flagship Store	Electricity savings1	Second purchase and its pending good results.	Jingdong prefecture level city in Shandong
Jinfeng Hongyuan Flagship Store	Electricity savings1	The appearance of the workmanship is exquisite and meticulous, beautiful appearance, just to see if there is no effect!	Jingdong prefecture level city in Shandong
Jinfeng Hongyuan Flagship Store	Electricity savings1	Let's see what happens in a month.	Jingdong prefecture level city in Shandong
Jinfeng Hongyuan Flagship Store	Electricity savings1	Trial, now short time not yet obvious effect	Jingdong prefecture level city in Shandong
Jinfeng Hongyuan Flagship Store	Electricity savings1	I'm using it now, don't know how well it works, it looks great!	Jingdong prefecture level city in Shandong
Jinfeng Hongyuan Flagship Store	Electricity savings1	There is no role! Now it's true that I don't know!	Jingdong prefecture level city in Shandong
Jinfeng Hongyuan Flagship Store	Electricity savings1	Purchased for the second time, power saving not sure if it's ok? Let's talk about it after using it	Jingdong prefecture level city in Shandong
Jinfeng Hongyuan Flagship Store	Electricity savings1	I just received it, I have to try it for a month to know how it works.	Jingdong prefecture level city in Shandong
Jinfeng Hongyuan Flagship Store	Electricity savings1	I just used it for an hour, and the meter's still spinning so fast it's not much use.	Jingdong prefecture level city in Shandong
Jinfeng Hongyuan Flagship Store	Electricity savings1	Not bad, logistics is fast	Jingdong prefecture level city in Shandong
Jinfeng Hongyuan Flagship Store	Electricity savings1	I don't know if it works. I'll find out next month when I pay the bill. It's easy. Just plug it in.	Jingdong prefecture level city in Shandong
Jinfeng Hongyuan Flagship Store	Electricity savings1	Logistics is particularly fast, received the goods are genuine, looking forward to power saving effect	Jingdong prefecture level city in Shandong
Jinfeng Hongyuan Flagship Store	Electricity savings1	First time buying this product like this one from this family use it and then evaluate.	Jingdong prefecture level city in Shandong
Jinfeng Hongyuan Flagship Store	Electricity savings1	Lightning arrived, the product is very good, before all do not know there is such a baby, in a friend's home has seen, and then bought to try, has been used for a period of time before coming to the evaluation, the work is very stable, so far looked very good, will introduce friends to buy!	Jingdong prefecture level city in Shandong
Jinfeng Hongyuan Flagship Store	Electricity savings1	I was a bit skeptical when I heard that I could save money on my electricity bill by installing this, I don't know if the power saving effect is really what it is said to be. But since I bought it, I just have to put it on and try it out. I hope it lives up to its name and saves me the money I spent on the device	Jingdong prefecture level city in Shandong
Jinfeng Hongyuan Flagship Store	Electricity savings1	The quality of the stuff is okay, but I'm not sure if it works, so I'll try using it first.	Jingdong prefecture level city in Shandong
Jinfeng Hongyuan Flagship Store	Electricity savings1	It should work. The current is much more stable.	Jingdong prefecture level city in Shandong
Jinfeng Hongyuan Flagship Store	Electricity savings1	The store has a great service attitude	Jingdong prefecture level city in Shandong

Jinfeng Hongyuan Flagship Store	Electricity savings1	Let's wait until we've used it for a month.	Jingdong prefecture level city in Shandong
Jinfeng Hongyuan Flagship Store	Electricity savings1	I'm using it and I don't know if it's a power saver or not	Jingdong prefecture level city in Shandong
Jinfeng Hongyuan Flagship Store	Electricity savings1	Original and authentic guarantee quality is good now.	Jingdong prefecture level city in Shandong
Jinfeng Hongyuan Flagship Store	Electricity savings1	Great, very happy with it, will buy a couple more next time	Jingdong prefecture level city in Shandong
Jinfeng Hongyuan Flagship Store	Electricity savings1	I just used it, I don't know if it works yet, I'll come back after using it...	Jingdong prefecture level city in Shandong
Jinfeng Hongyuan Flagship Store	Electricity savings1	It's still being tested.	Jingdong prefecture level city in Shandong
Jinfeng Hongyuan Flagship Store	Electricity savings1	I've been using it for a month and it's not working.	Jingdong prefecture level city in Shandong
Jinfeng Hongyuan Flagship Store	Electricity savings1	It's basically nothing!	Jingdong prefecture level city in Shandong
Jinfeng Hongyuan Flagship Store	Electricity savings1	It's not bad, it kind of works, support it.	Jingdong prefecture level city in Shandong
Jinfeng Hongyuan Flagship Store	Electricity savings1	I just received it last night, let's try it first to see how it works, it looks good!	Jingdong prefecture level city in Shandong
Jinfeng Hongyuan Flagship Store	Electricity savings1	The current is very stable, fine workmanship, small and exquisite, very satisfied	Jingdong prefecture level city in Shandong
Jinfeng Hongyuan Flagship Store	Electricity savings1	Not bad, exactly how much electricity can be saved; still have to follow up the evaluation	Jingdong prefecture level city in Shandong
Jinfeng Hongyuan Flagship Store	Electricity savings1	Logistics is also very fast, next time there is an opportunity to introduce friends to buy!	Jingdong prefecture level city in Shandong
Jinfeng Hongyuan Flagship Store	Electricity savings1	Voltage display and actual do not pay	Jingdong prefecture level city in Shandong
Jinfeng Hongyuan Flagship Store	Electricity savings1	Second purchase, still saves a little on the electric bill	Jingdong prefecture level city in Shandong
Jinfeng Hongyuan Flagship Store	Electricity savings1	Order received, currently trying it out, thanks a lot.	Jingdong prefecture level city in Shandong
Jinfeng Hongyuan Flagship Store	Electricity savings1	I haven't used it for a long time yet, I don't know if the effect is good or not, looking forward to it	Jingdong prefecture level city in Shandong
Jinfeng Hongyuan Flagship Store	Electricity savings1	Good, working, I just don't know if it will save electricity... 5 stars for now.	Jingdong prefecture level city in Shandong
Jinfeng Hongyuan Flagship Store	Electricity savings1	Great product, just as the owner said it would be.	Jingdong prefecture level city in Shandong
Jinfeng Hongyuan Flagship Store	Electricity savings1	Shipping was fast, I don't know if it works well or not, I have to use it for a while first.	Jingdong prefecture level city in Shandong
Jinfeng Hongyuan Flagship Store	Electricity savings1	I don't feel that it saves much electricity, but I'll give it a try. It's been more than a month now, and the electricity bill is still a bit high.	Jingdong prefecture level city in Shandong
Jinfeng Hongyuan Flagship Store	Electricity savings1	Got it. Just used it.	Jingdong prefecture level city in Shandong
Jinfeng Hongyuan Flagship Store	Electricity savings1	Use it for a while and then comment.	Jingdong prefecture level city in Shandong
Jinfeng Hongyuan Flagship Store	Electricity savings1	Used it, see how it works with it and then comment. The quality is very good!	Jingdong prefecture level city in Shandong
Jinfeng Hongyuan Flagship Store	Electricity savings1	I'll comment again after a while.	Jingdong prefecture level city in Shandong
Jinfeng Hongyuan Flagship Store	Electricity savings1	It's easy to operate, affordable and works well, love it.	Jingdong prefecture level city in Shandong
Jinfeng Hongyuan Flagship Store	Electricity savings1	It's good. You can try it. It's cheap.	Jingdong prefecture level city in Shandong
Jinfeng Hongyuan Flagship Store	Electricity savings1	It's in use, so we'll see how it works after a while!	Jingdong prefecture level city in Shandong
Jinfeng Hongyuan Flagship Store	Electricity savings1	Service is pretty good, not sure about the quality yet.	Jingdong prefecture level city in Shandong
Jinfeng Hongyuan Flagship Store	Electricity savings1	Just used it and will see the results in a month 😃	Jingdong prefecture level city in Shandong
Jinfeng Hongyuan Flagship Store	Electricity savings1	Saving electricity is expensive and good	Jingdong prefecture level city in Shandong
Jinfeng Hongyuan Flagship Store	Electricity savings1	Pretty good, 5 stars! Will buy again next time seller service is very good！！！！ Good quality! Will recommend to friends!	Jingdong prefecture level city in Shandong
Jinfeng Hongyuan Flagship Store	Electricity savings1	In use, with results naturally have follow-up reviews!	Jingdong prefecture level city in Shandong
Jinfeng Hongyuan Flagship Store	Electricity savings1	I'm using it now, I'll come back with results, it'll work.	Jingdong prefecture level city in Shandong
Jinfeng Hongyuan Flagship Store	Electricity savings1	Good, nice, fast shipping, cheap, worth having!	Jingdong prefecture level city in Shandong
Jinfeng Hongyuan Flagship Store	Electricity savings1	Logistics is fast.	Jingdong prefecture level city in Shandong
Jinfeng Hongyuan Flagship Store	Electricity savings1	I have just started using the product and will be reviewing it later.	Jingdong prefecture level city in Shandong
Jinfeng Hongyuan Flagship Store	Electricity savings1	I'll comment after I use it.	Jingdong prefecture level city in Shandong
Jinfeng Hongyuan Flagship Store	Electricity savings1	I don't know if it's working or not, though I'll try plugging it in.	Jingdong prefecture level city in Shandong
Jinfeng Hongyuan Flagship Store	Electricity savings1	It really saves electricity and contributes to energy savings in disguise	Jingdong prefecture level city in Shandong
Jinfeng Hongyuan Flagship Store	Electricity savings1	It feels good in the experience.	Jingdong prefecture level city in Shandong
Jinfeng Hongyuan Flagship Store	Electricity savings1	The product is better than I thought it would be, the quality looks great, the voltage is stable, and I'm looking forward to some great power savings!	Jingdong prefecture level city in Shandong
Jinfeng Hongyuan Flagship Store	Electricity savings1	I'm using it now looking forward to saving some points,, I'm feeding back	Jingdong prefecture level city in Shandong
Jinfeng Hongyuan Flagship Store	Electricity savings1	Displayed voltage below 200.	Jingdong prefecture level city in Shandong
Jinfeng Hongyuan Flagship Store	Electricity savings1	I haven't seen any great results, but it should last about a hundred or eighty years.	Jingdong prefecture level city in Shandong
Jinfeng Hongyuan Flagship Store	Electricity savings1	I don't know if it works or not, but I keep it plugged in anyway, so I hope it works!	Jingdong prefecture level city in Shandong
Jinfeng Hongyuan Flagship Store	Electricity savings1	Reasonable price, good quality, fast delivery, great service, already a repurchase.	Jingdong prefecture level city in Shandong
Jinfeng Hongyuan Flagship Store	Electricity savings1	Second purchase, looking forward to the results	Jingdong prefecture level city in Shandong
Jinfeng Hongyuan Flagship Store	Electricity savings1	The quality of this product feels like it should work in a use test! Just how many kilowatts of electricity must be wasted per day with the red light and digital display on for long periods of time?	Jingdong prefecture level city in Shandong
Jinfeng Hongyuan Flagship Store	Electricity savings1	It looks good, we'll see if we use it.	Jingdong prefecture level city in Shandong
Jinfeng Hongyuan Flagship Store	Electricity savings1	Great stuff, second purchase!	Jingdong prefecture level city in Shandong

Jinfeng Hongyuan Flagship Store	Electricity savings1	It is indeed very practical and saves electricity at home	Jingdong prefecture level city in Shandong
Jinfeng Hongyuan Flagship Store	Electricity savings1	Goods received, the physical and the same as the picture, logistics is also very fast!	Jingdong prefecture level city in Shandong
Jinfeng Hongyuan Flagship Store	Electricity savings1	I'll use it for a while and see how it goes. I'll buy it again if it's good.	Jingdong prefecture level city in Shandong
Jinfeng Hongyuan Flagship Store	Electricity savings1	In use, voltage monitoring, the effect of how to wait for next month's electricity consumption confirmation	Jingdong prefecture level city in Shandong
Jinfeng Hongyuan Flagship Store	Electricity savings1	It's still pretty good.	Jingdong prefecture level city in Shandong
Jinfeng Hongyuan Flagship Store	Electricity savings1	It's been plugged in for a month and there hasn't been a big change yet, since it usually uses very little electricity	Jingdong prefecture level city in Shandong
Jinfeng Hongyuan Flagship Store	Electricity savings1	Let's wait for a month to see if it works	Jingdong prefecture level city in Shandong
Jinfeng Hongyuan Flagship Store	Electricity savings1	Good, good, good, good, good, good, good, good, good, good, good, good, good, good, good.	Jingdong prefecture level city in Shandong
Jinfeng Hongyuan Flagship Store	Electricity savings1	The product received the trial a few days, feel good, praise!	Jingdong prefecture level city in Shandong
Jinfeng Hongyuan Flagship Store	Electricity savings1	After six or seven hours of use, 250 volts won't save you any power.	Jingdong prefecture level city in Shandong
Jinfeng Hongyuan Flagship Store	Electricity savings1	I haven't used it yet, I wonder if it saves electricity?	Jingdong prefecture level city in Shandong
Jinfeng Hongyuan Flagship Store	Electricity savings1	Baby value for money, love it, next time will come back is genuine, trust the business, baby texture is very good, the price is moderate, unlimited repurchase!	Jingdong prefecture level city in Shandong
Jinfeng Hongyuan Flagship Store	Electricity savings1	Power saving effect is obvious! Raising tropical fish daily power consumption, after using the daily power savings of more than 3 degrees, currently about 8 degrees / day!	Jingdong prefecture level city in Shandong
Jinfeng Hongyuan Flagship Store	Electricity savings1	Just used it, unknown results.	Jingdong prefecture level city in Shandong
Jinfeng Hongyuan Flagship Store	Electricity savings1	The goods received, well, look forward to the effect. Logistics is very fast, thanks to the platform, thanks to Jingdong. I hope it can save electricity.	Jingdong prefecture level city in Shandong
Jinfeng Hongyuan Flagship Store	Electricity savings1	Not sure 有 effect?	Jingdong prefecture level city in Shandong
Jinfeng Hongyuan Flagship Store	Electricity savings1	I'd say it's a 5% savings.	Jingdong prefecture level city in Shandong
Jinfeng Hongyuan Flagship Store	Electricity savings1	I received it and am using it, I don't know how effective it is, I'll evaluate it afterwards	Jingdong prefecture level city in Shandong
Jinfeng Hongyuan Flagship Store	Electricity savings1	Baby received not yet used, first give praise	Jingdong prefecture level city in Shandong
Jinfeng Hongyuan Flagship Store	Electricity savings1	Looks good, I'll evaluate it later	Jingdong prefecture level city in Shandong
Jinfeng Hongyuan Flagship Store	Electricity savings1	The goods have been received in use, the overall feeling is not bad!	Jingdong prefecture level city in Shandong
Jinfeng Hongyuan Flagship Store	Electricity savings1	It's working. This is the second one. This stuff has to be used over a long period of time to be effective. And it only works on inductive loads, i.e. appliances containing electric motors. Use it.	Jingdong prefecture level city in Shandong
Jinfeng Hongyuan Flagship Store	Electricity savings1	Use it for a while and see how it works.	Jingdong prefecture level city in Shandong
Jinfeng Hongyuan Flagship Store	Electricity savings1	Good courier service and the item is very nice and practical.	Jingdong prefecture level city in Shandong
Jinfeng Hongyuan Flagship Store	Electricity savings1	It doesn't do anything and it's a waste of electricity.	Jingdong prefecture level city in Shandong
Jinfeng Hongyuan Flagship Store	Electricity savings1	I haven't used it yet. It should be good.	Jingdong prefecture level city in Shandong
Jinfeng Hongyuan Flagship Store	Electricity savings1	That's good. OK, that's good.	Jingdong prefecture level city in Shandong
Jinfeng Hongyuan Flagship Store	Electricity savings1	It's a rip off, doesn't work, doesn't save electricity, does the job to well, and I've wasted a couple dozen dollars again.	Jingdong prefecture level city in Shandong
Jinfeng Hongyuan Flagship Store	Electricity savings1	Use it first, the effect is not as good as the road, then we will comment again	Jingdong prefecture level city in Shandong
Jinfeng Hongyuan Flagship Store	Electricity savings1	The price is affordable, the logistics is strong, just use four or five days, the effect will be added later!	Jingdong prefecture level city in Shandong
Jinfeng Hongyuan Flagship Store	Electricity savings1	It's easy to use and works fine.	Jingdong prefecture level city in Shandong
Jinfeng Hongyuan Flagship Store	Electricity savings1	It doesn't feel like it's working.	Jingdong prefecture level city in Shandong
Jinfeng Hongyuan Flagship Store	Electricity savings1	I'm using it. We'll see what happens in a couple months.	Jingdong prefecture level city in Shandong
Jinfeng Hongyuan Flagship Store	Electricity savings1	I just got it back to try it out and I'll comment later	Jingdong prefecture level city in Shandong
Jinfeng Hongyuan Flagship Store	Electricity savings1	Less than two months. Can't tell.	Jingdong prefecture level city in Shandong
Jinfeng Hongyuan Flagship Store	Electricity savings1	No power saving effect	Jingdong prefecture level city in Shandong
Jinfeng Hongyuan Flagship Store	Electricity savings1	Express Express Very quickly not only to the power saver can not save electricity, with two months, look at the evaluation put.	Jingdong prefecture level city in Shandong
Jinfeng Hongyuan Flagship Store	Electricity savings1	Genuine products are packaged well!	Jingdong prefecture level city in Shandong
Jinfeng Hongyuan Flagship Store	Electricity savings1	I've been using it for a while, but I don't know if it saves electricity. I hope it works.	Jingdong prefecture level city in Shandong
Jinfeng Hongyuan Flagship Store	Electricity savings1	Good service, fast logistics, the effect is to be used to know	Jingdong prefecture level city in Shandong
Jinfeng Hongyuan Flagship Store	Electricity savings1	Just used it, not sure about the power saving effect	Jingdong prefecture level city in Shandong
Jinfeng Hongyuan Flagship Store	Electricity savings1	The stuff is ok, save power or not, will comment after a month.	Jingdong prefecture level city in Shandong
Jinfeng Hongyuan Flagship Store	Electricity savings1	Used it for a month to save %25, normal is a little over 100, it's great! I want to buy one in.	Jingdong prefecture level city in Shandong
Jinfeng Hongyuan Flagship Store	Electricity savings1	I'm using it and looking forward to the results	Jingdong prefecture level city in Shandong
Jinfeng Hongyuan Flagship Store	Electricity savings1	The quality is very good and the workmanship is exquisite. The use effect is also very good.	Jingdong prefecture level city in Shandong
Jinfeng Hongyuan Flagship Store	Electricity savings1	Merchant service attitude is very good, logistics speed is super fast, online evaluation is very good, the first time to use, a month to know the specific effect, the effect is good, and then additional	Jingdong prefecture level city in Shandong
Jinfeng Hongyuan Flagship Store	Electricity savings1	Works great, solves the problem of high power usage in the house	Jingdong prefecture level city in Shandong
Jinfeng Hongyuan Flagship Store	Electricity savings1	To receive! Very delicate! It's not clear how effective it is, I won't know until I use it!!!!	Jingdong prefecture level city in Shandong
Jinfeng Hongyuan Flagship Store	Electricity savings1	I've just been using it for a while and I don't feel like it's too noticeable.	Jingdong prefecture level city in Shandong
Jinfeng Hongyuan Flagship Store	Electricity savings1	Already used, no visible effect for the time being	Jingdong prefecture level city in Shandong
Jinfeng Hongyuan Flagship Store	Electricity savings1	I can't feel the effect.	Jingdong prefecture level city in Shandong

Jinfeng Hongyuan Flagship Store	Electricity savings1	Use it for a while and see the results	Jingdong prefecture level city in Shandong
Jinfeng Hongyuan Flagship Store	Electricity savings1	The price is more reasonable, the power-saving effect is to be verified, the delivery is fast, and the courier service is good.	Jingdong prefecture level city in Shandong
Jinfeng Hongyuan Flagship Store	Electricity savings1	Quality is very good, consistent with the seller's description, very satisfied, very like, very fast shipping, packaging is very careful and strict, the logistics company has a very good service attitude, fast delivery, very satisfied with a shopping, very satisfied. Recommended to buy, cost-effective, the service attitude of the business is very enthusiastic, providing such high quality goods.	Jingdong prefecture level city in Shandong
Jinfeng Hongyuan Flagship Store	Electricity savings1	I don't know.	Jingdong prefecture level city in Shandong
Jinfeng Hongyuan Flagship Store	Electricity savings1	I didn't get the delivery.	Jingdong prefecture level city in Shandong
Jinfeng Hongyuan Flagship Store	Electricity savings1	It works, it does have power savings, better than expected, will repurchase.	Jingdong prefecture level city in Shandong
Jinfeng Hongyuan Flagship Store	Electricity savings1	That's where the five fuses are.	Jingdong prefecture level city in Shandong
Jinfeng Hongyuan Flagship Store	Electricity savings1	Don't buy it, it doesn't work and you can't return it.	Jingdong prefecture level city in Shandong
Jinfeng Hongyuan Flagship Store	Electricity savings1	I hope it works. Wait a month.	Jingdong prefecture level city in Shandong
Jinfeng Hongyuan Flagship Store	Electricity savings1	Nothing**, buy with caution	Jingdong prefecture level city in Shandong
Jinfeng Hongyuan Flagship Store	Electricity savings1	I'm using it and waiting for the results	Jingdong prefecture level city in Shandong
Jinfeng Hongyuan Flagship Store	Electricity savings1	Saver quality is very good, has always felt that the home power consumption is very large, this is the company's electrician recommended, said he bought this home is really able to save a little electricity, I also came to buy, the effect if it is good, then buy, a floor a...	Jingdong prefecture level city in Shandong
Jinfeng Hongyuan Flagship Store	Electricity savings1	Good product, satisfied with purchase	Jingdong prefecture level city in Shandong
Jinfeng Hongyuan Flagship Store	Electricity savings1	Occupying the time has not yet found the power saving, occupying not evaluation	Jingdong prefecture level city in Shandong
Jinfeng Hongyuan Flagship Store	Electricity savings1	Haven't tested the results yet. To be followed up with comments!	Jingdong prefecture level city in Shandong
Jinfeng Hongyuan Flagship Store	Electricity savings1	After experiencing it for a month, I don't seem to know how to save it, and I don't pay attention to it anyway	Jingdong prefecture level city in Shandong
Jinfeng Hongyuan Flagship Store	Electricity savings1	It's useless. It's a waste of electricity.	Jingdong prefecture level city in Shandong
Jinfeng Hongyuan Flagship Store	Electricity savings1	I don't know if it's a power saver. It's a couple bucks. I hope it works.	Jingdong prefecture level city in Shandong
Jinfeng Hongyuan Flagship Store	Electricity savings1	I don't know what to say about this stuff. I don't know if it works or not, it's kind of a psychological purchase. Dormitory use to see the meter can not be not so good-looking. You have to wait until the time to deduct the utility bill to know.	Jingdong prefecture level city in Shandong
Jinfeng Hongyuan Flagship Store	Electricity savings1	Energy savers don't work / they're all a bunch of hoaxes.	Jingdong prefecture level city in Shandong
Jinfeng Hongyuan Flagship Store	Electricity savings1	Received the goods, used for more than a week, it's okay, sure a little effect	Jingdong prefecture level city in Shandong
Jinfeng Hongyuan Flagship Store	Electricity savings1	Well made and compact! Looking forward to the effect!	Jingdong prefecture level city in Shandong
Jinfeng Hongyuan Flagship Store	Electricity savings1	It has arrived and we'll see how it works.	Jingdong prefecture level city in Shandong
Jinfeng Hongyuan Flagship Store	Electricity savings1	Good. Good. It's worth it.	Jingdong prefecture level city in Shandong
Jinfeng Hongyuan Flagship Store	Electricity savings1	How come there's no indicator light?	Jingdong prefecture level city in Shandong
Jinfeng Hongyuan Flagship Store	Electricity savings1	The machine that uses electricity works quite well. This saves	Jingdong prefecture level city in Shandong
Jinfeng Hongyuan Flagship Store	Electricity savings1	It's good for home use and feels quite energy efficient	Jingdong prefecture level city in Shandong
Jinfeng Hongyuan Flagship Store	Electricity savings1	I regret buying this product, after using it for two days it doesn't work at all, still so many kilowatts per day just like the original.	Jingdong prefecture level city in Shandong
Jinfeng Hongyuan Flagship Store	Electricity savings1	Works very well and has some scientific merit.	Jingdong prefecture level city in Shandong
Jinfeng Hongyuan Flagship Store	Electricity savings1	Logistics is also very fast this good power saving I use two months before commenting!	Jingdong prefecture level city in Shandong
Jinfeng Hongyuan Flagship Store	Electricity savings1	Repeat customer. Very good.	Jingdong prefecture level city in Shandong
Jinfeng Hongyuan Flagship Store	Electricity savings1	It's good stuff. I've been using it for a month now and it's a real power saver.	Jingdong prefecture level city in Shandong
Jinfeng Hongyuan Flagship Store	Electricity savings1	Jingdong platform to buy things, has been very assured, quality assurance. Jingdong Express is as good as ever, complete packaging, delivery, service attitude to go! The family has always wanted to buy an energy-saving and power-saving equipment, selected half a day to choose this one, the value is very good, used for a period of time to follow up, to see whether it can achieve the real purpose of power saving, I	Jingdong prefecture level city in Shandong
Jinfeng Hongyuan Flagship Store	Electricity savings1	Works great, inexpensive and will be back next time.	Jingdong prefecture level city in Shandong
Jinfeng Hongyuan Flagship Store	Electricity savings1	Baby received, looks good, wait for a month to see how it works?	Jingdong prefecture level city in Shandong
Jinfeng Hongyuan Flagship Store	Electricity savings1	This product is not bad quite good well customer service said quite power saving that look at the customer service to see if the accuracy of the look at all aspects of the color is also quite beautiful	Jingdong prefecture level city in Shandong
Jinfeng Hongyuan Flagship Store	Electricity savings1	Just got back and tried it feels pretty good, just don't know how the quality is, give a good comment first	Jingdong prefecture level city in Shandong
Jinfeng Hongyuan Flagship Store	Electricity savings1	Received the product, trying it out, hope it helps!	Jingdong prefecture level city in Shandong
Jinfeng Hongyuan Flagship Store	Electricity savings1	Works great, saves electricity a dollar a day and a night	Jingdong prefecture level city in Shandong
Jinfeng Hongyuan Flagship Store	Electricity savings1	Baby received, look at the workmanship is still very fine, is genuine. I hope that this summer to open the air conditioning electricity bill is no longer high scary!	Jingdong prefecture level city in Shandong
Jinfeng Hongyuan Flagship Store	Electricity savings1	It's here. I don't know how it's going to work, but I'm going to use it.	Jingdong prefecture level city in Shandong
Jinfeng Hongyuan Flagship Store	Electricity savings1	Power saver received, Jingdong logistics first-class, business customer service attitude is good, one by one to introduce the product performance, back on the horse plugged in, it is very simple, there is a voltage indicator, to be a few months to see how the effect of power-saving to come back to a good follow-up, but I believe that Jinfenghong products.	Jingdong prefecture level city in Shandong
Jinfeng Hongyuan Flagship Store	Electricity savings1	Took the third one...the super power-saving kind!	Jingdong prefecture level city in Shandong
Jinfeng Hongyuan Flagship Store	Electricity savings1	First time buyer, customer service is very polite and courteous, conscientious business, support! Baby has received 🎈, super like, high value and practical, super like! ❥❥❥❥❥ Very good quality, very satisfied with a shopping. Customer service has a question and answer, very professional finally received the goods, very, very much like, exquisite and beautiful workmanship, has been praised by many people, really too happy, is already a loyal fan of his family, will continue to buy buy buy buy!	Jingdong prefecture level city in Shandong
Jinfeng Hongyuan Flagship Store	Electricity savings1	That's good. That's good. That's really good.	Jingdong prefecture level city in Shandong
Jinfeng Hongyuan Flagship Store	Electricity savings1	Saver received, good-looking design, voltage at a glance, and the merchant description, save some about 12% or so	Jingdong prefecture level city in Shandong

Jinfeng Hongyuan Flagship Store	Electricity savings1	Intelligent power saver is small and does not take up space, very good, very practical, received and used, packaging is tight, logistics is also very fast, satisfied with a shopping!	Jingdong prefecture level city in Shandong
Jinfeng Hongyuan Flagship Store	Electricity savings1	The stuff is good! Well made, compact, alloy case, shows voltage! Very like! Kudos!	Jingdong prefecture level city in Shandong
Jinfeng Hongyuan Flagship Store	Electricity savings1	I just purchased this power saver from Jingdong, inserted in the socket shows the number 206, a moment and 207 back and forth display, I do not know what it means, my home voltage should be normal, because it is the owner of the community's own power plant to put, first use for a period of time in the positive feedback it, first use in the look at it!	Jingdong prefecture level city in Shandong
Jinfeng Hongyuan Flagship Store	Electricity savings1	Very delicate and compact, there is a convenient intelligent display prompts the voltage, you can know the voltage at a glance, there is also very easy to use, a plug can be used, you can save electricity	Jingdong prefecture level city in Shandong
Jinfeng Hongyuan Flagship Store	Electricity savings1	Logistics is fast, ask customer service said can save electricity, but only use two months to see the effect, use two months in the evaluation.	Jingdong prefecture level city in Shandong
Jinfeng Hongyuan Flagship Store	Electricity savings1	The quality is very good.	Jingdong prefecture level city in Shandong
Jinfeng Hongyuan Flagship Store	Electricity savings1	Smart Saver has been received and will be evaluated after use	Jingdong prefecture level city in Shandong
Jinfeng Hongyuan Flagship Store	Electricity savings1	Home electricity is too waste, a month more than 300 dollars of electricity, and then the family heard that this can save a lot of electricity, let me order to buy a, anyway, will not, buy on the buy, thought it has to be a pretty big box it, delivery to the home to see a very small, plug-and-play, you can really save electricity, it's worth it!	Jingdong prefecture level city in Shandong
Jinfeng Hongyuan Flagship Store	Electricity savings1	Smart Saver received. Unpacking the box is exquisite. The material workmanship is very good. Plugged in and tried the space. Did not open the air conditioning voltage display 206 air conditioning open up the air conditioning display for 194. whether to save electricity to a month later to pay the electricity bill will know. Logistics is also very fast service is also in place.	Jingdong prefecture level city in Shandong
Jinfeng Hongyuan Flagship Store	Electricity savings1	The product feels pretty good and I hope it works, as the store owner said it would. Works!!!	Jingdong prefecture level city in Shandong
Jinfeng Hongyuan Flagship Store	Electricity savings1	Baby received, physical very quickly, just plugged in hope that it can be as everyone said those power saving, with two months after additional comments, first give a five-star praise.	Jingdong prefecture level city in Shandong
Jinfeng Hongyuan Flagship Store	Electricity savings1	Good, very good, very good, you deserve it, whoever uses it knows, the stuff works great, will buy it again in the future.	Jingdong prefecture level city in Shandong
Jinfeng Hongyuan Flagship Store	Electricity savings1	Don't hesitate to buy wow! No cheap feeling, very high-end, especially great! It is genuine, and the same as the entity store, the price is also favorable, decisive turn powder 💗 catch up to do activities when you buy, the price of a penny for a penny ♡, from the outside looking very beautiful 💗, the material workmanship is good 💗, feel that the purchase is worth it, and the logistics is super	Jingdong prefecture level city in Shandong
Jinfeng Hongyuan Flagship Store	Electricity savings1	Logistics is fast! Very good quality! Very beautiful! Next time to patronize!	Jingdong prefecture level city in Shandong
Jinfeng Hongyuan Flagship Store	Electricity savings1	Really like it, completely exceeded expectations, the shipping speed is very fast, the packaging is very careful and strict, the delivery speed is very fast, very satisfied with a shopping Baby received, and the description is the same, love it!	Jingdong prefecture level city in Shandong
Jinfeng Hongyuan Flagship Store	Electricity savings1	Very good, if you need to order!	Jingdong prefecture level city in Shandong
Jinfeng Hongyuan Flagship Store	Electricity savings1	So far, plugged in the voltage is more stable, as for the effect of how to quietly wait until the payment of the electricity bill to compare, the appearance of the commodity design is very good, the real thing!	Jingdong prefecture level city in Shandong
Jinfeng Hongyuan Flagship Store	Electricity savings1	💌 I've been wanting it for a long time, I decided to go for it, I feel great after using it, it's genuine and the delivery is great, it lives up to its name👍	Jingdong prefecture level city in Shandong
Jinfeng Hongyuan Flagship Store	Electricity savings1	Looking forward to it!!! I don't know if it's working or not, let's look forward to it!!!! How much is saved ,,,,,,,,,, Saving a degree is a degree with long term use !!!!!!!	Jingdong prefecture level city in Shandong
Jinfeng Hongyuan Flagship Store	Electricity savings1	After using it for a while, I am seriously and responsibly saying that this saver is really very power efficient. Two rooms with the air conditioner on at night for one night, plus watching TV, charging cell phones, lights, etc. etc. only used one degree of electricity. So I bought several more and it's really	Jingdong prefecture level city in Shandong
Jinfeng Hongyuan Flagship Store	Electricity savings1	It arrived quickly, and the box is solid and delicate. Baby shell is thick aluminum and by multi-layer coating. The inner core is heavy, and the power cord and plug are genuine products from a large manufacturer. It is the best power saver I have found after choosing all of them!	Jingdong prefecture level city in Shandong
Jinfeng Hongyuan Flagship Store	Electricity savings1	The saver was received, genuine guarantee, good quality...	Jingdong prefecture level city in Shandong
Jinfeng Hongyuan Flagship Store	Electricity savings1	Logistics was fast! The product is easy to use!	Jingdong prefecture level city in Shandong
Jinfeng Hongyuan Flagship Store	Electricity savings1	Look appearance is okay, use a period of time to know how it is. Jingdong Mall, trustworthy! Home appliances, cell phones and computers are almost 100% bought by Jingdong. Ordered the night before yesterday, received yesterday. Jingdong logistics, the fastest in the network!	Jingdong prefecture level city in Shandong
Jinfeng Hongyuan Flagship Store	Electricity savings1	Very good, perfect packaging, home energy saver to determine the effect of power saving and also play a role in stabilizing the voltage Very good products	Jingdong prefecture level city in Shandong
Jinfeng Hongyuan Flagship Store	Electricity savings1	Smart Saver received, looks very exquisite. The method of use is simple, and it has been used. The later effect, with time to test.	Jingdong prefecture level city in Shandong
Jinfeng Hongyuan Flagship Store	Electricity savings1	Both the material and workmanship exceeded expectations, great value, and looks great on oh. The seller also shipped much earlier than expected, will come back. Buy it, you will not regret it! Full five points. Both the material and workmanship exceeded expectations, great value, wear it also looks good Oh. Seller delivery is also much earlier than expected, will come again. Buy it, will not regret it! Full	Jingdong prefecture level city in Shandong
Jinfeng Hongyuan Flagship Store	Electricity savings1	The appearance is clean and beautiful, the workmanship is very good, I didn't find any defects, I personally feel that the size is also quite suitable, I've used it for 2 months, I didn't find out exactly how energy efficient it is. Try using it again!	Jingdong prefecture level city in Shandong
Jinfeng Hongyuan Flagship Store	Electricity savings1	Power saving device received, product certification, accessories, etc. complete, very good workmanship, very practical power-saving effect is also very obvious.	Jingdong prefecture level city in Shandong
Jinfeng Hongyuan Flagship Store	Electricity savings1	Looked at a lot of think buy this one, see a lot of reviews say it works, a month with save nearly twenty degrees of electricity, there are sixty days of return guarantee, with a good and then come back to the	Jingdong prefecture level city in Shandong
Jinfeng Hongyuan Flagship Store	Electricity savings1	I received the power saving device, and I'm using it. I hope I can get the desired effect, and I'm trying to force it to work quickly.	Jingdong prefecture level city in Shandong
Jinfeng Hongyuan Flagship Store	Electricity savings1	Voltage is stable, already trying it out and waiting for the results to happen, saving electricity will follow up next month!	Jingdong prefecture level city in Shandong
Jinfeng Hongyuan Flagship Store	Electricity savings1	Jingdong Mall self-supporting goods of excellent quality, logistics is also very fast, worth recommending 👏👏👏👏 excellent!	Jingdong prefecture level city in Shandong
Jinfeng Hongyuan Flagship Store	Electricity savings1	Power saving has been received, the appearance of the beautiful, I have already been the second time to buy the use of down the effect is very good!	Jingdong prefecture level city in Shandong
Jinfeng Hongyuan Flagship Store	Electricity savings1	Energy saver received very happy, the color is also very good looking forward to a very good effect can save electricity	Jingdong prefecture level city in Shandong
Jinfeng Hongyuan Flagship Store	Electricity savings1	It's small and easy to plug in and use. It displays the voltage value when plugged in and	Jingdong prefecture level city in Shandong
Jinfeng Hongyuan Flagship Store	Electricity savings1	This saver really works well, because I installed one at home about seven or eight years ago, when there is no voltage display, now there is a voltage display, the appearance also looks much better, the electricity bill is also saved, the stability of the home appliances is also good, the best of both	Jingdong prefecture level city in Shandong
Jinfeng Hongyuan Flagship Store	Electricity savings1	Ordered yesterday, arrived today in use. Shipping was fast. The effect is looking forward to.	Jingdong prefecture level city in Shandong
Jinfeng Hongyuan Flagship Store	Electricity savings1	This thing, I don't know what the principle is. Why it can save electricity. Wait for a month to use it first, see if it will save electricity bill, then come back to evaluate. There is one thing that does not fit the principle ah. The law of conservation of energy.	Jingdong prefecture level city in Shandong
Jinfeng Hongyuan Flagship Store	Electricity savings1	Home mom listen to others say this can save electricity, specially let buy a, later a very large, go back to see a particularly small, directly plugged into the electricity can be, no need to install, the price is also very cheap, the first time with this product, do not know if it is not good to use, temporarily try for a period of time, if it is good to use, recommend that you buy this one, the	Jingdong prefecture level city in Shandong
Jinfeng Hongyuan Flagship Store	Electricity savings1	I've been using it for a few days, and I think the workmanship and materials are pretty good, so we'll have to look at the next bill for the real energy savings, and then we'll add more comments.	Jingdong prefecture level city in Shandong
Jinfeng Hongyuan Flagship Store	Electricity savings1	Energy saver is well made, energy saving and power saving, and anti-counterfeiting code, it is a regular commodity!	Jingdong prefecture level city in Shandong

Jinfeng Hongyuan Flagship Store	Electricity savings1	Jinfeng Hongyuan, buy back home, as a mouse, haha. First of all, normal electricity for 1 month, the power did not increase, the electricity bill feels smoother, year-on-year electricity costs in the low, whether it is very energy-saving still have to be used for a long time to know. All 5 stars ☆	Jingdong prefecture level city in Shandong
Jinfeng Hongyuan Flagship Store	Electricity savings1	The box is well packaged and beautiful! I hope to save electricity with it haha!	Jingdong prefecture level city in Shandong
Jinfeng Hongyuan Flagship Store	Electricity savings1	Ordered last night, arrived this morning, first try the effect before making comments	Jingdong prefecture level city in Shandong
Jinfeng Hongyuan Flagship Store	Electricity savings1	Looks good. Don't know the effect yet.	Jingdong prefecture level city in Shandong
Jinfeng Hongyuan Flagship Store	Electricity savings1	Jinfeng Hongyuan intelligent power saver has exquisite workmanship and modern fashionable design style. It is a good home economizer . I believe that the goods purchased from Jingdong will not disappoint our	Jingdong prefecture level city in Shandong
Jinfeng Hongyuan Flagship Store	Electricity savings1	Inadvertently flipped to this power saver, never heard of, feel very novel, the price is not expensive, so buy to try, see a month after the electricity bill will be less, recently the home air-conditioning open a lot of power consumption is very large.	Jingdong prefecture level city in Shandong
Jinfeng Hongyuan Flagship Store	Electricity savings1	The quality of the goods is very good workmanship, but also sent five fuse	Jingdong prefecture level city in Shandong
Jinfeng Hongyuan Flagship Store	Electricity savings1	I'm trying it out. I don't know if it'll work.	Jingdong prefecture level city in Shandong
Jinfeng Hongyuan Flagship Store	Electricity savings1	There is no effect yet do not know, is to look at the official sale, buy to try it! Use according to the instructions, plugged in, used for a period of time, anyway, do not know how, after a few months to see it, to see if the average month than before will be less electricity.	Jingdong prefecture level city in Shandong
Jinfeng Hongyuan Flagship Store	Electricity savings1	Received the goods, the first time to unpacking, the quality is very good, consistent with the description of the seller, completely exceeded my expectations, the packaging is very careful, strict, in general this time is very satisfied with a shopping, thanks to the seller.	Jingdong prefecture level city in Shandong
Jinfeng Hongyuan Flagship Store	Electricity savings1	The energy saver was received and used for a few days with good results.	Jingdong prefecture level city in Shandong
Jinfeng Hongyuan Flagship Store	Electricity savings1	Previously I have paid attention to this product, but I think that the monthly electricity consumption at home is not large, there is no need to save electricity savings. After a second thought, energy saving is the responsibility of each of our citizens, the winter is coming, this year's winter is cold, heating and all electric appliances, can save is to save, the price is not expensive, so buy to try. I analyze the principle of power saving, should be able to save electricity, but the larger the power saving effect is about good. To be tried after a month or two to see the effect and then evaluate. Jingdong trustworthy.	Jingdong prefecture level city in Shandong
Jinfeng Hongyuan Flagship Store	Electricity savings1	It just arrived and I can't wait to use it, and the customer service is great! There is also voltage showing up which is nice! I don't know if it can save a bit I won't know until I've used it for a month! When the time comes to come back to the follow-up review!	Jingdong prefecture level city in Shandong
Jinfeng Hongyuan Flagship Store	Electricity savings1	Checked out the reviews from numerous users. The shipment is still very large, so I decided to buy it anyway to use it and see the effect after a month. Jingdong Mall here to buy safe, convenient and fast, this price is very powerful 💪, very recommended.	Jingdong prefecture level city in Shandong
Jinfeng Hongyuan Flagship Store	Electricity savings1	Purchased two at the same time to use together. The other one works fine, the other has problems with the numbers not sure if the machine is faulty. Power saving effect can not be observed. Feel the effect is not very obvious like?	Jingdong prefecture level city in Shandong
Jinfeng Hongyuan Flagship Store	Electricity savings1	Looking forward to it!!! I don't know if it's working or not, let's look forward to it!!!! How much is saved ，，，，，，，，， Saving a degree is a degree with long term use ！！！！！！！	Jingdong prefecture level city in Shandong
Jinfeng Hongyuan Flagship Store	Electricity savings1	Received, directly on the use of the special convenient	Jingdong prefecture level city in Shandong
Jinfeng Hongyuan Flagship Store	Electricity savings1	Goods received a few days well-packed, the same day opened and plugged in to try to be normal is not sure what the power-saving effect. After a month with a follow-up evaluation.	Jingdong prefecture level city in Shandong
Jinfeng Hongyuan Flagship Store	Electricity savings1	I bought it and used it, I don't know how it works yet, we'll see later. Use the effect of it	Jingdong prefecture level city in Shandong
Jinfeng Hongyuan Flagship Store	Electricity savings1	Photographed 2, the old family also put a, with a small January, there should be some effect, with a long period of time to comment, with a long time to see the obvious effect, hard courier brother.	Jingdong prefecture level city in Shandong
Jinfeng Hongyuan Flagship Store	Electricity savings1	Experience: I am an electrician, this is the real use of electronic waves automatically connected to track the decomposition of ionized electrons, in situ compensation of reactive power, reduce effective power loss and waste, stabilize the voltage to achieve energy saving and power saving, the overall effect	Jingdong prefecture level city in Shandong
Jinfeng Hongyuan Flagship Store	Electricity savings1	Great looking and practical! Easy to use and save! Saves 15% in a month of comparison	Jingdong prefecture level city in Shandong
Jinfeng Hongyuan Flagship Store	Electricity savings1	Product quality is very good! Packaging is very nice, like a cell phone box, thick and well protected! Service is also great! Logistics is very fast! Thank you for your business! Very honest!	Jingdong prefecture level city in Shandong
Jinfeng Hongyuan Flagship Store	Electricity savings1	In several products inside the choice of this purchase for a period of time, but also need to use a few months to compare last year's electricity bill before you can analyze the savings in electricity, to the summer peak electricity consumption should be more obvious.	Jingdong prefecture level city in Shandong
Jinfeng Hongyuan Flagship Store	Electricity savings1	The goods have long been received, after more than a month to comment, the home electricity bill is indeed reduced, worth buying!	Jingdong prefecture level city in Shandong
Jinfeng Hongyuan Flagship Store	Electricity savings1	Baby received, the logistics is strong, the quality is reliable, it is worth having and recommending!	Jingdong prefecture level city in Shandong
Jinfeng Hongyuan Flagship Store	Electricity savings1	Using a conservation appliance, you can achieve a 10-15% savings in electricity usage.	Jingdong prefecture level city in Shandong
Jinfeng Hongyuan Flagship Store	Electricity savings1	Quality is very good, metal shell, touch is very good, very recommended Oh ~, the subsequent effect of continuous follow-up	Jingdong prefecture level city in Shandong
Jinfeng Hongyuan Flagship Store	Electricity savings1	The product is beautiful, practical, worth buying. The product is beautiful, practical, good quality, fast logistics product is beautiful, practical, beautiful and elegant, very classy, worth buying!	Jingdong prefecture level city in Shandong
Jinfeng Hongyuan Flagship Store	Electricity savings1	Just received the item and have already used it. Didn't know much about these products before. I hope it is as effective and useful as the product advertises.	Jingdong prefecture level city in Shandong
Jinfeng Hongyuan Flagship Store	Electricity savings1	A month two people used more than five hundred degrees of electricity, in a hurry and then add a power saver, I hope to have an effect, Jingdong shopping is very cool, logistics is very fast especially good service attitude delivery, this hot day is not easy, thank you!	Jingdong prefecture level city in Shandong
Jinfeng Hongyuan Flagship Store	Electricity savings1	Just received the baby, first plugged in to try to use a month and then additional evaluation it	Jingdong prefecture level city in Shandong
Jinfeng Hongyuan Flagship Store	Electricity savings1	Goods fast, the power saver has been received, a friend recommended to buy that can save electricity. Exquisite and compact is very convenient, plug and play. The effect is good, the price is affordable, the next need will patronize again!	Jingdong prefecture level city in Shandong
Jinfeng Hongyuan Flagship Store	Electricity savings1	Packaging is better than expected, sent a few fuse, power saving effect in the end do not know, try a period of time to say!	Jingdong prefecture level city in Shandong
Jinfeng Hongyuan Flagship Store	Electricity savings1	Shipped quickly, immediately installed, read countless reviews were not sure if this thing is effective, was going to buy three at once, ultimately decided to first use a try the effect, if the power ring significantly reduced, then will buy a few more!	Jingdong prefecture level city in Shandong
Jinfeng Hongyuan Flagship Store	Electricity savings1	Used for a period of time to evaluate, not to a month still do not know whether it can save electricity, the voltage has a display!	Jingdong prefecture level city in Shandong
Jinfeng Hongyuan Flagship Store	Electricity savings1	I don't know if it saves electricity or not, but I'm using it anyway, so I hope it saves a little bit, electricity is too expensive	Jingdong prefecture level city in Shandong
Jinfeng Hongyuan Flagship Store	Electricity savings1	The power saver has been received and I am very happy with it! Just waiting to see how the power saving works!	Jingdong prefecture level city in Shandong
Jinfeng Hongyuan Flagship Store	Electricity savings1	Saving power has been received, logistics is quite fast, the quality is not bad, small and a little weight, has been used to look forward to the effect of	Jingdong prefecture level city in Shandong
Jinfeng Hongyuan Flagship Store	Electricity savings1	The logistics is very fast, the effect is obvious and trustworthy.	Jingdong prefecture level city in Shandong
Jinfeng Hongyuan Flagship Store	Electricity savings1	Good quality, good quality, beautiful, beautiful, beautiful, beautiful, very worth buying, worth buying, worth buying, worth buying, worth customizing!	Jingdong prefecture level city in Shandong
Jinfeng Hongyuan Flagship Store	Electricity savings1	I've used it for now, I don't know if it saves electricity	Jingdong prefecture level city in Shandong
Jinfeng Hongyuan Flagship Store	Electricity savings1	Bought two at a time, logistics is very fast, the product is beautifully packaged, after two months to see the results	Jingdong prefecture level city in Shandong

Jinfeng Hongyuan Flagship Store	Electricity savings1	The workmanship is really fine, the packaging is also good, the regular factory production ~ use a break in the evaluation of the effectiveness of the	Jingdong prefecture level city in Shandong
Jinfeng Hongyuan Flagship Store	Electricity savings1	Sometimes 219. Sometimes 220 in this case	Jingdong prefecture level city in Shandong
Jinfeng Hongyuan Flagship Store	Electricity savings1	It is the ideal product, good quality, logistics is also very fast, worthy of praise!	Jingdong prefecture level city in Shandong
Jinfeng Hongyuan Flagship Store	Electricity savings1	Store service is very good, the courier received the next day. Product packaging is very good, just use the quality to be tested.	Jingdong prefecture level city in Shandong
Jinfeng Hongyuan Flagship Store	Electricity savings1	I've been using it for almost a month now and I feel like it's working.	Jingdong prefecture level city in Shandong
Jinfeng Hongyuan Flagship Store	Electricity savings1	Very satisfied, very restful, very pretty, very beautiful, very much enjoyed! Well worth it!	Jingdong prefecture level city in Shandong
Jinfeng Hongyuan Flagship Store	Electricity savings1	Baby especially like, the value of high-quality good, a very good shopping experience, attentive service, fast logistics, distribution is also very thoughtful, attitude is also very good, value for money ah, decisive praise! Quality is quite good, logistics is also very fast, praise!	Jingdong prefecture level city in Shandong
Jinfeng Hongyuan Flagship Store	Electricity savings1	The shape of the item is basically the same as ordered, the effect is to be examined.	Jingdong prefecture level city in Shandong
Jinfeng Hongyuan Flagship Store	Electricity savings1	Appearance style: good-looking workmanship material: very good, a small one, very delicate Experience: still in use, in the process of using the shopkeeper is very nice to have a careful explanation Use for a period of time in the look at the results	Jingdong prefecture level city in Shandong
Jinfeng Hongyuan Flagship Store	Electricity savings1	As described, compact and well packaged	Jingdong prefecture level city in Shandong
Jinfeng Hongyuan Flagship Store	Electricity savings1	Fine workmanship. Test in progress. Let's see how it works.	Jingdong prefecture level city in Shandong
Jinfeng Hongyuan Flagship Store	Electricity savings1	The goods have been received, I'm very satisfied and love it	Jingdong prefecture level city in Shandong
Jinfeng Hongyuan Flagship Store	Electricity savings1	Safety! Whether you can save electricity, you can roughly feel it in 2 months. The principle is simple. Before the electrical appliances and circuits inductive more, now the electrical appliances integrated, the home is not a lot of inductive circuits, but used certainly save no problem! Wiring according to the requirements of the manual is good, if it is not convenient, see the socket plug.	Jingdong prefecture level city in Shandong
Jinfeng Hongyuan Flagship Store	Electricity savings1	Great product, looking forward to how much power I can save, first time buyer!	Jingdong prefecture level city in Shandong
Jinfeng Hongyuan Flagship Store	Electricity savings1	The product is exquisite, beautifully packaged, and the customer service is excellent. Just waiting to see how the power saving effect is, looking forward to it!	Jingdong prefecture level city in Shandong
Jinfeng Hongyuan Flagship Store	Electricity savings1	Express very quickly very satisfied with the first use to see how the effect to know, if you can save 10 percent even if acceptable, if you can not meet this requirement is obviously not much effect. Obviously.	Jingdong prefecture level city in Shandong
Jinfeng Hongyuan Flagship Store	Electricity savings1	Very delicate and compact, there is a very convenient intelligent display prompts the voltage, you can know the voltage at a glance, there is very easy to use, a plug can be used, I hope to save money on	Jingdong prefecture level city in Shandong
Jinfeng Hongyuan Flagship Store	Electricity savings1	First time buyer is it really save after use	Jingdong prefecture level city in Shandong
Jinfeng Hongyuan Flagship Store	Electricity savings1	Purchased again and will be for a long time yet.	Jingdong prefecture level city in Shandong
Jinfeng Hongyuan Flagship Store	Electricity savings1	Bought it to stock up, came back and plugged it in, hope it works	Jingdong prefecture level city in Shandong
Jinfeng Hongyuan Flagship Store	Electricity savings1	Baby received the logistics very fast very like very satisfied praise a	Jingdong prefecture level city in Shandong
Jinfeng Hongyuan Flagship Store	Electricity savings1	Beginning the process of using, easy to evaluate more later.	Jingdong prefecture level city in Shandong
Jinfeng Hongyuan Flagship Store	Electricity savings1	The goods have been received, just do not know how effective	Jingdong prefecture level city in Shandong
Jinfeng Hongyuan Flagship Store	Electricity savings1	It feels okay. I wish there were more events.	Jingdong prefecture level city in Shandong
Jinfeng Hongyuan Flagship Store	Electricity savings1	Things have been received, just plugged in, I do not know if it works, wait a few days to have the effect of additional	Jingdong prefecture level city in Shandong
Jinfeng Hongyuan Flagship Store	Electricity savings1	The effect of this power saver is really good, the house more, a few air conditioners are open every day, not used before a month to say less than seven hundred eight hundred, used after three months only nine	Jingdong prefecture level city in Shandong
Jinfeng Hongyuan Flagship Store	Electricity savings1	Cheap, good and easy to use, recommend it.	Jingdong prefecture level city in Shandong
Jinfeng Hongyuan Flagship Store	Electricity savings1	Bought this device, to the home for a long time, wait to finish a month to see how, good can recommend friends to buy, we use the electricity every month scary, a month of electricity costs more than a thousand, can not afford to hurt ah! I hope this power saving can save me some money, the beginning of the shoot order did not pay attention to look at the address, sent elsewhere to go, that side called to find out, hurry to communicate with customer service, customer service is very careful to help me get it done, call to that side to help me turn around, thank you!	Jingdong prefecture level city in Shandong
Jinfeng Hongyuan Flagship Store	Electricity savings1	I don't know the effect if it, use it first to see.	Jingdong prefecture level city in Shandong
Jinfeng Hongyuan Flagship Store	Electricity savings1	Looks good. I won't know if it saves power until I use it.	Jingdong prefecture level city in Shandong
Jinfeng Hongyuan Flagship Store	Electricity savings1	Goods received, shipping speed is very fast, yesterday afternoon more than two o'clock shot, just express delivery, but also sent a few insurance, very good business, the display of the number do not understand what is the meaning of the display said voltage, do not understand this, try a month to see how, good and	Jingdong prefecture level city in Shandong
Jinfeng Hongyuan Flagship Store	Electricity savings1	What ** play also, save electricity more and more save more and more, save chicken hair electricity, are cheating, this is a night of air conditioning with electricity, a night with 11 electricity word, this is	Jingdong prefecture level city in Shandong
Jinfeng Hongyuan Flagship Store	Electricity savings1	Received the product, hope it works!	Jingdong prefecture level city in Shandong
Jinfeng Hongyuan Flagship Store	Electricity savings1	Bought home and used very convenient, easy to operate, quite good	Jingdong prefecture level city in Shandong
Jinfeng Hongyuan Flagship Store	Electricity savings1	It's really good, cost-effective and good quality, and the logistics are fast! It arrived in one day! Really good, cost-effective and good quality, logistics is fast ah! It arrived in one day!	Jingdong prefecture level city in Shandong
Jinfeng Hongyuan Flagship Store	Electricity savings1	Works well, stabilizes voltage and saves power	Jingdong prefecture level city in Shandong
Jinfeng Hongyuan Flagship Store	Electricity savings1	I'll use it right away and look forward to good results	Jingdong prefecture level city in Shandong
Jinfeng Hongyuan Flagship Store	Electricity savings1	Recommended by a friend, said it works well, so I bought one, the effect to be tested	Jingdong prefecture level city in Shandong
Jinfeng Hongyuan Flagship Store	Electricity savings1	I didn't know it would save electricity.	Jingdong prefecture level city in Shandong
Jinfeng Hongyuan Flagship Store	Electricity savings1	I've been using it for a week, I don't know what's going on yet I hope it works well and saves power I'll follow up after a month of use	Jingdong prefecture level city in Shandong
Jinfeng Hongyuan Flagship Store	Electricity savings1	Very well packaged, looks great, don't know how it works	Jingdong prefecture level city in Shandong
Jinfeng Hongyuan Flagship Store	Electricity savings1	Genuine, good quality, really saves energy.	Jingdong prefecture level city in Shandong
Jinfeng Hongyuan Flagship Store	Electricity savings1	I was worried that the stuff is not good, but when I got it, I realized that I was completely over-anxious, 5 stars!	Jingdong prefecture level city in Shandong
Jinfeng Hongyuan Flagship Store	Electricity savings1	Very good and satisfied trustworthy	Jingdong prefecture level city in Shandong
Jinfeng Hongyuan Flagship Store	Electricity savings1	I don't know if it saves power	Jingdong prefecture level city in Shandong
Jinfeng Hongyuan Flagship Store	Electricity savings1	The quality of the product is very good, beautiful and generous applicable fast delivery speed good service attitude	Jingdong prefecture level city in Shandong
Jinfeng Hongyuan Flagship Store	Electricity savings1	Let's use it for a while and then we'll follow up	Jingdong prefecture level city in Shandong

Jinfeng Hongyuan Flagship Store	Electricity savings1	I've only been using it for a month, I don't know if there's any change yet, I'll come back with a follow-up comment after checking the meter!	Jingdong prefecture level city in Shandong
Jinfeng Hongyuan Flagship Store	Electricity savings1	I don't know if it works, I read the instructions and online reviews, I hope I won't be disappointed, if I use it, I will buy it again. Jingdong Express, fast and guaranteed service.	Jingdong prefecture level city in Shandong
Jinfeng Hongyuan Flagship Store	Electricity savings1	It feels like it saves quite a bit huh?	Jingdong prefecture level city in Shandong
Jinfeng Hongyuan Flagship Store	Electricity savings1	I've been using it for a while now, and it still seems to work and have a power saving effect	Jingdong prefecture level city in Shandong
Jinfeng Hongyuan Flagship Store	Electricity savings1	Looks good. It says 235. The 222 bar voltage is shown as high. I don't know if that's right. We'll see.	Jingdong prefecture level city in Shandong
Jinfeng Hongyuan Flagship Store	Electricity savings1	Baby is really good, do really careful, worth to have, fast delivery!	Jingdong prefecture level city in Shandong
Jinfeng Hongyuan Flagship Store	Electricity savings1	Quite delicate and compact, simple and convenient to use. Things are still very good, bought two at a time. Very satisfied with an online purchase.	Jingdong prefecture level city in Shandong
Jinfeng Hongyuan Flagship Store	Electricity savings1	Feel very good, the use of unclear, ask customer service, timely explanation, big brand, trustworthy	Jingdong prefecture level city in Shandong
Jinfeng Hongyuan Flagship Store	Electricity savings1	Thrifty received a few days did not make a comment is to see how the quality of the product now feel the quality of good appearance is also powerful seller service is very good to buy the product is also attached to the 5 fuse thought very thoughtful!	Jingdong prefecture level city in Shandong
Jinfeng Hongyuan Flagship Store	Electricity savings1	Big brand, with a very assured, the quality is very good, in time for the event to buy very cost-effective, customer service is really very good Oh ~!	Jingdong prefecture level city in Shandong
Jinfeng Hongyuan Flagship Store	Electricity savings1	I ordered yesterday and received it this morning, I heard my friend say that it can save electricity, I don't know if it works, I'll come back to make a comment after using it.	Jingdong prefecture level city in Shandong
Jinfeng Hongyuan Flagship Store	Electricity savings1	I've been using it for over a month and it doesn't seem to be working!	Jingdong prefecture level city in Shandong
Jinfeng Hongyuan Flagship Store	Electricity savings1	Used it, the family has always been in the habit of using energy-saving devices, this is a new one. Digital display voltage is very good, can see that the voltage is more stable.	Jingdong prefecture level city in Shandong
Jinfeng Hongyuan Flagship Store	Electricity savings1	I haven't used it for more than a month, but it looks good. It's easy to plug into an outlet and it works.	Jingdong prefecture level city in Shandong
Jinfeng Hongyuan Flagship Store	Electricity savings1	Good Oh...appearance is very beautiful...just plugged in with...see the effect of it...with a period of time in the additional evaluation...	Jingdong prefecture level city in Shandong
Jinfeng Hongyuan Flagship Store	Electricity savings1	I've received the goods, whether it's good or not, I'll know after I use it.	Jingdong prefecture level city in Shandong
Jinfeng Hongyuan Flagship Store	Electricity savings1	Very good, logistics is fast, the stuff is very powerful and cost-effective! Can feel the meter slowing down	Jingdong prefecture level city in Shandong
Jinfeng Hongyuan Flagship Store	Electricity savings1	Logistics is very fast, get it immediately after use, easy to operate, plug in the power supply can be used, just do not know to the use of the effect of how.	Jingdong prefecture level city in Shandong
Jinfeng Hongyuan Flagship Store	Electricity savings1	Quality is very over the top, quality is guaranteed, quite a good choice, a lot of friends to see me use all want to buy, I recommend it!	Jingdong prefecture level city in Shandong
Jinfeng Hongyuan Flagship Store	Electricity savings1	The appearance is clean and beautiful, the workmanship is very good, did not find any flaws, I personally feel that the size is also quite suitable, looking for a long time to choose this one, big love! The price is very beautiful, things are still good, will continue to buy back! All five points! Ness Ness ~ logistics speed is really no words, is a word: fast. Commodities from the outer packaging is very high-grade, really a penny a penny, expensive has the reason for expensive	Jingdong prefecture level city in Shandong
Jinfeng Hongyuan Flagship Store	Electricity savings1	The logistics is very fast, the texture is very good, genuine, I like it very much.	Jingdong prefecture level city in Shandong
Jinfeng Hongyuan Flagship Store	Electricity savings1	It's been a few days since I put it on, I can't see any results now, waiting for a month to say, steady steady steady.	Jingdong prefecture level city in Shandong
Jinfeng Hongyuan Flagship Store	Electricity savings1	I've been using it for a while now and it should be fine, so I'm buying it for the second time.	Jingdong prefecture level city in Shandong
Jinfeng Hongyuan Flagship Store	Electricity savings1	Power Saving King has been received, looks good, quite delicate, looking forward to using it to save electricity	Jingdong prefecture level city in Shandong
Jinfeng Hongyuan Flagship Store	Electricity savings1	My mom said the home uses a little too much electricity, so I bought a Power Saver to try it out, I don't know how effective it is. Expect it to be a good thing!	Jingdong prefecture level city in Shandong
Jinfeng Hongyuan Flagship Store	Electricity savings1	Thank you Jingdong, logistics is very fast order to receipt only one day. Believe in Jingdong. Favorable comments	Jingdong prefecture level city in Shandong
Jinfeng Hongyuan Flagship Store	Electricity savings1	Saved some money on the electric bill compared to last month, watch this month again, no problems so far	Jingdong prefecture level city in Shandong
Jinfeng Hongyuan Flagship Store	Electricity savings1	Energy saver exquisite workmanship, beautiful style, more features, just bought, power saving effect to be used for a period of time, then evaluate!	Jingdong prefecture level city in Shandong
Jinfeng Hongyuan Flagship Store	Electricity savings1	This thing does save power is more compact to hang on the wall.	Jingdong prefecture level city in Shandong
Jinfeng Hongyuan Flagship Store	Electricity savings1	I've been buying it for a while now, I haven't actually measured it, but it still feels like it's effective	Jingdong prefecture level city in Shandong
Jinfeng Hongyuan Flagship Store	Electricity savings1	I've already used it since I bought it, I don't know how effective it is yet, I'll comment on it later.	Jingdong prefecture level city in Shandong
Jinfeng Hongyuan Flagship Store	Electricity savings1	Baby received, careful appearance, logistics is very fast, worth buying!	Jingdong prefecture level city in Shandong
Jinfeng Hongyuan Flagship Store	Electricity savings1	It's great, it's just not the same in hand, I bought it at that other cheaper online one for about the same price as this one. But then oops, doesn't work at all.	Jingdong prefecture level city in Shandong
Jinfeng Hongyuan Flagship Store	Electricity savings1	The product is exquisite, but you have to use it for a month to know how it works. I'll comment again after a month.	Jingdong prefecture level city in Shandong
Jinfeng Hongyuan Flagship Store	Electricity savings1	I've been using it for a while now, and it doesn't feel like it's working, and it doesn't save any power.	Jingdong prefecture level city in Shandong
Jinfeng Hongyuan Flagship Store	Electricity savings1	Affordable price, fast delivery, Jingdong self-supporting guarantee	Jingdong prefecture level city in Shandong
Jinfeng Hongyuan Flagship Store	Electricity savings1	Packaging is very good, no damage, good quality, fine workmanship Packaging is very good, no damage, good quality, fine workmanship Packaging is very good, no damage, good quality, fine workmanship Packaging is very good, no damage, good quality, fine workmanship	Jingdong prefecture level city in Shandong
Jinfeng Hongyuan Flagship Store	Electricity savings1	Big brand, with a very assured, the quality is very good, in time for the event to buy very cost-effective, customer service is really very good Oh ~!	Jingdong prefecture level city in Shandong
Jinfeng Hongyuan Flagship Store	Electricity savings1	Used a few days, the specific effect is not yet clear, looking forward to the effect as described by the merchant!	Jingdong prefecture level city in Shandong
Jinfeng Hongyuan Flagship Store	Electricity savings1	I just used it and don't know how it works, I'll follow up after using it!	Jingdong prefecture level city in Shandong
Jinfeng Hongyuan Flagship Store	Electricity savings1	Well, it's been installed and now I'm waiting to see the results, BOE shopping, hopefully I won't be disappointed!	Jingdong prefecture level city in Shandong
Jinfeng Hongyuan Flagship Store	Electricity savings1	Goods received, plugged in the voltage from 229 to 222, do not know whether to save electricity, that depends on the situation.	Jingdong prefecture level city in Shandong
Jinfeng Hongyuan Flagship Store	Electricity savings1	This is the second time I have bought the product and I feel it really saves electricity! Use it again for a while and then evaluate!	Jingdong prefecture level city in Shandong
Jinfeng Hongyuan Flagship Store	Electricity savings1	Overall, it's really good, and the customer service is great, you'll get a message back almost immediately when you speak, it's great value for money, I really recommend it!	Jingdong prefecture level city in Shandong
Jinfeng Hongyuan Flagship Store	Electricity savings1	✨ Have purchased many times, a must have for the family, affordable purchase price, fast delivery, 🎁 beautifully packaged!	Jingdong prefecture level city in Shandong
Jinfeng Hongyuan Flagship Store	Electricity savings1	I bought it after a friend introduced it, he said it is effective and can save electricity. I bought it to try, try a period of time and then follow up!	Jingdong prefecture level city in Shandong
Jinfeng Hongyuan Flagship Store	Electricity savings1	Received it, trying it out tonight, and bought one earlier.	Jingdong prefecture level city in Shandong
Jinfeng Hongyuan Flagship Store	Electricity savings1	It will take a month or two to see if it saves power or not.	Jingdong prefecture level city in Shandong
Jinfeng Hongyuan Flagship Store	Electricity savings1	Looked good in the instructions! Bought one and pressed it on! Use it and see! If it works! I'll buy some more!	Jingdong prefecture level city in Shandong

Jinfeng Hongyuan Flagship Store	Electricity savings1	Goods have been received packaging can also things are not bad appearance, with a use to know, now evaluation is nonsense.	Jingdong prefecture level city in Shandong
Jinfeng Hongyuan Flagship Store	Electricity savings1	Logistics is very fast, packaging is very good, the second unit, as always, looking forward to the effect of	Jingdong prefecture level city in Shandong
Jinfeng Hongyuan Flagship Store	Electricity savings1	I received the goods today, the quality of goods is very good, I am very satisfied ah.	Jingdong prefecture level city in Shandong
Jinfeng Hongyuan Flagship Store	Electricity savings1	Buy one and try it, I wonder if it has a power saving effect?	Jingdong prefecture level city in Shandong
Jinfeng Hongyuan Flagship Store	Electricity savings1	🎈 The item was received, well packaged, logistics is very fast, customer service attitude is very positive, there are questions answered in a timely manner! Overall very satisfied!	Jingdong prefecture level city in Shandong
Jinfeng Hongyuan Flagship Store	Electricity savings1	Baby packaging is very good, purchase submission goods quickly delivered to the home, use and then follow up.	Jingdong prefecture level city in Shandong
Jinfeng Hongyuan Flagship Store	Electricity savings1	My friend asked me to buy an extra one so he could try it too.	Jingdong prefecture level city in Shandong
Jinfeng Hongyuan Flagship Store	Electricity savings1	I've just put it on, I won't know the result until a month later if it will really save power.	Jingdong prefecture level city in Shandong
Jinfeng Hongyuan Flagship Store	Electricity savings1	It's good quality. Hopefully it'll help save power.	Jingdong prefecture level city in Shandong
Jinfeng Hongyuan Flagship Store	Electricity savings1	The appearance is clean and beautiful, the workmanship is very good, I did not find any flaws, I personally feel that the size is also quite suitable, I have been looking for a long time to choose this	Jingdong prefecture level city in Shandong
Jinfeng Hongyuan Flagship Store	Electricity savings1	Logistics is fast! Baby looks great! The price is fair! Wish the store business is prosperous!	Jingdong prefecture level city in Shandong
Jinfeng Hongyuan Flagship Store	Electricity savings1	Jingdong express fast, good, have used, sit and wait to save money...	Jingdong prefecture level city in Shandong
Jinfeng Hongyuan Flagship Store	Electricity savings1	In use, it didn't work as expected and there was nothing noticeable in terms of power savings.	Jingdong prefecture level city in Shandong
Jinfeng Hongyuan Flagship Store	Electricity savings1	Just started using it and looking forward to the results.	Jingdong prefecture level city in Shandong
Jinfeng Hongyuan Flagship Store	Electricity savings1	The quality is very good, the metal shell is very good, is genuine, energy saving and power saving, logistics is very fast, there is a need to buy o, recommend everyone, very satisfied with an online	Jingdong prefecture level city in Shandong
Jinfeng Hongyuan Flagship Store	Electricity savings1	Received, I do not know if the product is good, used to see if it is good to come back to buy!	Jingdong prefecture level city in Shandong
Jinfeng Hongyuan Flagship Store	Electricity savings1	Jinfeng source of intelligent power saving treasure also used less than three months, the last thunder may be burned, the home of the electric send gate is not on, only the old man at home, no way only their own in the field and run back to find out what is the problem with the circuit, the quality is still not very good, thunder is easy to burn!	Jingdong prefecture level city in Shandong
Jinfeng Hongyuan Flagship Store	Electricity savings1	Saving power just started to use, I do not know if there is no effect, but read a lot of reviews are said to be very good so I bought, baby All aspects are good There are spare 2 fuses, late good and then follow	Jingdong prefecture level city in Shandong
Jinfeng Hongyuan Flagship Store	Electricity savings1	Use it for a few days and see, the delivery is quite fast	Jingdong prefecture level city in Shandong
Jinfeng Hongyuan Flagship Store	Electricity savings1	Small, well made and easy to use.	Jingdong prefecture level city in Shandong
Jinfeng Hongyuan Flagship Store	Electricity savings1	Beautifully packaged, it is genuine. Whether it is power saving, use it to evaluate.	Jingdong prefecture level city in Shandong
Jinfeng Hongyuan Flagship Store	Electricity savings1	Power saver. Used two of them and they do save quite a bit of power.	Jingdong prefecture level city in Shandong
Jinfeng Hongyuan Flagship Store	Electricity savings1	Workmanship is not bad, small and does not take up space, is also do not know whether the power saving, buy a to try!	Jingdong prefecture level city in Shandong
Jinfeng Hongyuan Flagship Store	Electricity savings1	The logistics is very fast, the courier attitude is very good, worth buying	Jingdong prefecture level city in Shandong
Jinfeng Hongyuan Flagship Store	Electricity savings1	The effect is not known until it is used.	Jingdong prefecture level city in Shandong
Jinfeng Hongyuan Flagship Store	Electricity savings1	Just arrived plugged in won't know for a month	Jingdong prefecture level city in Shandong
Jinfeng Hongyuan Flagship Store	Electricity savings1	This is really good to use the pull to realize the savings ah think again for my sister's family to buy a	Jingdong prefecture level city in Shandong
Jinfeng Hongyuan Flagship Store	Electricity savings1	Lies, a month with a few hundred electricity are no savings, family use	Jingdong prefecture level city in Shandong
Jinfeng Hongyuan Flagship Store	Electricity savings1	Express very quickly, look at the fine workmanship also sent a few fuse, look forward to can really save electricity	Jingdong prefecture level city in Shandong
Jinfeng Hongyuan Flagship Store	Electricity savings1	I didn't realize it was so small.	Jingdong prefecture level city in Shandong
Jinfeng Hongyuan Flagship Store	Electricity savings1	Logistics courier is very fast, the courier service is very good	Jingdong prefecture level city in Shandong
Jinfeng Hongyuan Flagship Store	Electricity savings1	Used for a period of time the effect is quite good indeed than the previous power saving	Jingdong prefecture level city in Shandong
Jinfeng Hongyuan Flagship Store	Electricity savings1	Logistics is super fast, the product is in use, see the effect...	Jingdong prefecture level city in Shandong
Jinfeng Hongyuan Flagship Store	Electricity savings1	Baby got it, tried it out and it didn't work at all. How can I use the tips to save power?	Jingdong prefecture level city in Shandong
Jinfeng Hongyuan Flagship Store	Electricity savings1	Power Saving King received, immediately used has been very stable without any sound good, good service, fast logistics inexpensive and beautiful worth repurchasing!	Jingdong prefecture level city in Shandong
Jinfeng Hongyuan Flagship Store	Electricity savings1	Logistics courier is very fast, the courier service is good	Jingdong prefecture level city in Shandong
Jinfeng Hongyuan Flagship Store	Electricity savings1	Good to recommend, the first month of air conditioning only 1 week, the electricity bill has reduced about 100 yuan, may be to reduce the use of air conditioning is also related to the other or as usual electricity, estimated that the longer you use the more you can save electricity.	Jingdong prefecture level city in Shandong
Jinfeng Hongyuan Flagship Store	Electricity savings1	It doesn't work. I've been using it for 20 days and it doesn't work.	Jingdong prefecture level city in Shandong
Jinfeng Hongyuan Flagship Store	Electricity savings1	Fast shipping, good quality, affordable price, very satisfied with a shopping.	Jingdong prefecture level city in Shandong
Jinfeng Hongyuan Flagship Store	Electricity savings1	Try it for a month to see if it saves money.	Jingdong prefecture level city in Shandong
Jinfeng Hongyuan Flagship Store	Electricity savings1	It's in use. I'll comment again after a while.	Jingdong prefecture level city in Shandong
Jinfeng Hongyuan Flagship Store	Electricity savings1	Goods have received look good 👍👍👍👍, use a period of time and then look at the	Jingdong prefecture level city in Shandong
Jinfeng Hongyuan Flagship Store	Electricity savings1	No, it doesn't work. It's useless. It's garbage.	Jingdong prefecture level city in Shandong
Jinfeng Hongyuan Flagship Store	Electricity savings1	I've been using it for almost 2 months now and I don't feel any reduction in my electricity bill.	Jingdong prefecture level city in Shandong
Jinfeng Hongyuan Flagship Store	Electricity savings1	I've received the goods, the logistics is really fast, ordered yesterday, arrived today.	Jingdong prefecture level city in Shandong
Jinfeng Hongyuan Flagship Store	Electricity savings1	And the backup insurance.	Jingdong prefecture level city in Shandong
Jinfeng Hongyuan Flagship Store	Electricity savings1	The appearance of this product is good, easy to use and install, the effect of how there is no use to wait for time to verify that the voltage displayed is different from the voltage measured by other meters, the gap is not too large, the follow-up to add the effect.	Jingdong prefecture level city in Shandong
Jinfeng Hongyuan Flagship Store	Electricity savings1	Baby received it 👌, really great ah! 🌈The quality can't be beat, very satisfied! 🆗Happy happy happy 😃	Jingdong prefecture level city in Shandong
Jinfeng Hongyuan Flagship Store	Electricity savings1	I'll follow up with a review after using it for a while, hopefully it won't bring me back a disappointment!	Jingdong prefecture level city in Shandong

Jinfeng Hongyuan Flagship Store	Electricity savings1	In use and looking forward to results! Good review to send	Jingdong prefecture level city in Shandong
Jinfeng Hongyuan Flagship Store	Electricity savings1	Intelligent power saver received, the volume is not big, small and exquisite, quite good, Jingdong Express is also quite fast, the	Jingdong prefecture level city in Shandong
Jinfeng Hongyuan Flagship Store	Electricity savings1	I don't know how to use it, keep using it	Jingdong prefecture level city in Shandong
Jinfeng Hongyuan Flagship Store	Electricity savings1	Very good for saving electricity	Jingdong prefecture level city in Shandong
Jinfeng Hongyuan Flagship Store	Electricity savings1	Seriously, did not use, feel also more power consumption, the peak is bought back to use the first time, the next day unplugged, the power consumption is back to more than ten degrees (the frequency of power use in the past few days is pretty much the same), feel the IQ tax paid 😂	Jingdong prefecture level city in Shandong
Jinfeng Hongyuan Flagship Store	Electricity savings1	I haven't seen any effect on the power saving, but the voltage stabilization looks good.	Jingdong prefecture level city in Shandong
Jinfeng Hongyuan Flagship Store	Electricity savings1	It's very good, I'll come back to it after using it for a while	Jingdong prefecture level city in Shandong
Jinfeng Hongyuan Flagship Store	Electricity savings1	Already used it and see the results.	Jingdong prefecture level city in Shandong
Jinfeng Hongyuan Flagship Store	Electricity savings1	Fast shipping, beautiful packaging, attentive service, the use of the effect, to be followed up after the evaluation.	Jingdong prefecture level city in Shandong
Jinfeng Hongyuan Flagship Store	Electricity savings1	The process of using this product is more than satisfactory	Jingdong prefecture level city in Shandong
Jinfeng Hongyuan Flagship Store	Electricity savings1	Very good East East cost-effective! Every time so buy more assured, and the logistics is also very fast. It is also very good to use, so I will often come to buy in the future, and will also introduce to	Jingdong prefecture level city in Shandong
Jinfeng Hongyuan Flagship Store	Electricity savings1	But still understand how effective it is!	Jingdong prefecture level city in Shandong
Jinfeng Hongyuan Flagship Store	Electricity savings1	Fast shipping, great logistics, great stuff, great price.	Jingdong prefecture level city in Shandong
Jinfeng Hongyuan Flagship Store	Electricity savings1	I'll review it in a month and give it 5 stars if it's good	Jingdong prefecture level city in Shandong
Jinfeng Hongyuan Flagship Store	Electricity savings1	I've saved a lot of electricity in the past month, so I'm glad to hear that.	Jingdong prefecture level city in Shandong
Jinfeng Hongyuan Flagship Store	Electricity savings1	Baby just received and installed! Looks genuinely good, workmanship feels fine! Looking forward to the effect! Said to see the effect in two months! Waiting for if really have the effect must come to follow up! The boss is absolutely responsible! Asked and answered! Just for the boss this attitude must also	Jingdong prefecture level city in Shandong
Jinfeng Hongyuan Flagship Store	Electricity savings1	This baby is very delicate, I'll comment after I've used it	Jingdong prefecture level city in Shandong
Jinfeng Hongyuan Flagship Store	Electricity savings1	It's been over two months. It's not working. It's just like it was.	Jingdong prefecture level city in Shandong
Jinfeng Hongyuan Flagship Store	Electricity savings1	Fast shipping and logistics, let's see if it works.	Jingdong prefecture level city in Shandong
Jinfeng Hongyuan Flagship Store	Electricity savings1	Just received the goods, try it to know if it can really save electricity	Jingdong prefecture level city in Shandong
Jinfeng Hongyuan Flagship Store	Electricity savings1	Not bad. Not bad. Not bad.	Jingdong prefecture level city in Shandong
Jinfeng Hongyuan Flagship Store	Electricity savings1	Not sure if it works. I'll comment again if it works.	Jingdong prefecture level city in Shandong
Jinfeng Hongyuan Flagship Store	Electricity savings1	Safe and energy-saving helpers to use!	Jingdong prefecture level city in Shandong
Jinfeng Hongyuan Flagship Store	Electricity savings1	Good, first time buyer. Used good can	Jingdong prefecture level city in Shandong
Jinfeng Hongyuan Flagship Store	Electricity savings1	It feels okay, I guess! I won't know until I use it for a while!	Jingdong prefecture level city in Shandong
Jinfeng Hongyuan Flagship Store	Electricity savings1	The saver matches the description and has been used, waiting for a follow up review after two months.	Jingdong prefecture level city in Shandong
Jinfeng Hongyuan Flagship Store	Electricity savings1	Received, the appearance of the business as described, the effect of how to use a period of time to evaluate.	Jingdong prefecture level city in Shandong
Jinfeng Hongyuan Flagship Store	Electricity savings1	Still using it don't know how effective it is, I'll comment after three months	Jingdong prefecture level city in Shandong
Jinfeng Hongyuan Flagship Store	Electricity savings1	The stuff is very good, the packaging is also very good logistics is also very fast	Jingdong prefecture level city in Shandong
Jinfeng Hongyuan Flagship Store	Electricity savings1	I'll follow up with a review when I use it, hopefully I won't be disappointed	Jingdong prefecture level city in Shandong
Jinfeng Hongyuan Flagship Store	Electricity savings1	Cancel Houjiaying Logistics.	Jingdong prefecture level city in Shandong
Jinfeng Hongyuan Flagship Store	Electricity savings1	It's very good, it's worth buying.	Jingdong prefecture level city in Shandong
Jinfeng Hongyuan Flagship Store	Electricity savings1	My friend bought it to save electricity and referred me to buy it! I'll buy it and try it out! I will know how much electricity I can save next month, looking forward to it! 4-500 dollars a month electricity bill is horrible! I hope it can really help me save some electricity!	Jingdong prefecture level city in Shandong
Jinfeng Hongyuan Flagship Store	Electricity savings1	Just received it and installed it.	Jingdong prefecture level city in Shandong
Jinfeng Hongyuan Flagship Store	Electricity savings1	Very nice. This one is my favorite.	Jingdong prefecture level city in Shandong
Jinfeng Hongyuan Flagship Store	Electricity savings1	Good value for money worth buying very good	Jingdong prefecture level city in Shandong
Jinfeng Hongyuan Flagship Store	Electricity savings1	Very energy-saving, small and exquisite, does not take up space, very satisfied, complete packaging, no damage, customer service explanation is also very serious	Jingdong prefecture level city in Shandong
Jinfeng Hongyuan Flagship Store	Electricity savings1	Received immediately used, really effective, do not understand electricity, but looked at the meter every day, recommended that you can use, especially this weather every day to open the air conditioning, with him not afraid of the electricity bill.	Jingdong prefecture level city in Shandong
Jinfeng Hongyuan Flagship Store	Electricity savings1	Nice, good value for money, nice stuff.	Jingdong prefecture level city in Shandong
Jinfeng Hongyuan Flagship Store	Electricity savings1	The packaging of the product is very good, the logistics of the product is very fast, and the quality of the product will be evaluated after use!	Jingdong prefecture level city in Shandong
Jinfeng Hongyuan Flagship Store	Electricity savings1	Express is very, well very fast, give a compliment 👍	Jingdong prefecture level city in Shandong
Jinfeng Hongyuan Flagship Store	Electricity savings1	Packaging is very good, fast delivery, used a night and a day did not feel the power saving. Use a month to see if it saves power.	Jingdong prefecture level city in Shandong
Jinfeng Hongyuan Flagship Store	Electricity savings1	Seems to be a bit of a power saver.	Jingdong prefecture level city in Shandong
Jinfeng Hongyuan Flagship Store	Electricity savings1	Bought this for someone else and it came over so fast, ###!	Jingdong prefecture level city in Shandong
Jinfeng Hongyuan Flagship Store	Electricity savings1	Very delicate, just received a period of time to use in the follow-up evaluation	Jingdong prefecture level city in Shandong
Jinfeng Hongyuan Flagship Store	Electricity savings1	It's been installed for a while now, not sure if it's working or not	Jingdong prefecture level city in Shandong
Jinfeng Hongyuan Flagship Store	Electricity savings1	The item looks okay, I just don't know if it works or not.	Jingdong prefecture level city in Shandong
Jinfeng Hongyuan Flagship Store	Electricity savings1	The goods have been received, but there is a screw missing	Jingdong prefecture level city in Shandong
Jinfeng Hongyuan Flagship Store	Electricity savings1	I didn't believe it before, but after receiving the goods today and trying it out, it really works!	Jingdong prefecture level city in Shandong

Jinfeng Hongyuan Flagship Store	Electricity savings1	Received, this thing will not know whether it is the effect of saving electricity, no matter how to say that there should always be a little use it, otherwise why develop it I think there is still the value of	Jingdong prefecture level city in Shandong
Jinfeng Hongyuan Flagship Store	Electricity savings1	Another one just arrived.	Jingdong prefecture level city in Shandong
Jinfeng Hongyuan Flagship Store	Electricity savings1	I don't feel anything, ordinary farmers can't go to TEDA, just use it first, it's safe.	Jingdong prefecture level city in Shandong
Jinfeng Hongyuan Flagship Store	Electricity savings1	Just purchased and will follow up after using it for a while	Jingdong prefecture level city in Shandong
Jinfeng Hongyuan Flagship Store	Electricity savings1	I'm trying it out now. I'll comment after I use it.	Jingdong prefecture level city in Shandong
Jinfeng Hongyuan Flagship Store	Electricity savings1	Received the item, in use, hope it works.	Jingdong prefecture level city in Shandong
Jinfeng Hongyuan Flagship Store	Electricity savings1	It works well. Good review.	Jingdong prefecture level city in Shandong
Jinfeng Hongyuan Flagship Store	Electricity savings1	All a scam, no effect at all, I bought too many fakes in Poundland before, I did not think that Jingdong is also the same	Jingdong prefecture level city in Shandong
Jinfeng Hongyuan Flagship Store	Electricity savings1	I used the saver as soon as I received it, it's in good shape and still working!	Jingdong prefecture level city in Shandong
Jinfeng Hongyuan Flagship Store	Electricity savings1	It works. I don't know what to do with the picture, but it works.	Jingdong prefecture level city in Shandong
Jinfeng Hongyuan Flagship Store	Electricity savings1	A very good shopping experience, attentive service, fast logistics, delivery is also very thoughtful, attitude is also very good, value for money ah, decisive praise!	Jingdong prefecture level city in Shandong
Jinfeng Hongyuan Flagship Store	Electricity savings1	The product is consistent with the presentation picture.	Jingdong prefecture level city in Shandong
Jinfeng Hongyuan Flagship Store	Electricity savings1	Baby received, use a period of time to evaluate, wish the boss business prosperity!	Jingdong prefecture level city in Shandong
Jinfeng Hongyuan Flagship Store	Electricity savings1	The stuff is better! Recommended!	Jingdong prefecture level city in Shandong
Jinfeng Hongyuan Flagship Store	Electricity savings1	I don't know if it works or not, I'll use it for a while before I comment on it.	Jingdong prefecture level city in Shandong
Jinfeng Hongyuan Flagship Store	Electricity savings1	Cost-effective, I like it, next time will come back Baby received, very surprised, the quality is very good, very satisfied with a shopping, I will continue to buy back, the store is trustworthy	Jingdong prefecture level city in Shandong
Jinfeng Hongyuan Flagship Store	Electricity savings1	Plugged it in and used it for a few days, not sure if it saves power yet	Jingdong prefecture level city in Shandong
Jinfeng Hongyuan Flagship Store	Electricity savings1	I'm trying it out and will follow up if it seems to be working!	Jingdong prefecture level city in Shandong
Jinfeng Hongyuan Flagship Store	Electricity savings1	Works pretty well, big kudos 👍👍👍👍👍👍👍👍👍👍👍	Jingdong prefecture level city in Shandong
Jinfeng Hongyuan Flagship Store	Electricity savings1	Just put it on, we'll see how it looks in a month.	Jingdong prefecture level city in Shandong
Jinfeng Hongyuan Flagship Store	Electricity savings1	The baby is very nice and I've already recommended buying it for all my friends! The main thing is that it not only saves electricity, I was more concerned about the fact that it can adjust the current, usually wiring problems. Now I found that it is really good, it is the effect I want!	Jingdong prefecture level city in Shandong
Jinfeng Hongyuan Flagship Store	Electricity savings1	Baby arrived, looking forward to his results, hopefully it will be good	Jingdong prefecture level city in Shandong
Jinfeng Hongyuan Flagship Store	Electricity savings1	Things have been received, look at the workmanship and packaging are good, I do not know how the power-saving effect? Use a period of time to see the effect	Jingdong prefecture level city in Shandong
Jinfeng Hongyuan Flagship Store	Electricity savings1	The price is right, the shape is beautiful and does not take up space, use it for a while to see the effect	Jingdong prefecture level city in Shandong
Jinfeng Hongyuan Flagship Store	Electricity savings1	Can't save electricity, it doesn't work!	Jingdong prefecture level city in Shandong
Jinfeng Hongyuan Flagship Store	Electricity savings1	It's pretty convenient, just plug it in and it works. There's no noise, plug it in and leave it alone, it's very hassle free! Good workmanship and value for money.	Jingdong prefecture level city in Shandong
Jinfeng Hongyuan Flagship Store	Electricity savings1	Just received the goods have been installed, try two months later to comment!	Jingdong prefecture level city in Shandong
Jinfeng Hongyuan Flagship Store	Electricity savings1	I received the stuff, I haven't used it yet, and overall it's fine.	Jingdong prefecture level city in Shandong
Jinfeng Hongyuan Flagship Store	Electricity savings1	Couldn't use a return at all.	Jingdong prefecture level city in Shandong
Jinfeng Hongyuan Flagship Store	Electricity savings1	Second purchase.	Jingdong prefecture level city in Shandong
Jinfeng Hongyuan Flagship Store	Electricity savings1	Very good quality, high tech and practical.	Jingdong prefecture level city in Shandong
Jinfeng Hongyuan Flagship Store	Electricity savings1	Very good, very great! Very satisfied, thanks to the store!	Jingdong prefecture level city in Shandong
Jinfeng Hongyuan Flagship Store	Electricity savings1	Just used it, don't know if it's good or not, hope it works well	Jingdong prefecture level city in Shandong
Jinfeng Hongyuan Flagship Store	Electricity savings1	Saving power has been received, the product is good, good like.	Jingdong prefecture level city in Shandong
Jinfeng Hongyuan Flagship Store	Electricity savings1	Received the goods and used it for two months before evaluation	Jingdong prefecture level city in Shandong
Jinfeng Hongyuan Flagship Store	Electricity savings1	It looks a little small, I do not know how effective, first use to see the effect, there is a small adjustable button, I do not know this is to adjust to that position to work well	Jingdong prefecture level city in Shandong
Jinfeng Hongyuan Flagship Store	Electricity savings1	Simple, just plug it in, there are spare fuses, can't say how well it works, no comparison	Jingdong prefecture level city in Shandong
Jinfeng Hongyuan Flagship Store	Electricity savings1	It's been a few days since I used it, and the power's gone up.	Jingdong prefecture level city in Shandong
Jinfeng Hongyuan Flagship Store	Electricity savings1	The owner was very nice and sent a spare fuse.	Jingdong prefecture level city in Shandong
Jinfeng Hongyuan Flagship Store	Electricity savings1	It's authentic and it's not expensive and it's great to use, I use it all the time and it's great 🎉✨ just don't know if it really saves any points	Jingdong prefecture level city in Shandong
Jinfeng Hongyuan Flagship Store	Electricity savings1	The information is good, it is the child to buy, the child read it is also quite happy, I hope to be able to match the child's preparation for the midterm examination! Because of the epidemic, it is not too fast, but I also give full credit to the express delivery!	Jingdong prefecture level city in Shandong
Jinfeng Hongyuan Flagship Store	Electricity savings1	This power saver comes to the logistics very quickly, the business attitude is also very good, ** very comfortable, this brand is trustworthy, and buy in this store than in the physical store to buy cheaper,	Jingdong prefecture level city in Shandong
Jinfeng Hongyuan Flagship Store	Electricity savings1	It's very well made and the materials used are okay. This is just a few days old, so I don't know how it will work, only next month.	Jingdong prefecture level city in Shandong
Jinfeng Hongyuan Flagship Store	Electricity savings1	Already used on, have not tested the effect, wait for a couple of months to add comments.	Jingdong prefecture level city in Shandong
Jinfeng Hongyuan Flagship Store	Electricity savings1	Baby received, packaging is very thoughtful, already in use, should soon see the effect.	Jingdong prefecture level city in Shandong
Jinfeng Hongyuan Flagship Store	Electricity savings1	Just received in the office to try, to the home installed, the voltage are different, and do not know if there is no effect. Use a period of time and then follow up.	Jingdong prefecture level city in Shandong
Jinfeng Hongyuan Flagship Store	Electricity savings1	Just received the goods, is trying, looking forward to the effect, first give a favorable comment, two months later, then additional evaluation, please be patient.	Jingdong prefecture level city in Shandong
Jinfeng Hongyuan Flagship Store	Electricity savings1	The item has just been received, well packaged, results to be verified.	Jingdong prefecture level city in Shandong
Jinfeng Hongyuan Flagship Store	Electricity savings1	Packaging logistics are very good, the next look at how the effect, before I see others use the effect is good, as I bought this can also have good results.	Jingdong prefecture level city in Shandong
Jinfeng Hongyuan Flagship Store	Electricity savings1	I don't know if it's a real power saver or a fake power saver, but I've used it since I bought it, so I'll give you a good review.	Jingdong prefecture level city in Shandong

Jinfeng Hongyuan Flagship Store	Electricity savings1	Good quality, genuine goods, much better than imagined before purchase, next time will come back to care!	Jingdong prefecture level city in Shandong
Jinfeng Hongyuan Flagship Store	Electricity savings1	Logistics is very fast good use again	Jingdong prefecture level city in Shandong
Jinfeng Hongyuan Flagship Store	Electricity savings1	Received the goods. Let's see if it saves power in a month and then we'll follow up!	Jingdong prefecture level city in Shandong
Jinfeng Hongyuan Flagship Store	Electricity savings1	I bought it yesterday and it arrived today, I bought two of them, I hope they work well, I'll come back after a while to give a follow-up review	Jingdong prefecture level city in Shandong
Jinfeng Hongyuan Flagship Store	Electricity savings1	The home air conditioner consumes a lot of electricity, so I bought it and used it in the hope that there is A	Jingdong prefecture level city in Shandong
Jinfeng Hongyuan Flagship Store	Electricity savings1	The product is excellent and very current.	Jingdong prefecture level city in Shandong
Jinfeng Hongyuan Flagship Store	Electricity savings1	With the description, it is the most practical and good products that households can need, and it is an ideal shopping, the courier is also fast, and the service attitude is enthusiastic!	Jingdong prefecture level city in Shandong
Jinfeng Hongyuan Flagship Store	Electricity savings1	The Power Saver is easy to use, works well, is stable, and plugs into the top of the outlet	Jingdong prefecture level city in Shandong
Jinfeng Hongyuan Flagship Store	Electricity savings1	Overall still can, excellent craftsmanship, power saving do not know for the time being, after the use of the evaluation	Jingdong prefecture level city in Shandong
Jinfeng Hongyuan Flagship Store	Electricity savings1	Baby received on the plug, but also with four fuses, look at the quality is okay, I do not know whether it is useful, use and then follow up.	Jingdong prefecture level city in Shandong
Jinfeng Hongyuan Flagship Store	Electricity savings1	I'll evaluate it after a while, I don't know if it's good or not yet, and if it can save electricity	Jingdong prefecture level city in Shandong
Jinfeng Hongyuan Flagship Store	Electricity savings1	Jingdong self-operated purchase more assured delivery fast quality assurance	Jingdong prefecture level city in Shandong
Jinfeng Hongyuan Flagship Store	Electricity savings1	Shipping was fast, stuff was well packaged, great price, I'm happy with it 👍 and will repurchase!	Jingdong prefecture level city in Shandong
Jinfeng Hongyuan Flagship Store	Electricity savings1	The quality of the product is very good and I will definitely come back again next time.	Jingdong prefecture level city in Shandong
Jinfeng Hongyuan Flagship Store	Electricity savings1	I'll follow up with a review after I use it.	Jingdong prefecture level city in Shandong
Jinfeng Hongyuan Flagship Store	Electricity savings1	Hello, the goods are perfectly packaged, save power for a period of time to evaluate again!	Jingdong prefecture level city in Shandong
Jinfeng Hongyuan Flagship Store	Electricity savings1	Not bad, pretty good, good value for money, pretty good	Jingdong prefecture level city in Shandong
Jinfeng Hongyuan Flagship Store	Electricity savings1	The packaging is very good, received immediately used, looking forward to the effect in.	Jingdong prefecture level city in Shandong
Jinfeng Hongyuan Flagship Store	Electricity savings1	See how much power you save at the end of the month	Jingdong prefecture level city in Shandong
Jinfeng Hongyuan Flagship Store	Electricity savings1	Easy to use, affordable, beautiful color	Jingdong prefecture level city in Shandong
Jinfeng Hongyuan Flagship Store	Electricity savings1	It's taking too long to put together an order. It could be an epidemic.	Jingdong prefecture level city in Shandong
Jinfeng Hongyuan Flagship Store	Electricity savings1	I don't feel much after using it, no change in my electric bill 。。。。。。。	Jingdong prefecture level city in Shandong
Jinfeng Hongyuan Flagship Store	Electricity savings1	Logistics is very fast, the quality of the economizer is good, good quality and cheap, it is worth buying, I hope there is a power saving effect!	Jingdong prefecture level city in Shandong
Jinfeng Hongyuan Flagship Store	Electricity savings1	Feeling okay, in a couple of months to try to rely on it before making a comment. Let's give it a five-star review first!	Jingdong prefecture level city in Shandong
Jinfeng Hongyuan Flagship Store	Electricity savings1	Power saving device has been hesitating before in the end to buy or not to buy, after consideration, consideration, in consideration, or decided to buy a try. Things received can not wait to open a look, and the seller described the same, I hope to use 2 months to have the effect, two months to follow up on	Jingdong prefecture level city in Shandong
Jinfeng Hongyuan Flagship Store	Electricity savings1	Thank you Jingdong, logistics is very fast order to receipt only one day. Believe in Jingdong. Favorable comments	Jingdong prefecture level city in Shandong
Jinfeng Hongyuan Flagship Store	Electricity savings1	I used one to use it, but it didn't work very well, what to do	Jingdong prefecture level city in Shandong
Jinfeng Hongyuan Flagship Store	Electricity savings1	Quality and packaging are very good, fast shipping, good service, buy with confidence	Jingdong prefecture level city in Shandong
Jinfeng Hongyuan Flagship Store	Electricity savings1	Use a period of time to evaluate, the quality is very good, the price is affordable, logistics is also very fast	Jingdong prefecture level city in Shandong
Jinfeng Hongyuan Flagship Store	Electricity savings1	Goods received, the day of the order tomorrow delivery real God speed, delivery force, the product to be used after a period of time and then low comment!	Jingdong prefecture level city in Shandong
Jinfeng Hongyuan Flagship Store	Electricity savings1	I don't know, how effective it is, let's talk about it when the time comes, whether it saves power or not, how much, unknown.	Jingdong prefecture level city in Shandong
Jinfeng Hongyuan Flagship Store	Electricity savings1	It does save some electricity, and I don't want to use something that isn't legal! Used nearly ~ electricity last month, probably not much more this month with the AC on!	Jingdong prefecture level city in Shandong
Jinfeng Hongyuan Flagship Store	Electricity savings1	Well-packed, excellent quality, service satisfaction good value for money	Jingdong prefecture level city in Shandong
Jinfeng Hongyuan Flagship Store	Electricity savings1	Express very quickly, the goods received last night, today used a day of goods can not heat, looking forward to whether it can save electricity, with a month and then comment, is used well will buy again.	Jingdong prefecture level city in Shandong
Jinfeng Hongyuan Flagship Store	Electricity savings1	It's pretty good, hopefully it'll be a real power saver.	Jingdong prefecture level city in Shandong
Jinfeng Hongyuan Flagship Store	Electricity savings1	The goods received in use, can not see the effect of energy saving, after a month to know!	Jingdong prefecture level city in Shandong
Jinfeng Hongyuan Flagship Store	Electricity savings1	Received the goods, looking forward to better surprise, with the effect of the follow-up comment!	Jingdong prefecture level city in Shandong
Jinfeng Hongyuan Flagship Store	Electricity savings1	It's not bad, it's still in use and should save some	Jingdong prefecture level city in Shandong
Jinfeng Hongyuan Flagship Store	Electricity savings1	Logistics is very fast, easy to install, plugged in on it,, the effect of the use of the later to evaluate!	Jingdong prefecture level city in Shandong
Jinfeng Hongyuan Flagship Store	Electricity savings1	The stuff is recommendable to buy very good.	Jingdong prefecture level city in Shandong
Jinfeng Hongyuan Flagship Store	Electricity savings1	Voltmeter measurements of voltage are way off, aren't they?	Jingdong prefecture level city in Shandong
Jinfeng Hongyuan Flagship Store	Electricity savings1	Install it to the socket immediately after purchase and try to see if it can save electricity after one month.	Jingdong prefecture level city in Shandong
Jinfeng Hongyuan Flagship Store	Electricity savings1	I've been using it for almost a month now, and I won't know if it saves electricity until I pay the bill.	Jingdong prefecture level city in Shandong
Jinfeng Hongyuan Flagship Store	Electricity savings1	I bought one in the past and it was fine, so I'm buying it back.	Jingdong prefecture level city in Shandong
Jinfeng Hongyuan Flagship Store	Electricity savings1	Service is very good, this thing is small and exquisite to use, just waiting to use the effect of a period of time	Jingdong prefecture level city in Shandong
Jinfeng Hongyuan Flagship Store	Electricity savings1	Not sure how well it works yet	Jingdong prefecture level city in Shandong
Jinfeng Hongyuan Flagship Store	Electricity savings1	Kyodo goods habitually good reviews	Jingdong prefecture level city in Shandong
Jinfeng Hongyuan Flagship Store	Electricity savings1	Appearance is OK, whether it is power saving remains to be seen.	Jingdong prefecture level city in Shandong
Jinfeng Hongyuan Flagship Store	Electricity savings1	I bought it and used it, I just don't know if it really saves power	Jingdong prefecture level city in Shandong
Jinfeng Hongyuan Flagship Store	Electricity savings1	The saver is well made and of good quality, I will evaluate it after using it for a while.	Jingdong prefecture level city in Shandong
Jinfeng Hongyuan Flagship Store	Electricity savings1	Just received in use, use for a period of time to see if there is power saving	Jingdong prefecture level city in Shandong

Jinfeng Hongyuan Flagship Store	Electricity savings1	Used 3 days before commenting, it really saves electricity, I look at the "power calendar" after using one less degree of electricity per day, so I think it's worth it to buy this, I will introduce my friends	Jingdong prefecture level city in Shandong
Jinfeng Hongyuan Flagship Store	Electricity savings1	Reliable quality, very well packaged and matches the real thing.	Jingdong prefecture level city in Shandong
Jinfeng Hongyuan Flagship Store	Electricity savings1	Logistics is still quite fast, ha in the trial	Jingdong prefecture level city in Shandong
Jinfeng Hongyuan Flagship Store	Electricity savings1	Consultant service attitude is very good, the courier is also very powerful, the first day of the order the next day to arrive. Saver received used and then add comments!	Jingdong prefecture level city in Shandong
Jinfeng Hongyuan Flagship Store	Electricity savings1	I just got home to try it out and see how it goes.	Jingdong prefecture level city in Shandong
Jinfeng Hongyuan Flagship Store	Electricity savings1	Well made and weighted instrument. Kudos! Put it into use immediately, don't know if it saves power, will evaluate later!	Jingdong prefecture level city in Shandong
Jinfeng Hongyuan Flagship Store	Electricity savings1	I've just put it in place. I'll give you a review in ten days.	Jingdong prefecture level city in Shandong
Jinfeng Hongyuan Flagship Store	Electricity savings1	It looks very delicate, not bad. Well, I don't know if it can save power or not, I have to try it for a while and see. If it can really save electricity. Uh, if it works well. Then additional comments. Anyway, thanks for the logistics, thanks to Jingdong. Well, it deserves some praise.	Jingdong prefecture level city in Shandong
Jinfeng Hongyuan Flagship Store	Electricity savings1	Bought a month, the feeling is that there is a certain effect, a month to save a lot of money	Jingdong prefecture level city in Shandong
Jinfeng Hongyuan Flagship Store	Electricity savings1	Goods received, the physical and the same as the picture, logistics is also very fast, the service attitude is very good, it is worth buying!	Jingdong prefecture level city in Shandong
Jinfeng Hongyuan Flagship Store	Electricity savings1	I've used it, I don't know whether it saves electricity or not, I'll talk about it after a while.	Jingdong prefecture level city in Shandong
Jinfeng Hongyuan Flagship Store	Electricity savings1	August only began to use, October to know, have no power-saving effect, to the in to evaluate!	Jingdong prefecture level city in Shandong
Jinfeng Hongyuan Flagship Store	Electricity savings1	Goods received: I do not know to the factory products used to save electricity!	Jingdong prefecture level city in Shandong
Jinfeng Hongyuan Flagship Store	Electricity savings1	Don't know if it works, bought it anyway.	Jingdong prefecture level city in Shandong
Jinfeng Hongyuan Flagship Store	Electricity savings1	Still testing, will follow up next month to evaluate how much electricity is saved	Jingdong prefecture level city in Shandong
Jinfeng Hongyuan Flagship Store	Electricity savings1	I'm still testing it, I'll comment after I use it.	Jingdong prefecture level city in Shandong
Jinfeng Hongyuan Flagship Store	Electricity savings1	Fast service, praiseworthy. Good item pre-style, first praise, the use of the effect to be evaluated after use.	Jingdong prefecture level city in Shandong
Jinfeng Hongyuan Flagship Store	Electricity savings1	The power saving effect is more obvious through verified use.	Jingdong prefecture level city in Shandong
Jinfeng Hongyuan Flagship Store	Electricity savings1	Logistics is very fast yesterday order today arrived. The product was immediately used, and after a month to see its effect.	Jingdong prefecture level city in Shandong
Jinfeng Hongyuan Flagship Store	Electricity savings1	Power saving is not yet known, as far as the TV and audio are concerned, I feel that there is a significant quality improvement, and I recommend using a good product!	Jingdong prefecture level city in Shandong
Jinfeng Hongyuan Flagship Store	Electricity savings1	Good power savings, tens of dollars a month saved	Jingdong prefecture level city in Shandong
Jinfeng Hongyuan Flagship Store	Electricity savings1	Already took two, great value! You can save 30% on your electric bill in a month. A summer must-have!	Jingdong prefecture level city in Shandong
Jinfeng Hongyuan Flagship Store	Electricity savings1	Received the item, plugged it into a home outlet and it showed 230 volts (not that high with a multimeter). Let's use it first!	Jingdong prefecture level city in Shandong
Jinfeng Hongyuan Flagship Store	Electricity savings1	Expecting results in the experiment	Jingdong prefecture level city in Shandong
Jinfeng Hongyuan Flagship Store	Electricity savings1	I don't know if it really saves power.	Jingdong prefecture level city in Shandong
Jinfeng Hongyuan Flagship Store	Electricity savings1	It's very good and the efficacy is very visible.	Jingdong prefecture level city in Shandong
Jinfeng Hongyuan Flagship Store	Electricity savings1	It's good and worth buying with confidence	Jingdong prefecture level city in Shandong
Jinfeng Hongyuan Flagship Store	Electricity savings1	The goods have been received, just do not know if it is effective	Jingdong prefecture level city in Shandong
Jinfeng Hongyuan Flagship Store	Electricity savings1	It's already in use, but based on some of the descriptions, I suspect that to use it, you need to have one in series on each line, which seems to work for all appliances, that's my guess, but I feel as though the principle of the thing seems to require it.	Jingdong prefecture level city in Shandong
Jinfeng Hongyuan Flagship Store	Electricity savings1	Fast delivery, already in use, looks good!	Jingdong prefecture level city in Shandong
Jinfeng Hongyuan Flagship Store	Electricity savings1	Hoping to save 30% of power as presented in the graphic	Jingdong prefecture level city in Shandong
Jinfeng Hongyuan Flagship Store	Electricity savings1	Things look good, power saving do not know now, with a month after the continued evaluation	Jingdong prefecture level city in Shandong
Jinfeng Hongyuan Flagship Store	Electricity savings1	Already used it but not sure if it will top out!	Jingdong prefecture level city in Shandong
Jinfeng Hongyuan Flagship Store	Electricity savings1	Looking forward to using the results, the product quality looks okay.	Jingdong prefecture level city in Shandong
Jinfeng Hongyuan Flagship Store	Electricity savings1	It's somewhat effective and saves money more or less 💰	Jingdong prefecture level city in Shandong
Jinfeng Hongyuan Flagship Store	Electricity savings1	Got it, it's great, looking forward to saving power.	Jingdong prefecture level city in Shandong
Jinfeng Hongyuan Flagship Store	Electricity savings1	I don't know if it's working yet, I'll follow up when I get there	Jingdong prefecture level city in Shandong
Jinfeng Hongyuan Flagship Store	Electricity savings1	The product received, good workmanship, fast logistics, good inner packaging, the outer bag is damaged, but fortunately did not hurt the product, retrieved and inserted according to the instructions, looking forward to the effect is good, with a period of time to evaluate, I believe that the Jingdong.	Jingdong prefecture level city in Shandong
Jinfeng Hongyuan Flagship Store	Electricity savings1	Since it is a new product, it is said to work, so if it works well for a month, then I will give you a good review.	Jingdong prefecture level city in Shandong
Jinfeng Hongyuan Flagship Store	Electricity savings1	The quality of the product is very good, very well made, well packaged, easy to use and very convenient!	Jingdong prefecture level city in Shandong
Jinfeng Hongyuan Flagship Store	Electricity savings1	Good price for the item, good functionality	Jingdong prefecture level city in Shandong
Jinfeng Hongyuan Flagship Store	Electricity savings1	Received the first time to use it, the stuff is very good, specifics wait for a month to come back to feedback to see the effect.	Jingdong prefecture level city in Shandong
Jinfeng Hongyuan Flagship Store	Electricity savings1	Just received, the courier is too bad, not notified directly to put the Feng Nest courier cabinet	Jingdong prefecture level city in Shandong
Jinfeng Hongyuan Flagship Store	Electricity savings1	Baby received, the second time to buy, plugged in and used do not know if there is no effect. Baby received, the second time to buy, plugged in and used do not know if there is no effect. Baby received, the second time to buy, plugged in and used do not know if there is no effect.	Jingdong prefecture level city in Shandong
Jinfeng Hongyuan Flagship Store	Electricity savings1	It's okay if it saves power. It's not a bad product.	Jingdong prefecture level city in Shandong
Jinfeng Hongyuan Flagship Store	Electricity savings1	Received, very good, meticulous packaging, open a look is genuine, meticulous workmanship, the manual is very detailed, has been used, there is no effect on the only look at the next month's electricity bill, when I can I buy a few more to send people!	Jingdong prefecture level city in Shandong
Jinfeng Hongyuan Flagship Store	Electricity savings1	The quality is very good very delicate express delivery is too fast	Jingdong prefecture level city in Shandong
Jinfeng Hongyuan Flagship Store	Electricity savings1	I won't know until a month after I start using it today.	Jingdong prefecture level city in Shandong
Jinfeng Hongyuan Flagship Store	Electricity savings1	It doesn't work at all, the customer service attitude is very bad, use it or not!	Jingdong prefecture level city in Shandong

Jinfeng Hongyuan Flagship Store	Electricity savings1	Temporarily try, do not know the effect, Jingdong logistics delivery very quickly	Jingdong prefecture level city in Shandong
Jinfeng Hongyuan Flagship Store	Electricity savings1	It doesn't help. It's comforting.	Jingdong prefecture level city in Shandong
Jinfeng Hongyuan Flagship Store	Electricity savings1	It's great, use it before you say anything.	Jingdong prefecture level city in Shandong
Jinfeng Hongyuan Flagship Store	Electricity savings1	Received, already used, looking forward to a good a effect!	Jingdong prefecture level city in Shandong
Jinfeng Hongyuan Flagship Store	Electricity savings1	I don't know if it works yet, I won't know until I use it for a few months.	Jingdong prefecture level city in Shandong
Jinfeng Hongyuan Flagship Store	Electricity savings1	It takes a long time to get there. There's no change now.	Jingdong prefecture level city in Shandong
Jinfeng Hongyuan Flagship Store	Electricity savings1	Jingdong's delivery never disappoints, or so fast, and the stuff is great, next time you need to come back.	Jingdong prefecture level city in Shandong
Jinfeng Hongyuan Flagship Store	Electricity savings1	At first it was ok today, after using it for a while it didn't work anymore	Jingdong prefecture level city in Shandong
Jinfeng Hongyuan Flagship Store	Electricity savings1	Just received the goods, do not know the effect of using a few days before there may be effect	Jingdong prefecture level city in Shandong
Jinfeng Hongyuan Flagship Store	Electricity savings1	The logistics was fast, I received it the next day, I've purchased it for the second time and have been using it all along	Jingdong prefecture level city in Shandong
Jinfeng Hongyuan Flagship Store	Electricity savings1	Inexpensive, fast delivery, very pleased!	Jingdong prefecture level city in Shandong
Jinfeng Hongyuan Flagship Store	Electricity savings1	Received the goods immediately plugged directly into the use of power saving yet to be good news!	Jingdong prefecture level city in Shandong
Jinfeng Hongyuan Flagship Store	Electricity savings1	The service attitude is very good, and the Jingdong self-owned products can be trusted.	Jingdong prefecture level city in Shandong
Jinfeng Hongyuan Flagship Store	Electricity savings1	The product is good, I do not know if it can save play a power saving effect	Jingdong prefecture level city in Shandong
Jinfeng Hongyuan Flagship Store	Electricity savings1	We won't know how effective it is until we use it for a while, so I'll give you a good review now.	Jingdong prefecture level city in Shandong
Jinfeng Hongyuan Flagship Store	Electricity savings1	The quality is pretty good, just started using it, hopefully it will deliver results.	Jingdong prefecture level city in Shandong
Jinfeng Hongyuan Flagship Store	Electricity savings1	I've plugged it in first, and will follow up after I've used it to see how it works.	Jingdong prefecture level city in Shandong
Jinfeng Hongyuan Flagship Store	Electricity savings1	Things have arrived, the quality is good, the effect is also equivalent to can ah	Jingdong prefecture level city in Shandong
Jinfeng Hongyuan Flagship Store	Electricity savings1	It's not worth it to brag about power savings.	Jingdong prefecture level city in Shandong
Jinfeng Hongyuan Flagship Store	Electricity savings1	I don't know if it's an IQ tax, but I'll use it for now.	Jingdong prefecture level city in Shandong
Jinfeng Hongyuan Flagship Store	Electricity savings1	I don't feel the power saving yet, I can't know until I use it for 2 months.	Jingdong prefecture level city in Shandong
Jinfeng Hongyuan Flagship Store	Electricity savings1	There's nothing wrong with this at the moment.	Jingdong prefecture level city in Shandong
Jinfeng Hongyuan Flagship Store	Electricity savings1	It's authentic and worth buying	Jingdong prefecture level city in Shandong
Jinfeng Hongyuan Flagship Store	Electricity savings1	It's good. We'll see how it goes.	Jingdong prefecture level city in Shandong
Jinfeng Hongyuan Flagship Store	Electricity savings1	I don't know if it's working or not in the middle of using it, we'll see when the month is over	Jingdong prefecture level city in Shandong
Jinfeng Hongyuan Flagship Store	Electricity savings1	I'm not going to buy anything from Jingdong anymore, so I'll give it a good review	Jingdong prefecture level city in Shandong
Jinfeng Hongyuan Flagship Store	Electricity savings1	I'm not going to buy anything from Jingdong anymore, so I'll give it a good review	Jingdong prefecture level city in Shandong
Jinfeng Hongyuan Flagship Store	Electricity savings1	Just received the item and will see how it works in a month.	Jingdong prefecture level city in Shandong
Jinfeng Hongyuan Flagship Store	Electricity savings1	I don't know if it saves electricity, but it's plugged into an outlet every day anyway	Jingdong prefecture level city in Shandong
Jinfeng Hongyuan Flagship Store	Electricity savings1	I don't know if it works yet.	Jingdong prefecture level city in Shandong
Jinfeng Hongyuan Flagship Store	Electricity savings1	Plugged it in when I got back, waiting for a month to see the results	Jingdong prefecture level city in Shandong
Jinfeng Hongyuan Flagship Store	Electricity savings1	Effects remain to be seen, but it looks good quality and runs fine	Jingdong prefecture level city in Shandong
Jinfeng Hongyuan Flagship Store	Electricity savings1	The product received logistics very quickly, has not yet been used to test! After use in the evaluation!	Jingdong prefecture level city in Shandong
Jinfeng Hongyuan Flagship Store	Electricity savings1	The power saver is in use, and I don't know how much electricity has been saved after using it for more than a month, and the display voltmeter is very good.	Jingdong prefecture level city in Shandong
Jinfeng Hongyuan Flagship Store	Electricity savings1	It's so good, isn't it? You're a real Shanghainese, aren't you?	Jingdong prefecture level city in Shandong
Jinfeng Hongyuan Flagship Store	Electricity savings1	Great quality great great 👍👍👍👍	Jingdong prefecture level city in Shandong
Jinfeng Hongyuan Flagship Store	Electricity savings1	I'm using it, I don't know if it's working yet.	Jingdong prefecture level city in Shandong
Jinfeng Hongyuan Flagship Store	Electricity savings1	Just used it for more than 10 days and haven't seen any results yet	Jingdong prefecture level city in Shandong
Jinfeng Hongyuan Flagship Store	Electricity savings1	I don't know if it's a power saver. It's a couple bucks. I hope it works.	Jingdong prefecture level city in Shandong
Jinfeng Hongyuan Flagship Store	Electricity savings1	I don't know what to say about this stuff. I don't know if it works or not, it's kind of a psychological purchase. Dormitory use to see the meter can not be not so good-looking. You have to wait until the time to deduct the utility bill to know.	Jingdong prefecture level city in Shandong
Jinfeng Hongyuan Flagship Store	Electricity savings1	Energy savers don't work / they're all a bunch of hoaxes.	Jingdong prefecture level city in Shandong
Jinfeng Hongyuan Flagship Store	Electricity savings1	Received the goods, used for more than a week, it's okay, sure a little effect	Jingdong prefecture level city in Shandong
Jinfeng Hongyuan Flagship Store	Electricity savings1	Well made and compact! Looking forward to the effect!	Jingdong prefecture level city in Shandong
Jinfeng Hongyuan Flagship Store	Electricity savings1	It has arrived and we'll see how it works.	Jingdong prefecture level city in Shandong
Jinfeng Hongyuan Flagship Store	Electricity savings1	Good. Good. It's worth it.	Jingdong prefecture level city in Shandong
Jinfeng Hongyuan Flagship Store	Electricity savings1	How come there's no indicator light?	Jingdong prefecture level city in Shandong
Jinfeng Hongyuan Flagship Store	Electricity savings1	The machine that uses electricity works quite well. This saves	Jingdong prefecture level city in Shandong
Jinfeng Hongyuan Flagship Store	Electricity savings1	It's good for home use and feels quite energy efficient	Jingdong prefecture level city in Shandong
Jinfeng Hongyuan Flagship Store	Electricity savings1	I regret buying this product, after using it for two days it doesn't work at all, still so many kilowatts per day just like the original.	Jingdong prefecture level city in Shandong
Jinfeng Hongyuan Flagship Store	Electricity savings1	Works very well and has some scientific merit.	Jingdong prefecture level city in Shandong
Jinfeng Hongyuan Flagship Store	Electricity savings1	Logistics is also very fast this good power saving I use two months before commenting!	Jingdong prefecture level city in Shandong

Jinfeng Hongyuan Flagship Store	Electricity savings1	Repeat customer. Very good.	Jingdong prefecture level city in Shandong
Jinfeng Hongyuan Flagship Store	Electricity savings1	It's good stuff. I've been using it for a month now and it's a real power saver.	Jingdong prefecture level city in Shandong
Jinfeng Hongyuan Flagship Store	Electricity savings1	Jingdong platform to buy things, has been very assured, quality assurance. Jingdong Express is as good as ever, complete packaging, delivery, service attitude to go! The family has always wanted to buy an energy-saving and power-saving equipment, selected half a day to choose this one, the value is very good, used for a period of time to follow up, to see whether it can achieve the real purpose of power saving, I	Jingdong prefecture level city in Shandong
Jinfeng Hongyuan Flagship Store	Electricity savings1	Works great, inexpensive and will be back next time.	Jingdong prefecture level city in Shandong
Jinfeng Hongyuan Flagship Store	Electricity savings1	Baby received, looks good, wait for a month to see how it works?	Jingdong prefecture level city in Shandong
Jinfeng Hongyuan Flagship Store	Electricity savings1	This product is not bad quite good well customer service said quite power saving that look at the customer service to see if the accuracy of the look at all aspects of the color is also quite beautiful	Jingdong prefecture level city in Shandong
Jinfeng Hongyuan Flagship Store	Electricity savings1	Just got back and tried it feels pretty good, just don't know how the quality is, give a good comment first	Jingdong prefecture level city in Shandong
Jinfeng Hongyuan Flagship Store	Electricity savings1	Received the product, trying it out, hope it helps!	Jingdong prefecture level city in Shandong
Jinfeng Hongyuan Flagship Store	Electricity savings1	Works great, saves electricity a dollar a day and a night	Jingdong prefecture level city in Shandong
Jinfeng Hongyuan Flagship Store	Electricity savings1	Baby received, look at the workmanship is still very fine, is genuine. I hope that this summer to open the air conditioning electricity bill is no longer high scary!	Jingdong prefecture level city in Shandong
Jinfeng Hongyuan Flagship Store	Electricity savings1	It's here. I don't know how it's going to work, but I'm going to use it.	Jingdong prefecture level city in Shandong
Jinfeng Hongyuan Flagship Store	Electricity savings1	Power saver received, Jingdong logistics first-class, business customer service attitude is good, one by one to introduce the product performance, back on the horse plugged in, it is very simple, there is a voltage indicator, to be a few months to see how the effect of power-saving to come back to a good follow-up, but I believe that Jinfenghong products.	Jingdong prefecture level city in Shandong
Jinfeng Hongyuan Flagship Store	Electricity savings1	Took the third one...the super power-saving kind!	Jingdong prefecture level city in Shandong
Jinfeng Hongyuan Flagship Store	Electricity savings1	First time buyer, customer service is very polite and courteous, conscientious business, support! Baby has received 🎈, super like, high value and practical, super like! ❥❥❥❥❥ Very good quality, very satisfied with a shopping. Customer service has a question and answer, very professional finally received the goods, very, very much like, exquisite and beautiful workmanship, has been praised by many people, really too happy, is already a loyal fan of his family, will continue to buy buy buy buy!	Jingdong prefecture level city in Shandong
Jinfeng Hongyuan Flagship Store	Electricity savings1	That's good. That's good. That's really good.	Jingdong prefecture level city in Shandong
Jinfeng Hongyuan Flagship Store	Electricity savings1	Saver received, good-looking design, voltage at a glance, and the merchant description, save some about 12% or so	Jingdong prefecture level city in Shandong
Jinfeng Hongyuan Flagship Store	Electricity savings1	Intelligent power saver is small and does not take up space, very good, very practical, received and used, packaging is tight, logistics is also very fast, satisfied with a shopping!	Jingdong prefecture level city in Shandong
Jinfeng Hongyuan Flagship Store	Electricity savings1	The stuff is good! Well made, compact, alloy case, shows voltage! Very like! Kudos!	Jingdong prefecture level city in Shandong
Jinfeng Hongyuan Flagship Store	Electricity savings1	I just purchased this power saver from Jingdong, inserted in the socket shows the number 206, a moment and 207 back and forth display, I do not know what it means, my home voltage should be normal, because it is the owner of the community's own power plant to put, first use for a period of time in the positive feedback it, first use in the look at it!	Jingdong prefecture level city in Shandong
Jinfeng Hongyuan Flagship Store	Electricity savings1	Very delicate and compact, there is a convenient intelligent display prompts the voltage, you can know the voltage at a glance, there is also very easy to use, a plug can be used, you can save electricity	Jingdong prefecture level city in Shandong
Jinfeng Hongyuan Flagship Store	Electricity savings1	Logistics is fast, ask customer service said can save electricity, but only use two months to see the effect, use two months in the evaluation.	Jingdong prefecture level city in Shandong
Jinfeng Hongyuan Flagship Store	Electricity savings1	The quality is very good.	Jingdong prefecture level city in Shandong
Jinfeng Hongyuan Flagship Store	Electricity savings1	Smart Saver has been received and will be evaluated after use	Jingdong prefecture level city in Shandong
Jinfeng Hongyuan Flagship Store	Electricity savings1	Home electricity is too waste, a month more than 300 dollars of electricity, and then the family heard that this can save a lot of electricity, let me order to buy a, anyway, will not, buy on the buy, thought it has to be a pretty big box it, delivery to the home to see a very small, plug-and-play, you can really save electricity, it's worth it!	Jingdong prefecture level city in Shandong
Jinfeng Hongyuan Flagship Store	Electricity savings1	Smart Saver received. Unpacking the box is exquisite. The material workmanship is very good. Plugged in and tried the space. Did not open the air conditioning voltage display 206 air conditioning open up the air conditioning display for 194. whether to save electricity to a month later to pay the electricity bill will know. Logistics is also very fast service is also in place.	Jingdong prefecture level city in Shandong
Jinfeng Hongyuan Flagship Store	Electricity savings1	The product feels pretty good and I hope it works, as the store owner said it would. Works!!!	Jingdong prefecture level city in Shandong
Jinfeng Hongyuan Flagship Store	Electricity savings1	Baby received, physical very quickly, just plugged in hope that it can be as everyone said those power saving, with two months after additional comments, first give a five-star praise.	Jingdong prefecture level city in Shandong
Jinfeng Hongyuan Flagship Store	Electricity savings1	Good, very good, very good, you deserve it, whoever uses it knows, the stuff works great, will buy it again in the future.	Jingdong prefecture level city in Shandong
Jinfeng Hongyuan Flagship Store	Electricity savings1	Don't hesitate to buy wow! No cheap feeling, very high-end, especially great! It is genuine, and the same as the entity store, the price is also favorable, decisive turn powder 💗 catch up to do activities when you buy, the price of a penny for a penny ♡, from the outside looking very beautiful 💗, the material workmanship is good 💗, feel that the purchase is worth it, and the logistics is super	Jingdong prefecture level city in Shandong
Jinfeng Hongyuan Flagship Store	Electricity savings1	Logistics is fast! Very good quality! Very beautiful! Next time to patronize!	Jingdong prefecture level city in Shandong
Jinfeng Hongyuan Flagship Store	Electricity savings1	Really like it, completely exceeded expectations, the shipping speed is very fast, the packaging is very careful and strict, the delivery speed is very fast, very satisfied with a shopping Baby received, and the description is the same, love it!	Jingdong prefecture level city in Shandong
Jinfeng Hongyuan Flagship Store	Electricity savings1	Very good, if you need to order!	Jingdong prefecture level city in Shandong
Jinfeng Hongyuan Flagship Store	Electricity savings1	So far, plugged in the voltage is more stable, as for the effect of how to quietly wait until the payment of the electricity bill to compare, the appearance of the commodity design is very good, the real thing!	Jingdong prefecture level city in Shandong
Jinfeng Hongyuan Flagship Store	Electricity savings1	💌 I've been wanting it for a long time, I decided to go for it, I feel great after using it, it's genuine and the delivery is great, it lives up to its name👍	Jingdong prefecture level city in Shandong
Jinfeng Hongyuan Flagship Store	Electricity savings1	Looking forward to it!!! I don't know if it's working or not, let's look forward to it!!!! How much is saved ，，，，，，，，， Saving a degree is a degree with long term use ！！！！！！！	Jingdong prefecture level city in Shandong
Jinfeng Hongyuan Flagship Store	Electricity savings1	After using it for a while, I am seriously and responsibly saying that this saver is really very power efficient. Two rooms with the air conditioner on at night for one night, plus watching TV, charging cell phones, lights, etc. etc. only used one degree of electricity. So I bought several more and it's really	Jingdong prefecture level city in Shandong
Jinfeng Hongyuan Flagship Store	Electricity savings1	It arrived quickly, and the box is solid and delicate. Baby shell is thick aluminum and by multi-layer coating. The inner core is heavy, and the power cord and plug are genuine products from a large manufacturer. It is the best power saver I have found after choosing all of them!	Jingdong prefecture level city in Shandong
Jinfeng Hongyuan Flagship Store	Electricity savings1	The saver was received, genuine guarantee, good quality...	Jingdong prefecture level city in Shandong
Jinfeng Hongyuan Flagship Store	Electricity savings1	Logistics was fast! The product is easy to use!	Jingdong prefecture level city in Shandong
Jinfeng Hongyuan Flagship Store	Electricity savings1	Look appearance is okay, use a period of time to know how it is. Jingdong Mall, trustworthy! Home appliances, cell phones and computers are almost 100% bought by Jingdong. Ordered the night before yesterday, received yesterday. Jingdong logistics, the fastest in the network!	Jingdong prefecture level city in Shandong
Jinfeng Hongyuan Flagship Store	Electricity savings1	Very good, perfect packaging, home energy saver to determine the effect of power saving and also play a role in stabilizing the voltage Very good products	Jingdong prefecture level city in Shandong
Jinfeng Hongyuan Flagship Store	Electricity savings1	Smart Saver received, looks very exquisite. The method of use is simple, and it has been used. The later effect, with time to test.	Jingdong prefecture level city in Shandong

Jinfeng Hongyuan Flagship Store	Electricity savings1	Both the material and workmanship exceeded expectations, great value, and looks great on oh. The seller also shipped much earlier than expected, will come back. Buy it, you will not regret it! Full five points. Both the material and workmanship exceeded expectations, great value, wear it also looks good Oh. Seller delivery is also much earlier than expected, will come again. Buy it, will not regret it! Full	Jingdong prefecture level city in Shandong
Jinfeng Hongyuan Flagship Store	Electricity savings1	The appearance is clean and beautiful, the workmanship is very good, I didn't find any defects, I personally feel that the size is also quite suitable, I've used it for 2 months, I didn't find out exactly how energy efficient it is. Try using it again!	Jingdong prefecture level city in Shandong
Jinfeng Hongyuan Flagship Store	Electricity savings1	Power saving device received, product certification, accessories, etc. complete, very good workmanship, very practical power-saving effect is also very obvious.	Jingdong prefecture level city in Shandong
Jinfeng Hongyuan Flagship Store	Electricity savings1	Looked at a lot of think buy this one, see a lot of reviews say it works, a month with save nearly twenty degrees of electricity, there are sixty days of return guarantee, with a good and then come back to the	Jingdong prefecture level city in Shandong
Jinfeng Hongyuan Flagship Store	Electricity savings1	I received the power saving device, and I'm using it. I hope I can get the desired effect, and I'm trying to force it to work quickly.	Jingdong prefecture level city in Shandong
Jinfeng Hongyuan Flagship Store	Electricity savings1	Voltage is stable, already trying it out and waiting for the results to happen, saving electricity will follow up next month!	Jingdong prefecture level city in Shandong
Jinfeng Hongyuan Flagship Store	Electricity savings1	Jingdong Mall self-supporting goods of excellent quality, logistics is also very fast, worth recommending 👏👏👏👏 excellent!	Jingdong prefecture level city in Shandong
Jinfeng Hongyuan Flagship Store	Electricity savings1	Power saving has been received, the appearance of the beautiful, I have already been the second time to buy the use of down the effect is very good!	Jingdong prefecture level city in Shandong
Jinfeng Hongyuan Flagship Store	Electricity savings1	Energy saver received very happy, the color is also very good looking forward to a very good effect can save electricity	Jingdong prefecture level city in Shandong
Jinfeng Hongyuan Flagship Store	Electricity savings1	It's small and easy to plug in and use. It displays the voltage value when plugged in and	Jingdong prefecture level city in Shandong
Jinfeng Hongyuan Flagship Store	Electricity savings1	This saver really works well, because I installed one at home about seven or eight years ago, when there is no voltage display, now there is a voltage display, the appearance also looks much better, the electricity bill is also saved, the stability of the home appliances is also good, the best of both	Jingdong prefecture level city in Shandong
Jinfeng Hongyuan Flagship Store	Electricity savings1	Ordered yesterday, arrived today in use. Shipping was fast. The effect is looking forward to.	Jingdong prefecture level city in Shandong
Jinfeng Hongyuan Flagship Store	Electricity savings1	This thing, I don't know what the principle is. Why it can save electricity. Wait for a month to use it first, see if it will save electricity bill, then come back to evaluate. There is one thing that does not fit the principle ah. The law of conservation of energy.	Jingdong prefecture level city in Shandong
Jinfeng Hongyuan Flagship Store	Electricity savings1	Home mom listen to others say this can save electricity, specially let buy a, later a very large, go back to see a particularly small, directly plugged into the electricity can be, no need to install, the price is also very cheap, the first time with this product, do not know if it is not good to use, temporarily try for a period of time, if it is good to use, recommend that you buy this one, the	Jingdong prefecture level city in Shandong
Jinfeng Hongyuan Flagship Store	Electricity savings1	I've been using it for a few days, and I think the workmanship and materials are pretty good, so we'll have to look at the next bill for the real energy savings, and then we'll add more comments.	Jingdong prefecture level city in Shandong
Jinfeng Hongyuan Flagship Store	Electricity savings1	Energy saver is well made, energy saving and power saving, and anti-counterfeiting code, it is a regular commodity!	Jingdong prefecture level city in Shandong
Jinfeng Hongyuan Flagship Store	Electricity savings1	Jinfeng Hongyuan, buy back home, as a mouse, haha. First of all, normal electricity for 1 month, the power did not increase, the electricity bill feels smoother, year-on-year electricity costs in the low, whether it is very energy-saving still have to be used for a long time to know. All 5 stars ☆	Jingdong prefecture level city in Shandong
Jinfeng Hongyuan Flagship Store	Electricity savings1	The box is well packaged and beautiful! I hope to save electricity with it haha!	Jingdong prefecture level city in Shandong
Jinfeng Hongyuan Flagship Store	Electricity savings1	Ordered last night, arrived this morning, first try the effect before making comments	Jingdong prefecture level city in Shandong
Jinfeng Hongyuan Flagship Store	Electricity savings1	Looks good. Don't know the effect yet.	Jingdong prefecture level city in Shandong
Jinfeng Hongyuan Flagship Store	Electricity savings1	Jinfeng Hongyuan intelligent power saver has exquisite workmanship and modern fashionable design style. It is a good home economizer . I believe that the goods purchased from Jingdong will not disappoint our	Jingdong prefecture level city in Shandong
Jinfeng Hongyuan Flagship Store	Electricity savings1	Inadvertently flipped to this power saver, never heard of, feel very novel, the price is not expensive, so buy to try, see a month after the electricity bill will be less, recently the home air-conditioning open a lot of power consumption is very large.	Jingdong prefecture level city in Shandong
Jinfeng Hongyuan Flagship Store	Electricity savings1	The quality of the goods is very good workmanship, but also sent five fuse	Jingdong prefecture level city in Shandong
Jinfeng Hongyuan Flagship Store	Electricity savings1	I'm trying it out. I don't know if it'll work.	Jingdong prefecture level city in Shandong
Jinfeng Hongyuan Flagship Store	Electricity savings1	There is no effect yet do not know, is to look at the official sale, buy to try it! Use according to the instructions, plugged in, used for a period of time, anyway, do not know how, after a few months to see it, to see if the average month than before will be less electricity.	Jingdong prefecture level city in Shandong
Jinfeng Hongyuan Flagship Store	Electricity savings1	Received the goods, the first time to unpacking, the quality is very good, consistent with the description of the seller, completely exceeded my expectations, the packaging is very careful, strict, in general this time is very satisfied with a shopping, thanks to the seller.	Jingdong prefecture level city in Shandong
Jinfeng Hongyuan Flagship Store	Electricity savings1	The energy saver was received and used for a few days with good results.	Jingdong prefecture level city in Shandong
Jinfeng Hongyuan Flagship Store	Electricity savings1	Previously I have paid attention to this product, but I think that the monthly electricity consumption at home is not large, there is no need to save electricity savings. After a second thought, energy saving is the responsibility of each of our citizens, the winter is coming, this year's winter is cold, heating and all electric appliances, can save is to save, the price is not expensive, so buy to try. I analyze the principle of power saving, should be able to save electricity, but the larger the power saving effect is about good. To be tried after a month or two to see the effect and then evaluate. Jingdong trustworthy.	Jingdong prefecture level city in Shandong
Jinfeng Hongyuan Flagship Store	Electricity savings1	It just arrived and I can't wait to use it, and the customer service is great! There is also voltage showing up which is nice! I don't know if it can save a bit I won't know until I've used it for a month! When the time comes to come back to the follow-up review!	Jingdong prefecture level city in Shandong
Jinfeng Hongyuan Flagship Store	Electricity savings1	Checked out the reviews from numerous users. The shipment is still very large, so I decided to buy it anyway to use it and see the effect after a month. Jingdong Mall here to buy safe, convenient and fast, this price is very powerful 💪, very recommended.	Jingdong prefecture level city in Shandong
Jinfeng Hongyuan Flagship Store	Electricity savings1	Purchased two at the same time to use together. The other one works fine, the other has problems with the numbers not sure if the machine is faulty. Power saving effect can not be observed. Feel the effect is not very obvious like?	Jingdong prefecture level city in Shandong
Jinfeng Hongyuan Flagship Store	Electricity savings1	Looking forward to it!!! I don't know if it's working or not, let's look forward to it!!!! How much is saved ，，，，，，，，， Saving a degree is a degree with long term use ！！！！！！！	Jingdong prefecture level city in Shandong
Jinfeng Hongyuan Flagship Store	Electricity savings1	Received, directly on the use of the special convenient	Jingdong prefecture level city in Shandong
Jinfeng Hongyuan Flagship Store	Electricity savings1	Goods received a few days well-packed, the same day opened and plugged in to try to be normal is not sure what the power-saving effect. After a month with a follow-up evaluation.	Jingdong prefecture level city in Shandong
Jinfeng Hongyuan Flagship Store	Electricity savings1	I bought it and used it, I don't know how it works yet, we'll see later. Use the effect of it	Jingdong prefecture level city in Shandong
Jinfeng Hongyuan Flagship Store	Electricity savings1	Photographed 2, the old family also put a, with a small January, there should be some effect, with a long period of time to comment, with a long time to see the obvious effect, hard courier brother.	Jingdong prefecture level city in Shandong
Jinfeng Hongyuan Flagship Store	Electricity savings1	Experience: I am an electrician, this is the real use of electronic waves automatically connected to track the decomposition of ionized electrons, in situ compensation of reactive power, reduce effective power loss and waste, stabilize the voltage to achieve energy saving and power saving, the overall effect	Jingdong prefecture level city in Shandong
Jinfeng Hongyuan Flagship Store	Electricity savings1	Great looking and practical! Easy to use and save! Saves 15% in a month of comparison	Jingdong prefecture level city in Shandong
Jinfeng Hongyuan Flagship Store	Electricity savings1	Product quality is very good! Packaging is very nice, like a cell phone box, thick and well protected! Service is also great! Logistics is very fast! Thank you for your business! Very honest!	Jingdong prefecture level city in Shandong
Jinfeng Hongyuan Flagship Store	Electricity savings1	In several products inside the choice of this purchase for a period of time, but also need to use a few months to compare last year's electricity bill before you can analyze the savings in electricity, to the summer peak electricity consumption should be more obvious.	Jingdong prefecture level city in Shandong
Jinfeng Hongyuan Flagship Store	Electricity savings1	The goods have long been received, after more than a month to comment, the home electricity bill is indeed reduced, worth buying!	Jingdong prefecture level city in Shandong

Jinfeng Hongyuan Flagship Store	Electricity savings1	Baby received, the logistics is strong, the quality is reliable, it is worth having and recommending!	Jingdong prefecture level city in Shandong
Jinfeng Hongyuan Flagship Store	Electricity savings1	Using a conservation appliance, you can achieve a 10-15% savings in electricity usage.	Jingdong prefecture level city in Shandong
Jinfeng Hongyuan Flagship Store	Electricity savings1	Quality is very good, metal shell, touch is very good, very recommended Oh ~, the subsequent effect of continuous follow-up	Jingdong prefecture level city in Shandong
Jinfeng Hongyuan Flagship Store	Electricity savings1	The product is beautiful, practical, worth buying. The product is beautiful, practical, good quality, fast logistics product is beautiful, practical, beautiful and elegant, very classy, worth buying!	Jingdong prefecture level city in Shandong
Jinfeng Hongyuan Flagship Store	Electricity savings1	Just received the item and have already used it. Didn't know much about these products before. I hope it is as effective and useful as the product advertises.	Jingdong prefecture level city in Shandong
Jinfeng Hongyuan Flagship Store	Electricity savings1	A month two people used more than five hundred degrees of electricity, in a hurry and then add a power saver, I hope to have an effect, Jingdong shopping is very cool, logistics is very fast especially good service attitude delivery, this hot day is not easy, thank you!	Jingdong prefecture level city in Shandong
Jinfeng Hongyuan Flagship Store	Electricity savings1	Just received the baby, first plugged in to try to use a month and then additional evaluation it	Jingdong prefecture level city in Shandong
Jinfeng Hongyuan Flagship Store	Electricity savings1	Goods fast, the power saver has been received, a friend recommended to buy that can save electricity. Exquisite and compact is very convenient, plug and play. The effect is good, the price is affordable, the next need will patronize again!	Jingdong prefecture level city in Shandong
Jinfeng Hongyuan Flagship Store	Electricity savings1	Packaging is better than expected, sent a few fuse, power saving effect in the end do not know, try a period of time to say!	Jingdong prefecture level city in Shandong
Jinfeng Hongyuan Flagship Store	Electricity savings1	Shipped quickly, immediately installed, read countless reviews were not sure if this thing is effective, was going to buy three at once, ultimately decided to first use a try the effect, if the power ring significantly reduced, then will buy a few more!	Jingdong prefecture level city in Shandong
Jinfeng Hongyuan Flagship Store	Electricity savings1	Used for a period of time to evaluate, not to a month still do not know whether it can save electricity, the voltage has a display!	Jingdong prefecture level city in Shandong
Jinfeng Hongyuan Flagship Store	Electricity savings1	I don't know if it saves electricity or not, but I'm using it anyway, so I hope it saves a little bit, electricity is too expensive	Jingdong prefecture level city in Shandong
Jinfeng Hongyuan Flagship Store	Electricity savings1	The power saver has been received and I am very happy with it! Just waiting to see how the power saving works!	Jingdong prefecture level city in Shandong
Jinfeng Hongyuan Flagship Store	Electricity savings1	Saving power has been received, logistics is quite fast, the quality is not bad, small and a little weight, has been used to look forward to the effect of	Jingdong prefecture level city in Shandong
Jinfeng Hongyuan Flagship Store	Electricity savings1	The logistics is very fast, the effect is obvious and trustworthy.	Jingdong prefecture level city in Shandong
Jinfeng Hongyuan Flagship Store	Electricity savings1	Good quality, good quality, beautiful, beautiful, beautiful, beautiful, beautiful, very worth buying, worth buying, worth buying, worth buying, worth customizing!	Jingdong prefecture level city in Shandong
Jinfeng Hongyuan Flagship Store	Electricity savings1	I've used it for now, I don't know if it saves electricity	Jingdong prefecture level city in Shandong
Jinfeng Hongyuan Flagship Store	Electricity savings1	Bought two at a time, logistics is very fast, the product is beautifully packaged, after two months to see the results	Jingdong prefecture level city in Shandong
Jinfeng Hongyuan Flagship Store	Electricity savings1	The workmanship is really fine, the packaging is also good, the regular factory production ~ use a break in the evaluation of the effectiveness of the	Jingdong prefecture level city in Shandong
Jinfeng Hongyuan Flagship Store	Electricity savings1	Sometimes 219. Sometimes 220 in this case	Jingdong prefecture level city in Shandong
Jinfeng Hongyuan Flagship Store	Electricity savings1	It is the ideal product, good quality, logistics is also very fast, worthy of praise!	Jingdong prefecture level city in Shandong
Jinfeng Hongyuan Flagship Store	Electricity savings1	Store service is very good, the courier received the next day. Product packaging is very good, just use the quality to be tested.	Jingdong prefecture level city in Shandong
Jinfeng Hongyuan Flagship Store	Electricity savings1	I've been using it for almost a month now and I feel like it's working.	Jingdong prefecture level city in Shandong
Jinfeng Hongyuan Flagship Store	Electricity savings1	Very satisfied, very restful, very pretty, very beautiful, very much enjoyed! Well worth it!	Jingdong prefecture level city in Shandong
Jinfeng Hongyuan Flagship Store	Electricity savings1	Baby especially like, the value of high-quality good, a very good shopping experience, attentive service, fast logistics, distribution is also very thoughtful, attitude is also very good, value for money ah, decisive praise! Quality is quite good, logistics is also very fast, praise!	Jingdong prefecture level city in Shandong
Jinfeng Hongyuan Flagship Store	Electricity savings1	The shape of the item is basically the same as ordered, the effect is to be examined.	Jingdong prefecture level city in Shandong
Jinfeng Hongyuan Flagship Store	Electricity savings1	Appearance style: good-looking workmanship material: very good, a small one, very delicate Experience: still in use, in the process of using the shopkeeper is very nice to have a careful explanation Use for a period of time in the look at the results	Jingdong prefecture level city in Shandong
Jinfeng Hongyuan Flagship Store	Electricity savings1	As described, compact and well packaged	Jingdong prefecture level city in Shandong
Jinfeng Hongyuan Flagship Store	Electricity savings1	Fine workmanship. Test in progress. Let's see how it works.	Jingdong prefecture level city in Shandong
Jinfeng Hongyuan Flagship Store	Electricity savings1	The goods have been received, I'm very satisfied and love it	Jingdong prefecture level city in Shandong
Jinfeng Hongyuan Flagship Store	Electricity savings1	Safety! Whether you can save electricity, you can roughly feel it in 2 months. The principle is simple. Before the electrical appliances and circuits inductive more, now the electrical appliances integrated, the home is not a lot of inductive circuits, but used certainly save no problem! Wiring according to the requirements of the manual is good, if it is not convenient, see the socket plug.	Jingdong prefecture level city in Shandong
Jinfeng Hongyuan Flagship Store	Electricity savings1	Great product, looking forward to how much power I can save, first time buyer!	Jingdong prefecture level city in Shandong
Jinfeng Hongyuan Flagship Store	Electricity savings1	The product is exquisite, beautifully packaged, and the customer service is excellent. Just waiting to see how the power saving effect is, looking forward to it!	Jingdong prefecture level city in Shandong
Jinfeng Hongyuan Flagship Store	Electricity savings1	Express very quickly very satisfied with the first use to see how the effect to know, if you can save 10 percent even if acceptable, if you can not meet this requirement is obviously not much effect. Obviously.	Jingdong prefecture level city in Shandong
Jinfeng Hongyuan Flagship Store	Electricity savings1	Very delicate and compact, there is a very convenient intelligent display prompts the voltage, you can know the voltage at a glance, there is very easy to use, a plug can be used, I hope to save money on	Jingdong prefecture level city in Shandong
Jinfeng Hongyuan Flagship Store	Electricity savings1	First time buyer is it really save after use	Jingdong prefecture level city in Shandong
Jinfeng Hongyuan Flagship Store	Electricity savings1	Purchased again and will be for a long time yet.	Jingdong prefecture level city in Shandong
Jinfeng Hongyuan Flagship Store	Electricity savings1	Bought it to stock up, came back and plugged it in, hope it works	Jingdong prefecture level city in Shandong
Jinfeng Hongyuan Flagship Store	Electricity savings1	Baby received the logistics very fast very like very satisfied praise a	Jingdong prefecture level city in Shandong
Jinfeng Hongyuan Flagship Store	Electricity savings1	Beginning the process of using, easy to evaluate more later.	Jingdong prefecture level city in Shandong
Jinfeng Hongyuan Flagship Store	Electricity savings1	The goods have been received, just do not know how effective	Jingdong prefecture level city in Shandong
Jinfeng Hongyuan Flagship Store	Electricity savings1	It feels okay. I wish there were more events.	Jingdong prefecture level city in Shandong
Jinfeng Hongyuan Flagship Store	Electricity savings1	Things have been received, just plugged in, I do not know if it works, wait a few days to have the effect of additional	Jingdong prefecture level city in Shandong
Jinfeng Hongyuan Flagship Store	Electricity savings1	The effect of this power saver is really good, the house more, a few air conditioners are open every day, not used before a month to say less than seven hundred eight hundred, used after three months only nine	Jingdong prefecture level city in Shandong
Jinfeng Hongyuan Flagship Store	Electricity savings1	Cheap, good and easy to use, recommend it.	Jingdong prefecture level city in Shandong
Jinfeng Hongyuan Flagship Store	Electricity savings1	Bought this device, to the home for a long time, wait to finish a month to see how, good can recommend friends to buy, we use the electricity every month scary, a month of electricity costs more than a thousand, can not afford to hurt ah! I hope this power saving can save me some money, the beginning of the shoot order did not pay attention to look at the address, sent elsewhere to go, that side called to find out, hurry to communicate with customer service, customer service is very careful to help me get it done, call to that side to help me turn around, thank you!	Jingdong prefecture level city in Shandong

Jinfeng Hongyuan Flagship Store	Electricity savings1	I don't know the effect if it, use it first to see.	Jingdong prefecture level city in Shandong
Jinfeng Hongyuan Flagship Store	Electricity savings1	Looks good. I won't know if it saves power until I use it.	Jingdong prefecture level city in Shandong
Jinfeng Hongyuan Flagship Store	Electricity savings1	Goods received, shipping speed is very fast, yesterday afternoon more than two o'clock shot, just express delivery, but also sent a few insurance, very good business, the display of the number do not understand what is the meaning of the display said voltage, do not understand this, try a month to see how, good and	Jingdong prefecture level city in Shandong
Jinfeng Hongyuan Flagship Store	Electricity savings1	What ** play also, save electricity more and more save more and more, save chicken hair electricity, are cheating, this is a night of air conditioning with electricity, a night with 11 electricity word, this is	Jingdong prefecture level city in Shandong
Jinfeng Hongyuan Flagship Store	Electricity savings1	Received the product, hope it works!	Jingdong prefecture level city in Shandong
Jinfeng Hongyuan Flagship Store	Electricity savings1	Bought home and used very convenient, easy to operate, quite good	Jingdong prefecture level city in Shandong
Jinfeng Hongyuan Flagship Store	Electricity savings1	It's really good, cost-effective and good quality, and the logistics are fast! It arrived in one day! Really good, cost-effective and good quality, logistics is fast ah! It arrived in one day!	Jingdong prefecture level city in Shandong
Jinfeng Hongyuan Flagship Store	Electricity savings1	Works well, stabilizes voltage and saves power	Jingdong prefecture level city in Shandong
Jinfeng Hongyuan Flagship Store	Electricity savings1	I'll use it right away and look forward to good results	Jingdong prefecture level city in Shandong
Jinfeng Hongyuan Flagship Store	Electricity savings1	Recommended by a friend, said it works well, so I bought one, the effect to be tested	Jingdong prefecture level city in Shandong
Jinfeng Hongyuan Flagship Store	Electricity savings1	I didn't know it would save electricity.	Jingdong prefecture level city in Shandong
Jinfeng Hongyuan Flagship Store	Electricity savings1	I've been using it for a week, I don't know what's going on yet I hope it works well and saves power I'll follow up after a month of use	Jingdong prefecture level city in Shandong
Jinfeng Hongyuan Flagship Store	Electricity savings1	Very well packaged, looks great, don't know how it works	Jingdong prefecture level city in Shandong
Jinfeng Hongyuan Flagship Store	Electricity savings1	Genuine, good quality, really saves energy.	Jingdong prefecture level city in Shandong
Jinfeng Hongyuan Flagship Store	Electricity savings1	I was worried that the stuff is not good, but when I got it, I realized that I was completely over-anxious, 5 stars!	Jingdong prefecture level city in Shandong
Jinfeng Hongyuan Flagship Store	Electricity savings1	Very good and satisfied trustworthy	Jingdong prefecture level city in Shandong
Jinfeng Hongyuan Flagship Store	Electricity savings1	I don't know if it saves power	Jingdong prefecture level city in Shandong
Jinfeng Hongyuan Flagship Store	Electricity savings1	The quality of the product is very good, beautiful and generous applicable fast delivery speed good service attitude	Jingdong prefecture level city in Shandong
Jinfeng Hongyuan Flagship Store	Electricity savings1	Let's use it for a while and then we'll follow up	Jingdong prefecture level city in Shandong
Jinfeng Hongyuan Flagship Store	Electricity savings1	I've only been using it for a month, I don't know if there's any change yet, I'll come back with a follow-up comment after checking the meter!	Jingdong prefecture level city in Shandong
Jinfeng Hongyuan Flagship Store	Electricity savings1	I don't know if it works, I read the instructions and online reviews, I hope I won't be disappointed, if I use it, I will buy it again. Jingdong Express, fast and guaranteed service.	Jingdong prefecture level city in Shandong
Jinfeng Hongyuan Flagship Store	Electricity savings1	It feels like it saves quite a bit huh?	Jingdong prefecture level city in Shandong
Jinfeng Hongyuan Flagship Store	Electricity savings1	I've been using it for a while now, and it still seems to work and have a power saving effect	Jingdong prefecture level city in Shandong
Jinfeng Hongyuan Flagship Store	Electricity savings1	Looks good. It says 235. The 222 bar voltage is shown as high. I don't know if that's right. We'll see.	Jingdong prefecture level city in Shandong
Jinfeng Hongyuan Flagship Store	Electricity savings1	Baby is really good, do really careful, worth to have, fast delivery!	Jingdong prefecture level city in Shandong
Jinfeng Hongyuan Flagship Store	Electricity savings1	Quite delicate and compact, simple and convenient to use. Things are still very good, bought two at a time. Very satisfied with an online purchase.	Jingdong prefecture level city in Shandong
Jinfeng Hongyuan Flagship Store	Electricity savings1	Feel very good, the use of unclear, ask customer service, timely explanation, big brand, trustworthy	Jingdong prefecture level city in Shandong
Jinfeng Hongyuan Flagship Store	Electricity savings1	Thrifty received a few days did not make a comment is to see how the quality of the product now feel the quality of good appearance is also powerful seller service is very good to buy the product is also attached to the 5 fuse thought very thoughtful!	Jingdong prefecture level city in Shandong
Jinfeng Hongyuan Flagship Store	Electricity savings1	Big brand, with a very assured, the quality is very good, in time for the event to buy very cost-effective, customer service is really very good Oh ~!	Jingdong prefecture level city in Shandong
Jinfeng Hongyuan Flagship Store	Electricity savings1	I ordered yesterday and received it this morning, I heard my friend say that it can save electricity, I don't know if it works, I'll come back to make a comment after using it.	Jingdong prefecture level city in Shandong
Jinfeng Hongyuan Flagship Store	Electricity savings1	I've been using it for over a month and it doesn't seem to be working!	Jingdong prefecture level city in Shandong
Jinfeng Hongyuan Flagship Store	Electricity savings1	Used it, the family has always been in the habit of using energy-saving devices, this is a new one. Digital display voltage is very good, can see that the voltage is more stable.	Jingdong prefecture level city in Shandong
Jinfeng Hongyuan Flagship Store	Electricity savings1	I haven't used it for more than a month, but it looks good. It's easy to plug into an outlet and it works.	Jingdong prefecture level city in Shandong
Jinfeng Hongyuan Flagship Store	Electricity savings1	Good Oh...appearance is very beautiful...just plugged in with...see the effect of it...with a period of time in the additional evaluation...	Jingdong prefecture level city in Shandong
Jinfeng Hongyuan Flagship Store	Electricity savings1	I've received the goods, whether it's good or not, I'll know after I use it.	Jingdong prefecture level city in Shandong
Jinfeng Hongyuan Flagship Store	Electricity savings1	Very good, logistics is fast, the stuff is very powerful and cost-effective! Can feel the meter slowing down	Jingdong prefecture level city in Shandong
Jinfeng Hongyuan Flagship Store	Electricity savings1	Logistics is very fast, get it immediately after use, easy to operate, plug in the power supply can be used, just do not know to the use of the effect of how.	Jingdong prefecture level city in Shandong
Jinfeng Hongyuan Flagship Store	Electricity savings1	Quality is very over the top, quality is guaranteed, quite a good choice, a lot of friends to see me use all want to buy, I recommend it!	Jingdong prefecture level city in Shandong
Jinfeng Hongyuan Flagship Store	Electricity savings1	The appearance is clean and beautiful, the workmanship is very good, did not find any flaws, I personally feel that the size is also quite suitable, looking for a long time to choose this one, big love! The price is very beautiful, things are still good, will continue to buy back! All five points! Ness Ness ~ logistics speed is really no words, is a word: fast. Commodities from the outer packaging is very high-grade, really a penny a penny, expensive has the reason for expensive	Jingdong prefecture level city in Shandong
Jinfeng Hongyuan Flagship Store	Electricity savings1	The logistics is very fast, the texture is very good, genuine, I like it very much.	Jingdong prefecture level city in Shandong
Jinfeng Hongyuan Flagship Store	Electricity savings1	It's been a few days since I put it on, I can't see any results now, waiting for a month to say, steady steady steady.	Jingdong prefecture level city in Shandong
Jinfeng Hongyuan Flagship Store	Electricity savings1	I've been using it for a while now and it should be fine, so I'm buying it for the second time.	Jingdong prefecture level city in Shandong
Jinfeng Hongyuan Flagship Store	Electricity savings1	Power Saving King has been received, looks good, quite delicate, looking forward to using it to save electricity	Jingdong prefecture level city in Shandong
Jinfeng Hongyuan Flagship Store	Electricity savings1	My mom said the home uses a little too much electricity, so I bought a Power Saver to try it out, I don't know how effective it is. Expect it to be a good thing!	Jingdong prefecture level city in Shandong
Jinfeng Hongyuan Flagship Store	Electricity savings1	Thank you Jingdong, logistics is very fast order to receipt only one day. Believe in Jingdong. Favorable comments	Jingdong prefecture level city in Shandong
Jinfeng Hongyuan Flagship Store	Electricity savings1	Saved some money on the electric bill compared to last month, watch this month again, no problems so far	Jingdong prefecture level city in Shandong
Jinfeng Hongyuan Flagship Store	Electricity savings1	Energy saver exquisite workmanship, beautiful style, more features, just bought, power saving effect to be used for a period of time, then evaluate!	Jingdong prefecture level city in Shandong
Jinfeng Hongyuan Flagship Store	Electricity savings1	This thing does save power is more compact to hang on the wall.	Jingdong prefecture level city in Shandong
Jinfeng Hongyuan Flagship Store	Electricity savings1	I've been buying it for a while now, I haven't actually measured it, but it still feels like it's effective	Jingdong prefecture level city in Shandong

Jinfeng Hongyuan Flagship Store	Electricity savings1	I've already used it since I bought it, I don't know how effective it is yet, I'll comment on it later.	Jingdong prefecture level city in Shandong
Jinfeng Hongyuan Flagship Store	Electricity savings1	Baby received, careful appearance, logistics is very fast, worth buying!	Jingdong prefecture level city in Shandong
Jinfeng Hongyuan Flagship Store	Electricity savings1	It's great, it's just not the same in hand, I bought it at that other cheaper online one for about the same price as this one. But then oops, doesn't work at all.	Jingdong prefecture level city in Shandong
Jinfeng Hongyuan Flagship Store	Electricity savings1	The product is exquisite, but you have to use it for a month to know how it works. I'll comment again after a month.	Jingdong prefecture level city in Shandong
Jinfeng Hongyuan Flagship Store	Electricity savings1	I've been using it for a while now, and it doesn't feel like it's working, and it doesn't save any power.	Jingdong prefecture level city in Shandong
Jinfeng Hongyuan Flagship Store	Electricity savings1	Affordable price, fast delivery, Jingdong self-supporting guarantee	Jingdong prefecture level city in Shandong
Jinfeng Hongyuan Flagship Store	Electricity savings1	Packaging is very good, no damage, good quality, fine workmanship Packaging is very good, no damage, good quality, fine workmanship Packaging is very good, no damage, good quality, fine workmanship Packaging is very good, no damage, good quality, fine workmanship	Jingdong prefecture level city in Shandong
Jinfeng Hongyuan Flagship Store	Electricity savings1	Big brand, with a very assured, the quality is very good, in time for the event to buy very cost-effective, customer service is really very good Oh ~!	Jingdong prefecture level city in Shandong
Jinfeng Hongyuan Flagship Store	Electricity savings1	Used a few days, the specific effect is not yet clear, looking forward to the effect as described by the merchant!	Jingdong prefecture level city in Shandong
Jinfeng Hongyuan Flagship Store	Electricity savings1	I just used it and don't know how it works, I'll follow up after using it!	Jingdong prefecture level city in Shandong
Jinfeng Hongyuan Flagship Store	Electricity savings1	Well, it's been installed and now I'm waiting to see the results, BOE shopping, hopefully I won't be disappointed!	Jingdong prefecture level city in Shandong
Jinfeng Hongyuan Flagship Store	Electricity savings1	Goods received, plugged in the voltage from 229 to 222, do not know whether to save electricity, that depends on the situation.	Jingdong prefecture level city in Shandong
Jinfeng Hongyuan Flagship Store	Electricity savings1	This is the second time I have bought the product and I feel it really saves electricity! Use it again for a while and then evaluate!	Jingdong prefecture level city in Shandong
Jinfeng Hongyuan Flagship Store	Electricity savings1	Overall, it's really good, and the customer service is great, you'll get a message back almost immediately when you speak, it's great value for money, I really recommend it!	Jingdong prefecture level city in Shandong
Jinfeng Hongyuan Flagship Store	Electricity savings1	✨ Have purchased many times, a must have for the family, affordable purchase price, fast delivery, 🎁 beautifully packaged!	Jingdong prefecture level city in Shandong
Jinfeng Hongyuan Flagship Store	Electricity savings1	I bought it after a friend introduced it, he said it is effective and can save electricity. I bought it to try, try a period of time and then follow up!	Jingdong prefecture level city in Shandong
Jinfeng Hongyuan Flagship Store	Electricity savings1	Received it, trying it out tonight, and bought one earlier.	Jingdong prefecture level city in Shandong
Jinfeng Hongyuan Flagship Store	Electricity savings1	It will take a month or two to see if it saves power or not.	Jingdong prefecture level city in Shandong
Jinfeng Hongyuan Flagship Store	Electricity savings1	Looked good in the instructions! Bought one and pressed it on! Use it and see! If it works! I'll buy some more!	Jingdong prefecture level city in Shandong
Jinfeng Hongyuan Flagship Store	Electricity savings1	Goods have been received packaging can also things are not bad appearance, with a use to know, now evaluation is nonsense.	Jingdong prefecture level city in Shandong
Jinfeng Hongyuan Flagship Store	Electricity savings1	Logistics is very fast, packaging is very good, the second unit, as always, looking forward to the effect of	Jingdong prefecture level city in Shandong
Jinfeng Hongyuan Flagship Store	Electricity savings1	I received the goods today, the quality of goods is very good, I am very satisfied ah.	Jingdong prefecture level city in Shandong
Jinfeng Hongyuan Flagship Store	Electricity savings1	Buy one and try it, I wonder if it has a power saving effect?	Jingdong prefecture level city in Shandong
Jinfeng Hongyuan Flagship Store	Electricity savings1	🔍 The item was received, well packaged, logistics is very fast, customer service attitude is very positive, there are questions answered in a timely manner! Overall very satisfied!	Jingdong prefecture level city in Shandong
Jinfeng Hongyuan Flagship Store	Electricity savings1	Baby packaging is very good, purchase submission goods quickly delivered to the home, use and then follow up.	Jingdong prefecture level city in Shandong
Jinfeng Hongyuan Flagship Store	Electricity savings1	My friend asked me to buy an extra one so he could try it too.	Jingdong prefecture level city in Shandong
Jinfeng Hongyuan Flagship Store	Electricity savings1	I've just put it on, I won't know the result until a month later if it will really save power.	Jingdong prefecture level city in Shandong
Jinfeng Hongyuan Flagship Store	Electricity savings1	It's good quality. Hopefully it'll help save power.	Jingdong prefecture level city in Shandong
Jinfeng Hongyuan Flagship Store	Electricity savings1	The appearance is clean and beautiful, the workmanship is very good, I did not find any flaws, I personally feel that the size is also quite suitable, I have been looking for a long time to choose this	Jingdong prefecture level city in Shandong
Jinfeng Hongyuan Flagship Store	Electricity savings1	Logistics is fast! Baby looks great! The price is fair! Wish the store business is prosperous!	Jingdong prefecture level city in Shandong
Jinfeng Hongyuan Flagship Store	Electricity savings1	Jingdong express fast, good, have used, sit and wait to save money...	Jingdong prefecture level city in Shandong
Jinfeng Hongyuan Flagship Store	Electricity savings1	In use, it didn't work as expected and there was nothing noticeable in terms of power savings.	Jingdong prefecture level city in Shandong
Jinfeng Hongyuan Flagship Store	Electricity savings1	Just started using it and looking forward to the results.	Jingdong prefecture level city in Shandong
Jinfeng Hongyuan Flagship Store	Electricity savings1	The quality is very good, the metal shell is very good, is genuine, energy saving and power saving, logistics is very fast, there is a need to buy o, recommend everyone, very satisfied with an online	Jingdong prefecture level city in Shandong
Jinfeng Hongyuan Flagship Store	Electricity savings1	Received, I do not know if the product is good, used to see if it is good to come back to buy!	Jingdong prefecture level city in Shandong
Jinfeng Hongyuan Flagship Store	Electricity savings1	Jinfeng source of intelligent power saving treasure also used less than three months, the last thunder may be burned, the home of the electric send gate is not on, only the old man at home, no way only their own in the field and run back to find out what is the problem with the circuit, the quality is still not very good, thunder is easy to burn!	Jingdong prefecture level city in Shandong
Jinfeng Hongyuan Flagship Store	Electricity savings1	Saving power just started to use, I do not know if there is no effect, but read a lot of reviews are said to be very good so I bought, baby All aspects are good There are spare 2 fuses, late good and then follow	Jingdong prefecture level city in Shandong
Jinfeng Hongyuan Flagship Store	Electricity savings1	Use it for a few days and see, the delivery is quite fast	Jingdong prefecture level city in Shandong
Jinfeng Hongyuan Flagship Store	Electricity savings1	Small, well made and easy to use.	Jingdong prefecture level city in Shandong
Jinfeng Hongyuan Flagship Store	Electricity savings1	Beautifully packaged, it is genuine. Whether it is power saving, use it to evaluate.	Jingdong prefecture level city in Shandong
Jinfeng Hongyuan Flagship Store	Electricity savings1	Power saver. Used two of them and they do save quite a bit of power.	Jingdong prefecture level city in Shandong
Jinfeng Hongyuan Flagship Store	Electricity savings1	Workmanship is not bad, small and does not take up space, is also do not know whether the power saving, buy a to try!	Jingdong prefecture level city in Shandong
Jinfeng Hongyuan Flagship Store	Electricity savings1	The logistics is very fast, the courier attitude is very good, worth buying	Jingdong prefecture level city in Shandong
Jinfeng Hongyuan Flagship Store	Electricity savings1	The effect is not known until it is used.	Jingdong prefecture level city in Shandong
Jinfeng Hongyuan Flagship Store	Electricity savings1	Just arrived plugged in won't know for a month	Jingdong prefecture level city in Shandong
Jinfeng Hongyuan Flagship Store	Electricity savings1	This is really good to use the pull to realize the savings ah think again for my sister's family to buy a	Jingdong prefecture level city in Shandong
Jinfeng Hongyuan Flagship Store	Electricity savings1	Lies, a month with a few hundred electricity are no savings, family use	Jingdong prefecture level city in Shandong
Jinfeng Hongyuan Flagship Store	Electricity savings1	Express very quickly, look at the fine workmanship also sent a few fuse, look forward to can really save electricity	Jingdong prefecture level city in Shandong
Jinfeng Hongyuan Flagship Store	Electricity savings1	I didn't realize it was so small.	Jingdong prefecture level city in Shandong
Jinfeng Hongyuan Flagship Store	Electricity savings1	Logistics courier is very fast, the courier service is very good	Jingdong prefecture level city in Shandong

Jinfeng Hongyuan Flagship Store	Electricity savings1	Used for a period of time the effect is quite good indeed than the previous power saving	Jingdong prefecture level city in Shandong
Jinfeng Hongyuan Flagship Store	Electricity savings1	Logistics is super fast, the product is in use, see the effect...	Jingdong prefecture level city in Shandong
Jinfeng Hongyuan Flagship Store	Electricity savings1	Baby got it, tried it out and it didn't work at all. How can I use the tips to save power?	Jingdong prefecture level city in Shandong
Jinfeng Hongyuan Flagship Store	Electricity savings1	Power Saving King received, immediately used has been very stable without any sound good, good service, fast logistics inexpensive and beautiful worth repurchasing!	Jingdong prefecture level city in Shandong
Jinfeng Hongyuan Flagship Store	Electricity savings1	Logistics courier is very fast, the courier service is good	Jingdong prefecture level city in Shandong
Jinfeng Hongyuan Flagship Store	Electricity savings1	Good to recommend, the first month of air conditioning only 1 week, the electricity bill has reduced about 100 yuan, may be to reduce the use of air conditioning is also related to the other or as usual electricity, estimated that the longer you use the more you can save electricity.	Jingdong prefecture level city in Shandong
Jinfeng Hongyuan Flagship Store	Electricity savings1	It doesn't work. I've been using it for 20 days and it doesn't work.	Jingdong prefecture level city in Shandong
Jinfeng Hongyuan Flagship Store	Electricity savings1	Fast shipping, good quality, affordable price, very satisfied with a shopping.	Jingdong prefecture level city in Shandong
Jinfeng Hongyuan Flagship Store	Electricity savings1	Try it for a month to see if it saves money.	Jingdong prefecture level city in Shandong
Jinfeng Hongyuan Flagship Store	Electricity savings1	It's in use. I'll comment again after a while.	Jingdong prefecture level city in Shandong
Jinfeng Hongyuan Flagship Store	Electricity savings1	Goods have received look good 👍👍👍👍, use a period of time and then look at the	Jingdong prefecture level city in Shandong
Jinfeng Hongyuan Flagship Store	Electricity savings1	No, it doesn't work. It's useless. It's garbage.	Jingdong prefecture level city in Shandong
Jinfeng Hongyuan Flagship Store	Electricity savings1	I've been using it for almost 2 months now and I don't feel any reduction in my electricity bill.	Jingdong prefecture level city in Shandong
Jinfeng Hongyuan Flagship Store	Electricity savings1	I've received the goods, the logistics is really fast, ordered yesterday, arrived today.	Jingdong prefecture level city in Shandong
Jinfeng Hongyuan Flagship Store	Electricity savings1	And the backup insurance.	Jingdong prefecture level city in Shandong
Jinfeng Hongyuan Flagship Store	Electricity savings1	The appearance of this product is good, easy to use and install, the effect of how there is no use to wait for time to verify that the voltage displayed is different from the voltage measured by other meters, the gap is not too large, the follow-up to add the effect.	Jingdong prefecture level city in Shandong
Jinfeng Hongyuan Flagship Store	Electricity savings1	Baby received it 👌, really great ah! 🌈The quality can't be beat, very satisfied! 🆗Happy happy happy 😊	Jingdong prefecture level city in Shandong
Jinfeng Hongyuan Flagship Store	Electricity savings1	I'll follow up with a review after using it for a while, hopefully it won't bring me back a disappointment!	Jingdong prefecture level city in Shandong
Jinfeng Hongyuan Flagship Store	Electricity savings1	In use and looking forward to results! Good review to send	Jingdong prefecture level city in Shandong
Jinfeng Hongyuan Flagship Store	Electricity savings1	Intelligent power saver received, the volume is not big, small and exquisite, quite good, Jingdong Express is also quite fast, the	Jingdong prefecture level city in Shandong
Jinfeng Hongyuan Flagship Store	Electricity savings1	I don't know how to use it, keep using it	Jingdong prefecture level city in Shandong
Jinfeng Hongyuan Flagship Store	Electricity savings1	Very good for saving electricity	Jingdong prefecture level city in Shandong
Jinfeng Hongyuan Flagship Store	Electricity savings1	Seriously, did not use, feel also more power consumption, the peak is bought back to use the first time, the next day unplugged, the power consumption is back to more than ten degrees (the frequency of power use in the past few days is pretty much the same), feel the IQ tax paid 😂	Jingdong prefecture level city in Shandong
Jinfeng Hongyuan Flagship Store	Electricity savings1	I haven't seen any effect on the power saving, but the voltage stabilization looks good.	Jingdong prefecture level city in Shandong
Jinfeng Hongyuan Flagship Store	Electricity savings1	It's very good, I'll come back to it after using it for a while	Jingdong prefecture level city in Shandong
Jinfeng Hongyuan Flagship Store	Electricity savings1	Already used it and see the results.	Jingdong prefecture level city in Shandong
Jinfeng Hongyuan Flagship Store	Electricity savings1	Fast shipping, beautiful packaging, attentive service, the use of the effect, to be followed up after the evaluation.	Jingdong prefecture level city in Shandong
Jinfeng Hongyuan Flagship Store	Electricity savings1	The process of using this product is more than satisfactory	Jingdong prefecture level city in Shandong
Jinfeng Hongyuan Flagship Store	Electricity savings1	Very good East East cost-effective! Every time so buy more assured, and the logistics is also very fast. It is also very good to use, so I will often come to buy in the future, and will also introduce to	Jingdong prefecture level city in Shandong
Jinfeng Hongyuan Flagship Store	Electricity savings1	But still understand how effective it is!	Jingdong prefecture level city in Shandong
Jinfeng Hongyuan Flagship Store	Electricity savings1	Fast shipping, great logistics, great stuff, great price.	Jingdong prefecture level city in Shandong
Jinfeng Hongyuan Flagship Store	Electricity savings1	I'll review it in a month and give it 5 stars if it's good	Jingdong prefecture level city in Shandong
Jinfeng Hongyuan Flagship Store	Electricity savings1	I've saved a lot of electricity in the past month, so I'm glad to hear that.	Jingdong prefecture level city in Shandong
Jinfeng Hongyuan Flagship Store	Electricity savings1	Baby just received and installed! Looks genuinely good, workmanship feels fine! Looking forward to the effect! Said to see the effect in two months! Waiting for if really have the effect must come to follow up! The boss is absolutely responsible! Asked and answered! Just for the boss this attitude must also	Jingdong prefecture level city in Shandong
Jinfeng Hongyuan Flagship Store	Electricity savings1	This baby is very delicate, I'll comment after I've used it	Jingdong prefecture level city in Shandong
Jinfeng Hongyuan Flagship Store	Electricity savings1	It's been over two months. It's not working. It's just like it was.	Jingdong prefecture level city in Shandong
Jinfeng Hongyuan Flagship Store	Electricity savings1	Fast shipping and logistics, let's see if it works.	Jingdong prefecture level city in Shandong
Jinfeng Hongyuan Flagship Store	Electricity savings1	Just received the goods, try it to know if it can really save electricity	Jingdong prefecture level city in Shandong
Jinfeng Hongyuan Flagship Store	Electricity savings1	Not bad. Not bad. Not bad.	Jingdong prefecture level city in Shandong
Jinfeng Hongyuan Flagship Store	Electricity savings1	Not sure if it works. I'll comment again if it works.	Jingdong prefecture level city in Shandong
Jinfeng Hongyuan Flagship Store	Electricity savings1	Safe and energy-saving helpers to use!	Jingdong prefecture level city in Shandong
Jinfeng Hongyuan Flagship Store	Electricity savings1	Good, first time buyer. Used good can	Jingdong prefecture level city in Shandong
Jinfeng Hongyuan Flagship Store	Electricity savings1	It feels okay, I guess! I won't know until I use it for a while!	Jingdong prefecture level city in Shandong
Jinfeng Hongyuan Flagship Store	Electricity savings1	The saver matches the description and has been used, waiting for a follow up review after two months.	Jingdong prefecture level city in Shandong
Jinfeng Hongyuan Flagship Store	Electricity savings1	Received, the appearance of the business as described, the effect of how to use a period of time to evaluate.	Jingdong prefecture level city in Shandong
Jinfeng Hongyuan Flagship Store	Electricity savings1	Still using it don't know how effective it is, I'll comment after three months	Jingdong prefecture level city in Shandong
Jinfeng Hongyuan Flagship Store	Electricity savings1	The stuff is very good, the packaging is also very good logistics is also very fast	Jingdong prefecture level city in Shandong
Jinfeng Hongyuan Flagship Store	Electricity savings1	I'll follow up with a review when I use it, hopefully I won't be disappointed	Jingdong prefecture level city in Shandong
Jinfeng Hongyuan Flagship Store	Electricity savings1	Cancel Houjiaying Logistics.	Jingdong prefecture level city in Shandong

Jinfeng Hongyuan Flagship Store	Electricity savings1	It's very good, it's worth buying.	Jingdong prefecture level city in Shandong
Jinfeng Hongyuan Flagship Store	Electricity savings1	My friend bought it to save electricity and referred me to buy it! I'll buy it and try it out! I will know how much electricity I can save next month, looking forward to it! 4-500 dollars a month electricity bill is horrible! I hope it can really help me save some electricity!	Jingdong prefecture level city in Shandong
Jinfeng Hongyuan Flagship Store	Electricity savings1	Just received it and installed it.	Jingdong prefecture level city in Shandong
Jinfeng Hongyuan Flagship Store	Electricity savings1	Very nice. This one is my favorite.	Jingdong prefecture level city in Shandong
Jinfeng Hongyuan Flagship Store	Electricity savings1	Good value for money worth buying very good	Jingdong prefecture level city in Shandong
Jinfeng Hongyuan Flagship Store	Electricity savings1	Very energy-saving, small and exquisite, does not take up space, very satisfied, complete packaging, no damage, customer service explanation is also very serious	Jingdong prefecture level city in Shandong
Jinfeng Hongyuan Flagship Store	Electricity savings1	Received immediately used, really effective, do not understand electricity, but looked at the meter every day, recommended that you can use, especially this weather every day to open the air conditioning, with him not afraid of the electricity bill.	Jingdong prefecture level city in Shandong
Jinfeng Hongyuan Flagship Store	Electricity savings1	Nice, good value for money, nice stuff.	Jingdong prefecture level city in Shandong
Jinfeng Hongyuan Flagship Store	Electricity savings1	The packaging of the product is very good, the logistics of the product is very fast, and the quality of the product will be evaluated after use!	Jingdong prefecture level city in Shandong
Jinfeng Hongyuan Flagship Store	Electricity savings1	Express is very, well very fast, give a compliment 👍	Jingdong prefecture level city in Shandong
Jinfeng Hongyuan Flagship Store	Electricity savings1	Packaging is very good, fast delivery, used a night and a day did not feel the power saving. Use a month to see if it saves power.	Jingdong prefecture level city in Shandong
Jinfeng Hongyuan Flagship Store	Electricity savings1	Seems to be a bit of a power saver.	Jingdong prefecture level city in Shandong
Jinfeng Hongyuan Flagship Store	Electricity savings1	Bought this for someone else and it came over so fast, ###!	Jingdong prefecture level city in Shandong
Jinfeng Hongyuan Flagship Store	Electricity savings1	Very delicate, just received a period of time to use in the follow-up evaluation	Jingdong prefecture level city in Shandong
Jinfeng Hongyuan Flagship Store	Electricity savings1	It's been installed for a while now, not sure if it's working or not	Jingdong prefecture level city in Shandong
Jinfeng Hongyuan Flagship Store	Electricity savings1	The item looks okay, I just don't know if it works or not.	Jingdong prefecture level city in Shandong
Jinfeng Hongyuan Flagship Store	Electricity savings1	The goods have been received, but there is a screw missing	Jingdong prefecture level city in Shandong
Jinfeng Hongyuan Flagship Store	Electricity savings1	I didn't believe it before, but after receiving the goods today and trying it out, it really works!	Jingdong prefecture level city in Shandong
Jinfeng Hongyuan Flagship Store	Electricity savings1	Received, this thing will not know whether it is the effect of saving electricity, no matter how to say that there should always be a little use it, otherwise why develop it I think there is still the value of	Jingdong prefecture level city in Shandong
Jinfeng Hongyuan Flagship Store	Electricity savings1	Another one just arrived.	Jingdong prefecture level city in Shandong
Jinfeng Hongyuan Flagship Store	Electricity savings1	I don't feel anything, ordinary farmers can't go to TEDA, just use it first, it's safe.	Jingdong prefecture level city in Shandong
Jinfeng Hongyuan Flagship Store	Electricity savings1	Just purchased and will follow up after using it for a while	Jingdong prefecture level city in Shandong
Jinfeng Hongyuan Flagship Store	Electricity savings1	I'm trying it out now. I'll comment after I use it.	Jingdong prefecture level city in Shandong
Jinfeng Hongyuan Flagship Store	Electricity savings1	Received the item, in use, hope it works.	Jingdong prefecture level city in Shandong
Jinfeng Hongyuan Flagship Store	Electricity savings1	It works well. Good review.	Jingdong prefecture level city in Shandong
Jinfeng Hongyuan Flagship Store	Electricity savings1	All a scam, no effect at all, I bought too many fakes in Poundland before, I did not think that Jingdong is also the same	Jingdong prefecture level city in Shandong
Jinfeng Hongyuan Flagship Store	Electricity savings1	I used the saver as soon as I received it, it's in good shape and still working!	Jingdong prefecture level city in Shandong
Jinfeng Hongyuan Flagship Store	Electricity savings1	It works. I don't know what to do with the picture, but it works.	Jingdong prefecture level city in Shandong
Jinfeng Hongyuan Flagship Store	Electricity savings1	A very good shopping experience, attentive service, fast logistics, delivery is also very thoughtful, attitude is also very good, value for money ah, decisive praise!	Jingdong prefecture level city in Shandong
Jinfeng Hongyuan Flagship Store	Electricity savings1	The product is consistent with the presentation picture.	Jingdong prefecture level city in Shandong
Jinfeng Hongyuan Flagship Store	Electricity savings1	Baby received, use a period of time to evaluate, wish the boss business prosperity!	Jingdong prefecture level city in Shandong
Jinfeng Hongyuan Flagship Store	Electricity savings1	The stuff is better! Recommended!	Jingdong prefecture level city in Shandong
Jinfeng Hongyuan Flagship Store	Electricity savings1	I don't know if it works or not, I'll use it for a while before I comment on it.	Jingdong prefecture level city in Shandong
Jinfeng Hongyuan Flagship Store	Electricity savings1	Cost-effective, I like it, next time will come back Baby received, very surprised, the quality is very good, very satisfied with a shopping. I will continue to buy back, the store is trustworthy	Jingdong prefecture level city in Shandong
Jinfeng Hongyuan Flagship Store	Electricity savings1	Plugged it in and used it for a few days, not sure if it saves power yet	Jingdong prefecture level city in Shandong
Jinfeng Hongyuan Flagship Store	Electricity savings1	I'm trying it out and will follow up if it seems to be working!	Jingdong prefecture level city in Shandong
Jinfeng Hongyuan Flagship Store	Electricity savings1	Works pretty well, big kudos 👍👍👍👍👍👍👍👍👍👍👍	Jingdong prefecture level city in Shandong
Jinfeng Hongyuan Flagship Store	Electricity savings1	Just put it on, we'll see how it looks in a month.	Jingdong prefecture level city in Shandong
Jinfeng Hongyuan Flagship Store	Electricity savings1	The baby is very nice and I've already recommended buying it for all my friends! The main thing is that it not only saves electricity, I was more concerned about the fact that it can adjust the current, usually wiring problems. Now I found that it is really good, it is the effect I want!	Jingdong prefecture level city in Shandong
Jinfeng Hongyuan Flagship Store	Electricity savings1	Baby arrived, looking forward to his results, hopefully it will be good	Jingdong prefecture level city in Shandong
Jinfeng Hongyuan Flagship Store	Electricity savings1	Things have been received, look at the workmanship and packaging are good, I do not know how the power-saving effect? Use a period of time to see the effect	Jingdong prefecture level city in Shandong
Jinfeng Hongyuan Flagship Store	Electricity savings1	The price is right, the shape is beautiful and does not take up space, use it for a while to see the effect	Jingdong prefecture level city in Shandong
Jinfeng Hongyuan Flagship Store	Electricity savings1	Can't save electricity, it doesn't work!	Jingdong prefecture level city in Shandong
Jinfeng Hongyuan Flagship Store	Electricity savings1	It's pretty convenient, just plug it in and it works. There's no noise, plug it in and leave it alone, it's very hassle free! Good workmanship and value for money.	Jingdong prefecture level city in Shandong
Jinfeng Hongyuan Flagship Store	Electricity savings1	Just received the goods have been installed, try two months later to comment!	Jingdong prefecture level city in Shandong
Jinfeng Hongyuan Flagship Store	Electricity savings1	I received the stuff, I haven't used it yet, and overall it's fine.	Jingdong prefecture level city in Shandong
Jinfeng Hongyuan Flagship Store	Electricity savings1	Couldn't use a return at all.	Jingdong prefecture level city in Shandong
Jinfeng Hongyuan Flagship Store	Electricity savings1	Second purchase.	Jingdong prefecture level city in Shandong
Jinfeng Hongyuan Flagship Store	Electricity savings1	Very good quality, high tech and practical.	Jingdong prefecture level city in Shandong
Jinfeng Hongyuan Flagship Store	Electricity savings1	Very good, very great! Very satisfied, thanks to the store!	Jingdong prefecture level city in Shandong

Jinfeng Hongyuan Flagship Store	Electricity savings1	Just used it, don't know if it's good or not, hope it works well	Jingdong prefecture level city in Shandong
Jinfeng Hongyuan Flagship Store	Electricity savings1	Saving power has been received, the product is good, good like.	Jingdong prefecture level city in Shandong
Jinfeng Hongyuan Flagship Store	Electricity savings1	Received the goods and used it for two months before evaluation	Jingdong prefecture level city in Shandong
Jinfeng Hongyuan Flagship Store	Electricity savings1	It looks a little small, I do not know how effective, first use to see the effect, there is a small adjustable button, I do not know this is to adjust to that position to work well	Jingdong prefecture level city in Shandong
Jinfeng Hongyuan Flagship Store	Electricity savings1	Simple, just plug it in, there are spare fuses, can't say how well it works, no comparison	Jingdong prefecture level city in Shandong
Jinfeng Hongyuan Flagship Store	Electricity savings1	It's been a few days since I used it, and the power's gone up.	Jingdong prefecture level city in Shandong
Jinfeng Hongyuan Flagship Store	Electricity savings1	The owner was very nice and sent a spare fuse.	Jingdong prefecture level city in Shandong
Jinfeng Hongyuan Flagship Store	Electricity savings1	It's authentic and it's not expensive and it's great to use, I use it all the time and it's great 🎉✨ just don't know if it really saves any points	Jingdong prefecture level city in Shandong
Jinfeng Hongyuan Flagship Store	Electricity savings1	The information is good, it is the child to buy, the child read it is also quite happy, I hope to be able to match the child's preparation for the midterm examination! Because of the epidemic, it is not too fast, but I also give full credit to the express delivery!	Jingdong prefecture level city in Shandong
Jinfeng Hongyuan Flagship Store	Electricity savings1	This power saver comes to the logistics very quickly, the business attitude is also very good, ** very comfortable, this brand is trustworthy, and buy in this store than in the physical store to buy cheaper,	Jingdong prefecture level city in Shandong
Jinfeng Hongyuan Flagship Store	Electricity savings1	It's very well made and the materials used are okay. This is just a few days old, so I don't know how it will work, only next month.	Jingdong prefecture level city in Shandong
Jinfeng Hongyuan Flagship Store	Electricity savings1	Already used on, have not tested the effect, wait for a couple of months to add comments.	Jingdong prefecture level city in Shandong
Jinfeng Hongyuan Flagship Store	Electricity savings1	Baby received, packaging is very thoughtful, already in use, should soon see the effect.	Jingdong prefecture level city in Shandong
Jinfeng Hongyuan Flagship Store	Electricity savings1	Just received in the office to try, to the home installed, the voltage are different, and do not know if there is no effect. Use a period of time and then follow up.	Jingdong prefecture level city in Shandong
Jinfeng Hongyuan Flagship Store	Electricity savings1	Just received the goods, is trying, looking forward to the effect, first give a favorable comment, two months later, then additional evaluation, please be patient.	Jingdong prefecture level city in Shandong
Jinfeng Hongyuan Flagship Store	Electricity savings1	The item has just been received, well packaged, results to be verified.	Jingdong prefecture level city in Shandong
Jinfeng Hongyuan Flagship Store	Electricity savings1	Packaging logistics are very good, the next look at how the effect, before I see others use the effect is good, as I bought this can also have good results.	Jingdong prefecture level city in Shandong
Jinfeng Hongyuan Flagship Store	Electricity savings1	I don't know if it's a real power saver or a fake power saver, but I've used it since I bought it, so I'll give you a good review.	Jingdong prefecture level city in Shandong
Jinfeng Hongyuan Flagship Store	Electricity savings1	Good quality, genuine goods, much better than imagined before purchase, next time will come back to care!	Jingdong prefecture level city in Shandong
Jinfeng Hongyuan Flagship Store	Electricity savings1	Logistics is very fast good use again	Jingdong prefecture level city in Shandong
Jinfeng Hongyuan Flagship Store	Electricity savings1	Received the goods. Let's see if it saves power in a month and then we'll follow up!	Jingdong prefecture level city in Shandong
Jinfeng Hongyuan Flagship Store	Electricity savings1	I bought it yesterday and it arrived today, I bought two of them, I hope they work well, I'll come back after a while to give a follow-up review	Jingdong prefecture level city in Shandong
Jinfeng Hongyuan Flagship Store	Electricity savings1	The home air conditioner consumes a lot of electricity, so I bought it and used it in the hope that there is A	Jingdong prefecture level city in Shandong
Jinfeng Hongyuan Flagship Store	Electricity savings1	The product is excellent and very current.	Jingdong prefecture level city in Shandong
Jinfeng Hongyuan Flagship Store	Electricity savings1	With the description, it is the most practical and good products that households can need, and it is an ideal shopping, the courier is also fast, and the service attitude is enthusiastic!	Jingdong prefecture level city in Shandong
Jinfeng Hongyuan Flagship Store	Electricity savings1	The Power Saver is easy to use, works well, is stable, and plugs into the top of the outlet	Jingdong prefecture level city in Shandong
Jinfeng Hongyuan Flagship Store	Electricity savings1	Overall still can, excellent craftsmanship, power saving do not know for the time being, after the use of the evaluation	Jingdong prefecture level city in Shandong
Jinfeng Hongyuan Flagship Store	Electricity savings1	Baby received on the plug, but also with four fuses, look at the quality is okay, I do not know whether it is useful, use and then follow up.	Jingdong prefecture level city in Shandong
Jinfeng Hongyuan Flagship Store	Electricity savings1	I'll evaluate it after a while, I don't know if it's good or not yet, and if it can save electricity	Jingdong prefecture level city in Shandong
Jinfeng Hongyuan Flagship Store	Electricity savings1	Jingdong self-operated purchase more assured delivery fast quality assurance	Jingdong prefecture level city in Shandong
Jinfeng Hongyuan Flagship Store	Electricity savings1	Shipping was fast, stuff was well packaged, great price, I'm happy with it 👍 and will repurchase!	Jingdong prefecture level city in Shandong
Jinfeng Hongyuan Flagship Store	Electricity savings1	The quality of the product is very good and I will definitely come back again next time.	Jingdong prefecture level city in Shandong
Jinfeng Hongyuan Flagship Store	Electricity savings1	I'll follow up with a review after I use it.	Jingdong prefecture level city in Shandong
Jinfeng Hongyuan Flagship Store	Electricity savings1	Hello, the goods are perfectly packaged, save power for a period of time to evaluate again!	Jingdong prefecture level city in Shandong
Jinfeng Hongyuan Flagship Store	Electricity savings1	Not bad, pretty good, good value for money, pretty good	Jingdong prefecture level city in Shandong
Jinfeng Hongyuan Flagship Store	Electricity savings1	The packaging is very good, received immediately used, looking forward to the effect in.	Jingdong prefecture level city in Shandong
Jinfeng Hongyuan Flagship Store	Electricity savings1	See how much power you save at the end of the month	Jingdong prefecture level city in Shandong
Jinfeng Hongyuan Flagship Store	Electricity savings1	Easy to use, affordable, beautiful color	Jingdong prefecture level city in Shandong
Jinfeng Hongyuan Flagship Store	Electricity savings1	It's taking too long to put together an order. It could be an epidemic.	Jingdong prefecture level city in Shandong
Jinfeng Hongyuan Flagship Store	Electricity savings1	I don't feel much after using it, no change in my electric bill 。。。。。。。	Jingdong prefecture level city in Shandong
Jinfeng Hongyuan Flagship Store	Electricity savings1	Logistics is very fast, the quality of the economizer is good, good quality and cheap, it is worth buying, I hope there is a power saving effect!	Jingdong prefecture level city in Shandong
Jinfeng Hongyuan Flagship Store	Electricity savings1	Feeling okay, in a couple of months to try to rely on it before making a comment. Let's give it a five-star review first!	Jingdong prefecture level city in Shandong
Jinfeng Hongyuan Flagship Store	Electricity savings1	Power saving device has been hesitating before in the end to buy or not to buy, after consideration, consideration, in consideration, or decided to buy a try. Things received can not wait to open a look, and the seller described the same, I hope to use 2 months to have the effect, two months to follow up on	Jingdong prefecture level city in Shandong
Jinfeng Hongyuan Flagship Store	Electricity savings1	Thank you Jingdong, logistics is very fast order to receipt only one day. Believe in Jingdong. Favorable comments	Jingdong prefecture level city in Shandong
Jinfeng Hongyuan Flagship Store	Electricity savings1	I used one to use it, but it didn't work very well, what to do	Jingdong prefecture level city in Shandong
Jinfeng Hongyuan Flagship Store	Electricity savings1	Quality and packaging are very good, fast shipping, good service, buy with confidence	Jingdong prefecture level city in Shandong
Jinfeng Hongyuan Flagship Store	Electricity savings1	Use a period of time to evaluate, the quality is very good, the price is affordable, logistics is also very fast	Jingdong prefecture level city in Shandong
Jinfeng Hongyuan Flagship Store	Electricity savings1	Goods received, the day of the order tomorrow delivery real God speed, delivery force, the product to be used after a period of time and then low comment!	Jingdong prefecture level city in Shandong
Jinfeng Hongyuan Flagship Store	Electricity savings1	I don't know, how effective it is, let's talk about it when the time comes, whether it saves power or not, how much, unknown.	Jingdong prefecture level city in Shandong
Jinfeng Hongyuan Flagship Store	Electricity savings1	It does save some electricity, and I don't want to use something that isn't legal! Used nearly ~ electricity last month, probably not much more this month with the AC on!	Jingdong prefecture level city in Shandong

Jinfeng Hongyuan Flagship Store	Electricity savings1	Well-packed, excellent quality, service satisfaction good value for money	Jingdong prefecture level city in Shandong
Jinfeng Hongyuan Flagship Store	Electricity savings1	Express very quickly, the goods received last night, today used a day of goods can not heat, looking forward to whether it can save electricity, with a month and then comment, is used well will buy again.	Jingdong prefecture level city in Shandong
Jinfeng Hongyuan Flagship Store	Electricity savings1	It's pretty good, hopefully it'll be a real power saver.	Jingdong prefecture level city in Shandong
Jinfeng Hongyuan Flagship Store	Electricity savings1	The goods received in use, can not see the effect of energy saving, after a month to know!	Jingdong prefecture level city in Shandong
Jinfeng Hongyuan Flagship Store	Electricity savings1	Received the goods, looking forward to better surprise, with the effect of the follow-up comment!	Jingdong prefecture level city in Shandong
Jinfeng Hongyuan Flagship Store	Electricity savings1	It's not bad, it's still in use and should save some	Jingdong prefecture level city in Shandong
Jinfeng Hongyuan Flagship Store	Electricity savings1	Logistics is very fast, easy to install, plugged in on it,, the effect of the use of the later to evaluate!	Jingdong prefecture level city in Shandong
Jinfeng Hongyuan Flagship Store	Electricity savings1	The stuff is recommendable to buy very good.	Jingdong prefecture level city in Shandong
Jinfeng Hongyuan Flagship Store	Electricity savings1	Voltmeter measurements of voltage are way off, aren't they?	Jingdong prefecture level city in Shandong
Jinfeng Hongyuan Flagship Store	Electricity savings1	Install it to the socket immediately after purchase and try to see if it can save electricity after one month.	Jingdong prefecture level city in Shandong
Jinfeng Hongyuan Flagship Store	Electricity savings1	I've been using it for almost a month now, and I won't know if it saves electricity until I pay the bill.	Jingdong prefecture level city in Shandong
Jinfeng Hongyuan Flagship Store	Electricity savings1	I bought one in the past and it was fine, so I'm buying it back.	Jingdong prefecture level city in Shandong
Jinfeng Hongyuan Flagship Store	Electricity savings1	Service is very good, this thing is small and exquisite to use, just waiting to use the effect of a period of time	Jingdong prefecture level city in Shandong
Jinfeng Hongyuan Flagship Store	Electricity savings1	Not sure how well it works yet	Jingdong prefecture level city in Shandong
Jinfeng Hongyuan Flagship Store	Electricity savings1	Kyodo goods habitually good reviews	Jingdong prefecture level city in Shandong
Jinfeng Hongyuan Flagship Store	Electricity savings1	Appearance is OK, whether it is power saving remains to be seen.	Jingdong prefecture level city in Shandong
Jinfeng Hongyuan Flagship Store	Electricity savings1	I bought it and used it, I just don't know if it really saves power	Jingdong prefecture level city in Shandong
Jinfeng Hongyuan Flagship Store	Electricity savings1	The saver is well made and of good quality, I will evaluate it after using it for a while.	Jingdong prefecture level city in Shandong
Jinfeng Hongyuan Flagship Store	Electricity savings1	Just received in use, use for a period of time to see if there is power saving	Jingdong prefecture level city in Shandong
Jinfeng Hongyuan Flagship Store	Electricity savings1	Used 3 days before commenting, it really saves electricity, I look at the "power calendar" after using one less degree of electricity per day, so I think it's worth it to buy this, I will introduce my friends	Jingdong prefecture level city in Shandong
Jinfeng Hongyuan Flagship Store	Electricity savings1	Reliable quality, very well packaged and matches the real thing.	Jingdong prefecture level city in Shandong
Jinfeng Hongyuan Flagship Store	Electricity savings1	Logistics is still quite fast, ha in the trial	Jingdong prefecture level city in Shandong
Jinfeng Hongyuan Flagship Store	Electricity savings1	Consultant service attitude is very good, the courier is also very powerful, the first day of the order the next day to arrive. Saver received used and then add comments!	Jingdong prefecture level city in Shandong
Jinfeng Hongyuan Flagship Store	Electricity savings1	I just got home to try it out and see how it goes.	Jingdong prefecture level city in Shandong
Jinfeng Hongyuan Flagship Store	Electricity savings1	Well made and weighted instrument. Kudos! Put it into use immediately, don't know if it saves power, will evaluate later!	Jingdong prefecture level city in Shandong
Jinfeng Hongyuan Flagship Store	Electricity savings1	I've just put it in place. I'll give you a review in ten days.	Jingdong prefecture level city in Shandong
Jinfeng Hongyuan Flagship Store	Electricity savings1	It looks very delicate, not bad. Well, I don't know if it can save power or not, I have to try it for a while and see. If it can really save electricity. Uh, if it works well. Then additional comments. Anyway, thanks for the logistics, thanks to Jingdong. Well, it deserves some praise.	Jingdong prefecture level city in Shandong
Jinfeng Hongyuan Flagship Store	Electricity savings1	Bought a month, the feeling is that there is a certain effect, a month to save a lot of money	Jingdong prefecture level city in Shandong
Jinfeng Hongyuan Flagship Store	Electricity savings1	Goods received, the physical and the same as the picture, logistics is also very fast, the service attitude is very good, it is worth buying!	Jingdong prefecture level city in Shandong
Jinfeng Hongyuan Flagship Store	Electricity savings1	I've used it, I don't know whether it saves electricity or not, I'll talk about it after a while.	Jingdong prefecture level city in Shandong
Jinfeng Hongyuan Flagship Store	Electricity savings1	August only began to use, October to know, have no power-saving effect, to the in to evaluate!	Jingdong prefecture level city in Shandong
Jinfeng Hongyuan Flagship Store	Electricity savings1	Goods received: I do not know to the factory products used to save electricity!	Jingdong prefecture level city in Shandong
Jinfeng Hongyuan Flagship Store	Electricity savings1	Don't know if it works, bought it anyway.	Jingdong prefecture level city in Shandong
Jinfeng Hongyuan Flagship Store	Electricity savings1	Still testing, will follow up next month to evaluate how much electricity is saved	Jingdong prefecture level city in Shandong
Jinfeng Hongyuan Flagship Store	Electricity savings1	I'm still testing it, I'll comment after I use it.	Jingdong prefecture level city in Shandong
Jinfeng Hongyuan Flagship Store	Electricity savings1	Fast service, praiseworthy. Good item pre-style, first praise, the use of the effect to be evaluated after use.	Jingdong prefecture level city in Shandong
Jinfeng Hongyuan Flagship Store	Electricity savings1	The power saving effect is more obvious through verified use.	Jingdong prefecture level city in Shandong
Jinfeng Hongyuan Flagship Store	Electricity savings1	Logistics is very fast yesterday order today arrived. The product was immediately used, and after a month to see its effect.	Jingdong prefecture level city in Shandong
Jinfeng Hongyuan Flagship Store	Electricity savings1	Power saving is not yet known, as far as the TV and audio are concerned, I feel that there is a significant quality improvement, and I recommend using a good product!	Jingdong prefecture level city in Shandong
Jinfeng Hongyuan Flagship Store	Electricity savings1	Good power savings, tens of dollars a month saved	Jingdong prefecture level city in Shandong
Jinfeng Hongyuan Flagship Store	Electricity savings1	Already took two, great value! You can save 30% on your electric bill in a month. A summer must-have!	Jingdong prefecture level city in Shandong
Jinfeng Hongyuan Flagship Store	Electricity savings1	Received the item, plugged it into a home outlet and it showed 230 volts (not that high with a multimeter). Let's use it first!	Jingdong prefecture level city in Shandong
Jinfeng Hongyuan Flagship Store	Electricity savings1	Expecting results in the experiment	Jingdong prefecture level city in Shandong
Jinfeng Hongyuan Flagship Store	Electricity savings1	I don't know if it really saves power.	Jingdong prefecture level city in Shandong
Jinfeng Hongyuan Flagship Store	Electricity savings1	It's very good and the efficacy is very visible.	Jingdong prefecture level city in Shandong
Jinfeng Hongyuan Flagship Store	Electricity savings1	It's good and worth buying with confidence	Jingdong prefecture level city in Shandong
Jinfeng Hongyuan Flagship Store	Electricity savings1	The goods have been received, just do not know if it is effective	Jingdong prefecture level city in Shandong
Jinfeng Hongyuan Flagship Store	Electricity savings1	It's already in use, but based on some of the descriptions, I suspect that to use it, you need to have one in series on each line, which seems to work for all appliances, that's my guess, but I feel as though the principle of the thing seems to require it.	Jingdong prefecture level city in Shandong
Jinfeng Hongyuan Flagship Store	Electricity savings1	Fast delivery, already in use, looks good!	Jingdong prefecture level city in Shandong
Jinfeng Hongyuan Flagship Store	Electricity savings1	Hoping to save 30% of power as presented in the graphic	Jingdong prefecture level city in Shandong

Jinfeng Hongyuan Flagship Store	Electricity savings1	Things look good, power saving do not know now, with a month after the continued evaluation	Jingdong prefecture level city in Shandong
Jinfeng Hongyuan Flagship Store	Electricity savings1	Already used it but not sure if it will top out!	Jingdong prefecture level city in Shandong
Jinfeng Hongyuan Flagship Store	Electricity savings1	Looking forward to using the results, the product quality looks okay.	Jingdong prefecture level city in Shandong
Jinfeng Hongyuan Flagship Store	Electricity savings1	It's somewhat effective and saves money more or less 💰	Jingdong prefecture level city in Shandong
Jinfeng Hongyuan Flagship Store	Electricity savings1	Got it, it's great, looking forward to saving power.	Jingdong prefecture level city in Shandong
Jinfeng Hongyuan Flagship Store	Electricity savings1	I don't know if it's working yet, I'll follow up when I get there	Jingdong prefecture level city in Shandong
Jinfeng Hongyuan Flagship Store	Electricity savings1	The product received, good workmanship, fast logistics, good inner packaging, the outer bag is damaged, but fortunately did not hurt the product, retrieved and inserted according to the instructions, looking forward to the effect is good, with a period of time to evaluate, I believe that the Jingdong.	Jingdong prefecture level city in Shandong
Jinfeng Hongyuan Flagship Store	Electricity savings1	Since it is a new product, it is said to work, so if it works well for a month, then I will give you a good review.	Jingdong prefecture level city in Shandong
Jinfeng Hongyuan Flagship Store	Electricity savings1	The quality of the product is very good, very well made, well packaged, easy to use and very convenient!	Jingdong prefecture level city in Shandong
Jinfeng Hongyuan Flagship Store	Electricity savings1	Good price for the item, good functionality	Jingdong prefecture level city in Shandong
Jinfeng Hongyuan Flagship Store	Electricity savings1	Received the first time to use it, the stuff is very good, specifics wait for a month to come back to feedback to see the effect.	Jingdong prefecture level city in Shandong
Jinfeng Hongyuan Flagship Store	Electricity savings1	Just received, the courier is too bad, not notified directly to put the Feng Nest courier cabinet	Jingdong prefecture level city in Shandong
Jinfeng Hongyuan Flagship Store	Electricity savings1	Baby received, the second time to buy, plugged in and used do not know if there is no effect. Baby received, the second time to buy, plugged in and used do not know if there is no effect. Baby received, the second time to buy, plugged in and used do not know if there is no effect.	Jingdong prefecture level city in Shandong
Jinfeng Hongyuan Flagship Store	Electricity savings1	It's okay if it saves power. It's not a bad product.	Jingdong prefecture level city in Shandong
Jinfeng Hongyuan Flagship Store	Electricity savings1	Received, very good, meticulous packaging, open a look is genuine, meticulous workmanship, the manual is very detailed, has been used, there is no effect on the only look at the next month's electricity bill, when I can I buy a few more to send people!	Jingdong prefecture level city in Shandong
Jinfeng Hongyuan Flagship Store	Electricity savings1	The quality is very good very delicate express delivery is too fast	Jingdong prefecture level city in Shandong
Jinfeng Hongyuan Flagship Store	Electricity savings1	I won't know until a month after I start using it today.	Jingdong prefecture level city in Shandong
Jinfeng Hongyuan Flagship Store	Electricity savings1	It doesn't work at all, the customer service attitude is very bad, use it or not!	Jingdong prefecture level city in Shandong
Jinfeng Hongyuan Flagship Store	Electricity savings1	Temporarily try, do not know the effect, Jingdong logistics delivery very quickly	Jingdong prefecture level city in Shandong
Jinfeng Hongyuan Flagship Store	Electricity savings1	It doesn't help. It's comforting.	Jingdong prefecture level city in Shandong
Jinfeng Hongyuan Flagship Store	Electricity savings1	It's great, use it before you say anything.	Jingdong prefecture level city in Shandong
Jinfeng Hongyuan Flagship Store	Electricity savings1	Received, already used, looking forward to a good a effect!	Jingdong prefecture level city in Shandong
Jinfeng Hongyuan Flagship Store	Electricity savings1	I don't know if it works yet, I won't know until I use it for a few months.	Jingdong prefecture level city in Shandong
Jinfeng Hongyuan Flagship Store	Electricity savings1	It takes a long time to get there. There's no change now.	Jingdong prefecture level city in Shandong
Jinfeng Hongyuan Flagship Store	Electricity savings1	Jingdong's delivery never disappoints, or so fast, and the stuff is great, next time you need to come back.	Jingdong prefecture level city in Shandong
Jinfeng Hongyuan Flagship Store	Electricity savings1	At first it was ok today, after using it for a while it didn't work anymore	Jingdong prefecture level city in Shandong
Jinfeng Hongyuan Flagship Store	Electricity savings1	Just received the goods, do not know the effect of using a few days before there may be effect	Jingdong prefecture level city in Shandong
Jinfeng Hongyuan Flagship Store	Electricity savings1	The logistics was fast, I received it the next day, I've purchased it for the second time and have been using it all along	Jingdong prefecture level city in Shandong
Jinfeng Hongyuan Flagship Store	Electricity savings1	Inexpensive, fast delivery, very pleased!	Jingdong prefecture level city in Shandong
Jinfeng Hongyuan Flagship Store	Electricity savings1	Received the goods immediately plugged directly into the use of power saving yet to be good news!	Jingdong prefecture level city in Shandong
Jinfeng Hongyuan Flagship Store	Electricity savings1	The service attitude is very good, and the Jingdong self-owned products can be trusted.	Jingdong prefecture level city in Shandong
Jinfeng Hongyuan Flagship Store	Electricity savings1	The product is good, I do not know if it can save play a power saving effect	Jingdong prefecture level city in Shandong
Jinfeng Hongyuan Flagship Store	Electricity savings1	We won't know how effective it is until we use it for a while, so I'll give you a good review now.	Jingdong prefecture level city in Shandong
Jinfeng Hongyuan Flagship Store	Electricity savings1	The quality is pretty good, just started using it, hopefully it will deliver results.	Jingdong prefecture level city in Shandong
Jinfeng Hongyuan Flagship Store	Electricity savings1	I've plugged it in first, and will follow up after I've used it to see how it works.	Jingdong prefecture level city in Shandong
Jinfeng Hongyuan Flagship Store	Electricity savings1	Things have arrived, the quality is good, the effect is also equivalent to can ah	Jingdong prefecture level city in Shandong
Jinfeng Hongyuan Flagship Store	Electricity savings1	It's not worth it to brag about power savings.	Jingdong prefecture level city in Shandong
Jinfeng Hongyuan Flagship Store	Electricity savings1	I don't know if it's an IQ tax, but I'll use it for now.	Jingdong prefecture level city in Shandong
Jinfeng Hongyuan Flagship Store	Electricity savings1	I don't feel the power saving yet, I can't know until I use it for 2 months.	Jingdong prefecture level city in Shandong
Jinfeng Hongyuan Flagship Store	Electricity savings1	There's nothing wrong with this at the moment.	Jingdong prefecture level city in Shandong
Jinfeng Hongyuan Flagship Store	Electricity savings1	It's authentic and worth buying	Jingdong prefecture level city in Shandong
Jinfeng Hongyuan Flagship Store	Electricity savings1	It's good. We'll see how it goes.	Jingdong prefecture level city in Shandong
Jinfeng Hongyuan Flagship Store	Electricity savings1	I don't know if it's working or not in the middle of using it, we'll see when the month is over	Jingdong prefecture level city in Shandong
Jinfeng Hongyuan Flagship Store	Electricity savings1	I'm not going to buy anything from Jingdong anymore, so I'll give it a good review	Jingdong prefecture level city in Shandong
Jinfeng Hongyuan Flagship Store	Electricity savings1	I'm not going to buy anything from Jingdong anymore, so I'll give it a good review	Jingdong prefecture level city in Shandong
Jinfeng Hongyuan Flagship Store	Electricity savings1	Just received the item and will see how it works in a month.	Jingdong prefecture level city in Shandong
Jinfeng Hongyuan Flagship Store	Electricity savings1	I don't know if it saves electricity, but it's plugged into an outlet every day anyway	Jingdong prefecture level city in Shandong
Jinfeng Hongyuan Flagship Store	Electricity savings1	I don't know if it works yet.	Jingdong prefecture level city in Shandong
Jinfeng Hongyuan Flagship Store	Electricity savings1	Plugged it in when I got back, waiting for a month to see the results	Jingdong prefecture level city in Shandong
Jinfeng Hongyuan Flagship Store	Electricity savings1	Effects remain to be seen, but it looks good quality and runs fine	Jingdong prefecture level city in Shandong

Jinfeng Hongyuan Flagship Store	Electricity savings1	The product received logistics very quickly, has not yet been used to test! After use in the evaluation!	Jingdong prefecture level city in Shandong
Jinfeng Hongyuan Flagship Store	Electricity savings1	The power saver is in use, and I don't know how much electricity has been saved after using it for more than a month, and the display voltmeter is very good.	Jingdong prefecture level city in Shandong
Jinfeng Hongyuan Flagship Store	Electricity savings1	It's so good, isn't it? You're a real Shanghainese, aren't you?	Jingdong prefecture level city in Shandong
Jinfeng Hongyuan Flagship Store	Electricity savings1	Great quality great great great 👍👍👍👍	Jingdong prefecture level city in Shandong
Jinfeng Hongyuan Flagship Store	Electricity savings1	I'm using it, I don't know if it's working yet.	Jingdong prefecture level city in Shandong
Jinfeng Hongyuan Flagship Store	Electricity savings1	Just used it for more than 10 days and haven't seen any results yet	Jingdong prefecture level city in Shandong
Jinfeng Hongyuan Flagship Store	Electricity savings1	The product arrived home feeling like it should work	Jingdong prefecture level city in Shandong
Jinfeng Hongyuan Flagship Store	Electricity savings1	Just received the goods, use it and comment back	Jingdong prefecture level city in Shandong
Jinfeng Hongyuan Flagship Store	Electricity savings1	Try it for a month and then evaluate	Jingdong prefecture level city in Shandong
Jinfeng Hongyuan Flagship Store	Electricity savings1	I don't know if it saves power yet, but I'll comment again after using it for a while.	Jingdong prefecture level city in Shandong
Jinfeng Hongyuan Flagship Store	Electricity savings1	The product is better than I thought, the quality and appearance are very good, the voltage is stable, and I'm looking forward to a good power saving effect.	Jingdong prefecture level city in Shandong
Jinfeng Hongyuan Flagship Store	Electricity savings1	Received and scanned the QR code to check that it is genuine, have plugged it in, can it save power, looking forward to the advertised effect. Customary praise, 5 points!	Jingdong prefecture level city in Shandong
Jinfeng Hongyuan Flagship Store	Electricity savings1	The goods have been received and have not yet been used. Do not know how the effect.	Jingdong prefecture level city in Shandong
Jinfeng Hongyuan Flagship Store	Electricity savings1	Hopefully, you get what you pay for, and you get what you pay for!	Jingdong prefecture level city in Shandong
Jinfeng Hongyuan Flagship Store	Electricity savings1	Just put it on and it feels like it's turning as fast as a meter! Looking forward to the results!	Jingdong prefecture level city in Shandong
Jinfeng Hongyuan Flagship Store	Electricity savings1	Overall look on the product quality is good, I do not know is not the effect of power saving, wait to see and then follow up!	Jingdong prefecture level city in Shandong
Jinfeng Hongyuan Flagship Store	Electricity savings1	The logistics courier is very fast, the courier service attitude is very good	Jingdong prefecture level city in Shandong
Jinfeng Hongyuan Flagship Store	Electricity savings1	The product has been received, it works well worth buying	Jingdong prefecture level city in Shandong
Jinfeng Hongyuan Flagship Store	Electricity savings1	How did it not work? Is it purely an IQ tax?	Jingdong prefecture level city in Shandong
Jinfeng Hongyuan Flagship Store	Electricity savings1	It looks good, I don't know how well it works.	Jingdong prefecture level city in Shandong
Jinfeng Hongyuan Flagship Store	Electricity savings1	It does seem to be a bit of a power saver, I just don't know if the more expensive one is more power efficient?	Jingdong prefecture level city in Shandong
Jinfeng Hongyuan Flagship Store	Electricity savings1	Is genuine very good, the price is much cheaper, cost-effective, really special paddle just to do activities, the price is much cheaper, cost-effective, really special paddle counting, packaging is also very high-end atmospheric class, it is worth buying!	Jingdong prefecture level city in Shandong
Jinfeng Hongyuan Flagship Store	Electricity savings1	I received it yesterday and used it right away, I hope it works, I'll wait for next month to come back to the conclusion, I'm looking forward to it. Five hearts praise!	Jingdong prefecture level city in Shandong
Jinfeng Hongyuan Flagship Store	Electricity savings1	Looks good, packed properly, received it today, am using it, will see results in a month!	Jingdong prefecture level city in Shandong
Jinfeng Hongyuan Flagship Store	Electricity savings1	Regular product Jingdong self-support trustworthy. There is an effect Come back to the review. 😍😍😍😍	Jingdong prefecture level city in Shandong
Jinfeng Hongyuan Flagship Store	Electricity savings1	I haven't used it yet, hopefully it will work as a power saver, and will comment again if it works well.	Jingdong prefecture level city in Shandong
Jinfeng Hongyuan Flagship Store	Electricity savings1	Shipping was fast, logistics was fast, service was good, and I look forward to good results from the item!	Jingdong prefecture level city in Shandong
Jinfeng Hongyuan Flagship Store	Electricity savings1	This product is this compensator, it has to be at the main switch to be useful, haven't compared the final result, the	Jingdong prefecture level city in Shandong
Jinfeng Hongyuan Flagship Store	Electricity savings1	Packaging is perfect . I've already started using it, so I'm looking forward to the results.	Jingdong prefecture level city in Shandong
Jinfeng Hongyuan Flagship Store	Electricity savings1	I just got it back. I'll comment after I use it.	Jingdong prefecture level city in Shandong
Jinfeng Hongyuan Flagship Store	Electricity savings1	Big brand, with a very assured, very good quality, catch the event to buy very cost-effective to take advantage of the offer, hurry to get, it is genuine, the packaging is also very good, no breakage, the quality is not to speak of, great 👍 logistics super super fast! Quality is also very good, indeed value	Jingdong prefecture level city in Shandong
Jinfeng Hongyuan Flagship Store	Electricity savings1	The quality is pretty good. The price is also quite affordable.	Jingdong prefecture level city in Shandong
Jinfeng Hongyuan Flagship Store	Electricity savings1	I did not expect the courier so fast to arrive yesterday just ordered, the head is a little smaller than I thought, well, the picture looks bigger. The appearance still looks good, as for the practicality and effectiveness of the use of the only know.	Jingdong prefecture level city in Shandong
Jinfeng Hongyuan Flagship Store	Electricity savings1	I don't know. Looking forward to it.	Jingdong prefecture level city in Shandong
Jinfeng Hongyuan Flagship Store	Electricity savings1	Baby received, very delicate, very nice, like. Just don't know if it can really save electricity. Baby received, very delicate, very good-looking, like. Just don't know if it can really save electricity.	Jingdong prefecture level city in Shandong
Jinfeng Hongyuan Flagship Store	Electricity savings1	I'll use it for a month and then comment on the quality of the item	Jingdong prefecture level city in Shandong
Jinfeng Hongyuan Flagship Store	Electricity savings1	It feels ok, use it for a while and see how it works before adding more comments.	Jingdong prefecture level city in Shandong
Jinfeng Hongyuan Flagship Store	Electricity savings1	In use, see results in a month.	Jingdong prefecture level city in Shandong
Jinfeng Hongyuan Flagship Store	Electricity savings1	Jingdong's logistics and quality are very good, the service attitude is very good, the logistics is very fast	Jingdong prefecture level city in Shandong
Jinfeng Hongyuan Flagship Store	Electricity savings1	Intelligent power saver received, well-packed logistics is also quite fast, easy to use, looking forward to good results!	Jingdong prefecture level city in Shandong
Jinfeng Hongyuan Flagship Store	Electricity savings1	The quality is unknown at this point, I won't know until I use it.	Jingdong prefecture level city in Shandong
Jinfeng Hongyuan Flagship Store	Electricity savings1	It's a good product. It really saves power and doesn't take up much space.	Jingdong prefecture level city in Shandong
Jinfeng Hongyuan Flagship Store	Electricity savings1	Goods have been received, packaging is very good, good to see the effect of future use in the follow-up.	Jingdong prefecture level city in Shandong
Jinfeng Hongyuan Flagship Store	Electricity savings1	The product is in perfect shape and the light weight is worth it!	Jingdong prefecture level city in Shandong
Jinfeng Hongyuan Flagship Store	Electricity savings1	Just got home and plugged it in. It's good.	Jingdong prefecture level city in Shandong
Jinfeng Hongyuan Flagship Store	Electricity savings1	The logistics are quick, just to see the results.	Jingdong prefecture level city in Shandong
Jinfeng Hongyuan Flagship Store	Electricity savings1	Short time can not feel here to leave a message, to be followed up later!	Jingdong prefecture level city in Shandong
Jinfeng Hongyuan Flagship Store	Electricity savings1	This user thinks the product is good	Jingdong prefecture level city in Shandong
Jinfeng Hongyuan Flagship Store	Electricity savings1	Received the baby, to be used to see the effect of it!	Jingdong prefecture level city in Shandong
Jinfeng Hongyuan Flagship Store	Electricity savings1	You have to wait a month or two to see how it works.	Jingdong prefecture level city in Shandong

Jinfeng Hongyuan Flagship Store	Electricity savings1	Not bad, good, like it, don't know if it's good, try it first.	Jingdong prefecture level city in Shandong
Jinfeng Hongyuan Flagship Store	Electricity savings1	The goods are good value for money and the logistics is very fast and good service!	Jingdong prefecture level city in Shandong
Jinfeng Hongyuan Flagship Store	Electricity savings1	I don't know how well it works, we'll see in a month	Jingdong prefecture level city in Shandong
Jinfeng Hongyuan Flagship Store	Electricity savings1	The product is fine, but r saving power or not I won't know until I use it for two months. The display and lights take electricity too?	Jingdong prefecture level city in Shandong
Jinfeng Hongyuan Flagship Store	Electricity savings1	I've used it. I don't know how it works yet.	Jingdong prefecture level city in Shandong
Jinfeng Hongyuan Flagship Store	Electricity savings1	I can't say until I compare them.	Jingdong prefecture level city in Shandong
Jinfeng Hongyuan Flagship Store	Electricity savings1	It's ok in the process of using 。。。。	Jingdong prefecture level city in Shandong
Jinfeng Hongyuan Flagship Store	Electricity savings1	Jinfeng Hongyuan Intelligent Power Saver Household Electricity Meter Electricity Saving Appliance High Power Enhanced Power Saving King Energy Saving Treasure Butler Control Non Stealing Electricity Reversing Not Going Interfering Slow Turner Artifact King Gold Yellow. Haven't tested it accurately yet, really need to wait a few months to test it before I have a say.	Jingdong prefecture level city in Shandong
Jinfeng Hongyuan Flagship Store	Electricity savings1	Don't know how effective it is yet? I hope it works well and I'll come back next time	Jingdong prefecture level city in Shandong
Jinfeng Hongyuan Flagship Store	Electricity savings1	Saving device has been received, material workmanship are very good ah! Have not yet begun to use. First observe the daily electricity consumption and cost of the home meter, and then plug in the power saver to use, so as to compare the use of the effect is good!	Jingdong prefecture level city in Shandong
Jinfeng Hongyuan Flagship Store	Electricity savings1	Merchants shipping extremely fast, Jingdong logistics safe and fast, I believe that the product can play a safe use of electricity, the role of scientific power saving, first praise, the effect of how to be used for a period of time and then the pursuit of bubble!	Jingdong prefecture level city in Shandong
Jinfeng Hongyuan Flagship Store	Electricity savings1	I just bought it. I don't know yet.	Jingdong prefecture level city in Shandong
Jinfeng Hongyuan Flagship Store	Electricity savings1	It is genuine, good quality, and it is very reassuring to use. The logistics is very fast, and the business service attitude is on point.	Jingdong prefecture level city in Shandong
Jinfeng Hongyuan Flagship Store	Electricity savings1	The quality is fine, I don't know how effective it is.	Jingdong prefecture level city in Shandong
Jinfeng Hongyuan Flagship Store	Electricity savings1	The stuff is back and I expect it will save the electricity	Jingdong prefecture level city in Shandong
Jinfeng Hongyuan Flagship Store	Electricity savings1	I'll give it 4 stars first and see what happens in a couple of months and then follow up	Jingdong prefecture level city in Shandong
Jinfeng Hongyuan Flagship Store	Electricity savings1	It's not bad. It's in the mind.	Jingdong prefecture level city in Shandong
Jinfeng Hongyuan Flagship Store	Electricity savings1	Received the goods have not yet started to use do not know whether it is good	Jingdong prefecture level city in Shandong
Jinfeng Hongyuan Flagship Store	Electricity savings1	This user thinks the product is good	Jingdong prefecture level city in Shandong
Jinfeng Hongyuan Flagship Store	Electricity savings1	Hello! The saver was received, thank you! Not yet used, but from the packaging looks very good quality, exquisite workmanship. Logistics is quite fast, if it is really very power saving, I will come back to	Jingdong prefecture level city in Shandong
Jinfeng Hongyuan Flagship Store	Electricity savings1	I've been using it for a few days now, I'll comment again after a month or two of use	Jingdong prefecture level city in Shandong
Jinfeng Hongyuan Flagship Store	Electricity savings1	I don't know. Bought it for my father at a good price.	Jingdong prefecture level city in Shandong
Jinfeng Hongyuan Flagship Store	Electricity savings1	Plugged it in the same day I bought it and the voltage showed 223, 225 looking forward to the results	Jingdong prefecture level city in Shandong
Jinfeng Hongyuan Flagship Store	Electricity savings1	Still in use, the biggest role is to stabilize the current, reduce losses, product quality on the look is very good, with this feeling quite good, see colleagues are using, power saving effect should be good	Jingdong prefecture level city in Shandong
Jinfeng Hongyuan Flagship Store	Electricity savings1	How to receive goods but no invoice and warranty more than it ...... quality is the premise of reputation!	Jingdong prefecture level city in Shandong
Jinfeng Hongyuan Flagship Store	Electricity savings1	Received but not used Continued evaluation	Jingdong prefecture level city in Shandong
Jinfeng Hongyuan Flagship Store	Electricity savings1	I just received the product, I don't know if it works well yet	Jingdong prefecture level city in Shandong
Jinfeng Hongyuan Flagship Store	Electricity savings1	Received it and used it, not sure it really saves electricity use it and then evaluate it, first give a 5 star review!!!!	Jingdong prefecture level city in Shandong
Jinfeng Hongyuan Flagship Store	Electricity savings1	It's good to use, it's very good to use, and it smells OK too	Jingdong prefecture level city in Shandong
Jinfeng Hongyuan Flagship Store	Electricity savings1	Packaging is very good, feel the product is not bad, how much power saving with the only know	Jingdong prefecture level city in Shandong
Jinfeng Hongyuan Flagship Store	Electricity savings1	Additional comments to be made after use	Jingdong prefecture level city in Shandong
Jinfeng Hongyuan Flagship Store	Electricity savings1	The product has been installed, try a few months and then comment!	Jingdong prefecture level city in Shandong
Jinfeng Hongyuan Flagship Store	Electricity savings1	I've bought it, let's use it for now, I'll comment after a month	Jingdong prefecture level city in Shandong
Jinfeng Hongyuan Flagship Store	Electricity savings1	Try it out and then follow up	Jingdong prefecture level city in Shandong
Jinfeng Hongyuan Flagship Store	Electricity savings1	It's good in theory, it doesn't feel good in practice.	Jingdong prefecture level city in Shandong
Jinfeng Hongyuan Flagship Store	Electricity savings1	I haven't tried it yet. We'll see.	Jingdong prefecture level city in Shandong
Jinfeng Hongyuan Flagship Store	Electricity savings1	Great item, second purchase!	Jingdong prefecture level city in Shandong
Jinfeng Hongyuan Flagship Store	Electricity savings1	Goods received now use it, if it does save power, plus reevaluate.	Jingdong prefecture level city in Shandong
Jinfeng Hongyuan Flagship Store	Electricity savings1	The stuff is light and small, the stuff is good, the courier service is good	Jingdong prefecture level city in Shandong
Jinfeng Hongyuan Flagship Store	Electricity savings1	I haven't used it yet. I'm looking forward to it.	Jingdong prefecture level city in Shandong
Jinfeng Hongyuan Flagship Store	Electricity savings1	Express received, in use do not know if there is no power saving	Jingdong prefecture level city in Shandong
Jinfeng Hongyuan Flagship Store	Electricity savings1	The quality of the product is quite good! The power saving mode is still getting used to it, can't say yet, how much power it saves.	Jingdong prefecture level city in Shandong
Jinfeng Hongyuan Flagship Store	Electricity savings1	The goods have been received, just started to use, now I do not know how the quality, after a period of time to say.	Jingdong prefecture level city in Shandong
Jinfeng Hongyuan Flagship Store	Electricity savings1	I received the stuff, used it for a few days to evaluate, the stuff is very good and good, worth buying.	Jingdong prefecture level city in Shandong
Jinfeng Hongyuan Flagship Store	Electricity savings1	I've been using it for a while, but I don't know if it's good or if it saves electricity! What the heck, let's give it a good review first. And for the record, I'm not helping him**.	Jingdong prefecture level city in Shandong
Jinfeng Hongyuan Flagship Store	Electricity savings1	Haven't used it yet, looks good, try it for 60 days, might be good, great review!	Jingdong prefecture level city in Shandong
Jinfeng Hongyuan Flagship Store	Electricity savings1	Baby received, logistics is very fast. Ordered last night, received this morning. Received the goods immediately plugged in the power supply, save power to the first of next month to know.	Jingdong prefecture level city in Shandong
Jinfeng Hongyuan Flagship Store	Electricity savings1	First, I'll give you a good review and use it.	Jingdong prefecture level city in Shandong
Jinfeng Hongyuan Flagship Store	Electricity savings1	Just arrived to follow up with a genuine review	Jingdong prefecture level city in Shandong

Jinfeng Hongyuan Flagship Store	Electricity savings1	Not bad pretty fast, good quality.	Jingdong prefecture level city in Shandong
Jinfeng Hongyuan Flagship Store	Electricity savings1	Recommended, use it and hopefully it will save some electricity	Jingdong prefecture level city in Shandong
Jinfeng Hongyuan Flagship Store	Electricity savings1	The stuff looks ok, I don't know the effect if it.	Jingdong prefecture level city in Shandong
Jinfeng Hongyuan Flagship Store	Electricity savings1	The stuff looks OK, the stuff looks OK.	Jingdong prefecture level city in Shandong
Jinfeng Hongyuan Flagship Store	Electricity savings1	The item has been received, after using it in a follow-up review. Satisfied with the purchase.	Jingdong prefecture level city in Shandong
Jinfeng Hongyuan Flagship Store	Electricity savings1	Has been normal plug-in use, at present do not know how to see, use down to see and then evaluation	Jingdong prefecture level city in Shandong
Jinfeng Hongyuan Flagship Store	Electricity savings1	Commodities make noise.	Jingdong prefecture level city in Shandong
Jinfeng Hongyuan Flagship Store	Electricity savings1	After using it for a few days and observing it, the saving is about ten percent	Jingdong prefecture level city in Shandong
Jinfeng Hongyuan Flagship Store	Electricity savings1	The goods received, the quality looks quite OK. First use it, the effect is not good to say.	Jingdong prefecture level city in Shandong
Jinfeng Hongyuan Flagship Store	Electricity savings1	In use, follow up next month	Jingdong prefecture level city in Shandong
Jinfeng Hongyuan Flagship Store	Electricity savings1	I just received the goods and have not yet used it to comment after using it	Jingdong prefecture level city in Shandong
Jinfeng Hongyuan Flagship Store	Electricity savings1	So far I don't know if it really saves electricity yet! Spinning waiting a couple months to find out! Good reviews first!	Jingdong prefecture level city in Shandong
Jinfeng Hongyuan Flagship Store	Electricity savings1	Small and exquisite, looking forward to the effect, to be evaluated 6666666666666	Jingdong prefecture level city in Shandong
Jinfeng Hongyuan Flagship Store	Electricity savings1	I just used it, I don't know if it's effective, I'll comment again after a month	Jingdong prefecture level city in Shandong
Jinfeng Hongyuan Flagship Store	Electricity savings1	I just used it, I don't know if it's good or not, I'll comment later.	Jingdong prefecture level city in Shandong
Jinfeng Hongyuan Flagship Store	Electricity savings1	Baby received, Baobei quite good, use and then come back to comment!	Jingdong prefecture level city in Shandong
Jinfeng Hongyuan Flagship Store	Electricity savings1	Good quality power saving is fake	Jingdong prefecture level city in Shandong
Jinfeng Hongyuan Flagship Store	Electricity savings1	The packaging of the goods is atmospheric, the quality is exquisite, and I look forward to saving electricity.	Jingdong prefecture level city in Shandong
Jinfeng Hongyuan Flagship Store	Electricity savings1	The packaging is great and the quality is good!	Jingdong prefecture level city in Shandong
Jinfeng Hongyuan Flagship Store	Electricity savings1	I don't see any effect.	Jingdong prefecture level city in Shandong
Jinfeng Hongyuan Flagship Store	Electricity savings1	Already used, but did not see obvious results	Jingdong prefecture level city in Shandong
Jinfeng Hongyuan Flagship Store	Electricity savings1	It's good. I'm just using it. I don't know what it's like.	Jingdong prefecture level city in Shandong
Jinfeng Hongyuan Flagship Store	Electricity savings1	I just used it. I'll comment later.	Jingdong prefecture level city in Shandong
Jinfeng Hongyuan Flagship Store	Electricity savings1	I installed it when it arrived, try it for a month and then come back for a follow-up review	Jingdong prefecture level city in Shandong
Jinfeng Hongyuan Flagship Store	Electricity savings1	Goods arrived a few days fastidious does not call to pick up the goods, the quality is average	Jingdong prefecture level city in Shandong
Jinfeng Hongyuan Flagship Store	Electricity savings1	This user thinks the product is good	Jingdong prefecture level city in Shandong
Jinfeng Hongyuan Flagship Store	Electricity savings1	Roger that.	Jingdong prefecture level city in Shandong
Jinfeng Hongyuan Flagship Store	Electricity savings1	Second purchase was ok.	Jingdong prefecture level city in Shandong
Jinfeng Hongyuan Flagship Store	Electricity savings1	The merchant is very reputable and the product is good value for money. Logistics is fast!	Jingdong prefecture level city in Shandong
Jinfeng Hongyuan Flagship Store	Electricity savings1	The quality of the baby is very good, I have always trusted Kyodo	Jingdong prefecture level city in Shandong
Jinfeng Hongyuan Flagship Store	Electricity savings1	We'll see how it goes when we get it.	Jingdong prefecture level city in Shandong
Jinfeng Hongyuan Flagship Store	Electricity savings1	I just used it. I don't know if it works.	Jingdong prefecture level city in Shandong
Jinfeng Hongyuan Flagship Store	Electricity savings1	Good quality, used for a month to save about 20%, power saving effect is good	Jingdong prefecture level city in Shandong
Jinfeng Hongyuan Flagship Store	Electricity savings1	I'll give you a good review before I use it.	Jingdong prefecture level city in Shandong
Jinfeng Hongyuan Flagship Store	Electricity savings1	Received, satisfied with the follow-up evaluation	Jingdong prefecture level city in Shandong
Jinfeng Hongyuan Flagship Store	Electricity savings1	Immediate results, saving electricity and money	Jingdong prefecture level city in Shandong
Jinfeng Hongyuan Flagship Store	Electricity savings1	Measured to save a little	Jingdong prefecture level city in Shandong
Jinfeng Hongyuan Flagship Store	Electricity savings1	This user thinks the product is good	Jingdong prefecture level city in Shandong
Jinfeng Hongyuan Flagship Store	Electricity savings1	This user thinks the product is good	Jingdong prefecture level city in Shandong
Jinfeng Hongyuan Flagship Store	Electricity savings1	Product quality and logistics are very good, worth buying	Jingdong prefecture level city in Shandong
Jinfeng Hongyuan Flagship Store	Electricity savings1	Appearance looks ok! Express delivery was fast! Service is still relatively enthusiastic.	Jingdong prefecture level city in Shandong
Jinfeng Hongyuan Flagship Store	Electricity savings1	It doesn't feel like it's working.	Jingdong prefecture level city in Shandong
Jinfeng Hongyuan Flagship Store	Electricity savings1	Well made and looks good. Used it with other appliances and felt as if it had some effect.	Jingdong prefecture level city in Shandong
Jinfeng Hongyuan Flagship Store	Electricity savings1	Haven't used it yet, will follow up later	Jingdong prefecture level city in Shandong
Jinfeng Hongyuan Flagship Store	Electricity savings1	Things received, used a few days the plug is a little hot unplugged did not work	Jingdong prefecture level city in Shandong
Jinfeng Hongyuan Flagship Store	Electricity savings1	Look at the meter again in a month.	Jingdong prefecture level city in Shandong
Jinfeng Hongyuan Flagship Store	Electricity savings1	This one didn't work at all.	Jingdong prefecture level city in Shandong
Jinfeng Hongyuan Flagship Store	Electricity savings1	It's small. I've seen friends with much bigger ones.	Jingdong prefecture level city in Shandong
Jinfeng Hongyuan Flagship Store	Electricity savings1	The item was received and the quality is good, it will be nice to see how the power saving rate is down the road, the item is great.	Jingdong prefecture level city in Shandong
Jinfeng Hongyuan Flagship Store	Electricity savings1	Cheap and affordable quality is quite good, logistics is also very fast, praise! , baby good 😊, cost-effective very high, big brand trustworthy 👍	Jingdong prefecture level city in Shandong
Jinfeng Hongyuan Flagship Store	Electricity savings1	Used it for a while it really saves electricity ⚡ thanks 🈶 such a good product, compact and practical, every family should buy one ✨	Jingdong prefecture level city in Shandong

Jinfeng Hongyuan Flagship Store	Electricity savings1	Very satisfied with the purchase Next time I will come back	Jingdong prefecture level city in Shandong
Jinfeng Hongyuan Flagship Store	Electricity savings1	Logistics is fast. Good quality, used one before and it does save power	Jingdong prefecture level city in Shandong
Jinfeng Hongyuan Flagship Store	Electricity savings1	I don't know if it works.	Jingdong prefecture level city in Shandong
Jinfeng Hongyuan Flagship Store	Electricity savings1	Good goods, very solid, good.	Jingdong prefecture level city in Shandong
Jinfeng Hongyuan Flagship Store	Electricity savings1	This user thinks the product is good	Jingdong prefecture level city in Shandong
Jinfeng Hongyuan Flagship Store	Electricity savings1	I've been using it for half a month, my electricity bill is much lower, and the quality is very good, so don't worry about buying it!	Jingdong prefecture level city in Shandong
Jinfeng Hongyuan Flagship Store	Electricity savings1	I received the goods today and used it right away, looking forward to the effect	Jingdong prefecture level city in Shandong
Jinfeng Hongyuan Flagship Store	Electricity savings1	The merchandise is great. I am going to recommend it to my friends! Will buy again next time.	Jingdong prefecture level city in Shandong
Jinfeng Hongyuan Flagship Store	Electricity savings1	Let's give it a good review first, it's working fine so far, and I'll follow up with a review,...	Jingdong prefecture level city in Shandong
Jinfeng Hongyuan Flagship Store	Electricity savings1	Product quality is very good, looking forward to using the results	Jingdong prefecture level city in Shandong
Jinfeng Hongyuan Flagship Store	Electricity savings1	I just can't detect it. - Save it.	Jingdong prefecture level city in Shandong
Jinfeng Hongyuan Flagship Store	Electricity savings1	It arrived and is being used, so we'll see how it works!	Jingdong prefecture level city in Shandong
Jinfeng Hongyuan Flagship Store	Electricity savings1	Received yesterday thanks! This energy saver is available . Safe! But in the end how much energy can be saved must be used for a period of time to know, if the real energy saving next time to buy again.	Jingdong prefecture level city in Shandong
Jinfeng Hongyuan Flagship Store	Electricity savings1	Just used it, don't know if it saves power	Jingdong prefecture level city in Shandong
Jinfeng Hongyuan Flagship Store	Electricity savings1	I'll rate it first, is it a tease wait two months and see? It's been four days and it's not noticeable!	Jingdong prefecture level city in Shandong
Jinfeng Hongyuan Flagship Store	Electricity savings1	Workmanship is quite good, just started to use, the effect of the follow-up evaluation.	Jingdong prefecture level city in Shandong
Jinfeng Hongyuan Flagship Store	Electricity savings1	Received the goods used for a few days before evaluation, the effect is quite good	Jingdong prefecture level city in Shandong
Jinfeng Hongyuan Flagship Store	Electricity savings1	Try it for a month and then evaluate	Jingdong prefecture level city in Shandong
Jinfeng Hongyuan Flagship Store	Electricity savings1	Just received the goods, the packaging is very good, the appearance of the economizer feels good, I do not know how the effect of power saving, with a month or two and then follow up!	Jingdong prefecture level city in Shandong
Jinfeng Hongyuan Flagship Store	Electricity savings1	It's quite small and delicate, I don't know if it's useful yet.	Jingdong prefecture level city in Shandong
Jinfeng Hongyuan Flagship Store	Electricity savings1	Just received it, use it for a few days before commenting	Jingdong prefecture level city in Shandong
Jinfeng Hongyuan Flagship Store	Electricity savings1	Does this thing work! When it arrived, the button for the safety was loose. I'm a little afraid of fire!	Jingdong prefecture level city in Shandong
Jinfeng Hongyuan Flagship Store	Electricity savings1	I received it. I don't know how it works.	Jingdong prefecture level city in Shandong
Jinfeng Hongyuan Flagship Store	Electricity savings1	This user thinks the product is good	Jingdong prefecture level city in Shandong
Jinfeng Hongyuan Flagship Store	Electricity savings1	Customer service was great! Small i problem was taken care of in a timely manner! Effectiveness to be tested by time! No rush!!!	Jingdong prefecture level city in Shandong
Jinfeng Hongyuan Flagship Store	Electricity savings1	I put it on right after I received it and it looks compact. I believe the high technology should save electricity.	Jingdong prefecture level city in Shandong
Jinfeng Hongyuan Flagship Store	Electricity savings1	Logistics courier very quickly, the first day under the order the next day to receive, off pieces whether power saving is the most important thing, things are exquisite workmanship, a month later in the follow-up evaluation whether power saving	Jingdong prefecture level city in Shandong
Jinfeng Hongyuan Flagship Store	Electricity savings1	The delivery is pretty fast, I'm using it, have to have it for a while to see results, looking forward to it.	Jingdong prefecture level city in Shandong
Jinfeng Hongyuan Flagship Store	Electricity savings1	See pretty good just started to use a month later to give you a real evaluation, should be useful.	Jingdong prefecture level city in Shandong
Jinfeng Hongyuan Flagship Store	Electricity savings1	And then comment.	Jingdong prefecture level city in Shandong
Jinfeng Hongyuan Flagship Store	Electricity savings1	Received the goods, wait for the test to evaluate	Jingdong prefecture level city in Shandong
Jinfeng Hongyuan Flagship Store	Electricity savings1	This user thinks the product is good	Jingdong prefecture level city in Shandong
Jinfeng Hongyuan Flagship Store	Electricity savings1	I'm already using it, I just don't know how to judge the power savings. I won't know until I use it for a season.	Jingdong prefecture level city in Shandong
Jinfeng Hongyuan Flagship Store	Electricity savings1	Works. Says it works.	Jingdong prefecture level city in Shandong
Jinfeng Hongyuan Flagship Store	Electricity savings1	Very good. Satisfied.	Jingdong prefecture level city in Shandong
Jinfeng Hongyuan Flagship Store	Electricity savings1	This user thinks the product is good	Jingdong prefecture level city in Shandong
Jinfeng Hongyuan Flagship Store	Electricity savings1	It's working. Obviously, it's less expensive.	Jingdong prefecture level city in Shandong
Jinfeng Hongyuan Flagship Store	Electricity savings1	The second one is up, the first one still works! It's reactive power compensation! This is well understood if you want a professional!	Jingdong prefecture level city in Shandong
Jinfeng Hongyuan Flagship Store	Electricity savings1	It feels as if there are some nodes, but they need to be tested and observed over time, thanks.	Jingdong prefecture level city in Shandong
Jinfeng Hongyuan Flagship Store	Electricity savings1	I've received the goods, the energy saving effect will be evaluated after using it for a period of time, and all the services are very good, praise!	Jingdong prefecture level city in Shandong
Jinfeng Hongyuan Flagship Store	Electricity savings1	I don't know how well it works yet, I bought it and plugged it in, so we'll see if it saves some power or not!	Jingdong prefecture level city in Shandong
Jinfeng Hongyuan Flagship Store	Electricity savings1	Baby a bunch of knife quality is very good, previously bought a quality is not very good. It really works and has results.	Jingdong prefecture level city in Shandong
Jinfeng Hongyuan Flagship Store	Electricity savings1	Things have arrived, it looks good, just started to use, as to whether or not there is a business said the power-saving effect, but also to observe the effect, now can not evaluate	Jingdong prefecture level city in Shandong
Jinfeng Hongyuan Flagship Store	Electricity savings1	I haven't used it yet. I'll comment afterward.	Jingdong prefecture level city in Shandong
Jinfeng Hongyuan Flagship Store	Electricity savings1	I don't know if it's any good.	Jingdong prefecture level city in Shandong
Jinfeng Hongyuan Flagship Store	Electricity savings1	I don't know how well it works.	Jingdong prefecture level city in Shandong
Jinfeng Hongyuan Flagship Store	Electricity savings1	The baby has been received! Uh-huh! Good, quite exquisite, small and exquisite I thought it was big. Dare to tightly plugged in the electricity, just wait for the effect later!	Jingdong prefecture level city in Shandong
Jinfeng Hongyuan Flagship Store	Electricity savings1	The packaging is sturdy, I tried it right after I received it, so I'm looking forward to the results.	Jingdong prefecture level city in Shandong
Jinfeng Hongyuan Flagship Store	Electricity savings1	Received the item and installed it! Don't know if it works well! Will follow up with a review later! I hope it works!	Jingdong prefecture level city in Shandong
Jinfeng Hongyuan Flagship Store	Electricity savings1	Things used on the effect of waiting to use a little to say, but things are very good. Logistics is very fast, business service is very good.	Jingdong prefecture level city in Shandong
Jinfeng Hongyuan Flagship Store	Electricity savings1	This user thinks the product is good	Jingdong prefecture level city in Shandong

Jinfeng Hongyuan Flagship Store	Electricity savings1	I'm really using it. It feels good.	Jingdong prefecture level city in Shandong
Jinfeng Hongyuan Flagship Store	Electricity savings1	Good, Jingdong logistics is very fast, intelligent energy-saving appliances	Jingdong prefecture level city in Shandong
Jinfeng Hongyuan Flagship Store	Electricity savings1	It's not bad. It really saves energy.	Jingdong prefecture level city in Shandong
Jinfeng Hongyuan Flagship Store	Electricity savings1	The logistics is fast, praise	Jingdong prefecture level city in Shandong
Jinfeng Hongyuan Flagship Store	Electricity savings1	This user thinks the product is good	Jingdong prefecture level city in Shandong
Jinfeng Hongyuan Flagship Store	Electricity savings1	The first time I placed two, and today I placed another one, a friend introduced it, said it is particularly power-saving	Jingdong prefecture level city in Shandong
Jinfeng Hongyuan Flagship Store	Electricity savings1	I don't know how effective it is. Let's leave it at home for a while and see.	Jingdong prefecture level city in Shandong
Jinfeng Hongyuan Flagship Store	Electricity savings1	Dear customer service! I would like to ask you how to understand the digital display (increase/decrease jump) on the top of the saver? Thank you for your help.	Jingdong prefecture level city in Shandong
Jinfeng Hongyuan Flagship Store	Electricity savings1	This user thinks the product is good	Jingdong prefecture level city in Shandong
Jinfeng Hongyuan Flagship Store	Electricity savings1	The goods have been received, packaging exquisite, is using, later to see how the effect?	Jingdong prefecture level city in Shandong
Jinfeng Hongyuan Flagship Store	Electricity savings1	The saver received, the boss shipped very quickly, yesterday shot today arrived!	Jingdong prefecture level city in Shandong
Jinfeng Hongyuan Flagship Store	Electricity savings1	Beautiful appearance, well packaged. Recording data to see if I can lower my electric bill a bit.	Jingdong prefecture level city in Shandong
Jinfeng Hongyuan Flagship Store	Electricity savings1	Things received, the packaging is quite good, the effect of the use and then come back to evaluate.	Jingdong prefecture level city in Shandong
Jinfeng Hongyuan Flagship Store	Electricity savings1	Easy to use, just plug it in. But there is a current sound.	Jingdong prefecture level city in Shandong
Jinfeng Hongyuan Flagship Store	Electricity savings1	Fast logistics, easy to use and labor-saving	Jingdong prefecture level city in Shandong
Jinfeng Hongyuan Flagship Store	Electricity savings1	Received, thank you!	Jingdong prefecture level city in Shandong
Jinfeng Hongyuan Flagship Store	Electricity savings1	Second time I've bought it. It's good.	Jingdong prefecture level city in Shandong
Jinfeng Hongyuan Flagship Store	Electricity savings1	I've only been using it for a short time, so I'm looking forward to the results.	Jingdong prefecture level city in Shandong
Jinfeng Hongyuan Flagship Store	Electricity savings1	Good stuff, good stuff, good stuff good stuff.	Jingdong prefecture level city in Shandong
Jinfeng Hongyuan Flagship Store	Electricity savings1	Buy your stuff is not always good I have been using it for almost two months now, it does not save electricity at all the electricity bill is about ten dollars a day this is still called the king of power	Jingdong prefecture level city in Shandong
Jinfeng Hongyuan Flagship Store	Electricity savings1	Express arrived, the quality is very good, worth the purchase.	Jingdong prefecture level city in Shandong
Jinfeng Hongyuan Flagship Store	Electricity savings1	Energy saving has been received, now the voltage is more stable, energy-saving effect to see a few days and then chase flat, the service attitude is very good, the courier is relatively fast, I am satisfied.	Jingdong prefecture level city in Shandong
Jinfeng Hongyuan Flagship Store	Electricity savings1	Stuff installed, numbers are smaller than when installed, hopefully it works	Jingdong prefecture level city in Shandong
Jinfeng Hongyuan Flagship Store	Electricity savings1	Good appearance, fine workmanship, just bought, use up the feeling of power saving effect is good, worth buying!	Jingdong prefecture level city in Shandong
Jinfeng Hongyuan Flagship Store	Electricity savings1	Economical and practical 👍 Always use it	Jingdong prefecture level city in Shandong
Jinfeng Hongyuan Flagship Store	Electricity savings1	Just used it, don't know the effect yet, will come back to follow up after the effect. Hope it works well(▽)	Jingdong prefecture level city in Shandong
Jinfeng Hongyuan Flagship Store	Electricity savings1	The goods have been received, use it to see in the evaluation.	Jingdong prefecture level city in Shandong
Jinfeng Hongyuan Flagship Store	Electricity savings1	Doesn't it save electricity? The boil kettle still goes faster when you press the meter.	Jingdong prefecture level city in Shandong
Jinfeng Hongyuan Flagship Store	Electricity savings1	Already used. Will patronize again in the future	Jingdong prefecture level city in Shandong
Jinfeng Hongyuan Flagship Store	Electricity savings1	Works great, just plug it in, results to be checked, satisfied.	Jingdong prefecture level city in Shandong
Jinfeng Hongyuan Flagship Store	Electricity savings1	Second purchase. Works well 😍😍😍😍	Jingdong prefecture level city in Shandong
Jinfeng Hongyuan Flagship Store	Electricity savings1	Prompt shipment, very satisfied	Jingdong prefecture level city in Shandong
Jinfeng Hongyuan Flagship Store	Electricity savings1	It's been less than a month, let's see how it works, hopefully it's power efficient	Jingdong prefecture level city in Shandong
Jinfeng Hongyuan Flagship Store	Electricity savings1	Use a month in say.	Jingdong prefecture level city in Shandong
Jinfeng Hongyuan Flagship Store	Electricity savings1	I've saved a lot of electricity since I've been using it.	Jingdong prefecture level city in Shandong
Jinfeng Hongyuan Flagship Store	Electricity savings1	It's fast, looks and feels good on the outside, try it out and evaluate.	Jingdong prefecture level city in Shandong
Jinfeng Hongyuan Flagship Store	Electricity savings1	I haven't used it. I just used it. I don't know if it's any good.	Jingdong prefecture level city in Shandong
Jinfeng Hongyuan Flagship Store	Electricity savings1	Quality can be, power saving now can not see just use, after a while in the look at the power saving effect	Jingdong prefecture level city in Shandong
Jinfeng Hongyuan Flagship Store	Electricity savings1	The logistics is very fast, and it seems to have some effect	Jingdong prefecture level city in Shandong
Jinfeng Hongyuan Flagship Store	Electricity savings1	Good, but the effects remain to be seen	Jingdong prefecture level city in Shandong
Jinfeng Hongyuan Flagship Store	Electricity savings1	Baby is basically the same as the description, shipping quickly, worth a buy Good price, logistics is strong, worth repurchasing!	Jingdong prefecture level city in Shandong
Jinfeng Hongyuan Flagship Store	Electricity savings1	I bought it for my dad, I don't know much about it, if he thinks it's good then it must be good 👍	Jingdong prefecture level city in Shandong
Jinfeng Hongyuan Flagship Store	Electricity savings1	Goods received. It's working fine. Display voltage. After a period of time to see the effect in the addition to the level of theory	Jingdong prefecture level city in Shandong
Jinfeng Hongyuan Flagship Store	Electricity savings1	The stuff is pretty good, I just don't know how well it saves power.	Jingdong prefecture level city in Shandong
Jinfeng Hongyuan Flagship Store	Electricity savings1	Logistics is a little too fast 👍	Jingdong prefecture level city in Shandong
Jinfeng Hongyuan Flagship Store	Electricity savings1	Received the goods used for a period of time before coming, good, beautiful appearance, really stable flow	Jingdong prefecture level city in Shandong
Jinfeng Hongyuan Flagship Store	Electricity savings1	Things received, look at the packaging is very good, not yet, to the power saving in the follow-up evaluation	Jingdong prefecture level city in Shandong
Jinfeng Hongyuan Flagship Store	Electricity savings1	Baby is very good! Use a few days before to evaluate, I personally test really effective.	Jingdong prefecture level city in Shandong
Jinfeng Hongyuan Flagship Store	Electricity savings1	I received the goods are in use, I do not know how effective? Can it save energy? Let's see.	Jingdong prefecture level city in Shandong
Jinfeng Hongyuan Flagship Store	Electricity savings1	It's really good and has some power saving effect.	Jingdong prefecture level city in Shandong
Jinfeng Hongyuan Flagship Store	Electricity savings1	It's good quality, well made, and I hope it'll save on electricity over time.	Jingdong prefecture level city in Shandong

Jinfeng Hongyuan Flagship Store	Electricity savings1	I just used it, I don't know how it works yet, but I'll give it a good review first.	Jingdong prefecture level city in Shandong
Jinfeng Hongyuan Flagship Store	Electricity savings1	Second purchase, the delivery guy was fast and called ahead to deliver.	Jingdong prefecture level city in Shandong
Jinfeng Hongyuan Flagship Store	Electricity savings1	Goods have been received, but have not yet used, used to see how the effect, in the evaluation of fertilizer	Jingdong prefecture level city in Shandong
Jinfeng Hongyuan Flagship Store	Electricity savings1	The delivery is very fast. It's the same in kind. It's working.	Jingdong prefecture level city in Shandong
Jinfeng Hongyuan Flagship Store	Electricity savings1	Fast logistics, good quality, more satisfied	Jingdong prefecture level city in Shandong
Jinfeng Hongyuan Flagship Store	Electricity savings1	I've been here for almost a month now and there is a very noticeable difference than it is worth buying.	Jingdong prefecture level city in Shandong
Jinfeng Hongyuan Flagship Store	Electricity savings1	Fast shipping, received the next day, plug and play, but do not know how effective to not break down!	Jingdong prefecture level city in Shandong
Jinfeng Hongyuan Flagship Store	Electricity savings1	The logistics are great I've been using it for a while before commenting on it This saves electricity	Jingdong prefecture level city in Shandong
Jinfeng Hongyuan Flagship Store	Electricity savings1	Wait to use it for a while to know if it's energy efficient	Jingdong prefecture level city in Shandong
Jinfeng Hongyuan Flagship Store	Electricity savings1	Just received it, haven't used it yet don't know if it's any good.	Jingdong prefecture level city in Shandong
Jinfeng Hongyuan Flagship Store	Electricity savings1	It really doesn't work, and the electric bill is still the same amount every month.	Jingdong prefecture level city in Shandong
Jinfeng Hongyuan Flagship Store	Electricity savings1	It's well packaged and looks fine	Jingdong prefecture level city in Shandong
Jinfeng Hongyuan Flagship Store	Electricity savings1	Very good. Will patronize again if needed.	Jingdong prefecture level city in Shandong
Jinfeng Hongyuan Flagship Store	Electricity savings1	I have received the product and installed it, try it for a while and see how it works.	Jingdong prefecture level city in Shandong
Jinfeng Hongyuan Flagship Store	Electricity savings1	I've been using it for a month now...I don't feel like there's any noticeable power savings...last month it was $130 this month it's $123...let's see if I can use it for another month	Jingdong prefecture level city in Shandong
Jinfeng Hongyuan Flagship Store	Electricity savings1	Just received it, yet to try it and see if it works.	Jingdong prefecture level city in Shandong
Jinfeng Hongyuan Flagship Store	Electricity savings1	It's easy to use and has a power saving credit!	Jingdong prefecture level city in Shandong
Jinfeng Hongyuan Flagship Store	Electricity savings1	It's very good. I've already bought two of them.	Jingdong prefecture level city in Shandong
Jinfeng Hongyuan Flagship Store	Electricity savings1	It's very well made, packaged meticulously, and I hope it works!	Jingdong prefecture level city in Shandong
Jinfeng Hongyuan Flagship Store	Electricity savings1	Goods have been received have not yet used it, used to comment again	Jingdong prefecture level city in Shandong
Jinfeng Hongyuan Flagship Store	Electricity savings1	Looking forward to good results	Jingdong prefecture level city in Shandong
Jinfeng Hongyuan Flagship Store	Electricity savings1	Not sure if it saves power or not. Still trying it out.	Jingdong prefecture level city in Shandong
Jinfeng Hongyuan Flagship Store	Electricity savings1	It also really saves on electricity! Superb!	Jingdong prefecture level city in Shandong
Jinfeng Hongyuan Flagship Store	Electricity savings1	Didn't work at all, want to return it	Jingdong prefecture level city in Shandong
Jinfeng Hongyuan Flagship Store	Electricity savings1	Uh-huh! It's working, I've been using it for a month and it saves 20% of electricity compared to before.	Jingdong prefecture level city in Shandong
Jinfeng Hongyuan Flagship Store	Electricity savings1	Should be a little bit of a power saver, I wonder how long it will take to save back the money I spent on this one	Jingdong prefecture level city in Shandong
Jinfeng Hongyuan Flagship Store	Electricity savings1	Exquisite appearance and stable voltage	Jingdong prefecture level city in Shandong
Jinfeng Hongyuan Flagship Store	Electricity savings1	After using it for a few days it feels like it saves electricity.	Jingdong prefecture level city in Shandong
Jinfeng Hongyuan Flagship Store	Electricity savings1	Second purchase and its pending good results.	Jingdong prefecture level city in Shandong
Jinfeng Hongyuan Flagship Store	Electricity savings1	The appearance of the workmanship is exquisite and meticulous, beautiful appearance, just to see if there is no effect!	Jingdong prefecture level city in Shandong
Jinfeng Hongyuan Flagship Store	Electricity savings1	Let's see what happens in a month.	Jingdong prefecture level city in Shandong
Jinfeng Hongyuan Flagship Store	Electricity savings1	Trial, now short time not yet obvious effect	Jingdong prefecture level city in Shandong
Jinfeng Hongyuan Flagship Store	Electricity savings1	I'm using it now, don't know how well it works, it looks great!	Jingdong prefecture level city in Shandong
Jinfeng Hongyuan Flagship Store	Electricity savings1	There is no role! Now it's true that I don't know!	Jingdong prefecture level city in Shandong
Jinfeng Hongyuan Flagship Store	Electricity savings1	Purchased for the second time, power saving not sure if it's ok? Let's talk about it after using it	Jingdong prefecture level city in Shandong
Jinfeng Hongyuan Flagship Store	Electricity savings1	I just received it, I have to try it for a month to know how it works.	Jingdong prefecture level city in Shandong
Jinfeng Hongyuan Flagship Store	Electricity savings1	I just used it for an hour, and the meter's still spinning so fast it's not much use.	Jingdong prefecture level city in Shandong
Jinfeng Hongyuan Flagship Store	Electricity savings1	Not bad, logistics is fast	Jingdong prefecture level city in Shandong
Jinfeng Hongyuan Flagship Store	Electricity savings1	I don't know if it works. I'll find out next month when I pay the bill. It's easy. Just plug it in.	Jingdong prefecture level city in Shandong
Jinfeng Hongyuan Flagship Store	Electricity savings1	Logistics is particularly fast, received the goods are genuine, looking forward to power saving effect	Jingdong prefecture level city in Shandong
Jinfeng Hongyuan Flagship Store	Electricity savings1	First time buying this product like this one from this family use it and then evaluate.	Jingdong prefecture level city in Shandong
Jinfeng Hongyuan Flagship Store	Electricity savings1	Lightning arrived, the product is very good, before all do not know there is such a baby, in a friend's home has seen, and then bought to try, has been used for a period of time before coming to the evaluation, the work is very stable, so far looked very good, will introduce friends to buy!	Jingdong prefecture level city in Shandong
Jinfeng Hongyuan Flagship Store	Electricity savings1	I was a bit skeptical when I heard that I could save money on my electricity bill by installing this, I don't know if the power saving effect is really what it is said to be. But since I bought it, I just have to put it on and try it out. I hope it lives up to its name and saves me the money I spent on the device	Jingdong prefecture level city in Shandong
Jinfeng Hongyuan Flagship Store	Electricity savings1	The quality of the stuff is okay, but I'm not sure if it works, so I'll try using it first.	Jingdong prefecture level city in Shandong
Jinfeng Hongyuan Flagship Store	Electricity savings1	It should work. The current is much more stable.	Jingdong prefecture level city in Shandong
Jinfeng Hongyuan Flagship Store	Electricity savings1	The store has a great service attitude	Jingdong prefecture level city in Shandong
Jinfeng Hongyuan Flagship Store	Electricity savings1	Let's wait until we've used it for a month.	Jingdong prefecture level city in Shandong
Jinfeng Hongyuan Flagship Store	Electricity savings1	I'm using it and I don't know if it's a power saver or not	Jingdong prefecture level city in Shandong
Jinfeng Hongyuan Flagship Store	Electricity savings1	Original and authentic guarantee quality is good now.	Jingdong prefecture level city in Shandong
Jinfeng Hongyuan Flagship Store	Electricity savings1	Great, very happy with it, will buy a couple more next time	Jingdong prefecture level city in Shandong

Jinfeng Hongyuan Flagship Store	Electricity savings1	I just used it, I don't know if it works yet, I'll come back after using it...	Jingdong prefecture level city in Shandong
Jinfeng Hongyuan Flagship Store	Electricity savings1	It's still being tested.	Jingdong prefecture level city in Shandong
Jinfeng Hongyuan Flagship Store	Electricity savings1	I've been using it for a month and it's not working.	Jingdong prefecture level city in Shandong
Jinfeng Hongyuan Flagship Store	Electricity savings1	It's basically nothing!	Jingdong prefecture level city in Shandong
Jinfeng Hongyuan Flagship Store	Electricity savings1	It's not bad, it kind of works, support it.	Jingdong prefecture level city in Shandong
Jinfeng Hongyuan Flagship Store	Electricity savings1	I just received it last night, let's try it first to see how it works, it looks good!	Jingdong prefecture level city in Shandong
Jinfeng Hongyuan Flagship Store	Electricity savings1	The current is very stable, fine workmanship, small and exquisite, very satisfied	Jingdong prefecture level city in Shandong
Jinfeng Hongyuan Flagship Store	Electricity savings1	Not bad, exactly how much electricity can be saved; still have to follow up the evaluation	Jingdong prefecture level city in Shandong
Jinfeng Hongyuan Flagship Store	Electricity savings1	Logistics is also very fast, next time there is an opportunity to introduce friends to buy!	Jingdong prefecture level city in Shandong
Jinfeng Hongyuan Flagship Store	Electricity savings1	Voltage display and actual do not pay	Jingdong prefecture level city in Shandong
Jinfeng Hongyuan Flagship Store	Electricity savings1	Second purchase, still saves a little on the electric bill	Jingdong prefecture level city in Shandong
Jinfeng Hongyuan Flagship Store	Electricity savings1	Order received, currently trying it out, thanks a lot.	Jingdong prefecture level city in Shandong
Jinfeng Hongyuan Flagship Store	Electricity savings1	I haven't used it for a long time yet, I don't know if the effect is good or not, looking forward to it	Jingdong prefecture level city in Shandong
Jinfeng Hongyuan Flagship Store	Electricity savings1	Good, working, I just don't know if it will save electricity... 5 stars for now.	Jingdong prefecture level city in Shandong
Jinfeng Hongyuan Flagship Store	Electricity savings1	Great product, just as the owner said it would be.	Jingdong prefecture level city in Shandong
Jinfeng Hongyuan Flagship Store	Electricity savings1	Shipping was fast, I don't know if it works well or not, I have to use it for a while first.	Jingdong prefecture level city in Shandong
Jinfeng Hongyuan Flagship Store	Electricity savings1	I don't feel that it saves much electricity, but I'll give it a try. It's been more than a month now, and the electricity bill is still a bit high.	Jingdong prefecture level city in Shandong
Jinfeng Hongyuan Flagship Store	Electricity savings1	Got it. Just used it.	Jingdong prefecture level city in Shandong
Jinfeng Hongyuan Flagship Store	Electricity savings1	Use it for a while and then comment.	Jingdong prefecture level city in Shandong
Jinfeng Hongyuan Flagship Store	Electricity savings1	Used it, see how it works with it and then comment. The quality is very good!	Jingdong prefecture level city in Shandong
Jinfeng Hongyuan Flagship Store	Electricity savings1	I'll comment again after a while.	Jingdong prefecture level city in Shandong
Jinfeng Hongyuan Flagship Store	Electricity savings1	It's easy to operate, affordable and works well, love it.	Jingdong prefecture level city in Shandong
Jinfeng Hongyuan Flagship Store	Electricity savings1	It's good. You can try it. It's cheap.	Jingdong prefecture level city in Shandong
Jinfeng Hongyuan Flagship Store	Electricity savings1	It's in use, so we'll see how it works after a while!	Jingdong prefecture level city in Shandong
Jinfeng Hongyuan Flagship Store	Electricity savings1	Service is pretty good, not sure about the quality yet.	Jingdong prefecture level city in Shandong
Jinfeng Hongyuan Flagship Store	Electricity savings1	Just used it and will see the results in a month 😀	Jingdong prefecture level city in Shandong
Jinfeng Hongyuan Flagship Store	Electricity savings1	Saving electricity is expensive and good	Jingdong prefecture level city in Shandong
Jinfeng Hongyuan Flagship Store	Electricity savings1	Pretty good, 5 stars! Will buy again next time seller service is very good！！！！ Good quality! Will recommend to friends!	Jingdong prefecture level city in Shandong
Jinfeng Hongyuan Flagship Store	Electricity savings1	In use, with results naturally have follow-up reviews!	Jingdong prefecture level city in Shandong
Jinfeng Hongyuan Flagship Store	Electricity savings1	I'm using it now, I'll come back with results, it'll work.	Jingdong prefecture level city in Shandong
Jinfeng Hongyuan Flagship Store	Electricity savings1	Good, nice, fast shipping, cheap, worth having!	Jingdong prefecture level city in Shandong
Jinfeng Hongyuan Flagship Store	Electricity savings1	Logistics is fast.	Jingdong prefecture level city in Shandong
Jinfeng Hongyuan Flagship Store	Electricity savings1	I have just started using the product and will be reviewing it later.	Jingdong prefecture level city in Shandong
Jinfeng Hongyuan Flagship Store	Electricity savings1	I'll comment after I use it.	Jingdong prefecture level city in Shandong
Jinfeng Hongyuan Flagship Store	Electricity savings1	I don't know if it's working or not, though I'll try plugging it in.	Jingdong prefecture level city in Shandong
Jinfeng Hongyuan Flagship Store	Electricity savings1	It really saves electricity and contributes to energy savings in disguise	Jingdong prefecture level city in Shandong
Jinfeng Hongyuan Flagship Store	Electricity savings1	It feels good in the experience.	Jingdong prefecture level city in Shandong
Jinfeng Hongyuan Flagship Store	Electricity savings1	The product is better than I thought it would be, the quality looks great, the voltage is stable, and I'm looking forward to some great power savings!	Jingdong prefecture level city in Shandong
Jinfeng Hongyuan Flagship Store	Electricity savings1	I'm using it now looking forward to saving some points,, I'm feeding back	Jingdong prefecture level city in Shandong
Jinfeng Hongyuan Flagship Store	Electricity savings1	Displayed voltage below 200.	Jingdong prefecture level city in Shandong
Jinfeng Hongyuan Flagship Store	Electricity savings1	I haven't seen any great results, but it should last about a hundred or eighty years.	Jingdong prefecture level city in Shandong
Jinfeng Hongyuan Flagship Store	Electricity savings1	I don't know if it works or not, but I keep it plugged in anyway, so I hope it works!	Jingdong prefecture level city in Shandong
Jinfeng Hongyuan Flagship Store	Electricity savings1	Reasonable price, good quality, fast delivery, great service, already a repurchase.	Jingdong prefecture level city in Shandong
Jinfeng Hongyuan Flagship Store	Electricity savings1	Second purchase, looking forward to the results	Jingdong prefecture level city in Shandong
Jinfeng Hongyuan Flagship Store	Electricity savings1	The quality of this product feels like it should work in a use test! Just how many kilowatts of electricity must be wasted per day with the red light and digital display on for long periods of time?	Jingdong prefecture level city in Shandong
Jinfeng Hongyuan Flagship Store	Electricity savings1	It looks good, we'll see if we use it.	Jingdong prefecture level city in Shandong
Jinfeng Hongyuan Flagship Store	Electricity savings1	Great stuff, second purchase!	Jingdong prefecture level city in Shandong
Jinfeng Hongyuan Flagship Store	Electricity savings1	It is indeed very practical and saves electricity at home	Jingdong prefecture level city in Shandong
Jinfeng Hongyuan Flagship Store	Electricity savings1	Goods received, the physical and the same as the picture, logistics is also very fast!	Jingdong prefecture level city in Shandong
Jinfeng Hongyuan Flagship Store	Electricity savings1	I'll use it for a while and see how it goes. I'll buy it again if it's good.	Jingdong prefecture level city in Shandong
Jinfeng Hongyuan Flagship Store	Electricity savings1	In use, voltage monitoring, the effect of how to wait for next month's electricity consumption confirmation	Jingdong prefecture level city in Shandong

Jinfeng Hongyuan Flagship Store	Electricity savings1	It's still pretty good.	Jingdong prefecture level city in Shandong
Jinfeng Hongyuan Flagship Store	Electricity savings1	It's been plugged in for a month and there hasn't been a big change yet, since it usually uses very little electricity	Jingdong prefecture level city in Shandong
Jinfeng Hongyuan Flagship Store	Electricity savings1	Let's wait for a month to see if it works	Jingdong prefecture level city in Shandong
Jinfeng Hongyuan Flagship Store	Electricity savings1	Good, good, good, good, good, good, good, good, good, good, good, good, good, good, good.	Jingdong prefecture level city in Shandong
Jinfeng Hongyuan Flagship Store	Electricity savings1	The product received the trial a few days, feel good, praise!	Jingdong prefecture level city in Shandong
Jinfeng Hongyuan Flagship Store	Electricity savings1	After six or seven hours of use, 250 volts won't save you any power.	Jingdong prefecture level city in Shandong
Jinfeng Hongyuan Flagship Store	Electricity savings1	I haven't used it yet, I wonder if it saves electricity?	Jingdong prefecture level city in Shandong
Jinfeng Hongyuan Flagship Store	Electricity savings1	Baby value for money, love it, next time will come back is genuine, trust the business, baby texture is very good, the price is moderate, unlimited repurchase!	Jingdong prefecture level city in Shandong
Jinfeng Hongyuan Flagship Store	Electricity savings1	Power saving effect is obvious! Raising tropical fish daily power consumption, after using the daily power savings of more than 3 degrees, currently about 8 degrees / day!	Jingdong prefecture level city in Shandong
Jinfeng Hongyuan Flagship Store	Electricity savings1	Just used it, unknown results.	Jingdong prefecture level city in Shandong
Jinfeng Hongyuan Flagship Store	Electricity savings1	The goods received, well, look forward to the effect. Logistics is very fast, thanks to the platform, thanks to Jingdong. I hope it can save electricity.	Jingdong prefecture level city in Shandong
Jinfeng Hongyuan Flagship Store	Electricity savings1	Not sure 有 effect?	Jingdong prefecture level city in Shandong
Jinfeng Hongyuan Flagship Store	Electricity savings1	I'd say it's a 5% savings.	Jingdong prefecture level city in Shandong
Jinfeng Hongyuan Flagship Store	Electricity savings1	I received it and am using it, I don't know how effective it is, I'll evaluate it afterwards	Jingdong prefecture level city in Shandong
Jinfeng Hongyuan Flagship Store	Electricity savings1	Baby received not yet used, first give praise	Jingdong prefecture level city in Shandong
Jinfeng Hongyuan Flagship Store	Electricity savings1	Looks good, I'll evaluate it later	Jingdong prefecture level city in Shandong
Jinfeng Hongyuan Flagship Store	Electricity savings1	The goods have been received in use, the overall feeling is not bad!	Jingdong prefecture level city in Shandong
Jinfeng Hongyuan Flagship Store	Electricity savings1	It's working. This is the second one. This stuff has to be used over a long period of time to be effective. And it only works on inductive loads, i.e. appliances containing electric motors. Use it.	Jingdong prefecture level city in Shandong
Jinfeng Hongyuan Flagship Store	Electricity savings1	Use it for a while and see how it works.	Jingdong prefecture level city in Shandong
Jinfeng Hongyuan Flagship Store	Electricity savings1	Good courier service and the item is very nice and practical.	Jingdong prefecture level city in Shandong
Jinfeng Hongyuan Flagship Store	Electricity savings1	It doesn't do anything and it's a waste of electricity.	Jingdong prefecture level city in Shandong
Jinfeng Hongyuan Flagship Store	Electricity savings1	I haven't used it yet. It should be good.	Jingdong prefecture level city in Shandong
Jinfeng Hongyuan Flagship Store	Electricity savings1	That's good. OK, that's good.	Jingdong prefecture level city in Shandong
Jinfeng Hongyuan Flagship Store	Electricity savings1	It's a rip off, doesn't work, doesn't save electricity, does the job to well, and I've wasted a couple dozen dollars again.	Jingdong prefecture level city in Shandong
Jinfeng Hongyuan Flagship Store	Electricity savings1	Use it first, the effect is not as good as the road, then we will comment again	Jingdong prefecture level city in Shandong
Jinfeng Hongyuan Flagship Store	Electricity savings1	The price is affordable, the logistics is strong, just use four or five days, the effect will be added later!	Jingdong prefecture level city in Shandong
Jinfeng Hongyuan Flagship Store	Electricity savings1	It's easy to use and works fine.	Jingdong prefecture level city in Shandong
Jinfeng Hongyuan Flagship Store	Electricity savings1	It doesn't feel like it's working.	Jingdong prefecture level city in Shandong
Jinfeng Hongyuan Flagship Store	Electricity savings1	I'm using it. We'll see what happens in a couple months.	Jingdong prefecture level city in Shandong
Jinfeng Hongyuan Flagship Store	Electricity savings1	I just got it back to try it out and I'll comment later	Jingdong prefecture level city in Shandong
Jinfeng Hongyuan Flagship Store	Electricity savings1	Less than two months. Can't tell.	Jingdong prefecture level city in Shandong
Jinfeng Hongyuan Flagship Store	Electricity savings1	No power saving effect	Jingdong prefecture level city in Shandong
Jinfeng Hongyuan Flagship Store	Electricity savings1	Express Express Very quickly not only to the power saver can not save electricity, with two months, look at the evaluation put.	Jingdong prefecture level city in Shandong
Jinfeng Hongyuan Flagship Store	Electricity savings1	Genuine products are packaged well!	Jingdong prefecture level city in Shandong
Jinfeng Hongyuan Flagship Store	Electricity savings1	I've been using it for a while, but I don't know if it saves electricity. I hope it works.	Jingdong prefecture level city in Shandong
Jinfeng Hongyuan Flagship Store	Electricity savings1	Good service, fast logistics, the effect is to be used to know	Jingdong prefecture level city in Shandong
Jinfeng Hongyuan Flagship Store	Electricity savings1	Just used it, not sure about the power saving effect	Jingdong prefecture level city in Shandong
Jinfeng Hongyuan Flagship Store	Electricity savings1	The stuff is ok, save power or not, will comment after a month.	Jingdong prefecture level city in Shandong
Jinfeng Hongyuan Flagship Store	Electricity savings1	Used it for a month to save %25, normal is a little over 100, it's great! I want to buy one in.	Jingdong prefecture level city in Shandong
Jinfeng Hongyuan Flagship Store	Electricity savings1	I'm using it and looking forward to the results	Jingdong prefecture level city in Shandong
Jinfeng Hongyuan Flagship Store	Electricity savings1	The quality is very good and the workmanship is exquisite. The use effect is also very good.	Jingdong prefecture level city in Shandong
Jinfeng Hongyuan Flagship Store	Electricity savings1	Merchant service attitude is very good, logistics speed is super fast, online evaluation is very good, the first time to use, a month to know the specific effect, the effect is good, and then additional	Jingdong prefecture level city in Shandong
Jinfeng Hongyuan Flagship Store	Electricity savings1	Works great, solves the problem of high power usage in the house	Jingdong prefecture level city in Shandong
Jinfeng Hongyuan Flagship Store	Electricity savings1	To receive! Very delicate! It's not clear how effective it is, I won't know until I use it!!!!	Jingdong prefecture level city in Shandong
Jinfeng Hongyuan Flagship Store	Electricity savings1	I've just been using it for a while and I don't feel like it's too noticeable.	Jingdong prefecture level city in Shandong
Jinfeng Hongyuan Flagship Store	Electricity savings1	Already used, no visible effect for the time being	Jingdong prefecture level city in Shandong
Jinfeng Hongyuan Flagship Store	Electricity savings1	I can't feel the effect.	Jingdong prefecture level city in Shandong
Jinfeng Hongyuan Flagship Store	Electricity savings1	Use it for a while and see the results	Jingdong prefecture level city in Shandong
Jinfeng Hongyuan Flagship Store	Electricity savings1	The price is more reasonable, the power-saving effect is to be verified, the delivery is fast, and the courier service is good.	Jingdong prefecture level city in Shandong
Jinfeng Hongyuan Flagship Store	Electricity savings1	Quality is very good, consistent with the seller's description, very satisfied, very like, very fast shipping, packaging is very careful and strict, the logistics company has a very good service attitude, fast delivery, very satisfied with a shopping, very satisfied. Recommended to buy, cost-effective, the service attitude of the business is very enthusiastic, providing such high quality goods.	Jingdong prefecture level city in Shandong

Jinfeng Hongyuan Flagship Store	Electricity savings1	I don't know.	Jingdong prefecture level city in Shandong
Jinfeng Hongyuan Flagship Store	Electricity savings1	I didn't get the delivery.	Jingdong prefecture level city in Shandong
Jinfeng Hongyuan Flagship Store	Electricity savings1	It works, it does have power savings, better than expected, will repurchase.	Jingdong prefecture level city in Shandong
Jinfeng Hongyuan Flagship Store	Electricity savings1	That's where the five fuses are.	Jingdong prefecture level city in Shandong
Jinfeng Hongyuan Flagship Store	Electricity savings1	Don't buy it, it doesn't work and you can't return it.	Jingdong prefecture level city in Shandong
Jinfeng Hongyuan Flagship Store	Electricity savings1	I hope it works. Wait a month.	Jingdong prefecture level city in Shandong
Jinfeng Hongyuan Flagship Store	Electricity savings1	Nothing**, buy with caution	Jingdong prefecture level city in Shandong
Jinfeng Hongyuan Flagship Store	Electricity savings1	I'm using it and waiting for the results	Jingdong prefecture level city in Shandong
Jinfeng Hongyuan Flagship Store	Electricity savings1	Saver quality is very good, has always felt that the home power consumption is very large, this is the company's electrician recommended, said he bought this home is really able to save a little electricity, I also came to buy, the effect if it is good, then buy, a floor a...	Jingdong prefecture level city in Shandong
Jinfeng Hongyuan Flagship Store	Electricity savings1	Good product, satisfied with purchase	Jingdong prefecture level city in Shandong
Jinfeng Hongyuan Flagship Store	Electricity savings1	Occupying the time has not yet found the power saving, occupying not evaluation	Jingdong prefecture level city in Shandong
Jinfeng Hongyuan Flagship Store	Electricity savings1	Haven't tested the results yet. To be followed up with comments!	Jingdong prefecture level city in Shandong
Jinfeng Hongyuan Flagship Store	Electricity savings1	After experiencing it for a month, I don't seem to know how to save it, and I don't pay attention to it anyway	Jingdong prefecture level city in Shandong
Jinfeng Hongyuan Flagship Store	Electricity savings1	It's useless. It's a waste of electricity.	Jingdong prefecture level city in Shandong
Jinfeng Hongyuan Flagship Store	Electricity savings1	I don't know if it's a power saver. It's a couple bucks. I hope it works.	Jingdong prefecture level city in Shandong
Jinfeng Hongyuan Flagship Store	Electricity savings1	I don't know what to say about this stuff. I don't know if it works or not, it's kind of a psychological purchase. Dormitory use to see the meter can not be not so good-looking. You have to wait until the time to deduct the utility bill to know.	Jingdong prefecture level city in Shandong
Jinfeng Hongyuan Flagship Store	Electricity savings1	Energy savers don't work / they're all a bunch of hoaxes.	Jingdong prefecture level city in Shandong
Jinfeng Hongyuan Flagship Store	Electricity savings1	Received the goods, used for more than a week, it's okay, sure a little effect	Jingdong prefecture level city in Shandong
Jinfeng Hongyuan Flagship Store	Electricity savings1	Well made and compact! Looking forward to the effect!	Jingdong prefecture level city in Shandong
Jinfeng Hongyuan Flagship Store	Electricity savings1	It has arrived and we'll see how it works.	Jingdong prefecture level city in Shandong
Jinfeng Hongyuan Flagship Store	Electricity savings1	Good. Good. It's worth it.	Jingdong prefecture level city in Shandong
Jinfeng Hongyuan Flagship Store	Electricity savings1	How come there's no indicator light?	Jingdong prefecture level city in Shandong
Jinfeng Hongyuan Flagship Store	Electricity savings1	The machine that uses electricity works quite well. This saves	Jingdong prefecture level city in Shandong
Jinfeng Hongyuan Flagship Store	Electricity savings1	It's good for home use and feels quite energy efficient	Jingdong prefecture level city in Shandong
Jinfeng Hongyuan Flagship Store	Electricity savings1	I regret buying this product, after using it for two days it doesn't work at all, still so many kilowatts per day just like the original.	Jingdong prefecture level city in Shandong
Jinfeng Hongyuan Flagship Store	Electricity savings1	Works very well and has some scientific merit.	Jingdong prefecture level city in Shandong
Jinfeng Hongyuan Flagship Store	Electricity savings1	Logistics is also very fast this good power saving I use two months before commenting!	Jingdong prefecture level city in Shandong
Jinfeng Hongyuan Flagship Store	Electricity savings1	Repeat customer. Very good.	Jingdong prefecture level city in Shandong
Jinfeng Hongyuan Flagship Store	Electricity savings1	It's good stuff. I've been using it for a month now and it's a real power saver.	Jingdong prefecture level city in Shandong
Jinfeng Hongyuan Flagship Store	Electricity savings1	Jingdong platform to buy things, has been very assured, quality assurance. Jingdong Express is as good as ever, complete packaging, delivery, service attitude to go! The family has always wanted to buy an energy-saving and power-saving equipment, selected half a day to choose this one, the value is very good, used for a period of time to follow up, to see whether it can achieve the real purpose of power saving, I	Jingdong prefecture level city in Shandong
Jinfeng Hongyuan Flagship Store	Electricity savings1	Works great, inexpensive and will be back next time.	Jingdong prefecture level city in Shandong
Jinfeng Hongyuan Flagship Store	Electricity savings1	Baby received, looks good, wait for a month to see how it works?	Jingdong prefecture level city in Shandong
Jinfeng Hongyuan Flagship Store	Electricity savings1	This product is not bad quite good well customer service said quite power saving that look at the customer service to see if the accuracy of the look at all aspects of the color is also quite beautiful	Jingdong prefecture level city in Shandong
Jinfeng Hongyuan Flagship Store	Electricity savings1	Just got back and tried it feels pretty good, just don't know how the quality is, give a good comment first	Jingdong prefecture level city in Shandong
Jinfeng Hongyuan Flagship Store	Electricity savings1	Received the product, trying it out, hope it helps!	Jingdong prefecture level city in Shandong
Jinfeng Hongyuan Flagship Store	Electricity savings1	Works great, saves electricity a dollar a day and a night	Jingdong prefecture level city in Shandong
Jinfeng Hongyuan Flagship Store	Electricity savings1	Baby received, look at the workmanship is still very fine, is genuine. I hope that this summer to open the air conditioning electricity bill is no longer high scary!	Jingdong prefecture level city in Shandong
Jinfeng Hongyuan Flagship Store	Electricity savings1	It's here. I don't know how it's going to work, but I'm going to use it.	Jingdong prefecture level city in Shandong
Jinfeng Hongyuan Flagship Store	Electricity savings1	Power saver received, Jingdong logistics first-class, business customer service attitude is good, one by one to introduce the product performance, back on the horse plugged in, it is very simple, there is a voltage indicator, to be a few months to see how the effect of power-saving to come back to a good follow-up, but I believe that Jinfenghong products.	Jingdong prefecture level city in Shandong
Jinfeng Hongyuan Flagship Store	Electricity savings1	Took the third one...the super power-saving kind!	Jingdong prefecture level city in Shandong
Jinfeng Hongyuan Flagship Store	Electricity savings1	First time buyer, customer service is very polite and courteous, conscientious business, support! Baby has received 🎈, super like, high value and practical, super like! ❥❥❥❥❥ Very good quality, very satisfied with a shopping. Customer service has a question and answer, very professional finally received the goods, very, very much like, exquisite and beautiful workmanship, has been praised by many people, really too happy, is already a loyal fan of his family, will continue to buy buy buy buy!	Jingdong prefecture level city in Shandong
Jinfeng Hongyuan Flagship Store	Electricity savings1	That's good. That's good. That's really good.	Jingdong prefecture level city in Shandong
Jinfeng Hongyuan Flagship Store	Electricity savings1	Saver received, good-looking design, voltage at a glance, and the merchant description, save some about 12% or so	Jingdong prefecture level city in Shandong
Jinfeng Hongyuan Flagship Store	Electricity savings1	Intelligent power saver is small and does not take up space, very good, very practical, received and used, packaging is tight, logistics is also very fast, satisfied with a shopping!	Jingdong prefecture level city in Shandong
Jinfeng Hongyuan Flagship Store	Electricity savings1	The stuff is good! Well made, compact, alloy case, shows voltage! Very like! Kudos!	Jingdong prefecture level city in Shandong
Jinfeng Hongyuan Flagship Store	Electricity savings1	I just purchased this power saver from Jingdong, inserted in the socket shows the number 206, a moment and 207 back and forth display, I do not know what it means, my home voltage should be normal, because it is the owner of the community's own power plant to put, first use for a period of time in the positive feedback it, first use in the look at it!	Jingdong prefecture level city in Shandong

Jinfeng Hongyuan Flagship Store	Electricity savings1	Very delicate and compact, there is a convenient intelligent display prompts the voltage, you can know the voltage at a glance, there is also very easy to use, a plug can be used, you can save electricity	Jingdong prefecture level city in Shandong
Jinfeng Hongyuan Flagship Store	Electricity savings1	Logistics is fast, ask customer service said can save electricity, but only use two months to see the effect, use two months in the evaluation.	Jingdong prefecture level city in Shandong
Jinfeng Hongyuan Flagship Store	Electricity savings1	The quality is very good.	Jingdong prefecture level city in Shandong
Jinfeng Hongyuan Flagship Store	Electricity savings1	Smart Saver has been received and will be evaluated after use	Jingdong prefecture level city in Shandong
Jinfeng Hongyuan Flagship Store	Electricity savings1	Home electricity is too waste, a month more than 300 dollars of electricity, and then the family heard that this can save a lot of electricity, let me order to buy a, anyway, will not, buy on the buy, thought it has to be a pretty big box it, delivery to the home to see a very small, plug-and-play, you can really save electricity, it's worth it!	Jingdong prefecture level city in Shandong
Jinfeng Hongyuan Flagship Store	Electricity savings1	Smart Saver received. Unpacking the box is exquisite. The material workmanship is very good. Plugged in and tried the space. Did not open the air conditioning voltage display 206 air conditioning open up the air conditioning display for 194. whether to save electricity to a month later to pay the electricity bill will know. Logistics is also very fast service is also in place.	Jingdong prefecture level city in Shandong
Jinfeng Hongyuan Flagship Store	Electricity savings1	The product feels pretty good and I hope it works, as the store owner said it would. Works!!!	Jingdong prefecture level city in Shandong
Jinfeng Hongyuan Flagship Store	Electricity savings1	Baby received, physical very quickly, just plugged in hope that it can be as everyone said those power saving, with two months after additional comments, first give a five-star praise.	Jingdong prefecture level city in Shandong
Jinfeng Hongyuan Flagship Store	Electricity savings1	Good, very good, very good, you deserve it, whoever uses it knows, the stuff works great, will buy it again in the future.	Jingdong prefecture level city in Shandong
Jinfeng Hongyuan Flagship Store	Electricity savings1	Don't hesitate to buy wow! No cheap feeling, very high-end, especially great! It is genuine, and the same as the entity store, the price is also favorable, decisive turn powder 💗 catch up to do activities when you buy, the price of a penny for a penny ♡, from the outside looking very beautiful 💗, the material workmanship is good 💗, feel that the purchase is worth it, and the logistics is super	Jingdong prefecture level city in Shandong
Jinfeng Hongyuan Flagship Store	Electricity savings1	Logistics is fast! Very good quality! Very beautiful! Next time to patronize!	Jingdong prefecture level city in Shandong
Jinfeng Hongyuan Flagship Store	Electricity savings1	Really like it, completely exceeded expectations, the shipping speed is very fast, the packaging is very careful and strict, the delivery speed is very fast, very satisfied with a shopping Baby received, and the description is the same, love it!	Jingdong prefecture level city in Shandong
Jinfeng Hongyuan Flagship Store	Electricity savings1	Very good, if you need to order!	Jingdong prefecture level city in Shandong
Jinfeng Hongyuan Flagship Store	Electricity savings1	So far, plugged in the voltage is more stable, as for the effect of how to quietly wait until the payment of the electricity bill to compare, the appearance of the commodity design is very good, the real thing!	Jingdong prefecture level city in Shandong
Jinfeng Hongyuan Flagship Store	Electricity savings1	💌 I've been wanting it for a long time, I decided to go for it, I feel great after using it, it's genuine and the delivery is great, it lives up to its name👍	Jingdong prefecture level city in Shandong
Jinfeng Hongyuan Flagship Store	Electricity savings1	Looking forward to it!!! I don't know if it's working or not, let's look forward to it!!!! How much is saved ，，，，，，，，， Saving a degree is a degree with long term use ！！！！！！！	Jingdong prefecture level city in Shandong
Jinfeng Hongyuan Flagship Store	Electricity savings1	After using it for a while, I am seriously and responsibly saying that this saver is really very power efficient. Two rooms with the air conditioner on at night for one night, plus watching TV, charging cell phones, lights, etc. etc. only used one degree of electricity. So I bought several more and it's really	Jingdong prefecture level city in Shandong
Jinfeng Hongyuan Flagship Store	Electricity savings1	It arrived quickly, and the box is solid and delicate. Baby shell is thick aluminum and by multi-layer coating. The inner core is heavy, and the power cord and plug are genuine products from a large manufacturer. It is the best power saver I have found after choosing all of them!	Jingdong prefecture level city in Shandong
Jinfeng Hongyuan Flagship Store	Electricity savings1	The saver was received, genuine guarantee, good quality...	Jingdong prefecture level city in Shandong
Jinfeng Hongyuan Flagship Store	Electricity savings1	Logistics was fast! The product is easy to use!	Jingdong prefecture level city in Shandong
Jinfeng Hongyuan Flagship Store	Electricity savings1	Look appearance is okay, use a period of time to know how it is. Jingdong Mall, trustworthy! Home appliances, cell phones and computers are almost 100% bought by Jingdong. Ordered the night before yesterday, received yesterday. Jingdong logistics, the fastest in the network!	Jingdong prefecture level city in Shandong
Jinfeng Hongyuan Flagship Store	Electricity savings1	Very good, perfect packaging, home energy saver to determine the effect of power saving and also play a role in stabilizing the voltage Very good products	Jingdong prefecture level city in Shandong
Jinfeng Hongyuan Flagship Store	Electricity savings1	Smart Saver received, looks very exquisite. The method of use is simple, and it has been used. The later effect, with time to test.	Jingdong prefecture level city in Shandong
Jinfeng Hongyuan Flagship Store	Electricity savings1	Both the material and workmanship exceeded expectations, great value, and looks great on oh. The seller also shipped much earlier than expected, will come back. Buy it, you will not regret it! Full five points. Both the material and workmanship exceeded expectations, great value, wear it also looks good Oh. Seller delivery is also much earlier than expected, will come again. Buy it, will not regret it! Full	Jingdong prefecture level city in Shandong
Jinfeng Hongyuan Flagship Store	Electricity savings1	The appearance is clean and beautiful, the workmanship is very good, I didn't find any defects, I personally feel that the size is also quite suitable, I've used it for 2 months, I didn't find out exactly how energy efficient it is. Try using it again!	Jingdong prefecture level city in Shandong
Jinfeng Hongyuan Flagship Store	Electricity savings1	Power saving device received, product certification, accessories, etc. complete, very good workmanship, very practical power-saving effect is also very obvious.	Jingdong prefecture level city in Shandong
Jinfeng Hongyuan Flagship Store	Electricity savings1	Looked at a lot of think buy this one, see a lot of reviews say it works, a month with save nearly twenty degrees of electricity, there are sixty days of return guarantee, with a good and then come back to the	Jingdong prefecture level city in Shandong
Jinfeng Hongyuan Flagship Store	Electricity savings1	I received the power saving device, and I'm using it. I hope I can get the desired effect, and I'm trying to force it to work quickly.	Jingdong prefecture level city in Shandong
Jinfeng Hongyuan Flagship Store	Electricity savings1	Voltage is stable, already trying it out and waiting for the results to happen, saving electricity will follow up next month!	Jingdong prefecture level city in Shandong
Jinfeng Hongyuan Flagship Store	Electricity savings1	Jingdong Mall self-supporting goods of excellent quality, logistics is also very fast, worth recommending 👏👏👏👏 excellent!	Jingdong prefecture level city in Shandong
Jinfeng Hongyuan Flagship Store	Electricity savings1	Power saving has been received, the appearance of the beautiful, I have already been the second time to buy the use of down the effect is very good!	Jingdong prefecture level city in Shandong
Jinfeng Hongyuan Flagship Store	Electricity savings1	Energy saver received very happy, the color is also very good looking forward to a very good effect can save electricity	Jingdong prefecture level city in Shandong
Jinfeng Hongyuan Flagship Store	Electricity savings1	It's small and easy to plug in and use. It displays the voltage value when plugged in and	Jingdong prefecture level city in Shandong
Jinfeng Hongyuan Flagship Store	Electricity savings1	This saver really works well, because I installed one at home about seven or eight years ago, when there is no voltage display, now there is a voltage display, the appearance also looks much better, the electricity bill is also saved, the stability of the home appliances is also good, the best of both	Jingdong prefecture level city in Shandong
Jinfeng Hongyuan Flagship Store	Electricity savings1	Ordered yesterday, arrived today in use. Shipping was fast. The effect is looking forward to.	Jingdong prefecture level city in Shandong
Jinfeng Hongyuan Flagship Store	Electricity savings1	This thing, I don't know what the principle is. Why it can save electricity. Wait for a month to use it first, see if it will save electricity bill, then come back to evaluate. There is one thing that does not fit the principle ah. The law of conservation of energy.	Jingdong prefecture level city in Shandong
Jinfeng Hongyuan Flagship Store	Electricity savings1	Home mom listen to others say this can save electricity, specially let buy a, later a very large, go back to see a particularly small, directly plugged into the electricity can be, no need to install, the price is also very cheap, the first time with this product, do not know if it is not good to use, temporarily try for a period of time, if it is good to use, recommend that you buy this one, the	Jingdong prefecture level city in Shandong
Jinfeng Hongyuan Flagship Store	Electricity savings1	I've been using it for a few days, and I think the workmanship and materials are pretty good, so we'll have to look at the next bill for the real energy savings, and then we'll add more comments.	Jingdong prefecture level city in Shandong
Jinfeng Hongyuan Flagship Store	Electricity savings1	Energy saver is well made, energy saving and power saving, and anti-counterfeiting code, it is a regular commodity!	Jingdong prefecture level city in Shandong
Jinfeng Hongyuan Flagship Store	Electricity savings1	Jinfeng Hongyuan, buy back home, as a mouse, haha. First of all, normal electricity for 1 month, the power did not increase, the electricity bill feels smoother, year-on-year electricity costs in the low, whether it is energy-saving still have to be used for a long time to know. All 5 stars ☆	Jingdong prefecture level city in Shandong
Jinfeng Hongyuan Flagship Store	Electricity savings1	The box is well packaged and beautiful! I hope to save electricity with it haha!	Jingdong prefecture level city in Shandong
Jinfeng Hongyuan Flagship Store	Electricity savings1	Ordered last night, arrived this morning, first try the effect before making comments	Jingdong prefecture level city in Shandong

Jinfeng Hongyuan Flagship Store	Electricity savings1	Looks good. Don't know the effect yet.	Jingdong prefecture level city in Shandong
Jinfeng Hongyuan Flagship Store	Electricity savings1	Jinfeng Hongyuan intelligent power saver has exquisite workmanship and modern fashionable design style. It is a good home economizer . I believe that the goods purchased from Jingdong will not disappoint our	Jingdong prefecture level city in Shandong
Jinfeng Hongyuan Flagship Store	Electricity savings1	Inadvertently flipped to this power saver, never heard of, feel very novel, the price is not expensive, so buy to try, see a month after the electricity bill will be less, recently the home air-conditioning open a lot of power consumption is very large.	Jingdong prefecture level city in Shandong
Jinfeng Hongyuan Flagship Store	Electricity savings1	The quality of the goods is very good workmanship, but also sent five fuse	Jingdong prefecture level city in Shandong
Jinfeng Hongyuan Flagship Store	Electricity savings1	I'm trying it out. I don't know if it'll work.	Jingdong prefecture level city in Shandong
Jinfeng Hongyuan Flagship Store	Electricity savings1	There is no effect yet do not know, is to look at the official sale, buy to try it! Use according to the instructions, plugged in, used for a period of time, anyway, do not know how, after a few months to see it, to see if the average month than before will be less electricity.	Jingdong prefecture level city in Shandong
Jinfeng Hongyuan Flagship Store	Electricity savings1	Received the goods, the first time to unpacking, the quality is very good, consistent with the description of the seller, completely exceeded my expectations, the packaging is very careful, strict, in general this time is very satisfied with a shopping, thanks to the seller.	Jingdong prefecture level city in Shandong
Jinfeng Hongyuan Flagship Store	Electricity savings1	The energy saver was received and used for a few days with good results.	Jingdong prefecture level city in Shandong
Jinfeng Hongyuan Flagship Store	Electricity savings1	Previously I have paid attention to this product, but I think that the monthly electricity consumption at home is not large, there is no need to save electricity savings. After a second thought, energy saving is the responsibility of each of our citizens, the winter is coming, this year's winter is cold, heating and all electric appliances, can save is to save, the price is not expensive, so buy to try. I analyze the principle of power saving, should be able to save electricity, but the larger the power saving effect is about good. To be tried after a month or two to see the effect and then evaluate. Jingdong trustworthy.	Jingdong prefecture level city in Shandong
Jinfeng Hongyuan Flagship Store	Electricity savings1	It just arrived and I can't wait to use it, and the customer service is great! There is also voltage showing up which is nice! I don't know if it can save a bit I won't know until I've used it for a month! When the time comes to come back to the follow-up review!	Jingdong prefecture level city in Shandong
Jinfeng Hongyuan Flagship Store	Electricity savings1	Checked out the reviews from numerous users. The shipment is still very large, so I decided to buy it anyway to use it and see the effect after a month. Jingdong Mall here to buy safe, convenient and fast, this price is very powerful 💪, very recommended.	Jingdong prefecture level city in Shandong
Jinfeng Hongyuan Flagship Store	Electricity savings1	Purchased two at the same time to use together. The other one works fine, the other has problems with the numbers not sure if the machine is faulty. Power saving effect can not be observed. Feel the effect is not very obvious like?	Jingdong prefecture level city in Shandong
Jinfeng Hongyuan Flagship Store	Electricity savings1	Looking forward to it!!! I don't know if it's working or not, let's look forward to it!!!! How much is saved , , , , , , , , , Saving a degree is a degree with long term use ! ! ! ! ! ! !	Jingdong prefecture level city in Shandong
Jinfeng Hongyuan Flagship Store	Electricity savings1	Received, directly on the use of the special convenient	Jingdong prefecture level city in Shandong
Jinfeng Hongyuan Flagship Store	Electricity savings1	Goods received a few days well-packed, the same day opened and plugged in to try to be normal is not sure what the power-saving effect. After a month with a follow-up evaluation.	Jingdong prefecture level city in Shandong
Jinfeng Hongyuan Flagship Store	Electricity savings1	I bought it and used it, I don't know how it works yet, we'll see later. Use the effect of it	Jingdong prefecture level city in Shandong
Jinfeng Hongyuan Flagship Store	Electricity savings1	Photographed 2, the old family also put a, with a small January, there should be some effect, with a long period of time to comment, with a long time to see the obvious effect, hard courier brother.	Jingdong prefecture level city in Shandong
Jinfeng Hongyuan Flagship Store	Electricity savings1	Experience: I am an electrician, this is the real use of electronic waves automatically connected to track the decomposition of ionized electrons, in situ compensation of reactive power, reduce effective power loss and waste, stabilize the voltage to achieve energy saving and power saving, the overall effect	Jingdong prefecture level city in Shandong
Jinfeng Hongyuan Flagship Store	Electricity savings1	Great looking and practical! Easy to use and save! Saves 15% in a month of comparison	Jingdong prefecture level city in Shandong
Jinfeng Hongyuan Flagship Store	Electricity savings1	Product quality is very good! Packaging is very nice, like a cell phone box, thick and well protected! Service is also great! Logistics is very fast! Thank you for your business! Very honest!	Jingdong prefecture level city in Shandong
Jinfeng Hongyuan Flagship Store	Electricity savings1	In several products inside the choice of this purchase for a period of time, but also need to use a few months to compare last year's electricity bill before you can analyze the savings in electricity, to the summer peak electricity consumption should be more obvious.	Jingdong prefecture level city in Shandong
Jinfeng Hongyuan Flagship Store	Electricity savings1	The goods have long been received, after more than a month to comment, the home electricity bill is indeed reduced, worth buying!	Jingdong prefecture level city in Shandong
Jinfeng Hongyuan Flagship Store	Electricity savings1	Baby received, the logistics is strong, the quality is reliable, it is worth having and recommending!	Jingdong prefecture level city in Shandong
Jinfeng Hongyuan Flagship Store	Electricity savings1	Using a conservation appliance, you can achieve a 10-15% savings in electricity usage.	Jingdong prefecture level city in Shandong
Jinfeng Hongyuan Flagship Store	Electricity savings1	Quality is very good, metal shell, touch is very good, very recommended Oh ~, the subsequent effect of continuous follow-up	Jingdong prefecture level city in Shandong
Jinfeng Hongyuan Flagship Store	Electricity savings1	The product is beautiful, practical, worth buying. The product is beautiful, practical, good quality, fast logistics product is beautiful, practical, beautiful and elegant, very classy, worth buying!	Jingdong prefecture level city in Shandong
Jinfeng Hongyuan Flagship Store	Electricity savings1	Just received the item and have already used it. Didn't know much about these products before. I hope it is as effective and useful as the product advertises.	Jingdong prefecture level city in Shandong
Jinfeng Hongyuan Flagship Store	Electricity savings1	A month two people used more than five hundred degrees of electricity, in a hurry and then add a power saver, I hope to have an effect, Jingdong shopping is very cool, logistics is very fast especially good service attitude delivery, this hot day is not easy, thank you!	Jingdong prefecture level city in Shandong
Jinfeng Hongyuan Flagship Store	Electricity savings1	Just received the baby, first plugged in to try to use a month and then additional evaluation it	Jingdong prefecture level city in Shandong
Jinfeng Hongyuan Flagship Store	Electricity savings1	Goods fast, the power saver has been received, a friend recommended to buy that can save electricity. Exquisite and compact is very convenient, plug and play. The effect is good, the price is affordable, the next need will patronize again!	Jingdong prefecture level city in Shandong
Jinfeng Hongyuan Flagship Store	Electricity savings1	Packaging is better than expected, sent a few fuse, power saving effect in the end do not know, try a period of time to say!	Jingdong prefecture level city in Shandong
Jinfeng Hongyuan Flagship Store	Electricity savings1	Shipped quickly, immediately installed, read countless reviews were not sure if this thing is effective, was going to buy three at once, ultimately decided to first use a try the effect, if the power ring significantly reduced, then will buy a few more!	Jingdong prefecture level city in Shandong
Jinfeng Hongyuan Flagship Store	Electricity savings1	Used for a period of time to evaluate, not to a month still do not know whether it can save electricity, the voltage has a display!	Jingdong prefecture level city in Shandong
Jinfeng Hongyuan Flagship Store	Electricity savings1	I don't know if it saves electricity or not, but I'm using it anyway, so I hope it saves a little bit, electricity is too expensive	Jingdong prefecture level city in Shandong
Jinfeng Hongyuan Flagship Store	Electricity savings1	The power saver has been received and I am very happy with it! Just waiting to see how the power saving works!	Jingdong prefecture level city in Shandong
Jinfeng Hongyuan Flagship Store	Electricity savings1	Saving power has been received, logistics is quite fast, the quality is not bad, small and a little weight, has been used to look forward to the effect of	Jingdong prefecture level city in Shandong
Jinfeng Hongyuan Flagship Store	Electricity savings1	The logistics is very fast, the effect is obvious and trustworthy.	Jingdong prefecture level city in Shandong
Jinfeng Hongyuan Flagship Store	Electricity savings1	Good quality, good quality, beautiful, beautiful, beautiful, beautiful, beautiful, very worth buying, worth buying, worth buying, worth buying, worth customizing!	Jingdong prefecture level city in Shandong
Jinfeng Hongyuan Flagship Store	Electricity savings1	I've used it for now, I don't know if it saves electricity	Jingdong prefecture level city in Shandong
Jinfeng Hongyuan Flagship Store	Electricity savings1	Bought two at a time, logistics is very fast, the product is beautifully packaged, after two months to see the results	Jingdong prefecture level city in Shandong
Jinfeng Hongyuan Flagship Store	Electricity savings1	The workmanship is really fine, the packaging is also good, the regular factory production ~ use a break in the evaluation of the effectiveness of the	Jingdong prefecture level city in Shandong
Jinfeng Hongyuan Flagship Store	Electricity savings1	Sometimes 219. Sometimes 220 in this case	Jingdong prefecture level city in Shandong
Jinfeng Hongyuan Flagship Store	Electricity savings1	It is the ideal product, good quality, logistics is also very fast, worthy of praise!	Jingdong prefecture level city in Shandong
Jinfeng Hongyuan Flagship Store	Electricity savings1	Store service is very good, the courier received the next day. Product packaging is very good, just use the quality to be tested.	Jingdong prefecture level city in Shandong

Jinfeng Hongyuan Flagship Store	Electricity savings1	I've been using it for almost a month now and I feel like it's working.	Jingdong prefecture level city in Shandong
Jinfeng Hongyuan Flagship Store	Electricity savings1	Very satisfied, very restful, very pretty, very beautiful, very much enjoyed! Well worth it!	Jingdong prefecture level city in Shandong
Jinfeng Hongyuan Flagship Store	Electricity savings1	Baby especially like, the value of high-quality good, a very good shopping experience, attentive service, fast logistics, distribution is also very thoughtful, attitude is also very good, value for money ah, decisive praise! Quality is quite good, logistics is also very fast, praise!	Jingdong prefecture level city in Shandong
Jinfeng Hongyuan Flagship Store	Electricity savings1	The shape of the item is basically the same as ordered, the effect is to be examined.	Jingdong prefecture level city in Shandong
Jinfeng Hongyuan Flagship Store	Electricity savings1	Appearance style: good-looking workmanship material: very good, a small one, very delicate Experience: still in use, in the process of using the shopkeeper is very nice to have a careful explanation Use for a period of time in the look at the results	Jingdong prefecture level city in Shandong
Jinfeng Hongyuan Flagship Store	Electricity savings1	As described, compact and well packaged	Jingdong prefecture level city in Shandong
Jinfeng Hongyuan Flagship Store	Electricity savings1	Fine workmanship. Test in progress. Let's see how it works.	Jingdong prefecture level city in Shandong
Jinfeng Hongyuan Flagship Store	Electricity savings1	The goods have been received, I'm very satisfied and love it	Jingdong prefecture level city in Shandong
Jinfeng Hongyuan Flagship Store	Electricity savings1	Safety! Whether you can save electricity, you can roughly feel it in 2 months. The principle is simple. Before the electrical appliances and circuits inductive more, now the electrical appliances integrated, the home is not a lot of inductive circuits, but used certainly save no problem! Wiring according to the requirements of the manual is good, if it is not convenient, see the socket plug.	Jingdong prefecture level city in Shandong
Jinfeng Hongyuan Flagship Store	Electricity savings1	Great product, looking forward to how much power I can save, first time buyer!	Jingdong prefecture level city in Shandong
Jinfeng Hongyuan Flagship Store	Electricity savings1	The product is exquisite, beautifully packaged, and the customer service is excellent. Just waiting to see how the power saving effect is, looking forward to it!	Jingdong prefecture level city in Shandong
Jinfeng Hongyuan Flagship Store	Electricity savings1	Express very quickly very satisfied with the first use to see how the effect to know, if you can save 10 percent even if acceptable, if you can not meet this requirement is obviously not much effect. Obviously.	Jingdong prefecture level city in Shandong
Jinfeng Hongyuan Flagship Store	Electricity savings1	Very delicate and compact, there is a very convenient intelligent display prompts the voltage, you can know the voltage at a glance, there is very easy to use, a plug can be used, I hope to save money on	Jingdong prefecture level city in Shandong
Jinfeng Hongyuan Flagship Store	Electricity savings1	First time buyer is it really save after use	Jingdong prefecture level city in Shandong
Jinfeng Hongyuan Flagship Store	Electricity savings1	Purchased again and will be for a long time yet.	Jingdong prefecture level city in Shandong
Jinfeng Hongyuan Flagship Store	Electricity savings1	Bought it to stock up, came back and plugged it in, hope it works	Jingdong prefecture level city in Shandong
Jinfeng Hongyuan Flagship Store	Electricity savings1	Baby received the logistics very fast very like very satisfied praise a	Jingdong prefecture level city in Shandong
Jinfeng Hongyuan Flagship Store	Electricity savings1	Beginning the process of using, easy to evaluate more later.	Jingdong prefecture level city in Shandong
Jinfeng Hongyuan Flagship Store	Electricity savings1	The goods have been received, just do not know how effective	Jingdong prefecture level city in Shandong
Jinfeng Hongyuan Flagship Store	Electricity savings1	It feels okay. I wish there were more events.	Jingdong prefecture level city in Shandong
Jinfeng Hongyuan Flagship Store	Electricity savings1	Things have been received, just plugged in, I do not know if it works, wait a few days to have the effect of additional	Jingdong prefecture level city in Shandong
Jinfeng Hongyuan Flagship Store	Electricity savings1	The effect of this power saver is really good, the house more, a few air conditioners are open every day, not used before a month to say less than seven hundred eight hundred, used after three months only nine	Jingdong prefecture level city in Shandong
Jinfeng Hongyuan Flagship Store	Electricity savings1	Cheap, good and easy to use, recommend it.	Jingdong prefecture level city in Shandong
Jinfeng Hongyuan Flagship Store	Electricity savings1	Bought this device, to the home for a long time, wait to finish a month to see how, good can recommend friends to buy, we use the electricity every month scary, a month of electricity costs more than a thousand, can not afford to hurt ah! I hope this power saving can save me some money, the beginning of the shoot order did not pay attention to look at the address, sent elsewhere to go, that side called to find out, hurry to communicate with customer service, customer service is very careful to help me get it done, call to that side to help me turn around, thank you!	Jingdong prefecture level city in Shandong
Jinfeng Hongyuan Flagship Store	Electricity savings1	I don't know the effect if it, use it first to see.	Jingdong prefecture level city in Shandong
Jinfeng Hongyuan Flagship Store	Electricity savings1	Looks good. I won't know if it saves power until I use it.	Jingdong prefecture level city in Shandong
Jinfeng Hongyuan Flagship Store	Electricity savings1	Goods received, shipping speed is very fast, yesterday afternoon more than two o'clock shot, just express delivery, but also sent a few insurance, very good business, the display of the number do not understand what is the meaning of the display said voltage, do not understand this, try a month to see how, good and	Jingdong prefecture level city in Shandong
Jinfeng Hongyuan Flagship Store	Electricity savings1	What ** play also, save electricity more and more save more and more, save chicken hair electricity, are cheating, this is a night of air conditioning with electricity, a night with 11 electricity word, this is	Jingdong prefecture level city in Shandong
Jinfeng Hongyuan Flagship Store	Electricity savings1	Received the product, hope it works!	Jingdong prefecture level city in Shandong
Jinfeng Hongyuan Flagship Store	Electricity savings1	Bought home and used very convenient, easy to operate, quite good	Jingdong prefecture level city in Shandong
Jinfeng Hongyuan Flagship Store	Electricity savings1	It's really good, cost-effective and good quality, and the logistics are fast! It arrived in one day! Really good, cost-effective and good quality, logistics is fast ah! It arrived in one day!	Jingdong prefecture level city in Shandong
Jinfeng Hongyuan Flagship Store	Electricity savings1	Works well, stabilizes voltage and saves power	Jingdong prefecture level city in Shandong
Jinfeng Hongyuan Flagship Store	Electricity savings1	I'll use it right away and look forward to good results	Jingdong prefecture level city in Shandong
Jinfeng Hongyuan Flagship Store	Electricity savings1	Recommended by a friend, said it works well, so I bought one, the effect to be tested	Jingdong prefecture level city in Shandong
Jinfeng Hongyuan Flagship Store	Electricity savings1	I didn't know it would save electricity.	Jingdong prefecture level city in Shandong
Jinfeng Hongyuan Flagship Store	Electricity savings1	I've been using it for a week, I don't know what's going on yet I hope it works well and saves power I'll follow up after a month of use	Jingdong prefecture level city in Shandong
Jinfeng Hongyuan Flagship Store	Electricity savings1	Very well packaged, looks great, don't know how it works	Jingdong prefecture level city in Shandong
Jinfeng Hongyuan Flagship Store	Electricity savings1	Genuine, good quality, really saves energy.	Jingdong prefecture level city in Shandong
Jinfeng Hongyuan Flagship Store	Electricity savings1	I was worried that the stuff is not good, but when I got it, I realized that I was completely over-anxious, 5 stars!	Jingdong prefecture level city in Shandong
Jinfeng Hongyuan Flagship Store	Electricity savings1	Very good and satisfied trustworthy	Jingdong prefecture level city in Shandong
Jinfeng Hongyuan Flagship Store	Electricity savings1	I don't know if it saves power	Jingdong prefecture level city in Shandong
Jinfeng Hongyuan Flagship Store	Electricity savings1	The quality of the product is very good, beautiful and generous applicable fast delivery speed good service attitude	Jingdong prefecture level city in Shandong
Jinfeng Hongyuan Flagship Store	Electricity savings1	Let's use it for a while and then we'll follow up	Jingdong prefecture level city in Shandong
Jinfeng Hongyuan Flagship Store	Electricity savings1	I've only been using it for a month, I don't know if there's any change yet, I'll come back with a follow-up comment after checking the meter!	Jingdong prefecture level city in Shandong
Jinfeng Hongyuan Flagship Store	Electricity savings1	I don't know if it works, I read the instructions and online reviews, I hope I won't be disappointed, if I use it, I will buy it again. Jingdong Express, fast and guaranteed service.	Jingdong prefecture level city in Shandong
Jinfeng Hongyuan Flagship Store	Electricity savings1	It feels like it saves quite a bit huh?	Jingdong prefecture level city in Shandong
Jinfeng Hongyuan Flagship Store	Electricity savings1	I've been using it for a while now, and it still seems to work and have a power saving effect	Jingdong prefecture level city in Shandong

Jinfeng Hongyuan Flagship Store	Electricity savings1	Looks good. It says 235. The 222 bar voltage is shown as high. I don't know if that's right. We'll see.	Jingdong prefecture level city in Shandong
Jinfeng Hongyuan Flagship Store	Electricity savings1	Baby is really good, do really careful, worth to have, fast delivery!	Jingdong prefecture level city in Shandong
Jinfeng Hongyuan Flagship Store	Electricity savings1	Quite delicate and compact, simple and convenient to use. Things are still very good, bought two at a time. Very satisfied with an online purchase.	Jingdong prefecture level city in Shandong
Jinfeng Hongyuan Flagship Store	Electricity savings1	Feel very good, the use of unclear, ask customer service, timely explanation, big brand, trustworthy	Jingdong prefecture level city in Shandong
Jinfeng Hongyuan Flagship Store	Electricity savings1	Thrifty received a few days did not make a comment is to see how the quality of the product now feel the quality of good appearance is also powerful seller service is very good to buy the product is also attached to the 5 fuse thought very thoughtful!	Jingdong prefecture level city in Shandong
Jinfeng Hongyuan Flagship Store	Electricity savings1	Big brand, with a very assured, the quality is very good, in time for the event to buy very cost-effective, customer service is really very good Oh ~!	Jingdong prefecture level city in Shandong
Jinfeng Hongyuan Flagship Store	Electricity savings1	I ordered yesterday and received it this morning, I heard my friend say that it can save electricity, I don't know if it works, I'll come back to make a comment after using it.	Jingdong prefecture level city in Shandong
Jinfeng Hongyuan Flagship Store	Electricity savings1	I've been using it for over a month and it doesn't seem to be working!	Jingdong prefecture level city in Shandong
Jinfeng Hongyuan Flagship Store	Electricity savings1	Used it, the family has always been in the habit of using energy-saving devices, this is a new one. Digital display voltage is very good, can see that the voltage is more stable.	Jingdong prefecture level city in Shandong
Jinfeng Hongyuan Flagship Store	Electricity savings1	I haven't used it for more than a month, but it looks good. It's easy to plug into an outlet and it works.	Jingdong prefecture level city in Shandong
Jinfeng Hongyuan Flagship Store	Electricity savings1	Good Oh...appearance is very beautiful...just plugged in with...see the effect of it...with a period of time in the additional evaluation...	Jingdong prefecture level city in Shandong
Jinfeng Hongyuan Flagship Store	Electricity savings1	I've received the goods, whether it's good or not, I'll know after I use it.	Jingdong prefecture level city in Shandong
Jinfeng Hongyuan Flagship Store	Electricity savings1	Very good, logistics is fast, the stuff is very powerful and cost-effective! Can feel the meter slowing down	Jingdong prefecture level city in Shandong
Jinfeng Hongyuan Flagship Store	Electricity savings1	Logistics is very fast, get it immediately after use, easy to operate, plug in the power supply can be used, just do not know to the use of the effect of how.	Jingdong prefecture level city in Shandong
Jinfeng Hongyuan Flagship Store	Electricity savings1	Quality is very over the top, quality is guaranteed, quite a good choice, a lot of friends to see me use all want to buy, I recommend it!	Jingdong prefecture level city in Shandong
Jinfeng Hongyuan Flagship Store	Electricity savings1	The appearance is clean and beautiful, the workmanship is very good, did not find any flaws, I personally feel that the size is also quite suitable, looking for a long time to choose this one, big love! The price is very beautiful, things are still good, will continue to buy back! All five points! Ness Ness ~ logistics speed is really no words, is a word: fast. Commodities from the outer packaging is very high-grade, really a penny a penny, expensive has the reason for expensive	Jingdong prefecture level city in Shandong
Jinfeng Hongyuan Flagship Store	Electricity savings1	The logistics is very fast, the texture is very good, genuine, I like it very much.	Jingdong prefecture level city in Shandong
Jinfeng Hongyuan Flagship Store	Electricity savings1	It's been a few days since I put it on, I can't see any results now, waiting for a month to say, steady steady steady.	Jingdong prefecture level city in Shandong
Jinfeng Hongyuan Flagship Store	Electricity savings1	I've been using it for a while now and it should be fine, so I'm buying it for the second time.	Jingdong prefecture level city in Shandong
Jinfeng Hongyuan Flagship Store	Electricity savings1	Power Saving King has been received, looks good, quite delicate, looking forward to using it to save electricity	Jingdong prefecture level city in Shandong
Jinfeng Hongyuan Flagship Store	Electricity savings1	My mom said the home uses a little too much electricity, so I bought a Power Saver to try it out, I don't know how effective it is. Expect it to be a good thing!	Jingdong prefecture level city in Shandong
Jinfeng Hongyuan Flagship Store	Electricity savings1	Thank you Jingdong, logistics is very fast order to receipt only one day. Believe in Jingdong. Favorable comments	Jingdong prefecture level city in Shandong
Jinfeng Hongyuan Flagship Store	Electricity savings1	Saved some money on the electric bill compared to last month, watch this month again, no problems so far	Jingdong prefecture level city in Shandong
Jinfeng Hongyuan Flagship Store	Electricity savings1	Energy saver exquisite workmanship, beautiful style, more features, just bought, power saving effect to be used for a period of time, then evaluate!	Jingdong prefecture level city in Shandong
Jinfeng Hongyuan Flagship Store	Electricity savings1	This thing does save power is more compact to hang on the wall.	Jingdong prefecture level city in Shandong
Jinfeng Hongyuan Flagship Store	Electricity savings1	I've been buying it for a while now, I haven't actually measured it, but it still feels like it's effective	Jingdong prefecture level city in Shandong
Jinfeng Hongyuan Flagship Store	Electricity savings1	I've already used it since I bought it, I don't know how effective it is yet, I'll comment on it later.	Jingdong prefecture level city in Shandong
Jinfeng Hongyuan Flagship Store	Electricity savings1	Baby received, careful appearance, logistics is very fast, worth buying!	Jingdong prefecture level city in Shandong
Jinfeng Hongyuan Flagship Store	Electricity savings1	It's great, it's just not the same in hand, I bought it at that other cheaper online one for about the same price as this one. But then oops, doesn't work at all.	Jingdong prefecture level city in Shandong
Jinfeng Hongyuan Flagship Store	Electricity savings1	The product is exquisite, but you have to use it for a month to know how it works. I'll comment again after a month.	Jingdong prefecture level city in Shandong
Jinfeng Hongyuan Flagship Store	Electricity savings1	I've been using it for a while now, and it doesn't feel like it's working, and it doesn't save any power.	Jingdong prefecture level city in Shandong
Jinfeng Hongyuan Flagship Store	Electricity savings1	Affordable price, fast delivery, Jingdong self-supporting guarantee	Jingdong prefecture level city in Shandong
Jinfeng Hongyuan Flagship Store	Electricity savings1	Packaging is very good, no damage, good quality, fine workmanship Packaging is very good, no damage, good quality, fine workmanship Packaging is very good, no damage, good quality, fine workmanship Packaging is very good, no damage, good quality, fine workmanship	Jingdong prefecture level city in Shandong
Jinfeng Hongyuan Flagship Store	Electricity savings1	Big brand, with a very assured, the quality is very good, in time for the event to buy very cost-effective, customer service is really very good Oh ~!	Jingdong prefecture level city in Shandong
Jinfeng Hongyuan Flagship Store	Electricity savings1	Used a few days, the specific effect is not yet clear, looking forward to the effect as described by the merchant!	Jingdong prefecture level city in Shandong
Jinfeng Hongyuan Flagship Store	Electricity savings1	I just used it and don't know how it works, I'll follow up after using it!	Jingdong prefecture level city in Shandong
Jinfeng Hongyuan Flagship Store	Electricity savings1	Well, it's been installed and now I'm waiting to see the results, BOE shopping, hopefully I won't be disappointed!	Jingdong prefecture level city in Shandong
Jinfeng Hongyuan Flagship Store	Electricity savings1	Goods received, plugged in the voltage from 229 to 222, do not know whether to save electricity, that depends on the situation.	Jingdong prefecture level city in Shandong
Jinfeng Hongyuan Flagship Store	Electricity savings1	This is the second time I have bought the product and I feel it really saves electricity! Use it again for a while and then evaluate!	Jingdong prefecture level city in Shandong
Jinfeng Hongyuan Flagship Store	Electricity savings1	Overall, it's really good, and the customer service is great, you'll get a message back almost immediately when you speak, it's great value for money, I really recommend it!	Jingdong prefecture level city in Shandong
Jinfeng Hongyuan Flagship Store	Electricity savings1	✨ Have purchased many times, a must have for the family, affordable purchase price, fast delivery, 🎁 beautifully packaged!	Jingdong prefecture level city in Shandong
Jinfeng Hongyuan Flagship Store	Electricity savings1	I bought it after a friend introduced it, he said it is effective and can save electricity. I bought it to try, try a period of time and then follow up!	Jingdong prefecture level city in Shandong
Jinfeng Hongyuan Flagship Store	Electricity savings1	Received it, trying it out tonight, and bought one earlier.	Jingdong prefecture level city in Shandong
Jinfeng Hongyuan Flagship Store	Electricity savings1	It will take a month or two to see if it saves power or not.	Jingdong prefecture level city in Shandong
Jinfeng Hongyuan Flagship Store	Electricity savings1	Looked good in the instructions! Bought one and pressed it on! Use it and see! If it works! I'll buy some more!	Jingdong prefecture level city in Shandong
Jinfeng Hongyuan Flagship Store	Electricity savings1	Goods have been received packaging can also things are not bad appearance, with a use to know, now evaluation is nonsense.	Jingdong prefecture level city in Shandong
Jinfeng Hongyuan Flagship Store	Electricity savings1	Logistics is very fast, packaging is very good, the second unit, as always, looking forward to the effect of	Jingdong prefecture level city in Shandong
Jinfeng Hongyuan Flagship Store	Electricity savings1	I received the goods today, the quality of goods is very good, I am very satisfied ah.	Jingdong prefecture level city in Shandong
Jinfeng Hongyuan Flagship Store	Electricity savings1	Buy one and try it, I wonder if it has a power saving effect?	Jingdong prefecture level city in Shandong

Jinfeng Hongyuan Flagship Store	Electricity savings1	🔍 The item was received, well packaged, logistics is very fast, customer service attitude is very positive, there are questions answered in a timely manner! Overall very satisfied!	Jingdong prefecture level city in Shandong
Jinfeng Hongyuan Flagship Store	Electricity savings1	Baby packaging is very good, purchase submission goods quickly delivered to the home, use and then follow up.	Jingdong prefecture level city in Shandong
Jinfeng Hongyuan Flagship Store	Electricity savings1	My friend asked me to buy an extra one so he could try it too.	Jingdong prefecture level city in Shandong
Jinfeng Hongyuan Flagship Store	Electricity savings1	I've just put it on, I won't know the result until a month later if it will really save power.	Jingdong prefecture level city in Shandong
Jinfeng Hongyuan Flagship Store	Electricity savings1	It's good quality. Hopefully it'll help save power.	Jingdong prefecture level city in Shandong
Jinfeng Hongyuan Flagship Store	Electricity savings1	The appearance is clean and beautiful, the workmanship is very good, I did not find any flaws, I personally feel that the size is also quite suitable, I have been looking for a long time to choose this	Jingdong prefecture level city in Shandong
Jinfeng Hongyuan Flagship Store	Electricity savings1	Logistics is fast! Baby looks great! The price is fair! Wish the store business is prosperous!	Jingdong prefecture level city in Shandong
Jinfeng Hongyuan Flagship Store	Electricity savings1	Jingdong express fast, good, have used, sit and wait to save money...	Jingdong prefecture level city in Shandong
Jinfeng Hongyuan Flagship Store	Electricity savings1	In use, it didn't work as expected and there was nothing noticeable in terms of power savings.	Jingdong prefecture level city in Shandong
Jinfeng Hongyuan Flagship Store	Electricity savings1	Just started using it and looking forward to the results.	Jingdong prefecture level city in Shandong
Jinfeng Hongyuan Flagship Store	Electricity savings1	The quality is very good, the metal shell is very good, is genuine, energy saving and power saving, logistics is very fast, there is a need to buy o, recommend everyone, very satisfied with an online	Jingdong prefecture level city in Shandong
Jinfeng Hongyuan Flagship Store	Electricity savings1	Received, I do not know if the product is good, used to see if it is good to come back to buy!	Jingdong prefecture level city in Shandong
Jinfeng Hongyuan Flagship Store	Electricity savings1	Jinfeng source of intelligent power saving treasure also used less than three months, the last thunder may be burned, the home of the electric send gate is not on, only the old man at home, no way only their own in the field and run back to find out what is the problem with the circuit, the quality is still not very good, thunder is easy to burn!	Jingdong prefecture level city in Shandong
Jinfeng Hongyuan Flagship Store	Electricity savings1	Saving power just started to use, I do not know if there is no effect, but read a lot of reviews are said to be very good so I bought, baby All aspects are good There are spare 2 fuses, late good and then follow	Jingdong prefecture level city in Shandong
Jinfeng Hongyuan Flagship Store	Electricity savings1	Use it for a few days and see, the delivery is quite fast	Jingdong prefecture level city in Shandong
Jinfeng Hongyuan Flagship Store	Electricity savings1	Small, well made and easy to use.	Jingdong prefecture level city in Shandong
Jinfeng Hongyuan Flagship Store	Electricity savings1	Beautifully packaged, it is genuine. Whether it is power saving, use it to evaluate.	Jingdong prefecture level city in Shandong
Jinfeng Hongyuan Flagship Store	Electricity savings1	Power saver. Used two of them and they do save quite a bit of power.	Jingdong prefecture level city in Shandong
Jinfeng Hongyuan Flagship Store	Electricity savings1	Workmanship is not bad, small and does not take up space, is also do not know whether the power saving, buy a to try!	Jingdong prefecture level city in Shandong
Jinfeng Hongyuan Flagship Store	Electricity savings1	The logistics is very fast, the courier attitude is very good, worth buying	Jingdong prefecture level city in Shandong
Jinfeng Hongyuan Flagship Store	Electricity savings1	The effect is not known until it is used.	Jingdong prefecture level city in Shandong
Jinfeng Hongyuan Flagship Store	Electricity savings1	Just arrived plugged in won't know for a month	Jingdong prefecture level city in Shandong
Jinfeng Hongyuan Flagship Store	Electricity savings1	This is really good to use the pull to realize the savings ah think again for my sister's family to buy a	Jingdong prefecture level city in Shandong
Jinfeng Hongyuan Flagship Store	Electricity savings1	Lies, a month with a few hundred electricity are no savings, family use	Jingdong prefecture level city in Shandong
Jinfeng Hongyuan Flagship Store	Electricity savings1	Express very quickly, look at the fine workmanship also sent a few fuse, look forward to can really save electricity	Jingdong prefecture level city in Shandong
Jinfeng Hongyuan Flagship Store	Electricity savings1	I didn't realize it was so small.	Jingdong prefecture level city in Shandong
Jinfeng Hongyuan Flagship Store	Electricity savings1	Logistics courier is very fast, the courier service is very good	Jingdong prefecture level city in Shandong
Jinfeng Hongyuan Flagship Store	Electricity savings1	Used for a period of time the effect is quite good indeed than the previous power saving	Jingdong prefecture level city in Shandong
Jinfeng Hongyuan Flagship Store	Electricity savings1	Logistics is super fast, the product is in use, see the effect...	Jingdong prefecture level city in Shandong
Jinfeng Hongyuan Flagship Store	Electricity savings1	Baby got it, tried it out and it didn't work at all. How can I use the tips to save power?	Jingdong prefecture level city in Shandong
Jinfeng Hongyuan Flagship Store	Electricity savings1	Power Saving King received, immediately used has been very stable without any sound good, good service, fast logistics inexpensive and beautiful worth repurchasing!	Jingdong prefecture level city in Shandong
Jinfeng Hongyuan Flagship Store	Electricity savings1	Logistics courier is very fast, the courier service is good	Jingdong prefecture level city in Shandong
Jinfeng Hongyuan Flagship Store	Electricity savings1	Good to recommend, the first month of air conditioning only 1 week, the electricity bill has reduced about 100 yuan, may be to reduce the use of air conditioning is also related to the other or as usual electricity, estimated that the longer you use the more you can save electricity.	Jingdong prefecture level city in Shandong
Jinfeng Hongyuan Flagship Store	Electricity savings1	It doesn't work. I've been using it for 20 days and it doesn't work.	Jingdong prefecture level city in Shandong
Jinfeng Hongyuan Flagship Store	Electricity savings1	Fast shipping, good quality, affordable price, very satisfied with a shopping.	Jingdong prefecture level city in Shandong
Jinfeng Hongyuan Flagship Store	Electricity savings1	Try it for a month to see if it saves money.	Jingdong prefecture level city in Shandong
Jinfeng Hongyuan Flagship Store	Electricity savings1	It's in use. I'll comment again after a while.	Jingdong prefecture level city in Shandong
Jinfeng Hongyuan Flagship Store	Electricity savings1	Goods have received look good 👍👍👍👍, use a period of time and then look at the	Jingdong prefecture level city in Shandong
Jinfeng Hongyuan Flagship Store	Electricity savings1	No, it doesn't work. It's useless. It's garbage.	Jingdong prefecture level city in Shandong
Jinfeng Hongyuan Flagship Store	Electricity savings1	I've been using it for almost 2 months now and I don't feel any reduction in my electricity bill.	Jingdong prefecture level city in Shandong
Jinfeng Hongyuan Flagship Store	Electricity savings1	I've received the goods, the logistics is really fast, ordered yesterday, arrived today.	Jingdong prefecture level city in Shandong
Jinfeng Hongyuan Flagship Store	Electricity savings1	And the backup insurance.	Jingdong prefecture level city in Shandong
Jinfeng Hongyuan Flagship Store	Electricity savings1	The appearance of this product is good, easy to use and install, the effect of how there is no use to wait for time to verify that the voltage displayed is different from the voltage measured by other meters, the gap is not too large, the follow-up to add the effect.	Jingdong prefecture level city in Shandong
Jinfeng Hongyuan Flagship Store	Electricity savings1	Baby received it 👌, really great ah! 🌈The quality can't be beat, very satisfied! 🆗Happy happy happy ☺	Jingdong prefecture level city in Shandong
Jinfeng Hongyuan Flagship Store	Electricity savings1	I'll follow up with a review after using it for a while, hopefully it won't bring me back a disappointment!	Jingdong prefecture level city in Shandong
Jinfeng Hongyuan Flagship Store	Electricity savings1	In use and looking forward to results! Good review to send	Jingdong prefecture level city in Shandong
Jinfeng Hongyuan Flagship Store	Electricity savings1	Intelligent power saver received, the volume is not big, small and exquisite, quite good, Jingdong Express is also quite fast, the	Jingdong prefecture level city in Shandong
Jinfeng Hongyuan Flagship Store	Electricity savings1	I don't know how to use it, keep using it	Jingdong prefecture level city in Shandong
Jinfeng Hongyuan Flagship Store	Electricity savings1	Very good for saving electricity	Jingdong prefecture level city in Shandong

Jinfeng Hongyuan Flagship Store	Electricity savings1	Seriously, did not use, feel also more power consumption, the peak is bought back to use the first time, the next day unplugged, the power consumption is back to more than ten degrees (the frequency of power use in the past few days is pretty much the same), feel the IQ tax paid 😂	Jingdong prefecture level city in Shandong
Jinfeng Hongyuan Flagship Store	Electricity savings1	I haven't seen any effect on the power saving, but the voltage stabilization looks good.	Jingdong prefecture level city in Shandong
Jinfeng Hongyuan Flagship Store	Electricity savings1	It's very good, I'll come back to it after using it for a while	Jingdong prefecture level city in Shandong
Jinfeng Hongyuan Flagship Store	Electricity savings1	Already used it and see the results.	Jingdong prefecture level city in Shandong
Jinfeng Hongyuan Flagship Store	Electricity savings1	Fast shipping, beautiful packaging, attentive service, the use of the effect, to be followed up after the evaluation.	Jingdong prefecture level city in Shandong
Jinfeng Hongyuan Flagship Store	Electricity savings1	The process of using this product is more than satisfactory	Jingdong prefecture level city in Shandong
Jinfeng Hongyuan Flagship Store	Electricity savings1	Very good East East cost-effective! Every time so buy more assured, and the logistics is also very fast. It is also very good to use, so I will often come to buy in the future, and will also introduce to	Jingdong prefecture level city in Shandong
Jinfeng Hongyuan Flagship Store	Electricity savings1	But still understand how effective it is!	Jingdong prefecture level city in Shandong
Jinfeng Hongyuan Flagship Store	Electricity savings1	Fast shipping, great logistics, great stuff, great price.	Jingdong prefecture level city in Shandong
Jinfeng Hongyuan Flagship Store	Electricity savings1	I'll review it in a month and give it 5 stars if it's good	Jingdong prefecture level city in Shandong
Jinfeng Hongyuan Flagship Store	Electricity savings1	I've saved a lot of electricity in the past month, so I'm glad to hear that.	Jingdong prefecture level city in Shandong
Jinfeng Hongyuan Flagship Store	Electricity savings1	Baby just received and installed! Looks genuinely good, workmanship feels fine! Looking forward to the effect! Said to see the effect in two months! Waiting for if really have the effect must come to follow up! The boss is absolutely responsible! Asked and answered! Just for the boss this attitude must also	Jingdong prefecture level city in Shandong
Jinfeng Hongyuan Flagship Store	Electricity savings1	This baby is very delicate, I'll comment after I've used it	Jingdong prefecture level city in Shandong
Jinfeng Hongyuan Flagship Store	Electricity savings1	It's been over two months. It's not working. It's just like it was.	Jingdong prefecture level city in Shandong
Jinfeng Hongyuan Flagship Store	Electricity savings1	Fast shipping and logistics, let's see if it works.	Jingdong prefecture level city in Shandong
Jinfeng Hongyuan Flagship Store	Electricity savings1	Just received the goods, try it to know if it can really save electricity	Jingdong prefecture level city in Shandong
Jinfeng Hongyuan Flagship Store	Electricity savings1	Not bad. Not bad. Not bad.	Jingdong prefecture level city in Shandong
Jinfeng Hongyuan Flagship Store	Electricity savings1	Not sure if it works. I'll comment again if it works.	Jingdong prefecture level city in Shandong
Jinfeng Hongyuan Flagship Store	Electricity savings1	Safe and energy-saving helpers to use!	Jingdong prefecture level city in Shandong
Jinfeng Hongyuan Flagship Store	Electricity savings1	Good, first time buyer. Used good can	Jingdong prefecture level city in Shandong
Jinfeng Hongyuan Flagship Store	Electricity savings1	It feels okay, I guess! I won't know until I use it for a while!	Jingdong prefecture level city in Shandong
Jinfeng Hongyuan Flagship Store	Electricity savings1	The saver matches the description and has been used, waiting for a follow up review after two months.	Jingdong prefecture level city in Shandong
Jinfeng Hongyuan Flagship Store	Electricity savings1	Received, the appearance of the business as described, the effect of how to use a period of time to evaluate.	Jingdong prefecture level city in Shandong
Jinfeng Hongyuan Flagship Store	Electricity savings1	Still using it don't know how effective it is, I'll comment after three months	Jingdong prefecture level city in Shandong
Jinfeng Hongyuan Flagship Store	Electricity savings1	The stuff is very good, the packaging is also very good logistics is also very fast	Jingdong prefecture level city in Shandong
Jinfeng Hongyuan Flagship Store	Electricity savings1	I'll follow up with a review when I use it, hopefully I won't be disappointed	Jingdong prefecture level city in Shandong
Jinfeng Hongyuan Flagship Store	Electricity savings1	Cancel Houjiaying Logistics.	Jingdong prefecture level city in Shandong
Jinfeng Hongyuan Flagship Store	Electricity savings1	It's very good, it's worth buying.	Jingdong prefecture level city in Shandong
Jinfeng Hongyuan Flagship Store	Electricity savings1	My friend bought it to save electricity and referred me to buy it! I'll buy it and try it out! I will know how much electricity I can save next month, looking forward to it! 4-500 dollars a month electricity bill is horrible! I hope it can really help me save some electricity!	Jingdong prefecture level city in Shandong
Jinfeng Hongyuan Flagship Store	Electricity savings1	Just received it and installed it.	Jingdong prefecture level city in Shandong
Jinfeng Hongyuan Flagship Store	Electricity savings1	Very nice. This one is my favorite.	Jingdong prefecture level city in Shandong
Jinfeng Hongyuan Flagship Store	Electricity savings1	Good value for money worth buying very good	Jingdong prefecture level city in Shandong
Jinfeng Hongyuan Flagship Store	Electricity savings1	Very energy-saving, small and exquisite, does not take up space, very satisfied, complete packaging, no damage, customer service explanation is also very serious	Jingdong prefecture level city in Shandong
Jinfeng Hongyuan Flagship Store	Electricity savings1	Received immediately used, really effective, do not understand electricity, but looked at the meter every day, recommended that you can use, especially this weather every day to open the air conditioning, with him not afraid of the electricity bill.	Jingdong prefecture level city in Shandong
Jinfeng Hongyuan Flagship Store	Electricity savings1	Nice, good value for money, nice stuff.	Jingdong prefecture level city in Shandong
Jinfeng Hongyuan Flagship Store	Electricity savings1	The packaging of the product is very good, the logistics of the product is very fast, and the quality of the product will be evaluated after use!	Jingdong prefecture level city in Shandong
Jinfeng Hongyuan Flagship Store	Electricity savings1	Express is very, well very fast, give a compliment 👍	Jingdong prefecture level city in Shandong
Jinfeng Hongyuan Flagship Store	Electricity savings1	Packaging is very good, fast delivery, used a night and a day did not feel the power saving. Use a month to see if it saves power.	Jingdong prefecture level city in Shandong
Jinfeng Hongyuan Flagship Store	Electricity savings1	Seems to be a bit of a power saver.	Jingdong prefecture level city in Shandong
Jinfeng Hongyuan Flagship Store	Electricity savings1	Bought this for someone else and it came over so fast, ###!	Jingdong prefecture level city in Shandong
Jinfeng Hongyuan Flagship Store	Electricity savings1	Very delicate, just received a period of time to use in the follow-up evaluation	Jingdong prefecture level city in Shandong
Jinfeng Hongyuan Flagship Store	Electricity savings1	It's been installed for a while now, not sure if it's working or not	Jingdong prefecture level city in Shandong
Jinfeng Hongyuan Flagship Store	Electricity savings1	The item looks okay, I just don't know if it works or not.	Jingdong prefecture level city in Shandong
Jinfeng Hongyuan Flagship Store	Electricity savings1	The goods have been received, but there is a screw missing	Jingdong prefecture level city in Shandong
Jinfeng Hongyuan Flagship Store	Electricity savings1	I didn't believe it before, but after receiving the goods today and trying it out, it really works!	Jingdong prefecture level city in Shandong
Jinfeng Hongyuan Flagship Store	Electricity savings1	Received, this thing will not know whether it is the effect of saving electricity, no matter how to say that there should always be a little use it, otherwise why develop it I think there is still the value of	Jingdong prefecture level city in Shandong
Jinfeng Hongyuan Flagship Store	Electricity savings1	Another one just arrived.	Jingdong prefecture level city in Shandong
Jinfeng Hongyuan Flagship Store	Electricity savings1	I don't feel anything, ordinary farmers can't go to TEDA, just use it first, it's safe.	Jingdong prefecture level city in Shandong
Jinfeng Hongyuan Flagship Store	Electricity savings1	Just purchased and will follow up after using it for a while	Jingdong prefecture level city in Shandong

Jinfeng Hongyuan Flagship Store	Electricity savings1	I'm trying it out now. I'll comment after I use it.	Jingdong prefecture level city in Shandong
Jinfeng Hongyuan Flagship Store	Electricity savings1	Received the item, in use, hope it works.	Jingdong prefecture level city in Shandong
Jinfeng Hongyuan Flagship Store	Electricity savings1	It works well. Good review.	Jingdong prefecture level city in Shandong
Jinfeng Hongyuan Flagship Store	Electricity savings1	All a scam, no effect at all, I bought too many fakes in Poundland before, I did not think that Jingdong is also the same	Jingdong prefecture level city in Shandong
Jinfeng Hongyuan Flagship Store	Electricity savings1	I used the saver as soon as I received it, it's in good shape and still working!	Jingdong prefecture level city in Shandong
Jinfeng Hongyuan Flagship Store	Electricity savings1	It works. I don't know what to do with the picture, but it works.	Jingdong prefecture level city in Shandong
Jinfeng Hongyuan Flagship Store	Electricity savings1	A very good shopping experience, attentive service, fast logistics, delivery is also very thoughtful, attitude is also very good, value for money ah, decisive praise!	Jingdong prefecture level city in Shandong
Jinfeng Hongyuan Flagship Store	Electricity savings1	The product is consistent with the presentation picture.	Jingdong prefecture level city in Shandong
Jinfeng Hongyuan Flagship Store	Electricity savings1	Baby received, use a period of time to evaluate, wish the boss business prosperity!	Jingdong prefecture level city in Shandong
Jinfeng Hongyuan Flagship Store	Electricity savings1	The stuff is better! Recommended!	Jingdong prefecture level city in Shandong
Jinfeng Hongyuan Flagship Store	Electricity savings1	I don't know if it works or not, I'll use it for a while before I comment on it.	Jingdong prefecture level city in Shandong
Jinfeng Hongyuan Flagship Store	Electricity savings1	Cost-effective, I like it, next time will come back Baby received, very surprised, the quality is very good, very satisfied with a shopping. I will continue to buy back, the store is trustworthy	Jingdong prefecture level city in Shandong
Jinfeng Hongyuan Flagship Store	Electricity savings1	Plugged it in and used it for a few days, not sure if it saves power yet	Jingdong prefecture level city in Shandong
Jinfeng Hongyuan Flagship Store	Electricity savings1	I'm trying it out and will follow up if it seems to be working!	Jingdong prefecture level city in Shandong
Jinfeng Hongyuan Flagship Store	Electricity savings1	Works pretty well, big kudos 👍👍👍👍👍👍👍👍👍👍👍	Jingdong prefecture level city in Shandong
Jinfeng Hongyuan Flagship Store	Electricity savings1	Just put it on, we'll see how it looks in a month.	Jingdong prefecture level city in Shandong
Jinfeng Hongyuan Flagship Store	Electricity savings1	The baby is very nice and I've already recommended buying it for all my friends! The main thing is that it not only saves electricity, I was more concerned about the fact that it can adjust the current, usually wiring problems. Now I found that it is really good, it is the effect I want!	Jingdong prefecture level city in Shandong
Jinfeng Hongyuan Flagship Store	Electricity savings1	Baby arrived, looking forward to his results, hopefully it will be good	Jingdong prefecture level city in Shandong
Jinfeng Hongyuan Flagship Store	Electricity savings1	Things have been received, look at the workmanship and packaging are good, I do not know how the power-saving effect? Use a period of time to see the effect	Jingdong prefecture level city in Shandong
Jinfeng Hongyuan Flagship Store	Electricity savings1	The price is right, the shape is beautiful and does not take up space, use it for a while to see the effect	Jingdong prefecture level city in Shandong
Jinfeng Hongyuan Flagship Store	Electricity savings1	Can't save electricity, it doesn't work!	Jingdong prefecture level city in Shandong
Jinfeng Hongyuan Flagship Store	Electricity savings1	It's pretty convenient, just plug it in and it works. There's no noise, plug it in and leave it alone, it's very hassle free! Good workmanship and value for money.	Jingdong prefecture level city in Shandong
Jinfeng Hongyuan Flagship Store	Electricity savings1	Just received the goods have been installed, try two months later to comment!	Jingdong prefecture level city in Shandong
Jinfeng Hongyuan Flagship Store	Electricity savings1	I received the stuff, I haven't used it yet, and overall it's fine.	Jingdong prefecture level city in Shandong
Jinfeng Hongyuan Flagship Store	Electricity savings1	Couldn't use a return at all.	Jingdong prefecture level city in Shandong
Jinfeng Hongyuan Flagship Store	Electricity savings1	Second purchase.	Jingdong prefecture level city in Shandong
Jinfeng Hongyuan Flagship Store	Electricity savings1	Very good quality, high tech and practical.	Jingdong prefecture level city in Shandong
Jinfeng Hongyuan Flagship Store	Electricity savings1	Very good, very great! Very satisfied, thanks to the store!	Jingdong prefecture level city in Shandong
Jinfeng Hongyuan Flagship Store	Electricity savings1	Just used it, don't know if it's good or not, hope it works well	Jingdong prefecture level city in Shandong
Jinfeng Hongyuan Flagship Store	Electricity savings1	Saving power has been received, the product is good, good like.	Jingdong prefecture level city in Shandong
Jinfeng Hongyuan Flagship Store	Electricity savings1	Received the goods and used it for two months before evaluation	Jingdong prefecture level city in Shandong
Jinfeng Hongyuan Flagship Store	Electricity savings1	It looks a little small, I do not know how effective, first use to see the effect, there is a small adjustable button, I do not know this is to adjust to that position to work well	Jingdong prefecture level city in Shandong
Jinfeng Hongyuan Flagship Store	Electricity savings1	Simple, just plug it in, there are spare fuses, can't say how well it works, no comparison	Jingdong prefecture level city in Shandong
Jinfeng Hongyuan Flagship Store	Electricity savings1	It's been a few days since I used it, and the power's gone up.	Jingdong prefecture level city in Shandong
Jinfeng Hongyuan Flagship Store	Electricity savings1	The owner was very nice and sent a spare fuse.	Jingdong prefecture level city in Shandong
Jinfeng Hongyuan Flagship Store	Electricity savings1	It's authentic and it's not expensive and it's great to use, I use it all the time and it's great 🎉✨ just don't know if it really saves any points	Jingdong prefecture level city in Shandong
Jinfeng Hongyuan Flagship Store	Electricity savings1	The information is good, it is the child to buy, the child read it is also quite happy, I hope to be able to match the child's preparation for the midterm examination! Because of the epidemic, it is not too fast, but I also give full credit to the express delivery!	Jingdong prefecture level city in Shandong
Jinfeng Hongyuan Flagship Store	Electricity savings1	This power saver comes to the logistics very quickly, the business attitude is also very good, ** very comfortable, this brand is trustworthy, and buy in this store than in the physical store to buy cheaper,	Jingdong prefecture level city in Shandong
Jinfeng Hongyuan Flagship Store	Electricity savings1	It's very well made and the materials used are okay. This is just a few days old, so I don't know how it will work, only next month.	Jingdong prefecture level city in Shandong
Jinfeng Hongyuan Flagship Store	Electricity savings1	Already used on, have not tested the effect, wait for a couple of months to add comments.	Jingdong prefecture level city in Shandong
Jinfeng Hongyuan Flagship Store	Electricity savings1	Baby received, packaging is very thoughtful, already in use, should soon see the effect.	Jingdong prefecture level city in Shandong
Jinfeng Hongyuan Flagship Store	Electricity savings1	Just received in the office to try, to the home installed, the voltage are different, and do not know if there is no effect. Use a period of time and then follow up.	Jingdong prefecture level city in Shandong
Jinfeng Hongyuan Flagship Store	Electricity savings1	Just received the goods, is trying, looking forward to the effect, first give a favorable comment, two months later, then additional evaluation, please be patient.	Jingdong prefecture level city in Shandong
Jinfeng Hongyuan Flagship Store	Electricity savings1	The item has just been received, well packaged, results to be verified.	Jingdong prefecture level city in Shandong
Jinfeng Hongyuan Flagship Store	Electricity savings1	Packaging logistics are very good, the next look at how the effect, before I see others use the effect is good, as I bought this can also have good results.	Jingdong prefecture level city in Shandong
Jinfeng Hongyuan Flagship Store	Electricity savings1	I don't know if it's a real power saver or a fake power saver, but I've used it since I bought it, so I'll give you a good review.	Jingdong prefecture level city in Shandong
Jinfeng Hongyuan Flagship Store	Electricity savings1	Good quality, genuine goods, much better than imagined before purchase, next time will come back to care!	Jingdong prefecture level city in Shandong
Jinfeng Hongyuan Flagship Store	Electricity savings1	Logistics is very fast good use again	Jingdong prefecture level city in Shandong
Jinfeng Hongyuan Flagship Store	Electricity savings1	Received the goods. Let's see if it saves power in a month and then we'll follow up!	Jingdong prefecture level city in Shandong
Jinfeng Hongyuan Flagship Store	Electricity savings1	I bought it yesterday and it arrived today, I bought two of them, I hope they work well, I'll come back after a while to give a follow-up review	Jingdong prefecture level city in Shandong

Jinfeng Hongyuan Flagship Store	Electricity savings1	The home air conditioner consumes a lot of electricity, so I bought it and used it in the hope that there is A	Jingdong prefecture level city in Shandong
Jinfeng Hongyuan Flagship Store	Electricity savings1	The product is excellent and very current.	Jingdong prefecture level city in Shandong
Jinfeng Hongyuan Flagship Store	Electricity savings1	With the description, it is the most practical and good products that households can need, and it is an ideal shopping, the courier is also fast, and the service attitude is enthusiastic!	Jingdong prefecture level city in Shandong
Jinfeng Hongyuan Flagship Store	Electricity savings1	The Power Saver is easy to use, works well, is stable, and plugs into the top of the outlet	Jingdong prefecture level city in Shandong
Jinfeng Hongyuan Flagship Store	Electricity savings1	Overall still can, excellent craftsmanship, power saving do not know for the time being, after the use of the evaluation	Jingdong prefecture level city in Shandong
Jinfeng Hongyuan Flagship Store	Electricity savings1	Baby received on the plug, but also with four fuses, look at the quality is okay, I do not know whether it is useful, use and then follow up.	Jingdong prefecture level city in Shandong
Jinfeng Hongyuan Flagship Store	Electricity savings1	I'll evaluate it after a while, I don't know if it's good or not yet, and if it can save electricity	Jingdong prefecture level city in Shandong
Jinfeng Hongyuan Flagship Store	Electricity savings1	Jingdong self-operated purchase more assured delivery fast quality assurance	Jingdong prefecture level city in Shandong
Jinfeng Hongyuan Flagship Store	Electricity savings1	Shipping was fast, stuff was well packaged, great price, I'm happy with it 👍 and will repurchase!	Jingdong prefecture level city in Shandong
Jinfeng Hongyuan Flagship Store	Electricity savings1	The quality of the product is very good and I will definitely come back again next time.	Jingdong prefecture level city in Shandong
Jinfeng Hongyuan Flagship Store	Electricity savings1	I'll follow up with a review after I use it.	Jingdong prefecture level city in Shandong
Jinfeng Hongyuan Flagship Store	Electricity savings1	Hello, the goods are perfectly packaged, save power for a period of time to evaluate again!	Jingdong prefecture level city in Shandong
Jinfeng Hongyuan Flagship Store	Electricity savings1	Not bad, pretty good, good value for money, pretty good	Jingdong prefecture level city in Shandong
Jinfeng Hongyuan Flagship Store	Electricity savings1	The packaging is very good, received immediately used, looking forward to the effect in.	Jingdong prefecture level city in Shandong
Jinfeng Hongyuan Flagship Store	Electricity savings1	See how much power you save at the end of the month	Jingdong prefecture level city in Shandong
Jinfeng Hongyuan Flagship Store	Electricity savings1	Easy to use, affordable, beautiful color	Jingdong prefecture level city in Shandong
Jinfeng Hongyuan Flagship Store	Electricity savings1	It's taking too long to put together an order. It could be an epidemic.	Jingdong prefecture level city in Shandong
Jinfeng Hongyuan Flagship Store	Electricity savings1	I don't feel much after using it, no change in my electric bill 。。。。。。。	Jingdong prefecture level city in Shandong
Jinfeng Hongyuan Flagship Store	Electricity savings1	Logistics is very fast, the quality of the economizer is good, good quality and cheap, it is worth buying, I hope there is a power saving effect!	Jingdong prefecture level city in Shandong
Jinfeng Hongyuan Flagship Store	Electricity savings1	Feeling okay, in a couple of months to try to rely on it before making a comment. Let's give it a five-star review first!	Jingdong prefecture level city in Shandong
Jinfeng Hongyuan Flagship Store	Electricity savings1	Power saving device has been hesitating before in the end to buy or not to buy, after consideration, consideration, in consideration, or decided to buy a try. Things received can not wait to open a look, and the seller described the same, I hope to use 2 months to have the effect, two months to follow up on	Jingdong prefecture level city in Shandong
Jinfeng Hongyuan Flagship Store	Electricity savings1	Thank you Jingdong, logistics is very fast order to receipt only one day. Believe in Jingdong. Favorable comments	Jingdong prefecture level city in Shandong
Jinfeng Hongyuan Flagship Store	Electricity savings1	I used one to use it, but it didn't work very well, what to do	Jingdong prefecture level city in Shandong
Jinfeng Hongyuan Flagship Store	Electricity savings1	Quality and packaging are very good, fast shipping, good service, buy with confidence	Jingdong prefecture level city in Shandong
Jinfeng Hongyuan Flagship Store	Electricity savings1	Use a period of time to evaluate, the quality is very good, the price is affordable, logistics is also very fast	Jingdong prefecture level city in Shandong
Jinfeng Hongyuan Flagship Store	Electricity savings1	Goods received, the day of the order tomorrow delivery real God speed, delivery force, the product to be used after a period of time and then low comment!	Jingdong prefecture level city in Shandong
Jinfeng Hongyuan Flagship Store	Electricity savings1	I don't know, how effective it is, let's talk about it when the time comes, whether it saves power or not, how much, unknown.	Jingdong prefecture level city in Shandong
Jinfeng Hongyuan Flagship Store	Electricity savings1	It does save some electricity, and I don't want to use something that isn't legal! Used nearly ~ electricity last month, probably not much more this month with the AC on!	Jingdong prefecture level city in Shandong
Jinfeng Hongyuan Flagship Store	Electricity savings1	Well-packed, excellent quality, service satisfaction good value for money	Jingdong prefecture level city in Shandong
Jinfeng Hongyuan Flagship Store	Electricity savings1	Express very quickly, the goods received last night, today used a day of goods can not heat, looking forward to whether it can save electricity, with a month and then comment, is used well will buy again.	Jingdong prefecture level city in Shandong
Jinfeng Hongyuan Flagship Store	Electricity savings1	It's pretty good, hopefully it'll be a real power saver.	Jingdong prefecture level city in Shandong
Jinfeng Hongyuan Flagship Store	Electricity savings1	The goods received in use, can not see the effect of energy saving, after a month to know!	Jingdong prefecture level city in Shandong
Jinfeng Hongyuan Flagship Store	Electricity savings1	Received the goods, looking forward to better surprise, with the effect of the follow-up comment!	Jingdong prefecture level city in Shandong
Jinfeng Hongyuan Flagship Store	Electricity savings1	It's not bad, it's still in use and should save some	Jingdong prefecture level city in Shandong
Jinfeng Hongyuan Flagship Store	Electricity savings1	Logistics is very fast, easy to install, plugged in on it,, the effect of the use of the later to evaluate!	Jingdong prefecture level city in Shandong
Jinfeng Hongyuan Flagship Store	Electricity savings1	The stuff is recommendable to buy very good.	Jingdong prefecture level city in Shandong
Jinfeng Hongyuan Flagship Store	Electricity savings1	Voltmeter measurements of voltage are way off, aren't they?	Jingdong prefecture level city in Shandong
Jinfeng Hongyuan Flagship Store	Electricity savings1	Install it to the socket immediately after purchase and try to see if it can save electricity after one month.	Jingdong prefecture level city in Shandong
Jinfeng Hongyuan Flagship Store	Electricity savings1	I've been using it for almost a month now, and I won't know if it saves electricity until I pay the bill.	Jingdong prefecture level city in Shandong
Jinfeng Hongyuan Flagship Store	Electricity savings1	I bought one in the past and it was fine, so I'm buying it back.	Jingdong prefecture level city in Shandong
Jinfeng Hongyuan Flagship Store	Electricity savings1	Service is very good, this thing is small and exquisite to use, just waiting to use the effect of a period of time	Jingdong prefecture level city in Shandong
Jinfeng Hongyuan Flagship Store	Electricity savings1	Not sure how well it works yet	Jingdong prefecture level city in Shandong
Jinfeng Hongyuan Flagship Store	Electricity savings1	Kyodo goods habitually good reviews	Jingdong prefecture level city in Shandong
Jinfeng Hongyuan Flagship Store	Electricity savings1	Appearance is OK, whether it is power saving remains to be seen.	Jingdong prefecture level city in Shandong
Jinfeng Hongyuan Flagship Store	Electricity savings1	I bought it and used it, I just don't know if it really saves power	Jingdong prefecture level city in Shandong
Jinfeng Hongyuan Flagship Store	Electricity savings1	The saver is well made and of good quality, I will evaluate it after using it for a while.	Jingdong prefecture level city in Shandong
Jinfeng Hongyuan Flagship Store	Electricity savings1	Just received in use, use for a period of time to see if there is power saving	Jingdong prefecture level city in Shandong
Jinfeng Hongyuan Flagship Store	Electricity savings1	Used 3 days before commenting, it really saves electricity, I look at the "power calendar" after using one less degree of electricity per day, so I think it's worth it to buy this, I will introduce my friends	Jingdong prefecture level city in Shandong
Jinfeng Hongyuan Flagship Store	Electricity savings1	Reliable quality, very well packaged and matches the real thing.	Jingdong prefecture level city in Shandong
Jinfeng Hongyuan Flagship Store	Electricity savings1	Logistics is still quite fast, ha in the trial	Jingdong prefecture level city in Shandong
Jinfeng Hongyuan Flagship Store	Electricity savings1	Consultant service attitude is very good, the courier is also very powerful, the first day of the order the next day to arrive. Saver received used and then add comments!	Jingdong prefecture level city in Shandong

Jinfeng Hongyuan Flagship Store	Electricity savings1	I just got home to try it out and see how it goes.	Jingdong prefecture level city in Shandong
Jinfeng Hongyuan Flagship Store	Electricity savings1	Well made and weighted instrument. Kudos! Put it into use immediately, don't know if it saves power, will evaluate later!	Jingdong prefecture level city in Shandong
Jinfeng Hongyuan Flagship Store	Electricity savings1	I've just put it in place. I'll give you a review in ten days.	Jingdong prefecture level city in Shandong
Jinfeng Hongyuan Flagship Store	Electricity savings1	It looks very delicate, not bad. Well, I don't know if it can save power or not, I have to try it for a while and see. If it can really save electricity. Uh, if it works well. Then additional comments. Anyway, thanks for the logistics, thanks to Jingdong. Well, it deserves some praise.	Jingdong prefecture level city in Shandong
Jinfeng Hongyuan Flagship Store	Electricity savings1	Bought a month, the feeling is that there is a certain effect, a month to save a lot of money	Jingdong prefecture level city in Shandong
Jinfeng Hongyuan Flagship Store	Electricity savings1	Goods received, the physical and the same as the picture, logistics is also very fast, the service attitude is very good, it is worth buying!	Jingdong prefecture level city in Shandong
Jinfeng Hongyuan Flagship Store	Electricity savings1	I've used it, I don't know whether it saves electricity or not, I'll talk about it after a while.	Jingdong prefecture level city in Shandong
Jinfeng Hongyuan Flagship Store	Electricity savings1	August only began to use, October to know, have no power-saving effect, to the in to evaluate!	Jingdong prefecture level city in Shandong
Jinfeng Hongyuan Flagship Store	Electricity savings1	Goods received: I do not know to the factory products used to save electricity!	Jingdong prefecture level city in Shandong
Jinfeng Hongyuan Flagship Store	Electricity savings1	Don't know if it works, bought it anyway.	Jingdong prefecture level city in Shandong
Jinfeng Hongyuan Flagship Store	Electricity savings1	Still testing, will follow up next month to evaluate how much electricity is saved	Jingdong prefecture level city in Shandong
Jinfeng Hongyuan Flagship Store	Electricity savings1	I'm still testing it, I'll comment after I use it.	Jingdong prefecture level city in Shandong
Jinfeng Hongyuan Flagship Store	Electricity savings1	Fast service, praiseworthy. Good item pre-style, first praise, the use of the effect to be evaluated after use.	Jingdong prefecture level city in Shandong
Jinfeng Hongyuan Flagship Store	Electricity savings1	The power saving effect is more obvious through verified use.	Jingdong prefecture level city in Shandong
Jinfeng Hongyuan Flagship Store	Electricity savings1	Logistics is very fast yesterday order today arrived. The product was immediately used, and after a month to see its effect.	Jingdong prefecture level city in Shandong
Jinfeng Hongyuan Flagship Store	Electricity savings1	Power saving is not yet known, as far as the TV and audio are concerned, I feel that there is a significant quality improvement, and I recommend using a good product!	Jingdong prefecture level city in Shandong
Jinfeng Hongyuan Flagship Store	Electricity savings1	Good power savings, tens of dollars a month saved	Jingdong prefecture level city in Shandong
Jinfeng Hongyuan Flagship Store	Electricity savings1	Already took two, great value! You can save 30% on your electric bill in a month. A summer must-have!	Jingdong prefecture level city in Shandong
Jinfeng Hongyuan Flagship Store	Electricity savings1	Received the item, plugged it into a home outlet and it showed 230 volts (not that high with a multimeter). Let's use it first!	Jingdong prefecture level city in Shandong
Jinfeng Hongyuan Flagship Store	Electricity savings1	Expecting results in the experiment	Jingdong prefecture level city in Shandong
Jinfeng Hongyuan Flagship Store	Electricity savings1	I don't know if it really saves power.	Jingdong prefecture level city in Shandong
Jinfeng Hongyuan Flagship Store	Electricity savings1	It's very good and the efficacy is very visible.	Jingdong prefecture level city in Shandong
Jinfeng Hongyuan Flagship Store	Electricity savings1	It's good and worth buying with confidence	Jingdong prefecture level city in Shandong
Jinfeng Hongyuan Flagship Store	Electricity savings1	The goods have been received, just do not know if it is effective	Jingdong prefecture level city in Shandong
Jinfeng Hongyuan Flagship Store	Electricity savings1	It's already in use, but based on some of the descriptions, I suspect that to use it, you need to have one in series on each line, which seems to work for all appliances, that's my guess, but I feel as though the principle of the thing seems to require it.	Jingdong prefecture level city in Shandong
Jinfeng Hongyuan Flagship Store	Electricity savings1	Fast delivery, already in use, looks good!	Jingdong prefecture level city in Shandong
Jinfeng Hongyuan Flagship Store	Electricity savings1	Hoping to save 30% of power as presented in the graphic	Jingdong prefecture level city in Shandong
Jinfeng Hongyuan Flagship Store	Electricity savings1	Things look good, power saving do not know now, with a month after the continued evaluation	Jingdong prefecture level city in Shandong
Jinfeng Hongyuan Flagship Store	Electricity savings1	Already used it but not sure if it will top out!	Jingdong prefecture level city in Shandong
Jinfeng Hongyuan Flagship Store	Electricity savings1	Looking forward to using the results, the product quality looks okay.	Jingdong prefecture level city in Shandong
Jinfeng Hongyuan Flagship Store	Electricity savings1	It's somewhat effective and saves money more or less 💰	Jingdong prefecture level city in Shandong
Jinfeng Hongyuan Flagship Store	Electricity savings1	Got it, it's great, looking forward to saving power.	Jingdong prefecture level city in Shandong
Jinfeng Hongyuan Flagship Store	Electricity savings1	I don't know if it's working yet, I'll follow up when I get there	Jingdong prefecture level city in Shandong
Jinfeng Hongyuan Flagship Store	Electricity savings1	The product received, good workmanship, fast logistics, good inner packaging, the outer bag is damaged, but fortunately did not hurt the product, retrieved and inserted according to the instructions, looking forward to the effect is good, with a period of time to evaluate, I believe that the Jingdong.	Jingdong prefecture level city in Shandong
Jinfeng Hongyuan Flagship Store	Electricity savings1	Since it is a new product, it is said to work, so if it works well for a month, then I will give you a good review.	Jingdong prefecture level city in Shandong
Jinfeng Hongyuan Flagship Store	Electricity savings1	The quality of the product is very good, very well made, well packaged, easy to use and very convenient!	Jingdong prefecture level city in Shandong
Jinfeng Hongyuan Flagship Store	Electricity savings1	Good price for the item, good functionality	Jingdong prefecture level city in Shandong
Jinfeng Hongyuan Flagship Store	Electricity savings1	Received the first time to use it, the stuff is very good, specifics wait for a month to come back to feedback to see the effect.	Jingdong prefecture level city in Shandong
Jinfeng Hongyuan Flagship Store	Electricity savings1	Just received, the courier is too bad, not notified directly to put the Feng Nest courier cabinet	Jingdong prefecture level city in Shandong
Jinfeng Hongyuan Flagship Store	Electricity savings1	Baby received, the second time to buy, plugged in and used do not know if there is no effect. Baby received, the second time to buy, plugged in and used do not know if there is no effect. Baby received, the second time to buy, plugged in and used do not know if there is no effect.	Jingdong prefecture level city in Shandong
Jinfeng Hongyuan Flagship Store	Electricity savings1	It's okay if it saves power. It's not a bad product.	Jingdong prefecture level city in Shandong
Jinfeng Hongyuan Flagship Store	Electricity savings1	Received, very good, meticulous packaging, open a look is genuine, meticulous workmanship, the manual is very detailed, has been used, there is no effect on the only look at the next month's electricity bill, when I can I buy a few more to send people!	Jingdong prefecture level city in Shandong
Jinfeng Hongyuan Flagship Store	Electricity savings1	The quality is very good very delicate express delivery is too fast	Jingdong prefecture level city in Shandong
Jinfeng Hongyuan Flagship Store	Electricity savings1	I won't know until a month after I start using it today.	Jingdong prefecture level city in Shandong
Jinfeng Hongyuan Flagship Store	Electricity savings1	It doesn't work at all, the customer service attitude is very bad, use it or not!	Jingdong prefecture level city in Shandong
Jinfeng Hongyuan Flagship Store	Electricity savings1	Temporarily try, do not know the effect, Jingdong logistics delivery very quickly	Jingdong prefecture level city in Shandong
Jinfeng Hongyuan Flagship Store	Electricity savings1	It doesn't help. It's comforting.	Jingdong prefecture level city in Shandong
Jinfeng Hongyuan Flagship Store	Electricity savings1	It's great, use it before you say anything.	Jingdong prefecture level city in Shandong
Jinfeng Hongyuan Flagship Store	Electricity savings1	Received, already used, looking forward to a good a effect!	Jingdong prefecture level city in Shandong

Jinfeng Hongyuan Flagship Store	Electricity savings1	I don't know if it works yet, I won't know until I use it for a few months.	Jingdong prefecture level city in Shandong
Jinfeng Hongyuan Flagship Store	Electricity savings1	It takes a long time to get there. There's no change now.	Jingdong prefecture level city in Shandong
Jinfeng Hongyuan Flagship Store	Electricity savings1	Jingdong's delivery never disappoints, or so fast, and the stuff is great, next time you need to come back.	Jingdong prefecture level city in Shandong
Jinfeng Hongyuan Flagship Store	Electricity savings1	At first it was ok today, after using it for a while it didn't work anymore	Jingdong prefecture level city in Shandong
Jinfeng Hongyuan Flagship Store	Electricity savings1	Just received the goods, do not know the effect of using a few days before there may be effect	Jingdong prefecture level city in Shandong
Jinfeng Hongyuan Flagship Store	Electricity savings1	The logistics was fast, I received it the next day, I've purchased it for the second time and have been using it all along	Jingdong prefecture level city in Shandong
Jinfeng Hongyuan Flagship Store	Electricity savings1	Inexpensive, fast delivery, very pleased!	Jingdong prefecture level city in Shandong
Jinfeng Hongyuan Flagship Store	Electricity savings1	Received the goods immediately plugged directly into the use of power saving yet to be good news!	Jingdong prefecture level city in Shandong
Jinfeng Hongyuan Flagship Store	Electricity savings1	The service attitude is very good, and the Jingdong self-owned products can be trusted.	Jingdong prefecture level city in Shandong
Jinfeng Hongyuan Flagship Store	Electricity savings1	The product is good, I do not know if it can save play a power saving effect	Jingdong prefecture level city in Shandong
Jinfeng Hongyuan Flagship Store	Electricity savings1	We won't know how effective it is until we use it for a while, so I'll give you a good review now.	Jingdong prefecture level city in Shandong
Jinfeng Hongyuan Flagship Store	Electricity savings1	The quality is pretty good, just started using it, hopefully it will deliver results.	Jingdong prefecture level city in Shandong
Jinfeng Hongyuan Flagship Store	Electricity savings1	I've plugged it in first, and will follow up after I've used it to see how it works.	Jingdong prefecture level city in Shandong
Jinfeng Hongyuan Flagship Store	Electricity savings1	Things have arrived, the quality is good, the effect is also equivalent to can ah	Jingdong prefecture level city in Shandong
Jinfeng Hongyuan Flagship Store	Electricity savings1	It's not worth it to brag about power savings.	Jingdong prefecture level city in Shandong
Jinfeng Hongyuan Flagship Store	Electricity savings1	I don't know if it's an IQ tax, but I'll use it for now.	Jingdong prefecture level city in Shandong
Jinfeng Hongyuan Flagship Store	Electricity savings1	I don't feel the power saving yet, I can't know until I use it for 2 months.	Jingdong prefecture level city in Shandong
Jinfeng Hongyuan Flagship Store	Electricity savings1	There's nothing wrong with this at the moment.	Jingdong prefecture level city in Shandong
Jinfeng Hongyuan Flagship Store	Electricity savings1	It's authentic and worth buying	Jingdong prefecture level city in Shandong
Jinfeng Hongyuan Flagship Store	Electricity savings1	It's good. We'll see how it goes.	Jingdong prefecture level city in Shandong
Jinfeng Hongyuan Flagship Store	Electricity savings1	I don't know if it's working or not in the middle of using it, we'll see when the month is over	Jingdong prefecture level city in Shandong
Jinfeng Hongyuan Flagship Store	Electricity savings1	I'm not going to buy anything from Jingdong anymore, so I'll give it a good review	Jingdong prefecture level city in Shandong
Jinfeng Hongyuan Flagship Store	Electricity savings1	I'm not going to buy anything from Jingdong anymore, so I'll give it a good review	Jingdong prefecture level city in Shandong
Jinfeng Hongyuan Flagship Store	Electricity savings1	Just received the item and will see how it works in a month.	Jingdong prefecture level city in Shandong
Jinfeng Hongyuan Flagship Store	Electricity savings1	I don't know if it saves electricity, but it's plugged into an outlet every day anyway	Jingdong prefecture level city in Shandong
Jinfeng Hongyuan Flagship Store	Electricity savings1	I don't know if it works yet.	Jingdong prefecture level city in Shandong
Jinfeng Hongyuan Flagship Store	Electricity savings1	Plugged it in when I got back, waiting for a month to see the results	Jingdong prefecture level city in Shandong
Jinfeng Hongyuan Flagship Store	Electricity savings1	Effects remain to be seen, but it looks good quality and runs fine	Jingdong prefecture level city in Shandong
Jinfeng Hongyuan Flagship Store	Electricity savings1	The product received logistics very quickly, has not yet been used to test! After use in the evaluation!	Jingdong prefecture level city in Shandong
Jinfeng Hongyuan Flagship Store	Electricity savings1	The power saver is in use, and I don't know how much electricity has been saved after using it for more than a month, and the display voltmeter is very good.	Jingdong prefecture level city in Shandong
Jinfeng Hongyuan Flagship Store	Electricity savings1	It's so good, isn't it? You're a real Shanghainese, aren't you?	Jingdong prefecture level city in Shandong
Jinfeng Hongyuan Flagship Store	Electricity savings1	Great quality great great 👍👍👍👍	Jingdong prefecture level city in Shandong
Jinfeng Hongyuan Flagship Store	Electricity savings1	I'm using it, I don't know if it's working yet.	Jingdong prefecture level city in Shandong
Jinfeng Hongyuan Flagship Store	Electricity savings1	Just used it for more than 10 days and haven't seen any results yet	Jingdong prefecture level city in Shandong
Jinfeng Hongyuan Flagship Store	Electricity savings1	The product arrived home feeling like it should work	Jingdong prefecture level city in Shandong
Jinfeng Hongyuan Flagship Store	Electricity savings1	Just received the goods, use it and comment back	Jingdong prefecture level city in Shandong
Jinfeng Hongyuan Flagship Store	Electricity savings1	Try it for a month and then evaluate	Jingdong prefecture level city in Shandong
Jinfeng Hongyuan Flagship Store	Electricity savings1	I don't know if it saves power yet, but I'll comment again after using it for a while.	Jingdong prefecture level city in Shandong
Jinfeng Hongyuan Flagship Store	Electricity savings1	The product is better than I thought, the quality and appearance are very good, the voltage is stable, and I'm looking forward to a good power saving effect.	Jingdong prefecture level city in Shandong
Jinfeng Hongyuan Flagship Store	Electricity savings1	Received and scanned the QR code to check that it is genuine, have plugged it in, can it save power, looking forward to the advertised effect. Customary praise, 5 points!	Jingdong prefecture level city in Shandong
Jinfeng Hongyuan Flagship Store	Electricity savings1	The goods have been received and have not yet been used. Do not know how the effect.	Jingdong prefecture level city in Shandong
Jinfeng Hongyuan Flagship Store	Electricity savings1	Hopefully, you get what you pay for, and you get what you pay for!	Jingdong prefecture level city in Shandong
Jinfeng Hongyuan Flagship Store	Electricity savings1	Just put it on and it feels like it's turning as fast as a meter! Looking forward to the results!	Jingdong prefecture level city in Shandong
Jinfeng Hongyuan Flagship Store	Electricity savings1	Overall look on the product quality is good, I do not know is not the effect of power saving, wait to see and then follow up!	Jingdong prefecture level city in Shandong
Jinfeng Hongyuan Flagship Store	Electricity savings1	The logistics courier is very fast, the courier service attitude is very good	Jingdong prefecture level city in Shandong
Jinfeng Hongyuan Flagship Store	Electricity savings1	The product has been received, it works well worth buying	Jingdong prefecture level city in Shandong
Jinfeng Hongyuan Flagship Store	Electricity savings1	How did it not work? Is it purely an IQ tax?	Jingdong prefecture level city in Shandong
Jinfeng Hongyuan Flagship Store	Electricity savings1	It looks good, I don't know how well it works.	Jingdong prefecture level city in Shandong
Jinfeng Hongyuan Flagship Store	Electricity savings1	It does seem to be a bit of a power saver, I just don't know if the more expensive one is more power efficient?	Jingdong prefecture level city in Shandong
Jinfeng Hongyuan Flagship Store	Electricity savings1	Is genuine very good, the price is much cheaper, cost-effective, really special paddle just to do activities, the price is much cheaper, cost-effective, really special paddle counting, packaging is also very high-end atmospheric class, it is worth buying!	Jingdong prefecture level city in Shandong
Jinfeng Hongyuan Flagship Store	Electricity savings1	I received it yesterday and used it right away, I hope it works, I'll wait for next month to come back to the conclusion, I'm looking forward to it. Five hearts praise!	Jingdong prefecture level city in Shandong

Jinfeng Hongyuan Flagship Store	Electricity savings1	Looks good, packed properly, received it today, am using it, will see results in a month!	Jingdong prefecture level city in Shandong
Jinfeng Hongyuan Flagship Store	Electricity savings1	Regular product Jingdong self-support trustworthy. There is an effect Come back to the review. 😍😍😍😍	Jingdong prefecture level city in Shandong
Jinfeng Hongyuan Flagship Store	Electricity savings1	I haven't used it yet, hopefully it will work as a power saver, and will comment again if it works well.	Jingdong prefecture level city in Shandong
Jinfeng Hongyuan Flagship Store	Electricity savings1	Shipping was fast, logistics was fast, service was good, and I look forward to good results from the item!	Jingdong prefecture level city in Shandong
Jinfeng Hongyuan Flagship Store	Electricity savings1	This product is this compensator, it has to be at the main switch to be useful, haven't compared the final result, the	Jingdong prefecture level city in Shandong
Jinfeng Hongyuan Flagship Store	Electricity savings1	Packaging is perfect . I've already started using it, so I'm looking forward to the results.	Jingdong prefecture level city in Shandong
Jinfeng Hongyuan Flagship Store	Electricity savings1	I just got it back. I'll comment after I use it.	Jingdong prefecture level city in Shandong
Jinfeng Hongyuan Flagship Store	Electricity savings1	Big brand, with a very assured, very good quality, catch the event to buy very cost-effective to take advantage of the offer, hurry to get, it is genuine, the packaging is also very good, no breakage, the quality is not to speak of, great 👍 logistics super super fast! Quality is also very good, indeed value	Jingdong prefecture level city in Shandong
Jinfeng Hongyuan Flagship Store	Electricity savings1	The quality is pretty good. The price is also quite affordable.	Jingdong prefecture level city in Shandong
Jinfeng Hongyuan Flagship Store	Electricity savings1	I did not expect the courier so fast to arrive yesterday just ordered, the head is a little smaller than I thought, well, the picture looks bigger. The appearance still looks good, as for the practicality and effectiveness of the use of the only know.	Jingdong prefecture level city in Shandong
Jinfeng Hongyuan Flagship Store	Electricity savings1	I don't know. Looking forward to it.	Jingdong prefecture level city in Shandong
Jinfeng Hongyuan Flagship Store	Electricity savings1	Baby received, very delicate, very nice, like. Just don't know if it can really save electricity. Baby received, very delicate, very good-looking, like. Just don't know if it can really save electricity.	Jingdong prefecture level city in Shandong
Jinfeng Hongyuan Flagship Store	Electricity savings1	I'll use it for a month and then comment on the quality of the item	Jingdong prefecture level city in Shandong
Jinfeng Hongyuan Flagship Store	Electricity savings1	It feels ok, use it for a while and see how it works before adding more comments.	Jingdong prefecture level city in Shandong
Jinfeng Hongyuan Flagship Store	Electricity savings1	In use, see results in a month.	Jingdong prefecture level city in Shandong
Jinfeng Hongyuan Flagship Store	Electricity savings1	Jingdong's logistics and quality are very good, the service attitude is very good, the logistics is very fast	Jingdong prefecture level city in Shandong
Jinfeng Hongyuan Flagship Store	Electricity savings1	Intelligent power saver received, well-packed logistics is also quite fast, easy to use, looking forward to good results!	Jingdong prefecture level city in Shandong
Jinfeng Hongyuan Flagship Store	Electricity savings1	The quality is unknown at this point, I won't know until I use it.	Jingdong prefecture level city in Shandong
Jinfeng Hongyuan Flagship Store	Electricity savings1	It's a good product. It really saves power and doesn't take up much space.	Jingdong prefecture level city in Shandong
Jinfeng Hongyuan Flagship Store	Electricity savings1	Goods have been received, packaging is very good, good to see the effect of future use in the follow-up.	Jingdong prefecture level city in Shandong
Jinfeng Hongyuan Flagship Store	Electricity savings1	The product is in perfect shape and the light weight is worth it!	Jingdong prefecture level city in Shandong
Jinfeng Hongyuan Flagship Store	Electricity savings1	Just got home and plugged it in. It's good.	Jingdong prefecture level city in Shandong
Jinfeng Hongyuan Flagship Store	Electricity savings1	The logistics are quick, just to see the results.	Jingdong prefecture level city in Shandong
Jinfeng Hongyuan Flagship Store	Electricity savings1	Short time can not feel here to leave a message, to be followed up later!	Jingdong prefecture level city in Shandong
Jinfeng Hongyuan Flagship Store	Electricity savings1	This user thinks the product is good	Jingdong prefecture level city in Shandong
Jinfeng Hongyuan Flagship Store	Electricity savings1	Received the baby, to be used to see the effect of it!	Jingdong prefecture level city in Shandong
Jinfeng Hongyuan Flagship Store	Electricity savings1	You have to wait a month or two to see how it works.	Jingdong prefecture level city in Shandong
Jinfeng Hongyuan Flagship Store	Electricity savings1	Not bad, good, like it, don't know if it's good, try it first.	Jingdong prefecture level city in Shandong
Jinfeng Hongyuan Flagship Store	Electricity savings1	The goods are good value for money and the logistics is very fast and good service!	Jingdong prefecture level city in Shandong
Jinfeng Hongyuan Flagship Store	Electricity savings1	I don't know how well it works, we'll see in a month	Jingdong prefecture level city in Shandong
Jinfeng Hongyuan Flagship Store	Electricity savings1	The product is fine, but r saving power or not I won't know until I use it for two months. The display and lights take electricity too?	Jingdong prefecture level city in Shandong
Jinfeng Hongyuan Flagship Store	Electricity savings1	I've used it. I don't know how it works yet.	Jingdong prefecture level city in Shandong
Jinfeng Hongyuan Flagship Store	Electricity savings1	I can't say until I compare them.	Jingdong prefecture level city in Shandong
Jinfeng Hongyuan Flagship Store	Electricity savings1	It's ok in the process of using 。。。。	Jingdong prefecture level city in Shandong
Jinfeng Hongyuan Flagship Store	Electricity savings1	Jinfeng Hongyuan Intelligent Power Saver Household Electricity Meter Electricity Saving Appliance High Power Enhanced Power Saving King Energy Saving Treasure Butler Control Non Stealing Electricity Reversing Not Going Interfering Slow Turner Artifact King Gold Yellow. Haven't tested it accurately yet, really need to wait a few months to test it before I have a say.	Jingdong prefecture level city in Shandong
Jinfeng Hongyuan Flagship Store	Electricity savings1	Don't know how effective it is yet? I hope it works well and I'll come back next time	Jingdong prefecture level city in Shandong
Jinfeng Hongyuan Flagship Store	Electricity savings1	Saving device has been received, material workmanship are very good ah! Have not yet begun to use. First observe the daily electricity consumption and cost of the home meter, and then plug in the power saver to use, so as to compare the use of the effect is good!	Jingdong prefecture level city in Shandong
Jinfeng Hongyuan Flagship Store	Electricity savings1	Merchants shipping extremely fast, Jingdong logistics safe and fast, I believe that the product can play a safe use of electricity, the role of scientific power saving, first praise, the effect of how to be used for a period of time and then the pursuit of bubble!	Jingdong prefecture level city in Shandong
Jinfeng Hongyuan Flagship Store	Electricity savings1	I just bought it. I don't know yet.	Jingdong prefecture level city in Shandong
Jinfeng Hongyuan Flagship Store	Electricity savings1	It is genuine, good quality, and it is very reassuring to use. The logistics is very fast, and the business service attitude is on point.	Jingdong prefecture level city in Shandong
Jinfeng Hongyuan Flagship Store	Electricity savings1	The quality is fine, I don't know how effective it is.	Jingdong prefecture level city in Shandong
Jinfeng Hongyuan Flagship Store	Electricity savings1	The stuff is back and I expect it will save the electricity	Jingdong prefecture level city in Shandong
Jinfeng Hongyuan Flagship Store	Electricity savings1	I'll give it 4 stars first and see what happens in a couple of months and then follow up	Jingdong prefecture level city in Shandong
Jinfeng Hongyuan Flagship Store	Electricity savings1	It's not bad. It's in the mind.	Jingdong prefecture level city in Shandong
Jinfeng Hongyuan Flagship Store	Electricity savings1	Received the goods have not yet started to use do not know whether it is good	Jingdong prefecture level city in Shandong
Jinfeng Hongyuan Flagship Store	Electricity savings1	This user thinks the product is good	Jingdong prefecture level city in Shandong
Jinfeng Hongyuan Flagship Store	Electricity savings1	Hello! The saver was received, thank you! Not yet used, but from the packaging looks very good quality, exquisite workmanship. Logistics is quite fast, if it is really very power saving, I will come back to	Jingdong prefecture level city in Shandong
Jinfeng Hongyuan Flagship Store	Electricity savings1	I've been using it for a few days now, I'll comment again after a month or two of use	Jingdong prefecture level city in Shandong

Jinfeng Hongyuan Flagship Store	Electricity savings1	I don't know. Bought it for my father at a good price.	Jingdong prefecture level city in Shandong
Jinfeng Hongyuan Flagship Store	Electricity savings1	Plugged it in the same day I bought it and the voltage showed 223, 225 looking forward to the results	Jingdong prefecture level city in Shandong
Jinfeng Hongyuan Flagship Store	Electricity savings1	Still in use, the biggest role is to stabilize the current, reduce losses, product quality on the look is very good, with this feeling quite good, see colleagues are using, power saving effect should be good	Jingdong prefecture level city in Shandong
Jinfeng Hongyuan Flagship Store	Electricity savings1	How to receive goods but no invoice and warranty more than it ...... quality is the premise of reputation!	Jingdong prefecture level city in Shandong
Jinfeng Hongyuan Flagship Store	Electricity savings1	Received but not used Continued evaluation	Jingdong prefecture level city in Shandong
Jinfeng Hongyuan Flagship Store	Electricity savings1	I just received the product, I don't know if it works well yet	Jingdong prefecture level city in Shandong
Jinfeng Hongyuan Flagship Store	Electricity savings1	Received it and used it, not sure it really saves electricity use it and then evaluate it, first give a 5 star review!!!!	Jingdong prefecture level city in Shandong
Jinfeng Hongyuan Flagship Store	Electricity savings1	It's good to use, it's very good to use, and it smells OK too	Jingdong prefecture level city in Shandong
Jinfeng Hongyuan Flagship Store	Electricity savings1	Packaging is very good, feel the product is not bad, how much power saving with the only know	Jingdong prefecture level city in Shandong
Jinfeng Hongyuan Flagship Store	Electricity savings1	Additional comments to be made after use	Jingdong prefecture level city in Shandong
Jinfeng Hongyuan Flagship Store	Electricity savings1	The product has been installed, try a few months and then comment!	Jingdong prefecture level city in Shandong
Jinfeng Hongyuan Flagship Store	Electricity savings1	I've bought it, let's use it for now, I'll comment after a month	Jingdong prefecture level city in Shandong
Jinfeng Hongyuan Flagship Store	Electricity savings1	Try it out and then follow up	Jingdong prefecture level city in Shandong
Jinfeng Hongyuan Flagship Store	Electricity savings1	It's good in theory, it doesn't feel good in practice.	Jingdong prefecture level city in Shandong
Jinfeng Hongyuan Flagship Store	Electricity savings1	I haven't tried it yet. We'll see.	Jingdong prefecture level city in Shandong
Jinfeng Hongyuan Flagship Store	Electricity savings1	Great item, second purchase!	Jingdong prefecture level city in Shandong
Jinfeng Hongyuan Flagship Store	Electricity savings1	Goods received now use it, if it does save power, plus reevaluate.	Jingdong prefecture level city in Shandong
Jinfeng Hongyuan Flagship Store	Electricity savings1	The stuff is light and small, the stuff is good, the courier service is good	Jingdong prefecture level city in Shandong
Jinfeng Hongyuan Flagship Store	Electricity savings1	I haven't used it yet. I'm looking forward to it.	Jingdong prefecture level city in Shandong
Jinfeng Hongyuan Flagship Store	Electricity savings1	Express received, in use do not know if there is no power saving	Jingdong prefecture level city in Shandong
Jinfeng Hongyuan Flagship Store	Electricity savings1	The quality of the product is quite good! The power saving mode is still getting used to it, can't say yet, how much power it saves.	Jingdong prefecture level city in Shandong
Jinfeng Hongyuan Flagship Store	Electricity savings1	The goods have been received, just started to use, now I do not know how the quality, after a period of time to say.	Jingdong prefecture level city in Shandong
Jinfeng Hongyuan Flagship Store	Electricity savings1	I received the stuff, used it for a few days to evaluate, the stuff is very good and good, worth buying.	Jingdong prefecture level city in Shandong
Jinfeng Hongyuan Flagship Store	Electricity savings1	I've been using it for a while, but I don't know if it's good or if it saves electricity! What the heck, let's give it a good review first. And for the record, I'm not helping him**.	Jingdong prefecture level city in Shandong
Jinfeng Hongyuan Flagship Store	Electricity savings1	Haven't used it yet, looks good, try it for 60 days, might be good, great review!	Jingdong prefecture level city in Shandong
Jinfeng Hongyuan Flagship Store	Electricity savings1	Baby received, logistics is very fast. Ordered last night, received this morning. Received the goods immediately plugged in the power supply, save power to the first of next month to know.	Jingdong prefecture level city in Shandong
Jinfeng Hongyuan Flagship Store	Electricity savings1	First, I'll give you a good review and use it.	Jingdong prefecture level city in Shandong
Jinfeng Hongyuan Flagship Store	Electricity savings1	Just arrived to follow up with a genuine review	Jingdong prefecture level city in Shandong
Jinfeng Hongyuan Flagship Store	Electricity savings1	Not bad pretty fast, good quality.	Jingdong prefecture level city in Shandong
Jinfeng Hongyuan Flagship Store	Electricity savings1	Recommended, use it and hopefully it will save some electricity	Jingdong prefecture level city in Shandong
Jinfeng Hongyuan Flagship Store	Electricity savings1	The stuff looks ok, I don't know the effect if it.	Jingdong prefecture level city in Shandong
Jinfeng Hongyuan Flagship Store	Electricity savings1	The stuff looks OK, the stuff looks OK.	Jingdong prefecture level city in Shandong
Jinfeng Hongyuan Flagship Store	Electricity savings1	The item has been received, after using it in a follow-up review. Satisfied with the purchase.	Jingdong prefecture level city in Shandong
Jinfeng Hongyuan Flagship Store	Electricity savings1	Has been normal plug-in use, at present do not know how to see, use down to see and then evaluation	Jingdong prefecture level city in Shandong
Jinfeng Hongyuan Flagship Store	Electricity savings1	Commodities make noise.	Jingdong prefecture level city in Shandong
Jinfeng Hongyuan Flagship Store	Electricity savings1	After using it for a few days and observing it, the saving is about ten percent	Jingdong prefecture level city in Shandong
Jinfeng Hongyuan Flagship Store	Electricity savings1	The goods received, the quality looks quite OK. First use it, the effect is not good to say.	Jingdong prefecture level city in Shandong
Jinfeng Hongyuan Flagship Store	Electricity savings1	In use, follow up next month	Jingdong prefecture level city in Shandong
Jinfeng Hongyuan Flagship Store	Electricity savings1	I just received the goods and have not yet used it to comment after using it	Jingdong prefecture level city in Shandong
Jinfeng Hongyuan Flagship Store	Electricity savings1	So far I don't know if it really saves electricity yet! Spinning waiting a couple months to find out! Good reviews first!	Jingdong prefecture level city in Shandong
Jinfeng Hongyuan Flagship Store	Electricity savings1	Small and exquisite, looking forward to the effect, to be evaluated 6666666666666	Jingdong prefecture level city in Shandong
Jinfeng Hongyuan Flagship Store	Electricity savings1	I just used it, I don't know if it's effective, I'll comment again after a month	Jingdong prefecture level city in Shandong
Jinfeng Hongyuan Flagship Store	Electricity savings1	I just used it, I don't know if it's good or not, I'll comment later.	Jingdong prefecture level city in Shandong
Jinfeng Hongyuan Flagship Store	Electricity savings1	Baby received, Baobei quite good, use and then come back to comment!	Jingdong prefecture level city in Shandong
Jinfeng Hongyuan Flagship Store	Electricity savings1	Good quality power saving is fake	Jingdong prefecture level city in Shandong
Jinfeng Hongyuan Flagship Store	Electricity savings1	The packaging of the goods is atmospheric, the quality is exquisite, and I look forward to saving electricity.	Jingdong prefecture level city in Shandong
Jinfeng Hongyuan Flagship Store	Electricity savings1	The packaging is great and the quality is good!	Jingdong prefecture level city in Shandong
Jinfeng Hongyuan Flagship Store	Electricity savings1	I don't see any effect.	Jingdong prefecture level city in Shandong
Jinfeng Hongyuan Flagship Store	Electricity savings1	Already used, but did not see obvious results	Jingdong prefecture level city in Shandong
Jinfeng Hongyuan Flagship Store	Electricity savings1	It's good. I'm just using it. I don't know what it's like.	Jingdong prefecture level city in Shandong
Jinfeng Hongyuan Flagship Store	Electricity savings1	I just used it. I'll comment later.	Jingdong prefecture level city in Shandong

Jinfeng Hongyuan Flagship Store	Electricity savings1	I installed it when it arrived, try it for a month and then come back for a follow-up review	Jingdong prefecture level city in Shandong
Jinfeng Hongyuan Flagship Store	Electricity savings1	Goods arrived a few days fastidious does not call to pick up the goods, the quality is average	Jingdong prefecture level city in Shandong
Jinfeng Hongyuan Flagship Store	Electricity savings1	This user thinks the product is good	Jingdong prefecture level city in Shandong
Jinfeng Hongyuan Flagship Store	Electricity savings1	Roger that.	Jingdong prefecture level city in Shandong
Jinfeng Hongyuan Flagship Store	Electricity savings1	Second purchase was ok.	Jingdong prefecture level city in Shandong
Jinfeng Hongyuan Flagship Store	Electricity savings1	The merchant is very reputable and the product is good value for money. Logistics is fast!	Jingdong prefecture level city in Shandong
Jinfeng Hongyuan Flagship Store	Electricity savings1	The quality of the baby is very good, I have always trusted Kyodo	Jingdong prefecture level city in Shandong
Jinfeng Hongyuan Flagship Store	Electricity savings1	We'll see how it goes when we get it.	Jingdong prefecture level city in Shandong
Jinfeng Hongyuan Flagship Store	Electricity savings1	I just used it. I don't know if it works.	Jingdong prefecture level city in Shandong
Jinfeng Hongyuan Flagship Store	Electricity savings1	Good quality, used for a month to save about 20%, power saving effect is good	Jingdong prefecture level city in Shandong
Jinfeng Hongyuan Flagship Store	Electricity savings1	I'll give you a good review before I use it.	Jingdong prefecture level city in Shandong
Jinfeng Hongyuan Flagship Store	Electricity savings1	Received, satisfied with the follow-up evaluation	Jingdong prefecture level city in Shandong
Jinfeng Hongyuan Flagship Store	Electricity savings1	Immediate results, saving electricity and money	Jingdong prefecture level city in Shandong
Jinfeng Hongyuan Flagship Store	Electricity savings1	Measured to save a little	Jingdong prefecture level city in Shandong
Jinfeng Hongyuan Flagship Store	Electricity savings1	This user thinks the product is good	Jingdong prefecture level city in Shandong
Jinfeng Hongyuan Flagship Store	Electricity savings1	This user thinks the product is good	Jingdong prefecture level city in Shandong
Jinfeng Hongyuan Flagship Store	Electricity savings1	Product quality and logistics are very good, worth buying	Jingdong prefecture level city in Shandong
Jinfeng Hongyuan Flagship Store	Electricity savings1	Appearance looks ok! Express delivery was fast! Service is still relatively enthusiastic.	Jingdong prefecture level city in Shandong
Jinfeng Hongyuan Flagship Store	Electricity savings1	It doesn't feel like it's working.	Jingdong prefecture level city in Shandong
Jinfeng Hongyuan Flagship Store	Electricity savings1	Well made and looks good. Used it with other appliances and felt as if it had some effect.	Jingdong prefecture level city in Shandong
Jinfeng Hongyuan Flagship Store	Electricity savings1	Haven't used it yet, will follow up later	Jingdong prefecture level city in Shandong
Jinfeng Hongyuan Flagship Store	Electricity savings1	Things received, used a few days the plug is a little hot unplugged did not work	Jingdong prefecture level city in Shandong
Jinfeng Hongyuan Flagship Store	Electricity savings1	Look at the meter again in a month.	Jingdong prefecture level city in Shandong
Jinfeng Hongyuan Flagship Store	Electricity savings1	This one didn't work at all.	Jingdong prefecture level city in Shandong
Jinfeng Hongyuan Flagship Store	Electricity savings1	It's small. I've seen friends with much bigger ones.	Jingdong prefecture level city in Shandong
Jinfeng Hongyuan Flagship Store	Electricity savings1	The item was received and the quality is good, it will be nice to see how the power saving rate is down the road, the item is great.	Jingdong prefecture level city in Shandong
Jinfeng Hongyuan Flagship Store	Electricity savings1	Cheap and affordable quality is quite good, logistics is also very fast, praise! , baby good 😉, cost-effective very high, big brand trustworthy 👍	Jingdong prefecture level city in Shandong
Jinfeng Hongyuan Flagship Store	Electricity savings1	Used it for a while it really saves electricity ⚡ thanks 🈶 such a good product, compact and practical, every family should buy one 🌟	Jingdong prefecture level city in Shandong
Jinfeng Hongyuan Flagship Store	Electricity savings1	Very satisfied with the purchase Next time I will come back	Jingdong prefecture level city in Shandong
Jinfeng Hongyuan Flagship Store	Electricity savings1	Logistics is fast. Good quality, used one before and it does save power	Jingdong prefecture level city in Shandong
Jinfeng Hongyuan Flagship Store	Electricity savings1	I don't know if it works.	Jingdong prefecture level city in Shandong
Jinfeng Hongyuan Flagship Store	Electricity savings1	Good goods, very solid, good.	Jingdong prefecture level city in Shandong
Jinfeng Hongyuan Flagship Store	Electricity savings1	This user thinks the product is good	Jingdong prefecture level city in Shandong
Jinfeng Hongyuan Flagship Store	Electricity savings1	I've been using it for half a month, my electricity bill is much lower, and the quality is very good, so don't worry about buying it!	Jingdong prefecture level city in Shandong
Jinfeng Hongyuan Flagship Store	Electricity savings1	I received the goods today and used it right away, looking forward to the effect	Jingdong prefecture level city in Shandong
Jinfeng Hongyuan Flagship Store	Electricity savings1	The merchandise is great. I am going to recommend it to my friends! Will buy again next time.	Jingdong prefecture level city in Shandong
Jinfeng Hongyuan Flagship Store	Electricity savings1	Let's give it a good review first, it's working fine so far, and I'll follow up with a review,...	Jingdong prefecture level city in Shandong
Jinfeng Hongyuan Flagship Store	Electricity savings1	Product quality is very good, looking forward to using the results	Jingdong prefecture level city in Shandong
Jinfeng Hongyuan Flagship Store	Electricity savings1	I just can't detect it. - Save it.	Jingdong prefecture level city in Shandong
Jinfeng Hongyuan Flagship Store	Electricity savings1	It arrived and is being used, so we'll see how it works!	Jingdong prefecture level city in Shandong
Jinfeng Hongyuan Flagship Store	Electricity savings1	Received yesterday thanks! This energy saver is available . Safe! But in the end how much energy can be saved must be used for a period of time to know, if the real energy saving next time to buy again.	Jingdong prefecture level city in Shandong
Jinfeng Hongyuan Flagship Store	Electricity savings1	Just used it, don't know if it saves power	Jingdong prefecture level city in Shandong
Jinfeng Hongyuan Flagship Store	Electricity savings1	I'll rate it first, is it a tease wait two months and see? It's been four days and it's not noticeable!	Jingdong prefecture level city in Shandong
Jinfeng Hongyuan Flagship Store	Electricity savings1	Workmanship is quite good, just started to use, the effect of the follow-up evaluation.	Jingdong prefecture level city in Shandong
Jinfeng Hongyuan Flagship Store	Electricity savings1	Received the goods used for a few days before evaluation, the effect is quite good	Jingdong prefecture level city in Shandong
Jinfeng Hongyuan Flagship Store	Electricity savings1	Try it for a month and then evaluate	Jingdong prefecture level city in Shandong
Jinfeng Hongyuan Flagship Store	Electricity savings1	Just received the goods, the packaging is very good, the appearance of the economizer feels good, I do not know how the effect of power saving, with a month or two and then follow up!	Jingdong prefecture level city in Shandong
Jinfeng Hongyuan Flagship Store	Electricity savings1	It's quite small and delicate, I don't know if it's useful yet.	Jingdong prefecture level city in Shandong
Jinfeng Hongyuan Flagship Store	Electricity savings1	Just received it, use it for a few days before commenting	Jingdong prefecture level city in Shandong
Jinfeng Hongyuan Flagship Store	Electricity savings1	Does this thing work! When it arrived, the button for the safety was loose. I'm a little afraid of fire!	Jingdong prefecture level city in Shandong
Jinfeng Hongyuan Flagship Store	Electricity savings1	I received it. I don't know how it works.	Jingdong prefecture level city in Shandong

Jinfeng Hongyuan Flagship Store	Electricity savings1	This user thinks the product is good	Jingdong prefecture level city in Shandong
Jinfeng Hongyuan Flagship Store	Electricity savings1	Customer service was great! Small i problem was taken care of in a timely manner! Effectiveness to be tested by time! No rush!!!	Jingdong prefecture level city in Shandong
Jinfeng Hongyuan Flagship Store	Electricity savings1	I put it on right after I received it and it looks compact. I believe the high technology should save electricity.	Jingdong prefecture level city in Shandong
Jinfeng Hongyuan Flagship Store	Electricity savings1	Logistics courier very quickly, the first day under the order the next day to receive, off pieces whether power saving is the most important thing, things are exquisite workmanship, a month later in the follow-up evaluation whether power saving	Jingdong prefecture level city in Shandong
Jinfeng Hongyuan Flagship Store	Electricity savings1	The delivery is pretty fast, I'm using it, have to have it for a while to see results, looking forward to it.	Jingdong prefecture level city in Shandong
Jinfeng Hongyuan Flagship Store	Electricity savings1	See pretty good just started to use a month later to give you a real evaluation, should be useful.	Jingdong prefecture level city in Shandong
Jinfeng Hongyuan Flagship Store	Electricity savings1	And then comment.	Jingdong prefecture level city in Shandong
Jinfeng Hongyuan Flagship Store	Electricity savings1	Received the goods, wait for the test to evaluate	Jingdong prefecture level city in Shandong
Jinfeng Hongyuan Flagship Store	Electricity savings1	This user thinks the product is good	Jingdong prefecture level city in Shandong
Jinfeng Hongyuan Flagship Store	Electricity savings1	I'm already using it, I just don't know how to judge the power savings. I won't know until I use it for a season.	Jingdong prefecture level city in Shandong
Jinfeng Hongyuan Flagship Store	Electricity savings1	Works. Says it works.	Jingdong prefecture level city in Shandong
Jinfeng Hongyuan Flagship Store	Electricity savings1	Very good. Satisfied.	Jingdong prefecture level city in Shandong
Jinfeng Hongyuan Flagship Store	Electricity savings1	This user thinks the product is good	Jingdong prefecture level city in Shandong
Jinfeng Hongyuan Flagship Store	Electricity savings1	It's working. Obviously, it's less expensive.	Jingdong prefecture level city in Shandong
Jinfeng Hongyuan Flagship Store	Electricity savings1	The second one is up, the first one still works! It's reactive power compensation! This is well understood if you want a professional!	Jingdong prefecture level city in Shandong
Jinfeng Hongyuan Flagship Store	Electricity savings1	It feels as if there are some nodes, but they need to be tested and observed over time, thanks.	Jingdong prefecture level city in Shandong
Jinfeng Hongyuan Flagship Store	Electricity savings1	I've received the goods, the energy saving effect will be evaluated after using it for a period of time, and all the services are very good, praise!	Jingdong prefecture level city in Shandong
Jinfeng Hongyuan Flagship Store	Electricity savings1	I don't know how well it works yet, I bought it and plugged it in, so we'll see if it saves some power or not!	Jingdong prefecture level city in Shandong
Jinfeng Hongyuan Flagship Store	Electricity savings1	Baby a bunch of knife quality is very good, previously bought a quality is not very good. It really works and has results.	Jingdong prefecture level city in Shandong
Jinfeng Hongyuan Flagship Store	Electricity savings1	Things have arrived, it looks good, just started to use, as to whether or not there is a business said the power-saving effect, but also to observe the effect, now can not evaluate	Jingdong prefecture level city in Shandong
Jinfeng Hongyuan Flagship Store	Electricity savings1	I haven't used it yet. I'll comment afterward.	Jingdong prefecture level city in Shandong
Jinfeng Hongyuan Flagship Store	Electricity savings1	I don't know if it's any good.	Jingdong prefecture level city in Shandong
Jinfeng Hongyuan Flagship Store	Electricity savings1	I don't know how well it works.	Jingdong prefecture level city in Shandong
Jinfeng Hongyuan Flagship Store	Electricity savings1	The baby has been received! Uh-huh! Good, quite exquisite, small and exquisite I thought it was big. Dare to tightly plugged in the electricity, just wait for the effect later!	Jingdong prefecture level city in Shandong
Jinfeng Hongyuan Flagship Store	Electricity savings1	The packaging is sturdy, I tried it right after I received it, so I'm looking forward to the results.	Jingdong prefecture level city in Shandong
Jinfeng Hongyuan Flagship Store	Electricity savings1	Received the item and installed it! Don't know if it works well! Will follow up with a review later! I hope it works!	Jingdong prefecture level city in Shandong
Jinfeng Hongyuan Flagship Store	Electricity savings1	Things used on the effect of waiting to use a little to say, but things are very good. Logistics is very fast, business service is very good.	Jingdong prefecture level city in Shandong
Jinfeng Hongyuan Flagship Store	Electricity savings1	This user thinks the product is good	Jingdong prefecture level city in Shandong
Jinfeng Hongyuan Flagship Store	Electricity savings1	I'm really using it. It feels good.	Jingdong prefecture level city in Shandong
Jinfeng Hongyuan Flagship Store	Electricity savings1	Good, Jingdong logistics is very fast, intelligent energy-saving appliances	Jingdong prefecture level city in Shandong
Jinfeng Hongyuan Flagship Store	Electricity savings1	It's not bad. It really saves energy.	Jingdong prefecture level city in Shandong
Jinfeng Hongyuan Flagship Store	Electricity savings1	The logistics is fast, praise	Jingdong prefecture level city in Shandong
Jinfeng Hongyuan Flagship Store	Electricity savings1	This user thinks the product is good	Jingdong prefecture level city in Shandong
Jinfeng Hongyuan Flagship Store	Electricity savings1	The first time I placed two, and today I placed another one, a friend introduced it, said it is particularly power-saving	Jingdong prefecture level city in Shandong
Jinfeng Hongyuan Flagship Store	Electricity savings1	I don't know how effective it is. Let's leave it at home for a while and see.	Jingdong prefecture level city in Shandong
Jinfeng Hongyuan Flagship Store	Electricity savings1	Dear customer service! I would like to ask you how to understand the digital display (increase/decrease jump) on the top of the saver? Thank you for your help.	Jingdong prefecture level city in Shandong
Jinfeng Hongyuan Flagship Store	Electricity savings1	This user thinks the product is good	Jingdong prefecture level city in Shandong
Jinfeng Hongyuan Flagship Store	Electricity savings1	The goods have been received, packaging exquisite, is using, later to see how the effect?	Jingdong prefecture level city in Shandong
Jinfeng Hongyuan Flagship Store	Electricity savings1	The saver received, the boss shipped very quickly, yesterday shot today arrived!	Jingdong prefecture level city in Shandong
Jinfeng Hongyuan Flagship Store	Electricity savings1	Beautiful appearance, well packaged. Recording data to see if I can lower my electric bill a bit.	Jingdong prefecture level city in Shandong
Jinfeng Hongyuan Flagship Store	Electricity savings1	Things received, the packaging is quite good, the effect of the use and then come back to evaluate.	Jingdong prefecture level city in Shandong
Jinfeng Hongyuan Flagship Store	Electricity savings1	Easy to use, just plug it in. But there is a current sound.	Jingdong prefecture level city in Shandong
Jinfeng Hongyuan Flagship Store	Electricity savings1	Fast logistics, easy to use and labor-saving	Jingdong prefecture level city in Shandong
Jinfeng Hongyuan Flagship Store	Electricity savings1	Received, thank you!	Jingdong prefecture level city in Shandong
Jinfeng Hongyuan Flagship Store	Electricity savings1	Second time I've bought it. It's good.	Jingdong prefecture level city in Shandong
Jinfeng Hongyuan Flagship Store	Electricity savings1	I've only been using it for a short time, so I'm looking forward to the results.	Jingdong prefecture level city in Shandong
Jinfeng Hongyuan Flagship Store	Electricity savings1	Good stuff, good stuff, good stuff good stuff.	Jingdong prefecture level city in Shandong
Jinfeng Hongyuan Flagship Store	Electricity savings1	Buy your stuff is not always good I have been using it for almost two months now, it does not save electricity at all the electricity bill is about ten dollars a day this is still called the king of power	Jingdong prefecture level city in Shandong
Jinfeng Hongyuan Flagship Store	Electricity savings1	Express arrived, the quality is very good, worth the purchase.	Jingdong prefecture level city in Shandong
Jinfeng Hongyuan Flagship Store	Electricity savings1	Energy saving has been received, now the voltage is more stable, energy-saving effect to see a few days and then chase flat, the service attitude is very good, the courier is relatively fast, I am satisfied.	Jingdong prefecture level city in Shandong
Jinfeng Hongyuan Flagship Store	Electricity savings1	Stuff installed, numbers are smaller than when installed, hopefully it works	Jingdong prefecture level city in Shandong

Jinfeng Hongyuan Flagship Store	Electricity savings1	Good appearance, fine workmanship, just bought, use up the feeling of power saving effect is good, worth buying!	Jingdong prefecture level city in Shandong
Jinfeng Hongyuan Flagship Store	Electricity savings1	Economical and practical 👍 Always use it	Jingdong prefecture level city in Shandong
Jinfeng Hongyuan Flagship Store	Electricity savings1	Just used it, don't know the effect yet, will come back to follow up after the effect. Hope it works well(▽)	Jingdong prefecture level city in Shandong
Jinfeng Hongyuan Flagship Store	Electricity savings1	The goods have been received, use it to see in the evaluation.	Jingdong prefecture level city in Shandong
Jinfeng Hongyuan Flagship Store	Electricity savings1	Doesn't it save electricity? The boil kettle still goes faster when you press the meter.	Jingdong prefecture level city in Shandong
Jinfeng Hongyuan Flagship Store	Electricity savings1	Already used. Will patronize again in the future	Jingdong prefecture level city in Shandong
Jinfeng Hongyuan Flagship Store	Electricity savings1	Works great, just plug it in, results to be checked, satisfied.	Jingdong prefecture level city in Shandong
Jinfeng Hongyuan Flagship Store	Electricity savings1	Second purchase. Works well 😍😍😍😍	Jingdong prefecture level city in Shandong
Jinfeng Hongyuan Flagship Store	Electricity savings1	Prompt shipment, very satisfied	Jingdong prefecture level city in Shandong
Jinfeng Hongyuan Flagship Store	Electricity savings1	It's been less than a month, let's see how it works, hopefully it's power efficient	Jingdong prefecture level city in Shandong
Jinfeng Hongyuan Flagship Store	Electricity savings1	Use a month in say.	Jingdong prefecture level city in Shandong
Jinfeng Hongyuan Flagship Store	Electricity savings1	I've saved a lot of electricity since I've been using it.	Jingdong prefecture level city in Shandong
Jinfeng Hongyuan Flagship Store	Electricity savings1	It's fast, looks and feels good on the outside, try it out and evaluate.	Jingdong prefecture level city in Shandong
Jinfeng Hongyuan Flagship Store	Electricity savings1	I haven't used it. I just used it. I don't know if it's any good.	Jingdong prefecture level city in Shandong
Jinfeng Hongyuan Flagship Store	Electricity savings1	Quality can be, power saving now can not see just use, after a while in the look at the power saving effect	Jingdong prefecture level city in Shandong
Jinfeng Hongyuan Flagship Store	Electricity savings1	The logistics is very fast, and it seems to have some effect	Jingdong prefecture level city in Shandong
Jinfeng Hongyuan Flagship Store	Electricity savings1	Good, but the effects remain to be seen	Jingdong prefecture level city in Shandong
Jinfeng Hongyuan Flagship Store	Electricity savings1	Baby is basically the same as the description, shipping quickly, worth a buy Good price, logistics is strong, worth repurchasing!	Jingdong prefecture level city in Shandong
Jinfeng Hongyuan Flagship Store	Electricity savings1	I bought it for my dad, I don't know much about it, if he thinks it's good then it must be good 👍	Jingdong prefecture level city in Shandong
Jinfeng Hongyuan Flagship Store	Electricity savings1	Goods received. It's working fine. Display voltage. After a period of time to see the effect in the addition to the level of theory	Jingdong prefecture level city in Shandong
Jinfeng Hongyuan Flagship Store	Electricity savings1	The stuff is pretty good, I just don't know how well it saves power.	Jingdong prefecture level city in Shandong
Jinfeng Hongyuan Flagship Store	Electricity savings1	Logistics is a little too fast 👍	Jingdong prefecture level city in Shandong
Jinfeng Hongyuan Flagship Store	Electricity savings1	Received the goods used for a period of time before coming, good, beautiful appearance, really stable flow	Jingdong prefecture level city in Shandong
Jinfeng Hongyuan Flagship Store	Electricity savings1	Things received, look at the packaging is very good, not yet, to the power saving in the follow-up evaluation	Jingdong prefecture level city in Shandong
Jinfeng Hongyuan Flagship Store	Electricity savings1	Baby is very good! Use a few days before to evaluate, I personally test really effective.	Jingdong prefecture level city in Shandong
Jinfeng Hongyuan Flagship Store	Electricity savings1	I received the goods are in use, I do not know how effective? Can it save energy? Let's see.	Jingdong prefecture level city in Shandong
Jinfeng Hongyuan Flagship Store	Electricity savings1	It's really good and has some power saving effect.	Jingdong prefecture level city in Shandong
Jinfeng Hongyuan Flagship Store	Electricity savings1	It's good quality, well made, and I hope it'll save on electricity over time.	Jingdong prefecture level city in Shandong
Jinfeng Hongyuan Flagship Store	Electricity savings1	I just used it, I don't know how it works yet, but I'll give it a good review first.	Jingdong prefecture level city in Shandong
Jinfeng Hongyuan Flagship Store	Electricity savings1	Second purchase, the delivery guy was fast and called ahead to deliver.	Jingdong prefecture level city in Shandong
Jinfeng Hongyuan Flagship Store	Electricity savings1	Goods have been received, but have not yet used, used to see how the effect, in the evaluation of fertilizer	Jingdong prefecture level city in Shandong
Jinfeng Hongyuan Flagship Store	Electricity savings1	The delivery is very fast. It's the same in kind. It's working.	Jingdong prefecture level city in Shandong
Jinfeng Hongyuan Flagship Store	Electricity savings1	Fast logistics, good quality, more satisfied	Jingdong prefecture level city in Shandong
Jinfeng Hongyuan Flagship Store	Electricity savings1	I've been here for almost a month now and there is a very noticeable difference than it is worth buying.	Jingdong prefecture level city in Shandong
Jinfeng Hongyuan Flagship Store	Electricity savings1	Fast shipping, received the next day, plug and play, but do not know how effective to not break down!	Jingdong prefecture level city in Shandong
Jinfeng Hongyuan Flagship Store	Electricity savings1	The logistics are great I've been using it for a while before commenting on it This saves electricity	Jingdong prefecture level city in Shandong
Jinfeng Hongyuan Flagship Store	Electricity savings1	Wait to use it for a while to know if it's energy efficient	Jingdong prefecture level city in Shandong
Jinfeng Hongyuan Flagship Store	Electricity savings1	Just received it, haven't used it yet don't know if it's any good.	Jingdong prefecture level city in Shandong
Jinfeng Hongyuan Flagship Store	Electricity savings1	It really doesn't work, and the electric bill is still the same amount every month.	Jingdong prefecture level city in Shandong
Jinfeng Hongyuan Flagship Store	Electricity savings1	It's well packaged and looks fine	Jingdong prefecture level city in Shandong
Jinfeng Hongyuan Flagship Store	Electricity savings1	Very good. Will patronize again if needed.	Jingdong prefecture level city in Shandong
Jinfeng Hongyuan Flagship Store	Electricity savings1	I have received the product and installed it, try it for a while and see how it works.	Jingdong prefecture level city in Shandong
Jinfeng Hongyuan Flagship Store	Electricity savings1	I've been using it for a month now...I don't feel like there's any noticeable power savings...last month it was $130 this month it's $123...let's see if I can use it for another month	Jingdong prefecture level city in Shandong
Jinfeng Hongyuan Flagship Store	Electricity savings1	Just received it, yet to try it and see if it works.	Jingdong prefecture level city in Shandong
Jinfeng Hongyuan Flagship Store	Electricity savings1	It's easy to use and has a power saving credit!	Jingdong prefecture level city in Shandong
Jinfeng Hongyuan Flagship Store	Electricity savings1	It's very good. I've already bought two of them.	Jingdong prefecture level city in Shandong
Jinfeng Hongyuan Flagship Store	Electricity savings1	It's very well made, packaged meticulously, and I hope it works!	Jingdong prefecture level city in Shandong
Jinfeng Hongyuan Flagship Store	Electricity savings1	Goods have been received have not yet used it, used to comment again	Jingdong prefecture level city in Shandong
Jinfeng Hongyuan Flagship Store	Electricity savings1	Looking forward to good results	Jingdong prefecture level city in Shandong
Jinfeng Hongyuan Flagship Store	Electricity savings1	Not sure if it saves power or not. Still trying it out.	Jingdong prefecture level city in Shandong
Jinfeng Hongyuan Flagship Store	Electricity savings1	It also really saves on electricity! Superb!	Jingdong prefecture level city in Shandong

Jinfeng Hongyuan Flagship Store	Electricity savings1	Didn't work at all, want to return it	Jingdong prefecture level city in Shandong
Jinfeng Hongyuan Flagship Store	Electricity savings1	Uh-huh! It's working, I've been using it for a month and it saves 20% of electricity compared to before.	Jingdong prefecture level city in Shandong
Jinfeng Hongyuan Flagship Store	Electricity savings1	Should be a little bit of a power saver, I wonder how long it will take to save back the money I spent on this one	Jingdong prefecture level city in Shandong
Jinfeng Hongyuan Flagship Store	Electricity savings1	Exquisite appearance and stable voltage	Jingdong prefecture level city in Shandong
Jinfeng Hongyuan Flagship Store	Electricity savings1	After using it for a few days it feels like it saves electricity.	Jingdong prefecture level city in Shandong
Jinfeng Hongyuan Flagship Store	Electricity savings1	Second purchase and its pending good results.	Jingdong prefecture level city in Shandong
Jinfeng Hongyuan Flagship Store	Electricity savings1	The appearance of the workmanship is exquisite and meticulous, beautiful appearance, just to see if there is no effect!	Jingdong prefecture level city in Shandong
Jinfeng Hongyuan Flagship Store	Electricity savings1	Let's see what happens in a month.	Jingdong prefecture level city in Shandong
Jinfeng Hongyuan Flagship Store	Electricity savings1	Trial, now short time not yet obvious effect	Jingdong prefecture level city in Shandong
Jinfeng Hongyuan Flagship Store	Electricity savings1	I'm using it now, don't know how well it works, it looks great!	Jingdong prefecture level city in Shandong
Jinfeng Hongyuan Flagship Store	Electricity savings1	There is no role! Now it's true that I don't know!	Jingdong prefecture level city in Shandong
Jinfeng Hongyuan Flagship Store	Electricity savings1	Purchased for the second time, power saving not sure if it's ok? Let's talk about it after using it	Jingdong prefecture level city in Shandong
Jinfeng Hongyuan Flagship Store	Electricity savings1	I just received it, I have to try it for a month to know how it works.	Jingdong prefecture level city in Shandong
Jinfeng Hongyuan Flagship Store	Electricity savings1	I just used it for an hour, and the meter's still spinning so fast it's not much use.	Jingdong prefecture level city in Shandong
Jinfeng Hongyuan Flagship Store	Electricity savings1	Not bad, logistics is fast	Jingdong prefecture level city in Shandong
Jinfeng Hongyuan Flagship Store	Electricity savings1	I don't know if it works. I'll find out next month when I pay the bill. It's easy. Just plug it in.	Jingdong prefecture level city in Shandong
Jinfeng Hongyuan Flagship Store	Electricity savings1	Logistics is particularly fast, received the goods are genuine, looking forward to power saving effect	Jingdong prefecture level city in Shandong
Jinfeng Hongyuan Flagship Store	Electricity savings1	First time buying this product like this one from this family use it and then evaluate.	Jingdong prefecture level city in Shandong
Jinfeng Hongyuan Flagship Store	Electricity savings1	Lightning arrived, the product is very good, before all do not know there is such a baby, in a friend's home has seen, and then bought to try, has been used for a period of time before coming to the evaluation, the work is very stable, so far looked very good, will introduce friends to buy!	Jingdong prefecture level city in Shandong
Jinfeng Hongyuan Flagship Store	Electricity savings1	I was a bit skeptical when I heard that I could save money on my electricity bill by installing this, I don't know if the power saving effect is really what it is said to be. But since I bought it, I just have to put it on and try it out. I hope it lives up to its name and saves me the money I spent on the device	Jingdong prefecture level city in Shandong
Jinfeng Hongyuan Flagship Store	Electricity savings1	The quality of the stuff is okay, but I'm not sure if it works, so I'll try using it first.	Jingdong prefecture level city in Shandong
Jinfeng Hongyuan Flagship Store	Electricity savings1	It should work. The current is much more stable.	Jingdong prefecture level city in Shandong
Jinfeng Hongyuan Flagship Store	Electricity savings1	The store has a great service attitude	Jingdong prefecture level city in Shandong
Jinfeng Hongyuan Flagship Store	Electricity savings1	Let's wait until we've used it for a month.	Jingdong prefecture level city in Shandong
Jinfeng Hongyuan Flagship Store	Electricity savings1	I'm using it and I don't know if it's a power saver or not	Jingdong prefecture level city in Shandong
Jinfeng Hongyuan Flagship Store	Electricity savings1	Original and authentic guarantee quality is good now.	Jingdong prefecture level city in Shandong
Jinfeng Hongyuan Flagship Store	Electricity savings1	Great, very happy with it, will buy a couple more next time	Jingdong prefecture level city in Shandong
Jinfeng Hongyuan Flagship Store	Electricity savings1	I just used it, I don't know if it works yet, I'll come back after using it...	Jingdong prefecture level city in Shandong
Jinfeng Hongyuan Flagship Store	Electricity savings1	It's still being tested.	Jingdong prefecture level city in Shandong
Jinfeng Hongyuan Flagship Store	Electricity savings1	I've been using it for a month and it's not working.	Jingdong prefecture level city in Shandong
Jinfeng Hongyuan Flagship Store	Electricity savings1	It's basically nothing!	Jingdong prefecture level city in Shandong
Jinfeng Hongyuan Flagship Store	Electricity savings1	It's not bad, it kind of works, support it.	Jingdong prefecture level city in Shandong
Jinfeng Hongyuan Flagship Store	Electricity savings1	I just received it last night, let's try it first to see how it works, it looks good!	Jingdong prefecture level city in Shandong
Jinfeng Hongyuan Flagship Store	Electricity savings1	The current is very stable, fine workmanship, small and exquisite, very satisfied	Jingdong prefecture level city in Shandong
Jinfeng Hongyuan Flagship Store	Electricity savings1	Not bad, exactly how much electricity can be saved; still have to follow up the evaluation	Jingdong prefecture level city in Shandong
Jinfeng Hongyuan Flagship Store	Electricity savings1	Logistics is also very fast, next time there is an opportunity to introduce friends to buy!	Jingdong prefecture level city in Shandong
Jinfeng Hongyuan Flagship Store	Electricity savings1	Voltage display and actual do not pay	Jingdong prefecture level city in Shandong
Jinfeng Hongyuan Flagship Store	Electricity savings1	Second purchase, still saves a little on the electric bill	Jingdong prefecture level city in Shandong
Jinfeng Hongyuan Flagship Store	Electricity savings1	Order received, currently trying it out, thanks a lot.	Jingdong prefecture level city in Shandong
Jinfeng Hongyuan Flagship Store	Electricity savings1	I haven't used it for a long time yet, I don't know if the effect is good or not, looking forward to it	Jingdong prefecture level city in Shandong
Jinfeng Hongyuan Flagship Store	Electricity savings1	Good, working, I just don't know if it will save electricity... 5 stars for now.	Jingdong prefecture level city in Shandong
Jinfeng Hongyuan Flagship Store	Electricity savings1	Great product, just as the owner said it would be.	Jingdong prefecture level city in Shandong
Jinfeng Hongyuan Flagship Store	Electricity savings1	Shipping was fast, I don't know if it works well or not, I have to use it for a while first.	Jingdong prefecture level city in Shandong
Jinfeng Hongyuan Flagship Store	Electricity savings1	I don't feel that it saves much electricity, but I'll give it a try. It's been more than a month now, and the electricity bill is still a bit high.	Jingdong prefecture level city in Shandong
Jinfeng Hongyuan Flagship Store	Electricity savings1	Got it. Just used it.	Jingdong prefecture level city in Shandong
Jinfeng Hongyuan Flagship Store	Electricity savings1	Use it for a while and then comment.	Jingdong prefecture level city in Shandong
Jinfeng Hongyuan Flagship Store	Electricity savings1	Used it, see how it works with it and then comment. The quality is very good!	Jingdong prefecture level city in Shandong
Jinfeng Hongyuan Flagship Store	Electricity savings1	I'll comment again after a while.	Jingdong prefecture level city in Shandong
Jinfeng Hongyuan Flagship Store	Electricity savings1	It's easy to operate, affordable and works well, love it.	Jingdong prefecture level city in Shandong
Jinfeng Hongyuan Flagship Store	Electricity savings1	It's good. You can try it. It's cheap.	Jingdong prefecture level city in Shandong

Jinfeng Hongyuan Flagship Store	Electricity savings1	It's in use, so we'll see how it works after a while!	Jingdong prefecture level city in Shandong
Jinfeng Hongyuan Flagship Store	Electricity savings1	Service is pretty good, not sure about the quality yet.	Jingdong prefecture level city in Shandong
Jinfeng Hongyuan Flagship Store	Electricity savings1	Just used it and will see the results in a month 😊	Jingdong prefecture level city in Shandong
Jinfeng Hongyuan Flagship Store	Electricity savings1	Saving electricity is expensive and good	Jingdong prefecture level city in Shandong
Jinfeng Hongyuan Flagship Store	Electricity savings1	Pretty good, 5 stars! Will buy again next time seller service is very good ！！！！ Good quality! Will recommend to friends!	Jingdong prefecture level city in Shandong
Jinfeng Hongyuan Flagship Store	Electricity savings1	In use, with results naturally have follow-up reviews!	Jingdong prefecture level city in Shandong
Jinfeng Hongyuan Flagship Store	Electricity savings1	I'm using it now, I'll come back with results, it'll work.	Jingdong prefecture level city in Shandong
Jinfeng Hongyuan Flagship Store	Electricity savings1	Good, nice, fast shipping, cheap, worth having!	Jingdong prefecture level city in Shandong
Jinfeng Hongyuan Flagship Store	Electricity savings1	Logistics is fast.	Jingdong prefecture level city in Shandong
Jinfeng Hongyuan Flagship Store	Electricity savings1	I have just started using the product and will be reviewing it later.	Jingdong prefecture level city in Shandong
Jinfeng Hongyuan Flagship Store	Electricity savings1	I'll comment after I use it.	Jingdong prefecture level city in Shandong
Jinfeng Hongyuan Flagship Store	Electricity savings1	I don't know if it's working or not, though I'll try plugging it in.	Jingdong prefecture level city in Shandong
Jinfeng Hongyuan Flagship Store	Electricity savings1	It really saves electricity and contributes to energy savings in disguise	Jingdong prefecture level city in Shandong
Jinfeng Hongyuan Flagship Store	Electricity savings1	It feels good in the experience.	Jingdong prefecture level city in Shandong
Jinfeng Hongyuan Flagship Store	Electricity savings1	The product is better than I thought it would be, the quality looks great, the voltage is stable, and I'm looking forward to some great power savings!	Jingdong prefecture level city in Shandong
Jinfeng Hongyuan Flagship Store	Electricity savings1	I'm using it now looking forward to saving some points,, I'm feeding back	Jingdong prefecture level city in Shandong
Jinfeng Hongyuan Flagship Store	Electricity savings1	Displayed voltage below 200.	Jingdong prefecture level city in Shandong
Jinfeng Hongyuan Flagship Store	Electricity savings1	I haven't seen any great results, but it should last about a hundred or eighty years.	Jingdong prefecture level city in Shandong
Jinfeng Hongyuan Flagship Store	Electricity savings1	I don't know if it works or not, but I keep it plugged in anyway, so I hope it works!	Jingdong prefecture level city in Shandong
Jinfeng Hongyuan Flagship Store	Electricity savings1	Reasonable price, good quality, fast delivery, great service, already a repurchase.	Jingdong prefecture level city in Shandong
Jinfeng Hongyuan Flagship Store	Electricity savings1	Second purchase, looking forward to the results	Jingdong prefecture level city in Shandong
Jinfeng Hongyuan Flagship Store	Electricity savings1	The quality of this product feels like it should work in a use test! Just how many kilowatts of electricity must be wasted per day with the red light and digital display on for long periods of time?	Jingdong prefecture level city in Shandong
Jinfeng Hongyuan Flagship Store	Electricity savings1	It looks good, we'll see if we use it.	Jingdong prefecture level city in Shandong
Jinfeng Hongyuan Flagship Store	Electricity savings1	Great stuff, second purchase!	Jingdong prefecture level city in Shandong
Jinfeng Hongyuan Flagship Store	Electricity savings1	It is indeed very practical and saves electricity at home	Jingdong prefecture level city in Shandong
Jinfeng Hongyuan Flagship Store	Electricity savings1	Goods received, the physical and the same as the picture, logistics is also very fast!	Jingdong prefecture level city in Shandong
Jinfeng Hongyuan Flagship Store	Electricity savings1	I'll use it for a while and see how it goes. I'll buy it again if it's good.	Jingdong prefecture level city in Shandong
Jinfeng Hongyuan Flagship Store	Electricity savings1	In use, voltage monitoring, the effect of how to wait for next month's electricity consumption confirmation	Jingdong prefecture level city in Shandong
Jinfeng Hongyuan Flagship Store	Electricity savings1	It's still pretty good.	Jingdong prefecture level city in Shandong
Jinfeng Hongyuan Flagship Store	Electricity savings1	It's been plugged in for a month and there hasn't been a big change yet, since it usually uses very little electricity	Jingdong prefecture level city in Shandong
Jinfeng Hongyuan Flagship Store	Electricity savings1	Let's wait for a month to see if it works	Jingdong prefecture level city in Shandong
Jinfeng Hongyuan Flagship Store	Electricity savings1	Good, good, good, good, good, good, good, good, good, good, good, good, good, good, good.	Jingdong prefecture level city in Shandong
Jinfeng Hongyuan Flagship Store	Electricity savings1	The product received the trial a few days, feel good, praise!	Jingdong prefecture level city in Shandong
Jinfeng Hongyuan Flagship Store	Electricity savings1	After six or seven hours of use, 250 volts won't save you any power.	Jingdong prefecture level city in Shandong
Jinfeng Hongyuan Flagship Store	Electricity savings1	I haven't used it yet, I wonder if it saves electricity?	Jingdong prefecture level city in Shandong
Jinfeng Hongyuan Flagship Store	Electricity savings1	Baby value for money, love it, next time will come back is genuine, trust the business, baby texture is very good, the price is moderate, unlimited repurchase!	Jingdong prefecture level city in Shandong
Jinfeng Hongyuan Flagship Store	Electricity savings1	Power saving effect is obvious! Raising tropical fish daily power consumption, after using the daily power savings of more than 3 degrees, currently about 8 degrees / day!	Jingdong prefecture level city in Shandong
Jinfeng Hongyuan Flagship Store	Electricity savings1	Just used it, unknown results.	Jingdong prefecture level city in Shandong
Jinfeng Hongyuan Flagship Store	Electricity savings1	The goods received, well, look forward to the effect. Logistics is very fast, thanks to the platform, thanks to Jingdong. I hope it can save electricity.	Jingdong prefecture level city in Shandong
Jinfeng Hongyuan Flagship Store	Electricity savings1	Not sure 有 effect?	Jingdong prefecture level city in Shandong
Jinfeng Hongyuan Flagship Store	Electricity savings1	I'd say it's a 5% savings.	Jingdong prefecture level city in Shandong
Jinfeng Hongyuan Flagship Store	Electricity savings1	I received it and am using it, I don't know how effective it is, I'll evaluate it afterwards	Jingdong prefecture level city in Shandong
Jinfeng Hongyuan Flagship Store	Electricity savings1	Baby received not yet used, first give praise	Jingdong prefecture level city in Shandong
Jinfeng Hongyuan Flagship Store	Electricity savings1	Looks good, I'll evaluate it later	Jingdong prefecture level city in Shandong
Jinfeng Hongyuan Flagship Store	Electricity savings1	The goods have been received in use, the overall feeling is not bad!	Jingdong prefecture level city in Shandong
Jinfeng Hongyuan Flagship Store	Electricity savings1	It's working. This is the second one. This stuff has to be used over a long period of time to be effective. And it only works on inductive loads, i.e. appliances containing electric motors. Use it.	Jingdong prefecture level city in Shandong
Jinfeng Hongyuan Flagship Store	Electricity savings1	Use it for a while and see how it works.	Jingdong prefecture level city in Shandong
Jinfeng Hongyuan Flagship Store	Electricity savings1	Good courier service and the item is very nice and practical.	Jingdong prefecture level city in Shandong
Jinfeng Hongyuan Flagship Store	Electricity savings1	It doesn't do anything and it's a waste of electricity.	Jingdong prefecture level city in Shandong
Jinfeng Hongyuan Flagship Store	Electricity savings1	I haven't used it yet. It should be good.	Jingdong prefecture level city in Shandong
Jinfeng Hongyuan Flagship Store	Electricity savings1	That's good. OK, that's good.	Jingdong prefecture level city in Shandong

Jinfeng Hongyuan Flagship Store	Electricity savings1	It's a rip off, doesn't work, doesn't save electricity, does the job to well, and I've wasted a couple dozen dollars again.	Jingdong prefecture level city in Shandong
Jinfeng Hongyuan Flagship Store	Electricity savings1	Use it first, the effect is not as good as the road, then we will comment again	Jingdong prefecture level city in Shandong
Jinfeng Hongyuan Flagship Store	Electricity savings1	The price is affordable, the logistics is strong, just use four or five days, the effect will be added later!	Jingdong prefecture level city in Shandong
Jinfeng Hongyuan Flagship Store	Electricity savings1	It's easy to use and works fine.	Jingdong prefecture level city in Shandong
Jinfeng Hongyuan Flagship Store	Electricity savings1	It doesn't feel like it's working.	Jingdong prefecture level city in Shandong
Jinfeng Hongyuan Flagship Store	Electricity savings1	I'm using it. We'll see what happens in a couple months.	Jingdong prefecture level city in Shandong
Jinfeng Hongyuan Flagship Store	Electricity savings1	I just got it back to try it out and I'll comment later	Jingdong prefecture level city in Shandong
Jinfeng Hongyuan Flagship Store	Electricity savings1	Less than two months. Can't tell.	Jingdong prefecture level city in Shandong
Jinfeng Hongyuan Flagship Store	Electricity savings1	No power saving effect	Jingdong prefecture level city in Shandong
Jinfeng Hongyuan Flagship Store	Electricity savings1	Express Express Very quickly not only to the power saver can not save electricity, with two months, look at the evaluation put.	Jingdong prefecture level city in Shandong
Jinfeng Hongyuan Flagship Store	Electricity savings1	Genuine products are packaged well!	Jingdong prefecture level city in Shandong
Jinfeng Hongyuan Flagship Store	Electricity savings1	I've been using it for a while, but I don't know if it saves electricity. I hope it works.	Jingdong prefecture level city in Shandong
Jinfeng Hongyuan Flagship Store	Electricity savings1	Good service, fast logistics, the effect is to be used to know	Jingdong prefecture level city in Shandong
Jinfeng Hongyuan Flagship Store	Electricity savings1	Just used it, not sure about the power saving effect	Jingdong prefecture level city in Shandong
Jinfeng Hongyuan Flagship Store	Electricity savings1	The stuff is ok, save power or not, will comment after a month.	Jingdong prefecture level city in Shandong
Jinfeng Hongyuan Flagship Store	Electricity savings1	Used it for a month to save %25, normal is a little over 100, it's great! I want to buy one in.	Jingdong prefecture level city in Shandong
Jinfeng Hongyuan Flagship Store	Electricity savings1	I'm using it and looking forward to the results	Jingdong prefecture level city in Shandong
Jinfeng Hongyuan Flagship Store	Electricity savings1	The quality is very good and the workmanship is exquisite. The use effect is also very good.	Jingdong prefecture level city in Shandong
Jinfeng Hongyuan Flagship Store	Electricity savings1	Merchant service attitude is very good, logistics speed is super fast, online evaluation is very good, the first time to use, a month to know the specific effect, the effect is good, and then additional	Jingdong prefecture level city in Shandong
Jinfeng Hongyuan Flagship Store	Electricity savings1	Works great, solves the problem of high power usage in the house	Jingdong prefecture level city in Shandong
Jinfeng Hongyuan Flagship Store	Electricity savings1	To receive! Very delicate! It's not clear how effective it is, I won't know until I use it!!!!	Jingdong prefecture level city in Shandong
Jinfeng Hongyuan Flagship Store	Electricity savings1	I've just been using it for a while and I don't feel like it's too noticeable.	Jingdong prefecture level city in Shandong
Jinfeng Hongyuan Flagship Store	Electricity savings1	Already used, no visible effect for the time being	Jingdong prefecture level city in Shandong
Jinfeng Hongyuan Flagship Store	Electricity savings1	I can't feel the effect.	Jingdong prefecture level city in Shandong
Jinfeng Hongyuan Flagship Store	Electricity savings1	Use it for a while and see the results	Jingdong prefecture level city in Shandong
Jinfeng Hongyuan Flagship Store	Electricity savings1	The price is more reasonable, the power-saving effect is to be verified, the delivery is fast, and the courier service is good.	Jingdong prefecture level city in Shandong
Jinfeng Hongyuan Flagship Store	Electricity savings1	Quality is very good, consistent with the seller's description, very satisfied, very like, very fast shipping, packaging is very careful and strict, the logistics company has a very good service attitude, fast delivery, very satisfied with a shopping, very satisfied. Recommended to buy, cost-effective, the service attitude of the business is very enthusiastic, providing such high quality goods.	Jingdong prefecture level city in Shandong
Jinfeng Hongyuan Flagship Store	Electricity savings1	I don't know.	Jingdong prefecture level city in Shandong
Jinfeng Hongyuan Flagship Store	Electricity savings1	I didn't get the delivery.	Jingdong prefecture level city in Shandong
Jinfeng Hongyuan Flagship Store	Electricity savings1	It works, it does have power savings, better than expected, will repurchase.	Jingdong prefecture level city in Shandong
Jinfeng Hongyuan Flagship Store	Electricity savings1	That's where the five fuses are.	Jingdong prefecture level city in Shandong
Jinfeng Hongyuan Flagship Store	Electricity savings1	Don't buy it, it doesn't work and you can't return it.	Jingdong prefecture level city in Shandong
Jinfeng Hongyuan Flagship Store	Electricity savings1	I hope it works. Wait a month.	Jingdong prefecture level city in Shandong
Jinfeng Hongyuan Flagship Store	Electricity savings1	Nothing**, buy with caution	Jingdong prefecture level city in Shandong
Jinfeng Hongyuan Flagship Store	Electricity savings1	I'm using it and waiting for the results	Jingdong prefecture level city in Shandong
Jinfeng Hongyuan Flagship Store	Electricity savings1	Saver quality is very good, has always felt that the home power consumption is very large, this is the company's electrician recommended, said he bought this home is really able to save a little electricity, I also came to buy, the effect if it is good, then buy, a floor a...	Jingdong prefecture level city in Shandong
Jinfeng Hongyuan Flagship Store	Electricity savings1	Good product, satisfied with purchase	Jingdong prefecture level city in Shandong
Jinfeng Hongyuan Flagship Store	Electricity savings1	Occupying the time has not yet found the power saving, occupying not evaluation	Jingdong prefecture level city in Shandong
Jinfeng Hongyuan Flagship Store	Electricity savings1	Haven't tested the results yet. To be followed up with comments!	Jingdong prefecture level city in Shandong
Jinfeng Hongyuan Flagship Store	Electricity savings1	After experiencing it for a month, I don't seem to know how to save it, and I don't pay attention to it anyway	Jingdong prefecture level city in Shandong
Jinfeng Hongyuan Flagship Store	Electricity savings1	It's useless. It's a waste of electricity.	Jingdong prefecture level city in Shandong
Jinfeng Hongyuan Flagship Store	Electricity savings1	I don't know if it's a power saver. It's a couple bucks. I hope it works.	Jingdong prefecture level city in Shandong
Jinfeng Hongyuan Flagship Store	Electricity savings1	I don't know what to say about this stuff. I don't know if it works or not, it's kind of a psychological purchase. Dormitory use to see the meter can not be not so good-looking. You have to wait until the time to deduct the utility bill to know.	Jingdong prefecture level city in Shandong
Jinfeng Hongyuan Flagship Store	Electricity savings1	Energy savers don't work / they're all a bunch of hoaxes.	Jingdong prefecture level city in Shandong
Jinfeng Hongyuan Flagship Store	Electricity savings1	Received the goods, used for more than a week, it's okay, sure a little effect	Jingdong prefecture level city in Shandong
Jinfeng Hongyuan Flagship Store	Electricity savings1	Well made and compact! Looking forward to the effect!	Jingdong prefecture level city in Shandong
Jinfeng Hongyuan Flagship Store	Electricity savings1	It has arrived and we'll see how it works.	Jingdong prefecture level city in Shandong
Jinfeng Hongyuan Flagship Store	Electricity savings1	Good. Good. It's worth it.	Jingdong prefecture level city in Shandong
Jinfeng Hongyuan Flagship Store	Electricity savings1	How come there's no indicator light?	Jingdong prefecture level city in Shandong

Jinfeng Hongyuan Flagship Store	Electricity savings1	The machine that uses electricity works quite well. This saves	Jingdong prefecture level city in Shandong
Jinfeng Hongyuan Flagship Store	Electricity savings1	It's good for home use and feels quite energy efficient	Jingdong prefecture level city in Shandong
Jinfeng Hongyuan Flagship Store	Electricity savings1	I regret buying this product, after using it for two days it doesn't work at all, still so many kilowatts per day just like the original.	Jingdong prefecture level city in Shandong
Jinfeng Hongyuan Flagship Store	Electricity savings1	Works very well and has some scientific merit.	Jingdong prefecture level city in Shandong
Jinfeng Hongyuan Flagship Store	Electricity savings1	Logistics is also very fast this good power saving I use two months before commenting!	Jingdong prefecture level city in Shandong
Jinfeng Hongyuan Flagship Store	Electricity savings1	Repeat customer. Very good.	Jingdong prefecture level city in Shandong
Jinfeng Hongyuan Flagship Store	Electricity savings1	It's good stuff. I've been using it for a month now and it's a real power saver.	Jingdong prefecture level city in Shandong
Jinfeng Hongyuan Flagship Store	Electricity savings1	Jingdong platform to buy things, has been very assured, quality assurance. Jingdong Express is as good as ever, complete packaging, delivery, service attitude to go! The family has always wanted to buy an energy-saving and power-saving equipment, selected half a day to choose this one, the value is very good, used for a period of time to follow up, to see whether it can achieve the real purpose of power saving, I	Jingdong prefecture level city in Shandong
Jinfeng Hongyuan Flagship Store	Electricity savings1	Works great, inexpensive and will be back next time.	Jingdong prefecture level city in Shandong
Jinfeng Hongyuan Flagship Store	Electricity savings1	Baby received, looks good, wait for a month to see how it works?	Jingdong prefecture level city in Shandong
Jinfeng Hongyuan Flagship Store	Electricity savings1	This product is not bad quite good well customer service said quite power saving that look at the customer service to see if the accuracy of the look at all aspects of the color is also quite beautiful	Jingdong prefecture level city in Shandong
Jinfeng Hongyuan Flagship Store	Electricity savings1	Just got back and tried it feels pretty good, just don't know how the quality is, give a good comment first	Jingdong prefecture level city in Shandong
Jinfeng Hongyuan Flagship Store	Electricity savings1	Received the product, trying it out, hope it helps!	Jingdong prefecture level city in Shandong
Jinfeng Hongyuan Flagship Store	Electricity savings1	Works great, saves electricity a dollar a day and a night	Jingdong prefecture level city in Shandong
Jinfeng Hongyuan Flagship Store	Electricity savings1	Baby received, look at the workmanship is still very fine, is genuine. I hope that this summer to open the air conditioning electricity bill is no longer high scary!	Jingdong prefecture level city in Shandong
Jinfeng Hongyuan Flagship Store	Electricity savings1	It's here. I don't know how it's going to work, but I'm going to use it.	Jingdong prefecture level city in Shandong
Jinfeng Hongyuan Flagship Store	Electricity savings1	Power saver received, Jingdong logistics first-class, business customer service attitude is good, one by one to introduce the product performance, back on the horse plugged in, it is very simple, there is a voltage indicator, to be a few months to see how the effect of power-saving to come back to a good follow-up, but I believe that Jinfenghong products.	Jingdong prefecture level city in Shandong
Jinfeng Hongyuan Flagship Store	Electricity savings1	Took the third one...the super power-saving kind!	Jingdong prefecture level city in Shandong
Jinfeng Hongyuan Flagship Store	Electricity savings1	First time buyer, customer service is very polite and courteous, conscientious business, support! Baby has received 🎈, super like, high value and practical, super like! ❥❥❥❥❥ Very good quality, very satisfied with a shopping. Customer service has a question and answer, very professional finally received the goods, very, very much like, exquisite and beautiful workmanship, has been praised by many people, really too happy, is already a loyal fan of his family, will continue to buy buy buy buy!	Jingdong prefecture level city in Shandong
Jinfeng Hongyuan Flagship Store	Electricity savings1	That's good. That's good. That's really good.	Jingdong prefecture level city in Shandong
Jinfeng Hongyuan Flagship Store	Electricity savings1	Saver received, good-looking design, voltage at a glance, and the merchant description, save some about 12% or so	Jingdong prefecture level city in Shandong
Jinfeng Hongyuan Flagship Store	Electricity savings1	Intelligent power saver is small and does not take up space, very good, very practical, received and used, packaging is tight, logistics is also very fast, satisfied with a shopping!	Jingdong prefecture level city in Shandong
Jinfeng Hongyuan Flagship Store	Electricity savings1	The stuff is good! Well made, compact, alloy case, shows voltage! Very like! Kudos!	Jingdong prefecture level city in Shandong
Jinfeng Hongyuan Flagship Store	Electricity savings1	I just purchased this power saver from Jingdong, inserted in the socket shows the number 206, a moment and 207 back and forth display, I do not know what it means, my home voltage should be normal, because it is the owner of the community's own power plant to put, first use for a period of time in the positive feedback it, first use in the look at it!	Jingdong prefecture level city in Shandong
Jinfeng Hongyuan Flagship Store	Electricity savings1	Very delicate and compact, there is a convenient intelligent display prompts the voltage, you can know the voltage at a glance, there is also very easy to use, a plug can be used, you can save electricity	Jingdong prefecture level city in Shandong
Jinfeng Hongyuan Flagship Store	Electricity savings1	Logistics is fast, ask customer service said can save electricity, but only use two months to see the effect, use two months in the evaluation.	Jingdong prefecture level city in Shandong
Jinfeng Hongyuan Flagship Store	Electricity savings1	The quality is very good.	Jingdong prefecture level city in Shandong
Jinfeng Hongyuan Flagship Store	Electricity savings1	Smart Saver has been received and will be evaluated after use	Jingdong prefecture level city in Shandong
Jinfeng Hongyuan Flagship Store	Electricity savings1	Home electricity is too waste, a month more than 300 dollars of electricity, and then the family heard that this can save a lot of electricity, let me order to buy a, anyway, will not, buy on the buy, thought it has to be a pretty big box it, delivery to the home to see a very small, plug-and-play, you can really save electricity, it's worth it!	Jingdong prefecture level city in Shandong
Jinfeng Hongyuan Flagship Store	Electricity savings1	Smart Saver received. Unpacking the box is exquisite. The material workmanship is very good. Plugged in and tried the space. Did not open the air conditioning voltage display 206 air conditioning open up the air conditioning display for 194. whether to save electricity to a month later to pay the electricity bill will know. Logistics is also very fast service is also in place.	Jingdong prefecture level city in Shandong
Jinfeng Hongyuan Flagship Store	Electricity savings1	The product feels pretty good and I hope it works, as the store owner said it would. Works!!!	Jingdong prefecture level city in Shandong
Jinfeng Hongyuan Flagship Store	Electricity savings1	Baby received, physical very quickly, just plugged in hope that it can be as everyone said those power saving, with two months after additional comments, first give a five-star praise.	Jingdong prefecture level city in Shandong
Jinfeng Hongyuan Flagship Store	Electricity savings1	Good, very good, very good, you deserve it, whoever uses it knows, the stuff works great, will buy it again in the future.	Jingdong prefecture level city in Shandong
Jinfeng Hongyuan Flagship Store	Electricity savings1	Don't hesitate to buy wow! No cheap feeling, very high-end, especially great! It is genuine, and the same as the entity store, the price is also favorable, decisive turn powder 💗 catch up to do activities when you buy, the price of a penny for a penny ♡, from the outside looking very beautiful 💗, the material workmanship is good 💗, feel that the purchase is worth it, and the logistics is super	Jingdong prefecture level city in Shandong
Jinfeng Hongyuan Flagship Store	Electricity savings1	Logistics is fast! Very good quality! Very beautiful! Next time to patronize!	Jingdong prefecture level city in Shandong
Jinfeng Hongyuan Flagship Store	Electricity savings1	Really like it, completely exceeded expectations, the shipping speed is very fast, the packaging is very careful and strict, the delivery speed is very fast, very satisfied with a shopping Baby received, and the description is the same, love it!	Jingdong prefecture level city in Shandong
Jinfeng Hongyuan Flagship Store	Electricity savings1	Very good, if you need to order!	Jingdong prefecture level city in Shandong
Jinfeng Hongyuan Flagship Store	Electricity savings1	So far, plugged in the voltage is more stable, as for the effect of how to quietly wait until the payment of the electricity bill to compare, the appearance of the commodity design is very good, the real thing!	Jingdong prefecture level city in Shandong
Jinfeng Hongyuan Flagship Store	Electricity savings1	💌 I've been wanting it for a long time, I decided to go for it, I feel great after using it, it's genuine and the delivery is great, it lives up to its name👍	Jingdong prefecture level city in Shandong
Jinfeng Hongyuan Flagship Store	Electricity savings1	Looking forward to it!!! I don't know if it's working or not, let's look forward to it!!!! How much is saved , , , , , , , , , Saving a degree is a degree with long term use ! ! ! ! ! ! !	Jingdong prefecture level city in Shandong
Jinfeng Hongyuan Flagship Store	Electricity savings1	After using it for a while, I am seriously and responsibly saying that this saver is really very power efficient. Two rooms with the air conditioner on at night for one night, plus watching TV, charging cell phones, lights, etc. etc. only used one degree of electricity. So I bought several more and it's really	Jingdong prefecture level city in Shandong
Jinfeng Hongyuan Flagship Store	Electricity savings1	It arrived quickly, and the box is solid and delicate. Baby shell is thick aluminum and by multi-layer coating. The inner core is heavy, and the power cord and plug are genuine products from a large manufacturer. It is the best power saver I have found after choosing all of them!	Jingdong prefecture level city in Shandong

Jinfeng Hongyuan Flagship Store	Electricity savings1	The saver was received, genuine guarantee, good quality...	Jingdong prefecture level city in Shandong
Jinfeng Hongyuan Flagship Store	Electricity savings1	Logistics was fast! The product is easy to use!	Jingdong prefecture level city in Shandong
Jinfeng Hongyuan Flagship Store	Electricity savings1	Look appearance is okay, use a period of time to know how it is. Jingdong Mall, trustworthy! Home appliances, cell phones and computers are almost 100% bought by Jingdong. Ordered the night before yesterday, received yesterday. Jingdong logistics, the fastest in the network!	Jingdong prefecture level city in Shandong
Jinfeng Hongyuan Flagship Store	Electricity savings1	Very good, perfect packaging, home energy saver to determine the effect of power saving and also play a role in stabilizing the voltage Very good products	Jingdong prefecture level city in Shandong
Jinfeng Hongyuan Flagship Store	Electricity savings1	Smart Saver received, looks very exquisite. The method of use is simple, and it has been used. The later effect, with time to test.	Jingdong prefecture level city in Shandong
Jinfeng Hongyuan Flagship Store	Electricity savings1	Both the material and workmanship exceeded expectations, great value, and looks great on oh. The seller also shipped much earlier than expected, will come back. Buy it, you will not regret it! Full five points. Both the material and workmanship exceeded expectations, great value, wear it also looks good Oh. Seller delivery is also much earlier than expected, will come again. Buy it, will not regret it! Full	Jingdong prefecture level city in Shandong
Jinfeng Hongyuan Flagship Store	Electricity savings1	The appearance is clean and beautiful, the workmanship is very good, I didn't find any defects, I personally feel that the size is also quite suitable, I've used it for 2 months, I didn't find out exactly how energy efficient it is. Try using it again!	Jingdong prefecture level city in Shandong
Jinfeng Hongyuan Flagship Store	Electricity savings1	Power saving device received, product certification, accessories, etc. complete, very good workmanship, very practical power-saving effect is also very obvious.	Jingdong prefecture level city in Shandong
Jinfeng Hongyuan Flagship Store	Electricity savings1	Looked at a lot of think buy this one, see a lot of reviews say it works, a month with save nearly twenty degrees of electricity, there are sixty days of return guarantee, with a good and then come back to the	Jingdong prefecture level city in Shandong
Jinfeng Hongyuan Flagship Store	Electricity savings1	I received the power saving device, and I'm using it. I hope I can get the desired effect, and I'm trying to force it to work quickly.	Jingdong prefecture level city in Shandong
Jinfeng Hongyuan Flagship Store	Electricity savings1	Voltage is stable, already trying it out and waiting for the results to happen, saving electricity will follow up next month!	Jingdong prefecture level city in Shandong
Jinfeng Hongyuan Flagship Store	Electricity savings1	Jingdong Mall self-supporting goods of excellent quality, logistics is also very fast, worth recommending 👏👏👏👏 excellent!	Jingdong prefecture level city in Shandong
Jinfeng Hongyuan Flagship Store	Electricity savings1	Power saving has been received, the appearance of the beautiful, I have already been the second time to buy the use of down the effect is very good!	Jingdong prefecture level city in Shandong
Jinfeng Hongyuan Flagship Store	Electricity savings1	Energy saver received very happy, the color is also very good looking forward to a very good effect can save electricity	Jingdong prefecture level city in Shandong
Jinfeng Hongyuan Flagship Store	Electricity savings1	It's small and easy to plug in and use. It displays the voltage value when plugged in and	Jingdong prefecture level city in Shandong
Jinfeng Hongyuan Flagship Store	Electricity savings1	This saver really works well, because I installed one at home about seven or eight years ago, when there is no voltage display, now there is a voltage display, the appearance also looks much better, the electricity bill is also saved, the stability of the home appliances is also good, the best of both	Jingdong prefecture level city in Shandong
Jinfeng Hongyuan Flagship Store	Electricity savings1	Ordered yesterday, arrived today in use. Shipping was fast. The effect is looking forward to.	Jingdong prefecture level city in Shandong
Jinfeng Hongyuan Flagship Store	Electricity savings1	This thing, I don't know what the principle is. Why it can save electricity. Wait for a month to use it first, see if it will save electricity bill, then come back to evaluate. There is one thing that does not fit the principle ah. The law of conservation of energy.	Jingdong prefecture level city in Shandong
Jinfeng Hongyuan Flagship Store	Electricity savings1	Home mom listen to others say this can save electricity, specially let buy a, later a very large, go back to see a particularly small, directly plugged into the electricity can be, no need to install, the price is also very cheap, the first time with this product, do not know if it is not good to use, temporarily try for a period of time, if it is good to use, recommend that you buy this one, the	Jingdong prefecture level city in Shandong
Jinfeng Hongyuan Flagship Store	Electricity savings1	I've been using it for a few days, and I think the workmanship and materials are pretty good, so we'll have to look at the next bill for the real energy savings, and then we'll add more comments.	Jingdong prefecture level city in Shandong
Jinfeng Hongyuan Flagship Store	Electricity savings1	Energy saver is well made, energy saving and power saving, and anti-counterfeiting code, it is a regular commodity!	Jingdong prefecture level city in Shandong
Jinfeng Hongyuan Flagship Store	Electricity savings1	Jinfeng Hongyuan, buy back home, as a mouse, haha. First of all, normal electricity for 1 month, the power did not increase, the electricity bill feels smoother, year-on-year electricity costs in the low, whether it is very energy-saving still have to be used for a long time to know. All 5 stars ☆	Jingdong prefecture level city in Shandong
Jinfeng Hongyuan Flagship Store	Electricity savings1	The box is well packaged and beautiful! I hope to save electricity with it haha!	Jingdong prefecture level city in Shandong
Jinfeng Hongyuan Flagship Store	Electricity savings1	Ordered last night, arrived this morning, first try the effect before making comments	Jingdong prefecture level city in Shandong
Jinfeng Hongyuan Flagship Store	Electricity savings1	Looks good. Don't know the effect yet.	Jingdong prefecture level city in Shandong
Jinfeng Hongyuan Flagship Store	Electricity savings1	Jinfeng Hongyuan intelligent power saver has exquisite workmanship and modern fashionable design style. It is a good home economizer . I believe that the goods purchased from Jingdong will not disappoint our	Jingdong prefecture level city in Shandong
Jinfeng Hongyuan Flagship Store	Electricity savings1	Inadvertently flipped to this power saver, never heard of, feel very novel, the price is not expensive, so buy to try, see a month after the electricity bill will be less, recently the home air-conditioning open a lot of power consumption is very large.	Jingdong prefecture level city in Shandong
Jinfeng Hongyuan Flagship Store	Electricity savings1	The quality of the goods is very good workmanship, but also sent five fuse	Jingdong prefecture level city in Shandong
Jinfeng Hongyuan Flagship Store	Electricity savings1	I'm trying it out. I don't know if it'll work.	Jingdong prefecture level city in Shandong
Jinfeng Hongyuan Flagship Store	Electricity savings1	There is no effect yet do not know, is to look at the official sale, buy to try it! Use according to the instructions, plugged in, used for a period of time, anyway, do not know how, after a few months to see it, to see if the average month than before will be less electricity.	Jingdong prefecture level city in Shandong
Jinfeng Hongyuan Flagship Store	Electricity savings1	Received the goods, the first time to unpacking, the quality is very good, consistent with the description of the seller, completely exceeded my expectations, the packaging is very careful, strict, in general this time is very satisfied with a shopping, thanks to the seller.	Jingdong prefecture level city in Shandong
Jinfeng Hongyuan Flagship Store	Electricity savings1	The energy saver was received and used for a few days with good results.	Jingdong prefecture level city in Shandong
Jinfeng Hongyuan Flagship Store	Electricity savings1	Previously I have paid attention to this product, but I think that the monthly electricity consumption at home is not large, there is no need to save electricity savings. After a second thought, energy saving is the responsibility of each of our citizens, the winter is coming, this year's winter is cold, heating and all electric appliances, can save is to save, the price is not expensive, so buy to try. I analyze the principle of power saving, should be able to save electricity, but the larger the power saving effect is about good. To be tried after a month or two to see the effect and then evaluate. Jingdong trustworthy.	Jingdong prefecture level city in Shandong
Jinfeng Hongyuan Flagship Store	Electricity savings1	It just arrived and I can't wait to use it, and the customer service is great! There is also voltage showing up which is nice! I don't know if it can save a bit I won't know until I've used it for a month! When the time comes to come back to the follow-up review!	Jingdong prefecture level city in Shandong
Jinfeng Hongyuan Flagship Store	Electricity savings1	Checked out the reviews from numerous users. The shipment is still very large, so I decided to buy it anyway to use it and see the effect after a month. Jingdong Mall here to buy safe, convenient and fast, this price is very powerful 💪, very recommended.	Jingdong prefecture level city in Shandong
Jinfeng Hongyuan Flagship Store	Electricity savings1	Purchased two at the same time to use together. The other one works fine, the other has problems with the numbers not sure if the machine is faulty. Power saving effect can not be observed. Feel the effect is not very obvious like?	Jingdong prefecture level city in Shandong
Jinfeng Hongyuan Flagship Store	Electricity savings1	Looking forward to it!!! I don't know if it's working or not, let's look forward to it!!!! How much is saved , , , , , , , , , Saving a degree is a degree with long term use ! ! ! ! ! ! !	Jingdong prefecture level city in Shandong
Jinfeng Hongyuan Flagship Store	Electricity savings1	Received, directly on the use of the special convenient	Jingdong prefecture level city in Shandong
Jinfeng Hongyuan Flagship Store	Electricity savings1	Goods received a few days well-packed, the same day opened and plugged in to try to be normal is not sure what the power-saving effect. After a month with a follow-up evaluation.	Jingdong prefecture level city in Shandong
Jinfeng Hongyuan Flagship Store	Electricity savings1	I bought it and used it, I don't know how it works yet, we'll see later. Use the effect of it	Jingdong prefecture level city in Shandong
Jinfeng Hongyuan Flagship Store	Electricity savings1	Photographed 2, the old family also put a, with a small January, there should be some effect, with a long period of time to comment, with a long time to see the obvious effect, hard courier brother.	Jingdong prefecture level city in Shandong

Jinfeng Hongyuan Flagship Store	Electricity savings1	Experience: I am an electrician, this is the real use of electronic waves automatically connected to track the decomposition of ionized electrons, in situ compensation of reactive power, reduce effective power loss and waste, stabilize the voltage to achieve energy saving and power saving, the overall effect	Jingdong prefecture level city in Shandong
Jinfeng Hongyuan Flagship Store	Electricity savings1	Great looking and practical! Easy to use and save! Saves 15% in a month of comparison	Jingdong prefecture level city in Shandong
Jinfeng Hongyuan Flagship Store	Electricity savings1	Product quality is very good! Packaging is very nice, like a cell phone box, thick and well protected! Service is also great! Logistics is very fast! Thank you for your business! Very honest!	Jingdong prefecture level city in Shandong
Jinfeng Hongyuan Flagship Store	Electricity savings1	In several products inside the choice of this purchase for a period of time, but also need to use a few months to compare last year's electricity bill before you can analyze the savings in electricity, to the summer peak electricity consumption should be more obvious.	Jingdong prefecture level city in Shandong
Jinfeng Hongyuan Flagship Store	Electricity savings1	The goods have long been received, after more than a month to comment, the home electricity bill is indeed reduced, worth buying!	Jingdong prefecture level city in Shandong
Jinfeng Hongyuan Flagship Store	Electricity savings1	Baby received, the logistics is strong, the quality is reliable, it is worth having and recommending!	Jingdong prefecture level city in Shandong
Jinfeng Hongyuan Flagship Store	Electricity savings1	Using a conservation appliance, you can achieve a 10-15% savings in electricity usage.	Jingdong prefecture level city in Shandong
Jinfeng Hongyuan Flagship Store	Electricity savings1	Quality is very good, metal shell, touch is very good, very recommended Oh ~, the subsequent effect of continuous follow-up	Jingdong prefecture level city in Shandong
Jinfeng Hongyuan Flagship Store	Electricity savings1	The product is beautiful, practical, worth buying. The product is beautiful, practical, good quality, fast logistics product is beautiful, practical, beautiful and elegant, very classy, worth buying!	Jingdong prefecture level city in Shandong
Jinfeng Hongyuan Flagship Store	Electricity savings1	Just received the item and have already used it. Didn't know much about these products before. I hope it is as effective and useful as the product advertises.	Jingdong prefecture level city in Shandong
Jinfeng Hongyuan Flagship Store	Electricity savings1	A month two people used more than five hundred degrees of electricity, in a hurry and then add a power saver, I hope to have an effect, Jingdong shopping is very cool, logistics is very fast especially good service attitude delivery, this hot day is not easy, thank you!	Jingdong prefecture level city in Shandong
Jinfeng Hongyuan Flagship Store	Electricity savings1	Just received the baby, first plugged in to try to use a month and then additional evaluation it	Jingdong prefecture level city in Shandong
Jinfeng Hongyuan Flagship Store	Electricity savings1	Goods fast, the power saver has been received, a friend recommended to buy that can save electricity. Exquisite and compact is very convenient, plug and play. The effect is good, the price is affordable, the next need will patronize again!	Jingdong prefecture level city in Shandong
Jinfeng Hongyuan Flagship Store	Electricity savings1	Packaging is better than expected, sent a few fuse, power saving effect in the end do not know, try a period of time to say!	Jingdong prefecture level city in Shandong
Jinfeng Hongyuan Flagship Store	Electricity savings1	Shipped quickly, immediately installed, read countless reviews were not sure if this thing is effective, was going to buy three at once, ultimately decided to first use a try the effect, if the power ring significantly reduced, then will buy a few more!	Jingdong prefecture level city in Shandong
Jinfeng Hongyuan Flagship Store	Electricity savings1	Used for a period of time to evaluate, not to a month still do not know whether it can save electricity, the voltage has a display!	Jingdong prefecture level city in Shandong
Jinfeng Hongyuan Flagship Store	Electricity savings1	I don't know if it saves electricity or not, but I'm using it anyway, so I hope it saves a little bit, electricity is too expensive	Jingdong prefecture level city in Shandong
Jinfeng Hongyuan Flagship Store	Electricity savings1	The power saver has been received and I am very happy with it! Just waiting to see how the power saving works!	Jingdong prefecture level city in Shandong
Jinfeng Hongyuan Flagship Store	Electricity savings1	Saving power has been received, logistics is quite fast, the quality is not bad, small and a little weight, has been used to look forward to the effect of	Jingdong prefecture level city in Shandong
Jinfeng Hongyuan Flagship Store	Electricity savings1	The logistics is very fast, the effect is obvious and trustworthy.	Jingdong prefecture level city in Shandong
Jinfeng Hongyuan Flagship Store	Electricity savings1	Good quality, good quality, beautiful, beautiful, beautiful, beautiful, beautiful, very worth buying, worth buying, worth buying, worth buying, worth customizing!	Jingdong prefecture level city in Shandong
Jinfeng Hongyuan Flagship Store	Electricity savings1	I've used it for now, I don't know if it saves electricity	Jingdong prefecture level city in Shandong
Jinfeng Hongyuan Flagship Store	Electricity savings1	Bought two at a time, logistics is very fast, the product is beautifully packaged, after two months to see the results	Jingdong prefecture level city in Shandong
Jinfeng Hongyuan Flagship Store	Electricity savings1	The workmanship is really fine, the packaging is also good, the regular factory production ~ use a break in the evaluation of the effectiveness of the	Jingdong prefecture level city in Shandong
Jinfeng Hongyuan Flagship Store	Electricity savings1	Sometimes 219. Sometimes 220 in this case	Jingdong prefecture level city in Shandong
Jinfeng Hongyuan Flagship Store	Electricity savings1	It is the ideal product, good quality, logistics is also very fast, worthy of praise!	Jingdong prefecture level city in Shandong
Jinfeng Hongyuan Flagship Store	Electricity savings1	Store service is very good, the courier received the next day. Product packaging is very good, just use the quality to be tested.	Jingdong prefecture level city in Shandong
Jinfeng Hongyuan Flagship Store	Electricity savings1	I've been using it for almost a month now and I feel like it's working.	Jingdong prefecture level city in Shandong
Jinfeng Hongyuan Flagship Store	Electricity savings1	Very satisfied, very restful, very pretty, very beautiful, very much enjoyed! Well worth it!	Jingdong prefecture level city in Shandong
Jinfeng Hongyuan Flagship Store	Electricity savings1	Baby especially like, the value of high-quality good, a very good shopping experience, attentive service, fast logistics, distribution is also very thoughtful, attitude is also very good, value for money ah, decisive praise! Quality is quite good, logistics is also very fast, praise!	Jingdong prefecture level city in Shandong
Jinfeng Hongyuan Flagship Store	Electricity savings1	The shape of the item is basically the same as ordered, the effect is to be examined.	Jingdong prefecture level city in Shandong
Jinfeng Hongyuan Flagship Store	Electricity savings1	Appearance style: good-looking workmanship material: very good, a small one, very delicate Experience: still in use, in the process of using the shopkeeper is very nice to have a careful explanation Use for a period of time in the look at the results	Jingdong prefecture level city in Shandong
Jinfeng Hongyuan Flagship Store	Electricity savings1	As described, compact and well packaged	Jingdong prefecture level city in Shandong
Jinfeng Hongyuan Flagship Store	Electricity savings1	Fine workmanship. Test in progress. Let's see how it works.	Jingdong prefecture level city in Shandong
Jinfeng Hongyuan Flagship Store	Electricity savings1	The goods have been received, I'm very satisfied and love it	Jingdong prefecture level city in Shandong
Jinfeng Hongyuan Flagship Store	Electricity savings1	Safety! Whether you can save electricity, you can roughly feel it in 2 months. The principle is simple. Before the electrical appliances and circuits inductive more, now the electrical appliances integrated, the home is not a lot of inductive circuits, but used certainly save no problem! Wiring according to the requirements of the manual is good, if it is not convenient, see the socket plug.	Jingdong prefecture level city in Shandong
Jinfeng Hongyuan Flagship Store	Electricity savings1	Great product, looking forward to how much power I can save, first time buyer!	Jingdong prefecture level city in Shandong
Jinfeng Hongyuan Flagship Store	Electricity savings1	The product is exquisite, beautifully packaged, and the customer service is excellent. Just waiting to see how the power saving effect is, looking forward to it!	Jingdong prefecture level city in Shandong
Jinfeng Hongyuan Flagship Store	Electricity savings1	Express very quickly very satisfied with the first use to see how the effect to know, if you can save 10 percent even if acceptable, if you can not meet this requirement is obviously not much effect. Obviously.	Jingdong prefecture level city in Shandong
Jinfeng Hongyuan Flagship Store	Electricity savings1	Very delicate and compact, there is a very convenient intelligent display prompts the voltage, you can know the voltage at a glance, there is very easy to use, a plug can be used, I hope to save money on	Jingdong prefecture level city in Shandong
Jinfeng Hongyuan Flagship Store	Electricity savings1	First time buyer is it really save after use	Jingdong prefecture level city in Shandong
Jinfeng Hongyuan Flagship Store	Electricity savings1	Purchased again and will be for a long time yet.	Jingdong prefecture level city in Shandong
Jinfeng Hongyuan Flagship Store	Electricity savings1	Bought it to stock up, came back and plugged it in, hope it works	Jingdong prefecture level city in Shandong
Jinfeng Hongyuan Flagship Store	Electricity savings1	Baby received the logistics very fast very like very satisfied praise a	Jingdong prefecture level city in Shandong
Jinfeng Hongyuan Flagship Store	Electricity savings1	Beginning the process of using, easy to evaluate more later.	Jingdong prefecture level city in Shandong
Jinfeng Hongyuan Flagship Store	Electricity savings1	The goods have been received, just do not know how effective	Jingdong prefecture level city in Shandong
Jinfeng Hongyuan Flagship Store	Electricity savings1	It feels okay. I wish there were more events.	Jingdong prefecture level city in Shandong

Jinfeng Hongyuan Flagship Store	Electricity savings1	Things have been received, just plugged in, I do not know if it works, wait a few days to have the effect of additional	Jingdong prefecture level city in Shandong
Jinfeng Hongyuan Flagship Store	Electricity savings1	The effect of this power saver is really good, the house more, a few air conditioners are open every day, not used before a month to say less than seven hundred eight hundred, used after three months only nine	Jingdong prefecture level city in Shandong
Jinfeng Hongyuan Flagship Store	Electricity savings1	Cheap, good and easy to use, recommend it.	Jingdong prefecture level city in Shandong
Jinfeng Hongyuan Flagship Store	Electricity savings1	Bought this device, to the home for a long time, wait to finish a month to see how, good can recommend friends to buy, we use the electricity every month scary, a month of electricity costs more than a thousand, can not afford to hurt ah! I hope this power saving can save me some money, the beginning of the shoot order did not pay attention to look at the address, sent elsewhere to go, that side called to find out, hurry to communicate with customer service, customer service is very careful to help me get it done, call to that side to help me turn around, thank you!	Jingdong prefecture level city in Shandong
Jinfeng Hongyuan Flagship Store	Electricity savings1	I don't know the effect if it, use it first to see.	Jingdong prefecture level city in Shandong
Jinfeng Hongyuan Flagship Store	Electricity savings1	Looks good. I won't know if it saves power until I use it.	Jingdong prefecture level city in Shandong
Jinfeng Hongyuan Flagship Store	Electricity savings1	Goods received, shipping speed is very fast, yesterday afternoon more than two o'clock shot, just express delivery, but also sent a few insurance, very good business, the display of the number do not understand what is the meaning of the display said voltage, do not understand this, try a month to see how, good and	Jingdong prefecture level city in Shandong
Jinfeng Hongyuan Flagship Store	Electricity savings1	What ** play also, save electricity more and more save more and more, save chicken hair electricity, are cheating, this is a night of air conditioning with electricity, a night with 11 electricity word, this is	Jingdong prefecture level city in Shandong
Jinfeng Hongyuan Flagship Store	Electricity savings1	Received the product, hope it works!	Jingdong prefecture level city in Shandong
Jinfeng Hongyuan Flagship Store	Electricity savings1	Bought home and used very convenient, easy to operate, quite good	Jingdong prefecture level city in Shandong
Jinfeng Hongyuan Flagship Store	Electricity savings1	It's really good, cost-effective and good quality, and the logistics are fast! It arrived in one day! Really good, cost-effective and good quality, logistics is fast ah! It arrived in one day!	Jingdong prefecture level city in Shandong
Jinfeng Hongyuan Flagship Store	Electricity savings1	Works well, stabilizes voltage and saves power	Jingdong prefecture level city in Shandong
Jinfeng Hongyuan Flagship Store	Electricity savings1	I'll use it right away and look forward to good results	Jingdong prefecture level city in Shandong
Jinfeng Hongyuan Flagship Store	Electricity savings1	Recommended by a friend, said it works well, so I bought one, the effect to be tested	Jingdong prefecture level city in Shandong
Jinfeng Hongyuan Flagship Store	Electricity savings1	I didn't know it would save electricity.	Jingdong prefecture level city in Shandong
Jinfeng Hongyuan Flagship Store	Electricity savings1	I've been using it for a week, I don't know what's going on yet I hope it works well and saves power I'll follow up after a month of use	Jingdong prefecture level city in Shandong
Jinfeng Hongyuan Flagship Store	Electricity savings1	Very well packaged, looks great, don't know how it works	Jingdong prefecture level city in Shandong
Jinfeng Hongyuan Flagship Store	Electricity savings1	Genuine, good quality, really saves energy.	Jingdong prefecture level city in Shandong
Jinfeng Hongyuan Flagship Store	Electricity savings1	I was worried that the stuff is not good, but when I got it, I realized that I was completely over-anxious, 5 stars!	Jingdong prefecture level city in Shandong
Jinfeng Hongyuan Flagship Store	Electricity savings1	Very good and satisfied trustworthy	Jingdong prefecture level city in Shandong
Jinfeng Hongyuan Flagship Store	Electricity savings1	I don't know if it saves power	Jingdong prefecture level city in Shandong
Jinfeng Hongyuan Flagship Store	Electricity savings1	The quality of the product is very good, beautiful and generous applicable fast delivery speed good service attitude	Jingdong prefecture level city in Shandong
Jinfeng Hongyuan Flagship Store	Electricity savings1	Let's use it for a while and then we'll follow up	Jingdong prefecture level city in Shandong
Jinfeng Hongyuan Flagship Store	Electricity savings1	I've only been using it for a month, I don't know if there's any change yet, I'll come back with a follow-up comment after checking the meter!	Jingdong prefecture level city in Shandong
Jinfeng Hongyuan Flagship Store	Electricity savings1	I don't know if it works, I read the instructions and online reviews, I hope I won't be disappointed, if I use it, I will buy it again. Jingdong Express, fast and guaranteed service.	Jingdong prefecture level city in Shandong
Jinfeng Hongyuan Flagship Store	Electricity savings1	It feels like it saves quite a bit huh?	Jingdong prefecture level city in Shandong
Jinfeng Hongyuan Flagship Store	Electricity savings1	I've been using it for a while now, and it still seems to work and have a power saving effect	Jingdong prefecture level city in Shandong
Jinfeng Hongyuan Flagship Store	Electricity savings1	Looks good. It says 235. The 222 bar voltage is shown as high. I don't know if that's right. We'll see.	Jingdong prefecture level city in Shandong
Jinfeng Hongyuan Flagship Store	Electricity savings1	Baby is really good, do really careful, worth to have, fast delivery!	Jingdong prefecture level city in Shandong
Jinfeng Hongyuan Flagship Store	Electricity savings1	Quite delicate and compact, simple and convenient to use. Things are still very good, bought two at a time. Very satisfied with an online purchase.	Jingdong prefecture level city in Shandong
Jinfeng Hongyuan Flagship Store	Electricity savings1	Feel very good, the use of unclear, ask customer service, timely explanation, big brand, trustworthy	Jingdong prefecture level city in Shandong
Jinfeng Hongyuan Flagship Store	Electricity savings1	Thrifty received a few days did not make a comment is to see how the quality of the product now feel the quality of good appearance is also powerful seller service is very good to buy the product is also attached to the 5 fuse thought very thoughtful!	Jingdong prefecture level city in Shandong
Jinfeng Hongyuan Flagship Store	Electricity savings1	Big brand, with a very assured, the quality is very good, in time for the event to buy very cost-effective, customer service is really very good Oh ~!	Jingdong prefecture level city in Shandong
Jinfeng Hongyuan Flagship Store	Electricity savings1	I ordered yesterday and received it this morning, I heard my friend say that it can save electricity, I don't know if it works, I'll come back to make a comment after using it.	Jingdong prefecture level city in Shandong
Jinfeng Hongyuan Flagship Store	Electricity savings1	I've been using it for over a month and it doesn't seem to be working!	Jingdong prefecture level city in Shandong
Jinfeng Hongyuan Flagship Store	Electricity savings1	Used it, the family has always been in the habit of using energy-saving devices, this is a new one. Digital display voltage is very good, can see that the voltage is more stable.	Jingdong prefecture level city in Shandong
Jinfeng Hongyuan Flagship Store	Electricity savings1	I haven't used it for more than a month, but it looks good. It's easy to plug into an outlet and it works.	Jingdong prefecture level city in Shandong
Jinfeng Hongyuan Flagship Store	Electricity savings1	Good Oh...appearance is very beautiful...just plugged in with...see the effect of it...with a period of time in the additional evaluation...	Jingdong prefecture level city in Shandong
Jinfeng Hongyuan Flagship Store	Electricity savings1	I've received the goods, whether it's good or not, I'll know after I use it.	Jingdong prefecture level city in Shandong
Jinfeng Hongyuan Flagship Store	Electricity savings1	Very good, logistics is fast, the stuff is very powerful and cost-effective! Can feel the meter slowing down	Jingdong prefecture level city in Shandong
Jinfeng Hongyuan Flagship Store	Electricity savings1	Logistics is very fast, get it immediately after use, easy to operate, plug in the power supply can be used, just do not know to the use of the effect of how.	Jingdong prefecture level city in Shandong
Jinfeng Hongyuan Flagship Store	Electricity savings1	Quality is very over the top, quality is guaranteed, quite a good choice, a lot of friends to see me use all want to buy, I recommend it!	Jingdong prefecture level city in Shandong
Jinfeng Hongyuan Flagship Store	Electricity savings1	The appearance is clean and beautiful, the workmanship is very good, did not find any flaws, I personally feel that the size is also quite suitable, looking for a long time to choose this one, big love! The price is very beautiful, things are still good, will continue to buy back! All five points! Ness Ness ~ logistics speed is really no words, is a word: fast. Commodities from the outer packaging is very high-grade, really a penny a penny, expensive has the reason for expensive	Jingdong prefecture level city in Shandong
Jinfeng Hongyuan Flagship Store	Electricity savings1	The logistics is very fast, the texture is very good, genuine, I like it very much.	Jingdong prefecture level city in Shandong
Jinfeng Hongyuan Flagship Store	Electricity savings1	It's been a few days since I put it on, I can't see any results now, waiting for a month to say, steady steady steady.	Jingdong prefecture level city in Shandong
Jinfeng Hongyuan Flagship Store	Electricity savings1	I've been using it for a while now and it should be fine, so I'm buying it for the second time.	Jingdong prefecture level city in Shandong
Jinfeng Hongyuan Flagship Store	Electricity savings1	Power Saving King has been received, looks good, quite delicate, looking forward to using it to save electricity	Jingdong prefecture level city in Shandong

Jinfeng Hongyuan Flagship Store	Electricity savings1	My mom said the home uses a little too much electricity, so I bought a Power Saver to try it out, I don't know how effective it is. Expect it to be a good thing!	Jingdong prefecture level city in Shandong
Jinfeng Hongyuan Flagship Store	Electricity savings1	Thank you Jingdong, logistics is very fast order to receipt only one day. Believe in Jingdong. Favorable comments	Jingdong prefecture level city in Shandong
Jinfeng Hongyuan Flagship Store	Electricity savings1	Saved some money on the electric bill compared to last month, watch this month again, no problems so far	Jingdong prefecture level city in Shandong
Jinfeng Hongyuan Flagship Store	Electricity savings1	Energy saver exquisite workmanship, beautiful style, more features, just bought, power saving effect to be used for a period of time, then evaluate!	Jingdong prefecture level city in Shandong
Jinfeng Hongyuan Flagship Store	Electricity savings1	This thing does save power is more compact to hang on the wall.	Jingdong prefecture level city in Shandong
Jinfeng Hongyuan Flagship Store	Electricity savings1	I've been buying it for a while now, I haven't actually measured it, but it still feels like it's effective	Jingdong prefecture level city in Shandong
Jinfeng Hongyuan Flagship Store	Electricity savings1	I've already used it since I bought it, I don't know how effective it is yet, I'll comment on it later.	Jingdong prefecture level city in Shandong
Jinfeng Hongyuan Flagship Store	Electricity savings1	Baby received, careful appearance, logistics is very fast, worth buying!	Jingdong prefecture level city in Shandong
Jinfeng Hongyuan Flagship Store	Electricity savings1	It's great, it's just not the same in hand, I bought it at that other cheaper online one for about the same price as this one. But then oops, doesn't work at all.	Jingdong prefecture level city in Shandong
Jinfeng Hongyuan Flagship Store	Electricity savings1	The product is exquisite, but you have to use it for a month to know how it works. I'll comment again after a month.	Jingdong prefecture level city in Shandong
Jinfeng Hongyuan Flagship Store	Electricity savings1	I've been using it for a while now, and it doesn't feel like it's working, and it doesn't save any power.	Jingdong prefecture level city in Shandong
Jinfeng Hongyuan Flagship Store	Electricity savings1	Affordable price, fast delivery, Jingdong self-supporting guarantee	Jingdong prefecture level city in Shandong
Jinfeng Hongyuan Flagship Store	Electricity savings1	Packaging is very good, no damage, good quality, fine workmanship Packaging is very good, no damage, good quality, fine workmanship Packaging is very good, no damage, good quality, fine workmanship Packaging is very good, no damage, good quality, fine workmanship	Jingdong prefecture level city in Shandong
Jinfeng Hongyuan Flagship Store	Electricity savings1	Big brand, with a very assured, the quality is very good, in time for the event to buy very cost-effective, customer service is really very good Oh ~!	Jingdong prefecture level city in Shandong
Jinfeng Hongyuan Flagship Store	Electricity savings1	Used a few days, the specific effect is not yet clear, looking forward to the effect as described by the merchant!	Jingdong prefecture level city in Shandong
Jinfeng Hongyuan Flagship Store	Electricity savings1	I just used it and don't know how it works, I'll follow up after using it!	Jingdong prefecture level city in Shandong
Jinfeng Hongyuan Flagship Store	Electricity savings1	Well, it's been installed and now I'm waiting to see the results, BOE shopping, hopefully I won't be disappointed!	Jingdong prefecture level city in Shandong
Jinfeng Hongyuan Flagship Store	Electricity savings1	Goods received, plugged in the voltage from 229 to 222, do not know whether to save electricity, that depends on the situation.	Jingdong prefecture level city in Shandong
Jinfeng Hongyuan Flagship Store	Electricity savings1	This is the second time I have bought the product and I feel it really saves electricity! Use it again for a while and then evaluate!	Jingdong prefecture level city in Shandong
Jinfeng Hongyuan Flagship Store	Electricity savings1	Overall, it's really good, and the customer service is great, you'll get a message back almost immediately when you speak, it's great value for money, I really recommend it!	Jingdong prefecture level city in Shandong
Jinfeng Hongyuan Flagship Store	Electricity savings1	✨ Have purchased many times, a must have for the family, affordable purchase price, fast delivery, 🎁 beautifully packaged!	Jingdong prefecture level city in Shandong
Jinfeng Hongyuan Flagship Store	Electricity savings1	I bought it after a friend introduced it, he said it is effective and can save electricity. I bought it to try, try a period of time and then follow up!	Jingdong prefecture level city in Shandong
Jinfeng Hongyuan Flagship Store	Electricity savings1	Received it, trying it out tonight, and bought one earlier.	Jingdong prefecture level city in Shandong
Jinfeng Hongyuan Flagship Store	Electricity savings1	It will take a month or two to see if it saves power or not.	Jingdong prefecture level city in Shandong
Jinfeng Hongyuan Flagship Store	Electricity savings1	Looked good in the instructions! Bought one and pressed it on! Use it and see! If it works! I'll buy some more!	Jingdong prefecture level city in Shandong
Jinfeng Hongyuan Flagship Store	Electricity savings1	Goods have been received packaging can also things are not bad appearance, with a use to know, now evaluation is nonsense.	Jingdong prefecture level city in Shandong
Jinfeng Hongyuan Flagship Store	Electricity savings1	Logistics is very fast, packaging is very good, the second unit, as always, looking forward to the effect of	Jingdong prefecture level city in Shandong
Jinfeng Hongyuan Flagship Store	Electricity savings1	I received the goods today, the quality of goods is very good, I am very satisfied ah.	Jingdong prefecture level city in Shandong
Jinfeng Hongyuan Flagship Store	Electricity savings1	Buy one and try it, I wonder if it has a power saving effect?	Jingdong prefecture level city in Shandong
Jinfeng Hongyuan Flagship Store	Electricity savings1	🔍 The item was received, well packaged, logistics is very fast, customer service attitude is very positive, there are questions answered in a timely manner! Overall very satisfied!	Jingdong prefecture level city in Shandong
Jinfeng Hongyuan Flagship Store	Electricity savings1	Baby packaging is very good, purchase submission goods quickly delivered to the home, use and then follow up.	Jingdong prefecture level city in Shandong
Jinfeng Hongyuan Flagship Store	Electricity savings1	My friend asked me to buy an extra one so he could try it too.	Jingdong prefecture level city in Shandong
Jinfeng Hongyuan Flagship Store	Electricity savings1	I've just put it on, I won't know the result until a month later if it will really save power.	Jingdong prefecture level city in Shandong
Jinfeng Hongyuan Flagship Store	Electricity savings1	It's good quality. Hopefully it'll help save power.	Jingdong prefecture level city in Shandong
Jinfeng Hongyuan Flagship Store	Electricity savings1	The appearance is clean and beautiful, the workmanship is very good, I did not find any flaws, I personally feel that the size is also quite suitable, I have been looking for a long time to choose this	Jingdong prefecture level city in Shandong
Jinfeng Hongyuan Flagship Store	Electricity savings1	Logistics is fast! Baby looks great! The price is fair! Wish the store business is prosperous!	Jingdong prefecture level city in Shandong
Jinfeng Hongyuan Flagship Store	Electricity savings1	Jingdong express fast, good, have used, sit and wait to save money...	Jingdong prefecture level city in Shandong
Jinfeng Hongyuan Flagship Store	Electricity savings1	In use, it didn't work as expected and there was nothing noticeable in terms of power savings.	Jingdong prefecture level city in Shandong
Jinfeng Hongyuan Flagship Store	Electricity savings1	Just started using it and looking forward to the results.	Jingdong prefecture level city in Shandong
Jinfeng Hongyuan Flagship Store	Electricity savings1	The quality is very good, the metal shell is very good, it is genuine, energy saving and power saving, logistics is very fast, there is a need to buy o, recommend everyone, very satisfied with an online	Jingdong prefecture level city in Shandong
Jinfeng Hongyuan Flagship Store	Electricity savings1	Received, I do not know if the product is good, used to see if it is good to come back to buy!	Jingdong prefecture level city in Shandong
Jinfeng Hongyuan Flagship Store	Electricity savings1	Jinfeng source of intelligent power saving treasure also used less than three months, the last thunder may be burned, the home of the electric send gate is not on, only the old man at home, no way only their own in the field and run back to find out what is the problem with the circuit, the quality is still not very good, thunder is easy to burn!	Jingdong prefecture level city in Shandong
Jinfeng Hongyuan Flagship Store	Electricity savings1	Saving power just started to use, I do not know if there is no effect, but read a lot of reviews are said to be very good so I bought, baby All aspects are good There are spare 2 fuses, late good and then follow	Jingdong prefecture level city in Shandong
Jinfeng Hongyuan Flagship Store	Electricity savings1	Use it for a few days and see, the delivery is quite fast	Jingdong prefecture level city in Shandong
Jinfeng Hongyuan Flagship Store	Electricity savings1	Small, well made and easy to use.	Jingdong prefecture level city in Shandong
Jinfeng Hongyuan Flagship Store	Electricity savings1	Beautifully packaged, it is genuine. Whether it is power saving, use it to evaluate.	Jingdong prefecture level city in Shandong
Jinfeng Hongyuan Flagship Store	Electricity savings1	Power saver. Used two of them and they do save quite a bit of power.	Jingdong prefecture level city in Shandong
Jinfeng Hongyuan Flagship Store	Electricity savings1	Workmanship is not bad, small and does not take up space, is also do not know whether the power saving, buy a to try!	Jingdong prefecture level city in Shandong
Jinfeng Hongyuan Flagship Store	Electricity savings1	The logistics is very fast, the courier attitude is very good, worth buying	Jingdong prefecture level city in Shandong
Jinfeng Hongyuan Flagship Store	Electricity savings1	The effect is not known until it is used.	Jingdong prefecture level city in Shandong

Jinfeng Hongyuan Flagship Store	Electricity savings1	Just arrived plugged in won't know for a month	Jingdong prefecture level city in Shandong
Jinfeng Hongyuan Flagship Store	Electricity savings1	This is really good to use the pull to realize the savings ah think again for my sister's family to buy a	Jingdong prefecture level city in Shandong
Jinfeng Hongyuan Flagship Store	Electricity savings1	Lies, a month with a few hundred electricity are no savings, family use	Jingdong prefecture level city in Shandong
Jinfeng Hongyuan Flagship Store	Electricity savings1	Express very quickly, look at the fine workmanship also sent a few fuse, look forward to can really save electricity	Jingdong prefecture level city in Shandong
Jinfeng Hongyuan Flagship Store	Electricity savings1	I didn't realize it was so small.	Jingdong prefecture level city in Shandong
Jinfeng Hongyuan Flagship Store	Electricity savings1	Logistics courier is very fast, the courier service is very good	Jingdong prefecture level city in Shandong
Jinfeng Hongyuan Flagship Store	Electricity savings1	Used for a period of time the effect is quite good indeed than the previous power saving	Jingdong prefecture level city in Shandong
Jinfeng Hongyuan Flagship Store	Electricity savings1	Logistics is super fast, the product is in use, see the effect...	Jingdong prefecture level city in Shandong
Jinfeng Hongyuan Flagship Store	Electricity savings1	Baby got it, tried it out and it didn't work at all. How can I use the tips to save power?	Jingdong prefecture level city in Shandong
Jinfeng Hongyuan Flagship Store	Electricity savings1	Power Saving King received, immediately used has been very stable without any sound good, good service, fast logistics inexpensive and beautiful worth repurchasing!	Jingdong prefecture level city in Shandong
Jinfeng Hongyuan Flagship Store	Electricity savings1	Logistics courier is very fast, the courier service is good	Jingdong prefecture level city in Shandong
Jinfeng Hongyuan Flagship Store	Electricity savings1	Good to recommend, the first month of air conditioning only 1 week, the electricity bill has reduced about 100 yuan, may be to reduce the use of air conditioning is also related to the other or as usual electricity, estimated that the longer you use the more you can save electricity.	Jingdong prefecture level city in Shandong
Jinfeng Hongyuan Flagship Store	Electricity savings1	It doesn't work. I've been using it for 20 days and it doesn't work.	Jingdong prefecture level city in Shandong
Jinfeng Hongyuan Flagship Store	Electricity savings1	Fast shipping, good quality, affordable price, very satisfied with a shopping.	Jingdong prefecture level city in Shandong
Jinfeng Hongyuan Flagship Store	Electricity savings1	Try it for a month to see if it saves money.	Jingdong prefecture level city in Shandong
Jinfeng Hongyuan Flagship Store	Electricity savings1	It's in use. I'll comment again after a while.	Jingdong prefecture level city in Shandong
Jinfeng Hongyuan Flagship Store	Electricity savings1	Goods have received look good 👍👍👍👍, use a period of time and then look at the	Jingdong prefecture level city in Shandong
Jinfeng Hongyuan Flagship Store	Electricity savings1	No, it doesn't work. It's useless. It's garbage.	Jingdong prefecture level city in Shandong
Jinfeng Hongyuan Flagship Store	Electricity savings1	I've been using it for almost 2 months now and I don't feel any reduction in my electricity bill.	Jingdong prefecture level city in Shandong
Jinfeng Hongyuan Flagship Store	Electricity savings1	I've received the goods, the logistics is really fast, ordered yesterday, arrived today.	Jingdong prefecture level city in Shandong
Jinfeng Hongyuan Flagship Store	Electricity savings1	And the backup insurance.	Jingdong prefecture level city in Shandong
Jinfeng Hongyuan Flagship Store	Electricity savings1	The appearance of this product is good, easy to use and install, the effect of how there is no use to wait for time to verify that the voltage displayed is different from the voltage measured by other meters, the gap is not too large, the follow-up to add the effect.	Jingdong prefecture level city in Shandong
Jinfeng Hongyuan Flagship Store	Electricity savings1	Baby received it 👌, really great ah! 🌈The quality can't be beat, very satisfied! 🆗Happy happy happy 😊	Jingdong prefecture level city in Shandong
Jinfeng Hongyuan Flagship Store	Electricity savings1	I'll follow up with a review after using it for a while, hopefully it won't bring me back a disappointment!	Jingdong prefecture level city in Shandong
Jinfeng Hongyuan Flagship Store	Electricity savings1	In use and looking forward to results! Good review to send	Jingdong prefecture level city in Shandong
Jinfeng Hongyuan Flagship Store	Electricity savings1	Intelligent power saver received, the volume is not big, small and exquisite, quite good, Jingdong Express is also quite fast, the	Jingdong prefecture level city in Shandong
Jinfeng Hongyuan Flagship Store	Electricity savings1	I don't know how to use it, keep using it	Jingdong prefecture level city in Shandong
Jinfeng Hongyuan Flagship Store	Electricity savings1	Very good for saving electricity	Jingdong prefecture level city in Shandong
Jinfeng Hongyuan Flagship Store	Electricity savings1	Seriously, did not use, feel also more power consumption, the peak is bought back to use the first time, the next day unplugged, the power consumption is back to more than ten degrees (the frequency of power use in the past few days is pretty much the same), feel the IQ tax paid 😂	Jingdong prefecture level city in Shandong
Jinfeng Hongyuan Flagship Store	Electricity savings1	I haven't seen any effect on the power saving, but the voltage stabilization looks good.	Jingdong prefecture level city in Shandong
Jinfeng Hongyuan Flagship Store	Electricity savings1	It's very good, I'll come back to it after using it for a while	Jingdong prefecture level city in Shandong
Jinfeng Hongyuan Flagship Store	Electricity savings1	Already used it and see the results.	Jingdong prefecture level city in Shandong
Jinfeng Hongyuan Flagship Store	Electricity savings1	Fast shipping, beautiful packaging, attentive service, the use of the effect, to be followed up after the evaluation.	Jingdong prefecture level city in Shandong
Jinfeng Hongyuan Flagship Store	Electricity savings1	The process of using this product is more than satisfactory	Jingdong prefecture level city in Shandong
Jinfeng Hongyuan Flagship Store	Electricity savings1	Very good East East cost-effective! Every time so buy more assured, and the logistics is also very fast. It is also very good to use, so I will often come to buy in the future, and will also introduce to	Jingdong prefecture level city in Shandong
Jinfeng Hongyuan Flagship Store	Electricity savings1	But still understand how effective it is!	Jingdong prefecture level city in Shandong
Jinfeng Hongyuan Flagship Store	Electricity savings1	Fast shipping, great logistics, great stuff, great price.	Jingdong prefecture level city in Shandong
Jinfeng Hongyuan Flagship Store	Electricity savings1	I'll review it in a month and give it 5 stars if it's good	Jingdong prefecture level city in Shandong
Jinfeng Hongyuan Flagship Store	Electricity savings1	I've saved a lot of electricity in the past month, so I'm glad to hear that.	Jingdong prefecture level city in Shandong
Jinfeng Hongyuan Flagship Store	Electricity savings1	Baby just received and installed! Looks genuinely good, workmanship feels fine! Looking forward to the effect! Said to see the effect in two months! Waiting for if really have the effect must come to follow up! The boss is absolutely responsible! Asked and answered! Just for the boss this attitude must also	Jingdong prefecture level city in Shandong
Jinfeng Hongyuan Flagship Store	Electricity savings1	This baby is very delicate, I'll comment after I've used it	Jingdong prefecture level city in Shandong
Jinfeng Hongyuan Flagship Store	Electricity savings1	It's been over two months. It's not working. It's just like it was.	Jingdong prefecture level city in Shandong
Jinfeng Hongyuan Flagship Store	Electricity savings1	Fast shipping and logistics, let's see if it works.	Jingdong prefecture level city in Shandong
Jinfeng Hongyuan Flagship Store	Electricity savings1	Just received the goods, try it to know if it can really save electricity	Jingdong prefecture level city in Shandong
Jinfeng Hongyuan Flagship Store	Electricity savings1	Not bad. Not bad. Not bad.	Jingdong prefecture level city in Shandong
Jinfeng Hongyuan Flagship Store	Electricity savings1	Not sure if it works. I'll comment again if it works.	Jingdong prefecture level city in Shandong
Jinfeng Hongyuan Flagship Store	Electricity savings1	Safe and energy-saving helpers to use!	Jingdong prefecture level city in Shandong
Jinfeng Hongyuan Flagship Store	Electricity savings1	Good, first time buyer. Used good can	Jingdong prefecture level city in Shandong
Jinfeng Hongyuan Flagship Store	Electricity savings1	It feels okay, I guess! I won't know until I use it for a while!	Jingdong prefecture level city in Shandong

Jinfeng Hongyuan Flagship Store	Electricity savings1	The saver matches the description and has been used, waiting for a follow up review after two months.	Jingdong prefecture level city in Shandong
Jinfeng Hongyuan Flagship Store	Electricity savings1	Received, the appearance of the business as described, the effect of how to use a period of time to evaluate.	Jingdong prefecture level city in Shandong
Jinfeng Hongyuan Flagship Store	Electricity savings1	Still using it don't know how effective it is, I'll comment after three months	Jingdong prefecture level city in Shandong
Jinfeng Hongyuan Flagship Store	Electricity savings1	The stuff is very good, the packaging is also very good logistics is also very fast	Jingdong prefecture level city in Shandong
Jinfeng Hongyuan Flagship Store	Electricity savings1	I'll follow up with a review when I use it, hopefully I won't be disappointed	Jingdong prefecture level city in Shandong
Jinfeng Hongyuan Flagship Store	Electricity savings1	Cancel Houjiaying Logistics.	Jingdong prefecture level city in Shandong
Jinfeng Hongyuan Flagship Store	Electricity savings1	It's very good, it's worth buying.	Jingdong prefecture level city in Shandong
Jinfeng Hongyuan Flagship Store	Electricity savings1	My friend bought it to save electricity and referred me to buy it! I'll buy it and try it out! I will know how much electricity I can save next month, looking forward to it! 4-500 dollars a month electricity bill is horrible! I hope it can really help me save some electricity!	Jingdong prefecture level city in Shandong
Jinfeng Hongyuan Flagship Store	Electricity savings1	Just received it and installed it.	Jingdong prefecture level city in Shandong
Jinfeng Hongyuan Flagship Store	Electricity savings1	Very nice. This one is my favorite.	Jingdong prefecture level city in Shandong
Jinfeng Hongyuan Flagship Store	Electricity savings1	Good value for money worth buying very good	Jingdong prefecture level city in Shandong
Jinfeng Hongyuan Flagship Store	Electricity savings1	Very energy-saving, small and exquisite, does not take up space, very satisfied, complete packaging, no damage, customer service explanation is also very serious	Jingdong prefecture level city in Shandong
Jinfeng Hongyuan Flagship Store	Electricity savings1	Received immediately used, really effective, do not understand electricity, but looked at the meter every day, recommended that you can use, especially this weather every day to open the air conditioning, with him not afraid of the electricity bill.	Jingdong prefecture level city in Shandong
Jinfeng Hongyuan Flagship Store	Electricity savings1	Nice, good value for money, nice stuff.	Jingdong prefecture level city in Shandong
Jinfeng Hongyuan Flagship Store	Electricity savings1	The packaging of the product is very good, the logistics of the product is very fast, and the quality of the product will be evaluated after use!	Jingdong prefecture level city in Shandong
Jinfeng Hongyuan Flagship Store	Electricity savings1	Express is very, well very fast, give a compliment 👍	Jingdong prefecture level city in Shandong
Jinfeng Hongyuan Flagship Store	Electricity savings1	Packaging is very good, fast delivery, used a night and a day did not feel the power saving. Use a month to see if it saves power.	Jingdong prefecture level city in Shandong
Jinfeng Hongyuan Flagship Store	Electricity savings1	Seems to be a bit of a power saver.	Jingdong prefecture level city in Shandong
Jinfeng Hongyuan Flagship Store	Electricity savings1	Bought this for someone else and it came over so fast, ###!	Jingdong prefecture level city in Shandong
Jinfeng Hongyuan Flagship Store	Electricity savings1	Very delicate, just received a period of time to use in the follow-up evaluation	Jingdong prefecture level city in Shandong
Jinfeng Hongyuan Flagship Store	Electricity savings1	It's been installed for a while now, not sure if it's working or not	Jingdong prefecture level city in Shandong
Jinfeng Hongyuan Flagship Store	Electricity savings1	The item looks okay, I just don't know if it works or not.	Jingdong prefecture level city in Shandong
Jinfeng Hongyuan Flagship Store	Electricity savings1	The goods have been received, but there is a screw missing	Jingdong prefecture level city in Shandong
Jinfeng Hongyuan Flagship Store	Electricity savings1	I didn't believe it before, but after receiving the goods today and trying it out, it really works!	Jingdong prefecture level city in Shandong
Jinfeng Hongyuan Flagship Store	Electricity savings1	Received, this thing will not know whether it is the effect of saving electricity, no matter how to say that there should always be a little use it, otherwise why develop it I think there is still the value of	Jingdong prefecture level city in Shandong
Jinfeng Hongyuan Flagship Store	Electricity savings1	Another one just arrived.	Jingdong prefecture level city in Shandong
Jinfeng Hongyuan Flagship Store	Electricity savings1	I don't feel anything, ordinary farmers can't go to TEDA, just use it first, it's safe.	Jingdong prefecture level city in Shandong
Jinfeng Hongyuan Flagship Store	Electricity savings1	Just purchased and will follow up after using it for a while	Jingdong prefecture level city in Shandong
Jinfeng Hongyuan Flagship Store	Electricity savings1	I'm trying it out now. I'll comment after I use it.	Jingdong prefecture level city in Shandong
Jinfeng Hongyuan Flagship Store	Electricity savings1	Received the item, in use, hope it works.	Jingdong prefecture level city in Shandong
Jinfeng Hongyuan Flagship Store	Electricity savings1	It works well. Good review.	Jingdong prefecture level city in Shandong
Jinfeng Hongyuan Flagship Store	Electricity savings1	All a scam, no effect at all, I bought too many fakes in Poundland before, I did not think that Jingdong is also the same	Jingdong prefecture level city in Shandong
Jinfeng Hongyuan Flagship Store	Electricity savings1	I used the saver as soon as I received it, it's in good shape and still working!	Jingdong prefecture level city in Shandong
Jinfeng Hongyuan Flagship Store	Electricity savings1	It works. I don't know what to do with the picture, but it works.	Jingdong prefecture level city in Shandong
Jinfeng Hongyuan Flagship Store	Electricity savings1	A very good shopping experience, attentive service, fast logistics, delivery is also very thoughtful, attitude is also very good, value for money ah, decisive praise!	Jingdong prefecture level city in Shandong
Jinfeng Hongyuan Flagship Store	Electricity savings1	The product is consistent with the presentation picture.	Jingdong prefecture level city in Shandong
Jinfeng Hongyuan Flagship Store	Electricity savings1	Baby received, use a period of time to evaluate, wish the boss business prosperity!	Jingdong prefecture level city in Shandong
Jinfeng Hongyuan Flagship Store	Electricity savings1	The stuff is better! Recommended!	Jingdong prefecture level city in Shandong
Jinfeng Hongyuan Flagship Store	Electricity savings1	I don't know if it works or not, I'll use it for a while before I comment on it.	Jingdong prefecture level city in Shandong
Jinfeng Hongyuan Flagship Store	Electricity savings1	Cost-effective, I like it, next time will come back Baby received, very surprised, the quality is very good, very satisfied with a shopping, I will continue to buy back, the store is trustworthy	Jingdong prefecture level city in Shandong
Jinfeng Hongyuan Flagship Store	Electricity savings1	Plugged it in and used it for a few days, not sure if it saves power yet	Jingdong prefecture level city in Shandong
Jinfeng Hongyuan Flagship Store	Electricity savings1	I'm trying it out and will follow up if it seems to be working!	Jingdong prefecture level city in Shandong
Jinfeng Hongyuan Flagship Store	Electricity savings1	Works pretty well, big kudos 👍👍👍👍👍👍👍👍👍👍👍	Jingdong prefecture level city in Shandong
Jinfeng Hongyuan Flagship Store	Electricity savings1	Just put it on, we'll see how it looks in a month.	Jingdong prefecture level city in Shandong
Jinfeng Hongyuan Flagship Store	Electricity savings1	The baby is very nice and I've already recommended buying it for all my friends! The main thing is that it not only saves electricity, I was more concerned about the fact that it can adjust the current, usually wiring problems. Now I found that it is really good, it is the effect I want!	Jingdong prefecture level city in Shandong
Jinfeng Hongyuan Flagship Store	Electricity savings1	Baby arrived, looking forward to his results, hopefully it will be good	Jingdong prefecture level city in Shandong
Jinfeng Hongyuan Flagship Store	Electricity savings1	Things have been received, look at the workmanship and packaging are good, I do not know how the power-saving effect? Use a period of time to see the effect	Jingdong prefecture level city in Shandong
Jinfeng Hongyuan Flagship Store	Electricity savings1	The price is right, the shape is beautiful and does not take up space, use it for a while to see the effect	Jingdong prefecture level city in Shandong
Jinfeng Hongyuan Flagship Store	Electricity savings1	Can't save electricity, it doesn't work!	Jingdong prefecture level city in Shandong
Jinfeng Hongyuan Flagship Store	Electricity savings1	It's pretty convenient, just plug it in and it works. There's no noise, plug it in and leave it alone, it's very hassle free! Good workmanship and value for money.	Jingdong prefecture level city in Shandong

Jinfeng Hongyuan Flagship Store	Electricity savings1	Just received the goods have been installed, try two months later to comment!	Jingdong prefecture level city in Shandong
Jinfeng Hongyuan Flagship Store	Electricity savings1	I received the stuff, I haven't used it yet, and overall it's fine.	Jingdong prefecture level city in Shandong
Jinfeng Hongyuan Flagship Store	Electricity savings1	Couldn't use a return at all.	Jingdong prefecture level city in Shandong
Jinfeng Hongyuan Flagship Store	Electricity savings1	Second purchase.	Jingdong prefecture level city in Shandong
Jinfeng Hongyuan Flagship Store	Electricity savings1	Very good quality, high tech and practical.	Jingdong prefecture level city in Shandong
Jinfeng Hongyuan Flagship Store	Electricity savings1	Very good, very great! Very satisfied, thanks to the store!	Jingdong prefecture level city in Shandong
Jinfeng Hongyuan Flagship Store	Electricity savings1	Just used it, don't know if it's good or not, hope it works well	Jingdong prefecture level city in Shandong
Jinfeng Hongyuan Flagship Store	Electricity savings1	Saving power has been received, the product is good, good like.	Jingdong prefecture level city in Shandong
Jinfeng Hongyuan Flagship Store	Electricity savings1	Received the goods and used it for two months before evaluation	Jingdong prefecture level city in Shandong
Jinfeng Hongyuan Flagship Store	Electricity savings1	It looks a little small, I do not know how effective, first use to see the effect, there is a small adjustable button, I do not know this is to adjust to that position to work well	Jingdong prefecture level city in Shandong
Jinfeng Hongyuan Flagship Store	Electricity savings1	Simple, just plug it in, there are spare fuses, can't say how well it works, no comparison	Jingdong prefecture level city in Shandong
Jinfeng Hongyuan Flagship Store	Electricity savings1	It's been a few days since I used it, and the power's gone up.	Jingdong prefecture level city in Shandong
Jinfeng Hongyuan Flagship Store	Electricity savings1	The owner was very nice and sent a spare fuse.	Jingdong prefecture level city in Shandong
Jinfeng Hongyuan Flagship Store	Electricity savings1	It's authentic and it's not expensive and it's great to use, I use it all the time and it's great just don't know if it really saves any points	Jingdong prefecture level city in Shandong
Jinfeng Hongyuan Flagship Store	Electricity savings1	The information is good, it is the child to buy, the child read it is also quite happy, I hope to be able to match the child's preparation for the midterm examination! Because of the epidemic, it is not too fast, but I also give full credit to the express delivery!	Jingdong prefecture level city in Shandong
Jinfeng Hongyuan Flagship Store	Electricity savings1	This power saver comes to the logistics very quickly, the business attitude is also very good, ** very comfortable, this brand is trustworthy, and buy in this store than in the physical store to buy cheaper,	Jingdong prefecture level city in Shandong
Jinfeng Hongyuan Flagship Store	Electricity savings1	It's very well made and the materials used are okay. This is just a few days old, so I don't know how it will work, only next month.	Jingdong prefecture level city in Shandong
Jinfeng Hongyuan Flagship Store	Electricity savings1	Already used on, have not tested the effect, wait for a couple of months to add comments.	Jingdong prefecture level city in Shandong
Jinfeng Hongyuan Flagship Store	Electricity savings1	Baby received, packaging is very thoughtful, already in use, should soon see the effect.	Jingdong prefecture level city in Shandong
Jinfeng Hongyuan Flagship Store	Electricity savings1	Just received in the office to try, to the home installed, the voltage are different, and do not know if there is no effect. Use a period of time and then follow up.	Jingdong prefecture level city in Shandong
Jinfeng Hongyuan Flagship Store	Electricity savings1	Just received the goods, is trying, looking forward to the effect, first give a favorable comment, two months later, then additional evaluation, please be patient.	Jingdong prefecture level city in Shandong
Jinfeng Hongyuan Flagship Store	Electricity savings1	The item has just been received, well packaged, results to be verified.	Jingdong prefecture level city in Shandong
Jinfeng Hongyuan Flagship Store	Electricity savings1	Packaging logistics are very good, the next look at how the effect, before I see others use the effect is good, as I bought this can also have good results.	Jingdong prefecture level city in Shandong
Jinfeng Hongyuan Flagship Store	Electricity savings1	I don't know if it's a real power saver or a fake power saver, but I've used it since I bought it, so I'll give you a good review.	Jingdong prefecture level city in Shandong
Jinfeng Hongyuan Flagship Store	Electricity savings1	Good quality, genuine goods, much better than imagined before purchase, next time will come back to care!	Jingdong prefecture level city in Shandong
Jinfeng Hongyuan Flagship Store	Electricity savings1	Logistics is very fast good use again	Jingdong prefecture level city in Shandong
Jinfeng Hongyuan Flagship Store	Electricity savings1	Received the goods. Let's see if it saves power in a month and then we'll follow up!	Jingdong prefecture level city in Shandong
Jinfeng Hongyuan Flagship Store	Electricity savings1	I bought it yesterday and it arrived today, I bought two of them, I hope they work well, I'll come back after a while to give a follow-up review	Jingdong prefecture level city in Shandong
Jinfeng Hongyuan Flagship Store	Electricity savings1	The home air conditioner consumes a lot of electricity, so I bought it and used it in the hope that there is A	Jingdong prefecture level city in Shandong
Jinfeng Hongyuan Flagship Store	Electricity savings1	The product is excellent and very current.	Jingdong prefecture level city in Shandong
Jinfeng Hongyuan Flagship Store	Electricity savings1	With the description, it is the most practical and good products that households can need, and it is an ideal shopping, the courier is also fast, and the service attitude is enthusiastic!	Jingdong prefecture level city in Shandong
Jinfeng Hongyuan Flagship Store	Electricity savings1	The Power Saver is easy to use, works well, is stable, and plugs into the top of the outlet	Jingdong prefecture level city in Shandong
Jinfeng Hongyuan Flagship Store	Electricity savings1	Overall still can, excellent craftsmanship, power saving do not know for the time being, after the use of the evaluation	Jingdong prefecture level city in Shandong
Jinfeng Hongyuan Flagship Store	Electricity savings1	Baby received on the plug, but also with four fuses, look at the quality is okay, I do not know whether it is useful, use and then follow up.	Jingdong prefecture level city in Shandong
Jinfeng Hongyuan Flagship Store	Electricity savings1	I'll evaluate it after a while, I don't know if it's good or not yet, and if it can save electricity	Jingdong prefecture level city in Shandong
Jinfeng Hongyuan Flagship Store	Electricity savings1	Jingdong self-operated purchase more assured delivery fast quality assurance	Jingdong prefecture level city in Shandong
Jinfeng Hongyuan Flagship Store	Electricity savings1	Shipping was fast, stuff was well packaged, great price, I'm happy with it 👍 and will repurchase!	Jingdong prefecture level city in Shandong
Jinfeng Hongyuan Flagship Store	Electricity savings1	The quality of the product is very good and I will definitely come back again next time.	Jingdong prefecture level city in Shandong
Jinfeng Hongyuan Flagship Store	Electricity savings1	I'll follow up with a review after I use it.	Jingdong prefecture level city in Shandong
Jinfeng Hongyuan Flagship Store	Electricity savings1	Hello, the goods are perfectly packaged, save power for a period of time to evaluate again!	Jingdong prefecture level city in Shandong
Jinfeng Hongyuan Flagship Store	Electricity savings1	Not bad, pretty good, good value for money, pretty good	Jingdong prefecture level city in Shandong
Jinfeng Hongyuan Flagship Store	Electricity savings1	The packaging is very good, received immediately used, looking forward to the effect in.	Jingdong prefecture level city in Shandong
Jinfeng Hongyuan Flagship Store	Electricity savings1	See how much power you save at the end of the month	Jingdong prefecture level city in Shandong
Jinfeng Hongyuan Flagship Store	Electricity savings1	Easy to use, affordable, beautiful color	Jingdong prefecture level city in Shandong
Jinfeng Hongyuan Flagship Store	Electricity savings1	It's taking too long to put together an order. It could be an epidemic.	Jingdong prefecture level city in Shandong
Jinfeng Hongyuan Flagship Store	Electricity savings1	I don't feel much after using it, no change in my electric bill 。。。。。。。	Jingdong prefecture level city in Shandong
Jinfeng Hongyuan Flagship Store	Electricity savings1	Logistics is very fast, the quality of the economizer is good, good quality and cheap, it is worth buying, I hope there is a power saving effect!	Jingdong prefecture level city in Shandong
Jinfeng Hongyuan Flagship Store	Electricity savings1	Feeling okay, in a couple of months to try to rely on it before making a comment. Let's give it a five-star review first!	Jingdong prefecture level city in Shandong
Jinfeng Hongyuan Flagship Store	Electricity savings1	Power saving device has been hesitating before in the end to buy or not to buy, after consideration, consideration, in consideration, or decided to buy a try. Things received can not wait to open a look, and the seller described the same, I hope to use 2 months to have the effect, two months to follow up on	Jingdong prefecture level city in Shandong
Jinfeng Hongyuan Flagship Store	Electricity savings1	Thank you Jingdong, logistics is very fast order to receipt only one day. Believe in Jingdong. Favorable comments	Jingdong prefecture level city in Shandong

Jinfeng Hongyuan Flagship Store	Electricity savings1	I used one to use it, but it didn't work very well, what to do	Jingdong prefecture level city in Shandong
Jinfeng Hongyuan Flagship Store	Electricity savings1	Quality and packaging are very good, fast shipping, good service, buy with confidence	Jingdong prefecture level city in Shandong
Jinfeng Hongyuan Flagship Store	Electricity savings1	Use a period of time to evaluate, the quality is very good, the price is affordable, logistics is also very fast	Jingdong prefecture level city in Shandong
Jinfeng Hongyuan Flagship Store	Electricity savings1	Goods received, the day of the order tomorrow delivery real God speed, delivery force, the product to be used after a period of time and then low comment!	Jingdong prefecture level city in Shandong
Jinfeng Hongyuan Flagship Store	Electricity savings1	I don't know, how effective it is, let's talk about it when the time comes, whether it saves power or not, how much, unknown.	Jingdong prefecture level city in Shandong
Jinfeng Hongyuan Flagship Store	Electricity savings1	It does save some electricity, and I don't want to use something that isn't legal! Used nearly ~ electricity last month, probably not much more this month with the AC on!	Jingdong prefecture level city in Shandong
Jinfeng Hongyuan Flagship Store	Electricity savings1	Well-packed, excellent quality, service satisfaction good value for money	Jingdong prefecture level city in Shandong
Jinfeng Hongyuan Flagship Store	Electricity savings1	Express very quickly, the goods received last night, today used a day of goods can not heat, looking forward to whether it can save electricity, with a month and then comment, is used well will buy again.	Jingdong prefecture level city in Shandong
Jinfeng Hongyuan Flagship Store	Electricity savings1	It's pretty good, hopefully it'll be a real power saver.	Jingdong prefecture level city in Shandong
Jinfeng Hongyuan Flagship Store	Electricity savings1	The goods received in use, can not see the effect of energy saving, after a month to know!	Jingdong prefecture level city in Shandong
Jinfeng Hongyuan Flagship Store	Electricity savings1	Received the goods, looking forward to better surprise, with the effect of the follow-up comment!	Jingdong prefecture level city in Shandong
Jinfeng Hongyuan Flagship Store	Electricity savings1	It's not bad, it's still in use and should save some	Jingdong prefecture level city in Shandong
Jinfeng Hongyuan Flagship Store	Electricity savings1	Logistics is very fast, easy to install, plugged in on it,, the effect of the use of the later to evaluate!	Jingdong prefecture level city in Shandong
Jinfeng Hongyuan Flagship Store	Electricity savings1	The stuff is recommendable to buy very good.	Jingdong prefecture level city in Shandong
Jinfeng Hongyuan Flagship Store	Electricity savings1	Voltmeter measurements of voltage are way off, aren't they?	Jingdong prefecture level city in Shandong
Jinfeng Hongyuan Flagship Store	Electricity savings1	Install it to the socket immediately after purchase and try to see if it can save electricity after one month.	Jingdong prefecture level city in Shandong
Jinfeng Hongyuan Flagship Store	Electricity savings1	I've been using it for almost a month now, and I won't know if it saves electricity until I pay the bill.	Jingdong prefecture level city in Shandong
Jinfeng Hongyuan Flagship Store	Electricity savings1	I bought one in the past and it was fine, so I'm buying it back.	Jingdong prefecture level city in Shandong
Jinfeng Hongyuan Flagship Store	Electricity savings1	Service is very good, this thing is small and exquisite to use, just waiting to use the effect of a period of time	Jingdong prefecture level city in Shandong
Jinfeng Hongyuan Flagship Store	Electricity savings1	Not sure how well it works yet	Jingdong prefecture level city in Shandong
Jinfeng Hongyuan Flagship Store	Electricity savings1	Kyodo goods habitually good reviews	Jingdong prefecture level city in Shandong
Jinfeng Hongyuan Flagship Store	Electricity savings1	Appearance is OK, whether it is power saving remains to be seen.	Jingdong prefecture level city in Shandong
Jinfeng Hongyuan Flagship Store	Electricity savings1	I bought it and used it, I just don't know if it really saves power	Jingdong prefecture level city in Shandong
Jinfeng Hongyuan Flagship Store	Electricity savings1	The saver is well made and of good quality, I will evaluate it after using it for a while.	Jingdong prefecture level city in Shandong
Jinfeng Hongyuan Flagship Store	Electricity savings1	Just received in use, use for a period of time to see if there is power saving	Jingdong prefecture level city in Shandong
Jinfeng Hongyuan Flagship Store	Electricity savings1	Used 3 days before commenting, it really saves electricity, I look at the "power calendar" after using one less degree of electricity per day, so I think it's worth it to buy this, I will introduce my friends	Jingdong prefecture level city in Shandong
Jinfeng Hongyuan Flagship Store	Electricity savings1	Reliable quality, very well packaged and matches the real thing.	Jingdong prefecture level city in Shandong
Jinfeng Hongyuan Flagship Store	Electricity savings1	Logistics is still quite fast, ha in the trial	Jingdong prefecture level city in Shandong
Jinfeng Hongyuan Flagship Store	Electricity savings1	Consultant service attitude is very good, the courier is also very powerful, the first day of the order the next day to arrive. Saver received used and then add comments!	Jingdong prefecture level city in Shandong
Jinfeng Hongyuan Flagship Store	Electricity savings1	I just got home to try it out and see how it goes.	Jingdong prefecture level city in Shandong
Jinfeng Hongyuan Flagship Store	Electricity savings1	Well made and weighted instrument. Kudos! Put it into use immediately, don't know if it saves power, will evaluate later!	Jingdong prefecture level city in Shandong
Jinfeng Hongyuan Flagship Store	Electricity savings1	I've just put it in place. I'll give you a review in ten days.	Jingdong prefecture level city in Shandong
Jinfeng Hongyuan Flagship Store	Electricity savings1	It looks very delicate, not bad. Well, I don't know if it can save power or not, I have to try it for a while and see. If it can really save electricity. Uh, if it works well. Then additional comments. Anyway, thanks for the logistics, thanks to Jingdong. Well, it deserves some praise.	Jingdong prefecture level city in Shandong
Jinfeng Hongyuan Flagship Store	Electricity savings1	Bought a month, the feeling is that there is a certain effect, a month to save a lot of money	Jingdong prefecture level city in Shandong
Jinfeng Hongyuan Flagship Store	Electricity savings1	Goods received, the physical and the same as the picture, logistics is also very fast, the service attitude is very good, it is worth buying!	Jingdong prefecture level city in Shandong
Jinfeng Hongyuan Flagship Store	Electricity savings1	I've used it, I don't know whether it saves electricity or not, I'll talk about it after a while.	Jingdong prefecture level city in Shandong
Jinfeng Hongyuan Flagship Store	Electricity savings1	August only began to use, October to know, have no power-saving effect, to the in to evaluate!	Jingdong prefecture level city in Shandong
Jinfeng Hongyuan Flagship Store	Electricity savings1	Goods received: I do not know to the factory products used to save electricity!	Jingdong prefecture level city in Shandong
Jinfeng Hongyuan Flagship Store	Electricity savings1	Don't know if it works, bought it anyway.	Jingdong prefecture level city in Shandong
Jinfeng Hongyuan Flagship Store	Electricity savings1	Still testing, will follow up next month to evaluate how much electricity is saved	Jingdong prefecture level city in Shandong
Jinfeng Hongyuan Flagship Store	Electricity savings1	I'm still testing it, I'll comment after I use it.	Jingdong prefecture level city in Shandong
Jinfeng Hongyuan Flagship Store	Electricity savings1	Fast service, praiseworthy. Good item pre-style, first praise, the use of the effect to be evaluated after use.	Jingdong prefecture level city in Shandong
Jinfeng Hongyuan Flagship Store	Electricity savings1	The power saving effect is more obvious through verified use.	Jingdong prefecture level city in Shandong
Jinfeng Hongyuan Flagship Store	Electricity savings1	Logistics is very fast yesterday order today arrived. The product was immediately used, and after a month to see its effect.	Jingdong prefecture level city in Shandong
Jinfeng Hongyuan Flagship Store	Electricity savings1	Power saving is not yet known, as far as the TV and audio are concerned, I feel that there is a significant quality improvement, and I recommend using a good product!	Jingdong prefecture level city in Shandong
Jinfeng Hongyuan Flagship Store	Electricity savings1	Good power savings, tens of dollars a month saved	Jingdong prefecture level city in Shandong
Jinfeng Hongyuan Flagship Store	Electricity savings1	Already took two, great value! You can save 30% on your electric bill in a month. A summer must-have!	Jingdong prefecture level city in Shandong
Jinfeng Hongyuan Flagship Store	Electricity savings1	Received the item, plugged it into a home outlet and it showed 230 volts (not that high with a multimeter). Let's use it first!	Jingdong prefecture level city in Shandong
Jinfeng Hongyuan Flagship Store	Electricity savings1	Expecting results in the experiment	Jingdong prefecture level city in Shandong
Jinfeng Hongyuan Flagship Store	Electricity savings1	I don't know if it really saves power.	Jingdong prefecture level city in Shandong
Jinfeng Hongyuan Flagship Store	Electricity savings1	It's very good and the efficacy is very visible.	Jingdong prefecture level city in Shandong

Jinfeng Hongyuan Flagship Store	Electricity savings1	It's good and worth buying with confidence	Jingdong prefecture level city in Shandong
Jinfeng Hongyuan Flagship Store	Electricity savings1	The goods have been received, just do not know if it is effective	Jingdong prefecture level city in Shandong
Jinfeng Hongyuan Flagship Store	Electricity savings1	It's already in use, but based on some of the descriptions, I suspect that to use it, you need to have one in series on each line, which seems to work for all appliances, that's my guess, but I feel as though the principle of the thing seems to require it.	Jingdong prefecture level city in Shandong
Jinfeng Hongyuan Flagship Store	Electricity savings1	Fast delivery, already in use, looks good!	Jingdong prefecture level city in Shandong
Jinfeng Hongyuan Flagship Store	Electricity savings1	Hoping to save 30% of power as presented in the graphic	Jingdong prefecture level city in Shandong
Jinfeng Hongyuan Flagship Store	Electricity savings1	Things look good, power saving do not know now, with a month after the continued evaluation	Jingdong prefecture level city in Shandong
Jinfeng Hongyuan Flagship Store	Electricity savings1	Already used it but not sure if it will top out!	Jingdong prefecture level city in Shandong
Jinfeng Hongyuan Flagship Store	Electricity savings1	Looking forward to using the results, the product quality looks okay.	Jingdong prefecture level city in Shandong
Jinfeng Hongyuan Flagship Store	Electricity savings1	It's somewhat effective and saves money more or less 💰	Jingdong prefecture level city in Shandong
Jinfeng Hongyuan Flagship Store	Electricity savings1	Got it, it's great, looking forward to saving power.	Jingdong prefecture level city in Shandong
Jinfeng Hongyuan Flagship Store	Electricity savings1	I don't know if it's working yet, I'll follow up when I get there	Jingdong prefecture level city in Shandong
Jinfeng Hongyuan Flagship Store	Electricity savings1	The product received, good workmanship, fast logistics, good inner packaging, the outer bag is damaged, but fortunately did not hurt the product, retrieved and inserted according to the instructions, looking forward to the effect is good, with a period of time to evaluate, I believe that the Jingdong.	Jingdong prefecture level city in Shandong
Jinfeng Hongyuan Flagship Store	Electricity savings1	Since it is a new product, it is said to work, so if it works well for a month, then I will give you a good review.	Jingdong prefecture level city in Shandong
Jinfeng Hongyuan Flagship Store	Electricity savings1	The quality of the product is very good, very well made, well packaged, easy to use and very convenient!	Jingdong prefecture level city in Shandong
Jinfeng Hongyuan Flagship Store	Electricity savings1	Good price for the item, good functionality	Jingdong prefecture level city in Shandong
Jinfeng Hongyuan Flagship Store	Electricity savings1	Received the first time to use it, the stuff is very good, specifics wait for a month to come back to feedback to see the effect.	Jingdong prefecture level city in Shandong
Jinfeng Hongyuan Flagship Store	Electricity savings1	Just received, the courier is too bad, not notified directly to put the Feng Nest courier cabinet	Jingdong prefecture level city in Shandong
Jinfeng Hongyuan Flagship Store	Electricity savings1	Baby received, the second time to buy, plugged in and used do not know if there is no effect. Baby received, the second time to buy, plugged in and used do not know if there is no effect. Baby received, the second time to buy, plugged in and used do not know if there is no effect.	Jingdong prefecture level city in Shandong
Jinfeng Hongyuan Flagship Store	Electricity savings1	It's okay if it saves power. It's not a bad product.	Jingdong prefecture level city in Shandong
Jinfeng Hongyuan Flagship Store	Electricity savings1	Received, very good, meticulous packaging, open a look is genuine, meticulous workmanship, the manual is very detailed, has been used, there is no effect on the only look at the next month's electricity bill, when I can I buy a few more to send people!	Jingdong prefecture level city in Shandong
Jinfeng Hongyuan Flagship Store	Electricity savings1	The quality is very good very delicate express delivery is too fast	Jingdong prefecture level city in Shandong
Jinfeng Hongyuan Flagship Store	Electricity savings1	I won't know until a month after I start using it today.	Jingdong prefecture level city in Shandong
Jinfeng Hongyuan Flagship Store	Electricity savings1	It doesn't work at all, the customer service attitude is very bad, use it or not!	Jingdong prefecture level city in Shandong
Jinfeng Hongyuan Flagship Store	Electricity savings1	Temporarily try, do not know the effect, Jingdong logistics delivery very quickly	Jingdong prefecture level city in Shandong
Jinfeng Hongyuan Flagship Store	Electricity savings1	It doesn't help. It's comforting.	Jingdong prefecture level city in Shandong
Jinfeng Hongyuan Flagship Store	Electricity savings1	It's great, use it before you say anything.	Jingdong prefecture level city in Shandong
Jinfeng Hongyuan Flagship Store	Electricity savings1	Received, already used, looking forward to a good a effect!	Jingdong prefecture level city in Shandong
Jinfeng Hongyuan Flagship Store	Electricity savings1	I don't know if it works yet, I won't know until I use it for a few months.	Jingdong prefecture level city in Shandong
Jinfeng Hongyuan Flagship Store	Electricity savings1	It takes a long time to get there. There's no change now.	Jingdong prefecture level city in Shandong
Jinfeng Hongyuan Flagship Store	Electricity savings1	Jingdong's delivery never disappoints, or so fast, and the stuff is great, next time you need to come back.	Jingdong prefecture level city in Shandong
Jinfeng Hongyuan Flagship Store	Electricity savings1	At first it was ok today, after using it for a while it didn't work anymore	Jingdong prefecture level city in Shandong
Jinfeng Hongyuan Flagship Store	Electricity savings1	Just received the goods, do not know the effect of using a few days before there may be effect	Jingdong prefecture level city in Shandong
Jinfeng Hongyuan Flagship Store	Electricity savings1	The logistics was fast, I received it the next day, I've purchased it for the second time and have been using it all along	Jingdong prefecture level city in Shandong
Jinfeng Hongyuan Flagship Store	Electricity savings1	Inexpensive, fast delivery, very pleased!	Jingdong prefecture level city in Shandong
Jinfeng Hongyuan Flagship Store	Electricity savings1	Received the goods immediately plugged directly into the use of power saving yet to be good news!	Jingdong prefecture level city in Shandong
Jinfeng Hongyuan Flagship Store	Electricity savings1	The service attitude is very good, and the Jingdong self-owned products can be trusted.	Jingdong prefecture level city in Shandong
Jinfeng Hongyuan Flagship Store	Electricity savings1	The product is good, I do not know if it can save play a power saving effect	Jingdong prefecture level city in Shandong
Jinfeng Hongyuan Flagship Store	Electricity savings1	We won't know how effective it is until we use it for a while, so I'll give you a good review now.	Jingdong prefecture level city in Shandong
Jinfeng Hongyuan Flagship Store	Electricity savings1	The quality is pretty good, just started using it, hopefully it will deliver results.	Jingdong prefecture level city in Shandong
Jinfeng Hongyuan Flagship Store	Electricity savings1	I've plugged it in first, and will follow up after I've used it to see how it works.	Jingdong prefecture level city in Shandong
Jinfeng Hongyuan Flagship Store	Electricity savings1	Things have arrived, the quality is good, the effect is also equivalent to can ah	Jingdong prefecture level city in Shandong
Jinfeng Hongyuan Flagship Store	Electricity savings1	It's not worth it to brag about power savings.	Jingdong prefecture level city in Shandong
Jinfeng Hongyuan Flagship Store	Electricity savings1	I don't know if it's an IQ tax, but I'll use it for now.	Jingdong prefecture level city in Shandong
Jinfeng Hongyuan Flagship Store	Electricity savings1	I don't feel the power saving yet, I can't know until I use it for 2 months.	Jingdong prefecture level city in Shandong
Jinfeng Hongyuan Flagship Store	Electricity savings1	There's nothing wrong with this at the moment.	Jingdong prefecture level city in Shandong
Jinfeng Hongyuan Flagship Store	Electricity savings1	It's authentic and worth buying	Jingdong prefecture level city in Shandong
Jinfeng Hongyuan Flagship Store	Electricity savings1	It's good. We'll see how it goes.	Jingdong prefecture level city in Shandong
Jinfeng Hongyuan Flagship Store	Electricity savings1	I don't know if it's working or not in the middle of using it, we'll see when the month is over	Jingdong prefecture level city in Shandong
Jinfeng Hongyuan Flagship Store	Electricity savings1	I'm not going to buy anything from Jingdong anymore, so I'll give it a good review	Jingdong prefecture level city in Shandong

Jinfeng Hongyuan Flagship Store	Electricity savings1	I'm not going to buy anything from Jingdong anymore, so I'll give it a good review	Jingdong prefecture level city in Shandong
Jinfeng Hongyuan Flagship Store	Electricity savings1	Just received the item and will see how it works in a month.	Jingdong prefecture level city in Shandong
Jinfeng Hongyuan Flagship Store	Electricity savings1	I don't know if it saves electricity, but it's plugged into an outlet every day anyway	Jingdong prefecture level city in Shandong
Jinfeng Hongyuan Flagship Store	Electricity savings1	I don't know if it works yet.	Jingdong prefecture level city in Shandong
Jinfeng Hongyuan Flagship Store	Electricity savings1	Plugged it in when I got back, waiting for a month to see the results	Jingdong prefecture level city in Shandong
Jinfeng Hongyuan Flagship Store	Electricity savings1	Effects remain to be seen, but it looks good quality and runs fine	Jingdong prefecture level city in Shandong
Jinfeng Hongyuan Flagship Store	Electricity savings1	The product received logistics very quickly, has not yet been used to test! After use in the evaluation!	Jingdong prefecture level city in Shandong
Jinfeng Hongyuan Flagship Store	Electricity savings1	The power saver is in use, and I don't know how much electricity has been saved after using it for more than a month, and the display voltmeter is very good.	Jingdong prefecture level city in Shandong
Jinfeng Hongyuan Flagship Store	Electricity savings1	It's so good, isn't it? You're a real Shanghainese, aren't you?	Jingdong prefecture level city in Shandong
Jinfeng Hongyuan Flagship Store	Electricity savings1	Great quality great great great 👍👍👍👍	Jingdong prefecture level city in Shandong
Jinfeng Hongyuan Flagship Store	Electricity savings1	I'm using it, I don't know if it's working yet.	Jingdong prefecture level city in Shandong
Jinfeng Hongyuan Flagship Store	Electricity savings1	Just used it for more than 10 days and haven't seen any results yet	Jingdong prefecture level city in Shandong
Jinfeng Hongyuan Flagship Store	Electricity savings1	The product arrived home feeling like it should work	Jingdong prefecture level city in Shandong
Jinfeng Hongyuan Flagship Store	Electricity savings1	Just received the goods, use it and comment back	Jingdong prefecture level city in Shandong
Jinfeng Hongyuan Flagship Store	Electricity savings1	Try it for a month and then evaluate	Jingdong prefecture level city in Shandong
Jinfeng Hongyuan Flagship Store	Electricity savings1	I don't know if it saves power yet, but I'll comment again after using it for a while.	Jingdong prefecture level city in Shandong
Jinfeng Hongyuan Flagship Store	Electricity savings1	The product is better than I thought, the quality and appearance are very good, the voltage is stable, and I'm looking forward to a good power saving effect.	Jingdong prefecture level city in Shandong
Jinfeng Hongyuan Flagship Store	Electricity savings1	Received and scanned the QR code to check that it is genuine, have plugged it in, can it save power, looking forward to the advertised effect. Customary praise, 5 points!	Jingdong prefecture level city in Shandong
Jinfeng Hongyuan Flagship Store	Electricity savings1	The goods have been received and have not yet been used. Do not know how the effect.	Jingdong prefecture level city in Shandong
Jinfeng Hongyuan Flagship Store	Electricity savings1	Hopefully, you get what you pay for, and you get what you pay for!	Jingdong prefecture level city in Shandong
Jinfeng Hongyuan Flagship Store	Electricity savings1	Just put it on and it feels like it's turning as fast as a meter! Looking forward to the results!	Jingdong prefecture level city in Shandong
Jinfeng Hongyuan Flagship Store	Electricity savings1	Overall look on the product quality is good, I do not know is not the effect of power saving, wait to see and then follow up!	Jingdong prefecture level city in Shandong
Jinfeng Hongyuan Flagship Store	Electricity savings1	The logistics courier is very fast, the courier service attitude is very good	Jingdong prefecture level city in Shandong
Jinfeng Hongyuan Flagship Store	Electricity savings1	The product has been received, it works well worth buying	Jingdong prefecture level city in Shandong
Jinfeng Hongyuan Flagship Store	Electricity savings1	How did it not work? Is it purely an IQ tax?	Jingdong prefecture level city in Shandong
Jinfeng Hongyuan Flagship Store	Electricity savings1	It looks good, I don't know how well it works.	Jingdong prefecture level city in Shandong
Jinfeng Hongyuan Flagship Store	Electricity savings1	It does seem to be a bit of a power saver, I just don't know if the more expensive one is more power efficient?	Jingdong prefecture level city in Shandong
Jinfeng Hongyuan Flagship Store	Electricity savings1	Is genuine very good, the price is much cheaper, cost-effective, really special paddle just to do activities, the price is much cheaper, cost-effective, really special paddle counting, packaging is also very high-end atmospheric class, it is worth buying!	Jingdong prefecture level city in Shandong
Jinfeng Hongyuan Flagship Store	Electricity savings1	I received it yesterday and used it right away, I hope it works, I'll wait for next month to come back to the conclusion, I'm looking forward to it. Five hearts praise!	Jingdong prefecture level city in Shandong
Jinfeng Hongyuan Flagship Store	Electricity savings1	Looks good, packed properly, received it today, am using it, will see results in a month!	Jingdong prefecture level city in Shandong
Jinfeng Hongyuan Flagship Store	Electricity savings1	Regular product Jingdong self-support trustworthy. There is an effect Come back to the review. 😍😍😍😍	Jingdong prefecture level city in Shandong
Jinfeng Hongyuan Flagship Store	Electricity savings1	I haven't used it yet, hopefully it will work as a power saver, and will comment again if it works well.	Jingdong prefecture level city in Shandong
Jinfeng Hongyuan Flagship Store	Electricity savings1	Shipping was fast, logistics was fast, service was good, and I look forward to good results from the item!	Jingdong prefecture level city in Shandong
Jinfeng Hongyuan Flagship Store	Electricity savings1	This product is this compensator, it has to be at the main switch to be useful, haven't compared the final result, the	Jingdong prefecture level city in Shandong
Jinfeng Hongyuan Flagship Store	Electricity savings1	Packaging is perfect . I've already started using it, so I'm looking forward to the results.	Jingdong prefecture level city in Shandong
Jinfeng Hongyuan Flagship Store	Electricity savings1	I just got it back. I'll comment after I use it.	Jingdong prefecture level city in Shandong
Jinfeng Hongyuan Flagship Store	Electricity savings1	Big brand, with a very assured, very good quality, catch the event to buy very cost-effective to take advantage of the offer, hurry to get, it is genuine, the packaging is also very good, no breakage, the quality is not to speak of, great 👍 logistics super super fast! Quality is also very good, indeed value	Jingdong prefecture level city in Shandong
Jinfeng Hongyuan Flagship Store	Electricity savings1	The quality is pretty good. The price is also quite affordable.	Jingdong prefecture level city in Shandong
Jinfeng Hongyuan Flagship Store	Electricity savings1	I did not expect the courier so fast to arrive yesterday just ordered, the head is a little smaller than I thought, well, the picture looks bigger. The appearance still looks good, as for the practicality and effectiveness of the use of the only know.	Jingdong prefecture level city in Shandong
Jinfeng Hongyuan Flagship Store	Electricity savings1	I don't know. Looking forward to it.	Jingdong prefecture level city in Shandong
Jinfeng Hongyuan Flagship Store	Electricity savings1	Baby received, very delicate, very nice, like. Just don't know if it can really save electricity. Baby received, very delicate, very good-looking, like. Just don't know if it can really save electricity.	Jingdong prefecture level city in Shandong
Jinfeng Hongyuan Flagship Store	Electricity savings1	I'll use it for a month and then comment on the quality of the item	Jingdong prefecture level city in Shandong
Jinfeng Hongyuan Flagship Store	Electricity savings1	It feels ok, use it for a while and see how it works before adding more comments.	Jingdong prefecture level city in Shandong
Jinfeng Hongyuan Flagship Store	Electricity savings1	In use, see results in a month.	Jingdong prefecture level city in Shandong
Jinfeng Hongyuan Flagship Store	Electricity savings1	Jingdong's logistics and quality are very good, the service attitude is very good, the logistics is very fast	Jingdong prefecture level city in Shandong
Jinfeng Hongyuan Flagship Store	Electricity savings1	Intelligent power saver received, well-packed logistics is also quite fast, easy to use, looking forward to good results!	Jingdong prefecture level city in Shandong
Jinfeng Hongyuan Flagship Store	Electricity savings1	The quality is unknown at this point, I won't know until I use it.	Jingdong prefecture level city in Shandong
Jinfeng Hongyuan Flagship Store	Electricity savings1	It's a good product. It really saves power and doesn't take up much space.	Jingdong prefecture level city in Shandong
Jinfeng Hongyuan Flagship Store	Electricity savings1	Goods have been received, packaging is very good, good to see the effect of future use in the follow-up.	Jingdong prefecture level city in Shandong
Jinfeng Hongyuan Flagship Store	Electricity savings1	The product is in perfect shape and the light weight is worth it!	Jingdong prefecture level city in Shandong

Jinfeng Hongyuan Flagship Store	Electricity savings1	Just got home and plugged it in. It's good.	Jingdong prefecture level city in Shandong
Jinfeng Hongyuan Flagship Store	Electricity savings1	The logistics are quick, just to see the results.	Jingdong prefecture level city in Shandong
Jinfeng Hongyuan Flagship Store	Electricity savings1	Short time can not feel here to leave a message, to be followed up later!	Jingdong prefecture level city in Shandong
Jinfeng Hongyuan Flagship Store	Electricity savings1	This user thinks the product is good	Jingdong prefecture level city in Shandong
Jinfeng Hongyuan Flagship Store	Electricity savings1	Received the baby, to be used to see the effect of it!	Jingdong prefecture level city in Shandong
Jinfeng Hongyuan Flagship Store	Electricity savings1	You have to wait a month or two to see how it works.	Jingdong prefecture level city in Shandong
Jinfeng Hongyuan Flagship Store	Electricity savings1	Not bad, good, like it, don't know if it's good, try it first.	Jingdong prefecture level city in Shandong
Jinfeng Hongyuan Flagship Store	Electricity savings1	The goods are good value for money and the logistics is very fast and good service!	Jingdong prefecture level city in Shandong
Jinfeng Hongyuan Flagship Store	Electricity savings1	I don't know how well it works, we'll see in a month	Jingdong prefecture level city in Shandong
Jinfeng Hongyuan Flagship Store	Electricity savings1	The product is fine, but r saving power or not I won't know until I use it for two months. The display and lights take electricity too?	Jingdong prefecture level city in Shandong
Jinfeng Hongyuan Flagship Store	Electricity savings1	I've used it. I don't know how it works yet.	Jingdong prefecture level city in Shandong
Jinfeng Hongyuan Flagship Store	Electricity savings1	I can't say until I compare them.	Jingdong prefecture level city in Shandong
Jinfeng Hongyuan Flagship Store	Electricity savings1	It's ok in the process of using 。。。。	Jingdong prefecture level city in Shandong
Jinfeng Hongyuan Flagship Store	Electricity savings1	Jinfeng Hongyuan Intelligent Power Saver Household Electricity Meter Electricity Saving Appliance High Power Enhanced Power Saving King Energy Saving Treasure Butler Control Non Stealing Electricity Reversing Not Going Interfering Slow Turner Artifact King Gold Yellow. Haven't tested it accurately yet, really need to wait a few months to test it before I have a say.	Jingdong prefecture level city in Shandong
Jinfeng Hongyuan Flagship Store	Electricity savings1	Don't know how effective it is yet? I hope it works well and I'll come back next time	Jingdong prefecture level city in Shandong
Jinfeng Hongyuan Flagship Store	Electricity savings1	Saving device has been received, material workmanship are very good ah! Have not yet begun to use. First observe the daily electricity consumption and cost of the home meter, and then plug in the power saver to use, so as to compare the use of the effect is good!	Jingdong prefecture level city in Shandong
Jinfeng Hongyuan Flagship Store	Electricity savings1	Merchants shipping extremely fast, Jingdong logistics safe and fast, I believe that the product can play a safe use of electricity, the role of scientific power saving, first praise, the effect of how to be used for a period of time and then the pursuit of bubble!	Jingdong prefecture level city in Shandong
Jinfeng Hongyuan Flagship Store	Electricity savings1	I just bought it. I don't know yet.	Jingdong prefecture level city in Shandong
Jinfeng Hongyuan Flagship Store	Electricity savings1	It is genuine, good quality, and it is very reassuring to use. The logistics is very fast, and the business service attitude is on point.	Jingdong prefecture level city in Shandong
Jinfeng Hongyuan Flagship Store	Electricity savings1	The quality is fine, I don't know how effective it is.	Jingdong prefecture level city in Shandong
Jinfeng Hongyuan Flagship Store	Electricity savings1	The stuff is back and I expect it will save the electricity	Jingdong prefecture level city in Shandong
Jinfeng Hongyuan Flagship Store	Electricity savings1	I'll give it 4 stars first and see what happens in a couple of months and then follow up	Jingdong prefecture level city in Shandong
Jinfeng Hongyuan Flagship Store	Electricity savings1	It's not bad. It's in the mind.	Jingdong prefecture level city in Shandong
Jinfeng Hongyuan Flagship Store	Electricity savings1	Received the goods have not yet started to use do not know whether it is good	Jingdong prefecture level city in Shandong
Jinfeng Hongyuan Flagship Store	Electricity savings1	This user thinks the product is good	Jingdong prefecture level city in Shandong
Jinfeng Hongyuan Flagship Store	Electricity savings1	Hello! The saver was received, thank you! Not yet used, but from the packaging looks very good quality, exquisite workmanship. Logistics is quite fast, if it is really very power saving, I will come back to	Jingdong prefecture level city in Shandong
Jinfeng Hongyuan Flagship Store	Electricity savings1	I've been using it for a few days now, I'll comment again after a month or two of use	Jingdong prefecture level city in Shandong
Jinfeng Hongyuan Flagship Store	Electricity savings1	I don't know. Bought it for my father at a good price.	Jingdong prefecture level city in Shandong
Jinfeng Hongyuan Flagship Store	Electricity savings1	Plugged it in the same day I bought it and the voltage showed 223, 225 looking forward to the results	Jingdong prefecture level city in Shandong
Jinfeng Hongyuan Flagship Store	Electricity savings1	Still in use, the biggest role is to stabilize the current, reduce losses, product quality on the look is very good, with this feeling quite good, see colleagues are using, power saving effect should be good	Jingdong prefecture level city in Shandong
Jinfeng Hongyuan Flagship Store	Electricity savings1	How to receive goods but no invoice and warranty more than it ...... quality is the premise of reputation!	Jingdong prefecture level city in Shandong
Jinfeng Hongyuan Flagship Store	Electricity savings1	Received but not used Continued evaluation	Jingdong prefecture level city in Shandong
Jinfeng Hongyuan Flagship Store	Electricity savings1	I just received the product, I don't know if it works well yet	Jingdong prefecture level city in Shandong
Jinfeng Hongyuan Flagship Store	Electricity savings1	Received it and used it, not sure it really saves electricity use it and then evaluate it, first give a 5 star review!!!!	Jingdong prefecture level city in Shandong
Jinfeng Hongyuan Flagship Store	Electricity savings1	It's good to use, it's very good to use, and it smells OK too	Jingdong prefecture level city in Shandong
Jinfeng Hongyuan Flagship Store	Electricity savings1	Packaging is very good, feel the product is not bad, how much power saving with the only know	Jingdong prefecture level city in Shandong
Jinfeng Hongyuan Flagship Store	Electricity savings1	Additional comments to be made after use	Jingdong prefecture level city in Shandong
Jinfeng Hongyuan Flagship Store	Electricity savings1	The product has been installed, try a few months and then comment!	Jingdong prefecture level city in Shandong
Jinfeng Hongyuan Flagship Store	Electricity savings1	I've bought it, let's use it for now, I'll comment after a month	Jingdong prefecture level city in Shandong
Jinfeng Hongyuan Flagship Store	Electricity savings1	Try it out and then follow up	Jingdong prefecture level city in Shandong
Jinfeng Hongyuan Flagship Store	Electricity savings1	It's good in theory, it doesn't feel good in practice.	Jingdong prefecture level city in Shandong
Jinfeng Hongyuan Flagship Store	Electricity savings1	I haven't tried it yet. We'll see.	Jingdong prefecture level city in Shandong
Jinfeng Hongyuan Flagship Store	Electricity savings1	Great item, second purchase!	Jingdong prefecture level city in Shandong
Jinfeng Hongyuan Flagship Store	Electricity savings1	Goods received now use it, if it does save power, plus reevaluate.	Jingdong prefecture level city in Shandong
Jinfeng Hongyuan Flagship Store	Electricity savings1	The stuff is light and small, the stuff is good, the courier service is good	Jingdong prefecture level city in Shandong
Jinfeng Hongyuan Flagship Store	Electricity savings1	I haven't used it yet. I'm looking forward to it.	Jingdong prefecture level city in Shandong
Jinfeng Hongyuan Flagship Store	Electricity savings1	Express received, in use do not know if there is no power saving	Jingdong prefecture level city in Shandong
Jinfeng Hongyuan Flagship Store	Electricity savings1	The quality of the product is quite good! The power saving mode is still getting used to it, can't say yet, how much power it saves.	Jingdong prefecture level city in Shandong
Jinfeng Hongyuan Flagship Store	Electricity savings1	The goods have been received, just started to use, now I do not know how the quality, after a period of time to say.	Jingdong prefecture level city in Shandong

Jinfeng Hongyuan Flagship Store	Electricity savings1	I received the stuff, used it for a few days to evaluate, the stuff is very good and good, worth buying.	Jingdong prefecture level city in Shandong
Jinfeng Hongyuan Flagship Store	Electricity savings1	I've been using it for a while, but I don't know if it's good or if it saves electricity! What the heck, let's give it a good review first. And for the record, I'm not helping him**.	Jingdong prefecture level city in Shandong
Jinfeng Hongyuan Flagship Store	Electricity savings1	Haven't used it yet, looks good, try it for 60 days, might be good, great review!	Jingdong prefecture level city in Shandong
Jinfeng Hongyuan Flagship Store	Electricity savings1	Baby received, logistics is very fast. Ordered last night, received this morning. Received the goods immediately plugged in the power supply, save power to the first of next month to know.	Jingdong prefecture level city in Shandong
Jinfeng Hongyuan Flagship Store	Electricity savings1	First, I'll give you a good review and use it.	Jingdong prefecture level city in Shandong
Jinfeng Hongyuan Flagship Store	Electricity savings1	Just arrived to follow up with a genuine review	Jingdong prefecture level city in Shandong
Jinfeng Hongyuan Flagship Store	Electricity savings1	Not bad pretty fast, good quality.	Jingdong prefecture level city in Shandong
Jinfeng Hongyuan Flagship Store	Electricity savings1	Recommended, use it and hopefully it will save some electricity	Jingdong prefecture level city in Shandong
Jinfeng Hongyuan Flagship Store	Electricity savings1	The stuff looks ok, I don't know the effect if it.	Jingdong prefecture level city in Shandong
Jinfeng Hongyuan Flagship Store	Electricity savings1	The stuff looks OK, the stuff looks OK.	Jingdong prefecture level city in Shandong
Jinfeng Hongyuan Flagship Store	Electricity savings1	The item has been received, after using it in a follow-up review. Satisfied with the purchase.	Jingdong prefecture level city in Shandong
Jinfeng Hongyuan Flagship Store	Electricity savings1	Has been normal plug-in use, at present do not know how to see, use down to see and then evaluation	Jingdong prefecture level city in Shandong
Jinfeng Hongyuan Flagship Store	Electricity savings1	Commodities make noise.	Jingdong prefecture level city in Shandong
Jinfeng Hongyuan Flagship Store	Electricity savings1	After using it for a few days and observing it, the saving is about ten percent	Jingdong prefecture level city in Shandong
Jinfeng Hongyuan Flagship Store	Electricity savings1	The goods received, the quality looks quite OK. First use it, the effect is not good to say.	Jingdong prefecture level city in Shandong
Jinfeng Hongyuan Flagship Store	Electricity savings1	In use, follow up next month	Jingdong prefecture level city in Shandong
Jinfeng Hongyuan Flagship Store	Electricity savings1	I just received the goods and have not yet used it to comment after using it	Jingdong prefecture level city in Shandong
Jinfeng Hongyuan Flagship Store	Electricity savings1	So far I don't know if it really saves electricity yet! Spinning waiting a couple months to find out! Good reviews first!	Jingdong prefecture level city in Shandong
Jinfeng Hongyuan Flagship Store	Electricity savings1	Small and exquisite, looking forward to the effect, to be evaluated 6666666666666	Jingdong prefecture level city in Shandong
Jinfeng Hongyuan Flagship Store	Electricity savings1	I just used it, I don't know if it's effective, I'll comment again after a month	Jingdong prefecture level city in Shandong
Jinfeng Hongyuan Flagship Store	Electricity savings1	I just used it, I don't know if it's good or not, I'll comment later.	Jingdong prefecture level city in Shandong
Jinfeng Hongyuan Flagship Store	Electricity savings1	Baby received, Baobei quite good, use and then come back to comment!	Jingdong prefecture level city in Shandong
Jinfeng Hongyuan Flagship Store	Electricity savings1	Good quality power saving is fake	Jingdong prefecture level city in Shandong
Jinfeng Hongyuan Flagship Store	Electricity savings1	The packaging of the goods is atmospheric, the quality is exquisite, and I look forward to saving electricity.	Jingdong prefecture level city in Shandong
Jinfeng Hongyuan Flagship Store	Electricity savings1	The packaging is great and the quality is good!	Jingdong prefecture level city in Shandong
Jinfeng Hongyuan Flagship Store	Electricity savings1	I don't see any effect.	Jingdong prefecture level city in Shandong
Jinfeng Hongyuan Flagship Store	Electricity savings1	Already used, but did not see obvious results	Jingdong prefecture level city in Shandong
Jinfeng Hongyuan Flagship Store	Electricity savings1	It's good. I'm just using it. I don't know what it's like.	Jingdong prefecture level city in Shandong
Jinfeng Hongyuan Flagship Store	Electricity savings1	I just used it. I'll comment later.	Jingdong prefecture level city in Shandong
Jinfeng Hongyuan Flagship Store	Electricity savings1	I installed it when it arrived, try it for a month and then come back for a follow-up review	Jingdong prefecture level city in Shandong
Jinfeng Hongyuan Flagship Store	Electricity savings1	Goods arrived a few days fastidious does not call to pick up the goods, the quality is average	Jingdong prefecture level city in Shandong
Jinfeng Hongyuan Flagship Store	Electricity savings1	This user thinks the product is good	Jingdong prefecture level city in Shandong
Jinfeng Hongyuan Flagship Store	Electricity savings1	Roger that.	Jingdong prefecture level city in Shandong
Jinfeng Hongyuan Flagship Store	Electricity savings1	Second purchase was ok.	Jingdong prefecture level city in Shandong
Jinfeng Hongyuan Flagship Store	Electricity savings1	The merchant is very reputable and the product is good value for money. Logistics is fast!	Jingdong prefecture level city in Shandong
Jinfeng Hongyuan Flagship Store	Electricity savings1	The quality of the baby is very good, I have always trusted Kyodo	Jingdong prefecture level city in Shandong
Jinfeng Hongyuan Flagship Store	Electricity savings1	We'll see how it goes when we get it.	Jingdong prefecture level city in Shandong
Jinfeng Hongyuan Flagship Store	Electricity savings1	I just used it. I don't know if it works.	Jingdong prefecture level city in Shandong
Jinfeng Hongyuan Flagship Store	Electricity savings1	Good quality, used for a month to save about 20%, power saving effect is good	Jingdong prefecture level city in Shandong
Jinfeng Hongyuan Flagship Store	Electricity savings1	I'll give you a good review before I use it.	Jingdong prefecture level city in Shandong
Jinfeng Hongyuan Flagship Store	Electricity savings1	Received, satisfied with the follow-up evaluation	Jingdong prefecture level city in Shandong
Jinfeng Hongyuan Flagship Store	Electricity savings1	Immediate results, saving electricity and money	Jingdong prefecture level city in Shandong
Jinfeng Hongyuan Flagship Store	Electricity savings1	Measured to save a little	Jingdong prefecture level city in Shandong
Jinfeng Hongyuan Flagship Store	Electricity savings1	This user thinks the product is good	Jingdong prefecture level city in Shandong
Jinfeng Hongyuan Flagship Store	Electricity savings1	This user thinks the product is good	Jingdong prefecture level city in Shandong
Jinfeng Hongyuan Flagship Store	Electricity savings1	Product quality and logistics are very good, worth buying	Jingdong prefecture level city in Shandong
Jinfeng Hongyuan Flagship Store	Electricity savings1	Appearance looks ok! Express delivery was fast! Service is still relatively enthusiastic.	Jingdong prefecture level city in Shandong
Jinfeng Hongyuan Flagship Store	Electricity savings1	It doesn't feel like it's working.	Jingdong prefecture level city in Shandong
Jinfeng Hongyuan Flagship Store	Electricity savings1	Well made and looks good. Used it with other appliances and felt as if it had some effect.	Jingdong prefecture level city in Shandong
Jinfeng Hongyuan Flagship Store	Electricity savings1	Haven't used it yet, will follow up later	Jingdong prefecture level city in Shandong
Jinfeng Hongyuan Flagship Store	Electricity savings1	Things received, used a few days the plug is a little hot unplugged did not work	Jingdong prefecture level city in Shandong

Jinfeng Hongyuan Flagship Store	Electricity savings1	Look at the meter again in a month.	Jingdong prefecture level city in Shandong
Jinfeng Hongyuan Flagship Store	Electricity savings1	This one didn't work at all.	Jingdong prefecture level city in Shandong
Jinfeng Hongyuan Flagship Store	Electricity savings1	It's small. I've seen friends with much bigger ones.	Jingdong prefecture level city in Shandong
Jinfeng Hongyuan Flagship Store	Electricity savings1	The item was received and the quality is good, it will be nice to see how the power saving rate is down the road, the item is great.	Jingdong prefecture level city in Shandong
Jinfeng Hongyuan Flagship Store	Electricity savings1	Cheap and affordable quality is quite good, logistics is also very fast, praise! , baby good 😊, cost-effective very high, big brand trustworthy 👍	Jingdong prefecture level city in Shandong
Jinfeng Hongyuan Flagship Store	Electricity savings1	Used it for a while it really saves electricity ⚡ thanks 🈶 such a good product, compact and practical, every family should buy one ✨	Jingdong prefecture level city in Shandong
Jinfeng Hongyuan Flagship Store	Electricity savings1	Very satisfied with the purchase Next time I will come back	Jingdong prefecture level city in Shandong
Jinfeng Hongyuan Flagship Store	Electricity savings1	Logistics is fast. Good quality, used one before and it does save power	Jingdong prefecture level city in Shandong
Jinfeng Hongyuan Flagship Store	Electricity savings1	I don't know if it works.	Jingdong prefecture level city in Shandong
Jinfeng Hongyuan Flagship Store	Electricity savings1	Good goods, very solid, good.	Jingdong prefecture level city in Shandong
Jinfeng Hongyuan Flagship Store	Electricity savings1	This user thinks the product is good	Jingdong prefecture level city in Shandong
Jinfeng Hongyuan Flagship Store	Electricity savings1	I've been using it for half a month, my electricity bill is much lower, and the quality is very good, so don't worry about buying it!	Jingdong prefecture level city in Shandong
Jinfeng Hongyuan Flagship Store	Electricity savings1	I received the goods today and used it right away, looking forward to the effect	Jingdong prefecture level city in Shandong
Jinfeng Hongyuan Flagship Store	Electricity savings1	The merchandise is great. I am going to recommend it to my friends! Will buy again next time.	Jingdong prefecture level city in Shandong
Jinfeng Hongyuan Flagship Store	Electricity savings1	Let's give it a good review first, it's working fine so far, and I'll follow up with a review,...	Jingdong prefecture level city in Shandong
Jinfeng Hongyuan Flagship Store	Electricity savings1	Product quality is very good, looking forward to using the results	Jingdong prefecture level city in Shandong
Jinfeng Hongyuan Flagship Store	Electricity savings1	I just can't detect it. - Save it.	Jingdong prefecture level city in Shandong
Jinfeng Hongyuan Flagship Store	Electricity savings1	It arrived and is being used, so we'll see how it works!	Jingdong prefecture level city in Shandong
Jinfeng Hongyuan Flagship Store	Electricity savings1	Received yesterday thanks! This energy saver is available . Safe! But in the end how much energy can be saved must be used for a period of time to know, if the real energy saving next time to buy again.	Jingdong prefecture level city in Shandong
Jinfeng Hongyuan Flagship Store	Electricity savings1	Just used it, don't know if it saves power	Jingdong prefecture level city in Shandong
Jinfeng Hongyuan Flagship Store	Electricity savings1	I'll rate it first, is it a tease wait two months and see? It's been four days and it's not noticeable!	Jingdong prefecture level city in Shandong
Jinfeng Hongyuan Flagship Store	Electricity savings1	Workmanship is quite good, just started to use, the effect of the follow-up evaluation.	Jingdong prefecture level city in Shandong
Jinfeng Hongyuan Flagship Store	Electricity savings1	Received the goods used for a few days before evaluation, the effect is quite good	Jingdong prefecture level city in Shandong
Jinfeng Hongyuan Flagship Store	Electricity savings1	Try it for a month and then evaluate	Jingdong prefecture level city in Shandong
Jinfeng Hongyuan Flagship Store	Electricity savings1	Just received the goods, the packaging is very good, the appearance of the economizer feels good, I do not know how the effect of power saving, with a month or two and then follow up!	Jingdong prefecture level city in Shandong
Jinfeng Hongyuan Flagship Store	Electricity savings1	It's quite small and delicate, I don't know if it's useful yet.	Jingdong prefecture level city in Shandong
Jinfeng Hongyuan Flagship Store	Electricity savings1	Just received it, use it for a few days before commenting	Jingdong prefecture level city in Shandong
Jinfeng Hongyuan Flagship Store	Electricity savings1	Does this thing work! When it arrived, the button for the safety was loose. I'm a little afraid of fire!	Jingdong prefecture level city in Shandong
Jinfeng Hongyuan Flagship Store	Electricity savings1	I received it. I don't know how it works.	Jingdong prefecture level city in Shandong
Jinfeng Hongyuan Flagship Store	Electricity savings1	This user thinks the product is good	Jingdong prefecture level city in Shandong
Jinfeng Hongyuan Flagship Store	Electricity savings1	Customer service was great! Small i problem was taken care of in a timely manner! Effectiveness to be tested by time! No rush!!!	Jingdong prefecture level city in Shandong
Jinfeng Hongyuan Flagship Store	Electricity savings1	I put it on right after I received it and it looks compact. I believe the high technology should save electricity.	Jingdong prefecture level city in Shandong
Jinfeng Hongyuan Flagship Store	Electricity savings1	Logistics courier very quickly, the first day under the order the next day to receive, off pieces whether power saving is the most important thing, things are exquisite workmanship, a month later in the follow-up evaluation whether power saving	Jingdong prefecture level city in Shandong
Jinfeng Hongyuan Flagship Store	Electricity savings1	The delivery is pretty fast, I'm using it, have to have it for a while to see results, looking forward to it.	Jingdong prefecture level city in Shandong
Jinfeng Hongyuan Flagship Store	Electricity savings1	See pretty good just started to use a month later to give you a real evaluation, should be useful.	Jingdong prefecture level city in Shandong
Jinfeng Hongyuan Flagship Store	Electricity savings1	And then comment.	Jingdong prefecture level city in Shandong
Jinfeng Hongyuan Flagship Store	Electricity savings1	Received the goods, wait for the test to evaluate	Jingdong prefecture level city in Shandong
Jinfeng Hongyuan Flagship Store	Electricity savings1	This user thinks the product is good	Jingdong prefecture level city in Shandong
Jinfeng Hongyuan Flagship Store	Electricity savings1	I'm already using it, I just don't know how to judge the power savings. I won't know until I use it for a season.	Jingdong prefecture level city in Shandong
Jinfeng Hongyuan Flagship Store	Electricity savings1	Works. Says it works.	Jingdong prefecture level city in Shandong
Jinfeng Hongyuan Flagship Store	Electricity savings1	Very good. Satisfied.	Jingdong prefecture level city in Shandong
Jinfeng Hongyuan Flagship Store	Electricity savings1	This user thinks the product is good	Jingdong prefecture level city in Shandong
Jinfeng Hongyuan Flagship Store	Electricity savings1	It's working. Obviously, it's less expensive.	Jingdong prefecture level city in Shandong
Jinfeng Hongyuan Flagship Store	Electricity savings1	The second one is up, the first one still works! It's reactive power compensation! This is well understood if you want a professional!	Jingdong prefecture level city in Shandong
Jinfeng Hongyuan Flagship Store	Electricity savings1	It feels as if there are some nodes, but they need to be tested and observed over time, thanks.	Jingdong prefecture level city in Shandong
Jinfeng Hongyuan Flagship Store	Electricity savings1	I've received the goods, the energy saving effect will be evaluated after using it for a period of time, and all the services are very good, praise!	Jingdong prefecture level city in Shandong
Jinfeng Hongyuan Flagship Store	Electricity savings1	I don't know how well it works yet, I bought it and plugged it in, so we'll see if it saves some power or not!	Jingdong prefecture level city in Shandong
Jinfeng Hongyuan Flagship Store	Electricity savings1	Baby a bunch of knife quality is very good, previously bought a quality is not very good. It really works and has results.	Jingdong prefecture level city in Shandong
Jinfeng Hongyuan Flagship Store	Electricity savings1	Things have arrived, it looks good, just started to use, as to whether or not there is a business said the power-saving effect, but also to observe the effect, now can not evaluate	Jingdong prefecture level city in Shandong
Jinfeng Hongyuan Flagship Store	Electricity savings1	I haven't used it yet. I'll comment afterward.	Jingdong prefecture level city in Shandong
Jinfeng Hongyuan Flagship Store	Electricity savings1	I don't know if it's any good.	Jingdong prefecture level city in Shandong

Jinfeng Hongyuan Flagship Store	Electricity savings1	I don't know how well it works.	Jingdong prefecture level city in Shandong
Jinfeng Hongyuan Flagship Store	Electricity savings1	The baby has been received! Uh-huh! Good, quite exquisite, small and exquisite I thought it was big. Dare to tightly plugged in the electricity, just wait for the effect later!	Jingdong prefecture level city in Shandong
Jinfeng Hongyuan Flagship Store	Electricity savings1	The packaging is sturdy, I tried it right after I received it, so I'm looking forward to the results.	Jingdong prefecture level city in Shandong
Jinfeng Hongyuan Flagship Store	Electricity savings1	Received the item and installed it! Don't know if it works well! Will follow up with a review later! I hope it works!	Jingdong prefecture level city in Shandong
Jinfeng Hongyuan Flagship Store	Electricity savings1	Things used on the effect of waiting to use a little to say, but things are very good. Logistics is very fast, business service is very good.	Jingdong prefecture level city in Shandong
Jinfeng Hongyuan Flagship Store	Electricity savings1	This user thinks the product is good	Jingdong prefecture level city in Shandong
Jinfeng Hongyuan Flagship Store	Electricity savings1	I'm really using it. It feels good.	Jingdong prefecture level city in Shandong
Jinfeng Hongyuan Flagship Store	Electricity savings1	Good, Jingdong logistics is very fast, intelligent energy-saving appliances	Jingdong prefecture level city in Shandong
Jinfeng Hongyuan Flagship Store	Electricity savings1	It's not bad. It really saves energy.	Jingdong prefecture level city in Shandong
Jinfeng Hongyuan Flagship Store	Electricity savings1	The logistics is fast, praise	Jingdong prefecture level city in Shandong
Jinfeng Hongyuan Flagship Store	Electricity savings1	This user thinks the product is good	Jingdong prefecture level city in Shandong
Jinfeng Hongyuan Flagship Store	Electricity savings1	The first time I placed two, and today I placed another one, a friend introduced it, said it is particularly power-saving	Jingdong prefecture level city in Shandong
Jinfeng Hongyuan Flagship Store	Electricity savings1	I don't know how effective it is. Let's leave it at home for a while and see.	Jingdong prefecture level city in Shandong
Jinfeng Hongyuan Flagship Store	Electricity savings1	Dear customer service! I would like to ask you how to understand the digital display (increase/decrease jump) on the top of the saver? Thank you for your help.	Jingdong prefecture level city in Shandong
Jinfeng Hongyuan Flagship Store	Electricity savings1	This user thinks the product is good	Jingdong prefecture level city in Shandong
Jinfeng Hongyuan Flagship Store	Electricity savings1	The goods have been received, packaging exquisite, is using, later to see how the effect?	Jingdong prefecture level city in Shandong
Jinfeng Hongyuan Flagship Store	Electricity savings1	The saver received, the boss shipped very quickly, yesterday shot today arrived!	Jingdong prefecture level city in Shandong
Jinfeng Hongyuan Flagship Store	Electricity savings1	Beautiful appearance, well packaged. Recording data to see if I can lower my electric bill a bit.	Jingdong prefecture level city in Shandong
Jinfeng Hongyuan Flagship Store	Electricity savings1	Things received, the packaging is quite good, the effect of the use and then come back to evaluate.	Jingdong prefecture level city in Shandong
Jinfeng Hongyuan Flagship Store	Electricity savings1	Easy to use, just plug it in. But there is a current sound.	Jingdong prefecture level city in Shandong
Jinfeng Hongyuan Flagship Store	Electricity savings1	Fast logistics, easy to use and labor-saving	Jingdong prefecture level city in Shandong
Jinfeng Hongyuan Flagship Store	Electricity savings1	Received, thank you!	Jingdong prefecture level city in Shandong
Jinfeng Hongyuan Flagship Store	Electricity savings1	Second time I've bought it. It's good.	Jingdong prefecture level city in Shandong
Jinfeng Hongyuan Flagship Store	Electricity savings1	I've only been using it for a short time, so I'm looking forward to the results.	Jingdong prefecture level city in Shandong
Jinfeng Hongyuan Flagship Store	Electricity savings1	Good stuff, good stuff, good stuff good stuff.	Jingdong prefecture level city in Shandong
Jinfeng Hongyuan Flagship Store	Electricity savings1	Buy your stuff is not always good I have been using it for almost two months now, it does not save electricity at all the electricity bill is about ten dollars a day this is still called the king of power	Jingdong prefecture level city in Shandong
Jinfeng Hongyuan Flagship Store	Electricity savings1	Express arrived, the quality is very good, worth the purchase.	Jingdong prefecture level city in Shandong
Jinfeng Hongyuan Flagship Store	Electricity savings1	Energy saving has been received, now the voltage is more stable, energy-saving effect to see a few days and then chase flat, the service attitude is very good, the courier is relatively fast, I am satisfied.	Jingdong prefecture level city in Shandong
Jinfeng Hongyuan Flagship Store	Electricity savings1	Stuff installed, numbers are smaller than when installed, hopefully it works	Jingdong prefecture level city in Shandong
Jinfeng Hongyuan Flagship Store	Electricity savings1	Good appearance, fine workmanship, just bought, use up the feeling of power saving effect is good, worth buying!	Jingdong prefecture level city in Shandong
Jinfeng Hongyuan Flagship Store	Electricity savings1	Economical and practical 👍 Always use it	Jingdong prefecture level city in Shandong
Jinfeng Hongyuan Flagship Store	Electricity savings1	Just used it, don't know the effect yet, will come back to follow up after the effect. Hope it works well(▽)	Jingdong prefecture level city in Shandong
Jinfeng Hongyuan Flagship Store	Electricity savings1	The goods have been received, use it to see in the evaluation.	Jingdong prefecture level city in Shandong
Jinfeng Hongyuan Flagship Store	Electricity savings1	Doesn't it save electricity? The boil kettle still goes faster when you press the meter.	Jingdong prefecture level city in Shandong
Jinfeng Hongyuan Flagship Store	Electricity savings1	Already used. Will patronize again in the future	Jingdong prefecture level city in Shandong
Jinfeng Hongyuan Flagship Store	Electricity savings1	Works great, just plug it in, results to be checked, satisfied.	Jingdong prefecture level city in Shandong
Jinfeng Hongyuan Flagship Store	Electricity savings1	Second purchase. Works well 😍😍😍😍	Jingdong prefecture level city in Shandong
Jinfeng Hongyuan Flagship Store	Electricity savings1	Prompt shipment, very satisfied	Jingdong prefecture level city in Shandong
Jinfeng Hongyuan Flagship Store	Electricity savings1	It's been less than a month, let's see how it works, hopefully it's power efficient	Jingdong prefecture level city in Shandong
Jinfeng Hongyuan Flagship Store	Electricity savings1	Use a month in say.	Jingdong prefecture level city in Shandong
Jinfeng Hongyuan Flagship Store	Electricity savings1	I've saved a lot of electricity since I've been using it.	Jingdong prefecture level city in Shandong
Jinfeng Hongyuan Flagship Store	Electricity savings1	It's fast, looks and feels good on the outside, try it out and evaluate.	Jingdong prefecture level city in Shandong
Jinfeng Hongyuan Flagship Store	Electricity savings1	I haven't used it. I just used it. I don't know if it's any good.	Jingdong prefecture level city in Shandong
Jinfeng Hongyuan Flagship Store	Electricity savings1	Quality can be, power saving now can not see just use, after a while in the look at the power saving effect	Jingdong prefecture level city in Shandong
Jinfeng Hongyuan Flagship Store	Electricity savings1	The logistics is very fast, and it seems to have some effect	Jingdong prefecture level city in Shandong
Jinfeng Hongyuan Flagship Store	Electricity savings1	Good, but the effects remain to be seen	Jingdong prefecture level city in Shandong
Jinfeng Hongyuan Flagship Store	Electricity savings1	Baby is basically the same as the description, shipping quickly, worth a buy Good price, logistics is strong, worth repurchasing!	Jingdong prefecture level city in Shandong
Jinfeng Hongyuan Flagship Store	Electricity savings1	I bought it for my dad, I don't know much about it, if he thinks it's good then it must be good 👍	Jingdong prefecture level city in Shandong
Jinfeng Hongyuan Flagship Store	Electricity savings1	Goods received. It's working fine. Display voltage. After a period of time to see the effect in the addition to the level of theory	Jingdong prefecture level city in Shandong
Jinfeng Hongyuan Flagship Store	Electricity savings1	The stuff is pretty good, I just don't know how well it saves power.	Jingdong prefecture level city in Shandong
Jinfeng Hongyuan Flagship Store	Electricity savings1	Logistics is a little too fast 👍	Jingdong prefecture level city in Shandong

Jinfeng Hongyuan Flagship Store	Electricity savings1	Received the goods used for a period of time before coming, good, beautiful appearance, really stable flow	Jingdong prefecture level city in Shandong
Jinfeng Hongyuan Flagship Store	Electricity savings1	Things received, look at the packaging is very good, not yet, to the power saving in the follow-up evaluation	Jingdong prefecture level city in Shandong
Jinfeng Hongyuan Flagship Store	Electricity savings1	Baby is very good! Use a few days before to evaluate, I personally test really effective.	Jingdong prefecture level city in Shandong
Jinfeng Hongyuan Flagship Store	Electricity savings1	I received the goods are in use, I do not know how effective? Can it save energy? Let's see.	Jingdong prefecture level city in Shandong
Jinfeng Hongyuan Flagship Store	Electricity savings1	It's really good and has some power saving effect.	Jingdong prefecture level city in Shandong
Jinfeng Hongyuan Flagship Store	Electricity savings1	It's good quality, well made, and I hope it'll save on electricity over time.	Jingdong prefecture level city in Shandong
Jinfeng Hongyuan Flagship Store	Electricity savings1	I just used it, I don't know how it works yet, but I'll give it a good review first.	Jingdong prefecture level city in Shandong
Jinfeng Hongyuan Flagship Store	Electricity savings1	Second purchase, the delivery guy was fast and called ahead to deliver.	Jingdong prefecture level city in Shandong
Jinfeng Hongyuan Flagship Store	Electricity savings1	Goods have been received, but have not yet used, used to see how the effect, in the evaluation of fertilizer	Jingdong prefecture level city in Shandong
Jinfeng Hongyuan Flagship Store	Electricity savings1	The delivery is very fast. It's the same in kind. It's working.	Jingdong prefecture level city in Shandong
Jinfeng Hongyuan Flagship Store	Electricity savings1	Fast logistics, good quality, more satisfied	Jingdong prefecture level city in Shandong
Jinfeng Hongyuan Flagship Store	Electricity savings1	I've been here for almost a month now and there is a very noticeable difference than it is worth buying.	Jingdong prefecture level city in Shandong
Jinfeng Hongyuan Flagship Store	Electricity savings1	Fast shipping, received the next day, plug and play, but do not know how effective to not break down!	Jingdong prefecture level city in Shandong
Jinfeng Hongyuan Flagship Store	Electricity savings1	The logistics are great I've been using it for a while before commenting on it This saves electricity	Jingdong prefecture level city in Shandong
Jinfeng Hongyuan Flagship Store	Electricity savings1	Wait to use it for a while to know if it's energy efficient	Jingdong prefecture level city in Shandong
Jinfeng Hongyuan Flagship Store	Electricity savings1	Just received it, haven't used it yet don't know if it's any good.	Jingdong prefecture level city in Shandong
Jinfeng Hongyuan Flagship Store	Electricity savings1	It really doesn't work, and the electric bill is still the same amount every month.	Jingdong prefecture level city in Shandong
Jinfeng Hongyuan Flagship Store	Electricity savings1	It's well packaged and looks fine	Jingdong prefecture level city in Shandong
Jinfeng Hongyuan Flagship Store	Electricity savings1	Very good. Will patronize again if needed.	Jingdong prefecture level city in Shandong
Jinfeng Hongyuan Flagship Store	Electricity savings1	I have received the product and installed it, try it for a while and see how it works.	Jingdong prefecture level city in Shandong
Jinfeng Hongyuan Flagship Store	Electricity savings1	I've been using it for a month now...I don't feel like there's any noticeable power savings...last month it was $130 this month it's $123...let's see if I can use it for another month	Jingdong prefecture level city in Shandong
Jinfeng Hongyuan Flagship Store	Electricity savings1	Just received it, yet to try it and see if it works.	Jingdong prefecture level city in Shandong
Jinfeng Hongyuan Flagship Store	Electricity savings1	It's easy to use and has a power saving credit!	Jingdong prefecture level city in Shandong
Jinfeng Hongyuan Flagship Store	Electricity savings1	It's very good. I've already bought two of them.	Jingdong prefecture level city in Shandong
Jinfeng Hongyuan Flagship Store	Electricity savings1	It's very well made, packaged meticulously, and I hope it works!	Jingdong prefecture level city in Shandong
Jinfeng Hongyuan Flagship Store	Electricity savings1	Goods have been received have not yet used it, used to comment again	Jingdong prefecture level city in Shandong
Jinfeng Hongyuan Flagship Store	Electricity savings1	Looking forward to good results	Jingdong prefecture level city in Shandong
Jinfeng Hongyuan Flagship Store	Electricity savings1	Not sure if it saves power or not. Still trying it out.	Jingdong prefecture level city in Shandong
Jinfeng Hongyuan Flagship Store	Electricity savings1	It also really saves on electricity! Superb!	Jingdong prefecture level city in Shandong
Jinfeng Hongyuan Flagship Store	Electricity savings1	Didn't work at all, want to return it	Jingdong prefecture level city in Shandong
Jinfeng Hongyuan Flagship Store	Electricity savings1	Uh-huh! It's working, I've been using it for a month and it saves 20% of electricity compared to before.	Jingdong prefecture level city in Shandong
Jinfeng Hongyuan Flagship Store	Electricity savings1	Should be a little bit of a power saver, I wonder how long it will take to save back the money I spent on this one	Jingdong prefecture level city in Shandong
Jinfeng Hongyuan Flagship Store	Electricity savings1	Exquisite appearance and stable voltage	Jingdong prefecture level city in Shandong
Jinfeng Hongyuan Flagship Store	Electricity savings1	After using it for a few days it feels like it saves electricity.	Jingdong prefecture level city in Shandong
Jinfeng Hongyuan Flagship Store	Electricity savings1	Second purchase and its pending good results.	Jingdong prefecture level city in Shandong
Jinfeng Hongyuan Flagship Store	Electricity savings1	The appearance of the workmanship is exquisite and meticulous, beautiful appearance, just to see if there is no effect!	Jingdong prefecture level city in Shandong
Jinfeng Hongyuan Flagship Store	Electricity savings1	Let's see what happens in a month.	Jingdong prefecture level city in Shandong
Jinfeng Hongyuan Flagship Store	Electricity savings1	Trial, now short time not yet obvious effect	Jingdong prefecture level city in Shandong
Jinfeng Hongyuan Flagship Store	Electricity savings1	I'm using it now, don't know how well it works, it looks great!	Jingdong prefecture level city in Shandong
Jinfeng Hongyuan Flagship Store	Electricity savings1	There is no role! Now it's true that I don't know!	Jingdong prefecture level city in Shandong
Jinfeng Hongyuan Flagship Store	Electricity savings1	Purchased for the second time, power saving not sure if it's ok? Let's talk about it after using it	Jingdong prefecture level city in Shandong
Jinfeng Hongyuan Flagship Store	Electricity savings1	I just received it, I have to try it for a month to know how it works.	Jingdong prefecture level city in Shandong
Jinfeng Hongyuan Flagship Store	Electricity savings1	I just used it for an hour, and the meter's still spinning so fast it's not much use.	Jingdong prefecture level city in Shandong
Jinfeng Hongyuan Flagship Store	Electricity savings1	Not bad, logistics is fast	Jingdong prefecture level city in Shandong
Jinfeng Hongyuan Flagship Store	Electricity savings1	I don't know if it works. I'll find out next month when I pay the bill. It's easy. Just plug it in.	Jingdong prefecture level city in Shandong
Jinfeng Hongyuan Flagship Store	Electricity savings1	Logistics is particularly fast, received the goods are genuine, looking forward to power saving effect	Jingdong prefecture level city in Shandong
Jinfeng Hongyuan Flagship Store	Electricity savings1	First time buying this product like this one from this family use it and then evaluate.	Jingdong prefecture level city in Shandong
Jinfeng Hongyuan Flagship Store	Electricity savings1	Lightning arrived, the product is very good, before all do not know there is such a baby, in a friend's home has seen, and then bought to try, has been used for a period of time before coming to the evaluation, the work is very stable, so far looked very good, will introduce friends to buy!	Jingdong prefecture level city in Shandong
Jinfeng Hongyuan Flagship Store	Electricity savings1	I was a bit skeptical when I heard that I could save money on my electricity bill by installing this, I don't know if the power saving effect is really what it is said to be. But since I bought it, I just have to put it on and try it out. I hope it lives up to its name and saves me the money I spent on the device	Jingdong prefecture level city in Shandong
Jinfeng Hongyuan Flagship Store	Electricity savings1	The quality of the stuff is okay, but I'm not sure if it works, so I'll try using it first.	Jingdong prefecture level city in Shandong

Jinfeng Hongyuan Flagship Store	Electricity savings1	It should work. The current is much more stable.	Jingdong prefecture level city in Shandong
Jinfeng Hongyuan Flagship Store	Electricity savings1	The store has a great service attitude	Jingdong prefecture level city in Shandong
Jinfeng Hongyuan Flagship Store	Electricity savings1	Let's wait until we've used it for a month.	Jingdong prefecture level city in Shandong
Jinfeng Hongyuan Flagship Store	Electricity savings1	I'm using it and I don't know if it's a power saver or not	Jingdong prefecture level city in Shandong
Jinfeng Hongyuan Flagship Store	Electricity savings1	Original and authentic guarantee quality is good now.	Jingdong prefecture level city in Shandong
Jinfeng Hongyuan Flagship Store	Electricity savings1	Great, very happy with it, will buy a couple more next time	Jingdong prefecture level city in Shandong
Jinfeng Hongyuan Flagship Store	Electricity savings1	I just used it, I don't know if it works yet, I'll come back after using it...	Jingdong prefecture level city in Shandong
Jinfeng Hongyuan Flagship Store	Electricity savings1	It's still being tested.	Jingdong prefecture level city in Shandong
Jinfeng Hongyuan Flagship Store	Electricity savings1	I've been using it for a month and it's not working.	Jingdong prefecture level city in Shandong
Jinfeng Hongyuan Flagship Store	Electricity savings1	It's basically nothing!	Jingdong prefecture level city in Shandong
Jinfeng Hongyuan Flagship Store	Electricity savings1	It's not bad, it kind of works, support it.	Jingdong prefecture level city in Shandong
Jinfeng Hongyuan Flagship Store	Electricity savings1	I just received it last night, let's try it first to see how it works, it looks good!	Jingdong prefecture level city in Shandong
Jinfeng Hongyuan Flagship Store	Electricity savings1	The current is very stable, fine workmanship, small and exquisite, very satisfied	Jingdong prefecture level city in Shandong
Jinfeng Hongyuan Flagship Store	Electricity savings1	Not bad, exactly how much electricity can be saved; still have to follow up the evaluation	Jingdong prefecture level city in Shandong
Jinfeng Hongyuan Flagship Store	Electricity savings1	Logistics is also very fast, next time there is an opportunity to introduce friends to buy!	Jingdong prefecture level city in Shandong
Jinfeng Hongyuan Flagship Store	Electricity savings1	Voltage display and actual do not pay	Jingdong prefecture level city in Shandong
Jinfeng Hongyuan Flagship Store	Electricity savings1	Second purchase, still saves a little on the electric bill	Jingdong prefecture level city in Shandong
Jinfeng Hongyuan Flagship Store	Electricity savings1	Order received, currently trying it out, thanks a lot.	Jingdong prefecture level city in Shandong
Jinfeng Hongyuan Flagship Store	Electricity savings1	I haven't used it for a long time yet, I don't know if the effect is good or not, looking forward to it	Jingdong prefecture level city in Shandong
Jinfeng Hongyuan Flagship Store	Electricity savings1	Good, working, I just don't know if it will save electricity... 5 stars for now.	Jingdong prefecture level city in Shandong
Jinfeng Hongyuan Flagship Store	Electricity savings1	Great product, just as the owner said it would be.	Jingdong prefecture level city in Shandong
Jinfeng Hongyuan Flagship Store	Electricity savings1	Shipping was fast, I don't know if it works well or not, I have to use it for a while first.	Jingdong prefecture level city in Shandong
Jinfeng Hongyuan Flagship Store	Electricity savings1	I don't feel that it saves much electricity, but I'll give it a try. It's been more than a month now, and the electricity bill is still a bit high.	Jingdong prefecture level city in Shandong
Jinfeng Hongyuan Flagship Store	Electricity savings1	Got it. Just used it.	Jingdong prefecture level city in Shandong
Jinfeng Hongyuan Flagship Store	Electricity savings1	Use it for a while and then comment.	Jingdong prefecture level city in Shandong
Jinfeng Hongyuan Flagship Store	Electricity savings1	Used it, see how it works with it and then comment. The quality is very good!	Jingdong prefecture level city in Shandong
Jinfeng Hongyuan Flagship Store	Electricity savings1	I'll comment again after a while.	Jingdong prefecture level city in Shandong
Jinfeng Hongyuan Flagship Store	Electricity savings1	It's easy to operate, affordable and works well, love it.	Jingdong prefecture level city in Shandong
Jinfeng Hongyuan Flagship Store	Electricity savings1	It's good. You can try it. It's cheap.	Jingdong prefecture level city in Shandong
Jinfeng Hongyuan Flagship Store	Electricity savings1	It's in use, so we'll see how it works after a while!	Jingdong prefecture level city in Shandong
Jinfeng Hongyuan Flagship Store	Electricity savings1	Service is pretty good, not sure about the quality yet.	Jingdong prefecture level city in Shandong
Jinfeng Hongyuan Flagship Store	Electricity savings1	Just used it and will see the results in a month 😀	Jingdong prefecture level city in Shandong
Jinfeng Hongyuan Flagship Store	Electricity savings1	Saving electricity is expensive and good	Jingdong prefecture level city in Shandong
Jinfeng Hongyuan Flagship Store	Electricity savings1	Pretty good, 5 stars! Will buy again next time seller service is very good！！！！ Good quality! Will recommend to friends!	Jingdong prefecture level city in Shandong
Jinfeng Hongyuan Flagship Store	Electricity savings1	In use, with results naturally have follow-up reviews!	Jingdong prefecture level city in Shandong
Jinfeng Hongyuan Flagship Store	Electricity savings1	I'm using it now, I'll come back with results, it'll work.	Jingdong prefecture level city in Shandong
Jinfeng Hongyuan Flagship Store	Electricity savings1	Good, nice, fast shipping, cheap, worth having!	Jingdong prefecture level city in Shandong
Jinfeng Hongyuan Flagship Store	Electricity savings1	Logistics is fast.	Jingdong prefecture level city in Shandong
Jinfeng Hongyuan Flagship Store	Electricity savings1	I have just started using the product and will be reviewing it later.	Jingdong prefecture level city in Shandong
Jinfeng Hongyuan Flagship Store	Electricity savings1	I'll comment after I use it.	Jingdong prefecture level city in Shandong
Jinfeng Hongyuan Flagship Store	Electricity savings1	I don't know if it's working or not, though I'll try plugging it in.	Jingdong prefecture level city in Shandong
Jinfeng Hongyuan Flagship Store	Electricity savings1	It really saves electricity and contributes to energy savings in disguise	Jingdong prefecture level city in Shandong
Jinfeng Hongyuan Flagship Store	Electricity savings1	It feels good in the experience.	Jingdong prefecture level city in Shandong
Jinfeng Hongyuan Flagship Store	Electricity savings1	The product is better than I thought it would be, the quality looks great, the voltage is stable, and I'm looking forward to some great power savings!	Jingdong prefecture level city in Shandong
Jinfeng Hongyuan Flagship Store	Electricity savings1	I'm using it now looking forward to saving some points,, I'm feeding back	Jingdong prefecture level city in Shandong
Jinfeng Hongyuan Flagship Store	Electricity savings1	Displayed voltage below 200.	Jingdong prefecture level city in Shandong
Jinfeng Hongyuan Flagship Store	Electricity savings1	I haven't seen any great results, but it should last about a hundred or eighty years.	Jingdong prefecture level city in Shandong
Jinfeng Hongyuan Flagship Store	Electricity savings1	I don't know if it works or not, but I keep it plugged in anyway, so I hope it works!	Jingdong prefecture level city in Shandong
Jinfeng Hongyuan Flagship Store	Electricity savings1	Reasonable price, good quality, fast delivery, great service, already a repurchase.	Jingdong prefecture level city in Shandong
Jinfeng Hongyuan Flagship Store	Electricity savings1	Second purchase, looking forward to the results	Jingdong prefecture level city in Shandong
Jinfeng Hongyuan Flagship Store	Electricity savings1	The quality of this product feels like it should work in a use test! Just how many kilowatts of electricity must be wasted per day with the red light and digital display on for long periods of time?	Jingdong prefecture level city in Shandong

Jinfeng Hongyuan Flagship Store	Electricity savings1	It looks good, we'll see if we use it.	Jingdong prefecture level city in Shandong
Jinfeng Hongyuan Flagship Store	Electricity savings1	Great stuff, second purchase!	Jingdong prefecture level city in Shandong
Jinfeng Hongyuan Flagship Store	Electricity savings1	It is indeed very practical and saves electricity at home	Jingdong prefecture level city in Shandong
Jinfeng Hongyuan Flagship Store	Electricity savings1	Goods received, the physical and the same as the picture, logistics is also very fast!	Jingdong prefecture level city in Shandong
Jinfeng Hongyuan Flagship Store	Electricity savings1	I'll use it for a while and see how it goes. I'll buy it again if it's good.	Jingdong prefecture level city in Shandong
Jinfeng Hongyuan Flagship Store	Electricity savings1	In use, voltage monitoring, the effect of how to wait for next month's electricity consumption confirmation	Jingdong prefecture level city in Shandong
Jinfeng Hongyuan Flagship Store	Electricity savings1	It's still pretty good.	Jingdong prefecture level city in Shandong
Jinfeng Hongyuan Flagship Store	Electricity savings1	It's been plugged in for a month and there hasn't been a big change yet, since it usually uses very little electricity	Jingdong prefecture level city in Shandong
Jinfeng Hongyuan Flagship Store	Electricity savings1	Let's wait for a month to see if it works	Jingdong prefecture level city in Shandong
Jinfeng Hongyuan Flagship Store	Electricity savings1	Good, good, good, good, good, good, good, good, good, good, good, good, good, good, good.	Jingdong prefecture level city in Shandong
Jinfeng Hongyuan Flagship Store	Electricity savings1	The product received the trial a few days, feel good, praise!	Jingdong prefecture level city in Shandong
Jinfeng Hongyuan Flagship Store	Electricity savings1	After six or seven hours of use, 250 volts won't save you any power.	Jingdong prefecture level city in Shandong
Jinfeng Hongyuan Flagship Store	Electricity savings1	I haven't used it yet, I wonder if it saves electricity?	Jingdong prefecture level city in Shandong
Jinfeng Hongyuan Flagship Store	Electricity savings1	Baby value for money, love it, next time will come back is genuine, trust the business, baby texture is very good, the price is moderate, unlimited repurchase!	Jingdong prefecture level city in Shandong
Jinfeng Hongyuan Flagship Store	Electricity savings1	Power saving effect is obvious! Raising tropical fish daily power consumption, after using the daily power savings of more than 3 degrees, currently about 8 degrees / day!	Jingdong prefecture level city in Shandong
Jinfeng Hongyuan Flagship Store	Electricity savings1	Just used it, unknown results.	Jingdong prefecture level city in Shandong
Jinfeng Hongyuan Flagship Store	Electricity savings1	The goods received, well, look forward to the effect. Logistics is very fast, thanks to the platform, thanks to Jingdong. I hope it can save electricity.	Jingdong prefecture level city in Shandong
Jinfeng Hongyuan Flagship Store	Electricity savings1	Not sure 有 effect?	Jingdong prefecture level city in Shandong
Jinfeng Hongyuan Flagship Store	Electricity savings1	I'd say it's a 5% savings.	Jingdong prefecture level city in Shandong
Jinfeng Hongyuan Flagship Store	Electricity savings1	I received it and am using it, I don't know how effective it is, I'll evaluate it afterwards	Jingdong prefecture level city in Shandong
Jinfeng Hongyuan Flagship Store	Electricity savings1	Baby received not yet used, first give praise	Jingdong prefecture level city in Shandong
Jinfeng Hongyuan Flagship Store	Electricity savings1	Looks good, I'll evaluate it later	Jingdong prefecture level city in Shandong
Jinfeng Hongyuan Flagship Store	Electricity savings1	The goods have been received in use, the overall feeling is not bad!	Jingdong prefecture level city in Shandong
Jinfeng Hongyuan Flagship Store	Electricity savings1	It's working. This is the second one. This stuff has to be used over a long period of time to be effective. And it only works on inductive loads, i.e. appliances containing electric motors. Use it.	Jingdong prefecture level city in Shandong
Jinfeng Hongyuan Flagship Store	Electricity savings1	Use it for a while and see how it works.	Jingdong prefecture level city in Shandong
Jinfeng Hongyuan Flagship Store	Electricity savings1	Good courier service and the item is very nice and practical.	Jingdong prefecture level city in Shandong
Jinfeng Hongyuan Flagship Store	Electricity savings1	It doesn't do anything and it's a waste of electricity.	Jingdong prefecture level city in Shandong
Jinfeng Hongyuan Flagship Store	Electricity savings1	I haven't used it yet. It should be good.	Jingdong prefecture level city in Shandong
Jinfeng Hongyuan Flagship Store	Electricity savings1	That's good. OK, that's good.	Jingdong prefecture level city in Shandong
Jinfeng Hongyuan Flagship Store	Electricity savings1	It's a rip off, doesn't work, doesn't save electricity, does the job to well, and I've wasted a couple dozen dollars again.	Jingdong prefecture level city in Shandong
Jinfeng Hongyuan Flagship Store	Electricity savings1	Use it first, the effect is not as good as the road, then we will comment again	Jingdong prefecture level city in Shandong
Jinfeng Hongyuan Flagship Store	Electricity savings1	The price is affordable, the logistics is strong, just use four or five days, the effect will be added later!	Jingdong prefecture level city in Shandong
Jinfeng Hongyuan Flagship Store	Electricity savings1	It's easy to use and works fine.	Jingdong prefecture level city in Shandong
Jinfeng Hongyuan Flagship Store	Electricity savings1	It doesn't feel like it's working.	Jingdong prefecture level city in Shandong
Jinfeng Hongyuan Flagship Store	Electricity savings1	I'm using it. We'll see what happens in a couple months.	Jingdong prefecture level city in Shandong
Jinfeng Hongyuan Flagship Store	Electricity savings1	I just got it back to try it out and I'll comment later	Jingdong prefecture level city in Shandong
Jinfeng Hongyuan Flagship Store	Electricity savings1	Less than two months. Can't tell.	Jingdong prefecture level city in Shandong
Jinfeng Hongyuan Flagship Store	Electricity savings1	No power saving effect	Jingdong prefecture level city in Shandong
Jinfeng Hongyuan Flagship Store	Electricity savings1	Express Express Very quickly not only to the power saver can not save electricity, with two months, look at the evaluation put.	Jingdong prefecture level city in Shandong
Jinfeng Hongyuan Flagship Store	Electricity savings1	Genuine products are packaged well!	Jingdong prefecture level city in Shandong
Jinfeng Hongyuan Flagship Store	Electricity savings1	I've been using it for a while, but I don't know if it saves electricity. I hope it works.	Jingdong prefecture level city in Shandong
Jinfeng Hongyuan Flagship Store	Electricity savings1	Good service, fast logistics, the effect is to be used to know	Jingdong prefecture level city in Shandong
Jinfeng Hongyuan Flagship Store	Electricity savings1	Just used it, not sure about the power saving effect	Jingdong prefecture level city in Shandong
Jinfeng Hongyuan Flagship Store	Electricity savings1	The stuff is ok, save power or not, will comment after a month.	Jingdong prefecture level city in Shandong
Jinfeng Hongyuan Flagship Store	Electricity savings1	Used it for a month to save %25, normal is a little over 100, it's great! I want to buy one in.	Jingdong prefecture level city in Shandong
Jinfeng Hongyuan Flagship Store	Electricity savings1	I'm using it and looking forward to the results	Jingdong prefecture level city in Shandong
Jinfeng Hongyuan Flagship Store	Electricity savings1	The quality is very good and the workmanship is exquisite. The use effect is also very good.	Jingdong prefecture level city in Shandong
Jinfeng Hongyuan Flagship Store	Electricity savings1	Merchant service attitude is very good, logistics speed is super fast, online evaluation is very good, the first time to use, a month to know the specific effect, the effect is good, and then additional	Jingdong prefecture level city in Shandong
Jinfeng Hongyuan Flagship Store	Electricity savings1	Works great, solves the problem of high power usage in the house	Jingdong prefecture level city in Shandong
Jinfeng Hongyuan Flagship Store	Electricity savings1	To receive! Very delicate! It's not clear how effective it is, I won't know until I use it!!!!	Jingdong prefecture level city in Shandong
Jinfeng Hongyuan Flagship Store	Electricity savings1	I've just been using it for a while and I don't feel like it's too noticeable.	Jingdong prefecture level city in Shandong

Jinfeng Hongyuan Flagship Store	Electricity savings1	Already used, no visible effect for the time being	Jingdong prefecture level city in Shandong
Jinfeng Hongyuan Flagship Store	Electricity savings1	I can't feel the effect.	Jingdong prefecture level city in Shandong
Jinfeng Hongyuan Flagship Store	Electricity savings1	Use it for a while and see the results	Jingdong prefecture level city in Shandong
Jinfeng Hongyuan Flagship Store	Electricity savings1	The price is more reasonable, the power-saving effect is to be verified, the delivery is fast, and the courier service is good.	Jingdong prefecture level city in Shandong
Jinfeng Hongyuan Flagship Store	Electricity savings1	Quality is very good, consistent with the seller's description, very satisfied, very like, very fast shipping, packaging is very careful and strict, the logistics company has a very good service attitude, fast delivery, very satisfied with a shopping, very satisfied. Recommended to buy, cost-effective, the service attitude of the business is very enthusiastic, providing such high quality goods.	Jingdong prefecture level city in Shandong
Jinfeng Hongyuan Flagship Store	Electricity savings1	I don't know.	Jingdong prefecture level city in Shandong
Jinfeng Hongyuan Flagship Store	Electricity savings1	I didn't get the delivery.	Jingdong prefecture level city in Shandong
Jinfeng Hongyuan Flagship Store	Electricity savings1	It works, it does have power savings, better than expected, will repurchase.	Jingdong prefecture level city in Shandong
Jinfeng Hongyuan Flagship Store	Electricity savings1	That's where the five fuses are.	Jingdong prefecture level city in Shandong
Jinfeng Hongyuan Flagship Store	Electricity savings1	Don't buy it, it doesn't work and you can't return it.	Jingdong prefecture level city in Shandong
Jinfeng Hongyuan Flagship Store	Electricity savings1	I hope it works. Wait a month.	Jingdong prefecture level city in Shandong
Jinfeng Hongyuan Flagship Store	Electricity savings1	Nothing**, buy with caution	Jingdong prefecture level city in Shandong
Jinfeng Hongyuan Flagship Store	Electricity savings1	I'm using it and waiting for the results	Jingdong prefecture level city in Shandong
Jinfeng Hongyuan Flagship Store	Electricity savings1	Saver quality is very good, has always felt that the home power consumption is very large, this is the company's electrician recommended, said he bought this home is really able to save a little electricity, I also came to buy, the effect if it is good, then buy, a floor a...	Jingdong prefecture level city in Shandong
Jinfeng Hongyuan Flagship Store	Electricity savings1	Good product, satisfied with purchase	Jingdong prefecture level city in Shandong
Jinfeng Hongyuan Flagship Store	Electricity savings1	Occupying the time has not yet found the power saving, occupying not evaluation	Jingdong prefecture level city in Shandong
Jinfeng Hongyuan Flagship Store	Electricity savings1	Haven't tested the results yet. To be followed up with comments!	Jingdong prefecture level city in Shandong
Jinfeng Hongyuan Flagship Store	Electricity savings1	After experiencing it for a month, I don't seem to know how to save it, and I don't pay attention to it anyway	Jingdong prefecture level city in Shandong
Jinfeng Hongyuan Flagship Store	Electricity savings1	It's useless. It's a waste of electricity.	Jingdong prefecture level city in Shandong
Jinfeng Hongyuan Flagship Store	Electricity savings1	I don't know if it's a power saver. It's a couple bucks. I hope it works.	Jingdong prefecture level city in Shandong
Jinfeng Hongyuan Flagship Store	Electricity savings1	I don't know what to say about this stuff. I don't know if it works or not, it's kind of a psychological purchase. Dormitory use to see the meter can not be so good-looking. You have to wait until the time to deduct the utility bill to know.	Jingdong prefecture level city in Shandong
Jinfeng Hongyuan Flagship Store	Electricity savings1	Energy savers don't work / they're all a bunch of hoaxes.	Jingdong prefecture level city in Shandong
Jinfeng Hongyuan Flagship Store	Electricity savings1	Received the goods, used for more than a week, it's okay, sure a little effect	Jingdong prefecture level city in Shandong
Jinfeng Hongyuan Flagship Store	Electricity savings1	Well made and compact! Looking forward to the effect!	Jingdong prefecture level city in Shandong
Jinfeng Hongyuan Flagship Store	Electricity savings1	It has arrived and we'll see how it works.	Jingdong prefecture level city in Shandong
Jinfeng Hongyuan Flagship Store	Electricity savings1	Good. Good. It's worth it.	Jingdong prefecture level city in Shandong
Jinfeng Hongyuan Flagship Store	Electricity savings1	How come there's no indicator light?	Jingdong prefecture level city in Shandong
Jinfeng Hongyuan Flagship Store	Electricity savings1	The machine that uses electricity works quite well. This saves	Jingdong prefecture level city in Shandong
Jinfeng Hongyuan Flagship Store	Electricity savings1	It's good for home use and feels quite energy efficient	Jingdong prefecture level city in Shandong
Jinfeng Hongyuan Flagship Store	Electricity savings1	I regret buying this product, after using it for two days it doesn't work at all, still so many kilowatts per day just like the original.	Jingdong prefecture level city in Shandong
Jinfeng Hongyuan Flagship Store	Electricity savings1	Works very well and has some scientific merit.	Jingdong prefecture level city in Shandong
Jinfeng Hongyuan Flagship Store	Electricity savings1	Logistics is also very fast this good power saving I use two months before commenting!	Jingdong prefecture level city in Shandong
Jinfeng Hongyuan Flagship Store	Electricity savings1	Repeat customer. Very good.	Jingdong prefecture level city in Shandong
Jinfeng Hongyuan Flagship Store	Electricity savings1	It's good stuff. I've been using it for a month now and it's a real power saver.	Jingdong prefecture level city in Shandong
Jinfeng Hongyuan Flagship Store	Electricity savings1	Jingdong platform to buy things, has been very assured, quality assurance. Jingdong Express is as good as ever, complete packaging, delivery, service attitude to go! The family has always wanted to buy an energy-saving and power-saving equipment, selected half a day to choose this one, the value is very good, used for a period of time to follow up, to see whether it can achieve the real purpose of power saving, I	Jingdong prefecture level city in Shandong
Jinfeng Hongyuan Flagship Store	Electricity savings1	Works great, inexpensive and will be back next time.	Jingdong prefecture level city in Shandong
Jinfeng Hongyuan Flagship Store	Electricity savings1	Baby received, looks good, wait for a month to see how it works?	Jingdong prefecture level city in Shandong
Jinfeng Hongyuan Flagship Store	Electricity savings1	This product is not bad quite good well customer service said quite power saving that look at the customer service to see if the accuracy of the look at all aspects of the color is also quite beautiful	Jingdong prefecture level city in Shandong
Jinfeng Hongyuan Flagship Store	Electricity savings1	Just got back and tried it feels pretty good, just don't know how the quality is, give a good comment first	Jingdong prefecture level city in Shandong
Jinfeng Hongyuan Flagship Store	Electricity savings1	Received the product, trying it out, hope it helps!	Jingdong prefecture level city in Shandong
Jinfeng Hongyuan Flagship Store	Electricity savings1	Works great, saves electricity a dollar a day and a night	Jingdong prefecture level city in Shandong
Jinfeng Hongyuan Flagship Store	Electricity savings1	Baby received, look at the workmanship is still very fine, is genuine. I hope that this summer to open the air conditioning electricity bill is no longer high scary!	Jingdong prefecture level city in Shandong
Jinfeng Hongyuan Flagship Store	Electricity savings1	It's here. I don't know how it's going to work, but I'm going to use it.	Jingdong prefecture level city in Shandong
Jinfeng Hongyuan Flagship Store	Electricity savings1	Power saver received, Jingdong logistics first-class, business customer service attitude is good, one by one to introduce the product performance, back on the horse plugged in, it is very simple, there is a voltage indicator, to be a few months to see how the effect of power-saving to come back to a good follow-up, but I believe that Jinfenghong products.	Jingdong prefecture level city in Shandong
Jinfeng Hongyuan Flagship Store	Electricity savings1	Took the third one...the super power-saving kind!	Jingdong prefecture level city in Shandong
Jinfeng Hongyuan Flagship Store	Electricity savings1	First time buyer, customer service is very polite and courteous, conscientious business, support! Baby has received 🎈, super like, high value and practical, super like! ❥❥❥❥❥ Very good quality, very satisfied with a shopping. Customer service has a question and answer, very professional finally received the goods, very, very much like, exquisite and beautiful workmanship, has been praised by many people, really too happy, is already a loyal fan of his family, will continue to buy buy buy buy!	Jingdong prefecture level city in Shandong

Jinfeng Hongyuan Flagship Store	Electricity savings1	That's good. That's good. That's really good.	Jingdong prefecture level city in Shandong
Jinfeng Hongyuan Flagship Store	Electricity savings1	Saver received, good-looking design, voltage at a glance, and the merchant description, save some about 12% or so	Jingdong prefecture level city in Shandong
Jinfeng Hongyuan Flagship Store	Electricity savings1	Intelligent power saver is small and does not take up space, very good, very practical, received and used, packaging is tight, logistics is also very fast, satisfied with a shopping!	Jingdong prefecture level city in Shandong
Jinfeng Hongyuan Flagship Store	Electricity savings1	The stuff is good! Well made, compact, alloy case, shows voltage! Very like! Kudos!	Jingdong prefecture level city in Shandong
Jinfeng Hongyuan Flagship Store	Electricity savings1	I just purchased this power saver from Jingdong, inserted in the socket shows the number 206, a moment and 207 back and forth display, I do not know what it means, my home voltage should be normal, because it is the owner of the community's own power plant to put, first use for a period of time in the positive feedback it, first use in the look at it!	Jingdong prefecture level city in Shandong
Jinfeng Hongyuan Flagship Store	Electricity savings1	Very delicate and compact, there is a convenient intelligent display prompts the voltage, you can know the voltage at a glance, there is also very easy to use, a plug can be used, you can save electricity	Jingdong prefecture level city in Shandong
Jinfeng Hongyuan Flagship Store	Electricity savings1	Logistics is fast, ask customer service said can save electricity, but only use two months to see the effect, use two months in the evaluation.	Jingdong prefecture level city in Shandong
Jinfeng Hongyuan Flagship Store	Electricity savings1	The quality is very good.	Jingdong prefecture level city in Shandong
Jinfeng Hongyuan Flagship Store	Electricity savings1	Smart Saver has been received and will be evaluated after use	Jingdong prefecture level city in Shandong
Jinfeng Hongyuan Flagship Store	Electricity savings1	Home electricity is too waste, a month more than 300 dollars of electricity, and then the family heard that this can save a lot of electricity, let me order to buy a, anyway, will not, buy on the buy, thought it has to be a pretty big box it, delivery to the home to see a very small, plug-and-play, you can really save electricity, it's worth it!	Jingdong prefecture level city in Shandong
Jinfeng Hongyuan Flagship Store	Electricity savings1	Smart Saver received. Unpacking the box is exquisite. The material workmanship is very good. Plugged in and tried the space. Did not open the air conditioning voltage display 206 air conditioning open up the air conditioning display for 194. whether to save electricity to a month later to pay the electricity bill will know. Logistics is also very fast service is also in place.	Jingdong prefecture level city in Shandong
Jinfeng Hongyuan Flagship Store	Electricity savings1	The product feels pretty good and I hope it works, as the store owner said it would. Works!!!	Jingdong prefecture level city in Shandong
Jinfeng Hongyuan Flagship Store	Electricity savings1	Baby received, physical very quickly, just plugged in hope that it can be as everyone said those power saving, with two months after additional comments, first give a five-star praise.	Jingdong prefecture level city in Shandong
Jinfeng Hongyuan Flagship Store	Electricity savings1	Good, very good, very good, you deserve it, whoever uses it knows, the stuff works great, will buy it again in the future.	Jingdong prefecture level city in Shandong
Jinfeng Hongyuan Flagship Store	Electricity savings1	Don't hesitate to buy wow! No cheap feeling, very high-end, especially great! It is genuine, and the same as the entity store, the price is also favorable, decisive turn powder 💗 catch up to do activities when you buy, the price of a penny for a penny ♡, from the outside looking very beautiful 💗, the material workmanship is good 💗, feel that the purchase is worth it, and the logistics is super	Jingdong prefecture level city in Shandong
Jinfeng Hongyuan Flagship Store	Electricity savings1	Logistics is fast! Very good quality! Very beautiful! Next time to patronize!	Jingdong prefecture level city in Shandong
Jinfeng Hongyuan Flagship Store	Electricity savings1	Really like it, completely exceeded expectations, the shipping speed is very fast, the packaging is very careful and strict, the delivery speed is very fast, very satisfied with a shopping Baby received, and the description is the same, love it!	Jingdong prefecture level city in Shandong
Jinfeng Hongyuan Flagship Store	Electricity savings1	Very good, if you need to order!	Jingdong prefecture level city in Shandong
Jinfeng Hongyuan Flagship Store	Electricity savings1	So far, plugged in the voltage is more stable, as for the effect of how to quietly wait until the payment of the electricity bill to compare, the appearance of the commodity design is very good, the real thing!	Jingdong prefecture level city in Shandong
Jinfeng Hongyuan Flagship Store	Electricity savings1	💌 I've been wanting it for a long time, I decided to go for it, I feel great after using it, it's genuine and the delivery is great, it lives up to its name👍	Jingdong prefecture level city in Shandong
Jinfeng Hongyuan Flagship Store	Electricity savings1	Looking forward to it!!! I don't know if it's working or not, let's look forward to it!!!! How much is saved ，，，，，，，，，， Saving a degree is a degree with long term use ！！！！！！！	Jingdong prefecture level city in Shandong
Jinfeng Hongyuan Flagship Store	Electricity savings1	After using it for a while, I am seriously and responsibly saying that this saver is really very power efficient. Two rooms with the air conditioner on at night for one night, plus watching TV, charging cell phones, lights, etc. etc. only used one degree of electricity. So I bought several more and it's really	Jingdong prefecture level city in Shandong
Jinfeng Hongyuan Flagship Store	Electricity savings1	It arrived quickly, and the box is solid and delicate. Baby shell is thick aluminum and by multi-layer coating. The inner core is heavy, and the power cord and plug are genuine products from a large manufacturer. It is the best power saver I have found after choosing all of them!	Jingdong prefecture level city in Shandong
Jinfeng Hongyuan Flagship Store	Electricity savings1	The saver was received, genuine guarantee, good quality...	Jingdong prefecture level city in Shandong
Jinfeng Hongyuan Flagship Store	Electricity savings1	Logistics was fast! The product is easy to use!	Jingdong prefecture level city in Shandong
Jinfeng Hongyuan Flagship Store	Electricity savings1	Look appearance is okay, use a period of time to know how it is. Jingdong Mall, trustworthy! Home appliances, cell phones and computers are almost 100% bought by Jingdong. Ordered the night before yesterday, received yesterday. Jingdong logistics, the fastest in the network!	Jingdong prefecture level city in Shandong
Jinfeng Hongyuan Flagship Store	Electricity savings1	Very good, perfect packaging, home energy saver to determine the effect of power saving and also play a role in stabilizing the voltage Very good products	Jingdong prefecture level city in Shandong
Jinfeng Hongyuan Flagship Store	Electricity savings1	Smart Saver received, looks very exquisite. The method of use is simple, and it has been used. The later effect, with time to test.	Jingdong prefecture level city in Shandong
Jinfeng Hongyuan Flagship Store	Electricity savings1	Both the material and workmanship exceeded expectations, great value, and looks great on oh. The seller also shipped much earlier than expected, will come back. Buy it, you will not regret it! Full five points. Both the material and workmanship exceeded expectations, great value, wear it also looks good Oh. Seller delivery is also much earlier than expected, will come again. Buy it, will not regret it! Full	Jingdong prefecture level city in Shandong
Jinfeng Hongyuan Flagship Store	Electricity savings1	The appearance is clean and beautiful, the workmanship is very good, I didn't find any defects, I personally feel that the size is also quite suitable, I've used it for 2 months, I didn't find out exactly how energy efficient it is. Try using it again!	Jingdong prefecture level city in Shandong
Jinfeng Hongyuan Flagship Store	Electricity savings1	Power saving device received, product certification, accessories, etc. complete, very good workmanship, very practical power-saving effect is also very obvious.	Jingdong prefecture level city in Shandong
Jinfeng Hongyuan Flagship Store	Electricity savings1	Looked at a lot of think buy this one, see a lot of reviews say it works, a month with save nearly twenty degrees of electricity, there are sixty days of return guarantee, with a good and then come back to the	Jingdong prefecture level city in Shandong
Jinfeng Hongyuan Flagship Store	Electricity savings1	I received the power saving device, and I'm using it. I hope I can get the desired effect, and I'm trying to force it to work quickly.	Jingdong prefecture level city in Shandong
Jinfeng Hongyuan Flagship Store	Electricity savings1	Voltage is stable, already trying it out and waiting for the results to happen, saving electricity will follow up next month!	Jingdong prefecture level city in Shandong
Jinfeng Hongyuan Flagship Store	Electricity savings1	Jingdong Mall self-supporting goods of excellent quality, logistics is also very fast, worth recommending 👏👏👏👏 excellent!	Jingdong prefecture level city in Shandong
Jinfeng Hongyuan Flagship Store	Electricity savings1	Power saving has been received, the appearance of the beautiful, I have already been the second time to buy the use of down the effect is very good!	Jingdong prefecture level city in Shandong
Jinfeng Hongyuan Flagship Store	Electricity savings1	Energy saver received very happy, the color is also very good looking forward to a very good effect can save electricity	Jingdong prefecture level city in Shandong
Jinfeng Hongyuan Flagship Store	Electricity savings1	It's small and easy to plug in and use. It displays the voltage value when plugged in and	Jingdong prefecture level city in Shandong
Jinfeng Hongyuan Flagship Store	Electricity savings1	This saver really works well, because I installed one at home about seven or eight years ago, when there is no voltage display, now there is a voltage display, the appearance also looks much better, the electricity bill is also saved, the stability of the home appliances is also good, the best of both	Jingdong prefecture level city in Shandong
Jinfeng Hongyuan Flagship Store	Electricity savings1	Ordered yesterday, arrived today in use. Shipping was fast. The effect is looking forward to.	Jingdong prefecture level city in Shandong
Jinfeng Hongyuan Flagship Store	Electricity savings1	This thing, I don't know what the principle is. Why it can save electricity. Wait for a month to use it first, see if it will save electricity bill, then come back to evaluate. There is one thing that does not fit the principle ah. The law of conservation of energy.	Jingdong prefecture level city in Shandong
Jinfeng Hongyuan Flagship Store	Electricity savings1	Home mom listen to others say this can save electricity, specially let buy a, later a very large, go back to see a particularly small, directly plugged into the electricity can be, no need to install, the price is also very cheap, the first time with this product, do not know if it is not good to use, temporarily try for a period of time, if it is good to use, recommend that you buy this one, the	Jingdong prefecture level city in Shandong

Jinfeng Hongyuan Flagship Store	Electricity savings1	I've been using it for a few days, and I think the workmanship and materials are pretty good, so we'll have to look at the next bill for the real energy savings, and then we'll add more comments.	Jingdong prefecture level city in Shandong
Jinfeng Hongyuan Flagship Store	Electricity savings1	Energy saver is well made, energy saving and power saving, and anti-counterfeiting code, it is a regular commodity!	Jingdong prefecture level city in Shandong
Jinfeng Hongyuan Flagship Store	Electricity savings1	Jinfeng Hongyuan, buy back home, as a mouse, haha. First of all, normal electricity for 1 month, the power did not increase, the electricity bill feels smoother, year-on-year electricity costs in the low, whether it is very energy-saving still have to be used for a long time to know. All 5 stars ☆	Jingdong prefecture level city in Shandong
Jinfeng Hongyuan Flagship Store	Electricity savings1	The box is well packaged and beautiful! I hope to save electricity with it haha!	Jingdong prefecture level city in Shandong
Jinfeng Hongyuan Flagship Store	Electricity savings1	Ordered last night, arrived this morning, first try the effect before making comments	Jingdong prefecture level city in Shandong
Jinfeng Hongyuan Flagship Store	Electricity savings1	Looks good. Don't know the effect yet.	Jingdong prefecture level city in Shandong
Jinfeng Hongyuan Flagship Store	Electricity savings1	Jinfeng Hongyuan intelligent power saver has exquisite workmanship and modern fashionable design style. It is a good home economizer . I believe that the goods purchased from Jingdong will not disappoint our	Jingdong prefecture level city in Shandong
Jinfeng Hongyuan Flagship Store	Electricity savings1	Inadvertently flipped to this power saver, never heard of, feel very novel, the price is not expensive, so buy to try, see a month after the electricity bill will be less, recently the home air-conditioning open a lot of power consumption is very large.	Jingdong prefecture level city in Shandong
Jinfeng Hongyuan Flagship Store	Electricity savings1	The quality of the goods is very good workmanship, but also sent five fuse	Jingdong prefecture level city in Shandong
Jinfeng Hongyuan Flagship Store	Electricity savings1	I'm trying it out. I don't know if it'll work.	Jingdong prefecture level city in Shandong
Jinfeng Hongyuan Flagship Store	Electricity savings1	There is no effect yet do not know, is to look at the official sale, buy to try it! Use according to the instructions, plugged in, used for a period of time, anyway, do not know how, after a few months to see it, to see if the average month than before will be less electricity.	Jingdong prefecture level city in Shandong
Jinfeng Hongyuan Flagship Store	Electricity savings1	Received the goods, the first time to unpacking, the quality is very good, consistent with the description of the seller, completely exceeded my expectations, the packaging is very careful, strict, in general this time is very satisfied with a shopping, thanks to the seller.	Jingdong prefecture level city in Shandong
Jinfeng Hongyuan Flagship Store	Electricity savings1	The energy saver was received and used for a few days with good results.	Jingdong prefecture level city in Shandong
Jinfeng Hongyuan Flagship Store	Electricity savings1	Previously I have paid attention to this product, but I think that the monthly electricity consumption at home is not large, there is no need to save electricity savings. After a second thought, energy saving is the responsibility of each of our citizens, the winter is coming, this year's winter is cold, heating and all electric appliances, can save is to save, the price is not expensive, so buy to try. I analyze the principle of power saving, should be able to save electricity, but the larger the power saving effect is about good. To be tried after a month or two to see the effect and then evaluate. Jingdong trustworthy.	Jingdong prefecture level city in Shandong
Jinfeng Hongyuan Flagship Store	Electricity savings1	It just arrived and I can't wait to use it, and the customer service is great! There is also voltage showing up which is nice! I don't know if it can save a bit I won't know until I've used it for a month! When the time comes to come back to the follow-up review!	Jingdong prefecture level city in Shandong
Jinfeng Hongyuan Flagship Store	Electricity savings1	Checked out the reviews from numerous users. The shipment is still very large, so I decided to buy it anyway to use it and see the effect after a month. Jingdong Mall here to buy safe, convenient and fast, this price is very powerful 💪, very recommended.	Jingdong prefecture level city in Shandong
Jinfeng Hongyuan Flagship Store	Electricity savings1	Purchased two at the same time to use together. The other one works fine, the other has problems with the numbers not sure if the machine is faulty. Power saving effect can not be observed. Feel the effect is not very obvious like?	Jingdong prefecture level city in Shandong
Jinfeng Hongyuan Flagship Store	Electricity savings1	Looking forward to it!!! I don't know if it's working or not, let's look forward to it!!!! How much is saved , , , , , , , , , Saving a degree is a degree with long term use ! ! ! ! ! ! !	Jingdong prefecture level city in Shandong
Jinfeng Hongyuan Flagship Store	Electricity savings1	Received, directly on the use of the special convenient	Jingdong prefecture level city in Shandong
Jinfeng Hongyuan Flagship Store	Electricity savings1	Goods received a few days well-packed, the same day opened and plugged in to try to be normal is not sure what the power-saving effect. After a month with a follow-up evaluation.	Jingdong prefecture level city in Shandong
Jinfeng Hongyuan Flagship Store	Electricity savings1	I bought it and used it, I don't know how it works yet, we'll see later. Use the effect of it	Jingdong prefecture level city in Shandong
Jinfeng Hongyuan Flagship Store	Electricity savings1	Photographed 2, the old family also put a, with a small January, there should be some effect, with a long period of time to comment, with a long time to see the obvious effect, hard courier brother.	Jingdong prefecture level city in Shandong
Jinfeng Hongyuan Flagship Store	Electricity savings1	Experience: I am an electrician, this is the real use of electronic waves automatically connected to track the decomposition of ionized electrons, in situ compensation of reactive power, reduce effective power loss and waste, stabilize the voltage to achieve energy saving and power saving, the overall effect	Jingdong prefecture level city in Shandong
Jinfeng Hongyuan Flagship Store	Electricity savings1	Great looking and practical! Easy to use and save! Saves 15% in a month of comparison	Jingdong prefecture level city in Shandong
Jinfeng Hongyuan Flagship Store	Electricity savings1	Product quality is very good! Packaging is very nice, like a cell phone box, thick and well protected! Service is also great! Logistics is very fast! Thank you for your business! Very honest!	Jingdong prefecture level city in Shandong
Jinfeng Hongyuan Flagship Store	Electricity savings1	In several products inside the choice of this purchase for a period of time, but also need to use a few months to compare last year's electricity bill before you can analyze the savings in electricity, to the summer peak electricity consumption should be more obvious.	Jingdong prefecture level city in Shandong
Jinfeng Hongyuan Flagship Store	Electricity savings1	The goods have long been received, after more than a month to comment, the home electricity bill is indeed reduced, worth buying!	Jingdong prefecture level city in Shandong
Jinfeng Hongyuan Flagship Store	Electricity savings1	Baby received, the logistics is strong, the quality is reliable, it is worth having and recommending!	Jingdong prefecture level city in Shandong
Jinfeng Hongyuan Flagship Store	Electricity savings1	Using a conservation appliance, you can achieve a 10-15% savings in electricity usage.	Jingdong prefecture level city in Shandong
Jinfeng Hongyuan Flagship Store	Electricity savings1	Quality is very good, metal shell, touch is very good, very recommended Oh ~, the subsequent effect of continuous follow-up	Jingdong prefecture level city in Shandong
Jinfeng Hongyuan Flagship Store	Electricity savings1	The product is beautiful, practical, worth buying. The product is beautiful, practical, good quality, fast logistics product is beautiful, practical, beautiful and elegant, very classy, worth buying!	Jingdong prefecture level city in Shandong
Jinfeng Hongyuan Flagship Store	Electricity savings1	Just received the item and have already used it. Didn't know much about these products before. I hope it is as effective and useful as the product advertises.	Jingdong prefecture level city in Shandong
Jinfeng Hongyuan Flagship Store	Electricity savings1	A month two people used more than five hundred degrees of electricity, in a hurry and then add a power saver, I hope to have an effect, Jingdong shopping is very cool, logistics is very fast especially good service attitude delivery, this hot day is not easy, thank you!	Jingdong prefecture level city in Shandong
Jinfeng Hongyuan Flagship Store	Electricity savings1	Just received the baby, first plugged in to try to use a month and then additional evaluation it	Jingdong prefecture level city in Shandong
Jinfeng Hongyuan Flagship Store	Electricity savings1	Goods fast, the power saver has been received, a friend recommended to buy that can save electricity. Exquisite and compact is very convenient, plug and play. The effect is good, the price is affordable, the next need will patronize again!	Jingdong prefecture level city in Shandong
Jinfeng Hongyuan Flagship Store	Electricity savings1	Packaging is better than expected, sent a few fuse, power saving effect in the end do not know, try a period of time to say!	Jingdong prefecture level city in Shandong
Jinfeng Hongyuan Flagship Store	Electricity savings1	Shipped quickly, immediately installed, read countless reviews were not sure if this thing is effective, was going to buy three at once, ultimately decided to first use a try the effect, if the power ring significantly reduced, then will buy a few more!	Jingdong prefecture level city in Shandong
Jinfeng Hongyuan Flagship Store	Electricity savings1	Used for a period of time to evaluate, not to a month still do not know whether it can save electricity, the voltage has a display!	Jingdong prefecture level city in Shandong
Jinfeng Hongyuan Flagship Store	Electricity savings1	I don't know if it saves electricity or not, but I'm using it anyway, so I hope it saves a little bit, electricity is too expensive	Jingdong prefecture level city in Shandong
Jinfeng Hongyuan Flagship Store	Electricity savings1	The power saver has been received and I am very happy with it! Just waiting to see how the power saving works!	Jingdong prefecture level city in Shandong
Jinfeng Hongyuan Flagship Store	Electricity savings1	Saving power has been received, logistics is quite fast, the quality is not bad, small and a little weight, has been used to look forward to the effect of	Jingdong prefecture level city in Shandong
Jinfeng Hongyuan Flagship Store	Electricity savings1	The logistics is very fast, the effect is obvious and trustworthy.	Jingdong prefecture level city in Shandong
Jinfeng Hongyuan Flagship Store	Electricity savings1	Good quality, good quality, beautiful, beautiful, beautiful, beautiful, beautiful, very worth buying, worth buying, worth buying, worth buying, worth customizing!	Jingdong prefecture level city in Shandong

Jinfeng Hongyuan Flagship Store	Electricity savings1	I've used it for now, I don't know if it saves electricity	Jingdong prefecture level city in Shandong
Jinfeng Hongyuan Flagship Store	Electricity savings1	Bought two at a time, logistics is very fast, the product is beautifully packaged, after two months to see the results	Jingdong prefecture level city in Shandong
Jinfeng Hongyuan Flagship Store	Electricity savings1	The workmanship is really fine, the packaging is also good, the regular factory production ~ use a break in the evaluation of the effectiveness of the	Jingdong prefecture level city in Shandong
Jinfeng Hongyuan Flagship Store	Electricity savings1	Sometimes 219. Sometimes 220 in this case	Jingdong prefecture level city in Shandong
Jinfeng Hongyuan Flagship Store	Electricity savings1	It is the ideal product, good quality, logistics is also very fast, worthy of praise!	Jingdong prefecture level city in Shandong
Jinfeng Hongyuan Flagship Store	Electricity savings1	Store service is very good, the courier received the next day. Product packaging is very good, just use the quality to be tested.	Jingdong prefecture level city in Shandong
Jinfeng Hongyuan Flagship Store	Electricity savings1	I've been using it for almost a month now and I feel like it's working.	Jingdong prefecture level city in Shandong
Jinfeng Hongyuan Flagship Store	Electricity savings1	Very satisfied, very restful, very pretty, very beautiful, very much enjoyed! Well worth it!	Jingdong prefecture level city in Shandong
Jinfeng Hongyuan Flagship Store	Electricity savings1	Baby especially like, the value of high-quality good, a very good shopping experience, attentive service, fast logistics, distribution is also very thoughtful, attitude is also very good, value for money ah, decisive praise! Quality is quite good, logistics is also very fast, praise!	Jingdong prefecture level city in Shandong
Jinfeng Hongyuan Flagship Store	Electricity savings1	The shape of the item is basically the same as ordered, the effect is to be examined.	Jingdong prefecture level city in Shandong
Jinfeng Hongyuan Flagship Store	Electricity savings1	Appearance style: good-looking workmanship material: very good, a small one, very delicate Experience: still in use, in the process of using the shopkeeper is very nice to have a careful explanation Use for a period of time in the look at the results	Jingdong prefecture level city in Shandong
Jinfeng Hongyuan Flagship Store	Electricity savings1	As described, compact and well packaged	Jingdong prefecture level city in Shandong
Jinfeng Hongyuan Flagship Store	Electricity savings1	Fine workmanship. Test in progress. Let's see how it works.	Jingdong prefecture level city in Shandong
Jinfeng Hongyuan Flagship Store	Electricity savings1	The goods have been received, I'm very satisfied and love it	Jingdong prefecture level city in Shandong
Jinfeng Hongyuan Flagship Store	Electricity savings1	Safety! Whether you can save electricity, you can roughly feel it in 2 months. The principle is simple. Before the electrical appliances and circuits inductive more, now the electrical appliances integrated, the home is not a lot of inductive circuits, but used certainly save no problem! Wiring according to the requirements of the manual is good, if it is not convenient, see the socket plug.	Jingdong prefecture level city in Shandong
Jinfeng Hongyuan Flagship Store	Electricity savings1	Great product, looking forward to how much power I can save, first time buyer!	Jingdong prefecture level city in Shandong
Jinfeng Hongyuan Flagship Store	Electricity savings1	The product is exquisite, beautifully packaged, and the customer service is excellent. Just waiting to see how the power saving effect is, looking forward to it!	Jingdong prefecture level city in Shandong
Jinfeng Hongyuan Flagship Store	Electricity savings1	Express very quickly very satisfied with the first use to see how the effect to know, if you can save 10 percent even if acceptable, if you can not meet this requirement is obviously not much effect. Obviously.	Jingdong prefecture level city in Shandong
Jinfeng Hongyuan Flagship Store	Electricity savings1	Very delicate and compact, there is a very convenient intelligent display prompts the voltage, you can know the voltage at a glance, there is very easy to use, a plug can be used, I hope to save money on	Jingdong prefecture level city in Shandong
Jinfeng Hongyuan Flagship Store	Electricity savings1	First time buyer is it really save after use	Jingdong prefecture level city in Shandong
Jinfeng Hongyuan Flagship Store	Electricity savings1	Purchased again and will be for a long time yet.	Jingdong prefecture level city in Shandong
Jinfeng Hongyuan Flagship Store	Electricity savings1	Bought it to stock up, came back and plugged it in, hope it works	Jingdong prefecture level city in Shandong
Jinfeng Hongyuan Flagship Store	Electricity savings1	Baby received the logistics very fast very like very satisfied praise a	Jingdong prefecture level city in Shandong
Jinfeng Hongyuan Flagship Store	Electricity savings1	Beginning the process of using, easy to evaluate more later.	Jingdong prefecture level city in Shandong
Jinfeng Hongyuan Flagship Store	Electricity savings1	The goods have been received, just do not know how effective	Jingdong prefecture level city in Shandong
Jinfeng Hongyuan Flagship Store	Electricity savings1	It feels okay. I wish there were more events.	Jingdong prefecture level city in Shandong
Jinfeng Hongyuan Flagship Store	Electricity savings1	Things have been received, just plugged in, I do not know if it works, wait a few days to have the effect of additional	Jingdong prefecture level city in Shandong
Jinfeng Hongyuan Flagship Store	Electricity savings1	The effect of this power saver is really good, the house more, a few air conditioners are open every day, not used before a month to say less than seven hundred eight hundred, used after three months only nine	Jingdong prefecture level city in Shandong
Jinfeng Hongyuan Flagship Store	Electricity savings1	Cheap, good and easy to use, recommend it.	Jingdong prefecture level city in Shandong
Jinfeng Hongyuan Flagship Store	Electricity savings1	Bought this device, to the home for a long time, wait to finish a month to see how, good can recommend friends to buy, we use the electricity every month scary, a month of electricity costs more than a thousand, can not afford to hurt ah! I hope this power saving can save me some money, the beginning of the shoot order did not pay attention to look at the address, sent elsewhere to go, that side called to find out, hurry to communicate with customer service, customer service is very careful to help me get it done, call to that side to help me turn around, thank you!	Jingdong prefecture level city in Shandong
Jinfeng Hongyuan Flagship Store	Electricity savings1	I don't know the effect if it, use it first to see.	Jingdong prefecture level city in Shandong
Jinfeng Hongyuan Flagship Store	Electricity savings1	Looks good. I won't know if it saves power until I use it.	Jingdong prefecture level city in Shandong
Jinfeng Hongyuan Flagship Store	Electricity savings1	Goods received, shipping speed is very fast, yesterday afternoon more than two o'clock shot, just express delivery, but also sent a few insurance, very good business, the display of the number do not understand what is the meaning of the display said voltage, do not understand this, try a month to see how, good and	Jingdong prefecture level city in Shandong
Jinfeng Hongyuan Flagship Store	Electricity savings1	What ** play also, save electricity more and more save more and more, save chicken hair electricity, are cheating, this is a night of air conditioning with electricity, a night with 11 electricity word, this is	Jingdong prefecture level city in Shandong
Jinfeng Hongyuan Flagship Store	Electricity savings1	Received the product, hope it works!	Jingdong prefecture level city in Shandong
Jinfeng Hongyuan Flagship Store	Electricity savings1	Bought home and used very convenient, easy to operate, quite good	Jingdong prefecture level city in Shandong
Jinfeng Hongyuan Flagship Store	Electricity savings1	It's really good, cost-effective and good quality, and the logistics are fast! It arrived in one day! Really good, cost-effective and good quality, logistics is fast ah! It arrived in one day!	Jingdong prefecture level city in Shandong
Jinfeng Hongyuan Flagship Store	Electricity savings1	Works well, stabilizes voltage and saves power	Jingdong prefecture level city in Shandong
Jinfeng Hongyuan Flagship Store	Electricity savings1	I'll use it right away and look forward to good results	Jingdong prefecture level city in Shandong
Jinfeng Hongyuan Flagship Store	Electricity savings1	Recommended by a friend, said it works well, so I bought one, the effect to be tested	Jingdong prefecture level city in Shandong
Jinfeng Hongyuan Flagship Store	Electricity savings1	I didn't know it would save electricity.	Jingdong prefecture level city in Shandong
Jinfeng Hongyuan Flagship Store	Electricity savings1	I've been using it for a week, I don't know what's going on yet I hope it works well and saves power I'll follow up after a month of use	Jingdong prefecture level city in Shandong
Jinfeng Hongyuan Flagship Store	Electricity savings1	Very well packaged, looks great, don't know how it works	Jingdong prefecture level city in Shandong
Jinfeng Hongyuan Flagship Store	Electricity savings1	Genuine, good quality, really saves energy.	Jingdong prefecture level city in Shandong
Jinfeng Hongyuan Flagship Store	Electricity savings1	I was worried that the stuff is not good, but when I got it, I realized that I was completely over-anxious, 5 stars!	Jingdong prefecture level city in Shandong
Jinfeng Hongyuan Flagship Store	Electricity savings1	Very good and satisfied trustworthy	Jingdong prefecture level city in Shandong
Jinfeng Hongyuan Flagship Store	Electricity savings1	I don't know if it saves power	Jingdong prefecture level city in Shandong

Jinfeng Hongyuan Flagship Store	Electricity savings1	The quality of the product is very good, beautiful and generous applicable fast delivery speed good service attitude	Jingdong prefecture level city in Shandong
Jinfeng Hongyuan Flagship Store	Electricity savings1	Let's use it for a while and then we'll follow up	Jingdong prefecture level city in Shandong
Jinfeng Hongyuan Flagship Store	Electricity savings1	I've only been using it for a month, I don't know if there's any change yet, I'll come back with a follow-up comment after checking the meter!	Jingdong prefecture level city in Shandong
Jinfeng Hongyuan Flagship Store	Electricity savings1	I don't know if it works, I read the instructions and online reviews, I hope I won't be disappointed, if I use it, I will buy it again. Jingdong Express, fast and guaranteed service.	Jingdong prefecture level city in Shandong
Jinfeng Hongyuan Flagship Store	Electricity savings1	It feels like it saves quite a bit huh?	Jingdong prefecture level city in Shandong
Jinfeng Hongyuan Flagship Store	Electricity savings1	I've been using it for a while now, and it still seems to work and have a power saving effect	Jingdong prefecture level city in Shandong
Jinfeng Hongyuan Flagship Store	Electricity savings1	Looks good. It says 235. The 222 bar voltage is shown as high. I don't know if that's right. We'll see.	Jingdong prefecture level city in Shandong
Jinfeng Hongyuan Flagship Store	Electricity savings1	Baby is really good, do really careful, worth to have, fast delivery!	Jingdong prefecture level city in Shandong
Jinfeng Hongyuan Flagship Store	Electricity savings1	Quite delicate and compact, simple and convenient to use. Things are still very good, bought two at a time. Very satisfied with an online purchase.	Jingdong prefecture level city in Shandong
Jinfeng Hongyuan Flagship Store	Electricity savings1	Feel very good, the use of unclear, ask customer service, timely explanation, big brand, trustworthy	Jingdong prefecture level city in Shandong
Jinfeng Hongyuan Flagship Store	Electricity savings1	Thrifty received a few days did not make a comment is to see how the quality of the product now feel the quality of good appearance is also powerful seller service is very good to buy the product is also attached to the 5 fuse thought very thoughtful!	Jingdong prefecture level city in Shandong
Jinfeng Hongyuan Flagship Store	Electricity savings1	Big brand, with a very assured, the quality is very good, in time for the event to buy very cost-effective, customer service is really very good Oh ~!	Jingdong prefecture level city in Shandong
Jinfeng Hongyuan Flagship Store	Electricity savings1	I ordered yesterday and received it this morning, I heard my friend say that it can save electricity, I don't know if it works, I'll come back to make a comment after using it.	Jingdong prefecture level city in Shandong
Jinfeng Hongyuan Flagship Store	Electricity savings1	I've been using it for over a month and it doesn't seem to be working!	Jingdong prefecture level city in Shandong
Jinfeng Hongyuan Flagship Store	Electricity savings1	Used it, the family has always been in the habit of using energy-saving devices, this is a new one. Digital display voltage is very good, can see that the voltage is more stable.	Jingdong prefecture level city in Shandong
Jinfeng Hongyuan Flagship Store	Electricity savings1	I haven't used it for more than a month, but it looks good. It's easy to plug into an outlet and it works.	Jingdong prefecture level city in Shandong
Jinfeng Hongyuan Flagship Store	Electricity savings1	Good Oh...appearance is very beautiful...just plugged in with...see the effect of it...with a period of time in the additional evaluation...	Jingdong prefecture level city in Shandong
Jinfeng Hongyuan Flagship Store	Electricity savings1	I've received the goods, whether it's good or not, I'll know after I use it.	Jingdong prefecture level city in Shandong
Jinfeng Hongyuan Flagship Store	Electricity savings1	Very good, logistics is fast, the stuff is very powerful and cost-effective! Can feel the meter slowing down	Jingdong prefecture level city in Shandong
Jinfeng Hongyuan Flagship Store	Electricity savings1	Logistics is very fast, get it immediately after use, easy to operate, plug in the power supply can be used, just do not know to the use of the effect of how.	Jingdong prefecture level city in Shandong
Jinfeng Hongyuan Flagship Store	Electricity savings1	Quality is very over the top, quality is guaranteed, quite a good choice, a lot of friends to see me use all want to buy, I recommend it!	Jingdong prefecture level city in Shandong
Jinfeng Hongyuan Flagship Store	Electricity savings1	The appearance is clean and beautiful, the workmanship is very good, did not find any flaws, I personally feel that the size is also quite suitable, looking for a long time to choose this one, big love! The price is very beautiful, things are still good, will continue to buy back! All five points! Ness Ness ~ logistics speed is really no words, is a word: fast. Commodities from the outer packaging is very high-grade, really a penny a penny, expensive has the reason for expensive	Jingdong prefecture level city in Shandong
Jinfeng Hongyuan Flagship Store	Electricity savings1	The logistics is very fast, the texture is very good, genuine, I like it very much.	Jingdong prefecture level city in Shandong
Jinfeng Hongyuan Flagship Store	Electricity savings1	It's been a few days since I put it on, I can't see any results now, waiting for a month to say, steady steady steady.	Jingdong prefecture level city in Shandong
Jinfeng Hongyuan Flagship Store	Electricity savings1	I've been using it for a while now and it should be fine, so I'm buying it for the second time.	Jingdong prefecture level city in Shandong
Jinfeng Hongyuan Flagship Store	Electricity savings1	Power Saving King has been received, looks good, quite delicate, looking forward to using it to save electricity	Jingdong prefecture level city in Shandong
Jinfeng Hongyuan Flagship Store	Electricity savings1	My mom said the home uses a little too much electricity, so I bought a Power Saver to try it out, I don't know how effective it is. Expect it to be a good thing!	Jingdong prefecture level city in Shandong
Jinfeng Hongyuan Flagship Store	Electricity savings1	Thank you Jingdong, logistics is very fast order to receipt only one day. Believe in Jingdong. Favorable comments	Jingdong prefecture level city in Shandong
Jinfeng Hongyuan Flagship Store	Electricity savings1	Saved some money on the electric bill compared to last month, watch this month again, no problems so far	Jingdong prefecture level city in Shandong
Jinfeng Hongyuan Flagship Store	Electricity savings1	Energy saver exquisite workmanship, beautiful style, more features, just bought, power saving effect to be used for a period of time, then evaluate!	Jingdong prefecture level city in Shandong
Jinfeng Hongyuan Flagship Store	Electricity savings1	This thing does save power is more compact to hang on the wall.	Jingdong prefecture level city in Shandong
Jinfeng Hongyuan Flagship Store	Electricity savings1	I've been buying it for a while now, I haven't actually measured it, but it still feels like it's effective	Jingdong prefecture level city in Shandong
Jinfeng Hongyuan Flagship Store	Electricity savings1	I've already used it since I bought it, I don't know how effective it is yet, I'll comment on it later.	Jingdong prefecture level city in Shandong
Jinfeng Hongyuan Flagship Store	Electricity savings1	Baby received, careful appearance, logistics is very fast, worth buying!	Jingdong prefecture level city in Shandong
Jinfeng Hongyuan Flagship Store	Electricity savings1	It's great, it's just not the same in hand, I bought it at that other cheaper online one for about the same price as this one. But then oops, doesn't work at all.	Jingdong prefecture level city in Shandong
Jinfeng Hongyuan Flagship Store	Electricity savings1	The product is exquisite, but you have to use it for a month to know how it works. I'll comment again after a month.	Jingdong prefecture level city in Shandong
Jinfeng Hongyuan Flagship Store	Electricity savings1	I've been using it for a while now, and it doesn't feel like it's working, and it doesn't save any power.	Jingdong prefecture level city in Shandong
Jinfeng Hongyuan Flagship Store	Electricity savings1	Affordable price, fast delivery, Jingdong self-supporting guarantee	Jingdong prefecture level city in Shandong
Jinfeng Hongyuan Flagship Store	Electricity savings1	Packaging is very good, no damage, good quality, fine workmanship Packaging is very good, no damage, good quality, fine workmanship Packaging is very good, no damage, good quality, fine workmanship Packaging is very good, no damage, good quality, fine workmanship	Jingdong prefecture level city in Shandong
Jinfeng Hongyuan Flagship Store	Electricity savings1	Big brand, with a very assured, the quality is very good, in time for the event to buy very cost-effective, customer service is really very good Oh ~!	Jingdong prefecture level city in Shandong
Jinfeng Hongyuan Flagship Store	Electricity savings1	Used a few days, the specific effect is not yet clear, looking forward to the effect as described by the merchant!	Jingdong prefecture level city in Shandong
Jinfeng Hongyuan Flagship Store	Electricity savings1	I just used it and don't know how it works, I'll follow up after using it!	Jingdong prefecture level city in Shandong
Jinfeng Hongyuan Flagship Store	Electricity savings1	Well, it's been installed and now I'm waiting to see the results, BOE shopping, hopefully I won't be disappointed!	Jingdong prefecture level city in Shandong
Jinfeng Hongyuan Flagship Store	Electricity savings1	Goods received, plugged in the voltage from 229 to 222, do not know whether to save electricity, that depends on the situation.	Jingdong prefecture level city in Shandong
Jinfeng Hongyuan Flagship Store	Electricity savings1	This is the second time I have bought the product and I feel it really saves electricity! Use it again for a while and then evaluate!	Jingdong prefecture level city in Shandong
Jinfeng Hongyuan Flagship Store	Electricity savings1	Overall, it's really good, and the customer service is great, you'll get a message back almost immediately when you speak, it's great value for money, I really recommend it!	Jingdong prefecture level city in Shandong
Jinfeng Hongyuan Flagship Store	Electricity savings1	✨ Have purchased many times, a must have for the family, affordable purchase price, fast delivery, 🎁 beautifully packaged!	Jingdong prefecture level city in Shandong
Jinfeng Hongyuan Flagship Store	Electricity savings1	I bought it after a friend introduced it, he said it is effective and can save electricity. I bought it to try, try a period of time and then follow up!	Jingdong prefecture level city in Shandong
Jinfeng Hongyuan Flagship Store	Electricity savings1	Received it, trying it out tonight, and bought one earlier.	Jingdong prefecture level city in Shandong

Jinfeng Hongyuan Flagship Store	Electricity savings1	It will take a month or two to see if it saves power or not.	Jingdong prefecture level city in Shandong
Jinfeng Hongyuan Flagship Store	Electricity savings1	Looked good in the instructions! Bought one and pressed it on! Use it and see! If it works! I'll buy some more!	Jingdong prefecture level city in Shandong
Jinfeng Hongyuan Flagship Store	Electricity savings1	Goods have been received packaging can also things are not bad appearance, with a use to know, now evaluation is nonsense.	Jingdong prefecture level city in Shandong
Jinfeng Hongyuan Flagship Store	Electricity savings1	Logistics is very fast, packaging is very good, the second unit, as always, looking forward to the effect of	Jingdong prefecture level city in Shandong
Jinfeng Hongyuan Flagship Store	Electricity savings1	I received the goods today, the quality of goods is very good, I am very satisfied ah.	Jingdong prefecture level city in Shandong
Jinfeng Hongyuan Flagship Store	Electricity savings1	Buy one and try it, I wonder if it has a power saving effect?	Jingdong prefecture level city in Shandong
Jinfeng Hongyuan Flagship Store	Electricity savings1	🔍 The item was received, well packaged, logistics is very fast, customer service attitude is very positive, there are questions answered in a timely manner! Overall very satisfied!	Jingdong prefecture level city in Shandong
Jinfeng Hongyuan Flagship Store	Electricity savings1	Baby packaging is very good, purchase submission goods quickly delivered to the home, use and then follow up.	Jingdong prefecture level city in Shandong
Jinfeng Hongyuan Flagship Store	Electricity savings1	My friend asked me to buy an extra one so he could try it too.	Jingdong prefecture level city in Shandong
Jinfeng Hongyuan Flagship Store	Electricity savings1	I've just put it on, I won't know the result until a month later if it will really save power.	Jingdong prefecture level city in Shandong
Jinfeng Hongyuan Flagship Store	Electricity savings1	It's good quality. Hopefully it'll help save power.	Jingdong prefecture level city in Shandong
Jinfeng Hongyuan Flagship Store	Electricity savings1	The appearance is clean and beautiful, the workmanship is very good, I did not find any flaws, I personally feel that the size is also quite suitable, I have been looking for a long time to choose this	Jingdong prefecture level city in Shandong
Jinfeng Hongyuan Flagship Store	Electricity savings1	Logistics is fast! Baby looks great! The price is fair! Wish the store business is prosperous!	Jingdong prefecture level city in Shandong
Jinfeng Hongyuan Flagship Store	Electricity savings1	Jingdong express fast, good, have used, sit and wait to save money...	Jingdong prefecture level city in Shandong
Jinfeng Hongyuan Flagship Store	Electricity savings1	In use, it didn't work as expected and there was nothing noticeable in terms of power savings.	Jingdong prefecture level city in Shandong
Jinfeng Hongyuan Flagship Store	Electricity savings1	Just started using it and looking forward to the results.	Jingdong prefecture level city in Shandong
Jinfeng Hongyuan Flagship Store	Electricity savings1	The quality is very good, the metal shell is very good, is genuine, energy saving and power saving, logistics is very fast, there is a need to buy o, recommend everyone, very satisfied with an online	Jingdong prefecture level city in Shandong
Jinfeng Hongyuan Flagship Store	Electricity savings1	Received, I do not know if the product is good, used to see if it is good to come back to buy!	Jingdong prefecture level city in Shandong
Jinfeng Hongyuan Flagship Store	Electricity savings1	Jinfeng source of intelligent power saving treasure also used less than three months, the last thunder may be burned, the home of the electric send gate is not on, only the old man at home, no way only their own in the field and run back to find out what is the problem with the circuit, the quality is still not very good, thunder is easy to burn!	Jingdong prefecture level city in Shandong
Jinfeng Hongyuan Flagship Store	Electricity savings1	Saving power just started to use, I do not know if there is no effect, but read a lot of reviews are said to be very good so I bought, baby All aspects are good There are spare 2 fuses, late good and then follow	Jingdong prefecture level city in Shandong
Jinfeng Hongyuan Flagship Store	Electricity savings1	Use it for a few days and see, the delivery is quite fast	Jingdong prefecture level city in Shandong
Jinfeng Hongyuan Flagship Store	Electricity savings1	Small, well made and easy to use.	Jingdong prefecture level city in Shandong
Jinfeng Hongyuan Flagship Store	Electricity savings1	Beautifully packaged, it is genuine. Whether it is power saving, use it to evaluate.	Jingdong prefecture level city in Shandong
Jinfeng Hongyuan Flagship Store	Electricity savings1	Power saver. Used two of them and they do save quite a bit of power.	Jingdong prefecture level city in Shandong
Jinfeng Hongyuan Flagship Store	Electricity savings1	Workmanship is not bad, small and does not take up space, is also do not know whether the power saving, buy a to try!	Jingdong prefecture level city in Shandong
Jinfeng Hongyuan Flagship Store	Electricity savings1	The logistics is very fast, the courier attitude is very good, worth buying	Jingdong prefecture level city in Shandong
Jinfeng Hongyuan Flagship Store	Electricity savings1	The effect is not known until it is used.	Jingdong prefecture level city in Shandong
Jinfeng Hongyuan Flagship Store	Electricity savings1	Just arrived plugged in won't know for a month	Jingdong prefecture level city in Shandong
Jinfeng Hongyuan Flagship Store	Electricity savings1	This is really good to use the pull to realize the savings ah think again for my sister's family to buy a	Jingdong prefecture level city in Shandong
Jinfeng Hongyuan Flagship Store	Electricity savings1	Lies, a month with a few hundred electricity are no savings, family use	Jingdong prefecture level city in Shandong
Jinfeng Hongyuan Flagship Store	Electricity savings1	Express very quickly, look at the fine workmanship also sent a few fuse, look forward to can really save electricity	Jingdong prefecture level city in Shandong
Jinfeng Hongyuan Flagship Store	Electricity savings1	I didn't realize it was so small.	Jingdong prefecture level city in Shandong
Jinfeng Hongyuan Flagship Store	Electricity savings1	Logistics courier is very fast, the courier service is very good	Jingdong prefecture level city in Shandong
Jinfeng Hongyuan Flagship Store	Electricity savings1	Used for a period of time the effect is quite good indeed than the previous power saving	Jingdong prefecture level city in Shandong
Jinfeng Hongyuan Flagship Store	Electricity savings1	Logistics is super fast, the product is in use, see the effect...	Jingdong prefecture level city in Shandong
Jinfeng Hongyuan Flagship Store	Electricity savings1	Baby got it, tried it out and it didn't work at all. How can I use the tips to save power?	Jingdong prefecture level city in Shandong
Jinfeng Hongyuan Flagship Store	Electricity savings1	Power Saving King received, immediately used has been very stable without any sound good, good service, fast logistics inexpensive and beautiful worth repurchasing!	Jingdong prefecture level city in Shandong
Jinfeng Hongyuan Flagship Store	Electricity savings1	Logistics courier is very fast, the courier service is good	Jingdong prefecture level city in Shandong
Jinfeng Hongyuan Flagship Store	Electricity savings1	Good to recommend, the first month of air conditioning only 1 week, the electricity bill has reduced about 100 yuan, may be to reduce the use of air conditioning is also related to the other or as usual electricity, estimated that the longer you use the more you can save electricity.	Jingdong prefecture level city in Shandong
Jinfeng Hongyuan Flagship Store	Electricity savings1	It doesn't work. I've been using it for 20 days and it doesn't work.	Jingdong prefecture level city in Shandong
Jinfeng Hongyuan Flagship Store	Electricity savings1	Fast shipping, good quality, affordable price, very satisfied with a shopping.	Jingdong prefecture level city in Shandong
Jinfeng Hongyuan Flagship Store	Electricity savings1	Try it for a month to see if it saves money.	Jingdong prefecture level city in Shandong
Jinfeng Hongyuan Flagship Store	Electricity savings1	It's in use. I'll comment again after a while.	Jingdong prefecture level city in Shandong
Jinfeng Hongyuan Flagship Store	Electricity savings1	Goods have received look good 👍👍👍👍, use a period of time and then look at the	Jingdong prefecture level city in Shandong
Jinfeng Hongyuan Flagship Store	Electricity savings1	No, it doesn't work. It's useless. It's garbage.	Jingdong prefecture level city in Shandong
Jinfeng Hongyuan Flagship Store	Electricity savings1	I've been using it for almost 2 months now and I don't feel any reduction in my electricity bill.	Jingdong prefecture level city in Shandong
Jinfeng Hongyuan Flagship Store	Electricity savings1	I've received the goods, the logistics is really fast, ordered yesterday, arrived today.	Jingdong prefecture level city in Shandong
Jinfeng Hongyuan Flagship Store	Electricity savings1	And the backup insurance.	Jingdong prefecture level city in Shandong
Jinfeng Hongyuan Flagship Store	Electricity savings1	The appearance of this product is good, easy to use and install, the effect of how there is no use to wait for time to verify that the voltage displayed is different from the voltage measured by other meters, the gap is not too large, the follow-up to add the effect.	Jingdong prefecture level city in Shandong

Jinfeng Hongyuan Flagship Store	Electricity savings1	Baby received it 👌, really great ah! 🌈The quality can't be beat, very satisfied! 🆗Happy happy happy 😃	Jingdong prefecture level city in Shandong
Jinfeng Hongyuan Flagship Store	Electricity savings1	I'll follow up with a review after using it for a while, hopefully it won't bring me back a disappointment!	Jingdong prefecture level city in Shandong
Jinfeng Hongyuan Flagship Store	Electricity savings1	In use and looking forward to results! Good review to send	Jingdong prefecture level city in Shandong
Jinfeng Hongyuan Flagship Store	Electricity savings1	Intelligent power saver received, the volume is not big, small and exquisite, quite good, Jingdong Express is also quite fast, the	Jingdong prefecture level city in Shandong
Jinfeng Hongyuan Flagship Store	Electricity savings1	I don't know how to use it, keep using it	Jingdong prefecture level city in Shandong
Jinfeng Hongyuan Flagship Store	Electricity savings1	Very good for saving electricity	Jingdong prefecture level city in Shandong
Jinfeng Hongyuan Flagship Store	Electricity savings1	Seriously, did not use, feel also more power consumption, the peak is bought back to use the first time, the next day unplugged, the power consumption is back to more than ten degrees (the frequency of power use in the past few days is pretty much the same), feel the IQ tax paid 😂	Jingdong prefecture level city in Shandong
Jinfeng Hongyuan Flagship Store	Electricity savings1	I haven't seen any effect on the power saving, but the voltage stabilization looks good.	Jingdong prefecture level city in Shandong
Jinfeng Hongyuan Flagship Store	Electricity savings1	It's very good, I'll come back to it after using it for a while	Jingdong prefecture level city in Shandong
Jinfeng Hongyuan Flagship Store	Electricity savings1	Already used it and see the results.	Jingdong prefecture level city in Shandong
Jinfeng Hongyuan Flagship Store	Electricity savings1	Fast shipping, beautiful packaging, attentive service, the use of the effect, to be followed up after the evaluation.	Jingdong prefecture level city in Shandong
Jinfeng Hongyuan Flagship Store	Electricity savings1	The process of using this product is more than satisfactory	Jingdong prefecture level city in Shandong
Jinfeng Hongyuan Flagship Store	Electricity savings1	Very good East East cost-effective! Every time so buy more assured, and the logistics is also very fast. It is also very good to use, so I will often come to buy in the future, and will also introduce to	Jingdong prefecture level city in Shandong
Jinfeng Hongyuan Flagship Store	Electricity savings1	But still understand how effective it is!	Jingdong prefecture level city in Shandong
Jinfeng Hongyuan Flagship Store	Electricity savings1	Fast shipping, great logistics, great stuff, great price.	Jingdong prefecture level city in Shandong
Jinfeng Hongyuan Flagship Store	Electricity savings1	I'll review it in a month and give it 5 stars if it's good	Jingdong prefecture level city in Shandong
Jinfeng Hongyuan Flagship Store	Electricity savings1	I've saved a lot of electricity in the past month, so I'm glad to hear that.	Jingdong prefecture level city in Shandong
Jinfeng Hongyuan Flagship Store	Electricity savings1	Baby just received and installed! Looks genuinely good, workmanship feels fine! Looking forward to the effect! Said to see the effect in two months! Waiting for if really have the effect must come to follow up! The boss is absolutely responsible! Asked and answered! Just for the boss this attitude must also	Jingdong prefecture level city in Shandong
Jinfeng Hongyuan Flagship Store	Electricity savings1	This baby is very delicate, I'll comment after I've used it	Jingdong prefecture level city in Shandong
Jinfeng Hongyuan Flagship Store	Electricity savings1	It's been over two months. It's not working. It's just like it was.	Jingdong prefecture level city in Shandong
Jinfeng Hongyuan Flagship Store	Electricity savings1	Fast shipping and logistics, let's see if it works.	Jingdong prefecture level city in Shandong
Jinfeng Hongyuan Flagship Store	Electricity savings1	Just received the goods, try it to know if it can really save electricity	Jingdong prefecture level city in Shandong
Jinfeng Hongyuan Flagship Store	Electricity savings1	Not bad. Not bad. Not bad.	Jingdong prefecture level city in Shandong
Jinfeng Hongyuan Flagship Store	Electricity savings1	Not sure if it works. I'll comment again if it works.	Jingdong prefecture level city in Shandong
Jinfeng Hongyuan Flagship Store	Electricity savings1	Safe and energy-saving helpers to use!	Jingdong prefecture level city in Shandong
Jinfeng Hongyuan Flagship Store	Electricity savings1	Good, first time buyer. Used good can	Jingdong prefecture level city in Shandong
Jinfeng Hongyuan Flagship Store	Electricity savings1	It feels okay, I guess! I won't know until I use it for a while!	Jingdong prefecture level city in Shandong
Jinfeng Hongyuan Flagship Store	Electricity savings1	The saver matches the description and has been used, waiting for a follow up review after two months.	Jingdong prefecture level city in Shandong
Jinfeng Hongyuan Flagship Store	Electricity savings1	Received, the appearance of the business as described, the effect of how to use a period of time to evaluate.	Jingdong prefecture level city in Shandong
Jinfeng Hongyuan Flagship Store	Electricity savings1	Still using it don't know how effective it is, I'll comment after three months	Jingdong prefecture level city in Shandong
Jinfeng Hongyuan Flagship Store	Electricity savings1	The stuff is very good, the packaging is also very good logistics is also very fast	Jingdong prefecture level city in Shandong
Jinfeng Hongyuan Flagship Store	Electricity savings1	I'll follow up with a review when I use it, hopefully I won't be disappointed	Jingdong prefecture level city in Shandong
Jinfeng Hongyuan Flagship Store	Electricity savings1	Cancel Houjiaying Logistics.	Jingdong prefecture level city in Shandong
Jinfeng Hongyuan Flagship Store	Electricity savings1	It's very good, it's worth buying.	Jingdong prefecture level city in Shandong
Jinfeng Hongyuan Flagship Store	Electricity savings1	My friend bought it to save electricity and referred me to buy it! I'll buy it and try it out! I will know how much electricity I can save next month, looking forward to it! 4-500 dollars a month electricity bill is horrible! I hope it can really help me save some electricity!	Jingdong prefecture level city in Shandong
Jinfeng Hongyuan Flagship Store	Electricity savings1	Just received it and installed it.	Jingdong prefecture level city in Shandong
Jinfeng Hongyuan Flagship Store	Electricity savings1	Very nice. This one is my favorite.	Jingdong prefecture level city in Shandong
Jinfeng Hongyuan Flagship Store	Electricity savings1	Good value for money worth buying very good	Jingdong prefecture level city in Shandong
Jinfeng Hongyuan Flagship Store	Electricity savings1	Very energy-saving, small and exquisite, does not take up space, very satisfied, complete packaging, no damage, customer service explanation is also very serious	Jingdong prefecture level city in Shandong
Jinfeng Hongyuan Flagship Store	Electricity savings1	Received immediately used, really effective, do not understand electricity, but looked at the meter every day, recommended that you can use, especially this weather every day to open the air conditioning, with him not afraid of the electricity bill.	Jingdong prefecture level city in Shandong
Jinfeng Hongyuan Flagship Store	Electricity savings1	Nice, good value for money, nice stuff.	Jingdong prefecture level city in Shandong
Jinfeng Hongyuan Flagship Store	Electricity savings1	The packaging of the product is very good, the logistics of the product is very fast, and the quality of the product will be evaluated after use!	Jingdong prefecture level city in Shandong
Jinfeng Hongyuan Flagship Store	Electricity savings1	Express is very, well very fast, give a compliment 👍	Jingdong prefecture level city in Shandong
Jinfeng Hongyuan Flagship Store	Electricity savings1	Packaging is very good, fast delivery, used a night and a day did not feel the power saving. Use a month to see if it saves power.	Jingdong prefecture level city in Shandong
Jinfeng Hongyuan Flagship Store	Electricity savings1	Seems to be a bit of a power saver.	Jingdong prefecture level city in Shandong
Jinfeng Hongyuan Flagship Store	Electricity savings1	Bought this for someone else and it came over so fast, ###!	Jingdong prefecture level city in Shandong
Jinfeng Hongyuan Flagship Store	Electricity savings1	Very delicate, just received a period of time to use in the follow-up evaluation	Jingdong prefecture level city in Shandong
Jinfeng Hongyuan Flagship Store	Electricity savings1	It's been installed for a while now, not sure if it's working or not	Jingdong prefecture level city in Shandong
Jinfeng Hongyuan Flagship Store	Electricity savings1	The item looks okay, I just don't know if it works or not.	Jingdong prefecture level city in Shandong

Jinfeng Hongyuan Flagship Store	Electricity savings1	The goods have been received, but there is a screw missing	Jingdong prefecture level city in Shandong
Jinfeng Hongyuan Flagship Store	Electricity savings1	I didn't believe it before, but after receiving the goods today and trying it out, it really works!	Jingdong prefecture level city in Shandong
Jinfeng Hongyuan Flagship Store	Electricity savings1	Received, this thing will not know whether it is the effect of saving electricity, no matter how to say that there should always be a little use it, otherwise why develop it I think there is still the value of	Jingdong prefecture level city in Shandong
Jinfeng Hongyuan Flagship Store	Electricity savings1	Another one just arrived.	Jingdong prefecture level city in Shandong
Jinfeng Hongyuan Flagship Store	Electricity savings1	I don't feel anything, ordinary farmers can't go to TEDA, just use it first, it's safe.	Jingdong prefecture level city in Shandong
Jinfeng Hongyuan Flagship Store	Electricity savings1	Just purchased and will follow up after using it for a while	Jingdong prefecture level city in Shandong
Jinfeng Hongyuan Flagship Store	Electricity savings1	I'm trying it out now. I'll comment after I use it.	Jingdong prefecture level city in Shandong
Jinfeng Hongyuan Flagship Store	Electricity savings1	Received the item, in use, hope it works.	Jingdong prefecture level city in Shandong
Jinfeng Hongyuan Flagship Store	Electricity savings1	It works well. Good review.	Jingdong prefecture level city in Shandong
Jinfeng Hongyuan Flagship Store	Electricity savings1	All a scam, no effect at all, I bought too many fakes in Poundland before, I did not think that Jingdong is also the same	Jingdong prefecture level city in Shandong
Jinfeng Hongyuan Flagship Store	Electricity savings1	I used the saver as soon as I received it, it's in good shape and still working!	Jingdong prefecture level city in Shandong
Jinfeng Hongyuan Flagship Store	Electricity savings1	It works. I don't know what to do with the picture, but it works.	Jingdong prefecture level city in Shandong
Jinfeng Hongyuan Flagship Store	Electricity savings1	A very good shopping experience, attentive service, fast logistics, delivery is also very thoughtful, attitude is also very good, value for money ah, decisive praise!	Jingdong prefecture level city in Shandong
Jinfeng Hongyuan Flagship Store	Electricity savings1	The product is consistent with the presentation picture.	Jingdong prefecture level city in Shandong
Jinfeng Hongyuan Flagship Store	Electricity savings1	Baby received, use a period of time to evaluate, wish the boss business prosperity!	Jingdong prefecture level city in Shandong
Jinfeng Hongyuan Flagship Store	Electricity savings1	The stuff is better! Recommended!	Jingdong prefecture level city in Shandong
Jinfeng Hongyuan Flagship Store	Electricity savings1	I don't know if it works or not, I'll use it for a while before I comment on it.	Jingdong prefecture level city in Shandong
Jinfeng Hongyuan Flagship Store	Electricity savings1	Cost-effective, I like it, next time will come back Baby received, very surprised, the quality is very good, very satisfied with a shopping. I will continue to buy back, the store is trustworthy	Jingdong prefecture level city in Shandong
Jinfeng Hongyuan Flagship Store	Electricity savings1	Plugged it in and used it for a few days, not sure if it saves power yet	Jingdong prefecture level city in Shandong
Jinfeng Hongyuan Flagship Store	Electricity savings1	I'm trying it out and will follow up if it seems to be working!	Jingdong prefecture level city in Shandong
Jinfeng Hongyuan Flagship Store	Electricity savings1	Works pretty well, big kudos 👍👍👍👍👍👍👍👍👍👍👍	Jingdong prefecture level city in Shandong
Jinfeng Hongyuan Flagship Store	Electricity savings1	Just put it on, we'll see how it looks in a month.	Jingdong prefecture level city in Shandong
Jinfeng Hongyuan Flagship Store	Electricity savings1	The baby is very nice and I've already recommended buying it for all my friends! The main thing is that it not only saves electricity, I was more concerned about the fact that it can adjust the current, usually wiring problems. Now I found that it is really good, it is the effect I want!	Jingdong prefecture level city in Shandong
Jinfeng Hongyuan Flagship Store	Electricity savings1	Baby arrived, looking forward to his results, hopefully it will be good	Jingdong prefecture level city in Shandong
Jinfeng Hongyuan Flagship Store	Electricity savings1	Things have been received, look at the workmanship and packaging are good, I do not know how the power-saving effect? Use a period of time to see the effect	Jingdong prefecture level city in Shandong
Jinfeng Hongyuan Flagship Store	Electricity savings1	The price is right, the shape is beautiful and does not take up space, use it for a while to see the effect	Jingdong prefecture level city in Shandong
Jinfeng Hongyuan Flagship Store	Electricity savings1	Can't save electricity, it doesn't work!	Jingdong prefecture level city in Shandong
Jinfeng Hongyuan Flagship Store	Electricity savings1	It's pretty convenient, just plug it in and it works. There's no noise, plug it in and leave it alone, it's very hassle free! Good workmanship and value for money.	Jingdong prefecture level city in Shandong
Jinfeng Hongyuan Flagship Store	Electricity savings1	Just received the goods have been installed, try two months later to comment!	Jingdong prefecture level city in Shandong
Jinfeng Hongyuan Flagship Store	Electricity savings1	I received the stuff, I haven't used it yet, and overall it's fine.	Jingdong prefecture level city in Shandong
Jinfeng Hongyuan Flagship Store	Electricity savings1	Couldn't use a return at all.	Jingdong prefecture level city in Shandong
Jinfeng Hongyuan Flagship Store	Electricity savings1	Second purchase.	Jingdong prefecture level city in Shandong
Jinfeng Hongyuan Flagship Store	Electricity savings1	Very good quality, high tech and practical.	Jingdong prefecture level city in Shandong
Jinfeng Hongyuan Flagship Store	Electricity savings1	Very good, very great! Very satisfied, thanks to the store!	Jingdong prefecture level city in Shandong
Jinfeng Hongyuan Flagship Store	Electricity savings1	Just used it, don't know if it's good or not, hope it works well	Jingdong prefecture level city in Shandong
Jinfeng Hongyuan Flagship Store	Electricity savings1	Saving power has been received, the product is good, good like.	Jingdong prefecture level city in Shandong
Jinfeng Hongyuan Flagship Store	Electricity savings1	Received the goods and used it for two months before evaluation	Jingdong prefecture level city in Shandong
Jinfeng Hongyuan Flagship Store	Electricity savings1	It looks a little small, I do not know how effective, first use to see the effect, there is a small adjustable button, I do not know this is to adjust to that position to work well	Jingdong prefecture level city in Shandong
Jinfeng Hongyuan Flagship Store	Electricity savings1	Simple, just plug it in, there are spare fuses, can't say how well it works, no comparison	Jingdong prefecture level city in Shandong
Jinfeng Hongyuan Flagship Store	Electricity savings1	It's been a few days since I used it, and the power's gone up.	Jingdong prefecture level city in Shandong
Jinfeng Hongyuan Flagship Store	Electricity savings1	The owner was very nice and sent a spare fuse.	Jingdong prefecture level city in Shandong
Jinfeng Hongyuan Flagship Store	Electricity savings1	It's authentic and it's not expensive and it's great to use, I use it all the time and it's great 🎉✨ just don't know if it really saves any points	Jingdong prefecture level city in Shandong
Jinfeng Hongyuan Flagship Store	Electricity savings1	The information is good, it is the child to buy, the child read it is also quite happy, I hope to be able to match the child's preparation for the midterm examination! Because of the epidemic, it is not too fast, but I also give full credit to the express delivery!	Jingdong prefecture level city in Shandong
Jinfeng Hongyuan Flagship Store	Electricity savings1	This power saver comes to the logistics very quickly, the business attitude is also very good, ** very comfortable, this brand is trustworthy, and buy in this store than in the physical store to buy cheaper,	Jingdong prefecture level city in Shandong
Jinfeng Hongyuan Flagship Store	Electricity savings1	It's very well made and the materials used are okay. This is just a few days old, so I don't know how it will work, only next month.	Jingdong prefecture level city in Shandong
Jinfeng Hongyuan Flagship Store	Electricity savings1	Already used on, have not tested the effect, wait for a couple of months to add comments.	Jingdong prefecture level city in Shandong
Jinfeng Hongyuan Flagship Store	Electricity savings1	Baby received, packaging is very thoughtful, already in use, should soon see the effect.	Jingdong prefecture level city in Shandong
Jinfeng Hongyuan Flagship Store	Electricity savings1	Just received in the office to try, to the home installed, the voltage are different, and do not know if there is no effect. Use a period of time and then follow up.	Jingdong prefecture level city in Shandong
Jinfeng Hongyuan Flagship Store	Electricity savings1	Just received the goods, is trying, looking forward to the effect, first give a favorable comment, two months later, then additional evaluation, please be patient.	Jingdong prefecture level city in Shandong
Jinfeng Hongyuan Flagship Store	Electricity savings1	The item has just been received, well packaged, results to be verified.	Jingdong prefecture level city in Shandong

Jinfeng Hongyuan Flagship Store	Electricity savings1	Packaging logistics are very good, the next look at how the effect, before I see others use the effect is good, as I bought this can also have good results.	Jingdong prefecture level city in Shandong
Jinfeng Hongyuan Flagship Store	Electricity savings1	I don't know if it's a real power saver or a fake power saver, but I've used it since I bought it, so I'll give you a good review.	Jingdong prefecture level city in Shandong
Jinfeng Hongyuan Flagship Store	Electricity savings1	Good quality, genuine goods, much better than imagined before purchase, next time will come back to care!	Jingdong prefecture level city in Shandong
Jinfeng Hongyuan Flagship Store	Electricity savings1	Logistics is very fast good use again	Jingdong prefecture level city in Shandong
Jinfeng Hongyuan Flagship Store	Electricity savings1	Received the goods. Let's see if it saves power in a month and then we'll follow up!	Jingdong prefecture level city in Shandong
Jinfeng Hongyuan Flagship Store	Electricity savings1	I bought it yesterday and it arrived today, I bought two of them, I hope they work well, I'll come back after a while to give a follow-up review	Jingdong prefecture level city in Shandong
Jinfeng Hongyuan Flagship Store	Electricity savings1	The home air conditioner consumes a lot of electricity, so I bought it and used it in the hope that there is A	Jingdong prefecture level city in Shandong
Jinfeng Hongyuan Flagship Store	Electricity savings1	The product is excellent and very current.	Jingdong prefecture level city in Shandong
Jinfeng Hongyuan Flagship Store	Electricity savings1	With the description, it is the most practical and good products that households can need, and it is an ideal shopping, the courier is also fast, and the service attitude is enthusiastic!	Jingdong prefecture level city in Shandong
Jinfeng Hongyuan Flagship Store	Electricity savings1	The Power Saver is easy to use, works well, is stable, and plugs into the top of the outlet	Jingdong prefecture level city in Shandong
Jinfeng Hongyuan Flagship Store	Electricity savings1	Overall still can, excellent craftsmanship, power saving do not know for the time being, after the use of the evaluation	Jingdong prefecture level city in Shandong
Jinfeng Hongyuan Flagship Store	Electricity savings1	Baby received on the plug, but also with four fuses, look at the quality is okay, I do not know whether it is useful, use and then follow up.	Jingdong prefecture level city in Shandong
Jinfeng Hongyuan Flagship Store	Electricity savings1	I'll evaluate it after a while, I don't know if it's good or not yet, and if it can save electricity	Jingdong prefecture level city in Shandong
Jinfeng Hongyuan Flagship Store	Electricity savings1	Jingdong self-operated purchase more assured delivery fast quality assurance	Jingdong prefecture level city in Shandong
Jinfeng Hongyuan Flagship Store	Electricity savings1	Shipping was fast, stuff was well packaged, great price, I'm happy with it 👍 and will repurchase!	Jingdong prefecture level city in Shandong
Jinfeng Hongyuan Flagship Store	Electricity savings1	The quality of the product is very good and I will definitely come back again next time.	Jingdong prefecture level city in Shandong
Jinfeng Hongyuan Flagship Store	Electricity savings1	I'll follow up with a review after I use it.	Jingdong prefecture level city in Shandong
Jinfeng Hongyuan Flagship Store	Electricity savings1	Hello, the goods are perfectly packaged, save power for a period of time to evaluate again!	Jingdong prefecture level city in Shandong
Jinfeng Hongyuan Flagship Store	Electricity savings1	Not bad, pretty good, good value for money, pretty good	Jingdong prefecture level city in Shandong
Jinfeng Hongyuan Flagship Store	Electricity savings1	The packaging is very good, received immediately used, looking forward to the effect in.	Jingdong prefecture level city in Shandong
Jinfeng Hongyuan Flagship Store	Electricity savings1	See how much power you save at the end of the month	Jingdong prefecture level city in Shandong
Jinfeng Hongyuan Flagship Store	Electricity savings1	Easy to use, affordable, beautiful color	Jingdong prefecture level city in Shandong
Jinfeng Hongyuan Flagship Store	Electricity savings1	It's taking too long to put together an order. It could be an epidemic.	Jingdong prefecture level city in Shandong
Jinfeng Hongyuan Flagship Store	Electricity savings1	I don't feel much after using it, no change in my electric bill 。。。。。。。	Jingdong prefecture level city in Shandong
Jinfeng Hongyuan Flagship Store	Electricity savings1	Logistics is very fast, the quality of the economizer is good, good quality and cheap, it is worth buying, I hope there is a power saving effect!	Jingdong prefecture level city in Shandong
Jinfeng Hongyuan Flagship Store	Electricity savings1	Feeling okay, in a couple of months to try to rely on it before making a comment. Let's give it a five-star review first!	Jingdong prefecture level city in Shandong
Jinfeng Hongyuan Flagship Store	Electricity savings1	Power saving device has been hesitating before in the end to buy or not to buy, after consideration, consideration, in consideration, or decided to buy a try. Things received can not wait to open a look, and the seller described the same, I hope to use 2 months to have the effect, two months to follow up on	Jingdong prefecture level city in Shandong
Jinfeng Hongyuan Flagship Store	Electricity savings1	Thank you Jingdong, logistics is very fast order to receipt only one day. Believe in Jingdong. Favorable comments	Jingdong prefecture level city in Shandong
Jinfeng Hongyuan Flagship Store	Electricity savings1	I used one to use it, but it didn't work very well, what to do	Jingdong prefecture level city in Shandong
Jinfeng Hongyuan Flagship Store	Electricity savings1	Quality and packaging are very good, fast shipping, good service, buy with confidence	Jingdong prefecture level city in Shandong
Jinfeng Hongyuan Flagship Store	Electricity savings1	Use a period of time to evaluate, the quality is very good, the price is affordable, logistics is also very fast	Jingdong prefecture level city in Shandong
Jinfeng Hongyuan Flagship Store	Electricity savings1	Goods received, the day of the order tomorrow delivery real God speed, delivery force, the product to be used after a period of time and then low comment!	Jingdong prefecture level city in Shandong
Jinfeng Hongyuan Flagship Store	Electricity savings1	I don't know, how effective it is, let's talk about it when the time comes, whether it saves power or not, how much, unknown.	Jingdong prefecture level city in Shandong
Jinfeng Hongyuan Flagship Store	Electricity savings1	It does save some electricity, and I don't want to use something that isn't legal! Used nearly ~ electricity last month, probably not much more this month with the AC on!	Jingdong prefecture level city in Shandong
Jinfeng Hongyuan Flagship Store	Electricity savings1	Well-packed, excellent quality, service satisfaction good value for money	Jingdong prefecture level city in Shandong
Jinfeng Hongyuan Flagship Store	Electricity savings1	Express very quickly, the goods received last night, today used a day of goods can not heat, looking forward to whether it can save electricity, with a month and then comment, is used well will buy again.	Jingdong prefecture level city in Shandong
Jinfeng Hongyuan Flagship Store	Electricity savings1	It's pretty good, hopefully it'll be a real power saver.	Jingdong prefecture level city in Shandong
Jinfeng Hongyuan Flagship Store	Electricity savings1	The goods received in use, can not see the effect of energy saving, after a month to know!	Jingdong prefecture level city in Shandong
Jinfeng Hongyuan Flagship Store	Electricity savings1	Received the goods, looking forward to better surprise, with the effect of the follow-up comment!	Jingdong prefecture level city in Shandong
Jinfeng Hongyuan Flagship Store	Electricity savings1	It's not bad, it's still in use and should save some	Jingdong prefecture level city in Shandong
Jinfeng Hongyuan Flagship Store	Electricity savings1	Logistics is very fast, easy to install, plugged in on it,, the effect of the use of the later to evaluate!	Jingdong prefecture level city in Shandong
Jinfeng Hongyuan Flagship Store	Electricity savings1	The stuff is recommendable to buy very good.	Jingdong prefecture level city in Shandong
Jinfeng Hongyuan Flagship Store	Electricity savings1	Voltmeter measurements of voltage are way off, aren't they?	Jingdong prefecture level city in Shandong
Jinfeng Hongyuan Flagship Store	Electricity savings1	Install it to the socket immediately after purchase and try to see if it can save electricity after one month.	Jingdong prefecture level city in Shandong
Jinfeng Hongyuan Flagship Store	Electricity savings1	I've been using it for almost a month now, and I won't know if it saves electricity until I pay the bill.	Jingdong prefecture level city in Shandong
Jinfeng Hongyuan Flagship Store	Electricity savings1	I bought one in the past and it was fine, so I'm buying it back.	Jingdong prefecture level city in Shandong
Jinfeng Hongyuan Flagship Store	Electricity savings1	Service is very good, this thing is small and exquisite to use, just waiting to use the effect of a period of time	Jingdong prefecture level city in Shandong
Jinfeng Hongyuan Flagship Store	Electricity savings1	Not sure how well it works yet	Jingdong prefecture level city in Shandong
Jinfeng Hongyuan Flagship Store	Electricity savings1	Kyodo goods habitually good reviews	Jingdong prefecture level city in Shandong
Jinfeng Hongyuan Flagship Store	Electricity savings1	Appearance is OK, whether it is power saving remains to be seen.	Jingdong prefecture level city in Shandong
Jinfeng Hongyuan Flagship Store	Electricity savings1	I bought it and used it, I just don't know if it really saves power	Jingdong prefecture level city in Shandong

Jinfeng Hongyuan Flagship Store	Electricity savings1	The saver is well made and of good quality, I will evaluate it after using it for a while.	Jingdong prefecture level city in Shandong
Jinfeng Hongyuan Flagship Store	Electricity savings1	Just received in use, use for a period of time to see if there is power saving	Jingdong prefecture level city in Shandong
Jinfeng Hongyuan Flagship Store	Electricity savings1	Used 3 days before commenting, it really saves electricity, I look at the "power calendar" after using one less degree of electricity per day, so I think it's worth it to buy this, I will introduce my friends	Jingdong prefecture level city in Shandong
Jinfeng Hongyuan Flagship Store	Electricity savings1	Reliable quality, very well packaged and matches the real thing.	Jingdong prefecture level city in Shandong
Jinfeng Hongyuan Flagship Store	Electricity savings1	Logistics is still quite fast, ha in the trial	Jingdong prefecture level city in Shandong
Jinfeng Hongyuan Flagship Store	Electricity savings1	Consultant service attitude is very good, the courier is also very powerful, the first day of the order the next day to arrive. Saver received used and then add comments!	Jingdong prefecture level city in Shandong
Jinfeng Hongyuan Flagship Store	Electricity savings1	I just got home to try it out and see how it goes.	Jingdong prefecture level city in Shandong
Jinfeng Hongyuan Flagship Store	Electricity savings1	Well made and weighted instrument. Kudos! Put it into use immediately, don't know if it saves power, will evaluate later!	Jingdong prefecture level city in Shandong
Jinfeng Hongyuan Flagship Store	Electricity savings1	I've just put it in place. I'll give you a review in ten days.	Jingdong prefecture level city in Shandong
Jinfeng Hongyuan Flagship Store	Electricity savings1	It looks very delicate, not bad. Well, I don't know if it can save power or not, I have to try it for a while and see. If it can really save electricity. Uh, if it works well. Then additional comments. Anyway, thanks for the logistics, thanks to Jingdong. Well, it deserves some praise.	Jingdong prefecture level city in Shandong
Jinfeng Hongyuan Flagship Store	Electricity savings1	Bought a month, the feeling is that there is a certain effect, a month to save a lot of money	Jingdong prefecture level city in Shandong
Jinfeng Hongyuan Flagship Store	Electricity savings1	Goods received, the physical and the same as the picture, logistics is also very fast, the service attitude is very good, it is worth buying!	Jingdong prefecture level city in Shandong
Jinfeng Hongyuan Flagship Store	Electricity savings1	I've used it, I don't know whether it saves electricity or not, I'll talk about it after a while.	Jingdong prefecture level city in Shandong
Jinfeng Hongyuan Flagship Store	Electricity savings1	August only began to use, October to know, have no power-saving effect, to the in to evaluate!	Jingdong prefecture level city in Shandong
Jinfeng Hongyuan Flagship Store	Electricity savings1	Goods received: I do not know to the factory products used to save electricity!	Jingdong prefecture level city in Shandong
Jinfeng Hongyuan Flagship Store	Electricity savings1	Don't know if it works, bought it anyway.	Jingdong prefecture level city in Shandong
Jinfeng Hongyuan Flagship Store	Electricity savings1	Still testing, will follow up next month to evaluate how much electricity is saved	Jingdong prefecture level city in Shandong
Jinfeng Hongyuan Flagship Store	Electricity savings1	I'm still testing it, I'll comment after I use it.	Jingdong prefecture level city in Shandong
Jinfeng Hongyuan Flagship Store	Electricity savings1	Fast service, praiseworthy. Good item pre-style, first praise, the use of the effect to be evaluated after use.	Jingdong prefecture level city in Shandong
Jinfeng Hongyuan Flagship Store	Electricity savings1	The power saving effect is more obvious through verified use.	Jingdong prefecture level city in Shandong
Jinfeng Hongyuan Flagship Store	Electricity savings1	Logistics is very fast yesterday order today arrived. The product was immediately used, and after a month to see its effect.	Jingdong prefecture level city in Shandong
Jinfeng Hongyuan Flagship Store	Electricity savings1	Power saving is not yet known, as far as the TV and audio are concerned, I feel that there is a significant quality improvement, and I recommend using a good product!	Jingdong prefecture level city in Shandong
Jinfeng Hongyuan Flagship Store	Electricity savings1	Good power savings, tens of dollars a month saved	Jingdong prefecture level city in Shandong
Jinfeng Hongyuan Flagship Store	Electricity savings1	Already took two, great value! You can save 30% on your electric bill in a month. A summer must-have!	Jingdong prefecture level city in Shandong
Jinfeng Hongyuan Flagship Store	Electricity savings1	Received the item, plugged it into a home outlet and it showed 230 volts (not that high with a multimeter). Let's use it first!	Jingdong prefecture level city in Shandong
Jinfeng Hongyuan Flagship Store	Electricity savings1	Expecting results in the experiment	Jingdong prefecture level city in Shandong
Jinfeng Hongyuan Flagship Store	Electricity savings1	I don't know if it really saves power.	Jingdong prefecture level city in Shandong
Jinfeng Hongyuan Flagship Store	Electricity savings1	It's very good and the efficacy is very visible.	Jingdong prefecture level city in Shandong
Jinfeng Hongyuan Flagship Store	Electricity savings1	It's good and worth buying with confidence	Jingdong prefecture level city in Shandong
Jinfeng Hongyuan Flagship Store	Electricity savings1	The goods have been received, just do not know if it is effective	Jingdong prefecture level city in Shandong
Jinfeng Hongyuan Flagship Store	Electricity savings1	It's already in use, but based on some of the descriptions, I suspect that to use it, you need to have one in series on each line, which seems to work for all appliances, that's my guess, but I feel as though the principle of the thing seems to require it.	Jingdong prefecture level city in Shandong
Jinfeng Hongyuan Flagship Store	Electricity savings1	Fast delivery, already in use, looks good!	Jingdong prefecture level city in Shandong
Jinfeng Hongyuan Flagship Store	Electricity savings1	Hoping to save 30% of power as presented in the graphic	Jingdong prefecture level city in Shandong
Jinfeng Hongyuan Flagship Store	Electricity savings1	Things look good, power saving do not know now, with a month after the continued evaluation	Jingdong prefecture level city in Shandong
Jinfeng Hongyuan Flagship Store	Electricity savings1	Already used it but not sure if it will top out!	Jingdong prefecture level city in Shandong
Jinfeng Hongyuan Flagship Store	Electricity savings1	Looking forward to using the results, the product quality looks okay.	Jingdong prefecture level city in Shandong
Jinfeng Hongyuan Flagship Store	Electricity savings1	It's somewhat effective and saves money more or less 💰	Jingdong prefecture level city in Shandong
Jinfeng Hongyuan Flagship Store	Electricity savings1	Got it, it's great, looking forward to saving power.	Jingdong prefecture level city in Shandong
Jinfeng Hongyuan Flagship Store	Electricity savings1	I don't know if it's working yet, I'll follow up when I get there	Jingdong prefecture level city in Shandong
Jinfeng Hongyuan Flagship Store	Electricity savings1	The product received, good workmanship, fast logistics, good inner packaging, the outer bag is damaged, but fortunately did not hurt the product, retrieved and inserted according to the instructions, looking forward to the effect is good, with a period of time to evaluate, I believe that the Jingdong.	Jingdong prefecture level city in Shandong
Jinfeng Hongyuan Flagship Store	Electricity savings1	Since it is a new product, it is said to work, so if it works well for a month, then I will give you a good review.	Jingdong prefecture level city in Shandong
Jinfeng Hongyuan Flagship Store	Electricity savings1	The quality of the product is very good, very well made, well packaged, easy to use and very convenient!	Jingdong prefecture level city in Shandong
Jinfeng Hongyuan Flagship Store	Electricity savings1	Good price for the item, good functionality	Jingdong prefecture level city in Shandong
Jinfeng Hongyuan Flagship Store	Electricity savings1	Received the first time to use it, the stuff is very good, specifics wait for a month to come back to feedback to see the effect.	Jingdong prefecture level city in Shandong
Jinfeng Hongyuan Flagship Store	Electricity savings1	Just received, the courier is too bad, not notified directly to put the Feng Nest courier cabinet	Jingdong prefecture level city in Shandong
Jinfeng Hongyuan Flagship Store	Electricity savings1	Baby received, the second time to buy, plugged in and used do not know if there is no effect. Baby received, the second time to buy, plugged in and used do not know if there is no effect. Baby received, the second time to buy, plugged in and used do not know if there is no effect.	Jingdong prefecture level city in Shandong
Jinfeng Hongyuan Flagship Store	Electricity savings1	It's okay if it saves power. It's not a bad product.	Jingdong prefecture level city in Shandong
Jinfeng Hongyuan Flagship Store	Electricity savings1	Received, very good, meticulous packaging, open a look is genuine, meticulous workmanship, the manual is very detailed, has been used, there is no effect on the only look at the next month's electricity bill, when I can I buy a few more to send people!	Jingdong prefecture level city in Shandong
Jinfeng Hongyuan Flagship Store	Electricity savings1	The quality is very good very delicate express delivery is too fast	Jingdong prefecture level city in Shandong

Jinfeng Hongyuan Flagship Store	Electricity savings1	I won't know until a month after I start using it today.	Jingdong prefecture level city in Shandong
Jinfeng Hongyuan Flagship Store	Electricity savings1	It doesn't work at all, the customer service attitude is very bad, use it or not!	Jingdong prefecture level city in Shandong
Jinfeng Hongyuan Flagship Store	Electricity savings1	Temporarily try, do not know the effect, Jingdong logistics delivery very quickly	Jingdong prefecture level city in Shandong
Jinfeng Hongyuan Flagship Store	Electricity savings1	It doesn't help. It's comforting.	Jingdong prefecture level city in Shandong
Jinfeng Hongyuan Flagship Store	Electricity savings1	It's great, use it before you say anything.	Jingdong prefecture level city in Shandong
Jinfeng Hongyuan Flagship Store	Electricity savings1	Received, already used, looking forward to a good a effect!	Jingdong prefecture level city in Shandong
Jinfeng Hongyuan Flagship Store	Electricity savings1	I don't know if it works yet, I won't know until I use it for a few months.	Jingdong prefecture level city in Shandong
Jinfeng Hongyuan Flagship Store	Electricity savings1	It takes a long time to get there. There's no change now.	Jingdong prefecture level city in Shandong
Jinfeng Hongyuan Flagship Store	Electricity savings1	Jingdong's delivery never disappoints, or so fast, and the stuff is great, next time you need to come back.	Jingdong prefecture level city in Shandong
Jinfeng Hongyuan Flagship Store	Electricity savings1	At first it was ok today, after using it for a while it didn't work anymore	Jingdong prefecture level city in Shandong
Jinfeng Hongyuan Flagship Store	Electricity savings1	Just received the goods, do not know the effect of using a few days before there may be effect	Jingdong prefecture level city in Shandong
Jinfeng Hongyuan Flagship Store	Electricity savings1	The logistics was fast, I received it the next day, I've purchased it for the second time and have been using it all along	Jingdong prefecture level city in Shandong
Jinfeng Hongyuan Flagship Store	Electricity savings1	Inexpensive, fast delivery, very pleased!	Jingdong prefecture level city in Shandong
Jinfeng Hongyuan Flagship Store	Electricity savings1	Received the goods immediately plugged directly into the use of power saving yet to be good news!	Jingdong prefecture level city in Shandong
Jinfeng Hongyuan Flagship Store	Electricity savings1	The service attitude is very good, and the Jingdong self-owned products can be trusted.	Jingdong prefecture level city in Shandong
Jinfeng Hongyuan Flagship Store	Electricity savings1	The product is good, I do not know if it can save play a power saving effect	Jingdong prefecture level city in Shandong
Jinfeng Hongyuan Flagship Store	Electricity savings1	We won't know how effective it is until we use it for a while, so I'll give you a good review now.	Jingdong prefecture level city in Shandong
Jinfeng Hongyuan Flagship Store	Electricity savings1	The quality is pretty good, just started using it, hopefully it will deliver results.	Jingdong prefecture level city in Shandong
Jinfeng Hongyuan Flagship Store	Electricity savings1	I've plugged it in first, and will follow up after I've used it to see how it works.	Jingdong prefecture level city in Shandong
Jinfeng Hongyuan Flagship Store	Electricity savings1	Things have arrived, the quality is good, the effect is also equivalent to can ah	Jingdong prefecture level city in Shandong
Jinfeng Hongyuan Flagship Store	Electricity savings1	It's not worth it to brag about power savings.	Jingdong prefecture level city in Shandong
Jinfeng Hongyuan Flagship Store	Electricity savings1	I don't know if it's an IQ tax, but I'll use it for now.	Jingdong prefecture level city in Shandong
Jinfeng Hongyuan Flagship Store	Electricity savings1	I don't feel the power saving yet, I can't know until I use it for 2 months.	Jingdong prefecture level city in Shandong
Jinfeng Hongyuan Flagship Store	Electricity savings1	There's nothing wrong with this at the moment.	Jingdong prefecture level city in Shandong
Jinfeng Hongyuan Flagship Store	Electricity savings1	It's authentic and worth buying	Jingdong prefecture level city in Shandong
Jinfeng Hongyuan Flagship Store	Electricity savings1	It's good. We'll see how it goes.	Jingdong prefecture level city in Shandong
Jinfeng Hongyuan Flagship Store	Electricity savings1	I don't know if it's working or not in the middle of using it, we'll see when the month is over	Jingdong prefecture level city in Shandong
Jinfeng Hongyuan Flagship Store	Electricity savings1	I'm not going to buy anything from Jingdong anymore, so I'll give it a good review	Jingdong prefecture level city in Shandong
Jinfeng Hongyuan Flagship Store	Electricity savings1	I'm not going to buy anything from Jingdong anymore, so I'll give it a good review	Jingdong prefecture level city in Shandong
Jinfeng Hongyuan Flagship Store	Electricity savings1	Just received the item and will see how it works in a month.	Jingdong prefecture level city in Shandong
Jinfeng Hongyuan Flagship Store	Electricity savings1	I don't know if it saves electricity, but it's plugged into an outlet every day anyway	Jingdong prefecture level city in Shandong
Jinfeng Hongyuan Flagship Store	Electricity savings1	I don't know if it works yet.	Jingdong prefecture level city in Shandong
Jinfeng Hongyuan Flagship Store	Electricity savings1	Plugged it in when I got back, waiting for a month to see the results	Jingdong prefecture level city in Shandong
Jinfeng Hongyuan Flagship Store	Electricity savings1	Effects remain to be seen, but it looks good quality and runs fine	Jingdong prefecture level city in Shandong
Jinfeng Hongyuan Flagship Store	Electricity savings1	The product received logistics very quickly, has not yet been used to test! After use in the evaluation!	Jingdong prefecture level city in Shandong
Jinfeng Hongyuan Flagship Store	Electricity savings1	The power saver is in use, and I don't know how much electricity has been saved after using it for more than a month, and the display voltmeter is very good.	Jingdong prefecture level city in Shandong
Jinfeng Hongyuan Flagship Store	Electricity savings1	It's so good, isn't it? You're a real Shanghainese, aren't you?	Jingdong prefecture level city in Shandong
Jinfeng Hongyuan Flagship Store	Electricity savings1	Great quality great great great 👍👍👍👍	Jingdong prefecture level city in Shandong
Jinfeng Hongyuan Flagship Store	Electricity savings1	I'm using it, I don't know if it's working yet.	Jingdong prefecture level city in Shandong
Jinfeng Hongyuan Flagship Store	Electricity savings1	Just used it for more than 10 days and haven't seen any results yet	Jingdong prefecture level city in Shandong
Jinfeng Hongyuan Flagship Store	Electricity savings1	The product arrived home feeling like it should work	Jingdong prefecture level city in Shandong
Jinfeng Hongyuan Flagship Store	Electricity savings1	Just received the goods, use it and comment back	Jingdong prefecture level city in Shandong
Jinfeng Hongyuan Flagship Store	Electricity savings1	Try it for a month and then evaluate	Jingdong prefecture level city in Shandong
Jinfeng Hongyuan Flagship Store	Electricity savings1	I don't know if it saves power yet, but I'll comment again after using it for a while.	Jingdong prefecture level city in Shandong
Jinfeng Hongyuan Flagship Store	Electricity savings1	The product is better than I thought, the quality and appearance are very good, the voltage is stable, and I'm looking forward to a good power saving effect.	Jingdong prefecture level city in Shandong
Jinfeng Hongyuan Flagship Store	Electricity savings1	Received and scanned the QR code to check that it is genuine, have plugged it in, can it save power, looking forward to the advertised effect. Customary praise, 5 points!	Jingdong prefecture level city in Shandong
Jinfeng Hongyuan Flagship Store	Electricity savings1	The goods have been received and have not yet been used. Do not know how the effect.	Jingdong prefecture level city in Shandong
Jinfeng Hongyuan Flagship Store	Electricity savings1	Hopefully, you get what you pay for, and you get what you pay for!	Jingdong prefecture level city in Shandong
Jinfeng Hongyuan Flagship Store	Electricity savings1	Just put it on and it feels like it's turning as fast as a meter! Looking forward to the results!	Jingdong prefecture level city in Shandong
Jinfeng Hongyuan Flagship Store	Electricity savings1	Overall look on the product quality is good, I do not know is not the effect of power saving, wait to see and then follow up!	Jingdong prefecture level city in Shandong
Jinfeng Hongyuan Flagship Store	Electricity savings1	The logistics courier is very fast, the courier service attitude is very good	Jingdong prefecture level city in Shandong

Jinfeng Hongyuan Flagship Store	Electricity savings1	The product has been received, it works well worth buying	Jingdong prefecture level city in Shandong
Jinfeng Hongyuan Flagship Store	Electricity savings1	How did it not work? Is it purely an IQ tax?	Jingdong prefecture level city in Shandong
Jinfeng Hongyuan Flagship Store	Electricity savings1	It looks good, I don't know how well it works.	Jingdong prefecture level city in Shandong
Jinfeng Hongyuan Flagship Store	Electricity savings1	It does seem to be a bit of a power saver, I just don't know if the more expensive one is more power efficient?	Jingdong prefecture level city in Shandong
Jinfeng Hongyuan Flagship Store	Electricity savings1	Is genuine very good, the price is much cheaper, cost-effective, really special paddle just to do activities, the price is much cheaper, cost-effective, really special paddle counting, packaging is also very high-end atmospheric class, it is worth buying!	Jingdong prefecture level city in Shandong
Jinfeng Hongyuan Flagship Store	Electricity savings1	I received it yesterday and used it right away, I hope it works, I'll wait for next month to come back to the conclusion, I'm looking forward to it. Five hearts praise!	Jingdong prefecture level city in Shandong
Jinfeng Hongyuan Flagship Store	Electricity savings1	Looks good, packed properly, received it today, am using it, will see results in a month!	Jingdong prefecture level city in Shandong
Jinfeng Hongyuan Flagship Store	Electricity savings1	Regular product Jingdong self-support trustworthy. There is an effect Come back to the review. 😍😍😍😍	Jingdong prefecture level city in Shandong
Jinfeng Hongyuan Flagship Store	Electricity savings1	I haven't used it yet, hopefully it will work as a power saver, and will comment again if it works well.	Jingdong prefecture level city in Shandong
Jinfeng Hongyuan Flagship Store	Electricity savings1	Shipping was fast, logistics was fast, service was good, and I look forward to good results from the item!	Jingdong prefecture level city in Shandong
Jinfeng Hongyuan Flagship Store	Electricity savings1	This product is this compensator, it has to be at the main switch to be useful, haven't compared the final result, the	Jingdong prefecture level city in Shandong
Jinfeng Hongyuan Flagship Store	Electricity savings1	Packaging is perfect . I've already started using it, so I'm looking forward to the results.	Jingdong prefecture level city in Shandong
Jinfeng Hongyuan Flagship Store	Electricity savings1	I just got it back. I'll comment after I use it.	Jingdong prefecture level city in Shandong
Jinfeng Hongyuan Flagship Store	Electricity savings1	Big brand, with a very assured, very good quality, catch the event to buy very cost-effective to take advantage of the offer, hurry to get, it is genuine, the packaging is also very good, no breakage, the quality is not to speak of, great 👍 logistics super super fast! Quality is also very good, indeed value	Jingdong prefecture level city in Shandong
Jinfeng Hongyuan Flagship Store	Electricity savings1	The quality is pretty good. The price is also quite affordable.	Jingdong prefecture level city in Shandong
Jinfeng Hongyuan Flagship Store	Electricity savings1	I did not expect the courier so fast to arrive yesterday just ordered, the head is a little smaller than I thought, well, the picture looks bigger. The appearance still looks good, as for the practicality and effectiveness of the use of the only know.	Jingdong prefecture level city in Shandong
Jinfeng Hongyuan Flagship Store	Electricity savings1	I don't know. Looking forward to it.	Jingdong prefecture level city in Shandong
Jinfeng Hongyuan Flagship Store	Electricity savings1	Baby received, very delicate, very nice, like. Just don't know if it can really save electricity. Baby received, very delicate, very good-looking, like. Just don't know if it can really save electricity.	Jingdong prefecture level city in Shandong
Jinfeng Hongyuan Flagship Store	Electricity savings1	I'll use it for a month and then comment on the quality of the item	Jingdong prefecture level city in Shandong
Jinfeng Hongyuan Flagship Store	Electricity savings1	It feels ok, use it for a while and see how it works before adding more comments.	Jingdong prefecture level city in Shandong
Jinfeng Hongyuan Flagship Store	Electricity savings1	In use, see results in a month.	Jingdong prefecture level city in Shandong
Jinfeng Hongyuan Flagship Store	Electricity savings1	Jingdong's logistics and quality are very good, the service attitude is very good, the logistics is very fast	Jingdong prefecture level city in Shandong
Jinfeng Hongyuan Flagship Store	Electricity savings1	Intelligent power saver received, well-packed logistics is also quite fast, easy to use, looking forward to good results!	Jingdong prefecture level city in Shandong
Jinfeng Hongyuan Flagship Store	Electricity savings1	The quality is unknown at this point, I won't know until I use it.	Jingdong prefecture level city in Shandong
Jinfeng Hongyuan Flagship Store	Electricity savings1	It's a good product. It really saves power and doesn't take up much space.	Jingdong prefecture level city in Shandong
Jinfeng Hongyuan Flagship Store	Electricity savings1	Goods have been received, packaging is very good, good to see the effect of future use in the follow-up.	Jingdong prefecture level city in Shandong
Jinfeng Hongyuan Flagship Store	Electricity savings1	The product is in perfect shape and the light weight is worth it!	Jingdong prefecture level city in Shandong
Jinfeng Hongyuan Flagship Store	Electricity savings1	Just got home and plugged it in. It's good.	Jingdong prefecture level city in Shandong
Jinfeng Hongyuan Flagship Store	Electricity savings1	The logistics are quick, just to see the results.	Jingdong prefecture level city in Shandong
Jinfeng Hongyuan Flagship Store	Electricity savings1	Short time can not feel here to leave a message, to be followed up later!	Jingdong prefecture level city in Shandong
Jinfeng Hongyuan Flagship Store	Electricity savings1	This user thinks the product is good	Jingdong prefecture level city in Shandong
Jinfeng Hongyuan Flagship Store	Electricity savings1	Received the baby, to be used to see the effect of it!	Jingdong prefecture level city in Shandong
Jinfeng Hongyuan Flagship Store	Electricity savings1	You have to wait a month or two to see how it works.	Jingdong prefecture level city in Shandong
Jinfeng Hongyuan Flagship Store	Electricity savings1	Not bad, good, like it, don't know if it's good, try it first.	Jingdong prefecture level city in Shandong
Jinfeng Hongyuan Flagship Store	Electricity savings1	The goods are good value for money and the logistics is very fast and good service!	Jingdong prefecture level city in Shandong
Jinfeng Hongyuan Flagship Store	Electricity savings1	I don't know how well it works, we'll see in a month	Jingdong prefecture level city in Shandong
Jinfeng Hongyuan Flagship Store	Electricity savings1	The product is fine, but r saving power or not I won't know until I use it for two months. The display and lights take electricity too?	Jingdong prefecture level city in Shandong
Jinfeng Hongyuan Flagship Store	Electricity savings1	I've used it. I don't know how it works yet.	Jingdong prefecture level city in Shandong
Jinfeng Hongyuan Flagship Store	Electricity savings1	I can't say until I compare them.	Jingdong prefecture level city in Shandong
Jinfeng Hongyuan Flagship Store	Electricity savings1	It's ok in the process of using 。。。。	Jingdong prefecture level city in Shandong
Jinfeng Hongyuan Flagship Store	Electricity savings1	Jinfeng Hongyuan Intelligent Power Saver Household Electricity Meter Electricity Saving Appliance High Power Enhanced Power Saving King Energy Saving Treasure Butler Control Non Stealing Electricity Reversing Not Going Interfering Slow Turner Artifact King Gold Yellow. Haven't tested it accurately yet, really need to wait a few months to test it before I have a say.	Jingdong prefecture level city in Shandong
Jinfeng Hongyuan Flagship Store	Electricity savings1	Don't know how effective it is yet? I hope it works well and I'll come back next time	Jingdong prefecture level city in Shandong
Jinfeng Hongyuan Flagship Store	Electricity savings1	Saving device has been received, material workmanship are very good ah! Have not yet begun to use. First observe the daily electricity consumption and cost of the home meter, and then plug in the power saver to use, so as to compare the use of the effect is good!	Jingdong prefecture level city in Shandong
Jinfeng Hongyuan Flagship Store	Electricity savings1	Merchants shipping extremely fast, Jingdong logistics safe and fast, I believe that the product can play a safe use of electricity, the role of scientific power saving, first praise, the effect of how to be used for a period of time and then the pursuit of bubble!	Jingdong prefecture level city in Shandong
Jinfeng Hongyuan Flagship Store	Electricity savings1	I just bought it. I don't know yet.	Jingdong prefecture level city in Shandong
Jinfeng Hongyuan Flagship Store	Electricity savings1	It is genuine, good quality, and it is very reassuring to use. The logistics is very fast, and the business service attitude is on point.	Jingdong prefecture level city in Shandong
Jinfeng Hongyuan Flagship Store	Electricity savings1	The quality is fine, I don't know how effective it is.	Jingdong prefecture level city in Shandong
Jinfeng Hongyuan Flagship Store	Electricity savings1	The stuff is back and I expect it will save the electricity	Jingdong prefecture level city in Shandong

Jinfeng Hongyuan Flagship Store	Electricity savings1	I'll give it 4 stars first and see what happens in a couple of months and then follow up	Jingdong prefecture level city in Shandong
Jinfeng Hongyuan Flagship Store	Electricity savings1	It's not bad. It's in the mind.	Jingdong prefecture level city in Shandong
Jinfeng Hongyuan Flagship Store	Electricity savings1	Received the goods have not yet started to use do not know whether it is good	Jingdong prefecture level city in Shandong
Jinfeng Hongyuan Flagship Store	Electricity savings1	This user thinks the product is good	Jingdong prefecture level city in Shandong
Jinfeng Hongyuan Flagship Store	Electricity savings1	Hello! The saver was received, thank you! Not yet used, but from the packaging looks very good quality, exquisite workmanship. Logistics is quite fast, if it is really very power saving, I will come back to	Jingdong prefecture level city in Shandong
Jinfeng Hongyuan Flagship Store	Electricity savings1	I've been using it for a few days now, I'll comment again after a month or two of use	Jingdong prefecture level city in Shandong
Jinfeng Hongyuan Flagship Store	Electricity savings1	I don't know. Bought it for my father at a good price.	Jingdong prefecture level city in Shandong
Jinfeng Hongyuan Flagship Store	Electricity savings1	Plugged it in the same day I bought it and the voltage showed 223, 225 looking forward to the results	Jingdong prefecture level city in Shandong
Jinfeng Hongyuan Flagship Store	Electricity savings1	Still in use, the biggest role is to stabilize the current, reduce losses, product quality on the look is very good, with this feeling quite good, see colleagues are using, power saving effect should be good	Jingdong prefecture level city in Shandong
Jinfeng Hongyuan Flagship Store	Electricity savings1	How to receive goods but no invoice and warranty more than it ...... quality is the premise of reputation!	Jingdong prefecture level city in Shandong
Jinfeng Hongyuan Flagship Store	Electricity savings1	Received but not used Continued evaluation	Jingdong prefecture level city in Shandong
Jinfeng Hongyuan Flagship Store	Electricity savings1	I just received the product, I don't know if it works well yet	Jingdong prefecture level city in Shandong
Jinfeng Hongyuan Flagship Store	Electricity savings1	Received it and used it, not sure it really saves electricity use it and then evaluate it, first give a 5 star review!!!!	Jingdong prefecture level city in Shandong
Jinfeng Hongyuan Flagship Store	Electricity savings1	It's good to use, it's very good to use, and it smells OK too	Jingdong prefecture level city in Shandong
Jinfeng Hongyuan Flagship Store	Electricity savings1	Packaging is very good, feel the product is not bad, how much power saving with the only know	Jingdong prefecture level city in Shandong
Jinfeng Hongyuan Flagship Store	Electricity savings1	Additional comments to be made after use	Jingdong prefecture level city in Shandong
Jinfeng Hongyuan Flagship Store	Electricity savings1	The product has been installed, try a few months and then comment!	Jingdong prefecture level city in Shandong
Jinfeng Hongyuan Flagship Store	Electricity savings1	I've bought it, let's use it for now, I'll comment after a month	Jingdong prefecture level city in Shandong
Jinfeng Hongyuan Flagship Store	Electricity savings1	Try it out and then follow up	Jingdong prefecture level city in Shandong
Jinfeng Hongyuan Flagship Store	Electricity savings1	It's good in theory, it doesn't feel good in practice.	Jingdong prefecture level city in Shandong
Jinfeng Hongyuan Flagship Store	Electricity savings1	I haven't tried it yet. We'll see.	Jingdong prefecture level city in Shandong
Jinfeng Hongyuan Flagship Store	Electricity savings1	Great item, second purchase!	Jingdong prefecture level city in Shandong
Jinfeng Hongyuan Flagship Store	Electricity savings1	Goods received now use it, if it does save power, plus reevaluate.	Jingdong prefecture level city in Shandong
Jinfeng Hongyuan Flagship Store	Electricity savings1	The stuff is light and small, the stuff is good, the courier service is good	Jingdong prefecture level city in Shandong
Jinfeng Hongyuan Flagship Store	Electricity savings1	I haven't used it yet. I'm looking forward to it.	Jingdong prefecture level city in Shandong
Jinfeng Hongyuan Flagship Store	Electricity savings1	Express received, in use do not know if there is no power saving	Jingdong prefecture level city in Shandong
Jinfeng Hongyuan Flagship Store	Electricity savings1	The quality of the product is quite good! The power saving mode is still getting used to it, can't say yet, how much power it saves.	Jingdong prefecture level city in Shandong
Jinfeng Hongyuan Flagship Store	Electricity savings1	The goods have been received, just started to use, now I do not know how the quality, after a period of time to say.	Jingdong prefecture level city in Shandong
Jinfeng Hongyuan Flagship Store	Electricity savings1	I received the stuff, used it for a few days to evaluate, the stuff is very good and good, worth buying.	Jingdong prefecture level city in Shandong
Jinfeng Hongyuan Flagship Store	Electricity savings1	I've been using it for a while, but I don't know if it's good or if it saves electricity! What the heck, let's give it a good review first. And for the record, I'm not helping him**.	Jingdong prefecture level city in Shandong
Jinfeng Hongyuan Flagship Store	Electricity savings1	Haven't used it yet, looks good, try it for 60 days, might be good, great review!	Jingdong prefecture level city in Shandong
Jinfeng Hongyuan Flagship Store	Electricity savings1	Baby received, logistics is very fast. Ordered last night, received this morning. Received the goods immediately plugged in the power supply, save power to the first of next month to know.	Jingdong prefecture level city in Shandong
Jinfeng Hongyuan Flagship Store	Electricity savings1	First, I'll give you a good review and use it.	Jingdong prefecture level city in Shandong
Jinfeng Hongyuan Flagship Store	Electricity savings1	Just arrived to follow up with a genuine review	Jingdong prefecture level city in Shandong
Jinfeng Hongyuan Flagship Store	Electricity savings1	Not bad pretty fast, good quality.	Jingdong prefecture level city in Shandong
Jinfeng Hongyuan Flagship Store	Electricity savings1	Recommended, use it and hopefully it will save some electricity	Jingdong prefecture level city in Shandong
Jinfeng Hongyuan Flagship Store	Electricity savings1	The stuff looks ok, I don't know the effect if it.	Jingdong prefecture level city in Shandong
Jinfeng Hongyuan Flagship Store	Electricity savings1	The stuff looks OK, the stuff looks OK.	Jingdong prefecture level city in Shandong
Jinfeng Hongyuan Flagship Store	Electricity savings1	The item has been received, after using it in a follow-up review. Satisfied with the purchase.	Jingdong prefecture level city in Shandong
Jinfeng Hongyuan Flagship Store	Electricity savings1	Has been normal plug-in use, at present do not know how to see, use down to see and then evaluation	Jingdong prefecture level city in Shandong
Jinfeng Hongyuan Flagship Store	Electricity savings1	Commodities make noise.	Jingdong prefecture level city in Shandong
Jinfeng Hongyuan Flagship Store	Electricity savings1	After using it for a few days and observing it, the saving is about ten percent	Jingdong prefecture level city in Shandong
Jinfeng Hongyuan Flagship Store	Electricity savings1	The goods received, the quality looks quite OK. First use it, the effect is not good to say.	Jingdong prefecture level city in Shandong
Jinfeng Hongyuan Flagship Store	Electricity savings1	In use, follow up next month	Jingdong prefecture level city in Shandong
Jinfeng Hongyuan Flagship Store	Electricity savings1	I just received the goods and have not yet used it to comment after using it	Jingdong prefecture level city in Shandong
Jinfeng Hongyuan Flagship Store	Electricity savings1	So far I don't know if it really saves electricity yet! Spinning waiting a couple months to find out! Good reviews first!	Jingdong prefecture level city in Shandong
Jinfeng Hongyuan Flagship Store	Electricity savings1	Small and exquisite, looking forward to the effect, to be evaluated 6666666666666	Jingdong prefecture level city in Shandong
Jinfeng Hongyuan Flagship Store	Electricity savings1	I just used it, I don't know if it's effective, I'll comment again after a month	Jingdong prefecture level city in Shandong
Jinfeng Hongyuan Flagship Store	Electricity savings1	I just used it, I don't know if it's good or not, I'll comment later.	Jingdong prefecture level city in Shandong
Jinfeng Hongyuan Flagship Store	Electricity savings1	Baby received, Baobei quite good, use and then come back to comment!	Jingdong prefecture level city in Shandong
Jinfeng Hongyuan Flagship Store	Electricity savings1	Good quality power saving is fake	Jingdong prefecture level city in Shandong

Jinfeng Hongyuan Flagship Store	Electricity savings1	The packaging of the goods is atmospheric, the quality is exquisite, and I look forward to saving electricity.	Jingdong prefecture level city in Shandong
Jinfeng Hongyuan Flagship Store	Electricity savings1	The packaging is great and the quality is good!	Jingdong prefecture level city in Shandong
Jinfeng Hongyuan Flagship Store	Electricity savings1	I don't see any effect.	Jingdong prefecture level city in Shandong
Jinfeng Hongyuan Flagship Store	Electricity savings1	Already used, but did not see obvious results	Jingdong prefecture level city in Shandong
Jinfeng Hongyuan Flagship Store	Electricity savings1	It's good. I'm just using it. I don't know what it's like.	Jingdong prefecture level city in Shandong
Jinfeng Hongyuan Flagship Store	Electricity savings1	I just used it. I'll comment later.	Jingdong prefecture level city in Shandong
Jinfeng Hongyuan Flagship Store	Electricity savings1	I installed it when it arrived, try it for a month and then come back for a follow-up review	Jingdong prefecture level city in Shandong
Jinfeng Hongyuan Flagship Store	Electricity savings1	Goods arrived a few days fastidious does not call to pick up the goods, the quality is average	Jingdong prefecture level city in Shandong
Jinfeng Hongyuan Flagship Store	Electricity savings1	This user thinks the product is good	Jingdong prefecture level city in Shandong
Jinfeng Hongyuan Flagship Store	Electricity savings1	Roger that.	Jingdong prefecture level city in Shandong
Jinfeng Hongyuan Flagship Store	Electricity savings1	Second purchase was ok.	Jingdong prefecture level city in Shandong
Jinfeng Hongyuan Flagship Store	Electricity savings1	The merchant is very reputable and the product is good value for money. Logistics is fast!	Jingdong prefecture level city in Shandong
Jinfeng Hongyuan Flagship Store	Electricity savings1	The quality of the baby is very good, I have always trusted Kyodo	Jingdong prefecture level city in Shandong
Jinfeng Hongyuan Flagship Store	Electricity savings1	We'll see how it goes when we get it.	Jingdong prefecture level city in Shandong
Jinfeng Hongyuan Flagship Store	Electricity savings1	I just used it. I don't know if it works.	Jingdong prefecture level city in Shandong
Jinfeng Hongyuan Flagship Store	Electricity savings1	Good quality, used for a month to save about 20%, power saving effect is good	Jingdong prefecture level city in Shandong
Jinfeng Hongyuan Flagship Store	Electricity savings1	I'll give you a good review before I use it.	Jingdong prefecture level city in Shandong
Jinfeng Hongyuan Flagship Store	Electricity savings1	Received, satisfied with the follow-up evaluation	Jingdong prefecture level city in Shandong
Jinfeng Hongyuan Flagship Store	Electricity savings1	Immediate results, saving electricity and money	Jingdong prefecture level city in Shandong
Jinfeng Hongyuan Flagship Store	Electricity savings1	Measured to save a little	Jingdong prefecture level city in Shandong
Jinfeng Hongyuan Flagship Store	Electricity savings1	This user thinks the product is good	Jingdong prefecture level city in Shandong
Jinfeng Hongyuan Flagship Store	Electricity savings1	This user thinks the product is good	Jingdong prefecture level city in Shandong
Jinfeng Hongyuan Flagship Store	Electricity savings1	Product quality and logistics are very good, worth buying	Jingdong prefecture level city in Shandong
Jinfeng Hongyuan Flagship Store	Electricity savings1	Appearance looks ok! Express delivery was fast! Service is still relatively enthusiastic.	Jingdong prefecture level city in Shandong
Jinfeng Hongyuan Flagship Store	Electricity savings1	It doesn't feel like it's working.	Jingdong prefecture level city in Shandong
Jinfeng Hongyuan Flagship Store	Electricity savings1	Well made and looks good. Used it with other appliances and felt as if it had some effect.	Jingdong prefecture level city in Shandong
Jinfeng Hongyuan Flagship Store	Electricity savings1	Haven't used it yet, will follow up later	Jingdong prefecture level city in Shandong
Jinfeng Hongyuan Flagship Store	Electricity savings1	Things received, used a few days the plug is a little hot unplugged did not work	Jingdong prefecture level city in Shandong
Jinfeng Hongyuan Flagship Store	Electricity savings1	Look at the meter again in a month.	Jingdong prefecture level city in Shandong
Jinfeng Hongyuan Flagship Store	Electricity savings1	This one didn't work at all.	Jingdong prefecture level city in Shandong
Jinfeng Hongyuan Flagship Store	Electricity savings1	It's small. I've seen friends with much bigger ones.	Jingdong prefecture level city in Shandong
Jinfeng Hongyuan Flagship Store	Electricity savings1	The item was received and the quality is good, it will be nice to see how the power saving rate is down the road, the item is great.	Jingdong prefecture level city in Shandong
Jinfeng Hongyuan Flagship Store	Electricity savings1	Cheap and affordable quality is quite good, logistics is also very fast, praise! , baby good 😊, cost-effective very high, big brand trustworthy 👍	Jingdong prefecture level city in Shandong
Jinfeng Hongyuan Flagship Store	Electricity savings1	Used it for a while it really saves electricity ⚡ thanks 🈶 such a good product, compact and practical, every family should buy one ✨	Jingdong prefecture level city in Shandong
Jinfeng Hongyuan Flagship Store	Electricity savings1	Very satisfied with the purchase Next time I will come back	Jingdong prefecture level city in Shandong
Jinfeng Hongyuan Flagship Store	Electricity savings1	Logistics is fast. Good quality, used one before and it does save power	Jingdong prefecture level city in Shandong
Jinfeng Hongyuan Flagship Store	Electricity savings1	I don't know if it works.	Jingdong prefecture level city in Shandong
Jinfeng Hongyuan Flagship Store	Electricity savings1	Good goods, very solid, good.	Jingdong prefecture level city in Shandong
Jinfeng Hongyuan Flagship Store	Electricity savings1	This user thinks the product is good	Jingdong prefecture level city in Shandong
Jinfeng Hongyuan Flagship Store	Electricity savings1	I've been using it for half a month, my electricity bill is much lower, and the quality is very good, so don't worry about buying it!	Jingdong prefecture level city in Shandong
Jinfeng Hongyuan Flagship Store	Electricity savings1	I received the goods today and used it right away, looking forward to the effect	Jingdong prefecture level city in Shandong
Jinfeng Hongyuan Flagship Store	Electricity savings1	The merchandise is great. I am going to recommend it to my friends! Will buy again next time.	Jingdong prefecture level city in Shandong
Jinfeng Hongyuan Flagship Store	Electricity savings1	Let's give it a good review first, it's working fine so far, and I'll follow up with a review,...	Jingdong prefecture level city in Shandong
Jinfeng Hongyuan Flagship Store	Electricity savings1	Product quality is very good, looking forward to using the results	Jingdong prefecture level city in Shandong
Jinfeng Hongyuan Flagship Store	Electricity savings1	I just can't detect it. - Save it.	Jingdong prefecture level city in Shandong
Jinfeng Hongyuan Flagship Store	Electricity savings1	It arrived and is being used, so we'll see how it works!	Jingdong prefecture level city in Shandong
Jinfeng Hongyuan Flagship Store	Electricity savings1	Received yesterday thanks! This energy saver is available . Safe! But in the end how much energy can be saved must be used for a period of time to know, if the real energy saving next time to buy again.	Jingdong prefecture level city in Shandong
Jinfeng Hongyuan Flagship Store	Electricity savings1	Just used it, don't know if it saves power	Jingdong prefecture level city in Shandong
Jinfeng Hongyuan Flagship Store	Electricity savings1	I'll rate it first, is it a tease wait two months and see? It's been four days and it's not noticeable!	Jingdong prefecture level city in Shandong
Jinfeng Hongyuan Flagship Store	Electricity savings1	Workmanship is quite good, just started to use, the effect of the follow-up evaluation.	Jingdong prefecture level city in Shandong
Jinfeng Hongyuan Flagship Store	Electricity savings1	Received the goods used for a few days before evaluation, the effect is quite good	Jingdong prefecture level city in Shandong

Jinfeng Hongyuan Flagship Store	Electricity savings1	Try it for a month and then evaluate	Jingdong prefecture level city in Shandong
Jinfeng Hongyuan Flagship Store	Electricity savings1	Just received the goods, the packaging is very good, the appearance of the economizer feels good, I do not know how the effect of power saving, with a month or two and then follow up!	Jingdong prefecture level city in Shandong
Jinfeng Hongyuan Flagship Store	Electricity savings1	It's quite small and delicate, I don't know if it's useful yet.	Jingdong prefecture level city in Shandong
Jinfeng Hongyuan Flagship Store	Electricity savings1	Just received it, use it for a few days before commenting	Jingdong prefecture level city in Shandong
Jinfeng Hongyuan Flagship Store	Electricity savings1	Does this thing work! When it arrived, the button for the safety was loose. I'm a little afraid of fire!	Jingdong prefecture level city in Shandong
Jinfeng Hongyuan Flagship Store	Electricity savings1	I received it. I don't know how it works.	Jingdong prefecture level city in Shandong
Jinfeng Hongyuan Flagship Store	Electricity savings1	This user thinks the product is good	Jingdong prefecture level city in Shandong
Jinfeng Hongyuan Flagship Store	Electricity savings1	Customer service was great! Small i problem was taken care of in a timely manner! Effectiveness to be tested by time! No rush!!!	Jingdong prefecture level city in Shandong
Jinfeng Hongyuan Flagship Store	Electricity savings1	I put it on right after I received it and it looks compact. I believe the high technology should save electricity.	Jingdong prefecture level city in Shandong
Jinfeng Hongyuan Flagship Store	Electricity savings1	Logistics courier very quickly, the first day under the order the next day to receive, off pieces whether power saving is the most important thing, things are exquisite workmanship, a month later in the follow-up evaluation whether power saving	Jingdong prefecture level city in Shandong
Jinfeng Hongyuan Flagship Store	Electricity savings1	The delivery is pretty fast, I'm using it, have to have it for a while to see results, looking forward to it.	Jingdong prefecture level city in Shandong
Jinfeng Hongyuan Flagship Store	Electricity savings1	See pretty good just started to use a month later to give you a real evaluation, should be useful.	Jingdong prefecture level city in Shandong
Jinfeng Hongyuan Flagship Store	Electricity savings1	And then comment.	Jingdong prefecture level city in Shandong
Jinfeng Hongyuan Flagship Store	Electricity savings1	Received the goods, wait for the test to evaluate	Jingdong prefecture level city in Shandong
Jinfeng Hongyuan Flagship Store	Electricity savings1	This user thinks the product is good	Jingdong prefecture level city in Shandong
Jinfeng Hongyuan Flagship Store	Electricity savings1	I'm already using it, I just don't know how to judge the power savings. I won't know until I use it for a season.	Jingdong prefecture level city in Shandong
Jinfeng Hongyuan Flagship Store	Electricity savings1	Works. Says it works.	Jingdong prefecture level city in Shandong
Jinfeng Hongyuan Flagship Store	Electricity savings1	Very good. Satisfied.	Jingdong prefecture level city in Shandong
Jinfeng Hongyuan Flagship Store	Electricity savings1	This user thinks the product is good	Jingdong prefecture level city in Shandong
Jinfeng Hongyuan Flagship Store	Electricity savings1	It's working. Obviously, it's less expensive.	Jingdong prefecture level city in Shandong
Jinfeng Hongyuan Flagship Store	Electricity savings1	The second one is up, the first one still works! It's reactive power compensation! This is well understood if you want a professional!	Jingdong prefecture level city in Shandong
Jinfeng Hongyuan Flagship Store	Electricity savings1	It feels as if there are some nodes, but they need to be tested and observed over time, thanks.	Jingdong prefecture level city in Shandong
Jinfeng Hongyuan Flagship Store	Electricity savings1	I've received the goods, the energy saving effect will be evaluated after using it for a period of time, and all the services are very good, praise!	Jingdong prefecture level city in Shandong
Jinfeng Hongyuan Flagship Store	Electricity savings1	I don't know how well it works yet, I bought it and plugged it in, so we'll see if it saves some power or not!	Jingdong prefecture level city in Shandong
Jinfeng Hongyuan Flagship Store	Electricity savings1	Baby a bunch of knife quality is very good, previously bought a quality is not very good. It really works and has results.	Jingdong prefecture level city in Shandong
Jinfeng Hongyuan Flagship Store	Electricity savings1	Things have arrived, it looks good, just started to use, as to whether or not there is a business said the power-saving effect, but also to observe the effect, now can not evaluate	Jingdong prefecture level city in Shandong
Jinfeng Hongyuan Flagship Store	Electricity savings1	I haven't used it yet. I'll comment afterward.	Jingdong prefecture level city in Shandong
Jinfeng Hongyuan Flagship Store	Electricity savings1	I don't know if it's any good.	Jingdong prefecture level city in Shandong
Jinfeng Hongyuan Flagship Store	Electricity savings1	I don't know how well it works.	Jingdong prefecture level city in Shandong
Jinfeng Hongyuan Flagship Store	Electricity savings1	The baby has been received! Uh-huh! Good, quite exquisite, small and exquisite I thought it was big. Dare to tightly plugged in the electricity, just wait for the effect later!	Jingdong prefecture level city in Shandong
Jinfeng Hongyuan Flagship Store	Electricity savings1	The packaging is sturdy, I tried it right after I received it, so I'm looking forward to the results.	Jingdong prefecture level city in Shandong
Jinfeng Hongyuan Flagship Store	Electricity savings1	Received the item and installed it! Don't know if it works well! Will follow up with a review later! I hope it works!	Jingdong prefecture level city in Shandong
Jinfeng Hongyuan Flagship Store	Electricity savings1	Things used on the effect of waiting to use a little to say, but things are very good. Logistics is very fast, business service is very good.	Jingdong prefecture level city in Shandong
Jinfeng Hongyuan Flagship Store	Electricity savings1	This user thinks the product is good	Jingdong prefecture level city in Shandong
Jinfeng Hongyuan Flagship Store	Electricity savings1	I'm really using it. It feels good.	Jingdong prefecture level city in Shandong
Jinfeng Hongyuan Flagship Store	Electricity savings1	Good, Jingdong logistics is very fast, intelligent energy-saving appliances	Jingdong prefecture level city in Shandong
Jinfeng Hongyuan Flagship Store	Electricity savings1	It's not bad. It really saves energy.	Jingdong prefecture level city in Shandong
Jinfeng Hongyuan Flagship Store	Electricity savings1	The logistics is fast, praise	Jingdong prefecture level city in Shandong
Jinfeng Hongyuan Flagship Store	Electricity savings1	This user thinks the product is good	Jingdong prefecture level city in Shandong
Jinfeng Hongyuan Flagship Store	Electricity savings1	The first time I placed two, and today I placed another one, a friend introduced it, said it is particularly power-saving	Jingdong prefecture level city in Shandong
Jinfeng Hongyuan Flagship Store	Electricity savings1	I don't know how effective it is. Let's leave it at home for a while and see.	Jingdong prefecture level city in Shandong
Jinfeng Hongyuan Flagship Store	Electricity savings1	Dear customer service! I would like to ask you how to understand the digital display (increase/decrease jump) on the top of the saver? Thank you for your help.	Jingdong prefecture level city in Shandong
Jinfeng Hongyuan Flagship Store	Electricity savings1	This user thinks the product is good	Jingdong prefecture level city in Shandong
Jinfeng Hongyuan Flagship Store	Electricity savings1	The goods have been received, packaging exquisite, is using, later to see how the effect?	Jingdong prefecture level city in Shandong
Jinfeng Hongyuan Flagship Store	Electricity savings1	The saver received, the boss shipped very quickly, yesterday shot today arrived!	Jingdong prefecture level city in Shandong
Jinfeng Hongyuan Flagship Store	Electricity savings1	Beautiful appearance, well packaged. Recording data to see if I can lower my electric bill a bit.	Jingdong prefecture level city in Shandong
Jinfeng Hongyuan Flagship Store	Electricity savings1	Things received, the packaging is quite good, the effect of the use and then come back to evaluate.	Jingdong prefecture level city in Shandong
Jinfeng Hongyuan Flagship Store	Electricity savings1	Easy to use, just plug it in. But there is a current sound.	Jingdong prefecture level city in Shandong
Jinfeng Hongyuan Flagship Store	Electricity savings1	Fast logistics, easy to use and labor-saving	Jingdong prefecture level city in Shandong
Jinfeng Hongyuan Flagship Store	Electricity savings1	Received, thank you!	Jingdong prefecture level city in Shandong
Jinfeng Hongyuan Flagship Store	Electricity savings1	Second time I've bought it. It's good.	Jingdong prefecture level city in Shandong

Jinfeng Hongyuan Flagship Store	Electricity savings1	I've only been using it for a short time, so I'm looking forward to the results.	Jingdong prefecture level city in Shandong
Jinfeng Hongyuan Flagship Store	Electricity savings1	Good stuff, good stuff, good stuff good stuff.	Jingdong prefecture level city in Shandong
Jinfeng Hongyuan Flagship Store	Electricity savings1	Buy your stuff is not always good I have been using it for almost two months now, it does not save electricity at all the electricity bill is about ten dollars a day this is still called the king of power	Jingdong prefecture level city in Shandong
Jinfeng Hongyuan Flagship Store	Electricity savings1	Express arrived, the quality is very good, worth the purchase.	Jingdong prefecture level city in Shandong
Jinfeng Hongyuan Flagship Store	Electricity savings1	Energy saving has been received, now the voltage is more stable, energy-saving effect to see a few days and then chase flat, the service attitude is very good, the courier is relatively fast, I am satisfied.	Jingdong prefecture level city in Shandong
Jinfeng Hongyuan Flagship Store	Electricity savings1	Stuff installed, numbers are smaller than when installed, hopefully it works	Jingdong prefecture level city in Shandong
Jinfeng Hongyuan Flagship Store	Electricity savings1	Good appearance, fine workmanship, just bought, use up the feeling of power saving effect is good, worth buying!	Jingdong prefecture level city in Shandong
Jinfeng Hongyuan Flagship Store	Electricity savings1	Economical and practical 👍 Always use it	Jingdong prefecture level city in Shandong
Jinfeng Hongyuan Flagship Store	Electricity savings1	Just used it, don't know the effect yet, will come back to follow up after the effect. Hope it works well(▽)	Jingdong prefecture level city in Shandong
Jinfeng Hongyuan Flagship Store	Electricity savings1	The goods have been received, use it to see in the evaluation.	Jingdong prefecture level city in Shandong
Jinfeng Hongyuan Flagship Store	Electricity savings1	Doesn't it save electricity? The boil kettle still goes faster when you press the meter.	Jingdong prefecture level city in Shandong
Jinfeng Hongyuan Flagship Store	Electricity savings1	Already used. Will patronize again in the future	Jingdong prefecture level city in Shandong
Jinfeng Hongyuan Flagship Store	Electricity savings1	Works great, just plug it in, results to be checked, satisfied.	Jingdong prefecture level city in Shandong
Jinfeng Hongyuan Flagship Store	Electricity savings1	Second purchase. Works well 😍😍😍😍	Jingdong prefecture level city in Shandong
Jinfeng Hongyuan Flagship Store	Electricity savings1	Prompt shipment, very satisfied	Jingdong prefecture level city in Shandong
Jinfeng Hongyuan Flagship Store	Electricity savings1	It's been less than a month, let's see how it works, hopefully it's power efficient	Jingdong prefecture level city in Shandong
Jinfeng Hongyuan Flagship Store	Electricity savings1	Use a month in say.	Jingdong prefecture level city in Shandong
Jinfeng Hongyuan Flagship Store	Electricity savings1	I've saved a lot of electricity since I've been using it.	Jingdong prefecture level city in Shandong
Jinfeng Hongyuan Flagship Store	Electricity savings1	It's fast, looks and feels good on the outside, try it out and evaluate.	Jingdong prefecture level city in Shandong
Jinfeng Hongyuan Flagship Store	Electricity savings1	I haven't used it. I just used it. I don't know if it's any good.	Jingdong prefecture level city in Shandong
Jinfeng Hongyuan Flagship Store	Electricity savings1	Quality can be, power saving now can not see just use, after a while in the look at the power saving effect	Jingdong prefecture level city in Shandong
Jinfeng Hongyuan Flagship Store	Electricity savings1	The logistics is very fast, and it seems to have some effect	Jingdong prefecture level city in Shandong
Jinfeng Hongyuan Flagship Store	Electricity savings1	Good, but the effects remain to be seen	Jingdong prefecture level city in Shandong
Jinfeng Hongyuan Flagship Store	Electricity savings1	Baby is basically the same as the description, shipping quickly, worth a buy Good price, logistics is strong, worth repurchasing!	Jingdong prefecture level city in Shandong
Jinfeng Hongyuan Flagship Store	Electricity savings1	I bought it for my dad, I don't know much about it, if he thinks it's good then it must be good 👍	Jingdong prefecture level city in Shandong
Jinfeng Hongyuan Flagship Store	Electricity savings1	Goods received. It's working fine. Display voltage. After a period of time to see the effect in the addition to the level of theory	Jingdong prefecture level city in Shandong
Jinfeng Hongyuan Flagship Store	Electricity savings1	The stuff is pretty good, I just don't know how well it saves power.	Jingdong prefecture level city in Shandong
Jinfeng Hongyuan Flagship Store	Electricity savings1	Logistics is a little too fast 👍	Jingdong prefecture level city in Shandong
Jinfeng Hongyuan Flagship Store	Electricity savings1	Received the goods used for a period of time before coming, good, beautiful appearance, really stable flow	Jingdong prefecture level city in Shandong
Jinfeng Hongyuan Flagship Store	Electricity savings1	Things received, look at the packaging is very good, not yet, to the power saving in the follow-up evaluation	Jingdong prefecture level city in Shandong
Jinfeng Hongyuan Flagship Store	Electricity savings1	Baby is very good! Use a few days before to evaluate, I personally test really effective.	Jingdong prefecture level city in Shandong
Jinfeng Hongyuan Flagship Store	Electricity savings1	I received the goods are in use, I do not know how effective? Can it save energy? Let's see.	Jingdong prefecture level city in Shandong
Jinfeng Hongyuan Flagship Store	Electricity savings1	It's really good and has some power saving effect.	Jingdong prefecture level city in Shandong
Jinfeng Hongyuan Flagship Store	Electricity savings1	It's good quality, well made, and I hope it'll save on electricity over time.	Jingdong prefecture level city in Shandong
Jinfeng Hongyuan Flagship Store	Electricity savings1	I just used it, I don't know how it works yet, but I'll give it a good review first.	Jingdong prefecture level city in Shandong
Jinfeng Hongyuan Flagship Store	Electricity savings1	Second purchase, the delivery guy was fast and called ahead to deliver.	Jingdong prefecture level city in Shandong
Jinfeng Hongyuan Flagship Store	Electricity savings1	Goods have been received, but have not yet used, used to see how the effect, in the evaluation of fertilizer	Jingdong prefecture level city in Shandong
Jinfeng Hongyuan Flagship Store	Electricity savings1	The delivery is very fast. It's the same in kind. It's working.	Jingdong prefecture level city in Shandong
Jinfeng Hongyuan Flagship Store	Electricity savings1	Fast logistics, good quality, more satisfied	Jingdong prefecture level city in Shandong
Jinfeng Hongyuan Flagship Store	Electricity savings1	I've been here for almost a month now and there is a very noticeable difference than it is worth buying.	Jingdong prefecture level city in Shandong
Jinfeng Hongyuan Flagship Store	Electricity savings1	Fast shipping, received the next day, plug and play, but do not know how effective to not break down!	Jingdong prefecture level city in Shandong
Jinfeng Hongyuan Flagship Store	Electricity savings1	The logistics are great I've been using it for a while before commenting on it This saves electricity	Jingdong prefecture level city in Shandong
Jinfeng Hongyuan Flagship Store	Electricity savings1	Wait to use it for a while to know if it's energy efficient	Jingdong prefecture level city in Shandong
Jinfeng Hongyuan Flagship Store	Electricity savings1	Just received it, haven't used it yet don't know if it's any good.	Jingdong prefecture level city in Shandong
Jinfeng Hongyuan Flagship Store	Electricity savings1	It really doesn't work, and the electric bill is still the same amount every month.	Jingdong prefecture level city in Shandong
Jinfeng Hongyuan Flagship Store	Electricity savings1	It's well packaged and looks fine	Jingdong prefecture level city in Shandong
Jinfeng Hongyuan Flagship Store	Electricity savings1	Very good. Will patronize again if needed.	Jingdong prefecture level city in Shandong
Jinfeng Hongyuan Flagship Store	Electricity savings1	I have received the product and installed it, try it for a while and see how it works.	Jingdong prefecture level city in Shandong
Jinfeng Hongyuan Flagship Store	Electricity savings1	I've been using it for a month now...I don't feel like there's any noticeable power savings...last month it was $130 this month it's $123...let's see if I can use it for another month	Jingdong prefecture level city in Shandong
Jinfeng Hongyuan Flagship Store	Electricity savings1	Just received it, yet to try it and see if it works.	Jingdong prefecture level city in Shandong
Jinfeng Hongyuan Flagship Store	Electricity savings1	It's easy to use and has a power saving credit!	Jingdong prefecture level city in Shandong

Jinfeng Hongyuan Flagship Store	Electricity savings1	It's very good. I've already bought two of them.	Jingdong prefecture level city in Shandong
Jinfeng Hongyuan Flagship Store	Electricity savings1	It's very well made, packaged meticulously, and I hope it works!	Jingdong prefecture level city in Shandong
Jinfeng Hongyuan Flagship Store	Electricity savings1	Goods have been received have not yet used it, used to comment again	Jingdong prefecture level city in Shandong
Jinfeng Hongyuan Flagship Store	Electricity savings1	Looking forward to good results	Jingdong prefecture level city in Shandong
Jinfeng Hongyuan Flagship Store	Electricity savings1	Not sure if it saves power or not. Still trying it out.	Jingdong prefecture level city in Shandong
Jinfeng Hongyuan Flagship Store	Electricity savings1	It also really saves on electricity! Superb!	Jingdong prefecture level city in Shandong
Jinfeng Hongyuan Flagship Store	Electricity savings1	Didn't work at all, want to return it	Jingdong prefecture level city in Shandong
Jinfeng Hongyuan Flagship Store	Electricity savings1	Uh-huh! It's working, I've been using it for a month and it saves 20% of electricity compared to before.	Jingdong prefecture level city in Shandong
Jinfeng Hongyuan Flagship Store	Electricity savings1	Should be a little bit of a power saver, I wonder how long it will take to save back the money I spent on this one	Jingdong prefecture level city in Shandong
Jinfeng Hongyuan Flagship Store	Electricity savings1	Exquisite appearance and stable voltage	Jingdong prefecture level city in Shandong
Jinfeng Hongyuan Flagship Store	Electricity savings1	After using it for a few days it feels like it saves electricity.	Jingdong prefecture level city in Shandong
Jinfeng Hongyuan Flagship Store	Electricity savings1	Second purchase and its pending good results.	Jingdong prefecture level city in Shandong
Jinfeng Hongyuan Flagship Store	Electricity savings1	The appearance of the workmanship is exquisite and meticulous, beautiful appearance, just to see if there is no effect!	Jingdong prefecture level city in Shandong
Jinfeng Hongyuan Flagship Store	Electricity savings1	Let's see what happens in a month.	Jingdong prefecture level city in Shandong
Jinfeng Hongyuan Flagship Store	Electricity savings1	Trial, now short time not yet obvious effect	Jingdong prefecture level city in Shandong
Jinfeng Hongyuan Flagship Store	Electricity savings1	I'm using it now, don't know how well it works, it looks great!	Jingdong prefecture level city in Shandong
Jinfeng Hongyuan Flagship Store	Electricity savings1	There is no role! Now it's true that I don't know!	Jingdong prefecture level city in Shandong
Jinfeng Hongyuan Flagship Store	Electricity savings1	Purchased for the second time, power saving not sure if it's ok? Let's talk about it after using it	Jingdong prefecture level city in Shandong
Jinfeng Hongyuan Flagship Store	Electricity savings1	I just received it, I have to try it for a month to know how it works.	Jingdong prefecture level city in Shandong
Jinfeng Hongyuan Flagship Store	Electricity savings1	I just used it for an hour, and the meter's still spinning so fast it's not much use.	Jingdong prefecture level city in Shandong
Jinfeng Hongyuan Flagship Store	Electricity savings1	Not bad, logistics is fast	Jingdong prefecture level city in Shandong
Jinfeng Hongyuan Flagship Store	Electricity savings1	I don't know if it works. I'll find out next month when I pay the bill. It's easy. Just plug it in.	Jingdong prefecture level city in Shandong
Jinfeng Hongyuan Flagship Store	Electricity savings1	Logistics is particularly fast, received the goods are genuine, looking forward to power saving effect	Jingdong prefecture level city in Shandong
Jinfeng Hongyuan Flagship Store	Electricity savings1	First time buying this product like this one from this family use it and then evaluate.	Jingdong prefecture level city in Shandong
Jinfeng Hongyuan Flagship Store	Electricity savings1	Lightning arrived, the product is very good, before all do not know there is such a baby, in a friend's home has seen, and then bought to try, has been used for a period of time before coming to the evaluation, the work is very stable, so far looked very good, will introduce friends to buy!	Jingdong prefecture level city in Shandong
Jinfeng Hongyuan Flagship Store	Electricity savings1	I was a bit skeptical when I heard that I could save money on my electricity bill by installing this, I don't know if the power saving effect is really what it is said to be. But since I bought it, I just have to put it on and try it out. I hope it lives up to its name and saves me the money I spent on the device	Jingdong prefecture level city in Shandong
Jinfeng Hongyuan Flagship Store	Electricity savings1	The quality of the stuff is okay, but I'm not sure if it works, so I'll try using it first.	Jingdong prefecture level city in Shandong
Jinfeng Hongyuan Flagship Store	Electricity savings1	It should work. The current is much more stable.	Jingdong prefecture level city in Shandong
Jinfeng Hongyuan Flagship Store	Electricity savings1	The store has a great service attitude	Jingdong prefecture level city in Shandong
Jinfeng Hongyuan Flagship Store	Electricity savings1	Let's wait until we've used it for a month.	Jingdong prefecture level city in Shandong
Jinfeng Hongyuan Flagship Store	Electricity savings1	I'm using it and I don't know if it's a power saver or not	Jingdong prefecture level city in Shandong
Jinfeng Hongyuan Flagship Store	Electricity savings1	Original and authentic guarantee quality is good now.	Jingdong prefecture level city in Shandong
Jinfeng Hongyuan Flagship Store	Electricity savings1	Great, very happy with it, will buy a couple more next time	Jingdong prefecture level city in Shandong
Jinfeng Hongyuan Flagship Store	Electricity savings1	I just used it, I don't know if it works yet, I'll come back after using it...	Jingdong prefecture level city in Shandong
Jinfeng Hongyuan Flagship Store	Electricity savings1	It's still being tested.	Jingdong prefecture level city in Shandong
Jinfeng Hongyuan Flagship Store	Electricity savings1	I've been using it for a month and it's not working.	Jingdong prefecture level city in Shandong
Jinfeng Hongyuan Flagship Store	Electricity savings1	It's basically nothing!	Jingdong prefecture level city in Shandong
Jinfeng Hongyuan Flagship Store	Electricity savings1	It's not bad, it kind of works, support it.	Jingdong prefecture level city in Shandong
Jinfeng Hongyuan Flagship Store	Electricity savings1	I just received it last night, let's try it first to see how it works, it looks good!	Jingdong prefecture level city in Shandong
Jinfeng Hongyuan Flagship Store	Electricity savings1	The current is very stable, fine workmanship, small and exquisite, very satisfied	Jingdong prefecture level city in Shandong
Jinfeng Hongyuan Flagship Store	Electricity savings1	Not bad, exactly how much electricity can be saved; still have to follow up the evaluation	Jingdong prefecture level city in Shandong
Jinfeng Hongyuan Flagship Store	Electricity savings1	Logistics is also very fast, next time there is an opportunity to introduce friends to buy!	Jingdong prefecture level city in Shandong
Jinfeng Hongyuan Flagship Store	Electricity savings1	Voltage display and actual do not pay	Jingdong prefecture level city in Shandong
Jinfeng Hongyuan Flagship Store	Electricity savings1	Second purchase, still saves a little on the electric bill	Jingdong prefecture level city in Shandong
Jinfeng Hongyuan Flagship Store	Electricity savings1	Order received, currently trying it out, thanks a lot.	Jingdong prefecture level city in Shandong
Jinfeng Hongyuan Flagship Store	Electricity savings1	I haven't used it for a long time yet, I don't know if the effect is good or not, looking forward to it	Jingdong prefecture level city in Shandong
Jinfeng Hongyuan Flagship Store	Electricity savings1	Good, working, I just don't know if it will save electricity... 5 stars for now.	Jingdong prefecture level city in Shandong
Jinfeng Hongyuan Flagship Store	Electricity savings1	Great product, just as the owner said it would be.	Jingdong prefecture level city in Shandong
Jinfeng Hongyuan Flagship Store	Electricity savings1	Shipping was fast, I don't know if it works well or not, I have to use it for a while first.	Jingdong prefecture level city in Shandong
Jinfeng Hongyuan Flagship Store	Electricity savings1	I don't feel that it saves much electricity, but I'll give it a try. It's been more than a month now, and the electricity bill is still a bit high.	Jingdong prefecture level city in Shandong

Jinfeng Hongyuan Flagship Store	Electricity savings1	Got it. Just used it.	Jingdong prefecture level city in Shandong
Jinfeng Hongyuan Flagship Store	Electricity savings1	Use it for a while and then comment.	Jingdong prefecture level city in Shandong
Jinfeng Hongyuan Flagship Store	Electricity savings1	Used it, see how it works with it and then comment. The quality is very good!	Jingdong prefecture level city in Shandong
Jinfeng Hongyuan Flagship Store	Electricity savings1	I'll comment again after a while.	Jingdong prefecture level city in Shandong
Jinfeng Hongyuan Flagship Store	Electricity savings1	It's easy to operate, affordable and works well, love it.	Jingdong prefecture level city in Shandong
Jinfeng Hongyuan Flagship Store	Electricity savings1	It's good. You can try it. It's cheap.	Jingdong prefecture level city in Shandong
Jinfeng Hongyuan Flagship Store	Electricity savings1	It's in use, so we'll see how it works after a while!	Jingdong prefecture level city in Shandong
Jinfeng Hongyuan Flagship Store	Electricity savings1	Service is pretty good, not sure about the quality yet.	Jingdong prefecture level city in Shandong
Jinfeng Hongyuan Flagship Store	Electricity savings1	Just used it and will see the results in a month 😀	Jingdong prefecture level city in Shandong
Jinfeng Hongyuan Flagship Store	Electricity savings1	Saving electricity is expensive and good	Jingdong prefecture level city in Shandong
Jinfeng Hongyuan Flagship Store	Electricity savings1	Pretty good, 5 stars! Will buy again next time seller service is very good ！！！！ Good quality! Will recommend to friends!	Jingdong prefecture level city in Shandong
Jinfeng Hongyuan Flagship Store	Electricity savings1	In use, with results naturally have follow-up reviews!	Jingdong prefecture level city in Shandong
Jinfeng Hongyuan Flagship Store	Electricity savings1	I'm using it now, I'll come back with results, it'll work.	Jingdong prefecture level city in Shandong
Jinfeng Hongyuan Flagship Store	Electricity savings1	Good, nice, fast shipping, cheap, worth having!	Jingdong prefecture level city in Shandong
Jinfeng Hongyuan Flagship Store	Electricity savings1	Logistics is fast.	Jingdong prefecture level city in Shandong
Jinfeng Hongyuan Flagship Store	Electricity savings1	I have just started using the product and will be reviewing it later.	Jingdong prefecture level city in Shandong
Jinfeng Hongyuan Flagship Store	Electricity savings1	I'll comment after I use it.	Jingdong prefecture level city in Shandong
Jinfeng Hongyuan Flagship Store	Electricity savings1	I don't know if it's working or not, though I'll try plugging it in.	Jingdong prefecture level city in Shandong
Jinfeng Hongyuan Flagship Store	Electricity savings1	It really saves electricity and contributes to energy savings in disguise	Jingdong prefecture level city in Shandong
Jinfeng Hongyuan Flagship Store	Electricity savings1	It feels good in the experience.	Jingdong prefecture level city in Shandong
Jinfeng Hongyuan Flagship Store	Electricity savings1	The product is better than I thought it would be, the quality looks great, the voltage is stable, and I'm looking forward to some great power savings!	Jingdong prefecture level city in Shandong
Jinfeng Hongyuan Flagship Store	Electricity savings1	I'm using it now looking forward to saving some points,, I'm feeding back	Jingdong prefecture level city in Shandong
Jinfeng Hongyuan Flagship Store	Electricity savings1	Displayed voltage below 200.	Jingdong prefecture level city in Shandong
Jinfeng Hongyuan Flagship Store	Electricity savings1	I haven't seen any great results, but it should last about a hundred or eighty years.	Jingdong prefecture level city in Shandong
Jinfeng Hongyuan Flagship Store	Electricity savings1	I don't know if it works or not, but I keep it plugged in anyway, so I hope it works!	Jingdong prefecture level city in Shandong
Jinfeng Hongyuan Flagship Store	Electricity savings1	Reasonable price, good quality, fast delivery, great service, already a repurchase.	Jingdong prefecture level city in Shandong
Jinfeng Hongyuan Flagship Store	Electricity savings1	Second purchase, looking forward to the results	Jingdong prefecture level city in Shandong
Jinfeng Hongyuan Flagship Store	Electricity savings1	The quality of this product feels like it should work in a use test! Just how many kilowatts of electricity must be wasted per day with the red light and digital display on for long periods of time?	Jingdong prefecture level city in Shandong
Jinfeng Hongyuan Flagship Store	Electricity savings1	It looks good, we'll see if we use it.	Jingdong prefecture level city in Shandong
Jinfeng Hongyuan Flagship Store	Electricity savings1	Great stuff, second purchase!	Jingdong prefecture level city in Shandong
Jinfeng Hongyuan Flagship Store	Electricity savings1	It is indeed very practical and saves electricity at home	Jingdong prefecture level city in Shandong
Jinfeng Hongyuan Flagship Store	Electricity savings1	Goods received, the physical and the same as the picture, logistics is also very fast!	Jingdong prefecture level city in Shandong
Jinfeng Hongyuan Flagship Store	Electricity savings1	I'll use it for a while and see how it goes. I'll buy it again if it's good.	Jingdong prefecture level city in Shandong
Jinfeng Hongyuan Flagship Store	Electricity savings1	In use, voltage monitoring, the effect of how to wait for next month's electricity consumption confirmation	Jingdong prefecture level city in Shandong
Jinfeng Hongyuan Flagship Store	Electricity savings1	It's still pretty good.	Jingdong prefecture level city in Shandong
Jinfeng Hongyuan Flagship Store	Electricity savings1	It's been plugged in for a month and there hasn't been a big change yet, since it usually uses very little electricity	Jingdong prefecture level city in Shandong
Jinfeng Hongyuan Flagship Store	Electricity savings1	Let's wait for a month to see if it works	Jingdong prefecture level city in Shandong
Jinfeng Hongyuan Flagship Store	Electricity savings1	Good, good, good, good, good, good, good, good, good, good, good, good, good, good, good.	Jingdong prefecture level city in Shandong
Jinfeng Hongyuan Flagship Store	Electricity savings1	The product received the trial a few days, feel good, praise!	Jingdong prefecture level city in Shandong
Jinfeng Hongyuan Flagship Store	Electricity savings1	After six or seven hours of use, 250 volts won't save you any power.	Jingdong prefecture level city in Shandong
Jinfeng Hongyuan Flagship Store	Electricity savings1	I haven't used it yet, I wonder if it saves electricity?	Jingdong prefecture level city in Shandong
Jinfeng Hongyuan Flagship Store	Electricity savings1	Baby value for money, love it, next time will come back is genuine, trust the business, baby texture is very good, the price is moderate, unlimited repurchase!	Jingdong prefecture level city in Shandong
Jinfeng Hongyuan Flagship Store	Electricity savings1	Power saving effect is obvious! Raising tropical fish daily power consumption, after using the daily power savings of more than 3 degrees, currently about 8 degrees / day!	Jingdong prefecture level city in Shandong
Jinfeng Hongyuan Flagship Store	Electricity savings1	Just used it, unknown results.	Jingdong prefecture level city in Shandong
Jinfeng Hongyuan Flagship Store	Electricity savings1	The goods received, well, look forward to the effect. Logistics is very fast, thanks to the platform, thanks to Jingdong. I hope it can save electricity.	Jingdong prefecture level city in Shandong
Jinfeng Hongyuan Flagship Store	Electricity savings1	Not sure 有 effect?	Jingdong prefecture level city in Shandong
Jinfeng Hongyuan Flagship Store	Electricity savings1	I'd say it's a 5% savings.	Jingdong prefecture level city in Shandong
Jinfeng Hongyuan Flagship Store	Electricity savings1	I received it and am using it, I don't know how effective it is, I'll evaluate it afterwards	Jingdong prefecture level city in Shandong
Jinfeng Hongyuan Flagship Store	Electricity savings1	Baby received not yet used, first give praise	Jingdong prefecture level city in Shandong
Jinfeng Hongyuan Flagship Store	Electricity savings1	Looks good, I'll evaluate it later	Jingdong prefecture level city in Shandong
Jinfeng Hongyuan Flagship Store	Electricity savings1	The goods have been received in use, the overall feeling is not bad!	Jingdong prefecture level city in Shandong

Jinfeng Hongyuan Flagship Store	Electricity savings1	It's working. This is the second one. This stuff has to be used over a long period of time to be effective. And it only works on inductive loads, i.e. appliances containing electric motors. Use it.	Jingdong prefecture level city in Shandong
Jinfeng Hongyuan Flagship Store	Electricity savings1	Use it for a while and see how it works.	Jingdong prefecture level city in Shandong
Jinfeng Hongyuan Flagship Store	Electricity savings1	Good courier service and the item is very nice and practical.	Jingdong prefecture level city in Shandong
Jinfeng Hongyuan Flagship Store	Electricity savings1	It doesn't do anything and it's a waste of electricity.	Jingdong prefecture level city in Shandong
Jinfeng Hongyuan Flagship Store	Electricity savings1	I haven't used it yet. It should be good.	Jingdong prefecture level city in Shandong
Jinfeng Hongyuan Flagship Store	Electricity savings1	That's good. OK, that's good.	Jingdong prefecture level city in Shandong
Jinfeng Hongyuan Flagship Store	Electricity savings1	It's a rip off, doesn't work, doesn't save electricity, does the job to well, and I've wasted a couple dozen dollars again.	Jingdong prefecture level city in Shandong
Jinfeng Hongyuan Flagship Store	Electricity savings1	Use it first, the effect is not as good as the road, then we will comment again	Jingdong prefecture level city in Shandong
Jinfeng Hongyuan Flagship Store	Electricity savings1	The price is affordable, the logistics is strong, just use four or five days, the effect will be added later!	Jingdong prefecture level city in Shandong
Jinfeng Hongyuan Flagship Store	Electricity savings1	It's easy to use and works fine.	Jingdong prefecture level city in Shandong
Jinfeng Hongyuan Flagship Store	Electricity savings1	It doesn't feel like it's working.	Jingdong prefecture level city in Shandong
Jinfeng Hongyuan Flagship Store	Electricity savings1	I'm using it. We'll see what happens in a couple months.	Jingdong prefecture level city in Shandong
Jinfeng Hongyuan Flagship Store	Electricity savings1	I just got it back to try it out and I'll comment later	Jingdong prefecture level city in Shandong
Jinfeng Hongyuan Flagship Store	Electricity savings1	Less than two months. Can't tell.	Jingdong prefecture level city in Shandong
Jinfeng Hongyuan Flagship Store	Electricity savings1	No power saving effect	Jingdong prefecture level city in Shandong
Jinfeng Hongyuan Flagship Store	Electricity savings1	Express Express Very quickly not only to the power saver can not save electricity, with two months, look at the evaluation put.	Jingdong prefecture level city in Shandong
Jinfeng Hongyuan Flagship Store	Electricity savings1	Genuine products are packaged well!	Jingdong prefecture level city in Shandong
Jinfeng Hongyuan Flagship Store	Electricity savings1	I've been using it for a while, but I don't know if it saves electricity. I hope it works.	Jingdong prefecture level city in Shandong
Jinfeng Hongyuan Flagship Store	Electricity savings1	Good service, fast logistics, the effect is to be used to know	Jingdong prefecture level city in Shandong
Jinfeng Hongyuan Flagship Store	Electricity savings1	Just used it, not sure about the power saving effect	Jingdong prefecture level city in Shandong
Jinfeng Hongyuan Flagship Store	Electricity savings1	The stuff is ok, save power or not, will comment after a month.	Jingdong prefecture level city in Shandong
Jinfeng Hongyuan Flagship Store	Electricity savings1	Used it for a month to save %25, normal is a little over 100, it's great! I want to buy one in.	Jingdong prefecture level city in Shandong
Jinfeng Hongyuan Flagship Store	Electricity savings1	I'm using it and looking forward to the results	Jingdong prefecture level city in Shandong
Jinfeng Hongyuan Flagship Store	Electricity savings1	The quality is very good and the workmanship is exquisite. The use effect is also very good.	Jingdong prefecture level city in Shandong
Jinfeng Hongyuan Flagship Store	Electricity savings1	Merchant service attitude is very good, logistics speed is super fast, online evaluation is very good, the first time to use, a month to know the specific effect, the effect is good, and then additional	Jingdong prefecture level city in Shandong
Jinfeng Hongyuan Flagship Store	Electricity savings1	Works great, solves the problem of high power usage in the house	Jingdong prefecture level city in Shandong
Jinfeng Hongyuan Flagship Store	Electricity savings1	To receive! Very delicate! It's not clear how effective it is, I won't know until I use it!!!!	Jingdong prefecture level city in Shandong
Jinfeng Hongyuan Flagship Store	Electricity savings1	I've just been using it for a while and I don't feel like it's too noticeable.	Jingdong prefecture level city in Shandong
Jinfeng Hongyuan Flagship Store	Electricity savings1	Already used, no visible effect for the time being	Jingdong prefecture level city in Shandong
Jinfeng Hongyuan Flagship Store	Electricity savings1	I can't feel the effect.	Jingdong prefecture level city in Shandong
Jinfeng Hongyuan Flagship Store	Electricity savings1	Use it for a while and see the results	Jingdong prefecture level city in Shandong
Jinfeng Hongyuan Flagship Store	Electricity savings1	The price is more reasonable, the power-saving effect is to be verified, the delivery is fast, and the courier service is good.	Jingdong prefecture level city in Shandong
Jinfeng Hongyuan Flagship Store	Electricity savings1	Quality is very good, consistent with the seller's description, very satisfied, very like, very fast shipping, packaging is very careful and strict, the logistics company has a very good service attitude, fast delivery, very satisfied with a shopping, very satisfied. Recommended to buy, cost-effective, the service attitude of the business is very enthusiastic, providing such high quality goods.	Jingdong prefecture level city in Shandong
Jinfeng Hongyuan Flagship Store	Electricity savings1	I don't know.	Jingdong prefecture level city in Shandong
Jinfeng Hongyuan Flagship Store	Electricity savings1	I didn't get the delivery.	Jingdong prefecture level city in Shandong
Jinfeng Hongyuan Flagship Store	Electricity savings1	It works, it does have power savings, better than expected, will repurchase.	Jingdong prefecture level city in Shandong
Jinfeng Hongyuan Flagship Store	Electricity savings1	That's where the five fuses are.	Jingdong prefecture level city in Shandong
Jinfeng Hongyuan Flagship Store	Electricity savings1	Don't buy it, it doesn't work and you can't return it.	Jingdong prefecture level city in Shandong
Jinfeng Hongyuan Flagship Store	Electricity savings1	I hope it works. Wait a month.	Jingdong prefecture level city in Shandong
Jinfeng Hongyuan Flagship Store	Electricity savings1	Nothing**, buy with caution	Jingdong prefecture level city in Shandong
Jinfeng Hongyuan Flagship Store	Electricity savings1	I'm using it and waiting for the results	Jingdong prefecture level city in Shandong
Jinfeng Hongyuan Flagship Store	Electricity savings1	Saver quality is very good, has always felt that the home power consumption is very large, this is the company's electrician recommended, said he bought this home is really able to save a little electricity, I also came to buy, the effect if it is good, then buy, a floor a...	Jingdong prefecture level city in Shandong
Jinfeng Hongyuan Flagship Store	Electricity savings1	Good product, satisfied with purchase	Jingdong prefecture level city in Shandong
Jinfeng Hongyuan Flagship Store	Electricity savings1	Occupying the time has not yet found the power saving, occupying not evaluation	Jingdong prefecture level city in Shandong
Jinfeng Hongyuan Flagship Store	Electricity savings1	Haven't tested the results yet. To be followed up with comments!	Jingdong prefecture level city in Shandong
Jinfeng Hongyuan Flagship Store	Electricity savings1	After experiencing it for a month, I don't seem to know how to save it, and I don't pay attention to it anyway	Jingdong prefecture level city in Shandong
Jinfeng Hongyuan Flagship Store	Electricity savings1	It's useless. It's a waste of electricity.	Jingdong prefecture level city in Shandong
Jinfeng Hongyuan Flagship Store	Electricity savings1	I don't know if it's a power saver. It's a couple bucks. I hope it works.	Jingdong prefecture level city in Shandong
Jinfeng Hongyuan Flagship Store	Electricity savings1	I don't know what to say about this stuff. I don't know if it works or not, it's kind of a psychological purchase. Dormitory use to see the meter can not be not so good-looking. You have to wait until the time to deduct the utility bill to know.	Jingdong prefecture level city in Shandong

Jinfeng Hongyuan Flagship Store	Electricity savings1	Energy savers don't work / they're all a bunch of hoaxes.	Jingdong prefecture level city in Shandong
Jinfeng Hongyuan Flagship Store	Electricity savings1	Received the goods, used for more than a week, it's okay, sure a little effect	Jingdong prefecture level city in Shandong
Jinfeng Hongyuan Flagship Store	Electricity savings1	Well made and compact! Looking forward to the effect!	Jingdong prefecture level city in Shandong
Jinfeng Hongyuan Flagship Store	Electricity savings1	It has arrived and we'll see how it works.	Jingdong prefecture level city in Shandong
Jinfeng Hongyuan Flagship Store	Electricity savings1	Good. Good. It's worth it.	Jingdong prefecture level city in Shandong
Jinfeng Hongyuan Flagship Store	Electricity savings1	How come there's no indicator light?	Jingdong prefecture level city in Shandong
Jinfeng Hongyuan Flagship Store	Electricity savings1	The machine that uses electricity works quite well. This saves	Jingdong prefecture level city in Shandong
Jinfeng Hongyuan Flagship Store	Electricity savings1	It's good for home use and feels quite energy efficient	Jingdong prefecture level city in Shandong
Jinfeng Hongyuan Flagship Store	Electricity savings1	I regret buying this product, after using it for two days it doesn't work at all, still so many kilowatts per day just like the original.	Jingdong prefecture level city in Shandong
Jinfeng Hongyuan Flagship Store	Electricity savings1	Works very well and has some scientific merit.	Jingdong prefecture level city in Shandong
Jinfeng Hongyuan Flagship Store	Electricity savings1	Logistics is also very fast this good power saving I use two months before commenting!	Jingdong prefecture level city in Shandong
Jinfeng Hongyuan Flagship Store	Electricity savings1	Repeat customer. Very good.	Jingdong prefecture level city in Shandong
Jinfeng Hongyuan Flagship Store	Electricity savings1	It's good stuff. I've been using it for a month now and it's a real power saver.	Jingdong prefecture level city in Shandong
Jinfeng Hongyuan Flagship Store	Electricity savings1	Jingdong platform to buy things, has been very assured, quality assurance. Jingdong Express is as good as ever, complete packaging, delivery, service attitude to go! The family has always wanted to buy an energy-saving and power-saving equipment, selected half a day to choose this one, the value is very good, used for a period of time to follow up, to see whether it can achieve the real purpose of power saving, I	Jingdong prefecture level city in Shandong
Jinfeng Hongyuan Flagship Store	Electricity savings1	Works great, inexpensive and will be back next time.	Jingdong prefecture level city in Shandong
Jinfeng Hongyuan Flagship Store	Electricity savings1	Baby received, looks good, wait for a month to see how it works?	Jingdong prefecture level city in Shandong
Jinfeng Hongyuan Flagship Store	Electricity savings1	This product is not bad quite good well customer service said quite power saving that look at the customer service to see if the accuracy of the look at all aspects of the color is also quite beautiful	Jingdong prefecture level city in Shandong
Jinfeng Hongyuan Flagship Store	Electricity savings1	Just got back and tried it feels pretty good, just don't know how the quality is, give a good comment first	Jingdong prefecture level city in Shandong
Jinfeng Hongyuan Flagship Store	Electricity savings1	Received the product, trying it out, hope it helps!	Jingdong prefecture level city in Shandong
Jinfeng Hongyuan Flagship Store	Electricity savings1	Works great, saves electricity a dollar a day and a night	Jingdong prefecture level city in Shandong
Jinfeng Hongyuan Flagship Store	Electricity savings1	Baby received, look at the workmanship is still very fine, is genuine. I hope that this summer to open the air conditioning electricity bill is no longer high scary!	Jingdong prefecture level city in Shandong
Jinfeng Hongyuan Flagship Store	Electricity savings1	It's here. I don't know how it's going to work, but I'm going to use it.	Jingdong prefecture level city in Shandong
Jinfeng Hongyuan Flagship Store	Electricity savings1	Power saver received, Jingdong logistics first-class, business customer service attitude is good, one by one to introduce the product performance, back on the horse plugged in, it is very simple, there is a voltage indicator, to be a few months to see how the effect of power-saving to come back to a good follow-up, but I believe that Jinfenghong products.	Jingdong prefecture level city in Shandong
Jinfeng Hongyuan Flagship Store	Electricity savings1	Took the third one...the super power-saving kind!	Jingdong prefecture level city in Shandong
Jinfeng Hongyuan Flagship Store	Electricity savings1	First time buyer, customer service is very polite and courteous, conscientious business, support! Baby has received 🎈, super like, high value and practical, super like! ❥❥❥❥❥ Very good quality, very satisfied with a shopping. Customer service has a question and answer, very professional finally received the goods, very, very much like, exquisite and beautiful workmanship, has been praised by many people, really too happy, is already a loyal fan of his family, will continue to buy buy buy buy!	Jingdong prefecture level city in Shandong
Jinfeng Hongyuan Flagship Store	Electricity savings1	That's good. That's good. That's really good.	Jingdong prefecture level city in Shandong
Jinfeng Hongyuan Flagship Store	Electricity savings1	Saver received, good-looking design, voltage at a glance, and the merchant description, save some about 12% or so	Jingdong prefecture level city in Shandong
Jinfeng Hongyuan Flagship Store	Electricity savings1	Intelligent power saver is small and does not take up space, very good, very practical, received and used, packaging is tight, logistics is also very fast, satisfied with a shopping!	Jingdong prefecture level city in Shandong
Jinfeng Hongyuan Flagship Store	Electricity savings1	The stuff is good! Well made, compact, alloy case, shows voltage! Very like! Kudos!	Jingdong prefecture level city in Shandong
Jinfeng Hongyuan Flagship Store	Electricity savings1	I just purchased this power saver from Jingdong, inserted in the socket shows the number 206, a moment and 207 back and forth display, I do not know what it means, my home voltage should be normal, because it is the owner of the community's own power plant to put, first use for a period of time in the positive feedback it, first use in the look at it!	Jingdong prefecture level city in Shandong
Jinfeng Hongyuan Flagship Store	Electricity savings1	Very delicate and compact, there is a convenient intelligent display prompts the voltage, you can know the voltage at a glance, there is also very easy to use, a plug can be used, you can save electricity	Jingdong prefecture level city in Shandong
Jinfeng Hongyuan Flagship Store	Electricity savings1	Logistics is fast, ask customer service said can save electricity, but only use two months to see the effect, use two months in the evaluation.	Jingdong prefecture level city in Shandong
Jinfeng Hongyuan Flagship Store	Electricity savings1	The quality is very good.	Jingdong prefecture level city in Shandong
Jinfeng Hongyuan Flagship Store	Electricity savings1	Smart Saver has been received and will be evaluated after use	Jingdong prefecture level city in Shandong
Jinfeng Hongyuan Flagship Store	Electricity savings1	Home electricity is too waste, a month more than 300 dollars of electricity, and then the family heard that this can save a lot of electricity, let me order to buy a, anyway, will not, buy on the buy, thought it has to be a pretty big box it, delivery to the home to see a very small, plug-and-play, you can really save electricity, it's worth it!	Jingdong prefecture level city in Shandong
Jinfeng Hongyuan Flagship Store	Electricity savings1	Smart Saver received. Unpacking the box is exquisite. The material workmanship is very good. Plugged in and tried the space. Did not open the air conditioning voltage display 206 air conditioning open up the air conditioning display for 194. whether to save electricity to a month later to pay the electricity bill will know. Logistics is also very fast service is also in place.	Jingdong prefecture level city in Shandong
Jinfeng Hongyuan Flagship Store	Electricity savings1	The product feels pretty good and I hope it works, as the store owner said it would. Works!!!	Jingdong prefecture level city in Shandong
Jinfeng Hongyuan Flagship Store	Electricity savings1	Baby received, physical very quickly, just plugged in hope that it can be as everyone said those power saving, with two months after additional comments, first give a five-star praise.	Jingdong prefecture level city in Shandong
Jinfeng Hongyuan Flagship Store	Electricity savings1	Good, very good, very good, you deserve it, whoever uses it knows, the stuff works great, will buy it again in the future.	Jingdong prefecture level city in Shandong
Jinfeng Hongyuan Flagship Store	Electricity savings1	Don't hesitate to buy wow! No cheap feeling, very high-end, especially great! It is genuine, and the same as the entity store, the price is also favorable, decisive turn powder 💗 catch up to do activities when you buy, the price of a penny for a penny ♡, from the outside looking very beautiful 💗, the material workmanship is good 💗, feel that the purchase is worth it, and the logistics is super	Jingdong prefecture level city in Shandong
Jinfeng Hongyuan Flagship Store	Electricity savings1	Logistics is fast! Very good quality! Very beautiful! Next time to patronize!	Jingdong prefecture level city in Shandong
Jinfeng Hongyuan Flagship Store	Electricity savings1	Really like it, completely exceeded expectations, the shipping speed is very fast, the packaging is very careful and strict, the delivery speed is very fast, very satisfied with a shopping Baby received, and the description is the same, love it!	Jingdong prefecture level city in Shandong
Jinfeng Hongyuan Flagship Store	Electricity savings1	Very good, if you need to order!	Jingdong prefecture level city in Shandong

Jinfeng Hongyuan Flagship Store	Electricity savings1	So far, plugged in the voltage is more stable, as for the effect of how to quietly wait until the payment of the electricity bill to compare, the appearance of the commodity design is very good, the real thing!	Jingdong prefecture level city in Shandong
Jinfeng Hongyuan Flagship Store	Electricity savings1	📩 I've been wanting it for a long time, I decided to go for it, I feel great after using it, it's genuine and the delivery is great, it lives up to its name👍	Jingdong prefecture level city in Shandong
Jinfeng Hongyuan Flagship Store	Electricity savings1	Looking forward to it!!! I don't know if it's working or not, let's look forward to it!!!! How much is saved ，，，，，，，，， Saving a degree is a degree with long term use ！！！！！！！	Jingdong prefecture level city in Shandong
Jinfeng Hongyuan Flagship Store	Electricity savings1	After using it for a while, I am seriously and responsibly saying that this saver is really very power efficient. Two rooms with the air conditioner on at night for one night, plus watching TV, charging cell phones, lights, etc. etc. only used one degree of electricity. So I bought several more and it's really	Jingdong prefecture level city in Shandong
Jinfeng Hongyuan Flagship Store	Electricity savings1	It arrived quickly, and the box is solid and delicate. Baby shell is thick aluminum and by multi-layer coating. The inner core is heavy, and the power cord and plug are genuine products from a large manufacturer. It is the best power saver I have found after choosing all of them!	Jingdong prefecture level city in Shandong
Jinfeng Hongyuan Flagship Store	Electricity savings1	The saver was received, genuine guarantee, good quality...	Jingdong prefecture level city in Shandong
Jinfeng Hongyuan Flagship Store	Electricity savings1	Logistics was fast! The product is easy to use!	Jingdong prefecture level city in Shandong
Jinfeng Hongyuan Flagship Store	Electricity savings1	Look appearance is okay, use a period of time to know how it is. Jingdong Mall, trustworthy! Home appliances, cell phones and computers are almost 100% bought by Jingdong. Ordered the night before yesterday, received yesterday. Jingdong logistics, the fastest in the network!	Jingdong prefecture level city in Shandong
Jinfeng Hongyuan Flagship Store	Electricity savings1	Very good, perfect packaging, home energy saver to determine the effect of power saving and also play a role in stabilizing the voltage Very good products	Jingdong prefecture level city in Shandong
Jinfeng Hongyuan Flagship Store	Electricity savings1	Smart Saver received, looks very exquisite. The method of use is simple, and it has been used. The later effect, with time to test.	Jingdong prefecture level city in Shandong
Jinfeng Hongyuan Flagship Store	Electricity savings1	Both the material and workmanship exceeded expectations, great value, and looks great on oh. The seller also shipped much earlier than expected, will come back. Buy it, you will not regret it! Full five points. Both the material and workmanship exceeded expectations, great value, wear it also looks good Oh. Seller delivery is also much earlier than expected, will come again. Buy it, will not regret it! Full	Jingdong prefecture level city in Shandong
Jinfeng Hongyuan Flagship Store	Electricity savings1	The appearance is clean and beautiful, the workmanship is very good, I didn't find any defects, I personally feel that the size is also quite suitable, I've used it for 2 months, I didn't find out exactly how energy efficient it is. Try using it again!	Jingdong prefecture level city in Shandong
Jinfeng Hongyuan Flagship Store	Electricity savings1	Power saving device received, product certification, accessories, etc. complete, very good workmanship, very practical power-saving effect is also very obvious.	Jingdong prefecture level city in Shandong
Jinfeng Hongyuan Flagship Store	Electricity savings1	Looked at a lot of think buy this one, see a lot of reviews say it works, a month with save nearly twenty degrees of electricity, there are sixty days of return guarantee, with a good and then come back to the	Jingdong prefecture level city in Shandong
Jinfeng Hongyuan Flagship Store	Electricity savings1	I received the power saving device, and I'm using it. I hope I can get the desired effect, and I'm trying to force it to work quickly.	Jingdong prefecture level city in Shandong
Jinfeng Hongyuan Flagship Store	Electricity savings1	Voltage is stable, already trying it out and waiting for the results to happen, saving electricity will follow up next month!	Jingdong prefecture level city in Shandong
Jinfeng Hongyuan Flagship Store	Electricity savings1	Jingdong Mall self-supporting goods of excellent quality, logistics is also very fast, worth recommending 👏👏👏👏 excellent!	Jingdong prefecture level city in Shandong
Jinfeng Hongyuan Flagship Store	Electricity savings1	Power saving has been received, the appearance of the beautiful, I have already been the second time to buy the use of down the effect is very good!	Jingdong prefecture level city in Shandong
Jinfeng Hongyuan Flagship Store	Electricity savings1	Energy saver received very happy, the color is also very good looking forward to a very good effect can save electricity	Jingdong prefecture level city in Shandong
Jinfeng Hongyuan Flagship Store	Electricity savings1	It's small and easy to plug in and use. It displays the voltage value when plugged in and	Jingdong prefecture level city in Shandong
Jinfeng Hongyuan Flagship Store	Electricity savings1	This saver really works well, because I installed one at home about seven or eight years ago, when there is no voltage display, now there is a voltage display, the appearance also looks much better, the electricity bill is also saved, the stability of the home appliances is also good, the best of both	Jingdong prefecture level city in Shandong
Jinfeng Hongyuan Flagship Store	Electricity savings1	Ordered yesterday, arrived today in use. Shipping was fast. The effect is looking forward to.	Jingdong prefecture level city in Shandong
Jinfeng Hongyuan Flagship Store	Electricity savings1	This thing, I don't know what the principle is. Why it can save electricity. Wait for a month to use it first, see if it will save electricity bill, then come back to evaluate. There is one thing that does not fit the principle ah. The law of conservation of energy.	Jingdong prefecture level city in Shandong
Jinfeng Hongyuan Flagship Store	Electricity savings1	Home mom listen to others say this can save electricity, specially let buy a, later a very large, go back to see a particularly small, directly plugged into the electricity can be, no need to install, the price is also very cheap, the first time with this product, do not know if it is not good to use, temporarily try for a period of time, if it is good to use, recommend that you buy this one, the	Jingdong prefecture level city in Shandong
Jinfeng Hongyuan Flagship Store	Electricity savings1	I've been using it for a few days, and I think the workmanship and materials are pretty good, so we'll have to look at the next bill for the real energy savings, and then we'll add more comments.	Jingdong prefecture level city in Shandong
Jinfeng Hongyuan Flagship Store	Electricity savings1	Energy saver is well made, energy saving and power saving, and anti-counterfeiting code, it is a regular commodity!	Jingdong prefecture level city in Shandong
Jinfeng Hongyuan Flagship Store	Electricity savings1	Jinfeng Hongyuan, buy back home, as a mouse, haha. First of all, normal electricity for 1 month, the power did not increase, the electricity bill feels smoother, year-on-year electricity costs in the low, whether it is very energy-saving still have to be used for a long time to know. All 5 stars ☆	Jingdong prefecture level city in Shandong
Jinfeng Hongyuan Flagship Store	Electricity savings1	The box is well packaged and beautiful! I hope to save electricity with it haha!	Jingdong prefecture level city in Shandong
Jinfeng Hongyuan Flagship Store	Electricity savings1	Ordered last night, arrived this morning, first try the effect before making comments	Jingdong prefecture level city in Shandong
Jinfeng Hongyuan Flagship Store	Electricity savings1	Looks good. Don't know the effect yet.	Jingdong prefecture level city in Shandong
Jinfeng Hongyuan Flagship Store	Electricity savings1	Jinfeng Hongyuan intelligent power saver has exquisite workmanship and modern fashionable design style. It is a good home economizer . I believe that the goods purchased from Jingdong will not disappoint our	Jingdong prefecture level city in Shandong
Jinfeng Hongyuan Flagship Store	Electricity savings1	Inadvertently flipped to this power saver, never heard of, feel very novel, the price is not expensive, so buy to try, see a month after the electricity bill will be less, recently the home air-conditioning open a lot of power consumption is very large.	Jingdong prefecture level city in Shandong
Jinfeng Hongyuan Flagship Store	Electricity savings1	The quality of the goods is very good workmanship, but also sent five fuse	Jingdong prefecture level city in Shandong
Jinfeng Hongyuan Flagship Store	Electricity savings1	I'm trying it out. I don't know if it'll work.	Jingdong prefecture level city in Shandong
Jinfeng Hongyuan Flagship Store	Electricity savings1	There is no effect yet do not know, is to look at the official sale, buy to try it! Use according to the instructions, plugged in, used for a period of time, anyway, do not know how, after a few months to see it, to see if the average month than before will be less electricity.	Jingdong prefecture level city in Shandong
Jinfeng Hongyuan Flagship Store	Electricity savings1	Received the goods, the first time to unpacking, the quality is very good, consistent with the description of the seller, completely exceeded my expectations, the packaging is very careful, strict, in general this time is very satisfied with a shopping, thanks to the seller.	Jingdong prefecture level city in Shandong
Jinfeng Hongyuan Flagship Store	Electricity savings1	The energy saver was received and used for a few days with good results.	Jingdong prefecture level city in Shandong
Jinfeng Hongyuan Flagship Store	Electricity savings1	Previously I have paid attention to this product, but I think that the monthly electricity consumption at home is not large, there is no need to save electricity savings. After a second thought, energy saving is the responsibility of each of our citizens, the winter is coming, this year's winter is cold, heating and all electric appliances, can save is to save, the price is not expensive, so buy to try. I analyze the principle of power saving, should be able to save electricity, but the larger the power saving effect is about good. To be tried after a month or two to see the effect and then evaluate. Jingdong trustworthy.	Jingdong prefecture level city in Shandong
Jinfeng Hongyuan Flagship Store	Electricity savings1	It just arrived and I can't wait to use it, and the customer service is great! There is also voltage showing up which is nice! I don't know if it can save a bit I won't know until I've used it for a month! When the time comes to come back to the follow-up review!	Jingdong prefecture level city in Shandong
Jinfeng Hongyuan Flagship Store	Electricity savings1	Checked out the reviews from numerous users. The shipment is still very large, so I decided to buy it anyway to use it and see the effect after a month. Jingdong Mall here to buy safe, convenient and fast, this price is very powerful 💪, very recommended.	Jingdong prefecture level city in Shandong
Jinfeng Hongyuan Flagship Store	Electricity savings1	Purchased two at the same time to use together. The other one works fine, the other has problems with the numbers not sure if the machine is faulty. Power saving effect can not be observed. Feel the effect is not very obvious like?	Jingdong prefecture level city in Shandong

Jinfeng Hongyuan Flagship Store	Electricity savings1	Looking forward to it!!! I don't know if it's working or not, let's look forward to it!!!! How much is saved , , , , , , , , , Saving a degree is a degree with long term use ! ! ! ! ! ! !	Jingdong prefecture level city in Shandong
Jinfeng Hongyuan Flagship Store	Electricity savings1	Received, directly on the use of the special convenient	Jingdong prefecture level city in Shandong
Jinfeng Hongyuan Flagship Store	Electricity savings1	Goods received a few days well-packed, the same day opened and plugged in to try to be normal is not sure what the power-saving effect. After a month with a follow-up evaluation.	Jingdong prefecture level city in Shandong
Jinfeng Hongyuan Flagship Store	Electricity savings1	I bought it and used it, I don't know how it works yet, we'll see later. Use the effect of it	Jingdong prefecture level city in Shandong
Jinfeng Hongyuan Flagship Store	Electricity savings1	Photographed 2, the old family also put a, with a small January, there should be some effect, with a long period of time to comment, with a long time to see the obvious effect, hard courier brother.	Jingdong prefecture level city in Shandong
Jinfeng Hongyuan Flagship Store	Electricity savings1	Experience: I am an electrician, this is the real use of electronic waves automatically connected to track the decomposition of ionized electrons, in situ compensation of reactive power, reduce effective power loss and waste, stabilize the voltage to achieve energy saving and power saving, the overall effect	Jingdong prefecture level city in Shandong
Jinfeng Hongyuan Flagship Store	Electricity savings1	Great looking and practical! Easy to use and save! Saves 15% in a month of comparison	Jingdong prefecture level city in Shandong
Jinfeng Hongyuan Flagship Store	Electricity savings1	Product quality is very good! Packaging is very nice, like a cell phone box, thick and well protected! Service is also great! Logistics is very fast! Thank you for your business! Very honest!	Jingdong prefecture level city in Shandong
Jinfeng Hongyuan Flagship Store	Electricity savings1	In several products inside the choice of this purchase for a period of time, but also need to use a few months to compare last year's electricity bill before you can analyze the savings in electricity, to the summer peak electricity consumption should be more obvious.	Jingdong prefecture level city in Shandong
Jinfeng Hongyuan Flagship Store	Electricity savings1	The goods have long been received, after more than a month to comment, the home electricity bill is indeed reduced, worth buying!	Jingdong prefecture level city in Shandong
Jinfeng Hongyuan Flagship Store	Electricity savings1	Baby received, the logistics is strong, the quality is reliable, it is worth having and recommending!	Jingdong prefecture level city in Shandong
Jinfeng Hongyuan Flagship Store	Electricity savings1	Using a conservation appliance, you can achieve a 10-15% savings in electricity usage.	Jingdong prefecture level city in Shandong
Jinfeng Hongyuan Flagship Store	Electricity savings1	Quality is very good, metal shell, touch is very good, very recommended Oh ~, the subsequent effect of continuous follow-up	Jingdong prefecture level city in Shandong
Jinfeng Hongyuan Flagship Store	Electricity savings1	The product is beautiful, practical, worth buying. The product is beautiful, practical, good quality, fast logistics product is beautiful, practical, beautiful and elegant, very classy, worth buying!	Jingdong prefecture level city in Shandong
Jinfeng Hongyuan Flagship Store	Electricity savings1	Just received the item and have already used it. Didn't know much about these products before. I hope it is as effective and useful as the product advertises.	Jingdong prefecture level city in Shandong
Jinfeng Hongyuan Flagship Store	Electricity savings1	A month two people used more than five hundred degrees of electricity, in a hurry and then add a power saver, I hope to have an effect, Jingdong shopping is very cool, logistics is very fast especially good service attitude delivery, this hot day is not easy, thank you!	Jingdong prefecture level city in Shandong
Jinfeng Hongyuan Flagship Store	Electricity savings1	Just received the baby, first plugged in to try to use a month and then additional evaluation it	Jingdong prefecture level city in Shandong
Jinfeng Hongyuan Flagship Store	Electricity savings1	Goods fast, the power saver has been received, a friend recommended to buy that can save electricity. Exquisite and compact is very convenient, plug and play. The effect is good, the price is affordable, the next need will patronize again!	Jingdong prefecture level city in Shandong
Jinfeng Hongyuan Flagship Store	Electricity savings1	Packaging is better than expected, sent a few fuse, power saving effect in the end do not know, try a period of time to say!	Jingdong prefecture level city in Shandong
Jinfeng Hongyuan Flagship Store	Electricity savings1	Shipped quickly, immediately installed, read countless reviews were not sure if this thing is effective, was going to buy three at once, ultimately decided to first use a try the effect, if the power ring significantly reduced, then will buy a few more!	Jingdong prefecture level city in Shandong
Jinfeng Hongyuan Flagship Store	Electricity savings1	Used for a period of time to evaluate, not to a month still do not know whether it can save electricity, the voltage has a display!	Jingdong prefecture level city in Shandong
Jinfeng Hongyuan Flagship Store	Electricity savings1	I don't know if it saves electricity or not, but I'm using it anyway, so I hope it saves a little bit, electricity is too expensive	Jingdong prefecture level city in Shandong
Jinfeng Hongyuan Flagship Store	Electricity savings1	The power saver has been received and I am very happy with it! Just waiting to see how the power saving works!	Jingdong prefecture level city in Shandong
Jinfeng Hongyuan Flagship Store	Electricity savings1	Saving power has been received, logistics is quite fast, the quality is not bad, small and a little weight, has been used to look forward to the effect of	Jingdong prefecture level city in Shandong
Jinfeng Hongyuan Flagship Store	Electricity savings1	The logistics is very fast, the effect is obvious and trustworthy.	Jingdong prefecture level city in Shandong
Jinfeng Hongyuan Flagship Store	Electricity savings1	Good quality, good quality, beautiful, beautiful, beautiful, beautiful, beautiful, very worth buying, worth buying, worth buying, worth buying, worth customizing!	Jingdong prefecture level city in Shandong
Jinfeng Hongyuan Flagship Store	Electricity savings1	I've used it for now, I don't know if it saves electricity	Jingdong prefecture level city in Shandong
Jinfeng Hongyuan Flagship Store	Electricity savings1	Bought two at a time, logistics is very fast, the product is beautifully packaged, after two months to see the results	Jingdong prefecture level city in Shandong
Jinfeng Hongyuan Flagship Store	Electricity savings1	The workmanship is really fine, the packaging is also good, the regular factory production ~ use a break in the evaluation of the effectiveness of the	Jingdong prefecture level city in Shandong
Jinfeng Hongyuan Flagship Store	Electricity savings1	Sometimes 219. Sometimes 220 in this case	Jingdong prefecture level city in Shandong
Jinfeng Hongyuan Flagship Store	Electricity savings1	It is the ideal product, good quality, logistics is also very fast, worthy of praise!	Jingdong prefecture level city in Shandong
Jinfeng Hongyuan Flagship Store	Electricity savings1	Store service is very good, the courier received the next day. Product packaging is very good, just use the quality to be tested.	Jingdong prefecture level city in Shandong
Jinfeng Hongyuan Flagship Store	Electricity savings1	I've been using it for almost a month now and I feel like it's working.	Jingdong prefecture level city in Shandong
Jinfeng Hongyuan Flagship Store	Electricity savings1	Very satisfied, very restful, very pretty, very beautiful, very much enjoyed! Well worth it!	Jingdong prefecture level city in Shandong
Jinfeng Hongyuan Flagship Store	Electricity savings1	Baby especially like, the value of high-quality good, a very good shopping experience, attentive service, fast logistics, distribution is also very thoughtful, attitude is also very good, value for money ah, decisive praise! Quality is quite good, logistics is also very fast, praise!	Jingdong prefecture level city in Shandong
Jinfeng Hongyuan Flagship Store	Electricity savings1	The shape of the item is basically the same as ordered, the effect is to be examined.	Jingdong prefecture level city in Shandong
Jinfeng Hongyuan Flagship Store	Electricity savings1	Appearance style: good-looking workmanship material: very good, a small one, very delicate Experience: still in use, in the process of using the shopkeeper is very nice to have a careful explanation Use for a period of time in the look at the results	Jingdong prefecture level city in Shandong
Jinfeng Hongyuan Flagship Store	Electricity savings1	As described, compact and well packaged	Jingdong prefecture level city in Shandong
Jinfeng Hongyuan Flagship Store	Electricity savings1	Fine workmanship. Test in progress. Let's see how it works.	Jingdong prefecture level city in Shandong
Jinfeng Hongyuan Flagship Store	Electricity savings1	The goods have been received, I'm very satisfied and love it	Jingdong prefecture level city in Shandong
Jinfeng Hongyuan Flagship Store	Electricity savings1	Safety! Whether you can save electricity, you can roughly feel it in 2 months. The principle is simple. Before the electrical appliances and circuits inductive more, now the electrical appliances integrated, the home is not a lot of inductive circuits, but used certainly save no problem! Wiring according to the requirements of the manual is good, if it is not convenient, see the socket plug.	Jingdong prefecture level city in Shandong
Jinfeng Hongyuan Flagship Store	Electricity savings1	Great product, looking forward to how much power I can save, first time buyer!	Jingdong prefecture level city in Shandong
Jinfeng Hongyuan Flagship Store	Electricity savings1	The product is exquisite, beautifully packaged, and the customer service is excellent. Just waiting to see how the power saving effect is, looking forward to it!	Jingdong prefecture level city in Shandong
Jinfeng Hongyuan Flagship Store	Electricity savings1	Express very quickly very satisfied with the first use to see how the effect to know, if you can save 10 percent even if acceptable, if you can not meet this requirement is obviously not much effect. Obviously.	Jingdong prefecture level city in Shandong
Jinfeng Hongyuan Flagship Store	Electricity savings1	Very delicate and compact, there is a very convenient intelligent display prompts the voltage, you can know the voltage at a glance, there is very easy to use, a plug can be used, I hope to save money on	Jingdong prefecture level city in Shandong
Jinfeng Hongyuan Flagship Store	Electricity savings1	First time buyer is it really save after use	Jingdong prefecture level city in Shandong
Jinfeng Hongyuan Flagship Store	Electricity savings1	Purchased again and will be for a long time yet.	Jingdong prefecture level city in Shandong

Jinfeng Hongyuan Flagship Store	Electricity savings1	Bought it to stock up, came back and plugged it in, hope it works	Jingdong prefecture level city in Shandong
Jinfeng Hongyuan Flagship Store	Electricity savings1	Baby received the logistics very fast very like very satisfied praise a	Jingdong prefecture level city in Shandong
Jinfeng Hongyuan Flagship Store	Electricity savings1	Beginning the process of using, easy to evaluate more later.	Jingdong prefecture level city in Shandong
Jinfeng Hongyuan Flagship Store	Electricity savings1	The goods have been received, just do not know how effective	Jingdong prefecture level city in Shandong
Jinfeng Hongyuan Flagship Store	Electricity savings1	It feels okay. I wish there were more events.	Jingdong prefecture level city in Shandong
Jinfeng Hongyuan Flagship Store	Electricity savings1	Things have been received, just plugged in, I do not know if it works, wait a few days to have the effect of additional	Jingdong prefecture level city in Shandong
Jinfeng Hongyuan Flagship Store	Electricity savings1	The effect of this power saver is really good, the house more, a few air conditioners are open every day, not used before a month to say less than seven hundred eight hundred, used after three months only nine	Jingdong prefecture level city in Shandong
Jinfeng Hongyuan Flagship Store	Electricity savings1	Cheap, good and easy to use, recommend it.	Jingdong prefecture level city in Shandong
Jinfeng Hongyuan Flagship Store	Electricity savings1	Bought this device, to the home for a long time, wait to finish a month to see how, good can recommend friends to buy, we use the electricity every month scary, a month of electricity costs more than a thousand, can not afford to hurt ah! I hope this power saving can save me some money, the beginning of the shoot order did not pay attention to look at the address, sent elsewhere to go, that side called to find out, hurry to communicate with customer service, customer service is very careful to help me get it done, call to that side to help me turn around, thank you!	Jingdong prefecture level city in Shandong
Jinfeng Hongyuan Flagship Store	Electricity savings1	I don't know the effect if it, use it first to see.	Jingdong prefecture level city in Shandong
Jinfeng Hongyuan Flagship Store	Electricity savings1	Looks good. I won't know if it saves power until I use it.	Jingdong prefecture level city in Shandong
Jinfeng Hongyuan Flagship Store	Electricity savings1	Goods received, shipping speed is very fast, yesterday afternoon more than two o'clock shot, just express delivery, but also sent a few insurance, very good business, the display of the number do not understand what is the meaning of the display said voltage, do not understand this, try a month to see how, good and	Jingdong prefecture level city in Shandong
Jinfeng Hongyuan Flagship Store	Electricity savings1	What ** play also, save electricity more and more save more and more, save chicken hair electricity, are cheating, this is a night of air conditioning with electricity, a night with 11 electricity word, this is	Jingdong prefecture level city in Shandong
Jinfeng Hongyuan Flagship Store	Electricity savings1	Received the product, hope it works!	Jingdong prefecture level city in Shandong
Jinfeng Hongyuan Flagship Store	Electricity savings1	Bought home and used very convenient, easy to operate, quite good	Jingdong prefecture level city in Shandong
Jinfeng Hongyuan Flagship Store	Electricity savings1	It's really good, cost-effective and good quality, and the logistics are fast! It arrived in one day! Really good, cost-effective and good quality, logistics is fast ah! It arrived in one day!	Jingdong prefecture level city in Shandong
Jinfeng Hongyuan Flagship Store	Electricity savings1	Works well, stabilizes voltage and saves power	Jingdong prefecture level city in Shandong
Jinfeng Hongyuan Flagship Store	Electricity savings1	I'll use it right away and look forward to good results	Jingdong prefecture level city in Shandong
Jinfeng Hongyuan Flagship Store	Electricity savings1	Recommended by a friend, said it works well, so I bought one, the effect to be tested	Jingdong prefecture level city in Shandong
Jinfeng Hongyuan Flagship Store	Electricity savings1	I didn't know it would save electricity.	Jingdong prefecture level city in Shandong
Jinfeng Hongyuan Flagship Store	Electricity savings1	I've been using it for a week, I don't know what's going on yet I hope it works well and saves power I'll follow up after a month of use	Jingdong prefecture level city in Shandong
Jinfeng Hongyuan Flagship Store	Electricity savings1	Very well packaged, looks great, don't know how it works	Jingdong prefecture level city in Shandong
Jinfeng Hongyuan Flagship Store	Electricity savings1	Genuine, good quality, really saves energy.	Jingdong prefecture level city in Shandong
Jinfeng Hongyuan Flagship Store	Electricity savings1	I was worried that the stuff is not good, but when I got it, I realized that I was completely over-anxious, 5 stars!	Jingdong prefecture level city in Shandong
Jinfeng Hongyuan Flagship Store	Electricity savings1	Very good and satisfied trustworthy	Jingdong prefecture level city in Shandong
Jinfeng Hongyuan Flagship Store	Electricity savings1	I don't know if it saves power	Jingdong prefecture level city in Shandong
Jinfeng Hongyuan Flagship Store	Electricity savings1	The quality of the product is very good, beautiful and generous applicable fast delivery speed good service attitude	Jingdong prefecture level city in Shandong
Jinfeng Hongyuan Flagship Store	Electricity savings1	Let's use it for a while and then we'll follow up	Jingdong prefecture level city in Shandong
Jinfeng Hongyuan Flagship Store	Electricity savings1	I've only been using it for a month, I don't know if there's any change yet, I'll come back with a follow-up comment after checking the meter!	Jingdong prefecture level city in Shandong
Jinfeng Hongyuan Flagship Store	Electricity savings1	I don't know if it works, I read the instructions and online reviews, I hope I won't be disappointed, if I use it, I will buy it again. Jingdong Express, fast and guaranteed service.	Jingdong prefecture level city in Shandong
Jinfeng Hongyuan Flagship Store	Electricity savings1	It feels like it saves quite a bit huh?	Jingdong prefecture level city in Shandong
Jinfeng Hongyuan Flagship Store	Electricity savings1	I've been using it for a while now, and it still seems to work and have a power saving effect	Jingdong prefecture level city in Shandong
Jinfeng Hongyuan Flagship Store	Electricity savings1	Looks good. It says 235. The 222 bar voltage is shown as high. I don't know if that's right. We'll see.	Jingdong prefecture level city in Shandong
Jinfeng Hongyuan Flagship Store	Electricity savings1	Baby is really good, do really careful, worth to have, fast delivery!	Jingdong prefecture level city in Shandong
Jinfeng Hongyuan Flagship Store	Electricity savings1	Quite delicate and compact, simple and convenient to use. Things are still very good, bought two at a time. Very satisfied with an online purchase.	Jingdong prefecture level city in Shandong
Jinfeng Hongyuan Flagship Store	Electricity savings1	Feel very good, the use of unclear, ask customer service, timely explanation, big brand, trustworthy	Jingdong prefecture level city in Shandong
Jinfeng Hongyuan Flagship Store	Electricity savings1	Thrifty received a few days did not make a comment is to see how the quality of the product now feel the quality of good appearance is also powerful seller service is very good to buy the product is also attached to the 5 fuse thought very thoughtful!	Jingdong prefecture level city in Shandong
Jinfeng Hongyuan Flagship Store	Electricity savings1	Big brand, with a very assured, the quality is very good, in time for the event to buy very cost-effective, customer service is really very good Oh ~!	Jingdong prefecture level city in Shandong
Jinfeng Hongyuan Flagship Store	Electricity savings1	I ordered yesterday and received it this morning, I heard my friend say that it can save electricity, I don't know if it works, I'll come back to make a comment after using it.	Jingdong prefecture level city in Shandong
Jinfeng Hongyuan Flagship Store	Electricity savings1	I've been using it for over a month and it doesn't seem to be working!	Jingdong prefecture level city in Shandong
Jinfeng Hongyuan Flagship Store	Electricity savings1	Used it, the family has always been in the habit of using energy-saving devices, this is a new one. Digital display voltage is very good, can see that the voltage is more stable.	Jingdong prefecture level city in Shandong
Jinfeng Hongyuan Flagship Store	Electricity savings1	I haven't used it for more than a month, but it looks good. It's easy to plug into an outlet and it works.	Jingdong prefecture level city in Shandong
Jinfeng Hongyuan Flagship Store	Electricity savings1	Good Oh...appearance is very beautiful...just plugged in with...see the effect of it...with a period of time in the additional evaluation...	Jingdong prefecture level city in Shandong
Jinfeng Hongyuan Flagship Store	Electricity savings1	I've received the goods, whether it's good or not, I'll know after I use it.	Jingdong prefecture level city in Shandong
Jinfeng Hongyuan Flagship Store	Electricity savings1	Very good, logistics is fast, the stuff is very powerful and cost-effective! Can feel the meter slowing down	Jingdong prefecture level city in Shandong
Jinfeng Hongyuan Flagship Store	Electricity savings1	Logistics is very fast, get it immediately after use, easy to operate, plug in the power supply can be used, just do not know to the use of the effect of how.	Jingdong prefecture level city in Shandong
Jinfeng Hongyuan Flagship Store	Electricity savings1	Quality is very over the top, quality is guaranteed, quite a good choice, a lot of friends to see me use all want to buy, I recommend it!	Jingdong prefecture level city in Shandong

Jinfeng Hongyuan Flagship Store	Electricity savings1	The appearance is clean and beautiful, the workmanship is very good, did not find any flaws, I personally feel that the size is also quite suitable, looking for a long time to choose this one, big love! The price is very beautiful, things are still good, will continue to buy back! All five points! Ness Ness ~ logistics speed is really no words, is a word: fast. Commodities from the outer packaging is very high-grade, really a penny a penny, expensive has the reason for expensive	Jingdong prefecture level city in Shandong
Jinfeng Hongyuan Flagship Store	Electricity savings1	The logistics is very fast, the texture is very good, genuine, I like it very much.	Jingdong prefecture level city in Shandong
Jinfeng Hongyuan Flagship Store	Electricity savings1	It's been a few days since I put it on, I can't see any results now, waiting for a month to say, steady steady steady.	Jingdong prefecture level city in Shandong
Jinfeng Hongyuan Flagship Store	Electricity savings1	I've been using it for a while now and it should be fine, so I'm buying it for the second time.	Jingdong prefecture level city in Shandong
Jinfeng Hongyuan Flagship Store	Electricity savings1	Power Saving King has been received, looks good, quite delicate, looking forward to using it to save electricity	Jingdong prefecture level city in Shandong
Jinfeng Hongyuan Flagship Store	Electricity savings1	My mom said the home uses a little too much electricity, so I bought a Power Saver to try it out, I don't know how effective it is. Expect it to be a good thing!	Jingdong prefecture level city in Shandong
Jinfeng Hongyuan Flagship Store	Electricity savings1	Thank you Jingdong, logistics is very fast order to receipt only one day. Believe in Jingdong. Favorable comments	Jingdong prefecture level city in Shandong
Jinfeng Hongyuan Flagship Store	Electricity savings1	Saved some money on the electric bill compared to last month, watch this month again, no problems so far	Jingdong prefecture level city in Shandong
Jinfeng Hongyuan Flagship Store	Electricity savings1	Energy saver exquisite workmanship, beautiful style, more features, just bought, power saving effect to be used for a period of time, then evaluate!	Jingdong prefecture level city in Shandong
Jinfeng Hongyuan Flagship Store	Electricity savings1	This thing does save power is more compact to hang on the wall.	Jingdong prefecture level city in Shandong
Jinfeng Hongyuan Flagship Store	Electricity savings1	I've been buying it for a while now, I haven't actually measured it, but it still feels like it's effective	Jingdong prefecture level city in Shandong
Jinfeng Hongyuan Flagship Store	Electricity savings1	I've already used it since I bought it, I don't know how effective it is yet, I'll comment on it later.	Jingdong prefecture level city in Shandong
Jinfeng Hongyuan Flagship Store	Electricity savings1	Baby received, careful appearance, logistics is very fast, worth buying!	Jingdong prefecture level city in Shandong
Jinfeng Hongyuan Flagship Store	Electricity savings1	It's great, it's just not the same in hand, I bought it at that other cheaper online one for about the same price as this one. But then oops, doesn't work at all.	Jingdong prefecture level city in Shandong
Jinfeng Hongyuan Flagship Store	Electricity savings1	The product is exquisite, but you have to use it for a month to know how it works. I'll comment again after a month.	Jingdong prefecture level city in Shandong
Jinfeng Hongyuan Flagship Store	Electricity savings1	I've been using it for a while now, and it doesn't feel like it's working, and it doesn't save any power.	Jingdong prefecture level city in Shandong
Jinfeng Hongyuan Flagship Store	Electricity savings1	Affordable price, fast delivery, Jingdong self-supporting guarantee	Jingdong prefecture level city in Shandong
Jinfeng Hongyuan Flagship Store	Electricity savings1	Packaging is very good, no damage, good quality, fine workmanship Packaging is very good, no damage, good quality, fine workmanship Packaging is very good, no damage, good quality, fine workmanship Packaging is very good, no damage, good quality, fine workmanship	Jingdong prefecture level city in Shandong
Jinfeng Hongyuan Flagship Store	Electricity savings1	Big brand, with a very assured, the quality is very good, in time for the event to buy very cost-effective, customer service is really very good Oh ~!	Jingdong prefecture level city in Shandong
Jinfeng Hongyuan Flagship Store	Electricity savings1	Used a few days, the specific effect is not yet clear, looking forward to the effect as described by the merchant!	Jingdong prefecture level city in Shandong
Jinfeng Hongyuan Flagship Store	Electricity savings1	I just used it and don't know how it works, I'll follow up after using it!	Jingdong prefecture level city in Shandong
Jinfeng Hongyuan Flagship Store	Electricity savings1	Well, it's been installed and now I'm waiting to see the results, BOE shopping, hopefully I won't be disappointed!	Jingdong prefecture level city in Shandong
Jinfeng Hongyuan Flagship Store	Electricity savings1	Goods received, plugged in the voltage from 229 to 222, do not know whether to save electricity, that depends on the situation.	Jingdong prefecture level city in Shandong
Jinfeng Hongyuan Flagship Store	Electricity savings1	This is the second time I have bought the product and I feel it really saves electricity! Use it again for a while and then evaluate!	Jingdong prefecture level city in Shandong
Jinfeng Hongyuan Flagship Store	Electricity savings1	Overall, it's really good, and the customer service is great, you'll get a message back almost immediately when you speak, it's great value for money, I really recommend it!	Jingdong prefecture level city in Shandong
Jinfeng Hongyuan Flagship Store	Electricity savings1	Have purchased many times, a must have for the family, affordable purchase price, fast delivery, beautifully packaged!	Jingdong prefecture level city in Shandong
Jinfeng Hongyuan Flagship Store	Electricity savings1	I bought it after a friend introduced it, he said it is effective and can save electricity. I bought it to try, try a period of time and then follow up!	Jingdong prefecture level city in Shandong
Jinfeng Hongyuan Flagship Store	Electricity savings1	Received it, trying it out tonight, and bought one earlier.	Jingdong prefecture level city in Shandong
Jinfeng Hongyuan Flagship Store	Electricity savings1	It will take a month or two to see if it saves power or not.	Jingdong prefecture level city in Shandong
Jinfeng Hongyuan Flagship Store	Electricity savings1	Looked good in the instructions! Bought one and pressed it on! Use it and see! If it works! I'll buy some more!	Jingdong prefecture level city in Shandong
Jinfeng Hongyuan Flagship Store	Electricity savings1	Goods have been received packaging can also things are not bad appearance, with a use to know, now evaluation is nonsense.	Jingdong prefecture level city in Shandong
Jinfeng Hongyuan Flagship Store	Electricity savings1	Logistics is very fast, packaging is very good, the second unit, as always, looking forward to the effect of	Jingdong prefecture level city in Shandong
Jinfeng Hongyuan Flagship Store	Electricity savings1	I received the goods today, the quality of goods is very good, I am very satisfied ah.	Jingdong prefecture level city in Shandong
Jinfeng Hongyuan Flagship Store	Electricity savings1	Buy one and try it, I wonder if it has a power saving effect?	Jingdong prefecture level city in Shandong
Jinfeng Hongyuan Flagship Store	Electricity savings1	The item was received, well packaged, logistics is very fast, customer service attitude is very positive, there are questions answered in a timely manner! Overall very satisfied!	Jingdong prefecture level city in Shandong
Jinfeng Hongyuan Flagship Store	Electricity savings1	Baby packaging is very good, purchase submission goods quickly delivered to the home, use and then follow up.	Jingdong prefecture level city in Shandong
Jinfeng Hongyuan Flagship Store	Electricity savings1	My friend asked me to buy an extra one so he could try it too.	Jingdong prefecture level city in Shandong
Jinfeng Hongyuan Flagship Store	Electricity savings1	I've just put it on, I won't know the result until a month later if it will really save power.	Jingdong prefecture level city in Shandong
Jinfeng Hongyuan Flagship Store	Electricity savings1	It's good quality. Hopefully it'll help save power.	Jingdong prefecture level city in Shandong
Jinfeng Hongyuan Flagship Store	Electricity savings1	The appearance is clean and beautiful, the workmanship is very good, I did not find any flaws, I personally feel that the size is also quite suitable, I have been looking for a long time to choose this	Jingdong prefecture level city in Shandong
Jinfeng Hongyuan Flagship Store	Electricity savings1	Logistics is fast! Baby looks great! The price is fair! Wish the store business is prosperous!	Jingdong prefecture level city in Shandong
Jinfeng Hongyuan Flagship Store	Electricity savings1	Jingdong express fast, good, have used, sit and wait to save money...	Jingdong prefecture level city in Shandong
Jinfeng Hongyuan Flagship Store	Electricity savings1	In use, it didn't work as expected and there was nothing noticeable in terms of power savings.	Jingdong prefecture level city in Shandong
Jinfeng Hongyuan Flagship Store	Electricity savings1	Just started using it and looking forward to the results.	Jingdong prefecture level city in Shandong
Jinfeng Hongyuan Flagship Store	Electricity savings1	The quality is very good, the metal shell is very good, is genuine, energy saving and power saving, logistics is very fast, there is a need to buy o, recommend everyone, very satisfied with an online	Jingdong prefecture level city in Shandong
Jinfeng Hongyuan Flagship Store	Electricity savings1	Received, I do not know if the product is good, used to see if it is good to come back to buy!	Jingdong prefecture level city in Shandong
Jinfeng Hongyuan Flagship Store	Electricity savings1	Jinfeng source of intelligent power saving treasure also used less than three months, the last thunder may be burned, the home of the electric send gate is not on, only the old man at home, no way only their own in the field and run back to find out what is the problem with the circuit, the quality is still not very good, thunder is easy to burn!	Jingdong prefecture level city in Shandong
Jinfeng Hongyuan Flagship Store	Electricity savings1	Saving power just started to use, I do not know if there is no effect, but read a lot of reviews are said to be very good so I bought, baby All aspects are good There are spare 2 fuses, late good and then follow	Jingdong prefecture level city in Shandong

Jinfeng Hongyuan Flagship Store	Electricity savings1	Use it for a few days and see, the delivery is quite fast	Jingdong prefecture level city in Shandong
Jinfeng Hongyuan Flagship Store	Electricity savings1	Small, well made and easy to use.	Jingdong prefecture level city in Shandong
Jinfeng Hongyuan Flagship Store	Electricity savings1	Beautifully packaged, it is genuine. Whether it is power saving, use it to evaluate.	Jingdong prefecture level city in Shandong
Jinfeng Hongyuan Flagship Store	Electricity savings1	Power saver. Used two of them and they do save quite a bit of power.	Jingdong prefecture level city in Shandong
Jinfeng Hongyuan Flagship Store	Electricity savings1	Workmanship is not bad, small and does not take up space, is also do not know whether the power saving, buy a to try!	Jingdong prefecture level city in Shandong
Jinfeng Hongyuan Flagship Store	Electricity savings1	The logistics is very fast, the courier attitude is very good, worth buying	Jingdong prefecture level city in Shandong
Jinfeng Hongyuan Flagship Store	Electricity savings1	The effect is not known until it is used.	Jingdong prefecture level city in Shandong
Jinfeng Hongyuan Flagship Store	Electricity savings1	Just arrived plugged in won't know for a month	Jingdong prefecture level city in Shandong
Jinfeng Hongyuan Flagship Store	Electricity savings1	This is really good to use the pull to realize the savings ah think again for my sister's family to buy a	Jingdong prefecture level city in Shandong
Jinfeng Hongyuan Flagship Store	Electricity savings1	Lies, a month with a few hundred electricity are no savings, family use	Jingdong prefecture level city in Shandong
Jinfeng Hongyuan Flagship Store	Electricity savings1	Express very quickly, look at the fine workmanship also sent a few fuse, look forward to can really save electricity	Jingdong prefecture level city in Shandong
Jinfeng Hongyuan Flagship Store	Electricity savings1	I didn't realize it was so small.	Jingdong prefecture level city in Shandong
Jinfeng Hongyuan Flagship Store	Electricity savings1	Logistics courier is very fast, the courier service is very good	Jingdong prefecture level city in Shandong
Jinfeng Hongyuan Flagship Store	Electricity savings1	Used for a period of time the effect is quite good indeed than the previous power saving	Jingdong prefecture level city in Shandong
Jinfeng Hongyuan Flagship Store	Electricity savings1	Logistics is super fast, the product is in use, see the effect...	Jingdong prefecture level city in Shandong
Jinfeng Hongyuan Flagship Store	Electricity savings1	Baby got it, tried it out and it didn't work at all. How can I use the tips to save power?	Jingdong prefecture level city in Shandong
Jinfeng Hongyuan Flagship Store	Electricity savings1	Power Saving King received, immediately used has been very stable without any sound good, good service, fast logistics inexpensive and beautiful worth repurchasing!	Jingdong prefecture level city in Shandong
Jinfeng Hongyuan Flagship Store	Electricity savings1	Logistics courier is very fast, the courier service is good	Jingdong prefecture level city in Shandong
Jinfeng Hongyuan Flagship Store	Electricity savings1	Good to recommend, the first month of air conditioning only 1 week, the electricity bill has reduced about 100 yuan, may be to reduce the use of air conditioning is also related to the other or as usual electricity, estimated that the longer you use the more you can save electricity.	Jingdong prefecture level city in Shandong
Jinfeng Hongyuan Flagship Store	Electricity savings1	It doesn't work. I've been using it for 20 days and it doesn't work.	Jingdong prefecture level city in Shandong
Jinfeng Hongyuan Flagship Store	Electricity savings1	Fast shipping, good quality, affordable price, very satisfied with a shopping.	Jingdong prefecture level city in Shandong
Jinfeng Hongyuan Flagship Store	Electricity savings1	Try it for a month to see if it saves money.	Jingdong prefecture level city in Shandong
Jinfeng Hongyuan Flagship Store	Electricity savings1	It's in use. I'll comment again after a while.	Jingdong prefecture level city in Shandong
Jinfeng Hongyuan Flagship Store	Electricity savings1	Goods have received look good 👍👍👍👍, use a period of time and then look at the	Jingdong prefecture level city in Shandong
Jinfeng Hongyuan Flagship Store	Electricity savings1	No, it doesn't work. It's useless. It's garbage.	Jingdong prefecture level city in Shandong
Jinfeng Hongyuan Flagship Store	Electricity savings1	I've been using it for almost 2 months now and I don't feel any reduction in my electricity bill.	Jingdong prefecture level city in Shandong
Jinfeng Hongyuan Flagship Store	Electricity savings1	I've received the goods, the logistics is really fast, ordered yesterday, arrived today.	Jingdong prefecture level city in Shandong
Jinfeng Hongyuan Flagship Store	Electricity savings1	And the backup insurance.	Jingdong prefecture level city in Shandong
Jinfeng Hongyuan Flagship Store	Electricity savings1	The appearance of this product is good, easy to use and install, the effect of how there is no use to wait for time to verify that the voltage displayed is different from the voltage measured by other meters, the gap is not too large, the follow-up to add the effect.	Jingdong prefecture level city in Shandong
Jinfeng Hongyuan Flagship Store	Electricity savings1	Baby received it 👌, really great ah! 🌈The quality can't be beat, very satisfied! 🆗Happy happy happy 😃	Jingdong prefecture level city in Shandong
Jinfeng Hongyuan Flagship Store	Electricity savings1	I'll follow up with a review after using it for a while, hopefully it won't bring me back a disappointment!	Jingdong prefecture level city in Shandong
Jinfeng Hongyuan Flagship Store	Electricity savings1	In use and looking forward to results! Good review to send	Jingdong prefecture level city in Shandong
Jinfeng Hongyuan Flagship Store	Electricity savings1	Intelligent power saver received, the volume is not big, small and exquisite, quite good, Jingdong Express is also quite fast, the	Jingdong prefecture level city in Shandong
Jinfeng Hongyuan Flagship Store	Electricity savings1	I don't know how to use it, keep using it	Jingdong prefecture level city in Shandong
Jinfeng Hongyuan Flagship Store	Electricity savings1	Very good for saving electricity	Jingdong prefecture level city in Shandong
Jinfeng Hongyuan Flagship Store	Electricity savings1	Seriously, did not use, feel also more power consumption, the peak is bought back to use the first time, the next day unplugged, the power consumption is back to more than ten degrees (the frequency of power use in the past few days is pretty much the same), feel the IQ tax paid 😂	Jingdong prefecture level city in Shandong
Jinfeng Hongyuan Flagship Store	Electricity savings1	I haven't seen any effect on the power saving, but the voltage stabilization looks good.	Jingdong prefecture level city in Shandong
Jinfeng Hongyuan Flagship Store	Electricity savings1	It's very good, I'll come back to it after using it for a while	Jingdong prefecture level city in Shandong
Jinfeng Hongyuan Flagship Store	Electricity savings1	Already used it and see the results.	Jingdong prefecture level city in Shandong
Jinfeng Hongyuan Flagship Store	Electricity savings1	Fast shipping, beautiful packaging, attentive service, the use of the effect, to be followed up after the evaluation.	Jingdong prefecture level city in Shandong
Jinfeng Hongyuan Flagship Store	Electricity savings1	The process of using this product is more than satisfactory	Jingdong prefecture level city in Shandong
Jinfeng Hongyuan Flagship Store	Electricity savings1	Very good East East cost-effective! Every time so buy more assured, and the logistics is also very fast. It is also very good to use, so I will often come to buy in the future, and will also introduce to	Jingdong prefecture level city in Shandong
Jinfeng Hongyuan Flagship Store	Electricity savings1	But still understand how effective it is!	Jingdong prefecture level city in Shandong
Jinfeng Hongyuan Flagship Store	Electricity savings1	Fast shipping, great logistics, great stuff, great price.	Jingdong prefecture level city in Shandong
Jinfeng Hongyuan Flagship Store	Electricity savings1	I'll review it in a month and give it 5 stars if it's good	Jingdong prefecture level city in Shandong
Jinfeng Hongyuan Flagship Store	Electricity savings1	I've saved a lot of electricity in the past month, so I'm glad to hear that.	Jingdong prefecture level city in Shandong
Jinfeng Hongyuan Flagship Store	Electricity savings1	Baby just received and installed! Looks genuinely good, workmanship feels fine! Looking forward to the effect! Said to see the effect in two months! Waiting for if really have the effect must come to follow up! The boss is absolutely responsible! Asked and answered! Just for the boss this attitude must also	Jingdong prefecture level city in Shandong
Jinfeng Hongyuan Flagship Store	Electricity savings1	This baby is very delicate, I'll comment after I've used it	Jingdong prefecture level city in Shandong
Jinfeng Hongyuan Flagship Store	Electricity savings1	It's been over two months. It's not working. It's just like it was.	Jingdong prefecture level city in Shandong

Jinfeng Hongyuan Flagship Store	Electricity savings1	Fast shipping and logistics, let's see if it works.	Jingdong prefecture level city in Shandong
Jinfeng Hongyuan Flagship Store	Electricity savings1	Just received the goods, try it to know if it can really save electricity	Jingdong prefecture level city in Shandong
Jinfeng Hongyuan Flagship Store	Electricity savings1	Not bad. Not bad. Not bad.	Jingdong prefecture level city in Shandong
Jinfeng Hongyuan Flagship Store	Electricity savings1	Not sure if it works. I'll comment again if it works.	Jingdong prefecture level city in Shandong
Jinfeng Hongyuan Flagship Store	Electricity savings1	Safe and energy-saving helpers to use!	Jingdong prefecture level city in Shandong
Jinfeng Hongyuan Flagship Store	Electricity savings1	Good, first time buyer. Used good can	Jingdong prefecture level city in Shandong
Jinfeng Hongyuan Flagship Store	Electricity savings1	It feels okay, I guess! I won't know until I use it for a while!	Jingdong prefecture level city in Shandong
Jinfeng Hongyuan Flagship Store	Electricity savings1	The saver matches the description and has been used, waiting for a follow up review after two months.	Jingdong prefecture level city in Shandong
Jinfeng Hongyuan Flagship Store	Electricity savings1	Received, the appearance of the business as described, the effect of how to use a period of time to evaluate.	Jingdong prefecture level city in Shandong
Jinfeng Hongyuan Flagship Store	Electricity savings1	Still using it don't know how effective it is, I'll comment after three months	Jingdong prefecture level city in Shandong
Jinfeng Hongyuan Flagship Store	Electricity savings1	The stuff is very good, the packaging is also very good logistics is also very fast	Jingdong prefecture level city in Shandong
Jinfeng Hongyuan Flagship Store	Electricity savings1	I'll follow up with a review when I use it, hopefully I won't be disappointed	Jingdong prefecture level city in Shandong
Jinfeng Hongyuan Flagship Store	Electricity savings1	Cancel Houjiaying Logistics.	Jingdong prefecture level city in Shandong
Jinfeng Hongyuan Flagship Store	Electricity savings1	It's very good, it's worth buying.	Jingdong prefecture level city in Shandong
Jinfeng Hongyuan Flagship Store	Electricity savings1	My friend bought it to save electricity and referred me to buy it! I'll buy it and try it out! I will know how much electricity I can save next month, looking forward to it! 4-500 dollars a month electricity bill is horrible! I hope it can really help me save some electricity!	Jingdong prefecture level city in Shandong
Jinfeng Hongyuan Flagship Store	Electricity savings1	Just received it and installed it.	Jingdong prefecture level city in Shandong
Jinfeng Hongyuan Flagship Store	Electricity savings1	Very nice. This one is my favorite.	Jingdong prefecture level city in Shandong
Jinfeng Hongyuan Flagship Store	Electricity savings1	Good value for money worth buying very good	Jingdong prefecture level city in Shandong
Jinfeng Hongyuan Flagship Store	Electricity savings1	Very energy-saving, small and exquisite, does not take up space, very satisfied, complete packaging, no damage, customer service explanation is also very serious	Jingdong prefecture level city in Shandong
Jinfeng Hongyuan Flagship Store	Electricity savings1	Received immediately used, really effective, do not understand electricity, but looked at the meter every day, recommended that you can use, especially this weather every day to open the air conditioning, with him not afraid of the electricity bill.	Jingdong prefecture level city in Shandong
Jinfeng Hongyuan Flagship Store	Electricity savings1	Nice, good value for money, nice stuff.	Jingdong prefecture level city in Shandong
Jinfeng Hongyuan Flagship Store	Electricity savings1	The packaging of the product is very good, the logistics of the product is very fast, and the quality of the product will be evaluated after use!	Jingdong prefecture level city in Shandong
Jinfeng Hongyuan Flagship Store	Electricity savings1	Express is very, well very fast, give a compliment 👍	Jingdong prefecture level city in Shandong
Jinfeng Hongyuan Flagship Store	Electricity savings1	Packaging is very good, fast delivery, used a night and a day did not feel the power saving. Use a month to see if it saves power.	Jingdong prefecture level city in Shandong
Jinfeng Hongyuan Flagship Store	Electricity savings1	Seems to be a bit of a power saver.	Jingdong prefecture level city in Shandong
Jinfeng Hongyuan Flagship Store	Electricity savings1	Bought this for someone else and it came over so fast, ###!	Jingdong prefecture level city in Shandong
Jinfeng Hongyuan Flagship Store	Electricity savings1	Very delicate, just received a period of time to use in the follow-up evaluation	Jingdong prefecture level city in Shandong
Jinfeng Hongyuan Flagship Store	Electricity savings1	It's been installed for a while now, not sure if it's working or not	Jingdong prefecture level city in Shandong
Jinfeng Hongyuan Flagship Store	Electricity savings1	The item looks okay, I just don't know if it works or not.	Jingdong prefecture level city in Shandong
Jinfeng Hongyuan Flagship Store	Electricity savings1	The goods have been received, but there is a screw missing	Jingdong prefecture level city in Shandong
Jinfeng Hongyuan Flagship Store	Electricity savings1	I didn't believe it before, but after receiving the goods today and trying it out, it really works!	Jingdong prefecture level city in Shandong
Jinfeng Hongyuan Flagship Store	Electricity savings1	Received, this thing will not know whether it is the effect of saving electricity, no matter how to say that there should always be a little use it, otherwise why develop it I think there is still the value of	Jingdong prefecture level city in Shandong
Jinfeng Hongyuan Flagship Store	Electricity savings1	Another one just arrived.	Jingdong prefecture level city in Shandong
Jinfeng Hongyuan Flagship Store	Electricity savings1	I don't feel anything, ordinary farmers can't go to TEDA, just use it first, it's safe.	Jingdong prefecture level city in Shandong
Jinfeng Hongyuan Flagship Store	Electricity savings1	Just purchased and will follow up after using it for a while	Jingdong prefecture level city in Shandong
Jinfeng Hongyuan Flagship Store	Electricity savings1	I'm trying it out now. I'll comment after I use it.	Jingdong prefecture level city in Shandong
Jinfeng Hongyuan Flagship Store	Electricity savings1	Received the item, in use, hope it works.	Jingdong prefecture level city in Shandong
Jinfeng Hongyuan Flagship Store	Electricity savings1	It works well. Good review.	Jingdong prefecture level city in Shandong
Jinfeng Hongyuan Flagship Store	Electricity savings1	All a scam, no effect at all, I bought too many fakes in Poundland before, I did not think that Jingdong is also the same	Jingdong prefecture level city in Shandong
Jinfeng Hongyuan Flagship Store	Electricity savings1	I used the saver as soon as I received it, it's in good shape and still working!	Jingdong prefecture level city in Shandong
Jinfeng Hongyuan Flagship Store	Electricity savings1	It works. I don't know what to do with the picture, but it works.	Jingdong prefecture level city in Shandong
Jinfeng Hongyuan Flagship Store	Electricity savings1	A very good shopping experience, attentive service, fast logistics, delivery is also very thoughtful, attitude is also very good, value for money ah, decisive praise!	Jingdong prefecture level city in Shandong
Jinfeng Hongyuan Flagship Store	Electricity savings1	The product is consistent with the presentation picture.	Jingdong prefecture level city in Shandong
Jinfeng Hongyuan Flagship Store	Electricity savings1	Baby received, use a period of time to evaluate, wish the boss business prosperity!	Jingdong prefecture level city in Shandong
Jinfeng Hongyuan Flagship Store	Electricity savings1	The stuff is better! Recommended!	Jingdong prefecture level city in Shandong
Jinfeng Hongyuan Flagship Store	Electricity savings1	I don't know if it works or not, I'll use it for a while before I comment on it.	Jingdong prefecture level city in Shandong
Jinfeng Hongyuan Flagship Store	Electricity savings1	Cost-effective, I like it, next time will come back Baby received, very surprised, the quality is very good, very satisfied with a shopping, I will continue to buy back, the store is trustworthy	Jingdong prefecture level city in Shandong
Jinfeng Hongyuan Flagship Store	Electricity savings1	Plugged it in and used it for a few days, not sure if it saves power yet	Jingdong prefecture level city in Shandong
Jinfeng Hongyuan Flagship Store	Electricity savings1	I'm trying it out and will follow up if it seems to be working!	Jingdong prefecture level city in Shandong
Jinfeng Hongyuan Flagship Store	Electricity savings1	Works pretty well, big kudos 👍👍👍👍👍👍👍👍👍👍👍	Jingdong prefecture level city in Shandong

Jinfeng Hongyuan Flagship Store	Electricity savings1	Just put it on, we'll see how it looks in a month.	Jingdong prefecture level city in Shandong
Jinfeng Hongyuan Flagship Store	Electricity savings1	The baby is very nice and I've already recommended buying it for all my friends! The main thing is that it not only saves electricity, I was more concerned about the fact that it can adjust the current, usually wiring problems. Now I found that it is really good, it is the effect I want!	Jingdong prefecture level city in Shandong
Jinfeng Hongyuan Flagship Store	Electricity savings1	Baby arrived, looking forward to his results, hopefully it will be good	Jingdong prefecture level city in Shandong
Jinfeng Hongyuan Flagship Store	Electricity savings1	Things have been received, look at the workmanship and packaging are good, I do not know how the power-saving effect? Use a period of time to see the effect	Jingdong prefecture level city in Shandong
Jinfeng Hongyuan Flagship Store	Electricity savings1	The price is right, the shape is beautiful and does not take up space, use it for a while to see the effect	Jingdong prefecture level city in Shandong
Jinfeng Hongyuan Flagship Store	Electricity savings1	Can't save electricity, it doesn't work!	Jingdong prefecture level city in Shandong
Jinfeng Hongyuan Flagship Store	Electricity savings1	It's pretty convenient, just plug it in and it works. There's no noise, plug it in and leave it alone, it's very hassle free! Good workmanship and value for money.	Jingdong prefecture level city in Shandong
Jinfeng Hongyuan Flagship Store	Electricity savings1	Just received the goods have been installed, try two months later to comment!	Jingdong prefecture level city in Shandong
Jinfeng Hongyuan Flagship Store	Electricity savings1	I received the stuff, I haven't used it yet, and overall it's fine.	Jingdong prefecture level city in Shandong
Jinfeng Hongyuan Flagship Store	Electricity savings1	Couldn't use a return at all.	Jingdong prefecture level city in Shandong
Jinfeng Hongyuan Flagship Store	Electricity savings1	Second purchase.	Jingdong prefecture level city in Shandong
Jinfeng Hongyuan Flagship Store	Electricity savings1	Very good quality, high tech and practical.	Jingdong prefecture level city in Shandong
Jinfeng Hongyuan Flagship Store	Electricity savings1	Very good, very great! Very satisfied, thanks to the store!	Jingdong prefecture level city in Shandong
Jinfeng Hongyuan Flagship Store	Electricity savings1	Just used it, don't know if it's good or not, hope it works well	Jingdong prefecture level city in Shandong
Jinfeng Hongyuan Flagship Store	Electricity savings1	Saving power has been received, the product is good, good like.	Jingdong prefecture level city in Shandong
Jinfeng Hongyuan Flagship Store	Electricity savings1	Received the goods and used it for two months before evaluation	Jingdong prefecture level city in Shandong
Jinfeng Hongyuan Flagship Store	Electricity savings1	It looks a little small, I do not know how effective, first use to see the effect, there is a small adjustable button, I do not know this is to adjust to that position to work well	Jingdong prefecture level city in Shandong
Jinfeng Hongyuan Flagship Store	Electricity savings1	Simple, just plug it in, there are spare fuses, can't say how well it works, no comparison	Jingdong prefecture level city in Shandong
Jinfeng Hongyuan Flagship Store	Electricity savings1	It's been a few days since I used it, and the power's gone up.	Jingdong prefecture level city in Shandong
Jinfeng Hongyuan Flagship Store	Electricity savings1	The owner was very nice and sent a spare fuse.	Jingdong prefecture level city in Shandong
Jinfeng Hongyuan Flagship Store	Electricity savings1	It's authentic and it's not expensive and it's great to use, I use it all the time and it's great 🎉✨ just don't know if it really saves any points	Jingdong prefecture level city in Shandong
Jinfeng Hongyuan Flagship Store	Electricity savings1	The information is good, it is the child to buy, the child read it is also quite happy, I hope to be able to match the child's preparation for the midterm examination! Because of the epidemic, it is not too fast, but I also give full credit to the express delivery!	Jingdong prefecture level city in Shandong
Jinfeng Hongyuan Flagship Store	Electricity savings1	This power saver comes to the logistics very quickly, the business attitude is also very good, ** very comfortable, this brand is trustworthy, and buy in this store than in the physical store to buy cheaper,	Jingdong prefecture level city in Shandong
Jinfeng Hongyuan Flagship Store	Electricity savings1	It's very well made and the materials used are okay. This is just a few days old, so I don't know how it will work, only next month.	Jingdong prefecture level city in Shandong
Jinfeng Hongyuan Flagship Store	Electricity savings1	Already used on, have not tested the effect, wait for a couple of months to add comments.	Jingdong prefecture level city in Shandong
Jinfeng Hongyuan Flagship Store	Electricity savings1	Baby received, packaging is very thoughtful, already in use, should soon see the effect.	Jingdong prefecture level city in Shandong
Jinfeng Hongyuan Flagship Store	Electricity savings1	Just received in the office to try, to the home installed, the voltage are different, and do not know if there is no effect. Use a period of time and then follow up.	Jingdong prefecture level city in Shandong
Jinfeng Hongyuan Flagship Store	Electricity savings1	Just received the goods, is trying, looking forward to the effect, first give a favorable comment, two months later, then additional evaluation, please be patient.	Jingdong prefecture level city in Shandong
Jinfeng Hongyuan Flagship Store	Electricity savings1	The item has just been received, well packaged, results to be verified.	Jingdong prefecture level city in Shandong
Jinfeng Hongyuan Flagship Store	Electricity savings1	Packaging logistics are very good, the next look at how the effect, before I see others use the effect is good, as I bought this can also have good results.	Jingdong prefecture level city in Shandong
Jinfeng Hongyuan Flagship Store	Electricity savings1	I don't know if it's a real power saver or a fake power saver, but I've used it since I bought it, so I'll give you a good review.	Jingdong prefecture level city in Shandong
Jinfeng Hongyuan Flagship Store	Electricity savings1	Good quality, genuine goods, much better than imagined before purchase, next time will come back to care!	Jingdong prefecture level city in Shandong
Jinfeng Hongyuan Flagship Store	Electricity savings1	Logistics is very fast good use again	Jingdong prefecture level city in Shandong
Jinfeng Hongyuan Flagship Store	Electricity savings1	Received the goods. Let's see if it saves power in a month and then we'll follow up!	Jingdong prefecture level city in Shandong
Jinfeng Hongyuan Flagship Store	Electricity savings1	I bought it yesterday and it arrived today, I bought two of them, I hope they work well, I'll come back after a while to give a follow-up review	Jingdong prefecture level city in Shandong
Jinfeng Hongyuan Flagship Store	Electricity savings1	The home air conditioner consumes a lot of electricity, so I bought it and used it in the hope that there is A	Jingdong prefecture level city in Shandong
Jinfeng Hongyuan Flagship Store	Electricity savings1	The product is excellent and very current.	Jingdong prefecture level city in Shandong
Jinfeng Hongyuan Flagship Store	Electricity savings1	With the description, it is the most practical and good products that households can need, and it is an ideal shopping, the courier is also fast, and the service attitude is enthusiastic!	Jingdong prefecture level city in Shandong
Jinfeng Hongyuan Flagship Store	Electricity savings1	The Power Saver is easy to use, works well, is stable, and plugs into the top of the outlet	Jingdong prefecture level city in Shandong
Jinfeng Hongyuan Flagship Store	Electricity savings1	Overall still can, excellent craftsmanship, power saving do not know for the time being, after the use of the evaluation	Jingdong prefecture level city in Shandong
Jinfeng Hongyuan Flagship Store	Electricity savings1	Baby received on the plug, but also with four fuses, look at the quality is okay, I do not know whether it is useful, use and then follow up.	Jingdong prefecture level city in Shandong
Jinfeng Hongyuan Flagship Store	Electricity savings1	I'll evaluate it after a while, I don't know if it's good or not yet, and if it can save electricity	Jingdong prefecture level city in Shandong
Jinfeng Hongyuan Flagship Store	Electricity savings1	Jingdong self-operated purchase more assured delivery fast quality assurance	Jingdong prefecture level city in Shandong
Jinfeng Hongyuan Flagship Store	Electricity savings1	Shipping was fast, stuff was well packaged, great price, I'm happy with it 👍 and will repurchase!	Jingdong prefecture level city in Shandong
Jinfeng Hongyuan Flagship Store	Electricity savings1	The quality of the product is very good and I will definitely come back again next time.	Jingdong prefecture level city in Shandong
Jinfeng Hongyuan Flagship Store	Electricity savings1	I'll follow up with a review after I use it.	Jingdong prefecture level city in Shandong
Jinfeng Hongyuan Flagship Store	Electricity savings1	Hello, the goods are perfectly packaged, save power for a period of time to evaluate again!	Jingdong prefecture level city in Shandong
Jinfeng Hongyuan Flagship Store	Electricity savings1	Not bad, pretty good, good value for money, pretty good	Jingdong prefecture level city in Shandong
Jinfeng Hongyuan Flagship Store	Electricity savings1	The packaging is very good, received immediately used, looking forward to the effect in.	Jingdong prefecture level city in Shandong
Jinfeng Hongyuan Flagship Store	Electricity savings1	See how much power you save at the end of the month	Jingdong prefecture level city in Shandong

Jinfeng Hongyuan Flagship Store	Electricity savings1	Easy to use, affordable, beautiful color	Jingdong prefecture level city in Shandong
Jinfeng Hongyuan Flagship Store	Electricity savings1	It's taking too long to put together an order. It could be an epidemic.	Jingdong prefecture level city in Shandong
Jinfeng Hongyuan Flagship Store	Electricity savings1	I don't feel much after using it, no change in my electric bill 。。。。。。。	Jingdong prefecture level city in Shandong
Jinfeng Hongyuan Flagship Store	Electricity savings1	Logistics is very fast, the quality of the economizer is good, good quality and cheap, it is worth buying, I hope there is a power saving effect!	Jingdong prefecture level city in Shandong
Jinfeng Hongyuan Flagship Store	Electricity savings1	Feeling okay, in a couple of months to try to rely on it before making a comment. Let's give it a five-star review first!	Jingdong prefecture level city in Shandong
Jinfeng Hongyuan Flagship Store	Electricity savings1	Power saving device has been hesitating before in the end to buy or not to buy, after consideration, consideration, in consideration, or decided to buy a try. Things received can not wait to open a look, and the seller described the same, I hope to use 2 months to have the effect, two months to follow up on	Jingdong prefecture level city in Shandong
Jinfeng Hongyuan Flagship Store	Electricity savings1	Thank you Jingdong, logistics is very fast order to receipt only one day. Believe in Jingdong. Favorable comments	Jingdong prefecture level city in Shandong
Jinfeng Hongyuan Flagship Store	Electricity savings1	I used one to use it, but it didn't work very well, what to do	Jingdong prefecture level city in Shandong
Jinfeng Hongyuan Flagship Store	Electricity savings1	Quality and packaging are very good, fast shipping, good service, buy with confidence	Jingdong prefecture level city in Shandong
Jinfeng Hongyuan Flagship Store	Electricity savings1	Use a period of time to evaluate, the quality is very good, the price is affordable, logistics is also very fast	Jingdong prefecture level city in Shandong
Jinfeng Hongyuan Flagship Store	Electricity savings1	Goods received, the day of the order tomorrow delivery real God speed, delivery force, the product to be used after a period of time and then low comment!	Jingdong prefecture level city in Shandong
Jinfeng Hongyuan Flagship Store	Electricity savings1	I don't know, how effective it is, let's talk about it when the time comes, whether it saves power or not, how much, unknown.	Jingdong prefecture level city in Shandong
Jinfeng Hongyuan Flagship Store	Electricity savings1	It does save some electricity, and I don't want to use something that isn't legal! Used nearly ~ electricity last month, probably not much more this month with the AC on!	Jingdong prefecture level city in Shandong
Jinfeng Hongyuan Flagship Store	Electricity savings1	Well-packed, excellent quality, service satisfaction good value for money	Jingdong prefecture level city in Shandong
Jinfeng Hongyuan Flagship Store	Electricity savings1	Express very quickly, the goods received last night, today used a day of goods can not heat, looking forward to whether it can save electricity, with a month and then comment, is used well will buy again.	Jingdong prefecture level city in Shandong
Jinfeng Hongyuan Flagship Store	Electricity savings1	It's pretty good, hopefully it'll be a real power saver.	Jingdong prefecture level city in Shandong
Jinfeng Hongyuan Flagship Store	Electricity savings1	The goods received in use, can not see the effect of energy saving, after a month to know!	Jingdong prefecture level city in Shandong
Jinfeng Hongyuan Flagship Store	Electricity savings1	Received the goods, looking forward to better surprise, with the effect of the follow-up comment!	Jingdong prefecture level city in Shandong
Jinfeng Hongyuan Flagship Store	Electricity savings1	It's not bad, it's still in use and should save some	Jingdong prefecture level city in Shandong
Jinfeng Hongyuan Flagship Store	Electricity savings1	Logistics is very fast, easy to install, plugged in on it,, the effect of the use of the later to evaluate!	Jingdong prefecture level city in Shandong
Jinfeng Hongyuan Flagship Store	Electricity savings1	The stuff is recommendable to buy very good.	Jingdong prefecture level city in Shandong
Jinfeng Hongyuan Flagship Store	Electricity savings1	Voltmeter measurements of voltage are way off, aren't they?	Jingdong prefecture level city in Shandong
Jinfeng Hongyuan Flagship Store	Electricity savings1	Install it to the socket immediately after purchase and try to see if it can save electricity after one month.	Jingdong prefecture level city in Shandong
Jinfeng Hongyuan Flagship Store	Electricity savings1	I've been using it for almost a month now, and I won't know if it saves electricity until I pay the bill.	Jingdong prefecture level city in Shandong
Jinfeng Hongyuan Flagship Store	Electricity savings1	I bought one in the past and it was fine, so I'm buying it back.	Jingdong prefecture level city in Shandong
Jinfeng Hongyuan Flagship Store	Electricity savings1	Service is very good, this thing is small and exquisite to use, just waiting to use the effect of a period of time	Jingdong prefecture level city in Shandong
Jinfeng Hongyuan Flagship Store	Electricity savings1	Not sure how well it works yet	Jingdong prefecture level city in Shandong
Jinfeng Hongyuan Flagship Store	Electricity savings1	Kyodo goods habitually good reviews	Jingdong prefecture level city in Shandong
Jinfeng Hongyuan Flagship Store	Electricity savings1	Appearance is OK, whether it is power saving remains to be seen.	Jingdong prefecture level city in Shandong
Jinfeng Hongyuan Flagship Store	Electricity savings1	I bought it and used it, I just don't know if it really saves power	Jingdong prefecture level city in Shandong
Jinfeng Hongyuan Flagship Store	Electricity savings1	The saver is well made and of good quality, I will evaluate it after using it for a while.	Jingdong prefecture level city in Shandong
Jinfeng Hongyuan Flagship Store	Electricity savings1	Just received in use, use for a period of time to see if there is power saving	Jingdong prefecture level city in Shandong
Jinfeng Hongyuan Flagship Store	Electricity savings1	Used 3 days before commenting, it really saves electricity, I look at the "power calendar" after using one less degree of electricity per day, so I think it's worth it to buy this, I will introduce my friends	Jingdong prefecture level city in Shandong
Jinfeng Hongyuan Flagship Store	Electricity savings1	Reliable quality, very well packaged and matches the real thing.	Jingdong prefecture level city in Shandong
Jinfeng Hongyuan Flagship Store	Electricity savings1	Logistics is still quite fast, ha in the trial	Jingdong prefecture level city in Shandong
Jinfeng Hongyuan Flagship Store	Electricity savings1	Consultant service attitude is very good, the courier is also very powerful, the first day of the order the next day to arrive. Saver received used and then add comments!	Jingdong prefecture level city in Shandong
Jinfeng Hongyuan Flagship Store	Electricity savings1	I just got home to try it out and see how it goes.	Jingdong prefecture level city in Shandong
Jinfeng Hongyuan Flagship Store	Electricity savings1	Well made and weighted instrument. Kudos! Put it into use immediately, don't know if it saves power, will evaluate later!	Jingdong prefecture level city in Shandong
Jinfeng Hongyuan Flagship Store	Electricity savings1	I've just put it in place. I'll give you a review in ten days.	Jingdong prefecture level city in Shandong
Jinfeng Hongyuan Flagship Store	Electricity savings1	It looks very delicate, not bad. Well, I don't know if it can save power or not, I have to try it for a while and see. If it can really save electricity. Uh, if it works well. Then additional comments. Anyway, thanks for the logistics, thanks to Jingdong. Well, it deserves some praise.	Jingdong prefecture level city in Shandong
Jinfeng Hongyuan Flagship Store	Electricity savings1	Bought a month, the feeling is that there is a certain effect, a month to save a lot of money	Jingdong prefecture level city in Shandong
Jinfeng Hongyuan Flagship Store	Electricity savings1	Goods received, the physical and the same as the picture, logistics is also very fast, the service attitude is very good, it is worth buying!	Jingdong prefecture level city in Shandong
Jinfeng Hongyuan Flagship Store	Electricity savings1	I've used it, I don't know whether it saves electricity or not, I'll talk about it after a while.	Jingdong prefecture level city in Shandong
Jinfeng Hongyuan Flagship Store	Electricity savings1	August only began to use, October to know, have no power-saving effect, to the in to evaluate!	Jingdong prefecture level city in Shandong
Jinfeng Hongyuan Flagship Store	Electricity savings1	Goods received: I do not know to the factory products used to save electricity!	Jingdong prefecture level city in Shandong
Jinfeng Hongyuan Flagship Store	Electricity savings1	Don't know if it works, bought it anyway.	Jingdong prefecture level city in Shandong
Jinfeng Hongyuan Flagship Store	Electricity savings1	Still testing, will follow up next month to evaluate how much electricity is saved	Jingdong prefecture level city in Shandong
Jinfeng Hongyuan Flagship Store	Electricity savings1	I'm still testing it, I'll comment after I use it.	Jingdong prefecture level city in Shandong
Jinfeng Hongyuan Flagship Store	Electricity savings1	Fast service, praiseworthy. Good item pre-style, first praise, the use of the effect to be evaluated after use.	Jingdong prefecture level city in Shandong
Jinfeng Hongyuan Flagship Store	Electricity savings1	The power saving effect is more obvious through verified use.	Jingdong prefecture level city in Shandong

Jinfeng Hongyuan Flagship Store	Electricity savings1	Logistics is very fast yesterday order today arrived. The product was immediately used, and after a month to see its effect.	Jingdong prefecture level city in Shandong
Jinfeng Hongyuan Flagship Store	Electricity savings1	Power saving is not yet known, as far as the TV and audio are concerned, I feel that there is a significant quality improvement, and I recommend using a good product!	Jingdong prefecture level city in Shandong
Jinfeng Hongyuan Flagship Store	Electricity savings1	Good power savings, tens of dollars a month saved	Jingdong prefecture level city in Shandong
Jinfeng Hongyuan Flagship Store	Electricity savings1	Already took two, great value! You can save 30% on your electric bill in a month. A summer must-have!	Jingdong prefecture level city in Shandong
Jinfeng Hongyuan Flagship Store	Electricity savings1	Received the item, plugged it into a home outlet and it showed 230 volts (not that high with a multimeter). Let's use it first!	Jingdong prefecture level city in Shandong
Jinfeng Hongyuan Flagship Store	Electricity savings1	Expecting results in the experiment	Jingdong prefecture level city in Shandong
Jinfeng Hongyuan Flagship Store	Electricity savings1	I don't know if it really saves power.	Jingdong prefecture level city in Shandong
Jinfeng Hongyuan Flagship Store	Electricity savings1	It's very good and the efficacy is very visible.	Jingdong prefecture level city in Shandong
Jinfeng Hongyuan Flagship Store	Electricity savings1	It's good and worth buying with confidence	Jingdong prefecture level city in Shandong
Jinfeng Hongyuan Flagship Store	Electricity savings1	The goods have been received, just do not know if it is effective	Jingdong prefecture level city in Shandong
Jinfeng Hongyuan Flagship Store	Electricity savings1	It's already in use, but based on some of the descriptions, I suspect that to use it, you need to have one in series on each line, which seems to work for all appliances, that's my guess, but I feel as though the principle of the thing seems to require it.	Jingdong prefecture level city in Shandong
Jinfeng Hongyuan Flagship Store	Electricity savings1	Fast delivery, already in use, looks good!	Jingdong prefecture level city in Shandong
Jinfeng Hongyuan Flagship Store	Electricity savings1	Hoping to save 30% of power as presented in the graphic	Jingdong prefecture level city in Shandong
Jinfeng Hongyuan Flagship Store	Electricity savings1	Things look good, power saving do not know now, with a month after the continued evaluation	Jingdong prefecture level city in Shandong
Jinfeng Hongyuan Flagship Store	Electricity savings1	Already used it but not sure if it will top out!	Jingdong prefecture level city in Shandong
Jinfeng Hongyuan Flagship Store	Electricity savings1	Looking forward to using the results, the product quality looks okay.	Jingdong prefecture level city in Shandong
Jinfeng Hongyuan Flagship Store	Electricity savings1	It's somewhat effective and saves money more or less 💰	Jingdong prefecture level city in Shandong
Jinfeng Hongyuan Flagship Store	Electricity savings1	Got it, it's great, looking forward to saving power.	Jingdong prefecture level city in Shandong
Jinfeng Hongyuan Flagship Store	Electricity savings1	I don't know if it's working yet, I'll follow up when I get there	Jingdong prefecture level city in Shandong
Jinfeng Hongyuan Flagship Store	Electricity savings1	The product received, good workmanship, fast logistics, good inner packaging, the outer bag is damaged, but fortunately did not hurt the product, retrieved and inserted according to the instructions, looking forward to the effect is good, with a period of time to evaluate, I believe that the Jingdong.	Jingdong prefecture level city in Shandong
Jinfeng Hongyuan Flagship Store	Electricity savings1	Since it is a new product, it is said to work, so if it works well for a month, then I will give you a good review.	Jingdong prefecture level city in Shandong
Jinfeng Hongyuan Flagship Store	Electricity savings1	The quality of the product is very good, very well made, well packaged, easy to use and very convenient!	Jingdong prefecture level city in Shandong
Jinfeng Hongyuan Flagship Store	Electricity savings1	Good price for the item, good functionality	Jingdong prefecture level city in Shandong
Jinfeng Hongyuan Flagship Store	Electricity savings1	Received the first time to use it, the stuff is very good, specifics wait for a month to come back to feedback to see the effect.	Jingdong prefecture level city in Shandong
Jinfeng Hongyuan Flagship Store	Electricity savings1	Just received, the courier is too bad, not notified directly to put the Feng Nest courier cabinet	Jingdong prefecture level city in Shandong
Jinfeng Hongyuan Flagship Store	Electricity savings1	Baby received, the second time to buy, plugged in and used do not know if there is no effect. Baby received, the second time to buy, plugged in and used do not know if there is no effect. Baby received, the second time to buy, plugged in and used do not know if there is no effect.	Jingdong prefecture level city in Shandong
Jinfeng Hongyuan Flagship Store	Electricity savings1	It's okay if it saves power. It's not a bad product.	Jingdong prefecture level city in Shandong
Jinfeng Hongyuan Flagship Store	Electricity savings1	Received, very good, meticulous packaging, open a look is genuine, meticulous workmanship, the manual is very detailed, has been used, there is no effect on the only look at the next month's electricity bill, when I can I buy a few more to send people!	Jingdong prefecture level city in Shandong
Jinfeng Hongyuan Flagship Store	Electricity savings1	The quality is very good very delicate express delivery is too fast	Jingdong prefecture level city in Shandong
Jinfeng Hongyuan Flagship Store	Electricity savings1	I won't know until a month after I start using it today.	Jingdong prefecture level city in Shandong
Jinfeng Hongyuan Flagship Store	Electricity savings1	It doesn't work at all, the customer service attitude is very bad, use it or not!	Jingdong prefecture level city in Shandong
Jinfeng Hongyuan Flagship Store	Electricity savings1	Temporarily try, do not know the effect, Jingdong logistics delivery very quickly	Jingdong prefecture level city in Shandong
Jinfeng Hongyuan Flagship Store	Electricity savings1	It doesn't help. It's comforting.	Jingdong prefecture level city in Shandong
Jinfeng Hongyuan Flagship Store	Electricity savings1	It's great, use it before you say anything.	Jingdong prefecture level city in Shandong
Jinfeng Hongyuan Flagship Store	Electricity savings1	Received, already used, looking forward to a good a effect!	Jingdong prefecture level city in Shandong
Jinfeng Hongyuan Flagship Store	Electricity savings1	I don't know if it works yet, I won't know until I use it for a few months.	Jingdong prefecture level city in Shandong
Jinfeng Hongyuan Flagship Store	Electricity savings1	It takes a long time to get there. There's no change now.	Jingdong prefecture level city in Shandong
Jinfeng Hongyuan Flagship Store	Electricity savings1	Jingdong's delivery never disappoints, or so fast, and the stuff is great, next time you need to come back.	Jingdong prefecture level city in Shandong
Jinfeng Hongyuan Flagship Store	Electricity savings1	At first it was ok today, after using it for a while it didn't work anymore	Jingdong prefecture level city in Shandong
Jinfeng Hongyuan Flagship Store	Electricity savings1	Just received the goods, do not know the effect of using a few days before there may be effect	Jingdong prefecture level city in Shandong
Jinfeng Hongyuan Flagship Store	Electricity savings1	The logistics was fast, I received it the next day, I've purchased it for the second time and have been using it all along	Jingdong prefecture level city in Shandong
Jinfeng Hongyuan Flagship Store	Electricity savings1	Inexpensive, fast delivery, very pleased!	Jingdong prefecture level city in Shandong
Jinfeng Hongyuan Flagship Store	Electricity savings1	Received the goods immediately plugged directly into the use of power saving yet to be good news!	Jingdong prefecture level city in Shandong
Jinfeng Hongyuan Flagship Store	Electricity savings1	The service attitude is very good, and the Jingdong self-owned products can be trusted.	Jingdong prefecture level city in Shandong
Jinfeng Hongyuan Flagship Store	Electricity savings1	The product is good, I do not know if it can save play a power saving effect	Jingdong prefecture level city in Shandong
Jinfeng Hongyuan Flagship Store	Electricity savings1	We won't know how effective it is until we use it for a while, so I'll give you a good review now.	Jingdong prefecture level city in Shandong
Jinfeng Hongyuan Flagship Store	Electricity savings1	The quality is pretty good, just started using it, hopefully it will deliver results.	Jingdong prefecture level city in Shandong
Jinfeng Hongyuan Flagship Store	Electricity savings1	I've plugged it in first, and will follow up after I've used it to see how it works.	Jingdong prefecture level city in Shandong
Jinfeng Hongyuan Flagship Store	Electricity savings1	Things have arrived, the quality is good, the effect is also equivalent to can ah	Jingdong prefecture level city in Shandong

Jinfeng Hongyuan Flagship Store	Electricity savings1	It's not worth it to brag about power savings.	Jingdong prefecture level city in Shandong
Jinfeng Hongyuan Flagship Store	Electricity savings1	I don't know if it's an IQ tax, but I'll use it for now.	Jingdong prefecture level city in Shandong
Jinfeng Hongyuan Flagship Store	Electricity savings1	I don't feel the power saving yet, I can't know until I use it for 2 months.	Jingdong prefecture level city in Shandong
Jinfeng Hongyuan Flagship Store	Electricity savings1	There's nothing wrong with this at the moment.	Jingdong prefecture level city in Shandong
Jinfeng Hongyuan Flagship Store	Electricity savings1	It's authentic and worth buying	Jingdong prefecture level city in Shandong
Jinfeng Hongyuan Flagship Store	Electricity savings1	It's good. We'll see how it goes.	Jingdong prefecture level city in Shandong
Jinfeng Hongyuan Flagship Store	Electricity savings1	I don't know if it's working or not in the middle of using it, we'll see when the month is over	Jingdong prefecture level city in Shandong
Jinfeng Hongyuan Flagship Store	Electricity savings1	I'm not going to buy anything from Jingdong anymore, so I'll give it a good review	Jingdong prefecture level city in Shandong
Jinfeng Hongyuan Flagship Store	Electricity savings1	I'm not going to buy anything from Jingdong anymore, so I'll give it a good review	Jingdong prefecture level city in Shandong
Jinfeng Hongyuan Flagship Store	Electricity savings1	Just received the item and will see how it works in a month.	Jingdong prefecture level city in Shandong
Jinfeng Hongyuan Flagship Store	Electricity savings1	I don't know if it saves electricity, but it's plugged into an outlet every day anyway	Jingdong prefecture level city in Shandong
Jinfeng Hongyuan Flagship Store	Electricity savings1	I don't know if it works yet.	Jingdong prefecture level city in Shandong
Jinfeng Hongyuan Flagship Store	Electricity savings1	Plugged it in when I got back, waiting for a month to see the results	Jingdong prefecture level city in Shandong
Jinfeng Hongyuan Flagship Store	Electricity savings1	Effects remain to be seen, but it looks good quality and runs fine	Jingdong prefecture level city in Shandong
Jinfeng Hongyuan Flagship Store	Electricity savings1	The product received logistics very quickly, has not yet been used to test! After use in the evaluation!	Jingdong prefecture level city in Shandong
Jinfeng Hongyuan Flagship Store	Electricity savings1	The power saver is in use, and I don't know how much electricity has been saved after using it for more than a month, and the display voltmeter is very good.	Jingdong prefecture level city in Shandong
Jinfeng Hongyuan Flagship Store	Electricity savings1	It's so good, isn't it? You're a real Shanghainese, aren't you?	Jingdong prefecture level city in Shandong
Jinfeng Hongyuan Flagship Store	Electricity savings1	Great quality great great great 👍👍👍👍	Jingdong prefecture level city in Shandong
Jinfeng Hongyuan Flagship Store	Electricity savings1	I'm using it, I don't know if it's working yet.	Jingdong prefecture level city in Shandong
Jinfeng Hongyuan Flagship Store	Electricity savings1	Just used it for more than 10 days and haven't seen any results yet	Jingdong prefecture level city in Shandong
Jinfeng Hongyuan Flagship Store	Electricity savings1	The product arrived home feeling like it should work	Jingdong prefecture level city in Shandong
Jinfeng Hongyuan Flagship Store	Electricity savings1	Just received the goods, use it and comment back	Jingdong prefecture level city in Shandong
Jinfeng Hongyuan Flagship Store	Electricity savings1	Try it for a month and then evaluate	Jingdong prefecture level city in Shandong
Jinfeng Hongyuan Flagship Store	Electricity savings1	I don't know if it saves power yet, but I'll comment again after using it for a while.	Jingdong prefecture level city in Shandong
Jinfeng Hongyuan Flagship Store	Electricity savings1	The product is better than I thought, the quality and appearance are very good, the voltage is stable, and I'm looking forward to a good power saving effect.	Jingdong prefecture level city in Shandong
Jinfeng Hongyuan Flagship Store	Electricity savings1	Received and scanned the QR code to check that it is genuine, have plugged it in, can it save power, looking forward to the advertised effect. Customary praise, 5 points!	Jingdong prefecture level city in Shandong
Jinfeng Hongyuan Flagship Store	Electricity savings1	The goods have been received and have not yet been used. Do not know how the effect.	Jingdong prefecture level city in Shandong
Jinfeng Hongyuan Flagship Store	Electricity savings1	Hopefully, you get what you pay for, and you get what you pay for!	Jingdong prefecture level city in Shandong
Jinfeng Hongyuan Flagship Store	Electricity savings1	Just put it on and it feels like it's turning as fast as a meter! Looking forward to the results!	Jingdong prefecture level city in Shandong
Jinfeng Hongyuan Flagship Store	Electricity savings1	Overall look on the product quality is good, I do not know is not the effect of power saving, wait to see and then follow up!	Jingdong prefecture level city in Shandong
Jinfeng Hongyuan Flagship Store	Electricity savings1	The logistics courier is very fast, the courier service attitude is very good	Jingdong prefecture level city in Shandong
Jinfeng Hongyuan Flagship Store	Electricity savings1	The product has been received, it works well worth buying	Jingdong prefecture level city in Shandong
Jinfeng Hongyuan Flagship Store	Electricity savings1	How did it not work? Is it purely an IQ tax?	Jingdong prefecture level city in Shandong
Jinfeng Hongyuan Flagship Store	Electricity savings1	It looks good, I don't know how well it works.	Jingdong prefecture level city in Shandong
Jinfeng Hongyuan Flagship Store	Electricity savings1	It does seem to be a bit of a power saver, I just don't know if the more expensive one is more power efficient?	Jingdong prefecture level city in Shandong
Jinfeng Hongyuan Flagship Store	Electricity savings1	Is genuine very good, the price is much cheaper, cost-effective, really special paddle just to do activities, the price is much cheaper, cost-effective, really special paddle counting, packaging is also very high-end atmospheric class, it is worth buying!	Jingdong prefecture level city in Shandong
Jinfeng Hongyuan Flagship Store	Electricity savings1	I received it yesterday and used it right away, I hope it works, I'll wait for next month to come back to the conclusion, I'm looking forward to it. Five hearts praise!	Jingdong prefecture level city in Shandong
Jinfeng Hongyuan Flagship Store	Electricity savings1	Looks good, packed properly, received it today, am using it, will see results in a month!	Jingdong prefecture level city in Shandong
Jinfeng Hongyuan Flagship Store	Electricity savings1	Regular product Jingdong self-support trustworthy. There is an effect Come back to the review. 😍😍😍😍	Jingdong prefecture level city in Shandong
Jinfeng Hongyuan Flagship Store	Electricity savings1	I haven't used it yet, hopefully it will work as a power saver, and will comment again if it works well.	Jingdong prefecture level city in Shandong
Jinfeng Hongyuan Flagship Store	Electricity savings1	Shipping was fast, logistics was fast, service was good, and I look forward to good results from the item!	Jingdong prefecture level city in Shandong
Jinfeng Hongyuan Flagship Store	Electricity savings1	This product is this compensator, it has to be at the main switch to be useful, haven't compared the final result, the	Jingdong prefecture level city in Shandong
Jinfeng Hongyuan Flagship Store	Electricity savings1	Packaging is perfect . I've already started using it, so I'm looking forward to the results.	Jingdong prefecture level city in Shandong
Jinfeng Hongyuan Flagship Store	Electricity savings1	I just got it back. I'll comment after I use it.	Jingdong prefecture level city in Shandong
Jinfeng Hongyuan Flagship Store	Electricity savings1	Big brand, with a very assured, very good quality, catch the event to buy very cost-effective to take advantage of the offer, hurry to get, it is genuine, the packaging is also very good, no breakage, the quality is not to speak of, great 👍 logistics super super fast! Quality is also very good, indeed value	Jingdong prefecture level city in Shandong
Jinfeng Hongyuan Flagship Store	Electricity savings1	The quality is pretty good. The price is also quite affordable.	Jingdong prefecture level city in Shandong
Jinfeng Hongyuan Flagship Store	Electricity savings1	I did not expect the courier so fast to arrive yesterday just ordered, the head is a little smaller than I thought, well, the picture looks bigger. The appearance still looks good, as for the practicality and effectiveness of the use of the only know.	Jingdong prefecture level city in Shandong
Jinfeng Hongyuan Flagship Store	Electricity savings1	I don't know. Looking forward to it.	Jingdong prefecture level city in Shandong
Jinfeng Hongyuan Flagship Store	Electricity savings1	Baby received, very delicate, very nice, like. Just don't know if it can really save electricity. Baby received, very delicate, very good-looking, like. Just don't know if it can really save electricity.	Jingdong prefecture level city in Shandong
Jinfeng Hongyuan Flagship Store	Electricity savings1	I'll use it for a month and then comment on the quality of the item	Jingdong prefecture level city in Shandong

Jinfeng Hongyuan Flagship Store	Electricity savings1	It feels ok, use it for a while and see how it works before adding more comments.	Jingdong prefecture level city in Shandong
Jinfeng Hongyuan Flagship Store	Electricity savings1	In use, see results in a month.	Jingdong prefecture level city in Shandong
Jinfeng Hongyuan Flagship Store	Electricity savings1	Jingdong's logistics and quality are very good, the service attitude is very good, the logistics is very fast	Jingdong prefecture level city in Shandong
Jinfeng Hongyuan Flagship Store	Electricity savings1	Intelligent power saver received, well-packed logistics is also quite fast, easy to use, looking forward to good results!	Jingdong prefecture level city in Shandong
Jinfeng Hongyuan Flagship Store	Electricity savings1	The quality is unknown at this point, I won't know until I use it.	Jingdong prefecture level city in Shandong
Jinfeng Hongyuan Flagship Store	Electricity savings1	It's a good product. It really saves power and doesn't take up much space.	Jingdong prefecture level city in Shandong
Jinfeng Hongyuan Flagship Store	Electricity savings1	Goods have been received, packaging is very good, good to see the effect of future use in the follow-up.	Jingdong prefecture level city in Shandong
Jinfeng Hongyuan Flagship Store	Electricity savings1	The product is in perfect shape and the light weight is worth it!	Jingdong prefecture level city in Shandong
Jinfeng Hongyuan Flagship Store	Electricity savings1	Just got home and plugged it in. It's good.	Jingdong prefecture level city in Shandong
Jinfeng Hongyuan Flagship Store	Electricity savings1	The logistics are quick, just to see the results.	Jingdong prefecture level city in Shandong
Jinfeng Hongyuan Flagship Store	Electricity savings1	Short time can not feel here to leave a message, to be followed up later!	Jingdong prefecture level city in Shandong
Jinfeng Hongyuan Flagship Store	Electricity savings1	This user thinks the product is good	Jingdong prefecture level city in Shandong
Jinfeng Hongyuan Flagship Store	Electricity savings1	Received the baby, to be used to see the effect of it!	Jingdong prefecture level city in Shandong
Jinfeng Hongyuan Flagship Store	Electricity savings1	You have to wait a month or two to see how it works.	Jingdong prefecture level city in Shandong
Jinfeng Hongyuan Flagship Store	Electricity savings1	Not bad, good, like it, don't know if it's good, try it first.	Jingdong prefecture level city in Shandong
Jinfeng Hongyuan Flagship Store	Electricity savings1	The goods are good value for money and the logistics is very fast and good service!	Jingdong prefecture level city in Shandong
Jinfeng Hongyuan Flagship Store	Electricity savings1	I don't know how well it works, we'll see in a month	Jingdong prefecture level city in Shandong
Jinfeng Hongyuan Flagship Store	Electricity savings1	The product is fine, but r saving power or not I won't know until I use it for two months. The display and lights take electricity too?	Jingdong prefecture level city in Shandong
Jinfeng Hongyuan Flagship Store	Electricity savings1	I've used it. I don't know how it works yet.	Jingdong prefecture level city in Shandong
Jinfeng Hongyuan Flagship Store	Electricity savings1	I can't say until I compare them.	Jingdong prefecture level city in Shandong
Jinfeng Hongyuan Flagship Store	Electricity savings1	It's ok in the process of using 。。。。	Jingdong prefecture level city in Shandong
Jinfeng Hongyuan Flagship Store	Electricity savings1	Jinfeng Hongyuan Intelligent Power Saver Household Electricity Meter Electricity Saving Appliance High Power Enhanced Power Saving King Energy Saving Treasure Butler Control Non Stealing Electricity Reversing Not Going Interfering Slow Turner Artifact King Gold Yellow. Haven't tested it accurately yet, really need to wait a few months to test it before I have a say.	Jingdong prefecture level city in Shandong
Jinfeng Hongyuan Flagship Store	Electricity savings1	Don't know how effective it is yet? I hope it works well and I'll come back next time	Jingdong prefecture level city in Shandong
Jinfeng Hongyuan Flagship Store	Electricity savings1	Saving device has been received, material workmanship are very good ah! Have not yet begun to use. First observe the daily electricity consumption and cost of the home meter, and then plug in the power saver to use, so as to compare the use of the effect is good!	Jingdong prefecture level city in Shandong
Jinfeng Hongyuan Flagship Store	Electricity savings1	Merchants shipping extremely fast, Jingdong logistics safe and fast, I believe that the product can play a safe use of electricity, the role of scientific power saving, first praise, the effect of how to be used for a period of time and then the pursuit of bubble!	Jingdong prefecture level city in Shandong
Jinfeng Hongyuan Flagship Store	Electricity savings1	I just bought it. I don't know yet.	Jingdong prefecture level city in Shandong
Jinfeng Hongyuan Flagship Store	Electricity savings1	It is genuine, good quality, and it is very reassuring to use. The logistics is very fast, and the business service attitude is on point.	Jingdong prefecture level city in Shandong
Jinfeng Hongyuan Flagship Store	Electricity savings1	The quality is fine, I don't know how effective it is.	Jingdong prefecture level city in Shandong
Jinfeng Hongyuan Flagship Store	Electricity savings1	The stuff is back and I expect it will save the electricity	Jingdong prefecture level city in Shandong
Jinfeng Hongyuan Flagship Store	Electricity savings1	I'll give it 4 stars first and see what happens in a couple of months and then follow up	Jingdong prefecture level city in Shandong
Jinfeng Hongyuan Flagship Store	Electricity savings1	It's not bad. It's in the mind.	Jingdong prefecture level city in Shandong
Jinfeng Hongyuan Flagship Store	Electricity savings1	Received the goods have not yet started to use do not know whether it is good	Jingdong prefecture level city in Shandong
Jinfeng Hongyuan Flagship Store	Electricity savings1	This user thinks the product is good	Jingdong prefecture level city in Shandong
Jinfeng Hongyuan Flagship Store	Electricity savings1	Hello! The saver was received, thank you! Not yet used, but from the packaging looks very good quality, exquisite workmanship. Logistics is quite fast, if it is really very power saving, I will come back to	Jingdong prefecture level city in Shandong
Jinfeng Hongyuan Flagship Store	Electricity savings1	I've been using it for a few days now, I'll comment again after a month or two of use	Jingdong prefecture level city in Shandong
Jinfeng Hongyuan Flagship Store	Electricity savings1	I don't know. Bought it for my father at a good price.	Jingdong prefecture level city in Shandong
Jinfeng Hongyuan Flagship Store	Electricity savings1	Plugged it in the same day I bought it and the voltage showed 223, 225 looking forward to the results	Jingdong prefecture level city in Shandong
Jinfeng Hongyuan Flagship Store	Electricity savings1	Still in use, the biggest role is to stabilize the current, reduce losses, product quality on the look is very good, with this feeling quite good, see colleagues are using, power saving effect should be good	Jingdong prefecture level city in Shandong
Jinfeng Hongyuan Flagship Store	Electricity savings1	How to receive goods but no invoice and warranty more than it ...... quality is the premise of reputation!	Jingdong prefecture level city in Shandong
Jinfeng Hongyuan Flagship Store	Electricity savings1	Received but not used Continued evaluation	Jingdong prefecture level city in Shandong
Jinfeng Hongyuan Flagship Store	Electricity savings1	I just received the product, I don't know if it works well yet	Jingdong prefecture level city in Shandong
Jinfeng Hongyuan Flagship Store	Electricity savings1	Received it and used it, not sure it really saves electricity use it and then evaluate it, first give a 5 star review!!!!	Jingdong prefecture level city in Shandong
Jinfeng Hongyuan Flagship Store	Electricity savings1	It's good to use, it's very good to use, and it smells OK too	Jingdong prefecture level city in Shandong
Jinfeng Hongyuan Flagship Store	Electricity savings1	Packaging is very good, feel the product is not bad, how much power saving with the only know	Jingdong prefecture level city in Shandong
Jinfeng Hongyuan Flagship Store	Electricity savings1	Additional comments to be made after use	Jingdong prefecture level city in Shandong
Jinfeng Hongyuan Flagship Store	Electricity savings1	The product has been installed, try a few months and then comment!	Jingdong prefecture level city in Shandong
Jinfeng Hongyuan Flagship Store	Electricity savings1	I've bought it, let's use it for now, I'll comment after a month	Jingdong prefecture level city in Shandong
Jinfeng Hongyuan Flagship Store	Electricity savings1	Try it out and then follow up	Jingdong prefecture level city in Shandong
Jinfeng Hongyuan Flagship Store	Electricity savings1	It's good in theory, it doesn't feel good in practice.	Jingdong prefecture level city in Shandong

Jinfeng Hongyuan Flagship Store	Electricity savings1	I haven't tried it yet. We'll see.	Jingdong prefecture level city in Shandong
Jinfeng Hongyuan Flagship Store	Electricity savings1	Great item, second purchase!	Jingdong prefecture level city in Shandong
Jinfeng Hongyuan Flagship Store	Electricity savings1	Goods received now use it, if it does save power, plus reevaluate.	Jingdong prefecture level city in Shandong
Jinfeng Hongyuan Flagship Store	Electricity savings1	The stuff is light and small, the stuff is good, the courier service is good	Jingdong prefecture level city in Shandong
Jinfeng Hongyuan Flagship Store	Electricity savings1	I haven't used it yet. I'm looking forward to it.	Jingdong prefecture level city in Shandong
Jinfeng Hongyuan Flagship Store	Electricity savings1	Express received, in use do not know if there is no power saving	Jingdong prefecture level city in Shandong
Jinfeng Hongyuan Flagship Store	Electricity savings1	The quality of the product is quite good! The power saving mode is still getting used to it, can't say yet, how much power it saves.	Jingdong prefecture level city in Shandong
Jinfeng Hongyuan Flagship Store	Electricity savings1	The goods have been received, just started to use, now I do not know how the quality, after a period of time to say.	Jingdong prefecture level city in Shandong
Jinfeng Hongyuan Flagship Store	Electricity savings1	I received the stuff, used it for a few days to evaluate, the stuff is very good and good, worth buying.	Jingdong prefecture level city in Shandong
Jinfeng Hongyuan Flagship Store	Electricity savings1	I've been using it for a while, but I don't know if it's good or if it saves electricity! What the heck, let's give it a good review first. And for the record, I'm not helping him**.	Jingdong prefecture level city in Shandong
Jinfeng Hongyuan Flagship Store	Electricity savings1	Haven't used it yet, looks good, try it for 60 days, might be good, great review!	Jingdong prefecture level city in Shandong
Jinfeng Hongyuan Flagship Store	Electricity savings1	Baby received, logistics is very fast. Ordered last night, received this morning. Received the goods immediately plugged in the power supply, save power to the first of next month to know.	Jingdong prefecture level city in Shandong
Jinfeng Hongyuan Flagship Store	Electricity savings1	First, I'll give you a good review and use it.	Jingdong prefecture level city in Shandong
Jinfeng Hongyuan Flagship Store	Electricity savings1	Just arrived to follow up with a genuine review	Jingdong prefecture level city in Shandong
Jinfeng Hongyuan Flagship Store	Electricity savings1	Not bad pretty fast, good quality.	Jingdong prefecture level city in Shandong
Jinfeng Hongyuan Flagship Store	Electricity savings1	Recommended, use it and hopefully it will save some electricity	Jingdong prefecture level city in Shandong
Jinfeng Hongyuan Flagship Store	Electricity savings1	The stuff looks ok, I don't know the effect if it.	Jingdong prefecture level city in Shandong
Jinfeng Hongyuan Flagship Store	Electricity savings1	The stuff looks OK, the stuff looks OK.	Jingdong prefecture level city in Shandong
Jinfeng Hongyuan Flagship Store	Electricity savings1	The item has been received, after using it in a follow-up review. Satisfied with the purchase.	Jingdong prefecture level city in Shandong
Jinfeng Hongyuan Flagship Store	Electricity savings1	Has been normal plug-in use, at present do not know how to see, use down to see and then evaluation	Jingdong prefecture level city in Shandong
Jinfeng Hongyuan Flagship Store	Electricity savings1	Commodities make noise.	Jingdong prefecture level city in Shandong
Jinfeng Hongyuan Flagship Store	Electricity savings1	After using it for a few days and observing it, the saving is about ten percent	Jingdong prefecture level city in Shandong
Jinfeng Hongyuan Flagship Store	Electricity savings1	The goods received, the quality looks quite OK. First use it, the effect is not good to say.	Jingdong prefecture level city in Shandong
Jinfeng Hongyuan Flagship Store	Electricity savings1	In use, follow up next month	Jingdong prefecture level city in Shandong
Jinfeng Hongyuan Flagship Store	Electricity savings1	I just received the goods and have not yet used it to comment after using it	Jingdong prefecture level city in Shandong
Jinfeng Hongyuan Flagship Store	Electricity savings1	So far I don't know if it really saves electricity yet! Spinning waiting a couple months to find out! Good reviews first!	Jingdong prefecture level city in Shandong
Jinfeng Hongyuan Flagship Store	Electricity savings1	Small and exquisite, looking forward to the effect, to be evaluated 6666666666666	Jingdong prefecture level city in Shandong
Jinfeng Hongyuan Flagship Store	Electricity savings1	I just used it, I don't know if it's effective, I'll comment again after a month	Jingdong prefecture level city in Shandong
Jinfeng Hongyuan Flagship Store	Electricity savings1	I just used it, I don't know if it's good or not, I'll comment later.	Jingdong prefecture level city in Shandong
Jinfeng Hongyuan Flagship Store	Electricity savings1	Baby received, Baobei quite good, use and then come back to comment!	Jingdong prefecture level city in Shandong
Jinfeng Hongyuan Flagship Store	Electricity savings1	Good quality power saving is fake	Jingdong prefecture level city in Shandong
Jinfeng Hongyuan Flagship Store	Electricity savings1	The packaging of the goods is atmospheric, the quality is exquisite, and I look forward to saving electricity.	Jingdong prefecture level city in Shandong
Jinfeng Hongyuan Flagship Store	Electricity savings1	The packaging is great and the quality is good!	Jingdong prefecture level city in Shandong
Jinfeng Hongyuan Flagship Store	Electricity savings1	I don't see any effect.	Jingdong prefecture level city in Shandong
Jinfeng Hongyuan Flagship Store	Electricity savings1	Already used, but did not see obvious results	Jingdong prefecture level city in Shandong
Jinfeng Hongyuan Flagship Store	Electricity savings1	It's good. I'm just using it. I don't know what it's like.	Jingdong prefecture level city in Shandong
Jinfeng Hongyuan Flagship Store	Electricity savings1	I just used it. I'll comment later.	Jingdong prefecture level city in Shandong
Jinfeng Hongyuan Flagship Store	Electricity savings1	I installed it when it arrived, try it for a month and then come back for a follow-up review	Jingdong prefecture level city in Shandong
Jinfeng Hongyuan Flagship Store	Electricity savings1	Goods arrived a few days fastidious does not call to pick up the goods, the quality is average	Jingdong prefecture level city in Shandong
Jinfeng Hongyuan Flagship Store	Electricity savings1	This user thinks the product is good	Jingdong prefecture level city in Shandong
Jinfeng Hongyuan Flagship Store	Electricity savings1	Roger that.	Jingdong prefecture level city in Shandong
Jinfeng Hongyuan Flagship Store	Electricity savings1	Second purchase was ok.	Jingdong prefecture level city in Shandong
Jinfeng Hongyuan Flagship Store	Electricity savings1	The merchant is very reputable and the product is good value for money. Logistics is fast!	Jingdong prefecture level city in Shandong
Jinfeng Hongyuan Flagship Store	Electricity savings1	The quality of the baby is very good, I have always trusted Kyodo	Jingdong prefecture level city in Shandong
Jinfeng Hongyuan Flagship Store	Electricity savings1	We'll see how it goes when we get it.	Jingdong prefecture level city in Shandong
Jinfeng Hongyuan Flagship Store	Electricity savings1	I just used it. I don't know if it works.	Jingdong prefecture level city in Shandong
Jinfeng Hongyuan Flagship Store	Electricity savings1	Good quality, used for a month to save about 20%, power saving effect is good	Jingdong prefecture level city in Shandong
Jinfeng Hongyuan Flagship Store	Electricity savings1	I'll give you a good review before I use it.	Jingdong prefecture level city in Shandong
Jinfeng Hongyuan Flagship Store	Electricity savings1	Received, satisfied with the follow-up evaluation	Jingdong prefecture level city in Shandong
Jinfeng Hongyuan Flagship Store	Electricity savings1	Immediate results, saving electricity and money	Jingdong prefecture level city in Shandong
Jinfeng Hongyuan Flagship Store	Electricity savings1	Measured to save a little	Jingdong prefecture level city in Shandong

Jinfeng Hongyuan Flagship Store	Electricity savings1	This user thinks the product is good	Jingdong prefecture level city in Shandong
Jinfeng Hongyuan Flagship Store	Electricity savings1	This user thinks the product is good	Jingdong prefecture level city in Shandong
Jinfeng Hongyuan Flagship Store	Electricity savings1	Product quality and logistics are very good, worth buying	Jingdong prefecture level city in Shandong
Jinfeng Hongyuan Flagship Store	Electricity savings1	Appearance looks ok! Express delivery was fast! Service is still relatively enthusiastic.	Jingdong prefecture level city in Shandong
Jinfeng Hongyuan Flagship Store	Electricity savings1	It doesn't feel like it's working.	Jingdong prefecture level city in Shandong
Jinfeng Hongyuan Flagship Store	Electricity savings1	Well made and looks good. Used it with other appliances and felt as if it had some effect.	Jingdong prefecture level city in Shandong
Jinfeng Hongyuan Flagship Store	Electricity savings1	Haven't used it yet, will follow up later	Jingdong prefecture level city in Shandong
Jinfeng Hongyuan Flagship Store	Electricity savings1	Things received, used a few days the plug is a little hot unplugged did not work	Jingdong prefecture level city in Shandong
Jinfeng Hongyuan Flagship Store	Electricity savings1	Look at the meter again in a month.	Jingdong prefecture level city in Shandong
Jinfeng Hongyuan Flagship Store	Electricity savings1	This one didn't work at all.	Jingdong prefecture level city in Shandong
Jinfeng Hongyuan Flagship Store	Electricity savings1	It's small. I've seen friends with much bigger ones.	Jingdong prefecture level city in Shandong
Jinfeng Hongyuan Flagship Store	Electricity savings1	The item was received and the quality is good, it will be nice to see how the power saving rate is down the road, the item is great.	Jingdong prefecture level city in Shandong
Jinfeng Hongyuan Flagship Store	Electricity savings1	Cheap and affordable quality is quite good, logistics is also very fast, praise! , baby good 😊, cost-effective very high, big brand trustworthy 👍	Jingdong prefecture level city in Shandong
Jinfeng Hongyuan Flagship Store	Electricity savings1	Used it for a while it really saves electricity ⚡ thanks 🈶 such a good product, compact and practical, every family should buy one ✨	Jingdong prefecture level city in Shandong
Jinfeng Hongyuan Flagship Store	Electricity savings1	Very satisfied with the purchase Next time I will come back	Jingdong prefecture level city in Shandong
Jinfeng Hongyuan Flagship Store	Electricity savings1	Logistics is fast. Good quality, used one before and it does save power	Jingdong prefecture level city in Shandong
Jinfeng Hongyuan Flagship Store	Electricity savings1	I don't know if it works.	Jingdong prefecture level city in Shandong
Jinfeng Hongyuan Flagship Store	Electricity savings1	Good goods, very solid, good.	Jingdong prefecture level city in Shandong
Jinfeng Hongyuan Flagship Store	Electricity savings1	This user thinks the product is good	Jingdong prefecture level city in Shandong
Jinfeng Hongyuan Flagship Store	Electricity savings1	I've been using it for half a month, my electricity bill is much lower, and the quality is very good, so don't worry about buying it!	Jingdong prefecture level city in Shandong
Jinfeng Hongyuan Flagship Store	Electricity savings1	I received the goods today and used it right away, looking forward to the effect	Jingdong prefecture level city in Shandong
Jinfeng Hongyuan Flagship Store	Electricity savings1	The merchandise is great. I am going to recommend it to my friends! Will buy again next time.	Jingdong prefecture level city in Shandong
Jinfeng Hongyuan Flagship Store	Electricity savings1	Let's give it a good review first, it's working fine so far, and I'll follow up with a review,...	Jingdong prefecture level city in Shandong
Jinfeng Hongyuan Flagship Store	Electricity savings1	Product quality is very good, looking forward to using the results	Jingdong prefecture level city in Shandong
Jinfeng Hongyuan Flagship Store	Electricity savings1	I just can't detect it. - Save it.	Jingdong prefecture level city in Shandong
Jinfeng Hongyuan Flagship Store	Electricity savings1	It arrived and is being used, so we'll see how it works!	Jingdong prefecture level city in Shandong
Jinfeng Hongyuan Flagship Store	Electricity savings1	Received yesterday thanks! This energy saver is available . Safe! But in the end how much energy can be saved must be used for a period of time to know, if the real energy saving next time to buy again.	Jingdong prefecture level city in Shandong
Jinfeng Hongyuan Flagship Store	Electricity savings1	Just used it, don't know if it saves power	Jingdong prefecture level city in Shandong
Jinfeng Hongyuan Flagship Store	Electricity savings1	I'll rate it first, is it a tease wait two months and see? It's been four days and it's not noticeable!	Jingdong prefecture level city in Shandong
Jinfeng Hongyuan Flagship Store	Electricity savings1	Workmanship is quite good, just started to use, the effect of the follow-up evaluation.	Jingdong prefecture level city in Shandong
Jinfeng Hongyuan Flagship Store	Electricity savings1	Received the goods used for a few days before evaluation, the effect is quite good	Jingdong prefecture level city in Shandong
Jinfeng Hongyuan Flagship Store	Electricity savings1	Try it for a month and then evaluate	Jingdong prefecture level city in Shandong
Jinfeng Hongyuan Flagship Store	Electricity savings1	Just received the goods, the packaging is very good, the appearance of the economizer feels good, I do not know how the effect of power saving, with a month or two and then follow up!	Jingdong prefecture level city in Shandong
Jinfeng Hongyuan Flagship Store	Electricity savings1	It's quite small and delicate, I don't know if it's useful yet.	Jingdong prefecture level city in Shandong
Jinfeng Hongyuan Flagship Store	Electricity savings1	Just received it, use it for a few days before commenting	Jingdong prefecture level city in Shandong
Jinfeng Hongyuan Flagship Store	Electricity savings1	Does this thing work! When it arrived, the button for the safety was loose. I'm a little afraid of fire!	Jingdong prefecture level city in Shandong
Jinfeng Hongyuan Flagship Store	Electricity savings1	I received it. I don't know how it works.	Jingdong prefecture level city in Shandong
Jinfeng Hongyuan Flagship Store	Electricity savings1	This user thinks the product is good	Jingdong prefecture level city in Shandong
Jinfeng Hongyuan Flagship Store	Electricity savings1	Customer service was great! Small i problem was taken care of in a timely manner! Effectiveness to be tested by time! No rush!!!	Jingdong prefecture level city in Shandong
Jinfeng Hongyuan Flagship Store	Electricity savings1	I put it on right after I received it and it looks compact. I believe the high technology should save electricity.	Jingdong prefecture level city in Shandong
Jinfeng Hongyuan Flagship Store	Electricity savings1	Logistics courier very quickly, the first day under the order the next day to receive, off pieces whether power saving is the most important thing, things are exquisite workmanship, a month later in the follow-up evaluation whether power saving	Jingdong prefecture level city in Shandong
Jinfeng Hongyuan Flagship Store	Electricity savings1	The delivery is pretty fast, I'm using it, have to have it for a while to see results, looking forward to it.	Jingdong prefecture level city in Shandong
Jinfeng Hongyuan Flagship Store	Electricity savings1	See pretty good just started to use a month later to give you a real evaluation, should be useful.	Jingdong prefecture level city in Shandong
Jinfeng Hongyuan Flagship Store	Electricity savings1	And then comment.	Jingdong prefecture level city in Shandong
Jinfeng Hongyuan Flagship Store	Electricity savings1	Received the goods, wait for the test to evaluate	Jingdong prefecture level city in Shandong
Jinfeng Hongyuan Flagship Store	Electricity savings1	This user thinks the product is good	Jingdong prefecture level city in Shandong
Jinfeng Hongyuan Flagship Store	Electricity savings1	I'm already using it, I just don't know how to judge the power savings. I won't know until I use it for a season.	Jingdong prefecture level city in Shandong
Jinfeng Hongyuan Flagship Store	Electricity savings1	Works. Says it works.	Jingdong prefecture level city in Shandong
Jinfeng Hongyuan Flagship Store	Electricity savings1	Very good. Satisfied.	Jingdong prefecture level city in Shandong
Jinfeng Hongyuan Flagship Store	Electricity savings1	This user thinks the product is good	Jingdong prefecture level city in Shandong
Jinfeng Hongyuan Flagship Store	Electricity savings1	It's working. Obviously, it's less expensive.	Jingdong prefecture level city in Shandong

Jinfeng Hongyuan Flagship Store	Electricity savings1	The second one is up, the first one still works! It's reactive power compensation! This is well understood if you want a professional!	Jingdong prefecture level city in Shandong
Jinfeng Hongyuan Flagship Store	Electricity savings1	It feels as if there are some nodes, but they need to be tested and observed over time, thanks.	Jingdong prefecture level city in Shandong
Jinfeng Hongyuan Flagship Store	Electricity savings1	I've received the goods, the energy saving effect will be evaluated after using it for a period of time, and all the services are very good, praise!	Jingdong prefecture level city in Shandong
Jinfeng Hongyuan Flagship Store	Electricity savings1	I don't know how well it works yet, I bought it and plugged it in, so we'll see if it saves some power or not!	Jingdong prefecture level city in Shandong
Jinfeng Hongyuan Flagship Store	Electricity savings1	Baby a bunch of knife quality is very good, previously bought a quality is not very good. It really works and has results.	Jingdong prefecture level city in Shandong
Jinfeng Hongyuan Flagship Store	Electricity savings1	Things have arrived, it looks good, just started to use, as to whether or not there is a business said the power-saving effect, but also to observe the effect, now can not evaluate	Jingdong prefecture level city in Shandong
Jinfeng Hongyuan Flagship Store	Electricity savings1	I haven't used it yet. I'll comment afterward.	Jingdong prefecture level city in Shandong
Jinfeng Hongyuan Flagship Store	Electricity savings1	I don't know if it's any good.	Jingdong prefecture level city in Shandong
Jinfeng Hongyuan Flagship Store	Electricity savings1	I don't know how well it works.	Jingdong prefecture level city in Shandong
Jinfeng Hongyuan Flagship Store	Electricity savings1	The baby has been received! Uh-huh! Good, quite exquisite, small and exquisite I thought it was big. Dare to tightly plugged in the electricity, just wait for the effect later!	Jingdong prefecture level city in Shandong
Jinfeng Hongyuan Flagship Store	Electricity savings1	The packaging is sturdy, I tried it right after I received it, so I'm looking forward to the results.	Jingdong prefecture level city in Shandong
Jinfeng Hongyuan Flagship Store	Electricity savings1	Received the item and installed it! Don't know if it works well! Will follow up with a review later! I hope it works!	Jingdong prefecture level city in Shandong
Jinfeng Hongyuan Flagship Store	Electricity savings1	Things used on the effect of waiting to use a little to say, but things are very good. Logistics is very fast, business service is very good.	Jingdong prefecture level city in Shandong
Jinfeng Hongyuan Flagship Store	Electricity savings1	This user thinks the product is good	Jingdong prefecture level city in Shandong
Jinfeng Hongyuan Flagship Store	Electricity savings1	I'm really using it. It feels good.	Jingdong prefecture level city in Shandong
Jinfeng Hongyuan Flagship Store	Electricity savings1	Good, Jingdong logistics is very fast, intelligent energy-saving appliances	Jingdong prefecture level city in Shandong
Jinfeng Hongyuan Flagship Store	Electricity savings1	It's not bad. It really saves energy.	Jingdong prefecture level city in Shandong
Jinfeng Hongyuan Flagship Store	Electricity savings1	The logistics is fast, praise	Jingdong prefecture level city in Shandong
Jinfeng Hongyuan Flagship Store	Electricity savings1	This user thinks the product is good	Jingdong prefecture level city in Shandong
Jinfeng Hongyuan Flagship Store	Electricity savings1	The first time I placed two, and today I placed another one, a friend introduced it, said it is particularly power-saving	Jingdong prefecture level city in Shandong
Jinfeng Hongyuan Flagship Store	Electricity savings1	I don't know how effective it is. Let's leave it at home for a while and see.	Jingdong prefecture level city in Shandong
Jinfeng Hongyuan Flagship Store	Electricity savings1	Dear customer service! I would like to ask you how to understand the digital display (increase/decrease jump) on the top of the saver? Thank you for your help.	Jingdong prefecture level city in Shandong
Jinfeng Hongyuan Flagship Store	Electricity savings1	This user thinks the product is good	Jingdong prefecture level city in Shandong
Jinfeng Hongyuan Flagship Store	Electricity savings1	The goods have been received, packaging exquisite, is using, later to see how the effect?	Jingdong prefecture level city in Shandong
Jinfeng Hongyuan Flagship Store	Electricity savings1	The saver received, the boss shipped very quickly, yesterday shot today arrived!	Jingdong prefecture level city in Shandong
Jinfeng Hongyuan Flagship Store	Electricity savings1	Beautiful appearance, well packaged. Recording data to see if I can lower my electric bill a bit.	Jingdong prefecture level city in Shandong
Jinfeng Hongyuan Flagship Store	Electricity savings1	Things received, the packaging is quite good, the effect of the use and then come back to evaluate.	Jingdong prefecture level city in Shandong
Jinfeng Hongyuan Flagship Store	Electricity savings1	Easy to use, just plug it in. But there is a current sound.	Jingdong prefecture level city in Shandong
Jinfeng Hongyuan Flagship Store	Electricity savings1	Fast logistics, easy to use and labor-saving	Jingdong prefecture level city in Shandong
Jinfeng Hongyuan Flagship Store	Electricity savings1	Received, thank you!	Jingdong prefecture level city in Shandong
Jinfeng Hongyuan Flagship Store	Electricity savings1	Second time I've bought it. It's good.	Jingdong prefecture level city in Shandong
Jinfeng Hongyuan Flagship Store	Electricity savings1	I've only been using it for a short time, so I'm looking forward to the results.	Jingdong prefecture level city in Shandong
Jinfeng Hongyuan Flagship Store	Electricity savings1	Good stuff, good stuff, good stuff good stuff.	Jingdong prefecture level city in Shandong
Jinfeng Hongyuan Flagship Store	Electricity savings1	Buy your stuff is not always good I have been using it for almost two months now, it does not save electricity at all the electricity bill is about ten dollars a day this is still called the king of power	Jingdong prefecture level city in Shandong
Jinfeng Hongyuan Flagship Store	Electricity savings1	Express arrived, the quality is very good, worth the purchase.	Jingdong prefecture level city in Shandong
Jinfeng Hongyuan Flagship Store	Electricity savings1	Energy saving has been received, now the voltage is more stable, energy-saving effect to see a few days and then chase flat, the service attitude is very good, the courier is relatively fast, I am satisfied.	Jingdong prefecture level city in Shandong
Jinfeng Hongyuan Flagship Store	Electricity savings1	Stuff installed, numbers are smaller than when installed, hopefully it works	Jingdong prefecture level city in Shandong
Jinfeng Hongyuan Flagship Store	Electricity savings1	Good appearance, fine workmanship, just bought, use up the feeling of power saving effect is good, worth buying!	Jingdong prefecture level city in Shandong
Jinfeng Hongyuan Flagship Store	Electricity savings1	Economical and practical 👍 Always use it	Jingdong prefecture level city in Shandong
Jinfeng Hongyuan Flagship Store	Electricity savings1	Just used it, don't know the effect yet, will come back to follow up after the effect. Hope it works well(▽)	Jingdong prefecture level city in Shandong
Jinfeng Hongyuan Flagship Store	Electricity savings1	The goods have been received, use it to see in the evaluation.	Jingdong prefecture level city in Shandong
Jinfeng Hongyuan Flagship Store	Electricity savings1	Doesn't it save electricity? The boil kettle still goes faster when you press the meter.	Jingdong prefecture level city in Shandong
Jinfeng Hongyuan Flagship Store	Electricity savings1	Already used. Will patronize again in the future	Jingdong prefecture level city in Shandong
Jinfeng Hongyuan Flagship Store	Electricity savings1	Works great, just plug it in, results to be checked, satisfied.	Jingdong prefecture level city in Shandong
Jinfeng Hongyuan Flagship Store	Electricity savings1	Second purchase. Works well 😍😍😍😍	Jingdong prefecture level city in Shandong
Jinfeng Hongyuan Flagship Store	Electricity savings1	Prompt shipment, very satisfied	Jingdong prefecture level city in Shandong
Jinfeng Hongyuan Flagship Store	Electricity savings1	It's been less than a month, let's see how it works, hopefully it's power efficient	Jingdong prefecture level city in Shandong
Jinfeng Hongyuan Flagship Store	Electricity savings1	Use a month in say.	Jingdong prefecture level city in Shandong
Jinfeng Hongyuan Flagship Store	Electricity savings1	I've saved a lot of electricity since I've been using it.	Jingdong prefecture level city in Shandong
Jinfeng Hongyuan Flagship Store	Electricity savings1	It's fast, looks and feels good on the outside, try it out and evaluate.	Jingdong prefecture level city in Shandong
Jinfeng Hongyuan Flagship Store	Electricity savings1	I haven't used it. I just used it. I don't know if it's any good.	Jingdong prefecture level city in Shandong

Jinfeng Hongyuan Flagship Store	Electricity savings1	Quality can be, power saving now can not see just use, after a while in the look at the power saving effect	Jingdong prefecture level city in Shandong
Jinfeng Hongyuan Flagship Store	Electricity savings1	The logistics is very fast, and it seems to have some effect	Jingdong prefecture level city in Shandong
Jinfeng Hongyuan Flagship Store	Electricity savings1	Good, but the effects remain to be seen	Jingdong prefecture level city in Shandong
Jinfeng Hongyuan Flagship Store	Electricity savings1	Baby is basically the same as the description, shipping quickly, worth a buy Good price, logistics is strong, worth repurchasing!	Jingdong prefecture level city in Shandong
Jinfeng Hongyuan Flagship Store	Electricity savings1	I bought it for my dad, I don't know much about it, if he thinks it's good then it must be good 👍	Jingdong prefecture level city in Shandong
Jinfeng Hongyuan Flagship Store	Electricity savings1	Goods received. It's working fine. Display voltage. After a period of time to see the effect in the addition to the level of theory	Jingdong prefecture level city in Shandong
Jinfeng Hongyuan Flagship Store	Electricity savings1	The stuff is pretty good, I just don't know how well it saves power.	Jingdong prefecture level city in Shandong
Jinfeng Hongyuan Flagship Store	Electricity savings1	Logistics is a little too fast 👍	Jingdong prefecture level city in Shandong
Jinfeng Hongyuan Flagship Store	Electricity savings1	Received the goods used for a period of time before coming, good, beautiful appearance, really stable flow	Jingdong prefecture level city in Shandong
Jinfeng Hongyuan Flagship Store	Electricity savings1	Things received, look at the packaging is very good, not yet, to the power saving in the follow-up evaluation	Jingdong prefecture level city in Shandong
Jinfeng Hongyuan Flagship Store	Electricity savings1	Baby is very good! Use a few days before to evaluate, I personally test really effective.	Jingdong prefecture level city in Shandong
Jinfeng Hongyuan Flagship Store	Electricity savings1	I received the goods are in use, I do not know how effective? Can it save energy? Let's see.	Jingdong prefecture level city in Shandong
Jinfeng Hongyuan Flagship Store	Electricity savings1	It's really good and has some power saving effect.	Jingdong prefecture level city in Shandong
Jinfeng Hongyuan Flagship Store	Electricity savings1	It's good quality, well made, and I hope it'll save on electricity over time.	Jingdong prefecture level city in Shandong
Jinfeng Hongyuan Flagship Store	Electricity savings1	I just used it, I don't know how it works yet, but I'll give it a good review first.	Jingdong prefecture level city in Shandong
Jinfeng Hongyuan Flagship Store	Electricity savings1	Second purchase, the delivery guy was fast and called ahead to deliver.	Jingdong prefecture level city in Shandong
Jinfeng Hongyuan Flagship Store	Electricity savings1	Goods have been received, but have not yet used, used to see how the effect, in the evaluation of fertilizer	Jingdong prefecture level city in Shandong
Jinfeng Hongyuan Flagship Store	Electricity savings1	The delivery is very fast. It's the same in kind. It's working.	Jingdong prefecture level city in Shandong
Jinfeng Hongyuan Flagship Store	Electricity savings1	Fast logistics, good quality, more satisfied	Jingdong prefecture level city in Shandong
Jinfeng Hongyuan Flagship Store	Electricity savings1	I've been here for almost a month now and there is a very noticeable difference than it is worth buying.	Jingdong prefecture level city in Shandong
Jinfeng Hongyuan Flagship Store	Electricity savings1	Fast shipping, received the next day, plug and play, but do not know how effective to not break down!	Jingdong prefecture level city in Shandong
Jinfeng Hongyuan Flagship Store	Electricity savings1	The logistics are great I've been using it for a while before commenting on it This saves electricity	Jingdong prefecture level city in Shandong
Jinfeng Hongyuan Flagship Store	Electricity savings1	Wait to use it for a while to know if it's energy efficient	Jingdong prefecture level city in Shandong
Jinfeng Hongyuan Flagship Store	Electricity savings1	Just received it, haven't used it yet don't know if it's any good.	Jingdong prefecture level city in Shandong
Jinfeng Hongyuan Flagship Store	Electricity savings1	It really doesn't work, and the electric bill is still the same amount every month.	Jingdong prefecture level city in Shandong
Jinfeng Hongyuan Flagship Store	Electricity savings1	It's well packaged and looks fine	Jingdong prefecture level city in Shandong
Jinfeng Hongyuan Flagship Store	Electricity savings1	Very good. Will patronize again if needed.	Jingdong prefecture level city in Shandong
Jinfeng Hongyuan Flagship Store	Electricity savings1	I have received the product and installed it, try it for a while and see how it works.	Jingdong prefecture level city in Shandong
Jinfeng Hongyuan Flagship Store	Electricity savings1	I've been using it for a month now...I don't feel like there's any noticeable power savings...last month it was $130 this month it's $123...let's see if I can use it for another month	Jingdong prefecture level city in Shandong
Jinfeng Hongyuan Flagship Store	Electricity savings1	Just received it, yet to try it and see if it works.	Jingdong prefecture level city in Shandong
Jinfeng Hongyuan Flagship Store	Electricity savings1	It's easy to use and has a power saving credit!	Jingdong prefecture level city in Shandong
Jinfeng Hongyuan Flagship Store	Electricity savings1	It's very good. I've already bought two of them.	Jingdong prefecture level city in Shandong
Jinfeng Hongyuan Flagship Store	Electricity savings1	It's very well made, packaged meticulously, and I hope it works!	Jingdong prefecture level city in Shandong
Jinfeng Hongyuan Flagship Store	Electricity savings1	Goods have been received have not yet used it, used to comment again	Jingdong prefecture level city in Shandong
Jinfeng Hongyuan Flagship Store	Electricity savings1	Looking forward to good results	Jingdong prefecture level city in Shandong
Jinfeng Hongyuan Flagship Store	Electricity savings1	Not sure if it saves power or not. Still trying it out.	Jingdong prefecture level city in Shandong
Jinfeng Hongyuan Flagship Store	Electricity savings1	It also really saves on electricity! Superb!	Jingdong prefecture level city in Shandong
Jinfeng Hongyuan Flagship Store	Electricity savings1	Didn't work at all, want to return it	Jingdong prefecture level city in Shandong
Jinfeng Hongyuan Flagship Store	Electricity savings1	Uh-huh! It's working, I've been using it for a month and it saves 20% of electricity compared to before.	Jingdong prefecture level city in Shandong
Jinfeng Hongyuan Flagship Store	Electricity savings1	Should be a little bit of a power saver, I wonder how long it will take to save back the money I spent on this one	Jingdong prefecture level city in Shandong
Jinfeng Hongyuan Flagship Store	Electricity savings1	Exquisite appearance and stable voltage	Jingdong prefecture level city in Shandong
Jinfeng Hongyuan Flagship Store	Electricity savings1	After using it for a few days it feels like it saves electricity.	Jingdong prefecture level city in Shandong
Jinfeng Hongyuan Flagship Store	Electricity savings1	Second purchase and its pending good results.	Jingdong prefecture level city in Shandong
Jinfeng Hongyuan Flagship Store	Electricity savings1	The appearance of the workmanship is exquisite and meticulous, beautiful appearance, just to see if there is no effect!	Jingdong prefecture level city in Shandong
Jinfeng Hongyuan Flagship Store	Electricity savings1	Let's see what happens in a month.	Jingdong prefecture level city in Shandong
Jinfeng Hongyuan Flagship Store	Electricity savings1	Trial, now short time not yet obvious effect	Jingdong prefecture level city in Shandong
Jinfeng Hongyuan Flagship Store	Electricity savings1	I'm using it now, don't know how well it works, it looks great!	Jingdong prefecture level city in Shandong
Jinfeng Hongyuan Flagship Store	Electricity savings1	There is no role! Now it's true that I don't know!	Jingdong prefecture level city in Shandong
Jinfeng Hongyuan Flagship Store	Electricity savings1	Purchased for the second time, power saving not sure if it's ok? Let's talk about it after using it	Jingdong prefecture level city in Shandong
Jinfeng Hongyuan Flagship Store	Electricity savings1	I just received it, I have to try it for a month to know how it works.	Jingdong prefecture level city in Shandong
Jinfeng Hongyuan Flagship Store	Electricity savings1	I just used it for an hour, and the meter's still spinning so fast it's not much use.	Jingdong prefecture level city in Shandong

Jinfeng Hongyuan Flagship Store	Electricity savings1	Not bad, logistics is fast	Jingdong prefecture level city in Shandong
Jinfeng Hongyuan Flagship Store	Electricity savings1	I don't know if it works. I'll find out next month when I pay the bill. It's easy. Just plug it in.	Jingdong prefecture level city in Shandong
Jinfeng Hongyuan Flagship Store	Electricity savings1	Logistics is particularly fast, received the goods are genuine, looking forward to power saving effect	Jingdong prefecture level city in Shandong
Jinfeng Hongyuan Flagship Store	Electricity savings1	First time buying this product like this one from this family use it and then evaluate.	Jingdong prefecture level city in Shandong
Jinfeng Hongyuan Flagship Store	Electricity savings1	Lightning arrived, the product is very good, before all do not know there is such a baby, in a friend's home has seen, and then bought to try, has been used for a period of time before coming to the evaluation, the work is very stable, so far looked very good, will introduce friends to buy!	Jingdong prefecture level city in Shandong
Jinfeng Hongyuan Flagship Store	Electricity savings1	I was a bit skeptical when I heard that I could save money on my electricity bill by installing this, I don't know if the power saving effect is really what it is said to be. But since I bought it, I just have to put it on and try it out. I hope it lives up to its name and saves me the money I spent on the device	Jingdong prefecture level city in Shandong
Jinfeng Hongyuan Flagship Store	Electricity savings1	The quality of the stuff is okay, but I'm not sure if it works, so I'll try using it first.	Jingdong prefecture level city in Shandong
Jinfeng Hongyuan Flagship Store	Electricity savings1	It should work. The current is much more stable.	Jingdong prefecture level city in Shandong
Jinfeng Hongyuan Flagship Store	Electricity savings1	The store has a great service attitude	Jingdong prefecture level city in Shandong
Jinfeng Hongyuan Flagship Store	Electricity savings1	Let's wait until we've used it for a month.	Jingdong prefecture level city in Shandong
Jinfeng Hongyuan Flagship Store	Electricity savings1	I'm using it and I don't know if it's a power saver or not	Jingdong prefecture level city in Shandong
Jinfeng Hongyuan Flagship Store	Electricity savings1	Original and authentic guarantee quality is good now.	Jingdong prefecture level city in Shandong
Jinfeng Hongyuan Flagship Store	Electricity savings1	Great, very happy with it, will buy a couple more next time	Jingdong prefecture level city in Shandong
Jinfeng Hongyuan Flagship Store	Electricity savings1	I just used it, I don't know if it works yet, I'll come back after using it...	Jingdong prefecture level city in Shandong
Jinfeng Hongyuan Flagship Store	Electricity savings1	It's still being tested.	Jingdong prefecture level city in Shandong
Jinfeng Hongyuan Flagship Store	Electricity savings1	I've been using it for a month and it's not working.	Jingdong prefecture level city in Shandong
Jinfeng Hongyuan Flagship Store	Electricity savings1	It's basically nothing!	Jingdong prefecture level city in Shandong
Jinfeng Hongyuan Flagship Store	Electricity savings1	It's not bad, it kind of works, support it.	Jingdong prefecture level city in Shandong
Jinfeng Hongyuan Flagship Store	Electricity savings1	I just received it last night, let's try it first to see how it works, it looks good!	Jingdong prefecture level city in Shandong
Jinfeng Hongyuan Flagship Store	Electricity savings1	The current is very stable, fine workmanship, small and exquisite, very satisfied	Jingdong prefecture level city in Shandong
Jinfeng Hongyuan Flagship Store	Electricity savings1	Not bad, exactly how much electricity can be saved; still have to follow up the evaluation	Jingdong prefecture level city in Shandong
Jinfeng Hongyuan Flagship Store	Electricity savings1	Logistics is also very fast, next time there is an opportunity to introduce friends to buy!	Jingdong prefecture level city in Shandong
Jinfeng Hongyuan Flagship Store	Electricity savings1	Voltage display and actual do not pay	Jingdong prefecture level city in Shandong
Jinfeng Hongyuan Flagship Store	Electricity savings1	Second purchase, still saves a little on the electric bill	Jingdong prefecture level city in Shandong
Jinfeng Hongyuan Flagship Store	Electricity savings1	Order received, currently trying it out, thanks a lot.	Jingdong prefecture level city in Shandong
Jinfeng Hongyuan Flagship Store	Electricity savings1	I haven't used it for a long time yet, I don't know if the effect is good or not, looking forward to it	Jingdong prefecture level city in Shandong
Jinfeng Hongyuan Flagship Store	Electricity savings1	Good, working, I just don't know if it will save electricity... 5 stars for now.	Jingdong prefecture level city in Shandong
Jinfeng Hongyuan Flagship Store	Electricity savings1	Great product, just as the owner said it would be.	Jingdong prefecture level city in Shandong
Jinfeng Hongyuan Flagship Store	Electricity savings1	Shipping was fast, I don't know if it works well or not, I have to use it for a while first.	Jingdong prefecture level city in Shandong
Jinfeng Hongyuan Flagship Store	Electricity savings1	I don't feel that it saves much electricity, but I'll give it a try. It's been more than a month now, and the electricity bill is still a bit high.	Jingdong prefecture level city in Shandong
Jinfeng Hongyuan Flagship Store	Electricity savings1	Got it. Just used it.	Jingdong prefecture level city in Shandong
Jinfeng Hongyuan Flagship Store	Electricity savings1	Use it for a while and then comment.	Jingdong prefecture level city in Shandong
Jinfeng Hongyuan Flagship Store	Electricity savings1	Used it, see how it works with it and then comment. The quality is very good!	Jingdong prefecture level city in Shandong
Jinfeng Hongyuan Flagship Store	Electricity savings1	I'll comment again after a while.	Jingdong prefecture level city in Shandong
Jinfeng Hongyuan Flagship Store	Electricity savings1	It's easy to operate, affordable and works well, love it.	Jingdong prefecture level city in Shandong
Jinfeng Hongyuan Flagship Store	Electricity savings1	It's good. You can try it. It's cheap.	Jingdong prefecture level city in Shandong
Jinfeng Hongyuan Flagship Store	Electricity savings1	It's in use, so we'll see how it works after a while!	Jingdong prefecture level city in Shandong
Jinfeng Hongyuan Flagship Store	Electricity savings1	Service is pretty good, not sure about the quality yet.	Jingdong prefecture level city in Shandong
Jinfeng Hongyuan Flagship Store	Electricity savings1	Just used it and will see the results in a month 😀	Jingdong prefecture level city in Shandong
Jinfeng Hongyuan Flagship Store	Electricity savings1	Saving electricity is expensive and good	Jingdong prefecture level city in Shandong
Jinfeng Hongyuan Flagship Store	Electricity savings1	Pretty good, 5 stars! Will buy again next time seller service is very good ！！！！ Good quality! Will recommend to friends!	Jingdong prefecture level city in Shandong
Jinfeng Hongyuan Flagship Store	Electricity savings1	In use, with results naturally have follow-up reviews!	Jingdong prefecture level city in Shandong
Jinfeng Hongyuan Flagship Store	Electricity savings1	I'm using it now, I'll come back with results, it'll work.	Jingdong prefecture level city in Shandong
Jinfeng Hongyuan Flagship Store	Electricity savings1	Good, nice, fast shipping, cheap, worth having!	Jingdong prefecture level city in Shandong
Jinfeng Hongyuan Flagship Store	Electricity savings1	Logistics is fast.	Jingdong prefecture level city in Shandong
Jinfeng Hongyuan Flagship Store	Electricity savings1	I have just started using the product and will be reviewing it later.	Jingdong prefecture level city in Shandong
Jinfeng Hongyuan Flagship Store	Electricity savings1	I'll comment after I use it.	Jingdong prefecture level city in Shandong
Jinfeng Hongyuan Flagship Store	Electricity savings1	I don't know if it's working or not, though I'll try plugging it in.	Jingdong prefecture level city in Shandong
Jinfeng Hongyuan Flagship Store	Electricity savings1	It really saves electricity and contributes to energy savings in disguise	Jingdong prefecture level city in Shandong
Jinfeng Hongyuan Flagship Store	Electricity savings1	It feels good in the experience.	Jingdong prefecture level city in Shandong

Jinfeng Hongyuan Flagship Store	Electricity savings1	The product is better than I thought it would be, the quality looks great, the voltage is stable, and I'm looking forward to some great power savings!	Jingdong prefecture level city in Shandong
Jinfeng Hongyuan Flagship Store	Electricity savings1	I'm using it now looking forward to saving some points,, I'm feeding back	Jingdong prefecture level city in Shandong
Jinfeng Hongyuan Flagship Store	Electricity savings1	Displayed voltage below 200.	Jingdong prefecture level city in Shandong
Jinfeng Hongyuan Flagship Store	Electricity savings1	I haven't seen any great results, but it should last about a hundred or eighty years.	Jingdong prefecture level city in Shandong
Jinfeng Hongyuan Flagship Store	Electricity savings1	I don't know if it works or not, but I keep it plugged in anyway, so I hope it works!	Jingdong prefecture level city in Shandong
Jinfeng Hongyuan Flagship Store	Electricity savings1	Reasonable price, good quality, fast delivery, great service, already a repurchase.	Jingdong prefecture level city in Shandong
Jinfeng Hongyuan Flagship Store	Electricity savings1	Second purchase, looking forward to the results	Jingdong prefecture level city in Shandong
Jinfeng Hongyuan Flagship Store	Electricity savings1	The quality of this product feels like it should work in a use test! Just how many kilowatts of electricity must be wasted per day with the red light and digital display on for long periods of time?	Jingdong prefecture level city in Shandong
Jinfeng Hongyuan Flagship Store	Electricity savings1	It looks good, we'll see if we use it.	Jingdong prefecture level city in Shandong
Jinfeng Hongyuan Flagship Store	Electricity savings1	Great stuff, second purchase!	Jingdong prefecture level city in Shandong
Jinfeng Hongyuan Flagship Store	Electricity savings1	It is indeed very practical and saves electricity at home	Jingdong prefecture level city in Shandong
Jinfeng Hongyuan Flagship Store	Electricity savings1	Goods received, the physical and the same as the picture, logistics is also very fast!	Jingdong prefecture level city in Shandong
Jinfeng Hongyuan Flagship Store	Electricity savings1	I'll use it for a while and see how it goes. I'll buy it again if it's good.	Jingdong prefecture level city in Shandong
Jinfeng Hongyuan Flagship Store	Electricity savings1	In use, voltage monitoring, the effect of how to wait for next month's electricity consumption confirmation	Jingdong prefecture level city in Shandong
Jinfeng Hongyuan Flagship Store	Electricity savings1	It's still pretty good.	Jingdong prefecture level city in Shandong
Jinfeng Hongyuan Flagship Store	Electricity savings1	It's been plugged in for a month and there hasn't been a big change yet, since it usually uses very little electricity	Jingdong prefecture level city in Shandong
Jinfeng Hongyuan Flagship Store	Electricity savings1	Let's wait for a month to see if it works	Jingdong prefecture level city in Shandong
Jinfeng Hongyuan Flagship Store	Electricity savings1	Good, good, good, good, good, good, good, good, good, good, good, good, good, good, good.	Jingdong prefecture level city in Shandong
Jinfeng Hongyuan Flagship Store	Electricity savings1	The product received the trial a few days, feel good, praise!	Jingdong prefecture level city in Shandong
Jinfeng Hongyuan Flagship Store	Electricity savings1	After six or seven hours of use, 250 volts won't save you any power.	Jingdong prefecture level city in Shandong
Jinfeng Hongyuan Flagship Store	Electricity savings1	I haven't used it yet, I wonder if it saves electricity?	Jingdong prefecture level city in Shandong
Jinfeng Hongyuan Flagship Store	Electricity savings1	Baby value for money, love it, next time will come back is genuine, trust the business, baby texture is very good, the price is moderate, unlimited repurchase!	Jingdong prefecture level city in Shandong
Jinfeng Hongyuan Flagship Store	Electricity savings1	Power saving effect is obvious! Raising tropical fish daily power consumption, after using the daily power savings of more than 3 degrees, currently about 8 degrees / day!	Jingdong prefecture level city in Shandong
Jinfeng Hongyuan Flagship Store	Electricity savings1	Just used it, unknown results.	Jingdong prefecture level city in Shandong
Jinfeng Hongyuan Flagship Store	Electricity savings1	The goods received, well, look forward to the effect. Logistics is very fast, thanks to the platform, thanks to Jingdong. I hope it can save electricity.	Jingdong prefecture level city in Shandong
Jinfeng Hongyuan Flagship Store	Electricity savings1	Not sure 有 effect?	Jingdong prefecture level city in Shandong
Jinfeng Hongyuan Flagship Store	Electricity savings1	I'd say it's a 5% savings.	Jingdong prefecture level city in Shandong
Jinfeng Hongyuan Flagship Store	Electricity savings1	I received it and am using it, I don't know how effective it is, I'll evaluate it afterwards	Jingdong prefecture level city in Shandong
Jinfeng Hongyuan Flagship Store	Electricity savings1	Baby received not yet used, first give praise	Jingdong prefecture level city in Shandong
Jinfeng Hongyuan Flagship Store	Electricity savings1	Looks good, I'll evaluate it later	Jingdong prefecture level city in Shandong
Jinfeng Hongyuan Flagship Store	Electricity savings1	The goods have been received in use, the overall feeling is not bad!	Jingdong prefecture level city in Shandong
Jinfeng Hongyuan Flagship Store	Electricity savings1	It's working. This is the second one. This stuff has to be used over a long period of time to be effective. And it only works on inductive loads, i.e. appliances containing electric motors. Use it.	Jingdong prefecture level city in Shandong
Jinfeng Hongyuan Flagship Store	Electricity savings1	Use it for a while and see how it works.	Jingdong prefecture level city in Shandong
Jinfeng Hongyuan Flagship Store	Electricity savings1	Good courier service and the item is very nice and practical.	Jingdong prefecture level city in Shandong
Jinfeng Hongyuan Flagship Store	Electricity savings1	It doesn't do anything and it's a waste of electricity.	Jingdong prefecture level city in Shandong
Jinfeng Hongyuan Flagship Store	Electricity savings1	I haven't used it yet. It should be good.	Jingdong prefecture level city in Shandong
Jinfeng Hongyuan Flagship Store	Electricity savings1	That's good. OK, that's good.	Jingdong prefecture level city in Shandong
Jinfeng Hongyuan Flagship Store	Electricity savings1	It's a rip off, doesn't work, doesn't save electricity, does the job to well, and I've wasted a couple dozen dollars again.	Jingdong prefecture level city in Shandong
Jinfeng Hongyuan Flagship Store	Electricity savings1	Use it first, the effect is not as good as the road, then we will comment again	Jingdong prefecture level city in Shandong
Jinfeng Hongyuan Flagship Store	Electricity savings1	The price is affordable, the logistics is strong, just use four or five days, the effect will be added later!	Jingdong prefecture level city in Shandong
Jinfeng Hongyuan Flagship Store	Electricity savings1	It's easy to use and works fine.	Jingdong prefecture level city in Shandong
Jinfeng Hongyuan Flagship Store	Electricity savings1	It doesn't feel like it's working.	Jingdong prefecture level city in Shandong
Jinfeng Hongyuan Flagship Store	Electricity savings1	I'm using it. We'll see what happens in a couple months.	Jingdong prefecture level city in Shandong
Jinfeng Hongyuan Flagship Store	Electricity savings1	I just got it back to try it out and I'll comment later	Jingdong prefecture level city in Shandong
Jinfeng Hongyuan Flagship Store	Electricity savings1	Less than two months. Can't tell.	Jingdong prefecture level city in Shandong
Jinfeng Hongyuan Flagship Store	Electricity savings1	No power saving effect	Jingdong prefecture level city in Shandong
Jinfeng Hongyuan Flagship Store	Electricity savings1	Express Express Very quickly not only to the power saver can not save electricity, with two months, look at the evaluation put.	Jingdong prefecture level city in Shandong
Jinfeng Hongyuan Flagship Store	Electricity savings1	Genuine products are packaged well!	Jingdong prefecture level city in Shandong
Jinfeng Hongyuan Flagship Store	Electricity savings1	I've been using it for a while, but I don't know if it saves electricity. I hope it works.	Jingdong prefecture level city in Shandong
Jinfeng Hongyuan Flagship Store	Electricity savings1	Good service, fast logistics, the effect is to be used to know	Jingdong prefecture level city in Shandong
Jinfeng Hongyuan Flagship Store	Electricity savings1	Just used it, not sure about the power saving effect	Jingdong prefecture level city in Shandong

Jinfeng Hongyuan Flagship Store	Electricity savings1	The stuff is ok, save power or not, will comment after a month.	Jingdong prefecture level city in Shandong
Jinfeng Hongyuan Flagship Store	Electricity savings1	Used it for a month to save %25, normal is a little over 100, it's great! I want to buy one in.	Jingdong prefecture level city in Shandong
Jinfeng Hongyuan Flagship Store	Electricity savings1	I'm using it and looking forward to the results	Jingdong prefecture level city in Shandong
Jinfeng Hongyuan Flagship Store	Electricity savings1	The quality is very good and the workmanship is exquisite. The use effect is also very good.	Jingdong prefecture level city in Shandong
Jinfeng Hongyuan Flagship Store	Electricity savings1	Merchant service attitude is very good, logistics speed is super fast, online evaluation is very good, the first time to use, a month to know the specific effect, the effect is good, and then additional	Jingdong prefecture level city in Shandong
Jinfeng Hongyuan Flagship Store	Electricity savings1	Works great, solves the problem of high power usage in the house	Jingdong prefecture level city in Shandong
Jinfeng Hongyuan Flagship Store	Electricity savings1	To receive! Very delicate! It's not clear how effective it is, I won't know until I use it!!!!	Jingdong prefecture level city in Shandong
Jinfeng Hongyuan Flagship Store	Electricity savings1	I've just been using it for a while and I don't feel like it's too noticeable.	Jingdong prefecture level city in Shandong
Jinfeng Hongyuan Flagship Store	Electricity savings1	Already used, no visible effect for the time being	Jingdong prefecture level city in Shandong
Jinfeng Hongyuan Flagship Store	Electricity savings1	I can't feel the effect.	Jingdong prefecture level city in Shandong
Jinfeng Hongyuan Flagship Store	Electricity savings1	Use it for a while and see the results	Jingdong prefecture level city in Shandong
Jinfeng Hongyuan Flagship Store	Electricity savings1	The price is more reasonable, the power-saving effect is to be verified, the delivery is fast, and the courier service is good.	Jingdong prefecture level city in Shandong
Jinfeng Hongyuan Flagship Store	Electricity savings1	Quality is very good, consistent with the seller's description, very satisfied, very like, very fast shipping, packaging is very careful and strict, the logistics company has a very good service attitude, fast delivery, very satisfied with a shopping, very satisfied. Recommended to buy, cost-effective, the service attitude of the business is very enthusiastic, providing such high quality goods.	Jingdong prefecture level city in Shandong
Jinfeng Hongyuan Flagship Store	Electricity savings1	I don't know.	Jingdong prefecture level city in Shandong
Jinfeng Hongyuan Flagship Store	Electricity savings1	I didn't get the delivery.	Jingdong prefecture level city in Shandong
Jinfeng Hongyuan Flagship Store	Electricity savings1	It works, it does have power savings, better than expected, will repurchase.	Jingdong prefecture level city in Shandong
Jinfeng Hongyuan Flagship Store	Electricity savings1	That's where the five fuses are.	Jingdong prefecture level city in Shandong
Jinfeng Hongyuan Flagship Store	Electricity savings1	Don't buy it, it doesn't work and you can't return it.	Jingdong prefecture level city in Shandong
Jinfeng Hongyuan Flagship Store	Electricity savings1	I hope it works. Wait a month.	Jingdong prefecture level city in Shandong
Jinfeng Hongyuan Flagship Store	Electricity savings1	Nothing**, buy with caution	Jingdong prefecture level city in Shandong
Jinfeng Hongyuan Flagship Store	Electricity savings1	I'm using it and waiting for the results	Jingdong prefecture level city in Shandong
Jinfeng Hongyuan Flagship Store	Electricity savings1	Saver quality is very good, has always felt that the home power consumption is very large, this is the company's electrician recommended, said he bought this home is really able to save a little electricity, I also came to buy, the effect if it is good, then buy, a floor a...	Jingdong prefecture level city in Shandong
Jinfeng Hongyuan Flagship Store	Electricity savings1	Good product, satisfied with purchase	Jingdong prefecture level city in Shandong
Jinfeng Hongyuan Flagship Store	Electricity savings1	Occupying the time has not yet found the power saving, occupying not evaluation	Jingdong prefecture level city in Shandong
Jinfeng Hongyuan Flagship Store	Electricity savings1	Haven't tested the results yet. To be followed up with comments!	Jingdong prefecture level city in Shandong
Jinfeng Hongyuan Flagship Store	Electricity savings1	After experiencing it for a month, I don't seem to know how to save it, and I don't pay attention to it anyway	Jingdong prefecture level city in Shandong
Jinfeng Hongyuan Flagship Store	Electricity savings1	It's useless. It's a waste of electricity.	Jingdong prefecture level city in Shandong
Jinfeng Hongyuan Flagship Store	Electricity conservation2	Plugged it in first thing when I received it, very easy and good	Jingdong prefecture level city in Shandong
Jinfeng Hongyuan Flagship Store	Electricity conservation2	Just installed it, trying it out for a month to see how it works.	Jingdong prefecture level city in Shandong
Jinfeng Hongyuan Flagship Store	Electricity conservation2	Refrigerator every start a computer black screen, separate row plug or so, online inquiries about the cause, and then buy a power saver back to try, but also really useful, can balance the current, occasionally once or twice will be black, than the previous start a black once a lot of improvement, the power saving effect of the follow-up and then see	Jingdong prefecture level city in Shandong
Jinfeng Hongyuan Flagship Store	Electricity conservation2	Jinfeng Hongyuan power saver received, the appearance of the shape is very good 👍, a friend recommended that can save electricity, hold a try to see the mind to buy a, just use not a few days, wait for a period of time to say, useful will be back to buy.	Jingdong prefecture level city in Shandong
Jinfeng Hongyuan Flagship Store	Electricity conservation2	Ordered last night arrived today, fast enough, the quality of the way delicate and beautiful, looking forward to its effect as remarkable as the platform introduction, to be tried for a period of time, and then come back to the chase to comment on the summary to share with you!	Jingdong prefecture level city in Shandong
Jinfeng Hongyuan Flagship Store	Electricity conservation2	I could not imagine that it was received so quickly. JINFENGHONGYUAN (JINFENGHONGYUAN) power saver household intelligent power saving appliances home power saving artifact high-power enhanced version of the power use steward power saving expert light blue 99KW, very good.	Jingdong prefecture level city in Shandong
Jinfeng Hongyuan Flagship Store	Electricity conservation2	Energy saver is good ah, high value, aluminum alloy shell, LCD display, this workmanship, bar. Can save a lot of electricity ah	Jingdong prefecture level city in Shandong
Jinfeng Hongyuan Flagship Store	Electricity conservation2	Jingdong express delivery fast, well-packed, small, exquisite and beautiful electrical appliances, blue look upscale, product certificates and manuals are all complete, is a regular manufacturer. Plugged in the power to try, normal work, power-saving effect has to wait for a period of time to compare out.	Jingdong prefecture level city in Shandong
Jinfeng Hongyuan Flagship Store	Electricity conservation2	Listen to others say that this can save electricity, I bought with the idea of trying, used for a period of time before coming to the evaluation, will be a little bit of use, not a lot of savings just, long-term use of the words can save more.	Jingdong prefecture level city in Shandong
Jinfeng Hongyuan Flagship Store	Electricity conservation2	Logistics is fast, the next day arrived, workmanship is very delicate a power saver, looking forward to the late effect, customer service patiently explain the recommended which is suitable for, installed in what position is the best, look forward to using the effect after a period of time!	Jingdong prefecture level city in Shandong
Jinfeng Hongyuan Flagship Store	Electricity conservation2	It's good to use, I feel that it has been saving electricity, anyway, not a loss is, I used to feel that the power consumption is big, now I feel much better, my friends are also using, I hope to use a long time will be able to, I wish the boss a prosperous business	Jingdong prefecture level city in Shandong
Jinfeng Hongyuan Flagship Store	Electricity conservation2	Should be very good, to be able to plan the circuit, save the home of the circuit to the extent of the savings, waste of money on electricity, good if a month this money back, hope always hope to be able to	Jingdong prefecture level city in Shandong
Jinfeng Hongyuan Flagship Store	Electricity conservation2	Express delivery is very strong, the goods and I saw in the business consistent, the use of the effect of follow-up evaluation	Jingdong prefecture level city in Shandong
Jinfeng Hongyuan Flagship Store	Electricity conservation2	I have been using this brand of power saver for many years and this time I saw this upgraded version and got it again. The main purpose of this product is to reduce the loss of electricity for a long time, so as to achieve the purpose of saving electricity.	Jingdong prefecture level city in Shandong
Jinfeng Hongyuan Flagship Store	Electricity conservation2	Logistics is fast and the product looks beautiful, looking forward to the effect.	Jingdong prefecture level city in Shandong
Jinfeng Hongyuan Flagship Store	Electricity conservation2	Product quality is good, energy saving a little effect, but the price is a little expensive, fast arrival, good service.	Jingdong prefecture level city in Shandong
Jinfeng Hongyuan Flagship Store	Electricity conservation2	The quality is very good, inexpensive, Jingdong self-supporting guaranteed, worth having!	Jingdong prefecture level city in Shandong
Jinfeng Hongyuan Flagship Store	Electricity conservation2	I don't know if it's saving electricity yet, I'll find out next time I pay my electric bill	Jingdong prefecture level city in Shandong

Jinfeng Hongyuan Flagship Store	Electricity conservation2	Favorable price quality assurance appearance design is beautiful and generous, fine workmanship	Jingdong prefecture level city in Shandong
Jinfeng Hongyuan Flagship Store	Electricity conservation2	I bought it, I don't know how effective it is? I don't know how the quality is? After this summer results will know! Looking forward to it!	Jingdong prefecture level city in Shandong
Jinfeng Hongyuan Flagship Store	Electricity conservation2	Goods received Packaging intact, look at the shell workmanship is also good, high-power appliances open voltage has obvious changes in the effectiveness of the	Jingdong prefecture level city in Shandong
Jinfeng Hongyuan Flagship Store	Electricity conservation2	The goods have been received logistics is very fast, the product is similar to the line, just wait to use a month to see if it can save electricity, stay tuned.	Jingdong prefecture level city in Shandong
Jinfeng Hongyuan Flagship Store	Electricity conservation2	Electricity is plugged in, not sure if it will work, hopefully it will work.	Jingdong prefecture level city in Shandong
Jinfeng Hongyuan Flagship Store	Electricity conservation2	The shell is aluminum alloy, very good workmanship, the actual effect of waiting for the addition of evaluation	Jingdong prefecture level city in Shandong
Jinfeng Hongyuan Flagship Store	Electricity conservation2	Received, already plugged in, looking forward to the effect.	Jingdong prefecture level city in Shandong
Jinfeng Hongyuan Flagship Store	Electricity conservation2	Opened to see the regular manufacturer products, wait for a period of time to see it	Jingdong prefecture level city in Shandong
Jinfeng Hongyuan Flagship Store	Electricity conservation2	🉑 Baby received, very surprised ✨, reasonable design, fashionable and generous, continue to pay attention to the future will be repurchased ❥❥❥❥	Jingdong prefecture level city in Shandong
Jinfeng Hongyuan Flagship Store	Electricity conservation2	Baby received it 👌, really great ah! 🌈The quality can't be beat, very satisfied! 🆗Happy happy happy 😃	Jingdong prefecture level city in Shandong
Jinfeng Hongyuan Flagship Store	Electricity conservation2	Very good, power saving see, this is to help a friend to buy, I used to give my friend a say, my friend immediately let me order another one!	Jingdong prefecture level city in Shandong
Jinfeng Hongyuan Flagship Store	Electricity conservation2	The baby is just as introduced, the texture is very good, powerful, and it feels good to use it	Jingdong prefecture level city in Shandong
Jinfeng Hongyuan Flagship Store	Electricity conservation2	Value for money, very satisfied, seller service attitude is very good, very patient	Jingdong prefecture level city in Shandong
Jinfeng Hongyuan Flagship Store	Electricity conservation2	Power saving treasure design unique exquisite beautiful, voltage display clearly, plugged in that is good to use, I observed the meter, after using the meter is gone slow.	Jingdong prefecture level city in Shandong
Jinfeng Hongyuan Flagship Store	Electricity conservation2	Received on the use of the packaging is beautifully used for a period of time and then come back in the follow-up review!	Jingdong prefecture level city in Shandong
Jinfeng Hongyuan Flagship Store	Electricity conservation2	It should work. Not bad. Not bad. Not bad.	Jingdong prefecture level city in Shandong
Jinfeng Hongyuan Flagship Store	Electricity conservation2	I haven't used it yet, I'll plug it in and see if it saves power next month.	Jingdong prefecture level city in Shandong
Jinfeng Hongyuan Flagship Store	Electricity conservation2	The goods received and unpacked and used, the quality is very good, things and the official website to buy no difference!	Jingdong prefecture level city in Shandong
Jinfeng Hongyuan Flagship Store	Electricity conservation2	It's a rip off, doesn't help much at all, uses the same amount of power as before it was used.	Jingdong prefecture level city in Shandong
Jinfeng Hongyuan Flagship Store	Electricity conservation2	I've been using it for a while now and haven't noticed any unusual problems, so it's okay	Jingdong prefecture level city in Shandong
Jinfeng Hongyuan Flagship Store	Electricity conservation2	Received the goods and plugged in to use, good or not there is no use will have to wait until next month to find out	Jingdong prefecture level city in Shandong
Jinfeng Hongyuan Flagship Store	Electricity conservation2	It's very compact, it looks good, I'll introduce you to my clients when it's ready.	Jingdong prefecture level city in Shandong
Jinfeng Hongyuan Flagship Store	Electricity conservation2	Good workmanship, we'll have to see in a few months.	Jingdong prefecture level city in Shandong
Jinfeng Hongyuan Flagship Store	Electricity conservation2	Jingdong quality! Always trustworthy!	Jingdong prefecture level city in Shandong
Jinfeng Hongyuan Flagship Store	Electricity conservation2	This is the second time I've bought it, and the merchant said to give me five dollars out of the difference, so thank you merchant.	Jingdong prefecture level city in Shandong
Jinfeng Hongyuan Flagship Store	Electricity conservation2	Good quality and good service	Jingdong prefecture level city in Shandong
Jinfeng Hongyuan Flagship Store	Electricity conservation2	Feels good texture, aluminum alloy shell, voltage digital display clear.	Jingdong prefecture level city in Shandong
Jinfeng Hongyuan Flagship Store	Electricity conservation2	Good product, fast and great service, thanks!	Jingdong prefecture level city in Shandong
Jinfeng Hongyuan Flagship Store	Electricity conservation2	Have not yet used, first evaluation, later really good do not need to comment again.	Jingdong prefecture level city in Shandong
Jinfeng Hongyuan Flagship Store	Electricity conservation2	Works great. Pretty much as described by the merchant.	Jingdong prefecture level city in Shandong
Jinfeng Hongyuan Flagship Store	Electricity conservation2	Received it, looking forward to the results, hope it works	Jingdong prefecture level city in Shandong
Jinfeng Hongyuan Flagship Store	Electricity conservation2	Received it, looking forward to the results, hope it works	Jingdong prefecture level city in Shandong
Jinfeng Hongyuan Flagship Store	Electricity conservation2	Supposedly it's an IQ tax. Buy one and try it. Looks good. Well made.	Jingdong prefecture level city in Shandong
Jinfeng Hongyuan Flagship Store	Electricity conservation2	It just doesn't feel like it's working.	Jingdong prefecture level city in Shandong
Jinfeng Hongyuan Flagship Store	Electricity conservation2	This is a great product.	Jingdong prefecture level city in Shandong
Jinfeng Hongyuan Flagship Store	Electricity conservation2	I'm just using it. I'll tell you more.	Jingdong prefecture level city in Shandong
Jinfeng Hongyuan Flagship Store	Electricity conservation2	Second repurchase and it's working fine.	Jingdong prefecture level city in Shandong
Jinfeng Hongyuan Flagship Store	Electricity conservation2	I'll wait a few days before evaluating	Jingdong prefecture level city in Shandong
Jinfeng Hongyuan Flagship Store	Electricity conservation2	I'll comment after I use it hopefully the section will be useful.	Jingdong prefecture level city in Shandong
Jinfeng Hongyuan Flagship Store	Electricity conservation2	not very useful	Jingdong prefecture level city in Shandong
Jinfeng Hongyuan Flagship Store	Electricity conservation2	Not received	Jingdong prefecture level city in Shandong
Jinfeng Hongyuan Flagship Store	Electricity conservation2	rare	Jingdong prefecture level city in Shandong
Jinfeng Hongyuan Flagship Store	Electricity conservation2	It's good.	Jingdong prefecture level city in Shandong
Jinfeng Hongyuan Flagship Store	Electricity conservation2	just now	Jingdong prefecture level city in Shandong
Jinfeng Hongyuan Flagship Store	Electricity conservation2	serviceable	Jingdong prefecture level city in Shandong
Jinfeng Hongyuan Flagship Store	Electricity conservation2	desirable	Jingdong prefecture level city in Shandong
Jinfeng Hongyuan Flagship Store	Electricity conservation2	serviceable	Jingdong prefecture level city in Shandong
Jinfeng Hongyuan Flagship Store	Electricity conservation2	It doesn't save power and costs electricity.	Jingdong prefecture level city in Shandong
Jinfeng Hongyuan Flagship Store	Electricity conservation2	Not as good as I thought.	Jingdong prefecture level city in Shandong
Jinfeng Hongyuan Flagship Store	Electricity conservation2	Super bad review, it doesn't save power at all, but rather more power consumption	Jingdong prefecture level city in Shandong
Jinfeng Hongyuan Flagship Store	Electricity conservation2	Listen to others say that this can save electricity, I bought with the idea of trying, used for a period of time before coming to the evaluation, will be a little bit of use, not a lot of savings just, long-term use of the words can save more.	Jingdong prefecture level city in Shandong

Jinfeng Hongyuan Flagship Store	Electricity conservation2	Logistics is fast, the next day arrived, workmanship is very delicate a power saver, looking forward to the late effect, customer service patiently explain the recommended which is suitable for, installed in what position is the best, look forward to using the effect after a period of time!	Jingdong prefecture level city in Shandong
Jinfeng Hongyuan Flagship Store	Electricity conservation2	It's good to use, I feel that it has been saving electricity, anyway, not a loss is, I used to feel that the power consumption is big, now I feel much better, my friends are also using, I hope to use a long time will be able to, I wish the boss a prosperous business	Jingdong prefecture level city in Shandong
Jinfeng Hongyuan Flagship Store	Electricity conservation2	Should be very good, to be able to plan the circuit, save the home of the circuit to the extent of the savings, waste of money on electricity, good if a month this money back, hope always hope to be able to	Jingdong prefecture level city in Shandong
Jinfeng Hongyuan Flagship Store	Electricity conservation2	Express delivery is very strong, the goods and I saw in the business consistent, the use of the effect of follow-up evaluation	Jingdong prefecture level city in Shandong
Jinfeng Hongyuan Flagship Store	Electricity conservation2	I have been using this brand of power saver for many years and this time I saw this upgraded version and got it again. The main purpose of this product is to reduce the loss of electricity for a long time, so as to achieve the purpose of saving electricity.	Jingdong prefecture level city in Shandong
Jinfeng Hongyuan Flagship Store	Electricity conservation2	Logistics is fast and the product looks beautiful, looking forward to the effect.	Jingdong prefecture level city in Shandong
Jinfeng Hongyuan Flagship Store	Electricity conservation2	Product quality is good, energy saving a little effect, but the price is a little expensive, fast arrival, good service.	Jingdong prefecture level city in Shandong
Jinfeng Hongyuan Flagship Store	Electricity conservation2	The quality is very good, inexpensive, Jingdong self-supporting guaranteed, worth having!	Jingdong prefecture level city in Shandong
Jinfeng Hongyuan Flagship Store	Electricity conservation2	I don't know if it's saving electricity yet, I'll find out next time I pay my electric bill	Jingdong prefecture level city in Shandong
Jinfeng Hongyuan Flagship Store	Electricity conservation2	Favorable price quality assurance appearance design is beautiful and generous, fine workmanship	Jingdong prefecture level city in Shandong
Jinfeng Hongyuan Flagship Store	Electricity conservation2	I bought it, I don't know how effective it is? I don't know how the quality is? After this summer results will know! Looking forward to it!	Jingdong prefecture level city in Shandong
Jinfeng Hongyuan Flagship Store	Electricity conservation2	Goods received Packaging intact, look at the shell workmanship is also good, high-power appliances open voltage has obvious changes in the effectiveness of the	Jingdong prefecture level city in Shandong
Jinfeng Hongyuan Flagship Store	Electricity conservation2	The goods have been received logistics is very fast, the product is similar to the line, just wait to use a month to see if it can save electricity, stay tuned.	Jingdong prefecture level city in Shandong
Jinfeng Hongyuan Flagship Store	Electricity conservation2	Electricity is plugged in, not sure if it will work, hopefully it will work.	Jingdong prefecture level city in Shandong
Jinfeng Hongyuan Flagship Store	Electricity conservation2	The shell is aluminum alloy, very good workmanship, the actual effect of waiting for the addition of evaluation	Jingdong prefecture level city in Shandong
Jinfeng Hongyuan Flagship Store	Electricity conservation2	Received, already plugged in, looking forward to the effect.	Jingdong prefecture level city in Shandong
Jinfeng Hongyuan Flagship Store	Electricity conservation2	Opened to see the regular manufacturer products, wait for a period of time to see it	Jingdong prefecture level city in Shandong
Jinfeng Hongyuan Flagship Store	Electricity conservation2	🉑 Baby received, very surprised ✨, reasonable design, fashionable and generous, continue to pay attention to the future will be repurchased ❥❥❥❥	Jingdong prefecture level city in Shandong
Jinfeng Hongyuan Flagship Store	Electricity conservation2	Baby received it 👌, really great ah! 🌈The quality can't be beat, very satisfied! 🆗Happy happy happy 😃	Jingdong prefecture level city in Shandong
Jinfeng Hongyuan Flagship Store	Electricity conservation2	Very good, power saving see, this is to help a friend to buy, I used to give my friend a say, my friend immediately let me order another one!	Jingdong prefecture level city in Shandong
Jinfeng Hongyuan Flagship Store	Electricity conservation2	The baby is just as introduced, the texture is very good, powerful, and it feels good to use it	Jingdong prefecture level city in Shandong
Jinfeng Hongyuan Flagship Store	Electricity conservation2	Value for money, very satisfied, seller service attitude is very good, very patient	Jingdong prefecture level city in Shandong
Jinfeng Hongyuan Flagship Store	Electricity conservation2	Power saving treasure design unique exquisite beautiful, voltage display clearly, plugged in that is good to use, I observed the meter, after using the meter is gone slow.	Jingdong prefecture level city in Shandong
Jinfeng Hongyuan Flagship Store	Electricity conservation2	Received on the use of the packaging is beautifully used for a period of time and then come back in the follow-up review!	Jingdong prefecture level city in Shandong
Jinfeng Hongyuan Flagship Store	Electricity conservation2	It should work. Not bad. Not bad. Not bad.	Jingdong prefecture level city in Shandong
Jinfeng Hongyuan Flagship Store	Electricity conservation2	I haven't used it yet, I'll plug it in and see if it saves power next month.	Jingdong prefecture level city in Shandong
Jinfeng Hongyuan Flagship Store	Electricity conservation2	The goods received and unpacked and used, the quality is very good, things and the official website to buy no difference!	Jingdong prefecture level city in Shandong
Jinfeng Hongyuan Flagship Store	Electricity conservation2	It's a rip off, doesn't help much at all, uses the same amount of power as before it was used.	Jingdong prefecture level city in Shandong
Jinfeng Hongyuan Flagship Store	Electricity conservation2	I've been using it for a while now and haven't noticed any unusual problems, so it's okay	Jingdong prefecture level city in Shandong
Jinfeng Hongyuan Flagship Store	Electricity conservation2	Received the goods and plugged in to use, good or not there is no use will have to wait until next month to find out	Jingdong prefecture level city in Shandong
Jinfeng Hongyuan Flagship Store	Electricity conservation2	It's very compact, it looks good, I'll introduce you to my clients when it's ready.	Jingdong prefecture level city in Shandong
Jinfeng Hongyuan Flagship Store	Electricity conservation2	Good workmanship, we'll have to see in a few months.	Jingdong prefecture level city in Shandong
Jinfeng Hongyuan Flagship Store	Electricity conservation2	Jingdong quality! Always trustworthy!	Jingdong prefecture level city in Shandong
Jinfeng Hongyuan Flagship Store	Electricity conservation2	This is the second time I've bought it, and the merchant said to give me five dollars out of the difference, so thank you merchant.	Jingdong prefecture level city in Shandong
Jinfeng Hongyuan Flagship Store	Electricity conservation2	Good quality and good service	Jingdong prefecture level city in Shandong
Jinfeng Hongyuan Flagship Store	Electricity conservation2	Feels good texture, aluminum alloy shell, voltage digital display clear.	Jingdong prefecture level city in Shandong
Jinfeng Hongyuan Flagship Store	Electricity conservation2	Good product, fast and great service, thanks!	Jingdong prefecture level city in Shandong
Jinfeng Hongyuan Flagship Store	Electricity conservation2	Have not yet used, first evaluation, later really good do not need to comment again.	Jingdong prefecture level city in Shandong
Jinfeng Hongyuan Flagship Store	Electricity conservation2	Works great. Pretty much as described by the merchant.	Jingdong prefecture level city in Shandong
Jinfeng Hongyuan Flagship Store	Electricity conservation2	Received it, looking forward to the results, hope it works	Jingdong prefecture level city in Shandong
Jinfeng Hongyuan Flagship Store	Electricity conservation2	Received it, looking forward to the results, hope it works	Jingdong prefecture level city in Shandong
Jinfeng Hongyuan Flagship Store	Electricity conservation2	Supposedly it's an IQ tax. Buy one and try it. Looks good. Well made.	Jingdong prefecture level city in Shandong
Jinfeng Hongyuan Flagship Store	Electricity conservation2	It just doesn't feel like it's working.	Jingdong prefecture level city in Shandong
Jinfeng Hongyuan Flagship Store	Electricity conservation2	This is a great product.	Jingdong prefecture level city in Shandong
Jinfeng Hongyuan Flagship Store	Electricity conservation2	I'm just using it. I'll tell you more.	Jingdong prefecture level city in Shandong
Jinfeng Hongyuan Flagship Store	Electricity conservation2	Second repurchase and it's working fine.	Jingdong prefecture level city in Shandong
Jinfeng Hongyuan Flagship Store	Electricity conservation2	I'll wait a few days before evaluating	Jingdong prefecture level city in Shandong
Jinfeng Hongyuan Flagship Store	Electricity conservation2	I'll comment after I use it hopefully the section will be useful.	Jingdong prefecture level city in Shandong
Jinfeng Hongyuan Flagship Store	Electricity conservation2	not very useful	Jingdong prefecture level city in Shandong
Jinfeng Hongyuan Flagship Store	Electricity conservation2	Not received	Jingdong prefecture level city in Shandong

Jinfeng Hongyuan Flagship Store	Electricity conservation2	rare	Jingdong prefecture level city in Shandong
Jinfeng Hongyuan Flagship Store	Electricity conservation2	It's good.	Jingdong prefecture level city in Shandong
Jinfeng Hongyuan Flagship Store	Electricity conservation2	just now	Jingdong prefecture level city in Shandong
Jinfeng Hongyuan Flagship Store	Electricity conservation2	serviceable	Jingdong prefecture level city in Shandong
Jinfeng Hongyuan Flagship Store	Electricity conservation2	desirable	Jingdong prefecture level city in Shandong
Jinfeng Hongyuan Flagship Store	Electricity conservation2	serviceable	Jingdong prefecture level city in Shandong
Jinfeng Hongyuan Flagship Store	Electricity conservation2	It doesn't save power and costs electricity.	Jingdong prefecture level city in Shandong
Jinfeng Hongyuan Flagship Store	Electricity conservation2	Not as good as I thought.	Jingdong prefecture level city in Shandong
Jinfeng Hongyuan Flagship Store	Electricity conservation2	Super bad review, it doesn't save power at all, but rather more power consumption	Jingdong prefecture level city in Shandong
Jinfeng Hongyuan Flagship Store	Electricity conservation2	Listen to others say that this can save electricity, I bought with the idea of trying, used for a period of time before coming to the evaluation, will be a little bit of use, not a lot of savings just, long-term use of the words can save more.	Jingdong prefecture level city in Shandong
Jinfeng Hongyuan Flagship Store	Electricity conservation2	Logistics is fast, the next day arrived, workmanship is very delicate a power saver, looking forward to the late effect, customer service patiently explain the recommended which is suitable for, installed in what position is the best, look forward to using the effect after a period of time!	Jingdong prefecture level city in Shandong
Jinfeng Hongyuan Flagship Store	Electricity conservation2	It's good to use, I feel that it has been saving electricity, anyway, not a loss is, I used to feel that the power consumption is big, now I feel much better, my friends are also using, I hope to use a long time will be able to, I wish the boss a prosperous business	Jingdong prefecture level city in Shandong
Jinfeng Hongyuan Flagship Store	Electricity conservation2	Should be very good, to be able to plan the circuit, save the home of the circuit to the extent of the savings, waste of money on electricity, good if a month this money back, hope always hope to be able to	Jingdong prefecture level city in Shandong
Jinfeng Hongyuan Flagship Store	Electricity conservation2	Express delivery is very strong, the goods and I saw in the business consistent, the use of the effect of follow-up evaluation	Jingdong prefecture level city in Shandong
Jinfeng Hongyuan Flagship Store	Electricity conservation2	I have been using this brand of power saver for many years and this time I saw this upgraded version and got it again. The main purpose of this product is to reduce the loss of electricity for a long time, so as to achieve the purpose of saving electricity.	Jingdong prefecture level city in Shandong
Jinfeng Hongyuan Flagship Store	Electricity conservation2	Logistics is fast and the product looks beautiful, looking forward to the effect.	Jingdong prefecture level city in Shandong
Jinfeng Hongyuan Flagship Store	Electricity conservation2	Product quality is good, energy saving a little effect, but the price is a little expensive, fast arrival, good service.	Jingdong prefecture level city in Shandong
Jinfeng Hongyuan Flagship Store	Electricity conservation2	The quality is very good, inexpensive, Jingdong self-supporting guaranteed, worth having!	Jingdong prefecture level city in Shandong
Jinfeng Hongyuan Flagship Store	Electricity conservation2	I don't know if it's saving electricity yet, I'll find out next time I pay my electric bill	Jingdong prefecture level city in Shandong
Jinfeng Hongyuan Flagship Store	Electricity conservation2	Favorable price quality assurance appearance design is beautiful and generous, fine workmanship	Jingdong prefecture level city in Shandong
Jinfeng Hongyuan Flagship Store	Electricity conservation2	I bought it, I don't know how effective it is? I don't know how the quality is? After this summer results will know! Looking forward to it!	Jingdong prefecture level city in Shandong
Jinfeng Hongyuan Flagship Store	Electricity conservation2	Goods received Packaging intact, look at the shell workmanship is also good, high-power appliances open voltage has obvious changes in the effectiveness of the	Jingdong prefecture level city in Shandong
Jinfeng Hongyuan Flagship Store	Electricity conservation2	The goods have been received logistics is very fast, the product is similar to the line, just wait to use a month to see if it can save electricity, stay tuned.	Jingdong prefecture level city in Shandong
Jinfeng Hongyuan Flagship Store	Electricity conservation2	Electricity is plugged in, not sure if it will work, hopefully it will work.	Jingdong prefecture level city in Shandong
Jinfeng Hongyuan Flagship Store	Electricity conservation2	The shell is aluminum alloy, very good workmanship, the actual effect of waiting for the addition of evaluation	Jingdong prefecture level city in Shandong
Jinfeng Hongyuan Flagship Store	Electricity conservation2	Received, already plugged in, looking forward to the effect.	Jingdong prefecture level city in Shandong
Jinfeng Hongyuan Flagship Store	Electricity conservation2	Opened to see the regular manufacturer products, wait for a period of time to see it	Jingdong prefecture level city in Shandong
Jinfeng Hongyuan Flagship Store	Electricity conservation2	🉑 Baby received, very surprised ✨, reasonable design, fashionable and generous, continue to pay attention to the future will be repurchased ❤❤❤❤	Jingdong prefecture level city in Shandong
Jinfeng Hongyuan Flagship Store	Electricity conservation2	Baby received it 👌, really great ah! 🌈The quality can't be beat, very satisfied! 🆗Happy happy happy 😃	Jingdong prefecture level city in Shandong
Jinfeng Hongyuan Flagship Store	Electricity conservation2	Very good, power saving see, this is to help a friend to buy, I used to give my friend a say, my friend immediately let me order another one!	Jingdong prefecture level city in Shandong
Jinfeng Hongyuan Flagship Store	Electricity conservation2	The baby is just as introduced, the texture is very good, powerful, and it feels good to use it	Jingdong prefecture level city in Shandong
Jinfeng Hongyuan Flagship Store	Electricity conservation2	Value for money, very satisfied, seller service attitude is very good, very patient	Jingdong prefecture level city in Shandong
Jinfeng Hongyuan Flagship Store	Electricity conservation2	Power saving treasure design unique exquisite beautiful, voltage display clearly, plugged in that is good to use, I observed the meter, after using the meter is gone slow.	Jingdong prefecture level city in Shandong
Jinfeng Hongyuan Flagship Store	Electricity conservation2	Received on the use of the packaging is beautifully used for a period of time and then come back in the follow-up review!	Jingdong prefecture level city in Shandong
Jinfeng Hongyuan Flagship Store	Electricity conservation2	It should work. Not bad. Not bad. Not bad.	Jingdong prefecture level city in Shandong
Jinfeng Hongyuan Flagship Store	Electricity conservation2	I haven't used it yet, I'll plug it in and see if it saves power next month.	Jingdong prefecture level city in Shandong
Jinfeng Hongyuan Flagship Store	Electricity conservation2	The goods received and unpacked and used, the quality is very good, things and the official website to buy no difference!	Jingdong prefecture level city in Shandong
Jinfeng Hongyuan Flagship Store	Electricity conservation2	It's a rip off, doesn't help much at all, uses the same amount of power as before it was used.	Jingdong prefecture level city in Shandong
Jinfeng Hongyuan Flagship Store	Electricity conservation2	I've been using it for a while now and haven't noticed any unusual problems, so it's okay	Jingdong prefecture level city in Shandong
Jinfeng Hongyuan Flagship Store	Electricity conservation2	Received the goods and plugged in to use, good or not there is no use will have to wait until next month to find out	Jingdong prefecture level city in Shandong
Jinfeng Hongyuan Flagship Store	Electricity conservation2	It's very compact, it looks good, I'll introduce you to my clients when it's ready.	Jingdong prefecture level city in Shandong
Jinfeng Hongyuan Flagship Store	Electricity conservation2	Good workmanship, we'll have to see in a few months.	Jingdong prefecture level city in Shandong
Jinfeng Hongyuan Flagship Store	Electricity conservation2	Jingdong quality! Always trustworthy!	Jingdong prefecture level city in Shandong
Jinfeng Hongyuan Flagship Store	Electricity conservation2	This is the second time I've bought it, and the merchant said to give me five dollars out of the difference, so thank you merchant.	Jingdong prefecture level city in Shandong
Jinfeng Hongyuan Flagship Store	Electricity conservation2	Good quality and good service	Jingdong prefecture level city in Shandong
Jinfeng Hongyuan Flagship Store	Electricity conservation2	Feels good texture, aluminum alloy shell, voltage digital display clear.	Jingdong prefecture level city in Shandong
Jinfeng Hongyuan Flagship Store	Electricity conservation2	Good product, fast and great service, thanks!	Jingdong prefecture level city in Shandong
Jinfeng Hongyuan Flagship Store	Electricity conservation2	Have not yet used, first evaluation, later really good do not need to comment again.	Jingdong prefecture level city in Shandong
Jinfeng Hongyuan Flagship Store	Electricity conservation2	Works great. Pretty much as described by the merchant.	Jingdong prefecture level city in Shandong

Jinfeng Hongyuan Flagship Store	Electricity conservation2	Received it, looking forward to the results, hope it works	Jingdong prefecture level city in Shandong
Jinfeng Hongyuan Flagship Store	Electricity conservation2	Received it, looking forward to the results, hope it works	Jingdong prefecture level city in Shandong
Jinfeng Hongyuan Flagship Store	Electricity conservation2	Supposedly it's an IQ tax. Buy one and try it. Looks good. Well made.	Jingdong prefecture level city in Shandong
Jinfeng Hongyuan Flagship Store	Electricity conservation2	It just doesn't feel like it's working.	Jingdong prefecture level city in Shandong
Jinfeng Hongyuan Flagship Store	Electricity conservation2	This is a great product.	Jingdong prefecture level city in Shandong
Jinfeng Hongyuan Flagship Store	Electricity conservation2	I'm just using it. I'll tell you more.	Jingdong prefecture level city in Shandong
Jinfeng Hongyuan Flagship Store	Electricity conservation2	Second repurchase and it's working fine.	Jingdong prefecture level city in Shandong
Jinfeng Hongyuan Flagship Store	Electricity conservation2	I'll wait a few days before evaluating	Jingdong prefecture level city in Shandong
Jinfeng Hongyuan Flagship Store	Electricity conservation2	I'll comment after I use it hopefully the section will be useful.	Jingdong prefecture level city in Shandong
Jinfeng Hongyuan Flagship Store	Electricity conservation2	not very useful	Jingdong prefecture level city in Shandong
Jinfeng Hongyuan Flagship Store	Electricity conservation2	Not received	Jingdong prefecture level city in Shandong
Jinfeng Hongyuan Flagship Store	Electricity conservation2	rare	Jingdong prefecture level city in Shandong
Jinfeng Hongyuan Flagship Store	Electricity conservation2	It's good.	Jingdong prefecture level city in Shandong
Jinfeng Hongyuan Flagship Store	Electricity conservation2	just now	Jingdong prefecture level city in Shandong
Jinfeng Hongyuan Flagship Store	Electricity conservation2	serviceable	Jingdong prefecture level city in Shandong
Jinfeng Hongyuan Flagship Store	Electricity conservation2	desirable	Jingdong prefecture level city in Shandong
Jinfeng Hongyuan Flagship Store	Electricity conservation2	serviceable	Jingdong prefecture level city in Shandong
Jinfeng Hongyuan Flagship Store	Electricity conservation2	It doesn't save power and costs electricity.	Jingdong prefecture level city in Shandong
Jinfeng Hongyuan Flagship Store	Electricity conservation2	Not as good as I thought.	Jingdong prefecture level city in Shandong
Jinfeng Hongyuan Flagship Store	Electricity conservation2	Super bad review, it doesn't save power at all, but rather more power consumption	Jingdong prefecture level city in Shandong
Jinfeng Hongyuan Flagship Store	Electricity conservation2	Listen to others say that this can save electricity, I bought with the idea of trying, used for a period of time before coming to the evaluation, will be a little bit of use, not a lot of savings just, long-term use of the words can save more.	Jingdong prefecture level city in Shandong
Jinfeng Hongyuan Flagship Store	Electricity conservation2	Logistics is fast, the next day arrived, workmanship is very delicate a power saver, looking forward to the late effect, customer service patiently explain the recommended which is suitable for, installed in what position is the best, look forward to using the effect after a period of time!	Jingdong prefecture level city in Shandong
Jinfeng Hongyuan Flagship Store	Electricity conservation2	It's good to use, I feel that it has been saving electricity, anyway, not a loss is, I used to feel that the power consumption is big, now I feel much better, my friends are also using, I hope to use a long time will be able to, I wish the boss a prosperous business	Jingdong prefecture level city in Shandong
Jinfeng Hongyuan Flagship Store	Electricity conservation2	Should be very good, to be able to plan the circuit, save the home of the circuit to the extent of the savings, waste of money on electricity, good if a month this money back, hope always hope to be able to	Jingdong prefecture level city in Shandong
Jinfeng Hongyuan Flagship Store	Electricity conservation2	Express delivery is very strong, the goods and I saw in the business consistent, the use of the effect of follow-up evaluation	Jingdong prefecture level city in Shandong
Jinfeng Hongyuan Flagship Store	Electricity conservation2	I have been using this brand of power saver for many years and this time I saw this upgraded version and got it again. The main purpose of this product is to reduce the loss of electricity for a long time, so as to achieve the purpose of saving electricity.	Jingdong prefecture level city in Shandong
Jinfeng Hongyuan Flagship Store	Electricity conservation2	Logistics is fast and the product looks beautiful, looking forward to the effect.	Jingdong prefecture level city in Shandong
Jinfeng Hongyuan Flagship Store	Electricity conservation2	Product quality is good, energy saving a little effect, but the price is a little expensive, fast arrival, good service.	Jingdong prefecture level city in Shandong
Jinfeng Hongyuan Flagship Store	Electricity conservation2	The quality is very good, inexpensive, Jingdong self-supporting guaranteed, worth having!	Jingdong prefecture level city in Shandong
Jinfeng Hongyuan Flagship Store	Electricity conservation2	I don't know if it's saving electricity yet, I'll find out next time I pay my electric bill	Jingdong prefecture level city in Shandong
Jinfeng Hongyuan Flagship Store	Electricity conservation2	Favorable price quality assurance appearance design is beautiful and generous, fine workmanship	Jingdong prefecture level city in Shandong
Jinfeng Hongyuan Flagship Store	Electricity conservation2	I bought it, I don't know how effective it is? I don't know how the quality is? After this summer results will know! Looking forward to it!	Jingdong prefecture level city in Shandong
Jinfeng Hongyuan Flagship Store	Electricity conservation2	Goods received Packaging intact, look at the shell workmanship is also good, high-power appliances open voltage has obvious changes in the effectiveness of the	Jingdong prefecture level city in Shandong
Jinfeng Hongyuan Flagship Store	Electricity conservation2	The goods have been received logistics is very fast, the product is similar to the line, just wait to use a month to see if it can save electricity, stay tuned.	Jingdong prefecture level city in Shandong
Jinfeng Hongyuan Flagship Store	Electricity conservation2	Electricity is plugged in, not sure if it will work, hopefully it will work.	Jingdong prefecture level city in Shandong
Jinfeng Hongyuan Flagship Store	Electricity conservation2	The shell is aluminum alloy, very good workmanship, the actual effect of waiting for the addition of evaluation	Jingdong prefecture level city in Shandong
Jinfeng Hongyuan Flagship Store	Electricity conservation2	Received, already plugged in, looking forward to the effect.	Jingdong prefecture level city in Shandong
Jinfeng Hongyuan Flagship Store	Electricity conservation2	Opened to see the regular manufacturer products, wait for a period of time to see it	Jingdong prefecture level city in Shandong
Jinfeng Hongyuan Flagship Store	Electricity conservation2	🉑 Baby received, very surprised ✨, reasonable design, fashionable and generous, continue to pay attention to the future will be repurchased ❥❥❥❥	Jingdong prefecture level city in Shandong
Jinfeng Hongyuan Flagship Store	Electricity conservation2	Baby received it 👌, really great ah! 🌈The quality can't be beat, very satisfied! 🆗Happy happy happy 😃	Jingdong prefecture level city in Shandong
Jinfeng Hongyuan Flagship Store	Electricity conservation2	Very good, power saving see, this is to help a friend to buy, I used to give my friend a say, my friend immediately let me order another one!	Jingdong prefecture level city in Shandong
Jinfeng Hongyuan Flagship Store	Electricity conservation2	The baby is just as introduced, the texture is very good, powerful, and it feels good to use it	Jingdong prefecture level city in Shandong
Jinfeng Hongyuan Flagship Store	Electricity conservation2	Value for money, very satisfied, seller service attitude is very good, very patient	Jingdong prefecture level city in Shandong
Jinfeng Hongyuan Flagship Store	Electricity conservation2	Power saving treasure design unique exquisite beautiful, voltage display clearly, plugged in that is good to use, I observed the meter, after using the meter is gone slow.	Jingdong prefecture level city in Shandong
Jinfeng Hongyuan Flagship Store	Electricity conservation2	Received on the use of the packaging is beautifully used for a period of time and then come back in the follow-up review!	Jingdong prefecture level city in Shandong
Jinfeng Hongyuan Flagship Store	Electricity conservation2	It should work. Not bad. Not bad. Not bad.	Jingdong prefecture level city in Shandong
Jinfeng Hongyuan Flagship Store	Electricity conservation2	I haven't used it yet, I'll plug it in and see if it saves power next month.	Jingdong prefecture level city in Shandong
Jinfeng Hongyuan Flagship Store	Electricity conservation2	The goods received and unpacked and used, the quality is very good, things and the official website to buy no difference!	Jingdong prefecture level city in Shandong
Jinfeng Hongyuan Flagship Store	Electricity conservation2	It's a rip off, doesn't help much at all, uses the same amount of power as before it was used.	Jingdong prefecture level city in Shandong

Jinfeng Hongyuan Flagship Store	Electricity conservation2	I've been using it for a while now and haven't noticed any unusual problems, so it's okay	Jingdong prefecture level city in Shandong
Jinfeng Hongyuan Flagship Store	Electricity conservation2	Received the goods and plugged in to use, good or not there is no use will have to wait until next month to find out	Jingdong prefecture level city in Shandong
Jinfeng Hongyuan Flagship Store	Electricity conservation2	It's very compact, it looks good, I'll introduce you to my clients when it's ready.	Jingdong prefecture level city in Shandong
Jinfeng Hongyuan Flagship Store	Electricity conservation2	Good workmanship, we'll have to see in a few months.	Jingdong prefecture level city in Shandong
Jinfeng Hongyuan Flagship Store	Electricity conservation2	Jingdong quality! Always trustworthy!	Jingdong prefecture level city in Shandong
Jinfeng Hongyuan Flagship Store	Electricity conservation2	This is the second time I've bought it, and the merchant said to give me five dollars out of the difference, so thank you merchant.	Jingdong prefecture level city in Shandong
Jinfeng Hongyuan Flagship Store	Electricity conservation2	Good quality and good service	Jingdong prefecture level city in Shandong
Jinfeng Hongyuan Flagship Store	Electricity conservation2	Feels good texture, aluminum alloy shell, voltage digital display clear.	Jingdong prefecture level city in Shandong
Jinfeng Hongyuan Flagship Store	Electricity conservation2	Good product, fast and great service, thanks!	Jingdong prefecture level city in Shandong
Jinfeng Hongyuan Flagship Store	Electricity conservation2	Have not yet used, first evaluation, later really good do not need to comment again.	Jingdong prefecture level city in Shandong
Jinfeng Hongyuan Flagship Store	Electricity conservation2	Works great. Pretty much as described by the merchant.	Jingdong prefecture level city in Shandong
Jinfeng Hongyuan Flagship Store	Electricity conservation2	Received it, looking forward to the results, hope it works	Jingdong prefecture level city in Shandong
Jinfeng Hongyuan Flagship Store	Electricity conservation2	Received it, looking forward to the results, hope it works	Jingdong prefecture level city in Shandong
Jinfeng Hongyuan Flagship Store	Electricity conservation2	Supposedly it's an IQ tax. Buy one and try it. Looks good. Well made.	Jingdong prefecture level city in Shandong
Jinfeng Hongyuan Flagship Store	Electricity conservation2	It just doesn't feel like it's working.	Jingdong prefecture level city in Shandong
Jinfeng Hongyuan Flagship Store	Electricity conservation2	This is a great product.	Jingdong prefecture level city in Shandong
Jinfeng Hongyuan Flagship Store	Electricity conservation2	I'm just using it. I'll tell you more.	Jingdong prefecture level city in Shandong
Jinfeng Hongyuan Flagship Store	Electricity conservation2	Second repurchase and it's working fine.	Jingdong prefecture level city in Shandong
Jinfeng Hongyuan Flagship Store	Electricity conservation2	I'll wait a few days before evaluating	Jingdong prefecture level city in Shandong
Jinfeng Hongyuan Flagship Store	Electricity conservation2	I'll comment after I use it hopefully the section will be useful.	Jingdong prefecture level city in Shandong
Jinfeng Hongyuan Flagship Store	Electricity conservation2	not very useful	Jingdong prefecture level city in Shandong
Jinfeng Hongyuan Flagship Store	Electricity conservation2	Not received	Jingdong prefecture level city in Shandong
Jinfeng Hongyuan Flagship Store	Electricity conservation2	rare	Jingdong prefecture level city in Shandong
Jinfeng Hongyuan Flagship Store	Electricity conservation2	It's good.	Jingdong prefecture level city in Shandong
Jinfeng Hongyuan Flagship Store	Electricity conservation2	just now	Jingdong prefecture level city in Shandong
Jinfeng Hongyuan Flagship Store	Electricity conservation2	serviceable	Jingdong prefecture level city in Shandong
Jinfeng Hongyuan Flagship Store	Electricity conservation2	desirable	Jingdong prefecture level city in Shandong
Jinfeng Hongyuan Flagship Store	Electricity conservation2	serviceable	Jingdong prefecture level city in Shandong
Jinfeng Hongyuan Flagship Store	Electricity conservation2	It doesn't save power and costs electricity.	Jingdong prefecture level city in Shandong
Jinfeng Hongyuan Flagship Store	Electricity conservation2	Not as good as I thought.	Jingdong prefecture level city in Shandong
Jinfeng Hongyuan Flagship Store	Electricity conservation2	Super bad review, it doesn't save power at all, but rather more power consumption	Jingdong prefecture level city in Shandong
Jinfeng Hongyuan Flagship Store	Electricity conservation2	Listen to others say that this can save electricity, I bought with the idea of trying, used for a period of time before coming to the evaluation, will be a little bit of use, not a lot of savings just, long-term use of the words can save more.	Jingdong prefecture level city in Shandong
Jinfeng Hongyuan Flagship Store	Electricity conservation2	Logistics is fast, the next day arrived, workmanship is very delicate a power saver, looking forward to the late effect, customer service patiently explain the recommended which is suitable for, installed in what position is the best, look forward to using the effect after a period of time!	Jingdong prefecture level city in Shandong
Jinfeng Hongyuan Flagship Store	Electricity conservation2	It's good to use, I feel that it has been saving electricity, anyway, not a loss is, I used to feel that the power consumption is big, now I feel much better, my friends are also using, I hope to use a long time will be able to, I wish the boss a prosperous business	Jingdong prefecture level city in Shandong
Jinfeng Hongyuan Flagship Store	Electricity conservation2	Should be very good, to be able to plan the circuit, save the home of the circuit to the extent of the savings, waste of money on electricity, good if a month this money back, hope always hope to be able to	Jingdong prefecture level city in Shandong
Jinfeng Hongyuan Flagship Store	Electricity conservation2	Express delivery is very strong, the goods and I saw in the business consistent, the use of the effect of follow-up evaluation	Jingdong prefecture level city in Shandong
Jinfeng Hongyuan Flagship Store	Electricity conservation2	I have been using this brand of power saver for many years and this time I saw this upgraded version and got it again. The main purpose of this product is to reduce the loss of electricity for a long time, so as to achieve the purpose of saving electricity.	Jingdong prefecture level city in Shandong
Jinfeng Hongyuan Flagship Store	Electricity conservation2	Logistics is fast and the product looks beautiful, looking forward to the effect.	Jingdong prefecture level city in Shandong
Jinfeng Hongyuan Flagship Store	Electricity conservation2	Product quality is good, energy saving a little effect, but the price is a little expensive, fast arrival, good service.	Jingdong prefecture level city in Shandong
Jinfeng Hongyuan Flagship Store	Electricity conservation2	The quality is very good, inexpensive, Jingdong self-supporting guaranteed, worth having!	Jingdong prefecture level city in Shandong
Jinfeng Hongyuan Flagship Store	Electricity conservation2	I don't know if it's saving electricity yet, I'll find out next time I pay my electric bill	Jingdong prefecture level city in Shandong
Jinfeng Hongyuan Flagship Store	Electricity conservation2	Favorable price quality assurance appearance design is beautiful and generous, fine workmanship	Jingdong prefecture level city in Shandong
Jinfeng Hongyuan Flagship Store	Electricity conservation2	I bought it, I don't know how effective it is? I don't know how the quality is? After this summer results will know! Looking forward to it!	Jingdong prefecture level city in Shandong
Jinfeng Hongyuan Flagship Store	Electricity conservation2	Goods received Packaging intact, look at the shell workmanship is also good, high-power appliances open voltage has obvious changes in the effectiveness of the	Jingdong prefecture level city in Shandong
Jinfeng Hongyuan Flagship Store	Electricity conservation2	The goods have been received logistics is very fast, the product is similar to the line, just wait to use a month to see if it can save electricity, stay tuned.	Jingdong prefecture level city in Shandong
Jinfeng Hongyuan Flagship Store	Electricity conservation2	Electricity is plugged in, not sure if it will work, hopefully it will work.	Jingdong prefecture level city in Shandong
Jinfeng Hongyuan Flagship Store	Electricity conservation2	The shell is aluminum alloy, very good workmanship, the actual effect of waiting for the addition of evaluation	Jingdong prefecture level city in Shandong
Jinfeng Hongyuan Flagship Store	Electricity conservation2	Received, already plugged in, looking forward to the effect.	Jingdong prefecture level city in Shandong
Jinfeng Hongyuan Flagship Store	Electricity conservation2	Opened to see the regular manufacturer products, wait for a period of time to see it	Jingdong prefecture level city in Shandong

Jinfeng Hongyuan Flagship Store	Electricity conservation2	🉑 Baby received, very surprised ✨, reasonable design, fashionable and generous, continue to pay attention to the future will be repurchased ❤❤❤❤	Jingdong prefecture level city in Shandong
Jinfeng Hongyuan Flagship Store	Electricity conservation2	Baby received it 👌, really great ah! 🌈The quality can't be beat, very satisfied! 🆗Happy happy happy 😃	Jingdong prefecture level city in Shandong
Jinfeng Hongyuan Flagship Store	Electricity conservation2	Very good, power saving see, this is to help a friend to buy, I used to give my friend a say, my friend immediately let me order another one!	Jingdong prefecture level city in Shandong
Jinfeng Hongyuan Flagship Store	Electricity conservation2	The baby is just as introduced, the texture is very good, powerful, and it feels good to use it	Jingdong prefecture level city in Shandong
Jinfeng Hongyuan Flagship Store	Electricity conservation2	Value for money, very satisfied, seller service attitude is very good, very patient	Jingdong prefecture level city in Shandong
Jinfeng Hongyuan Flagship Store	Electricity conservation2	Power saving treasure design unique exquisite beautiful, voltage display clearly, plugged in that is good to use, I observed the meter, after using the meter is gone slow.	Jingdong prefecture level city in Shandong
Jinfeng Hongyuan Flagship Store	Electricity conservation2	Received on the use of the packaging is beautifully used for a period of time and then come back in the follow-up review!	Jingdong prefecture level city in Shandong
Jinfeng Hongyuan Flagship Store	Electricity conservation2	It should work. Not bad. Not bad. Not bad.	Jingdong prefecture level city in Shandong
Jinfeng Hongyuan Flagship Store	Electricity conservation2	I haven't used it yet, I'll plug it in and see if it saves power next month.	Jingdong prefecture level city in Shandong
Jinfeng Hongyuan Flagship Store	Electricity conservation2	The goods received and unpacked and used, the quality is very good, things and the official website to buy no difference!	Jingdong prefecture level city in Shandong
Jinfeng Hongyuan Flagship Store	Electricity conservation2	It's a rip off, doesn't help much at all, uses the same amount of power as before it was used.	Jingdong prefecture level city in Shandong
Jinfeng Hongyuan Flagship Store	Electricity conservation2	I've been using it for a while now and haven't noticed any unusual problems, so it's okay	Jingdong prefecture level city in Shandong
Jinfeng Hongyuan Flagship Store	Electricity conservation2	Received the goods and plugged in to use, good or not there is no use will have to wait until next month to find out	Jingdong prefecture level city in Shandong
Jinfeng Hongyuan Flagship Store	Electricity conservation2	It's very compact, it looks good, I'll introduce you to my clients when it's ready.	Jingdong prefecture level city in Shandong
Jinfeng Hongyuan Flagship Store	Electricity conservation2	Good workmanship, we'll have to see in a few months.	Jingdong prefecture level city in Shandong
Jinfeng Hongyuan Flagship Store	Electricity conservation2	Jingdong quality! Always trustworthy!	Jingdong prefecture level city in Shandong
Jinfeng Hongyuan Flagship Store	Electricity conservation2	This is the second time I've bought it, and the merchant said to give me five dollars out of the difference, so thank you merchant.	Jingdong prefecture level city in Shandong
Jinfeng Hongyuan Flagship Store	Electricity conservation2	Good quality and good service	Jingdong prefecture level city in Shandong
Jinfeng Hongyuan Flagship Store	Electricity conservation2	Feels good texture, aluminum alloy shell, voltage digital display clear.	Jingdong prefecture level city in Shandong
Jinfeng Hongyuan Flagship Store	Electricity conservation2	Good product, fast and great service, thanks!	Jingdong prefecture level city in Shandong
Jinfeng Hongyuan Flagship Store	Electricity conservation2	Have not yet used, first evaluation, later really good do not need to comment again.	Jingdong prefecture level city in Shandong
Jinfeng Hongyuan Flagship Store	Electricity conservation2	Works great. Pretty much as described by the merchant.	Jingdong prefecture level city in Shandong
Jinfeng Hongyuan Flagship Store	Electricity conservation2	Received it, looking forward to the results, hope it works	Jingdong prefecture level city in Shandong
Jinfeng Hongyuan Flagship Store	Electricity conservation2	Received it, looking forward to the results, hope it works	Jingdong prefecture level city in Shandong
Jinfeng Hongyuan Flagship Store	Electricity conservation2	Supposedly it's an IQ tax. Buy one and try it. Looks good. Well made.	Jingdong prefecture level city in Shandong
Jinfeng Hongyuan Flagship Store	Electricity conservation2	It just doesn't feel like it's working.	Jingdong prefecture level city in Shandong
Jinfeng Hongyuan Flagship Store	Electricity conservation2	This is a great product.	Jingdong prefecture level city in Shandong
Jinfeng Hongyuan Flagship Store	Electricity conservation2	I'm just using it. I'll tell you more.	Jingdong prefecture level city in Shandong
Jinfeng Hongyuan Flagship Store	Electricity conservation2	Second repurchase and it's working fine.	Jingdong prefecture level city in Shandong
Jinfeng Hongyuan Flagship Store	Electricity conservation2	I'll wait a few days before evaluating	Jingdong prefecture level city in Shandong
Jinfeng Hongyuan Flagship Store	Electricity conservation2	I'll comment after I use it hopefully the section will be useful.	Jingdong prefecture level city in Shandong
Jinfeng Hongyuan Flagship Store	Electricity conservation2	not very useful	Jingdong prefecture level city in Shandong
Jinfeng Hongyuan Flagship Store	Electricity conservation2	Not received	Jingdong prefecture level city in Shandong
Jinfeng Hongyuan Flagship Store	Electricity conservation2	rare	Jingdong prefecture level city in Shandong
Jinfeng Hongyuan Flagship Store	Electricity conservation2	It's good.	Jingdong prefecture level city in Shandong
Jinfeng Hongyuan Flagship Store	Electricity conservation2	just now	Jingdong prefecture level city in Shandong
Jinfeng Hongyuan Flagship Store	Electricity conservation2	serviceable	Jingdong prefecture level city in Shandong
Jinfeng Hongyuan Flagship Store	Electricity conservation2	desirable	Jingdong prefecture level city in Shandong
Jinfeng Hongyuan Flagship Store	Electricity conservation2	serviceable	Jingdong prefecture level city in Shandong
Jinfeng Hongyuan Flagship Store	Electricity conservation2	It doesn't save power and costs electricity.	Jingdong prefecture level city in Shandong
Jinfeng Hongyuan Flagship Store	Electricity conservation2	Not as good as I thought.	Jingdong prefecture level city in Shandong
Jinfeng Hongyuan Flagship Store	Electricity conservation2	Super bad review, it doesn't save power at all, but rather more power consumption	Jingdong prefecture level city in Shandong
Jinfeng Hongyuan Flagship Store	Electricity conservation2	Listen to others say that this can save electricity, I bought with the idea of trying, used for a period of time before coming to the evaluation, will be a little bit of use, not a lot of savings just, long-term use of the words can save more.	Jingdong prefecture level city in Shandong
Jinfeng Hongyuan Flagship Store	Electricity conservation2	Logistics is fast, the next day arrived, workmanship is very delicate a power saver, looking forward to the late effect, customer service patiently explain the recommended which is suitable for, installed in what position is the best, look forward to using the effect after a period of time!	Jingdong prefecture level city in Shandong
Jinfeng Hongyuan Flagship Store	Electricity conservation2	It's good to use, I feel that it has been saving electricity, anyway, not a loss is, I used to feel that the power consumption is big, now I feel much better, my friends are also using, I hope to use a long time will be able to, I wish the boss a prosperous business	Jingdong prefecture level city in Shandong
Jinfeng Hongyuan Flagship Store	Electricity conservation2	Should be very good, to be able to plan the circuit, save the home of the circuit to the extent of the savings, waste of money on electricity, good if a month this money back, hope always hope to be able to	Jingdong prefecture level city in Shandong
Jinfeng Hongyuan Flagship Store	Electricity conservation2	Express delivery is very strong, the goods and I saw in the business consistent, the use of the effect of follow-up evaluation	Jingdong prefecture level city in Shandong
Jinfeng Hongyuan Flagship Store	Electricity conservation2	I have been using this brand of power saver for many years and this time I saw this upgraded version and got it again. The main purpose of this product is to reduce the loss of electricity for a long time, so as to achieve the purpose of saving electricity.	Jingdong prefecture level city in Shandong
Jinfeng Hongyuan Flagship Store	Electricity conservation2	Logistics is fast and the product looks beautiful, looking forward to the effect.	Jingdong prefecture level city in Shandong

Jinfeng Hongyuan Flagship Store	Electricity conservation2	Product quality is good, energy saving a little effect, but the price is a little expensive, fast arrival, good service.	Jingdong prefecture level city in Shandong
Jinfeng Hongyuan Flagship Store	Electricity conservation2	The quality is very good, inexpensive, Jingdong self-supporting guaranteed, worth having!	Jingdong prefecture level city in Shandong
Jinfeng Hongyuan Flagship Store	Electricity conservation2	I don't know if it's saving electricity yet, I'll find out next time I pay my electric bill	Jingdong prefecture level city in Shandong
Jinfeng Hongyuan Flagship Store	Electricity conservation2	Favorable price quality assurance appearance design is beautiful and generous, fine workmanship	Jingdong prefecture level city in Shandong
Jinfeng Hongyuan Flagship Store	Electricity conservation2	I bought it, I don't know how effective it is? I don't know how the quality is? After this summer results will know! Looking forward to it!	Jingdong prefecture level city in Shandong
Jinfeng Hongyuan Flagship Store	Electricity conservation2	Goods received Packaging intact, look at the shell workmanship is also good, high-power appliances open voltage has obvious changes in the effectiveness of the	Jingdong prefecture level city in Shandong
Jinfeng Hongyuan Flagship Store	Electricity conservation2	The goods have been received logistics is very fast, the product is similar to the line, just wait to use a month to see if it can save electricity, stay tuned.	Jingdong prefecture level city in Shandong
Jinfeng Hongyuan Flagship Store	Electricity conservation2	Electricity is plugged in, not sure if it will work, hopefully it will work.	Jingdong prefecture level city in Shandong
Jinfeng Hongyuan Flagship Store	Electricity conservation2	The shell is aluminum alloy, very good workmanship, the actual effect of waiting for the addition of evaluation	Jingdong prefecture level city in Shandong
Jinfeng Hongyuan Flagship Store	Electricity conservation2	Received, already plugged in, looking forward to the effect.	Jingdong prefecture level city in Shandong
Jinfeng Hongyuan Flagship Store	Electricity conservation2	Opened to see the regular manufacturer products, wait for a period of time to see it	Jingdong prefecture level city in Shandong
Jinfeng Hongyuan Flagship Store	Electricity conservation2	🉑 Baby received, very surprised ✨, reasonable design, fashionable and generous, continue to pay attention to the future will be repurchased ❥❥❥❥	Jingdong prefecture level city in Shandong
Jinfeng Hongyuan Flagship Store	Electricity conservation2	Baby received it 👌, really great ah! 🌈The quality can't be beat, very satisfied! 🆗Happy happy happy 😃	Jingdong prefecture level city in Shandong
Jinfeng Hongyuan Flagship Store	Electricity conservation2	Very good, power saving see, this is to help a friend to buy, I used to give my friend a say, my friend immediately let me order another one!	Jingdong prefecture level city in Shandong
Jinfeng Hongyuan Flagship Store	Electricity conservation2	The baby is just as introduced, the texture is very good, powerful, and it feels good to use it	Jingdong prefecture level city in Shandong
Jinfeng Hongyuan Flagship Store	Electricity conservation2	Value for money, very satisfied, seller service attitude is very good, very patient	Jingdong prefecture level city in Shandong
Jinfeng Hongyuan Flagship Store	Electricity conservation2	Power saving treasure design unique exquisite beautiful, voltage display clearly, plugged in that is good to use, I observed the meter, after using the meter is gone slow.	Jingdong prefecture level city in Shandong
Jinfeng Hongyuan Flagship Store	Electricity conservation2	Received on the use of the packaging is beautifully used for a period of time and then come back in the follow-up review!	Jingdong prefecture level city in Shandong
Jinfeng Hongyuan Flagship Store	Electricity conservation2	It should work. Not bad. Not bad. Not bad.	Jingdong prefecture level city in Shandong
Jinfeng Hongyuan Flagship Store	Electricity conservation2	I haven't used it yet, I'll plug it in and see if it saves power next month.	Jingdong prefecture level city in Shandong
Jinfeng Hongyuan Flagship Store	Electricity conservation2	The goods received and unpacked and used, the quality is very good, things and the official website to buy no difference!	Jingdong prefecture level city in Shandong
Jinfeng Hongyuan Flagship Store	Electricity conservation2	It's a rip off, doesn't help much at all, uses the same amount of power as before it was used.	Jingdong prefecture level city in Shandong
Jinfeng Hongyuan Flagship Store	Electricity conservation2	I've been using it for a while now and haven't noticed any unusual problems, so it's okay	Jingdong prefecture level city in Shandong
Jinfeng Hongyuan Flagship Store	Electricity conservation2	Received the goods and plugged in to use, good or not there is no use will have to wait until next month to find out	Jingdong prefecture level city in Shandong
Jinfeng Hongyuan Flagship Store	Electricity conservation2	It's very compact, it looks good, I'll introduce you to my clients when it's ready.	Jingdong prefecture level city in Shandong
Jinfeng Hongyuan Flagship Store	Electricity conservation2	Good workmanship, we'll have to see in a few months.	Jingdong prefecture level city in Shandong
Jinfeng Hongyuan Flagship Store	Electricity conservation2	Jingdong quality! Always trustworthy!	Jingdong prefecture level city in Shandong
Jinfeng Hongyuan Flagship Store	Electricity conservation2	This is the second time I've bought it, and the merchant said to give me five dollars out of the difference, so thank you merchant.	Jingdong prefecture level city in Shandong
Jinfeng Hongyuan Flagship Store	Electricity conservation2	Good quality and good service	Jingdong prefecture level city in Shandong
Jinfeng Hongyuan Flagship Store	Electricity conservation2	Feels good texture, aluminum alloy shell, voltage digital display clear.	Jingdong prefecture level city in Shandong
Jinfeng Hongyuan Flagship Store	Electricity conservation2	Good product, fast and great service, thanks!	Jingdong prefecture level city in Shandong
Jinfeng Hongyuan Flagship Store	Electricity conservation2	Have not yet used, first evaluation, later really good do not need to comment again.	Jingdong prefecture level city in Shandong
Jinfeng Hongyuan Flagship Store	Electricity conservation2	Works great. Pretty much as described by the merchant.	Jingdong prefecture level city in Shandong
Jinfeng Hongyuan Flagship Store	Electricity conservation2	Received it, looking forward to the results, hope it works	Jingdong prefecture level city in Shandong
Jinfeng Hongyuan Flagship Store	Electricity conservation2	Received it, looking forward to the results, hope it works	Jingdong prefecture level city in Shandong
Jinfeng Hongyuan Flagship Store	Electricity conservation2	Supposedly it's an IQ tax. Buy one and try it. Looks good. Well made.	Jingdong prefecture level city in Shandong
Jinfeng Hongyuan Flagship Store	Electricity conservation2	It just doesn't feel like it's working.	Jingdong prefecture level city in Shandong
Jinfeng Hongyuan Flagship Store	Electricity conservation2	This is a great product.	Jingdong prefecture level city in Shandong
Jinfeng Hongyuan Flagship Store	Electricity conservation2	I'm just using it. I'll tell you more.	Jingdong prefecture level city in Shandong
Jinfeng Hongyuan Flagship Store	Electricity conservation2	Second repurchase and it's working fine.	Jingdong prefecture level city in Shandong
Jinfeng Hongyuan Flagship Store	Electricity conservation2	I'll wait a few days before evaluating	Jingdong prefecture level city in Shandong
Jinfeng Hongyuan Flagship Store	Electricity conservation2	I'll comment after I use it hopefully the section will be useful.	Jingdong prefecture level city in Shandong
Jinfeng Hongyuan Flagship Store	Electricity conservation2	not very useful	Jingdong prefecture level city in Shandong
Jinfeng Hongyuan Flagship Store	Electricity conservation2	Not received	Jingdong prefecture level city in Shandong
Jinfeng Hongyuan Flagship Store	Electricity conservation2	rare	Jingdong prefecture level city in Shandong
Jinfeng Hongyuan Flagship Store	Electricity conservation2	It's good.	Jingdong prefecture level city in Shandong
Jinfeng Hongyuan Flagship Store	Electricity conservation2	just now	Jingdong prefecture level city in Shandong
Jinfeng Hongyuan Flagship Store	Electricity conservation2	serviceable	Jingdong prefecture level city in Shandong
Jinfeng Hongyuan Flagship Store	Electricity conservation2	desirable	Jingdong prefecture level city in Shandong
Jinfeng Hongyuan Flagship Store	Electricity conservation2	serviceable	Jingdong prefecture level city in Shandong
Jinfeng Hongyuan Flagship Store	Electricity conservation2	It doesn't save power and costs electricity.	Jingdong prefecture level city in Shandong

Jinfeng Hongyuan Flagship Store	Electricity conservation2	Not as good as I thought.	Jingdong prefecture level city in Shandong
Jinfeng Hongyuan Flagship Store	Electricity conservation2	Super bad review, it doesn't save power at all, but rather more power consumption	Jingdong prefecture level city in Shandong
Jinfeng Hongyuan Flagship Store	Electricity conservation2	Listen to others say that this can save electricity, I bought with the idea of trying, used for a period of time before coming to the evaluation, will be a little bit of use, not a lot of savings just, long-term use of the words can save more.	Jingdong prefecture level city in Shandong
Jinfeng Hongyuan Flagship Store	Electricity conservation2	Logistics is fast, the next day arrived, workmanship is very delicate a power saver, looking forward to the late effect, customer service patiently explain the recommended which is suitable for, installed in what position is the best, look forward to using the effect after a period of time!	Jingdong prefecture level city in Shandong
Jinfeng Hongyuan Flagship Store	Electricity conservation2	It's good to use, I feel that it has been saving electricity, anyway, not a loss is, I used to feel that the power consumption is big, now I feel much better, my friends are also using, I hope to use a long time will be able to, I wish the boss a prosperous business	Jingdong prefecture level city in Shandong
Jinfeng Hongyuan Flagship Store	Electricity conservation2	Should be very good, to be able to plan the circuit, save the home of the circuit to the extent of the savings, waste of money on electricity, good if a month this money back, hope always hope to be able to	Jingdong prefecture level city in Shandong
Jinfeng Hongyuan Flagship Store	Electricity conservation2	Express delivery is very strong, the goods and I saw in the business consistent, the use of the effect of follow-up evaluation	Jingdong prefecture level city in Shandong
Jinfeng Hongyuan Flagship Store	Electricity conservation2	I have been using this brand of power saver for many years and this time I saw this upgraded version and got it again. The main purpose of this product is to reduce the loss of electricity for a long time, so as to achieve the purpose of saving electricity.	Jingdong prefecture level city in Shandong
Jinfeng Hongyuan Flagship Store	Electricity conservation2	Logistics is fast and the product looks beautiful, looking forward to the effect.	Jingdong prefecture level city in Shandong
Jinfeng Hongyuan Flagship Store	Electricity conservation2	Product quality is good, energy saving a little effect, but the price is a little expensive, fast arrival, good service.	Jingdong prefecture level city in Shandong
Jinfeng Hongyuan Flagship Store	Electricity conservation2	The quality is very good, inexpensive, Jingdong self-supporting guaranteed, worth having!	Jingdong prefecture level city in Shandong
Jinfeng Hongyuan Flagship Store	Electricity conservation2	I don't know if it's saving electricity yet, I'll find out next time I pay my electric bill	Jingdong prefecture level city in Shandong
Jinfeng Hongyuan Flagship Store	Electricity conservation2	Favorable price quality assurance appearance design is beautiful and generous, fine workmanship	Jingdong prefecture level city in Shandong
Jinfeng Hongyuan Flagship Store	Electricity conservation2	I bought it, I don't know how effective it is? I don't know how the quality is? After this summer results will know! Looking forward to it!	Jingdong prefecture level city in Shandong
Jinfeng Hongyuan Flagship Store	Electricity conservation2	Goods received Packaging intact, look at the shell workmanship is also good, high-power appliances open voltage has obvious changes in the effectiveness of the	Jingdong prefecture level city in Shandong
Jinfeng Hongyuan Flagship Store	Electricity conservation2	The goods have been received logistics is very fast, the product is similar to the line, just wait to use a month to see if it can save electricity, stay tuned.	Jingdong prefecture level city in Shandong
Jinfeng Hongyuan Flagship Store	Electricity conservation2	Electricity is plugged in, not sure if it will work, hopefully it will work.	Jingdong prefecture level city in Shandong
Jinfeng Hongyuan Flagship Store	Electricity conservation2	The shell is aluminum alloy, very good workmanship, the actual effect of waiting for the addition of evaluation	Jingdong prefecture level city in Shandong
Jinfeng Hongyuan Flagship Store	Electricity conservation2	Received, already plugged in, looking forward to the effect.	Jingdong prefecture level city in Shandong
Jinfeng Hongyuan Flagship Store	Electricity conservation2	Opened to see the regular manufacturer products, wait for a period of time to see it	Jingdong prefecture level city in Shandong
Jinfeng Hongyuan Flagship Store	Electricity conservation2	🉑 Baby received, very surprised ✨, reasonable design, fashionable and generous, continue to pay attention to the future will be repurchased ❤❤❤❤	Jingdong prefecture level city in Shandong
Jinfeng Hongyuan Flagship Store	Electricity conservation2	Baby received it 👌, really great ah! 🌈The quality can't be beat, very satisfied! 🆗Happy happy happy 😀	Jingdong prefecture level city in Shandong
Jinfeng Hongyuan Flagship Store	Electricity conservation2	Very good, power saving see, this is to help a friend to buy, I used to give my friend a say, my friend immediately let me order another one!	Jingdong prefecture level city in Shandong
Jinfeng Hongyuan Flagship Store	Electricity conservation2	The baby is just as introduced, the texture is very good, powerful, and it feels good to use it	Jingdong prefecture level city in Shandong
Jinfeng Hongyuan Flagship Store	Electricity conservation2	Value for money, very satisfied, seller service attitude is very good, very patient	Jingdong prefecture level city in Shandong
Jinfeng Hongyuan Flagship Store	Electricity conservation2	Power saving treasure design unique exquisite beautiful, voltage display clearly, plugged in that is good to use, I observed the meter, after using the meter is gone slow.	Jingdong prefecture level city in Shandong
Jinfeng Hongyuan Flagship Store	Electricity conservation2	Received on the use of the packaging is beautifully used for a period of time and then come back in the follow-up review!	Jingdong prefecture level city in Shandong
Jinfeng Hongyuan Flagship Store	Electricity conservation2	It should work. Not bad. Not bad. Not bad.	Jingdong prefecture level city in Shandong
Jinfeng Hongyuan Flagship Store	Electricity conservation2	I haven't used it yet, I'll plug it in and see if it saves power next month.	Jingdong prefecture level city in Shandong
Jinfeng Hongyuan Flagship Store	Electricity conservation2	The goods received and unpacked and used, the quality is very good, things and the official website to buy no difference!	Jingdong prefecture level city in Shandong
Jinfeng Hongyuan Flagship Store	Electricity conservation2	It's a rip off, doesn't help much at all, uses the same amount of power as before it was used.	Jingdong prefecture level city in Shandong
Jinfeng Hongyuan Flagship Store	Electricity conservation2	I've been using it for a while now and haven't noticed any unusual problems, so it's okay	Jingdong prefecture level city in Shandong
Jinfeng Hongyuan Flagship Store	Electricity conservation2	Received the goods and plugged in to use, good or not there is no use will have to wait until next month to find out	Jingdong prefecture level city in Shandong
Jinfeng Hongyuan Flagship Store	Electricity conservation2	It's very compact, it looks good, I'll introduce you to my clients when it's ready.	Jingdong prefecture level city in Shandong
Jinfeng Hongyuan Flagship Store	Electricity conservation2	Good workmanship, we'll have to see in a few months.	Jingdong prefecture level city in Shandong
Jinfeng Hongyuan Flagship Store	Electricity conservation2	Jingdong quality! Always trustworthy!	Jingdong prefecture level city in Shandong
Jinfeng Hongyuan Flagship Store	Electricity conservation2	This is the second time I've bought it, and the merchant said to give me five dollars out of the difference, so thank you merchant.	Jingdong prefecture level city in Shandong
Jinfeng Hongyuan Flagship Store	Electricity conservation2	Good quality and good service	Jingdong prefecture level city in Shandong
Jinfeng Hongyuan Flagship Store	Electricity conservation2	Feels good texture, aluminum alloy shell, voltage digital display clear.	Jingdong prefecture level city in Shandong
Jinfeng Hongyuan Flagship Store	Electricity conservation2	Good product, fast and great service, thanks!	Jingdong prefecture level city in Shandong
Jinfeng Hongyuan Flagship Store	Electricity conservation2	Have not yet used, first evaluation, later really good do not need to comment again.	Jingdong prefecture level city in Shandong
Jinfeng Hongyuan Flagship Store	Electricity conservation2	Works great. Pretty much as described by the merchant.	Jingdong prefecture level city in Shandong
Jinfeng Hongyuan Flagship Store	Electricity conservation2	Received it, looking forward to the results, hope it works	Jingdong prefecture level city in Shandong
Jinfeng Hongyuan Flagship Store	Electricity conservation2	Received it, looking forward to the results, hope it works	Jingdong prefecture level city in Shandong
Jinfeng Hongyuan Flagship Store	Electricity conservation2	Supposedly it's an IQ tax. Buy one and try it. Looks good. Well made.	Jingdong prefecture level city in Shandong
Jinfeng Hongyuan Flagship Store	Electricity conservation2	It just doesn't feel like it's working.	Jingdong prefecture level city in Shandong
Jinfeng Hongyuan Flagship Store	Electricity conservation2	This is a great product.	Jingdong prefecture level city in Shandong
Jinfeng Hongyuan Flagship Store	Electricity conservation2	I'm just using it. I'll tell you more.	Jingdong prefecture level city in Shandong
Jinfeng Hongyuan Flagship Store	Electricity conservation2	Second repurchase and it's working fine.	Jingdong prefecture level city in Shandong

Jinfeng Hongyuan Flagship Store	Electricity conservation2	I'll wait a few days before evaluating	Jingdong prefecture level city in Shandong
Jinfeng Hongyuan Flagship Store	Electricity conservation2	I'll comment after I use it hopefully the section will be useful.	Jingdong prefecture level city in Shandong
Jinfeng Hongyuan Flagship Store	Electricity conservation2	not very useful	Jingdong prefecture level city in Shandong
Jinfeng Hongyuan Flagship Store	Electricity conservation2	Not received	Jingdong prefecture level city in Shandong
Jinfeng Hongyuan Flagship Store	Electricity conservation2	rare	Jingdong prefecture level city in Shandong
Jinfeng Hongyuan Flagship Store	Electricity conservation2	It's good.	Jingdong prefecture level city in Shandong
Jinfeng Hongyuan Flagship Store	Electricity conservation2	just now	Jingdong prefecture level city in Shandong
Jinfeng Hongyuan Flagship Store	Electricity conservation2	serviceable	Jingdong prefecture level city in Shandong
Jinfeng Hongyuan Flagship Store	Electricity conservation2	desirable	Jingdong prefecture level city in Shandong
Jinfeng Hongyuan Flagship Store	Electricity conservation2	serviceable	Jingdong prefecture level city in Shandong
Jinfeng Hongyuan Flagship Store	Electricity conservation2	It doesn't save power and costs electricity.	Jingdong prefecture level city in Shandong
Jinfeng Hongyuan Flagship Store	Electricity conservation2	Not as good as I thought.	Jingdong prefecture level city in Shandong
Jinfeng Hongyuan Flagship Store	Electricity conservation2	Super bad review, it doesn't save power at all, but rather more power consumption	Jingdong prefecture level city in Shandong
Jinfeng Hongyuan Flagship Store	Electricity conservation2	Listen to others say that this can save electricity, I bought with the idea of trying, used for a period of time before coming to the evaluation, will be a little bit of use, not a lot of savings just, long-term use of the words can save more.	Jingdong prefecture level city in Shandong
Jinfeng Hongyuan Flagship Store	Electricity conservation2	Logistics is fast, the next day arrived, workmanship is very delicate a power saver, looking forward to the late effect, customer service patiently explain the recommended which is suitable for, installed in what position is the best, look forward to using the effect after a period of time!	Jingdong prefecture level city in Shandong
Jinfeng Hongyuan Flagship Store	Electricity conservation2	It's good to use, I feel that it has been saving electricity, anyway, not a loss is, I used to feel that the power consumption is big, now I feel much better, my friends are also using, I hope to use a long time will be able to, I wish the boss a prosperous business	Jingdong prefecture level city in Shandong
Jinfeng Hongyuan Flagship Store	Electricity conservation2	Should be very good, to be able to plan the circuit, save the home of the circuit to the extent of the savings, waste of money on electricity, good if a month this money back, hope always hope to be able to	Jingdong prefecture level city in Shandong
Jinfeng Hongyuan Flagship Store	Electricity conservation2	Express delivery is very strong, the goods and I saw in the business consistent, the use of the effect of follow-up evaluation	Jingdong prefecture level city in Shandong
Jinfeng Hongyuan Flagship Store	Electricity conservation2	I have been using this brand of power saver for many years and this time I saw this upgraded version and got it again. The main purpose of this product is to reduce the loss of electricity for a long time, so as to achieve the purpose of saving electricity.	Jingdong prefecture level city in Shandong
Jinfeng Hongyuan Flagship Store	Electricity conservation2	Logistics is fast and the product looks beautiful, looking forward to the effect.	Jingdong prefecture level city in Shandong
Jinfeng Hongyuan Flagship Store	Electricity conservation2	Product quality is good, energy saving a little effect, but the price is a little expensive, fast arrival, good service.	Jingdong prefecture level city in Shandong
Jinfeng Hongyuan Flagship Store	Electricity conservation2	The quality is very good, inexpensive, Jingdong self-supporting guaranteed, worth having!	Jingdong prefecture level city in Shandong
Jinfeng Hongyuan Flagship Store	Electricity conservation2	I don't know if it's saving electricity yet, I'll find out next time I pay my electric bill	Jingdong prefecture level city in Shandong
Jinfeng Hongyuan Flagship Store	Electricity conservation2	Favorable price quality assurance appearance design is beautiful and generous, fine workmanship	Jingdong prefecture level city in Shandong
Jinfeng Hongyuan Flagship Store	Electricity conservation2	I bought it, I don't know how effective it is? I don't know how the quality is? After this summer results will know! Looking forward to it!	Jingdong prefecture level city in Shandong
Jinfeng Hongyuan Flagship Store	Electricity conservation2	Goods received Packaging intact, look at the shell workmanship is also good, high-power appliances open voltage has obvious changes in the effectiveness of the	Jingdong prefecture level city in Shandong
Jinfeng Hongyuan Flagship Store	Electricity conservation2	The goods have been received logistics is very fast, the product is similar to the line, just wait to use a month to see if it can save electricity, stay tuned.	Jingdong prefecture level city in Shandong
Jinfeng Hongyuan Flagship Store	Electricity conservation2	Electricity is plugged in, not sure if it will work, hopefully it will work.	Jingdong prefecture level city in Shandong
Jinfeng Hongyuan Flagship Store	Electricity conservation2	The shell is aluminum alloy, very good workmanship, the actual effect of waiting for the addition of evaluation	Jingdong prefecture level city in Shandong
Jinfeng Hongyuan Flagship Store	Electricity conservation2	Received, already plugged in, looking forward to the effect.	Jingdong prefecture level city in Shandong
Jinfeng Hongyuan Flagship Store	Electricity conservation2	Opened to see the regular manufacturer products, wait for a period of time to see it	Jingdong prefecture level city in Shandong
Jinfeng Hongyuan Flagship Store	Electricity conservation2	🉑 Baby received, very surprised ✨, reasonable design, fashionable and generous, continue to pay attention to the future will be repurchased ❥❥❥❥	Jingdong prefecture level city in Shandong
Jinfeng Hongyuan Flagship Store	Electricity conservation2	Baby received it 👌, really great ah! 🌈The quality can't be beat, very satisfied! 🆗Happy happy happy 😀	Jingdong prefecture level city in Shandong
Jinfeng Hongyuan Flagship Store	Electricity conservation2	Very good, power saving see, this is to help a friend to buy, I used to give my friend a say, my friend immediately let me order another one!	Jingdong prefecture level city in Shandong
Jinfeng Hongyuan Flagship Store	Electricity conservation2	The baby is just as introduced, the texture is very good, powerful, and it feels good to use it	Jingdong prefecture level city in Shandong
Jinfeng Hongyuan Flagship Store	Electricity conservation2	Value for money, very satisfied, seller service attitude is very good, very patient	Jingdong prefecture level city in Shandong
Jinfeng Hongyuan Flagship Store	Electricity conservation2	Power saving treasure design unique exquisite beautiful, voltage display clearly, plugged in that is good to use, I observed the meter, after using the meter is gone slow.	Jingdong prefecture level city in Shandong
Jinfeng Hongyuan Flagship Store	Electricity conservation2	Received on the use of the packaging is beautifully used for a period of time and then come back in the follow-up review!	Jingdong prefecture level city in Shandong
Jinfeng Hongyuan Flagship Store	Electricity conservation2	It should work. Not bad. Not bad. Not bad.	Jingdong prefecture level city in Shandong
Jinfeng Hongyuan Flagship Store	Electricity conservation2	I haven't used it yet, I'll plug it in and see if it saves power next month.	Jingdong prefecture level city in Shandong
Jinfeng Hongyuan Flagship Store	Electricity conservation2	The goods received and unpacked and used, the quality is very good, things and the official website to buy no difference!	Jingdong prefecture level city in Shandong
Jinfeng Hongyuan Flagship Store	Electricity conservation2	It's a rip off, doesn't help much at all, uses the same amount of power as before it was used.	Jingdong prefecture level city in Shandong
Jinfeng Hongyuan Flagship Store	Electricity conservation2	I've been using it for a while now and haven't noticed any unusual problems, so it's okay	Jingdong prefecture level city in Shandong
Jinfeng Hongyuan Flagship Store	Electricity conservation2	Received the goods and plugged in to use, good or not there is no use will have to wait until next month to find out	Jingdong prefecture level city in Shandong
Jinfeng Hongyuan Flagship Store	Electricity conservation2	It's very compact, it looks good, I'll introduce you to my clients when it's ready.	Jingdong prefecture level city in Shandong
Jinfeng Hongyuan Flagship Store	Electricity conservation2	Good workmanship, we'll have to see in a few months.	Jingdong prefecture level city in Shandong
Jinfeng Hongyuan Flagship Store	Electricity conservation2	Jingdong quality! Always trustworthy!	Jingdong prefecture level city in Shandong
Jinfeng Hongyuan Flagship Store	Electricity conservation2	This is the second time I've bought it, and the merchant said to give me five dollars out of the difference, so thank you merchant.	Jingdong prefecture level city in Shandong
Jinfeng Hongyuan Flagship Store	Electricity conservation2	Good quality and good service	Jingdong prefecture level city in Shandong

Jinfeng Hongyuan Flagship Store	Electricity conservation2	Feels good texture, aluminum alloy shell, voltage digital display clear.	Jingdong prefecture level city in Shandong
Jinfeng Hongyuan Flagship Store	Electricity conservation2	Good product, fast and great service, thanks!	Jingdong prefecture level city in Shandong
Jinfeng Hongyuan Flagship Store	Electricity conservation2	Have not yet used, first evaluation, later really good do not need to comment again.	Jingdong prefecture level city in Shandong
Jinfeng Hongyuan Flagship Store	Electricity conservation2	Works great. Pretty much as described by the merchant.	Jingdong prefecture level city in Shandong
Jinfeng Hongyuan Flagship Store	Electricity conservation2	Received it, looking forward to the results, hope it works	Jingdong prefecture level city in Shandong
Jinfeng Hongyuan Flagship Store	Electricity conservation2	Received it, looking forward to the results, hope it works	Jingdong prefecture level city in Shandong
Jinfeng Hongyuan Flagship Store	Electricity conservation2	Supposedly it's an IQ tax. Buy one and try it. Looks good. Well made.	Jingdong prefecture level city in Shandong
Jinfeng Hongyuan Flagship Store	Electricity conservation2	It just doesn't feel like it's working.	Jingdong prefecture level city in Shandong
Jinfeng Hongyuan Flagship Store	Electricity conservation2	This is a great product.	Jingdong prefecture level city in Shandong
Jinfeng Hongyuan Flagship Store	Electricity conservation2	I'm just using it. I'll tell you more.	Jingdong prefecture level city in Shandong
Jinfeng Hongyuan Flagship Store	Electricity conservation2	Second repurchase and it's working fine.	Jingdong prefecture level city in Shandong
Jinfeng Hongyuan Flagship Store	Electricity conservation2	I'll wait a few days before evaluating	Jingdong prefecture level city in Shandong
Jinfeng Hongyuan Flagship Store	Electricity conservation2	I'll comment after I use it hopefully the section will be useful.	Jingdong prefecture level city in Shandong
Jinfeng Hongyuan Flagship Store	Electricity conservation2	not very useful	Jingdong prefecture level city in Shandong
Jinfeng Hongyuan Flagship Store	Electricity conservation2	Not received	Jingdong prefecture level city in Shandong
Jinfeng Hongyuan Flagship Store	Electricity conservation2	rare	Jingdong prefecture level city in Shandong
Jinfeng Hongyuan Flagship Store	Electricity conservation2	It's good.	Jingdong prefecture level city in Shandong
Jinfeng Hongyuan Flagship Store	Electricity conservation2	just now	Jingdong prefecture level city in Shandong
Jinfeng Hongyuan Flagship Store	Electricity conservation2	serviceable	Jingdong prefecture level city in Shandong
Jinfeng Hongyuan Flagship Store	Electricity conservation2	desirable	Jingdong prefecture level city in Shandong
Jinfeng Hongyuan Flagship Store	Electricity conservation2	serviceable	Jingdong prefecture level city in Shandong
Jinfeng Hongyuan Flagship Store	Electricity conservation2	It doesn't save power and costs electricity.	Jingdong prefecture level city in Shandong
Jinfeng Hongyuan Flagship Store	Electricity conservation2	Not as good as I thought.	Jingdong prefecture level city in Shandong
Jinfeng Hongyuan Flagship Store	Electricity conservation2	Super bad review, it doesn't save power at all, but rather more power consumption	Jingdong prefecture level city in Shandong
Jinfeng Hongyuan Flagship Store	Electricity conservation2	Listen to others say that this can save electricity, I bought with the idea of trying, used for a period of time before coming to the evaluation, will be a little bit of use, not a lot of savings just, long-term use of the words can save more.	Jingdong prefecture level city in Shandong
Jinfeng Hongyuan Flagship Store	Electricity conservation2	Logistics is fast, the next day arrived, workmanship is very delicate a power saver, looking forward to the late effect, customer service patiently explain the recommended which is suitable for, installed in what position is the best, look forward to using the effect after a period of time!	Jingdong prefecture level city in Shandong
Jinfeng Hongyuan Flagship Store	Electricity conservation2	It's good to use, I feel that it has been saving electricity, anyway, not a loss is, I used to feel that the power consumption is big, now I feel much better, my friends are also using, I hope to use a long time will be able to, I wish the boss a prosperous business	Jingdong prefecture level city in Shandong
Jinfeng Hongyuan Flagship Store	Electricity conservation2	Should be very good, to be able to plan the circuit, save the home of the circuit to the extent of the savings, waste of money on electricity, good if a month this money back, hope always hope to be able to	Jingdong prefecture level city in Shandong
Jinfeng Hongyuan Flagship Store	Electricity conservation2	Express delivery is very strong, the goods and I saw in the business consistent, the use of the effect of follow-up evaluation	Jingdong prefecture level city in Shandong
Jinfeng Hongyuan Flagship Store	Electricity conservation2	I have been using this brand of power saver for many years and this time I saw this upgraded version and got it again. The main purpose of this product is to reduce the loss of electricity for a long time, so as to achieve the purpose of saving electricity.	Jingdong prefecture level city in Shandong
Jinfeng Hongyuan Flagship Store	Electricity conservation2	Logistics is fast and the product looks beautiful, looking forward to the effect.	Jingdong prefecture level city in Shandong
Jinfeng Hongyuan Flagship Store	Electricity conservation2	Product quality is good, energy saving a little effect, but the price is a little expensive, fast arrival, good service.	Jingdong prefecture level city in Shandong
Jinfeng Hongyuan Flagship Store	Electricity conservation2	The quality is very good, inexpensive, Jingdong self-supporting guaranteed, worth having!	Jingdong prefecture level city in Shandong
Jinfeng Hongyuan Flagship Store	Electricity conservation2	I don't know if it's saving electricity yet, I'll find out next time I pay my electric bill	Jingdong prefecture level city in Shandong
Jinfeng Hongyuan Flagship Store	Electricity conservation2	Favorable price quality assurance appearance design is beautiful and generous, fine workmanship	Jingdong prefecture level city in Shandong
Jinfeng Hongyuan Flagship Store	Electricity conservation2	I bought it, I don't know how effective it is? I don't know how the quality is? After this summer results will know! Looking forward to it!	Jingdong prefecture level city in Shandong
Jinfeng Hongyuan Flagship Store	Electricity conservation2	Goods received Packaging intact, look at the shell workmanship is also good, high-power appliances open voltage has obvious changes in the effectiveness of the	Jingdong prefecture level city in Shandong
Jinfeng Hongyuan Flagship Store	Electricity conservation2	The goods have been received logistics is very fast, the product is similar to the line, just wait to use a month to see if it can save electricity, stay tuned.	Jingdong prefecture level city in Shandong
Jinfeng Hongyuan Flagship Store	Electricity conservation2	Electricity is plugged in, not sure if it will work, hopefully it will work.	Jingdong prefecture level city in Shandong
Jinfeng Hongyuan Flagship Store	Electricity conservation2	The shell is aluminum alloy, very good workmanship, the actual effect of waiting for the addition of evaluation	Jingdong prefecture level city in Shandong
Jinfeng Hongyuan Flagship Store	Electricity conservation2	Received, already plugged in, looking forward to the effect.	Jingdong prefecture level city in Shandong
Jinfeng Hongyuan Flagship Store	Electricity conservation2	Opened to see the regular manufacturer products, wait for a period of time to see it	Jingdong prefecture level city in Shandong
Jinfeng Hongyuan Flagship Store	Electricity conservation2	🉑 Baby received, very surprised ✨, reasonable design, fashionable and generous, continue to pay attention to the future will be repurchased ❥❥❥❥	Jingdong prefecture level city in Shandong
Jinfeng Hongyuan Flagship Store	Electricity conservation2	Baby received it 👌, really great ah! 🌈The quality can't be beat, very satisfied! 🆗Happy happy happy 😃	Jingdong prefecture level city in Shandong
Jinfeng Hongyuan Flagship Store	Electricity conservation2	Very good, power saving see, this is to help a friend to buy, I used to give my friend a say, my friend immediately let me order another one!	Jingdong prefecture level city in Shandong
Jinfeng Hongyuan Flagship Store	Electricity conservation2	The baby is just as introduced, the texture is very good, powerful, and it feels good to use it	Jingdong prefecture level city in Shandong
Jinfeng Hongyuan Flagship Store	Electricity conservation2	Value for money, very satisfied, seller service attitude is very good, very patient	Jingdong prefecture level city in Shandong
Jinfeng Hongyuan Flagship Store	Electricity conservation2	Power saving treasure design unique exquisite beautiful, voltage display clearly, plugged in that is good to use, I observed the meter, after using the meter is gone slow.	Jingdong prefecture level city in Shandong
Jinfeng Hongyuan Flagship Store	Electricity conservation2	Received on the use of the packaging is beautifully used for a period of time and then come back in the follow-up review!	Jingdong prefecture level city in Shandong

Jinfeng Hongyuan Flagship Store	Electricity conservation2	It should work. Not bad. Not bad. Not bad.	Jingdong prefecture level city in Shandong
Jinfeng Hongyuan Flagship Store	Electricity conservation2	I haven't used it yet, I'll plug it in and see if it saves power next month.	Jingdong prefecture level city in Shandong
Jinfeng Hongyuan Flagship Store	Electricity conservation2	The goods received and unpacked and used, the quality is very good, things and the official website to buy no difference!	Jingdong prefecture level city in Shandong
Jinfeng Hongyuan Flagship Store	Electricity conservation2	It's a rip off, doesn't help much at all, uses the same amount of power as before it was used.	Jingdong prefecture level city in Shandong
Jinfeng Hongyuan Flagship Store	Electricity conservation2	I've been using it for a while now and haven't noticed any unusual problems, so it's okay	Jingdong prefecture level city in Shandong
Jinfeng Hongyuan Flagship Store	Electricity conservation2	Received the goods and plugged in to use, good or not there is no use will have to wait until next month to find out	Jingdong prefecture level city in Shandong
Jinfeng Hongyuan Flagship Store	Electricity conservation2	It's very compact, it looks good, I'll introduce you to my clients when it's ready.	Jingdong prefecture level city in Shandong
Jinfeng Hongyuan Flagship Store	Electricity conservation2	Good workmanship, we'll have to see in a few months.	Jingdong prefecture level city in Shandong
Jinfeng Hongyuan Flagship Store	Electricity conservation2	Jingdong quality! Always trustworthy!	Jingdong prefecture level city in Shandong
Jinfeng Hongyuan Flagship Store	Electricity conservation2	This is the second time I've bought it, and the merchant said to give me five dollars out of the difference, so thank you merchant.	Jingdong prefecture level city in Shandong
Jinfeng Hongyuan Flagship Store	Electricity conservation2	Good quality and good service	Jingdong prefecture level city in Shandong
Jinfeng Hongyuan Flagship Store	Electricity conservation2	Feels good texture, aluminum alloy shell, voltage digital display clear.	Jingdong prefecture level city in Shandong
Jinfeng Hongyuan Flagship Store	Electricity conservation2	Good product, fast and great service, thanks!	Jingdong prefecture level city in Shandong
Jinfeng Hongyuan Flagship Store	Electricity conservation2	Have not yet used, first evaluation, later really good do not need to comment again.	Jingdong prefecture level city in Shandong
Jinfeng Hongyuan Flagship Store	Electricity conservation2	Works great. Pretty much as described by the merchant.	Jingdong prefecture level city in Shandong
Jinfeng Hongyuan Flagship Store	Electricity conservation2	Received it, looking forward to the results, hope it works	Jingdong prefecture level city in Shandong
Jinfeng Hongyuan Flagship Store	Electricity conservation2	Received it, looking forward to the results, hope it works	Jingdong prefecture level city in Shandong
Jinfeng Hongyuan Flagship Store	Electricity conservation2	Supposedly it's an IQ tax. Buy one and try it. Looks good. Well made.	Jingdong prefecture level city in Shandong
Jinfeng Hongyuan Flagship Store	Electricity conservation2	It just doesn't feel like it's working.	Jingdong prefecture level city in Shandong
Jinfeng Hongyuan Flagship Store	Electricity conservation2	This is a great product.	Jingdong prefecture level city in Shandong
Jinfeng Hongyuan Flagship Store	Electricity conservation2	I'm just using it. I'll tell you more.	Jingdong prefecture level city in Shandong
Jinfeng Hongyuan Flagship Store	Electricity conservation2	Second repurchase and it's working fine.	Jingdong prefecture level city in Shandong
Jinfeng Hongyuan Flagship Store	Electricity conservation2	I'll wait a few days before evaluating	Jingdong prefecture level city in Shandong
Jinfeng Hongyuan Flagship Store	Electricity conservation2	I'll comment after I use it hopefully the section will be useful.	Jingdong prefecture level city in Shandong
Jinfeng Hongyuan Flagship Store	Electricity conservation2	not very useful	Jingdong prefecture level city in Shandong
Jinfeng Hongyuan Flagship Store	Electricity conservation2	Not received	Jingdong prefecture level city in Shandong
Jinfeng Hongyuan Flagship Store	Electricity conservation2	rare	Jingdong prefecture level city in Shandong
Jinfeng Hongyuan Flagship Store	Electricity conservation2	It's good.	Jingdong prefecture level city in Shandong
Jinfeng Hongyuan Flagship Store	Electricity conservation2	just now	Jingdong prefecture level city in Shandong
Jinfeng Hongyuan Flagship Store	Electricity conservation2	serviceable	Jingdong prefecture level city in Shandong
Jinfeng Hongyuan Flagship Store	Electricity conservation2	desirable	Jingdong prefecture level city in Shandong
Jinfeng Hongyuan Flagship Store	Electricity conservation2	serviceable	Jingdong prefecture level city in Shandong
Jinfeng Hongyuan Flagship Store	Electricity conservation2	It doesn't save power and costs electricity.	Jingdong prefecture level city in Shandong
Jinfeng Hongyuan Flagship Store	Electricity conservation2	Not as good as I thought.	Jingdong prefecture level city in Shandong
Jinfeng Hongyuan Flagship Store	Electricity conservation2	Super bad review, it doesn't save power at all, but rather more power consumption	Jingdong prefecture level city in Shandong
Jinfeng Hongyuan Flagship Store	Electricity conservation2	Listen to others say that this can save electricity, I bought with the idea of trying, used for a period of time before coming to the evaluation, will be a little bit of use, not a lot of savings just, long-term use of the words can save more.	Jingdong prefecture level city in Shandong
Jinfeng Hongyuan Flagship Store	Electricity conservation2	Logistics is fast, the next day arrived, workmanship is very delicate a power saver, looking forward to the late effect, customer service patiently explain the recommended which is suitable for, installed in what position is the best, look forward to using the effect after a period of time!	Jingdong prefecture level city in Shandong
Jinfeng Hongyuan Flagship Store	Electricity conservation2	It's good to use, I feel that it has been saving electricity, anyway, not a loss is, I used to feel that the power consumption is big, now I feel much better, my friends are also using, I hope to use a long time will be able to, I wish the boss a prosperous business	Jingdong prefecture level city in Shandong
Jinfeng Hongyuan Flagship Store	Electricity conservation2	Should be very good, to be able to plan the circuit, save the home of the circuit to the extent of the savings, waste of money on electricity, good if a month this money back, hope always hope to be able to	Jingdong prefecture level city in Shandong
Jinfeng Hongyuan Flagship Store	Electricity conservation2	Express delivery is very strong, the goods and I saw in the business consistent, the use of the effect of follow-up evaluation	Jingdong prefecture level city in Shandong
Jinfeng Hongyuan Flagship Store	Electricity conservation2	I have been using this brand of power saver for many years and this time I saw this upgraded version and got it again. The main purpose of this product is to reduce the loss of electricity for a long time, so as to achieve the purpose of saving electricity.	Jingdong prefecture level city in Shandong
Jinfeng Hongyuan Flagship Store	Electricity conservation2	Logistics is fast and the product looks beautiful, looking forward to the effect.	Jingdong prefecture level city in Shandong
Jinfeng Hongyuan Flagship Store	Electricity conservation2	Product quality is good, energy saving a little effect, but the price is a little expensive, fast arrival, good service.	Jingdong prefecture level city in Shandong
Jinfeng Hongyuan Flagship Store	Electricity conservation2	The quality is very good, inexpensive, Jingdong self-supporting guaranteed, worth having!	Jingdong prefecture level city in Shandong
Jinfeng Hongyuan Flagship Store	Electricity conservation2	I don't know if it's saving electricity yet, I'll find out next time I pay my electric bill	Jingdong prefecture level city in Shandong
Jinfeng Hongyuan Flagship Store	Electricity conservation2	Favorable price quality assurance appearance design is beautiful and generous, fine workmanship	Jingdong prefecture level city in Shandong
Jinfeng Hongyuan Flagship Store	Electricity conservation2	I bought it, I don't know how effective it is? I don't know how the quality is? After this summer results will know! Looking forward to it!	Jingdong prefecture level city in Shandong
Jinfeng Hongyuan Flagship Store	Electricity conservation2	Goods received Packaging intact, look at the shell workmanship is also good, high-power appliances open voltage has obvious changes in the effectiveness of the	Jingdong prefecture level city in Shandong
Jinfeng Hongyuan Flagship Store	Electricity conservation2	The goods have been received logistics is very fast, the product is similar to the line, just wait to use a month to see if it can save electricity, stay tuned.	Jingdong prefecture level city in Shandong

Jinfeng Hongyuan Flagship Store	Electricity conservation2	Electricity is plugged in, not sure if it will work, hopefully it will work.	Jingdong prefecture level city in Shandong
Jinfeng Hongyuan Flagship Store	Electricity conservation2	The shell is aluminum alloy, very good workmanship, the actual effect of waiting for the addition of evaluation	Jingdong prefecture level city in Shandong
Jinfeng Hongyuan Flagship Store	Electricity conservation2	Received, already plugged in, looking forward to the effect.	Jingdong prefecture level city in Shandong
Jinfeng Hongyuan Flagship Store	Electricity conservation2	Opened to see the regular manufacturer products, wait for a period of time to see it	Jingdong prefecture level city in Shandong
Jinfeng Hongyuan Flagship Store	Electricity conservation2	Baby received, very surprised , reasonable design, fashionable and generous, continue to pay attention to the future will be repurchased	Jingdong prefecture level city in Shandong
Jinfeng Hongyuan Flagship Store	Electricity conservation2	Baby received it , really great ah! The quality can't be beat, very satisfied! Happy happy happy	Jingdong prefecture level city in Shandong
Jinfeng Hongyuan Flagship Store	Electricity conservation2	Very good, power saving see, this is to help a friend to buy, I used to give my friend a say, my friend immediately let me order another one!	Jingdong prefecture level city in Shandong
Jinfeng Hongyuan Flagship Store	Electricity conservation2	The baby is just as introduced, the texture is very good, powerful, and it feels good to use it	Jingdong prefecture level city in Shandong
Jinfeng Hongyuan Flagship Store	Electricity conservation2	Value for money, very satisfied, seller service attitude is very good, very patient	Jingdong prefecture level city in Shandong
Jinfeng Hongyuan Flagship Store	Electricity conservation2	Power saving treasure design unique exquisite beautiful, voltage display clearly, plugged in that is good to use, I observed the meter, after using the meter is gone slow.	Jingdong prefecture level city in Shandong
Jinfeng Hongyuan Flagship Store	Electricity conservation2	Received on the use of the packaging is beautifully used for a period of time and then come back in the follow-up review!	Jingdong prefecture level city in Shandong
Jinfeng Hongyuan Flagship Store	Electricity conservation2	It should work. Not bad. Not bad. Not bad.	Jingdong prefecture level city in Shandong
Jinfeng Hongyuan Flagship Store	Electricity conservation2	I haven't used it yet, I'll plug it in and see if it saves power next month.	Jingdong prefecture level city in Shandong
Jinfeng Hongyuan Flagship Store	Electricity conservation2	The goods received and unpacked and used, the quality is very good, things and the official website to buy no difference!	Jingdong prefecture level city in Shandong
Jinfeng Hongyuan Flagship Store	Electricity conservation2	It's a rip off, doesn't help much at all, uses the same amount of power as before it was used.	Jingdong prefecture level city in Shandong
Jinfeng Hongyuan Flagship Store	Electricity conservation2	I've been using it for a while now and haven't noticed any unusual problems, so it's okay	Jingdong prefecture level city in Shandong
Jinfeng Hongyuan Flagship Store	Electricity conservation2	Received the goods and plugged in to use, good or not there is no use will have to wait until next month to find out	Jingdong prefecture level city in Shandong
Jinfeng Hongyuan Flagship Store	Electricity conservation2	It's very compact, it looks good, I'll introduce you to my clients when it's ready.	Jingdong prefecture level city in Shandong
Jinfeng Hongyuan Flagship Store	Electricity conservation2	Good workmanship, we'll have to see in a few months.	Jingdong prefecture level city in Shandong
Jinfeng Hongyuan Flagship Store	Electricity conservation2	Jingdong quality! Always trustworthy!	Jingdong prefecture level city in Shandong
Jinfeng Hongyuan Flagship Store	Electricity conservation2	This is the second time I've bought it, and the merchant said to give me five dollars out of the difference, so thank you merchant.	Jingdong prefecture level city in Shandong
Jinfeng Hongyuan Flagship Store	Electricity conservation2	Good quality and good service	Jingdong prefecture level city in Shandong
Jinfeng Hongyuan Flagship Store	Electricity conservation2	Feels good texture, aluminum alloy shell, voltage digital display clear.	Jingdong prefecture level city in Shandong
Jinfeng Hongyuan Flagship Store	Electricity conservation2	Good product, fast and great service, thanks!	Jingdong prefecture level city in Shandong
Jinfeng Hongyuan Flagship Store	Electricity conservation2	Have not yet used, first evaluation, later really good do not need to comment again.	Jingdong prefecture level city in Shandong
Jinfeng Hongyuan Flagship Store	Electricity conservation2	Works great. Pretty much as described by the merchant.	Jingdong prefecture level city in Shandong
Jinfeng Hongyuan Flagship Store	Electricity conservation2	Received it, looking forward to the results, hope it works	Jingdong prefecture level city in Shandong
Jinfeng Hongyuan Flagship Store	Electricity conservation2	Received it, looking forward to the results, hope it works	Jingdong prefecture level city in Shandong
Jinfeng Hongyuan Flagship Store	Electricity conservation2	Supposedly it's an IQ tax. Buy one and try it. Looks good. Well made.	Jingdong prefecture level city in Shandong
Jinfeng Hongyuan Flagship Store	Electricity conservation2	It just doesn't feel like it's working.	Jingdong prefecture level city in Shandong
Jinfeng Hongyuan Flagship Store	Electricity conservation2	This is a great product.	Jingdong prefecture level city in Shandong
Jinfeng Hongyuan Flagship Store	Electricity conservation2	I'm just using it. I'll tell you more.	Jingdong prefecture level city in Shandong
Jinfeng Hongyuan Flagship Store	Electricity conservation2	Second repurchase and it's working fine.	Jingdong prefecture level city in Shandong
Jinfeng Hongyuan Flagship Store	Electricity conservation2	I'll wait a few days before evaluating	Jingdong prefecture level city in Shandong
Jinfeng Hongyuan Flagship Store	Electricity conservation2	I'll comment after I use it hopefully the section will be useful.	Jingdong prefecture level city in Shandong
Jinfeng Hongyuan Flagship Store	Electricity conservation2	not very useful	Jingdong prefecture level city in Shandong
Jinfeng Hongyuan Flagship Store	Electricity conservation2	Not received	Jingdong prefecture level city in Shandong
Jinfeng Hongyuan Flagship Store	Electricity conservation2	rare	Jingdong prefecture level city in Shandong
Jinfeng Hongyuan Flagship Store	Electricity conservation2	It's good.	Jingdong prefecture level city in Shandong
Jinfeng Hongyuan Flagship Store	Electricity conservation2	just now	Jingdong prefecture level city in Shandong
Jinfeng Hongyuan Flagship Store	Electricity conservation2	serviceable	Jingdong prefecture level city in Shandong
Jinfeng Hongyuan Flagship Store	Electricity conservation2	desirable	Jingdong prefecture level city in Shandong
Jinfeng Hongyuan Flagship Store	Electricity conservation2	serviceable	Jingdong prefecture level city in Shandong
Jinfeng Hongyuan Flagship Store	Electricity conservation2	It doesn't save power and costs electricity.	Jingdong prefecture level city in Shandong
Jinfeng Hongyuan Flagship Store	Electricity conservation2	Not as good as I thought.	Jingdong prefecture level city in Shandong
Jinfeng Hongyuan Flagship Store	Electricity conservation2	Super bad review, it doesn't save power at all, but rather more power consumption	Jingdong prefecture level city in Shandong
Jinfeng Hongyuan Flagship Store	Electricity conservation2	Listen to others say that this can save electricity, I bought with the idea of trying, used for a period of time before coming to the evaluation, will be a little bit of use, not a lot of savings just, long-term use of the words can save more.	Jingdong prefecture level city in Shandong
Jinfeng Hongyuan Flagship Store	Electricity conservation2	Logistics is fast, the next day arrived, workmanship is very delicate a power saver, looking forward to the late effect, customer service patiently explain the recommended which is suitable for, installed in what position is the best, look forward to using the effect after a period of time!	Jingdong prefecture level city in Shandong
Jinfeng Hongyuan Flagship Store	Electricity conservation2	It's good to use, I feel that it has been saving electricity, anyway, not a loss is, I used to feel that the power consumption is big, now I feel much better, my friends are also using, I hope to use a long time will be able to, I wish the boss a prosperous business	Jingdong prefecture level city in Shandong
Jinfeng Hongyuan Flagship Store	Electricity conservation2	Should be very good, to be able to plan the circuit, save the home of the circuit to the extent of the savings, waste of money on electricity, good if a month this money back, hope always hope to be able to	Jingdong prefecture level city in Shandong

Jinfeng Hongyuan Flagship Store	Electricity conservation2	Express delivery is very strong, the goods and I saw in the business consistent, the use of the effect of follow-up evaluation	Jingdong prefecture level city in Shandong
Jinfeng Hongyuan Flagship Store	Electricity conservation2	I have been using this brand of power saver for many years and this time I saw this upgraded version and got it again. The main purpose of this product is to reduce the loss of electricity for a long time, so as to achieve the purpose of saving electricity.	Jingdong prefecture level city in Shandong
Jinfeng Hongyuan Flagship Store	Electricity conservation2	Logistics is fast and the product looks beautiful, looking forward to the effect.	Jingdong prefecture level city in Shandong
Jinfeng Hongyuan Flagship Store	Electricity conservation2	Product quality is good, energy saving a little effect, but the price is a little expensive, fast arrival, good service.	Jingdong prefecture level city in Shandong
Jinfeng Hongyuan Flagship Store	Electricity conservation2	The quality is very good, inexpensive, Jingdong self-supporting guaranteed, worth having!	Jingdong prefecture level city in Shandong
Jinfeng Hongyuan Flagship Store	Electricity conservation2	I don't know if it's saving electricity yet, I'll find out next time I pay my electric bill	Jingdong prefecture level city in Shandong
Jinfeng Hongyuan Flagship Store	Electricity conservation2	Favorable price quality assurance appearance design is beautiful and generous, fine workmanship	Jingdong prefecture level city in Shandong
Jinfeng Hongyuan Flagship Store	Electricity conservation2	I bought it, I don't know how effective it is? I don't know how the quality is? After this summer results will know! Looking forward to it!	Jingdong prefecture level city in Shandong
Jinfeng Hongyuan Flagship Store	Electricity conservation2	Goods received Packaging intact, look at the shell workmanship is also good, high-power appliances open voltage has obvious changes in the effectiveness of the	Jingdong prefecture level city in Shandong
Jinfeng Hongyuan Flagship Store	Electricity conservation2	The goods have been received logistics is very fast, the product is similar to the line, just wait to use a month to see if it can save electricity, stay tuned.	Jingdong prefecture level city in Shandong
Jinfeng Hongyuan Flagship Store	Electricity conservation2	Electricity is plugged in, not sure if it will work, hopefully it will work.	Jingdong prefecture level city in Shandong
Jinfeng Hongyuan Flagship Store	Electricity conservation2	The shell is aluminum alloy, very good workmanship, the actual effect of waiting for the addition of evaluation	Jingdong prefecture level city in Shandong
Jinfeng Hongyuan Flagship Store	Electricity conservation2	Received, already plugged in, looking forward to the effect.	Jingdong prefecture level city in Shandong
Jinfeng Hongyuan Flagship Store	Electricity conservation2	Opened to see the regular manufacturer products, wait for a period of time to see it	Jingdong prefecture level city in Shandong
Jinfeng Hongyuan Flagship Store	Electricity conservation2	🉑 Baby received, very surprised ✨, reasonable design, fashionable and generous, continue to pay attention to the future will be repurchased ❥❥❥❥	Jingdong prefecture level city in Shandong
Jinfeng Hongyuan Flagship Store	Electricity conservation2	Baby received it 👌, really great ah! 🌈The quality can't be beat, very satisfied! 🆗Happy happy happy 😃	Jingdong prefecture level city in Shandong
Jinfeng Hongyuan Flagship Store	Electricity conservation2	Very good, power saving see, this is to help a friend to buy, I used to give my friend a say, my friend immediately let me order another one!	Jingdong prefecture level city in Shandong
Jinfeng Hongyuan Flagship Store	Electricity conservation2	The baby is just as introduced, the texture is very good, powerful, and it feels good to use it	Jingdong prefecture level city in Shandong
Jinfeng Hongyuan Flagship Store	Electricity conservation2	Value for money, very satisfied, seller service attitude is very good, very patient	Jingdong prefecture level city in Shandong
Jinfeng Hongyuan Flagship Store	Electricity conservation2	Power saving treasure design unique exquisite beautiful, voltage display clearly, plugged in that is good to use, I observed the meter, after using the meter is gone slow.	Jingdong prefecture level city in Shandong
Jinfeng Hongyuan Flagship Store	Electricity conservation2	Received on the use of the packaging is beautifully used for a period of time and then come back in the follow-up review!	Jingdong prefecture level city in Shandong
Jinfeng Hongyuan Flagship Store	Electricity conservation2	It should work. Not bad. Not bad. Not bad.	Jingdong prefecture level city in Shandong
Jinfeng Hongyuan Flagship Store	Electricity conservation2	I haven't used it yet, I'll plug it in and see if it saves power next month.	Jingdong prefecture level city in Shandong
Jinfeng Hongyuan Flagship Store	Electricity conservation2	The goods received and unpacked and used, the quality is very good, things and the official website to buy no difference!	Jingdong prefecture level city in Shandong
Jinfeng Hongyuan Flagship Store	Electricity conservation2	It's a rip off, doesn't help much at all, uses the same amount of power as before it was used.	Jingdong prefecture level city in Shandong
Jinfeng Hongyuan Flagship Store	Electricity conservation2	I've been using it for a while now and haven't noticed any unusual problems, so it's okay	Jingdong prefecture level city in Shandong
Jinfeng Hongyuan Flagship Store	Electricity conservation2	Received the goods and plugged in to use, good or not there is no use will have to wait until next month to find out	Jingdong prefecture level city in Shandong
Jinfeng Hongyuan Flagship Store	Electricity conservation2	It's very compact, it looks good, I'll introduce you to my clients when it's ready.	Jingdong prefecture level city in Shandong
Jinfeng Hongyuan Flagship Store	Electricity conservation2	Good workmanship, we'll have to see in a few months.	Jingdong prefecture level city in Shandong
Jinfeng Hongyuan Flagship Store	Electricity conservation2	Jingdong quality! Always trustworthy!	Jingdong prefecture level city in Shandong
Jinfeng Hongyuan Flagship Store	Electricity conservation2	This is the second time I've bought it, and the merchant said to give me five dollars out of the difference, so thank you merchant.	Jingdong prefecture level city in Shandong
Jinfeng Hongyuan Flagship Store	Electricity conservation2	Good quality and good service	Jingdong prefecture level city in Shandong
Jinfeng Hongyuan Flagship Store	Electricity conservation2	Feels good texture, aluminum alloy shell, voltage digital display clear.	Jingdong prefecture level city in Shandong
Jinfeng Hongyuan Flagship Store	Electricity conservation2	Good product, fast and great service, thanks!	Jingdong prefecture level city in Shandong
Jinfeng Hongyuan Flagship Store	Electricity conservation2	Have not yet used, first evaluation, later really good do not need to comment again.	Jingdong prefecture level city in Shandong
Jinfeng Hongyuan Flagship Store	Electricity conservation2	Works great. Pretty much as described by the merchant.	Jingdong prefecture level city in Shandong
Jinfeng Hongyuan Flagship Store	Electricity conservation2	Received it, looking forward to the results, hope it works	Jingdong prefecture level city in Shandong
Jinfeng Hongyuan Flagship Store	Electricity conservation2	Received it, looking forward to the results, hope it works	Jingdong prefecture level city in Shandong
Jinfeng Hongyuan Flagship Store	Electricity conservation2	Supposedly it's an IQ tax. Buy one and try it. Looks good. Well made.	Jingdong prefecture level city in Shandong
Jinfeng Hongyuan Flagship Store	Electricity conservation2	It just doesn't feel like it's working.	Jingdong prefecture level city in Shandong
Jinfeng Hongyuan Flagship Store	Electricity conservation2	This is a great product.	Jingdong prefecture level city in Shandong
Jinfeng Hongyuan Flagship Store	Electricity conservation2	I'm just using it. I'll tell you more.	Jingdong prefecture level city in Shandong
Jinfeng Hongyuan Flagship Store	Electricity conservation2	Second repurchase and it's working fine.	Jingdong prefecture level city in Shandong
Jinfeng Hongyuan Flagship Store	Electricity conservation2	I'll wait a few days before evaluating	Jingdong prefecture level city in Shandong
Jinfeng Hongyuan Flagship Store	Electricity conservation2	I'll comment after I use it hopefully the section will be useful.	Jingdong prefecture level city in Shandong
Jinfeng Hongyuan Flagship Store	Electricity conservation2	not very useful	Jingdong prefecture level city in Shandong
Jinfeng Hongyuan Flagship Store	Electricity conservation2	Not received	Jingdong prefecture level city in Shandong
Jinfeng Hongyuan Flagship Store	Electricity conservation2	rare	Jingdong prefecture level city in Shandong
Jinfeng Hongyuan Flagship Store	Electricity conservation2	It's good.	Jingdong prefecture level city in Shandong
Jinfeng Hongyuan Flagship Store	Electricity conservation2	just now	Jingdong prefecture level city in Shandong
Jinfeng Hongyuan Flagship Store	Electricity conservation2	serviceable	Jingdong prefecture level city in Shandong

Jinfeng Hongyuan Flagship Store	Electricity conservation2	desirable	Jingdong prefecture level city in Shandong
Jinfeng Hongyuan Flagship Store	Electricity conservation2	serviceable	Jingdong prefecture level city in Shandong
Jinfeng Hongyuan Flagship Store	Electricity conservation2	It doesn't save power and costs electricity.	Jingdong prefecture level city in Shandong
Jinfeng Hongyuan Flagship Store	Electricity conservation2	Not as good as I thought.	Jingdong prefecture level city in Shandong
Jinfeng Hongyuan Flagship Store	Electricity conservation2	Super bad review, it doesn't save power at all, but rather more power consumption	Jingdong prefecture level city in Shandong
Jinfeng Hongyuan Flagship Store	Electricity conservation2	Listen to others say that this can save electricity, I bought with the idea of trying, used for a period of time before coming to the evaluation, will be a little bit of use, not a lot of savings just, long-term use of the words can save more.	Jingdong prefecture level city in Shandong
Jinfeng Hongyuan Flagship Store	Electricity conservation2	Logistics is fast, the next day arrived, workmanship is very delicate a power saver, looking forward to the late effect, customer service patiently explain the recommended which is suitable for, installed in what position is the best, look forward to using the effect after a period of time!	Jingdong prefecture level city in Shandong
Jinfeng Hongyuan Flagship Store	Electricity conservation2	It's good to use, I feel that it has been saving electricity, anyway, not a loss is, I used to feel that the power consumption is big, now I feel much better, my friends are also using, I hope to use a long time will be able to, I wish the boss a prosperous business	Jingdong prefecture level city in Shandong
Jinfeng Hongyuan Flagship Store	Electricity conservation2	Should be very good, to be able to plan the circuit, save the home of the circuit to the extent of the savings, waste of money on electricity, good if a month this money back, hope always hope to be able to	Jingdong prefecture level city in Shandong
Jinfeng Hongyuan Flagship Store	Electricity conservation2	Express delivery is very strong, the goods and I saw in the business consistent, the use of the effect of follow-up evaluation	Jingdong prefecture level city in Shandong
Jinfeng Hongyuan Flagship Store	Electricity conservation2	I have been using this brand of power saver for many years and this time I saw this upgraded version and got it again. The main purpose of this product is to reduce the loss of electricity for a long time, so as to achieve the purpose of saving electricity.	Jingdong prefecture level city in Shandong
Jinfeng Hongyuan Flagship Store	Electricity conservation2	Logistics is fast and the product looks beautiful, looking forward to the effect.	Jingdong prefecture level city in Shandong
Jinfeng Hongyuan Flagship Store	Electricity conservation2	Product quality is good, energy saving a little effect, but the price is a little expensive, fast arrival, good service.	Jingdong prefecture level city in Shandong
Jinfeng Hongyuan Flagship Store	Electricity conservation2	The quality is very good, inexpensive, Jingdong self-supporting guaranteed, worth having!	Jingdong prefecture level city in Shandong
Jinfeng Hongyuan Flagship Store	Electricity conservation2	I don't know if it's saving electricity yet, I'll find out next time I pay my electric bill	Jingdong prefecture level city in Shandong
Jinfeng Hongyuan Flagship Store	Electricity conservation2	Favorable price quality assurance appearance design is beautiful and generous, fine workmanship	Jingdong prefecture level city in Shandong
Jinfeng Hongyuan Flagship Store	Electricity conservation2	I bought it, I don't know how effective it is? I don't know how the quality is? After this summer results will know! Looking forward to it!	Jingdong prefecture level city in Shandong
Jinfeng Hongyuan Flagship Store	Electricity conservation2	Goods received Packaging intact, look at the shell workmanship is also good, high-power appliances open voltage has obvious changes in the effectiveness of the	Jingdong prefecture level city in Shandong
Jinfeng Hongyuan Flagship Store	Electricity conservation2	The goods have been received logistics is very fast, the product is similar to the line, just wait to use a month to see if it can save electricity, stay tuned.	Jingdong prefecture level city in Shandong
Jinfeng Hongyuan Flagship Store	Electricity conservation2	Electricity is plugged in, not sure if it will work, hopefully it will work.	Jingdong prefecture level city in Shandong
Jinfeng Hongyuan Flagship Store	Electricity conservation2	The shell is aluminum alloy, very good workmanship, the actual effect of waiting for the addition of evaluation	Jingdong prefecture level city in Shandong
Jinfeng Hongyuan Flagship Store	Electricity conservation2	Received, already plugged in, looking forward to the effect.	Jingdong prefecture level city in Shandong
Jinfeng Hongyuan Flagship Store	Electricity conservation2	Opened to see the regular manufacturer products, wait for a period of time to see it	Jingdong prefecture level city in Shandong
Jinfeng Hongyuan Flagship Store	Electricity conservation2	🉑 Baby received, very surprised ✨, reasonable design, fashionable and generous, continue to pay attention to the future will be repurchased ❥❥❥❥	Jingdong prefecture level city in Shandong
Jinfeng Hongyuan Flagship Store	Electricity conservation2	Baby received it 👌, really great ah! 🌈The quality can't be beat, very satisfied! 🆗Happy happy happy 😀	Jingdong prefecture level city in Shandong
Jinfeng Hongyuan Flagship Store	Electricity conservation2	Very good, power saving see, this is to help a friend to buy, I used to give my friend a say, my friend immediately let me order another one!	Jingdong prefecture level city in Shandong
Jinfeng Hongyuan Flagship Store	Electricity conservation2	The baby is just as introduced, the texture is very good, powerful, and it feels good to use it	Jingdong prefecture level city in Shandong
Jinfeng Hongyuan Flagship Store	Electricity conservation2	Value for money, very satisfied, seller service attitude is very good, very patient	Jingdong prefecture level city in Shandong
Jinfeng Hongyuan Flagship Store	Electricity conservation2	Power saving treasure design unique exquisite beautiful, voltage display clearly, plugged in that is good to use, I observed the meter, after using the meter is gone slow.	Jingdong prefecture level city in Shandong
Jinfeng Hongyuan Flagship Store	Electricity conservation2	Received on the use of the packaging is beautifully used for a period of time and then come back in the follow-up review!	Jingdong prefecture level city in Shandong
Jinfeng Hongyuan Flagship Store	Electricity conservation2	It should work. Not bad. Not bad. Not bad.	Jingdong prefecture level city in Shandong
Jinfeng Hongyuan Flagship Store	Electricity conservation2	I haven't used it yet, I'll plug it in and see if it saves power next month.	Jingdong prefecture level city in Shandong
Jinfeng Hongyuan Flagship Store	Electricity conservation2	The goods received and unpacked and used, the quality is very good, things and the official website to buy no difference!	Jingdong prefecture level city in Shandong
Jinfeng Hongyuan Flagship Store	Electricity conservation2	It's a rip off, doesn't help much at all, uses the same amount of power as before it was used.	Jingdong prefecture level city in Shandong
Jinfeng Hongyuan Flagship Store	Electricity conservation2	I've been using it for a while now and haven't noticed any unusual problems, so it's okay	Jingdong prefecture level city in Shandong
Jinfeng Hongyuan Flagship Store	Electricity conservation2	Received the goods and plugged in to use, good or not there is no use will have to wait until next month to find out	Jingdong prefecture level city in Shandong
Jinfeng Hongyuan Flagship Store	Electricity conservation2	It's very compact, it looks good, I'll introduce you to my clients when it's ready.	Jingdong prefecture level city in Shandong
Jinfeng Hongyuan Flagship Store	Electricity conservation2	Good workmanship, we'll have to see in a few months.	Jingdong prefecture level city in Shandong
Jinfeng Hongyuan Flagship Store	Electricity conservation2	Jingdong quality! Always trustworthy!	Jingdong prefecture level city in Shandong
Jinfeng Hongyuan Flagship Store	Electricity conservation2	This is the second time I've bought it, and the merchant said to give me five dollars out of the difference, so thank you merchant.	Jingdong prefecture level city in Shandong
Jinfeng Hongyuan Flagship Store	Electricity conservation2	Good quality and good service	Jingdong prefecture level city in Shandong
Jinfeng Hongyuan Flagship Store	Electricity conservation2	Feels good texture, aluminum alloy shell, voltage digital display clear.	Jingdong prefecture level city in Shandong
Jinfeng Hongyuan Flagship Store	Electricity conservation2	Good product, fast and great service, thanks!	Jingdong prefecture level city in Shandong
Jinfeng Hongyuan Flagship Store	Electricity conservation2	Have not yet used, first evaluation, later really good do not need to comment again.	Jingdong prefecture level city in Shandong
Jinfeng Hongyuan Flagship Store	Electricity conservation2	Works great. Pretty much as described by the merchant.	Jingdong prefecture level city in Shandong
Jinfeng Hongyuan Flagship Store	Electricity conservation2	Received it, looking forward to the results, hope it works	Jingdong prefecture level city in Shandong
Jinfeng Hongyuan Flagship Store	Electricity conservation2	Received it, looking forward to the results, hope it works	Jingdong prefecture level city in Shandong
Jinfeng Hongyuan Flagship Store	Electricity conservation2	Supposedly it's an IQ tax. Buy one and try it. Looks good. Well made.	Jingdong prefecture level city in Shandong
Jinfeng Hongyuan Flagship Store	Electricity conservation2	It just doesn't feel like it's working.	Jingdong prefecture level city in Shandong

Jinfeng Hongyuan Flagship Store	Electricity conservation2	This is a great product.	Jingdong prefecture level city in Shandong
Jinfeng Hongyuan Flagship Store	Electricity conservation2	I'm just using it. I'll tell you more.	Jingdong prefecture level city in Shandong
Jinfeng Hongyuan Flagship Store	Electricity conservation2	Second repurchase and it's working fine.	Jingdong prefecture level city in Shandong
Jinfeng Hongyuan Flagship Store	Electricity conservation2	I'll wait a few days before evaluating	Jingdong prefecture level city in Shandong
Jinfeng Hongyuan Flagship Store	Electricity conservation2	I'll comment after I use it hopefully the section will be useful.	Jingdong prefecture level city in Shandong
Jinfeng Hongyuan Flagship Store	Electricity conservation2	not very useful	Jingdong prefecture level city in Shandong
Jinfeng Hongyuan Flagship Store	Electricity conservation2	Not received	Jingdong prefecture level city in Shandong
Jinfeng Hongyuan Flagship Store	Electricity conservation2	rare	Jingdong prefecture level city in Shandong
Jinfeng Hongyuan Flagship Store	Electricity conservation2	It's good.	Jingdong prefecture level city in Shandong
Jinfeng Hongyuan Flagship Store	Electricity conservation2	just now	Jingdong prefecture level city in Shandong
Jinfeng Hongyuan Flagship Store	Electricity conservation2	serviceable	Jingdong prefecture level city in Shandong
Jinfeng Hongyuan Flagship Store	Electricity conservation2	desirable	Jingdong prefecture level city in Shandong
Jinfeng Hongyuan Flagship Store	Electricity conservation2	serviceable	Jingdong prefecture level city in Shandong
Jinfeng Hongyuan Flagship Store	Electricity conservation2	It doesn't save power and costs electricity.	Jingdong prefecture level city in Shandong
Jinfeng Hongyuan Flagship Store	Electricity conservation2	Not as good as I thought.	Jingdong prefecture level city in Shandong
Jinfeng Hongyuan Flagship Store	Electricity conservation2	Super bad review, it doesn't save power at all, but rather more power consumption	Jingdong prefecture level city in Shandong
Jinfeng Hongyuan Flagship Store	Electricity conservation2	Listen to others say that this can save electricity, I bought with the idea of trying, used for a period of time before coming to the evaluation, will be a little bit of use, not a lot of savings just, long-term use of the words can save more.	Jingdong prefecture level city in Shandong
Jinfeng Hongyuan Flagship Store	Electricity conservation2	Logistics is fast, the next day arrived, workmanship is very delicate a power saver, looking forward to the late effect, customer service patiently explain the recommended which is suitable for, installed in what position is the best, look forward to using the effect after a period of time!	Jingdong prefecture level city in Shandong
Jinfeng Hongyuan Flagship Store	Electricity conservation2	It's good to use, I feel that it has been saving electricity, anyway, not a loss is, I used to feel that the power consumption is big, now I feel much better, my friends are also using, I hope to use a long time will be able to, I wish the boss a prosperous business	Jingdong prefecture level city in Shandong
Jinfeng Hongyuan Flagship Store	Electricity conservation2	Should be very good, to be able to plan the circuit, save the home of the circuit to the extent of the savings, waste of money on electricity, good if a month this money back, hope always hope to be able to	Jingdong prefecture level city in Shandong
Jinfeng Hongyuan Flagship Store	Electricity conservation2	Express delivery is very strong, the goods and I saw in the business consistent, the use of the effect of follow-up evaluation	Jingdong prefecture level city in Shandong
Jinfeng Hongyuan Flagship Store	Electricity conservation2	I have been using this brand of power saver for many years and this time I saw this upgraded version and got it again. The main purpose of this product is to reduce the loss of electricity for a long time, so as to achieve the purpose of saving electricity.	Jingdong prefecture level city in Shandong
Jinfeng Hongyuan Flagship Store	Electricity conservation2	Logistics is fast and the product looks beautiful, looking forward to the effect.	Jingdong prefecture level city in Shandong
Jinfeng Hongyuan Flagship Store	Electricity conservation2	Product quality is good, energy saving a little effect, but the price is a little expensive, fast arrival, good service.	Jingdong prefecture level city in Shandong
Jinfeng Hongyuan Flagship Store	Electricity conservation2	The quality is very good, inexpensive, Jingdong self-supporting guaranteed, worth having!	Jingdong prefecture level city in Shandong
Jinfeng Hongyuan Flagship Store	Electricity conservation2	I don't know if it's saving electricity yet, I'll find out next time I pay my electric bill	Jingdong prefecture level city in Shandong
Jinfeng Hongyuan Flagship Store	Electricity conservation2	Favorable price quality assurance appearance design is beautiful and generous, fine workmanship	Jingdong prefecture level city in Shandong
Jinfeng Hongyuan Flagship Store	Electricity conservation2	I bought it, I don't know how effective it is? I don't know how the quality is? After this summer results will know! Looking forward to it!	Jingdong prefecture level city in Shandong
Jinfeng Hongyuan Flagship Store	Electricity conservation2	Goods received Packaging intact, look at the shell workmanship is also good, high-power appliances open voltage has obvious changes in the effectiveness of the	Jingdong prefecture level city in Shandong
Jinfeng Hongyuan Flagship Store	Electricity conservation2	The goods have been received logistics is very fast, the product is similar to the line, just wait to use a month to see if it can save electricity, stay tuned.	Jingdong prefecture level city in Shandong
Jinfeng Hongyuan Flagship Store	Electricity conservation2	Electricity is plugged in, not sure if it will work, hopefully it will work.	Jingdong prefecture level city in Shandong
Jinfeng Hongyuan Flagship Store	Electricity conservation2	The shell is aluminum alloy, very good workmanship, the actual effect of waiting for the addition of evaluation	Jingdong prefecture level city in Shandong
Jinfeng Hongyuan Flagship Store	Electricity conservation2	Received, already plugged in, looking forward to the effect.	Jingdong prefecture level city in Shandong
Jinfeng Hongyuan Flagship Store	Electricity conservation2	Opened to see the regular manufacturer products, wait for a period of time to see it	Jingdong prefecture level city in Shandong
Jinfeng Hongyuan Flagship Store	Electricity conservation2	🉑 Baby received, very surprised ✨, reasonable design, fashionable and generous, continue to pay attention to the future will be repurchased ❤❤❤❤	Jingdong prefecture level city in Shandong
Jinfeng Hongyuan Flagship Store	Electricity conservation2	Baby received it 👌, really great ah! 🌈The quality can't be beat, very satisfied! 🆗Happy happy happy 😀	Jingdong prefecture level city in Shandong
Jinfeng Hongyuan Flagship Store	Electricity conservation2	Very good, power saving see, this is to help a friend to buy, I used to give my friend a say, my friend immediately let me order another one!	Jingdong prefecture level city in Shandong
Jinfeng Hongyuan Flagship Store	Electricity conservation2	The baby is just as introduced, the texture is very good, powerful, and it feels good to use it	Jingdong prefecture level city in Shandong
Jinfeng Hongyuan Flagship Store	Electricity conservation2	Value for money, very satisfied, seller service attitude is very good, very patient	Jingdong prefecture level city in Shandong
Jinfeng Hongyuan Flagship Store	Electricity conservation2	Power saving treasure design unique exquisite beautiful, voltage display clearly, plugged in that is good to use, I observed the meter, after using the meter is gone slow.	Jingdong prefecture level city in Shandong
Jinfeng Hongyuan Flagship Store	Electricity conservation2	Received on the use of the packaging is beautifully used for a period of time and then come back in the follow-up review!	Jingdong prefecture level city in Shandong
Jinfeng Hongyuan Flagship Store	Electricity conservation2	It should work. Not bad. Not bad. Not bad.	Jingdong prefecture level city in Shandong
Jinfeng Hongyuan Flagship Store	Electricity conservation2	I haven't used it yet, I'll plug it in and see if it saves power next month.	Jingdong prefecture level city in Shandong
Jinfeng Hongyuan Flagship Store	Electricity conservation2	The goods received and unpacked and used, the quality is very good, things and the official website to buy no difference!	Jingdong prefecture level city in Shandong
Jinfeng Hongyuan Flagship Store	Electricity conservation2	It's a rip off, doesn't help much at all, uses the same amount of power as before it was used.	Jingdong prefecture level city in Shandong
Jinfeng Hongyuan Flagship Store	Electricity conservation2	I've been using it for a while now and haven't noticed any unusual problems, so it's okay	Jingdong prefecture level city in Shandong
Jinfeng Hongyuan Flagship Store	Electricity conservation2	Received the goods and plugged in to use, good or not there is no use will have to wait until next month to find out	Jingdong prefecture level city in Shandong
Jinfeng Hongyuan Flagship Store	Electricity conservation2	It's very compact, it looks good, I'll introduce you to my clients when it's ready.	Jingdong prefecture level city in Shandong
Jinfeng Hongyuan Flagship Store	Electricity conservation2	Good workmanship, we'll have to see in a few months.	Jingdong prefecture level city in Shandong

Jinfeng Hongyuan Flagship Store	Electricity conservation2	Jingdong quality! Always trustworthy!	Jingdong prefecture level city in Shandong
Jinfeng Hongyuan Flagship Store	Electricity conservation2	This is the second time I've bought it, and the merchant said to give me five dollars out of the difference, so thank you merchant.	Jingdong prefecture level city in Shandong
Jinfeng Hongyuan Flagship Store	Electricity conservation2	Good quality and good service	Jingdong prefecture level city in Shandong
Jinfeng Hongyuan Flagship Store	Electricity conservation2	Feels good texture, aluminum alloy shell, voltage digital display clear.	Jingdong prefecture level city in Shandong
Jinfeng Hongyuan Flagship Store	Electricity conservation2	Good product, fast and great service, thanks!	Jingdong prefecture level city in Shandong
Jinfeng Hongyuan Flagship Store	Electricity conservation2	Have not yet used, first evaluation, later really good do not need to comment again.	Jingdong prefecture level city in Shandong
Jinfeng Hongyuan Flagship Store	Electricity conservation2	Works great. Pretty much as described by the merchant.	Jingdong prefecture level city in Shandong
Jinfeng Hongyuan Flagship Store	Electricity conservation2	Received it, looking forward to the results, hope it works	Jingdong prefecture level city in Shandong
Jinfeng Hongyuan Flagship Store	Electricity conservation2	Received it, looking forward to the results, hope it works	Jingdong prefecture level city in Shandong
Jinfeng Hongyuan Flagship Store	Electricity conservation2	Supposedly it's an IQ tax. Buy one and try it. Looks good. Well made.	Jingdong prefecture level city in Shandong
Jinfeng Hongyuan Flagship Store	Electricity conservation2	It just doesn't feel like it's working.	Jingdong prefecture level city in Shandong
Jinfeng Hongyuan Flagship Store	Electricity conservation2	This is a great product.	Jingdong prefecture level city in Shandong
Jinfeng Hongyuan Flagship Store	Electricity conservation2	I'm just using it. I'll tell you more.	Jingdong prefecture level city in Shandong
Jinfeng Hongyuan Flagship Store	Electricity conservation2	Second repurchase and it's working fine.	Jingdong prefecture level city in Shandong
Jinfeng Hongyuan Flagship Store	Electricity conservation2	I'll wait a few days before evaluating	Jingdong prefecture level city in Shandong
Jinfeng Hongyuan Flagship Store	Electricity conservation2	I'll comment after I use it hopefully the section will be useful.	Jingdong prefecture level city in Shandong
Jinfeng Hongyuan Flagship Store	Electricity conservation2	not very useful	Jingdong prefecture level city in Shandong
Jinfeng Hongyuan Flagship Store	Electricity conservation2	Not received	Jingdong prefecture level city in Shandong
Jinfeng Hongyuan Flagship Store	Electricity conservation2	rare	Jingdong prefecture level city in Shandong
Jinfeng Hongyuan Flagship Store	Electricity conservation2	It's good.	Jingdong prefecture level city in Shandong
Jinfeng Hongyuan Flagship Store	Electricity conservation2	just now	Jingdong prefecture level city in Shandong
Jinfeng Hongyuan Flagship Store	Electricity conservation2	serviceable	Jingdong prefecture level city in Shandong
Jinfeng Hongyuan Flagship Store	Electricity conservation2	desirable	Jingdong prefecture level city in Shandong
Jinfeng Hongyuan Flagship Store	Electricity conservation2	serviceable	Jingdong prefecture level city in Shandong
Jinfeng Hongyuan Flagship Store	Electricity conservation2	It doesn't save power and costs electricity.	Jingdong prefecture level city in Shandong
Jinfeng Hongyuan Flagship Store	Electricity conservation2	Not as good as I thought.	Jingdong prefecture level city in Shandong
Jinfeng Hongyuan Flagship Store	Electricity conservation2	Super bad review, it doesn't save power at all, but rather more power consumption	Jingdong prefecture level city in Shandong
Jinfeng Hongyuan Flagship Store	Electricity conservation2	Listen to others say that this can save electricity, I bought with the idea of trying, used for a period of time before coming to the evaluation, will be a little bit of use, not a lot of savings just, long-term use of the words can save more.	Jingdong prefecture level city in Shandong
Jinfeng Hongyuan Flagship Store	Electricity conservation2	Logistics is fast, the next day arrived, workmanship is very delicate a power saver, looking forward to the late effect, customer service patiently explain the recommended which is suitable for, installed in what position is the best, look forward to using the effect after a period of time!	Jingdong prefecture level city in Shandong
Jinfeng Hongyuan Flagship Store	Electricity conservation2	It's good to use, I feel that it has been saving electricity, anyway, not a loss is, I used to feel that the power consumption is big, now I feel much better, my friends are also using, I hope to use a long time will be able to, I wish the boss a prosperous business	Jingdong prefecture level city in Shandong
Jinfeng Hongyuan Flagship Store	Electricity conservation2	Should be very good, to be able to plan the circuit, save the home of the circuit to the extent of the savings, waste of money on electricity, good if a month this money back, hope always hope to be able to	Jingdong prefecture level city in Shandong
Jinfeng Hongyuan Flagship Store	Electricity conservation2	Express delivery is very strong, the goods and I saw in the business consistent, the use of the effect of follow-up evaluation	Jingdong prefecture level city in Shandong
Jinfeng Hongyuan Flagship Store	Electricity conservation2	I have been using this brand of power saver for many years and this time I saw this upgraded version and got it again. The main purpose of this product is to reduce the loss of electricity for a long time, so as to achieve the purpose of saving electricity.	Jingdong prefecture level city in Shandong
Jinfeng Hongyuan Flagship Store	Electricity conservation2	Logistics is fast and the product looks beautiful, looking forward to the effect.	Jingdong prefecture level city in Shandong
Jinfeng Hongyuan Flagship Store	Electricity conservation2	Product quality is good, energy saving a little effect, but the price is a little expensive, fast arrival, good service.	Jingdong prefecture level city in Shandong
Jinfeng Hongyuan Flagship Store	Electricity conservation2	The quality is very good, inexpensive, Jingdong self-supporting guaranteed, worth having!	Jingdong prefecture level city in Shandong
Jinfeng Hongyuan Flagship Store	Electricity conservation2	I don't know if it's saving electricity yet, I'll find out next time I pay my electric bill	Jingdong prefecture level city in Shandong
Jinfeng Hongyuan Flagship Store	Electricity conservation2	Favorable price quality assurance appearance design is beautiful and generous, fine workmanship	Jingdong prefecture level city in Shandong
Jinfeng Hongyuan Flagship Store	Electricity conservation2	I bought it, I don't know how effective it is? I don't know how the quality is? After this summer results will know! Looking forward to it!	Jingdong prefecture level city in Shandong
Jinfeng Hongyuan Flagship Store	Electricity conservation2	Goods received Packaging intact, look at the shell workmanship is also good, high-power appliances open voltage has obvious changes in the effectiveness of the	Jingdong prefecture level city in Shandong
Jinfeng Hongyuan Flagship Store	Electricity conservation2	The goods have been received logistics is very fast, the product is similar to the line, just wait to use a month to see if it can save electricity, stay tuned.	Jingdong prefecture level city in Shandong
Jinfeng Hongyuan Flagship Store	Electricity conservation2	Electricity is plugged in, not sure if it will work, hopefully it will work.	Jingdong prefecture level city in Shandong
Jinfeng Hongyuan Flagship Store	Electricity conservation2	The shell is aluminum alloy, very good workmanship, the actual effect of waiting for the addition of evaluation	Jingdong prefecture level city in Shandong
Jinfeng Hongyuan Flagship Store	Electricity conservation2	Received, already plugged in, looking forward to the effect.	Jingdong prefecture level city in Shandong
Jinfeng Hongyuan Flagship Store	Electricity conservation2	Opened to see the regular manufacturer products, wait for a period of time to see it	Jingdong prefecture level city in Shandong
Jinfeng Hongyuan Flagship Store	Electricity conservation2	🉑 Baby received, very surprised ✨, reasonable design, fashionable and generous, continue to pay attention to the future will be repurchased ❥❥❥❥	Jingdong prefecture level city in Shandong
Jinfeng Hongyuan Flagship Store	Electricity conservation2	Baby received it 👌, really great ah! 🌈The quality can't be beat, very satisfied! 🆗Happy happy happy 😃	Jingdong prefecture level city in Shandong
Jinfeng Hongyuan Flagship Store	Electricity conservation2	Very good, power saving see, this is to help a friend to buy, I used to give my friend a say, my friend immediately let me order another one!	Jingdong prefecture level city in Shandong
Jinfeng Hongyuan Flagship Store	Electricity conservation2	The baby is just as introduced, the texture is very good, powerful, and it feels good to use it	Jingdong prefecture level city in Shandong

Jinfeng Hongyuan Flagship Store	Electricity conservation2	Value for money, very satisfied, seller service attitude is very good, very patient	Jingdong prefecture level city in Shandong
Jinfeng Hongyuan Flagship Store	Electricity conservation2	Power saving treasure design unique exquisite beautiful, voltage display clearly, plugged in that is good to use, I observed the meter, after using the meter is gone slow.	Jingdong prefecture level city in Shandong
Jinfeng Hongyuan Flagship Store	Electricity conservation2	Received on the use of the packaging is beautifully used for a period of time and then come back in the follow-up review!	Jingdong prefecture level city in Shandong
Jinfeng Hongyuan Flagship Store	Electricity conservation2	It should work. Not bad. Not bad. Not bad.	Jingdong prefecture level city in Shandong
Jinfeng Hongyuan Flagship Store	Electricity conservation2	I haven't used it yet, I'll plug it in and see if it saves power next month.	Jingdong prefecture level city in Shandong
Jinfeng Hongyuan Flagship Store	Electricity conservation2	The goods received and unpacked and used, the quality is very good, things and the official website to buy no difference!	Jingdong prefecture level city in Shandong
Jinfeng Hongyuan Flagship Store	Electricity conservation2	It's a rip off, doesn't help much at all, uses the same amount of power as before it was used.	Jingdong prefecture level city in Shandong
Jinfeng Hongyuan Flagship Store	Electricity conservation2	I've been using it for a while now and haven't noticed any unusual problems, so it's okay	Jingdong prefecture level city in Shandong
Jinfeng Hongyuan Flagship Store	Electricity conservation2	Received the goods and plugged in to use, good or not there is no use will have to wait until next month to find out	Jingdong prefecture level city in Shandong
Jinfeng Hongyuan Flagship Store	Electricity conservation2	It's very compact, it looks good, I'll introduce you to my clients when it's ready.	Jingdong prefecture level city in Shandong
Jinfeng Hongyuan Flagship Store	Electricity conservation2	Good workmanship, we'll have to see in a few months.	Jingdong prefecture level city in Shandong
Jinfeng Hongyuan Flagship Store	Electricity conservation2	Jingdong quality! Always trustworthy!	Jingdong prefecture level city in Shandong
Jinfeng Hongyuan Flagship Store	Electricity conservation2	This is the second time I've bought it, and the merchant said to give me five dollars out of the difference, so thank you merchant.	Jingdong prefecture level city in Shandong
Jinfeng Hongyuan Flagship Store	Electricity conservation2	Good quality and good service	Jingdong prefecture level city in Shandong
Jinfeng Hongyuan Flagship Store	Electricity conservation2	Feels good texture, aluminum alloy shell, voltage digital display clear.	Jingdong prefecture level city in Shandong
Jinfeng Hongyuan Flagship Store	Electricity conservation2	Good product, fast and great service, thanks!	Jingdong prefecture level city in Shandong
Jinfeng Hongyuan Flagship Store	Electricity conservation2	Have not yet used, first evaluation, later really good do not need to comment again.	Jingdong prefecture level city in Shandong
Jinfeng Hongyuan Flagship Store	Electricity conservation2	Works great. Pretty much as described by the merchant.	Jingdong prefecture level city in Shandong
Jinfeng Hongyuan Flagship Store	Electricity conservation2	Received it, looking forward to the results, hope it works	Jingdong prefecture level city in Shandong
Jinfeng Hongyuan Flagship Store	Electricity conservation2	Received it, looking forward to the results, hope it works	Jingdong prefecture level city in Shandong
Jinfeng Hongyuan Flagship Store	Electricity conservation2	Supposedly it's an IQ tax. Buy one and try it. Looks good. Well made.	Jingdong prefecture level city in Shandong
Jinfeng Hongyuan Flagship Store	Electricity conservation2	It just doesn't feel like it's working.	Jingdong prefecture level city in Shandong
Jinfeng Hongyuan Flagship Store	Electricity conservation2	This is a great product.	Jingdong prefecture level city in Shandong
Jinfeng Hongyuan Flagship Store	Electricity conservation2	I'm just using it. I'll tell you more.	Jingdong prefecture level city in Shandong
Jinfeng Hongyuan Flagship Store	Electricity conservation2	Second repurchase and it's working fine.	Jingdong prefecture level city in Shandong
Jinfeng Hongyuan Flagship Store	Electricity conservation2	I'll wait a few days before evaluating	Jingdong prefecture level city in Shandong
Jinfeng Hongyuan Flagship Store	Electricity conservation2	I'll comment after I use it hopefully the section will be useful.	Jingdong prefecture level city in Shandong
Jinfeng Hongyuan Flagship Store	Electricity conservation2	not very useful	Jingdong prefecture level city in Shandong
Jinfeng Hongyuan Flagship Store	Electricity conservation2	Not received	Jingdong prefecture level city in Shandong
Jinfeng Hongyuan Flagship Store	Electricity conservation2	rare	Jingdong prefecture level city in Shandong
Jinfeng Hongyuan Flagship Store	Electricity conservation2	It's good.	Jingdong prefecture level city in Shandong
Jinfeng Hongyuan Flagship Store	Electricity conservation2	just now	Jingdong prefecture level city in Shandong
Jinfeng Hongyuan Flagship Store	Electricity conservation2	serviceable	Jingdong prefecture level city in Shandong
Jinfeng Hongyuan Flagship Store	Electricity conservation2	desirable	Jingdong prefecture level city in Shandong
Jinfeng Hongyuan Flagship Store	Electricity conservation2	serviceable	Jingdong prefecture level city in Shandong
Jinfeng Hongyuan Flagship Store	Electricity conservation2	It doesn't save power and costs electricity.	Jingdong prefecture level city in Shandong
Jinfeng Hongyuan Flagship Store	Electricity conservation2	Not as good as I thought.	Jingdong prefecture level city in Shandong
Jinfeng Hongyuan Flagship Store	Electricity conservation2	Super bad review, it doesn't save power at all, but rather more power consumption	Jingdong prefecture level city in Shandong
Jinfeng Hongyuan Flagship Store	Electricity conservation2	Listen to others say that this can save electricity, I bought with the idea of trying, used for a period of time before coming to the evaluation, will be a little bit of use, not a lot of savings just, long-term use of the words can save more.	Jingdong prefecture level city in Shandong
Jinfeng Hongyuan Flagship Store	Electricity conservation2	Logistics is fast, the next day arrived, workmanship is very delicate a power saver, looking forward to the late effect, customer service patiently explain the recommended which is suitable for, installed in what position is the best, look forward to using the effect after a period of time!	Jingdong prefecture level city in Shandong
Jinfeng Hongyuan Flagship Store	Electricity conservation2	It's good to use, I feel that it has been saving electricity, anyway, not a loss is, I used to feel that the power consumption is big, now I feel much better, my friends are also using, I hope to use a long time will be able to, I wish the boss a prosperous business	Jingdong prefecture level city in Shandong
Jinfeng Hongyuan Flagship Store	Electricity conservation2	Should be very good, to be able to plan the circuit, save the home of the circuit to the extent of the savings, waste of money on electricity, good if a month this money back, hope always hope to be able to	Jingdong prefecture level city in Shandong
Jinfeng Hongyuan Flagship Store	Electricity conservation2	Express delivery is very strong, the goods and I saw in the business consistent, the use of the effect of follow-up evaluation	Jingdong prefecture level city in Shandong
Jinfeng Hongyuan Flagship Store	Electricity conservation2	I have been using this brand of power saver for many years and this time I saw this upgraded version and got it again. The main purpose of this product is to reduce the loss of electricity for a long time, so as to achieve the purpose of saving electricity.	Jingdong prefecture level city in Shandong
Jinfeng Hongyuan Flagship Store	Electricity conservation2	Logistics is fast and the product looks beautiful, looking forward to the effect.	Jingdong prefecture level city in Shandong
Jinfeng Hongyuan Flagship Store	Electricity conservation2	Product quality is good, energy saving a little effect, but the price is a little expensive, fast arrival, good service.	Jingdong prefecture level city in Shandong
Jinfeng Hongyuan Flagship Store	Electricity conservation2	The quality is very good, inexpensive, Jingdong self-supporting guaranteed, worth having!	Jingdong prefecture level city in Shandong
Jinfeng Hongyuan Flagship Store	Electricity conservation2	I don't know if it's saving electricity yet, I'll find out next time I pay my electric bill	Jingdong prefecture level city in Shandong
Jinfeng Hongyuan Flagship Store	Electricity conservation2	Favorable price quality assurance appearance design is beautiful and generous, fine workmanship	Jingdong prefecture level city in Shandong

Jinfeng Hongyuan Flagship Store	Electricity conservation2	I bought it, I don't know how effective it is? I don't know how the quality is? After this summer results will know! Looking forward to it!	Jingdong prefecture level city in Shandong
Jinfeng Hongyuan Flagship Store	Electricity conservation2	Goods received Packaging intact, look at the shell workmanship is also good, high-power appliances open voltage has obvious changes in the effectiveness of the	Jingdong prefecture level city in Shandong
Jinfeng Hongyuan Flagship Store	Electricity conservation2	The goods have been received logistics is very fast, the product is similar to the line, just wait to use a month to see if it can save electricity, stay tuned.	Jingdong prefecture level city in Shandong
Jinfeng Hongyuan Flagship Store	Electricity conservation2	Electricity is plugged in, not sure if it will work, hopefully it will work.	Jingdong prefecture level city in Shandong
Jinfeng Hongyuan Flagship Store	Electricity conservation2	The shell is aluminum alloy, very good workmanship, the actual effect of waiting for the addition of evaluation	Jingdong prefecture level city in Shandong
Jinfeng Hongyuan Flagship Store	Electricity conservation2	Received, already plugged in, looking forward to the effect.	Jingdong prefecture level city in Shandong
Jinfeng Hongyuan Flagship Store	Electricity conservation2	Opened to see the regular manufacturer products, wait for a period of time to see it	Jingdong prefecture level city in Shandong
Jinfeng Hongyuan Flagship Store	Electricity conservation2	🉑 Baby received, very surprised ✨, reasonable design, fashionable and generous, continue to pay attention to the future will be repurchased ❥❥❥❥	Jingdong prefecture level city in Shandong
Jinfeng Hongyuan Flagship Store	Electricity conservation2	Baby received it 👌, really great ah! 🌈The quality can't be beat, very satisfied! 🆗Happy happy happy 😃	Jingdong prefecture level city in Shandong
Jinfeng Hongyuan Flagship Store	Electricity conservation2	Very good, power saving see, this is to help a friend to buy, I used to give my friend a say, my friend immediately let me order another one!	Jingdong prefecture level city in Shandong
Jinfeng Hongyuan Flagship Store	Electricity conservation2	The baby is just as introduced, the texture is very good, powerful, and it feels good to use it	Jingdong prefecture level city in Shandong
Jinfeng Hongyuan Flagship Store	Electricity conservation2	Value for money, very satisfied, seller service attitude is very good, very patient	Jingdong prefecture level city in Shandong
Jinfeng Hongyuan Flagship Store	Electricity conservation2	Power saving treasure design unique exquisite beautiful, voltage display clearly, plugged in that is good to use, I observed the meter, after using the meter is gone slow.	Jingdong prefecture level city in Shandong
Jinfeng Hongyuan Flagship Store	Electricity conservation2	Received on the use of the packaging is beautifully used for a period of time and then come back in the follow-up review!	Jingdong prefecture level city in Shandong
Jinfeng Hongyuan Flagship Store	Electricity conservation2	It should work. Not bad. Not bad. Not bad.	Jingdong prefecture level city in Shandong
Jinfeng Hongyuan Flagship Store	Electricity conservation2	I haven't used it yet, I'll plug it in and see if it saves power next month.	Jingdong prefecture level city in Shandong
Jinfeng Hongyuan Flagship Store	Electricity conservation2	The goods received and unpacked and used, the quality is very good, things and the official website to buy no difference!	Jingdong prefecture level city in Shandong
Jinfeng Hongyuan Flagship Store	Electricity conservation2	It's a rip off, doesn't help much at all, uses the same amount of power as before it was used.	Jingdong prefecture level city in Shandong
Jinfeng Hongyuan Flagship Store	Electricity conservation2	I've been using it for a while now and haven't noticed any unusual problems, so it's okay	Jingdong prefecture level city in Shandong
Jinfeng Hongyuan Flagship Store	Electricity conservation2	Received the goods and plugged in to use, good or not there is no use will have to wait until next month to find out	Jingdong prefecture level city in Shandong
Jinfeng Hongyuan Flagship Store	Electricity conservation2	It's very compact, it looks good, I'll introduce you to my clients when it's ready.	Jingdong prefecture level city in Shandong
Jinfeng Hongyuan Flagship Store	Electricity conservation2	Good workmanship, we'll have to see in a few months.	Jingdong prefecture level city in Shandong
Jinfeng Hongyuan Flagship Store	Electricity conservation2	Jingdong quality! Always trustworthy!	Jingdong prefecture level city in Shandong
Jinfeng Hongyuan Flagship Store	Electricity conservation2	This is the second time I've bought it, and the merchant said to give me five dollars out of the difference, so thank you merchant.	Jingdong prefecture level city in Shandong
Jinfeng Hongyuan Flagship Store	Electricity conservation2	Good quality and good service	Jingdong prefecture level city in Shandong
Jinfeng Hongyuan Flagship Store	Electricity conservation2	Feels good texture, aluminum alloy shell, voltage digital display clear.	Jingdong prefecture level city in Shandong
Jinfeng Hongyuan Flagship Store	Electricity conservation2	Good product, fast and great service, thanks!	Jingdong prefecture level city in Shandong
Jinfeng Hongyuan Flagship Store	Electricity conservation2	Have not yet used, first evaluation, later really good do not need to comment again.	Jingdong prefecture level city in Shandong
Jinfeng Hongyuan Flagship Store	Electricity conservation2	Works great. Pretty much as described by the merchant.	Jingdong prefecture level city in Shandong
Jinfeng Hongyuan Flagship Store	Electricity conservation2	Received it, looking forward to the results, hope it works	Jingdong prefecture level city in Shandong
Jinfeng Hongyuan Flagship Store	Electricity conservation2	Received it, looking forward to the results, hope it works	Jingdong prefecture level city in Shandong
Jinfeng Hongyuan Flagship Store	Electricity conservation2	Supposedly it's an IQ tax. Buy one and try it. Looks good. Well made.	Jingdong prefecture level city in Shandong
Jinfeng Hongyuan Flagship Store	Electricity conservation2	It just doesn't feel like it's working.	Jingdong prefecture level city in Shandong
Jinfeng Hongyuan Flagship Store	Electricity conservation2	This is a great product.	Jingdong prefecture level city in Shandong
Jinfeng Hongyuan Flagship Store	Electricity conservation2	I'm just using it. I'll tell you more.	Jingdong prefecture level city in Shandong
Jinfeng Hongyuan Flagship Store	Electricity conservation2	Second repurchase and it's working fine.	Jingdong prefecture level city in Shandong
Jinfeng Hongyuan Flagship Store	Electricity conservation2	I'll wait a few days before evaluating	Jingdong prefecture level city in Shandong
Jinfeng Hongyuan Flagship Store	Electricity conservation2	I'll comment after I use it hopefully the section will be useful.	Jingdong prefecture level city in Shandong
Jinfeng Hongyuan Flagship Store	Electricity conservation2	not very useful	Jingdong prefecture level city in Shandong
Jinfeng Hongyuan Flagship Store	Electricity conservation2	Not received	Jingdong prefecture level city in Shandong
Jinfeng Hongyuan Flagship Store	Electricity conservation2	rare	Jingdong prefecture level city in Shandong
Jinfeng Hongyuan Flagship Store	Electricity conservation2	It's good.	Jingdong prefecture level city in Shandong
Jinfeng Hongyuan Flagship Store	Electricity conservation2	just now	Jingdong prefecture level city in Shandong
Jinfeng Hongyuan Flagship Store	Electricity conservation2	serviceable	Jingdong prefecture level city in Shandong
Jinfeng Hongyuan Flagship Store	Electricity conservation2	desirable	Jingdong prefecture level city in Shandong
Jinfeng Hongyuan Flagship Store	Electricity conservation2	serviceable	Jingdong prefecture level city in Shandong
Jinfeng Hongyuan Flagship Store	Electricity conservation2	It doesn't save power and costs electricity.	Jingdong prefecture level city in Shandong
Jinfeng Hongyuan Flagship Store	Electricity conservation2	Not as good as I thought.	Jingdong prefecture level city in Shandong
Jinfeng Hongyuan Flagship Store	Electricity conservation2	Super bad review, it doesn't save power at all, but rather more power consumption	Jingdong prefecture level city in Shandong
Jinfeng Hongyuan Flagship Store	Electricity conservation2	Listen to others say that this can save electricity, I bought with the idea of trying, used for a period of time before coming to the evaluation, will be a little bit of use, not a lot of savings just, long-term use of the words can save more.	Jingdong prefecture level city in Shandong

Jinfeng Hongyuan Flagship Store	Electricity conservation2	Logistics is fast, the next day arrived, workmanship is very delicate a power saver, looking forward to the late effect, customer service patiently explain the recommended which is suitable for, installed in what position is the best, look forward to using the effect after a period of time!	Jingdong prefecture level city in Shandong
Jinfeng Hongyuan Flagship Store	Electricity conservation2	It's good to use, I feel that it has been saving electricity, anyway, not a loss is, I used to feel that the power consumption is big, now I feel much better, my friends are also using, I hope to use a long time will be able to, I wish the boss a prosperous business	Jingdong prefecture level city in Shandong
Jinfeng Hongyuan Flagship Store	Electricity conservation2	Should be very good, to be able to plan the circuit, save the home of the circuit to the extent of the savings, waste of money on electricity, good if a month this money back, hope always hope to be able to	Jingdong prefecture level city in Shandong
Jinfeng Hongyuan Flagship Store	Electricity conservation2	Express delivery is very strong, the goods and I saw in the business consistent, the use of the effect of follow-up evaluation	Jingdong prefecture level city in Shandong
Jinfeng Hongyuan Flagship Store	Electricity conservation2	I have been using this brand of power saver for many years and this time I saw this upgraded version and got it again. The main purpose of this product is to reduce the loss of electricity for a long time, so as to achieve the purpose of saving electricity.	Jingdong prefecture level city in Shandong
Jinfeng Hongyuan Flagship Store	Electricity conservation2	Logistics is fast and the product looks beautiful, looking forward to the effect.	Jingdong prefecture level city in Shandong
Jinfeng Hongyuan Flagship Store	Electricity conservation2	Product quality is good, energy saving a little effect, but the price is a little expensive, fast arrival, good service.	Jingdong prefecture level city in Shandong
Jinfeng Hongyuan Flagship Store	Electricity conservation2	The quality is very good, inexpensive, Jingdong self-supporting guaranteed, worth having!	Jingdong prefecture level city in Shandong
Jinfeng Hongyuan Flagship Store	Electricity conservation2	I don't know if it's saving electricity yet, I'll find out next time I pay my electric bill	Jingdong prefecture level city in Shandong
Jinfeng Hongyuan Flagship Store	Electricity conservation2	Favorable price quality assurance appearance design is beautiful and generous, fine workmanship	Jingdong prefecture level city in Shandong
Jinfeng Hongyuan Flagship Store	Electricity conservation2	I bought it, I don't know how effective it is? I don't know how the quality is? After this summer results will know! Looking forward to it!	Jingdong prefecture level city in Shandong
Jinfeng Hongyuan Flagship Store	Electricity conservation2	Goods received Packaging intact, look at the shell workmanship is also good, high-power appliances open voltage has obvious changes in the effectiveness of the	Jingdong prefecture level city in Shandong
Jinfeng Hongyuan Flagship Store	Electricity conservation2	The goods have been received logistics is very fast, the product is similar to the line, just wait to use a month to see if it can save electricity, stay tuned.	Jingdong prefecture level city in Shandong
Jinfeng Hongyuan Flagship Store	Electricity conservation2	Electricity is plugged in, not sure if it will work, hopefully it will work.	Jingdong prefecture level city in Shandong
Jinfeng Hongyuan Flagship Store	Electricity conservation2	The shell is aluminum alloy, very good workmanship, the actual effect of waiting for the addition of evaluation	Jingdong prefecture level city in Shandong
Jinfeng Hongyuan Flagship Store	Electricity conservation2	Received, already plugged in, looking forward to the effect.	Jingdong prefecture level city in Shandong
Jinfeng Hongyuan Flagship Store	Electricity conservation2	Opened to see the regular manufacturer products, wait for a period of time to see it	Jingdong prefecture level city in Shandong
Jinfeng Hongyuan Flagship Store	Electricity conservation2	🉑 Baby received, very surprised ✨, reasonable design, fashionable and generous, continue to pay attention to the future will be repurchased ❥❥❥❥	Jingdong prefecture level city in Shandong
Jinfeng Hongyuan Flagship Store	Electricity conservation2	Baby received it 👌, really great ah! 🌈The quality can't be beat, very satisfied! 🆗Happy happy happy 😃	Jingdong prefecture level city in Shandong
Jinfeng Hongyuan Flagship Store	Electricity conservation2	Very good, power saving see, this is to help a friend to buy, I used to give my friend a say, my friend immediately let me order another one!	Jingdong prefecture level city in Shandong
Jinfeng Hongyuan Flagship Store	Electricity conservation2	The baby is just as introduced, the texture is very good, powerful, and it feels good to use it	Jingdong prefecture level city in Shandong
Jinfeng Hongyuan Flagship Store	Electricity conservation2	Value for money, very satisfied, seller service attitude is very good, very patient	Jingdong prefecture level city in Shandong
Jinfeng Hongyuan Flagship Store	Electricity conservation2	Power saving treasure design unique exquisite beautiful, voltage display clearly, plugged in that is good to use, I observed the meter, after using the meter is gone slow.	Jingdong prefecture level city in Shandong
Jinfeng Hongyuan Flagship Store	Electricity conservation2	Received on the use of the packaging is beautifully used for a period of time and then come back in the follow-up review!	Jingdong prefecture level city in Shandong
Jinfeng Hongyuan Flagship Store	Electricity conservation2	It should work. Not bad. Not bad. Not bad.	Jingdong prefecture level city in Shandong
Jinfeng Hongyuan Flagship Store	Electricity conservation2	I haven't used it yet, I'll plug it in and see if it saves power next month.	Jingdong prefecture level city in Shandong
Jinfeng Hongyuan Flagship Store	Electricity conservation2	The goods received and unpacked and used, the quality is very good, things and the official website to buy no difference!	Jingdong prefecture level city in Shandong
Jinfeng Hongyuan Flagship Store	Electricity conservation2	It's a rip off, doesn't help much at all, uses the same amount of power as before it was used.	Jingdong prefecture level city in Shandong
Jinfeng Hongyuan Flagship Store	Electricity conservation2	I've been using it for a while now and haven't noticed any unusual problems, so it's okay	Jingdong prefecture level city in Shandong
Jinfeng Hongyuan Flagship Store	Electricity conservation2	Received the goods and plugged in to use, good or not there is no use will have to wait until next month to find out	Jingdong prefecture level city in Shandong
Jinfeng Hongyuan Flagship Store	Electricity conservation2	It's very compact, it looks good, I'll introduce you to my clients when it's ready.	Jingdong prefecture level city in Shandong
Jinfeng Hongyuan Flagship Store	Electricity conservation2	Good workmanship, we'll have to see in a few months.	Jingdong prefecture level city in Shandong
Jinfeng Hongyuan Flagship Store	Electricity conservation2	Jingdong quality! Always trustworthy!	Jingdong prefecture level city in Shandong
Jinfeng Hongyuan Flagship Store	Electricity conservation2	This is the second time I've bought it, and the merchant said to give me five dollars out of the difference, so thank you merchant.	Jingdong prefecture level city in Shandong
Jinfeng Hongyuan Flagship Store	Electricity conservation2	Good quality and good service	Jingdong prefecture level city in Shandong
Jinfeng Hongyuan Flagship Store	Electricity conservation2	Feels good texture, aluminum alloy shell, voltage digital display clear.	Jingdong prefecture level city in Shandong
Jinfeng Hongyuan Flagship Store	Electricity conservation2	Good product, fast and great service, thanks!	Jingdong prefecture level city in Shandong
Jinfeng Hongyuan Flagship Store	Electricity conservation2	Have not yet used, first evaluation, later really good do not need to comment again.	Jingdong prefecture level city in Shandong
Jinfeng Hongyuan Flagship Store	Electricity conservation2	Works great. Pretty much as described by the merchant.	Jingdong prefecture level city in Shandong
Jinfeng Hongyuan Flagship Store	Electricity conservation2	Received it, looking forward to the results, hope it works	Jingdong prefecture level city in Shandong
Jinfeng Hongyuan Flagship Store	Electricity conservation2	Received it, looking forward to the results, hope it works	Jingdong prefecture level city in Shandong
Jinfeng Hongyuan Flagship Store	Electricity conservation2	Supposedly it's an IQ tax. Buy one and try it. Looks good. Well made.	Jingdong prefecture level city in Shandong
Jinfeng Hongyuan Flagship Store	Electricity conservation2	It just doesn't feel like it's working.	Jingdong prefecture level city in Shandong
Jinfeng Hongyuan Flagship Store	Electricity conservation2	This is a great product.	Jingdong prefecture level city in Shandong
Jinfeng Hongyuan Flagship Store	Electricity conservation2	I'm just using it. I'll tell you more.	Jingdong prefecture level city in Shandong
Jinfeng Hongyuan Flagship Store	Electricity conservation2	Second repurchase and it's working fine.	Jingdong prefecture level city in Shandong
Jinfeng Hongyuan Flagship Store	Electricity conservation2	I'll wait a few days before evaluating	Jingdong prefecture level city in Shandong
Jinfeng Hongyuan Flagship Store	Electricity conservation2	I'll comment after I use it hopefully the section will be useful.	Jingdong prefecture level city in Shandong
Jinfeng Hongyuan Flagship Store	Electricity conservation2	not very useful	Jingdong prefecture level city in Shandong
Jinfeng Hongyuan Flagship Store	Electricity conservation2	Not received	Jingdong prefecture level city in Shandong

Jinfeng Hongyuan Flagship Store	Electricity conservation2	rare	Jingdong prefecture level city in Shandong
Jinfeng Hongyuan Flagship Store	Electricity conservation2	It's good.	Jingdong prefecture level city in Shandong
Jinfeng Hongyuan Flagship Store	Electricity conservation2	just now	Jingdong prefecture level city in Shandong
Jinfeng Hongyuan Flagship Store	Electricity conservation2	serviceable	Jingdong prefecture level city in Shandong
Jinfeng Hongyuan Flagship Store	Electricity conservation2	desirable	Jingdong prefecture level city in Shandong
Jinfeng Hongyuan Flagship Store	Electricity conservation2	serviceable	Jingdong prefecture level city in Shandong
Jinfeng Hongyuan Flagship Store	Electricity conservation2	It doesn't save power and costs electricity.	Jingdong prefecture level city in Shandong
Jinfeng Hongyuan Flagship Store	Electricity conservation2	Not as good as I thought.	Jingdong prefecture level city in Shandong
Jinfeng Hongyuan Flagship Store	Electricity conservation2	Super bad review, it doesn't save power at all, but rather more power consumption	Jingdong prefecture level city in Shandong
Jinfeng Hongyuan Flagship Store	Electricity conservation2	Listen to others say that this can save electricity, I bought with the idea of trying, used for a period of time before coming to the evaluation, will be a little bit of use, not a lot of savings just, long-term use of the words can save more.	Jingdong prefecture level city in Shandong
Jinfeng Hongyuan Flagship Store	Electricity conservation2	Logistics is fast, the next day arrived, workmanship is very delicate a power saver, looking forward to the late effect, customer service patiently explain the recommended which is suitable for, installed in what position is the best, look forward to using the effect after a period of time!	Jingdong prefecture level city in Shandong
Jinfeng Hongyuan Flagship Store	Electricity conservation2	It's good to use, I feel that it has been saving electricity, anyway, not a loss is, I used to feel that the power consumption is big, now I feel much better, my friends are also using, I hope to use a long time will be able to, I wish the boss a prosperous business	Jingdong prefecture level city in Shandong
Jinfeng Hongyuan Flagship Store	Electricity conservation2	Should be very good, to be able to plan the circuit, save the home of the circuit to the extent of the savings, waste of money on electricity, good if a month this money back, hope always hope to be able to	Jingdong prefecture level city in Shandong
Jinfeng Hongyuan Flagship Store	Electricity conservation2	Express delivery is very strong, the goods and I saw in the business consistent, the use of the effect of follow-up evaluation	Jingdong prefecture level city in Shandong
Jinfeng Hongyuan Flagship Store	Electricity conservation2	I have been using this brand of power saver for many years and this time I saw this upgraded version and got it again. The main purpose of this product is to reduce the loss of electricity for a long time, so as to achieve the purpose of saving electricity.	Jingdong prefecture level city in Shandong
Jinfeng Hongyuan Flagship Store	Electricity conservation2	Logistics is fast and the product looks beautiful, looking forward to the effect.	Jingdong prefecture level city in Shandong
Jinfeng Hongyuan Flagship Store	Electricity conservation2	Product quality is good, energy saving a little effect, but the price is a little expensive, fast arrival, good service.	Jingdong prefecture level city in Shandong
Jinfeng Hongyuan Flagship Store	Electricity conservation2	The quality is very good, inexpensive, Jingdong self-supporting guaranteed, worth having!	Jingdong prefecture level city in Shandong
Jinfeng Hongyuan Flagship Store	Electricity conservation2	I don't know if it's saving electricity yet, I'll find out next time I pay my electric bill	Jingdong prefecture level city in Shandong
Jinfeng Hongyuan Flagship Store	Electricity conservation2	Favorable price quality assurance appearance design is beautiful and generous, fine workmanship	Jingdong prefecture level city in Shandong
Jinfeng Hongyuan Flagship Store	Electricity conservation2	I bought it, I don't know how effective it is? I don't know how the quality is? After this summer results will know! Looking forward to it!	Jingdong prefecture level city in Shandong
Jinfeng Hongyuan Flagship Store	Electricity conservation2	Goods received Packaging intact, look at the shell workmanship is also good, high-power appliances open voltage has obvious changes in the effectiveness of the	Jingdong prefecture level city in Shandong
Jinfeng Hongyuan Flagship Store	Electricity conservation2	The goods have been received logistics is very fast, the product is similar to the line, just wait to use a month to see if it can save electricity, stay tuned.	Jingdong prefecture level city in Shandong
Jinfeng Hongyuan Flagship Store	Electricity conservation2	Electricity is plugged in, not sure if it will work, hopefully it will work.	Jingdong prefecture level city in Shandong
Jinfeng Hongyuan Flagship Store	Electricity conservation2	The shell is aluminum alloy, very good workmanship, the actual effect of waiting for the addition of evaluation	Jingdong prefecture level city in Shandong
Jinfeng Hongyuan Flagship Store	Electricity conservation2	Received, already plugged in, looking forward to the effect.	Jingdong prefecture level city in Shandong
Jinfeng Hongyuan Flagship Store	Electricity conservation2	Opened to see the regular manufacturer products, wait for a period of time to see it	Jingdong prefecture level city in Shandong
Jinfeng Hongyuan Flagship Store	Electricity conservation2	🉑 Baby received, very surprised ✨, reasonable design, fashionable and generous, continue to pay attention to the future will be repurchased ❥❥❥❥	Jingdong prefecture level city in Shandong
Jinfeng Hongyuan Flagship Store	Electricity conservation2	Baby received it 👌, really great ah! 🌈The quality can't be beat, very satisfied! 🆗Happy happy happy 😃	Jingdong prefecture level city in Shandong
Jinfeng Hongyuan Flagship Store	Electricity conservation2	Very good, power saving see, this is to help a friend to buy, I used to give my friend a say, my friend immediately let me order another one!	Jingdong prefecture level city in Shandong
Jinfeng Hongyuan Flagship Store	Electricity conservation2	The baby is just as introduced, the texture is very good, powerful, and it feels good to use it	Jingdong prefecture level city in Shandong
Jinfeng Hongyuan Flagship Store	Electricity conservation2	Value for money, very satisfied, seller service attitude is very good, very patient	Jingdong prefecture level city in Shandong
Jinfeng Hongyuan Flagship Store	Electricity conservation2	Power saving treasure design unique exquisite beautiful, voltage display clearly, plugged in that is good to use, I observed the meter, after using the meter is gone slow.	Jingdong prefecture level city in Shandong
Jinfeng Hongyuan Flagship Store	Electricity conservation2	Received on the use of the packaging is beautifully used for a period of time and then come back in the follow-up review!	Jingdong prefecture level city in Shandong
Jinfeng Hongyuan Flagship Store	Electricity conservation2	It should work. Not bad. Not bad. Not bad.	Jingdong prefecture level city in Shandong
Jinfeng Hongyuan Flagship Store	Electricity conservation2	I haven't used it yet, I'll plug it in and see if it saves power next month.	Jingdong prefecture level city in Shandong
Jinfeng Hongyuan Flagship Store	Electricity conservation2	The goods received and unpacked and used, the quality is very good, things and the official website to buy no difference!	Jingdong prefecture level city in Shandong
Jinfeng Hongyuan Flagship Store	Electricity conservation2	It's a rip off, doesn't help much at all, uses the same amount of power as before it was used.	Jingdong prefecture level city in Shandong
Jinfeng Hongyuan Flagship Store	Electricity conservation2	I've been using it for a while now and haven't noticed any unusual problems, so it's okay	Jingdong prefecture level city in Shandong
Jinfeng Hongyuan Flagship Store	Electricity conservation2	Received the goods and plugged in to use, good or not there is no use will have to wait until next month to find out	Jingdong prefecture level city in Shandong
Jinfeng Hongyuan Flagship Store	Electricity conservation2	It's very compact, it looks good, I'll introduce you to my clients when it's ready.	Jingdong prefecture level city in Shandong
Jinfeng Hongyuan Flagship Store	Electricity conservation2	Good workmanship, we'll have to see in a few months.	Jingdong prefecture level city in Shandong
Jinfeng Hongyuan Flagship Store	Electricity conservation2	Jingdong quality! Always trustworthy!	Jingdong prefecture level city in Shandong
Jinfeng Hongyuan Flagship Store	Electricity conservation2	This is the second time I've bought it, and the merchant said to give me five dollars out of the difference, so thank you merchant.	Jingdong prefecture level city in Shandong
Jinfeng Hongyuan Flagship Store	Electricity conservation2	Good quality and good service	Jingdong prefecture level city in Shandong
Jinfeng Hongyuan Flagship Store	Electricity conservation2	Feels good texture, aluminum alloy shell, voltage digital display clear.	Jingdong prefecture level city in Shandong
Jinfeng Hongyuan Flagship Store	Electricity conservation2	Good product, fast and great service, thanks!	Jingdong prefecture level city in Shandong
Jinfeng Hongyuan Flagship Store	Electricity conservation2	Have not yet used, first evaluation, later really good do not need to comment again.	Jingdong prefecture level city in Shandong
Jinfeng Hongyuan Flagship Store	Electricity conservation2	Works great. Pretty much as described by the merchant.	Jingdong prefecture level city in Shandong

Jinfeng Hongyuan Flagship Store	Electricity conservation2	Received it, looking forward to the results, hope it works	Jingdong prefecture level city in Shandong
Jinfeng Hongyuan Flagship Store	Electricity conservation2	Received it, looking forward to the results, hope it works	Jingdong prefecture level city in Shandong
Jinfeng Hongyuan Flagship Store	Electricity conservation2	Supposedly it's an IQ tax. Buy one and try it. Looks good. Well made.	Jingdong prefecture level city in Shandong
Jinfeng Hongyuan Flagship Store	Electricity conservation2	It just doesn't feel like it's working.	Jingdong prefecture level city in Shandong
Jinfeng Hongyuan Flagship Store	Electricity conservation2	This is a great product.	Jingdong prefecture level city in Shandong
Jinfeng Hongyuan Flagship Store	Electricity conservation2	I'm just using it. I'll tell you more.	Jingdong prefecture level city in Shandong
Jinfeng Hongyuan Flagship Store	Electricity conservation2	Second repurchase and it's working fine.	Jingdong prefecture level city in Shandong
Jinfeng Hongyuan Flagship Store	Electricity conservation2	I'll wait a few days before evaluating	Jingdong prefecture level city in Shandong
Jinfeng Hongyuan Flagship Store	Electricity conservation2	I'll comment after I use it hopefully the section will be useful.	Jingdong prefecture level city in Shandong
Jinfeng Hongyuan Flagship Store	Electricity conservation2	not very useful	Jingdong prefecture level city in Shandong
Jinfeng Hongyuan Flagship Store	Electricity conservation2	Not received	Jingdong prefecture level city in Shandong
Jinfeng Hongyuan Flagship Store	Electricity conservation2	rare	Jingdong prefecture level city in Shandong
Jinfeng Hongyuan Flagship Store	Electricity conservation2	It's good.	Jingdong prefecture level city in Shandong
Jinfeng Hongyuan Flagship Store	Electricity conservation2	just now	Jingdong prefecture level city in Shandong
Jinfeng Hongyuan Flagship Store	Electricity conservation2	serviceable	Jingdong prefecture level city in Shandong
Jinfeng Hongyuan Flagship Store	Electricity conservation2	desirable	Jingdong prefecture level city in Shandong
Jinfeng Hongyuan Flagship Store	Electricity conservation2	serviceable	Jingdong prefecture level city in Shandong
Jinfeng Hongyuan Flagship Store	Electricity conservation2	It doesn't save power and costs electricity.	Jingdong prefecture level city in Shandong
Jinfeng Hongyuan Flagship Store	Electricity conservation2	Not as good as I thought.	Jingdong prefecture level city in Shandong
Jinfeng Hongyuan Flagship Store	Electricity conservation2	Super bad review, it doesn't save power at all, but rather more power consumption	Jingdong prefecture level city in Shandong
Jinfeng Hongyuan Flagship Store	Electricity conservation2	Listen to others say that this can save electricity, I bought with the idea of trying, used for a period of time before coming to the evaluation, will be a little bit of use, not a lot of savings just, long-term use of the words can save more.	Jingdong prefecture level city in Shandong
Jinfeng Hongyuan Flagship Store	Electricity conservation2	Logistics is fast, the next day arrived, workmanship is very delicate a power saver, looking forward to the late effect, customer service patiently explain the recommended which is suitable for, installed in what position is the best, look forward to using the effect after a period of time!	Jingdong prefecture level city in Shandong
Jinfeng Hongyuan Flagship Store	Electricity conservation2	It's good to use, I feel that it has been saving electricity, anyway, not a loss is, I used to feel that the power consumption is big, now I feel much better, my friends are also using, I hope to use a long time will be able to, I wish the boss a prosperous business	Jingdong prefecture level city in Shandong
Jinfeng Hongyuan Flagship Store	Electricity conservation2	Should be very good, to be able to plan the circuit, save the home of the circuit to the extent of the savings, waste of money on electricity, good if a month this money back, hope always hope to be able to	Jingdong prefecture level city in Shandong
Jinfeng Hongyuan Flagship Store	Electricity conservation2	Express delivery is very strong, the goods and I saw in the business consistent, the use of the effect of follow-up evaluation	Jingdong prefecture level city in Shandong
Jinfeng Hongyuan Flagship Store	Electricity conservation2	I have been using this brand of power saver for many years and this time I saw this upgraded version and got it again. The main purpose of this product is to reduce the loss of electricity for a long time, so as to achieve the purpose of saving electricity.	Jingdong prefecture level city in Shandong
Jinfeng Hongyuan Flagship Store	Electricity conservation2	Logistics is fast and the product looks beautiful, looking forward to the effect.	Jingdong prefecture level city in Shandong
Jinfeng Hongyuan Flagship Store	Electricity conservation2	Product quality is good, energy saving a little effect, but the price is a little expensive, fast arrival, good service.	Jingdong prefecture level city in Shandong
Jinfeng Hongyuan Flagship Store	Electricity conservation2	The quality is very good, inexpensive, Jingdong self-supporting guaranteed, worth having!	Jingdong prefecture level city in Shandong
Jinfeng Hongyuan Flagship Store	Electricity conservation2	I don't know if it's saving electricity yet, I'll find out next time I pay my electric bill	Jingdong prefecture level city in Shandong
Jinfeng Hongyuan Flagship Store	Electricity conservation2	Favorable price quality assurance appearance design is beautiful and generous, fine workmanship	Jingdong prefecture level city in Shandong
Jinfeng Hongyuan Flagship Store	Electricity conservation2	I bought it, I don't know how effective it is? I don't know how the quality is? After this summer results will know! Looking forward to it!	Jingdong prefecture level city in Shandong
Jinfeng Hongyuan Flagship Store	Electricity conservation2	Goods received Packaging intact, look at the shell workmanship is also good, high-power appliances open voltage has obvious changes in the effectiveness of the	Jingdong prefecture level city in Shandong
Jinfeng Hongyuan Flagship Store	Electricity conservation2	The goods have been received logistics is very fast, the product is similar to the line, just wait to use a month to see if it can save electricity, stay tuned.	Jingdong prefecture level city in Shandong
Jinfeng Hongyuan Flagship Store	Electricity conservation2	Electricity is plugged in, not sure if it will work, hopefully it will work.	Jingdong prefecture level city in Shandong
Jinfeng Hongyuan Flagship Store	Electricity conservation2	The shell is aluminum alloy, very good workmanship, the actual effect of waiting for the addition of evaluation	Jingdong prefecture level city in Shandong
Jinfeng Hongyuan Flagship Store	Electricity conservation2	Received, already plugged in, looking forward to the effect.	Jingdong prefecture level city in Shandong
Jinfeng Hongyuan Flagship Store	Electricity conservation2	Opened to see the regular manufacturer products, wait for a period of time to see it	Jingdong prefecture level city in Shandong
Jinfeng Hongyuan Flagship Store	Electricity conservation2	🉑 Baby received, very surprised ✨, reasonable design, fashionable and generous, continue to pay attention to the future will be repurchased ❥❥❥❥	Jingdong prefecture level city in Shandong
Jinfeng Hongyuan Flagship Store	Electricity conservation2	Baby received it 👌, really great ah! 🌈The quality can't be beat, very satisfied! 🆗Happy happy happy 😃	Jingdong prefecture level city in Shandong
Jinfeng Hongyuan Flagship Store	Electricity conservation2	Very good, power saving see, this is to help a friend to buy, I used to give my friend a say, my friend immediately let me order another one!	Jingdong prefecture level city in Shandong
Jinfeng Hongyuan Flagship Store	Electricity conservation2	The baby is just as introduced, the texture is very good, powerful, and it feels good to use it	Jingdong prefecture level city in Shandong
Jinfeng Hongyuan Flagship Store	Electricity conservation2	Value for money, very satisfied, seller service attitude is very good, very patient	Jingdong prefecture level city in Shandong
Jinfeng Hongyuan Flagship Store	Electricity conservation2	Power saving treasure design unique exquisite beautiful, voltage display clearly, plugged in that is good to use, I observed the meter, after using the meter is gone slow.	Jingdong prefecture level city in Shandong
Jinfeng Hongyuan Flagship Store	Electricity conservation2	Received on the use of the packaging is beautifully used for a period of time and then come back in the follow-up review!	Jingdong prefecture level city in Shandong
Jinfeng Hongyuan Flagship Store	Electricity conservation2	It should work. Not bad. Not bad. Not bad.	Jingdong prefecture level city in Shandong
Jinfeng Hongyuan Flagship Store	Electricity conservation2	I haven't used it yet, I'll plug it in and see if it saves power next month.	Jingdong prefecture level city in Shandong
Jinfeng Hongyuan Flagship Store	Electricity conservation2	The goods received and unpacked and used, the quality is very good, things and the official website to buy no difference!	Jingdong prefecture level city in Shandong
Jinfeng Hongyuan Flagship Store	Electricity conservation2	It's a rip off, doesn't help much at all, uses the same amount of power as before it was used.	Jingdong prefecture level city in Shandong

Jinfeng Hongyuan Flagship Store	Electricity conservation2	I've been using it for a while now and haven't noticed any unusual problems, so it's okay	Jingdong prefecture level city in Shandong
Jinfeng Hongyuan Flagship Store	Electricity conservation2	Received the goods and plugged in to use, good or not there is no use will have to wait until next month to find out	Jingdong prefecture level city in Shandong
Jinfeng Hongyuan Flagship Store	Electricity conservation2	It's very compact, it looks good, I'll introduce you to my clients when it's ready.	Jingdong prefecture level city in Shandong
Jinfeng Hongyuan Flagship Store	Electricity conservation2	Good workmanship, we'll have to see in a few months.	Jingdong prefecture level city in Shandong
Jinfeng Hongyuan Flagship Store	Electricity conservation2	Jingdong quality! Always trustworthy!	Jingdong prefecture level city in Shandong
Jinfeng Hongyuan Flagship Store	Electricity conservation2	This is the second time I've bought it, and the merchant said to give me five dollars out of the difference, so thank you merchant.	Jingdong prefecture level city in Shandong
Jinfeng Hongyuan Flagship Store	Electricity conservation2	Good quality and good service	Jingdong prefecture level city in Shandong
Jinfeng Hongyuan Flagship Store	Electricity conservation2	Feels good texture, aluminum alloy shell, voltage digital display clear.	Jingdong prefecture level city in Shandong
Jinfeng Hongyuan Flagship Store	Electricity conservation2	Good product, fast and great service, thanks!	Jingdong prefecture level city in Shandong
Jinfeng Hongyuan Flagship Store	Electricity conservation2	Have not yet used, first evaluation, later really good do not need to comment again.	Jingdong prefecture level city in Shandong
Jinfeng Hongyuan Flagship Store	Electricity conservation2	Works great. Pretty much as described by the merchant.	Jingdong prefecture level city in Shandong
Jinfeng Hongyuan Flagship Store	Electricity conservation2	Received it, looking forward to the results, hope it works	Jingdong prefecture level city in Shandong
Jinfeng Hongyuan Flagship Store	Electricity conservation2	Received it, looking forward to the results, hope it works	Jingdong prefecture level city in Shandong
Jinfeng Hongyuan Flagship Store	Electricity conservation2	Supposedly it's an IQ tax. Buy one and try it. Looks good. Well made.	Jingdong prefecture level city in Shandong
Jinfeng Hongyuan Flagship Store	Electricity conservation2	It just doesn't feel like it's working.	Jingdong prefecture level city in Shandong
Jinfeng Hongyuan Flagship Store	Electricity conservation2	This is a great product.	Jingdong prefecture level city in Shandong
Jinfeng Hongyuan Flagship Store	Electricity conservation2	I'm just using it. I'll tell you more.	Jingdong prefecture level city in Shandong
Jinfeng Hongyuan Flagship Store	Electricity conservation2	Second repurchase and it's working fine.	Jingdong prefecture level city in Shandong
Jinfeng Hongyuan Flagship Store	Electricity conservation2	I'll wait a few days before evaluating	Jingdong prefecture level city in Shandong
Jinfeng Hongyuan Flagship Store	Electricity conservation2	I'll comment after I use it hopefully the section will be useful.	Jingdong prefecture level city in Shandong
Jinfeng Hongyuan Flagship Store	Electricity conservation2	not very useful	Jingdong prefecture level city in Shandong
Jinfeng Hongyuan Flagship Store	Electricity conservation2	Not received	Jingdong prefecture level city in Shandong
Jinfeng Hongyuan Flagship Store	Electricity conservation2	rare	Jingdong prefecture level city in Shandong
Jinfeng Hongyuan Flagship Store	Electricity conservation2	It's good.	Jingdong prefecture level city in Shandong
Jinfeng Hongyuan Flagship Store	Electricity conservation2	just now	Jingdong prefecture level city in Shandong
Jinfeng Hongyuan Flagship Store	Electricity conservation2	serviceable	Jingdong prefecture level city in Shandong
Jinfeng Hongyuan Flagship Store	Electricity conservation2	desirable	Jingdong prefecture level city in Shandong
Jinfeng Hongyuan Flagship Store	Electricity conservation2	serviceable	Jingdong prefecture level city in Shandong
Jinfeng Hongyuan Flagship Store	Electricity conservation2	It doesn't save power and costs electricity.	Jingdong prefecture level city in Shandong
Jinfeng Hongyuan Flagship Store	Electricity conservation2	Not as good as I thought.	Jingdong prefecture level city in Shandong
Jinfeng Hongyuan Flagship Store	Electricity conservation2	Super bad review, it doesn't save power at all, but rather more power consumption	Jingdong prefecture level city in Shandong
Jinfeng Hongyuan Flagship Store	Electricity conservation2	Listen to others say that this can save electricity, I bought with the idea of trying, used for a period of time before coming to the evaluation, will be a little bit of use, not a lot of savings just, long-term use of the words can save more.	Jingdong prefecture level city in Shandong
Jinfeng Hongyuan Flagship Store	Electricity conservation2	Logistics is fast, the next day arrived, workmanship is very delicate a power saver, looking forward to the late effect, customer service patiently explain the recommended which is suitable for, installed in what position is the best, look forward to using the effect after a period of time!	Jingdong prefecture level city in Shandong
Jinfeng Hongyuan Flagship Store	Electricity conservation2	It's good to use, I feel that it has been saving electricity, anyway, not a loss is, I used to feel that the power consumption is big, now I feel much better, my friends are also using, I hope to use a long time will be able to, I wish the boss a prosperous business	Jingdong prefecture level city in Shandong
Jinfeng Hongyuan Flagship Store	Electricity conservation2	Should be very good, to be able to plan the circuit, save the home of the circuit to the extent of the savings, waste of money on electricity, good if a month this money back, hope always hope to be able to	Jingdong prefecture level city in Shandong
Jinfeng Hongyuan Flagship Store	Electricity conservation2	Express delivery is very strong, the goods and I saw in the business consistent, the use of the effect of follow-up evaluation	Jingdong prefecture level city in Shandong
Jinfeng Hongyuan Flagship Store	Electricity conservation2	I have been using this brand of power saver for many years and this time I saw this upgraded version and got it again. The main purpose of this product is to reduce the loss of electricity for a long time, so as to achieve the purpose of saving electricity.	Jingdong prefecture level city in Shandong
Jinfeng Hongyuan Flagship Store	Electricity conservation2	Logistics is fast and the product looks beautiful, looking forward to the effect.	Jingdong prefecture level city in Shandong
Jinfeng Hongyuan Flagship Store	Electricity conservation2	Product quality is good, energy saving a little effect, but the price is a little expensive, fast arrival, good service.	Jingdong prefecture level city in Shandong
Jinfeng Hongyuan Flagship Store	Electricity conservation2	The quality is very good, inexpensive, Jingdong self-supporting guaranteed, worth having!	Jingdong prefecture level city in Shandong
Jinfeng Hongyuan Flagship Store	Electricity conservation2	I don't know if it's saving electricity yet, I'll find out next time I pay my electric bill	Jingdong prefecture level city in Shandong
Jinfeng Hongyuan Flagship Store	Electricity conservation2	Favorable price quality assurance appearance design is beautiful and generous, fine workmanship	Jingdong prefecture level city in Shandong
Jinfeng Hongyuan Flagship Store	Electricity conservation2	I bought it, I don't know how effective it is? I don't know how the quality is? After this summer results will know! Looking forward to it!	Jingdong prefecture level city in Shandong
Jinfeng Hongyuan Flagship Store	Electricity conservation2	Goods received Packaging intact, look at the shell workmanship is also good, high-power appliances open voltage has obvious changes in the effectiveness of the	Jingdong prefecture level city in Shandong
Jinfeng Hongyuan Flagship Store	Electricity conservation2	The goods have been received logistics is very fast, the product is similar to the line, just wait to use a month to see if it can save electricity, stay tuned.	Jingdong prefecture level city in Shandong
Jinfeng Hongyuan Flagship Store	Electricity conservation2	Electricity is plugged in, not sure if it will work, hopefully it will work.	Jingdong prefecture level city in Shandong
Jinfeng Hongyuan Flagship Store	Electricity conservation2	The shell is aluminum alloy, very good workmanship, the actual effect of waiting for the addition of evaluation	Jingdong prefecture level city in Shandong
Jinfeng Hongyuan Flagship Store	Electricity conservation2	Received, already plugged in, looking forward to the effect.	Jingdong prefecture level city in Shandong
Jinfeng Hongyuan Flagship Store	Electricity conservation2	Opened to see the regular manufacturer products, wait for a period of time to see it	Jingdong prefecture level city in Shandong

Jinfeng Hongyuan Flagship Store	Electricity conservation2	Baby received, very surprised, reasonable design, fashionable and generous, continue to pay attention to the future will be repurchased	Jingdong prefecture level city in Shandong
Jinfeng Hongyuan Flagship Store	Electricity conservation2	Baby received it, really great ah! The quality can't be beat, very satisfied! OK Happy happy happy	Jingdong prefecture level city in Shandong
Jinfeng Hongyuan Flagship Store	Electricity conservation2	Very good, power saving see, this is to help a friend to buy, I used to give my friend a say, my friend immediately let me order another one!	Jingdong prefecture level city in Shandong
Jinfeng Hongyuan Flagship Store	Electricity conservation2	The baby is just as introduced, the texture is very good, powerful, and it feels good to use it	Jingdong prefecture level city in Shandong
Jinfeng Hongyuan Flagship Store	Electricity conservation2	Value for money, very satisfied, seller service attitude is very good, very patient	Jingdong prefecture level city in Shandong
Jinfeng Hongyuan Flagship Store	Electricity conservation2	Power saving treasure design unique exquisite beautiful, voltage display clearly, plugged in that is good to use, I observed the meter, after using the meter is gone slow.	Jingdong prefecture level city in Shandong
Jinfeng Hongyuan Flagship Store	Electricity conservation2	Received on the use of the packaging is beautifully used for a period of time and then come back in the follow-up review!	Jingdong prefecture level city in Shandong
Jinfeng Hongyuan Flagship Store	Electricity conservation2	It should work. Not bad. Not bad. Not bad.	Jingdong prefecture level city in Shandong
Jinfeng Hongyuan Flagship Store	Electricity conservation2	I haven't used it yet, I'll plug it in and see if it saves power next month.	Jingdong prefecture level city in Shandong
Jinfeng Hongyuan Flagship Store	Electricity conservation2	The goods received and unpacked and used, the quality is very good, things and the official website to buy no difference!	Jingdong prefecture level city in Shandong
Jinfeng Hongyuan Flagship Store	Electricity conservation2	It's a rip off, doesn't help much at all, uses the same amount of power as before it was used.	Jingdong prefecture level city in Shandong
Jinfeng Hongyuan Flagship Store	Electricity conservation2	I've been using it for a while now and haven't noticed any unusual problems, so it's okay	Jingdong prefecture level city in Shandong
Jinfeng Hongyuan Flagship Store	Electricity conservation2	Received the goods and plugged in to use, good or not there is no use will have to wait until next month to find out	Jingdong prefecture level city in Shandong
Jinfeng Hongyuan Flagship Store	Electricity conservation2	It's very compact, it looks good, I'll introduce you to my clients when it's ready.	Jingdong prefecture level city in Shandong
Jinfeng Hongyuan Flagship Store	Electricity conservation2	Good workmanship, we'll have to see in a few months.	Jingdong prefecture level city in Shandong
Jinfeng Hongyuan Flagship Store	Electricity conservation2	Jingdong quality! Always trustworthy!	Jingdong prefecture level city in Shandong
Jinfeng Hongyuan Flagship Store	Electricity conservation2	This is the second time I've bought it, and the merchant said to give me five dollars out of the difference, so thank you merchant.	Jingdong prefecture level city in Shandong
Jinfeng Hongyuan Flagship Store	Electricity conservation2	Good quality and good service	Jingdong prefecture level city in Shandong
Jinfeng Hongyuan Flagship Store	Electricity conservation2	Feels good texture, aluminum alloy shell, voltage digital display clear.	Jingdong prefecture level city in Shandong
Jinfeng Hongyuan Flagship Store	Electricity conservation2	Good product, fast and great service, thanks!	Jingdong prefecture level city in Shandong
Jinfeng Hongyuan Flagship Store	Electricity conservation2	Have not yet used, first evaluation, later really good do not need to comment again.	Jingdong prefecture level city in Shandong
Jinfeng Hongyuan Flagship Store	Electricity conservation2	Works great. Pretty much as described by the merchant.	Jingdong prefecture level city in Shandong
Jinfeng Hongyuan Flagship Store	Electricity conservation2	Received it, looking forward to the results, hope it works	Jingdong prefecture level city in Shandong
Jinfeng Hongyuan Flagship Store	Electricity conservation2	Received it, looking forward to the results, hope it works	Jingdong prefecture level city in Shandong
Jinfeng Hongyuan Flagship Store	Electricity conservation2	Supposedly it's an IQ tax. Buy one and try it. Looks good. Well made.	Jingdong prefecture level city in Shandong
Jinfeng Hongyuan Flagship Store	Electricity conservation2	It just doesn't feel like it's working.	Jingdong prefecture level city in Shandong
Jinfeng Hongyuan Flagship Store	Electricity conservation2	This is a great product.	Jingdong prefecture level city in Shandong
Jinfeng Hongyuan Flagship Store	Electricity conservation2	I'm just using it. I'll tell you more.	Jingdong prefecture level city in Shandong
Jinfeng Hongyuan Flagship Store	Electricity conservation2	Second repurchase and it's working fine.	Jingdong prefecture level city in Shandong
Jinfeng Hongyuan Flagship Store	Electricity conservation2	I'll wait a few days before evaluating	Jingdong prefecture level city in Shandong
Jinfeng Hongyuan Flagship Store	Electricity conservation2	I'll comment after I use it hopefully the section will be useful.	Jingdong prefecture level city in Shandong
Jinfeng Hongyuan Flagship Store	Electricity conservation2	not very useful	Jingdong prefecture level city in Shandong
Jinfeng Hongyuan Flagship Store	Electricity conservation2	Not received	Jingdong prefecture level city in Shandong
Jinfeng Hongyuan Flagship Store	Electricity conservation2	rare	Jingdong prefecture level city in Shandong
Jinfeng Hongyuan Flagship Store	Electricity conservation2	It's good.	Jingdong prefecture level city in Shandong
Jinfeng Hongyuan Flagship Store	Electricity conservation2	just now	Jingdong prefecture level city in Shandong
Jinfeng Hongyuan Flagship Store	Electricity conservation2	serviceable	Jingdong prefecture level city in Shandong
Jinfeng Hongyuan Flagship Store	Electricity conservation2	desirable	Jingdong prefecture level city in Shandong
Jinfeng Hongyuan Flagship Store	Electricity conservation2	serviceable	Jingdong prefecture level city in Shandong
Jinfeng Hongyuan Flagship Store	Electricity conservation2	It doesn't save power and costs electricity.	Jingdong prefecture level city in Shandong
Jinfeng Hongyuan Flagship Store	Electricity conservation2	Not as good as I thought.	Jingdong prefecture level city in Shandong
Jinfeng Hongyuan Flagship Store	Electricity conservation2	Super bad review, it doesn't save power at all, but rather more power consumption	Jingdong prefecture level city in Shandong
Jinfeng Hongyuan Flagship Store	Electricity conservation2	Listen to others say that this can save electricity, I bought with the idea of trying, used for a period of time before coming to the evaluation, will be a little bit of use, not a lot of savings just, long-term use of the words can save more.	Jingdong prefecture level city in Shandong
Jinfeng Hongyuan Flagship Store	Electricity conservation2	Logistics is fast, the next day arrived, workmanship is very delicate a power saver, looking forward to the late effect, customer service patiently explain the recommended which is suitable for, installed in what position is the best, look forward to using the effect after a period of time!	Jingdong prefecture level city in Shandong
Jinfeng Hongyuan Flagship Store	Electricity conservation2	It's good to use, I feel that it has been saving electricity, anyway, not a loss is, I used to feel that the power consumption is big, now I feel much better, my friends are also using, I hope to use a long time will be able to, I wish the boss a prosperous business	Jingdong prefecture level city in Shandong
Jinfeng Hongyuan Flagship Store	Electricity conservation2	Should be very good, to be able to plan the circuit, save the home of the circuit to the extent of the savings, waste of money on electricity, good if a month this money back, hope always hope to be able to	Jingdong prefecture level city in Shandong
Jinfeng Hongyuan Flagship Store	Electricity conservation2	Express delivery is very strong, the goods and I saw in the business consistent, the use of the effect of follow-up evaluation	Jingdong prefecture level city in Shandong
Jinfeng Hongyuan Flagship Store	Electricity conservation2	I have been using this brand of power saver for many years and this time I saw this upgraded version and got it again. The main purpose of this product is to reduce the loss of electricity for a long time, so as to achieve the purpose of saving electricity.	Jingdong prefecture level city in Shandong
Jinfeng Hongyuan Flagship Store	Electricity conservation2	Logistics is fast and the product looks beautiful, looking forward to the effect.	Jingdong prefecture level city in Shandong

Jinfeng Hongyuan Flagship Store	Electricity conservation2	Product quality is good, energy saving a little effect, but the price is a little expensive, fast arrival, good service.	Jingdong prefecture level city in Shandong
Jinfeng Hongyuan Flagship Store	Electricity conservation2	The quality is very good, inexpensive, Jingdong self-supporting guaranteed, worth having!	Jingdong prefecture level city in Shandong
Jinfeng Hongyuan Flagship Store	Electricity conservation2	I don't know if it's saving electricity yet, I'll find out next time I pay my electric bill	Jingdong prefecture level city in Shandong
Jinfeng Hongyuan Flagship Store	Electricity conservation2	Favorable price quality assurance appearance design is beautiful and generous, fine workmanship	Jingdong prefecture level city in Shandong
Jinfeng Hongyuan Flagship Store	Electricity conservation2	I bought it, I don't know how effective it is? I don't know how the quality is? After this summer results will know! Looking forward to it!	Jingdong prefecture level city in Shandong
Jinfeng Hongyuan Flagship Store	Electricity conservation2	Goods received Packaging intact, look at the shell workmanship is also good, high-power appliances open voltage has obvious changes in the effectiveness of the	Jingdong prefecture level city in Shandong
Jinfeng Hongyuan Flagship Store	Electricity conservation2	The goods have been received logistics is very fast, the product is similar to the line, just wait to use a month to see if it can save electricity, stay tuned.	Jingdong prefecture level city in Shandong
Jinfeng Hongyuan Flagship Store	Electricity conservation2	Electricity is plugged in, not sure if it will work, hopefully it will work.	Jingdong prefecture level city in Shandong
Jinfeng Hongyuan Flagship Store	Electricity conservation2	The shell is aluminum alloy, very good workmanship, the actual effect of waiting for the addition of evaluation	Jingdong prefecture level city in Shandong
Jinfeng Hongyuan Flagship Store	Electricity conservation2	Received, already plugged in, looking forward to the effect.	Jingdong prefecture level city in Shandong
Jinfeng Hongyuan Flagship Store	Electricity conservation2	Opened to see the regular manufacturer products, wait for a period of time to see it	Jingdong prefecture level city in Shandong
Jinfeng Hongyuan Flagship Store	Electricity conservation2	🉑 Baby received, very surprised ✨, reasonable design, fashionable and generous, continue to pay attention to the future will be repurchased ❥❥❥❥	Jingdong prefecture level city in Shandong
Jinfeng Hongyuan Flagship Store	Electricity conservation2	Baby received it 👌, really great ah! 🌈The quality can't be beat, very satisfied! 🆗Happy happy happy 😃	Jingdong prefecture level city in Shandong
Jinfeng Hongyuan Flagship Store	Electricity conservation2	Very good, power saving see, this is to help a friend to buy, I used to give my friend a say, my friend immediately let me order another one!	Jingdong prefecture level city in Shandong
Jinfeng Hongyuan Flagship Store	Electricity conservation2	The baby is just as introduced, the texture is very good, powerful, and it feels good to use it	Jingdong prefecture level city in Shandong
Jinfeng Hongyuan Flagship Store	Electricity conservation2	Value for money, very satisfied, seller service attitude is very good, very patient	Jingdong prefecture level city in Shandong
Jinfeng Hongyuan Flagship Store	Electricity conservation2	Power saving treasure design unique exquisite beautiful, voltage display clearly, plugged in that is good to use, I observed the meter, after using the meter is gone slow.	Jingdong prefecture level city in Shandong
Jinfeng Hongyuan Flagship Store	Electricity conservation2	Received on the use of the packaging is beautifully used for a period of time and then come back in the follow-up review!	Jingdong prefecture level city in Shandong
Jinfeng Hongyuan Flagship Store	Electricity conservation2	It should work. Not bad. Not bad. Not bad.	Jingdong prefecture level city in Shandong
Jinfeng Hongyuan Flagship Store	Electricity conservation2	I haven't used it yet, I'll plug it in and see if it saves power next month.	Jingdong prefecture level city in Shandong
Jinfeng Hongyuan Flagship Store	Electricity conservation2	The goods received and unpacked and used, the quality is very good, things and the official website to buy no difference!	Jingdong prefecture level city in Shandong
Jinfeng Hongyuan Flagship Store	Electricity conservation2	It's a rip off, doesn't help much at all, uses the same amount of power as before it was used.	Jingdong prefecture level city in Shandong
Jinfeng Hongyuan Flagship Store	Electricity conservation2	I've been using it for a while now and haven't noticed any unusual problems, so it's okay	Jingdong prefecture level city in Shandong
Jinfeng Hongyuan Flagship Store	Electricity conservation2	Received the goods and plugged in to use, good or not there is no use will have to wait until next month to find out	Jingdong prefecture level city in Shandong
Jinfeng Hongyuan Flagship Store	Electricity conservation2	It's very compact, it looks good, I'll introduce you to my clients when it's ready.	Jingdong prefecture level city in Shandong
Jinfeng Hongyuan Flagship Store	Electricity conservation2	Good workmanship, we'll have to see in a few months.	Jingdong prefecture level city in Shandong
Jinfeng Hongyuan Flagship Store	Electricity conservation2	Jingdong quality! Always trustworthy!	Jingdong prefecture level city in Shandong
Jinfeng Hongyuan Flagship Store	Electricity conservation2	This is the second time I've bought it, and the merchant said to give me five dollars out of the difference, so thank you merchant.	Jingdong prefecture level city in Shandong
Jinfeng Hongyuan Flagship Store	Electricity conservation2	Good quality and good service	Jingdong prefecture level city in Shandong
Jinfeng Hongyuan Flagship Store	Electricity conservation2	Feels good texture, aluminum alloy shell, voltage digital display clear.	Jingdong prefecture level city in Shandong
Jinfeng Hongyuan Flagship Store	Electricity conservation2	Good product, fast and great service, thanks!	Jingdong prefecture level city in Shandong
Jinfeng Hongyuan Flagship Store	Electricity conservation2	Have not yet used, first evaluation, later really good do not need to comment again.	Jingdong prefecture level city in Shandong
Jinfeng Hongyuan Flagship Store	Electricity conservation2	Works great. Pretty much as described by the merchant.	Jingdong prefecture level city in Shandong
Jinfeng Hongyuan Flagship Store	Electricity conservation2	Received it, looking forward to the results, hope it works	Jingdong prefecture level city in Shandong
Jinfeng Hongyuan Flagship Store	Electricity conservation2	Received it, looking forward to the results, hope it works	Jingdong prefecture level city in Shandong
Jinfeng Hongyuan Flagship Store	Electricity conservation2	Supposedly it's an IQ tax. Buy one and try it. Looks good. Well made.	Jingdong prefecture level city in Shandong
Jinfeng Hongyuan Flagship Store	Electricity conservation2	It just doesn't feel like it's working.	Jingdong prefecture level city in Shandong
Jinfeng Hongyuan Flagship Store	Electricity conservation2	This is a great product.	Jingdong prefecture level city in Shandong
Jinfeng Hongyuan Flagship Store	Electricity conservation2	I'm just using it. I'll tell you more.	Jingdong prefecture level city in Shandong
Jinfeng Hongyuan Flagship Store	Electricity conservation2	Second repurchase and it's working fine.	Jingdong prefecture level city in Shandong
Jinfeng Hongyuan Flagship Store	Electricity conservation2	I'll wait a few days before evaluating	Jingdong prefecture level city in Shandong
Jinfeng Hongyuan Flagship Store	Electricity conservation2	I'll comment after I use it hopefully the section will be useful.	Jingdong prefecture level city in Shandong
Jinfeng Hongyuan Flagship Store	Electricity conservation2	not very useful	Jingdong prefecture level city in Shandong
Jinfeng Hongyuan Flagship Store	Electricity conservation2	Not received	Jingdong prefecture level city in Shandong
Jinfeng Hongyuan Flagship Store	Electricity conservation2	rare	Jingdong prefecture level city in Shandong
Jinfeng Hongyuan Flagship Store	Electricity conservation2	It's good.	Jingdong prefecture level city in Shandong
Jinfeng Hongyuan Flagship Store	Electricity conservation2	just now	Jingdong prefecture level city in Shandong
Jinfeng Hongyuan Flagship Store	Electricity conservation2	serviceable	Jingdong prefecture level city in Shandong
Jinfeng Hongyuan Flagship Store	Electricity conservation2	desirable	Jingdong prefecture level city in Shandong
Jinfeng Hongyuan Flagship Store	Electricity conservation2	serviceable	Jingdong prefecture level city in Shandong
Jinfeng Hongyuan Flagship Store	Electricity conservation2	It doesn't save power and costs electricity.	Jingdong prefecture level city in Shandong

Jinfeng Hongyuan Flagship Store	Electricity conservation2	Not as good as I thought.	Jingdong prefecture level city in Shandong
Jinfeng Hongyuan Flagship Store	Electricity conservation2	Super bad review, it doesn't save power at all, but rather more power consumption	Jingdong prefecture level city in Shandong
Jinfeng Hongyuan Flagship Store	Electricity conservation2	Listen to others say that this can save electricity, I bought with the idea of trying, used for a period of time before coming to the evaluation, will be a little bit of use, not a lot of savings just, long-term use of the words can save more.	Jingdong prefecture level city in Shandong
Jinfeng Hongyuan Flagship Store	Electricity conservation2	Logistics is fast, the next day arrived, workmanship is very delicate a power saver, looking forward to the late effect, customer service patiently explain the recommended which is suitable for, installed in what position is the best, look forward to using the effect after a period of time!	Jingdong prefecture level city in Shandong
Jinfeng Hongyuan Flagship Store	Electricity conservation2	It's good to use, I feel that it has been saving electricity, anyway, not a loss is, I used to feel that the power consumption is big, now I feel much better, my friends are also using, I hope to use a long time will be able to, I wish the boss a prosperous business	Jingdong prefecture level city in Shandong
Jinfeng Hongyuan Flagship Store	Electricity conservation2	Should be very good, to be able to plan the circuit, save the home of the circuit to the extent of the savings, waste of money on electricity, good if a month this money back, hope always hope to be able to	Jingdong prefecture level city in Shandong
Jinfeng Hongyuan Flagship Store	Electricity conservation2	Express delivery is very strong, the goods and I saw in the business consistent, the use of the effect of follow-up evaluation	Jingdong prefecture level city in Shandong
Jinfeng Hongyuan Flagship Store	Electricity conservation2	I have been using this brand of power saver for many years and this time I saw this upgraded version and got it again. The main purpose of this product is to reduce the loss of electricity for a long time, so as to achieve the purpose of saving electricity.	Jingdong prefecture level city in Shandong
Jinfeng Hongyuan Flagship Store	Electricity conservation2	Logistics is fast and the product looks beautiful, looking forward to the effect.	Jingdong prefecture level city in Shandong
Jinfeng Hongyuan Flagship Store	Electricity conservation2	Product quality is good, energy saving a little effect, but the price is a little expensive, fast arrival, good service.	Jingdong prefecture level city in Shandong
Jinfeng Hongyuan Flagship Store	Electricity conservation2	The quality is very good, inexpensive, Jingdong self-supporting guaranteed, worth having!	Jingdong prefecture level city in Shandong
Jinfeng Hongyuan Flagship Store	Electricity conservation2	I don't know if it's saving electricity yet, I'll find out next time I pay my electric bill	Jingdong prefecture level city in Shandong
Jinfeng Hongyuan Flagship Store	Electricity conservation2	Favorable price quality assurance appearance design is beautiful and generous, fine workmanship	Jingdong prefecture level city in Shandong
Jinfeng Hongyuan Flagship Store	Electricity conservation2	I bought it, I don't know how effective it is? I don't know how the quality is? After this summer results will know! Looking forward to it!	Jingdong prefecture level city in Shandong
Jinfeng Hongyuan Flagship Store	Electricity conservation2	Goods received Packaging intact, look at the shell workmanship is also good, high-power appliances open voltage has obvious changes in the effectiveness of the	Jingdong prefecture level city in Shandong
Jinfeng Hongyuan Flagship Store	Electricity conservation2	The goods have been received logistics is very fast, the product is similar to the line, just wait to use a month to see if it can save electricity, stay tuned.	Jingdong prefecture level city in Shandong
Jinfeng Hongyuan Flagship Store	Electricity conservation2	Electricity is plugged in, not sure if it will work, hopefully it will work.	Jingdong prefecture level city in Shandong
Jinfeng Hongyuan Flagship Store	Electricity conservation2	The shell is aluminum alloy, very good workmanship, the actual effect of waiting for the addition of evaluation	Jingdong prefecture level city in Shandong
Jinfeng Hongyuan Flagship Store	Electricity conservation2	Received, already plugged in, looking forward to the effect.	Jingdong prefecture level city in Shandong
Jinfeng Hongyuan Flagship Store	Electricity conservation2	Opened to see the regular manufacturer products, wait for a period of time to see it	Jingdong prefecture level city in Shandong
Jinfeng Hongyuan Flagship Store	Electricity conservation2	🉑 Baby received, very surprised ✨, reasonable design, fashionable and generous, continue to pay attention to the future will be repurchased ❥❥❥❥	Jingdong prefecture level city in Shandong
Jinfeng Hongyuan Flagship Store	Electricity conservation2	Baby received it 👌, really great ah! 🌈The quality can't be beat, very satisfied! 🆗Happy happy happy 😃	Jingdong prefecture level city in Shandong
Jinfeng Hongyuan Flagship Store	Electricity conservation2	Very good, power saving see, this is to help a friend to buy, I used to give my friend a say, my friend immediately let me order another one!	Jingdong prefecture level city in Shandong
Jinfeng Hongyuan Flagship Store	Electricity conservation2	The baby is just as introduced, the texture is very good, powerful, and it feels good to use it	Jingdong prefecture level city in Shandong
Jinfeng Hongyuan Flagship Store	Electricity conservation2	Value for money, very satisfied, seller service attitude is very good, very patient	Jingdong prefecture level city in Shandong
Jinfeng Hongyuan Flagship Store	Electricity conservation2	Power saving treasure design unique exquisite beautiful, voltage display clearly, plugged in that is good to use, I observed the meter, after using the meter is gone slow.	Jingdong prefecture level city in Shandong
Jinfeng Hongyuan Flagship Store	Electricity conservation2	Received on the use of the packaging is beautifully used for a period of time and then come back in the follow-up review!	Jingdong prefecture level city in Shandong
Jinfeng Hongyuan Flagship Store	Electricity conservation2	It should work. Not bad. Not bad. Not bad.	Jingdong prefecture level city in Shandong
Jinfeng Hongyuan Flagship Store	Electricity conservation2	I haven't used it yet, I'll plug it in and see if it saves power next month.	Jingdong prefecture level city in Shandong
Jinfeng Hongyuan Flagship Store	Electricity conservation2	The goods received and unpacked and used, the quality is very good, things and the official website to buy no difference!	Jingdong prefecture level city in Shandong
Jinfeng Hongyuan Flagship Store	Electricity conservation2	It's a rip off, doesn't help much at all, uses the same amount of power as before it was used.	Jingdong prefecture level city in Shandong
Jinfeng Hongyuan Flagship Store	Electricity conservation2	I've been using it for a while now and haven't noticed any unusual problems, so it's okay	Jingdong prefecture level city in Shandong
Jinfeng Hongyuan Flagship Store	Electricity conservation2	Received the goods and plugged in to use, good or not there is no use will have to wait until next month to find out	Jingdong prefecture level city in Shandong
Jinfeng Hongyuan Flagship Store	Electricity conservation2	It's very compact, it looks good, I'll introduce you to my clients when it's ready.	Jingdong prefecture level city in Shandong
Jinfeng Hongyuan Flagship Store	Electricity conservation2	Good workmanship, we'll have to see in a few months.	Jingdong prefecture level city in Shandong
Jinfeng Hongyuan Flagship Store	Electricity conservation2	Jingdong quality! Always trustworthy!	Jingdong prefecture level city in Shandong
Jinfeng Hongyuan Flagship Store	Electricity conservation2	This is the second time I've bought it, and the merchant said to give me five dollars out of the difference, so thank you merchant.	Jingdong prefecture level city in Shandong
Jinfeng Hongyuan Flagship Store	Electricity conservation2	Good quality and good service	Jingdong prefecture level city in Shandong
Jinfeng Hongyuan Flagship Store	Electricity conservation2	Feels good texture, aluminum alloy shell, voltage digital display clear.	Jingdong prefecture level city in Shandong
Jinfeng Hongyuan Flagship Store	Electricity conservation2	Good product, fast and great service, thanks!	Jingdong prefecture level city in Shandong
Jinfeng Hongyuan Flagship Store	Electricity conservation2	Have not yet used, first evaluation, later really good do not need to comment again.	Jingdong prefecture level city in Shandong
Jinfeng Hongyuan Flagship Store	Electricity conservation2	Works great. Pretty much as described by the merchant.	Jingdong prefecture level city in Shandong
Jinfeng Hongyuan Flagship Store	Electricity conservation2	Received it, looking forward to the results, hope it works	Jingdong prefecture level city in Shandong
Jinfeng Hongyuan Flagship Store	Electricity conservation2	Received it, looking forward to the results, hope it works	Jingdong prefecture level city in Shandong
Jinfeng Hongyuan Flagship Store	Electricity conservation2	Supposedly it's an IQ tax. Buy one and try it. Looks good. Well made.	Jingdong prefecture level city in Shandong
Jinfeng Hongyuan Flagship Store	Electricity conservation2	It just doesn't feel like it's working.	Jingdong prefecture level city in Shandong
Jinfeng Hongyuan Flagship Store	Electricity conservation2	This is a great product.	Jingdong prefecture level city in Shandong
Jinfeng Hongyuan Flagship Store	Electricity conservation2	I'm just using it. I'll tell you more.	Jingdong prefecture level city in Shandong
Jinfeng Hongyuan Flagship Store	Electricity conservation2	Second repurchase and it's working fine.	Jingdong prefecture level city in Shandong

Jinfeng Hongyuan Flagship Store	Electricity conservation2	I'll wait a few days before evaluating	Jingdong prefecture level city in Shandong
Jinfeng Hongyuan Flagship Store	Electricity conservation2	I'll comment after I use it hopefully the section will be useful.	Jingdong prefecture level city in Shandong
Jinfeng Hongyuan Flagship Store	Electricity conservation2	not very useful	Jingdong prefecture level city in Shandong
Jinfeng Hongyuan Flagship Store	Electricity conservation2	Not received	Jingdong prefecture level city in Shandong
Jinfeng Hongyuan Flagship Store	Electricity conservation2	rare	Jingdong prefecture level city in Shandong
Jinfeng Hongyuan Flagship Store	Electricity conservation2	It's good.	Jingdong prefecture level city in Shandong
Jinfeng Hongyuan Flagship Store	Electricity conservation2	just now	Jingdong prefecture level city in Shandong
Jinfeng Hongyuan Flagship Store	Electricity conservation2	serviceable	Jingdong prefecture level city in Shandong
Jinfeng Hongyuan Flagship Store	Electricity conservation2	desirable	Jingdong prefecture level city in Shandong
Jinfeng Hongyuan Flagship Store	Electricity conservation2	serviceable	Jingdong prefecture level city in Shandong
Jinfeng Hongyuan Flagship Store	Electricity conservation2	It doesn't save power and costs electricity.	Jingdong prefecture level city in Shandong
Jinfeng Hongyuan Flagship Store	Electricity conservation2	Not as good as I thought.	Jingdong prefecture level city in Shandong
Jinfeng Hongyuan Flagship Store	Electricity conservation2	Super bad review, it doesn't save power at all, but rather more power consumption	Jingdong prefecture level city in Shandong
Jinfeng Hongyuan Flagship Store	Electricity conservation2	Listen to others say that this can save electricity, I bought with the idea of trying, used for a period of time before coming to the evaluation, will be a little bit of use, not a lot of savings just, long-term use of the words can save more.	Jingdong prefecture level city in Shandong
Jinfeng Hongyuan Flagship Store	Electricity conservation2	Logistics is fast, the next day arrived, workmanship is very delicate a power saver, looking forward to the late effect, customer service patiently explain the recommended which is suitable for, installed in what position is the best, look forward to using the effect after a period of time!	Jingdong prefecture level city in Shandong
Jinfeng Hongyuan Flagship Store	Electricity conservation2	It's good to use, I feel that it has been saving electricity, anyway, not a loss is, I used to feel that the power consumption is big, now I feel much better, my friends are also using, I hope to use a long time will be able to, I wish the boss a prosperous business	Jingdong prefecture level city in Shandong
Jinfeng Hongyuan Flagship Store	Electricity conservation2	Should be very good, to be able to plan the circuit, save the home of the circuit to the extent of the savings, waste of money on electricity, good if a month this money back, hope always hope to be able to	Jingdong prefecture level city in Shandong
Jinfeng Hongyuan Flagship Store	Electricity conservation2	Express delivery is very strong, the goods and I saw in the business consistent, the use of the effect of follow-up evaluation	Jingdong prefecture level city in Shandong
Jinfeng Hongyuan Flagship Store	Electricity conservation2	I have been using this brand of power saver for many years and this time I saw this upgraded version and got it again. The main purpose of this product is to reduce the loss of electricity for a long time, so as to achieve the purpose of saving electricity.	Jingdong prefecture level city in Shandong
Jinfeng Hongyuan Flagship Store	Electricity conservation2	Logistics is fast and the product looks beautiful, looking forward to the effect.	Jingdong prefecture level city in Shandong
Jinfeng Hongyuan Flagship Store	Electricity conservation2	Product quality is good, energy saving a little effect, but the price is a little expensive, fast arrival, good service.	Jingdong prefecture level city in Shandong
Jinfeng Hongyuan Flagship Store	Electricity conservation2	The quality is very good, inexpensive, Jingdong self-supporting guaranteed, worth having!	Jingdong prefecture level city in Shandong
Jinfeng Hongyuan Flagship Store	Electricity conservation2	I don't know if it's saving electricity yet, I'll find out next time I pay my electric bill	Jingdong prefecture level city in Shandong
Jinfeng Hongyuan Flagship Store	Electricity conservation2	Favorable price quality assurance appearance design is beautiful and generous, fine workmanship	Jingdong prefecture level city in Shandong
Jinfeng Hongyuan Flagship Store	Electricity conservation2	I bought it, I don't know how effective it is? I don't know how the quality is? After this summer results will know! Looking forward to it!	Jingdong prefecture level city in Shandong
Jinfeng Hongyuan Flagship Store	Electricity conservation2	Goods received Packaging intact, look at the shell workmanship is also good, high-power appliances open voltage has obvious changes in the effectiveness of the	Jingdong prefecture level city in Shandong
Jinfeng Hongyuan Flagship Store	Electricity conservation2	The goods have been received logistics is very fast, the product is similar to the line, just wait to use a month to see if it can save electricity, stay tuned.	Jingdong prefecture level city in Shandong
Jinfeng Hongyuan Flagship Store	Electricity conservation2	Electricity is plugged in, not sure if it will work, hopefully it will work.	Jingdong prefecture level city in Shandong
Jinfeng Hongyuan Flagship Store	Electricity conservation2	The shell is aluminum alloy, very good workmanship, the actual effect of waiting for the addition of evaluation	Jingdong prefecture level city in Shandong
Jinfeng Hongyuan Flagship Store	Electricity conservation2	Received, already plugged in, looking forward to the effect.	Jingdong prefecture level city in Shandong
Jinfeng Hongyuan Flagship Store	Electricity conservation2	Opened to see the regular manufacturer products, wait for a period of time to see it	Jingdong prefecture level city in Shandong
Jinfeng Hongyuan Flagship Store	Electricity conservation2	🉑 Baby received, very surprised ✨, reasonable design, fashionable and generous, continue to pay attention to the future will be repurchased ❤❤❤❤	Jingdong prefecture level city in Shandong
Jinfeng Hongyuan Flagship Store	Electricity conservation2	Baby received it 👌, really great ah! 🌈The quality can't be beat, very satisfied! 🆗Happy happy happy 😀	Jingdong prefecture level city in Shandong
Jinfeng Hongyuan Flagship Store	Electricity conservation2	Very good, power saving see, this is to help a friend to buy, I used to give my friend a say, my friend immediately let me order another one!	Jingdong prefecture level city in Shandong
Jinfeng Hongyuan Flagship Store	Electricity conservation2	The baby is just as introduced, the texture is very good, powerful, and it feels good to use it	Jingdong prefecture level city in Shandong
Jinfeng Hongyuan Flagship Store	Electricity conservation2	Value for money, very satisfied, seller service attitude is very good, very patient	Jingdong prefecture level city in Shandong
Jinfeng Hongyuan Flagship Store	Electricity conservation2	Power saving treasure design unique exquisite beautiful, voltage display clearly, plugged in that is good to use, I observed the meter, after using the meter is gone slow.	Jingdong prefecture level city in Shandong
Jinfeng Hongyuan Flagship Store	Electricity conservation2	Received on the use of the packaging is beautifully used for a period of time and then come back in the follow-up review!	Jingdong prefecture level city in Shandong
Jinfeng Hongyuan Flagship Store	Electricity conservation2	It should work. Not bad. Not bad. Not bad.	Jingdong prefecture level city in Shandong
Jinfeng Hongyuan Flagship Store	Electricity conservation2	I haven't used it yet, I'll plug it in and see if it saves power next month.	Jingdong prefecture level city in Shandong
Jinfeng Hongyuan Flagship Store	Electricity conservation2	The goods received and unpacked and used, the quality is very good, things and the official website to buy no difference!	Jingdong prefecture level city in Shandong
Jinfeng Hongyuan Flagship Store	Electricity conservation2	It's a rip off, doesn't help much at all, uses the same amount of power as before it was used.	Jingdong prefecture level city in Shandong
Jinfeng Hongyuan Flagship Store	Electricity conservation2	I've been using it for a while now and haven't noticed any unusual problems, so it's okay	Jingdong prefecture level city in Shandong
Jinfeng Hongyuan Flagship Store	Electricity conservation2	Received the goods and plugged in to use, good or not there is no use will have to wait until next month to find out	Jingdong prefecture level city in Shandong
Jinfeng Hongyuan Flagship Store	Electricity conservation2	It's very compact, it looks good, I'll introduce you to my clients when it's ready.	Jingdong prefecture level city in Shandong
Jinfeng Hongyuan Flagship Store	Electricity conservation2	Good workmanship, we'll have to see in a few months.	Jingdong prefecture level city in Shandong
Jinfeng Hongyuan Flagship Store	Electricity conservation2	Jingdong quality! Always trustworthy!	Jingdong prefecture level city in Shandong
Jinfeng Hongyuan Flagship Store	Electricity conservation2	This is the second time I've bought it, and the merchant said to give me five dollars out of the difference, so thank you merchant.	Jingdong prefecture level city in Shandong
Jinfeng Hongyuan Flagship Store	Electricity conservation2	Good quality and good service	Jingdong prefecture level city in Shandong

Jinfeng Hongyuan Flagship Store	Electricity conservation2	Feels good texture, aluminum alloy shell, voltage digital display clear.	Jingdong prefecture level city in Shandong
Jinfeng Hongyuan Flagship Store	Electricity conservation2	Good product, fast and great service, thanks!	Jingdong prefecture level city in Shandong
Jinfeng Hongyuan Flagship Store	Electricity conservation2	Have not yet used, first evaluation, later really good do not need to comment again.	Jingdong prefecture level city in Shandong
Jinfeng Hongyuan Flagship Store	Electricity conservation2	Works great. Pretty much as described by the merchant.	Jingdong prefecture level city in Shandong
Jinfeng Hongyuan Flagship Store	Electricity conservation2	Received it, looking forward to the results, hope it works	Jingdong prefecture level city in Shandong
Jinfeng Hongyuan Flagship Store	Electricity conservation2	Received it, looking forward to the results, hope it works	Jingdong prefecture level city in Shandong
Jinfeng Hongyuan Flagship Store	Electricity conservation2	Supposedly it's an IQ tax. Buy one and try it. Looks good. Well made.	Jingdong prefecture level city in Shandong
Jinfeng Hongyuan Flagship Store	Electricity conservation2	It just doesn't feel like it's working.	Jingdong prefecture level city in Shandong
Jinfeng Hongyuan Flagship Store	Electricity conservation2	This is a great product.	Jingdong prefecture level city in Shandong
Jinfeng Hongyuan Flagship Store	Electricity conservation2	I'm just using it. I'll tell you more.	Jingdong prefecture level city in Shandong
Jinfeng Hongyuan Flagship Store	Electricity conservation2	Second repurchase and it's working fine.	Jingdong prefecture level city in Shandong
Jinfeng Hongyuan Flagship Store	Electricity conservation2	I'll wait a few days before evaluating	Jingdong prefecture level city in Shandong
Jinfeng Hongyuan Flagship Store	Electricity conservation2	I'll comment after I use it hopefully the section will be useful.	Jingdong prefecture level city in Shandong
Jinfeng Hongyuan Flagship Store	Electricity conservation2	not very useful	Jingdong prefecture level city in Shandong
Jinfeng Hongyuan Flagship Store	Electricity conservation2	Not received	Jingdong prefecture level city in Shandong
Jinfeng Hongyuan Flagship Store	Electricity conservation2	rare	Jingdong prefecture level city in Shandong
Jinfeng Hongyuan Flagship Store	Electricity conservation2	It's good.	Jingdong prefecture level city in Shandong
Jinfeng Hongyuan Flagship Store	Electricity conservation2	just now	Jingdong prefecture level city in Shandong
Jinfeng Hongyuan Flagship Store	Electricity conservation2	serviceable	Jingdong prefecture level city in Shandong
Jinfeng Hongyuan Flagship Store	Electricity conservation2	desirable	Jingdong prefecture level city in Shandong
Jinfeng Hongyuan Flagship Store	Electricity conservation2	serviceable	Jingdong prefecture level city in Shandong
Jinfeng Hongyuan Flagship Store	Electricity conservation2	It doesn't save power and costs electricity.	Jingdong prefecture level city in Shandong
Jinfeng Hongyuan Flagship Store	Electricity conservation2	Not as good as I thought.	Jingdong prefecture level city in Shandong
Jinfeng Hongyuan Flagship Store	Electricity conservation2	Super bad review, it doesn't save power at all, but rather more power consumption	Jingdong prefecture level city in Shandong
Jinfeng Hongyuan Flagship Store	Electricity conservation2	Listen to others say that this can save electricity, I bought with the idea of trying, used for a period of time before coming to the evaluation, will be a little bit of use, not a lot of savings just, long-term use of the words can save more.	Jingdong prefecture level city in Shandong
Jinfeng Hongyuan Flagship Store	Electricity conservation2	Logistics is fast, the next day arrived, workmanship is very delicate a power saver, looking forward to the late effect, customer service patiently explain the recommended which is suitable for, installed in what position is the best, look forward to using the effect after a period of time!	Jingdong prefecture level city in Shandong
Jinfeng Hongyuan Flagship Store	Electricity conservation2	It's good to use, I feel that it has been saving electricity, anyway, not a loss is, I used to feel that the power consumption is big, now I feel much better, my friends are also using, I hope to use a long time will be able to, I wish the boss a prosperous business	Jingdong prefecture level city in Shandong
Jinfeng Hongyuan Flagship Store	Electricity conservation2	Should be very good, to be able to plan the circuit, save the home of the circuit to the extent of the savings, waste of money on electricity, good if a month this money back, hope always hope to be able to	Jingdong prefecture level city in Shandong
Jinfeng Hongyuan Flagship Store	Electricity conservation2	Express delivery is very strong, the goods and I saw in the business consistent, the use of the effect of follow-up evaluation	Jingdong prefecture level city in Shandong
Jinfeng Hongyuan Flagship Store	Electricity conservation2	I have been using this brand of power saver for many years and this time I saw this upgraded version and got it again. The main purpose of this product is to reduce the loss of electricity for a long time, so as to achieve the purpose of saving electricity.	Jingdong prefecture level city in Shandong
Jinfeng Hongyuan Flagship Store	Electricity conservation2	Logistics is fast and the product looks beautiful, looking forward to the effect.	Jingdong prefecture level city in Shandong
Jinfeng Hongyuan Flagship Store	Electricity conservation2	Product quality is good, energy saving a little effect, but the price is a little expensive, fast arrival, good service.	Jingdong prefecture level city in Shandong
Jinfeng Hongyuan Flagship Store	Electricity conservation2	The quality is very good, inexpensive, Jingdong self-supporting guaranteed, worth having!	Jingdong prefecture level city in Shandong
Jinfeng Hongyuan Flagship Store	Electricity conservation2	I don't know if it's saving electricity yet, I'll find out next time I pay my electric bill	Jingdong prefecture level city in Shandong
Jinfeng Hongyuan Flagship Store	Electricity conservation2	Favorable price quality assurance appearance design is beautiful and generous, fine workmanship	Jingdong prefecture level city in Shandong
Jinfeng Hongyuan Flagship Store	Electricity conservation2	I bought it, I don't know how effective it is? I don't know how the quality is? After this summer results will know! Looking forward to it!	Jingdong prefecture level city in Shandong
Jinfeng Hongyuan Flagship Store	Electricity conservation2	Goods received Packaging intact, look at the shell workmanship is also good, high-power appliances open voltage has obvious changes in the effectiveness of the	Jingdong prefecture level city in Shandong
Jinfeng Hongyuan Flagship Store	Electricity conservation2	The goods have been received logistics is very fast, the product is similar to the line, just wait to use a month to see if it can save electricity, stay tuned.	Jingdong prefecture level city in Shandong
Jinfeng Hongyuan Flagship Store	Electricity conservation2	Electricity is plugged in, not sure if it will work, hopefully it will work.	Jingdong prefecture level city in Shandong
Jinfeng Hongyuan Flagship Store	Electricity conservation2	The shell is aluminum alloy, very good workmanship, the actual effect of waiting for the addition of evaluation	Jingdong prefecture level city in Shandong
Jinfeng Hongyuan Flagship Store	Electricity conservation2	Received, already plugged in, looking forward to the effect.	Jingdong prefecture level city in Shandong
Jinfeng Hongyuan Flagship Store	Electricity conservation2	Opened to see the regular manufacturer products, wait for a period of time to see it	Jingdong prefecture level city in Shandong
Jinfeng Hongyuan Flagship Store	Electricity conservation2	🉑 Baby received, very surprised ✨, reasonable design, fashionable and generous, continue to pay attention to the future will be repurchased ❤❤❤❤	Jingdong prefecture level city in Shandong
Jinfeng Hongyuan Flagship Store	Electricity conservation2	Baby received it 👌, really great ah! 🌈The quality can't be beat, very satisfied! 🆗Happy happy happy 😀	Jingdong prefecture level city in Shandong
Jinfeng Hongyuan Flagship Store	Electricity conservation2	Very good, power saving see, this is to help a friend to buy, I used to give my friend a say, my friend immediately let me order another one!	Jingdong prefecture level city in Shandong
Jinfeng Hongyuan Flagship Store	Electricity conservation2	The baby is just as introduced, the texture is very good, powerful, and it feels good to use it	Jingdong prefecture level city in Shandong
Jinfeng Hongyuan Flagship Store	Electricity conservation2	Value for money, very satisfied, seller service attitude is very good, very patient	Jingdong prefecture level city in Shandong
Jinfeng Hongyuan Flagship Store	Electricity conservation2	Power saving treasure design unique exquisite beautiful, voltage display clearly, plugged in that is good to use, I observed the meter, after using the meter is gone slow.	Jingdong prefecture level city in Shandong
Jinfeng Hongyuan Flagship Store	Electricity conservation2	Received on the use of the packaging is beautifully used for a period of time and then come back in the follow-up review!	Jingdong prefecture level city in Shandong

Jinfeng Hongyuan Flagship Store	Electricity conservation2	It should work. Not bad. Not bad. Not bad.	Jingdong prefecture level city in Shandong
Jinfeng Hongyuan Flagship Store	Electricity conservation2	I haven't used it yet, I'll plug it in and see if it saves power next month.	Jingdong prefecture level city in Shandong
Jinfeng Hongyuan Flagship Store	Electricity conservation2	The goods received and unpacked and used, the quality is very good, things and the official website to buy no difference!	Jingdong prefecture level city in Shandong
Jinfeng Hongyuan Flagship Store	Electricity conservation2	It's a rip off, doesn't help much at all, uses the same amount of power as before it was used.	Jingdong prefecture level city in Shandong
Jinfeng Hongyuan Flagship Store	Electricity conservation2	I've been using it for a while now and haven't noticed any unusual problems, so it's okay	Jingdong prefecture level city in Shandong
Jinfeng Hongyuan Flagship Store	Electricity conservation2	Received the goods and plugged in to use, good or not there is no use will have to wait until next month to find out	Jingdong prefecture level city in Shandong
Jinfeng Hongyuan Flagship Store	Electricity conservation2	It's very compact, it looks good, I'll introduce you to my clients when it's ready.	Jingdong prefecture level city in Shandong
Jinfeng Hongyuan Flagship Store	Electricity conservation2	Good workmanship, we'll have to see in a few months.	Jingdong prefecture level city in Shandong
Jinfeng Hongyuan Flagship Store	Electricity conservation2	Jingdong quality! Always trustworthy!	Jingdong prefecture level city in Shandong
Jinfeng Hongyuan Flagship Store	Electricity conservation2	This is the second time I've bought it, and the merchant said to give me five dollars out of the difference, so thank you merchant.	Jingdong prefecture level city in Shandong
Jinfeng Hongyuan Flagship Store	Electricity conservation2	Good quality and good service	Jingdong prefecture level city in Shandong
Jinfeng Hongyuan Flagship Store	Electricity conservation2	Feels good texture, aluminum alloy shell, voltage digital display clear.	Jingdong prefecture level city in Shandong
Jinfeng Hongyuan Flagship Store	Electricity conservation2	Good product, fast and great service, thanks!	Jingdong prefecture level city in Shandong
Jinfeng Hongyuan Flagship Store	Electricity conservation2	Have not yet used, first evaluation, later really good do not need to comment again.	Jingdong prefecture level city in Shandong
Jinfeng Hongyuan Flagship Store	Electricity conservation2	Works great. Pretty much as described by the merchant.	Jingdong prefecture level city in Shandong
Jinfeng Hongyuan Flagship Store	Electricity conservation2	Received it, looking forward to the results, hope it works	Jingdong prefecture level city in Shandong
Jinfeng Hongyuan Flagship Store	Electricity conservation2	Received it, looking forward to the results, hope it works	Jingdong prefecture level city in Shandong
Jinfeng Hongyuan Flagship Store	Electricity conservation2	Supposedly it's an IQ tax. Buy one and try it. Looks good. Well made.	Jingdong prefecture level city in Shandong
Jinfeng Hongyuan Flagship Store	Electricity conservation2	It just doesn't feel like it's working.	Jingdong prefecture level city in Shandong
Jinfeng Hongyuan Flagship Store	Electricity conservation2	This is a great product.	Jingdong prefecture level city in Shandong
Jinfeng Hongyuan Flagship Store	Electricity conservation2	I'm just using it. I'll tell you more.	Jingdong prefecture level city in Shandong
Jinfeng Hongyuan Flagship Store	Electricity conservation2	Second repurchase and it's working fine.	Jingdong prefecture level city in Shandong
Jinfeng Hongyuan Flagship Store	Electricity conservation2	I'll wait a few days before evaluating	Jingdong prefecture level city in Shandong
Jinfeng Hongyuan Flagship Store	Electricity conservation2	I'll comment after I use it hopefully the section will be useful.	Jingdong prefecture level city in Shandong
Jinfeng Hongyuan Flagship Store	Electricity conservation2	not very useful	Jingdong prefecture level city in Shandong
Jinfeng Hongyuan Flagship Store	Electricity conservation2	Not received	Jingdong prefecture level city in Shandong
Jinfeng Hongyuan Flagship Store	Electricity conservation2	rare	Jingdong prefecture level city in Shandong
Jinfeng Hongyuan Flagship Store	Electricity conservation2	It's good.	Jingdong prefecture level city in Shandong
Jinfeng Hongyuan Flagship Store	Electricity conservation2	just now	Jingdong prefecture level city in Shandong
Jinfeng Hongyuan Flagship Store	Electricity conservation2	serviceable	Jingdong prefecture level city in Shandong
Jinfeng Hongyuan Flagship Store	Electricity conservation2	desirable	Jingdong prefecture level city in Shandong
Jinfeng Hongyuan Flagship Store	Electricity conservation2	serviceable	Jingdong prefecture level city in Shandong
Jinfeng Hongyuan Flagship Store	Electricity conservation2	It doesn't save power and costs electricity.	Jingdong prefecture level city in Shandong
Jinfeng Hongyuan Flagship Store	Electricity conservation2	Not as good as I thought.	Jingdong prefecture level city in Shandong
Jinfeng Hongyuan Flagship Store	Electricity conservation2	Super bad review, it doesn't save power at all, but rather more power consumption	Jingdong prefecture level city in Shandong
Jinfeng Hongyuan Flagship Store	Electricity conservation2	Listen to others say that this can save electricity, I bought with the idea of trying, used for a period of time before coming to the evaluation, will be a little bit of use, not a lot of savings just, long-term use of the words can save more.	Jingdong prefecture level city in Shandong
Jinfeng Hongyuan Flagship Store	Electricity conservation2	Logistics is fast, the next day arrived, workmanship is very delicate a power saver, looking forward to the late effect, customer service patiently explain the recommended which is suitable for, installed in what position is the best, look forward to using the effect after a period of time!	Jingdong prefecture level city in Shandong
Jinfeng Hongyuan Flagship Store	Electricity conservation2	It's good to use, I feel that it has been saving electricity, anyway, not a loss is, I used to feel that the power consumption is big, now I feel much better, my friends are also using, I hope to use a long time will be able to, I wish the boss a prosperous business	Jingdong prefecture level city in Shandong
Jinfeng Hongyuan Flagship Store	Electricity conservation2	Should be very good, to be able to plan the circuit, save the home of the circuit to the extent of the savings, waste of money on electricity, good if a month this money back, hope always hope to be able to	Jingdong prefecture level city in Shandong
Jinfeng Hongyuan Flagship Store	Electricity conservation2	Express delivery is very strong, the goods and I saw in the business consistent, the use of the effect of follow-up evaluation	Jingdong prefecture level city in Shandong
Jinfeng Hongyuan Flagship Store	Electricity conservation2	I have been using this brand of power saver for many years and this time I saw this upgraded version and got it again. The main purpose of this product is to reduce the loss of electricity for a long time, so as to achieve the purpose of saving electricity.	Jingdong prefecture level city in Shandong
Jinfeng Hongyuan Flagship Store	Electricity conservation2	Logistics is fast and the product looks beautiful, looking forward to the effect.	Jingdong prefecture level city in Shandong
Jinfeng Hongyuan Flagship Store	Electricity conservation2	Product quality is good, energy saving a little effect, but the price is a little expensive, fast arrival, good service.	Jingdong prefecture level city in Shandong
Jinfeng Hongyuan Flagship Store	Electricity conservation2	The quality is very good, inexpensive, Jingdong self-supporting guaranteed, worth having!	Jingdong prefecture level city in Shandong
Jinfeng Hongyuan Flagship Store	Electricity conservation2	I don't know if it's saving electricity yet, I'll find out next time I pay my electric bill	Jingdong prefecture level city in Shandong
Jinfeng Hongyuan Flagship Store	Electricity conservation2	Favorable price quality assurance appearance design is beautiful and generous, fine workmanship	Jingdong prefecture level city in Shandong
Jinfeng Hongyuan Flagship Store	Electricity conservation2	I bought it, I don't know how effective it is? I don't know how the quality is? After this summer results will know! Looking forward to it!	Jingdong prefecture level city in Shandong
Jinfeng Hongyuan Flagship Store	Electricity conservation2	Goods received Packaging intact, look at the shell workmanship is also good, high-power appliances open voltage has obvious changes in the effectiveness of the	Jingdong prefecture level city in Shandong
Jinfeng Hongyuan Flagship Store	Electricity conservation2	The goods have been received logistics is very fast, the product is similar to the line, just wait to use a month to see if it can save electricity, stay tuned.	Jingdong prefecture level city in Shandong

Jinfeng Hongyuan Flagship Store	Electricity conservation2	Electricity is plugged in, not sure if it will work, hopefully it will work.	Jingdong prefecture level city in Shandong
Jinfeng Hongyuan Flagship Store	Electricity conservation2	The shell is aluminum alloy, very good workmanship, the actual effect of waiting for the addition of evaluation	Jingdong prefecture level city in Shandong
Jinfeng Hongyuan Flagship Store	Electricity conservation2	Received, already plugged in, looking forward to the effect.	Jingdong prefecture level city in Shandong
Jinfeng Hongyuan Flagship Store	Electricity conservation2	Opened to see the regular manufacturer products, wait for a period of time to see it	Jingdong prefecture level city in Shandong
Jinfeng Hongyuan Flagship Store	Electricity conservation2	🉑 Baby received, very surprised ✨, reasonable design, fashionable and generous, continue to pay attention to the future will be repurchased ❥❥❥❥	Jingdong prefecture level city in Shandong
Jinfeng Hongyuan Flagship Store	Electricity conservation2	Baby received it 👌, really great ah! 🌈The quality can't be beat, very satisfied! 🆗Happy happy happy 😃	Jingdong prefecture level city in Shandong
Jinfeng Hongyuan Flagship Store	Electricity conservation2	Very good, power saving see, this is to help a friend to buy, I used to give my friend a say, my friend immediately let me order another one!	Jingdong prefecture level city in Shandong
Jinfeng Hongyuan Flagship Store	Electricity conservation2	The baby is just as introduced, the texture is very good, powerful, and it feels good to use it	Jingdong prefecture level city in Shandong
Jinfeng Hongyuan Flagship Store	Electricity conservation2	Value for money, very satisfied, seller service attitude is very good, very patient	Jingdong prefecture level city in Shandong
Jinfeng Hongyuan Flagship Store	Electricity conservation2	Power saving treasure design unique exquisite beautiful, voltage display clearly, plugged in that is good to use, I observed the meter, after using the meter is gone slow.	Jingdong prefecture level city in Shandong
Jinfeng Hongyuan Flagship Store	Electricity conservation2	Received on the use of the packaging is beautifully used for a period of time and then come back in the follow-up review!	Jingdong prefecture level city in Shandong
Jinfeng Hongyuan Flagship Store	Electricity conservation2	It should work. Not bad. Not bad. Not bad.	Jingdong prefecture level city in Shandong
Jinfeng Hongyuan Flagship Store	Electricity conservation2	I haven't used it yet, I'll plug it in and see if it saves power next month.	Jingdong prefecture level city in Shandong
Jinfeng Hongyuan Flagship Store	Electricity conservation2	The goods received and unpacked and used, the quality is very good, things and the official website to buy no difference!	Jingdong prefecture level city in Shandong
Jinfeng Hongyuan Flagship Store	Electricity conservation2	It's a rip off, doesn't help much at all, uses the same amount of power as before it was used.	Jingdong prefecture level city in Shandong
Jinfeng Hongyuan Flagship Store	Electricity conservation2	I've been using it for a while now and haven't noticed any unusual problems, so it's okay	Jingdong prefecture level city in Shandong
Jinfeng Hongyuan Flagship Store	Electricity conservation2	Received the goods and plugged in to use, good or not there is no use will have to wait until next month to find out	Jingdong prefecture level city in Shandong
Jinfeng Hongyuan Flagship Store	Electricity conservation2	It's very compact, it looks good, I'll introduce you to my clients when it's ready.	Jingdong prefecture level city in Shandong
Jinfeng Hongyuan Flagship Store	Electricity conservation2	Good workmanship, we'll have to see in a few months.	Jingdong prefecture level city in Shandong
Jinfeng Hongyuan Flagship Store	Electricity conservation2	Jingdong quality! Always trustworthy!	Jingdong prefecture level city in Shandong
Jinfeng Hongyuan Flagship Store	Electricity conservation2	This is the second time I've bought it, and the merchant said to give me five dollars out of the difference, so thank you merchant.	Jingdong prefecture level city in Shandong
Jinfeng Hongyuan Flagship Store	Electricity conservation2	Good quality and good service	Jingdong prefecture level city in Shandong
Jinfeng Hongyuan Flagship Store	Electricity conservation2	Feels good texture, aluminum alloy shell, voltage digital display clear.	Jingdong prefecture level city in Shandong
Jinfeng Hongyuan Flagship Store	Electricity conservation2	Good product, fast and great service, thanks!	Jingdong prefecture level city in Shandong
Jinfeng Hongyuan Flagship Store	Electricity conservation2	Have not yet used, first evaluation, later really good do not need to comment again.	Jingdong prefecture level city in Shandong
Jinfeng Hongyuan Flagship Store	Electricity conservation2	Works great. Pretty much as described by the merchant.	Jingdong prefecture level city in Shandong
Jinfeng Hongyuan Flagship Store	Electricity conservation2	Received it, looking forward to the results, hope it works	Jingdong prefecture level city in Shandong
Jinfeng Hongyuan Flagship Store	Electricity conservation2	Received it, looking forward to the results, hope it works	Jingdong prefecture level city in Shandong
Jinfeng Hongyuan Flagship Store	Electricity conservation2	Supposedly it's an IQ tax. Buy one and try it. Looks good. Well made.	Jingdong prefecture level city in Shandong
Jinfeng Hongyuan Flagship Store	Electricity conservation2	It just doesn't feel like it's working.	Jingdong prefecture level city in Shandong
Jinfeng Hongyuan Flagship Store	Electricity conservation2	This is a great product.	Jingdong prefecture level city in Shandong
Jinfeng Hongyuan Flagship Store	Electricity conservation2	I'm just using it. I'll tell you more.	Jingdong prefecture level city in Shandong
Jinfeng Hongyuan Flagship Store	Electricity conservation2	Second repurchase and it's working fine.	Jingdong prefecture level city in Shandong
Jinfeng Hongyuan Flagship Store	Electricity conservation2	I'll wait a few days before evaluating	Jingdong prefecture level city in Shandong
Jinfeng Hongyuan Flagship Store	Electricity conservation2	I'll comment after I use it hopefully the section will be useful.	Jingdong prefecture level city in Shandong
Jinfeng Hongyuan Flagship Store	Electricity conservation2	not very useful	Jingdong prefecture level city in Shandong
Jinfeng Hongyuan Flagship Store	Electricity conservation2	Not received	Jingdong prefecture level city in Shandong
Jinfeng Hongyuan Flagship Store	Electricity conservation2	rare	Jingdong prefecture level city in Shandong
Jinfeng Hongyuan Flagship Store	Electricity conservation2	It's good.	Jingdong prefecture level city in Shandong
Jinfeng Hongyuan Flagship Store	Electricity conservation2	just now	Jingdong prefecture level city in Shandong
Jinfeng Hongyuan Flagship Store	Electricity conservation2	serviceable	Jingdong prefecture level city in Shandong
Jinfeng Hongyuan Flagship Store	Electricity conservation2	desirable	Jingdong prefecture level city in Shandong
Jinfeng Hongyuan Flagship Store	Electricity conservation2	serviceable	Jingdong prefecture level city in Shandong
Jinfeng Hongyuan Flagship Store	Electricity conservation2	It doesn't save power and costs electricity.	Jingdong prefecture level city in Shandong
Jinfeng Hongyuan Flagship Store	Electricity conservation2	Not as good as I thought.	Jingdong prefecture level city in Shandong
Jinfeng Hongyuan Flagship Store	Electricity conservation2	Super bad review, it doesn't save power at all, but rather more power consumption	Jingdong prefecture level city in Shandong
Jinfeng Hongyuan Flagship Store	Electricity conservation2	Listen to others say that this can save electricity, I bought with the idea of trying, used for a period of time before coming to the evaluation, will be a little bit of use, not a lot of savings just, long-term use of the words can save more.	Jingdong prefecture level city in Shandong
Jinfeng Hongyuan Flagship Store	Electricity conservation2	Logistics is fast, the next day arrived, workmanship is very delicate a power saver, looking forward to the late effect, customer service patiently explain the recommended which is suitable for, installed in what position is the best, look forward to using the effect after a period of time!	Jingdong prefecture level city in Shandong
Jinfeng Hongyuan Flagship Store	Electricity conservation2	It's good to use, I feel that it has been saving electricity, anyway, not a loss is, I used to feel that the power consumption is big, now I feel much better, my friends are also using, I hope to use a long time will be able to, I wish the boss a prosperous business	Jingdong prefecture level city in Shandong
Jinfeng Hongyuan Flagship Store	Electricity conservation2	Should be very good, to be able to plan the circuit, save the home of the circuit to the extent of the savings, waste of money on electricity, good if a month this money back, hope always hope to be able to	Jingdong prefecture level city in Shandong

Jinfeng Hongyuan Flagship Store	Electricity conservation2	Express delivery is very strong, the goods and I saw in the business consistent, the use of the effect of follow-up evaluation	Jingdong prefecture level city in Shandong
Jinfeng Hongyuan Flagship Store	Electricity conservation2	I have been using this brand of power saver for many years and this time I saw this upgraded version and got it again. The main purpose of this product is to reduce the loss of electricity for a long time, so as to achieve the purpose of saving electricity.	Jingdong prefecture level city in Shandong
Jinfeng Hongyuan Flagship Store	Electricity conservation2	Logistics is fast and the product looks beautiful, looking forward to the effect.	Jingdong prefecture level city in Shandong
Jinfeng Hongyuan Flagship Store	Electricity conservation2	Product quality is good, energy saving a little effect, but the price is a little expensive, fast arrival, good service.	Jingdong prefecture level city in Shandong
Jinfeng Hongyuan Flagship Store	Electricity conservation2	The quality is very good, inexpensive, Jingdong self-supporting guaranteed, worth having!	Jingdong prefecture level city in Shandong
Jinfeng Hongyuan Flagship Store	Electricity conservation2	I don't know if it's saving electricity yet, I'll find out next time I pay my electric bill	Jingdong prefecture level city in Shandong
Jinfeng Hongyuan Flagship Store	Electricity conservation2	Favorable price quality assurance appearance design is beautiful and generous, fine workmanship	Jingdong prefecture level city in Shandong
Jinfeng Hongyuan Flagship Store	Electricity conservation2	I bought it, I don't know how effective it is? I don't know how the quality is? After this summer results will know! Looking forward to it!	Jingdong prefecture level city in Shandong
Jinfeng Hongyuan Flagship Store	Electricity conservation2	Goods received Packaging intact, look at the shell workmanship is also good, high-power appliances open voltage has obvious changes in the effectiveness of the	Jingdong prefecture level city in Shandong
Jinfeng Hongyuan Flagship Store	Electricity conservation2	The goods have been received logistics is very fast, the product is similar to the line, just wait to use a month to see if it can save electricity, stay tuned.	Jingdong prefecture level city in Shandong
Jinfeng Hongyuan Flagship Store	Electricity conservation2	Electricity is plugged in, not sure if it will work, hopefully it will work.	Jingdong prefecture level city in Shandong
Jinfeng Hongyuan Flagship Store	Electricity conservation2	The shell is aluminum alloy, very good workmanship, the actual effect of waiting for the addition of evaluation	Jingdong prefecture level city in Shandong
Jinfeng Hongyuan Flagship Store	Electricity conservation2	Received, already plugged in, looking forward to the effect.	Jingdong prefecture level city in Shandong
Jinfeng Hongyuan Flagship Store	Electricity conservation2	Opened to see the regular manufacturer products, wait for a period of time to see it	Jingdong prefecture level city in Shandong
Jinfeng Hongyuan Flagship Store	Electricity conservation2	🉑 Baby received, very surprised ✨, reasonable design, fashionable and generous, continue to pay attention to the future will be repurchased ❤❤❤❤	Jingdong prefecture level city in Shandong
Jinfeng Hongyuan Flagship Store	Electricity conservation2	Baby received it 👌, really great ah! 🌈The quality can't be beat, very satisfied! 🆗Happy happy happy 😃	Jingdong prefecture level city in Shandong
Jinfeng Hongyuan Flagship Store	Electricity conservation2	Very good, power saving see, this is to help a friend to buy, I used to give my friend a say, my friend immediately let me order another one!	Jingdong prefecture level city in Shandong
Jinfeng Hongyuan Flagship Store	Electricity conservation2	The baby is just as introduced, the texture is very good, powerful, and it feels good to use it	Jingdong prefecture level city in Shandong
Jinfeng Hongyuan Flagship Store	Electricity conservation2	Value for money, very satisfied, seller service attitude is very good, very patient	Jingdong prefecture level city in Shandong
Jinfeng Hongyuan Flagship Store	Electricity conservation2	Power saving treasure design unique exquisite beautiful, voltage display clearly, plugged in that is good to use, I observed the meter, after using the meter is gone slow.	Jingdong prefecture level city in Shandong
Jinfeng Hongyuan Flagship Store	Electricity conservation2	Received on the use of the packaging is beautifully used for a period of time and then come back in the follow-up review!	Jingdong prefecture level city in Shandong
Jinfeng Hongyuan Flagship Store	Electricity conservation2	It should work. Not bad. Not bad. Not bad.	Jingdong prefecture level city in Shandong
Jinfeng Hongyuan Flagship Store	Electricity conservation2	I haven't used it yet, I'll plug it in and see if it saves power next month.	Jingdong prefecture level city in Shandong
Jinfeng Hongyuan Flagship Store	Electricity conservation2	The goods received and unpacked and used, the quality is very good, things and the official website to buy no difference!	Jingdong prefecture level city in Shandong
Jinfeng Hongyuan Flagship Store	Electricity conservation2	It's a rip off, doesn't help much at all, uses the same amount of power as before it was used.	Jingdong prefecture level city in Shandong
Jinfeng Hongyuan Flagship Store	Electricity conservation2	I've been using it for a while now and haven't noticed any unusual problems, so it's okay	Jingdong prefecture level city in Shandong
Jinfeng Hongyuan Flagship Store	Electricity conservation2	Received the goods and plugged in to use, good or not there is no use will have to wait until next month to find out	Jingdong prefecture level city in Shandong
Jinfeng Hongyuan Flagship Store	Electricity conservation2	It's very compact, it looks good, I'll introduce you to my clients when it's ready.	Jingdong prefecture level city in Shandong
Jinfeng Hongyuan Flagship Store	Electricity conservation2	Good workmanship, we'll have to see in a few months.	Jingdong prefecture level city in Shandong
Jinfeng Hongyuan Flagship Store	Electricity conservation2	Jingdong quality! Always trustworthy!	Jingdong prefecture level city in Shandong
Jinfeng Hongyuan Flagship Store	Electricity conservation2	This is the second time I've bought it, and the merchant said to give me five dollars out of the difference, so thank you merchant.	Jingdong prefecture level city in Shandong
Jinfeng Hongyuan Flagship Store	Electricity conservation2	Good quality and good service	Jingdong prefecture level city in Shandong
Jinfeng Hongyuan Flagship Store	Electricity conservation2	Feels good texture, aluminum alloy shell, voltage digital display clear.	Jingdong prefecture level city in Shandong
Jinfeng Hongyuan Flagship Store	Electricity conservation2	Good product, fast and great service, thanks!	Jingdong prefecture level city in Shandong
Jinfeng Hongyuan Flagship Store	Electricity conservation2	Have not yet used, first evaluation, later really good do not need to comment again.	Jingdong prefecture level city in Shandong
Jinfeng Hongyuan Flagship Store	Electricity conservation2	Works great. Pretty much as described by the merchant.	Jingdong prefecture level city in Shandong
Jinfeng Hongyuan Flagship Store	Electricity conservation2	Received it, looking forward to the results, hope it works	Jingdong prefecture level city in Shandong
Jinfeng Hongyuan Flagship Store	Electricity conservation2	Received it, looking forward to the results, hope it works	Jingdong prefecture level city in Shandong
Jinfeng Hongyuan Flagship Store	Electricity conservation2	Supposedly it's an IQ tax. Buy one and try it. Looks good. Well made.	Jingdong prefecture level city in Shandong
Jinfeng Hongyuan Flagship Store	Electricity conservation2	It just doesn't feel like it's working.	Jingdong prefecture level city in Shandong
Jinfeng Hongyuan Flagship Store	Electricity conservation2	This is a great product.	Jingdong prefecture level city in Shandong
Jinfeng Hongyuan Flagship Store	Electricity conservation2	I'm just using it. I'll tell you more.	Jingdong prefecture level city in Shandong
Jinfeng Hongyuan Flagship Store	Electricity conservation2	Second repurchase and it's working fine.	Jingdong prefecture level city in Shandong
Jinfeng Hongyuan Flagship Store	Electricity conservation2	I'll wait a few days before evaluating	Jingdong prefecture level city in Shandong
Jinfeng Hongyuan Flagship Store	Electricity conservation2	I'll comment after I use it hopefully the section will be useful.	Jingdong prefecture level city in Shandong
Jinfeng Hongyuan Flagship Store	Electricity conservation2	not very useful	Jingdong prefecture level city in Shandong
Jinfeng Hongyuan Flagship Store	Electricity conservation2	Not received	Jingdong prefecture level city in Shandong
Jinfeng Hongyuan Flagship Store	Electricity conservation2	rare	Jingdong prefecture level city in Shandong
Jinfeng Hongyuan Flagship Store	Electricity conservation2	It's good.	Jingdong prefecture level city in Shandong
Jinfeng Hongyuan Flagship Store	Electricity conservation2	just now	Jingdong prefecture level city in Shandong
Jinfeng Hongyuan Flagship Store	Electricity conservation2	serviceable	Jingdong prefecture level city in Shandong

Jinfeng Hongyuan Flagship Store	Electricity conservation2	desirable	Jingdong prefecture level city in Shandong
Jinfeng Hongyuan Flagship Store	Electricity conservation2	serviceable	Jingdong prefecture level city in Shandong
Jinfeng Hongyuan Flagship Store	Electricity conservation2	It doesn't save power and costs electricity.	Jingdong prefecture level city in Shandong
Jinfeng Hongyuan Flagship Store	Electricity conservation2	Not as good as I thought.	Jingdong prefecture level city in Shandong
Jinfeng Hongyuan Flagship Store	Electricity conservation2	Super bad review, it doesn't save power at all, but rather more power consumption	Jingdong prefecture level city in Shandong
Jinfeng Hongyuan Flagship Store	Electricity conservation2	Listen to others say that this can save electricity, I bought with the idea of trying, used for a period of time before coming to the evaluation, will be a little bit of use, not a lot of savings just, long-term use of the words can save more.	Jingdong prefecture level city in Shandong
Jinfeng Hongyuan Flagship Store	Electricity conservation2	Logistics is fast, the next day arrived, workmanship is very delicate a power saver, looking forward to the late effect, customer service patiently explain the recommended which is suitable for, installed in what position is the best, look forward to using the effect after a period of time!	Jingdong prefecture level city in Shandong
Jinfeng Hongyuan Flagship Store	Electricity conservation2	It's good to use, I feel that it has been saving electricity, anyway, not a loss is, I used to feel that the power consumption is big, now I feel much better, my friends are also using, I hope to use a long time will be able to, I wish the boss a prosperous business	Jingdong prefecture level city in Shandong
Jinfeng Hongyuan Flagship Store	Electricity conservation2	Should be very good, to be able to plan the circuit, save the home of the circuit to the extent of the savings, waste of money on electricity, good if a month this money back, hope always hope to be able to	Jingdong prefecture level city in Shandong
Jinfeng Hongyuan Flagship Store	Electricity conservation2	Express delivery is very strong, the goods and I saw in the business consistent, the use of the effect of follow-up evaluation	Jingdong prefecture level city in Shandong
Jinfeng Hongyuan Flagship Store	Electricity conservation2	I have been using this brand of power saver for many years and this time I saw this upgraded version and got it again. The main purpose of this product is to reduce the loss of electricity for a long time, so as to achieve the purpose of saving electricity.	Jingdong prefecture level city in Shandong
Jinfeng Hongyuan Flagship Store	Electricity conservation2	Logistics is fast and the product looks beautiful, looking forward to the effect.	Jingdong prefecture level city in Shandong
Jinfeng Hongyuan Flagship Store	Electricity conservation2	Product quality is good, energy saving a little effect, but the price is a little expensive, fast arrival, good service.	Jingdong prefecture level city in Shandong
Jinfeng Hongyuan Flagship Store	Electricity conservation2	The quality is very good, inexpensive, Jingdong self-supporting guaranteed, worth having!	Jingdong prefecture level city in Shandong
Jinfeng Hongyuan Flagship Store	Electricity conservation2	I don't know if it's saving electricity yet, I'll find out next time I pay my electric bill	Jingdong prefecture level city in Shandong
Jinfeng Hongyuan Flagship Store	Electricity conservation2	Favorable price quality assurance appearance design is beautiful and generous, fine workmanship	Jingdong prefecture level city in Shandong
Jinfeng Hongyuan Flagship Store	Electricity conservation2	I bought it, I don't know how effective it is? I don't know how the quality is? After this summer results will know! Looking forward to it!	Jingdong prefecture level city in Shandong
Jinfeng Hongyuan Flagship Store	Electricity conservation2	Goods received Packaging intact, look at the shell workmanship is also good, high-power appliances open voltage has obvious changes in the effectiveness of the	Jingdong prefecture level city in Shandong
Jinfeng Hongyuan Flagship Store	Electricity conservation2	The goods have been received logistics is very fast, the product is similar to the line, just wait to use a month to see if it can save electricity, stay tuned.	Jingdong prefecture level city in Shandong
Jinfeng Hongyuan Flagship Store	Electricity conservation2	Electricity is plugged in, not sure if it will work, hopefully it will work.	Jingdong prefecture level city in Shandong
Jinfeng Hongyuan Flagship Store	Electricity conservation2	The shell is aluminum alloy, very good workmanship, the actual effect of waiting for the addition of evaluation	Jingdong prefecture level city in Shandong
Jinfeng Hongyuan Flagship Store	Electricity conservation2	Received, already plugged in, looking forward to the effect.	Jingdong prefecture level city in Shandong
Jinfeng Hongyuan Flagship Store	Electricity conservation2	Opened to see the regular manufacturer products, wait for a period of time to see it	Jingdong prefecture level city in Shandong
Jinfeng Hongyuan Flagship Store	Electricity conservation2	🉑 Baby received, very surprised ✨, reasonable design, fashionable and generous, continue to pay attention to the future will be repurchased ❥❥❥❥	Jingdong prefecture level city in Shandong
Jinfeng Hongyuan Flagship Store	Electricity conservation2	Baby received it 👌, really great ah! 🌈The quality can't be beat, very satisfied! 🆗Happy happy happy 😃	Jingdong prefecture level city in Shandong
Jinfeng Hongyuan Flagship Store	Electricity conservation2	Very good, power saving see, this is to help a friend to buy, I used to give my friend a say, my friend immediately let me order another one!	Jingdong prefecture level city in Shandong
Jinfeng Hongyuan Flagship Store	Electricity conservation2	The baby is just as introduced, the texture is very good, powerful, and it feels good to use it	Jingdong prefecture level city in Shandong
Jinfeng Hongyuan Flagship Store	Electricity conservation2	Value for money, very satisfied, seller service attitude is very good, very patient	Jingdong prefecture level city in Shandong
Jinfeng Hongyuan Flagship Store	Electricity conservation2	Power saving treasure design unique exquisite beautiful, voltage display clearly, plugged in that is good to use, I observed the meter, after using the meter is gone slow.	Jingdong prefecture level city in Shandong
Jinfeng Hongyuan Flagship Store	Electricity conservation2	Received on the use of the packaging is beautifully used for a period of time and then come back in the follow-up review!	Jingdong prefecture level city in Shandong
Jinfeng Hongyuan Flagship Store	Electricity conservation2	It should work. Not bad. Not bad. Not bad.	Jingdong prefecture level city in Shandong
Jinfeng Hongyuan Flagship Store	Electricity conservation2	I haven't used it yet, I'll plug it in and see if it saves power next month.	Jingdong prefecture level city in Shandong
Jinfeng Hongyuan Flagship Store	Electricity conservation2	The goods received and unpacked and used, the quality is very good, things and the official website to buy no difference!	Jingdong prefecture level city in Shandong
Jinfeng Hongyuan Flagship Store	Electricity conservation2	It's a rip off, doesn't help much at all, uses the same amount of power as before it was used.	Jingdong prefecture level city in Shandong
Jinfeng Hongyuan Flagship Store	Electricity conservation2	I've been using it for a while now and haven't noticed any unusual problems, so it's okay	Jingdong prefecture level city in Shandong
Jinfeng Hongyuan Flagship Store	Electricity conservation2	Received the goods and plugged in to use, good or not there is no use will have to wait until next month to find out	Jingdong prefecture level city in Shandong
Jinfeng Hongyuan Flagship Store	Electricity conservation2	It's very compact, it looks good, I'll introduce you to my clients when it's ready.	Jingdong prefecture level city in Shandong
Jinfeng Hongyuan Flagship Store	Electricity conservation2	Good workmanship, we'll have to see in a few months.	Jingdong prefecture level city in Shandong
Jinfeng Hongyuan Flagship Store	Electricity conservation2	Jingdong quality! Always trustworthy!	Jingdong prefecture level city in Shandong
Jinfeng Hongyuan Flagship Store	Electricity conservation2	This is the second time I've bought it, and the merchant said to give me five dollars out of the difference, so thank you merchant.	Jingdong prefecture level city in Shandong
Jinfeng Hongyuan Flagship Store	Electricity conservation2	Good quality and good service	Jingdong prefecture level city in Shandong
Jinfeng Hongyuan Flagship Store	Electricity conservation2	Feels good texture, aluminum alloy shell, voltage digital display clear.	Jingdong prefecture level city in Shandong
Jinfeng Hongyuan Flagship Store	Electricity conservation2	Good product, fast and great service, thanks!	Jingdong prefecture level city in Shandong
Jinfeng Hongyuan Flagship Store	Electricity conservation2	Have not yet used, first evaluation, later really good do not need to comment again.	Jingdong prefecture level city in Shandong
Jinfeng Hongyuan Flagship Store	Electricity conservation2	Works great. Pretty much as described by the merchant.	Jingdong prefecture level city in Shandong
Jinfeng Hongyuan Flagship Store	Electricity conservation2	Received it, looking forward to the results, hope it works	Jingdong prefecture level city in Shandong
Jinfeng Hongyuan Flagship Store	Electricity conservation2	Received it, looking forward to the results, hope it works	Jingdong prefecture level city in Shandong
Jinfeng Hongyuan Flagship Store	Electricity conservation2	Supposedly it's an IQ tax. Buy one and try it. Looks good. Well made.	Jingdong prefecture level city in Shandong
Jinfeng Hongyuan Flagship Store	Electricity conservation2	It just doesn't feel like it's working.	Jingdong prefecture level city in Shandong

Jinfeng Hongyuan Flagship Store	Electricity conservation2	This is a great product.	Jingdong prefecture level city in Shandong
Jinfeng Hongyuan Flagship Store	Electricity conservation2	I'm just using it. I'll tell you more.	Jingdong prefecture level city in Shandong
Jinfeng Hongyuan Flagship Store	Electricity conservation2	Second repurchase and it's working fine.	Jingdong prefecture level city in Shandong
Jinfeng Hongyuan Flagship Store	Electricity conservation2	I'll wait a few days before evaluating	Jingdong prefecture level city in Shandong
Jinfeng Hongyuan Flagship Store	Electricity conservation2	I'll comment after I use it hopefully the section will be useful.	Jingdong prefecture level city in Shandong
Jinfeng Hongyuan Flagship Store	Electricity conservation2	not very useful	Jingdong prefecture level city in Shandong
Jinfeng Hongyuan Flagship Store	Electricity conservation2	Not received	Jingdong prefecture level city in Shandong
Jinfeng Hongyuan Flagship Store	Electricity conservation2	rare	Jingdong prefecture level city in Shandong
Jinfeng Hongyuan Flagship Store	Electricity conservation2	It's good.	Jingdong prefecture level city in Shandong
Jinfeng Hongyuan Flagship Store	Electricity conservation2	just now	Jingdong prefecture level city in Shandong
Jinfeng Hongyuan Flagship Store	Electricity conservation2	serviceable	Jingdong prefecture level city in Shandong
Jinfeng Hongyuan Flagship Store	Electricity conservation2	desirable	Jingdong prefecture level city in Shandong
Jinfeng Hongyuan Flagship Store	Electricity conservation2	serviceable	Jingdong prefecture level city in Shandong
Jinfeng Hongyuan Flagship Store	Electricity conservation2	It doesn't save power and costs electricity.	Jingdong prefecture level city in Shandong
Jinfeng Hongyuan Flagship Store	Electricity conservation2	Not as good as I thought.	Jingdong prefecture level city in Shandong
Jinfeng Hongyuan Flagship Store	Electricity conservation2	Super bad review, it doesn't save power at all, but rather more power consumption	Jingdong prefecture level city in Shandong
Jinfeng Hongyuan Flagship Store	Electricity conservation2	Listen to others say that this can save electricity, I bought with the idea of trying, used for a period of time before coming to the evaluation, will be a little bit of use, not a lot of savings just, long-term use of the words can save more.	Jingdong prefecture level city in Shandong
Jinfeng Hongyuan Flagship Store	Electricity conservation2	Logistics is fast, the next day arrived, workmanship is very delicate a power saver, looking forward to the late effect, customer service patiently explain the recommended which is suitable for, installed in what position is the best, look forward to using the effect after a period of time!	Jingdong prefecture level city in Shandong
Jinfeng Hongyuan Flagship Store	Electricity conservation2	It's good to use, I feel that it has been saving electricity, anyway, not a loss is, I used to feel that the power consumption is big, now I feel much better, my friends are also using, I hope to use a long time will be able to, I wish the boss a prosperous business	Jingdong prefecture level city in Shandong
Jinfeng Hongyuan Flagship Store	Electricity conservation2	Should be very good, to be able to plan the circuit, save the home of the circuit to the extent of the savings, waste of money on electricity, good if a month this money back, hope always hope to be able to	Jingdong prefecture level city in Shandong
Jinfeng Hongyuan Flagship Store	Electricity conservation2	Express delivery is very strong, the goods and I saw in the business consistent, the use of the effect of follow-up evaluation	Jingdong prefecture level city in Shandong
Jinfeng Hongyuan Flagship Store	Electricity conservation2	I have been using this brand of power saver for many years and this time I saw this upgraded version and got it again. The main purpose of this product is to reduce the loss of electricity for a long time, so as to achieve the purpose of saving electricity.	Jingdong prefecture level city in Shandong
Jinfeng Hongyuan Flagship Store	Electricity conservation2	Logistics is fast and the product looks beautiful, looking forward to the effect.	Jingdong prefecture level city in Shandong
Jinfeng Hongyuan Flagship Store	Electricity conservation2	Product quality is good, energy saving a little effect, but the price is a little expensive, fast arrival, good service.	Jingdong prefecture level city in Shandong
Jinfeng Hongyuan Flagship Store	Electricity conservation2	The quality is very good, inexpensive, Jingdong self-supporting guaranteed, worth having!	Jingdong prefecture level city in Shandong
Jinfeng Hongyuan Flagship Store	Electricity conservation2	I don't know if it's saving electricity yet, I'll find out next time I pay my electric bill	Jingdong prefecture level city in Shandong
Jinfeng Hongyuan Flagship Store	Electricity conservation2	Favorable price quality assurance appearance design is beautiful and generous, fine workmanship	Jingdong prefecture level city in Shandong
Jinfeng Hongyuan Flagship Store	Electricity conservation2	I bought it, I don't know how effective it is? I don't know how the quality is? After this summer results will know! Looking forward to it!	Jingdong prefecture level city in Shandong
Jinfeng Hongyuan Flagship Store	Electricity conservation2	Goods received Packaging intact, look at the shell workmanship is also good, high-power appliances open voltage has obvious changes in the effectiveness of the	Jingdong prefecture level city in Shandong
Jinfeng Hongyuan Flagship Store	Electricity conservation2	The goods have been received logistics is very fast, the product is similar to the line, just wait to use a month to see if it can save electricity, stay tuned.	Jingdong prefecture level city in Shandong
Jinfeng Hongyuan Flagship Store	Electricity conservation2	Electricity is plugged in, not sure if it will work, hopefully it will work.	Jingdong prefecture level city in Shandong
Jinfeng Hongyuan Flagship Store	Electricity conservation2	The shell is aluminum alloy, very good workmanship, the actual effect of waiting for the addition of evaluation	Jingdong prefecture level city in Shandong
Jinfeng Hongyuan Flagship Store	Electricity conservation2	Received, already plugged in, looking forward to the effect.	Jingdong prefecture level city in Shandong
Jinfeng Hongyuan Flagship Store	Electricity conservation2	Opened to see the regular manufacturer products, wait for a period of time to see it	Jingdong prefecture level city in Shandong
Jinfeng Hongyuan Flagship Store	Electricity conservation2	🉑 Baby received, very surprised ✨, reasonable design, fashionable and generous, continue to pay attention to the future will be repurchased ❤❤❤❤	Jingdong prefecture level city in Shandong
Jinfeng Hongyuan Flagship Store	Electricity conservation2	Baby received it 👌, really great ah! 🌈The quality can't be beat, very satisfied! 🆗Happy happy happy 😃	Jingdong prefecture level city in Shandong
Jinfeng Hongyuan Flagship Store	Electricity conservation2	Very good, power saving see, this is to help a friend to buy, I used to give my friend a say, my friend immediately let me order another one!	Jingdong prefecture level city in Shandong
Jinfeng Hongyuan Flagship Store	Electricity conservation2	The baby is just as introduced, the texture is very good, powerful, and it feels good to use it	Jingdong prefecture level city in Shandong
Jinfeng Hongyuan Flagship Store	Electricity conservation2	Value for money, very satisfied, seller service attitude is very good, very patient	Jingdong prefecture level city in Shandong
Jinfeng Hongyuan Flagship Store	Electricity conservation2	Power saving treasure design unique exquisite beautiful, voltage display clearly, plugged in that is good to use, I observed the meter, after using the meter is gone slow.	Jingdong prefecture level city in Shandong
Jinfeng Hongyuan Flagship Store	Electricity conservation2	Received on the use of the packaging is beautifully used for a period of time and then come back in the follow-up review!	Jingdong prefecture level city in Shandong
Jinfeng Hongyuan Flagship Store	Electricity conservation2	It should work. Not bad. Not bad. Not bad.	Jingdong prefecture level city in Shandong
Jinfeng Hongyuan Flagship Store	Electricity conservation2	I haven't used it yet, I'll plug it in and see if it saves power next month.	Jingdong prefecture level city in Shandong
Jinfeng Hongyuan Flagship Store	Electricity conservation2	The goods received and unpacked and used, the quality is very good, things and the official website to buy no difference!	Jingdong prefecture level city in Shandong
Jinfeng Hongyuan Flagship Store	Electricity conservation2	It's a rip off, doesn't help much at all, uses the same amount of power as before it was used.	Jingdong prefecture level city in Shandong
Jinfeng Hongyuan Flagship Store	Electricity conservation2	I've been using it for a while now and haven't noticed any unusual problems, so it's okay	Jingdong prefecture level city in Shandong
Jinfeng Hongyuan Flagship Store	Electricity conservation2	Received the goods and plugged in to use, good or not there is no use will have to wait until next month to find out	Jingdong prefecture level city in Shandong
Jinfeng Hongyuan Flagship Store	Electricity conservation2	It's very compact, it looks good, I'll introduce you to my clients when it's ready.	Jingdong prefecture level city in Shandong
Jinfeng Hongyuan Flagship Store	Electricity conservation2	Good workmanship, we'll have to see in a few months.	Jingdong prefecture level city in Shandong

Jinfeng Hongyuan Flagship Store	Electricity conservation2	Jingdong quality! Always trustworthy!	Jingdong prefecture level city in Shandong
Jinfeng Hongyuan Flagship Store	Electricity conservation2	This is the second time I've bought it, and the merchant said to give me five dollars out of the difference, so thank you merchant.	Jingdong prefecture level city in Shandong
Jinfeng Hongyuan Flagship Store	Electricity conservation2	Good quality and good service	Jingdong prefecture level city in Shandong
Jinfeng Hongyuan Flagship Store	Electricity conservation2	Feels good texture, aluminum alloy shell, voltage digital display clear.	Jingdong prefecture level city in Shandong
Jinfeng Hongyuan Flagship Store	Electricity conservation2	Good product, fast and great service, thanks!	Jingdong prefecture level city in Shandong
Jinfeng Hongyuan Flagship Store	Electricity conservation2	Have not yet used, first evaluation, later really good do not need to comment again.	Jingdong prefecture level city in Shandong
Jinfeng Hongyuan Flagship Store	Electricity conservation2	Works great. Pretty much as described by the merchant.	Jingdong prefecture level city in Shandong
Jinfeng Hongyuan Flagship Store	Electricity conservation2	Received it, looking forward to the results, hope it works	Jingdong prefecture level city in Shandong
Jinfeng Hongyuan Flagship Store	Electricity conservation2	Received it, looking forward to the results, hope it works	Jingdong prefecture level city in Shandong
Jinfeng Hongyuan Flagship Store	Electricity conservation2	Supposedly it's an IQ tax. Buy one and try it. Looks good. Well made.	Jingdong prefecture level city in Shandong
Jinfeng Hongyuan Flagship Store	Electricity conservation2	It just doesn't feel like it's working.	Jingdong prefecture level city in Shandong
Jinfeng Hongyuan Flagship Store	Electricity conservation2	This is a great product.	Jingdong prefecture level city in Shandong
Jinfeng Hongyuan Flagship Store	Electricity conservation2	I'm just using it. I'll tell you more.	Jingdong prefecture level city in Shandong
Jinfeng Hongyuan Flagship Store	Electricity conservation2	Second repurchase and it's working fine.	Jingdong prefecture level city in Shandong
Jinfeng Hongyuan Flagship Store	Electricity conservation2	I'll wait a few days before evaluating	Jingdong prefecture level city in Shandong
Jinfeng Hongyuan Flagship Store	Electricity conservation2	I'll comment after I use it hopefully the section will be useful.	Jingdong prefecture level city in Shandong
Jinfeng Hongyuan Flagship Store	Electricity conservation2	not very useful	Jingdong prefecture level city in Shandong
Jinfeng Hongyuan Flagship Store	Electricity conservation2	Not received	Jingdong prefecture level city in Shandong
Jinfeng Hongyuan Flagship Store	Electricity conservation2	rare	Jingdong prefecture level city in Shandong
Jinfeng Hongyuan Flagship Store	Electricity conservation2	It's good.	Jingdong prefecture level city in Shandong
Jinfeng Hongyuan Flagship Store	Electricity conservation2	just now	Jingdong prefecture level city in Shandong
Jinfeng Hongyuan Flagship Store	Electricity conservation2	serviceable	Jingdong prefecture level city in Shandong
Jinfeng Hongyuan Flagship Store	Electricity conservation2	desirable	Jingdong prefecture level city in Shandong
Jinfeng Hongyuan Flagship Store	Electricity conservation2	serviceable	Jingdong prefecture level city in Shandong
Jinfeng Hongyuan Flagship Store	Electricity conservation2	It doesn't save power and costs electricity.	Jingdong prefecture level city in Shandong
Jinfeng Hongyuan Flagship Store	Electricity conservation2	Not as good as I thought.	Jingdong prefecture level city in Shandong
Jinfeng Hongyuan Flagship Store	Electricity conservation2	Super bad review, it doesn't save power at all, but rather more power consumption	Jingdong prefecture level city in Shandong
Jinfeng Hongyuan Flagship Store	Electricity conservation2	Listen to others say that this can save electricity, I bought with the idea of trying, used for a period of time before coming to the evaluation, will be a little bit of use, not a lot of savings just, long-term use of the words can save more.	Jingdong prefecture level city in Shandong
Jinfeng Hongyuan Flagship Store	Electricity conservation2	Logistics is fast, the next day arrived, workmanship is very delicate a power saver, looking forward to the late effect, customer service patiently explain the recommended which is suitable for, installed in what position is the best, look forward to using the effect after a period of time!	Jingdong prefecture level city in Shandong
Jinfeng Hongyuan Flagship Store	Electricity conservation2	It's good to use, I feel that it has been saving electricity, anyway, not a loss is, I used to feel that the power consumption is big, now I feel much better, my friends are also using, I hope to use a long time will be able to, I wish the boss a prosperous business	Jingdong prefecture level city in Shandong
Jinfeng Hongyuan Flagship Store	Electricity conservation2	Should be very good, to be able to plan the circuit, save the home of the circuit to the extent of the savings, waste of money on electricity, good if a month this money back, hope always hope to be able to	Jingdong prefecture level city in Shandong
Jinfeng Hongyuan Flagship Store	Electricity conservation2	Express delivery is very strong, the goods and I saw in the business consistent, the use of the effect of follow-up evaluation	Jingdong prefecture level city in Shandong
Jinfeng Hongyuan Flagship Store	Electricity conservation2	I have been using this brand of power saver for many years and this time I saw this upgraded version and got it again. The main purpose of this product is to reduce the loss of electricity for a long time, so as to achieve the purpose of saving electricity.	Jingdong prefecture level city in Shandong
Jinfeng Hongyuan Flagship Store	Electricity conservation2	Logistics is fast and the product looks beautiful, looking forward to the effect.	Jingdong prefecture level city in Shandong
Jinfeng Hongyuan Flagship Store	Electricity conservation2	Product quality is good, energy saving a little effect, but the price is a little expensive, fast arrival, good service.	Jingdong prefecture level city in Shandong
Jinfeng Hongyuan Flagship Store	Electricity conservation2	The quality is very good, inexpensive, Jingdong self-supporting guaranteed, worth having!	Jingdong prefecture level city in Shandong
Jinfeng Hongyuan Flagship Store	Electricity conservation2	I don't know if it's saving electricity yet, I'll find out next time I pay my electric bill	Jingdong prefecture level city in Shandong
Jinfeng Hongyuan Flagship Store	Electricity conservation2	Favorable price quality assurance appearance design is beautiful and generous, fine workmanship	Jingdong prefecture level city in Shandong
Jinfeng Hongyuan Flagship Store	Electricity conservation2	I bought it, I don't know how effective it is? I don't know how the quality is? After this summer results will know! Looking forward to it!	Jingdong prefecture level city in Shandong
Jinfeng Hongyuan Flagship Store	Electricity conservation2	Goods received Packaging intact, look at the shell workmanship is also good, high-power appliances open voltage has obvious changes in the effectiveness of the	Jingdong prefecture level city in Shandong
Jinfeng Hongyuan Flagship Store	Electricity conservation2	The goods have been received logistics is very fast, the product is similar to the line, just wait to use a month to see if it can save electricity, stay tuned.	Jingdong prefecture level city in Shandong
Jinfeng Hongyuan Flagship Store	Electricity conservation2	Electricity is plugged in, not sure if it will work, hopefully it will work.	Jingdong prefecture level city in Shandong
Jinfeng Hongyuan Flagship Store	Electricity conservation2	The shell is aluminum alloy, very good workmanship, the actual effect of waiting for the addition of evaluation	Jingdong prefecture level city in Shandong
Jinfeng Hongyuan Flagship Store	Electricity conservation2	Received, already plugged in, looking forward to the effect.	Jingdong prefecture level city in Shandong
Jinfeng Hongyuan Flagship Store	Electricity conservation2	Opened to see the regular manufacturer products, wait for a period of time to see it	Jingdong prefecture level city in Shandong
Jinfeng Hongyuan Flagship Store	Electricity conservation2	🉑 Baby received, very surprised ✨, reasonable design, fashionable and generous, continue to pay attention to the future will be repurchased ❥❥❥❥	Jingdong prefecture level city in Shandong
Jinfeng Hongyuan Flagship Store	Electricity conservation2	Baby received it 👌, really great ah! 🌈The quality can't be beat, very satisfied! 🆗Happy happy happy 😃	Jingdong prefecture level city in Shandong
Jinfeng Hongyuan Flagship Store	Electricity conservation2	Very good, power saving see, this is to help a friend to buy, I used to give my friend a say, my friend immediately let me order another one!	Jingdong prefecture level city in Shandong
Jinfeng Hongyuan Flagship Store	Electricity conservation2	The baby is just as introduced, the texture is very good, powerful, and it feels good to use it	Jingdong prefecture level city in Shandong

Jinfeng Hongyuan Flagship Store	Electricity conservation2	Value for money, very satisfied, seller service attitude is very good, very patient	Jingdong prefecture level city in Shandong
Jinfeng Hongyuan Flagship Store	Electricity conservation2	Power saving treasure design unique exquisite beautiful, voltage display clearly, plugged in that is good to use, I observed the meter, after using the meter is gone slow.	Jingdong prefecture level city in Shandong
Jinfeng Hongyuan Flagship Store	Electricity conservation2	Received on the use of the packaging is beautifully used for a period of time and then come back in the follow-up review!	Jingdong prefecture level city in Shandong
Jinfeng Hongyuan Flagship Store	Electricity conservation2	It should work. Not bad. Not bad. Not bad.	Jingdong prefecture level city in Shandong
Jinfeng Hongyuan Flagship Store	Electricity conservation2	I haven't used it yet, I'll plug it in and see if it saves power next month.	Jingdong prefecture level city in Shandong
Jinfeng Hongyuan Flagship Store	Electricity conservation2	The goods received and unpacked and used, the quality is very good, things and the official website to buy no difference!	Jingdong prefecture level city in Shandong
Jinfeng Hongyuan Flagship Store	Electricity conservation2	It's a rip off, doesn't help much at all, uses the same amount of power as before it was used.	Jingdong prefecture level city in Shandong
Jinfeng Hongyuan Flagship Store	Electricity conservation2	I've been using it for a while now and haven't noticed any unusual problems, so it's okay	Jingdong prefecture level city in Shandong
Jinfeng Hongyuan Flagship Store	Electricity conservation2	Received the goods and plugged in to use, good or not there is no use will have to wait until next month to find out	Jingdong prefecture level city in Shandong
Jinfeng Hongyuan Flagship Store	Electricity conservation2	It's very compact, it looks good, I'll introduce you to my clients when it's ready.	Jingdong prefecture level city in Shandong
Jinfeng Hongyuan Flagship Store	Electricity conservation2	Good workmanship, we'll have to see in a few months.	Jingdong prefecture level city in Shandong
Jinfeng Hongyuan Flagship Store	Electricity conservation2	Jingdong quality! Always trustworthy!	Jingdong prefecture level city in Shandong
Jinfeng Hongyuan Flagship Store	Electricity conservation2	This is the second time I've bought it, and the merchant said to give me five dollars out of the difference, so thank you merchant.	Jingdong prefecture level city in Shandong
Jinfeng Hongyuan Flagship Store	Electricity conservation2	Good quality and good service	Jingdong prefecture level city in Shandong
Jinfeng Hongyuan Flagship Store	Electricity conservation2	Feels good texture, aluminum alloy shell, voltage digital display clear.	Jingdong prefecture level city in Shandong
Jinfeng Hongyuan Flagship Store	Electricity conservation2	Good product, fast and great service, thanks!	Jingdong prefecture level city in Shandong
Jinfeng Hongyuan Flagship Store	Electricity conservation2	Have not yet used, first evaluation, later really good do not need to comment again.	Jingdong prefecture level city in Shandong
Jinfeng Hongyuan Flagship Store	Electricity conservation2	Works great. Pretty much as described by the merchant.	Jingdong prefecture level city in Shandong
Jinfeng Hongyuan Flagship Store	Electricity conservation2	Received it, looking forward to the results, hope it works	Jingdong prefecture level city in Shandong
Jinfeng Hongyuan Flagship Store	Electricity conservation2	Received it, looking forward to the results, hope it works	Jingdong prefecture level city in Shandong
Jinfeng Hongyuan Flagship Store	Electricity conservation2	Supposedly it's an IQ tax. Buy one and try it. Looks good. Well made.	Jingdong prefecture level city in Shandong
Jinfeng Hongyuan Flagship Store	Electricity conservation2	It just doesn't feel like it's working.	Jingdong prefecture level city in Shandong
Jinfeng Hongyuan Flagship Store	Electricity conservation2	This is a great product.	Jingdong prefecture level city in Shandong
Jinfeng Hongyuan Flagship Store	Electricity conservation2	I'm just using it. I'll tell you more.	Jingdong prefecture level city in Shandong
Jinfeng Hongyuan Flagship Store	Electricity conservation2	Second repurchase and it's working fine.	Jingdong prefecture level city in Shandong
Jinfeng Hongyuan Flagship Store	Electricity conservation2	I'll wait a few days before evaluating	Jingdong prefecture level city in Shandong
Jinfeng Hongyuan Flagship Store	Electricity conservation2	I'll comment after I use it hopefully the section will be useful.	Jingdong prefecture level city in Shandong
Jinfeng Hongyuan Flagship Store	Electricity conservation2	not very useful	Jingdong prefecture level city in Shandong
Jinfeng Hongyuan Flagship Store	Electricity conservation2	Not received	Jingdong prefecture level city in Shandong
Jinfeng Hongyuan Flagship Store	Electricity conservation2	rare	Jingdong prefecture level city in Shandong
Jinfeng Hongyuan Flagship Store	Electricity conservation2	It's good.	Jingdong prefecture level city in Shandong
Jinfeng Hongyuan Flagship Store	Electricity conservation2	just now	Jingdong prefecture level city in Shandong
Jinfeng Hongyuan Flagship Store	Electricity conservation2	serviceable	Jingdong prefecture level city in Shandong
Jinfeng Hongyuan Flagship Store	Electricity conservation2	desirable	Jingdong prefecture level city in Shandong
Jinfeng Hongyuan Flagship Store	Electricity conservation2	serviceable	Jingdong prefecture level city in Shandong
Jinfeng Hongyuan Flagship Store	Electricity conservation2	It doesn't save power and costs electricity.	Jingdong prefecture level city in Shandong
Jinfeng Hongyuan Flagship Store	Electricity conservation2	Not as good as I thought.	Jingdong prefecture level city in Shandong
Jinfeng Hongyuan Flagship Store	Electricity conservation2	Super bad review, it doesn't save power at all, but rather more power consumption	Jingdong prefecture level city in Shandong
Jinfeng Hongyuan Flagship Store	Electricity conservation2	Listen to others say that this can save electricity, I bought with the idea of trying, used for a period of time before coming to the evaluation, will be a little bit of use, not a lot of savings just, long-term use of the words can save more.	Jingdong prefecture level city in Shandong
Jinfeng Hongyuan Flagship Store	Electricity conservation2	Logistics is fast, the next day arrived, workmanship is very delicate a power saver, looking forward to the late effect, customer service patiently explain the recommended which is suitable for, installed in what position is the best, look forward to using the effect after a period of time!	Jingdong prefecture level city in Shandong
Jinfeng Hongyuan Flagship Store	Electricity conservation2	It's good to use, I feel that it has been saving electricity, anyway, not a loss is, I used to feel that the power consumption is big, now I feel much better, my friends are also using, I hope to use a long time will be able to, I wish the boss a prosperous business	Jingdong prefecture level city in Shandong
Jinfeng Hongyuan Flagship Store	Electricity conservation2	Should be very good, to be able to plan the circuit, save the home of the circuit to the extent of the savings, waste of money on electricity, good if a month this money back, hope always hope to be able to	Jingdong prefecture level city in Shandong
Jinfeng Hongyuan Flagship Store	Electricity conservation2	Express delivery is very strong, the goods and I saw in the business consistent, the use of the effect of follow-up evaluation	Jingdong prefecture level city in Shandong
Jinfeng Hongyuan Flagship Store	Electricity conservation2	I have been using this brand of power saver for many years and this time I saw this upgraded version and got it again. The main purpose of this product is to reduce the loss of electricity for a long time, so as to achieve the purpose of saving electricity.	Jingdong prefecture level city in Shandong
Jinfeng Hongyuan Flagship Store	Electricity conservation2	Logistics is fast and the product looks beautiful, looking forward to the effect.	Jingdong prefecture level city in Shandong
Jinfeng Hongyuan Flagship Store	Electricity conservation2	Product quality is good, energy saving a little effect, but the price is a little expensive, fast arrival, good service.	Jingdong prefecture level city in Shandong
Jinfeng Hongyuan Flagship Store	Electricity conservation2	The quality is very good, inexpensive, Jingdong self-supporting guaranteed, worth having!	Jingdong prefecture level city in Shandong
Jinfeng Hongyuan Flagship Store	Electricity conservation2	I don't know if it's saving electricity yet, I'll find out next time I pay my electric bill	Jingdong prefecture level city in Shandong
Jinfeng Hongyuan Flagship Store	Electricity conservation2	Favorable price quality assurance appearance design is beautiful and generous, fine workmanship	Jingdong prefecture level city in Shandong

Jinfeng Hongyuan Flagship Store	Electricity conservation2	I bought it, I don't know how effective it is? I don't know how the quality is? After this summer results will know! Looking forward to it!	Jingdong prefecture level city in Shandong
Jinfeng Hongyuan Flagship Store	Electricity conservation2	Goods received Packaging intact, look at the shell workmanship is also good, high-power appliances open voltage has obvious changes in the effectiveness of the	Jingdong prefecture level city in Shandong
Jinfeng Hongyuan Flagship Store	Electricity conservation2	The goods have been received logistics is very fast, the product is similar to the line, just wait to use a month to see if it can save electricity, stay tuned.	Jingdong prefecture level city in Shandong
Jinfeng Hongyuan Flagship Store	Electricity conservation2	Electricity is plugged in, not sure if it will work, hopefully it will work.	Jingdong prefecture level city in Shandong
Jinfeng Hongyuan Flagship Store	Electricity conservation2	The shell is aluminum alloy, very good workmanship, the actual effect of waiting for the addition of evaluation	Jingdong prefecture level city in Shandong
Jinfeng Hongyuan Flagship Store	Electricity conservation2	Received, already plugged in, looking forward to the effect.	Jingdong prefecture level city in Shandong
Jinfeng Hongyuan Flagship Store	Electricity conservation2	Opened to see the regular manufacturer products, wait for a period of time to see it	Jingdong prefecture level city in Shandong
Jinfeng Hongyuan Flagship Store	Electricity conservation2	🉑 Baby received, very surprised ✨, reasonable design, fashionable and generous, continue to pay attention to the future will be repurchased ❥❥❥❥	Jingdong prefecture level city in Shandong
Jinfeng Hongyuan Flagship Store	Electricity conservation2	Baby received it 👌, really great ah! 🌈The quality can't be beat, very satisfied! 🆗Happy happy happy 😃	Jingdong prefecture level city in Shandong
Jinfeng Hongyuan Flagship Store	Electricity conservation2	Very good, power saving see, this is to help a friend to buy, I used to give my friend a say, my friend immediately let me order another one!	Jingdong prefecture level city in Shandong
Jinfeng Hongyuan Flagship Store	Electricity conservation2	The baby is just as introduced, the texture is very good, powerful, and it feels good to use it	Jingdong prefecture level city in Shandong
Jinfeng Hongyuan Flagship Store	Electricity conservation2	Value for money, very satisfied, seller service attitude is very good, very patient	Jingdong prefecture level city in Shandong
Jinfeng Hongyuan Flagship Store	Electricity conservation2	Power saving treasure design unique exquisite beautiful, voltage display clearly, plugged in that is good to use, I observed the meter, after using the meter is gone slow.	Jingdong prefecture level city in Shandong
Jinfeng Hongyuan Flagship Store	Electricity conservation2	Received on the use of the packaging is beautifully used for a period of time and then come back in the follow-up review!	Jingdong prefecture level city in Shandong
Jinfeng Hongyuan Flagship Store	Electricity conservation2	It should work. Not bad. Not bad. Not bad.	Jingdong prefecture level city in Shandong
Jinfeng Hongyuan Flagship Store	Electricity conservation2	I haven't used it yet, I'll plug it in and see if it saves power next month.	Jingdong prefecture level city in Shandong
Jinfeng Hongyuan Flagship Store	Electricity conservation2	The goods received and unpacked and used, the quality is very good, things and the official website to buy no difference!	Jingdong prefecture level city in Shandong
Jinfeng Hongyuan Flagship Store	Electricity conservation2	It's a rip off, doesn't help much at all, uses the same amount of power as before it was used.	Jingdong prefecture level city in Shandong
Jinfeng Hongyuan Flagship Store	Electricity conservation2	I've been using it for a while now and haven't noticed any unusual problems, so it's okay	Jingdong prefecture level city in Shandong
Jinfeng Hongyuan Flagship Store	Electricity conservation2	Received the goods and plugged in to use, good or not there is no use will have to wait until next month to find out	Jingdong prefecture level city in Shandong
Jinfeng Hongyuan Flagship Store	Electricity conservation2	It's very compact, it looks good, I'll introduce you to my clients when it's ready.	Jingdong prefecture level city in Shandong
Jinfeng Hongyuan Flagship Store	Electricity conservation2	Good workmanship, we'll have to see in a few months.	Jingdong prefecture level city in Shandong
Jinfeng Hongyuan Flagship Store	Electricity conservation2	Jingdong quality! Always trustworthy!	Jingdong prefecture level city in Shandong
Jinfeng Hongyuan Flagship Store	Electricity conservation2	This is the second time I've bought it, and the merchant said to give me five dollars out of the difference, so thank you merchant.	Jingdong prefecture level city in Shandong
Jinfeng Hongyuan Flagship Store	Electricity conservation2	Good quality and good service	Jingdong prefecture level city in Shandong
Jinfeng Hongyuan Flagship Store	Electricity conservation2	Feels good texture, aluminum alloy shell, voltage digital display clear.	Jingdong prefecture level city in Shandong
Jinfeng Hongyuan Flagship Store	Electricity conservation2	Good product, fast and great service, thanks!	Jingdong prefecture level city in Shandong
Jinfeng Hongyuan Flagship Store	Electricity conservation2	Have not yet used, first evaluation, later really good do not need to comment again.	Jingdong prefecture level city in Shandong
Jinfeng Hongyuan Flagship Store	Electricity conservation2	Works great. Pretty much as described by the merchant.	Jingdong prefecture level city in Shandong
Jinfeng Hongyuan Flagship Store	Electricity conservation2	Received it, looking forward to the results, hope it works	Jingdong prefecture level city in Shandong
Jinfeng Hongyuan Flagship Store	Electricity conservation2	Received it, looking forward to the results, hope it works	Jingdong prefecture level city in Shandong
Jinfeng Hongyuan Flagship Store	Electricity conservation2	Supposedly it's an IQ tax. Buy one and try it. Looks good. Well made.	Jingdong prefecture level city in Shandong
Jinfeng Hongyuan Flagship Store	Electricity conservation2	It just doesn't feel like it's working.	Jingdong prefecture level city in Shandong
Jinfeng Hongyuan Flagship Store	Electricity conservation2	This is a great product.	Jingdong prefecture level city in Shandong
Jinfeng Hongyuan Flagship Store	Electricity conservation2	I'm just using it. I'll tell you more.	Jingdong prefecture level city in Shandong
Jinfeng Hongyuan Flagship Store	Electricity conservation2	Second repurchase and it's working fine.	Jingdong prefecture level city in Shandong
Jinfeng Hongyuan Flagship Store	Electricity conservation2	I'll wait a few days before evaluating	Jingdong prefecture level city in Shandong
Jinfeng Hongyuan Flagship Store	Electricity conservation2	I'll comment after I use it hopefully the section will be useful.	Jingdong prefecture level city in Shandong
Jinfeng Hongyuan Flagship Store	Electricity conservation2	not very useful	Jingdong prefecture level city in Shandong
Jinfeng Hongyuan Flagship Store	Electricity conservation2	Not received	Jingdong prefecture level city in Shandong
Jinfeng Hongyuan Flagship Store	Electricity conservation2	rare	Jingdong prefecture level city in Shandong
Jinfeng Hongyuan Flagship Store	Electricity conservation2	It's good.	Jingdong prefecture level city in Shandong
Jinfeng Hongyuan Flagship Store	Electricity conservation2	just now	Jingdong prefecture level city in Shandong
Jinfeng Hongyuan Flagship Store	Electricity conservation2	serviceable	Jingdong prefecture level city in Shandong
Jinfeng Hongyuan Flagship Store	Electricity conservation2	desirable	Jingdong prefecture level city in Shandong
Jinfeng Hongyuan Flagship Store	Electricity conservation2	serviceable	Jingdong prefecture level city in Shandong
Jinfeng Hongyuan Flagship Store	Electricity conservation2	It doesn't save power and costs electricity.	Jingdong prefecture level city in Shandong
Jinfeng Hongyuan Flagship Store	Electricity conservation2	Not as good as I thought.	Jingdong prefecture level city in Shandong
Jinfeng Hongyuan Flagship Store	Electricity conservation2	Super bad review, it doesn't save power at all, but rather more power consumption	Jingdong prefecture level city in Shandong
Jinfeng Hongyuan Flagship Store	Electricity conservation2	Listen to others say that this can save electricity, I bought with the idea of trying, used for a period of time before coming to the evaluation, will be a little bit of use, not a lot of savings just, long-term use of the words can save more.	Jingdong prefecture level city in Shandong

Jinfeng Hongyuan Flagship Store	Electricity conservation2	Logistics is fast, the next day arrived, workmanship is very delicate a power saver, looking forward to the late effect, customer service patiently explain the recommended which is suitable for, installed in what position is the best, look forward to using the effect after a period of time!	Jingdong prefecture level city in Shandong
Jinfeng Hongyuan Flagship Store	Electricity conservation2	It's good to use, I feel that it has been saving electricity, anyway, not a loss is, I used to feel that the power consumption is big, now I feel much better, my friends are also using, I hope to use a long time will be able to, I wish the boss a prosperous business	Jingdong prefecture level city in Shandong
Jinfeng Hongyuan Flagship Store	Electricity conservation2	Should be very good, to be able to plan the circuit, save the home of the circuit to the extent of the savings, waste of money on electricity, good if a month this money back, hope always hope to be able to	Jingdong prefecture level city in Shandong
Jinfeng Hongyuan Flagship Store	Electricity conservation2	Express delivery is very strong, the goods and I saw in the business consistent, the use of the effect of follow-up evaluation	Jingdong prefecture level city in Shandong
Jinfeng Hongyuan Flagship Store	Electricity conservation2	I have been using this brand of power saver for many years and this time I saw this upgraded version and got it again. The main purpose of this product is to reduce the loss of electricity for a long time, so as to achieve the purpose of saving electricity.	Jingdong prefecture level city in Shandong
Jinfeng Hongyuan Flagship Store	Electricity conservation2	Logistics is fast and the product looks beautiful, looking forward to the effect.	Jingdong prefecture level city in Shandong
Jinfeng Hongyuan Flagship Store	Electricity conservation2	Product quality is good, energy saving a little effect, but the price is a little expensive, fast arrival, good service.	Jingdong prefecture level city in Shandong
Jinfeng Hongyuan Flagship Store	Electricity conservation2	The quality is very good, inexpensive, Jingdong self-supporting guaranteed, worth having!	Jingdong prefecture level city in Shandong
Jinfeng Hongyuan Flagship Store	Electricity conservation2	I don't know if it's saving electricity yet, I'll find out next time I pay my electric bill	Jingdong prefecture level city in Shandong
Jinfeng Hongyuan Flagship Store	Electricity conservation2	Favorable price quality assurance appearance design is beautiful and generous, fine workmanship	Jingdong prefecture level city in Shandong
Jinfeng Hongyuan Flagship Store	Electricity conservation2	I bought it, I don't know how effective it is? I don't know how the quality is? After this summer results will know! Looking forward to it!	Jingdong prefecture level city in Shandong
Jinfeng Hongyuan Flagship Store	Electricity conservation2	Goods received Packaging intact, look at the shell workmanship is also good, high-power appliances open voltage has obvious changes in the effectiveness of the	Jingdong prefecture level city in Shandong
Jinfeng Hongyuan Flagship Store	Electricity conservation2	The goods have been received logistics is very fast, the product is similar to the line, just wait to use a month to see if it can save electricity, stay tuned.	Jingdong prefecture level city in Shandong
Jinfeng Hongyuan Flagship Store	Electricity conservation2	Electricity is plugged in, not sure if it will work, hopefully it will work.	Jingdong prefecture level city in Shandong
Jinfeng Hongyuan Flagship Store	Electricity conservation2	The shell is aluminum alloy, very good workmanship, the actual effect of waiting for the addition of evaluation	Jingdong prefecture level city in Shandong
Jinfeng Hongyuan Flagship Store	Electricity conservation2	Received, already plugged in, looking forward to the effect.	Jingdong prefecture level city in Shandong
Jinfeng Hongyuan Flagship Store	Electricity conservation2	Opened to see the regular manufacturer products, wait for a period of time to see it	Jingdong prefecture level city in Shandong
Jinfeng Hongyuan Flagship Store	Electricity conservation2	🉑 Baby received, very surprised ✨, reasonable design, fashionable and generous, continue to pay attention to the future will be repurchased ❤❤❤❤	Jingdong prefecture level city in Shandong
Jinfeng Hongyuan Flagship Store	Electricity conservation2	Baby received it 👌, really great ah! 🌈The quality can't be beat, very satisfied! 🆗Happy happy happy 😃	Jingdong prefecture level city in Shandong
Jinfeng Hongyuan Flagship Store	Electricity conservation2	Very good, power saving see, this is to help a friend to buy, I used to give my friend a say, my friend immediately let me order another one!	Jingdong prefecture level city in Shandong
Jinfeng Hongyuan Flagship Store	Electricity conservation2	The baby is just as introduced, the texture is very good, powerful, and it feels good to use it	Jingdong prefecture level city in Shandong
Jinfeng Hongyuan Flagship Store	Electricity conservation2	Value for money, very satisfied, seller service attitude is very good, very patient	Jingdong prefecture level city in Shandong
Jinfeng Hongyuan Flagship Store	Electricity conservation2	Power saving treasure design unique exquisite beautiful, voltage display clearly, plugged in that is good to use, I observed the meter, after using the meter is gone slow.	Jingdong prefecture level city in Shandong
Jinfeng Hongyuan Flagship Store	Electricity conservation2	Received on the use of the packaging is beautifully used for a period of time and then come back in the follow-up review!	Jingdong prefecture level city in Shandong
Jinfeng Hongyuan Flagship Store	Electricity conservation2	It should work. Not bad. Not bad. Not bad.	Jingdong prefecture level city in Shandong
Jinfeng Hongyuan Flagship Store	Electricity conservation2	I haven't used it yet, I'll plug it in and see if it saves power next month.	Jingdong prefecture level city in Shandong
Jinfeng Hongyuan Flagship Store	Electricity conservation2	The goods received and unpacked and used, the quality is very good, things and the official website to buy no difference!	Jingdong prefecture level city in Shandong
Jinfeng Hongyuan Flagship Store	Electricity conservation2	It's a rip off, doesn't help much at all, uses the same amount of power as before it was used.	Jingdong prefecture level city in Shandong
Jinfeng Hongyuan Flagship Store	Electricity conservation2	I've been using it for a while now and haven't noticed any unusual problems, so it's okay	Jingdong prefecture level city in Shandong
Jinfeng Hongyuan Flagship Store	Electricity conservation2	Received the goods and plugged in to use, good or not there is no use will have to wait until next month to find out	Jingdong prefecture level city in Shandong
Jinfeng Hongyuan Flagship Store	Electricity conservation2	It's very compact, it looks good, I'll introduce you to my clients when it's ready.	Jingdong prefecture level city in Shandong
Jinfeng Hongyuan Flagship Store	Electricity conservation2	Good workmanship, we'll have to see in a few months.	Jingdong prefecture level city in Shandong
Jinfeng Hongyuan Flagship Store	Electricity conservation2	Jingdong quality! Always trustworthy!	Jingdong prefecture level city in Shandong
Jinfeng Hongyuan Flagship Store	Electricity conservation2	This is the second time I've bought it, and the merchant said to give me five dollars out of the difference, so thank you merchant.	Jingdong prefecture level city in Shandong
Jinfeng Hongyuan Flagship Store	Electricity conservation2	Good quality and good service	Jingdong prefecture level city in Shandong
Jinfeng Hongyuan Flagship Store	Electricity conservation2	Feels good texture, aluminum alloy shell, voltage digital display clear.	Jingdong prefecture level city in Shandong
Jinfeng Hongyuan Flagship Store	Electricity conservation2	Good product, fast and great service, thanks!	Jingdong prefecture level city in Shandong
Jinfeng Hongyuan Flagship Store	Electricity conservation2	Have not yet used, first evaluation, later really good do not need to comment again.	Jingdong prefecture level city in Shandong
Jinfeng Hongyuan Flagship Store	Electricity conservation2	Works great. Pretty much as described by the merchant.	Jingdong prefecture level city in Shandong
Jinfeng Hongyuan Flagship Store	Electricity conservation2	Received it, looking forward to the results, hope it works	Jingdong prefecture level city in Shandong
Jinfeng Hongyuan Flagship Store	Electricity conservation2	Received it, looking forward to the results, hope it works	Jingdong prefecture level city in Shandong
Jinfeng Hongyuan Flagship Store	Electricity conservation2	Supposedly it's an IQ tax. Buy one and try it. Looks good. Well made.	Jingdong prefecture level city in Shandong
Jinfeng Hongyuan Flagship Store	Electricity conservation2	It just doesn't feel like it's working.	Jingdong prefecture level city in Shandong
Jinfeng Hongyuan Flagship Store	Electricity conservation2	This is a great product.	Jingdong prefecture level city in Shandong
Jinfeng Hongyuan Flagship Store	Electricity conservation2	I'm just using it. I'll tell you more.	Jingdong prefecture level city in Shandong
Jinfeng Hongyuan Flagship Store	Electricity conservation2	Second repurchase and it's working fine.	Jingdong prefecture level city in Shandong
Jinfeng Hongyuan Flagship Store	Electricity conservation2	I'll wait a few days before evaluating	Jingdong prefecture level city in Shandong
Jinfeng Hongyuan Flagship Store	Electricity conservation2	I'll comment after I use it hopefully the section will be useful.	Jingdong prefecture level city in Shandong
Jinfeng Hongyuan Flagship Store	Electricity conservation2	not very useful	Jingdong prefecture level city in Shandong
Jinfeng Hongyuan Flagship Store	Electricity conservation2	Not received	Jingdong prefecture level city in Shandong

Jinfeng Hongyuan Flagship Store	Electricity conservation2	rare	Jingdong prefecture level city in Shandong
Jinfeng Hongyuan Flagship Store	Electricity conservation2	It's good.	Jingdong prefecture level city in Shandong
Jinfeng Hongyuan Flagship Store	Electricity conservation2	just now	Jingdong prefecture level city in Shandong
Jinfeng Hongyuan Flagship Store	Electricity conservation2	serviceable	Jingdong prefecture level city in Shandong
Jinfeng Hongyuan Flagship Store	Electricity conservation2	desirable	Jingdong prefecture level city in Shandong
Jinfeng Hongyuan Flagship Store	Electricity conservation2	serviceable	Jingdong prefecture level city in Shandong
Jinfeng Hongyuan Flagship Store	Electricity conservation2	It doesn't save power and costs electricity.	Jingdong prefecture level city in Shandong
Jinfeng Hongyuan Flagship Store	Electricity conservation2	Not as good as I thought.	Jingdong prefecture level city in Shandong
Jinfeng Hongyuan Flagship Store	Electricity conservation2	Super bad review, it doesn't save power at all, but rather more power consumption	Jingdong prefecture level city in Shandong
Jinfeng Hongyuan Flagship Store	Electricity conservation2	Listen to others say that this can save electricity, I bought with the idea of trying, used for a period of time before coming to the evaluation, will be a little bit of use, not a lot of savings just, long-term use of the words can save more.	Jingdong prefecture level city in Shandong
Jinfeng Hongyuan Flagship Store	Electricity conservation2	Logistics is fast, the next day arrived, workmanship is very delicate a power saver, looking forward to the late effect, customer service patiently explain the recommended which is suitable for, installed in what position is the best, look forward to using the effect after a period of time!	Jingdong prefecture level city in Shandong
Jinfeng Hongyuan Flagship Store	Electricity conservation2	It's good to use, I feel that it has been saving electricity, anyway, not a loss is, I used to feel that the power consumption is big, now I feel much better, my friends are also using, I hope to use a long time will be able to, I wish the boss a prosperous business	Jingdong prefecture level city in Shandong
Jinfeng Hongyuan Flagship Store	Electricity conservation2	Should be very good, to be able to plan the circuit, save the home of the circuit to the extent of the savings, waste of money on electricity, good if a month this money back, hope always hope to be able to	Jingdong prefecture level city in Shandong
Jinfeng Hongyuan Flagship Store	Electricity conservation2	Express delivery is very strong, the goods and I saw in the business consistent, the use of the effect of follow-up evaluation	Jingdong prefecture level city in Shandong
Jinfeng Hongyuan Flagship Store	Electricity conservation2	I have been using this brand of power saver for many years and this time I saw this upgraded version and got it again. The main purpose of this product is to reduce the loss of electricity for a long time, so as to achieve the purpose of saving electricity.	Jingdong prefecture level city in Shandong
Jinfeng Hongyuan Flagship Store	Electricity conservation2	Logistics is fast and the product looks beautiful, looking forward to the effect.	Jingdong prefecture level city in Shandong
Jinfeng Hongyuan Flagship Store	Electricity conservation2	Product quality is good, energy saving a little effect, but the price is a little expensive, fast arrival, good service.	Jingdong prefecture level city in Shandong
Jinfeng Hongyuan Flagship Store	Electricity conservation2	The quality is very good, inexpensive, Jingdong self-supporting guaranteed, worth having!	Jingdong prefecture level city in Shandong
Jinfeng Hongyuan Flagship Store	Electricity conservation2	I don't know if it's saving electricity yet, I'll find out next time I pay my electric bill	Jingdong prefecture level city in Shandong
Jinfeng Hongyuan Flagship Store	Electricity conservation2	Favorable price quality assurance appearance design is beautiful and generous, fine workmanship	Jingdong prefecture level city in Shandong
Jinfeng Hongyuan Flagship Store	Electricity conservation2	I bought it, I don't know how effective it is? I don't know how the quality is? After this summer results will know! Looking forward to it!	Jingdong prefecture level city in Shandong
Jinfeng Hongyuan Flagship Store	Electricity conservation2	Goods received Packaging intact, look at the shell workmanship is also good, high-power appliances open voltage has obvious changes in the effectiveness of the	Jingdong prefecture level city in Shandong
Jinfeng Hongyuan Flagship Store	Electricity conservation2	The goods have been received logistics is very fast, the product is similar to the line, just wait to use a month to see if it can save electricity, stay tuned.	Jingdong prefecture level city in Shandong
Jinfeng Hongyuan Flagship Store	Electricity conservation2	Electricity is plugged in, not sure if it will work, hopefully it will work.	Jingdong prefecture level city in Shandong
Jinfeng Hongyuan Flagship Store	Electricity conservation2	The shell is aluminum alloy, very good workmanship, the actual effect of waiting for the addition of evaluation	Jingdong prefecture level city in Shandong
Jinfeng Hongyuan Flagship Store	Electricity conservation2	Received, already plugged in, looking forward to the effect.	Jingdong prefecture level city in Shandong
Jinfeng Hongyuan Flagship Store	Electricity conservation2	Opened to see the regular manufacturer products, wait for a period of time to see it	Jingdong prefecture level city in Shandong
Jinfeng Hongyuan Flagship Store	Electricity conservation2	🉑 Baby received, very surprised ✨, reasonable design, fashionable and generous, continue to pay attention to the future will be repurchased ❤❤❤❤	Jingdong prefecture level city in Shandong
Jinfeng Hongyuan Flagship Store	Electricity conservation2	Baby received it 👌, really great ah! 🌈The quality can't be beat, very satisfied! 🆗Happy happy happy 😀	Jingdong prefecture level city in Shandong
Jinfeng Hongyuan Flagship Store	Electricity conservation2	Very good, power saving see, this is to help a friend to buy, I used to give my friend a say, my friend immediately let me order another one!	Jingdong prefecture level city in Shandong
Jinfeng Hongyuan Flagship Store	Electricity conservation2	The baby is just as introduced, the texture is very good, powerful, and it feels good to use it	Jingdong prefecture level city in Shandong
Jinfeng Hongyuan Flagship Store	Electricity conservation2	Value for money, very satisfied, seller service attitude is very good, very patient	Jingdong prefecture level city in Shandong
Jinfeng Hongyuan Flagship Store	Electricity conservation2	Power saving treasure design unique exquisite beautiful, voltage display clearly, plugged in that is good to use, I observed the meter, after using the meter is gone slow.	Jingdong prefecture level city in Shandong
Jinfeng Hongyuan Flagship Store	Electricity conservation2	Received on the use of the packaging is beautifully used for a period of time and then come back in the follow-up review!	Jingdong prefecture level city in Shandong
Jinfeng Hongyuan Flagship Store	Electricity conservation2	It should work. Not bad. Not bad. Not bad.	Jingdong prefecture level city in Shandong
Jinfeng Hongyuan Flagship Store	Electricity conservation2	I haven't used it yet, I'll plug it in and see if it saves power next month.	Jingdong prefecture level city in Shandong
Jinfeng Hongyuan Flagship Store	Electricity conservation2	The goods received and unpacked and used, the quality is very good, things and the official website to buy no difference!	Jingdong prefecture level city in Shandong
Jinfeng Hongyuan Flagship Store	Electricity conservation2	It's a rip off, doesn't help much at all, uses the same amount of power as before it was used.	Jingdong prefecture level city in Shandong
Jinfeng Hongyuan Flagship Store	Electricity conservation2	I've been using it for a while now and haven't noticed any unusual problems, so it's okay	Jingdong prefecture level city in Shandong
Jinfeng Hongyuan Flagship Store	Electricity conservation2	Received the goods and plugged in to use, good or not there is no use will have to wait until next month to find out	Jingdong prefecture level city in Shandong
Jinfeng Hongyuan Flagship Store	Electricity conservation2	It's very compact, it looks good, I'll introduce you to my clients when it's ready.	Jingdong prefecture level city in Shandong
Jinfeng Hongyuan Flagship Store	Electricity conservation2	Good workmanship, we'll have to see in a few months.	Jingdong prefecture level city in Shandong
Jinfeng Hongyuan Flagship Store	Electricity conservation2	Jingdong quality! Always trustworthy!	Jingdong prefecture level city in Shandong
Jinfeng Hongyuan Flagship Store	Electricity conservation2	This is the second time I've bought it, and the merchant said to give me five dollars out of the difference, so thank you merchant.	Jingdong prefecture level city in Shandong
Jinfeng Hongyuan Flagship Store	Electricity conservation2	Good quality and good service	Jingdong prefecture level city in Shandong
Jinfeng Hongyuan Flagship Store	Electricity conservation2	Feels good texture, aluminum alloy shell, voltage digital display clear.	Jingdong prefecture level city in Shandong
Jinfeng Hongyuan Flagship Store	Electricity conservation2	Good product, fast and great service, thanks!	Jingdong prefecture level city in Shandong
Jinfeng Hongyuan Flagship Store	Electricity conservation2	Have not yet used, first evaluation, later really good do not need to comment again.	Jingdong prefecture level city in Shandong
Jinfeng Hongyuan Flagship Store	Electricity conservation2	Works great. Pretty much as described by the merchant.	Jingdong prefecture level city in Shandong

Jinfeng Hongyuan Flagship Store	Electricity conservation2	Received it, looking forward to the results, hope it works	Jingdong prefecture level city in Shandong
Jinfeng Hongyuan Flagship Store	Electricity conservation2	Received it, looking forward to the results, hope it works	Jingdong prefecture level city in Shandong
Jinfeng Hongyuan Flagship Store	Electricity conservation2	Supposedly it's an IQ tax. Buy one and try it. Looks good. Well made.	Jingdong prefecture level city in Shandong
Jinfeng Hongyuan Flagship Store	Electricity conservation2	It just doesn't feel like it's working.	Jingdong prefecture level city in Shandong
Jinfeng Hongyuan Flagship Store	Electricity conservation2	This is a great product.	Jingdong prefecture level city in Shandong
Jinfeng Hongyuan Flagship Store	Electricity conservation2	I'm just using it. I'll tell you more.	Jingdong prefecture level city in Shandong
Jinfeng Hongyuan Flagship Store	Electricity conservation2	Second repurchase and it's working fine.	Jingdong prefecture level city in Shandong
Jinfeng Hongyuan Flagship Store	Electricity conservation2	I'll wait a few days before evaluating	Jingdong prefecture level city in Shandong
Jinfeng Hongyuan Flagship Store	Electricity conservation2	I'll comment after I use it hopefully the section will be useful.	Jingdong prefecture level city in Shandong
Jinfeng Hongyuan Flagship Store	Electricity conservation2	not very useful	Jingdong prefecture level city in Shandong
Jinfeng Hongyuan Flagship Store	Electricity conservation2	Not received	Jingdong prefecture level city in Shandong
Jinfeng Hongyuan Flagship Store	Electricity conservation2	rare	Jingdong prefecture level city in Shandong
Jinfeng Hongyuan Flagship Store	Electricity conservation2	It's good.	Jingdong prefecture level city in Shandong
Jinfeng Hongyuan Flagship Store	Electricity conservation2	just now	Jingdong prefecture level city in Shandong
Jinfeng Hongyuan Flagship Store	Electricity conservation2	serviceable	Jingdong prefecture level city in Shandong
Jinfeng Hongyuan Flagship Store	Electricity conservation2	desirable	Jingdong prefecture level city in Shandong
Jinfeng Hongyuan Flagship Store	Electricity conservation2	serviceable	Jingdong prefecture level city in Shandong
Jinfeng Hongyuan Flagship Store	Electricity conservation2	It doesn't save power and costs electricity.	Jingdong prefecture level city in Shandong
Jinfeng Hongyuan Flagship Store	Electricity conservation2	Not as good as I thought.	Jingdong prefecture level city in Shandong
Jinfeng Hongyuan Flagship Store	Electricity conservation2	Super bad review, it doesn't save power at all, but rather more power consumption	Jingdong prefecture level city in Shandong
Jinfeng Hongyuan Flagship Store	Electricity conservation2	Listen to others say that this can save electricity, I bought with the idea of trying, used for a period of time before coming to the evaluation, will be a little bit of use, not a lot of savings just, long-term use of the words can save more.	Jingdong prefecture level city in Shandong
Jinfeng Hongyuan Flagship Store	Electricity conservation2	Logistics is fast, the next day arrived, workmanship is very delicate a power saver, looking forward to the late effect, customer service patiently explain the recommended which is suitable for, installed in what position is the best, look forward to using the effect after a period of time!	Jingdong prefecture level city in Shandong
Jinfeng Hongyuan Flagship Store	Electricity conservation2	It's good to use, I feel that it has been saving electricity, anyway, not a loss is, I used to feel that the power consumption is big, now I feel much better, my friends are also using, I hope to use a long time will be able to, I wish the boss a prosperous business	Jingdong prefecture level city in Shandong
Jinfeng Hongyuan Flagship Store	Electricity conservation2	Should be very good, to be able to plan the circuit, save the home of the circuit to the extent of the savings, waste of money on electricity, good if a month this money back, hope always hope to be able to	Jingdong prefecture level city in Shandong
Jinfeng Hongyuan Flagship Store	Electricity conservation2	Express delivery is very strong, the goods and I saw in the business consistent, the use of the effect of follow-up evaluation	Jingdong prefecture level city in Shandong
Jinfeng Hongyuan Flagship Store	Electricity conservation2	I have been using this brand of power saver for many years and this time I saw this upgraded version and got it again. The main purpose of this product is to reduce the loss of electricity for a long time, so as to achieve the purpose of saving electricity.	Jingdong prefecture level city in Shandong
Jinfeng Hongyuan Flagship Store	Electricity conservation2	Logistics is fast and the product looks beautiful, looking forward to the effect.	Jingdong prefecture level city in Shandong
Jinfeng Hongyuan Flagship Store	Electricity conservation2	Product quality is good, energy saving a little effect, but the price is a little expensive, fast arrival, good service.	Jingdong prefecture level city in Shandong
Jinfeng Hongyuan Flagship Store	Electricity conservation2	The quality is very good, inexpensive, Jingdong self-supporting guaranteed, worth having!	Jingdong prefecture level city in Shandong
Jinfeng Hongyuan Flagship Store	Electricity conservation2	I don't know if it's saving electricity yet, I'll find out next time I pay my electric bill	Jingdong prefecture level city in Shandong
Jinfeng Hongyuan Flagship Store	Electricity conservation2	Favorable price quality assurance appearance design is beautiful and generous, fine workmanship	Jingdong prefecture level city in Shandong
Jinfeng Hongyuan Flagship Store	Electricity conservation2	I bought it, I don't know how effective it is? I don't know how the quality is? After this summer results will know! Looking forward to it!	Jingdong prefecture level city in Shandong
Jinfeng Hongyuan Flagship Store	Electricity conservation2	Goods received Packaging intact, look at the shell workmanship is also good, high-power appliances open voltage has obvious changes in the effectiveness of the	Jingdong prefecture level city in Shandong
Jinfeng Hongyuan Flagship Store	Electricity conservation2	The goods have been received logistics is very fast, the product is similar to the line, just wait to use a month to see if it can save electricity, stay tuned.	Jingdong prefecture level city in Shandong
Jinfeng Hongyuan Flagship Store	Electricity conservation2	Electricity is plugged in, not sure if it will work, hopefully it will work.	Jingdong prefecture level city in Shandong
Jinfeng Hongyuan Flagship Store	Electricity conservation2	The shell is aluminum alloy, very good workmanship, the actual effect of waiting for the addition of evaluation	Jingdong prefecture level city in Shandong
Jinfeng Hongyuan Flagship Store	Electricity conservation2	Received, already plugged in, looking forward to the effect.	Jingdong prefecture level city in Shandong
Jinfeng Hongyuan Flagship Store	Electricity conservation2	Opened to see the regular manufacturer products, wait for a period of time to see it	Jingdong prefecture level city in Shandong
Jinfeng Hongyuan Flagship Store	Electricity conservation2	🉑 Baby received, very surprised ✨, reasonable design, fashionable and generous, continue to pay attention to the future will be repurchased ❥❥❥❥	Jingdong prefecture level city in Shandong
Jinfeng Hongyuan Flagship Store	Electricity conservation2	Baby received it 👌, really great ah! 🌈The quality can't be beat, very satisfied! 🆗Happy happy happy 😃	Jingdong prefecture level city in Shandong
Jinfeng Hongyuan Flagship Store	Electricity conservation2	Very good, power saving see, this is to help a friend to buy, I used to give my friend a say, my friend immediately let me order another one!	Jingdong prefecture level city in Shandong
Jinfeng Hongyuan Flagship Store	Electricity conservation2	The baby is just as introduced, the texture is very good, powerful, and it feels good to use it	Jingdong prefecture level city in Shandong
Jinfeng Hongyuan Flagship Store	Electricity conservation2	Value for money, very satisfied, seller service attitude is very good, very patient	Jingdong prefecture level city in Shandong
Jinfeng Hongyuan Flagship Store	Electricity conservation2	Power saving treasure design unique exquisite beautiful, voltage display clearly, plugged in that is good to use, I observed the meter, after using the meter is gone slow.	Jingdong prefecture level city in Shandong
Jinfeng Hongyuan Flagship Store	Electricity conservation2	Received on the use of the packaging is beautifully used for a period of time and then come back in the follow-up review!	Jingdong prefecture level city in Shandong
Jinfeng Hongyuan Flagship Store	Electricity conservation2	It should work. Not bad. Not bad. Not bad.	Jingdong prefecture level city in Shandong
Jinfeng Hongyuan Flagship Store	Electricity conservation2	I haven't used it yet, I'll plug it in and see if it saves power next month.	Jingdong prefecture level city in Shandong
Jinfeng Hongyuan Flagship Store	Electricity conservation2	The goods received and unpacked and used, the quality is very good, things and the official website to buy no difference!	Jingdong prefecture level city in Shandong
Jinfeng Hongyuan Flagship Store	Electricity conservation2	It's a rip off, doesn't help much at all, uses the same amount of power as before it was used.	Jingdong prefecture level city in Shandong

Jinfeng Hongyuan Flagship Store	Electricity conservation2	I've been using it for a while now and haven't noticed any unusual problems, so it's okay	Jingdong prefecture level city in Shandong
Jinfeng Hongyuan Flagship Store	Electricity conservation2	Received the goods and plugged in to use, good or not there is no use will have to wait until next month to find out	Jingdong prefecture level city in Shandong
Jinfeng Hongyuan Flagship Store	Electricity conservation2	It's very compact, it looks good, I'll introduce you to my clients when it's ready.	Jingdong prefecture level city in Shandong
Jinfeng Hongyuan Flagship Store	Electricity conservation2	Good workmanship, we'll have to see in a few months.	Jingdong prefecture level city in Shandong
Jinfeng Hongyuan Flagship Store	Electricity conservation2	Jingdong quality! Always trustworthy!	Jingdong prefecture level city in Shandong
Jinfeng Hongyuan Flagship Store	Electricity conservation2	This is the second time I've bought it, and the merchant said to give me five dollars out of the difference, so thank you merchant.	Jingdong prefecture level city in Shandong
Jinfeng Hongyuan Flagship Store	Electricity conservation2	Good quality and good service	Jingdong prefecture level city in Shandong
Jinfeng Hongyuan Flagship Store	Electricity conservation2	Feels good texture, aluminum alloy shell, voltage digital display clear.	Jingdong prefecture level city in Shandong
Jinfeng Hongyuan Flagship Store	Electricity conservation2	Good product, fast and great service, thanks!	Jingdong prefecture level city in Shandong
Jinfeng Hongyuan Flagship Store	Electricity conservation2	Have not yet used, first evaluation, later really good do not need to comment again.	Jingdong prefecture level city in Shandong
Jinfeng Hongyuan Flagship Store	Electricity conservation2	Works great. Pretty much as described by the merchant.	Jingdong prefecture level city in Shandong
Jinfeng Hongyuan Flagship Store	Electricity conservation2	Received it, looking forward to the results, hope it works	Jingdong prefecture level city in Shandong
Jinfeng Hongyuan Flagship Store	Electricity conservation2	Received it, looking forward to the results, hope it works	Jingdong prefecture level city in Shandong
Jinfeng Hongyuan Flagship Store	Electricity conservation2	Supposedly it's an IQ tax. Buy one and try it. Looks good. Well made.	Jingdong prefecture level city in Shandong
Jinfeng Hongyuan Flagship Store	Electricity conservation2	It just doesn't feel like it's working.	Jingdong prefecture level city in Shandong
Jinfeng Hongyuan Flagship Store	Electricity conservation2	This is a great product.	Jingdong prefecture level city in Shandong
Jinfeng Hongyuan Flagship Store	Electricity conservation2	I'm just using it. I'll tell you more.	Jingdong prefecture level city in Shandong
Jinfeng Hongyuan Flagship Store	Electricity conservation2	Second repurchase and it's working fine.	Jingdong prefecture level city in Shandong
Jinfeng Hongyuan Flagship Store	Electricity conservation2	I'll wait a few days before evaluating	Jingdong prefecture level city in Shandong
Jinfeng Hongyuan Flagship Store	Electricity conservation2	I'll comment after I use it hopefully the section will be useful.	Jingdong prefecture level city in Shandong
Jinfeng Hongyuan Flagship Store	Electricity conservation2	not very useful	Jingdong prefecture level city in Shandong
Jinfeng Hongyuan Flagship Store	Electricity conservation2	Not received	Jingdong prefecture level city in Shandong
Jinfeng Hongyuan Flagship Store	Electricity conservation2	rare	Jingdong prefecture level city in Shandong
Jinfeng Hongyuan Flagship Store	Electricity conservation2	It's good.	Jingdong prefecture level city in Shandong
Jinfeng Hongyuan Flagship Store	Electricity conservation2	just now	Jingdong prefecture level city in Shandong
Jinfeng Hongyuan Flagship Store	Electricity conservation2	serviceable	Jingdong prefecture level city in Shandong
Jinfeng Hongyuan Flagship Store	Electricity conservation2	desirable	Jingdong prefecture level city in Shandong
Jinfeng Hongyuan Flagship Store	Electricity conservation2	serviceable	Jingdong prefecture level city in Shandong
Jinfeng Hongyuan Flagship Store	Electricity conservation2	It doesn't save power and costs electricity.	Jingdong prefecture level city in Shandong
Jinfeng Hongyuan Flagship Store	Electricity conservation2	Not as good as I thought.	Jingdong prefecture level city in Shandong
Jinfeng Hongyuan Flagship Store	Electricity conservation2	Super bad review, it doesn't save power at all, but rather more power consumption	Jingdong prefecture level city in Shandong
Jinfeng Hongyuan Flagship Store	Electricity conservation2	Listen to others say that this can save electricity, I bought with the idea of trying, used for a period of time before coming to the evaluation, will be a little bit of use, not a lot of savings just, long-term use of the words can save more.	Jingdong prefecture level city in Shandong
Jinfeng Hongyuan Flagship Store	Electricity conservation2	Logistics is fast, the next day arrived, workmanship is very delicate a power saver, looking forward to the late effect, customer service patiently explain the recommended which is suitable for, installed in what position is the best, look forward to using the effect after a period of time!	Jingdong prefecture level city in Shandong
Jinfeng Hongyuan Flagship Store	Electricity conservation2	It's good to use, I feel that it has been saving electricity, anyway, not a loss is, I used to feel that the power consumption is big, now I feel much better, my friends are also using, I hope to use a long time will be able to, I wish the boss a prosperous business	Jingdong prefecture level city in Shandong
Jinfeng Hongyuan Flagship Store	Electricity conservation2	Should be very good, to be able to plan the circuit, save the home of the circuit to the extent of the savings, waste of money on electricity, good if a month this money back, hope always hope to be able to	Jingdong prefecture level city in Shandong
Jinfeng Hongyuan Flagship Store	Electricity conservation2	Express delivery is very strong, the goods and I saw in the business consistent, the use of the effect of follow-up evaluation	Jingdong prefecture level city in Shandong
Jinfeng Hongyuan Flagship Store	Electricity conservation2	I have been using this brand of power saver for many years and this time I saw this upgraded version and got it again. The main purpose of this product is to reduce the loss of electricity for a long time, so as to achieve the purpose of saving electricity.	Jingdong prefecture level city in Shandong
Jinfeng Hongyuan Flagship Store	Electricity conservation2	Logistics is fast and the product looks beautiful, looking forward to the effect.	Jingdong prefecture level city in Shandong
Jinfeng Hongyuan Flagship Store	Electricity conservation2	Product quality is good, energy saving a little effect, but the price is a little expensive, fast arrival, good service.	Jingdong prefecture level city in Shandong
Jinfeng Hongyuan Flagship Store	Electricity conservation2	The quality is very good, inexpensive, Jingdong self-supporting guaranteed, worth having!	Jingdong prefecture level city in Shandong
Jinfeng Hongyuan Flagship Store	Electricity conservation2	I don't know if it's saving electricity yet, I'll find out next time I pay my electric bill	Jingdong prefecture level city in Shandong
Jinfeng Hongyuan Flagship Store	Electricity conservation2	Favorable price quality assurance appearance design is beautiful and generous, fine workmanship	Jingdong prefecture level city in Shandong
Jinfeng Hongyuan Flagship Store	Electricity conservation2	I bought it, I don't know how effective it is? I don't know how the quality is? After this summer results will know! Looking forward to it!	Jingdong prefecture level city in Shandong
Jinfeng Hongyuan Flagship Store	Electricity conservation2	Goods received Packaging intact, look at the shell workmanship is also good, high-power appliances open voltage has obvious changes in the effectiveness of the	Jingdong prefecture level city in Shandong
Jinfeng Hongyuan Flagship Store	Electricity conservation2	The goods have been received logistics is very fast, the product is similar to the line, just wait to use a month to see if it can save electricity, stay tuned.	Jingdong prefecture level city in Shandong
Jinfeng Hongyuan Flagship Store	Electricity conservation2	Electricity is plugged in, not sure if it will work, hopefully it will work.	Jingdong prefecture level city in Shandong
Jinfeng Hongyuan Flagship Store	Electricity conservation2	The shell is aluminum alloy, very good workmanship, the actual effect of waiting for the addition of evaluation	Jingdong prefecture level city in Shandong
Jinfeng Hongyuan Flagship Store	Electricity conservation2	Received, already plugged in, looking forward to the effect.	Jingdong prefecture level city in Shandong
Jinfeng Hongyuan Flagship Store	Electricity conservation2	Opened to see the regular manufacturer products, wait for a period of time to see it	Jingdong prefecture level city in Shandong

Jinfeng Hongyuan Flagship Store	Electricity conservation2	🉑 Baby received, very surprised ✨, reasonable design, fashionable and generous, continue to pay attention to the future will be repurchased ❤❤❤❤	Jingdong prefecture level city in Shandong
Jinfeng Hongyuan Flagship Store	Electricity conservation2	Baby received it 👌, really great ah! 🌈The quality can't be beat, very satisfied! 🆗Happy happy happy 😃	Jingdong prefecture level city in Shandong
Jinfeng Hongyuan Flagship Store	Electricity conservation2	Very good, power saving see, this is to help a friend to buy, I used to give my friend a say, my friend immediately let me order another one!	Jingdong prefecture level city in Shandong
Jinfeng Hongyuan Flagship Store	Electricity conservation2	The baby is just as introduced, the texture is very good, powerful, and it feels good to use it	Jingdong prefecture level city in Shandong
Jinfeng Hongyuan Flagship Store	Electricity conservation2	Value for money, very satisfied, seller service attitude is very good, very patient	Jingdong prefecture level city in Shandong
Jinfeng Hongyuan Flagship Store	Electricity conservation2	Power saving treasure design unique exquisite beautiful, voltage display clearly, plugged in that is good to use, I observed the meter, after using the meter is gone slow.	Jingdong prefecture level city in Shandong
Jinfeng Hongyuan Flagship Store	Electricity conservation2	Received on the use of the packaging is beautifully used for a period of time and then come back in the follow-up review!	Jingdong prefecture level city in Shandong
Jinfeng Hongyuan Flagship Store	Electricity conservation2	It should work. Not bad. Not bad. Not bad.	Jingdong prefecture level city in Shandong
Jinfeng Hongyuan Flagship Store	Electricity conservation2	I haven't used it yet, I'll plug it in and see if it saves power next month.	Jingdong prefecture level city in Shandong
Jinfeng Hongyuan Flagship Store	Electricity conservation2	The goods received and unpacked and used, the quality is very good, things and the official website to buy no difference!	Jingdong prefecture level city in Shandong
Jinfeng Hongyuan Flagship Store	Electricity conservation2	It's a rip off, doesn't help much at all, uses the same amount of power as before it was used.	Jingdong prefecture level city in Shandong
Jinfeng Hongyuan Flagship Store	Electricity conservation2	I've been using it for a while now and haven't noticed any unusual problems, so it's okay	Jingdong prefecture level city in Shandong
Jinfeng Hongyuan Flagship Store	Electricity conservation2	Received the goods and plugged in to use, good or not there is no use will have to wait until next month to find out	Jingdong prefecture level city in Shandong
Jinfeng Hongyuan Flagship Store	Electricity conservation2	It's very compact, it looks good, I'll introduce you to my clients when it's ready.	Jingdong prefecture level city in Shandong
Jinfeng Hongyuan Flagship Store	Electricity conservation2	Good workmanship, we'll have to see in a few months.	Jingdong prefecture level city in Shandong
Jinfeng Hongyuan Flagship Store	Electricity conservation2	Jingdong quality! Always trustworthy!	Jingdong prefecture level city in Shandong
Jinfeng Hongyuan Flagship Store	Electricity conservation2	This is the second time I've bought it, and the merchant said to give me five dollars out of the difference, so thank you merchant.	Jingdong prefecture level city in Shandong
Jinfeng Hongyuan Flagship Store	Electricity conservation2	Good quality and good service	Jingdong prefecture level city in Shandong
Jinfeng Hongyuan Flagship Store	Electricity conservation2	Feels good texture, aluminum alloy shell, voltage digital display clear.	Jingdong prefecture level city in Shandong
Jinfeng Hongyuan Flagship Store	Electricity conservation2	Good product, fast and great service, thanks!	Jingdong prefecture level city in Shandong
Jinfeng Hongyuan Flagship Store	Electricity conservation2	Have not yet used, first evaluation, later really good do not need to comment again.	Jingdong prefecture level city in Shandong
Jinfeng Hongyuan Flagship Store	Electricity conservation2	Works great. Pretty much as described by the merchant.	Jingdong prefecture level city in Shandong
Jinfeng Hongyuan Flagship Store	Electricity conservation2	Received it, looking forward to the results, hope it works	Jingdong prefecture level city in Shandong
Jinfeng Hongyuan Flagship Store	Electricity conservation2	Received it, looking forward to the results, hope it works	Jingdong prefecture level city in Shandong
Jinfeng Hongyuan Flagship Store	Electricity conservation2	Supposedly it's an IQ tax. Buy one and try it. Looks good. Well made.	Jingdong prefecture level city in Shandong
Jinfeng Hongyuan Flagship Store	Electricity conservation2	It just doesn't feel like it's working.	Jingdong prefecture level city in Shandong
Jinfeng Hongyuan Flagship Store	Electricity conservation2	This is a great product.	Jingdong prefecture level city in Shandong
Jinfeng Hongyuan Flagship Store	Electricity conservation2	I'm just using it. I'll tell you more.	Jingdong prefecture level city in Shandong
Jinfeng Hongyuan Flagship Store	Electricity conservation2	Second repurchase and it's working fine.	Jingdong prefecture level city in Shandong
Jinfeng Hongyuan Flagship Store	Electricity conservation2	I'll wait a few days before evaluating	Jingdong prefecture level city in Shandong
Jinfeng Hongyuan Flagship Store	Electricity conservation2	I'll comment after I use it hopefully the section will be useful.	Jingdong prefecture level city in Shandong
Jinfeng Hongyuan Flagship Store	Electricity conservation2	not very useful	Jingdong prefecture level city in Shandong
Jinfeng Hongyuan Flagship Store	Electricity conservation2	Not received	Jingdong prefecture level city in Shandong
Jinfeng Hongyuan Flagship Store	Electricity conservation2	rare	Jingdong prefecture level city in Shandong
Jinfeng Hongyuan Flagship Store	Electricity conservation2	It's good.	Jingdong prefecture level city in Shandong
Jinfeng Hongyuan Flagship Store	Electricity conservation2	just now	Jingdong prefecture level city in Shandong
Jinfeng Hongyuan Flagship Store	Electricity conservation2	serviceable	Jingdong prefecture level city in Shandong
Jinfeng Hongyuan Flagship Store	Electricity conservation2	desirable	Jingdong prefecture level city in Shandong
Jinfeng Hongyuan Flagship Store	Electricity conservation2	serviceable	Jingdong prefecture level city in Shandong
Jinfeng Hongyuan Flagship Store	Electricity conservation2	It doesn't save power and costs electricity.	Jingdong prefecture level city in Shandong
Jinfeng Hongyuan Flagship Store	Electricity conservation2	Not as good as I thought.	Jingdong prefecture level city in Shandong
Jinfeng Hongyuan Flagship Store	Electricity conservation2	Super bad review, it doesn't save power at all, but rather more power consumption	Jingdong prefecture level city in Shandong
Jinfeng Hongyuan Flagship Store	Electricity conservation2	Listen to others say that this can save electricity, I bought with the idea of trying, used for a period of time before coming to the evaluation, will be a little bit of use, not a lot of savings just, long-term use of the words can save more.	Jingdong prefecture level city in Shandong
Jinfeng Hongyuan Flagship Store	Electricity conservation2	Logistics is fast, the next day arrived, workmanship is very delicate a power saver, looking forward to the late effect, customer service patiently explain the recommended which is suitable for, installed in what position is the best, look forward to using the effect after a period of time!	Jingdong prefecture level city in Shandong
Jinfeng Hongyuan Flagship Store	Electricity conservation2	It's good to use, I feel that it has been saving electricity, anyway, not a loss is, I used to feel that the power consumption is big, now I feel much better, my friends are also using, I hope to use a long time will be able to, I wish the boss a prosperous business	Jingdong prefecture level city in Shandong
Jinfeng Hongyuan Flagship Store	Electricity conservation2	Should be very good, to be able to plan the circuit, save the home of the circuit to the extent of the savings, waste of money on electricity, good if a month this money back, hope always hope to be able to	Jingdong prefecture level city in Shandong
Jinfeng Hongyuan Flagship Store	Electricity conservation2	Express delivery is very strong, the goods and I saw in the business consistent, the use of the effect of follow-up evaluation	Jingdong prefecture level city in Shandong
Jinfeng Hongyuan Flagship Store	Electricity conservation2	I have been using this brand of power saver for many years and this time I saw this upgraded version and got it again. The main purpose of this product is to reduce the loss of electricity for a long time, so as to achieve the purpose of saving electricity.	Jingdong prefecture level city in Shandong
Jinfeng Hongyuan Flagship Store	Electricity conservation2	Logistics is fast and the product looks beautiful, looking forward to the effect.	Jingdong prefecture level city in Shandong

Jinfeng Hongyuan Flagship Store	Electricity conservation2	Product quality is good, energy saving a little effect, but the price is a little expensive, fast arrival, good service.	Jingdong prefecture level city in Shandong
Jinfeng Hongyuan Flagship Store	Electricity conservation2	The quality is very good, inexpensive, Jingdong self-supporting guaranteed, worth having!	Jingdong prefecture level city in Shandong
Jinfeng Hongyuan Flagship Store	Electricity conservation2	I don't know if it's saving electricity yet, I'll find out next time I pay my electric bill	Jingdong prefecture level city in Shandong
Jinfeng Hongyuan Flagship Store	Electricity conservation2	Favorable price quality assurance appearance design is beautiful and generous, fine workmanship	Jingdong prefecture level city in Shandong
Jinfeng Hongyuan Flagship Store	Electricity conservation2	I bought it, I don't know how effective it is? I don't know how the quality is? After this summer results will know! Looking forward to it!	Jingdong prefecture level city in Shandong
Jinfeng Hongyuan Flagship Store	Electricity conservation2	Goods received Packaging intact, look at the shell workmanship is also good, high-power appliances open voltage has obvious changes in the effectiveness of the	Jingdong prefecture level city in Shandong
Jinfeng Hongyuan Flagship Store	Electricity conservation2	The goods have been received logistics is very fast, the product is similar to the line, just wait to use a month to see if it can save electricity, stay tuned.	Jingdong prefecture level city in Shandong
Jinfeng Hongyuan Flagship Store	Electricity conservation2	Electricity is plugged in, not sure if it will work, hopefully it will work.	Jingdong prefecture level city in Shandong
Jinfeng Hongyuan Flagship Store	Electricity conservation2	The shell is aluminum alloy, very good workmanship, the actual effect of waiting for the addition of evaluation	Jingdong prefecture level city in Shandong
Jinfeng Hongyuan Flagship Store	Electricity conservation2	Received, already plugged in, looking forward to the effect.	Jingdong prefecture level city in Shandong
Jinfeng Hongyuan Flagship Store	Electricity conservation2	Opened to see the regular manufacturer products, wait for a period of time to see it	Jingdong prefecture level city in Shandong
Jinfeng Hongyuan Flagship Store	Electricity conservation2	🉑 Baby received, very surprised ✨, reasonable design, fashionable and generous, continue to pay attention to the future will be repurchased ❥❥❥❥	Jingdong prefecture level city in Shandong
Jinfeng Hongyuan Flagship Store	Electricity conservation2	Baby received it 👌, really great ah! 🌈The quality can't be beat, very satisfied! 🆗Happy happy happy 😃	Jingdong prefecture level city in Shandong
Jinfeng Hongyuan Flagship Store	Electricity conservation2	Very good, power saving see, this is to help a friend to buy, I used to give my friend a say, my friend immediately let me order another one!	Jingdong prefecture level city in Shandong
Jinfeng Hongyuan Flagship Store	Electricity conservation2	The baby is just as introduced, the texture is very good, powerful, and it feels good to use it	Jingdong prefecture level city in Shandong
Jinfeng Hongyuan Flagship Store	Electricity conservation2	Value for money, very satisfied, seller service attitude is very good, very patient	Jingdong prefecture level city in Shandong
Jinfeng Hongyuan Flagship Store	Electricity conservation2	Power saving treasure design unique exquisite beautiful, voltage display clearly, plugged in that is good to use, I observed the meter, after using the meter is gone slow.	Jingdong prefecture level city in Shandong
Jinfeng Hongyuan Flagship Store	Electricity conservation2	Received on the use of the packaging is beautifully used for a period of time and then come back in the follow-up review!	Jingdong prefecture level city in Shandong
Jinfeng Hongyuan Flagship Store	Electricity conservation2	It should work. Not bad. Not bad. Not bad.	Jingdong prefecture level city in Shandong
Jinfeng Hongyuan Flagship Store	Electricity conservation2	I haven't used it yet, I'll plug it in and see if it saves power next month.	Jingdong prefecture level city in Shandong
Jinfeng Hongyuan Flagship Store	Electricity conservation2	The goods received and unpacked and used, the quality is very good, things and the official website to buy no difference!	Jingdong prefecture level city in Shandong
Jinfeng Hongyuan Flagship Store	Electricity conservation2	It's a rip off, doesn't help much at all, uses the same amount of power as before it was used.	Jingdong prefecture level city in Shandong
Jinfeng Hongyuan Flagship Store	Electricity conservation2	I've been using it for a while now and haven't noticed any unusual problems, so it's okay	Jingdong prefecture level city in Shandong
Jinfeng Hongyuan Flagship Store	Electricity conservation2	Received the goods and plugged in to use, good or not there is no use will have to wait until next month to find out	Jingdong prefecture level city in Shandong
Jinfeng Hongyuan Flagship Store	Electricity conservation2	It's very compact, it looks good, I'll introduce you to my clients when it's ready.	Jingdong prefecture level city in Shandong
Jinfeng Hongyuan Flagship Store	Electricity conservation2	Good workmanship, we'll have to see in a few months.	Jingdong prefecture level city in Shandong
Jinfeng Hongyuan Flagship Store	Electricity conservation2	Jingdong quality! Always trustworthy!	Jingdong prefecture level city in Shandong
Jinfeng Hongyuan Flagship Store	Electricity conservation2	This is the second time I've bought it, and the merchant said to give me five dollars out of the difference, so thank you merchant.	Jingdong prefecture level city in Shandong
Jinfeng Hongyuan Flagship Store	Electricity conservation2	Good quality and good service	Jingdong prefecture level city in Shandong
Jinfeng Hongyuan Flagship Store	Electricity conservation2	Feels good texture, aluminum alloy shell, voltage digital display clear.	Jingdong prefecture level city in Shandong
Jinfeng Hongyuan Flagship Store	Electricity conservation2	Good product, fast and great service, thanks!	Jingdong prefecture level city in Shandong
Jinfeng Hongyuan Flagship Store	Electricity conservation2	Have not yet used, first evaluation, later really good do not need to comment again.	Jingdong prefecture level city in Shandong
Jinfeng Hongyuan Flagship Store	Electricity conservation2	Works great. Pretty much as described by the merchant.	Jingdong prefecture level city in Shandong
Jinfeng Hongyuan Flagship Store	Electricity conservation2	Received it, looking forward to the results, hope it works	Jingdong prefecture level city in Shandong
Jinfeng Hongyuan Flagship Store	Electricity conservation2	Received it, looking forward to the results, hope it works	Jingdong prefecture level city in Shandong
Jinfeng Hongyuan Flagship Store	Electricity conservation2	Supposedly it's an IQ tax. Buy one and try it. Looks good. Well made.	Jingdong prefecture level city in Shandong
Jinfeng Hongyuan Flagship Store	Electricity conservation2	It just doesn't feel like it's working.	Jingdong prefecture level city in Shandong
Jinfeng Hongyuan Flagship Store	Electricity conservation2	This is a great product.	Jingdong prefecture level city in Shandong
Jinfeng Hongyuan Flagship Store	Electricity conservation2	I'm just using it. I'll tell you more.	Jingdong prefecture level city in Shandong
Jinfeng Hongyuan Flagship Store	Electricity conservation2	Second repurchase and it's working fine.	Jingdong prefecture level city in Shandong
Jinfeng Hongyuan Flagship Store	Electricity conservation2	I'll wait a few days before evaluating	Jingdong prefecture level city in Shandong
Jinfeng Hongyuan Flagship Store	Electricity conservation2	I'll comment after I use it hopefully the section will be useful.	Jingdong prefecture level city in Shandong
Jinfeng Hongyuan Flagship Store	Electricity conservation2	not very useful	Jingdong prefecture level city in Shandong
Jinfeng Hongyuan Flagship Store	Electricity conservation2	Not received	Jingdong prefecture level city in Shandong
Jinfeng Hongyuan Flagship Store	Electricity conservation2	rare	Jingdong prefecture level city in Shandong
Jinfeng Hongyuan Flagship Store	Electricity conservation2	It's good.	Jingdong prefecture level city in Shandong
Jinfeng Hongyuan Flagship Store	Electricity conservation2	just now	Jingdong prefecture level city in Shandong
Jinfeng Hongyuan Flagship Store	Electricity conservation2	serviceable	Jingdong prefecture level city in Shandong
Jinfeng Hongyuan Flagship Store	Electricity conservation2	desirable	Jingdong prefecture level city in Shandong
Jinfeng Hongyuan Flagship Store	Electricity conservation2	serviceable	Jingdong prefecture level city in Shandong
Jinfeng Hongyuan Flagship Store	Electricity conservation2	It doesn't save power and costs electricity.	Jingdong prefecture level city in Shandong

Jinfeng Hongyuan Flagship Store	Electricity conservation2	Not as good as I thought.	Jingdong prefecture level city in Shandong
Jinfeng Hongyuan Flagship Store	Electricity conservation2	Super bad review, it doesn't save power at all, but rather more power consumption	Jingdong prefecture level city in Shandong
Jinfeng Hongyuan Flagship Store	Electricity conservation2	Listen to others say that this can save electricity, I bought with the idea of trying, used for a period of time before coming to the evaluation, will be a little bit of use, not a lot of savings just, long-term use of the words can save more.	Jingdong prefecture level city in Shandong
Jinfeng Hongyuan Flagship Store	Electricity conservation2	Logistics is fast, the next day arrived, workmanship is very delicate a power saver, looking forward to the late effect, customer service patiently explain the recommended which is suitable for, installed in what position is the best, look forward to using the effect after a period of time!	Jingdong prefecture level city in Shandong
Jinfeng Hongyuan Flagship Store	Electricity conservation2	It's good to use, I feel that it has been saving electricity, anyway, not a loss is, I used to feel that the power consumption is big, now I feel much better, my friends are also using, I hope to use a long time will be able to, I wish the boss a prosperous business	Jingdong prefecture level city in Shandong
Jinfeng Hongyuan Flagship Store	Electricity conservation2	Should be very good, to be able to plan the circuit, save the home of the circuit to the extent of the savings, waste of money on electricity, good if a month this money back, hope always hope to be able to	Jingdong prefecture level city in Shandong
Jinfeng Hongyuan Flagship Store	Electricity conservation2	Express delivery is very strong, the goods and I saw in the business consistent, the use of the effect of follow-up evaluation	Jingdong prefecture level city in Shandong
Jinfeng Hongyuan Flagship Store	Electricity conservation2	I have been using this brand of power saver for many years and this time I saw this upgraded version and got it again. The main purpose of this product is to reduce the loss of electricity for a long time, so as to achieve the purpose of saving electricity.	Jingdong prefecture level city in Shandong
Jinfeng Hongyuan Flagship Store	Electricity conservation2	Logistics is fast and the product looks beautiful, looking forward to the effect.	Jingdong prefecture level city in Shandong
Jinfeng Hongyuan Flagship Store	Electricity conservation2	Product quality is good, energy saving a little effect, but the price is a little expensive, fast arrival, good service.	Jingdong prefecture level city in Shandong
Jinfeng Hongyuan Flagship Store	Electricity conservation2	The quality is very good, inexpensive, Jingdong self-supporting guaranteed, worth having!	Jingdong prefecture level city in Shandong
Jinfeng Hongyuan Flagship Store	Electricity conservation2	I don't know if it's saving electricity yet, I'll find out next time I pay my electric bill	Jingdong prefecture level city in Shandong
Jinfeng Hongyuan Flagship Store	Electricity conservation2	Favorable price quality assurance appearance design is beautiful and generous, fine workmanship	Jingdong prefecture level city in Shandong
Jinfeng Hongyuan Flagship Store	Electricity conservation2	I bought it, I don't know how effective it is? I don't know how the quality is? After this summer results will know! Looking forward to it!	Jingdong prefecture level city in Shandong
Jinfeng Hongyuan Flagship Store	Electricity conservation2	Goods received Packaging intact, look at the shell workmanship is also good, high-power appliances open voltage has obvious changes in the effectiveness of the	Jingdong prefecture level city in Shandong
Jinfeng Hongyuan Flagship Store	Electricity conservation2	The goods have been received logistics is very fast, the product is similar to the line, just wait to use a month to see if it can save electricity, stay tuned.	Jingdong prefecture level city in Shandong
Jinfeng Hongyuan Flagship Store	Electricity conservation2	Electricity is plugged in, not sure if it will work, hopefully it will work.	Jingdong prefecture level city in Shandong
Jinfeng Hongyuan Flagship Store	Electricity conservation2	The shell is aluminum alloy, very good workmanship, the actual effect of waiting for the addition of evaluation	Jingdong prefecture level city in Shandong
Jinfeng Hongyuan Flagship Store	Electricity conservation2	Received, already plugged in, looking forward to the effect.	Jingdong prefecture level city in Shandong
Jinfeng Hongyuan Flagship Store	Electricity conservation2	Opened to see the regular manufacturer products, wait for a period of time to see it	Jingdong prefecture level city in Shandong
Jinfeng Hongyuan Flagship Store	Electricity conservation2	🉑 Baby received, very surprised ✨, reasonable design, fashionable and generous, continue to pay attention to the future will be repurchased ❥❥❥❥	Jingdong prefecture level city in Shandong
Jinfeng Hongyuan Flagship Store	Electricity conservation2	Baby received it 👌, really great ah! 🌈The quality can't be beat, very satisfied! 🆗Happy happy happy 😃	Jingdong prefecture level city in Shandong
Jinfeng Hongyuan Flagship Store	Electricity conservation2	Very good, power saving see, this is to help a friend to buy, I used to give my friend a say, my friend immediately let me order another one!	Jingdong prefecture level city in Shandong
Jinfeng Hongyuan Flagship Store	Electricity conservation2	The baby is just as introduced, the texture is very good, powerful, and it feels good to use it	Jingdong prefecture level city in Shandong
Jinfeng Hongyuan Flagship Store	Electricity conservation2	Value for money, very satisfied, seller service attitude is very good, very patient	Jingdong prefecture level city in Shandong
Jinfeng Hongyuan Flagship Store	Electricity conservation2	Power saving treasure design unique exquisite beautiful, voltage display clearly, plugged in that is good to use, I observed the meter, after using the meter is gone slow.	Jingdong prefecture level city in Shandong
Jinfeng Hongyuan Flagship Store	Electricity conservation2	Received on the use of the packaging is beautifully used for a period of time and then come back in the follow-up review!	Jingdong prefecture level city in Shandong
Jinfeng Hongyuan Flagship Store	Electricity conservation2	It should work. Not bad. Not bad. Not bad.	Jingdong prefecture level city in Shandong
Jinfeng Hongyuan Flagship Store	Electricity conservation2	I haven't used it yet, I'll plug it in and see if it saves power next month.	Jingdong prefecture level city in Shandong
Jinfeng Hongyuan Flagship Store	Electricity conservation2	The goods received and unpacked and used, the quality is very good, things and the official website to buy no difference!	Jingdong prefecture level city in Shandong
Jinfeng Hongyuan Flagship Store	Electricity conservation2	It's a rip off, doesn't help much at all, uses the same amount of power as before it was used.	Jingdong prefecture level city in Shandong
Jinfeng Hongyuan Flagship Store	Electricity conservation2	I've been using it for a while now and haven't noticed any unusual problems, so it's okay	Jingdong prefecture level city in Shandong
Jinfeng Hongyuan Flagship Store	Electricity conservation2	Received the goods and plugged in to use, good or not there is no use will have to wait until next month to find out	Jingdong prefecture level city in Shandong
Jinfeng Hongyuan Flagship Store	Electricity conservation2	It's very compact, it looks good, I'll introduce you to my clients when it's ready.	Jingdong prefecture level city in Shandong
Jinfeng Hongyuan Flagship Store	Electricity conservation2	Good workmanship, we'll have to see in a few months.	Jingdong prefecture level city in Shandong
Jinfeng Hongyuan Flagship Store	Electricity conservation2	Jingdong quality! Always trustworthy!	Jingdong prefecture level city in Shandong
Jinfeng Hongyuan Flagship Store	Electricity conservation2	This is the second time I've bought it, and the merchant said to give me five dollars out of the difference, so thank you merchant.	Jingdong prefecture level city in Shandong
Jinfeng Hongyuan Flagship Store	Electricity conservation2	Good quality and good service	Jingdong prefecture level city in Shandong
Jinfeng Hongyuan Flagship Store	Electricity conservation2	Feels good texture, aluminum alloy shell, voltage digital display clear.	Jingdong prefecture level city in Shandong
Jinfeng Hongyuan Flagship Store	Electricity conservation2	Good product, fast and great service, thanks!	Jingdong prefecture level city in Shandong
Jinfeng Hongyuan Flagship Store	Electricity conservation2	Have not yet used, first evaluation, later really good do not need to comment again.	Jingdong prefecture level city in Shandong
Jinfeng Hongyuan Flagship Store	Electricity conservation2	Works great. Pretty much as described by the merchant.	Jingdong prefecture level city in Shandong
Jinfeng Hongyuan Flagship Store	Electricity conservation2	Received it, looking forward to the results, hope it works	Jingdong prefecture level city in Shandong
Jinfeng Hongyuan Flagship Store	Electricity conservation2	Received it, looking forward to the results, hope it works	Jingdong prefecture level city in Shandong
Jinfeng Hongyuan Flagship Store	Electricity conservation2	Supposedly it's an IQ tax. Buy one and try it. Looks good. Well made.	Jingdong prefecture level city in Shandong
Jinfeng Hongyuan Flagship Store	Electricity conservation2	It just doesn't feel like it's working.	Jingdong prefecture level city in Shandong
Jinfeng Hongyuan Flagship Store	Electricity conservation2	This is a great product.	Jingdong prefecture level city in Shandong
Jinfeng Hongyuan Flagship Store	Electricity conservation2	I'm just using it. I'll tell you more.	Jingdong prefecture level city in Shandong
Jinfeng Hongyuan Flagship Store	Electricity conservation2	Second repurchase and it's working fine.	Jingdong prefecture level city in Shandong

Jinfeng Hongyuan Flagship Store	Electricity conservation2	I'll wait a few days before evaluating	Jingdong prefecture level city in Shandong
Jinfeng Hongyuan Flagship Store	Electricity conservation2	I'll comment after I use it hopefully the section will be useful.	Jingdong prefecture level city in Shandong
Jinfeng Hongyuan Flagship Store	Electricity conservation2	not very useful	Jingdong prefecture level city in Shandong
Jinfeng Hongyuan Flagship Store	Electricity conservation2	Not received	Jingdong prefecture level city in Shandong
Jinfeng Hongyuan Flagship Store	Electricity conservation2	rare	Jingdong prefecture level city in Shandong
Jinfeng Hongyuan Flagship Store	Electricity conservation2	It's good.	Jingdong prefecture level city in Shandong
Jinfeng Hongyuan Flagship Store	Electricity conservation2	just now	Jingdong prefecture level city in Shandong
Jinfeng Hongyuan Flagship Store	Electricity conservation2	serviceable	Jingdong prefecture level city in Shandong
Jinfeng Hongyuan Flagship Store	Electricity conservation2	desirable	Jingdong prefecture level city in Shandong
Jinfeng Hongyuan Flagship Store	Electricity conservation2	serviceable	Jingdong prefecture level city in Shandong
Jinfeng Hongyuan Flagship Store	Electricity conservation2	It doesn't save power and costs electricity.	Jingdong prefecture level city in Shandong
Jinfeng Hongyuan Flagship Store	Electricity conservation2	Not as good as I thought.	Jingdong prefecture level city in Shandong
Jinfeng Hongyuan Flagship Store	Electricity conservation2	Super bad review, it doesn't save power at all, but rather more power consumption	Jingdong prefecture level city in Shandong
Jinfeng Hongyuan Flagship Store	Electricity conservation2	Listen to others say that this can save electricity, I bought with the idea of trying, used for a period of time before coming to the evaluation, will be a little bit of use, not a lot of savings just, long-term use of the words can save more.	Jingdong prefecture level city in Shandong
Jinfeng Hongyuan Flagship Store	Electricity conservation2	Logistics is fast, the next day arrived, workmanship is very delicate a power saver, looking forward to the late effect, customer service patiently explain the recommended which is suitable for, installed in what position is the best, look forward to using the effect after a period of time!	Jingdong prefecture level city in Shandong
Jinfeng Hongyuan Flagship Store	Electricity conservation2	It's good to use, I feel that it has been saving electricity, anyway, not a loss is, I used to feel that the power consumption is big, now I feel much better, my friends are also using, I hope to use a long time will be able to, I wish the boss a prosperous business	Jingdong prefecture level city in Shandong
Jinfeng Hongyuan Flagship Store	Electricity conservation2	Should be very good, to be able to plan the circuit, save the home of the circuit to the extent of the savings, waste of money on electricity, good if a month this money back, hope always hope to be able to	Jingdong prefecture level city in Shandong
Jinfeng Hongyuan Flagship Store	Electricity conservation2	Express delivery is very strong, the goods and I saw in the business consistent, the use of the effect of follow-up evaluation	Jingdong prefecture level city in Shandong
Jinfeng Hongyuan Flagship Store	Electricity conservation2	I have been using this brand of power saver for many years and this time I saw this upgraded version and got it again. The main purpose of this product is to reduce the loss of electricity for a long time, so as to achieve the purpose of saving electricity.	Jingdong prefecture level city in Shandong
Jinfeng Hongyuan Flagship Store	Electricity conservation2	Logistics is fast and the product looks beautiful, looking forward to the effect.	Jingdong prefecture level city in Shandong
Jinfeng Hongyuan Flagship Store	Electricity conservation2	Product quality is good, energy saving a little effect, but the price is a little expensive, fast arrival, good service.	Jingdong prefecture level city in Shandong
Jinfeng Hongyuan Flagship Store	Electricity conservation2	The quality is very good, inexpensive, Jingdong self-supporting guaranteed, worth having!	Jingdong prefecture level city in Shandong
Jinfeng Hongyuan Flagship Store	Electricity conservation2	I don't know if it's saving electricity yet, I'll find out next time I pay my electric bill	Jingdong prefecture level city in Shandong
Jinfeng Hongyuan Flagship Store	Electricity conservation2	Favorable price quality assurance appearance design is beautiful and generous, fine workmanship	Jingdong prefecture level city in Shandong
Jinfeng Hongyuan Flagship Store	Electricity conservation2	I bought it, I don't know how effective it is? I don't know how the quality is? After this summer results will know! Looking forward to it!	Jingdong prefecture level city in Shandong
Jinfeng Hongyuan Flagship Store	Electricity conservation2	Goods received Packaging intact, look at the shell workmanship is also good, high-power appliances open voltage has obvious changes in the effectiveness of the	Jingdong prefecture level city in Shandong
Jinfeng Hongyuan Flagship Store	Electricity conservation2	The goods have been received logistics is very fast, the product is similar to the line, just wait to use a month to see if it can save electricity, stay tuned.	Jingdong prefecture level city in Shandong
Jinfeng Hongyuan Flagship Store	Electricity conservation2	Electricity is plugged in, not sure if it will work, hopefully it will work.	Jingdong prefecture level city in Shandong
Jinfeng Hongyuan Flagship Store	Electricity conservation2	The shell is aluminum alloy, very good workmanship, the actual effect of waiting for the addition of evaluation	Jingdong prefecture level city in Shandong
Jinfeng Hongyuan Flagship Store	Electricity conservation2	Received, already plugged in, looking forward to the effect.	Jingdong prefecture level city in Shandong
Jinfeng Hongyuan Flagship Store	Electricity conservation2	Opened to see the regular manufacturer products, wait for a period of time to see it	Jingdong prefecture level city in Shandong
Jinfeng Hongyuan Flagship Store	Electricity conservation2	🉑 Baby received, very surprised ✨, reasonable design, fashionable and generous, continue to pay attention to the future will be repurchased ❥❥❥❥	Jingdong prefecture level city in Shandong
Jinfeng Hongyuan Flagship Store	Electricity conservation2	Baby received it 👌, really great ah! 🌈The quality can't be beat, very satisfied! 🆗Happy happy happy 😀	Jingdong prefecture level city in Shandong
Jinfeng Hongyuan Flagship Store	Electricity conservation2	Very good, power saving see, this is to help a friend to buy, I used to give my friend a say, my friend immediately let me order another one!	Jingdong prefecture level city in Shandong
Jinfeng Hongyuan Flagship Store	Electricity conservation2	The baby is just as introduced, the texture is very good, powerful, and it feels good to use it	Jingdong prefecture level city in Shandong
Jinfeng Hongyuan Flagship Store	Electricity conservation2	Value for money, very satisfied, seller service attitude is very good, very patient	Jingdong prefecture level city in Shandong
Jinfeng Hongyuan Flagship Store	Electricity conservation2	Power saving treasure design unique exquisite beautiful, voltage display clearly, plugged in that is good to use, I observed the meter, after using the meter is gone slow.	Jingdong prefecture level city in Shandong
Jinfeng Hongyuan Flagship Store	Electricity conservation2	Received on the use of the packaging is beautifully used for a period of time and then come back in the follow-up review!	Jingdong prefecture level city in Shandong
Jinfeng Hongyuan Flagship Store	Electricity conservation2	It should work. Not bad. Not bad. Not bad.	Jingdong prefecture level city in Shandong
Jinfeng Hongyuan Flagship Store	Electricity conservation2	I haven't used it yet, I'll plug it in and see if it saves power next month.	Jingdong prefecture level city in Shandong
Jinfeng Hongyuan Flagship Store	Electricity conservation2	The goods received and unpacked and used, the quality is very good, things and the official website to buy no difference!	Jingdong prefecture level city in Shandong
Jinfeng Hongyuan Flagship Store	Electricity conservation2	It's a rip off, doesn't help much at all, uses the same amount of power as before it was used.	Jingdong prefecture level city in Shandong
Jinfeng Hongyuan Flagship Store	Electricity conservation2	I've been using it for a while now and haven't noticed any unusual problems, so it's okay	Jingdong prefecture level city in Shandong
Jinfeng Hongyuan Flagship Store	Electricity conservation2	Received the goods and plugged in to use, good or not there is no use will have to wait until next month to find out	Jingdong prefecture level city in Shandong
Jinfeng Hongyuan Flagship Store	Electricity conservation2	It's very compact, it looks good, I'll introduce you to my clients when it's ready.	Jingdong prefecture level city in Shandong
Jinfeng Hongyuan Flagship Store	Electricity conservation2	Good workmanship, we'll have to see in a few months.	Jingdong prefecture level city in Shandong
Jinfeng Hongyuan Flagship Store	Electricity conservation2	Jingdong quality! Always trustworthy!	Jingdong prefecture level city in Shandong
Jinfeng Hongyuan Flagship Store	Electricity conservation2	This is the second time I've bought it, and the merchant said to give me five dollars out of the difference, so thank you merchant.	Jingdong prefecture level city in Shandong
Jinfeng Hongyuan Flagship Store	Electricity conservation2	Good quality and good service	Jingdong prefecture level city in Shandong

Jinfeng Hongyuan Flagship Store	Electricity conservation2	Feels good texture, aluminum alloy shell, voltage digital display clear.	Jingdong prefecture level city in Shandong
Jinfeng Hongyuan Flagship Store	Electricity conservation2	Good product, fast and great service, thanks!	Jingdong prefecture level city in Shandong
Jinfeng Hongyuan Flagship Store	Electricity conservation2	Have not yet used, first evaluation, later really good do not need to comment again.	Jingdong prefecture level city in Shandong
Jinfeng Hongyuan Flagship Store	Electricity conservation2	Works great. Pretty much as described by the merchant.	Jingdong prefecture level city in Shandong
Jinfeng Hongyuan Flagship Store	Electricity conservation2	Received it, looking forward to the results, hope it works	Jingdong prefecture level city in Shandong
Jinfeng Hongyuan Flagship Store	Electricity conservation2	Received it, looking forward to the results, hope it works	Jingdong prefecture level city in Shandong
Jinfeng Hongyuan Flagship Store	Electricity conservation2	Supposedly it's an IQ tax. Buy one and try it. Looks good. Well made.	Jingdong prefecture level city in Shandong
Jinfeng Hongyuan Flagship Store	Electricity conservation2	It just doesn't feel like it's working.	Jingdong prefecture level city in Shandong
Jinfeng Hongyuan Flagship Store	Electricity conservation2	This is a great product.	Jingdong prefecture level city in Shandong
Jinfeng Hongyuan Flagship Store	Electricity conservation2	I'm just using it. I'll tell you more.	Jingdong prefecture level city in Shandong
Jinfeng Hongyuan Flagship Store	Electricity conservation2	Second repurchase and it's working fine.	Jingdong prefecture level city in Shandong
Jinfeng Hongyuan Flagship Store	Electricity conservation2	I'll wait a few days before evaluating	Jingdong prefecture level city in Shandong
Jinfeng Hongyuan Flagship Store	Electricity conservation2	I'll comment after I use it hopefully the section will be useful.	Jingdong prefecture level city in Shandong
Jinfeng Hongyuan Flagship Store	Electricity conservation2	not very useful	Jingdong prefecture level city in Shandong
Jinfeng Hongyuan Flagship Store	Electricity conservation2	Not received	Jingdong prefecture level city in Shandong
Jinfeng Hongyuan Flagship Store	Electricity conservation2	rare	Jingdong prefecture level city in Shandong
Jinfeng Hongyuan Flagship Store	Electricity conservation2	It's good.	Jingdong prefecture level city in Shandong
Jinfeng Hongyuan Flagship Store	Electricity conservation2	just now	Jingdong prefecture level city in Shandong
Jinfeng Hongyuan Flagship Store	Electricity conservation2	serviceable	Jingdong prefecture level city in Shandong
Jinfeng Hongyuan Flagship Store	Electricity conservation2	desirable	Jingdong prefecture level city in Shandong
Jinfeng Hongyuan Flagship Store	Electricity conservation2	serviceable	Jingdong prefecture level city in Shandong
Jinfeng Hongyuan Flagship Store	Electricity conservation2	It doesn't save power and costs electricity.	Jingdong prefecture level city in Shandong
Jinfeng Hongyuan Flagship Store	Electricity conservation2	Not as good as I thought.	Jingdong prefecture level city in Shandong
Jinfeng Hongyuan Flagship Store	Electricity conservation2	Super bad review, it doesn't save power at all, but rather more power consumption	Jingdong prefecture level city in Shandong
Jinfeng Hongyuan Flagship Store	Electricity conservation2	Listen to others say that this can save electricity, I bought with the idea of trying, used for a period of time before coming to the evaluation, will be a little bit of use, not a lot of savings just, long-term use of the words can save more.	Jingdong prefecture level city in Shandong
Jinfeng Hongyuan Flagship Store	Electricity conservation2	Logistics is fast, the next day arrived, workmanship is very delicate a power saver, looking forward to the late effect, customer service patiently explain the recommended which is suitable for, installed in what position is the best, look forward to using the effect after a period of time!	Jingdong prefecture level city in Shandong
Jinfeng Hongyuan Flagship Store	Electricity conservation2	It's good to use, I feel that it has been saving electricity, anyway, not a loss is, I used to feel that the power consumption is big, now I feel much better, my friends are also using, I hope to use a long time will be able to, I wish the boss a prosperous business	Jingdong prefecture level city in Shandong
Jinfeng Hongyuan Flagship Store	Electricity conservation2	Should be very good, to be able to plan the circuit, save the home of the circuit to the extent of the savings, waste of money on electricity, good if a month this money back, hope always hope to be able to	Jingdong prefecture level city in Shandong
Jinfeng Hongyuan Flagship Store	Electricity conservation2	Express delivery is very strong, the goods and I saw in the business consistent, the use of the effect of follow-up evaluation	Jingdong prefecture level city in Shandong
Jinfeng Hongyuan Flagship Store	Electricity conservation2	I have been using this brand of power saver for many years and this time I saw this upgraded version and got it again. The main purpose of this product is to reduce the loss of electricity for a long time, so as to achieve the purpose of saving electricity.	Jingdong prefecture level city in Shandong
Jinfeng Hongyuan Flagship Store	Electricity conservation2	Logistics is fast and the product looks beautiful, looking forward to the effect.	Jingdong prefecture level city in Shandong
Jinfeng Hongyuan Flagship Store	Electricity conservation2	Product quality is good, energy saving a little effect, but the price is a little expensive, fast arrival, good service.	Jingdong prefecture level city in Shandong
Jinfeng Hongyuan Flagship Store	Electricity conservation2	The quality is very good, inexpensive, Jingdong self-supporting guaranteed, worth having!	Jingdong prefecture level city in Shandong
Jinfeng Hongyuan Flagship Store	Electricity conservation2	I don't know if it's saving electricity yet, I'll find out next time I pay my electric bill	Jingdong prefecture level city in Shandong
Jinfeng Hongyuan Flagship Store	Electricity conservation2	Favorable price quality assurance appearance design is beautiful and generous, fine workmanship	Jingdong prefecture level city in Shandong
Jinfeng Hongyuan Flagship Store	Electricity conservation2	I bought it, I don't know how effective it is? I don't know how the quality is? After this summer results will know! Looking forward to it!	Jingdong prefecture level city in Shandong
Jinfeng Hongyuan Flagship Store	Electricity conservation2	Goods received Packaging intact, look at the shell workmanship is also good, high-power appliances open voltage has obvious changes in the effectiveness of the	Jingdong prefecture level city in Shandong
Jinfeng Hongyuan Flagship Store	Electricity conservation2	The goods have been received logistics is very fast, the product is similar to the line, just wait to use a month to see if it can save electricity, stay tuned.	Jingdong prefecture level city in Shandong
Jinfeng Hongyuan Flagship Store	Electricity conservation2	Electricity is plugged in, not sure if it will work, hopefully it will work.	Jingdong prefecture level city in Shandong
Jinfeng Hongyuan Flagship Store	Electricity conservation2	The shell is aluminum alloy, very good workmanship, the actual effect of waiting for the addition of evaluation	Jingdong prefecture level city in Shandong
Jinfeng Hongyuan Flagship Store	Electricity conservation2	Received, already plugged in, looking forward to the effect.	Jingdong prefecture level city in Shandong
Jinfeng Hongyuan Flagship Store	Electricity conservation2	Opened to see the regular manufacturer products, wait for a period of time to see it	Jingdong prefecture level city in Shandong
Jinfeng Hongyuan Flagship Store	Electricity conservation2	🉑 Baby received, very surprised ✨, reasonable design, fashionable and generous, continue to pay attention to the future will be repurchased ❤❤❤❤	Jingdong prefecture level city in Shandong
Jinfeng Hongyuan Flagship Store	Electricity conservation2	Baby received it 👌, really great ah! 🌈The quality can't be beat, very satisfied! 🆗Happy happy happy 😃	Jingdong prefecture level city in Shandong
Jinfeng Hongyuan Flagship Store	Electricity conservation2	Very good, power saving see, this is to help a friend to buy, I used to give my friend a say, my friend immediately let me order another one!	Jingdong prefecture level city in Shandong
Jinfeng Hongyuan Flagship Store	Electricity conservation2	The baby is just as introduced, the texture is very good, powerful, and it feels good to use it	Jingdong prefecture level city in Shandong
Jinfeng Hongyuan Flagship Store	Electricity conservation2	Value for money, very satisfied, seller service attitude is very good, very patient	Jingdong prefecture level city in Shandong
Jinfeng Hongyuan Flagship Store	Electricity conservation2	Power saving treasure design unique exquisite beautiful, voltage display clearly, plugged in that is good to use, I observed the meter, after using the meter is gone slow.	Jingdong prefecture level city in Shandong
Jinfeng Hongyuan Flagship Store	Electricity conservation2	Received on the use of the packaging is beautifully used for a period of time and then come back in the follow-up review!	Jingdong prefecture level city in Shandong

Jinfeng Hongyuan Flagship Store	Electricity conservation2	It should work. Not bad. Not bad. Not bad.	Jingdong prefecture level city in Shandong
Jinfeng Hongyuan Flagship Store	Electricity conservation2	I haven't used it yet, I'll plug it in and see if it saves power next month.	Jingdong prefecture level city in Shandong
Jinfeng Hongyuan Flagship Store	Electricity conservation2	The goods received and unpacked and used, the quality is very good, things and the official website to buy no difference!	Jingdong prefecture level city in Shandong
Jinfeng Hongyuan Flagship Store	Electricity conservation2	It's a rip off, doesn't help much at all, uses the same amount of power as before it was used.	Jingdong prefecture level city in Shandong
Jinfeng Hongyuan Flagship Store	Electricity conservation2	I've been using it for a while now and haven't noticed any unusual problems, so it's okay	Jingdong prefecture level city in Shandong
Jinfeng Hongyuan Flagship Store	Electricity conservation2	Received the goods and plugged in to use, good or not there is no use will have to wait until next month to find out	Jingdong prefecture level city in Shandong
Jinfeng Hongyuan Flagship Store	Electricity conservation2	It's very compact, it looks good, I'll introduce you to my clients when it's ready.	Jingdong prefecture level city in Shandong
Jinfeng Hongyuan Flagship Store	Electricity conservation2	Good workmanship, we'll have to see in a few months.	Jingdong prefecture level city in Shandong
Jinfeng Hongyuan Flagship Store	Electricity conservation2	Jingdong quality! Always trustworthy!	Jingdong prefecture level city in Shandong
Jinfeng Hongyuan Flagship Store	Electricity conservation2	This is the second time I've bought it, and the merchant said to give me five dollars out of the difference, so thank you merchant.	Jingdong prefecture level city in Shandong
Jinfeng Hongyuan Flagship Store	Electricity conservation2	Good quality and good service	Jingdong prefecture level city in Shandong
Jinfeng Hongyuan Flagship Store	Electricity conservation2	Feels good texture, aluminum alloy shell, voltage digital display clear.	Jingdong prefecture level city in Shandong
Jinfeng Hongyuan Flagship Store	Electricity conservation2	Good product, fast and great service, thanks!	Jingdong prefecture level city in Shandong
Jinfeng Hongyuan Flagship Store	Electricity conservation2	Have not yet used, first evaluation, later really good do not need to comment again.	Jingdong prefecture level city in Shandong
Jinfeng Hongyuan Flagship Store	Electricity conservation2	Works great. Pretty much as described by the merchant.	Jingdong prefecture level city in Shandong
Jinfeng Hongyuan Flagship Store	Electricity conservation2	Received it, looking forward to the results, hope it works	Jingdong prefecture level city in Shandong
Jinfeng Hongyuan Flagship Store	Electricity conservation2	Received it, looking forward to the results, hope it works	Jingdong prefecture level city in Shandong
Jinfeng Hongyuan Flagship Store	Electricity conservation2	Supposedly it's an IQ tax. Buy one and try it. Looks good. Well made.	Jingdong prefecture level city in Shandong
Jinfeng Hongyuan Flagship Store	Electricity conservation2	It just doesn't feel like it's working.	Jingdong prefecture level city in Shandong
Jinfeng Hongyuan Flagship Store	Electricity conservation2	This is a great product.	Jingdong prefecture level city in Shandong
Jinfeng Hongyuan Flagship Store	Electricity conservation2	I'm just using it. I'll tell you more.	Jingdong prefecture level city in Shandong
Jinfeng Hongyuan Flagship Store	Electricity conservation2	Second repurchase and it's working fine.	Jingdong prefecture level city in Shandong
Jinfeng Hongyuan Flagship Store	Electricity conservation2	I'll wait a few days before evaluating	Jingdong prefecture level city in Shandong
Jinfeng Hongyuan Flagship Store	Electricity conservation2	I'll comment after I use it hopefully the section will be useful.	Jingdong prefecture level city in Shandong
Jinfeng Hongyuan Flagship Store	Electricity conservation2	not very useful	Jingdong prefecture level city in Shandong
Jinfeng Hongyuan Flagship Store	Electricity conservation2	Not received	Jingdong prefecture level city in Shandong
Jinfeng Hongyuan Flagship Store	Electricity conservation2	rare	Jingdong prefecture level city in Shandong
Jinfeng Hongyuan Flagship Store	Electricity conservation2	It's good.	Jingdong prefecture level city in Shandong
Jinfeng Hongyuan Flagship Store	Electricity conservation2	just now	Jingdong prefecture level city in Shandong
Jinfeng Hongyuan Flagship Store	Electricity conservation2	serviceable	Jingdong prefecture level city in Shandong
Jinfeng Hongyuan Flagship Store	Electricity conservation2	desirable	Jingdong prefecture level city in Shandong
Jinfeng Hongyuan Flagship Store	Electricity conservation2	serviceable	Jingdong prefecture level city in Shandong
Jinfeng Hongyuan Flagship Store	Electricity conservation2	It doesn't save power and costs electricity.	Jingdong prefecture level city in Shandong
Jinfeng Hongyuan Flagship Store	Electricity conservation2	Not as good as I thought.	Jingdong prefecture level city in Shandong
Jinfeng Hongyuan Flagship Store	Electricity conservation2	Super bad review, it doesn't save power at all, but rather more power consumption	Jingdong prefecture level city in Shandong
Jinfeng Hongyuan Flagship Store	Electricity conservation2	Listen to others say that this can save electricity, I bought with the idea of trying, used for a period of time before coming to the evaluation, will be a little bit of use, not a lot of savings just, long-term use of the words can save more.	Jingdong prefecture level city in Shandong
Jinfeng Hongyuan Flagship Store	Electricity conservation2	Logistics is fast, the next day arrived, workmanship is very delicate a power saver, looking forward to the late effect, customer service patiently explain the recommended which is suitable for, installed in what position is the best, look forward to using the effect after a period of time!	Jingdong prefecture level city in Shandong
Jinfeng Hongyuan Flagship Store	Electricity conservation2	It's good to use, I feel that it has been saving electricity, anyway, not a loss is, I used to feel that the power consumption is big, now I feel much better, my friends are also using, I hope to use a long time will be able to, I wish the boss a prosperous business	Jingdong prefecture level city in Shandong
Jinfeng Hongyuan Flagship Store	Electricity conservation2	Should be very good, to be able to plan the circuit, save the home of the circuit to the extent of the savings, waste of money on electricity, good if a month this money back, hope always hope to be able to	Jingdong prefecture level city in Shandong
Jinfeng Hongyuan Flagship Store	Electricity conservation2	Express delivery is very strong, the goods and I saw in the business consistent, the use of the effect of follow-up evaluation	Jingdong prefecture level city in Shandong
Jinfeng Hongyuan Flagship Store	Electricity conservation2	I have been using this brand of power saver for many years and this time I saw this upgraded version and got it again. The main purpose of this product is to reduce the loss of electricity for a long time, so as to achieve the purpose of saving electricity.	Jingdong prefecture level city in Shandong
Jinfeng Hongyuan Flagship Store	Electricity conservation2	Logistics is fast and the product looks beautiful, looking forward to the effect.	Jingdong prefecture level city in Shandong
Jinfeng Hongyuan Flagship Store	Electricity conservation2	Product quality is good, energy saving a little effect, but the price is a little expensive, fast arrival, good service.	Jingdong prefecture level city in Shandong
Jinfeng Hongyuan Flagship Store	Electricity conservation2	The quality is very good, inexpensive, Jingdong self-supporting guaranteed, worth having!	Jingdong prefecture level city in Shandong
Jinfeng Hongyuan Flagship Store	Electricity conservation2	I don't know if it's saving electricity yet, I'll find out next time I pay my electric bill	Jingdong prefecture level city in Shandong
Jinfeng Hongyuan Flagship Store	Electricity conservation2	Favorable price quality assurance appearance design is beautiful and generous, fine workmanship	Jingdong prefecture level city in Shandong
Jinfeng Hongyuan Flagship Store	Electricity conservation2	I bought it, I don't know how effective it is? I don't know how the quality is? After this summer results will know! Looking forward to it!	Jingdong prefecture level city in Shandong
Jinfeng Hongyuan Flagship Store	Electricity conservation2	Goods received Packaging intact, look at the shell workmanship is also good, high-power appliances open voltage has obvious changes in the effectiveness of the	Jingdong prefecture level city in Shandong
Jinfeng Hongyuan Flagship Store	Electricity conservation2	The goods have been received logistics is very fast, the product is similar to the line, just wait to use a month to see if it can save electricity, stay tuned.	Jingdong prefecture level city in Shandong

Jinfeng Hongyuan Flagship Store	Electricity conservation2	Electricity is plugged in, not sure if it will work, hopefully it will work.	Jingdong prefecture level city in Shandong
Jinfeng Hongyuan Flagship Store	Electricity conservation2	The shell is aluminum alloy, very good workmanship, the actual effect of waiting for the addition of evaluation	Jingdong prefecture level city in Shandong
Jinfeng Hongyuan Flagship Store	Electricity conservation2	Received, already plugged in, looking forward to the effect.	Jingdong prefecture level city in Shandong
Jinfeng Hongyuan Flagship Store	Electricity conservation2	Opened to see the regular manufacturer products, wait for a period of time to see it	Jingdong prefecture level city in Shandong
Jinfeng Hongyuan Flagship Store	Electricity conservation2	🉑 Baby received, very surprised ✨, reasonable design, fashionable and generous, continue to pay attention to the future will be repurchased ❥❥❥❥	Jingdong prefecture level city in Shandong
Jinfeng Hongyuan Flagship Store	Electricity conservation2	Baby received it 👌, really great ah! 🌈The quality can't be beat, very satisfied! 🆗Happy happy happy 😃	Jingdong prefecture level city in Shandong
Jinfeng Hongyuan Flagship Store	Electricity conservation2	Very good, power saving see, this is to help a friend to buy, I used to give my friend a say, my friend immediately let me order another one!	Jingdong prefecture level city in Shandong
Jinfeng Hongyuan Flagship Store	Electricity conservation2	The baby is just as introduced, the texture is very good, powerful, and it feels good to use it	Jingdong prefecture level city in Shandong
Jinfeng Hongyuan Flagship Store	Electricity conservation2	Value for money, very satisfied, seller service attitude is very good, very patient	Jingdong prefecture level city in Shandong
Jinfeng Hongyuan Flagship Store	Electricity conservation2	Power saving treasure design unique exquisite beautiful, voltage display clearly, plugged in that is good to use, I observed the meter, after using the meter is gone slow.	Jingdong prefecture level city in Shandong
Jinfeng Hongyuan Flagship Store	Electricity conservation2	Received on the use of the packaging is beautifully used for a period of time and then come back in the follow-up review!	Jingdong prefecture level city in Shandong
Jinfeng Hongyuan Flagship Store	Electricity conservation2	It should work. Not bad. Not bad. Not bad.	Jingdong prefecture level city in Shandong
Jinfeng Hongyuan Flagship Store	Electricity conservation2	I haven't used it yet, I'll plug it in and see if it saves power next month.	Jingdong prefecture level city in Shandong
Jinfeng Hongyuan Flagship Store	Electricity conservation2	The goods received and unpacked and used, the quality is very good, things and the official website to buy no difference!	Jingdong prefecture level city in Shandong
Jinfeng Hongyuan Flagship Store	Electricity conservation2	It's a rip off, doesn't help much at all, uses the same amount of power as before it was used.	Jingdong prefecture level city in Shandong
Jinfeng Hongyuan Flagship Store	Electricity conservation2	I've been using it for a while now and haven't noticed any unusual problems, so it's okay	Jingdong prefecture level city in Shandong
Jinfeng Hongyuan Flagship Store	Electricity conservation2	Received the goods and plugged in to use, good or not there is no use will have to wait until next month to find out	Jingdong prefecture level city in Shandong
Jinfeng Hongyuan Flagship Store	Electricity conservation2	It's very compact, it looks good, I'll introduce you to my clients when it's ready.	Jingdong prefecture level city in Shandong
Jinfeng Hongyuan Flagship Store	Electricity conservation2	Good workmanship, we'll have to see in a few months.	Jingdong prefecture level city in Shandong
Jinfeng Hongyuan Flagship Store	Electricity conservation2	Jingdong quality! Always trustworthy!	Jingdong prefecture level city in Shandong
Jinfeng Hongyuan Flagship Store	Electricity conservation2	This is the second time I've bought it, and the merchant said to give me five dollars out of the difference, so thank you merchant.	Jingdong prefecture level city in Shandong
Jinfeng Hongyuan Flagship Store	Electricity conservation2	Good quality and good service	Jingdong prefecture level city in Shandong
Jinfeng Hongyuan Flagship Store	Electricity conservation2	Feels good texture, aluminum alloy shell, voltage digital display clear.	Jingdong prefecture level city in Shandong
Jinfeng Hongyuan Flagship Store	Electricity conservation2	Good product, fast and great service, thanks!	Jingdong prefecture level city in Shandong
Jinfeng Hongyuan Flagship Store	Electricity conservation2	Have not yet used, first evaluation, later really good do not need to comment again.	Jingdong prefecture level city in Shandong
Jinfeng Hongyuan Flagship Store	Electricity conservation2	Works great. Pretty much as described by the merchant.	Jingdong prefecture level city in Shandong
Jinfeng Hongyuan Flagship Store	Electricity conservation2	Received it, looking forward to the results, hope it works	Jingdong prefecture level city in Shandong
Jinfeng Hongyuan Flagship Store	Electricity conservation2	Received it, looking forward to the results, hope it works	Jingdong prefecture level city in Shandong
Jinfeng Hongyuan Flagship Store	Electricity conservation2	Supposedly it's an IQ tax. Buy one and try it. Looks good. Well made.	Jingdong prefecture level city in Shandong
Jinfeng Hongyuan Flagship Store	Electricity conservation2	It just doesn't feel like it's working.	Jingdong prefecture level city in Shandong
Jinfeng Hongyuan Flagship Store	Electricity conservation2	This is a great product.	Jingdong prefecture level city in Shandong
Jinfeng Hongyuan Flagship Store	Electricity conservation2	I'm just using it. I'll tell you more.	Jingdong prefecture level city in Shandong
Jinfeng Hongyuan Flagship Store	Electricity conservation2	Second repurchase and it's working fine.	Jingdong prefecture level city in Shandong
Jinfeng Hongyuan Flagship Store	Electricity conservation2	I'll wait a few days before evaluating	Jingdong prefecture level city in Shandong
Jinfeng Hongyuan Flagship Store	Electricity conservation2	I'll comment after I use it hopefully the section will be useful.	Jingdong prefecture level city in Shandong
Jinfeng Hongyuan Flagship Store	Electricity conservation2	not very useful	Jingdong prefecture level city in Shandong
Jinfeng Hongyuan Flagship Store	Electricity conservation2	Not received	Jingdong prefecture level city in Shandong
Jinfeng Hongyuan Flagship Store	Electricity conservation2	rare	Jingdong prefecture level city in Shandong
Jinfeng Hongyuan Flagship Store	Electricity conservation2	It's good.	Jingdong prefecture level city in Shandong
Jinfeng Hongyuan Flagship Store	Electricity conservation2	just now	Jingdong prefecture level city in Shandong
Jinfeng Hongyuan Flagship Store	Electricity conservation2	serviceable	Jingdong prefecture level city in Shandong
Jinfeng Hongyuan Flagship Store	Electricity conservation2	desirable	Jingdong prefecture level city in Shandong
Jinfeng Hongyuan Flagship Store	Electricity conservation2	serviceable	Jingdong prefecture level city in Shandong
Jinfeng Hongyuan Flagship Store	Electricity conservation2	It doesn't save power and costs electricity.	Jingdong prefecture level city in Shandong
Jinfeng Hongyuan Flagship Store	Electricity conservation2	Not as good as I thought.	Jingdong prefecture level city in Shandong
Jinfeng Hongyuan Flagship Store	Electricity conservation2	Super bad review, it doesn't save power at all, but rather more power consumption	Jingdong prefecture level city in Shandong
Jinfeng Hongyuan Flagship Store	Electricity conservation2	Listen to others say that this can save electricity, I bought with the idea of trying, used for a period of time before coming to the evaluation, will be a little bit of use, not a lot of savings just, long-term use of the words can save more.	Jingdong prefecture level city in Shandong
Jinfeng Hongyuan Flagship Store	Electricity conservation2	Logistics is fast, the next day arrived, workmanship is very delicate a power saver, looking forward to the late effect, customer service patiently explain the recommended which is suitable for, installed in what position is the best, look forward to using the effect after a period of time!	Jingdong prefecture level city in Shandong
Jinfeng Hongyuan Flagship Store	Electricity conservation2	It's good to use, I feel that it has been saving electricity, anyway, not a loss is, I used to feel that the power consumption is big, now I feel much better, my friends are also using, I hope to use a long time will be able to, I wish the boss a prosperous business	Jingdong prefecture level city in Shandong
Jinfeng Hongyuan Flagship Store	Electricity conservation2	Should be very good, to be able to plan the circuit, save the home of the circuit to the extent of the savings, waste of money on electricity, good if a month this money back, hope always hope to be able to	Jingdong prefecture level city in Shandong

Jinfeng Hongyuan Flagship Store	Electricity conservation2	Express delivery is very strong, the goods and I saw in the business consistent, the use of the effect of follow-up evaluation	Jingdong prefecture level city in Shandong
Jinfeng Hongyuan Flagship Store	Electricity conservation2	I have been using this brand of power saver for many years and this time I saw this upgraded version and got it again. The main purpose of this product is to reduce the loss of electricity for a long time, so as to achieve the purpose of saving electricity.	Jingdong prefecture level city in Shandong
Jinfeng Hongyuan Flagship Store	Electricity conservation2	Logistics is fast and the product looks beautiful, looking forward to the effect.	Jingdong prefecture level city in Shandong
Jinfeng Hongyuan Flagship Store	Electricity conservation2	Product quality is good, energy saving a little effect, but the price is a little expensive, fast arrival, good service.	Jingdong prefecture level city in Shandong
Jinfeng Hongyuan Flagship Store	Electricity conservation2	The quality is very good, inexpensive, Jingdong self-supporting guaranteed, worth having!	Jingdong prefecture level city in Shandong
Jinfeng Hongyuan Flagship Store	Electricity conservation2	I don't know if it's saving electricity yet, I'll find out next time I pay my electric bill	Jingdong prefecture level city in Shandong
Jinfeng Hongyuan Flagship Store	Electricity conservation2	Favorable price quality assurance appearance design is beautiful and generous, fine workmanship	Jingdong prefecture level city in Shandong
Jinfeng Hongyuan Flagship Store	Electricity conservation2	I bought it, I don't know how effective it is? I don't know how the quality is? After this summer results will know! Looking forward to it!	Jingdong prefecture level city in Shandong
Jinfeng Hongyuan Flagship Store	Electricity conservation2	Goods received Packaging intact, look at the shell workmanship is also good, high-power appliances open voltage has obvious changes in the effectiveness of the	Jingdong prefecture level city in Shandong
Jinfeng Hongyuan Flagship Store	Electricity conservation2	The goods have been received logistics is very fast, the product is similar to the line, just wait to use a month to see if it can save electricity, stay tuned.	Jingdong prefecture level city in Shandong
Jinfeng Hongyuan Flagship Store	Electricity conservation2	Electricity is plugged in, not sure if it will work, hopefully it will work.	Jingdong prefecture level city in Shandong
Jinfeng Hongyuan Flagship Store	Electricity conservation2	The shell is aluminum alloy, very good workmanship, the actual effect of waiting for the addition of evaluation	Jingdong prefecture level city in Shandong
Jinfeng Hongyuan Flagship Store	Electricity conservation2	Received, already plugged in, looking forward to the effect.	Jingdong prefecture level city in Shandong
Jinfeng Hongyuan Flagship Store	Electricity conservation2	Opened to see the regular manufacturer products, wait for a period of time to see it	Jingdong prefecture level city in Shandong
Jinfeng Hongyuan Flagship Store	Electricity conservation2	🉑 Baby received, very surprised ✨, reasonable design, fashionable and generous, continue to pay attention to the future will be repurchased ❥❥❥❥	Jingdong prefecture level city in Shandong
Jinfeng Hongyuan Flagship Store	Electricity conservation2	Baby received it 👌, really great ah! 🌈The quality can't be beat, very satisfied! 🆗Happy happy happy 😀	Jingdong prefecture level city in Shandong
Jinfeng Hongyuan Flagship Store	Electricity conservation2	Very good, power saving see, this is to help a friend to buy, I used to give my friend a say, my friend immediately let me order another one!	Jingdong prefecture level city in Shandong
Jinfeng Hongyuan Flagship Store	Electricity conservation2	The baby is just as introduced, the texture is very good, powerful, and it feels good to use it	Jingdong prefecture level city in Shandong
Jinfeng Hongyuan Flagship Store	Electricity conservation2	Value for money, very satisfied, seller service attitude is very good, very patient	Jingdong prefecture level city in Shandong
Jinfeng Hongyuan Flagship Store	Electricity conservation2	Power saving treasure design unique exquisite beautiful, voltage display clearly, plugged in that is good to use, I observed the meter, after using the meter is gone slow.	Jingdong prefecture level city in Shandong
Jinfeng Hongyuan Flagship Store	Electricity conservation2	Received on the use of the packaging is beautifully used for a period of time and then come back in the follow-up review!	Jingdong prefecture level city in Shandong
Jinfeng Hongyuan Flagship Store	Electricity conservation2	It should work. Not bad. Not bad. Not bad.	Jingdong prefecture level city in Shandong
Jinfeng Hongyuan Flagship Store	Electricity conservation2	I haven't used it yet, I'll plug it in and see if it saves power next month.	Jingdong prefecture level city in Shandong
Jinfeng Hongyuan Flagship Store	Electricity conservation2	The goods received and unpacked and used, the quality is very good, things and the official website to buy no difference!	Jingdong prefecture level city in Shandong
Jinfeng Hongyuan Flagship Store	Electricity conservation2	It's a rip off, doesn't help much at all, uses the same amount of power as before it was used.	Jingdong prefecture level city in Shandong
Jinfeng Hongyuan Flagship Store	Electricity conservation2	I've been using it for a while now and haven't noticed any unusual problems, so it's okay	Jingdong prefecture level city in Shandong
Jinfeng Hongyuan Flagship Store	Electricity conservation2	Received the goods and plugged in to use, good or not there is no use will have to wait until next month to find out	Jingdong prefecture level city in Shandong
Jinfeng Hongyuan Flagship Store	Electricity conservation2	It's very compact, it looks good, I'll introduce you to my clients when it's ready.	Jingdong prefecture level city in Shandong
Jinfeng Hongyuan Flagship Store	Electricity conservation2	Good workmanship, we'll have to see in a few months.	Jingdong prefecture level city in Shandong
Jinfeng Hongyuan Flagship Store	Electricity conservation2	Jingdong quality! Always trustworthy!	Jingdong prefecture level city in Shandong
Jinfeng Hongyuan Flagship Store	Electricity conservation2	This is the second time I've bought it, and the merchant said to give me five dollars out of the difference, so thank you merchant.	Jingdong prefecture level city in Shandong
Jinfeng Hongyuan Flagship Store	Electricity conservation2	Good quality and good service	Jingdong prefecture level city in Shandong
Jinfeng Hongyuan Flagship Store	Electricity conservation2	Feels good texture, aluminum alloy shell, voltage digital display clear.	Jingdong prefecture level city in Shandong
Jinfeng Hongyuan Flagship Store	Electricity conservation2	Good product, fast and great service, thanks!	Jingdong prefecture level city in Shandong
Jinfeng Hongyuan Flagship Store	Electricity conservation2	Have not yet used, first evaluation, later really good do not need to comment again.	Jingdong prefecture level city in Shandong
Jinfeng Hongyuan Flagship Store	Electricity conservation2	Works great. Pretty much as described by the merchant.	Jingdong prefecture level city in Shandong
Jinfeng Hongyuan Flagship Store	Electricity conservation2	Received it, looking forward to the results, hope it works	Jingdong prefecture level city in Shandong
Jinfeng Hongyuan Flagship Store	Electricity conservation2	Received it, looking forward to the results, hope it works	Jingdong prefecture level city in Shandong
Jinfeng Hongyuan Flagship Store	Electricity conservation2	Supposedly it's an IQ tax. Buy one and try it. Looks good. Well made.	Jingdong prefecture level city in Shandong
Jinfeng Hongyuan Flagship Store	Electricity conservation2	It just doesn't feel like it's working.	Jingdong prefecture level city in Shandong
Jinfeng Hongyuan Flagship Store	Electricity conservation2	This is a great product.	Jingdong prefecture level city in Shandong
Jinfeng Hongyuan Flagship Store	Electricity conservation2	I'm just using it. I'll tell you more.	Jingdong prefecture level city in Shandong
Jinfeng Hongyuan Flagship Store	Electricity conservation2	Second repurchase and it's working fine.	Jingdong prefecture level city in Shandong
Jinfeng Hongyuan Flagship Store	Electricity conservation2	I'll wait a few days before evaluating	Jingdong prefecture level city in Shandong
Jinfeng Hongyuan Flagship Store	Electricity conservation2	I'll comment after I use it hopefully the section will be useful.	Jingdong prefecture level city in Shandong
Jinfeng Hongyuan Flagship Store	Electricity conservation2	not very useful	Jingdong prefecture level city in Shandong
Jinfeng Hongyuan Flagship Store	Electricity conservation2	Not received	Jingdong prefecture level city in Shandong
Jinfeng Hongyuan Flagship Store	Electricity conservation2	rare	Jingdong prefecture level city in Shandong
Jinfeng Hongyuan Flagship Store	Electricity conservation2	It's good.	Jingdong prefecture level city in Shandong
Jinfeng Hongyuan Flagship Store	Electricity conservation2	just now	Jingdong prefecture level city in Shandong
Jinfeng Hongyuan Flagship Store	Electricity conservation2	serviceable	Jingdong prefecture level city in Shandong

Jinfeng Hongyuan Flagship Store	Electricity conservation2	desirable	Jingdong prefecture level city in Shandong
Jinfeng Hongyuan Flagship Store	Electricity conservation2	serviceable	Jingdong prefecture level city in Shandong
Jinfeng Hongyuan Flagship Store	Electricity conservation2	It doesn't save power and costs electricity.	Jingdong prefecture level city in Shandong
Jinfeng Hongyuan Flagship Store	Electricity conservation2	Not as good as I thought.	Jingdong prefecture level city in Shandong
Jinfeng Hongyuan Flagship Store	Electricity conservation2	Super bad review, it doesn't save power at all, but rather more power consumption	Jingdong prefecture level city in Shandong
Jinfeng Hongyuan Flagship Store	Electricity conservation2	Listen to others say that this can save electricity, I bought with the idea of trying, used for a period of time before coming to the evaluation, will be a little bit of use, not a lot of savings just, long-term use of the words can save more.	Jingdong prefecture level city in Shandong
Jinfeng Hongyuan Flagship Store	Electricity conservation2	Logistics is fast, the next day arrived, workmanship is very delicate a power saver, looking forward to the late effect, customer service patiently explain the recommended which is suitable for, installed in what position is the best, look forward to using the effect after a period of time!	Jingdong prefecture level city in Shandong
Jinfeng Hongyuan Flagship Store	Electricity conservation2	It's good to use, I feel that it has been saving electricity, anyway, not a loss is, I used to feel that the power consumption is big, now I feel much better, my friends are also using, I hope to use a long time will be able to, I wish the boss a prosperous business	Jingdong prefecture level city in Shandong
Jinfeng Hongyuan Flagship Store	Electricity conservation2	Should be very good, to be able to plan the circuit, save the home of the circuit to the extent of the savings, waste of money on electricity, good if a month this money back, hope always hope to be able to	Jingdong prefecture level city in Shandong
Jinfeng Hongyuan Flagship Store	Electricity conservation2	Express delivery is very strong, the goods and I saw in the business consistent, the use of the effect of follow-up evaluation	Jingdong prefecture level city in Shandong
Jinfeng Hongyuan Flagship Store	Electricity conservation2	I have been using this brand of power saver for many years and this time I saw this upgraded version and got it again. The main purpose of this product is to reduce the loss of electricity for a long time, so as to achieve the purpose of saving electricity.	Jingdong prefecture level city in Shandong
Jinfeng Hongyuan Flagship Store	Electricity conservation2	Logistics is fast and the product looks beautiful, looking forward to the effect.	Jingdong prefecture level city in Shandong
Jinfeng Hongyuan Flagship Store	Electricity conservation2	Product quality is good, energy saving a little effect, but the price is a little expensive, fast arrival, good service.	Jingdong prefecture level city in Shandong
Jinfeng Hongyuan Flagship Store	Electricity conservation2	The quality is very good, inexpensive, Jingdong self-supporting guaranteed, worth having!	Jingdong prefecture level city in Shandong
Jinfeng Hongyuan Flagship Store	Electricity conservation2	I don't know if it's saving electricity yet, I'll find out next time I pay my electric bill	Jingdong prefecture level city in Shandong
Jinfeng Hongyuan Flagship Store	Electricity conservation2	Favorable price quality assurance appearance design is beautiful and generous, fine workmanship	Jingdong prefecture level city in Shandong
Jinfeng Hongyuan Flagship Store	Electricity conservation2	I bought it, I don't know how effective it is? I don't know how the quality is? After this summer results will know! Looking forward to it!	Jingdong prefecture level city in Shandong
Jinfeng Hongyuan Flagship Store	Electricity conservation2	Goods received Packaging intact, look at the shell workmanship is also good, high-power appliances open voltage has obvious changes in the effectiveness of the	Jingdong prefecture level city in Shandong
Jinfeng Hongyuan Flagship Store	Electricity conservation2	The goods have been received logistics is very fast, the product is similar to the line, just wait to use a month to see if it can save electricity, stay tuned.	Jingdong prefecture level city in Shandong
Jinfeng Hongyuan Flagship Store	Electricity conservation2	Electricity is plugged in, not sure if it will work, hopefully it will work.	Jingdong prefecture level city in Shandong
Jinfeng Hongyuan Flagship Store	Electricity conservation2	The shell is aluminum alloy, very good workmanship, the actual effect of waiting for the addition of evaluation	Jingdong prefecture level city in Shandong
Jinfeng Hongyuan Flagship Store	Electricity conservation2	Received, already plugged in, looking forward to the effect.	Jingdong prefecture level city in Shandong
Jinfeng Hongyuan Flagship Store	Electricity conservation2	Opened to see the regular manufacturer products, wait for a period of time to see it	Jingdong prefecture level city in Shandong
Jinfeng Hongyuan Flagship Store	Electricity conservation2	🉑 Baby received, very surprised ✨, reasonable design, fashionable and generous, continue to pay attention to the future will be repurchased ❥❥❥❥	Jingdong prefecture level city in Shandong
Jinfeng Hongyuan Flagship Store	Electricity conservation2	Baby received it 👌, really great ah! 🌈The quality can't be beat, very satisfied! 🆗Happy happy happy 😃	Jingdong prefecture level city in Shandong
Jinfeng Hongyuan Flagship Store	Electricity conservation2	Very good, power saving see, this is to help a friend to buy, I used to give my friend a say, my friend immediately let me order another one!	Jingdong prefecture level city in Shandong
Jinfeng Hongyuan Flagship Store	Electricity conservation2	The baby is just as introduced, the texture is very good, powerful, and it feels good to use it	Jingdong prefecture level city in Shandong
Jinfeng Hongyuan Flagship Store	Electricity conservation2	Value for money, very satisfied, seller service attitude is very good, very patient	Jingdong prefecture level city in Shandong
Jinfeng Hongyuan Flagship Store	Electricity conservation2	Power saving treasure design unique exquisite beautiful, voltage display clearly, plugged in that is good to use, I observed the meter, after using the meter is gone slow.	Jingdong prefecture level city in Shandong
Jinfeng Hongyuan Flagship Store	Electricity conservation2	Received on the use of the packaging is beautifully used for a period of time and then come back in the follow-up review!	Jingdong prefecture level city in Shandong
Jinfeng Hongyuan Flagship Store	Electricity conservation2	It should work. Not bad. Not bad. Not bad.	Jingdong prefecture level city in Shandong
Jinfeng Hongyuan Flagship Store	Electricity conservation2	I haven't used it yet, I'll plug it in and see if it saves power next month.	Jingdong prefecture level city in Shandong
Jinfeng Hongyuan Flagship Store	Electricity conservation2	The goods received and unpacked and used, the quality is very good, things and the official website to buy no difference!	Jingdong prefecture level city in Shandong
Jinfeng Hongyuan Flagship Store	Electricity conservation2	It's a rip off, doesn't help much at all, uses the same amount of power as before it was used.	Jingdong prefecture level city in Shandong
Jinfeng Hongyuan Flagship Store	Electricity conservation2	I've been using it for a while now and haven't noticed any unusual problems, so it's okay	Jingdong prefecture level city in Shandong
Jinfeng Hongyuan Flagship Store	Electricity conservation2	Received the goods and plugged in to use, good or not there is no use will have to wait until next month to find out	Jingdong prefecture level city in Shandong
Jinfeng Hongyuan Flagship Store	Electricity conservation2	It's very compact, it looks good, I'll introduce you to my clients when it's ready.	Jingdong prefecture level city in Shandong
Jinfeng Hongyuan Flagship Store	Electricity conservation2	Good workmanship, we'll have to see in a few months.	Jingdong prefecture level city in Shandong
Jinfeng Hongyuan Flagship Store	Electricity conservation2	Jingdong quality! Always trustworthy!	Jingdong prefecture level city in Shandong
Jinfeng Hongyuan Flagship Store	Electricity conservation2	This is the second time I've bought it, and the merchant said to give me five dollars out of the difference, so thank you merchant.	Jingdong prefecture level city in Shandong
Jinfeng Hongyuan Flagship Store	Electricity conservation2	Good quality and good service	Jingdong prefecture level city in Shandong
Jinfeng Hongyuan Flagship Store	Electricity conservation2	Feels good texture, aluminum alloy shell, voltage digital display clear.	Jingdong prefecture level city in Shandong
Jinfeng Hongyuan Flagship Store	Electricity conservation2	Good product, fast and great service, thanks!	Jingdong prefecture level city in Shandong
Jinfeng Hongyuan Flagship Store	Electricity conservation2	Have not yet used, first evaluation, later really good do not need to comment again.	Jingdong prefecture level city in Shandong
Jinfeng Hongyuan Flagship Store	Electricity conservation2	Works great. Pretty much as described by the merchant.	Jingdong prefecture level city in Shandong
Jinfeng Hongyuan Flagship Store	Electricity conservation2	Received it, looking forward to the results, hope it works	Jingdong prefecture level city in Shandong
Jinfeng Hongyuan Flagship Store	Electricity conservation2	Received it, looking forward to the results, hope it works	Jingdong prefecture level city in Shandong
Jinfeng Hongyuan Flagship Store	Electricity conservation2	Supposedly it's an IQ tax. Buy one and try it. Looks good. Well made.	Jingdong prefecture level city in Shandong
Jinfeng Hongyuan Flagship Store	Electricity conservation2	It just doesn't feel like it's working.	Jingdong prefecture level city in Shandong

Jinfeng Hongyuan Flagship Store	Electricity conservation2	This is a great product.	Jingdong prefecture level city in Shandong
Jinfeng Hongyuan Flagship Store	Electricity conservation2	I'm just using it. I'll tell you more.	Jingdong prefecture level city in Shandong
Jinfeng Hongyuan Flagship Store	Electricity conservation2	Second repurchase and it's working fine.	Jingdong prefecture level city in Shandong
Jinfeng Hongyuan Flagship Store	Electricity conservation2	I'll wait a few days before evaluating	Jingdong prefecture level city in Shandong
Jinfeng Hongyuan Flagship Store	Electricity conservation2	I'll comment after I use it hopefully the section will be useful.	Jingdong prefecture level city in Shandong
Jinfeng Hongyuan Flagship Store	Electricity conservation2	not very useful	Jingdong prefecture level city in Shandong
Jinfeng Hongyuan Flagship Store	Electricity conservation2	Not received	Jingdong prefecture level city in Shandong
Jinfeng Hongyuan Flagship Store	Electricity conservation2	rare	Jingdong prefecture level city in Shandong
Jinfeng Hongyuan Flagship Store	Electricity conservation2	It's good.	Jingdong prefecture level city in Shandong
Jinfeng Hongyuan Flagship Store	Electricity conservation2	just now	Jingdong prefecture level city in Shandong
Jinfeng Hongyuan Flagship Store	Electricity conservation2	serviceable	Jingdong prefecture level city in Shandong
Jinfeng Hongyuan Flagship Store	Electricity conservation2	desirable	Jingdong prefecture level city in Shandong
Jinfeng Hongyuan Flagship Store	Electricity conservation2	serviceable	Jingdong prefecture level city in Shandong
Jinfeng Hongyuan Flagship Store	Electricity conservation2	It doesn't save power and costs electricity.	Jingdong prefecture level city in Shandong
Jinfeng Hongyuan Flagship Store	Electricity conservation2	Not as good as I thought.	Jingdong prefecture level city in Shandong
Jinfeng Hongyuan Flagship Store	Electricity conservation2	Super bad review, it doesn't save power at all, but rather more power consumption	Jingdong prefecture level city in Shandong
Jinfeng Hongyuan Flagship Store	Electricity conservation2	Listen to others say that this can save electricity, I bought with the idea of trying, used for a period of time before coming to the evaluation, will be a little bit of use, not a lot of savings just, long-term use of the words can save more.	Jingdong prefecture level city in Shandong
Jinfeng Hongyuan Flagship Store	Electricity conservation2	Logistics is fast, the next day arrived, workmanship is very delicate a power saver, looking forward to the late effect, customer service patiently explain the recommended which is suitable for, installed in what position is the best, look forward to using the effect after a period of time!	Jingdong prefecture level city in Shandong
Jinfeng Hongyuan Flagship Store	Electricity conservation2	It's good to use, I feel that it has been saving electricity, anyway, not a loss is, I used to feel that the power consumption is big, now I feel much better, my friends are also using, I hope to use a long time will be able to, I wish the boss a prosperous business	Jingdong prefecture level city in Shandong
Jinfeng Hongyuan Flagship Store	Electricity conservation2	Should be very good, to be able to plan the circuit, save the home of the circuit to the extent of the savings, waste of money on electricity, good if a month this money back, hope always hope to be able to	Jingdong prefecture level city in Shandong
Jinfeng Hongyuan Flagship Store	Electricity conservation2	Express delivery is very strong, the goods and I saw in the business consistent, the use of the effect of follow-up evaluation	Jingdong prefecture level city in Shandong
Jinfeng Hongyuan Flagship Store	Electricity conservation2	I have been using this brand of power saver for many years and this time I saw this upgraded version and got it again. The main purpose of this product is to reduce the loss of electricity for a long time, so as to achieve the purpose of saving electricity.	Jingdong prefecture level city in Shandong
Jinfeng Hongyuan Flagship Store	Electricity conservation2	Logistics is fast and the product looks beautiful, looking forward to the effect.	Jingdong prefecture level city in Shandong
Jinfeng Hongyuan Flagship Store	Electricity conservation2	Product quality is good, energy saving a little effect, but the price is a little expensive, fast arrival, good service.	Jingdong prefecture level city in Shandong
Jinfeng Hongyuan Flagship Store	Electricity conservation2	The quality is very good, inexpensive, Jingdong self-supporting guaranteed, worth having!	Jingdong prefecture level city in Shandong
Jinfeng Hongyuan Flagship Store	Electricity conservation2	I don't know if it's saving electricity yet, I'll find out next time I pay my electric bill	Jingdong prefecture level city in Shandong
Jinfeng Hongyuan Flagship Store	Electricity conservation2	Favorable price quality assurance appearance design is beautiful and generous, fine workmanship	Jingdong prefecture level city in Shandong
Jinfeng Hongyuan Flagship Store	Electricity conservation2	I bought it, I don't know how effective it is? I don't know how the quality is? After this summer results will know! Looking forward to it!	Jingdong prefecture level city in Shandong
Jinfeng Hongyuan Flagship Store	Electricity conservation2	Goods received Packaging intact, look at the shell workmanship is also good, high-power appliances open voltage has obvious changes in the effectiveness of the	Jingdong prefecture level city in Shandong
Jinfeng Hongyuan Flagship Store	Electricity conservation2	The goods have been received logistics is very fast, the product is similar to the line, just wait to use a month to see if it can save electricity, stay tuned.	Jingdong prefecture level city in Shandong
Jinfeng Hongyuan Flagship Store	Electricity conservation2	Electricity is plugged in, not sure if it will work, hopefully it will work.	Jingdong prefecture level city in Shandong
Jinfeng Hongyuan Flagship Store	Electricity conservation2	The shell is aluminum alloy, very good workmanship, the actual effect of waiting for the addition of evaluation	Jingdong prefecture level city in Shandong
Jinfeng Hongyuan Flagship Store	Electricity conservation2	Received, already plugged in, looking forward to the effect.	Jingdong prefecture level city in Shandong
Jinfeng Hongyuan Flagship Store	Electricity conservation2	Opened to see the regular manufacturer products, wait for a period of time to see it	Jingdong prefecture level city in Shandong
Jinfeng Hongyuan Flagship Store	Electricity conservation2	🉑 Baby received, very surprised ✨, reasonable design, fashionable and generous, continue to pay attention to the future will be repurchased ❥❥❥❥	Jingdong prefecture level city in Shandong
Jinfeng Hongyuan Flagship Store	Electricity conservation2	Baby received it 👌, really great ah! 🌈The quality can't be beat, very satisfied! 🆗Happy happy happy 😃	Jingdong prefecture level city in Shandong
Jinfeng Hongyuan Flagship Store	Electricity conservation2	Very good, power saving see, this is to help a friend to buy, I used to give my friend a say, my friend immediately let me order another one!	Jingdong prefecture level city in Shandong
Jinfeng Hongyuan Flagship Store	Electricity conservation2	The baby is just as introduced, the texture is very good, powerful, and it feels good to use it	Jingdong prefecture level city in Shandong
Jinfeng Hongyuan Flagship Store	Electricity conservation2	Value for money, very satisfied, seller service attitude is very good, very patient	Jingdong prefecture level city in Shandong
Jinfeng Hongyuan Flagship Store	Electricity conservation2	Power saving treasure design unique exquisite beautiful, voltage display clearly, plugged in that is good to use, I observed the meter, after using the meter is gone slow.	Jingdong prefecture level city in Shandong
Jinfeng Hongyuan Flagship Store	Electricity conservation2	Received on the use of the packaging is beautifully used for a period of time and then come back in the follow-up review!	Jingdong prefecture level city in Shandong
Jinfeng Hongyuan Flagship Store	Electricity conservation2	It should work. Not bad. Not bad. Not bad.	Jingdong prefecture level city in Shandong
Jinfeng Hongyuan Flagship Store	Electricity conservation2	I haven't used it yet, I'll plug it in and see if it saves power next month.	Jingdong prefecture level city in Shandong
Jinfeng Hongyuan Flagship Store	Electricity conservation2	The goods received and unpacked and used, the quality is very good, things and the official website to buy no difference!	Jingdong prefecture level city in Shandong
Jinfeng Hongyuan Flagship Store	Electricity conservation2	It's a rip off, doesn't help much at all, uses the same amount of power as before it was used.	Jingdong prefecture level city in Shandong
Jinfeng Hongyuan Flagship Store	Electricity conservation2	I've been using it for a while now and haven't noticed any unusual problems, so it's okay	Jingdong prefecture level city in Shandong
Jinfeng Hongyuan Flagship Store	Electricity conservation2	Received the goods and plugged in to use, good or not there is no use will have to wait until next month to find out	Jingdong prefecture level city in Shandong
Jinfeng Hongyuan Flagship Store	Electricity conservation2	It's very compact, it looks good, I'll introduce you to my clients when it's ready.	Jingdong prefecture level city in Shandong
Jinfeng Hongyuan Flagship Store	Electricity conservation2	Good workmanship, we'll have to see in a few months.	Jingdong prefecture level city in Shandong

Jinfeng Hongyuan Flagship Store	Electricity conservation2	Jingdong quality! Always trustworthy!	Jingdong prefecture level city in Shandong
Jinfeng Hongyuan Flagship Store	Electricity conservation2	This is the second time I've bought it, and the merchant said to give me five dollars out of the difference, so thank you merchant.	Jingdong prefecture level city in Shandong
Jinfeng Hongyuan Flagship Store	Electricity conservation2	Good quality and good service	Jingdong prefecture level city in Shandong
Jinfeng Hongyuan Flagship Store	Electricity conservation2	Feels good texture, aluminum alloy shell, voltage digital display clear.	Jingdong prefecture level city in Shandong
Jinfeng Hongyuan Flagship Store	Electricity conservation2	Good product, fast and great service, thanks!	Jingdong prefecture level city in Shandong
Jinfeng Hongyuan Flagship Store	Electricity conservation2	Have not yet used, first evaluation, later really good do not need to comment again.	Jingdong prefecture level city in Shandong
Jinfeng Hongyuan Flagship Store	Electricity conservation2	Works great. Pretty much as described by the merchant.	Jingdong prefecture level city in Shandong
Jinfeng Hongyuan Flagship Store	Electricity conservation2	Received it, looking forward to the results, hope it works	Jingdong prefecture level city in Shandong
Jinfeng Hongyuan Flagship Store	Electricity conservation2	Received it, looking forward to the results, hope it works	Jingdong prefecture level city in Shandong
Jinfeng Hongyuan Flagship Store	Electricity conservation2	Supposedly it's an IQ tax. Buy one and try it. Looks good. Well made.	Jingdong prefecture level city in Shandong
Jinfeng Hongyuan Flagship Store	Electricity conservation2	It just doesn't feel like it's working.	Jingdong prefecture level city in Shandong
Jinfeng Hongyuan Flagship Store	Electricity conservation2	This is a great product.	Jingdong prefecture level city in Shandong
Jinfeng Hongyuan Flagship Store	Electricity conservation2	I'm just using it. I'll tell you more.	Jingdong prefecture level city in Shandong
Jinfeng Hongyuan Flagship Store	Electricity conservation2	Second repurchase and it's working fine.	Jingdong prefecture level city in Shandong
Jinfeng Hongyuan Flagship Store	Electricity conservation2	I'll wait a few days before evaluating	Jingdong prefecture level city in Shandong
Jinfeng Hongyuan Flagship Store	Electricity conservation2	I'll comment after I use it hopefully the section will be useful.	Jingdong prefecture level city in Shandong
Jinfeng Hongyuan Flagship Store	Electricity conservation2	not very useful	Jingdong prefecture level city in Shandong
Jinfeng Hongyuan Flagship Store	Electricity conservation2	Not received	Jingdong prefecture level city in Shandong
Jinfeng Hongyuan Flagship Store	Electricity conservation2	rare	Jingdong prefecture level city in Shandong
Jinfeng Hongyuan Flagship Store	Electricity conservation2	It's good.	Jingdong prefecture level city in Shandong
Jinfeng Hongyuan Flagship Store	Electricity conservation2	just now	Jingdong prefecture level city in Shandong
Jinfeng Hongyuan Flagship Store	Electricity conservation2	serviceable	Jingdong prefecture level city in Shandong
Jinfeng Hongyuan Flagship Store	Electricity conservation2	desirable	Jingdong prefecture level city in Shandong
Jinfeng Hongyuan Flagship Store	Electricity conservation2	serviceable	Jingdong prefecture level city in Shandong
Jinfeng Hongyuan Flagship Store	Electricity conservation2	It doesn't save power and costs electricity.	Jingdong prefecture level city in Shandong
Jinfeng Hongyuan Flagship Store	Electricity conservation2	Not as good as I thought.	Jingdong prefecture level city in Shandong
Jinfeng Hongyuan Flagship Store	Electricity conservation2	Super bad review, it doesn't save power at all, but rather more power consumption	Jingdong prefecture level city in Shandong
Jinfeng Hongyuan Flagship Store	Electricity conservation2	Listen to others say that this can save electricity, I bought with the idea of trying, used for a period of time before coming to the evaluation, will be a little bit of use, not a lot of savings just, long-term use of the words can save more.	Jingdong prefecture level city in Shandong
Jinfeng Hongyuan Flagship Store	Electricity conservation2	Logistics is fast, the next day arrived, workmanship is very delicate a power saver, looking forward to the late effect, customer service patiently explain the recommended which is suitable for, installed in what position is the best, look forward to using the effect after a period of time!	Jingdong prefecture level city in Shandong
Jinfeng Hongyuan Flagship Store	Electricity conservation2	It's good to use, I feel that it has been saving electricity, anyway, not a loss is, I used to feel that the power consumption is big, now I feel much better, my friends are also using, I hope to use a long time will be able to, I wish the boss a prosperous business	Jingdong prefecture level city in Shandong
Jinfeng Hongyuan Flagship Store	Electricity conservation2	Should be very good, to be able to plan the circuit, save the home of the circuit to the extent of the savings, waste of money on electricity, good if a month this money back, hope always hope to be able to	Jingdong prefecture level city in Shandong
Jinfeng Hongyuan Flagship Store	Electricity conservation2	Express delivery is very strong, the goods and I saw in the business consistent, the use of the effect of follow-up evaluation	Jingdong prefecture level city in Shandong
Jinfeng Hongyuan Flagship Store	Electricity conservation2	I have been using this brand of power saver for many years and this time I saw this upgraded version and got it again. The main purpose of this product is to reduce the loss of electricity for a long time, so as to achieve the purpose of saving electricity.	Jingdong prefecture level city in Shandong
Jinfeng Hongyuan Flagship Store	Electricity conservation2	Logistics is fast and the product looks beautiful, looking forward to the effect.	Jingdong prefecture level city in Shandong
Jinfeng Hongyuan Flagship Store	Electricity conservation2	Product quality is good, energy saving a little effect, but the price is a little expensive, fast arrival, good service.	Jingdong prefecture level city in Shandong
Jinfeng Hongyuan Flagship Store	Electricity conservation2	The quality is very good, inexpensive, Jingdong self-supporting guaranteed, worth having!	Jingdong prefecture level city in Shandong
Jinfeng Hongyuan Flagship Store	Electricity conservation2	I don't know if it's saving electricity yet, I'll find out next time I pay my electric bill	Jingdong prefecture level city in Shandong
Jinfeng Hongyuan Flagship Store	Electricity conservation2	Favorable price quality assurance appearance design is beautiful and generous, fine workmanship	Jingdong prefecture level city in Shandong
Jinfeng Hongyuan Flagship Store	Electricity conservation2	I bought it, I don't know how effective it is? I don't know how the quality is? After this summer results will know! Looking forward to it!	Jingdong prefecture level city in Shandong
Jinfeng Hongyuan Flagship Store	Electricity conservation2	Goods received Packaging intact, look at the shell workmanship is also good, high-power appliances open voltage has obvious changes in the effectiveness of the	Jingdong prefecture level city in Shandong
Jinfeng Hongyuan Flagship Store	Electricity conservation2	The goods have been received logistics is very fast, the product is similar to the line, just wait to use a month to see if it can save electricity, stay tuned.	Jingdong prefecture level city in Shandong
Jinfeng Hongyuan Flagship Store	Electricity conservation2	Electricity is plugged in, not sure if it will work, hopefully it will work.	Jingdong prefecture level city in Shandong
Jinfeng Hongyuan Flagship Store	Electricity conservation2	The shell is aluminum alloy, very good workmanship, the actual effect of waiting for the addition of evaluation	Jingdong prefecture level city in Shandong
Jinfeng Hongyuan Flagship Store	Electricity conservation2	Received, already plugged in, looking forward to the effect.	Jingdong prefecture level city in Shandong
Jinfeng Hongyuan Flagship Store	Electricity conservation2	Opened to see the regular manufacturer products, wait for a period of time to see it	Jingdong prefecture level city in Shandong
Jinfeng Hongyuan Flagship Store	Electricity conservation2	Baby received, very surprised , reasonable design, fashionable and generous, continue to pay attention to the future will be repurchased	Jingdong prefecture level city in Shandong
Jinfeng Hongyuan Flagship Store	Electricity conservation2	Baby received it , really great ah! The quality can't be beat, very satisfied! OK Happy happy happy	Jingdong prefecture level city in Shandong
Jinfeng Hongyuan Flagship Store	Electricity conservation2	Very good, power saving see, this is to help a friend to buy, I used to give my friend a say, my friend immediately let me order another one!	Jingdong prefecture level city in Shandong
Jinfeng Hongyuan Flagship Store	Electricity conservation2	The baby is just as introduced, the texture is very good, powerful, and it feels good to use it	Jingdong prefecture level city in Shandong

Jinfeng Hongyuan Flagship Store	Electricity conservation2	Value for money, very satisfied, seller service attitude is very good, very patient	Jingdong prefecture level city in Shandong
Jinfeng Hongyuan Flagship Store	Electricity conservation2	Power saving treasure design unique exquisite beautiful, voltage display clearly, plugged in that is good to use, I observed the meter, after using the meter is gone slow.	Jingdong prefecture level city in Shandong
Jinfeng Hongyuan Flagship Store	Electricity conservation2	Received on the use of the packaging is beautifully used for a period of time and then come back in the follow-up review!	Jingdong prefecture level city in Shandong
Jinfeng Hongyuan Flagship Store	Electricity conservation2	It should work. Not bad. Not bad. Not bad.	Jingdong prefecture level city in Shandong
Jinfeng Hongyuan Flagship Store	Electricity conservation2	I haven't used it yet, I'll plug it in and see if it saves power next month.	Jingdong prefecture level city in Shandong
Jinfeng Hongyuan Flagship Store	Electricity conservation2	The goods received and unpacked and used, the quality is very good, things and the official website to buy no difference!	Jingdong prefecture level city in Shandong
Jinfeng Hongyuan Flagship Store	Electricity conservation2	It's a rip off, doesn't help much at all, uses the same amount of power as before it was used.	Jingdong prefecture level city in Shandong
Jinfeng Hongyuan Flagship Store	Electricity conservation2	I've been using it for a while now and haven't noticed any unusual problems, so it's okay	Jingdong prefecture level city in Shandong
Jinfeng Hongyuan Flagship Store	Electricity conservation2	Received the goods and plugged in to use, good or not there is no use will have to wait until next month to find out	Jingdong prefecture level city in Shandong
Jinfeng Hongyuan Flagship Store	Electricity conservation2	It's very compact, it looks good, I'll introduce you to my clients when it's ready.	Jingdong prefecture level city in Shandong
Jinfeng Hongyuan Flagship Store	Electricity conservation2	Good workmanship, we'll have to see in a few months.	Jingdong prefecture level city in Shandong
Jinfeng Hongyuan Flagship Store	Electricity conservation2	Jingdong quality! Always trustworthy!	Jingdong prefecture level city in Shandong
Jinfeng Hongyuan Flagship Store	Electricity conservation2	This is the second time I've bought it, and the merchant said to give me five dollars out of the difference, so thank you merchant.	Jingdong prefecture level city in Shandong
Jinfeng Hongyuan Flagship Store	Electricity conservation2	Good quality and good service	Jingdong prefecture level city in Shandong
Jinfeng Hongyuan Flagship Store	Electricity conservation2	Feels good texture, aluminum alloy shell, voltage digital display clear.	Jingdong prefecture level city in Shandong
Jinfeng Hongyuan Flagship Store	Electricity conservation2	Good product, fast and great service, thanks!	Jingdong prefecture level city in Shandong
Jinfeng Hongyuan Flagship Store	Electricity conservation2	Have not yet used, first evaluation, later really good do not need to comment again.	Jingdong prefecture level city in Shandong
Jinfeng Hongyuan Flagship Store	Electricity conservation2	Works great. Pretty much as described by the merchant.	Jingdong prefecture level city in Shandong
Jinfeng Hongyuan Flagship Store	Electricity conservation2	Received it, looking forward to the results, hope it works	Jingdong prefecture level city in Shandong
Jinfeng Hongyuan Flagship Store	Electricity conservation2	Received it, looking forward to the results, hope it works	Jingdong prefecture level city in Shandong
Jinfeng Hongyuan Flagship Store	Electricity conservation2	Supposedly it's an IQ tax. Buy one and try it. Looks good. Well made.	Jingdong prefecture level city in Shandong
Jinfeng Hongyuan Flagship Store	Electricity conservation2	It just doesn't feel like it's working.	Jingdong prefecture level city in Shandong
Jinfeng Hongyuan Flagship Store	Electricity conservation2	This is a great product.	Jingdong prefecture level city in Shandong
Jinfeng Hongyuan Flagship Store	Electricity conservation2	I'm just using it. I'll tell you more.	Jingdong prefecture level city in Shandong
Jinfeng Hongyuan Flagship Store	Electricity conservation2	Second repurchase and it's working fine.	Jingdong prefecture level city in Shandong
Jinfeng Hongyuan Flagship Store	Electricity conservation2	I'll wait a few days before evaluating	Jingdong prefecture level city in Shandong
Jinfeng Hongyuan Flagship Store	Electricity conservation2	I'll comment after I use it hopefully the section will be useful.	Jingdong prefecture level city in Shandong
Jinfeng Hongyuan Flagship Store	Electricity conservation2	not very useful	Jingdong prefecture level city in Shandong
Jinfeng Hongyuan Flagship Store	Electricity conservation2	Not received	Jingdong prefecture level city in Shandong
Jinfeng Hongyuan Flagship Store	Electricity conservation2	rare	Jingdong prefecture level city in Shandong
Jinfeng Hongyuan Flagship Store	Electricity conservation2	It's good.	Jingdong prefecture level city in Shandong
Jinfeng Hongyuan Flagship Store	Electricity conservation2	just now	Jingdong prefecture level city in Shandong
Jinfeng Hongyuan Flagship Store	Electricity conservation2	serviceable	Jingdong prefecture level city in Shandong
Jinfeng Hongyuan Flagship Store	Electricity conservation2	desirable	Jingdong prefecture level city in Shandong
Jinfeng Hongyuan Flagship Store	Electricity conservation2	serviceable	Jingdong prefecture level city in Shandong
Jinfeng Hongyuan Flagship Store	Electricity conservation2	It doesn't save power and costs electricity.	Jingdong prefecture level city in Shandong
Jinfeng Hongyuan Flagship Store	Electricity conservation2	Not as good as I thought.	Jingdong prefecture level city in Shandong
Jinfeng Hongyuan Flagship Store	Electricity conservation2	Super bad review, it doesn't save power at all, but rather more power consumption	Jingdong prefecture level city in Shandong
Jinfeng Hongyuan Flagship Store	Electricity conservation2	Listen to others say that this can save electricity, I bought with the idea of trying, used for a period of time before coming to the evaluation, will be a little bit of use, not a lot of savings just, long-term use of the words can save more.	Jingdong prefecture level city in Shandong
Jinfeng Hongyuan Flagship Store	Electricity conservation2	Logistics is fast, the next day arrived, workmanship is very delicate a power saver, looking forward to the late effect, customer service patiently explain the recommended which is suitable for, installed in what position is the best, look forward to using the effect after a period of time!	Jingdong prefecture level city in Shandong
Jinfeng Hongyuan Flagship Store	Electricity conservation2	It's good to use, I feel that it has been saving electricity, anyway, not a loss is, I used to feel that the power consumption is big, now I feel much better, my friends are also using, I hope to use a long time will be able to, I wish the boss a prosperous business	Jingdong prefecture level city in Shandong
Jinfeng Hongyuan Flagship Store	Electricity conservation2	Should be very good, to be able to plan the circuit, save the home of the circuit to the extent of the savings, waste of money on electricity, good if a month this money back, hope always hope to be able to	Jingdong prefecture level city in Shandong
Jinfeng Hongyuan Flagship Store	Electricity conservation2	Express delivery is very strong, the goods and I saw in the business consistent, the use of the effect of follow-up evaluation	Jingdong prefecture level city in Shandong
Jinfeng Hongyuan Flagship Store	Electricity conservation2	I have been using this brand of power saver for many years and this time I saw this upgraded version and got it again. The main purpose of this product is to reduce the loss of electricity for a long time, so as to achieve the purpose of saving electricity.	Jingdong prefecture level city in Shandong
Jinfeng Hongyuan Flagship Store	Electricity conservation2	Logistics is fast and the product looks beautiful, looking forward to the effect.	Jingdong prefecture level city in Shandong
Jinfeng Hongyuan Flagship Store	Electricity conservation2	Product quality is good, energy saving a little effect, but the price is a little expensive, fast arrival, good service.	Jingdong prefecture level city in Shandong
Jinfeng Hongyuan Flagship Store	Electricity conservation2	The quality is very good, inexpensive, Jingdong self-supporting guaranteed, worth having!	Jingdong prefecture level city in Shandong
Jinfeng Hongyuan Flagship Store	Electricity conservation2	I don't know if it's saving electricity yet, I'll find out next time I pay my electric bill	Jingdong prefecture level city in Shandong
Jinfeng Hongyuan Flagship Store	Electricity conservation2	Favorable price quality assurance appearance design is beautiful and generous, fine workmanship	Jingdong prefecture level city in Shandong

Jinfeng Hongyuan Flagship Store	Electricity conservation2	I bought it, I don't know how effective it is? I don't know how the quality is? After this summer results will know! Looking forward to it!	Jingdong prefecture level city in Shandong
Jinfeng Hongyuan Flagship Store	Electricity conservation2	Goods received Packaging intact, look at the shell workmanship is also good, high-power appliances open voltage has obvious changes in the effectiveness of the	Jingdong prefecture level city in Shandong
Jinfeng Hongyuan Flagship Store	Electricity conservation2	The goods have been received logistics is very fast, the product is similar to the line, just wait to use a month to see if it can save electricity, stay tuned.	Jingdong prefecture level city in Shandong
Jinfeng Hongyuan Flagship Store	Electricity conservation2	Electricity is plugged in, not sure if it will work, hopefully it will work.	Jingdong prefecture level city in Shandong
Jinfeng Hongyuan Flagship Store	Electricity conservation2	The shell is aluminum alloy, very good workmanship, the actual effect of waiting for the addition of evaluation	Jingdong prefecture level city in Shandong
Jinfeng Hongyuan Flagship Store	Electricity conservation2	Received, already plugged in, looking forward to the effect.	Jingdong prefecture level city in Shandong
Jinfeng Hongyuan Flagship Store	Electricity conservation2	Opened to see the regular manufacturer products, wait for a period of time to see it	Jingdong prefecture level city in Shandong
Jinfeng Hongyuan Flagship Store	Electricity conservation2	🉑 Baby received, very surprised ✨, reasonable design, fashionable and generous, continue to pay attention to the future will be repurchased ❥❥❥❥	Jingdong prefecture level city in Shandong
Jinfeng Hongyuan Flagship Store	Electricity conservation2	Baby received it 👌, really great ah! 🌈The quality can't be beat, very satisfied! 🆗Happy happy happy 😃	Jingdong prefecture level city in Shandong
Jinfeng Hongyuan Flagship Store	Electricity conservation2	Very good, power saving see, this is to help a friend to buy, I used to give my friend a say, my friend immediately let me order another one!	Jingdong prefecture level city in Shandong
Jinfeng Hongyuan Flagship Store	Electricity conservation2	The baby is just as introduced, the texture is very good, powerful, and it feels good to use it	Jingdong prefecture level city in Shandong
Jinfeng Hongyuan Flagship Store	Electricity conservation2	Value for money, very satisfied, seller service attitude is very good, very patient	Jingdong prefecture level city in Shandong
Jinfeng Hongyuan Flagship Store	Electricity conservation2	Power saving treasure design unique exquisite beautiful, voltage display clearly, plugged in that is good to use, I observed the meter, after using the meter is gone slow.	Jingdong prefecture level city in Shandong
Jinfeng Hongyuan Flagship Store	Electricity conservation2	Received on the use of the packaging is beautifully used for a period of time and then come back in the follow-up review!	Jingdong prefecture level city in Shandong
Jinfeng Hongyuan Flagship Store	Electricity conservation2	It should work. Not bad. Not bad. Not bad.	Jingdong prefecture level city in Shandong
Jinfeng Hongyuan Flagship Store	Electricity conservation2	I haven't used it yet, I'll plug it in and see if it saves power next month.	Jingdong prefecture level city in Shandong
Jinfeng Hongyuan Flagship Store	Electricity conservation2	The goods received and unpacked and used, the quality is very good, things and the official website to buy no difference!	Jingdong prefecture level city in Shandong
Jinfeng Hongyuan Flagship Store	Electricity conservation2	It's a rip off, doesn't help much at all, uses the same amount of power as before it was used.	Jingdong prefecture level city in Shandong
Jinfeng Hongyuan Flagship Store	Electricity conservation2	I've been using it for a while now and haven't noticed any unusual problems, so it's okay	Jingdong prefecture level city in Shandong
Jinfeng Hongyuan Flagship Store	Electricity conservation2	Received the goods and plugged in to use, good or not there is no use will have to wait until next month to find out	Jingdong prefecture level city in Shandong
Jinfeng Hongyuan Flagship Store	Electricity conservation2	It's very compact, it looks good, I'll introduce you to my clients when it's ready.	Jingdong prefecture level city in Shandong
Jinfeng Hongyuan Flagship Store	Electricity conservation2	Good workmanship, we'll have to see in a few months.	Jingdong prefecture level city in Shandong
Jinfeng Hongyuan Flagship Store	Electricity conservation2	Jingdong quality! Always trustworthy!	Jingdong prefecture level city in Shandong
Jinfeng Hongyuan Flagship Store	Electricity conservation2	This is the second time I've bought it, and the merchant said to give me five dollars out of the difference, so thank you merchant.	Jingdong prefecture level city in Shandong
Jinfeng Hongyuan Flagship Store	Electricity conservation2	Good quality and good service	Jingdong prefecture level city in Shandong
Jinfeng Hongyuan Flagship Store	Electricity conservation2	Feels good texture, aluminum alloy shell, voltage digital display clear.	Jingdong prefecture level city in Shandong
Jinfeng Hongyuan Flagship Store	Electricity conservation2	Good product, fast and great service, thanks!	Jingdong prefecture level city in Shandong
Jinfeng Hongyuan Flagship Store	Electricity conservation2	Have not yet used, first evaluation, later really good do not need to comment again.	Jingdong prefecture level city in Shandong
Jinfeng Hongyuan Flagship Store	Electricity conservation2	Works great. Pretty much as described by the merchant.	Jingdong prefecture level city in Shandong
Jinfeng Hongyuan Flagship Store	Electricity conservation2	Received it, looking forward to the results, hope it works	Jingdong prefecture level city in Shandong
Jinfeng Hongyuan Flagship Store	Electricity conservation2	Received it, looking forward to the results, hope it works	Jingdong prefecture level city in Shandong
Jinfeng Hongyuan Flagship Store	Electricity conservation2	Supposedly it's an IQ tax. Buy one and try it. Looks good. Well made.	Jingdong prefecture level city in Shandong
Jinfeng Hongyuan Flagship Store	Electricity conservation2	It just doesn't feel like it's working.	Jingdong prefecture level city in Shandong
Jinfeng Hongyuan Flagship Store	Electricity conservation2	This is a great product.	Jingdong prefecture level city in Shandong
Jinfeng Hongyuan Flagship Store	Electricity conservation2	I'm just using it. I'll tell you more.	Jingdong prefecture level city in Shandong
Jinfeng Hongyuan Flagship Store	Electricity conservation2	Second repurchase and it's working fine.	Jingdong prefecture level city in Shandong
Jinfeng Hongyuan Flagship Store	Electricity conservation2	I'll wait a few days before evaluating	Jingdong prefecture level city in Shandong
Jinfeng Hongyuan Flagship Store	Electricity conservation2	I'll comment after I use it hopefully the section will be useful.	Jingdong prefecture level city in Shandong
Jinfeng Hongyuan Flagship Store	Electricity conservation2	not very useful	Jingdong prefecture level city in Shandong
Jinfeng Hongyuan Flagship Store	Electricity conservation2	Not received	Jingdong prefecture level city in Shandong
Jinfeng Hongyuan Flagship Store	Electricity conservation2	rare	Jingdong prefecture level city in Shandong
Jinfeng Hongyuan Flagship Store	Electricity conservation2	It's good.	Jingdong prefecture level city in Shandong
Jinfeng Hongyuan Flagship Store	Electricity conservation2	just now	Jingdong prefecture level city in Shandong
Jinfeng Hongyuan Flagship Store	Electricity conservation2	serviceable	Jingdong prefecture level city in Shandong
Jinfeng Hongyuan Flagship Store	Electricity conservation2	desirable	Jingdong prefecture level city in Shandong
Jinfeng Hongyuan Flagship Store	Electricity conservation2	serviceable	Jingdong prefecture level city in Shandong
Jinfeng Hongyuan Flagship Store	Electricity conservation2	It doesn't save power and costs electricity.	Jingdong prefecture level city in Shandong
Jinfeng Hongyuan Flagship Store	Electricity conservation2	Not as good as I thought.	Jingdong prefecture level city in Shandong
Jinfeng Hongyuan Flagship Store	Electricity conservation2	Super bad review, it doesn't save power at all, but rather more power consumption	Jingdong prefecture level city in Shandong
Jinfeng Hongyuan Flagship Store	Electricity conservation2	Listen to others say that this can save electricity, I bought with the idea of trying, used for a period of time before coming to the evaluation, will be a little bit of use, not a lot of savings just, long-term use of the words can save more.	Jingdong prefecture level city in Shandong

Jinfeng Hongyuan Flagship Store	Electricity conservation2	Logistics is fast, the next day arrived, workmanship is very delicate a power saver, looking forward to the late effect, customer service patiently explain the recommended which is suitable for, installed in what position is the best, look forward to using the effect after a period of time!	Jingdong prefecture level city in Shandong
Jinfeng Hongyuan Flagship Store	Electricity conservation2	It's good to use, I feel that it has been saving electricity, anyway, not a loss is, I used to feel that the power consumption is big, now I feel much better, my friends are also using, I hope to use a long time will be able to, I wish the boss a prosperous business	Jingdong prefecture level city in Shandong
Jinfeng Hongyuan Flagship Store	Electricity conservation2	Should be very good, to be able to plan the circuit, save the home of the circuit to the extent of the savings, waste of money on electricity, good if a month this money back, hope always hope to be able to	Jingdong prefecture level city in Shandong
Jinfeng Hongyuan Flagship Store	Electricity conservation2	Express delivery is very strong, the goods and I saw in the business consistent, the use of the effect of follow-up evaluation	Jingdong prefecture level city in Shandong
Jinfeng Hongyuan Flagship Store	Electricity conservation2	I have been using this brand of power saver for many years and this time I saw this upgraded version and got it again. The main purpose of this product is to reduce the loss of electricity for a long time, so as to achieve the purpose of saving electricity.	Jingdong prefecture level city in Shandong
Jinfeng Hongyuan Flagship Store	Electricity conservation2	Logistics is fast and the product looks beautiful, looking forward to the effect.	Jingdong prefecture level city in Shandong
Jinfeng Hongyuan Flagship Store	Electricity conservation2	Product quality is good, energy saving a little effect, but the price is a little expensive, fast arrival, good service.	Jingdong prefecture level city in Shandong
Jinfeng Hongyuan Flagship Store	Electricity conservation2	The quality is very good, inexpensive, Jingdong self-supporting guaranteed, worth having!	Jingdong prefecture level city in Shandong
Jinfeng Hongyuan Flagship Store	Electricity conservation2	I don't know if it's saving electricity yet, I'll find out next time I pay my electric bill	Jingdong prefecture level city in Shandong
Jinfeng Hongyuan Flagship Store	Electricity conservation2	Favorable price quality assurance appearance design is beautiful and generous, fine workmanship	Jingdong prefecture level city in Shandong
Jinfeng Hongyuan Flagship Store	Electricity conservation2	I bought it, I don't know how effective it is? I don't know how the quality is? After this summer results will know! Looking forward to it!	Jingdong prefecture level city in Shandong
Jinfeng Hongyuan Flagship Store	Electricity conservation2	Goods received Packaging intact, look at the shell workmanship is also good, high-power appliances open voltage has obvious changes in the effectiveness of the	Jingdong prefecture level city in Shandong
Jinfeng Hongyuan Flagship Store	Electricity conservation2	The goods have been received logistics is very fast, the product is similar to the line, just wait to use a month to see if it can save electricity, stay tuned.	Jingdong prefecture level city in Shandong
Jinfeng Hongyuan Flagship Store	Electricity conservation2	Electricity is plugged in, not sure if it will work, hopefully it will work.	Jingdong prefecture level city in Shandong
Jinfeng Hongyuan Flagship Store	Electricity conservation2	The shell is aluminum alloy, very good workmanship, the actual effect of waiting for the addition of evaluation	Jingdong prefecture level city in Shandong
Jinfeng Hongyuan Flagship Store	Electricity conservation2	Received, already plugged in, looking forward to the effect.	Jingdong prefecture level city in Shandong
Jinfeng Hongyuan Flagship Store	Electricity conservation2	Opened to see the regular manufacturer products, wait for a period of time to see it	Jingdong prefecture level city in Shandong
Jinfeng Hongyuan Flagship Store	Electricity conservation2	🉑 Baby received, very surprised ✨, reasonable design, fashionable and generous, continue to pay attention to the future will be repurchased ❥❥❥❥	Jingdong prefecture level city in Shandong
Jinfeng Hongyuan Flagship Store	Electricity conservation2	Baby received it 👌, really great ah! 🌈The quality can't be beat, very satisfied! 🆗Happy happy happy 😃	Jingdong prefecture level city in Shandong
Jinfeng Hongyuan Flagship Store	Electricity conservation2	Very good, power saving see, this is to help a friend to buy, I used to give my friend a say, my friend immediately let me order another one!	Jingdong prefecture level city in Shandong
Jinfeng Hongyuan Flagship Store	Electricity conservation2	The baby is just as introduced, the texture is very good, powerful, and it feels good to use it	Jingdong prefecture level city in Shandong
Jinfeng Hongyuan Flagship Store	Electricity conservation2	Value for money, very satisfied, seller service attitude is very good, very patient	Jingdong prefecture level city in Shandong
Jinfeng Hongyuan Flagship Store	Electricity conservation2	Power saving treasure design unique exquisite beautiful, voltage display clearly, plugged in that is good to use, I observed the meter, after using the meter is gone slow.	Jingdong prefecture level city in Shandong
Jinfeng Hongyuan Flagship Store	Electricity conservation2	Received on the use of the packaging is beautifully used for a period of time and then come back in the follow-up review!	Jingdong prefecture level city in Shandong
Jinfeng Hongyuan Flagship Store	Electricity conservation2	It should work. Not bad. Not bad. Not bad.	Jingdong prefecture level city in Shandong
Jinfeng Hongyuan Flagship Store	Electricity conservation2	I haven't used it yet, I'll plug it in and see if it saves power next month.	Jingdong prefecture level city in Shandong
Jinfeng Hongyuan Flagship Store	Electricity conservation2	The goods received and unpacked and used, the quality is very good, things and the official website to buy no difference!	Jingdong prefecture level city in Shandong
Jinfeng Hongyuan Flagship Store	Electricity conservation2	It's a rip off, doesn't help much at all, uses the same amount of power as before it was used.	Jingdong prefecture level city in Shandong
Jinfeng Hongyuan Flagship Store	Electricity conservation2	I've been using it for a while now and haven't noticed any unusual problems, so it's okay	Jingdong prefecture level city in Shandong
Jinfeng Hongyuan Flagship Store	Electricity conservation2	Received the goods and plugged in to use, good or not there is no use will have to wait until next month to find out	Jingdong prefecture level city in Shandong
Jinfeng Hongyuan Flagship Store	Electricity conservation2	It's very compact, it looks good, I'll introduce you to my clients when it's ready.	Jingdong prefecture level city in Shandong
Jinfeng Hongyuan Flagship Store	Electricity conservation2	Good workmanship, we'll have to see in a few months.	Jingdong prefecture level city in Shandong
Jinfeng Hongyuan Flagship Store	Electricity conservation2	Jingdong quality! Always trustworthy!	Jingdong prefecture level city in Shandong
Jinfeng Hongyuan Flagship Store	Electricity conservation2	This is the second time I've bought it, and the merchant said to give me five dollars out of the difference, so thank you merchant.	Jingdong prefecture level city in Shandong
Jinfeng Hongyuan Flagship Store	Electricity conservation2	Good quality and good service	Jingdong prefecture level city in Shandong
Jinfeng Hongyuan Flagship Store	Electricity conservation2	Feels good texture, aluminum alloy shell, voltage digital display clear.	Jingdong prefecture level city in Shandong
Jinfeng Hongyuan Flagship Store	Electricity conservation2	Good product, fast and great service, thanks!	Jingdong prefecture level city in Shandong
Jinfeng Hongyuan Flagship Store	Electricity conservation2	Have not yet used, first evaluation, later really good do not need to comment again.	Jingdong prefecture level city in Shandong
Jinfeng Hongyuan Flagship Store	Electricity conservation2	Works great. Pretty much as described by the merchant.	Jingdong prefecture level city in Shandong
Jinfeng Hongyuan Flagship Store	Electricity conservation2	Received it, looking forward to the results, hope it works	Jingdong prefecture level city in Shandong
Jinfeng Hongyuan Flagship Store	Electricity conservation2	Received it, looking forward to the results, hope it works	Jingdong prefecture level city in Shandong
Jinfeng Hongyuan Flagship Store	Electricity conservation2	Supposedly it's an IQ tax. Buy one and try it. Looks good. Well made.	Jingdong prefecture level city in Shandong
Jinfeng Hongyuan Flagship Store	Electricity conservation2	It just doesn't feel like it's working.	Jingdong prefecture level city in Shandong
Jinfeng Hongyuan Flagship Store	Electricity conservation2	This is a great product.	Jingdong prefecture level city in Shandong
Jinfeng Hongyuan Flagship Store	Electricity conservation2	I'm just using it. I'll tell you more.	Jingdong prefecture level city in Shandong
Jinfeng Hongyuan Flagship Store	Electricity conservation2	Second repurchase and it's working fine.	Jingdong prefecture level city in Shandong
Jinfeng Hongyuan Flagship Store	Electricity conservation2	I'll wait a few days before evaluating	Jingdong prefecture level city in Shandong
Jinfeng Hongyuan Flagship Store	Electricity conservation2	I'll comment after I use it hopefully the section will be useful.	Jingdong prefecture level city in Shandong
Jinfeng Hongyuan Flagship Store	Electricity conservation2	not very useful	Jingdong prefecture level city in Shandong
Jinfeng Hongyuan Flagship Store	Electricity conservation2	Not received	Jingdong prefecture level city in Shandong

Jinfeng Hongyuan Flagship Store	Electricity conservation2	rare	Jingdong prefecture level city in Shandong
Jinfeng Hongyuan Flagship Store	Electricity conservation2	It's good.	Jingdong prefecture level city in Shandong
Jinfeng Hongyuan Flagship Store	Electricity conservation2	just now	Jingdong prefecture level city in Shandong
Jinfeng Hongyuan Flagship Store	Electricity conservation2	serviceable	Jingdong prefecture level city in Shandong
Jinfeng Hongyuan Flagship Store	Electricity conservation2	desirable	Jingdong prefecture level city in Shandong
Jinfeng Hongyuan Flagship Store	Electricity conservation2	serviceable	Jingdong prefecture level city in Shandong
Jinfeng Hongyuan Flagship Store	Electricity conservation2	It doesn't save power and costs electricity.	Jingdong prefecture level city in Shandong
Jinfeng Hongyuan Flagship Store	Electricity conservation2	Not as good as I thought.	Jingdong prefecture level city in Shandong
Jinfeng Hongyuan Flagship Store	Electricity conservation2	Super bad review, it doesn't save power at all, but rather more power consumption	Jingdong prefecture level city in Shandong
Jinfeng Hongyuan Flagship Store	Electricity conservation2	Listen to others say that this can save electricity, I bought with the idea of trying, used for a period of time before coming to the evaluation, will be a little bit of use, not a lot of savings just, long-term use of the words can save more.	Jingdong prefecture level city in Shandong
Jinfeng Hongyuan Flagship Store	Electricity conservation2	Logistics is fast, the next day arrived, workmanship is very delicate a power saver, looking forward to the late effect, customer service patiently explain the recommended which is suitable for, installed in what position is the best, look forward to using the effect after a period of time!	Jingdong prefecture level city in Shandong
Jinfeng Hongyuan Flagship Store	Electricity conservation2	It's good to use, I feel that it has been saving electricity, anyway, not a loss is, I used to feel that the power consumption is big, now I feel much better, my friends are also using, I hope to use a long time will be able to, I wish the boss a prosperous business	Jingdong prefecture level city in Shandong
Jinfeng Hongyuan Flagship Store	Electricity conservation2	Should be very good, to be able to plan the circuit, save the home of the circuit to the extent of the savings, waste of money on electricity, good if a month this money back, hope always hope to be able to	Jingdong prefecture level city in Shandong
Jinfeng Hongyuan Flagship Store	Electricity conservation2	Express delivery is very strong, the goods and I saw in the business consistent, the use of the effect of follow-up evaluation	Jingdong prefecture level city in Shandong
Jinfeng Hongyuan Flagship Store	Electricity conservation2	I have been using this brand of power saver for many years and this time I saw this upgraded version and got it again. The main purpose of this product is to reduce the loss of electricity for a long time, so as to achieve the purpose of saving electricity.	Jingdong prefecture level city in Shandong
Jinfeng Hongyuan Flagship Store	Electricity conservation2	Logistics is fast and the product looks beautiful, looking forward to the effect.	Jingdong prefecture level city in Shandong
Jinfeng Hongyuan Flagship Store	Electricity conservation2	Product quality is good, energy saving a little effect, but the price is a little expensive, fast arrival, good service.	Jingdong prefecture level city in Shandong
Jinfeng Hongyuan Flagship Store	Electricity conservation2	The quality is very good, inexpensive, Jingdong self-supporting guaranteed, worth having!	Jingdong prefecture level city in Shandong
Jinfeng Hongyuan Flagship Store	Electricity conservation2	I don't know if it's saving electricity yet, I'll find out next time I pay my electric bill	Jingdong prefecture level city in Shandong
Jinfeng Hongyuan Flagship Store	Electricity conservation2	Favorable price quality assurance appearance design is beautiful and generous, fine workmanship	Jingdong prefecture level city in Shandong
Jinfeng Hongyuan Flagship Store	Electricity conservation2	I bought it, I don't know how effective it is? I don't know how the quality is? After this summer results will know! Looking forward to it!	Jingdong prefecture level city in Shandong
Jinfeng Hongyuan Flagship Store	Electricity conservation2	Goods received Packaging intact, look at the shell workmanship is also good, high-power appliances open voltage has obvious changes in the effectiveness of the	Jingdong prefecture level city in Shandong
Jinfeng Hongyuan Flagship Store	Electricity conservation2	The goods have been received logistics is very fast, the product is similar to the line, just wait to use a month to see if it can save electricity, stay tuned.	Jingdong prefecture level city in Shandong
Jinfeng Hongyuan Flagship Store	Electricity conservation2	Electricity is plugged in, not sure if it will work, hopefully it will work.	Jingdong prefecture level city in Shandong
Jinfeng Hongyuan Flagship Store	Electricity conservation2	The shell is aluminum alloy, very good workmanship, the actual effect of waiting for the addition of evaluation	Jingdong prefecture level city in Shandong
Jinfeng Hongyuan Flagship Store	Electricity conservation2	Received, already plugged in, looking forward to the effect.	Jingdong prefecture level city in Shandong
Jinfeng Hongyuan Flagship Store	Electricity conservation2	Opened to see the regular manufacturer products, wait for a period of time to see it	Jingdong prefecture level city in Shandong
Jinfeng Hongyuan Flagship Store	Electricity conservation2	🉑 Baby received, very surprised ✨, reasonable design, fashionable and generous, continue to pay attention to the future will be repurchased ❥❥❥❥	Jingdong prefecture level city in Shandong
Jinfeng Hongyuan Flagship Store	Electricity conservation2	Baby received it 👌, really great ah! 🌈The quality can't be beat, very satisfied! 🆗Happy happy happy 😃	Jingdong prefecture level city in Shandong
Jinfeng Hongyuan Flagship Store	Electricity conservation2	Very good, power saving see, this is to help a friend to buy, I used to give my friend a say, my friend immediately let me order another one!	Jingdong prefecture level city in Shandong
Jinfeng Hongyuan Flagship Store	Electricity conservation2	The baby is just as introduced, the texture is very good, powerful, and it feels good to use it	Jingdong prefecture level city in Shandong
Jinfeng Hongyuan Flagship Store	Electricity conservation2	Value for money, very satisfied, seller service attitude is very good, very patient	Jingdong prefecture level city in Shandong
Jinfeng Hongyuan Flagship Store	Electricity conservation2	Power saving treasure design unique exquisite beautiful, voltage display clearly, plugged in that is good to use, I observed the meter, after using the meter is gone slow.	Jingdong prefecture level city in Shandong
Jinfeng Hongyuan Flagship Store	Electricity conservation2	Received on the use of the packaging is beautifully used for a period of time and then come back in the follow-up review!	Jingdong prefecture level city in Shandong
Jinfeng Hongyuan Flagship Store	Electricity conservation2	It should work. Not bad. Not bad. Not bad.	Jingdong prefecture level city in Shandong
Jinfeng Hongyuan Flagship Store	Electricity conservation2	I haven't used it yet, I'll plug it in and see if it saves power next month.	Jingdong prefecture level city in Shandong
Jinfeng Hongyuan Flagship Store	Electricity conservation2	The goods received and unpacked and used, the quality is very good, things and the official website to buy no difference!	Jingdong prefecture level city in Shandong
Jinfeng Hongyuan Flagship Store	Electricity conservation2	It's a rip off, doesn't help much at all, uses the same amount of power as before it was used.	Jingdong prefecture level city in Shandong
Jinfeng Hongyuan Flagship Store	Electricity conservation2	I've been using it for a while now and haven't noticed any unusual problems, so it's okay	Jingdong prefecture level city in Shandong
Jinfeng Hongyuan Flagship Store	Electricity conservation2	Received the goods and plugged in to use, good or not there is no use will have to wait until next month to find out	Jingdong prefecture level city in Shandong
Jinfeng Hongyuan Flagship Store	Electricity conservation2	It's very compact, it looks good, I'll introduce you to my clients when it's ready.	Jingdong prefecture level city in Shandong
Jinfeng Hongyuan Flagship Store	Electricity conservation2	Good workmanship, we'll have to see in a few months.	Jingdong prefecture level city in Shandong
Jinfeng Hongyuan Flagship Store	Electricity conservation2	Jingdong quality! Always trustworthy!	Jingdong prefecture level city in Shandong
Jinfeng Hongyuan Flagship Store	Electricity conservation2	This is the second time I've bought it, and the merchant said to give me five dollars out of the difference, so thank you merchant.	Jingdong prefecture level city in Shandong
Jinfeng Hongyuan Flagship Store	Electricity conservation2	Good quality and good service	Jingdong prefecture level city in Shandong
Jinfeng Hongyuan Flagship Store	Electricity conservation2	Feels good texture, aluminum alloy shell, voltage digital display clear.	Jingdong prefecture level city in Shandong
Jinfeng Hongyuan Flagship Store	Electricity conservation2	Good product, fast and great service, thanks!	Jingdong prefecture level city in Shandong
Jinfeng Hongyuan Flagship Store	Electricity conservation2	Have not yet used, first evaluation, later really good do not need to comment again.	Jingdong prefecture level city in Shandong
Jinfeng Hongyuan Flagship Store	Electricity conservation2	Works great. Pretty much as described by the merchant.	Jingdong prefecture level city in Shandong

Jinfeng Hongyuan Flagship Store	Electricity conservation2	Received it, looking forward to the results, hope it works	Jingdong prefecture level city in Shandong
Jinfeng Hongyuan Flagship Store	Electricity conservation2	Received it, looking forward to the results, hope it works	Jingdong prefecture level city in Shandong
Jinfeng Hongyuan Flagship Store	Electricity conservation2	Supposedly it's an IQ tax. Buy one and try it. Looks good. Well made.	Jingdong prefecture level city in Shandong
Jinfeng Hongyuan Flagship Store	Electricity conservation2	It just doesn't feel like it's working.	Jingdong prefecture level city in Shandong
Jinfeng Hongyuan Flagship Store	Electricity conservation2	This is a great product.	Jingdong prefecture level city in Shandong
Jinfeng Hongyuan Flagship Store	Electricity conservation2	I'm just using it. I'll tell you more.	Jingdong prefecture level city in Shandong
Jinfeng Hongyuan Flagship Store	Electricity conservation2	Second repurchase and it's working fine.	Jingdong prefecture level city in Shandong
Jinfeng Hongyuan Flagship Store	Electricity conservation2	I'll wait a few days before evaluating	Jingdong prefecture level city in Shandong
Jinfeng Hongyuan Flagship Store	Electricity conservation2	I'll comment after I use it hopefully the section will be useful.	Jingdong prefecture level city in Shandong
Jinfeng Hongyuan Flagship Store	Electricity conservation2	not very useful	Jingdong prefecture level city in Shandong
Jinfeng Hongyuan Flagship Store	Electricity conservation2	Not received	Jingdong prefecture level city in Shandong
Jinfeng Hongyuan Flagship Store	Electricity conservation2	rare	Jingdong prefecture level city in Shandong
Jinfeng Hongyuan Flagship Store	Electricity conservation2	It's good.	Jingdong prefecture level city in Shandong
Jinfeng Hongyuan Flagship Store	Electricity conservation2	just now	Jingdong prefecture level city in Shandong
Jinfeng Hongyuan Flagship Store	Electricity conservation2	serviceable	Jingdong prefecture level city in Shandong
Jinfeng Hongyuan Flagship Store	Electricity conservation2	desirable	Jingdong prefecture level city in Shandong
Jinfeng Hongyuan Flagship Store	Electricity conservation2	serviceable	Jingdong prefecture level city in Shandong
Jinfeng Hongyuan Flagship Store	Electricity conservation2	It doesn't save power and costs electricity.	Jingdong prefecture level city in Shandong
Jinfeng Hongyuan Flagship Store	Electricity conservation2	Not as good as I thought.	Jingdong prefecture level city in Shandong
Jinfeng Hongyuan Flagship Store	Electricity conservation2	Super bad review, it doesn't save power at all, but rather more power consumption	Jingdong prefecture level city in Shandong
Jinfeng Hongyuan Flagship Store	Electricity conservation2	Listen to others say that this can save electricity, I bought with the idea of trying, used for a period of time before coming to the evaluation, will be a little bit of use, not a lot of savings just, long-term use of the words can save more.	Jingdong prefecture level city in Shandong
Jinfeng Hongyuan Flagship Store	Electricity conservation2	Logistics is fast, the next day arrived, workmanship is very delicate a power saver, looking forward to the late effect, customer service patiently explain the recommended which is suitable for, installed in what position is the best, look forward to using the effect after a period of time!	Jingdong prefecture level city in Shandong
Jinfeng Hongyuan Flagship Store	Electricity conservation2	It's good to use, I feel that it has been saving electricity, anyway, not a loss is, I used to feel that the power consumption is big, now I feel much better, my friends are also using, I hope to use a long time will be able to, I wish the boss a prosperous business	Jingdong prefecture level city in Shandong
Jinfeng Hongyuan Flagship Store	Electricity conservation2	Should be very good, to be able to plan the circuit, save the home of the circuit to the extent of the savings, waste of money on electricity, good if a month this money back, hope always hope to be able to	Jingdong prefecture level city in Shandong
Jinfeng Hongyuan Flagship Store	Electricity conservation2	Express delivery is very strong, the goods and I saw in the business consistent, the use of the effect of follow-up evaluation	Jingdong prefecture level city in Shandong
Jinfeng Hongyuan Flagship Store	Electricity conservation2	I have been using this brand of power saver for many years and this time I saw this upgraded version and got it again. The main purpose of this product is to reduce the loss of electricity for a long time, so as to achieve the purpose of saving electricity.	Jingdong prefecture level city in Shandong
Jinfeng Hongyuan Flagship Store	Electricity conservation2	Logistics is fast and the product looks beautiful, looking forward to the effect.	Jingdong prefecture level city in Shandong
Jinfeng Hongyuan Flagship Store	Electricity conservation2	Product quality is good, energy saving a little effect, but the price is a little expensive, fast arrival, good service.	Jingdong prefecture level city in Shandong
Jinfeng Hongyuan Flagship Store	Electricity conservation2	The quality is very good, inexpensive, Jingdong self-supporting guaranteed, worth having!	Jingdong prefecture level city in Shandong
Jinfeng Hongyuan Flagship Store	Electricity conservation2	I don't know if it's saving electricity yet, I'll find out next time I pay my electric bill	Jingdong prefecture level city in Shandong
Jinfeng Hongyuan Flagship Store	Electricity conservation2	Favorable price quality assurance appearance design is beautiful and generous, fine workmanship	Jingdong prefecture level city in Shandong
Jinfeng Hongyuan Flagship Store	Electricity conservation2	I bought it, I don't know how effective it is? I don't know how the quality is? After this summer results will know! Looking forward to it!	Jingdong prefecture level city in Shandong
Jinfeng Hongyuan Flagship Store	Electricity conservation2	Goods received Packaging intact, look at the shell workmanship is also good, high-power appliances open voltage has obvious changes in the effectiveness of the	Jingdong prefecture level city in Shandong
Jinfeng Hongyuan Flagship Store	Electricity conservation2	The goods have been received logistics is very fast, the product is similar to the line, just wait to use a month to see if it can save electricity, stay tuned.	Jingdong prefecture level city in Shandong
Jinfeng Hongyuan Flagship Store	Electricity conservation2	Electricity is plugged in, not sure if it will work, hopefully it will work.	Jingdong prefecture level city in Shandong
Jinfeng Hongyuan Flagship Store	Electricity conservation2	The shell is aluminum alloy, very good workmanship, the actual effect of waiting for the addition of evaluation	Jingdong prefecture level city in Shandong
Jinfeng Hongyuan Flagship Store	Electricity conservation2	Received, already plugged in, looking forward to the effect.	Jingdong prefecture level city in Shandong
Jinfeng Hongyuan Flagship Store	Electricity conservation2	Opened to see the regular manufacturer products, wait for a period of time to see it	Jingdong prefecture level city in Shandong
Jinfeng Hongyuan Flagship Store	Electricity conservation2	🉑 Baby received, very surprised ✨, reasonable design, fashionable and generous, continue to pay attention to the future will be repurchased ❤❤❤❤	Jingdong prefecture level city in Shandong
Jinfeng Hongyuan Flagship Store	Electricity conservation2	Baby received it 👌, really great ah! 🌈The quality can't be beat, very satisfied! 🆗Happy happy happy 😀	Jingdong prefecture level city in Shandong
Jinfeng Hongyuan Flagship Store	Electricity conservation2	Very good, power saving see, this is to help a friend to buy, I used to give my friend a say, my friend immediately let me order another one!	Jingdong prefecture level city in Shandong
Jinfeng Hongyuan Flagship Store	Electricity conservation2	The baby is just as introduced, the texture is very good, powerful, and it feels good to use it	Jingdong prefecture level city in Shandong
Jinfeng Hongyuan Flagship Store	Electricity conservation2	Value for money, very satisfied, seller service attitude is very good, very patient	Jingdong prefecture level city in Shandong
Jinfeng Hongyuan Flagship Store	Electricity conservation2	Power saving treasure design unique exquisite beautiful, voltage display clearly, plugged in that is good to use, I observed the meter, after using the meter is gone slow.	Jingdong prefecture level city in Shandong
Jinfeng Hongyuan Flagship Store	Electricity conservation2	Received on the use of the packaging is beautifully used for a period of time and then come back in the follow-up review!	Jingdong prefecture level city in Shandong
Jinfeng Hongyuan Flagship Store	Electricity conservation2	It should work. Not bad. Not bad. Not bad.	Jingdong prefecture level city in Shandong
Jinfeng Hongyuan Flagship Store	Electricity conservation2	I haven't used it yet, I'll plug it in and see if it saves power next month.	Jingdong prefecture level city in Shandong
Jinfeng Hongyuan Flagship Store	Electricity conservation2	The goods received and unpacked and used, the quality is very good, things and the official website to buy no difference!	Jingdong prefecture level city in Shandong
Jinfeng Hongyuan Flagship Store	Electricity conservation2	It's a rip off, doesn't help much at all, uses the same amount of power as before it was used.	Jingdong prefecture level city in Shandong

Jinfeng Hongyuan Flagship Store	Electricity conservation2	I've been using it for a while now and haven't noticed any unusual problems, so it's okay	Jingdong prefecture level city in Shandong
Jinfeng Hongyuan Flagship Store	Electricity conservation2	Received the goods and plugged in to use, good or not there is no use will have to wait until next month to find out	Jingdong prefecture level city in Shandong
Jinfeng Hongyuan Flagship Store	Electricity conservation2	It's very compact, it looks good, I'll introduce you to my clients when it's ready.	Jingdong prefecture level city in Shandong
Jinfeng Hongyuan Flagship Store	Electricity conservation2	Good workmanship, we'll have to see in a few months.	Jingdong prefecture level city in Shandong
Jinfeng Hongyuan Flagship Store	Electricity conservation2	Jingdong quality! Always trustworthy!	Jingdong prefecture level city in Shandong
Jinfeng Hongyuan Flagship Store	Electricity conservation2	This is the second time I've bought it, and the merchant said to give me five dollars out of the difference, so thank you merchant.	Jingdong prefecture level city in Shandong
Jinfeng Hongyuan Flagship Store	Electricity conservation2	Good quality and good service	Jingdong prefecture level city in Shandong
Jinfeng Hongyuan Flagship Store	Electricity conservation2	Feels good texture, aluminum alloy shell, voltage digital display clear.	Jingdong prefecture level city in Shandong
Jinfeng Hongyuan Flagship Store	Electricity conservation2	Good product, fast and great service, thanks!	Jingdong prefecture level city in Shandong
Jinfeng Hongyuan Flagship Store	Electricity conservation2	Have not yet used, first evaluation, later really good do not need to comment again.	Jingdong prefecture level city in Shandong
Jinfeng Hongyuan Flagship Store	Electricity conservation2	Works great. Pretty much as described by the merchant.	Jingdong prefecture level city in Shandong
Jinfeng Hongyuan Flagship Store	Electricity conservation2	Received it, looking forward to the results, hope it works	Jingdong prefecture level city in Shandong
Jinfeng Hongyuan Flagship Store	Electricity conservation2	Received it, looking forward to the results, hope it works	Jingdong prefecture level city in Shandong
Jinfeng Hongyuan Flagship Store	Electricity conservation2	Supposedly it's an IQ tax. Buy one and try it. Looks good. Well made.	Jingdong prefecture level city in Shandong
Jinfeng Hongyuan Flagship Store	Electricity conservation2	It just doesn't feel like it's working.	Jingdong prefecture level city in Shandong
Jinfeng Hongyuan Flagship Store	Electricity conservation2	This is a great product.	Jingdong prefecture level city in Shandong
Jinfeng Hongyuan Flagship Store	Electricity conservation2	I'm just using it. I'll tell you more.	Jingdong prefecture level city in Shandong
Jinfeng Hongyuan Flagship Store	Electricity conservation2	Second repurchase and it's working fine.	Jingdong prefecture level city in Shandong
Jinfeng Hongyuan Flagship Store	Electricity conservation2	I'll wait a few days before evaluating	Jingdong prefecture level city in Shandong
Jinfeng Hongyuan Flagship Store	Electricity conservation2	I'll comment after I use it hopefully the section will be useful.	Jingdong prefecture level city in Shandong
Jinfeng Hongyuan Flagship Store	Electricity conservation2	not very useful	Jingdong prefecture level city in Shandong
Jinfeng Hongyuan Flagship Store	Electricity conservation2	Not received	Jingdong prefecture level city in Shandong
Jinfeng Hongyuan Flagship Store	Electricity conservation2	rare	Jingdong prefecture level city in Shandong
Jinfeng Hongyuan Flagship Store	Electricity conservation2	It's good.	Jingdong prefecture level city in Shandong
Jinfeng Hongyuan Flagship Store	Electricity conservation2	just now	Jingdong prefecture level city in Shandong
Jinfeng Hongyuan Flagship Store	Electricity conservation2	serviceable	Jingdong prefecture level city in Shandong
Jinfeng Hongyuan Flagship Store	Electricity conservation2	desirable	Jingdong prefecture level city in Shandong
Jinfeng Hongyuan Flagship Store	Electricity conservation2	serviceable	Jingdong prefecture level city in Shandong
Jinfeng Hongyuan Flagship Store	Electricity conservation2	It doesn't save power and costs electricity.	Jingdong prefecture level city in Shandong
Jinfeng Hongyuan Flagship Store	Electricity conservation2	Not as good as I thought.	Jingdong prefecture level city in Shandong
Jinfeng Hongyuan Flagship Store	Electricity conservation2	Super bad review, it doesn't save power at all, but rather more power consumption	Jingdong prefecture level city in Shandong
Jinfeng Hongyuan Flagship Store	Electricity conservation2	Listen to others say that this can save electricity, I bought with the idea of trying, used for a period of time before coming to the evaluation, will be a little bit of use, not a lot of savings just, long-term use of the words can save more.	Jingdong prefecture level city in Shandong
Jinfeng Hongyuan Flagship Store	Electricity conservation2	Logistics is fast, the next day arrived, workmanship is very delicate a power saver, looking forward to the late effect, customer service patiently explain the recommended which is suitable for, installed in what position is the best, look forward to using the effect after a period of time!	Jingdong prefecture level city in Shandong
Jinfeng Hongyuan Flagship Store	Electricity conservation2	It's good to use, I feel that it has been saving electricity, anyway, not a loss is, I used to feel that the power consumption is big, now I feel much better, my friends are also using, I hope to use a long time will be able to, I wish the boss a prosperous business	Jingdong prefecture level city in Shandong
Jinfeng Hongyuan Flagship Store	Electricity conservation2	Should be very good, to be able to plan the circuit, save the home of the circuit to the extent of the savings, waste of money on electricity, good if a month this money back, hope always hope to be able to	Jingdong prefecture level city in Shandong
Jinfeng Hongyuan Flagship Store	Electricity conservation2	Express delivery is very strong, the goods and I saw in the business consistent, the use of the effect of follow-up evaluation	Jingdong prefecture level city in Shandong
Jinfeng Hongyuan Flagship Store	Electricity conservation2	I have been using this brand of power saver for many years and this time I saw this upgraded version and got it again. The main purpose of this product is to reduce the loss of electricity for a long time, so as to achieve the purpose of saving electricity.	Jingdong prefecture level city in Shandong
Jinfeng Hongyuan Flagship Store	Electricity conservation2	Logistics is fast and the product looks beautiful, looking forward to the effect.	Jingdong prefecture level city in Shandong
Jinfeng Hongyuan Flagship Store	Electricity conservation2	Product quality is good, energy saving a little effect, but the price is a little expensive, fast arrival, good service.	Jingdong prefecture level city in Shandong
Jinfeng Hongyuan Flagship Store	Electricity conservation2	The quality is very good, inexpensive, Jingdong self-supporting guaranteed, worth having!	Jingdong prefecture level city in Shandong
Jinfeng Hongyuan Flagship Store	Electricity conservation2	I don't know if it's saving electricity yet, I'll find out next time I pay my electric bill	Jingdong prefecture level city in Shandong
Jinfeng Hongyuan Flagship Store	Electricity conservation2	Favorable price quality assurance appearance design is beautiful and generous, fine workmanship	Jingdong prefecture level city in Shandong
Jinfeng Hongyuan Flagship Store	Electricity conservation2	I bought it, I don't know how effective it is? I don't know how the quality is? After this summer results will know! Looking forward to it!	Jingdong prefecture level city in Shandong
Jinfeng Hongyuan Flagship Store	Electricity conservation2	Goods received Packaging intact, look at the shell workmanship is also good, high-power appliances open voltage has obvious changes in the effectiveness of the	Jingdong prefecture level city in Shandong
Jinfeng Hongyuan Flagship Store	Electricity conservation2	The goods have been received logistics is very fast, the product is similar to the line, just wait to use a month to see if it can save electricity, stay tuned.	Jingdong prefecture level city in Shandong
Jinfeng Hongyuan Flagship Store	Electricity conservation2	Electricity is plugged in, not sure if it will work, hopefully it will work.	Jingdong prefecture level city in Shandong
Jinfeng Hongyuan Flagship Store	Electricity conservation2	The shell is aluminum alloy, very good workmanship, the actual effect of waiting for the addition of evaluation	Jingdong prefecture level city in Shandong
Jinfeng Hongyuan Flagship Store	Electricity conservation2	Received, already plugged in, looking forward to the effect.	Jingdong prefecture level city in Shandong
Jinfeng Hongyuan Flagship Store	Electricity conservation2	Opened to see the regular manufacturer products, wait for a period of time to see it	Jingdong prefecture level city in Shandong

Jinfeng Hongyuan Flagship Store	Electricity conservation2	🉑 Baby received, very surprised ✨, reasonable design, fashionable and generous, continue to pay attention to the future will be repurchased ❤❤❤❤	Jingdong prefecture level city in Shandong
Jinfeng Hongyuan Flagship Store	Electricity conservation2	Baby received it 👌, really great ah! 🌈The quality can't be beat, very satisfied! 🆗Happy happy happy 😀	Jingdong prefecture level city in Shandong
Jinfeng Hongyuan Flagship Store	Electricity conservation2	Very good, power saving see, this is to help a friend to buy, I used to give my friend a say, my friend immediately let me order another one!	Jingdong prefecture level city in Shandong
Jinfeng Hongyuan Flagship Store	Electricity conservation2	The baby is just as introduced, the texture is very good, powerful, and it feels good to use it	Jingdong prefecture level city in Shandong
Jinfeng Hongyuan Flagship Store	Electricity conservation2	Value for money, very satisfied, seller service attitude is very good, very patient	Jingdong prefecture level city in Shandong
Jinfeng Hongyuan Flagship Store	Electricity conservation2	Power saving treasure design unique exquisite beautiful, voltage display clearly, plugged in that is good to use, I observed the meter, after using the meter is gone slow.	Jingdong prefecture level city in Shandong
Jinfeng Hongyuan Flagship Store	Electricity conservation2	Received on the use of the packaging is beautifully used for a period of time and then come back in the follow-up review!	Jingdong prefecture level city in Shandong
Jinfeng Hongyuan Flagship Store	Electricity conservation2	It should work. Not bad. Not bad. Not bad.	Jingdong prefecture level city in Shandong
Jinfeng Hongyuan Flagship Store	Electricity conservation2	I haven't used it yet, I'll plug it in and see if it saves power next month.	Jingdong prefecture level city in Shandong
Jinfeng Hongyuan Flagship Store	Electricity conservation2	The goods received and unpacked and used, the quality is very good, things and the official website to buy no difference!	Jingdong prefecture level city in Shandong
Jinfeng Hongyuan Flagship Store	Electricity conservation2	It's a rip off, doesn't help much at all, uses the same amount of power as before it was used.	Jingdong prefecture level city in Shandong
Jinfeng Hongyuan Flagship Store	Electricity conservation2	I've been using it for a while now and haven't noticed any unusual problems, so it's okay	Jingdong prefecture level city in Shandong
Jinfeng Hongyuan Flagship Store	Electricity conservation2	Received the goods and plugged in to use, good or not there is no use will have to wait until next month to find out	Jingdong prefecture level city in Shandong
Jinfeng Hongyuan Flagship Store	Electricity conservation2	It's very compact, it looks good, I'll introduce you to my clients when it's ready.	Jingdong prefecture level city in Shandong
Jinfeng Hongyuan Flagship Store	Electricity conservation2	Good workmanship, we'll have to see in a few months.	Jingdong prefecture level city in Shandong
Jinfeng Hongyuan Flagship Store	Electricity conservation2	Jingdong quality! Always trustworthy!	Jingdong prefecture level city in Shandong
Jinfeng Hongyuan Flagship Store	Electricity conservation2	This is the second time I've bought it, and the merchant said to give me five dollars out of the difference, so thank you merchant.	Jingdong prefecture level city in Shandong
Jinfeng Hongyuan Flagship Store	Electricity conservation2	Good quality and good service	Jingdong prefecture level city in Shandong
Jinfeng Hongyuan Flagship Store	Electricity conservation2	Feels good texture, aluminum alloy shell, voltage digital display clear.	Jingdong prefecture level city in Shandong
Jinfeng Hongyuan Flagship Store	Electricity conservation2	Good product, fast and great service, thanks!	Jingdong prefecture level city in Shandong
Jinfeng Hongyuan Flagship Store	Electricity conservation2	Have not yet used, first evaluation, later really good do not need to comment again.	Jingdong prefecture level city in Shandong
Jinfeng Hongyuan Flagship Store	Electricity conservation2	Works great. Pretty much as described by the merchant.	Jingdong prefecture level city in Shandong
Jinfeng Hongyuan Flagship Store	Electricity conservation2	Received it, looking forward to the results, hope it works	Jingdong prefecture level city in Shandong
Jinfeng Hongyuan Flagship Store	Electricity conservation2	Received it, looking forward to the results, hope it works	Jingdong prefecture level city in Shandong
Jinfeng Hongyuan Flagship Store	Electricity conservation2	Supposedly it's an IQ tax. Buy one and try it. Looks good. Well made.	Jingdong prefecture level city in Shandong
Jinfeng Hongyuan Flagship Store	Electricity conservation2	It just doesn't feel like it's working.	Jingdong prefecture level city in Shandong
Jinfeng Hongyuan Flagship Store	Electricity conservation2	This is a great product.	Jingdong prefecture level city in Shandong
Jinfeng Hongyuan Flagship Store	Electricity conservation2	I'm just using it. I'll tell you more.	Jingdong prefecture level city in Shandong
Jinfeng Hongyuan Flagship Store	Electricity conservation2	Second repurchase and it's working fine.	Jingdong prefecture level city in Shandong
Jinfeng Hongyuan Flagship Store	Electricity conservation2	I'll wait a few days before evaluating	Jingdong prefecture level city in Shandong
Jinfeng Hongyuan Flagship Store	Electricity conservation2	I'll comment after I use it hopefully the section will be useful.	Jingdong prefecture level city in Shandong
Jinfeng Hongyuan Flagship Store	Electricity conservation2	not very useful	Jingdong prefecture level city in Shandong
Jinfeng Hongyuan Flagship Store	Electricity conservation2	Not received	Jingdong prefecture level city in Shandong
Jinfeng Hongyuan Flagship Store	Electricity conservation2	rare	Jingdong prefecture level city in Shandong
Jinfeng Hongyuan Flagship Store	Electricity conservation2	It's good.	Jingdong prefecture level city in Shandong
Jinfeng Hongyuan Flagship Store	Electricity conservation2	just now	Jingdong prefecture level city in Shandong
Jinfeng Hongyuan Flagship Store	Electricity conservation2	serviceable	Jingdong prefecture level city in Shandong
Jinfeng Hongyuan Flagship Store	Electricity conservation2	desirable	Jingdong prefecture level city in Shandong
Jinfeng Hongyuan Flagship Store	Electricity conservation2	serviceable	Jingdong prefecture level city in Shandong
Jinfeng Hongyuan Flagship Store	Electricity conservation2	It doesn't save power and costs electricity.	Jingdong prefecture level city in Shandong
Jinfeng Hongyuan Flagship Store	Electricity conservation2	Not as good as I thought.	Jingdong prefecture level city in Shandong
Jinfeng Hongyuan Flagship Store	Electricity conservation2	Super bad review, it doesn't save power at all, but rather more power consumption	Jingdong prefecture level city in Shandong
Jinfeng Hongyuan Flagship Store	Electricity conservation2	Listen to others say that this can save electricity, I bought with the idea of trying, used for a period of time before coming to the evaluation, will be a little bit of use, not a lot of savings just, long-term use of the words can save more.	Jingdong prefecture level city in Shandong
Jinfeng Hongyuan Flagship Store	Electricity conservation2	Logistics is fast, the next day arrived, workmanship is very delicate a power saver, looking forward to the late effect, customer service patiently explain the recommended which is suitable for, installed in what position is the best, look forward to using the effect after a period of time!	Jingdong prefecture level city in Shandong
Jinfeng Hongyuan Flagship Store	Electricity conservation2	It's good to use, I feel that it has been saving electricity, anyway, not a loss is, I used to feel that the power consumption is big, now I feel much better, my friends are also using, I hope to use a long time will be able to, I wish the boss a prosperous business	Jingdong prefecture level city in Shandong
Jinfeng Hongyuan Flagship Store	Electricity conservation2	Should be very good, to be able to plan the circuit, save the home of the circuit to the extent of the savings, waste of money on electricity, good if a month this money back, hope always hope to be able to	Jingdong prefecture level city in Shandong
Jinfeng Hongyuan Flagship Store	Electricity conservation2	Express delivery is very strong, the goods and I saw in the business consistent, the use of the effect of follow-up evaluation	Jingdong prefecture level city in Shandong
Jinfeng Hongyuan Flagship Store	Electricity conservation2	I have been using this brand of power saver for many years and this time I saw this upgraded version and got it again. The main purpose of this product is to reduce the loss of electricity for a long time, so as to achieve the purpose of saving electricity.	Jingdong prefecture level city in Shandong
Jinfeng Hongyuan Flagship Store	Electricity conservation2	Logistics is fast and the product looks beautiful, looking forward to the effect.	Jingdong prefecture level city in Shandong

Jinfeng Hongyuan Flagship Store	Electricity conservation2	Product quality is good, energy saving a little effect, but the price is a little expensive, fast arrival, good service.	Jingdong prefecture level city in Shandong
Jinfeng Hongyuan Flagship Store	Electricity conservation2	The quality is very good, inexpensive, Jingdong self-supporting guaranteed, worth having!	Jingdong prefecture level city in Shandong
Jinfeng Hongyuan Flagship Store	Electricity conservation2	I don't know if it's saving electricity yet, I'll find out next time I pay my electric bill	Jingdong prefecture level city in Shandong
Jinfeng Hongyuan Flagship Store	Electricity conservation2	Favorable price quality assurance appearance design is beautiful and generous, fine workmanship	Jingdong prefecture level city in Shandong
Jinfeng Hongyuan Flagship Store	Electricity conservation2	I bought it, I don't know how effective it is? I don't know how the quality is? After this summer results will know! Looking forward to it!	Jingdong prefecture level city in Shandong
Jinfeng Hongyuan Flagship Store	Electricity conservation2	Goods received Packaging intact, look at the shell workmanship is also good, high-power appliances open voltage has obvious changes in the effectiveness of the	Jingdong prefecture level city in Shandong
Jinfeng Hongyuan Flagship Store	Electricity conservation2	The goods have been received logistics is very fast, the product is similar to the line, just wait to use a month to see if it can save electricity, stay tuned.	Jingdong prefecture level city in Shandong
Jinfeng Hongyuan Flagship Store	Electricity conservation2	Electricity is plugged in, not sure if it will work, hopefully it will work.	Jingdong prefecture level city in Shandong
Jinfeng Hongyuan Flagship Store	Electricity conservation2	The shell is aluminum alloy, very good workmanship, the actual effect of waiting for the addition of evaluation	Jingdong prefecture level city in Shandong
Jinfeng Hongyuan Flagship Store	Electricity conservation2	Received, already plugged in, looking forward to the effect.	Jingdong prefecture level city in Shandong
Jinfeng Hongyuan Flagship Store	Electricity conservation2	Opened to see the regular manufacturer products, wait for a period of time to see it	Jingdong prefecture level city in Shandong
Jinfeng Hongyuan Flagship Store	Electricity conservation2	🉑 Baby received, very surprised ✨, reasonable design, fashionable and generous, continue to pay attention to the future will be repurchased ❤❤❤❤	Jingdong prefecture level city in Shandong
Jinfeng Hongyuan Flagship Store	Electricity conservation2	Baby received it 👌, really great ah! 🌈The quality can't be beat, very satisfied! 🆗Happy happy happy 😃	Jingdong prefecture level city in Shandong
Jinfeng Hongyuan Flagship Store	Electricity conservation2	Very good, power saving see, this is to help a friend to buy, I used to give my friend a say, my friend immediately let me order another one!	Jingdong prefecture level city in Shandong
Jinfeng Hongyuan Flagship Store	Electricity conservation2	The baby is just as introduced, the texture is very good, powerful, and it feels good to use it	Jingdong prefecture level city in Shandong
Jinfeng Hongyuan Flagship Store	Electricity conservation2	Value for money, very satisfied, seller service attitude is very good, very patient	Jingdong prefecture level city in Shandong
Jinfeng Hongyuan Flagship Store	Electricity conservation2	Power saving treasure design unique exquisite beautiful, voltage display clearly, plugged in that is good to use, I observed the meter, after using the meter is gone slow.	Jingdong prefecture level city in Shandong
Jinfeng Hongyuan Flagship Store	Electricity conservation2	Received on the use of the packaging is beautifully used for a period of time and then come back in the follow-up review!	Jingdong prefecture level city in Shandong
Jinfeng Hongyuan Flagship Store	Electricity conservation2	It should work. Not bad. Not bad. Not bad.	Jingdong prefecture level city in Shandong
Jinfeng Hongyuan Flagship Store	Electricity conservation2	I haven't used it yet, I'll plug it in and see if it saves power next month.	Jingdong prefecture level city in Shandong
Jinfeng Hongyuan Flagship Store	Electricity conservation2	The goods received and unpacked and used, the quality is very good, things and the official website to buy no difference!	Jingdong prefecture level city in Shandong
Jinfeng Hongyuan Flagship Store	Electricity conservation2	It's a rip off, doesn't help much at all, uses the same amount of power as before it was used.	Jingdong prefecture level city in Shandong
Jinfeng Hongyuan Flagship Store	Electricity conservation2	I've been using it for a while now and haven't noticed any unusual problems, so it's okay	Jingdong prefecture level city in Shandong
Jinfeng Hongyuan Flagship Store	Electricity conservation2	Received the goods and plugged in to use, good or not there is no use will have to wait until next month to find out	Jingdong prefecture level city in Shandong
Jinfeng Hongyuan Flagship Store	Electricity conservation2	It's very compact, it looks good, I'll introduce you to my clients when it's ready.	Jingdong prefecture level city in Shandong
Jinfeng Hongyuan Flagship Store	Electricity conservation2	Good workmanship, we'll have to see in a few months.	Jingdong prefecture level city in Shandong
Jinfeng Hongyuan Flagship Store	Electricity conservation2	Jingdong quality! Always trustworthy!	Jingdong prefecture level city in Shandong
Jinfeng Hongyuan Flagship Store	Electricity conservation2	This is the second time I've bought it, and the merchant said to give me five dollars out of the difference, so thank you merchant.	Jingdong prefecture level city in Shandong
Jinfeng Hongyuan Flagship Store	Electricity conservation2	Good quality and good service	Jingdong prefecture level city in Shandong
Jinfeng Hongyuan Flagship Store	Electricity conservation2	Feels good texture, aluminum alloy shell, voltage digital display clear.	Jingdong prefecture level city in Shandong
Jinfeng Hongyuan Flagship Store	Electricity conservation2	Good product, fast and great service, thanks!	Jingdong prefecture level city in Shandong
Jinfeng Hongyuan Flagship Store	Electricity conservation2	Have not yet used, first evaluation, later really good do not need to comment again.	Jingdong prefecture level city in Shandong
Jinfeng Hongyuan Flagship Store	Electricity conservation2	Works great. Pretty much as described by the merchant.	Jingdong prefecture level city in Shandong
Jinfeng Hongyuan Flagship Store	Electricity conservation2	Received it, looking forward to the results, hope it works	Jingdong prefecture level city in Shandong
Jinfeng Hongyuan Flagship Store	Electricity conservation2	Received it, looking forward to the results, hope it works	Jingdong prefecture level city in Shandong
Jinfeng Hongyuan Flagship Store	Electricity conservation2	Supposedly it's an IQ tax. Buy one and try it. Looks good. Well made.	Jingdong prefecture level city in Shandong
Jinfeng Hongyuan Flagship Store	Electricity conservation2	It just doesn't feel like it's working.	Jingdong prefecture level city in Shandong
Jinfeng Hongyuan Flagship Store	Electricity conservation2	This is a great product.	Jingdong prefecture level city in Shandong
Jinfeng Hongyuan Flagship Store	Electricity conservation2	I'm just using it. I'll tell you more.	Jingdong prefecture level city in Shandong
Jinfeng Hongyuan Flagship Store	Electricity conservation2	Second repurchase and it's working fine.	Jingdong prefecture level city in Shandong
Jinfeng Hongyuan Flagship Store	Electricity conservation2	I'll wait a few days before evaluating	Jingdong prefecture level city in Shandong
Jinfeng Hongyuan Flagship Store	Electricity conservation2	I'll comment after I use it hopefully the section will be useful.	Jingdong prefecture level city in Shandong
Jinfeng Hongyuan Flagship Store	Electricity conservation2	not very useful	Jingdong prefecture level city in Shandong
Jinfeng Hongyuan Flagship Store	Electricity conservation2	Not received	Jingdong prefecture level city in Shandong
Jinfeng Hongyuan Flagship Store	Electricity conservation2	rare	Jingdong prefecture level city in Shandong
Jinfeng Hongyuan Flagship Store	Electricity conservation2	It's good.	Jingdong prefecture level city in Shandong
Jinfeng Hongyuan Flagship Store	Electricity conservation2	just now	Jingdong prefecture level city in Shandong
Jinfeng Hongyuan Flagship Store	Electricity conservation2	serviceable	Jingdong prefecture level city in Shandong
Jinfeng Hongyuan Flagship Store	Electricity conservation2	desirable	Jingdong prefecture level city in Shandong
Jinfeng Hongyuan Flagship Store	Electricity conservation2	serviceable	Jingdong prefecture level city in Shandong
Jinfeng Hongyuan Flagship Store	Electricity conservation2	It doesn't save power and costs electricity.	Jingdong prefecture level city in Shandong

Jinfeng Hongyuan Flagship Store	Electricity conservation2	Not as good as I thought.	Jingdong prefecture level city in Shandong
Jinfeng Hongyuan Flagship Store	Electricity conservation2	Super bad review, it doesn't save power at all, but rather more power consumption	Jingdong prefecture level city in Shandong
Jinfeng Hongyuan Flagship Store	Electricity conservation2	Listen to others say that this can save electricity, I bought with the idea of trying, used for a period of time before coming to the evaluation, will be a little bit of use, not a lot of savings just, long-term use of the words can save more.	Jingdong prefecture level city in Shandong
Jinfeng Hongyuan Flagship Store	Electricity conservation2	Logistics is fast, the next day arrived, workmanship is very delicate a power saver, looking forward to the late effect, customer service patiently explain the recommended which is suitable for, installed in what position is the best, look forward to using the effect after a period of time!	Jingdong prefecture level city in Shandong
Jinfeng Hongyuan Flagship Store	Electricity conservation2	It's good to use, I feel that it has been saving electricity, anyway, not a loss is, I used to feel that the power consumption is big, now I feel much better, my friends are also using, I hope to use a long time will be able to, I wish the boss a prosperous business	Jingdong prefecture level city in Shandong
Jinfeng Hongyuan Flagship Store	Electricity conservation2	Should be very good, to be able to plan the circuit, save the home of the circuit to the extent of the savings, waste of money on electricity, good if a month this money back, hope always hope to be able to	Jingdong prefecture level city in Shandong
Jinfeng Hongyuan Flagship Store	Electricity conservation2	Express delivery is very strong, the goods and I saw in the business consistent, the use of the effect of follow-up evaluation	Jingdong prefecture level city in Shandong
Jinfeng Hongyuan Flagship Store	Electricity conservation2	I have been using this brand of power saver for many years and this time I saw this upgraded version and got it again. The main purpose of this product is to reduce the loss of electricity for a long time, so as to achieve the purpose of saving electricity.	Jingdong prefecture level city in Shandong
Jinfeng Hongyuan Flagship Store	Electricity conservation2	Logistics is fast and the product looks beautiful, looking forward to the effect.	Jingdong prefecture level city in Shandong
Jinfeng Hongyuan Flagship Store	Electricity conservation2	Product quality is good, energy saving a little effect, but the price is a little expensive, fast arrival, good service.	Jingdong prefecture level city in Shandong
Jinfeng Hongyuan Flagship Store	Electricity conservation2	The quality is very good, inexpensive, Jingdong self-supporting guaranteed, worth having!	Jingdong prefecture level city in Shandong
Jinfeng Hongyuan Flagship Store	Electricity conservation2	I don't know if it's saving electricity yet, I'll find out next time I pay my electric bill	Jingdong prefecture level city in Shandong
Jinfeng Hongyuan Flagship Store	Electricity conservation2	Favorable price quality assurance appearance design is beautiful and generous, fine workmanship	Jingdong prefecture level city in Shandong
Jinfeng Hongyuan Flagship Store	Electricity conservation2	I bought it, I don't know how effective it is? I don't know how the quality is? After this summer results will know! Looking forward to it!	Jingdong prefecture level city in Shandong
Jinfeng Hongyuan Flagship Store	Electricity conservation2	Goods received Packaging intact, look at the shell workmanship is also good, high-power appliances open voltage has obvious changes in the effectiveness of the	Jingdong prefecture level city in Shandong
Jinfeng Hongyuan Flagship Store	Electricity conservation2	The goods have been received logistics is very fast, the product is similar to the line, just wait to use a month to see if it can save electricity, stay tuned.	Jingdong prefecture level city in Shandong
Jinfeng Hongyuan Flagship Store	Electricity conservation2	Electricity is plugged in, not sure if it will work, hopefully it will work.	Jingdong prefecture level city in Shandong
Jinfeng Hongyuan Flagship Store	Electricity conservation2	The shell is aluminum alloy, very good workmanship, the actual effect of waiting for the addition of evaluation	Jingdong prefecture level city in Shandong
Jinfeng Hongyuan Flagship Store	Electricity conservation2	Received, already plugged in, looking forward to the effect.	Jingdong prefecture level city in Shandong
Jinfeng Hongyuan Flagship Store	Electricity conservation2	Opened to see the regular manufacturer products, wait for a period of time to see it	Jingdong prefecture level city in Shandong
Jinfeng Hongyuan Flagship Store	Electricity conservation2	🉑 Baby received, very surprised ✨, reasonable design, fashionable and generous, continue to pay attention to the future will be repurchased ❥❥❥❥	Jingdong prefecture level city in Shandong
Jinfeng Hongyuan Flagship Store	Electricity conservation2	Baby received it 👌, really great ah! 🌈The quality can't be beat, very satisfied! 🆗Happy happy happy 😀	Jingdong prefecture level city in Shandong
Jinfeng Hongyuan Flagship Store	Electricity conservation2	Very good, power saving see, this is to help a friend to buy, I used to give my friend a say, my friend immediately let me order another one!	Jingdong prefecture level city in Shandong
Jinfeng Hongyuan Flagship Store	Electricity conservation2	The baby is just as introduced, the texture is very good, powerful, and it feels good to use it	Jingdong prefecture level city in Shandong
Jinfeng Hongyuan Flagship Store	Electricity conservation2	Value for money, very satisfied, seller service attitude is very good, very patient	Jingdong prefecture level city in Shandong
Jinfeng Hongyuan Flagship Store	Electricity conservation2	Power saving treasure design unique exquisite beautiful, voltage display clearly, plugged in that is good to use, I observed the meter, after using the meter is gone slow.	Jingdong prefecture level city in Shandong
Jinfeng Hongyuan Flagship Store	Electricity conservation2	Received on the use of the packaging is beautifully used for a period of time and then come back in the follow-up review!	Jingdong prefecture level city in Shandong
Jinfeng Hongyuan Flagship Store	Electricity conservation2	It should work. Not bad. Not bad. Not bad.	Jingdong prefecture level city in Shandong
Jinfeng Hongyuan Flagship Store	Electricity conservation2	I haven't used it yet, I'll plug it in and see if it saves power next month.	Jingdong prefecture level city in Shandong
Jinfeng Hongyuan Flagship Store	Electricity conservation2	The goods received and unpacked and used, the quality is very good, things and the official website to buy no difference!	Jingdong prefecture level city in Shandong
Jinfeng Hongyuan Flagship Store	Electricity conservation2	It's a rip off, doesn't help much at all, uses the same amount of power as before it was used.	Jingdong prefecture level city in Shandong
Jinfeng Hongyuan Flagship Store	Electricity conservation2	I've been using it for a while now and haven't noticed any unusual problems, so it's okay	Jingdong prefecture level city in Shandong
Jinfeng Hongyuan Flagship Store	Electricity conservation2	Received the goods and plugged in to use, good or not there is no use will have to wait until next month to find out	Jingdong prefecture level city in Shandong
Jinfeng Hongyuan Flagship Store	Electricity conservation2	It's very compact, it looks good, I'll introduce you to my clients when it's ready.	Jingdong prefecture level city in Shandong
Jinfeng Hongyuan Flagship Store	Electricity conservation2	Good workmanship, we'll have to see in a few months.	Jingdong prefecture level city in Shandong
Jinfeng Hongyuan Flagship Store	Electricity conservation2	Jingdong quality! Always trustworthy!	Jingdong prefecture level city in Shandong
Jinfeng Hongyuan Flagship Store	Electricity conservation2	This is the second time I've bought it, and the merchant said to give me five dollars out of the difference, so thank you merchant.	Jingdong prefecture level city in Shandong
Jinfeng Hongyuan Flagship Store	Electricity conservation2	Good quality and good service	Jingdong prefecture level city in Shandong
Jinfeng Hongyuan Flagship Store	Electricity conservation2	Feels good texture, aluminum alloy shell, voltage digital display clear.	Jingdong prefecture level city in Shandong
Jinfeng Hongyuan Flagship Store	Electricity conservation2	Good product, fast and great service, thanks!	Jingdong prefecture level city in Shandong
Jinfeng Hongyuan Flagship Store	Electricity conservation2	Have not yet used, first evaluation, later really good do not need to comment again.	Jingdong prefecture level city in Shandong
Jinfeng Hongyuan Flagship Store	Electricity conservation2	Works great. Pretty much as described by the merchant.	Jingdong prefecture level city in Shandong
Jinfeng Hongyuan Flagship Store	Electricity conservation2	Received it, looking forward to the results, hope it works	Jingdong prefecture level city in Shandong
Jinfeng Hongyuan Flagship Store	Electricity conservation2	Received it, looking forward to the results, hope it works	Jingdong prefecture level city in Shandong
Jinfeng Hongyuan Flagship Store	Electricity conservation2	Supposedly it's an IQ tax. Buy one and try it. Looks good. Well made.	Jingdong prefecture level city in Shandong
Jinfeng Hongyuan Flagship Store	Electricity conservation2	It just doesn't feel like it's working.	Jingdong prefecture level city in Shandong
Jinfeng Hongyuan Flagship Store	Electricity conservation2	This is a great product.	Jingdong prefecture level city in Shandong
Jinfeng Hongyuan Flagship Store	Electricity conservation2	I'm just using it. I'll tell you more.	Jingdong prefecture level city in Shandong
Jinfeng Hongyuan Flagship Store	Electricity conservation2	Second repurchase and it's working fine.	Jingdong prefecture level city in Shandong

Jinfeng Hongyuan Flagship Store	Electricity conservation2	I'll wait a few days before evaluating	Jingdong prefecture level city in Shandong
Jinfeng Hongyuan Flagship Store	Electricity conservation2	I'll comment after I use it hopefully the section will be useful.	Jingdong prefecture level city in Shandong
Jinfeng Hongyuan Flagship Store	Electricity conservation2	not very useful	Jingdong prefecture level city in Shandong
Jinfeng Hongyuan Flagship Store	Electricity conservation2	Not received	Jingdong prefecture level city in Shandong
Jinfeng Hongyuan Flagship Store	Electricity conservation2	rare	Jingdong prefecture level city in Shandong
Jinfeng Hongyuan Flagship Store	Electricity conservation2	It's good.	Jingdong prefecture level city in Shandong
Jinfeng Hongyuan Flagship Store	Electricity conservation2	just now	Jingdong prefecture level city in Shandong
Jinfeng Hongyuan Flagship Store	Electricity conservation2	serviceable	Jingdong prefecture level city in Shandong
Jinfeng Hongyuan Flagship Store	Electricity conservation2	desirable	Jingdong prefecture level city in Shandong
Jinfeng Hongyuan Flagship Store	Electricity conservation2	serviceable	Jingdong prefecture level city in Shandong
Jinfeng Hongyuan Flagship Store	Electricity conservation2	It doesn't save power and costs electricity.	Jingdong prefecture level city in Shandong
Jinfeng Hongyuan Flagship Store	Electricity conservation2	Not as good as I thought.	Jingdong prefecture level city in Shandong
Jinfeng Hongyuan Flagship Store	Electricity conservation2	Super bad review, it doesn't save power at all, but rather more power consumption	Jingdong prefecture level city in Shandong
Jinfeng Hongyuan Flagship Store	Electricity conservation2	Listen to others say that this can save electricity, I bought with the idea of trying, used for a period of time before coming to the evaluation, will be a little bit of use, not a lot of savings just, long-term use of the words can save more.	Jingdong prefecture level city in Shandong
Jinfeng Hongyuan Flagship Store	Electricity conservation2	Logistics is fast, the next day arrived, workmanship is very delicate a power saver, looking forward to the late effect, customer service patiently explain the recommended which is suitable for, installed in what position is the best, look forward to using the effect after a period of time!	Jingdong prefecture level city in Shandong
Jinfeng Hongyuan Flagship Store	Electricity conservation2	It's good to use, I feel that it has been saving electricity, anyway, not a loss is, I used to feel that the power consumption is big, now I feel much better, my friends are also using, I hope to use a long time will be able to, I wish the boss a prosperous business	Jingdong prefecture level city in Shandong
Jinfeng Hongyuan Flagship Store	Electricity conservation2	Should be very good, to be able to plan the circuit, save the home of the circuit to the extent of the savings, waste of money on electricity, good if a month this money back, hope always hope to be able to	Jingdong prefecture level city in Shandong
Jinfeng Hongyuan Flagship Store	Electricity conservation2	Express delivery is very strong, the goods and I saw in the business consistent, the use of the effect of follow-up evaluation	Jingdong prefecture level city in Shandong
Jinfeng Hongyuan Flagship Store	Electricity conservation2	I have been using this brand of power saver for many years and this time I saw this upgraded version and got it again. The main purpose of this product is to reduce the loss of electricity for a long time, so as to achieve the purpose of saving electricity.	Jingdong prefecture level city in Shandong
Jinfeng Hongyuan Flagship Store	Electricity conservation2	Logistics is fast and the product looks beautiful, looking forward to the effect.	Jingdong prefecture level city in Shandong
Jinfeng Hongyuan Flagship Store	Electricity conservation2	Product quality is good, energy saving a little effect, but the price is a little expensive, fast arrival, good service.	Jingdong prefecture level city in Shandong
Jinfeng Hongyuan Flagship Store	Electricity conservation2	The quality is very good, inexpensive, Jingdong self-supporting guaranteed, worth having!	Jingdong prefecture level city in Shandong
Jinfeng Hongyuan Flagship Store	Electricity conservation2	I don't know if it's saving electricity yet, I'll find out next time I pay my electric bill	Jingdong prefecture level city in Shandong
Jinfeng Hongyuan Flagship Store	Electricity conservation2	Favorable price quality assurance appearance design is beautiful and generous, fine workmanship	Jingdong prefecture level city in Shandong
Jinfeng Hongyuan Flagship Store	Electricity conservation2	I bought it, I don't know how effective it is? I don't know how the quality is? After this summer results will know! Looking forward to it!	Jingdong prefecture level city in Shandong
Jinfeng Hongyuan Flagship Store	Electricity conservation2	Goods received Packaging intact, look at the shell workmanship is also good, high-power appliances open voltage has obvious changes in the effectiveness of the	Jingdong prefecture level city in Shandong
Jinfeng Hongyuan Flagship Store	Electricity conservation2	The goods have been received logistics is very fast, the product is similar to the line, just wait to use a month to see if it can save electricity, stay tuned.	Jingdong prefecture level city in Shandong
Jinfeng Hongyuan Flagship Store	Electricity conservation2	Electricity is plugged in, not sure if it will work, hopefully it will work.	Jingdong prefecture level city in Shandong
Jinfeng Hongyuan Flagship Store	Electricity conservation2	The shell is aluminum alloy, very good workmanship, the actual effect of waiting for the addition of evaluation	Jingdong prefecture level city in Shandong
Jinfeng Hongyuan Flagship Store	Electricity conservation2	Received, already plugged in, looking forward to the effect.	Jingdong prefecture level city in Shandong
Jinfeng Hongyuan Flagship Store	Electricity conservation2	Opened to see the regular manufacturer products, wait for a period of time to see it	Jingdong prefecture level city in Shandong
Jinfeng Hongyuan Flagship Store	Electricity conservation2	🉑 Baby received, very surprised 🎉, reasonable design, fashionable and generous, continue to pay attention to the future will be repurchased ❥❥❥❥	Jingdong prefecture level city in Shandong
Jinfeng Hongyuan Flagship Store	Electricity conservation2	Baby received it 👌, really great ah! 🌈The quality can't be beat, very satisfied! 🆗Happy happy happy 😊	Jingdong prefecture level city in Shandong
Jinfeng Hongyuan Flagship Store	Electricity conservation2	Very good, power saving see, this is to help a friend to buy, I used to give my friend a say, my friend immediately let me order another one!	Jingdong prefecture level city in Shandong
Jinfeng Hongyuan Flagship Store	Electricity conservation2	The baby is just as introduced, the texture is very good, powerful, and it feels good to use it	Jingdong prefecture level city in Shandong
Jinfeng Hongyuan Flagship Store	Electricity conservation2	Value for money, very satisfied, seller service attitude is very good, very patient	Jingdong prefecture level city in Shandong
Jinfeng Hongyuan Flagship Store	Electricity conservation2	Power saving treasure design unique exquisite beautiful, voltage display clearly, plugged in that is good to use, I observed the meter, after using the meter is gone slow.	Jingdong prefecture level city in Shandong
Jinfeng Hongyuan Flagship Store	Electricity conservation2	Received on the use of the packaging is beautifully used for a period of time and then come back in the follow-up review!	Jingdong prefecture level city in Shandong
Jinfeng Hongyuan Flagship Store	Electricity conservation2	It should work. Not bad. Not bad. Not bad.	Jingdong prefecture level city in Shandong
Jinfeng Hongyuan Flagship Store	Electricity conservation2	I haven't used it yet, I'll plug it in and see if it saves power next month.	Jingdong prefecture level city in Shandong
Jinfeng Hongyuan Flagship Store	Electricity conservation2	The goods received and unpacked and used, the quality is very good, things and the official website to buy no difference!	Jingdong prefecture level city in Shandong
Jinfeng Hongyuan Flagship Store	Electricity conservation2	It's a rip off, doesn't help much at all, uses the same amount of power as before it was used.	Jingdong prefecture level city in Shandong
Jinfeng Hongyuan Flagship Store	Electricity conservation2	I've been using it for a while now and haven't noticed any unusual problems, so it's okay	Jingdong prefecture level city in Shandong
Jinfeng Hongyuan Flagship Store	Electricity conservation2	Received the goods and plugged in to use, good or not there is no use will have to wait until next month to find out	Jingdong prefecture level city in Shandong
Jinfeng Hongyuan Flagship Store	Electricity conservation2	It's very compact, it looks good, I'll introduce you to my clients when it's ready.	Jingdong prefecture level city in Shandong
Jinfeng Hongyuan Flagship Store	Electricity conservation2	Good workmanship, we'll have to see in a few months.	Jingdong prefecture level city in Shandong
Jinfeng Hongyuan Flagship Store	Electricity conservation2	Jingdong quality! Always trustworthy!	Jingdong prefecture level city in Shandong
Jinfeng Hongyuan Flagship Store	Electricity conservation2	This is the second time I've bought it, and the merchant said to give me five dollars out of the difference, so thank you merchant.	Jingdong prefecture level city in Shandong
Jinfeng Hongyuan Flagship Store	Electricity conservation2	Good quality and good service	Jingdong prefecture level city in Shandong

Jinfeng Hongyuan Flagship Store	Electricity conservation2	Feels good texture, aluminum alloy shell, voltage digital display clear.	Jingdong prefecture level city in Shandong
Jinfeng Hongyuan Flagship Store	Electricity conservation2	Good product, fast and great service, thanks!	Jingdong prefecture level city in Shandong
Jinfeng Hongyuan Flagship Store	Electricity conservation2	Have not yet used, first evaluation, later really good do not need to comment again.	Jingdong prefecture level city in Shandong
Jinfeng Hongyuan Flagship Store	Electricity conservation2	Works great. Pretty much as described by the merchant.	Jingdong prefecture level city in Shandong
Jinfeng Hongyuan Flagship Store	Electricity conservation2	Received it, looking forward to the results, hope it works	Jingdong prefecture level city in Shandong
Jinfeng Hongyuan Flagship Store	Electricity conservation2	Received it, looking forward to the results, hope it works	Jingdong prefecture level city in Shandong
Jinfeng Hongyuan Flagship Store	Electricity conservation2	Supposedly it's an IQ tax. Buy one and try it. Looks good. Well made.	Jingdong prefecture level city in Shandong
Jinfeng Hongyuan Flagship Store	Electricity conservation2	It just doesn't feel like it's working.	Jingdong prefecture level city in Shandong
Jinfeng Hongyuan Flagship Store	Electricity conservation2	This is a great product.	Jingdong prefecture level city in Shandong
Jinfeng Hongyuan Flagship Store	Electricity conservation2	I'm just using it. I'll tell you more.	Jingdong prefecture level city in Shandong
Jinfeng Hongyuan Flagship Store	Electricity conservation2	Second repurchase and it's working fine.	Jingdong prefecture level city in Shandong
Jinfeng Hongyuan Flagship Store	Electricity conservation2	I'll wait a few days before evaluating	Jingdong prefecture level city in Shandong
Jinfeng Hongyuan Flagship Store	Electricity conservation2	I'll comment after I use it hopefully the section will be useful.	Jingdong prefecture level city in Shandong
Jinfeng Hongyuan Flagship Store	Electricity conservation2	not very useful	Jingdong prefecture level city in Shandong
Jinfeng Hongyuan Flagship Store	Electricity conservation2	Not received	Jingdong prefecture level city in Shandong
Jinfeng Hongyuan Flagship Store	Electricity conservation2	rare	Jingdong prefecture level city in Shandong
Jinfeng Hongyuan Flagship Store	Electricity conservation2	It's good.	Jingdong prefecture level city in Shandong
Jinfeng Hongyuan Flagship Store	Electricity conservation2	just now	Jingdong prefecture level city in Shandong
Jinfeng Hongyuan Flagship Store	Electricity conservation2	serviceable	Jingdong prefecture level city in Shandong
Jinfeng Hongyuan Flagship Store	Electricity conservation2	desirable	Jingdong prefecture level city in Shandong
Jinfeng Hongyuan Flagship Store	Electricity conservation2	serviceable	Jingdong prefecture level city in Shandong
Jinfeng Hongyuan Flagship Store	Electricity conservation2	It doesn't save power and costs electricity.	Jingdong prefecture level city in Shandong
Jinfeng Hongyuan Flagship Store	Electricity conservation2	Not as good as I thought.	Jingdong prefecture level city in Shandong
Jinfeng Hongyuan Flagship Store	Electricity conservation2	Super bad review, it doesn't save power at all, but rather more power consumption	Jingdong prefecture level city in Shandong
Jinfeng Hongyuan Flagship Store	Electricity conservation2	Listen to others say that this can save electricity, I bought with the idea of trying, used for a period of time before coming to the evaluation, will be a little bit of use, not a lot of savings just, long-term use of the words can save more.	Jingdong prefecture level city in Shandong
Jinfeng Hongyuan Flagship Store	Electricity conservation2	Logistics is fast, the next day arrived, workmanship is very delicate a power saver, looking forward to the late effect, customer service patiently explain the recommended which is suitable for, installed in what position is the best, look forward to using the effect after a period of time!	Jingdong prefecture level city in Shandong
Jinfeng Hongyuan Flagship Store	Electricity conservation2	It's good to use, I feel that it has been saving electricity, anyway, not a loss is, I used to feel that the power consumption is big, now I feel much better, my friends are also using, I hope to use a long time will be able to, I wish the boss a prosperous business	Jingdong prefecture level city in Shandong
Jinfeng Hongyuan Flagship Store	Electricity conservation2	Should be very good, to be able to plan the circuit, save the home of the circuit to the extent of the savings, waste of money on electricity, good if a month this money back, hope always hope to be able to	Jingdong prefecture level city in Shandong
Jinfeng Hongyuan Flagship Store	Electricity conservation2	Express delivery is very strong, the goods and I saw in the business consistent, the use of the effect of follow-up evaluation	Jingdong prefecture level city in Shandong
Jinfeng Hongyuan Flagship Store	Electricity conservation2	I have been using this brand of power saver for many years and this time I saw this upgraded version and got it again. The main purpose of this product is to reduce the loss of electricity for a long time, so as to achieve the purpose of saving electricity.	Jingdong prefecture level city in Shandong
Jinfeng Hongyuan Flagship Store	Electricity conservation2	Logistics is fast and the product looks beautiful, looking forward to the effect.	Jingdong prefecture level city in Shandong
Jinfeng Hongyuan Flagship Store	Electricity conservation2	Product quality is good, energy saving a little effect, but the price is a little expensive, fast arrival, good service.	Jingdong prefecture level city in Shandong
Jinfeng Hongyuan Flagship Store	Electricity conservation2	The quality is very good, inexpensive, Jingdong self-supporting guaranteed, worth having!	Jingdong prefecture level city in Shandong
Jinfeng Hongyuan Flagship Store	Electricity conservation2	I don't know if it's saving electricity yet, I'll find out next time I pay my electric bill	Jingdong prefecture level city in Shandong
Jinfeng Hongyuan Flagship Store	Electricity conservation2	Favorable price quality assurance appearance design is beautiful and generous, fine workmanship	Jingdong prefecture level city in Shandong
Jinfeng Hongyuan Flagship Store	Electricity conservation2	I bought it, I don't know how effective it is? I don't know how the quality is? After this summer results will know! Looking forward to it!	Jingdong prefecture level city in Shandong
Jinfeng Hongyuan Flagship Store	Electricity conservation2	Goods received Packaging intact, look at the shell workmanship is also good, high-power appliances open voltage has obvious changes in the effectiveness of the	Jingdong prefecture level city in Shandong
Jinfeng Hongyuan Flagship Store	Electricity conservation2	The goods have been received logistics is very fast, the product is similar to the line, just wait to use a month to see if it can save electricity, stay tuned.	Jingdong prefecture level city in Shandong
Jinfeng Hongyuan Flagship Store	Electricity conservation2	Electricity is plugged in, not sure if it will work, hopefully it will work.	Jingdong prefecture level city in Shandong
Jinfeng Hongyuan Flagship Store	Electricity conservation2	The shell is aluminum alloy, very good workmanship, the actual effect of waiting for the addition of evaluation	Jingdong prefecture level city in Shandong
Jinfeng Hongyuan Flagship Store	Electricity conservation2	Received, already plugged in, looking forward to the effect.	Jingdong prefecture level city in Shandong
Jinfeng Hongyuan Flagship Store	Electricity conservation2	Opened to see the regular manufacturer products, wait for a period of time to see it	Jingdong prefecture level city in Shandong
Jinfeng Hongyuan Flagship Store	Electricity conservation2	🉑 Baby received, very surprised ✨, reasonable design, fashionable and generous, continue to pay attention to the future will be repurchased ❤❤❤❤	Jingdong prefecture level city in Shandong
Jinfeng Hongyuan Flagship Store	Electricity conservation2	Baby received it 👌, really great ah! 🌈The quality can't be beat, very satisfied! 🆗Happy happy happy 😃	Jingdong prefecture level city in Shandong
Jinfeng Hongyuan Flagship Store	Electricity conservation2	Very good, power saving see, this is to help a friend to buy, I used to give my friend a say, my friend immediately let me order another one!	Jingdong prefecture level city in Shandong
Jinfeng Hongyuan Flagship Store	Electricity conservation2	The baby is just as introduced, the texture is very good, powerful, and it feels good to use it	Jingdong prefecture level city in Shandong
Jinfeng Hongyuan Flagship Store	Electricity conservation2	Value for money, very satisfied, seller service attitude is very good, very patient	Jingdong prefecture level city in Shandong
Jinfeng Hongyuan Flagship Store	Electricity conservation2	Power saving treasure design unique exquisite beautiful, voltage display clearly, plugged in that is good to use, I observed the meter, after using the meter is gone slow.	Jingdong prefecture level city in Shandong
Jinfeng Hongyuan Flagship Store	Electricity conservation2	Received on the use of the packaging is beautifully used for a period of time and then come back in the follow-up review!	Jingdong prefecture level city in Shandong

Jinfeng Hongyuan Flagship Store	Electricity conservation2	It should work. Not bad. Not bad. Not bad.	Jingdong prefecture level city in Shandong
Jinfeng Hongyuan Flagship Store	Electricity conservation2	I haven't used it yet, I'll plug it in and see if it saves power next month.	Jingdong prefecture level city in Shandong
Jinfeng Hongyuan Flagship Store	Electricity conservation2	The goods received and unpacked and used, the quality is very good, things and the official website to buy no difference!	Jingdong prefecture level city in Shandong
Jinfeng Hongyuan Flagship Store	Electricity conservation2	It's a rip off, doesn't help much at all, uses the same amount of power as before it was used.	Jingdong prefecture level city in Shandong
Jinfeng Hongyuan Flagship Store	Electricity conservation2	I've been using it for a while now and haven't noticed any unusual problems, so it's okay	Jingdong prefecture level city in Shandong
Jinfeng Hongyuan Flagship Store	Electricity conservation2	Received the goods and plugged in to use, good or not there is no use will have to wait until next month to find out	Jingdong prefecture level city in Shandong
Jinfeng Hongyuan Flagship Store	Electricity conservation2	It's very compact, it looks good, I'll introduce you to my clients when it's ready.	Jingdong prefecture level city in Shandong
Jinfeng Hongyuan Flagship Store	Electricity conservation2	Good workmanship, we'll have to see in a few months.	Jingdong prefecture level city in Shandong
Jinfeng Hongyuan Flagship Store	Electricity conservation2	Jingdong quality! Always trustworthy!	Jingdong prefecture level city in Shandong
Jinfeng Hongyuan Flagship Store	Electricity conservation2	This is the second time I've bought it, and the merchant said to give me five dollars out of the difference, so thank you merchant.	Jingdong prefecture level city in Shandong
Jinfeng Hongyuan Flagship Store	Electricity conservation2	Good quality and good service	Jingdong prefecture level city in Shandong
Jinfeng Hongyuan Flagship Store	Electricity conservation2	Feels good texture, aluminum alloy shell, voltage digital display clear.	Jingdong prefecture level city in Shandong
Jinfeng Hongyuan Flagship Store	Electricity conservation2	Good product, fast and great service, thanks!	Jingdong prefecture level city in Shandong
Jinfeng Hongyuan Flagship Store	Electricity conservation2	Have not yet used, first evaluation, later really good do not need to comment again.	Jingdong prefecture level city in Shandong
Jinfeng Hongyuan Flagship Store	Electricity conservation2	Works great. Pretty much as described by the merchant.	Jingdong prefecture level city in Shandong
Jinfeng Hongyuan Flagship Store	Electricity conservation2	Received it, looking forward to the results, hope it works	Jingdong prefecture level city in Shandong
Jinfeng Hongyuan Flagship Store	Electricity conservation2	Received it, looking forward to the results, hope it works	Jingdong prefecture level city in Shandong
Jinfeng Hongyuan Flagship Store	Electricity conservation2	Supposedly it's an IQ tax. Buy one and try it. Looks good. Well made.	Jingdong prefecture level city in Shandong
Jinfeng Hongyuan Flagship Store	Electricity conservation2	It just doesn't feel like it's working.	Jingdong prefecture level city in Shandong
Jinfeng Hongyuan Flagship Store	Electricity conservation2	This is a great product.	Jingdong prefecture level city in Shandong
Jinfeng Hongyuan Flagship Store	Electricity conservation2	I'm just using it. I'll tell you more.	Jingdong prefecture level city in Shandong
Jinfeng Hongyuan Flagship Store	Electricity conservation2	Second repurchase and it's working fine.	Jingdong prefecture level city in Shandong
Jinfeng Hongyuan Flagship Store	Electricity conservation2	I'll wait a few days before evaluating	Jingdong prefecture level city in Shandong
Jinfeng Hongyuan Flagship Store	Electricity conservation2	I'll comment after I use it hopefully the section will be useful.	Jingdong prefecture level city in Shandong
Jinfeng Hongyuan Flagship Store	Electricity conservation2	not very useful	Jingdong prefecture level city in Shandong
Jinfeng Hongyuan Flagship Store	Electricity conservation2	Not received	Jingdong prefecture level city in Shandong
Jinfeng Hongyuan Flagship Store	Electricity conservation2	rare	Jingdong prefecture level city in Shandong
Jinfeng Hongyuan Flagship Store	Electricity conservation2	It's good.	Jingdong prefecture level city in Shandong
Jinfeng Hongyuan Flagship Store	Electricity conservation2	just now	Jingdong prefecture level city in Shandong
Jinfeng Hongyuan Flagship Store	Electricity conservation2	serviceable	Jingdong prefecture level city in Shandong
Jinfeng Hongyuan Flagship Store	Electricity conservation2	desirable	Jingdong prefecture level city in Shandong
Jinfeng Hongyuan Flagship Store	Electricity conservation2	serviceable	Jingdong prefecture level city in Shandong
Jinfeng Hongyuan Flagship Store	Electricity conservation2	It doesn't save power and costs electricity.	Jingdong prefecture level city in Shandong
Jinfeng Hongyuan Flagship Store	Electricity conservation2	Not as good as I thought.	Jingdong prefecture level city in Shandong
Jinfeng Hongyuan Flagship Store	Electricity conservation2	Super bad review, it doesn't save power at all, but rather more power consumption	Jingdong prefecture level city in Shandong
Jinfeng Hongyuan Flagship Store	Electricity conservation2	Listen to others say that this can save electricity, I bought with the idea of trying, used for a period of time before coming to the evaluation, will be a little bit of use, not a lot of savings just, long-term use of the words can save more.	Jingdong prefecture level city in Shandong
Jinfeng Hongyuan Flagship Store	Electricity conservation2	Logistics is fast, the next day arrived, workmanship is very delicate a power saver, looking forward to the late effect, customer service patiently explain the recommended which is suitable for, installed in what position is the best, look forward to using the effect after a period of time!	Jingdong prefecture level city in Shandong
Jinfeng Hongyuan Flagship Store	Electricity conservation2	It's good to use, I feel that it has been saving electricity, anyway, not a loss is, I used to feel that the power consumption is big, now I feel much better, my friends are also using, I hope to use a long time will be able to, I wish the boss a prosperous business	Jingdong prefecture level city in Shandong
Jinfeng Hongyuan Flagship Store	Electricity conservation2	Should be very good, to be able to plan the circuit, save the home of the circuit to the extent of the savings, waste of money on electricity, good if a month this money back, hope always hope to be able to	Jingdong prefecture level city in Shandong
Jinfeng Hongyuan Flagship Store	Electricity conservation2	Express delivery is very strong, the goods and I saw in the business consistent, the use of the effect of follow-up evaluation	Jingdong prefecture level city in Shandong
Jinfeng Hongyuan Flagship Store	Electricity conservation2	I have been using this brand of power saver for many years and this time I saw this upgraded version and got it again. The main purpose of this product is to reduce the loss of electricity for a long time, so as to achieve the purpose of saving electricity.	Jingdong prefecture level city in Shandong
Jinfeng Hongyuan Flagship Store	Electricity conservation2	Logistics is fast and the product looks beautiful, looking forward to the effect.	Jingdong prefecture level city in Shandong
Jinfeng Hongyuan Flagship Store	Electricity conservation2	Product quality is good, energy saving a little effect, but the price is a little expensive, fast arrival, good service.	Jingdong prefecture level city in Shandong
Jinfeng Hongyuan Flagship Store	Electricity conservation2	The quality is very good, inexpensive, Jingdong self-supporting guaranteed, worth having!	Jingdong prefecture level city in Shandong
Jinfeng Hongyuan Flagship Store	Electricity conservation2	I don't know if it's saving electricity yet, I'll find out next time I pay my electric bill	Jingdong prefecture level city in Shandong
Jinfeng Hongyuan Flagship Store	Electricity conservation2	Favorable price quality assurance appearance design is beautiful and generous, fine workmanship	Jingdong prefecture level city in Shandong
Jinfeng Hongyuan Flagship Store	Electricity conservation2	I bought it, I don't know how effective it is? I don't know how the quality is? After this summer results will know! Looking forward to it!	Jingdong prefecture level city in Shandong
Jinfeng Hongyuan Flagship Store	Electricity conservation2	Goods received Packaging intact, look at the shell workmanship is also good, high-power appliances open voltage has obvious changes in the effectiveness of the	Jingdong prefecture level city in Shandong
Jinfeng Hongyuan Flagship Store	Electricity conservation2	The goods have been received logistics is very fast, the product is similar to the line, just wait to use a month to see if it can save electricity, stay tuned.	Jingdong prefecture level city in Shandong

Jinfeng Hongyuan Flagship Store	Electricity conservation2	Electricity is plugged in, not sure if it will work, hopefully it will work.	Jingdong prefecture level city in Shandong
Jinfeng Hongyuan Flagship Store	Electricity conservation2	The shell is aluminum alloy, very good workmanship, the actual effect of waiting for the addition of evaluation	Jingdong prefecture level city in Shandong
Jinfeng Hongyuan Flagship Store	Electricity conservation2	Received, already plugged in, looking forward to the effect.	Jingdong prefecture level city in Shandong
Jinfeng Hongyuan Flagship Store	Electricity conservation2	Opened to see the regular manufacturer products, wait for a period of time to see it	Jingdong prefecture level city in Shandong
Jinfeng Hongyuan Flagship Store	Electricity conservation2	🉑 Baby received, very surprised ✨, reasonable design, fashionable and generous, continue to pay attention to the future will be repurchased ❥❥❥❥	Jingdong prefecture level city in Shandong
Jinfeng Hongyuan Flagship Store	Electricity conservation2	Baby received it 👌, really great ah! 🌈The quality can't be beat, very satisfied! 🆗Happy happy happy 😃	Jingdong prefecture level city in Shandong
Jinfeng Hongyuan Flagship Store	Electricity conservation2	Very good, power saving see, this is to help a friend to buy, I used to give my friend a say, my friend immediately let me order another one!	Jingdong prefecture level city in Shandong
Jinfeng Hongyuan Flagship Store	Electricity conservation2	The baby is just as introduced, the texture is very good, powerful, and it feels good to use it	Jingdong prefecture level city in Shandong
Jinfeng Hongyuan Flagship Store	Electricity conservation2	Value for money, very satisfied, seller service attitude is very good, very patient	Jingdong prefecture level city in Shandong
Jinfeng Hongyuan Flagship Store	Electricity conservation2	Power saving treasure design unique exquisite beautiful, voltage display clearly, plugged in that is good to use, I observed the meter, after using the meter is gone slow.	Jingdong prefecture level city in Shandong
Jinfeng Hongyuan Flagship Store	Electricity conservation2	Received on the use of the packaging is beautifully used for a period of time and then come back in the follow-up review!	Jingdong prefecture level city in Shandong
Jinfeng Hongyuan Flagship Store	Electricity conservation2	It should work. Not bad. Not bad. Not bad.	Jingdong prefecture level city in Shandong
Jinfeng Hongyuan Flagship Store	Electricity conservation2	I haven't used it yet, I'll plug it in and see if it saves power next month.	Jingdong prefecture level city in Shandong
Jinfeng Hongyuan Flagship Store	Electricity conservation2	The goods received and unpacked and used, the quality is very good, things and the official website to buy no difference!	Jingdong prefecture level city in Shandong
Jinfeng Hongyuan Flagship Store	Electricity conservation2	It's a rip off, doesn't help much at all, uses the same amount of power as before it was used.	Jingdong prefecture level city in Shandong
Jinfeng Hongyuan Flagship Store	Electricity conservation2	I've been using it for a while now and haven't noticed any unusual problems, so it's okay	Jingdong prefecture level city in Shandong
Jinfeng Hongyuan Flagship Store	Electricity conservation2	Received the goods and plugged in to use, good or not there is no use will have to wait until next month to find out	Jingdong prefecture level city in Shandong
Jinfeng Hongyuan Flagship Store	Electricity conservation2	It's very compact, it looks good, I'll introduce you to my clients when it's ready.	Jingdong prefecture level city in Shandong
Jinfeng Hongyuan Flagship Store	Electricity conservation2	Good workmanship, we'll have to see in a few months.	Jingdong prefecture level city in Shandong
Jinfeng Hongyuan Flagship Store	Electricity conservation2	Jingdong quality! Always trustworthy!	Jingdong prefecture level city in Shandong
Jinfeng Hongyuan Flagship Store	Electricity conservation2	This is the second time I've bought it, and the merchant said to give me five dollars out of the difference, so thank you merchant.	Jingdong prefecture level city in Shandong
Jinfeng Hongyuan Flagship Store	Electricity conservation2	Good quality and good service	Jingdong prefecture level city in Shandong
Jinfeng Hongyuan Flagship Store	Electricity conservation2	Feels good texture, aluminum alloy shell, voltage digital display clear.	Jingdong prefecture level city in Shandong
Jinfeng Hongyuan Flagship Store	Electricity conservation2	Good product, fast and great service, thanks!	Jingdong prefecture level city in Shandong
Jinfeng Hongyuan Flagship Store	Electricity conservation2	Have not yet used, first evaluation, later really good do not need to comment again.	Jingdong prefecture level city in Shandong
Jinfeng Hongyuan Flagship Store	Electricity conservation2	Works great. Pretty much as described by the merchant.	Jingdong prefecture level city in Shandong
Jinfeng Hongyuan Flagship Store	Electricity conservation2	Received it, looking forward to the results, hope it works	Jingdong prefecture level city in Shandong
Jinfeng Hongyuan Flagship Store	Electricity conservation2	Received it, looking forward to the results, hope it works	Jingdong prefecture level city in Shandong
Jinfeng Hongyuan Flagship Store	Electricity conservation2	Supposedly it's an IQ tax. Buy one and try it. Looks good. Well made.	Jingdong prefecture level city in Shandong
Jinfeng Hongyuan Flagship Store	Electricity conservation2	It just doesn't feel like it's working.	Jingdong prefecture level city in Shandong
Jinfeng Hongyuan Flagship Store	Electricity conservation2	This is a great product.	Jingdong prefecture level city in Shandong
Jinfeng Hongyuan Flagship Store	Electricity conservation2	I'm just using it. I'll tell you more.	Jingdong prefecture level city in Shandong
Jinfeng Hongyuan Flagship Store	Electricity conservation2	Second repurchase and it's working fine.	Jingdong prefecture level city in Shandong
Jinfeng Hongyuan Flagship Store	Electricity conservation2	I'll wait a few days before evaluating	Jingdong prefecture level city in Shandong
Jinfeng Hongyuan Flagship Store	Electricity conservation2	I'll comment after I use it hopefully the section will be useful.	Jingdong prefecture level city in Shandong
Jinfeng Hongyuan Flagship Store	Electricity conservation2	not very useful	Jingdong prefecture level city in Shandong
Jinfeng Hongyuan Flagship Store	Electricity conservation2	Not received	Jingdong prefecture level city in Shandong
Jinfeng Hongyuan Flagship Store	Electricity conservation2	rare	Jingdong prefecture level city in Shandong
Jinfeng Hongyuan Flagship Store	Electricity conservation2	It's good.	Jingdong prefecture level city in Shandong
Jinfeng Hongyuan Flagship Store	Electricity conservation2	just now	Jingdong prefecture level city in Shandong
Jinfeng Hongyuan Flagship Store	Electricity conservation2	serviceable	Jingdong prefecture level city in Shandong
Jinfeng Hongyuan Flagship Store	Electricity conservation2	desirable	Jingdong prefecture level city in Shandong
Jinfeng Hongyuan Flagship Store	Electricity conservation2	serviceable	Jingdong prefecture level city in Shandong
Jinfeng Hongyuan Flagship Store	Electricity conservation2	It doesn't save power and costs electricity.	Jingdong prefecture level city in Shandong
Jinfeng Hongyuan Flagship Store	Electricity conservation2	Not as good as I thought.	Jingdong prefecture level city in Shandong
Jinfeng Hongyuan Flagship Store	Electricity conservation2	Super bad review, it doesn't save power at all, but rather more power consumption	Jingdong prefecture level city in Shandong
Jinfeng Hongyuan Flagship Store	Electricity conservation2	Listen to others say that this can save electricity, I bought with the idea of trying, used for a period of time before coming to the evaluation, will be a little bit of use, not a lot of savings just, long-term use of the words can save more.	Jingdong prefecture level city in Shandong
Jinfeng Hongyuan Flagship Store	Electricity conservation2	Logistics is fast, the next day arrived, workmanship is very delicate a power saver, looking forward to the late effect, customer service patiently explain the recommended which is suitable for, installed in what position is the best, look forward to using the effect after a period of time!	Jingdong prefecture level city in Shandong
Jinfeng Hongyuan Flagship Store	Electricity conservation2	It's good to use, I feel that it has been saving electricity, anyway, not a loss is, I used to feel that the power consumption is big, now I feel much better, my friends are also using, I hope to use a long time will be able to, I wish the boss a prosperous business	Jingdong prefecture level city in Shandong
Jinfeng Hongyuan Flagship Store	Electricity conservation2	Should be very good, to be able to plan the circuit, save the home of the circuit to the extent of the savings, waste of money on electricity, good if a month this money back, hope always hope to be able to	Jingdong prefecture level city in Shandong

Jinfeng Hongyuan Flagship Store	Electricity conservation2	Express delivery is very strong, the goods and I saw in the business consistent, the use of the effect of follow-up evaluation	Jingdong prefecture level city in Shandong
Jinfeng Hongyuan Flagship Store	Electricity conservation2	I have been using this brand of power saver for many years and this time I saw this upgraded version and got it again. The main purpose of this product is to reduce the loss of electricity for a long time, so as to achieve the purpose of saving electricity.	Jingdong prefecture level city in Shandong
Jinfeng Hongyuan Flagship Store	Electricity conservation2	Logistics is fast and the product looks beautiful, looking forward to the effect.	Jingdong prefecture level city in Shandong
Jinfeng Hongyuan Flagship Store	Electricity conservation2	Product quality is good, energy saving a little effect, but the price is a little expensive, fast arrival, good service.	Jingdong prefecture level city in Shandong
Jinfeng Hongyuan Flagship Store	Electricity conservation2	The quality is very good, inexpensive, Jingdong self-supporting guaranteed, worth having!	Jingdong prefecture level city in Shandong
Jinfeng Hongyuan Flagship Store	Electricity conservation2	I don't know if it's saving electricity yet, I'll find out next time I pay my electric bill	Jingdong prefecture level city in Shandong
Jinfeng Hongyuan Flagship Store	Electricity conservation2	Favorable price quality assurance appearance design is beautiful and generous, fine workmanship	Jingdong prefecture level city in Shandong
Jinfeng Hongyuan Flagship Store	Electricity conservation2	I bought it, I don't know how effective it is? I don't know how the quality is? After this summer results will know! Looking forward to it!	Jingdong prefecture level city in Shandong
Jinfeng Hongyuan Flagship Store	Electricity conservation2	Goods received Packaging intact, look at the shell workmanship is also good, high-power appliances open voltage has obvious changes in the effectiveness of the	Jingdong prefecture level city in Shandong
Jinfeng Hongyuan Flagship Store	Electricity conservation2	The goods have been received logistics is very fast, the product is similar to the line, just wait to use a month to see if it can save electricity, stay tuned.	Jingdong prefecture level city in Shandong
Jinfeng Hongyuan Flagship Store	Electricity conservation2	Electricity is plugged in, not sure if it will work, hopefully it will work.	Jingdong prefecture level city in Shandong
Jinfeng Hongyuan Flagship Store	Electricity conservation2	The shell is aluminum alloy, very good workmanship, the actual effect of waiting for the addition of evaluation	Jingdong prefecture level city in Shandong
Jinfeng Hongyuan Flagship Store	Electricity conservation2	Received, already plugged in, looking forward to the effect.	Jingdong prefecture level city in Shandong
Jinfeng Hongyuan Flagship Store	Electricity conservation2	Opened to see the regular manufacturer products, wait for a period of time to see it	Jingdong prefecture level city in Shandong
Jinfeng Hongyuan Flagship Store	Electricity conservation2	🉑 Baby received, very surprised ✨, reasonable design, fashionable and generous, continue to pay attention to the future will be repurchased ❥❥❥❥	Jingdong prefecture level city in Shandong
Jinfeng Hongyuan Flagship Store	Electricity conservation2	Baby received it 👌, really great ah! 🌈The quality can't be beat, very satisfied! 🆗Happy happy happy 😃	Jingdong prefecture level city in Shandong
Jinfeng Hongyuan Flagship Store	Electricity conservation2	Very good, power saving see, this is to help a friend to buy, I used to give my friend a say, my friend immediately let me order another one!	Jingdong prefecture level city in Shandong
Jinfeng Hongyuan Flagship Store	Electricity conservation2	The baby is just as introduced, the texture is very good, powerful, and it feels good to use it	Jingdong prefecture level city in Shandong
Jinfeng Hongyuan Flagship Store	Electricity conservation2	Value for money, very satisfied, seller service attitude is very good, very patient	Jingdong prefecture level city in Shandong
Jinfeng Hongyuan Flagship Store	Electricity conservation2	Power saving treasure design unique exquisite beautiful, voltage display clearly, plugged in that is good to use, I observed the meter, after using the meter is gone slow.	Jingdong prefecture level city in Shandong
Jinfeng Hongyuan Flagship Store	Electricity conservation2	Received on the use of the packaging is beautifully used for a period of time and then come back in the follow-up review!	Jingdong prefecture level city in Shandong
Jinfeng Hongyuan Flagship Store	Electricity conservation2	It should work. Not bad. Not bad. Not bad.	Jingdong prefecture level city in Shandong
Jinfeng Hongyuan Flagship Store	Electricity conservation2	I haven't used it yet, I'll plug it in and see if it saves power next month.	Jingdong prefecture level city in Shandong
Jinfeng Hongyuan Flagship Store	Electricity conservation2	The goods received and unpacked and used, the quality is very good, things and the official website to buy no difference!	Jingdong prefecture level city in Shandong
Jinfeng Hongyuan Flagship Store	Electricity conservation2	It's a rip off, doesn't help much at all, uses the same amount of power as before it was used.	Jingdong prefecture level city in Shandong
Jinfeng Hongyuan Flagship Store	Electricity conservation2	I've been using it for a while now and haven't noticed any unusual problems, so it's okay	Jingdong prefecture level city in Shandong
Jinfeng Hongyuan Flagship Store	Electricity conservation2	Received the goods and plugged in to use, good or not there is no use will have to wait until next month to find out	Jingdong prefecture level city in Shandong
Jinfeng Hongyuan Flagship Store	Electricity conservation2	It's very compact, it looks good, I'll introduce you to my clients when it's ready.	Jingdong prefecture level city in Shandong
Jinfeng Hongyuan Flagship Store	Electricity conservation2	Good workmanship, we'll have to see in a few months.	Jingdong prefecture level city in Shandong
Jinfeng Hongyuan Flagship Store	Electricity conservation2	Jingdong quality! Always trustworthy!	Jingdong prefecture level city in Shandong
Jinfeng Hongyuan Flagship Store	Electricity conservation2	This is the second time I've bought it, and the merchant said to give me five dollars out of the difference, so thank you merchant.	Jingdong prefecture level city in Shandong
Jinfeng Hongyuan Flagship Store	Electricity conservation2	Good quality and good service	Jingdong prefecture level city in Shandong
Jinfeng Hongyuan Flagship Store	Electricity conservation2	Feels good texture, aluminum alloy shell, voltage digital display clear.	Jingdong prefecture level city in Shandong
Jinfeng Hongyuan Flagship Store	Electricity conservation2	Good product, fast and great service, thanks!	Jingdong prefecture level city in Shandong
Jinfeng Hongyuan Flagship Store	Electricity conservation2	Have not yet used, first evaluation, later really good do not need to comment again.	Jingdong prefecture level city in Shandong
Jinfeng Hongyuan Flagship Store	Electricity conservation2	Works great. Pretty much as described by the merchant.	Jingdong prefecture level city in Shandong
Jinfeng Hongyuan Flagship Store	Electricity conservation2	Received it, looking forward to the results, hope it works	Jingdong prefecture level city in Shandong
Jinfeng Hongyuan Flagship Store	Electricity conservation2	Received it, looking forward to the results, hope it works	Jingdong prefecture level city in Shandong
Jinfeng Hongyuan Flagship Store	Electricity conservation2	Supposedly it's an IQ tax. Buy one and try it. Looks good. Well made.	Jingdong prefecture level city in Shandong
Jinfeng Hongyuan Flagship Store	Electricity conservation2	It just doesn't feel like it's working.	Jingdong prefecture level city in Shandong
Jinfeng Hongyuan Flagship Store	Electricity conservation2	This is a great product.	Jingdong prefecture level city in Shandong
Jinfeng Hongyuan Flagship Store	Electricity conservation2	I'm just using it. I'll tell you more.	Jingdong prefecture level city in Shandong
Jinfeng Hongyuan Flagship Store	Electricity conservation2	Second repurchase and it's working fine.	Jingdong prefecture level city in Shandong
Jinfeng Hongyuan Flagship Store	Electricity conservation2	I'll wait a few days before evaluating	Jingdong prefecture level city in Shandong
Jinfeng Hongyuan Flagship Store	Electricity conservation2	I'll comment after I use it hopefully the section will be useful.	Jingdong prefecture level city in Shandong
Jinfeng Hongyuan Flagship Store	Electricity conservation2	not very useful	Jingdong prefecture level city in Shandong
Jinfeng Hongyuan Flagship Store	Electricity conservation2	Not received	Jingdong prefecture level city in Shandong
Jinfeng Hongyuan Flagship Store	Electricity conservation2	rare	Jingdong prefecture level city in Shandong
Jinfeng Hongyuan Flagship Store	Electricity conservation2	It's good.	Jingdong prefecture level city in Shandong
Jinfeng Hongyuan Flagship Store	Electricity conservation2	just now	Jingdong prefecture level city in Shandong
Jinfeng Hongyuan Flagship Store	Electricity conservation2	serviceable	Jingdong prefecture level city in Shandong

Jinfeng Hongyuan Flagship Store	Electricity conservation2	desirable	Jingdong prefecture level city in Shandong
Jinfeng Hongyuan Flagship Store	Electricity conservation2	serviceable	Jingdong prefecture level city in Shandong
Jinfeng Hongyuan Flagship Store	Electricity conservation2	It doesn't save power and costs electricity.	Jingdong prefecture level city in Shandong
Jinfeng Hongyuan Flagship Store	Electricity conservation2	Not as good as I thought.	Jingdong prefecture level city in Shandong
Jinfeng Hongyuan Flagship Store	Electricity conservation2	Super bad review, it doesn't save power at all, but rather more power consumption	Jingdong prefecture level city in Shandong
Jinfeng Hongyuan Flagship Store	Electricity conservation2	Listen to others say that this can save electricity, I bought with the idea of trying, used for a period of time before coming to the evaluation, will be a little bit of use, not a lot of savings just, long-term use of the words can save more.	Jingdong prefecture level city in Shandong
Jinfeng Hongyuan Flagship Store	Electricity conservation2	Logistics is fast, the next day arrived, workmanship is very delicate a power saver, looking forward to the late effect, customer service patiently explain the recommended which is suitable for, installed in what position is the best, look forward to using the effect after a period of time!	Jingdong prefecture level city in Shandong
Jinfeng Hongyuan Flagship Store	Electricity conservation2	It's good to use, I feel that it has been saving electricity, anyway, not a loss is, I used to feel that the power consumption is big, now I feel much better, my friends are also using, I hope to use a long time will be able to, I wish the boss a prosperous business	Jingdong prefecture level city in Shandong
Jinfeng Hongyuan Flagship Store	Electricity conservation2	Should be very good, to be able to plan the circuit, save the home of the circuit to the extent of the savings, waste of money on electricity, good if a month this money back, hope always hope to be able to	Jingdong prefecture level city in Shandong
Jinfeng Hongyuan Flagship Store	Electricity conservation2	Express delivery is very strong, the goods and I saw in the business consistent, the use of the effect of follow-up evaluation	Jingdong prefecture level city in Shandong
Jinfeng Hongyuan Flagship Store	Electricity conservation2	I have been using this brand of power saver for many years and this time I saw this upgraded version and got it again. The main purpose of this product is to reduce the loss of electricity for a long time, so as to achieve the purpose of saving electricity.	Jingdong prefecture level city in Shandong
Jinfeng Hongyuan Flagship Store	Electricity conservation2	Logistics is fast and the product looks beautiful, looking forward to the effect.	Jingdong prefecture level city in Shandong
Jinfeng Hongyuan Flagship Store	Electricity conservation2	Product quality is good, energy saving a little effect, but the price is a little expensive, fast arrival, good service.	Jingdong prefecture level city in Shandong
Jinfeng Hongyuan Flagship Store	Electricity conservation2	The quality is very good, inexpensive, Jingdong self-supporting guaranteed, worth having!	Jingdong prefecture level city in Shandong
Jinfeng Hongyuan Flagship Store	Electricity conservation2	I don't know if it's saving electricity yet, I'll find out next time I pay my electric bill	Jingdong prefecture level city in Shandong
Jinfeng Hongyuan Flagship Store	Electricity conservation2	Favorable price quality assurance appearance design is beautiful and generous, fine workmanship	Jingdong prefecture level city in Shandong
Jinfeng Hongyuan Flagship Store	Electricity conservation2	I bought it, I don't know how effective it is? I don't know how the quality is? After this summer results will know! Looking forward to it!	Jingdong prefecture level city in Shandong
Jinfeng Hongyuan Flagship Store	Electricity conservation2	Goods received Packaging intact, look at the shell workmanship is also good, high-power appliances open voltage has obvious changes in the effectiveness of the	Jingdong prefecture level city in Shandong
Jinfeng Hongyuan Flagship Store	Electricity conservation2	The goods have been received logistics is very fast, the product is similar to the line, just wait to use a month to see if it can save electricity, stay tuned.	Jingdong prefecture level city in Shandong
Jinfeng Hongyuan Flagship Store	Electricity conservation2	Electricity is plugged in, not sure if it will work, hopefully it will work.	Jingdong prefecture level city in Shandong
Jinfeng Hongyuan Flagship Store	Electricity conservation2	The shell is aluminum alloy, very good workmanship, the actual effect of waiting for the addition of evaluation	Jingdong prefecture level city in Shandong
Jinfeng Hongyuan Flagship Store	Electricity conservation2	Received, already plugged in, looking forward to the effect.	Jingdong prefecture level city in Shandong
Jinfeng Hongyuan Flagship Store	Electricity conservation2	Opened to see the regular manufacturer products, wait for a period of time to see it	Jingdong prefecture level city in Shandong
Jinfeng Hongyuan Flagship Store	Electricity conservation2	🉑 Baby received, very surprised ✨, reasonable design, fashionable and generous, continue to pay attention to the future will be repurchased ❥❥❥❥	Jingdong prefecture level city in Shandong
Jinfeng Hongyuan Flagship Store	Electricity conservation2	Baby received it 👌, really great ah! 🌈The quality can't be beat, very satisfied! 🆗Happy happy happy 😃	Jingdong prefecture level city in Shandong
Jinfeng Hongyuan Flagship Store	Electricity conservation2	Very good, power saving see, this is to help a friend to buy, I used to give my friend a say, my friend immediately let me order another one!	Jingdong prefecture level city in Shandong
Jinfeng Hongyuan Flagship Store	Electricity conservation2	The baby is just as introduced, the texture is very good, powerful, and it feels good to use it	Jingdong prefecture level city in Shandong
Jinfeng Hongyuan Flagship Store	Electricity conservation2	Value for money, very satisfied, seller service attitude is very good, very patient	Jingdong prefecture level city in Shandong
Jinfeng Hongyuan Flagship Store	Electricity conservation2	Power saving treasure design unique exquisite beautiful, voltage display clearly, plugged in that is good to use, I observed the meter, after using the meter is gone slow.	Jingdong prefecture level city in Shandong
Jinfeng Hongyuan Flagship Store	Electricity conservation2	Received on the use of the packaging is beautifully used for a period of time and then come back in the follow-up review!	Jingdong prefecture level city in Shandong
Jinfeng Hongyuan Flagship Store	Electricity conservation2	It should work. Not bad. Not bad. Not bad.	Jingdong prefecture level city in Shandong
Jinfeng Hongyuan Flagship Store	Electricity conservation2	I haven't used it yet, I'll plug it in and see if it saves power next month.	Jingdong prefecture level city in Shandong
Jinfeng Hongyuan Flagship Store	Electricity conservation2	The goods received and unpacked and used, the quality is very good, things and the official website to buy no difference!	Jingdong prefecture level city in Shandong
Jinfeng Hongyuan Flagship Store	Electricity conservation2	It's a rip off, doesn't help much at all, uses the same amount of power as before it was used.	Jingdong prefecture level city in Shandong
Jinfeng Hongyuan Flagship Store	Electricity conservation2	I've been using it for a while now and haven't noticed any unusual problems, so it's okay	Jingdong prefecture level city in Shandong
Jinfeng Hongyuan Flagship Store	Electricity conservation2	Received the goods and plugged in to use, good or not there is no use will have to wait until next month to find out	Jingdong prefecture level city in Shandong
Jinfeng Hongyuan Flagship Store	Electricity conservation2	It's very compact, it looks good, I'll introduce you to my clients when it's ready.	Jingdong prefecture level city in Shandong
Jinfeng Hongyuan Flagship Store	Electricity conservation2	Good workmanship, we'll have to see in a few months.	Jingdong prefecture level city in Shandong
Jinfeng Hongyuan Flagship Store	Electricity conservation2	Jingdong quality! Always trustworthy!	Jingdong prefecture level city in Shandong
Jinfeng Hongyuan Flagship Store	Electricity conservation2	This is the second time I've bought it, and the merchant said to give me five dollars out of the difference, so thank you merchant.	Jingdong prefecture level city in Shandong
Jinfeng Hongyuan Flagship Store	Electricity conservation2	Good quality and good service	Jingdong prefecture level city in Shandong
Jinfeng Hongyuan Flagship Store	Electricity conservation2	Feels good texture, aluminum alloy shell, voltage digital display clear.	Jingdong prefecture level city in Shandong
Jinfeng Hongyuan Flagship Store	Electricity conservation2	Good product, fast and great service, thanks!	Jingdong prefecture level city in Shandong
Jinfeng Hongyuan Flagship Store	Electricity conservation2	Have not yet used, first evaluation, later really good do not need to comment again.	Jingdong prefecture level city in Shandong
Jinfeng Hongyuan Flagship Store	Electricity conservation2	Works great. Pretty much as described by the merchant.	Jingdong prefecture level city in Shandong
Jinfeng Hongyuan Flagship Store	Electricity conservation2	Received it, looking forward to the results, hope it works	Jingdong prefecture level city in Shandong
Jinfeng Hongyuan Flagship Store	Electricity conservation2	Received it, looking forward to the results, hope it works	Jingdong prefecture level city in Shandong
Jinfeng Hongyuan Flagship Store	Electricity conservation2	Supposedly it's an IQ tax. Buy one and try it. Looks good. Well made.	Jingdong prefecture level city in Shandong
Jinfeng Hongyuan Flagship Store	Electricity conservation2	It just doesn't feel like it's working.	Jingdong prefecture level city in Shandong

Jinfeng Hongyuan Flagship Store	Electricity conservation2	This is a great product.	Jingdong prefecture level city in Shandong
Jinfeng Hongyuan Flagship Store	Electricity conservation2	I'm just using it. I'll tell you more.	Jingdong prefecture level city in Shandong
Jinfeng Hongyuan Flagship Store	Electricity conservation2	Second repurchase and it's working fine.	Jingdong prefecture level city in Shandong
Jinfeng Hongyuan Flagship Store	Electricity conservation2	I'll wait a few days before evaluating	Jingdong prefecture level city in Shandong
Jinfeng Hongyuan Flagship Store	Electricity conservation2	I'll comment after I use it hopefully the section will be useful.	Jingdong prefecture level city in Shandong
Jinfeng Hongyuan Flagship Store	Electricity conservation2	not very useful	Jingdong prefecture level city in Shandong
Jinfeng Hongyuan Flagship Store	Electricity conservation2	Not received	Jingdong prefecture level city in Shandong
Jinfeng Hongyuan Flagship Store	Electricity conservation2	rare	Jingdong prefecture level city in Shandong
Jinfeng Hongyuan Flagship Store	Electricity conservation2	It's good.	Jingdong prefecture level city in Shandong
Jinfeng Hongyuan Flagship Store	Electricity conservation2	just now	Jingdong prefecture level city in Shandong
Jinfeng Hongyuan Flagship Store	Electricity conservation2	serviceable	Jingdong prefecture level city in Shandong
Jinfeng Hongyuan Flagship Store	Electricity conservation2	desirable	Jingdong prefecture level city in Shandong
Jinfeng Hongyuan Flagship Store	Electricity conservation2	serviceable	Jingdong prefecture level city in Shandong
Jinfeng Hongyuan Flagship Store	Electricity conservation2	It doesn't save power and costs electricity.	Jingdong prefecture level city in Shandong
Jinfeng Hongyuan Flagship Store	Electricity conservation2	Not as good as I thought.	Jingdong prefecture level city in Shandong
Jinfeng Hongyuan Flagship Store	Electricity conservation2	Super bad review, it doesn't save power at all, but rather more power consumption	Jingdong prefecture level city in Shandong
Jinfeng Hongyuan Flagship Store	Electricity conservation2	Listen to others say that this can save electricity, I bought with the idea of trying, used for a period of time before coming to the evaluation, will be a little bit of use, not a lot of savings just, long-term use of the words can save more.	Jingdong prefecture level city in Shandong
Jinfeng Hongyuan Flagship Store	Electricity conservation2	Logistics is fast, the next day arrived, workmanship is very delicate a power saver, looking forward to the late effect, customer service patiently explain the recommended which is suitable for, installed in what position is the best, look forward to using the effect after a period of time!	Jingdong prefecture level city in Shandong
Jinfeng Hongyuan Flagship Store	Electricity conservation2	It's good to use, I feel that it has been saving electricity, anyway, not a loss is, I used to feel that the power consumption is big, now I feel much better, my friends are also using, I hope to use a long time will be able to, I wish the boss a prosperous business	Jingdong prefecture level city in Shandong
Jinfeng Hongyuan Flagship Store	Electricity conservation2	Should be very good, to be able to plan the circuit, save the home of the circuit to the extent of the savings, waste of money on electricity, good if a month this money back, hope always hope to be able to	Jingdong prefecture level city in Shandong
Jinfeng Hongyuan Flagship Store	Electricity conservation2	Express delivery is very strong, the goods and I saw in the business consistent, the use of the effect of follow-up evaluation	Jingdong prefecture level city in Shandong
Jinfeng Hongyuan Flagship Store	Electricity conservation2	I have been using this brand of power saver for many years and this time I saw this upgraded version and got it again. The main purpose of this product is to reduce the loss of electricity for a long time, so as to achieve the purpose of saving electricity.	Jingdong prefecture level city in Shandong
Jinfeng Hongyuan Flagship Store	Electricity conservation2	Logistics is fast and the product looks beautiful, looking forward to the effect.	Jingdong prefecture level city in Shandong
Jinfeng Hongyuan Flagship Store	Electricity conservation2	Product quality is good, energy saving a little effect, but the price is a little expensive, fast arrival, good service.	Jingdong prefecture level city in Shandong
Jinfeng Hongyuan Flagship Store	Electricity conservation2	The quality is very good, inexpensive, Jingdong self-supporting guaranteed, worth having!	Jingdong prefecture level city in Shandong
Jinfeng Hongyuan Flagship Store	Electricity conservation2	I don't know if it's saving electricity yet, I'll find out next time I pay my electric bill	Jingdong prefecture level city in Shandong
Jinfeng Hongyuan Flagship Store	Electricity conservation2	Favorable price quality assurance appearance design is beautiful and generous, fine workmanship	Jingdong prefecture level city in Shandong
Jinfeng Hongyuan Flagship Store	Electricity conservation2	I bought it, I don't know how effective it is? I don't know how the quality is? After this summer results will know! Looking forward to it!	Jingdong prefecture level city in Shandong
Jinfeng Hongyuan Flagship Store	Electricity conservation2	Goods received Packaging intact, look at the shell workmanship is also good, high-power appliances open voltage has obvious changes in the effectiveness of the	Jingdong prefecture level city in Shandong
Jinfeng Hongyuan Flagship Store	Electricity conservation2	The goods have been received logistics is very fast, the product is similar to the line, just wait to use a month to see if it can save electricity, stay tuned.	Jingdong prefecture level city in Shandong
Jinfeng Hongyuan Flagship Store	Electricity conservation2	Electricity is plugged in, not sure if it will work, hopefully it will work.	Jingdong prefecture level city in Shandong
Jinfeng Hongyuan Flagship Store	Electricity conservation2	The shell is aluminum alloy, very good workmanship, the actual effect of waiting for the addition of evaluation	Jingdong prefecture level city in Shandong
Jinfeng Hongyuan Flagship Store	Electricity conservation2	Received, already plugged in, looking forward to the effect.	Jingdong prefecture level city in Shandong
Jinfeng Hongyuan Flagship Store	Electricity conservation2	Opened to see the regular manufacturer products, wait for a period of time to see it	Jingdong prefecture level city in Shandong
Jinfeng Hongyuan Flagship Store	Electricity conservation2	🉑 Baby received, very surprised ✨, reasonable design, fashionable and generous, continue to pay attention to the future will be repurchased ❥❥❥❥	Jingdong prefecture level city in Shandong
Jinfeng Hongyuan Flagship Store	Electricity conservation2	Baby received it 👌, really great ah! 🌈The quality can't be beat, very satisfied! 🆗Happy happy happy 😃	Jingdong prefecture level city in Shandong
Jinfeng Hongyuan Flagship Store	Electricity conservation2	Very good, power saving see, this is to help a friend to buy, I used to give my friend a say, my friend immediately let me order another one!	Jingdong prefecture level city in Shandong
Jinfeng Hongyuan Flagship Store	Electricity conservation2	The baby is just as introduced, the texture is very good, powerful, and it feels good to use it	Jingdong prefecture level city in Shandong
Jinfeng Hongyuan Flagship Store	Electricity conservation2	Value for money, very satisfied, seller service attitude is very good, very patient	Jingdong prefecture level city in Shandong
Jinfeng Hongyuan Flagship Store	Electricity conservation2	Power saving treasure design unique exquisite beautiful, voltage display clearly, plugged in that is good to use, I observed the meter, after using the meter is gone slow.	Jingdong prefecture level city in Shandong
Jinfeng Hongyuan Flagship Store	Electricity conservation2	Received on the use of the packaging is beautifully used for a period of time and then come back in the follow-up review!	Jingdong prefecture level city in Shandong
Jinfeng Hongyuan Flagship Store	Electricity conservation2	It should work. Not bad. Not bad. Not bad.	Jingdong prefecture level city in Shandong
Jinfeng Hongyuan Flagship Store	Electricity conservation2	I haven't used it yet, I'll plug it in and see if it saves power next month.	Jingdong prefecture level city in Shandong
Jinfeng Hongyuan Flagship Store	Electricity conservation2	The goods received and unpacked and used, the quality is very good, things and the official website to buy no difference!	Jingdong prefecture level city in Shandong
Jinfeng Hongyuan Flagship Store	Electricity conservation2	It's a rip off, doesn't help much at all, uses the same amount of power as before it was used.	Jingdong prefecture level city in Shandong
Jinfeng Hongyuan Flagship Store	Electricity conservation2	I've been using it for a while now and haven't noticed any unusual problems, so it's okay	Jingdong prefecture level city in Shandong
Jinfeng Hongyuan Flagship Store	Electricity conservation2	Received the goods and plugged in to use, good or not there is no use will have to wait until next month to find out	Jingdong prefecture level city in Shandong
Jinfeng Hongyuan Flagship Store	Electricity conservation2	It's very compact, it looks good, I'll introduce you to my clients when it's ready.	Jingdong prefecture level city in Shandong
Jinfeng Hongyuan Flagship Store	Electricity conservation2	Good workmanship, we'll have to see in a few months.	Jingdong prefecture level city in Shandong

Jinfeng Hongyuan Flagship Store	Electricity conservation2	Jingdong quality! Always trustworthy!	Jingdong prefecture level city in Shandong
Jinfeng Hongyuan Flagship Store	Electricity conservation2	This is the second time I've bought it, and the merchant said to give me five dollars out of the difference, so thank you merchant.	Jingdong prefecture level city in Shandong
Jinfeng Hongyuan Flagship Store	Electricity conservation2	Good quality and good service	Jingdong prefecture level city in Shandong
Jinfeng Hongyuan Flagship Store	Electricity conservation2	Feels good texture, aluminum alloy shell, voltage digital display clear.	Jingdong prefecture level city in Shandong
Jinfeng Hongyuan Flagship Store	Electricity conservation2	Good product, fast and great service, thanks!	Jingdong prefecture level city in Shandong
Jinfeng Hongyuan Flagship Store	Electricity conservation2	Have not yet used, first evaluation, later really good do not need to comment again.	Jingdong prefecture level city in Shandong
Jinfeng Hongyuan Flagship Store	Electricity conservation2	Works great. Pretty much as described by the merchant.	Jingdong prefecture level city in Shandong
Jinfeng Hongyuan Flagship Store	Electricity conservation2	Received it, looking forward to the results, hope it works	Jingdong prefecture level city in Shandong
Jinfeng Hongyuan Flagship Store	Electricity conservation2	Received it, looking forward to the results, hope it works	Jingdong prefecture level city in Shandong
Jinfeng Hongyuan Flagship Store	Electricity conservation2	Supposedly it's an IQ tax. Buy one and try it. Looks good. Well made.	Jingdong prefecture level city in Shandong
Jinfeng Hongyuan Flagship Store	Electricity conservation2	It just doesn't feel like it's working.	Jingdong prefecture level city in Shandong
Jinfeng Hongyuan Flagship Store	Electricity conservation2	This is a great product.	Jingdong prefecture level city in Shandong
Jinfeng Hongyuan Flagship Store	Electricity conservation2	I'm just using it. I'll tell you more.	Jingdong prefecture level city in Shandong
Jinfeng Hongyuan Flagship Store	Electricity conservation2	Second repurchase and it's working fine.	Jingdong prefecture level city in Shandong
Jinfeng Hongyuan Flagship Store	Electricity conservation2	I'll wait a few days before evaluating	Jingdong prefecture level city in Shandong
Jinfeng Hongyuan Flagship Store	Electricity conservation2	I'll comment after I use it hopefully the section will be useful.	Jingdong prefecture level city in Shandong
Jinfeng Hongyuan Flagship Store	Electricity conservation2	not very useful	Jingdong prefecture level city in Shandong
Jinfeng Hongyuan Flagship Store	Electricity conservation2	Not received	Jingdong prefecture level city in Shandong
Jinfeng Hongyuan Flagship Store	Electricity conservation2	rare	Jingdong prefecture level city in Shandong
Jinfeng Hongyuan Flagship Store	Electricity conservation2	It's good.	Jingdong prefecture level city in Shandong
Jinfeng Hongyuan Flagship Store	Electricity conservation2	just now	Jingdong prefecture level city in Shandong
Jinfeng Hongyuan Flagship Store	Electricity conservation2	serviceable	Jingdong prefecture level city in Shandong
Jinfeng Hongyuan Flagship Store	Electricity conservation2	desirable	Jingdong prefecture level city in Shandong
Jinfeng Hongyuan Flagship Store	Electricity conservation2	serviceable	Jingdong prefecture level city in Shandong
Jinfeng Hongyuan Flagship Store	Electricity conservation2	It doesn't save power and costs electricity.	Jingdong prefecture level city in Shandong
Jinfeng Hongyuan Flagship Store	Electricity conservation2	Not as good as I thought.	Jingdong prefecture level city in Shandong
Jinfeng Hongyuan Flagship Store	Electricity conservation2	Super bad review, it doesn't save power at all, but rather more power consumption	Jingdong prefecture level city in Shandong
Jinfeng Hongyuan Flagship Store	Electricity conservation2	Listen to others say that this can save electricity, I bought with the idea of trying, used for a period of time before coming to the evaluation, will be a little bit of use, not a lot of savings just, long-term use of the words can save more.	Jingdong prefecture level city in Shandong
Jinfeng Hongyuan Flagship Store	Electricity conservation2	Logistics is fast, the next day arrived, workmanship is very delicate a power saver, looking forward to the late effect, customer service patiently explain the recommended which is suitable for, installed in what position is the best, look forward to using the effect after a period of time!	Jingdong prefecture level city in Shandong
Jinfeng Hongyuan Flagship Store	Electricity conservation2	It's good to use, I feel that it has been saving electricity, anyway, not a loss is, I used to feel that the power consumption is big, now I feel much better, my friends are also using, I hope to use a long time will be able to, I wish the boss a prosperous business	Jingdong prefecture level city in Shandong
Jinfeng Hongyuan Flagship Store	Electricity conservation2	Should be very good, to be able to plan the circuit, save the home of the circuit to the extent of the savings, waste of money on electricity, good if a month this money back, hope always hope to be able to	Jingdong prefecture level city in Shandong
Jinfeng Hongyuan Flagship Store	Electricity conservation2	Express delivery is very strong, the goods and I saw in the business consistent, the use of the effect of follow-up evaluation	Jingdong prefecture level city in Shandong
Jinfeng Hongyuan Flagship Store	Electricity conservation2	I have been using this brand of power saver for many years and this time I saw this upgraded version and got it again. The main purpose of this product is to reduce the loss of electricity for a long time, so as to achieve the purpose of saving electricity.	Jingdong prefecture level city in Shandong
Jinfeng Hongyuan Flagship Store	Electricity conservation2	Logistics is fast and the product looks beautiful, looking forward to the effect.	Jingdong prefecture level city in Shandong
Jinfeng Hongyuan Flagship Store	Electricity conservation2	Product quality is good, energy saving a little effect, but the price is a little expensive, fast arrival, good service.	Jingdong prefecture level city in Shandong
Jinfeng Hongyuan Flagship Store	Electricity conservation2	The quality is very good, inexpensive, Jingdong self-supporting guaranteed, worth having!	Jingdong prefecture level city in Shandong
Jinfeng Hongyuan Flagship Store	Electricity conservation2	I don't know if it's saving electricity yet, I'll find out next time I pay my electric bill	Jingdong prefecture level city in Shandong
Jinfeng Hongyuan Flagship Store	Electricity conservation2	Favorable price quality assurance appearance design is beautiful and generous, fine workmanship	Jingdong prefecture level city in Shandong
Jinfeng Hongyuan Flagship Store	Electricity conservation2	I bought it, I don't know how effective it is? I don't know how the quality is? After this summer results will know! Looking forward to it!	Jingdong prefecture level city in Shandong
Jinfeng Hongyuan Flagship Store	Electricity conservation2	Goods received Packaging intact, look at the shell workmanship is also good, high-power appliances open voltage has obvious changes in the effectiveness of the	Jingdong prefecture level city in Shandong
Jinfeng Hongyuan Flagship Store	Electricity conservation2	The goods have been received logistics is very fast, the product is similar to the line, just wait to use a month to see if it can save electricity, stay tuned.	Jingdong prefecture level city in Shandong
Jinfeng Hongyuan Flagship Store	Electricity conservation2	Electricity is plugged in, not sure if it will work, hopefully it will work.	Jingdong prefecture level city in Shandong
Jinfeng Hongyuan Flagship Store	Electricity conservation2	The shell is aluminum alloy, very good workmanship, the actual effect of waiting for the addition of evaluation	Jingdong prefecture level city in Shandong
Jinfeng Hongyuan Flagship Store	Electricity conservation2	Received, already plugged in, looking forward to the effect.	Jingdong prefecture level city in Shandong
Jinfeng Hongyuan Flagship Store	Electricity conservation2	Opened to see the regular manufacturer products, wait for a period of time to see it	Jingdong prefecture level city in Shandong
Jinfeng Hongyuan Flagship Store	Electricity conservation2	🉑 Baby received, very surprised ✨, reasonable design, fashionable and generous, continue to pay attention to the future will be repurchased ❥❥❥❥	Jingdong prefecture level city in Shandong
Jinfeng Hongyuan Flagship Store	Electricity conservation2	Baby received it 👌, really great ah! 🌈The quality can't be beat, very satisfied! 🆗Happy happy happy 😃	Jingdong prefecture level city in Shandong
Jinfeng Hongyuan Flagship Store	Electricity conservation2	Very good, power saving see, this is to help a friend to buy, I used to give my friend a say, my friend immediately let me order another one!	Jingdong prefecture level city in Shandong
Jinfeng Hongyuan Flagship Store	Electricity conservation2	The baby is just as introduced, the texture is very good, powerful, and it feels good to use it	Jingdong prefecture level city in Shandong

Jinfeng Hongyuan Flagship Store	Electricity conservation2	Value for money, very satisfied, seller service attitude is very good, very patient	Jingdong prefecture level city in Shandong
Jinfeng Hongyuan Flagship Store	Electricity conservation2	Power saving treasure design unique exquisite beautiful, voltage display clearly, plugged in that is good to use, I observed the meter, after using the meter is gone slow.	Jingdong prefecture level city in Shandong
Jinfeng Hongyuan Flagship Store	Electricity conservation2	Received on the use of the packaging is beautifully used for a period of time and then come back in the follow-up review!	Jingdong prefecture level city in Shandong
Jinfeng Hongyuan Flagship Store	Electricity conservation2	It should work. Not bad. Not bad. Not bad.	Jingdong prefecture level city in Shandong
Jinfeng Hongyuan Flagship Store	Electricity conservation2	I haven't used it yet, I'll plug it in and see if it saves power next month.	Jingdong prefecture level city in Shandong
Jinfeng Hongyuan Flagship Store	Electricity conservation2	The goods received and unpacked and used, the quality is very good, things and the official website to buy no difference!	Jingdong prefecture level city in Shandong
Jinfeng Hongyuan Flagship Store	Electricity conservation2	It's a rip off, doesn't help much at all, uses the same amount of power as before it was used.	Jingdong prefecture level city in Shandong
Jinfeng Hongyuan Flagship Store	Electricity conservation2	I've been using it for a while now and haven't noticed any unusual problems, so it's okay	Jingdong prefecture level city in Shandong
Jinfeng Hongyuan Flagship Store	Electricity conservation2	Received the goods and plugged in to use, good or not there is no use will have to wait until next month to find out	Jingdong prefecture level city in Shandong
Jinfeng Hongyuan Flagship Store	Electricity conservation2	It's very compact, it looks good, I'll introduce you to my clients when it's ready.	Jingdong prefecture level city in Shandong
Jinfeng Hongyuan Flagship Store	Electricity conservation2	Good workmanship, we'll have to see in a few months.	Jingdong prefecture level city in Shandong
Jinfeng Hongyuan Flagship Store	Electricity conservation2	Jingdong quality! Always trustworthy!	Jingdong prefecture level city in Shandong
Jinfeng Hongyuan Flagship Store	Electricity conservation2	This is the second time I've bought it, and the merchant said to give me five dollars out of the difference, so thank you merchant.	Jingdong prefecture level city in Shandong
Jinfeng Hongyuan Flagship Store	Electricity conservation2	Good quality and good service	Jingdong prefecture level city in Shandong
Jinfeng Hongyuan Flagship Store	Electricity conservation2	Feels good texture, aluminum alloy shell, voltage digital display clear.	Jingdong prefecture level city in Shandong
Jinfeng Hongyuan Flagship Store	Electricity conservation2	Good product, fast and great service, thanks!	Jingdong prefecture level city in Shandong
Jinfeng Hongyuan Flagship Store	Electricity conservation2	Have not yet used, first evaluation, later really good do not need to comment again.	Jingdong prefecture level city in Shandong
Jinfeng Hongyuan Flagship Store	Electricity conservation2	Works great. Pretty much as described by the merchant.	Jingdong prefecture level city in Shandong
Jinfeng Hongyuan Flagship Store	Electricity conservation2	Received it, looking forward to the results, hope it works	Jingdong prefecture level city in Shandong
Jinfeng Hongyuan Flagship Store	Electricity conservation2	Received it, looking forward to the results, hope it works	Jingdong prefecture level city in Shandong
Jinfeng Hongyuan Flagship Store	Electricity conservation2	Supposedly it's an IQ tax. Buy one and try it. Looks good. Well made.	Jingdong prefecture level city in Shandong
Jinfeng Hongyuan Flagship Store	Electricity conservation2	It just doesn't feel like it's working.	Jingdong prefecture level city in Shandong
Jinfeng Hongyuan Flagship Store	Electricity conservation2	This is a great product.	Jingdong prefecture level city in Shandong
Jinfeng Hongyuan Flagship Store	Electricity conservation2	I'm just using it. I'll tell you more.	Jingdong prefecture level city in Shandong
Jinfeng Hongyuan Flagship Store	Electricity conservation2	Second repurchase and it's working fine.	Jingdong prefecture level city in Shandong
Jinfeng Hongyuan Flagship Store	Electricity conservation2	I'll wait a few days before evaluating	Jingdong prefecture level city in Shandong
Jinfeng Hongyuan Flagship Store	Electricity conservation2	I'll comment after I use it hopefully the section will be useful.	Jingdong prefecture level city in Shandong
Jinfeng Hongyuan Flagship Store	Electricity conservation2	not very useful	Jingdong prefecture level city in Shandong
Jinfeng Hongyuan Flagship Store	Electricity conservation2	Not received	Jingdong prefecture level city in Shandong
Jinfeng Hongyuan Flagship Store	Electricity conservation2	rare	Jingdong prefecture level city in Shandong
Jinfeng Hongyuan Flagship Store	Electricity conservation2	It's good.	Jingdong prefecture level city in Shandong
Jinfeng Hongyuan Flagship Store	Electricity conservation2	just now	Jingdong prefecture level city in Shandong
Jinfeng Hongyuan Flagship Store	Electricity conservation2	serviceable	Jingdong prefecture level city in Shandong
Jinfeng Hongyuan Flagship Store	Electricity conservation2	desirable	Jingdong prefecture level city in Shandong
Jinfeng Hongyuan Flagship Store	Electricity conservation2	serviceable	Jingdong prefecture level city in Shandong
Jinfeng Hongyuan Flagship Store	Electricity conservation2	It doesn't save power and costs electricity.	Jingdong prefecture level city in Shandong
Jinfeng Hongyuan Flagship Store	Electricity conservation2	Not as good as I thought.	Jingdong prefecture level city in Shandong
Jinfeng Hongyuan Flagship Store	Electricity conservation2	Super bad review, it doesn't save power at all, but rather more power consumption	Jingdong prefecture level city in Shandong
Jinfeng Hongyuan Flagship Store	Electricity conservation2	Listen to others say that this can save electricity, I bought with the idea of trying, used for a period of time before coming to the evaluation, will be a little bit of use, not a lot of savings just, long-term use of the words can save more.	Jingdong prefecture level city in Shandong
Jinfeng Hongyuan Flagship Store	Electricity conservation2	Logistics is fast, the next day arrived, workmanship is very delicate a power saver, looking forward to the late effect, customer service patiently explain the recommended which is suitable for, installed in what position is the best, look forward to using the effect after a period of time!	Jingdong prefecture level city in Shandong
Jinfeng Hongyuan Flagship Store	Electricity conservation2	It's good to use, I feel that it has been saving electricity, anyway, not a loss is, I used to feel that the power consumption is big, now I feel much better, my friends are also using, I hope to use a long time will be able to, I wish the boss a prosperous business	Jingdong prefecture level city in Shandong
Jinfeng Hongyuan Flagship Store	Electricity conservation2	Should be very good, to be able to plan the circuit, save the home of the circuit to the extent of the savings, waste of money on electricity, good if a month this money back, hope always hope to be able to	Jingdong prefecture level city in Shandong
Jinfeng Hongyuan Flagship Store	Electricity conservation2	Express delivery is very strong, the goods and I saw in the business consistent, the use of the effect of follow-up evaluation	Jingdong prefecture level city in Shandong
Jinfeng Hongyuan Flagship Store	Electricity conservation2	I have been using this brand of power saver for many years and this time I saw this upgraded version and got it again. The main purpose of this product is to reduce the loss of electricity for a long time, so as to achieve the purpose of saving electricity.	Jingdong prefecture level city in Shandong
Jinfeng Hongyuan Flagship Store	Electricity conservation2	Logistics is fast and the product looks beautiful, looking forward to the effect.	Jingdong prefecture level city in Shandong
Jinfeng Hongyuan Flagship Store	Electricity conservation2	Product quality is good, energy saving a little effect, but the price is a little expensive, fast arrival, good service.	Jingdong prefecture level city in Shandong
Jinfeng Hongyuan Flagship Store	Electricity conservation2	The quality is very good, inexpensive, Jingdong self-supporting guaranteed, worth having!	Jingdong prefecture level city in Shandong
Jinfeng Hongyuan Flagship Store	Electricity conservation2	I don't know if it's saving electricity yet, I'll find out next time I pay my electric bill	Jingdong prefecture level city in Shandong
Jinfeng Hongyuan Flagship Store	Electricity conservation2	Favorable price quality assurance appearance design is beautiful and generous, fine workmanship	Jingdong prefecture level city in Shandong

Jinfeng Hongyuan Flagship Store	Electricity conservation2	I bought it, I don't know how effective it is? I don't know how the quality is? After this summer results will know! Looking forward to it!	Jingdong prefecture level city in Shandong
Jinfeng Hongyuan Flagship Store	Electricity conservation2	Goods received Packaging intact, look at the shell workmanship is also good, high-power appliances open voltage has obvious changes in the effectiveness of the	Jingdong prefecture level city in Shandong
Jinfeng Hongyuan Flagship Store	Electricity conservation2	The goods have been received logistics is very fast, the product is similar to the line, just wait to use a month to see if it can save electricity, stay tuned.	Jingdong prefecture level city in Shandong
Jinfeng Hongyuan Flagship Store	Electricity conservation2	Electricity is plugged in, not sure if it will work, hopefully it will work.	Jingdong prefecture level city in Shandong
Jinfeng Hongyuan Flagship Store	Electricity conservation2	The shell is aluminum alloy, very good workmanship, the actual effect of waiting for the addition of evaluation	Jingdong prefecture level city in Shandong
Jinfeng Hongyuan Flagship Store	Electricity conservation2	Received, already plugged in, looking forward to the effect.	Jingdong prefecture level city in Shandong
Jinfeng Hongyuan Flagship Store	Electricity conservation2	Opened to see the regular manufacturer products, wait for a period of time to see it	Jingdong prefecture level city in Shandong
Jinfeng Hongyuan Flagship Store	Electricity conservation2	🉑 Baby received, very surprised ✨, reasonable design, fashionable and generous, continue to pay attention to the future will be repurchased ❤❤❤❤	Jingdong prefecture level city in Shandong
Jinfeng Hongyuan Flagship Store	Electricity conservation2	Baby received it 👌, really great ah! 🌈The quality can't be beat, very satisfied! 🆗Happy happy happy 😃	Jingdong prefecture level city in Shandong
Jinfeng Hongyuan Flagship Store	Electricity conservation2	Very good, power saving see, this is to help a friend to buy, I used to give my friend a say, my friend immediately let me order another one!	Jingdong prefecture level city in Shandong
Jinfeng Hongyuan Flagship Store	Electricity conservation2	The baby is just as introduced, the texture is very good, powerful, and it feels good to use it	Jingdong prefecture level city in Shandong
Jinfeng Hongyuan Flagship Store	Electricity conservation2	Value for money, very satisfied, seller service attitude is very good, very patient	Jingdong prefecture level city in Shandong
Jinfeng Hongyuan Flagship Store	Electricity conservation2	Power saving treasure design unique exquisite beautiful, voltage display clearly, plugged in that is good to use, I observed the meter, after using the meter is gone slow.	Jingdong prefecture level city in Shandong
Jinfeng Hongyuan Flagship Store	Electricity conservation2	Received on the use of the packaging is beautifully used for a period of time and then come back in the follow-up review!	Jingdong prefecture level city in Shandong
Jinfeng Hongyuan Flagship Store	Electricity conservation2	It should work. Not bad. Not bad. Not bad.	Jingdong prefecture level city in Shandong
Jinfeng Hongyuan Flagship Store	Electricity conservation2	I haven't used it yet, I'll plug it in and see if it saves power next month.	Jingdong prefecture level city in Shandong
Jinfeng Hongyuan Flagship Store	Electricity conservation2	The goods received and unpacked and used, the quality is very good, things and the official website to buy no difference!	Jingdong prefecture level city in Shandong
Jinfeng Hongyuan Flagship Store	Electricity conservation2	It's a rip off, doesn't help much at all, uses the same amount of power as before it was used.	Jingdong prefecture level city in Shandong
Jinfeng Hongyuan Flagship Store	Electricity conservation2	I've been using it for a while now and haven't noticed any unusual problems, so it's okay	Jingdong prefecture level city in Shandong
Jinfeng Hongyuan Flagship Store	Electricity conservation2	Received the goods and plugged in to use, good or not there is no use will have to wait until next month to find out	Jingdong prefecture level city in Shandong
Jinfeng Hongyuan Flagship Store	Electricity conservation2	It's very compact, it looks good, I'll introduce you to my clients when it's ready.	Jingdong prefecture level city in Shandong
Jinfeng Hongyuan Flagship Store	Electricity conservation2	Good workmanship, we'll have to see in a few months.	Jingdong prefecture level city in Shandong
Jinfeng Hongyuan Flagship Store	Electricity conservation2	Jingdong quality! Always trustworthy!	Jingdong prefecture level city in Shandong
Jinfeng Hongyuan Flagship Store	Electricity conservation2	This is the second time I've bought it, and the merchant said to give me five dollars out of the difference, so thank you merchant.	Jingdong prefecture level city in Shandong
Jinfeng Hongyuan Flagship Store	Electricity conservation2	Good quality and good service	Jingdong prefecture level city in Shandong
Jinfeng Hongyuan Flagship Store	Electricity conservation2	Feels good texture, aluminum alloy shell, voltage digital display clear.	Jingdong prefecture level city in Shandong
Jinfeng Hongyuan Flagship Store	Electricity conservation2	Good product, fast and great service, thanks!	Jingdong prefecture level city in Shandong
Jinfeng Hongyuan Flagship Store	Electricity conservation2	Have not yet used, first evaluation, later really good do not need to comment again.	Jingdong prefecture level city in Shandong
Jinfeng Hongyuan Flagship Store	Electricity conservation2	Works great. Pretty much as described by the merchant.	Jingdong prefecture level city in Shandong
Jinfeng Hongyuan Flagship Store	Electricity conservation2	Received it, looking forward to the results, hope it works	Jingdong prefecture level city in Shandong
Jinfeng Hongyuan Flagship Store	Electricity conservation2	Received it, looking forward to the results, hope it works	Jingdong prefecture level city in Shandong
Jinfeng Hongyuan Flagship Store	Electricity conservation2	Supposedly it's an IQ tax. Buy one and try it. Looks good. Well made.	Jingdong prefecture level city in Shandong
Jinfeng Hongyuan Flagship Store	Electricity conservation2	It just doesn't feel like it's working.	Jingdong prefecture level city in Shandong
Jinfeng Hongyuan Flagship Store	Electricity conservation2	This is a great product.	Jingdong prefecture level city in Shandong
Jinfeng Hongyuan Flagship Store	Electricity conservation2	I'm just using it. I'll tell you more.	Jingdong prefecture level city in Shandong
Jinfeng Hongyuan Flagship Store	Electricity conservation2	Second repurchase and it's working fine.	Jingdong prefecture level city in Shandong
Jinfeng Hongyuan Flagship Store	Electricity conservation2	I'll wait a few days before evaluating	Jingdong prefecture level city in Shandong
Jinfeng Hongyuan Flagship Store	Electricity conservation2	I'll comment after I use it hopefully the section will be useful.	Jingdong prefecture level city in Shandong
Jinfeng Hongyuan Flagship Store	Electricity conservation2	not very useful	Jingdong prefecture level city in Shandong
Jinfeng Hongyuan Flagship Store	Electricity conservation2	Not received	Jingdong prefecture level city in Shandong
Jinfeng Hongyuan Flagship Store	Electricity conservation2	rare	Jingdong prefecture level city in Shandong
Jinfeng Hongyuan Flagship Store	Electricity conservation2	It's good.	Jingdong prefecture level city in Shandong
Jinfeng Hongyuan Flagship Store	Electricity conservation2	just now	Jingdong prefecture level city in Shandong
Jinfeng Hongyuan Flagship Store	Electricity conservation2	serviceable	Jingdong prefecture level city in Shandong
Jinfeng Hongyuan Flagship Store	Electricity conservation2	desirable	Jingdong prefecture level city in Shandong
Jinfeng Hongyuan Flagship Store	Electricity conservation2	serviceable	Jingdong prefecture level city in Shandong
Jinfeng Hongyuan Flagship Store	Electricity conservation2	It doesn't save power and costs electricity.	Jingdong prefecture level city in Shandong
Jinfeng Hongyuan Flagship Store	Electricity conservation2	Not as good as I thought.	Jingdong prefecture level city in Shandong
Jinfeng Hongyuan Flagship Store	Electricity conservation2	Super bad review, it doesn't save power at all, but rather more power consumption	Jingdong prefecture level city in Shandong
Jinfeng Hongyuan Flagship Store	Electricity conservation2	Listen to others say that this can save electricity, I bought with the idea of trying, used for a period of time before coming to the evaluation, will be a little bit of use, not a lot of savings just, long-term use of the words can save more.	Jingdong prefecture level city in Shandong

Jinfeng Hongyuan Flagship Store	Electricity conservation2	Logistics is fast, the next day arrived, workmanship is very delicate a power saver, looking forward to the late effect, customer service patiently explain the recommended which is suitable for, installed in what position is the best, look forward to using the effect after a period of time!	Jingdong prefecture level city in Shandong
Jinfeng Hongyuan Flagship Store	Electricity conservation2	It's good to use, I feel that it has been saving electricity, anyway, not a loss is, I used to feel that the power consumption is big, now I feel much better, my friends are also using, I hope to use a long time will be able to, I wish the boss a prosperous business	Jingdong prefecture level city in Shandong
Jinfeng Hongyuan Flagship Store	Electricity conservation2	Should be very good, to be able to plan the circuit, save the home of the circuit to the extent of the savings, waste of money on electricity, good if a month this money back, hope always hope to be able to	Jingdong prefecture level city in Shandong
Jinfeng Hongyuan Flagship Store	Electricity conservation2	Express delivery is very strong, the goods and I saw in the business consistent, the use of the effect of follow-up evaluation	Jingdong prefecture level city in Shandong
Jinfeng Hongyuan Flagship Store	Electricity conservation2	I have been using this brand of power saver for many years and this time I saw this upgraded version and got it again. The main purpose of this product is to reduce the loss of electricity for a long time, so as to achieve the purpose of saving electricity.	Jingdong prefecture level city in Shandong
Jinfeng Hongyuan Flagship Store	Electricity conservation2	Logistics is fast and the product looks beautiful, looking forward to the effect.	Jingdong prefecture level city in Shandong
Jinfeng Hongyuan Flagship Store	Electricity conservation2	Product quality is good, energy saving a little effect, but the price is a little expensive, fast arrival, good service.	Jingdong prefecture level city in Shandong
Jinfeng Hongyuan Flagship Store	Electricity conservation2	The quality is very good, inexpensive, Jingdong self-supporting guaranteed, worth having!	Jingdong prefecture level city in Shandong
Jinfeng Hongyuan Flagship Store	Electricity conservation2	I don't know if it's saving electricity yet, I'll find out next time I pay my electric bill	Jingdong prefecture level city in Shandong
Jinfeng Hongyuan Flagship Store	Electricity conservation2	Favorable price quality assurance appearance design is beautiful and generous, fine workmanship	Jingdong prefecture level city in Shandong
Jinfeng Hongyuan Flagship Store	Electricity conservation2	I bought it, I don't know how effective it is? I don't know how the quality is? After this summer results will know! Looking forward to it!	Jingdong prefecture level city in Shandong
Jinfeng Hongyuan Flagship Store	Electricity conservation2	Goods received Packaging intact, look at the shell workmanship is also good, high-power appliances open voltage has obvious changes in the effectiveness of the	Jingdong prefecture level city in Shandong
Jinfeng Hongyuan Flagship Store	Electricity conservation2	The goods have been received logistics is very fast, the product is similar to the line, just wait to use a month to see if it can save electricity, stay tuned.	Jingdong prefecture level city in Shandong
Jinfeng Hongyuan Flagship Store	Electricity conservation2	Electricity is plugged in, not sure if it will work, hopefully it will work.	Jingdong prefecture level city in Shandong
Jinfeng Hongyuan Flagship Store	Electricity conservation2	The shell is aluminum alloy, very good workmanship, the actual effect of waiting for the addition of evaluation	Jingdong prefecture level city in Shandong
Jinfeng Hongyuan Flagship Store	Electricity conservation2	Received, already plugged in, looking forward to the effect.	Jingdong prefecture level city in Shandong
Jinfeng Hongyuan Flagship Store	Electricity conservation2	Opened to see the regular manufacturer products, wait for a period of time to see it	Jingdong prefecture level city in Shandong
Jinfeng Hongyuan Flagship Store	Electricity conservation2	🉑 Baby received, very surprised ✨, reasonable design, fashionable and generous, continue to pay attention to the future will be repurchased ❥❥❥❥	Jingdong prefecture level city in Shandong
Jinfeng Hongyuan Flagship Store	Electricity conservation2	Baby received it 👌, really great ah! 🌈The quality can't be beat, very satisfied! 🆗Happy happy happy 😃	Jingdong prefecture level city in Shandong
Jinfeng Hongyuan Flagship Store	Electricity conservation2	Very good, power saving see, this is to help a friend to buy, I used to give my friend a say, my friend immediately let me order another one!	Jingdong prefecture level city in Shandong
Jinfeng Hongyuan Flagship Store	Electricity conservation2	The baby is just as introduced, the texture is very good, powerful, and it feels good to use it	Jingdong prefecture level city in Shandong
Jinfeng Hongyuan Flagship Store	Electricity conservation2	Value for money, very satisfied, seller service attitude is very good, very patient	Jingdong prefecture level city in Shandong
Jinfeng Hongyuan Flagship Store	Electricity conservation2	Power saving treasure design unique exquisite beautiful, voltage display clearly, plugged in that is good to use, I observed the meter, after using the meter is gone slow.	Jingdong prefecture level city in Shandong
Jinfeng Hongyuan Flagship Store	Electricity conservation2	Received on the use of the packaging is beautifully used for a period of time and then come back in the follow-up review!	Jingdong prefecture level city in Shandong
Jinfeng Hongyuan Flagship Store	Electricity conservation2	It should work. Not bad. Not bad. Not bad.	Jingdong prefecture level city in Shandong
Jinfeng Hongyuan Flagship Store	Electricity conservation2	I haven't used it yet, I'll plug it in and see if it saves power next month.	Jingdong prefecture level city in Shandong
Jinfeng Hongyuan Flagship Store	Electricity conservation2	The goods received and unpacked and used, the quality is very good, things and the official website to buy no difference!	Jingdong prefecture level city in Shandong
Jinfeng Hongyuan Flagship Store	Electricity conservation2	It's a rip off, doesn't help much at all, uses the same amount of power as before it was used.	Jingdong prefecture level city in Shandong
Jinfeng Hongyuan Flagship Store	Electricity conservation2	I've been using it for a while now and haven't noticed any unusual problems, so it's okay	Jingdong prefecture level city in Shandong
Jinfeng Hongyuan Flagship Store	Electricity conservation2	Received the goods and plugged in to use, good or not there is no use will have to wait until next month to find out	Jingdong prefecture level city in Shandong
Jinfeng Hongyuan Flagship Store	Electricity conservation2	It's very compact, it looks good, I'll introduce you to my clients when it's ready.	Jingdong prefecture level city in Shandong
Jinfeng Hongyuan Flagship Store	Electricity conservation2	Good workmanship, we'll have to see in a few months.	Jingdong prefecture level city in Shandong
Jinfeng Hongyuan Flagship Store	Electricity conservation2	Jingdong quality! Always trustworthy!	Jingdong prefecture level city in Shandong
Jinfeng Hongyuan Flagship Store	Electricity conservation2	This is the second time I've bought it, and the merchant said to give me five dollars out of the difference, so thank you merchant.	Jingdong prefecture level city in Shandong
Jinfeng Hongyuan Flagship Store	Electricity conservation2	Good quality and good service	Jingdong prefecture level city in Shandong
Jinfeng Hongyuan Flagship Store	Electricity conservation2	Feels good texture, aluminum alloy shell, voltage digital display clear.	Jingdong prefecture level city in Shandong
Jinfeng Hongyuan Flagship Store	Electricity conservation2	Good product, fast and great service, thanks!	Jingdong prefecture level city in Shandong
Jinfeng Hongyuan Flagship Store	Electricity conservation2	Have not yet used, first evaluation, later really good do not need to comment again.	Jingdong prefecture level city in Shandong
Jinfeng Hongyuan Flagship Store	Electricity conservation2	Works great. Pretty much as described by the merchant.	Jingdong prefecture level city in Shandong
Jinfeng Hongyuan Flagship Store	Electricity conservation2	Received it, looking forward to the results, hope it works	Jingdong prefecture level city in Shandong
Jinfeng Hongyuan Flagship Store	Electricity conservation2	Received it, looking forward to the results, hope it works	Jingdong prefecture level city in Shandong
Jinfeng Hongyuan Flagship Store	Electricity conservation2	Supposedly it's an IQ tax. Buy one and try it. Looks good. Well made.	Jingdong prefecture level city in Shandong
Jinfeng Hongyuan Flagship Store	Electricity conservation2	It just doesn't feel like it's working.	Jingdong prefecture level city in Shandong
Jinfeng Hongyuan Flagship Store	Electricity conservation2	This is a great product.	Jingdong prefecture level city in Shandong
Jinfeng Hongyuan Flagship Store	Electricity conservation2	I'm just using it. I'll tell you more.	Jingdong prefecture level city in Shandong
Jinfeng Hongyuan Flagship Store	Electricity conservation2	Second repurchase and it's working fine.	Jingdong prefecture level city in Shandong
Jinfeng Hongyuan Flagship Store	Electricity conservation2	I'll wait a few days before evaluating	Jingdong prefecture level city in Shandong
Jinfeng Hongyuan Flagship Store	Electricity conservation2	I'll comment after I use it hopefully the section will be useful.	Jingdong prefecture level city in Shandong
Jinfeng Hongyuan Flagship Store	Electricity conservation2	not very useful	Jingdong prefecture level city in Shandong
Jinfeng Hongyuan Flagship Store	Electricity conservation2	Not received	Jingdong prefecture level city in Shandong

Jinfeng Hongyuan Flagship Store	Electricity conservation2	rare	Jingdong prefecture level city in Shandong
Jinfeng Hongyuan Flagship Store	Electricity conservation2	It's good.	Jingdong prefecture level city in Shandong
Jinfeng Hongyuan Flagship Store	Electricity conservation2	just now	Jingdong prefecture level city in Shandong
Jinfeng Hongyuan Flagship Store	Electricity conservation2	serviceable	Jingdong prefecture level city in Shandong
Jinfeng Hongyuan Flagship Store	Electricity conservation2	desirable	Jingdong prefecture level city in Shandong
Jinfeng Hongyuan Flagship Store	Electricity conservation2	serviceable	Jingdong prefecture level city in Shandong
Jinfeng Hongyuan Flagship Store	Electricity conservation2	It doesn't save power and costs electricity.	Jingdong prefecture level city in Shandong
Jinfeng Hongyuan Flagship Store	Electricity conservation2	Not as good as I thought.	Jingdong prefecture level city in Shandong
Jinfeng Hongyuan Flagship Store	Electricity conservation2	Super bad review, it doesn't save power at all, but rather more power consumption	Jingdong prefecture level city in Shandong
Jinfeng Hongyuan Flagship Store	Electricity conservation2	Listen to others say that this can save electricity, I bought with the idea of trying, used for a period of time before coming to the evaluation, will be a little bit of use, not a lot of savings just, long-term use of the words can save more.	Jingdong prefecture level city in Shandong
Jinfeng Hongyuan Flagship Store	Electricity conservation2	Logistics is fast, the next day arrived, workmanship is very delicate a power saver, looking forward to the late effect, customer service patiently explain the recommended which is suitable for, installed in what position is the best, look forward to using the effect after a period of time!	Jingdong prefecture level city in Shandong
Jinfeng Hongyuan Flagship Store	Electricity conservation2	It's good to use, I feel that it has been saving electricity, anyway, not a loss is, I used to feel that the power consumption is big, now I feel much better, my friends are also using, I hope to use a long time will be able to, I wish the boss a prosperous business	Jingdong prefecture level city in Shandong
Jinfeng Hongyuan Flagship Store	Electricity conservation2	Should be very good, to be able to plan the circuit, save the home of the circuit to the extent of the savings, waste of money on electricity, good if a month this money back, hope always hope to be able to	Jingdong prefecture level city in Shandong
Jinfeng Hongyuan Flagship Store	Electricity conservation2	Express delivery is very strong, the goods and I saw in the business consistent, the use of the effect of follow-up evaluation	Jingdong prefecture level city in Shandong
Jinfeng Hongyuan Flagship Store	Electricity conservation2	I have been using this brand of power saver for many years and this time I saw this upgraded version and got it again. The main purpose of this product is to reduce the loss of electricity for a long time, so as to achieve the purpose of saving electricity.	Jingdong prefecture level city in Shandong
Jinfeng Hongyuan Flagship Store	Electricity conservation2	Logistics is fast and the product looks beautiful, looking forward to the effect.	Jingdong prefecture level city in Shandong
Jinfeng Hongyuan Flagship Store	Electricity conservation2	Product quality is good, energy saving a little effect, but the price is a little expensive, fast arrival, good service.	Jingdong prefecture level city in Shandong
Jinfeng Hongyuan Flagship Store	Electricity conservation2	The quality is very good, inexpensive, Jingdong self-supporting guaranteed, worth having!	Jingdong prefecture level city in Shandong
Jinfeng Hongyuan Flagship Store	Electricity conservation2	I don't know if it's saving electricity yet, I'll find out next time I pay my electric bill	Jingdong prefecture level city in Shandong
Jinfeng Hongyuan Flagship Store	Electricity conservation2	Favorable price quality assurance appearance design is beautiful and generous, fine workmanship	Jingdong prefecture level city in Shandong
Jinfeng Hongyuan Flagship Store	Electricity conservation2	I bought it, I don't know how effective it is? I don't know how the quality is? After this summer results will know! Looking forward to it!	Jingdong prefecture level city in Shandong
Jinfeng Hongyuan Flagship Store	Electricity conservation2	Goods received Packaging intact, look at the shell workmanship is also good, high-power appliances open voltage has obvious changes in the effectiveness of the	Jingdong prefecture level city in Shandong
Jinfeng Hongyuan Flagship Store	Electricity conservation2	The goods have been received logistics is very fast, the product is similar to the line, just wait to use a month to see if it can save electricity, stay tuned.	Jingdong prefecture level city in Shandong
Jinfeng Hongyuan Flagship Store	Electricity conservation2	Electricity is plugged in, not sure if it will work, hopefully it will work.	Jingdong prefecture level city in Shandong
Jinfeng Hongyuan Flagship Store	Electricity conservation2	The shell is aluminum alloy, very good workmanship, the actual effect of waiting for the addition of evaluation	Jingdong prefecture level city in Shandong
Jinfeng Hongyuan Flagship Store	Electricity conservation2	Received, already plugged in, looking forward to the effect.	Jingdong prefecture level city in Shandong
Jinfeng Hongyuan Flagship Store	Electricity conservation2	Opened to see the regular manufacturer products, wait for a period of time to see it	Jingdong prefecture level city in Shandong
Jinfeng Hongyuan Flagship Store	Electricity conservation2	🉑 Baby received, very surprised ✨, reasonable design, fashionable and generous, continue to pay attention to the future will be repurchased 💗💗💗💗	Jingdong prefecture level city in Shandong
Jinfeng Hongyuan Flagship Store	Electricity conservation2	Baby received it 👌, really great ah! 🌈The quality can't be beat, very satisfied! 🆗Happy happy happy 😃	Jingdong prefecture level city in Shandong
Jinfeng Hongyuan Flagship Store	Electricity conservation2	Very good, power saving see, this is to help a friend to buy, I used to give my friend a say, my friend immediately let me order another one!	Jingdong prefecture level city in Shandong
Jinfeng Hongyuan Flagship Store	Electricity conservation2	The baby is just as introduced, the texture is very good, powerful, and it feels good to use it	Jingdong prefecture level city in Shandong
Jinfeng Hongyuan Flagship Store	Electricity conservation2	Value for money, very satisfied, seller service attitude is very good, very patient	Jingdong prefecture level city in Shandong
Jinfeng Hongyuan Flagship Store	Electricity conservation2	Power saving treasure design unique exquisite beautiful, voltage display clearly, plugged in that is good to use, I observed the meter, after using the meter is gone slow.	Jingdong prefecture level city in Shandong
Jinfeng Hongyuan Flagship Store	Electricity conservation2	Received on the use of the packaging is beautifully used for a period of time and then come back in the follow-up review!	Jingdong prefecture level city in Shandong
Jinfeng Hongyuan Flagship Store	Electricity conservation2	It should work. Not bad. Not bad. Not bad.	Jingdong prefecture level city in Shandong
Jinfeng Hongyuan Flagship Store	Electricity conservation2	I haven't used it yet, I'll plug it in and see if it saves power next month.	Jingdong prefecture level city in Shandong
Jinfeng Hongyuan Flagship Store	Electricity conservation2	The goods received and unpacked and used, the quality is very good, things and the official website to buy no difference!	Jingdong prefecture level city in Shandong
Jinfeng Hongyuan Flagship Store	Electricity conservation2	It's a rip off, doesn't help much at all, uses the same amount of power as before it was used.	Jingdong prefecture level city in Shandong
Jinfeng Hongyuan Flagship Store	Electricity conservation2	I've been using it for a while now and haven't noticed any unusual problems, so it's okay	Jingdong prefecture level city in Shandong
Jinfeng Hongyuan Flagship Store	Electricity conservation2	Received the goods and plugged in to use, good or not there is no use will have to wait until next month to find out	Jingdong prefecture level city in Shandong
Jinfeng Hongyuan Flagship Store	Electricity conservation2	It's very compact, it looks good, I'll introduce you to my clients when it's ready.	Jingdong prefecture level city in Shandong
Jinfeng Hongyuan Flagship Store	Electricity conservation2	Good workmanship, we'll have to see in a few months.	Jingdong prefecture level city in Shandong
Jinfeng Hongyuan Flagship Store	Electricity conservation2	Jingdong quality! Always trustworthy!	Jingdong prefecture level city in Shandong
Jinfeng Hongyuan Flagship Store	Electricity conservation2	This is the second time I've bought it, and the merchant said to give me five dollars out of the difference, so thank you merchant.	Jingdong prefecture level city in Shandong
Jinfeng Hongyuan Flagship Store	Electricity conservation2	Good quality and good service	Jingdong prefecture level city in Shandong
Jinfeng Hongyuan Flagship Store	Electricity conservation2	Feels good texture, aluminum alloy shell, voltage digital display clear.	Jingdong prefecture level city in Shandong
Jinfeng Hongyuan Flagship Store	Electricity conservation2	Good product, fast and great service, thanks!	Jingdong prefecture level city in Shandong
Jinfeng Hongyuan Flagship Store	Electricity conservation2	Have not yet used, first evaluation, later really good do not need to comment again.	Jingdong prefecture level city in Shandong
Jinfeng Hongyuan Flagship Store	Electricity conservation2	Works great. Pretty much as described by the merchant.	Jingdong prefecture level city in Shandong

Jinfeng Hongyuan Flagship Store	Electricity conservation2	Received it, looking forward to the results, hope it works	Jingdong prefecture level city in Shandong
Jinfeng Hongyuan Flagship Store	Electricity conservation2	Received it, looking forward to the results, hope it works	Jingdong prefecture level city in Shandong
Jinfeng Hongyuan Flagship Store	Electricity conservation2	Supposedly it's an IQ tax. Buy one and try it. Looks good. Well made.	Jingdong prefecture level city in Shandong
Jinfeng Hongyuan Flagship Store	Electricity conservation2	It just doesn't feel like it's working.	Jingdong prefecture level city in Shandong
Jinfeng Hongyuan Flagship Store	Electricity conservation2	This is a great product.	Jingdong prefecture level city in Shandong
Jinfeng Hongyuan Flagship Store	Electricity conservation2	I'm just using it. I'll tell you more.	Jingdong prefecture level city in Shandong
Jinfeng Hongyuan Flagship Store	Electricity conservation2	Second repurchase and it's working fine.	Jingdong prefecture level city in Shandong
Jinfeng Hongyuan Flagship Store	Electricity conservation2	I'll wait a few days before evaluating	Jingdong prefecture level city in Shandong
Jinfeng Hongyuan Flagship Store	Electricity conservation2	I'll comment after I use it hopefully the section will be useful.	Jingdong prefecture level city in Shandong
Jinfeng Hongyuan Flagship Store	Electricity conservation2	not very useful	Jingdong prefecture level city in Shandong
Jinfeng Hongyuan Flagship Store	Electricity conservation2	Not received	Jingdong prefecture level city in Shandong
Jinfeng Hongyuan Flagship Store	Electricity conservation2	rare	Jingdong prefecture level city in Shandong
Jinfeng Hongyuan Flagship Store	Electricity conservation2	It's good.	Jingdong prefecture level city in Shandong
Jinfeng Hongyuan Flagship Store	Electricity conservation2	just now	Jingdong prefecture level city in Shandong
Jinfeng Hongyuan Flagship Store	Electricity conservation2	serviceable	Jingdong prefecture level city in Shandong
Jinfeng Hongyuan Flagship Store	Electricity conservation2	desirable	Jingdong prefecture level city in Shandong
Jinfeng Hongyuan Flagship Store	Electricity conservation2	serviceable	Jingdong prefecture level city in Shandong
Jinfeng Hongyuan Flagship Store	Electricity conservation2	It doesn't save power and costs electricity.	Jingdong prefecture level city in Shandong
Jinfeng Hongyuan Flagship Store	Electricity conservation2	Not as good as I thought.	Jingdong prefecture level city in Shandong
Jinfeng Hongyuan Flagship Store	Electricity conservation2	Super bad review, it doesn't save power at all, but rather more power consumption	Jingdong prefecture level city in Shandong
Jinfeng Hongyuan Flagship Store	Electricity conservation2	Listen to others say that this can save electricity, I bought with the idea of trying, used for a period of time before coming to the evaluation, will be a little bit of use, not a lot of savings just, long-term use of the words can save more.	Jingdong prefecture level city in Shandong
Jinfeng Hongyuan Flagship Store	Electricity conservation2	Logistics is fast, the next day arrived, workmanship is very delicate a power saver, looking forward to the late effect, customer service patiently explain the recommended which is suitable for, installed in what position is the best, look forward to using the effect after a period of time!	Jingdong prefecture level city in Shandong
Jinfeng Hongyuan Flagship Store	Electricity conservation2	It's good to use, I feel that it has been saving electricity, anyway, not a loss is, I used to feel that the power consumption is big, now I feel much better, my friends are also using, I hope to use a long time will be able to, I wish the boss a prosperous business	Jingdong prefecture level city in Shandong
Jinfeng Hongyuan Flagship Store	Electricity conservation2	Should be very good, to be able to plan the circuit, save the home of the circuit to the extent of the savings, waste of money on electricity, good if a month this money back, hope always hope to be able to	Jingdong prefecture level city in Shandong
Jinfeng Hongyuan Flagship Store	Electricity conservation2	Express delivery is very strong, the goods and I saw in the business consistent, the use of the effect of follow-up evaluation	Jingdong prefecture level city in Shandong
Jinfeng Hongyuan Flagship Store	Electricity conservation2	I have been using this brand of power saver for many years and this time I saw this upgraded version and got it again. The main purpose of this product is to reduce the loss of electricity for a long time, so as to achieve the purpose of saving electricity.	Jingdong prefecture level city in Shandong
Jinfeng Hongyuan Flagship Store	Electricity conservation2	Logistics is fast and the product looks beautiful, looking forward to the effect.	Jingdong prefecture level city in Shandong
Jinfeng Hongyuan Flagship Store	Electricity conservation2	Product quality is good, energy saving a little effect, but the price is a little expensive, fast arrival, good service.	Jingdong prefecture level city in Shandong
Jinfeng Hongyuan Flagship Store	Electricity conservation2	The quality is very good, inexpensive, Jingdong self-supporting guaranteed, worth having!	Jingdong prefecture level city in Shandong
Jinfeng Hongyuan Flagship Store	Electricity conservation2	I don't know if it's saving electricity yet, I'll find out next time I pay my electric bill	Jingdong prefecture level city in Shandong
Jinfeng Hongyuan Flagship Store	Electricity conservation2	Favorable price quality assurance appearance design is beautiful and generous, fine workmanship	Jingdong prefecture level city in Shandong
Jinfeng Hongyuan Flagship Store	Electricity conservation2	I bought it, I don't know how effective it is? I don't know how the quality is? After this summer results will know! Looking forward to it!	Jingdong prefecture level city in Shandong
Jinfeng Hongyuan Flagship Store	Electricity conservation2	Goods received Packaging intact, look at the shell workmanship is also good, high-power appliances open voltage has obvious changes in the effectiveness of the	Jingdong prefecture level city in Shandong
Jinfeng Hongyuan Flagship Store	Electricity conservation2	The goods have been received logistics is very fast, the product is similar to the line, just wait to use a month to see if it can save electricity, stay tuned.	Jingdong prefecture level city in Shandong
Jinfeng Hongyuan Flagship Store	Electricity conservation2	Electricity is plugged in, not sure if it will work, hopefully it will work.	Jingdong prefecture level city in Shandong
Jinfeng Hongyuan Flagship Store	Electricity conservation2	The shell is aluminum alloy, very good workmanship, the actual effect of waiting for the addition of evaluation	Jingdong prefecture level city in Shandong
Jinfeng Hongyuan Flagship Store	Electricity conservation2	Received, already plugged in, looking forward to the effect.	Jingdong prefecture level city in Shandong
Jinfeng Hongyuan Flagship Store	Electricity conservation2	Opened to see the regular manufacturer products, wait for a period of time to see it	Jingdong prefecture level city in Shandong
Jinfeng Hongyuan Flagship Store	Electricity conservation2	🉑 Baby received, very surprised ✨, reasonable design, fashionable and generous, continue to pay attention to the future will be repurchased ❥❥❥❥	Jingdong prefecture level city in Shandong
Jinfeng Hongyuan Flagship Store	Electricity conservation2	Baby received it 👌, really great ah! 🌈The quality can't be beat, very satisfied! 🆗Happy happy happy 😃	Jingdong prefecture level city in Shandong
Jinfeng Hongyuan Flagship Store	Electricity conservation2	Very good, power saving see, this is to help a friend to buy, I used to give my friend a say, my friend immediately let me order another one!	Jingdong prefecture level city in Shandong
Jinfeng Hongyuan Flagship Store	Electricity conservation2	The baby is just as introduced, the texture is very good, powerful, and it feels good to use it	Jingdong prefecture level city in Shandong
Jinfeng Hongyuan Flagship Store	Electricity conservation2	Value for money, very satisfied, seller service attitude is very good, very patient	Jingdong prefecture level city in Shandong
Jinfeng Hongyuan Flagship Store	Electricity conservation2	Power saving treasure design unique exquisite beautiful, voltage display clearly, plugged in that is good to use, I observed the meter, after using the meter is gone slow.	Jingdong prefecture level city in Shandong
Jinfeng Hongyuan Flagship Store	Electricity conservation2	Received on the use of the packaging is beautifully used for a period of time and then come back in the follow-up review!	Jingdong prefecture level city in Shandong
Jinfeng Hongyuan Flagship Store	Electricity conservation2	It should work. Not bad. Not bad. Not bad.	Jingdong prefecture level city in Shandong
Jinfeng Hongyuan Flagship Store	Electricity conservation2	I haven't used it yet, I'll plug it in and see if it saves power next month.	Jingdong prefecture level city in Shandong
Jinfeng Hongyuan Flagship Store	Electricity conservation2	The goods received and unpacked and used, the quality is very good, things and the official website to buy no difference!	Jingdong prefecture level city in Shandong
Jinfeng Hongyuan Flagship Store	Electricity conservation2	It's a rip off, doesn't help much at all, uses the same amount of power as before it was used.	Jingdong prefecture level city in Shandong

Jinfeng Hongyuan Flagship Store	Electricity conservation2	I've been using it for a while now and haven't noticed any unusual problems, so it's okay	Jingdong prefecture level city in Shandong
Jinfeng Hongyuan Flagship Store	Electricity conservation2	Received the goods and plugged in to use, good or not there is no use will have to wait until next month to find out	Jingdong prefecture level city in Shandong
Jinfeng Hongyuan Flagship Store	Electricity conservation2	It's very compact, it looks good, I'll introduce you to my clients when it's ready.	Jingdong prefecture level city in Shandong
Jinfeng Hongyuan Flagship Store	Electricity conservation2	Good workmanship, we'll have to see in a few months.	Jingdong prefecture level city in Shandong
Jinfeng Hongyuan Flagship Store	Electricity conservation2	Jingdong quality! Always trustworthy!	Jingdong prefecture level city in Shandong
Jinfeng Hongyuan Flagship Store	Electricity conservation2	This is the second time I've bought it, and the merchant said to give me five dollars out of the difference, so thank you merchant.	Jingdong prefecture level city in Shandong
Jinfeng Hongyuan Flagship Store	Electricity conservation2	Good quality and good service	Jingdong prefecture level city in Shandong
Jinfeng Hongyuan Flagship Store	Electricity conservation2	Feels good texture, aluminum alloy shell, voltage digital display clear.	Jingdong prefecture level city in Shandong
Jinfeng Hongyuan Flagship Store	Electricity conservation2	Good product, fast and great service, thanks!	Jingdong prefecture level city in Shandong
Jinfeng Hongyuan Flagship Store	Electricity conservation2	Have not yet used, first evaluation, later really good do not need to comment again.	Jingdong prefecture level city in Shandong
Jinfeng Hongyuan Flagship Store	Electricity conservation2	Works great. Pretty much as described by the merchant.	Jingdong prefecture level city in Shandong
Jinfeng Hongyuan Flagship Store	Electricity conservation2	Received it, looking forward to the results, hope it works	Jingdong prefecture level city in Shandong
Jinfeng Hongyuan Flagship Store	Electricity conservation2	Received it, looking forward to the results, hope it works	Jingdong prefecture level city in Shandong
Jinfeng Hongyuan Flagship Store	Electricity conservation2	Supposedly it's an IQ tax. Buy one and try it. Looks good. Well made.	Jingdong prefecture level city in Shandong
Jinfeng Hongyuan Flagship Store	Electricity conservation2	It just doesn't feel like it's working.	Jingdong prefecture level city in Shandong
Jinfeng Hongyuan Flagship Store	Electricity conservation2	This is a great product.	Jingdong prefecture level city in Shandong
Jinfeng Hongyuan Flagship Store	Electricity conservation2	I'm just using it. I'll tell you more.	Jingdong prefecture level city in Shandong
Jinfeng Hongyuan Flagship Store	Electricity conservation2	Second repurchase and it's working fine.	Jingdong prefecture level city in Shandong
Jinfeng Hongyuan Flagship Store	Electricity conservation2	I'll wait a few days before evaluating	Jingdong prefecture level city in Shandong
Jinfeng Hongyuan Flagship Store	Electricity conservation2	I'll comment after I use it hopefully the section will be useful.	Jingdong prefecture level city in Shandong
Jinfeng Hongyuan Flagship Store	Electricity conservation2	not very useful	Jingdong prefecture level city in Shandong
Jinfeng Hongyuan Flagship Store	Electricity conservation2	Not received	Jingdong prefecture level city in Shandong
Jinfeng Hongyuan Flagship Store	Electricity conservation2	rare	Jingdong prefecture level city in Shandong
Jinfeng Hongyuan Flagship Store	Electricity conservation2	It's good.	Jingdong prefecture level city in Shandong
Jinfeng Hongyuan Flagship Store	Electricity conservation2	just now	Jingdong prefecture level city in Shandong
Jinfeng Hongyuan Flagship Store	Electricity conservation2	serviceable	Jingdong prefecture level city in Shandong
Jinfeng Hongyuan Flagship Store	Electricity conservation2	desirable	Jingdong prefecture level city in Shandong
Jinfeng Hongyuan Flagship Store	Electricity conservation2	serviceable	Jingdong prefecture level city in Shandong
Jinfeng Hongyuan Flagship Store	Electricity conservation2	It doesn't save power and costs electricity.	Jingdong prefecture level city in Shandong
Jinfeng Hongyuan Flagship Store	Electricity conservation2	Not as good as I thought.	Jingdong prefecture level city in Shandong
Jinfeng Hongyuan Flagship Store	Electricity conservation2	Super bad review, it doesn't save power at all, but rather more power consumption	Jingdong prefecture level city in Shandong
Jinfeng Hongyuan Flagship Store	Electricity conservation2	Listen to others say that this can save electricity, I bought with the idea of trying, used for a period of time before coming to the evaluation, will be a little bit of use, not a lot of savings just, long-term use of the words can save more.	Jingdong prefecture level city in Shandong
Jinfeng Hongyuan Flagship Store	Electricity conservation2	Logistics is fast, the next day arrived, workmanship is very delicate a power saver, looking forward to the late effect, customer service patiently explain the recommended which is suitable for, installed in what position is the best, look forward to using the effect after a period of time!	Jingdong prefecture level city in Shandong
Jinfeng Hongyuan Flagship Store	Electricity conservation2	It's good to use, I feel that it has been saving electricity, anyway, not a loss is, I used to feel that the power consumption is big, now I feel much better, my friends are also using, I hope to use a long time will be able to, I wish the boss a prosperous business	Jingdong prefecture level city in Shandong
Jinfeng Hongyuan Flagship Store	Electricity conservation2	Should be very good, to be able to plan the circuit, save the home of the circuit to the extent of the savings, waste of money on electricity, good if a month this money back, hope always hope to be able to	Jingdong prefecture level city in Shandong
Jinfeng Hongyuan Flagship Store	Electricity conservation2	Express delivery is very strong, the goods and I saw in the business consistent, the use of the effect of follow-up evaluation	Jingdong prefecture level city in Shandong
Jinfeng Hongyuan Flagship Store	Electricity conservation2	I have been using this brand of power saver for many years and this time I saw this upgraded version and got it again. The main purpose of this product is to reduce the loss of electricity for a long time, so as to achieve the purpose of saving electricity.	Jingdong prefecture level city in Shandong
Jinfeng Hongyuan Flagship Store	Electricity conservation2	Logistics is fast and the product looks beautiful, looking forward to the effect.	Jingdong prefecture level city in Shandong
Jinfeng Hongyuan Flagship Store	Electricity conservation2	Product quality is good, energy saving a little effect, but the price is a little expensive, fast arrival, good service.	Jingdong prefecture level city in Shandong
Jinfeng Hongyuan Flagship Store	Electricity conservation2	The quality is very good, inexpensive, Jingdong self-supporting guaranteed, worth having!	Jingdong prefecture level city in Shandong
Jinfeng Hongyuan Flagship Store	Electricity conservation2	I don't know if it's saving electricity yet, I'll find out next time I pay my electric bill	Jingdong prefecture level city in Shandong
Jinfeng Hongyuan Flagship Store	Electricity conservation2	Favorable price quality assurance appearance design is beautiful and generous, fine workmanship	Jingdong prefecture level city in Shandong
Jinfeng Hongyuan Flagship Store	Electricity conservation2	I bought it, I don't know how effective it is? I don't know how the quality is? After this summer results will know! Looking forward to it!	Jingdong prefecture level city in Shandong
Jinfeng Hongyuan Flagship Store	Electricity conservation2	Goods received Packaging intact, look at the shell workmanship is also good, high-power appliances open voltage has obvious changes in the effectiveness of the	Jingdong prefecture level city in Shandong
Jinfeng Hongyuan Flagship Store	Electricity conservation2	The goods have been received logistics is very fast, the product is similar to the line, just wait to use a month to see if it can save electricity, stay tuned.	Jingdong prefecture level city in Shandong
Jinfeng Hongyuan Flagship Store	Electricity conservation2	Electricity is plugged in, not sure if it will work, hopefully it will work.	Jingdong prefecture level city in Shandong
Jinfeng Hongyuan Flagship Store	Electricity conservation2	The shell is aluminum alloy, very good workmanship, the actual effect of waiting for the addition of evaluation	Jingdong prefecture level city in Shandong
Jinfeng Hongyuan Flagship Store	Electricity conservation2	Received, already plugged in, looking forward to the effect.	Jingdong prefecture level city in Shandong
Jinfeng Hongyuan Flagship Store	Electricity conservation2	Opened to see the regular manufacturer products, wait for a period of time to see it	Jingdong prefecture level city in Shandong

Jinfeng Hongyuan Flagship Store	Electricity conservation2	🉑 Baby received, very surprised ✨, reasonable design, fashionable and generous, continue to pay attention to the future will be repurchased ❤❤❤❤	Jingdong prefecture level city in Shandong
Jinfeng Hongyuan Flagship Store	Electricity conservation2	Baby received it 👌, really great ah! 🌈The quality can't be beat, very satisfied! 🆗Happy happy happy 😃	Jingdong prefecture level city in Shandong
Jinfeng Hongyuan Flagship Store	Electricity conservation2	Very good, power saving see, this is to help a friend to buy, I used to give my friend a say, my friend immediately let me order another one!	Jingdong prefecture level city in Shandong
Jinfeng Hongyuan Flagship Store	Electricity conservation2	The baby is just as introduced, the texture is very good, powerful, and it feels good to use it	Jingdong prefecture level city in Shandong
Jinfeng Hongyuan Flagship Store	Electricity conservation2	Value for money, very satisfied, seller service attitude is very good, very patient	Jingdong prefecture level city in Shandong
Jinfeng Hongyuan Flagship Store	Electricity conservation2	Power saving treasure design unique exquisite beautiful, voltage display clearly, plugged in that is good to use, I observed the meter, after using the meter is gone slow.	Jingdong prefecture level city in Shandong
Jinfeng Hongyuan Flagship Store	Electricity conservation2	Received on the use of the packaging is beautifully used for a period of time and then come back in the follow-up review!	Jingdong prefecture level city in Shandong
Jinfeng Hongyuan Flagship Store	Electricity conservation2	It should work. Not bad. Not bad. Not bad.	Jingdong prefecture level city in Shandong
Jinfeng Hongyuan Flagship Store	Electricity conservation2	I haven't used it yet, I'll plug it in and see if it saves power next month.	Jingdong prefecture level city in Shandong
Jinfeng Hongyuan Flagship Store	Electricity conservation2	The goods received and unpacked and used, the quality is very good, things and the official website to buy no difference!	Jingdong prefecture level city in Shandong
Jinfeng Hongyuan Flagship Store	Electricity conservation2	It's a rip off, doesn't help much at all, uses the same amount of power as before it was used.	Jingdong prefecture level city in Shandong
Jinfeng Hongyuan Flagship Store	Electricity conservation2	I've been using it for a while now and haven't noticed any unusual problems, so it's okay	Jingdong prefecture level city in Shandong
Jinfeng Hongyuan Flagship Store	Electricity conservation2	Received the goods and plugged in to use, good or not there is no use will have to wait until next month to find out	Jingdong prefecture level city in Shandong
Jinfeng Hongyuan Flagship Store	Electricity conservation2	It's very compact, it looks good, I'll introduce you to my clients when it's ready.	Jingdong prefecture level city in Shandong
Jinfeng Hongyuan Flagship Store	Electricity conservation2	Good workmanship, we'll have to see in a few months.	Jingdong prefecture level city in Shandong
Jinfeng Hongyuan Flagship Store	Electricity conservation2	Jingdong quality! Always trustworthy!	Jingdong prefecture level city in Shandong
Jinfeng Hongyuan Flagship Store	Electricity conservation2	This is the second time I've bought it, and the merchant said to give me five dollars out of the difference, so thank you merchant.	Jingdong prefecture level city in Shandong
Jinfeng Hongyuan Flagship Store	Electricity conservation2	Good quality and good service	Jingdong prefecture level city in Shandong
Jinfeng Hongyuan Flagship Store	Electricity conservation2	Feels good texture, aluminum alloy shell, voltage digital display clear.	Jingdong prefecture level city in Shandong
Jinfeng Hongyuan Flagship Store	Electricity conservation2	Good product, fast and great service, thanks!	Jingdong prefecture level city in Shandong
Jinfeng Hongyuan Flagship Store	Electricity conservation2	Have not yet used, first evaluation, later really good do not need to comment again.	Jingdong prefecture level city in Shandong
Jinfeng Hongyuan Flagship Store	Electricity conservation2	Works great. Pretty much as described by the merchant.	Jingdong prefecture level city in Shandong
Jinfeng Hongyuan Flagship Store	Electricity conservation2	Received it, looking forward to the results, hope it works	Jingdong prefecture level city in Shandong
Jinfeng Hongyuan Flagship Store	Electricity conservation2	Received it, looking forward to the results, hope it works	Jingdong prefecture level city in Shandong
Jinfeng Hongyuan Flagship Store	Electricity conservation2	Supposedly it's an IQ tax. Buy one and try it. Looks good. Well made.	Jingdong prefecture level city in Shandong
Jinfeng Hongyuan Flagship Store	Electricity conservation2	It just doesn't feel like it's working.	Jingdong prefecture level city in Shandong
Jinfeng Hongyuan Flagship Store	Electricity conservation2	This is a great product.	Jingdong prefecture level city in Shandong
Jinfeng Hongyuan Flagship Store	Electricity conservation2	I'm just using it. I'll tell you more.	Jingdong prefecture level city in Shandong
Jinfeng Hongyuan Flagship Store	Electricity conservation2	Second repurchase and it's working fine.	Jingdong prefecture level city in Shandong
Jinfeng Hongyuan Flagship Store	Electricity conservation2	I'll wait a few days before evaluating	Jingdong prefecture level city in Shandong
Jinfeng Hongyuan Flagship Store	Electricity conservation2	I'll comment after I use it hopefully the section will be useful.	Jingdong prefecture level city in Shandong
Jinfeng Hongyuan Flagship Store	Electricity conservation2	not very useful	Jingdong prefecture level city in Shandong
Jinfeng Hongyuan Flagship Store	Electricity conservation2	Not received	Jingdong prefecture level city in Shandong
Jinfeng Hongyuan Flagship Store	Electricity conservation2	rare	Jingdong prefecture level city in Shandong
Jinfeng Hongyuan Flagship Store	Electricity conservation2	It's good.	Jingdong prefecture level city in Shandong
Jinfeng Hongyuan Flagship Store	Electricity conservation2	just now	Jingdong prefecture level city in Shandong
Jinfeng Hongyuan Flagship Store	Electricity conservation2	serviceable	Jingdong prefecture level city in Shandong
Jinfeng Hongyuan Flagship Store	Electricity conservation2	desirable	Jingdong prefecture level city in Shandong
Jinfeng Hongyuan Flagship Store	Electricity conservation2	serviceable	Jingdong prefecture level city in Shandong
Jinfeng Hongyuan Flagship Store	Electricity conservation2	It doesn't save power and costs electricity.	Jingdong prefecture level city in Shandong
Jinfeng Hongyuan Flagship Store	Electricity conservation2	Not as good as I thought.	Jingdong prefecture level city in Shandong
Jinfeng Hongyuan Flagship Store	Electricity conservation2	Super bad review, it doesn't save power at all, but rather more power consumption	Jingdong prefecture level city in Shandong
Jinfeng Hongyuan Flagship Store	Electricity conservation2	Listen to others say that this can save electricity, I bought with the idea of trying, used for a period of time before coming to the evaluation, will be a little bit of use, not a lot of savings just, long-term use of the words can save more.	Jingdong prefecture level city in Shandong
Jinfeng Hongyuan Flagship Store	Electricity conservation2	Logistics is fast, the next day arrived, workmanship is very delicate a power saver, looking forward to the late effect, customer service patiently explain the recommended which is suitable for, installed in what position is the best, look forward to using the effect after a period of time!	Jingdong prefecture level city in Shandong
Jinfeng Hongyuan Flagship Store	Electricity conservation2	It's good to use, I feel that it has been saving electricity, anyway, not a loss is, I used to feel that the power consumption is big, now I feel much better, my friends are also using, I hope to use a long time will be able to, I wish the boss a prosperous business	Jingdong prefecture level city in Shandong
Jinfeng Hongyuan Flagship Store	Electricity conservation2	Should be very good, to be able to plan the circuit, save the home of the circuit to the extent of the savings, waste of money on electricity, good if a month this money back, hope always hope to be able to	Jingdong prefecture level city in Shandong
Jinfeng Hongyuan Flagship Store	Electricity conservation2	Express delivery is very strong, the goods and I saw in the business consistent, the use of the effect of follow-up evaluation	Jingdong prefecture level city in Shandong
Jinfeng Hongyuan Flagship Store	Electricity conservation2	I have been using this brand of power saver for many years and this time I saw this upgraded version and got it again. The main purpose of this product is to reduce the loss of electricity for a long time, so as to achieve the purpose of saving electricity.	Jingdong prefecture level city in Shandong
Jinfeng Hongyuan Flagship Store	Electricity conservation2	Logistics is fast and the product looks beautiful, looking forward to the effect.	Jingdong prefecture level city in Shandong

Jinfeng Hongyuan Flagship Store	Electricity conservation2	Product quality is good, energy saving a little effect, but the price is a little expensive, fast arrival, good service.	Jingdong prefecture level city in Shandong
Jinfeng Hongyuan Flagship Store	Electricity conservation2	The quality is very good, inexpensive, Jingdong self-supporting guaranteed, worth having!	Jingdong prefecture level city in Shandong
Jinfeng Hongyuan Flagship Store	Electricity conservation2	I don't know if it's saving electricity yet, I'll find out next time I pay my electric bill	Jingdong prefecture level city in Shandong
Jinfeng Hongyuan Flagship Store	Electricity conservation2	Favorable price quality assurance appearance design is beautiful and generous, fine workmanship	Jingdong prefecture level city in Shandong
Jinfeng Hongyuan Flagship Store	Electricity conservation2	I bought it, I don't know how effective it is? I don't know how the quality is? After this summer results will know! Looking forward to it!	Jingdong prefecture level city in Shandong
Jinfeng Hongyuan Flagship Store	Electricity conservation2	Goods received Packaging intact, look at the shell workmanship is also good, high-power appliances open voltage has obvious changes in the effectiveness of the	Jingdong prefecture level city in Shandong
Jinfeng Hongyuan Flagship Store	Electricity conservation2	The goods have been received logistics is very fast, the product is similar to the line, just wait to use a month to see if it can save electricity, stay tuned.	Jingdong prefecture level city in Shandong
Jinfeng Hongyuan Flagship Store	Electricity conservation2	Electricity is plugged in, not sure if it will work, hopefully it will work.	Jingdong prefecture level city in Shandong
Jinfeng Hongyuan Flagship Store	Electricity conservation2	The shell is aluminum alloy, very good workmanship, the actual effect of waiting for the addition of evaluation	Jingdong prefecture level city in Shandong
Jinfeng Hongyuan Flagship Store	Electricity conservation2	Received, already plugged in, looking forward to the effect.	Jingdong prefecture level city in Shandong
Jinfeng Hongyuan Flagship Store	Electricity conservation2	Opened to see the regular manufacturer products, wait for a period of time to see it	Jingdong prefecture level city in Shandong
Jinfeng Hongyuan Flagship Store	Electricity conservation2	🉑 Baby received, very surprised ✨, reasonable design, fashionable and generous, continue to pay attention to the future will be repurchased ❥❥❥❥	Jingdong prefecture level city in Shandong
Jinfeng Hongyuan Flagship Store	Electricity conservation2	Baby received it 👌, really great ah! 🌈The quality can't be beat, very satisfied! 🆗Happy happy happy 😃	Jingdong prefecture level city in Shandong
Jinfeng Hongyuan Flagship Store	Electricity conservation2	Very good, power saving see, this is to help a friend to buy, I used to give my friend a say, my friend immediately let me order another one!	Jingdong prefecture level city in Shandong
Jinfeng Hongyuan Flagship Store	Electricity conservation2	The baby is just as introduced, the texture is very good, powerful, and it feels good to use it	Jingdong prefecture level city in Shandong
Jinfeng Hongyuan Flagship Store	Electricity conservation2	Value for money, very satisfied, seller service attitude is very good, very patient	Jingdong prefecture level city in Shandong
Jinfeng Hongyuan Flagship Store	Electricity conservation2	Power saving treasure design unique exquisite beautiful, voltage display clearly, plugged in that is good to use, I observed the meter, after using the meter is gone slow.	Jingdong prefecture level city in Shandong
Jinfeng Hongyuan Flagship Store	Electricity conservation2	Received on the use of the packaging is beautifully used for a period of time and then come back in the follow-up review!	Jingdong prefecture level city in Shandong
Jinfeng Hongyuan Flagship Store	Electricity conservation2	It should work. Not bad. Not bad. Not bad.	Jingdong prefecture level city in Shandong
Jinfeng Hongyuan Flagship Store	Electricity conservation2	I haven't used it yet, I'll plug it in and see if it saves power next month.	Jingdong prefecture level city in Shandong
Jinfeng Hongyuan Flagship Store	Electricity conservation2	The goods received and unpacked and used, the quality is very good, things and the official website to buy no difference!	Jingdong prefecture level city in Shandong
Jinfeng Hongyuan Flagship Store	Electricity conservation2	It's a rip off, doesn't help much at all, uses the same amount of power as before it was used.	Jingdong prefecture level city in Shandong
Jinfeng Hongyuan Flagship Store	Electricity conservation2	I've been using it for a while now and haven't noticed any unusual problems, so it's okay	Jingdong prefecture level city in Shandong
Jinfeng Hongyuan Flagship Store	Electricity conservation2	Received the goods and plugged in to use, good or not there is no use will have to wait until next month to find out	Jingdong prefecture level city in Shandong
Jinfeng Hongyuan Flagship Store	Electricity conservation2	It's very compact, it looks good, I'll introduce you to my clients when it's ready.	Jingdong prefecture level city in Shandong
Jinfeng Hongyuan Flagship Store	Electricity conservation2	Good workmanship, we'll have to see in a few months.	Jingdong prefecture level city in Shandong
Jinfeng Hongyuan Flagship Store	Electricity conservation2	Jingdong quality! Always trustworthy!	Jingdong prefecture level city in Shandong
Jinfeng Hongyuan Flagship Store	Electricity conservation2	This is the second time I've bought it, and the merchant said to give me five dollars out of the difference, so thank you merchant.	Jingdong prefecture level city in Shandong
Jinfeng Hongyuan Flagship Store	Electricity conservation2	Good quality and good service	Jingdong prefecture level city in Shandong
Jinfeng Hongyuan Flagship Store	Electricity conservation2	Feels good texture, aluminum alloy shell, voltage digital display clear.	Jingdong prefecture level city in Shandong
Jinfeng Hongyuan Flagship Store	Electricity conservation2	Good product, fast and great service, thanks!	Jingdong prefecture level city in Shandong
Jinfeng Hongyuan Flagship Store	Electricity conservation2	Have not yet used, first evaluation, later really good do not need to comment again.	Jingdong prefecture level city in Shandong
Jinfeng Hongyuan Flagship Store	Electricity conservation2	Works great. Pretty much as described by the merchant.	Jingdong prefecture level city in Shandong
Jinfeng Hongyuan Flagship Store	Electricity conservation2	Received it, looking forward to the results, hope it works	Jingdong prefecture level city in Shandong
Jinfeng Hongyuan Flagship Store	Electricity conservation2	Received it, looking forward to the results, hope it works	Jingdong prefecture level city in Shandong
Jinfeng Hongyuan Flagship Store	Electricity conservation2	Supposedly it's an IQ tax. Buy one and try it. Looks good. Well made.	Jingdong prefecture level city in Shandong
Jinfeng Hongyuan Flagship Store	Electricity conservation2	It just doesn't feel like it's working.	Jingdong prefecture level city in Shandong
Jinfeng Hongyuan Flagship Store	Electricity conservation2	This is a great product.	Jingdong prefecture level city in Shandong
Jinfeng Hongyuan Flagship Store	Electricity conservation2	I'm just using it. I'll tell you more.	Jingdong prefecture level city in Shandong
Jinfeng Hongyuan Flagship Store	Electricity conservation2	Second repurchase and it's working fine.	Jingdong prefecture level city in Shandong
Jinfeng Hongyuan Flagship Store	Electricity conservation2	I'll wait a few days before evaluating	Jingdong prefecture level city in Shandong
Jinfeng Hongyuan Flagship Store	Electricity conservation2	I'll comment after I use it hopefully the section will be useful.	Jingdong prefecture level city in Shandong
Jinfeng Hongyuan Flagship Store	Electricity conservation2	not very useful	Jingdong prefecture level city in Shandong
Jinfeng Hongyuan Flagship Store	Electricity conservation2	Not received	Jingdong prefecture level city in Shandong
Jinfeng Hongyuan Flagship Store	Electricity conservation2	rare	Jingdong prefecture level city in Shandong
Jinfeng Hongyuan Flagship Store	Electricity conservation2	It's good.	Jingdong prefecture level city in Shandong
Jinfeng Hongyuan Flagship Store	Electricity conservation2	just now	Jingdong prefecture level city in Shandong
Jinfeng Hongyuan Flagship Store	Electricity conservation2	serviceable	Jingdong prefecture level city in Shandong
Jinfeng Hongyuan Flagship Store	Electricity conservation2	desirable	Jingdong prefecture level city in Shandong
Jinfeng Hongyuan Flagship Store	Electricity conservation2	serviceable	Jingdong prefecture level city in Shandong
Jinfeng Hongyuan Flagship Store	Electricity conservation2	It doesn't save power and costs electricity.	Jingdong prefecture level city in Shandong

Jinfeng Hongyuan Flagship Store	Electricity conservation2	Not as good as I thought.	Jingdong prefecture level city in Shandong
Jinfeng Hongyuan Flagship Store	Electricity conservation2	Super bad review, it doesn't save power at all, but rather more power consumption	Jingdong prefecture level city in Shandong
Jinfeng Hongyuan Flagship Store	Electricity conservation2	Listen to others say that this can save electricity, I bought with the idea of trying, used for a period of time before coming to the evaluation, will be a little bit of use, not a lot of savings just, long-term use of the words can save more.	Jingdong prefecture level city in Shandong
Jinfeng Hongyuan Flagship Store	Electricity conservation2	Logistics is fast, the next day arrived, workmanship is very delicate a power saver, looking forward to the late effect, customer service patiently explain the recommended which is suitable for, installed in what position is the best, look forward to using the effect after a period of time!	Jingdong prefecture level city in Shandong
Jinfeng Hongyuan Flagship Store	Electricity conservation2	It's good to use, I feel that it has been saving electricity, anyway, not a loss is, I used to feel that the power consumption is big, now I feel much better, my friends are also using, I hope to use a long time will be able to, I wish the boss a prosperous business	Jingdong prefecture level city in Shandong
Jinfeng Hongyuan Flagship Store	Electricity conservation2	Should be very good, to be able to plan the circuit, save the home of the circuit to the extent of the savings, waste of money on electricity, good if a month this money back, hope always hope to be able to	Jingdong prefecture level city in Shandong
Jinfeng Hongyuan Flagship Store	Electricity conservation2	Express delivery is very strong, the goods and I saw in the business consistent, the use of the effect of follow-up evaluation	Jingdong prefecture level city in Shandong
Jinfeng Hongyuan Flagship Store	Electricity conservation2	I have been using this brand of power saver for many years and this time I saw this upgraded version and got it again. The main purpose of this product is to reduce the loss of electricity for a long time, so as to achieve the purpose of saving electricity.	Jingdong prefecture level city in Shandong
Jinfeng Hongyuan Flagship Store	Electricity conservation2	Logistics is fast and the product looks beautiful, looking forward to the effect.	Jingdong prefecture level city in Shandong
Jinfeng Hongyuan Flagship Store	Electricity conservation2	Product quality is good, energy saving a little effect, but the price is a little expensive, fast arrival, good service.	Jingdong prefecture level city in Shandong
Jinfeng Hongyuan Flagship Store	Electricity conservation2	The quality is very good, inexpensive, Jingdong self-supporting guaranteed, worth having!	Jingdong prefecture level city in Shandong
Jinfeng Hongyuan Flagship Store	Electricity conservation2	I don't know if it's saving electricity yet, I'll find out next time I pay my electric bill	Jingdong prefecture level city in Shandong
Jinfeng Hongyuan Flagship Store	Electricity conservation2	Favorable price quality assurance appearance design is beautiful and generous, fine workmanship	Jingdong prefecture level city in Shandong
Jinfeng Hongyuan Flagship Store	Electricity conservation2	I bought it, I don't know how effective it is? I don't know how the quality is? After this summer results will know! Looking forward to it!	Jingdong prefecture level city in Shandong
Jinfeng Hongyuan Flagship Store	Electricity conservation2	Goods received Packaging intact, look at the shell workmanship is also good, high-power appliances open voltage has obvious changes in the effectiveness of the	Jingdong prefecture level city in Shandong
Jinfeng Hongyuan Flagship Store	Electricity conservation2	The goods have been received logistics is very fast, the product is similar to the line, just wait to use a month to see if it can save electricity, stay tuned.	Jingdong prefecture level city in Shandong
Jinfeng Hongyuan Flagship Store	Electricity conservation2	Electricity is plugged in, not sure if it will work, hopefully it will work.	Jingdong prefecture level city in Shandong
Jinfeng Hongyuan Flagship Store	Electricity conservation2	The shell is aluminum alloy, very good workmanship, the actual effect of waiting for the addition of evaluation	Jingdong prefecture level city in Shandong
Jinfeng Hongyuan Flagship Store	Electricity conservation2	Received, already plugged in, looking forward to the effect.	Jingdong prefecture level city in Shandong
Jinfeng Hongyuan Flagship Store	Electricity conservation2	Opened to see the regular manufacturer products, wait for a period of time to see it	Jingdong prefecture level city in Shandong
Jinfeng Hongyuan Flagship Store	Electricity conservation2	🉑 Baby received, very surprised ✨, reasonable design, fashionable and generous, continue to pay attention to the future will be repurchased ❤❤❤❤	Jingdong prefecture level city in Shandong
Jinfeng Hongyuan Flagship Store	Electricity conservation2	Baby received it 👌, really great ah! 🌈The quality can't be beat, very satisfied! 🆗Happy happy happy 😃	Jingdong prefecture level city in Shandong
Jinfeng Hongyuan Flagship Store	Electricity conservation2	Very good, power saving see, this is to help a friend to buy, I used to give my friend a say, my friend immediately let me order another one!	Jingdong prefecture level city in Shandong
Jinfeng Hongyuan Flagship Store	Electricity conservation2	The baby is just as introduced, the texture is very good, powerful, and it feels good to use it	Jingdong prefecture level city in Shandong
Jinfeng Hongyuan Flagship Store	Electricity conservation2	Value for money, very satisfied, seller service attitude is very good, very patient	Jingdong prefecture level city in Shandong
Jinfeng Hongyuan Flagship Store	Electricity conservation2	Power saving treasure design unique exquisite beautiful, voltage display clearly, plugged in that is good to use, I observed the meter, after using the meter is gone slow.	Jingdong prefecture level city in Shandong
Jinfeng Hongyuan Flagship Store	Electricity conservation2	Received on the use of the packaging is beautifully used for a period of time and then come back in the follow-up review!	Jingdong prefecture level city in Shandong
Jinfeng Hongyuan Flagship Store	Electricity conservation2	It should work. Not bad. Not bad. Not bad.	Jingdong prefecture level city in Shandong
Jinfeng Hongyuan Flagship Store	Electricity conservation2	I haven't used it yet, I'll plug it in and see if it saves power next month.	Jingdong prefecture level city in Shandong
Jinfeng Hongyuan Flagship Store	Electricity conservation2	The goods received and unpacked and used, the quality is very good, things and the official website to buy no difference!	Jingdong prefecture level city in Shandong
Jinfeng Hongyuan Flagship Store	Electricity conservation2	It's a rip off, doesn't help much at all, uses the same amount of power as before it was used.	Jingdong prefecture level city in Shandong
Jinfeng Hongyuan Flagship Store	Electricity conservation2	I've been using it for a while now and haven't noticed any unusual problems, so it's okay	Jingdong prefecture level city in Shandong
Jinfeng Hongyuan Flagship Store	Electricity conservation2	Received the goods and plugged in to use, good or not there is no use will have to wait until next month to find out	Jingdong prefecture level city in Shandong
Jinfeng Hongyuan Flagship Store	Electricity conservation2	It's very compact, it looks good, I'll introduce you to my clients when it's ready.	Jingdong prefecture level city in Shandong
Jinfeng Hongyuan Flagship Store	Electricity conservation2	Good workmanship, we'll have to see in a few months.	Jingdong prefecture level city in Shandong
Jinfeng Hongyuan Flagship Store	Electricity conservation2	Jingdong quality! Always trustworthy!	Jingdong prefecture level city in Shandong
Jinfeng Hongyuan Flagship Store	Electricity conservation2	This is the second time I've bought it, and the merchant said to give me five dollars out of the difference, so thank you merchant.	Jingdong prefecture level city in Shandong
Jinfeng Hongyuan Flagship Store	Electricity conservation2	Good quality and good service	Jingdong prefecture level city in Shandong
Jinfeng Hongyuan Flagship Store	Electricity conservation2	Feels good texture, aluminum alloy shell, voltage digital display clear.	Jingdong prefecture level city in Shandong
Jinfeng Hongyuan Flagship Store	Electricity conservation2	Good product, fast and great service, thanks!	Jingdong prefecture level city in Shandong
Jinfeng Hongyuan Flagship Store	Electricity conservation2	Have not yet used, first evaluation, later really good do not need to comment again.	Jingdong prefecture level city in Shandong
Jinfeng Hongyuan Flagship Store	Electricity conservation2	Works great. Pretty much as described by the merchant.	Jingdong prefecture level city in Shandong
Jinfeng Hongyuan Flagship Store	Electricity conservation2	Received it, looking forward to the results, hope it works	Jingdong prefecture level city in Shandong
Jinfeng Hongyuan Flagship Store	Electricity conservation2	Received it, looking forward to the results, hope it works	Jingdong prefecture level city in Shandong
Jinfeng Hongyuan Flagship Store	Electricity conservation2	Supposedly it's an IQ tax. Buy one and try it. Looks good. Well made.	Jingdong prefecture level city in Shandong
Jinfeng Hongyuan Flagship Store	Electricity conservation2	It just doesn't feel like it's working.	Jingdong prefecture level city in Shandong
Jinfeng Hongyuan Flagship Store	Electricity conservation2	This is a great product.	Jingdong prefecture level city in Shandong
Jinfeng Hongyuan Flagship Store	Electricity conservation2	I'm just using it. I'll tell you more.	Jingdong prefecture level city in Shandong
Jinfeng Hongyuan Flagship Store	Electricity conservation2	Second repurchase and it's working fine.	Jingdong prefecture level city in Shandong

Jinfeng Hongyuan Flagship Store	Electricity conservation2	I'll wait a few days before evaluating	Jingdong prefecture level city in Shandong
Jinfeng Hongyuan Flagship Store	Electricity conservation2	I'll comment after I use it hopefully the section will be useful.	Jingdong prefecture level city in Shandong
Jinfeng Hongyuan Flagship Store	Electricity conservation2	not very useful	Jingdong prefecture level city in Shandong
Jinfeng Hongyuan Flagship Store	Electricity conservation2	Not received	Jingdong prefecture level city in Shandong
Jinfeng Hongyuan Flagship Store	Electricity conservation2	rare	Jingdong prefecture level city in Shandong
Jinfeng Hongyuan Flagship Store	Electricity conservation2	It's good.	Jingdong prefecture level city in Shandong
Jinfeng Hongyuan Flagship Store	Electricity conservation2	just now	Jingdong prefecture level city in Shandong
Jinfeng Hongyuan Flagship Store	Electricity conservation2	serviceable	Jingdong prefecture level city in Shandong
Jinfeng Hongyuan Flagship Store	Electricity conservation2	desirable	Jingdong prefecture level city in Shandong
Jinfeng Hongyuan Flagship Store	Electricity conservation2	serviceable	Jingdong prefecture level city in Shandong
Jinfeng Hongyuan Flagship Store	Electricity conservation2	It doesn't save power and costs electricity.	Jingdong prefecture level city in Shandong
Jinfeng Hongyuan Flagship Store	Electricity conservation2	Not as good as I thought.	Jingdong prefecture level city in Shandong
Jinfeng Hongyuan Flagship Store	Electricity conservation2	Super bad review, it doesn't save power at all, but rather more power consumption	Jingdong prefecture level city in Shandong
Jinfeng Hongyuan Flagship Store	Electricity conservation2	Listen to others say that this can save electricity, I bought with the idea of trying, used for a period of time before coming to the evaluation, will be a little bit of use, not a lot of savings just, long-term use of the words can save more.	Jingdong prefecture level city in Shandong
Jinfeng Hongyuan Flagship Store	Electricity conservation2	Logistics is fast, the next day arrived, workmanship is very delicate a power saver, looking forward to the late effect, customer service patiently explain the recommended which is suitable for, installed in what position is the best, look forward to using the effect after a period of time!	Jingdong prefecture level city in Shandong
Jinfeng Hongyuan Flagship Store	Electricity conservation2	It's good to use, I feel that it has been saving electricity, anyway, not a loss is, I used to feel that the power consumption is big, now I feel much better, my friends are also using, I hope to use a long time will be able to, I wish the boss a prosperous business	Jingdong prefecture level city in Shandong
Jinfeng Hongyuan Flagship Store	Electricity conservation2	Should be very good, to be able to plan the circuit, save the home of the circuit to the extent of the savings, waste of money on electricity, good if a month this money back, hope always hope to be able to	Jingdong prefecture level city in Shandong
Jinfeng Hongyuan Flagship Store	Electricity conservation2	Express delivery is very strong, the goods and I saw in the business consistent, the use of the effect of follow-up evaluation	Jingdong prefecture level city in Shandong
Jinfeng Hongyuan Flagship Store	Electricity conservation2	I have been using this brand of power saver for many years and this time I saw this upgraded version and got it again. The main purpose of this product is to reduce the loss of electricity for a long time, so as to achieve the purpose of saving electricity.	Jingdong prefecture level city in Shandong
Jinfeng Hongyuan Flagship Store	Electricity conservation2	Logistics is fast and the product looks beautiful, looking forward to the effect.	Jingdong prefecture level city in Shandong
Jinfeng Hongyuan Flagship Store	Electricity conservation2	Product quality is good, energy saving a little effect, but the price is a little expensive, fast arrival, good service.	Jingdong prefecture level city in Shandong
Jinfeng Hongyuan Flagship Store	Electricity conservation2	The quality is very good, inexpensive, Jingdong self-supporting guaranteed, worth having!	Jingdong prefecture level city in Shandong
Jinfeng Hongyuan Flagship Store	Electricity conservation2	I don't know if it's saving electricity yet, I'll find out next time I pay my electric bill	Jingdong prefecture level city in Shandong
Jinfeng Hongyuan Flagship Store	Electricity conservation2	Favorable price quality assurance appearance design is beautiful and generous, fine workmanship	Jingdong prefecture level city in Shandong
Jinfeng Hongyuan Flagship Store	Electricity conservation2	I bought it, I don't know how effective it is? I don't know how the quality is? After this summer results will know! Looking forward to it!	Jingdong prefecture level city in Shandong
Jinfeng Hongyuan Flagship Store	Electricity conservation2	Goods received Packaging intact, look at the shell workmanship is also good, high-power appliances open voltage has obvious changes in the effectiveness of the	Jingdong prefecture level city in Shandong
Jinfeng Hongyuan Flagship Store	Electricity conservation2	The goods have been received logistics is very fast, the product is similar to the line, just wait to use a month to see if it can save electricity, stay tuned.	Jingdong prefecture level city in Shandong
Jinfeng Hongyuan Flagship Store	Electricity conservation2	Electricity is plugged in, not sure if it will work, hopefully it will work.	Jingdong prefecture level city in Shandong
Jinfeng Hongyuan Flagship Store	Electricity conservation2	The shell is aluminum alloy, very good workmanship, the actual effect of waiting for the addition of evaluation	Jingdong prefecture level city in Shandong
Jinfeng Hongyuan Flagship Store	Electricity conservation2	Received, already plugged in, looking forward to the effect.	Jingdong prefecture level city in Shandong
Jinfeng Hongyuan Flagship Store	Electricity conservation2	Opened to see the regular manufacturer products, wait for a period of time to see it	Jingdong prefecture level city in Shandong
Jinfeng Hongyuan Flagship Store	Electricity conservation2	🉑 Baby received, very surprised ✨, reasonable design, fashionable and generous, continue to pay attention to the future will be repurchased ❤❤❤❤	Jingdong prefecture level city in Shandong
Jinfeng Hongyuan Flagship Store	Electricity conservation2	Baby received it 👌, really great ah! 🌈The quality can't be beat, very satisfied! 🆗Happy happy happy 😃	Jingdong prefecture level city in Shandong
Jinfeng Hongyuan Flagship Store	Electricity conservation2	Very good, power saving see, this is to help a friend to buy, I used to give my friend a say, my friend immediately let me order another one!	Jingdong prefecture level city in Shandong
Jinfeng Hongyuan Flagship Store	Electricity conservation2	The baby is just as introduced, the texture is very good, powerful, and it feels good to use it	Jingdong prefecture level city in Shandong
Jinfeng Hongyuan Flagship Store	Electricity conservation2	Value for money, very satisfied, seller service attitude is very good, very patient	Jingdong prefecture level city in Shandong
Jinfeng Hongyuan Flagship Store	Electricity conservation2	Power saving treasure design unique exquisite beautiful, voltage display clearly, plugged in that is good to use, I observed the meter, after using the meter is gone slow.	Jingdong prefecture level city in Shandong
Jinfeng Hongyuan Flagship Store	Electricity conservation2	Received on the use of the packaging is beautifully used for a period of time and then come back in the follow-up review!	Jingdong prefecture level city in Shandong
Jinfeng Hongyuan Flagship Store	Electricity conservation2	It should work. Not bad. Not bad. Not bad.	Jingdong prefecture level city in Shandong
Jinfeng Hongyuan Flagship Store	Electricity conservation2	I haven't used it yet, I'll plug it in and see if it saves power next month.	Jingdong prefecture level city in Shandong
Jinfeng Hongyuan Flagship Store	Electricity conservation2	The goods received and unpacked and used, the quality is very good, things and the official website to buy no difference!	Jingdong prefecture level city in Shandong
Jinfeng Hongyuan Flagship Store	Electricity conservation2	It's a rip off, doesn't help much at all, uses the same amount of power as before it was used.	Jingdong prefecture level city in Shandong
Jinfeng Hongyuan Flagship Store	Electricity conservation2	I've been using it for a while now and haven't noticed any unusual problems, so it's okay	Jingdong prefecture level city in Shandong
Jinfeng Hongyuan Flagship Store	Electricity conservation2	Received the goods and plugged in to use, good or not there is no use will have to wait until next month to find out	Jingdong prefecture level city in Shandong
Jinfeng Hongyuan Flagship Store	Electricity conservation2	It's very compact, it looks good, I'll introduce you to my clients when it's ready.	Jingdong prefecture level city in Shandong
Jinfeng Hongyuan Flagship Store	Electricity conservation2	Good workmanship, we'll have to see in a few months.	Jingdong prefecture level city in Shandong
Jinfeng Hongyuan Flagship Store	Electricity conservation2	Jingdong quality! Always trustworthy!	Jingdong prefecture level city in Shandong
Jinfeng Hongyuan Flagship Store	Electricity conservation2	This is the second time I've bought it, and the merchant said to give me five dollars out of the difference, so thank you merchant.	Jingdong prefecture level city in Shandong
Jinfeng Hongyuan Flagship Store	Electricity conservation2	Good quality and good service	Jingdong prefecture level city in Shandong

Jinfeng Hongyuan Flagship Store	Electricity conservation2	Feels good texture, aluminum alloy shell, voltage digital display clear.	Jingdong prefecture level city in Shandong
Jinfeng Hongyuan Flagship Store	Electricity conservation2	Good product, fast and great service, thanks!	Jingdong prefecture level city in Shandong
Jinfeng Hongyuan Flagship Store	Electricity conservation2	Have not yet used, first evaluation, later really good do not need to comment again.	Jingdong prefecture level city in Shandong
Jinfeng Hongyuan Flagship Store	Electricity conservation2	Works great. Pretty much as described by the merchant.	Jingdong prefecture level city in Shandong
Jinfeng Hongyuan Flagship Store	Electricity conservation2	Received it, looking forward to the results, hope it works	Jingdong prefecture level city in Shandong
Jinfeng Hongyuan Flagship Store	Electricity conservation2	Received it, looking forward to the results, hope it works	Jingdong prefecture level city in Shandong
Jinfeng Hongyuan Flagship Store	Electricity conservation2	Supposedly it's an IQ tax. Buy one and try it. Looks good. Well made.	Jingdong prefecture level city in Shandong
Jinfeng Hongyuan Flagship Store	Electricity conservation2	It just doesn't feel like it's working.	Jingdong prefecture level city in Shandong
Jinfeng Hongyuan Flagship Store	Electricity conservation2	This is a great product.	Jingdong prefecture level city in Shandong
Jinfeng Hongyuan Flagship Store	Electricity conservation2	I'm just using it. I'll tell you more.	Jingdong prefecture level city in Shandong
Jinfeng Hongyuan Flagship Store	Electricity conservation2	Second repurchase and it's working fine.	Jingdong prefecture level city in Shandong
Jinfeng Hongyuan Flagship Store	Electricity conservation2	I'll wait a few days before evaluating	Jingdong prefecture level city in Shandong
Jinfeng Hongyuan Flagship Store	Electricity conservation2	I'll comment after I use it hopefully the section will be useful.	Jingdong prefecture level city in Shandong
Jinfeng Hongyuan Flagship Store	Electricity conservation2	not very useful	Jingdong prefecture level city in Shandong
Jinfeng Hongyuan Flagship Store	Electricity conservation2	Not received	Jingdong prefecture level city in Shandong
Jinfeng Hongyuan Flagship Store	Electricity conservation2	rare	Jingdong prefecture level city in Shandong
Jinfeng Hongyuan Flagship Store	Electricity conservation2	It's good.	Jingdong prefecture level city in Shandong
Jinfeng Hongyuan Flagship Store	Electricity conservation2	just now	Jingdong prefecture level city in Shandong
Jinfeng Hongyuan Flagship Store	Electricity conservation2	serviceable	Jingdong prefecture level city in Shandong
Jinfeng Hongyuan Flagship Store	Electricity conservation2	desirable	Jingdong prefecture level city in Shandong
Jinfeng Hongyuan Flagship Store	Electricity conservation2	serviceable	Jingdong prefecture level city in Shandong
Jinfeng Hongyuan Flagship Store	Electricity conservation2	It doesn't save power and costs electricity.	Jingdong prefecture level city in Shandong
Jinfeng Hongyuan Flagship Store	Electricity conservation2	Not as good as I thought.	Jingdong prefecture level city in Shandong
Jinfeng Hongyuan Flagship Store	Electricity conservation2	Super bad review, it doesn't save power at all, but rather more power consumption	Jingdong prefecture level city in Shandong
Jinfeng Hongyuan Flagship Store	Electricity conservation2	Listen to others say that this can save electricity, I bought with the idea of trying, used for a period of time before coming to the evaluation, will be a little bit of use, not a lot of savings just, long-term use of the words can save more.	Jingdong prefecture level city in Shandong
Jinfeng Hongyuan Flagship Store	Electricity conservation2	Logistics is fast, the next day arrived, workmanship is very delicate a power saver, looking forward to the late effect, customer service patiently explain the recommended which is suitable for, installed in what position is the best, look forward to using the effect after a period of time!	Jingdong prefecture level city in Shandong
Jinfeng Hongyuan Flagship Store	Electricity conservation2	It's good to use, I feel that it has been saving electricity, anyway, not a loss is, I used to feel that the power consumption is big, now I feel much better, my friends are also using, I hope to use a long time will be able to, I wish the boss a prosperous business	Jingdong prefecture level city in Shandong
Jinfeng Hongyuan Flagship Store	Electricity conservation2	Should be very good, to be able to plan the circuit, save the home of the circuit to the extent of the savings, waste of money on electricity, good if a month this money back, hope always hope to be able to	Jingdong prefecture level city in Shandong
Jinfeng Hongyuan Flagship Store	Electricity conservation2	Express delivery is very strong, the goods and I saw in the business consistent, the use of the effect of follow-up evaluation	Jingdong prefecture level city in Shandong
Jinfeng Hongyuan Flagship Store	Electricity conservation2	I have been using this brand of power saver for many years and this time I saw this upgraded version and got it again. The main purpose of this product is to reduce the loss of electricity for a long time, so as to achieve the purpose of saving electricity.	Jingdong prefecture level city in Shandong
Jinfeng Hongyuan Flagship Store	Electricity conservation2	Logistics is fast and the product looks beautiful, looking forward to the effect.	Jingdong prefecture level city in Shandong
Jinfeng Hongyuan Flagship Store	Electricity conservation2	Product quality is good, energy saving a little effect, but the price is a little expensive, fast arrival, good service.	Jingdong prefecture level city in Shandong
Jinfeng Hongyuan Flagship Store	Electricity conservation2	The quality is very good, inexpensive, Jingdong self-supporting guaranteed, worth having!	Jingdong prefecture level city in Shandong
Jinfeng Hongyuan Flagship Store	Electricity conservation2	I don't know if it's saving electricity yet, I'll find out next time I pay my electric bill	Jingdong prefecture level city in Shandong
Jinfeng Hongyuan Flagship Store	Electricity conservation2	Favorable price quality assurance appearance design is beautiful and generous, fine workmanship	Jingdong prefecture level city in Shandong
Jinfeng Hongyuan Flagship Store	Electricity conservation2	I bought it, I don't know how effective it is? I don't know how the quality is? After this summer results will know! Looking forward to it!	Jingdong prefecture level city in Shandong
Jinfeng Hongyuan Flagship Store	Electricity conservation2	Goods received Packaging intact, look at the shell workmanship is also good, high-power appliances open voltage has obvious changes in the effectiveness of the	Jingdong prefecture level city in Shandong
Jinfeng Hongyuan Flagship Store	Electricity conservation2	The goods have been received logistics is very fast, the product is similar to the line, just wait to use a month to see if it can save electricity, stay tuned.	Jingdong prefecture level city in Shandong
Jinfeng Hongyuan Flagship Store	Electricity conservation2	Electricity is plugged in, not sure if it will work, hopefully it will work.	Jingdong prefecture level city in Shandong
Jinfeng Hongyuan Flagship Store	Electricity conservation2	The shell is aluminum alloy, very good workmanship, the actual effect of waiting for the addition of evaluation	Jingdong prefecture level city in Shandong
Jinfeng Hongyuan Flagship Store	Electricity conservation2	Received, already plugged in, looking forward to the effect.	Jingdong prefecture level city in Shandong
Jinfeng Hongyuan Flagship Store	Electricity conservation2	Opened to see the regular manufacturer products, wait for a period of time to see it	Jingdong prefecture level city in Shandong
Jinfeng Hongyuan Flagship Store	Electricity conservation2	🉑 Baby received, very surprised ✨, reasonable design, fashionable and generous, continue to pay attention to the future will be repurchased ❥❥❥❥	Jingdong prefecture level city in Shandong
Jinfeng Hongyuan Flagship Store	Electricity conservation2	Baby received it 👌, really great ah! 🌈The quality can't be beat, very satisfied! 🆗Happy happy happy 😃	Jingdong prefecture level city in Shandong
Jinfeng Hongyuan Flagship Store	Electricity conservation2	Very good, power saving see, this is to help a friend to buy, I used to give my friend a say, my friend immediately let me order another one!	Jingdong prefecture level city in Shandong
Jinfeng Hongyuan Flagship Store	Electricity conservation2	The baby is just as introduced, the texture is very good, powerful, and it feels good to use it	Jingdong prefecture level city in Shandong
Jinfeng Hongyuan Flagship Store	Electricity conservation2	Value for money, very satisfied, seller service attitude is very good, very patient	Jingdong prefecture level city in Shandong
Jinfeng Hongyuan Flagship Store	Electricity conservation2	Power saving treasure design unique exquisite beautiful, voltage display clearly, plugged in that is good to use, I observed the meter, after using the meter is gone slow.	Jingdong prefecture level city in Shandong
Jinfeng Hongyuan Flagship Store	Electricity conservation2	Received on the use of the packaging is beautifully used for a period of time and then come back in the follow-up review!	Jingdong prefecture level city in Shandong

Jinfeng Hongyuan Flagship Store	Electricity conservation2	It should work. Not bad. Not bad. Not bad.	Jingdong prefecture level city in Shandong
Jinfeng Hongyuan Flagship Store	Electricity conservation2	I haven't used it yet, I'll plug it in and see if it saves power next month.	Jingdong prefecture level city in Shandong
Jinfeng Hongyuan Flagship Store	Electricity conservation2	The goods received and unpacked and used, the quality is very good, things and the official website to buy no difference!	Jingdong prefecture level city in Shandong
Jinfeng Hongyuan Flagship Store	Electricity conservation2	It's a rip off, doesn't help much at all, uses the same amount of power as before it was used.	Jingdong prefecture level city in Shandong
Jinfeng Hongyuan Flagship Store	Electricity conservation2	I've been using it for a while now and haven't noticed any unusual problems, so it's okay	Jingdong prefecture level city in Shandong
Jinfeng Hongyuan Flagship Store	Electricity conservation2	Received the goods and plugged in to use, good or not there is no use will have to wait until next month to find out	Jingdong prefecture level city in Shandong
Jinfeng Hongyuan Flagship Store	Electricity conservation2	It's very compact, it looks good, I'll introduce you to my clients when it's ready.	Jingdong prefecture level city in Shandong
Jinfeng Hongyuan Flagship Store	Electricity conservation2	Good workmanship, we'll have to see in a few months.	Jingdong prefecture level city in Shandong
Jinfeng Hongyuan Flagship Store	Electricity conservation2	Jingdong quality! Always trustworthy!	Jingdong prefecture level city in Shandong
Jinfeng Hongyuan Flagship Store	Electricity conservation2	This is the second time I've bought it, and the merchant said to give me five dollars out of the difference, so thank you merchant.	Jingdong prefecture level city in Shandong
Jinfeng Hongyuan Flagship Store	Electricity conservation2	Good quality and good service	Jingdong prefecture level city in Shandong
Jinfeng Hongyuan Flagship Store	Electricity conservation2	Feels good texture, aluminum alloy shell, voltage digital display clear.	Jingdong prefecture level city in Shandong
Jinfeng Hongyuan Flagship Store	Electricity conservation2	Good product, fast and great service, thanks!	Jingdong prefecture level city in Shandong
Jinfeng Hongyuan Flagship Store	Electricity conservation2	Have not yet used, first evaluation, later really good do not need to comment again.	Jingdong prefecture level city in Shandong
Jinfeng Hongyuan Flagship Store	Electricity conservation2	Works great. Pretty much as described by the merchant.	Jingdong prefecture level city in Shandong
Jinfeng Hongyuan Flagship Store	Electricity conservation2	Received it, looking forward to the results, hope it works	Jingdong prefecture level city in Shandong
Jinfeng Hongyuan Flagship Store	Electricity conservation2	Received it, looking forward to the results, hope it works	Jingdong prefecture level city in Shandong
Jinfeng Hongyuan Flagship Store	Electricity conservation2	Supposedly it's an IQ tax. Buy one and try it. Looks good. Well made.	Jingdong prefecture level city in Shandong
Jinfeng Hongyuan Flagship Store	Electricity conservation2	It just doesn't feel like it's working.	Jingdong prefecture level city in Shandong
Jinfeng Hongyuan Flagship Store	Electricity conservation2	This is a great product.	Jingdong prefecture level city in Shandong
Jinfeng Hongyuan Flagship Store	Electricity conservation2	I'm just using it. I'll tell you more.	Jingdong prefecture level city in Shandong
Jinfeng Hongyuan Flagship Store	Electricity conservation2	Second repurchase and it's working fine.	Jingdong prefecture level city in Shandong
Jinfeng Hongyuan Flagship Store	Electricity conservation2	I'll wait a few days before evaluating	Jingdong prefecture level city in Shandong
Jinfeng Hongyuan Flagship Store	Electricity conservation2	I'll comment after I use it hopefully the section will be useful.	Jingdong prefecture level city in Shandong
Jinfeng Hongyuan Flagship Store	Electricity conservation2	not very useful	Jingdong prefecture level city in Shandong
Jinfeng Hongyuan Flagship Store	Electricity conservation2	Not received	Jingdong prefecture level city in Shandong
Jinfeng Hongyuan Flagship Store	Electricity conservation2	rare	Jingdong prefecture level city in Shandong
Jinfeng Hongyuan Flagship Store	Electricity conservation2	It's good.	Jingdong prefecture level city in Shandong
Jinfeng Hongyuan Flagship Store	Electricity conservation2	just now	Jingdong prefecture level city in Shandong
Jinfeng Hongyuan Flagship Store	Electricity conservation2	serviceable	Jingdong prefecture level city in Shandong
Jinfeng Hongyuan Flagship Store	Electricity conservation2	desirable	Jingdong prefecture level city in Shandong
Jinfeng Hongyuan Flagship Store	Electricity conservation2	serviceable	Jingdong prefecture level city in Shandong
Jinfeng Hongyuan Flagship Store	Electricity conservation2	It doesn't save power and costs electricity.	Jingdong prefecture level city in Shandong
Jinfeng Hongyuan Flagship Store	Electricity conservation2	Not as good as I thought.	Jingdong prefecture level city in Shandong
Jinfeng Hongyuan Flagship Store	Electricity conservation2	Super bad review, it doesn't save power at all, but rather more power consumption	Jingdong prefecture level city in Shandong
Jinfeng Hongyuan Flagship Store	Electricity conservation2	Listen to others say that this can save electricity, I bought with the idea of trying, used for a period of time before coming to the evaluation, will be a little bit of use, not a lot of savings just, long-term use of the words can save more.	Jingdong prefecture level city in Shandong
Jinfeng Hongyuan Flagship Store	Electricity conservation2	Logistics is fast, the next day arrived, workmanship is very delicate a power saver, looking forward to the late effect, customer service patiently explain the recommended which is suitable for, installed in what position is the best, look forward to using the effect after a period of time!	Jingdong prefecture level city in Shandong
Jinfeng Hongyuan Flagship Store	Electricity conservation2	It's good to use, I feel that it has been saving electricity, anyway, not a loss is, I used to feel that the power consumption is big, now I feel much better, my friends are also using, I hope to use a long time will be able to, I wish the boss a prosperous business	Jingdong prefecture level city in Shandong
Jinfeng Hongyuan Flagship Store	Electricity conservation2	Should be very good, to be able to plan the circuit, save the home of the circuit to the extent of the savings, waste of money on electricity, good if a month this money back, hope always hope to be able to	Jingdong prefecture level city in Shandong
Jinfeng Hongyuan Flagship Store	Electricity conservation2	Express delivery is very strong, the goods and I saw in the business consistent, the use of the effect of follow-up evaluation	Jingdong prefecture level city in Shandong
Jinfeng Hongyuan Flagship Store	Electricity conservation2	I have been using this brand of power saver for many years and this time I saw this upgraded version and got it again. The main purpose of this product is to reduce the loss of electricity for a long time, so as to achieve the purpose of saving electricity.	Jingdong prefecture level city in Shandong
Jinfeng Hongyuan Flagship Store	Electricity conservation2	Logistics is fast and the product looks beautiful, looking forward to the effect.	Jingdong prefecture level city in Shandong
Jinfeng Hongyuan Flagship Store	Electricity conservation2	Product quality is good, energy saving a little effect, but the price is a little expensive, fast arrival, good service.	Jingdong prefecture level city in Shandong
Jinfeng Hongyuan Flagship Store	Electricity conservation2	The quality is very good, inexpensive, Jingdong self-supporting guaranteed, worth having!	Jingdong prefecture level city in Shandong
Jinfeng Hongyuan Flagship Store	Electricity conservation2	I don't know if it's saving electricity yet, I'll find out next time I pay my electric bill	Jingdong prefecture level city in Shandong
Jinfeng Hongyuan Flagship Store	Electricity conservation2	Favorable price quality assurance appearance design is beautiful and generous, fine workmanship	Jingdong prefecture level city in Shandong
Jinfeng Hongyuan Flagship Store	Electricity conservation2	I bought it, I don't know how effective it is? I don't know how the quality is? After this summer results will know! Looking forward to it!	Jingdong prefecture level city in Shandong
Jinfeng Hongyuan Flagship Store	Electricity conservation2	Goods received Packaging intact, look at the shell workmanship is also good, high-power appliances open voltage has obvious changes in the effectiveness of the	Jingdong prefecture level city in Shandong
Jinfeng Hongyuan Flagship Store	Electricity conservation2	The goods have been received logistics is very fast, the product is similar to the line, just wait to use a month to see if it can save electricity, stay tuned.	Jingdong prefecture level city in Shandong

Jinfeng Hongyuan Flagship Store	Electricity conservation2	Electricity is plugged in, not sure if it will work, hopefully it will work.	Jingdong prefecture level city in Shandong
Jinfeng Hongyuan Flagship Store	Electricity conservation2	The shell is aluminum alloy, very good workmanship, the actual effect of waiting for the addition of evaluation	Jingdong prefecture level city in Shandong
Jinfeng Hongyuan Flagship Store	Electricity conservation2	Received, already plugged in, looking forward to the effect.	Jingdong prefecture level city in Shandong
Jinfeng Hongyuan Flagship Store	Electricity conservation2	Opened to see the regular manufacturer products, wait for a period of time to see it	Jingdong prefecture level city in Shandong
Jinfeng Hongyuan Flagship Store	Electricity conservation2	Baby received, very surprised, reasonable design, fashionable and generous, continue to pay attention to the future will be repurchased	Jingdong prefecture level city in Shandong
Jinfeng Hongyuan Flagship Store	Electricity conservation2	Baby received it, really great ah! The quality can't be beat, very satisfied! OK Happy happy happy	Jingdong prefecture level city in Shandong
Jinfeng Hongyuan Flagship Store	Electricity conservation2	Very good, power saving see, this is to help a friend to buy, I used to give my friend a say, my friend immediately let me order another one!	Jingdong prefecture level city in Shandong
Jinfeng Hongyuan Flagship Store	Electricity conservation2	The baby is just as introduced, the texture is very good, powerful, and it feels good to use it	Jingdong prefecture level city in Shandong
Jinfeng Hongyuan Flagship Store	Electricity conservation2	Value for money, very satisfied, seller service attitude is very good, very patient	Jingdong prefecture level city in Shandong
Jinfeng Hongyuan Flagship Store	Electricity conservation2	Power saving treasure design unique exquisite beautiful, voltage display clearly, plugged in that is good to use, I observed the meter, after using the meter is gone slow.	Jingdong prefecture level city in Shandong
Jinfeng Hongyuan Flagship Store	Electricity conservation2	Received on the use of the packaging is beautifully used for a period of time and then come back in the follow-up review!	Jingdong prefecture level city in Shandong
Jinfeng Hongyuan Flagship Store	Electricity conservation2	It should work. Not bad. Not bad. Not bad.	Jingdong prefecture level city in Shandong
Jinfeng Hongyuan Flagship Store	Electricity conservation2	I haven't used it yet, I'll plug it in and see if it saves power next month.	Jingdong prefecture level city in Shandong
Jinfeng Hongyuan Flagship Store	Electricity conservation2	The goods received and unpacked and used, the quality is very good, things and the official website to buy no difference!	Jingdong prefecture level city in Shandong
Jinfeng Hongyuan Flagship Store	Electricity conservation2	It's a rip off, doesn't help much at all, uses the same amount of power as before it was used.	Jingdong prefecture level city in Shandong
Jinfeng Hongyuan Flagship Store	Electricity conservation2	I've been using it for a while now and haven't noticed any unusual problems, so it's okay	Jingdong prefecture level city in Shandong
Jinfeng Hongyuan Flagship Store	Electricity conservation2	Received the goods and plugged in to use, good or not there is no use will have to wait until next month to find out	Jingdong prefecture level city in Shandong
Jinfeng Hongyuan Flagship Store	Electricity conservation2	It's very compact, it looks good, I'll introduce you to my clients when it's ready.	Jingdong prefecture level city in Shandong
Jinfeng Hongyuan Flagship Store	Electricity conservation2	Good workmanship, we'll have to see in a few months.	Jingdong prefecture level city in Shandong
Jinfeng Hongyuan Flagship Store	Electricity conservation2	Jingdong quality! Always trustworthy!	Jingdong prefecture level city in Shandong
Jinfeng Hongyuan Flagship Store	Electricity conservation2	This is the second time I've bought it, and the merchant said to give me five dollars out of the difference, so thank you merchant.	Jingdong prefecture level city in Shandong
Jinfeng Hongyuan Flagship Store	Electricity conservation2	Good quality and good service	Jingdong prefecture level city in Shandong
Jinfeng Hongyuan Flagship Store	Electricity conservation2	Feels good texture, aluminum alloy shell, voltage digital display clear.	Jingdong prefecture level city in Shandong
Jinfeng Hongyuan Flagship Store	Electricity conservation2	Good product, fast and great service, thanks!	Jingdong prefecture level city in Shandong
Jinfeng Hongyuan Flagship Store	Electricity conservation2	Have not yet used, first evaluation, later really good do not need to comment again.	Jingdong prefecture level city in Shandong
Jinfeng Hongyuan Flagship Store	Electricity conservation2	Works great. Pretty much as described by the merchant.	Jingdong prefecture level city in Shandong
Jinfeng Hongyuan Flagship Store	Electricity conservation2	Received it, looking forward to the results, hope it works	Jingdong prefecture level city in Shandong
Jinfeng Hongyuan Flagship Store	Electricity conservation2	Received it, looking forward to the results, hope it works	Jingdong prefecture level city in Shandong
Jinfeng Hongyuan Flagship Store	Electricity conservation2	Supposedly it's an IQ tax. Buy one and try it. Looks good. Well made.	Jingdong prefecture level city in Shandong
Jinfeng Hongyuan Flagship Store	Electricity conservation2	It just doesn't feel like it's working.	Jingdong prefecture level city in Shandong
Jinfeng Hongyuan Flagship Store	Electricity conservation2	This is a great product.	Jingdong prefecture level city in Shandong
Jinfeng Hongyuan Flagship Store	Electricity conservation2	I'm just using it. I'll tell you more.	Jingdong prefecture level city in Shandong
Jinfeng Hongyuan Flagship Store	Electricity conservation2	Second repurchase and it's working fine.	Jingdong prefecture level city in Shandong
Jinfeng Hongyuan Flagship Store	Electricity conservation2	I'll wait a few days before evaluating	Jingdong prefecture level city in Shandong
Jinfeng Hongyuan Flagship Store	Electricity conservation2	I'll comment after I use it hopefully the section will be useful.	Jingdong prefecture level city in Shandong
Jinfeng Hongyuan Flagship Store	Electricity conservation2	not very useful	Jingdong prefecture level city in Shandong
Jinfeng Hongyuan Flagship Store	Electricity conservation2	Not received	Jingdong prefecture level city in Shandong
Jinfeng Hongyuan Flagship Store	Electricity conservation2	rare	Jingdong prefecture level city in Shandong
Jinfeng Hongyuan Flagship Store	Electricity conservation2	It's good.	Jingdong prefecture level city in Shandong
Jinfeng Hongyuan Flagship Store	Electricity conservation2	just now	Jingdong prefecture level city in Shandong
Jinfeng Hongyuan Flagship Store	Electricity conservation2	serviceable	Jingdong prefecture level city in Shandong
Jinfeng Hongyuan Flagship Store	Electricity conservation2	desirable	Jingdong prefecture level city in Shandong
Jinfeng Hongyuan Flagship Store	Electricity conservation2	serviceable	Jingdong prefecture level city in Shandong
Jinfeng Hongyuan Flagship Store	Electricity conservation2	It doesn't save power and costs electricity.	Jingdong prefecture level city in Shandong
Jinfeng Hongyuan Flagship Store	Electricity conservation2	Not as good as I thought.	Jingdong prefecture level city in Shandong
Jinfeng Hongyuan Flagship Store	Electricity conservation2	Super bad review, it doesn't save power at all, but rather more power consumption	Jingdong prefecture level city in Shandong
Jinfeng Hongyuan Flagship Store	Electricity conservation2	Listen to others say that this can save electricity, I bought with the idea of trying, used for a period of time before coming to the evaluation, will be a little bit of use, not a lot of savings just, long-term use of the words can save more.	Jingdong prefecture level city in Shandong
Jinfeng Hongyuan Flagship Store	Electricity conservation2	Logistics is fast, the next day arrived, workmanship is very delicate a power saver, looking forward to the late effect, customer service patiently explain the recommended which is suitable for, installed in what position is the best, look forward to using the effect after a period of time!	Jingdong prefecture level city in Shandong
Jinfeng Hongyuan Flagship Store	Electricity conservation2	It's good to use, I feel that it has been saving electricity, anyway, not a loss is, I used to feel that the power consumption is big, now I feel much better, my friends are also using, I hope to use a long time will be able to, I wish the boss a prosperous business	Jingdong prefecture level city in Shandong
Jinfeng Hongyuan Flagship Store	Electricity conservation2	Should be very good, to be able to plan the circuit, save the home of the circuit to the extent of the savings, waste of money on electricity, good if a month this money back, hope always hope to be able to	Jingdong prefecture level city in Shandong

Jinfeng Hongyuan Flagship Store	Electricity conservation2	Express delivery is very strong, the goods and I saw in the business consistent, the use of the effect of follow-up evaluation	Jingdong prefecture level city in Shandong
Jinfeng Hongyuan Flagship Store	Electricity conservation2	I have been using this brand of power saver for many years and this time I saw this upgraded version and got it again. The main purpose of this product is to reduce the loss of electricity for a long time, so as to achieve the purpose of saving electricity.	Jingdong prefecture level city in Shandong
Jinfeng Hongyuan Flagship Store	Electricity conservation2	Logistics is fast and the product looks beautiful, looking forward to the effect.	Jingdong prefecture level city in Shandong
Jinfeng Hongyuan Flagship Store	Electricity conservation2	Product quality is good, energy saving a little effect, but the price is a little expensive, fast arrival, good service.	Jingdong prefecture level city in Shandong
Jinfeng Hongyuan Flagship Store	Electricity conservation2	The quality is very good, inexpensive, Jingdong self-supporting guaranteed, worth having!	Jingdong prefecture level city in Shandong
Jinfeng Hongyuan Flagship Store	Electricity conservation2	I don't know if it's saving electricity yet, I'll find out next time I pay my electric bill	Jingdong prefecture level city in Shandong
Jinfeng Hongyuan Flagship Store	Electricity conservation2	Favorable price quality assurance appearance design is beautiful and generous, fine workmanship	Jingdong prefecture level city in Shandong
Jinfeng Hongyuan Flagship Store	Electricity conservation2	I bought it, I don't know how effective it is? I don't know how the quality is? After this summer results will know! Looking forward to it!	Jingdong prefecture level city in Shandong
Jinfeng Hongyuan Flagship Store	Electricity conservation2	Goods received Packaging intact, look at the shell workmanship is also good, high-power appliances open voltage has obvious changes in the effectiveness of the	Jingdong prefecture level city in Shandong
Jinfeng Hongyuan Flagship Store	Electricity conservation2	The goods have been received logistics is very fast, the product is similar to the line, just wait to use a month to see if it can save electricity, stay tuned.	Jingdong prefecture level city in Shandong
Jinfeng Hongyuan Flagship Store	Electricity conservation2	Electricity is plugged in, not sure if it will work, hopefully it will work.	Jingdong prefecture level city in Shandong
Jinfeng Hongyuan Flagship Store	Electricity conservation2	The shell is aluminum alloy, very good workmanship, the actual effect of waiting for the addition of evaluation	Jingdong prefecture level city in Shandong
Jinfeng Hongyuan Flagship Store	Electricity conservation2	Received, already plugged in, looking forward to the effect.	Jingdong prefecture level city in Shandong
Jinfeng Hongyuan Flagship Store	Electricity conservation2	Opened to see the regular manufacturer products, wait for a period of time to see it	Jingdong prefecture level city in Shandong
Jinfeng Hongyuan Flagship Store	Electricity conservation2	🉑 Baby received, very surprised ✨, reasonable design, fashionable and generous, continue to pay attention to the future will be repurchased ❥❥❥❥	Jingdong prefecture level city in Shandong
Jinfeng Hongyuan Flagship Store	Electricity conservation2	Baby received it 👌, really great ah! 🌈The quality can't be beat, very satisfied! 🆗Happy happy happy 😀	Jingdong prefecture level city in Shandong
Jinfeng Hongyuan Flagship Store	Electricity conservation2	Very good, power saving see, this is to help a friend to buy, I used to give my friend a say, my friend immediately let me order another one!	Jingdong prefecture level city in Shandong
Jinfeng Hongyuan Flagship Store	Electricity conservation2	The baby is just as introduced, the texture is very good, powerful, and it feels good to use it	Jingdong prefecture level city in Shandong
Jinfeng Hongyuan Flagship Store	Electricity conservation2	Value for money, very satisfied, seller service attitude is very good, very patient	Jingdong prefecture level city in Shandong
Jinfeng Hongyuan Flagship Store	Electricity conservation2	Power saving treasure design unique exquisite beautiful, voltage display clearly, plugged in that is good to use, I observed the meter, after using the meter is gone slow.	Jingdong prefecture level city in Shandong
Jinfeng Hongyuan Flagship Store	Electricity conservation2	Received on the use of the packaging is beautifully used for a period of time and then come back in the follow-up review!	Jingdong prefecture level city in Shandong
Jinfeng Hongyuan Flagship Store	Electricity conservation2	It should work. Not bad. Not bad. Not bad.	Jingdong prefecture level city in Shandong
Jinfeng Hongyuan Flagship Store	Electricity conservation2	I haven't used it yet, I'll plug it in and see if it saves power next month.	Jingdong prefecture level city in Shandong
Jinfeng Hongyuan Flagship Store	Electricity conservation2	The goods received and unpacked and used, the quality is very good, things and the official website to buy no difference!	Jingdong prefecture level city in Shandong
Jinfeng Hongyuan Flagship Store	Electricity conservation2	It's a rip off, doesn't help much at all, uses the same amount of power as before it was used.	Jingdong prefecture level city in Shandong
Jinfeng Hongyuan Flagship Store	Electricity conservation2	I've been using it for a while now and haven't noticed any unusual problems, so it's okay	Jingdong prefecture level city in Shandong
Jinfeng Hongyuan Flagship Store	Electricity conservation2	Received the goods and plugged in to use, good or not there is no use will have to wait until next month to find out	Jingdong prefecture level city in Shandong
Jinfeng Hongyuan Flagship Store	Electricity conservation2	It's very compact, it looks good, I'll introduce you to my clients when it's ready.	Jingdong prefecture level city in Shandong
Jinfeng Hongyuan Flagship Store	Electricity conservation2	Good workmanship, we'll have to see in a few months.	Jingdong prefecture level city in Shandong
Jinfeng Hongyuan Flagship Store	Electricity conservation2	Jingdong quality! Always trustworthy!	Jingdong prefecture level city in Shandong
Jinfeng Hongyuan Flagship Store	Electricity conservation2	This is the second time I've bought it, and the merchant said to give me five dollars out of the difference, so thank you merchant.	Jingdong prefecture level city in Shandong
Jinfeng Hongyuan Flagship Store	Electricity conservation2	Good quality and good service	Jingdong prefecture level city in Shandong
Jinfeng Hongyuan Flagship Store	Electricity conservation2	Feels good texture, aluminum alloy shell, voltage digital display clear.	Jingdong prefecture level city in Shandong
Jinfeng Hongyuan Flagship Store	Electricity conservation2	Good product, fast and great service, thanks!	Jingdong prefecture level city in Shandong
Jinfeng Hongyuan Flagship Store	Electricity conservation2	Have not yet used, first evaluation, later really good do not need to comment again.	Jingdong prefecture level city in Shandong
Jinfeng Hongyuan Flagship Store	Electricity conservation2	Works great. Pretty much as described by the merchant.	Jingdong prefecture level city in Shandong
Jinfeng Hongyuan Flagship Store	Electricity conservation2	Received it, looking forward to the results, hope it works	Jingdong prefecture level city in Shandong
Jinfeng Hongyuan Flagship Store	Electricity conservation2	Received it, looking forward to the results, hope it works	Jingdong prefecture level city in Shandong
Jinfeng Hongyuan Flagship Store	Electricity conservation2	Supposedly it's an IQ tax. Buy one and try it. Looks good. Well made.	Jingdong prefecture level city in Shandong
Jinfeng Hongyuan Flagship Store	Electricity conservation2	It just doesn't feel like it's working.	Jingdong prefecture level city in Shandong
Jinfeng Hongyuan Flagship Store	Electricity conservation2	This is a great product.	Jingdong prefecture level city in Shandong
Jinfeng Hongyuan Flagship Store	Electricity conservation2	I'm just using it. I'll tell you more.	Jingdong prefecture level city in Shandong
Jinfeng Hongyuan Flagship Store	Electricity conservation2	Second repurchase and it's working fine.	Jingdong prefecture level city in Shandong
Jinfeng Hongyuan Flagship Store	Electricity conservation2	I'll wait a few days before evaluating	Jingdong prefecture level city in Shandong
Jinfeng Hongyuan Flagship Store	Electricity conservation2	I'll comment after I use it hopefully the section will be useful.	Jingdong prefecture level city in Shandong
Jinfeng Hongyuan Flagship Store	Electricity conservation2	not very useful	Jingdong prefecture level city in Shandong
Jinfeng Hongyuan Flagship Store	Electricity conservation2	Not received	Jingdong prefecture level city in Shandong
Jinfeng Hongyuan Flagship Store	Electricity conservation2	rare	Jingdong prefecture level city in Shandong
Jinfeng Hongyuan Flagship Store	Electricity conservation2	It's good.	Jingdong prefecture level city in Shandong
Jinfeng Hongyuan Flagship Store	Electricity conservation2	just now	Jingdong prefecture level city in Shandong
Jinfeng Hongyuan Flagship Store	Electricity conservation2	serviceable	Jingdong prefecture level city in Shandong

Jinfeng Hongyuan Flagship Store	Electricity conservation2	desirable	Jingdong prefecture level city in Shandong
Jinfeng Hongyuan Flagship Store	Electricity conservation2	serviceable	Jingdong prefecture level city in Shandong
Jinfeng Hongyuan Flagship Store	Electricity conservation2	It doesn't save power and costs electricity.	Jingdong prefecture level city in Shandong
Jinfeng Hongyuan Flagship Store	Electricity conservation2	Not as good as I thought.	Jingdong prefecture level city in Shandong
Jinfeng Hongyuan Flagship Store	Electricity conservation2	Super bad review, it doesn't save power at all, but rather more power consumption	Jingdong prefecture level city in Shandong
Jinfeng Hongyuan Flagship Store	Electricity conservation2	Listen to others say that this can save electricity, I bought with the idea of trying, used for a period of time before coming to the evaluation, will be a little bit of use, not a lot of savings just, long-term use of the words can save more.	Jingdong prefecture level city in Shandong
Jinfeng Hongyuan Flagship Store	Electricity conservation2	Logistics is fast, the next day arrived, workmanship is very delicate a power saver, looking forward to the late effect, customer service patiently explain the recommended which is suitable for, installed in what position is the best, look forward to using the effect after a period of time!	Jingdong prefecture level city in Shandong
Jinfeng Hongyuan Flagship Store	Electricity conservation2	It's good to use, I feel that it has been saving electricity, anyway, not a loss is, I used to feel that the power consumption is big, now I feel much better, my friends are also using, I hope to use a long time will be able to, I wish the boss a prosperous business	Jingdong prefecture level city in Shandong
Jinfeng Hongyuan Flagship Store	Electricity conservation2	Should be very good, to be able to plan the circuit, save the home of the circuit to the extent of the savings, waste of money on electricity, good if a month this money back, hope always hope to be able to	Jingdong prefecture level city in Shandong
Jinfeng Hongyuan Flagship Store	Electricity conservation2	Express delivery is very strong, the goods and I saw in the business consistent, the use of the effect of follow-up evaluation	Jingdong prefecture level city in Shandong
Jinfeng Hongyuan Flagship Store	Electricity conservation2	I have been using this brand of power saver for many years and this time I saw this upgraded version and got it again. The main purpose of this product is to reduce the loss of electricity for a long time, so as to achieve the purpose of saving electricity.	Jingdong prefecture level city in Shandong
Jinfeng Hongyuan Flagship Store	Electricity conservation2	Logistics is fast and the product looks beautiful, looking forward to the effect.	Jingdong prefecture level city in Shandong
Jinfeng Hongyuan Flagship Store	Electricity conservation2	Product quality is good, energy saving a little effect, but the price is a little expensive, fast arrival, good service.	Jingdong prefecture level city in Shandong
Jinfeng Hongyuan Flagship Store	Electricity conservation2	The quality is very good, inexpensive, Jingdong self-supporting guaranteed, worth having!	Jingdong prefecture level city in Shandong
Jinfeng Hongyuan Flagship Store	Electricity conservation2	I don't know if it's saving electricity yet, I'll find out next time I pay my electric bill	Jingdong prefecture level city in Shandong
Jinfeng Hongyuan Flagship Store	Electricity conservation2	Favorable price quality assurance appearance design is beautiful and generous, fine workmanship	Jingdong prefecture level city in Shandong
Jinfeng Hongyuan Flagship Store	Electricity conservation2	I bought it, I don't know how effective it is? I don't know how the quality is? After this summer results will know! Looking forward to it!	Jingdong prefecture level city in Shandong
Jinfeng Hongyuan Flagship Store	Electricity conservation2	Goods received Packaging intact, look at the shell workmanship is also good, high-power appliances open voltage has obvious changes in the effectiveness of the	Jingdong prefecture level city in Shandong
Jinfeng Hongyuan Flagship Store	Electricity conservation2	The goods have been received logistics is very fast, the product is similar to the line, just wait to use a month to see if it can save electricity, stay tuned.	Jingdong prefecture level city in Shandong
Jinfeng Hongyuan Flagship Store	Electricity conservation2	Electricity is plugged in, not sure if it will work, hopefully it will work.	Jingdong prefecture level city in Shandong
Jinfeng Hongyuan Flagship Store	Electricity conservation2	The shell is aluminum alloy, very good workmanship, the actual effect of waiting for the addition of evaluation	Jingdong prefecture level city in Shandong
Jinfeng Hongyuan Flagship Store	Electricity conservation2	Received, already plugged in, looking forward to the effect.	Jingdong prefecture level city in Shandong
Jinfeng Hongyuan Flagship Store	Electricity conservation2	Opened to see the regular manufacturer products, wait for a period of time to see it	Jingdong prefecture level city in Shandong
Jinfeng Hongyuan Flagship Store	Electricity conservation2	🉑 Baby received, very surprised ✨, reasonable design, fashionable and generous, continue to pay attention to the future will be repurchased ❤❤❤❤	Jingdong prefecture level city in Shandong
Jinfeng Hongyuan Flagship Store	Electricity conservation2	Baby received it 👌, really great ah! 🌈The quality can't be beat, very satisfied! 🆗Happy happy happy 😃	Jingdong prefecture level city in Shandong
Jinfeng Hongyuan Flagship Store	Electricity conservation2	Very good, power saving see, this is to help a friend to buy, I used to give my friend a say, my friend immediately let me order another one!	Jingdong prefecture level city in Shandong
Jinfeng Hongyuan Flagship Store	Electricity conservation2	The baby is just as introduced, the texture is very good, powerful, and it feels good to use it	Jingdong prefecture level city in Shandong
Jinfeng Hongyuan Flagship Store	Electricity conservation2	Value for money, very satisfied, seller service attitude is very good, very patient	Jingdong prefecture level city in Shandong
Jinfeng Hongyuan Flagship Store	Electricity conservation2	Power saving treasure design unique exquisite beautiful, voltage display clearly, plugged in that is good to use, I observed the meter, after using the meter is gone slow.	Jingdong prefecture level city in Shandong
Jinfeng Hongyuan Flagship Store	Electricity conservation2	Received on the use of the packaging is beautifully used for a period of time and then come back in the follow-up review!	Jingdong prefecture level city in Shandong
Jinfeng Hongyuan Flagship Store	Electricity conservation2	It should work. Not bad. Not bad. Not bad.	Jingdong prefecture level city in Shandong
Jinfeng Hongyuan Flagship Store	Electricity conservation2	I haven't used it yet, I'll plug it in and see if it saves power next month.	Jingdong prefecture level city in Shandong
Jinfeng Hongyuan Flagship Store	Electricity conservation2	The goods received and unpacked and used, the quality is very good, things and the official website to buy no difference!	Jingdong prefecture level city in Shandong
Jinfeng Hongyuan Flagship Store	Electricity conservation2	It's a rip off, doesn't help much at all, uses the same amount of power as before it was used.	Jingdong prefecture level city in Shandong
Jinfeng Hongyuan Flagship Store	Electricity conservation2	I've been using it for a while now and haven't noticed any unusual problems, so it's okay	Jingdong prefecture level city in Shandong
Jinfeng Hongyuan Flagship Store	Electricity conservation2	Received the goods and plugged in to use, good or not there is no use will have to wait until next month to find out	Jingdong prefecture level city in Shandong
Jinfeng Hongyuan Flagship Store	Electricity conservation2	It's very compact, it looks good, I'll introduce you to my clients when it's ready.	Jingdong prefecture level city in Shandong
Jinfeng Hongyuan Flagship Store	Electricity conservation2	Good workmanship, we'll have to see in a few months.	Jingdong prefecture level city in Shandong
Jinfeng Hongyuan Flagship Store	Electricity conservation2	Jingdong quality! Always trustworthy!	Jingdong prefecture level city in Shandong
Jinfeng Hongyuan Flagship Store	Electricity conservation2	This is the second time I've bought it, and the merchant said to give me five dollars out of the difference, so thank you merchant.	Jingdong prefecture level city in Shandong
Jinfeng Hongyuan Flagship Store	Electricity conservation2	Good quality and good service	Jingdong prefecture level city in Shandong
Jinfeng Hongyuan Flagship Store	Electricity conservation2	Feels good texture, aluminum alloy shell, voltage digital display clear.	Jingdong prefecture level city in Shandong
Jinfeng Hongyuan Flagship Store	Electricity conservation2	Good product, fast and great service, thanks!	Jingdong prefecture level city in Shandong
Jinfeng Hongyuan Flagship Store	Electricity conservation2	Have not yet used, first evaluation, later really good do not need to comment again.	Jingdong prefecture level city in Shandong
Jinfeng Hongyuan Flagship Store	Electricity conservation2	Works great. Pretty much as described by the merchant.	Jingdong prefecture level city in Shandong
Jinfeng Hongyuan Flagship Store	Electricity conservation2	Received it, looking forward to the results, hope it works	Jingdong prefecture level city in Shandong
Jinfeng Hongyuan Flagship Store	Electricity conservation2	Received it, looking forward to the results, hope it works	Jingdong prefecture level city in Shandong
Jinfeng Hongyuan Flagship Store	Electricity conservation2	Supposedly it's an IQ tax. Buy one and try it. Looks good. Well made.	Jingdong prefecture level city in Shandong
Jinfeng Hongyuan Flagship Store	Electricity conservation2	It just doesn't feel like it's working.	Jingdong prefecture level city in Shandong

Jinfeng Hongyuan Flagship Store	Electricity conservation2	This is a great product.	Jingdong prefecture level city in Shandong
Jinfeng Hongyuan Flagship Store	Electricity conservation2	I'm just using it. I'll tell you more.	Jingdong prefecture level city in Shandong
Jinfeng Hongyuan Flagship Store	Electricity conservation2	Second repurchase and it's working fine.	Jingdong prefecture level city in Shandong
Jinfeng Hongyuan Flagship Store	Electricity conservation2	I'll wait a few days before evaluating	Jingdong prefecture level city in Shandong
Jinfeng Hongyuan Flagship Store	Electricity conservation2	I'll comment after I use it hopefully the section will be useful.	Jingdong prefecture level city in Shandong
Jinfeng Hongyuan Flagship Store	Electricity conservation2	not very useful	Jingdong prefecture level city in Shandong
Jinfeng Hongyuan Flagship Store	Electricity conservation2	Not received	Jingdong prefecture level city in Shandong
Jinfeng Hongyuan Flagship Store	Electricity conservation2	rare	Jingdong prefecture level city in Shandong
Jinfeng Hongyuan Flagship Store	Electricity conservation2	It's good.	Jingdong prefecture level city in Shandong
Jinfeng Hongyuan Flagship Store	Electricity conservation2	just now	Jingdong prefecture level city in Shandong
Jinfeng Hongyuan Flagship Store	Electricity conservation2	serviceable	Jingdong prefecture level city in Shandong
Jinfeng Hongyuan Flagship Store	Electricity conservation2	desirable	Jingdong prefecture level city in Shandong
Jinfeng Hongyuan Flagship Store	Electricity conservation2	serviceable	Jingdong prefecture level city in Shandong
Jinfeng Hongyuan Flagship Store	Electricity conservation2	It doesn't save power and costs electricity.	Jingdong prefecture level city in Shandong
Jinfeng Hongyuan Flagship Store	Electricity conservation2	Not as good as I thought.	Jingdong prefecture level city in Shandong
Jinfeng Hongyuan Flagship Store	Electricity conservation2	Super bad review, it doesn't save power at all, but rather more power consumption	Jingdong prefecture level city in Shandong
Jinfeng Hongyuan Flagship Store	Electricity conservation2	Listen to others say that this can save electricity, I bought with the idea of trying, used for a period of time before coming to the evaluation, will be a little bit of use, not a lot of savings just, long-term use of the words can save more.	Jingdong prefecture level city in Shandong
Jinfeng Hongyuan Flagship Store	Electricity conservation2	Logistics is fast, the next day arrived, workmanship is very delicate a power saver, looking forward to the late effect, customer service patiently explain the recommended which is suitable for, installed in what position is the best, look forward to using the effect after a period of time!	Jingdong prefecture level city in Shandong
Jinfeng Hongyuan Flagship Store	Electricity conservation2	It's good to use, I feel that it has been saving electricity, anyway, not a loss is, I used to feel that the power consumption is big, now I feel much better, my friends are also using, I hope to use a long time will be able to, I wish the boss a prosperous business	Jingdong prefecture level city in Shandong
Jinfeng Hongyuan Flagship Store	Electricity conservation2	Should be very good, to be able to plan the circuit, save the home of the circuit to the extent of the savings, waste of money on electricity, good if a month this money back, hope always hope to be able to	Jingdong prefecture level city in Shandong
Jinfeng Hongyuan Flagship Store	Electricity conservation2	Express delivery is very strong, the goods and I saw in the business consistent, the use of the effect of follow-up evaluation	Jingdong prefecture level city in Shandong
Jinfeng Hongyuan Flagship Store	Electricity conservation2	I have been using this brand of power saver for many years and this time I saw this upgraded version and got it again. The main purpose of this product is to reduce the loss of electricity for a long time, so as to achieve the purpose of saving electricity.	Jingdong prefecture level city in Shandong
Jinfeng Hongyuan Flagship Store	Electricity conservation2	Logistics is fast and the product looks beautiful, looking forward to the effect.	Jingdong prefecture level city in Shandong
Jinfeng Hongyuan Flagship Store	Electricity conservation2	Product quality is good, energy saving a little effect, but the price is a little expensive, fast arrival, good service.	Jingdong prefecture level city in Shandong
Jinfeng Hongyuan Flagship Store	Electricity conservation2	The quality is very good, inexpensive, Jingdong self-supporting guaranteed, worth having!	Jingdong prefecture level city in Shandong
Jinfeng Hongyuan Flagship Store	Electricity conservation2	I don't know if it's saving electricity yet, I'll find out next time I pay my electric bill	Jingdong prefecture level city in Shandong
Jinfeng Hongyuan Flagship Store	Electricity conservation2	Favorable price quality assurance appearance design is beautiful and generous, fine workmanship	Jingdong prefecture level city in Shandong
Jinfeng Hongyuan Flagship Store	Electricity conservation2	I bought it, I don't know how effective it is? I don't know how the quality is? After this summer results will know! Looking forward to it!	Jingdong prefecture level city in Shandong
Jinfeng Hongyuan Flagship Store	Electricity conservation2	Goods received Packaging intact, look at the shell workmanship is also good, high-power appliances open voltage has obvious changes in the effectiveness of the	Jingdong prefecture level city in Shandong
Jinfeng Hongyuan Flagship Store	Electricity conservation2	The goods have been received logistics is very fast, the product is similar to the line, just wait to use a month to see if it can save electricity, stay tuned.	Jingdong prefecture level city in Shandong
Jinfeng Hongyuan Flagship Store	Electricity conservation2	Electricity is plugged in, not sure if it will work, hopefully it will work.	Jingdong prefecture level city in Shandong
Jinfeng Hongyuan Flagship Store	Electricity conservation2	The shell is aluminum alloy, very good workmanship, the actual effect of waiting for the addition of evaluation	Jingdong prefecture level city in Shandong
Jinfeng Hongyuan Flagship Store	Electricity conservation2	Received, already plugged in, looking forward to the effect.	Jingdong prefecture level city in Shandong
Jinfeng Hongyuan Flagship Store	Electricity conservation2	Opened to see the regular manufacturer products, wait for a period of time to see it	Jingdong prefecture level city in Shandong
Jinfeng Hongyuan Flagship Store	Electricity conservation2	🉑 Baby received, very surprised ✨, reasonable design, fashionable and generous, continue to pay attention to the future will be repurchased ❥❥❥❥	Jingdong prefecture level city in Shandong
Jinfeng Hongyuan Flagship Store	Electricity conservation2	Baby received it 👌, really great ah! 🌈The quality can't be beat, very satisfied! 🆗Happy happy happy 😃	Jingdong prefecture level city in Shandong
Jinfeng Hongyuan Flagship Store	Electricity conservation2	Very good, power saving see, this is to help a friend to buy, I used to give my friend a say, my friend immediately let me order another one!	Jingdong prefecture level city in Shandong
Jinfeng Hongyuan Flagship Store	Electricity conservation2	The baby is just as introduced, the texture is very good, powerful, and it feels good to use it	Jingdong prefecture level city in Shandong
Jinfeng Hongyuan Flagship Store	Electricity conservation2	Value for money, very satisfied, seller service attitude is very good, very patient	Jingdong prefecture level city in Shandong
Jinfeng Hongyuan Flagship Store	Electricity conservation2	Power saving treasure design unique exquisite beautiful, voltage display clearly, plugged in that is good to use, I observed the meter, after using the meter is gone slow.	Jingdong prefecture level city in Shandong
Jinfeng Hongyuan Flagship Store	Electricity conservation2	Received on the use of the packaging is beautifully used for a period of time and then come back in the follow-up review!	Jingdong prefecture level city in Shandong
Jinfeng Hongyuan Flagship Store	Electricity conservation2	It should work. Not bad. Not bad. Not bad.	Jingdong prefecture level city in Shandong
Jinfeng Hongyuan Flagship Store	Electricity conservation2	I haven't used it yet, I'll plug it in and see if it saves power next month.	Jingdong prefecture level city in Shandong
Jinfeng Hongyuan Flagship Store	Electricity conservation2	The goods received and unpacked and used, the quality is very good, things and the official website to buy no difference!	Jingdong prefecture level city in Shandong
Jinfeng Hongyuan Flagship Store	Electricity conservation2	It's a rip off, doesn't help much at all, uses the same amount of power as before it was used.	Jingdong prefecture level city in Shandong
Jinfeng Hongyuan Flagship Store	Electricity conservation2	I've been using it for a while now and haven't noticed any unusual problems, so it's okay	Jingdong prefecture level city in Shandong
Jinfeng Hongyuan Flagship Store	Electricity conservation2	Received the goods and plugged in to use, good or not there is no use will have to wait until next month to find out	Jingdong prefecture level city in Shandong
Jinfeng Hongyuan Flagship Store	Electricity conservation2	It's very compact, it looks good, I'll introduce you to my clients when it's ready.	Jingdong prefecture level city in Shandong
Jinfeng Hongyuan Flagship Store	Electricity conservation2	Good workmanship, we'll have to see in a few months.	Jingdong prefecture level city in Shandong

Jinfeng Hongyuan Flagship Store	Electricity conservation2	Jingdong quality! Always trustworthy!	Jingdong prefecture level city in Shandong
Jinfeng Hongyuan Flagship Store	Electricity conservation2	This is the second time I've bought it, and the merchant said to give me five dollars out of the difference, so thank you merchant.	Jingdong prefecture level city in Shandong
Jinfeng Hongyuan Flagship Store	Electricity conservation2	Good quality and good service	Jingdong prefecture level city in Shandong
Jinfeng Hongyuan Flagship Store	Electricity conservation2	Feels good texture, aluminum alloy shell, voltage digital display clear.	Jingdong prefecture level city in Shandong
Jinfeng Hongyuan Flagship Store	Electricity conservation2	Good product, fast and great service, thanks!	Jingdong prefecture level city in Shandong
Jinfeng Hongyuan Flagship Store	Electricity conservation2	Have not yet used, first evaluation, later really good do not need to comment again.	Jingdong prefecture level city in Shandong
Jinfeng Hongyuan Flagship Store	Electricity conservation2	Works great. Pretty much as described by the merchant.	Jingdong prefecture level city in Shandong
Jinfeng Hongyuan Flagship Store	Electricity conservation2	Received it, looking forward to the results, hope it works	Jingdong prefecture level city in Shandong
Jinfeng Hongyuan Flagship Store	Electricity conservation2	Received it, looking forward to the results, hope it works	Jingdong prefecture level city in Shandong
Jinfeng Hongyuan Flagship Store	Electricity conservation2	Supposedly it's an IQ tax. Buy one and try it. Looks good. Well made.	Jingdong prefecture level city in Shandong
Jinfeng Hongyuan Flagship Store	Electricity conservation2	It just doesn't feel like it's working.	Jingdong prefecture level city in Shandong
Jinfeng Hongyuan Flagship Store	Electricity conservation2	This is a great product.	Jingdong prefecture level city in Shandong
Jinfeng Hongyuan Flagship Store	Electricity conservation2	I'm just using it. I'll tell you more.	Jingdong prefecture level city in Shandong
Jinfeng Hongyuan Flagship Store	Electricity conservation2	Second repurchase and it's working fine.	Jingdong prefecture level city in Shandong
Jinfeng Hongyuan Flagship Store	Electricity conservation2	I'll wait a few days before evaluating	Jingdong prefecture level city in Shandong
Jinfeng Hongyuan Flagship Store	Electricity conservation2	I'll comment after I use it hopefully the section will be useful.	Jingdong prefecture level city in Shandong
Jinfeng Hongyuan Flagship Store	Electricity conservation2	not very useful	Jingdong prefecture level city in Shandong
Jinfeng Hongyuan Flagship Store	Electricity conservation2	Not received	Jingdong prefecture level city in Shandong
Jinfeng Hongyuan Flagship Store	Electricity conservation2	rare	Jingdong prefecture level city in Shandong
Jinfeng Hongyuan Flagship Store	Electricity conservation2	It's good.	Jingdong prefecture level city in Shandong
Jinfeng Hongyuan Flagship Store	Electricity conservation2	just now	Jingdong prefecture level city in Shandong
Jinfeng Hongyuan Flagship Store	Electricity conservation2	serviceable	Jingdong prefecture level city in Shandong
Jinfeng Hongyuan Flagship Store	Electricity conservation2	desirable	Jingdong prefecture level city in Shandong
Jinfeng Hongyuan Flagship Store	Electricity conservation2	serviceable	Jingdong prefecture level city in Shandong
Jinfeng Hongyuan Flagship Store	Electricity conservation2	It doesn't save power and costs electricity.	Jingdong prefecture level city in Shandong
Jinfeng Hongyuan Flagship Store	Electricity conservation2	Not as good as I thought.	Jingdong prefecture level city in Shandong
Jinfeng Hongyuan Flagship Store	Electricity conservation2	Super bad review, it doesn't save power at all, but rather more power consumption	Jingdong prefecture level city in Shandong
Jinfeng Hongyuan Flagship Store	Electricity conservation2	Listen to others say that this can save electricity, I bought with the idea of trying, used for a period of time before coming to the evaluation, will be a little bit of use, not a lot of savings just, long-term use of the words can save more.	Jingdong prefecture level city in Shandong
Jinfeng Hongyuan Flagship Store	Electricity conservation2	Logistics is fast, the next day arrived, workmanship is very delicate a power saver, looking forward to the late effect, customer service patiently explain the recommended which is suitable for, installed in what position is the best, look forward to using the effect after a period of time!	Jingdong prefecture level city in Shandong
Jinfeng Hongyuan Flagship Store	Electricity conservation2	It's good to use, I feel that it has been saving electricity, anyway, not a loss is, I used to feel that the power consumption is big, now I feel much better, my friends are also using, I hope to use a long time will be able to, I wish the boss a prosperous business	Jingdong prefecture level city in Shandong
Jinfeng Hongyuan Flagship Store	Electricity conservation2	Should be very good, to be able to plan the circuit, save the home of the circuit to the extent of the savings, waste of money on electricity, good if a month this money back, hope always hope to be able to	Jingdong prefecture level city in Shandong
Jinfeng Hongyuan Flagship Store	Electricity conservation2	Express delivery is very strong, the goods and I saw in the business consistent, the use of the effect of follow-up evaluation	Jingdong prefecture level city in Shandong
Jinfeng Hongyuan Flagship Store	Electricity conservation2	I have been using this brand of power saver for many years and this time I saw this upgraded version and got it again. The main purpose of this product is to reduce the loss of electricity for a long time, so as to achieve the purpose of saving electricity.	Jingdong prefecture level city in Shandong
Jinfeng Hongyuan Flagship Store	Electricity conservation2	Logistics is fast and the product looks beautiful, looking forward to the effect.	Jingdong prefecture level city in Shandong
Jinfeng Hongyuan Flagship Store	Electricity conservation2	Product quality is good, energy saving a little effect, but the price is a little expensive, fast arrival, good service.	Jingdong prefecture level city in Shandong
Jinfeng Hongyuan Flagship Store	Electricity conservation2	The quality is very good, inexpensive, Jingdong self-supporting guaranteed, worth having!	Jingdong prefecture level city in Shandong
Jinfeng Hongyuan Flagship Store	Electricity conservation2	I don't know if it's saving electricity yet, I'll find out next time I pay my electric bill	Jingdong prefecture level city in Shandong
Jinfeng Hongyuan Flagship Store	Electricity conservation2	Favorable price quality assurance appearance design is beautiful and generous, fine workmanship	Jingdong prefecture level city in Shandong
Jinfeng Hongyuan Flagship Store	Electricity conservation2	I bought it, I don't know how effective it is? I don't know how the quality is? After this summer results will know! Looking forward to it!	Jingdong prefecture level city in Shandong
Jinfeng Hongyuan Flagship Store	Electricity conservation2	Goods received Packaging intact, look at the shell workmanship is also good, high-power appliances open voltage has obvious changes in the effectiveness of the	Jingdong prefecture level city in Shandong
Jinfeng Hongyuan Flagship Store	Electricity conservation2	The goods have been received logistics is very fast, the product is similar to the line, just wait to use a month to see if it can save electricity, stay tuned.	Jingdong prefecture level city in Shandong
Jinfeng Hongyuan Flagship Store	Electricity conservation2	Electricity is plugged in, not sure if it will work, hopefully it will work.	Jingdong prefecture level city in Shandong
Jinfeng Hongyuan Flagship Store	Electricity conservation2	The shell is aluminum alloy, very good workmanship, the actual effect of waiting for the addition of evaluation	Jingdong prefecture level city in Shandong
Jinfeng Hongyuan Flagship Store	Electricity conservation2	Received, already plugged in, looking forward to the effect.	Jingdong prefecture level city in Shandong
Jinfeng Hongyuan Flagship Store	Electricity conservation2	Opened to see the regular manufacturer products, wait for a period of time to see it	Jingdong prefecture level city in Shandong
Jinfeng Hongyuan Flagship Store	Electricity conservation2	🉑 Baby received, very surprised ✨, reasonable design, fashionable and generous, continue to pay attention to the future will be repurchased ❤❤❤❤	Jingdong prefecture level city in Shandong
Jinfeng Hongyuan Flagship Store	Electricity conservation2	Baby received it 👌, really great ah! 🌈The quality can't be beat, very satisfied! 🆗Happy happy happy 😀	Jingdong prefecture level city in Shandong
Jinfeng Hongyuan Flagship Store	Electricity conservation2	Very good, power saving see, this is to help a friend to buy, I used to give my friend a say, my friend immediately let me order another one!	Jingdong prefecture level city in Shandong
Jinfeng Hongyuan Flagship Store	Electricity conservation2	The baby is just as introduced, the texture is very good, powerful, and it feels good to use it	Jingdong prefecture level city in Shandong

Jinfeng Hongyuan Flagship Store	Electricity conservation2	Value for money, very satisfied, seller service attitude is very good, very patient	Jingdong prefecture level city in Shandong
Jinfeng Hongyuan Flagship Store	Electricity conservation2	Power saving treasure design unique exquisite beautiful, voltage display clearly, plugged in that is good to use, I observed the meter, after using the meter is gone slow.	Jingdong prefecture level city in Shandong
Jinfeng Hongyuan Flagship Store	Electricity conservation2	Received on the use of the packaging is beautifully used for a period of time and then come back in the follow-up review!	Jingdong prefecture level city in Shandong
Jinfeng Hongyuan Flagship Store	Electricity conservation2	It should work. Not bad. Not bad. Not bad.	Jingdong prefecture level city in Shandong
Jinfeng Hongyuan Flagship Store	Electricity conservation2	I haven't used it yet, I'll plug it in and see if it saves power next month.	Jingdong prefecture level city in Shandong
Jinfeng Hongyuan Flagship Store	Electricity conservation2	The goods received and unpacked and used, the quality is very good, things and the official website to buy no difference!	Jingdong prefecture level city in Shandong
Jinfeng Hongyuan Flagship Store	Electricity conservation2	It's a rip off, doesn't help much at all, uses the same amount of power as before it was used.	Jingdong prefecture level city in Shandong
Jinfeng Hongyuan Flagship Store	Electricity conservation2	I've been using it for a while now and haven't noticed any unusual problems, so it's okay	Jingdong prefecture level city in Shandong
Jinfeng Hongyuan Flagship Store	Electricity conservation2	Received the goods and plugged in to use, good or not there is no use will have to wait until next month to find out	Jingdong prefecture level city in Shandong
Jinfeng Hongyuan Flagship Store	Electricity conservation2	It's very compact, it looks good, I'll introduce you to my clients when it's ready.	Jingdong prefecture level city in Shandong
Jinfeng Hongyuan Flagship Store	Electricity conservation2	Good workmanship, we'll have to see in a few months.	Jingdong prefecture level city in Shandong
Jinfeng Hongyuan Flagship Store	Electricity conservation2	Jingdong quality! Always trustworthy!	Jingdong prefecture level city in Shandong
Jinfeng Hongyuan Flagship Store	Electricity conservation2	This is the second time I've bought it, and the merchant said to give me five dollars out of the difference, so thank you merchant.	Jingdong prefecture level city in Shandong
Jinfeng Hongyuan Flagship Store	Electricity conservation2	Good quality and good service	Jingdong prefecture level city in Shandong
Jinfeng Hongyuan Flagship Store	Electricity conservation2	Feels good texture, aluminum alloy shell, voltage digital display clear.	Jingdong prefecture level city in Shandong
Jinfeng Hongyuan Flagship Store	Electricity conservation2	Good product, fast and great service, thanks!	Jingdong prefecture level city in Shandong
Jinfeng Hongyuan Flagship Store	Electricity conservation2	Have not yet used, first evaluation, later really good do not need to comment again.	Jingdong prefecture level city in Shandong
Jinfeng Hongyuan Flagship Store	Electricity conservation2	Works great. Pretty much as described by the merchant.	Jingdong prefecture level city in Shandong
Jinfeng Hongyuan Flagship Store	Electricity conservation2	Received it, looking forward to the results, hope it works	Jingdong prefecture level city in Shandong
Jinfeng Hongyuan Flagship Store	Electricity conservation2	Received it, looking forward to the results, hope it works	Jingdong prefecture level city in Shandong
Jinfeng Hongyuan Flagship Store	Electricity conservation2	Supposedly it's an IQ tax. Buy one and try it. Looks good. Well made.	Jingdong prefecture level city in Shandong
Jinfeng Hongyuan Flagship Store	Electricity conservation2	It just doesn't feel like it's working.	Jingdong prefecture level city in Shandong
Jinfeng Hongyuan Flagship Store	Electricity conservation2	This is a great product.	Jingdong prefecture level city in Shandong
Jinfeng Hongyuan Flagship Store	Electricity conservation2	I'm just using it. I'll tell you more.	Jingdong prefecture level city in Shandong
Jinfeng Hongyuan Flagship Store	Electricity conservation2	Second repurchase and it's working fine.	Jingdong prefecture level city in Shandong
Jinfeng Hongyuan Flagship Store	Electricity conservation2	I'll wait a few days before evaluating	Jingdong prefecture level city in Shandong
Jinfeng Hongyuan Flagship Store	Electricity conservation2	I'll comment after I use it hopefully the section will be useful.	Jingdong prefecture level city in Shandong
Jinfeng Hongyuan Flagship Store	Electricity conservation2	not very useful	Jingdong prefecture level city in Shandong
Jinfeng Hongyuan Flagship Store	Electricity conservation2	Not received	Jingdong prefecture level city in Shandong
Jinfeng Hongyuan Flagship Store	Electricity conservation2	rare	Jingdong prefecture level city in Shandong
Jinfeng Hongyuan Flagship Store	Electricity conservation2	It's good.	Jingdong prefecture level city in Shandong
Jinfeng Hongyuan Flagship Store	Electricity conservation2	just now	Jingdong prefecture level city in Shandong
Jinfeng Hongyuan Flagship Store	Electricity conservation2	serviceable	Jingdong prefecture level city in Shandong
Jinfeng Hongyuan Flagship Store	Electricity conservation2	desirable	Jingdong prefecture level city in Shandong
Jinfeng Hongyuan Flagship Store	Electricity conservation2	serviceable	Jingdong prefecture level city in Shandong
Jinfeng Hongyuan Flagship Store	Electricity conservation2	It doesn't save power and costs electricity.	Jingdong prefecture level city in Shandong
Jinfeng Hongyuan Flagship Store	Electricity conservation2	Not as good as I thought.	Jingdong prefecture level city in Shandong
Jinfeng Hongyuan Flagship Store	Electricity conservation2	Super bad review, it doesn't save power at all, but rather more power consumption	Jingdong prefecture level city in Shandong
Jinfeng Hongyuan Flagship Store	Electricity conservation2	Listen to others say that this can save electricity, I bought with the idea of trying, used for a period of time before coming to the evaluation, will be a little bit of use, not a lot of savings just, long-term use of the words can save more.	Jingdong prefecture level city in Shandong
Jinfeng Hongyuan Flagship Store	Electricity conservation2	Logistics is fast, the next day arrived, workmanship is very delicate a power saver, looking forward to the late effect, customer service patiently explain the recommended which is suitable for, installed in what position is the best, look forward to using the effect after a period of time!	Jingdong prefecture level city in Shandong
Jinfeng Hongyuan Flagship Store	Electricity conservation2	It's good to use, I feel that it has been saving electricity, anyway, not a loss is, I used to feel that the power consumption is big, now I feel much better, my friends are also using, I hope to use a long time will be able to, I wish the boss a prosperous business	Jingdong prefecture level city in Shandong
Jinfeng Hongyuan Flagship Store	Electricity conservation2	Should be very good, to be able to plan the circuit, save the home of the circuit to the extent of the savings, waste of money on electricity, good if a month this money back, hope always hope to be able to	Jingdong prefecture level city in Shandong
Jinfeng Hongyuan Flagship Store	Electricity conservation2	Express delivery is very strong, the goods and I saw in the business consistent, the use of the effect of follow-up evaluation	Jingdong prefecture level city in Shandong
Jinfeng Hongyuan Flagship Store	Electricity conservation2	I have been using this brand of power saver for many years and this time I saw this upgraded version and got it again. The main purpose of this product is to reduce the loss of electricity for a long time, so as to achieve the purpose of saving electricity.	Jingdong prefecture level city in Shandong
Jinfeng Hongyuan Flagship Store	Electricity conservation2	Logistics is fast and the product looks beautiful, looking forward to the effect.	Jingdong prefecture level city in Shandong
Jinfeng Hongyuan Flagship Store	Electricity conservation2	Product quality is good, energy saving a little effect, but the price is a little expensive, fast arrival, good service.	Jingdong prefecture level city in Shandong
Jinfeng Hongyuan Flagship Store	Electricity conservation2	The quality is very good, inexpensive, Jingdong self-supporting guaranteed, worth having!	Jingdong prefecture level city in Shandong
Jinfeng Hongyuan Flagship Store	Electricity conservation2	I don't know if it's saving electricity yet, I'll find out next time I pay my electric bill	Jingdong prefecture level city in Shandong
Jinfeng Hongyuan Flagship Store	Electricity conservation2	Favorable price quality assurance appearance design is beautiful and generous, fine workmanship	Jingdong prefecture level city in Shandong

Jinfeng Hongyuan Flagship Store	Electricity conservation2	I bought it, I don't know how effective it is? I don't know how the quality is? After this summer results will know! Looking forward to it!	Jingdong prefecture level city in Shandong
Jinfeng Hongyuan Flagship Store	Electricity conservation2	Goods received Packaging intact, look at the shell workmanship is also good, high-power appliances open voltage has obvious changes in the effectiveness of the	Jingdong prefecture level city in Shandong
Jinfeng Hongyuan Flagship Store	Electricity conservation2	The goods have been received logistics is very fast, the product is similar to the line, just wait to use a month to see if it can save electricity, stay tuned.	Jingdong prefecture level city in Shandong
Jinfeng Hongyuan Flagship Store	Electricity conservation2	Electricity is plugged in, not sure if it will work, hopefully it will work.	Jingdong prefecture level city in Shandong
Jinfeng Hongyuan Flagship Store	Electricity conservation2	The shell is aluminum alloy, very good workmanship, the actual effect of waiting for the addition of evaluation	Jingdong prefecture level city in Shandong
Jinfeng Hongyuan Flagship Store	Electricity conservation2	Received, already plugged in, looking forward to the effect.	Jingdong prefecture level city in Shandong
Jinfeng Hongyuan Flagship Store	Electricity conservation2	Opened to see the regular manufacturer products, wait for a period of time to see it	Jingdong prefecture level city in Shandong
Jinfeng Hongyuan Flagship Store	Electricity conservation2	🉑 Baby received, very surprised ✨, reasonable design, fashionable and generous, continue to pay attention to the future will be repurchased ❤❤❤❤	Jingdong prefecture level city in Shandong
Jinfeng Hongyuan Flagship Store	Electricity conservation2	Baby received it 👌, really great ah! 🌈The quality can't be beat, very satisfied! 🆗Happy happy happy 😃	Jingdong prefecture level city in Shandong
Jinfeng Hongyuan Flagship Store	Electricity conservation2	Very good, power saving see, this is to help a friend to buy, I used to give my friend a say, my friend immediately let me order another one!	Jingdong prefecture level city in Shandong
Jinfeng Hongyuan Flagship Store	Electricity conservation2	The baby is just as introduced, the texture is very good, powerful, and it feels good to use it	Jingdong prefecture level city in Shandong
Jinfeng Hongyuan Flagship Store	Electricity conservation2	Value for money, very satisfied, seller service attitude is very good, very patient	Jingdong prefecture level city in Shandong
Jinfeng Hongyuan Flagship Store	Electricity conservation2	Power saving treasure design unique exquisite beautiful, voltage display clearly, plugged in that is good to use, I observed the meter, after using the meter is gone slow.	Jingdong prefecture level city in Shandong
Jinfeng Hongyuan Flagship Store	Electricity conservation2	Received on the use of the packaging is beautifully used for a period of time and then come back in the follow-up review!	Jingdong prefecture level city in Shandong
Jinfeng Hongyuan Flagship Store	Electricity conservation2	It should work. Not bad. Not bad. Not bad.	Jingdong prefecture level city in Shandong
Jinfeng Hongyuan Flagship Store	Electricity conservation2	I haven't used it yet, I'll plug it in and see if it saves power next month.	Jingdong prefecture level city in Shandong
Jinfeng Hongyuan Flagship Store	Electricity conservation2	The goods received and unpacked and used, the quality is very good, things and the official website to buy no difference!	Jingdong prefecture level city in Shandong
Jinfeng Hongyuan Flagship Store	Electricity conservation2	It's a rip off, doesn't help much at all, uses the same amount of power as before it was used.	Jingdong prefecture level city in Shandong
Jinfeng Hongyuan Flagship Store	Electricity conservation2	I've been using it for a while now and haven't noticed any unusual problems, so it's okay	Jingdong prefecture level city in Shandong
Jinfeng Hongyuan Flagship Store	Electricity conservation2	Received the goods and plugged in to use, good or not there is no use will have to wait until next month to find out	Jingdong prefecture level city in Shandong
Jinfeng Hongyuan Flagship Store	Electricity conservation2	It's very compact, it looks good, I'll introduce you to my clients when it's ready.	Jingdong prefecture level city in Shandong
Jinfeng Hongyuan Flagship Store	Electricity conservation2	Good workmanship, we'll have to see in a few months.	Jingdong prefecture level city in Shandong
Jinfeng Hongyuan Flagship Store	Electricity conservation2	Jingdong quality! Always trustworthy!	Jingdong prefecture level city in Shandong
Jinfeng Hongyuan Flagship Store	Electricity conservation2	This is the second time I've bought it, and the merchant said to give me five dollars out of the difference, so thank you merchant.	Jingdong prefecture level city in Shandong
Jinfeng Hongyuan Flagship Store	Electricity conservation2	Good quality and good service	Jingdong prefecture level city in Shandong
Jinfeng Hongyuan Flagship Store	Electricity conservation2	Feels good texture, aluminum alloy shell, voltage digital display clear.	Jingdong prefecture level city in Shandong
Jinfeng Hongyuan Flagship Store	Electricity conservation2	Good product, fast and great service, thanks!	Jingdong prefecture level city in Shandong
Jinfeng Hongyuan Flagship Store	Electricity conservation2	Have not yet used, first evaluation, later really good do not need to comment again.	Jingdong prefecture level city in Shandong
Jinfeng Hongyuan Flagship Store	Electricity conservation2	Works great. Pretty much as described by the merchant.	Jingdong prefecture level city in Shandong
Jinfeng Hongyuan Flagship Store	Electricity conservation2	Received it, looking forward to the results, hope it works	Jingdong prefecture level city in Shandong
Jinfeng Hongyuan Flagship Store	Electricity conservation2	Received it, looking forward to the results, hope it works	Jingdong prefecture level city in Shandong
Jinfeng Hongyuan Flagship Store	Electricity conservation2	Supposedly it's an IQ tax. Buy one and try it. Looks good. Well made.	Jingdong prefecture level city in Shandong
Jinfeng Hongyuan Flagship Store	Electricity conservation2	It just doesn't feel like it's working.	Jingdong prefecture level city in Shandong
Jinfeng Hongyuan Flagship Store	Electricity conservation2	This is a great product.	Jingdong prefecture level city in Shandong
Jinfeng Hongyuan Flagship Store	Electricity conservation2	I'm just using it. I'll tell you more.	Jingdong prefecture level city in Shandong
Jinfeng Hongyuan Flagship Store	Electricity conservation2	Second repurchase and it's working fine.	Jingdong prefecture level city in Shandong
Jinfeng Hongyuan Flagship Store	Electricity conservation2	I'll wait a few days before evaluating	Jingdong prefecture level city in Shandong
Jinfeng Hongyuan Flagship Store	Electricity conservation2	I'll comment after I use it hopefully the section will be useful.	Jingdong prefecture level city in Shandong
Jinfeng Hongyuan Flagship Store	Electricity conservation2	not very useful	Jingdong prefecture level city in Shandong
Jinfeng Hongyuan Flagship Store	Electricity conservation2	Not received	Jingdong prefecture level city in Shandong
Jinfeng Hongyuan Flagship Store	Electricity conservation2	rare	Jingdong prefecture level city in Shandong
Jinfeng Hongyuan Flagship Store	Electricity conservation2	It's good.	Jingdong prefecture level city in Shandong
Jinfeng Hongyuan Flagship Store	Electricity conservation2	just now	Jingdong prefecture level city in Shandong
Jinfeng Hongyuan Flagship Store	Electricity conservation2	serviceable	Jingdong prefecture level city in Shandong
Jinfeng Hongyuan Flagship Store	Electricity conservation2	desirable	Jingdong prefecture level city in Shandong
Jinfeng Hongyuan Flagship Store	Electricity conservation2	serviceable	Jingdong prefecture level city in Shandong
Jinfeng Hongyuan Flagship Store	Electricity conservation2	It doesn't save power and costs electricity.	Jingdong prefecture level city in Shandong
Jinfeng Hongyuan Flagship Store	Electricity conservation2	Not as good as I thought.	Jingdong prefecture level city in Shandong
Jinfeng Hongyuan Flagship Store	Electricity conservation2	Super bad review, it doesn't save power at all, but rather more power consumption	Jingdong prefecture level city in Shandong
Jinfeng Hongyuan Flagship Store	Electricity conservation2	Listen to others say that this can save electricity, I bought with the idea of trying, used for a period of time before coming to the evaluation, will be a little bit of use, not a lot of savings just, long-term use of the words can save more.	Jingdong prefecture level city in Shandong

Jinfeng Hongyuan Flagship Store	Electricity conservation2	Logistics is fast, the next day arrived, workmanship is very delicate a power saver, looking forward to the late effect, customer service patiently explain the recommended which is suitable for, installed in what position is the best, look forward to using the effect after a period of time!	Jingdong prefecture level city in Shandong
Jinfeng Hongyuan Flagship Store	Electricity conservation2	It's good to use, I feel that it has been saving electricity, anyway, not a loss is, I used to feel that the power consumption is big, now I feel much better, my friends are also using, I hope to use a long time will be able to, I wish the boss a prosperous business	Jingdong prefecture level city in Shandong
Jinfeng Hongyuan Flagship Store	Electricity conservation2	Should be very good, to be able to plan the circuit, save the home of the circuit to the extent of the savings, waste of money on electricity, good if a month this money back, hope always hope to be able to	Jingdong prefecture level city in Shandong
Jinfeng Hongyuan Flagship Store	Electricity conservation2	Express delivery is very strong, the goods and I saw in the business consistent, the use of the effect of follow-up evaluation	Jingdong prefecture level city in Shandong
Jinfeng Hongyuan Flagship Store	Electricity conservation2	I have been using this brand of power saver for many years and this time I saw this upgraded version and got it again. The main purpose of this product is to reduce the loss of electricity for a long time, so as to achieve the purpose of saving electricity.	Jingdong prefecture level city in Shandong
Jinfeng Hongyuan Flagship Store	Electricity conservation2	Logistics is fast and the product looks beautiful, looking forward to the effect.	Jingdong prefecture level city in Shandong
Jinfeng Hongyuan Flagship Store	Electricity conservation2	Product quality is good, energy saving a little effect, but the price is a little expensive, fast arrival, good service.	Jingdong prefecture level city in Shandong
Jinfeng Hongyuan Flagship Store	Electricity conservation2	The quality is very good, inexpensive, Jingdong self-supporting guaranteed, worth having!	Jingdong prefecture level city in Shandong
Jinfeng Hongyuan Flagship Store	Electricity conservation2	I don't know if it's saving electricity yet, I'll find out next time I pay my electric bill	Jingdong prefecture level city in Shandong
Jinfeng Hongyuan Flagship Store	Electricity conservation2	Favorable price quality assurance appearance design is beautiful and generous, fine workmanship	Jingdong prefecture level city in Shandong
Jinfeng Hongyuan Flagship Store	Electricity conservation2	I bought it, I don't know how effective it is? I don't know how the quality is? After this summer results will know! Looking forward to it!	Jingdong prefecture level city in Shandong
Jinfeng Hongyuan Flagship Store	Electricity conservation2	Goods received Packaging intact, look at the shell workmanship is also good, high-power appliances open voltage has obvious changes in the effectiveness of the	Jingdong prefecture level city in Shandong
Jinfeng Hongyuan Flagship Store	Electricity conservation2	The goods have been received logistics is very fast, the product is similar to the line, just wait to use a month to see if it can save electricity, stay tuned.	Jingdong prefecture level city in Shandong
Jinfeng Hongyuan Flagship Store	Electricity conservation2	Electricity is plugged in, not sure if it will work, hopefully it will work.	Jingdong prefecture level city in Shandong
Jinfeng Hongyuan Flagship Store	Electricity conservation2	The shell is aluminum alloy, very good workmanship, the actual effect of waiting for the addition of evaluation	Jingdong prefecture level city in Shandong
Jinfeng Hongyuan Flagship Store	Electricity conservation2	Received, already plugged in, looking forward to the effect.	Jingdong prefecture level city in Shandong
Jinfeng Hongyuan Flagship Store	Electricity conservation2	Opened to see the regular manufacturer products, wait for a period of time to see it	Jingdong prefecture level city in Shandong
Jinfeng Hongyuan Flagship Store	Electricity conservation2	🉑 Baby received, very surprised ✨, reasonable design, fashionable and generous, continue to pay attention to the future will be repurchased ❥❥❥❥	Jingdong prefecture level city in Shandong
Jinfeng Hongyuan Flagship Store	Electricity conservation2	Baby received it 👌, really great ah! 🌈The quality can't be beat, very satisfied! 🆗Happy happy happy 😃	Jingdong prefecture level city in Shandong
Jinfeng Hongyuan Flagship Store	Electricity conservation2	Very good, power saving see, this is to help a friend to buy, I used to give my friend a say, my friend immediately let me order another one!	Jingdong prefecture level city in Shandong
Jinfeng Hongyuan Flagship Store	Electricity conservation2	The baby is just as introduced, the texture is very good, powerful, and it feels good to use it	Jingdong prefecture level city in Shandong
Jinfeng Hongyuan Flagship Store	Electricity conservation2	Value for money, very satisfied, seller service attitude is very good, very patient	Jingdong prefecture level city in Shandong
Jinfeng Hongyuan Flagship Store	Electricity conservation2	Power saving treasure design unique exquisite beautiful, voltage display clearly, plugged in that is good to use, I observed the meter, after using the meter is gone slow.	Jingdong prefecture level city in Shandong
Jinfeng Hongyuan Flagship Store	Electricity conservation2	Received on the use of the packaging is beautifully used for a period of time and then come back in the follow-up review!	Jingdong prefecture level city in Shandong
Jinfeng Hongyuan Flagship Store	Electricity conservation2	It should work. Not bad. Not bad. Not bad.	Jingdong prefecture level city in Shandong
Jinfeng Hongyuan Flagship Store	Electricity conservation2	I haven't used it yet, I'll plug it in and see if it saves power next month.	Jingdong prefecture level city in Shandong
Jinfeng Hongyuan Flagship Store	Electricity conservation2	The goods received and unpacked and used, the quality is very good, things and the official website to buy no difference!	Jingdong prefecture level city in Shandong
Jinfeng Hongyuan Flagship Store	Electricity conservation2	It's a rip off, doesn't help much at all, uses the same amount of power as before it was used.	Jingdong prefecture level city in Shandong
Jinfeng Hongyuan Flagship Store	Electricity conservation2	I've been using it for a while now and haven't noticed any unusual problems, so it's okay	Jingdong prefecture level city in Shandong
Jinfeng Hongyuan Flagship Store	Electricity conservation2	Received the goods and plugged in to use, good or not there is no use will have to wait until next month to find out	Jingdong prefecture level city in Shandong
Jinfeng Hongyuan Flagship Store	Electricity conservation2	It's very compact, it looks good, I'll introduce you to my clients when it's ready.	Jingdong prefecture level city in Shandong
Jinfeng Hongyuan Flagship Store	Electricity conservation2	Good workmanship, we'll have to see in a few months.	Jingdong prefecture level city in Shandong
Jinfeng Hongyuan Flagship Store	Electricity conservation2	Jingdong quality! Always trustworthy!	Jingdong prefecture level city in Shandong
Jinfeng Hongyuan Flagship Store	Electricity conservation2	This is the second time I've bought it, and the merchant said to give me five dollars out of the difference, so thank you merchant.	Jingdong prefecture level city in Shandong
Jinfeng Hongyuan Flagship Store	Electricity conservation2	Good quality and good service	Jingdong prefecture level city in Shandong
Jinfeng Hongyuan Flagship Store	Electricity conservation2	Feels good texture, aluminum alloy shell, voltage digital display clear.	Jingdong prefecture level city in Shandong
Jinfeng Hongyuan Flagship Store	Electricity conservation2	Good product, fast and great service, thanks!	Jingdong prefecture level city in Shandong
Jinfeng Hongyuan Flagship Store	Electricity conservation2	Have not yet used, first evaluation, later really good do not need to comment again.	Jingdong prefecture level city in Shandong
Jinfeng Hongyuan Flagship Store	Electricity conservation2	Works great. Pretty much as described by the merchant.	Jingdong prefecture level city in Shandong
Jinfeng Hongyuan Flagship Store	Electricity conservation2	Received it, looking forward to the results, hope it works	Jingdong prefecture level city in Shandong
Jinfeng Hongyuan Flagship Store	Electricity conservation2	Received it, looking forward to the results, hope it works	Jingdong prefecture level city in Shandong
Jinfeng Hongyuan Flagship Store	Electricity conservation2	Supposedly it's an IQ tax. Buy one and try it. Looks good. Well made.	Jingdong prefecture level city in Shandong
Jinfeng Hongyuan Flagship Store	Electricity conservation2	It just doesn't feel like it's working.	Jingdong prefecture level city in Shandong
Jinfeng Hongyuan Flagship Store	Electricity conservation2	This is a great product.	Jingdong prefecture level city in Shandong
Jinfeng Hongyuan Flagship Store	Electricity conservation2	I'm just using it. I'll tell you more.	Jingdong prefecture level city in Shandong
Jinfeng Hongyuan Flagship Store	Electricity conservation2	Second repurchase and it's working fine.	Jingdong prefecture level city in Shandong
Jinfeng Hongyuan Flagship Store	Electricity conservation2	I'll wait a few days before evaluating	Jingdong prefecture level city in Shandong
Jinfeng Hongyuan Flagship Store	Electricity conservation2	I'll comment after I use it hopefully the section will be useful.	Jingdong prefecture level city in Shandong
Jinfeng Hongyuan Flagship Store	Electricity conservation2	not very useful	Jingdong prefecture level city in Shandong
Jinfeng Hongyuan Flagship Store	Electricity conservation2	Not received	Jingdong prefecture level city in Shandong

Jinfeng Hongyuan Flagship Store	Electricity conservation2	rare	Jingdong prefecture level city in Shandong
Jinfeng Hongyuan Flagship Store	Electricity conservation2	It's good.	Jingdong prefecture level city in Shandong
Jinfeng Hongyuan Flagship Store	Electricity conservation2	just now	Jingdong prefecture level city in Shandong
Jinfeng Hongyuan Flagship Store	Electricity conservation2	serviceable	Jingdong prefecture level city in Shandong
Jinfeng Hongyuan Flagship Store	Electricity conservation2	desirable	Jingdong prefecture level city in Shandong
Jinfeng Hongyuan Flagship Store	Electricity conservation2	serviceable	Jingdong prefecture level city in Shandong
Jinfeng Hongyuan Flagship Store	Electricity conservation2	It doesn't save power and costs electricity.	Jingdong prefecture level city in Shandong
Jinfeng Hongyuan Flagship Store	Electricity conservation2	Not as good as I thought.	Jingdong prefecture level city in Shandong
Jinfeng Hongyuan Flagship Store	Electricity conservation2	Super bad review, it doesn't save power at all, but rather more power consumption	Jingdong prefecture level city in Shandong
Jinfeng Hongyuan Flagship Store	Electricity conservation2	Listen to others say that this can save electricity, I bought with the idea of trying, used for a period of time before coming to the evaluation, will be a little bit of use, not a lot of savings just, long-term use of the words can save more.	Jingdong prefecture level city in Shandong
Jinfeng Hongyuan Flagship Store	Electricity conservation2	Logistics is fast, the next day arrived, workmanship is very delicate a power saver, looking forward to the late effect, customer service patiently explain the recommended which is suitable for, installed in what position is the best, look forward to using the effect after a period of time!	Jingdong prefecture level city in Shandong
Jinfeng Hongyuan Flagship Store	Electricity conservation2	It's good to use, I feel that it has been saving electricity, anyway, not a loss is, I used to feel that the power consumption is big, now I feel much better, my friends are also using, I hope to use a long time will be able to, I wish the boss a prosperous business	Jingdong prefecture level city in Shandong
Jinfeng Hongyuan Flagship Store	Electricity conservation2	Should be very good, to be able to plan the circuit, save the home of the circuit to the extent of the savings, waste of money on electricity, good if a month this money back, hope always hope to be able to	Jingdong prefecture level city in Shandong
Jinfeng Hongyuan Flagship Store	Electricity conservation2	Express delivery is very strong, the goods and I saw in the business consistent, the use of the effect of follow-up evaluation	Jingdong prefecture level city in Shandong
Jinfeng Hongyuan Flagship Store	Electricity conservation2	I have been using this brand of power saver for many years and this time I saw this upgraded version and got it again. The main purpose of this product is to reduce the loss of electricity for a long time, so as to achieve the purpose of saving electricity.	Jingdong prefecture level city in Shandong
Jinfeng Hongyuan Flagship Store	Electricity conservation2	Logistics is fast and the product looks beautiful, looking forward to the effect.	Jingdong prefecture level city in Shandong
Jinfeng Hongyuan Flagship Store	Electricity conservation2	Product quality is good, energy saving a little effect, but the price is a little expensive, fast arrival, good service.	Jingdong prefecture level city in Shandong
Jinfeng Hongyuan Flagship Store	Electricity conservation2	The quality is very good, inexpensive, Jingdong self-supporting guaranteed, worth having!	Jingdong prefecture level city in Shandong
Jinfeng Hongyuan Flagship Store	Electricity conservation2	I don't know if it's saving electricity yet, I'll find out next time I pay my electric bill	Jingdong prefecture level city in Shandong
Jinfeng Hongyuan Flagship Store	Electricity conservation2	Favorable price quality assurance appearance design is beautiful and generous, fine workmanship	Jingdong prefecture level city in Shandong
Jinfeng Hongyuan Flagship Store	Electricity conservation2	I bought it, I don't know how effective it is? I don't know how the quality is? After this summer results will know! Looking forward to it!	Jingdong prefecture level city in Shandong
Jinfeng Hongyuan Flagship Store	Electricity conservation2	Goods received Packaging intact, look at the shell workmanship is also good, high-power appliances open voltage has obvious changes in the effectiveness of the	Jingdong prefecture level city in Shandong
Jinfeng Hongyuan Flagship Store	Electricity conservation2	The goods have been received logistics is very fast, the product is similar to the line, just wait to use a month to see if it can save electricity, stay tuned.	Jingdong prefecture level city in Shandong
Jinfeng Hongyuan Flagship Store	Electricity conservation2	Electricity is plugged in, not sure if it will work, hopefully it will work.	Jingdong prefecture level city in Shandong
Jinfeng Hongyuan Flagship Store	Electricity conservation2	The shell is aluminum alloy, very good workmanship, the actual effect of waiting for the addition of evaluation	Jingdong prefecture level city in Shandong
Jinfeng Hongyuan Flagship Store	Electricity conservation2	Received, already plugged in, looking forward to the effect.	Jingdong prefecture level city in Shandong
Jinfeng Hongyuan Flagship Store	Electricity conservation2	Opened to see the regular manufacturer products, wait for a period of time to see it	Jingdong prefecture level city in Shandong
Jinfeng Hongyuan Flagship Store	Electricity conservation2	🉑 Baby received, very surprised ✨, reasonable design, fashionable and generous, continue to pay attention to the future will be repurchased ❥❥❥❥	Jingdong prefecture level city in Shandong
Jinfeng Hongyuan Flagship Store	Electricity conservation2	Baby received it 👌, really great ah! 🌈The quality can't be beat, very satisfied! 🆗Happy happy happy 😃	Jingdong prefecture level city in Shandong
Jinfeng Hongyuan Flagship Store	Electricity conservation2	Very good, power saving see, this is to help a friend to buy, I used to give my friend a say, my friend immediately let me order another one!	Jingdong prefecture level city in Shandong
Jinfeng Hongyuan Flagship Store	Electricity conservation2	The baby is just as introduced, the texture is very good, powerful, and it feels good to use it	Jingdong prefecture level city in Shandong
Jinfeng Hongyuan Flagship Store	Electricity conservation2	Value for money, very satisfied, seller service attitude is very good, very patient	Jingdong prefecture level city in Shandong
Jinfeng Hongyuan Flagship Store	Electricity conservation2	Power saving treasure design unique exquisite beautiful, voltage display clearly, plugged in that is good to use, I observed the meter, after using the meter is gone slow.	Jingdong prefecture level city in Shandong
Jinfeng Hongyuan Flagship Store	Electricity conservation2	Received on the use of the packaging is beautifully used for a period of time and then come back in the follow-up review!	Jingdong prefecture level city in Shandong
Jinfeng Hongyuan Flagship Store	Electricity conservation2	It should work. Not bad. Not bad. Not bad.	Jingdong prefecture level city in Shandong
Jinfeng Hongyuan Flagship Store	Electricity conservation2	I haven't used it yet, I'll plug it in and see if it saves power next month.	Jingdong prefecture level city in Shandong
Jinfeng Hongyuan Flagship Store	Electricity conservation2	The goods received and unpacked and used, the quality is very good, things and the official website to buy no difference!	Jingdong prefecture level city in Shandong
Jinfeng Hongyuan Flagship Store	Electricity conservation2	It's a rip off, doesn't help much at all, uses the same amount of power as before it was used.	Jingdong prefecture level city in Shandong
Jinfeng Hongyuan Flagship Store	Electricity conservation2	I've been using it for a while now and haven't noticed any unusual problems, so it's okay	Jingdong prefecture level city in Shandong
Jinfeng Hongyuan Flagship Store	Electricity conservation2	Received the goods and plugged in to use, good or not there is no use will have to wait until next month to find out	Jingdong prefecture level city in Shandong
Jinfeng Hongyuan Flagship Store	Electricity conservation2	It's very compact, it looks good, I'll introduce you to my clients when it's ready.	Jingdong prefecture level city in Shandong
Jinfeng Hongyuan Flagship Store	Electricity conservation2	Good workmanship, we'll have to see in a few months.	Jingdong prefecture level city in Shandong
Jinfeng Hongyuan Flagship Store	Electricity conservation2	Jingdong quality! Always trustworthy!	Jingdong prefecture level city in Shandong
Jinfeng Hongyuan Flagship Store	Electricity conservation2	This is the second time I've bought it, and the merchant said to give me five dollars out of the difference, so thank you merchant.	Jingdong prefecture level city in Shandong
Jinfeng Hongyuan Flagship Store	Electricity conservation2	Good quality and good service	Jingdong prefecture level city in Shandong
Jinfeng Hongyuan Flagship Store	Electricity conservation2	Feels good texture, aluminum alloy shell, voltage digital display clear.	Jingdong prefecture level city in Shandong
Jinfeng Hongyuan Flagship Store	Electricity conservation2	Good product, fast and great service, thanks!	Jingdong prefecture level city in Shandong
Jinfeng Hongyuan Flagship Store	Electricity conservation2	Have not yet used, first evaluation, later really good do not need to comment again.	Jingdong prefecture level city in Shandong
Jinfeng Hongyuan Flagship Store	Electricity conservation2	Works great. Pretty much as described by the merchant.	Jingdong prefecture level city in Shandong

Jinfeng Hongyuan Flagship Store	Electricity conservation2	Received it, looking forward to the results, hope it works	Jingdong prefecture level city in Shandong
Jinfeng Hongyuan Flagship Store	Electricity conservation2	Received it, looking forward to the results, hope it works	Jingdong prefecture level city in Shandong
Jinfeng Hongyuan Flagship Store	Electricity conservation2	Supposedly it's an IQ tax. Buy one and try it. Looks good. Well made.	Jingdong prefecture level city in Shandong
Jinfeng Hongyuan Flagship Store	Electricity conservation2	It just doesn't feel like it's working.	Jingdong prefecture level city in Shandong
Jinfeng Hongyuan Flagship Store	Electricity conservation2	This is a great product.	Jingdong prefecture level city in Shandong
Jinfeng Hongyuan Flagship Store	Electricity conservation2	I'm just using it. I'll tell you more.	Jingdong prefecture level city in Shandong
Jinfeng Hongyuan Flagship Store	Electricity conservation2	Second repurchase and it's working fine.	Jingdong prefecture level city in Shandong
Jinfeng Hongyuan Flagship Store	Electricity conservation2	I'll wait a few days before evaluating	Jingdong prefecture level city in Shandong
Jinfeng Hongyuan Flagship Store	Electricity conservation2	I'll comment after I use it hopefully the section will be useful.	Jingdong prefecture level city in Shandong
Jinfeng Hongyuan Flagship Store	Electricity conservation2	not very useful	Jingdong prefecture level city in Shandong
Jinfeng Hongyuan Flagship Store	Electricity conservation2	Not received	Jingdong prefecture level city in Shandong
Jinfeng Hongyuan Flagship Store	Electricity conservation2	rare	Jingdong prefecture level city in Shandong
Jinfeng Hongyuan Flagship Store	Electricity conservation2	It's good.	Jingdong prefecture level city in Shandong
Jinfeng Hongyuan Flagship Store	Electricity conservation2	just now	Jingdong prefecture level city in Shandong
Jinfeng Hongyuan Flagship Store	Electricity conservation2	serviceable	Jingdong prefecture level city in Shandong
Jinfeng Hongyuan Flagship Store	Electricity conservation2	desirable	Jingdong prefecture level city in Shandong
Jinfeng Hongyuan Flagship Store	Electricity conservation2	serviceable	Jingdong prefecture level city in Shandong
Jinfeng Hongyuan Flagship Store	Electricity conservation2	It doesn't save power and costs electricity.	Jingdong prefecture level city in Shandong
Jinfeng Hongyuan Flagship Store	Electricity conservation2	Not as good as I thought.	Jingdong prefecture level city in Shandong
Jinfeng Hongyuan Flagship Store	Electricity conservation2	Super bad review, it doesn't save power at all, but rather more power consumption	Jingdong prefecture level city in Shandong
Jinfeng Hongyuan Flagship Store	Electricity conservation2	Listen to others say that this can save electricity, I bought with the idea of trying, used for a period of time before coming to the evaluation, will be a little bit of use, not a lot of savings just, long-term use of the words can save more.	Jingdong prefecture level city in Shandong
Jinfeng Hongyuan Flagship Store	Electricity conservation2	Logistics is fast, the next day arrived, workmanship is very delicate a power saver, looking forward to the late effect, customer service patiently explain the recommended which is suitable for, installed in what position is the best, look forward to using the effect after a period of time!	Jingdong prefecture level city in Shandong
Jinfeng Hongyuan Flagship Store	Electricity conservation2	It's good to use, I feel that it has been saving electricity, anyway, not a loss is, I used to feel that the power consumption is big, now I feel much better, my friends are also using, I hope to use a long time will be able to, I wish the boss a prosperous business	Jingdong prefecture level city in Shandong
Jinfeng Hongyuan Flagship Store	Electricity conservation2	Should be very good, to be able to plan the circuit, save the home of the circuit to the extent of the savings, waste of money on electricity, good if a month this money back, hope always hope to be able to	Jingdong prefecture level city in Shandong
Jinfeng Hongyuan Flagship Store	Electricity conservation2	Express delivery is very strong, the goods and I saw in the business consistent, the use of the effect of follow-up evaluation	Jingdong prefecture level city in Shandong
Jinfeng Hongyuan Flagship Store	Electricity conservation2	I have been using this brand of power saver for many years and this time I saw this upgraded version and got it again. The main purpose of this product is to reduce the loss of electricity for a long time, so as to achieve the purpose of saving electricity.	Jingdong prefecture level city in Shandong
Jinfeng Hongyuan Flagship Store	Electricity conservation2	Logistics is fast and the product looks beautiful, looking forward to the effect.	Jingdong prefecture level city in Shandong
Jinfeng Hongyuan Flagship Store	Electricity conservation2	Product quality is good, energy saving a little effect, but the price is a little expensive, fast arrival, good service.	Jingdong prefecture level city in Shandong
Jinfeng Hongyuan Flagship Store	Electricity conservation2	The quality is very good, inexpensive, Jingdong self-supporting guaranteed, worth having!	Jingdong prefecture level city in Shandong
Jinfeng Hongyuan Flagship Store	Electricity conservation2	I don't know if it's saving electricity yet, I'll find out next time I pay my electric bill	Jingdong prefecture level city in Shandong
Jinfeng Hongyuan Flagship Store	Electricity conservation2	Favorable price quality assurance appearance design is beautiful and generous, fine workmanship	Jingdong prefecture level city in Shandong
Jinfeng Hongyuan Flagship Store	Electricity conservation2	I bought it, I don't know how effective it is? I don't know how the quality is? After this summer results will know! Looking forward to it!	Jingdong prefecture level city in Shandong
Jinfeng Hongyuan Flagship Store	Electricity conservation2	Goods received Packaging intact, look at the shell workmanship is also good, high-power appliances open voltage has obvious changes in the effectiveness of the	Jingdong prefecture level city in Shandong
Jinfeng Hongyuan Flagship Store	Electricity conservation2	The goods have been received logistics is very fast, the product is similar to the line, just wait to use a month to see if it can save electricity, stay tuned.	Jingdong prefecture level city in Shandong
Jinfeng Hongyuan Flagship Store	Electricity conservation2	Electricity is plugged in, not sure if it will work, hopefully it will work.	Jingdong prefecture level city in Shandong
Jinfeng Hongyuan Flagship Store	Electricity conservation2	The shell is aluminum alloy, very good workmanship, the actual effect of waiting for the addition of evaluation	Jingdong prefecture level city in Shandong
Jinfeng Hongyuan Flagship Store	Electricity conservation2	Received, already plugged in, looking forward to the effect.	Jingdong prefecture level city in Shandong
Jinfeng Hongyuan Flagship Store	Electricity conservation2	Opened to see the regular manufacturer products, wait for a period of time to see it	Jingdong prefecture level city in Shandong
Jinfeng Hongyuan Flagship Store	Electricity conservation2	🉑 Baby received, very surprised ✨, reasonable design, fashionable and generous, continue to pay attention to the future will be repurchased ❥❥❥❥	Jingdong prefecture level city in Shandong
Jinfeng Hongyuan Flagship Store	Electricity conservation2	Baby received it 👌, really great ah! 🌈The quality can't be beat, very satisfied! 🆗Happy happy happy 😃	Jingdong prefecture level city in Shandong
Jinfeng Hongyuan Flagship Store	Electricity conservation2	Very good, power saving see, this is to help a friend to buy, I used to give my friend a say, my friend immediately let me order another one!	Jingdong prefecture level city in Shandong
Jinfeng Hongyuan Flagship Store	Electricity conservation2	The baby is just as introduced, the texture is very good, powerful, and it feels good to use it	Jingdong prefecture level city in Shandong
Jinfeng Hongyuan Flagship Store	Electricity conservation2	Value for money, very satisfied, seller service attitude is very good, very patient	Jingdong prefecture level city in Shandong
Jinfeng Hongyuan Flagship Store	Electricity conservation2	Power saving treasure design unique exquisite beautiful, voltage display clearly, plugged in that is good to use, I observed the meter, after using the meter is gone slow.	Jingdong prefecture level city in Shandong
Jinfeng Hongyuan Flagship Store	Electricity conservation2	Received on the use of the packaging is beautifully used for a period of time and then come back in the follow-up review!	Jingdong prefecture level city in Shandong
Jinfeng Hongyuan Flagship Store	Electricity conservation2	It should work. Not bad. Not bad. Not bad.	Jingdong prefecture level city in Shandong
Jinfeng Hongyuan Flagship Store	Electricity conservation2	I haven't used it yet, I'll plug it in and see if it saves power next month.	Jingdong prefecture level city in Shandong
Jinfeng Hongyuan Flagship Store	Electricity conservation2	The goods received and unpacked and used, the quality is very good, things and the official website to buy no difference!	Jingdong prefecture level city in Shandong
Jinfeng Hongyuan Flagship Store	Electricity conservation2	It's a rip off, doesn't help much at all, uses the same amount of power as before it was used.	Jingdong prefecture level city in Shandong

Jinfeng Hongyuan Flagship Store	Electricity conservation2	I've been using it for a while now and haven't noticed any unusual problems, so it's okay	Jingdong prefecture level city in Shandong
Jinfeng Hongyuan Flagship Store	Electricity conservation2	Received the goods and plugged in to use, good or not there is no use will have to wait until next month to find out	Jingdong prefecture level city in Shandong
Jinfeng Hongyuan Flagship Store	Electricity conservation2	It's very compact, it looks good, I'll introduce you to my clients when it's ready.	Jingdong prefecture level city in Shandong
Jinfeng Hongyuan Flagship Store	Electricity conservation2	Good workmanship, we'll have to see in a few months.	Jingdong prefecture level city in Shandong
Jinfeng Hongyuan Flagship Store	Electricity conservation2	Jingdong quality! Always trustworthy!	Jingdong prefecture level city in Shandong
Jinfeng Hongyuan Flagship Store	Electricity conservation2	This is the second time I've bought it, and the merchant said to give me five dollars out of the difference, so thank you merchant.	Jingdong prefecture level city in Shandong
Jinfeng Hongyuan Flagship Store	Electricity conservation2	Good quality and good service	Jingdong prefecture level city in Shandong
Jinfeng Hongyuan Flagship Store	Electricity conservation2	Feels good texture, aluminum alloy shell, voltage digital display clear.	Jingdong prefecture level city in Shandong
Jinfeng Hongyuan Flagship Store	Electricity conservation2	Good product, fast and great service, thanks!	Jingdong prefecture level city in Shandong
Jinfeng Hongyuan Flagship Store	Electricity conservation2	Have not yet used, first evaluation, later really good do not need to comment again.	Jingdong prefecture level city in Shandong
Jinfeng Hongyuan Flagship Store	Electricity conservation2	Works great. Pretty much as described by the merchant.	Jingdong prefecture level city in Shandong
Jinfeng Hongyuan Flagship Store	Electricity conservation2	Received it, looking forward to the results, hope it works	Jingdong prefecture level city in Shandong
Jinfeng Hongyuan Flagship Store	Electricity conservation2	Received it, looking forward to the results, hope it works	Jingdong prefecture level city in Shandong
Jinfeng Hongyuan Flagship Store	Electricity conservation2	Supposedly it's an IQ tax. Buy one and try it. Looks good. Well made.	Jingdong prefecture level city in Shandong
Jinfeng Hongyuan Flagship Store	Electricity conservation2	It just doesn't feel like it's working.	Jingdong prefecture level city in Shandong
Jinfeng Hongyuan Flagship Store	Electricity conservation2	This is a great product.	Jingdong prefecture level city in Shandong
Jinfeng Hongyuan Flagship Store	Electricity conservation2	I'm just using it. I'll tell you more.	Jingdong prefecture level city in Shandong
Jinfeng Hongyuan Flagship Store	Electricity conservation2	Second repurchase and it's working fine.	Jingdong prefecture level city in Shandong
Jinfeng Hongyuan Flagship Store	Electricity conservation2	I'll wait a few days before evaluating	Jingdong prefecture level city in Shandong
Jinfeng Hongyuan Flagship Store	Electricity conservation2	I'll comment after I use it hopefully the section will be useful.	Jingdong prefecture level city in Shandong
Jinfeng Hongyuan Flagship Store	Electricity conservation2	not very useful	Jingdong prefecture level city in Shandong
Jinfeng Hongyuan Flagship Store	Electricity conservation2	Not received	Jingdong prefecture level city in Shandong
Jinfeng Hongyuan Flagship Store	Electricity conservation2	rare	Jingdong prefecture level city in Shandong
Jinfeng Hongyuan Flagship Store	Electricity conservation2	It's good.	Jingdong prefecture level city in Shandong
Jinfeng Hongyuan Flagship Store	Electricity conservation2	just now	Jingdong prefecture level city in Shandong
Jinfeng Hongyuan Flagship Store	Electricity conservation2	serviceable	Jingdong prefecture level city in Shandong
Jinfeng Hongyuan Flagship Store	Electricity conservation2	desirable	Jingdong prefecture level city in Shandong
Jinfeng Hongyuan Flagship Store	Electricity conservation2	serviceable	Jingdong prefecture level city in Shandong
Jinfeng Hongyuan Flagship Store	Electricity conservation2	It doesn't save power and costs electricity.	Jingdong prefecture level city in Shandong
Jinfeng Hongyuan Flagship Store	Electricity conservation2	Not as good as I thought.	Jingdong prefecture level city in Shandong
Jinfeng Hongyuan Flagship Store	Electricity conservation2	Super bad review, it doesn't save power at all, but rather more power consumption	Jingdong prefecture level city in Shandong
Jinfeng Hongyuan Flagship Store	Electricity conservation2	Listen to others say that this can save electricity, I bought with the idea of trying, used for a period of time before coming to the evaluation, will be a little bit of use, not a lot of savings just, long-term use of the words can save more.	Jingdong prefecture level city in Shandong
Jinfeng Hongyuan Flagship Store	Electricity conservation2	Logistics is fast, the next day arrived, workmanship is very delicate a power saver, looking forward to the late effect, customer service patiently explain the recommended which is suitable for, installed in what position is the best, look forward to using the effect after a period of time!	Jingdong prefecture level city in Shandong
Jinfeng Hongyuan Flagship Store	Electricity conservation2	It's good to use, I feel that it has been saving electricity, anyway, not a loss is, I used to feel that the power consumption is big, now I feel much better, my friends are also using, I hope to use a long time will be able to, I wish the boss a prosperous business	Jingdong prefecture level city in Shandong
Jinfeng Hongyuan Flagship Store	Electricity conservation2	Should be very good, to be able to plan the circuit, save the home of the circuit to the extent of the savings, waste of money on electricity, good if a month this money back, hope always hope to be able to	Jingdong prefecture level city in Shandong
Jinfeng Hongyuan Flagship Store	Electricity conservation2	Express delivery is very strong, the goods and I saw in the business consistent, the use of the effect of follow-up evaluation	Jingdong prefecture level city in Shandong
Jinfeng Hongyuan Flagship Store	Electricity conservation2	I have been using this brand of power saver for many years and this time I saw this upgraded version and got it again. The main purpose of this product is to reduce the loss of electricity for a long time, so as to achieve the purpose of saving electricity.	Jingdong prefecture level city in Shandong
Jinfeng Hongyuan Flagship Store	Electricity conservation2	Logistics is fast and the product looks beautiful, looking forward to the effect.	Jingdong prefecture level city in Shandong
Jinfeng Hongyuan Flagship Store	Electricity conservation2	Product quality is good, energy saving a little effect, but the price is a little expensive, fast arrival, good service.	Jingdong prefecture level city in Shandong
Jinfeng Hongyuan Flagship Store	Electricity conservation2	The quality is very good, inexpensive, Jingdong self-supporting guaranteed, worth having!	Jingdong prefecture level city in Shandong
Jinfeng Hongyuan Flagship Store	Electricity conservation2	I don't know if it's saving electricity yet, I'll find out next time I pay my electric bill	Jingdong prefecture level city in Shandong
Jinfeng Hongyuan Flagship Store	Electricity conservation2	Favorable price quality assurance appearance design is beautiful and generous, fine workmanship	Jingdong prefecture level city in Shandong
Jinfeng Hongyuan Flagship Store	Electricity conservation2	I bought it, I don't know how effective it is? I don't know how the quality is? After this summer results will know! Looking forward to it!	Jingdong prefecture level city in Shandong
Jinfeng Hongyuan Flagship Store	Electricity conservation2	Goods received Packaging intact, look at the shell workmanship is also good, high-power appliances open voltage has obvious changes in the effectiveness of the	Jingdong prefecture level city in Shandong
Jinfeng Hongyuan Flagship Store	Electricity conservation2	The goods have been received logistics is very fast, the product is similar to the line, just wait to use a month to see if it can save electricity, stay tuned.	Jingdong prefecture level city in Shandong
Jinfeng Hongyuan Flagship Store	Electricity conservation2	Electricity is plugged in, not sure if it will work, hopefully it will work.	Jingdong prefecture level city in Shandong
Jinfeng Hongyuan Flagship Store	Electricity conservation2	The shell is aluminum alloy, very good workmanship, the actual effect of waiting for the addition of evaluation	Jingdong prefecture level city in Shandong
Jinfeng Hongyuan Flagship Store	Electricity conservation2	Received, already plugged in, looking forward to the effect.	Jingdong prefecture level city in Shandong
Jinfeng Hongyuan Flagship Store	Electricity conservation2	Opened to see the regular manufacturer products, wait for a period of time to see it	Jingdong prefecture level city in Shandong

Jinfeng Hongyuan Flagship Store	Electricity conservation2	🉑 Baby received, very surprised ✨, reasonable design, fashionable and generous, continue to pay attention to the future will be repurchased ❤❤❤❤	Jingdong prefecture level city in Shandong
Jinfeng Hongyuan Flagship Store	Electricity conservation2	Baby received it 👌, really great ah! 🌈The quality can't be beat, very satisfied! 🆗Happy happy happy 😃	Jingdong prefecture level city in Shandong
Jinfeng Hongyuan Flagship Store	Electricity conservation2	Very good, power saving see, this is to help a friend to buy, I used to give my friend a say, my friend immediately let me order another one!	Jingdong prefecture level city in Shandong
Jinfeng Hongyuan Flagship Store	Electricity conservation2	The baby is just as introduced, the texture is very good, powerful, and it feels good to use it	Jingdong prefecture level city in Shandong
Jinfeng Hongyuan Flagship Store	Electricity conservation2	Value for money, very satisfied, seller service attitude is very good, very patient	Jingdong prefecture level city in Shandong
Jinfeng Hongyuan Flagship Store	Electricity conservation2	Power saving treasure design unique exquisite beautiful, voltage display clearly, plugged in that is good to use, I observed the meter, after using the meter is gone slow.	Jingdong prefecture level city in Shandong
Jinfeng Hongyuan Flagship Store	Electricity conservation2	Received on the use of the packaging is beautifully used for a period of time and then come back in the follow-up review!	Jingdong prefecture level city in Shandong
Jinfeng Hongyuan Flagship Store	Electricity conservation2	It should work. Not bad. Not bad. Not bad.	Jingdong prefecture level city in Shandong
Jinfeng Hongyuan Flagship Store	Electricity conservation2	I haven't used it yet, I'll plug it in and see if it saves power next month.	Jingdong prefecture level city in Shandong
Jinfeng Hongyuan Flagship Store	Electricity conservation2	The goods received and unpacked and used, the quality is very good, things and the official website to buy no difference!	Jingdong prefecture level city in Shandong
Jinfeng Hongyuan Flagship Store	Electricity conservation2	It's a rip off, doesn't help much at all, uses the same amount of power as before it was used.	Jingdong prefecture level city in Shandong
Jinfeng Hongyuan Flagship Store	Electricity conservation2	I've been using it for a while now and haven't noticed any unusual problems, so it's okay	Jingdong prefecture level city in Shandong
Jinfeng Hongyuan Flagship Store	Electricity conservation2	Received the goods and plugged in to use, good or not there is no use will have to wait until next month to find out	Jingdong prefecture level city in Shandong
Jinfeng Hongyuan Flagship Store	Electricity conservation2	It's very compact, it looks good, I'll introduce you to my clients when it's ready.	Jingdong prefecture level city in Shandong
Jinfeng Hongyuan Flagship Store	Electricity conservation2	Good workmanship, we'll have to see in a few months.	Jingdong prefecture level city in Shandong
Jinfeng Hongyuan Flagship Store	Electricity conservation2	Jingdong quality! Always trustworthy!	Jingdong prefecture level city in Shandong
Jinfeng Hongyuan Flagship Store	Electricity conservation2	This is the second time I've bought it, and the merchant said to give me five dollars out of the difference, so thank you merchant.	Jingdong prefecture level city in Shandong
Jinfeng Hongyuan Flagship Store	Electricity conservation2	Good quality and good service	Jingdong prefecture level city in Shandong
Jinfeng Hongyuan Flagship Store	Electricity conservation2	Feels good texture, aluminum alloy shell, voltage digital display clear.	Jingdong prefecture level city in Shandong
Jinfeng Hongyuan Flagship Store	Electricity conservation2	Good product, fast and great service, thanks!	Jingdong prefecture level city in Shandong
Jinfeng Hongyuan Flagship Store	Electricity conservation2	Have not yet used, first evaluation, later really good do not need to comment again.	Jingdong prefecture level city in Shandong
Jinfeng Hongyuan Flagship Store	Electricity conservation2	Works great. Pretty much as described by the merchant.	Jingdong prefecture level city in Shandong
Jinfeng Hongyuan Flagship Store	Electricity conservation2	Received it, looking forward to the results, hope it works	Jingdong prefecture level city in Shandong
Jinfeng Hongyuan Flagship Store	Electricity conservation2	Received it, looking forward to the results, hope it works	Jingdong prefecture level city in Shandong
Jinfeng Hongyuan Flagship Store	Electricity conservation2	Supposedly it's an IQ tax. Buy one and try it. Looks good. Well made.	Jingdong prefecture level city in Shandong
Jinfeng Hongyuan Flagship Store	Electricity conservation2	It just doesn't feel like it's working.	Jingdong prefecture level city in Shandong
Jinfeng Hongyuan Flagship Store	Electricity conservation2	This is a great product.	Jingdong prefecture level city in Shandong
Jinfeng Hongyuan Flagship Store	Electricity conservation2	I'm just using it. I'll tell you more.	Jingdong prefecture level city in Shandong
Jinfeng Hongyuan Flagship Store	Electricity conservation2	Second repurchase and it's working fine.	Jingdong prefecture level city in Shandong
Jinfeng Hongyuan Flagship Store	Electricity conservation2	I'll wait a few days before evaluating	Jingdong prefecture level city in Shandong
Jinfeng Hongyuan Flagship Store	Electricity conservation2	I'll comment after I use it hopefully the section will be useful.	Jingdong prefecture level city in Shandong
Jinfeng Hongyuan Flagship Store	Electricity conservation2	not very useful	Jingdong prefecture level city in Shandong
Jinfeng Hongyuan Flagship Store	Electricity conservation2	Not received	Jingdong prefecture level city in Shandong
Jinfeng Hongyuan Flagship Store	Electricity conservation2	rare	Jingdong prefecture level city in Shandong
Jinfeng Hongyuan Flagship Store	Electricity conservation2	It's good.	Jingdong prefecture level city in Shandong
Jinfeng Hongyuan Flagship Store	Electricity conservation2	just now	Jingdong prefecture level city in Shandong
Jinfeng Hongyuan Flagship Store	Electricity conservation2	serviceable	Jingdong prefecture level city in Shandong
Jinfeng Hongyuan Flagship Store	Electricity conservation2	desirable	Jingdong prefecture level city in Shandong
Jinfeng Hongyuan Flagship Store	Electricity conservation2	serviceable	Jingdong prefecture level city in Shandong
Jinfeng Hongyuan Flagship Store	Electricity conservation2	It doesn't save power and costs electricity.	Jingdong prefecture level city in Shandong
Jinfeng Hongyuan Flagship Store	Electricity conservation2	Not as good as I thought.	Jingdong prefecture level city in Shandong
Jinfeng Hongyuan Flagship Store	Electricity conservation2	Super bad review, it doesn't save power at all, but rather more power consumption	Jingdong prefecture level city in Shandong
Jinfeng Hongyuan Flagship Store	Electricity conservation2	Listen to others say that this can save electricity, I bought with the idea of trying, used for a period of time before coming to the evaluation, will be a little bit of use, not a lot of savings just, long-term use of the words can save more.	Jingdong prefecture level city in Shandong
Jinfeng Hongyuan Flagship Store	Electricity conservation2	Logistics is fast, the next day arrived, workmanship is very delicate a power saver, looking forward to the late effect, customer service patiently explain the recommended which is suitable for, installed in what position is the best, look forward to using the effect after a period of time!	Jingdong prefecture level city in Shandong
Jinfeng Hongyuan Flagship Store	Electricity conservation2	It's good to use, I feel that it has been saving electricity, anyway, not a loss is, I used to feel that the power consumption is big, now I feel much better, my friends are also using, I hope to use a long time will be able to, I wish the boss a prosperous business	Jingdong prefecture level city in Shandong
Jinfeng Hongyuan Flagship Store	Electricity conservation2	Should be very good, to be able to plan the circuit, save the home of the circuit to the extent of the savings, waste of money on electricity, good if a month this money back, hope always hope to be able to	Jingdong prefecture level city in Shandong
Jinfeng Hongyuan Flagship Store	Electricity conservation2	Express delivery is very strong, the goods and I saw in the business consistent, the use of the effect of follow-up evaluation	Jingdong prefecture level city in Shandong
Jinfeng Hongyuan Flagship Store	Electricity conservation2	I have been using this brand of power saver for many years and this time I saw this upgraded version and got it again. The main purpose of this product is to reduce the loss of electricity for a long time, so as to achieve the purpose of saving electricity.	Jingdong prefecture level city in Shandong
Jinfeng Hongyuan Flagship Store	Electricity conservation2	Logistics is fast and the product looks beautiful, looking forward to the effect.	Jingdong prefecture level city in Shandong

Jinfeng Hongyuan Flagship Store	Electricity conservation2	Product quality is good, energy saving a little effect, but the price is a little expensive, fast arrival, good service.	Jingdong prefecture level city in Shandong
Jinfeng Hongyuan Flagship Store	Electricity conservation2	The quality is very good, inexpensive, Jingdong self-supporting guaranteed, worth having!	Jingdong prefecture level city in Shandong
Jinfeng Hongyuan Flagship Store	Electricity conservation2	I don't know if it's saving electricity yet, I'll find out next time I pay my electric bill	Jingdong prefecture level city in Shandong
Jinfeng Hongyuan Flagship Store	Electricity conservation2	Favorable price quality assurance appearance design is beautiful and generous, fine workmanship	Jingdong prefecture level city in Shandong
Jinfeng Hongyuan Flagship Store	Electricity conservation2	I bought it, I don't know how effective it is? I don't know how the quality is? After this summer results will know! Looking forward to it!	Jingdong prefecture level city in Shandong
Jinfeng Hongyuan Flagship Store	Electricity conservation2	Goods received Packaging intact, look at the shell workmanship is also good, high-power appliances open voltage has obvious changes in the effectiveness of the	Jingdong prefecture level city in Shandong
Jinfeng Hongyuan Flagship Store	Electricity conservation2	The goods have been received logistics is very fast, the product is similar to the line, just wait to use a month to see if it can save electricity, stay tuned.	Jingdong prefecture level city in Shandong
Jinfeng Hongyuan Flagship Store	Electricity conservation2	Electricity is plugged in, not sure if it will work, hopefully it will work.	Jingdong prefecture level city in Shandong
Jinfeng Hongyuan Flagship Store	Electricity conservation2	The shell is aluminum alloy, very good workmanship, the actual effect of waiting for the addition of evaluation	Jingdong prefecture level city in Shandong
Jinfeng Hongyuan Flagship Store	Electricity conservation2	Received, already plugged in, looking forward to the effect.	Jingdong prefecture level city in Shandong
Jinfeng Hongyuan Flagship Store	Electricity conservation2	Opened to see the regular manufacturer products, wait for a period of time to see it	Jingdong prefecture level city in Shandong
Jinfeng Hongyuan Flagship Store	Electricity conservation2	🉑 Baby received, very surprised ✨, reasonable design, fashionable and generous, continue to pay attention to the future will be repurchased ❤❤❤❤	Jingdong prefecture level city in Shandong
Jinfeng Hongyuan Flagship Store	Electricity conservation2	Baby received it 👌, really great ah! 🌈The quality can't be beat, very satisfied! 🆗Happy happy happy 😃	Jingdong prefecture level city in Shandong
Jinfeng Hongyuan Flagship Store	Electricity conservation2	Very good, power saving see, this is to help a friend to buy, I used to give my friend a say, my friend immediately let me order another one!	Jingdong prefecture level city in Shandong
Jinfeng Hongyuan Flagship Store	Electricity conservation2	The baby is just as introduced, the texture is very good, powerful, and it feels good to use it	Jingdong prefecture level city in Shandong
Jinfeng Hongyuan Flagship Store	Electricity conservation2	Value for money, very satisfied, seller service attitude is very good, very patient	Jingdong prefecture level city in Shandong
Jinfeng Hongyuan Flagship Store	Electricity conservation2	Power saving treasure design unique exquisite beautiful, voltage display clearly, plugged in that is good to use, I observed the meter, after using the meter is gone slow.	Jingdong prefecture level city in Shandong
Jinfeng Hongyuan Flagship Store	Electricity conservation2	Received on the use of the packaging is beautifully used for a period of time and then come back in the follow-up review!	Jingdong prefecture level city in Shandong
Jinfeng Hongyuan Flagship Store	Electricity conservation2	It should work. Not bad. Not bad. Not bad.	Jingdong prefecture level city in Shandong
Jinfeng Hongyuan Flagship Store	Electricity conservation2	I haven't used it yet, I'll plug it in and see if it saves power next month.	Jingdong prefecture level city in Shandong
Jinfeng Hongyuan Flagship Store	Electricity conservation2	The goods received and unpacked and used, the quality is very good, things and the official website to buy no difference!	Jingdong prefecture level city in Shandong
Jinfeng Hongyuan Flagship Store	Electricity conservation2	It's a rip off, doesn't help much at all, uses the same amount of power as before it was used.	Jingdong prefecture level city in Shandong
Jinfeng Hongyuan Flagship Store	Electricity conservation2	I've been using it for a while now and haven't noticed any unusual problems, so it's okay	Jingdong prefecture level city in Shandong
Jinfeng Hongyuan Flagship Store	Electricity conservation2	Received the goods and plugged in to use, good or not there is no use will have to wait until next month to find out	Jingdong prefecture level city in Shandong
Jinfeng Hongyuan Flagship Store	Electricity conservation2	It's very compact, it looks good, I'll introduce you to my clients when it's ready.	Jingdong prefecture level city in Shandong
Jinfeng Hongyuan Flagship Store	Electricity conservation2	Good workmanship, we'll have to see in a few months.	Jingdong prefecture level city in Shandong
Jinfeng Hongyuan Flagship Store	Electricity conservation2	Jingdong quality! Always trustworthy!	Jingdong prefecture level city in Shandong
Jinfeng Hongyuan Flagship Store	Electricity conservation2	This is the second time I've bought it, and the merchant said to give me five dollars out of the difference, so thank you merchant.	Jingdong prefecture level city in Shandong
Jinfeng Hongyuan Flagship Store	Electricity conservation2	Good quality and good service	Jingdong prefecture level city in Shandong
Jinfeng Hongyuan Flagship Store	Electricity conservation2	Feels good texture, aluminum alloy shell, voltage digital display clear.	Jingdong prefecture level city in Shandong
Jinfeng Hongyuan Flagship Store	Electricity conservation2	Good product, fast and great service, thanks!	Jingdong prefecture level city in Shandong
Jinfeng Hongyuan Flagship Store	Electricity conservation2	Have not yet used, first evaluation, later really good do not need to comment again.	Jingdong prefecture level city in Shandong
Jinfeng Hongyuan Flagship Store	Electricity conservation2	Works great. Pretty much as described by the merchant.	Jingdong prefecture level city in Shandong
Jinfeng Hongyuan Flagship Store	Electricity conservation2	Received it, looking forward to the results, hope it works	Jingdong prefecture level city in Shandong
Jinfeng Hongyuan Flagship Store	Electricity conservation2	Received it, looking forward to the results, hope it works	Jingdong prefecture level city in Shandong
Jinfeng Hongyuan Flagship Store	Electricity conservation2	Supposedly it's an IQ tax. Buy one and try it. Looks good. Well made.	Jingdong prefecture level city in Shandong
Jinfeng Hongyuan Flagship Store	Electricity conservation2	It just doesn't feel like it's working.	Jingdong prefecture level city in Shandong
Jinfeng Hongyuan Flagship Store	Electricity conservation2	This is a great product.	Jingdong prefecture level city in Shandong
Jinfeng Hongyuan Flagship Store	Electricity conservation2	I'm just using it. I'll tell you more.	Jingdong prefecture level city in Shandong
Jinfeng Hongyuan Flagship Store	Electricity conservation2	Second repurchase and it's working fine.	Jingdong prefecture level city in Shandong
Jinfeng Hongyuan Flagship Store	Electricity conservation2	I'll wait a few days before evaluating	Jingdong prefecture level city in Shandong
Jinfeng Hongyuan Flagship Store	Electricity conservation2	I'll comment after I use it hopefully the section will be useful.	Jingdong prefecture level city in Shandong
Jinfeng Hongyuan Flagship Store	Electricity conservation2	not very useful	Jingdong prefecture level city in Shandong
Jinfeng Hongyuan Flagship Store	Electricity conservation2	Not received	Jingdong prefecture level city in Shandong
Jinfeng Hongyuan Flagship Store	Electricity conservation2	rare	Jingdong prefecture level city in Shandong
Jinfeng Hongyuan Flagship Store	Electricity conservation2	It's good.	Jingdong prefecture level city in Shandong
Jinfeng Hongyuan Flagship Store	Electricity conservation2	just now	Jingdong prefecture level city in Shandong
Jinfeng Hongyuan Flagship Store	Electricity conservation2	serviceable	Jingdong prefecture level city in Shandong
Jinfeng Hongyuan Flagship Store	Electricity conservation2	desirable	Jingdong prefecture level city in Shandong
Jinfeng Hongyuan Flagship Store	Electricity conservation2	serviceable	Jingdong prefecture level city in Shandong
Jinfeng Hongyuan Flagship Store	Electricity conservation2	It doesn't save power and costs electricity.	Jingdong prefecture level city in Shandong

Jinfeng Hongyuan Flagship Store	Electricity conservation2	Not as good as I thought.	Jingdong prefecture level city in Shandong
Jinfeng Hongyuan Flagship Store	Electricity conservation2	Super bad review, it doesn't save power at all, but rather more power consumption	Jingdong prefecture level city in Shandong
Jinfeng Hongyuan Flagship Store	Electricity conservation2	Listen to others say that this can save electricity, I bought with the idea of trying, used for a period of time before coming to the evaluation, will be a little bit of use, not a lot of savings just, long-term use of the words can save more.	Jingdong prefecture level city in Shandong
Jinfeng Hongyuan Flagship Store	Electricity conservation2	Logistics is fast, the next day arrived, workmanship is very delicate a power saver, looking forward to the late effect, customer service patiently explain the recommended which is suitable for, installed in what position is the best, look forward to using the effect after a period of time!	Jingdong prefecture level city in Shandong
Jinfeng Hongyuan Flagship Store	Electricity conservation2	It's good to use, I feel that it has been saving electricity, anyway, not a loss is, I used to feel that the power consumption is big, now I feel much better, my friends are also using, I hope to use a long time will be able to, I wish the boss a prosperous business	Jingdong prefecture level city in Shandong
Jinfeng Hongyuan Flagship Store	Electricity conservation2	Should be very good, to be able to plan the circuit, save the home of the circuit to the extent of the savings, waste of money on electricity, good if a month this money back, hope always hope to be able to	Jingdong prefecture level city in Shandong
Jinfeng Hongyuan Flagship Store	Electricity conservation2	Express delivery is very strong, the goods and I saw in the business consistent, the use of the effect of follow-up evaluation	Jingdong prefecture level city in Shandong
Jinfeng Hongyuan Flagship Store	Electricity conservation2	I have been using this brand of power saver for many years and this time I saw this upgraded version and got it again. The main purpose of this product is to reduce the loss of electricity for a long time, so as to achieve the purpose of saving electricity.	Jingdong prefecture level city in Shandong
Jinfeng Hongyuan Flagship Store	Electricity conservation2	Logistics is fast and the product looks beautiful, looking forward to the effect.	Jingdong prefecture level city in Shandong
Jinfeng Hongyuan Flagship Store	Electricity conservation2	Product quality is good, energy saving a little effect, but the price is a little expensive, fast arrival, good service.	Jingdong prefecture level city in Shandong
Jinfeng Hongyuan Flagship Store	Electricity conservation2	The quality is very good, inexpensive, Jingdong self-supporting guaranteed, worth having!	Jingdong prefecture level city in Shandong
Jinfeng Hongyuan Flagship Store	Electricity conservation2	I don't know if it's saving electricity yet, I'll find out next time I pay my electric bill	Jingdong prefecture level city in Shandong
Jinfeng Hongyuan Flagship Store	Electricity conservation2	Favorable price quality assurance appearance design is beautiful and generous, fine workmanship	Jingdong prefecture level city in Shandong
Jinfeng Hongyuan Flagship Store	Electricity conservation2	I bought it, I don't know how effective it is? I don't know how the quality is? After this summer results will know! Looking forward to it!	Jingdong prefecture level city in Shandong
Jinfeng Hongyuan Flagship Store	Electricity conservation2	Goods received Packaging intact, look at the shell workmanship is also good, high-power appliances open voltage has obvious changes in the effectiveness of the	Jingdong prefecture level city in Shandong
Jinfeng Hongyuan Flagship Store	Electricity conservation2	The goods have been received logistics is very fast, the product is similar to the line, just wait to use a month to see if it can save electricity, stay tuned.	Jingdong prefecture level city in Shandong
Jinfeng Hongyuan Flagship Store	Electricity conservation2	Electricity is plugged in, not sure if it will work, hopefully it will work.	Jingdong prefecture level city in Shandong
Jinfeng Hongyuan Flagship Store	Electricity conservation2	The shell is aluminum alloy, very good workmanship, the actual effect of waiting for the addition of evaluation	Jingdong prefecture level city in Shandong
Jinfeng Hongyuan Flagship Store	Electricity conservation2	Received, already plugged in, looking forward to the effect.	Jingdong prefecture level city in Shandong
Jinfeng Hongyuan Flagship Store	Electricity conservation2	Opened to see the regular manufacturer products, wait for a period of time to see it	Jingdong prefecture level city in Shandong
Jinfeng Hongyuan Flagship Store	Electricity conservation2	🉑 Baby received, very surprised ✨, reasonable design, fashionable and generous, continue to pay attention to the future will be repurchased ❥❥❥❥	Jingdong prefecture level city in Shandong
Jinfeng Hongyuan Flagship Store	Electricity conservation2	Baby received it 👌, really great ah! 🌈The quality can't be beat, very satisfied! 🆗Happy happy happy 😃	Jingdong prefecture level city in Shandong
Jinfeng Hongyuan Flagship Store	Electricity conservation2	Very good, power saving see, this is to help a friend to buy, I used to give my friend a say, my friend immediately let me order another one!	Jingdong prefecture level city in Shandong
Jinfeng Hongyuan Flagship Store	Electricity conservation2	The baby is just as introduced, the texture is very good, powerful, and it feels good to use it	Jingdong prefecture level city in Shandong
Jinfeng Hongyuan Flagship Store	Electricity conservation2	Value for money, very satisfied, seller service attitude is very good, very patient	Jingdong prefecture level city in Shandong
Jinfeng Hongyuan Flagship Store	Electricity conservation2	Power saving treasure design unique exquisite beautiful, voltage display clearly, plugged in that is good to use, I observed the meter, after using the meter is gone slow.	Jingdong prefecture level city in Shandong
Jinfeng Hongyuan Flagship Store	Electricity conservation2	Received on the use of the packaging is beautifully used for a period of time and then come back in the follow-up review!	Jingdong prefecture level city in Shandong
Jinfeng Hongyuan Flagship Store	Electricity conservation2	It should work. Not bad. Not bad. Not bad.	Jingdong prefecture level city in Shandong
Jinfeng Hongyuan Flagship Store	Electricity conservation2	I haven't used it yet, I'll plug it in and see if it saves power next month.	Jingdong prefecture level city in Shandong
Jinfeng Hongyuan Flagship Store	Electricity conservation2	The goods received and unpacked and used, the quality is very good, things and the official website to buy no difference!	Jingdong prefecture level city in Shandong
Jinfeng Hongyuan Flagship Store	Electricity conservation2	It's a rip off, doesn't help much at all, uses the same amount of power as before it was used.	Jingdong prefecture level city in Shandong
Jinfeng Hongyuan Flagship Store	Electricity conservation2	I've been using it for a while now and haven't noticed any unusual problems, so it's okay	Jingdong prefecture level city in Shandong
Jinfeng Hongyuan Flagship Store	Electricity conservation2	Received the goods and plugged in to use, good or not there is no use will have to wait until next month to find out	Jingdong prefecture level city in Shandong
Jinfeng Hongyuan Flagship Store	Electricity conservation2	It's very compact, it looks good, I'll introduce you to my clients when it's ready.	Jingdong prefecture level city in Shandong
Jinfeng Hongyuan Flagship Store	Electricity conservation2	Good workmanship, we'll have to see in a few months.	Jingdong prefecture level city in Shandong
Jinfeng Hongyuan Flagship Store	Electricity conservation2	Jingdong quality! Always trustworthy!	Jingdong prefecture level city in Shandong
Jinfeng Hongyuan Flagship Store	Electricity conservation2	This is the second time I've bought it, and the merchant said to give me five dollars out of the difference, so thank you merchant.	Jingdong prefecture level city in Shandong
Jinfeng Hongyuan Flagship Store	Electricity conservation2	Good quality and good service	Jingdong prefecture level city in Shandong
Jinfeng Hongyuan Flagship Store	Electricity conservation2	Feels good texture, aluminum alloy shell, voltage digital display clear.	Jingdong prefecture level city in Shandong
Jinfeng Hongyuan Flagship Store	Electricity conservation2	Good product, fast and great service, thanks!	Jingdong prefecture level city in Shandong
Jinfeng Hongyuan Flagship Store	Electricity conservation2	Have not yet used, first evaluation, later really good do not need to comment again.	Jingdong prefecture level city in Shandong
Jinfeng Hongyuan Flagship Store	Electricity conservation2	Works great. Pretty much as described by the merchant.	Jingdong prefecture level city in Shandong
Jinfeng Hongyuan Flagship Store	Electricity conservation2	Received it, looking forward to the results, hope it works	Jingdong prefecture level city in Shandong
Jinfeng Hongyuan Flagship Store	Electricity conservation2	Received it, looking forward to the results, hope it works	Jingdong prefecture level city in Shandong
Jinfeng Hongyuan Flagship Store	Electricity conservation2	Supposedly it's an IQ tax. Buy one and try it. Looks good. Well made.	Jingdong prefecture level city in Shandong
Jinfeng Hongyuan Flagship Store	Electricity conservation2	It just doesn't feel like it's working.	Jingdong prefecture level city in Shandong
Jinfeng Hongyuan Flagship Store	Electricity conservation2	This is a great product.	Jingdong prefecture level city in Shandong
Jinfeng Hongyuan Flagship Store	Electricity conservation2	I'm just using it. I'll tell you more.	Jingdong prefecture level city in Shandong
Jinfeng Hongyuan Flagship Store	Electricity conservation2	Second repurchase and it's working fine.	Jingdong prefecture level city in Shandong

Jinfeng Hongyuan Flagship Store	Electricity conservation2	I'll wait a few days before evaluating	Jingdong prefecture level city in Shandong
Jinfeng Hongyuan Flagship Store	Electricity conservation2	I'll comment after I use it hopefully the section will be useful.	Jingdong prefecture level city in Shandong
Jinfeng Hongyuan Flagship Store	Electricity conservation2	not very useful	Jingdong prefecture level city in Shandong
Jinfeng Hongyuan Flagship Store	Electricity conservation2	Not received	Jingdong prefecture level city in Shandong
Jinfeng Hongyuan Flagship Store	Electricity conservation2	rare	Jingdong prefecture level city in Shandong
Jinfeng Hongyuan Flagship Store	Electricity conservation2	It's good.	Jingdong prefecture level city in Shandong
Jinfeng Hongyuan Flagship Store	Electricity conservation2	just now	Jingdong prefecture level city in Shandong
Jinfeng Hongyuan Flagship Store	Electricity conservation2	serviceable	Jingdong prefecture level city in Shandong
Jinfeng Hongyuan Flagship Store	Electricity conservation2	desirable	Jingdong prefecture level city in Shandong
Jinfeng Hongyuan Flagship Store	Electricity conservation2	serviceable	Jingdong prefecture level city in Shandong
Jinfeng Hongyuan Flagship Store	Electricity conservation2	It doesn't save power and costs electricity.	Jingdong prefecture level city in Shandong
Jinfeng Hongyuan Flagship Store	Electricity conservation2	Not as good as I thought.	Jingdong prefecture level city in Shandong
Jinfeng Hongyuan Flagship Store	Electricity conservation2	Super bad review, it doesn't save power at all, but rather more power consumption	Jingdong prefecture level city in Shandong
Jinfeng Hongyuan Flagship Store	Electricity conservation2	Listen to others say that this can save electricity, I bought with the idea of trying, used for a period of time before coming to the evaluation, will be a little bit of use, not a lot of savings just, long-term use of the words can save more.	Jingdong prefecture level city in Shandong
Jinfeng Hongyuan Flagship Store	Electricity conservation2	Logistics is fast, the next day arrived, workmanship is very delicate a power saver, looking forward to the late effect, customer service patiently explain the recommended which is suitable for, installed in what position is the best, look forward to using the effect after a period of time!	Jingdong prefecture level city in Shandong
Jinfeng Hongyuan Flagship Store	Electricity conservation2	It's good to use, I feel that it has been saving electricity, anyway, not a loss is, I used to feel that the power consumption is big, now I feel much better, my friends are also using, I hope to use a long time will be able to, I wish the boss a prosperous business	Jingdong prefecture level city in Shandong
Jinfeng Hongyuan Flagship Store	Electricity conservation2	Should be very good, to be able to plan the circuit, save the home of the circuit to the extent of the savings, waste of money on electricity, good if a month this money back, hope always hope to be able to	Jingdong prefecture level city in Shandong
Jinfeng Hongyuan Flagship Store	Electricity conservation2	Express delivery is very strong, the goods and I saw in the business consistent, the use of the effect of follow-up evaluation	Jingdong prefecture level city in Shandong
Jinfeng Hongyuan Flagship Store	Electricity conservation2	I have been using this brand of power saver for many years and this time I saw this upgraded version and got it again. The main purpose of this product is to reduce the loss of electricity for a long time, so as to achieve the purpose of saving electricity.	Jingdong prefecture level city in Shandong
Jinfeng Hongyuan Flagship Store	Electricity conservation2	Logistics is fast and the product looks beautiful, looking forward to the effect.	Jingdong prefecture level city in Shandong
Jinfeng Hongyuan Flagship Store	Electricity conservation2	Product quality is good, energy saving a little effect, but the price is a little expensive, fast arrival, good service.	Jingdong prefecture level city in Shandong
Jinfeng Hongyuan Flagship Store	Electricity conservation2	The quality is very good, inexpensive, Jingdong self-supporting guaranteed, worth having!	Jingdong prefecture level city in Shandong
Jinfeng Hongyuan Flagship Store	Electricity conservation2	I don't know if it's saving electricity yet, I'll find out next time I pay my electric bill	Jingdong prefecture level city in Shandong
Jinfeng Hongyuan Flagship Store	Electricity conservation2	Favorable price quality assurance appearance design is beautiful and generous, fine workmanship	Jingdong prefecture level city in Shandong
Jinfeng Hongyuan Flagship Store	Electricity conservation2	I bought it, I don't know how effective it is? I don't know how the quality is? After this summer results will know! Looking forward to it!	Jingdong prefecture level city in Shandong
Jinfeng Hongyuan Flagship Store	Electricity conservation2	Goods received Packaging intact, look at the shell workmanship is also good, high-power appliances open voltage has obvious changes in the effectiveness of the	Jingdong prefecture level city in Shandong
Jinfeng Hongyuan Flagship Store	Electricity conservation2	The goods have been received logistics is very fast, the product is similar to the line, just wait to use a month to see if it can save electricity, stay tuned.	Jingdong prefecture level city in Shandong
Jinfeng Hongyuan Flagship Store	Electricity conservation2	Electricity is plugged in, not sure if it will work, hopefully it will work.	Jingdong prefecture level city in Shandong
Jinfeng Hongyuan Flagship Store	Electricity conservation2	The shell is aluminum alloy, very good workmanship, the actual effect of waiting for the addition of evaluation	Jingdong prefecture level city in Shandong
Jinfeng Hongyuan Flagship Store	Electricity conservation2	Received, already plugged in, looking forward to the effect.	Jingdong prefecture level city in Shandong
Jinfeng Hongyuan Flagship Store	Electricity conservation2	Opened to see the regular manufacturer products, wait for a period of time to see it	Jingdong prefecture level city in Shandong
Jinfeng Hongyuan Flagship Store	Electricity conservation2	🉑 Baby received, very surprised ✨, reasonable design, fashionable and generous, continue to pay attention to the future will be repurchased ❥❥❥❥	Jingdong prefecture level city in Shandong
Jinfeng Hongyuan Flagship Store	Electricity conservation2	Baby received it 👌, really great ah! 🌈The quality can't be beat, very satisfied! 🆗Happy happy happy 😃	Jingdong prefecture level city in Shandong
Jinfeng Hongyuan Flagship Store	Electricity conservation2	Very good, power saving see, this is to help a friend to buy, I used to give my friend a say, my friend immediately let me order another one!	Jingdong prefecture level city in Shandong
Jinfeng Hongyuan Flagship Store	Electricity conservation2	The baby is just as introduced, the texture is very good, powerful, and it feels good to use it	Jingdong prefecture level city in Shandong
Jinfeng Hongyuan Flagship Store	Electricity conservation2	Value for money, very satisfied, seller service attitude is very good, very patient	Jingdong prefecture level city in Shandong
Jinfeng Hongyuan Flagship Store	Electricity conservation2	Power saving treasure design unique exquisite beautiful, voltage display clearly, plugged in that is good to use, I observed the meter, after using the meter is gone slow.	Jingdong prefecture level city in Shandong
Jinfeng Hongyuan Flagship Store	Electricity conservation2	Received on the use of the packaging is beautifully used for a period of time and then come back in the follow-up review!	Jingdong prefecture level city in Shandong
Jinfeng Hongyuan Flagship Store	Electricity conservation2	It should work. Not bad. Not bad. Not bad.	Jingdong prefecture level city in Shandong
Jinfeng Hongyuan Flagship Store	Electricity conservation2	I haven't used it yet, I'll plug it in and see if it saves power next month.	Jingdong prefecture level city in Shandong
Jinfeng Hongyuan Flagship Store	Electricity conservation2	The goods received and unpacked and used, the quality is very good, things and the official website to buy no difference!	Jingdong prefecture level city in Shandong
Jinfeng Hongyuan Flagship Store	Electricity conservation2	It's a rip off, doesn't help much at all, uses the same amount of power as before it was used.	Jingdong prefecture level city in Shandong
Jinfeng Hongyuan Flagship Store	Electricity conservation2	I've been using it for a while now and haven't noticed any unusual problems, so it's okay	Jingdong prefecture level city in Shandong
Jinfeng Hongyuan Flagship Store	Electricity conservation2	Received the goods and plugged in to use, good or not there is no use will have to wait until next month to find out	Jingdong prefecture level city in Shandong
Jinfeng Hongyuan Flagship Store	Electricity conservation2	It's very compact, it looks good, I'll introduce you to my clients when it's ready.	Jingdong prefecture level city in Shandong
Jinfeng Hongyuan Flagship Store	Electricity conservation2	Good workmanship, we'll have to see in a few months.	Jingdong prefecture level city in Shandong
Jinfeng Hongyuan Flagship Store	Electricity conservation2	Jingdong quality! Always trustworthy!	Jingdong prefecture level city in Shandong
Jinfeng Hongyuan Flagship Store	Electricity conservation2	This is the second time I've bought it, and the merchant said to give me five dollars out of the difference, so thank you merchant.	Jingdong prefecture level city in Shandong
Jinfeng Hongyuan Flagship Store	Electricity conservation2	Good quality and good service	Jingdong prefecture level city in Shandong

Jinfeng Hongyuan Flagship Store	Electricity conservation2	Feels good texture, aluminum alloy shell, voltage digital display clear.	Jingdong prefecture level city in Shandong
Jinfeng Hongyuan Flagship Store	Electricity conservation2	Good product, fast and great service, thanks!	Jingdong prefecture level city in Shandong
Jinfeng Hongyuan Flagship Store	Electricity conservation2	Have not yet used, first evaluation, later really good do not need to comment again.	Jingdong prefecture level city in Shandong
Jinfeng Hongyuan Flagship Store	Electricity conservation2	Works great. Pretty much as described by the merchant.	Jingdong prefecture level city in Shandong
Jinfeng Hongyuan Flagship Store	Electricity conservation2	Received it, looking forward to the results, hope it works	Jingdong prefecture level city in Shandong
Jinfeng Hongyuan Flagship Store	Electricity conservation2	Received it, looking forward to the results, hope it works	Jingdong prefecture level city in Shandong
Jinfeng Hongyuan Flagship Store	Electricity conservation2	Supposedly it's an IQ tax. Buy one and try it. Looks good. Well made.	Jingdong prefecture level city in Shandong
Jinfeng Hongyuan Flagship Store	Electricity conservation2	It just doesn't feel like it's working.	Jingdong prefecture level city in Shandong
Jinfeng Hongyuan Flagship Store	Electricity conservation2	This is a great product.	Jingdong prefecture level city in Shandong
Jinfeng Hongyuan Flagship Store	Electricity conservation2	I'm just using it. I'll tell you more.	Jingdong prefecture level city in Shandong
Jinfeng Hongyuan Flagship Store	Electricity conservation2	Second repurchase and it's working fine.	Jingdong prefecture level city in Shandong
Jinfeng Hongyuan Flagship Store	Electricity conservation2	I'll wait a few days before evaluating	Jingdong prefecture level city in Shandong
Jinfeng Hongyuan Flagship Store	Electricity conservation2	I'll comment after I use it hopefully the section will be useful.	Jingdong prefecture level city in Shandong
Jinfeng Hongyuan Flagship Store	Electricity conservation2	not very useful	Jingdong prefecture level city in Shandong
Jinfeng Hongyuan Flagship Store	Electricity conservation2	Not received	Jingdong prefecture level city in Shandong
Jinfeng Hongyuan Flagship Store	Electricity conservation2	rare	Jingdong prefecture level city in Shandong
Jinfeng Hongyuan Flagship Store	Electricity conservation2	It's good.	Jingdong prefecture level city in Shandong
Jinfeng Hongyuan Flagship Store	Electricity conservation2	just now	Jingdong prefecture level city in Shandong
Jinfeng Hongyuan Flagship Store	Electricity conservation2	serviceable	Jingdong prefecture level city in Shandong
Jinfeng Hongyuan Flagship Store	Electricity conservation2	desirable	Jingdong prefecture level city in Shandong
Jinfeng Hongyuan Flagship Store	Electricity conservation2	serviceable	Jingdong prefecture level city in Shandong
Jinfeng Hongyuan Flagship Store	Electricity conservation2	It doesn't save power and costs electricity.	Jingdong prefecture level city in Shandong
Jinfeng Hongyuan Flagship Store	Electricity conservation2	Not as good as I thought.	Jingdong prefecture level city in Shandong
Jinfeng Hongyuan Flagship Store	Electricity conservation2	Super bad review, it doesn't save power at all, but rather more power consumption	Jingdong prefecture level city in Shandong
Jinfeng Hongyuan Flagship Store	Electricity conservation2	Listen to others say that this can save electricity, I bought with the idea of trying, used for a period of time before coming to the evaluation, will be a little bit of use, not a lot of savings just, long-term use of the words can save more.	Jingdong prefecture level city in Shandong
Jinfeng Hongyuan Flagship Store	Electricity conservation2	Logistics is fast, the next day arrived, workmanship is very delicate a power saver, looking forward to the late effect, customer service patiently explain the recommended which is suitable for, installed in what position is the best, look forward to using the effect after a period of time!	Jingdong prefecture level city in Shandong
Jinfeng Hongyuan Flagship Store	Electricity conservation2	It's good to use, I feel that it has been saving electricity, anyway, not a loss is, I used to feel that the power consumption is big, now I feel much better, my friends are also using, I hope to use a long time will be able to, I wish the boss a prosperous business	Jingdong prefecture level city in Shandong
Jinfeng Hongyuan Flagship Store	Electricity conservation2	Should be very good, to be able to plan the circuit, save the home of the circuit to the extent of the savings, waste of money on electricity, good if a month this money back, hope always hope to be able to	Jingdong prefecture level city in Shandong
Jinfeng Hongyuan Flagship Store	Electricity conservation2	Express delivery is very strong, the goods and I saw in the business consistent, the use of the effect of follow-up evaluation	Jingdong prefecture level city in Shandong
Jinfeng Hongyuan Flagship Store	Electricity conservation2	I have been using this brand of power saver for many years and this time I saw this upgraded version and got it again. The main purpose of this product is to reduce the loss of electricity for a long time, so as to achieve the purpose of saving electricity.	Jingdong prefecture level city in Shandong
Jinfeng Hongyuan Flagship Store	Electricity conservation2	Logistics is fast and the product looks beautiful, looking forward to the effect.	Jingdong prefecture level city in Shandong
Jinfeng Hongyuan Flagship Store	Electricity conservation2	Product quality is good, energy saving a little effect, but the price is a little expensive, fast arrival, good service.	Jingdong prefecture level city in Shandong
Jinfeng Hongyuan Flagship Store	Electricity conservation2	The quality is very good, inexpensive, Jingdong self-supporting guaranteed, worth having!	Jingdong prefecture level city in Shandong
Jinfeng Hongyuan Flagship Store	Electricity conservation2	I don't know if it's saving electricity yet, I'll find out next time I pay my electric bill	Jingdong prefecture level city in Shandong
Jinfeng Hongyuan Flagship Store	Electricity conservation2	Favorable price quality assurance appearance design is beautiful and generous, fine workmanship	Jingdong prefecture level city in Shandong
Jinfeng Hongyuan Flagship Store	Electricity conservation2	I bought it, I don't know how effective it is? I don't know how the quality is? After this summer results will know! Looking forward to it!	Jingdong prefecture level city in Shandong
Jinfeng Hongyuan Flagship Store	Electricity conservation2	Goods received Packaging intact, look at the shell workmanship is also good, high-power appliances open voltage has obvious changes in the effectiveness of the	Jingdong prefecture level city in Shandong
Jinfeng Hongyuan Flagship Store	Electricity conservation2	The goods have been received logistics is very fast, the product is similar to the line, just wait to use a month to see if it can save electricity, stay tuned.	Jingdong prefecture level city in Shandong
Jinfeng Hongyuan Flagship Store	Electricity conservation2	Electricity is plugged in, not sure if it will work, hopefully it will work.	Jingdong prefecture level city in Shandong
Jinfeng Hongyuan Flagship Store	Electricity conservation2	The shell is aluminum alloy, very good workmanship, the actual effect of waiting for the addition of evaluation	Jingdong prefecture level city in Shandong
Jinfeng Hongyuan Flagship Store	Electricity conservation2	Received, already plugged in, looking forward to the effect.	Jingdong prefecture level city in Shandong
Jinfeng Hongyuan Flagship Store	Electricity conservation2	Opened to see the regular manufacturer products, wait for a period of time to see it	Jingdong prefecture level city in Shandong
Jinfeng Hongyuan Flagship Store	Electricity conservation2	🉑 Baby received, very surprised ✨, reasonable design, fashionable and generous, continue to pay attention to the future will be repurchased ❤❤❤❤	Jingdong prefecture level city in Shandong
Jinfeng Hongyuan Flagship Store	Electricity conservation2	Baby received it 👌, really great ah! 🌈The quality can't be beat, very satisfied! 🆗Happy happy happy 😃	Jingdong prefecture level city in Shandong
Jinfeng Hongyuan Flagship Store	Electricity conservation2	Very good, power saving see, this is to help a friend to buy, I used to give my friend a say, my friend immediately let me order another one!	Jingdong prefecture level city in Shandong
Jinfeng Hongyuan Flagship Store	Electricity conservation2	The baby is just as introduced, the texture is very good, powerful, and it feels good to use it	Jingdong prefecture level city in Shandong
Jinfeng Hongyuan Flagship Store	Electricity conservation2	Value for money, very satisfied, seller service attitude is very good, very patient	Jingdong prefecture level city in Shandong
Jinfeng Hongyuan Flagship Store	Electricity conservation2	Power saving treasure design unique exquisite beautiful, voltage display clearly, plugged in that is good to use, I observed the meter, after using the meter is gone slow.	Jingdong prefecture level city in Shandong
Jinfeng Hongyuan Flagship Store	Electricity conservation2	Received on the use of the packaging is beautifully used for a period of time and then come back in the follow-up review!	Jingdong prefecture level city in Shandong

Jinfeng Hongyuan Flagship Store	Electricity conservation2	It should work. Not bad. Not bad. Not bad.	Jingdong prefecture level city in Shandong
Jinfeng Hongyuan Flagship Store	Electricity conservation2	I haven't used it yet, I'll plug it in and see if it saves power next month.	Jingdong prefecture level city in Shandong
Jinfeng Hongyuan Flagship Store	Electricity conservation2	The goods received and unpacked and used, the quality is very good, things and the official website to buy no difference!	Jingdong prefecture level city in Shandong
Jinfeng Hongyuan Flagship Store	Electricity conservation2	It's a rip off, doesn't help much at all, uses the same amount of power as before it was used.	Jingdong prefecture level city in Shandong
Jinfeng Hongyuan Flagship Store	Electricity conservation2	I've been using it for a while now and haven't noticed any unusual problems, so it's okay	Jingdong prefecture level city in Shandong
Jinfeng Hongyuan Flagship Store	Electricity conservation2	Received the goods and plugged in to use, good or not there is no use will have to wait until next month to find out	Jingdong prefecture level city in Shandong
Jinfeng Hongyuan Flagship Store	Electricity conservation2	It's very compact, it looks good, I'll introduce you to my clients when it's ready.	Jingdong prefecture level city in Shandong
Jinfeng Hongyuan Flagship Store	Electricity conservation2	Good workmanship, we'll have to see in a few months.	Jingdong prefecture level city in Shandong
Jinfeng Hongyuan Flagship Store	Electricity conservation2	Jingdong quality! Always trustworthy!	Jingdong prefecture level city in Shandong
Jinfeng Hongyuan Flagship Store	Electricity conservation2	This is the second time I've bought it, and the merchant said to give me five dollars out of the difference, so thank you merchant.	Jingdong prefecture level city in Shandong
Jinfeng Hongyuan Flagship Store	Electricity conservation2	Good quality and good service	Jingdong prefecture level city in Shandong
Jinfeng Hongyuan Flagship Store	Electricity conservation2	Feels good texture, aluminum alloy shell, voltage digital display clear.	Jingdong prefecture level city in Shandong
Jinfeng Hongyuan Flagship Store	Electricity conservation2	Good product, fast and great service, thanks!	Jingdong prefecture level city in Shandong
Jinfeng Hongyuan Flagship Store	Electricity conservation2	Have not yet used, first evaluation, later really good do not need to comment again.	Jingdong prefecture level city in Shandong
Jinfeng Hongyuan Flagship Store	Electricity conservation2	Works great. Pretty much as described by the merchant.	Jingdong prefecture level city in Shandong
Jinfeng Hongyuan Flagship Store	Electricity conservation2	Received it, looking forward to the results, hope it works	Jingdong prefecture level city in Shandong
Jinfeng Hongyuan Flagship Store	Electricity conservation2	Received it, looking forward to the results, hope it works	Jingdong prefecture level city in Shandong
Jinfeng Hongyuan Flagship Store	Electricity conservation2	Supposedly it's an IQ tax. Buy one and try it. Looks good. Well made.	Jingdong prefecture level city in Shandong
Jinfeng Hongyuan Flagship Store	Electricity conservation2	It just doesn't feel like it's working.	Jingdong prefecture level city in Shandong
Jinfeng Hongyuan Flagship Store	Electricity conservation2	This is a great product.	Jingdong prefecture level city in Shandong
Jinfeng Hongyuan Flagship Store	Electricity conservation2	I'm just using it. I'll tell you more.	Jingdong prefecture level city in Shandong
Jinfeng Hongyuan Flagship Store	Electricity conservation2	Second repurchase and it's working fine.	Jingdong prefecture level city in Shandong
Jinfeng Hongyuan Flagship Store	Electricity conservation2	I'll wait a few days before evaluating	Jingdong prefecture level city in Shandong
Jinfeng Hongyuan Flagship Store	Electricity conservation2	I'll comment after I use it hopefully the section will be useful.	Jingdong prefecture level city in Shandong
Jinfeng Hongyuan Flagship Store	Electricity conservation2	not very useful	Jingdong prefecture level city in Shandong
Jinfeng Hongyuan Flagship Store	Electricity conservation2	Not received	Jingdong prefecture level city in Shandong
Jinfeng Hongyuan Flagship Store	Electricity conservation2	rare	Jingdong prefecture level city in Shandong
Jinfeng Hongyuan Flagship Store	Electricity conservation2	It's good.	Jingdong prefecture level city in Shandong
Jinfeng Hongyuan Flagship Store	Electricity conservation2	just now	Jingdong prefecture level city in Shandong
Jinfeng Hongyuan Flagship Store	Electricity conservation2	serviceable	Jingdong prefecture level city in Shandong
Jinfeng Hongyuan Flagship Store	Electricity conservation2	desirable	Jingdong prefecture level city in Shandong
Jinfeng Hongyuan Flagship Store	Electricity conservation2	serviceable	Jingdong prefecture level city in Shandong
Jinfeng Hongyuan Flagship Store	Electricity conservation2	It doesn't save power and costs electricity.	Jingdong prefecture level city in Shandong
Jinfeng Hongyuan Flagship Store	Electricity conservation2	Not as good as I thought.	Jingdong prefecture level city in Shandong
Jinfeng Hongyuan Flagship Store	Electricity conservation2	Super bad review, it doesn't save power at all, but rather more power consumption	Jingdong prefecture level city in Shandong
Jinfeng Hongyuan Flagship Store	Electricity conservation2	Listen to others say that this can save electricity, I bought with the idea of trying, used for a period of time before coming to the evaluation, will be a little bit of use, not a lot of savings just, long-term use of the words can save more.	Jingdong prefecture level city in Shandong
Jinfeng Hongyuan Flagship Store	Electricity conservation2	Logistics is fast, the next day arrived, workmanship is very delicate a power saver, looking forward to the late effect, customer service patiently explain the recommended which is suitable for, installed in what position is the best, look forward to using the effect after a period of time!	Jingdong prefecture level city in Shandong
Jinfeng Hongyuan Flagship Store	Electricity conservation2	It's good to use, I feel that it has been saving electricity, anyway, not a loss is, I used to feel that the power consumption is big, now I feel much better, my friends are also using, I hope to use a long time will be able to, I wish the boss a prosperous business	Jingdong prefecture level city in Shandong
Jinfeng Hongyuan Flagship Store	Electricity conservation2	Should be very good, to be able to plan the circuit, save the home of the circuit to the extent of the savings, waste of money on electricity, good if a month this money back, hope always hope to be able to	Jingdong prefecture level city in Shandong
Jinfeng Hongyuan Flagship Store	Electricity conservation2	Express delivery is very strong, the goods and I saw in the business consistent, the use of the effect of follow-up evaluation	Jingdong prefecture level city in Shandong
Jinfeng Hongyuan Flagship Store	Electricity conservation2	I have been using this brand of power saver for many years and this time I saw this upgraded version and got it again. The main purpose of this product is to reduce the loss of electricity for a long time, so as to achieve the purpose of saving electricity.	Jingdong prefecture level city in Shandong
Jinfeng Hongyuan Flagship Store	Electricity conservation2	Logistics is fast and the product looks beautiful, looking forward to the effect.	Jingdong prefecture level city in Shandong
Jinfeng Hongyuan Flagship Store	Electricity conservation2	Product quality is good, energy saving a little effect, but the price is a little expensive, fast arrival, good service.	Jingdong prefecture level city in Shandong
Jinfeng Hongyuan Flagship Store	Electricity conservation2	The quality is very good, inexpensive, Jingdong self-supporting guaranteed, worth having!	Jingdong prefecture level city in Shandong
Jinfeng Hongyuan Flagship Store	Electricity conservation2	I don't know if it's saving electricity yet, I'll find out next time I pay my electric bill	Jingdong prefecture level city in Shandong
Jinfeng Hongyuan Flagship Store	Electricity conservation2	Favorable price quality assurance appearance design is beautiful and generous, fine workmanship	Jingdong prefecture level city in Shandong
Jinfeng Hongyuan Flagship Store	Electricity conservation2	I bought it, I don't know how effective it is? I don't know how the quality is? After this summer results will know! Looking forward to it!	Jingdong prefecture level city in Shandong
Jinfeng Hongyuan Flagship Store	Electricity conservation2	Goods received Packaging intact, look at the shell workmanship is also good, high-power appliances open voltage has obvious changes in the effectiveness of the	Jingdong prefecture level city in Shandong
Jinfeng Hongyuan Flagship Store	Electricity conservation2	The goods have been received logistics is very fast, the product is similar to the line, just wait to use a month to see if it can save electricity, stay tuned.	Jingdong prefecture level city in Shandong

Jinfeng Hongyuan Flagship Store	Electricity conservation2	Electricity is plugged in, not sure if it will work, hopefully it will work.	Jingdong prefecture level city in Shandong
Jinfeng Hongyuan Flagship Store	Electricity conservation2	The shell is aluminum alloy, very good workmanship, the actual effect of waiting for the addition of evaluation	Jingdong prefecture level city in Shandong
Jinfeng Hongyuan Flagship Store	Electricity conservation2	Received, already plugged in, looking forward to the effect.	Jingdong prefecture level city in Shandong
Jinfeng Hongyuan Flagship Store	Electricity conservation2	Opened to see the regular manufacturer products, wait for a period of time to see it	Jingdong prefecture level city in Shandong
Jinfeng Hongyuan Flagship Store	Electricity conservation2	🉑 Baby received, very surprised ✨, reasonable design, fashionable and generous, continue to pay attention to the future will be repurchased ❥❥❥❥	Jingdong prefecture level city in Shandong
Jinfeng Hongyuan Flagship Store	Electricity conservation2	Baby received it 👌, really great ah! 🌈The quality can't be beat, very satisfied! 🆗Happy happy happy 😀	Jingdong prefecture level city in Shandong
Jinfeng Hongyuan Flagship Store	Electricity conservation2	Very good, power saving see, this is to help a friend to buy, I used to give my friend a say, my friend immediately let me order another one!	Jingdong prefecture level city in Shandong
Jinfeng Hongyuan Flagship Store	Electricity conservation2	The baby is just as introduced, the texture is very good, powerful, and it feels good to use it	Jingdong prefecture level city in Shandong
Jinfeng Hongyuan Flagship Store	Electricity conservation2	Value for money, very satisfied, seller service attitude is very good, very patient	Jingdong prefecture level city in Shandong
Jinfeng Hongyuan Flagship Store	Electricity conservation2	Power saving treasure design unique exquisite beautiful, voltage display clearly, plugged in that is good to use, I observed the meter, after using the meter is gone slow.	Jingdong prefecture level city in Shandong
Jinfeng Hongyuan Flagship Store	Electricity conservation2	Received on the use of the packaging is beautifully used for a period of time and then come back in the follow-up review!	Jingdong prefecture level city in Shandong
Jinfeng Hongyuan Flagship Store	Electricity conservation2	It should work. Not bad. Not bad. Not bad.	Jingdong prefecture level city in Shandong
Jinfeng Hongyuan Flagship Store	Electricity conservation2	I haven't used it yet, I'll plug it in and see if it saves power next month.	Jingdong prefecture level city in Shandong
Jinfeng Hongyuan Flagship Store	Electricity conservation2	The goods received and unpacked and used, the quality is very good, things and the official website to buy no difference!	Jingdong prefecture level city in Shandong
Jinfeng Hongyuan Flagship Store	Electricity conservation2	It's a rip off, doesn't help much at all, uses the same amount of power as before it was used.	Jingdong prefecture level city in Shandong
Jinfeng Hongyuan Flagship Store	Electricity conservation2	I've been using it for a while now and haven't noticed any unusual problems, so it's okay	Jingdong prefecture level city in Shandong
Jinfeng Hongyuan Flagship Store	Electricity conservation2	Received the goods and plugged in to use, good or not there is no use will have to wait until next month to find out	Jingdong prefecture level city in Shandong
Jinfeng Hongyuan Flagship Store	Electricity conservation2	It's very compact, it looks good, I'll introduce you to my clients when it's ready.	Jingdong prefecture level city in Shandong
Jinfeng Hongyuan Flagship Store	Electricity conservation2	Good workmanship, we'll have to see in a few months.	Jingdong prefecture level city in Shandong
Jinfeng Hongyuan Flagship Store	Electricity conservation2	Jingdong quality! Always trustworthy!	Jingdong prefecture level city in Shandong
Jinfeng Hongyuan Flagship Store	Electricity conservation2	This is the second time I've bought it, and the merchant said to give me five dollars out of the difference, so thank you merchant.	Jingdong prefecture level city in Shandong
Jinfeng Hongyuan Flagship Store	Electricity conservation2	Good quality and good service	Jingdong prefecture level city in Shandong
Jinfeng Hongyuan Flagship Store	Electricity conservation2	Feels good texture, aluminum alloy shell, voltage digital display clear.	Jingdong prefecture level city in Shandong
Jinfeng Hongyuan Flagship Store	Electricity conservation2	Good product, fast and great service, thanks!	Jingdong prefecture level city in Shandong
Jinfeng Hongyuan Flagship Store	Electricity conservation2	Have not yet used, first evaluation, later really good do not need to comment again.	Jingdong prefecture level city in Shandong
Jinfeng Hongyuan Flagship Store	Electricity conservation2	Works great. Pretty much as described by the merchant.	Jingdong prefecture level city in Shandong
Jinfeng Hongyuan Flagship Store	Electricity conservation2	Received it, looking forward to the results, hope it works	Jingdong prefecture level city in Shandong
Jinfeng Hongyuan Flagship Store	Electricity conservation2	Received it, looking forward to the results, hope it works	Jingdong prefecture level city in Shandong
Jinfeng Hongyuan Flagship Store	Electricity conservation2	Supposedly it's an IQ tax. Buy one and try it. Looks good. Well made.	Jingdong prefecture level city in Shandong
Jinfeng Hongyuan Flagship Store	Electricity conservation2	It just doesn't feel like it's working.	Jingdong prefecture level city in Shandong
Jinfeng Hongyuan Flagship Store	Electricity conservation2	This is a great product.	Jingdong prefecture level city in Shandong
Jinfeng Hongyuan Flagship Store	Electricity conservation2	I'm just using it. I'll tell you more.	Jingdong prefecture level city in Shandong
Jinfeng Hongyuan Flagship Store	Electricity conservation2	Second repurchase and it's working fine.	Jingdong prefecture level city in Shandong
Jinfeng Hongyuan Flagship Store	Electricity conservation2	I'll wait a few days before evaluating	Jingdong prefecture level city in Shandong
Jinfeng Hongyuan Flagship Store	Electricity conservation2	I'll comment after I use it hopefully the section will be useful.	Jingdong prefecture level city in Shandong
Jinfeng Hongyuan Flagship Store	Electricity conservation2	not very useful	Jingdong prefecture level city in Shandong
Jinfeng Hongyuan Flagship Store	Electricity conservation2	Not received	Jingdong prefecture level city in Shandong
Jinfeng Hongyuan Flagship Store	Electricity conservation2	rare	Jingdong prefecture level city in Shandong
Jinfeng Hongyuan Flagship Store	Electricity conservation2	It's good.	Jingdong prefecture level city in Shandong
Jinfeng Hongyuan Flagship Store	Electricity conservation2	just now	Jingdong prefecture level city in Shandong
Jinfeng Hongyuan Flagship Store	Electricity conservation2	serviceable	Jingdong prefecture level city in Shandong
Jinfeng Hongyuan Flagship Store	Electricity conservation2	desirable	Jingdong prefecture level city in Shandong
Jinfeng Hongyuan Flagship Store	Electricity conservation2	serviceable	Jingdong prefecture level city in Shandong
Jinfeng Hongyuan Flagship Store	Electricity conservation2	It doesn't save power and costs electricity.	Jingdong prefecture level city in Shandong
Jinfeng Hongyuan Flagship Store	Electricity conservation2	Not as good as I thought.	Jingdong prefecture level city in Shandong
Jinfeng Hongyuan Flagship Store	Electricity conservation2	Super bad review, it doesn't save power at all, but rather more power consumption	Jingdong prefecture level city in Shandong
Jinfeng Hongyuan Flagship Store	Electricity savings3	Jingdong logistics is very fast oh, thoughtful service, praise, next time to come back to patronize!	Jingdong prefecture level city in Shandong
Jinfeng Hongyuan Flagship Store	Electricity savings3	Installation is complete, customer service is very patient to explain, praise, there is no effect a month later to add	Jingdong prefecture level city in Shandong
Jinfeng Hongyuan Flagship Store	Electricity savings3	Appearance design is beautiful, the size of the right size, packaging carefully and completely, baby feel good, feel good, fast shipping, first-class service attitude, give! 5 stars praise! Quite a good product	Jingdong prefecture level city in Shandong
Jinfeng Hongyuan Flagship Store	Electricity savings3	I purchased one before, and I purchased another one this time, and this one is smaller and more beautiful than the previous one, which was $368, and I bought it to protect the appliances, and since I've been using the saver, when I've been playing with the computer or watching TV, when I turn on the lights, the computer and the TV don't have flickering or black screens anymore, so I purchased another one, and I put it in an outlet that's closer to the incoming wires.	Jingdong prefecture level city in Shandong

Jinfeng Hongyuan Flagship Store	Electricity savings3	Logistics is very fast, after-sales service is also very good, the quality of the product is also very good Oh, I look forward to it can bring me the joy of energy saving! Good products also look forward to recommending to everyone ha, but the overall look is quite high.	Jingdong prefecture level city in Shandong
Jinfeng Hongyuan Flagship Store	Electricity savings3	The box was very intact and the logistics were very fast. The instructions for use are also very simple.	Jingdong prefecture level city in Shandong
Jinfeng Hongyuan Flagship Store	Electricity savings3	Jinfeng Hongyuan power saver has received, small east east blue is very beautiful, very good workmanship, today installed, energy-saving effect, after a period of time to be actually used to evaluate. I believe it will not be wrong.	Jingdong prefecture level city in Shandong
Jinfeng Hongyuan Flagship Store	Electricity savings3	Shipped very quickly, packaged very well, the price is particularly affordable, there are so many efficacy is also good, it is worth buying worth buying the product, the workmanship is very fine, better than imagined, recommended that everyone try not yet used, after the use of the follow-up comment	Jingdong prefecture level city in Shandong
Jinfeng Hongyuan Flagship Store	Electricity savings3	It looks regular. It's working now.	Jingdong prefecture level city in Shandong
Jinfeng Hongyuan Flagship Store	Electricity savings3	It arrived pretty fast and the owner is very responsible. Have not used it yet, will see after use	Jingdong prefecture level city in Shandong
Jinfeng Hongyuan Flagship Store	Electricity savings3	🎁 baby quality is very good, the first time I received the goods to open the first time to see, it is very good 👍, very satisfied ☺, customer service also have a question and answer, thank you 🙏	Jingdong prefecture level city in Shandong
Jinfeng Hongyuan Flagship Store	Electricity savings3	I don't know if it's any good 。。。。。。	Jingdong prefecture level city in Shandong
Jinfeng Hongyuan Flagship Store	Electricity savings3	Buy electronic products! Still trust the things of the Jingdong self-owned store! Energy-saving effect to evaluate after heating!	Jingdong prefecture level city in Shandong
Jinfeng Hongyuan Flagship Store	Electricity savings3	The goods have been received, Blu is very beautiful, energy saving effect, to be confirmed only after actual use.	Jingdong prefecture level city in Shandong
Jinfeng Hongyuan Flagship Store	Electricity savings3	Jingdong's speed is so fast, has received, easy to operate. The display is quite good, how many volts what mode is quite understandable.	Jingdong prefecture level city in Shandong
Jinfeng Hongyuan Flagship Store	Electricity savings3	The first use, the effect is good to recommend it to colleagues to use, a month of electricity to save a lot or can be	Jingdong prefecture level city in Shandong
Jinfeng Hongyuan Flagship Store	Electricity savings3	I don't know if it's useful. Mom's is a store. I'll return it if it doesn't work.	Jingdong prefecture level city in Shandong
Jinfeng Hongyuan Flagship Store	Electricity savings3	I don't know if it works or not, I'll wait for two months to use it and then come back to comment!	Jingdong prefecture level city in Shandong
Jinfeng Hongyuan Flagship Store	Electricity savings3	I bought the second one now, and I've been using it for more than half a year, and I feel that it really saves electricity. Now it is summer, the weather is hot, sometimes the fan and air conditioning on all day, if not this power saver, the monthly electricity bill is quite high, but as long as the installation of this power saver, not afraid of its high electricity bill, absolutely let you wantonly use	Jingdong prefecture level city in Shandong
Jinfeng Hongyuan Flagship Store	Electricity savings3	The product is better than I thought it would be, the quality looks great, the voltage is stable, and I'm looking forward to some great power savings!	Jingdong prefecture level city in Shandong
Jinfeng Hongyuan Flagship Store	Electricity savings3	Inadvertently see bought a try, delivery is also quite timely, just do not know how to use, customer service patience to answer, with a pretty good, value for money!	Jingdong prefecture level city in Shandong
Jinfeng Hongyuan Flagship Store	Electricity savings3	It's better than nothing! Hopefully this will help save slightly.	Jingdong prefecture level city in Shandong
Jinfeng Hongyuan Flagship Store	Electricity savings3	Still trying it out, follow up	Jingdong prefecture level city in Shandong
Jinfeng Hongyuan Flagship Store	Electricity savings3	I've just started using it, I don't know how well it works yet, I hope it's good.	Jingdong prefecture level city in Shandong
Jinfeng Hongyuan Flagship Store	Electricity savings3	Fourth purchase, great baby!	Jingdong prefecture level city in Shandong
Jinfeng Hongyuan Flagship Store	Electricity savings3	Shipped really fast, just got it and use it to see what happens and then come back for a review!	Jingdong prefecture level city in Shandong
Jinfeng Hongyuan Flagship Store	Electricity savings3	Timely delivery **** good attitude	Jingdong prefecture level city in Shandong
Jinfeng Hongyuan Flagship Store	Electricity savings3	Home large and small things are purchased in the Jingdong Mall, have been accustomed to shopping in the Jingdong, many times can buy good quality and cheap products, and courier service attitude is particularly good, sometimes too many couriers are to help me to the home, so many years have been in the Jingdong Mall shopping, trust and support! Will also support the Jingdong as always!	Jingdong prefecture level city in Shandong
Jinfeng Hongyuan Flagship Store	Electricity savings3	Modified a pump and the modification worked.	Jingdong prefecture level city in Shandong
Jinfeng Hongyuan Flagship Store	Electricity savings3	Already installed, next time you pay the electric bill to see the results	Jingdong prefecture level city in Shandong
Jinfeng Hongyuan Flagship Store	Electricity savings3	The product is very good to use and practical to look at	Jingdong prefecture level city in Shandong
Jinfeng Hongyuan Flagship Store	Electricity savings3	Works great, great service, waiting for results.	Jingdong prefecture level city in Shandong
Jinfeng Hongyuan Flagship Store	Electricity savings3	I just used it and I'm not sure about the efficacy yet, I'll come back to evaluate it after a while.	Jingdong prefecture level city in Shandong
Jinfeng Hongyuan Flagship Store	Electricity savings3	Just received the goods, use a period of time to evaluate	Jingdong prefecture level city in Shandong
Jinfeng Hongyuan Flagship Store	Electricity savings3	Opened and tried very good, use it to see if there are results	Jingdong prefecture level city in Shandong
Jinfeng Hongyuan Flagship Store	Electricity savings3	Arrived today! Still trying it out!	Jingdong prefecture level city in Shandong
Jinfeng Hongyuan Flagship Store	Electricity savings3	Shipped quickly, well packaged, already installed, hope there is a power saving effect	Jingdong prefecture level city in Shandong
Jinfeng Hongyuan Flagship Store	Electricity savings3	I got it. It's great.	Jingdong prefecture level city in Shandong
Jinfeng Hongyuan Flagship Store	Electricity savings3	Haven't used it yet, will come back in a month	Jingdong prefecture level city in Shandong
Jinfeng Hongyuan Flagship Store	Electricity savings3	It looks good. It's good. Try it first.	Jingdong prefecture level city in Shandong
Jinfeng Hongyuan Flagship Store	Electricity savings3	It works so well that I have relatives, friends, coworkers, and 6 families that have purchased it for them through me.	Jingdong prefecture level city in Shandong
Jinfeng Hongyuan Flagship Store	Electricity savings3	In use	Jingdong prefecture level city in Shandong
Jinfeng Hongyuan Flagship Store	Electricity savings3	Received the goods today, power-saving effect do not know being used, the boss has a very good attitude, praise!	Jingdong prefecture level city in Shandong
Jinfeng Hongyuan Flagship Store	Electricity savings3	The product is compact and doesn't take up much space, but hopefully it's power efficient.	Jingdong prefecture level city in Shandong
Jinfeng Hongyuan Flagship Store	Electricity savings3	Power saving is indeed effective, I used for a period of time before coming to evaluate, the electricity bill is obviously less, the quality is good, the shopkeeper service is also very good, very patient,	Jingdong prefecture level city in Shandong
Jinfeng Hongyuan Flagship Store	Electricity savings3	It feels fine, I'll comment after I use it	Jingdong prefecture level city in Shandong
Jinfeng Hongyuan Flagship Store	Electricity savings3	I just received it and I don't know the effect yet! I will comment again after I use it and have results!	Jingdong prefecture level city in Shandong
Jinfeng Hongyuan Flagship Store	Electricity savings3	The goods look fine, I hope they really work!	Jingdong prefecture level city in Shandong
Jinfeng Hongyuan Flagship Store	Electricity savings3	I just don't know if it saves power	Jingdong prefecture level city in Shandong
Jinfeng Hongyuan Flagship Store	Electricity savings3	My friend introduced me to use this product, it is professional and I am looking forward to the long term effect~!	Jingdong prefecture level city in Shandong
Jinfeng Hongyuan Flagship Store	Electricity savings3	Try it before you judge.	Jingdong prefecture level city in Shandong
Jinfeng Hongyuan Flagship Store	Electricity savings3	It's great, the power savings are obvious! Kudos!	Jingdong prefecture level city in Shandong

Jinfeng Hongyuan Flagship Store	Electricity savings3	The quality is excellent, stabilizes the voltage and saves electricity, very satisfied.	Jingdong prefecture level city in Shandong
Jinfeng Hongyuan Flagship Store	Electricity savings3	You have to use it for a while to know the effect	Jingdong prefecture level city in Shandong
Jinfeng Hongyuan Flagship Store	Electricity savings3	It's on trial.	Jingdong prefecture level city in Shandong
Jinfeng Hongyuan Flagship Store	Electricity savings3	The stuff works great.	Jingdong prefecture level city in Shandong
Jinfeng Hongyuan Flagship Store	Electricity savings3	Doesn't seem to work, not as god as other reviews	Jingdong prefecture level city in Shandong
Jinfeng Hongyuan Flagship Store	Electricity savings3	It's not good quality, it doesn't work, it stops working after a few days, it doesn't work, the customer service doesn't care, it's a bit irritating.	Jingdong prefecture level city in Shandong
Jinfeng Hongyuan Flagship Store	Electricity savings3	It doesn't do anything.	Jingdong prefecture level city in Shandong
Jinfeng Hongyuan Flagship Store	Electricity savings3	It's good.	Jingdong prefecture level city in Shandong
Jinfeng Hongyuan Flagship Store	Electricity savings3	Used a month effect is not obvious, are psychological effects, feel are not less, did not save to the electricity, there are details of the problem, the store said to buy to send 5 fuse, in fact, received only three, small problems are not committed to in place, I believe that the product is not good enough	Jingdong prefecture level city in Shandong
Jinfeng Hongyuan Flagship Store	Electricity savings3	Well received quickly, looking quite high, but also sent 5 fuses. Customer service is very patient to teach the operation, in fact, quite simple, lazy to read the instructions ha!	Jingdong prefecture level city in Shandong
Jinfeng Hongyuan Flagship Store	Electricity savings3	You have to use it for a while to know the effect	Jingdong prefecture level city in Shandong
Jinfeng Hongyuan Flagship Store	Electricity savings3	Shipped very quickly, packaged very well, the price is particularly affordable, there are so many efficacy is also good, it is worth buying worth buying the product, the workmanship is very fine, better than imagined, recommended that everyone try not yet used, after the use of the follow-up comment	Jingdong prefecture level city in Shandong
Jinfeng Hongyuan Flagship Store	Electricity savings3	It looks regular. It's working now.	Jingdong prefecture level city in Shandong
Jinfeng Hongyuan Flagship Store	Electricity savings3	It arrived pretty fast and the owner is very responsible. Have not used it yet, will see after use	Jingdong prefecture level city in Shandong
Jinfeng Hongyuan Flagship Store	Electricity savings3	🎁 baby quality is very good, the first time I received the goods to open the first time to see, it is very good 👍, very satisfied 😊, customer service also have a question and answer, thank you 🙏	Jingdong prefecture level city in Shandong
Jinfeng Hongyuan Flagship Store	Electricity savings3	I don't know if it's any good 。。。。。。	Jingdong prefecture level city in Shandong
Jinfeng Hongyuan Flagship Store	Electricity savings3	Buy electronic products! Still trust the things of the Jingdong self-owned store! Energy-saving effect to evaluate after heating!	Jingdong prefecture level city in Shandong
Jinfeng Hongyuan Flagship Store	Electricity savings3	The goods have been received, Blu is very beautiful, energy saving effect, to be confirmed only after actual use.	Jingdong prefecture level city in Shandong
Jinfeng Hongyuan Flagship Store	Electricity savings3	Jingdong's speed is so fast, has received, easy to operate. The display is quite good, how many volts what mode is quite understandable.	Jingdong prefecture level city in Shandong
Jinfeng Hongyuan Flagship Store	Electricity savings3	The first use, the effect is good to recommend it to colleagues to use, a month of electricity to save a lot or can be	Jingdong prefecture level city in Shandong
Jinfeng Hongyuan Flagship Store	Electricity savings3	I don't know if it's useful. Mom's is a store. I'll return it if it doesn't work.	Jingdong prefecture level city in Shandong
Jinfeng Hongyuan Flagship Store	Electricity savings3	I don't know if it works or not, I'll wait for two months to use it and then come back to comment!	Jingdong prefecture level city in Shandong
Jinfeng Hongyuan Flagship Store	Electricity savings3	I bought the second one now, and I've been using it for more than half a year, and I feel that it really saves electricity. Now it is summer, the weather is hot, sometimes the fan and air conditioning on all day, if not this power saver, the monthly electricity bill is quite high, but as long as the installation of this power saver, not afraid of its high electricity bill, absolutely let you wantonly use	Jingdong prefecture level city in Shandong
Jinfeng Hongyuan Flagship Store	Electricity savings3	The product is better than I thought it would be, the quality looks great, the voltage is stable, and I'm looking forward to some great power savings!	Jingdong prefecture level city in Shandong
Jinfeng Hongyuan Flagship Store	Electricity savings3	Inadvertently see bought a try, delivery is also quite timely, just do not know how to use, customer service patience to answer, with a pretty good, value for money!	Jingdong prefecture level city in Shandong
Jinfeng Hongyuan Flagship Store	Electricity savings3	It's better than nothing! Hopefully this will help save slightly.	Jingdong prefecture level city in Shandong
Jinfeng Hongyuan Flagship Store	Electricity savings3	Still trying it out, follow up	Jingdong prefecture level city in Shandong
Jinfeng Hongyuan Flagship Store	Electricity savings3	I've just started using it, I don't know how well it works yet, I hope it's good.	Jingdong prefecture level city in Shandong
Jinfeng Hongyuan Flagship Store	Electricity savings3	Fourth purchase, great baby!	Jingdong prefecture level city in Shandong
Jinfeng Hongyuan Flagship Store	Electricity savings3	Shipped really fast, just got it and use it to see what happens and then come back for a review!	Jingdong prefecture level city in Shandong
Jinfeng Hongyuan Flagship Store	Electricity savings3	Timely delivery **** good attitude	Jingdong prefecture level city in Shandong
Jinfeng Hongyuan Flagship Store	Electricity savings3	Home large and small things are purchased in the Jingdong Mall, have been accustomed to shopping in the Jingdong, many times can buy good quality and cheap products, and courier service attitude is particularly good, sometimes too many couriers are to help me to the home, so many years have been in the Jingdong Mall shopping, trust and support! Will also support the Jingdong as always!	Jingdong prefecture level city in Shandong
Jinfeng Hongyuan Flagship Store	Electricity savings3	Modified a pump and the modification worked.	Jingdong prefecture level city in Shandong
Jinfeng Hongyuan Flagship Store	Electricity savings3	Already installed, next time you pay the electric bill to see the results	Jingdong prefecture level city in Shandong
Jinfeng Hongyuan Flagship Store	Electricity savings3	The product is very good to use and practical to look at	Jingdong prefecture level city in Shandong
Jinfeng Hongyuan Flagship Store	Electricity savings3	Works great, great service, waiting for results.	Jingdong prefecture level city in Shandong
Jinfeng Hongyuan Flagship Store	Electricity savings3	I just used it and I'm not sure about the efficacy yet, I'll come back to evaluate it after a while.	Jingdong prefecture level city in Shandong
Jinfeng Hongyuan Flagship Store	Electricity savings3	Just received the goods, use a period of time to evaluate	Jingdong prefecture level city in Shandong
Jinfeng Hongyuan Flagship Store	Electricity savings3	Opened and tried very good, use it to see if there are results	Jingdong prefecture level city in Shandong
Jinfeng Hongyuan Flagship Store	Electricity savings3	Arrived today! Still trying it out!	Jingdong prefecture level city in Shandong
Jinfeng Hongyuan Flagship Store	Electricity savings3	Shipped quickly, well packaged, already installed, hope there is a power saving effect	Jingdong prefecture level city in Shandong
Jinfeng Hongyuan Flagship Store	Electricity savings3	I got it. It's great.	Jingdong prefecture level city in Shandong
Jinfeng Hongyuan Flagship Store	Electricity savings3	Haven't used it yet, will come back in a month	Jingdong prefecture level city in Shandong
Jinfeng Hongyuan Flagship Store	Electricity savings3	It looks good. It's good. Try it first.	Jingdong prefecture level city in Shandong
Jinfeng Hongyuan Flagship Store	Electricity savings3	It works so well that I have relatives, friends, coworkers, and 6 families that have purchased it for them through me.	Jingdong prefecture level city in Shandong
Jinfeng Hongyuan Flagship Store	Electricity savings3	In use	Jingdong prefecture level city in Shandong
Jinfeng Hongyuan Flagship Store	Electricity savings3	Received the goods today, power-saving effect do not know being used, the boss has a very good attitude, praise!	Jingdong prefecture level city in Shandong
Jinfeng Hongyuan Flagship Store	Electricity savings3	The product is compact and doesn't take up much space, but hopefully it's power efficient.	Jingdong prefecture level city in Shandong

Jinfeng Hongyuan Flagship Store	Electricity savings3	Power saving is indeed effective, I used for a period of time before coming to evaluate, the electricity bill is obviously less, the quality is good, the shopkeeper service is also very good, very patient,	Jingdong prefecture level city in Shandong
Jinfeng Hongyuan Flagship Store	Electricity savings3	It feels fine, I'll comment after I use it	Jingdong prefecture level city in Shandong
Jinfeng Hongyuan Flagship Store	Electricity savings3	I just received it and I don't know the effect yet! I will comment again after I use it and have results!	Jingdong prefecture level city in Shandong
Jinfeng Hongyuan Flagship Store	Electricity savings3	The goods look fine, I hope they really work!	Jingdong prefecture level city in Shandong
Jinfeng Hongyuan Flagship Store	Electricity savings3	I just don't know if it saves power	Jingdong prefecture level city in Shandong
Jinfeng Hongyuan Flagship Store	Electricity savings3	My friend introduced me to use this product, it is professional and I am looking forward to the long term effect~!	Jingdong prefecture level city in Shandong
Jinfeng Hongyuan Flagship Store	Electricity savings3	Try it before you judge.	Jingdong prefecture level city in Shandong
Jinfeng Hongyuan Flagship Store	Electricity savings3	It's great, the power savings are obvious! Kudos!	Jingdong prefecture level city in Shandong
Jinfeng Hongyuan Flagship Store	Electricity savings3	The quality is excellent, stabilizes the voltage and saves electricity, very satisfied.	Jingdong prefecture level city in Shandong
Jinfeng Hongyuan Flagship Store	Electricity savings3	You have to use it for a while to know the effect	Jingdong prefecture level city in Shandong
Jinfeng Hongyuan Flagship Store	Electricity savings3	It's on trial.	Jingdong prefecture level city in Shandong
Jinfeng Hongyuan Flagship Store	Electricity savings3	The stuff works great.	Jingdong prefecture level city in Shandong
Jinfeng Hongyuan Flagship Store	Electricity savings3	Doesn't seem to work, not as god as other reviews	Jingdong prefecture level city in Shandong
Jinfeng Hongyuan Flagship Store	Electricity savings3	It's not good quality, it doesn't work, it stops working after a few days, it doesn't work, the customer service doesn't care, it's a bit irritating.	Jingdong prefecture level city in Shandong
Jinfeng Hongyuan Flagship Store	Electricity savings3	It doesn't do anything.	Jingdong prefecture level city in Shandong
Jinfeng Hongyuan Flagship Store	Electricity savings3	It's good.	Jingdong prefecture level city in Shandong
Jinfeng Hongyuan Flagship Store	Electricity savings3	Used a month effect is not obvious, are psychological effects, feel are not less, did not save to the electricity, there are details of the problem, the store said to buy to send 5 fuse, in fact, received only three, small problems are not committed to in place, I believe that the product is not good enough	Jingdong prefecture level city in Shandong
Jinfeng Hongyuan Flagship Store	Electricity savings3	Well received quickly, looking quite high, but also sent 5 fuses. Customer service is very patient to teach the operation, in fact, quite simple, lazy to read the instructions ha!	Jingdong prefecture level city in Shandong
Jinfeng Hongyuan Flagship Store	Electricity savings3	You have to use it for a while to know the effect	Jingdong prefecture level city in Shandong
Jinfeng Hongyuan Flagship Store	Electricity savings3	Shipped very quickly, packaged very well, the price is particularly affordable, there are so many efficacy is also good, it is worth buying worth buying the product, the workmanship is very fine, better than imagined, recommended that everyone try not yet used, after the use of the follow-up comment	Jingdong prefecture level city in Shandong
Jinfeng Hongyuan Flagship Store	Electricity savings3	It looks regular. It's working now.	Jingdong prefecture level city in Shandong
Jinfeng Hongyuan Flagship Store	Electricity savings3	It arrived pretty fast and the owner is very responsible. Have not used it yet, will see after use	Jingdong prefecture level city in Shandong
Jinfeng Hongyuan Flagship Store	Electricity savings3	🎁 baby quality is very good, the first time I received the goods to open the first time to see, it is very good 👍, very satisfied 😊, customer service also have a question and answer, thank you 🙏	Jingdong prefecture level city in Shandong
Jinfeng Hongyuan Flagship Store	Electricity savings3	I don't know if it's any good 。。。。。。	Jingdong prefecture level city in Shandong
Jinfeng Hongyuan Flagship Store	Electricity savings3	Buy electronic products! Still trust the things of the Jingdong self-owned store! Energy-saving effect to evaluate after heating!	Jingdong prefecture level city in Shandong
Jinfeng Hongyuan Flagship Store	Electricity savings3	The goods have been received, Blu is very beautiful, energy saving effect, to be confirmed only after actual use.	Jingdong prefecture level city in Shandong
Jinfeng Hongyuan Flagship Store	Electricity savings3	Jingdong's speed is so fast, has received, easy to operate. The display is quite good, how many volts what mode is quite understandable.	Jingdong prefecture level city in Shandong
Jinfeng Hongyuan Flagship Store	Electricity savings3	The first use, the effect is good to recommend it to colleagues to use, a month of electricity to save a lot or can be	Jingdong prefecture level city in Shandong
Jinfeng Hongyuan Flagship Store	Electricity savings3	I don't know if it's useful. Mom's is a store. I'll return it if it doesn't work.	Jingdong prefecture level city in Shandong
Jinfeng Hongyuan Flagship Store	Electricity savings3	I don't know if it works or not, I'll wait for two months to use it and then come back to comment!	Jingdong prefecture level city in Shandong
Jinfeng Hongyuan Flagship Store	Electricity savings3	I bought the second one now, and I've been using it for more than half a year, and I feel that it really saves electricity. Now it is summer, the weather is hot, sometimes the fan and air conditioning on all day, if not this power saver, the monthly electricity bill is quite high, but as long as the installation of this power saver, not afraid of its high electricity bill, absolutely let you wantonly use	Jingdong prefecture level city in Shandong
Jinfeng Hongyuan Flagship Store	Electricity savings3	The product is better than I thought it would be, the quality looks great, the voltage is stable, and I'm looking forward to some great power savings!	Jingdong prefecture level city in Shandong
Jinfeng Hongyuan Flagship Store	Electricity savings3	Inadvertently see bought a try, delivery is also quite timely, just do not know how to use, customer service patience to answer, with a pretty good, value for money!	Jingdong prefecture level city in Shandong
Jinfeng Hongyuan Flagship Store	Electricity savings3	It's better than nothing! Hopefully this will help save slightly.	Jingdong prefecture level city in Shandong
Jinfeng Hongyuan Flagship Store	Electricity savings3	Still trying it out, follow up	Jingdong prefecture level city in Shandong
Jinfeng Hongyuan Flagship Store	Electricity savings3	I've just started using it, I don't know how well it works yet, I hope it's good.	Jingdong prefecture level city in Shandong
Jinfeng Hongyuan Flagship Store	Electricity savings3	Fourth purchase, great baby!	Jingdong prefecture level city in Shandong
Jinfeng Hongyuan Flagship Store	Electricity savings3	Shipped really fast, just got it and use it to see what happens and then come back for a review!	Jingdong prefecture level city in Shandong
Jinfeng Hongyuan Flagship Store	Electricity savings3	Timely delivery **** good attitude	Jingdong prefecture level city in Shandong
Jinfeng Hongyuan Flagship Store	Electricity savings3	Home large and small things are purchased in the Jingdong Mall, have been accustomed to shopping in the Jingdong, many times can buy good quality and cheap products, and courier service attitude is particularly good, sometimes too many couriers are to help me to the home, so many years have been in the Jingdong Mall shopping, trust and support! Will also support the Jingdong as always!	Jingdong prefecture level city in Shandong
Jinfeng Hongyuan Flagship Store	Electricity savings3	Modified a pump and the modification worked.	Jingdong prefecture level city in Shandong
Jinfeng Hongyuan Flagship Store	Electricity savings3	Already installed, next time you pay the electric bill to see the results	Jingdong prefecture level city in Shandong
Jinfeng Hongyuan Flagship Store	Electricity savings3	The product is very good to use and practical to look at	Jingdong prefecture level city in Shandong
Jinfeng Hongyuan Flagship Store	Electricity savings3	Works great, great service, waiting for results.	Jingdong prefecture level city in Shandong
Jinfeng Hongyuan Flagship Store	Electricity savings3	I just used it and I'm not sure about the efficacy yet, I'll come back to evaluate it after a while.	Jingdong prefecture level city in Shandong
Jinfeng Hongyuan Flagship Store	Electricity savings3	Just received the goods, use a period of time to evaluate	Jingdong prefecture level city in Shandong
Jinfeng Hongyuan Flagship Store	Electricity savings3	Opened and tried very good, use it to see if there are results	Jingdong prefecture level city in Shandong
Jinfeng Hongyuan Flagship Store	Electricity savings3	Arrived today! Still trying it out!	Jingdong prefecture level city in Shandong

Jinfeng Hongyuan Flagship Store	Electricity savings3	Shipped quickly, well packaged, already installed, hope there is a power saving effect	Jingdong prefecture level city in Shandong
Jinfeng Hongyuan Flagship Store	Electricity savings3	I got it. It's great.	Jingdong prefecture level city in Shandong
Jinfeng Hongyuan Flagship Store	Electricity savings3	Haven't used it yet, will come back in a month	Jingdong prefecture level city in Shandong
Jinfeng Hongyuan Flagship Store	Electricity savings3	It looks good. It's good. Try it first.	Jingdong prefecture level city in Shandong
Jinfeng Hongyuan Flagship Store	Electricity savings3	It works so well that I have relatives, friends, coworkers, and 6 families that have purchased it for them through me.	Jingdong prefecture level city in Shandong
Jinfeng Hongyuan Flagship Store	Electricity savings3	In use	Jingdong prefecture level city in Shandong
Jinfeng Hongyuan Flagship Store	Electricity savings3	Received the goods today, power-saving effect do not know being used, the boss has a very good attitude, praise!	Jingdong prefecture level city in Shandong
Jinfeng Hongyuan Flagship Store	Electricity savings3	The product is compact and doesn't take up much space, but hopefully it's power efficient.	Jingdong prefecture level city in Shandong
Jinfeng Hongyuan Flagship Store	Electricity savings3	Power saving is indeed effective, I used for a period of time before coming to evaluate, the electricity bill is obviously less, the quality is good, the shopkeeper service is also very good, very patient,	Jingdong prefecture level city in Shandong
Jinfeng Hongyuan Flagship Store	Electricity savings3	It feels fine, I'll comment after I use it	Jingdong prefecture level city in Shandong
Jinfeng Hongyuan Flagship Store	Electricity savings3	I just received it and I don't know the effect yet! I will comment again after I use it and have results!	Jingdong prefecture level city in Shandong
Jinfeng Hongyuan Flagship Store	Electricity savings3	The goods look fine, I hope they really work!	Jingdong prefecture level city in Shandong
Jinfeng Hongyuan Flagship Store	Electricity savings3	I just don't know if it saves power	Jingdong prefecture level city in Shandong
Jinfeng Hongyuan Flagship Store	Electricity savings3	My friend introduced me to use this product, it is professional and I am looking forward to the long term effect~!	Jingdong prefecture level city in Shandong
Jinfeng Hongyuan Flagship Store	Electricity savings3	Try it before you judge.	Jingdong prefecture level city in Shandong
Jinfeng Hongyuan Flagship Store	Electricity savings3	It's great, the power savings are obvious! Kudos!	Jingdong prefecture level city in Shandong
Jinfeng Hongyuan Flagship Store	Electricity savings3	The quality is excellent, stabilizes the voltage and saves electricity, very satisfied.	Jingdong prefecture level city in Shandong
Jinfeng Hongyuan Flagship Store	Electricity savings3	You have to use it for a while to know the effect	Jingdong prefecture level city in Shandong
Jinfeng Hongyuan Flagship Store	Electricity savings3	It's on trial.	Jingdong prefecture level city in Shandong
Jinfeng Hongyuan Flagship Store	Electricity savings3	The stuff works great.	Jingdong prefecture level city in Shandong
Jinfeng Hongyuan Flagship Store	Electricity savings3	Doesn't seem to work, not as god as other reviews	Jingdong prefecture level city in Shandong
Jinfeng Hongyuan Flagship Store	Electricity savings3	It's not good quality, it doesn't work, it stops working after a few days, it doesn't work, the customer service doesn't care, it's a bit irritating.	Jingdong prefecture level city in Shandong
Jinfeng Hongyuan Flagship Store	Electricity savings3	It doesn't do anything.	Jingdong prefecture level city in Shandong
Jinfeng Hongyuan Flagship Store	Electricity savings3	It's good.	Jingdong prefecture level city in Shandong
Jinfeng Hongyuan Flagship Store	Electricity savings3	Used a month effect is not obvious, are psychological effects, feel are not less, did not save to the electricity, there are details of the problem, the store said to buy to send 5 fuse, in fact, received only three, small problems are not committed to in place, I believe that the product is not good enough	Jingdong prefecture level city in Shandong
Jinfeng Hongyuan Flagship Store	Electricity savings3	Well received quickly, looking quite high, but also sent 5 fuses. Customer service is very patient to teach the operation, in fact, quite simple, lazy to read the instructions ha!	Jingdong prefecture level city in Shandong
Jinfeng Hongyuan Flagship Store	Electricity savings3	You have to use it for a while to know the effect	Jingdong prefecture level city in Shandong
Jinfeng Hongyuan Flagship Store	Electricity savings3	Shipped very quickly, packaged very well, the price is particularly affordable, there are so many efficacy is also good, it is worth buying worth buying the product, the workmanship is very fine, better than imagined, recommended that everyone try not yet used, after the use of the follow-up comment	Jingdong prefecture level city in Shandong
Jinfeng Hongyuan Flagship Store	Electricity savings3	It looks regular. It's working now.	Jingdong prefecture level city in Shandong
Jinfeng Hongyuan Flagship Store	Electricity savings3	It arrived pretty fast and the owner is very responsible. Have not used it yet, will see after use	Jingdong prefecture level city in Shandong
Jinfeng Hongyuan Flagship Store	Electricity savings3	🎁 baby quality is very good, the first time I received the goods to open the first time to see, it is very good 👍, very satisfied 😊, customer service also have a question and answer, thank you 🙏	Jingdong prefecture level city in Shandong
Jinfeng Hongyuan Flagship Store	Electricity savings3	I don't know if it's any good 。。。。。。	Jingdong prefecture level city in Shandong
Jinfeng Hongyuan Flagship Store	Electricity savings3	Buy electronic products! Still trust the things of the Jingdong self-owned store! Energy-saving effect to evaluate after heating!	Jingdong prefecture level city in Shandong
Jinfeng Hongyuan Flagship Store	Electricity savings3	The goods have been received, Blu is very beautiful, energy saving effect, to be confirmed only after actual use.	Jingdong prefecture level city in Shandong
Jinfeng Hongyuan Flagship Store	Electricity savings3	Jingdong's speed is so fast, has received, easy to operate. The display is quite good, how many volts what mode is quite understandable.	Jingdong prefecture level city in Shandong
Jinfeng Hongyuan Flagship Store	Electricity savings3	The first use, the effect is good to recommend it to colleagues to use, a month of electricity to save a lot or can be	Jingdong prefecture level city in Shandong
Jinfeng Hongyuan Flagship Store	Electricity savings3	I don't know if it's useful. Mom's is a store. I'll return it if it doesn't work.	Jingdong prefecture level city in Shandong
Jinfeng Hongyuan Flagship Store	Electricity savings3	I don't know if it works or not, I'll wait for two months to use it and then come back to comment!	Jingdong prefecture level city in Shandong
Jinfeng Hongyuan Flagship Store	Electricity savings3	I bought the second one now, and I've been using it for more than half a year, and I feel that it really saves electricity. Now it is summer, the weather is hot, sometimes the fan and air conditioning on all day, if not this power saver, the monthly electricity bill is quite high, but as long as the installation of this power saver, not afraid of its high electricity bill, absolutely let you wantonly use	Jingdong prefecture level city in Shandong
Jinfeng Hongyuan Flagship Store	Electricity savings3	The product is better than I thought it would be, the quality looks great, the voltage is stable, and I'm looking forward to some great power savings!	Jingdong prefecture level city in Shandong
Jinfeng Hongyuan Flagship Store	Electricity savings3	Inadvertently see bought a try, delivery is also quite timely, just do not know how to use, customer service patience to answer, with a pretty good, value for money!	Jingdong prefecture level city in Shandong
Jinfeng Hongyuan Flagship Store	Electricity savings3	It's better than nothing! Hopefully this will help save slightly.	Jingdong prefecture level city in Shandong
Jinfeng Hongyuan Flagship Store	Electricity savings3	Still trying it out, follow up	Jingdong prefecture level city in Shandong
Jinfeng Hongyuan Flagship Store	Electricity savings3	I've just started using it, I don't know how well it works yet, I hope it's good.	Jingdong prefecture level city in Shandong
Jinfeng Hongyuan Flagship Store	Electricity savings3	Fourth purchase, great baby!	Jingdong prefecture level city in Shandong
Jinfeng Hongyuan Flagship Store	Electricity savings3	Shipped really fast, just got it and use it to see what happens and then come back for a review!	Jingdong prefecture level city in Shandong
Jinfeng Hongyuan Flagship Store	Electricity savings3	Timely delivery **** good attitude	Jingdong prefecture level city in Shandong
Jinfeng Hongyuan Flagship Store	Electricity savings3	Home large and small things are purchased in the Jingdong Mall, have been accustomed to shopping in the Jingdong, many times can buy good quality and cheap products, and courier service attitude is particularly good, sometimes too many couriers are to help me to the home, so many years have been in the Jingdong Mall shopping, trust and support! Will also support the Jingdong as always!	Jingdong prefecture level city in Shandong

Jinfeng Hongyuan Flagship Store	Electricity savings3	Modified a pump and the modification worked.	Jingdong prefecture level city in Shandong
Jinfeng Hongyuan Flagship Store	Electricity savings3	Already installed, next time you pay the electric bill to see the results	Jingdong prefecture level city in Shandong
Jinfeng Hongyuan Flagship Store	Electricity savings3	The product is very good to use and practical to look at	Jingdong prefecture level city in Shandong
Jinfeng Hongyuan Flagship Store	Electricity savings3	Works great, great service, waiting for results.	Jingdong prefecture level city in Shandong
Jinfeng Hongyuan Flagship Store	Electricity savings3	I just used it and I'm not sure about the efficacy yet, I'll come back to evaluate it after a while.	Jingdong prefecture level city in Shandong
Jinfeng Hongyuan Flagship Store	Electricity savings3	Just received the goods, use a period of time to evaluate	Jingdong prefecture level city in Shandong
Jinfeng Hongyuan Flagship Store	Electricity savings3	Opened and tried very good, use it to see if there are results	Jingdong prefecture level city in Shandong
Jinfeng Hongyuan Flagship Store	Electricity savings3	Arrived today! Still trying it out!	Jingdong prefecture level city in Shandong
Jinfeng Hongyuan Flagship Store	Electricity savings3	Shipped quickly, well packaged, already installed, hope there is a power saving effect	Jingdong prefecture level city in Shandong
Jinfeng Hongyuan Flagship Store	Electricity savings3	I got it. It's great.	Jingdong prefecture level city in Shandong
Jinfeng Hongyuan Flagship Store	Electricity savings3	Haven't used it yet, will come back in a month	Jingdong prefecture level city in Shandong
Jinfeng Hongyuan Flagship Store	Electricity savings3	It looks good. It's good. Try it first.	Jingdong prefecture level city in Shandong
Jinfeng Hongyuan Flagship Store	Electricity savings3	It works so well that I have relatives, friends, coworkers, and 6 families that have purchased it for them through me.	Jingdong prefecture level city in Shandong
Jinfeng Hongyuan Flagship Store	Electricity savings3	In use	Jingdong prefecture level city in Shandong
Jinfeng Hongyuan Flagship Store	Electricity savings3	Received the goods today, power-saving effect do not know being used, the boss has a very good attitude, praise!	Jingdong prefecture level city in Shandong
Jinfeng Hongyuan Flagship Store	Electricity savings3	The product is compact and doesn't take up much space, but hopefully it's power efficient.	Jingdong prefecture level city in Shandong
Jinfeng Hongyuan Flagship Store	Electricity savings3	Power saving is indeed effective, I used for a period of time before coming to evaluate, the electricity bill is obviously less, the quality is good, the shopkeeper service is also very good, very patient,	Jingdong prefecture level city in Shandong
Jinfeng Hongyuan Flagship Store	Electricity savings3	It feels fine, I'll comment after I use it	Jingdong prefecture level city in Shandong
Jinfeng Hongyuan Flagship Store	Electricity savings3	I just received it and I don't know the effect yet! I will comment again after I use it and have results!	Jingdong prefecture level city in Shandong
Jinfeng Hongyuan Flagship Store	Electricity savings3	The goods look fine, I hope they really work!	Jingdong prefecture level city in Shandong
Jinfeng Hongyuan Flagship Store	Electricity savings3	I just don't know if it saves power	Jingdong prefecture level city in Shandong
Jinfeng Hongyuan Flagship Store	Electricity savings3	My friend introduced me to use this product, it is professional and I am looking forward to the long term effect~!	Jingdong prefecture level city in Shandong
Jinfeng Hongyuan Flagship Store	Electricity savings3	Try it before you judge.	Jingdong prefecture level city in Shandong
Jinfeng Hongyuan Flagship Store	Electricity savings3	It's great, the power savings are obvious! Kudos!	Jingdong prefecture level city in Shandong
Jinfeng Hongyuan Flagship Store	Electricity savings3	The quality is excellent, stabilizes the voltage and saves electricity, very satisfied.	Jingdong prefecture level city in Shandong
Jinfeng Hongyuan Flagship Store	Electricity savings3	You have to use it for a while to know the effect	Jingdong prefecture level city in Shandong
Jinfeng Hongyuan Flagship Store	Electricity savings3	It's on trial.	Jingdong prefecture level city in Shandong
Jinfeng Hongyuan Flagship Store	Electricity savings3	The stuff works great.	Jingdong prefecture level city in Shandong
Jinfeng Hongyuan Flagship Store	Electricity savings3	Doesn't seem to work, not as god as other reviews	Jingdong prefecture level city in Shandong
Jinfeng Hongyuan Flagship Store	Electricity savings3	It's not good quality, it doesn't work, it stops working after a few days, it doesn't work, the customer service doesn't care, it's a bit irritating.	Jingdong prefecture level city in Shandong
Jinfeng Hongyuan Flagship Store	Electricity savings3	It doesn't do anything.	Jingdong prefecture level city in Shandong
Jinfeng Hongyuan Flagship Store	Electricity savings3	It's good.	Jingdong prefecture level city in Shandong
Jinfeng Hongyuan Flagship Store	Electricity savings3	Used a month effect is not obvious, are psychological effects, feel are not less, did not save to the electricity, there are details of the problem, the store said to buy to send 5 fuse, in fact, received only three, small problems are not committed to in place, I believe that the product is not good enough	Jingdong prefecture level city in Shandong
Jinfeng Hongyuan Flagship Store	Electricity savings3	Well received quickly, looking quite high, but also sent 5 fuses. Customer service is very patient to teach the operation, in fact, quite simple, lazy to read the instructions ha!	Jingdong prefecture level city in Shandong
Jinfeng Hongyuan Flagship Store	Electricity savings3	You have to use it for a while to know the effect	Jingdong prefecture level city in Shandong
Jinfeng Hongyuan Flagship Store	Electricity savings3	Shipped very quickly, packaged very well, the price is particularly affordable, there are so many efficacy is also good, it is worth buying worth buying the product, the workmanship is very fine, better than imagined, recommended that everyone try not yet used, after the use of the follow-up comment	Jingdong prefecture level city in Shandong
Jinfeng Hongyuan Flagship Store	Electricity savings3	It looks regular. It's working now.	Jingdong prefecture level city in Shandong
Jinfeng Hongyuan Flagship Store	Electricity savings3	It arrived pretty fast and the owner is very responsible. Have not used it yet, will see after use	Jingdong prefecture level city in Shandong
Jinfeng Hongyuan Flagship Store	Electricity savings3	🎁 baby quality is very good, the first time I received the goods to open the first time to see, it is very good 👍, very satisfied 😊, customer service also have a question and answer, thank you 🙏	Jingdong prefecture level city in Shandong
Jinfeng Hongyuan Flagship Store	Electricity savings3	I don't know if it's any good 。。。。。。	Jingdong prefecture level city in Shandong
Jinfeng Hongyuan Flagship Store	Electricity savings3	Buy electronic products! Still trust the things of the Jingdong self-owned store! Energy-saving effect to evaluate after heating!	Jingdong prefecture level city in Shandong
Jinfeng Hongyuan Flagship Store	Electricity savings3	The goods have been received, Blu is very beautiful, energy saving effect, to be confirmed only after actual use.	Jingdong prefecture level city in Shandong
Jinfeng Hongyuan Flagship Store	Electricity savings3	Jingdong's speed is so fast, has received, easy to operate. The display is quite good, how many volts what mode is quite understandable.	Jingdong prefecture level city in Shandong
Jinfeng Hongyuan Flagship Store	Electricity savings3	The first use, the effect is good to recommend it to colleagues to use, a month of electricity to save a lot or can be	Jingdong prefecture level city in Shandong
Jinfeng Hongyuan Flagship Store	Electricity savings3	I don't know if it's useful. Mom's is a store. I'll return it if it doesn't work.	Jingdong prefecture level city in Shandong
Jinfeng Hongyuan Flagship Store	Electricity savings3	I don't know if it works or not, I'll wait for two months to use it and then come back to comment!	Jingdong prefecture level city in Shandong
Jinfeng Hongyuan Flagship Store	Electricity savings3	I bought the second one now, and I've been using it for more than half a year, and I feel that it really saves electricity. Now it is summer, the weather is hot, sometimes the fan and air conditioning on all day, if not this power saver, the monthly electricity bill is quite high, but as long as the installation of this power saver, not afraid of its high electricity bill, absolutely let you wantonly use	Jingdong prefecture level city in Shandong
Jinfeng Hongyuan Flagship Store	Electricity savings3	The product is better than I thought it would be, the quality looks great, the voltage is stable, and I'm looking forward to some great power savings!	Jingdong prefecture level city in Shandong
Jinfeng Hongyuan Flagship Store	Electricity savings3	Inadvertently see bought a try, delivery is also quite timely, just do not know how to use, customer service patience to answer, with a pretty good, value for money!	Jingdong prefecture level city in Shandong

Jinfeng Hongyuan Flagship Store	Electricity savings3	It's better than nothing! Hopefully this will help save slightly.	Jingdong prefecture level city in Shandong
Jinfeng Hongyuan Flagship Store	Electricity savings3	Still trying it out, follow up	Jingdong prefecture level city in Shandong
Jinfeng Hongyuan Flagship Store	Electricity savings3	I've just started using it, I don't know how well it works yet, I hope it's good.	Jingdong prefecture level city in Shandong
Jinfeng Hongyuan Flagship Store	Electricity savings3	Fourth purchase, great baby!	Jingdong prefecture level city in Shandong
Jinfeng Hongyuan Flagship Store	Electricity savings3	Shipped really fast, just got it and use it to see what happens and then come back for a review!	Jingdong prefecture level city in Shandong
Jinfeng Hongyuan Flagship Store	Electricity savings3	Timely delivery **** good attitude	Jingdong prefecture level city in Shandong
Jinfeng Hongyuan Flagship Store	Electricity savings3	Home large and small things are purchased in the Jingdong Mall, have been accustomed to shopping in the Jingdong, many times can buy good quality and cheap products, and courier service attitude is particularly good, sometimes too many couriers are to help me to the home, so many years have been in the Jingdong Mall shopping, trust and support! Will also support the Jingdong as always!	Jingdong prefecture level city in Shandong
Jinfeng Hongyuan Flagship Store	Electricity savings3	Modified a pump and the modification worked.	Jingdong prefecture level city in Shandong
Jinfeng Hongyuan Flagship Store	Electricity savings3	Already installed, next time you pay the electric bill to see the results	Jingdong prefecture level city in Shandong
Jinfeng Hongyuan Flagship Store	Electricity savings3	The product is very good to use and practical to look at	Jingdong prefecture level city in Shandong
Jinfeng Hongyuan Flagship Store	Electricity savings3	Works great, great service, waiting for results.	Jingdong prefecture level city in Shandong
Jinfeng Hongyuan Flagship Store	Electricity savings3	I just used it and I'm not sure about the efficacy yet, I'll come back to evaluate it after a while.	Jingdong prefecture level city in Shandong
Jinfeng Hongyuan Flagship Store	Electricity savings3	Just received the goods, use a period of time to evaluate	Jingdong prefecture level city in Shandong
Jinfeng Hongyuan Flagship Store	Electricity savings3	Opened and tried very good, use it to see if there are results	Jingdong prefecture level city in Shandong
Jinfeng Hongyuan Flagship Store	Electricity savings3	Arrived today! Still trying it out!	Jingdong prefecture level city in Shandong
Jinfeng Hongyuan Flagship Store	Electricity savings3	Shipped quickly, well packaged, already installed, hope there is a power saving effect	Jingdong prefecture level city in Shandong
Jinfeng Hongyuan Flagship Store	Electricity savings3	I got it. It's great.	Jingdong prefecture level city in Shandong
Jinfeng Hongyuan Flagship Store	Electricity savings3	Haven't used it yet, will come back in a month	Jingdong prefecture level city in Shandong
Jinfeng Hongyuan Flagship Store	Electricity savings3	It looks good. It's good. Try it first.	Jingdong prefecture level city in Shandong
Jinfeng Hongyuan Flagship Store	Electricity savings3	It works so well that I have relatives, friends, coworkers, and 6 families that have purchased it for them through me.	Jingdong prefecture level city in Shandong
Jinfeng Hongyuan Flagship Store	Electricity savings3	In use	Jingdong prefecture level city in Shandong
Jinfeng Hongyuan Flagship Store	Electricity savings3	Received the goods today, power-saving effect do not know being used, the boss has a very good attitude, praise!	Jingdong prefecture level city in Shandong
Jinfeng Hongyuan Flagship Store	Electricity savings3	The product is compact and doesn't take up much space, but hopefully it's power efficient.	Jingdong prefecture level city in Shandong
Jinfeng Hongyuan Flagship Store	Electricity savings3	Power saving is indeed effective, I used for a period of time before coming to evaluate, the electricity bill is obviously less, the quality is good, the shopkeeper service is also very good, very patient,	Jingdong prefecture level city in Shandong
Jinfeng Hongyuan Flagship Store	Electricity savings3	It feels fine, I'll comment after I use it	Jingdong prefecture level city in Shandong
Jinfeng Hongyuan Flagship Store	Electricity savings3	I just received it and I don't know the effect yet! I will comment again after I use it and have results!	Jingdong prefecture level city in Shandong
Jinfeng Hongyuan Flagship Store	Electricity savings3	The goods look fine, I hope they really work!	Jingdong prefecture level city in Shandong
Jinfeng Hongyuan Flagship Store	Electricity savings3	I just don't know if it saves power	Jingdong prefecture level city in Shandong
Jinfeng Hongyuan Flagship Store	Electricity savings3	My friend introduced me to use this product, it is professional and I am looking forward to the long term effect~!	Jingdong prefecture level city in Shandong
Jinfeng Hongyuan Flagship Store	Electricity savings3	Try it before you judge.	Jingdong prefecture level city in Shandong
Jinfeng Hongyuan Flagship Store	Electricity savings3	It's great, the power savings are obvious! Kudos!	Jingdong prefecture level city in Shandong
Jinfeng Hongyuan Flagship Store	Electricity savings3	The quality is excellent, stabilizes the voltage and saves electricity, very satisfied.	Jingdong prefecture level city in Shandong
Jinfeng Hongyuan Flagship Store	Electricity savings3	You have to use it for a while to know the effect	Jingdong prefecture level city in Shandong
Jinfeng Hongyuan Flagship Store	Electricity savings3	It's on trial.	Jingdong prefecture level city in Shandong
Jinfeng Hongyuan Flagship Store	Electricity savings3	The stuff works great.	Jingdong prefecture level city in Shandong
Jinfeng Hongyuan Flagship Store	Electricity savings3	Doesn't seem to work, not as god as other reviews	Jingdong prefecture level city in Shandong
Jinfeng Hongyuan Flagship Store	Electricity savings3	It's not good quality, it doesn't work, it stops working after a few days, it doesn't work, the customer service doesn't care, it's a bit irritating.	Jingdong prefecture level city in Shandong
Jinfeng Hongyuan Flagship Store	Electricity savings3	It doesn't do anything.	Jingdong prefecture level city in Shandong
Jinfeng Hongyuan Flagship Store	Electricity savings3	It's good.	Jingdong prefecture level city in Shandong
Jinfeng Hongyuan Flagship Store	Electricity savings3	Used a month effect is not obvious, are psychological effects, feel are not less, did not save to the electricity, there are details of the problem, the store said to buy to send 5 fuse, in fact, received only three, small problems are not committed to in place, I believe that the product is not good enough	Jingdong prefecture level city in Shandong
Jinfeng Hongyuan Flagship Store	Electricity savings3	Well received quickly, looking quite high, but also sent 5 fuses. Customer service is very patient to teach the operation, in fact, quite simple, lazy to read the instructions ha!	Jingdong prefecture level city in Shandong
Jinfeng Hongyuan Flagship Store	Electricity savings3	You have to use it for a while to know the effect	Jingdong prefecture level city in Shandong
Jinfeng Hongyuan Flagship Store	Electricity savings3	Shipped very quickly, packaged very well, the price is particularly affordable, there are so many efficacy is also good, it is worth buying worth buying the product, the workmanship is very fine, better than imagined, recommended that everyone try not yet used, after the use of the follow-up comment	Jingdong prefecture level city in Shandong
Jinfeng Hongyuan Flagship Store	Electricity savings3	It looks regular. It's working now.	Jingdong prefecture level city in Shandong
Jinfeng Hongyuan Flagship Store	Electricity savings3	It arrived pretty fast and the owner is very responsible. Have not used it yet, will see after use	Jingdong prefecture level city in Shandong
Jinfeng Hongyuan Flagship Store	Electricity savings3	🎁 baby quality is very good, the first time I received the goods to open the first time to see, it is very good 👍, very satisfied 😉, customer service also have a question and answer, thank you 🙏	Jingdong prefecture level city in Shandong
Jinfeng Hongyuan Flagship Store	Electricity savings3	I don't know if it's any good 。。。。。。	Jingdong prefecture level city in Shandong
Jinfeng Hongyuan Flagship Store	Electricity savings3	Buy electronic products! Still trust the things of the Jingdong self-owned store! Energy-saving effect to evaluate after heating!	Jingdong prefecture level city in Shandong
Jinfeng Hongyuan Flagship Store	Electricity savings3	The goods have been received, Blu is very beautiful, energy saving effect, to be confirmed only after actual use.	Jingdong prefecture level city in Shandong

Jinfeng Hongyuan Flagship Store	Electricity savings3	Jingdong's speed is so fast, has received, easy to operate. The display is quite good, how many volts what mode is quite understandable.	Jingdong prefecture level city in Shandong
Jinfeng Hongyuan Flagship Store	Electricity savings3	The first use, the effect is good to recommend it to colleagues to use, a month of electricity to save a lot or can be	Jingdong prefecture level city in Shandong
Jinfeng Hongyuan Flagship Store	Electricity savings3	I don't know if it's useful. Mom's is a store. I'll return it if it doesn't work.	Jingdong prefecture level city in Shandong
Jinfeng Hongyuan Flagship Store	Electricity savings3	I don't know if it works or not, I'll wait for two months to use it and then come back to comment!	Jingdong prefecture level city in Shandong
Jinfeng Hongyuan Flagship Store	Electricity savings3	I bought the second one now, and I've been using it for more than half a year, and I feel that it really saves electricity. Now it is summer, the weather is hot, sometimes the fan and air conditioning on all day, if not this power saver, the monthly electricity bill is quite high, but as long as the installation of this power saver, not afraid of its high electricity bill, absolutely let you wantonly use	Jingdong prefecture level city in Shandong
Jinfeng Hongyuan Flagship Store	Electricity savings3	The product is better than I thought it would be, the quality looks great, the voltage is stable, and I'm looking forward to some great power savings!	Jingdong prefecture level city in Shandong
Jinfeng Hongyuan Flagship Store	Electricity savings3	Inadvertently see bought a try, delivery is also quite timely, just do not know how to use, customer service patience to answer, with a pretty good, value for money!	Jingdong prefecture level city in Shandong
Jinfeng Hongyuan Flagship Store	Electricity savings3	It's better than nothing! Hopefully this will help save slightly.	Jingdong prefecture level city in Shandong
Jinfeng Hongyuan Flagship Store	Electricity savings3	Still trying it out, follow up	Jingdong prefecture level city in Shandong
Jinfeng Hongyuan Flagship Store	Electricity savings3	I've just started using it, I don't know how well it works yet, I hope it's good.	Jingdong prefecture level city in Shandong
Jinfeng Hongyuan Flagship Store	Electricity savings3	Fourth purchase, great baby!	Jingdong prefecture level city in Shandong
Jinfeng Hongyuan Flagship Store	Electricity savings3	Shipped really fast, just got it and use it to see what happens and then come back for a review!	Jingdong prefecture level city in Shandong
Jinfeng Hongyuan Flagship Store	Electricity savings3	Timely delivery **** good attitude	Jingdong prefecture level city in Shandong
Jinfeng Hongyuan Flagship Store	Electricity savings3	Home large and small things are purchased in the Jingdong Mall, have been accustomed to shopping in the Jingdong, many times can buy good quality and cheap products, and courier service attitude is particularly good, sometimes too many couriers are to help me to the home, so many years have been in the Jingdong Mall shopping, trust and support! Will also support the Jingdong as always!	Jingdong prefecture level city in Shandong
Jinfeng Hongyuan Flagship Store	Electricity savings3	Modified a pump and the modification worked.	Jingdong prefecture level city in Shandong
Jinfeng Hongyuan Flagship Store	Electricity savings3	Already installed, next time you pay the electric bill to see the results	Jingdong prefecture level city in Shandong
Jinfeng Hongyuan Flagship Store	Electricity savings3	The product is very good to use and practical to look at	Jingdong prefecture level city in Shandong
Jinfeng Hongyuan Flagship Store	Electricity savings3	Works great, great service, waiting for results.	Jingdong prefecture level city in Shandong
Jinfeng Hongyuan Flagship Store	Electricity savings3	I just used it and I'm not sure about the efficacy yet, I'll come back to evaluate it after a while.	Jingdong prefecture level city in Shandong
Jinfeng Hongyuan Flagship Store	Electricity savings3	Just received the goods, use a period of time to evaluate	Jingdong prefecture level city in Shandong
Jinfeng Hongyuan Flagship Store	Electricity savings3	Opened and tried very good, use it to see if there are results	Jingdong prefecture level city in Shandong
Jinfeng Hongyuan Flagship Store	Electricity savings3	Arrived today! Still trying it out!	Jingdong prefecture level city in Shandong
Jinfeng Hongyuan Flagship Store	Electricity savings3	Shipped quickly, well packaged, already installed, hope there is a power saving effect	Jingdong prefecture level city in Shandong
Jinfeng Hongyuan Flagship Store	Electricity savings3	I got it. It's great.	Jingdong prefecture level city in Shandong
Jinfeng Hongyuan Flagship Store	Electricity savings3	Haven't used it yet, will come back in a month	Jingdong prefecture level city in Shandong
Jinfeng Hongyuan Flagship Store	Electricity savings3	It looks good. It's good. Try it first.	Jingdong prefecture level city in Shandong
Jinfeng Hongyuan Flagship Store	Electricity savings3	It works so well that I have relatives, friends, coworkers, and 6 families that have purchased it for them through me.	Jingdong prefecture level city in Shandong
Jinfeng Hongyuan Flagship Store	Electricity savings3	In use	Jingdong prefecture level city in Shandong
Jinfeng Hongyuan Flagship Store	Electricity savings3	Received the goods today, power-saving effect do not know being used, the boss has a very good attitude, praise!	Jingdong prefecture level city in Shandong
Jinfeng Hongyuan Flagship Store	Electricity savings3	The product is compact and doesn't take up much space, but hopefully it's power efficient.	Jingdong prefecture level city in Shandong
Jinfeng Hongyuan Flagship Store	Electricity savings3	Power saving is indeed effective, I used for a period of time before coming to evaluate, the electricity bill is obviously less, the quality is good, the shopkeeper service is also very good, very patient,	Jingdong prefecture level city in Shandong
Jinfeng Hongyuan Flagship Store	Electricity savings3	It feels fine, I'll comment after I use it	Jingdong prefecture level city in Shandong
Jinfeng Hongyuan Flagship Store	Electricity savings3	I just received it and I don't know the effect yet! I will comment again after I use it and have results!	Jingdong prefecture level city in Shandong
Jinfeng Hongyuan Flagship Store	Electricity savings3	The goods look fine, I hope they really work!	Jingdong prefecture level city in Shandong
Jinfeng Hongyuan Flagship Store	Electricity savings3	I just don't know if it saves power	Jingdong prefecture level city in Shandong
Jinfeng Hongyuan Flagship Store	Electricity savings3	My friend introduced me to use this product, it is professional and I am looking forward to the long term effect~!	Jingdong prefecture level city in Shandong
Jinfeng Hongyuan Flagship Store	Electricity savings3	Try it before you judge.	Jingdong prefecture level city in Shandong
Jinfeng Hongyuan Flagship Store	Electricity savings3	It's great, the power savings are obvious! Kudos!	Jingdong prefecture level city in Shandong
Jinfeng Hongyuan Flagship Store	Electricity savings3	The quality is excellent, stabilizes the voltage and saves electricity, very satisfied.	Jingdong prefecture level city in Shandong
Jinfeng Hongyuan Flagship Store	Electricity savings3	You have to use it for a while to know the effect	Jingdong prefecture level city in Shandong
Jinfeng Hongyuan Flagship Store	Electricity savings3	It's on trial.	Jingdong prefecture level city in Shandong
Jinfeng Hongyuan Flagship Store	Electricity savings3	The stuff works great.	Jingdong prefecture level city in Shandong
Jinfeng Hongyuan Flagship Store	Electricity savings3	Doesn't seem to work, not as god as other reviews	Jingdong prefecture level city in Shandong
Jinfeng Hongyuan Flagship Store	Electricity savings3	It's not good quality, it doesn't work, it stops working after a few days, it doesn't work, the customer service doesn't care, it's a bit irritating.	Jingdong prefecture level city in Shandong
Jinfeng Hongyuan Flagship Store	Electricity savings3	It doesn't do anything.	Jingdong prefecture level city in Shandong
Jinfeng Hongyuan Flagship Store	Electricity savings3	It's good.	Jingdong prefecture level city in Shandong
Jinfeng Hongyuan Flagship Store	Electricity savings3	Used a month effect is not obvious, are psychological effects, feel are not less, did not save to the electricity, there are details of the problem, the store said to buy to send 5 fuse, in fact, received only three, small problems are not committed to in place, I believe that the product is not good enough	Jingdong prefecture level city in Shandong
Jinfeng Hongyuan Flagship Store	Electricity savings3	Well received quickly, looking quite high, but also sent 5 fuses. Customer service is very patient to teach the operation, in fact, quite simple, lazy to read the instructions ha!	Jingdong prefecture level city in Shandong
Jinfeng Hongyuan Flagship Store	Electricity savings3	You have to use it for a while to know the effect	Jingdong prefecture level city in Shandong

Jinfeng Hongyuan Flagship Store	Electricity savings3	Shipped very quickly, packaged very well, the price is particularly affordable, there are so many efficacy is also good, it is worth buying worth buying the product, the workmanship is very fine, better than imagined, recommended that everyone try not yet used, after the use of the follow-up comment	Jingdong prefecture level city in Shandong
Jinfeng Hongyuan Flagship Store	Electricity savings3	It looks regular. It's working now.	Jingdong prefecture level city in Shandong
Jinfeng Hongyuan Flagship Store	Electricity savings3	It arrived pretty fast and the owner is very responsible. Have not used it yet, will see after use	Jingdong prefecture level city in Shandong
Jinfeng Hongyuan Flagship Store	Electricity savings3	🎁 baby quality is very good, the first time I received the goods to open the first time to see, it is very good 👍, very satisfied 😊, customer service also have a question and answer, thank you 🙏	Jingdong prefecture level city in Shandong
Jinfeng Hongyuan Flagship Store	Electricity savings3	I don't know if it's any good 。。。。。。	Jingdong prefecture level city in Shandong
Jinfeng Hongyuan Flagship Store	Electricity savings3	Buy electronic products! Still trust the things of the Jingdong self-owned store! Energy-saving effect to evaluate after heating!	Jingdong prefecture level city in Shandong
Jinfeng Hongyuan Flagship Store	Electricity savings3	The goods have been received, Blu is very beautiful, energy saving effect, to be confirmed only after actual use.	Jingdong prefecture level city in Shandong
Jinfeng Hongyuan Flagship Store	Electricity savings3	Jingdong's speed is so fast, has received, easy to operate. The display is quite good, how many volts what mode is quite understandable.	Jingdong prefecture level city in Shandong
Jinfeng Hongyuan Flagship Store	Electricity savings3	The first use, the effect is good to recommend it to colleagues to use, a month of electricity to save a lot or can be	Jingdong prefecture level city in Shandong
Jinfeng Hongyuan Flagship Store	Electricity savings3	I don't know if it's useful. Mom's is a store. I'll return it if it doesn't work.	Jingdong prefecture level city in Shandong
Jinfeng Hongyuan Flagship Store	Electricity savings3	I don't know if it works or not, I'll wait for two months to use it and then come back to comment!	Jingdong prefecture level city in Shandong
Jinfeng Hongyuan Flagship Store	Electricity savings3	I bought the second one now, and I've been using it for more than half a year, and I feel that it really saves electricity. Now it is summer, the weather is hot, sometimes the fan and air conditioning on all day, if not this power saver, the monthly electricity bill is quite high, but as long as the installation of this power saver, not afraid of its high electricity bill, absolutely let you wantonly use	Jingdong prefecture level city in Shandong
Jinfeng Hongyuan Flagship Store	Electricity savings3	The product is better than I thought it would be, the quality looks great, the voltage is stable, and I'm looking forward to some great power savings!	Jingdong prefecture level city in Shandong
Jinfeng Hongyuan Flagship Store	Electricity savings3	Inadvertently see bought a try, delivery is also quite timely, just do not know how to use, customer service patience to answer, with a pretty good, value for money!	Jingdong prefecture level city in Shandong
Jinfeng Hongyuan Flagship Store	Electricity savings3	It's better than nothing! Hopefully this will help save slightly.	Jingdong prefecture level city in Shandong
Jinfeng Hongyuan Flagship Store	Electricity savings3	Still trying it out, follow up	Jingdong prefecture level city in Shandong
Jinfeng Hongyuan Flagship Store	Electricity savings3	I've just started using it, I don't know how well it works yet, I hope it's good.	Jingdong prefecture level city in Shandong
Jinfeng Hongyuan Flagship Store	Electricity savings3	Fourth purchase, great baby!	Jingdong prefecture level city in Shandong
Jinfeng Hongyuan Flagship Store	Electricity savings3	Shipped really fast, just got it and use it to see what happens and then come back for a review!	Jingdong prefecture level city in Shandong
Jinfeng Hongyuan Flagship Store	Electricity savings3	Timely delivery **** good attitude	Jingdong prefecture level city in Shandong
Jinfeng Hongyuan Flagship Store	Electricity savings3	Home large and small things are purchased in the Jingdong Mall, have been accustomed to shopping in the Jingdong, many times can buy good quality and cheap products, and courier service attitude is particularly good, sometimes too many couriers are to help me to the home, so many years have been in the Jingdong Mall shopping, trust and support! Will also support the Jingdong as always!	Jingdong prefecture level city in Shandong
Jinfeng Hongyuan Flagship Store	Electricity savings3	Modified a pump and the modification worked.	Jingdong prefecture level city in Shandong
Jinfeng Hongyuan Flagship Store	Electricity savings3	Already installed, next time you pay the electric bill to see the results	Jingdong prefecture level city in Shandong
Jinfeng Hongyuan Flagship Store	Electricity savings3	The product is very good to use and practical to look at	Jingdong prefecture level city in Shandong
Jinfeng Hongyuan Flagship Store	Electricity savings3	Works great, great service, waiting for results.	Jingdong prefecture level city in Shandong
Jinfeng Hongyuan Flagship Store	Electricity savings3	I just used it and I'm not sure about the efficacy yet, I'll come back to evaluate it after a while.	Jingdong prefecture level city in Shandong
Jinfeng Hongyuan Flagship Store	Electricity savings3	Just received the goods, use a period of time to evaluate	Jingdong prefecture level city in Shandong
Jinfeng Hongyuan Flagship Store	Electricity savings3	Opened and tried very good, use it to see if there are results	Jingdong prefecture level city in Shandong
Jinfeng Hongyuan Flagship Store	Electricity savings3	Arrived today! Still trying it out!	Jingdong prefecture level city in Shandong
Jinfeng Hongyuan Flagship Store	Electricity savings3	Shipped quickly, well packaged, already installed, hope there is a power saving effect	Jingdong prefecture level city in Shandong
Jinfeng Hongyuan Flagship Store	Electricity savings3	I got it. It's great.	Jingdong prefecture level city in Shandong
Jinfeng Hongyuan Flagship Store	Electricity savings3	Haven't used it yet, will come back in a month	Jingdong prefecture level city in Shandong
Jinfeng Hongyuan Flagship Store	Electricity savings3	It looks good. It's good. Try it first.	Jingdong prefecture level city in Shandong
Jinfeng Hongyuan Flagship Store	Electricity savings3	It works so well that I have relatives, friends, coworkers, and 6 families that have purchased it for them through me.	Jingdong prefecture level city in Shandong
Jinfeng Hongyuan Flagship Store	Electricity savings3	In use	Jingdong prefecture level city in Shandong
Jinfeng Hongyuan Flagship Store	Electricity savings3	Received the goods today, power-saving effect do not know being used, the boss has a very good attitude, praise!	Jingdong prefecture level city in Shandong
Jinfeng Hongyuan Flagship Store	Electricity savings3	The product is compact and doesn't take up much space, but hopefully it's power efficient.	Jingdong prefecture level city in Shandong
Jinfeng Hongyuan Flagship Store	Electricity savings3	Power saving is indeed effective, I used for a period of time before coming to evaluate, the electricity bill is obviously less, the quality is good, the shopkeeper service is also very good, very patient,	Jingdong prefecture level city in Shandong
Jinfeng Hongyuan Flagship Store	Electricity savings3	It feels fine, I'll comment after I use it	Jingdong prefecture level city in Shandong
Jinfeng Hongyuan Flagship Store	Electricity savings3	I just received it and I don't know the effect yet! I will comment again after I use it and have results!	Jingdong prefecture level city in Shandong
Jinfeng Hongyuan Flagship Store	Electricity savings3	The goods look fine, I hope they really work!	Jingdong prefecture level city in Shandong
Jinfeng Hongyuan Flagship Store	Electricity savings3	I just don't know if it saves power	Jingdong prefecture level city in Shandong
Jinfeng Hongyuan Flagship Store	Electricity savings3	My friend introduced me to use this product, it is professional and I am looking forward to the long term effect~!	Jingdong prefecture level city in Shandong
Jinfeng Hongyuan Flagship Store	Electricity savings3	Try it before you judge.	Jingdong prefecture level city in Shandong
Jinfeng Hongyuan Flagship Store	Electricity savings3	It's great, the power savings are obvious! Kudos!	Jingdong prefecture level city in Shandong
Jinfeng Hongyuan Flagship Store	Electricity savings3	The quality is excellent, stabilizes the voltage and saves electricity, very satisfied.	Jingdong prefecture level city in Shandong
Jinfeng Hongyuan Flagship Store	Electricity savings3	You have to use it for a while to know the effect	Jingdong prefecture level city in Shandong
Jinfeng Hongyuan Flagship Store	Electricity savings3	It's on trial.	Jingdong prefecture level city in Shandong
Jinfeng Hongyuan Flagship Store	Electricity savings3	The stuff works great.	Jingdong prefecture level city in Shandong

Jinfeng Hongyuan Flagship Store	Electricity savings3	Doesn't seem to work, not as god as other reviews	Jingdong prefecture level city in Shandong
Jinfeng Hongyuan Flagship Store	Electricity savings3	It's not good quality, it doesn't work, it stops working after a few days, it doesn't work, the customer service doesn't care, it's a bit irritating.	Jingdong prefecture level city in Shandong
Jinfeng Hongyuan Flagship Store	Electricity savings3	It doesn't do anything.	Jingdong prefecture level city in Shandong
Jinfeng Hongyuan Flagship Store	Electricity savings3	It's good.	Jingdong prefecture level city in Shandong
Jinfeng Hongyuan Flagship Store	Electricity savings3	Used a month effect is not obvious, are psychological effects, feel are not less, did not save to the electricity, there are details of the problem, the store said to buy to send 5 fuse, in fact, received only three, small problems are not committed to in place, I believe that the product is not good enough	Jingdong prefecture level city in Shandong
Jinfeng Hongyuan Flagship Store	Electricity savings3	Well received quickly, looking quite high, but also sent 5 fuses. Customer service is very patient to teach the operation, in fact, quite simple, lazy to read the instructions ha!	Jingdong prefecture level city in Shandong
Jinfeng Hongyuan Flagship Store	Electricity savings3	You have to use it for a while to know the effect	Jingdong prefecture level city in Shandong
Jinfeng Hongyuan Flagship Store	Electricity savings3	Shipped very quickly, packaged very well, the price is particularly affordable, there are so many efficacy is also good, it is worth buying worth buying the product, the workmanship is very fine, better than imagined, recommended that everyone try not yet used, after the use of the follow-up comment	Jingdong prefecture level city in Shandong
Jinfeng Hongyuan Flagship Store	Electricity savings3	It looks regular. It's working now.	Jingdong prefecture level city in Shandong
Jinfeng Hongyuan Flagship Store	Electricity savings3	It arrived pretty fast and the owner is very responsible. Have not used it yet, will see after use	Jingdong prefecture level city in Shandong
Jinfeng Hongyuan Flagship Store	Electricity savings3	🎁 baby quality is very good, the first time I received the goods to open the first time to see, it is very good 👍, very satisfied 😊, customer service also have a question and answer, thank you 🙏	Jingdong prefecture level city in Shandong
Jinfeng Hongyuan Flagship Store	Electricity savings3	I don't know if it's any good 。。。。。。	Jingdong prefecture level city in Shandong
Jinfeng Hongyuan Flagship Store	Electricity savings3	Buy electronic products! Still trust the things of the Jingdong self-owned store! Energy-saving effect to evaluate after heating!	Jingdong prefecture level city in Shandong
Jinfeng Hongyuan Flagship Store	Electricity savings3	The goods have been received, Blu is very beautiful, energy saving effect, to be confirmed only after actual use.	Jingdong prefecture level city in Shandong
Jinfeng Hongyuan Flagship Store	Electricity savings3	Jingdong's speed is so fast, has received, easy to operate. The display is quite good, how many volts what mode is quite understandable.	Jingdong prefecture level city in Shandong
Jinfeng Hongyuan Flagship Store	Electricity savings3	The first use, the effect is good to recommend it to colleagues to use, a month of electricity to save a lot or can be	Jingdong prefecture level city in Shandong
Jinfeng Hongyuan Flagship Store	Electricity savings3	I don't know if it's useful. Mom's is a store. I'll return it if it doesn't work.	Jingdong prefecture level city in Shandong
Jinfeng Hongyuan Flagship Store	Electricity savings3	I don't know if it works or not, I'll wait for two months to use it and then come back to comment!	Jingdong prefecture level city in Shandong
Jinfeng Hongyuan Flagship Store	Electricity savings3	I bought the second one now, and I've been using it for more than half a year, and I feel that it really saves electricity. Now it is summer, the weather is hot, sometimes the fan and air conditioning on all day, if not this power saver, the monthly electricity bill is quite high, but as long as the installation of this power saver, not afraid of its high electricity bill, absolutely let you wantonly use	Jingdong prefecture level city in Shandong
Jinfeng Hongyuan Flagship Store	Electricity savings3	The product is better than I thought it would be, the quality looks great, the voltage is stable, and I'm looking forward to some great power savings!	Jingdong prefecture level city in Shandong
Jinfeng Hongyuan Flagship Store	Electricity savings3	Inadvertently see bought a try, delivery is also quite timely, just do not know how to use, customer service patience to answer, with a pretty good, value for money!	Jingdong prefecture level city in Shandong
Jinfeng Hongyuan Flagship Store	Electricity savings3	It's better than nothing! Hopefully this will help save slightly.	Jingdong prefecture level city in Shandong
Jinfeng Hongyuan Flagship Store	Electricity savings3	Still trying it out, follow up	Jingdong prefecture level city in Shandong
Jinfeng Hongyuan Flagship Store	Electricity savings3	I've just started using it, I don't know how well it works yet, I hope it's good.	Jingdong prefecture level city in Shandong
Jinfeng Hongyuan Flagship Store	Electricity savings3	Fourth purchase, great baby!	Jingdong prefecture level city in Shandong
Jinfeng Hongyuan Flagship Store	Electricity savings3	Shipped really fast, just got it and use it to see what happens and then come back for a review!	Jingdong prefecture level city in Shandong
Jinfeng Hongyuan Flagship Store	Electricity savings3	Timely delivery **** good attitude	Jingdong prefecture level city in Shandong
Jinfeng Hongyuan Flagship Store	Electricity savings3	Home large and small things are purchased in the Jingdong Mall, have been accustomed to shopping in the Jingdong, many times can buy good quality and cheap products, and courier service attitude is particularly good, sometimes too many couriers are to help me to the home, so many years have been in the Jingdong Mall shopping, trust and support! Will also support the Jingdong as always!	Jingdong prefecture level city in Shandong
Jinfeng Hongyuan Flagship Store	Electricity savings3	Modified a pump and the modification worked.	Jingdong prefecture level city in Shandong
Jinfeng Hongyuan Flagship Store	Electricity savings3	Already installed, next time you pay the electric bill to see the results	Jingdong prefecture level city in Shandong
Jinfeng Hongyuan Flagship Store	Electricity savings3	The product is very good to use and practical to look at	Jingdong prefecture level city in Shandong
Jinfeng Hongyuan Flagship Store	Electricity savings3	Works great, great service, waiting for results.	Jingdong prefecture level city in Shandong
Jinfeng Hongyuan Flagship Store	Electricity savings3	I just used it and I'm not sure about the efficacy yet, I'll come back to evaluate it after a while.	Jingdong prefecture level city in Shandong
Jinfeng Hongyuan Flagship Store	Electricity savings3	Just received the goods, use a period of time to evaluate	Jingdong prefecture level city in Shandong
Jinfeng Hongyuan Flagship Store	Electricity savings3	Opened and tried very good, use it to see if there are results	Jingdong prefecture level city in Shandong
Jinfeng Hongyuan Flagship Store	Electricity savings3	Arrived today! Still trying it out!	Jingdong prefecture level city in Shandong
Jinfeng Hongyuan Flagship Store	Electricity savings3	Shipped quickly, well packaged, already installed, hope there is a power saving effect	Jingdong prefecture level city in Shandong
Jinfeng Hongyuan Flagship Store	Electricity savings3	I got it. It's great.	Jingdong prefecture level city in Shandong
Jinfeng Hongyuan Flagship Store	Electricity savings3	Haven't used it yet, will come back in a month	Jingdong prefecture level city in Shandong
Jinfeng Hongyuan Flagship Store	Electricity savings3	It looks good. It's good. Try it first.	Jingdong prefecture level city in Shandong
Jinfeng Hongyuan Flagship Store	Electricity savings3	It works so well that I have relatives, friends, coworkers, and 6 families that have purchased it for them through me.	Jingdong prefecture level city in Shandong
Jinfeng Hongyuan Flagship Store	Electricity savings3	In use	Jingdong prefecture level city in Shandong
Jinfeng Hongyuan Flagship Store	Electricity savings3	Received the goods today, power-saving effect do not know being used, the boss has a very good attitude, praise!	Jingdong prefecture level city in Shandong
Jinfeng Hongyuan Flagship Store	Electricity savings3	The product is compact and doesn't take up much space, but hopefully it's power efficient.	Jingdong prefecture level city in Shandong
Jinfeng Hongyuan Flagship Store	Electricity savings3	Power saving is indeed effective, I used for a period of time before coming to evaluate, the electricity bill is obviously less, the quality is good, the shopkeeper service is also very good, very patient,	Jingdong prefecture level city in Shandong
Jinfeng Hongyuan Flagship Store	Electricity savings3	It feels fine, I'll comment after I use it	Jingdong prefecture level city in Shandong
Jinfeng Hongyuan Flagship Store	Electricity savings3	I just received it and I don't know the effect yet! I will comment again after I use it and have results!	Jingdong prefecture level city in Shandong
Jinfeng Hongyuan Flagship Store	Electricity savings3	The goods look fine, I hope they really work!	Jingdong prefecture level city in Shandong

Jinfeng Hongyuan Flagship Store	Electricity savings3	I just don't know if it saves power	Jingdong prefecture level city in Shandong
Jinfeng Hongyuan Flagship Store	Electricity savings3	My friend introduced me to use this product, it is professional and I am looking forward to the long term effect~!	Jingdong prefecture level city in Shandong
Jinfeng Hongyuan Flagship Store	Electricity savings3	Try it before you judge.	Jingdong prefecture level city in Shandong
Jinfeng Hongyuan Flagship Store	Electricity savings3	It's great, the power savings are obvious! Kudos!	Jingdong prefecture level city in Shandong
Jinfeng Hongyuan Flagship Store	Electricity savings3	The quality is excellent, stabilizes the voltage and saves electricity, very satisfied.	Jingdong prefecture level city in Shandong
Jinfeng Hongyuan Flagship Store	Electricity savings3	You have to use it for a while to know the effect	Jingdong prefecture level city in Shandong
Jinfeng Hongyuan Flagship Store	Electricity savings3	It's on trial.	Jingdong prefecture level city in Shandong
Jinfeng Hongyuan Flagship Store	Electricity savings3	The stuff works great.	Jingdong prefecture level city in Shandong
Jinfeng Hongyuan Flagship Store	Electricity savings3	Doesn't seem to work, not as god as other reviews	Jingdong prefecture level city in Shandong
Jinfeng Hongyuan Flagship Store	Electricity savings3	It's not good quality, it doesn't work, it stops working after a few days, it doesn't work, the customer service doesn't care, it's a bit irritating.	Jingdong prefecture level city in Shandong
Jinfeng Hongyuan Flagship Store	Electricity savings3	It doesn't do anything.	Jingdong prefecture level city in Shandong
Jinfeng Hongyuan Flagship Store	Electricity savings3	It's good.	Jingdong prefecture level city in Shandong
Jinfeng Hongyuan Flagship Store	Electricity savings3	Used a month effect is not obvious, are psychological effects, feel are not less, did not save to the electricity, there are details of the problem, the store said to buy to send 5 fuse, in fact, received only three, small problems are not committed to in place, I believe that the product is not good enough	Jingdong prefecture level city in Shandong
Jinfeng Hongyuan Flagship Store	Electricity savings3	Well received quickly, looking quite high, but also sent 5 fuses. Customer service is very patient to teach the operation, in fact, quite simple, lazy to read the instructions ha!	Jingdong prefecture level city in Shandong
Jinfeng Hongyuan Flagship Store	Electricity savings3	You have to use it for a while to know the effect	Jingdong prefecture level city in Shandong
Jinfeng Hongyuan Flagship Store	Electricity savings3	Shipped very quickly, packaged very well, the price is particularly affordable, there are so many efficacy is also good, it is worth buying worth buying the product, the workmanship is very fine, better than imagined, recommended that everyone try not yet used, after the use of the follow-up comment	Jingdong prefecture level city in Shandong
Jinfeng Hongyuan Flagship Store	Electricity savings3	It looks regular. It's working now.	Jingdong prefecture level city in Shandong
Jinfeng Hongyuan Flagship Store	Electricity savings3	It arrived pretty fast and the owner is very responsible. Have not used it yet, will see after use	Jingdong prefecture level city in Shandong
Jinfeng Hongyuan Flagship Store	Electricity savings3	🎁 baby quality is very good, the first time I received the goods to open the first time to see, it is very good 👍, very satisfied 😊, customer service also have a question and answer, thank you 🙏	Jingdong prefecture level city in Shandong
Jinfeng Hongyuan Flagship Store	Electricity savings3	I don't know if it's any good 。。。。。。	Jingdong prefecture level city in Shandong
Jinfeng Hongyuan Flagship Store	Electricity savings3	Buy electronic products! Still trust the things of the Jingdong self-owned store! Energy-saving effect to evaluate after heating!	Jingdong prefecture level city in Shandong
Jinfeng Hongyuan Flagship Store	Electricity savings3	The goods have been received, Blu is very beautiful, energy saving effect, to be confirmed only after actual use.	Jingdong prefecture level city in Shandong
Jinfeng Hongyuan Flagship Store	Electricity savings3	Jingdong's speed is so fast, has received, easy to operate. The display is quite good, how many volts what mode is quite understandable.	Jingdong prefecture level city in Shandong
Jinfeng Hongyuan Flagship Store	Electricity savings3	The first use, the effect is good to recommend it to colleagues to use, a month of electricity to save a lot or can be	Jingdong prefecture level city in Shandong
Jinfeng Hongyuan Flagship Store	Electricity savings3	I don't know if it's useful. Mom's is a store. I'll return it if it doesn't work.	Jingdong prefecture level city in Shandong
Jinfeng Hongyuan Flagship Store	Electricity savings3	I don't know if it works or not, I'll wait for two months to use it and then come back to comment!	Jingdong prefecture level city in Shandong
Jinfeng Hongyuan Flagship Store	Electricity savings3	I bought the second one now, and I've been using it for more than half a year, and I feel that it really saves electricity. Now it is summer, the weather is hot, sometimes the fan and air conditioning on all day, if not this power saver, the monthly electricity bill is quite high, but as long as the installation of this power saver, not afraid of its high electricity bill, absolutely let you wantonly use	Jingdong prefecture level city in Shandong
Jinfeng Hongyuan Flagship Store	Electricity savings3	The product is better than I thought it would be, the quality looks great, the voltage is stable, and I'm looking forward to some great power savings!	Jingdong prefecture level city in Shandong
Jinfeng Hongyuan Flagship Store	Electricity savings3	Inadvertently see bought a try, delivery is also quite timely, just do not know how to use, customer service patience to answer, with a pretty good, value for money!	Jingdong prefecture level city in Shandong
Jinfeng Hongyuan Flagship Store	Electricity savings3	It's better than nothing! Hopefully this will help save slightly.	Jingdong prefecture level city in Shandong
Jinfeng Hongyuan Flagship Store	Electricity savings3	Still trying it out, follow up	Jingdong prefecture level city in Shandong
Jinfeng Hongyuan Flagship Store	Electricity savings3	I've just started using it, I don't know how well it works yet, I hope it's good.	Jingdong prefecture level city in Shandong
Jinfeng Hongyuan Flagship Store	Electricity savings3	Fourth purchase, great baby!	Jingdong prefecture level city in Shandong
Jinfeng Hongyuan Flagship Store	Electricity savings3	Shipped really fast, just got it and use it to see what happens and then come back for a review!	Jingdong prefecture level city in Shandong
Jinfeng Hongyuan Flagship Store	Electricity savings3	Timely delivery **** good attitude	Jingdong prefecture level city in Shandong
Jinfeng Hongyuan Flagship Store	Electricity savings3	Home large and small things are purchased in the Jingdong Mall, have been accustomed to shopping in the Jingdong, many times can buy good quality and cheap products, and courier service attitude is particularly good, sometimes too many couriers are to help me to the home, so many years have been in the Jingdong Mall shopping, trust and support! Will also support the Jingdong as always!	Jingdong prefecture level city in Shandong
Jinfeng Hongyuan Flagship Store	Electricity savings3	Modified a pump and the modification worked.	Jingdong prefecture level city in Shandong
Jinfeng Hongyuan Flagship Store	Electricity savings3	Already installed, next time you pay the electric bill to see the results	Jingdong prefecture level city in Shandong
Jinfeng Hongyuan Flagship Store	Electricity savings3	The product is very good to use and practical to look at	Jingdong prefecture level city in Shandong
Jinfeng Hongyuan Flagship Store	Electricity savings3	Works great, great service, waiting for results.	Jingdong prefecture level city in Shandong
Jinfeng Hongyuan Flagship Store	Electricity savings3	I just used it and I'm not sure about the efficacy yet, I'll come back to evaluate it after a while.	Jingdong prefecture level city in Shandong
Jinfeng Hongyuan Flagship Store	Electricity savings3	Just received the goods, use a period of time to evaluate	Jingdong prefecture level city in Shandong
Jinfeng Hongyuan Flagship Store	Electricity savings3	Opened and tried very good, use it to see if there are results	Jingdong prefecture level city in Shandong
Jinfeng Hongyuan Flagship Store	Electricity savings3	Arrived today! Still trying it out!	Jingdong prefecture level city in Shandong
Jinfeng Hongyuan Flagship Store	Electricity savings3	Shipped quickly, well packaged, already installed, hope there is a power saving effect	Jingdong prefecture level city in Shandong
Jinfeng Hongyuan Flagship Store	Electricity savings3	I got it. It's great.	Jingdong prefecture level city in Shandong
Jinfeng Hongyuan Flagship Store	Electricity savings3	Haven't used it yet, will come back in a month	Jingdong prefecture level city in Shandong
Jinfeng Hongyuan Flagship Store	Electricity savings3	It looks good. It's good. Try it first.	Jingdong prefecture level city in Shandong

Jinfeng Hongyuan Flagship Store	Electricity savings3	It works so well that I have relatives, friends, coworkers, and 6 families that have purchased it for them through me.	Jingdong prefecture level city in Shandong
Jinfeng Hongyuan Flagship Store	Electricity savings3	In use	Jingdong prefecture level city in Shandong
Jinfeng Hongyuan Flagship Store	Electricity savings3	Received the goods today, power-saving effect do not know being used, the boss has a very good attitude, praise!	Jingdong prefecture level city in Shandong
Jinfeng Hongyuan Flagship Store	Electricity savings3	The product is compact and doesn't take up much space, but hopefully it's power efficient.	Jingdong prefecture level city in Shandong
Jinfeng Hongyuan Flagship Store	Electricity savings3	Power saving is indeed effective, I used for a period of time before coming to evaluate, the electricity bill is obviously less, the quality is good, the shopkeeper service is also very good, very patient,	Jingdong prefecture level city in Shandong
Jinfeng Hongyuan Flagship Store	Electricity savings3	It feels fine, I'll comment after I use it	Jingdong prefecture level city in Shandong
Jinfeng Hongyuan Flagship Store	Electricity savings3	I just received it and I don't know the effect yet! I will comment again after I use it and have results!	Jingdong prefecture level city in Shandong
Jinfeng Hongyuan Flagship Store	Electricity savings3	The goods look fine, I hope they really work!	Jingdong prefecture level city in Shandong
Jinfeng Hongyuan Flagship Store	Electricity savings3	I just don't know if it saves power	Jingdong prefecture level city in Shandong
Jinfeng Hongyuan Flagship Store	Electricity savings3	My friend introduced me to use this product, it is professional and I am looking forward to the long term effect~!	Jingdong prefecture level city in Shandong
Jinfeng Hongyuan Flagship Store	Electricity savings3	Try it before you judge.	Jingdong prefecture level city in Shandong
Jinfeng Hongyuan Flagship Store	Electricity savings3	It's great, the power savings are obvious! Kudos!	Jingdong prefecture level city in Shandong
Jinfeng Hongyuan Flagship Store	Electricity savings3	The quality is excellent, stabilizes the voltage and saves electricity, very satisfied.	Jingdong prefecture level city in Shandong
Jinfeng Hongyuan Flagship Store	Electricity savings3	You have to use it for a while to know the effect	Jingdong prefecture level city in Shandong
Jinfeng Hongyuan Flagship Store	Electricity savings3	It's on trial.	Jingdong prefecture level city in Shandong
Jinfeng Hongyuan Flagship Store	Electricity savings3	The stuff works great.	Jingdong prefecture level city in Shandong
Jinfeng Hongyuan Flagship Store	Electricity savings3	Doesn't seem to work, not as god as other reviews	Jingdong prefecture level city in Shandong
Jinfeng Hongyuan Flagship Store	Electricity savings3	It's not good quality, it doesn't work, it stops working after a few days, it doesn't work, the customer service doesn't care, it's a bit irritating.	Jingdong prefecture level city in Shandong
Jinfeng Hongyuan Flagship Store	Electricity savings3	It doesn't do anything.	Jingdong prefecture level city in Shandong
Jinfeng Hongyuan Flagship Store	Electricity savings3	It's good.	Jingdong prefecture level city in Shandong
Jinfeng Hongyuan Flagship Store	Electricity savings3	Used a month effect is not obvious, are psychological effects, feel are not less, did not save to the electricity, there are details of the problem, the store said to buy to send 5 fuse, in fact, received only three, small problems are not committed to in place, I believe that the product is not good enough	Jingdong prefecture level city in Shandong
Jinfeng Hongyuan Flagship Store	Electricity savings3	Well received quickly, looking quite high, but also sent 5 fuses. Customer service is very patient to teach the operation, in fact, quite simple, lazy to read the instructions ha!	Jingdong prefecture level city in Shandong
Jinfeng Hongyuan Flagship Store	Electricity savings3	You have to use it for a while to know the effect	Jingdong prefecture level city in Shandong
Jinfeng Hongyuan Flagship Store	Electricity savings3	Shipped very quickly, packaged very well, the price is particularly affordable, there are so many efficacy is also good, it is worth buying worth buying the product, the workmanship is very fine, better than imagined, recommended that everyone try not yet used, after the use of the follow-up comment	Jingdong prefecture level city in Shandong
Jinfeng Hongyuan Flagship Store	Electricity savings3	It looks regular. It's working now.	Jingdong prefecture level city in Shandong
Jinfeng Hongyuan Flagship Store	Electricity savings3	It arrived pretty fast and the owner is very responsible. Have not used it yet, will see after use	Jingdong prefecture level city in Shandong
Jinfeng Hongyuan Flagship Store	Electricity savings3	🎁 baby quality is very good, the first time I received the goods to open the first time to see, it is very good 👍, very satisfied 😊, customer service also have a question and answer, thank you 🙏	Jingdong prefecture level city in Shandong
Jinfeng Hongyuan Flagship Store	Electricity savings3	I don't know if it's any good 。。。。。。	Jingdong prefecture level city in Shandong
Jinfeng Hongyuan Flagship Store	Electricity savings3	Buy electronic products! Still trust the things of the Jingdong self-owned store! Energy-saving effect to evaluate after heating!	Jingdong prefecture level city in Shandong
Jinfeng Hongyuan Flagship Store	Electricity savings3	The goods have been received, Blu is very beautiful, energy saving effect, to be confirmed only after actual use.	Jingdong prefecture level city in Shandong
Jinfeng Hongyuan Flagship Store	Electricity savings3	Jingdong's speed is so fast, has received, easy to operate. The display is quite good, how many volts what mode is quite understandable.	Jingdong prefecture level city in Shandong
Jinfeng Hongyuan Flagship Store	Electricity savings3	The first use, the effect is good to recommend it to colleagues to use, a month of electricity to save a lot or can be	Jingdong prefecture level city in Shandong
Jinfeng Hongyuan Flagship Store	Electricity savings3	I don't know if it's useful. Mom's is a store. I'll return it if it doesn't work.	Jingdong prefecture level city in Shandong
Jinfeng Hongyuan Flagship Store	Electricity savings3	I don't know if it works or not, I'll wait for two months to use it and then come back to comment!	Jingdong prefecture level city in Shandong
Jinfeng Hongyuan Flagship Store	Electricity savings3	I bought the second one now, and I've been using it for more than half a year, and I feel that it really saves electricity. Now it is summer, the weather is hot, sometimes the fan and air conditioning on all day, if not this power saver, the monthly electricity bill is quite high, but as long as the installation of this power saver, not afraid of its high electricity bill, absolutely let you wantonly use	Jingdong prefecture level city in Shandong
Jinfeng Hongyuan Flagship Store	Electricity savings3	The product is better than I thought it would be, the quality looks great, the voltage is stable, and I'm looking forward to some great power savings!	Jingdong prefecture level city in Shandong
Jinfeng Hongyuan Flagship Store	Electricity savings3	Inadvertently see bought a try, delivery is also quite timely, just do not know how to use, customer service patience to answer, with a pretty good, value for money!	Jingdong prefecture level city in Shandong
Jinfeng Hongyuan Flagship Store	Electricity savings3	It's better than nothing! Hopefully this will help save slightly.	Jingdong prefecture level city in Shandong
Jinfeng Hongyuan Flagship Store	Electricity savings3	Still trying it out, follow up	Jingdong prefecture level city in Shandong
Jinfeng Hongyuan Flagship Store	Electricity savings3	I've just started using it, I don't know how well it works yet, I hope it's good.	Jingdong prefecture level city in Shandong
Jinfeng Hongyuan Flagship Store	Electricity savings3	Fourth purchase, great baby!	Jingdong prefecture level city in Shandong
Jinfeng Hongyuan Flagship Store	Electricity savings3	Shipped really fast, just got it and use it to see what happens and then come back for a review!	Jingdong prefecture level city in Shandong
Jinfeng Hongyuan Flagship Store	Electricity savings3	Timely delivery **** good attitude	Jingdong prefecture level city in Shandong
Jinfeng Hongyuan Flagship Store	Electricity savings3	Home large and small things are purchased in the Jingdong Mall, have been accustomed to shopping in the Jingdong, many times can buy good quality and cheap products, and courier service attitude is particularly good, sometimes too many couriers are to help me to the home, so many years have been in the Jingdong Mall shopping, trust and support! Will also support the Jingdong as always!	Jingdong prefecture level city in Shandong
Jinfeng Hongyuan Flagship Store	Electricity savings3	Modified a pump and the modification worked.	Jingdong prefecture level city in Shandong
Jinfeng Hongyuan Flagship Store	Electricity savings3	Already installed, next time you pay the electric bill to see the results	Jingdong prefecture level city in Shandong
Jinfeng Hongyuan Flagship Store	Electricity savings3	The product is very good to use and practical to look at	Jingdong prefecture level city in Shandong
Jinfeng Hongyuan Flagship Store	Electricity savings3	Works great, great service, waiting for results.	Jingdong prefecture level city in Shandong

Jinfeng Hongyuan Flagship Store	Electricity savings3	I just used it and I'm not sure about the efficacy yet, I'll come back to evaluate it after a while.	Jingdong prefecture level city in Shandong
Jinfeng Hongyuan Flagship Store	Electricity savings3	Just received the goods, use a period of time to evaluate	Jingdong prefecture level city in Shandong
Jinfeng Hongyuan Flagship Store	Electricity savings3	Opened and tried very good, use it to see if there are results	Jingdong prefecture level city in Shandong
Jinfeng Hongyuan Flagship Store	Electricity savings3	Arrived today! Still trying it out!	Jingdong prefecture level city in Shandong
Jinfeng Hongyuan Flagship Store	Electricity savings3	Shipped quickly, well packaged, already installed, hope there is a power saving effect	Jingdong prefecture level city in Shandong
Jinfeng Hongyuan Flagship Store	Electricity savings3	I got it. It's great.	Jingdong prefecture level city in Shandong
Jinfeng Hongyuan Flagship Store	Electricity savings3	Haven't used it yet, will come back in a month	Jingdong prefecture level city in Shandong
Jinfeng Hongyuan Flagship Store	Electricity savings3	It looks good. It's good. Try it first.	Jingdong prefecture level city in Shandong
Jinfeng Hongyuan Flagship Store	Electricity savings3	It works so well that I have relatives, friends, coworkers, and 6 families that have purchased it for them through me.	Jingdong prefecture level city in Shandong
Jinfeng Hongyuan Flagship Store	Electricity savings3	In use	Jingdong prefecture level city in Shandong
Jinfeng Hongyuan Flagship Store	Electricity savings3	Received the goods today, power-saving effect do not know being used, the boss has a very good attitude, praise!	Jingdong prefecture level city in Shandong
Jinfeng Hongyuan Flagship Store	Electricity savings3	The product is compact and doesn't take up much space, but hopefully it's power efficient.	Jingdong prefecture level city in Shandong
Jinfeng Hongyuan Flagship Store	Electricity savings3	Power saving is indeed effective, I used for a period of time before coming to evaluate, the electricity bill is obviously less, the quality is good, the shopkeeper service is also very good, very patient,	Jingdong prefecture level city in Shandong
Jinfeng Hongyuan Flagship Store	Electricity savings3	It feels fine, I'll comment after I use it	Jingdong prefecture level city in Shandong
Jinfeng Hongyuan Flagship Store	Electricity savings3	I just received it and I don't know the effect yet! I will comment again after I use it and have results!	Jingdong prefecture level city in Shandong
Jinfeng Hongyuan Flagship Store	Electricity savings3	The goods look fine, I hope they really work!	Jingdong prefecture level city in Shandong
Jinfeng Hongyuan Flagship Store	Electricity savings3	I just don't know if it saves power	Jingdong prefecture level city in Shandong
Jinfeng Hongyuan Flagship Store	Electricity savings3	My friend introduced me to use this product, it is professional and I am looking forward to the long term effect~!	Jingdong prefecture level city in Shandong
Jinfeng Hongyuan Flagship Store	Electricity savings3	Try it before you judge.	Jingdong prefecture level city in Shandong
Jinfeng Hongyuan Flagship Store	Electricity savings3	It's great, the power savings are obvious! Kudos!	Jingdong prefecture level city in Shandong
Jinfeng Hongyuan Flagship Store	Electricity savings3	The quality is excellent, stabilizes the voltage and saves electricity, very satisfied.	Jingdong prefecture level city in Shandong
Jinfeng Hongyuan Flagship Store	Electricity savings3	You have to use it for a while to know the effect	Jingdong prefecture level city in Shandong
Jinfeng Hongyuan Flagship Store	Electricity savings3	It's on trial.	Jingdong prefecture level city in Shandong
Jinfeng Hongyuan Flagship Store	Electricity savings3	The stuff works great.	Jingdong prefecture level city in Shandong
Jinfeng Hongyuan Flagship Store	Electricity savings3	Doesn't seem to work, not as god as other reviews	Jingdong prefecture level city in Shandong
Jinfeng Hongyuan Flagship Store	Electricity savings3	It's not good quality, it doesn't work, it stops working after a few days, it doesn't work, the customer service doesn't care, it's a bit irritating.	Jingdong prefecture level city in Shandong
Jinfeng Hongyuan Flagship Store	Electricity savings3	It doesn't do anything.	Jingdong prefecture level city in Shandong
Jinfeng Hongyuan Flagship Store	Electricity savings3	It's good.	Jingdong prefecture level city in Shandong
Jinfeng Hongyuan Flagship Store	Electricity savings3	Used a month effect is not obvious, are psychological effects, feel are not less, did not save to the electricity, there are details of the problem, the store said to buy to send 5 fuse, in fact, received only three, small problems are not committed to in place, I believe that the product is not good enough	Jingdong prefecture level city in Shandong
Jinfeng Hongyuan Flagship Store	Electricity savings3	Well received quickly, looking quite high, but also sent 5 fuses. Customer service is very patient to teach the operation, in fact, quite simple, lazy to read the instructions ha!	Jingdong prefecture level city in Shandong
Jinfeng Hongyuan Flagship Store	Electricity savings3	You have to use it for a while to know the effect	Jingdong prefecture level city in Shandong
Jinfeng Hongyuan Flagship Store	Electricity savings3	Shipped very quickly, packaged very well, the price is particularly affordable, there are so many efficacy is also good, it is worth buying worth buying the product, the workmanship is very fine, better than imagined, recommended that everyone try not yet used, after the use of the follow-up comment	Jingdong prefecture level city in Shandong
Jinfeng Hongyuan Flagship Store	Electricity savings3	It looks regular. It's working now.	Jingdong prefecture level city in Shandong
Jinfeng Hongyuan Flagship Store	Electricity savings3	It arrived pretty fast and the owner is very responsible. Have not used it yet, will see after use	Jingdong prefecture level city in Shandong
Jinfeng Hongyuan Flagship Store	Electricity savings3	🎁 baby quality is very good, the first time I received the goods to open the first time to see, it is very good 👍, very satisfied 😊, customer service also have a question and answer, thank you 🙏	Jingdong prefecture level city in Shandong
Jinfeng Hongyuan Flagship Store	Electricity savings3	I don't know if it's any good 。。。。。。	Jingdong prefecture level city in Shandong
Jinfeng Hongyuan Flagship Store	Electricity savings3	Buy electronic products! Still trust the things of the Jingdong self-owned store! Energy-saving effect to evaluate after heating!	Jingdong prefecture level city in Shandong
Jinfeng Hongyuan Flagship Store	Electricity savings3	The goods have been received, Blu is very beautiful, energy saving effect, to be confirmed only after actual use.	Jingdong prefecture level city in Shandong
Jinfeng Hongyuan Flagship Store	Electricity savings3	Jingdong's speed is so fast, has received, easy to operate. The display is quite good, how many volts what mode is quite understandable.	Jingdong prefecture level city in Shandong
Jinfeng Hongyuan Flagship Store	Electricity savings3	The first use, the effect is good to recommend it to colleagues to use, a month of electricity to save a lot or can be	Jingdong prefecture level city in Shandong
Jinfeng Hongyuan Flagship Store	Electricity savings3	I don't know if it's useful. Mom's is a store. I'll return it if it doesn't work.	Jingdong prefecture level city in Shandong
Jinfeng Hongyuan Flagship Store	Electricity savings3	I don't know if it works or not, I'll wait for two months to use it and then come back to comment!	Jingdong prefecture level city in Shandong
Jinfeng Hongyuan Flagship Store	Electricity savings3	I bought the second one now, and I've been using it for more than half a year, and I feel that it really saves electricity. Now it is summer, the weather is hot, sometimes the fan and air conditioning on all day, if not this power saver, the monthly electricity bill is quite high, but as long as the installation of this power saver, not afraid of its high electricity bill, absolutely let you wantonly use	Jingdong prefecture level city in Shandong
Jinfeng Hongyuan Flagship Store	Electricity savings3	The product is better than I thought it would be, the quality looks great, the voltage is stable, and I'm looking forward to some great power savings!	Jingdong prefecture level city in Shandong
Jinfeng Hongyuan Flagship Store	Electricity savings3	Inadvertently see bought a try, delivery is also quite timely, just do not know how to use, customer service patience to answer, with a pretty good, value for money!	Jingdong prefecture level city in Shandong
Jinfeng Hongyuan Flagship Store	Electricity savings3	It's better than nothing! Hopefully this will help save slightly.	Jingdong prefecture level city in Shandong
Jinfeng Hongyuan Flagship Store	Electricity savings3	Still trying it out, follow up	Jingdong prefecture level city in Shandong
Jinfeng Hongyuan Flagship Store	Electricity savings3	I've just started using it, I don't know how well it works yet, I hope it's good.	Jingdong prefecture level city in Shandong
Jinfeng Hongyuan Flagship Store	Electricity savings3	Fourth purchase, great baby!	Jingdong prefecture level city in Shandong

Jinfeng Hongyuan Flagship Store	Electricity savings3	Shipped really fast, just got it and use it to see what happens and then come back for a review!	Jingdong prefecture level city in Shandong
Jinfeng Hongyuan Flagship Store	Electricity savings3	Timely delivery **** good attitude	Jingdong prefecture level city in Shandong
Jinfeng Hongyuan Flagship Store	Electricity savings3	Home large and small things are purchased in the Jingdong Mall, have been accustomed to shopping in the Jingdong, many times can buy good quality and cheap products, and courier service attitude is particularly good, sometimes too many couriers are to help me to the home, so many years have been in the Jingdong Mall shopping, trust and support! Will also support the Jingdong as always!	Jingdong prefecture level city in Shandong
Jinfeng Hongyuan Flagship Store	Electricity savings3	Modified a pump and the modification worked.	Jingdong prefecture level city in Shandong
Jinfeng Hongyuan Flagship Store	Electricity savings3	Already installed, next time you pay the electric bill to see the results	Jingdong prefecture level city in Shandong
Jinfeng Hongyuan Flagship Store	Electricity savings3	The product is very good to use and practical to look at	Jingdong prefecture level city in Shandong
Jinfeng Hongyuan Flagship Store	Electricity savings3	Works great, great service, waiting for results.	Jingdong prefecture level city in Shandong
Jinfeng Hongyuan Flagship Store	Electricity savings3	I just used it and I'm not sure about the efficacy yet, I'll come back to evaluate it after a while.	Jingdong prefecture level city in Shandong
Jinfeng Hongyuan Flagship Store	Electricity savings3	Just received the goods, use a period of time to evaluate	Jingdong prefecture level city in Shandong
Jinfeng Hongyuan Flagship Store	Electricity savings3	Opened and tried very good, use it to see if there are results	Jingdong prefecture level city in Shandong
Jinfeng Hongyuan Flagship Store	Electricity savings3	Arrived today! Still trying it out!	Jingdong prefecture level city in Shandong
Jinfeng Hongyuan Flagship Store	Electricity savings3	Shipped quickly, well packaged, already installed, hope there is a power saving effect	Jingdong prefecture level city in Shandong
Jinfeng Hongyuan Flagship Store	Electricity savings3	I got it. It's great.	Jingdong prefecture level city in Shandong
Jinfeng Hongyuan Flagship Store	Electricity savings3	Haven't used it yet, will come back in a month	Jingdong prefecture level city in Shandong
Jinfeng Hongyuan Flagship Store	Electricity savings3	It looks good. It's good. Try it first.	Jingdong prefecture level city in Shandong
Jinfeng Hongyuan Flagship Store	Electricity savings3	It works so well that I have relatives, friends, coworkers, and 6 families that have purchased it for them through me.	Jingdong prefecture level city in Shandong
Jinfeng Hongyuan Flagship Store	Electricity savings3	In use	Jingdong prefecture level city in Shandong
Jinfeng Hongyuan Flagship Store	Electricity savings3	Received the goods today, power-saving effect do not know being used, the boss has a very good attitude, praise!	Jingdong prefecture level city in Shandong
Jinfeng Hongyuan Flagship Store	Electricity savings3	The product is compact and doesn't take up much space, but hopefully it's power efficient.	Jingdong prefecture level city in Shandong
Jinfeng Hongyuan Flagship Store	Electricity savings3	Power saving is indeed effective, I used for a period of time before coming to evaluate, the electricity bill is obviously less, the quality is good, the shopkeeper service is also very good, very patient,	Jingdong prefecture level city in Shandong
Jinfeng Hongyuan Flagship Store	Electricity savings3	It feels fine, I'll comment after I use it	Jingdong prefecture level city in Shandong
Jinfeng Hongyuan Flagship Store	Electricity savings3	I just received it and I don't know the effect yet! I will comment again after I use it and have results!	Jingdong prefecture level city in Shandong
Jinfeng Hongyuan Flagship Store	Electricity savings3	The goods look fine, I hope they really work!	Jingdong prefecture level city in Shandong
Jinfeng Hongyuan Flagship Store	Electricity savings3	I just don't know if it saves power	Jingdong prefecture level city in Shandong
Jinfeng Hongyuan Flagship Store	Electricity savings3	My friend introduced me to use this product, it is professional and I am looking forward to the long term effect~!	Jingdong prefecture level city in Shandong
Jinfeng Hongyuan Flagship Store	Electricity savings3	Try it before you judge.	Jingdong prefecture level city in Shandong
Jinfeng Hongyuan Flagship Store	Electricity savings3	It's great, the power savings are obvious! Kudos!	Jingdong prefecture level city in Shandong
Jinfeng Hongyuan Flagship Store	Electricity savings3	The quality is excellent, stabilizes the voltage and saves electricity, very satisfied.	Jingdong prefecture level city in Shandong
Jinfeng Hongyuan Flagship Store	Electricity savings3	You have to use it for a while to know the effect	Jingdong prefecture level city in Shandong
Jinfeng Hongyuan Flagship Store	Electricity savings3	It's on trial.	Jingdong prefecture level city in Shandong
Jinfeng Hongyuan Flagship Store	Electricity savings3	The stuff works great.	Jingdong prefecture level city in Shandong
Jinfeng Hongyuan Flagship Store	Electricity savings3	Doesn't seem to work, not as god as other reviews	Jingdong prefecture level city in Shandong
Jinfeng Hongyuan Flagship Store	Electricity savings3	It's not good quality, it doesn't work, it stops working after a few days, it doesn't work, the customer service doesn't care, it's a bit irritating.	Jingdong prefecture level city in Shandong
Jinfeng Hongyuan Flagship Store	Electricity savings3	It doesn't do anything.	Jingdong prefecture level city in Shandong
Jinfeng Hongyuan Flagship Store	Electricity savings3	It's good.	Jingdong prefecture level city in Shandong
Jinfeng Hongyuan Flagship Store	Electricity savings3	Used a month effect is not obvious, are psychological effects, feel are not less, did not save to the electricity, there are details of the problem, the store said to buy to send 5 fuse, in fact, received only three, small problems are not committed to in place, I believe that the product is not good enough	Jingdong prefecture level city in Shandong
Jinfeng Hongyuan Flagship Store	Electricity savings3	Well received quickly, looking quite high, but also sent 5 fuses. Customer service is very patient to teach the operation, in fact, quite simple, lazy to read the instructions ha!	Jingdong prefecture level city in Shandong
Jinfeng Hongyuan Flagship Store	Electricity savings3	You have to use it for a while to know the effect	Jingdong prefecture level city in Shandong
Jinfeng Hongyuan Flagship Store	Electricity savings3	Shipped very quickly, packaged very well, the price is particularly affordable, there are so many efficacy is also good, it is worth buying worth buying the product, the workmanship is very fine, better than imagined, recommended that everyone try not yet used, after the use of the follow-up comment	Jingdong prefecture level city in Shandong
Jinfeng Hongyuan Flagship Store	Electricity savings3	It looks regular. It's working now.	Jingdong prefecture level city in Shandong
Jinfeng Hongyuan Flagship Store	Electricity savings3	It arrived pretty fast and the owner is very responsible. Have not used it yet, will see after use	Jingdong prefecture level city in Shandong
Jinfeng Hongyuan Flagship Store	Electricity savings3	🎁 baby quality is very good, the first time I received the goods to open the first time to see, it is very good 👍, very satisfied 😊, customer service also have a question and answer, thank you 🙏	Jingdong prefecture level city in Shandong
Jinfeng Hongyuan Flagship Store	Electricity savings3	I don't know if it's any good 。。。。。。	Jingdong prefecture level city in Shandong
Jinfeng Hongyuan Flagship Store	Electricity savings3	Buy electronic products! Still trust the things of the Jingdong self-owned store! Energy-saving effect to evaluate after heating!	Jingdong prefecture level city in Shandong
Jinfeng Hongyuan Flagship Store	Electricity savings3	The goods have been received, Blu is very beautiful, energy saving effect, to be confirmed only after actual use.	Jingdong prefecture level city in Shandong
Jinfeng Hongyuan Flagship Store	Electricity savings3	Jingdong's speed is so fast, has received, easy to operate. The display is quite good, how many volts what mode is quite understandable.	Jingdong prefecture level city in Shandong
Jinfeng Hongyuan Flagship Store	Electricity savings3	The first use, the effect is good to recommend it to colleagues to use, a month of electricity to save a lot or can be	Jingdong prefecture level city in Shandong
Jinfeng Hongyuan Flagship Store	Electricity savings3	I don't know if it's useful. Mom's is a store. I'll return it if it doesn't work.	Jingdong prefecture level city in Shandong
Jinfeng Hongyuan Flagship Store	Electricity savings3	I don't know if it works or not, I'll wait for two months to use it and then come back to comment!	Jingdong prefecture level city in Shandong

Jinfeng Hongyuan Flagship Store	Electricity savings3	I bought the second one now, and I've been using it for more than half a year, and I feel that it really saves electricity. Now it is summer, the weather is hot, sometimes the fan and air conditioning on all day, if not this power saver, the monthly electricity bill is quite high, but as long as the installation of this power saver, not afraid of its high electricity bill, absolutely let you wantonly use	Jingdong prefecture level city in Shandong
Jinfeng Hongyuan Flagship Store	Electricity savings3	The product is better than I thought it would be, the quality looks great, the voltage is stable, and I'm looking forward to some great power savings!	Jingdong prefecture level city in Shandong
Jinfeng Hongyuan Flagship Store	Electricity savings3	Inadvertently see bought a try, delivery is also quite timely, just do not know how to use, customer service patience to answer, with a pretty good, value for money!	Jingdong prefecture level city in Shandong
Jinfeng Hongyuan Flagship Store	Electricity savings3	It's better than nothing! Hopefully this will help save slightly.	Jingdong prefecture level city in Shandong
Jinfeng Hongyuan Flagship Store	Electricity savings3	Still trying it out, follow up	Jingdong prefecture level city in Shandong
Jinfeng Hongyuan Flagship Store	Electricity savings3	I've just started using it, I don't know how well it works yet, I hope it's good.	Jingdong prefecture level city in Shandong
Jinfeng Hongyuan Flagship Store	Electricity savings3	Fourth purchase, great baby!	Jingdong prefecture level city in Shandong
Jinfeng Hongyuan Flagship Store	Electricity savings3	Shipped really fast, just got it and use it to see what happens and then come back for a review!	Jingdong prefecture level city in Shandong
Jinfeng Hongyuan Flagship Store	Electricity savings3	Timely delivery **** good attitude	Jingdong prefecture level city in Shandong
Jinfeng Hongyuan Flagship Store	Electricity savings3	Home large and small things are purchased in the Jingdong Mall, have been accustomed to shopping in the Jingdong, many times can buy good quality and cheap products, and courier service attitude is particularly good, sometimes too many couriers are to help me to the home, so many years have been in the Jingdong Mall shopping, trust and support! Will also support the Jingdong as always!	Jingdong prefecture level city in Shandong
Jinfeng Hongyuan Flagship Store	Electricity savings3	Modified a pump and the modification worked.	Jingdong prefecture level city in Shandong
Jinfeng Hongyuan Flagship Store	Electricity savings3	Already installed, next time you pay the electric bill to see the results	Jingdong prefecture level city in Shandong
Jinfeng Hongyuan Flagship Store	Electricity savings3	The product is very good to use and practical to look at	Jingdong prefecture level city in Shandong
Jinfeng Hongyuan Flagship Store	Electricity savings3	Works great, great service, waiting for results.	Jingdong prefecture level city in Shandong
Jinfeng Hongyuan Flagship Store	Electricity savings3	I just used it and I'm not sure about the efficacy yet, I'll come back to evaluate it after a while.	Jingdong prefecture level city in Shandong
Jinfeng Hongyuan Flagship Store	Electricity savings3	Just received the goods, use a period of time to evaluate	Jingdong prefecture level city in Shandong
Jinfeng Hongyuan Flagship Store	Electricity savings3	Opened and tried very good, use it to see if there are results	Jingdong prefecture level city in Shandong
Jinfeng Hongyuan Flagship Store	Electricity savings3	Arrived today! Still trying it out!	Jingdong prefecture level city in Shandong
Jinfeng Hongyuan Flagship Store	Electricity savings3	Shipped quickly, well packaged, already installed, hope there is a power saving effect	Jingdong prefecture level city in Shandong
Jinfeng Hongyuan Flagship Store	Electricity savings3	I got it. It's great.	Jingdong prefecture level city in Shandong
Jinfeng Hongyuan Flagship Store	Electricity savings3	Haven't used it yet, will come back in a month	Jingdong prefecture level city in Shandong
Jinfeng Hongyuan Flagship Store	Electricity savings3	It looks good. It's good. Try it first.	Jingdong prefecture level city in Shandong
Jinfeng Hongyuan Flagship Store	Electricity savings3	It works so well that I have relatives, friends, coworkers, and 6 families that have purchased it for them through me.	Jingdong prefecture level city in Shandong
Jinfeng Hongyuan Flagship Store	Electricity savings3	In use	Jingdong prefecture level city in Shandong
Jinfeng Hongyuan Flagship Store	Electricity savings3	Received the goods today, power-saving effect do not know being used, the boss has a very good attitude, praise!	Jingdong prefecture level city in Shandong
Jinfeng Hongyuan Flagship Store	Electricity savings3	The product is compact and doesn't take up much space, but hopefully it's power efficient.	Jingdong prefecture level city in Shandong
Jinfeng Hongyuan Flagship Store	Electricity savings3	Power saving is indeed effective, I used for a period of time before coming to evaluate, the electricity bill is obviously less, the quality is good, the shopkeeper service is also very good, very patient,	Jingdong prefecture level city in Shandong
Jinfeng Hongyuan Flagship Store	Electricity savings3	It feels fine, I'll comment after I use it	Jingdong prefecture level city in Shandong
Jinfeng Hongyuan Flagship Store	Electricity savings3	I just received it and I don't know the effect yet! I will comment again after I use it and have results!	Jingdong prefecture level city in Shandong
Jinfeng Hongyuan Flagship Store	Electricity savings3	The goods look fine, I hope they really work!	Jingdong prefecture level city in Shandong
Jinfeng Hongyuan Flagship Store	Electricity savings3	I just don't know if it saves power	Jingdong prefecture level city in Shandong
Jinfeng Hongyuan Flagship Store	Electricity savings3	My friend introduced me to use this product, it is professional and I am looking forward to the long term effect~!	Jingdong prefecture level city in Shandong
Jinfeng Hongyuan Flagship Store	Electricity savings3	Try it before you judge.	Jingdong prefecture level city in Shandong
Jinfeng Hongyuan Flagship Store	Electricity savings3	It's great, the power savings are obvious! Kudos!	Jingdong prefecture level city in Shandong
Jinfeng Hongyuan Flagship Store	Electricity savings3	The quality is excellent, stabilizes the voltage and saves electricity, very satisfied.	Jingdong prefecture level city in Shandong
Jinfeng Hongyuan Flagship Store	Electricity savings3	You have to use it for a while to know the effect	Jingdong prefecture level city in Shandong
Jinfeng Hongyuan Flagship Store	Electricity savings3	It's on trial.	Jingdong prefecture level city in Shandong
Jinfeng Hongyuan Flagship Store	Electricity savings3	The stuff works great.	Jingdong prefecture level city in Shandong
Jinfeng Hongyuan Flagship Store	Electricity savings3	Doesn't seem to work, not as god as other reviews	Jingdong prefecture level city in Shandong
Jinfeng Hongyuan Flagship Store	Electricity savings3	It's not good quality, it doesn't work, it stops working after a few days, it doesn't work, the customer service doesn't care, it's a bit irritating.	Jingdong prefecture level city in Shandong
Jinfeng Hongyuan Flagship Store	Electricity savings3	It doesn't do anything.	Jingdong prefecture level city in Shandong
Jinfeng Hongyuan Flagship Store	Electricity savings3	It's good.	Jingdong prefecture level city in Shandong
Jinfeng Hongyuan Flagship Store	Electricity savings3	Used a month effect is not obvious, are psychological effects, feel are not less, did not save to the electricity, there are details of the problem, the store said to buy to send 5 fuse, in fact, received only three, small problems are not committed to in place, I believe that the product is not good enough	Jingdong prefecture level city in Shandong
Jinfeng Hongyuan Flagship Store	Electricity savings3	Well received quickly, looking quite high, but also sent 5 fuses. Customer service is very patient to teach the operation, in fact, quite simple, lazy to read the instructions ha!	Jingdong prefecture level city in Shandong
Jinfeng Hongyuan Flagship Store	Electricity savings3	You have to use it for a while to know the effect	Jingdong prefecture level city in Shandong
Jinfeng Hongyuan Flagship Store	Electricity savings3	Shipped very quickly, packaged very well, the price is particularly affordable, there are so many efficacy is also good, it is worth buying worth buying the product, the workmanship is very fine, better than imagined, recommended that everyone try not yet used, after the use of the follow-up comment	Jingdong prefecture level city in Shandong
Jinfeng Hongyuan Flagship Store	Electricity savings3	It looks regular. It's working now.	Jingdong prefecture level city in Shandong
Jinfeng Hongyuan Flagship Store	Electricity savings3	It arrived pretty fast and the owner is very responsible. Have not used it yet, will see after use	Jingdong prefecture level city in Shandong

Jinfeng Hongyuan Flagship Store	Electricity savings3	🎁 baby quality is very good, the first time I received the goods to open the first time to see, it is very good 👍, very satisfied 😃, customer service also have a question and answer, thank you 🙏	Jingdong prefecture level city in Shandong
Jinfeng Hongyuan Flagship Store	Electricity savings3	I don't know if it's any good 。。。。。。	Jingdong prefecture level city in Shandong
Jinfeng Hongyuan Flagship Store	Electricity savings3	Buy electronic products! Still trust the things of the Jingdong self-owned store! Energy-saving effect to evaluate after heating!	Jingdong prefecture level city in Shandong
Jinfeng Hongyuan Flagship Store	Electricity savings3	The goods have been received, Blu is very beautiful, energy saving effect, to be confirmed only after actual use.	Jingdong prefecture level city in Shandong
Jinfeng Hongyuan Flagship Store	Electricity savings3	Jingdong's speed is so fast, has received, easy to operate. The display is quite good, how many volts what mode is quite understandable.	Jingdong prefecture level city in Shandong
Jinfeng Hongyuan Flagship Store	Electricity savings3	The first use, the effect is good to recommend it to colleagues to use, a month of electricity to save a lot or can be	Jingdong prefecture level city in Shandong
Jinfeng Hongyuan Flagship Store	Electricity savings3	I don't know if it's useful. Mom's is a store. I'll return it if it doesn't work.	Jingdong prefecture level city in Shandong
Jinfeng Hongyuan Flagship Store	Electricity savings3	I don't know if it works or not, I'll wait for two months to use it and then come back to comment!	Jingdong prefecture level city in Shandong
Jinfeng Hongyuan Flagship Store	Electricity savings3	I bought the second one now, and I've been using it for more than half a year, and I feel that it really saves electricity. Now it is summer, the weather is hot, sometimes the fan and air conditioning on all day, if not this power saver, the monthly electricity bill is quite high, but as long as the installation of this power saver, not afraid of its high electricity bill, absolutely let you wantonly use	Jingdong prefecture level city in Shandong
Jinfeng Hongyuan Flagship Store	Electricity savings3	The product is better than I thought it would be, the quality looks great, the voltage is stable, and I'm looking forward to some great power savings!	Jingdong prefecture level city in Shandong
Jinfeng Hongyuan Flagship Store	Electricity savings3	Inadvertently see bought a try, delivery is also quite timely, just do not know how to use, customer service patience to answer, with a pretty good, value for money!	Jingdong prefecture level city in Shandong
Jinfeng Hongyuan Flagship Store	Electricity savings3	It's better than nothing! Hopefully this will help save slightly.	Jingdong prefecture level city in Shandong
Jinfeng Hongyuan Flagship Store	Electricity savings3	Still trying it out, follow up	Jingdong prefecture level city in Shandong
Jinfeng Hongyuan Flagship Store	Electricity savings3	I've just started using it, I don't know how well it works yet, I hope it's good.	Jingdong prefecture level city in Shandong
Jinfeng Hongyuan Flagship Store	Electricity savings3	Fourth purchase, great baby!	Jingdong prefecture level city in Shandong
Jinfeng Hongyuan Flagship Store	Electricity savings3	Shipped really fast, just got it and use it to see what happens and then come back for a review!	Jingdong prefecture level city in Shandong
Jinfeng Hongyuan Flagship Store	Electricity savings3	Timely delivery **** good attitude	Jingdong prefecture level city in Shandong
Jinfeng Hongyuan Flagship Store	Electricity savings3	Home large and small things are purchased in the Jingdong Mall, have been accustomed to shopping in the Jingdong, many times can buy good quality and cheap products, and courier service attitude is particularly good, sometimes too many couriers are to help me to the home, so many years have been in the Jingdong Mall shopping, trust and support! Will also support the Jingdong as always!	Jingdong prefecture level city in Shandong
Jinfeng Hongyuan Flagship Store	Electricity savings3	Modified a pump and the modification worked.	Jingdong prefecture level city in Shandong
Jinfeng Hongyuan Flagship Store	Electricity savings3	Already installed, next time you pay the electric bill to see the results	Jingdong prefecture level city in Shandong
Jinfeng Hongyuan Flagship Store	Electricity savings3	The product is very good to use and practical to look at	Jingdong prefecture level city in Shandong
Jinfeng Hongyuan Flagship Store	Electricity savings3	Works great, great service, waiting for results.	Jingdong prefecture level city in Shandong
Jinfeng Hongyuan Flagship Store	Electricity savings3	I just used it and I'm not sure about the efficacy yet, I'll come back to evaluate it after a while.	Jingdong prefecture level city in Shandong
Jinfeng Hongyuan Flagship Store	Electricity savings3	Just received the goods, use a period of time to evaluate	Jingdong prefecture level city in Shandong
Jinfeng Hongyuan Flagship Store	Electricity savings3	Opened and tried very good, use it to see if there are results	Jingdong prefecture level city in Shandong
Jinfeng Hongyuan Flagship Store	Electricity savings3	Arrived today! Still trying it out!	Jingdong prefecture level city in Shandong
Jinfeng Hongyuan Flagship Store	Electricity savings3	Shipped quickly, well packaged, already installed, hope there is a power saving effect	Jingdong prefecture level city in Shandong
Jinfeng Hongyuan Flagship Store	Electricity savings3	I got it. It's great.	Jingdong prefecture level city in Shandong
Jinfeng Hongyuan Flagship Store	Electricity savings3	Haven't used it yet, will come back in a month	Jingdong prefecture level city in Shandong
Jinfeng Hongyuan Flagship Store	Electricity savings3	It looks good. It's good. Try it first.	Jingdong prefecture level city in Shandong
Jinfeng Hongyuan Flagship Store	Electricity savings3	It works so well that I have relatives, friends, coworkers, and 6 families that have purchased it for them through me.	Jingdong prefecture level city in Shandong
Jinfeng Hongyuan Flagship Store	Electricity savings3	In use	Jingdong prefecture level city in Shandong
Jinfeng Hongyuan Flagship Store	Electricity savings3	Received the goods today, power-saving effect do not know being used, the boss has a very good attitude, praise!	Jingdong prefecture level city in Shandong
Jinfeng Hongyuan Flagship Store	Electricity savings3	The product is compact and doesn't take up much space, but hopefully it's power efficient.	Jingdong prefecture level city in Shandong
Jinfeng Hongyuan Flagship Store	Electricity savings3	Power saving is indeed effective, I used for a period of time before coming to evaluate, the electricity bill is obviously less, the quality is good, the shopkeeper service is also very good, very patient,	Jingdong prefecture level city in Shandong
Jinfeng Hongyuan Flagship Store	Electricity savings3	It feels fine, I'll comment after I use it	Jingdong prefecture level city in Shandong
Jinfeng Hongyuan Flagship Store	Electricity savings3	I just received it and I don't know the effect yet! I will comment again after I use it and have results!	Jingdong prefecture level city in Shandong
Jinfeng Hongyuan Flagship Store	Electricity savings3	The goods look fine, I hope they really work!	Jingdong prefecture level city in Shandong
Jinfeng Hongyuan Flagship Store	Electricity savings3	I just don't know if it saves power	Jingdong prefecture level city in Shandong
Jinfeng Hongyuan Flagship Store	Electricity savings3	My friend introduced me to use this product, it is professional and I am looking forward to the long term effect~!	Jingdong prefecture level city in Shandong
Jinfeng Hongyuan Flagship Store	Electricity savings3	Try it before you judge.	Jingdong prefecture level city in Shandong
Jinfeng Hongyuan Flagship Store	Electricity savings3	It's great, the power savings are obvious! Kudos!	Jingdong prefecture level city in Shandong
Jinfeng Hongyuan Flagship Store	Electricity savings3	The quality is excellent, stabilizes the voltage and saves electricity, very satisfied.	Jingdong prefecture level city in Shandong
Jinfeng Hongyuan Flagship Store	Electricity savings3	You have to use it for a while to know the effect	Jingdong prefecture level city in Shandong
Jinfeng Hongyuan Flagship Store	Electricity savings3	It's on trial.	Jingdong prefecture level city in Shandong
Jinfeng Hongyuan Flagship Store	Electricity savings3	The stuff works great.	Jingdong prefecture level city in Shandong
Jinfeng Hongyuan Flagship Store	Electricity savings3	Doesn't seem to work, not as god as other reviews	Jingdong prefecture level city in Shandong
Jinfeng Hongyuan Flagship Store	Electricity savings3	It's not good quality, it doesn't work, it stops working after a few days, it doesn't work, the customer service doesn't care, it's a bit irritating.	Jingdong prefecture level city in Shandong
Jinfeng Hongyuan Flagship Store	Electricity savings3	It doesn't do anything.	Jingdong prefecture level city in Shandong

Jinfeng Hongyuan Flagship Store	Electricity savings3	It's good.	Jingdong prefecture level city in Shandong
Jinfeng Hongyuan Flagship Store	Electricity savings3	Used a month effect is not obvious, are psychological effects, feel are not less, did not save to the electricity, there are details of the problem, the store said to buy to send 5 fuse, in fact, received only three, small problems are not committed to in place, I believe that the product is not good enough	Jingdong prefecture level city in Shandong
Jinfeng Hongyuan Flagship Store	Electricity savings3	Well received quickly, looking quite high, but also sent 5 fuses. Customer service is very patient to teach the operation, in fact, quite simple, lazy to read the instructions ha!	Jingdong prefecture level city in Shandong
Jinfeng Hongyuan Flagship Store	Electricity savings3	You have to use it for a while to know the effect	Jingdong prefecture level city in Shandong
Jinfeng Hongyuan Flagship Store	Electricity savings3	Shipped very quickly, packaged very well, the price is particularly affordable, there are so many efficacy is also good, it is worth buying worth buying the product, the workmanship is very fine, better than imagined, recommended that everyone try not yet used, after the use of the follow-up comment	Jingdong prefecture level city in Shandong
Jinfeng Hongyuan Flagship Store	Electricity savings3	It looks regular. It's working now.	Jingdong prefecture level city in Shandong
Jinfeng Hongyuan Flagship Store	Electricity savings3	It arrived pretty fast and the owner is very responsible. Have not used it yet, will see after use	Jingdong prefecture level city in Shandong
Jinfeng Hongyuan Flagship Store	Electricity savings3	🎁 baby quality is very good, the first time I received the goods to open the first time to see, it is very good 👍, very satisfied 😃, customer service also have a question and answer, thank you 🙏	Jingdong prefecture level city in Shandong
Jinfeng Hongyuan Flagship Store	Electricity savings3	I don't know if it's any good 。。。。。。	Jingdong prefecture level city in Shandong
Jinfeng Hongyuan Flagship Store	Electricity savings3	Buy electronic products! Still trust the things of the Jingdong self-owned store! Energy-saving effect to evaluate after heating!	Jingdong prefecture level city in Shandong
Jinfeng Hongyuan Flagship Store	Electricity savings3	The goods have been received, Blu is very beautiful, energy saving effect, to be confirmed only after actual use.	Jingdong prefecture level city in Shandong
Jinfeng Hongyuan Flagship Store	Electricity savings3	Jingdong's speed is so fast, has received, easy to operate. The display is quite good, how many volts what mode is quite understandable.	Jingdong prefecture level city in Shandong
Jinfeng Hongyuan Flagship Store	Electricity savings3	The first use, the effect is good to recommend it to colleagues to use, a month of electricity to save a lot or can be	Jingdong prefecture level city in Shandong
Jinfeng Hongyuan Flagship Store	Electricity savings3	I don't know if it's useful. Mom's is a store. I'll return it if it doesn't work.	Jingdong prefecture level city in Shandong
Jinfeng Hongyuan Flagship Store	Electricity savings3	I don't know if it works or not, I'll wait for two months to use it and then come back to comment!	Jingdong prefecture level city in Shandong
Jinfeng Hongyuan Flagship Store	Electricity savings3	I bought the second one now, and I've been using it for more than half a year, and I feel that it really saves electricity. Now it is summer, the weather is hot, sometimes the fan and air conditioning on all day, if not this power saver, the monthly electricity bill is quite high, but as long as the installation of this power saver, not afraid of its high electricity bill, absolutely let you wantonly use	Jingdong prefecture level city in Shandong
Jinfeng Hongyuan Flagship Store	Electricity savings3	The product is better than I thought it would be, the quality looks great, the voltage is stable, and I'm looking forward to some great power savings!	Jingdong prefecture level city in Shandong
Jinfeng Hongyuan Flagship Store	Electricity savings3	Inadvertently see bought a try, delivery is also quite timely, just do not know how to use, customer service patience to answer, with a pretty good, value for money!	Jingdong prefecture level city in Shandong
Jinfeng Hongyuan Flagship Store	Electricity savings3	It's better than nothing! Hopefully this will help save slightly.	Jingdong prefecture level city in Shandong
Jinfeng Hongyuan Flagship Store	Electricity savings3	Still trying it out, follow up	Jingdong prefecture level city in Shandong
Jinfeng Hongyuan Flagship Store	Electricity savings3	I've just started using it, I don't know how well it works yet, I hope it's good.	Jingdong prefecture level city in Shandong
Jinfeng Hongyuan Flagship Store	Electricity savings3	Fourth purchase, great baby!	Jingdong prefecture level city in Shandong
Jinfeng Hongyuan Flagship Store	Electricity savings3	Shipped really fast, just got it and use it to see what happens and then come back for a review!	Jingdong prefecture level city in Shandong
Jinfeng Hongyuan Flagship Store	Electricity savings3	Timely delivery **** good attitude	Jingdong prefecture level city in Shandong
Jinfeng Hongyuan Flagship Store	Electricity savings3	Home large and small things are purchased in the Jingdong Mall, have been accustomed to shopping in the Jingdong, many times can buy good quality and cheap products, and courier service attitude is particularly good, sometimes too many couriers are to help me to the home, so many years have been in the Jingdong Mall shopping, trust and support! Will also support the Jingdong as always!	Jingdong prefecture level city in Shandong
Jinfeng Hongyuan Flagship Store	Electricity savings3	Modified a pump and the modification worked.	Jingdong prefecture level city in Shandong
Jinfeng Hongyuan Flagship Store	Electricity savings3	Already installed, next time you pay the electric bill to see the results	Jingdong prefecture level city in Shandong
Jinfeng Hongyuan Flagship Store	Electricity savings3	The product is very good to use and practical to look at	Jingdong prefecture level city in Shandong
Jinfeng Hongyuan Flagship Store	Electricity savings3	Works great, great service, waiting for results.	Jingdong prefecture level city in Shandong
Jinfeng Hongyuan Flagship Store	Electricity savings3	I just used it and I'm not sure about the efficacy yet, I'll come back to evaluate it after a while.	Jingdong prefecture level city in Shandong
Jinfeng Hongyuan Flagship Store	Electricity savings3	Just received the goods, use a period of time to evaluate	Jingdong prefecture level city in Shandong
Jinfeng Hongyuan Flagship Store	Electricity savings3	Opened and tried very good, use it to see if there are results	Jingdong prefecture level city in Shandong
Jinfeng Hongyuan Flagship Store	Electricity savings3	Arrived today! Still trying it out!	Jingdong prefecture level city in Shandong
Jinfeng Hongyuan Flagship Store	Electricity savings3	Shipped quickly, well packaged, already installed, hope there is a power saving effect	Jingdong prefecture level city in Shandong
Jinfeng Hongyuan Flagship Store	Electricity savings3	I got it. It's great.	Jingdong prefecture level city in Shandong
Jinfeng Hongyuan Flagship Store	Electricity savings3	Haven't used it yet, will come back in a month	Jingdong prefecture level city in Shandong
Jinfeng Hongyuan Flagship Store	Electricity savings3	It looks good. It's good. Try it first.	Jingdong prefecture level city in Shandong
Jinfeng Hongyuan Flagship Store	Electricity savings3	It works so well that I have relatives, friends, coworkers, and 6 families that have purchased it for them through me.	Jingdong prefecture level city in Shandong
Jinfeng Hongyuan Flagship Store	Electricity savings3	In use	Jingdong prefecture level city in Shandong
Jinfeng Hongyuan Flagship Store	Electricity savings3	Received the goods today, power-saving effect do not know being used, the boss has a very good attitude, praise!	Jingdong prefecture level city in Shandong
Jinfeng Hongyuan Flagship Store	Electricity savings3	The product is compact and doesn't take up much space, but hopefully it's power efficient.	Jingdong prefecture level city in Shandong
Jinfeng Hongyuan Flagship Store	Electricity savings3	Power saving is indeed effective, I used for a period of time before coming to evaluate, the electricity bill is obviously less, the quality is good, the shopkeeper service is also very good, very patient,	Jingdong prefecture level city in Shandong
Jinfeng Hongyuan Flagship Store	Electricity savings3	It feels fine, I'll comment after I use it	Jingdong prefecture level city in Shandong
Jinfeng Hongyuan Flagship Store	Electricity savings3	I just received it and I don't know the effect yet! I will comment again after I use it and have results!	Jingdong prefecture level city in Shandong
Jinfeng Hongyuan Flagship Store	Electricity savings3	The goods look fine, I hope they really work!	Jingdong prefecture level city in Shandong
Jinfeng Hongyuan Flagship Store	Electricity savings3	I just don't know if it saves power	Jingdong prefecture level city in Shandong
Jinfeng Hongyuan Flagship Store	Electricity savings3	My friend introduced me to use this product, it is professional and I am looking forward to the long term effect~!	Jingdong prefecture level city in Shandong
Jinfeng Hongyuan Flagship Store	Electricity savings3	Try it before you judge.	Jingdong prefecture level city in Shandong

Jinfeng Hongyuan Flagship Store	Electricity savings3	It's great, the power savings are obvious! Kudos!	Jingdong prefecture level city in Shandong
Jinfeng Hongyuan Flagship Store	Electricity savings3	The quality is excellent, stabilizes the voltage and saves electricity, very satisfied.	Jingdong prefecture level city in Shandong
Jinfeng Hongyuan Flagship Store	Electricity savings3	You have to use it for a while to know the effect	Jingdong prefecture level city in Shandong
Jinfeng Hongyuan Flagship Store	Electricity savings3	It's on trial.	Jingdong prefecture level city in Shandong
Jinfeng Hongyuan Flagship Store	Electricity savings3	The stuff works great.	Jingdong prefecture level city in Shandong
Jinfeng Hongyuan Flagship Store	Electricity savings3	Doesn't seem to work, not as god as other reviews	Jingdong prefecture level city in Shandong
Jinfeng Hongyuan Flagship Store	Electricity savings3	It's not good quality, it doesn't work, it stops working after a few days, it doesn't work, the customer service doesn't care, it's a bit irritating.	Jingdong prefecture level city in Shandong
Jinfeng Hongyuan Flagship Store	Electricity savings3	It doesn't do anything.	Jingdong prefecture level city in Shandong
Jinfeng Hongyuan Flagship Store	Electricity savings3	It's good.	Jingdong prefecture level city in Shandong
Jinfeng Hongyuan Flagship Store	Electricity savings3	Used a month effect is not obvious, are psychological effects, feel are not less, did not save to the electricity, there are details of the problem, the store said to buy to send 5 fuse, in fact, received only three, small problems are not committed to in place, I believe that the product is not good enough	Jingdong prefecture level city in Shandong
Jinfeng Hongyuan Flagship Store	Electricity savings3	Well received quickly, looking quite high, but also sent 5 fuses. Customer service is very patient to teach the operation, in fact, quite simple, lazy to read the instructions ha!	Jingdong prefecture level city in Shandong
Jinfeng Hongyuan Flagship Store	Electricity savings3	You have to use it for a while to know the effect	Jingdong prefecture level city in Shandong
Jinfeng Hongyuan Flagship Store	Electricity savings3	Shipped very quickly, packaged very well, the price is particularly affordable, there are so many efficacy is also good, it is worth buying worth buying the product, the workmanship is very fine, better than imagined, recommended that everyone try not yet used, after the use of the follow-up comment	Jingdong prefecture level city in Shandong
Jinfeng Hongyuan Flagship Store	Electricity savings3	It looks regular. It's working now.	Jingdong prefecture level city in Shandong
Jinfeng Hongyuan Flagship Store	Electricity savings3	It arrived pretty fast and the owner is very responsible. Have not used it yet, will see after use	Jingdong prefecture level city in Shandong
Jinfeng Hongyuan Flagship Store	Electricity savings3	🎁 baby quality is very good, the first time I received the goods to open the first time to see, it is very good 👍, very satisfied 😀, customer service also have a question and answer, thank you 🙏	Jingdong prefecture level city in Shandong
Jinfeng Hongyuan Flagship Store	Electricity savings3	I don't know if it's any good 。。。。。。	Jingdong prefecture level city in Shandong
Jinfeng Hongyuan Flagship Store	Electricity savings3	Buy electronic products! Still trust the things of the Jingdong self-owned store! Energy-saving effect to evaluate after heating!	Jingdong prefecture level city in Shandong
Jinfeng Hongyuan Flagship Store	Electricity savings3	The goods have been received, Blu is very beautiful, energy saving effect, to be confirmed only after actual use.	Jingdong prefecture level city in Shandong
Jinfeng Hongyuan Flagship Store	Electricity savings3	Jingdong's speed is so fast, has received, easy to operate. The display is quite good, how many volts what mode is quite understandable.	Jingdong prefecture level city in Shandong
Jinfeng Hongyuan Flagship Store	Electricity savings3	The first use, the effect is good to recommend it to colleagues to use, a month of electricity to save a lot or can be	Jingdong prefecture level city in Shandong
Jinfeng Hongyuan Flagship Store	Electricity savings3	I don't know if it's useful. Mom's is a store. I'll return it if it doesn't work.	Jingdong prefecture level city in Shandong
Jinfeng Hongyuan Flagship Store	Electricity savings3	I don't know if it works or not, I'll wait for two months to use it and then come back to comment!	Jingdong prefecture level city in Shandong
Jinfeng Hongyuan Flagship Store	Electricity savings3	I bought the second one now, and I've been using it for more than half a year, and I feel that it really saves electricity. Now it is summer, the weather is hot, sometimes the fan and air conditioning on all day, if not this power saver, the monthly electricity bill is quite high, but as long as the installation of this power saver, not afraid of its high electricity bill, absolutely let you wantonly use	Jingdong prefecture level city in Shandong
Jinfeng Hongyuan Flagship Store	Electricity savings3	The product is better than I thought it would be, the quality looks great, the voltage is stable, and I'm looking forward to some great power savings!	Jingdong prefecture level city in Shandong
Jinfeng Hongyuan Flagship Store	Electricity savings3	Inadvertently see bought a try, delivery is also quite timely, just do not know how to use, customer service patience to answer, with a pretty good, value for money!	Jingdong prefecture level city in Shandong
Jinfeng Hongyuan Flagship Store	Electricity savings3	It's better than nothing! Hopefully this will help save slightly.	Jingdong prefecture level city in Shandong
Jinfeng Hongyuan Flagship Store	Electricity savings3	Still trying it out, follow up	Jingdong prefecture level city in Shandong
Jinfeng Hongyuan Flagship Store	Electricity savings3	I've just started using it, I don't know how well it works yet, I hope it's good.	Jingdong prefecture level city in Shandong
Jinfeng Hongyuan Flagship Store	Electricity savings3	Fourth purchase, great baby!	Jingdong prefecture level city in Shandong
Jinfeng Hongyuan Flagship Store	Electricity savings3	Shipped really fast, just got it and use it to see what happens and then come back for a review!	Jingdong prefecture level city in Shandong
Jinfeng Hongyuan Flagship Store	Electricity savings3	Timely delivery **** good attitude	Jingdong prefecture level city in Shandong
Jinfeng Hongyuan Flagship Store	Electricity savings3	Home large and small things are purchased in the Jingdong Mall, have been accustomed to shopping in the Jingdong, many times can buy good quality and cheap products, and courier service attitude is particularly good, sometimes too many couriers are to help me to the home, so many years have been in the Jingdong Mall shopping, trust and support! Will also support the Jingdong as always!	Jingdong prefecture level city in Shandong
Jinfeng Hongyuan Flagship Store	Electricity savings3	Modified a pump and the modification worked.	Jingdong prefecture level city in Shandong
Jinfeng Hongyuan Flagship Store	Electricity savings3	Already installed, next time you pay the electric bill to see the results	Jingdong prefecture level city in Shandong
Jinfeng Hongyuan Flagship Store	Electricity savings3	The product is very good to use and practical to look at	Jingdong prefecture level city in Shandong
Jinfeng Hongyuan Flagship Store	Electricity savings3	Works great, great service, waiting for results.	Jingdong prefecture level city in Shandong
Jinfeng Hongyuan Flagship Store	Electricity savings3	I just used it and I'm not sure about the efficacy yet, I'll come back to evaluate it after a while.	Jingdong prefecture level city in Shandong
Jinfeng Hongyuan Flagship Store	Electricity savings3	Just received the goods, use a period of time to evaluate	Jingdong prefecture level city in Shandong
Jinfeng Hongyuan Flagship Store	Electricity savings3	Opened and tried very good, use it to see if there are results	Jingdong prefecture level city in Shandong
Jinfeng Hongyuan Flagship Store	Electricity savings3	Arrived today! Still trying it out!	Jingdong prefecture level city in Shandong
Jinfeng Hongyuan Flagship Store	Electricity savings3	Shipped quickly, well packaged, already installed, hope there is a power saving effect	Jingdong prefecture level city in Shandong
Jinfeng Hongyuan Flagship Store	Electricity savings3	I got it. It's great.	Jingdong prefecture level city in Shandong
Jinfeng Hongyuan Flagship Store	Electricity savings3	Haven't used it yet, will come back in a month	Jingdong prefecture level city in Shandong
Jinfeng Hongyuan Flagship Store	Electricity savings3	It looks good. It's good. Try it first.	Jingdong prefecture level city in Shandong
Jinfeng Hongyuan Flagship Store	Electricity savings3	It works so well that I have relatives, friends, coworkers, and 6 families that have purchased it for them through me.	Jingdong prefecture level city in Shandong
Jinfeng Hongyuan Flagship Store	Electricity savings3	In use	Jingdong prefecture level city in Shandong
Jinfeng Hongyuan Flagship Store	Electricity savings3	Received the goods today, power-saving effect do not know being used, the boss has a very good attitude, praise!	Jingdong prefecture level city in Shandong

Jinfeng Hongyuan Flagship Store	Electricity savings3	The product is compact and doesn't take up much space, but hopefully it's power efficient.	Jingdong prefecture level city in Shandong
Jinfeng Hongyuan Flagship Store	Electricity savings3	Power saving is indeed effective, I used for a period of time before coming to evaluate, the electricity bill is obviously less, the quality is good, the shopkeeper service is also very good, very patient,	Jingdong prefecture level city in Shandong
Jinfeng Hongyuan Flagship Store	Electricity savings3	It feels fine, I'll comment after I use it	Jingdong prefecture level city in Shandong
Jinfeng Hongyuan Flagship Store	Electricity savings3	I just received it and I don't know the effect yet! I will comment again after I use it and have results!	Jingdong prefecture level city in Shandong
Jinfeng Hongyuan Flagship Store	Electricity savings3	The goods look fine, I hope they really work!	Jingdong prefecture level city in Shandong
Jinfeng Hongyuan Flagship Store	Electricity savings3	I just don't know if it saves power	Jingdong prefecture level city in Shandong
Jinfeng Hongyuan Flagship Store	Electricity savings3	My friend introduced me to use this product, it is professional and I am looking forward to the long term effect~!	Jingdong prefecture level city in Shandong
Jinfeng Hongyuan Flagship Store	Electricity savings3	Try it before you judge.	Jingdong prefecture level city in Shandong
Jinfeng Hongyuan Flagship Store	Electricity savings3	It's great, the power savings are obvious! Kudos!	Jingdong prefecture level city in Shandong
Jinfeng Hongyuan Flagship Store	Electricity savings3	The quality is excellent, stabilizes the voltage and saves electricity, very satisfied.	Jingdong prefecture level city in Shandong
Jinfeng Hongyuan Flagship Store	Electricity savings3	You have to use it for a while to know the effect	Jingdong prefecture level city in Shandong
Jinfeng Hongyuan Flagship Store	Electricity savings3	It's on trial.	Jingdong prefecture level city in Shandong
Jinfeng Hongyuan Flagship Store	Electricity savings3	The stuff works great.	Jingdong prefecture level city in Shandong
Jinfeng Hongyuan Flagship Store	Electricity savings3	Doesn't seem to work, not as god as other reviews	Jingdong prefecture level city in Shandong
Jinfeng Hongyuan Flagship Store	Electricity savings3	It's not good quality, it doesn't work, it stops working after a few days, it doesn't work, the customer service doesn't care, it's a bit irritating.	Jingdong prefecture level city in Shandong
Jinfeng Hongyuan Flagship Store	Electricity savings3	It doesn't do anything.	Jingdong prefecture level city in Shandong
Jinfeng Hongyuan Flagship Store	Electricity savings3	It's good.	Jingdong prefecture level city in Shandong
Jinfeng Hongyuan Flagship Store	Electricity savings3	Used a month effect is not obvious, are psychological effects, feel are not less, did not save to the electricity, there are details of the problem, the store said to buy to send 5 fuse, in fact, received only three, small problems are not committed to in place, I believe that the product is not good enough	Jingdong prefecture level city in Shandong
Jinfeng Hongyuan Flagship Store	Electricity savings3	Well received quickly, looking quite high, but also sent 5 fuses. Customer service is very patient to teach the operation, in fact, quite simple, lazy to read the instructions ha!	Jingdong prefecture level city in Shandong
Jinfeng Hongyuan Flagship Store	Electricity savings3	You have to use it for a while to know the effect	Jingdong prefecture level city in Shandong
Jinfeng Hongyuan Flagship Store	Electricity savings3	Shipped very quickly, packaged very well, the price is particularly affordable, there are so many efficacy is also good, it is worth buying worth buying the product, the workmanship is very fine, better than imagined, recommended that everyone try not yet used, after the use of the follow-up comment	Jingdong prefecture level city in Shandong
Jinfeng Hongyuan Flagship Store	Electricity savings3	It looks regular. It's working now.	Jingdong prefecture level city in Shandong
Jinfeng Hongyuan Flagship Store	Electricity savings3	It arrived pretty fast and the owner is very responsible. Have not used it yet, will see after use	Jingdong prefecture level city in Shandong
Jinfeng Hongyuan Flagship Store	Electricity savings3	🎁 baby quality is very good, the first time I received the goods to open the first time to see, it is very good 👍, very satisfied 😊, customer service also have a question and answer, thank you 🙏	Jingdong prefecture level city in Shandong
Jinfeng Hongyuan Flagship Store	Electricity savings3	I don't know if it's any good 。。。。。。	Jingdong prefecture level city in Shandong
Jinfeng Hongyuan Flagship Store	Electricity savings3	Buy electronic products! Still trust the things of the Jingdong self-owned store! Energy-saving effect to evaluate after heating!	Jingdong prefecture level city in Shandong
Jinfeng Hongyuan Flagship Store	Electricity savings3	The goods have been received, Blu is very beautiful, energy saving effect, to be confirmed only after actual use.	Jingdong prefecture level city in Shandong
Jinfeng Hongyuan Flagship Store	Electricity savings3	Jingdong's speed is so fast, has received, easy to operate. The display is quite good, how many volts what mode is quite understandable.	Jingdong prefecture level city in Shandong
Jinfeng Hongyuan Flagship Store	Electricity savings3	The first use, the effect is good to recommend it to colleagues to use, a month of electricity to save a lot or can be	Jingdong prefecture level city in Shandong
Jinfeng Hongyuan Flagship Store	Electricity savings3	I don't know if it's useful. Mom's is a store. I'll return it if it doesn't work.	Jingdong prefecture level city in Shandong
Jinfeng Hongyuan Flagship Store	Electricity savings3	I don't know if it works or not, I'll wait for two months to use it and then come back to comment!	Jingdong prefecture level city in Shandong
Jinfeng Hongyuan Flagship Store	Electricity savings3	I bought the second one now, and I've been using it for more than half a year, and I feel that it really saves electricity. Now it is summer, the weather is hot, sometimes the fan and air conditioning on all day, if not this power saver, the monthly electricity bill is quite high, but as long as the installation of this power saver, not afraid of its high electricity bill, absolutely let you wantonly use	Jingdong prefecture level city in Shandong
Jinfeng Hongyuan Flagship Store	Electricity savings3	The product is better than I thought it would be, the quality looks great, the voltage is stable, and I'm looking forward to some great power savings!	Jingdong prefecture level city in Shandong
Jinfeng Hongyuan Flagship Store	Electricity savings3	Inadvertently see bought a try, delivery is also quite timely, just do not know how to use, customer service patience to answer, with a pretty good, value for money!	Jingdong prefecture level city in Shandong
Jinfeng Hongyuan Flagship Store	Electricity savings3	It's better than nothing! Hopefully this will help save slightly.	Jingdong prefecture level city in Shandong
Jinfeng Hongyuan Flagship Store	Electricity savings3	Still trying it out, follow up	Jingdong prefecture level city in Shandong
Jinfeng Hongyuan Flagship Store	Electricity savings3	I've just started using it, I don't know how well it works yet, I hope it's good.	Jingdong prefecture level city in Shandong
Jinfeng Hongyuan Flagship Store	Electricity savings3	Fourth purchase, great baby!	Jingdong prefecture level city in Shandong
Jinfeng Hongyuan Flagship Store	Electricity savings3	Shipped really fast, just got it and use it to see what happens and then come back for a review!	Jingdong prefecture level city in Shandong
Jinfeng Hongyuan Flagship Store	Electricity savings3	Timely delivery **** good attitude	Jingdong prefecture level city in Shandong
Jinfeng Hongyuan Flagship Store	Electricity savings3	Home large and small things are purchased in the Jingdong Mall, have been accustomed to shopping in the Jingdong, many times can buy good quality and cheap products, and courier service attitude is particularly good, sometimes too many couriers are to help me to the home, so many years have been in the Jingdong Mall shopping, trust and support! Will also support the Jingdong as always!	Jingdong prefecture level city in Shandong
Jinfeng Hongyuan Flagship Store	Electricity savings3	Modified a pump and the modification worked.	Jingdong prefecture level city in Shandong
Jinfeng Hongyuan Flagship Store	Electricity savings3	Already installed, next time you pay the electric bill to see the results	Jingdong prefecture level city in Shandong
Jinfeng Hongyuan Flagship Store	Electricity savings3	The product is very good to use and practical to look at	Jingdong prefecture level city in Shandong
Jinfeng Hongyuan Flagship Store	Electricity savings3	Works great, great service, waiting for results.	Jingdong prefecture level city in Shandong
Jinfeng Hongyuan Flagship Store	Electricity savings3	I just used it and I'm not sure about the efficacy yet, I'll come back to evaluate it after a while.	Jingdong prefecture level city in Shandong
Jinfeng Hongyuan Flagship Store	Electricity savings3	Just received the goods, use a period of time to evaluate	Jingdong prefecture level city in Shandong
Jinfeng Hongyuan Flagship Store	Electricity savings3	Opened and tried very good, use it to see if there are results	Jingdong prefecture level city in Shandong

Jinfeng Hongyuan Flagship Store	Electricity savings3	Arrived today! Still trying it out!	Jingdong prefecture level city in Shandong
Jinfeng Hongyuan Flagship Store	Electricity savings3	Shipped quickly, well packaged, already installed, hope there is a power saving effect	Jingdong prefecture level city in Shandong
Jinfeng Hongyuan Flagship Store	Electricity savings3	I got it. It's great.	Jingdong prefecture level city in Shandong
Jinfeng Hongyuan Flagship Store	Electricity savings3	Haven't used it yet, will come back in a month	Jingdong prefecture level city in Shandong
Jinfeng Hongyuan Flagship Store	Electricity savings3	It looks good. It's good. Try it first.	Jingdong prefecture level city in Shandong
Jinfeng Hongyuan Flagship Store	Electricity savings3	It works so well that I have relatives, friends, coworkers, and 6 families that have purchased it for them through me.	Jingdong prefecture level city in Shandong
Jinfeng Hongyuan Flagship Store	Electricity savings3	In use	Jingdong prefecture level city in Shandong
Jinfeng Hongyuan Flagship Store	Electricity savings3	Received the goods today, power-saving effect do not know being used, the boss has a very good attitude, praise!	Jingdong prefecture level city in Shandong
Jinfeng Hongyuan Flagship Store	Electricity savings3	The product is compact and doesn't take up much space, but hopefully it's power efficient.	Jingdong prefecture level city in Shandong
Jinfeng Hongyuan Flagship Store	Electricity savings3	Power saving is indeed effective, I used for a period of time before coming to evaluate, the electricity bill is obviously less, the quality is good, the shopkeeper service is also very good, very patient,	Jingdong prefecture level city in Shandong
Jinfeng Hongyuan Flagship Store	Electricity savings3	It feels fine, I'll comment after I use it	Jingdong prefecture level city in Shandong
Jinfeng Hongyuan Flagship Store	Electricity savings3	I just received it and I don't know the effect yet! I will comment again after I use it and have results!	Jingdong prefecture level city in Shandong
Jinfeng Hongyuan Flagship Store	Electricity savings3	The goods look fine, I hope they really work!	Jingdong prefecture level city in Shandong
Jinfeng Hongyuan Flagship Store	Electricity savings3	I just don't know if it saves power	Jingdong prefecture level city in Shandong
Jinfeng Hongyuan Flagship Store	Electricity savings3	My friend introduced me to use this product, it is professional and I am looking forward to the long term effect~!	Jingdong prefecture level city in Shandong
Jinfeng Hongyuan Flagship Store	Electricity savings3	Try it before you judge.	Jingdong prefecture level city in Shandong
Jinfeng Hongyuan Flagship Store	Electricity savings3	It's great, the power savings are obvious! Kudos!	Jingdong prefecture level city in Shandong
Jinfeng Hongyuan Flagship Store	Electricity savings3	The quality is excellent, stabilizes the voltage and saves electricity, very satisfied.	Jingdong prefecture level city in Shandong
Jinfeng Hongyuan Flagship Store	Electricity savings3	You have to use it for a while to know the effect	Jingdong prefecture level city in Shandong
Jinfeng Hongyuan Flagship Store	Electricity savings3	It's on trial.	Jingdong prefecture level city in Shandong
Jinfeng Hongyuan Flagship Store	Electricity savings3	The stuff works great.	Jingdong prefecture level city in Shandong
Jinfeng Hongyuan Flagship Store	Electricity savings3	Doesn't seem to work, not as god as other reviews	Jingdong prefecture level city in Shandong
Jinfeng Hongyuan Flagship Store	Electricity savings3	It's not good quality, it doesn't work, it stops working after a few days, it doesn't work, the customer service doesn't care, it's a bit irritating.	Jingdong prefecture level city in Shandong
Jinfeng Hongyuan Flagship Store	Electricity savings3	It doesn't do anything.	Jingdong prefecture level city in Shandong
Jinfeng Hongyuan Flagship Store	Electricity savings3	It's good.	Jingdong prefecture level city in Shandong
Jinfeng Hongyuan Flagship Store	Electricity savings3	Used a month effect is not obvious, are psychological effects, feel are not less, did not save to the electricity, there are details of the problem, the store said to buy to send 5 fuse, in fact, received only three, small problems are not committed to in place, I believe that the product is not good enough	Jingdong prefecture level city in Shandong
Jinfeng Hongyuan Flagship Store	Electricity savings3	Well received quickly, looking quite high, but also sent 5 fuses. Customer service is very patient to teach the operation, in fact, quite simple, lazy to read the instructions ha!	Jingdong prefecture level city in Shandong
Jinfeng Hongyuan Flagship Store	Electricity savings3	You have to use it for a while to know the effect	Jingdong prefecture level city in Shandong
Jinfeng Hongyuan Flagship Store	Electricity savings3	Shipped very quickly, packaged very well, the price is particularly affordable, there are so many efficacy is also good, it is worth buying worth buying the product, the workmanship is very fine, better than imagined, recommended that everyone try not yet used, after the use of the follow-up comment	Jingdong prefecture level city in Shandong
Jinfeng Hongyuan Flagship Store	Electricity savings3	It looks regular. It's working now.	Jingdong prefecture level city in Shandong
Jinfeng Hongyuan Flagship Store	Electricity savings3	It arrived pretty fast and the owner is very responsible. Have not used it yet, will see after use	Jingdong prefecture level city in Shandong
Jinfeng Hongyuan Flagship Store	Electricity savings3	🎁 baby quality is very good, the first time I received the goods to open the first time to see, it is very good 👍, very satisfied 😊, customer service also have a question and answer, thank you 🙏	Jingdong prefecture level city in Shandong
Jinfeng Hongyuan Flagship Store	Electricity savings3	I don't know if it's any good 。。。。。。	Jingdong prefecture level city in Shandong
Jinfeng Hongyuan Flagship Store	Electricity savings3	Buy electronic products! Still trust the things of the Jingdong self-owned store! Energy-saving effect to evaluate after heating!	Jingdong prefecture level city in Shandong
Jinfeng Hongyuan Flagship Store	Electricity savings3	The goods have been received, Blu is very beautiful, energy saving effect, to be confirmed only after actual use.	Jingdong prefecture level city in Shandong
Jinfeng Hongyuan Flagship Store	Electricity savings3	Jingdong's speed is so fast, has received, easy to operate. The display is quite good, how many volts what mode is quite understandable.	Jingdong prefecture level city in Shandong
Jinfeng Hongyuan Flagship Store	Electricity savings3	The first use, the effect is good to recommend it to colleagues to use, a month of electricity to save a lot or can be	Jingdong prefecture level city in Shandong
Jinfeng Hongyuan Flagship Store	Electricity savings3	I don't know if it's useful. Mom's is a store. I'll return it if it doesn't work.	Jingdong prefecture level city in Shandong
Jinfeng Hongyuan Flagship Store	Electricity savings3	I don't know if it works or not, I'll wait for two months to use it and then come back to comment!	Jingdong prefecture level city in Shandong
Jinfeng Hongyuan Flagship Store	Electricity savings3	I bought the second one now, and I've been using it for more than half a year, and I feel that it really saves electricity. Now it is summer, the weather is hot, sometimes the fan and air conditioning on all day, if not this power saver, the monthly electricity bill is quite high, but as long as the installation of this power saver, not afraid of its high electricity bill, absolutely let you wantonly use	Jingdong prefecture level city in Shandong
Jinfeng Hongyuan Flagship Store	Electricity savings3	The product is better than I thought it would be, the quality looks great, the voltage is stable, and I'm looking forward to some great power savings!	Jingdong prefecture level city in Shandong
Jinfeng Hongyuan Flagship Store	Electricity savings3	Inadvertently see bought a try, delivery is also quite timely, just do not know how to use, customer service patience to answer, with a pretty good, value for money!	Jingdong prefecture level city in Shandong
Jinfeng Hongyuan Flagship Store	Electricity savings3	It's better than nothing! Hopefully this will help save slightly.	Jingdong prefecture level city in Shandong
Jinfeng Hongyuan Flagship Store	Electricity savings3	Still trying it out, follow up	Jingdong prefecture level city in Shandong
Jinfeng Hongyuan Flagship Store	Electricity savings3	I've just started using it, I don't know how well it works yet, I hope it's good.	Jingdong prefecture level city in Shandong
Jinfeng Hongyuan Flagship Store	Electricity savings3	Fourth purchase, great baby!	Jingdong prefecture level city in Shandong
Jinfeng Hongyuan Flagship Store	Electricity savings3	Shipped really fast, just got it and use it to see what happens and then come back for a review!	Jingdong prefecture level city in Shandong
Jinfeng Hongyuan Flagship Store	Electricity savings3	Timely delivery **** good attitude	Jingdong prefecture level city in Shandong

Jinfeng Hongyuan Flagship Store	Electricity savings3	Home large and small things are purchased in the Jingdong Mall, have been accustomed to shopping in the Jingdong, many times can buy good quality and cheap products, and courier service attitude is particularly good, sometimes too many couriers are to help me to the home, so many years have been in the Jingdong Mall shopping, trust and support! Will also support the Jingdong as always!	Jingdong prefecture level city in Shandong
Jinfeng Hongyuan Flagship Store	Electricity savings3	Modified a pump and the modification worked.	Jingdong prefecture level city in Shandong
Jinfeng Hongyuan Flagship Store	Electricity savings3	Already installed, next time you pay the electric bill to see the results	Jingdong prefecture level city in Shandong
Jinfeng Hongyuan Flagship Store	Electricity savings3	The product is very good to use and practical to look at	Jingdong prefecture level city in Shandong
Jinfeng Hongyuan Flagship Store	Electricity savings3	Works great, great service, waiting for results.	Jingdong prefecture level city in Shandong
Jinfeng Hongyuan Flagship Store	Electricity savings3	I just used it and I'm not sure about the efficacy yet, I'll come back to evaluate it after a while.	Jingdong prefecture level city in Shandong
Jinfeng Hongyuan Flagship Store	Electricity savings3	Just received the goods, use a period of time to evaluate	Jingdong prefecture level city in Shandong
Jinfeng Hongyuan Flagship Store	Electricity savings3	Opened and tried very good, use it to see if there are results	Jingdong prefecture level city in Shandong
Jinfeng Hongyuan Flagship Store	Electricity savings3	Arrived today! Still trying it out!	Jingdong prefecture level city in Shandong
Jinfeng Hongyuan Flagship Store	Electricity savings3	Shipped quickly, well packaged, already installed, hope there is a power saving effect	Jingdong prefecture level city in Shandong
Jinfeng Hongyuan Flagship Store	Electricity savings3	I got it. It's great.	Jingdong prefecture level city in Shandong
Jinfeng Hongyuan Flagship Store	Electricity savings3	Haven't used it yet, will come back in a month	Jingdong prefecture level city in Shandong
Jinfeng Hongyuan Flagship Store	Electricity savings3	It looks good. It's good. Try it first.	Jingdong prefecture level city in Shandong
Jinfeng Hongyuan Flagship Store	Electricity savings3	It works so well that I have relatives, friends, coworkers, and 6 families that have purchased it for them through me.	Jingdong prefecture level city in Shandong
Jinfeng Hongyuan Flagship Store	Electricity savings3	In use	Jingdong prefecture level city in Shandong
Jinfeng Hongyuan Flagship Store	Electricity savings3	Received the goods today, power-saving effect do not know being used, the boss has a very good attitude, praise!	Jingdong prefecture level city in Shandong
Jinfeng Hongyuan Flagship Store	Electricity savings3	The product is compact and doesn't take up much space, but hopefully it's power efficient.	Jingdong prefecture level city in Shandong
Jinfeng Hongyuan Flagship Store	Electricity savings3	Power saving is indeed effective, I used for a period of time before coming to evaluate, the electricity bill is obviously less, the quality is good, the shopkeeper service is also very good, very patient,	Jingdong prefecture level city in Shandong
Jinfeng Hongyuan Flagship Store	Electricity savings3	It feels fine, I'll comment after I use it	Jingdong prefecture level city in Shandong
Jinfeng Hongyuan Flagship Store	Electricity savings3	I just received it and I don't know the effect yet! I will comment again after I use it and have results!	Jingdong prefecture level city in Shandong
Jinfeng Hongyuan Flagship Store	Electricity savings3	The goods look fine, I hope they really work!	Jingdong prefecture level city in Shandong
Jinfeng Hongyuan Flagship Store	Electricity savings3	I just don't know if it saves power	Jingdong prefecture level city in Shandong
Jinfeng Hongyuan Flagship Store	Electricity savings3	My friend introduced me to use this product, it is professional and I am looking forward to the long term effect~!	Jingdong prefecture level city in Shandong
Jinfeng Hongyuan Flagship Store	Electricity savings3	Try it before you judge.	Jingdong prefecture level city in Shandong
Jinfeng Hongyuan Flagship Store	Electricity savings3	It's great, the power savings are obvious! Kudos!	Jingdong prefecture level city in Shandong
Jinfeng Hongyuan Flagship Store	Electricity savings3	The quality is excellent, stabilizes the voltage and saves electricity, very satisfied.	Jingdong prefecture level city in Shandong
Jinfeng Hongyuan Flagship Store	Electricity savings3	You have to use it for a while to know the effect	Jingdong prefecture level city in Shandong
Jinfeng Hongyuan Flagship Store	Electricity savings3	It's on trial.	Jingdong prefecture level city in Shandong
Jinfeng Hongyuan Flagship Store	Electricity savings3	The stuff works great.	Jingdong prefecture level city in Shandong
Jinfeng Hongyuan Flagship Store	Electricity savings3	Doesn't seem to work, not as god as other reviews	Jingdong prefecture level city in Shandong
Jinfeng Hongyuan Flagship Store	Electricity savings3	It's not good quality, it doesn't work, it stops working after a few days, it doesn't work, the customer service doesn't care, it's a bit irritating.	Jingdong prefecture level city in Shandong
Jinfeng Hongyuan Flagship Store	Electricity savings3	It doesn't do anything.	Jingdong prefecture level city in Shandong
Jinfeng Hongyuan Flagship Store	Electricity savings3	It's good.	Jingdong prefecture level city in Shandong
Jinfeng Hongyuan Flagship Store	Electricity savings3	Used a month effect is not obvious, are psychological effects, feel are not less, did not save to the electricity, there are details of the problem, the store said to buy to send 5 fuse, in fact, received only three, small problems are not committed to in place, I believe that the product is not good enough	Jingdong prefecture level city in Shandong
Jinfeng Hongyuan Flagship Store	Electricity savings3	Well received quickly, looking quite high, but also sent 5 fuses. Customer service is very patient to teach the operation, in fact, quite simple, lazy to read the instructions ha!	Jingdong prefecture level city in Shandong
Jinfeng Hongyuan Flagship Store	Electricity savings3	You have to use it for a while to know the effect	Jingdong prefecture level city in Shandong
Jinfeng Hongyuan Flagship Store	Electricity savings3	Shipped very quickly, packaged very well, the price is particularly affordable, there are so many efficacy is also good, it is worth buying worth buying the product, the workmanship is very fine, better than imagined, recommended that everyone try not yet used, after the use of the follow-up comment	Jingdong prefecture level city in Shandong
Jinfeng Hongyuan Flagship Store	Electricity savings3	It looks regular. It's working now.	Jingdong prefecture level city in Shandong
Jinfeng Hongyuan Flagship Store	Electricity savings3	It arrived pretty fast and the owner is very responsible. Have not used it yet, will see after use	Jingdong prefecture level city in Shandong
Jinfeng Hongyuan Flagship Store	Electricity savings3	🎁 baby quality is very good, the first time I received the goods to open the first time to see, it is very good 👍, very satisfied ☺, customer service also have a question and answer, thank you 🙏	Jingdong prefecture level city in Shandong
Jinfeng Hongyuan Flagship Store	Electricity savings3	I don't know if it's any good 。。。。。。	Jingdong prefecture level city in Shandong
Jinfeng Hongyuan Flagship Store	Electricity savings3	Buy electronic products! Still trust the things of the Jingdong self-owned store! Energy-saving effect to evaluate after heating!	Jingdong prefecture level city in Shandong
Jinfeng Hongyuan Flagship Store	Electricity savings3	The goods have been received, Blu is very beautiful, energy saving effect, to be confirmed only after actual use.	Jingdong prefecture level city in Shandong
Jinfeng Hongyuan Flagship Store	Electricity savings3	Jingdong's speed is so fast, has received, easy to operate. The display is quite good, how many volts what mode is quite understandable.	Jingdong prefecture level city in Shandong
Jinfeng Hongyuan Flagship Store	Electricity savings3	The first use, the effect is good to recommend it to colleagues to use, a month of electricity to save a lot or can be	Jingdong prefecture level city in Shandong
Jinfeng Hongyuan Flagship Store	Electricity savings3	I don't know if it's useful. Mom's is a store. I'll return it if it doesn't work.	Jingdong prefecture level city in Shandong
Jinfeng Hongyuan Flagship Store	Electricity savings3	I don't know if it works or not, I'll wait for two months to use it and then come back to comment!	Jingdong prefecture level city in Shandong
Jinfeng Hongyuan Flagship Store	Electricity savings3	I bought the second one now, and I've been using it for more than half a year, and I feel that it really saves electricity. Now it is summer, the weather is hot, sometimes the fan and air conditioning on all day, if not this power saver, the monthly electricity bill is quite high, but as long as the installation of this power saver, not afraid of its high electricity bill, absolutely let you wantonly use	Jingdong prefecture level city in Shandong

Jinfeng Hongyuan Flagship Store	Electricity savings3	The product is better than I thought it would be, the quality looks great, the voltage is stable, and I'm looking forward to some great power savings!	Jingdong prefecture level city in Shandong
Jinfeng Hongyuan Flagship Store	Electricity savings3	Inadvertently see bought a try, delivery is also quite timely, just do not know how to use, customer service patience to answer, with a pretty good, value for money!	Jingdong prefecture level city in Shandong
Jinfeng Hongyuan Flagship Store	Electricity savings3	It's better than nothing! Hopefully this will help save slightly.	Jingdong prefecture level city in Shandong
Jinfeng Hongyuan Flagship Store	Electricity savings3	Still trying it out, follow up	Jingdong prefecture level city in Shandong
Jinfeng Hongyuan Flagship Store	Electricity savings3	I've just started using it, I don't know how well it works yet, I hope it's good.	Jingdong prefecture level city in Shandong
Jinfeng Hongyuan Flagship Store	Electricity savings3	Fourth purchase, great baby!	Jingdong prefecture level city in Shandong
Jinfeng Hongyuan Flagship Store	Electricity savings3	Shipped really fast, just got it and use it to see what happens and then come back for a review!	Jingdong prefecture level city in Shandong
Jinfeng Hongyuan Flagship Store	Electricity savings3	Timely delivery **** good attitude	Jingdong prefecture level city in Shandong
Jinfeng Hongyuan Flagship Store	Electricity savings3	Home large and small things are purchased in the Jingdong Mall, have been accustomed to shopping in the Jingdong, many times can buy good quality and cheap products, and courier service attitude is particularly good, sometimes too many couriers are to help me to the home, so many years have been in the Jingdong Mall shopping, trust and support! Will also support the Jingdong as always!	Jingdong prefecture level city in Shandong
Jinfeng Hongyuan Flagship Store	Electricity savings3	Modified a pump and the modification worked.	Jingdong prefecture level city in Shandong
Jinfeng Hongyuan Flagship Store	Electricity savings3	Already installed, next time you pay the electric bill to see the results	Jingdong prefecture level city in Shandong
Jinfeng Hongyuan Flagship Store	Electricity savings3	The product is very good to use and practical to look at	Jingdong prefecture level city in Shandong
Jinfeng Hongyuan Flagship Store	Electricity savings3	Works great, great service, waiting for results.	Jingdong prefecture level city in Shandong
Jinfeng Hongyuan Flagship Store	Electricity savings3	I just used it and I'm not sure about the efficacy yet, I'll come back to evaluate it after a while.	Jingdong prefecture level city in Shandong
Jinfeng Hongyuan Flagship Store	Electricity savings3	Just received the goods, use a period of time to evaluate	Jingdong prefecture level city in Shandong
Jinfeng Hongyuan Flagship Store	Electricity savings3	Opened and tried very good, use it to see if there are results	Jingdong prefecture level city in Shandong
Jinfeng Hongyuan Flagship Store	Electricity savings3	Arrived today! Still trying it out!	Jingdong prefecture level city in Shandong
Jinfeng Hongyuan Flagship Store	Electricity savings3	Shipped quickly, well packaged, already installed, hope there is a power saving effect	Jingdong prefecture level city in Shandong
Jinfeng Hongyuan Flagship Store	Electricity savings3	I got it. It's great.	Jingdong prefecture level city in Shandong
Jinfeng Hongyuan Flagship Store	Electricity savings3	Haven't used it yet, will come back in a month	Jingdong prefecture level city in Shandong
Jinfeng Hongyuan Flagship Store	Electricity savings3	It looks good. It's good. Try it first.	Jingdong prefecture level city in Shandong
Jinfeng Hongyuan Flagship Store	Electricity savings3	It works so well that I have relatives, friends, coworkers, and 6 families that have purchased it for them through me.	Jingdong prefecture level city in Shandong
Jinfeng Hongyuan Flagship Store	Electricity savings3	In use	Jingdong prefecture level city in Shandong
Jinfeng Hongyuan Flagship Store	Electricity savings3	Received the goods today, power-saving effect do not know being used, the boss has a very good attitude, praise!	Jingdong prefecture level city in Shandong
Jinfeng Hongyuan Flagship Store	Electricity savings3	The product is compact and doesn't take up much space, but hopefully it's power efficient.	Jingdong prefecture level city in Shandong
Jinfeng Hongyuan Flagship Store	Electricity savings3	Power saving is indeed effective, I used for a period of time before coming to evaluate, the electricity bill is obviously less, the quality is good, the shopkeeper service is also very good, very patient,	Jingdong prefecture level city in Shandong
Jinfeng Hongyuan Flagship Store	Electricity savings3	It feels fine, I'll comment after I use it	Jingdong prefecture level city in Shandong
Jinfeng Hongyuan Flagship Store	Electricity savings3	I just received it and I don't know the effect yet! I will comment again after I use it and have results!	Jingdong prefecture level city in Shandong
Jinfeng Hongyuan Flagship Store	Electricity savings3	The goods look fine, I hope they really work!	Jingdong prefecture level city in Shandong
Jinfeng Hongyuan Flagship Store	Electricity savings3	I just don't know if it saves power	Jingdong prefecture level city in Shandong
Jinfeng Hongyuan Flagship Store	Electricity savings3	My friend introduced me to use this product, it is professional and I am looking forward to the long term effect~!	Jingdong prefecture level city in Shandong
Jinfeng Hongyuan Flagship Store	Electricity savings3	Try it before you judge.	Jingdong prefecture level city in Shandong
Jinfeng Hongyuan Flagship Store	Electricity savings3	It's great, the power savings are obvious! Kudos!	Jingdong prefecture level city in Shandong
Jinfeng Hongyuan Flagship Store	Electricity savings3	The quality is excellent, stabilizes the voltage and saves electricity, very satisfied.	Jingdong prefecture level city in Shandong
Jinfeng Hongyuan Flagship Store	Electricity savings3	You have to use it for a while to know the effect	Jingdong prefecture level city in Shandong
Jinfeng Hongyuan Flagship Store	Electricity savings3	It's on trial.	Jingdong prefecture level city in Shandong
Jinfeng Hongyuan Flagship Store	Electricity savings3	The stuff works great.	Jingdong prefecture level city in Shandong
Jinfeng Hongyuan Flagship Store	Electricity savings3	Doesn't seem to work, not as god as other reviews	Jingdong prefecture level city in Shandong
Jinfeng Hongyuan Flagship Store	Electricity savings3	It's not good quality, it doesn't work, it stops working after a few days, it doesn't work, the customer service doesn't care, it's a bit irritating.	Jingdong prefecture level city in Shandong
Jinfeng Hongyuan Flagship Store	Electricity savings3	It doesn't do anything.	Jingdong prefecture level city in Shandong
Jinfeng Hongyuan Flagship Store	Electricity savings3	It's good.	Jingdong prefecture level city in Shandong
Jinfeng Hongyuan Flagship Store	Electricity savings3	Used a month effect is not obvious, are psychological effects, feel are not less, did not save to the electricity, there are details of the problem, the store said to buy to send 5 fuse, in fact, received only three, small problems are not committed to in place, I believe that the product is not good enough	Jingdong prefecture level city in Shandong
Jinfeng Hongyuan Flagship Store	Electricity savings3	Well received quickly, looking quite high, but also sent 5 fuses. Customer service is very patient to teach the operation, in fact, quite simple, lazy to read the instructions ha!	Jingdong prefecture level city in Shandong
Jinfeng Hongyuan Flagship Store	Electricity savings3	You have to use it for a while to know the effect	Jingdong prefecture level city in Shandong
Jinfeng Hongyuan Flagship Store	Electricity savings3	Shipped very quickly, packaged very well, the price is particularly affordable, there are so many efficacy is also good, it is worth buying worth buying the product, the workmanship is very fine, better than imagined, recommended that everyone try not yet used, after the use of the follow-up comment	Jingdong prefecture level city in Shandong
Jinfeng Hongyuan Flagship Store	Electricity savings3	It looks regular. It's working now.	Jingdong prefecture level city in Shandong
Jinfeng Hongyuan Flagship Store	Electricity savings3	It arrived pretty fast and the owner is very responsible. Have not used it yet, will see after use	Jingdong prefecture level city in Shandong
Jinfeng Hongyuan Flagship Store	Electricity savings3	🎁 baby quality is very good, the first time I received the goods to open the first time to see, it is very good 👍, very satisfied 😊, customer service also have a question and answer, thank you 🙏	Jingdong prefecture level city in Shandong
Jinfeng Hongyuan Flagship Store	Electricity savings3	I don't know if it's any good 。。。。。。	Jingdong prefecture level city in Shandong

Jinfeng Hongyuan Flagship Store	Electricity savings3	Buy electronic products! Still trust the things of the Jingdong self-owned store! Energy-saving effect to evaluate after heating!	Jingdong prefecture level city in Shandong
Jinfeng Hongyuan Flagship Store	Electricity savings3	The goods have been received, Blu is very beautiful, energy saving effect, to be confirmed only after actual use.	Jingdong prefecture level city in Shandong
Jinfeng Hongyuan Flagship Store	Electricity savings3	Jingdong's speed is so fast, has received, easy to operate. The display is quite good, how many volts what mode is quite understandable.	Jingdong prefecture level city in Shandong
Jinfeng Hongyuan Flagship Store	Electricity savings3	The first use, the effect is good to recommend it to colleagues to use, a month of electricity to save a lot or can be	Jingdong prefecture level city in Shandong
Jinfeng Hongyuan Flagship Store	Electricity savings3	I don't know if it's useful. Mom's is a store. I'll return it if it doesn't work.	Jingdong prefecture level city in Shandong
Jinfeng Hongyuan Flagship Store	Electricity savings3	I don't know if it works or not, I'll wait for two months to use it and then come back to comment!	Jingdong prefecture level city in Shandong
Jinfeng Hongyuan Flagship Store	Electricity savings3	I bought the second one now, and I've been using it for more than half a year, and I feel that it really saves electricity. Now it is summer, the weather is hot, sometimes the fan and air conditioning on all day, if not this power saver, the monthly electricity bill is quite high, but as long as the installation of this power saver, not afraid of its high electricity bill, absolutely let you wantonly use	Jingdong prefecture level city in Shandong
Jinfeng Hongyuan Flagship Store	Electricity savings3	The product is better than I thought it would be, the quality looks great, the voltage is stable, and I'm looking forward to some great power savings!	Jingdong prefecture level city in Shandong
Jinfeng Hongyuan Flagship Store	Electricity savings3	Inadvertently see bought a try, delivery is also quite timely, just do not know how to use, customer service patience to answer, with a pretty good, value for money!	Jingdong prefecture level city in Shandong
Jinfeng Hongyuan Flagship Store	Electricity savings3	It's better than nothing! Hopefully this will help save slightly.	Jingdong prefecture level city in Shandong
Jinfeng Hongyuan Flagship Store	Electricity savings3	Still trying it out, follow up	Jingdong prefecture level city in Shandong
Jinfeng Hongyuan Flagship Store	Electricity savings3	I've just started using it, I don't know how well it works yet, I hope it's good.	Jingdong prefecture level city in Shandong
Jinfeng Hongyuan Flagship Store	Electricity savings3	Fourth purchase, great baby!	Jingdong prefecture level city in Shandong
Jinfeng Hongyuan Flagship Store	Electricity savings3	Shipped really fast, just got it and use it to see what happens and then come back for a review!	Jingdong prefecture level city in Shandong
Jinfeng Hongyuan Flagship Store	Electricity savings3	Timely delivery **** good attitude	Jingdong prefecture level city in Shandong
Jinfeng Hongyuan Flagship Store	Electricity savings3	Home large and small things are purchased in the Jingdong Mall, have been accustomed to shopping in the Jingdong, many times can buy good quality and cheap products, and courier service attitude is particularly good, sometimes too many couriers are to help me to the home, so many years have been in the Jingdong Mall shopping, trust and support! Will also support the Jingdong as always!	Jingdong prefecture level city in Shandong
Jinfeng Hongyuan Flagship Store	Electricity savings3	Modified a pump and the modification worked.	Jingdong prefecture level city in Shandong
Jinfeng Hongyuan Flagship Store	Electricity savings3	Already installed, next time you pay the electric bill to see the results	Jingdong prefecture level city in Shandong
Jinfeng Hongyuan Flagship Store	Electricity savings3	The product is very good to use and practical to look at	Jingdong prefecture level city in Shandong
Jinfeng Hongyuan Flagship Store	Electricity savings3	Works great, great service, waiting for results.	Jingdong prefecture level city in Shandong
Jinfeng Hongyuan Flagship Store	Electricity savings3	I just used it and I'm not sure about the efficacy yet, I'll come back to evaluate it after a while.	Jingdong prefecture level city in Shandong
Jinfeng Hongyuan Flagship Store	Electricity savings3	Just received the goods, use a period of time to evaluate	Jingdong prefecture level city in Shandong
Jinfeng Hongyuan Flagship Store	Electricity savings3	Opened and tried very good, use it to see if there are results	Jingdong prefecture level city in Shandong
Jinfeng Hongyuan Flagship Store	Electricity savings3	Arrived today! Still trying it out!	Jingdong prefecture level city in Shandong
Jinfeng Hongyuan Flagship Store	Electricity savings3	Shipped quickly, well packaged, already installed, hope there is a power saving effect	Jingdong prefecture level city in Shandong
Jinfeng Hongyuan Flagship Store	Electricity savings3	I got it. It's great.	Jingdong prefecture level city in Shandong
Jinfeng Hongyuan Flagship Store	Electricity savings3	Haven't used it yet, will come back in a month	Jingdong prefecture level city in Shandong
Jinfeng Hongyuan Flagship Store	Electricity savings3	It looks good. It's good. Try it first.	Jingdong prefecture level city in Shandong
Jinfeng Hongyuan Flagship Store	Electricity savings3	It works so well that I have relatives, friends, coworkers, and 6 families that have purchased it for them through me.	Jingdong prefecture level city in Shandong
Jinfeng Hongyuan Flagship Store	Electricity savings3	In use	Jingdong prefecture level city in Shandong
Jinfeng Hongyuan Flagship Store	Electricity savings3	Received the goods today, power-saving effect do not know being used, the boss has a very good attitude, praise!	Jingdong prefecture level city in Shandong
Jinfeng Hongyuan Flagship Store	Electricity savings3	The product is compact and doesn't take up much space, but hopefully it's power efficient.	Jingdong prefecture level city in Shandong
Jinfeng Hongyuan Flagship Store	Electricity savings3	Power saving is indeed effective, I used for a period of time before coming to evaluate, the electricity bill is obviously less, the quality is good, the shopkeeper service is also very good, very patient,	Jingdong prefecture level city in Shandong
Jinfeng Hongyuan Flagship Store	Electricity savings3	It feels fine, I'll comment after I use it	Jingdong prefecture level city in Shandong
Jinfeng Hongyuan Flagship Store	Electricity savings3	I just received it and I don't know the effect yet! I will comment again after I use it and have results!	Jingdong prefecture level city in Shandong
Jinfeng Hongyuan Flagship Store	Electricity savings3	The goods look fine, I hope they really work!	Jingdong prefecture level city in Shandong
Jinfeng Hongyuan Flagship Store	Electricity savings3	I just don't know if it saves power	Jingdong prefecture level city in Shandong
Jinfeng Hongyuan Flagship Store	Electricity savings3	My friend introduced me to use this product, it is professional and I am looking forward to the long term effect~!	Jingdong prefecture level city in Shandong
Jinfeng Hongyuan Flagship Store	Electricity savings3	Try it before you judge.	Jingdong prefecture level city in Shandong
Jinfeng Hongyuan Flagship Store	Electricity savings3	It's great, the power savings are obvious! Kudos!	Jingdong prefecture level city in Shandong
Jinfeng Hongyuan Flagship Store	Electricity savings3	The quality is excellent, stabilizes the voltage and saves electricity, very satisfied.	Jingdong prefecture level city in Shandong
Jinfeng Hongyuan Flagship Store	Electricity savings3	You have to use it for a while to know the effect	Jingdong prefecture level city in Shandong
Jinfeng Hongyuan Flagship Store	Electricity savings3	It's on trial.	Jingdong prefecture level city in Shandong
Jinfeng Hongyuan Flagship Store	Electricity savings3	The stuff works great.	Jingdong prefecture level city in Shandong
Jinfeng Hongyuan Flagship Store	Electricity savings3	Doesn't seem to work, not as god as other reviews	Jingdong prefecture level city in Shandong
Jinfeng Hongyuan Flagship Store	Electricity savings3	It's not good quality, it doesn't work, it stops working after a few days, it doesn't work, the customer service doesn't care, it's a bit irritating.	Jingdong prefecture level city in Shandong
Jinfeng Hongyuan Flagship Store	Electricity savings3	It doesn't do anything.	Jingdong prefecture level city in Shandong
Jinfeng Hongyuan Flagship Store	Electricity savings3	It's good.	Jingdong prefecture level city in Shandong
Jinfeng Hongyuan Flagship Store	Electricity savings3	Used a month effect is not obvious, are psychological effects, feel are not less, did not save to the electricity, there are details of the problem, the store said to buy to send 5 fuse, in fact, received only three, small problems are not committed to in place, I believe that the product is not good enough	Jingdong prefecture level city in Shandong

Jinfeng Hongyuan Flagship Store	Electricity savings3	Well received quickly, looking quite high, but also sent 5 fuses. Customer service is very patient to teach the operation, in fact, quite simple, lazy to read the instructions ha!	Jingdong prefecture level city in Shandong
Jinfeng Hongyuan Flagship Store	Electricity savings3	You have to use it for a while to know the effect	Jingdong prefecture level city in Shandong
Jinfeng Hongyuan Flagship Store	Electricity savings3	Shipped very quickly, packaged very well, the price is particularly affordable, there are so many efficacy is also good, it is worth buying worth buying the product, the workmanship is very fine, better than imagined, recommended that everyone try not yet used, after the use of the follow-up comment	Jingdong prefecture level city in Shandong
Jinfeng Hongyuan Flagship Store	Electricity savings3	It looks regular. It's working now.	Jingdong prefecture level city in Shandong
Jinfeng Hongyuan Flagship Store	Electricity savings3	It arrived pretty fast and the owner is very responsible. Have not used it yet, will see after use	Jingdong prefecture level city in Shandong
Jinfeng Hongyuan Flagship Store	Electricity savings3	🎁 baby quality is very good, the first time I received the goods to open the first time to see, it is very good 👍, very satisfied ☺, customer service also have a question and answer, thank you 🙏	Jingdong prefecture level city in Shandong
Jinfeng Hongyuan Flagship Store	Electricity savings3	I don't know if it's any good 。。。。。。	Jingdong prefecture level city in Shandong
Jinfeng Hongyuan Flagship Store	Electricity savings3	Buy electronic products! Still trust the things of the Jingdong self-owned store! Energy-saving effect to evaluate after heating!	Jingdong prefecture level city in Shandong
Jinfeng Hongyuan Flagship Store	Electricity savings3	The goods have been received, Blu is very beautiful, energy saving effect, to be confirmed only after actual use.	Jingdong prefecture level city in Shandong
Jinfeng Hongyuan Flagship Store	Electricity savings3	Jingdong's speed is so fast, has received, easy to operate. The display is quite good, how many volts what mode is quite understandable.	Jingdong prefecture level city in Shandong
Jinfeng Hongyuan Flagship Store	Electricity savings3	The first use, the effect is good to recommend it to colleagues to use, a month of electricity to save a lot or can be	Jingdong prefecture level city in Shandong
Jinfeng Hongyuan Flagship Store	Electricity savings3	I don't know if it's useful. Mom's is a store. I'll return it if it doesn't work.	Jingdong prefecture level city in Shandong
Jinfeng Hongyuan Flagship Store	Electricity savings3	I don't know if it works or not, I'll wait for two months to use it and then come back to comment!	Jingdong prefecture level city in Shandong
Jinfeng Hongyuan Flagship Store	Electricity savings3	I bought the second one now, and I've been using it for more than half a year, and I feel that it really saves electricity. Now it is summer, the weather is hot, sometimes the fan and air conditioning on all day, if not this power saver, the monthly electricity bill is quite high, but as long as the installation of this power saver, not afraid of its high electricity bill, absolutely let you wantonly use	Jingdong prefecture level city in Shandong
Jinfeng Hongyuan Flagship Store	Electricity savings3	The product is better than I thought it would be, the quality looks great, the voltage is stable, and I'm looking forward to some great power savings!	Jingdong prefecture level city in Shandong
Jinfeng Hongyuan Flagship Store	Electricity savings3	Inadvertently see bought a try, delivery is also quite timely, just do not know how to use, customer service patience to answer, with a pretty good, value for money!	Jingdong prefecture level city in Shandong
Jinfeng Hongyuan Flagship Store	Electricity savings3	It's better than nothing! Hopefully this will help save slightly.	Jingdong prefecture level city in Shandong
Jinfeng Hongyuan Flagship Store	Electricity savings3	Still trying it out, follow up	Jingdong prefecture level city in Shandong
Jinfeng Hongyuan Flagship Store	Electricity savings3	I've just started using it, I don't know how well it works yet, I hope it's good.	Jingdong prefecture level city in Shandong
Jinfeng Hongyuan Flagship Store	Electricity savings3	Fourth purchase, great baby!	Jingdong prefecture level city in Shandong
Jinfeng Hongyuan Flagship Store	Electricity savings3	Shipped really fast, just got it and use it to see what happens and then come back for a review!	Jingdong prefecture level city in Shandong
Jinfeng Hongyuan Flagship Store	Electricity savings3	Timely delivery **** good attitude	Jingdong prefecture level city in Shandong
Jinfeng Hongyuan Flagship Store	Electricity savings3	Home large and small things are purchased in the Jingdong Mall, have been accustomed to shopping in the Jingdong, many times can buy good quality and cheap products, and courier service attitude is particularly good, sometimes too many couriers are to help me to the home, so many years have been in the Jingdong Mall shopping, trust and support! Will also support the Jingdong as always!	Jingdong prefecture level city in Shandong
Jinfeng Hongyuan Flagship Store	Electricity savings3	Modified a pump and the modification worked.	Jingdong prefecture level city in Shandong
Jinfeng Hongyuan Flagship Store	Electricity savings3	Already installed, next time you pay the electric bill to see the results	Jingdong prefecture level city in Shandong
Jinfeng Hongyuan Flagship Store	Electricity savings3	The product is very good to use and practical to look at	Jingdong prefecture level city in Shandong
Jinfeng Hongyuan Flagship Store	Electricity savings3	Works great, great service, waiting for results.	Jingdong prefecture level city in Shandong
Jinfeng Hongyuan Flagship Store	Electricity savings3	I just used it and I'm not sure about the efficacy yet, I'll come back to evaluate it after a while.	Jingdong prefecture level city in Shandong
Jinfeng Hongyuan Flagship Store	Electricity savings3	Just received the goods, use a period of time to evaluate	Jingdong prefecture level city in Shandong
Jinfeng Hongyuan Flagship Store	Electricity savings3	Opened and tried very good, use it to see if there are results	Jingdong prefecture level city in Shandong
Jinfeng Hongyuan Flagship Store	Electricity savings3	Arrived today! Still trying it out!	Jingdong prefecture level city in Shandong
Jinfeng Hongyuan Flagship Store	Electricity savings3	Shipped quickly, well packaged, already installed, hope there is a power saving effect	Jingdong prefecture level city in Shandong
Jinfeng Hongyuan Flagship Store	Electricity savings3	I got it. It's great.	Jingdong prefecture level city in Shandong
Jinfeng Hongyuan Flagship Store	Electricity savings3	Haven't used it yet, will come back in a month	Jingdong prefecture level city in Shandong
Jinfeng Hongyuan Flagship Store	Electricity savings3	It looks good. It's good. Try it first.	Jingdong prefecture level city in Shandong
Jinfeng Hongyuan Flagship Store	Electricity savings3	It works so well that I have relatives, friends, coworkers, and 6 families that have purchased it for them through me.	Jingdong prefecture level city in Shandong
Jinfeng Hongyuan Flagship Store	Electricity savings3	In use	Jingdong prefecture level city in Shandong
Jinfeng Hongyuan Flagship Store	Electricity savings3	Received the goods today, power-saving effect do not know being used, the boss has a very good attitude, praise!	Jingdong prefecture level city in Shandong
Jinfeng Hongyuan Flagship Store	Electricity savings3	The product is compact and doesn't take up much space, but hopefully it's power efficient.	Jingdong prefecture level city in Shandong
Jinfeng Hongyuan Flagship Store	Electricity savings3	Power saving is indeed effective, I used for a period of time before coming to evaluate, the electricity bill is obviously less, the quality is good, the shopkeeper service is also very good, very patient,	Jingdong prefecture level city in Shandong
Jinfeng Hongyuan Flagship Store	Electricity savings3	It feels fine, I'll comment after I use it	Jingdong prefecture level city in Shandong
Jinfeng Hongyuan Flagship Store	Electricity savings3	I just received it and I don't know the effect yet! I will comment again after I use it and have results!	Jingdong prefecture level city in Shandong
Jinfeng Hongyuan Flagship Store	Electricity savings3	The goods look fine, I hope they really work!	Jingdong prefecture level city in Shandong
Jinfeng Hongyuan Flagship Store	Electricity savings3	I just don't know if it saves power	Jingdong prefecture level city in Shandong
Jinfeng Hongyuan Flagship Store	Electricity savings3	My friend introduced me to use this product, it is professional and I am looking forward to the long term effect~!	Jingdong prefecture level city in Shandong
Jinfeng Hongyuan Flagship Store	Electricity savings3	Try it before you judge.	Jingdong prefecture level city in Shandong
Jinfeng Hongyuan Flagship Store	Electricity savings3	It's great, the power savings are obvious! Kudos!	Jingdong prefecture level city in Shandong
Jinfeng Hongyuan Flagship Store	Electricity savings3	The quality is excellent, stabilizes the voltage and saves electricity, very satisfied.	Jingdong prefecture level city in Shandong
Jinfeng Hongyuan Flagship Store	Electricity savings3	You have to use it for a while to know the effect	Jingdong prefecture level city in Shandong

Jinfeng Hongyuan Flagship Store	Electricity savings3	It's on trial.	Jingdong prefecture level city in Shandong
Jinfeng Hongyuan Flagship Store	Electricity savings3	The stuff works great.	Jingdong prefecture level city in Shandong
Jinfeng Hongyuan Flagship Store	Electricity savings3	Doesn't seem to work, not as god as other reviews	Jingdong prefecture level city in Shandong
Jinfeng Hongyuan Flagship Store	Electricity savings3	It's not good quality, it doesn't work, it stops working after a few days, it doesn't work, the customer service doesn't care, it's a bit irritating.	Jingdong prefecture level city in Shandong
Jinfeng Hongyuan Flagship Store	Electricity savings3	It doesn't do anything.	Jingdong prefecture level city in Shandong
Jinfeng Hongyuan Flagship Store	Electricity savings3	It's good.	Jingdong prefecture level city in Shandong
Jinfeng Hongyuan Flagship Store	Electricity savings3	Used a month effect is not obvious, are psychological effects, feel are not less, did not save to the electricity, there are details of the problem, the store said to buy to send 5 fuse, in fact, received only three, small problems are not committed to in place, I believe that the product is not good enough	Jingdong prefecture level city in Shandong
Jinfeng Hongyuan Flagship Store	Electricity savings3	Well received quickly, looking quite high, but also sent 5 fuses. Customer service is very patient to teach the operation, in fact, quite simple, lazy to read the instructions ha!	Jingdong prefecture level city in Shandong
Jinfeng Hongyuan Flagship Store	Electricity savings3	You have to use it for a while to know the effect	Jingdong prefecture level city in Shandong
Jinfeng Hongyuan Flagship Store	Electricity savings3	Shipped very quickly, packaged very well, the price is particularly affordable, there are so many efficacy is also good, it is worth buying worth buying the product, the workmanship is very fine, better than imagined, recommended that everyone try not yet used, after the use of the follow-up comment	Jingdong prefecture level city in Shandong
Jinfeng Hongyuan Flagship Store	Electricity savings3	It looks regular. It's working now.	Jingdong prefecture level city in Shandong
Jinfeng Hongyuan Flagship Store	Electricity savings3	It arrived pretty fast and the owner is very responsible. Have not used it yet, will see after use	Jingdong prefecture level city in Shandong
Jinfeng Hongyuan Flagship Store	Electricity savings3	🎁 baby quality is very good, the first time I received the goods to open the first time to see, it is very good 👍, very satisfied 😊, customer service also have a question and answer, thank you 🙏	Jingdong prefecture level city in Shandong
Jinfeng Hongyuan Flagship Store	Electricity savings3	I don't know if it's any good 。。。。。。	Jingdong prefecture level city in Shandong
Jinfeng Hongyuan Flagship Store	Electricity savings3	Buy electronic products! Still trust the things of the Jingdong self-owned store! Energy-saving effect to evaluate after heating!	Jingdong prefecture level city in Shandong
Jinfeng Hongyuan Flagship Store	Electricity savings3	The goods have been received, Blu is very beautiful, energy saving effect, to be confirmed only after actual use.	Jingdong prefecture level city in Shandong
Jinfeng Hongyuan Flagship Store	Electricity savings3	Jingdong's speed is so fast, has received, easy to operate. The display is quite good, how many volts what mode is quite understandable.	Jingdong prefecture level city in Shandong
Jinfeng Hongyuan Flagship Store	Electricity savings3	The first use, the effect is good to recommend it to colleagues to use, a month of electricity to save a lot or can be	Jingdong prefecture level city in Shandong
Jinfeng Hongyuan Flagship Store	Electricity savings3	I don't know if it's useful. Mom's is a store. I'll return it if it doesn't work.	Jingdong prefecture level city in Shandong
Jinfeng Hongyuan Flagship Store	Electricity savings3	I don't know if it works or not, I'll wait for two months to use it and then come back to comment!	Jingdong prefecture level city in Shandong
Jinfeng Hongyuan Flagship Store	Electricity savings3	I bought the second one now, and I've been using it for more than half a year, and I feel that it really saves electricity. Now it is summer, the weather is hot, sometimes the fan and air conditioning on all day, if not this power saver, the monthly electricity bill is quite high, but as long as the installation of this power saver, not afraid of its high electricity bill, absolutely let you wantonly use	Jingdong prefecture level city in Shandong
Jinfeng Hongyuan Flagship Store	Electricity savings3	The product is better than I thought it would be, the quality looks great, the voltage is stable, and I'm looking forward to some great power savings!	Jingdong prefecture level city in Shandong
Jinfeng Hongyuan Flagship Store	Electricity savings3	Inadvertently see bought a try, delivery is also quite timely, just do not know how to use, customer service patience to answer, with a pretty good, value for money!	Jingdong prefecture level city in Shandong
Jinfeng Hongyuan Flagship Store	Electricity savings3	It's better than nothing! Hopefully this will help save slightly.	Jingdong prefecture level city in Shandong
Jinfeng Hongyuan Flagship Store	Electricity savings3	Still trying it out, follow up	Jingdong prefecture level city in Shandong
Jinfeng Hongyuan Flagship Store	Electricity savings3	I've just started using it, I don't know how well it works yet, I hope it's good.	Jingdong prefecture level city in Shandong
Jinfeng Hongyuan Flagship Store	Electricity savings3	Fourth purchase, great baby!	Jingdong prefecture level city in Shandong
Jinfeng Hongyuan Flagship Store	Electricity savings3	Shipped really fast, just got it and use it to see what happens and then come back for a review!	Jingdong prefecture level city in Shandong
Jinfeng Hongyuan Flagship Store	Electricity savings3	Timely delivery **** good attitude	Jingdong prefecture level city in Shandong
Jinfeng Hongyuan Flagship Store	Electricity savings3	Home large and small things are purchased in the Jingdong Mall, have been accustomed to shopping in the Jingdong, many times can buy good quality and cheap products, and courier service attitude is particularly good, sometimes too many couriers are to help me to the home, so many years have been in the Jingdong Mall shopping, trust and support! Will also support the Jingdong as always!	Jingdong prefecture level city in Shandong
Jinfeng Hongyuan Flagship Store	Electricity savings3	Modified a pump and the modification worked.	Jingdong prefecture level city in Shandong
Jinfeng Hongyuan Flagship Store	Electricity savings3	Already installed, next time you pay the electric bill to see the results	Jingdong prefecture level city in Shandong
Jinfeng Hongyuan Flagship Store	Electricity savings3	The product is very good to use and practical to look at	Jingdong prefecture level city in Shandong
Jinfeng Hongyuan Flagship Store	Electricity savings3	Works great, great service, waiting for results.	Jingdong prefecture level city in Shandong
Jinfeng Hongyuan Flagship Store	Electricity savings3	I just used it and I'm not sure about the efficacy yet, I'll come back to evaluate it after a while.	Jingdong prefecture level city in Shandong
Jinfeng Hongyuan Flagship Store	Electricity savings3	Just received the goods, use a period of time to evaluate	Jingdong prefecture level city in Shandong
Jinfeng Hongyuan Flagship Store	Electricity savings3	Opened and tried very good, use it to see if there are results	Jingdong prefecture level city in Shandong
Jinfeng Hongyuan Flagship Store	Electricity savings3	Arrived today! Still trying it out!	Jingdong prefecture level city in Shandong
Jinfeng Hongyuan Flagship Store	Electricity savings3	Shipped quickly, well packaged, already installed, hope there is a power saving effect	Jingdong prefecture level city in Shandong
Jinfeng Hongyuan Flagship Store	Electricity savings3	I got it. It's great.	Jingdong prefecture level city in Shandong
Jinfeng Hongyuan Flagship Store	Electricity savings3	Haven't used it yet, will come back in a month	Jingdong prefecture level city in Shandong
Jinfeng Hongyuan Flagship Store	Electricity savings3	It looks good. It's good. Try it first.	Jingdong prefecture level city in Shandong
Jinfeng Hongyuan Flagship Store	Electricity savings3	It works so well that I have relatives, friends, coworkers, and 6 families that have purchased it for them through me.	Jingdong prefecture level city in Shandong
Jinfeng Hongyuan Flagship Store	Electricity savings3	In use	Jingdong prefecture level city in Shandong
Jinfeng Hongyuan Flagship Store	Electricity savings3	Received the goods today, power-saving effect do not know being used, the boss has a very good attitude, praise!	Jingdong prefecture level city in Shandong
Jinfeng Hongyuan Flagship Store	Electricity savings3	The product is compact and doesn't take up much space, but hopefully it's power efficient.	Jingdong prefecture level city in Shandong
Jinfeng Hongyuan Flagship Store	Electricity savings3	Power saving is indeed effective, I used for a period of time before coming to evaluate, the electricity bill is obviously less, the quality is good, the shopkeeper service is also very good, very patient,	Jingdong prefecture level city in Shandong
Jinfeng Hongyuan Flagship Store	Electricity savings3	It feels fine, I'll comment after I use it	Jingdong prefecture level city in Shandong

Jinfeng Hongyuan Flagship Store	Electricity savings3	I just received it and I don't know the effect yet! I will comment again after I use it and have results!	Jingdong prefecture level city in Shandong
Jinfeng Hongyuan Flagship Store	Electricity savings3	The goods look fine, I hope they really work!	Jingdong prefecture level city in Shandong
Jinfeng Hongyuan Flagship Store	Electricity savings3	I just don't know if it saves power	Jingdong prefecture level city in Shandong
Jinfeng Hongyuan Flagship Store	Electricity savings3	My friend introduced me to use this product, it is professional and I am looking forward to the long term effect~!	Jingdong prefecture level city in Shandong
Jinfeng Hongyuan Flagship Store	Electricity savings3	Try it before you judge.	Jingdong prefecture level city in Shandong
Jinfeng Hongyuan Flagship Store	Electricity savings3	It's great, the power savings are obvious! Kudos!	Jingdong prefecture level city in Shandong
Jinfeng Hongyuan Flagship Store	Electricity savings3	The quality is excellent, stabilizes the voltage and saves electricity, very satisfied.	Jingdong prefecture level city in Shandong
Jinfeng Hongyuan Flagship Store	Electricity savings3	You have to use it for a while to know the effect	Jingdong prefecture level city in Shandong
Jinfeng Hongyuan Flagship Store	Electricity savings3	It's on trial.	Jingdong prefecture level city in Shandong
Jinfeng Hongyuan Flagship Store	Electricity savings3	The stuff works great.	Jingdong prefecture level city in Shandong
Jinfeng Hongyuan Flagship Store	Electricity savings3	Doesn't seem to work, not as god as other reviews	Jingdong prefecture level city in Shandong
Jinfeng Hongyuan Flagship Store	Electricity savings3	It's not good quality, it doesn't work, it stops working after a few days, it doesn't work, the customer service doesn't care, it's a bit irritating.	Jingdong prefecture level city in Shandong
Jinfeng Hongyuan Flagship Store	Electricity savings3	It doesn't do anything.	Jingdong prefecture level city in Shandong
Jinfeng Hongyuan Flagship Store	Electricity savings3	It's good.	Jingdong prefecture level city in Shandong
Jinfeng Hongyuan Flagship Store	Electricity savings3	Used a month effect is not obvious, are psychological effects, feel are not less, did not save to the electricity, there are details of the problem, the store said to buy to send 5 fuse, in fact, received only three, small problems are not committed to in place, I believe that the product is not good enough	Jingdong prefecture level city in Shandong
Jinfeng Hongyuan Flagship Store	Electricity savings3	Well received quickly, looking quite high, but also sent 5 fuses. Customer service is very patient to teach the operation, in fact, quite simple, lazy to read the instructions ha!	Jingdong prefecture level city in Shandong
Jinfeng Hongyuan Flagship Store	Electricity savings3	You have to use it for a while to know the effect	Jingdong prefecture level city in Shandong
Jinfeng Hongyuan Flagship Store	Electricity savings3	Shipped very quickly, packaged very well, the price is particularly affordable, there are so many efficacy is also good, it is worth buying worth buying the product, the workmanship is very fine, better than imagined, recommended that everyone try not yet used, after the use of the follow-up comment	Jingdong prefecture level city in Shandong
Jinfeng Hongyuan Flagship Store	Electricity savings3	It looks regular. It's working now.	Jingdong prefecture level city in Shandong
Jinfeng Hongyuan Flagship Store	Electricity savings3	It arrived pretty fast and the owner is very responsible. Have not used it yet, will see after use	Jingdong prefecture level city in Shandong
Jinfeng Hongyuan Flagship Store	Electricity savings3	🎁 baby quality is very good, the first time I received the goods to open the first time to see, it is very good 👍, very satisfied 😉, customer service also have a question and answer, thank you 🙏	Jingdong prefecture level city in Shandong
Jinfeng Hongyuan Flagship Store	Electricity savings3	I don't know if it's any good 。。。。。。	Jingdong prefecture level city in Shandong
Jinfeng Hongyuan Flagship Store	Electricity savings3	Buy electronic products! Still trust the things of the Jingdong self-owned store! Energy-saving effect to evaluate after heating!	Jingdong prefecture level city in Shandong
Jinfeng Hongyuan Flagship Store	Electricity savings3	The goods have been received, Blu is very beautiful, energy saving effect, to be confirmed only after actual use.	Jingdong prefecture level city in Shandong
Jinfeng Hongyuan Flagship Store	Electricity savings3	Jingdong's speed is so fast, has received, easy to operate. The display is quite good, how many volts what mode is quite understandable.	Jingdong prefecture level city in Shandong
Jinfeng Hongyuan Flagship Store	Electricity savings3	The first use, the effect is good to recommend it to colleagues to use, a month of electricity to save a lot or can be	Jingdong prefecture level city in Shandong
Jinfeng Hongyuan Flagship Store	Electricity savings3	I don't know if it's useful. Mom's is a store. I'll return it if it doesn't work.	Jingdong prefecture level city in Shandong
Jinfeng Hongyuan Flagship Store	Electricity savings3	I don't know if it works or not, I'll wait for two months to use it and then come back to comment!	Jingdong prefecture level city in Shandong
Jinfeng Hongyuan Flagship Store	Electricity savings3	I bought the second one now, and I've been using it for more than half a year, and I feel that it really saves electricity. Now it is summer, the weather is hot, sometimes the fan and air conditioning on all day, if not this power saver, the monthly electricity bill is quite high, but as long as the installation of this power saver, not afraid of its high electricity bill, absolutely let you wantonly use	Jingdong prefecture level city in Shandong
Jinfeng Hongyuan Flagship Store	Electricity savings3	The product is better than I thought it would be, the quality looks great, the voltage is stable, and I'm looking forward to some great power savings!	Jingdong prefecture level city in Shandong
Jinfeng Hongyuan Flagship Store	Electricity savings3	Inadvertently see bought a try, delivery is also quite timely, just do not know how to use, customer service patience to answer, with a pretty good, value for money!	Jingdong prefecture level city in Shandong
Jinfeng Hongyuan Flagship Store	Electricity savings3	It's better than nothing! Hopefully this will help save slightly.	Jingdong prefecture level city in Shandong
Jinfeng Hongyuan Flagship Store	Electricity savings3	Still trying it out, follow up	Jingdong prefecture level city in Shandong
Jinfeng Hongyuan Flagship Store	Electricity savings3	I've just started using it, I don't know how well it works yet, I hope it's good.	Jingdong prefecture level city in Shandong
Jinfeng Hongyuan Flagship Store	Electricity savings3	Fourth purchase, great baby!	Jingdong prefecture level city in Shandong
Jinfeng Hongyuan Flagship Store	Electricity savings3	Shipped really fast, just got it and use it to see what happens and then come back for a review!	Jingdong prefecture level city in Shandong
Jinfeng Hongyuan Flagship Store	Electricity savings3	Timely delivery **** good attitude	Jingdong prefecture level city in Shandong
Jinfeng Hongyuan Flagship Store	Electricity savings3	Home large and small things are purchased in the Jingdong Mall, have been accustomed to shopping in the Jingdong, many times can buy good quality and cheap products, and courier service attitude is particularly good, sometimes too many couriers are to help me to the home, so many years have been in the Jingdong Mall shopping, trust and support! Will also support the Jingdong as always!	Jingdong prefecture level city in Shandong
Jinfeng Hongyuan Flagship Store	Electricity savings3	Modified a pump and the modification worked.	Jingdong prefecture level city in Shandong
Jinfeng Hongyuan Flagship Store	Electricity savings3	Already installed, next time you pay the electric bill to see the results	Jingdong prefecture level city in Shandong
Jinfeng Hongyuan Flagship Store	Electricity savings3	The product is very good to use and practical to look at	Jingdong prefecture level city in Shandong
Jinfeng Hongyuan Flagship Store	Electricity savings3	Works great, great service, waiting for results.	Jingdong prefecture level city in Shandong
Jinfeng Hongyuan Flagship Store	Electricity savings3	I just used it and I'm not sure about the efficacy yet, I'll come back to evaluate it after a while.	Jingdong prefecture level city in Shandong
Jinfeng Hongyuan Flagship Store	Electricity savings3	Just received the goods, use a period of time to evaluate	Jingdong prefecture level city in Shandong
Jinfeng Hongyuan Flagship Store	Electricity savings3	Opened and tried very good, use it to see if there are results	Jingdong prefecture level city in Shandong
Jinfeng Hongyuan Flagship Store	Electricity savings3	Arrived today! Still trying it out!	Jingdong prefecture level city in Shandong
Jinfeng Hongyuan Flagship Store	Electricity savings3	Shipped quickly, well packaged, already installed, hope there is a power saving effect	Jingdong prefecture level city in Shandong
Jinfeng Hongyuan Flagship Store	Electricity savings3	I got it. It's great.	Jingdong prefecture level city in Shandong

Jinfeng Hongyuan Flagship Store	Electricity savings3	Haven't used it yet, will come back in a month	Jingdong prefecture level city in Shandong
Jinfeng Hongyuan Flagship Store	Electricity savings3	It looks good. It's good. Try it first.	Jingdong prefecture level city in Shandong
Jinfeng Hongyuan Flagship Store	Electricity savings3	It works so well that I have relatives, friends, coworkers, and 6 families that have purchased it for them through me.	Jingdong prefecture level city in Shandong
Jinfeng Hongyuan Flagship Store	Electricity savings3	In use	Jingdong prefecture level city in Shandong
Jinfeng Hongyuan Flagship Store	Electricity savings3	Received the goods today, power-saving effect do not know being used, the boss has a very good attitude, praise!	Jingdong prefecture level city in Shandong
Jinfeng Hongyuan Flagship Store	Electricity savings3	The product is compact and doesn't take up much space, but hopefully it's power efficient.	Jingdong prefecture level city in Shandong
Jinfeng Hongyuan Flagship Store	Electricity savings3	Power saving is indeed effective, I used for a period of time before coming to evaluate, the electricity bill is obviously less, the quality is good, the shopkeeper service is also very good, very patient,	Jingdong prefecture level city in Shandong
Jinfeng Hongyuan Flagship Store	Electricity savings3	It feels fine, I'll comment after I use it	Jingdong prefecture level city in Shandong
Jinfeng Hongyuan Flagship Store	Electricity savings3	I just received it and I don't know the effect yet! I will comment again after I use it and have results!	Jingdong prefecture level city in Shandong
Jinfeng Hongyuan Flagship Store	Electricity savings3	The goods look fine, I hope they really work!	Jingdong prefecture level city in Shandong
Jinfeng Hongyuan Flagship Store	Electricity savings3	I just don't know if it saves power	Jingdong prefecture level city in Shandong
Jinfeng Hongyuan Flagship Store	Electricity savings3	My friend introduced me to use this product, it is professional and I am looking forward to the long term effect~!	Jingdong prefecture level city in Shandong
Jinfeng Hongyuan Flagship Store	Electricity savings3	Try it before you judge.	Jingdong prefecture level city in Shandong
Jinfeng Hongyuan Flagship Store	Electricity savings3	It's great, the power savings are obvious! Kudos!	Jingdong prefecture level city in Shandong
Jinfeng Hongyuan Flagship Store	Electricity savings3	The quality is excellent, stabilizes the voltage and saves electricity, very satisfied.	Jingdong prefecture level city in Shandong
Jinfeng Hongyuan Flagship Store	Electricity savings3	You have to use it for a while to know the effect	Jingdong prefecture level city in Shandong
Jinfeng Hongyuan Flagship Store	Electricity savings3	It's on trial.	Jingdong prefecture level city in Shandong
Jinfeng Hongyuan Flagship Store	Electricity savings3	The stuff works great.	Jingdong prefecture level city in Shandong
Jinfeng Hongyuan Flagship Store	Electricity savings3	Doesn't seem to work, not as god as other reviews	Jingdong prefecture level city in Shandong
Jinfeng Hongyuan Flagship Store	Electricity savings3	It's not good quality, it doesn't work, it stops working after a few days, it doesn't work, the customer service doesn't care, it's a bit irritating.	Jingdong prefecture level city in Shandong
Jinfeng Hongyuan Flagship Store	Electricity savings3	It doesn't do anything.	Jingdong prefecture level city in Shandong
Jinfeng Hongyuan Flagship Store	Electricity savings3	It's good.	Jingdong prefecture level city in Shandong
Jinfeng Hongyuan Flagship Store	Electricity savings3	Used a month effect is not obvious, are psychological effects, feel are not less, did not save to the electricity, there are details of the problem, the store said to buy to send 5 fuse, in fact, received only three, small problems are not committed to in place, I believe that the product is not good enough	Jingdong prefecture level city in Shandong
Jinfeng Hongyuan Flagship Store	Electricity savings3	Well received quickly, looking quite high, but also sent 5 fuses. Customer service is very patient to teach the operation, in fact, quite simple, lazy to read the instructions ha!	Jingdong prefecture level city in Shandong
Jinfeng Hongyuan Flagship Store	Electricity savings3	You have to use it for a while to know the effect	Jingdong prefecture level city in Shandong
Jinfeng Hongyuan Flagship Store	Electricity savings3	Shipped very quickly, packaged very well, the price is particularly affordable, there are so many efficacy is also good, it is worth buying worth buying the product, the workmanship is very fine, better than imagined, recommended that everyone try not yet used, after the use of the follow-up comment	Jingdong prefecture level city in Shandong
Jinfeng Hongyuan Flagship Store	Electricity savings3	It looks regular. It's working now.	Jingdong prefecture level city in Shandong
Jinfeng Hongyuan Flagship Store	Electricity savings3	It arrived pretty fast and the owner is very responsible. Have not used it yet, will see after use	Jingdong prefecture level city in Shandong
Jinfeng Hongyuan Flagship Store	Electricity savings3	🎁 baby quality is very good, the first time I received the goods to open the first time to see, it is very good 👍, very satisfied 😊, customer service also have a question and answer, thank you 🙏	Jingdong prefecture level city in Shandong
Jinfeng Hongyuan Flagship Store	Electricity savings3	I don't know if it's any good 。。。。。。	Jingdong prefecture level city in Shandong
Jinfeng Hongyuan Flagship Store	Electricity savings3	Buy electronic products! Still trust the things of the Jingdong self-owned store! Energy-saving effect to evaluate after heating!	Jingdong prefecture level city in Shandong
Jinfeng Hongyuan Flagship Store	Electricity savings3	The goods have been received, Blu is very beautiful, energy saving effect, to be confirmed only after actual use.	Jingdong prefecture level city in Shandong
Jinfeng Hongyuan Flagship Store	Electricity savings3	Jingdong's speed is so fast, has received, easy to operate. The display is quite good, how many volts what mode is quite understandable.	Jingdong prefecture level city in Shandong
Jinfeng Hongyuan Flagship Store	Electricity savings3	The first use, the effect is good to recommend it to colleagues to use, a month of electricity to save a lot or can be	Jingdong prefecture level city in Shandong
Jinfeng Hongyuan Flagship Store	Electricity savings3	I don't know if it's useful. Mom's is a store. I'll return it if it doesn't work.	Jingdong prefecture level city in Shandong
Jinfeng Hongyuan Flagship Store	Electricity savings3	I don't know if it works or not, I'll wait for two months to use it and then come back to comment!	Jingdong prefecture level city in Shandong
Jinfeng Hongyuan Flagship Store	Electricity savings3	I bought the second one now, and I've been using it for more than half a year, and I feel that it really saves electricity. Now it is summer, the weather is hot, sometimes the fan and air conditioning on all day, if not this power saver, the monthly electricity bill is quite high, but as long as the installation of this power saver, not afraid of its high electricity bill, absolutely let you wantonly use	Jingdong prefecture level city in Shandong
Jinfeng Hongyuan Flagship Store	Electricity savings3	The product is better than I thought it would be, the quality looks great, the voltage is stable, and I'm looking forward to some great power savings!	Jingdong prefecture level city in Shandong
Jinfeng Hongyuan Flagship Store	Electricity savings3	Inadvertently see bought a try, delivery is also quite timely, just do not know how to use, customer service patience to answer, with a pretty good, value for money!	Jingdong prefecture level city in Shandong
Jinfeng Hongyuan Flagship Store	Electricity savings3	It's better than nothing! Hopefully this will help save slightly.	Jingdong prefecture level city in Shandong
Jinfeng Hongyuan Flagship Store	Electricity savings3	Still trying it out, follow up	Jingdong prefecture level city in Shandong
Jinfeng Hongyuan Flagship Store	Electricity savings3	I've just started using it, I don't know how well it works yet, I hope it's good.	Jingdong prefecture level city in Shandong
Jinfeng Hongyuan Flagship Store	Electricity savings3	Fourth purchase, great baby!	Jingdong prefecture level city in Shandong
Jinfeng Hongyuan Flagship Store	Electricity savings3	Shipped really fast, just got it and use it to see what happens and then come back for a review!	Jingdong prefecture level city in Shandong
Jinfeng Hongyuan Flagship Store	Electricity savings3	Timely delivery **** good attitude	Jingdong prefecture level city in Shandong
Jinfeng Hongyuan Flagship Store	Electricity savings3	Home large and small things are purchased in the Jingdong Mall, have been accustomed to shopping in the Jingdong, many times can buy good quality and cheap products, and courier service attitude is particularly good, sometimes too many couriers are to help me to the home, so many years have been in the Jingdong Mall shopping, trust and support! Will also support the Jingdong as always!	Jingdong prefecture level city in Shandong
Jinfeng Hongyuan Flagship Store	Electricity savings3	Modified a pump and the modification worked.	Jingdong prefecture level city in Shandong
Jinfeng Hongyuan Flagship Store	Electricity savings3	Already installed, next time you pay the electric bill to see the results	Jingdong prefecture level city in Shandong

Jinfeng Hongyuan Flagship Store	Electricity savings3	The product is very good to use and practical to look at	Jingdong prefecture level city in Shandong
Jinfeng Hongyuan Flagship Store	Electricity savings3	Works great, great service, waiting for results.	Jingdong prefecture level city in Shandong
Jinfeng Hongyuan Flagship Store	Electricity savings3	I just used it and I'm not sure about the efficacy yet, I'll come back to evaluate it after a while.	Jingdong prefecture level city in Shandong
Jinfeng Hongyuan Flagship Store	Electricity savings3	Just received the goods, use a period of time to evaluate	Jingdong prefecture level city in Shandong
Jinfeng Hongyuan Flagship Store	Electricity savings3	Opened and tried very good, use it to see if there are results	Jingdong prefecture level city in Shandong
Jinfeng Hongyuan Flagship Store	Electricity savings3	Arrived today! Still trying it out!	Jingdong prefecture level city in Shandong
Jinfeng Hongyuan Flagship Store	Electricity savings3	Shipped quickly, well packaged, already installed, hope there is a power saving effect	Jingdong prefecture level city in Shandong
Jinfeng Hongyuan Flagship Store	Electricity savings3	I got it. It's great.	Jingdong prefecture level city in Shandong
Jinfeng Hongyuan Flagship Store	Electricity savings3	Haven't used it yet, will come back in a month	Jingdong prefecture level city in Shandong
Jinfeng Hongyuan Flagship Store	Electricity savings3	It looks good. It's good. Try it first.	Jingdong prefecture level city in Shandong
Jinfeng Hongyuan Flagship Store	Electricity savings3	It works so well that I have relatives, friends, coworkers, and 6 families that have purchased it for them through me.	Jingdong prefecture level city in Shandong
Jinfeng Hongyuan Flagship Store	Electricity savings3	In use	Jingdong prefecture level city in Shandong
Jinfeng Hongyuan Flagship Store	Electricity savings3	Received the goods today, power-saving effect do not know being used, the boss has a very good attitude, praise!	Jingdong prefecture level city in Shandong
Jinfeng Hongyuan Flagship Store	Electricity savings3	The product is compact and doesn't take up much space, but hopefully it's power efficient.	Jingdong prefecture level city in Shandong
Jinfeng Hongyuan Flagship Store	Electricity savings3	Power saving is indeed effective, I used for a period of time before coming to evaluate, the electricity bill is obviously less, the quality is good, the shopkeeper service is also very good, very patient,	Jingdong prefecture level city in Shandong
Jinfeng Hongyuan Flagship Store	Electricity savings3	It feels fine, I'll comment after I use it	Jingdong prefecture level city in Shandong
Jinfeng Hongyuan Flagship Store	Electricity savings3	I just received it and I don't know the effect yet! I will comment again after I use it and have results!	Jingdong prefecture level city in Shandong
Jinfeng Hongyuan Flagship Store	Electricity savings3	The goods look fine, I hope they really work!	Jingdong prefecture level city in Shandong
Jinfeng Hongyuan Flagship Store	Electricity savings3	I just don't know if it saves power	Jingdong prefecture level city in Shandong
Jinfeng Hongyuan Flagship Store	Electricity savings3	My friend introduced me to use this product, it is professional and I am looking forward to the long term effect~!	Jingdong prefecture level city in Shandong
Jinfeng Hongyuan Flagship Store	Electricity savings3	Try it before you judge.	Jingdong prefecture level city in Shandong
Jinfeng Hongyuan Flagship Store	Electricity savings3	It's great, the power savings are obvious! Kudos!	Jingdong prefecture level city in Shandong
Jinfeng Hongyuan Flagship Store	Electricity savings3	The quality is excellent, stabilizes the voltage and saves electricity, very satisfied.	Jingdong prefecture level city in Shandong
Jinfeng Hongyuan Flagship Store	Electricity savings3	You have to use it for a while to know the effect	Jingdong prefecture level city in Shandong
Jinfeng Hongyuan Flagship Store	Electricity savings3	It's on trial.	Jingdong prefecture level city in Shandong
Jinfeng Hongyuan Flagship Store	Electricity savings3	The stuff works great.	Jingdong prefecture level city in Shandong
Jinfeng Hongyuan Flagship Store	Electricity savings3	Doesn't seem to work, not as god as other reviews	Jingdong prefecture level city in Shandong
Jinfeng Hongyuan Flagship Store	Electricity savings3	It's not good quality, it doesn't work, it stops working after a few days, it doesn't work, the customer service doesn't care, it's a bit irritating.	Jingdong prefecture level city in Shandong
Jinfeng Hongyuan Flagship Store	Electricity savings3	It doesn't do anything.	Jingdong prefecture level city in Shandong
Jinfeng Hongyuan Flagship Store	Electricity savings3	It's good.	Jingdong prefecture level city in Shandong
Jinfeng Hongyuan Flagship Store	Electricity savings3	Used a month effect is not obvious, are psychological effects, feel are not less, did not save to the electricity, there are details of the problem, the store said to buy to send 5 fuse, in fact, received only three, small problems are not committed to in place, I believe that the product is not good enough	Jingdong prefecture level city in Shandong
Jinfeng Hongyuan Flagship Store	Electricity savings3	Well received quickly, looking quite high, but also sent 5 fuses. Customer service is very patient to teach the operation, in fact, quite simple, lazy to read the instructions ha!	Jingdong prefecture level city in Shandong
Jinfeng Hongyuan Flagship Store	Electricity savings3	You have to use it for a while to know the effect	Jingdong prefecture level city in Shandong
Jinfeng Hongyuan Flagship Store	Electricity savings3	Shipped very quickly, packaged very well, the price is particularly affordable, there are so many efficacy is also good, it is worth buying worth buying the product, the workmanship is very fine, better than imagined, recommended that everyone try not yet used, after the use of the follow-up comment	Jingdong prefecture level city in Shandong
Jinfeng Hongyuan Flagship Store	Electricity savings3	It looks regular. It's working now.	Jingdong prefecture level city in Shandong
Jinfeng Hongyuan Flagship Store	Electricity savings3	It arrived pretty fast and the owner is very responsible. Have not used it yet, will see after use	Jingdong prefecture level city in Shandong
Jinfeng Hongyuan Flagship Store	Electricity savings3	🎁 baby quality is very good, the first time I received the goods to open the first time to see, it is very good 👍, very satisfied 😀, customer service also have a question and answer, thank you 🙏	Jingdong prefecture level city in Shandong
Jinfeng Hongyuan Flagship Store	Electricity savings3	I don't know if it's any good 。。。。。。	Jingdong prefecture level city in Shandong
Jinfeng Hongyuan Flagship Store	Electricity savings3	Buy electronic products! Still trust the things of the Jingdong self-owned store! Energy-saving effect to evaluate after heating!	Jingdong prefecture level city in Shandong
Jinfeng Hongyuan Flagship Store	Electricity savings3	The goods have been received, Blu is very beautiful, energy saving effect, to be confirmed only after actual use.	Jingdong prefecture level city in Shandong
Jinfeng Hongyuan Flagship Store	Electricity savings3	Jingdong's speed is so fast, has received, easy to operate. The display is quite good, how many volts what mode is quite understandable.	Jingdong prefecture level city in Shandong
Jinfeng Hongyuan Flagship Store	Electricity savings3	The first use, the effect is good to recommend it to colleagues to use, a month of electricity to save a lot or can be	Jingdong prefecture level city in Shandong
Jinfeng Hongyuan Flagship Store	Electricity savings3	I don't know if it's useful. Mom's is a store. I'll return it if it doesn't work.	Jingdong prefecture level city in Shandong
Jinfeng Hongyuan Flagship Store	Electricity savings3	I don't know if it works or not, I'll wait for two months to use it and then come back to comment!	Jingdong prefecture level city in Shandong
Jinfeng Hongyuan Flagship Store	Electricity savings3	I bought the second one now, and I've been using it for more than half a year, and I feel that it really saves electricity. Now it is summer, the weather is hot, sometimes the fan and air conditioning on all day, if not this power saver, the monthly electricity bill is quite high, but as long as the installation of this power saver, not afraid of its high electricity bill, absolutely let you wantonly use	Jingdong prefecture level city in Shandong
Jinfeng Hongyuan Flagship Store	Electricity savings3	The product is better than I thought it would be, the quality looks great, the voltage is stable, and I'm looking forward to some great power savings!	Jingdong prefecture level city in Shandong
Jinfeng Hongyuan Flagship Store	Electricity savings3	Inadvertently see bought a try, delivery is also quite timely, just do not know how to use, customer service patience to answer, with a pretty good, value for money!	Jingdong prefecture level city in Shandong
Jinfeng Hongyuan Flagship Store	Electricity savings3	It's better than nothing! Hopefully this will help save slightly.	Jingdong prefecture level city in Shandong
Jinfeng Hongyuan Flagship Store	Electricity savings3	Still trying it out, follow up	Jingdong prefecture level city in Shandong

Jinfeng Hongyuan Flagship Store	Electricity savings3	I've just started using it, I don't know how well it works yet, I hope it's good.	Jingdong prefecture level city in Shandong
Jinfeng Hongyuan Flagship Store	Electricity savings3	Fourth purchase, great baby!	Jingdong prefecture level city in Shandong
Jinfeng Hongyuan Flagship Store	Electricity savings3	Shipped really fast, just got it and use it to see what happens and then come back for a review!	Jingdong prefecture level city in Shandong
Jinfeng Hongyuan Flagship Store	Electricity savings3	Timely delivery **** good attitude	Jingdong prefecture level city in Shandong
Jinfeng Hongyuan Flagship Store	Electricity savings3	Home large and small things are purchased in the Jingdong Mall, have been accustomed to shopping in the Jingdong, many times can buy good quality and cheap products, and courier service attitude is particularly good, sometimes too many couriers are to help me to the home, so many years have been in the Jingdong Mall shopping, trust and support! Will also support the Jingdong as always!	Jingdong prefecture level city in Shandong
Jinfeng Hongyuan Flagship Store	Electricity savings3	Modified a pump and the modification worked.	Jingdong prefecture level city in Shandong
Jinfeng Hongyuan Flagship Store	Electricity savings3	Already installed, next time you pay the electric bill to see the results	Jingdong prefecture level city in Shandong
Jinfeng Hongyuan Flagship Store	Electricity savings3	The product is very good to use and practical to look at	Jingdong prefecture level city in Shandong
Jinfeng Hongyuan Flagship Store	Electricity savings3	Works great, great service, waiting for results.	Jingdong prefecture level city in Shandong
Jinfeng Hongyuan Flagship Store	Electricity savings3	I just used it and I'm not sure about the efficacy yet, I'll come back to evaluate it after a while.	Jingdong prefecture level city in Shandong
Jinfeng Hongyuan Flagship Store	Electricity savings3	Just received the goods, use a period of time to evaluate	Jingdong prefecture level city in Shandong
Jinfeng Hongyuan Flagship Store	Electricity savings3	Opened and tried very good, use it to see if there are results	Jingdong prefecture level city in Shandong
Jinfeng Hongyuan Flagship Store	Electricity savings3	Arrived today! Still trying it out!	Jingdong prefecture level city in Shandong
Jinfeng Hongyuan Flagship Store	Electricity savings3	Shipped quickly, well packaged, already installed, hope there is a power saving effect	Jingdong prefecture level city in Shandong
Jinfeng Hongyuan Flagship Store	Electricity savings3	I got it. It's great.	Jingdong prefecture level city in Shandong
Jinfeng Hongyuan Flagship Store	Electricity savings3	Haven't used it yet, will come back in a month	Jingdong prefecture level city in Shandong
Jinfeng Hongyuan Flagship Store	Electricity savings3	It looks good. It's good. Try it first.	Jingdong prefecture level city in Shandong
Jinfeng Hongyuan Flagship Store	Electricity savings3	It works so well that I have relatives, friends, coworkers, and 6 families that have purchased it for them through me.	Jingdong prefecture level city in Shandong
Jinfeng Hongyuan Flagship Store	Electricity savings3	In use	Jingdong prefecture level city in Shandong
Jinfeng Hongyuan Flagship Store	Electricity savings3	Received the goods today, power-saving effect do not know being used, the boss has a very good attitude, praise!	Jingdong prefecture level city in Shandong
Jinfeng Hongyuan Flagship Store	Electricity savings3	The product is compact and doesn't take up much space, but hopefully it's power efficient.	Jingdong prefecture level city in Shandong
Jinfeng Hongyuan Flagship Store	Electricity savings3	Power saving is indeed effective, I used for a period of time before coming to evaluate, the electricity bill is obviously less, the quality is good, the shopkeeper service is also very good, very patient,	Jingdong prefecture level city in Shandong
Jinfeng Hongyuan Flagship Store	Electricity savings3	It feels fine, I'll comment after I use it	Jingdong prefecture level city in Shandong
Jinfeng Hongyuan Flagship Store	Electricity savings3	I just received it and I don't know the effect yet! I will comment again after I use it and have results!	Jingdong prefecture level city in Shandong
Jinfeng Hongyuan Flagship Store	Electricity savings3	The goods look fine, I hope they really work!	Jingdong prefecture level city in Shandong
Jinfeng Hongyuan Flagship Store	Electricity savings3	I just don't know if it saves power	Jingdong prefecture level city in Shandong
Jinfeng Hongyuan Flagship Store	Electricity savings3	My friend introduced me to use this product, it is professional and I am looking forward to the long term effect~!	Jingdong prefecture level city in Shandong
Jinfeng Hongyuan Flagship Store	Electricity savings3	Try it before you judge.	Jingdong prefecture level city in Shandong
Jinfeng Hongyuan Flagship Store	Electricity savings3	It's great, the power savings are obvious! Kudos!	Jingdong prefecture level city in Shandong
Jinfeng Hongyuan Flagship Store	Electricity savings3	The quality is excellent, stabilizes the voltage and saves electricity, very satisfied.	Jingdong prefecture level city in Shandong
Jinfeng Hongyuan Flagship Store	Electricity savings3	You have to use it for a while to know the effect	Jingdong prefecture level city in Shandong
Jinfeng Hongyuan Flagship Store	Electricity savings3	It's on trial.	Jingdong prefecture level city in Shandong
Jinfeng Hongyuan Flagship Store	Electricity savings3	The stuff works great.	Jingdong prefecture level city in Shandong
Jinfeng Hongyuan Flagship Store	Electricity savings3	Doesn't seem to work, not as god as other reviews	Jingdong prefecture level city in Shandong
Jinfeng Hongyuan Flagship Store	Electricity savings3	It's not good quality, it doesn't work, it stops working after a few days, it doesn't work, the customer service doesn't care, it's a bit irritating.	Jingdong prefecture level city in Shandong
Jinfeng Hongyuan Flagship Store	Electricity savings3	It doesn't do anything.	Jingdong prefecture level city in Shandong
Jinfeng Hongyuan Flagship Store	Electricity savings3	It's good.	Jingdong prefecture level city in Shandong
Jinfeng Hongyuan Flagship Store	Electricity savings3	Used a month effect is not obvious, are psychological effects, feel are not less, did not save to the electricity, there are details of the problem, the store said to buy to send 5 fuse, in fact, received only three, small problems are not committed to in place, I believe that the product is not good enough	Jingdong prefecture level city in Shandong
Jinfeng Hongyuan Flagship Store	Electricity savings3	Well received quickly, looking quite high, but also sent 5 fuses. Customer service is very patient to teach the operation, in fact, quite simple, lazy to read the instructions ha!	Jingdong prefecture level city in Shandong
Jinfeng Hongyuan Flagship Store	Electricity savings3	You have to use it for a while to know the effect	Jingdong prefecture level city in Shandong
Jinfeng Hongyuan Flagship Store	Electricity savings3	Shipped very quickly, packaged very well, the price is particularly affordable, there are so many efficacy is also good, it is worth buying worth buying the product, the workmanship is very fine, better than imagined, recommended that everyone try not yet used, after the use of the follow-up comment	Jingdong prefecture level city in Shandong
Jinfeng Hongyuan Flagship Store	Electricity savings3	It looks regular. It's working now.	Jingdong prefecture level city in Shandong
Jinfeng Hongyuan Flagship Store	Electricity savings3	It arrived pretty fast and the owner is very responsible. Have not used it yet, will see after use	Jingdong prefecture level city in Shandong
Jinfeng Hongyuan Flagship Store	Electricity savings3	🎁 baby quality is very good, the first time I received the goods to open the first time to see, it is very good 👍, very satisfied 😊, customer service also have a question and answer, thank you 🙏	Jingdong prefecture level city in Shandong
Jinfeng Hongyuan Flagship Store	Electricity savings3	I don't know if it's any good 。。。。。。	Jingdong prefecture level city in Shandong
Jinfeng Hongyuan Flagship Store	Electricity savings3	Buy electronic products! Still trust the things of the Jingdong self-owned store! Energy-saving effect to evaluate after heating!	Jingdong prefecture level city in Shandong
Jinfeng Hongyuan Flagship Store	Electricity savings3	The goods have been received, Blu is very beautiful, energy saving effect, to be confirmed only after actual use.	Jingdong prefecture level city in Shandong
Jinfeng Hongyuan Flagship Store	Electricity savings3	Jingdong's speed is so fast, has received, easy to operate. The display is quite good, how many volts what mode is quite understandable.	Jingdong prefecture level city in Shandong
Jinfeng Hongyuan Flagship Store	Electricity savings3	The first use, the effect is good to recommend it to colleagues to use, a month of electricity to save a lot or can be	Jingdong prefecture level city in Shandong

Jinfeng Hongyuan Flagship Store	Electricity savings3	I don't know if it's useful. Mom's is a store. I'll return it if it doesn't work.	Jingdong prefecture level city in Shandong
Jinfeng Hongyuan Flagship Store	Electricity savings3	I don't know if it works or not, I'll wait for two months to use it and then come back to comment!	Jingdong prefecture level city in Shandong
Jinfeng Hongyuan Flagship Store	Electricity savings3	I bought the second one now, and I've been using it for more than half a year, and I feel that it really saves electricity. Now it is summer, the weather is hot, sometimes the fan and air conditioning on all day, if not this power saver, the monthly electricity bill is quite high, but as long as the installation of this power saver, not afraid of its high electricity bill, absolutely let you wantonly use	Jingdong prefecture level city in Shandong
Jinfeng Hongyuan Flagship Store	Electricity savings3	The product is better than I thought it would be, the quality looks great, the voltage is stable, and I'm looking forward to some great power savings!	Jingdong prefecture level city in Shandong
Jinfeng Hongyuan Flagship Store	Electricity savings3	Inadvertently see bought a try, delivery is also quite timely, just do not know how to use, customer service patience to answer, with a pretty good, value for money!	Jingdong prefecture level city in Shandong
Jinfeng Hongyuan Flagship Store	Electricity savings3	It's better than nothing! Hopefully this will help save slightly.	Jingdong prefecture level city in Shandong
Jinfeng Hongyuan Flagship Store	Electricity savings3	Still trying it out, follow up	Jingdong prefecture level city in Shandong
Jinfeng Hongyuan Flagship Store	Electricity savings3	I've just started using it, I don't know how well it works yet, I hope it's good.	Jingdong prefecture level city in Shandong
Jinfeng Hongyuan Flagship Store	Electricity savings3	Fourth purchase, great baby!	Jingdong prefecture level city in Shandong
Jinfeng Hongyuan Flagship Store	Electricity savings3	Shipped really fast, just got it and use it to see what happens and then come back for a review!	Jingdong prefecture level city in Shandong
Jinfeng Hongyuan Flagship Store	Electricity savings3	Timely delivery **** good attitude	Jingdong prefecture level city in Shandong
Jinfeng Hongyuan Flagship Store	Electricity savings3	Home large and small things are purchased in the Jingdong Mall, have been accustomed to shopping in the Jingdong, many times can buy good quality and cheap products, and courier service attitude is particularly good, sometimes too many couriers are to help me to the home, so many years have been in the Jingdong Mall shopping, trust and support! Will also support the Jingdong as always!	Jingdong prefecture level city in Shandong
Jinfeng Hongyuan Flagship Store	Electricity savings3	Modified a pump and the modification worked.	Jingdong prefecture level city in Shandong
Jinfeng Hongyuan Flagship Store	Electricity savings3	Already installed, next time you pay the electric bill to see the results	Jingdong prefecture level city in Shandong
Jinfeng Hongyuan Flagship Store	Electricity savings3	The product is very good to use and practical to look at	Jingdong prefecture level city in Shandong
Jinfeng Hongyuan Flagship Store	Electricity savings3	Works great, great service, waiting for results.	Jingdong prefecture level city in Shandong
Jinfeng Hongyuan Flagship Store	Electricity savings3	I just used it and I'm not sure about the efficacy yet, I'll come back to evaluate it after a while.	Jingdong prefecture level city in Shandong
Jinfeng Hongyuan Flagship Store	Electricity savings3	Just received the goods, use a period of time to evaluate	Jingdong prefecture level city in Shandong
Jinfeng Hongyuan Flagship Store	Electricity savings3	Opened and tried very good, use it to see if there are results	Jingdong prefecture level city in Shandong
Jinfeng Hongyuan Flagship Store	Electricity savings3	Arrived today! Still trying it out!	Jingdong prefecture level city in Shandong
Jinfeng Hongyuan Flagship Store	Electricity savings3	Shipped quickly, well packaged, already installed, hope there is a power saving effect	Jingdong prefecture level city in Shandong
Jinfeng Hongyuan Flagship Store	Electricity savings3	I got it. It's great.	Jingdong prefecture level city in Shandong
Jinfeng Hongyuan Flagship Store	Electricity savings3	Haven't used it yet, will come back in a month	Jingdong prefecture level city in Shandong
Jinfeng Hongyuan Flagship Store	Electricity savings3	It looks good. It's good. Try it first.	Jingdong prefecture level city in Shandong
Jinfeng Hongyuan Flagship Store	Electricity savings3	It works so well that I have relatives, friends, coworkers, and 6 families that have purchased it for them through me.	Jingdong prefecture level city in Shandong
Jinfeng Hongyuan Flagship Store	Electricity savings3	In use	Jingdong prefecture level city in Shandong
Jinfeng Hongyuan Flagship Store	Electricity savings3	Received the goods today, power-saving effect do not know being used, the boss has a very good attitude, praise!	Jingdong prefecture level city in Shandong
Jinfeng Hongyuan Flagship Store	Electricity savings3	The product is compact and doesn't take up much space, but hopefully it's power efficient.	Jingdong prefecture level city in Shandong
Jinfeng Hongyuan Flagship Store	Electricity savings3	Power saving is indeed effective, I used for a period of time before coming to evaluate, the electricity bill is obviously less, the quality is good, the shopkeeper service is also very good, very patient,	Jingdong prefecture level city in Shandong
Jinfeng Hongyuan Flagship Store	Electricity savings3	It feels fine, I'll comment after I use it	Jingdong prefecture level city in Shandong
Jinfeng Hongyuan Flagship Store	Electricity savings3	I just received it and I don't know the effect yet! I will comment again after I use it and have results!	Jingdong prefecture level city in Shandong
Jinfeng Hongyuan Flagship Store	Electricity savings3	The goods look fine, I hope they really work!	Jingdong prefecture level city in Shandong
Jinfeng Hongyuan Flagship Store	Electricity savings3	I just don't know if it saves power	Jingdong prefecture level city in Shandong
Jinfeng Hongyuan Flagship Store	Electricity savings3	My friend introduced me to use this product, it is professional and I am looking forward to the long term effect~!	Jingdong prefecture level city in Shandong
Jinfeng Hongyuan Flagship Store	Electricity savings3	Try it before you judge.	Jingdong prefecture level city in Shandong
Jinfeng Hongyuan Flagship Store	Electricity savings3	It's great, the power savings are obvious! Kudos!	Jingdong prefecture level city in Shandong
Jinfeng Hongyuan Flagship Store	Electricity savings3	The quality is excellent, stabilizes the voltage and saves electricity, very satisfied.	Jingdong prefecture level city in Shandong
Jinfeng Hongyuan Flagship Store	Electricity savings3	You have to use it for a while to know the effect	Jingdong prefecture level city in Shandong
Jinfeng Hongyuan Flagship Store	Electricity savings3	It's on trial.	Jingdong prefecture level city in Shandong
Jinfeng Hongyuan Flagship Store	Electricity savings3	The stuff works great.	Jingdong prefecture level city in Shandong
Jinfeng Hongyuan Flagship Store	Electricity savings3	Doesn't seem to work, not as god as other reviews	Jingdong prefecture level city in Shandong
Jinfeng Hongyuan Flagship Store	Electricity savings3	It's not good quality, it doesn't work, it stops working after a few days, it doesn't work, the customer service doesn't care, it's a bit irritating.	Jingdong prefecture level city in Shandong
Jinfeng Hongyuan Flagship Store	Electricity savings3	It doesn't do anything.	Jingdong prefecture level city in Shandong
Jinfeng Hongyuan Flagship Store	Electricity savings3	It's good.	Jingdong prefecture level city in Shandong
Jinfeng Hongyuan Flagship Store	Electricity savings3	Used a month effect is not obvious, are psychological effects, feel are not less, did not save to the electricity, there are details of the problem, the store said to buy to send 5 fuse, in fact, received only three, small problems are not committed to in place, I believe that the product is not good enough	Jingdong prefecture level city in Shandong
Jinfeng Hongyuan Flagship Store	Electricity savings3	Well received quickly, looking quite high, but also sent 5 fuses. Customer service is very patient to teach the operation, in fact, quite simple, lazy to read the instructions ha!	Jingdong prefecture level city in Shandong
Jinfeng Hongyuan Flagship Store	Electricity savings3	You have to use it for a while to know the effect	Jingdong prefecture level city in Shandong
Jinfeng Hongyuan Flagship Store	Electricity savings3	Shipped very quickly, packaged very well, the price is particularly affordable, there are so many efficacy is also good, it is worth buying worth buying the product, the workmanship is very fine, better than imagined, recommended that everyone try not yet used, after the use of the follow-up comment	Jingdong prefecture level city in Shandong

Jinfeng Hongyuan Flagship Store	Electricity savings3	It looks regular. It's working now.	Jingdong prefecture level city in Shandong
Jinfeng Hongyuan Flagship Store	Electricity savings3	It arrived pretty fast and the owner is very responsible. Have not used it yet, will see after use	Jingdong prefecture level city in Shandong
Jinfeng Hongyuan Flagship Store	Electricity savings3	🎁 baby quality is very good, the first time I received the goods to open the first time to see, it is very good 👍, very satisfied 😊, customer service also have a question and answer, thank you 🙏	Jingdong prefecture level city in Shandong
Jinfeng Hongyuan Flagship Store	Electricity savings3	I don't know if it's any good 。。。。。。	Jingdong prefecture level city in Shandong
Jinfeng Hongyuan Flagship Store	Electricity savings3	Buy electronic products! Still trust the things of the Jingdong self-owned store! Energy-saving effect to evaluate after heating!	Jingdong prefecture level city in Shandong
Jinfeng Hongyuan Flagship Store	Electricity savings3	The goods have been received, Blu is very beautiful, energy saving effect, to be confirmed only after actual use.	Jingdong prefecture level city in Shandong
Jinfeng Hongyuan Flagship Store	Electricity savings3	Jingdong's speed is so fast, has received, easy to operate. The display is quite good, how many volts what mode is quite understandable.	Jingdong prefecture level city in Shandong
Jinfeng Hongyuan Flagship Store	Electricity savings3	The first use, the effect is good to recommend it to colleagues to use, a month of electricity to save a lot or can be	Jingdong prefecture level city in Shandong
Jinfeng Hongyuan Flagship Store	Electricity savings3	I don't know if it's useful. Mom's is a store. I'll return it if it doesn't work.	Jingdong prefecture level city in Shandong
Jinfeng Hongyuan Flagship Store	Electricity savings3	I don't know if it works or not, I'll wait for two months to use it and then come back to comment!	Jingdong prefecture level city in Shandong
Jinfeng Hongyuan Flagship Store	Electricity savings3	I bought the second one now, and I've been using it for more than half a year, and I feel that it really saves electricity. Now it is summer, the weather is hot, sometimes the fan and air conditioning on all day, if not this power saver, the monthly electricity bill is quite high, but as long as the installation of this power saver, not afraid of its high electricity bill, absolutely let you wantonly use	Jingdong prefecture level city in Shandong
Jinfeng Hongyuan Flagship Store	Electricity savings3	The product is better than I thought it would be, the quality looks great, the voltage is stable, and I'm looking forward to some great power savings!	Jingdong prefecture level city in Shandong
Jinfeng Hongyuan Flagship Store	Electricity savings3	Inadvertently see bought a try, delivery is also quite timely, just do not know how to use, customer service patience to answer, with a pretty good, value for money!	Jingdong prefecture level city in Shandong
Jinfeng Hongyuan Flagship Store	Electricity savings3	It's better than nothing! Hopefully this will help save slightly.	Jingdong prefecture level city in Shandong
Jinfeng Hongyuan Flagship Store	Electricity savings3	Still trying it out, follow up	Jingdong prefecture level city in Shandong
Jinfeng Hongyuan Flagship Store	Electricity savings3	I've just started using it, I don't know how well it works yet, I hope it's good.	Jingdong prefecture level city in Shandong
Jinfeng Hongyuan Flagship Store	Electricity savings3	Fourth purchase, great baby!	Jingdong prefecture level city in Shandong
Jinfeng Hongyuan Flagship Store	Electricity savings3	Shipped really fast, just got it and use it to see what happens and then come back for a review!	Jingdong prefecture level city in Shandong
Jinfeng Hongyuan Flagship Store	Electricity savings3	Timely delivery **** good attitude	Jingdong prefecture level city in Shandong
Jinfeng Hongyuan Flagship Store	Electricity savings3	Home large and small things are purchased in the Jingdong Mall, have been accustomed to shopping in the Jingdong, many times can buy good quality and cheap products, and courier service attitude is particularly good, sometimes too many couriers are to help me to the home, so many years have been in the Jingdong Mall shopping, trust and support! Will also support the Jingdong as always!	Jingdong prefecture level city in Shandong
Jinfeng Hongyuan Flagship Store	Electricity savings3	Modified a pump and the modification worked.	Jingdong prefecture level city in Shandong
Jinfeng Hongyuan Flagship Store	Electricity savings3	Already installed, next time you pay the electric bill to see the results	Jingdong prefecture level city in Shandong
Jinfeng Hongyuan Flagship Store	Electricity savings3	The product is very good to use and practical to look at	Jingdong prefecture level city in Shandong
Jinfeng Hongyuan Flagship Store	Electricity savings3	Works great, great service, waiting for results.	Jingdong prefecture level city in Shandong
Jinfeng Hongyuan Flagship Store	Electricity savings3	I just used it and I'm not sure about the efficacy yet, I'll come back to evaluate it after a while.	Jingdong prefecture level city in Shandong
Jinfeng Hongyuan Flagship Store	Electricity savings3	Just received the goods, use a period of time to evaluate	Jingdong prefecture level city in Shandong
Jinfeng Hongyuan Flagship Store	Electricity savings3	Opened and tried very good, use it to see if there are results	Jingdong prefecture level city in Shandong
Jinfeng Hongyuan Flagship Store	Electricity savings3	Arrived today! Still trying it out!	Jingdong prefecture level city in Shandong
Jinfeng Hongyuan Flagship Store	Electricity savings3	Shipped quickly, well packaged, already installed, hope there is a power saving effect	Jingdong prefecture level city in Shandong
Jinfeng Hongyuan Flagship Store	Electricity savings3	I got it. It's great.	Jingdong prefecture level city in Shandong
Jinfeng Hongyuan Flagship Store	Electricity savings3	Haven't used it yet, will come back in a month	Jingdong prefecture level city in Shandong
Jinfeng Hongyuan Flagship Store	Electricity savings3	It looks good. It's good. Try it first.	Jingdong prefecture level city in Shandong
Jinfeng Hongyuan Flagship Store	Electricity savings3	It works so well that I have relatives, friends, coworkers, and 6 families that have purchased it for them through me.	Jingdong prefecture level city in Shandong
Jinfeng Hongyuan Flagship Store	Electricity savings3	In use	Jingdong prefecture level city in Shandong
Jinfeng Hongyuan Flagship Store	Electricity savings3	Received the goods today, power-saving effect do not know being used, the boss has a very good attitude, praise!	Jingdong prefecture level city in Shandong
Jinfeng Hongyuan Flagship Store	Electricity savings3	The product is compact and doesn't take up much space, but hopefully it's power efficient.	Jingdong prefecture level city in Shandong
Jinfeng Hongyuan Flagship Store	Electricity savings3	Power saving is indeed effective, I used for a period of time before coming to evaluate, the electricity bill is obviously less, the quality is good, the shopkeeper service is also very good, very patient,	Jingdong prefecture level city in Shandong
Jinfeng Hongyuan Flagship Store	Electricity savings3	It feels fine, I'll comment after I use it	Jingdong prefecture level city in Shandong
Jinfeng Hongyuan Flagship Store	Electricity savings3	I just received it and I don't know the effect yet! I will comment again after I use it and have results!	Jingdong prefecture level city in Shandong
Jinfeng Hongyuan Flagship Store	Electricity savings3	The goods look fine, I hope they really work!	Jingdong prefecture level city in Shandong
Jinfeng Hongyuan Flagship Store	Electricity savings3	I just don't know if it saves power	Jingdong prefecture level city in Shandong
Jinfeng Hongyuan Flagship Store	Electricity savings3	My friend introduced me to use this product, it is professional and I am looking forward to the long term effect~!	Jingdong prefecture level city in Shandong
Jinfeng Hongyuan Flagship Store	Electricity savings3	Try it before you judge.	Jingdong prefecture level city in Shandong
Jinfeng Hongyuan Flagship Store	Electricity savings3	It's great, the power savings are obvious! Kudos!	Jingdong prefecture level city in Shandong
Jinfeng Hongyuan Flagship Store	Electricity savings3	The quality is excellent, stabilizes the voltage and saves electricity, very satisfied.	Jingdong prefecture level city in Shandong
Jinfeng Hongyuan Flagship Store	Electricity savings3	You have to use it for a while to know the effect	Jingdong prefecture level city in Shandong
Jinfeng Hongyuan Flagship Store	Electricity savings3	It's on trial.	Jingdong prefecture level city in Shandong
Jinfeng Hongyuan Flagship Store	Electricity savings3	The stuff works great.	Jingdong prefecture level city in Shandong
Jinfeng Hongyuan Flagship Store	Electricity savings3	Doesn't seem to work, not as god as other reviews	Jingdong prefecture level city in Shandong

Jinfeng Hongyuan Flagship Store	Electricity savings3	It's not good quality, it doesn't work, it stops working after a few days, it doesn't work, the customer service doesn't care, it's a bit irritating.	Jingdong prefecture level city in Shandong
Jinfeng Hongyuan Flagship Store	Electricity savings3	It doesn't do anything.	Jingdong prefecture level city in Shandong
Jinfeng Hongyuan Flagship Store	Electricity savings3	It's good.	Jingdong prefecture level city in Shandong
Jinfeng Hongyuan Flagship Store	Electricity savings3	Used a month effect is not obvious, are psychological effects, feel are not less, did not save to the electricity, there are details of the problem, the store said to buy to send 5 fuse, in fact, received only three, small problems are not committed to in place, I believe that the product is not good enough	Jingdong prefecture level city in Shandong
Jinfeng Hongyuan Flagship Store	Electricity savings3	Well received quickly, looking quite high, but also sent 5 fuses. Customer service is very patient to teach the operation, in fact, quite simple, lazy to read the instructions ha!	Jingdong prefecture level city in Shandong
Jinfeng Hongyuan Flagship Store	Electricity savings3	You have to use it for a while to know the effect	Jingdong prefecture level city in Shandong
Jinfeng Hongyuan Flagship Store	Electricity savings3	Shipped very quickly, packaged very well, the price is particularly affordable, there are so many efficacy is also good, it is worth buying worth buying the product, the workmanship is very fine, better than imagined, recommended that everyone try not yet used, after the use of the follow-up comment	Jingdong prefecture level city in Shandong
Jinfeng Hongyuan Flagship Store	Electricity savings3	It looks regular. It's working now.	Jingdong prefecture level city in Shandong
Jinfeng Hongyuan Flagship Store	Electricity savings3	It arrived pretty fast and the owner is very responsible. Have not used it yet, will see after use	Jingdong prefecture level city in Shandong
Jinfeng Hongyuan Flagship Store	Electricity savings3	🎁 baby quality is very good, the first time I received the goods to open the first time to see, it is very good 👍, very satisfied 😃, customer service also have a question and answer, thank you 🙏	Jingdong prefecture level city in Shandong
Jinfeng Hongyuan Flagship Store	Electricity savings3	I don't know if it's any good 。。。。。。	Jingdong prefecture level city in Shandong
Jinfeng Hongyuan Flagship Store	Electricity savings3	Buy electronic products! Still trust the things of the Jingdong self-owned store! Energy-saving effect to evaluate after heating!	Jingdong prefecture level city in Shandong
Jinfeng Hongyuan Flagship Store	Electricity savings3	The goods have been received, Blu is very beautiful, energy saving effect, to be confirmed only after actual use.	Jingdong prefecture level city in Shandong
Jinfeng Hongyuan Flagship Store	Electricity savings3	Jingdong's speed is so fast, has received, easy to operate. The display is quite good, how many volts what mode is quite understandable.	Jingdong prefecture level city in Shandong
Jinfeng Hongyuan Flagship Store	Electricity savings3	The first use, the effect is good to recommend it to colleagues to use, a month of electricity to save a lot or can be	Jingdong prefecture level city in Shandong
Jinfeng Hongyuan Flagship Store	Electricity savings3	I don't know if it's useful. Mom's is a store. I'll return it if it doesn't work.	Jingdong prefecture level city in Shandong
Jinfeng Hongyuan Flagship Store	Electricity savings3	I don't know if it works or not, I'll wait for two months to use it and then come back to comment!	Jingdong prefecture level city in Shandong
Jinfeng Hongyuan Flagship Store	Electricity savings3	I bought the second one now, and I've been using it for more than half a year, and I feel that it really saves electricity. Now it is summer, the weather is hot, sometimes the fan and air conditioning on all day, if not this power saver, the monthly electricity bill is quite high, but as long as the installation of this power saver, not afraid of its high electricity bill, absolutely let you wantonly use	Jingdong prefecture level city in Shandong
Jinfeng Hongyuan Flagship Store	Electricity savings3	The product is better than I thought it would be, the quality looks great, the voltage is stable, and I'm looking forward to some great power savings!	Jingdong prefecture level city in Shandong
Jinfeng Hongyuan Flagship Store	Electricity savings3	Inadvertently see bought a try, delivery is also quite timely, just do not know how to use, customer service patience to answer, with a pretty good, value for money!	Jingdong prefecture level city in Shandong
Jinfeng Hongyuan Flagship Store	Electricity savings3	It's better than nothing! Hopefully this will help save slightly.	Jingdong prefecture level city in Shandong
Jinfeng Hongyuan Flagship Store	Electricity savings3	Still trying it out, follow up	Jingdong prefecture level city in Shandong
Jinfeng Hongyuan Flagship Store	Electricity savings3	I've just started using it, I don't know how well it works yet, I hope it's good.	Jingdong prefecture level city in Shandong
Jinfeng Hongyuan Flagship Store	Electricity savings3	Fourth purchase, great baby!	Jingdong prefecture level city in Shandong
Jinfeng Hongyuan Flagship Store	Electricity savings3	Shipped really fast, just got it and use it to see what happens and then come back for a review!	Jingdong prefecture level city in Shandong
Jinfeng Hongyuan Flagship Store	Electricity savings3	Timely delivery **** good attitude	Jingdong prefecture level city in Shandong
Jinfeng Hongyuan Flagship Store	Electricity savings3	Home large and small things are purchased in the Jingdong Mall, have been accustomed to shopping in the Jingdong, many times can buy good quality and cheap products, and courier service attitude is particularly good, sometimes too many couriers are to help me to the home, so many years have been in the Jingdong Mall shopping, trust and support! Will also support the Jingdong as always!	Jingdong prefecture level city in Shandong
Jinfeng Hongyuan Flagship Store	Electricity savings3	Modified a pump and the modification worked.	Jingdong prefecture level city in Shandong
Jinfeng Hongyuan Flagship Store	Electricity savings3	Already installed, next time you pay the electric bill to see the results	Jingdong prefecture level city in Shandong
Jinfeng Hongyuan Flagship Store	Electricity savings3	The product is very good to use and practical to look at	Jingdong prefecture level city in Shandong
Jinfeng Hongyuan Flagship Store	Electricity savings3	Works great, great service, waiting for results.	Jingdong prefecture level city in Shandong
Jinfeng Hongyuan Flagship Store	Electricity savings3	I just used it and I'm not sure about the efficacy yet, I'll come back to evaluate it after a while.	Jingdong prefecture level city in Shandong
Jinfeng Hongyuan Flagship Store	Electricity savings3	Just received the goods, use a period of time to evaluate	Jingdong prefecture level city in Shandong
Jinfeng Hongyuan Flagship Store	Electricity savings3	Opened and tried very good, use it to see if there are results	Jingdong prefecture level city in Shandong
Jinfeng Hongyuan Flagship Store	Electricity savings3	Arrived today! Still trying it out!	Jingdong prefecture level city in Shandong
Jinfeng Hongyuan Flagship Store	Electricity savings3	Shipped quickly, well packaged, already installed, hope there is a power saving effect	Jingdong prefecture level city in Shandong
Jinfeng Hongyuan Flagship Store	Electricity savings3	I got it. It's great.	Jingdong prefecture level city in Shandong
Jinfeng Hongyuan Flagship Store	Electricity savings3	Haven't used it yet, will come back in a month	Jingdong prefecture level city in Shandong
Jinfeng Hongyuan Flagship Store	Electricity savings3	It looks good. It's good. Try it first.	Jingdong prefecture level city in Shandong
Jinfeng Hongyuan Flagship Store	Electricity savings3	It works so well that I have relatives, friends, coworkers, and 6 families that have purchased it for them through me.	Jingdong prefecture level city in Shandong
Jinfeng Hongyuan Flagship Store	Electricity savings3	In use	Jingdong prefecture level city in Shandong
Jinfeng Hongyuan Flagship Store	Electricity savings3	Received the goods today, power-saving effect do not know being used, the boss has a very good attitude, praise!	Jingdong prefecture level city in Shandong
Jinfeng Hongyuan Flagship Store	Electricity savings3	The product is compact and doesn't take up much space, but hopefully it's power efficient.	Jingdong prefecture level city in Shandong
Jinfeng Hongyuan Flagship Store	Electricity savings3	Power saving is indeed effective, I used for a period of time before coming to evaluate, the electricity bill is obviously less, the quality is good, the shopkeeper service is also very good, very patient,	Jingdong prefecture level city in Shandong
Jinfeng Hongyuan Flagship Store	Electricity savings3	It feels fine, I'll comment after I use it	Jingdong prefecture level city in Shandong
Jinfeng Hongyuan Flagship Store	Electricity savings3	I just received it and I don't know the effect yet! I will comment again after I use it and have results!	Jingdong prefecture level city in Shandong
Jinfeng Hongyuan Flagship Store	Electricity savings3	The goods look fine, I hope they really work!	Jingdong prefecture level city in Shandong
Jinfeng Hongyuan Flagship Store	Electricity savings3	I just don't know if it saves power	Jingdong prefecture level city in Shandong

Jinfeng Hongyuan Flagship Store	Electricity savings3	My friend introduced me to use this product, it is professional and I am looking forward to the long term effect~!	Jingdong prefecture level city in Shandong
Jinfeng Hongyuan Flagship Store	Electricity savings3	Try it before you judge.	Jingdong prefecture level city in Shandong
Jinfeng Hongyuan Flagship Store	Electricity savings3	It's great, the power savings are obvious! Kudos!	Jingdong prefecture level city in Shandong
Jinfeng Hongyuan Flagship Store	Electricity savings3	The quality is excellent, stabilizes the voltage and saves electricity, very satisfied.	Jingdong prefecture level city in Shandong
Jinfeng Hongyuan Flagship Store	Electricity savings3	You have to use it for a while to know the effect	Jingdong prefecture level city in Shandong
Jinfeng Hongyuan Flagship Store	Electricity savings3	It's on trial.	Jingdong prefecture level city in Shandong
Jinfeng Hongyuan Flagship Store	Electricity savings3	The stuff works great.	Jingdong prefecture level city in Shandong
Jinfeng Hongyuan Flagship Store	Electricity savings3	Doesn't seem to work, not as god as other reviews	Jingdong prefecture level city in Shandong
Jinfeng Hongyuan Flagship Store	Electricity savings3	It's not good quality, it doesn't work, it stops working after a few days, it doesn't work, the customer service doesn't care, it's a bit irritating.	Jingdong prefecture level city in Shandong
Jinfeng Hongyuan Flagship Store	Electricity savings3	It doesn't do anything.	Jingdong prefecture level city in Shandong
Jinfeng Hongyuan Flagship Store	Electricity savings3	It's good.	Jingdong prefecture level city in Shandong
Jinfeng Hongyuan Flagship Store	Electricity savings3	Used a month effect is not obvious, are psychological effects, feel are not less, did not save to the electricity, there are details of the problem, the store said to buy to send 5 fuse, in fact, received only three, small problems are not committed to in place, I believe that the product is not good enough	Jingdong prefecture level city in Shandong
Jinfeng Hongyuan Flagship Store	Electricity savings3	Well received quickly, looking quite high, but also sent 5 fuses. Customer service is very patient to teach the operation, in fact, quite simple, lazy to read the instructions ha!	Jingdong prefecture level city in Shandong
Jinfeng Hongyuan Flagship Store	Electricity savings3	You have to use it for a while to know the effect	Jingdong prefecture level city in Shandong
Jinfeng Hongyuan Flagship Store	Electricity savings3	Shipped very quickly, packaged very well, the price is particularly affordable, there are so many efficacy is also good, it is worth buying worth buying the product, the workmanship is very fine, better than imagined, recommended that everyone try not yet used, after the use of the follow-up comment	Jingdong prefecture level city in Shandong
Jinfeng Hongyuan Flagship Store	Electricity savings3	It looks regular. It's working now.	Jingdong prefecture level city in Shandong
Jinfeng Hongyuan Flagship Store	Electricity savings3	It arrived pretty fast and the owner is very responsible. Have not used it yet, will see after use	Jingdong prefecture level city in Shandong
Jinfeng Hongyuan Flagship Store	Electricity savings3	🎁 baby quality is very good, the first time I received the goods to open the first time to see, it is very good 👍, very satisfied 😊, customer service also have a question and answer, thank you 🙏	Jingdong prefecture level city in Shandong
Jinfeng Hongyuan Flagship Store	Electricity savings3	I don't know if it's any good 。。。。。。	Jingdong prefecture level city in Shandong
Jinfeng Hongyuan Flagship Store	Electricity savings3	Buy electronic products! Still trust the things of the Jingdong self-owned store! Energy-saving effect to evaluate after heating!	Jingdong prefecture level city in Shandong
Jinfeng Hongyuan Flagship Store	Electricity savings3	The goods have been received, Blu is very beautiful, energy saving effect, to be confirmed only after actual use.	Jingdong prefecture level city in Shandong
Jinfeng Hongyuan Flagship Store	Electricity savings3	Jingdong's speed is so fast, has received, easy to operate. The display is quite good, how many volts what mode is quite understandable.	Jingdong prefecture level city in Shandong
Jinfeng Hongyuan Flagship Store	Electricity savings3	The first use, the effect is good to recommend it to colleagues to use, a month of electricity to save a lot or can be	Jingdong prefecture level city in Shandong
Jinfeng Hongyuan Flagship Store	Electricity savings3	I don't know if it's useful. Mom's is a store. I'll return it if it doesn't work.	Jingdong prefecture level city in Shandong
Jinfeng Hongyuan Flagship Store	Electricity savings3	I don't know if it works or not, I'll wait for two months to use it and then come back to comment!	Jingdong prefecture level city in Shandong
Jinfeng Hongyuan Flagship Store	Electricity savings3	I bought the second one now, and I've been using it for more than half a year, and I feel that it really saves electricity. Now it is summer, the weather is hot, sometimes the fan and air conditioning on all day, if not this power saver, the monthly electricity bill is quite high, but as long as the installation of this power saver, not afraid of its high electricity bill, absolutely let you wantonly use	Jingdong prefecture level city in Shandong
Jinfeng Hongyuan Flagship Store	Electricity savings3	The product is better than I thought it would be, the quality looks great, the voltage is stable, and I'm looking forward to some great power savings!	Jingdong prefecture level city in Shandong
Jinfeng Hongyuan Flagship Store	Electricity savings3	Inadvertently see bought a try, delivery is also quite timely, just do not know how to use, customer service patience to answer, with a pretty good, value for money!	Jingdong prefecture level city in Shandong
Jinfeng Hongyuan Flagship Store	Electricity savings3	It's better than nothing! Hopefully this will help save slightly.	Jingdong prefecture level city in Shandong
Jinfeng Hongyuan Flagship Store	Electricity savings3	Still trying it out, follow up	Jingdong prefecture level city in Shandong
Jinfeng Hongyuan Flagship Store	Electricity savings3	I've just started using it, I don't know how well it works yet, I hope it's good.	Jingdong prefecture level city in Shandong
Jinfeng Hongyuan Flagship Store	Electricity savings3	Fourth purchase, great baby!	Jingdong prefecture level city in Shandong
Jinfeng Hongyuan Flagship Store	Electricity savings3	Shipped really fast, just got it and use it to see what happens and then come back for a review!	Jingdong prefecture level city in Shandong
Jinfeng Hongyuan Flagship Store	Electricity savings3	Timely delivery **** good attitude	Jingdong prefecture level city in Shandong
Jinfeng Hongyuan Flagship Store	Electricity savings3	Home large and small things are purchased in the Jingdong Mall, have been accustomed to shopping in the Jingdong, many times can buy good quality and cheap products, and courier service attitude is particularly good, sometimes too many couriers are to help me to the home, so many years have been in the Jingdong Mall shopping, trust and support! Will also support the Jingdong as always!	Jingdong prefecture level city in Shandong
Jinfeng Hongyuan Flagship Store	Electricity savings3	Modified a pump and the modification worked.	Jingdong prefecture level city in Shandong
Jinfeng Hongyuan Flagship Store	Electricity savings3	Already installed, next time you pay the electric bill to see the results	Jingdong prefecture level city in Shandong
Jinfeng Hongyuan Flagship Store	Electricity savings3	The product is very good to use and practical to look at	Jingdong prefecture level city in Shandong
Jinfeng Hongyuan Flagship Store	Electricity savings3	Works great, great service, waiting for results.	Jingdong prefecture level city in Shandong
Jinfeng Hongyuan Flagship Store	Electricity savings3	I just used it and I'm not sure about the efficacy yet, I'll come back to evaluate it after a while.	Jingdong prefecture level city in Shandong
Jinfeng Hongyuan Flagship Store	Electricity savings3	Just received the goods, use a period of time to evaluate	Jingdong prefecture level city in Shandong
Jinfeng Hongyuan Flagship Store	Electricity savings3	Opened and tried very good, use it to see if there are results	Jingdong prefecture level city in Shandong
Jinfeng Hongyuan Flagship Store	Electricity savings3	Arrived today! Still trying it out!	Jingdong prefecture level city in Shandong
Jinfeng Hongyuan Flagship Store	Electricity savings3	Shipped quickly, well packaged, already installed, hope there is a power saving effect	Jingdong prefecture level city in Shandong
Jinfeng Hongyuan Flagship Store	Electricity savings3	I got it. It's great.	Jingdong prefecture level city in Shandong
Jinfeng Hongyuan Flagship Store	Electricity savings3	Haven't used it yet, will come back in a month	Jingdong prefecture level city in Shandong
Jinfeng Hongyuan Flagship Store	Electricity savings3	It looks good. It's good. Try it first.	Jingdong prefecture level city in Shandong
Jinfeng Hongyuan Flagship Store	Electricity savings3	It works so well that I have relatives, friends, coworkers, and 6 families that have purchased it for them through me.	Jingdong prefecture level city in Shandong

Jinfeng Hongyuan Flagship Store	Electricity savings3	In use	Jingdong prefecture level city in Shandong
Jinfeng Hongyuan Flagship Store	Electricity savings3	Received the goods today, power-saving effect do not know being used, the boss has a very good attitude, praise!	Jingdong prefecture level city in Shandong
Jinfeng Hongyuan Flagship Store	Electricity savings3	The product is compact and doesn't take up much space, but hopefully it's power efficient.	Jingdong prefecture level city in Shandong
Jinfeng Hongyuan Flagship Store	Electricity savings3	Power saving is indeed effective, I used for a period of time before coming to evaluate, the electricity bill is obviously less, the quality is good, the shopkeeper service is also very good, very patient,	Jingdong prefecture level city in Shandong
Jinfeng Hongyuan Flagship Store	Electricity savings3	It feels fine, I'll comment after I use it	Jingdong prefecture level city in Shandong
Jinfeng Hongyuan Flagship Store	Electricity savings3	I just received it and I don't know the effect yet! I will comment again after I use it and have results!	Jingdong prefecture level city in Shandong
Jinfeng Hongyuan Flagship Store	Electricity savings3	The goods look fine, I hope they really work!	Jingdong prefecture level city in Shandong
Jinfeng Hongyuan Flagship Store	Electricity savings3	I just don't know if it saves power	Jingdong prefecture level city in Shandong
Jinfeng Hongyuan Flagship Store	Electricity savings3	My friend introduced me to use this product, it is professional and I am looking forward to the long term effect~!	Jingdong prefecture level city in Shandong
Jinfeng Hongyuan Flagship Store	Electricity savings3	Try it before you judge.	Jingdong prefecture level city in Shandong
Jinfeng Hongyuan Flagship Store	Electricity savings3	It's great, the power savings are obvious! Kudos!	Jingdong prefecture level city in Shandong
Jinfeng Hongyuan Flagship Store	Electricity savings3	The quality is excellent, stabilizes the voltage and saves electricity, very satisfied.	Jingdong prefecture level city in Shandong
Jinfeng Hongyuan Flagship Store	Electricity savings3	You have to use it for a while to know the effect	Jingdong prefecture level city in Shandong
Jinfeng Hongyuan Flagship Store	Electricity savings3	It's on trial.	Jingdong prefecture level city in Shandong
Jinfeng Hongyuan Flagship Store	Electricity savings3	The stuff works great.	Jingdong prefecture level city in Shandong
Jinfeng Hongyuan Flagship Store	Electricity savings3	Doesn't seem to work, not as god as other reviews	Jingdong prefecture level city in Shandong
Jinfeng Hongyuan Flagship Store	Electricity savings3	It's not good quality, it doesn't work, it stops working after a few days, it doesn't work, the customer service doesn't care, it's a bit irritating.	Jingdong prefecture level city in Shandong
Jinfeng Hongyuan Flagship Store	Electricity savings3	It doesn't do anything.	Jingdong prefecture level city in Shandong
Jinfeng Hongyuan Flagship Store	Electricity savings3	It's good.	Jingdong prefecture level city in Shandong
Jinfeng Hongyuan Flagship Store	Electricity savings3	Used a month effect is not obvious, are psychological effects, feel are not less, did not save to the electricity, there are details of the problem, the store said to buy to send 5 fuse, in fact, received only three, small problems are not committed to in place, I believe that the product is not good enough	Jingdong prefecture level city in Shandong
Jinfeng Hongyuan Flagship Store	Electricity savings3	Well received quickly, looking quite high, but also sent 5 fuses. Customer service is very patient to teach the operation, in fact, quite simple, lazy to read the instructions ha!	Jingdong prefecture level city in Shandong
Jinfeng Hongyuan Flagship Store	Electricity savings3	You have to use it for a while to know the effect	Jingdong prefecture level city in Shandong
Jinfeng Hongyuan Flagship Store	Electricity savings3	Shipped very quickly, packaged very well, the price is particularly affordable, there are so many efficacy is also good, it is worth buying worth buying the product, the workmanship is very fine, better than imagined, recommended that everyone try not yet used, after the use of the follow-up comment	Jingdong prefecture level city in Shandong
Jinfeng Hongyuan Flagship Store	Electricity savings3	It looks regular. It's working now.	Jingdong prefecture level city in Shandong
Jinfeng Hongyuan Flagship Store	Electricity savings3	It arrived pretty fast and the owner is very responsible. Have not used it yet, will see after use	Jingdong prefecture level city in Shandong
Jinfeng Hongyuan Flagship Store	Electricity savings3	🎁 baby quality is very good, the first time I received the goods to open the first time to see, it is very good 👍, very satisfied 😊, customer service also have a question and answer, thank you 🙏	Jingdong prefecture level city in Shandong
Jinfeng Hongyuan Flagship Store	Electricity savings3	I don't know if it's any good 。。。。。。	Jingdong prefecture level city in Shandong
Jinfeng Hongyuan Flagship Store	Electricity savings3	Buy electronic products! Still trust the things of the Jingdong self-owned store! Energy-saving effect to evaluate after heating!	Jingdong prefecture level city in Shandong
Jinfeng Hongyuan Flagship Store	Electricity savings3	The goods have been received, Blu is very beautiful, energy saving effect, to be confirmed only after actual use.	Jingdong prefecture level city in Shandong
Jinfeng Hongyuan Flagship Store	Electricity savings3	Jingdong's speed is so fast, has received, easy to operate. The display is quite good, how many volts what mode is quite understandable.	Jingdong prefecture level city in Shandong
Jinfeng Hongyuan Flagship Store	Electricity savings3	The first use, the effect is good to recommend it to colleagues to use, a month of electricity to save a lot or can be	Jingdong prefecture level city in Shandong
Jinfeng Hongyuan Flagship Store	Electricity savings3	I don't know if it's useful. Mom's is a store. I'll return it if it doesn't work.	Jingdong prefecture level city in Shandong
Jinfeng Hongyuan Flagship Store	Electricity savings3	I don't know if it works or not, I'll wait for two months to use it and then come back to comment!	Jingdong prefecture level city in Shandong
Jinfeng Hongyuan Flagship Store	Electricity savings3	I bought the second one now, and I've been using it for more than half a year, and I feel that it really saves electricity. Now it is summer, the weather is hot, sometimes the fan and air conditioning on all day, if not this power saver, the monthly electricity bill is quite high, but as long as the installation of this power saver, not afraid of its high electricity bill, absolutely let you wantonly use	Jingdong prefecture level city in Shandong
Jinfeng Hongyuan Flagship Store	Electricity savings3	The product is better than I thought it would be, the quality looks great, the voltage is stable, and I'm looking forward to some great power savings!	Jingdong prefecture level city in Shandong
Jinfeng Hongyuan Flagship Store	Electricity savings3	Inadvertently see bought a try, delivery is also quite timely, just do not know how to use, customer service patience to answer, with a pretty good, value for money!	Jingdong prefecture level city in Shandong
Jinfeng Hongyuan Flagship Store	Electricity savings3	It's better than nothing! Hopefully this will help save slightly.	Jingdong prefecture level city in Shandong
Jinfeng Hongyuan Flagship Store	Electricity savings3	Still trying it out, follow up	Jingdong prefecture level city in Shandong
Jinfeng Hongyuan Flagship Store	Electricity savings3	I've just started using it, I don't know how well it works yet, I hope it's good.	Jingdong prefecture level city in Shandong
Jinfeng Hongyuan Flagship Store	Electricity savings3	Fourth purchase, great baby!	Jingdong prefecture level city in Shandong
Jinfeng Hongyuan Flagship Store	Electricity savings3	Shipped really fast, just got it and use it to see what happens and then come back for a review!	Jingdong prefecture level city in Shandong
Jinfeng Hongyuan Flagship Store	Electricity savings3	Timely delivery **** good attitude	Jingdong prefecture level city in Shandong
Jinfeng Hongyuan Flagship Store	Electricity savings3	Home large and small things are purchased in the Jingdong Mall, have been accustomed to shopping in the Jingdong, many times can buy good quality and cheap products, and courier service attitude is particularly good, sometimes too many couriers are to help me to the home, so many years have been in the Jingdong Mall shopping, trust and support! Will also support the Jingdong as always!	Jingdong prefecture level city in Shandong
Jinfeng Hongyuan Flagship Store	Electricity savings3	Modified a pump and the modification worked.	Jingdong prefecture level city in Shandong
Jinfeng Hongyuan Flagship Store	Electricity savings3	Already installed, next time you pay the electric bill to see the results	Jingdong prefecture level city in Shandong
Jinfeng Hongyuan Flagship Store	Electricity savings3	The product is very good to use and practical to look at	Jingdong prefecture level city in Shandong
Jinfeng Hongyuan Flagship Store	Electricity savings3	Works great, great service, waiting for results.	Jingdong prefecture level city in Shandong
Jinfeng Hongyuan Flagship Store	Electricity savings3	I just used it and I'm not sure about the efficacy yet, I'll come back to evaluate it after a while.	Jingdong prefecture level city in Shandong

Jinfeng Hongyuan Flagship Store	Electricity savings3	Just received the goods, use a period of time to evaluate	Jingdong prefecture level city in Shandong
Jinfeng Hongyuan Flagship Store	Electricity savings3	Opened and tried very good, use it to see if there are results	Jingdong prefecture level city in Shandong
Jinfeng Hongyuan Flagship Store	Electricity savings3	Arrived today! Still trying it out!	Jingdong prefecture level city in Shandong
Jinfeng Hongyuan Flagship Store	Electricity savings3	Shipped quickly, well packaged, already installed, hope there is a power saving effect	Jingdong prefecture level city in Shandong
Jinfeng Hongyuan Flagship Store	Electricity savings3	I got it. It's great.	Jingdong prefecture level city in Shandong
Jinfeng Hongyuan Flagship Store	Electricity savings3	Haven't used it yet, will come back in a month	Jingdong prefecture level city in Shandong
Jinfeng Hongyuan Flagship Store	Electricity savings3	It looks good. It's good. Try it first.	Jingdong prefecture level city in Shandong
Jinfeng Hongyuan Flagship Store	Electricity savings3	It works so well that I have relatives, friends, coworkers, and 6 families that have purchased it for them through me.	Jingdong prefecture level city in Shandong
Jinfeng Hongyuan Flagship Store	Electricity savings3	In use	Jingdong prefecture level city in Shandong
Jinfeng Hongyuan Flagship Store	Electricity savings3	Received the goods today, power-saving effect do not know being used, the boss has a very good attitude, praise!	Jingdong prefecture level city in Shandong
Jinfeng Hongyuan Flagship Store	Electricity savings3	The product is compact and doesn't take up much space, but hopefully it's power efficient.	Jingdong prefecture level city in Shandong
Jinfeng Hongyuan Flagship Store	Electricity savings3	Power saving is indeed effective, I used for a period of time before coming to evaluate, the electricity bill is obviously less, the quality is good, the shopkeeper service is also very good, very patient,	Jingdong prefecture level city in Shandong
Jinfeng Hongyuan Flagship Store	Electricity savings3	It feels fine, I'll comment after I use it	Jingdong prefecture level city in Shandong
Jinfeng Hongyuan Flagship Store	Electricity savings3	I just received it and I don't know the effect yet! I will comment again after I use it and have results!	Jingdong prefecture level city in Shandong
Jinfeng Hongyuan Flagship Store	Electricity savings3	The goods look fine, I hope they really work!	Jingdong prefecture level city in Shandong
Jinfeng Hongyuan Flagship Store	Electricity savings3	I just don't know if it saves power	Jingdong prefecture level city in Shandong
Jinfeng Hongyuan Flagship Store	Electricity savings3	My friend introduced me to use this product, it is professional and I am looking forward to the long term effect~!	Jingdong prefecture level city in Shandong
Jinfeng Hongyuan Flagship Store	Electricity savings3	Try it before you judge.	Jingdong prefecture level city in Shandong
Jinfeng Hongyuan Flagship Store	Electricity savings3	It's great, the power savings are obvious! Kudos!	Jingdong prefecture level city in Shandong
Jinfeng Hongyuan Flagship Store	Electricity savings3	The quality is excellent, stabilizes the voltage and saves electricity, very satisfied.	Jingdong prefecture level city in Shandong
Jinfeng Hongyuan Flagship Store	Electricity savings3	You have to use it for a while to know the effect	Jingdong prefecture level city in Shandong
Jinfeng Hongyuan Flagship Store	Electricity savings3	It's on trial.	Jingdong prefecture level city in Shandong
Jinfeng Hongyuan Flagship Store	Electricity savings3	The stuff works great.	Jingdong prefecture level city in Shandong
Jinfeng Hongyuan Flagship Store	Electricity savings3	Doesn't seem to work, not as god as other reviews	Jingdong prefecture level city in Shandong
Jinfeng Hongyuan Flagship Store	Electricity savings3	It's not good quality, it doesn't work, it stops working after a few days, it doesn't work, the customer service doesn't care, it's a bit irritating.	Jingdong prefecture level city in Shandong
Jinfeng Hongyuan Flagship Store	Electricity savings3	It doesn't do anything.	Jingdong prefecture level city in Shandong
Jinfeng Hongyuan Flagship Store	Electricity savings3	It's good.	Jingdong prefecture level city in Shandong
Jinfeng Hongyuan Flagship Store	Electricity savings3	Used a month effect is not obvious, are psychological effects, feel are not less, did not save to the electricity, there are details of the problem, the store said to buy to send 5 fuse, in fact, received only three, small problems are not committed to in place, I believe that the product is not good enough	Jingdong prefecture level city in Shandong
Jinfeng Hongyuan Flagship Store	Electricity savings3	Well received quickly, looking quite high, but also sent 5 fuses. Customer service is very patient to teach the operation, in fact, quite simple, lazy to read the instructions ha!	Jingdong prefecture level city in Shandong
Jinfeng Hongyuan Flagship Store	Electricity savings3	You have to use it for a while to know the effect	Jingdong prefecture level city in Shandong
Jinfeng Hongyuan Flagship Store	Electricity savings3	Shipped very quickly, packaged very well, the price is particularly affordable, there are so many efficacy is also good, it is worth buying worth buying the product, the workmanship is very fine, better than imagined, recommended that everyone try not yet used, after the use of the follow-up comment	Jingdong prefecture level city in Shandong
Jinfeng Hongyuan Flagship Store	Electricity savings3	It looks regular. It's working now.	Jingdong prefecture level city in Shandong
Jinfeng Hongyuan Flagship Store	Electricity savings3	It arrived pretty fast and the owner is very responsible. Have not used it yet, will see after use	Jingdong prefecture level city in Shandong
Jinfeng Hongyuan Flagship Store	Electricity savings3	🎁 baby quality is very good, the first time I received the goods to open the first time to see, it is very good 👍, very satisfied 😊, customer service also have a question and answer, thank you 🙏	Jingdong prefecture level city in Shandong
Jinfeng Hongyuan Flagship Store	Electricity savings3	I don't know if it's any good 。。。。。。	Jingdong prefecture level city in Shandong
Jinfeng Hongyuan Flagship Store	Electricity savings3	Buy electronic products! Still trust the things of the Jingdong self-owned store! Energy-saving effect to evaluate after heating!	Jingdong prefecture level city in Shandong
Jinfeng Hongyuan Flagship Store	Electricity savings3	The goods have been received, Blu is very beautiful, energy saving effect, to be confirmed only after actual use.	Jingdong prefecture level city in Shandong
Jinfeng Hongyuan Flagship Store	Electricity savings3	Jingdong's speed is so fast, has received, easy to operate. The display is quite good, how many volts what mode is quite understandable.	Jingdong prefecture level city in Shandong
Jinfeng Hongyuan Flagship Store	Electricity savings3	The first use, the effect is good to recommend it to colleagues to use, a month of electricity to save a lot or can be	Jingdong prefecture level city in Shandong
Jinfeng Hongyuan Flagship Store	Electricity savings3	I don't know if it's useful. Mom's is a store. I'll return it if it doesn't work.	Jingdong prefecture level city in Shandong
Jinfeng Hongyuan Flagship Store	Electricity savings3	I don't know if it works or not, I'll wait for two months to use it and then come back to comment!	Jingdong prefecture level city in Shandong
Jinfeng Hongyuan Flagship Store	Electricity savings3	I bought the second one now, and I've been using it for more than half a year, and I feel that it really saves electricity. Now it is summer, the weather is hot, sometimes the fan and air conditioning on all day, if not this power saver, the monthly electricity bill is quite high, but as long as the installation of this power saver, not afraid of its high electricity bill, absolutely let you wantonly use	Jingdong prefecture level city in Shandong
Jinfeng Hongyuan Flagship Store	Electricity savings3	The product is better than I thought it would be, the quality looks great, the voltage is stable, and I'm looking forward to some great power savings!	Jingdong prefecture level city in Shandong
Jinfeng Hongyuan Flagship Store	Electricity savings3	Inadvertently see bought a try, delivery is also quite timely, just do not know how to use, customer service patience to answer, with a pretty good, value for money!	Jingdong prefecture level city in Shandong
Jinfeng Hongyuan Flagship Store	Electricity savings3	It's better than nothing! Hopefully this will help save slightly.	Jingdong prefecture level city in Shandong
Jinfeng Hongyuan Flagship Store	Electricity savings3	Still trying it out, follow up	Jingdong prefecture level city in Shandong
Jinfeng Hongyuan Flagship Store	Electricity savings3	I've just started using it, I don't know how well it works yet, I hope it's good.	Jingdong prefecture level city in Shandong
Jinfeng Hongyuan Flagship Store	Electricity savings3	Fourth purchase, great baby!	Jingdong prefecture level city in Shandong
Jinfeng Hongyuan Flagship Store	Electricity savings3	Shipped really fast, just got it and use it to see what happens and then come back for a review!	Jingdong prefecture level city in Shandong

Jinfeng Hongyuan Flagship Store	Electricity savings3	Timely delivery **** good attitude	Jingdong prefecture level city in Shandong
Jinfeng Hongyuan Flagship Store	Electricity savings3	Home large and small things are purchased in the Jingdong Mall, have been accustomed to shopping in the Jingdong, many times can buy good quality and cheap products, and courier service attitude is particularly good, sometimes too many couriers are to help me to the home, so many years have been in the Jingdong Mall shopping, trust and support! Will also support the Jingdong as always!	Jingdong prefecture level city in Shandong
Jinfeng Hongyuan Flagship Store	Electricity savings3	Modified a pump and the modification worked.	Jingdong prefecture level city in Shandong
Jinfeng Hongyuan Flagship Store	Electricity savings3	Already installed, next time you pay the electric bill to see the results	Jingdong prefecture level city in Shandong
Jinfeng Hongyuan Flagship Store	Electricity savings3	The product is very good to use and practical to look at	Jingdong prefecture level city in Shandong
Jinfeng Hongyuan Flagship Store	Electricity savings3	Works great, great service, waiting for results.	Jingdong prefecture level city in Shandong
Jinfeng Hongyuan Flagship Store	Electricity savings3	I just used it and I'm not sure about the efficacy yet, I'll come back to evaluate it after a while.	Jingdong prefecture level city in Shandong
Jinfeng Hongyuan Flagship Store	Electricity savings3	Just received the goods, use a period of time to evaluate	Jingdong prefecture level city in Shandong
Jinfeng Hongyuan Flagship Store	Electricity savings3	Opened and tried very good, use it to see if there are results	Jingdong prefecture level city in Shandong
Jinfeng Hongyuan Flagship Store	Electricity savings3	Arrived today! Still trying it out!	Jingdong prefecture level city in Shandong
Jinfeng Hongyuan Flagship Store	Electricity savings3	Shipped quickly, well packaged, already installed, hope there is a power saving effect	Jingdong prefecture level city in Shandong
Jinfeng Hongyuan Flagship Store	Electricity savings3	I got it. It's great.	Jingdong prefecture level city in Shandong
Jinfeng Hongyuan Flagship Store	Electricity savings3	Haven't used it yet, will come back in a month	Jingdong prefecture level city in Shandong
Jinfeng Hongyuan Flagship Store	Electricity savings3	It looks good. It's good. Try it first.	Jingdong prefecture level city in Shandong
Jinfeng Hongyuan Flagship Store	Electricity savings3	It works so well that I have relatives, friends, coworkers, and 6 families that have purchased it for them through me.	Jingdong prefecture level city in Shandong
Jinfeng Hongyuan Flagship Store	Electricity savings3	In use	Jingdong prefecture level city in Shandong
Jinfeng Hongyuan Flagship Store	Electricity savings3	Received the goods today, power-saving effect do not know being used, the boss has a very good attitude, praise!	Jingdong prefecture level city in Shandong
Jinfeng Hongyuan Flagship Store	Electricity savings3	The product is compact and doesn't take up much space, but hopefully it's power efficient.	Jingdong prefecture level city in Shandong
Jinfeng Hongyuan Flagship Store	Electricity savings3	Power saving is indeed effective, I used for a period of time before coming to evaluate, the electricity bill is obviously less, the quality is good, the shopkeeper service is also very good, very patient,	Jingdong prefecture level city in Shandong
Jinfeng Hongyuan Flagship Store	Electricity savings3	It feels fine, I'll comment after I use it	Jingdong prefecture level city in Shandong
Jinfeng Hongyuan Flagship Store	Electricity savings3	I just received it and I don't know the effect yet! I will comment again after I use it and have results!	Jingdong prefecture level city in Shandong
Jinfeng Hongyuan Flagship Store	Electricity savings3	The goods look fine, I hope they really work!	Jingdong prefecture level city in Shandong
Jinfeng Hongyuan Flagship Store	Electricity savings3	I just don't know if it saves power	Jingdong prefecture level city in Shandong
Jinfeng Hongyuan Flagship Store	Electricity savings3	My friend introduced me to use this product, it is professional and I am looking forward to the long term effect~!	Jingdong prefecture level city in Shandong
Jinfeng Hongyuan Flagship Store	Electricity savings3	Try it before you judge.	Jingdong prefecture level city in Shandong
Jinfeng Hongyuan Flagship Store	Electricity savings3	It's great, the power savings are obvious! Kudos!	Jingdong prefecture level city in Shandong
Jinfeng Hongyuan Flagship Store	Electricity savings3	The quality is excellent, stabilizes the voltage and saves electricity, very satisfied.	Jingdong prefecture level city in Shandong
Jinfeng Hongyuan Flagship Store	Electricity savings3	You have to use it for a while to know the effect	Jingdong prefecture level city in Shandong
Jinfeng Hongyuan Flagship Store	Electricity savings3	It's on trial.	Jingdong prefecture level city in Shandong
Jinfeng Hongyuan Flagship Store	Electricity savings3	The stuff works great.	Jingdong prefecture level city in Shandong
Jinfeng Hongyuan Flagship Store	Electricity savings3	Doesn't seem to work, not as god as other reviews	Jingdong prefecture level city in Shandong
Jinfeng Hongyuan Flagship Store	Electricity savings3	It's not good quality, it doesn't work, it stops working after a few days, it doesn't work, the customer service doesn't care, it's a bit irritating.	Jingdong prefecture level city in Shandong
Jinfeng Hongyuan Flagship Store	Electricity savings3	It doesn't do anything.	Jingdong prefecture level city in Shandong
Jinfeng Hongyuan Flagship Store	Electricity savings3	It's good.	Jingdong prefecture level city in Shandong
Jinfeng Hongyuan Flagship Store	Electricity savings3	Used a month effect is not obvious, are psychological effects, feel are not less, did not save to the electricity, there are details of the problem, the store said to buy to send 5 fuse, in fact, received only three, small problems are not committed to in place, I believe that the product is not good enough	Jingdong prefecture level city in Shandong
Jinfeng Hongyuan Flagship Store	Electricity savings3	Well received quickly, looking quite high, but also sent 5 fuses. Customer service is very patient to teach the operation, in fact, quite simple, lazy to read the instructions ha!	Jingdong prefecture level city in Shandong
Jinfeng Hongyuan Flagship Store	Electricity savings3	You have to use it for a while to know the effect	Jingdong prefecture level city in Shandong
Jinfeng Hongyuan Flagship Store	Electricity savings3	Shipped very quickly, packaged very well, the price is particularly affordable, there are so many efficacy is also good, it is worth buying worth buying the product, the workmanship is very fine, better than imagined, recommended that everyone try not yet used, after the use of the follow-up comment	Jingdong prefecture level city in Shandong
Jinfeng Hongyuan Flagship Store	Electricity savings3	It looks regular. It's working now.	Jingdong prefecture level city in Shandong
Jinfeng Hongyuan Flagship Store	Electricity savings3	It arrived pretty fast and the owner is very responsible. Have not used it yet, will see after use	Jingdong prefecture level city in Shandong
Jinfeng Hongyuan Flagship Store	Electricity savings3	🎁 baby quality is very good, the first time I received the goods to open the first time to see, it is very good 👍, very satisfied 😃, customer service also have a question and answer, thank you 🙏	Jingdong prefecture level city in Shandong
Jinfeng Hongyuan Flagship Store	Electricity savings3	I don't know if it's any good 。。。。。。	Jingdong prefecture level city in Shandong
Jinfeng Hongyuan Flagship Store	Electricity savings3	Buy electronic products! Still trust the things of the Jingdong self-owned store! Energy-saving effect to evaluate after heating!	Jingdong prefecture level city in Shandong
Jinfeng Hongyuan Flagship Store	Electricity savings3	The goods have been received, Blu is very beautiful, energy saving effect, to be confirmed only after actual use.	Jingdong prefecture level city in Shandong
Jinfeng Hongyuan Flagship Store	Electricity savings3	Jingdong's speed is so fast, has received, easy to operate. The display is quite good, how many volts what mode is quite understandable.	Jingdong prefecture level city in Shandong
Jinfeng Hongyuan Flagship Store	Electricity savings3	The first use, the effect is good to recommend it to colleagues to use, a month of electricity to save a lot or can be	Jingdong prefecture level city in Shandong
Jinfeng Hongyuan Flagship Store	Electricity savings3	I don't know if it's useful. Mom's is a store. I'll return it if it doesn't work.	Jingdong prefecture level city in Shandong
Jinfeng Hongyuan Flagship Store	Electricity savings3	I don't know if it works or not, I'll wait for two months to use it and then come back to comment!	Jingdong prefecture level city in Shandong

Jinfeng Hongyuan Flagship Store	Electricity savings3	I bought the second one now, and I've been using it for more than half a year, and I feel that it really saves electricity. Now it is summer, the weather is hot, sometimes the fan and air conditioning on all day, if not this power saver, the monthly electricity bill is quite high, but as long as the installation of this power saver, not afraid of its high electricity bill, absolutely let you wantonly use	Jingdong prefecture level city in Shandong
Jinfeng Hongyuan Flagship Store	Electricity savings3	The product is better than I thought it would be, the quality looks great, the voltage is stable, and I'm looking forward to some great power savings!	Jingdong prefecture level city in Shandong
Jinfeng Hongyuan Flagship Store	Electricity savings3	Inadvertently see bought a try, delivery is also quite timely, just do not know how to use, customer service patience to answer, with a pretty good, value for money!	Jingdong prefecture level city in Shandong
Jinfeng Hongyuan Flagship Store	Electricity savings3	It's better than nothing! Hopefully this will help save slightly.	Jingdong prefecture level city in Shandong
Jinfeng Hongyuan Flagship Store	Electricity savings3	Still trying it out, follow up	Jingdong prefecture level city in Shandong
Jinfeng Hongyuan Flagship Store	Electricity savings3	I've just started using it, I don't know how well it works yet, I hope it's good.	Jingdong prefecture level city in Shandong
Jinfeng Hongyuan Flagship Store	Electricity savings3	Fourth purchase, great baby!	Jingdong prefecture level city in Shandong
Jinfeng Hongyuan Flagship Store	Electricity savings3	Shipped really fast, just got it and use it to see what happens and then come back for a review!	Jingdong prefecture level city in Shandong
Jinfeng Hongyuan Flagship Store	Electricity savings3	Timely delivery **** good attitude	Jingdong prefecture level city in Shandong
Jinfeng Hongyuan Flagship Store	Electricity savings3	Home large and small things are purchased in the Jingdong Mall, have been accustomed to shopping in the Jingdong, many times can buy good quality and cheap products, and courier service attitude is particularly good, sometimes too many couriers are to help me to the home, so many years have been in the Jingdong Mall shopping, trust and support! Will also support the Jingdong as always!	Jingdong prefecture level city in Shandong
Jinfeng Hongyuan Flagship Store	Electricity savings3	Modified a pump and the modification worked.	Jingdong prefecture level city in Shandong
Jinfeng Hongyuan Flagship Store	Electricity savings3	Already installed, next time you pay the electric bill to see the results	Jingdong prefecture level city in Shandong
Jinfeng Hongyuan Flagship Store	Electricity savings3	The product is very good to use and practical to look at	Jingdong prefecture level city in Shandong
Jinfeng Hongyuan Flagship Store	Electricity savings3	Works great, great service, waiting for results.	Jingdong prefecture level city in Shandong
Jinfeng Hongyuan Flagship Store	Electricity savings3	I just used it and I'm not sure about the efficacy yet, I'll come back to evaluate it after a while.	Jingdong prefecture level city in Shandong
Jinfeng Hongyuan Flagship Store	Electricity savings3	Just received the goods, use a period of time to evaluate	Jingdong prefecture level city in Shandong
Jinfeng Hongyuan Flagship Store	Electricity savings3	Opened and tried very good, use it to see if there are results	Jingdong prefecture level city in Shandong
Jinfeng Hongyuan Flagship Store	Electricity savings3	Arrived today! Still trying it out!	Jingdong prefecture level city in Shandong
Jinfeng Hongyuan Flagship Store	Electricity savings3	Shipped quickly, well packaged, already installed, hope there is a power saving effect	Jingdong prefecture level city in Shandong
Jinfeng Hongyuan Flagship Store	Electricity savings3	I got it. It's great.	Jingdong prefecture level city in Shandong
Jinfeng Hongyuan Flagship Store	Electricity savings3	Haven't used it yet, will come back in a month	Jingdong prefecture level city in Shandong
Jinfeng Hongyuan Flagship Store	Electricity savings3	It looks good. It's good. Try it first.	Jingdong prefecture level city in Shandong
Jinfeng Hongyuan Flagship Store	Electricity savings3	It works so well that I have relatives, friends, coworkers, and 6 families that have purchased it for them through me.	Jingdong prefecture level city in Shandong
Jinfeng Hongyuan Flagship Store	Electricity savings3	In use	Jingdong prefecture level city in Shandong
Jinfeng Hongyuan Flagship Store	Electricity savings3	Received the goods today, power-saving effect do not know being used, the boss has a very good attitude, praise!	Jingdong prefecture level city in Shandong
Jinfeng Hongyuan Flagship Store	Electricity savings3	The product is compact and doesn't take up much space, but hopefully it's power efficient.	Jingdong prefecture level city in Shandong
Jinfeng Hongyuan Flagship Store	Electricity savings3	Power saving is indeed effective, I used for a period of time before coming to evaluate, the electricity bill is obviously less, the quality is good, the shopkeeper service is also very good, very patient,	Jingdong prefecture level city in Shandong
Jinfeng Hongyuan Flagship Store	Electricity savings3	It feels fine, I'll comment after I use it	Jingdong prefecture level city in Shandong
Jinfeng Hongyuan Flagship Store	Electricity savings3	I just received it and I don't know the effect yet! I will comment again after I use it and have results!	Jingdong prefecture level city in Shandong
Jinfeng Hongyuan Flagship Store	Electricity savings3	The goods look fine, I hope they really work!	Jingdong prefecture level city in Shandong
Jinfeng Hongyuan Flagship Store	Electricity savings3	I just don't know if it saves power	Jingdong prefecture level city in Shandong
Jinfeng Hongyuan Flagship Store	Electricity savings3	My friend introduced me to use this product, it is professional and I am looking forward to the long term effect~!	Jingdong prefecture level city in Shandong
Jinfeng Hongyuan Flagship Store	Electricity savings3	Try it before you judge.	Jingdong prefecture level city in Shandong
Jinfeng Hongyuan Flagship Store	Electricity savings3	It's great, the power savings are obvious! Kudos!	Jingdong prefecture level city in Shandong
Jinfeng Hongyuan Flagship Store	Electricity savings3	The quality is excellent, stabilizes the voltage and saves electricity, very satisfied.	Jingdong prefecture level city in Shandong
Jinfeng Hongyuan Flagship Store	Electricity savings3	You have to use it for a while to know the effect	Jingdong prefecture level city in Shandong
Jinfeng Hongyuan Flagship Store	Electricity savings3	It's on trial.	Jingdong prefecture level city in Shandong
Jinfeng Hongyuan Flagship Store	Electricity savings3	The stuff works great.	Jingdong prefecture level city in Shandong
Jinfeng Hongyuan Flagship Store	Electricity savings3	Doesn't seem to work, not as god as other reviews	Jingdong prefecture level city in Shandong
Jinfeng Hongyuan Flagship Store	Electricity savings3	It's not good quality, it doesn't work, it stops working after a few days, it doesn't work, the customer service doesn't care, it's a bit irritating.	Jingdong prefecture level city in Shandong
Jinfeng Hongyuan Flagship Store	Electricity savings3	It doesn't do anything.	Jingdong prefecture level city in Shandong
Jinfeng Hongyuan Flagship Store	Electricity savings3	It's good.	Jingdong prefecture level city in Shandong
Jinfeng Hongyuan Flagship Store	Electricity savings3	Used a month effect is not obvious, are psychological effects, feel are not less, did not save to the electricity, there are details of the problem, the store said to buy to send 5 fuse, in fact, received only three, small problems are not committed to in place, I believe that the product is not good enough	Jingdong prefecture level city in Shandong
Jinfeng Hongyuan Flagship Store	Electricity savings3	Well received quickly, looking quite high, but also sent 5 fuses. Customer service is very patient to teach the operation, in fact, quite simple, lazy to read the instructions ha!	Jingdong prefecture level city in Shandong
Jinfeng Hongyuan Flagship Store	Electricity savings3	You have to use it for a while to know the effect	Jingdong prefecture level city in Shandong
Jinfeng Hongyuan Flagship Store	Electricity savings3	Shipped very quickly, packaged very well, the price is particularly affordable, there are so many efficacy is also good, it is worth buying worth buying the product, the workmanship is very fine, better than imagined, recommended that everyone try not yet used, after the use of the follow-up comment	Jingdong prefecture level city in Shandong
Jinfeng Hongyuan Flagship Store	Electricity savings3	It looks regular. It's working now.	Jingdong prefecture level city in Shandong
Jinfeng Hongyuan Flagship Store	Electricity savings3	It arrived pretty fast and the owner is very responsible. Have not used it yet, will see after use	Jingdong prefecture level city in Shandong

Jinfeng Hongyuan Flagship Store	Electricity savings3	🎁 baby quality is very good, the first time I received the goods to open the first time to see, it is very good 👍, very satisfied 😊, customer service also have a question and answer, thank you 🙏	Jingdong prefecture level city in Shandong
Jinfeng Hongyuan Flagship Store	Electricity savings3	I don't know if it's any good 。。。。。。	Jingdong prefecture level city in Shandong
Jinfeng Hongyuan Flagship Store	Electricity savings3	Buy electronic products! Still trust the things of the Jingdong self-owned store! Energy-saving effect to evaluate after heating!	Jingdong prefecture level city in Shandong
Jinfeng Hongyuan Flagship Store	Electricity savings3	The goods have been received, Blu is very beautiful, energy saving effect, to be confirmed only after actual use.	Jingdong prefecture level city in Shandong
Jinfeng Hongyuan Flagship Store	Electricity savings3	Jingdong's speed is so fast, has received, easy to operate. The display is quite good, how many volts what mode is quite understandable.	Jingdong prefecture level city in Shandong
Jinfeng Hongyuan Flagship Store	Electricity savings3	The first use, the effect is good to recommend it to colleagues to use, a month of electricity to save a lot or can be	Jingdong prefecture level city in Shandong
Jinfeng Hongyuan Flagship Store	Electricity savings3	I don't know if it's useful. Mom's is a store. I'll return it if it doesn't work.	Jingdong prefecture level city in Shandong
Jinfeng Hongyuan Flagship Store	Electricity savings3	I don't know if it works or not, I'll wait for two months to use it and then come back to comment!	Jingdong prefecture level city in Shandong
Jinfeng Hongyuan Flagship Store	Electricity savings3	I bought the second one now, and I've been using it for more than half a year, and I feel that it really saves electricity. Now it is summer, the weather is hot, sometimes the fan and air conditioning on all day, if not this power saver, the monthly electricity bill is quite high, but as long as the installation of this power saver, not afraid of its high electricity bill, absolutely let you wantonly use	Jingdong prefecture level city in Shandong
Jinfeng Hongyuan Flagship Store	Electricity savings3	The product is better than I thought it would be, the quality looks great, the voltage is stable, and I'm looking forward to some great power savings!	Jingdong prefecture level city in Shandong
Jinfeng Hongyuan Flagship Store	Electricity savings3	Inadvertently see bought a try, delivery is also quite timely, just do not know how to use, customer service patience to answer, with a pretty good, value for money!	Jingdong prefecture level city in Shandong
Jinfeng Hongyuan Flagship Store	Electricity savings3	It's better than nothing! Hopefully this will help save slightly.	Jingdong prefecture level city in Shandong
Jinfeng Hongyuan Flagship Store	Electricity savings3	Still trying it out, follow up	Jingdong prefecture level city in Shandong
Jinfeng Hongyuan Flagship Store	Electricity savings3	I've just started using it, I don't know how well it works yet, I hope it's good.	Jingdong prefecture level city in Shandong
Jinfeng Hongyuan Flagship Store	Electricity savings3	Fourth purchase, great baby!	Jingdong prefecture level city in Shandong
Jinfeng Hongyuan Flagship Store	Electricity savings3	Shipped really fast, just got it and use it to see what happens and then come back for a review!	Jingdong prefecture level city in Shandong
Jinfeng Hongyuan Flagship Store	Electricity savings3	Timely delivery **** good attitude	Jingdong prefecture level city in Shandong
Jinfeng Hongyuan Flagship Store	Electricity savings3	Home large and small things are purchased in the Jingdong Mall, have been accustomed to shopping in the Jingdong, many times can buy good quality and cheap products, and courier service attitude is particularly good, sometimes too many couriers are to help me to the home, so many years have been in the Jingdong Mall shopping, trust and support! Will also support the Jingdong as always!	Jingdong prefecture level city in Shandong
Jinfeng Hongyuan Flagship Store	Electricity savings3	Modified a pump and the modification worked.	Jingdong prefecture level city in Shandong
Jinfeng Hongyuan Flagship Store	Electricity savings3	Already installed, next time you pay the electric bill to see the results	Jingdong prefecture level city in Shandong
Jinfeng Hongyuan Flagship Store	Electricity savings3	The product is very good to use and practical to look at	Jingdong prefecture level city in Shandong
Jinfeng Hongyuan Flagship Store	Electricity savings3	Works great, great service, waiting for results.	Jingdong prefecture level city in Shandong
Jinfeng Hongyuan Flagship Store	Electricity savings3	I just used it and I'm not sure about the efficacy yet, I'll come back to evaluate it after a while.	Jingdong prefecture level city in Shandong
Jinfeng Hongyuan Flagship Store	Electricity savings3	Just received the goods, use a period of time to evaluate	Jingdong prefecture level city in Shandong
Jinfeng Hongyuan Flagship Store	Electricity savings3	Opened and tried very good, use it to see if there are results	Jingdong prefecture level city in Shandong
Jinfeng Hongyuan Flagship Store	Electricity savings3	Arrived today! Still trying it out!	Jingdong prefecture level city in Shandong
Jinfeng Hongyuan Flagship Store	Electricity savings3	Shipped quickly, well packaged, already installed, hope there is a power saving effect	Jingdong prefecture level city in Shandong
Jinfeng Hongyuan Flagship Store	Electricity savings3	I got it. It's great.	Jingdong prefecture level city in Shandong
Jinfeng Hongyuan Flagship Store	Electricity savings3	Haven't used it yet, will come back in a month	Jingdong prefecture level city in Shandong
Jinfeng Hongyuan Flagship Store	Electricity savings3	It looks good. It's good. Try it first.	Jingdong prefecture level city in Shandong
Jinfeng Hongyuan Flagship Store	Electricity savings3	It works so well that I have relatives, friends, coworkers, and 6 families that have purchased it for them through me.	Jingdong prefecture level city in Shandong
Jinfeng Hongyuan Flagship Store	Electricity savings3	In use	Jingdong prefecture level city in Shandong
Jinfeng Hongyuan Flagship Store	Electricity savings3	Received the goods today, power-saving effect do not know being used, the boss has a very good attitude, praise!	Jingdong prefecture level city in Shandong
Jinfeng Hongyuan Flagship Store	Electricity savings3	The product is compact and doesn't take up much space, but hopefully it's power efficient.	Jingdong prefecture level city in Shandong
Jinfeng Hongyuan Flagship Store	Electricity savings3	Power saving is indeed effective, I used for a period of time before coming to evaluate, the electricity bill is obviously less, the quality is good, the shopkeeper service is also very good, very patient,	Jingdong prefecture level city in Shandong
Jinfeng Hongyuan Flagship Store	Electricity savings3	It feels fine, I'll comment after I use it	Jingdong prefecture level city in Shandong
Jinfeng Hongyuan Flagship Store	Electricity savings3	I just received it and I don't know the effect yet! I will comment again after I use it and have results!	Jingdong prefecture level city in Shandong
Jinfeng Hongyuan Flagship Store	Electricity savings3	The goods look fine, I hope they really work!	Jingdong prefecture level city in Shandong
Jinfeng Hongyuan Flagship Store	Electricity savings3	I just don't know if it saves power	Jingdong prefecture level city in Shandong
Jinfeng Hongyuan Flagship Store	Electricity savings3	My friend introduced me to use this product, it is professional and I am looking forward to the long term effect~!	Jingdong prefecture level city in Shandong
Jinfeng Hongyuan Flagship Store	Electricity savings3	Try it before you judge.	Jingdong prefecture level city in Shandong
Jinfeng Hongyuan Flagship Store	Electricity savings3	It's great, the power savings are obvious! Kudos!	Jingdong prefecture level city in Shandong
Jinfeng Hongyuan Flagship Store	Electricity savings3	The quality is excellent, stabilizes the voltage and saves electricity, very satisfied.	Jingdong prefecture level city in Shandong
Jinfeng Hongyuan Flagship Store	Electricity savings3	You have to use it for a while to know the effect	Jingdong prefecture level city in Shandong
Jinfeng Hongyuan Flagship Store	Electricity savings3	It's on trial.	Jingdong prefecture level city in Shandong
Jinfeng Hongyuan Flagship Store	Electricity savings3	The stuff works great.	Jingdong prefecture level city in Shandong
Jinfeng Hongyuan Flagship Store	Electricity savings3	Doesn't seem to work, not as god as other reviews	Jingdong prefecture level city in Shandong
Jinfeng Hongyuan Flagship Store	Electricity savings3	It's not good quality, it doesn't work, it stops working after a few days, it doesn't work, the customer service doesn't care, it's a bit irritating.	Jingdong prefecture level city in Shandong
Jinfeng Hongyuan Flagship Store	Electricity savings3	It doesn't do anything.	Jingdong prefecture level city in Shandong

Jinfeng Hongyuan Flagship Store	Electricity savings3	It's good.	Jingdong prefecture level city in Shandong
Jinfeng Hongyuan Flagship Store	Electricity savings3	Used a month effect is not obvious, are psychological effects, feel are not less, did not save to the electricity, there are details of the problem, the store said to buy to send 5 fuse, in fact, received only three, small problems are not committed to in place, I believe that the product is not good enough	Jingdong prefecture level city in Shandong
Jinfeng Hongyuan Flagship Store	Electricity savings3	Well received quickly, looking quite high, but also sent 5 fuses. Customer service is very patient to teach the operation, in fact, quite simple, lazy to read the instructions ha!	Jingdong prefecture level city in Shandong
Jinfeng Hongyuan Flagship Store	Electricity savings3	You have to use it for a while to know the effect	Jingdong prefecture level city in Shandong
Jinfeng Hongyuan Flagship Store	Electricity savings3	Shipped very quickly, packaged very well, the price is particularly affordable, there are so many efficacy is also good, it is worth buying worth buying the product, the workmanship is very fine, better than imagined, recommended that everyone try not yet used, after the use of the follow-up comment	Jingdong prefecture level city in Shandong
Jinfeng Hongyuan Flagship Store	Electricity savings3	It looks regular. It's working now.	Jingdong prefecture level city in Shandong
Jinfeng Hongyuan Flagship Store	Electricity savings3	It arrived pretty fast and the owner is very responsible. Have not used it yet, will see after use	Jingdong prefecture level city in Shandong
Jinfeng Hongyuan Flagship Store	Electricity savings3	🎁 baby quality is very good, the first time I received the goods to open the first time to see, it is very good 👍, very satisfied 😊, customer service also have a question and answer, thank you 🙏	Jingdong prefecture level city in Shandong
Jinfeng Hongyuan Flagship Store	Electricity savings3	I don't know if it's any good 。。。。。。	Jingdong prefecture level city in Shandong
Jinfeng Hongyuan Flagship Store	Electricity savings3	Buy electronic products! Still trust the things of the Jingdong self-owned store! Energy-saving effect to evaluate after heating!	Jingdong prefecture level city in Shandong
Jinfeng Hongyuan Flagship Store	Electricity savings3	The goods have been received, Blu is very beautiful, energy saving effect, to be confirmed only after actual use.	Jingdong prefecture level city in Shandong
Jinfeng Hongyuan Flagship Store	Electricity savings3	Jingdong's speed is so fast, has received, easy to operate. The display is quite good, how many volts what mode is quite understandable.	Jingdong prefecture level city in Shandong
Jinfeng Hongyuan Flagship Store	Electricity savings3	The first use, the effect is good to recommend it to colleagues to use, a month of electricity to save a lot or can be	Jingdong prefecture level city in Shandong
Jinfeng Hongyuan Flagship Store	Electricity savings3	I don't know if it's useful. Mom's is a store. I'll return it if it doesn't work.	Jingdong prefecture level city in Shandong
Jinfeng Hongyuan Flagship Store	Electricity savings3	I don't know if it works or not, I'll wait for two months to use it and then come back to comment!	Jingdong prefecture level city in Shandong
Jinfeng Hongyuan Flagship Store	Electricity savings3	I bought the second one now, and I've been using it for more than half a year, and I feel that it really saves electricity. Now it is summer, the weather is hot, sometimes the fan and air conditioning on all day, if not this power saver, the monthly electricity bill is quite high, but as long as the installation of this power saver, not afraid of its high electricity bill, absolutely let you wantonly use	Jingdong prefecture level city in Shandong
Jinfeng Hongyuan Flagship Store	Electricity savings3	The product is better than I thought it would be, the quality looks great, the voltage is stable, and I'm looking forward to some great power savings!	Jingdong prefecture level city in Shandong
Jinfeng Hongyuan Flagship Store	Electricity savings3	Inadvertently see bought a try, delivery is also quite timely, just do not know how to use, customer service patience to answer, with a pretty good, value for money!	Jingdong prefecture level city in Shandong
Jinfeng Hongyuan Flagship Store	Electricity savings3	It's better than nothing! Hopefully this will help save slightly.	Jingdong prefecture level city in Shandong
Jinfeng Hongyuan Flagship Store	Electricity savings3	Still trying it out, follow up	Jingdong prefecture level city in Shandong
Jinfeng Hongyuan Flagship Store	Electricity savings3	I've just started using it, I don't know how well it works yet, I hope it's good.	Jingdong prefecture level city in Shandong
Jinfeng Hongyuan Flagship Store	Electricity savings3	Fourth purchase, great baby!	Jingdong prefecture level city in Shandong
Jinfeng Hongyuan Flagship Store	Electricity savings3	Shipped really fast, just got it and use it to see what happens and then come back for a review!	Jingdong prefecture level city in Shandong
Jinfeng Hongyuan Flagship Store	Electricity savings3	Timely delivery **** good attitude	Jingdong prefecture level city in Shandong
Jinfeng Hongyuan Flagship Store	Electricity savings3	Home large and small things are purchased in the Jingdong Mall, have been accustomed to shopping in the Jingdong, many times can buy good quality and cheap products, and courier service attitude is particularly good, sometimes too many couriers are to help me to the home, so many years have been in the Jingdong Mall shopping, trust and support! Will also support the Jingdong as always!	Jingdong prefecture level city in Shandong
Jinfeng Hongyuan Flagship Store	Electricity savings3	Modified a pump and the modification worked.	Jingdong prefecture level city in Shandong
Jinfeng Hongyuan Flagship Store	Electricity savings3	Already installed, next time you pay the electric bill to see the results	Jingdong prefecture level city in Shandong
Jinfeng Hongyuan Flagship Store	Electricity savings3	The product is very good to use and practical to look at	Jingdong prefecture level city in Shandong
Jinfeng Hongyuan Flagship Store	Electricity savings3	Works great, great service, waiting for results.	Jingdong prefecture level city in Shandong
Jinfeng Hongyuan Flagship Store	Electricity savings3	I just used it and I'm not sure about the efficacy yet, I'll come back to evaluate it after a while.	Jingdong prefecture level city in Shandong
Jinfeng Hongyuan Flagship Store	Electricity savings3	Just received the goods, use a period of time to evaluate	Jingdong prefecture level city in Shandong
Jinfeng Hongyuan Flagship Store	Electricity savings3	Opened and tried very good, use it to see if there are results	Jingdong prefecture level city in Shandong
Jinfeng Hongyuan Flagship Store	Electricity savings3	Arrived today! Still trying it out!	Jingdong prefecture level city in Shandong
Jinfeng Hongyuan Flagship Store	Electricity savings3	Shipped quickly, well packaged, already installed, hope there is a power saving effect	Jingdong prefecture level city in Shandong
Jinfeng Hongyuan Flagship Store	Electricity savings3	I got it. It's great.	Jingdong prefecture level city in Shandong
Jinfeng Hongyuan Flagship Store	Electricity savings3	Haven't used it yet, will come back in a month	Jingdong prefecture level city in Shandong
Jinfeng Hongyuan Flagship Store	Electricity savings3	It looks good. It's good. Try it first.	Jingdong prefecture level city in Shandong
Jinfeng Hongyuan Flagship Store	Electricity savings3	It works so well that I have relatives, friends, coworkers, and 6 families that have purchased it for them through me.	Jingdong prefecture level city in Shandong
Jinfeng Hongyuan Flagship Store	Electricity savings3	In use	Jingdong prefecture level city in Shandong
Jinfeng Hongyuan Flagship Store	Electricity savings3	Received the goods today, power-saving effect do not know being used, the boss has a very good attitude, praise!	Jingdong prefecture level city in Shandong
Jinfeng Hongyuan Flagship Store	Electricity savings3	The product is compact and doesn't take up much space, but hopefully it's power efficient.	Jingdong prefecture level city in Shandong
Jinfeng Hongyuan Flagship Store	Electricity savings3	Power saving is indeed effective, I used for a period of time before coming to evaluate, the electricity bill is obviously less, the quality is good, the shopkeeper service is also very good, very patient,	Jingdong prefecture level city in Shandong
Jinfeng Hongyuan Flagship Store	Electricity savings3	It feels fine, I'll comment after I use it	Jingdong prefecture level city in Shandong
Jinfeng Hongyuan Flagship Store	Electricity savings3	I just received it and I don't know the effect yet! I will comment again after I use it and have results!	Jingdong prefecture level city in Shandong
Jinfeng Hongyuan Flagship Store	Electricity savings3	The goods look fine, I hope they really work!	Jingdong prefecture level city in Shandong
Jinfeng Hongyuan Flagship Store	Electricity savings3	I just don't know if it saves power	Jingdong prefecture level city in Shandong
Jinfeng Hongyuan Flagship Store	Electricity savings3	My friend introduced me to use this product, it is professional and I am looking forward to the long term effect~!	Jingdong prefecture level city in Shandong
Jinfeng Hongyuan Flagship Store	Electricity savings3	Try it before you judge.	Jingdong prefecture level city in Shandong

Jinfeng Hongyuan Flagship Store	Electricity savings3	It's great, the power savings are obvious! Kudos!	Jingdong prefecture level city in Shandong
Jinfeng Hongyuan Flagship Store	Electricity savings3	The quality is excellent, stabilizes the voltage and saves electricity, very satisfied.	Jingdong prefecture level city in Shandong
Jinfeng Hongyuan Flagship Store	Electricity savings3	You have to use it for a while to know the effect	Jingdong prefecture level city in Shandong
Jinfeng Hongyuan Flagship Store	Electricity savings3	It's on trial.	Jingdong prefecture level city in Shandong
Jinfeng Hongyuan Flagship Store	Electricity savings3	The stuff works great.	Jingdong prefecture level city in Shandong
Jinfeng Hongyuan Flagship Store	Electricity savings3	Doesn't seem to work, not as god as other reviews	Jingdong prefecture level city in Shandong
Jinfeng Hongyuan Flagship Store	Electricity savings3	It's not good quality, it doesn't work, it stops working after a few days, it doesn't work, the customer service doesn't care, it's a bit irritating.	Jingdong prefecture level city in Shandong
Jinfeng Hongyuan Flagship Store	Electricity savings3	It doesn't do anything.	Jingdong prefecture level city in Shandong
Jinfeng Hongyuan Flagship Store	Electricity savings3	It's good.	Jingdong prefecture level city in Shandong
Jinfeng Hongyuan Flagship Store	Electricity savings3	Used a month effect is not obvious, are psychological effects, feel are not less, did not save to the electricity, there are details of the problem, the store said to buy to send 5 fuse, in fact, received only three, small problems are not committed to in place, I believe that the product is not good enough	Jingdong prefecture level city in Shandong
Jinfeng Hongyuan Flagship Store	Electricity savings3	Well received quickly, looking quite high, but also sent 5 fuses. Customer service is very patient to teach the operation, in fact, quite simple, lazy to read the instructions ha!	Jingdong prefecture level city in Shandong
Jinfeng Hongyuan Flagship Store	Electricity savings3	You have to use it for a while to know the effect	Jingdong prefecture level city in Shandong
Jinfeng Hongyuan Flagship Store	Electricity savings3	Shipped very quickly, packaged very well, the price is particularly affordable, there are so many efficacy is also good, it is worth buying worth buying the product, the workmanship is very fine, better than imagined, recommended that everyone try not yet used, after the use of the follow-up comment	Jingdong prefecture level city in Shandong
Jinfeng Hongyuan Flagship Store	Electricity savings3	It looks regular. It's working now.	Jingdong prefecture level city in Shandong
Jinfeng Hongyuan Flagship Store	Electricity savings3	It arrived pretty fast and the owner is very responsible. Have not used it yet, will see after use	Jingdong prefecture level city in Shandong
Jinfeng Hongyuan Flagship Store	Electricity savings3	🎁 baby quality is very good, the first time I received the goods to open the first time to see, it is very good 👍, very satisfied 😃, customer service also have a question and answer, thank you 🙏	Jingdong prefecture level city in Shandong
Jinfeng Hongyuan Flagship Store	Electricity savings3	I don't know if it's any good 。。。。。。	Jingdong prefecture level city in Shandong
Jinfeng Hongyuan Flagship Store	Electricity savings3	Buy electronic products! Still trust the things of the Jingdong self-owned store! Energy-saving effect to evaluate after heating!	Jingdong prefecture level city in Shandong
Jinfeng Hongyuan Flagship Store	Electricity savings3	The goods have been received, Blu is very beautiful, energy saving effect, to be confirmed only after actual use.	Jingdong prefecture level city in Shandong
Jinfeng Hongyuan Flagship Store	Electricity savings3	Jingdong's speed is so fast, has received, easy to operate. The display is quite good, how many volts what mode is quite understandable.	Jingdong prefecture level city in Shandong
Jinfeng Hongyuan Flagship Store	Electricity savings3	The first use, the effect is good to recommend it to colleagues to use, a month of electricity to save a lot or can be	Jingdong prefecture level city in Shandong
Jinfeng Hongyuan Flagship Store	Electricity savings3	I don't know if it's useful. Mom's is a store. I'll return it if it doesn't work.	Jingdong prefecture level city in Shandong
Jinfeng Hongyuan Flagship Store	Electricity savings3	I don't know if it works or not, I'll wait for two months to use it and then come back to comment!	Jingdong prefecture level city in Shandong
Jinfeng Hongyuan Flagship Store	Electricity savings3	I bought the second one now, and I've been using it for more than half a year, and I feel that it really saves electricity. Now it is summer, the weather is hot, sometimes the fan and air conditioning on all day, if not this power saver, the monthly electricity bill is quite high, but as long as the installation of this power saver, not afraid of its high electricity bill, absolutely let you wantonly use	Jingdong prefecture level city in Shandong
Jinfeng Hongyuan Flagship Store	Electricity savings3	The product is better than I thought it would be, the quality looks great, the voltage is stable, and I'm looking forward to some great power savings!	Jingdong prefecture level city in Shandong
Jinfeng Hongyuan Flagship Store	Electricity savings3	Inadvertently see bought a try, delivery is also quite timely, just do not know how to use, customer service patience to answer, with a pretty good, value for money!	Jingdong prefecture level city in Shandong
Jinfeng Hongyuan Flagship Store	Electricity savings3	It's better than nothing! Hopefully this will help save slightly.	Jingdong prefecture level city in Shandong
Jinfeng Hongyuan Flagship Store	Electricity savings3	Still trying it out, follow up	Jingdong prefecture level city in Shandong
Jinfeng Hongyuan Flagship Store	Electricity savings3	I've just started using it, I don't know how well it works yet, I hope it's good.	Jingdong prefecture level city in Shandong
Jinfeng Hongyuan Flagship Store	Electricity savings3	Fourth purchase, great baby!	Jingdong prefecture level city in Shandong
Jinfeng Hongyuan Flagship Store	Electricity savings3	Shipped really fast, just got it and use it to see what happens and then come back for a review!	Jingdong prefecture level city in Shandong
Jinfeng Hongyuan Flagship Store	Electricity savings3	Timely delivery **** good attitude	Jingdong prefecture level city in Shandong
Jinfeng Hongyuan Flagship Store	Electricity savings3	Home large and small things are purchased in the Jingdong Mall, have been accustomed to shopping in the Jingdong, many times can buy good quality and cheap products, and courier service attitude is particularly good, sometimes too many couriers are to help me to the home, so many years have been in the Jingdong Mall shopping, trust and support! Will also support the Jingdong as always!	Jingdong prefecture level city in Shandong
Jinfeng Hongyuan Flagship Store	Electricity savings3	Modified a pump and the modification worked.	Jingdong prefecture level city in Shandong
Jinfeng Hongyuan Flagship Store	Electricity savings3	Already installed, next time you pay the electric bill to see the results	Jingdong prefecture level city in Shandong
Jinfeng Hongyuan Flagship Store	Electricity savings3	The product is very good to use and practical to look at	Jingdong prefecture level city in Shandong
Jinfeng Hongyuan Flagship Store	Electricity savings3	Works great, great service, waiting for results.	Jingdong prefecture level city in Shandong
Jinfeng Hongyuan Flagship Store	Electricity savings3	I just used it and I'm not sure about the efficacy yet, I'll come back to evaluate it after a while.	Jingdong prefecture level city in Shandong
Jinfeng Hongyuan Flagship Store	Electricity savings3	Just received the goods, use a period of time to evaluate	Jingdong prefecture level city in Shandong
Jinfeng Hongyuan Flagship Store	Electricity savings3	Opened and tried very good, use it to see if there are results	Jingdong prefecture level city in Shandong
Jinfeng Hongyuan Flagship Store	Electricity savings3	Arrived today! Still trying it out!	Jingdong prefecture level city in Shandong
Jinfeng Hongyuan Flagship Store	Electricity savings3	Shipped quickly, well packaged, already installed, hope there is a power saving effect	Jingdong prefecture level city in Shandong
Jinfeng Hongyuan Flagship Store	Electricity savings3	I got it. It's great.	Jingdong prefecture level city in Shandong
Jinfeng Hongyuan Flagship Store	Electricity savings3	Haven't used it yet, will come back in a month	Jingdong prefecture level city in Shandong
Jinfeng Hongyuan Flagship Store	Electricity savings3	It looks good. It's good. Try it first.	Jingdong prefecture level city in Shandong
Jinfeng Hongyuan Flagship Store	Electricity savings3	It works so well that I have relatives, friends, coworkers, and 6 families that have purchased it for them through me.	Jingdong prefecture level city in Shandong
Jinfeng Hongyuan Flagship Store	Electricity savings3	In use	Jingdong prefecture level city in Shandong
Jinfeng Hongyuan Flagship Store	Electricity savings3	Received the goods today, power-saving effect do not know being used, the boss has a very good attitude, praise!	Jingdong prefecture level city in Shandong

Jinfeng Hongyuan Flagship Store	Electricity savings3	The product is compact and doesn't take up much space, but hopefully it's power efficient.	Jingdong prefecture level city in Shandong
Jinfeng Hongyuan Flagship Store	Electricity savings3	Power saving is indeed effective, I used for a period of time before coming to evaluate, the electricity bill is obviously less, the quality is good, the shopkeeper service is also very good, very patient,	Jingdong prefecture level city in Shandong
Jinfeng Hongyuan Flagship Store	Electricity savings3	It feels fine, I'll comment after I use it	Jingdong prefecture level city in Shandong
Jinfeng Hongyuan Flagship Store	Electricity savings3	I just received it and I don't know the effect yet! I will comment again after I use it and have results!	Jingdong prefecture level city in Shandong
Jinfeng Hongyuan Flagship Store	Electricity savings3	The goods look fine, I hope they really work!	Jingdong prefecture level city in Shandong
Jinfeng Hongyuan Flagship Store	Electricity savings3	I just don't know if it saves power	Jingdong prefecture level city in Shandong
Jinfeng Hongyuan Flagship Store	Electricity savings3	My friend introduced me to use this product, it is professional and I am looking forward to the long term effect~!	Jingdong prefecture level city in Shandong
Jinfeng Hongyuan Flagship Store	Electricity savings3	Try it before you judge.	Jingdong prefecture level city in Shandong
Jinfeng Hongyuan Flagship Store	Electricity savings3	It's great, the power savings are obvious! Kudos!	Jingdong prefecture level city in Shandong
Jinfeng Hongyuan Flagship Store	Electricity savings3	The quality is excellent, stabilizes the voltage and saves electricity, very satisfied.	Jingdong prefecture level city in Shandong
Jinfeng Hongyuan Flagship Store	Electricity savings3	You have to use it for a while to know the effect	Jingdong prefecture level city in Shandong
Jinfeng Hongyuan Flagship Store	Electricity savings3	It's on trial.	Jingdong prefecture level city in Shandong
Jinfeng Hongyuan Flagship Store	Electricity savings3	The stuff works great.	Jingdong prefecture level city in Shandong
Jinfeng Hongyuan Flagship Store	Electricity savings3	Doesn't seem to work, not as god as other reviews	Jingdong prefecture level city in Shandong
Jinfeng Hongyuan Flagship Store	Electricity savings3	It's not good quality, it doesn't work, it stops working after a few days, it doesn't work, the customer service doesn't care, it's a bit irritating.	Jingdong prefecture level city in Shandong
Jinfeng Hongyuan Flagship Store	Electricity savings3	It doesn't do anything.	Jingdong prefecture level city in Shandong
Jinfeng Hongyuan Flagship Store	Electricity savings3	It's good.	Jingdong prefecture level city in Shandong
Jinfeng Hongyuan Flagship Store	Electricity savings3	Used a month effect is not obvious, are psychological effects, feel are not less, did not save to the electricity, there are details of the problem, the store said to buy to send 5 fuse, in fact, received only three, small problems are not committed to in place, I believe that the product is not good enough	Jingdong prefecture level city in Shandong
Jinfeng Hongyuan Flagship Store	Electricity savings3	Well received quickly, looking quite high, but also sent 5 fuses. Customer service is very patient to teach the operation, in fact, quite simple, lazy to read the instructions ha!	Jingdong prefecture level city in Shandong
Jinfeng Hongyuan Flagship Store	Electricity savings3	You have to use it for a while to know the effect	Jingdong prefecture level city in Shandong
Jinfeng Hongyuan Flagship Store	Electricity savings3	Shipped very quickly, packaged very well, the price is particularly affordable, there are so many efficacy is also good, it is worth buying worth buying the product, the workmanship is very fine, better than imagined, recommended that everyone try not yet used, after the use of the follow-up comment	Jingdong prefecture level city in Shandong
Jinfeng Hongyuan Flagship Store	Electricity savings3	It looks regular. It's working now.	Jingdong prefecture level city in Shandong
Jinfeng Hongyuan Flagship Store	Electricity savings3	It arrived pretty fast and the owner is very responsible. Have not used it yet, will see after use	Jingdong prefecture level city in Shandong
Jinfeng Hongyuan Flagship Store	Electricity savings3	🎁 baby quality is very good, the first time I received the goods to open the first time to see, it is very good 👍, very satisfied 😊, customer service also have a question and answer, thank you 🙏	Jingdong prefecture level city in Shandong
Jinfeng Hongyuan Flagship Store	Electricity savings3	I don't know if it's any good 。。。。。。	Jingdong prefecture level city in Shandong
Jinfeng Hongyuan Flagship Store	Electricity savings3	Buy electronic products! Still trust the things of the Jingdong self-owned store! Energy-saving effect to evaluate after heating!	Jingdong prefecture level city in Shandong
Jinfeng Hongyuan Flagship Store	Electricity savings3	The goods have been received, Blu is very beautiful, energy saving effect, to be confirmed only after actual use.	Jingdong prefecture level city in Shandong
Jinfeng Hongyuan Flagship Store	Electricity savings3	Jingdong's speed is so fast, has received, easy to operate. The display is quite good, how many volts what mode is quite understandable.	Jingdong prefecture level city in Shandong
Jinfeng Hongyuan Flagship Store	Electricity savings3	The first use, the effect is good to recommend it to colleagues to use, a month of electricity to save a lot or can be	Jingdong prefecture level city in Shandong
Jinfeng Hongyuan Flagship Store	Electricity savings3	I don't know if it's useful. Mom's is a store. I'll return it if it doesn't work.	Jingdong prefecture level city in Shandong
Jinfeng Hongyuan Flagship Store	Electricity savings3	I don't know if it works or not, I'll wait for two months to use it and then come back to comment!	Jingdong prefecture level city in Shandong
Jinfeng Hongyuan Flagship Store	Electricity savings3	I bought the second one now, and I've been using it for more than half a year, and I feel that it really saves electricity. Now it is summer, the weather is hot, sometimes the fan and air conditioning on all day, if not this power saver, the monthly electricity bill is quite high, but as long as the installation of this power saver, not afraid of its high electricity bill, absolutely let you wantonly use	Jingdong prefecture level city in Shandong
Jinfeng Hongyuan Flagship Store	Electricity savings3	The product is better than I thought it would be, the quality looks great, the voltage is stable, and I'm looking forward to some great power savings!	Jingdong prefecture level city in Shandong
Jinfeng Hongyuan Flagship Store	Electricity savings3	Inadvertently see bought a try, delivery is also quite timely, just do not know how to use, customer service patience to answer, with a pretty good, value for money!	Jingdong prefecture level city in Shandong
Jinfeng Hongyuan Flagship Store	Electricity savings3	It's better than nothing! Hopefully this will help save slightly.	Jingdong prefecture level city in Shandong
Jinfeng Hongyuan Flagship Store	Electricity savings3	Still trying it out, follow up	Jingdong prefecture level city in Shandong
Jinfeng Hongyuan Flagship Store	Electricity savings3	I've just started using it, I don't know how well it works yet, I hope it's good.	Jingdong prefecture level city in Shandong
Jinfeng Hongyuan Flagship Store	Electricity savings3	Fourth purchase, great baby!	Jingdong prefecture level city in Shandong
Jinfeng Hongyuan Flagship Store	Electricity savings3	Shipped really fast, just got it and use it to see what happens and then come back for a review!	Jingdong prefecture level city in Shandong
Jinfeng Hongyuan Flagship Store	Electricity savings3	Timely delivery **** good attitude	Jingdong prefecture level city in Shandong
Jinfeng Hongyuan Flagship Store	Electricity savings3	Home large and small things are purchased in the Jingdong Mall, have been accustomed to shopping in the Jingdong, many times can buy good quality and cheap products, and courier service attitude is particularly good, sometimes too many couriers are to help me to the home, so many years have been in the Jingdong Mall shopping, trust and support! Will also support the Jingdong as always!	Jingdong prefecture level city in Shandong
Jinfeng Hongyuan Flagship Store	Electricity savings3	Modified a pump and the modification worked.	Jingdong prefecture level city in Shandong
Jinfeng Hongyuan Flagship Store	Electricity savings3	Already installed, next time you pay the electric bill to see the results	Jingdong prefecture level city in Shandong
Jinfeng Hongyuan Flagship Store	Electricity savings3	The product is very good to use and practical to look at	Jingdong prefecture level city in Shandong
Jinfeng Hongyuan Flagship Store	Electricity savings3	Works great, great service, waiting for results.	Jingdong prefecture level city in Shandong
Jinfeng Hongyuan Flagship Store	Electricity savings3	I just used it and I'm not sure about the efficacy yet, I'll come back to evaluate it after a while.	Jingdong prefecture level city in Shandong
Jinfeng Hongyuan Flagship Store	Electricity savings3	Just received the goods, use a period of time to evaluate	Jingdong prefecture level city in Shandong
Jinfeng Hongyuan Flagship Store	Electricity savings3	Opened and tried very good, use it to see if there are results	Jingdong prefecture level city in Shandong

Jinfeng Hongyuan Flagship Store	Electricity savings3	Arrived today! Still trying it out!	Jingdong prefecture level city in Shandong
Jinfeng Hongyuan Flagship Store	Electricity savings3	Shipped quickly, well packaged, already installed, hope there is a power saving effect	Jingdong prefecture level city in Shandong
Jinfeng Hongyuan Flagship Store	Electricity savings3	I got it. It's great.	Jingdong prefecture level city in Shandong
Jinfeng Hongyuan Flagship Store	Electricity savings3	Haven't used it yet, will come back in a month	Jingdong prefecture level city in Shandong
Jinfeng Hongyuan Flagship Store	Electricity savings3	It looks good. It's good. Try it first.	Jingdong prefecture level city in Shandong
Jinfeng Hongyuan Flagship Store	Electricity savings3	It works so well that I have relatives, friends, coworkers, and 6 families that have purchased it for them through me.	Jingdong prefecture level city in Shandong
Jinfeng Hongyuan Flagship Store	Electricity savings3	In use	Jingdong prefecture level city in Shandong
Jinfeng Hongyuan Flagship Store	Electricity savings3	Received the goods today, power-saving effect do not know being used, the boss has a very good attitude, praise!	Jingdong prefecture level city in Shandong
Jinfeng Hongyuan Flagship Store	Electricity savings3	The product is compact and doesn't take up much space, but hopefully it's power efficient.	Jingdong prefecture level city in Shandong
Jinfeng Hongyuan Flagship Store	Electricity savings3	Power saving is indeed effective, I used for a period of time before coming to evaluate, the electricity bill is obviously less, the quality is good, the shopkeeper service is also very good, very patient,	Jingdong prefecture level city in Shandong
Jinfeng Hongyuan Flagship Store	Electricity savings3	It feels fine, I'll comment after I use it	Jingdong prefecture level city in Shandong
Jinfeng Hongyuan Flagship Store	Electricity savings3	I just received it and I don't know the effect yet! I will comment again after I use it and have results!	Jingdong prefecture level city in Shandong
Jinfeng Hongyuan Flagship Store	Electricity savings3	The goods look fine, I hope they really work!	Jingdong prefecture level city in Shandong
Jinfeng Hongyuan Flagship Store	Electricity savings3	I just don't know if it saves power	Jingdong prefecture level city in Shandong
Jinfeng Hongyuan Flagship Store	Electricity savings3	My friend introduced me to use this product, it is professional and I am looking forward to the long term effect~!	Jingdong prefecture level city in Shandong
Jinfeng Hongyuan Flagship Store	Electricity savings3	Try it before you judge.	Jingdong prefecture level city in Shandong
Jinfeng Hongyuan Flagship Store	Electricity savings3	It's great, the power savings are obvious! Kudos!	Jingdong prefecture level city in Shandong
Jinfeng Hongyuan Flagship Store	Electricity savings3	The quality is excellent, stabilizes the voltage and saves electricity, very satisfied.	Jingdong prefecture level city in Shandong
Jinfeng Hongyuan Flagship Store	Electricity savings3	You have to use it for a while to know the effect	Jingdong prefecture level city in Shandong
Jinfeng Hongyuan Flagship Store	Electricity savings3	It's on trial.	Jingdong prefecture level city in Shandong
Jinfeng Hongyuan Flagship Store	Electricity savings3	The stuff works great.	Jingdong prefecture level city in Shandong
Jinfeng Hongyuan Flagship Store	Electricity savings3	Doesn't seem to work, not as god as other reviews	Jingdong prefecture level city in Shandong
Jinfeng Hongyuan Flagship Store	Electricity savings3	It's not good quality, it doesn't work, it stops working after a few days, it doesn't work, the customer service doesn't care, it's a bit irritating.	Jingdong prefecture level city in Shandong
Jinfeng Hongyuan Flagship Store	Electricity savings3	It doesn't do anything.	Jingdong prefecture level city in Shandong
Jinfeng Hongyuan Flagship Store	Electricity savings3	It's good.	Jingdong prefecture level city in Shandong
Jinfeng Hongyuan Flagship Store	Electricity savings3	Used a month effect is not obvious, are psychological effects, feel are not less, did not save to the electricity, there are details of the problem, the store said to buy to send 5 fuse, in fact, received only three, small problems are not committed to in place, I believe that the product is not good enough	Jingdong prefecture level city in Shandong
Jinfeng Hongyuan Flagship Store	Electricity savings3	Well received quickly, looking quite high, but also sent 5 fuses. Customer service is very patient to teach the operation, in fact, quite simple, lazy to read the instructions ha!	Jingdong prefecture level city in Shandong
Jinfeng Hongyuan Flagship Store	Electricity savings3	You have to use it for a while to know the effect	Jingdong prefecture level city in Shandong
Jinfeng Hongyuan Flagship Store	Electricity savings3	Shipped very quickly, packaged very well, the price is particularly affordable, there are so many efficacy is also good, it is worth buying worth buying the product, the workmanship is very fine, better than imagined, recommended that everyone try not yet used, after the use of the follow-up comment	Jingdong prefecture level city in Shandong
Jinfeng Hongyuan Flagship Store	Electricity savings3	It looks regular. It's working now.	Jingdong prefecture level city in Shandong
Jinfeng Hongyuan Flagship Store	Electricity savings3	It arrived pretty fast and the owner is very responsible. Have not used it yet, will see after use	Jingdong prefecture level city in Shandong
Jinfeng Hongyuan Flagship Store	Electricity savings3	🎁 baby quality is very good, the first time I received the goods to open the first time to see, it is very good 👍, very satisfied 😊, customer service also have a question and answer, thank you 🙏	Jingdong prefecture level city in Shandong
Jinfeng Hongyuan Flagship Store	Electricity savings3	I don't know if it's any good 。。。。。。	Jingdong prefecture level city in Shandong
Jinfeng Hongyuan Flagship Store	Electricity savings3	Buy electronic products! Still trust the things of the Jingdong self-owned store! Energy-saving effect to evaluate after heating!	Jingdong prefecture level city in Shandong
Jinfeng Hongyuan Flagship Store	Electricity savings3	The goods have been received, Blu is very beautiful, energy saving effect, to be confirmed only after actual use.	Jingdong prefecture level city in Shandong
Jinfeng Hongyuan Flagship Store	Electricity savings3	Jingdong's speed is so fast, has received, easy to operate. The display is quite good, how many volts what mode is quite understandable.	Jingdong prefecture level city in Shandong
Jinfeng Hongyuan Flagship Store	Electricity savings3	The first use, the effect is good to recommend it to colleagues to use, a month of electricity to save a lot or can be	Jingdong prefecture level city in Shandong
Jinfeng Hongyuan Flagship Store	Electricity savings3	I don't know if it's useful. Mom's is a store. I'll return it if it doesn't work.	Jingdong prefecture level city in Shandong
Jinfeng Hongyuan Flagship Store	Electricity savings3	I don't know if it works or not, I'll wait for two months to use it and then come back to comment!	Jingdong prefecture level city in Shandong
Jinfeng Hongyuan Flagship Store	Electricity savings3	I bought the second one now, and I've been using it for more than half a year, and I feel that it really saves electricity. Now it is summer, the weather is hot, sometimes the fan and air conditioning on all day, if not this power saver, the monthly electricity bill is quite high, but as long as the installation of this power saver, not afraid of its high electricity bill, absolutely let you wantonly use	Jingdong prefecture level city in Shandong
Jinfeng Hongyuan Flagship Store	Electricity savings3	The product is better than I thought it would be, the quality looks great, the voltage is stable, and I'm looking forward to some great power savings!	Jingdong prefecture level city in Shandong
Jinfeng Hongyuan Flagship Store	Electricity savings3	Inadvertently see bought a try, delivery is also quite timely, just do not know how to use, customer service patience to answer, with a pretty good, value for money!	Jingdong prefecture level city in Shandong
Jinfeng Hongyuan Flagship Store	Electricity savings3	It's better than nothing! Hopefully this will help save slightly.	Jingdong prefecture level city in Shandong
Jinfeng Hongyuan Flagship Store	Electricity savings3	Still trying it out, follow up	Jingdong prefecture level city in Shandong
Jinfeng Hongyuan Flagship Store	Electricity savings3	I've just started using it, I don't know how well it works yet, I hope it's good.	Jingdong prefecture level city in Shandong
Jinfeng Hongyuan Flagship Store	Electricity savings3	Fourth purchase, great baby!	Jingdong prefecture level city in Shandong
Jinfeng Hongyuan Flagship Store	Electricity savings3	Shipped really fast, just got it and use it to see what happens and then come back for a review!	Jingdong prefecture level city in Shandong
Jinfeng Hongyuan Flagship Store	Electricity savings3	Timely delivery **** good attitude	Jingdong prefecture level city in Shandong

Jinfeng Hongyuan Flagship Store	Electricity savings3	Home large and small things are purchased in the Jingdong Mall, have been accustomed to shopping in the Jingdong, many times can buy good quality and cheap products, and courier service attitude is particularly good, sometimes too many couriers are to help me to the home, so many years have been in the Jingdong Mall shopping, trust and support! Will also support the Jingdong as always!	Jingdong prefecture level city in Shandong
Jinfeng Hongyuan Flagship Store	Electricity savings3	Modified a pump and the modification worked.	Jingdong prefecture level city in Shandong
Jinfeng Hongyuan Flagship Store	Electricity savings3	Already installed, next time you pay the electric bill to see the results	Jingdong prefecture level city in Shandong
Jinfeng Hongyuan Flagship Store	Electricity savings3	The product is very good to use and practical to look at	Jingdong prefecture level city in Shandong
Jinfeng Hongyuan Flagship Store	Electricity savings3	Works great, great service, waiting for results.	Jingdong prefecture level city in Shandong
Jinfeng Hongyuan Flagship Store	Electricity savings3	I just used it and I'm not sure about the efficacy yet, I'll come back to evaluate it after a while.	Jingdong prefecture level city in Shandong
Jinfeng Hongyuan Flagship Store	Electricity savings3	Just received the goods, use a period of time to evaluate	Jingdong prefecture level city in Shandong
Jinfeng Hongyuan Flagship Store	Electricity savings3	Opened and tried very good, use it to see if there are results	Jingdong prefecture level city in Shandong
Jinfeng Hongyuan Flagship Store	Electricity savings3	Arrived today! Still trying it out!	Jingdong prefecture level city in Shandong
Jinfeng Hongyuan Flagship Store	Electricity savings3	Shipped quickly, well packaged, already installed, hope there is a power saving effect	Jingdong prefecture level city in Shandong
Jinfeng Hongyuan Flagship Store	Electricity savings3	I got it. It's great.	Jingdong prefecture level city in Shandong
Jinfeng Hongyuan Flagship Store	Electricity savings3	Haven't used it yet, will come back in a month	Jingdong prefecture level city in Shandong
Jinfeng Hongyuan Flagship Store	Electricity savings3	It looks good. It's good. Try it first.	Jingdong prefecture level city in Shandong
Jinfeng Hongyuan Flagship Store	Electricity savings3	It works so well that I have relatives, friends, coworkers, and 6 families that have purchased it for them through me.	Jingdong prefecture level city in Shandong
Jinfeng Hongyuan Flagship Store	Electricity savings3	In use	Jingdong prefecture level city in Shandong
Jinfeng Hongyuan Flagship Store	Electricity savings3	Received the goods today, power-saving effect do not know being used, the boss has a very good attitude, praise!	Jingdong prefecture level city in Shandong
Jinfeng Hongyuan Flagship Store	Electricity savings3	The product is compact and doesn't take up much space, but hopefully it's power efficient.	Jingdong prefecture level city in Shandong
Jinfeng Hongyuan Flagship Store	Electricity savings3	Power saving is indeed effective, I used for a period of time before coming to evaluate, the electricity bill is obviously less, the quality is good, the shopkeeper service is also very good, very patient,	Jingdong prefecture level city in Shandong
Jinfeng Hongyuan Flagship Store	Electricity savings3	It feels fine, I'll comment after I use it	Jingdong prefecture level city in Shandong
Jinfeng Hongyuan Flagship Store	Electricity savings3	I just received it and I don't know the effect yet! I will comment again after I use it and have results!	Jingdong prefecture level city in Shandong
Jinfeng Hongyuan Flagship Store	Electricity savings3	The goods look fine, I hope they really work!	Jingdong prefecture level city in Shandong
Jinfeng Hongyuan Flagship Store	Electricity savings3	I just don't know if it saves power	Jingdong prefecture level city in Shandong
Jinfeng Hongyuan Flagship Store	Electricity savings3	My friend introduced me to use this product, it is professional and I am looking forward to the long term effect~!	Jingdong prefecture level city in Shandong
Jinfeng Hongyuan Flagship Store	Electricity savings3	Try it before you judge.	Jingdong prefecture level city in Shandong
Jinfeng Hongyuan Flagship Store	Electricity savings3	It's great, the power savings are obvious! Kudos!	Jingdong prefecture level city in Shandong
Jinfeng Hongyuan Flagship Store	Electricity savings3	The quality is excellent, stabilizes the voltage and saves electricity, very satisfied.	Jingdong prefecture level city in Shandong
Jinfeng Hongyuan Flagship Store	Electricity savings3	You have to use it for a while to know the effect	Jingdong prefecture level city in Shandong
Jinfeng Hongyuan Flagship Store	Electricity savings3	It's on trial.	Jingdong prefecture level city in Shandong
Jinfeng Hongyuan Flagship Store	Electricity savings3	The stuff works great.	Jingdong prefecture level city in Shandong
Jinfeng Hongyuan Flagship Store	Electricity savings3	Doesn't seem to work, not as god as other reviews	Jingdong prefecture level city in Shandong
Jinfeng Hongyuan Flagship Store	Electricity savings3	It's not good quality, it doesn't work, it stops working after a few days, it doesn't work, the customer service doesn't care, it's a bit irritating.	Jingdong prefecture level city in Shandong
Jinfeng Hongyuan Flagship Store	Electricity savings3	It doesn't do anything.	Jingdong prefecture level city in Shandong
Jinfeng Hongyuan Flagship Store	Electricity savings3	It's good.	Jingdong prefecture level city in Shandong
Jinfeng Hongyuan Flagship Store	Electricity savings3	Used a month effect is not obvious, are psychological effects, feel are not less, did not save to the electricity, there are details of the problem, the store said to buy to send 5 fuse, in fact, received only three, small problems are not committed to in place, I believe that the product is not good enough	Jingdong prefecture level city in Shandong
Jinfeng Hongyuan Flagship Store	Electricity savings3	Well received quickly, looking quite high, but also sent 5 fuses. Customer service is very patient to teach the operation, in fact, quite simple, lazy to read the instructions ha!	Jingdong prefecture level city in Shandong
Jinfeng Hongyuan Flagship Store	Electricity savings3	You have to use it for a while to know the effect	Jingdong prefecture level city in Shandong
Jinfeng Hongyuan Flagship Store	Electricity savings3	Shipped very quickly, packaged very well, the price is particularly affordable, there are so many efficacy is also good, it is worth buying worth buying the product, the workmanship is very fine, better than imagined, recommended that everyone try not yet used, after the use of the follow-up comment	Jingdong prefecture level city in Shandong
Jinfeng Hongyuan Flagship Store	Electricity savings3	It looks regular. It's working now.	Jingdong prefecture level city in Shandong
Jinfeng Hongyuan Flagship Store	Electricity savings3	It arrived pretty fast and the owner is very responsible. Have not used it yet, will see after use	Jingdong prefecture level city in Shandong
Jinfeng Hongyuan Flagship Store	Electricity savings3	🎁 baby quality is very good, the first time I received the goods to open the first time to see, it is very good 👍, very satisfied 😊, customer service also have a question and answer, thank you 🙏	Jingdong prefecture level city in Shandong
Jinfeng Hongyuan Flagship Store	Electricity savings3	I don't know if it's any good 。。。。。。	Jingdong prefecture level city in Shandong
Jinfeng Hongyuan Flagship Store	Electricity savings3	Buy electronic products! Still trust the things of the Jingdong self-owned store! Energy-saving effect to evaluate after heating!	Jingdong prefecture level city in Shandong
Jinfeng Hongyuan Flagship Store	Electricity savings3	The goods have been received, Blu is very beautiful, energy saving effect, to be confirmed only after actual use.	Jingdong prefecture level city in Shandong
Jinfeng Hongyuan Flagship Store	Electricity savings3	Jingdong's speed is so fast, has received, easy to operate. The display is quite good, how many volts what mode is quite understandable.	Jingdong prefecture level city in Shandong
Jinfeng Hongyuan Flagship Store	Electricity savings3	The first use, the effect is good to recommend it to colleagues to use, a month of electricity to save a lot or can be	Jingdong prefecture level city in Shandong
Jinfeng Hongyuan Flagship Store	Electricity savings3	I don't know if it's useful. Mom's is a store. I'll return it if it doesn't work.	Jingdong prefecture level city in Shandong
Jinfeng Hongyuan Flagship Store	Electricity savings3	I don't know if it works or not, I'll wait for two months to use it and then come back to comment!	Jingdong prefecture level city in Shandong
Jinfeng Hongyuan Flagship Store	Electricity savings3	I bought the second one now, and I've been using it for more than half a year, and I feel that it really saves electricity. Now it is summer, the weather is hot, sometimes the fan and air conditioning on all day, if not this power saver, the monthly electricity bill is quite high, but as long as the installation of this power saver, not afraid of its high electricity bill, absolutely let you wantonly use	Jingdong prefecture level city in Shandong

Jinfeng Hongyuan Flagship Store	Electricity savings3	The product is better than I thought it would be, the quality looks great, the voltage is stable, and I'm looking forward to some great power savings!	Jingdong prefecture level city in Shandong
Jinfeng Hongyuan Flagship Store	Electricity savings3	Inadvertently see bought a try, delivery is also quite timely, just do not know how to use, customer service patience to answer, with a pretty good, value for money!	Jingdong prefecture level city in Shandong
Jinfeng Hongyuan Flagship Store	Electricity savings3	It's better than nothing! Hopefully this will help save slightly.	Jingdong prefecture level city in Shandong
Jinfeng Hongyuan Flagship Store	Electricity savings3	Still trying it out, follow up	Jingdong prefecture level city in Shandong
Jinfeng Hongyuan Flagship Store	Electricity savings3	I've just started using it, I don't know how well it works yet, I hope it's good.	Jingdong prefecture level city in Shandong
Jinfeng Hongyuan Flagship Store	Electricity savings3	Fourth purchase, great baby!	Jingdong prefecture level city in Shandong
Jinfeng Hongyuan Flagship Store	Electricity savings3	Shipped really fast, just got it and use it to see what happens and then come back for a review!	Jingdong prefecture level city in Shandong
Jinfeng Hongyuan Flagship Store	Electricity savings3	Timely delivery **** good attitude	Jingdong prefecture level city in Shandong
Jinfeng Hongyuan Flagship Store	Electricity savings3	Home large and small things are purchased in the Jingdong Mall, have been accustomed to shopping in the Jingdong, many times can buy good quality and cheap products, and courier service attitude is particularly good, sometimes too many couriers are to help me to the home, so many years have been in the Jingdong Mall shopping, trust and support! Will also support the Jingdong as always!	Jingdong prefecture level city in Shandong
Jinfeng Hongyuan Flagship Store	Electricity savings3	Modified a pump and the modification worked.	Jingdong prefecture level city in Shandong
Jinfeng Hongyuan Flagship Store	Electricity savings3	Already installed, next time you pay the electric bill to see the results	Jingdong prefecture level city in Shandong
Jinfeng Hongyuan Flagship Store	Electricity savings3	The product is very good to use and practical to look at	Jingdong prefecture level city in Shandong
Jinfeng Hongyuan Flagship Store	Electricity savings3	Works great, great service, waiting for results.	Jingdong prefecture level city in Shandong
Jinfeng Hongyuan Flagship Store	Electricity savings3	I just used it and I'm not sure about the efficacy yet, I'll come back to evaluate it after a while.	Jingdong prefecture level city in Shandong
Jinfeng Hongyuan Flagship Store	Electricity savings3	Just received the goods, use a period of time to evaluate	Jingdong prefecture level city in Shandong
Jinfeng Hongyuan Flagship Store	Electricity savings3	Opened and tried very good, use it to see if there are results	Jingdong prefecture level city in Shandong
Jinfeng Hongyuan Flagship Store	Electricity savings3	Arrived today! Still trying it out!	Jingdong prefecture level city in Shandong
Jinfeng Hongyuan Flagship Store	Electricity savings3	Shipped quickly, well packaged, already installed, hope there is a power saving effect	Jingdong prefecture level city in Shandong
Jinfeng Hongyuan Flagship Store	Electricity savings3	I got it. It's great.	Jingdong prefecture level city in Shandong
Jinfeng Hongyuan Flagship Store	Electricity savings3	Haven't used it yet, will come back in a month	Jingdong prefecture level city in Shandong
Jinfeng Hongyuan Flagship Store	Electricity savings3	It looks good. It's good. Try it first.	Jingdong prefecture level city in Shandong
Jinfeng Hongyuan Flagship Store	Electricity savings3	It works so well that I have relatives, friends, coworkers, and 6 families that have purchased it for them through me.	Jingdong prefecture level city in Shandong
Jinfeng Hongyuan Flagship Store	Electricity savings3	In use	Jingdong prefecture level city in Shandong
Jinfeng Hongyuan Flagship Store	Electricity savings3	Received the goods today, power-saving effect do not know being used, the boss has a very good attitude, praise!	Jingdong prefecture level city in Shandong
Jinfeng Hongyuan Flagship Store	Electricity savings3	The product is compact and doesn't take up much space, but hopefully it's power efficient.	Jingdong prefecture level city in Shandong
Jinfeng Hongyuan Flagship Store	Electricity savings3	Power saving is indeed effective, I used for a period of time before coming to evaluate, the electricity bill is obviously less, the quality is good, the shopkeeper service is also very good, very patient,	Jingdong prefecture level city in Shandong
Jinfeng Hongyuan Flagship Store	Electricity savings3	It feels fine, I'll comment after I use it	Jingdong prefecture level city in Shandong
Jinfeng Hongyuan Flagship Store	Electricity savings3	I just received it and I don't know the effect yet! I will comment again after I use it and have results!	Jingdong prefecture level city in Shandong
Jinfeng Hongyuan Flagship Store	Electricity savings3	The goods look fine, I hope they really work!	Jingdong prefecture level city in Shandong
Jinfeng Hongyuan Flagship Store	Electricity savings3	I just don't know if it saves power	Jingdong prefecture level city in Shandong
Jinfeng Hongyuan Flagship Store	Electricity savings3	My friend introduced me to use this product, it is professional and I am looking forward to the long term effect~!	Jingdong prefecture level city in Shandong
Jinfeng Hongyuan Flagship Store	Electricity savings3	Try it before you judge.	Jingdong prefecture level city in Shandong
Jinfeng Hongyuan Flagship Store	Electricity savings3	It's great, the power savings are obvious! Kudos!	Jingdong prefecture level city in Shandong
Jinfeng Hongyuan Flagship Store	Electricity savings3	The quality is excellent, stabilizes the voltage and saves electricity, very satisfied.	Jingdong prefecture level city in Shandong
Jinfeng Hongyuan Flagship Store	Electricity savings3	You have to use it for a while to know the effect	Jingdong prefecture level city in Shandong
Jinfeng Hongyuan Flagship Store	Electricity savings3	It's on trial.	Jingdong prefecture level city in Shandong
Jinfeng Hongyuan Flagship Store	Electricity savings3	The stuff works great.	Jingdong prefecture level city in Shandong
Jinfeng Hongyuan Flagship Store	Electricity savings3	Doesn't seem to work, not as god as other reviews	Jingdong prefecture level city in Shandong
Jinfeng Hongyuan Flagship Store	Electricity savings3	It's not good quality, it doesn't work, it stops working after a few days, it doesn't work, the customer service doesn't care, it's a bit irritating.	Jingdong prefecture level city in Shandong
Jinfeng Hongyuan Flagship Store	Electricity savings3	It doesn't do anything.	Jingdong prefecture level city in Shandong
Jinfeng Hongyuan Flagship Store	Electricity savings3	It's good.	Jingdong prefecture level city in Shandong
Jinfeng Hongyuan Flagship Store	Electricity savings3	Used a month effect is not obvious, are psychological effects, feel are not less, did not save to the electricity, there are details of the problem, the store said to buy to send 5 fuse, in fact, received only three, small problems are not committed to in place, I believe that the product is not good enough	Jingdong prefecture level city in Shandong
Jinfeng Hongyuan Flagship Store	Electricity savings3	Well received quickly, looking quite high, but also sent 5 fuses. Customer service is very patient to teach the operation, in fact, quite simple, lazy to read the instructions ha!	Jingdong prefecture level city in Shandong
Jinfeng Hongyuan Flagship Store	Electricity savings3	You have to use it for a while to know the effect	Jingdong prefecture level city in Shandong
Jinfeng Hongyuan Flagship Store	Electricity savings3	Shipped very quickly, packaged very well, the price is particularly affordable, there are so many efficacy is also good, it is worth buying worth buying the product, the workmanship is very fine, better than imagined, recommended that everyone try not yet used, after the use of the follow-up comment	Jingdong prefecture level city in Shandong
Jinfeng Hongyuan Flagship Store	Electricity savings3	It looks regular. It's working now.	Jingdong prefecture level city in Shandong
Jinfeng Hongyuan Flagship Store	Electricity savings3	It arrived pretty fast and the owner is very responsible. Have not used it yet, will see after use	Jingdong prefecture level city in Shandong
Jinfeng Hongyuan Flagship Store	Electricity savings3	🎁 baby quality is very good, the first time I received the goods to open the first time to see, it is very good 👍, very satisfied 😊, customer service also have a question and answer, thank you 🙏	Jingdong prefecture level city in Shandong
Jinfeng Hongyuan Flagship Store	Electricity savings3	I don't know if it's any good 。。。。。。	Jingdong prefecture level city in Shandong

Jinfeng Hongyuan Flagship Store	Electricity savings3	Buy electronic products! Still trust the things of the Jingdong self-owned store! Energy-saving effect to evaluate after heating!	Jingdong prefecture level city in Shandong
Jinfeng Hongyuan Flagship Store	Electricity savings3	The goods have been received, Blu is very beautiful, energy saving effect, to be confirmed only after actual use.	Jingdong prefecture level city in Shandong
Jinfeng Hongyuan Flagship Store	Electricity savings3	Jingdong's speed is so fast, has received, easy to operate. The display is quite good, how many volts what mode is quite understandable.	Jingdong prefecture level city in Shandong
Jinfeng Hongyuan Flagship Store	Electricity savings3	The first use, the effect is good to recommend it to colleagues to use, a month of electricity to save a lot or can be	Jingdong prefecture level city in Shandong
Jinfeng Hongyuan Flagship Store	Electricity savings3	I don't know if it's useful. Mom's is a store. I'll return it if it doesn't work.	Jingdong prefecture level city in Shandong
Jinfeng Hongyuan Flagship Store	Electricity savings3	I don't know if it works or not, I'll wait for two months to use it and then come back to comment!	Jingdong prefecture level city in Shandong
Jinfeng Hongyuan Flagship Store	Electricity savings3	I bought the second one now, and I've been using it for more than half a year, and I feel that it really saves electricity. Now it is summer, the weather is hot, sometimes the fan and air conditioning on all day, if not this power saver, the monthly electricity bill is quite high, but as long as the installation of this power saver, not afraid of its high electricity bill, absolutely let you wantonly use	Jingdong prefecture level city in Shandong
Jinfeng Hongyuan Flagship Store	Electricity savings3	The product is better than I thought it would be, the quality looks great, the voltage is stable, and I'm looking forward to some great power savings!	Jingdong prefecture level city in Shandong
Jinfeng Hongyuan Flagship Store	Electricity savings3	Inadvertently see bought a try, delivery is also quite timely, just do not know how to use, customer service patience to answer, with a pretty good, value for money!	Jingdong prefecture level city in Shandong
Jinfeng Hongyuan Flagship Store	Electricity savings3	It's better than nothing! Hopefully this will help save slightly.	Jingdong prefecture level city in Shandong
Jinfeng Hongyuan Flagship Store	Electricity savings3	Still trying it out, follow up	Jingdong prefecture level city in Shandong
Jinfeng Hongyuan Flagship Store	Electricity savings3	I've just started using it, I don't know how well it works yet, I hope it's good.	Jingdong prefecture level city in Shandong
Jinfeng Hongyuan Flagship Store	Electricity savings3	Fourth purchase, great baby!	Jingdong prefecture level city in Shandong
Jinfeng Hongyuan Flagship Store	Electricity savings3	Shipped really fast, just got it and use it to see what happens and then come back for a review!	Jingdong prefecture level city in Shandong
Jinfeng Hongyuan Flagship Store	Electricity savings3	Timely delivery **** good attitude	Jingdong prefecture level city in Shandong
Jinfeng Hongyuan Flagship Store	Electricity savings3	Home large and small things are purchased in the Jingdong Mall, have been accustomed to shopping in the Jingdong, many times can buy good quality and cheap products, and courier service attitude is particularly good, sometimes too many couriers are to help me to the home, so many years have been in the Jingdong Mall shopping, trust and support! Will also support the Jingdong as always!	Jingdong prefecture level city in Shandong
Jinfeng Hongyuan Flagship Store	Electricity savings3	Modified a pump and the modification worked.	Jingdong prefecture level city in Shandong
Jinfeng Hongyuan Flagship Store	Electricity savings3	Already installed, next time you pay the electric bill to see the results	Jingdong prefecture level city in Shandong
Jinfeng Hongyuan Flagship Store	Electricity savings3	The product is very good to use and practical to look at	Jingdong prefecture level city in Shandong
Jinfeng Hongyuan Flagship Store	Electricity savings3	Works great, great service, waiting for results.	Jingdong prefecture level city in Shandong
Jinfeng Hongyuan Flagship Store	Electricity savings3	I just used it and I'm not sure about the efficacy yet, I'll come back to evaluate it after a while.	Jingdong prefecture level city in Shandong
Jinfeng Hongyuan Flagship Store	Electricity savings3	Just received the goods, use a period of time to evaluate	Jingdong prefecture level city in Shandong
Jinfeng Hongyuan Flagship Store	Electricity savings3	Opened and tried very good, use it to see if there are results	Jingdong prefecture level city in Shandong
Jinfeng Hongyuan Flagship Store	Electricity savings3	Arrived today! Still trying it out!	Jingdong prefecture level city in Shandong
Jinfeng Hongyuan Flagship Store	Electricity savings3	Shipped quickly, well packaged, already installed, hope there is a power saving effect	Jingdong prefecture level city in Shandong
Jinfeng Hongyuan Flagship Store	Electricity savings3	I got it. It's great.	Jingdong prefecture level city in Shandong
Jinfeng Hongyuan Flagship Store	Electricity savings3	Haven't used it yet, will come back in a month	Jingdong prefecture level city in Shandong
Jinfeng Hongyuan Flagship Store	Electricity savings3	It looks good. It's good. Try it first.	Jingdong prefecture level city in Shandong
Jinfeng Hongyuan Flagship Store	Electricity savings3	It works so well that I have relatives, friends, coworkers, and 6 families that have purchased it for them through me.	Jingdong prefecture level city in Shandong
Jinfeng Hongyuan Flagship Store	Electricity savings3	In use	Jingdong prefecture level city in Shandong
Jinfeng Hongyuan Flagship Store	Electricity savings3	Received the goods today, power-saving effect do not know being used, the boss has a very good attitude, praise!	Jingdong prefecture level city in Shandong
Jinfeng Hongyuan Flagship Store	Electricity savings3	The product is compact and doesn't take up much space, but hopefully it's power efficient.	Jingdong prefecture level city in Shandong
Jinfeng Hongyuan Flagship Store	Electricity savings3	Power saving is indeed effective, I used for a period of time before coming to evaluate, the electricity bill is obviously less, the quality is good, the shopkeeper service is also very good, very patient,	Jingdong prefecture level city in Shandong
Jinfeng Hongyuan Flagship Store	Electricity savings3	It feels fine, I'll comment after I use it	Jingdong prefecture level city in Shandong
Jinfeng Hongyuan Flagship Store	Electricity savings3	I just received it and I don't know the effect yet! I will comment again after I use it and have results!	Jingdong prefecture level city in Shandong
Jinfeng Hongyuan Flagship Store	Electricity savings3	The goods look fine, I hope they really work!	Jingdong prefecture level city in Shandong
Jinfeng Hongyuan Flagship Store	Electricity savings3	I just don't know if it saves power	Jingdong prefecture level city in Shandong
Jinfeng Hongyuan Flagship Store	Electricity savings3	My friend introduced me to use this product, it is professional and I am looking forward to the long term effect~!	Jingdong prefecture level city in Shandong
Jinfeng Hongyuan Flagship Store	Electricity savings3	Try it before you judge.	Jingdong prefecture level city in Shandong
Jinfeng Hongyuan Flagship Store	Electricity savings3	It's great, the power savings are obvious! Kudos!	Jingdong prefecture level city in Shandong
Jinfeng Hongyuan Flagship Store	Electricity savings3	The quality is excellent, stabilizes the voltage and saves electricity, very satisfied.	Jingdong prefecture level city in Shandong
Jinfeng Hongyuan Flagship Store	Electricity savings3	You have to use it for a while to know the effect	Jingdong prefecture level city in Shandong
Jinfeng Hongyuan Flagship Store	Electricity savings3	It's on trial.	Jingdong prefecture level city in Shandong
Jinfeng Hongyuan Flagship Store	Electricity savings3	The stuff works great.	Jingdong prefecture level city in Shandong
Jinfeng Hongyuan Flagship Store	Electricity savings3	Doesn't seem to work, not as god as other reviews	Jingdong prefecture level city in Shandong
Jinfeng Hongyuan Flagship Store	Electricity savings3	It's not good quality, it doesn't work, it stops working after a few days, it doesn't work, the customer service doesn't care, it's a bit irritating.	Jingdong prefecture level city in Shandong
Jinfeng Hongyuan Flagship Store	Electricity savings3	It doesn't do anything.	Jingdong prefecture level city in Shandong
Jinfeng Hongyuan Flagship Store	Electricity savings3	It's good.	Jingdong prefecture level city in Shandong
Jinfeng Hongyuan Flagship Store	Electricity savings3	Used a month effect is not obvious, are psychological effects, feel are not less, did not save to the electricity, there are details of the problem, the store said to buy to send 5 fuse, in fact, received only three, small problems are not committed to in place, I believe that the product is not good enough	Jingdong prefecture level city in Shandong

Jinfeng Hongyuan Flagship Store	Electricity savings3	Well received quickly, looking quite high, but also sent 5 fuses. Customer service is very patient to teach the operation, in fact, quite simple, lazy to read the instructions ha!	Jingdong prefecture level city in Shandong
Jinfeng Hongyuan Flagship Store	Electricity savings3	You have to use it for a while to know the effect	Jingdong prefecture level city in Shandong
Jinfeng Hongyuan Flagship Store	Electricity savings3	Shipped very quickly, packaged very well, the price is particularly affordable, there are so many efficacy is also good, it is worth buying worth buying the product, the workmanship is very fine, better than imagined, recommended that everyone try not yet used, after the use of the follow-up comment	Jingdong prefecture level city in Shandong
Jinfeng Hongyuan Flagship Store	Electricity savings3	It looks regular. It's working now.	Jingdong prefecture level city in Shandong
Jinfeng Hongyuan Flagship Store	Electricity savings3	It arrived pretty fast and the owner is very responsible. Have not used it yet, will see after use	Jingdong prefecture level city in Shandong
Jinfeng Hongyuan Flagship Store	Electricity savings3	🎁 baby quality is very good, the first time I received the goods to open the first time to see, it is very good 👍, very satisfied 😊, customer service also have a question and answer, thank you 🙏	Jingdong prefecture level city in Shandong
Jinfeng Hongyuan Flagship Store	Electricity savings3	I don't know if it's any good 。。。。。。	Jingdong prefecture level city in Shandong
Jinfeng Hongyuan Flagship Store	Electricity savings3	Buy electronic products! Still trust the things of the Jingdong self-owned store! Energy-saving effect to evaluate after heating!	Jingdong prefecture level city in Shandong
Jinfeng Hongyuan Flagship Store	Electricity savings3	The goods have been received, Blu is very beautiful, energy saving effect, to be confirmed only after actual use.	Jingdong prefecture level city in Shandong
Jinfeng Hongyuan Flagship Store	Electricity savings3	Jingdong's speed is so fast, has received, easy to operate. The display is quite good, how many volts what mode is quite understandable.	Jingdong prefecture level city in Shandong
Jinfeng Hongyuan Flagship Store	Electricity savings3	The first use, the effect is good to recommend it to colleagues to use, a month of electricity to save a lot or can be	Jingdong prefecture level city in Shandong
Jinfeng Hongyuan Flagship Store	Electricity savings3	I don't know if it's useful. Mom's is a store. I'll return it if it doesn't work.	Jingdong prefecture level city in Shandong
Jinfeng Hongyuan Flagship Store	Electricity savings3	I don't know if it works or not, I'll wait for two months to use it and then come back to comment!	Jingdong prefecture level city in Shandong
Jinfeng Hongyuan Flagship Store	Electricity savings3	I bought the second one now, and I've been using it for more than half a year, and I feel that it really saves electricity. Now it is summer, the weather is hot, sometimes the fan and air conditioning on all day, if not this power saver, the monthly electricity bill is quite high, but as long as the installation of this power saver, not afraid of its high electricity bill, absolutely let you wantonly use	Jingdong prefecture level city in Shandong
Jinfeng Hongyuan Flagship Store	Electricity savings3	The product is better than I thought it would be, the quality looks great, the voltage is stable, and I'm looking forward to some great power savings!	Jingdong prefecture level city in Shandong
Jinfeng Hongyuan Flagship Store	Electricity savings3	Inadvertently see bought a try, delivery is also quite timely, just do not know how to use, customer service patience to answer, with a pretty good, value for money!	Jingdong prefecture level city in Shandong
Jinfeng Hongyuan Flagship Store	Electricity savings3	It's better than nothing! Hopefully this will help save slightly.	Jingdong prefecture level city in Shandong
Jinfeng Hongyuan Flagship Store	Electricity savings3	Still trying it out, follow up	Jingdong prefecture level city in Shandong
Jinfeng Hongyuan Flagship Store	Electricity savings3	I've just started using it, I don't know how well it works yet, I hope it's good.	Jingdong prefecture level city in Shandong
Jinfeng Hongyuan Flagship Store	Electricity savings3	Fourth purchase, great baby!	Jingdong prefecture level city in Shandong
Jinfeng Hongyuan Flagship Store	Electricity savings3	Shipped really fast, just got it and use it to see what happens and then come back for a review!	Jingdong prefecture level city in Shandong
Jinfeng Hongyuan Flagship Store	Electricity savings3	Timely delivery **** good attitude	Jingdong prefecture level city in Shandong
Jinfeng Hongyuan Flagship Store	Electricity savings3	Home large and small things are purchased in the Jingdong Mall, have been accustomed to shopping in the Jingdong, many times can buy good quality and cheap products, and courier service attitude is particularly good, sometimes too many couriers are to help me to the home, so many years have been in the Jingdong Mall shopping, trust and support! Will also support the Jingdong as always!	Jingdong prefecture level city in Shandong
Jinfeng Hongyuan Flagship Store	Electricity savings3	Modified a pump and the modification worked.	Jingdong prefecture level city in Shandong
Jinfeng Hongyuan Flagship Store	Electricity savings3	Already installed, next time you pay the electric bill to see the results	Jingdong prefecture level city in Shandong
Jinfeng Hongyuan Flagship Store	Electricity savings3	The product is very good to use and practical to look at	Jingdong prefecture level city in Shandong
Jinfeng Hongyuan Flagship Store	Electricity savings3	Works great, great service, waiting for results.	Jingdong prefecture level city in Shandong
Jinfeng Hongyuan Flagship Store	Electricity savings3	I just used it and I'm not sure about the efficacy yet, I'll come back to evaluate it after a while.	Jingdong prefecture level city in Shandong
Jinfeng Hongyuan Flagship Store	Electricity savings3	Just received the goods, use a period of time to evaluate	Jingdong prefecture level city in Shandong
Jinfeng Hongyuan Flagship Store	Electricity savings3	Opened and tried very good, use it to see if there are results	Jingdong prefecture level city in Shandong
Jinfeng Hongyuan Flagship Store	Electricity savings3	Arrived today! Still trying it out!	Jingdong prefecture level city in Shandong
Jinfeng Hongyuan Flagship Store	Electricity savings3	Shipped quickly, well packaged, already installed, hope there is a power saving effect	Jingdong prefecture level city in Shandong
Jinfeng Hongyuan Flagship Store	Electricity savings3	I got it. It's great.	Jingdong prefecture level city in Shandong
Jinfeng Hongyuan Flagship Store	Electricity savings3	Haven't used it yet, will come back in a month	Jingdong prefecture level city in Shandong
Jinfeng Hongyuan Flagship Store	Electricity savings3	It looks good. It's good. Try it first.	Jingdong prefecture level city in Shandong
Jinfeng Hongyuan Flagship Store	Electricity savings3	It works so well that I have relatives, friends, coworkers, and 6 families that have purchased it for them through me.	Jingdong prefecture level city in Shandong
Jinfeng Hongyuan Flagship Store	Electricity savings3	In use	Jingdong prefecture level city in Shandong
Jinfeng Hongyuan Flagship Store	Electricity savings3	Received the goods today, power-saving effect do not know being used, the boss has a very good attitude, praise!	Jingdong prefecture level city in Shandong
Jinfeng Hongyuan Flagship Store	Electricity savings3	The product is compact and doesn't take up much space, but hopefully it's power efficient.	Jingdong prefecture level city in Shandong
Jinfeng Hongyuan Flagship Store	Electricity savings3	Power saving is indeed effective, I used for a period of time before coming to evaluate, the electricity bill is obviously less, the quality is good, the shopkeeper service is also very good, very patient,	Jingdong prefecture level city in Shandong
Jinfeng Hongyuan Flagship Store	Electricity savings3	It feels fine, I'll comment after I use it	Jingdong prefecture level city in Shandong
Jinfeng Hongyuan Flagship Store	Electricity savings3	I just received it and I don't know the effect yet! I will comment again after I use it and have results!	Jingdong prefecture level city in Shandong
Jinfeng Hongyuan Flagship Store	Electricity savings3	The goods look fine, I hope they really work!	Jingdong prefecture level city in Shandong
Jinfeng Hongyuan Flagship Store	Electricity savings3	I just don't know if it saves power	Jingdong prefecture level city in Shandong
Jinfeng Hongyuan Flagship Store	Electricity savings3	My friend introduced me to use this product, it is professional and I am looking forward to the long term effect~!	Jingdong prefecture level city in Shandong
Jinfeng Hongyuan Flagship Store	Electricity savings3	Try it before you judge.	Jingdong prefecture level city in Shandong
Jinfeng Hongyuan Flagship Store	Electricity savings3	It's great, the power savings are obvious! Kudos!	Jingdong prefecture level city in Shandong
Jinfeng Hongyuan Flagship Store	Electricity savings3	The quality is excellent, stabilizes the voltage and saves electricity, very satisfied.	Jingdong prefecture level city in Shandong
Jinfeng Hongyuan Flagship Store	Electricity savings3	You have to use it for a while to know the effect	Jingdong prefecture level city in Shandong

Jinfeng Hongyuan Flagship Store	Electricity savings3	It's on trial.	Jingdong prefecture level city in Shandong
Jinfeng Hongyuan Flagship Store	Electricity savings3	The stuff works great.	Jingdong prefecture level city in Shandong
Jinfeng Hongyuan Flagship Store	Electricity savings3	Doesn't seem to work, not as god as other reviews	Jingdong prefecture level city in Shandong
Jinfeng Hongyuan Flagship Store	Electricity savings3	It's not good quality, it doesn't work, it stops working after a few days, it doesn't work, the customer service doesn't care, it's a bit irritating.	Jingdong prefecture level city in Shandong
Jinfeng Hongyuan Flagship Store	Electricity savings3	It doesn't do anything.	Jingdong prefecture level city in Shandong
Jinfeng Hongyuan Flagship Store	Electricity savings3	It's good.	Jingdong prefecture level city in Shandong
Jinfeng Hongyuan Flagship Store	Electricity savings3	Used a month effect is not obvious, are psychological effects, feel are not less, did not save to the electricity, there are details of the problem, the store said to buy to send 5 fuse, in fact, received only three, small problems are not committed to in place, I believe that the product is not good enough	Jingdong prefecture level city in Shandong
Jinfeng Hongyuan Flagship Store	Electricity savings3	Well received quickly, looking quite high, but also sent 5 fuses. Customer service is very patient to teach the operation, in fact, quite simple, lazy to read the instructions ha!	Jingdong prefecture level city in Shandong
Jinfeng Hongyuan Flagship Store	Electricity savings3	You have to use it for a while to know the effect	Jingdong prefecture level city in Shandong
Jinfeng Hongyuan Flagship Store	Electricity savings3	Shipped very quickly, packaged very well, the price is particularly affordable, there are so many efficacy is also good, it is worth buying worth buying the product, the workmanship is very fine, better than imagined, recommended that everyone try not yet used, after the use of the follow-up comment	Jingdong prefecture level city in Shandong
Jinfeng Hongyuan Flagship Store	Electricity savings3	It looks regular. It's working now.	Jingdong prefecture level city in Shandong
Jinfeng Hongyuan Flagship Store	Electricity savings3	It arrived pretty fast and the owner is very responsible. Have not used it yet, will see after use	Jingdong prefecture level city in Shandong
Jinfeng Hongyuan Flagship Store	Electricity savings3	🎁 baby quality is very good, the first time I received the goods to open the first time to see, it is very good 👍, very satisfied 🙂, customer service also have a question and answer, thank you 🙏	Jingdong prefecture level city in Shandong
Jinfeng Hongyuan Flagship Store	Electricity savings3	I don't know if it's any good 。。。。。。	Jingdong prefecture level city in Shandong
Jinfeng Hongyuan Flagship Store	Electricity savings3	Buy electronic products! Still trust the things of the Jingdong self-owned store! Energy-saving effect to evaluate after heating!	Jingdong prefecture level city in Shandong
Jinfeng Hongyuan Flagship Store	Electricity savings3	The goods have been received, Blu is very beautiful, energy saving effect, to be confirmed only after actual use.	Jingdong prefecture level city in Shandong
Jinfeng Hongyuan Flagship Store	Electricity savings3	Jingdong's speed is so fast, has received, easy to operate. The display is quite good, how many volts what mode is quite understandable.	Jingdong prefecture level city in Shandong
Jinfeng Hongyuan Flagship Store	Electricity savings3	The first use, the effect is good to recommend it to colleagues to use, a month of electricity to save a lot or can be	Jingdong prefecture level city in Shandong
Jinfeng Hongyuan Flagship Store	Electricity savings3	I don't know if it's useful. Mom's is a store. I'll return it if it doesn't work.	Jingdong prefecture level city in Shandong
Jinfeng Hongyuan Flagship Store	Electricity savings3	I don't know if it works or not, I'll wait for two months to use it and then come back to comment!	Jingdong prefecture level city in Shandong
Jinfeng Hongyuan Flagship Store	Electricity savings3	I bought the second one now, and I've been using it for more than half a year, and I feel that it really saves electricity. Now it is summer, the weather is hot, sometimes the fan and air conditioning on all day, if not this power saver, the monthly electricity bill is quite high, but as long as the installation of this power saver, not afraid of its high electricity bill, absolutely let you wantonly use	Jingdong prefecture level city in Shandong
Jinfeng Hongyuan Flagship Store	Electricity savings3	The product is better than I thought it would be, the quality looks great, the voltage is stable, and I'm looking forward to some great power savings!	Jingdong prefecture level city in Shandong
Jinfeng Hongyuan Flagship Store	Electricity savings3	Inadvertently see bought a try, delivery is also quite timely, just do not know how to use, customer service patience to answer, with a pretty good, value for money!	Jingdong prefecture level city in Shandong
Jinfeng Hongyuan Flagship Store	Electricity savings3	It's better than nothing! Hopefully this will help save slightly.	Jingdong prefecture level city in Shandong
Jinfeng Hongyuan Flagship Store	Electricity savings3	Still trying it out, follow up	Jingdong prefecture level city in Shandong
Jinfeng Hongyuan Flagship Store	Electricity savings3	I've just started using it, I don't know how well it works yet, I hope it's good.	Jingdong prefecture level city in Shandong
Jinfeng Hongyuan Flagship Store	Electricity savings3	Fourth purchase, great baby!	Jingdong prefecture level city in Shandong
Jinfeng Hongyuan Flagship Store	Electricity savings3	Shipped really fast, just got it and use it to see what happens and then come back for a review!	Jingdong prefecture level city in Shandong
Jinfeng Hongyuan Flagship Store	Electricity savings3	Timely delivery **** good attitude	Jingdong prefecture level city in Shandong
Jinfeng Hongyuan Flagship Store	Electricity savings3	Home large and small things are purchased in the Jingdong Mall, have been accustomed to shopping in the Jingdong, many times can buy good quality and cheap products, and courier service attitude is particularly good, sometimes too many couriers are to help me to the home, so many years have been in the Jingdong Mall shopping, trust and support! Will also support the Jingdong as always!	Jingdong prefecture level city in Shandong
Jinfeng Hongyuan Flagship Store	Electricity savings3	Modified a pump and the modification worked.	Jingdong prefecture level city in Shandong
Jinfeng Hongyuan Flagship Store	Electricity savings3	Already installed, next time you pay the electric bill to see the results	Jingdong prefecture level city in Shandong
Jinfeng Hongyuan Flagship Store	Electricity savings3	The product is very good to use and practical to look at	Jingdong prefecture level city in Shandong
Jinfeng Hongyuan Flagship Store	Electricity savings3	Works great, great service, waiting for results.	Jingdong prefecture level city in Shandong
Jinfeng Hongyuan Flagship Store	Electricity savings3	I just used it and I'm not sure about the efficacy yet, I'll come back to evaluate it after a while.	Jingdong prefecture level city in Shandong
Jinfeng Hongyuan Flagship Store	Electricity savings3	Just received the goods, use a period of time to evaluate	Jingdong prefecture level city in Shandong
Jinfeng Hongyuan Flagship Store	Electricity savings3	Opened and tried very good, use it to see if there are results	Jingdong prefecture level city in Shandong
Jinfeng Hongyuan Flagship Store	Electricity savings3	Arrived today! Still trying it out!	Jingdong prefecture level city in Shandong
Jinfeng Hongyuan Flagship Store	Electricity savings3	Shipped quickly, well packaged, already installed, hope there is a power saving effect	Jingdong prefecture level city in Shandong
Jinfeng Hongyuan Flagship Store	Electricity savings3	I got it. It's great.	Jingdong prefecture level city in Shandong
Jinfeng Hongyuan Flagship Store	Electricity savings3	Haven't used it yet, will come back in a month	Jingdong prefecture level city in Shandong
Jinfeng Hongyuan Flagship Store	Electricity savings3	It looks good. It's good. Try it first.	Jingdong prefecture level city in Shandong
Jinfeng Hongyuan Flagship Store	Electricity savings3	It works so well that I have relatives, friends, coworkers, and 6 families that have purchased it for them through me.	Jingdong prefecture level city in Shandong
Jinfeng Hongyuan Flagship Store	Electricity savings3	In use	Jingdong prefecture level city in Shandong
Jinfeng Hongyuan Flagship Store	Electricity savings3	Received the goods today, power-saving effect do not know being used, the boss has a very good attitude, praise!	Jingdong prefecture level city in Shandong
Jinfeng Hongyuan Flagship Store	Electricity savings3	The product is compact and doesn't take up much space, but hopefully it's power efficient.	Jingdong prefecture level city in Shandong
Jinfeng Hongyuan Flagship Store	Electricity savings3	Power saving is indeed effective, I used for a period of time before coming to evaluate, the electricity bill is obviously less, the quality is good, the shopkeeper service is also very good, very patient,	Jingdong prefecture level city in Shandong
Jinfeng Hongyuan Flagship Store	Electricity savings3	It feels fine, I'll comment after I use it	Jingdong prefecture level city in Shandong

Jinfeng Hongyuan Flagship Store	Electricity savings3	I just received it and I don't know the effect yet! I will comment again after I use it and have results!	Jingdong prefecture level city in Shandong
Jinfeng Hongyuan Flagship Store	Electricity savings3	The goods look fine, I hope they really work!	Jingdong prefecture level city in Shandong
Jinfeng Hongyuan Flagship Store	Electricity savings3	I just don't know if it saves power	Jingdong prefecture level city in Shandong
Jinfeng Hongyuan Flagship Store	Electricity savings3	My friend introduced me to use this product, it is professional and I am looking forward to the long term effect~!	Jingdong prefecture level city in Shandong
Jinfeng Hongyuan Flagship Store	Electricity savings3	Try it before you judge.	Jingdong prefecture level city in Shandong
Jinfeng Hongyuan Flagship Store	Electricity savings3	It's great, the power savings are obvious! Kudos!	Jingdong prefecture level city in Shandong
Jinfeng Hongyuan Flagship Store	Electricity savings3	The quality is excellent, stabilizes the voltage and saves electricity, very satisfied.	Jingdong prefecture level city in Shandong
Jinfeng Hongyuan Flagship Store	Electricity savings3	You have to use it for a while to know the effect	Jingdong prefecture level city in Shandong
Jinfeng Hongyuan Flagship Store	Electricity savings3	It's on trial.	Jingdong prefecture level city in Shandong
Jinfeng Hongyuan Flagship Store	Electricity savings3	The stuff works great.	Jingdong prefecture level city in Shandong
Jinfeng Hongyuan Flagship Store	Electricity savings3	Doesn't seem to work, not as god as other reviews	Jingdong prefecture level city in Shandong
Jinfeng Hongyuan Flagship Store	Electricity savings3	It's not good quality, it doesn't work, it stops working after a few days, it doesn't work, the customer service doesn't care, it's a bit irritating.	Jingdong prefecture level city in Shandong
Jinfeng Hongyuan Flagship Store	Electricity savings3	It doesn't do anything.	Jingdong prefecture level city in Shandong
Jinfeng Hongyuan Flagship Store	Electricity savings3	It's good.	Jingdong prefecture level city in Shandong
Jinfeng Hongyuan Flagship Store	Electricity savings3	Used a month effect is not obvious, are psychological effects, feel are not less, did not save to the electricity, there are details of the problem, the store said to buy to send 5 fuse, in fact, received only three, small problems are not committed to in place, I believe that the product is not good enough	Jingdong prefecture level city in Shandong
Jinfeng Hongyuan Flagship Store	Electricity savings3	Well received quickly, looking quite high, but also sent 5 fuses. Customer service is very patient to teach the operation, in fact, quite simple, lazy to read the instructions ha!	Jingdong prefecture level city in Shandong
Jinfeng Hongyuan Flagship Store	Electricity savings3	You have to use it for a while to know the effect	Jingdong prefecture level city in Shandong
Jinfeng Hongyuan Flagship Store	Electricity savings3	Shipped very quickly, packaged very well, the price is particularly affordable, there are so many efficacy is also good, it is worth buying worth buying the product, the workmanship is very fine, better than imagined, recommended that everyone try not yet used, after the use of the follow-up comment	Jingdong prefecture level city in Shandong
Jinfeng Hongyuan Flagship Store	Electricity savings3	It looks regular. It's working now.	Jingdong prefecture level city in Shandong
Jinfeng Hongyuan Flagship Store	Electricity savings3	It arrived pretty fast and the owner is very responsible. Have not used it yet, will see after use	Jingdong prefecture level city in Shandong
Jinfeng Hongyuan Flagship Store	Electricity savings3	🎁 baby quality is very good, the first time I received the goods to open the first time to see, it is very good 👍, very satisfied 😊, customer service also have a question and answer, thank you 🙏	Jingdong prefecture level city in Shandong
Jinfeng Hongyuan Flagship Store	Electricity savings3	I don't know if it's any good 。。。。。。	Jingdong prefecture level city in Shandong
Jinfeng Hongyuan Flagship Store	Electricity savings3	Buy electronic products! Still trust the things of the Jingdong self-owned store! Energy-saving effect to evaluate after heating!	Jingdong prefecture level city in Shandong
Jinfeng Hongyuan Flagship Store	Electricity savings3	The goods have been received, Blu is very beautiful, energy saving effect, to be confirmed only after actual use.	Jingdong prefecture level city in Shandong
Jinfeng Hongyuan Flagship Store	Electricity savings3	Jingdong's speed is so fast, has received, easy to operate. The display is quite good, how many volts what mode is quite understandable.	Jingdong prefecture level city in Shandong
Jinfeng Hongyuan Flagship Store	Electricity savings3	The first use, the effect is good to recommend it to colleagues to use, a month of electricity to save a lot or can be	Jingdong prefecture level city in Shandong
Jinfeng Hongyuan Flagship Store	Electricity savings3	I don't know if it's useful. Mom's is a store. I'll return it if it doesn't work.	Jingdong prefecture level city in Shandong
Jinfeng Hongyuan Flagship Store	Electricity savings3	I don't know if it works or not, I'll wait for two months to use it and then come back to comment!	Jingdong prefecture level city in Shandong
Jinfeng Hongyuan Flagship Store	Electricity savings3	I bought the second one now, and I've been using it for more than half a year, and I feel that it really saves electricity. Now it is summer, the weather is hot, sometimes the fan and air conditioning on all day, if not this power saver, the monthly electricity bill is quite high, but as long as the installation of this power saver, not afraid of its high electricity bill, absolutely let you wantonly use	Jingdong prefecture level city in Shandong
Jinfeng Hongyuan Flagship Store	Electricity savings3	The product is better than I thought it would be, the quality looks great, the voltage is stable, and I'm looking forward to some great power savings!	Jingdong prefecture level city in Shandong
Jinfeng Hongyuan Flagship Store	Electricity savings3	Inadvertently see bought a try, delivery is also quite timely, just do not know how to use, customer service patience to answer, with a pretty good, value for money!	Jingdong prefecture level city in Shandong
Jinfeng Hongyuan Flagship Store	Electricity savings3	It's better than nothing! Hopefully this will help save slightly.	Jingdong prefecture level city in Shandong
Jinfeng Hongyuan Flagship Store	Electricity savings3	Still trying it out, follow up	Jingdong prefecture level city in Shandong
Jinfeng Hongyuan Flagship Store	Electricity savings3	I've just started using it, I don't know how well it works yet, I hope it's good.	Jingdong prefecture level city in Shandong
Jinfeng Hongyuan Flagship Store	Electricity savings3	Fourth purchase, great baby!	Jingdong prefecture level city in Shandong
Jinfeng Hongyuan Flagship Store	Electricity savings3	Shipped really fast, just got it and use it to see what happens and then come back for a review!	Jingdong prefecture level city in Shandong
Jinfeng Hongyuan Flagship Store	Electricity savings3	Timely delivery **** good attitude	Jingdong prefecture level city in Shandong
Jinfeng Hongyuan Flagship Store	Electricity savings3	Home large and small things are purchased in the Jingdong Mall, have been accustomed to shopping in the Jingdong, many times can buy good quality and cheap products, and courier service attitude is particularly good, sometimes too many couriers are to help me to the home, so many years have been in the Jingdong Mall shopping, trust and support! Will also support the Jingdong as always!	Jingdong prefecture level city in Shandong
Jinfeng Hongyuan Flagship Store	Electricity savings3	Modified a pump and the modification worked.	Jingdong prefecture level city in Shandong
Jinfeng Hongyuan Flagship Store	Electricity savings3	Already installed, next time you pay the electric bill to see the results	Jingdong prefecture level city in Shandong
Jinfeng Hongyuan Flagship Store	Electricity savings3	The product is very good to use and practical to look at	Jingdong prefecture level city in Shandong
Jinfeng Hongyuan Flagship Store	Electricity savings3	Works great, great service, waiting for results.	Jingdong prefecture level city in Shandong
Jinfeng Hongyuan Flagship Store	Electricity savings3	I just used it and I'm not sure about the efficacy yet, I'll come back to evaluate it after a while.	Jingdong prefecture level city in Shandong
Jinfeng Hongyuan Flagship Store	Electricity savings3	Just received the goods, use a period of time to evaluate	Jingdong prefecture level city in Shandong
Jinfeng Hongyuan Flagship Store	Electricity savings3	Opened and tried very good, use it to see if there are results	Jingdong prefecture level city in Shandong
Jinfeng Hongyuan Flagship Store	Electricity savings3	Arrived today! Still trying it out!	Jingdong prefecture level city in Shandong
Jinfeng Hongyuan Flagship Store	Electricity savings3	Shipped quickly, well packaged, already installed, hope there is a power saving effect	Jingdong prefecture level city in Shandong
Jinfeng Hongyuan Flagship Store	Electricity savings3	I got it. It's great.	Jingdong prefecture level city in Shandong

Jinfeng Hongyuan Flagship Store	Electricity savings3	Haven't used it yet, will come back in a month	Jingdong prefecture level city in Shandong
Jinfeng Hongyuan Flagship Store	Electricity savings3	It looks good. It's good. Try it first.	Jingdong prefecture level city in Shandong
Jinfeng Hongyuan Flagship Store	Electricity savings3	It works so well that I have relatives, friends, coworkers, and 6 families that have purchased it for them through me.	Jingdong prefecture level city in Shandong
Jinfeng Hongyuan Flagship Store	Electricity savings3	In use	Jingdong prefecture level city in Shandong
Jinfeng Hongyuan Flagship Store	Electricity savings3	Received the goods today, power-saving effect do not know being used, the boss has a very good attitude, praise!	Jingdong prefecture level city in Shandong
Jinfeng Hongyuan Flagship Store	Electricity savings3	The product is compact and doesn't take up much space, but hopefully it's power efficient.	Jingdong prefecture level city in Shandong
Jinfeng Hongyuan Flagship Store	Electricity savings3	Power saving is indeed effective, I used for a period of time before coming to evaluate, the electricity bill is obviously less, the quality is good, the shopkeeper service is also very good, very patient,	Jingdong prefecture level city in Shandong
Jinfeng Hongyuan Flagship Store	Electricity savings3	It feels fine, I'll comment after I use it	Jingdong prefecture level city in Shandong
Jinfeng Hongyuan Flagship Store	Electricity savings3	I just received it and I don't know the effect yet! I will comment again after I use it and have results!	Jingdong prefecture level city in Shandong
Jinfeng Hongyuan Flagship Store	Electricity savings3	The goods look fine, I hope they really work!	Jingdong prefecture level city in Shandong
Jinfeng Hongyuan Flagship Store	Electricity savings3	I just don't know if it saves power	Jingdong prefecture level city in Shandong
Jinfeng Hongyuan Flagship Store	Electricity savings3	My friend introduced me to use this product, it is professional and I am looking forward to the long term effect~!	Jingdong prefecture level city in Shandong
Jinfeng Hongyuan Flagship Store	Electricity savings3	Try it before you judge.	Jingdong prefecture level city in Shandong
Jinfeng Hongyuan Flagship Store	Electricity savings3	It's great, the power savings are obvious! Kudos!	Jingdong prefecture level city in Shandong
Jinfeng Hongyuan Flagship Store	Electricity savings3	The quality is excellent, stabilizes the voltage and saves electricity, very satisfied.	Jingdong prefecture level city in Shandong
Jinfeng Hongyuan Flagship Store	Electricity savings3	You have to use it for a while to know the effect	Jingdong prefecture level city in Shandong
Jinfeng Hongyuan Flagship Store	Electricity savings3	It's on trial.	Jingdong prefecture level city in Shandong
Jinfeng Hongyuan Flagship Store	Electricity savings3	The stuff works great.	Jingdong prefecture level city in Shandong
Jinfeng Hongyuan Flagship Store	Electricity savings3	Doesn't seem to work, not as god as other reviews	Jingdong prefecture level city in Shandong
Jinfeng Hongyuan Flagship Store	Electricity savings3	It's not good quality, it doesn't work, it stops working after a few days, it doesn't work, the customer service doesn't care, it's a bit irritating.	Jingdong prefecture level city in Shandong
Jinfeng Hongyuan Flagship Store	Electricity savings3	It doesn't do anything.	Jingdong prefecture level city in Shandong
Jinfeng Hongyuan Flagship Store	Electricity savings3	It's good.	Jingdong prefecture level city in Shandong
Jinfeng Hongyuan Flagship Store	Electricity savings3	Used a month effect is not obvious, are psychological effects, feel are not less, did not save to the electricity, there are details of the problem, the store said to buy to send 5 fuse, in fact, received only three, small problems are not committed to in place, I believe that the product is not good enough	Jingdong prefecture level city in Shandong
Jinfeng Hongyuan Flagship Store	Electricity savings3	Well received quickly, looking quite high, but also sent 5 fuses. Customer service is very patient to teach the operation, in fact, quite simple, lazy to read the instructions ha!	Jingdong prefecture level city in Shandong
Jinfeng Hongyuan Flagship Store	Electricity savings3	You have to use it for a while to know the effect	Jingdong prefecture level city in Shandong
Jinfeng Hongyuan Flagship Store	Electricity savings3	Shipped very quickly, packaged very well, the price is particularly affordable, there are so many efficacy is also good, it is worth buying worth buying the product, the workmanship is very fine, better than imagined, recommended that everyone try not yet used, after the use of the follow-up comment	Jingdong prefecture level city in Shandong
Jinfeng Hongyuan Flagship Store	Electricity savings3	It looks regular. It's working now.	Jingdong prefecture level city in Shandong
Jinfeng Hongyuan Flagship Store	Electricity savings3	It arrived pretty fast and the owner is very responsible. Have not used it yet, will see after use	Jingdong prefecture level city in Shandong
Jinfeng Hongyuan Flagship Store	Electricity savings3	🎁 baby quality is very good, the first time I received the goods to open the first time to see, it is very good 👍, very satisfied 😊, customer service also have a question and answer, thank you 🙏	Jingdong prefecture level city in Shandong
Jinfeng Hongyuan Flagship Store	Electricity savings3	I don't know if it's any good 。。。。。。	Jingdong prefecture level city in Shandong
Jinfeng Hongyuan Flagship Store	Electricity savings3	Buy electronic products! Still trust the things of the Jingdong self-owned store! Energy-saving effect to evaluate after heating!	Jingdong prefecture level city in Shandong
Jinfeng Hongyuan Flagship Store	Electricity savings3	The goods have been received, Blu is very beautiful, energy saving effect, to be confirmed only after actual use.	Jingdong prefecture level city in Shandong
Jinfeng Hongyuan Flagship Store	Electricity savings3	Jingdong's speed is so fast, has received, easy to operate. The display is quite good, how many volts what mode is quite understandable.	Jingdong prefecture level city in Shandong
Jinfeng Hongyuan Flagship Store	Electricity savings3	The first use, the effect is good to recommend it to colleagues to use, a month of electricity to save a lot or can be	Jingdong prefecture level city in Shandong
Jinfeng Hongyuan Flagship Store	Electricity savings3	I don't know if it's useful. Mom's is a store. I'll return it if it doesn't work.	Jingdong prefecture level city in Shandong
Jinfeng Hongyuan Flagship Store	Electricity savings3	I don't know if it works or not, I'll wait for two months to use it and then come back to comment!	Jingdong prefecture level city in Shandong
Jinfeng Hongyuan Flagship Store	Electricity savings3	I bought the second one now, and I've been using it for more than half a year, and I feel that it really saves electricity. Now it is summer, the weather is hot, sometimes the fan and air conditioning on all day, if not this power saver, the monthly electricity bill is quite high, but as long as the installation of this power saver, not afraid of its high electricity bill, absolutely let you wantonly use	Jingdong prefecture level city in Shandong
Jinfeng Hongyuan Flagship Store	Electricity savings3	The product is better than I thought it would be, the quality looks great, the voltage is stable, and I'm looking forward to some great power savings!	Jingdong prefecture level city in Shandong
Jinfeng Hongyuan Flagship Store	Electricity savings3	Inadvertently see bought a try, delivery is also quite timely, just do not know how to use, customer service patience to answer, with a pretty good, value for money!	Jingdong prefecture level city in Shandong
Jinfeng Hongyuan Flagship Store	Electricity savings3	It's better than nothing! Hopefully this will help save slightly.	Jingdong prefecture level city in Shandong
Jinfeng Hongyuan Flagship Store	Electricity savings3	Still trying it out, follow up	Jingdong prefecture level city in Shandong
Jinfeng Hongyuan Flagship Store	Electricity savings3	I've just started using it, I don't know how well it works yet, I hope it's good.	Jingdong prefecture level city in Shandong
Jinfeng Hongyuan Flagship Store	Electricity savings3	Fourth purchase, great baby!	Jingdong prefecture level city in Shandong
Jinfeng Hongyuan Flagship Store	Electricity savings3	Shipped really fast, just got it and use it to see what happens and then come back for a review!	Jingdong prefecture level city in Shandong
Jinfeng Hongyuan Flagship Store	Electricity savings3	Timely delivery **** good attitude	Jingdong prefecture level city in Shandong
Jinfeng Hongyuan Flagship Store	Electricity savings3	Home large and small things are purchased in the Jingdong Mall, have been accustomed to shopping in the Jingdong, many times can buy good quality and cheap products, and courier service attitude is particularly good, sometimes too many couriers are to help me to the home, so many years have been in the Jingdong Mall shopping, trust and support! Will also support the Jingdong as always!	Jingdong prefecture level city in Shandong
Jinfeng Hongyuan Flagship Store	Electricity savings3	Modified a pump and the modification worked.	Jingdong prefecture level city in Shandong
Jinfeng Hongyuan Flagship Store	Electricity savings3	Already installed, next time you pay the electric bill to see the results	Jingdong prefecture level city in Shandong

Jinfeng Hongyuan Flagship Store	Electricity savings3	The product is very good to use and practical to look at	Jingdong prefecture level city in Shandong
Jinfeng Hongyuan Flagship Store	Electricity savings3	Works great, great service, waiting for results.	Jingdong prefecture level city in Shandong
Jinfeng Hongyuan Flagship Store	Electricity savings3	I just used it and I'm not sure about the efficacy yet, I'll come back to evaluate it after a while.	Jingdong prefecture level city in Shandong
Jinfeng Hongyuan Flagship Store	Electricity savings3	Just received the goods, use a period of time to evaluate	Jingdong prefecture level city in Shandong
Jinfeng Hongyuan Flagship Store	Electricity savings3	Opened and tried very good, use it to see if there are results	Jingdong prefecture level city in Shandong
Jinfeng Hongyuan Flagship Store	Electricity savings3	Arrived today! Still trying it out!	Jingdong prefecture level city in Shandong
Jinfeng Hongyuan Flagship Store	Electricity savings3	Shipped quickly, well packaged, already installed, hope there is a power saving effect	Jingdong prefecture level city in Shandong
Jinfeng Hongyuan Flagship Store	Electricity savings3	I got it. It's great.	Jingdong prefecture level city in Shandong
Jinfeng Hongyuan Flagship Store	Electricity savings3	Haven't used it yet, will come back in a month	Jingdong prefecture level city in Shandong
Jinfeng Hongyuan Flagship Store	Electricity savings3	It looks good. It's good. Try it first.	Jingdong prefecture level city in Shandong
Jinfeng Hongyuan Flagship Store	Electricity savings3	It works so well that I have relatives, friends, coworkers, and 6 families that have purchased it for them through me.	Jingdong prefecture level city in Shandong
Jinfeng Hongyuan Flagship Store	Electricity savings3	In use	Jingdong prefecture level city in Shandong
Jinfeng Hongyuan Flagship Store	Electricity savings3	Received the goods today, power-saving effect do not know being used, the boss has a very good attitude, praise!	Jingdong prefecture level city in Shandong
Jinfeng Hongyuan Flagship Store	Electricity savings3	The product is compact and doesn't take up much space, but hopefully it's power efficient.	Jingdong prefecture level city in Shandong
Jinfeng Hongyuan Flagship Store	Electricity savings3	Power saving is indeed effective, I used for a period of time before coming to evaluate, the electricity bill is obviously less, the quality is good, the shopkeeper service is also very good, very patient,	Jingdong prefecture level city in Shandong
Jinfeng Hongyuan Flagship Store	Electricity savings3	It feels fine, I'll comment after I use it	Jingdong prefecture level city in Shandong
Jinfeng Hongyuan Flagship Store	Electricity savings3	I just received it and I don't know the effect yet! I will comment again after I use it and have results!	Jingdong prefecture level city in Shandong
Jinfeng Hongyuan Flagship Store	Electricity savings3	The goods look fine, I hope they really work!	Jingdong prefecture level city in Shandong
Jinfeng Hongyuan Flagship Store	Electricity savings3	I just don't know if it saves power	Jingdong prefecture level city in Shandong
Jinfeng Hongyuan Flagship Store	Electricity savings3	My friend introduced me to use this product, it is professional and I am looking forward to the long term effect~!	Jingdong prefecture level city in Shandong
Jinfeng Hongyuan Flagship Store	Electricity savings3	Try it before you judge.	Jingdong prefecture level city in Shandong
Jinfeng Hongyuan Flagship Store	Electricity savings3	It's great, the power savings are obvious! Kudos!	Jingdong prefecture level city in Shandong
Jinfeng Hongyuan Flagship Store	Electricity savings3	The quality is excellent, stabilizes the voltage and saves electricity, very satisfied.	Jingdong prefecture level city in Shandong
Jinfeng Hongyuan Flagship Store	Electricity savings3	You have to use it for a while to know the effect	Jingdong prefecture level city in Shandong
Jinfeng Hongyuan Flagship Store	Electricity savings3	It's on trial.	Jingdong prefecture level city in Shandong
Jinfeng Hongyuan Flagship Store	Electricity savings3	The stuff works great.	Jingdong prefecture level city in Shandong
Jinfeng Hongyuan Flagship Store	Electricity savings3	Doesn't seem to work, not as god as other reviews	Jingdong prefecture level city in Shandong
Jinfeng Hongyuan Flagship Store	Electricity savings3	It's not good quality, it doesn't work, it stops working after a few days, it doesn't work, the customer service doesn't care, it's a bit irritating.	Jingdong prefecture level city in Shandong
Jinfeng Hongyuan Flagship Store	Electricity savings3	It doesn't do anything.	Jingdong prefecture level city in Shandong
Jinfeng Hongyuan Flagship Store	Electricity savings3	It's good.	Jingdong prefecture level city in Shandong
Jinfeng Hongyuan Flagship Store	Electricity savings3	Used a month effect is not obvious, are psychological effects, feel are not less, did not save to the electricity, there are details of the problem, the store said to buy to send 5 fuse, in fact, received only three, small problems are not committed to in place, I believe that the product is not good enough	Jingdong prefecture level city in Shandong
Jinfeng Hongyuan Flagship Store	Electricity savings3	Well received quickly, looking quite high, but also sent 5 fuses. Customer service is very patient to teach the operation, in fact, quite simple, lazy to read the instructions ha!	Jingdong prefecture level city in Shandong
Jinfeng Hongyuan Flagship Store	Electricity savings3	You have to use it for a while to know the effect	Jingdong prefecture level city in Shandong
Jinfeng Hongyuan Flagship Store	Electricity savings3	Shipped very quickly, packaged very well, the price is particularly affordable, there are so many efficacy is also good, it is worth buying worth buying the product, the workmanship is very fine, better than imagined, recommended that everyone try not yet used, after the use of the follow-up comment	Jingdong prefecture level city in Shandong
Jinfeng Hongyuan Flagship Store	Electricity savings3	It looks regular. It's working now.	Jingdong prefecture level city in Shandong
Jinfeng Hongyuan Flagship Store	Electricity savings3	It arrived pretty fast and the owner is very responsible. Have not used it yet, will see after use	Jingdong prefecture level city in Shandong
Jinfeng Hongyuan Flagship Store	Electricity savings3	🎁 baby quality is very good, the first time I received the goods to open the first time to see, it is very good 👍, very satisfied 😊, customer service also have a question and answer, thank you 🙏	Jingdong prefecture level city in Shandong
Jinfeng Hongyuan Flagship Store	Electricity savings3	I don't know if it's any good 。。。。。。	Jingdong prefecture level city in Shandong
Jinfeng Hongyuan Flagship Store	Electricity savings3	Buy electronic products! Still trust the things of the Jingdong self-owned store! Energy-saving effect to evaluate after heating!	Jingdong prefecture level city in Shandong
Jinfeng Hongyuan Flagship Store	Electricity savings3	The goods have been received, Blu is very beautiful, energy saving effect, to be confirmed only after actual use.	Jingdong prefecture level city in Shandong
Jinfeng Hongyuan Flagship Store	Electricity savings3	Jingdong's speed is so fast, has received, easy to operate. The display is quite good, how many volts what mode is quite understandable.	Jingdong prefecture level city in Shandong
Jinfeng Hongyuan Flagship Store	Electricity savings3	The first use, the effect is good to recommend it to colleagues to use, a month of electricity to save a lot or can be	Jingdong prefecture level city in Shandong
Jinfeng Hongyuan Flagship Store	Electricity savings3	I don't know if it's useful. Mom's is a store. I'll return it if it doesn't work.	Jingdong prefecture level city in Shandong
Jinfeng Hongyuan Flagship Store	Electricity savings3	I don't know if it works or not, I'll wait for two months to use it and then come back to comment!	Jingdong prefecture level city in Shandong
Jinfeng Hongyuan Flagship Store	Electricity savings3	I bought the second one now, and I've been using it for more than half a year, and I feel that it really saves electricity. Now it is summer, the weather is hot, sometimes the fan and air conditioning on all day, if not this power saver, the monthly electricity bill is quite high, but as long as the installation of this power saver, not afraid of its high electricity bill, absolutely let you wantonly use	Jingdong prefecture level city in Shandong
Jinfeng Hongyuan Flagship Store	Electricity savings3	The product is better than I thought it would be, the quality looks great, the voltage is stable, and I'm looking forward to some great power savings!	Jingdong prefecture level city in Shandong
Jinfeng Hongyuan Flagship Store	Electricity savings3	Inadvertently see bought a try, delivery is also quite timely, just do not know how to use, customer service patience to answer, with a pretty good, value for money!	Jingdong prefecture level city in Shandong
Jinfeng Hongyuan Flagship Store	Electricity savings3	It's better than nothing! Hopefully this will help save slightly.	Jingdong prefecture level city in Shandong
Jinfeng Hongyuan Flagship Store	Electricity savings3	Still trying it out, follow up	Jingdong prefecture level city in Shandong

Jinfeng Hongyuan Flagship Store	Electricity savings3	I've just started using it, I don't know how well it works yet, I hope it's good.	Jingdong prefecture level city in Shandong
Jinfeng Hongyuan Flagship Store	Electricity savings3	Fourth purchase, great baby!	Jingdong prefecture level city in Shandong
Jinfeng Hongyuan Flagship Store	Electricity savings3	Shipped really fast, just got it and use it to see what happens and then come back for a review!	Jingdong prefecture level city in Shandong
Jinfeng Hongyuan Flagship Store	Electricity savings3	Timely delivery **** good attitude	Jingdong prefecture level city in Shandong
Jinfeng Hongyuan Flagship Store	Electricity savings3	Home large and small things are purchased in the Jingdong Mall, have been accustomed to shopping in the Jingdong, many times can buy good quality and cheap products, and courier service attitude is particularly good, sometimes too many couriers are to help me to the home, so many years have been in the Jingdong Mall shopping, trust and support! Will also support the Jingdong as always!	Jingdong prefecture level city in Shandong
Jinfeng Hongyuan Flagship Store	Electricity savings3	Modified a pump and the modification worked.	Jingdong prefecture level city in Shandong
Jinfeng Hongyuan Flagship Store	Electricity savings3	Already installed, next time you pay the electric bill to see the results	Jingdong prefecture level city in Shandong
Jinfeng Hongyuan Flagship Store	Electricity savings3	The product is very good to use and practical to look at	Jingdong prefecture level city in Shandong
Jinfeng Hongyuan Flagship Store	Electricity savings3	Works great, great service, waiting for results.	Jingdong prefecture level city in Shandong
Jinfeng Hongyuan Flagship Store	Electricity savings3	I just used it and I'm not sure about the efficacy yet, I'll come back to evaluate it after a while.	Jingdong prefecture level city in Shandong
Jinfeng Hongyuan Flagship Store	Electricity savings3	Just received the goods, use a period of time to evaluate	Jingdong prefecture level city in Shandong
Jinfeng Hongyuan Flagship Store	Electricity savings3	Opened and tried very good, use it to see if there are results	Jingdong prefecture level city in Shandong
Jinfeng Hongyuan Flagship Store	Electricity savings3	Arrived today! Still trying it out!	Jingdong prefecture level city in Shandong
Jinfeng Hongyuan Flagship Store	Electricity savings3	Shipped quickly, well packaged, already installed, hope there is a power saving effect	Jingdong prefecture level city in Shandong
Jinfeng Hongyuan Flagship Store	Electricity savings3	I got it. It's great.	Jingdong prefecture level city in Shandong
Jinfeng Hongyuan Flagship Store	Electricity savings3	Haven't used it yet, will come back in a month	Jingdong prefecture level city in Shandong
Jinfeng Hongyuan Flagship Store	Electricity savings3	It looks good. It's good. Try it first.	Jingdong prefecture level city in Shandong
Jinfeng Hongyuan Flagship Store	Electricity savings3	It works so well that I have relatives, friends, coworkers, and 6 families that have purchased it for them through me.	Jingdong prefecture level city in Shandong
Jinfeng Hongyuan Flagship Store	Electricity savings3	In use	Jingdong prefecture level city in Shandong
Jinfeng Hongyuan Flagship Store	Electricity savings3	Received the goods today, power-saving effect do not know being used, the boss has a very good attitude, praise!	Jingdong prefecture level city in Shandong
Jinfeng Hongyuan Flagship Store	Electricity savings3	The product is compact and doesn't take up much space, but hopefully it's power efficient.	Jingdong prefecture level city in Shandong
Jinfeng Hongyuan Flagship Store	Electricity savings3	Power saving is indeed effective, I used for a period of time before coming to evaluate, the electricity bill is obviously less, the quality is good, the shopkeeper service is also very good, very patient,	Jingdong prefecture level city in Shandong
Jinfeng Hongyuan Flagship Store	Electricity savings3	It feels fine, I'll comment after I use it	Jingdong prefecture level city in Shandong
Jinfeng Hongyuan Flagship Store	Electricity savings3	I just received it and I don't know the effect yet! I will comment again after I use it and have results!	Jingdong prefecture level city in Shandong
Jinfeng Hongyuan Flagship Store	Electricity savings3	The goods look fine, I hope they really work!	Jingdong prefecture level city in Shandong
Jinfeng Hongyuan Flagship Store	Electricity savings3	I just don't know if it saves power	Jingdong prefecture level city in Shandong
Jinfeng Hongyuan Flagship Store	Electricity savings3	My friend introduced me to use this product, it is professional and I am looking forward to the long term effect~!	Jingdong prefecture level city in Shandong
Jinfeng Hongyuan Flagship Store	Electricity savings3	Try it before you judge.	Jingdong prefecture level city in Shandong
Jinfeng Hongyuan Flagship Store	Electricity savings3	It's great, the power savings are obvious! Kudos!	Jingdong prefecture level city in Shandong
Jinfeng Hongyuan Flagship Store	Electricity savings3	The quality is excellent, stabilizes the voltage and saves electricity, very satisfied.	Jingdong prefecture level city in Shandong
Jinfeng Hongyuan Flagship Store	Electricity savings3	You have to use it for a while to know the effect	Jingdong prefecture level city in Shandong
Jinfeng Hongyuan Flagship Store	Electricity savings3	It's on trial.	Jingdong prefecture level city in Shandong
Jinfeng Hongyuan Flagship Store	Electricity savings3	The stuff works great.	Jingdong prefecture level city in Shandong
Jinfeng Hongyuan Flagship Store	Electricity savings3	Doesn't seem to work, not as god as other reviews	Jingdong prefecture level city in Shandong
Jinfeng Hongyuan Flagship Store	Electricity savings3	It's not good quality, it doesn't work, it stops working after a few days, it doesn't work, the customer service doesn't care, it's a bit irritating.	Jingdong prefecture level city in Shandong
Jinfeng Hongyuan Flagship Store	Electricity savings3	It doesn't do anything.	Jingdong prefecture level city in Shandong
Jinfeng Hongyuan Flagship Store	Electricity savings3	It's good.	Jingdong prefecture level city in Shandong
Jinfeng Hongyuan Flagship Store	Electricity savings3	Used a month effect is not obvious, are psychological effects, feel are not less, did not save to the electricity, there are details of the problem, the store said to buy to send 5 fuse, in fact, received only three, small problems are not committed to in place, I believe that the product is not good enough	Jingdong prefecture level city in Shandong
Jinfeng Hongyuan Flagship Store	Electricity savings3	Well received quickly, looking quite high, but also sent 5 fuses. Customer service is very patient to teach the operation, in fact, quite simple, lazy to read the instructions ha!	Jingdong prefecture level city in Shandong
Jinfeng Hongyuan Flagship Store	Electricity savings3	You have to use it for a while to know the effect	Jingdong prefecture level city in Shandong
Jinfeng Hongyuan Flagship Store	Electricity savings3	Shipped very quickly, packaged very well, the price is particularly affordable, there are so many efficacy is also good, it is worth buying worth buying the product, the workmanship is very fine, better than imagined, recommended that everyone try not yet used, after the use of the follow-up comment	Jingdong prefecture level city in Shandong
Jinfeng Hongyuan Flagship Store	Electricity savings3	It looks regular. It's working now.	Jingdong prefecture level city in Shandong
Jinfeng Hongyuan Flagship Store	Electricity savings3	It arrived pretty fast and the owner is very responsible. Have not used it yet, will see after use	Jingdong prefecture level city in Shandong
Jinfeng Hongyuan Flagship Store	Electricity savings3	🎁 baby quality is very good, the first time I received the goods to open the first time to see, it is very good 👍, very satisfied 😊, customer service also have a question and answer, thank you 🙏	Jingdong prefecture level city in Shandong
Jinfeng Hongyuan Flagship Store	Electricity savings3	I don't know if it's any good 。。。。。。	Jingdong prefecture level city in Shandong
Jinfeng Hongyuan Flagship Store	Electricity savings3	Buy electronic products! Still trust the things of the Jingdong self-owned store! Energy-saving effect to evaluate after heating!	Jingdong prefecture level city in Shandong
Jinfeng Hongyuan Flagship Store	Electricity savings3	The goods have been received, Blu is very beautiful, energy saving effect, to be confirmed only after actual use.	Jingdong prefecture level city in Shandong
Jinfeng Hongyuan Flagship Store	Electricity savings3	Jingdong's speed is so fast, has received, easy to operate. The display is quite good, how many volts what mode is quite understandable.	Jingdong prefecture level city in Shandong
Jinfeng Hongyuan Flagship Store	Electricity savings3	The first use, the effect is good to recommend it to colleagues to use, a month of electricity to save a lot or can be	Jingdong prefecture level city in Shandong

Jinfeng Hongyuan Flagship Store	Electricity savings3	I don't know if it's useful. Mom's is a store. I'll return it if it doesn't work.	Jingdong prefecture level city in Shandong
Jinfeng Hongyuan Flagship Store	Electricity savings3	I don't know if it works or not, I'll wait for two months to use it and then come back to comment!	Jingdong prefecture level city in Shandong
Jinfeng Hongyuan Flagship Store	Electricity savings3	I bought the second one now, and I've been using it for more than half a year, and I feel that it really saves electricity. Now it is summer, the weather is hot, sometimes the fan and air conditioning on all day, if not this power saver, the monthly electricity bill is quite high, but as long as the installation of this power saver, not afraid of its high electricity bill, absolutely let you wantonly use	Jingdong prefecture level city in Shandong
Jinfeng Hongyuan Flagship Store	Electricity savings3	The product is better than I thought it would be, the quality looks great, the voltage is stable, and I'm looking forward to some great power savings!	Jingdong prefecture level city in Shandong
Jinfeng Hongyuan Flagship Store	Electricity savings3	Inadvertently see bought a try, delivery is also quite timely, just do not know how to use, customer service patience to answer, with a pretty good, value for money!	Jingdong prefecture level city in Shandong
Jinfeng Hongyuan Flagship Store	Electricity savings3	It's better than nothing! Hopefully this will help save slightly.	Jingdong prefecture level city in Shandong
Jinfeng Hongyuan Flagship Store	Electricity savings3	Still trying it out, follow up	Jingdong prefecture level city in Shandong
Jinfeng Hongyuan Flagship Store	Electricity savings3	I've just started using it, I don't know how well it works yet, I hope it's good.	Jingdong prefecture level city in Shandong
Jinfeng Hongyuan Flagship Store	Electricity savings3	Fourth purchase, great baby!	Jingdong prefecture level city in Shandong
Jinfeng Hongyuan Flagship Store	Electricity savings3	Shipped really fast, just got it and use it to see what happens and then come back for a review!	Jingdong prefecture level city in Shandong
Jinfeng Hongyuan Flagship Store	Electricity savings3	Timely delivery **** good attitude	Jingdong prefecture level city in Shandong
Jinfeng Hongyuan Flagship Store	Electricity savings3	Home large and small things are purchased in the Jingdong Mall, have been accustomed to shopping in the Jingdong, many times can buy good quality and cheap products, and courier service attitude is particularly good, sometimes too many couriers are to help me to the home, so many years have been in the Jingdong Mall shopping, trust and support! Will also support the Jingdong as always!	Jingdong prefecture level city in Shandong
Jinfeng Hongyuan Flagship Store	Electricity savings3	Modified a pump and the modification worked.	Jingdong prefecture level city in Shandong
Jinfeng Hongyuan Flagship Store	Electricity savings3	Already installed, next time you pay the electric bill to see the results	Jingdong prefecture level city in Shandong
Jinfeng Hongyuan Flagship Store	Electricity savings3	The product is very good to use and practical to look at	Jingdong prefecture level city in Shandong
Jinfeng Hongyuan Flagship Store	Electricity savings3	Works great, great service, waiting for results.	Jingdong prefecture level city in Shandong
Jinfeng Hongyuan Flagship Store	Electricity savings3	I just used it and I'm not sure about the efficacy yet, I'll come back to evaluate it after a while.	Jingdong prefecture level city in Shandong
Jinfeng Hongyuan Flagship Store	Electricity savings3	Just received the goods, use a period of time to evaluate	Jingdong prefecture level city in Shandong
Jinfeng Hongyuan Flagship Store	Electricity savings3	Opened and tried very good, use it to see if there are results	Jingdong prefecture level city in Shandong
Jinfeng Hongyuan Flagship Store	Electricity savings3	Arrived today! Still trying it out!	Jingdong prefecture level city in Shandong
Jinfeng Hongyuan Flagship Store	Electricity savings3	Shipped quickly, well packaged, already installed, hope there is a power saving effect	Jingdong prefecture level city in Shandong
Jinfeng Hongyuan Flagship Store	Electricity savings3	I got it. It's great.	Jingdong prefecture level city in Shandong
Jinfeng Hongyuan Flagship Store	Electricity savings3	Haven't used it yet, will come back in a month	Jingdong prefecture level city in Shandong
Jinfeng Hongyuan Flagship Store	Electricity savings3	It looks good. It's good. Try it first.	Jingdong prefecture level city in Shandong
Jinfeng Hongyuan Flagship Store	Electricity savings3	It works so well that I have relatives, friends, coworkers, and 6 families that have purchased it for them through me.	Jingdong prefecture level city in Shandong
Jinfeng Hongyuan Flagship Store	Electricity savings3	In use	Jingdong prefecture level city in Shandong
Jinfeng Hongyuan Flagship Store	Electricity savings3	Received the goods today, power-saving effect do not know being used, the boss has a very good attitude, praise!	Jingdong prefecture level city in Shandong
Jinfeng Hongyuan Flagship Store	Electricity savings3	The product is compact and doesn't take up much space, but hopefully it's power efficient.	Jingdong prefecture level city in Shandong
Jinfeng Hongyuan Flagship Store	Electricity savings3	Power saving is indeed effective, I used for a period of time before coming to evaluate, the electricity bill is obviously less, the quality is good, the shopkeeper service is also very good, very patient,	Jingdong prefecture level city in Shandong
Jinfeng Hongyuan Flagship Store	Electricity savings3	It feels fine, I'll comment after I use it	Jingdong prefecture level city in Shandong
Jinfeng Hongyuan Flagship Store	Electricity savings3	I just received it and I don't know the effect yet! I will comment again after I use it and have results!	Jingdong prefecture level city in Shandong
Jinfeng Hongyuan Flagship Store	Electricity savings3	The goods look fine, I hope they really work!	Jingdong prefecture level city in Shandong
Jinfeng Hongyuan Flagship Store	Electricity savings3	I just don't know if it saves power	Jingdong prefecture level city in Shandong
Jinfeng Hongyuan Flagship Store	Electricity savings3	My friend introduced me to use this product, it is professional and I am looking forward to the long term effect~!	Jingdong prefecture level city in Shandong
Jinfeng Hongyuan Flagship Store	Electricity savings3	Try it before you judge.	Jingdong prefecture level city in Shandong
Jinfeng Hongyuan Flagship Store	Electricity savings3	It's great, the power savings are obvious! Kudos!	Jingdong prefecture level city in Shandong
Jinfeng Hongyuan Flagship Store	Electricity savings3	The quality is excellent, stabilizes the voltage and saves electricity, very satisfied.	Jingdong prefecture level city in Shandong
Jinfeng Hongyuan Flagship Store	Electricity savings3	You have to use it for a while to know the effect	Jingdong prefecture level city in Shandong
Jinfeng Hongyuan Flagship Store	Electricity savings3	It's on trial.	Jingdong prefecture level city in Shandong
Jinfeng Hongyuan Flagship Store	Electricity savings3	The stuff works great.	Jingdong prefecture level city in Shandong
Jinfeng Hongyuan Flagship Store	Electricity savings3	Doesn't seem to work, not as god as other reviews	Jingdong prefecture level city in Shandong
Jinfeng Hongyuan Flagship Store	Electricity savings3	It's not good quality, it doesn't work, it stops working after a few days, it doesn't work, the customer service doesn't care, it's a bit irritating.	Jingdong prefecture level city in Shandong
Jinfeng Hongyuan Flagship Store	Electricity savings3	It doesn't do anything.	Jingdong prefecture level city in Shandong
Jinfeng Hongyuan Flagship Store	Electricity savings3	It's good.	Jingdong prefecture level city in Shandong
Jinfeng Hongyuan Flagship Store	Electricity savings3	Used a month effect is not obvious, are psychological effects, feel are not less, did not save to the electricity, there are details of the problem, the store said to buy to send 5 fuse, in fact, received only three, small problems are not committed to in place, I believe that the product is not good enough	Jingdong prefecture level city in Shandong
Jinfeng Hongyuan Flagship Store	Electricity savings3	Well received quickly, looking quite high, but also sent 5 fuses. Customer service is very patient to teach the operation, in fact, quite simple, lazy to read the instructions ha!	Jingdong prefecture level city in Shandong
Jinfeng Hongyuan Flagship Store	Electricity savings3	You have to use it for a while to know the effect	Jingdong prefecture level city in Shandong
Jinfeng Hongyuan Flagship Store	Electricity savings3	Shipped very quickly, packaged very well, the price is particularly affordable, there are so many efficacy is also good, it is worth buying worth buying the product, the workmanship is very fine, better than imagined, recommended that everyone try not yet used, after the use of the follow-up comment	Jingdong prefecture level city in Shandong

Jinfeng Hongyuan Flagship Store	Electricity savings3	It looks regular. It's working now.	Jingdong prefecture level city in Shandong
Jinfeng Hongyuan Flagship Store	Electricity savings3	It arrived pretty fast and the owner is very responsible. Have not used it yet, will see after use	Jingdong prefecture level city in Shandong
Jinfeng Hongyuan Flagship Store	Electricity savings3	🎁 baby quality is very good, the first time I received the goods to open the first time to see, it is very good 👍, very satisfied 🙂, customer service also have a question and answer, thank you 🙏	Jingdong prefecture level city in Shandong
Jinfeng Hongyuan Flagship Store	Electricity savings3	I don't know if it's any good 。。。。。。	Jingdong prefecture level city in Shandong
Jinfeng Hongyuan Flagship Store	Electricity savings3	Buy electronic products! Still trust the things of the Jingdong self-owned store! Energy-saving effect to evaluate after heating!	Jingdong prefecture level city in Shandong
Jinfeng Hongyuan Flagship Store	Electricity savings3	The goods have been received, Blu is very beautiful, energy saving effect, to be confirmed only after actual use.	Jingdong prefecture level city in Shandong
Jinfeng Hongyuan Flagship Store	Electricity savings3	Jingdong's speed is so fast, has received, easy to operate. The display is quite good, how many volts what mode is quite understandable.	Jingdong prefecture level city in Shandong
Jinfeng Hongyuan Flagship Store	Electricity savings3	The first use, the effect is good to recommend it to colleagues to use, a month of electricity to save a lot or can be	Jingdong prefecture level city in Shandong
Jinfeng Hongyuan Flagship Store	Electricity savings3	I don't know if it's useful. Mom's is a store. I'll return it if it doesn't work.	Jingdong prefecture level city in Shandong
Jinfeng Hongyuan Flagship Store	Electricity savings3	I don't know if it works or not, I'll wait for two months to use it and then come back to comment!	Jingdong prefecture level city in Shandong
Jinfeng Hongyuan Flagship Store	Electricity savings3	I bought the second one now, and I've been using it for more than half a year, and I feel that it really saves electricity. Now it is summer, the weather is hot, sometimes the fan and air conditioning on all day, if not this power saver, the monthly electricity bill is quite high, but as long as the installation of this power saver, not afraid of its high electricity bill, absolutely let you wantonly use	Jingdong prefecture level city in Shandong
Jinfeng Hongyuan Flagship Store	Electricity savings3	The product is better than I thought it would be, the quality looks great, the voltage is stable, and I'm looking forward to some great power savings!	Jingdong prefecture level city in Shandong
Jinfeng Hongyuan Flagship Store	Electricity savings3	Inadvertently see bought a try, delivery is also quite timely, just do not know how to use, customer service patience to answer, with a pretty good, value for money!	Jingdong prefecture level city in Shandong
Jinfeng Hongyuan Flagship Store	Electricity savings3	It's better than nothing! Hopefully this will help save slightly.	Jingdong prefecture level city in Shandong
Jinfeng Hongyuan Flagship Store	Electricity savings3	Still trying it out, follow up	Jingdong prefecture level city in Shandong
Jinfeng Hongyuan Flagship Store	Electricity savings3	I've just started using it, I don't know how well it works yet, I hope it's good.	Jingdong prefecture level city in Shandong
Jinfeng Hongyuan Flagship Store	Electricity savings3	Fourth purchase, great baby!	Jingdong prefecture level city in Shandong
Jinfeng Hongyuan Flagship Store	Electricity savings3	Shipped really fast, just got it and use it to see what happens and then come back for a review!	Jingdong prefecture level city in Shandong
Jinfeng Hongyuan Flagship Store	Electricity savings3	Timely delivery **** good attitude	Jingdong prefecture level city in Shandong
Jinfeng Hongyuan Flagship Store	Electricity savings3	Home large and small things are purchased in the Jingdong Mall, have been accustomed to shopping in the Jingdong, many times can buy good quality and cheap products, and courier service attitude is particularly good, sometimes too many couriers are to help me to the home, so many years have been in the Jingdong Mall shopping, trust and support! Will also support the Jingdong as always!	Jingdong prefecture level city in Shandong
Jinfeng Hongyuan Flagship Store	Electricity savings3	Modified a pump and the modification worked.	Jingdong prefecture level city in Shandong
Jinfeng Hongyuan Flagship Store	Electricity savings3	Already installed, next time you pay the electric bill to see the results	Jingdong prefecture level city in Shandong
Jinfeng Hongyuan Flagship Store	Electricity savings3	The product is very good to use and practical to look at	Jingdong prefecture level city in Shandong
Jinfeng Hongyuan Flagship Store	Electricity savings3	Works great, great service, waiting for results.	Jingdong prefecture level city in Shandong
Jinfeng Hongyuan Flagship Store	Electricity savings3	I just used it and I'm not sure about the efficacy yet, I'll come back to evaluate it after a while.	Jingdong prefecture level city in Shandong
Jinfeng Hongyuan Flagship Store	Electricity savings3	Just received the goods, use a period of time to evaluate	Jingdong prefecture level city in Shandong
Jinfeng Hongyuan Flagship Store	Electricity savings3	Opened and tried very good, use it to see if there are results	Jingdong prefecture level city in Shandong
Jinfeng Hongyuan Flagship Store	Electricity savings3	Arrived today! Still trying it out!	Jingdong prefecture level city in Shandong
Jinfeng Hongyuan Flagship Store	Electricity savings3	Shipped quickly, well packaged, already installed, hope there is a power saving effect	Jingdong prefecture level city in Shandong
Jinfeng Hongyuan Flagship Store	Electricity savings3	I got it. It's great.	Jingdong prefecture level city in Shandong
Jinfeng Hongyuan Flagship Store	Electricity savings3	Haven't used it yet, will come back in a month	Jingdong prefecture level city in Shandong
Jinfeng Hongyuan Flagship Store	Electricity savings3	It looks good. It's good. Try it first.	Jingdong prefecture level city in Shandong
Jinfeng Hongyuan Flagship Store	Electricity savings3	It works so well that I have relatives, friends, coworkers, and 6 families that have purchased it for them through me.	Jingdong prefecture level city in Shandong
Jinfeng Hongyuan Flagship Store	Electricity savings3	In use	Jingdong prefecture level city in Shandong
Jinfeng Hongyuan Flagship Store	Electricity savings3	Received the goods today, power-saving effect do not know being used, the boss has a very good attitude, praise!	Jingdong prefecture level city in Shandong
Jinfeng Hongyuan Flagship Store	Electricity savings3	The product is compact and doesn't take up much space, but hopefully it's power efficient.	Jingdong prefecture level city in Shandong
Jinfeng Hongyuan Flagship Store	Electricity savings3	Power saving is indeed effective, I used for a period of time before coming to evaluate, the electricity bill is obviously less, the quality is good, the shopkeeper service is also very good, very patient,	Jingdong prefecture level city in Shandong
Jinfeng Hongyuan Flagship Store	Electricity savings3	It feels fine, I'll comment after I use it	Jingdong prefecture level city in Shandong
Jinfeng Hongyuan Flagship Store	Electricity savings3	I just received it and I don't know the effect yet! I will comment again after I use it and have results!	Jingdong prefecture level city in Shandong
Jinfeng Hongyuan Flagship Store	Electricity savings3	The goods look fine, I hope they really work!	Jingdong prefecture level city in Shandong
Jinfeng Hongyuan Flagship Store	Electricity savings3	I just don't know if it saves power	Jingdong prefecture level city in Shandong
Jinfeng Hongyuan Flagship Store	Electricity savings3	My friend introduced me to use this product, it is professional and I am looking forward to the long term effect~!	Jingdong prefecture level city in Shandong
Jinfeng Hongyuan Flagship Store	Electricity savings3	Try it before you judge.	Jingdong prefecture level city in Shandong
Jinfeng Hongyuan Flagship Store	Electricity savings3	It's great, the power savings are obvious! Kudos!	Jingdong prefecture level city in Shandong
Jinfeng Hongyuan Flagship Store	Electricity savings3	The quality is excellent, stabilizes the voltage and saves electricity, very satisfied.	Jingdong prefecture level city in Shandong
Jinfeng Hongyuan Flagship Store	Electricity savings3	You have to use it for a while to know the effect	Jingdong prefecture level city in Shandong
Jinfeng Hongyuan Flagship Store	Electricity savings3	It's on trial.	Jingdong prefecture level city in Shandong
Jinfeng Hongyuan Flagship Store	Electricity savings3	The stuff works great.	Jingdong prefecture level city in Shandong
Jinfeng Hongyuan Flagship Store	Electricity savings3	Doesn't seem to work, not as god as other reviews	Jingdong prefecture level city in Shandong

Jinfeng Hongyuan Flagship Store	Electricity savings3	It's not good quality, it doesn't work, it stops working after a few days, it doesn't work, the customer service doesn't care, it's a bit irritating.	Jingdong prefecture level city in Shandong
Jinfeng Hongyuan Flagship Store	Electricity savings3	It doesn't do anything.	Jingdong prefecture level city in Shandong
Jinfeng Hongyuan Flagship Store	Electricity savings3	It's good.	Jingdong prefecture level city in Shandong
Jinfeng Hongyuan Flagship Store	Electricity savings3	Used a month effect is not obvious, are psychological effects, feel are not less, did not save to the electricity, there are details of the problem, the store said to buy to send 5 fuse, in fact, received only three, small problems are not committed to in place, I believe that the product is not good enough	Jingdong prefecture level city in Shandong
Jinfeng Hongyuan Flagship Store	Electricity savings3	Well received quickly, looking quite high, but also sent 5 fuses. Customer service is very patient to teach the operation, in fact, quite simple, lazy to read the instructions ha!	Jingdong prefecture level city in Shandong
Jinfeng Hongyuan Flagship Store	Electricity savings3	You have to use it for a while to know the effect	Jingdong prefecture level city in Shandong
Jinfeng Hongyuan Flagship Store	Electricity savings3	Shipped very quickly, packaged very well, the price is particularly affordable, there are so many efficacy is also good, it is worth buying worth buying the product, the workmanship is very fine, better than imagined, recommended that everyone try not yet used, after the use of the follow-up comment	Jingdong prefecture level city in Shandong
Jinfeng Hongyuan Flagship Store	Electricity savings3	It looks regular. It's working now.	Jingdong prefecture level city in Shandong
Jinfeng Hongyuan Flagship Store	Electricity savings3	It arrived pretty fast and the owner is very responsible. Have not used it yet, will see after use	Jingdong prefecture level city in Shandong
Jinfeng Hongyuan Flagship Store	Electricity savings3	🎁 baby quality is very good, the first time I received the goods to open the first time to see, it is very good 👍, very satisfied 😊, customer service also have a question and answer, thank you 🙏	Jingdong prefecture level city in Shandong
Jinfeng Hongyuan Flagship Store	Electricity savings3	I don't know if it's any good 。。。。。。	Jingdong prefecture level city in Shandong
Jinfeng Hongyuan Flagship Store	Electricity savings3	Buy electronic products! Still trust the things of the Jingdong self-owned store! Energy-saving effect to evaluate after heating!	Jingdong prefecture level city in Shandong
Jinfeng Hongyuan Flagship Store	Electricity savings3	The goods have been received, Blu is very beautiful, energy saving effect, to be confirmed only after actual use.	Jingdong prefecture level city in Shandong
Jinfeng Hongyuan Flagship Store	Electricity savings3	Jingdong's speed is so fast, has received, easy to operate. The display is quite good, how many volts what mode is quite understandable.	Jingdong prefecture level city in Shandong
Jinfeng Hongyuan Flagship Store	Electricity savings3	The first use, the effect is good to recommend it to colleagues to use, a month of electricity to save a lot or can be	Jingdong prefecture level city in Shandong
Jinfeng Hongyuan Flagship Store	Electricity savings3	I don't know if it's useful. Mom's is a store. I'll return it if it doesn't work.	Jingdong prefecture level city in Shandong
Jinfeng Hongyuan Flagship Store	Electricity savings3	I don't know if it works or not, I'll wait for two months to use it and then come back to comment!	Jingdong prefecture level city in Shandong
Jinfeng Hongyuan Flagship Store	Electricity savings3	I bought the second one now, and I've been using it for more than half a year, and I feel that it really saves electricity. Now it is summer, the weather is hot, sometimes the fan and air conditioning on all day, if not this power saver, the monthly electricity bill is quite high, but as long as the installation of this power saver, not afraid of its high electricity bill, absolutely let you wantonly use	Jingdong prefecture level city in Shandong
Jinfeng Hongyuan Flagship Store	Electricity savings3	The product is better than I thought it would be, the quality looks great, the voltage is stable, and I'm looking forward to some great power savings!	Jingdong prefecture level city in Shandong
Jinfeng Hongyuan Flagship Store	Electricity savings3	Inadvertently see bought a try, delivery is also quite timely, just do not know how to use, customer service patience to answer, with a pretty good, value for money!	Jingdong prefecture level city in Shandong
Jinfeng Hongyuan Flagship Store	Electricity savings3	It's better than nothing! Hopefully this will help save slightly.	Jingdong prefecture level city in Shandong
Jinfeng Hongyuan Flagship Store	Electricity savings3	Still trying it out, follow up	Jingdong prefecture level city in Shandong
Jinfeng Hongyuan Flagship Store	Electricity savings3	I've just started using it, I don't know how well it works yet, I hope it's good.	Jingdong prefecture level city in Shandong
Jinfeng Hongyuan Flagship Store	Electricity savings3	Fourth purchase, great baby!	Jingdong prefecture level city in Shandong
Jinfeng Hongyuan Flagship Store	Electricity savings3	Shipped really fast, just got it and use it to see what happens and then come back for a review!	Jingdong prefecture level city in Shandong
Jinfeng Hongyuan Flagship Store	Electricity savings3	Timely delivery **** good attitude	Jingdong prefecture level city in Shandong
Jinfeng Hongyuan Flagship Store	Electricity savings3	Home large and small things are purchased in the Jingdong Mall, have been accustomed to shopping in the Jingdong, many times can buy good quality and cheap products, and courier service attitude is particularly good, sometimes too many couriers are to help me to the home, so many years have been in the Jingdong Mall shopping, trust and support! Will also support the Jingdong as always!	Jingdong prefecture level city in Shandong
Jinfeng Hongyuan Flagship Store	Electricity savings3	Modified a pump and the modification worked.	Jingdong prefecture level city in Shandong
Jinfeng Hongyuan Flagship Store	Electricity savings3	Already installed, next time you pay the electric bill to see the results	Jingdong prefecture level city in Shandong
Jinfeng Hongyuan Flagship Store	Electricity savings3	The product is very good to use and practical to look at	Jingdong prefecture level city in Shandong
Jinfeng Hongyuan Flagship Store	Electricity savings3	Works great, great service, waiting for results.	Jingdong prefecture level city in Shandong
Jinfeng Hongyuan Flagship Store	Electricity savings3	I just used it and I'm not sure about the efficacy yet, I'll come back to evaluate it after a while.	Jingdong prefecture level city in Shandong
Jinfeng Hongyuan Flagship Store	Electricity savings3	Just received the goods, use a period of time to evaluate	Jingdong prefecture level city in Shandong
Jinfeng Hongyuan Flagship Store	Electricity savings3	Opened and tried very good, use it to see if there are results	Jingdong prefecture level city in Shandong
Jinfeng Hongyuan Flagship Store	Electricity savings3	Arrived today! Still trying it out!	Jingdong prefecture level city in Shandong
Jinfeng Hongyuan Flagship Store	Electricity savings3	Shipped quickly, well packaged, already installed, hope there is a power saving effect	Jingdong prefecture level city in Shandong
Jinfeng Hongyuan Flagship Store	Electricity savings3	I got it. It's great.	Jingdong prefecture level city in Shandong
Jinfeng Hongyuan Flagship Store	Electricity savings3	Haven't used it yet, will come back in a month	Jingdong prefecture level city in Shandong
Jinfeng Hongyuan Flagship Store	Electricity savings3	It looks good. It's good. Try it first.	Jingdong prefecture level city in Shandong
Jinfeng Hongyuan Flagship Store	Electricity savings3	It works so well that I have relatives, friends, coworkers, and 6 families that have purchased it for them through me.	Jingdong prefecture level city in Shandong
Jinfeng Hongyuan Flagship Store	Electricity savings3	In use	Jingdong prefecture level city in Shandong
Jinfeng Hongyuan Flagship Store	Electricity savings3	Received the goods today, power-saving effect do not know being used, the boss has a very good attitude, praise!	Jingdong prefecture level city in Shandong
Jinfeng Hongyuan Flagship Store	Electricity savings3	The product is compact and doesn't take up much space, but hopefully it's power efficient.	Jingdong prefecture level city in Shandong
Jinfeng Hongyuan Flagship Store	Electricity savings3	Power saving is indeed effective, I used for a period of time before coming to evaluate, the electricity bill is obviously less, the quality is good, the shopkeeper service is also very good, very patient,	Jingdong prefecture level city in Shandong
Jinfeng Hongyuan Flagship Store	Electricity savings3	It feels fine, I'll comment after I use it	Jingdong prefecture level city in Shandong
Jinfeng Hongyuan Flagship Store	Electricity savings3	I just received it and I don't know the effect yet! I will comment again after I use it and have results!	Jingdong prefecture level city in Shandong
Jinfeng Hongyuan Flagship Store	Electricity savings3	The goods look fine, I hope they really work!	Jingdong prefecture level city in Shandong
Jinfeng Hongyuan Flagship Store	Electricity savings3	I just don't know if it saves power	Jingdong prefecture level city in Shandong

Jinfeng Hongyuan Flagship Store	Electricity savings3	My friend introduced me to use this product, it is professional and I am looking forward to the long term effect~!	Jingdong prefecture level city in Shandong
Jinfeng Hongyuan Flagship Store	Electricity savings3	Try it before you judge.	Jingdong prefecture level city in Shandong
Jinfeng Hongyuan Flagship Store	Electricity savings3	It's great, the power savings are obvious! Kudos!	Jingdong prefecture level city in Shandong
Jinfeng Hongyuan Flagship Store	Electricity savings3	The quality is excellent, stabilizes the voltage and saves electricity, very satisfied.	Jingdong prefecture level city in Shandong
Jinfeng Hongyuan Flagship Store	Electricity savings3	You have to use it for a while to know the effect	Jingdong prefecture level city in Shandong
Jinfeng Hongyuan Flagship Store	Electricity savings3	It's on trial.	Jingdong prefecture level city in Shandong
Jinfeng Hongyuan Flagship Store	Electricity savings3	The stuff works great.	Jingdong prefecture level city in Shandong
Jinfeng Hongyuan Flagship Store	Electricity savings3	Doesn't seem to work, not as god as other reviews	Jingdong prefecture level city in Shandong
Jinfeng Hongyuan Flagship Store	Electricity savings3	It's not good quality, it doesn't work, it stops working after a few days, it doesn't work, the customer service doesn't care, it's a bit irritating.	Jingdong prefecture level city in Shandong
Jinfeng Hongyuan Flagship Store	Electricity savings3	It doesn't do anything.	Jingdong prefecture level city in Shandong
Jinfeng Hongyuan Flagship Store	Electricity savings3	It's good.	Jingdong prefecture level city in Shandong
Jinfeng Hongyuan Flagship Store	Electricity savings3	Used a month effect is not obvious, are psychological effects, feel are not less, did not save to the electricity, there are details of the problem, the store said to buy to send 5 fuse, in fact, received only three, small problems are not committed to in place, I believe that the product is not good enough	Jingdong prefecture level city in Shandong
Jinfeng Hongyuan Flagship Store	Electricity savings3	Well received quickly, looking quite high, but also sent 5 fuses. Customer service is very patient to teach the operation, in fact, quite simple, lazy to read the instructions ha!	Jingdong prefecture level city in Shandong
Jinfeng Hongyuan Flagship Store	Electricity savings3	You have to use it for a while to know the effect	Jingdong prefecture level city in Shandong
Jinfeng Hongyuan Flagship Store	Electricity savings3	Shipped very quickly, packaged very well, the price is particularly affordable, there are so many efficacy is also good, it is worth buying worth buying the product, the workmanship is very fine, better than imagined, recommended that everyone try not yet used, after the use of the follow-up comment	Jingdong prefecture level city in Shandong
Jinfeng Hongyuan Flagship Store	Electricity savings3	It looks regular. It's working now.	Jingdong prefecture level city in Shandong
Jinfeng Hongyuan Flagship Store	Electricity savings3	It arrived pretty fast and the owner is very responsible. Have not used it yet, will see after use	Jingdong prefecture level city in Shandong
Jinfeng Hongyuan Flagship Store	Electricity savings3	🎁 baby quality is very good, the first time I received the goods to open the first time to see, it is very good 👍, very satisfied 🙂, customer service also have a question and answer, thank you 🙏	Jingdong prefecture level city in Shandong
Jinfeng Hongyuan Flagship Store	Electricity savings3	I don't know if it's any good 。。。。。。	Jingdong prefecture level city in Shandong
Jinfeng Hongyuan Flagship Store	Electricity savings3	Buy electronic products! Still trust the things of the Jingdong self-owned store! Energy-saving effect to evaluate after heating!	Jingdong prefecture level city in Shandong
Jinfeng Hongyuan Flagship Store	Electricity savings3	The goods have been received, Blu is very beautiful, energy saving effect, to be confirmed only after actual use.	Jingdong prefecture level city in Shandong
Jinfeng Hongyuan Flagship Store	Electricity savings3	Jingdong's speed is so fast, has received, easy to operate. The display is quite good, how many volts what mode is quite understandable.	Jingdong prefecture level city in Shandong
Jinfeng Hongyuan Flagship Store	Electricity savings3	The first use, the effect is good to recommend it to colleagues to use, a month of electricity to save a lot or can be	Jingdong prefecture level city in Shandong
Jinfeng Hongyuan Flagship Store	Electricity savings3	I don't know if it's useful. Mom's is a store. I'll return it if it doesn't work.	Jingdong prefecture level city in Shandong
Jinfeng Hongyuan Flagship Store	Electricity savings3	I don't know if it works or not, I'll wait for two months to use it and then come back to comment!	Jingdong prefecture level city in Shandong
Jinfeng Hongyuan Flagship Store	Electricity savings3	I bought the second one now, and I've been using it for more than half a year, and I feel that it really saves electricity. Now it is summer, the weather is hot, sometimes the fan and air conditioning on all day, if not this power saver, the monthly electricity bill is quite high, but as long as the installation of this power saver, not afraid of its high electricity bill, absolutely let you wantonly use	Jingdong prefecture level city in Shandong
Jinfeng Hongyuan Flagship Store	Electricity savings3	The product is better than I thought it would be, the quality looks great, the voltage is stable, and I'm looking forward to some great power savings!	Jingdong prefecture level city in Shandong
Jinfeng Hongyuan Flagship Store	Electricity savings3	Inadvertently see bought a try, delivery is also quite timely, just do not know how to use, customer service patience to answer, with a pretty good, value for money!	Jingdong prefecture level city in Shandong
Jinfeng Hongyuan Flagship Store	Electricity savings3	It's better than nothing! Hopefully this will help save slightly.	Jingdong prefecture level city in Shandong
Jinfeng Hongyuan Flagship Store	Electricity savings3	Still trying it out, follow up	Jingdong prefecture level city in Shandong
Jinfeng Hongyuan Flagship Store	Electricity savings3	I've just started using it, I don't know how well it works yet, I hope it's good.	Jingdong prefecture level city in Shandong
Jinfeng Hongyuan Flagship Store	Electricity savings3	Fourth purchase, great baby!	Jingdong prefecture level city in Shandong
Jinfeng Hongyuan Flagship Store	Electricity savings3	Shipped really fast, just got it and use it to see what happens and then come back for a review!	Jingdong prefecture level city in Shandong
Jinfeng Hongyuan Flagship Store	Electricity savings3	Timely delivery **** good attitude	Jingdong prefecture level city in Shandong
Jinfeng Hongyuan Flagship Store	Electricity savings3	Home large and small things are purchased in the Jingdong Mall, have been accustomed to shopping in the Jingdong, many times can buy good quality and cheap products, and courier service attitude is particularly good, sometimes too many couriers are to help me to the home, so many years have been in the Jingdong Mall shopping, trust and support! Will also support the Jingdong as always!	Jingdong prefecture level city in Shandong
Jinfeng Hongyuan Flagship Store	Electricity savings3	Modified a pump and the modification worked.	Jingdong prefecture level city in Shandong
Jinfeng Hongyuan Flagship Store	Electricity savings3	Already installed, next time you pay the electric bill to see the results	Jingdong prefecture level city in Shandong
Jinfeng Hongyuan Flagship Store	Electricity savings3	The product is very good to use and practical to look at	Jingdong prefecture level city in Shandong
Jinfeng Hongyuan Flagship Store	Electricity savings3	Works great, great service, waiting for results.	Jingdong prefecture level city in Shandong
Jinfeng Hongyuan Flagship Store	Electricity savings3	I just used it and I'm not sure about the efficacy yet, I'll come back to evaluate it after a while.	Jingdong prefecture level city in Shandong
Jinfeng Hongyuan Flagship Store	Electricity savings3	Just received the goods, use a period of time to evaluate	Jingdong prefecture level city in Shandong
Jinfeng Hongyuan Flagship Store	Electricity savings3	Opened and tried very good, use it to see if there are results	Jingdong prefecture level city in Shandong
Jinfeng Hongyuan Flagship Store	Electricity savings3	Arrived today! Still trying it out!	Jingdong prefecture level city in Shandong
Jinfeng Hongyuan Flagship Store	Electricity savings3	Shipped quickly, well packaged, already installed, hope there is a power saving effect	Jingdong prefecture level city in Shandong
Jinfeng Hongyuan Flagship Store	Electricity savings3	I got it. It's great.	Jingdong prefecture level city in Shandong
Jinfeng Hongyuan Flagship Store	Electricity savings3	Haven't used it yet, will come back in a month	Jingdong prefecture level city in Shandong
Jinfeng Hongyuan Flagship Store	Electricity savings3	It looks good. It's good. Try it first.	Jingdong prefecture level city in Shandong
Jinfeng Hongyuan Flagship Store	Electricity savings3	It works so well that I have relatives, friends, coworkers, and 6 families that have purchased it for them through me.	Jingdong prefecture level city in Shandong

Jinfeng Hongyuan Flagship Store	Electricity savings3	In use	Jingdong prefecture level city in Shandong
Jinfeng Hongyuan Flagship Store	Electricity savings3	Received the goods today, power-saving effect do not know being used, the boss has a very good attitude, praise!	Jingdong prefecture level city in Shandong
Jinfeng Hongyuan Flagship Store	Electricity savings3	The product is compact and doesn't take up much space, but hopefully it's power efficient.	Jingdong prefecture level city in Shandong
Jinfeng Hongyuan Flagship Store	Electricity savings3	Power saving is indeed effective, I used for a period of time before coming to evaluate, the electricity bill is obviously less, the quality is good, the shopkeeper service is also very good, very patient,	Jingdong prefecture level city in Shandong
Jinfeng Hongyuan Flagship Store	Electricity savings3	It feels fine, I'll comment after I use it	Jingdong prefecture level city in Shandong
Jinfeng Hongyuan Flagship Store	Electricity savings3	I just received it and I don't know the effect yet! I will comment again after I use it and have results!	Jingdong prefecture level city in Shandong
Jinfeng Hongyuan Flagship Store	Electricity savings3	The goods look fine, I hope they really work!	Jingdong prefecture level city in Shandong
Jinfeng Hongyuan Flagship Store	Electricity savings3	I just don't know if it saves power	Jingdong prefecture level city in Shandong
Jinfeng Hongyuan Flagship Store	Electricity savings3	My friend introduced me to use this product, it is professional and I am looking forward to the long term effect~!	Jingdong prefecture level city in Shandong
Jinfeng Hongyuan Flagship Store	Electricity savings3	Try it before you judge.	Jingdong prefecture level city in Shandong
Jinfeng Hongyuan Flagship Store	Electricity savings3	It's great, the power savings are obvious! Kudos!	Jingdong prefecture level city in Shandong
Jinfeng Hongyuan Flagship Store	Electricity savings3	The quality is excellent, stabilizes the voltage and saves electricity, very satisfied.	Jingdong prefecture level city in Shandong
Jinfeng Hongyuan Flagship Store	Electricity savings3	You have to use it for a while to know the effect	Jingdong prefecture level city in Shandong
Jinfeng Hongyuan Flagship Store	Electricity savings3	It's on trial.	Jingdong prefecture level city in Shandong
Jinfeng Hongyuan Flagship Store	Electricity savings3	The stuff works great.	Jingdong prefecture level city in Shandong
Jinfeng Hongyuan Flagship Store	Electricity savings3	Doesn't seem to work, not as god as other reviews	Jingdong prefecture level city in Shandong
Jinfeng Hongyuan Flagship Store	Electricity savings3	It's not good quality, it doesn't work, it stops working after a few days, it doesn't work, the customer service doesn't care, it's a bit irritating.	Jingdong prefecture level city in Shandong
Jinfeng Hongyuan Flagship Store	Electricity savings3	It doesn't do anything.	Jingdong prefecture level city in Shandong
Jinfeng Hongyuan Flagship Store	Electricity savings3	It's good.	Jingdong prefecture level city in Shandong
Jinfeng Hongyuan Flagship Store	Electricity savings3	Used a month effect is not obvious, are psychological effects, feel are not less, did not save to the electricity, there are details of the problem, the store said to buy to send 5 fuse, in fact, received only three, small problems are not committed to in place, I believe that the product is not good enough	Jingdong prefecture level city in Shandong
Jinfeng Hongyuan Flagship Store	Electricity savings3	Well received quickly, looking quite high, but also sent 5 fuses. Customer service is very patient to teach the operation, in fact, quite simple, lazy to read the instructions ha!	Jingdong prefecture level city in Shandong
Jinfeng Hongyuan Flagship Store	Electricity savings3	You have to use it for a while to know the effect	Jingdong prefecture level city in Shandong
Jinfeng Hongyuan Flagship Store	Electricity savings3	Shipped very quickly, packaged very well, the price is particularly affordable, there are so many efficacy is also good, it is worth buying worth buying the product, the workmanship is very fine, better than imagined, recommended that everyone try not yet used, after the use of the follow-up comment	Jingdong prefecture level city in Shandong
Jinfeng Hongyuan Flagship Store	Electricity savings3	It looks regular. It's working now.	Jingdong prefecture level city in Shandong
Jinfeng Hongyuan Flagship Store	Electricity savings3	It arrived pretty fast and the owner is very responsible. Have not used it yet, will see after use	Jingdong prefecture level city in Shandong
Jinfeng Hongyuan Flagship Store	Electricity savings3	🎁 baby quality is very good, the first time I received the goods to open the first time to see, it is very good 👍, very satisfied 😃, customer service also have a question and answer, thank you 🙏	Jingdong prefecture level city in Shandong
Jinfeng Hongyuan Flagship Store	Electricity savings3	I don't know if it's any good 。。。。。。	Jingdong prefecture level city in Shandong
Jinfeng Hongyuan Flagship Store	Electricity savings3	Buy electronic products! Still trust the things of the Jingdong self-owned store! Energy-saving effect to evaluate after heating!	Jingdong prefecture level city in Shandong
Jinfeng Hongyuan Flagship Store	Electricity savings3	The goods have been received, Blu is very beautiful, energy saving effect, to be confirmed only after actual use.	Jingdong prefecture level city in Shandong
Jinfeng Hongyuan Flagship Store	Electricity savings3	Jingdong's speed is so fast, has received, easy to operate. The display is quite good, how many volts what mode is quite understandable.	Jingdong prefecture level city in Shandong
Jinfeng Hongyuan Flagship Store	Electricity savings3	The first use, the effect is good to recommend it to colleagues to use, a month of electricity to save a lot or can be	Jingdong prefecture level city in Shandong
Jinfeng Hongyuan Flagship Store	Electricity savings3	I don't know if it's useful. Mom's is a store. I'll return it if it doesn't work.	Jingdong prefecture level city in Shandong
Jinfeng Hongyuan Flagship Store	Electricity savings3	I don't know if it works or not, I'll wait for two months to use it and then come back to comment!	Jingdong prefecture level city in Shandong
Jinfeng Hongyuan Flagship Store	Electricity savings3	I bought the second one now, and I've been using it for more than half a year, and I feel that it really saves electricity. Now it is summer, the weather is hot, sometimes the fan and air conditioning on all day, if not this power saver, the monthly electricity bill is quite high, but as long as the installation of this power saver, not afraid of its high electricity bill, absolutely let you wantonly use	Jingdong prefecture level city in Shandong
Jinfeng Hongyuan Flagship Store	Electricity savings3	The product is better than I thought it would be, the quality looks great, the voltage is stable, and I'm looking forward to some great power savings!	Jingdong prefecture level city in Shandong
Jinfeng Hongyuan Flagship Store	Electricity savings3	Inadvertently see bought a try, delivery is also quite timely, just do not know how to use, customer service patience to answer, with a pretty good, value for money!	Jingdong prefecture level city in Shandong
Jinfeng Hongyuan Flagship Store	Electricity savings3	It's better than nothing! Hopefully this will help save slightly.	Jingdong prefecture level city in Shandong
Jinfeng Hongyuan Flagship Store	Electricity savings3	Still trying it out, follow up	Jingdong prefecture level city in Shandong
Jinfeng Hongyuan Flagship Store	Electricity savings3	I've just started using it, I don't know how well it works yet, I hope it's good.	Jingdong prefecture level city in Shandong
Jinfeng Hongyuan Flagship Store	Electricity savings3	Fourth purchase, great baby!	Jingdong prefecture level city in Shandong
Jinfeng Hongyuan Flagship Store	Electricity savings3	Shipped really fast, just got it and use it to see what happens and then come back for a review!	Jingdong prefecture level city in Shandong
Jinfeng Hongyuan Flagship Store	Electricity savings3	Timely delivery **** good attitude	Jingdong prefecture level city in Shandong
Jinfeng Hongyuan Flagship Store	Electricity savings3	Home large and small things are purchased in the Jingdong Mall, have been accustomed to shopping in the Jingdong, many times can buy good quality and cheap products, and courier service attitude is particularly good, sometimes too many couriers are to help me to the home, so many years have been in the Jingdong Mall shopping, trust and support! Will also support the Jingdong as always!	Jingdong prefecture level city in Shandong
Jinfeng Hongyuan Flagship Store	Electricity savings3	Modified a pump and the modification worked.	Jingdong prefecture level city in Shandong
Jinfeng Hongyuan Flagship Store	Electricity savings3	Already installed, next time you pay the electric bill to see the results	Jingdong prefecture level city in Shandong
Jinfeng Hongyuan Flagship Store	Electricity savings3	The product is very good to use and practical to look at	Jingdong prefecture level city in Shandong
Jinfeng Hongyuan Flagship Store	Electricity savings3	Works great, great service, waiting for results.	Jingdong prefecture level city in Shandong
Jinfeng Hongyuan Flagship Store	Electricity savings3	I just used it and I'm not sure about the efficacy yet, I'll come back to evaluate it after a while.	Jingdong prefecture level city in Shandong

Jinfeng Hongyuan Flagship Store	Electricity savings3	Just received the goods, use a period of time to evaluate	Jingdong prefecture level city in Shandong
Jinfeng Hongyuan Flagship Store	Electricity savings3	Opened and tried very good, use it to see if there are results	Jingdong prefecture level city in Shandong
Jinfeng Hongyuan Flagship Store	Electricity savings3	Arrived today! Still trying it out!	Jingdong prefecture level city in Shandong
Jinfeng Hongyuan Flagship Store	Electricity savings3	Shipped quickly, well packaged, already installed, hope there is a power saving effect	Jingdong prefecture level city in Shandong
Jinfeng Hongyuan Flagship Store	Electricity savings3	I got it. It's great.	Jingdong prefecture level city in Shandong
Jinfeng Hongyuan Flagship Store	Electricity savings3	Haven't used it yet, will come back in a month	Jingdong prefecture level city in Shandong
Jinfeng Hongyuan Flagship Store	Electricity savings3	It looks good. It's good. Try it first.	Jingdong prefecture level city in Shandong
Jinfeng Hongyuan Flagship Store	Electricity savings3	It works so well that I have relatives, friends, coworkers, and 6 families that have purchased it for them through me.	Jingdong prefecture level city in Shandong
Jinfeng Hongyuan Flagship Store	Electricity savings3	In use	Jingdong prefecture level city in Shandong
Jinfeng Hongyuan Flagship Store	Electricity savings3	Received the goods today, power-saving effect do not know being used, the boss has a very good attitude, praise!	Jingdong prefecture level city in Shandong
Jinfeng Hongyuan Flagship Store	Electricity savings3	The product is compact and doesn't take up much space, but hopefully it's power efficient.	Jingdong prefecture level city in Shandong
Jinfeng Hongyuan Flagship Store	Electricity savings3	Power saving is indeed effective, I used for a period of time before coming to evaluate, the electricity bill is obviously less, the quality is good, the shopkeeper service is also very good, very patient,	Jingdong prefecture level city in Shandong
Jinfeng Hongyuan Flagship Store	Electricity savings3	It feels fine, I'll comment after I use it	Jingdong prefecture level city in Shandong
Jinfeng Hongyuan Flagship Store	Electricity savings3	I just received it and I don't know the effect yet! I will comment again after I use it and have results!	Jingdong prefecture level city in Shandong
Jinfeng Hongyuan Flagship Store	Electricity savings3	The goods look fine, I hope they really work!	Jingdong prefecture level city in Shandong
Jinfeng Hongyuan Flagship Store	Electricity savings3	I just don't know if it saves power	Jingdong prefecture level city in Shandong
Jinfeng Hongyuan Flagship Store	Electricity savings3	My friend introduced me to use this product, it is professional and I am looking forward to the long term effect~!	Jingdong prefecture level city in Shandong
Jinfeng Hongyuan Flagship Store	Electricity savings3	Try it before you judge.	Jingdong prefecture level city in Shandong
Jinfeng Hongyuan Flagship Store	Electricity savings3	It's great, the power savings are obvious! Kudos!	Jingdong prefecture level city in Shandong
Jinfeng Hongyuan Flagship Store	Electricity savings3	The quality is excellent, stabilizes the voltage and saves electricity, very satisfied.	Jingdong prefecture level city in Shandong
Jinfeng Hongyuan Flagship Store	Electricity savings3	You have to use it for a while to know the effect	Jingdong prefecture level city in Shandong
Jinfeng Hongyuan Flagship Store	Electricity savings3	It's on trial.	Jingdong prefecture level city in Shandong
Jinfeng Hongyuan Flagship Store	Electricity savings3	The stuff works great.	Jingdong prefecture level city in Shandong
Jinfeng Hongyuan Flagship Store	Electricity savings3	Doesn't seem to work, not as god as other reviews	Jingdong prefecture level city in Shandong
Jinfeng Hongyuan Flagship Store	Electricity savings3	It's not good quality, it doesn't work, it stops working after a few days, it doesn't work, the customer service doesn't care, it's a bit irritating.	Jingdong prefecture level city in Shandong
Jinfeng Hongyuan Flagship Store	Electricity savings3	It doesn't do anything.	Jingdong prefecture level city in Shandong
Jinfeng Hongyuan Flagship Store	Electricity savings3	It's good.	Jingdong prefecture level city in Shandong
Jinfeng Hongyuan Flagship Store	Electricity savings3	Used a month effect is not obvious, are psychological effects, feel are not less, did not save to the electricity, there are details of the problem, the store said to buy to send 5 fuse, in fact, received only three, small problems are not committed to in place, I believe that the product is not good enough	Jingdong prefecture level city in Shandong
Jinfeng Hongyuan Flagship Store	Electricity savings3	Well received quickly, looking quite high, but also sent 5 fuses. Customer service is very patient to teach the operation, in fact, quite simple, lazy to read the instructions ha!	Jingdong prefecture level city in Shandong
Jinfeng Hongyuan Flagship Store	Electricity savings3	You have to use it for a while to know the effect	Jingdong prefecture level city in Shandong
Jinfeng Hongyuan Flagship Store	Electricity savings3	Shipped very quickly, packaged very well, the price is particularly affordable, there are so many efficacy is also good, it is worth buying worth buying the product, the workmanship is very fine, better than imagined, recommended that everyone try not yet used, after the use of the follow-up comment	Jingdong prefecture level city in Shandong
Jinfeng Hongyuan Flagship Store	Electricity savings3	It looks regular. It's working now.	Jingdong prefecture level city in Shandong
Jinfeng Hongyuan Flagship Store	Electricity savings3	It arrived pretty fast and the owner is very responsible. Have not used it yet, will see after use	Jingdong prefecture level city in Shandong
Jinfeng Hongyuan Flagship Store	Electricity savings3	🎁 baby quality is very good, the first time I received the goods to open the first time to see, it is very good 👍, very satisfied 😊, customer service also have a question and answer, thank you 🙏	Jingdong prefecture level city in Shandong
Jinfeng Hongyuan Flagship Store	Electricity savings3	I don't know if it's any good 。。。。。。	Jingdong prefecture level city in Shandong
Jinfeng Hongyuan Flagship Store	Electricity savings3	Buy electronic products! Still trust the things of the Jingdong self-owned store! Energy-saving effect to evaluate after heating!	Jingdong prefecture level city in Shandong
Jinfeng Hongyuan Flagship Store	Electricity savings3	The goods have been received, Blu is very beautiful, energy saving effect, to be confirmed only after actual use.	Jingdong prefecture level city in Shandong
Jinfeng Hongyuan Flagship Store	Electricity savings3	Jingdong's speed is so fast, has received, easy to operate. The display is quite good, how many volts what mode is quite understandable.	Jingdong prefecture level city in Shandong
Jinfeng Hongyuan Flagship Store	Electricity savings3	The first use, the effect is good to recommend it to colleagues to use, a month of electricity to save a lot or can be	Jingdong prefecture level city in Shandong
Jinfeng Hongyuan Flagship Store	Electricity savings3	I don't know if it's useful. Mom's is a store. I'll return it if it doesn't work.	Jingdong prefecture level city in Shandong
Jinfeng Hongyuan Flagship Store	Electricity savings3	I don't know if it works or not, I'll wait for two months to use it and then come back to comment!	Jingdong prefecture level city in Shandong
Jinfeng Hongyuan Flagship Store	Electricity savings3	I bought the second one now, and I've been using it for more than half a year, and I feel that it really saves electricity. Now it is summer, the weather is hot, sometimes the fan and air conditioning on all day, if not this power saver, the monthly electricity bill is quite high, but as long as the installation of this power saver, not afraid of its high electricity bill, absolutely let you wantonly use	Jingdong prefecture level city in Shandong
Jinfeng Hongyuan Flagship Store	Electricity savings3	The product is better than I thought it would be, the quality looks great, the voltage is stable, and I'm looking forward to some great power savings!	Jingdong prefecture level city in Shandong
Jinfeng Hongyuan Flagship Store	Electricity savings3	Inadvertently see bought a try, delivery is also quite timely, just do not know how to use, customer service patience to answer, with a pretty good, value for money!	Jingdong prefecture level city in Shandong
Jinfeng Hongyuan Flagship Store	Electricity savings3	It's better than nothing! Hopefully this will help save slightly.	Jingdong prefecture level city in Shandong
Jinfeng Hongyuan Flagship Store	Electricity savings3	Still trying it out, follow up	Jingdong prefecture level city in Shandong
Jinfeng Hongyuan Flagship Store	Electricity savings3	I've just started using it, I don't know how well it works yet, I hope it's good.	Jingdong prefecture level city in Shandong
Jinfeng Hongyuan Flagship Store	Electricity savings3	Fourth purchase, great baby!	Jingdong prefecture level city in Shandong
Jinfeng Hongyuan Flagship Store	Electricity savings3	Shipped really fast, just got it and use it to see what happens and then come back for a review!	Jingdong prefecture level city in Shandong

Jinfeng Hongyuan Flagship Store	Electricity savings3	Timely delivery **** good attitude	Jingdong prefecture level city in Shandong
Jinfeng Hongyuan Flagship Store	Electricity savings3	Home large and small things are purchased in the Jingdong Mall, have been accustomed to shopping in the Jingdong, many times can buy good quality and cheap products, and courier service attitude is particularly good, sometimes too many couriers are to help me to the home, so many years have been in the Jingdong Mall shopping, trust and support! Will also support the Jingdong as always!	Jingdong prefecture level city in Shandong
Jinfeng Hongyuan Flagship Store	Electricity savings3	Modified a pump and the modification worked.	Jingdong prefecture level city in Shandong
Jinfeng Hongyuan Flagship Store	Electricity savings3	Already installed, next time you pay the electric bill to see the results	Jingdong prefecture level city in Shandong
Jinfeng Hongyuan Flagship Store	Electricity savings3	The product is very good to use and practical to look at	Jingdong prefecture level city in Shandong
Jinfeng Hongyuan Flagship Store	Electricity savings3	Works great, great service, waiting for results.	Jingdong prefecture level city in Shandong
Jinfeng Hongyuan Flagship Store	Electricity savings3	I just used it and I'm not sure about the efficacy yet, I'll come back to evaluate it after a while.	Jingdong prefecture level city in Shandong
Jinfeng Hongyuan Flagship Store	Electricity savings3	Just received the goods, use a period of time to evaluate	Jingdong prefecture level city in Shandong
Jinfeng Hongyuan Flagship Store	Electricity savings3	Opened and tried very good, use it to see if there are results	Jingdong prefecture level city in Shandong
Jinfeng Hongyuan Flagship Store	Electricity savings3	Arrived today! Still trying it out!	Jingdong prefecture level city in Shandong
Jinfeng Hongyuan Flagship Store	Electricity savings3	Shipped quickly, well packaged, already installed, hope there is a power saving effect	Jingdong prefecture level city in Shandong
Jinfeng Hongyuan Flagship Store	Electricity savings3	I got it. It's great.	Jingdong prefecture level city in Shandong
Jinfeng Hongyuan Flagship Store	Electricity savings3	Haven't used it yet, will come back in a month	Jingdong prefecture level city in Shandong
Jinfeng Hongyuan Flagship Store	Electricity savings3	It looks good. It's good. Try it first.	Jingdong prefecture level city in Shandong
Jinfeng Hongyuan Flagship Store	Electricity savings3	It works so well that I have relatives, friends, coworkers, and 6 families that have purchased it for them through me.	Jingdong prefecture level city in Shandong
Jinfeng Hongyuan Flagship Store	Electricity savings3	In use	Jingdong prefecture level city in Shandong
Jinfeng Hongyuan Flagship Store	Electricity savings3	Received the goods today, power-saving effect do not know being used, the boss has a very good attitude, praise!	Jingdong prefecture level city in Shandong
Jinfeng Hongyuan Flagship Store	Electricity savings3	The product is compact and doesn't take up much space, but hopefully it's power efficient.	Jingdong prefecture level city in Shandong
Jinfeng Hongyuan Flagship Store	Electricity savings3	Power saving is indeed effective, I used for a period of time before coming to evaluate, the electricity bill is obviously less, the quality is good, the shopkeeper service is also very good, very patient,	Jingdong prefecture level city in Shandong
Jinfeng Hongyuan Flagship Store	Electricity savings3	It feels fine, I'll comment after I use it	Jingdong prefecture level city in Shandong
Jinfeng Hongyuan Flagship Store	Electricity savings3	I just received it and I don't know the effect yet! I will comment again after I use it and have results!	Jingdong prefecture level city in Shandong
Jinfeng Hongyuan Flagship Store	Electricity savings3	The goods look fine, I hope they really work!	Jingdong prefecture level city in Shandong
Jinfeng Hongyuan Flagship Store	Electricity savings3	I just don't know if it saves power	Jingdong prefecture level city in Shandong
Jinfeng Hongyuan Flagship Store	Electricity savings3	My friend introduced me to use this product, it is professional and I am looking forward to the long term effect~!	Jingdong prefecture level city in Shandong
Jinfeng Hongyuan Flagship Store	Electricity savings3	Try it before you judge.	Jingdong prefecture level city in Shandong
Jinfeng Hongyuan Flagship Store	Electricity savings3	It's great, the power savings are obvious! Kudos!	Jingdong prefecture level city in Shandong
Jinfeng Hongyuan Flagship Store	Electricity savings3	The quality is excellent, stabilizes the voltage and saves electricity, very satisfied.	Jingdong prefecture level city in Shandong
Jinfeng Hongyuan Flagship Store	Electricity savings3	You have to use it for a while to know the effect	Jingdong prefecture level city in Shandong
Jinfeng Hongyuan Flagship Store	Electricity savings3	It's on trial.	Jingdong prefecture level city in Shandong
Jinfeng Hongyuan Flagship Store	Electricity savings3	The stuff works great.	Jingdong prefecture level city in Shandong
Jinfeng Hongyuan Flagship Store	Electricity savings3	Doesn't seem to work, not as god as other reviews	Jingdong prefecture level city in Shandong
Jinfeng Hongyuan Flagship Store	Electricity savings3	It's not good quality, it doesn't work, it stops working after a few days, it doesn't work, the customer service doesn't care, it's a bit irritating.	Jingdong prefecture level city in Shandong
Jinfeng Hongyuan Flagship Store	Electricity savings3	It doesn't do anything.	Jingdong prefecture level city in Shandong
Jinfeng Hongyuan Flagship Store	Electricity savings3	It's good.	Jingdong prefecture level city in Shandong
Jinfeng Hongyuan Flagship Store	Electricity savings3	Used a month effect is not obvious, are psychological effects, feel are not less, did not save to the electricity, there are details of the problem, the store said to buy to send 5 fuse, in fact, received only three, small problems are not committed to in place, I believe that the product is not good enough	Jingdong prefecture level city in Shandong
Jinfeng Hongyuan Flagship Store	Electricity savings3	Well received quickly, looking quite high, but also sent 5 fuses. Customer service is very patient to teach the operation, in fact, quite simple, lazy to read the instructions ha!	Jingdong prefecture level city in Shandong
Jinfeng Hongyuan Flagship Store	Electricity savings3	You have to use it for a while to know the effect	Jingdong prefecture level city in Shandong
Jinfeng Hongyuan Flagship Store	Electricity savings3	Shipped very quickly, packaged very well, the price is particularly affordable, there are so many efficacy is also good, it is worth buying worth buying the product, the workmanship is very fine, better than imagined, recommended that everyone try not yet used, after the use of the follow-up comment	Jingdong prefecture level city in Shandong
Jinfeng Hongyuan Flagship Store	Electricity savings3	It looks regular. It's working now.	Jingdong prefecture level city in Shandong
Jinfeng Hongyuan Flagship Store	Electricity savings3	It arrived pretty fast and the owner is very responsible. Have not used it yet, will see after use	Jingdong prefecture level city in Shandong
Jinfeng Hongyuan Flagship Store	Electricity savings3	🎁 baby quality is very good, the first time I received the goods to open the first time to see, it is very good 👍, very satisfied 😀, customer service also have a question and answer, thank you 🙏	Jingdong prefecture level city in Shandong
Jinfeng Hongyuan Flagship Store	Electricity savings3	I don't know if it's any good 。。。。。。	Jingdong prefecture level city in Shandong
Jinfeng Hongyuan Flagship Store	Electricity savings3	Buy electronic products! Still trust the things of the Jingdong self-owned store! Energy-saving effect to evaluate after heating!	Jingdong prefecture level city in Shandong
Jinfeng Hongyuan Flagship Store	Electricity savings3	The goods have been received, Blu is very beautiful, energy saving effect, to be confirmed only after actual use.	Jingdong prefecture level city in Shandong
Jinfeng Hongyuan Flagship Store	Electricity savings3	Jingdong's speed is so fast, has received, easy to operate. The display is quite good, how many volts what mode is quite understandable.	Jingdong prefecture level city in Shandong
Jinfeng Hongyuan Flagship Store	Electricity savings3	The first use, the effect is good to recommend it to colleagues to use, a month of electricity to save a lot or can be	Jingdong prefecture level city in Shandong
Jinfeng Hongyuan Flagship Store	Electricity savings3	I don't know if it's useful. Mom's is a store. I'll return it if it doesn't work.	Jingdong prefecture level city in Shandong
Jinfeng Hongyuan Flagship Store	Electricity savings3	I don't know if it works or not, I'll wait for two months to use it and then come back to comment!	Jingdong prefecture level city in Shandong

Jinfeng Hongyuan Flagship Store	Electricity savings3	I bought the second one now, and I've been using it for more than half a year, and I feel that it really saves electricity. Now it is summer, the weather is hot, sometimes the fan and air conditioning on all day, if not this power saver, the monthly electricity bill is quite high, but as long as the installation of this power saver, not afraid of its high electricity bill, absolutely let you wantonly use	Jingdong prefecture level city in Shandong
Jinfeng Hongyuan Flagship Store	Electricity savings3	The product is better than I thought it would be, the quality looks great, the voltage is stable, and I'm looking forward to some great power savings!	Jingdong prefecture level city in Shandong
Jinfeng Hongyuan Flagship Store	Electricity savings3	Inadvertently see bought a try, delivery is also quite timely, just do not know how to use, customer service patience to answer, with a pretty good, value for money!	Jingdong prefecture level city in Shandong
Jinfeng Hongyuan Flagship Store	Electricity savings3	It's better than nothing! Hopefully this will help save slightly.	Jingdong prefecture level city in Shandong
Jinfeng Hongyuan Flagship Store	Electricity savings3	Still trying it out, follow up	Jingdong prefecture level city in Shandong
Jinfeng Hongyuan Flagship Store	Electricity savings3	I've just started using it, I don't know how well it works yet, I hope it's good.	Jingdong prefecture level city in Shandong
Jinfeng Hongyuan Flagship Store	Electricity savings3	Fourth purchase, great baby!	Jingdong prefecture level city in Shandong
Jinfeng Hongyuan Flagship Store	Electricity savings3	Shipped really fast, just got it and use it to see what happens and then come back for a review!	Jingdong prefecture level city in Shandong
Jinfeng Hongyuan Flagship Store	Electricity savings3	Timely delivery **** good attitude	Jingdong prefecture level city in Shandong
Jinfeng Hongyuan Flagship Store	Electricity savings3	Home large and small things are purchased in the Jingdong Mall, have been accustomed to shopping in the Jingdong, many times can buy good quality and cheap products, and courier service attitude is particularly good, sometimes too many couriers are to help me to the home, so many years have been in the Jingdong Mall shopping, trust and support! Will also support the Jingdong as always!	Jingdong prefecture level city in Shandong
Jinfeng Hongyuan Flagship Store	Electricity savings3	Modified a pump and the modification worked.	Jingdong prefecture level city in Shandong
Jinfeng Hongyuan Flagship Store	Electricity savings3	Already installed, next time you pay the electric bill to see the results	Jingdong prefecture level city in Shandong
Jinfeng Hongyuan Flagship Store	Electricity savings3	The product is very good to use and practical to look at	Jingdong prefecture level city in Shandong
Jinfeng Hongyuan Flagship Store	Electricity savings3	Works great, great service, waiting for results.	Jingdong prefecture level city in Shandong
Jinfeng Hongyuan Flagship Store	Electricity savings3	I just used it and I'm not sure about the efficacy yet, I'll come back to evaluate it after a while.	Jingdong prefecture level city in Shandong
Jinfeng Hongyuan Flagship Store	Electricity savings3	Just received the goods, use a period of time to evaluate	Jingdong prefecture level city in Shandong
Jinfeng Hongyuan Flagship Store	Electricity savings3	Opened and tried very good, use it to see if there are results	Jingdong prefecture level city in Shandong
Jinfeng Hongyuan Flagship Store	Electricity savings3	Arrived today! Still trying it out!	Jingdong prefecture level city in Shandong
Jinfeng Hongyuan Flagship Store	Electricity savings3	Shipped quickly, well packaged, already installed, hope there is a power saving effect	Jingdong prefecture level city in Shandong
Jinfeng Hongyuan Flagship Store	Electricity savings3	I got it. It's great.	Jingdong prefecture level city in Shandong
Jinfeng Hongyuan Flagship Store	Electricity savings3	Haven't used it yet, will come back in a month	Jingdong prefecture level city in Shandong
Jinfeng Hongyuan Flagship Store	Electricity savings3	It looks good. It's good. Try it first.	Jingdong prefecture level city in Shandong
Jinfeng Hongyuan Flagship Store	Electricity savings3	It works so well that I have relatives, friends, coworkers, and 6 families that have purchased it for them through me.	Jingdong prefecture level city in Shandong
Jinfeng Hongyuan Flagship Store	Electricity savings3	In use	Jingdong prefecture level city in Shandong
Jinfeng Hongyuan Flagship Store	Electricity savings3	Received the goods today, power-saving effect do not know being used, the boss has a very good attitude, praise!	Jingdong prefecture level city in Shandong
Jinfeng Hongyuan Flagship Store	Electricity savings3	The product is compact and doesn't take up much space, but hopefully it's power efficient.	Jingdong prefecture level city in Shandong
Jinfeng Hongyuan Flagship Store	Electricity savings3	Power saving is indeed effective, I used for a period of time before coming to evaluate, the electricity bill is obviously less, the quality is good, the shopkeeper service is also very good, very patient,	Jingdong prefecture level city in Shandong
Jinfeng Hongyuan Flagship Store	Electricity savings3	It feels fine, I'll comment after I use it	Jingdong prefecture level city in Shandong
Jinfeng Hongyuan Flagship Store	Electricity savings3	I just received it and I don't know the effect yet! I will comment again after I use it and have results!	Jingdong prefecture level city in Shandong
Jinfeng Hongyuan Flagship Store	Electricity savings3	The goods look fine, I hope they really work!	Jingdong prefecture level city in Shandong
Jinfeng Hongyuan Flagship Store	Electricity savings3	I just don't know if it saves power	Jingdong prefecture level city in Shandong
Jinfeng Hongyuan Flagship Store	Electricity savings3	My friend introduced me to use this product, it is professional and I am looking forward to the long term effect~!	Jingdong prefecture level city in Shandong
Jinfeng Hongyuan Flagship Store	Electricity savings3	Try it before you judge.	Jingdong prefecture level city in Shandong
Jinfeng Hongyuan Flagship Store	Electricity savings3	It's great, the power savings are obvious! Kudos!	Jingdong prefecture level city in Shandong
Jinfeng Hongyuan Flagship Store	Electricity savings3	The quality is excellent, stabilizes the voltage and saves electricity, very satisfied.	Jingdong prefecture level city in Shandong
Jinfeng Hongyuan Flagship Store	Electricity savings3	You have to use it for a while to know the effect	Jingdong prefecture level city in Shandong
Jinfeng Hongyuan Flagship Store	Electricity savings3	It's on trial.	Jingdong prefecture level city in Shandong
Jinfeng Hongyuan Flagship Store	Electricity savings3	The stuff works great.	Jingdong prefecture level city in Shandong
Jinfeng Hongyuan Flagship Store	Electricity savings3	Doesn't seem to work, not as god as other reviews	Jingdong prefecture level city in Shandong
Jinfeng Hongyuan Flagship Store	Electricity savings3	It's not good quality, it doesn't work, it stops working after a few days, it doesn't work, the customer service doesn't care, it's a bit irritating.	Jingdong prefecture level city in Shandong
Jinfeng Hongyuan Flagship Store	Electricity savings3	It doesn't do anything.	Jingdong prefecture level city in Shandong
Jinfeng Hongyuan Flagship Store	Electricity savings3	It's good.	Jingdong prefecture level city in Shandong
Jinfeng Hongyuan Flagship Store	Electricity savings3	Used a month effect is not obvious, are psychological effects, feel are not less, did not save to the electricity, there are details of the problem, the store said to buy to send 5 fuse, in fact, received only three, small problems are not committed to in place, I believe that the product is not good enough	Jingdong prefecture level city in Shandong
Jinfeng Hongyuan Flagship Store	Electricity savings3	Well received quickly, looking quite high, but also sent 5 fuses. Customer service is very patient to teach the operation, in fact, quite simple, lazy to read the instructions ha!	Jingdong prefecture level city in Shandong
Jinfeng Hongyuan Flagship Store	Electricity savings3	You have to use it for a while to know the effect	Jingdong prefecture level city in Shandong
Jinfeng Hongyuan Flagship Store	Electricity savings3	Shipped very quickly, packaged very well, the price is particularly affordable, there are so many efficacy is also good, it is worth buying worth buying the product, the workmanship is very fine, better than imagined, recommended that everyone try not yet used, after the use of the follow-up comment	Jingdong prefecture level city in Shandong
Jinfeng Hongyuan Flagship Store	Electricity savings3	It looks regular. It's working now.	Jingdong prefecture level city in Shandong
Jinfeng Hongyuan Flagship Store	Electricity savings3	It arrived pretty fast and the owner is very responsible. Have not used it yet, will see after use	Jingdong prefecture level city in Shandong

Jinfeng Hongyuan Flagship Store	Electricity savings3	🎁 baby quality is very good, the first time I received the goods to open the first time to see, it is very good 👍, very satisfied 😀, customer service also have a question and answer, thank you 🙏	Jingdong prefecture level city in Shandong
Jinfeng Hongyuan Flagship Store	Electricity savings3	I don't know if it's any good 。。。。。。	Jingdong prefecture level city in Shandong
Jinfeng Hongyuan Flagship Store	Electricity savings3	Buy electronic products! Still trust the things of the Jingdong self-owned store! Energy-saving effect to evaluate after heating!	Jingdong prefecture level city in Shandong
Jinfeng Hongyuan Flagship Store	Electricity savings3	The goods have been received, Blu is very beautiful, energy saving effect, to be confirmed only after actual use.	Jingdong prefecture level city in Shandong
Jinfeng Hongyuan Flagship Store	Electricity savings3	Jingdong's speed is so fast, has received, easy to operate. The display is quite good, how many volts what mode is quite understandable.	Jingdong prefecture level city in Shandong
Jinfeng Hongyuan Flagship Store	Electricity savings3	The first use, the effect is good to recommend it to colleagues to use, a month of electricity to save a lot or can be	Jingdong prefecture level city in Shandong
Jinfeng Hongyuan Flagship Store	Electricity savings3	I don't know if it's useful. Mom's is a store. I'll return it if it doesn't work.	Jingdong prefecture level city in Shandong
Jinfeng Hongyuan Flagship Store	Electricity savings3	I don't know if it works or not, I'll wait for two months to use it and then come back to comment!	Jingdong prefecture level city in Shandong
Jinfeng Hongyuan Flagship Store	Electricity savings3	I bought the second one now, and I've been using it for more than half a year, and I feel that it really saves electricity. Now it is summer, the weather is hot, sometimes the fan and air conditioning on all day, if not this power saver, the monthly electricity bill is quite high, but as long as the installation of this power saver, not afraid of its high electricity bill, absolutely let you wantonly use	Jingdong prefecture level city in Shandong
Jinfeng Hongyuan Flagship Store	Electricity savings3	The product is better than I thought it would be, the quality looks great, the voltage is stable, and I'm looking forward to some great power savings!	Jingdong prefecture level city in Shandong
Jinfeng Hongyuan Flagship Store	Electricity savings3	Inadvertently see bought a try, delivery is also quite timely, just do not know how to use, customer service patience to answer, with a pretty good, value for money!	Jingdong prefecture level city in Shandong
Jinfeng Hongyuan Flagship Store	Electricity savings3	It's better than nothing! Hopefully this will help save slightly.	Jingdong prefecture level city in Shandong
Jinfeng Hongyuan Flagship Store	Electricity savings3	Still trying it out, follow up	Jingdong prefecture level city in Shandong
Jinfeng Hongyuan Flagship Store	Electricity savings3	I've just started using it, I don't know how well it works yet, I hope it's good.	Jingdong prefecture level city in Shandong
Jinfeng Hongyuan Flagship Store	Electricity savings3	Fourth purchase, great baby!	Jingdong prefecture level city in Shandong
Jinfeng Hongyuan Flagship Store	Electricity savings3	Shipped really fast, just got it and use it to see what happens and then come back for a review!	Jingdong prefecture level city in Shandong
Jinfeng Hongyuan Flagship Store	Electricity savings3	Timely delivery **** good attitude	Jingdong prefecture level city in Shandong
Jinfeng Hongyuan Flagship Store	Electricity savings3	Home large and small things are purchased in the Jingdong Mall, have been accustomed to shopping in the Jingdong, many times can buy good quality and cheap products, and courier service attitude is particularly good, sometimes too many couriers are to help me to the home, so many years have been in the Jingdong Mall shopping, trust and support! Will also support the Jingdong as always!	Jingdong prefecture level city in Shandong
Jinfeng Hongyuan Flagship Store	Electricity savings3	Modified a pump and the modification worked.	Jingdong prefecture level city in Shandong
Jinfeng Hongyuan Flagship Store	Electricity savings3	Already installed, next time you pay the electric bill to see the results	Jingdong prefecture level city in Shandong
Jinfeng Hongyuan Flagship Store	Electricity savings3	The product is very good to use and practical to look at	Jingdong prefecture level city in Shandong
Jinfeng Hongyuan Flagship Store	Electricity savings3	Works great, great service, waiting for results.	Jingdong prefecture level city in Shandong
Jinfeng Hongyuan Flagship Store	Electricity savings3	I just used it and I'm not sure about the efficacy yet, I'll come back to evaluate it after a while.	Jingdong prefecture level city in Shandong
Jinfeng Hongyuan Flagship Store	Electricity savings3	Just received the goods, use a period of time to evaluate	Jingdong prefecture level city in Shandong
Jinfeng Hongyuan Flagship Store	Electricity savings3	Opened and tried very good, use it to see if there are results	Jingdong prefecture level city in Shandong
Jinfeng Hongyuan Flagship Store	Electricity savings3	Arrived today! Still trying it out!	Jingdong prefecture level city in Shandong
Jinfeng Hongyuan Flagship Store	Electricity savings3	Shipped quickly, well packaged, already installed, hope there is a power saving effect	Jingdong prefecture level city in Shandong
Jinfeng Hongyuan Flagship Store	Electricity savings3	I got it. It's great.	Jingdong prefecture level city in Shandong
Jinfeng Hongyuan Flagship Store	Electricity savings3	Haven't used it yet, will come back in a month	Jingdong prefecture level city in Shandong
Jinfeng Hongyuan Flagship Store	Electricity savings3	It looks good. It's good. Try it first.	Jingdong prefecture level city in Shandong
Jinfeng Hongyuan Flagship Store	Electricity savings3	It works so well that I have relatives, friends, coworkers, and 6 families that have purchased it for them through me.	Jingdong prefecture level city in Shandong
Jinfeng Hongyuan Flagship Store	Electricity savings3	In use	Jingdong prefecture level city in Shandong
Jinfeng Hongyuan Flagship Store	Electricity savings3	Received the goods today, power-saving effect do not know being used, the boss has a very good attitude, praise!	Jingdong prefecture level city in Shandong
Jinfeng Hongyuan Flagship Store	Electricity savings3	The product is compact and doesn't take up much space, but hopefully it's power efficient.	Jingdong prefecture level city in Shandong
Jinfeng Hongyuan Flagship Store	Electricity savings3	Power saving is indeed effective, I used for a period of time before coming to evaluate, the electricity bill is obviously less, the quality is good, the shopkeeper service is also very good, very patient,	Jingdong prefecture level city in Shandong
Jinfeng Hongyuan Flagship Store	Electricity savings3	It feels fine, I'll comment after I use it	Jingdong prefecture level city in Shandong
Jinfeng Hongyuan Flagship Store	Electricity savings3	I just received it and I don't know the effect yet! I will comment again after I use it and have results!	Jingdong prefecture level city in Shandong
Jinfeng Hongyuan Flagship Store	Electricity savings3	The goods look fine, I hope they really work!	Jingdong prefecture level city in Shandong
Jinfeng Hongyuan Flagship Store	Electricity savings3	I just don't know if it saves power	Jingdong prefecture level city in Shandong
Jinfeng Hongyuan Flagship Store	Electricity savings3	My friend introduced me to use this product, it is professional and I am looking forward to the long term effect~!	Jingdong prefecture level city in Shandong
Jinfeng Hongyuan Flagship Store	Electricity savings3	Try it before you judge.	Jingdong prefecture level city in Shandong
Jinfeng Hongyuan Flagship Store	Electricity savings3	It's great, the power savings are obvious! Kudos!	Jingdong prefecture level city in Shandong
Jinfeng Hongyuan Flagship Store	Electricity savings3	The quality is excellent, stabilizes the voltage and saves electricity, very satisfied.	Jingdong prefecture level city in Shandong
Jinfeng Hongyuan Flagship Store	Electricity savings3	You have to use it for a while to know the effect	Jingdong prefecture level city in Shandong
Jinfeng Hongyuan Flagship Store	Electricity savings3	It's on trial.	Jingdong prefecture level city in Shandong
Jinfeng Hongyuan Flagship Store	Electricity savings3	The stuff works great.	Jingdong prefecture level city in Shandong
Jinfeng Hongyuan Flagship Store	Electricity savings3	Doesn't seem to work, not as god as other reviews	Jingdong prefecture level city in Shandong
Jinfeng Hongyuan Flagship Store	Electricity savings3	It's not good quality, it doesn't work, it stops working after a few days, it doesn't work, the customer service doesn't care, it's a bit irritating.	Jingdong prefecture level city in Shandong
Jinfeng Hongyuan Flagship Store	Electricity savings3	It doesn't do anything.	Jingdong prefecture level city in Shandong

Jinfeng Hongyuan Flagship Store	Electricity savings3	It's good.	Jingdong prefecture level city in Shandong
Jinfeng Hongyuan Flagship Store	Electricity savings3	Used a month effect is not obvious, are psychological effects, feel are not less, did not save to the electricity, there are details of the problem, the store said to buy to send 5 fuse, in fact, received only three, small problems are not committed to in place, I believe that the product is not good enough	Jingdong prefecture level city in Shandong
Jinfeng Hongyuan Flagship Store	Electricity savings3	Well received quickly, looking quite high, but also sent 5 fuses. Customer service is very patient to teach the operation, in fact, quite simple, lazy to read the instructions ha!	Jingdong prefecture level city in Shandong
Jinfeng Hongyuan Flagship Store	Electricity savings3	You have to use it for a while to know the effect	Jingdong prefecture level city in Shandong
Jinfeng Hongyuan Flagship Store	Electricity savings3	Shipped very quickly, packaged very well, the price is particularly affordable, there are so many efficacy is also good, it is worth buying worth buying the product, the workmanship is very fine, better than imagined, recommended that everyone try not yet used, after the use of the follow-up comment	Jingdong prefecture level city in Shandong
Jinfeng Hongyuan Flagship Store	Electricity savings3	It looks regular. It's working now.	Jingdong prefecture level city in Shandong
Jinfeng Hongyuan Flagship Store	Electricity savings3	It arrived pretty fast and the owner is very responsible. Have not used it yet, will see after use	Jingdong prefecture level city in Shandong
Jinfeng Hongyuan Flagship Store	Electricity savings3	🎁 baby quality is very good, the first time I received the goods to open the first time to see, it is very good 👍, very satisfied 😊, customer service also have a question and answer, thank you 🙏	Jingdong prefecture level city in Shandong
Jinfeng Hongyuan Flagship Store	Electricity savings3	I don't know if it's any good 。。。。。。	Jingdong prefecture level city in Shandong
Jinfeng Hongyuan Flagship Store	Electricity savings3	Buy electronic products! Still trust the things of the Jingdong self-owned store! Energy-saving effect to evaluate after heating!	Jingdong prefecture level city in Shandong
Jinfeng Hongyuan Flagship Store	Electricity savings3	The goods have been received, Blu is very beautiful, energy saving effect, to be confirmed only after actual use.	Jingdong prefecture level city in Shandong
Jinfeng Hongyuan Flagship Store	Electricity savings3	Jingdong's speed is so fast, has received, easy to operate. The display is quite good, how many volts what mode is quite understandable.	Jingdong prefecture level city in Shandong
Jinfeng Hongyuan Flagship Store	Electricity savings3	The first use, the effect is good to recommend it to colleagues to use, a month of electricity to save a lot or can be	Jingdong prefecture level city in Shandong
Jinfeng Hongyuan Flagship Store	Electricity savings3	I don't know if it's useful. Mom's is a store. I'll return it if it doesn't work.	Jingdong prefecture level city in Shandong
Jinfeng Hongyuan Flagship Store	Electricity savings3	I don't know if it works or not, I'll wait for two months to use it and then come back to comment!	Jingdong prefecture level city in Shandong
Jinfeng Hongyuan Flagship Store	Electricity savings3	I bought the second one now, and I've been using it for more than half a year, and I feel that it really saves electricity. Now it is summer, the weather is hot, sometimes the fan and air conditioning on all day, if not this power saver, the monthly electricity bill is quite high, but as long as the installation of this power saver, not afraid of its high electricity bill, absolutely let you wantonly use	Jingdong prefecture level city in Shandong
Jinfeng Hongyuan Flagship Store	Electricity savings3	The product is better than I thought it would be, the quality looks great, the voltage is stable, and I'm looking forward to some great power savings!	Jingdong prefecture level city in Shandong
Jinfeng Hongyuan Flagship Store	Electricity savings3	Inadvertently see bought a try, delivery is also quite timely, just do not know how to use, customer service patience to answer, with a pretty good, value for money!	Jingdong prefecture level city in Shandong
Jinfeng Hongyuan Flagship Store	Electricity savings3	It's better than nothing! Hopefully this will help save slightly.	Jingdong prefecture level city in Shandong
Jinfeng Hongyuan Flagship Store	Electricity savings3	Still trying it out, follow up	Jingdong prefecture level city in Shandong
Jinfeng Hongyuan Flagship Store	Electricity savings3	I've just started using it, I don't know how well it works yet, I hope it's good.	Jingdong prefecture level city in Shandong
Jinfeng Hongyuan Flagship Store	Electricity savings3	Fourth purchase, great baby!	Jingdong prefecture level city in Shandong
Jinfeng Hongyuan Flagship Store	Electricity savings3	Shipped really fast, just got it and use it to see what happens and then come back for a review!	Jingdong prefecture level city in Shandong
Jinfeng Hongyuan Flagship Store	Electricity savings3	Timely delivery **** good attitude	Jingdong prefecture level city in Shandong
Jinfeng Hongyuan Flagship Store	Electricity savings3	Home large and small things are purchased in the Jingdong Mall, have been accustomed to shopping in the Jingdong, many times can buy good quality and cheap products, and courier service attitude is particularly good, sometimes too many couriers are to help me to the home, so many years have been in the Jingdong Mall shopping, trust and support! Will also support the Jingdong as always!	Jingdong prefecture level city in Shandong
Jinfeng Hongyuan Flagship Store	Electricity savings3	Modified a pump and the modification worked.	Jingdong prefecture level city in Shandong
Jinfeng Hongyuan Flagship Store	Electricity savings3	Already installed, next time you pay the electric bill to see the results	Jingdong prefecture level city in Shandong
Jinfeng Hongyuan Flagship Store	Electricity savings3	The product is very good to use and practical to look at	Jingdong prefecture level city in Shandong
Jinfeng Hongyuan Flagship Store	Electricity savings3	Works great, great service, waiting for results.	Jingdong prefecture level city in Shandong
Jinfeng Hongyuan Flagship Store	Electricity savings3	I just used it and I'm not sure about the efficacy yet, I'll come back to evaluate it after a while.	Jingdong prefecture level city in Shandong
Jinfeng Hongyuan Flagship Store	Electricity savings3	Just received the goods, use a period of time to evaluate	Jingdong prefecture level city in Shandong
Jinfeng Hongyuan Flagship Store	Electricity savings3	Opened and tried very good, use it to see if there are results	Jingdong prefecture level city in Shandong
Jinfeng Hongyuan Flagship Store	Electricity savings3	Arrived today! Still trying it out!	Jingdong prefecture level city in Shandong
Jinfeng Hongyuan Flagship Store	Electricity savings3	Shipped quickly, well packaged, already installed, hope there is a power saving effect	Jingdong prefecture level city in Shandong
Jinfeng Hongyuan Flagship Store	Electricity savings3	I got it. It's great.	Jingdong prefecture level city in Shandong
Jinfeng Hongyuan Flagship Store	Electricity savings3	Haven't used it yet, will come back in a month	Jingdong prefecture level city in Shandong
Jinfeng Hongyuan Flagship Store	Electricity savings3	It looks good. It's good. Try it first.	Jingdong prefecture level city in Shandong
Jinfeng Hongyuan Flagship Store	Electricity savings3	It works so well that I have relatives, friends, coworkers, and 6 families that have purchased it for them through me.	Jingdong prefecture level city in Shandong
Jinfeng Hongyuan Flagship Store	Electricity savings3	In use	Jingdong prefecture level city in Shandong
Jinfeng Hongyuan Flagship Store	Electricity savings3	Received the goods today, power-saving effect do not know being used, the boss has a very good attitude, praise!	Jingdong prefecture level city in Shandong
Jinfeng Hongyuan Flagship Store	Electricity savings3	The product is compact and doesn't take up much space, but hopefully it's power efficient.	Jingdong prefecture level city in Shandong
Jinfeng Hongyuan Flagship Store	Electricity savings3	Power saving is indeed effective, I used for a period of time before coming to evaluate, the electricity bill is obviously less, the quality is good, the shopkeeper service is also very good, very patient,	Jingdong prefecture level city in Shandong
Jinfeng Hongyuan Flagship Store	Electricity savings3	It feels fine, I'll comment after I use it	Jingdong prefecture level city in Shandong
Jinfeng Hongyuan Flagship Store	Electricity savings3	I just received it and I don't know the effect yet! I will comment again after I use it and have results!	Jingdong prefecture level city in Shandong
Jinfeng Hongyuan Flagship Store	Electricity savings3	The goods look fine, I hope they really work!	Jingdong prefecture level city in Shandong
Jinfeng Hongyuan Flagship Store	Electricity savings3	I just don't know if it saves power	Jingdong prefecture level city in Shandong
Jinfeng Hongyuan Flagship Store	Electricity savings3	My friend introduced me to use this product, it is professional and I am looking forward to the long term effect~!	Jingdong prefecture level city in Shandong
Jinfeng Hongyuan Flagship Store	Electricity savings3	Try it before you judge.	Jingdong prefecture level city in Shandong

Jinfeng Hongyuan Flagship Store	Electricity savings3	It's great, the power savings are obvious! Kudos!	Jingdong prefecture level city in Shandong
Jinfeng Hongyuan Flagship Store	Electricity savings3	The quality is excellent, stabilizes the voltage and saves electricity, very satisfied.	Jingdong prefecture level city in Shandong
Jinfeng Hongyuan Flagship Store	Electricity savings3	You have to use it for a while to know the effect	Jingdong prefecture level city in Shandong
Jinfeng Hongyuan Flagship Store	Electricity savings3	It's on trial.	Jingdong prefecture level city in Shandong
Jinfeng Hongyuan Flagship Store	Electricity savings3	The stuff works great.	Jingdong prefecture level city in Shandong
Jinfeng Hongyuan Flagship Store	Electricity savings3	Doesn't seem to work, not as god as other reviews	Jingdong prefecture level city in Shandong
Jinfeng Hongyuan Flagship Store	Electricity savings3	It's not good quality, it doesn't work, it stops working after a few days, it doesn't work, the customer service doesn't care, it's a bit irritating.	Jingdong prefecture level city in Shandong
Jinfeng Hongyuan Flagship Store	Electricity savings3	It doesn't do anything.	Jingdong prefecture level city in Shandong
Jinfeng Hongyuan Flagship Store	Electricity savings3	It's good.	Jingdong prefecture level city in Shandong
Jinfeng Hongyuan Flagship Store	Electricity savings3	Used a month effect is not obvious, are psychological effects, feel are not less, did not save to the electricity, there are details of the problem, the store said to buy to send 5 fuse, in fact, received only three, small problems are not committed to in place, I believe that the product is not good enough	Jingdong prefecture level city in Shandong
Jinfeng Hongyuan Flagship Store	Electricity savings3	Well received quickly, looking quite high, but also sent 5 fuses. Customer service is very patient to teach the operation, in fact, quite simple, lazy to read the instructions ha!	Jingdong prefecture level city in Shandong
Jinfeng Hongyuan Flagship Store	Electricity savings3	You have to use it for a while to know the effect	Jingdong prefecture level city in Shandong
Jinfeng Hongyuan Flagship Store	Electricity savings3	Shipped very quickly, packaged very well, the price is particularly affordable, there are so many efficacy is also good, it is worth buying worth buying the product, the workmanship is very fine, better than imagined, recommended that everyone try not yet used, after the use of the follow-up comment	Jingdong prefecture level city in Shandong
Jinfeng Hongyuan Flagship Store	Electricity savings3	It looks regular. It's working now.	Jingdong prefecture level city in Shandong
Jinfeng Hongyuan Flagship Store	Electricity savings3	It arrived pretty fast and the owner is very responsible. Have not used it yet, will see after use	Jingdong prefecture level city in Shandong
Jinfeng Hongyuan Flagship Store	Electricity savings3	🎁 baby quality is very good, the first time I received the goods to open the first time to see, it is very good 👍, very satisfied 🙂, customer service also have a question and answer, thank you 🙏	Jingdong prefecture level city in Shandong
Jinfeng Hongyuan Flagship Store	Electricity savings3	I don't know if it's any good 。。。。。。	Jingdong prefecture level city in Shandong
Jinfeng Hongyuan Flagship Store	Electricity savings3	Buy electronic products! Still trust the things of the Jingdong self-owned store! Energy-saving effect to evaluate after heating!	Jingdong prefecture level city in Shandong
Jinfeng Hongyuan Flagship Store	Electricity savings3	The goods have been received, Blu is very beautiful, energy saving effect, to be confirmed only after actual use.	Jingdong prefecture level city in Shandong
Jinfeng Hongyuan Flagship Store	Electricity savings3	Jingdong's speed is so fast, has received, easy to operate. The display is quite good, how many volts what mode is quite understandable.	Jingdong prefecture level city in Shandong
Jinfeng Hongyuan Flagship Store	Electricity savings3	The first use, the effect is good to recommend it to colleagues to use, a month of electricity to save a lot or can be	Jingdong prefecture level city in Shandong
Jinfeng Hongyuan Flagship Store	Electricity savings3	I don't know if it's useful. Mom's is a store. I'll return it if it doesn't work.	Jingdong prefecture level city in Shandong
Jinfeng Hongyuan Flagship Store	Electricity savings3	I don't know if it works or not, I'll wait for two months to use it and then come back to comment!	Jingdong prefecture level city in Shandong
Jinfeng Hongyuan Flagship Store	Electricity savings3	I bought the second one now, and I've been using it for more than half a year, and I feel that it really saves electricity. Now it is summer, the weather is hot, sometimes the fan and air conditioning on all day, if not this power saver, the monthly electricity bill is quite high, but as long as the installation of this power saver, not afraid of its high electricity bill, absolutely let you wantonly use	Jingdong prefecture level city in Shandong
Jinfeng Hongyuan Flagship Store	Electricity savings3	The product is better than I thought it would be, the quality looks great, the voltage is stable, and I'm looking forward to some great power savings!	Jingdong prefecture level city in Shandong
Jinfeng Hongyuan Flagship Store	Electricity savings3	Inadvertently see bought a try, delivery is also quite timely, just do not know how to use, customer service patience to answer, with a pretty good, value for money!	Jingdong prefecture level city in Shandong
Jinfeng Hongyuan Flagship Store	Electricity savings3	It's better than nothing! Hopefully this will help save slightly.	Jingdong prefecture level city in Shandong
Jinfeng Hongyuan Flagship Store	Electricity savings3	Still trying it out, follow up	Jingdong prefecture level city in Shandong
Jinfeng Hongyuan Flagship Store	Electricity savings3	I've just started using it, I don't know how well it works yet, I hope it's good.	Jingdong prefecture level city in Shandong
Jinfeng Hongyuan Flagship Store	Electricity savings3	Fourth purchase, great baby!	Jingdong prefecture level city in Shandong
Jinfeng Hongyuan Flagship Store	Electricity savings3	Shipped really fast, just got it and use it to see what happens and then come back for a review!	Jingdong prefecture level city in Shandong
Jinfeng Hongyuan Flagship Store	Electricity savings3	Timely delivery **** good attitude	Jingdong prefecture level city in Shandong
Jinfeng Hongyuan Flagship Store	Electricity savings3	Home large and small things are purchased in the Jingdong Mall, have been accustomed to shopping in the Jingdong, many times can buy good quality and cheap products, and courier service attitude is particularly good, sometimes too many couriers are to help me to the home, so many years have been in the Jingdong Mall shopping, trust and support! Will also support the Jingdong as always!	Jingdong prefecture level city in Shandong
Jinfeng Hongyuan Flagship Store	Electricity savings3	Modified a pump and the modification worked.	Jingdong prefecture level city in Shandong
Jinfeng Hongyuan Flagship Store	Electricity savings3	Already installed, next time you pay the electric bill to see the results	Jingdong prefecture level city in Shandong
Jinfeng Hongyuan Flagship Store	Electricity savings3	The product is very good to use and practical to look at	Jingdong prefecture level city in Shandong
Jinfeng Hongyuan Flagship Store	Electricity savings3	Works great, great service, waiting for results.	Jingdong prefecture level city in Shandong
Jinfeng Hongyuan Flagship Store	Electricity savings3	I just used it and I'm not sure about the efficacy yet, I'll come back to evaluate it after a while.	Jingdong prefecture level city in Shandong
Jinfeng Hongyuan Flagship Store	Electricity savings3	Just received the goods, use a period of time to evaluate	Jingdong prefecture level city in Shandong
Jinfeng Hongyuan Flagship Store	Electricity savings3	Opened and tried very good, use it to see if there are results	Jingdong prefecture level city in Shandong
Jinfeng Hongyuan Flagship Store	Electricity savings3	Arrived today! Still trying it out!	Jingdong prefecture level city in Shandong
Jinfeng Hongyuan Flagship Store	Electricity savings3	Shipped quickly, well packaged, already installed, hope there is a power saving effect	Jingdong prefecture level city in Shandong
Jinfeng Hongyuan Flagship Store	Electricity savings3	I got it. It's great.	Jingdong prefecture level city in Shandong
Jinfeng Hongyuan Flagship Store	Electricity savings3	Haven't used it yet, will come back in a month	Jingdong prefecture level city in Shandong
Jinfeng Hongyuan Flagship Store	Electricity savings3	It looks good. It's good. Try it first.	Jingdong prefecture level city in Shandong
Jinfeng Hongyuan Flagship Store	Electricity savings3	It works so well that I have relatives, friends, coworkers, and 6 families that have purchased it for them through me.	Jingdong prefecture level city in Shandong
Jinfeng Hongyuan Flagship Store	Electricity savings3	In use	Jingdong prefecture level city in Shandong
Jinfeng Hongyuan Flagship Store	Electricity savings3	Received the goods today, power-saving effect do not know being used, the boss has a very good attitude, praise!	Jingdong prefecture level city in Shandong

Jinfeng Hongyuan Flagship Store	Electricity savings3	The product is compact and doesn't take up much space, but hopefully it's power efficient.	Jingdong prefecture level city in Shandong
Jinfeng Hongyuan Flagship Store	Electricity savings3	Power saving is indeed effective, I used for a period of time before coming to evaluate, the electricity bill is obviously less, the quality is good, the shopkeeper service is also very good, very patient,	Jingdong prefecture level city in Shandong
Jinfeng Hongyuan Flagship Store	Electricity savings3	It feels fine, I'll comment after I use it	Jingdong prefecture level city in Shandong
Jinfeng Hongyuan Flagship Store	Electricity savings3	I just received it and I don't know the effect yet! I will comment again after I use it and have results!	Jingdong prefecture level city in Shandong
Jinfeng Hongyuan Flagship Store	Electricity savings3	The goods look fine, I hope they really work!	Jingdong prefecture level city in Shandong
Jinfeng Hongyuan Flagship Store	Electricity savings3	I just don't know if it saves power	Jingdong prefecture level city in Shandong
Jinfeng Hongyuan Flagship Store	Electricity savings3	My friend introduced me to use this product, it is professional and I am looking forward to the long term effect~!	Jingdong prefecture level city in Shandong
Jinfeng Hongyuan Flagship Store	Electricity savings3	Try it before you judge.	Jingdong prefecture level city in Shandong
Jinfeng Hongyuan Flagship Store	Electricity savings3	It's great, the power savings are obvious! Kudos!	Jingdong prefecture level city in Shandong
Jinfeng Hongyuan Flagship Store	Electricity savings3	The quality is excellent, stabilizes the voltage and saves electricity, very satisfied.	Jingdong prefecture level city in Shandong
Jinfeng Hongyuan Flagship Store	Electricity savings3	You have to use it for a while to know the effect	Jingdong prefecture level city in Shandong
Jinfeng Hongyuan Flagship Store	Electricity savings3	It's on trial.	Jingdong prefecture level city in Shandong
Jinfeng Hongyuan Flagship Store	Electricity savings3	The stuff works great.	Jingdong prefecture level city in Shandong
Jinfeng Hongyuan Flagship Store	Electricity savings3	Doesn't seem to work, not as god as other reviews	Jingdong prefecture level city in Shandong
Jinfeng Hongyuan Flagship Store	Electricity savings3	It's not good quality, it doesn't work, it stops working after a few days, it doesn't work, the customer service doesn't care, it's a bit irritating.	Jingdong prefecture level city in Shandong
Jinfeng Hongyuan Flagship Store	Electricity savings3	It doesn't do anything.	Jingdong prefecture level city in Shandong
Jinfeng Hongyuan Flagship Store	Electricity savings3	It's good.	Jingdong prefecture level city in Shandong
Jinfeng Hongyuan Flagship Store	Electricity savings3	Used a month effect is not obvious, are psychological effects, feel are not less, did not save to the electricity, there are details of the problem, the store said to buy to send 5 fuse, in fact, received only three, small problems are not committed to in place, I believe that the product is not good enough	Jingdong prefecture level city in Shandong
Jinfeng Hongyuan Flagship Store	Electricity savings3	Well received quickly, looking quite high, but also sent 5 fuses. Customer service is very patient to teach the operation, in fact, quite simple, lazy to read the instructions ha!	Jingdong prefecture level city in Shandong
Jinfeng Hongyuan Flagship Store	Electricity savings3	You have to use it for a while to know the effect	Jingdong prefecture level city in Shandong
Jinfeng Hongyuan Flagship Store	Electricity savings3	Shipped very quickly, packaged very well, the price is particularly affordable, there are so many efficacy is also good, it is worth buying worth buying the product, the workmanship is very fine, better than imagined, recommended that everyone try not yet used, after the use of the follow-up comment	Jingdong prefecture level city in Shandong
Jinfeng Hongyuan Flagship Store	Electricity savings3	It looks regular. It's working now.	Jingdong prefecture level city in Shandong
Jinfeng Hongyuan Flagship Store	Electricity savings3	It arrived pretty fast and the owner is very responsible. Have not used it yet, will see after use	Jingdong prefecture level city in Shandong
Jinfeng Hongyuan Flagship Store	Electricity savings3	🎁 baby quality is very good, the first time I received the goods to open the first time to see, it is very good 👍, very satisfied 😊, customer service also have a question and answer, thank you 🙏	Jingdong prefecture level city in Shandong
Jinfeng Hongyuan Flagship Store	Electricity savings3	I don't know if it's any good 。。。。。。	Jingdong prefecture level city in Shandong
Jinfeng Hongyuan Flagship Store	Electricity savings3	Buy electronic products! Still trust the things of the Jingdong self-owned store! Energy-saving effect to evaluate after heating!	Jingdong prefecture level city in Shandong
Jinfeng Hongyuan Flagship Store	Electricity savings3	The goods have been received, Blu is very beautiful, energy saving effect, to be confirmed only after actual use.	Jingdong prefecture level city in Shandong
Jinfeng Hongyuan Flagship Store	Electricity savings3	Jingdong's speed is so fast, has received, easy to operate. The display is quite good, how many volts what mode is quite understandable.	Jingdong prefecture level city in Shandong
Jinfeng Hongyuan Flagship Store	Electricity savings3	The first use, the effect is good to recommend it to colleagues to use, a month of electricity to save a lot or can be	Jingdong prefecture level city in Shandong
Jinfeng Hongyuan Flagship Store	Electricity savings3	I don't know if it's useful. Mom's is a store. I'll return it if it doesn't work.	Jingdong prefecture level city in Shandong
Jinfeng Hongyuan Flagship Store	Electricity savings3	I don't know if it works or not, I'll wait for two months to use it and then come back to comment!	Jingdong prefecture level city in Shandong
Jinfeng Hongyuan Flagship Store	Electricity savings3	I bought the second one now, and I've been using it for more than half a year, and I feel that it really saves electricity. Now it is summer, the weather is hot, sometimes the fan and air conditioning on all day, if not this power saver, the monthly electricity bill is quite high, but as long as the installation of this power saver, not afraid of its high electricity bill, absolutely let you wantonly use	Jingdong prefecture level city in Shandong
Jinfeng Hongyuan Flagship Store	Electricity savings3	The product is better than I thought it would be, the quality looks great, the voltage is stable, and I'm looking forward to some great power savings!	Jingdong prefecture level city in Shandong
Jinfeng Hongyuan Flagship Store	Electricity savings3	Inadvertently see bought a try, delivery is also quite timely, just do not know how to use, customer service patience to answer, with a pretty good, value for money!	Jingdong prefecture level city in Shandong
Jinfeng Hongyuan Flagship Store	Electricity savings3	It's better than nothing! Hopefully this will help save slightly.	Jingdong prefecture level city in Shandong
Jinfeng Hongyuan Flagship Store	Electricity savings3	Still trying it out, follow up	Jingdong prefecture level city in Shandong
Jinfeng Hongyuan Flagship Store	Electricity savings3	I've just started using it, I don't know how well it works yet, I hope it's good.	Jingdong prefecture level city in Shandong
Jinfeng Hongyuan Flagship Store	Electricity savings3	Fourth purchase, great baby!	Jingdong prefecture level city in Shandong
Jinfeng Hongyuan Flagship Store	Electricity savings3	Shipped really fast, just got it and use it to see what happens and then come back for a review!	Jingdong prefecture level city in Shandong
Jinfeng Hongyuan Flagship Store	Electricity savings3	Timely delivery **** good attitude	Jingdong prefecture level city in Shandong
Jinfeng Hongyuan Flagship Store	Electricity savings3	Home large and small things are purchased in the Jingdong Mall, have been accustomed to shopping in the Jingdong, many times can buy good quality and cheap products, and courier service attitude is particularly good, sometimes too many couriers are to help me to the home, so many years have been in the Jingdong Mall shopping, trust and support! Will also support the Jingdong as always!	Jingdong prefecture level city in Shandong
Jinfeng Hongyuan Flagship Store	Electricity savings3	Modified a pump and the modification worked.	Jingdong prefecture level city in Shandong
Jinfeng Hongyuan Flagship Store	Electricity savings3	Already installed, next time you pay the electric bill to see the results	Jingdong prefecture level city in Shandong
Jinfeng Hongyuan Flagship Store	Electricity savings3	The product is very good to use and practical to look at	Jingdong prefecture level city in Shandong
Jinfeng Hongyuan Flagship Store	Electricity savings3	Works great, great service, waiting for results.	Jingdong prefecture level city in Shandong
Jinfeng Hongyuan Flagship Store	Electricity savings3	I just used it and I'm not sure about the efficacy yet, I'll come back to evaluate it after a while.	Jingdong prefecture level city in Shandong
Jinfeng Hongyuan Flagship Store	Electricity savings3	Just received the goods, use a period of time to evaluate	Jingdong prefecture level city in Shandong
Jinfeng Hongyuan Flagship Store	Electricity savings3	Opened and tried very good, use it to see if there are results	Jingdong prefecture level city in Shandong

Jinfeng Hongyuan Flagship Store	Electricity savings3	Arrived today! Still trying it out!	Jingdong prefecture level city in Shandong
Jinfeng Hongyuan Flagship Store	Electricity savings3	Shipped quickly, well packaged, already installed, hope there is a power saving effect	Jingdong prefecture level city in Shandong
Jinfeng Hongyuan Flagship Store	Electricity savings3	I got it. It's great.	Jingdong prefecture level city in Shandong
Jinfeng Hongyuan Flagship Store	Electricity savings3	Haven't used it yet, will come back in a month	Jingdong prefecture level city in Shandong
Jinfeng Hongyuan Flagship Store	Electricity savings3	It looks good. It's good. Try it first.	Jingdong prefecture level city in Shandong
Jinfeng Hongyuan Flagship Store	Electricity savings3	It works so well that I have relatives, friends, coworkers, and 6 families that have purchased it for them through me.	Jingdong prefecture level city in Shandong
Jinfeng Hongyuan Flagship Store	Electricity savings3	In use	Jingdong prefecture level city in Shandong
Jinfeng Hongyuan Flagship Store	Electricity savings3	Received the goods today, power-saving effect do not know being used, the boss has a very good attitude, praise!	Jingdong prefecture level city in Shandong
Jinfeng Hongyuan Flagship Store	Electricity savings3	The product is compact and doesn't take up much space, but hopefully it's power efficient.	Jingdong prefecture level city in Shandong
Jinfeng Hongyuan Flagship Store	Electricity savings3	Power saving is indeed effective, I used for a period of time before coming to evaluate, the electricity bill is obviously less, the quality is good, the shopkeeper service is also very good, very patient,	Jingdong prefecture level city in Shandong
Jinfeng Hongyuan Flagship Store	Electricity savings3	It feels fine, I'll comment after I use it	Jingdong prefecture level city in Shandong
Jinfeng Hongyuan Flagship Store	Electricity savings3	I just received it and I don't know the effect yet! I will comment again after I use it and have results!	Jingdong prefecture level city in Shandong
Jinfeng Hongyuan Flagship Store	Electricity savings3	The goods look fine, I hope they really work!	Jingdong prefecture level city in Shandong
Jinfeng Hongyuan Flagship Store	Electricity savings3	I just don't know if it saves power	Jingdong prefecture level city in Shandong
Jinfeng Hongyuan Flagship Store	Electricity savings3	My friend introduced me to use this product, it is professional and I am looking forward to the long term effect~!	Jingdong prefecture level city in Shandong
Jinfeng Hongyuan Flagship Store	Electricity savings3	Try it before you judge.	Jingdong prefecture level city in Shandong
Jinfeng Hongyuan Flagship Store	Electricity savings3	It's great, the power savings are obvious! Kudos!	Jingdong prefecture level city in Shandong
Jinfeng Hongyuan Flagship Store	Electricity savings3	The quality is excellent, stabilizes the voltage and saves electricity, very satisfied.	Jingdong prefecture level city in Shandong
Jinfeng Hongyuan Flagship Store	Electricity savings3	You have to use it for a while to know the effect	Jingdong prefecture level city in Shandong
Jinfeng Hongyuan Flagship Store	Electricity savings3	It's on trial.	Jingdong prefecture level city in Shandong
Jinfeng Hongyuan Flagship Store	Electricity savings3	The stuff works great.	Jingdong prefecture level city in Shandong
Jinfeng Hongyuan Flagship Store	Electricity savings3	Doesn't seem to work, not as god as other reviews	Jingdong prefecture level city in Shandong
Jinfeng Hongyuan Flagship Store	Electricity savings3	It's not good quality, it doesn't work, it stops working after a few days, it doesn't work, the customer service doesn't care, it's a bit irritating.	Jingdong prefecture level city in Shandong
Jinfeng Hongyuan Flagship Store	Electricity savings3	It doesn't do anything.	Jingdong prefecture level city in Shandong
Jinfeng Hongyuan Flagship Store	Electricity savings3	It's good.	Jingdong prefecture level city in Shandong
Jinfeng Hongyuan Flagship Store	Electricity savings3	Used a month effect is not obvious, are psychological effects, feel are not less, did not save to the electricity, there are details of the problem, the store said to buy to send 5 fuse, in fact, received only three, small problems are not committed to in place, I believe that the product is not good enough	Jingdong prefecture level city in Shandong
Jinfeng Hongyuan Flagship Store	Electricity savings3	Well received quickly, looking quite high, but also sent 5 fuses. Customer service is very patient to teach the operation, in fact, quite simple, lazy to read the instructions ha!	Jingdong prefecture level city in Shandong
Jinfeng Hongyuan Flagship Store	Electricity savings3	You have to use it for a while to know the effect	Jingdong prefecture level city in Shandong
Jinfeng Hongyuan Flagship Store	Electricity savings3	Shipped very quickly, packaged very well, the price is particularly affordable, there are so many efficacy is also good, it is worth buying worth buying the product, the workmanship is very fine, better than imagined, recommended that everyone try not yet used, after the use of the follow-up comment	Jingdong prefecture level city in Shandong
Jinfeng Hongyuan Flagship Store	Electricity savings3	It looks regular. It's working now.	Jingdong prefecture level city in Shandong
Jinfeng Hongyuan Flagship Store	Electricity savings3	It arrived pretty fast and the owner is very responsible. Have not used it yet, will see after use	Jingdong prefecture level city in Shandong
Jinfeng Hongyuan Flagship Store	Electricity savings3	🎁 baby quality is very good, the first time I received the goods to open the first time to see, it is very good 👍, very satisfied 😃, customer service also have a question and answer, thank you 🙏	Jingdong prefecture level city in Shandong
Jinfeng Hongyuan Flagship Store	Electricity savings3	I don't know if it's any good 。。。。。。	Jingdong prefecture level city in Shandong
Jinfeng Hongyuan Flagship Store	Electricity savings3	Buy electronic products! Still trust the things of the Jingdong self-owned store! Energy-saving effect to evaluate after heating!	Jingdong prefecture level city in Shandong
Jinfeng Hongyuan Flagship Store	Electricity savings3	The goods have been received, Blu is very beautiful, energy saving effect, to be confirmed only after actual use.	Jingdong prefecture level city in Shandong
Jinfeng Hongyuan Flagship Store	Electricity savings3	Jingdong's speed is so fast, has received, easy to operate. The display is quite good, how many volts what mode is quite understandable.	Jingdong prefecture level city in Shandong
Jinfeng Hongyuan Flagship Store	Electricity savings3	The first use, the effect is good to recommend it to colleagues to use, a month of electricity to save a lot or can be	Jingdong prefecture level city in Shandong
Jinfeng Hongyuan Flagship Store	Electricity savings3	I don't know if it's useful. Mom's is a store. I'll return it if it doesn't work.	Jingdong prefecture level city in Shandong
Jinfeng Hongyuan Flagship Store	Electricity savings3	I don't know if it works or not, I'll wait for two months to use it and then come back to comment!	Jingdong prefecture level city in Shandong
Jinfeng Hongyuan Flagship Store	Electricity savings3	I bought the second one now, and I've been using it for more than half a year, and I feel that it really saves electricity. Now it is summer, the weather is hot, sometimes the fan and air conditioning on all day, if not this power saver, the monthly electricity bill is quite high, but as long as the installation of this power saver, not afraid of its high electricity bill, absolutely let you wantonly use	Jingdong prefecture level city in Shandong
Jinfeng Hongyuan Flagship Store	Electricity savings3	The product is better than I thought it would be, the quality looks great, the voltage is stable, and I'm looking forward to some great power savings!	Jingdong prefecture level city in Shandong
Jinfeng Hongyuan Flagship Store	Electricity savings3	Inadvertently see bought a try, delivery is also quite timely, just do not know how to use, customer service patience to answer, with a pretty good, value for money!	Jingdong prefecture level city in Shandong
Jinfeng Hongyuan Flagship Store	Electricity savings3	It's better than nothing! Hopefully this will help save slightly.	Jingdong prefecture level city in Shandong
Jinfeng Hongyuan Flagship Store	Electricity savings3	Still trying it out, follow up	Jingdong prefecture level city in Shandong
Jinfeng Hongyuan Flagship Store	Electricity savings3	I've just started using it, I don't know how well it works yet, I hope it's good.	Jingdong prefecture level city in Shandong
Jinfeng Hongyuan Flagship Store	Electricity savings3	Fourth purchase, great baby!	Jingdong prefecture level city in Shandong
Jinfeng Hongyuan Flagship Store	Electricity savings3	Shipped really fast, just got it and use it to see what happens and then come back for a review!	Jingdong prefecture level city in Shandong
Jinfeng Hongyuan Flagship Store	Electricity savings3	Timely delivery **** good attitude	Jingdong prefecture level city in Shandong

Jinfeng Hongyuan Flagship Store	Electricity savings3	Home large and small things are purchased in the Jingdong Mall, have been accustomed to shopping in the Jingdong, many times can buy good quality and cheap products, and courier service attitude is particularly good, sometimes too many couriers are to help me to the home, so many years have been in the Jingdong Mall shopping, trust and support! Will also support the Jingdong as always!	Jingdong prefecture level city in Shandong
Jinfeng Hongyuan Flagship Store	Electricity savings3	Modified a pump and the modification worked.	Jingdong prefecture level city in Shandong
Jinfeng Hongyuan Flagship Store	Electricity savings3	Already installed, next time you pay the electric bill to see the results	Jingdong prefecture level city in Shandong
Jinfeng Hongyuan Flagship Store	Electricity savings3	The product is very good to use and practical to look at	Jingdong prefecture level city in Shandong
Jinfeng Hongyuan Flagship Store	Electricity savings3	Works great, great service, waiting for results.	Jingdong prefecture level city in Shandong
Jinfeng Hongyuan Flagship Store	Electricity savings3	I just used it and I'm not sure about the efficacy yet, I'll come back to evaluate it after a while.	Jingdong prefecture level city in Shandong
Jinfeng Hongyuan Flagship Store	Electricity savings3	Just received the goods, use a period of time to evaluate	Jingdong prefecture level city in Shandong
Jinfeng Hongyuan Flagship Store	Electricity savings3	Opened and tried very good, use it to see if there are results	Jingdong prefecture level city in Shandong
Jinfeng Hongyuan Flagship Store	Electricity savings3	Arrived today! Still trying it out!	Jingdong prefecture level city in Shandong
Jinfeng Hongyuan Flagship Store	Electricity savings3	Shipped quickly, well packaged, already installed, hope there is a power saving effect	Jingdong prefecture level city in Shandong
Jinfeng Hongyuan Flagship Store	Electricity savings3	I got it. It's great.	Jingdong prefecture level city in Shandong
Jinfeng Hongyuan Flagship Store	Electricity savings3	Haven't used it yet, will come back in a month	Jingdong prefecture level city in Shandong
Jinfeng Hongyuan Flagship Store	Electricity savings3	It looks good. It's good. Try it first.	Jingdong prefecture level city in Shandong
Jinfeng Hongyuan Flagship Store	Electricity savings3	It works so well that I have relatives, friends, coworkers, and 6 families that have purchased it for them through me.	Jingdong prefecture level city in Shandong
Jinfeng Hongyuan Flagship Store	Electricity savings3	In use	Jingdong prefecture level city in Shandong
Jinfeng Hongyuan Flagship Store	Electricity savings3	Received the goods today, power-saving effect do not know being used, the boss has a very good attitude, praise!	Jingdong prefecture level city in Shandong
Jinfeng Hongyuan Flagship Store	Electricity savings3	The product is compact and doesn't take up much space, but hopefully it's power efficient.	Jingdong prefecture level city in Shandong
Jinfeng Hongyuan Flagship Store	Electricity savings3	Power saving is indeed effective, I used for a period of time before coming to evaluate, the electricity bill is obviously less, the quality is good, the shopkeeper service is also very good, very patient,	Jingdong prefecture level city in Shandong
Jinfeng Hongyuan Flagship Store	Electricity savings3	It feels fine, I'll comment after I use it	Jingdong prefecture level city in Shandong
Jinfeng Hongyuan Flagship Store	Electricity savings3	I just received it and I don't know the effect yet! I will comment again after I use it and have results!	Jingdong prefecture level city in Shandong
Jinfeng Hongyuan Flagship Store	Electricity savings3	The goods look fine, I hope they really work!	Jingdong prefecture level city in Shandong
Jinfeng Hongyuan Flagship Store	Electricity savings3	I just don't know if it saves power	Jingdong prefecture level city in Shandong
Jinfeng Hongyuan Flagship Store	Electricity savings3	My friend introduced me to use this product, it is professional and I am looking forward to the long term effect~!	Jingdong prefecture level city in Shandong
Jinfeng Hongyuan Flagship Store	Electricity savings3	Try it before you judge.	Jingdong prefecture level city in Shandong
Jinfeng Hongyuan Flagship Store	Electricity savings3	It's great, the power savings are obvious! Kudos!	Jingdong prefecture level city in Shandong
Jinfeng Hongyuan Flagship Store	Electricity savings3	The quality is excellent, stabilizes the voltage and saves electricity, very satisfied.	Jingdong prefecture level city in Shandong
Jinfeng Hongyuan Flagship Store	Electricity savings3	You have to use it for a while to know the effect	Jingdong prefecture level city in Shandong
Jinfeng Hongyuan Flagship Store	Electricity savings3	It's on trial.	Jingdong prefecture level city in Shandong
Jinfeng Hongyuan Flagship Store	Electricity savings3	The stuff works great.	Jingdong prefecture level city in Shandong
Jinfeng Hongyuan Flagship Store	Electricity savings3	Doesn't seem to work, not as god as other reviews	Jingdong prefecture level city in Shandong
Jinfeng Hongyuan Flagship Store	Electricity savings3	It's not good quality, it doesn't work, it stops working after a few days, it doesn't work, the customer service doesn't care, it's a bit irritating.	Jingdong prefecture level city in Shandong
Jinfeng Hongyuan Flagship Store	Electricity savings3	It doesn't do anything.	Jingdong prefecture level city in Shandong
Jinfeng Hongyuan Flagship Store	Electricity savings3	It's good.	Jingdong prefecture level city in Shandong
Jinfeng Hongyuan Flagship Store	Electricity savings3	Used a month effect is not obvious, are psychological effects, feel are not less, did not save to the electricity, there are details of the problem, the store said to buy to send 5 fuse, in fact, received only three, small problems are not committed to in place, I believe that the product is not good enough	Jingdong prefecture level city in Shandong
Jinfeng Hongyuan Flagship Store	Electricity savings3	Well received quickly, looking quite high, but also sent 5 fuses. Customer service is very patient to teach the operation, in fact, quite simple, lazy to read the instructions ha!	Jingdong prefecture level city in Shandong
Jinfeng Hongyuan Flagship Store	Electricity savings3	You have to use it for a while to know the effect	Jingdong prefecture level city in Shandong
Jinfeng Hongyuan Flagship Store	Electricity savings3	Shipped very quickly, packaged very well, the price is particularly affordable, there are so many efficacy is also good, it is worth buying worth buying the product, the workmanship is very fine, better than imagined, recommended that everyone try not yet used, after the use of the follow-up comment	Jingdong prefecture level city in Shandong
Jinfeng Hongyuan Flagship Store	Electricity savings3	It looks regular. It's working now.	Jingdong prefecture level city in Shandong
Jinfeng Hongyuan Flagship Store	Electricity savings3	It arrived pretty fast and the owner is very responsible. Have not used it yet, will see after use	Jingdong prefecture level city in Shandong
Jinfeng Hongyuan Flagship Store	Electricity savings3	🎁 baby quality is very good, the first time I received the goods to open the first time to see, it is very good 👍, very satisfied 😊, customer service also have a question and answer, thank you 🙏	Jingdong prefecture level city in Shandong
Jinfeng Hongyuan Flagship Store	Electricity savings3	I don't know if it's any good 。。。。。。	Jingdong prefecture level city in Shandong
Jinfeng Hongyuan Flagship Store	Electricity savings3	Buy electronic products! Still trust the things of the Jingdong self-owned store! Energy-saving effect to evaluate after heating!	Jingdong prefecture level city in Shandong
Jinfeng Hongyuan Flagship Store	Electricity savings3	The goods have been received, Blu is very beautiful, energy saving effect, to be confirmed only after actual use.	Jingdong prefecture level city in Shandong
Jinfeng Hongyuan Flagship Store	Electricity savings3	Jingdong's speed is so fast, has received, easy to operate. The display is quite good, how many volts what mode is quite understandable.	Jingdong prefecture level city in Shandong
Jinfeng Hongyuan Flagship Store	Electricity savings3	The first use, the effect is good to recommend it to colleagues to use, a month of electricity to save a lot or can be	Jingdong prefecture level city in Shandong
Jinfeng Hongyuan Flagship Store	Electricity savings3	I don't know if it's useful. Mom's is a store. I'll return it if it doesn't work.	Jingdong prefecture level city in Shandong
Jinfeng Hongyuan Flagship Store	Electricity savings3	I don't know if it works or not, I'll wait for two months to use it and then come back to comment!	Jingdong prefecture level city in Shandong
Jinfeng Hongyuan Flagship Store	Electricity savings3	I bought the second one now, and I've been using it for more than half a year, and I feel that it really saves electricity. Now it is summer, the weather is hot, sometimes the fan and air conditioning on all day, if not this power saver, the monthly electricity bill is quite high, but as long as the installation of this power saver, not afraid of its high electricity bill, absolutely let you wantonly use	Jingdong prefecture level city in Shandong

Jinfeng Hongyuan Flagship Store	Electricity savings3	The product is better than I thought it would be, the quality looks great, the voltage is stable, and I'm looking forward to some great power savings!	Jingdong prefecture level city in Shandong
Jinfeng Hongyuan Flagship Store	Electricity savings3	Inadvertently see bought a try, delivery is also quite timely, just do not know how to use, customer service patience to answer, with a pretty good, value for money!	Jingdong prefecture level city in Shandong
Jinfeng Hongyuan Flagship Store	Electricity savings3	It's better than nothing! Hopefully this will help save slightly.	Jingdong prefecture level city in Shandong
Jinfeng Hongyuan Flagship Store	Electricity savings3	Still trying it out, follow up	Jingdong prefecture level city in Shandong
Jinfeng Hongyuan Flagship Store	Electricity savings3	I've just started using it, I don't know how well it works yet, I hope it's good.	Jingdong prefecture level city in Shandong
Jinfeng Hongyuan Flagship Store	Electricity savings3	Fourth purchase, great baby!	Jingdong prefecture level city in Shandong
Jinfeng Hongyuan Flagship Store	Electricity savings3	Shipped really fast, just got it and use it to see what happens and then come back for a review!	Jingdong prefecture level city in Shandong
Jinfeng Hongyuan Flagship Store	Electricity savings3	Timely delivery **** good attitude	Jingdong prefecture level city in Shandong
Jinfeng Hongyuan Flagship Store	Electricity savings3	Home large and small things are purchased in the Jingdong Mall, have been accustomed to shopping in the Jingdong, many times can buy good quality and cheap products, and courier service attitude is particularly good, sometimes too many couriers are to help me to the home, so many years have been in the Jingdong Mall shopping, trust and support! Will also support the Jingdong as always!	Jingdong prefecture level city in Shandong
Jinfeng Hongyuan Flagship Store	Electricity savings3	Modified a pump and the modification worked.	Jingdong prefecture level city in Shandong
Jinfeng Hongyuan Flagship Store	Electricity savings3	Already installed, next time you pay the electric bill to see the results	Jingdong prefecture level city in Shandong
Jinfeng Hongyuan Flagship Store	Electricity savings3	The product is very good to use and practical to look at	Jingdong prefecture level city in Shandong
Jinfeng Hongyuan Flagship Store	Electricity savings3	Works great, great service, waiting for results.	Jingdong prefecture level city in Shandong
Jinfeng Hongyuan Flagship Store	Electricity savings3	I just used it and I'm not sure about the efficacy yet, I'll come back to evaluate it after a while.	Jingdong prefecture level city in Shandong
Jinfeng Hongyuan Flagship Store	Electricity savings3	Just received the goods, use a period of time to evaluate	Jingdong prefecture level city in Shandong
Jinfeng Hongyuan Flagship Store	Electricity savings3	Opened and tried very good, use it to see if there are results	Jingdong prefecture level city in Shandong
Jinfeng Hongyuan Flagship Store	Electricity savings3	Arrived today! Still trying it out!	Jingdong prefecture level city in Shandong
Jinfeng Hongyuan Flagship Store	Electricity savings3	Shipped quickly, well packaged, already installed, hope there is a power saving effect	Jingdong prefecture level city in Shandong
Jinfeng Hongyuan Flagship Store	Electricity savings3	I got it. It's great.	Jingdong prefecture level city in Shandong
Jinfeng Hongyuan Flagship Store	Electricity savings3	Haven't used it yet, will come back in a month	Jingdong prefecture level city in Shandong
Jinfeng Hongyuan Flagship Store	Electricity savings3	It looks good. It's good. Try it first.	Jingdong prefecture level city in Shandong
Jinfeng Hongyuan Flagship Store	Electricity savings3	It works so well that I have relatives, friends, coworkers, and 6 families that have purchased it for them through me.	Jingdong prefecture level city in Shandong
Jinfeng Hongyuan Flagship Store	Electricity savings3	In use	Jingdong prefecture level city in Shandong
Jinfeng Hongyuan Flagship Store	Electricity savings3	Received the goods today, power-saving effect do not know being used, the boss has a very good attitude, praise!	Jingdong prefecture level city in Shandong
Jinfeng Hongyuan Flagship Store	Electricity savings3	The product is compact and doesn't take up much space, but hopefully it's power efficient.	Jingdong prefecture level city in Shandong
Jinfeng Hongyuan Flagship Store	Electricity savings3	Power saving is indeed effective, I used for a period of time before coming to evaluate, the electricity bill is obviously less, the quality is good, the shopkeeper service is also very good, very patient,	Jingdong prefecture level city in Shandong
Jinfeng Hongyuan Flagship Store	Electricity savings3	It feels fine, I'll comment after I use it	Jingdong prefecture level city in Shandong
Jinfeng Hongyuan Flagship Store	Electricity savings3	I just received it and I don't know the effect yet! I will comment again after I use it and have results!	Jingdong prefecture level city in Shandong
Jinfeng Hongyuan Flagship Store	Electricity savings3	The goods look fine, I hope they really work!	Jingdong prefecture level city in Shandong
Jinfeng Hongyuan Flagship Store	Electricity savings3	I just don't know if it saves power	Jingdong prefecture level city in Shandong
Jinfeng Hongyuan Flagship Store	Electricity savings3	My friend introduced me to use this product, it is professional and I am looking forward to the long term effect~!	Jingdong prefecture level city in Shandong
Jinfeng Hongyuan Flagship Store	Electricity savings3	Try it before you judge.	Jingdong prefecture level city in Shandong
Jinfeng Hongyuan Flagship Store	Electricity savings3	It's great, the power savings are obvious! Kudos!	Jingdong prefecture level city in Shandong
Jinfeng Hongyuan Flagship Store	Electricity savings3	The quality is excellent, stabilizes the voltage and saves electricity, very satisfied.	Jingdong prefecture level city in Shandong
Jinfeng Hongyuan Flagship Store	Electricity savings3	You have to use it for a while to know the effect	Jingdong prefecture level city in Shandong
Jinfeng Hongyuan Flagship Store	Electricity savings3	It's on trial.	Jingdong prefecture level city in Shandong
Jinfeng Hongyuan Flagship Store	Electricity savings3	The stuff works great.	Jingdong prefecture level city in Shandong
Jinfeng Hongyuan Flagship Store	Electricity savings3	Doesn't seem to work, not as god as other reviews	Jingdong prefecture level city in Shandong
Jinfeng Hongyuan Flagship Store	Electricity savings3	It's not good quality, it doesn't work, it stops working after a few days, it doesn't work, the customer service doesn't care, it's a bit irritating.	Jingdong prefecture level city in Shandong
Jinfeng Hongyuan Flagship Store	Electricity savings3	It doesn't do anything.	Jingdong prefecture level city in Shandong
Jinfeng Hongyuan Flagship Store	Electricity savings3	It's good.	Jingdong prefecture level city in Shandong
Jinfeng Hongyuan Flagship Store	Electricity savings3	Used a month effect is not obvious, are psychological effects, feel are not less, did not save to the electricity, there are details of the problem, the store said to buy to send 5 fuse, in fact, received only three, small problems are not committed to in place, I believe that the product is not good enough	Jingdong prefecture level city in Shandong
Jinfeng Hongyuan Flagship Store	Electricity savings3	Well received quickly, looking quite high, but also sent 5 fuses. Customer service is very patient to teach the operation, in fact, quite simple, lazy to read the instructions ha!	Jingdong prefecture level city in Shandong
Jinfeng Hongyuan Flagship Store	Electricity savings3	You have to use it for a while to know the effect	Jingdong prefecture level city in Shandong
Jinfeng Hongyuan Flagship Store	Electricity savings3	Shipped very quickly, packaged very well, the price is particularly affordable, there are so many efficacy is also good, it is worth buying worth buying the product, the workmanship is very fine, better than imagined, recommended that everyone try not yet used, after the use of the follow-up comment	Jingdong prefecture level city in Shandong
Jinfeng Hongyuan Flagship Store	Electricity savings3	It looks regular. It's working now.	Jingdong prefecture level city in Shandong
Jinfeng Hongyuan Flagship Store	Electricity savings3	It arrived pretty fast and the owner is very responsible. Have not used it yet, will see after use	Jingdong prefecture level city in Shandong
Jinfeng Hongyuan Flagship Store	Electricity savings3	🎁 baby quality is very good, the first time I received the goods to open the first time to see, it is very good 👍, very satisfied ☺, customer service also have a question and answer, thank you 🙏	Jingdong prefecture level city in Shandong
Jinfeng Hongyuan Flagship Store	Electricity savings3	I don't know if it's any good 。。。。。。	Jingdong prefecture level city in Shandong

Jinfeng Hongyuan Flagship Store	Electricity savings3	Buy electronic products! Still trust the things of the Jingdong self-owned store! Energy-saving effect to evaluate after heating!	Jingdong prefecture level city in Shandong
Jinfeng Hongyuan Flagship Store	Electricity savings3	The goods have been received, Blu is very beautiful, energy saving effect, to be confirmed only after actual use.	Jingdong prefecture level city in Shandong
Jinfeng Hongyuan Flagship Store	Electricity savings3	Jingdong's speed is so fast, has received, easy to operate. The display is quite good, how many volts what mode is quite understandable.	Jingdong prefecture level city in Shandong
Jinfeng Hongyuan Flagship Store	Electricity savings3	The first use, the effect is good to recommend it to colleagues to use, a month of electricity to save a lot or can be	Jingdong prefecture level city in Shandong
Jinfeng Hongyuan Flagship Store	Electricity savings3	I don't know if it's useful. Mom's is a store. I'll return it if it doesn't work.	Jingdong prefecture level city in Shandong
Jinfeng Hongyuan Flagship Store	Electricity savings3	I don't know if it works or not, I'll wait for two months to use it and then come back to comment!	Jingdong prefecture level city in Shandong
Jinfeng Hongyuan Flagship Store	Electricity savings3	I bought the second one now, and I've been using it for more than half a year, and I feel that it really saves electricity. Now it is summer, the weather is hot, sometimes the fan and air conditioning on all day, if not this power saver, the monthly electricity bill is quite high, but as long as the installation of this power saver, not afraid of its high electricity bill, absolutely let you wantonly use	Jingdong prefecture level city in Shandong
Jinfeng Hongyuan Flagship Store	Electricity savings3	The product is better than I thought it would be, the quality looks great, the voltage is stable, and I'm looking forward to some great power savings!	Jingdong prefecture level city in Shandong
Jinfeng Hongyuan Flagship Store	Electricity savings3	Inadvertently see bought a try, delivery is also quite timely, just do not know how to use, customer service patience to answer, with a pretty good, value for money!	Jingdong prefecture level city in Shandong
Jinfeng Hongyuan Flagship Store	Electricity savings3	It's better than nothing! Hopefully this will help save slightly.	Jingdong prefecture level city in Shandong
Jinfeng Hongyuan Flagship Store	Electricity savings3	Still trying it out, follow up	Jingdong prefecture level city in Shandong
Jinfeng Hongyuan Flagship Store	Electricity savings3	I've just started using it, I don't know how well it works yet, I hope it's good.	Jingdong prefecture level city in Shandong
Jinfeng Hongyuan Flagship Store	Electricity savings3	Fourth purchase, great baby!	Jingdong prefecture level city in Shandong
Jinfeng Hongyuan Flagship Store	Electricity savings3	Shipped really fast, just got it and use it to see what happens and then come back for a review!	Jingdong prefecture level city in Shandong
Jinfeng Hongyuan Flagship Store	Electricity savings3	Timely delivery **** good attitude	Jingdong prefecture level city in Shandong
Jinfeng Hongyuan Flagship Store	Electricity savings3	Home large and small things are purchased in the Jingdong Mall, have been accustomed to shopping in the Jingdong, many times can buy good quality and cheap products, and courier service attitude is particularly good, sometimes too many couriers are to help me to the home, so many years have been in the Jingdong Mall shopping, trust and support! Will also support the Jingdong as always!	Jingdong prefecture level city in Shandong
Jinfeng Hongyuan Flagship Store	Electricity savings3	Modified a pump and the modification worked.	Jingdong prefecture level city in Shandong
Jinfeng Hongyuan Flagship Store	Electricity savings3	Already installed, next time you pay the electric bill to see the results	Jingdong prefecture level city in Shandong
Jinfeng Hongyuan Flagship Store	Electricity savings3	The product is very good to use and practical to look at	Jingdong prefecture level city in Shandong
Jinfeng Hongyuan Flagship Store	Electricity savings3	Works great, great service, waiting for results.	Jingdong prefecture level city in Shandong
Jinfeng Hongyuan Flagship Store	Electricity savings3	I just used it and I'm not sure about the efficacy yet, I'll come back to evaluate it after a while.	Jingdong prefecture level city in Shandong
Jinfeng Hongyuan Flagship Store	Electricity savings3	Just received the goods, use a period of time to evaluate	Jingdong prefecture level city in Shandong
Jinfeng Hongyuan Flagship Store	Electricity savings3	Opened and tried very good, use it to see if there are results	Jingdong prefecture level city in Shandong
Jinfeng Hongyuan Flagship Store	Electricity savings3	Arrived today! Still trying it out!	Jingdong prefecture level city in Shandong
Jinfeng Hongyuan Flagship Store	Electricity savings3	Shipped quickly, well packaged, already installed, hope there is a power saving effect	Jingdong prefecture level city in Shandong
Jinfeng Hongyuan Flagship Store	Electricity savings3	I got it. It's great.	Jingdong prefecture level city in Shandong
Jinfeng Hongyuan Flagship Store	Electricity savings3	Haven't used it yet, will come back in a month	Jingdong prefecture level city in Shandong
Jinfeng Hongyuan Flagship Store	Electricity savings3	It looks good. It's good. Try it first.	Jingdong prefecture level city in Shandong
Jinfeng Hongyuan Flagship Store	Electricity savings3	It works so well that I have relatives, friends, coworkers, and 6 families that have purchased it for them through me.	Jingdong prefecture level city in Shandong
Jinfeng Hongyuan Flagship Store	Electricity savings3	In use	Jingdong prefecture level city in Shandong
Jinfeng Hongyuan Flagship Store	Electricity savings3	Received the goods today, power-saving effect do not know being used, the boss has a very good attitude, praise!	Jingdong prefecture level city in Shandong
Jinfeng Hongyuan Flagship Store	Electricity savings3	The product is compact and doesn't take up much space, but hopefully it's power efficient.	Jingdong prefecture level city in Shandong
Jinfeng Hongyuan Flagship Store	Electricity savings3	Power saving is indeed effective, I used for a period of time before coming to evaluate, the electricity bill is obviously less, the quality is good, the shopkeeper service is also very good, very patient,	Jingdong prefecture level city in Shandong
Jinfeng Hongyuan Flagship Store	Electricity savings3	It feels fine, I'll comment after I use it	Jingdong prefecture level city in Shandong
Jinfeng Hongyuan Flagship Store	Electricity savings3	I just received it and I don't know the effect yet! I will comment again after I use it and have results!	Jingdong prefecture level city in Shandong
Jinfeng Hongyuan Flagship Store	Electricity savings3	The goods look fine, I hope they really work!	Jingdong prefecture level city in Shandong
Jinfeng Hongyuan Flagship Store	Electricity savings3	I just don't know if it saves power	Jingdong prefecture level city in Shandong
Jinfeng Hongyuan Flagship Store	Electricity savings3	My friend introduced me to use this product, it is professional and I am looking forward to the long term effect~!	Jingdong prefecture level city in Shandong
Jinfeng Hongyuan Flagship Store	Electricity savings3	Try it before you judge.	Jingdong prefecture level city in Shandong
Jinfeng Hongyuan Flagship Store	Electricity savings3	It's great, the power savings are obvious! Kudos!	Jingdong prefecture level city in Shandong
Jinfeng Hongyuan Flagship Store	Electricity savings3	The quality is excellent, stabilizes the voltage and saves electricity, very satisfied.	Jingdong prefecture level city in Shandong
Jinfeng Hongyuan Flagship Store	Electricity savings3	You have to use it for a while to know the effect	Jingdong prefecture level city in Shandong
Jinfeng Hongyuan Flagship Store	Electricity savings3	It's on trial.	Jingdong prefecture level city in Shandong
Jinfeng Hongyuan Flagship Store	Electricity savings3	The stuff works great.	Jingdong prefecture level city in Shandong
Jinfeng Hongyuan Flagship Store	Electricity savings3	Doesn't seem to work, not as god as other reviews	Jingdong prefecture level city in Shandong
Jinfeng Hongyuan Flagship Store	Electricity savings3	It's not good quality, it doesn't work, it stops working after a few days, it doesn't work, the customer service doesn't care, it's a bit irritating.	Jingdong prefecture level city in Shandong
Jinfeng Hongyuan Flagship Store	Electricity savings3	It doesn't do anything.	Jingdong prefecture level city in Shandong
Jinfeng Hongyuan Flagship Store	Electricity savings3	It's good.	Jingdong prefecture level city in Shandong
Jinfeng Hongyuan Flagship Store	Electricity savings3	Used a month effect is not obvious, are psychological effects, feel are not less, did not save to the electricity, there are details of the problem, the store said to buy to send 5 fuse, in fact, received only three, small problems are not committed to in place, I believe that the product is not good enough	Jingdong prefecture level city in Shandong

Jinfeng Hongyuan Flagship Store	Electricity savings3	Well received quickly, looking quite high, but also sent 5 fuses. Customer service is very patient to teach the operation, in fact, quite simple, lazy to read the instructions ha!	Jingdong prefecture level city in Shandong
Jinfeng Hongyuan Flagship Store	Electricity savings3	You have to use it for a while to know the effect	Jingdong prefecture level city in Shandong
Jinfeng Hongyuan Flagship Store	Electricity savings3	Shipped very quickly, packaged very well, the price is particularly affordable, there are so many efficacy is also good, it is worth buying worth buying the product, the workmanship is very fine, better than imagined, recommended that everyone try not yet used, after the use of the follow-up comment	Jingdong prefecture level city in Shandong
Jinfeng Hongyuan Flagship Store	Electricity savings3	It looks regular. It's working now.	Jingdong prefecture level city in Shandong
Jinfeng Hongyuan Flagship Store	Electricity savings3	It arrived pretty fast and the owner is very responsible. Have not used it yet, will see after use	Jingdong prefecture level city in Shandong
Jinfeng Hongyuan Flagship Store	Electricity savings3	🎁 baby quality is very good, the first time I received the goods to open the first time to see, it is very good 👍, very satisfied 🙂, customer service also have a question and answer, thank you 🙏	Jingdong prefecture level city in Shandong
Jinfeng Hongyuan Flagship Store	Electricity savings3	I don't know if it's any good 。。。。。。	Jingdong prefecture level city in Shandong
Jinfeng Hongyuan Flagship Store	Electricity savings3	Buy electronic products! Still trust the things of the Jingdong self-owned store! Energy-saving effect to evaluate after heating!	Jingdong prefecture level city in Shandong
Jinfeng Hongyuan Flagship Store	Electricity savings3	The goods have been received, Blu is very beautiful, energy saving effect, to be confirmed only after actual use.	Jingdong prefecture level city in Shandong
Jinfeng Hongyuan Flagship Store	Electricity savings3	Jingdong's speed is so fast, has received, easy to operate. The display is quite good, how many volts what mode is quite understandable.	Jingdong prefecture level city in Shandong
Jinfeng Hongyuan Flagship Store	Electricity savings3	The first use, the effect is good to recommend it to colleagues to use, a month of electricity to save a lot or can be	Jingdong prefecture level city in Shandong
Jinfeng Hongyuan Flagship Store	Electricity savings3	I don't know if it's useful. Mom's is a store. I'll return it if it doesn't work.	Jingdong prefecture level city in Shandong
Jinfeng Hongyuan Flagship Store	Electricity savings3	I don't know if it works or not, I'll wait for two months to use it and then come back to comment!	Jingdong prefecture level city in Shandong
Jinfeng Hongyuan Flagship Store	Electricity savings3	I bought the second one now, and I've been using it for more than half a year, and I feel that it really saves electricity. Now it is summer, the weather is hot, sometimes the fan and air conditioning on all day, if not this power saver, the monthly electricity bill is quite high, but as long as the installation of this power saver, not afraid of its high electricity bill, absolutely let you wantonly use	Jingdong prefecture level city in Shandong
Jinfeng Hongyuan Flagship Store	Electricity savings3	The product is better than I thought it would be, the quality looks great, the voltage is stable, and I'm looking forward to some great power savings!	Jingdong prefecture level city in Shandong
Jinfeng Hongyuan Flagship Store	Electricity savings3	Inadvertently see bought a try, delivery is also quite timely, just do not know how to use, customer service patience to answer, with a pretty good, value for money!	Jingdong prefecture level city in Shandong
Jinfeng Hongyuan Flagship Store	Electricity savings3	It's better than nothing! Hopefully this will help save slightly.	Jingdong prefecture level city in Shandong
Jinfeng Hongyuan Flagship Store	Electricity savings3	Still trying it out, follow up	Jingdong prefecture level city in Shandong
Jinfeng Hongyuan Flagship Store	Electricity savings3	I've just started using it, I don't know how well it works yet, I hope it's good.	Jingdong prefecture level city in Shandong
Jinfeng Hongyuan Flagship Store	Electricity savings3	Fourth purchase, great baby!	Jingdong prefecture level city in Shandong
Jinfeng Hongyuan Flagship Store	Electricity savings3	Shipped really fast, just got it and use it to see what happens and then come back for a review!	Jingdong prefecture level city in Shandong
Jinfeng Hongyuan Flagship Store	Electricity savings3	Timely delivery **** good attitude	Jingdong prefecture level city in Shandong
Jinfeng Hongyuan Flagship Store	Electricity savings3	Home large and small things are purchased in the Jingdong Mall, have been accustomed to shopping in the Jingdong, many times can buy good quality and cheap products, and courier service attitude is particularly good, sometimes too many couriers are to help me to the home, so many years have been in the Jingdong Mall shopping, trust and support! Will also support the Jingdong as always!	Jingdong prefecture level city in Shandong
Jinfeng Hongyuan Flagship Store	Electricity savings3	Modified a pump and the modification worked.	Jingdong prefecture level city in Shandong
Jinfeng Hongyuan Flagship Store	Electricity savings3	Already installed, next time you pay the electric bill to see the results	Jingdong prefecture level city in Shandong
Jinfeng Hongyuan Flagship Store	Electricity savings3	The product is very good to use and practical to look at	Jingdong prefecture level city in Shandong
Jinfeng Hongyuan Flagship Store	Electricity savings3	Works great, great service, waiting for results.	Jingdong prefecture level city in Shandong
Jinfeng Hongyuan Flagship Store	Electricity savings3	I just used it and I'm not sure about the efficacy yet, I'll come back to evaluate it after a while.	Jingdong prefecture level city in Shandong
Jinfeng Hongyuan Flagship Store	Electricity savings3	Just received the goods, use a period of time to evaluate	Jingdong prefecture level city in Shandong
Jinfeng Hongyuan Flagship Store	Electricity savings3	Opened and tried very good, use it to see if there are results	Jingdong prefecture level city in Shandong
Jinfeng Hongyuan Flagship Store	Electricity savings3	Arrived today! Still trying it out!	Jingdong prefecture level city in Shandong
Jinfeng Hongyuan Flagship Store	Electricity savings3	Shipped quickly, well packaged, already installed, hope there is a power saving effect	Jingdong prefecture level city in Shandong
Jinfeng Hongyuan Flagship Store	Electricity savings3	I got it. It's great.	Jingdong prefecture level city in Shandong
Jinfeng Hongyuan Flagship Store	Electricity savings3	Haven't used it yet, will come back in a month	Jingdong prefecture level city in Shandong
Jinfeng Hongyuan Flagship Store	Electricity savings3	It looks good. It's good. Try it first.	Jingdong prefecture level city in Shandong
Jinfeng Hongyuan Flagship Store	Electricity savings3	It works so well that I have relatives, friends, coworkers, and 6 families that have purchased it for them through me.	Jingdong prefecture level city in Shandong
Jinfeng Hongyuan Flagship Store	Electricity savings3	In use	Jingdong prefecture level city in Shandong
Jinfeng Hongyuan Flagship Store	Electricity savings3	Received the goods today, power-saving effect do not know being used, the boss has a very good attitude, praise!	Jingdong prefecture level city in Shandong
Jinfeng Hongyuan Flagship Store	Electricity savings3	The product is compact and doesn't take up much space, but hopefully it's power efficient.	Jingdong prefecture level city in Shandong
Jinfeng Hongyuan Flagship Store	Electricity savings3	Power saving is indeed effective, I used for a period of time before coming to evaluate, the electricity bill is obviously less, the quality is good, the shopkeeper service is also very good, very patient,	Jingdong prefecture level city in Shandong
Jinfeng Hongyuan Flagship Store	Electricity savings3	It feels fine, I'll comment after I use it	Jingdong prefecture level city in Shandong
Jinfeng Hongyuan Flagship Store	Electricity savings3	I just received it and I don't know the effect yet! I will comment again after I use it and have results!	Jingdong prefecture level city in Shandong
Jinfeng Hongyuan Flagship Store	Electricity savings3	The goods look fine, I hope they really work!	Jingdong prefecture level city in Shandong
Jinfeng Hongyuan Flagship Store	Electricity savings3	I just don't know if it saves power	Jingdong prefecture level city in Shandong
Jinfeng Hongyuan Flagship Store	Electricity savings3	My friend introduced me to use this product, it is professional and I am looking forward to the long term effect~!	Jingdong prefecture level city in Shandong
Jinfeng Hongyuan Flagship Store	Electricity savings3	Try it before you judge.	Jingdong prefecture level city in Shandong
Jinfeng Hongyuan Flagship Store	Electricity savings3	It's great, the power savings are obvious! Kudos!	Jingdong prefecture level city in Shandong
Jinfeng Hongyuan Flagship Store	Electricity savings3	The quality is excellent, stabilizes the voltage and saves electricity, very satisfied.	Jingdong prefecture level city in Shandong
Jinfeng Hongyuan Flagship Store	Electricity savings3	You have to use it for a while to know the effect	Jingdong prefecture level city in Shandong

Jinfeng Hongyuan Flagship Store	Electricity savings3	It's on trial.	Jingdong prefecture level city in Shandong
Jinfeng Hongyuan Flagship Store	Electricity savings3	The stuff works great.	Jingdong prefecture level city in Shandong
Jinfeng Hongyuan Flagship Store	Electricity savings3	Doesn't seem to work, not as god as other reviews	Jingdong prefecture level city in Shandong
Jinfeng Hongyuan Flagship Store	Electricity savings3	It's not good quality, it doesn't work, it stops working after a few days, it doesn't work, the customer service doesn't care, it's a bit irritating.	Jingdong prefecture level city in Shandong
Jinfeng Hongyuan Flagship Store	Electricity savings3	It doesn't do anything.	Jingdong prefecture level city in Shandong
Jinfeng Hongyuan Flagship Store	Electricity savings3	It's good.	Jingdong prefecture level city in Shandong
Jinfeng Hongyuan Flagship Store	Electricity savings3	Used a month effect is not obvious, are psychological effects, feel are not less, did not save to the electricity, there are details of the problem, the store said to buy to send 5 fuse, in fact, received only three, small problems are not committed to in place, I believe that the product is not good enough	Jingdong prefecture level city in Shandong
Jinfeng Hongyuan Flagship Store	Electricity savings3	Well received quickly, looking quite high, but also sent 5 fuses. Customer service is very patient to teach the operation, in fact, quite simple, lazy to read the instructions ha!	Jingdong prefecture level city in Shandong
Jinfeng Hongyuan Flagship Store	Electricity savings3	You have to use it for a while to know the effect	Jingdong prefecture level city in Shandong
Jinfeng Hongyuan Flagship Store	Electricity savings3	Shipped very quickly, packaged very well, the price is particularly affordable, there are so many efficacy is also good, it is worth buying worth buying the product, the workmanship is very fine, better than imagined, recommended that everyone try not yet used, after the use of the follow-up comment	Jingdong prefecture level city in Shandong
Jinfeng Hongyuan Flagship Store	Electricity savings3	It looks regular. It's working now.	Jingdong prefecture level city in Shandong
Jinfeng Hongyuan Flagship Store	Electricity savings3	It arrived pretty fast and the owner is very responsible. Have not used it yet, will see after use	Jingdong prefecture level city in Shandong
Jinfeng Hongyuan Flagship Store	Electricity savings3	🎁 baby quality is very good, the first time I received the goods to open the first time to see, it is very good 👍, very satisfied ☺, customer service also have a question and answer, thank you 🙏	Jingdong prefecture level city in Shandong
Jinfeng Hongyuan Flagship Store	Electricity savings3	I don't know if it's any good 。。。。。。	Jingdong prefecture level city in Shandong
Jinfeng Hongyuan Flagship Store	Electricity savings3	Buy electronic products! Still trust the things of the Jingdong self-owned store! Energy-saving effect to evaluate after heating!	Jingdong prefecture level city in Shandong
Jinfeng Hongyuan Flagship Store	Electricity savings3	The goods have been received, Blu is very beautiful, energy saving effect, to be confirmed only after actual use.	Jingdong prefecture level city in Shandong
Jinfeng Hongyuan Flagship Store	Electricity savings3	Jingdong's speed is so fast, has received, easy to operate. The display is quite good, how many volts what mode is quite understandable.	Jingdong prefecture level city in Shandong
Jinfeng Hongyuan Flagship Store	Electricity savings3	The first use, the effect is good to recommend it to colleagues to use, a month of electricity to save a lot or can be	Jingdong prefecture level city in Shandong
Jinfeng Hongyuan Flagship Store	Electricity savings3	I don't know if it's useful. Mom's is a store. I'll return it if it doesn't work.	Jingdong prefecture level city in Shandong
Jinfeng Hongyuan Flagship Store	Electricity savings3	I don't know if it works or not, I'll wait for two months to use it and then come back to comment!	Jingdong prefecture level city in Shandong
Jinfeng Hongyuan Flagship Store	Electricity savings3	I bought the second one now, and I've been using it for more than half a year, and I feel that it really saves electricity. Now it is summer, the weather is hot, sometimes the fan and air conditioning on all day, if not this power saver, the monthly electricity bill is quite high, but as long as the installation of this power saver, not afraid of its high electricity bill, absolutely let you wantonly use	Jingdong prefecture level city in Shandong
Jinfeng Hongyuan Flagship Store	Electricity savings3	The product is better than I thought it would be, the quality looks great, the voltage is stable, and I'm looking forward to some great power savings!	Jingdong prefecture level city in Shandong
Jinfeng Hongyuan Flagship Store	Electricity savings3	Inadvertently see bought a try, delivery is also quite timely, just do not know how to use, customer service patience to answer, with a pretty good, value for money!	Jingdong prefecture level city in Shandong
Jinfeng Hongyuan Flagship Store	Electricity savings3	It's better than nothing! Hopefully this will help save slightly.	Jingdong prefecture level city in Shandong
Jinfeng Hongyuan Flagship Store	Electricity savings3	Still trying it out, follow up	Jingdong prefecture level city in Shandong
Jinfeng Hongyuan Flagship Store	Electricity savings3	I've just started using it, I don't know how well it works yet, I hope it's good.	Jingdong prefecture level city in Shandong
Jinfeng Hongyuan Flagship Store	Electricity savings3	Fourth purchase, great baby!	Jingdong prefecture level city in Shandong
Jinfeng Hongyuan Flagship Store	Electricity savings3	Shipped really fast, just got it and use it to see what happens and then come back for a review!	Jingdong prefecture level city in Shandong
Jinfeng Hongyuan Flagship Store	Electricity savings3	Timely delivery **** good attitude	Jingdong prefecture level city in Shandong
Jinfeng Hongyuan Flagship Store	Electricity savings3	Home large and small things are purchased in the Jingdong Mall, have been accustomed to shopping in the Jingdong, many times can buy good quality and cheap products, and courier service attitude is particularly good, sometimes too many couriers are to help me to the home, so many years have been in the Jingdong Mall shopping, trust and support! Will also support the Jingdong as always!	Jingdong prefecture level city in Shandong
Jinfeng Hongyuan Flagship Store	Electricity savings3	Modified a pump and the modification worked.	Jingdong prefecture level city in Shandong
Jinfeng Hongyuan Flagship Store	Electricity savings3	Already installed, next time you pay the electric bill to see the results	Jingdong prefecture level city in Shandong
Jinfeng Hongyuan Flagship Store	Electricity savings3	The product is very good to use and practical to look at	Jingdong prefecture level city in Shandong
Jinfeng Hongyuan Flagship Store	Electricity savings3	Works great, great service, waiting for results.	Jingdong prefecture level city in Shandong
Jinfeng Hongyuan Flagship Store	Electricity savings3	I just used it and I'm not sure about the efficacy yet, I'll come back to evaluate it after a while.	Jingdong prefecture level city in Shandong
Jinfeng Hongyuan Flagship Store	Electricity savings3	Just received the goods, use a period of time to evaluate	Jingdong prefecture level city in Shandong
Jinfeng Hongyuan Flagship Store	Electricity savings3	Opened and tried very good, use it to see if there are results	Jingdong prefecture level city in Shandong
Jinfeng Hongyuan Flagship Store	Electricity savings3	Arrived today! Still trying it out!	Jingdong prefecture level city in Shandong
Jinfeng Hongyuan Flagship Store	Electricity savings3	Shipped quickly, well packaged, already installed, hope there is a power saving effect	Jingdong prefecture level city in Shandong
Jinfeng Hongyuan Flagship Store	Electricity savings3	I got it. It's great.	Jingdong prefecture level city in Shandong
Jinfeng Hongyuan Flagship Store	Electricity savings3	Haven't used it yet, will come back in a month	Jingdong prefecture level city in Shandong
Jinfeng Hongyuan Flagship Store	Electricity savings3	It looks good. It's good. Try it first.	Jingdong prefecture level city in Shandong
Jinfeng Hongyuan Flagship Store	Electricity savings3	It works so well that I have relatives, friends, coworkers, and 6 families that have purchased it for them through me.	Jingdong prefecture level city in Shandong
Jinfeng Hongyuan Flagship Store	Electricity savings3	In use	Jingdong prefecture level city in Shandong
Jinfeng Hongyuan Flagship Store	Electricity savings3	Received the goods today, power-saving effect do not know being used, the boss has a very good attitude, praise!	Jingdong prefecture level city in Shandong
Jinfeng Hongyuan Flagship Store	Electricity savings3	The product is compact and doesn't take up much space, but hopefully it's power efficient.	Jingdong prefecture level city in Shandong
Jinfeng Hongyuan Flagship Store	Electricity savings3	Power saving is indeed effective, I used for a period of time before coming to evaluate, the electricity bill is obviously less, the quality is good, the shopkeeper service is also very good, very patient,	Jingdong prefecture level city in Shandong
Jinfeng Hongyuan Flagship Store	Electricity savings3	It feels fine, I'll comment after I use it	Jingdong prefecture level city in Shandong

Jinfeng Hongyuan Flagship Store	Electricity savings3	I just received it and I don't know the effect yet! I will comment again after I use it and have results!	Jingdong prefecture level city in Shandong
Jinfeng Hongyuan Flagship Store	Electricity savings3	The goods look fine, I hope they really work!	Jingdong prefecture level city in Shandong
Jinfeng Hongyuan Flagship Store	Electricity savings3	I just don't know if it saves power	Jingdong prefecture level city in Shandong
Jinfeng Hongyuan Flagship Store	Electricity savings3	My friend introduced me to use this product, it is professional and I am looking forward to the long term effect~!	Jingdong prefecture level city in Shandong
Jinfeng Hongyuan Flagship Store	Electricity savings3	Try it before you judge.	Jingdong prefecture level city in Shandong
Jinfeng Hongyuan Flagship Store	Electricity savings3	It's great, the power savings are obvious! Kudos!	Jingdong prefecture level city in Shandong
Jinfeng Hongyuan Flagship Store	Electricity savings3	The quality is excellent, stabilizes the voltage and saves electricity, very satisfied.	Jingdong prefecture level city in Shandong
Jinfeng Hongyuan Flagship Store	Electricity savings3	You have to use it for a while to know the effect	Jingdong prefecture level city in Shandong
Jinfeng Hongyuan Flagship Store	Electricity savings3	It's on trial.	Jingdong prefecture level city in Shandong
Jinfeng Hongyuan Flagship Store	Electricity savings3	The stuff works great.	Jingdong prefecture level city in Shandong
Jinfeng Hongyuan Flagship Store	Electricity savings3	Doesn't seem to work, not as god as other reviews	Jingdong prefecture level city in Shandong
Jinfeng Hongyuan Flagship Store	Electricity savings3	It's not good quality, it doesn't work, it stops working after a few days, it doesn't work, the customer service doesn't care, it's a bit irritating.	Jingdong prefecture level city in Shandong
Jinfeng Hongyuan Flagship Store	Electricity savings3	It doesn't do anything.	Jingdong prefecture level city in Shandong
Jinfeng Hongyuan Flagship Store	Electricity savings3	It's good.	Jingdong prefecture level city in Shandong
Jinfeng Hongyuan Flagship Store	Electricity savings3	Used a month effect is not obvious, are psychological effects, feel are not less, did not save to the electricity, there are details of the problem, the store said to buy to send 5 fuse, in fact, received only three, small problems are not committed to in place, I believe that the product is not good enough	Jingdong prefecture level city in Shandong
Jinfeng Hongyuan Flagship Store	Electricity savings3	Well received quickly, looking quite high, but also sent 5 fuses. Customer service is very patient to teach the operation, in fact, quite simple, lazy to read the instructions ha!	Jingdong prefecture level city in Shandong
Jinfeng Hongyuan Flagship Store	Electricity savings3	You have to use it for a while to know the effect	Jingdong prefecture level city in Shandong
Jinfeng Hongyuan Flagship Store	Electricity savings3	Shipped very quickly, packaged very well, the price is particularly affordable, there are so many efficacy is also good, it is worth buying worth buying the product, the workmanship is very fine, better than imagined, recommended that everyone try not yet used, after the use of the follow-up comment	Jingdong prefecture level city in Shandong
Jinfeng Hongyuan Flagship Store	Electricity savings3	It looks regular. It's working now.	Jingdong prefecture level city in Shandong
Jinfeng Hongyuan Flagship Store	Electricity savings3	It arrived pretty fast and the owner is very responsible. Have not used it yet, will see after use	Jingdong prefecture level city in Shandong
Jinfeng Hongyuan Flagship Store	Electricity savings3	🎁 baby quality is very good, the first time I received the goods to open the first time to see, it is very good 👍, very satisfied 🙂, customer service also have a question and answer, thank you 🙏	Jingdong prefecture level city in Shandong
Jinfeng Hongyuan Flagship Store	Electricity savings3	I don't know if it's any good 。。。。。。	Jingdong prefecture level city in Shandong
Jinfeng Hongyuan Flagship Store	Electricity savings3	Buy electronic products! Still trust the things of the Jingdong self-owned store! Energy-saving effect to evaluate after heating!	Jingdong prefecture level city in Shandong
Jinfeng Hongyuan Flagship Store	Electricity savings3	The goods have been received, Blu is very beautiful, energy saving effect, to be confirmed only after actual use.	Jingdong prefecture level city in Shandong
Jinfeng Hongyuan Flagship Store	Electricity savings3	Jingdong's speed is so fast, has received, easy to operate. The display is quite good, how many volts what mode is quite understandable.	Jingdong prefecture level city in Shandong
Jinfeng Hongyuan Flagship Store	Electricity savings3	The first use, the effect is good to recommend it to colleagues to use, a month of electricity to save a lot or can be	Jingdong prefecture level city in Shandong
Jinfeng Hongyuan Flagship Store	Electricity savings3	I don't know if it's useful. Mom's is a store. I'll return it if it doesn't work.	Jingdong prefecture level city in Shandong
Jinfeng Hongyuan Flagship Store	Electricity savings3	I don't know if it works or not, I'll wait for two months to use it and then come back to comment!	Jingdong prefecture level city in Shandong
Jinfeng Hongyuan Flagship Store	Electricity savings3	I bought the second one now, and I've been using it for more than half a year, and I feel that it really saves electricity. Now it is summer, the weather is hot, sometimes the fan and air conditioning on all day, if not this power saver, the monthly electricity bill is quite high, but as long as the installation of this power saver, not afraid of its high electricity bill, absolutely let you wantonly use	Jingdong prefecture level city in Shandong
Jinfeng Hongyuan Flagship Store	Electricity savings3	The product is better than I thought it would be, the quality looks great, the voltage is stable, and I'm looking forward to some great power savings!	Jingdong prefecture level city in Shandong
Jinfeng Hongyuan Flagship Store	Electricity savings3	Inadvertently see bought a try, delivery is also quite timely, just do not know how to use, customer service patience to answer, with a pretty good, value for money!	Jingdong prefecture level city in Shandong
Jinfeng Hongyuan Flagship Store	Electricity savings3	It's better than nothing! Hopefully this will help save slightly.	Jingdong prefecture level city in Shandong
Jinfeng Hongyuan Flagship Store	Electricity savings3	Still trying it out, follow up	Jingdong prefecture level city in Shandong
Jinfeng Hongyuan Flagship Store	Electricity savings3	I've just started using it, I don't know how well it works yet, I hope it's good.	Jingdong prefecture level city in Shandong
Jinfeng Hongyuan Flagship Store	Electricity savings3	Fourth purchase, great baby!	Jingdong prefecture level city in Shandong
Jinfeng Hongyuan Flagship Store	Electricity savings3	Shipped really fast, just got it and use it to see what happens and then come back for a review!	Jingdong prefecture level city in Shandong
Jinfeng Hongyuan Flagship Store	Electricity savings3	Timely delivery **** good attitude	Jingdong prefecture level city in Shandong
Jinfeng Hongyuan Flagship Store	Electricity savings3	Home large and small things are purchased in the Jingdong Mall, have been accustomed to shopping in the Jingdong, many times can buy good quality and cheap products, and courier service attitude is particularly good, sometimes too many couriers are to help me to the home, so many years have been in the Jingdong Mall shopping, trust and support! Will also support the Jingdong as always!	Jingdong prefecture level city in Shandong
Jinfeng Hongyuan Flagship Store	Electricity savings3	Modified a pump and the modification worked.	Jingdong prefecture level city in Shandong
Jinfeng Hongyuan Flagship Store	Electricity savings3	Already installed, next time you pay the electric bill to see the results	Jingdong prefecture level city in Shandong
Jinfeng Hongyuan Flagship Store	Electricity savings3	The product is very good to use and practical to look at	Jingdong prefecture level city in Shandong
Jinfeng Hongyuan Flagship Store	Electricity savings3	Works great, great service, waiting for results.	Jingdong prefecture level city in Shandong
Jinfeng Hongyuan Flagship Store	Electricity savings3	I just used it and I'm not sure about the efficacy yet, I'll come back to evaluate it after a while.	Jingdong prefecture level city in Shandong
Jinfeng Hongyuan Flagship Store	Electricity savings3	Just received the goods, use a period of time to evaluate	Jingdong prefecture level city in Shandong
Jinfeng Hongyuan Flagship Store	Electricity savings3	Opened and tried very good, use it to see if there are results	Jingdong prefecture level city in Shandong
Jinfeng Hongyuan Flagship Store	Electricity savings3	Arrived today! Still trying it out!	Jingdong prefecture level city in Shandong
Jinfeng Hongyuan Flagship Store	Electricity savings3	Shipped quickly, well packaged, already installed, hope there is a power saving effect	Jingdong prefecture level city in Shandong
Jinfeng Hongyuan Flagship Store	Electricity savings3	I got it. It's great.	Jingdong prefecture level city in Shandong

Jinfeng Hongyuan Flagship Store	Electricity savings3	Haven't used it yet, will come back in a month	Jingdong prefecture level city in Shandong
Jinfeng Hongyuan Flagship Store	Electricity savings3	It looks good. It's good. Try it first.	Jingdong prefecture level city in Shandong
Jinfeng Hongyuan Flagship Store	Electricity savings3	It works so well that I have relatives, friends, coworkers, and 6 families that have purchased it for them through me.	Jingdong prefecture level city in Shandong
Jinfeng Hongyuan Flagship Store	Electricity savings3	In use	Jingdong prefecture level city in Shandong
Jinfeng Hongyuan Flagship Store	Electricity savings3	Received the goods today, power-saving effect do not know being used, the boss has a very good attitude, praise!	Jingdong prefecture level city in Shandong
Jinfeng Hongyuan Flagship Store	Electricity savings3	The product is compact and doesn't take up much space, but hopefully it's power efficient.	Jingdong prefecture level city in Shandong
Jinfeng Hongyuan Flagship Store	Electricity savings3	Power saving is indeed effective, I used for a period of time before coming to evaluate, the electricity bill is obviously less, the quality is good, the shopkeeper service is also very good, very patient,	Jingdong prefecture level city in Shandong
Jinfeng Hongyuan Flagship Store	Electricity savings3	It feels fine, I'll comment after I use it	Jingdong prefecture level city in Shandong
Jinfeng Hongyuan Flagship Store	Electricity savings3	I just received it and I don't know the effect yet! I will comment again after I use it and have results!	Jingdong prefecture level city in Shandong
Jinfeng Hongyuan Flagship Store	Electricity savings3	The goods look fine, I hope they really work!	Jingdong prefecture level city in Shandong
Jinfeng Hongyuan Flagship Store	Electricity savings3	I just don't know if it saves power	Jingdong prefecture level city in Shandong
Jinfeng Hongyuan Flagship Store	Electricity savings3	My friend introduced me to use this product, it is professional and I am looking forward to the long term effect~!	Jingdong prefecture level city in Shandong
Jinfeng Hongyuan Flagship Store	Electricity savings3	Try it before you judge.	Jingdong prefecture level city in Shandong
Jinfeng Hongyuan Flagship Store	Electricity savings3	It's great, the power savings are obvious! Kudos!	Jingdong prefecture level city in Shandong
Jinfeng Hongyuan Flagship Store	Electricity savings3	The quality is excellent, stabilizes the voltage and saves electricity, very satisfied.	Jingdong prefecture level city in Shandong
Jinfeng Hongyuan Flagship Store	Electricity savings3	You have to use it for a while to know the effect	Jingdong prefecture level city in Shandong
Jinfeng Hongyuan Flagship Store	Electricity savings3	It's on trial.	Jingdong prefecture level city in Shandong
Jinfeng Hongyuan Flagship Store	Electricity savings3	The stuff works great.	Jingdong prefecture level city in Shandong
Jinfeng Hongyuan Flagship Store	Electricity savings3	Doesn't seem to work, not as god as other reviews	Jingdong prefecture level city in Shandong
Jinfeng Hongyuan Flagship Store	Electricity savings3	It's not good quality, it doesn't work, it stops working after a few days, it doesn't work, the customer service doesn't care, it's a bit irritating.	Jingdong prefecture level city in Shandong
Jinfeng Hongyuan Flagship Store	Electricity savings3	It doesn't do anything.	Jingdong prefecture level city in Shandong
Jinfeng Hongyuan Flagship Store	Electricity savings3	It's good.	Jingdong prefecture level city in Shandong
Jinfeng Hongyuan Flagship Store	Electricity savings3	Used a month effect is not obvious, are psychological effects, feel are not less, did not save to the electricity, there are details of the problem, the store said to buy to send 5 fuse, in fact, received only three, small problems are not committed to in place, I believe that the product is not good enough	Jingdong prefecture level city in Shandong
Jinfeng Hongyuan Flagship Store	Electricity savings3	Well received quickly, looking quite high, but also sent 5 fuses. Customer service is very patient to teach the operation, in fact, quite simple, lazy to read the instructions ha!	Jingdong prefecture level city in Shandong
Jinfeng Hongyuan Flagship Store	Electricity savings3	You have to use it for a while to know the effect	Jingdong prefecture level city in Shandong
Jinfeng Hongyuan Flagship Store	Electricity savings3	Shipped very quickly, packaged very well, the price is particularly affordable, there are so many efficacy is also good, it is worth buying worth buying the product, the workmanship is very fine, better than imagined, recommended that everyone try not yet used, after the use of the follow-up comment	Jingdong prefecture level city in Shandong
Jinfeng Hongyuan Flagship Store	Electricity savings3	It looks regular. It's working now.	Jingdong prefecture level city in Shandong
Jinfeng Hongyuan Flagship Store	Electricity savings3	It arrived pretty fast and the owner is very responsible. Have not used it yet, will see after use	Jingdong prefecture level city in Shandong
Jinfeng Hongyuan Flagship Store	Electricity savings3	🎁 baby quality is very good, the first time I received the goods to open the first time to see, it is very good 👍, very satisfied 😊, customer service also have a question and answer, thank you 🙏	Jingdong prefecture level city in Shandong
Jinfeng Hongyuan Flagship Store	Electricity savings3	I don't know if it's any good 。。。。。。	Jingdong prefecture level city in Shandong
Jinfeng Hongyuan Flagship Store	Electricity savings3	Buy electronic products! Still trust the things of the Jingdong self-owned store! Energy-saving effect to evaluate after heating!	Jingdong prefecture level city in Shandong
Jinfeng Hongyuan Flagship Store	Electricity savings3	The goods have been received, Blu is very beautiful, energy saving effect, to be confirmed only after actual use.	Jingdong prefecture level city in Shandong
Jinfeng Hongyuan Flagship Store	Electricity savings3	Jingdong's speed is so fast, has received, easy to operate. The display is quite good, how many volts what mode is quite understandable.	Jingdong prefecture level city in Shandong
Jinfeng Hongyuan Flagship Store	Electricity savings3	The first use, the effect is good to recommend it to colleagues to use, a month of electricity to save a lot or can be	Jingdong prefecture level city in Shandong
Jinfeng Hongyuan Flagship Store	Electricity savings3	I don't know if it's useful. Mom's is a store. I'll return it if it doesn't work.	Jingdong prefecture level city in Shandong
Jinfeng Hongyuan Flagship Store	Electricity savings3	I don't know if it works or not, I'll wait for two months to use it and then come back to comment!	Jingdong prefecture level city in Shandong
Jinfeng Hongyuan Flagship Store	Electricity savings3	I bought the second one now, and I've been using it for more than half a year, and I feel that it really saves electricity. Now it is summer, the weather is hot, sometimes the fan and air conditioning on all day, if not this power saver, the monthly electricity bill is quite high, but as long as the installation of this power saver, not afraid of its high electricity bill, absolutely let you wantonly use	Jingdong prefecture level city in Shandong
Jinfeng Hongyuan Flagship Store	Electricity savings3	The product is better than I thought it would be, the quality looks great, the voltage is stable, and I'm looking forward to some great power savings!	Jingdong prefecture level city in Shandong
Jinfeng Hongyuan Flagship Store	Electricity savings3	Inadvertently see bought a try, delivery is also quite timely, just do not know how to use, customer service patience to answer, with a pretty good, value for money!	Jingdong prefecture level city in Shandong
Jinfeng Hongyuan Flagship Store	Electricity savings3	It's better than nothing! Hopefully this will help save slightly.	Jingdong prefecture level city in Shandong
Jinfeng Hongyuan Flagship Store	Electricity savings3	Still trying it out, follow up	Jingdong prefecture level city in Shandong
Jinfeng Hongyuan Flagship Store	Electricity savings3	I've just started using it, I don't know how well it works yet, I hope it's good.	Jingdong prefecture level city in Shandong
Jinfeng Hongyuan Flagship Store	Electricity savings3	Fourth purchase, great baby!	Jingdong prefecture level city in Shandong
Jinfeng Hongyuan Flagship Store	Electricity savings3	Shipped really fast, just got it and use it to see what happens and then come back for a review!	Jingdong prefecture level city in Shandong
Jinfeng Hongyuan Flagship Store	Electricity savings3	Timely delivery **** good attitude	Jingdong prefecture level city in Shandong
Jinfeng Hongyuan Flagship Store	Electricity savings3	Home large and small things are purchased in the Jingdong Mall, have been accustomed to shopping in the Jingdong, many times can buy good quality and cheap products, and courier service attitude is particularly good, sometimes too many couriers are to help me to the home, so many years have been in the Jingdong Mall shopping, trust and support! Will also support the Jingdong as always!	Jingdong prefecture level city in Shandong
Jinfeng Hongyuan Flagship Store	Electricity savings3	Modified a pump and the modification worked.	Jingdong prefecture level city in Shandong
Jinfeng Hongyuan Flagship Store	Electricity savings3	Already installed, next time you pay the electric bill to see the results	Jingdong prefecture level city in Shandong

Jinfeng Hongyuan Flagship Store	Electricity savings3	The product is very good to use and practical to look at	Jingdong prefecture level city in Shandong
Jinfeng Hongyuan Flagship Store	Electricity savings3	Works great, great service, waiting for results.	Jingdong prefecture level city in Shandong
Jinfeng Hongyuan Flagship Store	Electricity savings3	I just used it and I'm not sure about the efficacy yet, I'll come back to evaluate it after a while.	Jingdong prefecture level city in Shandong
Jinfeng Hongyuan Flagship Store	Electricity savings3	Just received the goods, use a period of time to evaluate	Jingdong prefecture level city in Shandong
Jinfeng Hongyuan Flagship Store	Electricity savings3	Opened and tried very good, use it to see if there are results	Jingdong prefecture level city in Shandong
Jinfeng Hongyuan Flagship Store	Electricity savings3	Arrived today! Still trying it out!	Jingdong prefecture level city in Shandong
Jinfeng Hongyuan Flagship Store	Electricity savings3	Shipped quickly, well packaged, already installed, hope there is a power saving effect	Jingdong prefecture level city in Shandong
Jinfeng Hongyuan Flagship Store	Electricity savings3	I got it. It's great.	Jingdong prefecture level city in Shandong
Jinfeng Hongyuan Flagship Store	Electricity savings3	Haven't used it yet, will come back in a month	Jingdong prefecture level city in Shandong
Jinfeng Hongyuan Flagship Store	Electricity savings3	It looks good. It's good. Try it first.	Jingdong prefecture level city in Shandong
Jinfeng Hongyuan Flagship Store	Electricity savings3	It works so well that I have relatives, friends, coworkers, and 6 families that have purchased it for them through me.	Jingdong prefecture level city in Shandong
Jinfeng Hongyuan Flagship Store	Electricity savings3	In use	Jingdong prefecture level city in Shandong
Jinfeng Hongyuan Flagship Store	Electricity savings3	Received the goods today, power-saving effect do not know being used, the boss has a very good attitude, praise!	Jingdong prefecture level city in Shandong
Jinfeng Hongyuan Flagship Store	Electricity savings3	The product is compact and doesn't take up much space, but hopefully it's power efficient.	Jingdong prefecture level city in Shandong
Jinfeng Hongyuan Flagship Store	Electricity savings3	Power saving is indeed effective, I used for a period of time before coming to evaluate, the electricity bill is obviously less, the quality is good, the shopkeeper service is also very good, very patient,	Jingdong prefecture level city in Shandong
Jinfeng Hongyuan Flagship Store	Electricity savings3	It feels fine, I'll comment after I use it	Jingdong prefecture level city in Shandong
Jinfeng Hongyuan Flagship Store	Electricity savings3	I just received it and I don't know the effect yet! I will comment again after I use it and have results!	Jingdong prefecture level city in Shandong
Jinfeng Hongyuan Flagship Store	Electricity savings3	The goods look fine, I hope they really work!	Jingdong prefecture level city in Shandong
Jinfeng Hongyuan Flagship Store	Electricity savings3	I just don't know if it saves power	Jingdong prefecture level city in Shandong
Jinfeng Hongyuan Flagship Store	Electricity savings3	My friend introduced me to use this product, it is professional and I am looking forward to the long term effect~!	Jingdong prefecture level city in Shandong
Jinfeng Hongyuan Flagship Store	Electricity savings3	Try it before you judge.	Jingdong prefecture level city in Shandong
Jinfeng Hongyuan Flagship Store	Electricity savings3	It's great, the power savings are obvious! Kudos!	Jingdong prefecture level city in Shandong
Jinfeng Hongyuan Flagship Store	Electricity savings3	The quality is excellent, stabilizes the voltage and saves electricity, very satisfied.	Jingdong prefecture level city in Shandong
Jinfeng Hongyuan Flagship Store	Electricity savings3	You have to use it for a while to know the effect	Jingdong prefecture level city in Shandong
Jinfeng Hongyuan Flagship Store	Electricity savings3	It's on trial.	Jingdong prefecture level city in Shandong
Jinfeng Hongyuan Flagship Store	Electricity savings3	The stuff works great.	Jingdong prefecture level city in Shandong
Jinfeng Hongyuan Flagship Store	Electricity savings3	Doesn't seem to work, not as god as other reviews	Jingdong prefecture level city in Shandong
Jinfeng Hongyuan Flagship Store	Electricity savings3	It's not good quality, it doesn't work, it stops working after a few days, it doesn't work, the customer service doesn't care, it's a bit irritating.	Jingdong prefecture level city in Shandong
Jinfeng Hongyuan Flagship Store	Electricity savings3	It doesn't do anything.	Jingdong prefecture level city in Shandong
Jinfeng Hongyuan Flagship Store	Electricity savings3	It's good.	Jingdong prefecture level city in Shandong
Jinfeng Hongyuan Flagship Store	Electricity savings3	Used a month effect is not obvious, are psychological effects, feel are not less, did not save to the electricity, there are details of the problem, the store said to buy to send 5 fuse, in fact, received only three, small problems are not committed to in place, I believe that the product is not good enough	Jingdong prefecture level city in Shandong
Jinfeng Hongyuan Flagship Store	Electricity savings3	Well received quickly, looking quite high, but also sent 5 fuses. Customer service is very patient to teach the operation, in fact, quite simple, lazy to read the instructions ha!	Jingdong prefecture level city in Shandong
Jinfeng Hongyuan Flagship Store	Electricity savings3	You have to use it for a while to know the effect	Jingdong prefecture level city in Shandong
Jinfeng Hongyuan Flagship Store	Electricity savings3	Shipped very quickly, packaged very well, the price is particularly affordable, there are so many efficacy is also good, it is worth buying worth buying the product, the workmanship is very fine, better than imagined, recommended that everyone try not yet used, after the use of the follow-up comment	Jingdong prefecture level city in Shandong
Jinfeng Hongyuan Flagship Store	Electricity savings3	It looks regular. It's working now.	Jingdong prefecture level city in Shandong
Jinfeng Hongyuan Flagship Store	Electricity savings3	It arrived pretty fast and the owner is very responsible. Have not used it yet, will see after use	Jingdong prefecture level city in Shandong
Jinfeng Hongyuan Flagship Store	Electricity savings3	🎁 baby quality is very good, the first time I received the goods to open the first time to see, it is very good 👍, very satisfied 😊, customer service also have a question and answer, thank you 🙏	Jingdong prefecture level city in Shandong
Jinfeng Hongyuan Flagship Store	Electricity savings3	I don't know if it's any good 。。。。。。	Jingdong prefecture level city in Shandong
Jinfeng Hongyuan Flagship Store	Electricity savings3	Buy electronic products! Still trust the things of the Jingdong self-owned store! Energy-saving effect to evaluate after heating!	Jingdong prefecture level city in Shandong
Jinfeng Hongyuan Flagship Store	Electricity savings3	The goods have been received, Blu is very beautiful, energy saving effect, to be confirmed only after actual use.	Jingdong prefecture level city in Shandong
Jinfeng Hongyuan Flagship Store	Electricity savings3	Jingdong's speed is so fast, has received, easy to operate. The display is quite good, how many volts what mode is quite understandable.	Jingdong prefecture level city in Shandong
Jinfeng Hongyuan Flagship Store	Electricity savings3	The first use, the effect is good to recommend it to colleagues to use, a month of electricity to save a lot or can be	Jingdong prefecture level city in Shandong
Jinfeng Hongyuan Flagship Store	Electricity savings3	I don't know if it's useful. Mom's is a store. I'll return it if it doesn't work.	Jingdong prefecture level city in Shandong
Jinfeng Hongyuan Flagship Store	Electricity savings3	I don't know if it works or not, I'll wait for two months to use it and then come back to comment!	Jingdong prefecture level city in Shandong
Jinfeng Hongyuan Flagship Store	Electricity savings3	I bought the second one now, and I've been using it for more than half a year, and I feel that it really saves electricity. Now it is summer, the weather is hot, sometimes the fan and air conditioning on all day, if not this power saver, the monthly electricity bill is quite high, but as long as the installation of this power saver, not afraid of its high electricity bill, absolutely let you wantonly use	Jingdong prefecture level city in Shandong
Jinfeng Hongyuan Flagship Store	Electricity savings3	The product is better than I thought it would be, the quality looks great, the voltage is stable, and I'm looking forward to some great power savings!	Jingdong prefecture level city in Shandong
Jinfeng Hongyuan Flagship Store	Electricity savings3	Inadvertently see bought a try, delivery is also quite timely, just do not know how to use, customer service patience to answer, with a pretty good, value for money!	Jingdong prefecture level city in Shandong
Jinfeng Hongyuan Flagship Store	Electricity savings3	It's better than nothing! Hopefully this will help save slightly.	Jingdong prefecture level city in Shandong
Jinfeng Hongyuan Flagship Store	Electricity savings3	Still trying it out, follow up	Jingdong prefecture level city in Shandong

Jinfeng Hongyuan Flagship Store	Electricity savings3	I've just started using it, I don't know how well it works yet, I hope it's good.	Jingdong prefecture level city in Shandong
Jinfeng Hongyuan Flagship Store	Electricity savings3	Fourth purchase, great baby!	Jingdong prefecture level city in Shandong
Jinfeng Hongyuan Flagship Store	Electricity savings3	Shipped really fast, just got it and use it to see what happens and then come back for a review!	Jingdong prefecture level city in Shandong
Jinfeng Hongyuan Flagship Store	Electricity savings3	Timely delivery **** good attitude	Jingdong prefecture level city in Shandong
Jinfeng Hongyuan Flagship Store	Electricity savings3	Home large and small things are purchased in the Jingdong Mall, have been accustomed to shopping in the Jingdong, many times can buy good quality and cheap products, and courier service attitude is particularly good, sometimes too many couriers are to help me to the home, so many years have been in the Jingdong Mall shopping, trust and support! Will also support the Jingdong as always!	Jingdong prefecture level city in Shandong
Jinfeng Hongyuan Flagship Store	Electricity savings3	Modified a pump and the modification worked.	Jingdong prefecture level city in Shandong
Jinfeng Hongyuan Flagship Store	Electricity savings3	Already installed, next time you pay the electric bill to see the results	Jingdong prefecture level city in Shandong
Jinfeng Hongyuan Flagship Store	Electricity savings3	The product is very good to use and practical to look at	Jingdong prefecture level city in Shandong
Jinfeng Hongyuan Flagship Store	Electricity savings3	Works great, great service, waiting for results.	Jingdong prefecture level city in Shandong
Jinfeng Hongyuan Flagship Store	Electricity savings3	I just used it and I'm not sure about the efficacy yet, I'll come back to evaluate it after a while.	Jingdong prefecture level city in Shandong
Jinfeng Hongyuan Flagship Store	Electricity savings3	Just received the goods, use a period of time to evaluate	Jingdong prefecture level city in Shandong
Jinfeng Hongyuan Flagship Store	Electricity savings3	Opened and tried very good, use it to see if there are results	Jingdong prefecture level city in Shandong
Jinfeng Hongyuan Flagship Store	Electricity savings3	Arrived today! Still trying it out!	Jingdong prefecture level city in Shandong
Jinfeng Hongyuan Flagship Store	Electricity savings3	Shipped quickly, well packaged, already installed, hope there is a power saving effect	Jingdong prefecture level city in Shandong
Jinfeng Hongyuan Flagship Store	Electricity savings3	I got it. It's great.	Jingdong prefecture level city in Shandong
Jinfeng Hongyuan Flagship Store	Electricity savings3	Haven't used it yet, will come back in a month	Jingdong prefecture level city in Shandong
Jinfeng Hongyuan Flagship Store	Electricity savings3	It looks good. It's good. Try it first.	Jingdong prefecture level city in Shandong
Jinfeng Hongyuan Flagship Store	Electricity savings3	It works so well that I have relatives, friends, coworkers, and 6 families that have purchased it for them through me.	Jingdong prefecture level city in Shandong
Jinfeng Hongyuan Flagship Store	Electricity savings3	In use	Jingdong prefecture level city in Shandong
Jinfeng Hongyuan Flagship Store	Electricity savings3	Received the goods today, power-saving effect do not know being used, the boss has a very good attitude, praise!	Jingdong prefecture level city in Shandong
Jinfeng Hongyuan Flagship Store	Electricity savings3	The product is compact and doesn't take up much space, but hopefully it's power efficient.	Jingdong prefecture level city in Shandong
Jinfeng Hongyuan Flagship Store	Electricity savings3	Power saving is indeed effective, I used for a period of time before coming to evaluate, the electricity bill is obviously less, the quality is good, the shopkeeper service is also very good, very patient,	Jingdong prefecture level city in Shandong
Jinfeng Hongyuan Flagship Store	Electricity savings3	It feels fine, I'll comment after I use it	Jingdong prefecture level city in Shandong
Jinfeng Hongyuan Flagship Store	Electricity savings3	I just received it and I don't know the effect yet! I will comment again after I use it and have results!	Jingdong prefecture level city in Shandong
Jinfeng Hongyuan Flagship Store	Electricity savings3	The goods look fine, I hope they really work!	Jingdong prefecture level city in Shandong
Jinfeng Hongyuan Flagship Store	Electricity savings3	I just don't know if it saves power	Jingdong prefecture level city in Shandong
Jinfeng Hongyuan Flagship Store	Electricity savings3	My friend introduced me to use this product, it is professional and I am looking forward to the long term effect~!	Jingdong prefecture level city in Shandong
Jinfeng Hongyuan Flagship Store	Electricity savings3	Try it before you judge.	Jingdong prefecture level city in Shandong
Jinfeng Hongyuan Flagship Store	Electricity savings3	It's great, the power savings are obvious! Kudos!	Jingdong prefecture level city in Shandong
Jinfeng Hongyuan Flagship Store	Electricity savings3	The quality is excellent, stabilizes the voltage and saves electricity, very satisfied.	Jingdong prefecture level city in Shandong
Jinfeng Hongyuan Flagship Store	Electricity savings3	You have to use it for a while to know the effect	Jingdong prefecture level city in Shandong
Jinfeng Hongyuan Flagship Store	Electricity savings3	It's on trial.	Jingdong prefecture level city in Shandong
Jinfeng Hongyuan Flagship Store	Electricity savings3	The stuff works great.	Jingdong prefecture level city in Shandong
Jinfeng Hongyuan Flagship Store	Electricity savings3	Doesn't seem to work, not as god as other reviews	Jingdong prefecture level city in Shandong
Jinfeng Hongyuan Flagship Store	Electricity savings3	It's not good quality, it doesn't work, it stops working after a few days, it doesn't work, the customer service doesn't care, it's a bit irritating.	Jingdong prefecture level city in Shandong
Jinfeng Hongyuan Flagship Store	Electricity savings3	It doesn't do anything.	Jingdong prefecture level city in Shandong
Jinfeng Hongyuan Flagship Store	Electricity savings3	It's good.	Jingdong prefecture level city in Shandong
Jinfeng Hongyuan Flagship Store	Electricity savings3	Used a month effect is not obvious, are psychological effects, feel are not less, did not save to the electricity, there are details of the problem, the store said to buy to send 5 fuse, in fact, received only three, small problems are not committed to in place, I believe that the product is not good enough	Jingdong prefecture level city in Shandong
Jinfeng Hongyuan Flagship Store	Electricity savings3	Well received quickly, looking quite high, but also sent 5 fuses. Customer service is very patient to teach the operation, in fact, quite simple, lazy to read the instructions ha!	Jingdong prefecture level city in Shandong
Jinfeng Hongyuan Flagship Store	Electricity savings3	You have to use it for a while to know the effect	Jingdong prefecture level city in Shandong
Jinfeng Hongyuan Flagship Store	Electricity savings3	Shipped very quickly, packaged very well, the price is particularly affordable, there are so many efficacy is also good, it is worth buying worth buying the product, the workmanship is very fine, better than imagined, recommended that everyone try not yet used, after the use of the follow-up comment	Jingdong prefecture level city in Shandong
Jinfeng Hongyuan Flagship Store	Electricity savings3	It looks regular. It's working now.	Jingdong prefecture level city in Shandong
Jinfeng Hongyuan Flagship Store	Electricity savings3	It arrived pretty fast and the owner is very responsible. Have not used it yet, will see after use	Jingdong prefecture level city in Shandong
Jinfeng Hongyuan Flagship Store	Electricity savings3	🎁 baby quality is very good, the first time I received the goods to open the first time to see, it is very good 👍, very satisfied 😊, customer service also have a question and answer, thank you 🙏	Jingdong prefecture level city in Shandong
Jinfeng Hongyuan Flagship Store	Electricity savings3	I don't know if it's any good 。。。。。。	Jingdong prefecture level city in Shandong
Jinfeng Hongyuan Flagship Store	Electricity savings3	Buy electronic products! Still trust the things of the Jingdong self-owned store! Energy-saving effect to evaluate after heating!	Jingdong prefecture level city in Shandong
Jinfeng Hongyuan Flagship Store	Electricity savings3	The goods have been received, Blu is very beautiful, energy saving effect, to be confirmed only after actual use.	Jingdong prefecture level city in Shandong
Jinfeng Hongyuan Flagship Store	Electricity savings3	Jingdong's speed is so fast, has received, easy to operate. The display is quite good, how many volts what mode is quite understandable.	Jingdong prefecture level city in Shandong
Jinfeng Hongyuan Flagship Store	Electricity savings3	The first use, the effect is good to recommend it to colleagues to use, a month of electricity to save a lot or can be	Jingdong prefecture level city in Shandong

Jinfeng Hongyuan Flagship Store	Electricity savings3	I don't know if it's useful. Mom's is a store. I'll return it if it doesn't work.	Jingdong prefecture level city in Shandong
Jinfeng Hongyuan Flagship Store	Electricity savings3	I don't know if it works or not, I'll wait for two months to use it and then come back to comment!	Jingdong prefecture level city in Shandong
Jinfeng Hongyuan Flagship Store	Electricity savings3	I bought the second one now, and I've been using it for more than half a year, and I feel that it really saves electricity. Now it is summer, the weather is hot, sometimes the fan and air conditioning on all day, if not this power saver, the monthly electricity bill is quite high, but as long as the installation of this power saver, not afraid of its high electricity bill, absolutely let you wantonly use	Jingdong prefecture level city in Shandong
Jinfeng Hongyuan Flagship Store	Electricity savings3	The product is better than I thought it would be, the quality looks great, the voltage is stable, and I'm looking forward to some great power savings!	Jingdong prefecture level city in Shandong
Jinfeng Hongyuan Flagship Store	Electricity savings3	Inadvertently see bought a try, delivery is also quite timely, just do not know how to use, customer service patience to answer, with a pretty good, value for money!	Jingdong prefecture level city in Shandong
Jinfeng Hongyuan Flagship Store	Electricity savings3	It's better than nothing! Hopefully this will help save slightly.	Jingdong prefecture level city in Shandong
Jinfeng Hongyuan Flagship Store	Electricity savings3	Still trying it out, follow up	Jingdong prefecture level city in Shandong
Jinfeng Hongyuan Flagship Store	Electricity savings3	I've just started using it, I don't know how well it works yet, I hope it's good.	Jingdong prefecture level city in Shandong
Jinfeng Hongyuan Flagship Store	Electricity savings3	Fourth purchase, great baby!	Jingdong prefecture level city in Shandong
Jinfeng Hongyuan Flagship Store	Electricity savings3	Shipped really fast, just got it and use it to see what happens and then come back for a review!	Jingdong prefecture level city in Shandong
Jinfeng Hongyuan Flagship Store	Electricity savings3	Timely delivery **** good attitude	Jingdong prefecture level city in Shandong
Jinfeng Hongyuan Flagship Store	Electricity savings3	Home large and small things are purchased in the Jingdong Mall, have been accustomed to shopping in the Jingdong, many times can buy good quality and cheap products, and courier service attitude is particularly good, sometimes too many couriers are to help me to the home, so many years have been in the Jingdong Mall shopping, trust and support! Will also support the Jingdong as always!	Jingdong prefecture level city in Shandong
Jinfeng Hongyuan Flagship Store	Electricity savings3	Modified a pump and the modification worked.	Jingdong prefecture level city in Shandong
Jinfeng Hongyuan Flagship Store	Electricity savings3	Already installed, next time you pay the electric bill to see the results	Jingdong prefecture level city in Shandong
Jinfeng Hongyuan Flagship Store	Electricity savings3	The product is very good to use and practical to look at	Jingdong prefecture level city in Shandong
Jinfeng Hongyuan Flagship Store	Electricity savings3	Works great, great service, waiting for results.	Jingdong prefecture level city in Shandong
Jinfeng Hongyuan Flagship Store	Electricity savings3	I just used it and I'm not sure about the efficacy yet, I'll come back to evaluate it after a while.	Jingdong prefecture level city in Shandong
Jinfeng Hongyuan Flagship Store	Electricity savings3	Just received the goods, use a period of time to evaluate	Jingdong prefecture level city in Shandong
Jinfeng Hongyuan Flagship Store	Electricity savings3	Opened and tried very good, use it to see if there are results	Jingdong prefecture level city in Shandong
Jinfeng Hongyuan Flagship Store	Electricity savings3	Arrived today! Still trying it out!	Jingdong prefecture level city in Shandong
Jinfeng Hongyuan Flagship Store	Electricity savings3	Shipped quickly, well packaged, already installed, hope there is a power saving effect	Jingdong prefecture level city in Shandong
Jinfeng Hongyuan Flagship Store	Electricity savings3	I got it. It's great.	Jingdong prefecture level city in Shandong
Jinfeng Hongyuan Flagship Store	Electricity savings3	Haven't used it yet, will come back in a month	Jingdong prefecture level city in Shandong
Jinfeng Hongyuan Flagship Store	Electricity savings3	It looks good. It's good. Try it first.	Jingdong prefecture level city in Shandong
Jinfeng Hongyuan Flagship Store	Electricity savings3	It works so well that I have relatives, friends, coworkers, and 6 families that have purchased it for them through me.	Jingdong prefecture level city in Shandong
Jinfeng Hongyuan Flagship Store	Electricity savings3	In use	Jingdong prefecture level city in Shandong
Jinfeng Hongyuan Flagship Store	Electricity savings3	Received the goods today, power-saving effect do not know being used, the boss has a very good attitude, praise!	Jingdong prefecture level city in Shandong
Jinfeng Hongyuan Flagship Store	Electricity savings3	The product is compact and doesn't take up much space, but hopefully it's power efficient.	Jingdong prefecture level city in Shandong
Jinfeng Hongyuan Flagship Store	Electricity savings3	Power saving is indeed effective, I used for a period of time before coming to evaluate, the electricity bill is obviously less, the quality is good, the shopkeeper service is also very good, very patient,	Jingdong prefecture level city in Shandong
Jinfeng Hongyuan Flagship Store	Electricity savings3	It feels fine, I'll comment after I use it	Jingdong prefecture level city in Shandong
Jinfeng Hongyuan Flagship Store	Electricity savings3	I just received it and I don't know the effect yet! I will comment again after I use it and have results!	Jingdong prefecture level city in Shandong
Jinfeng Hongyuan Flagship Store	Electricity savings3	The goods look fine, I hope they really work!	Jingdong prefecture level city in Shandong
Jinfeng Hongyuan Flagship Store	Electricity savings3	I just don't know if it saves power	Jingdong prefecture level city in Shandong
Jinfeng Hongyuan Flagship Store	Electricity savings3	My friend introduced me to use this product, it is professional and I am looking forward to the long term effect~!	Jingdong prefecture level city in Shandong
Jinfeng Hongyuan Flagship Store	Electricity savings3	Try it before you judge.	Jingdong prefecture level city in Shandong
Jinfeng Hongyuan Flagship Store	Electricity savings3	It's great, the power savings are obvious! Kudos!	Jingdong prefecture level city in Shandong
Jinfeng Hongyuan Flagship Store	Electricity savings3	The quality is excellent, stabilizes the voltage and saves electricity, very satisfied.	Jingdong prefecture level city in Shandong
Jinfeng Hongyuan Flagship Store	Electricity savings3	You have to use it for a while to know the effect	Jingdong prefecture level city in Shandong
Jinfeng Hongyuan Flagship Store	Electricity savings3	It's on trial.	Jingdong prefecture level city in Shandong
Jinfeng Hongyuan Flagship Store	Electricity savings3	The stuff works great.	Jingdong prefecture level city in Shandong
Jinfeng Hongyuan Flagship Store	Electricity savings3	Doesn't seem to work, not as god as other reviews	Jingdong prefecture level city in Shandong
Jinfeng Hongyuan Flagship Store	Electricity savings3	It's not good quality, it doesn't work, it stops working after a few days, it doesn't work, the customer service doesn't care, it's a bit irritating.	Jingdong prefecture level city in Shandong
Jinfeng Hongyuan Flagship Store	Electricity savings3	It doesn't do anything.	Jingdong prefecture level city in Shandong
Jinfeng Hongyuan Flagship Store	Electricity savings3	It's good.	Jingdong prefecture level city in Shandong
Jinfeng Hongyuan Flagship Store	Electricity savings3	Used a month effect is not obvious, are psychological effects, feel are not less, did not save to the electricity, there are details of the problem, the store said to buy to send 5 fuse, in fact, received only three, small problems are not committed to in place, I believe that the product is not good enough	Jingdong prefecture level city in Shandong
Jinfeng Hongyuan Flagship Store	Electricity savings3	Well received quickly, looking quite high, but also sent 5 fuses. Customer service is very patient to teach the operation, in fact, quite simple, lazy to read the instructions ha!	Jingdong prefecture level city in Shandong
Jinfeng Hongyuan Flagship Store	Electricity savings3	You have to use it for a while to know the effect	Jingdong prefecture level city in Shandong
Jinfeng Hongyuan Flagship Store	Electricity savings3	Shipped very quickly, packaged very well, the price is particularly affordable, there are so many efficacy is also good, it is worth buying worth buying the product, the workmanship is very fine, better than imagined, recommended that everyone try not yet used, after the use of the follow-up comment	Jingdong prefecture level city in Shandong

Jinfeng Hongyuan Flagship Store	Electricity savings3	It looks regular. It's working now.	Jingdong prefecture level city in Shandong
Jinfeng Hongyuan Flagship Store	Electricity savings3	It arrived pretty fast and the owner is very responsible. Have not used it yet, will see after use	Jingdong prefecture level city in Shandong
Jinfeng Hongyuan Flagship Store	Electricity savings3	🎁 baby quality is very good, the first time I received the goods to open the first time to see, it is very good 👍, very satisfied 😊, customer service also have a question and answer, thank you 🙏	Jingdong prefecture level city in Shandong
Jinfeng Hongyuan Flagship Store	Electricity savings3	I don't know if it's any good 。。。。。。	Jingdong prefecture level city in Shandong
Jinfeng Hongyuan Flagship Store	Electricity savings3	Buy electronic products! Still trust the things of the Jingdong self-owned store! Energy-saving effect to evaluate after heating!	Jingdong prefecture level city in Shandong
Jinfeng Hongyuan Flagship Store	Electricity savings3	The goods have been received, Blu is very beautiful, energy saving effect, to be confirmed only after actual use.	Jingdong prefecture level city in Shandong
Jinfeng Hongyuan Flagship Store	Electricity savings3	Jingdong's speed is so fast, has received, easy to operate. The display is quite good, how many volts what mode is quite understandable.	Jingdong prefecture level city in Shandong
Jinfeng Hongyuan Flagship Store	Electricity savings3	The first use, the effect is good to recommend it to colleagues to use, a month of electricity to save a lot or can be	Jingdong prefecture level city in Shandong
Jinfeng Hongyuan Flagship Store	Electricity savings3	I don't know if it's useful. Mom's is a store. I'll return it if it doesn't work.	Jingdong prefecture level city in Shandong
Jinfeng Hongyuan Flagship Store	Electricity savings3	I don't know if it works or not, I'll wait for two months to use it and then come back to comment!	Jingdong prefecture level city in Shandong
Jinfeng Hongyuan Flagship Store	Electricity savings3	I bought the second one now, and I've been using it for more than half a year, and I feel that it really saves electricity. Now it is summer, the weather is hot, sometimes the fan and air conditioning on all day, if not this power saver, the monthly electricity bill is quite high, but as long as the installation of this power saver, not afraid of its high electricity bill, absolutely let you wantonly use	Jingdong prefecture level city in Shandong
Jinfeng Hongyuan Flagship Store	Electricity savings3	The product is better than I thought it would be, the quality looks great, the voltage is stable, and I'm looking forward to some great power savings!	Jingdong prefecture level city in Shandong
Jinfeng Hongyuan Flagship Store	Electricity savings3	Inadvertently see bought a try, delivery is also quite timely, just do not know how to use, customer service patience to answer, with a pretty good, value for money!	Jingdong prefecture level city in Shandong
Jinfeng Hongyuan Flagship Store	Electricity savings3	It's better than nothing! Hopefully this will help save slightly.	Jingdong prefecture level city in Shandong
Jinfeng Hongyuan Flagship Store	Electricity savings3	Still trying it out, follow up	Jingdong prefecture level city in Shandong
Jinfeng Hongyuan Flagship Store	Electricity savings3	I've just started using it, I don't know how well it works yet, I hope it's good.	Jingdong prefecture level city in Shandong
Jinfeng Hongyuan Flagship Store	Electricity savings3	Fourth purchase, great baby!	Jingdong prefecture level city in Shandong
Jinfeng Hongyuan Flagship Store	Electricity savings3	Shipped really fast, just got it and use it to see what happens and then come back for a review!	Jingdong prefecture level city in Shandong
Jinfeng Hongyuan Flagship Store	Electricity savings3	Timely delivery **** good attitude	Jingdong prefecture level city in Shandong
Jinfeng Hongyuan Flagship Store	Electricity savings3	Home large and small things are purchased in the Jingdong Mall, have been accustomed to shopping in the Jingdong, many times can buy good quality and cheap products, and courier service attitude is particularly good, sometimes too many couriers are to help me to the home, so many years have been in the Jingdong Mall shopping, trust and support! Will also support the Jingdong as always!	Jingdong prefecture level city in Shandong
Jinfeng Hongyuan Flagship Store	Electricity savings3	Modified a pump and the modification worked.	Jingdong prefecture level city in Shandong
Jinfeng Hongyuan Flagship Store	Electricity savings3	Already installed, next time you pay the electric bill to see the results	Jingdong prefecture level city in Shandong
Jinfeng Hongyuan Flagship Store	Electricity savings3	The product is very good to use and practical to look at	Jingdong prefecture level city in Shandong
Jinfeng Hongyuan Flagship Store	Electricity savings3	Works great, great service, waiting for results.	Jingdong prefecture level city in Shandong
Jinfeng Hongyuan Flagship Store	Electricity savings3	I just used it and I'm not sure about the efficacy yet, I'll come back to evaluate it after a while.	Jingdong prefecture level city in Shandong
Jinfeng Hongyuan Flagship Store	Electricity savings3	Just received the goods, use a period of time to evaluate	Jingdong prefecture level city in Shandong
Jinfeng Hongyuan Flagship Store	Electricity savings3	Opened and tried very good, use it to see if there are results	Jingdong prefecture level city in Shandong
Jinfeng Hongyuan Flagship Store	Electricity savings3	Arrived today! Still trying it out!	Jingdong prefecture level city in Shandong
Jinfeng Hongyuan Flagship Store	Electricity savings3	Shipped quickly, well packaged, already installed, hope there is a power saving effect	Jingdong prefecture level city in Shandong
Jinfeng Hongyuan Flagship Store	Electricity savings3	I got it. It's great.	Jingdong prefecture level city in Shandong
Jinfeng Hongyuan Flagship Store	Electricity savings3	Haven't used it yet, will come back in a month	Jingdong prefecture level city in Shandong
Jinfeng Hongyuan Flagship Store	Electricity savings3	It looks good. It's good. Try it first.	Jingdong prefecture level city in Shandong
Jinfeng Hongyuan Flagship Store	Electricity savings3	It works so well that I have relatives, friends, coworkers, and 6 families that have purchased it for them through me.	Jingdong prefecture level city in Shandong
Jinfeng Hongyuan Flagship Store	Electricity savings3	In use	Jingdong prefecture level city in Shandong
Jinfeng Hongyuan Flagship Store	Electricity savings3	Received the goods today, power-saving effect do not know being used, the boss has a very good attitude, praise!	Jingdong prefecture level city in Shandong
Jinfeng Hongyuan Flagship Store	Electricity savings3	The product is compact and doesn't take up much space, but hopefully it's power efficient.	Jingdong prefecture level city in Shandong
Jinfeng Hongyuan Flagship Store	Electricity savings3	Power saving is indeed effective, I used for a period of time before coming to evaluate, the electricity bill is obviously less, the quality is good, the shopkeeper service is also very good, very patient,	Jingdong prefecture level city in Shandong
Jinfeng Hongyuan Flagship Store	Electricity savings3	It feels fine, I'll comment after I use it	Jingdong prefecture level city in Shandong
Jinfeng Hongyuan Flagship Store	Electricity savings3	I just received it and I don't know the effect yet! I will comment again after I use it and have results!	Jingdong prefecture level city in Shandong
Jinfeng Hongyuan Flagship Store	Electricity savings3	The goods look fine, I hope they really work!	Jingdong prefecture level city in Shandong
Jinfeng Hongyuan Flagship Store	Electricity savings3	I just don't know if it saves power	Jingdong prefecture level city in Shandong
Jinfeng Hongyuan Flagship Store	Electricity savings3	My friend introduced me to use this product, it is professional and I am looking forward to the long term effect~!	Jingdong prefecture level city in Shandong
Jinfeng Hongyuan Flagship Store	Electricity savings3	Try it before you judge.	Jingdong prefecture level city in Shandong
Jinfeng Hongyuan Flagship Store	Electricity savings3	It's great, the power savings are obvious! Kudos!	Jingdong prefecture level city in Shandong
Jinfeng Hongyuan Flagship Store	Electricity savings3	The quality is excellent, stabilizes the voltage and saves electricity, very satisfied.	Jingdong prefecture level city in Shandong
Jinfeng Hongyuan Flagship Store	Electricity savings3	You have to use it for a while to know the effect	Jingdong prefecture level city in Shandong
Jinfeng Hongyuan Flagship Store	Electricity savings3	It's on trial.	Jingdong prefecture level city in Shandong
Jinfeng Hongyuan Flagship Store	Electricity savings3	The stuff works great.	Jingdong prefecture level city in Shandong
Jinfeng Hongyuan Flagship Store	Electricity savings3	Doesn't seem to work, not as god as other reviews	Jingdong prefecture level city in Shandong

Jinfeng Hongyuan Flagship Store	Electricity savings3	It's not good quality, it doesn't work, it stops working after a few days, it doesn't work, the customer service doesn't care, it's a bit irritating.	Jingdong prefecture level city in Shandong
Jinfeng Hongyuan Flagship Store	Electricity savings3	It doesn't do anything.	Jingdong prefecture level city in Shandong
Jinfeng Hongyuan Flagship Store	Electricity savings3	It's good.	Jingdong prefecture level city in Shandong
Jinfeng Hongyuan Flagship Store	Electricity savings3	Used a month effect is not obvious, are psychological effects, feel are not less, did not save to the electricity, there are details of the problem, the store said to buy to send 5 fuse, in fact, received only three, small problems are not committed to in place, I believe that the product is not good enough	Jingdong prefecture level city in Shandong
Jinfeng Hongyuan Flagship Store	Electricity savings3	Well received quickly, looking quite high, but also sent 5 fuses. Customer service is very patient to teach the operation, in fact, quite simple, lazy to read the instructions ha!	Jingdong prefecture level city in Shandong
Jinfeng Hongyuan Flagship Store	Electricity savings3	You have to use it for a while to know the effect	Jingdong prefecture level city in Shandong
Jinfeng Hongyuan Flagship Store	Electricity savings3	Shipped very quickly, packaged very well, the price is particularly affordable, there are so many efficacy is also good, it is worth buying worth buying the product, the workmanship is very fine, better than imagined, recommended that everyone try not yet used, after the use of the follow-up comment	Jingdong prefecture level city in Shandong
Jinfeng Hongyuan Flagship Store	Electricity savings3	It looks regular. It's working now.	Jingdong prefecture level city in Shandong
Jinfeng Hongyuan Flagship Store	Electricity savings3	It arrived pretty fast and the owner is very responsible. Have not used it yet, will see after use	Jingdong prefecture level city in Shandong
Jinfeng Hongyuan Flagship Store	Electricity savings3	🎁 baby quality is very good, the first time I received the goods to open the first time to see, it is very good 👍, very satisfied 😃, customer service also have a question and answer, thank you 🙏	Jingdong prefecture level city in Shandong
Jinfeng Hongyuan Flagship Store	Electricity savings3	I don't know if it's any good 。。。。。。	Jingdong prefecture level city in Shandong
Jinfeng Hongyuan Flagship Store	Electricity savings3	Buy electronic products! Still trust the things of the Jingdong self-owned store! Energy-saving effect to evaluate after heating!	Jingdong prefecture level city in Shandong
Jinfeng Hongyuan Flagship Store	Electricity savings3	The goods have been received, Blu is very beautiful, energy saving effect, to be confirmed only after actual use.	Jingdong prefecture level city in Shandong
Jinfeng Hongyuan Flagship Store	Electricity savings3	Jingdong's speed is so fast, has received, easy to operate. The display is quite good, how many volts what mode is quite understandable.	Jingdong prefecture level city in Shandong
Jinfeng Hongyuan Flagship Store	Electricity savings3	The first use, the effect is good to recommend it to colleagues to use, a month of electricity to save a lot or can be	Jingdong prefecture level city in Shandong
Jinfeng Hongyuan Flagship Store	Electricity savings3	I don't know if it's useful. Mom's is a store. I'll return it if it doesn't work.	Jingdong prefecture level city in Shandong
Jinfeng Hongyuan Flagship Store	Electricity savings3	I don't know if it works or not, I'll wait for two months to use it and then come back to comment!	Jingdong prefecture level city in Shandong
Jinfeng Hongyuan Flagship Store	Electricity savings3	I bought the second one now, and I've been using it for more than half a year, and I feel that it really saves electricity. Now it is summer, the weather is hot, sometimes the fan and air conditioning on all day, if not this power saver, the monthly electricity bill is quite high, but as long as the installation of this power saver, not afraid of its high electricity bill, absolutely let you wantonly use	Jingdong prefecture level city in Shandong
Jinfeng Hongyuan Flagship Store	Electricity savings3	The product is better than I thought it would be, the quality looks great, the voltage is stable, and I'm looking forward to some great power savings!	Jingdong prefecture level city in Shandong
Jinfeng Hongyuan Flagship Store	Electricity savings3	Inadvertently see bought a try, delivery is also quite timely, just do not know how to use, customer service patience to answer, with a pretty good, value for money!	Jingdong prefecture level city in Shandong
Jinfeng Hongyuan Flagship Store	Electricity savings3	It's better than nothing! Hopefully this will help save slightly.	Jingdong prefecture level city in Shandong
Jinfeng Hongyuan Flagship Store	Electricity savings3	Still trying it out, follow up	Jingdong prefecture level city in Shandong
Jinfeng Hongyuan Flagship Store	Electricity savings3	I've just started using it, I don't know how well it works yet, I hope it's good.	Jingdong prefecture level city in Shandong
Jinfeng Hongyuan Flagship Store	Electricity savings3	Fourth purchase, great baby!	Jingdong prefecture level city in Shandong
Jinfeng Hongyuan Flagship Store	Electricity savings3	Shipped really fast, just got it and use it to see what happens and then come back for a review!	Jingdong prefecture level city in Shandong
Jinfeng Hongyuan Flagship Store	Electricity savings3	Timely delivery **** good attitude	Jingdong prefecture level city in Shandong
Jinfeng Hongyuan Flagship Store	Electricity savings3	Home large and small things are purchased in the Jingdong Mall, have been accustomed to shopping in the Jingdong, many times can buy good quality and cheap products, and courier service attitude is particularly good, sometimes too many couriers are to help me to the home, so many years have been in the Jingdong Mall shopping, trust and support! Will also support the Jingdong as always!	Jingdong prefecture level city in Shandong
Jinfeng Hongyuan Flagship Store	Electricity savings3	Modified a pump and the modification worked.	Jingdong prefecture level city in Shandong
Jinfeng Hongyuan Flagship Store	Electricity savings3	Already installed, next time you pay the electric bill to see the results	Jingdong prefecture level city in Shandong
Jinfeng Hongyuan Flagship Store	Electricity savings3	The product is very good to use and practical to look at	Jingdong prefecture level city in Shandong
Jinfeng Hongyuan Flagship Store	Electricity savings3	Works great, great service, waiting for results.	Jingdong prefecture level city in Shandong
Jinfeng Hongyuan Flagship Store	Electricity savings3	I just used it and I'm not sure about the efficacy yet, I'll come back to evaluate it after a while.	Jingdong prefecture level city in Shandong
Jinfeng Hongyuan Flagship Store	Electricity savings3	Just received the goods, use a period of time to evaluate	Jingdong prefecture level city in Shandong
Jinfeng Hongyuan Flagship Store	Electricity savings3	Opened and tried very good, use it to see if there are results	Jingdong prefecture level city in Shandong
Jinfeng Hongyuan Flagship Store	Electricity savings3	Arrived today! Still trying it out!	Jingdong prefecture level city in Shandong
Jinfeng Hongyuan Flagship Store	Electricity savings3	Shipped quickly, well packaged, already installed, hope there is a power saving effect	Jingdong prefecture level city in Shandong
Jinfeng Hongyuan Flagship Store	Electricity savings3	I got it. It's great.	Jingdong prefecture level city in Shandong
Jinfeng Hongyuan Flagship Store	Electricity savings3	Haven't used it yet, will come back in a month	Jingdong prefecture level city in Shandong
Jinfeng Hongyuan Flagship Store	Electricity savings3	It looks good. It's good. Try it first.	Jingdong prefecture level city in Shandong
Jinfeng Hongyuan Flagship Store	Electricity savings3	It works so well that I have relatives, friends, coworkers, and 6 families that have purchased it for them through me.	Jingdong prefecture level city in Shandong
Jinfeng Hongyuan Flagship Store	Electricity savings3	In use	Jingdong prefecture level city in Shandong
Jinfeng Hongyuan Flagship Store	Electricity savings3	Received the goods today, power-saving effect do not know being used, the boss has a very good attitude, praise!	Jingdong prefecture level city in Shandong
Jinfeng Hongyuan Flagship Store	Electricity savings3	The product is compact and doesn't take up much space, but hopefully it's power efficient.	Jingdong prefecture level city in Shandong
Jinfeng Hongyuan Flagship Store	Electricity savings3	Power saving is indeed effective, I used for a period of time before coming to evaluate, the electricity bill is obviously less, the quality is good, the shopkeeper service is also very good, very patient,	Jingdong prefecture level city in Shandong
Jinfeng Hongyuan Flagship Store	Electricity savings3	It feels fine, I'll comment after I use it	Jingdong prefecture level city in Shandong
Jinfeng Hongyuan Flagship Store	Electricity savings3	I just received it and I don't know the effect yet! I will comment again after I use it and have results!	Jingdong prefecture level city in Shandong
Jinfeng Hongyuan Flagship Store	Electricity savings3	The goods look fine, I hope they really work!	Jingdong prefecture level city in Shandong
Jinfeng Hongyuan Flagship Store	Electricity savings3	I just don't know if it saves power	Jingdong prefecture level city in Shandong

Jinfeng Hongyuan Flagship Store	Electricity savings3	My friend introduced me to use this product, it is professional and I am looking forward to the long term effect~!	Jingdong prefecture level city in Shandong
Jinfeng Hongyuan Flagship Store	Electricity savings3	Try it before you judge.	Jingdong prefecture level city in Shandong
Jinfeng Hongyuan Flagship Store	Electricity savings3	It's great, the power savings are obvious! Kudos!	Jingdong prefecture level city in Shandong
Jinfeng Hongyuan Flagship Store	Electricity savings3	The quality is excellent, stabilizes the voltage and saves electricity, very satisfied.	Jingdong prefecture level city in Shandong
Jinfeng Hongyuan Flagship Store	Electricity savings3	You have to use it for a while to know the effect	Jingdong prefecture level city in Shandong
Jinfeng Hongyuan Flagship Store	Electricity savings3	It's on trial.	Jingdong prefecture level city in Shandong
Jinfeng Hongyuan Flagship Store	Electricity savings3	The stuff works great.	Jingdong prefecture level city in Shandong
Jinfeng Hongyuan Flagship Store	Electricity savings3	Doesn't seem to work, not as god as other reviews	Jingdong prefecture level city in Shandong
Jinfeng Hongyuan Flagship Store	Electricity savings3	It's not good quality, it doesn't work, it stops working after a few days, it doesn't work, the customer service doesn't care, it's a bit irritating.	Jingdong prefecture level city in Shandong
Jinfeng Hongyuan Flagship Store	Electricity savings3	It doesn't do anything.	Jingdong prefecture level city in Shandong
Jinfeng Hongyuan Flagship Store	Electricity savings3	It's good.	Jingdong prefecture level city in Shandong
Jinfeng Hongyuan Flagship Store	Electricity savings3	Used a month effect is not obvious, are psychological effects, feel are not less, did not save to the electricity, there are details of the problem, the store said to buy to send 5 fuse, in fact, received only three, small problems are not committed to in place, I believe that the product is not good enough	Jingdong prefecture level city in Shandong
Jinfeng Hongyuan Flagship Store	Electricity savings3	Well received quickly, looking quite high, but also sent 5 fuses. Customer service is very patient to teach the operation, in fact, quite simple, lazy to read the instructions ha!	Jingdong prefecture level city in Shandong
Jinfeng Hongyuan Flagship Store	Electricity savings3	You have to use it for a while to know the effect	Jingdong prefecture level city in Shandong
Jinfeng Hongyuan Flagship Store	Electricity savings3	Shipped very quickly, packaged very well, the price is particularly affordable, there are so many efficacy is also good, it is worth buying worth buying the product, the workmanship is very fine, better than imagined, recommended that everyone try not yet used, after the use of the follow-up comment	Jingdong prefecture level city in Shandong
Jinfeng Hongyuan Flagship Store	Electricity savings3	It looks regular. It's working now.	Jingdong prefecture level city in Shandong
Jinfeng Hongyuan Flagship Store	Electricity savings3	It arrived pretty fast and the owner is very responsible. Have not used it yet, will see after use	Jingdong prefecture level city in Shandong
Jinfeng Hongyuan Flagship Store	Electricity savings3	🎁 baby quality is very good, the first time I received the goods to open the first time to see, it is very good 👍, very satisfied 😊, customer service also have a question and answer, thank you 🙏	Jingdong prefecture level city in Shandong
Jinfeng Hongyuan Flagship Store	Electricity savings3	I don't know if it's any good 。。。。。。	Jingdong prefecture level city in Shandong
Jinfeng Hongyuan Flagship Store	Electricity savings3	Buy electronic products! Still trust the things of the Jingdong self-owned store! Energy-saving effect to evaluate after heating!	Jingdong prefecture level city in Shandong
Jinfeng Hongyuan Flagship Store	Electricity savings3	The goods have been received, Blu is very beautiful, energy saving effect, to be confirmed only after actual use.	Jingdong prefecture level city in Shandong
Jinfeng Hongyuan Flagship Store	Electricity savings3	Jingdong's speed is so fast, has received, easy to operate. The display is quite good, how many volts what mode is quite understandable.	Jingdong prefecture level city in Shandong
Jinfeng Hongyuan Flagship Store	Electricity savings3	The first use, the effect is good to recommend it to colleagues to use, a month of electricity to save a lot or can be	Jingdong prefecture level city in Shandong
Jinfeng Hongyuan Flagship Store	Electricity savings3	I don't know if it's useful. Mom's is a store. I'll return it if it doesn't work.	Jingdong prefecture level city in Shandong
Jinfeng Hongyuan Flagship Store	Electricity savings3	I don't know if it works or not, I'll wait for two months to use it and then come back to comment!	Jingdong prefecture level city in Shandong
Jinfeng Hongyuan Flagship Store	Electricity savings3	I bought the second one now, and I've been using it for more than half a year, and I feel that it really saves electricity. Now it is summer, the weather is hot, sometimes the fan and air conditioning on all day, if not this power saver, the monthly electricity bill is quite high, but as long as the installation of this power saver, not afraid of its high electricity bill, absolutely let you wantonly use	Jingdong prefecture level city in Shandong
Jinfeng Hongyuan Flagship Store	Electricity savings3	The product is better than I thought it would be, the quality looks great, the voltage is stable, and I'm looking forward to some great power savings!	Jingdong prefecture level city in Shandong
Jinfeng Hongyuan Flagship Store	Electricity savings3	Inadvertently see bought a try, delivery is also quite timely, just do not know how to use, customer service patience to answer, with a pretty good, value for money!	Jingdong prefecture level city in Shandong
Jinfeng Hongyuan Flagship Store	Electricity savings3	It's better than nothing! Hopefully this will help save slightly.	Jingdong prefecture level city in Shandong
Jinfeng Hongyuan Flagship Store	Electricity savings3	Still trying it out, follow up	Jingdong prefecture level city in Shandong
Jinfeng Hongyuan Flagship Store	Electricity savings3	I've just started using it, I don't know how well it works yet, I hope it's good.	Jingdong prefecture level city in Shandong
Jinfeng Hongyuan Flagship Store	Electricity savings3	Fourth purchase, great baby!	Jingdong prefecture level city in Shandong
Jinfeng Hongyuan Flagship Store	Electricity savings3	Shipped really fast, just got it and use it to see what happens and then come back for a review!	Jingdong prefecture level city in Shandong
Jinfeng Hongyuan Flagship Store	Electricity savings3	Timely delivery **** good attitude	Jingdong prefecture level city in Shandong
Jinfeng Hongyuan Flagship Store	Electricity savings3	Home large and small things are purchased in the Jingdong Mall, have been accustomed to shopping in the Jingdong, many times can buy good quality and cheap products, and courier service attitude is particularly good, sometimes too many couriers are to help me to the home, so many years have been in the Jingdong Mall shopping, trust and support! Will also support the Jingdong as always!	Jingdong prefecture level city in Shandong
Jinfeng Hongyuan Flagship Store	Electricity savings3	Modified a pump and the modification worked.	Jingdong prefecture level city in Shandong
Jinfeng Hongyuan Flagship Store	Electricity savings3	Already installed, next time you pay the electric bill to see the results	Jingdong prefecture level city in Shandong
Jinfeng Hongyuan Flagship Store	Electricity savings3	The product is very good to use and practical to look at	Jingdong prefecture level city in Shandong
Jinfeng Hongyuan Flagship Store	Electricity savings3	Works great, great service, waiting for results.	Jingdong prefecture level city in Shandong
Jinfeng Hongyuan Flagship Store	Electricity savings3	I just used it and I'm not sure about the efficacy yet, I'll come back to evaluate it after a while.	Jingdong prefecture level city in Shandong
Jinfeng Hongyuan Flagship Store	Electricity savings3	Just received the goods, use a period of time to evaluate	Jingdong prefecture level city in Shandong
Jinfeng Hongyuan Flagship Store	Electricity savings3	Opened and tried very good, use it to see if there are results	Jingdong prefecture level city in Shandong
Jinfeng Hongyuan Flagship Store	Electricity savings3	Arrived today! Still trying it out!	Jingdong prefecture level city in Shandong
Jinfeng Hongyuan Flagship Store	Electricity savings3	Shipped quickly, well packaged, already installed, hope there is a power saving effect	Jingdong prefecture level city in Shandong
Jinfeng Hongyuan Flagship Store	Electricity savings3	I got it. It's great.	Jingdong prefecture level city in Shandong
Jinfeng Hongyuan Flagship Store	Electricity savings3	Haven't used it yet, will come back in a month	Jingdong prefecture level city in Shandong
Jinfeng Hongyuan Flagship Store	Electricity savings3	It looks good. It's good. Try it first.	Jingdong prefecture level city in Shandong
Jinfeng Hongyuan Flagship Store	Electricity savings3	It works so well that I have relatives, friends, coworkers, and 6 families that have purchased it for them through me.	Jingdong prefecture level city in Shandong

Jinfeng Hongyuan Flagship Store	Electricity savings3	In use	Jingdong prefecture level city in Shandong
Jinfeng Hongyuan Flagship Store	Electricity savings3	Received the goods today, power-saving effect do not know being used, the boss has a very good attitude, praise!	Jingdong prefecture level city in Shandong
Jinfeng Hongyuan Flagship Store	Electricity savings3	The product is compact and doesn't take up much space, but hopefully it's power efficient.	Jingdong prefecture level city in Shandong
Jinfeng Hongyuan Flagship Store	Electricity savings3	Power saving is indeed effective, I used for a period of time before coming to evaluate, the electricity bill is obviously less, the quality is good, the shopkeeper service is also very good, very patient,	Jingdong prefecture level city in Shandong
Jinfeng Hongyuan Flagship Store	Electricity savings3	It feels fine, I'll comment after I use it	Jingdong prefecture level city in Shandong
Jinfeng Hongyuan Flagship Store	Electricity savings3	I just received it and I don't know the effect yet! I will comment again after I use it and have results!	Jingdong prefecture level city in Shandong
Jinfeng Hongyuan Flagship Store	Electricity savings3	The goods look fine, I hope they really work!	Jingdong prefecture level city in Shandong
Jinfeng Hongyuan Flagship Store	Electricity savings3	I just don't know if it saves power	Jingdong prefecture level city in Shandong
Jinfeng Hongyuan Flagship Store	Electricity savings3	My friend introduced me to use this product, it is professional and I am looking forward to the long term effect~!	Jingdong prefecture level city in Shandong
Jinfeng Hongyuan Flagship Store	Electricity savings3	Try it before you judge.	Jingdong prefecture level city in Shandong
Jinfeng Hongyuan Flagship Store	Electricity savings3	It's great, the power savings are obvious! Kudos!	Jingdong prefecture level city in Shandong
Jinfeng Hongyuan Flagship Store	Electricity savings3	The quality is excellent, stabilizes the voltage and saves electricity, very satisfied.	Jingdong prefecture level city in Shandong
Jinfeng Hongyuan Flagship Store	Electricity savings3	You have to use it for a while to know the effect	Jingdong prefecture level city in Shandong
Jinfeng Hongyuan Flagship Store	Electricity savings3	It's on trial.	Jingdong prefecture level city in Shandong
Jinfeng Hongyuan Flagship Store	Electricity savings3	The stuff works great.	Jingdong prefecture level city in Shandong
Jinfeng Hongyuan Flagship Store	Electricity savings3	Doesn't seem to work, not as god as other reviews	Jingdong prefecture level city in Shandong
Jinfeng Hongyuan Flagship Store	Electricity savings3	It's not good quality, it doesn't work, it stops working after a few days, it doesn't work, the customer service doesn't care, it's a bit irritating.	Jingdong prefecture level city in Shandong
Jinfeng Hongyuan Flagship Store	Electricity savings3	It doesn't do anything.	Jingdong prefecture level city in Shandong
Jinfeng Hongyuan Flagship Store	Electricity savings3	It's good.	Jingdong prefecture level city in Shandong
Jinfeng Hongyuan Flagship Store	Electricity savings3	Used a month effect is not obvious, are psychological effects, feel are not less, did not save to the electricity, there are details of the problem, the store said to buy to send 5 fuse, in fact, received only three, small problems are not committed to in place, I believe that the product is not good enough	Jingdong prefecture level city in Shandong
Jinfeng Hongyuan Flagship Store	Electricity savings3	Well received quickly, looking quite high, but also sent 5 fuses. Customer service is very patient to teach the operation, in fact, quite simple, lazy to read the instructions ha!	Jingdong prefecture level city in Shandong
Jinfeng Hongyuan Flagship Store	Electricity savings3	You have to use it for a while to know the effect	Jingdong prefecture level city in Shandong
Jinfeng Hongyuan Flagship Store	Electricity savings3	Shipped very quickly, packaged very well, the price is particularly affordable, there are so many efficacy is also good, it is worth buying worth buying the product, the workmanship is very fine, better than imagined, recommended that everyone try not yet used, after the use of the follow-up comment	Jingdong prefecture level city in Shandong
Jinfeng Hongyuan Flagship Store	Electricity savings3	It looks regular. It's working now.	Jingdong prefecture level city in Shandong
Jinfeng Hongyuan Flagship Store	Electricity savings3	It arrived pretty fast and the owner is very responsible. Have not used it yet, will see after use	Jingdong prefecture level city in Shandong
Jinfeng Hongyuan Flagship Store	Electricity savings3	🎁 baby quality is very good, the first time I received the goods to open the first time to see, it is very good 👍, very satisfied 😊, customer service also have a question and answer, thank you 🙏	Jingdong prefecture level city in Shandong
Jinfeng Hongyuan Flagship Store	Electricity savings3	I don't know if it's any good 。。。。。。	Jingdong prefecture level city in Shandong
Jinfeng Hongyuan Flagship Store	Electricity savings3	Buy electronic products! Still trust the things of the Jingdong self-owned store! Energy-saving effect to evaluate after heating!	Jingdong prefecture level city in Shandong
Jinfeng Hongyuan Flagship Store	Electricity savings3	The goods have been received, Blu is very beautiful, energy saving effect, to be confirmed only after actual use.	Jingdong prefecture level city in Shandong
Jinfeng Hongyuan Flagship Store	Electricity savings3	Jingdong's speed is so fast, has received, easy to operate. The display is quite good, how many volts what mode is quite understandable.	Jingdong prefecture level city in Shandong
Jinfeng Hongyuan Flagship Store	Electricity savings3	The first use, the effect is good to recommend it to colleagues to use, a month of electricity to save a lot or can be	Jingdong prefecture level city in Shandong
Jinfeng Hongyuan Flagship Store	Electricity savings3	I don't know if it's useful. Mom's is a store. I'll return it if it doesn't work.	Jingdong prefecture level city in Shandong
Jinfeng Hongyuan Flagship Store	Electricity savings3	I don't know if it works or not, I'll wait for two months to use it and then come back to comment!	Jingdong prefecture level city in Shandong
Jinfeng Hongyuan Flagship Store	Electricity savings3	I bought the second one now, and I've been using it for more than half a year, and I feel that it really saves electricity. Now it is summer, the weather is hot, sometimes the fan and air conditioning on all day, if not this power saver, the monthly electricity bill is quite high, but as long as the installation of this power saver, not afraid of its high electricity bill, absolutely let you wantonly use	Jingdong prefecture level city in Shandong
Jinfeng Hongyuan Flagship Store	Electricity savings3	The product is better than I thought it would be, the quality looks great, the voltage is stable, and I'm looking forward to some great power savings!	Jingdong prefecture level city in Shandong
Jinfeng Hongyuan Flagship Store	Electricity savings3	Inadvertently see bought a try, delivery is also quite timely, just do not know how to use, customer service patience to answer, with a pretty good, value for money!	Jingdong prefecture level city in Shandong
Jinfeng Hongyuan Flagship Store	Electricity savings3	It's better than nothing! Hopefully this will help save slightly.	Jingdong prefecture level city in Shandong
Jinfeng Hongyuan Flagship Store	Electricity savings3	Still trying it out, follow up	Jingdong prefecture level city in Shandong
Jinfeng Hongyuan Flagship Store	Electricity savings3	I've just started using it, I don't know how well it works yet, I hope it's good.	Jingdong prefecture level city in Shandong
Jinfeng Hongyuan Flagship Store	Electricity savings3	Fourth purchase, great baby!	Jingdong prefecture level city in Shandong
Jinfeng Hongyuan Flagship Store	Electricity savings3	Shipped really fast, just got it and use it to see what happens and then come back for a review!	Jingdong prefecture level city in Shandong
Jinfeng Hongyuan Flagship Store	Electricity savings3	Timely delivery **** good attitude	Jingdong prefecture level city in Shandong
Jinfeng Hongyuan Flagship Store	Electricity savings3	Home large and small things are purchased in the Jingdong Mall, have been accustomed to shopping in the Jingdong, many times can buy good quality and cheap products, and courier service attitude is particularly good, sometimes too many couriers are to help me to the home, so many years have been in the Jingdong Mall shopping, trust and support! Will also support the Jingdong as always!	Jingdong prefecture level city in Shandong
Jinfeng Hongyuan Flagship Store	Electricity savings3	Modified a pump and the modification worked.	Jingdong prefecture level city in Shandong
Jinfeng Hongyuan Flagship Store	Electricity savings3	Already installed, next time you pay the electric bill to see the results	Jingdong prefecture level city in Shandong
Jinfeng Hongyuan Flagship Store	Electricity savings3	The product is very good to use and practical to look at	Jingdong prefecture level city in Shandong
Jinfeng Hongyuan Flagship Store	Electricity savings3	Works great, great service, waiting for results.	Jingdong prefecture level city in Shandong
Jinfeng Hongyuan Flagship Store	Electricity savings3	I just used it and I'm not sure about the efficacy yet, I'll come back to evaluate it after a while.	Jingdong prefecture level city in Shandong

Jinfeng Hongyuan Flagship Store	Electricity savings3	Just received the goods, use a period of time to evaluate	Jingdong prefecture level city in Shandong
Jinfeng Hongyuan Flagship Store	Electricity savings3	Opened and tried very good, use it to see if there are results	Jingdong prefecture level city in Shandong
Jinfeng Hongyuan Flagship Store	Electricity savings3	Arrived today! Still trying it out!	Jingdong prefecture level city in Shandong
Jinfeng Hongyuan Flagship Store	Electricity savings3	Shipped quickly, well packaged, already installed, hope there is a power saving effect	Jingdong prefecture level city in Shandong
Jinfeng Hongyuan Flagship Store	Electricity savings3	I got it. It's great.	Jingdong prefecture level city in Shandong
Jinfeng Hongyuan Flagship Store	Electricity savings3	Haven't used it yet, will come back in a month	Jingdong prefecture level city in Shandong
Jinfeng Hongyuan Flagship Store	Electricity savings3	It looks good. It's good. Try it first.	Jingdong prefecture level city in Shandong
Jinfeng Hongyuan Flagship Store	Electricity savings3	It works so well that I have relatives, friends, coworkers, and 6 families that have purchased it for them through me.	Jingdong prefecture level city in Shandong
Jinfeng Hongyuan Flagship Store	Electricity savings3	In use	Jingdong prefecture level city in Shandong
Jinfeng Hongyuan Flagship Store	Electricity savings3	Received the goods today, power-saving effect do not know being used, the boss has a very good attitude, praise!	Jingdong prefecture level city in Shandong
Jinfeng Hongyuan Flagship Store	Electricity savings3	The product is compact and doesn't take up much space, but hopefully it's power efficient.	Jingdong prefecture level city in Shandong
Jinfeng Hongyuan Flagship Store	Electricity savings3	Power saving is indeed effective, I used for a period of time before coming to evaluate, the electricity bill is obviously less, the quality is good, the shopkeeper service is also very good, very patient,	Jingdong prefecture level city in Shandong
Jinfeng Hongyuan Flagship Store	Electricity savings3	It feels fine, I'll comment after I use it	Jingdong prefecture level city in Shandong
Jinfeng Hongyuan Flagship Store	Electricity savings3	I just received it and I don't know the effect yet! I will comment again after I use it and have results!	Jingdong prefecture level city in Shandong
Jinfeng Hongyuan Flagship Store	Electricity savings3	The goods look fine, I hope they really work!	Jingdong prefecture level city in Shandong
Jinfeng Hongyuan Flagship Store	Electricity savings3	I just don't know if it saves power	Jingdong prefecture level city in Shandong
Jinfeng Hongyuan Flagship Store	Electricity savings3	My friend introduced me to use this product, it is professional and I am looking forward to the long term effect~!	Jingdong prefecture level city in Shandong
Jinfeng Hongyuan Flagship Store	Electricity savings3	Try it before you judge.	Jingdong prefecture level city in Shandong
Jinfeng Hongyuan Flagship Store	Electricity savings3	It's great, the power savings are obvious! Kudos!	Jingdong prefecture level city in Shandong
Jinfeng Hongyuan Flagship Store	Electricity savings3	The quality is excellent, stabilizes the voltage and saves electricity, very satisfied.	Jingdong prefecture level city in Shandong
Jinfeng Hongyuan Flagship Store	Electricity savings3	You have to use it for a while to know the effect	Jingdong prefecture level city in Shandong
Jinfeng Hongyuan Flagship Store	Electricity savings3	It's on trial.	Jingdong prefecture level city in Shandong
Jinfeng Hongyuan Flagship Store	Electricity savings3	The stuff works great.	Jingdong prefecture level city in Shandong
Jinfeng Hongyuan Flagship Store	Electricity savings3	Doesn't seem to work, not as god as other reviews	Jingdong prefecture level city in Shandong
Jinfeng Hongyuan Flagship Store	Electricity savings3	It's not good quality, it doesn't work, it stops working after a few days, it doesn't work, the customer service doesn't care, it's a bit irritating.	Jingdong prefecture level city in Shandong
Jinfeng Hongyuan Flagship Store	Electricity savings3	It doesn't do anything.	Jingdong prefecture level city in Shandong
Jinfeng Hongyuan Flagship Store	Electricity savings3	It's good.	Jingdong prefecture level city in Shandong
Jinfeng Hongyuan Flagship Store	Electricity savings3	Used a month effect is not obvious, are psychological effects, feel are not less, did not save to the electricity, there are details of the problem, the store said to buy to send 5 fuse, in fact, received only three, small problems are not committed to in place, I believe that the product is not good enough	Jingdong prefecture level city in Shandong
Jinfeng Hongyuan Flagship Store	Electricity savings3	Well received quickly, looking quite high, but also sent 5 fuses. Customer service is very patient to teach the operation, in fact, quite simple, lazy to read the instructions ha!	Jingdong prefecture level city in Shandong
Jinfeng Hongyuan Flagship Store	Electricity savings3	You have to use it for a while to know the effect	Jingdong prefecture level city in Shandong
Jinfeng Hongyuan Flagship Store	Electricity savings3	Shipped very quickly, packaged very well, the price is particularly affordable, there are so many efficacy is also good, it is worth buying worth buying the product, the workmanship is very fine, better than imagined, recommended that everyone try not yet used, after the use of the follow-up comment	Jingdong prefecture level city in Shandong
Jinfeng Hongyuan Flagship Store	Electricity savings3	It looks regular. It's working now.	Jingdong prefecture level city in Shandong
Jinfeng Hongyuan Flagship Store	Electricity savings3	It arrived pretty fast and the owner is very responsible. Have not used it yet, will see after use	Jingdong prefecture level city in Shandong
Jinfeng Hongyuan Flagship Store	Electricity savings3	🎁 baby quality is very good, the first time I received the goods to open the first time to see, it is very good 👍, very satisfied 😊, customer service also have a question and answer, thank you 🙏	Jingdong prefecture level city in Shandong
Jinfeng Hongyuan Flagship Store	Electricity savings3	I don't know if it's any good 。。。。。。	Jingdong prefecture level city in Shandong
Jinfeng Hongyuan Flagship Store	Electricity savings3	Buy electronic products! Still trust the things of the Jingdong self-owned store! Energy-saving effect to evaluate after heating!	Jingdong prefecture level city in Shandong
Jinfeng Hongyuan Flagship Store	Electricity savings3	The goods have been received, Blu is very beautiful, energy saving effect, to be confirmed only after actual use.	Jingdong prefecture level city in Shandong
Jinfeng Hongyuan Flagship Store	Electricity savings3	Jingdong's speed is so fast, has received, easy to operate. The display is quite good, how many volts what mode is quite understandable.	Jingdong prefecture level city in Shandong
Jinfeng Hongyuan Flagship Store	Electricity savings3	The first use, the effect is good to recommend it to colleagues to use, a month of electricity to save a lot or can be	Jingdong prefecture level city in Shandong
Jinfeng Hongyuan Flagship Store	Electricity savings3	I don't know if it's useful. Mom's is a store. I'll return it if it doesn't work.	Jingdong prefecture level city in Shandong
Jinfeng Hongyuan Flagship Store	Electricity savings3	I don't know if it works or not, I'll wait for two months to use it and then come back to comment!	Jingdong prefecture level city in Shandong
Jinfeng Hongyuan Flagship Store	Electricity savings3	I bought the second one now, and I've been using it for more than half a year, and I feel that it really saves electricity. Now it is summer, the weather is hot, sometimes the fan and air conditioning on all day, if not this power saver, the monthly electricity bill is quite high, but as long as the installation of this power saver, not afraid of its high electricity bill, absolutely let you wantonly use	Jingdong prefecture level city in Shandong
Jinfeng Hongyuan Flagship Store	Electricity savings3	The product is better than I thought it would be, the quality looks great, the voltage is stable, and I'm looking forward to some great power savings!	Jingdong prefecture level city in Shandong
Jinfeng Hongyuan Flagship Store	Electricity savings3	Inadvertently see bought a try, delivery is also quite timely, just do not know how to use, customer service patience to answer, with a pretty good, value for money!	Jingdong prefecture level city in Shandong
Jinfeng Hongyuan Flagship Store	Electricity savings3	It's better than nothing! Hopefully this will help save slightly.	Jingdong prefecture level city in Shandong
Jinfeng Hongyuan Flagship Store	Electricity savings3	Still trying it out, follow up	Jingdong prefecture level city in Shandong
Jinfeng Hongyuan Flagship Store	Electricity savings3	I've just started using it, I don't know how well it works yet, I hope it's good.	Jingdong prefecture level city in Shandong
Jinfeng Hongyuan Flagship Store	Electricity savings3	Fourth purchase, great baby!	Jingdong prefecture level city in Shandong
Jinfeng Hongyuan Flagship Store	Electricity savings3	Shipped really fast, just got it and use it to see what happens and then come back for a review!	Jingdong prefecture level city in Shandong

Jinfeng Hongyuan Flagship Store	Electricity savings3	Timely delivery **** good attitude	Jingdong prefecture level city in Shandong
Jinfeng Hongyuan Flagship Store	Electricity savings3	Home large and small things are purchased in the Jingdong Mall, have been accustomed to shopping in the Jingdong, many times can buy good quality and cheap products, and courier service attitude is particularly good, sometimes too many couriers are to help me to the home, so many years have been in the Jingdong Mall shopping, trust and support! Will also support the Jingdong as always!	Jingdong prefecture level city in Shandong
Jinfeng Hongyuan Flagship Store	Electricity savings3	Modified a pump and the modification worked.	Jingdong prefecture level city in Shandong
Jinfeng Hongyuan Flagship Store	Electricity savings3	Already installed, next time you pay the electric bill to see the results	Jingdong prefecture level city in Shandong
Jinfeng Hongyuan Flagship Store	Electricity savings3	The product is very good to use and practical to look at	Jingdong prefecture level city in Shandong
Jinfeng Hongyuan Flagship Store	Electricity savings3	Works great, great service, waiting for results.	Jingdong prefecture level city in Shandong
Jinfeng Hongyuan Flagship Store	Electricity savings3	I just used it and I'm not sure about the efficacy yet, I'll come back to evaluate it after a while.	Jingdong prefecture level city in Shandong
Jinfeng Hongyuan Flagship Store	Electricity savings3	Just received the goods, use a period of time to evaluate	Jingdong prefecture level city in Shandong
Jinfeng Hongyuan Flagship Store	Electricity savings3	Opened and tried very good, use it to see if there are results	Jingdong prefecture level city in Shandong
Jinfeng Hongyuan Flagship Store	Electricity savings3	Arrived today! Still trying it out!	Jingdong prefecture level city in Shandong
Jinfeng Hongyuan Flagship Store	Electricity savings3	Shipped quickly, well packaged, already installed, hope there is a power saving effect	Jingdong prefecture level city in Shandong
Jinfeng Hongyuan Flagship Store	Electricity savings3	I got it. It's great.	Jingdong prefecture level city in Shandong
Jinfeng Hongyuan Flagship Store	Electricity savings3	Haven't used it yet, will come back in a month	Jingdong prefecture level city in Shandong
Jinfeng Hongyuan Flagship Store	Electricity savings3	It looks good. It's good. Try it first.	Jingdong prefecture level city in Shandong
Jinfeng Hongyuan Flagship Store	Electricity savings3	It works so well that I have relatives, friends, coworkers, and 6 families that have purchased it for them through me.	Jingdong prefecture level city in Shandong
Jinfeng Hongyuan Flagship Store	Electricity savings3	In use	Jingdong prefecture level city in Shandong
Jinfeng Hongyuan Flagship Store	Electricity savings3	Received the goods today, power-saving effect do not know being used, the boss has a very good attitude, praise!	Jingdong prefecture level city in Shandong
Jinfeng Hongyuan Flagship Store	Electricity savings3	The product is compact and doesn't take up much space, but hopefully it's power efficient.	Jingdong prefecture level city in Shandong
Jinfeng Hongyuan Flagship Store	Electricity savings3	Power saving is indeed effective, I used for a period of time before coming to evaluate, the electricity bill is obviously less, the quality is good, the shopkeeper service is also very good, very patient,	Jingdong prefecture level city in Shandong
Jinfeng Hongyuan Flagship Store	Electricity savings3	It feels fine, I'll comment after I use it	Jingdong prefecture level city in Shandong
Jinfeng Hongyuan Flagship Store	Electricity savings3	I just received it and I don't know the effect yet! I will comment again after I use it and have results!	Jingdong prefecture level city in Shandong
Jinfeng Hongyuan Flagship Store	Electricity savings3	The goods look fine, I hope they really work!	Jingdong prefecture level city in Shandong
Jinfeng Hongyuan Flagship Store	Electricity savings3	I just don't know if it saves power	Jingdong prefecture level city in Shandong
Jinfeng Hongyuan Flagship Store	Electricity savings3	My friend introduced me to use this product, it is professional and I am looking forward to the long term effect~!	Jingdong prefecture level city in Shandong
Jinfeng Hongyuan Flagship Store	Electricity savings3	Try it before you judge.	Jingdong prefecture level city in Shandong
Jinfeng Hongyuan Flagship Store	Electricity savings3	It's great, the power savings are obvious! Kudos!	Jingdong prefecture level city in Shandong
Jinfeng Hongyuan Flagship Store	Electricity savings3	The quality is excellent, stabilizes the voltage and saves electricity, very satisfied.	Jingdong prefecture level city in Shandong
Jinfeng Hongyuan Flagship Store	Electricity savings3	You have to use it for a while to know the effect	Jingdong prefecture level city in Shandong
Jinfeng Hongyuan Flagship Store	Electricity savings3	It's on trial.	Jingdong prefecture level city in Shandong
Jinfeng Hongyuan Flagship Store	Electricity savings3	The stuff works great.	Jingdong prefecture level city in Shandong
Jinfeng Hongyuan Flagship Store	Electricity savings3	Doesn't seem to work, not as god as other reviews	Jingdong prefecture level city in Shandong
Jinfeng Hongyuan Flagship Store	Electricity savings3	It's not good quality, it doesn't work, it stops working after a few days, it doesn't work, the customer service doesn't care, it's a bit irritating.	Jingdong prefecture level city in Shandong
Jinfeng Hongyuan Flagship Store	Electricity savings3	It doesn't do anything.	Jingdong prefecture level city in Shandong
Jinfeng Hongyuan Flagship Store	Electricity savings3	It's good.	Jingdong prefecture level city in Shandong
Jinfeng Hongyuan Flagship Store	Electricity savings3	Used a month effect is not obvious, are psychological effects, feel are not less, did not save to the electricity, there are details of the problem, the store said to buy to send 5 fuse, in fact, received only three, small problems are not committed to in place, I believe that the product is not good enough	Jingdong prefecture level city in Shandong
Jinfeng Hongyuan Flagship Store	Electricity savings3	Well received quickly, looking quite high, but also sent 5 fuses. Customer service is very patient to teach the operation, in fact, quite simple, lazy to read the instructions ha!	Jingdong prefecture level city in Shandong
Jinfeng Hongyuan Flagship Store	Electricity savings3	You have to use it for a while to know the effect	Jingdong prefecture level city in Shandong
Jinfeng Hongyuan Flagship Store	Electricity savings3	Shipped very quickly, packaged very well, the price is particularly affordable, there are so many efficacy is also good, it is worth buying worth buying the product, the workmanship is very fine, better than imagined, recommended that everyone try not yet used, after the use of the follow-up comment	Jingdong prefecture level city in Shandong
Jinfeng Hongyuan Flagship Store	Electricity savings3	It looks regular. It's working now.	Jingdong prefecture level city in Shandong
Jinfeng Hongyuan Flagship Store	Electricity savings3	It arrived pretty fast and the owner is very responsible. Have not used it yet, will see after use	Jingdong prefecture level city in Shandong
Jinfeng Hongyuan Flagship Store	Electricity savings3	🎁 baby quality is very good, the first time I received the goods to open the first time to see, it is very good 👍, very satisfied 😊, customer service also have a question and answer, thank you 🙏	Jingdong prefecture level city in Shandong
Jinfeng Hongyuan Flagship Store	Electricity savings3	I don't know if it's any good 。。。。。。	Jingdong prefecture level city in Shandong
Jinfeng Hongyuan Flagship Store	Electricity savings3	Buy electronic products! Still trust the things of the Jingdong self-owned store! Energy-saving effect to evaluate after heating!	Jingdong prefecture level city in Shandong
Jinfeng Hongyuan Flagship Store	Electricity savings3	The goods have been received, Blu is very beautiful, energy saving effect, to be confirmed only after actual use.	Jingdong prefecture level city in Shandong
Jinfeng Hongyuan Flagship Store	Electricity savings3	Jingdong's speed is so fast, has received, easy to operate. The display is quite good, how many volts what mode is quite understandable.	Jingdong prefecture level city in Shandong
Jinfeng Hongyuan Flagship Store	Electricity savings3	The first use, the effect is good to recommend it to colleagues to use, a month of electricity to save a lot or can be	Jingdong prefecture level city in Shandong
Jinfeng Hongyuan Flagship Store	Electricity savings3	I don't know if it's useful. Mom's is a store. I'll return it if it doesn't work.	Jingdong prefecture level city in Shandong
Jinfeng Hongyuan Flagship Store	Electricity savings3	I don't know if it works or not, I'll wait for two months to use it and then come back to comment!	Jingdong prefecture level city in Shandong

Jinfeng Hongyuan Flagship Store	Electricity savings3	I bought the second one now, and I've been using it for more than half a year, and I feel that it really saves electricity. Now it is summer, the weather is hot, sometimes the fan and air conditioning on all day, if not this power saver, the monthly electricity bill is quite high, but as long as the installation of this power saver, not afraid of its high electricity bill, absolutely let you wantonly use	Jingdong prefecture level city in Shandong
Jinfeng Hongyuan Flagship Store	Electricity savings3	The product is better than I thought it would be, the quality looks great, the voltage is stable, and I'm looking forward to some great power savings!	Jingdong prefecture level city in Shandong
Jinfeng Hongyuan Flagship Store	Electricity savings3	Inadvertently see bought a try, delivery is also quite timely, just do not know how to use, customer service patience to answer, with a pretty good, value for money!	Jingdong prefecture level city in Shandong
Jinfeng Hongyuan Flagship Store	Electricity savings3	It's better than nothing! Hopefully this will help save slightly.	Jingdong prefecture level city in Shandong
Jinfeng Hongyuan Flagship Store	Electricity savings3	Still trying it out, follow up	Jingdong prefecture level city in Shandong
Jinfeng Hongyuan Flagship Store	Electricity savings3	I've just started using it, I don't know how well it works yet, I hope it's good.	Jingdong prefecture level city in Shandong
Jinfeng Hongyuan Flagship Store	Electricity savings3	Fourth purchase, great baby!	Jingdong prefecture level city in Shandong
Jinfeng Hongyuan Flagship Store	Electricity savings3	Shipped really fast, just got it and use it to see what happens and then come back for a review!	Jingdong prefecture level city in Shandong
Jinfeng Hongyuan Flagship Store	Electricity savings3	Timely delivery **** good attitude	Jingdong prefecture level city in Shandong
Jinfeng Hongyuan Flagship Store	Electricity savings3	Home large and small things are purchased in the Jingdong Mall, have been accustomed to shopping in the Jingdong, many times can buy good quality and cheap products, and courier service attitude is particularly good, sometimes too many couriers are to help me to the home, so many years have been in the Jingdong Mall shopping, trust and support! Will also support the Jingdong as always!	Jingdong prefecture level city in Shandong
Jinfeng Hongyuan Flagship Store	Electricity savings3	Modified a pump and the modification worked.	Jingdong prefecture level city in Shandong
Jinfeng Hongyuan Flagship Store	Electricity savings3	Already installed, next time you pay the electric bill to see the results	Jingdong prefecture level city in Shandong
Jinfeng Hongyuan Flagship Store	Electricity savings3	The product is very good to use and practical to look at	Jingdong prefecture level city in Shandong
Jinfeng Hongyuan Flagship Store	Electricity savings3	Works great, great service, waiting for results.	Jingdong prefecture level city in Shandong
Jinfeng Hongyuan Flagship Store	Electricity savings3	I just used it and I'm not sure about the efficacy yet, I'll come back to evaluate it after a while.	Jingdong prefecture level city in Shandong
Jinfeng Hongyuan Flagship Store	Electricity savings3	Just received the goods, use a period of time to evaluate	Jingdong prefecture level city in Shandong
Jinfeng Hongyuan Flagship Store	Electricity savings3	Opened and tried very good, use it to see if there are results	Jingdong prefecture level city in Shandong
Jinfeng Hongyuan Flagship Store	Electricity savings3	Arrived today! Still trying it out!	Jingdong prefecture level city in Shandong
Jinfeng Hongyuan Flagship Store	Electricity savings3	Shipped quickly, well packaged, already installed, hope there is a power saving effect	Jingdong prefecture level city in Shandong
Jinfeng Hongyuan Flagship Store	Electricity savings3	I got it. It's great.	Jingdong prefecture level city in Shandong
Jinfeng Hongyuan Flagship Store	Electricity savings3	Haven't used it yet, will come back in a month	Jingdong prefecture level city in Shandong
Jinfeng Hongyuan Flagship Store	Electricity savings3	It looks good. It's good. Try it first.	Jingdong prefecture level city in Shandong
Jinfeng Hongyuan Flagship Store	Electricity savings3	It works so well that I have relatives, friends, coworkers, and 6 families that have purchased it for them through me.	Jingdong prefecture level city in Shandong
Jinfeng Hongyuan Flagship Store	Electricity savings3	In use	Jingdong prefecture level city in Shandong
Jinfeng Hongyuan Flagship Store	Electricity savings3	Received the goods today, power-saving effect do not know being used, the boss has a very good attitude, praise!	Jingdong prefecture level city in Shandong
Jinfeng Hongyuan Flagship Store	Electricity savings3	The product is compact and doesn't take up much space, but hopefully it's power efficient.	Jingdong prefecture level city in Shandong
Jinfeng Hongyuan Flagship Store	Electricity savings3	Power saving is indeed effective, I used for a period of time before coming to evaluate, the electricity bill is obviously less, the quality is good, the shopkeeper service is also very good, very patient,	Jingdong prefecture level city in Shandong
Jinfeng Hongyuan Flagship Store	Electricity savings3	It feels fine, I'll comment after I use it	Jingdong prefecture level city in Shandong
Jinfeng Hongyuan Flagship Store	Electricity savings3	I just received it and I don't know the effect yet! I will comment again after I use it and have results!	Jingdong prefecture level city in Shandong
Jinfeng Hongyuan Flagship Store	Electricity savings3	The goods look fine, I hope they really work!	Jingdong prefecture level city in Shandong
Jinfeng Hongyuan Flagship Store	Electricity savings3	I just don't know if it saves power	Jingdong prefecture level city in Shandong
Jinfeng Hongyuan Flagship Store	Electricity savings3	My friend introduced me to use this product, it is professional and I am looking forward to the long term effect~!	Jingdong prefecture level city in Shandong
Jinfeng Hongyuan Flagship Store	Electricity savings3	Try it before you judge.	Jingdong prefecture level city in Shandong
Jinfeng Hongyuan Flagship Store	Electricity savings3	It's great, the power savings are obvious! Kudos!	Jingdong prefecture level city in Shandong
Jinfeng Hongyuan Flagship Store	Electricity savings3	The quality is excellent, stabilizes the voltage and saves electricity, very satisfied.	Jingdong prefecture level city in Shandong
Jinfeng Hongyuan Flagship Store	Electricity savings3	You have to use it for a while to know the effect	Jingdong prefecture level city in Shandong
Jinfeng Hongyuan Flagship Store	Electricity savings3	It's on trial.	Jingdong prefecture level city in Shandong
Jinfeng Hongyuan Flagship Store	Electricity savings3	The stuff works great.	Jingdong prefecture level city in Shandong
Jinfeng Hongyuan Flagship Store	Electricity savings3	Doesn't seem to work, not as god as other reviews	Jingdong prefecture level city in Shandong
Jinfeng Hongyuan Flagship Store	Electricity savings3	It's not good quality, it doesn't work, it stops working after a few days, it doesn't work, the customer service doesn't care, it's a bit irritating.	Jingdong prefecture level city in Shandong
Jinfeng Hongyuan Flagship Store	Electricity savings3	It doesn't do anything.	Jingdong prefecture level city in Shandong
Jinfeng Hongyuan Flagship Store	Electricity savings3	It's good.	Jingdong prefecture level city in Shandong
Jinfeng Hongyuan Flagship Store	Electricity savings3	Used a month effect is not obvious, are psychological effects, feel are not less, did not save to the electricity, there are details of the problem, the store said to buy to send 5 fuse, in fact, received only three, small problems are not committed to in place, I believe that the product is not good enough	Jingdong prefecture level city in Shandong
Jinfeng Hongyuan Flagship Store	Electricity savings3	Well received quickly, looking quite high, but also sent 5 fuses. Customer service is very patient to teach the operation, in fact, quite simple, lazy to read the instructions ha!	Jingdong prefecture level city in Shandong
Jinfeng Hongyuan Flagship Store	Electricity savings3	You have to use it for a while to know the effect	Jingdong prefecture level city in Shandong
Jinfeng Hongyuan Flagship Store	Electricity savings3	Shipped very quickly, packaged very well, the price is particularly affordable, there are so many efficacy is also good, it is worth buying worth buying the product, the workmanship is very fine, better than imagined, recommended that everyone try not yet used, after the use of the follow-up comment	Jingdong prefecture level city in Shandong
Jinfeng Hongyuan Flagship Store	Electricity savings3	It looks regular. It's working now.	Jingdong prefecture level city in Shandong
Jinfeng Hongyuan Flagship Store	Electricity savings3	It arrived pretty fast and the owner is very responsible. Have not used it yet, will see after use	Jingdong prefecture level city in Shandong

Jinfeng Hongyuan Flagship Store	Electricity savings3	🎁 baby quality is very good, the first time I received the goods to open the first time to see, it is very good 👍, very satisfied 😀, customer service also have a question and answer, thank you 🙏	Jingdong prefecture level city in Shandong
Jinfeng Hongyuan Flagship Store	Electricity savings3	I don't know if it's any good 。。。。。。	Jingdong prefecture level city in Shandong
Jinfeng Hongyuan Flagship Store	Electricity savings3	Buy electronic products! Still trust the things of the Jingdong self-owned store! Energy-saving effect to evaluate after heating!	Jingdong prefecture level city in Shandong
Jinfeng Hongyuan Flagship Store	Electricity savings3	The goods have been received, Blu is very beautiful, energy saving effect, to be confirmed only after actual use.	Jingdong prefecture level city in Shandong
Jinfeng Hongyuan Flagship Store	Electricity savings3	Jingdong's speed is so fast, has received, easy to operate. The display is quite good, how many volts what mode is quite understandable.	Jingdong prefecture level city in Shandong
Jinfeng Hongyuan Flagship Store	Electricity savings3	The first use, the effect is good to recommend it to colleagues to use, a month of electricity to save a lot or can be	Jingdong prefecture level city in Shandong
Jinfeng Hongyuan Flagship Store	Electricity savings3	I don't know if it's useful. Mom's is a store. I'll return it if it doesn't work.	Jingdong prefecture level city in Shandong
Jinfeng Hongyuan Flagship Store	Electricity savings3	I don't know if it works or not, I'll wait for two months to use it and then come back to comment!	Jingdong prefecture level city in Shandong
Jinfeng Hongyuan Flagship Store	Electricity savings3	I bought the second one now, and I've been using it for more than half a year, and I feel that it really saves electricity. Now it is summer, the weather is hot, sometimes the fan and air conditioning on all day, if not this power saver, the monthly electricity bill is quite high, but as long as the installation of this power saver, not afraid of its high electricity bill, absolutely let you wantonly use	Jingdong prefecture level city in Shandong
Jinfeng Hongyuan Flagship Store	Electricity savings3	The product is better than I thought it would be, the quality looks great, the voltage is stable, and I'm looking forward to some great power savings!	Jingdong prefecture level city in Shandong
Jinfeng Hongyuan Flagship Store	Electricity savings3	Inadvertently see bought a try, delivery is also quite timely, just do not know how to use, customer service patience to answer, with a pretty good, value for money!	Jingdong prefecture level city in Shandong
Jinfeng Hongyuan Flagship Store	Electricity savings3	It's better than nothing! Hopefully this will help save slightly.	Jingdong prefecture level city in Shandong
Jinfeng Hongyuan Flagship Store	Electricity savings3	Still trying it out, follow up	Jingdong prefecture level city in Shandong
Jinfeng Hongyuan Flagship Store	Electricity savings3	I've just started using it, I don't know how well it works yet, I hope it's good.	Jingdong prefecture level city in Shandong
Jinfeng Hongyuan Flagship Store	Electricity savings3	Fourth purchase, great baby!	Jingdong prefecture level city in Shandong
Jinfeng Hongyuan Flagship Store	Electricity savings3	Shipped really fast, just got it and use it to see what happens and then come back for a review!	Jingdong prefecture level city in Shandong
Jinfeng Hongyuan Flagship Store	Electricity savings3	Timely delivery **** good attitude	Jingdong prefecture level city in Shandong
Jinfeng Hongyuan Flagship Store	Electricity savings3	Home large and small things are purchased in the Jingdong Mall, have been accustomed to shopping in the Jingdong, many times can buy good quality and cheap products, and courier service attitude is particularly good, sometimes too many couriers are to help me to the home, so many years have been in the Jingdong Mall shopping, trust and support! Will also support the Jingdong as always!	Jingdong prefecture level city in Shandong
Jinfeng Hongyuan Flagship Store	Electricity savings3	Modified a pump and the modification worked.	Jingdong prefecture level city in Shandong
Jinfeng Hongyuan Flagship Store	Electricity savings3	Already installed, next time you pay the electric bill to see the results	Jingdong prefecture level city in Shandong
Jinfeng Hongyuan Flagship Store	Electricity savings3	The product is very good to use and practical to look at	Jingdong prefecture level city in Shandong
Jinfeng Hongyuan Flagship Store	Electricity savings3	Works great, great service, waiting for results.	Jingdong prefecture level city in Shandong
Jinfeng Hongyuan Flagship Store	Electricity savings3	I just used it and I'm not sure about the efficacy yet, I'll come back to evaluate it after a while.	Jingdong prefecture level city in Shandong
Jinfeng Hongyuan Flagship Store	Electricity savings3	Just received the goods, use a period of time to evaluate	Jingdong prefecture level city in Shandong
Jinfeng Hongyuan Flagship Store	Electricity savings3	Opened and tried very good, use it to see if there are results	Jingdong prefecture level city in Shandong
Jinfeng Hongyuan Flagship Store	Electricity savings3	Arrived today! Still trying it out!	Jingdong prefecture level city in Shandong
Jinfeng Hongyuan Flagship Store	Electricity savings3	Shipped quickly, well packaged, already installed, hope there is a power saving effect	Jingdong prefecture level city in Shandong
Jinfeng Hongyuan Flagship Store	Electricity savings3	I got it. It's great.	Jingdong prefecture level city in Shandong
Jinfeng Hongyuan Flagship Store	Electricity savings3	Haven't used it yet, will come back in a month	Jingdong prefecture level city in Shandong
Jinfeng Hongyuan Flagship Store	Electricity savings3	It looks good. It's good. Try it first.	Jingdong prefecture level city in Shandong
Jinfeng Hongyuan Flagship Store	Electricity savings3	It works so well that I have relatives, friends, coworkers, and 6 families that have purchased it for them through me.	Jingdong prefecture level city in Shandong
Jinfeng Hongyuan Flagship Store	Electricity savings3	In use	Jingdong prefecture level city in Shandong
Jinfeng Hongyuan Flagship Store	Electricity savings3	Received the goods today, power-saving effect do not know being used, the boss has a very good attitude, praise!	Jingdong prefecture level city in Shandong
Jinfeng Hongyuan Flagship Store	Electricity savings3	The product is compact and doesn't take up much space, but hopefully it's power efficient.	Jingdong prefecture level city in Shandong
Jinfeng Hongyuan Flagship Store	Electricity savings3	Power saving is indeed effective, I used for a period of time before coming to evaluate, the electricity bill is obviously less, the quality is good, the shopkeeper service is also very good, very patient,	Jingdong prefecture level city in Shandong
Jinfeng Hongyuan Flagship Store	Electricity savings3	It feels fine, I'll comment after I use it	Jingdong prefecture level city in Shandong
Jinfeng Hongyuan Flagship Store	Electricity savings3	I just received it and I don't know the effect yet! I will comment again after I use it and have results!	Jingdong prefecture level city in Shandong
Jinfeng Hongyuan Flagship Store	Electricity savings3	The goods look fine, I hope they really work!	Jingdong prefecture level city in Shandong
Jinfeng Hongyuan Flagship Store	Electricity savings3	I just don't know if it saves power	Jingdong prefecture level city in Shandong
Jinfeng Hongyuan Flagship Store	Electricity savings3	My friend introduced me to use this product, it is professional and I am looking forward to the long term effect~!	Jingdong prefecture level city in Shandong
Jinfeng Hongyuan Flagship Store	Electricity savings3	Try it before you judge.	Jingdong prefecture level city in Shandong
Jinfeng Hongyuan Flagship Store	Electricity savings3	It's great, the power savings are obvious! Kudos!	Jingdong prefecture level city in Shandong
Jinfeng Hongyuan Flagship Store	Electricity savings3	The quality is excellent, stabilizes the voltage and saves electricity, very satisfied.	Jingdong prefecture level city in Shandong
Jinfeng Hongyuan Flagship Store	Electricity savings3	You have to use it for a while to know the effect	Jingdong prefecture level city in Shandong
Jinfeng Hongyuan Flagship Store	Electricity savings3	It's on trial.	Jingdong prefecture level city in Shandong
Jinfeng Hongyuan Flagship Store	Electricity savings3	The stuff works great.	Jingdong prefecture level city in Shandong
Jinfeng Hongyuan Flagship Store	Electricity savings3	Doesn't seem to work, not as god as other reviews	Jingdong prefecture level city in Shandong
Jinfeng Hongyuan Flagship Store	Electricity savings3	It's not good quality, it doesn't work, it stops working after a few days, it doesn't work, the customer service doesn't care, it's a bit irritating.	Jingdong prefecture level city in Shandong
Jinfeng Hongyuan Flagship Store	Electricity savings3	It doesn't do anything.	Jingdong prefecture level city in Shandong

Jinfeng Hongyuan Flagship Store	Electricity savings3	It's good.	Jingdong prefecture level city in Shandong
Jinfeng Hongyuan Flagship Store	Electricity savings3	Used a month effect is not obvious, are psychological effects, feel are not less, did not save to the electricity, there are details of the problem, the store said to buy to send 5 fuse, in fact, received only three, small problems are not committed to in place, I believe that the product is not good enough	Jingdong prefecture level city in Shandong
Jinfeng Hongyuan Flagship Store	Electricity savings3	Well received quickly, looking quite high, but also sent 5 fuses. Customer service is very patient to teach the operation, in fact, quite simple, lazy to read the instructions ha!	Jingdong prefecture level city in Shandong
Jinfeng Hongyuan Flagship Store	Electricity savings3	You have to use it for a while to know the effect	Jingdong prefecture level city in Shandong
Jinfeng Hongyuan Flagship Store	Electricity savings3	Shipped very quickly, packaged very well, the price is particularly affordable, there are so many efficacy is also good, it is worth buying worth buying the product, the workmanship is very fine, better than imagined, recommended that everyone try not yet used, after the use of the follow-up comment	Jingdong prefecture level city in Shandong
Jinfeng Hongyuan Flagship Store	Electricity savings3	It looks regular. It's working now.	Jingdong prefecture level city in Shandong
Jinfeng Hongyuan Flagship Store	Electricity savings3	It arrived pretty fast and the owner is very responsible. Have not used it yet, will see after use	Jingdong prefecture level city in Shandong
Jinfeng Hongyuan Flagship Store	Electricity savings3	🎁 baby quality is very good, the first time I received the goods to open the first time to see, it is very good 👍, very satisfied 😊, customer service also have a question and answer, thank you 🙏	Jingdong prefecture level city in Shandong
Jinfeng Hongyuan Flagship Store	Electricity savings3	I don't know if it's any good 。。。。。。	Jingdong prefecture level city in Shandong
Jinfeng Hongyuan Flagship Store	Electricity savings3	Buy electronic products! Still trust the things of the Jingdong self-owned store! Energy-saving effect to evaluate after heating!	Jingdong prefecture level city in Shandong
Jinfeng Hongyuan Flagship Store	Electricity savings3	The goods have been received, Blu is very beautiful, energy saving effect, to be confirmed only after actual use.	Jingdong prefecture level city in Shandong
Jinfeng Hongyuan Flagship Store	Electricity savings3	Jingdong's speed is so fast, has received, easy to operate. The display is quite good, how many volts what mode is quite understandable.	Jingdong prefecture level city in Shandong
Jinfeng Hongyuan Flagship Store	Electricity savings3	The first use, the effect is good to recommend it to colleagues to use, a month of electricity to save a lot or can be	Jingdong prefecture level city in Shandong
Jinfeng Hongyuan Flagship Store	Electricity savings3	I don't know if it's useful. Mom's is a store. I'll return it if it doesn't work.	Jingdong prefecture level city in Shandong
Jinfeng Hongyuan Flagship Store	Electricity savings3	I don't know if it works or not, I'll wait for two months to use it and then come back to comment!	Jingdong prefecture level city in Shandong
Jinfeng Hongyuan Flagship Store	Electricity savings3	I bought the second one now, and I've been using it for more than half a year, and I feel that it really saves electricity. Now it is summer, the weather is hot, sometimes the fan and air conditioning on all day, if not this power saver, the monthly electricity bill is quite high, but as long as the installation of this power saver, not afraid of its high electricity bill, absolutely let you wantonly use	Jingdong prefecture level city in Shandong
Jinfeng Hongyuan Flagship Store	Electricity savings3	The product is better than I thought it would be, the quality looks great, the voltage is stable, and I'm looking forward to some great power savings!	Jingdong prefecture level city in Shandong
Jinfeng Hongyuan Flagship Store	Electricity savings3	Inadvertently see bought a try, delivery is also quite timely, just do not know how to use, customer service patience to answer, with a pretty good, value for money!	Jingdong prefecture level city in Shandong
Jinfeng Hongyuan Flagship Store	Electricity savings3	It's better than nothing! Hopefully this will help save slightly.	Jingdong prefecture level city in Shandong
Jinfeng Hongyuan Flagship Store	Electricity savings3	Still trying it out, follow up	Jingdong prefecture level city in Shandong
Jinfeng Hongyuan Flagship Store	Electricity savings3	I've just started using it, I don't know how well it works yet, I hope it's good.	Jingdong prefecture level city in Shandong
Jinfeng Hongyuan Flagship Store	Electricity savings3	Fourth purchase, great baby!	Jingdong prefecture level city in Shandong
Jinfeng Hongyuan Flagship Store	Electricity savings3	Shipped really fast, just got it and use it to see what happens and then come back for a review!	Jingdong prefecture level city in Shandong
Jinfeng Hongyuan Flagship Store	Electricity savings3	Timely delivery **** good attitude	Jingdong prefecture level city in Shandong
Jinfeng Hongyuan Flagship Store	Electricity savings3	Home large and small things are purchased in the Jingdong Mall, have been accustomed to shopping in the Jingdong, many times can buy good quality and cheap products, and courier service attitude is particularly good, sometimes too many couriers are to help me to the home, so many years have been in the Jingdong Mall shopping, trust and support! Will also support the Jingdong as always!	Jingdong prefecture level city in Shandong
Jinfeng Hongyuan Flagship Store	Electricity savings3	Modified a pump and the modification worked.	Jingdong prefecture level city in Shandong
Jinfeng Hongyuan Flagship Store	Electricity savings3	Already installed, next time you pay the electric bill to see the results	Jingdong prefecture level city in Shandong
Jinfeng Hongyuan Flagship Store	Electricity savings3	The product is very good to use and practical to look at	Jingdong prefecture level city in Shandong
Jinfeng Hongyuan Flagship Store	Electricity savings3	Works great, great service, waiting for results.	Jingdong prefecture level city in Shandong
Jinfeng Hongyuan Flagship Store	Electricity savings3	I just used it and I'm not sure about the efficacy yet, I'll come back to evaluate it after a while.	Jingdong prefecture level city in Shandong
Jinfeng Hongyuan Flagship Store	Electricity savings3	Just received the goods, use a period of time to evaluate	Jingdong prefecture level city in Shandong
Jinfeng Hongyuan Flagship Store	Electricity savings3	Opened and tried very good, use it to see if there are results	Jingdong prefecture level city in Shandong
Jinfeng Hongyuan Flagship Store	Electricity savings3	Arrived today! Still trying it out!	Jingdong prefecture level city in Shandong
Jinfeng Hongyuan Flagship Store	Electricity savings3	Shipped quickly, well packaged, already installed, hope there is a power saving effect	Jingdong prefecture level city in Shandong
Jinfeng Hongyuan Flagship Store	Electricity savings3	I got it. It's great.	Jingdong prefecture level city in Shandong
Jinfeng Hongyuan Flagship Store	Electricity savings3	Haven't used it yet, will come back in a month	Jingdong prefecture level city in Shandong
Jinfeng Hongyuan Flagship Store	Electricity savings3	It looks good. It's good. Try it first.	Jingdong prefecture level city in Shandong
Jinfeng Hongyuan Flagship Store	Electricity savings3	It works so well that I have relatives, friends, coworkers, and 6 families that have purchased it for them through me.	Jingdong prefecture level city in Shandong
Jinfeng Hongyuan Flagship Store	Electricity savings3	In use	Jingdong prefecture level city in Shandong
Jinfeng Hongyuan Flagship Store	Electricity savings3	Received the goods today, power-saving effect do not know being used, the boss has a very good attitude, praise!	Jingdong prefecture level city in Shandong
Jinfeng Hongyuan Flagship Store	Electricity savings3	The product is compact and doesn't take up much space, but hopefully it's power efficient.	Jingdong prefecture level city in Shandong
Jinfeng Hongyuan Flagship Store	Electricity savings3	Power saving is indeed effective, I used for a period of time before coming to evaluate, the electricity bill is obviously less, the quality is good, the shopkeeper service is also very good, very patient,	Jingdong prefecture level city in Shandong
Jinfeng Hongyuan Flagship Store	Electricity savings3	It feels fine, I'll comment after I use it	Jingdong prefecture level city in Shandong
Jinfeng Hongyuan Flagship Store	Electricity savings3	I just received it and I don't know the effect yet! I will comment again after I use it and have results!	Jingdong prefecture level city in Shandong
Jinfeng Hongyuan Flagship Store	Electricity savings3	The goods look fine, I hope they really work!	Jingdong prefecture level city in Shandong
Jinfeng Hongyuan Flagship Store	Electricity savings3	I just don't know if it saves power	Jingdong prefecture level city in Shandong
Jinfeng Hongyuan Flagship Store	Electricity savings3	My friend introduced me to use this product, it is professional and I am looking forward to the long term effect~!	Jingdong prefecture level city in Shandong
Jinfeng Hongyuan Flagship Store	Electricity savings3	Try it before you judge.	Jingdong prefecture level city in Shandong

Jinfeng Hongyuan Flagship Store	Electricity savings3	It's great, the power savings are obvious! Kudos!	Jingdong prefecture level city in Shandong
Jinfeng Hongyuan Flagship Store	Electricity savings3	The quality is excellent, stabilizes the voltage and saves electricity, very satisfied.	Jingdong prefecture level city in Shandong
Jinfeng Hongyuan Flagship Store	Electricity savings3	You have to use it for a while to know the effect	Jingdong prefecture level city in Shandong
Jinfeng Hongyuan Flagship Store	Electricity savings3	It's on trial.	Jingdong prefecture level city in Shandong
Jinfeng Hongyuan Flagship Store	Electricity savings3	The stuff works great.	Jingdong prefecture level city in Shandong
Jinfeng Hongyuan Flagship Store	Electricity savings3	Doesn't seem to work, not as god as other reviews	Jingdong prefecture level city in Shandong
Jinfeng Hongyuan Flagship Store	Electricity savings3	It's not good quality, it doesn't work, it stops working after a few days, it doesn't work, the customer service doesn't care, it's a bit irritating.	Jingdong prefecture level city in Shandong
Jinfeng Hongyuan Flagship Store	Electricity savings3	It doesn't do anything.	Jingdong prefecture level city in Shandong
Jinfeng Hongyuan Flagship Store	Electricity savings3	It's good.	Jingdong prefecture level city in Shandong
Jinfeng Hongyuan Flagship Store	Electricity savings3	Used a month effect is not obvious, are psychological effects, feel are not less, did not save to the electricity, there are details of the problem, the store said to buy to send 5 fuse, in fact, received only three, small problems are not committed to in place, I believe that the product is not good enough	Jingdong prefecture level city in Shandong
Jinfeng Hongyuan Flagship Store	Electricity savings3	Well received quickly, looking quite high, but also sent 5 fuses. Customer service is very patient to teach the operation, in fact, quite simple, lazy to read the instructions ha!	Jingdong prefecture level city in Shandong
Jinfeng Hongyuan Flagship Store	Electricity savings3	You have to use it for a while to know the effect	Jingdong prefecture level city in Shandong
Jinfeng Hongyuan Flagship Store	Electricity savings3	Shipped very quickly, packaged very well, the price is particularly affordable, there are so many efficacy is also good, it is worth buying worth buying the product, the workmanship is very fine, better than imagined, recommended that everyone try not yet used, after the use of the follow-up comment	Jingdong prefecture level city in Shandong
Jinfeng Hongyuan Flagship Store	Electricity savings3	It looks regular. It's working now.	Jingdong prefecture level city in Shandong
Jinfeng Hongyuan Flagship Store	Electricity savings3	It arrived pretty fast and the owner is very responsible. Have not used it yet, will see after use	Jingdong prefecture level city in Shandong
Jinfeng Hongyuan Flagship Store	Electricity savings3	🎁 baby quality is very good, the first time I received the goods to open the first time to see, it is very good 👍, very satisfied 😀, customer service also have a question and answer, thank you 🙏	Jingdong prefecture level city in Shandong
Jinfeng Hongyuan Flagship Store	Electricity savings3	I don't know if it's any good 。。。。。。	Jingdong prefecture level city in Shandong
Jinfeng Hongyuan Flagship Store	Electricity savings3	Buy electronic products! Still trust the things of the Jingdong self-owned store! Energy-saving effect to evaluate after heating!	Jingdong prefecture level city in Shandong
Jinfeng Hongyuan Flagship Store	Electricity savings3	The goods have been received, Blu is very beautiful, energy saving effect, to be confirmed only after actual use.	Jingdong prefecture level city in Shandong
Jinfeng Hongyuan Flagship Store	Electricity savings3	Jingdong's speed is so fast, has received, easy to operate. The display is quite good, how many volts what mode is quite understandable.	Jingdong prefecture level city in Shandong
Jinfeng Hongyuan Flagship Store	Electricity savings3	The first use, the effect is good to recommend it to colleagues to use, a month of electricity to save a lot or can be	Jingdong prefecture level city in Shandong
Jinfeng Hongyuan Flagship Store	Electricity savings3	I don't know if it's useful. Mom's is a store. I'll return it if it doesn't work.	Jingdong prefecture level city in Shandong
Jinfeng Hongyuan Flagship Store	Electricity savings3	I don't know if it works or not, I'll wait for two months to use it and then come back to comment!	Jingdong prefecture level city in Shandong
Jinfeng Hongyuan Flagship Store	Electricity savings3	I bought the second one now, and I've been using it for more than half a year, and I feel that it really saves electricity. Now it is summer, the weather is hot, sometimes the fan and air conditioning on all day, if not this power saver, the monthly electricity bill is quite high, but as long as the installation of this power saver, not afraid of its high electricity bill, absolutely let you wantonly use	Jingdong prefecture level city in Shandong
Jinfeng Hongyuan Flagship Store	Electricity savings3	The product is better than I thought it would be, the quality looks great, the voltage is stable, and I'm looking forward to some great power savings!	Jingdong prefecture level city in Shandong
Jinfeng Hongyuan Flagship Store	Electricity savings3	Inadvertently see bought a try, delivery is also quite timely, just do not know how to use, customer service patience to answer, with a pretty good, value for money!	Jingdong prefecture level city in Shandong
Jinfeng Hongyuan Flagship Store	Electricity savings3	It's better than nothing! Hopefully this will help save slightly.	Jingdong prefecture level city in Shandong
Jinfeng Hongyuan Flagship Store	Electricity savings3	Still trying it out, follow up	Jingdong prefecture level city in Shandong
Jinfeng Hongyuan Flagship Store	Electricity savings3	I've just started using it, I don't know how well it works yet, I hope it's good.	Jingdong prefecture level city in Shandong
Jinfeng Hongyuan Flagship Store	Electricity savings3	Fourth purchase, great baby!	Jingdong prefecture level city in Shandong
Jinfeng Hongyuan Flagship Store	Electricity savings3	Shipped really fast, just got it and use it to see what happens and then come back for a review!	Jingdong prefecture level city in Shandong
Jinfeng Hongyuan Flagship Store	Electricity savings3	Timely delivery **** good attitude	Jingdong prefecture level city in Shandong
Jinfeng Hongyuan Flagship Store	Electricity savings3	Home large and small things are purchased in the Jingdong Mall, have been accustomed to shopping in the Jingdong, many times can buy good quality and cheap products, and courier service attitude is particularly good, sometimes too many couriers are to help me to the home, so many years have been in the Jingdong Mall shopping, trust and support! Will also support the Jingdong as always!	Jingdong prefecture level city in Shandong
Jinfeng Hongyuan Flagship Store	Electricity savings3	Modified a pump and the modification worked.	Jingdong prefecture level city in Shandong
Jinfeng Hongyuan Flagship Store	Electricity savings3	Already installed, next time you pay the electric bill to see the results	Jingdong prefecture level city in Shandong
Jinfeng Hongyuan Flagship Store	Electricity savings3	The product is very good to use and practical to look at	Jingdong prefecture level city in Shandong
Jinfeng Hongyuan Flagship Store	Electricity savings3	Works great, great service, waiting for results.	Jingdong prefecture level city in Shandong
Jinfeng Hongyuan Flagship Store	Electricity savings3	I just used it and I'm not sure about the efficacy yet, I'll come back to evaluate it after a while.	Jingdong prefecture level city in Shandong
Jinfeng Hongyuan Flagship Store	Electricity savings3	Just received the goods, use a period of time to evaluate	Jingdong prefecture level city in Shandong
Jinfeng Hongyuan Flagship Store	Electricity savings3	Opened and tried very good, use it to see if there are results	Jingdong prefecture level city in Shandong
Jinfeng Hongyuan Flagship Store	Electricity savings3	Arrived today! Still trying it out!	Jingdong prefecture level city in Shandong
Jinfeng Hongyuan Flagship Store	Electricity savings3	Shipped quickly, well packaged, already installed, hope there is a power saving effect	Jingdong prefecture level city in Shandong
Jinfeng Hongyuan Flagship Store	Electricity savings3	I got it. It's great.	Jingdong prefecture level city in Shandong
Jinfeng Hongyuan Flagship Store	Electricity savings3	Haven't used it yet, will come back in a month	Jingdong prefecture level city in Shandong
Jinfeng Hongyuan Flagship Store	Electricity savings3	It looks good. It's good. Try it first.	Jingdong prefecture level city in Shandong
Jinfeng Hongyuan Flagship Store	Electricity savings3	It works so well that I have relatives, friends, coworkers, and 6 families that have purchased it for them through me.	Jingdong prefecture level city in Shandong
Jinfeng Hongyuan Flagship Store	Electricity savings3	In use	Jingdong prefecture level city in Shandong
Jinfeng Hongyuan Flagship Store	Electricity savings3	Received the goods today, power-saving effect do not know being used, the boss has a very good attitude, praise!	Jingdong prefecture level city in Shandong

Jinfeng Hongyuan Flagship Store	Electricity savings3	The product is compact and doesn't take up much space, but hopefully it's power efficient.	Jingdong prefecture level city in Shandong
Jinfeng Hongyuan Flagship Store	Electricity savings3	Power saving is indeed effective, I used for a period of time before coming to evaluate, the electricity bill is obviously less, the quality is good, the shopkeeper service is also very good, very patient,	Jingdong prefecture level city in Shandong
Jinfeng Hongyuan Flagship Store	Electricity savings3	It feels fine, I'll comment after I use it	Jingdong prefecture level city in Shandong
Jinfeng Hongyuan Flagship Store	Electricity savings3	I just received it and I don't know the effect yet! I will comment again after I use it and have results!	Jingdong prefecture level city in Shandong
Jinfeng Hongyuan Flagship Store	Electricity savings3	The goods look fine, I hope they really work!	Jingdong prefecture level city in Shandong
Jinfeng Hongyuan Flagship Store	Electricity savings3	I just don't know if it saves power	Jingdong prefecture level city in Shandong
Jinfeng Hongyuan Flagship Store	Electricity savings3	My friend introduced me to use this product, it is professional and I am looking forward to the long term effect~!	Jingdong prefecture level city in Shandong
Jinfeng Hongyuan Flagship Store	Electricity savings3	Try it before you judge.	Jingdong prefecture level city in Shandong
Jinfeng Hongyuan Flagship Store	Electricity savings3	It's great, the power savings are obvious! Kudos!	Jingdong prefecture level city in Shandong
Jinfeng Hongyuan Flagship Store	Electricity savings3	The quality is excellent, stabilizes the voltage and saves electricity, very satisfied.	Jingdong prefecture level city in Shandong
Jinfeng Hongyuan Flagship Store	Electricity savings3	You have to use it for a while to know the effect	Jingdong prefecture level city in Shandong
Jinfeng Hongyuan Flagship Store	Electricity savings3	It's on trial.	Jingdong prefecture level city in Shandong
Jinfeng Hongyuan Flagship Store	Electricity savings3	The stuff works great.	Jingdong prefecture level city in Shandong
Jinfeng Hongyuan Flagship Store	Electricity savings3	Doesn't seem to work, not as god as other reviews	Jingdong prefecture level city in Shandong
Jinfeng Hongyuan Flagship Store	Electricity savings3	It's not good quality, it doesn't work, it stops working after a few days, it doesn't work, the customer service doesn't care, it's a bit irritating.	Jingdong prefecture level city in Shandong
Jinfeng Hongyuan Flagship Store	Electricity savings3	It doesn't do anything.	Jingdong prefecture level city in Shandong
Jinfeng Hongyuan Flagship Store	Electricity savings3	It's good.	Jingdong prefecture level city in Shandong
Jinfeng Hongyuan Flagship Store	Electricity savings3	Used a month effect is not obvious, are psychological effects, feel are not less, did not save to the electricity, there are details of the problem, the store said to buy to send 5 fuse, in fact, received only three, small problems are not committed to in place, I believe that the product is not good enough	Jingdong prefecture level city in Shandong
Jinfeng Hongyuan Flagship Store	Electricity savings3	Well received quickly, looking quite high, but also sent 5 fuses. Customer service is very patient to teach the operation, in fact, quite simple, lazy to read the instructions ha!	Jingdong prefecture level city in Shandong
Jinfeng Hongyuan Flagship Store	Electricity savings3	You have to use it for a while to know the effect	Jingdong prefecture level city in Shandong
Jinfeng Hongyuan Flagship Store	Electricity savings3	Shipped very quickly, packaged very well, the price is particularly affordable, there are so many efficacy is also good, it is worth buying worth buying the product, the workmanship is very fine, better than imagined, recommended that everyone try not yet used, after the use of the follow-up comment	Jingdong prefecture level city in Shandong
Jinfeng Hongyuan Flagship Store	Electricity savings3	It looks regular. It's working now.	Jingdong prefecture level city in Shandong
Jinfeng Hongyuan Flagship Store	Electricity savings3	It arrived pretty fast and the owner is very responsible. Have not used it yet, will see after use	Jingdong prefecture level city in Shandong
Jinfeng Hongyuan Flagship Store	Electricity savings3	🎁 baby quality is very good, the first time I received the goods to open the first time to see, it is very good 👍, very satisfied 😊, customer service also have a question and answer, thank you 🙏	Jingdong prefecture level city in Shandong
Jinfeng Hongyuan Flagship Store	Electricity savings3	I don't know if it's any good 。。。。。。	Jingdong prefecture level city in Shandong
Jinfeng Hongyuan Flagship Store	Electricity savings3	Buy electronic products! Still trust the things of the Jingdong self-owned store! Energy-saving effect to evaluate after heating!	Jingdong prefecture level city in Shandong
Jinfeng Hongyuan Flagship Store	Electricity savings3	The goods have been received, Blu is very beautiful, energy saving effect, to be confirmed only after actual use.	Jingdong prefecture level city in Shandong
Jinfeng Hongyuan Flagship Store	Electricity savings3	Jingdong's speed is so fast, has received, easy to operate. The display is quite good, how many volts what mode is quite understandable.	Jingdong prefecture level city in Shandong
Jinfeng Hongyuan Flagship Store	Electricity savings3	The first use, the effect is good to recommend it to colleagues to use, a month of electricity to save a lot or can be	Jingdong prefecture level city in Shandong
Jinfeng Hongyuan Flagship Store	Electricity savings3	I don't know if it's useful. Mom's is a store. I'll return it if it doesn't work.	Jingdong prefecture level city in Shandong
Jinfeng Hongyuan Flagship Store	Electricity savings3	I don't know if it works or not, I'll wait for two months to use it and then come back to comment!	Jingdong prefecture level city in Shandong
Jinfeng Hongyuan Flagship Store	Electricity savings3	I bought the second one now, and I've been using it for more than half a year, and I feel that it really saves electricity. Now it is summer, the weather is hot, sometimes the fan and air conditioning on all day, if not this power saver, the monthly electricity bill is quite high, but as long as the installation of this power saver, not afraid of its high electricity bill, absolutely let you wantonly use	Jingdong prefecture level city in Shandong
Jinfeng Hongyuan Flagship Store	Electricity savings3	The product is better than I thought it would be, the quality looks great, the voltage is stable, and I'm looking forward to some great power savings!	Jingdong prefecture level city in Shandong
Jinfeng Hongyuan Flagship Store	Electricity savings3	Inadvertently see bought a try, delivery is also quite timely, just do not know how to use, customer service patience to answer, with a pretty good, value for money!	Jingdong prefecture level city in Shandong
Jinfeng Hongyuan Flagship Store	Electricity savings3	It's better than nothing! Hopefully this will help save slightly.	Jingdong prefecture level city in Shandong
Jinfeng Hongyuan Flagship Store	Electricity savings3	Still trying it out, follow up	Jingdong prefecture level city in Shandong
Jinfeng Hongyuan Flagship Store	Electricity savings3	I've just started using it, I don't know how well it works yet, I hope it's good.	Jingdong prefecture level city in Shandong
Jinfeng Hongyuan Flagship Store	Electricity savings3	Fourth purchase, great baby!	Jingdong prefecture level city in Shandong
Jinfeng Hongyuan Flagship Store	Electricity savings3	Shipped really fast, just got it and use it to see what happens and then come back for a review!	Jingdong prefecture level city in Shandong
Jinfeng Hongyuan Flagship Store	Electricity savings3	Timely delivery **** good attitude	Jingdong prefecture level city in Shandong
Jinfeng Hongyuan Flagship Store	Electricity savings3	Home large and small things are purchased in the Jingdong Mall, have been accustomed to shopping in the Jingdong, many times can buy good quality and cheap products, and courier service attitude is particularly good, sometimes too many couriers are to help me to the home, so many years have been in the Jingdong Mall shopping, trust and support! Will also support the Jingdong as always!	Jingdong prefecture level city in Shandong
Jinfeng Hongyuan Flagship Store	Electricity savings3	Modified a pump and the modification worked.	Jingdong prefecture level city in Shandong
Jinfeng Hongyuan Flagship Store	Electricity savings3	Already installed, next time you pay the electric bill to see the results	Jingdong prefecture level city in Shandong
Jinfeng Hongyuan Flagship Store	Electricity savings3	The product is very good to use and practical to look at	Jingdong prefecture level city in Shandong
Jinfeng Hongyuan Flagship Store	Electricity savings3	Works great, great service, waiting for results.	Jingdong prefecture level city in Shandong
Jinfeng Hongyuan Flagship Store	Electricity savings3	I just used it and I'm not sure about the efficacy yet, I'll come back to evaluate it after a while.	Jingdong prefecture level city in Shandong
Jinfeng Hongyuan Flagship Store	Electricity savings3	Just received the goods, use a period of time to evaluate	Jingdong prefecture level city in Shandong
Jinfeng Hongyuan Flagship Store	Electricity savings3	Opened and tried very good, use it to see if there are results	Jingdong prefecture level city in Shandong

Jinfeng Hongyuan Flagship Store	Electricity savings3	Arrived today! Still trying it out!	Jingdong prefecture level city in Shandong
Jinfeng Hongyuan Flagship Store	Electricity savings3	Shipped quickly, well packaged, already installed, hope there is a power saving effect	Jingdong prefecture level city in Shandong
Jinfeng Hongyuan Flagship Store	Electricity savings3	I got it. It's great.	Jingdong prefecture level city in Shandong
Jinfeng Hongyuan Flagship Store	Electricity savings3	Haven't used it yet, will come back in a month	Jingdong prefecture level city in Shandong
Jinfeng Hongyuan Flagship Store	Electricity savings3	It looks good. It's good. Try it first.	Jingdong prefecture level city in Shandong
Jinfeng Hongyuan Flagship Store	Electricity savings3	It works so well that I have relatives, friends, coworkers, and 6 families that have purchased it for them through me.	Jingdong prefecture level city in Shandong
Jinfeng Hongyuan Flagship Store	Electricity savings3	In use	Jingdong prefecture level city in Shandong
Jinfeng Hongyuan Flagship Store	Electricity savings3	Received the goods today, power-saving effect do not know being used, the boss has a very good attitude, praise!	Jingdong prefecture level city in Shandong
Jinfeng Hongyuan Flagship Store	Electricity savings3	The product is compact and doesn't take up much space, but hopefully it's power efficient.	Jingdong prefecture level city in Shandong
Jinfeng Hongyuan Flagship Store	Electricity savings3	Power saving is indeed effective, I used for a period of time before coming to evaluate, the electricity bill is obviously less, the quality is good, the shopkeeper service is also very good, very patient,	Jingdong prefecture level city in Shandong
Jinfeng Hongyuan Flagship Store	Electricity savings3	It feels fine, I'll comment after I use it	Jingdong prefecture level city in Shandong
Jinfeng Hongyuan Flagship Store	Electricity savings3	I just received it and I don't know the effect yet! I will comment again after I use it and have results!	Jingdong prefecture level city in Shandong
Jinfeng Hongyuan Flagship Store	Electricity savings3	The goods look fine, I hope they really work!	Jingdong prefecture level city in Shandong
Jinfeng Hongyuan Flagship Store	Electricity savings3	I just don't know if it saves power	Jingdong prefecture level city in Shandong
Jinfeng Hongyuan Flagship Store	Electricity savings3	My friend introduced me to use this product, it is professional and I am looking forward to the long term effect~!	Jingdong prefecture level city in Shandong
Jinfeng Hongyuan Flagship Store	Electricity savings3	Try it before you judge.	Jingdong prefecture level city in Shandong
Jinfeng Hongyuan Flagship Store	Electricity savings3	It's great, the power savings are obvious! Kudos!	Jingdong prefecture level city in Shandong
Jinfeng Hongyuan Flagship Store	Electricity savings3	The quality is excellent, stabilizes the voltage and saves electricity, very satisfied.	Jingdong prefecture level city in Shandong
Jinfeng Hongyuan Flagship Store	Electricity savings3	You have to use it for a while to know the effect	Jingdong prefecture level city in Shandong
Jinfeng Hongyuan Flagship Store	Electricity savings3	It's on trial.	Jingdong prefecture level city in Shandong
Jinfeng Hongyuan Flagship Store	Electricity savings3	The stuff works great.	Jingdong prefecture level city in Shandong
Jinfeng Hongyuan Flagship Store	Electricity savings3	Doesn't seem to work, not as god as other reviews	Jingdong prefecture level city in Shandong
Jinfeng Hongyuan Flagship Store	Electricity savings3	It's not good quality, it doesn't work, it stops working after a few days, it doesn't work, the customer service doesn't care, it's a bit irritating.	Jingdong prefecture level city in Shandong
Jinfeng Hongyuan Flagship Store	Electricity savings3	It doesn't do anything.	Jingdong prefecture level city in Shandong
Jinfeng Hongyuan Flagship Store	Electricity savings3	It's good.	Jingdong prefecture level city in Shandong
Jinfeng Hongyuan Flagship Store	Electricity savings3	Used a month effect is not obvious, are psychological effects, feel are not less, did not save to the electricity, there are details of the problem, the store said to buy to send 5 fuse, in fact, received only three, small problems are not committed to in place, I believe that the product is not good enough	Jingdong prefecture level city in Shandong
Jinfeng Hongyuan Flagship Store	Electricity savings3	Well received quickly, looking quite high, but also sent 5 fuses. Customer service is very patient to teach the operation, in fact, quite simple, lazy to read the instructions ha!	Jingdong prefecture level city in Shandong
Jinfeng Hongyuan Flagship Store	Electricity savings3	You have to use it for a while to know the effect	Jingdong prefecture level city in Shandong
Jinfeng Hongyuan Flagship Store	Electricity savings3	Shipped very quickly, packaged very well, the price is particularly affordable, there are so many efficacy is also good, it is worth buying worth buying the product, the workmanship is very fine, better than imagined, recommended that everyone try not yet used, after the use of the follow-up comment	Jingdong prefecture level city in Shandong
Jinfeng Hongyuan Flagship Store	Electricity savings3	It looks regular. It's working now.	Jingdong prefecture level city in Shandong
Jinfeng Hongyuan Flagship Store	Electricity savings3	It arrived pretty fast and the owner is very responsible. Have not used it yet, will see after use	Jingdong prefecture level city in Shandong
Jinfeng Hongyuan Flagship Store	Electricity savings3	🎁 baby quality is very good, the first time I received the goods to open the first time to see, it is very good 👍, very satisfied 😊, customer service also have a question and answer, thank you 🙏	Jingdong prefecture level city in Shandong
Jinfeng Hongyuan Flagship Store	Electricity savings3	I don't know if it's any good 。。。。。。	Jingdong prefecture level city in Shandong
Jinfeng Hongyuan Flagship Store	Electricity savings3	Buy electronic products! Still trust the things of the Jingdong self-owned store! Energy-saving effect to evaluate after heating!	Jingdong prefecture level city in Shandong
Jinfeng Hongyuan Flagship Store	Electricity savings3	The goods have been received, Blu is very beautiful, energy saving effect, to be confirmed only after actual use.	Jingdong prefecture level city in Shandong
Jinfeng Hongyuan Flagship Store	Electricity savings3	Jingdong's speed is so fast, has received, easy to operate. The display is quite good, how many volts what mode is quite understandable.	Jingdong prefecture level city in Shandong
Jinfeng Hongyuan Flagship Store	Electricity savings3	The first use, the effect is good to recommend it to colleagues to use, a month of electricity to save a lot or can be	Jingdong prefecture level city in Shandong
Jinfeng Hongyuan Flagship Store	Electricity savings3	I don't know if it's useful. Mom's is a store. I'll return it if it doesn't work.	Jingdong prefecture level city in Shandong
Jinfeng Hongyuan Flagship Store	Electricity savings3	I don't know if it works or not, I'll wait for two months to use it and then come back to comment!	Jingdong prefecture level city in Shandong
Jinfeng Hongyuan Flagship Store	Electricity savings3	I bought the second one now, and I've been using it for more than half a year, and I feel that it really saves electricity. Now it is summer, the weather is hot, sometimes the fan and air conditioning on all day, if not this power saver, the monthly electricity bill is quite high, but as long as the installation of this power saver, not afraid of its high electricity bill, absolutely let you wantonly use	Jingdong prefecture level city in Shandong
Jinfeng Hongyuan Flagship Store	Electricity savings3	The product is better than I thought it would be, the quality looks great, the voltage is stable, and I'm looking forward to some great power savings!	Jingdong prefecture level city in Shandong
Jinfeng Hongyuan Flagship Store	Electricity savings3	Inadvertently see bought a try, delivery is also quite timely, just do not know how to use, customer service patience to answer, with a pretty good, value for money!	Jingdong prefecture level city in Shandong
Jinfeng Hongyuan Flagship Store	Electricity savings3	It's better than nothing! Hopefully this will help save slightly.	Jingdong prefecture level city in Shandong
Jinfeng Hongyuan Flagship Store	Electricity savings3	Still trying it out, follow up	Jingdong prefecture level city in Shandong
Jinfeng Hongyuan Flagship Store	Electricity savings3	I've just started using it, I don't know how well it works yet, I hope it's good.	Jingdong prefecture level city in Shandong
Jinfeng Hongyuan Flagship Store	Electricity savings3	Fourth purchase, great baby!	Jingdong prefecture level city in Shandong
Jinfeng Hongyuan Flagship Store	Electricity savings3	Shipped really fast, just got it and use it to see what happens and then come back for a review!	Jingdong prefecture level city in Shandong
Jinfeng Hongyuan Flagship Store	Electricity savings3	Timely delivery **** good attitude	Jingdong prefecture level city in Shandong

Jinfeng Hongyuan Flagship Store	Electricity savings3	Home large and small things are purchased in the Jingdong Mall, have been accustomed to shopping in the Jingdong, many times can buy good quality and cheap products, and courier service attitude is particularly good, sometimes too many couriers are to help me to the home, so many years have been in the Jingdong Mall shopping, trust and support! Will also support the Jingdong as always!	Jingdong prefecture level city in Shandong
Jinfeng Hongyuan Flagship Store	Electricity savings3	Modified a pump and the modification worked.	Jingdong prefecture level city in Shandong
Jinfeng Hongyuan Flagship Store	Electricity savings3	Already installed, next time you pay the electric bill to see the results	Jingdong prefecture level city in Shandong
Jinfeng Hongyuan Flagship Store	Electricity savings3	The product is very good to use and practical to look at	Jingdong prefecture level city in Shandong
Jinfeng Hongyuan Flagship Store	Electricity savings3	Works great, great service, waiting for results.	Jingdong prefecture level city in Shandong
Jinfeng Hongyuan Flagship Store	Electricity savings3	I just used it and I'm not sure about the efficacy yet, I'll come back to evaluate it after a while.	Jingdong prefecture level city in Shandong
Jinfeng Hongyuan Flagship Store	Electricity savings3	Just received the goods, use a period of time to evaluate	Jingdong prefecture level city in Shandong
Jinfeng Hongyuan Flagship Store	Electricity savings3	Opened and tried very good, use it to see if there are results	Jingdong prefecture level city in Shandong
Jinfeng Hongyuan Flagship Store	Electricity savings3	Arrived today! Still trying it out!	Jingdong prefecture level city in Shandong
Jinfeng Hongyuan Flagship Store	Electricity savings3	Shipped quickly, well packaged, already installed, hope there is a power saving effect	Jingdong prefecture level city in Shandong
Jinfeng Hongyuan Flagship Store	Electricity savings3	I got it. It's great.	Jingdong prefecture level city in Shandong
Jinfeng Hongyuan Flagship Store	Electricity savings3	Haven't used it yet, will come back in a month	Jingdong prefecture level city in Shandong
Jinfeng Hongyuan Flagship Store	Electricity savings3	It looks good. It's good. Try it first.	Jingdong prefecture level city in Shandong
Jinfeng Hongyuan Flagship Store	Electricity savings3	It works so well that I have relatives, friends, coworkers, and 6 families that have purchased it for them through me.	Jingdong prefecture level city in Shandong
Jinfeng Hongyuan Flagship Store	Electricity savings3	In use	Jingdong prefecture level city in Shandong
Jinfeng Hongyuan Flagship Store	Electricity savings3	Received the goods today, power-saving effect do not know being used, the boss has a very good attitude, praise!	Jingdong prefecture level city in Shandong
Jinfeng Hongyuan Flagship Store	Electricity savings3	The product is compact and doesn't take up much space, but hopefully it's power efficient.	Jingdong prefecture level city in Shandong
Jinfeng Hongyuan Flagship Store	Electricity savings3	Power saving is indeed effective, I used for a period of time before coming to evaluate, the electricity bill is obviously less, the quality is good, the shopkeeper service is also very good, very patient,	Jingdong prefecture level city in Shandong
Jinfeng Hongyuan Flagship Store	Electricity savings3	It feels fine, I'll comment after I use it	Jingdong prefecture level city in Shandong
Jinfeng Hongyuan Flagship Store	Electricity savings3	I just received it and I don't know the effect yet! I will comment again after I use it and have results!	Jingdong prefecture level city in Shandong
Jinfeng Hongyuan Flagship Store	Electricity savings3	The goods look fine, I hope they really work!	Jingdong prefecture level city in Shandong
Jinfeng Hongyuan Flagship Store	Electricity savings3	I just don't know if it saves power	Jingdong prefecture level city in Shandong
Jinfeng Hongyuan Flagship Store	Electricity savings3	My friend introduced me to use this product, it is professional and I am looking forward to the long term effect~!	Jingdong prefecture level city in Shandong
Jinfeng Hongyuan Flagship Store	Electricity savings3	Try it before you judge.	Jingdong prefecture level city in Shandong
Jinfeng Hongyuan Flagship Store	Electricity savings3	It's great, the power savings are obvious! Kudos!	Jingdong prefecture level city in Shandong
Jinfeng Hongyuan Flagship Store	Electricity savings3	The quality is excellent, stabilizes the voltage and saves electricity, very satisfied.	Jingdong prefecture level city in Shandong
Jinfeng Hongyuan Flagship Store	Electricity savings3	You have to use it for a while to know the effect	Jingdong prefecture level city in Shandong
Jinfeng Hongyuan Flagship Store	Electricity savings3	It's on trial.	Jingdong prefecture level city in Shandong
Jinfeng Hongyuan Flagship Store	Electricity savings3	The stuff works great.	Jingdong prefecture level city in Shandong
Jinfeng Hongyuan Flagship Store	Electricity savings3	Doesn't seem to work, not as god as other reviews	Jingdong prefecture level city in Shandong
Jinfeng Hongyuan Flagship Store	Electricity savings3	It's not good quality, it doesn't work, it stops working after a few days, it doesn't work, the customer service doesn't care, it's a bit irritating.	Jingdong prefecture level city in Shandong
Jinfeng Hongyuan Flagship Store	Electricity savings3	It doesn't do anything.	Jingdong prefecture level city in Shandong
Jinfeng Hongyuan Flagship Store	Electricity savings3	It's good.	Jingdong prefecture level city in Shandong
Jinfeng Hongyuan Flagship Store	Electricity savings3	Used a month effect is not obvious, are psychological effects, feel are not less, did not save to the electricity, there are details of the problem, the store said to buy to send 5 fuse, in fact, received only three, small problems are not committed to in place, I believe that the product is not good enough	Jingdong prefecture level city in Shandong
Jinfeng Hongyuan Flagship Store	Electricity savings3	Well received quickly, looking quite high, but also sent 5 fuses. Customer service is very patient to teach the operation, in fact, quite simple, lazy to read the instructions ha!	Jingdong prefecture level city in Shandong
Jinfeng Hongyuan Flagship Store	Electricity savings3	You have to use it for a while to know the effect	Jingdong prefecture level city in Shandong
Jinfeng Hongyuan Flagship Store	Electricity savings3	Shipped very quickly, packaged very well, the price is particularly affordable, there are so many efficacy is also good, it is worth buying worth buying the product, the workmanship is very fine, better than imagined, recommended that everyone try not yet used, after the use of the follow-up comment	Jingdong prefecture level city in Shandong
Jinfeng Hongyuan Flagship Store	Electricity savings3	It looks regular. It's working now.	Jingdong prefecture level city in Shandong
Jinfeng Hongyuan Flagship Store	Electricity savings3	It arrived pretty fast and the owner is very responsible. Have not used it yet, will see after use	Jingdong prefecture level city in Shandong
Jinfeng Hongyuan Flagship Store	Electricity savings3	🎁 baby quality is very good, the first time I received the goods to open the first time to see, it is very good 👍, very satisfied 😊, customer service also have a question and answer, thank you 🙏	Jingdong prefecture level city in Shandong
Jinfeng Hongyuan Flagship Store	Electricity savings3	I don't know if it's any good 。。。。。。	Jingdong prefecture level city in Shandong
Jinfeng Hongyuan Flagship Store	Electricity savings3	Buy electronic products! Still trust the things of the Jingdong self-owned store! Energy-saving effect to evaluate after heating!	Jingdong prefecture level city in Shandong
Jinfeng Hongyuan Flagship Store	Electricity savings3	The goods have been received, Blu is very beautiful, energy saving effect, to be confirmed only after actual use.	Jingdong prefecture level city in Shandong
Jinfeng Hongyuan Flagship Store	Electricity savings3	Jingdong's speed is so fast, has received, easy to operate. The display is quite good, how many volts what mode is quite understandable.	Jingdong prefecture level city in Shandong
Jinfeng Hongyuan Flagship Store	Electricity savings3	The first use, the effect is good to recommend it to colleagues to use, a month of electricity to save a lot or can be	Jingdong prefecture level city in Shandong
Jinfeng Hongyuan Flagship Store	Electricity savings3	I don't know if it's useful. Mom's is a store. I'll return it if it doesn't work.	Jingdong prefecture level city in Shandong
Jinfeng Hongyuan Flagship Store	Electricity savings3	I don't know if it works or not, I'll wait for two months to use it and then come back to comment!	Jingdong prefecture level city in Shandong
Jinfeng Hongyuan Flagship Store	Electricity savings3	I bought the second one now, and I've been using it for more than half a year, and I feel that it really saves electricity. Now it is summer, the weather is hot, sometimes the fan and air conditioning on all day, if not this power saver, the monthly electricity bill is quite high, but as long as the installation of this power saver, not afraid of its high electricity bill, absolutely let you wantonly use	Jingdong prefecture level city in Shandong

Jinfeng Hongyuan Flagship Store	Electricity savings3	The product is better than I thought it would be, the quality looks great, the voltage is stable, and I'm looking forward to some great power savings!	Jingdong prefecture level city in Shandong
Jinfeng Hongyuan Flagship Store	Electricity savings3	Inadvertently see bought a try, delivery is also quite timely, just do not know how to use, customer service patience to answer, with a pretty good, value for money!	Jingdong prefecture level city in Shandong
Jinfeng Hongyuan Flagship Store	Electricity savings3	It's better than nothing! Hopefully this will help save slightly.	Jingdong prefecture level city in Shandong
Jinfeng Hongyuan Flagship Store	Electricity savings3	Still trying it out, follow up	Jingdong prefecture level city in Shandong
Jinfeng Hongyuan Flagship Store	Electricity savings3	I've just started using it, I don't know how well it works yet, I hope it's good.	Jingdong prefecture level city in Shandong
Jinfeng Hongyuan Flagship Store	Electricity savings3	Fourth purchase, great baby!	Jingdong prefecture level city in Shandong
Jinfeng Hongyuan Flagship Store	Electricity savings3	Shipped really fast, just got it and use it to see what happens and then come back for a review!	Jingdong prefecture level city in Shandong
Jinfeng Hongyuan Flagship Store	Electricity savings3	Timely delivery **** good attitude	Jingdong prefecture level city in Shandong
Jinfeng Hongyuan Flagship Store	Electricity savings3	Home large and small things are purchased in the Jingdong Mall, have been accustomed to shopping in the Jingdong, many times can buy good quality and cheap products, and courier service attitude is particularly good, sometimes too many couriers are to help me to the home, so many years have been in the Jingdong Mall shopping, trust and support! Will also support the Jingdong as always!	Jingdong prefecture level city in Shandong
Jinfeng Hongyuan Flagship Store	Electricity savings3	Modified a pump and the modification worked.	Jingdong prefecture level city in Shandong
Jinfeng Hongyuan Flagship Store	Electricity savings3	Already installed, next time you pay the electric bill to see the results	Jingdong prefecture level city in Shandong
Jinfeng Hongyuan Flagship Store	Electricity savings3	The product is very good to use and practical to look at	Jingdong prefecture level city in Shandong
Jinfeng Hongyuan Flagship Store	Electricity savings3	Works great, great service, waiting for results.	Jingdong prefecture level city in Shandong
Jinfeng Hongyuan Flagship Store	Electricity savings3	I just used it and I'm not sure about the efficacy yet, I'll come back to evaluate it after a while.	Jingdong prefecture level city in Shandong
Jinfeng Hongyuan Flagship Store	Electricity savings3	Just received the goods, use a period of time to evaluate	Jingdong prefecture level city in Shandong
Jinfeng Hongyuan Flagship Store	Electricity savings3	Opened and tried very good, use it to see if there are results	Jingdong prefecture level city in Shandong
Jinfeng Hongyuan Flagship Store	Electricity savings3	Arrived today! Still trying it out!	Jingdong prefecture level city in Shandong
Jinfeng Hongyuan Flagship Store	Electricity savings3	Shipped quickly, well packaged, already installed, hope there is a power saving effect	Jingdong prefecture level city in Shandong
Jinfeng Hongyuan Flagship Store	Electricity savings3	I got it. It's great.	Jingdong prefecture level city in Shandong
Jinfeng Hongyuan Flagship Store	Electricity savings3	Haven't used it yet, will come back in a month	Jingdong prefecture level city in Shandong
Jinfeng Hongyuan Flagship Store	Electricity savings3	It looks good. It's good. Try it first.	Jingdong prefecture level city in Shandong
Jinfeng Hongyuan Flagship Store	Electricity savings3	It works so well that I have relatives, friends, coworkers, and 6 families that have purchased it for them through me.	Jingdong prefecture level city in Shandong
Jinfeng Hongyuan Flagship Store	Electricity savings3	In use	Jingdong prefecture level city in Shandong
Jinfeng Hongyuan Flagship Store	Electricity savings3	Received the goods today, power-saving effect do not know being used, the boss has a very good attitude, praise!	Jingdong prefecture level city in Shandong
Jinfeng Hongyuan Flagship Store	Electricity savings3	The product is compact and doesn't take up much space, but hopefully it's power efficient.	Jingdong prefecture level city in Shandong
Jinfeng Hongyuan Flagship Store	Electricity savings3	Power saving is indeed effective, I used for a period of time before coming to evaluate, the electricity bill is obviously less, the quality is good, the shopkeeper service is also very good, very patient,	Jingdong prefecture level city in Shandong
Jinfeng Hongyuan Flagship Store	Electricity savings3	It feels fine, I'll comment after I use it	Jingdong prefecture level city in Shandong
Jinfeng Hongyuan Flagship Store	Electricity savings3	I just received it and I don't know the effect yet! I will comment again after I use it and have results!	Jingdong prefecture level city in Shandong
Jinfeng Hongyuan Flagship Store	Electricity savings3	The goods look fine, I hope they really work!	Jingdong prefecture level city in Shandong
Jinfeng Hongyuan Flagship Store	Electricity savings3	I just don't know if it saves power	Jingdong prefecture level city in Shandong
Jinfeng Hongyuan Flagship Store	Electricity savings3	My friend introduced me to use this product, it is professional and I am looking forward to the long term effect~!	Jingdong prefecture level city in Shandong
Jinfeng Hongyuan Flagship Store	Electricity savings3	Try it before you judge.	Jingdong prefecture level city in Shandong
Jinfeng Hongyuan Flagship Store	Electricity savings3	It's great, the power savings are obvious! Kudos!	Jingdong prefecture level city in Shandong
Jinfeng Hongyuan Flagship Store	Electricity savings3	The quality is excellent, stabilizes the voltage and saves electricity, very satisfied.	Jingdong prefecture level city in Shandong
Jinfeng Hongyuan Flagship Store	Electricity savings3	You have to use it for a while to know the effect	Jingdong prefecture level city in Shandong
Jinfeng Hongyuan Flagship Store	Electricity savings3	It's on trial.	Jingdong prefecture level city in Shandong
Jinfeng Hongyuan Flagship Store	Electricity savings3	The stuff works great.	Jingdong prefecture level city in Shandong
Jinfeng Hongyuan Flagship Store	Electricity savings3	Doesn't seem to work, not as god as other reviews	Jingdong prefecture level city in Shandong
Jinfeng Hongyuan Flagship Store	Electricity savings3	It's not good quality, it doesn't work, it stops working after a few days, it doesn't work, the customer service doesn't care, it's a bit irritating.	Jingdong prefecture level city in Shandong
Jinfeng Hongyuan Flagship Store	Electricity savings3	It doesn't do anything.	Jingdong prefecture level city in Shandong
Jinfeng Hongyuan Flagship Store	Electricity savings3	It's good.	Jingdong prefecture level city in Shandong
Jinfeng Hongyuan Flagship Store	Electricity savings3	Used a month effect is not obvious, are psychological effects, feel are not less, did not save to the electricity, there are details of the problem, the store said to buy to send 5 fuse, in fact, received only three, small problems are not committed to in place, I believe that the product is not good enough	Jingdong prefecture level city in Shandong
Jinfeng Hongyuan Flagship Store	Electricity savings3	Well received quickly, looking quite high, but also sent 5 fuses. Customer service is very patient to teach the operation, in fact, quite simple, lazy to read the instructions ha!	Jingdong prefecture level city in Shandong
Jinfeng Hongyuan Flagship Store	Electricity savings3	You have to use it for a while to know the effect	Jingdong prefecture level city in Shandong
Jinfeng Hongyuan Flagship Store	Electricity savings3	Shipped very quickly, packaged very well, the price is particularly affordable, there are so many efficacy is also good, it is worth buying worth buying the product, the workmanship is very fine, better than imagined, recommended that everyone try not yet used, after the use of the follow-up comment	Jingdong prefecture level city in Shandong
Jinfeng Hongyuan Flagship Store	Electricity savings3	It looks regular. It's working now.	Jingdong prefecture level city in Shandong
Jinfeng Hongyuan Flagship Store	Electricity savings3	It arrived pretty fast and the owner is very responsible. Have not used it yet, will see after use	Jingdong prefecture level city in Shandong
Jinfeng Hongyuan Flagship Store	Electricity savings3	🎁 baby quality is very good, the first time I received the goods to open the first time to see, it is very good 👍, very satisfied 😊, customer service also have a question and answer, thank you 🙏	Jingdong prefecture level city in Shandong
Jinfeng Hongyuan Flagship Store	Electricity savings3	I don't know if it's any good 。。。。。。	Jingdong prefecture level city in Shandong

Jinfeng Hongyuan Flagship Store	Electricity savings3	Buy electronic products! Still trust the things of the Jingdong self-owned store! Energy-saving effect to evaluate after heating!	Jingdong prefecture level city in Shandong
Jinfeng Hongyuan Flagship Store	Electricity savings3	The goods have been received, Blu is very beautiful, energy saving effect, to be confirmed only after actual use.	Jingdong prefecture level city in Shandong
Jinfeng Hongyuan Flagship Store	Electricity savings3	Jingdong's speed is so fast, has received, easy to operate. The display is quite good, how many volts what mode is quite understandable.	Jingdong prefecture level city in Shandong
Jinfeng Hongyuan Flagship Store	Electricity savings3	The first use, the effect is good to recommend it to colleagues to use, a month of electricity to save a lot or can be	Jingdong prefecture level city in Shandong
Jinfeng Hongyuan Flagship Store	Electricity savings3	I don't know if it's useful. Mom's is a store. I'll return it if it doesn't work.	Jingdong prefecture level city in Shandong
Jinfeng Hongyuan Flagship Store	Electricity savings3	I don't know if it works or not, I'll wait for two months to use it and then come back to comment!	Jingdong prefecture level city in Shandong
Jinfeng Hongyuan Flagship Store	Electricity savings3	I bought the second one now, and I've been using it for more than half a year, and I feel that it really saves electricity. Now it is summer, the weather is hot, sometimes the fan and air conditioning on all day, if not this power saver, the monthly electricity bill is quite high, but as long as the installation of this power saver, not afraid of its high electricity bill, absolutely let you wantonly use	Jingdong prefecture level city in Shandong
Jinfeng Hongyuan Flagship Store	Electricity savings3	The product is better than I thought it would be, the quality looks great, the voltage is stable, and I'm looking forward to some great power savings!	Jingdong prefecture level city in Shandong
Jinfeng Hongyuan Flagship Store	Electricity savings3	Inadvertently see bought a try, delivery is also quite timely, just do not know how to use, customer service patience to answer, with a pretty good, value for money!	Jingdong prefecture level city in Shandong
Jinfeng Hongyuan Flagship Store	Electricity savings3	It's better than nothing! Hopefully this will help save slightly.	Jingdong prefecture level city in Shandong
Jinfeng Hongyuan Flagship Store	Electricity savings3	Still trying it out, follow up	Jingdong prefecture level city in Shandong
Jinfeng Hongyuan Flagship Store	Electricity savings3	I've just started using it, I don't know how well it works yet, I hope it's good.	Jingdong prefecture level city in Shandong
Jinfeng Hongyuan Flagship Store	Electricity savings3	Fourth purchase, great baby!	Jingdong prefecture level city in Shandong
Jinfeng Hongyuan Flagship Store	Electricity savings3	Shipped really fast, just got it and use it to see what happens and then come back for a review!	Jingdong prefecture level city in Shandong
Jinfeng Hongyuan Flagship Store	Electricity savings3	Timely delivery **** good attitude	Jingdong prefecture level city in Shandong
Jinfeng Hongyuan Flagship Store	Electricity savings3	Home large and small things are purchased in the Jingdong Mall, have been accustomed to shopping in the Jingdong, many times can buy good quality and cheap products, and courier service attitude is particularly good, sometimes too many couriers are to help me to the home, so many years have been in the Jingdong Mall shopping, trust and support! Will also support the Jingdong as always!	Jingdong prefecture level city in Shandong
Jinfeng Hongyuan Flagship Store	Electricity savings3	Modified a pump and the modification worked.	Jingdong prefecture level city in Shandong
Jinfeng Hongyuan Flagship Store	Electricity savings3	Already installed, next time you pay the electric bill to see the results	Jingdong prefecture level city in Shandong
Jinfeng Hongyuan Flagship Store	Electricity savings3	The product is very good to use and practical to look at	Jingdong prefecture level city in Shandong
Jinfeng Hongyuan Flagship Store	Electricity savings3	Works great, great service, waiting for results.	Jingdong prefecture level city in Shandong
Jinfeng Hongyuan Flagship Store	Electricity savings3	I just used it and I'm not sure about the efficacy yet, I'll come back to evaluate it after a while.	Jingdong prefecture level city in Shandong
Jinfeng Hongyuan Flagship Store	Electricity savings3	Just received the goods, use a period of time to evaluate	Jingdong prefecture level city in Shandong
Jinfeng Hongyuan Flagship Store	Electricity savings3	Opened and tried very good, use it to see if there are results	Jingdong prefecture level city in Shandong
Jinfeng Hongyuan Flagship Store	Electricity savings3	Arrived today! Still trying it out!	Jingdong prefecture level city in Shandong
Jinfeng Hongyuan Flagship Store	Electricity savings3	Shipped quickly, well packaged, already installed, hope there is a power saving effect	Jingdong prefecture level city in Shandong
Jinfeng Hongyuan Flagship Store	Electricity savings3	I got it. It's great.	Jingdong prefecture level city in Shandong
Jinfeng Hongyuan Flagship Store	Electricity savings3	Haven't used it yet, will come back in a month	Jingdong prefecture level city in Shandong
Jinfeng Hongyuan Flagship Store	Electricity savings3	It looks good. It's good. Try it first.	Jingdong prefecture level city in Shandong
Jinfeng Hongyuan Flagship Store	Electricity savings3	It works so well that I have relatives, friends, coworkers, and 6 families that have purchased it for them through me.	Jingdong prefecture level city in Shandong
Jinfeng Hongyuan Flagship Store	Electricity savings3	In use	Jingdong prefecture level city in Shandong
Jinfeng Hongyuan Flagship Store	Electricity savings3	Received the goods today, power-saving effect do not know being used, the boss has a very good attitude, praise!	Jingdong prefecture level city in Shandong
Jinfeng Hongyuan Flagship Store	Electricity savings3	The product is compact and doesn't take up much space, but hopefully it's power efficient.	Jingdong prefecture level city in Shandong
Jinfeng Hongyuan Flagship Store	Electricity savings3	Power saving is indeed effective, I used for a period of time before coming to evaluate, the electricity bill is obviously less, the quality is good, the shopkeeper service is also very good, very patient,	Jingdong prefecture level city in Shandong
Jinfeng Hongyuan Flagship Store	Electricity savings3	It feels fine, I'll comment after I use it	Jingdong prefecture level city in Shandong
Jinfeng Hongyuan Flagship Store	Electricity savings3	I just received it and I don't know the effect yet! I will comment again after I use it and have results!	Jingdong prefecture level city in Shandong
Jinfeng Hongyuan Flagship Store	Electricity savings3	The goods look fine, I hope they really work!	Jingdong prefecture level city in Shandong
Jinfeng Hongyuan Flagship Store	Electricity savings3	I just don't know if it saves power	Jingdong prefecture level city in Shandong
Jinfeng Hongyuan Flagship Store	Electricity savings3	My friend introduced me to use this product, it is professional and I am looking forward to the long term effect~!	Jingdong prefecture level city in Shandong
Jinfeng Hongyuan Flagship Store	Electricity savings3	Try it before you judge.	Jingdong prefecture level city in Shandong
Jinfeng Hongyuan Flagship Store	Electricity savings3	It's great, the power savings are obvious! Kudos!	Jingdong prefecture level city in Shandong
Jinfeng Hongyuan Flagship Store	Electricity savings3	The quality is excellent, stabilizes the voltage and saves electricity, very satisfied.	Jingdong prefecture level city in Shandong
Jinfeng Hongyuan Flagship Store	Electricity savings3	You have to use it for a while to know the effect	Jingdong prefecture level city in Shandong
Jinfeng Hongyuan Flagship Store	Electricity savings3	It's on trial.	Jingdong prefecture level city in Shandong
Jinfeng Hongyuan Flagship Store	Electricity savings3	The stuff works great.	Jingdong prefecture level city in Shandong
Jinfeng Hongyuan Flagship Store	Electricity savings3	Doesn't seem to work, not as god as other reviews	Jingdong prefecture level city in Shandong
Jinfeng Hongyuan Flagship Store	Electricity savings3	It's not good quality, it doesn't work, it stops working after a few days, it doesn't work, the customer service doesn't care, it's a bit irritating.	Jingdong prefecture level city in Shandong
Jinfeng Hongyuan Flagship Store	Electricity savings3	It doesn't do anything.	Jingdong prefecture level city in Shandong
Jinfeng Hongyuan Flagship Store	Electricity savings3	It's good.	Jingdong prefecture level city in Shandong
Jinfeng Hongyuan Flagship Store	Electricity savings3	Used a month effect is not obvious, are psychological effects, feel are not less, did not save to the electricity, there are details of the problem, the store said to buy to send 5 fuse, in fact, received only three, small problems are not committed to in place, I believe that the product is not good enough	Jingdong prefecture level city in Shandong

Jinfeng Hongyuan Flagship Store	Electricity savings3	Well received quickly, looking quite high, but also sent 5 fuses. Customer service is very patient to teach the operation, in fact, quite simple, lazy to read the instructions ha!	Jingdong prefecture level city in Shandong
Jinfeng Hongyuan Flagship Store	Electricity savings3	You have to use it for a while to know the effect	Jingdong prefecture level city in Shandong
Jinfeng Hongyuan Flagship Store	Electricity savings3	Shipped very quickly, packaged very well, the price is particularly affordable, there are so many efficacy is also good, it is worth buying worth buying the product, the workmanship is very fine, better than imagined, recommended that everyone try not yet used, after the use of the follow-up comment	Jingdong prefecture level city in Shandong
Jinfeng Hongyuan Flagship Store	Electricity savings3	It looks regular. It's working now.	Jingdong prefecture level city in Shandong
Jinfeng Hongyuan Flagship Store	Electricity savings3	It arrived pretty fast and the owner is very responsible. Have not used it yet, will see after use	Jingdong prefecture level city in Shandong
Jinfeng Hongyuan Flagship Store	Electricity savings3	🎁 baby quality is very good, the first time I received the goods to open the first time to see, it is very good 👍, very satisfied 😊, customer service also have a question and answer, thank you 🙏	Jingdong prefecture level city in Shandong
Jinfeng Hongyuan Flagship Store	Electricity savings3	I don't know if it's any good 。。。。。。	Jingdong prefecture level city in Shandong
Jinfeng Hongyuan Flagship Store	Electricity savings3	Buy electronic products! Still trust the things of the Jingdong self-owned store! Energy-saving effect to evaluate after heating!	Jingdong prefecture level city in Shandong
Jinfeng Hongyuan Flagship Store	Electricity savings3	The goods have been received, Blu is very beautiful, energy saving effect, to be confirmed only after actual use.	Jingdong prefecture level city in Shandong
Jinfeng Hongyuan Flagship Store	Electricity savings3	Jingdong's speed is so fast, has received, easy to operate. The display is quite good, how many volts what mode is quite understandable.	Jingdong prefecture level city in Shandong
Jinfeng Hongyuan Flagship Store	Electricity savings3	The first use, the effect is good to recommend it to colleagues to use, a month of electricity to save a lot or can be	Jingdong prefecture level city in Shandong
Jinfeng Hongyuan Flagship Store	Electricity savings3	I don't know if it's useful. Mom's is a store. I'll return it if it doesn't work.	Jingdong prefecture level city in Shandong
Jinfeng Hongyuan Flagship Store	Electricity savings3	I don't know if it works or not, I'll wait for two months to use it and then come back to comment!	Jingdong prefecture level city in Shandong
Jinfeng Hongyuan Flagship Store	Electricity savings3	I bought the second one now, and I've been using it for more than half a year, and I feel that it really saves electricity. Now it is summer, the weather is hot, sometimes the fan and air conditioning on all day, if not this power saver, the monthly electricity bill is quite high, but as long as the installation of this power saver, not afraid of its high electricity bill, absolutely let you wantonly use	Jingdong prefecture level city in Shandong
Jinfeng Hongyuan Flagship Store	Electricity savings3	The product is better than I thought it would be, the quality looks great, the voltage is stable, and I'm looking forward to some great power savings!	Jingdong prefecture level city in Shandong
Jinfeng Hongyuan Flagship Store	Electricity savings3	Inadvertently see bought a try, delivery is also quite timely, just do not know how to use, customer service patience to answer, with a pretty good, value for money!	Jingdong prefecture level city in Shandong
Jinfeng Hongyuan Flagship Store	Electricity savings3	It's better than nothing! Hopefully this will help save slightly.	Jingdong prefecture level city in Shandong
Jinfeng Hongyuan Flagship Store	Electricity savings3	Still trying it out, follow up	Jingdong prefecture level city in Shandong
Jinfeng Hongyuan Flagship Store	Electricity savings3	I've just started using it, I don't know how well it works yet, I hope it's good.	Jingdong prefecture level city in Shandong
Jinfeng Hongyuan Flagship Store	Electricity savings3	Fourth purchase, great baby!	Jingdong prefecture level city in Shandong
Jinfeng Hongyuan Flagship Store	Electricity savings3	Shipped really fast, just got it and use it to see what happens and then come back for a review!	Jingdong prefecture level city in Shandong
Jinfeng Hongyuan Flagship Store	Electricity savings3	Timely delivery **** good attitude	Jingdong prefecture level city in Shandong
Jinfeng Hongyuan Flagship Store	Electricity savings3	Home large and small things are purchased in the Jingdong Mall, have been accustomed to shopping in the Jingdong, many times can buy good quality and cheap products, and courier service attitude is particularly good, sometimes too many couriers are to help me to the home, so many years have been in the Jingdong Mall shopping, trust and support! Will also support the Jingdong as always!	Jingdong prefecture level city in Shandong
Jinfeng Hongyuan Flagship Store	Electricity savings3	Modified a pump and the modification worked.	Jingdong prefecture level city in Shandong
Jinfeng Hongyuan Flagship Store	Electricity savings3	Already installed, next time you pay the electric bill to see the results	Jingdong prefecture level city in Shandong
Jinfeng Hongyuan Flagship Store	Electricity savings3	The product is very good to use and practical to look at	Jingdong prefecture level city in Shandong
Jinfeng Hongyuan Flagship Store	Electricity savings3	Works great, great service, waiting for results.	Jingdong prefecture level city in Shandong
Jinfeng Hongyuan Flagship Store	Electricity savings3	I just used it and I'm not sure about the efficacy yet, I'll come back to evaluate it after a while.	Jingdong prefecture level city in Shandong
Jinfeng Hongyuan Flagship Store	Electricity savings3	Just received the goods, use a period of time to evaluate	Jingdong prefecture level city in Shandong
Jinfeng Hongyuan Flagship Store	Electricity savings3	Opened and tried very good, use it to see if there are results	Jingdong prefecture level city in Shandong
Jinfeng Hongyuan Flagship Store	Electricity savings3	Arrived today! Still trying it out!	Jingdong prefecture level city in Shandong
Jinfeng Hongyuan Flagship Store	Electricity savings3	Shipped quickly, well packaged, already installed, hope there is a power saving effect	Jingdong prefecture level city in Shandong
Jinfeng Hongyuan Flagship Store	Electricity savings3	I got it. It's great.	Jingdong prefecture level city in Shandong
Jinfeng Hongyuan Flagship Store	Electricity savings3	Haven't used it yet, will come back in a month	Jingdong prefecture level city in Shandong
Jinfeng Hongyuan Flagship Store	Electricity savings3	It looks good. It's good. Try it first.	Jingdong prefecture level city in Shandong
Jinfeng Hongyuan Flagship Store	Electricity savings3	It works so well that I have relatives, friends, coworkers, and 6 families that have purchased it for them through me.	Jingdong prefecture level city in Shandong
Jinfeng Hongyuan Flagship Store	Electricity savings3	In use	Jingdong prefecture level city in Shandong
Jinfeng Hongyuan Flagship Store	Electricity savings3	Received the goods today, power-saving effect do not know being used, the boss has a very good attitude, praise!	Jingdong prefecture level city in Shandong
Jinfeng Hongyuan Flagship Store	Electricity savings3	The product is compact and doesn't take up much space, but hopefully it's power efficient.	Jingdong prefecture level city in Shandong
Jinfeng Hongyuan Flagship Store	Electricity savings3	Power saving is indeed effective, I used for a period of time before coming to evaluate, the electricity bill is obviously less, the quality is good, the shopkeeper service is also very good, very patient,	Jingdong prefecture level city in Shandong
Jinfeng Hongyuan Flagship Store	Electricity savings3	It feels fine, I'll comment after I use it	Jingdong prefecture level city in Shandong
Jinfeng Hongyuan Flagship Store	Electricity savings3	I just received it and I don't know the effect yet! I will comment again after I use it and have results!	Jingdong prefecture level city in Shandong
Jinfeng Hongyuan Flagship Store	Electricity savings3	The goods look fine, I hope they really work!	Jingdong prefecture level city in Shandong
Jinfeng Hongyuan Flagship Store	Electricity savings3	I just don't know if it saves power	Jingdong prefecture level city in Shandong
Jinfeng Hongyuan Flagship Store	Electricity savings3	My friend introduced me to use this product, it is professional and I am looking forward to the long term effect~!	Jingdong prefecture level city in Shandong
Jinfeng Hongyuan Flagship Store	Electricity savings3	Try it before you judge.	Jingdong prefecture level city in Shandong
Jinfeng Hongyuan Flagship Store	Electricity savings3	It's great, the power savings are obvious! Kudos!	Jingdong prefecture level city in Shandong
Jinfeng Hongyuan Flagship Store	Electricity savings3	The quality is excellent, stabilizes the voltage and saves electricity, very satisfied.	Jingdong prefecture level city in Shandong
Jinfeng Hongyuan Flagship Store	Electricity savings3	You have to use it for a while to know the effect	Jingdong prefecture level city in Shandong

Jinfeng Hongyuan Flagship Store	Electricity savings3	It's on trial.	Jingdong prefecture level city in Shandong
Jinfeng Hongyuan Flagship Store	Electricity savings3	The stuff works great.	Jingdong prefecture level city in Shandong
Jinfeng Hongyuan Flagship Store	Electricity savings3	Doesn't seem to work, not as god as other reviews	Jingdong prefecture level city in Shandong
Jinfeng Hongyuan Flagship Store	Electricity savings3	It's not good quality, it doesn't work, it stops working after a few days, it doesn't work, the customer service doesn't care, it's a bit irritating.	Jingdong prefecture level city in Shandong
Jinfeng Hongyuan Flagship Store	Electricity savings3	It doesn't do anything.	Jingdong prefecture level city in Shandong
Jinfeng Hongyuan Flagship Store	Electricity savings3	It's good.	Jingdong prefecture level city in Shandong
Jinfeng Hongyuan Flagship Store	Electricity savings3	Used a month effect is not obvious, are psychological effects, feel are not less, did not save to the electricity, there are details of the problem, the store said to buy to send 5 fuse, in fact, received only three, small problems are not committed to in place, I believe that the product is not good enough	Jingdong prefecture level city in Shandong
Jinfeng Hongyuan Flagship Store	Electricity savings3	Well received quickly, looking quite high, but also sent 5 fuses. Customer service is very patient to teach the operation, in fact, quite simple, lazy to read the instructions ha!	Jingdong prefecture level city in Shandong
Jinfeng Hongyuan Flagship Store	Electricity savings3	You have to use it for a while to know the effect	Jingdong prefecture level city in Shandong
Jinfeng Hongyuan Flagship Store	Electricity savings3	Shipped very quickly, packaged very well, the price is particularly affordable, there are so many efficacy is also good, it is worth buying worth buying the product, the workmanship is very fine, better than imagined, recommended that everyone try not yet used, after the use of the follow-up comment	Jingdong prefecture level city in Shandong
Jinfeng Hongyuan Flagship Store	Electricity savings3	It looks regular. It's working now.	Jingdong prefecture level city in Shandong
Jinfeng Hongyuan Flagship Store	Electricity savings3	It arrived pretty fast and the owner is very responsible. Have not used it yet, will see after use	Jingdong prefecture level city in Shandong
Jinfeng Hongyuan Flagship Store	Electricity savings3	🎁 baby quality is very good, the first time I received the goods to open the first time to see, it is very good 👍, very satisfied 😊, customer service also have a question and answer, thank you 🙏	Jingdong prefecture level city in Shandong
Jinfeng Hongyuan Flagship Store	Electricity savings3	I don't know if it's any good 。。。。。。	Jingdong prefecture level city in Shandong
Jinfeng Hongyuan Flagship Store	Electricity savings3	Buy electronic products! Still trust the things of the Jingdong self-owned store! Energy-saving effect to evaluate after heating!	Jingdong prefecture level city in Shandong
Jinfeng Hongyuan Flagship Store	Electricity savings3	The goods have been received, Blu is very beautiful, energy saving effect, to be confirmed only after actual use.	Jingdong prefecture level city in Shandong
Jinfeng Hongyuan Flagship Store	Electricity savings3	Jingdong's speed is so fast, has received, easy to operate. The display is quite good, how many volts what mode is quite understandable.	Jingdong prefecture level city in Shandong
Jinfeng Hongyuan Flagship Store	Electricity savings3	The first use, the effect is good to recommend it to colleagues to use, a month of electricity to save a lot or can be	Jingdong prefecture level city in Shandong
Jinfeng Hongyuan Flagship Store	Electricity savings3	I don't know if it's useful. Mom's is a store. I'll return it if it doesn't work.	Jingdong prefecture level city in Shandong
Jinfeng Hongyuan Flagship Store	Electricity savings3	I don't know if it works or not, I'll wait for two months to use it and then come back to comment!	Jingdong prefecture level city in Shandong
Jinfeng Hongyuan Flagship Store	Electricity savings3	I bought the second one now, and I've been using it for more than half a year, and I feel that it really saves electricity. Now it is summer, the weather is hot, sometimes the fan and air conditioning on all day, if not this power saver, the monthly electricity bill is quite high, but as long as the installation of this power saver, not afraid of its high electricity bill, absolutely let you wantonly use	Jingdong prefecture level city in Shandong
Jinfeng Hongyuan Flagship Store	Electricity savings3	The product is better than I thought it would be, the quality looks great, the voltage is stable, and I'm looking forward to some great power savings!	Jingdong prefecture level city in Shandong
Jinfeng Hongyuan Flagship Store	Electricity savings3	Inadvertently see bought a try, delivery is also quite timely, just do not know how to use, customer service patience to answer, with a pretty good, value for money!	Jingdong prefecture level city in Shandong
Jinfeng Hongyuan Flagship Store	Electricity savings3	It's better than nothing! Hopefully this will help save slightly.	Jingdong prefecture level city in Shandong
Jinfeng Hongyuan Flagship Store	Electricity savings3	Still trying it out, follow up	Jingdong prefecture level city in Shandong
Jinfeng Hongyuan Flagship Store	Electricity savings3	I've just started using it, I don't know how well it works yet, I hope it's good.	Jingdong prefecture level city in Shandong
Jinfeng Hongyuan Flagship Store	Electricity savings3	Fourth purchase, great baby!	Jingdong prefecture level city in Shandong
Jinfeng Hongyuan Flagship Store	Electricity savings3	Shipped really fast, just got it and use it to see what happens and then come back for a review!	Jingdong prefecture level city in Shandong
Jinfeng Hongyuan Flagship Store	Electricity savings3	Timely delivery **** good attitude	Jingdong prefecture level city in Shandong
Jinfeng Hongyuan Flagship Store	Electricity savings3	Home large and small things are purchased in the Jingdong Mall, have been accustomed to shopping in the Jingdong, many times can buy good quality and cheap products, and courier service attitude is particularly good, sometimes too many couriers are to help me to the home, so many years have been in the Jingdong Mall shopping, trust and support! Will also support the Jingdong as always!	Jingdong prefecture level city in Shandong
Jinfeng Hongyuan Flagship Store	Electricity savings3	Modified a pump and the modification worked.	Jingdong prefecture level city in Shandong
Jinfeng Hongyuan Flagship Store	Electricity savings3	Already installed, next time you pay the electric bill to see the results	Jingdong prefecture level city in Shandong
Jinfeng Hongyuan Flagship Store	Electricity savings3	The product is very good to use and practical to look at	Jingdong prefecture level city in Shandong
Jinfeng Hongyuan Flagship Store	Electricity savings3	Works great, great service, waiting for results.	Jingdong prefecture level city in Shandong
Jinfeng Hongyuan Flagship Store	Electricity savings3	I just used it and I'm not sure about the efficacy yet, I'll come back to evaluate it after a while.	Jingdong prefecture level city in Shandong
Jinfeng Hongyuan Flagship Store	Electricity savings3	Just received the goods, use a period of time to evaluate	Jingdong prefecture level city in Shandong
Jinfeng Hongyuan Flagship Store	Electricity savings3	Opened and tried very good, use it to see if there are results	Jingdong prefecture level city in Shandong
Jinfeng Hongyuan Flagship Store	Electricity savings3	Arrived today! Still trying it out!	Jingdong prefecture level city in Shandong
Jinfeng Hongyuan Flagship Store	Electricity savings3	Shipped quickly, well packaged, already installed, hope there is a power saving effect	Jingdong prefecture level city in Shandong
Jinfeng Hongyuan Flagship Store	Electricity savings3	I got it. It's great.	Jingdong prefecture level city in Shandong
Jinfeng Hongyuan Flagship Store	Electricity savings3	Haven't used it yet, will come back in a month	Jingdong prefecture level city in Shandong
Jinfeng Hongyuan Flagship Store	Electricity savings3	It looks good. It's good. Try it first.	Jingdong prefecture level city in Shandong
Jinfeng Hongyuan Flagship Store	Electricity savings3	It works so well that I have relatives, friends, coworkers, and 6 families that have purchased it for them through me.	Jingdong prefecture level city in Shandong
Jinfeng Hongyuan Flagship Store	Electricity savings3	In use	Jingdong prefecture level city in Shandong
Jinfeng Hongyuan Flagship Store	Electricity savings3	Received the goods today, power-saving effect do not know being used, the boss has a very good attitude, praise!	Jingdong prefecture level city in Shandong
Jinfeng Hongyuan Flagship Store	Electricity savings3	The product is compact and doesn't take up much space, but hopefully it's power efficient.	Jingdong prefecture level city in Shandong
Jinfeng Hongyuan Flagship Store	Electricity savings3	Power saving is indeed effective, I used for a period of time before coming to evaluate, the electricity bill is obviously less, the quality is good, the shopkeeper service is also very good, very patient,	Jingdong prefecture level city in Shandong
Jinfeng Hongyuan Flagship Store	Electricity savings3	It feels fine, I'll comment after I use it	Jingdong prefecture level city in Shandong

Jinfeng Hongyuan Flagship Store	Electricity savings3	I just received it and I don't know the effect yet! I will comment again after I use it and have results!	Jingdong prefecture level city in Shandong
Jinfeng Hongyuan Flagship Store	Electricity savings3	The goods look fine, I hope they really work!	Jingdong prefecture level city in Shandong
Jinfeng Hongyuan Flagship Store	Electricity savings3	I just don't know if it saves power	Jingdong prefecture level city in Shandong
Jinfeng Hongyuan Flagship Store	Electricity savings3	My friend introduced me to use this product, it is professional and I am looking forward to the long term effect~!	Jingdong prefecture level city in Shandong
Jinfeng Hongyuan Flagship Store	Electricity savings3	Try it before you judge.	Jingdong prefecture level city in Shandong
Jinfeng Hongyuan Flagship Store	Electricity savings3	It's great, the power savings are obvious! Kudos!	Jingdong prefecture level city in Shandong
Jinfeng Hongyuan Flagship Store	Electricity savings3	The quality is excellent, stabilizes the voltage and saves electricity, very satisfied.	Jingdong prefecture level city in Shandong
Jinfeng Hongyuan Flagship Store	Electricity savings3	You have to use it for a while to know the effect	Jingdong prefecture level city in Shandong
Jinfeng Hongyuan Flagship Store	Electricity savings3	It's on trial.	Jingdong prefecture level city in Shandong
Jinfeng Hongyuan Flagship Store	Electricity savings3	The stuff works great.	Jingdong prefecture level city in Shandong
Jinfeng Hongyuan Flagship Store	Electricity savings3	Doesn't seem to work, not as god as other reviews	Jingdong prefecture level city in Shandong
Jinfeng Hongyuan Flagship Store	Electricity savings3	It's not good quality, it doesn't work, it stops working after a few days, it doesn't work, the customer service doesn't care, it's a bit irritating.	Jingdong prefecture level city in Shandong
Jinfeng Hongyuan Flagship Store	Electricity savings3	It doesn't do anything.	Jingdong prefecture level city in Shandong
Jinfeng Hongyuan Flagship Store	Electricity savings3	It's good.	Jingdong prefecture level city in Shandong
Jinfeng Hongyuan Flagship Store	Electricity savings3	Used a month effect is not obvious, are psychological effects, feel are not less, did not save to the electricity, there are details of the problem, the store said to buy to send 5 fuse, in fact, received only three, small problems are not committed to in place, I believe that the product is not good enough	Jingdong prefecture level city in Shandong
Jinfeng Hongyuan Flagship Store	Electricity savings3	Well received quickly, looking quite high, but also sent 5 fuses. Customer service is very patient to teach the operation, in fact, quite simple, lazy to read the instructions ha!	Jingdong prefecture level city in Shandong
Jinfeng Hongyuan Flagship Store	Electricity savings3	You have to use it for a while to know the effect	Jingdong prefecture level city in Shandong
Jinfeng Hongyuan Flagship Store	Electricity savings3	Shipped very quickly, packaged very well, the price is particularly affordable, there are so many efficacy is also good, it is worth buying worth buying the product, the workmanship is very fine, better than imagined, recommended that everyone try not yet used, after the use of the follow-up comment	Jingdong prefecture level city in Shandong
Jinfeng Hongyuan Flagship Store	Electricity savings3	It looks regular. It's working now.	Jingdong prefecture level city in Shandong
Jinfeng Hongyuan Flagship Store	Electricity savings3	It arrived pretty fast and the owner is very responsible. Have not used it yet, will see after use	Jingdong prefecture level city in Shandong
Jinfeng Hongyuan Flagship Store	Electricity savings3	🎁 baby quality is very good, the first time I received the goods to open the first time to see, it is very good 👍, very satisfied 🙂, customer service also have a question and answer, thank you 🙏	Jingdong prefecture level city in Shandong
Jinfeng Hongyuan Flagship Store	Electricity savings3	I don't know if it's any good 。。。。。。	Jingdong prefecture level city in Shandong
Jinfeng Hongyuan Flagship Store	Electricity savings3	Buy electronic products! Still trust the things of the Jingdong self-owned store! Energy-saving effect to evaluate after heating!	Jingdong prefecture level city in Shandong
Jinfeng Hongyuan Flagship Store	Electricity savings3	The goods have been received, Blu is very beautiful, energy saving effect, to be confirmed only after actual use.	Jingdong prefecture level city in Shandong
Jinfeng Hongyuan Flagship Store	Electricity savings3	Jingdong's speed is so fast, has received, easy to operate. The display is quite good, how many volts what mode is quite understandable.	Jingdong prefecture level city in Shandong
Jinfeng Hongyuan Flagship Store	Electricity savings3	The first use, the effect is good to recommend it to colleagues to use, a month of electricity to save a lot or can be	Jingdong prefecture level city in Shandong
Jinfeng Hongyuan Flagship Store	Electricity savings3	I don't know if it's useful. Mom's is a store. I'll return it if it doesn't work.	Jingdong prefecture level city in Shandong
Jinfeng Hongyuan Flagship Store	Electricity savings3	I don't know if it works or not, I'll wait for two months to use it and then come back to comment!	Jingdong prefecture level city in Shandong
Jinfeng Hongyuan Flagship Store	Electricity savings3	I bought the second one now, and I've been using it for more than half a year, and I feel that it really saves electricity. Now it is summer, the weather is hot, sometimes the fan and air conditioning on all day, if not this power saver, the monthly electricity bill is quite high, but as long as the installation of this power saver, not afraid of its high electricity bill, absolutely let you wantonly use	Jingdong prefecture level city in Shandong
Jinfeng Hongyuan Flagship Store	Electricity savings3	The product is better than I thought it would be, the quality looks great, the voltage is stable, and I'm looking forward to some great power savings!	Jingdong prefecture level city in Shandong
Jinfeng Hongyuan Flagship Store	Electricity savings3	Inadvertently see bought a try, delivery is also quite timely, just do not know how to use, customer service patience to answer, with a pretty good, value for money!	Jingdong prefecture level city in Shandong
Jinfeng Hongyuan Flagship Store	Electricity savings3	It's better than nothing! Hopefully this will help save slightly.	Jingdong prefecture level city in Shandong
Jinfeng Hongyuan Flagship Store	Electricity savings3	Still trying it out, follow up	Jingdong prefecture level city in Shandong
Jinfeng Hongyuan Flagship Store	Electricity savings3	I've just started using it, I don't know how well it works yet, I hope it's good.	Jingdong prefecture level city in Shandong
Jinfeng Hongyuan Flagship Store	Electricity savings3	Fourth purchase, great baby!	Jingdong prefecture level city in Shandong
Jinfeng Hongyuan Flagship Store	Electricity savings3	Shipped really fast, just got it and use it to see what happens and then come back for a review!	Jingdong prefecture level city in Shandong
Jinfeng Hongyuan Flagship Store	Electricity savings3	Timely delivery **** good attitude	Jingdong prefecture level city in Shandong
Jinfeng Hongyuan Flagship Store	Electricity savings3	Home large and small things are purchased in the Jingdong Mall, have been accustomed to shopping in the Jingdong, many times can buy good quality and cheap products, and courier service attitude is particularly good, sometimes too many couriers are to help me to the home, so many years have been in the Jingdong Mall shopping, trust and support! Will also support the Jingdong as always!	Jingdong prefecture level city in Shandong
Jinfeng Hongyuan Flagship Store	Electricity savings3	Modified a pump and the modification worked.	Jingdong prefecture level city in Shandong
Jinfeng Hongyuan Flagship Store	Electricity savings3	Already installed, next time you pay the electric bill to see the results	Jingdong prefecture level city in Shandong
Jinfeng Hongyuan Flagship Store	Electricity savings3	The product is very good to use and practical to look at	Jingdong prefecture level city in Shandong
Jinfeng Hongyuan Flagship Store	Electricity savings3	Works great, great service, waiting for results.	Jingdong prefecture level city in Shandong
Jinfeng Hongyuan Flagship Store	Electricity savings3	I just used it and I'm not sure about the efficacy yet, I'll come back to evaluate it after a while.	Jingdong prefecture level city in Shandong
Jinfeng Hongyuan Flagship Store	Electricity savings3	Just received the goods, use a period of time to evaluate	Jingdong prefecture level city in Shandong
Jinfeng Hongyuan Flagship Store	Electricity savings3	Opened and tried very good, use it to see if there are results	Jingdong prefecture level city in Shandong
Jinfeng Hongyuan Flagship Store	Electricity savings3	Arrived today! Still trying it out!	Jingdong prefecture level city in Shandong
Jinfeng Hongyuan Flagship Store	Electricity savings3	Shipped quickly, well packaged, already installed, hope there is a power saving effect	Jingdong prefecture level city in Shandong
Jinfeng Hongyuan Flagship Store	Electricity savings3	I got it. It's great.	Jingdong prefecture level city in Shandong

Jinfeng Hongyuan Flagship Store	Electricity savings3	Haven't used it yet, will come back in a month	Jingdong prefecture level city in Shandong
Jinfeng Hongyuan Flagship Store	Electricity savings3	It looks good. It's good. Try it first.	Jingdong prefecture level city in Shandong
Jinfeng Hongyuan Flagship Store	Electricity savings3	It works so well that I have relatives, friends, coworkers, and 6 families that have purchased it for them through me.	Jingdong prefecture level city in Shandong
Jinfeng Hongyuan Flagship Store	Electricity savings3	In use	Jingdong prefecture level city in Shandong
Jinfeng Hongyuan Flagship Store	Electricity savings3	Received the goods today, power-saving effect do not know being used, the boss has a very good attitude, praise!	Jingdong prefecture level city in Shandong
Jinfeng Hongyuan Flagship Store	Electricity savings3	The product is compact and doesn't take up much space, but hopefully it's power efficient.	Jingdong prefecture level city in Shandong
Jinfeng Hongyuan Flagship Store	Electricity savings3	Power saving is indeed effective, I used for a period of time before coming to evaluate, the electricity bill is obviously less, the quality is good, the shopkeeper service is also very good, very patient,	Jingdong prefecture level city in Shandong
Jinfeng Hongyuan Flagship Store	Electricity savings3	It feels fine, I'll comment after I use it	Jingdong prefecture level city in Shandong
Jinfeng Hongyuan Flagship Store	Electricity savings3	I just received it and I don't know the effect yet! I will comment again after I use it and have results!	Jingdong prefecture level city in Shandong
Jinfeng Hongyuan Flagship Store	Electricity savings3	The goods look fine, I hope they really work!	Jingdong prefecture level city in Shandong
Jinfeng Hongyuan Flagship Store	Electricity savings3	I just don't know if it saves power	Jingdong prefecture level city in Shandong
Jinfeng Hongyuan Flagship Store	Electricity savings3	My friend introduced me to use this product, it is professional and I am looking forward to the long term effect~!	Jingdong prefecture level city in Shandong
Jinfeng Hongyuan Flagship Store	Electricity savings3	Try it before you judge.	Jingdong prefecture level city in Shandong
Jinfeng Hongyuan Flagship Store	Electricity savings3	It's great, the power savings are obvious! Kudos!	Jingdong prefecture level city in Shandong
Jinfeng Hongyuan Flagship Store	Electricity savings3	The quality is excellent, stabilizes the voltage and saves electricity, very satisfied.	Jingdong prefecture level city in Shandong
Jinfeng Hongyuan Flagship Store	Electricity savings3	You have to use it for a while to know the effect	Jingdong prefecture level city in Shandong
Jinfeng Hongyuan Flagship Store	Electricity savings3	It's on trial.	Jingdong prefecture level city in Shandong
Jinfeng Hongyuan Flagship Store	Electricity savings3	The stuff works great.	Jingdong prefecture level city in Shandong
Jinfeng Hongyuan Flagship Store	Electricity savings3	Doesn't seem to work, not as god as other reviews	Jingdong prefecture level city in Shandong
Jinfeng Hongyuan Flagship Store	Electricity savings3	It's not good quality, it doesn't work, it stops working after a few days, it doesn't work, the customer service doesn't care, it's a bit irritating.	Jingdong prefecture level city in Shandong
Jinfeng Hongyuan Flagship Store	Electricity savings3	It doesn't do anything.	Jingdong prefecture level city in Shandong
Jinfeng Hongyuan Flagship Store	Electricity savings3	It's good.	Jingdong prefecture level city in Shandong
Jinfeng Hongyuan Flagship Store	Electricity savings3	Used a month effect is not obvious, are psychological effects, feel are not less, did not save to the electricity, there are details of the problem, the store said to buy to send 5 fuse, in fact, received only three, small problems are not committed to in place, I believe that the product is not good enough	Jingdong prefecture level city in Shandong
Jinfeng Hongyuan Flagship Store	Electricity savings3	Well received quickly, looking quite high, but also sent 5 fuses. Customer service is very patient to teach the operation, in fact, quite simple, lazy to read the instructions ha!	Jingdong prefecture level city in Shandong
Jinfeng Hongyuan Flagship Store	Electricity savings3	You have to use it for a while to know the effect	Jingdong prefecture level city in Shandong
Jinfeng Hongyuan Flagship Store	Electricity savings3	Shipped very quickly, packaged very well, the price is particularly affordable, there are so many efficacy is also good, it is worth buying worth buying the product, the workmanship is very fine, better than imagined, recommended that everyone try not yet used, after the use of the follow-up comment	Jingdong prefecture level city in Shandong
Jinfeng Hongyuan Flagship Store	Electricity savings3	It looks regular. It's working now.	Jingdong prefecture level city in Shandong
Jinfeng Hongyuan Flagship Store	Electricity savings3	It arrived pretty fast and the owner is very responsible. Have not used it yet, will see after use	Jingdong prefecture level city in Shandong
Jinfeng Hongyuan Flagship Store	Electricity savings3	🎁 baby quality is very good, the first time I received the goods to open the first time to see, it is very good 👍, very satisfied 😊, customer service also have a question and answer, thank you 🙏	Jingdong prefecture level city in Shandong
Jinfeng Hongyuan Flagship Store	Electricity savings3	I don't know if it's any good 。。。。。。	Jingdong prefecture level city in Shandong
Jinfeng Hongyuan Flagship Store	Electricity savings3	Buy electronic products! Still trust the things of the Jingdong self-owned store! Energy-saving effect to evaluate after heating!	Jingdong prefecture level city in Shandong
Jinfeng Hongyuan Flagship Store	Electricity savings3	The goods have been received, Blu is very beautiful, energy saving effect, to be confirmed only after actual use.	Jingdong prefecture level city in Shandong
Jinfeng Hongyuan Flagship Store	Electricity savings3	Jingdong's speed is so fast, has received, easy to operate. The display is quite good, how many volts what mode is quite understandable.	Jingdong prefecture level city in Shandong
Jinfeng Hongyuan Flagship Store	Electricity savings3	The first use, the effect is good to recommend it to colleagues to use, a month of electricity to save a lot or can be	Jingdong prefecture level city in Shandong
Jinfeng Hongyuan Flagship Store	Electricity savings3	I don't know if it's useful. Mom's is a store. I'll return it if it doesn't work.	Jingdong prefecture level city in Shandong
Jinfeng Hongyuan Flagship Store	Electricity savings3	I don't know if it works or not, I'll wait for two months to use it and then come back to comment!	Jingdong prefecture level city in Shandong
Jinfeng Hongyuan Flagship Store	Electricity savings3	I bought the second one now, and I've been using it for more than half a year, and I feel that it really saves electricity. Now it is summer, the weather is hot, sometimes the fan and air conditioning on all day, if not this power saver, the monthly electricity bill is quite high, but as long as the installation of this power saver, not afraid of its high electricity bill, absolutely let you wantonly use	Jingdong prefecture level city in Shandong
Jinfeng Hongyuan Flagship Store	Electricity savings3	The product is better than I thought it would be, the quality looks great, the voltage is stable, and I'm looking forward to some great power savings!	Jingdong prefecture level city in Shandong
Jinfeng Hongyuan Flagship Store	Electricity savings3	Inadvertently see bought a try, delivery is also quite timely, just do not know how to use, customer service patience to answer, with a pretty good, value for money!	Jingdong prefecture level city in Shandong
Jinfeng Hongyuan Flagship Store	Electricity savings3	It's better than nothing! Hopefully this will help save slightly.	Jingdong prefecture level city in Shandong
Jinfeng Hongyuan Flagship Store	Electricity savings3	Still trying it out, follow up	Jingdong prefecture level city in Shandong
Jinfeng Hongyuan Flagship Store	Electricity savings3	I've just started using it, I don't know how well it works yet, I hope it's good.	Jingdong prefecture level city in Shandong
Jinfeng Hongyuan Flagship Store	Electricity savings3	Fourth purchase, great baby!	Jingdong prefecture level city in Shandong
Jinfeng Hongyuan Flagship Store	Electricity savings3	Shipped really fast, just got it and use it to see what happens and then come back for a review!	Jingdong prefecture level city in Shandong
Jinfeng Hongyuan Flagship Store	Electricity savings3	Timely delivery **** good attitude	Jingdong prefecture level city in Shandong
Jinfeng Hongyuan Flagship Store	Electricity savings3	Home large and small things are purchased in the Jingdong Mall, have been accustomed to shopping in the Jingdong, many times can buy good quality and cheap products, and courier service attitude is particularly good, sometimes too many couriers are to help me to the home, so many years have been in the Jingdong Mall shopping, trust and support! Will also support the Jingdong as always!	Jingdong prefecture level city in Shandong
Jinfeng Hongyuan Flagship Store	Electricity savings3	Modified a pump and the modification worked.	Jingdong prefecture level city in Shandong
Jinfeng Hongyuan Flagship Store	Electricity savings3	Already installed, next time you pay the electric bill to see the results	Jingdong prefecture level city in Shandong

Jinfeng Hongyuan Flagship Store	Electricity savings3	The product is very good to use and practical to look at	Jingdong prefecture level city in Shandong
Jinfeng Hongyuan Flagship Store	Electricity savings3	Works great, great service, waiting for results.	Jingdong prefecture level city in Shandong
Jinfeng Hongyuan Flagship Store	Electricity savings3	I just used it and I'm not sure about the efficacy yet, I'll come back to evaluate it after a while.	Jingdong prefecture level city in Shandong
Jinfeng Hongyuan Flagship Store	Electricity savings3	Just received the goods, use a period of time to evaluate	Jingdong prefecture level city in Shandong
Jinfeng Hongyuan Flagship Store	Electricity savings3	Opened and tried very good, use it to see if there are results	Jingdong prefecture level city in Shandong
Jinfeng Hongyuan Flagship Store	Electricity savings3	Arrived today! Still trying it out!	Jingdong prefecture level city in Shandong
Jinfeng Hongyuan Flagship Store	Electricity savings3	Shipped quickly, well packaged, already installed, hope there is a power saving effect	Jingdong prefecture level city in Shandong
Jinfeng Hongyuan Flagship Store	Electricity savings3	I got it. It's great.	Jingdong prefecture level city in Shandong
Jinfeng Hongyuan Flagship Store	Electricity savings3	Haven't used it yet, will come back in a month	Jingdong prefecture level city in Shandong
Jinfeng Hongyuan Flagship Store	Electricity savings3	It looks good. It's good. Try it first.	Jingdong prefecture level city in Shandong
Jinfeng Hongyuan Flagship Store	Electricity savings3	It works so well that I have relatives, friends, coworkers, and 6 families that have purchased it for them through me.	Jingdong prefecture level city in Shandong
Jinfeng Hongyuan Flagship Store	Electricity savings3	In use	Jingdong prefecture level city in Shandong
Jinfeng Hongyuan Flagship Store	Electricity savings3	Received the goods today, power-saving effect do not know being used, the boss has a very good attitude, praise!	Jingdong prefecture level city in Shandong
Jinfeng Hongyuan Flagship Store	Electricity savings3	The product is compact and doesn't take up much space, but hopefully it's power efficient.	Jingdong prefecture level city in Shandong
Jinfeng Hongyuan Flagship Store	Electricity savings3	Power saving is indeed effective, I used for a period of time before coming to evaluate, the electricity bill is obviously less, the quality is good, the shopkeeper service is also very good, very patient,	Jingdong prefecture level city in Shandong
Jinfeng Hongyuan Flagship Store	Electricity savings3	It feels fine, I'll comment after I use it	Jingdong prefecture level city in Shandong
Jinfeng Hongyuan Flagship Store	Electricity savings3	I just received it and I don't know the effect yet! I will comment again after I use it and have results!	Jingdong prefecture level city in Shandong
Jinfeng Hongyuan Flagship Store	Electricity savings3	The goods look fine, I hope they really work!	Jingdong prefecture level city in Shandong
Jinfeng Hongyuan Flagship Store	Electricity savings3	I just don't know if it saves power	Jingdong prefecture level city in Shandong
Jinfeng Hongyuan Flagship Store	Electricity savings3	My friend introduced me to use this product, it is professional and I am looking forward to the long term effect~!	Jingdong prefecture level city in Shandong
Jinfeng Hongyuan Flagship Store	Electricity savings3	Try it before you judge.	Jingdong prefecture level city in Shandong
Jinfeng Hongyuan Flagship Store	Electricity savings3	It's great, the power savings are obvious! Kudos!	Jingdong prefecture level city in Shandong
Jinfeng Hongyuan Flagship Store	Electricity savings3	The quality is excellent, stabilizes the voltage and saves electricity, very satisfied.	Jingdong prefecture level city in Shandong
Jinfeng Hongyuan Flagship Store	Electricity savings3	You have to use it for a while to know the effect	Jingdong prefecture level city in Shandong
Jinfeng Hongyuan Flagship Store	Electricity savings3	It's on trial.	Jingdong prefecture level city in Shandong
Jinfeng Hongyuan Flagship Store	Electricity savings3	The stuff works great.	Jingdong prefecture level city in Shandong
Jinfeng Hongyuan Flagship Store	Electricity savings3	Doesn't seem to work, not as god as other reviews	Jingdong prefecture level city in Shandong
Jinfeng Hongyuan Flagship Store	Electricity savings3	It's not good quality, it doesn't work, it stops working after a few days, it doesn't work, the customer service doesn't care, it's a bit irritating.	Jingdong prefecture level city in Shandong
Jinfeng Hongyuan Flagship Store	Electricity savings3	It doesn't do anything.	Jingdong prefecture level city in Shandong
Jinfeng Hongyuan Flagship Store	Electricity savings3	It's good.	Jingdong prefecture level city in Shandong
Jinfeng Hongyuan Flagship Store	Electricity savings3	Used a month effect is not obvious, are psychological effects, feel are not less, did not save to the electricity, there are details of the problem, the store said to buy to send 5 fuse, in fact, received only three, small problems are not committed to in place, I believe that the product is not good enough	Jingdong prefecture level city in Shandong
Jinfeng Hongyuan Flagship Store	Electricity savings3	Well received quickly, looking quite high, but also sent 5 fuses. Customer service is very patient to teach the operation, in fact, quite simple, lazy to read the instructions ha!	Jingdong prefecture level city in Shandong
Jinfeng Hongyuan Flagship Store	Electricity savings3	You have to use it for a while to know the effect	Jingdong prefecture level city in Shandong
Jinfeng Hongyuan Flagship Store	Electricity savings3	Shipped very quickly, packaged very well, the price is particularly affordable, there are so many efficacy is also good, it is worth buying worth buying the product, the workmanship is very fine, better than imagined, recommended that everyone try not yet used, after the use of the follow-up comment	Jingdong prefecture level city in Shandong
Jinfeng Hongyuan Flagship Store	Electricity savings3	It looks regular. It's working now.	Jingdong prefecture level city in Shandong
Jinfeng Hongyuan Flagship Store	Electricity savings3	It arrived pretty fast and the owner is very responsible. Have not used it yet, will see after use	Jingdong prefecture level city in Shandong
Jinfeng Hongyuan Flagship Store	Electricity savings3	🎁 baby quality is very good, the first time I received the goods to open the first time to see, it is very good 👍, very satisfied 😊, customer service also have a question and answer, thank you 🙏	Jingdong prefecture level city in Shandong
Jinfeng Hongyuan Flagship Store	Electricity savings3	I don't know if it's any good 。。。。。。	Jingdong prefecture level city in Shandong
Jinfeng Hongyuan Flagship Store	Electricity savings3	Buy electronic products! Still trust the things of the Jingdong self-owned store! Energy-saving effect to evaluate after heating!	Jingdong prefecture level city in Shandong
Jinfeng Hongyuan Flagship Store	Electricity savings3	The goods have been received, Blu is very beautiful, energy saving effect, to be confirmed only after actual use.	Jingdong prefecture level city in Shandong
Jinfeng Hongyuan Flagship Store	Electricity savings3	Jingdong's speed is so fast, has received, easy to operate. The display is quite good, how many volts what mode is quite understandable.	Jingdong prefecture level city in Shandong
Jinfeng Hongyuan Flagship Store	Electricity savings3	The first use, the effect is good to recommend it to colleagues to use, a month of electricity to save a lot or can be	Jingdong prefecture level city in Shandong
Jinfeng Hongyuan Flagship Store	Electricity savings3	I don't know if it's useful. Mom's is a store. I'll return it if it doesn't work.	Jingdong prefecture level city in Shandong
Jinfeng Hongyuan Flagship Store	Electricity savings3	I don't know if it works or not, I'll wait for two months to use it and then come back to comment!	Jingdong prefecture level city in Shandong
Jinfeng Hongyuan Flagship Store	Electricity savings3	I bought the second one now, and I've been using it for more than half a year, and I feel that it really saves electricity. Now it is summer, the weather is hot, sometimes the fan and air conditioning on all day, if not this power saver, the monthly electricity bill is quite high, but as long as the installation of this power saver, not afraid of its high electricity bill, absolutely let you wantonly use	Jingdong prefecture level city in Shandong
Jinfeng Hongyuan Flagship Store	Electricity savings3	The product is better than I thought it would be, the quality looks great, the voltage is stable, and I'm looking forward to some great power savings!	Jingdong prefecture level city in Shandong
Jinfeng Hongyuan Flagship Store	Electricity savings3	Inadvertently see bought a try, delivery is also quite timely, just do not know how to use, customer service patience to answer, with a pretty good, value for money!	Jingdong prefecture level city in Shandong
Jinfeng Hongyuan Flagship Store	Electricity savings3	It's better than nothing! Hopefully this will help save slightly.	Jingdong prefecture level city in Shandong
Jinfeng Hongyuan Flagship Store	Electricity savings3	Still trying it out, follow up	Jingdong prefecture level city in Shandong

Jinfeng Hongyuan Flagship Store	Electricity savings3	I've just started using it, I don't know how well it works yet, I hope it's good.	Jingdong prefecture level city in Shandong
Jinfeng Hongyuan Flagship Store	Electricity savings3	Fourth purchase, great baby!	Jingdong prefecture level city in Shandong
Jinfeng Hongyuan Flagship Store	Electricity savings3	Shipped really fast, just got it and use it to see what happens and then come back for a review!	Jingdong prefecture level city in Shandong
Jinfeng Hongyuan Flagship Store	Electricity savings3	Timely delivery **** good attitude	Jingdong prefecture level city in Shandong
Jinfeng Hongyuan Flagship Store	Electricity savings3	Home large and small things are purchased in the Jingdong Mall, have been accustomed to shopping in the Jingdong, many times can buy good quality and cheap products, and courier service attitude is particularly good, sometimes too many couriers are to help me to the home, so many years have been in the Jingdong Mall shopping, trust and support! Will also support the Jingdong as always!	Jingdong prefecture level city in Shandong
Jinfeng Hongyuan Flagship Store	Electricity savings3	Modified a pump and the modification worked.	Jingdong prefecture level city in Shandong
Jinfeng Hongyuan Flagship Store	Electricity savings3	Already installed, next time you pay the electric bill to see the results	Jingdong prefecture level city in Shandong
Jinfeng Hongyuan Flagship Store	Electricity savings3	The product is very good to use and practical to look at	Jingdong prefecture level city in Shandong
Jinfeng Hongyuan Flagship Store	Electricity savings3	Works great, great service, waiting for results.	Jingdong prefecture level city in Shandong
Jinfeng Hongyuan Flagship Store	Electricity savings3	I just used it and I'm not sure about the efficacy yet, I'll come back to evaluate it after a while.	Jingdong prefecture level city in Shandong
Jinfeng Hongyuan Flagship Store	Electricity savings3	Just received the goods, use a period of time to evaluate	Jingdong prefecture level city in Shandong
Jinfeng Hongyuan Flagship Store	Electricity savings3	Opened and tried very good, use it to see if there are results	Jingdong prefecture level city in Shandong
Jinfeng Hongyuan Flagship Store	Electricity savings3	Arrived today! Still trying it out!	Jingdong prefecture level city in Shandong
Jinfeng Hongyuan Flagship Store	Electricity savings3	Shipped quickly, well packaged, already installed, hope there is a power saving effect	Jingdong prefecture level city in Shandong
Jinfeng Hongyuan Flagship Store	Electricity savings3	I got it. It's great.	Jingdong prefecture level city in Shandong
Jinfeng Hongyuan Flagship Store	Electricity savings3	Haven't used it yet, will come back in a month	Jingdong prefecture level city in Shandong
Jinfeng Hongyuan Flagship Store	Electricity savings3	It looks good. It's good. Try it first.	Jingdong prefecture level city in Shandong
Jinfeng Hongyuan Flagship Store	Electricity savings3	It works so well that I have relatives, friends, coworkers, and 6 families that have purchased it for them through me.	Jingdong prefecture level city in Shandong
Jinfeng Hongyuan Flagship Store	Electricity savings3	In use	Jingdong prefecture level city in Shandong
Jinfeng Hongyuan Flagship Store	Electricity savings3	Received the goods today, power-saving effect do not know being used, the boss has a very good attitude, praise!	Jingdong prefecture level city in Shandong
Jinfeng Hongyuan Flagship Store	Electricity savings3	The product is compact and doesn't take up much space, but hopefully it's power efficient.	Jingdong prefecture level city in Shandong
Jinfeng Hongyuan Flagship Store	Electricity savings3	Power saving is indeed effective, I used for a period of time before coming to evaluate, the electricity bill is obviously less, the quality is good, the shopkeeper service is also very good, very patient,	Jingdong prefecture level city in Shandong
Jinfeng Hongyuan Flagship Store	Electricity savings3	It feels fine, I'll comment after I use it	Jingdong prefecture level city in Shandong
Jinfeng Hongyuan Flagship Store	Electricity savings3	I just received it and I don't know the effect yet! I will comment again after I use it and have results!	Jingdong prefecture level city in Shandong
Jinfeng Hongyuan Flagship Store	Electricity savings3	The goods look fine, I hope they really work!	Jingdong prefecture level city in Shandong
Jinfeng Hongyuan Flagship Store	Electricity savings3	I just don't know if it saves power	Jingdong prefecture level city in Shandong
Jinfeng Hongyuan Flagship Store	Electricity savings3	My friend introduced me to use this product, it is professional and I am looking forward to the long term effect~!	Jingdong prefecture level city in Shandong
Jinfeng Hongyuan Flagship Store	Electricity savings3	Try it before you judge.	Jingdong prefecture level city in Shandong
Jinfeng Hongyuan Flagship Store	Electricity savings3	It's great, the power savings are obvious! Kudos!	Jingdong prefecture level city in Shandong
Jinfeng Hongyuan Flagship Store	Electricity savings3	The quality is excellent, stabilizes the voltage and saves electricity, very satisfied.	Jingdong prefecture level city in Shandong
Jinfeng Hongyuan Flagship Store	Electricity savings3	You have to use it for a while to know the effect	Jingdong prefecture level city in Shandong
Jinfeng Hongyuan Flagship Store	Electricity savings3	It's on trial.	Jingdong prefecture level city in Shandong
Jinfeng Hongyuan Flagship Store	Electricity savings3	The stuff works great.	Jingdong prefecture level city in Shandong
Jinfeng Hongyuan Flagship Store	Electricity savings3	Doesn't seem to work, not as god as other reviews	Jingdong prefecture level city in Shandong
Jinfeng Hongyuan Flagship Store	Electricity savings3	It's not good quality, it doesn't work, it stops working after a few days, it doesn't work, the customer service doesn't care, it's a bit irritating.	Jingdong prefecture level city in Shandong
Jinfeng Hongyuan Flagship Store	Electricity savings3	It doesn't do anything.	Jingdong prefecture level city in Shandong
Jinfeng Hongyuan Flagship Store	Electricity savings3	It's good.	Jingdong prefecture level city in Shandong
Jinfeng Hongyuan Flagship Store	Electricity savings3	Used a month effect is not obvious, are psychological effects, feel are not less, did not save to the electricity, there are details of the problem, the store said to buy to send 5 fuse, in fact, received only three, small problems are not committed to in place, I believe that the product is not good enough	Jingdong prefecture level city in Shandong
Jinfeng Hongyuan Flagship Store	Electricity savings3	Well received quickly, looking quite high, but also sent 5 fuses. Customer service is very patient to teach the operation, in fact, quite simple, lazy to read the instructions ha!	Jingdong prefecture level city in Shandong
Jinfeng Hongyuan Flagship Store	Electricity savings3	You have to use it for a while to know the effect	Jingdong prefecture level city in Shandong
Jinfeng Hongyuan Flagship Store	Electricity savings3	Shipped very quickly, packaged very well, the price is particularly affordable, there are so many efficacy is also good, it is worth buying worth buying the product, the workmanship is very fine, better than imagined, recommended that everyone try not yet used, after the use of the follow-up comment	Jingdong prefecture level city in Shandong
Jinfeng Hongyuan Flagship Store	Electricity savings3	It looks regular. It's working now.	Jingdong prefecture level city in Shandong
Jinfeng Hongyuan Flagship Store	Electricity savings3	It arrived pretty fast and the owner is very responsible. Have not used it yet, will see after use	Jingdong prefecture level city in Shandong
Jinfeng Hongyuan Flagship Store	Electricity savings3	🎁 baby quality is very good, the first time I received the goods to open the first time to see, it is very good 👍, very satisfied 😊, customer service also have a question and answer, thank you 🙏	Jingdong prefecture level city in Shandong
Jinfeng Hongyuan Flagship Store	Electricity savings3	I don't know if it's any good 。。。。。。	Jingdong prefecture level city in Shandong
Jinfeng Hongyuan Flagship Store	Electricity savings3	Buy electronic products! Still trust the things of the Jingdong self-owned store! Energy-saving effect to evaluate after heating!	Jingdong prefecture level city in Shandong
Jinfeng Hongyuan Flagship Store	Electricity savings3	The goods have been received, Blu is very beautiful, energy saving effect, to be confirmed only after actual use.	Jingdong prefecture level city in Shandong
Jinfeng Hongyuan Flagship Store	Electricity savings3	Jingdong's speed is so fast, has received, easy to operate. The display is quite good, how many volts what mode is quite understandable.	Jingdong prefecture level city in Shandong
Jinfeng Hongyuan Flagship Store	Electricity savings3	The first use, the effect is good to recommend it to colleagues to use, a month of electricity to save a lot or can be	Jingdong prefecture level city in Shandong

Jinfeng Hongyuan Flagship Store	Electricity savings3	I don't know if it's useful. Mom's is a store. I'll return it if it doesn't work.	Jingdong prefecture level city in Shandong
Jinfeng Hongyuan Flagship Store	Electricity savings3	I don't know if it works or not, I'll wait for two months to use it and then come back to comment!	Jingdong prefecture level city in Shandong
Jinfeng Hongyuan Flagship Store	Electricity savings3	I bought the second one now, and I've been using it for more than half a year, and I feel that it really saves electricity. Now it is summer, the weather is hot, sometimes the fan and air conditioning on all day, if not this power saver, the monthly electricity bill is quite high, but as long as the installation of this power saver, not afraid of its high electricity bill, absolutely let you wantonly use	Jingdong prefecture level city in Shandong
Jinfeng Hongyuan Flagship Store	Electricity savings3	The product is better than I thought it would be, the quality looks great, the voltage is stable, and I'm looking forward to some great power savings!	Jingdong prefecture level city in Shandong
Jinfeng Hongyuan Flagship Store	Electricity savings3	Inadvertently see bought a try, delivery is also quite timely, just do not know how to use, customer service patience to answer, with a pretty good, value for money!	Jingdong prefecture level city in Shandong
Jinfeng Hongyuan Flagship Store	Electricity savings3	It's better than nothing! Hopefully this will help save slightly.	Jingdong prefecture level city in Shandong
Jinfeng Hongyuan Flagship Store	Electricity savings3	Still trying it out, follow up	Jingdong prefecture level city in Shandong
Jinfeng Hongyuan Flagship Store	Electricity savings3	I've just started using it, I don't know how well it works yet, I hope it's good.	Jingdong prefecture level city in Shandong
Jinfeng Hongyuan Flagship Store	Electricity savings3	Fourth purchase, great baby!	Jingdong prefecture level city in Shandong
Jinfeng Hongyuan Flagship Store	Electricity savings3	Shipped really fast, just got it and use it to see what happens and then come back for a review!	Jingdong prefecture level city in Shandong
Jinfeng Hongyuan Flagship Store	Electricity savings3	Timely delivery **** good attitude	Jingdong prefecture level city in Shandong
Jinfeng Hongyuan Flagship Store	Electricity savings3	Home large and small things are purchased in the Jingdong Mall, have been accustomed to shopping in the Jingdong, many times can buy good quality and cheap products, and courier service attitude is particularly good, sometimes too many couriers are to help me to the home, so many years have been in the Jingdong Mall shopping, trust and support! Will also support the Jingdong as always!	Jingdong prefecture level city in Shandong
Jinfeng Hongyuan Flagship Store	Electricity savings3	Modified a pump and the modification worked.	Jingdong prefecture level city in Shandong
Jinfeng Hongyuan Flagship Store	Electricity savings3	Already installed, next time you pay the electric bill to see the results	Jingdong prefecture level city in Shandong
Jinfeng Hongyuan Flagship Store	Electricity savings3	The product is very good to use and practical to look at	Jingdong prefecture level city in Shandong
Jinfeng Hongyuan Flagship Store	Electricity savings3	Works great, great service, waiting for results.	Jingdong prefecture level city in Shandong
Jinfeng Hongyuan Flagship Store	Electricity savings3	I just used it and I'm not sure about the efficacy yet, I'll come back to evaluate it after a while.	Jingdong prefecture level city in Shandong
Jinfeng Hongyuan Flagship Store	Electricity savings3	Just received the goods, use a period of time to evaluate	Jingdong prefecture level city in Shandong
Jinfeng Hongyuan Flagship Store	Electricity savings3	Opened and tried very good, use it to see if there are results	Jingdong prefecture level city in Shandong
Jinfeng Hongyuan Flagship Store	Electricity savings3	Arrived today! Still trying it out!	Jingdong prefecture level city in Shandong
Jinfeng Hongyuan Flagship Store	Electricity savings3	Shipped quickly, well packaged, already installed, hope there is a power saving effect	Jingdong prefecture level city in Shandong
Jinfeng Hongyuan Flagship Store	Electricity savings3	I got it. It's great.	Jingdong prefecture level city in Shandong
Jinfeng Hongyuan Flagship Store	Electricity savings3	Haven't used it yet, will come back in a month	Jingdong prefecture level city in Shandong
Jinfeng Hongyuan Flagship Store	Electricity savings3	It looks good. It's good. Try it first.	Jingdong prefecture level city in Shandong
Jinfeng Hongyuan Flagship Store	Electricity savings3	It works so well that I have relatives, friends, coworkers, and 6 families that have purchased it for them through me.	Jingdong prefecture level city in Shandong
Jinfeng Hongyuan Flagship Store	Electricity savings3	In use	Jingdong prefecture level city in Shandong
Jinfeng Hongyuan Flagship Store	Electricity savings3	Received the goods today, power-saving effect do not know being used, the boss has a very good attitude, praise!	Jingdong prefecture level city in Shandong
Jinfeng Hongyuan Flagship Store	Electricity savings3	The product is compact and doesn't take up much space, but hopefully it's power efficient.	Jingdong prefecture level city in Shandong
Jinfeng Hongyuan Flagship Store	Electricity savings3	Power saving is indeed effective, I used for a period of time before coming to evaluate, the electricity bill is obviously less, the quality is good, the shopkeeper service is also very good, very patient,	Jingdong prefecture level city in Shandong
Jinfeng Hongyuan Flagship Store	Electricity savings3	It feels fine, I'll comment after I use it	Jingdong prefecture level city in Shandong
Jinfeng Hongyuan Flagship Store	Electricity savings3	I just received it and I don't know the effect yet! I will comment again after I use it and have results!	Jingdong prefecture level city in Shandong
Jinfeng Hongyuan Flagship Store	Electricity savings3	The goods look fine, I hope they really work!	Jingdong prefecture level city in Shandong
Jinfeng Hongyuan Flagship Store	Electricity savings3	I just don't know if it saves power	Jingdong prefecture level city in Shandong
Jinfeng Hongyuan Flagship Store	Electricity savings3	My friend introduced me to use this product, it is professional and I am looking forward to the long term effect~!	Jingdong prefecture level city in Shandong
Jinfeng Hongyuan Flagship Store	Electricity savings3	Try it before you judge.	Jingdong prefecture level city in Shandong
Jinfeng Hongyuan Flagship Store	Electricity savings3	It's great, the power savings are obvious! Kudos!	Jingdong prefecture level city in Shandong
Jinfeng Hongyuan Flagship Store	Electricity savings3	The quality is excellent, stabilizes the voltage and saves electricity, very satisfied.	Jingdong prefecture level city in Shandong
Jinfeng Hongyuan Flagship Store	Electricity savings3	You have to use it for a while to know the effect	Jingdong prefecture level city in Shandong
Jinfeng Hongyuan Flagship Store	Electricity savings3	It's on trial.	Jingdong prefecture level city in Shandong
Jinfeng Hongyuan Flagship Store	Electricity savings3	The stuff works great.	Jingdong prefecture level city in Shandong
Jinfeng Hongyuan Flagship Store	Electricity savings3	Doesn't seem to work, not as god as other reviews	Jingdong prefecture level city in Shandong
Jinfeng Hongyuan Flagship Store	Electricity savings3	It's not good quality, it doesn't work, it stops working after a few days, it doesn't work, the customer service doesn't care, it's a bit irritating.	Jingdong prefecture level city in Shandong
Jinfeng Hongyuan Flagship Store	Electricity savings3	It doesn't do anything.	Jingdong prefecture level city in Shandong
Jinfeng Hongyuan Flagship Store	Electricity savings3	It's good.	Jingdong prefecture level city in Shandong
Jinfeng Hongyuan Flagship Store	Electricity savings3	Used a month effect is not obvious, are psychological effects, feel are not less, did not save to the electricity, there are details of the problem, the store said to buy to send 5 fuse, in fact, received only three, small problems are not committed to in place, I believe that the product is not good enough	Jingdong prefecture level city in Shandong
Jinfeng Hongyuan Flagship Store	Electricity savings3	Well received quickly, looking quite high, but also sent 5 fuses. Customer service is very patient to teach the operation, in fact, quite simple, lazy to read the instructions ha!	Jingdong prefecture level city in Shandong
Jinfeng Hongyuan Flagship Store	Electricity savings3	You have to use it for a while to know the effect	Jingdong prefecture level city in Shandong
Jinfeng Hongyuan Flagship Store	Electricity savings3	Shipped very quickly, packaged very well, the price is particularly affordable, there are so many efficacy is also good, it is worth buying worth buying the product, the workmanship is very fine, better than imagined, recommended that everyone try not yet used, after the use of the follow-up comment	Jingdong prefecture level city in Shandong

Jinfeng Hongyuan Flagship Store	Electricity savings3	It looks regular. It's working now.	Jingdong prefecture level city in Shandong
Jinfeng Hongyuan Flagship Store	Electricity savings3	It arrived pretty fast and the owner is very responsible. Have not used it yet, will see after use	Jingdong prefecture level city in Shandong
Jinfeng Hongyuan Flagship Store	Electricity savings3	🎁 baby quality is very good, the first time I received the goods to open the first time to see, it is very good 👍, very satisfied 😊, customer service also have a question and answer, thank you 🙏	Jingdong prefecture level city in Shandong
Jinfeng Hongyuan Flagship Store	Electricity savings3	I don't know if it's any good 。。。。。。	Jingdong prefecture level city in Shandong
Jinfeng Hongyuan Flagship Store	Electricity savings3	Buy electronic products! Still trust the things of the Jingdong self-owned store! Energy-saving effect to evaluate after heating!	Jingdong prefecture level city in Shandong
Jinfeng Hongyuan Flagship Store	Electricity savings3	The goods have been received, Blu is very beautiful, energy saving effect, to be confirmed only after actual use.	Jingdong prefecture level city in Shandong
Jinfeng Hongyuan Flagship Store	Electricity savings3	Jingdong's speed is so fast, has received, easy to operate. The display is quite good, how many volts what mode is quite understandable.	Jingdong prefecture level city in Shandong
Jinfeng Hongyuan Flagship Store	Electricity savings3	The first use, the effect is good to recommend it to colleagues to use, a month of electricity to save a lot or can be	Jingdong prefecture level city in Shandong
Jinfeng Hongyuan Flagship Store	Electricity savings3	I don't know if it's useful. Mom's is a store. I'll return it if it doesn't work.	Jingdong prefecture level city in Shandong
Jinfeng Hongyuan Flagship Store	Electricity savings3	I don't know if it works or not, I'll wait for two months to use it and then come back to comment!	Jingdong prefecture level city in Shandong
Jinfeng Hongyuan Flagship Store	Electricity savings3	I bought the second one now, and I've been using it for more than half a year, and I feel that it really saves electricity. Now it is summer, the weather is hot, sometimes the fan and air conditioning on all day, if not this power saver, the monthly electricity bill is quite high, but as long as the installation of this power saver, not afraid of its high electricity bill, absolutely let you wantonly use	Jingdong prefecture level city in Shandong
Jinfeng Hongyuan Flagship Store	Electricity savings3	The product is better than I thought it would be, the quality looks great, the voltage is stable, and I'm looking forward to some great power savings!	Jingdong prefecture level city in Shandong
Jinfeng Hongyuan Flagship Store	Electricity savings3	Inadvertently see bought a try, delivery is also quite timely, just do not know how to use, customer service patience to answer, with a pretty good, value for money!	Jingdong prefecture level city in Shandong
Jinfeng Hongyuan Flagship Store	Electricity savings3	It's better than nothing! Hopefully this will help save slightly.	Jingdong prefecture level city in Shandong
Jinfeng Hongyuan Flagship Store	Electricity savings3	Still trying it out, follow up	Jingdong prefecture level city in Shandong
Jinfeng Hongyuan Flagship Store	Electricity savings3	I've just started using it, I don't know how well it works yet, I hope it's good.	Jingdong prefecture level city in Shandong
Jinfeng Hongyuan Flagship Store	Electricity savings3	Fourth purchase, great baby!	Jingdong prefecture level city in Shandong
Jinfeng Hongyuan Flagship Store	Electricity savings3	Shipped really fast, just got it and use it to see what happens and then come back for a review!	Jingdong prefecture level city in Shandong
Jinfeng Hongyuan Flagship Store	Electricity savings3	Timely delivery **** good attitude	Jingdong prefecture level city in Shandong
Jinfeng Hongyuan Flagship Store	Electricity savings3	Home large and small things are purchased in the Jingdong Mall, have been accustomed to shopping in the Jingdong, many times can buy good quality and cheap products, and courier service attitude is particularly good, sometimes too many couriers are to help me to the home, so many years have been in the Jingdong Mall shopping, trust and support! Will also support the Jingdong as always!	Jingdong prefecture level city in Shandong
Jinfeng Hongyuan Flagship Store	Electricity savings3	Modified a pump and the modification worked.	Jingdong prefecture level city in Shandong
Jinfeng Hongyuan Flagship Store	Electricity savings3	Already installed, next time you pay the electric bill to see the results	Jingdong prefecture level city in Shandong
Jinfeng Hongyuan Flagship Store	Electricity savings3	The product is very good to use and practical to look at	Jingdong prefecture level city in Shandong
Jinfeng Hongyuan Flagship Store	Electricity savings3	Works great, great service, waiting for results.	Jingdong prefecture level city in Shandong
Jinfeng Hongyuan Flagship Store	Electricity savings3	I just used it and I'm not sure about the efficacy yet, I'll come back to evaluate it after a while.	Jingdong prefecture level city in Shandong
Jinfeng Hongyuan Flagship Store	Electricity savings3	Just received the goods, use a period of time to evaluate	Jingdong prefecture level city in Shandong
Jinfeng Hongyuan Flagship Store	Electricity savings3	Opened and tried very good, use it to see if there are results	Jingdong prefecture level city in Shandong
Jinfeng Hongyuan Flagship Store	Electricity savings3	Arrived today! Still trying it out!	Jingdong prefecture level city in Shandong
Jinfeng Hongyuan Flagship Store	Electricity savings3	Shipped quickly, well packaged, already installed, hope there is a power saving effect	Jingdong prefecture level city in Shandong
Jinfeng Hongyuan Flagship Store	Electricity savings3	I got it. It's great.	Jingdong prefecture level city in Shandong
Jinfeng Hongyuan Flagship Store	Electricity savings3	Haven't used it yet, will come back in a month	Jingdong prefecture level city in Shandong
Jinfeng Hongyuan Flagship Store	Electricity savings3	It looks good. It's good. Try it first.	Jingdong prefecture level city in Shandong
Jinfeng Hongyuan Flagship Store	Electricity savings3	It works so well that I have relatives, friends, coworkers, and 6 families that have purchased it for them through me.	Jingdong prefecture level city in Shandong
Jinfeng Hongyuan Flagship Store	Electricity savings3	In use	Jingdong prefecture level city in Shandong
Jinfeng Hongyuan Flagship Store	Electricity savings3	Received the goods today, power-saving effect do not know being used, the boss has a very good attitude, praise!	Jingdong prefecture level city in Shandong
Jinfeng Hongyuan Flagship Store	Electricity savings3	The product is compact and doesn't take up much space, but hopefully it's power efficient.	Jingdong prefecture level city in Shandong
Jinfeng Hongyuan Flagship Store	Electricity savings3	Power saving is indeed effective, I used for a period of time before coming to evaluate, the electricity bill is obviously less, the quality is good, the shopkeeper service is also very good, very patient,	Jingdong prefecture level city in Shandong
Jinfeng Hongyuan Flagship Store	Electricity savings3	It feels fine, I'll comment after I use it	Jingdong prefecture level city in Shandong
Jinfeng Hongyuan Flagship Store	Electricity savings3	I just received it and I don't know the effect yet! I will comment again after I use it and have results!	Jingdong prefecture level city in Shandong
Jinfeng Hongyuan Flagship Store	Electricity savings3	The goods look fine, I hope they really work!	Jingdong prefecture level city in Shandong
Jinfeng Hongyuan Flagship Store	Electricity savings3	I just don't know if it saves power	Jingdong prefecture level city in Shandong
Jinfeng Hongyuan Flagship Store	Electricity savings3	My friend introduced me to use this product, it is professional and I am looking forward to the long term effect~!	Jingdong prefecture level city in Shandong
Jinfeng Hongyuan Flagship Store	Electricity savings3	Try it before you judge.	Jingdong prefecture level city in Shandong
Jinfeng Hongyuan Flagship Store	Electricity savings3	It's great, the power savings are obvious! Kudos!	Jingdong prefecture level city in Shandong
Jinfeng Hongyuan Flagship Store	Electricity savings3	The quality is excellent, stabilizes the voltage and saves electricity, very satisfied.	Jingdong prefecture level city in Shandong
Jinfeng Hongyuan Flagship Store	Electricity savings3	You have to use it for a while to know the effect	Jingdong prefecture level city in Shandong
Jinfeng Hongyuan Flagship Store	Electricity savings3	It's on trial.	Jingdong prefecture level city in Shandong
Jinfeng Hongyuan Flagship Store	Electricity savings3	The stuff works great.	Jingdong prefecture level city in Shandong
Jinfeng Hongyuan Flagship Store	Electricity savings3	Doesn't seem to work, not as god as other reviews	Jingdong prefecture level city in Shandong

Jinfeng Hongyuan Flagship Store	Electricity savings3	It's not good quality, it doesn't work, it stops working after a few days, it doesn't work, the customer service doesn't care, it's a bit irritating.	Jingdong prefecture level city in Shandong
Jinfeng Hongyuan Flagship Store	Electricity savings3	It doesn't do anything.	Jingdong prefecture level city in Shandong
Jinfeng Hongyuan Flagship Store	Electricity savings3	It's good.	Jingdong prefecture level city in Shandong
Jinfeng Hongyuan Flagship Store	Electricity savings3	Used a month effect is not obvious, are psychological effects, feel are not less, did not save to the electricity, there are details of the problem, the store said to buy to send 5 fuse, in fact, received only three, small problems are not committed to in place, I believe that the product is not good enough	Jingdong prefecture level city in Shandong
Jinfeng Hongyuan Flagship Store	Electricity savings3	Well received quickly, looking quite high, but also sent 5 fuses. Customer service is very patient to teach the operation, in fact, quite simple, lazy to read the instructions ha!	Jingdong prefecture level city in Shandong
Jinfeng Hongyuan Flagship Store	Electricity savings3	You have to use it for a while to know the effect	Jingdong prefecture level city in Shandong
Jinfeng Hongyuan Flagship Store	Electricity savings4	The power saver is able to save electricity. The economizer is through real-time online detection of the motor's working status, and then change the input voltage. When the motor is detected in the light load or load is constantly changing, the use of thyristors can be within one hundredth of a second to adjust the input voltage of the motor, so that the output power of the motor and the real-time load just to match, thus effectively reducing the power loss of the motor, improve the motor startup, shutdown performance, to achieve the effect of power saving. When using, just plug in any idle socket (generally used in the line or large electrical appliances that use a lot of electricity by the side, you can enter the whole house into the energy-saving [1] state; no need for specialized maintenance). Through voltage stabilization, balancing current, reducing line loss, reactive power in situ compensation, improve power factor to achieve power saving effect. Relatively balance the supply voltage, reduce line loss, increase the effective power of the load, reduce the excess loss of excess active power, so as to achieve the	Jingdong prefecture level city in Shandong
Jinfeng Hongyuan Flagship Store	Savings in electricity consumption5	This power-saving steward energy-saving device is very good to use simple and very aspects plugged into the power supply can be If there are a lot of household appliances it is recommended to buy a is the best choice I use a multimeter to do a balanced voltage test The results are really as energy-saving steward shows balanced voltage and energy-saving current environmental protection and no noise and does not take up much space appearance blue good-looking good quality is very good and durable a long time is very	Jingdong prefecture level city in Shandong
Jinfeng Hongyuan Flagship Store	Electricity savings6	Intelligent ultra-high-power three-stage power saver, Jingdong logistics delivery quickly received, well-packed, tried the quality, the adjustment function is more satisfactory, plugged in the power supply can be used, convenient	Jingdong prefecture level city in Shandong
Jinfeng Hongyuan Flagship Store	Electricity savings7	We'll have to see how it works in a month's time.	Jingdong prefecture level city in Shandong
Jinfeng Hongyuan Flagship Store	Electricity savings8	This saver is good, high value aluminum alloy shell, the key is also with LCD display, workmanship and performance are very good, I hope to save a lot of electricity!	Jingdong prefecture level city in Shandong
Jinfeng Hongyuan Flagship Store	Electricity savings9	Nice stuff, good product, fast logistics.	Jingdong prefecture level city in Shandong
Jinfeng Hongyuan Flagship Store	Electricity savings of 10	The quality is very good, Jingdong shipping speed is very fast, packaging is very good, easy to use, can save electricity to save money	Jingdong prefecture level city in Shandong
Jinfeng Hongyuan Flagship Store	Electricity savings 11	Looking forward to the results	Jingdong prefecture level city in Shandong
Jinfeng Hongyuan Flagship Store	Savings in electricity consumption12	The product is barely ok, not perfect yet, use it for a while and see what happens!	Jingdong prefecture level city in Shandong
Jinfeng Hongyuan Flagship Store	Power Saving Specialist 1	Looked at the stuff, quite satisfied, now do not know how the effect, first sun a picture	Jingdong prefecture level city in Shandong
Jinfeng Hongyuan Flagship Store	Power Saving Specialist 1	The quality is good, the price is not expensive, it does save electricity, the logistics is also fast, customer service is very good	Jingdong prefecture level city in Shandong
Jinfeng Hongyuan Flagship Store	Power Saving Specialist 1	The product is well packaged, the logistics is fast, and the voltage is stable.	Jingdong prefecture level city in Shandong
Jinfeng Hongyuan Flagship Store	Power Saving Specialist 1	The baby is just as introduced, the texture is very good, powerful, and it feels good to use it	Jingdong prefecture level city in Shandong
Jinfeng Hongyuan Flagship Store	Power Saving Specialist 1	This is the first time this product has been purchased for use and it is not clear how effective it is. Hopefully it will save a lot of electricity.	Jingdong prefecture level city in Shandong
Jinfeng Hongyuan Flagship Store	Power Saving Specialist 1	Big brand, good stuff, worth buying.	Jingdong prefecture level city in Shandong
Jinfeng Hongyuan Flagship Store	Power Saving Specialist 1	Very good, looking forward to the results, worth buying	Jingdong prefecture level city in Shandong
Jinfeng Hongyuan Flagship Store	Power Saving Specialist 1	What is the problem when I get it back from the CTS, it is plugged into the wall and the voltage shows 206v.	Jingdong prefecture level city in Shandong
Jinfeng Hongyuan Flagship Store	Power Saving Specialist 1	The logistics was fast and the stuff was perfectly packaged. It is in the process of trial.	Jingdong prefecture level city in Shandong
Jinfeng Hongyuan Flagship Store	Power Saving Specialist 1	This saver is particularly good to use can really save electricity, with two months and then look at the line if you buy one for the family	Jingdong prefecture level city in Shandong
Jinfeng Hongyuan Flagship Store	Power Saving Specialist 1	Novel appearance design, all aluminum alloy profile, also with a fuse device, voltage monitoring display. After receiving the goods, I directly cut the plug and connect it to the main power box under the leakage switch, hoping to save electricity, and then follow up later. Logistics is very fast, arrived the next	Jingdong prefecture level city in Shandong
Jinfeng Hongyuan Flagship Store	Power Saving Specialist 1	Received, very good and like, if necessary next time to buy ha ha	Jingdong prefecture level city in Shandong
Jinfeng Hongyuan Flagship Store	Power Saving Specialist 1	It's good. It's worth it. Get it if you need it.	Jingdong prefecture level city in Shandong
Jinfeng Hongyuan Flagship Store	Power Saving Specialist 1	It feels more than good and is worth having.	Jingdong prefecture level city in Shandong
Jinfeng Hongyuan Flagship Store	Power Saving Specialist 1	The goods arrived very good and fast first time to buy	Jingdong prefecture level city in Shandong
Jinfeng Hongyuan Flagship Store	Power Saving Specialist 1	The power saving is very good, I can save so much money every month, I will buy another one next time.	Jingdong prefecture level city in Shandong
Jinfeng Hongyuan Flagship Store	Power Saving Specialist 1	It's not what it says.	Jingdong prefecture level city in Shandong
Jinfeng Hongyuan Flagship Store	Power Saving Specialist 1	I have been using it for many days now and it saves electricity, it really does I have an 8 kW electric snail stove and it saves about $3.50 a day.	Jingdong prefecture level city in Shandong
Jinfeng Hongyuan Flagship Store	Power Saving Specialist 1	Express delivery is very fast, the quality is also quite good, worth buying!	Jingdong prefecture level city in Shandong
Jinfeng Hongyuan Flagship Store	Power Saving Specialist 1	Just received expecting the baby to work well.	Jingdong prefecture level city in Shandong
Jinfeng Hongyuan Flagship Store	Power Saving Specialist 1	Very good, there are more fruit, has bought a second!	Jingdong prefecture level city in Shandong
Jinfeng Hongyuan Flagship Store	Power Saving Specialist 1	Good is good So far, no power savings have been noticed Maybe it's too little to notice.	Jingdong prefecture level city in Shandong
Jinfeng Hongyuan Flagship Store	Power Saving Specialist 1	I just received the item and plugged it in to see what happens later.	Jingdong prefecture level city in Shandong
Jinfeng Hongyuan Flagship Store	Power Saving Specialist 1	Baby received, especially good, packaging is also good, the courier brother is very strong, thank you.	Jingdong prefecture level city in Shandong
Jinfeng Hongyuan Flagship Store	Power Saving Specialist 1	Just received the goods have not yet used it, do not know how, three months later in the follow-up comment!	Jingdong prefecture level city in Shandong
Jinfeng Hongyuan Flagship Store	Power Saving Specialist 1	The item was received, it is quite good, recommended to buy.	Jingdong prefecture level city in Shandong
Jinfeng Hongyuan Flagship Store	Power Saving Specialist 1	This energy saver works very well and I recommend everyone to buy it!	Jingdong prefecture level city in Shandong
Jinfeng Hongyuan Flagship Store	Power Saving Specialist 1	Voltage stabilization and current reduction nice power saving product.	Jingdong prefecture level city in Shandong
Jinfeng Hongyuan Flagship Store	Power Saving Specialist 1	Voltage stabilization and current reduction nice power saving product.	Jingdong prefecture level city in Shandong

Jinfeng Hongyuan Flagship Store	Power Saving Specialist 1	A good power saver for stabilizing and lowering current.	Jingdong prefecture level city in Shandong
Jinfeng Hongyuan Flagship Store	Power Saving Specialist 1	You'll know if it saves power after you use it.	Jingdong prefecture level city in Shandong
Jinfeng Hongyuan Flagship Store	Power Saving Specialist 1	The quality is great. The quality is very good. The service is very good. Express delivery arrived in two days.	Jingdong prefecture level city in Shandong
Jinfeng Hongyuan Flagship Store	Power Saving Specialist 1	It works great.	Jingdong prefecture level city in Shandong
Jinfeng Hongyuan Flagship Store	Power Saving Specialist 1	Logistics is very fast, not yet used, used to comment again.	Jingdong prefecture level city in Shandong
Jinfeng Hongyuan Flagship Store	Power Saving Specialist 1	Logistics is very fast things are also very good, worthy of friends to buy	Jingdong prefecture level city in Shandong
Jinfeng Hongyuan Flagship Store	Power Saving Specialist 1	It works. It really works.	Jingdong prefecture level city in Shandong
Jinfeng Hongyuan Flagship Store	Power Saving Specialist 1	LANGUAGE COMMENTS: just received the shipment. I immediately plugged it in and used it. If you ask how effective. It depends on whether the next month's electricity bill has been reduced. If there is a decrease, I am buying more than one to use.	Jingdong prefecture level city in Shandong
Jinfeng Hongyuan Flagship Store	Power Saving Specialist 1	Well made, power saving, good and convenient!	Jingdong prefecture level city in Shandong
Jinfeng Hongyuan Flagship Store	Power Saving Specialist 1	I'm using it. I'll rate it next month.	Jingdong prefecture level city in Shandong
Jinfeng Hongyuan Flagship Store	Power Saving Specialist 1	Baby received Oh, the courier delivery really fast, with a good and then come back to the evaluation	Jingdong prefecture level city in Shandong
Jinfeng Hongyuan Flagship Store	Power Saving Specialist 1	Fast logistics, good packaging, worth it!	Jingdong prefecture level city in Shandong
Jinfeng Hongyuan Flagship Store	Power Saving Specialist 1	Customer service is okay. It's just that.	Jingdong prefecture level city in Shandong
Jinfeng Hongyuan Flagship Store	Power Saving Specialist 1	Poor power saving	Jingdong prefecture level city in Shandong
Jinfeng Hongyuan Flagship Store	Power Saving Specialist 1	I'll tell you when I've used it.	Jingdong prefecture level city in Shandong
Jinfeng Hongyuan Flagship Store	Power Saving Specialist 1	I think it's good.	Jingdong prefecture level city in Shandong
Jinfeng Hongyuan Flagship Store	Power Saving Specialist 1	This energy saver works very well and I recommend everyone to buy it!	Jingdong prefecture level city in Shandong
Jinfeng Hongyuan Flagship Store	Power Saving Specialist 1	I do not know how effective, just use half a month on the burn out, good business reissued a, and then use it after the follow-up comment.	Jingdong prefecture level city in Shandong
Jinfeng Hongyuan Flagship Store	Power Saving Specialist 1	in use	Jingdong prefecture level city in Shandong
Jinfeng Hongyuan Flagship Store	Power Saving Specialist 1	This thing is very poor. It's already only been used for ten days and it's broken. Now waiting for you to return it.	Jingdong prefecture level city in Shandong
Jinfeng Hongyuan Flagship Store	Power Saving Specialist 1	It's not the same as the seller's show.	Jingdong prefecture level city in Shandong
Jinfeng Hongyuan Flagship Store	Power Saving Specialist 1	What is the problem when I get it back from the CTS, it is plugged into the wall and the voltage shows 206v.	Jingdong prefecture level city in Shandong
Jinfeng Hongyuan Flagship Store	Power Saving Specialist 1	The logistics was fast and the stuff was perfectly packaged. It is in the process of trial.	Jingdong prefecture level city in Shandong
Jinfeng Hongyuan Flagship Store	Power Saving Specialist 1	This saver is particularly good to use can really save electricity, with two months and then look at the line if you buy one for the family	Jingdong prefecture level city in Shandong
Jinfeng Hongyuan Flagship Store	Power Saving Specialist 1	Novel appearance design, all aluminum alloy profile, also with a fuse device, voltage monitoring display. After receiving the goods, I directly cut the plug and connect it to the main power box under the leakage switch, hoping to save electricity, and then follow up later. Logistics is very fast, arrived the next	Jingdong prefecture level city in Shandong
Jinfeng Hongyuan Flagship Store	Power Saving Specialist 1	Received, very good and like, if necessary next time to buy ha ha	Jingdong prefecture level city in Shandong
Jinfeng Hongyuan Flagship Store	Power Saving Specialist 1	It's good. It's worth it. Get it if you need it.	Jingdong prefecture level city in Shandong
Jinfeng Hongyuan Flagship Store	Power Saving Specialist 1	It feels more than good and is worth having.	Jingdong prefecture level city in Shandong
Jinfeng Hongyuan Flagship Store	Power Saving Specialist 1	The goods arrived very good and fast first time to buy	Jingdong prefecture level city in Shandong
Jinfeng Hongyuan Flagship Store	Power Saving Specialist 1	The power saving is very good, I can save so much money every month, I will buy another one next time.	Jingdong prefecture level city in Shandong
Jinfeng Hongyuan Flagship Store	Power Saving Specialist 1	It's not what it says.	Jingdong prefecture level city in Shandong
Jinfeng Hongyuan Flagship Store	Power Saving Specialist 1	I have been using it for many days now and it saves electricity, it really does I have an 8 kW electric snail stove and it saves about $3.50 a day.	Jingdong prefecture level city in Shandong
Jinfeng Hongyuan Flagship Store	Power Saving Specialist 1	Express delivery is very fast, the quality is also quite good, worth buying!	Jingdong prefecture level city in Shandong
Jinfeng Hongyuan Flagship Store	Power Saving Specialist 1	Just received expecting the baby to work well.	Jingdong prefecture level city in Shandong
Jinfeng Hongyuan Flagship Store	Power Saving Specialist 1	Very good, there are more fruit, has bought a second!	Jingdong prefecture level city in Shandong
Jinfeng Hongyuan Flagship Store	Power Saving Specialist 1	Good is good So far, no power savings have been noticed Maybe it's too little to notice.	Jingdong prefecture level city in Shandong
Jinfeng Hongyuan Flagship Store	Power Saving Specialist 1	I just received the item and plugged it in to see what happens later.	Jingdong prefecture level city in Shandong
Jinfeng Hongyuan Flagship Store	Power Saving Specialist 1	Baby received, especially good, packaging is also good, the courier brother is very strong, thank you.	Jingdong prefecture level city in Shandong
Jinfeng Hongyuan Flagship Store	Power Saving Specialist 1	Just received the goods have not yet used it, do not know how, three months later in the follow-up comment!	Jingdong prefecture level city in Shandong
Jinfeng Hongyuan Flagship Store	Power Saving Specialist 1	The item was received, it is quite good, recommended to buy.	Jingdong prefecture level city in Shandong
Jinfeng Hongyuan Flagship Store	Power Saving Specialist 1	This energy saver works very well and I recommend everyone to buy it!	Jingdong prefecture level city in Shandong
Jinfeng Hongyuan Flagship Store	Power Saving Specialist 1	Voltage stabilization and current reduction nice power saving product.	Jingdong prefecture level city in Shandong
Jinfeng Hongyuan Flagship Store	Power Saving Specialist 1	Voltage stabilization and current reduction nice power saving product.	Jingdong prefecture level city in Shandong
Jinfeng Hongyuan Flagship Store	Power Saving Specialist 1	A good power saver for stabilizing and lowering current.	Jingdong prefecture level city in Shandong
Jinfeng Hongyuan Flagship Store	Power Saving Specialist 1	You'll know if it saves power after you use it.	Jingdong prefecture level city in Shandong
Jinfeng Hongyuan Flagship Store	Power Saving Specialist 1	The quality is great. The quality is very good. The service is very good. Express delivery arrived in two days.	Jingdong prefecture level city in Shandong
Jinfeng Hongyuan Flagship Store	Power Saving Specialist 1	It works great.	Jingdong prefecture level city in Shandong
Jinfeng Hongyuan Flagship Store	Power Saving Specialist 1	Logistics is very fast, not yet used, used to comment again.	Jingdong prefecture level city in Shandong
Jinfeng Hongyuan Flagship Store	Power Saving Specialist 1	Logistics is very fast things are also very good, worthy of friends to buy	Jingdong prefecture level city in Shandong
Jinfeng Hongyuan Flagship Store	Power Saving Specialist 1	It works. It really works.	Jingdong prefecture level city in Shandong

Jinfeng Hongyuan Flagship Store	Power Saving Specialist 1	LANGUAGE COMMENTS: just received the shipment. I immediately plugged it in and used it. If you ask how effective. It depends on whether the next month's electricity bill has been reduced. If there is a decrease. I am buying more than one to use.	Jingdong prefecture level city in Shandong
Jinfeng Hongyuan Flagship Store	Power Saving Specialist 1	Well made, power saving, good and convenient!	Jingdong prefecture level city in Shandong
Jinfeng Hongyuan Flagship Store	Power Saving Specialist 1	I'm using it. I'll rate it next month.	Jingdong prefecture level city in Shandong
Jinfeng Hongyuan Flagship Store	Power Saving Specialist 1	Baby received Oh, the courier delivery really fast, with a good and then come back to the evaluation	Jingdong prefecture level city in Shandong
Jinfeng Hongyuan Flagship Store	Power Saving Specialist 1	Fast logistics, good packaging, worth it!	Jingdong prefecture level city in Shandong
Jinfeng Hongyuan Flagship Store	Power Saving Specialist 1	Customer service is okay. It's just that.	Jingdong prefecture level city in Shandong
Jinfeng Hongyuan Flagship Store	Power Saving Specialist 1	Poor power saving	Jingdong prefecture level city in Shandong
Jinfeng Hongyuan Flagship Store	Power Saving Specialist 1	I'll tell you when I've used it.	Jingdong prefecture level city in Shandong
Jinfeng Hongyuan Flagship Store	Power Saving Specialist 1	I think it's good.	Jingdong prefecture level city in Shandong
Jinfeng Hongyuan Flagship Store	Power Saving Specialist 1	This energy saver works very well and I recommend everyone to buy it!	Jingdong prefecture level city in Shandong
Jinfeng Hongyuan Flagship Store	Power Saving Specialist 1	I do not know how effective, just use half a month on the burn out, good business reissued a, and then use it after the follow-up comment.	Jingdong prefecture level city in Shandong
Jinfeng Hongyuan Flagship Store	Power Saving Specialist 1	in use	Jingdong prefecture level city in Shandong
Jinfeng Hongyuan Flagship Store	Power Saving Specialist 1	This thing is very poor. It's already only been used for ten days and it's broken. Now waiting for you to return it.	Jingdong prefecture level city in Shandong
Jinfeng Hongyuan Flagship Store	Power Saving Specialist 1	It's not the same as the seller's show.	Jingdong prefecture level city in Shandong
Jinfeng Hongyuan Flagship Store	Power Saving Specialist 1	What is the problem when I get it back from the CTS, it is plugged into the wall and the voltage shows 206v.	Jingdong prefecture level city in Shandong
Jinfeng Hongyuan Flagship Store	Power Saving Specialist 1	The logistics was fast and the stuff was perfectly packaged. It is in the process of trial.	Jingdong prefecture level city in Shandong
Jinfeng Hongyuan Flagship Store	Power Saving Specialist 1	This saver is particularly good to use can really save electricity, with two months and then look at the line if you buy one for the family	Jingdong prefecture level city in Shandong
Jinfeng Hongyuan Flagship Store	Power Saving Specialist 1	Novel appearance design, all aluminum alloy profile, also with a fuse device, voltage monitoring display. After receiving the goods, I directly cut the plug and connect it to the main power box under the leakage switch, hoping to save electricity, and then follow up later. Logistics is very fast, arrived the next	Jingdong prefecture level city in Shandong
Jinfeng Hongyuan Flagship Store	Power Saving Specialist 1	Received, very good and like, if necessary next time to buy ha ha	Jingdong prefecture level city in Shandong
Jinfeng Hongyuan Flagship Store	Power Saving Specialist 1	It's good. It's worth it. Get it if you need it.	Jingdong prefecture level city in Shandong
Jinfeng Hongyuan Flagship Store	Power Saving Specialist 1	It feels more than good and is worth having.	Jingdong prefecture level city in Shandong
Jinfeng Hongyuan Flagship Store	Power Saving Specialist 1	The goods arrived very good and fast first time to buy	Jingdong prefecture level city in Shandong
Jinfeng Hongyuan Flagship Store	Power Saving Specialist 1	The power saving is very good, I can save so much money every month, I will buy another one next time.	Jingdong prefecture level city in Shandong
Jinfeng Hongyuan Flagship Store	Power Saving Specialist 1	It's not what it says.	Jingdong prefecture level city in Shandong
Jinfeng Hongyuan Flagship Store	Power Saving Specialist 1	I have been using it for many days now and it saves electricity, it really does I have an 8 kW electric snail stove and it saves about $3.50 a day.	Jingdong prefecture level city in Shandong
Jinfeng Hongyuan Flagship Store	Power Saving Specialist 1	Express delivery is very fast, the quality is also quite good, worth buying!	Jingdong prefecture level city in Shandong
Jinfeng Hongyuan Flagship Store	Power Saving Specialist 1	Just received expecting the baby to work well.	Jingdong prefecture level city in Shandong
Jinfeng Hongyuan Flagship Store	Power Saving Specialist 1	Very good, there are more fruit, has bought a second!	Jingdong prefecture level city in Shandong
Jinfeng Hongyuan Flagship Store	Power Saving Specialist 1	Good is good So far, no power savings have been noticed Maybe it's too little to notice.	Jingdong prefecture level city in Shandong
Jinfeng Hongyuan Flagship Store	Power Saving Specialist 1	I just received the item and plugged it in to see what happens later.	Jingdong prefecture level city in Shandong
Jinfeng Hongyuan Flagship Store	Power Saving Specialist 1	Baby received, especially good, packaging is also good, the courier brother is very strong, thank you.	Jingdong prefecture level city in Shandong
Jinfeng Hongyuan Flagship Store	Power Saving Specialist 1	Just received the goods have not yet used it, do not know how, three months later in the follow-up comment!	Jingdong prefecture level city in Shandong
Jinfeng Hongyuan Flagship Store	Power Saving Specialist 1	The item was received, it is quite good, recommended to buy.	Jingdong prefecture level city in Shandong
Jinfeng Hongyuan Flagship Store	Power Saving Specialist 1	This energy saver works very well and I recommend everyone to buy it!	Jingdong prefecture level city in Shandong
Jinfeng Hongyuan Flagship Store	Power Saving Specialist 1	Voltage stabilization and current reduction nice power saving product.	Jingdong prefecture level city in Shandong
Jinfeng Hongyuan Flagship Store	Power Saving Specialist 1	Voltage stabilization and current reduction nice power saving product.	Jingdong prefecture level city in Shandong
Jinfeng Hongyuan Flagship Store	Power Saving Specialist 1	A good power saver for stabilizing and lowering current.	Jingdong prefecture level city in Shandong
Jinfeng Hongyuan Flagship Store	Power Saving Specialist 1	You'll know if it saves power after you use it.	Jingdong prefecture level city in Shandong
Jinfeng Hongyuan Flagship Store	Power Saving Specialist 1	The quality is great. The quality is very good. The service is very good. Express delivery arrived in two days.	Jingdong prefecture level city in Shandong
Jinfeng Hongyuan Flagship Store	Power Saving Specialist 1	It works great.	Jingdong prefecture level city in Shandong
Jinfeng Hongyuan Flagship Store	Power Saving Specialist 1	Logistics is very fast, not yet used, used to comment again.	Jingdong prefecture level city in Shandong
Jinfeng Hongyuan Flagship Store	Power Saving Specialist 1	Logistics is very fast things are also very good, worthy of friends to buy	Jingdong prefecture level city in Shandong
Jinfeng Hongyuan Flagship Store	Power Saving Specialist 1	It works. It really works.	Jingdong prefecture level city in Shandong
Jinfeng Hongyuan Flagship Store	Power Saving Specialist 1	LANGUAGE COMMENTS: just received the shipment. I immediately plugged it in and used it. If you ask how effective. It depends on whether the next month's electricity bill has been reduced. If there is a decrease. I am buying more than one to use.	Jingdong prefecture level city in Shandong
Jinfeng Hongyuan Flagship Store	Power Saving Specialist 1	Well made, power saving, good and convenient!	Jingdong prefecture level city in Shandong
Jinfeng Hongyuan Flagship Store	Power Saving Specialist 1	I'm using it. I'll rate it next month.	Jingdong prefecture level city in Shandong
Jinfeng Hongyuan Flagship Store	Power Saving Specialist 1	Baby received Oh, the courier delivery really fast, with a good and then come back to the evaluation	Jingdong prefecture level city in Shandong
Jinfeng Hongyuan Flagship Store	Power Saving Specialist 1	Fast logistics, good packaging, worth it!	Jingdong prefecture level city in Shandong
Jinfeng Hongyuan Flagship Store	Power Saving Specialist 1	Customer service is okay. It's just that.	Jingdong prefecture level city in Shandong
Jinfeng Hongyuan Flagship Store	Power Saving Specialist 1	Poor power saving	Jingdong prefecture level city in Shandong

Jinfeng Hongyuan Flagship Store	Power Saving Specialist 1	I'll tell you when I've used it.	Jingdong prefecture level city in Shandong
Jinfeng Hongyuan Flagship Store	Power Saving Specialist 1	I think it's good.	Jingdong prefecture level city in Shandong
Jinfeng Hongyuan Flagship Store	Power Saving Specialist 1	This energy saver works very well and I recommend everyone to buy it!	Jingdong prefecture level city in Shandong
Jinfeng Hongyuan Flagship Store	Power Saving Specialist 1	I do not know how effective, just use half a month on the burn out, good business reissued a, and then use it after the follow-up comment.	Jingdong prefecture level city in Shandong
Jinfeng Hongyuan Flagship Store	Power Saving Specialist 1	in use	Jingdong prefecture level city in Shandong
Jinfeng Hongyuan Flagship Store	Power Saving Specialist 1	This thing is very poor. It's already only been used for ten days and it's broken. Now waiting for you to return it.	Jingdong prefecture level city in Shandong
Jinfeng Hongyuan Flagship Store	Power Saving Specialist 1	It's not the same as the seller's show.	Jingdong prefecture level city in Shandong
Jinfeng Hongyuan Flagship Store	Power Saving Specialist 1	What is the problem when I get it back from the CTS, it is plugged into the wall and the voltage shows 206v.	Jingdong prefecture level city in Shandong
Jinfeng Hongyuan Flagship Store	Power Saving Specialist 1	The logistics was fast and the stuff was perfectly packaged. It is in the process of trial.	Jingdong prefecture level city in Shandong
Jinfeng Hongyuan Flagship Store	Power Saving Specialist 1	This saver is particularly good to use can really save electricity, with two months and then look at the line if you buy one for the family	Jingdong prefecture level city in Shandong
Jinfeng Hongyuan Flagship Store	Power Saving Specialist 1	Novel appearance design, all aluminum alloy profile, also with a fuse device, voltage monitoring display. After receiving the goods, I directly cut the plug and connect it to the main power box under the leakage switch, hoping to save electricity, and then follow up later. Logistics is very fast, arrived the next	Jingdong prefecture level city in Shandong
Jinfeng Hongyuan Flagship Store	Power Saving Specialist 1	Received, very good and like, if necessary next time to buy ha ha	Jingdong prefecture level city in Shandong
Jinfeng Hongyuan Flagship Store	Power Saving Specialist 1	It's good. It's worth it. Get it if you need it.	Jingdong prefecture level city in Shandong
Jinfeng Hongyuan Flagship Store	Power Saving Specialist 1	It feels more than good and is worth having.	Jingdong prefecture level city in Shandong
Jinfeng Hongyuan Flagship Store	Power Saving Specialist 1	The goods arrived very good and fast first time to buy	Jingdong prefecture level city in Shandong
Jinfeng Hongyuan Flagship Store	Power Saving Specialist 1	The power saving is very good, I can save so much money every month, I will buy another one next time.	Jingdong prefecture level city in Shandong
Jinfeng Hongyuan Flagship Store	Power Saving Specialist 1	It's not what it says.	Jingdong prefecture level city in Shandong
Jinfeng Hongyuan Flagship Store	Power Saving Specialist 1	I have been using it for many days now and it saves electricity, it really does I have an 8 kW electric snail stove and it saves about $3.50 a day.	Jingdong prefecture level city in Shandong
Jinfeng Hongyuan Flagship Store	Power Saving Specialist 1	Express delivery is very fast, the quality is also quite good, worth buying!	Jingdong prefecture level city in Shandong
Jinfeng Hongyuan Flagship Store	Power Saving Specialist 1	Just received expecting the baby to work well.	Jingdong prefecture level city in Shandong
Jinfeng Hongyuan Flagship Store	Power Saving Specialist 1	Very good, there are more fruit, has bought a second!	Jingdong prefecture level city in Shandong
Jinfeng Hongyuan Flagship Store	Power Saving Specialist 1	Good is good So far, no power savings have been noticed Maybe it's too little to notice.	Jingdong prefecture level city in Shandong
Jinfeng Hongyuan Flagship Store	Power Saving Specialist 1	I just received the item and plugged it in to see what happens later.	Jingdong prefecture level city in Shandong
Jinfeng Hongyuan Flagship Store	Power Saving Specialist 1	Baby received, especially good, packaging is also good, the courier brother is very strong, thank you.	Jingdong prefecture level city in Shandong
Jinfeng Hongyuan Flagship Store	Power Saving Specialist 1	Just received the goods have not yet used it, do not know how, three months later in the follow-up comment!	Jingdong prefecture level city in Shandong
Jinfeng Hongyuan Flagship Store	Power Saving Specialist 1	The item was received, it is quite good, recommended to buy.	Jingdong prefecture level city in Shandong
Jinfeng Hongyuan Flagship Store	Power Saving Specialist 1	This energy saver works very well and I recommend everyone to buy it!	Jingdong prefecture level city in Shandong
Jinfeng Hongyuan Flagship Store	Power Saving Specialist 1	Voltage stabilization and current reduction nice power saving product.	Jingdong prefecture level city in Shandong
Jinfeng Hongyuan Flagship Store	Power Saving Specialist 1	Voltage stabilization and current reduction nice power saving product.	Jingdong prefecture level city in Shandong
Jinfeng Hongyuan Flagship Store	Power Saving Specialist 1	A good power saver for stabilizing and lowering current.	Jingdong prefecture level city in Shandong
Jinfeng Hongyuan Flagship Store	Power Saving Specialist 1	You'll know if it saves power after you use it.	Jingdong prefecture level city in Shandong
Jinfeng Hongyuan Flagship Store	Power Saving Specialist 1	The quality is great. The quality is very good. The service is very good. Express delivery arrived in two days.	Jingdong prefecture level city in Shandong
Jinfeng Hongyuan Flagship Store	Power Saving Specialist 1	It works great.	Jingdong prefecture level city in Shandong
Jinfeng Hongyuan Flagship Store	Power Saving Specialist 1	Logistics is very fast, not yet used, used to comment again.	Jingdong prefecture level city in Shandong
Jinfeng Hongyuan Flagship Store	Power Saving Specialist 1	Logistics is very fast things are also very good, worthy of friends to buy	Jingdong prefecture level city in Shandong
Jinfeng Hongyuan Flagship Store	Power Saving Specialist 1	It works. It really works.	Jingdong prefecture level city in Shandong
Jinfeng Hongyuan Flagship Store	Power Saving Specialist 1	LANGUAGE COMMENTS: just received the shipment. I immediately plugged it in and used it. If you ask how effective. It depends on whether the next month's electricity bill has been reduced. If there is a decrease. I am buying more than one to use.	Jingdong prefecture level city in Shandong
Jinfeng Hongyuan Flagship Store	Power Saving Specialist 1	Well made, power saving, good and convenient!	Jingdong prefecture level city in Shandong
Jinfeng Hongyuan Flagship Store	Power Saving Specialist 1	I'm using it. I'll rate it next month.	Jingdong prefecture level city in Shandong
Jinfeng Hongyuan Flagship Store	Power Saving Specialist 1	Baby received Oh, the courier delivery really fast, with a good and then come back to the evaluation	Jingdong prefecture level city in Shandong
Jinfeng Hongyuan Flagship Store	Power Saving Specialist 1	Fast logistics, good packaging, worth it!	Jingdong prefecture level city in Shandong
Jinfeng Hongyuan Flagship Store	Power Saving Specialist 1	Customer service is okay. It's just that.	Jingdong prefecture level city in Shandong
Jinfeng Hongyuan Flagship Store	Power Saving Specialist 1	Poor power saving	Jingdong prefecture level city in Shandong
Jinfeng Hongyuan Flagship Store	Power Saving Specialist 1	I'll tell you when I've used it.	Jingdong prefecture level city in Shandong
Jinfeng Hongyuan Flagship Store	Power Saving Specialist 1	I think it's good.	Jingdong prefecture level city in Shandong
Jinfeng Hongyuan Flagship Store	Power Saving Specialist 1	This energy saver works very well and I recommend everyone to buy it!	Jingdong prefecture level city in Shandong
Jinfeng Hongyuan Flagship Store	Power Saving Specialist 1	I do not know how effective, just use half a month on the burn out, good business reissued a, and then use it after the follow-up comment.	Jingdong prefecture level city in Shandong
Jinfeng Hongyuan Flagship Store	Power Saving Specialist 1	in use	Jingdong prefecture level city in Shandong
Jinfeng Hongyuan Flagship Store	Power Saving Specialist 1	This thing is very poor. It's already only been used for ten days and it's broken. Now waiting for you to return it.	Jingdong prefecture level city in Shandong
Jinfeng Hongyuan Flagship Store	Power Saving Specialist 1	It's not the same as the seller's show.	Jingdong prefecture level city in Shandong

Jinfeng Hongyuan Flagship Store	Power Saving Specialist 1	What is the problem when I get it back from the CTS, it is plugged into the wall and the voltage shows 206v.	Jingdong prefecture level city in Shandong
Jinfeng Hongyuan Flagship Store	Power Saving Specialist 1	The logistics was fast and the stuff was perfectly packaged. It is in the process of trial.	Jingdong prefecture level city in Shandong
Jinfeng Hongyuan Flagship Store	Power Saving Specialist 1	This saver is particularly good to use can really save electricity, with two months and then look at the line if you buy one for the family	Jingdong prefecture level city in Shandong
Jinfeng Hongyuan Flagship Store	Power Saving Specialist 1	Novel appearance design, all aluminum alloy profile, also with a fuse device, voltage monitoring display. After receiving the goods, I directly cut the plug and connect it to the main power box under the leakage switch, hoping to save electricity, and then follow up later. Logistics is very fast, arrived the next	Jingdong prefecture level city in Shandong
Jinfeng Hongyuan Flagship Store	Power Saving Specialist 1	Received, very good and like, if necessary next time to buy ha ha	Jingdong prefecture level city in Shandong
Jinfeng Hongyuan Flagship Store	Power Saving Specialist 1	It's good. It's worth it. Get it if you need it.	Jingdong prefecture level city in Shandong
Jinfeng Hongyuan Flagship Store	Power Saving Specialist 1	It feels more than good and is worth having.	Jingdong prefecture level city in Shandong
Jinfeng Hongyuan Flagship Store	Power Saving Specialist 1	The goods arrived very good and fast first time to buy	Jingdong prefecture level city in Shandong
Jinfeng Hongyuan Flagship Store	Power Saving Specialist 1	The power saving is very good, I can save so much money every month, I will buy another one next time.	Jingdong prefecture level city in Shandong
Jinfeng Hongyuan Flagship Store	Power Saving Specialist 1	It's not what it says.	Jingdong prefecture level city in Shandong
Jinfeng Hongyuan Flagship Store	Power Saving Specialist 1	I have been using it for many days now and it saves electricity, it really does I have an 8 kW electric snail stove and it saves about $3.50 a day.	Jingdong prefecture level city in Shandong
Jinfeng Hongyuan Flagship Store	Power Saving Specialist 1	Express delivery is very fast, the quality is also quite good, worth buying!	Jingdong prefecture level city in Shandong
Jinfeng Hongyuan Flagship Store	Power Saving Specialist 1	Just received expecting the baby to work well.	Jingdong prefecture level city in Shandong
Jinfeng Hongyuan Flagship Store	Power Saving Specialist 1	Very good, there are more fruit, has bought a second!	Jingdong prefecture level city in Shandong
Jinfeng Hongyuan Flagship Store	Power Saving Specialist 1	Good is good So far, no power savings have been noticed Maybe it's too little to notice.	Jingdong prefecture level city in Shandong
Jinfeng Hongyuan Flagship Store	Power Saving Specialist 1	I just received the item and plugged it in to see what happens later.	Jingdong prefecture level city in Shandong
Jinfeng Hongyuan Flagship Store	Power Saving Specialist 1	Baby received, especially good, packaging is also good, the courier brother is very strong, thank you.	Jingdong prefecture level city in Shandong
Jinfeng Hongyuan Flagship Store	Power Saving Specialist 1	Just received the goods have not yet used it, do not know how, three months later in the follow-up comment!	Jingdong prefecture level city in Shandong
Jinfeng Hongyuan Flagship Store	Power Saving Specialist 1	The item was received, it is quite good, recommended to buy.	Jingdong prefecture level city in Shandong
Jinfeng Hongyuan Flagship Store	Power Saving Specialist 1	This energy saver works very well and I recommend everyone to buy it!	Jingdong prefecture level city in Shandong
Jinfeng Hongyuan Flagship Store	Power Saving Specialist 1	Voltage stabilization and current reduction nice power saving product.	Jingdong prefecture level city in Shandong
Jinfeng Hongyuan Flagship Store	Power Saving Specialist 1	Voltage stabilization and current reduction nice power saving product.	Jingdong prefecture level city in Shandong
Jinfeng Hongyuan Flagship Store	Power Saving Specialist 1	A good power saver for stabilizing and lowering current.	Jingdong prefecture level city in Shandong
Jinfeng Hongyuan Flagship Store	Power Saving Specialist 1	You'll know if it saves power after you use it.	Jingdong prefecture level city in Shandong
Jinfeng Hongyuan Flagship Store	Power Saving Specialist 1	The quality is great. The quality is very good. The service is very good. Express delivery arrived in two days.	Jingdong prefecture level city in Shandong
Jinfeng Hongyuan Flagship Store	Power Saving Specialist 1	It works great.	Jingdong prefecture level city in Shandong
Jinfeng Hongyuan Flagship Store	Power Saving Specialist 1	Logistics is very fast, not yet used, used to comment again.	Jingdong prefecture level city in Shandong
Jinfeng Hongyuan Flagship Store	Power Saving Specialist 1	Logistics is very fast things are also very good, worthy of friends to buy	Jingdong prefecture level city in Shandong
Jinfeng Hongyuan Flagship Store	Power Saving Specialist 1	It works. It really works.	Jingdong prefecture level city in Shandong
Jinfeng Hongyuan Flagship Store	Power Saving Specialist 1	LANGUAGE COMMENTS: just received the shipment. I immediately plugged it in and used it. If you ask how effective. It depends on whether the next month's electricity bill has been reduced. If there is a decrease. I am buying more than one to use.	Jingdong prefecture level city in Shandong
Jinfeng Hongyuan Flagship Store	Power Saving Specialist 1	Well made, power saving, good and convenient!	Jingdong prefecture level city in Shandong
Jinfeng Hongyuan Flagship Store	Power Saving Specialist 1	I'm using it. I'll rate it next month.	Jingdong prefecture level city in Shandong
Jinfeng Hongyuan Flagship Store	Power Saving Specialist 1	Baby received Oh, the courier delivery really fast, with a good and then come back to the evaluation	Jingdong prefecture level city in Shandong
Jinfeng Hongyuan Flagship Store	Power Saving Specialist 1	Fast logistics, good packaging, worth it!	Jingdong prefecture level city in Shandong
Jinfeng Hongyuan Flagship Store	Power Saving Specialist 1	Customer service is okay. It's just that.	Jingdong prefecture level city in Shandong
Jinfeng Hongyuan Flagship Store	Power Saving Specialist 1	Poor power saving	Jingdong prefecture level city in Shandong
Jinfeng Hongyuan Flagship Store	Power Saving Specialist 1	I'll tell you when I've used it.	Jingdong prefecture level city in Shandong
Jinfeng Hongyuan Flagship Store	Power Saving Specialist 1	I think it's good.	Jingdong prefecture level city in Shandong
Jinfeng Hongyuan Flagship Store	Power Saving Specialist 1	This energy saver works very well and I recommend everyone to buy it!	Jingdong prefecture level city in Shandong
Jinfeng Hongyuan Flagship Store	Power Saving Specialist 1	I do not know how effective, just use half a month on the burn out, good business reissued a, and then use it after the follow-up comment.	Jingdong prefecture level city in Shandong
Jinfeng Hongyuan Flagship Store	Power Saving Specialist 1	in use	Jingdong prefecture level city in Shandong
Jinfeng Hongyuan Flagship Store	Power Saving Specialist 1	This thing is very poor. It's already only been used for ten days and it's broken. Now waiting for you to return it.	Jingdong prefecture level city in Shandong
Jinfeng Hongyuan Flagship Store	Power Saving Specialist 1	It's not the same as the seller's show.	Jingdong prefecture level city in Shandong
Jinfeng Hongyuan Flagship Store	Power Saving Specialist 1	What is the problem when I get it back from the CTS, it is plugged into the wall and the voltage shows 206v.	Jingdong prefecture level city in Shandong
Jinfeng Hongyuan Flagship Store	Power Saving Specialist 1	The logistics was fast and the stuff was perfectly packaged. It is in the process of trial.	Jingdong prefecture level city in Shandong
Jinfeng Hongyuan Flagship Store	Power Saving Specialist 1	This saver is particularly good to use can really save electricity, with two months and then look at the line if you buy one for the family	Jingdong prefecture level city in Shandong
Jinfeng Hongyuan Flagship Store	Power Saving Specialist 1	Novel appearance design, all aluminum alloy profile, also with a fuse device, voltage monitoring display. After receiving the goods, I directly cut the plug and connect it to the main power box under the leakage switch, hoping to save electricity, and then follow up later. Logistics is very fast, arrived the next	Jingdong prefecture level city in Shandong
Jinfeng Hongyuan Flagship Store	Power Saving Specialist 1	Received, very good and like, if necessary next time to buy ha ha	Jingdong prefecture level city in Shandong
Jinfeng Hongyuan Flagship Store	Power Saving Specialist 1	It's good. It's worth it. Get it if you need it.	Jingdong prefecture level city in Shandong
Jinfeng Hongyuan Flagship Store	Power Saving Specialist 1	It feels more than good and is worth having.	Jingdong prefecture level city in Shandong

Jinfeng Hongyuan Flagship Store	Power Saving Specialist 1	The goods arrived very good and fast first time to buy	Jingdong prefecture level city in Shandong
Jinfeng Hongyuan Flagship Store	Power Saving Specialist 1	The power saving is very good, I can save so much money every month, I will buy another one next time.	Jingdong prefecture level city in Shandong
Jinfeng Hongyuan Flagship Store	Power Saving Specialist 1	It's not what it says.	Jingdong prefecture level city in Shandong
Jinfeng Hongyuan Flagship Store	Power Saving Specialist 1	I have been using it for many days now and it saves electricity, it really does I have an 8 kW electric snail stove and it saves about $3.50 a day.	Jingdong prefecture level city in Shandong
Jinfeng Hongyuan Flagship Store	Power Saving Specialist 1	Express delivery is very fast, the quality is also quite good, worth buying!	Jingdong prefecture level city in Shandong
Jinfeng Hongyuan Flagship Store	Power Saving Specialist 1	Just received expecting the baby to work well.	Jingdong prefecture level city in Shandong
Jinfeng Hongyuan Flagship Store	Power Saving Specialist 1	Very good, there are more fruit, has bought a second!	Jingdong prefecture level city in Shandong
Jinfeng Hongyuan Flagship Store	Power Saving Specialist 1	Good is good So far, no power savings have been noticed Maybe it's too little to notice.	Jingdong prefecture level city in Shandong
Jinfeng Hongyuan Flagship Store	Power Saving Specialist 1	I just received the item and plugged it in to see what happens later.	Jingdong prefecture level city in Shandong
Jinfeng Hongyuan Flagship Store	Power Saving Specialist 1	Baby received, especially good, packaging is also good, the courier brother is very strong, thank you.	Jingdong prefecture level city in Shandong
Jinfeng Hongyuan Flagship Store	Power Saving Specialist 1	Just received the goods have not yet used it, do not know how, three months later in the follow-up comment!	Jingdong prefecture level city in Shandong
Jinfeng Hongyuan Flagship Store	Power Saving Specialist 1	The item was received, it is quite good, recommended to buy.	Jingdong prefecture level city in Shandong
Jinfeng Hongyuan Flagship Store	Power Saving Specialist 1	This energy saver works very well and I recommend everyone to buy it!	Jingdong prefecture level city in Shandong
Jinfeng Hongyuan Flagship Store	Power Saving Specialist 1	Voltage stabilization and current reduction nice power saving product.	Jingdong prefecture level city in Shandong
Jinfeng Hongyuan Flagship Store	Power Saving Specialist 1	Voltage stabilization and current reduction nice power saving product.	Jingdong prefecture level city in Shandong
Jinfeng Hongyuan Flagship Store	Power Saving Specialist 1	A good power saver for stabilizing and lowering current.	Jingdong prefecture level city in Shandong
Jinfeng Hongyuan Flagship Store	Power Saving Specialist 1	You'll know if it saves power after you use it.	Jingdong prefecture level city in Shandong
Jinfeng Hongyuan Flagship Store	Power Saving Specialist 1	The quality is great. The quality is very good. The service is very good. Express delivery arrived in two days.	Jingdong prefecture level city in Shandong
Jinfeng Hongyuan Flagship Store	Power Saving Specialist 1	It works great.	Jingdong prefecture level city in Shandong
Jinfeng Hongyuan Flagship Store	Power Saving Specialist 1	Logistics is very fast, not yet used, used to comment again.	Jingdong prefecture level city in Shandong
Jinfeng Hongyuan Flagship Store	Power Saving Specialist 1	Logistics is very fast things are also very good, worthy of friends to buy	Jingdong prefecture level city in Shandong
Jinfeng Hongyuan Flagship Store	Power Saving Specialist 1	It works. It really works.	Jingdong prefecture level city in Shandong
Jinfeng Hongyuan Flagship Store	Power Saving Specialist 1	LANGUAGE COMMENTS: just received the shipment. I immediately plugged it in and used it. If you ask how effective. It depends on whether the next month's electricity bill has been reduced. If there is a decrease. I am buying more than one to use.	Jingdong prefecture level city in Shandong
Jinfeng Hongyuan Flagship Store	Power Saving Specialist 1	Well made, power saving, good and convenient!	Jingdong prefecture level city in Shandong
Jinfeng Hongyuan Flagship Store	Power Saving Specialist 1	I'm using it. I'll rate it next month.	Jingdong prefecture level city in Shandong
Jinfeng Hongyuan Flagship Store	Power Saving Specialist 1	Baby received Oh, the courier delivery really fast, with a good and then come back to the evaluation	Jingdong prefecture level city in Shandong
Jinfeng Hongyuan Flagship Store	Power Saving Specialist 1	Fast logistics, good packaging, worth it!	Jingdong prefecture level city in Shandong
Jinfeng Hongyuan Flagship Store	Power Saving Specialist 1	Customer service is okay. It's just that.	Jingdong prefecture level city in Shandong
Jinfeng Hongyuan Flagship Store	Power Saving Specialist 1	Poor power saving	Jingdong prefecture level city in Shandong
Jinfeng Hongyuan Flagship Store	Power Saving Specialist 1	I'll tell you when I've used it.	Jingdong prefecture level city in Shandong
Jinfeng Hongyuan Flagship Store	Power Saving Specialist 1	I think it's good.	Jingdong prefecture level city in Shandong
Jinfeng Hongyuan Flagship Store	Power Saving Specialist 1	This energy saver works very well and I recommend everyone to buy it!	Jingdong prefecture level city in Shandong
Jinfeng Hongyuan Flagship Store	Power Saving Specialist 1	I do not know how effective, just use half a month on the burn out, good business reissued a, and then use it after the follow-up comment.	Jingdong prefecture level city in Shandong
Jinfeng Hongyuan Flagship Store	Power Saving Specialist 1	in use	Jingdong prefecture level city in Shandong
Jinfeng Hongyuan Flagship Store	Power Saving Specialist 1	This thing is very poor. It's already only been used for ten days and it's broken. Now waiting for you to return it.	Jingdong prefecture level city in Shandong
Jinfeng Hongyuan Flagship Store	Power Saving Specialist 1	It's not the same as the seller's show.	Jingdong prefecture level city in Shandong
Jinfeng Hongyuan Flagship Store	Power Saving Specialist 1	What is the problem when I get it back from the CTS, it is plugged into the wall and the voltage shows 206v.	Jingdong prefecture level city in Shandong
Jinfeng Hongyuan Flagship Store	Power Saving Specialist 1	The logistics was fast and the stuff was perfectly packaged. It is in the process of trial.	Jingdong prefecture level city in Shandong
Jinfeng Hongyuan Flagship Store	Power Saving Specialist 1	This saver is particularly good to use can really save electricity, with two months and then look at the line if you buy one for the family	Jingdong prefecture level city in Shandong
Jinfeng Hongyuan Flagship Store	Power Saving Specialist 1	Novel appearance design, all aluminum alloy profile, also with a fuse device, voltage monitoring display. After receiving the goods, I directly cut the plug and connect it to the main power box under the leakage switch, hoping to save electricity, and then follow up later. Logistics is very fast, arrived the next	Jingdong prefecture level city in Shandong
Jinfeng Hongyuan Flagship Store	Power Saving Specialist 1	Received, very good and like, if necessary next time to buy ha ha	Jingdong prefecture level city in Shandong
Jinfeng Hongyuan Flagship Store	Power Saving Specialist 1	It's good. It's worth it. Get it if you need it.	Jingdong prefecture level city in Shandong
Jinfeng Hongyuan Flagship Store	Power Saving Specialist 1	It feels more than good and is worth having.	Jingdong prefecture level city in Shandong
Jinfeng Hongyuan Flagship Store	Power Saving Specialist 1	The goods arrived very good and fast first time to buy	Jingdong prefecture level city in Shandong
Jinfeng Hongyuan Flagship Store	Power Saving Specialist 1	The power saving is very good, I can save so much money every month, I will buy another one next time.	Jingdong prefecture level city in Shandong
Jinfeng Hongyuan Flagship Store	Power Saving Specialist 1	It's not what it says.	Jingdong prefecture level city in Shandong
Jinfeng Hongyuan Flagship Store	Power Saving Specialist 1	I have been using it for many days now and it saves electricity, it really does I have an 8 kW electric snail stove and it saves about $3.50 a day.	Jingdong prefecture level city in Shandong
Jinfeng Hongyuan Flagship Store	Power Saving Specialist 1	Express delivery is very fast, the quality is also quite good, worth buying!	Jingdong prefecture level city in Shandong
Jinfeng Hongyuan Flagship Store	Power Saving Specialist 1	Just received expecting the baby to work well.	Jingdong prefecture level city in Shandong
Jinfeng Hongyuan Flagship Store	Power Saving Specialist 1	Very good, there are more fruit, has bought a second!	Jingdong prefecture level city in Shandong

Jinfeng Hongyuan Flagship Store	Power Saving Specialist 1	Good is good So far, no power savings have been noticed Maybe it's too little to notice.	Jingdong prefecture level city in Shandong
Jinfeng Hongyuan Flagship Store	Power Saving Specialist 1	I just received the item and plugged it in to see what happens later.	Jingdong prefecture level city in Shandong
Jinfeng Hongyuan Flagship Store	Power Saving Specialist 1	Baby received, especially good, packaging is also good, the courier brother is very strong, thank you.	Jingdong prefecture level city in Shandong
Jinfeng Hongyuan Flagship Store	Power Saving Specialist 1	Just received the goods have not yet used it, do not know how, three months later in the follow-up comment!	Jingdong prefecture level city in Shandong
Jinfeng Hongyuan Flagship Store	Power Saving Specialist 1	The item was received, it is quite good, recommended to buy.	Jingdong prefecture level city in Shandong
Jinfeng Hongyuan Flagship Store	Power Saving Specialist 1	This energy saver works very well and I recommend everyone to buy it!	Jingdong prefecture level city in Shandong
Jinfeng Hongyuan Flagship Store	Power Saving Specialist 1	Voltage stabilization and current reduction nice power saving product.	Jingdong prefecture level city in Shandong
Jinfeng Hongyuan Flagship Store	Power Saving Specialist 1	Voltage stabilization and current reduction nice power saving product.	Jingdong prefecture level city in Shandong
Jinfeng Hongyuan Flagship Store	Power Saving Specialist 1	A good power saver for stabilizing and lowering current.	Jingdong prefecture level city in Shandong
Jinfeng Hongyuan Flagship Store	Power Saving Specialist 1	You'll know if it saves power after you use it.	Jingdong prefecture level city in Shandong
Jinfeng Hongyuan Flagship Store	Power Saving Specialist 1	The quality is great. The quality is very good. The service is very good. Express delivery arrived in two days.	Jingdong prefecture level city in Shandong
Jinfeng Hongyuan Flagship Store	Power Saving Specialist 1	It works great.	Jingdong prefecture level city in Shandong
Jinfeng Hongyuan Flagship Store	Power Saving Specialist 1	Logistics is very fast, not yet used, used to comment again.	Jingdong prefecture level city in Shandong
Jinfeng Hongyuan Flagship Store	Power Saving Specialist 1	Logistics is very fast things are also very good, worthy of friends to buy	Jingdong prefecture level city in Shandong
Jinfeng Hongyuan Flagship Store	Power Saving Specialist 1	It works. It really works.	Jingdong prefecture level city in Shandong
Jinfeng Hongyuan Flagship Store	Power Saving Specialist 1	LANGUAGE COMMENTS: just received the shipment. I immediately plugged it in and used it. If you ask how effective. It depends on whether the next month's electricity bill has been reduced. If there is a decrease. I am buying more than one to use.	Jingdong prefecture level city in Shandong
Jinfeng Hongyuan Flagship Store	Power Saving Specialist 1	Well made, power saving, good and convenient!	Jingdong prefecture level city in Shandong
Jinfeng Hongyuan Flagship Store	Power Saving Specialist 1	I'm using it. I'll rate it next month.	Jingdong prefecture level city in Shandong
Jinfeng Hongyuan Flagship Store	Power Saving Specialist 1	Baby received Oh, the courier delivery really fast, with a good and then come back to the evaluation	Jingdong prefecture level city in Shandong
Jinfeng Hongyuan Flagship Store	Power Saving Specialist 1	Fast logistics, good packaging, worth it!	Jingdong prefecture level city in Shandong
Jinfeng Hongyuan Flagship Store	Power Saving Specialist 1	Customer service is okay. It's just that.	Jingdong prefecture level city in Shandong
Jinfeng Hongyuan Flagship Store	Power Saving Specialist 1	Poor power saving	Jingdong prefecture level city in Shandong
Jinfeng Hongyuan Flagship Store	Power Saving Specialist 1	I'll tell you when I've used it.	Jingdong prefecture level city in Shandong
Jinfeng Hongyuan Flagship Store	Power Saving Specialist 1	I think it's good.	Jingdong prefecture level city in Shandong
Jinfeng Hongyuan Flagship Store	Power Saving Specialist 1	This energy saver works very well and I recommend everyone to buy it!	Jingdong prefecture level city in Shandong
Jinfeng Hongyuan Flagship Store	Power Saving Specialist 1	I do not know how effective, just use half a month on the burn out, good business reissued a, and then use it after the follow-up comment.	Jingdong prefecture level city in Shandong
Jinfeng Hongyuan Flagship Store	Power Saving Specialist 1	in use	Jingdong prefecture level city in Shandong
Jinfeng Hongyuan Flagship Store	Power Saving Specialist 1	This thing is very poor. It's already only been used for ten days and it's broken. Now waiting for you to return it.	Jingdong prefecture level city in Shandong
Jinfeng Hongyuan Flagship Store	Power Saving Specialist 1	It's not the same as the seller's show.	Jingdong prefecture level city in Shandong
Jinfeng Hongyuan Flagship Store	Power Saving Specialist 1	What is the problem when I get it back from the CTS, it is plugged into the wall and the voltage shows 206v.	Jingdong prefecture level city in Shandong
Jinfeng Hongyuan Flagship Store	Power Saving Specialist 1	The logistics was fast and the stuff was perfectly packaged. It is in the process of trial.	Jingdong prefecture level city in Shandong
Jinfeng Hongyuan Flagship Store	Power Saving Specialist 1	This saver is particularly good to use can really save electricity, with two months and then look at the line if you buy one for the family	Jingdong prefecture level city in Shandong
Jinfeng Hongyuan Flagship Store	Power Saving Specialist 1	Novel appearance design, all aluminum alloy profile, also with a fuse device, voltage monitoring display. After receiving the goods, I directly cut the plug and connect it to the main power box under the leakage switch, hoping to save electricity, and then follow up later. Logistics is very fast, arrived the next	Jingdong prefecture level city in Shandong
Jinfeng Hongyuan Flagship Store	Power Saving Specialist 1	Received, very good and like, if necessary next time to buy ha ha	Jingdong prefecture level city in Shandong
Jinfeng Hongyuan Flagship Store	Power Saving Specialist 1	It's good. It's worth it. Get it if you need it.	Jingdong prefecture level city in Shandong
Jinfeng Hongyuan Flagship Store	Power Saving Specialist 1	It feels more than good and is worth having.	Jingdong prefecture level city in Shandong
Jinfeng Hongyuan Flagship Store	Power Saving Specialist 1	The goods arrived very good and fast first time to buy	Jingdong prefecture level city in Shandong
Jinfeng Hongyuan Flagship Store	Power Saving Specialist 1	The power saving is very good, I can save so much money every month, I will buy another one next time.	Jingdong prefecture level city in Shandong
Jinfeng Hongyuan Flagship Store	Power Saving Specialist 1	It's not what it says.	Jingdong prefecture level city in Shandong
Jinfeng Hongyuan Flagship Store	Power Saving Specialist 1	I have been using it for many days now and it saves electricity, it really does I have an 8 kW electric snail stove and it saves about $3.50 a day.	Jingdong prefecture level city in Shandong
Jinfeng Hongyuan Flagship Store	Power Saving Specialist 1	Express delivery is very fast, the quality is also quite good, worth buying!	Jingdong prefecture level city in Shandong
Jinfeng Hongyuan Flagship Store	Power Saving Specialist 1	Just received expecting the baby to work well.	Jingdong prefecture level city in Shandong
Jinfeng Hongyuan Flagship Store	Power Saving Specialist 1	Very good, there are more fruit, has bought a second!	Jingdong prefecture level city in Shandong
Jinfeng Hongyuan Flagship Store	Power Saving Specialist 1	Good is good So far, no power savings have been noticed Maybe it's too little to notice.	Jingdong prefecture level city in Shandong
Jinfeng Hongyuan Flagship Store	Power Saving Specialist 1	I just received the item and plugged it in to see what happens later.	Jingdong prefecture level city in Shandong
Jinfeng Hongyuan Flagship Store	Power Saving Specialist 1	Baby received, especially good, packaging is also good, the courier brother is very strong, thank you.	Jingdong prefecture level city in Shandong
Jinfeng Hongyuan Flagship Store	Power Saving Specialist 1	Just received the goods have not yet used it, do not know how, three months later in the follow-up comment!	Jingdong prefecture level city in Shandong
Jinfeng Hongyuan Flagship Store	Power Saving Specialist 1	The item was received, it is quite good, recommended to buy.	Jingdong prefecture level city in Shandong
Jinfeng Hongyuan Flagship Store	Power Saving Specialist 1	This energy saver works very well and I recommend everyone to buy it!	Jingdong prefecture level city in Shandong
Jinfeng Hongyuan Flagship Store	Power Saving Specialist 1	Voltage stabilization and current reduction nice power saving product.	Jingdong prefecture level city in Shandong

Jinfeng Hongyuan Flagship Store	Power Saving Specialist 1	Voltage stabilization and current reduction nice power saving product.	Jingdong prefecture level city in Shandong
Jinfeng Hongyuan Flagship Store	Power Saving Specialist 1	A good power saver for stabilizing and lowering current.	Jingdong prefecture level city in Shandong
Jinfeng Hongyuan Flagship Store	Power Saving Specialist 1	You'll know if it saves power after you use it.	Jingdong prefecture level city in Shandong
Jinfeng Hongyuan Flagship Store	Power Saving Specialist 1	The quality is great. The quality is very good. The service is very good. Express delivery arrived in two days.	Jingdong prefecture level city in Shandong
Jinfeng Hongyuan Flagship Store	Power Saving Specialist 1	It works great.	Jingdong prefecture level city in Shandong
Jinfeng Hongyuan Flagship Store	Power Saving Specialist 1	Logistics is very fast, not yet used, used to comment again.	Jingdong prefecture level city in Shandong
Jinfeng Hongyuan Flagship Store	Power Saving Specialist 1	Logistics is very fast things are also very good, worthy of friends to buy	Jingdong prefecture level city in Shandong
Jinfeng Hongyuan Flagship Store	Power Saving Specialist 1	It works. It really works.	Jingdong prefecture level city in Shandong
Jinfeng Hongyuan Flagship Store	Power Saving Specialist 1	LANGUAGE COMMENTS: just received the shipment. I immediately plugged it in and used it. If you ask how effective. It depends on whether the next month's electricity bill has been reduced. If there is a decrease. I am buying more than one to use.	Jingdong prefecture level city in Shandong
Jinfeng Hongyuan Flagship Store	Power Saving Specialist 1	Well made, power saving, good and convenient!	Jingdong prefecture level city in Shandong
Jinfeng Hongyuan Flagship Store	Power Saving Specialist 1	I'm using it. I'll rate it next month.	Jingdong prefecture level city in Shandong
Jinfeng Hongyuan Flagship Store	Power Saving Specialist 1	Baby received Oh, the courier delivery really fast, with a good and then come back to the evaluation	Jingdong prefecture level city in Shandong
Jinfeng Hongyuan Flagship Store	Power Saving Specialist 1	Fast logistics, good packaging, worth it!	Jingdong prefecture level city in Shandong
Jinfeng Hongyuan Flagship Store	Power Saving Specialist 1	Customer service is okay. It's just that.	Jingdong prefecture level city in Shandong
Jinfeng Hongyuan Flagship Store	Power Saving Specialist 1	Poor power saving	Jingdong prefecture level city in Shandong
Jinfeng Hongyuan Flagship Store	Power Saving Specialist 1	I'll tell you when I've used it.	Jingdong prefecture level city in Shandong
Jinfeng Hongyuan Flagship Store	Power Saving Specialist 1	I think it's good.	Jingdong prefecture level city in Shandong
Jinfeng Hongyuan Flagship Store	Power Saving Specialist 1	This energy saver works very well and I recommend everyone to buy it!	Jingdong prefecture level city in Shandong
Jinfeng Hongyuan Flagship Store	Power Saving Specialist 1	I do not know how effective, just use half a month on the burn out, good business reissued a, and then use it after the follow-up comment.	Jingdong prefecture level city in Shandong
Jinfeng Hongyuan Flagship Store	Power Saving Specialist 1	in use	Jingdong prefecture level city in Shandong
Jinfeng Hongyuan Flagship Store	Power Saving Specialist 1	This thing is very poor. It's already only been used for ten days and it's broken. Now waiting for you to return it.	Jingdong prefecture level city in Shandong
Jinfeng Hongyuan Flagship Store	Power Saving Specialist 1	It's not the same as the seller's show.	Jingdong prefecture level city in Shandong
Jinfeng Hongyuan Flagship Store	Power Saving Specialist 1	What is the problem when I get it back from the CTS, it is plugged into the wall and the voltage shows 206v.	Jingdong prefecture level city in Shandong
Jinfeng Hongyuan Flagship Store	Power Saving Specialist 1	The logistics was fast and the stuff was perfectly packaged. It is in the process of trial.	Jingdong prefecture level city in Shandong
Jinfeng Hongyuan Flagship Store	Power Saving Specialist 1	This saver is particularly good to use can really save electricity, with two months and then look at the line if you buy one for the family	Jingdong prefecture level city in Shandong
Jinfeng Hongyuan Flagship Store	Power Saving Specialist 1	Novel appearance design, all aluminum alloy profile, also with a fuse device, voltage monitoring display. After receiving the goods, I directly cut the plug and connect it to the main power box under the leakage switch, hoping to save electricity, and then follow up later. Logistics is very fast, arrived the next	Jingdong prefecture level city in Shandong
Jinfeng Hongyuan Flagship Store	Power Saving Specialist 1	Received, very good and like, if necessary next time to buy ha ha	Jingdong prefecture level city in Shandong
Jinfeng Hongyuan Flagship Store	Power Saving Specialist 1	It's good. It's worth it. Get it if you need it.	Jingdong prefecture level city in Shandong
Jinfeng Hongyuan Flagship Store	Power Saving Specialist 1	It feels more than good and is worth having.	Jingdong prefecture level city in Shandong
Jinfeng Hongyuan Flagship Store	Power Saving Specialist 1	The goods arrived very good and fast first time to buy	Jingdong prefecture level city in Shandong
Jinfeng Hongyuan Flagship Store	Power Saving Specialist 1	The power saving is very good, I can save so much money every month, I will buy another one next time.	Jingdong prefecture level city in Shandong
Jinfeng Hongyuan Flagship Store	Power Saving Specialist 1	It's not what it says.	Jingdong prefecture level city in Shandong
Jinfeng Hongyuan Flagship Store	Power Saving Specialist 1	I have been using it for many days now and it saves electricity, it really does I have an 8 kW electric snail stove and it saves about $3.50 a day.	Jingdong prefecture level city in Shandong
Jinfeng Hongyuan Flagship Store	Power Saving Specialist 1	Express delivery is very fast, the quality is also quite good, worth buying!	Jingdong prefecture level city in Shandong
Jinfeng Hongyuan Flagship Store	Power Saving Specialist 1	Just received expecting the baby to work well.	Jingdong prefecture level city in Shandong
Jinfeng Hongyuan Flagship Store	Power Saving Specialist 1	Very good, there are more fruit, has bought a second!	Jingdong prefecture level city in Shandong
Jinfeng Hongyuan Flagship Store	Power Saving Specialist 1	Good is good So far, no power savings have been noticed Maybe it's too little to notice.	Jingdong prefecture level city in Shandong
Jinfeng Hongyuan Flagship Store	Power Saving Specialist 1	I just received the item and plugged it in to see what happens later.	Jingdong prefecture level city in Shandong
Jinfeng Hongyuan Flagship Store	Power Saving Specialist 1	Baby received, especially good, packaging is also good, the courier brother is very strong, thank you.	Jingdong prefecture level city in Shandong
Jinfeng Hongyuan Flagship Store	Power Saving Specialist 1	Just received the goods have not yet used it, do not know how, three months later in the follow-up comment!	Jingdong prefecture level city in Shandong
Jinfeng Hongyuan Flagship Store	Power Saving Specialist 1	The item was received, it is quite good, recommended to buy.	Jingdong prefecture level city in Shandong
Jinfeng Hongyuan Flagship Store	Power Saving Specialist 1	This energy saver works very well and I recommend everyone to buy it!	Jingdong prefecture level city in Shandong
Jinfeng Hongyuan Flagship Store	Power Saving Specialist 1	Voltage stabilization and current reduction nice power saving product.	Jingdong prefecture level city in Shandong
Jinfeng Hongyuan Flagship Store	Power Saving Specialist 1	Voltage stabilization and current reduction nice power saving product.	Jingdong prefecture level city in Shandong
Jinfeng Hongyuan Flagship Store	Power Saving Specialist 1	A good power saver for stabilizing and lowering current.	Jingdong prefecture level city in Shandong
Jinfeng Hongyuan Flagship Store	Power Saving Specialist 1	You'll know if it saves power after you use it.	Jingdong prefecture level city in Shandong
Jinfeng Hongyuan Flagship Store	Power Saving Specialist 1	The quality is great. The quality is very good. The service is very good. Express delivery arrived in two days.	Jingdong prefecture level city in Shandong
Jinfeng Hongyuan Flagship Store	Power Saving Specialist 1	It works great.	Jingdong prefecture level city in Shandong
Jinfeng Hongyuan Flagship Store	Power Saving Specialist 1	Logistics is very fast, not yet used, used to comment again.	Jingdong prefecture level city in Shandong
Jinfeng Hongyuan Flagship Store	Power Saving Specialist 1	Logistics is very fast things are also very good, worthy of friends to buy	Jingdong prefecture level city in Shandong

Jinfeng Hongyuan Flagship Store	Power Saving Specialist 1	It works. It really works.	Jingdong prefecture level city in Shandong
Jinfeng Hongyuan Flagship Store	Power Saving Specialist 1	LANGUAGE COMMENTS: just received the shipment. I immediately plugged it in and used it. If you ask how effective. It depends on whether the next month's electricity bill has been reduced. If there is a decrease. I am buying more than one to use.	Jingdong prefecture level city in Shandong
Jinfeng Hongyuan Flagship Store	Power Saving Specialist 1	Well made, power saving, good and convenient!	Jingdong prefecture level city in Shandong
Jinfeng Hongyuan Flagship Store	Power Saving Specialist 1	I'm using it. I'll rate it next month.	Jingdong prefecture level city in Shandong
Jinfeng Hongyuan Flagship Store	Power Saving Specialist 1	Baby received Oh, the courier delivery really fast, with a good and then come back to the evaluation	Jingdong prefecture level city in Shandong
Jinfeng Hongyuan Flagship Store	Power Saving Specialist 1	Fast logistics, good packaging, worth it!	Jingdong prefecture level city in Shandong
Jinfeng Hongyuan Flagship Store	Power Saving Specialist 1	Customer service is okay. It's just that.	Jingdong prefecture level city in Shandong
Jinfeng Hongyuan Flagship Store	Power Saving Specialist 1	Poor power saving	Jingdong prefecture level city in Shandong
Jinfeng Hongyuan Flagship Store	Power Saving Specialist 1	I'll tell you when I've used it.	Jingdong prefecture level city in Shandong
Jinfeng Hongyuan Flagship Store	Power Saving Specialist 1	I think it's good.	Jingdong prefecture level city in Shandong
Jinfeng Hongyuan Flagship Store	Power Saving Specialist 1	This energy saver works very well and I recommend everyone to buy it!	Jingdong prefecture level city in Shandong
Jinfeng Hongyuan Flagship Store	Power Saving Specialist 1	I do not know how effective, just use half a month on the burn out, good business reissued a, and then use it after the follow-up comment.	Jingdong prefecture level city in Shandong
Jinfeng Hongyuan Flagship Store	Power Saving Specialist 1	in use	Jingdong prefecture level city in Shandong
Jinfeng Hongyuan Flagship Store	Power Saving Specialist 1	This thing is very poor. It's already only been used for ten days and it's broken. Now waiting for you to return it.	Jingdong prefecture level city in Shandong
Jinfeng Hongyuan Flagship Store	Power Saving Specialist 1	It's not the same as the seller's show.	Jingdong prefecture level city in Shandong
Jinfeng Hongyuan Flagship Store	Power Saving Specialist 1	What is the problem when I get it back from the CTS, it is plugged into the wall and the voltage shows 206v.	Jingdong prefecture level city in Shandong
Jinfeng Hongyuan Flagship Store	Power Saving Specialist 1	The logistics was fast and the stuff was perfectly packaged. It is in the process of trial.	Jingdong prefecture level city in Shandong
Jinfeng Hongyuan Flagship Store	Power Saving Specialist 1	This saver is particularly good to use can really save electricity, with two months and then look at the line if you buy one for the family	Jingdong prefecture level city in Shandong
Jinfeng Hongyuan Flagship Store	Power Saving Specialist 1	Novel appearance design, all aluminum alloy profile, also with a fuse device, voltage monitoring display. After receiving the goods, I directly cut the plug and connect it to the main power box under the leakage switch, hoping to save electricity, and then follow up later. Logistics is very fast, arrived the next	Jingdong prefecture level city in Shandong
Jinfeng Hongyuan Flagship Store	Power Saving Specialist 1	Received, very good and like, if necessary next time to buy ha ha	Jingdong prefecture level city in Shandong
Jinfeng Hongyuan Flagship Store	Power Saving Specialist 1	It's good. It's worth it. Get it if you need it.	Jingdong prefecture level city in Shandong
Jinfeng Hongyuan Flagship Store	Power Saving Specialist 1	It feels more than good and is worth having.	Jingdong prefecture level city in Shandong
Jinfeng Hongyuan Flagship Store	Power Saving Specialist 1	The goods arrived very good and fast first time to buy	Jingdong prefecture level city in Shandong
Jinfeng Hongyuan Flagship Store	Power Saving Specialist 1	The power saving is very good, I can save so much money every month, I will buy another one next time.	Jingdong prefecture level city in Shandong
Jinfeng Hongyuan Flagship Store	Power Saving Specialist 1	It's not what it says.	Jingdong prefecture level city in Shandong
Jinfeng Hongyuan Flagship Store	Power Saving Specialist 1	I have been using it for many days now and it saves electricity, it really does I have an 8 kW electric snail stove and it saves about $3.50 a day.	Jingdong prefecture level city in Shandong
Jinfeng Hongyuan Flagship Store	Power Saving Specialist 1	Express delivery is very fast, the quality is also quite good, worth buying!	Jingdong prefecture level city in Shandong
Jinfeng Hongyuan Flagship Store	Power Saving Specialist 1	Just received expecting the baby to work well.	Jingdong prefecture level city in Shandong
Jinfeng Hongyuan Flagship Store	Power Saving Specialist 1	Very good, there are more fruit, has bought a second!	Jingdong prefecture level city in Shandong
Jinfeng Hongyuan Flagship Store	Power Saving Specialist 1	Good is good So far, no power savings have been noticed Maybe it's too little to notice.	Jingdong prefecture level city in Shandong
Jinfeng Hongyuan Flagship Store	Power Saving Specialist 1	I just received the item and plugged it in to see what happens later.	Jingdong prefecture level city in Shandong
Jinfeng Hongyuan Flagship Store	Power Saving Specialist 1	Baby received, especially good, packaging is also good, the courier brother is very strong, thank you.	Jingdong prefecture level city in Shandong
Jinfeng Hongyuan Flagship Store	Power Saving Specialist 1	Just received the goods have not yet used it, do not know how, three months later in the follow-up comment!	Jingdong prefecture level city in Shandong
Jinfeng Hongyuan Flagship Store	Power Saving Specialist 1	The item was received, it is quite good, recommended to buy.	Jingdong prefecture level city in Shandong
Jinfeng Hongyuan Flagship Store	Power Saving Specialist 1	This energy saver works very well and I recommend everyone to buy it!	Jingdong prefecture level city in Shandong
Jinfeng Hongyuan Flagship Store	Power Saving Specialist 1	Voltage stabilization and current reduction nice power saving product.	Jingdong prefecture level city in Shandong
Jinfeng Hongyuan Flagship Store	Power Saving Specialist 1	Voltage stabilization and current reduction nice power saving product.	Jingdong prefecture level city in Shandong
Jinfeng Hongyuan Flagship Store	Power Saving Specialist 1	A good power saver for stabilizing and lowering current.	Jingdong prefecture level city in Shandong
Jinfeng Hongyuan Flagship Store	Power Saving Specialist 1	You'll know if it saves power after you use it.	Jingdong prefecture level city in Shandong
Jinfeng Hongyuan Flagship Store	Power Saving Specialist 1	The quality is great. The quality is very good. The service is very good. Express delivery arrived in two days.	Jingdong prefecture level city in Shandong
Jinfeng Hongyuan Flagship Store	Power Saving Specialist 1	It works great.	Jingdong prefecture level city in Shandong
Jinfeng Hongyuan Flagship Store	Power Saving Specialist 1	Logistics is very fast, not yet used, used to comment again.	Jingdong prefecture level city in Shandong
Jinfeng Hongyuan Flagship Store	Power Saving Specialist 1	Logistics is very fast things are also very good, worthy of friends to buy	Jingdong prefecture level city in Shandong
Jinfeng Hongyuan Flagship Store	Power Saving Specialist 1	It works. It really works.	Jingdong prefecture level city in Shandong
Jinfeng Hongyuan Flagship Store	Power Saving Specialist 1	LANGUAGE COMMENTS: just received the shipment. I immediately plugged it in and used it. If you ask how effective. It depends on whether the next month's electricity bill has been reduced. If there is a decrease. I am buying more than one to use.	Jingdong prefecture level city in Shandong
Jinfeng Hongyuan Flagship Store	Power Saving Specialist 1	Well made, power saving, good and convenient!	Jingdong prefecture level city in Shandong
Jinfeng Hongyuan Flagship Store	Power Saving Specialist 1	I'm using it. I'll rate it next month.	Jingdong prefecture level city in Shandong
Jinfeng Hongyuan Flagship Store	Power Saving Specialist 1	Baby received Oh, the courier delivery really fast, with a good and then come back to the evaluation	Jingdong prefecture level city in Shandong
Jinfeng Hongyuan Flagship Store	Power Saving Specialist 1	Fast logistics, good packaging, worth it!	Jingdong prefecture level city in Shandong
Jinfeng Hongyuan Flagship Store	Power Saving Specialist 1	Customer service is okay. It's just that.	Jingdong prefecture level city in Shandong

Jinfeng Hongyuan Flagship Store	Power Saving Specialist 1	Poor power saving	Jingdong prefecture level city in Shandong
Jinfeng Hongyuan Flagship Store	Power Saving Specialist 1	I'll tell you when I've used it.	Jingdong prefecture level city in Shandong
Jinfeng Hongyuan Flagship Store	Power Saving Specialist 1	I think it's good.	Jingdong prefecture level city in Shandong
Jinfeng Hongyuan Flagship Store	Power Saving Specialist 1	This energy saver works very well and I recommend everyone to buy it!	Jingdong prefecture level city in Shandong
Jinfeng Hongyuan Flagship Store	Power Saving Specialist 1	I do not know how effective, just use half a month on the burn out, good business reissued a, and then use it after the follow-up comment.	Jingdong prefecture level city in Shandong
Jinfeng Hongyuan Flagship Store	Power Saving Specialist 1	in use	Jingdong prefecture level city in Shandong
Jinfeng Hongyuan Flagship Store	Power Saving Specialist 1	This thing is very poor. It's already only been used for ten days and it's broken. Now waiting for you to return it.	Jingdong prefecture level city in Shandong
Jinfeng Hongyuan Flagship Store	Power Saving Specialist 1	It's not the same as the seller's show.	Jingdong prefecture level city in Shandong
Jinfeng Hongyuan Flagship Store	Power Saving Specialist 1	What is the problem when I get it back from the CTS, it is plugged into the wall and the voltage shows 206v.	Jingdong prefecture level city in Shandong
Jinfeng Hongyuan Flagship Store	Power Saving Specialist 1	The logistics was fast and the stuff was perfectly packaged. It is in the process of trial.	Jingdong prefecture level city in Shandong
Jinfeng Hongyuan Flagship Store	Power Saving Specialist 1	This saver is particularly good to use can really save electricity, with two months and then look at the line if you buy one for the family	Jingdong prefecture level city in Shandong
Jinfeng Hongyuan Flagship Store	Power Saving Specialist 1	Novel appearance design, all aluminum alloy profile, also with a fuse device, voltage monitoring display. After receiving the goods, I directly cut the plug and connect it to the main power box under the leakage switch, hoping to save electricity, and then follow up later. Logistics is very fast, arrived the next	Jingdong prefecture level city in Shandong
Jinfeng Hongyuan Flagship Store	Power Saving Specialist 1	Received, very good and like, if necessary next time to buy ha ha	Jingdong prefecture level city in Shandong
Jinfeng Hongyuan Flagship Store	Power Saving Specialist 1	It's good. It's worth it. Get it if you need it.	Jingdong prefecture level city in Shandong
Jinfeng Hongyuan Flagship Store	Power Saving Specialist 1	It feels more than good and is worth having.	Jingdong prefecture level city in Shandong
Jinfeng Hongyuan Flagship Store	Power Saving Specialist 1	The goods arrived very good and fast first time to buy	Jingdong prefecture level city in Shandong
Jinfeng Hongyuan Flagship Store	Power Saving Specialist 1	The power saving is very good, I can save so much money every month, I will buy another one next time.	Jingdong prefecture level city in Shandong
Jinfeng Hongyuan Flagship Store	Power Saving Specialist 1	It's not what it says.	Jingdong prefecture level city in Shandong
Jinfeng Hongyuan Flagship Store	Power Saving Specialist 1	I have been using it for many days now and it saves electricity, it really does I have an 8 kW electric snail stove and it saves about $3.50 a day.	Jingdong prefecture level city in Shandong
Jinfeng Hongyuan Flagship Store	Power Saving Specialist 1	Express delivery is very fast, the quality is also quite good, worth buying!	Jingdong prefecture level city in Shandong
Jinfeng Hongyuan Flagship Store	Power Saving Specialist 1	Just received expecting the baby to work well.	Jingdong prefecture level city in Shandong
Jinfeng Hongyuan Flagship Store	Power Saving Specialist 1	Very good, there are more fruit, has bought a second!	Jingdong prefecture level city in Shandong
Jinfeng Hongyuan Flagship Store	Power Saving Specialist 1	Good is good So far, no power savings have been noticed Maybe it's too little to notice.	Jingdong prefecture level city in Shandong
Jinfeng Hongyuan Flagship Store	Power Saving Specialist 1	I just received the item and plugged it in to see what happens later.	Jingdong prefecture level city in Shandong
Jinfeng Hongyuan Flagship Store	Power Saving Specialist 1	Baby received, especially good, packaging is also good, the courier brother is very strong, thank you.	Jingdong prefecture level city in Shandong
Jinfeng Hongyuan Flagship Store	Power Saving Specialist 1	Just received the goods have not yet used it, do not know how, three months later in the follow-up comment!	Jingdong prefecture level city in Shandong
Jinfeng Hongyuan Flagship Store	Power Saving Specialist 1	The item was received, it is quite good, recommended to buy.	Jingdong prefecture level city in Shandong
Jinfeng Hongyuan Flagship Store	Power Saving Specialist 1	This energy saver works very well and I recommend everyone to buy it!	Jingdong prefecture level city in Shandong
Jinfeng Hongyuan Flagship Store	Power Saving Specialist 1	Voltage stabilization and current reduction nice power saving product.	Jingdong prefecture level city in Shandong
Jinfeng Hongyuan Flagship Store	Power Saving Specialist 1	Voltage stabilization and current reduction nice power saving product.	Jingdong prefecture level city in Shandong
Jinfeng Hongyuan Flagship Store	Power Saving Specialist 1	A good power saver for stabilizing and lowering current.	Jingdong prefecture level city in Shandong
Jinfeng Hongyuan Flagship Store	Power Saving Specialist 1	You'll know if it saves power after you use it.	Jingdong prefecture level city in Shandong
Jinfeng Hongyuan Flagship Store	Power Saving Specialist 1	The quality is great. The quality is very good. The service is very good. Express delivery arrived in two days.	Jingdong prefecture level city in Shandong
Jinfeng Hongyuan Flagship Store	Power Saving Specialist 1	It works great.	Jingdong prefecture level city in Shandong
Jinfeng Hongyuan Flagship Store	Power Saving Specialist 1	Logistics is very fast, not yet used, used to comment again.	Jingdong prefecture level city in Shandong
Jinfeng Hongyuan Flagship Store	Power Saving Specialist 1	Logistics is very fast things are also very good, worthy of friends to buy	Jingdong prefecture level city in Shandong
Jinfeng Hongyuan Flagship Store	Power Saving Specialist 1	It works. It really works.	Jingdong prefecture level city in Shandong
Jinfeng Hongyuan Flagship Store	Power Saving Specialist 1	LANGUAGE COMMENTS: just received the shipment. I immediately plugged it in and used it. If you ask how effective. It depends on whether the next month's electricity bill has been reduced. If there is a decrease. I am buying more than one to use.	Jingdong prefecture level city in Shandong
Jinfeng Hongyuan Flagship Store	Power Saving Specialist 1	Well made, power saving, good and convenient!	Jingdong prefecture level city in Shandong
Jinfeng Hongyuan Flagship Store	Power Saving Specialist 1	I'm using it. I'll rate it next month.	Jingdong prefecture level city in Shandong
Jinfeng Hongyuan Flagship Store	Power Saving Specialist 1	Baby received Oh, the courier delivery really fast, with a good and then come back to the evaluation	Jingdong prefecture level city in Shandong
Jinfeng Hongyuan Flagship Store	Power Saving Specialist 1	Fast logistics, good packaging, worth it!	Jingdong prefecture level city in Shandong
Jinfeng Hongyuan Flagship Store	Power Saving Specialist 1	Customer service is okay. It's just that.	Jingdong prefecture level city in Shandong
Jinfeng Hongyuan Flagship Store	Power Saving Specialist 1	Poor power saving	Jingdong prefecture level city in Shandong
Jinfeng Hongyuan Flagship Store	Power Saving Specialist 1	I'll tell you when I've used it.	Jingdong prefecture level city in Shandong
Jinfeng Hongyuan Flagship Store	Power Saving Specialist 1	I think it's good.	Jingdong prefecture level city in Shandong
Jinfeng Hongyuan Flagship Store	Power Saving Specialist 1	This energy saver works very well and I recommend everyone to buy it!	Jingdong prefecture level city in Shandong
Jinfeng Hongyuan Flagship Store	Power Saving Specialist 1	I do not know how effective, just use half a month on the burn out, good business reissued a, and then use it after the follow-up comment.	Jingdong prefecture level city in Shandong
Jinfeng Hongyuan Flagship Store	Power Saving Specialist 1	in use	Jingdong prefecture level city in Shandong
Jinfeng Hongyuan Flagship Store	Power Saving Specialist 1	This thing is very poor. It's already only been used for ten days and it's broken. Now waiting for you to return it.	Jingdong prefecture level city in Shandong

Jinfeng Hongyuan Flagship Store	Power Saving Specialist 1	It's not the same as the seller's show.	Jingdong prefecture level city in Shandong
Jinfeng Hongyuan Flagship Store	Power Saving Specialist 1	What is the problem when I get it back from the CTS, it is plugged into the wall and the voltage shows 206v.	Jingdong prefecture level city in Shandong
Jinfeng Hongyuan Flagship Store	Power Saving Specialist 1	The logistics was fast and the stuff was perfectly packaged. It is in the process of trial.	Jingdong prefecture level city in Shandong
Jinfeng Hongyuan Flagship Store	Power Saving Specialist 1	This saver is particularly good to use can really save electricity, with two months and then look at the line if you buy one for the family	Jingdong prefecture level city in Shandong
Jinfeng Hongyuan Flagship Store	Power Saving Specialist 1	Novel appearance design, all aluminum alloy profile, also with a fuse device, voltage monitoring display. After receiving the goods, I directly cut the plug and connect it to the main power box under the leakage switch, hoping to save electricity, and then follow up later. Logistics is very fast, arrived the next	Jingdong prefecture level city in Shandong
Jinfeng Hongyuan Flagship Store	Power Saving Specialist 1	Received, very good and like, if necessary next time to buy ha ha	Jingdong prefecture level city in Shandong
Jinfeng Hongyuan Flagship Store	Power Saving Specialist 1	It's good. It's worth it. Get it if you need it.	Jingdong prefecture level city in Shandong
Jinfeng Hongyuan Flagship Store	Power Saving Specialist 1	It feels more than good and is worth having.	Jingdong prefecture level city in Shandong
Jinfeng Hongyuan Flagship Store	Power Saving Specialist 1	The power saving is very good, I can save so much money every month, I will buy another one next time.	Jingdong prefecture level city in Shandong
Jinfeng Hongyuan Flagship Store	Power Saving Specialist 1	The goods arrived very good and fast first time to buy	Jingdong prefecture level city in Shandong
Jinfeng Hongyuan Flagship Store	Power Saving Specialist 1	It's not what it says.	Jingdong prefecture level city in Shandong
Jinfeng Hongyuan Flagship Store	Power Saving Specialist 1	I have been using it for many days now and it saves electricity, it really does I have an 8 kW electric snail stove and it saves about $3.50 a day.	Jingdong prefecture level city in Shandong
Jinfeng Hongyuan Flagship Store	Power Saving Specialist 1	Express delivery is very fast, the quality is also quite good, worth buying!	Jingdong prefecture level city in Shandong
Jinfeng Hongyuan Flagship Store	Power Saving Specialist 1	Just received expecting the baby to work well.	Jingdong prefecture level city in Shandong
Jinfeng Hongyuan Flagship Store	Power Saving Specialist 1	Very good, there are more fruit, has bought a second!	Jingdong prefecture level city in Shandong
Jinfeng Hongyuan Flagship Store	Power Saving Specialist 1	Good is good So far, no power savings have been noticed Maybe it's too little to notice.	Jingdong prefecture level city in Shandong
Jinfeng Hongyuan Flagship Store	Power Saving Specialist 1	I just received the item and plugged it in to see what happens later.	Jingdong prefecture level city in Shandong
Jinfeng Hongyuan Flagship Store	Power Saving Specialist 1	Baby received, especially good, packaging is also good, the courier brother is very strong, thank you.	Jingdong prefecture level city in Shandong
Jinfeng Hongyuan Flagship Store	Power Saving Specialist 1	Just received the goods have not yet used it, do not know how, three months later in the follow-up comment!	Jingdong prefecture level city in Shandong
Jinfeng Hongyuan Flagship Store	Power Saving Specialist 1	The item was received, it is quite good, recommended to buy.	Jingdong prefecture level city in Shandong
Jinfeng Hongyuan Flagship Store	Power Saving Specialist 1	This energy saver works very well and I recommend everyone to buy it!	Jingdong prefecture level city in Shandong
Jinfeng Hongyuan Flagship Store	Power Saving Specialist 1	Voltage stabilization and current reduction nice power saving product.	Jingdong prefecture level city in Shandong
Jinfeng Hongyuan Flagship Store	Power Saving Specialist 1	Voltage stabilization and current reduction nice power saving product.	Jingdong prefecture level city in Shandong
Jinfeng Hongyuan Flagship Store	Power Saving Specialist 1	A good power saver for stabilizing and lowering current.	Jingdong prefecture level city in Shandong
Jinfeng Hongyuan Flagship Store	Power Saving Specialist 1	You'll know if it saves power after you use it.	Jingdong prefecture level city in Shandong
Jinfeng Hongyuan Flagship Store	Power Saving Specialist 1	The quality is great. The quality is very good. The service is very good. Express delivery arrived in two days.	Jingdong prefecture level city in Shandong
Jinfeng Hongyuan Flagship Store	Power Saving Specialist 1	It works great.	Jingdong prefecture level city in Shandong
Jinfeng Hongyuan Flagship Store	Power Saving Specialist 1	Logistics is very fast, not yet used, used to comment again.	Jingdong prefecture level city in Shandong
Jinfeng Hongyuan Flagship Store	Power Saving Specialist 1	Logistics is very fast things are also very good, worthy of friends to buy	Jingdong prefecture level city in Shandong
Jinfeng Hongyuan Flagship Store	Power Saving Specialist 1	It works. It really works.	Jingdong prefecture level city in Shandong
Jinfeng Hongyuan Flagship Store	Power Saving Specialist 1	LANGUAGE COMMENTS: just received the shipment. I immediately plugged it in and used it. If you ask how effective. It depends on whether the next month's electricity bill has been reduced. If there is a decrease. I am buying more than one to use.	Jingdong prefecture level city in Shandong
Jinfeng Hongyuan Flagship Store	Power Saving Specialist 1	Well made, power saving, good and convenient!	Jingdong prefecture level city in Shandong
Jinfeng Hongyuan Flagship Store	Power Saving Specialist 1	I'm using it. I'll rate it next month.	Jingdong prefecture level city in Shandong
Jinfeng Hongyuan Flagship Store	Power Saving Specialist 1	Baby received Oh, the courier delivery really fast, with a good and then come back to the evaluation	Jingdong prefecture level city in Shandong
Jinfeng Hongyuan Flagship Store	Power Saving Specialist 1	Fast logistics, good packaging, worth it!	Jingdong prefecture level city in Shandong
Jinfeng Hongyuan Flagship Store	Power Saving Specialist 1	Customer service is okay. It's just that.	Jingdong prefecture level city in Shandong
Jinfeng Hongyuan Flagship Store	Power Saving Specialist 1	Poor power saving	Jingdong prefecture level city in Shandong
Jinfeng Hongyuan Flagship Store	Power Saving Specialist 1	I'll tell you when I've used it.	Jingdong prefecture level city in Shandong
Jinfeng Hongyuan Flagship Store	Power Saving Specialist 1	I think it's good.	Jingdong prefecture level city in Shandong
Jinfeng Hongyuan Flagship Store	Power Saving Specialist 1	This energy saver works very well and I recommend everyone to buy it!	Jingdong prefecture level city in Shandong
Jinfeng Hongyuan Flagship Store	Power Saving Specialist 1	I do not know how effective, just use half a month on the burn out, good business reissued a, and then use it after the follow-up comment.	Jingdong prefecture level city in Shandong
Jinfeng Hongyuan Flagship Store	Power Saving Specialist 1	in use	Jingdong prefecture level city in Shandong
Jinfeng Hongyuan Flagship Store	Power Saving Specialist 1	This thing is very poor. It's already only been used for ten days and it's broken. Now waiting for you to return it.	Jingdong prefecture level city in Shandong
Jinfeng Hongyuan Flagship Store	Power Saving Specialist 1	It's not the same as the seller's show.	Jingdong prefecture level city in Shandong
Jinfeng Hongyuan Flagship Store	Power Saving Specialist 1	What is the problem when I get it back from the CTS, it is plugged into the wall and the voltage shows 206v.	Jingdong prefecture level city in Shandong
Jinfeng Hongyuan Flagship Store	Power Saving Specialist 1	The logistics was fast and the stuff was perfectly packaged. It is in the process of trial.	Jingdong prefecture level city in Shandong
Jinfeng Hongyuan Flagship Store	Power Saving Specialist 1	This saver is particularly good to use can really save electricity, with two months and then look at the line if you buy one for the family	Jingdong prefecture level city in Shandong
Jinfeng Hongyuan Flagship Store	Power Saving Specialist 1	Novel appearance design, all aluminum alloy profile, also with a fuse device, voltage monitoring display. After receiving the goods, I directly cut the plug and connect it to the main power box under the leakage switch, hoping to save electricity, and then follow up later. Logistics is very fast, arrived the next	Jingdong prefecture level city in Shandong
Jinfeng Hongyuan Flagship Store	Power Saving Specialist 1	Received, very good and like, if necessary next time to buy ha ha	Jingdong prefecture level city in Shandong
Jinfeng Hongyuan Flagship Store	Power Saving Specialist 1	It's good. It's worth it. Get it if you need it.	Jingdong prefecture level city in Shandong

Jinfeng Hongyuan Flagship Store	Power Saving Specialist 1	It feels more than good and is worth having.	Jingdong prefecture level city in Shandong
Jinfeng Hongyuan Flagship Store	Power Saving Specialist 1	The goods arrived very good and fast first time to buy	Jingdong prefecture level city in Shandong
Jinfeng Hongyuan Flagship Store	Power Saving Specialist 1	The power saving is very good, I can save so much money every month, I will buy another one next time.	Jingdong prefecture level city in Shandong
Jinfeng Hongyuan Flagship Store	Power Saving Specialist 1	It's not what it says.	Jingdong prefecture level city in Shandong
Jinfeng Hongyuan Flagship Store	Power Saving Specialist 1	I have been using it for many days now and it saves electricity, it really does I have an 8 kW electric snail stove and it saves about $3.50 a day.	Jingdong prefecture level city in Shandong
Jinfeng Hongyuan Flagship Store	Power Saving Specialist 1	Express delivery is very fast, the quality is also quite good, worth buying!	Jingdong prefecture level city in Shandong
Jinfeng Hongyuan Flagship Store	Power Saving Specialist 1	Just received expecting the baby to work well.	Jingdong prefecture level city in Shandong
Jinfeng Hongyuan Flagship Store	Power Saving Specialist 1	Very good, there are more fruit, has bought a second!	Jingdong prefecture level city in Shandong
Jinfeng Hongyuan Flagship Store	Power Saving Specialist 1	Good is good So far, no power savings have been noticed Maybe it's too little to notice.	Jingdong prefecture level city in Shandong
Jinfeng Hongyuan Flagship Store	Power Saving Specialist 1	I just received the item and plugged it in to see what happens later.	Jingdong prefecture level city in Shandong
Jinfeng Hongyuan Flagship Store	Power Saving Specialist 1	Baby received, especially good, packaging is also good, the courier brother is very strong, thank you.	Jingdong prefecture level city in Shandong
Jinfeng Hongyuan Flagship Store	Power Saving Specialist 1	Just received the goods have not yet used it, do not know how, three months later in the follow-up comment!	Jingdong prefecture level city in Shandong
Jinfeng Hongyuan Flagship Store	Power Saving Specialist 1	The item was received, it is quite good, recommended to buy.	Jingdong prefecture level city in Shandong
Jinfeng Hongyuan Flagship Store	Power Saving Specialist 1	This energy saver works very well and I recommend everyone to buy it!	Jingdong prefecture level city in Shandong
Jinfeng Hongyuan Flagship Store	Power Saving Specialist 1	Voltage stabilization and current reduction nice power saving product.	Jingdong prefecture level city in Shandong
Jinfeng Hongyuan Flagship Store	Power Saving Specialist 1	Voltage stabilization and current reduction nice power saving product.	Jingdong prefecture level city in Shandong
Jinfeng Hongyuan Flagship Store	Power Saving Specialist 1	A good power saver for stabilizing and lowering current.	Jingdong prefecture level city in Shandong
Jinfeng Hongyuan Flagship Store	Power Saving Specialist 1	You'll know if it saves power after you use it.	Jingdong prefecture level city in Shandong
Jinfeng Hongyuan Flagship Store	Power Saving Specialist 1	The quality is great. The quality is very good. The service is very good. Express delivery arrived in two days.	Jingdong prefecture level city in Shandong
Jinfeng Hongyuan Flagship Store	Power Saving Specialist 1	It works great.	Jingdong prefecture level city in Shandong
Jinfeng Hongyuan Flagship Store	Power Saving Specialist 1	Logistics is very fast, not yet used, used to comment again.	Jingdong prefecture level city in Shandong
Jinfeng Hongyuan Flagship Store	Power Saving Specialist 1	Logistics is very fast things are also very good, worthy of friends to buy	Jingdong prefecture level city in Shandong
Jinfeng Hongyuan Flagship Store	Power Saving Specialist 1	It works. It really works.	Jingdong prefecture level city in Shandong
Jinfeng Hongyuan Flagship Store	Power Saving Specialist 1	LANGUAGE COMMENTS: just received the shipment. I immediately plugged it in and used it. If you ask how effective. It depends on whether the next month's electricity bill has been reduced. If there is a decrease. I am buying more than one to use.	Jingdong prefecture level city in Shandong
Jinfeng Hongyuan Flagship Store	Power Saving Specialist 1	Well made, power saving, good and convenient!	Jingdong prefecture level city in Shandong
Jinfeng Hongyuan Flagship Store	Power Saving Specialist 1	I'm using it. I'll rate it next month.	Jingdong prefecture level city in Shandong
Jinfeng Hongyuan Flagship Store	Power Saving Specialist 1	Baby received Oh, the courier delivery really fast, with a good and then come back to the evaluation	Jingdong prefecture level city in Shandong
Jinfeng Hongyuan Flagship Store	Power Saving Specialist 1	Fast logistics, good packaging, worth it!	Jingdong prefecture level city in Shandong
Jinfeng Hongyuan Flagship Store	Power Saving Specialist 1	Customer service is okay. It's just that.	Jingdong prefecture level city in Shandong
Jinfeng Hongyuan Flagship Store	Power Saving Specialist 1	Poor power saving	Jingdong prefecture level city in Shandong
Jinfeng Hongyuan Flagship Store	Power Saving Specialist 1	I'll tell you when I've used it.	Jingdong prefecture level city in Shandong
Jinfeng Hongyuan Flagship Store	Power Saving Specialist 1	I think it's good.	Jingdong prefecture level city in Shandong
Jinfeng Hongyuan Flagship Store	Power Saving Specialist 1	This energy saver works very well and I recommend everyone to buy it!	Jingdong prefecture level city in Shandong
Jinfeng Hongyuan Flagship Store	Power Saving Specialist 1	I do not know how effective, just use half a month on the burn out, good business reissued a, and then use it after the follow-up comment.	Jingdong prefecture level city in Shandong
Jinfeng Hongyuan Flagship Store	Power Saving Specialist 1	in use	Jingdong prefecture level city in Shandong
Jinfeng Hongyuan Flagship Store	Power Saving Specialist 1	This thing is very poor. It's already only been used for ten days and it's broken. Now waiting for you to return it.	Jingdong prefecture level city in Shandong
Jinfeng Hongyuan Flagship Store	Power Saving Specialist 1	It's not the same as the seller's show.	Jingdong prefecture level city in Shandong
Jinfeng Hongyuan Flagship Store	Power Saving Specialist 1	What is the problem when I get it back from the CTS, it is plugged into the wall and the voltage shows 206v.	Jingdong prefecture level city in Shandong
Jinfeng Hongyuan Flagship Store	Power Saving Specialist 1	The logistics was fast and the stuff was perfectly packaged. It is in the process of trial.	Jingdong prefecture level city in Shandong
Jinfeng Hongyuan Flagship Store	Power Saving Specialist 1	This saver is particularly good to use can really save electricity, with two months and then look at the line if you buy one for the family	Jingdong prefecture level city in Shandong
Jinfeng Hongyuan Flagship Store	Power Saving Specialist 1	Novel appearance design, all aluminum alloy profile, also with a fuse device, voltage monitoring display. After receiving the goods, I directly cut the plug and connect it to the main power box under the leakage switch, hoping to save electricity, and then follow up later. Logistics is very fast, arrived the next	Jingdong prefecture level city in Shandong
Jinfeng Hongyuan Flagship Store	Power Saving Specialist 1	Received, very good and like, if necessary next time to buy ha ha	Jingdong prefecture level city in Shandong
Jinfeng Hongyuan Flagship Store	Power Saving Specialist 1	It's good. It's worth it. Get it if you need it.	Jingdong prefecture level city in Shandong
Jinfeng Hongyuan Flagship Store	Power Saving Specialist 1	It feels more than good and is worth having.	Jingdong prefecture level city in Shandong
Jinfeng Hongyuan Flagship Store	Power Saving Specialist 1	The goods arrived very good and fast first time to buy	Jingdong prefecture level city in Shandong
Jinfeng Hongyuan Flagship Store	Power Saving Specialist 1	The power saving is very good, I can save so much money every month, I will buy another one next time.	Jingdong prefecture level city in Shandong
Jinfeng Hongyuan Flagship Store	Power Saving Specialist 1	It's not what it says.	Jingdong prefecture level city in Shandong
Jinfeng Hongyuan Flagship Store	Power Saving Specialist 1	I have been using it for many days now and it saves electricity, it really does I have an 8 kW electric snail stove and it saves about $3.50 a day.	Jingdong prefecture level city in Shandong
Jinfeng Hongyuan Flagship Store	Power Saving Specialist 1	Express delivery is very fast, the quality is also quite good, worth buying!	Jingdong prefecture level city in Shandong
Jinfeng Hongyuan Flagship Store	Power Saving Specialist 1	Just received expecting the baby to work well.	Jingdong prefecture level city in Shandong

Jinfeng Hongyuan Flagship Store	Power Saving Specialist 1	Very good, there are more fruit, has bought a second!	Jingdong prefecture level city in Shandong
Jinfeng Hongyuan Flagship Store	Power Saving Specialist 1	Good is good So far, no power savings have been noticed Maybe it's too little to notice.	Jingdong prefecture level city in Shandong
Jinfeng Hongyuan Flagship Store	Power Saving Specialist 1	I just received the item and plugged it in to see what happens later.	Jingdong prefecture level city in Shandong
Jinfeng Hongyuan Flagship Store	Power Saving Specialist 1	Baby received, especially good, packaging is also good, the courier brother is very strong, thank you.	Jingdong prefecture level city in Shandong
Jinfeng Hongyuan Flagship Store	Power Saving Specialist 1	Just received the goods have not yet used it, do not know how, three months later in the follow-up comment!	Jingdong prefecture level city in Shandong
Jinfeng Hongyuan Flagship Store	Power Saving Specialist 1	The item was received, it is quite good, recommended to buy.	Jingdong prefecture level city in Shandong
Jinfeng Hongyuan Flagship Store	Power Saving Specialist 1	This energy saver works very well and I recommend everyone to buy it!	Jingdong prefecture level city in Shandong
Jinfeng Hongyuan Flagship Store	Power Saving Specialist 1	Voltage stabilization and current reduction nice power saving product.	Jingdong prefecture level city in Shandong
Jinfeng Hongyuan Flagship Store	Power Saving Specialist 1	Voltage stabilization and current reduction nice power saving product.	Jingdong prefecture level city in Shandong
Jinfeng Hongyuan Flagship Store	Power Saving Specialist 1	A good power saver for stabilizing and lowering current.	Jingdong prefecture level city in Shandong
Jinfeng Hongyuan Flagship Store	Power Saving Specialist 1	You'll know if it saves power after you use it.	Jingdong prefecture level city in Shandong
Jinfeng Hongyuan Flagship Store	Power Saving Specialist 1	The quality is great. The quality is very good. The service is very good. Express delivery arrived in two days.	Jingdong prefecture level city in Shandong
Jinfeng Hongyuan Flagship Store	Power Saving Specialist 1	It works great.	Jingdong prefecture level city in Shandong
Jinfeng Hongyuan Flagship Store	Power Saving Specialist 1	Logistics is very fast, not yet used, used to comment again.	Jingdong prefecture level city in Shandong
Jinfeng Hongyuan Flagship Store	Power Saving Specialist 1	Logistics is very fast things are also very good, worthy of friends to buy	Jingdong prefecture level city in Shandong
Jinfeng Hongyuan Flagship Store	Power Saving Specialist 1	It works. It really works.	Jingdong prefecture level city in Shandong
Jinfeng Hongyuan Flagship Store	Power Saving Specialist 1	LANGUAGE COMMENTS: just received the shipment. I immediately plugged it in and used it. If you ask how effective. It depends on whether the next month's electricity bill has been reduced. If there is a decrease. I am buying more than one to use.	Jingdong prefecture level city in Shandong
Jinfeng Hongyuan Flagship Store	Power Saving Specialist 1	Well made, power saving, good and convenient!	Jingdong prefecture level city in Shandong
Jinfeng Hongyuan Flagship Store	Power Saving Specialist 1	I'm using it. I'll rate it next month.	Jingdong prefecture level city in Shandong
Jinfeng Hongyuan Flagship Store	Power Saving Specialist 1	Baby received Oh, the courier delivery really fast, with a good and then come back to the evaluation	Jingdong prefecture level city in Shandong
Jinfeng Hongyuan Flagship Store	Power Saving Specialist 1	Fast logistics, good packaging, worth it!	Jingdong prefecture level city in Shandong
Jinfeng Hongyuan Flagship Store	Power Saving Specialist 1	Customer service is okay. It's just that.	Jingdong prefecture level city in Shandong
Jinfeng Hongyuan Flagship Store	Power Saving Specialist 1	Poor power saving	Jingdong prefecture level city in Shandong
Jinfeng Hongyuan Flagship Store	Power Saving Specialist 1	I'll tell you when I've used it.	Jingdong prefecture level city in Shandong
Jinfeng Hongyuan Flagship Store	Power Saving Specialist 1	I think it's good.	Jingdong prefecture level city in Shandong
Jinfeng Hongyuan Flagship Store	Power Saving Specialist 1	This energy saver works very well and I recommend everyone to buy it!	Jingdong prefecture level city in Shandong
Jinfeng Hongyuan Flagship Store	Power Saving Specialist 1	I do not know how effective, just use half a month on the burn out, good business reissued a, and then use it after the follow-up comment.	Jingdong prefecture level city in Shandong
Jinfeng Hongyuan Flagship Store	Power Saving Specialist 1	in use	Jingdong prefecture level city in Shandong
Jinfeng Hongyuan Flagship Store	Power Saving Specialist 1	This thing is very poor. It's already only been used for ten days and it's broken. Now waiting for you to return it.	Jingdong prefecture level city in Shandong
Jinfeng Hongyuan Flagship Store	Power Saving Specialist 1	It's not the same as the seller's show.	Jingdong prefecture level city in Shandong
Jinfeng Hongyuan Flagship Store	Power Saving Specialist 1	What is the problem when I get it back from the CTS, it is plugged into the wall and the voltage shows 206v.	Jingdong prefecture level city in Shandong
Jinfeng Hongyuan Flagship Store	Power Saving Specialist 1	The logistics was fast and the stuff was perfectly packaged. It is in the process of trial.	Jingdong prefecture level city in Shandong
Jinfeng Hongyuan Flagship Store	Power Saving Specialist 1	This saver is particularly good to use can really save electricity, with two months and then look at the line if you buy one for the family	Jingdong prefecture level city in Shandong
Jinfeng Hongyuan Flagship Store	Power Saving Specialist 1	Novel appearance design, all aluminum alloy profile, also with a fuse device, voltage monitoring display. After receiving the goods, I directly cut the plug and connect it to the main power box under the leakage switch, hoping to save electricity, and then follow up later. Logistics is very fast, arrived the next	Jingdong prefecture level city in Shandong
Jinfeng Hongyuan Flagship Store	Power Saving Specialist 1	Received, very good and like, if necessary next time to buy ha ha	Jingdong prefecture level city in Shandong
Jinfeng Hongyuan Flagship Store	Power Saving Specialist 1	It's good. It's worth it. Get it if you need it.	Jingdong prefecture level city in Shandong
Jinfeng Hongyuan Flagship Store	Power Saving Specialist 1	It feels more than good and is worth having.	Jingdong prefecture level city in Shandong
Jinfeng Hongyuan Flagship Store	Power Saving Specialist 1	The goods arrived very good and fast first time to buy	Jingdong prefecture level city in Shandong
Jinfeng Hongyuan Flagship Store	Power Saving Specialist 1	The power saving is very good, I can save so much money every month, I will buy another one next time.	Jingdong prefecture level city in Shandong
Jinfeng Hongyuan Flagship Store	Power Saving Specialist 1	It's not what it says.	Jingdong prefecture level city in Shandong
Jinfeng Hongyuan Flagship Store	Power Saving Specialist 1	I have been using it for many days now and it saves electricity, it really does I have an 8 kW electric snail stove and it saves about $3.50 a day.	Jingdong prefecture level city in Shandong
Jinfeng Hongyuan Flagship Store	Power Saving Specialist 1	Express delivery is very fast, the quality is also quite good, worth buying!	Jingdong prefecture level city in Shandong
Jinfeng Hongyuan Flagship Store	Power Saving Specialist 1	Just received expecting the baby to work well.	Jingdong prefecture level city in Shandong
Jinfeng Hongyuan Flagship Store	Power Saving Specialist 1	Very good, there are more fruit, has bought a second!	Jingdong prefecture level city in Shandong
Jinfeng Hongyuan Flagship Store	Power Saving Specialist 1	Good is good So far, no power savings have been noticed Maybe it's too little to notice.	Jingdong prefecture level city in Shandong
Jinfeng Hongyuan Flagship Store	Power Saving Specialist 1	I just received the item and plugged it in to see what happens later.	Jingdong prefecture level city in Shandong
Jinfeng Hongyuan Flagship Store	Power Saving Specialist 1	Baby received, especially good, packaging is also good, the courier brother is very strong, thank you.	Jingdong prefecture level city in Shandong
Jinfeng Hongyuan Flagship Store	Power Saving Specialist 1	Just received the goods have not yet used it, do not know how, three months later in the follow-up comment!	Jingdong prefecture level city in Shandong
Jinfeng Hongyuan Flagship Store	Power Saving Specialist 1	The item was received, it is quite good, recommended to buy.	Jingdong prefecture level city in Shandong
Jinfeng Hongyuan Flagship Store	Power Saving Specialist 1	This energy saver works very well and I recommend everyone to buy it!	Jingdong prefecture level city in Shandong

Jinfeng Hongyuan Flagship Store	Power Saving Specialist 1	Voltage stabilization and current reduction nice power saving product.	Jingdong prefecture level city in Shandong
Jinfeng Hongyuan Flagship Store	Power Saving Specialist 1	Voltage stabilization and current reduction nice power saving product.	Jingdong prefecture level city in Shandong
Jinfeng Hongyuan Flagship Store	Power Saving Specialist 1	A good power saver for stabilizing and lowering current.	Jingdong prefecture level city in Shandong
Jinfeng Hongyuan Flagship Store	Power Saving Specialist 1	You'll know if it saves power after you use it.	Jingdong prefecture level city in Shandong
Jinfeng Hongyuan Flagship Store	Power Saving Specialist 1	The quality is great. The quality is very good. The service is very good. Express delivery arrived in two days.	Jingdong prefecture level city in Shandong
Jinfeng Hongyuan Flagship Store	Power Saving Specialist 1	It works great.	Jingdong prefecture level city in Shandong
Jinfeng Hongyuan Flagship Store	Power Saving Specialist 1	Logistics is very fast, not yet used, used to comment again.	Jingdong prefecture level city in Shandong
Jinfeng Hongyuan Flagship Store	Power Saving Specialist 1	Logistics is very fast things are also very good, worthy of friends to buy	Jingdong prefecture level city in Shandong
Jinfeng Hongyuan Flagship Store	Power Saving Specialist 1	It works. It really works.	Jingdong prefecture level city in Shandong
Jinfeng Hongyuan Flagship Store	Power Saving Specialist 1	LANGUAGE COMMENTS: just received the shipment. I immediately plugged it in and used it. If you ask how effective. It depends on whether the next month's electricity bill has been reduced. If there is a decrease. I am buying more than one to use.	Jingdong prefecture level city in Shandong
Jinfeng Hongyuan Flagship Store	Power Saving Specialist 1	Well made, power saving, good and convenient!	Jingdong prefecture level city in Shandong
Jinfeng Hongyuan Flagship Store	Power Saving Specialist 1	I'm using it. I'll rate it next month.	Jingdong prefecture level city in Shandong
Jinfeng Hongyuan Flagship Store	Power Saving Specialist 1	Baby received Oh, the courier delivery really fast, with a good and then come back to the evaluation	Jingdong prefecture level city in Shandong
Jinfeng Hongyuan Flagship Store	Power Saving Specialist 1	Fast logistics, good packaging, worth it!	Jingdong prefecture level city in Shandong
Jinfeng Hongyuan Flagship Store	Power Saving Specialist 1	Customer service is okay. It's just that.	Jingdong prefecture level city in Shandong
Jinfeng Hongyuan Flagship Store	Power Saving Specialist 1	Poor power saving	Jingdong prefecture level city in Shandong
Jinfeng Hongyuan Flagship Store	Power Saving Specialist 1	I'll tell you when I've used it.	Jingdong prefecture level city in Shandong
Jinfeng Hongyuan Flagship Store	Power Saving Specialist 1	I think it's good.	Jingdong prefecture level city in Shandong
Jinfeng Hongyuan Flagship Store	Power Saving Specialist 1	This energy saver works very well and I recommend everyone to buy it!	Jingdong prefecture level city in Shandong
Jinfeng Hongyuan Flagship Store	Power Saving Specialist 1	I do not know how effective, just use half a month on the burn out, good business reissued a, and then use it after the follow-up comment.	Jingdong prefecture level city in Shandong
Jinfeng Hongyuan Flagship Store	Power Saving Specialist 1	in use	Jingdong prefecture level city in Shandong
Jinfeng Hongyuan Flagship Store	Power Saving Specialist 1	This thing is very poor. It's already only been used for ten days and it's broken. Now waiting for you to return it.	Jingdong prefecture level city in Shandong
Jinfeng Hongyuan Flagship Store	Power Saving Specialist 1	It's not the same as the seller's show.	Jingdong prefecture level city in Shandong
Jinfeng Hongyuan Flagship Store	Power Saving Specialist 1	What is the problem when I get it back from the CTS, it is plugged into the wall and the voltage shows 206v.	Jingdong prefecture level city in Shandong
Jinfeng Hongyuan Flagship Store	Power Saving Specialist 1	The logistics was fast and the stuff was perfectly packaged. It is in the process of trial.	Jingdong prefecture level city in Shandong
Jinfeng Hongyuan Flagship Store	Power Saving Specialist 1	This saver is particularly good to use can really save electricity, with two months and then look at the line if you buy one for the family	Jingdong prefecture level city in Shandong
Jinfeng Hongyuan Flagship Store	Power Saving Specialist 1	Novel appearance design, all aluminum alloy profile, also with a fuse device, voltage monitoring display. After receiving the goods, I directly cut the plug and connect it to the main power box under the leakage switch, hoping to save electricity, and then follow up later. Logistics is very fast, arrived the next	Jingdong prefecture level city in Shandong
Jinfeng Hongyuan Flagship Store	Power Saving Specialist 1	Received, very good and like, if necessary next time to buy ha ha	Jingdong prefecture level city in Shandong
Jinfeng Hongyuan Flagship Store	Power Saving Specialist 1	It's good. It's worth it. Get it if you need it.	Jingdong prefecture level city in Shandong
Jinfeng Hongyuan Flagship Store	Power Saving Specialist 1	It feels more than good and is worth having.	Jingdong prefecture level city in Shandong
Jinfeng Hongyuan Flagship Store	Power Saving Specialist 1	The goods arrived very good and fast first time to buy	Jingdong prefecture level city in Shandong
Jinfeng Hongyuan Flagship Store	Power Saving Specialist 1	The power saving is very good, I can save so much money every month, I will buy another one next time.	Jingdong prefecture level city in Shandong
Jinfeng Hongyuan Flagship Store	Power Saving Specialist 1	It's not what it says.	Jingdong prefecture level city in Shandong
Jinfeng Hongyuan Flagship Store	Power Saving Specialist 1	I have been using it for many days now and it saves electricity, it really does I have an 8 kW electric snail stove and it saves about $3.50 a day.	Jingdong prefecture level city in Shandong
Jinfeng Hongyuan Flagship Store	Power Saving Specialist 1	Express delivery is very fast, the quality is also quite good, worth buying!	Jingdong prefecture level city in Shandong
Jinfeng Hongyuan Flagship Store	Power Saving Specialist 1	Just received expecting the baby to work well.	Jingdong prefecture level city in Shandong
Jinfeng Hongyuan Flagship Store	Power Saving Specialist 1	Very good, there are more fruit, has bought a second!	Jingdong prefecture level city in Shandong
Jinfeng Hongyuan Flagship Store	Power Saving Specialist 1	Good is good So far, no power savings have been noticed Maybe it's too little to notice.	Jingdong prefecture level city in Shandong
Jinfeng Hongyuan Flagship Store	Power Saving Specialist 1	I just received the item and plugged it in to see what happens later.	Jingdong prefecture level city in Shandong
Jinfeng Hongyuan Flagship Store	Power Saving Specialist 1	Baby received, especially good, packaging is also good, the courier brother is very strong, thank you.	Jingdong prefecture level city in Shandong
Jinfeng Hongyuan Flagship Store	Power Saving Specialist 1	Just received the goods have not yet used it, do not know how, three months later in the follow-up comment!	Jingdong prefecture level city in Shandong
Jinfeng Hongyuan Flagship Store	Power Saving Specialist 1	The item was received, it is quite good, recommended to buy.	Jingdong prefecture level city in Shandong
Jinfeng Hongyuan Flagship Store	Power Saving Specialist 1	This energy saver works very well and I recommend everyone to buy it!	Jingdong prefecture level city in Shandong
Jinfeng Hongyuan Flagship Store	Power Saving Specialist 1	Voltage stabilization and current reduction nice power saving product.	Jingdong prefecture level city in Shandong
Jinfeng Hongyuan Flagship Store	Power Saving Specialist 1	Voltage stabilization and current reduction nice power saving product.	Jingdong prefecture level city in Shandong
Jinfeng Hongyuan Flagship Store	Power Saving Specialist 1	A good power saver for stabilizing and lowering current.	Jingdong prefecture level city in Shandong
Jinfeng Hongyuan Flagship Store	Power Saving Specialist 1	You'll know if it saves power after you use it.	Jingdong prefecture level city in Shandong
Jinfeng Hongyuan Flagship Store	Power Saving Specialist 1	The quality is great. The quality is very good. The service is very good. Express delivery arrived in two days.	Jingdong prefecture level city in Shandong
Jinfeng Hongyuan Flagship Store	Power Saving Specialist 1	It works great.	Jingdong prefecture level city in Shandong
Jinfeng Hongyuan Flagship Store	Power Saving Specialist 1	Logistics is very fast, not yet used, used to comment again.	Jingdong prefecture level city in Shandong

Jinfeng Hongyuan Flagship Store	Power Saving Specialist 1	Logistics is very fast things are also very good, worthy of friends to buy	Jingdong prefecture level city in Shandong
Jinfeng Hongyuan Flagship Store	Power Saving Specialist 1	It works. It really works.	Jingdong prefecture level city in Shandong
Jinfeng Hongyuan Flagship Store	Power Saving Specialist 1	LANGUAGE COMMENTS: just received the shipment. I immediately plugged it in and used it. If you ask how effective. It depends on whether the next month's electricity bill has been reduced. If there is a decrease. I am buying more than one to use.	Jingdong prefecture level city in Shandong
Jinfeng Hongyuan Flagship Store	Power Saving Specialist 1	Well made, power saving, good and convenient!	Jingdong prefecture level city in Shandong
Jinfeng Hongyuan Flagship Store	Power Saving Specialist 1	I'm using it. I'll rate it next month.	Jingdong prefecture level city in Shandong
Jinfeng Hongyuan Flagship Store	Power Saving Specialist 1	Baby received Oh, the courier delivery really fast, with a good and then come back to the evaluation	Jingdong prefecture level city in Shandong
Jinfeng Hongyuan Flagship Store	Power Saving Specialist 1	Fast logistics, good packaging, worth it!	Jingdong prefecture level city in Shandong
Jinfeng Hongyuan Flagship Store	Power Saving Specialist 1	Customer service is okay. It's just that.	Jingdong prefecture level city in Shandong
Jinfeng Hongyuan Flagship Store	Power Saving Specialist 1	Poor power saving	Jingdong prefecture level city in Shandong
Jinfeng Hongyuan Flagship Store	Power Saving Specialist 1	I'll tell you when I've used it.	Jingdong prefecture level city in Shandong
Jinfeng Hongyuan Flagship Store	Power Saving Specialist 1	I think it's good.	Jingdong prefecture level city in Shandong
Jinfeng Hongyuan Flagship Store	Power Saving Specialist 1	This energy saver works very well and I recommend everyone to buy it!	Jingdong prefecture level city in Shandong
Jinfeng Hongyuan Flagship Store	Power Saving Specialist 1	I do not know how effective, just use half a month on the burn out, good business reissued a, and then use it after the follow-up comment.	Jingdong prefecture level city in Shandong
Jinfeng Hongyuan Flagship Store	Power Saving Specialist 1	in use	Jingdong prefecture level city in Shandong
Jinfeng Hongyuan Flagship Store	Power Saving Specialist 1	This thing is very poor. It's already only been used for ten days and it's broken. Now waiting for you to return it.	Jingdong prefecture level city in Shandong
Jinfeng Hongyuan Flagship Store	Power Saving Specialist 1	It's not the same as the seller's show.	Jingdong prefecture level city in Shandong
Jinfeng Hongyuan Flagship Store	Power Saving Specialist 1	What is the problem when I get it back from the CTS, it is plugged into the wall and the voltage shows 206v.	Jingdong prefecture level city in Shandong
Jinfeng Hongyuan Flagship Store	Power Saving Specialist 1	The logistics was fast and the stuff was perfectly packaged. It is in the process of trial.	Jingdong prefecture level city in Shandong
Jinfeng Hongyuan Flagship Store	Power Saving Specialist 1	This saver is particularly good to use can really save electricity, with two months and then look at the line if you buy one for the family	Jingdong prefecture level city in Shandong
Jinfeng Hongyuan Flagship Store	Power Saving Specialist 1	Novel appearance design, all aluminum alloy profile, also with a fuse device, voltage monitoring display. After receiving the goods, I directly cut the plug and connect it to the main power box under the leakage switch, hoping to save electricity, and then follow up later. Logistics is very fast, arrived the next	Jingdong prefecture level city in Shandong
Jinfeng Hongyuan Flagship Store	Power Saving Specialist 1	Received, very good and like, if necessary next time to buy ha ha	Jingdong prefecture level city in Shandong
Jinfeng Hongyuan Flagship Store	Power Saving Specialist 1	It's good. It's worth it. Get it if you need it.	Jingdong prefecture level city in Shandong
Jinfeng Hongyuan Flagship Store	Power Saving Specialist 1	It feels more than good and is worth having.	Jingdong prefecture level city in Shandong
Jinfeng Hongyuan Flagship Store	Power Saving Specialist 1	The goods arrived very good and fast first time to buy	Jingdong prefecture level city in Shandong
Jinfeng Hongyuan Flagship Store	Power Saving Specialist 1	The power saving is very good, I can save so much money every month, I will buy another one next time.	Jingdong prefecture level city in Shandong
Jinfeng Hongyuan Flagship Store	Power Saving Specialist 1	It's not what it says.	Jingdong prefecture level city in Shandong
Jinfeng Hongyuan Flagship Store	Power Saving Specialist 1	I have been using it for many days now and it saves electricity, it really does I have an 8 kW electric snail stove and it saves about $3.50 a day.	Jingdong prefecture level city in Shandong
Jinfeng Hongyuan Flagship Store	Power Saving Specialist 1	Express delivery is very fast, the quality is also quite good, worth buying!	Jingdong prefecture level city in Shandong
Jinfeng Hongyuan Flagship Store	Power Saving Specialist 1	Just received expecting the baby to work well.	Jingdong prefecture level city in Shandong
Jinfeng Hongyuan Flagship Store	Power Saving Specialist 1	Very good, there are more fruit, has bought a second!	Jingdong prefecture level city in Shandong
Jinfeng Hongyuan Flagship Store	Power Saving Specialist 1	Good is good So far, no power savings have been noticed Maybe it's too little to notice.	Jingdong prefecture level city in Shandong
Jinfeng Hongyuan Flagship Store	Power Saving Specialist 1	I just received the item and plugged it in to see what happens later.	Jingdong prefecture level city in Shandong
Jinfeng Hongyuan Flagship Store	Power Saving Specialist 1	Baby received, especially good, packaging is also good, the courier brother is very strong, thank you.	Jingdong prefecture level city in Shandong
Jinfeng Hongyuan Flagship Store	Power Saving Specialist 1	Just received the goods have not yet used it, do not know how, three months later in the follow-up comment!	Jingdong prefecture level city in Shandong
Jinfeng Hongyuan Flagship Store	Power Saving Specialist 1	The item was received, it is quite good, recommended to buy.	Jingdong prefecture level city in Shandong
Jinfeng Hongyuan Flagship Store	Power Saving Specialist 1	This energy saver works very well and I recommend everyone to buy it!	Jingdong prefecture level city in Shandong
Jinfeng Hongyuan Flagship Store	Power Saving Specialist 1	Voltage stabilization and current reduction nice power saving product.	Jingdong prefecture level city in Shandong
Jinfeng Hongyuan Flagship Store	Power Saving Specialist 1	Voltage stabilization and current reduction nice power saving product.	Jingdong prefecture level city in Shandong
Jinfeng Hongyuan Flagship Store	Power Saving Specialist 1	A good power saver for stabilizing and lowering current.	Jingdong prefecture level city in Shandong
Jinfeng Hongyuan Flagship Store	Power Saving Specialist 1	You'll know if it saves power after you use it.	Jingdong prefecture level city in Shandong
Jinfeng Hongyuan Flagship Store	Power Saving Specialist 1	The quality is great. The quality is very good. The service is very good. Express delivery arrived in two days.	Jingdong prefecture level city in Shandong
Jinfeng Hongyuan Flagship Store	Power Saving Specialist 1	It works great.	Jingdong prefecture level city in Shandong
Jinfeng Hongyuan Flagship Store	Power Saving Specialist 1	Logistics is very fast, not yet used, used to comment again.	Jingdong prefecture level city in Shandong
Jinfeng Hongyuan Flagship Store	Power Saving Specialist 1	Logistics is very fast things are also very good, worthy of friends to buy	Jingdong prefecture level city in Shandong
Jinfeng Hongyuan Flagship Store	Power Saving Specialist 1	It works. It really works.	Jingdong prefecture level city in Shandong
Jinfeng Hongyuan Flagship Store	Power Saving Specialist 1	LANGUAGE COMMENTS: just received the shipment. I immediately plugged it in and used it. If you ask how effective. It depends on whether the next month's electricity bill has been reduced. If there is a decrease. I am buying more than one to use.	Jingdong prefecture level city in Shandong
Jinfeng Hongyuan Flagship Store	Power Saving Specialist 1	Well made, power saving, good and convenient!	Jingdong prefecture level city in Shandong
Jinfeng Hongyuan Flagship Store	Power Saving Specialist 1	I'm using it. I'll rate it next month.	Jingdong prefecture level city in Shandong
Jinfeng Hongyuan Flagship Store	Power Saving Specialist 1	Baby received Oh, the courier delivery really fast, with a good and then come back to the evaluation	Jingdong prefecture level city in Shandong
Jinfeng Hongyuan Flagship Store	Power Saving Specialist 1	Fast logistics, good packaging, worth it!	Jingdong prefecture level city in Shandong

Jinfeng Hongyuan Flagship Store	Power Saving Specialist 1	Customer service is okay. It's just that.	Jingdong prefecture level city in Shandong
Jinfeng Hongyuan Flagship Store	Power Saving Specialist 1	Poor power saving	Jingdong prefecture level city in Shandong
Jinfeng Hongyuan Flagship Store	Power Saving Specialist 1	I'll tell you when I've used it.	Jingdong prefecture level city in Shandong
Jinfeng Hongyuan Flagship Store	Power Saving Specialist 1	I think it's good.	Jingdong prefecture level city in Shandong
Jinfeng Hongyuan Flagship Store	Power Saving Specialist 1	This energy saver works very well and I recommend everyone to buy it!	Jingdong prefecture level city in Shandong
Jinfeng Hongyuan Flagship Store	Power Saving Specialist 1	I do not know how effective, just use half a month on the burn out, good business reissued a, and then use it after the follow-up comment.	Jingdong prefecture level city in Shandong
Jinfeng Hongyuan Flagship Store	Power Saving Specialist 1	in use	Jingdong prefecture level city in Shandong
Jinfeng Hongyuan Flagship Store	Power Saving Specialist 1	This thing is very poor. It's already only been used for ten days and it's broken. Now waiting for you to return it.	Jingdong prefecture level city in Shandong
Jinfeng Hongyuan Flagship Store	Power Saving Specialist 1	It's not the same as the seller's show.	Jingdong prefecture level city in Shandong
Jinfeng Hongyuan Flagship Store	Power Saving Specialist 1	What is the problem when I get it back from the CTS, it is plugged into the wall and the voltage shows 206v.	Jingdong prefecture level city in Shandong
Jinfeng Hongyuan Flagship Store	Power Saving Specialist 1	The logistics was fast and the stuff was perfectly packaged. It is in the process of trial.	Jingdong prefecture level city in Shandong
Jinfeng Hongyuan Flagship Store	Power Saving Specialist 1	This saver is particularly good to use can really save electricity, with two months and then look at the line if you buy one for the family	Jingdong prefecture level city in Shandong
Jinfeng Hongyuan Flagship Store	Power Saving Specialist 1	Novel appearance design, all aluminum alloy profile, also with a fuse device, voltage monitoring display. After receiving the goods, I directly cut the plug and connect it to the main power box under the leakage switch, hoping to save electricity, and then follow up later. Logistics is very fast, arrived the next	Jingdong prefecture level city in Shandong
Jinfeng Hongyuan Flagship Store	Power Saving Specialist 1	Received, very good and like, if necessary next time to buy ha ha	Jingdong prefecture level city in Shandong
Jinfeng Hongyuan Flagship Store	Power Saving Specialist 1	It's good. It's worth it. Get it if you need it.	Jingdong prefecture level city in Shandong
Jinfeng Hongyuan Flagship Store	Power Saving Specialist 1	It feels more than good and is worth having.	Jingdong prefecture level city in Shandong
Jinfeng Hongyuan Flagship Store	Power Saving Specialist 1	The goods arrived very good and fast first time to buy	Jingdong prefecture level city in Shandong
Jinfeng Hongyuan Flagship Store	Power Saving Specialist 1	The power saving is very good, I can save so much money every month, I will buy another one next time.	Jingdong prefecture level city in Shandong
Jinfeng Hongyuan Flagship Store	Power Saving Specialist 1	It's not what it says.	Jingdong prefecture level city in Shandong
Jinfeng Hongyuan Flagship Store	Power Saving Specialist 1	I have been using it for many days now and it saves electricity, it really does I have an 8 kW electric snail stove and it saves about $3.50 a day.	Jingdong prefecture level city in Shandong
Jinfeng Hongyuan Flagship Store	Power Saving Specialist 1	Express delivery is very fast, the quality is also quite good, worth buying!	Jingdong prefecture level city in Shandong
Jinfeng Hongyuan Flagship Store	Power Saving Specialist 1	Just received expecting the baby to work well.	Jingdong prefecture level city in Shandong
Jinfeng Hongyuan Flagship Store	Power Saving Specialist 1	Very good, there are more fruit, has bought a second!	Jingdong prefecture level city in Shandong
Jinfeng Hongyuan Flagship Store	Power Saving Specialist 1	Good is good So far, no power savings have been noticed Maybe it's too little to notice.	Jingdong prefecture level city in Shandong
Jinfeng Hongyuan Flagship Store	Power Saving Specialist 1	I just received the item and plugged it in to see what happens later.	Jingdong prefecture level city in Shandong
Jinfeng Hongyuan Flagship Store	Power Saving Specialist 1	Baby received, especially good, packaging is also good, the courier brother is very strong, thank you.	Jingdong prefecture level city in Shandong
Jinfeng Hongyuan Flagship Store	Power Saving Specialist 1	Just received the goods have not yet used it, do not know how, three months later in the follow-up comment!	Jingdong prefecture level city in Shandong
Jinfeng Hongyuan Flagship Store	Power Saving Specialist 1	The item was received, it is quite good, recommended to buy.	Jingdong prefecture level city in Shandong
Jinfeng Hongyuan Flagship Store	Power Saving Specialist 1	This energy saver works very well and I recommend everyone to buy it!	Jingdong prefecture level city in Shandong
Jinfeng Hongyuan Flagship Store	Power Saving Specialist 1	Voltage stabilization and current reduction nice power saving product.	Jingdong prefecture level city in Shandong
Jinfeng Hongyuan Flagship Store	Power Saving Specialist 1	Voltage stabilization and current reduction nice power saving product.	Jingdong prefecture level city in Shandong
Jinfeng Hongyuan Flagship Store	Power Saving Specialist 1	A good power saver for stabilizing and lowering current.	Jingdong prefecture level city in Shandong
Jinfeng Hongyuan Flagship Store	Power Saving Specialist 1	You'll know if it saves power after you use it.	Jingdong prefecture level city in Shandong
Jinfeng Hongyuan Flagship Store	Power Saving Specialist 1	The quality is great. The quality is very good. The service is very good. Express delivery arrived in two days.	Jingdong prefecture level city in Shandong
Jinfeng Hongyuan Flagship Store	Power Saving Specialist 1	It works great.	Jingdong prefecture level city in Shandong
Jinfeng Hongyuan Flagship Store	Power Saving Specialist 1	Logistics is very fast, not yet used, used to comment again.	Jingdong prefecture level city in Shandong
Jinfeng Hongyuan Flagship Store	Power Saving Specialist 1	Logistics is very fast things are also very good, worthy of friends to buy	Jingdong prefecture level city in Shandong
Jinfeng Hongyuan Flagship Store	Power Saving Specialist 1	It works. It really works.	Jingdong prefecture level city in Shandong
Jinfeng Hongyuan Flagship Store	Power Saving Specialist 1	LANGUAGE COMMENTS: just received the shipment. I immediately plugged it in and used it. If you ask how effective. It depends on whether the next month's electricity bill has been reduced. If there is a decrease. I am buying more than one to use.	Jingdong prefecture level city in Shandong
Jinfeng Hongyuan Flagship Store	Power Saving Specialist 1	Well made, power saving, good and convenient!	Jingdong prefecture level city in Shandong
Jinfeng Hongyuan Flagship Store	Power Saving Specialist 1	I'm using it. I'll rate it next month.	Jingdong prefecture level city in Shandong
Jinfeng Hongyuan Flagship Store	Power Saving Specialist 1	Baby received Oh, the courier delivery really fast, with a good and then come back to the evaluation	Jingdong prefecture level city in Shandong
Jinfeng Hongyuan Flagship Store	Power Saving Specialist 1	Fast logistics, good packaging, worth it!	Jingdong prefecture level city in Shandong
Jinfeng Hongyuan Flagship Store	Power Saving Specialist 1	Customer service is okay. It's just that.	Jingdong prefecture level city in Shandong
Jinfeng Hongyuan Flagship Store	Power Saving Specialist 1	Poor power saving	Jingdong prefecture level city in Shandong
Jinfeng Hongyuan Flagship Store	Power Saving Specialist 1	I'll tell you when I've used it.	Jingdong prefecture level city in Shandong
Jinfeng Hongyuan Flagship Store	Power Saving Specialist 1	I think it's good.	Jingdong prefecture level city in Shandong
Jinfeng Hongyuan Flagship Store	Power Saving Specialist 1	This energy saver works very well and I recommend everyone to buy it!	Jingdong prefecture level city in Shandong
Jinfeng Hongyuan Flagship Store	Power Saving Specialist 1	I do not know how effective, just use half a month on the burn out, good business reissued a, and then use it after the follow-up comment.	Jingdong prefecture level city in Shandong
Jinfeng Hongyuan Flagship Store	Power Saving Specialist 1	in use	Jingdong prefecture level city in Shandong

Jinfeng Hongyuan Flagship Store	Power Saving Specialist 1	This thing is very poor. It's already only been used for ten days and it's broken. Now waiting for you to return it.	Jingdong prefecture level city in Shandong
Jinfeng Hongyuan Flagship Store	Power Saving Specialist 1	It's not the same as the seller's show.	Jingdong prefecture level city in Shandong
Jinfeng Hongyuan Flagship Store	Power Saving Specialist 1	What is the problem when I get it back from the CTS, it is plugged into the wall and the voltage shows 206v.	Jingdong prefecture level city in Shandong
Jinfeng Hongyuan Flagship Store	Power Saving Specialist 1	The logistics was fast and the stuff was perfectly packaged. It is in the process of trial.	Jingdong prefecture level city in Shandong
Jinfeng Hongyuan Flagship Store	Power Saving Specialist 1	This saver is particularly good to use can really save electricity, with two months and then look at the line if you buy one for the family	Jingdong prefecture level city in Shandong
Jinfeng Hongyuan Flagship Store	Power Saving Specialist 1	Novel appearance design, all aluminum alloy profile, also with a fuse device, voltage monitoring display. After receiving the goods, I directly cut the plug and connect it to the main power box under the leakage switch, hoping to save electricity, and then follow up later. Logistics is very fast, arrived the next	Jingdong prefecture level city in Shandong
Jinfeng Hongyuan Flagship Store	Power Saving Specialist 1	Received, very good and like, if necessary next time to buy ha ha	Jingdong prefecture level city in Shandong
Jinfeng Hongyuan Flagship Store	Power Saving Specialist 1	It's good. It's worth it. Get it if you need it.	Jingdong prefecture level city in Shandong
Jinfeng Hongyuan Flagship Store	Power Saving Specialist 1	It feels more than good and is worth having.	Jingdong prefecture level city in Shandong
Jinfeng Hongyuan Flagship Store	Power Saving Specialist 1	The goods arrived very good and fast first time to buy	Jingdong prefecture level city in Shandong
Jinfeng Hongyuan Flagship Store	Power Saving Specialist 1	The power saving is very good, I can save so much money every month, I will buy another one next time.	Jingdong prefecture level city in Shandong
Jinfeng Hongyuan Flagship Store	Power Saving Specialist 1	It's not what it says.	Jingdong prefecture level city in Shandong
Jinfeng Hongyuan Flagship Store	Power Saving Specialist 1	I have been using it for many days now and it saves electricity, it really does I have an 8 kW electric snail stove and it saves about $3.50 a day.	Jingdong prefecture level city in Shandong
Jinfeng Hongyuan Flagship Store	Power Saving Specialist 1	Express delivery is very fast, the quality is also quite good, worth buying!	Jingdong prefecture level city in Shandong
Jinfeng Hongyuan Flagship Store	Power Saving Specialist 1	Just received expecting the baby to work well.	Jingdong prefecture level city in Shandong
Jinfeng Hongyuan Flagship Store	Power Saving Specialist 1	Very good, there are more fruit, has bought a second!	Jingdong prefecture level city in Shandong
Jinfeng Hongyuan Flagship Store	Power Saving Specialist 1	Good is good So far, no power savings have been noticed Maybe it's too little to notice.	Jingdong prefecture level city in Shandong
Jinfeng Hongyuan Flagship Store	Power Saving Specialist 1	I just received the item and plugged it in to see what happens later.	Jingdong prefecture level city in Shandong
Jinfeng Hongyuan Flagship Store	Power Saving Specialist 1	Baby received, especially good, packaging is also good, the courier brother is very strong, thank you.	Jingdong prefecture level city in Shandong
Jinfeng Hongyuan Flagship Store	Power Saving Specialist 1	Just received the goods have not yet used it, do not know how, three months later in the follow-up comment!	Jingdong prefecture level city in Shandong
Jinfeng Hongyuan Flagship Store	Power Saving Specialist 1	The item was received, it is quite good, recommended to buy.	Jingdong prefecture level city in Shandong
Jinfeng Hongyuan Flagship Store	Power Saving Specialist 1	This energy saver works very well and I recommend everyone to buy it!	Jingdong prefecture level city in Shandong
Jinfeng Hongyuan Flagship Store	Power Saving Specialist 1	Voltage stabilization and current reduction nice power saving product.	Jingdong prefecture level city in Shandong
Jinfeng Hongyuan Flagship Store	Power Saving Specialist 1	Voltage stabilization and current reduction nice power saving product.	Jingdong prefecture level city in Shandong
Jinfeng Hongyuan Flagship Store	Power Saving Specialist 1	A good power saver for stabilizing and lowering current.	Jingdong prefecture level city in Shandong
Jinfeng Hongyuan Flagship Store	Power Saving Specialist 1	You'll know if it saves power after you use it.	Jingdong prefecture level city in Shandong
Jinfeng Hongyuan Flagship Store	Power Saving Specialist 1	The quality is great. The quality is very good. The service is very good. Express delivery arrived in two days.	Jingdong prefecture level city in Shandong
Jinfeng Hongyuan Flagship Store	Power Saving Specialist 1	It works great.	Jingdong prefecture level city in Shandong
Jinfeng Hongyuan Flagship Store	Power Saving Specialist 1	Logistics is very fast, not yet used, used to comment again.	Jingdong prefecture level city in Shandong
Jinfeng Hongyuan Flagship Store	Power Saving Specialist 1	Logistics is very fast things are also very good, worthy of friends to buy	Jingdong prefecture level city in Shandong
Jinfeng Hongyuan Flagship Store	Power Saving Specialist 1	It works. It really works.	Jingdong prefecture level city in Shandong
Jinfeng Hongyuan Flagship Store	Power Saving Specialist 1	LANGUAGE COMMENTS: just received the shipment. I immediately plugged it in and used it. If you ask how effective. It depends on whether the next month's electricity bill has been reduced. If there is a decrease. I am buying more than one to use.	Jingdong prefecture level city in Shandong
Jinfeng Hongyuan Flagship Store	Power Saving Specialist 1	Well made, power saving, good and convenient!	Jingdong prefecture level city in Shandong
Jinfeng Hongyuan Flagship Store	Power Saving Specialist 1	I'm using it. I'll rate it next month.	Jingdong prefecture level city in Shandong
Jinfeng Hongyuan Flagship Store	Power Saving Specialist 1	Baby received Oh, the courier delivery really fast, with a good and then come back to the evaluation	Jingdong prefecture level city in Shandong
Jinfeng Hongyuan Flagship Store	Power Saving Specialist 1	Fast logistics, good packaging, worth it!	Jingdong prefecture level city in Shandong
Jinfeng Hongyuan Flagship Store	Power Saving Specialist 1	Customer service is okay. It's just that.	Jingdong prefecture level city in Shandong
Jinfeng Hongyuan Flagship Store	Power Saving Specialist 1	Poor power saving	Jingdong prefecture level city in Shandong
Jinfeng Hongyuan Flagship Store	Power Saving Specialist 1	I'll tell you when I've used it.	Jingdong prefecture level city in Shandong
Jinfeng Hongyuan Flagship Store	Power Saving Specialist 1	I think it's good.	Jingdong prefecture level city in Shandong
Jinfeng Hongyuan Flagship Store	Power Saving Specialist 1	This energy saver works very well and I recommend everyone to buy it!	Jingdong prefecture level city in Shandong
Jinfeng Hongyuan Flagship Store	Power Saving Specialist 1	I do not know how effective, just use half a month on the burn out, good business reissued a, and then use it after the follow-up comment.	Jingdong prefecture level city in Shandong
Jinfeng Hongyuan Flagship Store	Power Saving Specialist 1	This thing is very poor. It's already only been used for ten days and it's broken. Now waiting for you to return it.	Jingdong prefecture level city in Shandong
Jinfeng Hongyuan Flagship Store	Power Saving Specialist 1	in use	Jingdong prefecture level city in Shandong
Jinfeng Hongyuan Flagship Store	Power Saving Specialist 1	It's not the same as the seller's show.	Jingdong prefecture level city in Shandong
Jinfeng Hongyuan Flagship Store	Power Saving Specialist 1	What is the problem when I get it back from the CTS, it is plugged into the wall and the voltage shows 206v.	Jingdong prefecture level city in Shandong
Jinfeng Hongyuan Flagship Store	Power Saving Specialist 1	The logistics was fast and the stuff was perfectly packaged. It is in the process of trial.	Jingdong prefecture level city in Shandong
Jinfeng Hongyuan Flagship Store	Power Saving Specialist 1	This saver is particularly good to use can really save electricity, with two months and then look at the line if you buy one for the family	Jingdong prefecture level city in Shandong
Jinfeng Hongyuan Flagship Store	Power Saving Specialist 1	Received, very good and like, if necessary next time to buy ha ha	Jingdong prefecture level city in Shandong
Jinfeng Hongyuan Flagship Store	Power Saving Specialist 1	Novel appearance design, all aluminum alloy profile, also with a fuse device, voltage monitoring display. After receiving the goods, I directly cut the plug and connect it to the main power box under the leakage switch, hoping to save electricity, and then follow up later. Logistics is very fast, arrived the next	Jingdong prefecture level city in Shandong

Jinfeng Hongyuan Flagship Store	Power Saving Specialist 1	It's good. It's worth it. Get it if you need it.	Jingdong prefecture level city in Shandong
Jinfeng Hongyuan Flagship Store	Power Saving Specialist 1	It feels more than good and is worth having.	Jingdong prefecture level city in Shandong
Jinfeng Hongyuan Flagship Store	Power Saving Specialist 1	The goods arrived very good and fast first time to buy	Jingdong prefecture level city in Shandong
Jinfeng Hongyuan Flagship Store	Power Saving Specialist 1	The power saving is very good, I can save so much money every month, I will buy another one next time.	Jingdong prefecture level city in Shandong
Jinfeng Hongyuan Flagship Store	Power Saving Specialist 1	It's not what it says.	Jingdong prefecture level city in Shandong
Jinfeng Hongyuan Flagship Store	Power Saving Specialist 1	I have been using it for many days now and it saves electricity, it really does I have an 8 kW electric snail stove and it saves about $3.50 a day.	Jingdong prefecture level city in Shandong
Jinfeng Hongyuan Flagship Store	Power Saving Specialist 1	Express delivery is very fast, the quality is also quite good, worth buying!	Jingdong prefecture level city in Shandong
Jinfeng Hongyuan Flagship Store	Power Saving Specialist 1	Just received expecting the baby to work well.	Jingdong prefecture level city in Shandong
Jinfeng Hongyuan Flagship Store	Power Saving Specialist 1	Very good, there are more fruit, has bought a second!	Jingdong prefecture level city in Shandong
Jinfeng Hongyuan Flagship Store	Power Saving Specialist 1	Good is good So far, no power savings have been noticed Maybe it's too little to notice.	Jingdong prefecture level city in Shandong
Jinfeng Hongyuan Flagship Store	Power Saving Specialist 1	I just received the item and plugged it in to see what happens later.	Jingdong prefecture level city in Shandong
Jinfeng Hongyuan Flagship Store	Power Saving Specialist 1	Baby received, especially good, packaging is also good, the courier brother is very strong, thank you.	Jingdong prefecture level city in Shandong
Jinfeng Hongyuan Flagship Store	Power Saving Specialist 1	Just received the goods have not yet used it, do not know how, three months later in the follow-up comment!	Jingdong prefecture level city in Shandong
Jinfeng Hongyuan Flagship Store	Power Saving Specialist 1	The item was received, it is quite good, recommended to buy.	Jingdong prefecture level city in Shandong
Jinfeng Hongyuan Flagship Store	Power Saving Specialist 1	This energy saver works very well and I recommend everyone to buy it!	Jingdong prefecture level city in Shandong
Jinfeng Hongyuan Flagship Store	Power Saving Specialist 1	Voltage stabilization and current reduction nice power saving product.	Jingdong prefecture level city in Shandong
Jinfeng Hongyuan Flagship Store	Power Saving Specialist 1	Voltage stabilization and current reduction nice power saving product.	Jingdong prefecture level city in Shandong
Jinfeng Hongyuan Flagship Store	Power Saving Specialist 1	A good power saver for stabilizing and lowering current.	Jingdong prefecture level city in Shandong
Jinfeng Hongyuan Flagship Store	Power Saving Specialist 1	You'll know if it saves power after you use it.	Jingdong prefecture level city in Shandong
Jinfeng Hongyuan Flagship Store	Power Saving Specialist 1	The quality is great. The quality is very good. The service is very good. Express delivery arrived in two days.	Jingdong prefecture level city in Shandong
Jinfeng Hongyuan Flagship Store	Power Saving Specialist 1	It works great.	Jingdong prefecture level city in Shandong
Jinfeng Hongyuan Flagship Store	Power Saving Specialist 1	Logistics is very fast, not yet used, used to comment again.	Jingdong prefecture level city in Shandong
Jinfeng Hongyuan Flagship Store	Power Saving Specialist 1	Logistics is very fast things are also very good, worthy of friends to buy	Jingdong prefecture level city in Shandong
Jinfeng Hongyuan Flagship Store	Power Saving Specialist 1	It works. It really works.	Jingdong prefecture level city in Shandong
Jinfeng Hongyuan Flagship Store	Power Saving Specialist 1	LANGUAGE COMMENTS: just received the shipment. I immediately plugged it in and used it. If you ask how effective. It depends on whether the next month's electricity bill has been reduced. If there is a decrease. I am buying more than one to use.	Jingdong prefecture level city in Shandong
Jinfeng Hongyuan Flagship Store	Power Saving Specialist 1	Well made, power saving, good and convenient!	Jingdong prefecture level city in Shandong
Jinfeng Hongyuan Flagship Store	Power Saving Specialist 1	I'm using it. I'll rate it next month.	Jingdong prefecture level city in Shandong
Jinfeng Hongyuan Flagship Store	Power Saving Specialist 1	Baby received Oh, the courier delivery really fast, with a good and then come back to the evaluation	Jingdong prefecture level city in Shandong
Jinfeng Hongyuan Flagship Store	Power Saving Specialist 1	Fast logistics, good packaging, worth it!	Jingdong prefecture level city in Shandong
Jinfeng Hongyuan Flagship Store	Power Saving Specialist 1	Customer service is okay. It's just that.	Jingdong prefecture level city in Shandong
Jinfeng Hongyuan Flagship Store	Power Saving Specialist 1	Poor power saving	Jingdong prefecture level city in Shandong
Jinfeng Hongyuan Flagship Store	Power Saving Specialist 1	I'll tell you when I've used it.	Jingdong prefecture level city in Shandong
Jinfeng Hongyuan Flagship Store	Power Saving Specialist 1	I think it's good.	Jingdong prefecture level city in Shandong
Jinfeng Hongyuan Flagship Store	Power Saving Specialist 1	This energy saver works very well and I recommend everyone to buy it!	Jingdong prefecture level city in Shandong
Jinfeng Hongyuan Flagship Store	Power Saving Specialist 1	I do not know how effective, just use half a month on the burn out, good business reissued a, and then use it after the follow-up comment.	Jingdong prefecture level city in Shandong
Jinfeng Hongyuan Flagship Store	Power Saving Specialist 1	in use	Jingdong prefecture level city in Shandong
Jinfeng Hongyuan Flagship Store	Power Saving Specialist 1	This thing is very poor. It's already only been used for ten days and it's broken. Now waiting for you to return it.	Jingdong prefecture level city in Shandong
Jinfeng Hongyuan Flagship Store	Power Saving Specialist 1	It's not the same as the seller's show.	Jingdong prefecture level city in Shandong
Jinfeng Hongyuan Flagship Store	Power Saving Specialist 1	What is the problem when I get it back from the CTS, it is plugged into the wall and the voltage shows 206v.	Jingdong prefecture level city in Shandong
Jinfeng Hongyuan Flagship Store	Power Saving Specialist 1	The logistics was fast and the stuff was perfectly packaged. It is in the process of trial.	Jingdong prefecture level city in Shandong
Jinfeng Hongyuan Flagship Store	Power Saving Specialist 1	This saver is particularly good to use can really save electricity, with two months and then look at the line if you buy one for the family	Jingdong prefecture level city in Shandong
Jinfeng Hongyuan Flagship Store	Power Saving Specialist 1	Novel appearance design, all aluminum alloy profile, also with a fuse device, voltage monitoring display. After receiving the goods, I directly cut the plug and connect it to the main power box under the leakage switch, hoping to save electricity, and then follow up later. Logistics is very fast, arrived the next	Jingdong prefecture level city in Shandong
Jinfeng Hongyuan Flagship Store	Power Saving Specialist 1	Received, very good and like, if necessary next time to buy ha ha	Jingdong prefecture level city in Shandong
Jinfeng Hongyuan Flagship Store	Power Saving Specialist 1	It's good. It's worth it. Get it if you need it.	Jingdong prefecture level city in Shandong
Jinfeng Hongyuan Flagship Store	Power Saving Specialist 1	It feels more than good and is worth having.	Jingdong prefecture level city in Shandong
Jinfeng Hongyuan Flagship Store	Power Saving Specialist 1	The goods arrived very good and fast first time to buy	Jingdong prefecture level city in Shandong
Jinfeng Hongyuan Flagship Store	Power Saving Specialist 1	The power saving is very good, I can save so much money every month, I will buy another one next time.	Jingdong prefecture level city in Shandong
Jinfeng Hongyuan Flagship Store	Power Saving Specialist 1	It's not what it says.	Jingdong prefecture level city in Shandong
Jinfeng Hongyuan Flagship Store	Power Saving Specialist 1	I have been using it for many days now and it saves electricity, it really does I have an 8 kW electric snail stove and it saves about $3.50 a day.	Jingdong prefecture level city in Shandong
Jinfeng Hongyuan Flagship Store	Power Saving Specialist 1	Express delivery is very fast, the quality is also quite good, worth buying!	Jingdong prefecture level city in Shandong

Jinfeng Hongyuan Flagship Store	Power Saving Specialist 1	Just received expecting the baby to work well.	Jingdong prefecture level city in Shandong
Jinfeng Hongyuan Flagship Store	Power Saving Specialist 1	Very good, there are more fruit, has bought a second!	Jingdong prefecture level city in Shandong
Jinfeng Hongyuan Flagship Store	Power Saving Specialist 1	Good is good So far, no power savings have been noticed Maybe it's too little to notice.	Jingdong prefecture level city in Shandong
Jinfeng Hongyuan Flagship Store	Power Saving Specialist 1	I just received the item and plugged it in to see what happens later.	Jingdong prefecture level city in Shandong
Jinfeng Hongyuan Flagship Store	Power Saving Specialist 1	Baby received, especially good, packaging is also good, the courier brother is very strong, thank you.	Jingdong prefecture level city in Shandong
Jinfeng Hongyuan Flagship Store	Power Saving Specialist 1	Just received the goods have not yet used it, do not know how, three months later in the follow-up comment!	Jingdong prefecture level city in Shandong
Jinfeng Hongyuan Flagship Store	Power Saving Specialist 1	The item was received, it is quite good, recommended to buy.	Jingdong prefecture level city in Shandong
Jinfeng Hongyuan Flagship Store	Power Saving Specialist 1	This energy saver works very well and I recommend everyone to buy it!	Jingdong prefecture level city in Shandong
Jinfeng Hongyuan Flagship Store	Power Saving Specialist 1	Voltage stabilization and current reduction nice power saving product.	Jingdong prefecture level city in Shandong
Jinfeng Hongyuan Flagship Store	Power Saving Specialist 1	Voltage stabilization and current reduction nice power saving product.	Jingdong prefecture level city in Shandong
Jinfeng Hongyuan Flagship Store	Power Saving Specialist 1	A good power saver for stabilizing and lowering current.	Jingdong prefecture level city in Shandong
Jinfeng Hongyuan Flagship Store	Power Saving Specialist 1	You'll know if it saves power after you use it.	Jingdong prefecture level city in Shandong
Jinfeng Hongyuan Flagship Store	Power Saving Specialist 1	The quality is great. The quality is very good. The service is very good. Express delivery arrived in two days.	Jingdong prefecture level city in Shandong
Jinfeng Hongyuan Flagship Store	Power Saving Specialist 1	It works great.	Jingdong prefecture level city in Shandong
Jinfeng Hongyuan Flagship Store	Power Saving Specialist 1	Logistics is very fast, not yet used, used to comment again.	Jingdong prefecture level city in Shandong
Jinfeng Hongyuan Flagship Store	Power Saving Specialist 1	Logistics is very fast things are also very good, worthy of friends to buy	Jingdong prefecture level city in Shandong
Jinfeng Hongyuan Flagship Store	Power Saving Specialist 1	It works. It really works.	Jingdong prefecture level city in Shandong
Jinfeng Hongyuan Flagship Store	Power Saving Specialist 1	LANGUAGE COMMENTS: just received the shipment. I immediately plugged it in and used it. If you ask how effective. It depends on whether the next month's electricity bill has been reduced. If there is a decrease. I am buying more than one to use.	Jingdong prefecture level city in Shandong
Jinfeng Hongyuan Flagship Store	Power Saving Specialist 1	Well made, power saving, good and convenient!	Jingdong prefecture level city in Shandong
Jinfeng Hongyuan Flagship Store	Power Saving Specialist 1	I'm using it. I'll rate it next month.	Jingdong prefecture level city in Shandong
Jinfeng Hongyuan Flagship Store	Power Saving Specialist 1	Baby received Oh, the courier delivery really fast, with a good and then come back to the evaluation	Jingdong prefecture level city in Shandong
Jinfeng Hongyuan Flagship Store	Power Saving Specialist 1	Fast logistics, good packaging, worth it!	Jingdong prefecture level city in Shandong
Jinfeng Hongyuan Flagship Store	Power Saving Specialist 1	Customer service is okay. It's just that.	Jingdong prefecture level city in Shandong
Jinfeng Hongyuan Flagship Store	Power Saving Specialist 1	Poor power saving	Jingdong prefecture level city in Shandong
Jinfeng Hongyuan Flagship Store	Power Saving Specialist 1	I'll tell you when I've used it.	Jingdong prefecture level city in Shandong
Jinfeng Hongyuan Flagship Store	Power Saving Specialist 1	I think it's good.	Jingdong prefecture level city in Shandong
Jinfeng Hongyuan Flagship Store	Power Saving Specialist 1	This energy saver works very well and I recommend everyone to buy it!	Jingdong prefecture level city in Shandong
Jinfeng Hongyuan Flagship Store	Power Saving Specialist 1	I do not know how effective, just use half a month on the burn out, good business reissued a, and then use it after the follow-up comment.	Jingdong prefecture level city in Shandong
Jinfeng Hongyuan Flagship Store	Power Saving Specialist 1	in use	Jingdong prefecture level city in Shandong
Jinfeng Hongyuan Flagship Store	Power Saving Specialist 1	This thing is very poor. It's already only been used for ten days and it's broken. Now waiting for you to return it.	Jingdong prefecture level city in Shandong
Jinfeng Hongyuan Flagship Store	Power Saving Specialist 1	It's not the same as the seller's show.	Jingdong prefecture level city in Shandong
Jinfeng Hongyuan Flagship Store	Power Saving Specialist 1	What is the problem when I get it back from the CTS, it is plugged into the wall and the voltage shows 206v.	Jingdong prefecture level city in Shandong
Jinfeng Hongyuan Flagship Store	Power Saving Specialist 1	The logistics was fast and the stuff was perfectly packaged. It is in the process of trial.	Jingdong prefecture level city in Shandong
Jinfeng Hongyuan Flagship Store	Power Saving Specialist 1	This saver is particularly good to use can really save electricity, with two months and then look at the line if you buy one for the family	Jingdong prefecture level city in Shandong
Jinfeng Hongyuan Flagship Store	Power Saving Specialist 1	Novel appearance design, all aluminum alloy profile, also with a fuse device, voltage monitoring display. After receiving the goods, I directly cut the plug and connect it to the main power box under the leakage switch, hoping to save electricity, and then follow up later. Logistics is very fast, arrived the next	Jingdong prefecture level city in Shandong
Jinfeng Hongyuan Flagship Store	Power Saving Specialist 1	Received, very good and like, if necessary next time to buy ha ha	Jingdong prefecture level city in Shandong
Jinfeng Hongyuan Flagship Store	Power Saving Specialist 1	It's good. It's worth it. Get it if you need it.	Jingdong prefecture level city in Shandong
Jinfeng Hongyuan Flagship Store	Power Saving Specialist 1	It feels more than good and is worth having.	Jingdong prefecture level city in Shandong
Jinfeng Hongyuan Flagship Store	Power Saving Specialist 1	The goods arrived very good and fast first time to buy	Jingdong prefecture level city in Shandong
Jinfeng Hongyuan Flagship Store	Power Saving Specialist 1	The power saving is very good, I can save so much money every month, I will buy another one next time.	Jingdong prefecture level city in Shandong
Jinfeng Hongyuan Flagship Store	Power Saving Specialist 1	It's not what it says.	Jingdong prefecture level city in Shandong
Jinfeng Hongyuan Flagship Store	Power Saving Specialist 1	I have been using it for many days now and it saves electricity, it really does I have an 8 kW electric snail stove and it saves about $3.50 a day.	Jingdong prefecture level city in Shandong
Jinfeng Hongyuan Flagship Store	Power Saving Specialist 1	Express delivery is very fast, the quality is also quite good, worth buying!	Jingdong prefecture level city in Shandong
Jinfeng Hongyuan Flagship Store	Power Saving Specialist 1	Just received expecting the baby to work well.	Jingdong prefecture level city in Shandong
Jinfeng Hongyuan Flagship Store	Power Saving Specialist 1	Very good, there are more fruit, has bought a second!	Jingdong prefecture level city in Shandong
Jinfeng Hongyuan Flagship Store	Power Saving Specialist 1	Good is good So far, no power savings have been noticed Maybe it's too little to notice.	Jingdong prefecture level city in Shandong
Jinfeng Hongyuan Flagship Store	Power Saving Specialist 1	I just received the item and plugged it in to see what happens later.	Jingdong prefecture level city in Shandong
Jinfeng Hongyuan Flagship Store	Power Saving Specialist 1	Baby received, especially good, packaging is also good, the courier brother is very strong, thank you.	Jingdong prefecture level city in Shandong
Jinfeng Hongyuan Flagship Store	Power Saving Specialist 1	Just received the goods have not yet used it, do not know how, three months later in the follow-up comment!	Jingdong prefecture level city in Shandong
Jinfeng Hongyuan Flagship Store	Power Saving Specialist 1	The item was received, it is quite good, recommended to buy.	Jingdong prefecture level city in Shandong

Jinfeng Hongyuan Flagship Store	Power Saving Specialist 1	This energy saver works very well and I recommend everyone to buy it!	Jingdong prefecture level city in Shandong
Jinfeng Hongyuan Flagship Store	Power Saving Specialist 1	Voltage stabilization and current reduction nice power saving product.	Jingdong prefecture level city in Shandong
Jinfeng Hongyuan Flagship Store	Power Saving Specialist 1	Voltage stabilization and current reduction nice power saving product.	Jingdong prefecture level city in Shandong
Jinfeng Hongyuan Flagship Store	Power Saving Specialist 1	A good power saver for stabilizing and lowering current.	Jingdong prefecture level city in Shandong
Jinfeng Hongyuan Flagship Store	Power Saving Specialist 1	You'll know if it saves power after you use it.	Jingdong prefecture level city in Shandong
Jinfeng Hongyuan Flagship Store	Power Saving Specialist 1	The quality is great. The quality is very good. The service is very good. Express delivery arrived in two days.	Jingdong prefecture level city in Shandong
Jinfeng Hongyuan Flagship Store	Power Saving Specialist 1	It works great.	Jingdong prefecture level city in Shandong
Jinfeng Hongyuan Flagship Store	Power Saving Specialist 1	Logistics is very fast, not yet used, used to comment again.	Jingdong prefecture level city in Shandong
Jinfeng Hongyuan Flagship Store	Power Saving Specialist 1	Logistics is very fast things are also very good, worthy of friends to buy	Jingdong prefecture level city in Shandong
Jinfeng Hongyuan Flagship Store	Power Saving Specialist 1	It works. It really works.	Jingdong prefecture level city in Shandong
Jinfeng Hongyuan Flagship Store	Power Saving Specialist 1	LANGUAGE COMMENTS: just received the shipment. I immediately plugged it in and used it. If you ask how effective. It depends on whether the next month's electricity bill has been reduced. If there is a decrease. I am buying more than one to use.	Jingdong prefecture level city in Shandong
Jinfeng Hongyuan Flagship Store	Power Saving Specialist 1	Well made, power saving, good and convenient!	Jingdong prefecture level city in Shandong
Jinfeng Hongyuan Flagship Store	Power Saving Specialist 1	I'm using it. I'll rate it next month.	Jingdong prefecture level city in Shandong
Jinfeng Hongyuan Flagship Store	Power Saving Specialist 1	Baby received Oh, the courier delivery really fast, with a good and then come back to the evaluation	Jingdong prefecture level city in Shandong
Jinfeng Hongyuan Flagship Store	Power Saving Specialist 1	Fast logistics, good packaging, worth it!	Jingdong prefecture level city in Shandong
Jinfeng Hongyuan Flagship Store	Power Saving Specialist 1	Customer service is okay. It's just that.	Jingdong prefecture level city in Shandong
Jinfeng Hongyuan Flagship Store	Power Saving Specialist 1	Poor power saving	Jingdong prefecture level city in Shandong
Jinfeng Hongyuan Flagship Store	Power Saving Specialist 1	I'll tell you when I've used it.	Jingdong prefecture level city in Shandong
Jinfeng Hongyuan Flagship Store	Power Saving Specialist 1	I think it's good.	Jingdong prefecture level city in Shandong
Jinfeng Hongyuan Flagship Store	Power Saving Specialist 1	This energy saver works very well and I recommend everyone to buy it!	Jingdong prefecture level city in Shandong
Jinfeng Hongyuan Flagship Store	Power Saving Specialist 1	I do not know how effective, just use half a month on the burn out, good business reissued a, and then use it after the follow-up comment.	Jingdong prefecture level city in Shandong
Jinfeng Hongyuan Flagship Store	Power Saving Specialist 1	in use	Jingdong prefecture level city in Shandong
Jinfeng Hongyuan Flagship Store	Power Saving Specialist 1	This thing is very poor. It's already only been used for ten days and it's broken. Now waiting for you to return it.	Jingdong prefecture level city in Shandong
Jinfeng Hongyuan Flagship Store	Power Saving Specialist 1	It's not the same as the seller's show.	Jingdong prefecture level city in Shandong
Jinfeng Hongyuan Flagship Store	Power Saving Specialist 1	What is the problem when I get it back from the CTS, it is plugged into the wall and the voltage shows 206v.	Jingdong prefecture level city in Shandong
Jinfeng Hongyuan Flagship Store	Power Saving Specialist 1	The logistics was fast and the stuff was perfectly packaged. It is in the process of trial.	Jingdong prefecture level city in Shandong
Jinfeng Hongyuan Flagship Store	Power Saving Specialist 1	This saver is particularly good to use can really save electricity, with two months and then look at the line if you buy one for the family	Jingdong prefecture level city in Shandong
Jinfeng Hongyuan Flagship Store	Power Saving Specialist 1	Novel appearance design, all aluminum alloy profile, also with a fuse device, voltage monitoring display. After receiving the goods, I directly cut the plug and connect it to the main power box under the leakage switch, hoping to save electricity, and then follow up later. Logistics is very fast, arrived the next	Jingdong prefecture level city in Shandong
Jinfeng Hongyuan Flagship Store	Power Saving Specialist 1	Received, very good and like, if necessary next time to buy ha ha	Jingdong prefecture level city in Shandong
Jinfeng Hongyuan Flagship Store	Power Saving Specialist 1	It's good. It's worth it. Get it if you need it.	Jingdong prefecture level city in Shandong
Jinfeng Hongyuan Flagship Store	Power Saving Specialist 1	It feels more than good and is worth having.	Jingdong prefecture level city in Shandong
Jinfeng Hongyuan Flagship Store	Power Saving Specialist 1	The goods arrived very good and fast first time to buy	Jingdong prefecture level city in Shandong
Jinfeng Hongyuan Flagship Store	Power Saving Specialist 1	The power saving is very good, I can save so much money every month, I will buy another one next time.	Jingdong prefecture level city in Shandong
Jinfeng Hongyuan Flagship Store	Power Saving Specialist 1	It's not what it says.	Jingdong prefecture level city in Shandong
Jinfeng Hongyuan Flagship Store	Power Saving Specialist 1	I have been using it for many days now and it saves electricity, it really does I have an 8 kW electric snail stove and it saves about $3.50 a day.	Jingdong prefecture level city in Shandong
Jinfeng Hongyuan Flagship Store	Power Saving Specialist 1	Express delivery is very fast, the quality is also quite good, worth buying!	Jingdong prefecture level city in Shandong
Jinfeng Hongyuan Flagship Store	Power Saving Specialist 1	Just received expecting the baby to work well.	Jingdong prefecture level city in Shandong
Jinfeng Hongyuan Flagship Store	Power Saving Specialist 1	Very good, there are more fruit, has bought a second!	Jingdong prefecture level city in Shandong
Jinfeng Hongyuan Flagship Store	Power Saving Specialist 1	Good is good So far, no power savings have been noticed Maybe it's too little to notice.	Jingdong prefecture level city in Shandong
Jinfeng Hongyuan Flagship Store	Power Saving Specialist 1	I just received the item and plugged it in to see what happens later.	Jingdong prefecture level city in Shandong
Jinfeng Hongyuan Flagship Store	Power Saving Specialist 1	Baby received, especially good, packaging is also good, the courier brother is very strong, thank you.	Jingdong prefecture level city in Shandong
Jinfeng Hongyuan Flagship Store	Power Saving Specialist 1	Just received the goods have not yet used it, do not know how, three months later in the follow-up comment!	Jingdong prefecture level city in Shandong
Jinfeng Hongyuan Flagship Store	Power Saving Specialist 1	The item was received, it is quite good, recommended to buy.	Jingdong prefecture level city in Shandong
Jinfeng Hongyuan Flagship Store	Power Saving Specialist 1	This energy saver works very well and I recommend everyone to buy it!	Jingdong prefecture level city in Shandong
Jinfeng Hongyuan Flagship Store	Power Saving Specialist 1	Voltage stabilization and current reduction nice power saving product.	Jingdong prefecture level city in Shandong
Jinfeng Hongyuan Flagship Store	Power Saving Specialist 1	Voltage stabilization and current reduction nice power saving product.	Jingdong prefecture level city in Shandong
Jinfeng Hongyuan Flagship Store	Power Saving Specialist 1	A good power saver for stabilizing and lowering current.	Jingdong prefecture level city in Shandong
Jinfeng Hongyuan Flagship Store	Power Saving Specialist 1	You'll know if it saves power after you use it.	Jingdong prefecture level city in Shandong
Jinfeng Hongyuan Flagship Store	Power Saving Specialist 1	The quality is great. The quality is very good. The service is very good. Express delivery arrived in two days.	Jingdong prefecture level city in Shandong
Jinfeng Hongyuan Flagship Store	Power Saving Specialist 1	It works great.	Jingdong prefecture level city in Shandong

Jinfeng Hongyuan Flagship Store	Power Saving Specialist 1	Logistics is very fast, not yet used, used to comment again.	Jingdong prefecture level city in Shandong
Jinfeng Hongyuan Flagship Store	Power Saving Specialist 1	Logistics is very fast things are also very good, worthy of friends to buy	Jingdong prefecture level city in Shandong
Jinfeng Hongyuan Flagship Store	Power Saving Specialist 1	It works. It really works.	Jingdong prefecture level city in Shandong
Jinfeng Hongyuan Flagship Store	Power Saving Specialist 1	LANGUAGE COMMENTS: just received the shipment. I immediately plugged it in and used it. If you ask how effective. It depends on whether the next month's electricity bill has been reduced. If there is a decrease. I am buying more than one to use.	Jingdong prefecture level city in Shandong
Jinfeng Hongyuan Flagship Store	Power Saving Specialist 1	Well made, power saving, good and convenient!	Jingdong prefecture level city in Shandong
Jinfeng Hongyuan Flagship Store	Power Saving Specialist 1	I'm using it. I'll rate it next month.	Jingdong prefecture level city in Shandong
Jinfeng Hongyuan Flagship Store	Power Saving Specialist 1	Baby received Oh, the courier delivery really fast, with a good and then come back to the evaluation	Jingdong prefecture level city in Shandong
Jinfeng Hongyuan Flagship Store	Power Saving Specialist 1	Fast logistics, good packaging, worth it!	Jingdong prefecture level city in Shandong
Jinfeng Hongyuan Flagship Store	Power Saving Specialist 1	Customer service is okay. It's just that.	Jingdong prefecture level city in Shandong
Jinfeng Hongyuan Flagship Store	Power Saving Specialist 1	Poor power saving	Jingdong prefecture level city in Shandong
Jinfeng Hongyuan Flagship Store	Power Saving Specialist 1	I'll tell you when I've used it.	Jingdong prefecture level city in Shandong
Jinfeng Hongyuan Flagship Store	Power Saving Specialist 1	I think it's good.	Jingdong prefecture level city in Shandong
Jinfeng Hongyuan Flagship Store	Power Saving Specialist 1	This energy saver works very well and I recommend everyone to buy it!	Jingdong prefecture level city in Shandong
Jinfeng Hongyuan Flagship Store	Power Saving Specialist 1	I do not know how effective, just use half a month on the burn out, good business reissued a, and then use it after the follow-up comment.	Jingdong prefecture level city in Shandong
Jinfeng Hongyuan Flagship Store	Power Saving Specialist 1	in use	Jingdong prefecture level city in Shandong
Jinfeng Hongyuan Flagship Store	Power Saving Specialist 1	This thing is very poor. It's already only been used for ten days and it's broken. Now waiting for you to return it.	Jingdong prefecture level city in Shandong
Jinfeng Hongyuan Flagship Store	Power Saving Specialist 1	It's not the same as the seller's show.	Jingdong prefecture level city in Shandong
Jinfeng Hongyuan Flagship Store	Power Saving Specialist 1	What is the problem when I get it back from the CTS, it is plugged into the wall and the voltage shows 206v.	Jingdong prefecture level city in Shandong
Jinfeng Hongyuan Flagship Store	Power Saving Specialist 1	The logistics was fast and the stuff was perfectly packaged. It is in the process of trial.	Jingdong prefecture level city in Shandong
Jinfeng Hongyuan Flagship Store	Power Saving Specialist 1	This saver is particularly good to use can really save electricity, with two months and then look at the line if you buy one for the family	Jingdong prefecture level city in Shandong
Jinfeng Hongyuan Flagship Store	Power Saving Specialist 1	Novel appearance design, all aluminum alloy profile, also with a fuse device, voltage monitoring display. After receiving the goods, I directly cut the plug and connect it to the main power box under the leakage switch, hoping to save electricity, and then follow up later. Logistics is very fast, arrived the next	Jingdong prefecture level city in Shandong
Jinfeng Hongyuan Flagship Store	Power Saving Specialist 1	Received, very good and like, if necessary next time to buy ha ha	Jingdong prefecture level city in Shandong
Jinfeng Hongyuan Flagship Store	Power Saving Specialist 1	It's good. It's worth it. Get it if you need it.	Jingdong prefecture level city in Shandong
Jinfeng Hongyuan Flagship Store	Power Saving Specialist 1	It feels more than good and is worth having.	Jingdong prefecture level city in Shandong
Jinfeng Hongyuan Flagship Store	Power Saving Specialist 1	The goods arrived very good and fast first time to buy	Jingdong prefecture level city in Shandong
Jinfeng Hongyuan Flagship Store	Power Saving Specialist 1	The power saving is very good, I can save so much money every month, I will buy another one next time.	Jingdong prefecture level city in Shandong
Jinfeng Hongyuan Flagship Store	Power Saving Specialist 1	It's not what it says.	Jingdong prefecture level city in Shandong
Jinfeng Hongyuan Flagship Store	Power Saving Specialist 1	I have been using it for many days now and it saves electricity, it really does I have an 8 kW electric snail stove and it saves about $3.50 a day.	Jingdong prefecture level city in Shandong
Jinfeng Hongyuan Flagship Store	Power Saving Specialist 1	Express delivery is very fast, the quality is also quite good, worth buying!	Jingdong prefecture level city in Shandong
Jinfeng Hongyuan Flagship Store	Power Saving Specialist 1	Just received expecting the baby to work well.	Jingdong prefecture level city in Shandong
Jinfeng Hongyuan Flagship Store	Power Saving Specialist 1	Very good, there are more fruit, has bought a second!	Jingdong prefecture level city in Shandong
Jinfeng Hongyuan Flagship Store	Power Saving Specialist 1	Good is good So far, no power savings have been noticed Maybe it's too little to notice.	Jingdong prefecture level city in Shandong
Jinfeng Hongyuan Flagship Store	Power Saving Specialist 1	I just received the item and plugged it in to see what happens later.	Jingdong prefecture level city in Shandong
Jinfeng Hongyuan Flagship Store	Power Saving Specialist 1	Baby received, especially good, packaging is also good, the courier brother is very strong, thank you.	Jingdong prefecture level city in Shandong
Jinfeng Hongyuan Flagship Store	Power Saving Specialist 1	Just received the goods have not yet used it, do not know how, three months later in the follow-up comment!	Jingdong prefecture level city in Shandong
Jinfeng Hongyuan Flagship Store	Power Saving Specialist 1	The item was received, it is quite good, recommended to buy.	Jingdong prefecture level city in Shandong
Jinfeng Hongyuan Flagship Store	Power Saving Specialist 1	This energy saver works very well and I recommend everyone to buy it!	Jingdong prefecture level city in Shandong
Jinfeng Hongyuan Flagship Store	Power Saving Specialist 1	Voltage stabilization and current reduction nice power saving product.	Jingdong prefecture level city in Shandong
Jinfeng Hongyuan Flagship Store	Power Saving Specialist 1	Voltage stabilization and current reduction nice power saving product.	Jingdong prefecture level city in Shandong
Jinfeng Hongyuan Flagship Store	Power Saving Specialist 1	A good power saver for stabilizing and lowering current.	Jingdong prefecture level city in Shandong
Jinfeng Hongyuan Flagship Store	Power Saving Specialist 1	You'll know if it saves power after you use it.	Jingdong prefecture level city in Shandong
Jinfeng Hongyuan Flagship Store	Power Saving Specialist 1	The quality is great. The quality is very good. The service is very good. Express delivery arrived in two days.	Jingdong prefecture level city in Shandong
Jinfeng Hongyuan Flagship Store	Power Saving Specialist 1	It works great.	Jingdong prefecture level city in Shandong
Jinfeng Hongyuan Flagship Store	Power Saving Specialist 1	Logistics is very fast, not yet used, used to comment again.	Jingdong prefecture level city in Shandong
Jinfeng Hongyuan Flagship Store	Power Saving Specialist 1	Logistics is very fast things are also very good, worthy of friends to buy	Jingdong prefecture level city in Shandong
Jinfeng Hongyuan Flagship Store	Power Saving Specialist 1	It works. It really works.	Jingdong prefecture level city in Shandong
Jinfeng Hongyuan Flagship Store	Power Saving Specialist 1	LANGUAGE COMMENTS: just received the shipment. I immediately plugged it in and used it. If you ask how effective. It depends on whether the next month's electricity bill has been reduced. If there is a decrease. I am buying more than one to use.	Jingdong prefecture level city in Shandong
Jinfeng Hongyuan Flagship Store	Power Saving Specialist 1	Well made, power saving, good and convenient!	Jingdong prefecture level city in Shandong
Jinfeng Hongyuan Flagship Store	Power Saving Specialist 1	I'm using it. I'll rate it next month.	Jingdong prefecture level city in Shandong
Jinfeng Hongyuan Flagship Store	Power Saving Specialist 1	Baby received Oh, the courier delivery really fast, with a good and then come back to the evaluation	Jingdong prefecture level city in Shandong

Jinfeng Hongyuan Flagship Store	Power Saving Specialist 1	Fast logistics, good packaging, worth it!	Jingdong prefecture level city in Shandong
Jinfeng Hongyuan Flagship Store	Power Saving Specialist 1	Customer service is okay. It's just that.	Jingdong prefecture level city in Shandong
Jinfeng Hongyuan Flagship Store	Power Saving Specialist 1	Poor power saving	Jingdong prefecture level city in Shandong
Jinfeng Hongyuan Flagship Store	Power Saving Specialist 1	I'll tell you when I've used it.	Jingdong prefecture level city in Shandong
Jinfeng Hongyuan Flagship Store	Power Saving Specialist 1	I think it's good.	Jingdong prefecture level city in Shandong
Jinfeng Hongyuan Flagship Store	Power Saving Specialist 1	This energy saver works very well and I recommend everyone to buy it!	Jingdong prefecture level city in Shandong
Jinfeng Hongyuan Flagship Store	Power Saving Specialist 1	I do not know how effective, just use half a month on the burn out, good business reissued a, and then use it after the follow-up comment.	Jingdong prefecture level city in Shandong
Jinfeng Hongyuan Flagship Store	Power Saving Specialist 1	in use	Jingdong prefecture level city in Shandong
Jinfeng Hongyuan Flagship Store	Power Saving Specialist 1	This thing is very poor. It's already only been used for ten days and it's broken. Now waiting for you to return it.	Jingdong prefecture level city in Shandong
Jinfeng Hongyuan Flagship Store	Power Saving Specialist 1	It's not the same as the seller's show.	Jingdong prefecture level city in Shandong
Jinfeng Hongyuan Flagship Store	Power Saving Specialist 1	What is the problem when I get it back from the CTS, it is plugged into the wall and the voltage shows 206v.	Jingdong prefecture level city in Shandong
Jinfeng Hongyuan Flagship Store	Power Saving Specialist 1	The logistics was fast and the stuff was perfectly packaged. It is in the process of trial.	Jingdong prefecture level city in Shandong
Jinfeng Hongyuan Flagship Store	Power Saving Specialist 1	This saver is particularly good to use can really save electricity, with two months and then look at the line if you buy one for the family	Jingdong prefecture level city in Shandong
Jinfeng Hongyuan Flagship Store	Power Saving Specialist 1	Novel appearance design, all aluminum alloy profile, also with a fuse device, voltage monitoring display. After receiving the goods, I directly cut the plug and connect it to the main power box under the leakage switch, hoping to save electricity, and then follow up later. Logistics is very fast, arrived the next	Jingdong prefecture level city in Shandong
Jinfeng Hongyuan Flagship Store	Power Saving Specialist 1	Received, very good and like, if necessary next time to buy ha ha	Jingdong prefecture level city in Shandong
Jinfeng Hongyuan Flagship Store	Power Saving Specialist 1	It's good. It's worth it. Get it if you need it.	Jingdong prefecture level city in Shandong
Jinfeng Hongyuan Flagship Store	Power Saving Specialist 1	It feels more than good and is worth having.	Jingdong prefecture level city in Shandong
Jinfeng Hongyuan Flagship Store	Power Saving Specialist 1	The goods arrived very good and fast first time to buy	Jingdong prefecture level city in Shandong
Jinfeng Hongyuan Flagship Store	Power Saving Specialist 1	The power saving is very good, I can save so much money every month, I will buy another one next time.	Jingdong prefecture level city in Shandong
Jinfeng Hongyuan Flagship Store	Power Saving Specialist 1	It's not what it says.	Jingdong prefecture level city in Shandong
Jinfeng Hongyuan Flagship Store	Power Saving Specialist 1	I have been using it for many days now and it saves electricity, it really does I have an 8 kW electric snail stove and it saves about $3.50 a day.	Jingdong prefecture level city in Shandong
Jinfeng Hongyuan Flagship Store	Power Saving Specialist 1	Express delivery is very fast, the quality is also quite good, worth buying!	Jingdong prefecture level city in Shandong
Jinfeng Hongyuan Flagship Store	Power Saving Specialist 1	Just received expecting the baby to work well.	Jingdong prefecture level city in Shandong
Jinfeng Hongyuan Flagship Store	Power Saving Specialist 1	Very good, there are more fruit, has bought a second!	Jingdong prefecture level city in Shandong
Jinfeng Hongyuan Flagship Store	Power Saving Specialist 1	Good is good So far, no power savings have been noticed Maybe it's too little to notice.	Jingdong prefecture level city in Shandong
Jinfeng Hongyuan Flagship Store	Power Saving Specialist 1	I just received the item and plugged it in to see what happens later.	Jingdong prefecture level city in Shandong
Jinfeng Hongyuan Flagship Store	Power Saving Specialist 1	Baby received, especially good, packaging is also good, the courier brother is very strong, thank you.	Jingdong prefecture level city in Shandong
Jinfeng Hongyuan Flagship Store	Power Saving Specialist 1	Just received the goods have not yet used it, do not know how, three months later in the follow-up comment!	Jingdong prefecture level city in Shandong
Jinfeng Hongyuan Flagship Store	Power Saving Specialist 1	The item was received, it is quite good, recommended to buy.	Jingdong prefecture level city in Shandong
Jinfeng Hongyuan Flagship Store	Power Saving Specialist 1	This energy saver works very well and I recommend everyone to buy it!	Jingdong prefecture level city in Shandong
Jinfeng Hongyuan Flagship Store	Power Saving Specialist 1	Voltage stabilization and current reduction nice power saving product.	Jingdong prefecture level city in Shandong
Jinfeng Hongyuan Flagship Store	Power Saving Specialist 1	Voltage stabilization and current reduction nice power saving product.	Jingdong prefecture level city in Shandong
Jinfeng Hongyuan Flagship Store	Power Saving Specialist 1	A good power saver for stabilizing and lowering current.	Jingdong prefecture level city in Shandong
Jinfeng Hongyuan Flagship Store	Power Saving Specialist 1	You'll know if it saves power after you use it.	Jingdong prefecture level city in Shandong
Jinfeng Hongyuan Flagship Store	Power Saving Specialist 1	The quality is great. The quality is very good. The service is very good. Express delivery arrived in two days.	Jingdong prefecture level city in Shandong
Jinfeng Hongyuan Flagship Store	Power Saving Specialist 1	It works great.	Jingdong prefecture level city in Shandong
Jinfeng Hongyuan Flagship Store	Power Saving Specialist 1	Logistics is very fast, not yet used, used to comment again.	Jingdong prefecture level city in Shandong
Jinfeng Hongyuan Flagship Store	Power Saving Specialist 1	Logistics is very fast things are also very good, worthy of friends to buy	Jingdong prefecture level city in Shandong
Jinfeng Hongyuan Flagship Store	Power Saving Specialist 1	It works. It really works.	Jingdong prefecture level city in Shandong
Jinfeng Hongyuan Flagship Store	Power Saving Specialist 1	LANGUAGE COMMENTS: just received the shipment. I immediately plugged it in and used it. If you ask how effective. It depends on whether the next month's electricity bill has been reduced. If there is a decrease. I am buying more than one to use.	Jingdong prefecture level city in Shandong
Jinfeng Hongyuan Flagship Store	Power Saving Specialist 1	Well made, power saving, good and convenient!	Jingdong prefecture level city in Shandong
Jinfeng Hongyuan Flagship Store	Power Saving Specialist 1	I'm using it. I'll rate it next month.	Jingdong prefecture level city in Shandong
Jinfeng Hongyuan Flagship Store	Power Saving Specialist 1	Baby received Oh, the courier delivery really fast, with a good and then come back to the evaluation	Jingdong prefecture level city in Shandong
Jinfeng Hongyuan Flagship Store	Power Saving Specialist 1	Fast logistics, good packaging, worth it!	Jingdong prefecture level city in Shandong
Jinfeng Hongyuan Flagship Store	Power Saving Specialist 1	Customer service is okay. It's just that.	Jingdong prefecture level city in Shandong
Jinfeng Hongyuan Flagship Store	Power Saving Specialist 1	Poor power saving	Jingdong prefecture level city in Shandong
Jinfeng Hongyuan Flagship Store	Power Saving Specialist 1	I'll tell you when I've used it.	Jingdong prefecture level city in Shandong
Jinfeng Hongyuan Flagship Store	Power Saving Specialist 1	I think it's good.	Jingdong prefecture level city in Shandong
Jinfeng Hongyuan Flagship Store	Power Saving Specialist 1	This energy saver works very well and I recommend everyone to buy it!	Jingdong prefecture level city in Shandong
Jinfeng Hongyuan Flagship Store	Power Saving Specialist 1	I do not know how effective, just use half a month on the burn out, good business reissued a, and then use it after the follow-up comment.	Jingdong prefecture level city in Shandong

Jinfeng Hongyuan Flagship Store	Power Saving Specialist 1	in use	Jingdong prefecture level city in Shandong
Jinfeng Hongyuan Flagship Store	Power Saving Specialist 1	This thing is very poor. It's already only been used for ten days and it's broken. Now waiting for you to return it.	Jingdong prefecture level city in Shandong
Jinfeng Hongyuan Flagship Store	Power Saving Specialist 1	It's not the same as the seller's show.	Jingdong prefecture level city in Shandong
Jinfeng Hongyuan Flagship Store	Power Saving Specialist 1	What is the problem when I get it back from the CTS, it is plugged into the wall and the voltage shows 206v.	Jingdong prefecture level city in Shandong
Jinfeng Hongyuan Flagship Store	Power Saving Specialist 1	The logistics was fast and the stuff was perfectly packaged. It is in the process of trial.	Jingdong prefecture level city in Shandong
Jinfeng Hongyuan Flagship Store	Power Saving Specialist 1	This saver is particularly good to use can really save electricity, with two months and then look at the line if you buy one for the family	Jingdong prefecture level city in Shandong
Jinfeng Hongyuan Flagship Store	Power Saving Specialist 1	Novel appearance design, all aluminum alloy profile, also with a fuse device, voltage monitoring display. After receiving the goods, I directly cut the plug and connect it to the main power box under the leakage switch, hoping to save electricity, and then follow up later. Logistics is very fast, arrived the next	Jingdong prefecture level city in Shandong
Jinfeng Hongyuan Flagship Store	Power Saving Specialist 1	Received, very good and like, if necessary next time to buy ha ha	Jingdong prefecture level city in Shandong
Jinfeng Hongyuan Flagship Store	Power Saving Specialist 1	It's good. It's worth it. Get it if you need it.	Jingdong prefecture level city in Shandong
Jinfeng Hongyuan Flagship Store	Power Saving Specialist 1	It feels more than good and is worth having.	Jingdong prefecture level city in Shandong
Jinfeng Hongyuan Flagship Store	Power Saving Specialist 1	The goods arrived very good and fast first time to buy	Jingdong prefecture level city in Shandong
Jinfeng Hongyuan Flagship Store	Power Saving Specialist 1	The power saving is very good, I can save so much money every month, I will buy another one next time.	Jingdong prefecture level city in Shandong
Jinfeng Hongyuan Flagship Store	Power Saving Specialist 1	It's not what it says.	Jingdong prefecture level city in Shandong
Jinfeng Hongyuan Flagship Store	Power Saving Specialist 1	I have been using it for many days now and it saves electricity, it really does I have an 8 kW electric snail stove and it saves about $3.50 a day.	Jingdong prefecture level city in Shandong
Jinfeng Hongyuan Flagship Store	Power Saving Specialist 1	Express delivery is very fast, the quality is also quite good, worth buying!	Jingdong prefecture level city in Shandong
Jinfeng Hongyuan Flagship Store	Power Saving Specialist 1	Just received expecting the baby to work well.	Jingdong prefecture level city in Shandong
Jinfeng Hongyuan Flagship Store	Power Saving Specialist 1	Very good, there are more fruit, has bought a second!	Jingdong prefecture level city in Shandong
Jinfeng Hongyuan Flagship Store	Power Saving Specialist 1	Good is good So far, no power savings have been noticed Maybe it's too little to notice.	Jingdong prefecture level city in Shandong
Jinfeng Hongyuan Flagship Store	Power Saving Specialist 1	I just received the item and plugged it in to see what happens later.	Jingdong prefecture level city in Shandong
Jinfeng Hongyuan Flagship Store	Power Saving Specialist 1	Baby received, especially good, packaging is also good, the courier brother is very strong, thank you.	Jingdong prefecture level city in Shandong
Jinfeng Hongyuan Flagship Store	Power Saving Specialist 1	Just received the goods have not yet used it, do not know how, three months later in the follow-up comment!	Jingdong prefecture level city in Shandong
Jinfeng Hongyuan Flagship Store	Power Saving Specialist 1	The item was received, it is quite good, recommended to buy.	Jingdong prefecture level city in Shandong
Jinfeng Hongyuan Flagship Store	Power Saving Specialist 1	This energy saver works very well and I recommend everyone to buy it!	Jingdong prefecture level city in Shandong
Jinfeng Hongyuan Flagship Store	Power Saving Specialist 1	Voltage stabilization and current reduction nice power saving product.	Jingdong prefecture level city in Shandong
Jinfeng Hongyuan Flagship Store	Power Saving Specialist 1	Voltage stabilization and current reduction nice power saving product.	Jingdong prefecture level city in Shandong
Jinfeng Hongyuan Flagship Store	Power Saving Specialist 1	A good power saver for stabilizing and lowering current.	Jingdong prefecture level city in Shandong
Jinfeng Hongyuan Flagship Store	Power Saving Specialist 1	You'll know if it saves power after you use it.	Jingdong prefecture level city in Shandong
Jinfeng Hongyuan Flagship Store	Power Saving Specialist 1	The quality is great. The quality is very good. The service is very good. Express delivery arrived in two days.	Jingdong prefecture level city in Shandong
Jinfeng Hongyuan Flagship Store	Power Saving Specialist 1	It works great.	Jingdong prefecture level city in Shandong
Jinfeng Hongyuan Flagship Store	Power Saving Specialist 1	Logistics is very fast, not yet used, used to comment again.	Jingdong prefecture level city in Shandong
Jinfeng Hongyuan Flagship Store	Power Saving Specialist 1	Logistics is very fast things are also very good, worthy of friends to buy	Jingdong prefecture level city in Shandong
Jinfeng Hongyuan Flagship Store	Power Saving Specialist 1	It works. It really works.	Jingdong prefecture level city in Shandong
Jinfeng Hongyuan Flagship Store	Power Saving Specialist 1	LANGUAGE COMMENTS: just received the shipment. I immediately plugged it in and used it. If you ask how effective. It depends on whether the next month's electricity bill has been reduced. If there is a decrease. I am buying more than one to use.	Jingdong prefecture level city in Shandong
Jinfeng Hongyuan Flagship Store	Power Saving Specialist 1	Well made, power saving, good and convenient!	Jingdong prefecture level city in Shandong
Jinfeng Hongyuan Flagship Store	Power Saving Specialist 1	I'm using it. I'll rate it next month.	Jingdong prefecture level city in Shandong
Jinfeng Hongyuan Flagship Store	Power Saving Specialist 1	Baby received Oh, the courier delivery really fast, with a good and then come back to the evaluation	Jingdong prefecture level city in Shandong
Jinfeng Hongyuan Flagship Store	Power Saving Specialist 1	Fast logistics, good packaging, worth it!	Jingdong prefecture level city in Shandong
Jinfeng Hongyuan Flagship Store	Power Saving Specialist 1	Customer service is okay. It's just that.	Jingdong prefecture level city in Shandong
Jinfeng Hongyuan Flagship Store	Power Saving Specialist 1	Poor power saving	Jingdong prefecture level city in Shandong
Jinfeng Hongyuan Flagship Store	Power Saving Specialist 1	I'll tell you when I've used it.	Jingdong prefecture level city in Shandong
Jinfeng Hongyuan Flagship Store	Power Saving Specialist 1	I think it's good.	Jingdong prefecture level city in Shandong
Jinfeng Hongyuan Flagship Store	Power Saving Specialist 1	This energy saver works very well and I recommend everyone to buy it!	Jingdong prefecture level city in Shandong
Jinfeng Hongyuan Flagship Store	Power Saving Specialist 1	I do not know how effective, just use half a month on the burn out, good business reissued a, and then use it after the follow-up comment.	Jingdong prefecture level city in Shandong
Jinfeng Hongyuan Flagship Store	Power Saving Specialist 1	in use	Jingdong prefecture level city in Shandong
Jinfeng Hongyuan Flagship Store	Power Saving Specialist 1	This thing is very poor. It's already only been used for ten days and it's broken. Now waiting for you to return it.	Jingdong prefecture level city in Shandong
Jinfeng Hongyuan Flagship Store	Power Saving Specialist 1	It's not the same as the seller's show.	Jingdong prefecture level city in Shandong
Jinfeng Hongyuan Flagship Store	Power Saving Specialist 1	What is the problem when I get it back from the CTS, it is plugged into the wall and the voltage shows 206v.	Jingdong prefecture level city in Shandong
Jinfeng Hongyuan Flagship Store	Power Saving Specialist 1	The logistics was fast and the stuff was perfectly packaged. It is in the process of trial.	Jingdong prefecture level city in Shandong
Jinfeng Hongyuan Flagship Store	Power Saving Specialist 1	This saver is particularly good to use can really save electricity, with two months and then look at the line if you buy one for the family	Jingdong prefecture level city in Shandong
Jinfeng Hongyuan Flagship Store	Power Saving Specialist 1	Novel appearance design, all aluminum alloy profile, also with a fuse device, voltage monitoring display. After receiving the goods, I directly cut the plug and connect it to the main power box under the leakage switch, hoping to save electricity, and then follow up later. Logistics is very fast, arrived the next	Jingdong prefecture level city in Shandong

Jinfeng Hongyuan Flagship Store	Power Saving Specialist 1	Received, very good and like, if necessary next time to buy ha ha	Jingdong prefecture level city in Shandong
Jinfeng Hongyuan Flagship Store	Power Saving Specialist 1	It's good. It's worth it. Get it if you need it.	Jingdong prefecture level city in Shandong
Jinfeng Hongyuan Flagship Store	Power Saving Specialist 1	It feels more than good and is worth having.	Jingdong prefecture level city in Shandong
Jinfeng Hongyuan Flagship Store	Power Saving Specialist 1	The goods arrived very good and fast first time to buy	Jingdong prefecture level city in Shandong
Jinfeng Hongyuan Flagship Store	Power Saving Specialist 1	The power saving is very good, I can save so much money every month, I will buy another one next time.	Jingdong prefecture level city in Shandong
Jinfeng Hongyuan Flagship Store	Power Saving Specialist 1	It's not what it says.	Jingdong prefecture level city in Shandong
Jinfeng Hongyuan Flagship Store	Power Saving Specialist 1	I have been using it for many days now and it saves electricity, it really does I have an 8 kW electric snail stove and it saves about $3.50 a day.	Jingdong prefecture level city in Shandong
Jinfeng Hongyuan Flagship Store	Power Saving Specialist 1	Express delivery is very fast, the quality is also quite good, worth buying!	Jingdong prefecture level city in Shandong
Jinfeng Hongyuan Flagship Store	Power Saving Specialist 1	Just received expecting the baby to work well.	Jingdong prefecture level city in Shandong
Jinfeng Hongyuan Flagship Store	Power Saving Specialist 1	Very good, there are more fruit, has bought a second!	Jingdong prefecture level city in Shandong
Jinfeng Hongyuan Flagship Store	Power Saving Specialist 1	Good is good So far, no power savings have been noticed Maybe it's too little to notice.	Jingdong prefecture level city in Shandong
Jinfeng Hongyuan Flagship Store	Power Saving Specialist 1	I just received the item and plugged it in to see what happens later.	Jingdong prefecture level city in Shandong
Jinfeng Hongyuan Flagship Store	Power Saving Specialist 1	Baby received, especially good, packaging is also good, the courier brother is very strong, thank you.	Jingdong prefecture level city in Shandong
Jinfeng Hongyuan Flagship Store	Power Saving Specialist 1	Just received the goods have not yet used it, do not know how, three months later in the follow-up comment!	Jingdong prefecture level city in Shandong
Jinfeng Hongyuan Flagship Store	Power Saving Specialist 1	The item was received, it is quite good, recommended to buy.	Jingdong prefecture level city in Shandong
Jinfeng Hongyuan Flagship Store	Power Saving Specialist 1	This energy saver works very well and I recommend everyone to buy it!	Jingdong prefecture level city in Shandong
Jinfeng Hongyuan Flagship Store	Power Saving Specialist 1	Voltage stabilization and current reduction nice power saving product.	Jingdong prefecture level city in Shandong
Jinfeng Hongyuan Flagship Store	Power Saving Specialist 1	Voltage stabilization and current reduction nice power saving product.	Jingdong prefecture level city in Shandong
Jinfeng Hongyuan Flagship Store	Power Saving Specialist 1	A good power saver for stabilizing and lowering current.	Jingdong prefecture level city in Shandong
Jinfeng Hongyuan Flagship Store	Power Saving Specialist 1	You'll know if it saves power after you use it.	Jingdong prefecture level city in Shandong
Jinfeng Hongyuan Flagship Store	Power Saving Specialist 1	The quality is great. The quality is very good. The service is very good. Express delivery arrived in two days.	Jingdong prefecture level city in Shandong
Jinfeng Hongyuan Flagship Store	Power Saving Specialist 1	It works great.	Jingdong prefecture level city in Shandong
Jinfeng Hongyuan Flagship Store	Power Saving Specialist 1	Logistics is very fast, not yet used, used to comment again.	Jingdong prefecture level city in Shandong
Jinfeng Hongyuan Flagship Store	Power Saving Specialist 1	Logistics is very fast things are also very good, worthy of friends to buy	Jingdong prefecture level city in Shandong
Jinfeng Hongyuan Flagship Store	Power Saving Specialist 1	It works. It really works.	Jingdong prefecture level city in Shandong
Jinfeng Hongyuan Flagship Store	Power Saving Specialist 1	LANGUAGE COMMENTS: just received the shipment. I immediately plugged it in and used it. If you ask how effective. It depends on whether the next month's electricity bill has been reduced. If there is a decrease. I am buying more than one to use.	Jingdong prefecture level city in Shandong
Jinfeng Hongyuan Flagship Store	Power Saving Specialist 1	Well made, power saving, good and convenient!	Jingdong prefecture level city in Shandong
Jinfeng Hongyuan Flagship Store	Power Saving Specialist 1	I'm using it. I'll rate it next month.	Jingdong prefecture level city in Shandong
Jinfeng Hongyuan Flagship Store	Power Saving Specialist 1	Baby received Oh, the courier delivery really fast, with a good and then come back to the evaluation	Jingdong prefecture level city in Shandong
Jinfeng Hongyuan Flagship Store	Power Saving Specialist 1	Fast logistics, good packaging, worth it!	Jingdong prefecture level city in Shandong
Jinfeng Hongyuan Flagship Store	Power Saving Specialist 1	Customer service is okay. It's just that.	Jingdong prefecture level city in Shandong
Jinfeng Hongyuan Flagship Store	Power Saving Specialist 1	Poor power saving	Jingdong prefecture level city in Shandong
Jinfeng Hongyuan Flagship Store	Power Saving Specialist 1	I'll tell you when I've used it.	Jingdong prefecture level city in Shandong
Jinfeng Hongyuan Flagship Store	Power Saving Specialist 1	I think it's good.	Jingdong prefecture level city in Shandong
Jinfeng Hongyuan Flagship Store	Power Saving Specialist 1	This energy saver works very well and I recommend everyone to buy it!	Jingdong prefecture level city in Shandong
Jinfeng Hongyuan Flagship Store	Power Saving Specialist 1	I do not know how effective, just use half a month on the burn out, good business reissued a, and then use it after the follow-up comment.	Jingdong prefecture level city in Shandong
Jinfeng Hongyuan Flagship Store	Power Saving Specialist 1	in use	Jingdong prefecture level city in Shandong
Jinfeng Hongyuan Flagship Store	Power Saving Specialist 1	This thing is very poor. It's already only been used for ten days and it's broken. Now waiting for you to return it.	Jingdong prefecture level city in Shandong
Jinfeng Hongyuan Flagship Store	Power Saving Specialist 1	It's not the same as the seller's show.	Jingdong prefecture level city in Shandong
Jinfeng Hongyuan Flagship Store	Power Saving Specialist 1	What is the problem when I get it back from the CTS, it is plugged into the wall and the voltage shows 206v.	Jingdong prefecture level city in Shandong
Jinfeng Hongyuan Flagship Store	Power Saving Specialist 1	The logistics was fast and the stuff was perfectly packaged. It is in the process of trial.	Jingdong prefecture level city in Shandong
Jinfeng Hongyuan Flagship Store	Power Saving Specialist 1	This saver is particularly good to use can really save electricity, with two months and then look at the line if you buy one for the family	Jingdong prefecture level city in Shandong
Jinfeng Hongyuan Flagship Store	Power Saving Specialist 1	Novel appearance design, all aluminum alloy profile, also with a fuse device, voltage monitoring display. After receiving the goods, I directly cut the plug and connect it to the main power box under the leakage switch, hoping to save electricity, and then follow up later. Logistics is very fast, arrived the next	Jingdong prefecture level city in Shandong
Jinfeng Hongyuan Flagship Store	Power Saving Specialist 1	Received, very good and like, if necessary next time to buy ha ha	Jingdong prefecture level city in Shandong
Jinfeng Hongyuan Flagship Store	Power Saving Specialist 1	It's good. It's worth it. Get it if you need it.	Jingdong prefecture level city in Shandong
Jinfeng Hongyuan Flagship Store	Power Saving Specialist 1	It feels more than good and is worth having.	Jingdong prefecture level city in Shandong
Jinfeng Hongyuan Flagship Store	Power Saving Specialist 1	The goods arrived very good and fast first time to buy	Jingdong prefecture level city in Shandong
Jinfeng Hongyuan Flagship Store	Power Saving Specialist 1	The power saving is very good, I can save so much money every month, I will buy another one next time.	Jingdong prefecture level city in Shandong
Jinfeng Hongyuan Flagship Store	Power Saving Specialist 1	It's not what it says.	Jingdong prefecture level city in Shandong
Jinfeng Hongyuan Flagship Store	Power Saving Specialist 1	I have been using it for many days now and it saves electricity, it really does I have an 8 kW electric snail stove and it saves about $3.50 a day.	Jingdong prefecture level city in Shandong

Jinfeng Hongyuan Flagship Store	Power Saving Specialist 1	Express delivery is very fast, the quality is also quite good, worth buying!	Jingdong prefecture level city in Shandong
Jinfeng Hongyuan Flagship Store	Power Saving Specialist 1	Just received expecting the baby to work well.	Jingdong prefecture level city in Shandong
Jinfeng Hongyuan Flagship Store	Power Saving Specialist 1	Very good, there are more fruit, has bought a second!	Jingdong prefecture level city in Shandong
Jinfeng Hongyuan Flagship Store	Power Saving Specialist 1	Good is good So far, no power savings have been noticed Maybe it's too little to notice.	Jingdong prefecture level city in Shandong
Jinfeng Hongyuan Flagship Store	Power Saving Specialist 1	I just received the item and plugged it in to see what happens later.	Jingdong prefecture level city in Shandong
Jinfeng Hongyuan Flagship Store	Power Saving Specialist 1	Baby received, especially good, packaging is also good, the courier brother is very strong, thank you.	Jingdong prefecture level city in Shandong
Jinfeng Hongyuan Flagship Store	Power Saving Specialist 1	Just received the goods have not yet used it, do not know how, three months later in the follow-up comment!	Jingdong prefecture level city in Shandong
Jinfeng Hongyuan Flagship Store	Power Saving Specialist 1	The item was received, it is quite good, recommended to buy.	Jingdong prefecture level city in Shandong
Jinfeng Hongyuan Flagship Store	Power Saving Specialist 1	This energy saver works very well and I recommend everyone to buy it!	Jingdong prefecture level city in Shandong
Jinfeng Hongyuan Flagship Store	Power Saving Specialist 1	Voltage stabilization and current reduction nice power saving product.	Jingdong prefecture level city in Shandong
Jinfeng Hongyuan Flagship Store	Power Saving Specialist 1	Voltage stabilization and current reduction nice power saving product.	Jingdong prefecture level city in Shandong
Jinfeng Hongyuan Flagship Store	Power Saving Specialist 1	A good power saver for stabilizing and lowering current.	Jingdong prefecture level city in Shandong
Jinfeng Hongyuan Flagship Store	Power Saving Specialist 1	You'll know if it saves power after you use it.	Jingdong prefecture level city in Shandong
Jinfeng Hongyuan Flagship Store	Power Saving Specialist 1	The quality is great. The quality is very good. The service is very good. Express delivery arrived in two days.	Jingdong prefecture level city in Shandong
Jinfeng Hongyuan Flagship Store	Power Saving Specialist 1	It works great.	Jingdong prefecture level city in Shandong
Jinfeng Hongyuan Flagship Store	Power Saving Specialist 1	Logistics is very fast, not yet used, used to comment again.	Jingdong prefecture level city in Shandong
Jinfeng Hongyuan Flagship Store	Power Saving Specialist 1	Logistics is very fast things are also very good, worthy of friends to buy	Jingdong prefecture level city in Shandong
Jinfeng Hongyuan Flagship Store	Power Saving Specialist 1	It works. It really works.	Jingdong prefecture level city in Shandong
Jinfeng Hongyuan Flagship Store	Power Saving Specialist 1	LANGUAGE COMMENTS: just received the shipment. I immediately plugged it in and used it. If you ask how effective. It depends on whether the next month's electricity bill has been reduced. If there is a decrease. I am buying more than one to use.	Jingdong prefecture level city in Shandong
Jinfeng Hongyuan Flagship Store	Power Saving Specialist 1	Well made, power saving, good and convenient!	Jingdong prefecture level city in Shandong
Jinfeng Hongyuan Flagship Store	Power Saving Specialist 1	I'm using it. I'll rate it next month.	Jingdong prefecture level city in Shandong
Jinfeng Hongyuan Flagship Store	Power Saving Specialist 1	Baby received Oh, the courier delivery really fast, with a good and then come back to the evaluation	Jingdong prefecture level city in Shandong
Jinfeng Hongyuan Flagship Store	Power Saving Specialist 1	Fast logistics, good packaging, worth it!	Jingdong prefecture level city in Shandong
Jinfeng Hongyuan Flagship Store	Power Saving Specialist 1	Customer service is okay. It's just that.	Jingdong prefecture level city in Shandong
Jinfeng Hongyuan Flagship Store	Power Saving Specialist 1	Poor power saving	Jingdong prefecture level city in Shandong
Jinfeng Hongyuan Flagship Store	Power Saving Specialist 1	I'll tell you when I've used it.	Jingdong prefecture level city in Shandong
Jinfeng Hongyuan Flagship Store	Power Saving Specialist 1	I think it's good.	Jingdong prefecture level city in Shandong
Jinfeng Hongyuan Flagship Store	Power Saving Specialist 1	This energy saver works very well and I recommend everyone to buy it!	Jingdong prefecture level city in Shandong
Jinfeng Hongyuan Flagship Store	Power Saving Specialist 1	I do not know how effective, just use half a month on the burn out, good business reissued a, and then use it after the follow-up comment.	Jingdong prefecture level city in Shandong
Jinfeng Hongyuan Flagship Store	Power Saving Specialist 1	in use	Jingdong prefecture level city in Shandong
Jinfeng Hongyuan Flagship Store	Power Saving Specialist 1	This thing is very poor. It's already only been used for ten days and it's broken. Now waiting for you to return it.	Jingdong prefecture level city in Shandong
Jinfeng Hongyuan Flagship Store	Power Saving Specialist 1	It's not the same as the seller's show.	Jingdong prefecture level city in Shandong
Jinfeng Hongyuan Flagship Store	Power Saving Specialist 1	What is the problem when I get it back from the CTS, it is plugged into the wall and the voltage shows 206v.	Jingdong prefecture level city in Shandong
Jinfeng Hongyuan Flagship Store	Power Saving Specialist 1	The logistics was fast and the stuff was perfectly packaged. It is in the process of trial.	Jingdong prefecture level city in Shandong
Jinfeng Hongyuan Flagship Store	Power Saving Specialist 1	This saver is particularly good to use can really save electricity, with two months and then look at the line if you buy one for the family	Jingdong prefecture level city in Shandong
Jinfeng Hongyuan Flagship Store	Power Saving Specialist 1	Novel appearance design, all aluminum alloy profile, also with a fuse device, voltage monitoring display. After receiving the goods, I directly cut the plug and connect it to the main power box under the leakage switch, hoping to save electricity, and then follow up later. Logistics is very fast, arrived the next	Jingdong prefecture level city in Shandong
Jinfeng Hongyuan Flagship Store	Power Saving Specialist 1	Received, very good and like, if necessary next time to buy ha ha	Jingdong prefecture level city in Shandong
Jinfeng Hongyuan Flagship Store	Power Saving Specialist 1	It's good. It's worth it. Get it if you need it.	Jingdong prefecture level city in Shandong
Jinfeng Hongyuan Flagship Store	Power Saving Specialist 1	It feels more than good and is worth having.	Jingdong prefecture level city in Shandong
Jinfeng Hongyuan Flagship Store	Power Saving Specialist 1	The goods arrived very good and fast first time to buy	Jingdong prefecture level city in Shandong
Jinfeng Hongyuan Flagship Store	Power Saving Specialist 1	The power saving is very good, I can save so much money every month, I will buy another one next time.	Jingdong prefecture level city in Shandong
Jinfeng Hongyuan Flagship Store	Power Saving Specialist 1	It's not what it says.	Jingdong prefecture level city in Shandong
Jinfeng Hongyuan Flagship Store	Power Saving Specialist 1	I have been using it for many days now and it saves electricity, it really does I have an 8 kW electric snail stove and it saves about $3.50 a day.	Jingdong prefecture level city in Shandong
Jinfeng Hongyuan Flagship Store	Power Saving Specialist 1	Express delivery is very fast, the quality is also quite good, worth buying!	Jingdong prefecture level city in Shandong
Jinfeng Hongyuan Flagship Store	Power Saving Specialist 1	Just received expecting the baby to work well.	Jingdong prefecture level city in Shandong
Jinfeng Hongyuan Flagship Store	Power Saving Specialist 1	Very good, there are more fruit, has bought a second!	Jingdong prefecture level city in Shandong
Jinfeng Hongyuan Flagship Store	Power Saving Specialist 1	Good is good So far, no power savings have been noticed Maybe it's too little to notice.	Jingdong prefecture level city in Shandong
Jinfeng Hongyuan Flagship Store	Power Saving Specialist 1	I just received the item and plugged it in to see what happens later.	Jingdong prefecture level city in Shandong
Jinfeng Hongyuan Flagship Store	Power Saving Specialist 1	Baby received, especially good, packaging is also good, the courier brother is very strong, thank you.	Jingdong prefecture level city in Shandong
Jinfeng Hongyuan Flagship Store	Power Saving Specialist 1	Just received the goods have not yet used it, do not know how, three months later in the follow-up comment!	Jingdong prefecture level city in Shandong

Jinfeng Hongyuan Flagship Store	Power Saving Specialist 1	The item was received, it is quite good, recommended to buy.	Jingdong prefecture level city in Shandong
Jinfeng Hongyuan Flagship Store	Power Saving Specialist 1	This energy saver works very well and I recommend everyone to buy it!	Jingdong prefecture level city in Shandong
Jinfeng Hongyuan Flagship Store	Power Saving Specialist 1	Voltage stabilization and current reduction nice power saving product.	Jingdong prefecture level city in Shandong
Jinfeng Hongyuan Flagship Store	Power Saving Specialist 1	Voltage stabilization and current reduction nice power saving product.	Jingdong prefecture level city in Shandong
Jinfeng Hongyuan Flagship Store	Power Saving Specialist 1	A good power saver for stabilizing and lowering current.	Jingdong prefecture level city in Shandong
Jinfeng Hongyuan Flagship Store	Power Saving Specialist 1	You'll know if it saves power after you use it.	Jingdong prefecture level city in Shandong
Jinfeng Hongyuan Flagship Store	Power Saving Specialist 1	The quality is great. The quality is very good. The service is very good. Express delivery arrived in two days.	Jingdong prefecture level city in Shandong
Jinfeng Hongyuan Flagship Store	Power Saving Specialist 1	It works great.	Jingdong prefecture level city in Shandong
Jinfeng Hongyuan Flagship Store	Power Saving Specialist 1	Logistics is very fast, not yet used, used to comment again.	Jingdong prefecture level city in Shandong
Jinfeng Hongyuan Flagship Store	Power Saving Specialist 1	Logistics is very fast things are also very good, worthy of friends to buy	Jingdong prefecture level city in Shandong
Jinfeng Hongyuan Flagship Store	Power Saving Specialist 1	It works. It really works.	Jingdong prefecture level city in Shandong
Jinfeng Hongyuan Flagship Store	Power Saving Specialist 1	LANGUAGE COMMENTS: just received the shipment. I immediately plugged it in and used it. If you ask how effective. It depends on whether the next month's electricity bill has been reduced. If there is a decrease. I am buying more than one to use.	Jingdong prefecture level city in Shandong
Jinfeng Hongyuan Flagship Store	Power Saving Specialist 1	Well made, power saving, good and convenient!	Jingdong prefecture level city in Shandong
Jinfeng Hongyuan Flagship Store	Power Saving Specialist 1	I'm using it. I'll rate it next month.	Jingdong prefecture level city in Shandong
Jinfeng Hongyuan Flagship Store	Power Saving Specialist 1	Baby received Oh, the courier delivery really fast, with a good and then come back to the evaluation	Jingdong prefecture level city in Shandong
Jinfeng Hongyuan Flagship Store	Power Saving Specialist 1	Fast logistics, good packaging, worth it!	Jingdong prefecture level city in Shandong
Jinfeng Hongyuan Flagship Store	Power Saving Specialist 1	Customer service is okay. It's just that.	Jingdong prefecture level city in Shandong
Jinfeng Hongyuan Flagship Store	Power Saving Specialist 1	Poor power saving	Jingdong prefecture level city in Shandong
Jinfeng Hongyuan Flagship Store	Power Saving Specialist 1	I'll tell you when I've used it.	Jingdong prefecture level city in Shandong
Jinfeng Hongyuan Flagship Store	Power Saving Specialist 1	I think it's good.	Jingdong prefecture level city in Shandong
Jinfeng Hongyuan Flagship Store	Power Saving Specialist 1	This energy saver works very well and I recommend everyone to buy it!	Jingdong prefecture level city in Shandong
Jinfeng Hongyuan Flagship Store	Power Saving Specialist 1	I do not know how effective, just use half a month on the burn out, good business reissued a, and then use it after the follow-up comment.	Jingdong prefecture level city in Shandong
Jinfeng Hongyuan Flagship Store	Power Saving Specialist 1	in use	Jingdong prefecture level city in Shandong
Jinfeng Hongyuan Flagship Store	Power Saving Specialist 1	This thing is very poor. It's already only been used for ten days and it's broken. Now waiting for you to return it.	Jingdong prefecture level city in Shandong
Jinfeng Hongyuan Flagship Store	Power Saving Specialist 1	It's not the same as the seller's show.	Jingdong prefecture level city in Shandong
Jinfeng Hongyuan Flagship Store	Power Saving Specialist 1	What is the problem when I get it back from the CTS, it is plugged into the wall and the voltage shows 206v.	Jingdong prefecture level city in Shandong
Jinfeng Hongyuan Flagship Store	Power Saving Specialist 1	The logistics was fast and the stuff was perfectly packaged. It is in the process of trial.	Jingdong prefecture level city in Shandong
Jinfeng Hongyuan Flagship Store	Power Saving Specialist 1	This saver is particularly good to use can really save electricity, with two months and then look at the line if you buy one for the family	Jingdong prefecture level city in Shandong
Jinfeng Hongyuan Flagship Store	Power Saving Specialist 1	Novel appearance design, all aluminum alloy profile, also with a fuse device, voltage monitoring display. After receiving the goods, I directly cut the plug and connect it to the main power box under the leakage switch, hoping to save electricity, and then follow up later. Logistics is very fast, arrived the next	Jingdong prefecture level city in Shandong
Jinfeng Hongyuan Flagship Store	Power Saving Specialist 1	Received, very good and like, if necessary next time to buy ha ha	Jingdong prefecture level city in Shandong
Jinfeng Hongyuan Flagship Store	Power Saving Specialist 1	It's good. It's worth it. Get it if you need it.	Jingdong prefecture level city in Shandong
Jinfeng Hongyuan Flagship Store	Power Saving Specialist 1	It feels more than good and is worth having.	Jingdong prefecture level city in Shandong
Jinfeng Hongyuan Flagship Store	Power Saving Specialist 1	The goods arrived very good and fast first time to buy	Jingdong prefecture level city in Shandong
Jinfeng Hongyuan Flagship Store	Power Saving Specialist 1	The power saving is very good, I can save so much money every month, I will buy another one next time.	Jingdong prefecture level city in Shandong
Jinfeng Hongyuan Flagship Store	Power Saving Specialist 1	It's not what it says.	Jingdong prefecture level city in Shandong
Jinfeng Hongyuan Flagship Store	Power Saving Specialist 1	I have been using it for many days now and it saves electricity, it really does I have an 8 kW electric snail stove and it saves about $3.50 a day.	Jingdong prefecture level city in Shandong
Jinfeng Hongyuan Flagship Store	Power Saving Specialist 1	Express delivery is very fast, the quality is also quite good, worth buying!	Jingdong prefecture level city in Shandong
Jinfeng Hongyuan Flagship Store	Power Saving Specialist 1	Just received expecting the baby to work well.	Jingdong prefecture level city in Shandong
Jinfeng Hongyuan Flagship Store	Power Saving Specialist 1	Very good, there are more fruit, has bought a second!	Jingdong prefecture level city in Shandong
Jinfeng Hongyuan Flagship Store	Power Saving Specialist 1	Good is good So far, no power savings have been noticed Maybe it's too little to notice.	Jingdong prefecture level city in Shandong
Jinfeng Hongyuan Flagship Store	Power Saving Specialist 1	I just received the item and plugged it in to see what happens later.	Jingdong prefecture level city in Shandong
Jinfeng Hongyuan Flagship Store	Power Saving Specialist 1	Baby received, especially good, packaging is also good, the courier brother is very strong, thank you.	Jingdong prefecture level city in Shandong
Jinfeng Hongyuan Flagship Store	Power Saving Specialist 1	Just received the goods have not yet used it, do not know how, three months later in the follow-up comment!	Jingdong prefecture level city in Shandong
Jinfeng Hongyuan Flagship Store	Power Saving Specialist 1	The item was received, it is quite good, recommended to buy.	Jingdong prefecture level city in Shandong
Jinfeng Hongyuan Flagship Store	Power Saving Specialist 1	This energy saver works very well and I recommend everyone to buy it!	Jingdong prefecture level city in Shandong
Jinfeng Hongyuan Flagship Store	Power Saving Specialist 1	Voltage stabilization and current reduction nice power saving product.	Jingdong prefecture level city in Shandong
Jinfeng Hongyuan Flagship Store	Power Saving Specialist 1	Voltage stabilization and current reduction nice power saving product.	Jingdong prefecture level city in Shandong
Jinfeng Hongyuan Flagship Store	Power Saving Specialist 1	A good power saver for stabilizing and lowering current.	Jingdong prefecture level city in Shandong
Jinfeng Hongyuan Flagship Store	Power Saving Specialist 1	You'll know if it saves power after you use it.	Jingdong prefecture level city in Shandong
Jinfeng Hongyuan Flagship Store	Power Saving Specialist 1	The quality is great. The quality is very good. The service is very good. Express delivery arrived in two days.	Jingdong prefecture level city in Shandong

Jinfeng Hongyuan Flagship Store	Power Saving Specialist 1	It works great.	Jingdong prefecture level city in Shandong
Jinfeng Hongyuan Flagship Store	Power Saving Specialist 1	Logistics is very fast, not yet used, used to comment again.	Jingdong prefecture level city in Shandong
Jinfeng Hongyuan Flagship Store	Power Saving Specialist 1	Logistics is very fast things are also very good, worthy of friends to buy	Jingdong prefecture level city in Shandong
Jinfeng Hongyuan Flagship Store	Power Saving Specialist 1	It works. It really works.	Jingdong prefecture level city in Shandong
Jinfeng Hongyuan Flagship Store	Power Saving Specialist 1	LANGUAGE COMMENTS: just received the shipment. I immediately plugged it in and used it. If you ask how effective. It depends on whether the next month's electricity bill has been reduced. If there is a decrease. I am buying more than one to use.	Jingdong prefecture level city in Shandong
Jinfeng Hongyuan Flagship Store	Power Saving Specialist 1	Well made, power saving, good and convenient!	Jingdong prefecture level city in Shandong
Jinfeng Hongyuan Flagship Store	Power Saving Specialist 1	I'm using it. I'll rate it next month.	Jingdong prefecture level city in Shandong
Jinfeng Hongyuan Flagship Store	Power Saving Specialist 1	Baby received Oh, the courier delivery really fast, with a good and then come back to the evaluation	Jingdong prefecture level city in Shandong
Jinfeng Hongyuan Flagship Store	Power Saving Specialist 1	Fast logistics, good packaging, worth it!	Jingdong prefecture level city in Shandong
Jinfeng Hongyuan Flagship Store	Power Saving Specialist 1	Customer service is okay. It's just that.	Jingdong prefecture level city in Shandong
Jinfeng Hongyuan Flagship Store	Power Saving Specialist 1	Poor power saving	Jingdong prefecture level city in Shandong
Jinfeng Hongyuan Flagship Store	Power Saving Specialist 1	I'll tell you when I've used it.	Jingdong prefecture level city in Shandong
Jinfeng Hongyuan Flagship Store	Power Saving Specialist 1	I think it's good.	Jingdong prefecture level city in Shandong
Jinfeng Hongyuan Flagship Store	Power Saving Specialist 1	This energy saver works very well and I recommend everyone to buy it!	Jingdong prefecture level city in Shandong
Jinfeng Hongyuan Flagship Store	Power Saving Specialist 1	I do not know how effective, just use half a month on the burn out, good business reissued a, and then use it after the follow-up comment.	Jingdong prefecture level city in Shandong
Jinfeng Hongyuan Flagship Store	Power Saving Specialist 1	in use	Jingdong prefecture level city in Shandong
Jinfeng Hongyuan Flagship Store	Power Saving Specialist 1	This thing is very poor. It's already only been used for ten days and it's broken. Now waiting for you to return it.	Jingdong prefecture level city in Shandong
Jinfeng Hongyuan Flagship Store	Power Saving Specialist 1	It's not the same as the seller's show.	Jingdong prefecture level city in Shandong
Jinfeng Hongyuan Flagship Store	Power Saving Specialist 1	What is the problem when I get it back from the CTS, it is plugged into the wall and the voltage shows 206v.	Jingdong prefecture level city in Shandong
Jinfeng Hongyuan Flagship Store	Power Saving Specialist 1	The logistics was fast and the stuff was perfectly packaged. It is in the process of trial.	Jingdong prefecture level city in Shandong
Jinfeng Hongyuan Flagship Store	Power Saving Specialist 1	This saver is particularly good to use can really save electricity, with two months and then look at the line if you buy one for the family	Jingdong prefecture level city in Shandong
Jinfeng Hongyuan Flagship Store	Power Saving Specialist 1	Novel appearance design, all aluminum alloy profile, also with a fuse device, voltage monitoring display. After receiving the goods, I directly cut the plug and connect it to the main power box under the leakage switch, hoping to save electricity, and then follow up later. Logistics is very fast, arrived the next	Jingdong prefecture level city in Shandong
Jinfeng Hongyuan Flagship Store	Power Saving Specialist 1	Received, very good and like, if necessary next time to buy ha ha	Jingdong prefecture level city in Shandong
Jinfeng Hongyuan Flagship Store	Power Saving Specialist 1	It's good. It's worth it. Get it if you need it.	Jingdong prefecture level city in Shandong
Jinfeng Hongyuan Flagship Store	Power Saving Specialist 1	It feels more than good and is worth having.	Jingdong prefecture level city in Shandong
Jinfeng Hongyuan Flagship Store	Power Saving Specialist 1	The goods arrived very good and fast first time to buy	Jingdong prefecture level city in Shandong
Jinfeng Hongyuan Flagship Store	Power Saving Specialist 1	The power saving is very good, I can save so much money every month, I will buy another one next time.	Jingdong prefecture level city in Shandong
Jinfeng Hongyuan Flagship Store	Power Saving Specialist 1	It's not what it says.	Jingdong prefecture level city in Shandong
Jinfeng Hongyuan Flagship Store	Power Saving Specialist 1	I have been using it for many days now and it saves electricity, it really does I have an 8 kW electric snail stove and it saves about $3.50 a day.	Jingdong prefecture level city in Shandong
Jinfeng Hongyuan Flagship Store	Power Saving Specialist 1	Express delivery is very fast, the quality is also quite good, worth buying!	Jingdong prefecture level city in Shandong
Jinfeng Hongyuan Flagship Store	Power Saving Specialist 1	Just received expecting the baby to work well.	Jingdong prefecture level city in Shandong
Jinfeng Hongyuan Flagship Store	Power Saving Specialist 1	Very good, there are more fruit, has bought a second!	Jingdong prefecture level city in Shandong
Jinfeng Hongyuan Flagship Store	Power Saving Specialist 1	Good is good So far, no power savings have been noticed Maybe it's too little to notice.	Jingdong prefecture level city in Shandong
Jinfeng Hongyuan Flagship Store	Power Saving Specialist 1	I just received the item and plugged it in to see what happens later.	Jingdong prefecture level city in Shandong
Jinfeng Hongyuan Flagship Store	Power Saving Specialist 1	Baby received, especially good, packaging is also good, the courier brother is very strong, thank you.	Jingdong prefecture level city in Shandong
Jinfeng Hongyuan Flagship Store	Power Saving Specialist 1	Just received the goods have not yet used it, do not know how, three months later in the follow-up comment!	Jingdong prefecture level city in Shandong
Jinfeng Hongyuan Flagship Store	Power Saving Specialist 1	The item was received, it is quite good, recommended to buy.	Jingdong prefecture level city in Shandong
Jinfeng Hongyuan Flagship Store	Power Saving Specialist 1	This energy saver works very well and I recommend everyone to buy it!	Jingdong prefecture level city in Shandong
Jinfeng Hongyuan Flagship Store	Power Saving Specialist 1	Voltage stabilization and current reduction nice power saving product.	Jingdong prefecture level city in Shandong
Jinfeng Hongyuan Flagship Store	Power Saving Specialist 1	Voltage stabilization and current reduction nice power saving product.	Jingdong prefecture level city in Shandong
Jinfeng Hongyuan Flagship Store	Power Saving Specialist 1	A good power saver for stabilizing and lowering current.	Jingdong prefecture level city in Shandong
Jinfeng Hongyuan Flagship Store	Power Saving Specialist 1	You'll know if it saves power after you use it.	Jingdong prefecture level city in Shandong
Jinfeng Hongyuan Flagship Store	Power Saving Specialist 1	The quality is great. The quality is very good. The service is very good. Express delivery arrived in two days.	Jingdong prefecture level city in Shandong
Jinfeng Hongyuan Flagship Store	Power Saving Specialist 1	It works great.	Jingdong prefecture level city in Shandong
Jinfeng Hongyuan Flagship Store	Power Saving Specialist 1	Logistics is very fast, not yet used, used to comment again.	Jingdong prefecture level city in Shandong
Jinfeng Hongyuan Flagship Store	Power Saving Specialist 1	Logistics is very fast things are also very good, worthy of friends to buy	Jingdong prefecture level city in Shandong
Jinfeng Hongyuan Flagship Store	Power Saving Specialist 1	It works. It really works.	Jingdong prefecture level city in Shandong
Jinfeng Hongyuan Flagship Store	Power Saving Specialist 1	LANGUAGE COMMENTS: just received the shipment. I immediately plugged it in and used it. If you ask how effective. It depends on whether the next month's electricity bill has been reduced. If there is a decrease. I am buying more than one to use.	Jingdong prefecture level city in Shandong
Jinfeng Hongyuan Flagship Store	Power Saving Specialist 1	Well made, power saving, good and convenient!	Jingdong prefecture level city in Shandong
Jinfeng Hongyuan Flagship Store	Power Saving Specialist 1	I'm using it. I'll rate it next month.	Jingdong prefecture level city in Shandong

Jinfeng Hongyuan Flagship Store	Power Saving Specialist 1	Baby received Oh, the courier delivery really fast, with a good and then come back to the evaluation	Jingdong prefecture level city in Shandong
Jinfeng Hongyuan Flagship Store	Power Saving Specialist 1	Fast logistics, good packaging, worth it!	Jingdong prefecture level city in Shandong
Jinfeng Hongyuan Flagship Store	Power Saving Specialist 1	Customer service is okay. It's just that.	Jingdong prefecture level city in Shandong
Jinfeng Hongyuan Flagship Store	Power Saving Specialist 1	Poor power saving	Jingdong prefecture level city in Shandong
Jinfeng Hongyuan Flagship Store	Power Saving Specialist 1	I'll tell you when I've used it.	Jingdong prefecture level city in Shandong
Jinfeng Hongyuan Flagship Store	Power Saving Specialist 1	I think it's good.	Jingdong prefecture level city in Shandong
Jinfeng Hongyuan Flagship Store	Power Saving Specialist 1	This energy saver works very well and I recommend everyone to buy it!	Jingdong prefecture level city in Shandong
Jinfeng Hongyuan Flagship Store	Power Saving Specialist 1	I do not know how effective, just use half a month on the burn out, good business reissued a, and then use it after the follow-up comment.	Jingdong prefecture level city in Shandong
Jinfeng Hongyuan Flagship Store	Power Saving Specialist 1	in use	Jingdong prefecture level city in Shandong
Jinfeng Hongyuan Flagship Store	Power Saving Specialist 1	This thing is very poor. It's already only been used for ten days and it's broken. Now waiting for you to return it.	Jingdong prefecture level city in Shandong
Jinfeng Hongyuan Flagship Store	Power Saving Specialist 1	It's not the same as the seller's show.	Jingdong prefecture level city in Shandong
Jinfeng Hongyuan Flagship Store	Power Saving Specialist 1	What is the problem when I get it back from the CTS, it is plugged into the wall and the voltage shows 206v.	Jingdong prefecture level city in Shandong
Jinfeng Hongyuan Flagship Store	Power Saving Specialist 1	The logistics was fast and the stuff was perfectly packaged. It is in the process of trial.	Jingdong prefecture level city in Shandong
Jinfeng Hongyuan Flagship Store	Power Saving Specialist 1	This saver is particularly good to use can really save electricity, with two months and then look at the line if you buy one for the family	Jingdong prefecture level city in Shandong
Jinfeng Hongyuan Flagship Store	Power Saving Specialist 1	Novel appearance design, all aluminum alloy profile, also with a fuse device, voltage monitoring display. After receiving the goods, I directly cut the plug and connect it to the main power box under the leakage switch, hoping to save electricity, and then follow up later. Logistics is very fast, arrived the next	Jingdong prefecture level city in Shandong
Jinfeng Hongyuan Flagship Store	Power Saving Specialist 1	Received, very good and like, if necessary next time to buy ha ha	Jingdong prefecture level city in Shandong
Jinfeng Hongyuan Flagship Store	Power Saving Specialist 1	It's good. It's worth it. Get it if you need it.	Jingdong prefecture level city in Shandong
Jinfeng Hongyuan Flagship Store	Power Saving Specialist 1	It feels more than good and is worth having.	Jingdong prefecture level city in Shandong
Jinfeng Hongyuan Flagship Store	Power Saving Specialist 1	The goods arrived very good and fast first time to buy	Jingdong prefecture level city in Shandong
Jinfeng Hongyuan Flagship Store	Power Saving Specialist 1	The power saving is very good, I can save so much money every month, I will buy another one next time.	Jingdong prefecture level city in Shandong
Jinfeng Hongyuan Flagship Store	Power Saving Specialist 1	It's not what it says.	Jingdong prefecture level city in Shandong
Jinfeng Hongyuan Flagship Store	Power Saving Specialist 1	I have been using it for many days now and it saves electricity, it really does I have an 8 kW electric snail stove and it saves about $3.50 a day.	Jingdong prefecture level city in Shandong
Jinfeng Hongyuan Flagship Store	Power Saving Specialist 1	Express delivery is very fast, the quality is also quite good, worth buying!	Jingdong prefecture level city in Shandong
Jinfeng Hongyuan Flagship Store	Power Saving Specialist 1	Just received expecting the baby to work well.	Jingdong prefecture level city in Shandong
Jinfeng Hongyuan Flagship Store	Power Saving Specialist 1	Very good, there are more fruit, has bought a second!	Jingdong prefecture level city in Shandong
Jinfeng Hongyuan Flagship Store	Power Saving Specialist 1	Good is good So far, no power savings have been noticed Maybe it's too little to notice.	Jingdong prefecture level city in Shandong
Jinfeng Hongyuan Flagship Store	Power Saving Specialist 1	I just received the item and plugged it in to see what happens later.	Jingdong prefecture level city in Shandong
Jinfeng Hongyuan Flagship Store	Power Saving Specialist 1	Baby received, especially good, packaging is also good, the courier brother is very strong, thank you.	Jingdong prefecture level city in Shandong
Jinfeng Hongyuan Flagship Store	Power Saving Specialist 1	Just received the goods have not yet used it, do not know how, three months later in the follow-up comment!	Jingdong prefecture level city in Shandong
Jinfeng Hongyuan Flagship Store	Power Saving Specialist 1	The item was received, it is quite good, recommended to buy.	Jingdong prefecture level city in Shandong
Jinfeng Hongyuan Flagship Store	Power Saving Specialist 1	This energy saver works very well and I recommend everyone to buy it!	Jingdong prefecture level city in Shandong
Jinfeng Hongyuan Flagship Store	Power Saving Specialist 1	Voltage stabilization and current reduction nice power saving product.	Jingdong prefecture level city in Shandong
Jinfeng Hongyuan Flagship Store	Power Saving Specialist 1	Voltage stabilization and current reduction nice power saving product.	Jingdong prefecture level city in Shandong
Jinfeng Hongyuan Flagship Store	Power Saving Specialist 1	A good power saver for stabilizing and lowering current.	Jingdong prefecture level city in Shandong
Jinfeng Hongyuan Flagship Store	Power Saving Specialist 1	You'll know if it saves power after you use it.	Jingdong prefecture level city in Shandong
Jinfeng Hongyuan Flagship Store	Power Saving Specialist 1	The quality is great. The quality is very good. The service is very good. Express delivery arrived in two days.	Jingdong prefecture level city in Shandong
Jinfeng Hongyuan Flagship Store	Power Saving Specialist 1	It works great.	Jingdong prefecture level city in Shandong
Jinfeng Hongyuan Flagship Store	Power Saving Specialist 1	Logistics is very fast, not yet used, used to comment again.	Jingdong prefecture level city in Shandong
Jinfeng Hongyuan Flagship Store	Power Saving Specialist 1	Logistics is very fast things are also very good, worthy of friends to buy	Jingdong prefecture level city in Shandong
Jinfeng Hongyuan Flagship Store	Power Saving Specialist 1	It works. It really works.	Jingdong prefecture level city in Shandong
Jinfeng Hongyuan Flagship Store	Power Saving Specialist 1	LANGUAGE COMMENTS: just received the shipment. I immediately plugged it in and used it. If you ask how effective. It depends on whether the next month's electricity bill has been reduced. If there is a decrease. I am buying more than one to use.	Jingdong prefecture level city in Shandong
Jinfeng Hongyuan Flagship Store	Power Saving Specialist 1	Well made, power saving, good and convenient!	Jingdong prefecture level city in Shandong
Jinfeng Hongyuan Flagship Store	Power Saving Specialist 1	I'm using it. I'll rate it next month.	Jingdong prefecture level city in Shandong
Jinfeng Hongyuan Flagship Store	Power Saving Specialist 1	Baby received Oh, the courier delivery really fast, with a good and then come back to the evaluation	Jingdong prefecture level city in Shandong
Jinfeng Hongyuan Flagship Store	Power Saving Specialist 1	Fast logistics, good packaging, worth it!	Jingdong prefecture level city in Shandong
Jinfeng Hongyuan Flagship Store	Power Saving Specialist 1	Customer service is okay. It's just that.	Jingdong prefecture level city in Shandong
Jinfeng Hongyuan Flagship Store	Power Saving Specialist 1	Poor power saving	Jingdong prefecture level city in Shandong
Jinfeng Hongyuan Flagship Store	Power Saving Specialist 1	I'll tell you when I've used it.	Jingdong prefecture level city in Shandong
Jinfeng Hongyuan Flagship Store	Power Saving Specialist 1	I think it's good.	Jingdong prefecture level city in Shandong
Jinfeng Hongyuan Flagship Store	Power Saving Specialist 1	This energy saver works very well and I recommend everyone to buy it!	Jingdong prefecture level city in Shandong

Jinfeng Hongyuan Flagship Store	Power Saving Specialist 1	I do not know how effective, just use half a month on the burn out, good business reissued a, and then use it after the follow-up comment.	Jingdong prefecture level city in Shandong
Jinfeng Hongyuan Flagship Store	Power Saving Specialist 1	in use	Jingdong prefecture level city in Shandong
Jinfeng Hongyuan Flagship Store	Power Saving Specialist 1	This thing is very poor. It's already only been used for ten days and it's broken. Now waiting for you to return it.	Jingdong prefecture level city in Shandong
Jinfeng Hongyuan Flagship Store	Power Saving Specialist 1	It's not the same as the seller's show.	Jingdong prefecture level city in Shandong
Jinfeng Hongyuan Flagship Store	Power Saving Specialist 1	What is the problem when I get it back from the CTS, it is plugged into the wall and the voltage shows 206v.	Jingdong prefecture level city in Shandong
Jinfeng Hongyuan Flagship Store	Power Saving Specialist 1	The logistics was fast and the stuff was perfectly packaged. It is in the process of trial.	Jingdong prefecture level city in Shandong
Jinfeng Hongyuan Flagship Store	Power Saving Specialist 1	This saver is particularly good to use can really save electricity, with two months and then look at the line if you buy one for the family	Jingdong prefecture level city in Shandong
Jinfeng Hongyuan Flagship Store	Power Saving Specialist 1	Novel appearance design, all aluminum alloy profile, also with a fuse device, voltage monitoring display. After receiving the goods, I directly cut the plug and connect it to the main power box under the leakage switch, hoping to save electricity, and then follow up later. Logistics is very fast, arrived the next	Jingdong prefecture level city in Shandong
Jinfeng Hongyuan Flagship Store	Power Saving Specialist 1	Received, very good and like, if necessary next time to buy ha ha	Jingdong prefecture level city in Shandong
Jinfeng Hongyuan Flagship Store	Power Saving Specialist 1	It's good. It's worth it. Get it if you need it.	Jingdong prefecture level city in Shandong
Jinfeng Hongyuan Flagship Store	Power Saving Specialist 1	It feels more than good and is worth having.	Jingdong prefecture level city in Shandong
Jinfeng Hongyuan Flagship Store	Power Saving Specialist 1	The goods arrived very good and fast first time to buy	Jingdong prefecture level city in Shandong
Jinfeng Hongyuan Flagship Store	Power Saving Specialist 1	The power saving is very good, I can save so much money every month, I will buy another one next time.	Jingdong prefecture level city in Shandong
Jinfeng Hongyuan Flagship Store	Power Saving Specialist 1	It's not what it says.	Jingdong prefecture level city in Shandong
Jinfeng Hongyuan Flagship Store	Power Saving Specialist 1	I have been using it for many days now and it saves electricity, it really does I have an 8 kW electric snail stove and it saves about $3.50 a day.	Jingdong prefecture level city in Shandong
Jinfeng Hongyuan Flagship Store	Power Saving Specialist 1	Express delivery is very fast, the quality is also quite good, worth buying!	Jingdong prefecture level city in Shandong
Jinfeng Hongyuan Flagship Store	Power Saving Specialist 1	Just received expecting the baby to work well.	Jingdong prefecture level city in Shandong
Jinfeng Hongyuan Flagship Store	Power Saving Specialist 1	Very good, there are more fruit, has bought a second!	Jingdong prefecture level city in Shandong
Jinfeng Hongyuan Flagship Store	Power Saving Specialist 1	Good is good So far, no power savings have been noticed Maybe it's too little to notice.	Jingdong prefecture level city in Shandong
Jinfeng Hongyuan Flagship Store	Power Saving Specialist 1	I just received the item and plugged it in to see what happens later.	Jingdong prefecture level city in Shandong
Jinfeng Hongyuan Flagship Store	Power Saving Specialist 1	Baby received, especially good, packaging is also good, the courier brother is very strong, thank you.	Jingdong prefecture level city in Shandong
Jinfeng Hongyuan Flagship Store	Power Saving Specialist 1	Just received the goods have not yet used it, do not know how, three months later in the follow-up comment!	Jingdong prefecture level city in Shandong
Jinfeng Hongyuan Flagship Store	Power Saving Specialist 1	The item was received, it is quite good, recommended to buy.	Jingdong prefecture level city in Shandong
Jinfeng Hongyuan Flagship Store	Power Saving Specialist 1	This energy saver works very well and I recommend everyone to buy it!	Jingdong prefecture level city in Shandong
Jinfeng Hongyuan Flagship Store	Power Saving Specialist 1	Voltage stabilization and current reduction nice power saving product.	Jingdong prefecture level city in Shandong
Jinfeng Hongyuan Flagship Store	Power Saving Specialist 1	Voltage stabilization and current reduction nice power saving product.	Jingdong prefecture level city in Shandong
Jinfeng Hongyuan Flagship Store	Power Saving Specialist 1	A good power saver for stabilizing and lowering current.	Jingdong prefecture level city in Shandong
Jinfeng Hongyuan Flagship Store	Power Saving Specialist 1	You'll know if it saves power after you use it.	Jingdong prefecture level city in Shandong
Jinfeng Hongyuan Flagship Store	Power Saving Specialist 1	The quality is great. The quality is very good. The service is very good. Express delivery arrived in two days.	Jingdong prefecture level city in Shandong
Jinfeng Hongyuan Flagship Store	Power Saving Specialist 1	It works great.	Jingdong prefecture level city in Shandong
Jinfeng Hongyuan Flagship Store	Power Saving Specialist 1	Logistics is very fast, not yet used, used to comment again.	Jingdong prefecture level city in Shandong
Jinfeng Hongyuan Flagship Store	Power Saving Specialist 1	Logistics is very fast things are also very good, worthy of friends to buy	Jingdong prefecture level city in Shandong
Jinfeng Hongyuan Flagship Store	Power Saving Specialist 1	It works. It really works.	Jingdong prefecture level city in Shandong
Jinfeng Hongyuan Flagship Store	Power Saving Specialist 1	LANGUAGE COMMENTS: just received the shipment. I immediately plugged it in and used it. If you ask how effective. It depends on whether the next month's electricity bill has been reduced. If there is a decrease. I am buying more than one to use.	Jingdong prefecture level city in Shandong
Jinfeng Hongyuan Flagship Store	Power Saving Specialist 1	Well made, power saving, good and convenient!	Jingdong prefecture level city in Shandong
Jinfeng Hongyuan Flagship Store	Power Saving Specialist 1	I'm using it. I'll rate it next month.	Jingdong prefecture level city in Shandong
Jinfeng Hongyuan Flagship Store	Power Saving Specialist 1	Baby received Oh, the courier delivery really fast, with a good and then come back to the evaluation	Jingdong prefecture level city in Shandong
Jinfeng Hongyuan Flagship Store	Power Saving Specialist 1	Fast logistics, good packaging, worth it!	Jingdong prefecture level city in Shandong
Jinfeng Hongyuan Flagship Store	Power Saving Specialist 1	Customer service is okay. It's just that.	Jingdong prefecture level city in Shandong
Jinfeng Hongyuan Flagship Store	Power Saving Specialist 1	Poor power saving	Jingdong prefecture level city in Shandong
Jinfeng Hongyuan Flagship Store	Power Saving Specialist 1	I'll tell you when I've used it.	Jingdong prefecture level city in Shandong
Jinfeng Hongyuan Flagship Store	Power Saving Specialist 1	I think it's good.	Jingdong prefecture level city in Shandong
Jinfeng Hongyuan Flagship Store	Power Saving Specialist 1	This energy saver works very well and I recommend everyone to buy it!	Jingdong prefecture level city in Shandong
Jinfeng Hongyuan Flagship Store	Power Saving Specialist 1	I do not know how effective, just use half a month on the burn out, good business reissued a, and then use it after the follow-up comment.	Jingdong prefecture level city in Shandong
Jinfeng Hongyuan Flagship Store	Power Saving Specialist 1	in use	Jingdong prefecture level city in Shandong
Jinfeng Hongyuan Flagship Store	Power Saving Specialist 1	This thing is very poor. It's already only been used for ten days and it's broken. Now waiting for you to return it.	Jingdong prefecture level city in Shandong
Jinfeng Hongyuan Flagship Store	Power Saving Specialist 1	It's not the same as the seller's show.	Jingdong prefecture level city in Shandong
Jinfeng Hongyuan Flagship Store	Power Saving Specialist 1	What is the problem when I get it back from the CTS, it is plugged into the wall and the voltage shows 206v.	Jingdong prefecture level city in Shandong
Jinfeng Hongyuan Flagship Store	Power Saving Specialist 1	The logistics was fast and the stuff was perfectly packaged. It is in the process of trial.	Jingdong prefecture level city in Shandong
Jinfeng Hongyuan Flagship Store	Power Saving Specialist 1	This saver is particularly good to use can really save electricity, with two months and then look at the line if you buy one for the family	Jingdong prefecture level city in Shandong

Jinfeng Hongyuan Flagship Store	Power Saving Specialist 1	Novel appearance design, all aluminum alloy profile, also with a fuse device, voltage monitoring display. After receiving the goods, I directly cut the plug and connect it to the main power box under the leakage switch, hoping to save electricity, and then follow up later. Logistics is very fast, arrived the next	Jingdong prefecture level city in Shandong
Jinfeng Hongyuan Flagship Store	Power Saving Specialist 1	Received, very good and like, if necessary next time to buy ha ha	Jingdong prefecture level city in Shandong
Jinfeng Hongyuan Flagship Store	Power Saving Specialist 1	It's good. It's worth it. Get it if you need it.	Jingdong prefecture level city in Shandong
Jinfeng Hongyuan Flagship Store	Power Saving Specialist 1	It feels more than good and is worth having.	Jingdong prefecture level city in Shandong
Jinfeng Hongyuan Flagship Store	Power Saving Specialist 1	The goods arrived very good and fast first time to buy	Jingdong prefecture level city in Shandong
Jinfeng Hongyuan Flagship Store	Power Saving Specialist 1	The power saving is very good, I can save so much money every month, I will buy another one next time.	Jingdong prefecture level city in Shandong
Jinfeng Hongyuan Flagship Store	Power Saving Specialist 1	It's not what it says.	Jingdong prefecture level city in Shandong
Jinfeng Hongyuan Flagship Store	Power Saving Specialist 1	I have been using it for many days now and it saves electricity, it really does I have an 8 kW electric snail stove and it saves about $3.50 a day.	Jingdong prefecture level city in Shandong
Jinfeng Hongyuan Flagship Store	Power Saving Specialist 1	Express delivery is very fast, the quality is also quite good, worth buying!	Jingdong prefecture level city in Shandong
Jinfeng Hongyuan Flagship Store	Power Saving Specialist 1	Just received expecting the baby to work well.	Jingdong prefecture level city in Shandong
Jinfeng Hongyuan Flagship Store	Power Saving Specialist 1	Very good, there are more fruit, has bought a second!	Jingdong prefecture level city in Shandong
Jinfeng Hongyuan Flagship Store	Power Saving Specialist 1	Good is good So far, no power savings have been noticed Maybe it's too little to notice.	Jingdong prefecture level city in Shandong
Jinfeng Hongyuan Flagship Store	Power Saving Specialist 1	I just received the item and plugged it in to see what happens later.	Jingdong prefecture level city in Shandong
Jinfeng Hongyuan Flagship Store	Power Saving Specialist 1	Baby received, especially good, packaging is also good, the courier brother is very strong, thank you.	Jingdong prefecture level city in Shandong
Jinfeng Hongyuan Flagship Store	Power Saving Specialist 1	Just received the goods have not yet used it, do not know how, three months later in the follow-up comment!	Jingdong prefecture level city in Shandong
Jinfeng Hongyuan Flagship Store	Power Saving Specialist 1	The item was received, it is quite good, recommended to buy.	Jingdong prefecture level city in Shandong
Jinfeng Hongyuan Flagship Store	Power Saving Specialist 1	This energy saver works very well and I recommend everyone to buy it!	Jingdong prefecture level city in Shandong
Jinfeng Hongyuan Flagship Store	Power Saving Specialist 1	Voltage stabilization and current reduction nice power saving product.	Jingdong prefecture level city in Shandong
Jinfeng Hongyuan Flagship Store	Power Saving Specialist 1	Voltage stabilization and current reduction nice power saving product.	Jingdong prefecture level city in Shandong
Jinfeng Hongyuan Flagship Store	Power Saving Specialist 1	A good power saver for stabilizing and lowering current.	Jingdong prefecture level city in Shandong
Jinfeng Hongyuan Flagship Store	Power Saving Specialist 1	You'll know if it saves power after you use it.	Jingdong prefecture level city in Shandong
Jinfeng Hongyuan Flagship Store	Power Saving Specialist 1	The quality is great. The quality is very good. The service is very good. Express delivery arrived in two days.	Jingdong prefecture level city in Shandong
Jinfeng Hongyuan Flagship Store	Power Saving Specialist 1	It works great.	Jingdong prefecture level city in Shandong
Jinfeng Hongyuan Flagship Store	Power Saving Specialist 1	Logistics is very fast, not yet used, used to comment again.	Jingdong prefecture level city in Shandong
Jinfeng Hongyuan Flagship Store	Power Saving Specialist 1	Logistics is very fast things are also very good, worthy of friends to buy	Jingdong prefecture level city in Shandong
Jinfeng Hongyuan Flagship Store	Power Saving Specialist 1	It works. It really works.	Jingdong prefecture level city in Shandong
Jinfeng Hongyuan Flagship Store	Power Saving Specialist 1	LANGUAGE COMMENTS: just received the shipment. I immediately plugged it in and used it. If you ask how effective. It depends on whether the next month's electricity bill has been reduced. If there is a decrease. I am buying more than one to use.	Jingdong prefecture level city in Shandong
Jinfeng Hongyuan Flagship Store	Power Saving Specialist 1	Well made, power saving, good and convenient!	Jingdong prefecture level city in Shandong
Jinfeng Hongyuan Flagship Store	Power Saving Specialist 1	I'm using it. I'll rate it next month.	Jingdong prefecture level city in Shandong
Jinfeng Hongyuan Flagship Store	Power Saving Specialist 1	Baby received Oh, the courier delivery really fast, with a good and then come back to the evaluation	Jingdong prefecture level city in Shandong
Jinfeng Hongyuan Flagship Store	Power Saving Specialist 1	Fast logistics, good packaging, worth it!	Jingdong prefecture level city in Shandong
Jinfeng Hongyuan Flagship Store	Power Saving Specialist 1	Customer service is okay. It's just that.	Jingdong prefecture level city in Shandong
Jinfeng Hongyuan Flagship Store	Power Saving Specialist 1	Poor power saving	Jingdong prefecture level city in Shandong
Jinfeng Hongyuan Flagship Store	Power Saving Specialist 1	I'll tell you when I've used it.	Jingdong prefecture level city in Shandong
Jinfeng Hongyuan Flagship Store	Power Saving Specialist 1	I think it's good.	Jingdong prefecture level city in Shandong
Jinfeng Hongyuan Flagship Store	Power Saving Specialist 1	This energy saver works very well and I recommend everyone to buy it!	Jingdong prefecture level city in Shandong
Jinfeng Hongyuan Flagship Store	Power Saving Specialist 1	I do not know how effective, just use half a month on the burn out, good business reissued a, and then use it after the follow-up comment.	Jingdong prefecture level city in Shandong
Jinfeng Hongyuan Flagship Store	Power Saving Specialist 1	in use	Jingdong prefecture level city in Shandong
Jinfeng Hongyuan Flagship Store	Power Saving Specialist 1	This thing is very poor. It's already only been used for ten days and it's broken. Now waiting for you to return it.	Jingdong prefecture level city in Shandong
Jinfeng Hongyuan Flagship Store	Power Saving Specialist 1	It's not the same as the seller's show.	Jingdong prefecture level city in Shandong
Jinfeng Hongyuan Flagship Store	Power Saving Specialist 1	What is the problem when I get it back from the CTS, it is plugged into the wall and the voltage shows 206v.	Jingdong prefecture level city in Shandong
Jinfeng Hongyuan Flagship Store	Power Saving Specialist 1	The logistics was fast and the stuff was perfectly packaged. It is in the process of trial.	Jingdong prefecture level city in Shandong
Jinfeng Hongyuan Flagship Store	Power Saving Specialist 1	This saver is particularly good to use can really save electricity, with two months and then look at the line if you buy one for the family	Jingdong prefecture level city in Shandong
Jinfeng Hongyuan Flagship Store	Power Saving Specialist 1	Novel appearance design, all aluminum alloy profile, also with a fuse device, voltage monitoring display. After receiving the goods, I directly cut the plug and connect it to the main power box under the leakage switch, hoping to save electricity, and then follow up later. Logistics is very fast, arrived the next	Jingdong prefecture level city in Shandong
Jinfeng Hongyuan Flagship Store	Power Saving Specialist 1	Received, very good and like, if necessary next time to buy ha ha	Jingdong prefecture level city in Shandong
Jinfeng Hongyuan Flagship Store	Power Saving Specialist 1	It's good. It's worth it. Get it if you need it.	Jingdong prefecture level city in Shandong
Jinfeng Hongyuan Flagship Store	Power Saving Specialist 1	It feels more than good and is worth having.	Jingdong prefecture level city in Shandong
Jinfeng Hongyuan Flagship Store	Power Saving Specialist 1	The goods arrived very good and fast first time to buy	Jingdong prefecture level city in Shandong
Jinfeng Hongyuan Flagship Store	Power Saving Specialist 1	The power saving is very good, I can save so much money every month, I will buy another one next time.	Jingdong prefecture level city in Shandong
Jinfeng Hongyuan Flagship Store	Power Saving Specialist 1	It's not what it says.	Jingdong prefecture level city in Shandong

Jinfeng Hongyuan Flagship Store	Power Saving Specialist 1	I have been using it for many days now and it saves electricity, it really does I have an 8 kW electric snail stove and it saves about $3.50 a day.	Jingdong prefecture level city in Shandong
Jinfeng Hongyuan Flagship Store	Power Saving Specialist 1	Express delivery is very fast, the quality is also quite good, worth buying!	Jingdong prefecture level city in Shandong
Jinfeng Hongyuan Flagship Store	Power Saving Specialist 1	Just received expecting the baby to work well.	Jingdong prefecture level city in Shandong
Jinfeng Hongyuan Flagship Store	Power Saving Specialist 1	Very good, there are more fruit, has bought a second!	Jingdong prefecture level city in Shandong
Jinfeng Hongyuan Flagship Store	Power Saving Specialist 1	Good is good So far, no power savings have been noticed Maybe it's too little to notice.	Jingdong prefecture level city in Shandong
Jinfeng Hongyuan Flagship Store	Power Saving Specialist 1	I just received the item and plugged it in to see what happens later.	Jingdong prefecture level city in Shandong
Jinfeng Hongyuan Flagship Store	Power Saving Specialist 1	Baby received, especially good, packaging is also good, the courier brother is very strong, thank you.	Jingdong prefecture level city in Shandong
Jinfeng Hongyuan Flagship Store	Power Saving Specialist 1	Just received the goods have not yet used it, do not know how, three months later in the follow-up comment!	Jingdong prefecture level city in Shandong
Jinfeng Hongyuan Flagship Store	Power Saving Specialist 1	The item was received, it is quite good, recommended to buy.	Jingdong prefecture level city in Shandong
Jinfeng Hongyuan Flagship Store	Power Saving Specialist 1	This energy saver works very well and I recommend everyone to buy it!	Jingdong prefecture level city in Shandong
Jinfeng Hongyuan Flagship Store	Power Saving Specialist 1	Voltage stabilization and current reduction nice power saving product.	Jingdong prefecture level city in Shandong
Jinfeng Hongyuan Flagship Store	Power Saving Specialist 1	Voltage stabilization and current reduction nice power saving product.	Jingdong prefecture level city in Shandong
Jinfeng Hongyuan Flagship Store	Power Saving Specialist 1	A good power saver for stabilizing and lowering current.	Jingdong prefecture level city in Shandong
Jinfeng Hongyuan Flagship Store	Power Saving Specialist 1	You'll know if it saves power after you use it.	Jingdong prefecture level city in Shandong
Jinfeng Hongyuan Flagship Store	Power Saving Specialist 1	The quality is great. The quality is very good. The service is very good. Express delivery arrived in two days.	Jingdong prefecture level city in Shandong
Jinfeng Hongyuan Flagship Store	Power Saving Specialist 1	It works great.	Jingdong prefecture level city in Shandong
Jinfeng Hongyuan Flagship Store	Power Saving Specialist 1	Logistics is very fast, not yet used, used to comment again.	Jingdong prefecture level city in Shandong
Jinfeng Hongyuan Flagship Store	Power Saving Specialist 1	Logistics is very fast things are also very good, worthy of friends to buy	Jingdong prefecture level city in Shandong
Jinfeng Hongyuan Flagship Store	Power Saving Specialist 1	It works. It really works.	Jingdong prefecture level city in Shandong
Jinfeng Hongyuan Flagship Store	Power Saving Specialist 1	LANGUAGE COMMENTS: just received the shipment. I immediately plugged it in and used it. If you ask how effective. It depends on whether the next month's electricity bill has been reduced. If there is a decrease. I am buying more than one to use.	Jingdong prefecture level city in Shandong
Jinfeng Hongyuan Flagship Store	Power Saving Specialist 1	Well made, power saving, good and convenient!	Jingdong prefecture level city in Shandong
Jinfeng Hongyuan Flagship Store	Power Saving Specialist 1	I'm using it. I'll rate it next month.	Jingdong prefecture level city in Shandong
Jinfeng Hongyuan Flagship Store	Power Saving Specialist 1	Baby received Oh, the courier delivery really fast, with a good and then come back to the evaluation	Jingdong prefecture level city in Shandong
Jinfeng Hongyuan Flagship Store	Power Saving Specialist 1	Fast logistics, good packaging, worth it!	Jingdong prefecture level city in Shandong
Jinfeng Hongyuan Flagship Store	Power Saving Specialist 1	Customer service is okay. It's just that.	Jingdong prefecture level city in Shandong
Jinfeng Hongyuan Flagship Store	Power Saving Specialist 1	Poor power saving	Jingdong prefecture level city in Shandong
Jinfeng Hongyuan Flagship Store	Power Saving Specialist 1	I'll tell you when I've used it.	Jingdong prefecture level city in Shandong
Jinfeng Hongyuan Flagship Store	Power Saving Specialist 1	I think it's good.	Jingdong prefecture level city in Shandong
Jinfeng Hongyuan Flagship Store	Power Saving Specialist 1	This energy saver works very well and I recommend everyone to buy it!	Jingdong prefecture level city in Shandong
Jinfeng Hongyuan Flagship Store	Power Saving Specialist 1	I do not know how effective, just use half a month on the burn out, good business reissued a, and then use it after the follow-up comment.	Jingdong prefecture level city in Shandong
Jinfeng Hongyuan Flagship Store	Power Saving Specialist 1	in use	Jingdong prefecture level city in Shandong
Jinfeng Hongyuan Flagship Store	Power Saving Specialist 1	This thing is very poor. It's already only been used for ten days and it's broken. Now waiting for you to return it.	Jingdong prefecture level city in Shandong
Jinfeng Hongyuan Flagship Store	Power Saving Specialist 1	It's not the same as the seller's show.	Jingdong prefecture level city in Shandong
Jinfeng Hongyuan Flagship Store	Power Saving Specialist 1	What is the problem when I get it back from the CTS, it is plugged into the wall and the voltage shows 206v.	Jingdong prefecture level city in Shandong
Jinfeng Hongyuan Flagship Store	Power Saving Specialist 1	The logistics was fast and the stuff was perfectly packaged. It is in the process of trial.	Jingdong prefecture level city in Shandong
Jinfeng Hongyuan Flagship Store	Power Saving Specialist 1	This saver is particularly good to use can really save electricity, with two months and then look at the line if you buy one for the family	Jingdong prefecture level city in Shandong
Jinfeng Hongyuan Flagship Store	Power Saving Specialist 1	Novel appearance design, all aluminum alloy profile, also with a fuse device, voltage monitoring display. After receiving the goods, I directly cut the plug and connect it to the main power box under the leakage switch, hoping to save electricity, and then follow up later. Logistics is very fast, arrived the next	Jingdong prefecture level city in Shandong
Jinfeng Hongyuan Flagship Store	Power Saving Specialist 1	Received, very good and like, if necessary next time to buy ha ha	Jingdong prefecture level city in Shandong
Jinfeng Hongyuan Flagship Store	Power Saving Specialist 1	It's good. It's worth it. Get it if you need it.	Jingdong prefecture level city in Shandong
Jinfeng Hongyuan Flagship Store	Power Saving Specialist 1	It feels more than good and is worth having.	Jingdong prefecture level city in Shandong
Jinfeng Hongyuan Flagship Store	Power Saving Specialist 1	The goods arrived very good and fast first time to buy	Jingdong prefecture level city in Shandong
Jinfeng Hongyuan Flagship Store	Power Saving Specialist 1	The power saving is very good, I can save so much money every month, I will buy another one next time.	Jingdong prefecture level city in Shandong
Jinfeng Hongyuan Flagship Store	Power Saving Specialist 1	It's not what it says.	Jingdong prefecture level city in Shandong
Jinfeng Hongyuan Flagship Store	Power Saving Specialist 1	I have been using it for many days now and it saves electricity, it really does I have an 8 kW electric snail stove and it saves about $3.50 a day.	Jingdong prefecture level city in Shandong
Jinfeng Hongyuan Flagship Store	Power Saving Specialist 1	Express delivery is very fast, the quality is also quite good, worth buying!	Jingdong prefecture level city in Shandong
Jinfeng Hongyuan Flagship Store	Power Saving Specialist 1	Just received expecting the baby to work well.	Jingdong prefecture level city in Shandong
Jinfeng Hongyuan Flagship Store	Power Saving Specialist 1	Very good, there are more fruit, has bought a second!	Jingdong prefecture level city in Shandong
Jinfeng Hongyuan Flagship Store	Power Saving Specialist 1	Good is good So far, no power savings have been noticed Maybe it's too little to notice.	Jingdong prefecture level city in Shandong
Jinfeng Hongyuan Flagship Store	Power Saving Specialist 1	I just received the item and plugged it in to see what happens later.	Jingdong prefecture level city in Shandong
Jinfeng Hongyuan Flagship Store	Power Saving Specialist 1	Baby received, especially good, packaging is also good, the courier brother is very strong, thank you.	Jingdong prefecture level city in Shandong

Jinfeng Hongyuan Flagship Store	Power Saving Specialist 1	Just received the goods have not yet used it, do not know how, three months later in the follow-up comment!	Jingdong prefecture level city in Shandong
Jinfeng Hongyuan Flagship Store	Power Saving Specialist 1	The item was received, it is quite good, recommended to buy.	Jingdong prefecture level city in Shandong
Jinfeng Hongyuan Flagship Store	Power Saving Specialist 1	This energy saver works very well and I recommend everyone to buy it!	Jingdong prefecture level city in Shandong
Jinfeng Hongyuan Flagship Store	Power Saving Specialist 1	Voltage stabilization and current reduction nice power saving product.	Jingdong prefecture level city in Shandong
Jinfeng Hongyuan Flagship Store	Power Saving Specialist 1	Voltage stabilization and current reduction nice power saving product.	Jingdong prefecture level city in Shandong
Jinfeng Hongyuan Flagship Store	Power Saving Specialist 1	A good power saver for stabilizing and lowering current.	Jingdong prefecture level city in Shandong
Jinfeng Hongyuan Flagship Store	Power Saving Specialist 1	You'll know if it saves power after you use it.	Jingdong prefecture level city in Shandong
Jinfeng Hongyuan Flagship Store	Power Saving Specialist 1	The quality is great. The quality is very good. The service is very good. Express delivery arrived in two days.	Jingdong prefecture level city in Shandong
Jinfeng Hongyuan Flagship Store	Power Saving Specialist 1	It works great.	Jingdong prefecture level city in Shandong
Jinfeng Hongyuan Flagship Store	Power Saving Specialist 1	Logistics is very fast, not yet used, used to comment again.	Jingdong prefecture level city in Shandong
Jinfeng Hongyuan Flagship Store	Power Saving Specialist 1	Logistics is very fast things are also very good, worthy of friends to buy	Jingdong prefecture level city in Shandong
Jinfeng Hongyuan Flagship Store	Power Saving Specialist 1	It works. It really works.	Jingdong prefecture level city in Shandong
Jinfeng Hongyuan Flagship Store	Power Saving Specialist 1	LANGUAGE COMMENTS: just received the shipment. I immediately plugged it in and used it. If you ask how effective. It depends on whether the next month's electricity bill has been reduced. If there is a decrease. I am buying more than one to use.	Jingdong prefecture level city in Shandong
Jinfeng Hongyuan Flagship Store	Power Saving Specialist 1	Well made, power saving, good and convenient!	Jingdong prefecture level city in Shandong
Jinfeng Hongyuan Flagship Store	Power Saving Specialist 1	I'm using it. I'll rate it next month.	Jingdong prefecture level city in Shandong
Jinfeng Hongyuan Flagship Store	Power Saving Specialist 1	Baby received Oh, the courier delivery really fast, with a good and then come back to the evaluation	Jingdong prefecture level city in Shandong
Jinfeng Hongyuan Flagship Store	Power Saving Specialist 1	Fast logistics, good packaging, worth it!	Jingdong prefecture level city in Shandong
Jinfeng Hongyuan Flagship Store	Power Saving Specialist 1	Customer service is okay. It's just that.	Jingdong prefecture level city in Shandong
Jinfeng Hongyuan Flagship Store	Power Saving Specialist 1	Poor power saving	Jingdong prefecture level city in Shandong
Jinfeng Hongyuan Flagship Store	Power Saving Specialist 1	I'll tell you when I've used it.	Jingdong prefecture level city in Shandong
Jinfeng Hongyuan Flagship Store	Power Saving Specialist 1	I think it's good.	Jingdong prefecture level city in Shandong
Jinfeng Hongyuan Flagship Store	Power Saving Specialist 1	This energy saver works very well and I recommend everyone to buy it!	Jingdong prefecture level city in Shandong
Jinfeng Hongyuan Flagship Store	Power Saving Specialist 1	I do not know how effective, just use half a month on the burn out, good business reissued a, and then use it after the follow-up comment.	Jingdong prefecture level city in Shandong
Jinfeng Hongyuan Flagship Store	Power Saving Specialist 1	in use	Jingdong prefecture level city in Shandong
Jinfeng Hongyuan Flagship Store	Power Saving Specialist 1	This thing is very poor. It's already only been used for ten days and it's broken. Now waiting for you to return it.	Jingdong prefecture level city in Shandong
Jinfeng Hongyuan Flagship Store	Power Saving Specialist 1	It's not the same as the seller's show.	Jingdong prefecture level city in Shandong
Jinfeng Hongyuan Flagship Store	Power Saving Specialist 1	What is the problem when I get it back from the CTS, it is plugged into the wall and the voltage shows 206v.	Jingdong prefecture level city in Shandong
Jinfeng Hongyuan Flagship Store	Power Saving Specialist 1	The logistics was fast and the stuff was perfectly packaged. It is in the process of trial.	Jingdong prefecture level city in Shandong
Jinfeng Hongyuan Flagship Store	Power Saving Specialist 1	This saver is particularly good to use can really save electricity, with two months and then look at the line if you buy one for the family	Jingdong prefecture level city in Shandong
Jinfeng Hongyuan Flagship Store	Power Saving Specialist 1	Novel appearance design, all aluminum alloy profile, also with a fuse device, voltage monitoring display. After receiving the goods, I directly cut the plug and connect it to the main power box under the leakage switch, hoping to save electricity, and then follow up later. Logistics is very fast, arrived the next	Jingdong prefecture level city in Shandong
Jinfeng Hongyuan Flagship Store	Power Saving Specialist 1	Received, very good and like, if necessary next time to buy ha ha	Jingdong prefecture level city in Shandong
Jinfeng Hongyuan Flagship Store	Power Saving Specialist 1	It's good. It's worth it. Get it if you need it.	Jingdong prefecture level city in Shandong
Jinfeng Hongyuan Flagship Store	Power Saving Specialist 1	It feels more than good and is worth having.	Jingdong prefecture level city in Shandong
Jinfeng Hongyuan Flagship Store	Power Saving Specialist 1	The goods arrived very good and fast first time to buy	Jingdong prefecture level city in Shandong
Jinfeng Hongyuan Flagship Store	Power Saving Specialist 1	The power saving is very good, I can save so much money every month, I will buy another one next time.	Jingdong prefecture level city in Shandong
Jinfeng Hongyuan Flagship Store	Power Saving Specialist 1	It's not what it says.	Jingdong prefecture level city in Shandong
Jinfeng Hongyuan Flagship Store	Power Saving Specialist 1	I have been using it for many days now and it saves electricity, it really does I have an 8 kW electric snail stove and it saves about $3.50 a day.	Jingdong prefecture level city in Shandong
Jinfeng Hongyuan Flagship Store	Power Saving Specialist 1	Express delivery is very fast, the quality is also quite good, worth buying!	Jingdong prefecture level city in Shandong
Jinfeng Hongyuan Flagship Store	Power Saving Specialist 1	Just received expecting the baby to work well.	Jingdong prefecture level city in Shandong
Jinfeng Hongyuan Flagship Store	Power Saving Specialist 1	Very good, there are more fruit, has bought a second!	Jingdong prefecture level city in Shandong
Jinfeng Hongyuan Flagship Store	Power Saving Specialist 1	Good is good So far, no power savings have been noticed Maybe it's too little to notice.	Jingdong prefecture level city in Shandong
Jinfeng Hongyuan Flagship Store	Power Saving Specialist 1	I just received the item and plugged it in to see what happens later.	Jingdong prefecture level city in Shandong
Jinfeng Hongyuan Flagship Store	Power Saving Specialist 1	Baby received, especially good, packaging is also good, the courier brother is very strong, thank you.	Jingdong prefecture level city in Shandong
Jinfeng Hongyuan Flagship Store	Power Saving Specialist 1	Just received the goods have not yet used it, do not know how, three months later in the follow-up comment!	Jingdong prefecture level city in Shandong
Jinfeng Hongyuan Flagship Store	Power Saving Specialist 1	The item was received, it is quite good, recommended to buy.	Jingdong prefecture level city in Shandong
Jinfeng Hongyuan Flagship Store	Power Saving Specialist 1	This energy saver works very well and I recommend everyone to buy it!	Jingdong prefecture level city in Shandong
Jinfeng Hongyuan Flagship Store	Power Saving Specialist 1	Voltage stabilization and current reduction nice power saving product.	Jingdong prefecture level city in Shandong
Jinfeng Hongyuan Flagship Store	Power Saving Specialist 1	Voltage stabilization and current reduction nice power saving product.	Jingdong prefecture level city in Shandong
Jinfeng Hongyuan Flagship Store	Power Saving Specialist 1	A good power saver for stabilizing and lowering current.	Jingdong prefecture level city in Shandong
Jinfeng Hongyuan Flagship Store	Power Saving Specialist 1	You'll know if it saves power after you use it.	Jingdong prefecture level city in Shandong

Jinfeng Hongyuan Flagship Store	Power Saving Specialist 1	The quality is great. The quality is very good. The service is very good. Express delivery arrived in two days.	Jingdong prefecture level city in Shandong
Jinfeng Hongyuan Flagship Store	Power Saving Specialist 1	It works great.	Jingdong prefecture level city in Shandong
Jinfeng Hongyuan Flagship Store	Power Saving Specialist 1	Logistics is very fast, not yet used, used to comment again.	Jingdong prefecture level city in Shandong
Jinfeng Hongyuan Flagship Store	Power Saving Specialist 1	Logistics is very fast things are also very good, worthy of friends to buy	Jingdong prefecture level city in Shandong
Jinfeng Hongyuan Flagship Store	Power Saving Specialist 1	It works. It really works.	Jingdong prefecture level city in Shandong
Jinfeng Hongyuan Flagship Store	Power Saving Specialist 1	LANGUAGE COMMENTS: just received the shipment. I immediately plugged it in and used it. If you ask how effective. It depends on whether the next month's electricity bill has been reduced. If there is a decrease. I am buying more than one to use.	Jingdong prefecture level city in Shandong
Jinfeng Hongyuan Flagship Store	Power Saving Specialist 1	Well made, power saving, good and convenient!	Jingdong prefecture level city in Shandong
Jinfeng Hongyuan Flagship Store	Power Saving Specialist 1	I'm using it. I'll rate it next month.	Jingdong prefecture level city in Shandong
Jinfeng Hongyuan Flagship Store	Power Saving Specialist 1	Baby received Oh, the courier delivery really fast, with a good and then come back to the evaluation	Jingdong prefecture level city in Shandong
Jinfeng Hongyuan Flagship Store	Power Saving Specialist 1	Fast logistics, good packaging, worth it!	Jingdong prefecture level city in Shandong
Jinfeng Hongyuan Flagship Store	Power Saving Specialist 1	Customer service is okay. It's just that.	Jingdong prefecture level city in Shandong
Jinfeng Hongyuan Flagship Store	Power Saving Specialist 1	Poor power saving	Jingdong prefecture level city in Shandong
Jinfeng Hongyuan Flagship Store	Power Saving Specialist 1	I'll tell you when I've used it.	Jingdong prefecture level city in Shandong
Jinfeng Hongyuan Flagship Store	Power Saving Specialist 1	I think it's good.	Jingdong prefecture level city in Shandong
Jinfeng Hongyuan Flagship Store	Power Saving Specialist 1	This energy saver works very well and I recommend everyone to buy it!	Jingdong prefecture level city in Shandong
Jinfeng Hongyuan Flagship Store	Power Saving Specialist 1	I do not know how effective, just use half a month on the burn out, good business reissued a, and then use it after the follow-up comment.	Jingdong prefecture level city in Shandong
Jinfeng Hongyuan Flagship Store	Power Saving Specialist 1	in use	Jingdong prefecture level city in Shandong
Jinfeng Hongyuan Flagship Store	Power Saving Specialist 1	This thing is very poor. It's already only been used for ten days and it's broken. Now waiting for you to return it.	Jingdong prefecture level city in Shandong
Jinfeng Hongyuan Flagship Store	Power Saving Specialist 1	It's not the same as the seller's show.	Jingdong prefecture level city in Shandong
Jinfeng Hongyuan Flagship Store	Power Saving Specialist 1	What is the problem when I get it back from the CTS, it is plugged into the wall and the voltage shows 206v.	Jingdong prefecture level city in Shandong
Jinfeng Hongyuan Flagship Store	Power Saving Specialist 1	The logistics was fast and the stuff was perfectly packaged. It is in the process of trial.	Jingdong prefecture level city in Shandong
Jinfeng Hongyuan Flagship Store	Power Saving Specialist 1	This saver is particularly good to use can really save electricity, with two months and then look at the line if you buy one for the family	Jingdong prefecture level city in Shandong
Jinfeng Hongyuan Flagship Store	Power Saving Specialist 1	Novel appearance design, all aluminum alloy profile, also with a fuse device, voltage monitoring display. After receiving the goods, I directly cut the plug and connect it to the main power box under the leakage switch, hoping to save electricity, and then follow up later. Logistics is very fast, arrived the next	Jingdong prefecture level city in Shandong
Jinfeng Hongyuan Flagship Store	Power Saving Specialist 1	Received, very good and like, if necessary next time to buy ha ha	Jingdong prefecture level city in Shandong
Jinfeng Hongyuan Flagship Store	Power Saving Specialist 1	It's good. It's worth it. Get it if you need it.	Jingdong prefecture level city in Shandong
Jinfeng Hongyuan Flagship Store	Power Saving Specialist 1	It feels more than good and is worth having.	Jingdong prefecture level city in Shandong
Jinfeng Hongyuan Flagship Store	Power Saving Specialist 1	The goods arrived very good and fast first time to buy	Jingdong prefecture level city in Shandong
Jinfeng Hongyuan Flagship Store	Power Saving Specialist 1	The power saving is very good, I can save so much money every month, I will buy another one next time.	Jingdong prefecture level city in Shandong
Jinfeng Hongyuan Flagship Store	Power Saving Specialist 1	It's not what it says.	Jingdong prefecture level city in Shandong
Jinfeng Hongyuan Flagship Store	Power Saving Specialist 1	I have been using it for many days now and it saves electricity, it really does I have an 8 kW electric snail stove and it saves about $3.50 a day.	Jingdong prefecture level city in Shandong
Jinfeng Hongyuan Flagship Store	Power Saving Specialist 1	Express delivery is very fast, the quality is also quite good, worth buying!	Jingdong prefecture level city in Shandong
Jinfeng Hongyuan Flagship Store	Power Saving Specialist 1	Just received expecting the baby to work well.	Jingdong prefecture level city in Shandong
Jinfeng Hongyuan Flagship Store	Power Saving Specialist 1	Very good, there are more fruit, has bought a second!	Jingdong prefecture level city in Shandong
Jinfeng Hongyuan Flagship Store	Power Saving Specialist 1	Good is good So far, no power savings have been noticed Maybe it's too little to notice.	Jingdong prefecture level city in Shandong
Jinfeng Hongyuan Flagship Store	Power Saving Specialist 1	I just received the item and plugged it in to see what happens later.	Jingdong prefecture level city in Shandong
Jinfeng Hongyuan Flagship Store	Power Saving Specialist 1	Baby received, especially good, packaging is also good, the courier brother is very strong, thank you.	Jingdong prefecture level city in Shandong
Jinfeng Hongyuan Flagship Store	Power Saving Specialist 1	Just received the goods have not yet used it, do not know how, three months later in the follow-up comment!	Jingdong prefecture level city in Shandong
Jinfeng Hongyuan Flagship Store	Power Saving Specialist 1	The item was received, it is quite good, recommended to buy.	Jingdong prefecture level city in Shandong
Jinfeng Hongyuan Flagship Store	Power Saving Specialist 1	This energy saver works very well and I recommend everyone to buy it!	Jingdong prefecture level city in Shandong
Jinfeng Hongyuan Flagship Store	Power Saving Specialist 1	Voltage stabilization and current reduction nice power saving product.	Jingdong prefecture level city in Shandong
Jinfeng Hongyuan Flagship Store	Power Saving Specialist 1	Voltage stabilization and current reduction nice power saving product.	Jingdong prefecture level city in Shandong
Jinfeng Hongyuan Flagship Store	Power Saving Specialist 1	A good power saver for stabilizing and lowering current.	Jingdong prefecture level city in Shandong
Jinfeng Hongyuan Flagship Store	Power Saving Specialist 1	You'll know if it saves power after you use it.	Jingdong prefecture level city in Shandong
Jinfeng Hongyuan Flagship Store	Power Saving Specialist 1	The quality is great. The quality is very good. The service is very good. Express delivery arrived in two days.	Jingdong prefecture level city in Shandong
Jinfeng Hongyuan Flagship Store	Power Saving Specialist 1	It works great.	Jingdong prefecture level city in Shandong
Jinfeng Hongyuan Flagship Store	Power Saving Specialist 1	Logistics is very fast, not yet used, used to comment again.	Jingdong prefecture level city in Shandong
Jinfeng Hongyuan Flagship Store	Power Saving Specialist 1	Logistics is very fast things are also very good, worthy of friends to buy	Jingdong prefecture level city in Shandong
Jinfeng Hongyuan Flagship Store	Power Saving Specialist 1	It works. It really works.	Jingdong prefecture level city in Shandong
Jinfeng Hongyuan Flagship Store	Power Saving Specialist 1	LANGUAGE COMMENTS: just received the shipment. I immediately plugged it in and used it. If you ask how effective. It depends on whether the next month's electricity bill has been reduced. If there is a decrease. I am buying more than one to use.	Jingdong prefecture level city in Shandong
Jinfeng Hongyuan Flagship Store	Power Saving Specialist 1	Well made, power saving, good and convenient!	Jingdong prefecture level city in Shandong

Jinfeng Hongyuan Flagship Store	Power Saving Specialist 1	I’m using it. I’ll rate it next month.	Jingdong prefecture level city in Shandong
Jinfeng Hongyuan Flagship Store	Power Saving Specialist 1	Baby received Oh, the courier delivery really fast, with a good and then come back to the evaluation	Jingdong prefecture level city in Shandong
Jinfeng Hongyuan Flagship Store	Power Saving Specialist 1	Fast logistics, good packaging, worth it!	Jingdong prefecture level city in Shandong
Jinfeng Hongyuan Flagship Store	Power Saving Specialist 1	Customer service is okay. It’s just that.	Jingdong prefecture level city in Shandong
Jinfeng Hongyuan Flagship Store	Power Saving Specialist 1	Poor power saving	Jingdong prefecture level city in Shandong
Jinfeng Hongyuan Flagship Store	Power Saving Specialist 1	I’ll tell you when I’ve used it.	Jingdong prefecture level city in Shandong
Jinfeng Hongyuan Flagship Store	Power Saving Specialist 1	I think it’s good.	Jingdong prefecture level city in Shandong
Jinfeng Hongyuan Flagship Store	Power Saving Specialist 1	This energy saver works very well and I recommend everyone to buy it!	Jingdong prefecture level city in Shandong
Jinfeng Hongyuan Flagship Store	Power Saving Specialist 1	I do not know how effective, just use half a month on the burn out, good business reissued a, and then use it after the follow-up comment.	Jingdong prefecture level city in Shandong
Jinfeng Hongyuan Flagship Store	Power Saving Specialist 1	in use	Jingdong prefecture level city in Shandong
Jinfeng Hongyuan Flagship Store	Power Saving Specialist 1	This thing is very poor. It’s already only been used for ten days and it’s broken. Now waiting for you to return it.	Jingdong prefecture level city in Shandong
Jinfeng Hongyuan Flagship Store	Power Saving Specialist 1	It’s not the same as the seller’s show.	Jingdong prefecture level city in Shandong
Jinfeng Hongyuan Flagship Store	Power Saving Specialist 1	What is the problem when I get it back from the CTS, it is plugged into the wall and the voltage shows 206v.	Jingdong prefecture level city in Shandong
Jinfeng Hongyuan Flagship Store	Power Saving Specialist 1	The logistics was fast and the stuff was perfectly packaged. It is in the process of trial.	Jingdong prefecture level city in Shandong
Jinfeng Hongyuan Flagship Store	Power Saving Specialist 1	This saver is particularly good to use can really save electricity, with two months and then look at the line if you buy one for the family	Jingdong prefecture level city in Shandong
Jinfeng Hongyuan Flagship Store	Power Saving Specialist 1	Novel appearance design, all aluminum alloy profile, also with a fuse device, voltage monitoring display. After receiving the goods, I directly cut the plug and connect it to the main power box under the leakage switch, hoping to save electricity, and then follow up later. Logistics is very fast, arrived the next	Jingdong prefecture level city in Shandong
Jinfeng Hongyuan Flagship Store	Power Saving Specialist 1	Received, very good and like, if necessary next time to buy ha ha	Jingdong prefecture level city in Shandong
Jinfeng Hongyuan Flagship Store	Power Saving Specialist 1	It’s good. It’s worth it. Get it if you need it.	Jingdong prefecture level city in Shandong
Jinfeng Hongyuan Flagship Store	Power Saving Specialist 1	It feels more than good and is worth having.	Jingdong prefecture level city in Shandong
Jinfeng Hongyuan Flagship Store	Power Saving Specialist 1	The goods arrived very good and fast first time to buy	Jingdong prefecture level city in Shandong
Jinfeng Hongyuan Flagship Store	Power Saving Specialist 1	The power saving is very good, I can save so much money every month, I will buy another one next time.	Jingdong prefecture level city in Shandong
Jinfeng Hongyuan Flagship Store	Power Saving Specialist 1	It’s not what it says.	Jingdong prefecture level city in Shandong
Jinfeng Hongyuan Flagship Store	Power Saving Specialist 1	I have been using it for many days now and it saves electricity, it really does I have an 8 kW electric snail stove and it saves about $3.50 a day.	Jingdong prefecture level city in Shandong
Jinfeng Hongyuan Flagship Store	Power Saving Specialist 1	Express delivery is very fast, the quality is also quite good, worth buying!	Jingdong prefecture level city in Shandong
Jinfeng Hongyuan Flagship Store	Power Saving Specialist 1	Just received expecting the baby to work well.	Jingdong prefecture level city in Shandong
Jinfeng Hongyuan Flagship Store	Power Saving Specialist 1	Very good, there are more fruit, has bought a second!	Jingdong prefecture level city in Shandong
Jinfeng Hongyuan Flagship Store	Power Saving Specialist 1	Good is good So far, no power savings have been noticed Maybe it’s too little to notice.	Jingdong prefecture level city in Shandong
Jinfeng Hongyuan Flagship Store	Power Saving Specialist 1	I just received the item and plugged it in to see what happens later.	Jingdong prefecture level city in Shandong
Jinfeng Hongyuan Flagship Store	Power Saving Specialist 1	Baby received, especially good, packaging is also good, the courier brother is very strong, thank you.	Jingdong prefecture level city in Shandong
Jinfeng Hongyuan Flagship Store	Power Saving Specialist 1	Just received the goods have not yet used it, do not know how, three months later in the follow-up comment!	Jingdong prefecture level city in Shandong
Jinfeng Hongyuan Flagship Store	Power Saving Specialist 1	The item was received, it is quite good, recommended to buy.	Jingdong prefecture level city in Shandong
Jinfeng Hongyuan Flagship Store	Power Saving Specialist 1	This energy saver works very well and I recommend everyone to buy it!	Jingdong prefecture level city in Shandong
Jinfeng Hongyuan Flagship Store	Power Saving Specialist 1	Voltage stabilization and current reduction nice power saving product.	Jingdong prefecture level city in Shandong
Jinfeng Hongyuan Flagship Store	Power Saving Specialist 1	Voltage stabilization and current reduction nice power saving product.	Jingdong prefecture level city in Shandong
Jinfeng Hongyuan Flagship Store	Power Saving Specialist 1	A good power saver for stabilizing and lowering current.	Jingdong prefecture level city in Shandong
Jinfeng Hongyuan Flagship Store	Power Saving Specialist 1	You’ll know if it saves power after you use it.	Jingdong prefecture level city in Shandong
Jinfeng Hongyuan Flagship Store	Power Saving Specialist 1	The quality is great. The quality is very good. The service is very good. Express delivery arrived in two days.	Jingdong prefecture level city in Shandong
Jinfeng Hongyuan Flagship Store	Power Saving Specialist 1	It works great.	Jingdong prefecture level city in Shandong
Jinfeng Hongyuan Flagship Store	Power Saving Specialist 1	Logistics is very fast, not yet used, used to comment again.	Jingdong prefecture level city in Shandong
Jinfeng Hongyuan Flagship Store	Power Saving Specialist 1	Logistics is very fast things are also very good, worthy of friends to buy	Jingdong prefecture level city in Shandong
Jinfeng Hongyuan Flagship Store	Power Saving Specialist 1	It works. It really works.	Jingdong prefecture level city in Shandong
Jinfeng Hongyuan Flagship Store	Power Saving Specialist 1	LANGUAGE COMMENTS: just received the shipment. I immediately plugged it in and used it. If you ask how effective. It depends on whether the next month’s electricity bill has been reduced. If there is a decrease. I am buying more than one to use.	Jingdong prefecture level city in Shandong
Jinfeng Hongyuan Flagship Store	Power Saving Specialist 1	Well made, power saving, good and convenient!	Jingdong prefecture level city in Shandong
Jinfeng Hongyuan Flagship Store	Power Saving Specialist 1	I’m using it. I’ll rate it next month.	Jingdong prefecture level city in Shandong
Jinfeng Hongyuan Flagship Store	Power Saving Specialist 1	Baby received Oh, the courier delivery really fast, with a good and then come back to the evaluation	Jingdong prefecture level city in Shandong
Jinfeng Hongyuan Flagship Store	Power Saving Specialist 1	Fast logistics, good packaging, worth it!	Jingdong prefecture level city in Shandong
Jinfeng Hongyuan Flagship Store	Power Saving Specialist 1	Customer service is okay. It’s just that.	Jingdong prefecture level city in Shandong
Jinfeng Hongyuan Flagship Store	Power Saving Specialist 1	Poor power saving	Jingdong prefecture level city in Shandong
Jinfeng Hongyuan Flagship Store	Power Saving Specialist 1	I’ll tell you when I’ve used it.	Jingdong prefecture level city in Shandong
Jinfeng Hongyuan Flagship Store	Power Saving Specialist 1	I think it’s good.	Jingdong prefecture level city in Shandong

Jinfeng Hongyuan Flagship Store	Power Saving Specialist 1	This energy saver works very well and I recommend everyone to buy it!	Jingdong prefecture level city in Shandong
Jinfeng Hongyuan Flagship Store	Power Saving Specialist 1	I do not know how effective, just use half a month on the burn out, good business reissued a, and then use it after the follow-up comment.	Jingdong prefecture level city in Shandong
Jinfeng Hongyuan Flagship Store	Power Saving Specialist 1	in use	Jingdong prefecture level city in Shandong
Jinfeng Hongyuan Flagship Store	Power Saving Specialist 1	This thing is very poor. It's already only been used for ten days and it's broken. Now waiting for you to return it.	Jingdong prefecture level city in Shandong
Jinfeng Hongyuan Flagship Store	Power Saving Specialist 1	It's not the same as the seller's show.	Jingdong prefecture level city in Shandong
Jinfeng Hongyuan Flagship Store	Power Saving Specialist 1	What is the problem when I get it back from the CTS, it is plugged into the wall and the voltage shows 206v.	Jingdong prefecture level city in Shandong
Jinfeng Hongyuan Flagship Store	Power Saving Specialist 1	The logistics was fast and the stuff was perfectly packaged. It is in the process of trial.	Jingdong prefecture level city in Shandong
Jinfeng Hongyuan Flagship Store	Power Saving Specialist 1	This saver is particularly good to use can really save electricity, with two months and then look at the line if you buy one for the family	Jingdong prefecture level city in Shandong
Jinfeng Hongyuan Flagship Store	Power Saving Specialist 1	Novel appearance design, all aluminum alloy profile, also with a fuse device, voltage monitoring display. After receiving the goods, I directly cut the plug and connect it to the main power box under the leakage switch, hoping to save electricity, and then follow up later. Logistics is very fast, arrived the next	Jingdong prefecture level city in Shandong
Jinfeng Hongyuan Flagship Store	Power Saving Specialist 1	Received, very good and like, if necessary next time to buy ha ha	Jingdong prefecture level city in Shandong
Jinfeng Hongyuan Flagship Store	Power Saving Specialist 1	It's good. It's worth it. Get it if you need it.	Jingdong prefecture level city in Shandong
Jinfeng Hongyuan Flagship Store	Power Saving Specialist 1	It feels more than good and is worth having.	Jingdong prefecture level city in Shandong
Jinfeng Hongyuan Flagship Store	Power Saving Specialist 1	The goods arrived very good and fast first time to buy	Jingdong prefecture level city in Shandong
Jinfeng Hongyuan Flagship Store	Power Saving Specialist 1	The power saving is very good, I can save so much money every month, I will buy another one next time.	Jingdong prefecture level city in Shandong
Jinfeng Hongyuan Flagship Store	Power Saving Specialist 1	It's not what it says.	Jingdong prefecture level city in Shandong
Jinfeng Hongyuan Flagship Store	Power Saving Specialist 1	I have been using it for many days now and it saves electricity, it really does I have an 8 kW electric snail stove and it saves about $3.50 a day.	Jingdong prefecture level city in Shandong
Jinfeng Hongyuan Flagship Store	Power Saving Specialist 1	Express delivery is very fast, the quality is also quite good, worth buying!	Jingdong prefecture level city in Shandong
Jinfeng Hongyuan Flagship Store	Power Saving Specialist 1	Just received expecting the baby to work well.	Jingdong prefecture level city in Shandong
Jinfeng Hongyuan Flagship Store	Power Saving Specialist 1	Very good, there are more fruit, has bought a second!	Jingdong prefecture level city in Shandong
Jinfeng Hongyuan Flagship Store	Power Saving Specialist 1	Good is good So far, no power savings have been noticed Maybe it's too little to notice.	Jingdong prefecture level city in Shandong
Jinfeng Hongyuan Flagship Store	Power Saving Specialist 1	I just received the item and plugged it in to see what happens later.	Jingdong prefecture level city in Shandong
Jinfeng Hongyuan Flagship Store	Power Saving Specialist 1	Baby received, especially good, packaging is also good, the courier brother is very strong, thank you.	Jingdong prefecture level city in Shandong
Jinfeng Hongyuan Flagship Store	Power Saving Specialist 1	Just received the goods have not yet used it, do not know how, three months later in the follow-up comment!	Jingdong prefecture level city in Shandong
Jinfeng Hongyuan Flagship Store	Power Saving Specialist 1	The item was received, it is quite good, recommended to buy.	Jingdong prefecture level city in Shandong
Jinfeng Hongyuan Flagship Store	Power Saving Specialist 1	This energy saver works very well and I recommend everyone to buy it!	Jingdong prefecture level city in Shandong
Jinfeng Hongyuan Flagship Store	Power Saving Specialist 1	Voltage stabilization and current reduction nice power saving product.	Jingdong prefecture level city in Shandong
Jinfeng Hongyuan Flagship Store	Power Saving Specialist 1	Voltage stabilization and current reduction nice power saving product.	Jingdong prefecture level city in Shandong
Jinfeng Hongyuan Flagship Store	Power Saving Specialist 1	A good power saver for stabilizing and lowering current.	Jingdong prefecture level city in Shandong
Jinfeng Hongyuan Flagship Store	Power Saving Specialist 1	You'll know if it saves power after you use it.	Jingdong prefecture level city in Shandong
Jinfeng Hongyuan Flagship Store	Power Saving Specialist 1	The quality is great. The quality is very good. The service is very good. Express delivery arrived in two days.	Jingdong prefecture level city in Shandong
Jinfeng Hongyuan Flagship Store	Power Saving Specialist 1	It works great.	Jingdong prefecture level city in Shandong
Jinfeng Hongyuan Flagship Store	Power Saving Specialist 1	Logistics is very fast, not yet used, used to comment again.	Jingdong prefecture level city in Shandong
Jinfeng Hongyuan Flagship Store	Power Saving Specialist 1	Logistics is very fast things are also very good, worthy of friends to buy	Jingdong prefecture level city in Shandong
Jinfeng Hongyuan Flagship Store	Power Saving Specialist 1	It works. It really works.	Jingdong prefecture level city in Shandong
Jinfeng Hongyuan Flagship Store	Power Saving Specialist 1	LANGUAGE COMMENTS: just received the shipment. I immediately plugged it in and used it. If you ask how effective. It depends on whether the next month's electricity bill has been reduced. If there is a decrease. I am buying more than one to use.	Jingdong prefecture level city in Shandong
Jinfeng Hongyuan Flagship Store	Power Saving Specialist 1	Well made, power saving, good and convenient!	Jingdong prefecture level city in Shandong
Jinfeng Hongyuan Flagship Store	Power Saving Specialist 1	I'm using it. I'll rate it next month.	Jingdong prefecture level city in Shandong
Jinfeng Hongyuan Flagship Store	Power Saving Specialist 1	Baby received Oh, the courier delivery really fast, with a good and then come back to the evaluation	Jingdong prefecture level city in Shandong
Jinfeng Hongyuan Flagship Store	Power Saving Specialist 1	Fast logistics, good packaging, worth it!	Jingdong prefecture level city in Shandong
Jinfeng Hongyuan Flagship Store	Power Saving Specialist 1	Customer service is okay. It's just that.	Jingdong prefecture level city in Shandong
Jinfeng Hongyuan Flagship Store	Power Saving Specialist 1	Poor power saving	Jingdong prefecture level city in Shandong
Jinfeng Hongyuan Flagship Store	Power Saving Specialist 1	I'll tell you when I've used it.	Jingdong prefecture level city in Shandong
Jinfeng Hongyuan Flagship Store	Power Saving Specialist 1	I think it's good.	Jingdong prefecture level city in Shandong
Jinfeng Hongyuan Flagship Store	Power Saving Specialist 1	This energy saver works very well and I recommend everyone to buy it!	Jingdong prefecture level city in Shandong
Jinfeng Hongyuan Flagship Store	Power Saving Specialist 1	I do not know how effective, just use half a month on the burn out, good business reissued a, and then use it after the follow-up comment.	Jingdong prefecture level city in Shandong
Jinfeng Hongyuan Flagship Store	Power Saving Specialist 1	in use	Jingdong prefecture level city in Shandong
Jinfeng Hongyuan Flagship Store	Power Saving Specialist 1	This thing is very poor. It's already only been used for ten days and it's broken. Now waiting for you to return it.	Jingdong prefecture level city in Shandong
Jinfeng Hongyuan Flagship Store	Power Saving Specialist 1	It's not the same as the seller's show.	Jingdong prefecture level city in Shandong
Jinfeng Hongyuan Flagship Store	Power Saving Specialist 1	What is the problem when I get it back from the CTS, it is plugged into the wall and the voltage shows 206v.	Jingdong prefecture level city in Shandong
Jinfeng Hongyuan Flagship Store	Power Saving Specialist 1	The logistics was fast and the stuff was perfectly packaged. It is in the process of trial.	Jingdong prefecture level city in Shandong

Jinfeng Hongyuan Flagship Store	Power Saving Specialist 1	This saver is particularly good to use can really save electricity, with two months and then look at the line if you buy one for the family	Jingdong prefecture level city in Shandong
Jinfeng Hongyuan Flagship Store	Power Saving Specialist 1	Novel appearance design, all aluminum alloy profile, also with a fuse device, voltage monitoring display. After receiving the goods, I directly cut the plug and connect it to the main power box under the leakage switch, hoping to save electricity, and then follow up later. Logistics is very fast, arrived the next	Jingdong prefecture level city in Shandong
Jinfeng Hongyuan Flagship Store	Power Saving Specialist 1	Received, very good and like, if necessary next time to buy ha ha	Jingdong prefecture level city in Shandong
Jinfeng Hongyuan Flagship Store	Power Saving Specialist 1	It's good. It's worth it. Get it if you need it.	Jingdong prefecture level city in Shandong
Jinfeng Hongyuan Flagship Store	Power Saving Specialist 1	It feels more than good and is worth having.	Jingdong prefecture level city in Shandong
Jinfeng Hongyuan Flagship Store	Power Saving Specialist 1	The goods arrived very good and fast first time to buy	Jingdong prefecture level city in Shandong
Jinfeng Hongyuan Flagship Store	Power Saving Specialist 1	The power saving is very good, I can save so much money every month, I will buy another one next time.	Jingdong prefecture level city in Shandong
Jinfeng Hongyuan Flagship Store	Power Saving Specialist 1	It's not what it says.	Jingdong prefecture level city in Shandong
Jinfeng Hongyuan Flagship Store	Power Saving Specialist 1	I have been using it for many days now and it saves electricity, it really does I have an 8 kW electric snail stove and it saves about $3.50 a day.	Jingdong prefecture level city in Shandong
Jinfeng Hongyuan Flagship Store	Power Saving Specialist 1	Express delivery is very fast, the quality is also quite good, worth buying!	Jingdong prefecture level city in Shandong
Jinfeng Hongyuan Flagship Store	Power Saving Specialist 1	Just received expecting the baby to work well.	Jingdong prefecture level city in Shandong
Jinfeng Hongyuan Flagship Store	Power Saving Specialist 1	Very good, there are more fruit, has bought a second!	Jingdong prefecture level city in Shandong
Jinfeng Hongyuan Flagship Store	Power Saving Specialist 1	Good is good So far, no power savings have been noticed Maybe it's too little to notice.	Jingdong prefecture level city in Shandong
Jinfeng Hongyuan Flagship Store	Power Saving Specialist 1	I just received the item and plugged it in to see what happens later.	Jingdong prefecture level city in Shandong
Jinfeng Hongyuan Flagship Store	Power Saving Specialist 1	Baby received, especially good, packaging is also good, the courier brother is very strong, thank you.	Jingdong prefecture level city in Shandong
Jinfeng Hongyuan Flagship Store	Power Saving Specialist 1	Just received the goods have not yet used it, do not know how, three months later in the follow-up comment!	Jingdong prefecture level city in Shandong
Jinfeng Hongyuan Flagship Store	Power Saving Specialist 1	The item was received, it is quite good, recommended to buy.	Jingdong prefecture level city in Shandong
Jinfeng Hongyuan Flagship Store	Power Saving Specialist 1	This energy saver works very well and I recommend everyone to buy it!	Jingdong prefecture level city in Shandong
Jinfeng Hongyuan Flagship Store	Power Saving Specialist 1	Voltage stabilization and current reduction nice power saving product.	Jingdong prefecture level city in Shandong
Jinfeng Hongyuan Flagship Store	Power Saving Specialist 1	Voltage stabilization and current reduction nice power saving product.	Jingdong prefecture level city in Shandong
Jinfeng Hongyuan Flagship Store	Power Saving Specialist 1	A good power saver for stabilizing and lowering current.	Jingdong prefecture level city in Shandong
Jinfeng Hongyuan Flagship Store	Power Saving Specialist 1	You'll know if it saves power after you use it.	Jingdong prefecture level city in Shandong
Jinfeng Hongyuan Flagship Store	Power Saving Specialist 1	The quality is great. The quality is very good. The service is very good. Express delivery arrived in two days.	Jingdong prefecture level city in Shandong
Jinfeng Hongyuan Flagship Store	Power Saving Specialist 1	It works great.	Jingdong prefecture level city in Shandong
Jinfeng Hongyuan Flagship Store	Power Saving Specialist 1	Logistics is very fast, not yet used, used to comment again.	Jingdong prefecture level city in Shandong
Jinfeng Hongyuan Flagship Store	Power Saving Specialist 1	Logistics is very fast things are also very good, worthy of friends to buy	Jingdong prefecture level city in Shandong
Jinfeng Hongyuan Flagship Store	Power Saving Specialist 1	It works. It really works.	Jingdong prefecture level city in Shandong
Jinfeng Hongyuan Flagship Store	Power Saving Specialist 1	LANGUAGE COMMENTS: just received the shipment. I immediately plugged it in and used it. If you ask how effective. It depends on whether the next month's electricity bill has been reduced. If there is a decrease. I am buying more than one to use.	Jingdong prefecture level city in Shandong
Jinfeng Hongyuan Flagship Store	Power Saving Specialist 1	Well made, power saving, good and convenient!	Jingdong prefecture level city in Shandong
Jinfeng Hongyuan Flagship Store	Power Saving Specialist 1	I'm using it. I'll rate it next month.	Jingdong prefecture level city in Shandong
Jinfeng Hongyuan Flagship Store	Power Saving Specialist 1	Baby received Oh, the courier delivery really fast, with a good and then come back to the evaluation	Jingdong prefecture level city in Shandong
Jinfeng Hongyuan Flagship Store	Power Saving Specialist 1	Fast logistics, good packaging, worth it!	Jingdong prefecture level city in Shandong
Jinfeng Hongyuan Flagship Store	Power Saving Specialist 1	Customer service is okay. It's just that.	Jingdong prefecture level city in Shandong
Jinfeng Hongyuan Flagship Store	Power Saving Specialist 1	Poor power saving	Jingdong prefecture level city in Shandong
Jinfeng Hongyuan Flagship Store	Power Saving Specialist 1	I'll tell you when I've used it.	Jingdong prefecture level city in Shandong
Jinfeng Hongyuan Flagship Store	Power Saving Specialist 1	I think it's good.	Jingdong prefecture level city in Shandong
Jinfeng Hongyuan Flagship Store	Power Saving Specialist 1	This energy saver works very well and I recommend everyone to buy it!	Jingdong prefecture level city in Shandong
Jinfeng Hongyuan Flagship Store	Power Saving Specialist 1	I do not know how effective, just use half a month on the burn out, good business reissued a, and then use it after the follow-up comment.	Jingdong prefecture level city in Shandong
Jinfeng Hongyuan Flagship Store	Power Saving Specialist 1	in use	Jingdong prefecture level city in Shandong
Jinfeng Hongyuan Flagship Store	Power Saving Specialist 1	This thing is very poor. It's already only been used for ten days and it's broken. Now waiting for you to return it.	Jingdong prefecture level city in Shandong
Jinfeng Hongyuan Flagship Store	Power Saving Specialist 1	It's not the same as the seller's show.	Jingdong prefecture level city in Shandong
Jinfeng Hongyuan Flagship Store	Power Saving Specialist 1	What is the problem when I get it back from the CTS, it is plugged into the wall and the voltage shows 206v.	Jingdong prefecture level city in Shandong
Jinfeng Hongyuan Flagship Store	Power Saving Specialist 1	The logistics was fast and the stuff was perfectly packaged. It is in the process of trial.	Jingdong prefecture level city in Shandong
Jinfeng Hongyuan Flagship Store	Power Saving Specialist 1	This saver is particularly good to use can really save electricity, with two months and then look at the line if you buy one for the family	Jingdong prefecture level city in Shandong
Jinfeng Hongyuan Flagship Store	Power Saving Specialist 1	Novel appearance design, all aluminum alloy profile, also with a fuse device, voltage monitoring display. After receiving the goods, I directly cut the plug and connect it to the main power box under the leakage switch, hoping to save electricity, and then follow up later. Logistics is very fast, arrived the next	Jingdong prefecture level city in Shandong
Jinfeng Hongyuan Flagship Store	Power Saving Specialist 1	Received, very good and like, if necessary next time to buy ha ha	Jingdong prefecture level city in Shandong
Jinfeng Hongyuan Flagship Store	Power Saving Specialist 1	It's good. It's worth it. Get it if you need it.	Jingdong prefecture level city in Shandong
Jinfeng Hongyuan Flagship Store	Power Saving Specialist 1	It feels more than good and is worth having.	Jingdong prefecture level city in Shandong
Jinfeng Hongyuan Flagship Store	Power Saving Specialist 1	The goods arrived very good and fast first time to buy	Jingdong prefecture level city in Shandong
Jinfeng Hongyuan Flagship Store	Power Saving Specialist 1	The power saving is very good, I can save so much money every month, I will buy another one next time.	Jingdong prefecture level city in Shandong

Jinfeng Hongyuan Flagship Store	Power Saving Specialist 1	It's not what it says.	Jingdong prefecture level city in Shandong
Jinfeng Hongyuan Flagship Store	Power Saving Specialist 1	I have been using it for many days now and it saves electricity, it really does I have an 8 kW electric snail stove and it saves about $3.50 a day.	Jingdong prefecture level city in Shandong
Jinfeng Hongyuan Flagship Store	Power Saving Specialist 1	Express delivery is very fast, the quality is also quite good, worth buying!	Jingdong prefecture level city in Shandong
Jinfeng Hongyuan Flagship Store	Power Saving Specialist 1	Just received expecting the baby to work well.	Jingdong prefecture level city in Shandong
Jinfeng Hongyuan Flagship Store	Power Saving Specialist 1	Very good, there are more fruit, has bought a second!	Jingdong prefecture level city in Shandong
Jinfeng Hongyuan Flagship Store	Power Saving Specialist 1	Good is good So far, no power savings have been noticed Maybe it's too little to notice.	Jingdong prefecture level city in Shandong
Jinfeng Hongyuan Flagship Store	Power Saving Specialist 1	I just received the item and plugged it in to see what happens later.	Jingdong prefecture level city in Shandong
Jinfeng Hongyuan Flagship Store	Power Saving Specialist 1	Baby received, especially good, packaging is also good, the courier brother is very strong, thank you.	Jingdong prefecture level city in Shandong
Jinfeng Hongyuan Flagship Store	Power Saving Specialist 1	Just received the goods have not yet used it, do not know how, three months later in the follow-up comment!	Jingdong prefecture level city in Shandong
Jinfeng Hongyuan Flagship Store	Power Saving Specialist 1	The item was received, it is quite good, recommended to buy.	Jingdong prefecture level city in Shandong
Jinfeng Hongyuan Flagship Store	Power Saving Specialist 1	This energy saver works very well and I recommend everyone to buy it!	Jingdong prefecture level city in Shandong
Jinfeng Hongyuan Flagship Store	Power Saving Specialist 1	Voltage stabilization and current reduction nice power saving product.	Jingdong prefecture level city in Shandong
Jinfeng Hongyuan Flagship Store	Power Saving Specialist 1	Voltage stabilization and current reduction nice power saving product.	Jingdong prefecture level city in Shandong
Jinfeng Hongyuan Flagship Store	Power Saving Specialist 1	A good power saver for stabilizing and lowering current.	Jingdong prefecture level city in Shandong
Jinfeng Hongyuan Flagship Store	Power Saving Specialist 1	You'll know if it saves power after you use it.	Jingdong prefecture level city in Shandong
Jinfeng Hongyuan Flagship Store	Power Saving Specialist 1	The quality is great. The quality is very good. The service is very good. Express delivery arrived in two days.	Jingdong prefecture level city in Shandong
Jinfeng Hongyuan Flagship Store	Power Saving Specialist 1	It works great.	Jingdong prefecture level city in Shandong
Jinfeng Hongyuan Flagship Store	Power Saving Specialist 1	Logistics is very fast, not yet used, used to comment again.	Jingdong prefecture level city in Shandong
Jinfeng Hongyuan Flagship Store	Power Saving Specialist 1	Logistics is very fast things are also very good, worthy of friends to buy	Jingdong prefecture level city in Shandong
Jinfeng Hongyuan Flagship Store	Power Saving Specialist 1	It works. It really works.	Jingdong prefecture level city in Shandong
Jinfeng Hongyuan Flagship Store	Power Saving Specialist 1	LANGUAGE COMMENTS: just received the shipment. I immediately plugged it in and used it. If you ask how effective. It depends on whether the next month's electricity bill has been reduced. If there is a decrease. I am buying more than one to use.	Jingdong prefecture level city in Shandong
Jinfeng Hongyuan Flagship Store	Power Saving Specialist 1	Well made, power saving, good and convenient!	Jingdong prefecture level city in Shandong
Jinfeng Hongyuan Flagship Store	Power Saving Specialist 1	I'm using it. I'll rate it next month.	Jingdong prefecture level city in Shandong
Jinfeng Hongyuan Flagship Store	Power Saving Specialist 1	Baby received Oh, the courier delivery really fast, with a good and then come back to the evaluation	Jingdong prefecture level city in Shandong
Jinfeng Hongyuan Flagship Store	Power Saving Specialist 1	Fast logistics, good packaging, worth it!	Jingdong prefecture level city in Shandong
Jinfeng Hongyuan Flagship Store	Power Saving Specialist 1	Customer service is okay. It's just that.	Jingdong prefecture level city in Shandong
Jinfeng Hongyuan Flagship Store	Power Saving Specialist 1	Poor power saving	Jingdong prefecture level city in Shandong
Jinfeng Hongyuan Flagship Store	Power Saving Specialist 1	I'll tell you when I've used it.	Jingdong prefecture level city in Shandong
Jinfeng Hongyuan Flagship Store	Power Saving Specialist 1	I think it's good.	Jingdong prefecture level city in Shandong
Jinfeng Hongyuan Flagship Store	Power Saving Specialist 1	This energy saver works very well and I recommend everyone to buy it!	Jingdong prefecture level city in Shandong
Jinfeng Hongyuan Flagship Store	Power Saving Specialist 1	I do not know how effective, just use half a month on the burn out, good business reissued a, and then use it after the follow-up comment.	Jingdong prefecture level city in Shandong
Jinfeng Hongyuan Flagship Store	Power Saving Specialist 1	in use	Jingdong prefecture level city in Shandong
Jinfeng Hongyuan Flagship Store	Power Saving Specialist 1	This thing is very poor. It's already only been used for ten days and it's broken. Now waiting for you to return it.	Jingdong prefecture level city in Shandong
Jinfeng Hongyuan Flagship Store	Power Saving Specialist 1	It's not the same as the seller's show.	Jingdong prefecture level city in Shandong
Jinfeng Hongyuan Flagship Store	Power Saving Specialist 1	What is the problem when I get it back from the CTS, it is plugged into the wall and the voltage shows 206v.	Jingdong prefecture level city in Shandong
Jinfeng Hongyuan Flagship Store	Power Saving Specialist 1	The logistics was fast and the stuff was perfectly packaged. It is in the process of trial.	Jingdong prefecture level city in Shandong
Jinfeng Hongyuan Flagship Store	Power Saving Specialist 1	This saver is particularly good to use can really save electricity, with two months and then look at the line if you buy one for the family	Jingdong prefecture level city in Shandong
Jinfeng Hongyuan Flagship Store	Power Saving Specialist 1	Novel appearance design, all aluminum alloy profile, also with a fuse device, voltage monitoring display. After receiving the goods, I directly cut the plug and connect it to the main power box under the leakage switch, hoping to save electricity, and then follow up later. Logistics is very fast, arrived the next	Jingdong prefecture level city in Shandong
Jinfeng Hongyuan Flagship Store	Power Saving Specialist 1	Received, very good and like, if necessary next time to buy ha ha	Jingdong prefecture level city in Shandong
Jinfeng Hongyuan Flagship Store	Power Saving Specialist 1	It's good. It's worth it. Get it if you need it.	Jingdong prefecture level city in Shandong
Jinfeng Hongyuan Flagship Store	Power Saving Specialist 1	It feels more than good and is worth having.	Jingdong prefecture level city in Shandong
Jinfeng Hongyuan Flagship Store	Power Saving Specialist 1	The goods arrived very good and fast first time to buy	Jingdong prefecture level city in Shandong
Jinfeng Hongyuan Flagship Store	Power Saving Specialist 1	The power saving is very good, I can save so much money every month, I will buy another one next time.	Jingdong prefecture level city in Shandong
Jinfeng Hongyuan Flagship Store	Power Saving Specialist 1	It's not what it says.	Jingdong prefecture level city in Shandong
Jinfeng Hongyuan Flagship Store	Power Saving Specialist 1	I have been using it for many days now and it saves electricity, it really does I have an 8 kW electric snail stove and it saves about $3.50 a day.	Jingdong prefecture level city in Shandong
Jinfeng Hongyuan Flagship Store	Power Saving Specialist 1	Express delivery is very fast, the quality is also quite good, worth buying!	Jingdong prefecture level city in Shandong
Jinfeng Hongyuan Flagship Store	Power Saving Specialist 1	Just received expecting the baby to work well.	Jingdong prefecture level city in Shandong
Jinfeng Hongyuan Flagship Store	Power Saving Specialist 1	Very good, there are more fruit, has bought a second!	Jingdong prefecture level city in Shandong
Jinfeng Hongyuan Flagship Store	Power Saving Specialist 1	Good is good So far, no power savings have been noticed Maybe it's too little to notice.	Jingdong prefecture level city in Shandong
Jinfeng Hongyuan Flagship Store	Power Saving Specialist 1	I just received the item and plugged it in to see what happens later.	Jingdong prefecture level city in Shandong

Jinfeng Hongyuan Flagship Store	Power Saving Specialist 1	Baby received, especially good, packaging is also good, the courier brother is very strong, thank you.	Jingdong prefecture level city in Shandong
Jinfeng Hongyuan Flagship Store	Power Saving Specialist 1	Just received the goods have not yet used it, do not know how, three months later in the follow-up comment!	Jingdong prefecture level city in Shandong
Jinfeng Hongyuan Flagship Store	Power Saving Specialist 1	The item was received, it is quite good, recommended to buy.	Jingdong prefecture level city in Shandong
Jinfeng Hongyuan Flagship Store	Power Saving Specialist 1	This energy saver works very well and I recommend everyone to buy it!	Jingdong prefecture level city in Shandong
Jinfeng Hongyuan Flagship Store	Power Saving Specialist 1	Voltage stabilization and current reduction nice power saving product.	Jingdong prefecture level city in Shandong
Jinfeng Hongyuan Flagship Store	Power Saving Specialist 1	Voltage stabilization and current reduction nice power saving product.	Jingdong prefecture level city in Shandong
Jinfeng Hongyuan Flagship Store	Power Saving Specialist 1	A good power saver for stabilizing and lowering current.	Jingdong prefecture level city in Shandong
Jinfeng Hongyuan Flagship Store	Power Saving Specialist 1	You'll know if it saves power after you use it.	Jingdong prefecture level city in Shandong
Jinfeng Hongyuan Flagship Store	Power Saving Specialist 1	The quality is great. The quality is very good. The service is very good. Express delivery arrived in two days.	Jingdong prefecture level city in Shandong
Jinfeng Hongyuan Flagship Store	Power Saving Specialist 1	It works great.	Jingdong prefecture level city in Shandong
Jinfeng Hongyuan Flagship Store	Power Saving Specialist 1	Logistics is very fast, not yet used, used to comment again.	Jingdong prefecture level city in Shandong
Jinfeng Hongyuan Flagship Store	Power Saving Specialist 1	Logistics is very fast things are also very good, worthy of friends to buy	Jingdong prefecture level city in Shandong
Jinfeng Hongyuan Flagship Store	Power Saving Specialist 1	It works. It really works.	Jingdong prefecture level city in Shandong
Jinfeng Hongyuan Flagship Store	Power Saving Specialist 1	LANGUAGE COMMENTS: just received the shipment. I immediately plugged it in and used it. If you ask how effective. It depends on whether the next month's electricity bill has been reduced. If there is a decrease. I am buying more than one to use.	Jingdong prefecture level city in Shandong
Jinfeng Hongyuan Flagship Store	Power Saving Specialist 1	Well made, power saving, good and convenient!	Jingdong prefecture level city in Shandong
Jinfeng Hongyuan Flagship Store	Power Saving Specialist 1	I'm using it. I'll rate it next month.	Jingdong prefecture level city in Shandong
Jinfeng Hongyuan Flagship Store	Power Saving Specialist 1	Baby received Oh, the courier delivery really fast, with a good and then come back to the evaluation	Jingdong prefecture level city in Shandong
Jinfeng Hongyuan Flagship Store	Power Saving Specialist 1	Fast logistics, good packaging, worth it!	Jingdong prefecture level city in Shandong
Jinfeng Hongyuan Flagship Store	Power Saving Specialist 1	Customer service is okay. It's just that.	Jingdong prefecture level city in Shandong
Jinfeng Hongyuan Flagship Store	Power Saving Specialist 1	Poor power saving	Jingdong prefecture level city in Shandong
Jinfeng Hongyuan Flagship Store	Power Saving Specialist 1	I'll tell you when I've used it.	Jingdong prefecture level city in Shandong
Jinfeng Hongyuan Flagship Store	Power Saving Specialist 1	I think it's good.	Jingdong prefecture level city in Shandong
Jinfeng Hongyuan Flagship Store	Power Saving Specialist 1	This energy saver works very well and I recommend everyone to buy it!	Jingdong prefecture level city in Shandong
Jinfeng Hongyuan Flagship Store	Power Saving Specialist 1	I do not know how effective, just use half a month on the burn out, good business reissued a, and then use it after the follow-up comment.	Jingdong prefecture level city in Shandong
Jinfeng Hongyuan Flagship Store	Power Saving Specialist 1	in use	Jingdong prefecture level city in Shandong
Jinfeng Hongyuan Flagship Store	Power Saving Specialist 1	This thing is very poor. It's already only been used for ten days and it's broken. Now waiting for you to return it.	Jingdong prefecture level city in Shandong
Jinfeng Hongyuan Flagship Store	Power Saving Specialist 1	It's not the same as the seller's show.	Jingdong prefecture level city in Shandong
Jinfeng Hongyuan Flagship Store	Power Saving Specialist 1	What is the problem when I get it back from the CTS, it is plugged into the wall and the voltage shows 206v.	Jingdong prefecture level city in Shandong
Jinfeng Hongyuan Flagship Store	Power Saving Specialist 1	The logistics was fast and the stuff was perfectly packaged. It is in the process of trial.	Jingdong prefecture level city in Shandong
Jinfeng Hongyuan Flagship Store	Power Saving Specialist 1	This saver is particularly good to use can really save electricity, with two months and then look at the line if you buy one for the family	Jingdong prefecture level city in Shandong
Jinfeng Hongyuan Flagship Store	Power Saving Specialist 1	Novel appearance design, all aluminum alloy profile, also with a fuse device, voltage monitoring display. After receiving the goods, I directly cut the plug and connect it to the main power box under the leakage switch, hoping to save electricity, and then follow up later. Logistics is very fast, arrived the next	Jingdong prefecture level city in Shandong
Jinfeng Hongyuan Flagship Store	Power Saving Specialist 1	Received, very good and like, if necessary next time to buy ha ha	Jingdong prefecture level city in Shandong
Jinfeng Hongyuan Flagship Store	Power Saving Specialist 1	It's good. It's worth it. Get it if you need it.	Jingdong prefecture level city in Shandong
Jinfeng Hongyuan Flagship Store	Power Saving Specialist 1	It feels more than good and is worth having.	Jingdong prefecture level city in Shandong
Jinfeng Hongyuan Flagship Store	Power Saving Specialist 1	The goods arrived very good and fast first time to buy	Jingdong prefecture level city in Shandong
Jinfeng Hongyuan Flagship Store	Power Saving Specialist 1	The power saving is very good, I can save so much money every month, I will buy another one next time.	Jingdong prefecture level city in Shandong
Jinfeng Hongyuan Flagship Store	Power Saving Specialist 1	It's not what it says.	Jingdong prefecture level city in Shandong
Jinfeng Hongyuan Flagship Store	Power Saving Specialist 1	I have been using it for many days now and it saves electricity, it really does I have an 8 kW electric snail stove and it saves about $3.50 a day.	Jingdong prefecture level city in Shandong
Jinfeng Hongyuan Flagship Store	Power Saving Specialist 1	Express delivery is very fast, the quality is also quite good, worth buying!	Jingdong prefecture level city in Shandong
Jinfeng Hongyuan Flagship Store	Power Saving Specialist 1	Just received expecting the baby to work well.	Jingdong prefecture level city in Shandong
Jinfeng Hongyuan Flagship Store	Power Saving Specialist 1	Very good, there are more fruit, has bought a second!	Jingdong prefecture level city in Shandong
Jinfeng Hongyuan Flagship Store	Power Saving Specialist 1	Good is good So far, no power savings have been noticed Maybe it's too little to notice.	Jingdong prefecture level city in Shandong
Jinfeng Hongyuan Flagship Store	Power Saving Specialist 1	I just received the item and plugged it in to see what happens later.	Jingdong prefecture level city in Shandong
Jinfeng Hongyuan Flagship Store	Power Saving Specialist 1	Baby received, especially good, packaging is also good, the courier brother is very strong, thank you.	Jingdong prefecture level city in Shandong
Jinfeng Hongyuan Flagship Store	Power Saving Specialist 1	Just received the goods have not yet used it, do not know how, three months later in the follow-up comment!	Jingdong prefecture level city in Shandong
Jinfeng Hongyuan Flagship Store	Power Saving Specialist 1	The item was received, it is quite good, recommended to buy.	Jingdong prefecture level city in Shandong
Jinfeng Hongyuan Flagship Store	Power Saving Specialist 1	This energy saver works very well and I recommend everyone to buy it!	Jingdong prefecture level city in Shandong
Jinfeng Hongyuan Flagship Store	Power Saving Specialist 1	Voltage stabilization and current reduction nice power saving product.	Jingdong prefecture level city in Shandong
Jinfeng Hongyuan Flagship Store	Power Saving Specialist 1	Voltage stabilization and current reduction nice power saving product.	Jingdong prefecture level city in Shandong
Jinfeng Hongyuan Flagship Store	Power Saving Specialist 1	A good power saver for stabilizing and lowering current.	Jingdong prefecture level city in Shandong

Jinfeng Hongyuan Flagship Store	Power Saving Specialist 1	You'll know if it saves power after you use it.	Jingdong prefecture level city in Shandong
Jinfeng Hongyuan Flagship Store	Power Saving Specialist 1	The quality is great. The quality is very good. The service is very good. Express delivery arrived in two days.	Jingdong prefecture level city in Shandong
Jinfeng Hongyuan Flagship Store	Power Saving Specialist 1	It works great.	Jingdong prefecture level city in Shandong
Jinfeng Hongyuan Flagship Store	Power Saving Specialist 1	Logistics is very fast, not yet used, used to comment again.	Jingdong prefecture level city in Shandong
Jinfeng Hongyuan Flagship Store	Power Saving Specialist 1	Logistics is very fast things are also very good, worthy of friends to buy	Jingdong prefecture level city in Shandong
Jinfeng Hongyuan Flagship Store	Power Saving Specialist 1	It works. It really works.	Jingdong prefecture level city in Shandong
Jinfeng Hongyuan Flagship Store	Power Saving Specialist 1	LANGUAGE COMMENTS: just received the shipment. I immediately plugged it in and used it. If you ask how effective. It depends on whether the next month's electricity bill has been reduced. If there is a decrease. I am buying more than one to use.	Jingdong prefecture level city in Shandong
Jinfeng Hongyuan Flagship Store	Power Saving Specialist 1	Well made, power saving, good and convenient!	Jingdong prefecture level city in Shandong
Jinfeng Hongyuan Flagship Store	Power Saving Specialist 1	I'm using it. I'll rate it next month.	Jingdong prefecture level city in Shandong
Jinfeng Hongyuan Flagship Store	Power Saving Specialist 1	Baby received Oh, the courier delivery really fast, with a good and then come back to the evaluation	Jingdong prefecture level city in Shandong
Jinfeng Hongyuan Flagship Store	Power Saving Specialist 1	Fast logistics, good packaging, worth it!	Jingdong prefecture level city in Shandong
Jinfeng Hongyuan Flagship Store	Power Saving Specialist 1	Customer service is okay. It's just that.	Jingdong prefecture level city in Shandong
Jinfeng Hongyuan Flagship Store	Power Saving Specialist 1	Poor power saving	Jingdong prefecture level city in Shandong
Jinfeng Hongyuan Flagship Store	Power Saving Specialist 1	I'll tell you when I've used it.	Jingdong prefecture level city in Shandong
Jinfeng Hongyuan Flagship Store	Power Saving Specialist 1	I think it's good.	Jingdong prefecture level city in Shandong
Jinfeng Hongyuan Flagship Store	Power Saving Specialist 1	This energy saver works very well and I recommend everyone to buy it!	Jingdong prefecture level city in Shandong
Jinfeng Hongyuan Flagship Store	Power Saving Specialist 1	I do not know how effective, just use half a month on the burn out, good business reissued a, and then use it after the follow-up comment.	Jingdong prefecture level city in Shandong
Jinfeng Hongyuan Flagship Store	Power Saving Specialist 1	in use	Jingdong prefecture level city in Shandong
Jinfeng Hongyuan Flagship Store	Power Saving Specialist 1	This thing is very poor. It's already only been used for ten days and it's broken. Now waiting for you to return it.	Jingdong prefecture level city in Shandong
Jinfeng Hongyuan Flagship Store	Power Saving Specialist 1	It's not the same as the seller's show.	Jingdong prefecture level city in Shandong
Jinfeng Hongyuan Flagship Store	Power Saving Specialist 1	What is the problem when I get it back from the CTS, it is plugged into the wall and the voltage shows 206v.	Jingdong prefecture level city in Shandong
Jinfeng Hongyuan Flagship Store	Power Saving Specialist 1	The logistics was fast and the stuff was perfectly packaged. It is in the process of trial.	Jingdong prefecture level city in Shandong
Jinfeng Hongyuan Flagship Store	Power Saving Specialist 1	This saver is particularly good to use can really save electricity, with two months and then look at the line if you buy one for the family	Jingdong prefecture level city in Shandong
Jinfeng Hongyuan Flagship Store	Power Saving Specialist 1	Novel appearance design, all aluminum alloy profile, also with a fuse device, voltage monitoring display. After receiving the goods, I directly cut the plug and connect it to the main power box under the leakage switch, hoping to save electricity, and then follow up later. Logistics is very fast, arrived the next	Jingdong prefecture level city in Shandong
Jinfeng Hongyuan Flagship Store	Power Saving Specialist 1	Received, very good and like, if necessary next time to buy ha ha	Jingdong prefecture level city in Shandong
Jinfeng Hongyuan Flagship Store	Power Saving Specialist 1	It's good. It's worth it. Get it if you need it.	Jingdong prefecture level city in Shandong
Jinfeng Hongyuan Flagship Store	Power Saving Specialist 1	It feels more than good and is worth having.	Jingdong prefecture level city in Shandong
Jinfeng Hongyuan Flagship Store	Power Saving Specialist 1	The goods arrived very good and fast first time to buy	Jingdong prefecture level city in Shandong
Jinfeng Hongyuan Flagship Store	Power Saving Specialist 1	The power saving is very good, I can save so much money every month, I will buy another one next time.	Jingdong prefecture level city in Shandong
Jinfeng Hongyuan Flagship Store	Power Saving Specialist 1	It's not what it says.	Jingdong prefecture level city in Shandong
Jinfeng Hongyuan Flagship Store	Power Saving Specialist 1	I have been using it for many days now and it saves electricity, it really does I have an 8 kW electric snail stove and it saves about $3.50 a day.	Jingdong prefecture level city in Shandong
Jinfeng Hongyuan Flagship Store	Power Saving Specialist 1	Express delivery is very fast, the quality is also quite good, worth buying!	Jingdong prefecture level city in Shandong
Jinfeng Hongyuan Flagship Store	Power Saving Specialist 1	Just received expecting the baby to work well.	Jingdong prefecture level city in Shandong
Jinfeng Hongyuan Flagship Store	Power Saving Specialist 1	Very good, there are more fruit, has bought a second!	Jingdong prefecture level city in Shandong
Jinfeng Hongyuan Flagship Store	Power Saving Specialist 1	Good is good So far, no power savings have been noticed Maybe it's too little to notice.	Jingdong prefecture level city in Shandong
Jinfeng Hongyuan Flagship Store	Power Saving Specialist 1	I just received the item and plugged it in to see what happens later.	Jingdong prefecture level city in Shandong
Jinfeng Hongyuan Flagship Store	Power Saving Specialist 1	Baby received, especially good, packaging is also good, the courier brother is very strong, thank you.	Jingdong prefecture level city in Shandong
Jinfeng Hongyuan Flagship Store	Power Saving Specialist 1	Just received the goods have not yet used it, do not know how, three months later in the follow-up comment!	Jingdong prefecture level city in Shandong
Jinfeng Hongyuan Flagship Store	Power Saving Specialist 1	The item was received, it is quite good, recommended to buy.	Jingdong prefecture level city in Shandong
Jinfeng Hongyuan Flagship Store	Power Saving Specialist 1	This energy saver works very well and I recommend everyone to buy it!	Jingdong prefecture level city in Shandong
Jinfeng Hongyuan Flagship Store	Power Saving Specialist 1	Voltage stabilization and current reduction nice power saving product.	Jingdong prefecture level city in Shandong
Jinfeng Hongyuan Flagship Store	Power Saving Specialist 1	Voltage stabilization and current reduction nice power saving product.	Jingdong prefecture level city in Shandong
Jinfeng Hongyuan Flagship Store	Power Saving Specialist 1	A good power saver for stabilizing and lowering current.	Jingdong prefecture level city in Shandong
Jinfeng Hongyuan Flagship Store	Power Saving Specialist 1	You'll know if it saves power after you use it.	Jingdong prefecture level city in Shandong
Jinfeng Hongyuan Flagship Store	Power Saving Specialist 1	The quality is great. The quality is very good. The service is very good. Express delivery arrived in two days.	Jingdong prefecture level city in Shandong
Jinfeng Hongyuan Flagship Store	Power Saving Specialist 1	It works great.	Jingdong prefecture level city in Shandong
Jinfeng Hongyuan Flagship Store	Power Saving Specialist 1	Logistics is very fast, not yet used, used to comment again.	Jingdong prefecture level city in Shandong
Jinfeng Hongyuan Flagship Store	Power Saving Specialist 1	Logistics is very fast things are also very good, worthy of friends to buy	Jingdong prefecture level city in Shandong
Jinfeng Hongyuan Flagship Store	Power Saving Specialist 1	It works. It really works.	Jingdong prefecture level city in Shandong
Jinfeng Hongyuan Flagship Store	Power Saving Specialist 1	LANGUAGE COMMENTS: just received the shipment. I immediately plugged it in and used it. If you ask how effective. It depends on whether the next month's electricity bill has been reduced. If there is a decrease. I am buying more than one to use.	Jingdong prefecture level city in Shandong

Jinfeng Hongyuan Flagship Store	Power Saving Specialist 1	Well made, power saving, good and convenient!	Jingdong prefecture level city in Shandong
Jinfeng Hongyuan Flagship Store	Power Saving Specialist 1	I'm using it. I'll rate it next month.	Jingdong prefecture level city in Shandong
Jinfeng Hongyuan Flagship Store	Power Saving Specialist 1	Baby received Oh, the courier delivery really fast, with a good and then come back to the evaluation	Jingdong prefecture level city in Shandong
Jinfeng Hongyuan Flagship Store	Power Saving Specialist 1	Fast logistics, good packaging, worth it!	Jingdong prefecture level city in Shandong
Jinfeng Hongyuan Flagship Store	Power Saving Specialist 1	Customer service is okay. It's just that.	Jingdong prefecture level city in Shandong
Jinfeng Hongyuan Flagship Store	Power Saving Specialist 1	Poor power saving	Jingdong prefecture level city in Shandong
Jinfeng Hongyuan Flagship Store	Power Saving Specialist 1	I'll tell you when I've used it.	Jingdong prefecture level city in Shandong
Jinfeng Hongyuan Flagship Store	Power Saving Specialist 1	I think it's good.	Jingdong prefecture level city in Shandong
Jinfeng Hongyuan Flagship Store	Power Saving Specialist 1	This energy saver works very well and I recommend everyone to buy it!	Jingdong prefecture level city in Shandong
Jinfeng Hongyuan Flagship Store	Power Saving Specialist 1	I do not know how effective, just use half a month on the burn out, good business reissued a, and then use it after the follow-up comment.	Jingdong prefecture level city in Shandong
Jinfeng Hongyuan Flagship Store	Power Saving Specialist 1	in use	Jingdong prefecture level city in Shandong
Jinfeng Hongyuan Flagship Store	Power Saving Specialist 1	This thing is very poor. It's already only been used for ten days and it's broken. Now waiting for you to return it.	Jingdong prefecture level city in Shandong
Jinfeng Hongyuan Flagship Store	Power Saving Specialist 1	It's not the same as the seller's show.	Jingdong prefecture level city in Shandong
Jinfeng Hongyuan Flagship Store	Power Saving Specialist 1	What is the problem when I get it back from the CTS, it is plugged into the wall and the voltage shows 206v.	Jingdong prefecture level city in Shandong
Jinfeng Hongyuan Flagship Store	Power Saving Specialist 1	The logistics was fast and the stuff was perfectly packaged. It is in the process of trial.	Jingdong prefecture level city in Shandong
Jinfeng Hongyuan Flagship Store	Power Saving Specialist 1	This saver is particularly good to use can really save electricity, with two months and then look at the line if you buy one for the family	Jingdong prefecture level city in Shandong
Jinfeng Hongyuan Flagship Store	Power Saving Specialist 1	Novel appearance design, all aluminum alloy profile, also with a fuse device, voltage monitoring display. After receiving the goods, I directly cut the plug and connect it to the main power box under the leakage switch, hoping to save electricity, and then follow up later. Logistics is very fast, arrived the next	Jingdong prefecture level city in Shandong
Jinfeng Hongyuan Flagship Store	Power Saving Specialist 1	Received, very good and like, if necessary next time to buy ha ha	Jingdong prefecture level city in Shandong
Jinfeng Hongyuan Flagship Store	Power Saving Specialist 1	It's good. It's worth it. Get it if you need it.	Jingdong prefecture level city in Shandong
Jinfeng Hongyuan Flagship Store	Power Saving Specialist 1	It feels more than good and is worth having.	Jingdong prefecture level city in Shandong
Jinfeng Hongyuan Flagship Store	Power Saving Specialist 1	The goods arrived very good and fast first time to buy	Jingdong prefecture level city in Shandong
Jinfeng Hongyuan Flagship Store	Power Saving Specialist 1	The power saving is very good, I can save so much money every month, I will buy another one next time.	Jingdong prefecture level city in Shandong
Jinfeng Hongyuan Flagship Store	Power Saving Specialist 1	It's not what it says.	Jingdong prefecture level city in Shandong
Jinfeng Hongyuan Flagship Store	Power Saving Specialist 1	I have been using it for many days now and it saves electricity, it really does I have an 8 kW electric snail stove and it saves about $3.50 a day.	Jingdong prefecture level city in Shandong
Jinfeng Hongyuan Flagship Store	Power Saving Specialist 1	Express delivery is very fast, the quality is also quite good, worth buying!	Jingdong prefecture level city in Shandong
Jinfeng Hongyuan Flagship Store	Power Saving Specialist 1	Just received expecting the baby to work well.	Jingdong prefecture level city in Shandong
Jinfeng Hongyuan Flagship Store	Power Saving Specialist 1	Very good, there are more fruit, has bought a second!	Jingdong prefecture level city in Shandong
Jinfeng Hongyuan Flagship Store	Power Saving Specialist 1	Good is good So far, no power savings have been noticed Maybe it's too little to notice.	Jingdong prefecture level city in Shandong
Jinfeng Hongyuan Flagship Store	Power Saving Specialist 1	I just received the item and plugged it in to see what happens later.	Jingdong prefecture level city in Shandong
Jinfeng Hongyuan Flagship Store	Power Saving Specialist 1	Baby received, especially good, packaging is also good, the courier brother is very strong, thank you.	Jingdong prefecture level city in Shandong
Jinfeng Hongyuan Flagship Store	Power Saving Specialist 1	Just received the goods have not yet used it, do not know how, three months later in the follow-up comment!	Jingdong prefecture level city in Shandong
Jinfeng Hongyuan Flagship Store	Power Saving Specialist 1	The item was received, it is quite good, recommended to buy.	Jingdong prefecture level city in Shandong
Jinfeng Hongyuan Flagship Store	Power Saving Specialist 1	This energy saver works very well and I recommend everyone to buy it!	Jingdong prefecture level city in Shandong
Jinfeng Hongyuan Flagship Store	Power Saving Specialist 1	Voltage stabilization and current reduction nice power saving product.	Jingdong prefecture level city in Shandong
Jinfeng Hongyuan Flagship Store	Power Saving Specialist 1	Voltage stabilization and current reduction nice power saving product.	Jingdong prefecture level city in Shandong
Jinfeng Hongyuan Flagship Store	Power Saving Specialist 1	A good power saver for stabilizing and lowering current.	Jingdong prefecture level city in Shandong
Jinfeng Hongyuan Flagship Store	Power Saving Specialist 1	You'll know if it saves power after you use it.	Jingdong prefecture level city in Shandong
Jinfeng Hongyuan Flagship Store	Power Saving Specialist 1	The quality is great. The quality is very good. The service is very good. Express delivery arrived in two days.	Jingdong prefecture level city in Shandong
Jinfeng Hongyuan Flagship Store	Power Saving Specialist 1	It works great.	Jingdong prefecture level city in Shandong
Jinfeng Hongyuan Flagship Store	Power Saving Specialist 1	Logistics is very fast, not yet used, used to comment again.	Jingdong prefecture level city in Shandong
Jinfeng Hongyuan Flagship Store	Power Saving Specialist 1	Logistics is very fast things are also very good, worthy of friends to buy	Jingdong prefecture level city in Shandong
Jinfeng Hongyuan Flagship Store	Power Saving Specialist 1	It works. It really works.	Jingdong prefecture level city in Shandong
Jinfeng Hongyuan Flagship Store	Power Saving Specialist 1	LANGUAGE COMMENTS: just received the shipment. I immediately plugged it in and used it. If you ask how effective. It depends on whether the next month's electricity bill has been reduced. If there is a decrease. I am buying more than one to use.	Jingdong prefecture level city in Shandong
Jinfeng Hongyuan Flagship Store	Power Saving Specialist 1	Well made, power saving, good and convenient!	Jingdong prefecture level city in Shandong
Jinfeng Hongyuan Flagship Store	Power Saving Specialist 1	I'm using it. I'll rate it next month.	Jingdong prefecture level city in Shandong
Jinfeng Hongyuan Flagship Store	Power Saving Specialist 1	Baby received Oh, the courier delivery really fast, with a good and then come back to the evaluation	Jingdong prefecture level city in Shandong
Jinfeng Hongyuan Flagship Store	Power Saving Specialist 1	Fast logistics, good packaging, worth it!	Jingdong prefecture level city in Shandong
Jinfeng Hongyuan Flagship Store	Power Saving Specialist 1	Customer service is okay. It's just that.	Jingdong prefecture level city in Shandong
Jinfeng Hongyuan Flagship Store	Power Saving Specialist 1	Poor power saving	Jingdong prefecture level city in Shandong
Jinfeng Hongyuan Flagship Store	Power Saving Specialist 1	I'll tell you when I've used it.	Jingdong prefecture level city in Shandong

Jinfeng Hongyuan Flagship Store	Power Saving Specialist 1	I think it's good.	Jingdong prefecture level city in Shandong
Jinfeng Hongyuan Flagship Store	Power Saving Specialist 1	This energy saver works very well and I recommend everyone to buy it!	Jingdong prefecture level city in Shandong
Jinfeng Hongyuan Flagship Store	Power Saving Specialist 1	I do not know how effective, just use half a month on the burn out, good business reissued a, and then use it after the follow-up comment.	Jingdong prefecture level city in Shandong
Jinfeng Hongyuan Flagship Store	Power Saving Specialist 1	in use	Jingdong prefecture level city in Shandong
Jinfeng Hongyuan Flagship Store	Power Saving Specialist 1	This thing is very poor. It's already only been used for ten days and it's broken. Now waiting for you to return it.	Jingdong prefecture level city in Shandong
Jinfeng Hongyuan Flagship Store	Power Saving Specialist 1	It's not the same as the seller's show.	Jingdong prefecture level city in Shandong
Jinfeng Hongyuan Flagship Store	Power Saving Specialist 1	What is the problem when I get it back from the CTS, it is plugged into the wall and the voltage shows 206v.	Jingdong prefecture level city in Shandong
Jinfeng Hongyuan Flagship Store	Power Saving Specialist 1	The logistics was fast and the stuff was perfectly packaged. It is in the process of trial.	Jingdong prefecture level city in Shandong
Jinfeng Hongyuan Flagship Store	Power Saving Specialist 1	This saver is particularly good to use can really save electricity, with two months and then look at the line if you buy one for the family	Jingdong prefecture level city in Shandong
Jinfeng Hongyuan Flagship Store	Power Saving Specialist 1	Novel appearance design, all aluminum alloy profile, also with a fuse device, voltage monitoring display. After receiving the goods, I directly cut the plug and connect it to the main power box under the leakage switch, hoping to save electricity, and then follow up later. Logistics is very fast, arrived the next	Jingdong prefecture level city in Shandong
Jinfeng Hongyuan Flagship Store	Power Saving Specialist 1	Received, very good and like, if necessary next time to buy ha ha	Jingdong prefecture level city in Shandong
Jinfeng Hongyuan Flagship Store	Power Saving Specialist 1	It's good. It's worth it. Get it if you need it.	Jingdong prefecture level city in Shandong
Jinfeng Hongyuan Flagship Store	Power Saving Specialist 1	It feels more than good and is worth having.	Jingdong prefecture level city in Shandong
Jinfeng Hongyuan Flagship Store	Power Saving Specialist 1	The goods arrived very good and fast first time to buy	Jingdong prefecture level city in Shandong
Jinfeng Hongyuan Flagship Store	Power Saving Specialist 1	The power saving is very good, I can save so much money every month, I will buy another one next time.	Jingdong prefecture level city in Shandong
Jinfeng Hongyuan Flagship Store	Power Saving Specialist 1	It's not what it says.	Jingdong prefecture level city in Shandong
Jinfeng Hongyuan Flagship Store	Power Saving Specialist 1	I have been using it for many days now and it saves electricity, it really does I have an 8 kW electric snail stove and it saves about $3.50 a day.	Jingdong prefecture level city in Shandong
Jinfeng Hongyuan Flagship Store	Power Saving Specialist 1	Express delivery is very fast, the quality is also quite good, worth buying!	Jingdong prefecture level city in Shandong
Jinfeng Hongyuan Flagship Store	Power Saving Specialist 1	Just received expecting the baby to work well.	Jingdong prefecture level city in Shandong
Jinfeng Hongyuan Flagship Store	Power Saving Specialist 1	Very good, there are more fruit, has bought a second!	Jingdong prefecture level city in Shandong
Jinfeng Hongyuan Flagship Store	Power Saving Specialist 1	Good is good So far, no power savings have been noticed Maybe it's too little to notice.	Jingdong prefecture level city in Shandong
Jinfeng Hongyuan Flagship Store	Power Saving Specialist 1	I just received the item and plugged it in to see what happens later.	Jingdong prefecture level city in Shandong
Jinfeng Hongyuan Flagship Store	Power Saving Specialist 1	Baby received, especially good, packaging is also good, the courier brother is very strong, thank you.	Jingdong prefecture level city in Shandong
Jinfeng Hongyuan Flagship Store	Power Saving Specialist 1	Just received the goods have not yet used it, do not know how, three months later in the follow-up comment!	Jingdong prefecture level city in Shandong
Jinfeng Hongyuan Flagship Store	Power Saving Specialist 1	The item was received, it is quite good, recommended to buy.	Jingdong prefecture level city in Shandong
Jinfeng Hongyuan Flagship Store	Power Saving Specialist 1	This energy saver works very well and I recommend everyone to buy it!	Jingdong prefecture level city in Shandong
Jinfeng Hongyuan Flagship Store	Power Saving Specialist 1	Voltage stabilization and current reduction nice power saving product.	Jingdong prefecture level city in Shandong
Jinfeng Hongyuan Flagship Store	Power Saving Specialist 1	Voltage stabilization and current reduction nice power saving product.	Jingdong prefecture level city in Shandong
Jinfeng Hongyuan Flagship Store	Power Saving Specialist 1	A good power saver for stabilizing and lowering current.	Jingdong prefecture level city in Shandong
Jinfeng Hongyuan Flagship Store	Power Saving Specialist 1	You'll know if it saves power after you use it.	Jingdong prefecture level city in Shandong
Jinfeng Hongyuan Flagship Store	Power Saving Specialist 1	The quality is great. The quality is very good. The service is very good. Express delivery arrived in two days.	Jingdong prefecture level city in Shandong
Jinfeng Hongyuan Flagship Store	Power Saving Specialist 1	It works great.	Jingdong prefecture level city in Shandong
Jinfeng Hongyuan Flagship Store	Power Saving Specialist 1	Logistics is very fast, not yet used, used to comment again.	Jingdong prefecture level city in Shandong
Jinfeng Hongyuan Flagship Store	Power Saving Specialist 1	Logistics is very fast things are also very good, worthy of friends to buy	Jingdong prefecture level city in Shandong
Jinfeng Hongyuan Flagship Store	Power Saving Specialist 1	It works. It really works.	Jingdong prefecture level city in Shandong
Jinfeng Hongyuan Flagship Store	Power Saving Specialist 1	LANGUAGE COMMENTS: just received the shipment. I immediately plugged it in and used it. If you ask how effective. It depends on whether the next month's electricity bill has been reduced. If there is a decrease. I am buying more than one to use.	Jingdong prefecture level city in Shandong
Jinfeng Hongyuan Flagship Store	Power Saving Specialist 1	Well made, power saving, good and convenient!	Jingdong prefecture level city in Shandong
Jinfeng Hongyuan Flagship Store	Power Saving Specialist 1	I'm using it. I'll rate it next month.	Jingdong prefecture level city in Shandong
Jinfeng Hongyuan Flagship Store	Power Saving Specialist 1	Baby received Oh, the courier delivery really fast, with a good and then come back to the evaluation	Jingdong prefecture level city in Shandong
Jinfeng Hongyuan Flagship Store	Power Saving Specialist 1	Fast logistics, good packaging, worth it!	Jingdong prefecture level city in Shandong
Jinfeng Hongyuan Flagship Store	Power Saving Specialist 1	Customer service is okay. It's just that.	Jingdong prefecture level city in Shandong
Jinfeng Hongyuan Flagship Store	Power Saving Specialist 1	Poor power saving	Jingdong prefecture level city in Shandong
Jinfeng Hongyuan Flagship Store	Power Saving Specialist 1	I'll tell you when I've used it.	Jingdong prefecture level city in Shandong
Jinfeng Hongyuan Flagship Store	Power Saving Specialist 1	I think it's good.	Jingdong prefecture level city in Shandong
Jinfeng Hongyuan Flagship Store	Power Saving Specialist 1	This energy saver works very well and I recommend everyone to buy it!	Jingdong prefecture level city in Shandong
Jinfeng Hongyuan Flagship Store	Power Saving Specialist 1	I do not know how effective, just use half a month on the burn out, good business reissued a, and then use it after the follow-up comment.	Jingdong prefecture level city in Shandong
Jinfeng Hongyuan Flagship Store	Power Saving Specialist 1	in use	Jingdong prefecture level city in Shandong
Jinfeng Hongyuan Flagship Store	Power Saving Specialist 1	This thing is very poor. It's already only been used for ten days and it's broken. Now waiting for you to return it.	Jingdong prefecture level city in Shandong
Jinfeng Hongyuan Flagship Store	Power Saving Specialist 1	It's not the same as the seller's show.	Jingdong prefecture level city in Shandong
Jinfeng Hongyuan Flagship Store	Power Saving Specialist 1	What is the problem when I get it back from the CTS, it is plugged into the wall and the voltage shows 206v.	Jingdong prefecture level city in Shandong

Jinfeng Hongyuan Flagship Store	Power Saving Specialist 1	The logistics was fast and the stuff was perfectly packaged. It is in the process of trial.	Jingdong prefecture level city in Shandong
Jinfeng Hongyuan Flagship Store	Power Saving Specialist 1	This saver is particularly good to use can really save electricity, with two months and then look at the line if you buy one for the family	Jingdong prefecture level city in Shandong
Jinfeng Hongyuan Flagship Store	Power Saving Specialist 1	Novel appearance design, all aluminum alloy profile, also with a fuse device, voltage monitoring display. After receiving the goods, I directly cut the plug and connect it to the main power box under the leakage switch, hoping to save electricity, and then follow up later. Logistics is very fast, arrived the next	Jingdong prefecture level city in Shandong
Jinfeng Hongyuan Flagship Store	Power Saving Specialist 1	Received, very good and like, if necessary next time to buy ha ha	Jingdong prefecture level city in Shandong
Jinfeng Hongyuan Flagship Store	Power Saving Specialist 1	It's good. It's worth it. Get it if you need it.	Jingdong prefecture level city in Shandong
Jinfeng Hongyuan Flagship Store	Power Saving Specialist 1	It feels more than good and is worth having.	Jingdong prefecture level city in Shandong
Jinfeng Hongyuan Flagship Store	Power Saving Specialist 1	The goods arrived very good and fast first time to buy	Jingdong prefecture level city in Shandong
Jinfeng Hongyuan Flagship Store	Power Saving Specialist 1	The power saving is very good, I can save so much money every month, I will buy another one next time.	Jingdong prefecture level city in Shandong
Jinfeng Hongyuan Flagship Store	Power Saving Specialist 1	It's not what it says.	Jingdong prefecture level city in Shandong
Jinfeng Hongyuan Flagship Store	Power Saving Specialist 1	I have been using it for many days now and it saves electricity, it really does I have an 8 kW electric snail stove and it saves about $3.50 a day.	Jingdong prefecture level city in Shandong
Jinfeng Hongyuan Flagship Store	Power Saving Specialist 1	Express delivery is very fast, the quality is also quite good, worth buying!	Jingdong prefecture level city in Shandong
Jinfeng Hongyuan Flagship Store	Power Saving Specialist 1	Just received expecting the baby to work well.	Jingdong prefecture level city in Shandong
Jinfeng Hongyuan Flagship Store	Power Saving Specialist 1	Very good, there are more fruit, has bought a second!	Jingdong prefecture level city in Shandong
Jinfeng Hongyuan Flagship Store	Power Saving Specialist 1	Good is good So far, no power savings have been noticed Maybe it's too little to notice.	Jingdong prefecture level city in Shandong
Jinfeng Hongyuan Flagship Store	Power Saving Specialist 1	I just received the item and plugged it in to see what happens later.	Jingdong prefecture level city in Shandong
Jinfeng Hongyuan Flagship Store	Power Saving Specialist 1	Baby received, especially good, packaging is also good, the courier brother is very strong, thank you.	Jingdong prefecture level city in Shandong
Jinfeng Hongyuan Flagship Store	Power Saving Specialist 1	Just received the goods have not yet used it, do not know how, three months later in the follow-up comment!	Jingdong prefecture level city in Shandong
Jinfeng Hongyuan Flagship Store	Power Saving Specialist 1	The item was received, it is quite good, recommended to buy.	Jingdong prefecture level city in Shandong
Jinfeng Hongyuan Flagship Store	Power Saving Specialist 1	This energy saver works very well and I recommend everyone to buy it!	Jingdong prefecture level city in Shandong
Jinfeng Hongyuan Flagship Store	Power Saving Specialist 1	Voltage stabilization and current reduction nice power saving product.	Jingdong prefecture level city in Shandong
Jinfeng Hongyuan Flagship Store	Power Saving Specialist 1	Voltage stabilization and current reduction nice power saving product.	Jingdong prefecture level city in Shandong
Jinfeng Hongyuan Flagship Store	Power Saving Specialist 1	A good power saver for stabilizing and lowering current.	Jingdong prefecture level city in Shandong
Jinfeng Hongyuan Flagship Store	Power Saving Specialist 1	You'll know if it saves power after you use it.	Jingdong prefecture level city in Shandong
Jinfeng Hongyuan Flagship Store	Power Saving Specialist 1	The quality is great. The quality is very good. The service is very good. Express delivery arrived in two days.	Jingdong prefecture level city in Shandong
Jinfeng Hongyuan Flagship Store	Power Saving Specialist 1	It works great.	Jingdong prefecture level city in Shandong
Jinfeng Hongyuan Flagship Store	Power Saving Specialist 1	Logistics is very fast, not yet used, used to comment again.	Jingdong prefecture level city in Shandong
Jinfeng Hongyuan Flagship Store	Power Saving Specialist 1	Logistics is very fast things are also very good, worthy of friends to buy	Jingdong prefecture level city in Shandong
Jinfeng Hongyuan Flagship Store	Power Saving Specialist 1	It works. It really works.	Jingdong prefecture level city in Shandong
Jinfeng Hongyuan Flagship Store	Power Saving Specialist 1	LANGUAGE COMMENTS: just received the shipment. I immediately plugged it in and used it. If you ask how effective. It depends on whether the next month's electricity bill has been reduced. If there is a decrease. I am buying more than one to use.	Jingdong prefecture level city in Shandong
Jinfeng Hongyuan Flagship Store	Power Saving Specialist 1	Well made, power saving, good and convenient!	Jingdong prefecture level city in Shandong
Jinfeng Hongyuan Flagship Store	Power Saving Specialist 1	I'm using it. I'll rate it next month.	Jingdong prefecture level city in Shandong
Jinfeng Hongyuan Flagship Store	Power Saving Specialist 1	Baby received Oh, the courier delivery really fast, with a good and then come back to the evaluation	Jingdong prefecture level city in Shandong
Jinfeng Hongyuan Flagship Store	Power Saving Specialist 1	Fast logistics, good packaging, worth it!	Jingdong prefecture level city in Shandong
Jinfeng Hongyuan Flagship Store	Power Saving Specialist 1	Customer service is okay. It's just that.	Jingdong prefecture level city in Shandong
Jinfeng Hongyuan Flagship Store	Power Saving Specialist 1	Poor power saving	Jingdong prefecture level city in Shandong
Jinfeng Hongyuan Flagship Store	Power Saving Specialist 1	I'll tell you when I've used it.	Jingdong prefecture level city in Shandong
Jinfeng Hongyuan Flagship Store	Power Saving Specialist 1	I think it's good.	Jingdong prefecture level city in Shandong
Jinfeng Hongyuan Flagship Store	Power Saving Specialist 1	This energy saver works very well and I recommend everyone to buy it!	Jingdong prefecture level city in Shandong
Jinfeng Hongyuan Flagship Store	Power Saving Specialist 1	I do not know how effective, just use half a month on the burn out, good business reissued a, and then use it after the follow-up comment.	Jingdong prefecture level city in Shandong
Jinfeng Hongyuan Flagship Store	Power Saving Specialist 1	in use	Jingdong prefecture level city in Shandong
Jinfeng Hongyuan Flagship Store	Power Saving Specialist 1	This thing is very poor. It's already only been used for ten days and it's broken. Now waiting for you to return it.	Jingdong prefecture level city in Shandong
Jinfeng Hongyuan Flagship Store	Power Saving Specialist 1	It's not the same as the seller's show.	Jingdong prefecture level city in Shandong
Jinfeng Hongyuan Flagship Store	Power Saving Specialist 1	What is the problem when I get it back from the CTS, it is plugged into the wall and the voltage shows 206v.	Jingdong prefecture level city in Shandong
Jinfeng Hongyuan Flagship Store	Power Saving Specialist 1	The logistics was fast and the stuff was perfectly packaged. It is in the process of trial.	Jingdong prefecture level city in Shandong
Jinfeng Hongyuan Flagship Store	Power Saving Specialist 1	This saver is particularly good to use can really save electricity, with two months and then look at the line if you buy one for the family	Jingdong prefecture level city in Shandong
Jinfeng Hongyuan Flagship Store	Power Saving Specialist 1	Novel appearance design, all aluminum alloy profile, also with a fuse device, voltage monitoring display. After receiving the goods, I directly cut the plug and connect it to the main power box under the leakage switch, hoping to save electricity, and then follow up later. Logistics is very fast, arrived the next	Jingdong prefecture level city in Shandong
Jinfeng Hongyuan Flagship Store	Power Saving Specialist 1	Received, very good and like, if necessary next time to buy ha ha	Jingdong prefecture level city in Shandong
Jinfeng Hongyuan Flagship Store	Power Saving Specialist 1	It's good. It's worth it. Get it if you need it.	Jingdong prefecture level city in Shandong
Jinfeng Hongyuan Flagship Store	Power Saving Specialist 1	It feels more than good and is worth having.	Jingdong prefecture level city in Shandong
Jinfeng Hongyuan Flagship Store	Power Saving Specialist 1	The goods arrived very good and fast first time to buy	Jingdong prefecture level city in Shandong

Jinfeng Hongyuan Flagship Store	Power Saving Specialist 1	The power saving is very good, I can save so much money every month, I will buy another one next time.	Jingdong prefecture level city in Shandong
Jinfeng Hongyuan Flagship Store	Power Saving Specialist 1	It’s not what it says.	Jingdong prefecture level city in Shandong
Jinfeng Hongyuan Flagship Store	Power Saving Specialist 1	I have been using it for many days now and it saves electricity, it really does I have an 8 kW electric snail stove and it saves about $3.50 a day.	Jingdong prefecture level city in Shandong
Jinfeng Hongyuan Flagship Store	Power Saving Specialist 1	Express delivery is very fast, the quality is also quite good, worth buying!	Jingdong prefecture level city in Shandong
Jinfeng Hongyuan Flagship Store	Power Saving Specialist 1	Just received expecting the baby to work well.	Jingdong prefecture level city in Shandong
Jinfeng Hongyuan Flagship Store	Power Saving Specialist 1	Very good, there are more fruit, has bought a second!	Jingdong prefecture level city in Shandong
Jinfeng Hongyuan Flagship Store	Power Saving Specialist 1	Good is good So far, no power savings have been noticed Maybe it’s too little to notice.	Jingdong prefecture level city in Shandong
Jinfeng Hongyuan Flagship Store	Power Saving Specialist 1	I just received the item and plugged it in to see what happens later.	Jingdong prefecture level city in Shandong
Jinfeng Hongyuan Flagship Store	Power Saving Specialist 1	Baby received, especially good, packaging is also good, the courier brother is very strong, thank you.	Jingdong prefecture level city in Shandong
Jinfeng Hongyuan Flagship Store	Power Saving Specialist 1	Just received the goods have not yet used it, do not know how, three months later in the follow-up comment!	Jingdong prefecture level city in Shandong
Jinfeng Hongyuan Flagship Store	Power Saving Specialist 1	The item was received, it is quite good, recommended to buy.	Jingdong prefecture level city in Shandong
Jinfeng Hongyuan Flagship Store	Power Saving Specialist 1	This energy saver works very well and I recommend everyone to buy it!	Jingdong prefecture level city in Shandong
Jinfeng Hongyuan Flagship Store	Power Saving Specialist 1	Voltage stabilization and current reduction nice power saving product.	Jingdong prefecture level city in Shandong
Jinfeng Hongyuan Flagship Store	Power Saving Specialist 1	Voltage stabilization and current reduction nice power saving product.	Jingdong prefecture level city in Shandong
Jinfeng Hongyuan Flagship Store	Power Saving Specialist 1	A good power saver for stabilizing and lowering current.	Jingdong prefecture level city in Shandong
Jinfeng Hongyuan Flagship Store	Power Saving Specialist 1	You’ll know if it saves power after you use it.	Jingdong prefecture level city in Shandong
Jinfeng Hongyuan Flagship Store	Power Saving Specialist 1	The quality is great. The quality is very good. The service is very good. Express delivery arrived in two days.	Jingdong prefecture level city in Shandong
Jinfeng Hongyuan Flagship Store	Power Saving Specialist 1	It works great.	Jingdong prefecture level city in Shandong
Jinfeng Hongyuan Flagship Store	Power Saving Specialist 1	Logistics is very fast, not yet used, used to comment again.	Jingdong prefecture level city in Shandong
Jinfeng Hongyuan Flagship Store	Power Saving Specialist 1	Logistics is very fast things are also very good, worthy of friends to buy	Jingdong prefecture level city in Shandong
Jinfeng Hongyuan Flagship Store	Power Saving Specialist 1	It works. It really works.	Jingdong prefecture level city in Shandong
Jinfeng Hongyuan Flagship Store	Power Saving Specialist 1	LANGUAGE COMMENTS: just received the shipment. I immediately plugged it in and used it. If you ask how effective. It depends on whether the next month’s electricity bill has been reduced. If there is a decrease. I am buying more than one to use.	Jingdong prefecture level city in Shandong
Jinfeng Hongyuan Flagship Store	Power Saving Specialist 1	Well made, power saving, good and convenient!	Jingdong prefecture level city in Shandong
Jinfeng Hongyuan Flagship Store	Power Saving Specialist 1	I’m using it. I’ll rate it next month.	Jingdong prefecture level city in Shandong
Jinfeng Hongyuan Flagship Store	Power Saving Specialist 1	Baby received Oh, the courier delivery really fast, with a good and then come back to the evaluation	Jingdong prefecture level city in Shandong
Jinfeng Hongyuan Flagship Store	Power Saving Specialist 1	Fast logistics, good packaging, worth it!	Jingdong prefecture level city in Shandong
Jinfeng Hongyuan Flagship Store	Power Saving Specialist 1	Customer service is okay. It’s just that.	Jingdong prefecture level city in Shandong
Jinfeng Hongyuan Flagship Store	Power Saving Specialist 1	Poor power saving	Jingdong prefecture level city in Shandong
Jinfeng Hongyuan Flagship Store	Power Saving Specialist 1	I’ll tell you when I’ve used it.	Jingdong prefecture level city in Shandong
Jinfeng Hongyuan Flagship Store	Power Saving Specialist 1	I think it’s good.	Jingdong prefecture level city in Shandong
Jinfeng Hongyuan Flagship Store	Power Saving Specialist 1	This energy saver works very well and I recommend everyone to buy it!	Jingdong prefecture level city in Shandong
Jinfeng Hongyuan Flagship Store	Power Saving Specialist 1	I do not know how effective, just use half a month on the burn out, good business reissued a, and then use it after the follow-up comment.	Jingdong prefecture level city in Shandong
Jinfeng Hongyuan Flagship Store	Power Saving Specialist 1	in use	Jingdong prefecture level city in Shandong
Jinfeng Hongyuan Flagship Store	Power Saving Specialist 1	This thing is very poor. It’s already only been used for ten days and it’s broken. Now waiting for you to return it.	Jingdong prefecture level city in Shandong
Jinfeng Hongyuan Flagship Store	Power Saving Specialist 1	It’s not the same as the seller’s show.	Jingdong prefecture level city in Shandong
Jinfeng Hongyuan Flagship Store	Power Saving Specialist 1	What is the problem when I get it back from the CTS, it is plugged into the wall and the voltage shows 206v.	Jingdong prefecture level city in Shandong
Jinfeng Hongyuan Flagship Store	Power Saving Specialist 1	The logistics was fast and the stuff was perfectly packaged. It is in the process of trial.	Jingdong prefecture level city in Shandong
Jinfeng Hongyuan Flagship Store	Power Saving Specialist 1	This saver is particularly good to use can really save electricity, with two months and then look at the line if you buy one for the family	Jingdong prefecture level city in Shandong
Jinfeng Hongyuan Flagship Store	Power Saving Specialist 1	Novel appearance design, all aluminum alloy profile, also with a fuse device, voltage monitoring display. After receiving the goods, I directly cut the plug and connect it to the main power box under the leakage switch, hoping to save electricity, and then follow up later. Logistics is very fast, arrived the next	Jingdong prefecture level city in Shandong
Jinfeng Hongyuan Flagship Store	Power Saving Specialist 1	Received, very good and like, if necessary next time to buy ha ha	Jingdong prefecture level city in Shandong
Jinfeng Hongyuan Flagship Store	Power Saving Specialist 1	It’s good. It’s worth it. Get it if you need it.	Jingdong prefecture level city in Shandong
Jinfeng Hongyuan Flagship Store	Power Saving Specialist 1	It feels more than good and is worth having.	Jingdong prefecture level city in Shandong
Jinfeng Hongyuan Flagship Store	Power Saving Specialist 1	The goods arrived very good and fast first time to buy	Jingdong prefecture level city in Shandong
Jinfeng Hongyuan Flagship Store	Power Saving Specialist 1	The power saving is very good, I can save so much money every month, I will buy another one next time.	Jingdong prefecture level city in Shandong
Jinfeng Hongyuan Flagship Store	Power Saving Specialist 1	It’s not what it says.	Jingdong prefecture level city in Shandong
Jinfeng Hongyuan Flagship Store	Power Saving Specialist 1	I have been using it for many days now and it saves electricity, it really does I have an 8 kW electric snail stove and it saves about $3.50 a day.	Jingdong prefecture level city in Shandong
Jinfeng Hongyuan Flagship Store	Power Saving Specialist 1	Express delivery is very fast, the quality is also quite good, worth buying!	Jingdong prefecture level city in Shandong
Jinfeng Hongyuan Flagship Store	Power Saving Specialist 1	Just received expecting the baby to work well.	Jingdong prefecture level city in Shandong
Jinfeng Hongyuan Flagship Store	Power Saving Specialist 1	Very good, there are more fruit, has bought a second!	Jingdong prefecture level city in Shandong
Jinfeng Hongyuan Flagship Store	Power Saving Specialist 1	Good is good So far, no power savings have been noticed Maybe it’s too little to notice.	Jingdong prefecture level city in Shandong

Jinfeng Hongyuan Flagship Store	Power Saving Specialist 1	I just received the item and plugged it in to see what happens later.	Jingdong prefecture level city in Shandong
Jinfeng Hongyuan Flagship Store	Power Saving Specialist 1	Baby received, especially good, packaging is also good, the courier brother is very strong, thank you.	Jingdong prefecture level city in Shandong
Jinfeng Hongyuan Flagship Store	Power Saving Specialist 1	Just received the goods have not yet used it, do not know how, three months later in the follow-up comment!	Jingdong prefecture level city in Shandong
Jinfeng Hongyuan Flagship Store	Power Saving Specialist 1	The item was received, it is quite good, recommended to buy.	Jingdong prefecture level city in Shandong
Jinfeng Hongyuan Flagship Store	Power Saving Specialist 1	This energy saver works very well and I recommend everyone to buy it!	Jingdong prefecture level city in Shandong
Jinfeng Hongyuan Flagship Store	Power Saving Specialist 1	Voltage stabilization and current reduction nice power saving product.	Jingdong prefecture level city in Shandong
Jinfeng Hongyuan Flagship Store	Power Saving Specialist 1	Voltage stabilization and current reduction nice power saving product.	Jingdong prefecture level city in Shandong
Jinfeng Hongyuan Flagship Store	Power Saving Specialist 1	A good power saver for stabilizing and lowering current.	Jingdong prefecture level city in Shandong
Jinfeng Hongyuan Flagship Store	Power Saving Specialist 1	You'll know if it saves power after you use it.	Jingdong prefecture level city in Shandong
Jinfeng Hongyuan Flagship Store	Power Saving Specialist 1	The quality is great. The quality is very good. The service is very good. Express delivery arrived in two days.	Jingdong prefecture level city in Shandong
Jinfeng Hongyuan Flagship Store	Power Saving Specialist 1	It works great.	Jingdong prefecture level city in Shandong
Jinfeng Hongyuan Flagship Store	Power Saving Specialist 1	Logistics is very fast, not yet used, used to comment again.	Jingdong prefecture level city in Shandong
Jinfeng Hongyuan Flagship Store	Power Saving Specialist 1	Logistics is very fast things are also very good, worthy of friends to buy	Jingdong prefecture level city in Shandong
Jinfeng Hongyuan Flagship Store	Power Saving Specialist 1	It works. It really works.	Jingdong prefecture level city in Shandong
Jinfeng Hongyuan Flagship Store	Power Saving Specialist 1	LANGUAGE COMMENTS: just received the shipment. I immediately plugged it in and used it. If you ask how effective. It depends on whether the next month's electricity bill has been reduced. If there is a decrease. I am buying more than one to use.	Jingdong prefecture level city in Shandong
Jinfeng Hongyuan Flagship Store	Power Saving Specialist 1	Well made, power saving, good and convenient!	Jingdong prefecture level city in Shandong
Jinfeng Hongyuan Flagship Store	Power Saving Specialist 1	I'm using it. I'll rate it next month.	Jingdong prefecture level city in Shandong
Jinfeng Hongyuan Flagship Store	Power Saving Specialist 1	Baby received Oh, the courier delivery really fast, with a good and then come back to the evaluation	Jingdong prefecture level city in Shandong
Jinfeng Hongyuan Flagship Store	Power Saving Specialist 1	Fast logistics, good packaging, worth it!	Jingdong prefecture level city in Shandong
Jinfeng Hongyuan Flagship Store	Power Saving Specialist 1	Customer service is okay. It's just that.	Jingdong prefecture level city in Shandong
Jinfeng Hongyuan Flagship Store	Power Saving Specialist 1	Poor power saving	Jingdong prefecture level city in Shandong
Jinfeng Hongyuan Flagship Store	Power Saving Specialist 1	I'll tell you when I've used it.	Jingdong prefecture level city in Shandong
Jinfeng Hongyuan Flagship Store	Power Saving Specialist 1	I think it's good.	Jingdong prefecture level city in Shandong
Jinfeng Hongyuan Flagship Store	Power Saving Specialist 1	This energy saver works very well and I recommend everyone to buy it!	Jingdong prefecture level city in Shandong
Jinfeng Hongyuan Flagship Store	Power Saving Specialist 1	I do not know how effective, just use half a month on the burn out, good business reissued a, and then use it after the follow-up comment.	Jingdong prefecture level city in Shandong
Jinfeng Hongyuan Flagship Store	Power Saving Specialist 1	in use	Jingdong prefecture level city in Shandong
Jinfeng Hongyuan Flagship Store	Power Saving Specialist 1	This thing is very poor. It's already only been used for ten days and it's broken. Now waiting for you to return it.	Jingdong prefecture level city in Shandong
Jinfeng Hongyuan Flagship Store	Power Saving Specialist 1	It's not the same as the seller's show.	Jingdong prefecture level city in Shandong
Jinfeng Hongyuan Flagship Store	Power Saving Specialist 1	What is the problem when I get it back from the CTS, it is plugged into the wall and the voltage shows 206v.	Jingdong prefecture level city in Shandong
Jinfeng Hongyuan Flagship Store	Power Saving Specialist 1	The logistics was fast and the stuff was perfectly packaged. It is in the process of trial.	Jingdong prefecture level city in Shandong
Jinfeng Hongyuan Flagship Store	Power Saving Specialist 1	This saver is particularly good to use can really save electricity, with two months and then look at the line if you buy one for the family	Jingdong prefecture level city in Shandong
Jinfeng Hongyuan Flagship Store	Power Saving Specialist 1	Novel appearance design, all aluminum alloy profile, also with a fuse device, voltage monitoring display. After receiving the goods, I directly cut the plug and connect it to the main power box under the leakage switch, hoping to save electricity, and then follow up later. Logistics is very fast, arrived the next	Jingdong prefecture level city in Shandong
Jinfeng Hongyuan Flagship Store	Power Saving Specialist 1	Received, very good and like, if necessary next time to buy ha ha	Jingdong prefecture level city in Shandong
Jinfeng Hongyuan Flagship Store	Power Saving Specialist 1	It's good. It's worth it. Get it if you need it.	Jingdong prefecture level city in Shandong
Jinfeng Hongyuan Flagship Store	Power Saving Specialist 1	It feels more than good and is worth having.	Jingdong prefecture level city in Shandong
Jinfeng Hongyuan Flagship Store	Power Saving Specialist 1	The goods arrived very good and fast first time to buy	Jingdong prefecture level city in Shandong
Jinfeng Hongyuan Flagship Store	Power Saving Specialist 1	The power saving is very good, I can save so much money every month, I will buy another one next time.	Jingdong prefecture level city in Shandong
Jinfeng Hongyuan Flagship Store	Power Saving Specialist 1	It's not what it says.	Jingdong prefecture level city in Shandong
Jinfeng Hongyuan Flagship Store	Power Saving Specialist 1	I have been using it for many days now and it saves electricity, it really does I have an 8 kW electric snail stove and it saves about $3.50 a day.	Jingdong prefecture level city in Shandong
Jinfeng Hongyuan Flagship Store	Power Saving Specialist 1	Express delivery is very fast, the quality is also quite good, worth buying!	Jingdong prefecture level city in Shandong
Jinfeng Hongyuan Flagship Store	Power Saving Specialist 1	Just received expecting the baby to work well.	Jingdong prefecture level city in Shandong
Jinfeng Hongyuan Flagship Store	Power Saving Specialist 1	Very good, there are more fruit, has bought a second!	Jingdong prefecture level city in Shandong
Jinfeng Hongyuan Flagship Store	Power Saving Specialist 1	Good is good So far, no power savings have been noticed Maybe it's too little to notice.	Jingdong prefecture level city in Shandong
Jinfeng Hongyuan Flagship Store	Power Saving Specialist 1	I just received the item and plugged it in to see what happens later.	Jingdong prefecture level city in Shandong
Jinfeng Hongyuan Flagship Store	Power Saving Specialist 1	Baby received, especially good, packaging is also good, the courier brother is very strong, thank you.	Jingdong prefecture level city in Shandong
Jinfeng Hongyuan Flagship Store	Power Saving Specialist 1	Just received the goods have not yet used it, do not know how, three months later in the follow-up comment!	Jingdong prefecture level city in Shandong
Jinfeng Hongyuan Flagship Store	Power Saving Specialist 1	The item was received, it is quite good, recommended to buy.	Jingdong prefecture level city in Shandong
Jinfeng Hongyuan Flagship Store	Power Saving Specialist 1	This energy saver works very well and I recommend everyone to buy it!	Jingdong prefecture level city in Shandong
Jinfeng Hongyuan Flagship Store	Power Saving Specialist 1	Voltage stabilization and current reduction nice power saving product.	Jingdong prefecture level city in Shandong
Jinfeng Hongyuan Flagship Store	Power Saving Specialist 1	Voltage stabilization and current reduction nice power saving product.	Jingdong prefecture level city in Shandong

Jinfeng Hongyuan Flagship Store	Power Saving Specialist 1	A good power saver for stabilizing and lowering current.	Jingdong prefecture level city in Shandong
Jinfeng Hongyuan Flagship Store	Power Saving Specialist 1	You'll know if it saves power after you use it.	Jingdong prefecture level city in Shandong
Jinfeng Hongyuan Flagship Store	Power Saving Specialist 1	The quality is great. The quality is very good. The service is very good. Express delivery arrived in two days.	Jingdong prefecture level city in Shandong
Jinfeng Hongyuan Flagship Store	Power Saving Specialist 1	It works great.	Jingdong prefecture level city in Shandong
Jinfeng Hongyuan Flagship Store	Power Saving Specialist 1	Logistics is very fast, not yet used, used to comment again.	Jingdong prefecture level city in Shandong
Jinfeng Hongyuan Flagship Store	Power Saving Specialist 1	Logistics is very fast things are also very good, worthy of friends to buy	Jingdong prefecture level city in Shandong
Jinfeng Hongyuan Flagship Store	Power Saving Specialist 1	It works. It really works.	Jingdong prefecture level city in Shandong
Jinfeng Hongyuan Flagship Store	Power Saving Specialist 1	LANGUAGE COMMENTS: just received the shipment. I immediately plugged it in and used it. If you ask how effective. It depends on whether the next month's electricity bill has been reduced. If there is a decrease. I am buying more than one to use.	Jingdong prefecture level city in Shandong
Jinfeng Hongyuan Flagship Store	Power Saving Specialist 1	Well made, power saving, good and convenient!	Jingdong prefecture level city in Shandong
Jinfeng Hongyuan Flagship Store	Power Saving Specialist 1	I'm using it. I'll rate it next month.	Jingdong prefecture level city in Shandong
Jinfeng Hongyuan Flagship Store	Power Saving Specialist 1	Baby received Oh, the courier delivery really fast, with a good and then come back to the evaluation	Jingdong prefecture level city in Shandong
Jinfeng Hongyuan Flagship Store	Power Saving Specialist 1	Fast logistics, good packaging, worth it!	Jingdong prefecture level city in Shandong
Jinfeng Hongyuan Flagship Store	Power Saving Specialist 1	Customer service is okay. It's just that.	Jingdong prefecture level city in Shandong
Jinfeng Hongyuan Flagship Store	Power Saving Specialist 1	Poor power saving	Jingdong prefecture level city in Shandong
Jinfeng Hongyuan Flagship Store	Power Saving Specialist 1	I'll tell you when I've used it.	Jingdong prefecture level city in Shandong
Jinfeng Hongyuan Flagship Store	Power Saving Specialist 1	I think it's good.	Jingdong prefecture level city in Shandong
Jinfeng Hongyuan Flagship Store	Power Saving Specialist 1	This energy saver works very well and I recommend everyone to buy it!	Jingdong prefecture level city in Shandong
Jinfeng Hongyuan Flagship Store	Power Saving Specialist 1	I do not know how effective, just use half a month on the burn out, good business reissued a, and then use it after the follow-up comment.	Jingdong prefecture level city in Shandong
Jinfeng Hongyuan Flagship Store	Power Saving Specialist 1	in use	Jingdong prefecture level city in Shandong
Jinfeng Hongyuan Flagship Store	Power Saving Specialist 1	This thing is very poor. It's already only been used for ten days and it's broken. Now waiting for you to return it.	Jingdong prefecture level city in Shandong
Jinfeng Hongyuan Flagship Store	Power Saving Specialist 1	It's not the same as the seller's show.	Jingdong prefecture level city in Shandong
Jinfeng Hongyuan Flagship Store	Power Saving Specialist 1	What is the problem when I get it back from the CTS, it is plugged into the wall and the voltage shows 206v.	Jingdong prefecture level city in Shandong
Jinfeng Hongyuan Flagship Store	Power Saving Specialist 1	The logistics was fast and the stuff was perfectly packaged. It is in the process of trial.	Jingdong prefecture level city in Shandong
Jinfeng Hongyuan Flagship Store	Power Saving Specialist 1	This saver is particularly good to use can really save electricity, with two months and then look at the line if you buy one for the family	Jingdong prefecture level city in Shandong
Jinfeng Hongyuan Flagship Store	Power Saving Specialist 1	Novel appearance design, all aluminum alloy profile, also with a fuse device, voltage monitoring display. After receiving the goods, I directly cut the plug and connect it to the main power box under the leakage switch, hoping to save electricity, and then follow up later. Logistics is very fast, arrived the next	Jingdong prefecture level city in Shandong
Jinfeng Hongyuan Flagship Store	Power Saving Specialist 1	Received, very good and like, if necessary next time to buy ha ha	Jingdong prefecture level city in Shandong
Jinfeng Hongyuan Flagship Store	Power Saving Specialist 1	It's good. It's worth it. Get it if you need it.	Jingdong prefecture level city in Shandong
Jinfeng Hongyuan Flagship Store	Power Saving Specialist 1	It feels more than good and is worth having.	Jingdong prefecture level city in Shandong
Jinfeng Hongyuan Flagship Store	Power Saving Specialist 1	The goods arrived very good and fast first time to buy	Jingdong prefecture level city in Shandong
Jinfeng Hongyuan Flagship Store	Power Saving Specialist 1	The power saving is very good, I can save so much money every month, I will buy another one next time.	Jingdong prefecture level city in Shandong
Jinfeng Hongyuan Flagship Store	Power Saving Specialist 1	It's not what it says.	Jingdong prefecture level city in Shandong
Jinfeng Hongyuan Flagship Store	Power Saving Specialist 1	I have been using it for many days now and it saves electricity, it really does I have an 8 kW electric snail stove and it saves about $3.50 a day.	Jingdong prefecture level city in Shandong
Jinfeng Hongyuan Flagship Store	Power Saving Specialist 1	Express delivery is very fast, the quality is also quite good, worth buying!	Jingdong prefecture level city in Shandong
Jinfeng Hongyuan Flagship Store	Power Saving Specialist 1	Just received expecting the baby to work well.	Jingdong prefecture level city in Shandong
Jinfeng Hongyuan Flagship Store	Power Saving Specialist 1	Very good, there are more fruit, has bought a second!	Jingdong prefecture level city in Shandong
Jinfeng Hongyuan Flagship Store	Power Saving Specialist 1	Good is good So far, no power savings have been noticed Maybe it's too little to notice.	Jingdong prefecture level city in Shandong
Jinfeng Hongyuan Flagship Store	Power Saving Specialist 1	I just received the item and plugged it in to see what happens later.	Jingdong prefecture level city in Shandong
Jinfeng Hongyuan Flagship Store	Power Saving Specialist 1	Baby received, especially good, packaging is also good, the courier brother is very strong, thank you.	Jingdong prefecture level city in Shandong
Jinfeng Hongyuan Flagship Store	Power Saving Specialist 1	Just received the goods have not yet used it, do not know how, three months later in the follow-up comment!	Jingdong prefecture level city in Shandong
Jinfeng Hongyuan Flagship Store	Power Saving Specialist 1	The item was received, it is quite good, recommended to buy.	Jingdong prefecture level city in Shandong
Jinfeng Hongyuan Flagship Store	Power Saving Specialist 1	This energy saver works very well and I recommend everyone to buy it!	Jingdong prefecture level city in Shandong
Jinfeng Hongyuan Flagship Store	Power Saving Specialist 1	Voltage stabilization and current reduction nice power saving product.	Jingdong prefecture level city in Shandong
Jinfeng Hongyuan Flagship Store	Power Saving Specialist 1	Voltage stabilization and current reduction nice power saving product.	Jingdong prefecture level city in Shandong
Jinfeng Hongyuan Flagship Store	Power Saving Specialist 1	A good power saver for stabilizing and lowering current.	Jingdong prefecture level city in Shandong
Jinfeng Hongyuan Flagship Store	Power Saving Specialist 1	You'll know if it saves power after you use it.	Jingdong prefecture level city in Shandong
Jinfeng Hongyuan Flagship Store	Power Saving Specialist 1	The quality is great. The quality is very good. The service is very good. Express delivery arrived in two days.	Jingdong prefecture level city in Shandong
Jinfeng Hongyuan Flagship Store	Power Saving Specialist 1	It works great.	Jingdong prefecture level city in Shandong
Jinfeng Hongyuan Flagship Store	Power Saving Specialist 1	Logistics is very fast, not yet used, used to comment again.	Jingdong prefecture level city in Shandong
Jinfeng Hongyuan Flagship Store	Power Saving Specialist 1	Logistics is very fast things are also very good, worthy of friends to buy	Jingdong prefecture level city in Shandong
Jinfeng Hongyuan Flagship Store	Power Saving Specialist 1	It works. It really works.	Jingdong prefecture level city in Shandong

Jinfeng Hongyuan Flagship Store	Power Saving Specialist 1	LANGUAGE COMMENTS: just received the shipment. I immediately plugged it in and used it. If you ask how effective. It depends on whether the next month's electricity bill has been reduced. If there is a decrease. I am buying more than one to use.	Jingdong prefecture level city in Shandong
Jinfeng Hongyuan Flagship Store	Power Saving Specialist 1	Well made, power saving, good and convenient!	Jingdong prefecture level city in Shandong
Jinfeng Hongyuan Flagship Store	Power Saving Specialist 1	I'm using it. I'll rate it next month.	Jingdong prefecture level city in Shandong
Jinfeng Hongyuan Flagship Store	Power Saving Specialist 1	Baby received Oh, the courier delivery really fast, with a good and then come back to the evaluation	Jingdong prefecture level city in Shandong
Jinfeng Hongyuan Flagship Store	Power Saving Specialist 1	Fast logistics, good packaging, worth it!	Jingdong prefecture level city in Shandong
Jinfeng Hongyuan Flagship Store	Power Saving Specialist 1	Customer service is okay. It's just that.	Jingdong prefecture level city in Shandong
Jinfeng Hongyuan Flagship Store	Power Saving Specialist 1	Poor power saving	Jingdong prefecture level city in Shandong
Jinfeng Hongyuan Flagship Store	Power Saving Specialist 1	I'll tell you when I've used it.	Jingdong prefecture level city in Shandong
Jinfeng Hongyuan Flagship Store	Power Saving Specialist 1	I think it's good.	Jingdong prefecture level city in Shandong
Jinfeng Hongyuan Flagship Store	Power Saving Specialist 1	This energy saver works very well and I recommend everyone to buy it!	Jingdong prefecture level city in Shandong
Jinfeng Hongyuan Flagship Store	Power Saving Specialist 1	I do not know how effective, just use half a month on the burn out, good business reissued a, and then use it after the follow-up comment.	Jingdong prefecture level city in Shandong
Jinfeng Hongyuan Flagship Store	Power Saving Specialist 1	in use	Jingdong prefecture level city in Shandong
Jinfeng Hongyuan Flagship Store	Power Saving Specialist 1	This thing is very poor. It's already only been used for ten days and it's broken. Now waiting for you to return it.	Jingdong prefecture level city in Shandong
Jinfeng Hongyuan Flagship Store	Power Saving Specialist 1	It's not the same as the seller's show.	Jingdong prefecture level city in Shandong
Jinfeng Hongyuan Flagship Store	Power Saving Specialist 1	What is the problem when I get it back from the CTS, it is plugged into the wall and the voltage shows 206v.	Jingdong prefecture level city in Shandong
Jinfeng Hongyuan Flagship Store	Power Saving Specialist 1	The logistics was fast and the stuff was perfectly packaged. It is in the process of trial.	Jingdong prefecture level city in Shandong
Jinfeng Hongyuan Flagship Store	Power Saving Specialist 1	This saver is particularly good to use can really save electricity, with two months and then look at the line if you buy one for the family	Jingdong prefecture level city in Shandong
Jinfeng Hongyuan Flagship Store	Power Saving Specialist 1	Novel appearance design, all aluminum alloy profile, also with a fuse device, voltage monitoring display. After receiving the goods, I directly cut the plug and connect it to the main power box under the leakage switch, hoping to save electricity, and then follow up later. Logistics is very fast, arrived the next	Jingdong prefecture level city in Shandong
Jinfeng Hongyuan Flagship Store	Power Saving Specialist 1	Received, very good and like, if necessary next time to buy ha ha	Jingdong prefecture level city in Shandong
Jinfeng Hongyuan Flagship Store	Power Saving Specialist 1	It's good. It's worth it. Get it if you need it.	Jingdong prefecture level city in Shandong
Jinfeng Hongyuan Flagship Store	Power Saving Specialist 1	It feels more than good and is worth having.	Jingdong prefecture level city in Shandong
Jinfeng Hongyuan Flagship Store	Power Saving Specialist 1	The goods arrived very good and fast first time to buy	Jingdong prefecture level city in Shandong
Jinfeng Hongyuan Flagship Store	Power Saving Specialist 1	The power saving is very good, I can save so much money every month, I will buy another one next time.	Jingdong prefecture level city in Shandong
Jinfeng Hongyuan Flagship Store	Power Saving Specialist 1	It's not what it says.	Jingdong prefecture level city in Shandong
Jinfeng Hongyuan Flagship Store	Power Saving Specialist 1	I have been using it for many days now and it saves electricity, it really does I have an 8 kW electric snail stove and it saves about $3.50 a day.	Jingdong prefecture level city in Shandong
Jinfeng Hongyuan Flagship Store	Power Saving Specialist 1	Express delivery is very fast, the quality is also quite good, worth buying!	Jingdong prefecture level city in Shandong
Jinfeng Hongyuan Flagship Store	Power Saving Specialist 1	Just received expecting the baby to work well.	Jingdong prefecture level city in Shandong
Jinfeng Hongyuan Flagship Store	Power Saving Specialist 1	Very good, there are more fruit, has bought a second!	Jingdong prefecture level city in Shandong
Jinfeng Hongyuan Flagship Store	Power Saving Specialist 1	Good is good So far, no power savings have been noticed Maybe it's too little to notice.	Jingdong prefecture level city in Shandong
Jinfeng Hongyuan Flagship Store	Power Saving Specialist 1	I just received the item and plugged it in to see what happens later.	Jingdong prefecture level city in Shandong
Jinfeng Hongyuan Flagship Store	Power Saving Specialist 1	Baby received, especially good, packaging is also good, the courier brother is very strong, thank you.	Jingdong prefecture level city in Shandong
Jinfeng Hongyuan Flagship Store	Power Saving Specialist 1	Just received the goods have not yet used it, do not know how, three months later in the follow-up comment!	Jingdong prefecture level city in Shandong
Jinfeng Hongyuan Flagship Store	Power Saving Specialist 1	The item was received, it is quite good, recommended to buy.	Jingdong prefecture level city in Shandong
Jinfeng Hongyuan Flagship Store	Power Saving Specialist 1	This energy saver works very well and I recommend everyone to buy it!	Jingdong prefecture level city in Shandong
Jinfeng Hongyuan Flagship Store	Power Saving Specialist 1	Voltage stabilization and current reduction nice power saving product.	Jingdong prefecture level city in Shandong
Jinfeng Hongyuan Flagship Store	Power Saving Specialist 1	Voltage stabilization and current reduction nice power saving product.	Jingdong prefecture level city in Shandong
Jinfeng Hongyuan Flagship Store	Power Saving Specialist 1	A good power saver for stabilizing and lowering current.	Jingdong prefecture level city in Shandong
Jinfeng Hongyuan Flagship Store	Power Saving Specialist 1	You'll know if it saves power after you use it.	Jingdong prefecture level city in Shandong
Jinfeng Hongyuan Flagship Store	Power Saving Specialist 1	The quality is great. The quality is very good. The service is very good. Express delivery arrived in two days.	Jingdong prefecture level city in Shandong
Jinfeng Hongyuan Flagship Store	Power Saving Specialist 1	It works great.	Jingdong prefecture level city in Shandong
Jinfeng Hongyuan Flagship Store	Power Saving Specialist 1	Logistics is very fast, not yet used, used to comment again.	Jingdong prefecture level city in Shandong
Jinfeng Hongyuan Flagship Store	Power Saving Specialist 1	Logistics is very fast things are also very good, worthy of friends to buy	Jingdong prefecture level city in Shandong
Jinfeng Hongyuan Flagship Store	Power Saving Specialist 1	It works. It really works.	Jingdong prefecture level city in Shandong
Jinfeng Hongyuan Flagship Store	Power Saving Specialist 1	LANGUAGE COMMENTS: just received the shipment. I immediately plugged it in and used it. If you ask how effective. It depends on whether the next month's electricity bill has been reduced. If there is a decrease. I am buying more than one to use.	Jingdong prefecture level city in Shandong
Jinfeng Hongyuan Flagship Store	Power Saving Specialist 1	Well made, power saving, good and convenient!	Jingdong prefecture level city in Shandong
Jinfeng Hongyuan Flagship Store	Power Saving Specialist 1	I'm using it. I'll rate it next month.	Jingdong prefecture level city in Shandong
Jinfeng Hongyuan Flagship Store	Power Saving Specialist 1	Baby received Oh, the courier delivery really fast, with a good and then come back to the evaluation	Jingdong prefecture level city in Shandong
Jinfeng Hongyuan Flagship Store	Power Saving Specialist 1	Fast logistics, good packaging, worth it!	Jingdong prefecture level city in Shandong
Jinfeng Hongyuan Flagship Store	Power Saving Specialist 1	Customer service is okay. It's just that.	Jingdong prefecture level city in Shandong
Jinfeng Hongyuan Flagship Store	Power Saving Specialist 1	Poor power saving	Jingdong prefecture level city in Shandong

Jinfeng Hongyuan Flagship Store	Power Saving Specialist 1	I'll tell you when I've used it.	Jingdong prefecture level city in Shandong
Jinfeng Hongyuan Flagship Store	Power Saving Specialist 1	I think it's good.	Jingdong prefecture level city in Shandong
Jinfeng Hongyuan Flagship Store	Power Saving Specialist 1	This energy saver works very well and I recommend everyone to buy it!	Jingdong prefecture level city in Shandong
Jinfeng Hongyuan Flagship Store	Power Saving Specialist 1	I do not know how effective, just use half a month on the burn out, good business reissued a, and then use it after the follow-up comment.	Jingdong prefecture level city in Shandong
Jinfeng Hongyuan Flagship Store	Power Saving Specialist 1	in use	Jingdong prefecture level city in Shandong
Jinfeng Hongyuan Flagship Store	Power Saving Specialist 1	This thing is very poor. It's already only been used for ten days and it's broken. Now waiting for you to return it.	Jingdong prefecture level city in Shandong
Jinfeng Hongyuan Flagship Store	Power Saving Specialist 1	It's not the same as the seller's show.	Jingdong prefecture level city in Shandong
Jinfeng Hongyuan Flagship Store	Power Saving Specialist 1	What is the problem when I get it back from the CTS, it is plugged into the wall and the voltage shows 206v.	Jingdong prefecture level city in Shandong
Jinfeng Hongyuan Flagship Store	Power Saving Specialist 1	The logistics was fast and the stuff was perfectly packaged. It is in the process of trial.	Jingdong prefecture level city in Shandong
Jinfeng Hongyuan Flagship Store	Power Saving Specialist 1	This saver is particularly good to use can really save electricity, with two months and then look at the line if you buy one for the family	Jingdong prefecture level city in Shandong
Jinfeng Hongyuan Flagship Store	Power Saving Specialist 1	Novel appearance design, all aluminum alloy profile, also with a fuse device, voltage monitoring display. After receiving the goods, I directly cut the plug and connect it to the main power box under the leakage switch, hoping to save electricity, and then follow up later. Logistics is very fast, arrived the next	Jingdong prefecture level city in Shandong
Jinfeng Hongyuan Flagship Store	Power Saving Specialist 1	Received, very good and like, if necessary next time to buy ha ha	Jingdong prefecture level city in Shandong
Jinfeng Hongyuan Flagship Store	Power Saving Specialist 1	It's good. It's worth it. Get it if you need it.	Jingdong prefecture level city in Shandong
Jinfeng Hongyuan Flagship Store	Power Saving Specialist 1	It feels more than good and is worth having.	Jingdong prefecture level city in Shandong
Jinfeng Hongyuan Flagship Store	Power Saving Specialist 1	The goods arrived very good and fast first time to buy	Jingdong prefecture level city in Shandong
Jinfeng Hongyuan Flagship Store	Power Saving Specialist 1	The power saving is very good, I can save so much money every month, I will buy another one next time.	Jingdong prefecture level city in Shandong
Jinfeng Hongyuan Flagship Store	Power Saving Specialist 1	It's not what it says.	Jingdong prefecture level city in Shandong
Jinfeng Hongyuan Flagship Store	Power Saving Specialist 1	I have been using it for many days now and it really does I have an 8 kW electric snail stove and it saves about $3.50 a day.	Jingdong prefecture level city in Shandong
Jinfeng Hongyuan Flagship Store	Power Saving Specialist 1	Express delivery is very fast, the quality is also quite good, worth buying!	Jingdong prefecture level city in Shandong
Jinfeng Hongyuan Flagship Store	Power Saving Specialist 1	Just received expecting the baby to work well.	Jingdong prefecture level city in Shandong
Jinfeng Hongyuan Flagship Store	Power Saving Specialist 1	Very good, there are more fruit, has bought a second!	Jingdong prefecture level city in Shandong
Jinfeng Hongyuan Flagship Store	Power Saving Specialist 1	Good is good So far, no power savings have been noticed Maybe it's too little to notice.	Jingdong prefecture level city in Shandong
Jinfeng Hongyuan Flagship Store	Power Saving Specialist 1	I just received the item and plugged it in to see what happens later.	Jingdong prefecture level city in Shandong
Jinfeng Hongyuan Flagship Store	Power Saving Specialist 1	Baby received, especially good, packaging is also good, the courier brother is very strong, thank you.	Jingdong prefecture level city in Shandong
Jinfeng Hongyuan Flagship Store	Power Saving Specialist 1	Just received the goods have not yet used it, do not know how, three months later in the follow-up comment!	Jingdong prefecture level city in Shandong
Jinfeng Hongyuan Flagship Store	Power Saving Specialist 1	The item was received, it is quite good, recommended to buy.	Jingdong prefecture level city in Shandong
Jinfeng Hongyuan Flagship Store	Power Saving Specialist 1	This energy saver works very well and I recommend everyone to buy it!	Jingdong prefecture level city in Shandong
Jinfeng Hongyuan Flagship Store	Power Saving Specialist 1	Voltage stabilization and current reduction nice power saving product.	Jingdong prefecture level city in Shandong
Jinfeng Hongyuan Flagship Store	Power Saving Specialist 1	Voltage stabilization and current reduction nice power saving product.	Jingdong prefecture level city in Shandong
Jinfeng Hongyuan Flagship Store	Power Saving Specialist 1	A good power saver for stabilizing and lowering current.	Jingdong prefecture level city in Shandong
Jinfeng Hongyuan Flagship Store	Power Saving Specialist 1	You'll know if it saves power after you use it.	Jingdong prefecture level city in Shandong
Jinfeng Hongyuan Flagship Store	Power Saving Specialist 1	The quality is great. The quality is very good. The service is very good. Express delivery arrived in two days.	Jingdong prefecture level city in Shandong
Jinfeng Hongyuan Flagship Store	Power Saving Specialist 1	It works great.	Jingdong prefecture level city in Shandong
Jinfeng Hongyuan Flagship Store	Power Saving Specialist 1	Logistics is very fast, not yet used, used to comment again.	Jingdong prefecture level city in Shandong
Jinfeng Hongyuan Flagship Store	Power Saving Specialist 1	Logistics is very fast things are also very good, worthy of friends to buy	Jingdong prefecture level city in Shandong
Jinfeng Hongyuan Flagship Store	Power Saving Specialist 1	It works. It really works.	Jingdong prefecture level city in Shandong
Jinfeng Hongyuan Flagship Store	Power Saving Specialist 1	LANGUAGE COMMENTS: just received the shipment. I immediately plugged it in and used it. If you ask how effective. It depends on whether the next month's electricity bill has been reduced. If there is a decrease. I am buying more than one to use.	Jingdong prefecture level city in Shandong
Jinfeng Hongyuan Flagship Store	Power Saving Specialist 1	Well made, power saving, good and convenient!	Jingdong prefecture level city in Shandong
Jinfeng Hongyuan Flagship Store	Power Saving Specialist 1	I'm using it. I'll rate it next month.	Jingdong prefecture level city in Shandong
Jinfeng Hongyuan Flagship Store	Power Saving Specialist 1	Baby received Oh, the courier delivery really fast, with a good and then come back to the evaluation	Jingdong prefecture level city in Shandong
Jinfeng Hongyuan Flagship Store	Power Saving Specialist 1	Fast logistics, good packaging, worth it!	Jingdong prefecture level city in Shandong
Jinfeng Hongyuan Flagship Store	Power Saving Specialist 1	Customer service is okay. It's just that.	Jingdong prefecture level city in Shandong
Jinfeng Hongyuan Flagship Store	Power Saving Specialist 1	Poor power saving	Jingdong prefecture level city in Shandong
Jinfeng Hongyuan Flagship Store	Power Saving Specialist 1	I'll tell you when I've used it.	Jingdong prefecture level city in Shandong
Jinfeng Hongyuan Flagship Store	Power Saving Specialist 1	I think it's good.	Jingdong prefecture level city in Shandong
Jinfeng Hongyuan Flagship Store	Power Saving Specialist 1	This energy saver works very well and I recommend everyone to buy it!	Jingdong prefecture level city in Shandong
Jinfeng Hongyuan Flagship Store	Power Saving Specialist 1	I do not know how effective, just use half a month on the burn out, good business reissued a, and then use it after the follow-up comment.	Jingdong prefecture level city in Shandong
Jinfeng Hongyuan Flagship Store	Power Saving Specialist 1	in use	Jingdong prefecture level city in Shandong
Jinfeng Hongyuan Flagship Store	Power Saving Specialist 1	This thing is very poor. It's already only been used for ten days and it's broken. Now waiting for you to return it.	Jingdong prefecture level city in Shandong
Jinfeng Hongyuan Flagship Store	Power Saving Specialist 1	It's not the same as the seller's show.	Jingdong prefecture level city in Shandong

Jinfeng Hongyuan Flagship Store	Power Saving Specialist 1	What is the problem when I get it back from the CTS, it is plugged into the wall and the voltage shows 206v.	Jingdong prefecture level city in Shandong
Jinfeng Hongyuan Flagship Store	Power Saving Specialist 1	The logistics was fast and the stuff was perfectly packaged. It is in the process of trial.	Jingdong prefecture level city in Shandong
Jinfeng Hongyuan Flagship Store	Power Saving Specialist 1	This saver is particularly good to use can really save electricity, with two months and then look at the line if you buy one for the family	Jingdong prefecture level city in Shandong
Jinfeng Hongyuan Flagship Store	Power Saving Specialist 1	Novel appearance design, all aluminum alloy profile, also with a fuse device, voltage monitoring display. After receiving the goods, I directly cut the plug and connect it to the main power box under the leakage switch, hoping to save electricity, and then follow up later. Logistics is very fast, arrived the next	Jingdong prefecture level city in Shandong
Jinfeng Hongyuan Flagship Store	Power Saving Specialist 1	Received, very good and like, if necessary next time to buy ha ha	Jingdong prefecture level city in Shandong
Jinfeng Hongyuan Flagship Store	Power Saving Specialist 1	It's good. It's worth it. Get it if you need it.	Jingdong prefecture level city in Shandong
Jinfeng Hongyuan Flagship Store	Power Saving Specialist 1	It feels more than good and is worth having.	Jingdong prefecture level city in Shandong
Jinfeng Hongyuan Flagship Store	Power Saving Specialist 1	The goods arrived very good and fast first time to buy	Jingdong prefecture level city in Shandong
Jinfeng Hongyuan Flagship Store	Power Saving Specialist 1	The power saving is very good, I can save so much money every month, I will buy another one next time.	Jingdong prefecture level city in Shandong
Jinfeng Hongyuan Flagship Store	Power Saving Specialist 1	It's not what it says.	Jingdong prefecture level city in Shandong
Jinfeng Hongyuan Flagship Store	Power Saving Specialist 1	I have been using it for many days now and it saves electricity, it really does I have an 8 kW electric snail stove and it saves about $3.50 a day.	Jingdong prefecture level city in Shandong
Jinfeng Hongyuan Flagship Store	Power Saving Specialist 1	Express delivery is very fast, the quality is also quite good, worth buying!	Jingdong prefecture level city in Shandong
Jinfeng Hongyuan Flagship Store	Power Saving Specialist 1	Just received expecting the baby to work well.	Jingdong prefecture level city in Shandong
Jinfeng Hongyuan Flagship Store	Power Saving Specialist 1	Very good, there are more fruit, has bought a second!	Jingdong prefecture level city in Shandong
Jinfeng Hongyuan Flagship Store	Power Saving Specialist 1	Good is good So far, no power savings have been noticed Maybe it's too little to notice.	Jingdong prefecture level city in Shandong
Jinfeng Hongyuan Flagship Store	Power Saving Specialist 1	I just received the item and plugged it in to see what happens later.	Jingdong prefecture level city in Shandong
Jinfeng Hongyuan Flagship Store	Power Saving Specialist 1	Baby received, especially good, packaging is also good, the courier brother is very strong, thank you.	Jingdong prefecture level city in Shandong
Jinfeng Hongyuan Flagship Store	Power Saving Specialist 1	Just received the goods have not yet used it, do not know how, three months later in the follow-up comment!	Jingdong prefecture level city in Shandong
Jinfeng Hongyuan Flagship Store	Power Saving Specialist 1	The item was received, it is quite good, recommended to buy.	Jingdong prefecture level city in Shandong
Jinfeng Hongyuan Flagship Store	Power Saving Specialist 1	This energy saver works very well and I recommend everyone to buy it!	Jingdong prefecture level city in Shandong
Jinfeng Hongyuan Flagship Store	Power Saving Specialist 1	Voltage stabilization and current reduction nice power saving product.	Jingdong prefecture level city in Shandong
Jinfeng Hongyuan Flagship Store	Power Saving Specialist 1	Voltage stabilization and current reduction nice power saving product.	Jingdong prefecture level city in Shandong
Jinfeng Hongyuan Flagship Store	Power Saving Specialist 1	A good power saver for stabilizing and lowering current.	Jingdong prefecture level city in Shandong
Jinfeng Hongyuan Flagship Store	Power Saving Specialist 1	You'll know if it saves power after you use it.	Jingdong prefecture level city in Shandong
Jinfeng Hongyuan Flagship Store	Power Saving Specialist 1	The quality is great. The quality is very good. The service is very good. Express delivery arrived in two days.	Jingdong prefecture level city in Shandong
Jinfeng Hongyuan Flagship Store	Power Saving Specialist 1	It works great.	Jingdong prefecture level city in Shandong
Jinfeng Hongyuan Flagship Store	Power Saving Specialist 1	Logistics is very fast, not yet used, used to comment again.	Jingdong prefecture level city in Shandong
Jinfeng Hongyuan Flagship Store	Power Saving Specialist 1	Logistics is very fast things are also very good, worthy of friends to buy	Jingdong prefecture level city in Shandong
Jinfeng Hongyuan Flagship Store	Power Saving Specialist 1	It works. It really works.	Jingdong prefecture level city in Shandong
Jinfeng Hongyuan Flagship Store	Power Saving Specialist 1	LANGUAGE COMMENTS: just received the shipment. I immediately plugged it in and used it. If you ask how effective. It depends on whether the next month's electricity bill has been reduced. If there is a decrease. I am buying more than one to use.	Jingdong prefecture level city in Shandong
Jinfeng Hongyuan Flagship Store	Power Saving Specialist 1	Well made, power saving, good and convenient!	Jingdong prefecture level city in Shandong
Jinfeng Hongyuan Flagship Store	Power Saving Specialist 1	I'm using it. I'll rate it next month.	Jingdong prefecture level city in Shandong
Jinfeng Hongyuan Flagship Store	Power Saving Specialist 1	Baby received Oh, the courier delivery really fast, with a good and then come back to the evaluation	Jingdong prefecture level city in Shandong
Jinfeng Hongyuan Flagship Store	Power Saving Specialist 1	Fast logistics, good packaging, worth it!	Jingdong prefecture level city in Shandong
Jinfeng Hongyuan Flagship Store	Power Saving Specialist 1	Customer service is okay. It's just that.	Jingdong prefecture level city in Shandong
Jinfeng Hongyuan Flagship Store	Power Saving Specialist 1	Poor power saving	Jingdong prefecture level city in Shandong
Jinfeng Hongyuan Flagship Store	Power Saving Specialist 1	I'll tell you when I've used it.	Jingdong prefecture level city in Shandong
Jinfeng Hongyuan Flagship Store	Power Saving Specialist 1	I think it's good.	Jingdong prefecture level city in Shandong
Jinfeng Hongyuan Flagship Store	Power Saving Specialist 1	This energy saver works very well and I recommend everyone to buy it!	Jingdong prefecture level city in Shandong
Jinfeng Hongyuan Flagship Store	Power Saving Specialist 1	I do not know how effective, just use half a month on the burn out, good business reissued a, and then use it after the follow-up comment.	Jingdong prefecture level city in Shandong
Jinfeng Hongyuan Flagship Store	Power Saving Specialist 1	in use	Jingdong prefecture level city in Shandong
Jinfeng Hongyuan Flagship Store	Power Saving Specialist 1	This thing is very poor. It's already only been used for ten days and it's broken. Now waiting for you to return it.	Jingdong prefecture level city in Shandong
Jinfeng Hongyuan Flagship Store	Power Saving Specialist 1	It's not the same as the seller's show.	Jingdong prefecture level city in Shandong
Jinfeng Hongyuan Flagship Store	Power Saving Specialist 1	What is the problem when I get it back from the CTS, it is plugged into the wall and the voltage shows 206v.	Jingdong prefecture level city in Shandong
Jinfeng Hongyuan Flagship Store	Power Saving Specialist 1	The logistics was fast and the stuff was perfectly packaged. It is in the process of trial.	Jingdong prefecture level city in Shandong
Jinfeng Hongyuan Flagship Store	Power Saving Specialist 1	This saver is particularly good to use can really save electricity, with two months and then look at the line if you buy one for the family	Jingdong prefecture level city in Shandong
Jinfeng Hongyuan Flagship Store	Power Saving Specialist 1	Novel appearance design, all aluminum alloy profile, also with a fuse device, voltage monitoring display. After receiving the goods, I directly cut the plug and connect it to the main power box under the leakage switch, hoping to save electricity, and then follow up later. Logistics is very fast, arrived the next	Jingdong prefecture level city in Shandong
Jinfeng Hongyuan Flagship Store	Power Saving Specialist 1	Received, very good and like, if necessary next time to buy ha ha	Jingdong prefecture level city in Shandong
Jinfeng Hongyuan Flagship Store	Power Saving Specialist 1	It's good. It's worth it. Get it if you need it.	Jingdong prefecture level city in Shandong
Jinfeng Hongyuan Flagship Store	Power Saving Specialist 1	It feels more than good and is worth having.	Jingdong prefecture level city in Shandong

Jinfeng Hongyuan Flagship Store	Power Saving Specialist 1	The goods arrived very good and fast first time to buy	Jingdong prefecture level city in Shandong
Jinfeng Hongyuan Flagship Store	Power Saving Specialist 1	The power saving is very good, I can save so much money every month, I will buy another one next time.	Jingdong prefecture level city in Shandong
Jinfeng Hongyuan Flagship Store	Power Saving Specialist 1	It's not what it says.	Jingdong prefecture level city in Shandong
Jinfeng Hongyuan Flagship Store	Power Saving Specialist 1	I have been using it for many days now and it saves electricity, it really does I have an 8 kW electric snail stove and it saves about $3.50 a day.	Jingdong prefecture level city in Shandong
Jinfeng Hongyuan Flagship Store	Power Saving Specialist 1	Express delivery is very fast, the quality is also quite good, worth buying!	Jingdong prefecture level city in Shandong
Jinfeng Hongyuan Flagship Store	Power Saving Specialist 1	Just received expecting the baby to work well.	Jingdong prefecture level city in Shandong
Jinfeng Hongyuan Flagship Store	Power Saving Specialist 1	Very good, there are more fruit, has bought a second!	Jingdong prefecture level city in Shandong
Jinfeng Hongyuan Flagship Store	Power Saving Specialist 1	Good is good So far, no power savings have been noticed Maybe it's too little to notice.	Jingdong prefecture level city in Shandong
Jinfeng Hongyuan Flagship Store	Power Saving Specialist 1	I just received the item and plugged it in to see what happens later.	Jingdong prefecture level city in Shandong
Jinfeng Hongyuan Flagship Store	Power Saving Specialist 1	Baby received, especially good, packaging is also good, the courier brother is very strong, thank you.	Jingdong prefecture level city in Shandong
Jinfeng Hongyuan Flagship Store	Power Saving Specialist 1	Just received the goods have not yet used it, do not know how, three months later in the follow-up comment!	Jingdong prefecture level city in Shandong
Jinfeng Hongyuan Flagship Store	Power Saving Specialist 1	The item was received, it is quite good, recommended to buy.	Jingdong prefecture level city in Shandong
Jinfeng Hongyuan Flagship Store	Power Saving Specialist 1	This energy saver works very well and I recommend everyone to buy it!	Jingdong prefecture level city in Shandong
Jinfeng Hongyuan Flagship Store	Power Saving Specialist 1	Voltage stabilization and current reduction nice power saving product.	Jingdong prefecture level city in Shandong
Jinfeng Hongyuan Flagship Store	Power Saving Specialist 1	Voltage stabilization and current reduction nice power saving product.	Jingdong prefecture level city in Shandong
Jinfeng Hongyuan Flagship Store	Power Saving Specialist 1	A good power saver for stabilizing and lowering current.	Jingdong prefecture level city in Shandong
Jinfeng Hongyuan Flagship Store	Power Saving Specialist 1	You'll know if it saves power after you use it.	Jingdong prefecture level city in Shandong
Jinfeng Hongyuan Flagship Store	Power Saving Specialist 1	The quality is great. The quality is very good. The service is very good. Express delivery arrived in two days.	Jingdong prefecture level city in Shandong
Jinfeng Hongyuan Flagship Store	Power Saving Specialist 1	It works great.	Jingdong prefecture level city in Shandong
Jinfeng Hongyuan Flagship Store	Power Saving Specialist 1	Logistics is very fast, not yet used, used to comment again.	Jingdong prefecture level city in Shandong
Jinfeng Hongyuan Flagship Store	Power Saving Specialist 1	Logistics is very fast things are also very good, worthy of friends to buy	Jingdong prefecture level city in Shandong
Jinfeng Hongyuan Flagship Store	Power Saving Specialist 1	It works. It really works.	Jingdong prefecture level city in Shandong
Jinfeng Hongyuan Flagship Store	Power Saving Specialist 1	LANGUAGE COMMENTS: just received the shipment. I immediately plugged it in and used it. If you ask how effective. It depends on whether the next month's electricity bill has been reduced. If there is a decrease. I am buying more than one to use.	Jingdong prefecture level city in Shandong
Jinfeng Hongyuan Flagship Store	Power Saving Specialist 1	Well made, power saving, good and convenient!	Jingdong prefecture level city in Shandong
Jinfeng Hongyuan Flagship Store	Power Saving Specialist 1	I'm using it. I'll rate it next month.	Jingdong prefecture level city in Shandong
Jinfeng Hongyuan Flagship Store	Power Saving Specialist 1	Baby received Oh, the courier delivery really fast, with a good and then come back to the evaluation	Jingdong prefecture level city in Shandong
Jinfeng Hongyuan Flagship Store	Power Saving Specialist 1	Fast logistics, good packaging, worth it!	Jingdong prefecture level city in Shandong
Jinfeng Hongyuan Flagship Store	Power Saving Specialist 1	Customer service is okay. It's just that.	Jingdong prefecture level city in Shandong
Jinfeng Hongyuan Flagship Store	Power Saving Specialist 1	Poor power saving	Jingdong prefecture level city in Shandong
Jinfeng Hongyuan Flagship Store	Power Saving Specialist 1	I'll tell you when I've used it.	Jingdong prefecture level city in Shandong
Jinfeng Hongyuan Flagship Store	Power Saving Specialist 1	I think it's good.	Jingdong prefecture level city in Shandong
Jinfeng Hongyuan Flagship Store	Power Saving Specialist 1	This energy saver works very well and I recommend everyone to buy it!	Jingdong prefecture level city in Shandong
Jinfeng Hongyuan Flagship Store	Power Saving Specialist 1	I do not know how effective, just use half a month on the burn out, good business reissued a, and then use it after the follow-up comment.	Jingdong prefecture level city in Shandong
Jinfeng Hongyuan Flagship Store	Power Saving Specialist 1	in use	Jingdong prefecture level city in Shandong
Jinfeng Hongyuan Flagship Store	Power Saving Specialist 1	This thing is very poor. It's already only been used for ten days and it's broken. Now waiting for you to return it.	Jingdong prefecture level city in Shandong
Jinfeng Hongyuan Flagship Store	Power Saving Specialist 1	It's not the same as the seller's show.	Jingdong prefecture level city in Shandong
Jinfeng Hongyuan Flagship Store	Power Saving Specialist 1	What is the problem when I get it back from the CTS, it is plugged into the wall and the voltage shows 206v.	Jingdong prefecture level city in Shandong
Jinfeng Hongyuan Flagship Store	Power Saving Specialist 1	The logistics was fast and the stuff was perfectly packaged. It is in the process of trial.	Jingdong prefecture level city in Shandong
Jinfeng Hongyuan Flagship Store	Power Saving Specialist 1	This saver is particularly good to use can really save electricity, with two months and then look at the line if you buy one for the family	Jingdong prefecture level city in Shandong
Jinfeng Hongyuan Flagship Store	Power Saving Specialist 1	Novel appearance design, all aluminum alloy profile, also with a fuse device, voltage monitoring display. After receiving the goods, I directly cut the plug and connect it to the main power box under the leakage switch, hoping to save electricity, and then follow up later. Logistics is very fast, arrived the next	Jingdong prefecture level city in Shandong
Jinfeng Hongyuan Flagship Store	Power Saving Specialist 1	Received, very good and like, if necessary next time to buy ha ha	Jingdong prefecture level city in Shandong
Jinfeng Hongyuan Flagship Store	Power Saving Specialist 1	It's good. It's worth it. Get it if you need it.	Jingdong prefecture level city in Shandong
Jinfeng Hongyuan Flagship Store	Power Saving Specialist 1	It feels more than good and is worth having.	Jingdong prefecture level city in Shandong
Jinfeng Hongyuan Flagship Store	Power Saving Specialist 1	The goods arrived very good and fast first time to buy	Jingdong prefecture level city in Shandong
Jinfeng Hongyuan Flagship Store	Power Saving Specialist 1	The power saving is very good, I can save so much money every month, I will buy another one next time.	Jingdong prefecture level city in Shandong
Jinfeng Hongyuan Flagship Store	Power Saving Specialist 1	It's not what it says.	Jingdong prefecture level city in Shandong
Jinfeng Hongyuan Flagship Store	Power Saving Specialist 1	I have been using it for many days now and it saves electricity, it really does I have an 8 kW electric snail stove and it saves about $3.50 a day.	Jingdong prefecture level city in Shandong
Jinfeng Hongyuan Flagship Store	Power Saving Specialist 1	Express delivery is very fast, the quality is also quite good, worth buying!	Jingdong prefecture level city in Shandong
Jinfeng Hongyuan Flagship Store	Power Saving Specialist 1	Just received expecting the baby to work well.	Jingdong prefecture level city in Shandong
Jinfeng Hongyuan Flagship Store	Power Saving Specialist 1	Very good, there are more fruit, has bought a second!	Jingdong prefecture level city in Shandong

Jinfeng Hongyuan Flagship Store	Power Saving Specialist 1	Good is good So far, no power savings have been noticed Maybe it's too little to notice.	Jingdong prefecture level city in Shandong
Jinfeng Hongyuan Flagship Store	Power Saving Specialist 1	I just received the item and plugged it in to see what happens later.	Jingdong prefecture level city in Shandong
Jinfeng Hongyuan Flagship Store	Power Saving Specialist 1	Baby received, especially good, packaging is also good, the courier brother is very strong, thank you.	Jingdong prefecture level city in Shandong
Jinfeng Hongyuan Flagship Store	Power Saving Specialist 1	Just received the goods have not yet used it, do not know how, three months later in the follow-up comment!	Jingdong prefecture level city in Shandong
Jinfeng Hongyuan Flagship Store	Power Saving Specialist 1	The item was received, it is quite good, recommended to buy.	Jingdong prefecture level city in Shandong
Jinfeng Hongyuan Flagship Store	Power Saving Specialist 1	This energy saver works very well and I recommend everyone to buy it!	Jingdong prefecture level city in Shandong
Jinfeng Hongyuan Flagship Store	Power Saving Specialist 1	Voltage stabilization and current reduction nice power saving product.	Jingdong prefecture level city in Shandong
Jinfeng Hongyuan Flagship Store	Power Saving Specialist 1	Voltage stabilization and current reduction nice power saving product.	Jingdong prefecture level city in Shandong
Jinfeng Hongyuan Flagship Store	Power Saving Specialist 1	A good power saver for stabilizing and lowering current.	Jingdong prefecture level city in Shandong
Jinfeng Hongyuan Flagship Store	Power Saving Specialist 1	You'll know if it saves power after you use it.	Jingdong prefecture level city in Shandong
Jinfeng Hongyuan Flagship Store	Power Saving Specialist 1	The quality is great. The quality is very good. The service is very good. Express delivery arrived in two days.	Jingdong prefecture level city in Shandong
Jinfeng Hongyuan Flagship Store	Power Saving Specialist 1	It works great.	Jingdong prefecture level city in Shandong
Jinfeng Hongyuan Flagship Store	Power Saving Specialist 1	Logistics is very fast, not yet used, used to comment again.	Jingdong prefecture level city in Shandong
Jinfeng Hongyuan Flagship Store	Power Saving Specialist 1	Logistics is very fast things are also very good, worthy of friends to buy	Jingdong prefecture level city in Shandong
Jinfeng Hongyuan Flagship Store	Power Saving Specialist 1	It works. It really works.	Jingdong prefecture level city in Shandong
Jinfeng Hongyuan Flagship Store	Power Saving Specialist 1	LANGUAGE COMMENTS: just received the shipment. I immediately plugged it in and used it. If you ask how effective. It depends on whether the next month's electricity bill has been reduced. If there is a decrease. I am buying more than one to use.	Jingdong prefecture level city in Shandong
Jinfeng Hongyuan Flagship Store	Power Saving Specialist 1	Well made, power saving, good and convenient!	Jingdong prefecture level city in Shandong
Jinfeng Hongyuan Flagship Store	Power Saving Specialist 1	I'm using it. I'll rate it next month.	Jingdong prefecture level city in Shandong
Jinfeng Hongyuan Flagship Store	Power Saving Specialist 1	Baby received Oh, the courier delivery really fast, with a good and then come back to the evaluation	Jingdong prefecture level city in Shandong
Jinfeng Hongyuan Flagship Store	Power Saving Specialist 1	Fast logistics, good packaging, worth it!	Jingdong prefecture level city in Shandong
Jinfeng Hongyuan Flagship Store	Power Saving Specialist 1	Customer service is okay. It's just that.	Jingdong prefecture level city in Shandong
Jinfeng Hongyuan Flagship Store	Power Saving Specialist 1	Poor power saving	Jingdong prefecture level city in Shandong
Jinfeng Hongyuan Flagship Store	Power Saving Specialist 1	I'll tell you when I've used it.	Jingdong prefecture level city in Shandong
Jinfeng Hongyuan Flagship Store	Power Saving Specialist 1	I think it's good.	Jingdong prefecture level city in Shandong
Jinfeng Hongyuan Flagship Store	Power Saving Specialist 1	This energy saver works very well and I recommend everyone to buy it!	Jingdong prefecture level city in Shandong
Jinfeng Hongyuan Flagship Store	Power Saving Specialist 1	I do not know how effective, just use half a month on the burn out, good business reissued a, and then use it after the follow-up comment.	Jingdong prefecture level city in Shandong
Jinfeng Hongyuan Flagship Store	Power Saving Specialist 1	in use	Jingdong prefecture level city in Shandong
Jinfeng Hongyuan Flagship Store	Power Saving Specialist 1	This thing is very poor. It's already only been used for ten days and it's broken. Now waiting for you to return it.	Jingdong prefecture level city in Shandong
Jinfeng Hongyuan Flagship Store	Power Saving Specialist 1	It's not the same as the seller's show.	Jingdong prefecture level city in Shandong
Jinfeng Hongyuan Flagship Store	Power Saving Specialist 1	What is the problem when I get it back from the CTS, it is plugged into the wall and the voltage shows 206v.	Jingdong prefecture level city in Shandong
Jinfeng Hongyuan Flagship Store	Power Saving Specialist 1	The logistics was fast and the stuff was perfectly packaged. It is in the process of trial.	Jingdong prefecture level city in Shandong
Jinfeng Hongyuan Flagship Store	Power Saving Specialist 1	This saver is particularly good to use can really save electricity, with two months and then look at the line if you buy one for the family	Jingdong prefecture level city in Shandong
Jinfeng Hongyuan Flagship Store	Power Saving Specialist 1	Novel appearance design, all aluminum alloy profile, also with a fuse device, voltage monitoring display. After receiving the goods, I directly cut the plug and connect it to the main power box under the leakage switch, hoping to save electricity, and then follow up later. Logistics is very fast, arrived the next	Jingdong prefecture level city in Shandong
Jinfeng Hongyuan Flagship Store	Power Saving Specialist 1	Received, very good and like, if necessary next time to buy ha ha	Jingdong prefecture level city in Shandong
Jinfeng Hongyuan Flagship Store	Power Saving Specialist 1	It's good. It's worth it. Get it if you need it.	Jingdong prefecture level city in Shandong
Jinfeng Hongyuan Flagship Store	Power Saving Specialist 1	It feels more than good and is worth having.	Jingdong prefecture level city in Shandong
Jinfeng Hongyuan Flagship Store	Power Saving Specialist 1	The goods arrived very good and fast first time to buy	Jingdong prefecture level city in Shandong
Jinfeng Hongyuan Flagship Store	Power Saving Specialist 1	The power saving is very good, I can save so much money every month, I will buy another one next time.	Jingdong prefecture level city in Shandong
Jinfeng Hongyuan Flagship Store	Power Saving Specialist 1	It's not what it says.	Jingdong prefecture level city in Shandong
Jinfeng Hongyuan Flagship Store	Power Saving Specialist 1	I have been using it for many days now and it saves electricity, it really does I have an 8 kW electric snail stove and it saves about $3.50 a day.	Jingdong prefecture level city in Shandong
Jinfeng Hongyuan Flagship Store	Power Saving Specialist 1	Express delivery is very fast, the quality is also quite good, worth buying!	Jingdong prefecture level city in Shandong
Jinfeng Hongyuan Flagship Store	Power Saving Specialist 1	Just received expecting the baby to work well.	Jingdong prefecture level city in Shandong
Jinfeng Hongyuan Flagship Store	Power Saving Specialist 1	Very good, there are more fruit, has bought a second!	Jingdong prefecture level city in Shandong
Jinfeng Hongyuan Flagship Store	Power Saving Specialist 1	Good is good So far, no power savings have been noticed Maybe it's too little to notice.	Jingdong prefecture level city in Shandong
Jinfeng Hongyuan Flagship Store	Power Saving Specialist 1	I just received the item and plugged it in to see what happens later.	Jingdong prefecture level city in Shandong
Jinfeng Hongyuan Flagship Store	Power Saving Specialist 1	Baby received, especially good, packaging is also good, the courier brother is very strong, thank you.	Jingdong prefecture level city in Shandong
Jinfeng Hongyuan Flagship Store	Power Saving Specialist 1	Just received the goods have not yet used it, do not know how, three months later in the follow-up comment!	Jingdong prefecture level city in Shandong
Jinfeng Hongyuan Flagship Store	Power Saving Specialist 1	The item was received, it is quite good, recommended to buy.	Jingdong prefecture level city in Shandong
Jinfeng Hongyuan Flagship Store	Power Saving Specialist 1	This energy saver works very well and I recommend everyone to buy it!	Jingdong prefecture level city in Shandong
Jinfeng Hongyuan Flagship Store	Power Saving Specialist 1	Voltage stabilization and current reduction nice power saving product.	Jingdong prefecture level city in Shandong

Jinfeng Hongyuan Flagship Store	Power Saving Specialist 1	Voltage stabilization and current reduction nice power saving product.	Jingdong prefecture level city in Shandong
Jinfeng Hongyuan Flagship Store	Power Saving Specialist 1	A good power saver for stabilizing and lowering current.	Jingdong prefecture level city in Shandong
Jinfeng Hongyuan Flagship Store	Power Saving Specialist 1	You'll know if it saves power after you use it.	Jingdong prefecture level city in Shandong
Jinfeng Hongyuan Flagship Store	Power Saving Specialist 1	The quality is great. The quality is very good. The service is very good. Express delivery arrived in two days.	Jingdong prefecture level city in Shandong
Jinfeng Hongyuan Flagship Store	Power Saving Specialist 1	It works great.	Jingdong prefecture level city in Shandong
Jinfeng Hongyuan Flagship Store	Power Saving Specialist 1	Logistics is very fast, not yet used, used to comment again.	Jingdong prefecture level city in Shandong
Jinfeng Hongyuan Flagship Store	Power Saving Specialist 1	Logistics is very fast things are also very good, worthy of friends to buy	Jingdong prefecture level city in Shandong
Jinfeng Hongyuan Flagship Store	Power Saving Specialist 1	It works. It really works.	Jingdong prefecture level city in Shandong
Jinfeng Hongyuan Flagship Store	Power Saving Specialist 1	LANGUAGE COMMENTS: just received the shipment. I immediately plugged it in and used it. If you ask how effective. It depends on whether the next month's electricity bill has been reduced. If there is a decrease. I am buying more than one to use.	Jingdong prefecture level city in Shandong
Jinfeng Hongyuan Flagship Store	Power Saving Specialist 1	Well made, power saving, good and convenient!	Jingdong prefecture level city in Shandong
Jinfeng Hongyuan Flagship Store	Power Saving Specialist 1	I'm using it. I'll rate it next month.	Jingdong prefecture level city in Shandong
Jinfeng Hongyuan Flagship Store	Power Saving Specialist 1	Baby received Oh, the courier delivery really fast, with a good and then come back to the evaluation	Jingdong prefecture level city in Shandong
Jinfeng Hongyuan Flagship Store	Power Saving Specialist 1	Fast logistics, good packaging, worth it!	Jingdong prefecture level city in Shandong
Jinfeng Hongyuan Flagship Store	Power Saving Specialist 1	Customer service is okay. It's just that.	Jingdong prefecture level city in Shandong
Jinfeng Hongyuan Flagship Store	Power Saving Specialist 1	Poor power saving	Jingdong prefecture level city in Shandong
Jinfeng Hongyuan Flagship Store	Power Saving Specialist 1	I'll tell you when I've used it.	Jingdong prefecture level city in Shandong
Jinfeng Hongyuan Flagship Store	Power Saving Specialist 1	I think it's good.	Jingdong prefecture level city in Shandong
Jinfeng Hongyuan Flagship Store	Power Saving Specialist 1	This energy saver works very well and I recommend everyone to buy it!	Jingdong prefecture level city in Shandong
Jinfeng Hongyuan Flagship Store	Power Saving Specialist 1	I do not know how effective, just use half a month on the burn out, good business reissued a, and then use it after the follow-up comment.	Jingdong prefecture level city in Shandong
Jinfeng Hongyuan Flagship Store	Power Saving Specialist 1	in use	Jingdong prefecture level city in Shandong
Jinfeng Hongyuan Flagship Store	Power Saving Specialist 1	This thing is very poor. It's already only been used for ten days and it's broken. Now waiting for you to return it.	Jingdong prefecture level city in Shandong
Jinfeng Hongyuan Flagship Store	Power Saving Specialist 1	It's not the same as the seller's show.	Jingdong prefecture level city in Shandong
Jinfeng Hongyuan Flagship Store	Power Saving Specialist 1	What is the problem when I get it back from the CTS, it is plugged into the wall and the voltage shows 206v.	Jingdong prefecture level city in Shandong
Jinfeng Hongyuan Flagship Store	Power Saving Specialist 1	The logistics was fast and the stuff was perfectly packaged. It is in the process of trial.	Jingdong prefecture level city in Shandong
Jinfeng Hongyuan Flagship Store	Power Saving Specialist 1	This saver is particularly good to use can really save electricity, with two months and then look at the line if you buy one for the family	Jingdong prefecture level city in Shandong
Jinfeng Hongyuan Flagship Store	Power Saving Specialist 1	Novel appearance design, all aluminum alloy profile, also with a fuse device, voltage monitoring display. After receiving the goods, I directly cut the plug and connect it to the main power box under the leakage switch, hoping to save electricity, and then follow up later. Logistics is very fast, arrived the next	Jingdong prefecture level city in Shandong
Jinfeng Hongyuan Flagship Store	Power Saving Specialist 1	Received, very good and like, if necessary next time to buy ha ha	Jingdong prefecture level city in Shandong
Jinfeng Hongyuan Flagship Store	Power Saving Specialist 1	It's good. It's worth it. Get it if you need it.	Jingdong prefecture level city in Shandong
Jinfeng Hongyuan Flagship Store	Power Saving Specialist 1	It feels more than good and is worth having.	Jingdong prefecture level city in Shandong
Jinfeng Hongyuan Flagship Store	Power Saving Specialist 1	The goods arrived very good and fast first time to buy	Jingdong prefecture level city in Shandong
Jinfeng Hongyuan Flagship Store	Power Saving Specialist 1	The power saving is very good, I can save so much money every month, I will buy another one next time.	Jingdong prefecture level city in Shandong
Jinfeng Hongyuan Flagship Store	Power Saving Specialist 1	It's not what it says.	Jingdong prefecture level city in Shandong
Jinfeng Hongyuan Flagship Store	Power Saving Specialist 1	I have been using it for many days now and it saves electricity, it really does I have an 8 kW electric snail stove and it saves about $3.50 a day.	Jingdong prefecture level city in Shandong
Jinfeng Hongyuan Flagship Store	Power Saving Specialist 1	Express delivery is very fast, the quality is also quite good, worth buying!	Jingdong prefecture level city in Shandong
Jinfeng Hongyuan Flagship Store	Power Saving Specialist 1	Just received expecting the baby to work well.	Jingdong prefecture level city in Shandong
Jinfeng Hongyuan Flagship Store	Power Saving Specialist 1	Very good, there are more fruit, has bought a second!	Jingdong prefecture level city in Shandong
Jinfeng Hongyuan Flagship Store	Power Saving Specialist 1	Good is good So far, no power savings have been noticed Maybe it's too little to notice.	Jingdong prefecture level city in Shandong
Jinfeng Hongyuan Flagship Store	Power Saving Specialist 1	I just received the item and plugged it in to see what happens later.	Jingdong prefecture level city in Shandong
Jinfeng Hongyuan Flagship Store	Power Saving Specialist 1	Baby received, especially good, packaging is also good, the courier brother is very strong, thank you.	Jingdong prefecture level city in Shandong
Jinfeng Hongyuan Flagship Store	Power Saving Specialist 1	Just received the goods have not yet used it, do not know how, three months later in the follow-up comment!	Jingdong prefecture level city in Shandong
Jinfeng Hongyuan Flagship Store	Power Saving Specialist 1	The item was received, it is quite good, recommended to buy.	Jingdong prefecture level city in Shandong
Jinfeng Hongyuan Flagship Store	Power Saving Specialist 1	This energy saver works very well and I recommend everyone to buy it!	Jingdong prefecture level city in Shandong
Jinfeng Hongyuan Flagship Store	Power Saving Specialist 1	Voltage stabilization and current reduction nice power saving product.	Jingdong prefecture level city in Shandong
Jinfeng Hongyuan Flagship Store	Power Saving Specialist 1	Voltage stabilization and current reduction nice power saving product.	Jingdong prefecture level city in Shandong
Jinfeng Hongyuan Flagship Store	Power Saving Specialist 1	A good power saver for stabilizing and lowering current.	Jingdong prefecture level city in Shandong
Jinfeng Hongyuan Flagship Store	Power Saving Specialist 1	You'll know if it saves power after you use it.	Jingdong prefecture level city in Shandong
Jinfeng Hongyuan Flagship Store	Power Saving Specialist 1	The quality is great. The quality is very good. The service is very good. Express delivery arrived in two days.	Jingdong prefecture level city in Shandong
Jinfeng Hongyuan Flagship Store	Power Saving Specialist 1	It works great.	Jingdong prefecture level city in Shandong
Jinfeng Hongyuan Flagship Store	Power Saving Specialist 1	Logistics is very fast, not yet used, used to comment again.	Jingdong prefecture level city in Shandong
Jinfeng Hongyuan Flagship Store	Power Saving Specialist 1	Logistics is very fast things are also very good, worthy of friends to buy	Jingdong prefecture level city in Shandong

Jinfeng Hongyuan Flagship Store	Power Saving Specialist 1	It works. It really works.	Jingdong prefecture level city in Shandong
Jinfeng Hongyuan Flagship Store	Power Saving Specialist 1	LANGUAGE COMMENTS: just received the shipment. I immediately plugged it in and used it. If you ask how effective. It depends on whether the next month's electricity bill has been reduced. If there is a decrease. I am buying more than one to use.	Jingdong prefecture level city in Shandong
Jinfeng Hongyuan Flagship Store	Power Saving Specialist 1	Well made, power saving, good and convenient!	Jingdong prefecture level city in Shandong
Jinfeng Hongyuan Flagship Store	Power Saving Specialist 1	I'm using it. I'll rate it next month.	Jingdong prefecture level city in Shandong
Jinfeng Hongyuan Flagship Store	Power Saving Specialist 1	Baby received Oh, the courier delivery really fast, with a good and then come back to the evaluation	Jingdong prefecture level city in Shandong
Jinfeng Hongyuan Flagship Store	Power Saving Specialist 1	Fast logistics, good packaging, worth it!	Jingdong prefecture level city in Shandong
Jinfeng Hongyuan Flagship Store	Power Saving Specialist 1	Customer service is okay. It's just that.	Jingdong prefecture level city in Shandong
Jinfeng Hongyuan Flagship Store	Power Saving Specialist 1	Poor power saving	Jingdong prefecture level city in Shandong
Jinfeng Hongyuan Flagship Store	Power Saving Specialist 1	I'll tell you when I've used it.	Jingdong prefecture level city in Shandong
Jinfeng Hongyuan Flagship Store	Power Saving Specialist 1	I think it's good.	Jingdong prefecture level city in Shandong
Jinfeng Hongyuan Flagship Store	Power Saving Specialist 1	This energy saver works very well and I recommend everyone to buy it!	Jingdong prefecture level city in Shandong
Jinfeng Hongyuan Flagship Store	Power Saving Specialist 1	I do not know how effective, just use half a month on the burn out, good business reissued a, and then use it after the follow-up comment.	Jingdong prefecture level city in Shandong
Jinfeng Hongyuan Flagship Store	Power Saving Specialist 1	in use	Jingdong prefecture level city in Shandong
Jinfeng Hongyuan Flagship Store	Power Saving Specialist 1	This thing is very poor. It's already only been used for ten days and it's broken. Now waiting for you to return it.	Jingdong prefecture level city in Shandong
Jinfeng Hongyuan Flagship Store	Power Saving Specialist 1	It's not the same as the seller's show.	Jingdong prefecture level city in Shandong
Jinfeng Hongyuan Flagship Store	Power Saving Specialist 1	What is the problem when I get it back from the CTS, it is plugged into the wall and the voltage shows 206v.	Jingdong prefecture level city in Shandong
Jinfeng Hongyuan Flagship Store	Power Saving Specialist 1	The logistics was fast and the stuff was perfectly packaged. It is in the process of trial.	Jingdong prefecture level city in Shandong
Jinfeng Hongyuan Flagship Store	Power Saving Specialist 1	This saver is particularly good to use can really save electricity, with two months and then look at the line if you buy one for the family	Jingdong prefecture level city in Shandong
Jinfeng Hongyuan Flagship Store	Power Saving Specialist 1	Novel appearance design, all aluminum alloy profile, also with a fuse device, voltage monitoring display. After receiving the goods, I directly cut the plug and connect it to the main power box under the leakage switch, hoping to save electricity, and then follow up later. Logistics is very fast, arrived the next	Jingdong prefecture level city in Shandong
Jinfeng Hongyuan Flagship Store	Power Saving Specialist 1	Received, very good and like, if necessary next time to buy ha ha	Jingdong prefecture level city in Shandong
Jinfeng Hongyuan Flagship Store	Power Saving Specialist 1	It's good. It's worth it. Get it if you need it.	Jingdong prefecture level city in Shandong
Jinfeng Hongyuan Flagship Store	Power Saving Specialist 1	It feels more than good and is worth having.	Jingdong prefecture level city in Shandong
Jinfeng Hongyuan Flagship Store	Power Saving Specialist 1	The goods arrived very good and fast first time to buy	Jingdong prefecture level city in Shandong
Jinfeng Hongyuan Flagship Store	Power Saving Specialist 1	The power saving is very good, I can save so much money every month, I will buy another one next time.	Jingdong prefecture level city in Shandong
Jinfeng Hongyuan Flagship Store	Power Saving Specialist 1	It's not what it says.	Jingdong prefecture level city in Shandong
Jinfeng Hongyuan Flagship Store	Power Saving Specialist 1	I have been using it for many days now and it saves electricity, it really does I have an 8 kW electric snail stove and it saves about $3.50 a day.	Jingdong prefecture level city in Shandong
Jinfeng Hongyuan Flagship Store	Power Saving Specialist 1	Express delivery is very fast, the quality is also quite good, worth buying!	Jingdong prefecture level city in Shandong
Jinfeng Hongyuan Flagship Store	Power Saving Specialist 1	Just received expecting the baby to work well.	Jingdong prefecture level city in Shandong
Jinfeng Hongyuan Flagship Store	Power Saving Specialist 1	Very good, there are more fruit, has bought a second!	Jingdong prefecture level city in Shandong
Jinfeng Hongyuan Flagship Store	Power Saving Specialist 1	Good is good So far, no power savings have been noticed Maybe it's too little to notice.	Jingdong prefecture level city in Shandong
Jinfeng Hongyuan Flagship Store	Power Saving Specialist 1	I just received the item and plugged it in to see what happens later.	Jingdong prefecture level city in Shandong
Jinfeng Hongyuan Flagship Store	Power Saving Specialist 1	Baby received, especially good, packaging is also good, the courier brother is very strong, thank you.	Jingdong prefecture level city in Shandong
Jinfeng Hongyuan Flagship Store	Power Saving Specialist 1	Just received the goods have not yet used it, do not know how, three months later in the follow-up comment!	Jingdong prefecture level city in Shandong
Jinfeng Hongyuan Flagship Store	Power Saving Specialist 1	The item was received, it is quite good, recommended to buy.	Jingdong prefecture level city in Shandong
Jinfeng Hongyuan Flagship Store	Power Saving Specialist 1	This energy saver works very well and I recommend everyone to buy it!	Jingdong prefecture level city in Shandong
Jinfeng Hongyuan Flagship Store	Power Saving Specialist 1	Voltage stabilization and current reduction nice power saving product.	Jingdong prefecture level city in Shandong
Jinfeng Hongyuan Flagship Store	Power Saving Specialist 1	Voltage stabilization and current reduction nice power saving product.	Jingdong prefecture level city in Shandong
Jinfeng Hongyuan Flagship Store	Power Saving Specialist 1	A good power saver for stabilizing and lowering current.	Jingdong prefecture level city in Shandong
Jinfeng Hongyuan Flagship Store	Power Saving Specialist 1	You'll know if it saves power after you use it.	Jingdong prefecture level city in Shandong
Jinfeng Hongyuan Flagship Store	Power Saving Specialist 1	The quality is great. The quality is very good. The service is very good. Express delivery arrived in two days.	Jingdong prefecture level city in Shandong
Jinfeng Hongyuan Flagship Store	Power Saving Specialist 1	It works great.	Jingdong prefecture level city in Shandong
Jinfeng Hongyuan Flagship Store	Power Saving Specialist 1	Logistics is very fast, not yet used, used to comment again.	Jingdong prefecture level city in Shandong
Jinfeng Hongyuan Flagship Store	Power Saving Specialist 1	Logistics is very fast things are also very good, worthy of friends to buy	Jingdong prefecture level city in Shandong
Jinfeng Hongyuan Flagship Store	Power Saving Specialist 1	It works. It really works.	Jingdong prefecture level city in Shandong
Jinfeng Hongyuan Flagship Store	Power Saving Specialist 1	LANGUAGE COMMENTS: just received the shipment. I immediately plugged it in and used it. If you ask how effective. It depends on whether the next month's electricity bill has been reduced. If there is a decrease. I am buying more than one to use.	Jingdong prefecture level city in Shandong
Jinfeng Hongyuan Flagship Store	Power Saving Specialist 1	Well made, power saving, good and convenient!	Jingdong prefecture level city in Shandong
Jinfeng Hongyuan Flagship Store	Power Saving Specialist 1	I'm using it. I'll rate it next month.	Jingdong prefecture level city in Shandong
Jinfeng Hongyuan Flagship Store	Power Saving Specialist 1	Baby received Oh, the courier delivery really fast, with a good and then come back to the evaluation	Jingdong prefecture level city in Shandong
Jinfeng Hongyuan Flagship Store	Power Saving Specialist 1	Fast logistics, good packaging, worth it!	Jingdong prefecture level city in Shandong
Jinfeng Hongyuan Flagship Store	Power Saving Specialist 1	Customer service is okay. It's just that.	Jingdong prefecture level city in Shandong

Jinfeng Hongyuan Flagship Store	Power Saving Specialist 1	Poor power saving	Jingdong prefecture level city in Shandong
Jinfeng Hongyuan Flagship Store	Power Saving Specialist 1	I'll tell you when I've used it.	Jingdong prefecture level city in Shandong
Jinfeng Hongyuan Flagship Store	Power Saving Specialist 1	I think it's good.	Jingdong prefecture level city in Shandong
Jinfeng Hongyuan Flagship Store	Power Saving Specialist 1	This energy saver works very well and I recommend everyone to buy it!	Jingdong prefecture level city in Shandong
Jinfeng Hongyuan Flagship Store	Power Saving Specialist 1	I do not know how effective, just use half a month on the burn out, good business reissued a, and then use it after the follow-up comment.	Jingdong prefecture level city in Shandong
Jinfeng Hongyuan Flagship Store	Power Saving Specialist 1	in use	Jingdong prefecture level city in Shandong
Jinfeng Hongyuan Flagship Store	Power Saving Specialist 1	This thing is very poor. It's already only been used for ten days and it's broken. Now waiting for you to return it.	Jingdong prefecture level city in Shandong
Jinfeng Hongyuan Flagship Store	Power Saving Specialist 1	It's not the same as the seller's show.	Jingdong prefecture level city in Shandong
Jinfeng Hongyuan Flagship Store	Power Saving Specialist 1	What is the problem when I get it back from the CTS, it is plugged into the wall and the voltage shows 206v.	Jingdong prefecture level city in Shandong
Jinfeng Hongyuan Flagship Store	Power Saving Specialist 1	The logistics was fast and the stuff was perfectly packaged. It is in the process of trial.	Jingdong prefecture level city in Shandong
Jinfeng Hongyuan Flagship Store	Power Saving Specialist 1	This saver is particularly good to use can really save electricity, with two months and then look at the line if you buy one for the family	Jingdong prefecture level city in Shandong
Jinfeng Hongyuan Flagship Store	Power Saving Specialist 1	Novel appearance design, all aluminum alloy profile, also with a fuse device, voltage monitoring display. After receiving the goods, I directly cut the plug and connect it to the main power box under the leakage switch, hoping to save electricity, and then follow up later. Logistics is very fast, arrived the next	Jingdong prefecture level city in Shandong
Jinfeng Hongyuan Flagship Store	Power Saving Specialist 1	Received, very good and like, if necessary next time to buy ha ha	Jingdong prefecture level city in Shandong
Jinfeng Hongyuan Flagship Store	Power Saving Specialist 1	It's good. It's worth it. Get it if you need it.	Jingdong prefecture level city in Shandong
Jinfeng Hongyuan Flagship Store	Power Saving Specialist 1	It feels more than good and is worth having.	Jingdong prefecture level city in Shandong
Jinfeng Hongyuan Flagship Store	Power Saving Specialist 1	The goods arrived very good and fast first time to buy	Jingdong prefecture level city in Shandong
Jinfeng Hongyuan Flagship Store	Power Saving Specialist 1	The power saving is very good, I can save so much money every month, I will buy another one next time.	Jingdong prefecture level city in Shandong
Jinfeng Hongyuan Flagship Store	Power Saving Specialist 1	It's not what it says.	Jingdong prefecture level city in Shandong
Jinfeng Hongyuan Flagship Store	Power Saving Specialist 1	I have been using it for many days now and it saves electricity, it really does I have an 8 kW electric snail stove and it saves about $3.50 a day.	Jingdong prefecture level city in Shandong
Jinfeng Hongyuan Flagship Store	Power Saving Specialist 1	Express delivery is very fast, the quality is also quite good, worth buying!	Jingdong prefecture level city in Shandong
Jinfeng Hongyuan Flagship Store	Power Saving Specialist 1	Just received expecting the baby to work well.	Jingdong prefecture level city in Shandong
Jinfeng Hongyuan Flagship Store	Power Saving Specialist 1	Very good, there are more fruit, has bought a second!	Jingdong prefecture level city in Shandong
Jinfeng Hongyuan Flagship Store	Power Saving Specialist 1	Good is good So far, no power savings have been noticed Maybe it's too little to notice.	Jingdong prefecture level city in Shandong
Jinfeng Hongyuan Flagship Store	Power Saving Specialist 1	I just received the item and plugged it in to see what happens later.	Jingdong prefecture level city in Shandong
Jinfeng Hongyuan Flagship Store	Power Saving Specialist 1	Baby received, especially good, packaging is also good, the courier brother is very strong, thank you.	Jingdong prefecture level city in Shandong
Jinfeng Hongyuan Flagship Store	Power Saving Specialist 1	Just received the goods have not yet used it, do not know how, three months later in the follow-up comment!	Jingdong prefecture level city in Shandong
Jinfeng Hongyuan Flagship Store	Power Saving Specialist 1	The item was received, it is quite good, recommended to buy.	Jingdong prefecture level city in Shandong
Jinfeng Hongyuan Flagship Store	Power Saving Specialist 1	This energy saver works very well and I recommend everyone to buy it!	Jingdong prefecture level city in Shandong
Jinfeng Hongyuan Flagship Store	Power Saving Specialist 1	Voltage stabilization and current reduction nice power saving product.	Jingdong prefecture level city in Shandong
Jinfeng Hongyuan Flagship Store	Power Saving Specialist 1	Voltage stabilization and current reduction nice power saving product.	Jingdong prefecture level city in Shandong
Jinfeng Hongyuan Flagship Store	Power Saving Specialist 1	A good power saver for stabilizing and lowering current.	Jingdong prefecture level city in Shandong
Jinfeng Hongyuan Flagship Store	Power Saving Specialist 1	You'll know if it saves power after you use it.	Jingdong prefecture level city in Shandong
Jinfeng Hongyuan Flagship Store	Power Saving Specialist 1	The quality is great. The quality is very good. The service is very good. Express delivery arrived in two days.	Jingdong prefecture level city in Shandong
Jinfeng Hongyuan Flagship Store	Power Saving Specialist 1	It works great.	Jingdong prefecture level city in Shandong
Jinfeng Hongyuan Flagship Store	Power Saving Specialist 1	Logistics is very fast, not yet used, used to comment again.	Jingdong prefecture level city in Shandong
Jinfeng Hongyuan Flagship Store	Power Saving Specialist 1	Logistics is very fast things are also very good, worthy of friends to buy	Jingdong prefecture level city in Shandong
Jinfeng Hongyuan Flagship Store	Power Saving Specialist 1	It works. It really works.	Jingdong prefecture level city in Shandong
Jinfeng Hongyuan Flagship Store	Power Saving Specialist 1	LANGUAGE COMMENTS: just received the shipment. I immediately plugged it in and used it. If you ask how effective. It depends on whether the next month's electricity bill has been reduced. If there is a decrease. I am buying more than one to use.	Jingdong prefecture level city in Shandong
Jinfeng Hongyuan Flagship Store	Power Saving Specialist 1	Well made, power saving, good and convenient!	Jingdong prefecture level city in Shandong
Jinfeng Hongyuan Flagship Store	Power Saving Specialist 1	I'm using it. I'll rate it next month.	Jingdong prefecture level city in Shandong
Jinfeng Hongyuan Flagship Store	Power Saving Specialist 1	Baby received Oh, the courier delivery really fast, with a good and then come back to the evaluation	Jingdong prefecture level city in Shandong
Jinfeng Hongyuan Flagship Store	Power Saving Specialist 1	Fast logistics, good packaging, worth it!	Jingdong prefecture level city in Shandong
Jinfeng Hongyuan Flagship Store	Power Saving Specialist 1	Customer service is okay. It's just that.	Jingdong prefecture level city in Shandong
Jinfeng Hongyuan Flagship Store	Power Saving Specialist 1	Poor power saving	Jingdong prefecture level city in Shandong
Jinfeng Hongyuan Flagship Store	Power Saving Specialist 1	I'll tell you when I've used it.	Jingdong prefecture level city in Shandong
Jinfeng Hongyuan Flagship Store	Power Saving Specialist 1	I think it's good.	Jingdong prefecture level city in Shandong
Jinfeng Hongyuan Flagship Store	Power Saving Specialist 1	This energy saver works very well and I recommend everyone to buy it!	Jingdong prefecture level city in Shandong
Jinfeng Hongyuan Flagship Store	Power Saving Specialist 1	I do not know how effective, just use half a month on the burn out, good business reissued a, and then use it after the follow-up comment.	Jingdong prefecture level city in Shandong
Jinfeng Hongyuan Flagship Store	Power Saving Specialist 1	in use	Jingdong prefecture level city in Shandong
Jinfeng Hongyuan Flagship Store	Power Saving Specialist 1	This thing is very poor. It's already only been used for ten days and it's broken. Now waiting for you to return it.	Jingdong prefecture level city in Shandong

Jinfeng Hongyuan Flagship Store	Power Saving Specialist 1	It's not the same as the seller's show.	Jingdong prefecture level city in Shandong
Jinfeng Hongyuan Flagship Store	Power Saving Specialist 1	What is the problem when I get it back from the CTS, it is plugged into the wall and the voltage shows 206v.	Jingdong prefecture level city in Shandong
Jinfeng Hongyuan Flagship Store	Power Saving Specialist 1	The logistics was fast and the stuff was perfectly packaged. It is in the process of trial.	Jingdong prefecture level city in Shandong
Jinfeng Hongyuan Flagship Store	Power Saving Specialist 1	This saver is particularly good to use can really save electricity, with two months and then look at the line if you buy one for the family	Jingdong prefecture level city in Shandong
Jinfeng Hongyuan Flagship Store	Power Saving Specialist 1	Novel appearance design, all aluminum alloy profile, also with a fuse device, voltage monitoring display. After receiving the goods, I directly cut the plug and connect it to the main power box under the leakage switch, hoping to save electricity, and then follow up later. Logistics is very fast, arrived the next	Jingdong prefecture level city in Shandong
Jinfeng Hongyuan Flagship Store	Power Saving Specialist 1	Received, very good and like, if necessary next time to buy ha ha	Jingdong prefecture level city in Shandong
Jinfeng Hongyuan Flagship Store	Power Saving Specialist 1	It's good. It's worth it. Get it if you need it.	Jingdong prefecture level city in Shandong
Jinfeng Hongyuan Flagship Store	Power Saving Specialist 1	It feels more than good and is worth having.	Jingdong prefecture level city in Shandong
Jinfeng Hongyuan Flagship Store	Power Saving Specialist 1	The goods arrived very good and fast first time to buy	Jingdong prefecture level city in Shandong
Jinfeng Hongyuan Flagship Store	Power Saving Specialist 1	The power saving is very good, I can save so much money every month, I will buy another one next time.	Jingdong prefecture level city in Shandong
Jinfeng Hongyuan Flagship Store	Power Saving Specialist 1	It's not what it says.	Jingdong prefecture level city in Shandong
Jinfeng Hongyuan Flagship Store	Power Saving Specialist 1	I have been using it for many days now and it saves electricity, it really does I have an 8 kW electric snail stove and it saves about $3.50 a day.	Jingdong prefecture level city in Shandong
Jinfeng Hongyuan Flagship Store	Power Saving Specialist 1	Express delivery is very fast, the quality is also quite good, worth buying!	Jingdong prefecture level city in Shandong
Jinfeng Hongyuan Flagship Store	Power Saving Specialist 1	Just received expecting the baby to work well.	Jingdong prefecture level city in Shandong
Jinfeng Hongyuan Flagship Store	Power Saving Specialist 1	Very good, there are more fruit, has bought a second!	Jingdong prefecture level city in Shandong
Jinfeng Hongyuan Flagship Store	Power Saving Specialist 1	Good is good So far, no power savings have been noticed Maybe it's too little to notice.	Jingdong prefecture level city in Shandong
Jinfeng Hongyuan Flagship Store	Power Saving Specialist 1	I just received the item and plugged it in to see what happens later.	Jingdong prefecture level city in Shandong
Jinfeng Hongyuan Flagship Store	Power Saving Specialist 1	Baby received, especially good, packaging is also good, the courier brother is very strong, thank you.	Jingdong prefecture level city in Shandong
Jinfeng Hongyuan Flagship Store	Power Saving Specialist 1	Just received the goods have not yet used it, do not know how, three months later in the follow-up comment!	Jingdong prefecture level city in Shandong
Jinfeng Hongyuan Flagship Store	Power Saving Specialist 1	The item was received, it is quite good, recommended to buy.	Jingdong prefecture level city in Shandong
Jinfeng Hongyuan Flagship Store	Power Saving Specialist 1	This energy saver works very well and I recommend everyone to buy it!	Jingdong prefecture level city in Shandong
Jinfeng Hongyuan Flagship Store	Power Saving Specialist 1	Voltage stabilization and current reduction nice power saving product.	Jingdong prefecture level city in Shandong
Jinfeng Hongyuan Flagship Store	Power Saving Specialist 1	Voltage stabilization and current reduction nice power saving product.	Jingdong prefecture level city in Shandong
Jinfeng Hongyuan Flagship Store	Power Saving Specialist 1	A good power saver for stabilizing and lowering current.	Jingdong prefecture level city in Shandong
Jinfeng Hongyuan Flagship Store	Power Saving Specialist 1	You'll know if it saves power after you use it.	Jingdong prefecture level city in Shandong
Jinfeng Hongyuan Flagship Store	Power Saving Specialist 1	The quality is great. The quality is very good. The service is very good. Express delivery arrived in two days.	Jingdong prefecture level city in Shandong
Jinfeng Hongyuan Flagship Store	Power Saving Specialist 1	It works great.	Jingdong prefecture level city in Shandong
Jinfeng Hongyuan Flagship Store	Power Saving Specialist 1	Logistics is very fast, not yet used, used to comment again.	Jingdong prefecture level city in Shandong
Jinfeng Hongyuan Flagship Store	Power Saving Specialist 1	Logistics is very fast things are also very good, worthy of friends to buy	Jingdong prefecture level city in Shandong
Jinfeng Hongyuan Flagship Store	Power Saving Specialist 1	It works. It really works.	Jingdong prefecture level city in Shandong
Jinfeng Hongyuan Flagship Store	Power Saving Specialist 1	LANGUAGE COMMENTS: just received the shipment. I immediately plugged it in and used it. If you ask how effective. It depends on whether the next month's electricity bill has been reduced. If there is a decrease. I am buying more than one to use.	Jingdong prefecture level city in Shandong
Jinfeng Hongyuan Flagship Store	Power Saving Specialist 1	Well made, power saving, good and convenient!	Jingdong prefecture level city in Shandong
Jinfeng Hongyuan Flagship Store	Power Saving Specialist 1	I'm using it. I'll rate it next month.	Jingdong prefecture level city in Shandong
Jinfeng Hongyuan Flagship Store	Power Saving Specialist 1	Baby received Oh, the courier delivery really fast, with a good and then come back to the evaluation	Jingdong prefecture level city in Shandong
Jinfeng Hongyuan Flagship Store	Power Saving Specialist 1	Fast logistics, good packaging, worth it!	Jingdong prefecture level city in Shandong
Jinfeng Hongyuan Flagship Store	Power Saving Specialist 1	Customer service is okay. It's just that.	Jingdong prefecture level city in Shandong
Jinfeng Hongyuan Flagship Store	Power Saving Specialist 1	Poor power saving	Jingdong prefecture level city in Shandong
Jinfeng Hongyuan Flagship Store	Power Saving Specialist 1	I'll tell you when I've used it.	Jingdong prefecture level city in Shandong
Jinfeng Hongyuan Flagship Store	Power Saving Specialist 1	I think it's good.	Jingdong prefecture level city in Shandong
Jinfeng Hongyuan Flagship Store	Power Saving Specialist 1	This energy saver works very well and I recommend everyone to buy it!	Jingdong prefecture level city in Shandong
Jinfeng Hongyuan Flagship Store	Power Saving Specialist 1	I do not know how effective, just use half a month on the burn out, good business reissued a, and then use it after the follow-up comment.	Jingdong prefecture level city in Shandong
Jinfeng Hongyuan Flagship Store	Power Saving Specialist 1	in use	Jingdong prefecture level city in Shandong
Jinfeng Hongyuan Flagship Store	Power Saving Specialist 1	This thing is very poor. It's already only been used for ten days and it's broken. Now waiting for you to return it.	Jingdong prefecture level city in Shandong
Jinfeng Hongyuan Flagship Store	Power Saving Specialist 1	It's not the same as the seller's show.	Jingdong prefecture level city in Shandong
Jinfeng Hongyuan Flagship Store	Power Saving Specialist 1	What is the problem when I get it back from the CTS, it is plugged into the wall and the voltage shows 206v.	Jingdong prefecture level city in Shandong
Jinfeng Hongyuan Flagship Store	Power Saving Specialist 1	The logistics was fast and the stuff was perfectly packaged. It is in the process of trial.	Jingdong prefecture level city in Shandong
Jinfeng Hongyuan Flagship Store	Power Saving Specialist 1	This saver is particularly good to use can really save electricity, with two months and then look at the line if you buy one for the family	Jingdong prefecture level city in Shandong
Jinfeng Hongyuan Flagship Store	Power Saving Specialist 1	Novel appearance design, all aluminum alloy profile, also with a fuse device, voltage monitoring display. After receiving the goods, I directly cut the plug and connect it to the main power box under the leakage switch, hoping to save electricity, and then follow up later. Logistics is very fast, arrived the next	Jingdong prefecture level city in Shandong
Jinfeng Hongyuan Flagship Store	Power Saving Specialist 1	Received, very good and like, if necessary next time to buy ha ha	Jingdong prefecture level city in Shandong
Jinfeng Hongyuan Flagship Store	Power Saving Specialist 1	It's good. It's worth it. Get it if you need it.	Jingdong prefecture level city in Shandong

Jinfeng Hongyuan Flagship Store	Power Saving Specialist 1	It feels more than good and is worth having.	Jingdong prefecture level city in Shandong
Jinfeng Hongyuan Flagship Store	Power Saving Specialist 1	The goods arrived very good and fast first time to buy	Jingdong prefecture level city in Shandong
Jinfeng Hongyuan Flagship Store	Power Saving Specialist 1	The power saving is very good, I can save so much money every month, I will buy another one next time.	Jingdong prefecture level city in Shandong
Jinfeng Hongyuan Flagship Store	Power Saving Specialist 1	It's not what it says.	Jingdong prefecture level city in Shandong
Jinfeng Hongyuan Flagship Store	Power Saving Specialist 1	I have been using it for many days now and it saves electricity, it really does I have an 8 kW electric snail stove and it saves about $3.50 a day.	Jingdong prefecture level city in Shandong
Jinfeng Hongyuan Flagship Store	Power Saving Specialist 1	Express delivery is very fast, the quality is also quite good, worth buying!	Jingdong prefecture level city in Shandong
Jinfeng Hongyuan Flagship Store	Power Saving Specialist 1	Just received expecting the baby to work well.	Jingdong prefecture level city in Shandong
Jinfeng Hongyuan Flagship Store	Power Saving Specialist 1	Very good, there are more fruit, has bought a second!	Jingdong prefecture level city in Shandong
Jinfeng Hongyuan Flagship Store	Power Saving Specialist 1	Good is good So far, no power savings have been noticed Maybe it's too little to notice.	Jingdong prefecture level city in Shandong
Jinfeng Hongyuan Flagship Store	Power Saving Specialist 1	I just received the item and plugged it in to see what happens later.	Jingdong prefecture level city in Shandong
Jinfeng Hongyuan Flagship Store	Power Saving Specialist 1	Baby received, especially good, packaging is also good, the courier brother is very strong, thank you.	Jingdong prefecture level city in Shandong
Jinfeng Hongyuan Flagship Store	Power Saving Specialist 1	Just received the goods have not yet used it, do not know how, three months later in the follow-up comment!	Jingdong prefecture level city in Shandong
Jinfeng Hongyuan Flagship Store	Power Saving Specialist 1	The item was received, it is quite good, recommended to buy.	Jingdong prefecture level city in Shandong
Jinfeng Hongyuan Flagship Store	Power Saving Specialist 1	This energy saver works very well and I recommend everyone to buy it!	Jingdong prefecture level city in Shandong
Jinfeng Hongyuan Flagship Store	Power Saving Specialist 1	Voltage stabilization and current reduction nice power saving product.	Jingdong prefecture level city in Shandong
Jinfeng Hongyuan Flagship Store	Power Saving Specialist 1	Voltage stabilization and current reduction nice power saving product.	Jingdong prefecture level city in Shandong
Jinfeng Hongyuan Flagship Store	Power Saving Specialist 1	A good power saver for stabilizing and lowering current.	Jingdong prefecture level city in Shandong
Jinfeng Hongyuan Flagship Store	Power Saving Specialist 1	You'll know if it saves power after you use it.	Jingdong prefecture level city in Shandong
Jinfeng Hongyuan Flagship Store	Power Saving Specialist 1	The quality is great. The quality is very good. The service is very good. Express delivery arrived in two days.	Jingdong prefecture level city in Shandong
Jinfeng Hongyuan Flagship Store	Power Saving Specialist 1	It works great.	Jingdong prefecture level city in Shandong
Jinfeng Hongyuan Flagship Store	Power Saving Specialist 1	Logistics is very fast, not yet used, used to comment again.	Jingdong prefecture level city in Shandong
Jinfeng Hongyuan Flagship Store	Power Saving Specialist 1	Logistics is very fast things are also very good, worthy of friends to buy	Jingdong prefecture level city in Shandong
Jinfeng Hongyuan Flagship Store	Power Saving Specialist 1	It works. It really works.	Jingdong prefecture level city in Shandong
Jinfeng Hongyuan Flagship Store	Power Saving Specialist 1	LANGUAGE COMMENTS: just received the shipment. I immediately plugged it in and used it. If you ask how effective. It depends on whether the next month's electricity bill has been reduced. If there is a decrease. I am buying more than one to use.	Jingdong prefecture level city in Shandong
Jinfeng Hongyuan Flagship Store	Power Saving Specialist 1	Well made, power saving, good and convenient!	Jingdong prefecture level city in Shandong
Jinfeng Hongyuan Flagship Store	Power Saving Specialist 1	I'm using it. I'll rate it next month.	Jingdong prefecture level city in Shandong
Jinfeng Hongyuan Flagship Store	Power Saving Specialist 1	Baby received Oh, the courier delivery really fast, with a good and then come back to the evaluation	Jingdong prefecture level city in Shandong
Jinfeng Hongyuan Flagship Store	Power Saving Specialist 1	Fast logistics, good packaging, worth it!	Jingdong prefecture level city in Shandong
Jinfeng Hongyuan Flagship Store	Power Saving Specialist 1	Customer service is okay. It's just that.	Jingdong prefecture level city in Shandong
Jinfeng Hongyuan Flagship Store	Power Saving Specialist 1	Poor power saving	Jingdong prefecture level city in Shandong
Jinfeng Hongyuan Flagship Store	Power Saving Specialist 1	I'll tell you when I've used it.	Jingdong prefecture level city in Shandong
Jinfeng Hongyuan Flagship Store	Power Saving Specialist 1	I think it's good.	Jingdong prefecture level city in Shandong
Jinfeng Hongyuan Flagship Store	Power Saving Specialist 1	This energy saver works very well and I recommend everyone to buy it!	Jingdong prefecture level city in Shandong
Jinfeng Hongyuan Flagship Store	Power Saving Specialist 1	I do not know how effective, just use half a month on the burn out, good business reissued a, and then use it after the follow-up comment.	Jingdong prefecture level city in Shandong
Jinfeng Hongyuan Flagship Store	Power Saving Specialist 1	in use	Jingdong prefecture level city in Shandong
Jinfeng Hongyuan Flagship Store	Power Saving Specialist 1	This thing is very poor. It's already only been used for ten days and it's broken. Now waiting for you to return it.	Jingdong prefecture level city in Shandong
Jinfeng Hongyuan Flagship Store	Power Saving Specialist 1	It's not the same as the seller's show.	Jingdong prefecture level city in Shandong
Jinfeng Hongyuan Flagship Store	Power Saving Specialist 1	What is the problem when I get it back from the CTS, it is plugged into the wall and the voltage shows 206v.	Jingdong prefecture level city in Shandong
Jinfeng Hongyuan Flagship Store	Power Saving Specialist 1	The logistics was fast and the stuff was perfectly packaged. It is in the process of trial.	Jingdong prefecture level city in Shandong
Jinfeng Hongyuan Flagship Store	Power Saving Specialist 1	This saver is particularly good to use can really save electricity, with two months and then look at the line if you buy one for the family	Jingdong prefecture level city in Shandong
Jinfeng Hongyuan Flagship Store	Power Saving Specialist 1	Novel appearance design, all aluminum alloy profile, also with a fuse device, voltage monitoring display. After receiving the goods, I directly cut the plug and connect it to the main power box under the leakage switch, hoping to save electricity, and then follow up later. Logistics is very fast, arrived the next	Jingdong prefecture level city in Shandong
Jinfeng Hongyuan Flagship Store	Power Saving Specialist 1	Received, very good and like, if necessary next time to buy ha ha	Jingdong prefecture level city in Shandong
Jinfeng Hongyuan Flagship Store	Power Saving Specialist 1	It's good. It's worth it. Get it if you need it.	Jingdong prefecture level city in Shandong
Jinfeng Hongyuan Flagship Store	Power Saving Specialist 1	It feels more than good and is worth having.	Jingdong prefecture level city in Shandong
Jinfeng Hongyuan Flagship Store	Power Saving Specialist 1	The goods arrived very good and fast first time to buy	Jingdong prefecture level city in Shandong
Jinfeng Hongyuan Flagship Store	Power Saving Specialist 1	The power saving is very good, I can save so much money every month, I will buy another one next time.	Jingdong prefecture level city in Shandong
Jinfeng Hongyuan Flagship Store	Power Saving Specialist 1	It's not what it says.	Jingdong prefecture level city in Shandong
Jinfeng Hongyuan Flagship Store	Power Saving Specialist 1	I have been using it for many days now and it saves electricity, it really does I have an 8 kW electric snail stove and it saves about $3.50 a day.	Jingdong prefecture level city in Shandong
Jinfeng Hongyuan Flagship Store	Power Saving Specialist 1	Express delivery is very fast, the quality is also quite good, worth buying!	Jingdong prefecture level city in Shandong
Jinfeng Hongyuan Flagship Store	Power Saving Specialist 1	Just received expecting the baby to work well.	Jingdong prefecture level city in Shandong

Jinfeng Hongyuan Flagship Store	Power Saving Specialist 1	Very good, there are more fruit, has bought a second!	Jingdong prefecture level city in Shandong
Jinfeng Hongyuan Flagship Store	Power Saving Specialist 1	Good is good So far, no power savings have been noticed Maybe it's too little to notice.	Jingdong prefecture level city in Shandong
Jinfeng Hongyuan Flagship Store	Power Saving Specialist 1	I just received the item and plugged it in to see what happens later.	Jingdong prefecture level city in Shandong
Jinfeng Hongyuan Flagship Store	Power Saving Specialist 1	Baby received, especially good, packaging is also good, the courier brother is very strong, thank you.	Jingdong prefecture level city in Shandong
Jinfeng Hongyuan Flagship Store	Power Saving Specialist 1	Just received the goods have not yet used it, do not know how, three months later in the follow-up comment!	Jingdong prefecture level city in Shandong
Jinfeng Hongyuan Flagship Store	Power Saving Specialist 1	The item was received, it is quite good, recommended to buy.	Jingdong prefecture level city in Shandong
Jinfeng Hongyuan Flagship Store	Power Saving Specialist 1	This energy saver works very well and I recommend everyone to buy it!	Jingdong prefecture level city in Shandong
Jinfeng Hongyuan Flagship Store	Power Saving Specialist 1	Voltage stabilization and current reduction nice power saving product.	Jingdong prefecture level city in Shandong
Jinfeng Hongyuan Flagship Store	Power Saving Specialist 1	Voltage stabilization and current reduction nice power saving product.	Jingdong prefecture level city in Shandong
Jinfeng Hongyuan Flagship Store	Power Saving Specialist 1	A good power saver for stabilizing and lowering current.	Jingdong prefecture level city in Shandong
Jinfeng Hongyuan Flagship Store	Power Saving Specialist 1	You'll know if it saves power after you use it.	Jingdong prefecture level city in Shandong
Jinfeng Hongyuan Flagship Store	Power Saving Specialist 1	The quality is great. The quality is very good. The service is very good. Express delivery arrived in two days.	Jingdong prefecture level city in Shandong
Jinfeng Hongyuan Flagship Store	Power Saving Specialist 1	It works great.	Jingdong prefecture level city in Shandong
Jinfeng Hongyuan Flagship Store	Power Saving Specialist 1	Logistics is very fast, not yet used, used to comment again.	Jingdong prefecture level city in Shandong
Jinfeng Hongyuan Flagship Store	Power Saving Specialist 1	Logistics is very fast things are also very good, worthy of friends to buy	Jingdong prefecture level city in Shandong
Jinfeng Hongyuan Flagship Store	Power Saving Specialist 1	It works. It really works.	Jingdong prefecture level city in Shandong
Jinfeng Hongyuan Flagship Store	Power Saving Specialist 1	LANGUAGE COMMENTS: just received the shipment. I immediately plugged it in and used it. If you ask how effective. It depends on whether the next month's electricity bill has been reduced. If there is a decrease. I am buying more than one to use.	Jingdong prefecture level city in Shandong
Jinfeng Hongyuan Flagship Store	Power Saving Specialist 1	Well made, power saving, good and convenient!	Jingdong prefecture level city in Shandong
Jinfeng Hongyuan Flagship Store	Power Saving Specialist 1	I'm using it. I'll rate it next month.	Jingdong prefecture level city in Shandong
Jinfeng Hongyuan Flagship Store	Power Saving Specialist 1	Baby received Oh, the courier delivery really fast, with a good and then come back to the evaluation	Jingdong prefecture level city in Shandong
Jinfeng Hongyuan Flagship Store	Power Saving Specialist 1	Fast logistics, good packaging, worth it!	Jingdong prefecture level city in Shandong
Jinfeng Hongyuan Flagship Store	Power Saving Specialist 1	Customer service is okay. It's just that.	Jingdong prefecture level city in Shandong
Jinfeng Hongyuan Flagship Store	Power Saving Specialist 1	Poor power saving	Jingdong prefecture level city in Shandong
Jinfeng Hongyuan Flagship Store	Power Saving Specialist 1	I'll tell you when I've used it.	Jingdong prefecture level city in Shandong
Jinfeng Hongyuan Flagship Store	Power Saving Specialist 1	I think it's good.	Jingdong prefecture level city in Shandong
Jinfeng Hongyuan Flagship Store	Power Saving Specialist 1	This energy saver works very well and I recommend everyone to buy it!	Jingdong prefecture level city in Shandong
Jinfeng Hongyuan Flagship Store	Power Saving Specialist 1	I do not know how effective, just use half a month on the burn out, good business reissued a, and then use it after the follow-up comment.	Jingdong prefecture level city in Shandong
Jinfeng Hongyuan Flagship Store	Power Saving Specialist 1	in use	Jingdong prefecture level city in Shandong
Jinfeng Hongyuan Flagship Store	Power Saving Specialist 1	This thing is very poor. It's already only been used for ten days and it's broken. Now waiting for you to return it.	Jingdong prefecture level city in Shandong
Jinfeng Hongyuan Flagship Store	Power Saving Specialist 1	It's not the same as the seller's show.	Jingdong prefecture level city in Shandong
Jinfeng Hongyuan Flagship Store	Power Saving Specialist 1	What is the problem when I get it back from the CTS, it is plugged into the wall and the voltage shows 206v.	Jingdong prefecture level city in Shandong
Jinfeng Hongyuan Flagship Store	Power Saving Specialist 1	The logistics was fast and the stuff was perfectly packaged. It is in the process of trial.	Jingdong prefecture level city in Shandong
Jinfeng Hongyuan Flagship Store	Power Saving Specialist 1	This saver is particularly good to use can really save electricity, with two months and then look at the line if you buy one for the family	Jingdong prefecture level city in Shandong
Jinfeng Hongyuan Flagship Store	Power Saving Specialist 1	Novel appearance design, all aluminum alloy profile, also with a fuse device, voltage monitoring display. After receiving the goods, I directly cut the plug and connect it to the main power box under the leakage switch, hoping to save electricity, and then follow up later. Logistics is very fast, arrived the next	Jingdong prefecture level city in Shandong
Jinfeng Hongyuan Flagship Store	Power Saving Specialist 1	Received, very good and like, if necessary next time to buy ha ha	Jingdong prefecture level city in Shandong
Jinfeng Hongyuan Flagship Store	Power Saving Specialist 1	It's good. It's worth it. Get it if you need it.	Jingdong prefecture level city in Shandong
Jinfeng Hongyuan Flagship Store	Power Saving Specialist 1	It feels more than good and is worth having.	Jingdong prefecture level city in Shandong
Jinfeng Hongyuan Flagship Store	Power Saving Specialist 1	The goods arrived very good and fast first time to buy	Jingdong prefecture level city in Shandong
Jinfeng Hongyuan Flagship Store	Power Saving Specialist 1	The power saving is very good, I can save so much money every month, I will buy another one next time.	Jingdong prefecture level city in Shandong
Jinfeng Hongyuan Flagship Store	Power Saving Specialist 1	It's not what it says.	Jingdong prefecture level city in Shandong
Jinfeng Hongyuan Flagship Store	Power Saving Specialist 1	I have been using it for many days now and it saves electricity, it really does I have an 8 kW electric snail stove and it saves about $3.50 a day.	Jingdong prefecture level city in Shandong
Jinfeng Hongyuan Flagship Store	Power Saving Specialist 1	Express delivery is very fast, the quality is also quite good, worth buying!	Jingdong prefecture level city in Shandong
Jinfeng Hongyuan Flagship Store	Power Saving Specialist 1	Just received expecting the baby to work well.	Jingdong prefecture level city in Shandong
Jinfeng Hongyuan Flagship Store	Power Saving Specialist 1	Very good, there are more fruit, has bought a second!	Jingdong prefecture level city in Shandong
Jinfeng Hongyuan Flagship Store	Power Saving Specialist 1	Good is good So far, no power savings have been noticed Maybe it's too little to notice.	Jingdong prefecture level city in Shandong
Jinfeng Hongyuan Flagship Store	Power Saving Specialist 1	I just received the item and plugged it in to see what happens later.	Jingdong prefecture level city in Shandong
Jinfeng Hongyuan Flagship Store	Power Saving Specialist 1	Baby received, especially good, packaging is also good, the courier brother is very strong, thank you.	Jingdong prefecture level city in Shandong
Jinfeng Hongyuan Flagship Store	Power Saving Specialist 1	Just received the goods have not yet used it, do not know how, three months later in the follow-up comment!	Jingdong prefecture level city in Shandong
Jinfeng Hongyuan Flagship Store	Power Saving Specialist 1	The item was received, it is quite good, recommended to buy.	Jingdong prefecture level city in Shandong
Jinfeng Hongyuan Flagship Store	Power Saving Specialist 1	This energy saver works very well and I recommend everyone to buy it!	Jingdong prefecture level city in Shandong

Jinfeng Hongyuan Flagship Store	Power Saving Specialist 1	Voltage stabilization and current reduction nice power saving product.	Jingdong prefecture level city in Shandong
Jinfeng Hongyuan Flagship Store	Power Saving Specialist 1	Voltage stabilization and current reduction nice power saving product.	Jingdong prefecture level city in Shandong
Jinfeng Hongyuan Flagship Store	Power Saving Specialist 1	A good power saver for stabilizing and lowering current.	Jingdong prefecture level city in Shandong
Jinfeng Hongyuan Flagship Store	Power Saving Specialist 1	You'll know if it saves power after you use it.	Jingdong prefecture level city in Shandong
Jinfeng Hongyuan Flagship Store	Power Saving Specialist 1	The quality is great. The quality is very good. The service is very good. Express delivery arrived in two days.	Jingdong prefecture level city in Shandong
Jinfeng Hongyuan Flagship Store	Power Saving Specialist 1	It works great.	Jingdong prefecture level city in Shandong
Jinfeng Hongyuan Flagship Store	Power Saving Specialist 1	Logistics is very fast, not yet used, used to comment again.	Jingdong prefecture level city in Shandong
Jinfeng Hongyuan Flagship Store	Power Saving Specialist 1	Logistics is very fast things are also very good, worthy of friends to buy	Jingdong prefecture level city in Shandong
Jinfeng Hongyuan Flagship Store	Power Saving Specialist 1	It works. It really works.	Jingdong prefecture level city in Shandong
Jinfeng Hongyuan Flagship Store	Power Saving Specialist 1	LANGUAGE COMMENTS: just received the shipment. I immediately plugged it in and used it. If you ask how effective. It depends on whether the next month's electricity bill has been reduced. If there is a decrease. I am buying more than one to use.	Jingdong prefecture level city in Shandong
Jinfeng Hongyuan Flagship Store	Power Saving Specialist 1	Well made, power saving, good and convenient!	Jingdong prefecture level city in Shandong
Jinfeng Hongyuan Flagship Store	Power Saving Specialist 1	I'm using it. I'll rate it next month.	Jingdong prefecture level city in Shandong
Jinfeng Hongyuan Flagship Store	Power Saving Specialist 1	Baby received Oh, the courier delivery really fast, with a good and then come back to the evaluation	Jingdong prefecture level city in Shandong
Jinfeng Hongyuan Flagship Store	Power Saving Specialist 1	Fast logistics, good packaging, worth it!	Jingdong prefecture level city in Shandong
Jinfeng Hongyuan Flagship Store	Power Saving Specialist 1	Customer service is okay. It's just that.	Jingdong prefecture level city in Shandong
Jinfeng Hongyuan Flagship Store	Power Saving Specialist 1	Poor power saving	Jingdong prefecture level city in Shandong
Jinfeng Hongyuan Flagship Store	Power Saving Specialist 1	I'll tell you when I've used it.	Jingdong prefecture level city in Shandong
Jinfeng Hongyuan Flagship Store	Power Saving Specialist 1	I think it's good.	Jingdong prefecture level city in Shandong
Jinfeng Hongyuan Flagship Store	Power Saving Specialist 1	This energy saver works very well and I recommend everyone to buy it!	Jingdong prefecture level city in Shandong
Jinfeng Hongyuan Flagship Store	Power Saving Specialist 1	I do not know how effective, just use half a month on the burn out, good business reissued a, and then use it after the follow-up comment.	Jingdong prefecture level city in Shandong
Jinfeng Hongyuan Flagship Store	Power Saving Specialist 1	in use	Jingdong prefecture level city in Shandong
Jinfeng Hongyuan Flagship Store	Power Saving Specialist 1	This thing is very poor. It's already only been used for ten days and it's broken. Now waiting for you to return it.	Jingdong prefecture level city in Shandong
Jinfeng Hongyuan Flagship Store	Power Saving Specialist 1	It's not the same as the seller's show.	Jingdong prefecture level city in Shandong
Jinfeng Hongyuan Flagship Store	Power Saving Specialist 1	What is the problem when I get it back from the CTS, it is plugged into the wall and the voltage shows 206v.	Jingdong prefecture level city in Shandong
Jinfeng Hongyuan Flagship Store	Power Saving Specialist 1	The logistics was fast and the stuff was perfectly packaged. It is in the process of trial.	Jingdong prefecture level city in Shandong
Jinfeng Hongyuan Flagship Store	Power Saving Specialist 1	This saver is particularly good to use can really save electricity, with two months and then look at the line if you buy one for the family	Jingdong prefecture level city in Shandong
Jinfeng Hongyuan Flagship Store	Power Saving Specialist 1	Novel appearance design, all aluminum alloy profile, also with a fuse device, voltage monitoring display. After receiving the goods, I directly cut the plug and connect it to the main power box under the leakage switch, hoping to save electricity, and then follow up later. Logistics is very fast, arrived the next	Jingdong prefecture level city in Shandong
Jinfeng Hongyuan Flagship Store	Power Saving Specialist 1	Received, very good and like, if necessary next time to buy ha ha	Jingdong prefecture level city in Shandong
Jinfeng Hongyuan Flagship Store	Power Saving Specialist 1	It's good. It's worth it. Get it if you need it.	Jingdong prefecture level city in Shandong
Jinfeng Hongyuan Flagship Store	Power Saving Specialist 1	It feels more than good and is worth having.	Jingdong prefecture level city in Shandong
Jinfeng Hongyuan Flagship Store	Power Saving Specialist 1	The goods arrived very good and fast first time to buy	Jingdong prefecture level city in Shandong
Jinfeng Hongyuan Flagship Store	Power Saving Specialist 1	The power saving is very good, I can save so much money every month, I will buy another one next time.	Jingdong prefecture level city in Shandong
Jinfeng Hongyuan Flagship Store	Power Saving Specialist 1	It's not what it says.	Jingdong prefecture level city in Shandong
Jinfeng Hongyuan Flagship Store	Power Saving Specialist 1	I have been using it for many days now and it saves electricity, it really does I have an 8 kW electric snail stove and it saves about $3.50 a day.	Jingdong prefecture level city in Shandong
Jinfeng Hongyuan Flagship Store	Power Saving Specialist 1	Express delivery is very fast, the quality is also quite good, worth buying!	Jingdong prefecture level city in Shandong
Jinfeng Hongyuan Flagship Store	Power Saving Specialist 1	Just received expecting the baby to work well.	Jingdong prefecture level city in Shandong
Jinfeng Hongyuan Flagship Store	Power Saving Specialist 1	Very good, there are more fruit, has bought a second!	Jingdong prefecture level city in Shandong
Jinfeng Hongyuan Flagship Store	Power Saving Specialist 1	Good is good So far, no power savings have been noticed Maybe it's too little to notice.	Jingdong prefecture level city in Shandong
Jinfeng Hongyuan Flagship Store	Power Saving Specialist 1	I just received the item and plugged it in to see what happens later.	Jingdong prefecture level city in Shandong
Jinfeng Hongyuan Flagship Store	Power Saving Specialist 1	Baby received, especially good, packaging is also good, the courier brother is very strong, thank you.	Jingdong prefecture level city in Shandong
Jinfeng Hongyuan Flagship Store	Power Saving Specialist 1	Just received the goods have not yet used it, do not know how, three months later in the follow-up comment!	Jingdong prefecture level city in Shandong
Jinfeng Hongyuan Flagship Store	Power Saving Specialist 1	The item was received, it is quite good, recommended to buy.	Jingdong prefecture level city in Shandong
Jinfeng Hongyuan Flagship Store	Power Saving Specialist 1	This energy saver works very well and I recommend everyone to buy it!	Jingdong prefecture level city in Shandong
Jinfeng Hongyuan Flagship Store	Power Saving Specialist 1	Voltage stabilization and current reduction nice power saving product.	Jingdong prefecture level city in Shandong
Jinfeng Hongyuan Flagship Store	Power Saving Specialist 1	Voltage stabilization and current reduction nice power saving product.	Jingdong prefecture level city in Shandong
Jinfeng Hongyuan Flagship Store	Power Saving Specialist 1	A good power saver for stabilizing and lowering current.	Jingdong prefecture level city in Shandong
Jinfeng Hongyuan Flagship Store	Power Saving Specialist 1	You'll know if it saves power after you use it.	Jingdong prefecture level city in Shandong
Jinfeng Hongyuan Flagship Store	Power Saving Specialist 1	The quality is great. The quality is very good. The service is very good. Express delivery arrived in two days.	Jingdong prefecture level city in Shandong
Jinfeng Hongyuan Flagship Store	Power Saving Specialist 1	It works great.	Jingdong prefecture level city in Shandong
Jinfeng Hongyuan Flagship Store	Power Saving Specialist 1	Logistics is very fast, not yet used, used to comment again.	Jingdong prefecture level city in Shandong

Jinfeng Hongyuan Flagship Store	Power Saving Specialist 1	Logistics is very fast things are also very good, worthy of friends to buy	Jingdong prefecture level city in Shandong
Jinfeng Hongyuan Flagship Store	Power Saving Specialist 1	It works. It really works.	Jingdong prefecture level city in Shandong
Jinfeng Hongyuan Flagship Store	Power Saving Specialist 1	LANGUAGE COMMENTS: just received the shipment. I immediately plugged it in and used it. If you ask how effective. It depends on whether the next month's electricity bill has been reduced. If there is a decrease. I am buying more than one to use.	Jingdong prefecture level city in Shandong
Jinfeng Hongyuan Flagship Store	Power Saving Specialist 1	Well made, power saving, good and convenient!	Jingdong prefecture level city in Shandong
Jinfeng Hongyuan Flagship Store	Power Saving Specialist 1	I'm using it. I'll rate it next month.	Jingdong prefecture level city in Shandong
Jinfeng Hongyuan Flagship Store	Power Saving Specialist 1	Baby received Oh, the courier delivery really fast, with a good and then come back to the evaluation	Jingdong prefecture level city in Shandong
Jinfeng Hongyuan Flagship Store	Power Saving Specialist 1	Fast logistics, good packaging, worth it!	Jingdong prefecture level city in Shandong
Jinfeng Hongyuan Flagship Store	Power Saving Specialist 1	Customer service is okay. It's just that.	Jingdong prefecture level city in Shandong
Jinfeng Hongyuan Flagship Store	Power Saving Specialist 1	Poor power saving	Jingdong prefecture level city in Shandong
Jinfeng Hongyuan Flagship Store	Power Saving Specialist 1	I'll tell you when I've used it.	Jingdong prefecture level city in Shandong
Jinfeng Hongyuan Flagship Store	Power Saving Specialist 1	I think it's good.	Jingdong prefecture level city in Shandong
Jinfeng Hongyuan Flagship Store	Power Saving Specialist 1	This energy saver works very well and I recommend everyone to buy it!	Jingdong prefecture level city in Shandong
Jinfeng Hongyuan Flagship Store	Power Saving Specialist 1	I do not know how effective, just use half a month on the burn out, good business reissued a, and then use it after the follow-up comment.	Jingdong prefecture level city in Shandong
Jinfeng Hongyuan Flagship Store	Power Saving Specialist 1	in use	Jingdong prefecture level city in Shandong
Jinfeng Hongyuan Flagship Store	Power Saving Specialist 1	This thing is very poor. It's already only been used for ten days and it's broken. Now waiting for you to return it.	Jingdong prefecture level city in Shandong
Jinfeng Hongyuan Flagship Store	Power Saving Specialist 1	It's not the same as the seller's show.	Jingdong prefecture level city in Shandong
Jinfeng Hongyuan Flagship Store	Power Saving Specialist 1	What is the problem when I get it back from the CTS, it is plugged into the wall and the voltage shows 206v.	Jingdong prefecture level city in Shandong
Jinfeng Hongyuan Flagship Store	Power Saving Specialist 1	The logistics was fast and the stuff was perfectly packaged. It is in the process of trial.	Jingdong prefecture level city in Shandong
Jinfeng Hongyuan Flagship Store	Power Saving Specialist 1	This saver is particularly good to use can really save electricity, with two months and then look at the line if you buy one for the family	Jingdong prefecture level city in Shandong
Jinfeng Hongyuan Flagship Store	Power Saving Specialist 1	Novel appearance design, all aluminum alloy profile, also with a fuse device, voltage monitoring display. After receiving the goods, I directly cut the plug and connect it to the main power box under the leakage switch, hoping to save electricity, and then follow up later. Logistics is very fast, arrived the next	Jingdong prefecture level city in Shandong
Jinfeng Hongyuan Flagship Store	Power Saving Specialist 1	Received, very good and like, if necessary next time to buy ha ha	Jingdong prefecture level city in Shandong
Jinfeng Hongyuan Flagship Store	Power Saving Specialist 1	It's good. It's worth it. Get it if you need it.	Jingdong prefecture level city in Shandong
Jinfeng Hongyuan Flagship Store	Power Saving Specialist 1	It feels more than good and is worth having.	Jingdong prefecture level city in Shandong
Jinfeng Hongyuan Flagship Store	Power Saving Specialist 1	The goods arrived very good and fast first time to buy	Jingdong prefecture level city in Shandong
Jinfeng Hongyuan Flagship Store	Power Saving Specialist 1	The power saving is very good, I can save so much money every month, I will buy another one next time.	Jingdong prefecture level city in Shandong
Jinfeng Hongyuan Flagship Store	Power Saving Specialist 1	It's not what it says.	Jingdong prefecture level city in Shandong
Jinfeng Hongyuan Flagship Store	Power Saving Specialist 1	I have been using it for many days now and it saves electricity, it really does I have an 8 kW electric snail stove and it saves about $3.50 a day.	Jingdong prefecture level city in Shandong
Jinfeng Hongyuan Flagship Store	Power Saving Specialist 1	Express delivery is very fast, the quality is also quite good, worth buying!	Jingdong prefecture level city in Shandong
Jinfeng Hongyuan Flagship Store	Power Saving Specialist 1	Just received expecting the baby to work well.	Jingdong prefecture level city in Shandong
Jinfeng Hongyuan Flagship Store	Power Saving Specialist 1	Very good, there are more fruit, has bought a second!	Jingdong prefecture level city in Shandong
Jinfeng Hongyuan Flagship Store	Power Saving Specialist 1	Good is good So far, no power savings have been noticed Maybe it's too little to notice.	Jingdong prefecture level city in Shandong
Jinfeng Hongyuan Flagship Store	Power Saving Specialist 1	I just received the item and plugged it in to see what happens later.	Jingdong prefecture level city in Shandong
Jinfeng Hongyuan Flagship Store	Power Saving Specialist 1	Baby received, especially good, packaging is also good, the courier brother is very strong, thank you.	Jingdong prefecture level city in Shandong
Jinfeng Hongyuan Flagship Store	Power Saving Specialist 1	Just received the goods have not yet used it, do not know how, three months later in the follow-up comment!	Jingdong prefecture level city in Shandong
Jinfeng Hongyuan Flagship Store	Power Saving Specialist 1	The item was received, it is quite good, recommended to buy.	Jingdong prefecture level city in Shandong
Jinfeng Hongyuan Flagship Store	Power Saving Specialist 1	This energy saver works very well and I recommend everyone to buy it!	Jingdong prefecture level city in Shandong
Jinfeng Hongyuan Flagship Store	Power Saving Specialist 1	Voltage stabilization and current reduction nice power saving product.	Jingdong prefecture level city in Shandong
Jinfeng Hongyuan Flagship Store	Power Saving Specialist 1	Voltage stabilization and current reduction nice power saving product.	Jingdong prefecture level city in Shandong
Jinfeng Hongyuan Flagship Store	Power Saving Specialist 1	A good power saver for stabilizing and lowering current.	Jingdong prefecture level city in Shandong
Jinfeng Hongyuan Flagship Store	Power Saving Specialist 1	You'll know if it saves power after you use it.	Jingdong prefecture level city in Shandong
Jinfeng Hongyuan Flagship Store	Power Saving Specialist 1	The quality is great. The quality is very good. The service is very good. Express delivery arrived in two days.	Jingdong prefecture level city in Shandong
Jinfeng Hongyuan Flagship Store	Power Saving Specialist 1	It works great.	Jingdong prefecture level city in Shandong
Jinfeng Hongyuan Flagship Store	Power Saving Specialist 1	Logistics is very fast, not yet used, used to comment again.	Jingdong prefecture level city in Shandong
Jinfeng Hongyuan Flagship Store	Power Saving Specialist 1	Logistics is very fast things are also very good, worthy of friends to buy	Jingdong prefecture level city in Shandong
Jinfeng Hongyuan Flagship Store	Power Saving Specialist 1	It works. It really works.	Jingdong prefecture level city in Shandong
Jinfeng Hongyuan Flagship Store	Power Saving Specialist 1	LANGUAGE COMMENTS: just received the shipment. I immediately plugged it in and used it. If you ask how effective. It depends on whether the next month's electricity bill has been reduced. If there is a decrease. I am buying more than one to use.	Jingdong prefecture level city in Shandong
Jinfeng Hongyuan Flagship Store	Power Saving Specialist 1	Well made, power saving, good and convenient!	Jingdong prefecture level city in Shandong
Jinfeng Hongyuan Flagship Store	Power Saving Specialist 1	I'm using it. I'll rate it next month.	Jingdong prefecture level city in Shandong
Jinfeng Hongyuan Flagship Store	Power Saving Specialist 1	Baby received Oh, the courier delivery really fast, with a good and then come back to the evaluation	Jingdong prefecture level city in Shandong
Jinfeng Hongyuan Flagship Store	Power Saving Specialist 1	Fast logistics, good packaging, worth it!	Jingdong prefecture level city in Shandong

Jinfeng Hongyuan Flagship Store	Power Saving Specialist 1	Customer service is okay. It's just that.	Jingdong prefecture level city in Shandong
Jinfeng Hongyuan Flagship Store	Power Saving Specialist 1	Poor power saving	Jingdong prefecture level city in Shandong
Jinfeng Hongyuan Flagship Store	Power Saving Specialist 1	I'll tell you when I've used it.	Jingdong prefecture level city in Shandong
Jinfeng Hongyuan Flagship Store	Power Saving Specialist 1	I think it's good.	Jingdong prefecture level city in Shandong
Jinfeng Hongyuan Flagship Store	Power Saving Specialist 1	This energy saver works very well and I recommend everyone to buy it!	Jingdong prefecture level city in Shandong
Jinfeng Hongyuan Flagship Store	Power Saving Specialist 1	I do not know how effective, just use half a month on the burn out, good business reissued a, and then use it after the follow-up comment.	Jingdong prefecture level city in Shandong
Jinfeng Hongyuan Flagship Store	Power Saving Specialist 1	in use	Jingdong prefecture level city in Shandong
Jinfeng Hongyuan Flagship Store	Power Saving Specialist 1	This thing is very poor. It's already only been used for ten days and it's broken. Now waiting for you to return it.	Jingdong prefecture level city in Shandong
Jinfeng Hongyuan Flagship Store	Power Saving Specialist 1	It's not the same as the seller's show.	Jingdong prefecture level city in Shandong
Jinfeng Hongyuan Flagship Store	Power Saving Specialist 1	What is the problem when I get it back from the CTS, it is plugged into the wall and the voltage shows 206v.	Jingdong prefecture level city in Shandong
Jinfeng Hongyuan Flagship Store	Power Saving Specialist 1	The logistics was fast and the stuff was perfectly packaged. It is in the process of trial.	Jingdong prefecture level city in Shandong
Jinfeng Hongyuan Flagship Store	Power Saving Specialist 1	This saver is particularly good to use can really save electricity, with two months and then look at the line if you buy one for the family	Jingdong prefecture level city in Shandong
Jinfeng Hongyuan Flagship Store	Power Saving Specialist 1	Novel appearance design, all aluminum alloy profile, also with a fuse device, voltage monitoring display. After receiving the goods, I directly cut the plug and connect it to the main power box under the leakage switch, hoping to save electricity, and then follow up later. Logistics is very fast, arrived the next	Jingdong prefecture level city in Shandong
Jinfeng Hongyuan Flagship Store	Power Saving Specialist 1	Received, very good and like, if necessary next time to buy ha ha	Jingdong prefecture level city in Shandong
Jinfeng Hongyuan Flagship Store	Power Saving Specialist 1	It's good. It's worth it. Get it if you need it.	Jingdong prefecture level city in Shandong
Jinfeng Hongyuan Flagship Store	Power Saving Specialist 1	It feels more than good and is worth having.	Jingdong prefecture level city in Shandong
Jinfeng Hongyuan Flagship Store	Power Saving Specialist 1	The goods arrived very good and fast first time to buy	Jingdong prefecture level city in Shandong
Jinfeng Hongyuan Flagship Store	Power Saving Specialist 1	The power saving is very good, I can save so much money every month, I will buy another one next time.	Jingdong prefecture level city in Shandong
Jinfeng Hongyuan Flagship Store	Power Saving Specialist 1	It's not what it says.	Jingdong prefecture level city in Shandong
Jinfeng Hongyuan Flagship Store	Power Saving Specialist 1	I have been using it for many days now and it saves electricity, it really does I have an 8 kW electric snail stove and it saves about $3.50 a day.	Jingdong prefecture level city in Shandong
Jinfeng Hongyuan Flagship Store	Power Saving Specialist 1	Express delivery is very fast, the quality is also quite good, worth buying!	Jingdong prefecture level city in Shandong
Jinfeng Hongyuan Flagship Store	Power Saving Specialist 1	Just received expecting the baby to work well.	Jingdong prefecture level city in Shandong
Jinfeng Hongyuan Flagship Store	Power Saving Specialist 1	Very good, there are more fruit, has bought a second!	Jingdong prefecture level city in Shandong
Jinfeng Hongyuan Flagship Store	Power Saving Specialist 1	Good is good So far, no power savings have been noticed Maybe it's too little to notice.	Jingdong prefecture level city in Shandong
Jinfeng Hongyuan Flagship Store	Power Saving Specialist 1	I just received the item and plugged it in to see what happens later.	Jingdong prefecture level city in Shandong
Jinfeng Hongyuan Flagship Store	Power Saving Specialist 1	Baby received, especially good, packaging is also good, the courier brother is very strong, thank you.	Jingdong prefecture level city in Shandong
Jinfeng Hongyuan Flagship Store	Power Saving Specialist 1	Just received the goods have not yet used it, do not know how, three months later in the follow-up comment!	Jingdong prefecture level city in Shandong
Jinfeng Hongyuan Flagship Store	Power Saving Specialist 1	The item was received, it is quite good, recommended to buy.	Jingdong prefecture level city in Shandong
Jinfeng Hongyuan Flagship Store	Power Saving Specialist 1	This energy saver works very well and I recommend everyone to buy it!	Jingdong prefecture level city in Shandong
Jinfeng Hongyuan Flagship Store	Power Saving Specialist 1	Voltage stabilization and current reduction nice power saving product.	Jingdong prefecture level city in Shandong
Jinfeng Hongyuan Flagship Store	Power Saving Specialist 1	Voltage stabilization and current reduction nice power saving product.	Jingdong prefecture level city in Shandong
Jinfeng Hongyuan Flagship Store	Power Saving Specialist 1	A good power saver for stabilizing and lowering current.	Jingdong prefecture level city in Shandong
Jinfeng Hongyuan Flagship Store	Power Saving Specialist 1	You'll know if it saves power after you use it.	Jingdong prefecture level city in Shandong
Jinfeng Hongyuan Flagship Store	Power Saving Specialist 1	The quality is great. The quality is very good. The service is very good. Express delivery arrived in two days.	Jingdong prefecture level city in Shandong
Jinfeng Hongyuan Flagship Store	Power Saving Specialist 1	It works great.	Jingdong prefecture level city in Shandong
Jinfeng Hongyuan Flagship Store	Power Saving Specialist 1	Logistics is very fast, not yet used, used to comment again.	Jingdong prefecture level city in Shandong
Jinfeng Hongyuan Flagship Store	Power Saving Specialist 1	Logistics is very fast things are also very good, worthy of friends to buy	Jingdong prefecture level city in Shandong
Jinfeng Hongyuan Flagship Store	Power Saving Specialist 1	It works. It really works.	Jingdong prefecture level city in Shandong
Jinfeng Hongyuan Flagship Store	Power Saving Specialist 1	LANGUAGE COMMENTS: just received the shipment. I immediately plugged it in and used it. If you ask how effective. It depends on whether the next month's electricity bill has been reduced. If there is a decrease. I am buying more than one to use.	Jingdong prefecture level city in Shandong
Jinfeng Hongyuan Flagship Store	Power Saving Specialist 1	Well made, power saving, good and convenient!	Jingdong prefecture level city in Shandong
Jinfeng Hongyuan Flagship Store	Power Saving Specialist 1	I'm using it. I'll rate it next month.	Jingdong prefecture level city in Shandong
Jinfeng Hongyuan Flagship Store	Power Saving Specialist 1	Baby received Oh, the courier delivery really fast, with a good and then come back to the evaluation	Jingdong prefecture level city in Shandong
Jinfeng Hongyuan Flagship Store	Power Saving Specialist 1	Fast logistics, good packaging, worth it!	Jingdong prefecture level city in Shandong
Jinfeng Hongyuan Flagship Store	Power Saving Specialist 1	Customer service is okay. It's just that.	Jingdong prefecture level city in Shandong
Jinfeng Hongyuan Flagship Store	Power Saving Specialist 1	Poor power saving	Jingdong prefecture level city in Shandong
Jinfeng Hongyuan Flagship Store	Power Saving Specialist 1	I'll tell you when I've used it.	Jingdong prefecture level city in Shandong
Jinfeng Hongyuan Flagship Store	Power Saving Specialist 1	I think it's good.	Jingdong prefecture level city in Shandong
Jinfeng Hongyuan Flagship Store	Power Saving Specialist 1	This energy saver works very well and I recommend everyone to buy it!	Jingdong prefecture level city in Shandong
Jinfeng Hongyuan Flagship Store	Power Saving Specialist 1	I do not know how effective, just use half a month on the burn out, good business reissued a, and then use it after the follow-up comment.	Jingdong prefecture level city in Shandong
Jinfeng Hongyuan Flagship Store	Power Saving Specialist 1	in use	Jingdong prefecture level city in Shandong

Jinfeng Hongyuan Flagship Store	Power Saving Specialist 1	This thing is very poor. It's already only been used for ten days and it's broken. Now waiting for you to return it.	Jingdong prefecture level city in Shandong
Jinfeng Hongyuan Flagship Store	Power Saving Specialist 1	It's not the same as the seller's show.	Jingdong prefecture level city in Shandong
Jinfeng Hongyuan Flagship Store	Power Saving Specialist 1	What is the problem when I get it back from the CTS, it is plugged into the wall and the voltage shows 206v.	Jingdong prefecture level city in Shandong
Jinfeng Hongyuan Flagship Store	Power Saving Specialist 1	The logistics was fast and the stuff was perfectly packaged. It is in the process of trial.	Jingdong prefecture level city in Shandong
Jinfeng Hongyuan Flagship Store	Power Saving Specialist 1	This saver is particularly good to use can really save electricity, with two months and then look at the line if you buy one for the family	Jingdong prefecture level city in Shandong
Jinfeng Hongyuan Flagship Store	Power Saving Specialist 1	Novel appearance design, all aluminum alloy profile, also with a fuse device, voltage monitoring display. After receiving the goods, I directly cut the plug and connect it to the main power box under the leakage switch, hoping to save electricity, and then follow up later. Logistics is very fast, arrived the next	Jingdong prefecture level city in Shandong
Jinfeng Hongyuan Flagship Store	Power Saving Specialist 1	Received, very good and like, if necessary next time to buy ha ha	Jingdong prefecture level city in Shandong
Jinfeng Hongyuan Flagship Store	Power Saving Specialist 1	It's good. It's worth it. Get it if you need it.	Jingdong prefecture level city in Shandong
Jinfeng Hongyuan Flagship Store	Power Saving Specialist 1	It feels more than good and is worth having.	Jingdong prefecture level city in Shandong
Jinfeng Hongyuan Flagship Store	Power Saving Specialist 1	The goods arrived very good and fast first time to buy	Jingdong prefecture level city in Shandong
Jinfeng Hongyuan Flagship Store	Power Saving Specialist 1	The power saving is very good, I can save so much money every month, I will buy another one next time.	Jingdong prefecture level city in Shandong
Jinfeng Hongyuan Flagship Store	Power Saving Specialist 1	It's not what it says.	Jingdong prefecture level city in Shandong
Jinfeng Hongyuan Flagship Store	Power Saving Specialist 1	I have been using it for many days now and it saves electricity, it really does I have an 8 kW electric snail stove and it saves about $3.50 a day.	Jingdong prefecture level city in Shandong
Jinfeng Hongyuan Flagship Store	Power Saving Specialist 1	Express delivery is very fast, the quality is also quite good, worth buying!	Jingdong prefecture level city in Shandong
Jinfeng Hongyuan Flagship Store	Power Saving Specialist 1	Just received expecting the baby to work well.	Jingdong prefecture level city in Shandong
Jinfeng Hongyuan Flagship Store	Power Saving Specialist 1	Very good, there are more fruit, has bought a second!	Jingdong prefecture level city in Shandong
Jinfeng Hongyuan Flagship Store	Power Saving Specialist 1	Good is good So far, no power savings have been noticed Maybe it's too little to notice.	Jingdong prefecture level city in Shandong
Jinfeng Hongyuan Flagship Store	Power Saving Specialist 1	I just received the item and plugged it in to see what happens later.	Jingdong prefecture level city in Shandong
Jinfeng Hongyuan Flagship Store	Power Saving Specialist 1	Baby received, especially good, packaging is also good, the courier brother is very strong, thank you.	Jingdong prefecture level city in Shandong
Jinfeng Hongyuan Flagship Store	Power Saving Specialist 1	Just received the goods have not yet used it, do not know how, three months later in the follow-up comment!	Jingdong prefecture level city in Shandong
Jinfeng Hongyuan Flagship Store	Power Saving Specialist 1	The item was received, it is quite good, recommended to buy.	Jingdong prefecture level city in Shandong
Jinfeng Hongyuan Flagship Store	Power Saving Specialist 1	This energy saver works very well and I recommend everyone to buy it!	Jingdong prefecture level city in Shandong
Jinfeng Hongyuan Flagship Store	Power Saving Specialist 1	Voltage stabilization and current reduction nice power saving product.	Jingdong prefecture level city in Shandong
Jinfeng Hongyuan Flagship Store	Power Saving Specialist 1	Voltage stabilization and current reduction nice power saving product.	Jingdong prefecture level city in Shandong
Jinfeng Hongyuan Flagship Store	Power Saving Specialist 1	A good power saver for stabilizing and lowering current.	Jingdong prefecture level city in Shandong
Jinfeng Hongyuan Flagship Store	Power Saving Specialist 1	You'll know if it saves power after you use it.	Jingdong prefecture level city in Shandong
Jinfeng Hongyuan Flagship Store	Power Saving Specialist 1	The quality is great. The quality is very good. The service is very good. Express delivery arrived in two days.	Jingdong prefecture level city in Shandong
Jinfeng Hongyuan Flagship Store	Power Saving Specialist 1	It works great.	Jingdong prefecture level city in Shandong
Jinfeng Hongyuan Flagship Store	Power Saving Specialist 1	Logistics is very fast, not yet used, used to comment again.	Jingdong prefecture level city in Shandong
Jinfeng Hongyuan Flagship Store	Power Saving Specialist 1	Logistics is very fast things are also very good, worthy of friends to buy	Jingdong prefecture level city in Shandong
Jinfeng Hongyuan Flagship Store	Power Saving Specialist 1	It works. It really works.	Jingdong prefecture level city in Shandong
Jinfeng Hongyuan Flagship Store	Power Saving Specialist 1	LANGUAGE COMMENTS: just received the shipment. I immediately plugged it in and used it. If you ask how effective. It depends on whether the next month's electricity bill has been reduced. If there is a decrease. I am buying more than one to use.	Jingdong prefecture level city in Shandong
Jinfeng Hongyuan Flagship Store	Power Saving Specialist 1	Well made, power saving, good and convenient!	Jingdong prefecture level city in Shandong
Jinfeng Hongyuan Flagship Store	Power Saving Specialist 1	I'm using it. I'll rate it next month.	Jingdong prefecture level city in Shandong
Jinfeng Hongyuan Flagship Store	Power Saving Specialist 1	Baby received Oh, the courier delivery really fast, with a good and then come back to the evaluation	Jingdong prefecture level city in Shandong
Jinfeng Hongyuan Flagship Store	Power Saving Specialist 1	Fast logistics, good packaging, worth it!	Jingdong prefecture level city in Shandong
Jinfeng Hongyuan Flagship Store	Power Saving Specialist 1	Customer service is okay. It's just that.	Jingdong prefecture level city in Shandong
Jinfeng Hongyuan Flagship Store	Power Saving Specialist 1	Poor power saving	Jingdong prefecture level city in Shandong
Jinfeng Hongyuan Flagship Store	Power Saving Specialist 1	I'll tell you when I've used it.	Jingdong prefecture level city in Shandong
Jinfeng Hongyuan Flagship Store	Power Saving Specialist 1	I think it's good.	Jingdong prefecture level city in Shandong
Jinfeng Hongyuan Flagship Store	Power Saving Specialist 1	This energy saver works very well and I recommend everyone to buy it!	Jingdong prefecture level city in Shandong
Jinfeng Hongyuan Flagship Store	Power Saving Specialist 1	I do not know how effective, just use half a month on the burn out, good business reissued a, and then use it after the follow-up comment.	Jingdong prefecture level city in Shandong
Jinfeng Hongyuan Flagship Store	Power Saving Specialist 1	in use	Jingdong prefecture level city in Shandong
Jinfeng Hongyuan Flagship Store	Power Saving Specialist 1	This thing is very poor. It's already only been used for ten days and it's broken. Now waiting for you to return it.	Jingdong prefecture level city in Shandong
Jinfeng Hongyuan Flagship Store	Power Saving Specialist 1	It's not the same as the seller's show.	Jingdong prefecture level city in Shandong
Jinfeng Hongyuan Flagship Store	Power Saving Specialist 1	What is the problem when I get it back from the CTS, it is plugged into the wall and the voltage shows 206v.	Jingdong prefecture level city in Shandong
Jinfeng Hongyuan Flagship Store	Power Saving Specialist 1	The logistics was fast and the stuff was perfectly packaged. It is in the process of trial.	Jingdong prefecture level city in Shandong
Jinfeng Hongyuan Flagship Store	Power Saving Specialist 1	This saver is particularly good to use can really save electricity, with two months and then look at the line if you buy one for the family	Jingdong prefecture level city in Shandong
Jinfeng Hongyuan Flagship Store	Power Saving Specialist 1	Novel appearance design, all aluminum alloy profile, also with a fuse device, voltage monitoring display. After receiving the goods, I directly cut the plug and connect it to the main power box under the leakage switch, hoping to save electricity, and then follow up later. Logistics is very fast, arrived the next	Jingdong prefecture level city in Shandong
Jinfeng Hongyuan Flagship Store	Power Saving Specialist 1	Received, very good and like, if necessary next time to buy ha ha	Jingdong prefecture level city in Shandong

Jinfeng Hongyuan Flagship Store	Power Saving Specialist 1	It's good. It's worth it. Get it if you need it.	Jingdong prefecture level city in Shandong
Jinfeng Hongyuan Flagship Store	Power Saving Specialist 1	It feels more than good and is worth having.	Jingdong prefecture level city in Shandong
Jinfeng Hongyuan Flagship Store	Power Saving Specialist 1	The goods arrived very good and fast first time to buy	Jingdong prefecture level city in Shandong
Jinfeng Hongyuan Flagship Store	Power Saving Specialist 1	The power saving is very good, I can save so much money every month, I will buy another one next time.	Jingdong prefecture level city in Shandong
Jinfeng Hongyuan Flagship Store	Power Saving Specialist 1	It's not what it says.	Jingdong prefecture level city in Shandong
Jinfeng Hongyuan Flagship Store	Power Saving Specialist 1	I have been using it for many days now and it saves electricity, it really does I have an 8 kW electric snail stove and it saves about $3.50 a day.	Jingdong prefecture level city in Shandong
Jinfeng Hongyuan Flagship Store	Power Saving Specialist 1	Express delivery is very fast, the quality is also quite good, worth buying!	Jingdong prefecture level city in Shandong
Jinfeng Hongyuan Flagship Store	Power Saving Specialist 1	Just received expecting the baby to work well.	Jingdong prefecture level city in Shandong
Jinfeng Hongyuan Flagship Store	Power Saving Specialist 1	Very good, there are more fruit, has bought a second!	Jingdong prefecture level city in Shandong
Jinfeng Hongyuan Flagship Store	Power Saving Specialist 1	Good is good So far, no power savings have been noticed Maybe it's too little to notice.	Jingdong prefecture level city in Shandong
Jinfeng Hongyuan Flagship Store	Power Saving Specialist 1	I just received the item and plugged it in to see what happens later.	Jingdong prefecture level city in Shandong
Jinfeng Hongyuan Flagship Store	Power Saving Specialist 1	Baby received, especially good, packaging is also good, the courier brother is very strong, thank you.	Jingdong prefecture level city in Shandong
Jinfeng Hongyuan Flagship Store	Power Saving Specialist 1	Just received the goods have not yet used it, do not know how, three months later in the follow-up comment!	Jingdong prefecture level city in Shandong
Jinfeng Hongyuan Flagship Store	Power Saving Specialist 1	The item was received, it is quite good, recommended to buy.	Jingdong prefecture level city in Shandong
Jinfeng Hongyuan Flagship Store	Power Saving Specialist 1	This energy saver works very well and I recommend everyone to buy it!	Jingdong prefecture level city in Shandong
Jinfeng Hongyuan Flagship Store	Power Saving Specialist 1	Voltage stabilization and current reduction nice power saving product.	Jingdong prefecture level city in Shandong
Jinfeng Hongyuan Flagship Store	Power Saving Specialist 1	Voltage stabilization and current reduction nice power saving product.	Jingdong prefecture level city in Shandong
Jinfeng Hongyuan Flagship Store	Power Saving Specialist 1	A good power saver for stabilizing and lowering current.	Jingdong prefecture level city in Shandong
Jinfeng Hongyuan Flagship Store	Power Saving Specialist 1	You'll know if it saves power after you use it.	Jingdong prefecture level city in Shandong
Jinfeng Hongyuan Flagship Store	Power Saving Specialist 1	The quality is great. The quality is very good. The service is very good. Express delivery arrived in two days.	Jingdong prefecture level city in Shandong
Jinfeng Hongyuan Flagship Store	Power Saving Specialist 1	It works great.	Jingdong prefecture level city in Shandong
Jinfeng Hongyuan Flagship Store	Power Saving Specialist 1	Logistics is very fast, not yet used, used to comment again.	Jingdong prefecture level city in Shandong
Jinfeng Hongyuan Flagship Store	Power Saving Specialist 1	Logistics is very fast things are also very good, worthy of friends to buy	Jingdong prefecture level city in Shandong
Jinfeng Hongyuan Flagship Store	Power Saving Specialist 1	It works. It really works.	Jingdong prefecture level city in Shandong
Jinfeng Hongyuan Flagship Store	Power Saving Specialist 1	LANGUAGE COMMENTS: just received the shipment. I immediately plugged it in and used it. If you ask how effective. It depends on whether the next month's electricity bill has been reduced. If there is a decrease. I am buying more than one to use.	Jingdong prefecture level city in Shandong
Jinfeng Hongyuan Flagship Store	Power Saving Specialist 1	Well made, power saving, good and convenient!	Jingdong prefecture level city in Shandong
Jinfeng Hongyuan Flagship Store	Power Saving Specialist 1	I'm using it. I'll rate it next month.	Jingdong prefecture level city in Shandong
Jinfeng Hongyuan Flagship Store	Power Saving Specialist 1	Baby received Oh, the courier delivery really fast, with a good and then come back to the evaluation	Jingdong prefecture level city in Shandong
Jinfeng Hongyuan Flagship Store	Power Saving Specialist 1	Fast logistics, good packaging, worth it!	Jingdong prefecture level city in Shandong
Jinfeng Hongyuan Flagship Store	Power Saving Specialist 1	Customer service is okay. It's just that.	Jingdong prefecture level city in Shandong
Jinfeng Hongyuan Flagship Store	Power Saving Specialist 1	Poor power saving	Jingdong prefecture level city in Shandong
Jinfeng Hongyuan Flagship Store	Power Saving Specialist 1	I'll tell you when I've used it.	Jingdong prefecture level city in Shandong
Jinfeng Hongyuan Flagship Store	Power Saving Specialist 1	I think it's good.	Jingdong prefecture level city in Shandong
Jinfeng Hongyuan Flagship Store	Power Saving Specialist 1	This energy saver works very well and I recommend everyone to buy it!	Jingdong prefecture level city in Shandong
Jinfeng Hongyuan Flagship Store	Power Saving Specialist 1	I do not know how effective, just use half a month on the burn out, good business reissued a, and then use it after the follow-up comment.	Jingdong prefecture level city in Shandong
Jinfeng Hongyuan Flagship Store	Power Saving Specialist 1	in use	Jingdong prefecture level city in Shandong
Jinfeng Hongyuan Flagship Store	Power Saving Specialist 1	This thing is very poor. It's already only been used for ten days and it's broken. Now waiting for you to return it.	Jingdong prefecture level city in Shandong
Jinfeng Hongyuan Flagship Store	Power Saving Specialist 1	It's not the same as the seller's show.	Jingdong prefecture level city in Shandong
Jinfeng Hongyuan Flagship Store	Power Saving Specialist 1	What is the problem when I get it back from the CTS, it is plugged into the wall and the voltage shows 206v.	Jingdong prefecture level city in Shandong
Jinfeng Hongyuan Flagship Store	Power Saving Specialist 1	The logistics was fast and the stuff was perfectly packaged. It is in the process of trial.	Jingdong prefecture level city in Shandong
Jinfeng Hongyuan Flagship Store	Power Saving Specialist 1	This saver is particularly good to use can really save electricity, with two months and then look at the line if you buy one for the family	Jingdong prefecture level city in Shandong
Jinfeng Hongyuan Flagship Store	Power Saving Specialist 1	Novel appearance design, all aluminum alloy profile, also with a fuse device, voltage monitoring display. After receiving the goods, I directly cut the plug and connect it to the main power box under the leakage switch, hoping to save electricity, and then follow up later. Logistics is very fast, arrived the next	Jingdong prefecture level city in Shandong
Jinfeng Hongyuan Flagship Store	Power Saving Specialist 1	Received, very good and like, if necessary next time to buy ha ha	Jingdong prefecture level city in Shandong
Jinfeng Hongyuan Flagship Store	Power Saving Specialist 1	It's good. It's worth it. Get it if you need it.	Jingdong prefecture level city in Shandong
Jinfeng Hongyuan Flagship Store	Power Saving Specialist 1	It feels more than good and is worth having.	Jingdong prefecture level city in Shandong
Jinfeng Hongyuan Flagship Store	Power Saving Specialist 1	The goods arrived very good and fast first time to buy	Jingdong prefecture level city in Shandong
Jinfeng Hongyuan Flagship Store	Power Saving Specialist 1	The power saving is very good, I can save so much money every month, I will buy another one next time.	Jingdong prefecture level city in Shandong
Jinfeng Hongyuan Flagship Store	Power Saving Specialist 1	It's not what it says.	Jingdong prefecture level city in Shandong
Jinfeng Hongyuan Flagship Store	Power Saving Specialist 1	I have been using it for many days now and it saves electricity, it really does I have an 8 kW electric snail stove and it saves about $3.50 a day.	Jingdong prefecture level city in Shandong
Jinfeng Hongyuan Flagship Store	Power Saving Specialist 1	Express delivery is very fast, the quality is also quite good, worth buying!	Jingdong prefecture level city in Shandong

Jinfeng Hongyuan Flagship Store	Power Saving Specialist 1	Just received expecting the baby to work well.	Jingdong prefecture level city in Shandong
Jinfeng Hongyuan Flagship Store	Power Saving Specialist 1	Very good, there are more fruit, has bought a second!	Jingdong prefecture level city in Shandong
Jinfeng Hongyuan Flagship Store	Power Saving Specialist 1	Good is good So far, no power savings have been noticed Maybe it's too little to notice.	Jingdong prefecture level city in Shandong
Jinfeng Hongyuan Flagship Store	Power Saving Specialist 1	I just received the item and plugged it in to see what happens later.	Jingdong prefecture level city in Shandong
Jinfeng Hongyuan Flagship Store	Power Saving Specialist 1	Baby received, especially good, packaging is also good, the courier brother is very strong, thank you.	Jingdong prefecture level city in Shandong
Jinfeng Hongyuan Flagship Store	Power Saving Specialist 1	Just received the goods have not yet used it, do not know how, three months later in the follow-up comment!	Jingdong prefecture level city in Shandong
Jinfeng Hongyuan Flagship Store	Power Saving Specialist 1	The item was received, it is quite good, recommended to buy.	Jingdong prefecture level city in Shandong
Jinfeng Hongyuan Flagship Store	Power Saving Specialist 1	This energy saver works very well and I recommend everyone to buy it!	Jingdong prefecture level city in Shandong
Jinfeng Hongyuan Flagship Store	Power Saving Specialist 1	Voltage stabilization and current reduction nice power saving product.	Jingdong prefecture level city in Shandong
Jinfeng Hongyuan Flagship Store	Power Saving Specialist 1	Voltage stabilization and current reduction nice power saving product.	Jingdong prefecture level city in Shandong
Jinfeng Hongyuan Flagship Store	Power Saving Specialist 1	A good power saver for stabilizing and lowering current.	Jingdong prefecture level city in Shandong
Jinfeng Hongyuan Flagship Store	Power Saving Specialist 1	You'll know if it saves power after you use it.	Jingdong prefecture level city in Shandong
Jinfeng Hongyuan Flagship Store	Power Saving Specialist 1	The quality is great. The quality is very good. The service is very good. Express delivery arrived in two days.	Jingdong prefecture level city in Shandong
Jinfeng Hongyuan Flagship Store	Power Saving Specialist 1	It works great.	Jingdong prefecture level city in Shandong
Jinfeng Hongyuan Flagship Store	Power Saving Specialist 1	Logistics is very fast, not yet used, used to comment again.	Jingdong prefecture level city in Shandong
Jinfeng Hongyuan Flagship Store	Power Saving Specialist 1	Logistics is very fast things are also very good, worthy of friends to buy	Jingdong prefecture level city in Shandong
Jinfeng Hongyuan Flagship Store	Power Saving Specialist 1	It works. It really works.	Jingdong prefecture level city in Shandong
Jinfeng Hongyuan Flagship Store	Power Saving Specialist 1	LANGUAGE COMMENTS: just received the shipment. I immediately plugged it in and used it. If you ask how effective. It depends on whether the next month's electricity bill has been reduced. If there is a decrease. I am buying more than one to use.	Jingdong prefecture level city in Shandong
Jinfeng Hongyuan Flagship Store	Power Saving Specialist 1	Well made, power saving, good and convenient!	Jingdong prefecture level city in Shandong
Jinfeng Hongyuan Flagship Store	Power Saving Specialist 1	I'm using it. I'll rate it next month.	Jingdong prefecture level city in Shandong
Jinfeng Hongyuan Flagship Store	Power Saving Specialist 1	Baby received Oh, the courier delivery really fast, with a good and then come back to the evaluation	Jingdong prefecture level city in Shandong
Jinfeng Hongyuan Flagship Store	Power Saving Specialist 1	Fast logistics, good packaging, worth it!	Jingdong prefecture level city in Shandong
Jinfeng Hongyuan Flagship Store	Power Saving Specialist 1	Customer service is okay. It's just that.	Jingdong prefecture level city in Shandong
Jinfeng Hongyuan Flagship Store	Power Saving Specialist 1	Poor power saving	Jingdong prefecture level city in Shandong
Jinfeng Hongyuan Flagship Store	Power Saving Specialist 1	I'll tell you when I've used it.	Jingdong prefecture level city in Shandong
Jinfeng Hongyuan Flagship Store	Power Saving Specialist 1	I think it's good.	Jingdong prefecture level city in Shandong
Jinfeng Hongyuan Flagship Store	Power Saving Specialist 1	This energy saver works very well and I recommend everyone to buy it!	Jingdong prefecture level city in Shandong
Jinfeng Hongyuan Flagship Store	Power Saving Specialist 1	I do not know how effective, just use half a month on the burn out, good business reissued a, and then use it after the follow-up comment.	Jingdong prefecture level city in Shandong
Jinfeng Hongyuan Flagship Store	Power Saving Specialist 1	in use	Jingdong prefecture level city in Shandong
Jinfeng Hongyuan Flagship Store	Power Saving Specialist 1	This thing is very poor. It's already only been used for ten days and it's broken. Now waiting for you to return it.	Jingdong prefecture level city in Shandong
Jinfeng Hongyuan Flagship Store	Power Saving Specialist 1	It's not the same as the seller's show.	Jingdong prefecture level city in Shandong
Jinfeng Hongyuan Flagship Store	Power Saving Specialist 1	What is the problem when I get it back from the CTS, it is plugged into the wall and the voltage shows 206v.	Jingdong prefecture level city in Shandong
Jinfeng Hongyuan Flagship Store	Power Saving Specialist 1	The logistics was fast and the stuff was perfectly packaged. It is in the process of trial.	Jingdong prefecture level city in Shandong
Jinfeng Hongyuan Flagship Store	Power Saving Specialist 1	This saver is particularly good to use can really save electricity, with two months and then look at the line if you buy one for the family	Jingdong prefecture level city in Shandong
Jinfeng Hongyuan Flagship Store	Power Saving Specialist 1	Novel appearance design, all aluminum alloy profile, also with a fuse device, voltage monitoring display. After receiving the goods, I directly cut the plug and connect it to the main power box under the leakage switch, hoping to save electricity, and then follow up later. Logistics is very fast, arrived the next	Jingdong prefecture level city in Shandong
Jinfeng Hongyuan Flagship Store	Power Saving Specialist 1	Received, very good and like, if necessary next time to buy ha ha	Jingdong prefecture level city in Shandong
Jinfeng Hongyuan Flagship Store	Power Saving Specialist 1	It's good. It's worth it. Get it if you need it.	Jingdong prefecture level city in Shandong
Jinfeng Hongyuan Flagship Store	Power Saving Specialist 1	It feels more than good and is worth having.	Jingdong prefecture level city in Shandong
Jinfeng Hongyuan Flagship Store	Power Saving Specialist 1	The goods arrived very good and fast first time to buy	Jingdong prefecture level city in Shandong
Jinfeng Hongyuan Flagship Store	Power Saving Specialist 1	The power saving is very good, I can save so much money every month, I will buy another one next time.	Jingdong prefecture level city in Shandong
Jinfeng Hongyuan Flagship Store	Power Saving Specialist 1	It's not what it says.	Jingdong prefecture level city in Shandong
Jinfeng Hongyuan Flagship Store	Power Saving Specialist 1	I have been using it for many days now and it saves electricity, it really does I have an 8 kW electric snail stove and it saves about $3.50 a day.	Jingdong prefecture level city in Shandong
Jinfeng Hongyuan Flagship Store	Power Saving Specialist 1	Express delivery is very fast, the quality is also quite good, worth buying!	Jingdong prefecture level city in Shandong
Jinfeng Hongyuan Flagship Store	Power Saving Specialist 1	Just received expecting the baby to work well.	Jingdong prefecture level city in Shandong
Jinfeng Hongyuan Flagship Store	Power Saving Specialist 1	Very good, there are more fruit, has bought a second!	Jingdong prefecture level city in Shandong
Jinfeng Hongyuan Flagship Store	Power Saving Specialist 1	Good is good So far, no power savings have been noticed Maybe it's too little to notice.	Jingdong prefecture level city in Shandong
Jinfeng Hongyuan Flagship Store	Power Saving Specialist 1	I just received the item and plugged it in to see what happens later.	Jingdong prefecture level city in Shandong
Jinfeng Hongyuan Flagship Store	Power Saving Specialist 1	Baby received, especially good, packaging is also good, the courier brother is very strong, thank you.	Jingdong prefecture level city in Shandong
Jinfeng Hongyuan Flagship Store	Power Saving Specialist 1	Just received the goods have not yet used it, do not know how, three months later in the follow-up comment!	Jingdong prefecture level city in Shandong
Jinfeng Hongyuan Flagship Store	Power Saving Specialist 1	The item was received, it is quite good, recommended to buy.	Jingdong prefecture level city in Shandong

Jinfeng Hongyuan Flagship Store	Power Saving Specialist 1	This energy saver works very well and I recommend everyone to buy it!	Jingdong prefecture level city in Shandong
Jinfeng Hongyuan Flagship Store	Power Saving Specialist 1	Voltage stabilization and current reduction nice power saving product.	Jingdong prefecture level city in Shandong
Jinfeng Hongyuan Flagship Store	Power Saving Specialist 1	Voltage stabilization and current reduction nice power saving product.	Jingdong prefecture level city in Shandong
Jinfeng Hongyuan Flagship Store	Power Saving Specialist 1	A good power saver for stabilizing and lowering current.	Jingdong prefecture level city in Shandong
Jinfeng Hongyuan Flagship Store	Power Saving Specialist 1	You'll know if it saves power after you use it.	Jingdong prefecture level city in Shandong
Jinfeng Hongyuan Flagship Store	Power Saving Specialist 1	The quality is great. The quality is very good. The service is very good. Express delivery arrived in two days.	Jingdong prefecture level city in Shandong
Jinfeng Hongyuan Flagship Store	Power Saving Specialist 1	It works great.	Jingdong prefecture level city in Shandong
Jinfeng Hongyuan Flagship Store	Power Saving Specialist 1	Logistics is very fast, not yet used, used to comment again.	Jingdong prefecture level city in Shandong
Jinfeng Hongyuan Flagship Store	Power Saving Specialist 1	Logistics is very fast things are also very good, worthy of friends to buy	Jingdong prefecture level city in Shandong
Jinfeng Hongyuan Flagship Store	Power Saving Specialist 1	It works. It really works.	Jingdong prefecture level city in Shandong
Jinfeng Hongyuan Flagship Store	Power Saving Specialist 1	LANGUAGE COMMENTS: just received the shipment. I immediately plugged it in and used it. If you ask how effective. It depends on whether the next month's electricity bill has been reduced. If there is a decrease. I am buying more than one to use.	Jingdong prefecture level city in Shandong
Jinfeng Hongyuan Flagship Store	Power Saving Specialist 1	Well made, power saving, good and convenient!	Jingdong prefecture level city in Shandong
Jinfeng Hongyuan Flagship Store	Power Saving Specialist 1	I'm using it. I'll rate it next month.	Jingdong prefecture level city in Shandong
Jinfeng Hongyuan Flagship Store	Power Saving Specialist 1	Baby received Oh, the courier delivery really fast, with a good and then come back to the evaluation	Jingdong prefecture level city in Shandong
Jinfeng Hongyuan Flagship Store	Power Saving Specialist 1	Fast logistics, good packaging, worth it!	Jingdong prefecture level city in Shandong
Jinfeng Hongyuan Flagship Store	Power Saving Specialist 1	Customer service is okay. It's just that.	Jingdong prefecture level city in Shandong
Jinfeng Hongyuan Flagship Store	Power Saving Specialist 1	Poor power saving	Jingdong prefecture level city in Shandong
Jinfeng Hongyuan Flagship Store	Power Saving Specialist 1	I'll tell you when I've used it.	Jingdong prefecture level city in Shandong
Jinfeng Hongyuan Flagship Store	Power Saving Specialist 1	I think it's good.	Jingdong prefecture level city in Shandong
Jinfeng Hongyuan Flagship Store	Power Saving Specialist 1	This energy saver works very well and I recommend everyone to buy it!	Jingdong prefecture level city in Shandong
Jinfeng Hongyuan Flagship Store	Power Saving Specialist 1	I do not know how effective, just use half a month on the burn out, good business reissued a, and then use it after the follow-up comment.	Jingdong prefecture level city in Shandong
Jinfeng Hongyuan Flagship Store	Power Saving Specialist 1	in use	Jingdong prefecture level city in Shandong
Jinfeng Hongyuan Flagship Store	Power Saving Specialist 1	This thing is very poor. It's already only been used for ten days and it's broken. Now waiting for you to return it.	Jingdong prefecture level city in Shandong
Jinfeng Hongyuan Flagship Store	Power Saving Specialist 1	It's not the same as the seller's show.	Jingdong prefecture level city in Shandong
Jinfeng Hongyuan Flagship Store	Power Saving Specialist 1	What is the problem when I get it back from the CTS, it is plugged into the wall and the voltage shows 206v.	Jingdong prefecture level city in Shandong
Jinfeng Hongyuan Flagship Store	Power Saving Specialist 1	The logistics was fast and the stuff was perfectly packaged. It is in the process of trial.	Jingdong prefecture level city in Shandong
Jinfeng Hongyuan Flagship Store	Power Saving Specialist 1	This saver is particularly good to use can really save electricity, with two months and then look at the line if you buy one for the family	Jingdong prefecture level city in Shandong
Jinfeng Hongyuan Flagship Store	Power Saving Specialist 1	Novel appearance design, all aluminum alloy profile, also with a fuse device, voltage monitoring display. After receiving the goods, I directly cut the plug and connect it to the main power box under the leakage switch, hoping to save electricity, and then follow up later. Logistics is very fast, arrived the next	Jingdong prefecture level city in Shandong
Jinfeng Hongyuan Flagship Store	Power Saving Specialist 1	Received, very good and like, if necessary next time to buy ha ha	Jingdong prefecture level city in Shandong
Jinfeng Hongyuan Flagship Store	Power Saving Specialist 1	It's good. It's worth it. Get it if you need it.	Jingdong prefecture level city in Shandong
Jinfeng Hongyuan Flagship Store	Power Saving Specialist 1	It feels more than good and is worth having.	Jingdong prefecture level city in Shandong
Jinfeng Hongyuan Flagship Store	Power Saving Specialist 1	The goods arrived very good and fast first time to buy	Jingdong prefecture level city in Shandong
Jinfeng Hongyuan Flagship Store	Power Saving Specialist 1	The power saving is very good, I can save so much money every month, I will buy another one next time.	Jingdong prefecture level city in Shandong
Jinfeng Hongyuan Flagship Store	Power Saving Specialist 1	It's not what it says.	Jingdong prefecture level city in Shandong
Jinfeng Hongyuan Flagship Store	Power Saving Specialist 1	I have been using it for many days now and it saves electricity, it really does I have an 8 kW electric snail stove and it saves about $3.50 a day.	Jingdong prefecture level city in Shandong
Jinfeng Hongyuan Flagship Store	Power Saving Specialist 1	Express delivery is very fast, the quality is also quite good, worth buying!	Jingdong prefecture level city in Shandong
Jinfeng Hongyuan Flagship Store	Power Saving Specialist 1	Just received expecting the baby to work well.	Jingdong prefecture level city in Shandong
Jinfeng Hongyuan Flagship Store	Power Saving Specialist 1	Very good, there are more fruit, has bought a second!	Jingdong prefecture level city in Shandong
Jinfeng Hongyuan Flagship Store	Power Saving Specialist 1	Good is good So far, no power savings have been noticed Maybe it's too little to notice.	Jingdong prefecture level city in Shandong
Jinfeng Hongyuan Flagship Store	Power Saving Specialist 1	I just received the item and plugged it in to see what happens later.	Jingdong prefecture level city in Shandong
Jinfeng Hongyuan Flagship Store	Power Saving Specialist 1	Baby received, especially good, packaging is also good, the courier brother is very strong, thank you.	Jingdong prefecture level city in Shandong
Jinfeng Hongyuan Flagship Store	Power Saving Specialist 1	Just received the goods have not yet used it, do not know how, three months later in the follow-up comment!	Jingdong prefecture level city in Shandong
Jinfeng Hongyuan Flagship Store	Power Saving Specialist 1	The item was received, it is quite good, recommended to buy.	Jingdong prefecture level city in Shandong
Jinfeng Hongyuan Flagship Store	Power Saving Specialist 1	This energy saver works very well and I recommend everyone to buy it!	Jingdong prefecture level city in Shandong
Jinfeng Hongyuan Flagship Store	Power Saving Specialist 1	Voltage stabilization and current reduction nice power saving product.	Jingdong prefecture level city in Shandong
Jinfeng Hongyuan Flagship Store	Power Saving Specialist 1	Voltage stabilization and current reduction nice power saving product.	Jingdong prefecture level city in Shandong
Jinfeng Hongyuan Flagship Store	Power Saving Specialist 1	A good power saver for stabilizing and lowering current.	Jingdong prefecture level city in Shandong
Jinfeng Hongyuan Flagship Store	Power Saving Specialist 1	You'll know if it saves power after you use it.	Jingdong prefecture level city in Shandong
Jinfeng Hongyuan Flagship Store	Power Saving Specialist 1	The quality is great. The quality is very good. The service is very good. Express delivery arrived in two days.	Jingdong prefecture level city in Shandong
Jinfeng Hongyuan Flagship Store	Power Saving Specialist 1	It works great.	Jingdong prefecture level city in Shandong

Jinfeng Hongyuan Flagship Store	Power Saving Specialist 1	Logistics is very fast, not yet used, used to comment again.	Jingdong prefecture level city in Shandong
Jinfeng Hongyuan Flagship Store	Power Saving Specialist 1	Logistics is very fast things are also very good, worthy of friends to buy	Jingdong prefecture level city in Shandong
Jinfeng Hongyuan Flagship Store	Power Saving Specialist 1	It works. It really works.	Jingdong prefecture level city in Shandong
Jinfeng Hongyuan Flagship Store	Power Saving Specialist 1	LANGUAGE COMMENTS: just received the shipment. I immediately plugged it in and used it. If you ask how effective. It depends on whether the next month's electricity bill has been reduced. If there is a decrease. I am buying more than one to use.	Jingdong prefecture level city in Shandong
Jinfeng Hongyuan Flagship Store	Power Saving Specialist 1	Well made, power saving, good and convenient!	Jingdong prefecture level city in Shandong
Jinfeng Hongyuan Flagship Store	Power Saving Specialist 1	I'm using it. I'll rate it next month.	Jingdong prefecture level city in Shandong
Jinfeng Hongyuan Flagship Store	Power Saving Specialist 1	Baby received Oh, the courier delivery really fast, with a good and then come back to the evaluation	Jingdong prefecture level city in Shandong
Jinfeng Hongyuan Flagship Store	Power Saving Specialist 1	Fast logistics, good packaging, worth it!	Jingdong prefecture level city in Shandong
Jinfeng Hongyuan Flagship Store	Power Saving Specialist 1	Customer service is okay. It's just that.	Jingdong prefecture level city in Shandong
Jinfeng Hongyuan Flagship Store	Power Saving Specialist 1	Poor power saving	Jingdong prefecture level city in Shandong
Jinfeng Hongyuan Flagship Store	Power Saving Specialist 1	I'll tell you when I've used it.	Jingdong prefecture level city in Shandong
Jinfeng Hongyuan Flagship Store	Power Saving Specialist 1	I think it's good.	Jingdong prefecture level city in Shandong
Jinfeng Hongyuan Flagship Store	Power Saving Specialist 1	This energy saver works very well and I recommend everyone to buy it!	Jingdong prefecture level city in Shandong
Jinfeng Hongyuan Flagship Store	Power Saving Specialist 1	I do not know how effective, just use half a month on the burn out, good business reissued a, and then use it after the follow-up comment.	Jingdong prefecture level city in Shandong
Jinfeng Hongyuan Flagship Store	Power Saving Specialist 1	in use	Jingdong prefecture level city in Shandong
Jinfeng Hongyuan Flagship Store	Power Saving Specialist 1	This thing is very poor. It's already only been used for ten days and it's broken. Now waiting for you to return it.	Jingdong prefecture level city in Shandong
Jinfeng Hongyuan Flagship Store	Power Saving Specialist 1	It's not the same as the seller's show.	Jingdong prefecture level city in Shandong
Jinfeng Hongyuan Flagship Store	Power Saving Specialist 1	What is the problem when I get it back from the CTS, it is plugged into the wall and the voltage shows 206v.	Jingdong prefecture level city in Shandong
Jinfeng Hongyuan Flagship Store	Power Saving Specialist 1	The logistics was fast and the stuff was perfectly packaged. It is in the process of trial.	Jingdong prefecture level city in Shandong
Jinfeng Hongyuan Flagship Store	Power Saving Specialist 1	This saver is particularly good to use can really save electricity, with two months and then look at the line if you buy one for the family	Jingdong prefecture level city in Shandong
Jinfeng Hongyuan Flagship Store	Power Saving Specialist 1	Novel appearance design, all aluminum alloy profile, also with a fuse device, voltage monitoring display. After receiving the goods, I directly cut the plug and connect it to the main power box under the leakage switch, hoping to save electricity, and then follow up later. Logistics is very fast, arrived the next	Jingdong prefecture level city in Shandong
Jinfeng Hongyuan Flagship Store	Power Saving Specialist 1	Received, very good and like, if necessary next time to buy ha ha	Jingdong prefecture level city in Shandong
Jinfeng Hongyuan Flagship Store	Power Saving Specialist 1	It's good. It's worth it. Get it if you need it.	Jingdong prefecture level city in Shandong
Jinfeng Hongyuan Flagship Store	Power Saving Specialist 1	It feels more than good and is worth having.	Jingdong prefecture level city in Shandong
Jinfeng Hongyuan Flagship Store	Power Saving Specialist 1	The goods arrived very good and fast first time to buy	Jingdong prefecture level city in Shandong
Jinfeng Hongyuan Flagship Store	Power Saving Specialist 1	The power saving is very good, I can save so much money every month, I will buy another one next time.	Jingdong prefecture level city in Shandong
Jinfeng Hongyuan Flagship Store	Power Saving Specialist 1	It's not what it says.	Jingdong prefecture level city in Shandong
Jinfeng Hongyuan Flagship Store	Power Saving Specialist 1	I have been using it for many days now and it saves electricity, it really does I have an 8 kW electric snail stove and it saves about $3.50 a day.	Jingdong prefecture level city in Shandong
Jinfeng Hongyuan Flagship Store	Power Saving Specialist 1	Express delivery is very fast, the quality is also quite good, worth buying!	Jingdong prefecture level city in Shandong
Jinfeng Hongyuan Flagship Store	Power Saving Specialist 1	Just received expecting the baby to work well.	Jingdong prefecture level city in Shandong
Jinfeng Hongyuan Flagship Store	Power Saving Specialist 1	Very good, there are more fruit, has bought a second!	Jingdong prefecture level city in Shandong
Jinfeng Hongyuan Flagship Store	Power Saving Specialist 1	Good is good So far, no power savings have been noticed Maybe it's too little to notice.	Jingdong prefecture level city in Shandong
Jinfeng Hongyuan Flagship Store	Power Saving Specialist 1	I just received the item and plugged it in to see what happens later.	Jingdong prefecture level city in Shandong
Jinfeng Hongyuan Flagship Store	Power Saving Specialist 1	Baby received, especially good, packaging is also good, the courier brother is very strong, thank you.	Jingdong prefecture level city in Shandong
Jinfeng Hongyuan Flagship Store	Power Saving Specialist 1	Just received the goods have not yet used it, do not know how, three months later in the follow-up comment!	Jingdong prefecture level city in Shandong
Jinfeng Hongyuan Flagship Store	Power Saving Specialist 1	The item was received, it is quite good, recommended to buy.	Jingdong prefecture level city in Shandong
Jinfeng Hongyuan Flagship Store	Power Saving Specialist 1	This energy saver works very well and I recommend everyone to buy it!	Jingdong prefecture level city in Shandong
Jinfeng Hongyuan Flagship Store	Power Saving Specialist 1	Voltage stabilization and current reduction nice power saving product.	Jingdong prefecture level city in Shandong
Jinfeng Hongyuan Flagship Store	Power Saving Specialist 1	Voltage stabilization and current reduction nice power saving product.	Jingdong prefecture level city in Shandong
Jinfeng Hongyuan Flagship Store	Power Saving Specialist 1	A good power saver for stabilizing and lowering current.	Jingdong prefecture level city in Shandong
Jinfeng Hongyuan Flagship Store	Power Saving Specialist 1	You'll know if it saves power after you use it.	Jingdong prefecture level city in Shandong
Jinfeng Hongyuan Flagship Store	Power Saving Specialist 1	The quality is great. The quality is very good. The service is very good. Express delivery arrived in two days.	Jingdong prefecture level city in Shandong
Jinfeng Hongyuan Flagship Store	Power Saving Specialist 1	It works great.	Jingdong prefecture level city in Shandong
Jinfeng Hongyuan Flagship Store	Power Saving Specialist 1	Logistics is very fast, not yet used, used to comment again.	Jingdong prefecture level city in Shandong
Jinfeng Hongyuan Flagship Store	Power Saving Specialist 1	Logistics is very fast things are also very good, worthy of friends to buy	Jingdong prefecture level city in Shandong
Jinfeng Hongyuan Flagship Store	Power Saving Specialist 1	It works. It really works.	Jingdong prefecture level city in Shandong
Jinfeng Hongyuan Flagship Store	Power Saving Specialist 1	LANGUAGE COMMENTS: just received the shipment. I immediately plugged it in and used it. If you ask how effective. It depends on whether the next month's electricity bill has been reduced. If there is a decrease. I am buying more than one to use.	Jingdong prefecture level city in Shandong
Jinfeng Hongyuan Flagship Store	Power Saving Specialist 1	Well made, power saving, good and convenient!	Jingdong prefecture level city in Shandong
Jinfeng Hongyuan Flagship Store	Power Saving Specialist 1	I'm using it. I'll rate it next month.	Jingdong prefecture level city in Shandong
Jinfeng Hongyuan Flagship Store	Power Saving Specialist 1	Baby received Oh, the courier delivery really fast, with a good and then come back to the evaluation	Jingdong prefecture level city in Shandong

Jinfeng Hongyuan Flagship Store	Power Saving Specialist 1	Fast logistics, good packaging, worth it!	Jingdong prefecture level city in Shandong
Jinfeng Hongyuan Flagship Store	Power Saving Specialist 1	Customer service is okay. It's just that.	Jingdong prefecture level city in Shandong
Jinfeng Hongyuan Flagship Store	Power Saving Specialist 1	Poor power saving	Jingdong prefecture level city in Shandong
Jinfeng Hongyuan Flagship Store	Power Saving Specialist 1	I'll tell you when I've used it.	Jingdong prefecture level city in Shandong
Jinfeng Hongyuan Flagship Store	Power Saving Specialist 1	I think it's good.	Jingdong prefecture level city in Shandong
Jinfeng Hongyuan Flagship Store	Power Saving Specialist 1	This energy saver works very well and I recommend everyone to buy it!	Jingdong prefecture level city in Shandong
Jinfeng Hongyuan Flagship Store	Power Saving Specialist 1	I do not know how effective, just use half a month on the burn out, good business reissued a, and then use it after the follow-up comment.	Jingdong prefecture level city in Shandong
Jinfeng Hongyuan Flagship Store	Power Saving Specialist 1	in use	Jingdong prefecture level city in Shandong
Jinfeng Hongyuan Flagship Store	Power Saving Specialist 1	This thing is very poor. It's already only been used for ten days and it's broken. Now waiting for you to return it.	Jingdong prefecture level city in Shandong
Jinfeng Hongyuan Flagship Store	Power Saving Specialist 1	It's not the same as the seller's show.	Jingdong prefecture level city in Shandong
Jinfeng Hongyuan Flagship Store	Power Saving Specialist 1	What is the problem when I get it back from the CTS, it is plugged into the wall and the voltage shows 206v.	Jingdong prefecture level city in Shandong
Jinfeng Hongyuan Flagship Store	Power Saving Specialist 1	The logistics was fast and the stuff was perfectly packaged. It is in the process of trial.	Jingdong prefecture level city in Shandong
Jinfeng Hongyuan Flagship Store	Power Saving Specialist 1	This saver is particularly good to use can really save electricity, with two months and then look at the line if you buy one for the family	Jingdong prefecture level city in Shandong
Jinfeng Hongyuan Flagship Store	Power Saving Specialist 1	Novel appearance design, all aluminum alloy profile, also with a fuse device, voltage monitoring display. After receiving the goods, I directly cut the plug and connect it to the main power box under the leakage switch, hoping to save electricity, and then follow up later. Logistics is very fast, arrived the next	Jingdong prefecture level city in Shandong
Jinfeng Hongyuan Flagship Store	Power Saving Specialist 1	Received, very good and like, if necessary next time to buy ha ha	Jingdong prefecture level city in Shandong
Jinfeng Hongyuan Flagship Store	Power Saving Specialist 1	It's good. It's worth it. Get it if you need it.	Jingdong prefecture level city in Shandong
Jinfeng Hongyuan Flagship Store	Power Saving Specialist 1	It feels more than good and is worth having.	Jingdong prefecture level city in Shandong
Jinfeng Hongyuan Flagship Store	Power Saving Specialist 1	The goods arrived very good and fast first time to buy	Jingdong prefecture level city in Shandong
Jinfeng Hongyuan Flagship Store	Power Saving Specialist 1	The power saving is very good, I can save so much money every month, I will buy another one next time.	Jingdong prefecture level city in Shandong
Jinfeng Hongyuan Flagship Store	Power Saving Specialist 1	It's not what it says.	Jingdong prefecture level city in Shandong
Jinfeng Hongyuan Flagship Store	Power Saving Specialist 1	I have been using it for many days now and it saves electricity, it really does I have an 8 kW electric snail stove and it saves about $3.50 a day.	Jingdong prefecture level city in Shandong
Jinfeng Hongyuan Flagship Store	Power Saving Specialist 1	Express delivery is very fast, the quality is also quite good, worth buying!	Jingdong prefecture level city in Shandong
Jinfeng Hongyuan Flagship Store	Power Saving Specialist 1	Just received expecting the baby to work well.	Jingdong prefecture level city in Shandong
Jinfeng Hongyuan Flagship Store	Power Saving Specialist 1	Very good, there are more fruit, has bought a second!	Jingdong prefecture level city in Shandong
Jinfeng Hongyuan Flagship Store	Power Saving Specialist 1	Good is good So far, no power savings have been noticed Maybe it's too little to notice.	Jingdong prefecture level city in Shandong
Jinfeng Hongyuan Flagship Store	Power Saving Specialist 1	I just received the item and plugged it in to see what happens later.	Jingdong prefecture level city in Shandong
Jinfeng Hongyuan Flagship Store	Power Saving Specialist 1	Baby received, especially good, packaging is also good, the courier brother is very strong, thank you.	Jingdong prefecture level city in Shandong
Jinfeng Hongyuan Flagship Store	Power Saving Specialist 1	Just received the goods have not yet used it, do not know how, three months later in the follow-up comment!	Jingdong prefecture level city in Shandong
Jinfeng Hongyuan Flagship Store	Power Saving Specialist 1	The item was received, it is quite good, recommended to buy.	Jingdong prefecture level city in Shandong
Jinfeng Hongyuan Flagship Store	Power Saving Specialist 1	This energy saver works very well and I recommend everyone to buy it!	Jingdong prefecture level city in Shandong
Jinfeng Hongyuan Flagship Store	Power Saving Specialist 1	Voltage stabilization and current reduction nice power saving product.	Jingdong prefecture level city in Shandong
Jinfeng Hongyuan Flagship Store	Power Saving Specialist 1	Voltage stabilization and current reduction nice power saving product.	Jingdong prefecture level city in Shandong
Jinfeng Hongyuan Flagship Store	Power Saving Specialist 1	A good power saver for stabilizing and lowering current.	Jingdong prefecture level city in Shandong
Jinfeng Hongyuan Flagship Store	Power Saving Specialist 1	You'll know if it saves power after you use it.	Jingdong prefecture level city in Shandong
Jinfeng Hongyuan Flagship Store	Power Saving Specialist 1	The quality is great. The quality is very good. The service is very good. Express delivery arrived in two days.	Jingdong prefecture level city in Shandong
Jinfeng Hongyuan Flagship Store	Power Saving Specialist 1	It works great.	Jingdong prefecture level city in Shandong
Jinfeng Hongyuan Flagship Store	Power Saving Specialist 1	Logistics is very fast, not yet used, used to comment again.	Jingdong prefecture level city in Shandong
Jinfeng Hongyuan Flagship Store	Power Saving Specialist 1	Logistics is very fast things are also very good, worthy of friends to buy	Jingdong prefecture level city in Shandong
Jinfeng Hongyuan Flagship Store	Power Saving Specialist 1	It works. It really works.	Jingdong prefecture level city in Shandong
Jinfeng Hongyuan Flagship Store	Power Saving Specialist 1	LANGUAGE COMMENTS: just received the shipment. I immediately plugged it in and used it. If you ask how effective. It depends on whether the next month's electricity bill has been reduced. If there is a decrease. I am buying more than one to use.	Jingdong prefecture level city in Shandong
Jinfeng Hongyuan Flagship Store	Power Saving Specialist 1	Well made, power saving, good and convenient!	Jingdong prefecture level city in Shandong
Jinfeng Hongyuan Flagship Store	Power Saving Specialist 1	I'm using it. I'll rate it next month.	Jingdong prefecture level city in Shandong
Jinfeng Hongyuan Flagship Store	Power Saving Specialist 1	Baby received Oh, the courier delivery really fast, with a good and then come back to the evaluation	Jingdong prefecture level city in Shandong
Jinfeng Hongyuan Flagship Store	Power Saving Specialist 1	Fast logistics, good packaging, worth it!	Jingdong prefecture level city in Shandong
Jinfeng Hongyuan Flagship Store	Power Saving Specialist 1	Customer service is okay. It's just that.	Jingdong prefecture level city in Shandong
Jinfeng Hongyuan Flagship Store	Power Saving Specialist 1	Poor power saving	Jingdong prefecture level city in Shandong
Jinfeng Hongyuan Flagship Store	Power Saving Specialist 1	I'll tell you when I've used it.	Jingdong prefecture level city in Shandong
Jinfeng Hongyuan Flagship Store	Power Saving Specialist 1	I think it's good.	Jingdong prefecture level city in Shandong
Jinfeng Hongyuan Flagship Store	Power Saving Specialist 1	This energy saver works very well and I recommend everyone to buy it!	Jingdong prefecture level city in Shandong
Jinfeng Hongyuan Flagship Store	Power Saving Specialist 1	I do not know how effective, just use half a month on the burn out, good business reissued a, and then use it after the follow-up comment.	Jingdong prefecture level city in Shandong

Jinfeng Hongyuan Flagship Store	Power Saving Specialist 1	in use	Jingdong prefecture level city in Shandong
Jinfeng Hongyuan Flagship Store	Power Saving Specialist 1	This thing is very poor. It's already only been used for ten days and it's broken. Now waiting for you to return it.	Jingdong prefecture level city in Shandong
Jinfeng Hongyuan Flagship Store	Power Saving Specialist 1	It's not the same as the seller's show.	Jingdong prefecture level city in Shandong
Jinfeng Hongyuan Flagship Store	Power Saving Specialist 1	What is the problem when I get it back from the CTS, it is plugged into the wall and the voltage shows 206v.	Jingdong prefecture level city in Shandong
Jinfeng Hongyuan Flagship Store	Power Saving Specialist 1	The logistics was fast and the stuff was perfectly packaged. It is in the process of trial.	Jingdong prefecture level city in Shandong
Jinfeng Hongyuan Flagship Store	Power Saving Specialist 1	This saver is particularly good to use can really save electricity, with two months and then look at the line if you buy one for the family	Jingdong prefecture level city in Shandong
Jinfeng Hongyuan Flagship Store	Power Saving Specialist 1	Novel appearance design, all aluminum alloy profile, also with a fuse device, voltage monitoring display. After receiving the goods, I directly cut the plug and connect it to the main power box under the leakage switch, hoping to save electricity, and then follow up later. Logistics is very fast, arrived the next	Jingdong prefecture level city in Shandong
Jinfeng Hongyuan Flagship Store	Power Saving Specialist 1	Received, very good and like, if necessary next time to buy ha ha	Jingdong prefecture level city in Shandong
Jinfeng Hongyuan Flagship Store	Power Saving Specialist 1	It's good. It's worth it. Get it if you need it.	Jingdong prefecture level city in Shandong
Jinfeng Hongyuan Flagship Store	Power Saving Specialist 1	It feels more than good and is worth having.	Jingdong prefecture level city in Shandong
Jinfeng Hongyuan Flagship Store	Power Saving Specialist 1	The goods arrived very good and fast first time to buy	Jingdong prefecture level city in Shandong
Jinfeng Hongyuan Flagship Store	Power Saving Specialist 1	The power saving is very good, I can save so much money every month, I will buy another one next time.	Jingdong prefecture level city in Shandong
Jinfeng Hongyuan Flagship Store	Power Saving Specialist 1	It's not what it says.	Jingdong prefecture level city in Shandong
Jinfeng Hongyuan Flagship Store	Power Saving Specialist 1	I have been using it for many days now and it saves electricity, it really does I have an 8 kW electric snail stove and it saves about $3.50 a day.	Jingdong prefecture level city in Shandong
Jinfeng Hongyuan Flagship Store	Power Saving Specialist 1	Express delivery is very fast, the quality is also quite good, worth buying!	Jingdong prefecture level city in Shandong
Jinfeng Hongyuan Flagship Store	Power Saving Specialist 1	Just received expecting the baby to work well.	Jingdong prefecture level city in Shandong
Jinfeng Hongyuan Flagship Store	Power Saving Specialist 1	Very good, there are more fruit, has bought a second!	Jingdong prefecture level city in Shandong
Jinfeng Hongyuan Flagship Store	Power Saving Specialist 1	Good is good So far, no power savings have been noticed Maybe it's too little to notice.	Jingdong prefecture level city in Shandong
Jinfeng Hongyuan Flagship Store	Power Saving Specialist 1	I just received the item and plugged it in to see what happens later.	Jingdong prefecture level city in Shandong
Jinfeng Hongyuan Flagship Store	Power Saving Specialist 1	Baby received, especially good, packaging is also good, the courier brother is very strong, thank you.	Jingdong prefecture level city in Shandong
Jinfeng Hongyuan Flagship Store	Power Saving Specialist 1	Just received the goods have not yet used it, do not know how, three months later in the follow-up comment!	Jingdong prefecture level city in Shandong
Jinfeng Hongyuan Flagship Store	Power Saving Specialist 1	The item was received, it is quite good, recommended to buy.	Jingdong prefecture level city in Shandong
Jinfeng Hongyuan Flagship Store	Power Saving Specialist 1	This energy saver works very well and I recommend everyone to buy it!	Jingdong prefecture level city in Shandong
Jinfeng Hongyuan Flagship Store	Power Saving Specialist 1	Voltage stabilization and current reduction nice power saving product.	Jingdong prefecture level city in Shandong
Jinfeng Hongyuan Flagship Store	Power Saving Specialist 1	Voltage stabilization and current reduction nice power saving product.	Jingdong prefecture level city in Shandong
Jinfeng Hongyuan Flagship Store	Power Saving Specialist 1	A good power saver for stabilizing and lowering current.	Jingdong prefecture level city in Shandong
Jinfeng Hongyuan Flagship Store	Power Saving Specialist 1	You'll know if it saves power after you use it.	Jingdong prefecture level city in Shandong
Jinfeng Hongyuan Flagship Store	Power Saving Specialist 1	The quality is great. The quality is very good. The service is very good. Express delivery arrived in two days.	Jingdong prefecture level city in Shandong
Jinfeng Hongyuan Flagship Store	Power Saving Specialist 1	It works great.	Jingdong prefecture level city in Shandong
Jinfeng Hongyuan Flagship Store	Power Saving Specialist 1	Logistics is very fast, not yet used, used to comment again.	Jingdong prefecture level city in Shandong
Jinfeng Hongyuan Flagship Store	Power Saving Specialist 1	Logistics is very fast things are also very good, worthy of friends to buy	Jingdong prefecture level city in Shandong
Jinfeng Hongyuan Flagship Store	Power Saving Specialist 1	It works. It really works.	Jingdong prefecture level city in Shandong
Jinfeng Hongyuan Flagship Store	Power Saving Specialist 1	LANGUAGE COMMENTS: just received the shipment. I immediately plugged it in and used it. If you ask how effective. It depends on whether the next month's electricity bill has been reduced. If there is a decrease. I am buying more than one to use.	Jingdong prefecture level city in Shandong
Jinfeng Hongyuan Flagship Store	Power Saving Specialist 1	Well made, power saving, good and convenient!	Jingdong prefecture level city in Shandong
Jinfeng Hongyuan Flagship Store	Power Saving Specialist 1	I'm using it. I'll rate it next month.	Jingdong prefecture level city in Shandong
Jinfeng Hongyuan Flagship Store	Power Saving Specialist 1	Baby received Oh, the courier delivery really fast, with a good and then come back to the evaluation	Jingdong prefecture level city in Shandong
Jinfeng Hongyuan Flagship Store	Power Saving Specialist 1	Fast logistics, good packaging, worth it!	Jingdong prefecture level city in Shandong
Jinfeng Hongyuan Flagship Store	Power Saving Specialist 1	Customer service is okay. It's just that.	Jingdong prefecture level city in Shandong
Jinfeng Hongyuan Flagship Store	Power Saving Specialist 1	Poor power saving	Jingdong prefecture level city in Shandong
Jinfeng Hongyuan Flagship Store	Power Saving Specialist 1	I'll tell you when I've used it.	Jingdong prefecture level city in Shandong
Jinfeng Hongyuan Flagship Store	Power Saving Specialist 1	I think it's good.	Jingdong prefecture level city in Shandong
Jinfeng Hongyuan Flagship Store	Power Saving Specialist 1	This energy saver works very well and I recommend everyone to buy it!	Jingdong prefecture level city in Shandong
Jinfeng Hongyuan Flagship Store	Power Saving Specialist 1	I do not know how effective, just use half a month on the burn out, good business reissued a, and then use it after the follow-up comment.	Jingdong prefecture level city in Shandong
Jinfeng Hongyuan Flagship Store	Power Saving Specialist 1	in use	Jingdong prefecture level city in Shandong
Jinfeng Hongyuan Flagship Store	Power Saving Specialist 1	This thing is very poor. It's already only been used for ten days and it's broken. Now waiting for you to return it.	Jingdong prefecture level city in Shandong
Jinfeng Hongyuan Flagship Store	Power Saving Specialist 1	It's not the same as the seller's show.	Jingdong prefecture level city in Shandong
Jinfeng Hongyuan Flagship Store	Power Saving Specialist 1	What is the problem when I get it back from the CTS, it is plugged into the wall and the voltage shows 206v.	Jingdong prefecture level city in Shandong
Jinfeng Hongyuan Flagship Store	Power Saving Specialist 1	The logistics was fast and the stuff was perfectly packaged. It is in the process of trial.	Jingdong prefecture level city in Shandong
Jinfeng Hongyuan Flagship Store	Power Saving Specialist 1	This saver is particularly good to use can really save electricity, with two months and then look at the line if you buy one for the family	Jingdong prefecture level city in Shandong
Jinfeng Hongyuan Flagship Store	Power Saving Specialist 1	Novel appearance design, all aluminum alloy profile, also with a fuse device, voltage monitoring display. After receiving the goods, I directly cut the plug and connect it to the main power box under the leakage switch, hoping to save electricity, and then follow up later. Logistics is very fast, arrived the next	Jingdong prefecture level city in Shandong

Jinfeng Hongyuan Flagship Store	Power Saving Specialist 1	Received, very good and like, if necessary next time to buy ha ha	Jingdong prefecture level city in Shandong
Jinfeng Hongyuan Flagship Store	Power Saving Specialist 1	It's good. It's worth it. Get it if you need it.	Jingdong prefecture level city in Shandong
Jinfeng Hongyuan Flagship Store	Power Saving Specialist 1	It feels more than good and is worth having.	Jingdong prefecture level city in Shandong
Jinfeng Hongyuan Flagship Store	Power Saving Specialist 1	The goods arrived very good and fast first time to buy	Jingdong prefecture level city in Shandong
Jinfeng Hongyuan Flagship Store	Power Saving Specialist 1	The power saving is very good, I can save so much money every month, I will buy another one next time.	Jingdong prefecture level city in Shandong
Jinfeng Hongyuan Flagship Store	Power Saving Specialist 1	It's not what it says.	Jingdong prefecture level city in Shandong
Jinfeng Hongyuan Flagship Store	Power Saving Specialist 1	I have been using it for many days now and it saves electricity, it really does I have an 8 kW electric snail stove and it saves about $3.50 a day.	Jingdong prefecture level city in Shandong
Jinfeng Hongyuan Flagship Store	Power Saving Specialist 1	Express delivery is very fast, the quality is also quite good, worth buying!	Jingdong prefecture level city in Shandong
Jinfeng Hongyuan Flagship Store	Power Saving Specialist 1	Just received expecting the baby to work well.	Jingdong prefecture level city in Shandong
Jinfeng Hongyuan Flagship Store	Power Saving Specialist 1	Very good, there are more fruit, has bought a second!	Jingdong prefecture level city in Shandong
Jinfeng Hongyuan Flagship Store	Power Saving Specialist 1	Good is good So far, no power savings have been noticed Maybe it's too little to notice.	Jingdong prefecture level city in Shandong
Jinfeng Hongyuan Flagship Store	Power Saving Specialist 1	I just received the item and plugged it in to see what happens later.	Jingdong prefecture level city in Shandong
Jinfeng Hongyuan Flagship Store	Power Saving Specialist 1	Baby received, especially good, packaging is also good, the courier brother is very strong, thank you.	Jingdong prefecture level city in Shandong
Jinfeng Hongyuan Flagship Store	Power Saving Specialist 1	Just received the goods have not yet used it, do not know how, three months later in the follow-up comment!	Jingdong prefecture level city in Shandong
Jinfeng Hongyuan Flagship Store	Power Saving Specialist 1	The item was received, it is quite good, recommended to buy.	Jingdong prefecture level city in Shandong
Jinfeng Hongyuan Flagship Store	Power Saving Specialist 1	This energy saver works very well and I recommend everyone to buy it!	Jingdong prefecture level city in Shandong
Jinfeng Hongyuan Flagship Store	Power Saving Specialist 1	Voltage stabilization and current reduction nice power saving product.	Jingdong prefecture level city in Shandong
Jinfeng Hongyuan Flagship Store	Power Saving Specialist 1	Voltage stabilization and current reduction nice power saving product.	Jingdong prefecture level city in Shandong
Jinfeng Hongyuan Flagship Store	Power Saving Specialist 1	A good power saver for stabilizing and lowering current.	Jingdong prefecture level city in Shandong
Jinfeng Hongyuan Flagship Store	Power Saving Specialist 1	You'll know if it saves power after you use it.	Jingdong prefecture level city in Shandong
Jinfeng Hongyuan Flagship Store	Power Saving Specialist 1	The quality is great. The quality is very good. The service is very good. Express delivery arrived in two days.	Jingdong prefecture level city in Shandong
Jinfeng Hongyuan Flagship Store	Power Saving Specialist 1	It works great.	Jingdong prefecture level city in Shandong
Jinfeng Hongyuan Flagship Store	Power Saving Specialist 1	Logistics is very fast, not yet used, used to comment again.	Jingdong prefecture level city in Shandong
Jinfeng Hongyuan Flagship Store	Power Saving Specialist 1	Logistics is very fast things are also very good, worthy of friends to buy	Jingdong prefecture level city in Shandong
Jinfeng Hongyuan Flagship Store	Power Saving Specialist 1	It works. It really works.	Jingdong prefecture level city in Shandong
Jinfeng Hongyuan Flagship Store	Power Saving Specialist 1	LANGUAGE COMMENTS: just received the shipment. I immediately plugged it in and used it. If you ask how effective. It depends on whether the next month's electricity bill has been reduced. If there is a decrease. I am buying more than one to use.	Jingdong prefecture level city in Shandong
Jinfeng Hongyuan Flagship Store	Power Saving Specialist 1	Well made, power saving, good and convenient!	Jingdong prefecture level city in Shandong
Jinfeng Hongyuan Flagship Store	Power Saving Specialist 1	I'm using it. I'll rate it next month.	Jingdong prefecture level city in Shandong
Jinfeng Hongyuan Flagship Store	Power Saving Specialist 1	Baby received Oh, the courier delivery really fast, with a good and then come back to the evaluation	Jingdong prefecture level city in Shandong
Jinfeng Hongyuan Flagship Store	Power Saving Specialist 1	Fast logistics, good packaging, worth it!	Jingdong prefecture level city in Shandong
Jinfeng Hongyuan Flagship Store	Power Saving Specialist 1	Customer service is okay. It's just that.	Jingdong prefecture level city in Shandong
Jinfeng Hongyuan Flagship Store	Power Saving Specialist 1	Poor power saving	Jingdong prefecture level city in Shandong
Jinfeng Hongyuan Flagship Store	Power Saving Specialist 1	I'll tell you when I've used it.	Jingdong prefecture level city in Shandong
Jinfeng Hongyuan Flagship Store	Power Saving Specialist 1	I think it's good.	Jingdong prefecture level city in Shandong
Jinfeng Hongyuan Flagship Store	Power Saving Specialist 1	This energy saver works very well and I recommend everyone to buy it!	Jingdong prefecture level city in Shandong
Jinfeng Hongyuan Flagship Store	Power Saving Specialist 1	I do not know how effective, just use half a month on the burn out, good business reissued a, and then use it after the follow-up comment.	Jingdong prefecture level city in Shandong
Jinfeng Hongyuan Flagship Store	Power Saving Specialist 1	in use	Jingdong prefecture level city in Shandong
Jinfeng Hongyuan Flagship Store	Power Saving Specialist 1	This thing is very poor. It's already only been used for ten days and it's broken. Now waiting for you to return it.	Jingdong prefecture level city in Shandong
Jinfeng Hongyuan Flagship Store	Power Saving Specialist 1	It's not the same as the seller's show.	Jingdong prefecture level city in Shandong
Jinfeng Hongyuan Flagship Store	Power Saving Specialist 1	What is the problem when I get it back from the CTS, it is plugged into the wall and the voltage shows 206v.	Jingdong prefecture level city in Shandong
Jinfeng Hongyuan Flagship Store	Power Saving Specialist 1	The logistics was fast and the stuff was perfectly packaged. It is in the process of trial.	Jingdong prefecture level city in Shandong
Jinfeng Hongyuan Flagship Store	Power Saving Specialist 1	This saver is particularly good to use can really save electricity, with two months and then look at the line if you buy one for the family	Jingdong prefecture level city in Shandong
Jinfeng Hongyuan Flagship Store	Power Saving Specialist 1	Novel appearance design, all aluminum alloy profile, also with a fuse device, voltage monitoring display. After receiving the goods, I directly cut the plug and connect it to the main power box under the leakage switch, hoping to save electricity, and then follow up later. Logistics is very fast, arrived the next	Jingdong prefecture level city in Shandong
Jinfeng Hongyuan Flagship Store	Power Saving Specialist 1	Received, very good and like, if necessary next time to buy ha ha	Jingdong prefecture level city in Shandong
Jinfeng Hongyuan Flagship Store	Power Saving Specialist 1	It's good. It's worth it. Get it if you need it.	Jingdong prefecture level city in Shandong
Jinfeng Hongyuan Flagship Store	Power Saving Specialist 1	It feels more than good and is worth having.	Jingdong prefecture level city in Shandong
Jinfeng Hongyuan Flagship Store	Power Saving Specialist 1	The goods arrived very good and fast first time to buy	Jingdong prefecture level city in Shandong
Jinfeng Hongyuan Flagship Store	Power Saving Specialist 1	The power saving is very good, I can save so much money every month, I will buy another one next time.	Jingdong prefecture level city in Shandong
Jinfeng Hongyuan Flagship Store	Power Saving Specialist 1	It's not what it says.	Jingdong prefecture level city in Shandong
Jinfeng Hongyuan Flagship Store	Power Saving Specialist 1	I have been using it for many days now and it saves electricity, it really does I have an 8 kW electric snail stove and it saves about $3.50 a day.	Jingdong prefecture level city in Shandong

Jinfeng Hongyuan Flagship Store	Power Saving Specialist 1	Express delivery is very fast, the quality is also quite good, worth buying!	Jingdong prefecture level city in Shandong
Jinfeng Hongyuan Flagship Store	Power Saving Specialist 1	Just received expecting the baby to work well.	Jingdong prefecture level city in Shandong
Jinfeng Hongyuan Flagship Store	Power Saving Specialist 1	Very good, there are more fruit, has bought a second!	Jingdong prefecture level city in Shandong
Jinfeng Hongyuan Flagship Store	Power Saving Specialist 1	Good is good So far, no power savings have been noticed Maybe it's too little to notice.	Jingdong prefecture level city in Shandong
Jinfeng Hongyuan Flagship Store	Power Saving Specialist 1	I just received the item and plugged it in to see what happens later.	Jingdong prefecture level city in Shandong
Jinfeng Hongyuan Flagship Store	Power Saving Specialist 1	Baby received, especially good, packaging is also good, the courier brother is very strong, thank you.	Jingdong prefecture level city in Shandong
Jinfeng Hongyuan Flagship Store	Power Saving Specialist 1	Just received the goods have not yet used it, do not know how, three months later in the follow-up comment!	Jingdong prefecture level city in Shandong
Jinfeng Hongyuan Flagship Store	Power Saving Specialist 1	The item was received, it is quite good, recommended to buy.	Jingdong prefecture level city in Shandong
Jinfeng Hongyuan Flagship Store	Power Saving Specialist 1	This energy saver works very well and I recommend everyone to buy it!	Jingdong prefecture level city in Shandong
Jinfeng Hongyuan Flagship Store	Power Saving Specialist 1	Voltage stabilization and current reduction nice power saving product.	Jingdong prefecture level city in Shandong
Jinfeng Hongyuan Flagship Store	Power Saving Specialist 1	Voltage stabilization and current reduction nice power saving product.	Jingdong prefecture level city in Shandong
Jinfeng Hongyuan Flagship Store	Power Saving Specialist 1	A good power saver for stabilizing and lowering current.	Jingdong prefecture level city in Shandong
Jinfeng Hongyuan Flagship Store	Power Saving Specialist 1	You'll know if it saves power after you use it.	Jingdong prefecture level city in Shandong
Jinfeng Hongyuan Flagship Store	Power Saving Specialist 1	The quality is great. The quality is very good. The service is very good. Express delivery arrived in two days.	Jingdong prefecture level city in Shandong
Jinfeng Hongyuan Flagship Store	Power Saving Specialist 1	It works great.	Jingdong prefecture level city in Shandong
Jinfeng Hongyuan Flagship Store	Power Saving Specialist 1	Logistics is very fast, not yet used, used to comment again.	Jingdong prefecture level city in Shandong
Jinfeng Hongyuan Flagship Store	Power Saving Specialist 1	Logistics is very fast things are also very good, worthy of friends to buy	Jingdong prefecture level city in Shandong
Jinfeng Hongyuan Flagship Store	Power Saving Specialist 1	It works. It really works.	Jingdong prefecture level city in Shandong
Jinfeng Hongyuan Flagship Store	Power Saving Specialist 1	LANGUAGE COMMENTS: just received the shipment. I immediately plugged it in and used it. If you ask how effective. It depends on whether the next month's electricity bill has been reduced. If there is a decrease. I am buying more than one to use.	Jingdong prefecture level city in Shandong
Jinfeng Hongyuan Flagship Store	Power Saving Specialist 1	Well made, power saving, good and convenient!	Jingdong prefecture level city in Shandong
Jinfeng Hongyuan Flagship Store	Power Saving Specialist 1	I'm using it. I'll rate it next month.	Jingdong prefecture level city in Shandong
Jinfeng Hongyuan Flagship Store	Power Saving Specialist 1	Baby received Oh, the courier delivery really fast, with a good and then come back to the evaluation	Jingdong prefecture level city in Shandong
Jinfeng Hongyuan Flagship Store	Power Saving Specialist 1	Fast logistics, good packaging, worth it!	Jingdong prefecture level city in Shandong
Jinfeng Hongyuan Flagship Store	Power Saving Specialist 1	Customer service is okay. It's just that.	Jingdong prefecture level city in Shandong
Jinfeng Hongyuan Flagship Store	Power Saving Specialist 1	Poor power saving	Jingdong prefecture level city in Shandong
Jinfeng Hongyuan Flagship Store	Power Saving Specialist 1	I'll tell you when I've used it.	Jingdong prefecture level city in Shandong
Jinfeng Hongyuan Flagship Store	Power Saving Specialist 1	I think it's good.	Jingdong prefecture level city in Shandong
Jinfeng Hongyuan Flagship Store	Power Saving Specialist 1	This energy saver works very well and I recommend everyone to buy it!	Jingdong prefecture level city in Shandong
Jinfeng Hongyuan Flagship Store	Power Saving Specialist 1	I do not know how effective, just use half a month on the burn out, good business reissued a, and then use it after the follow-up comment.	Jingdong prefecture level city in Shandong
Jinfeng Hongyuan Flagship Store	Power Saving Specialist 1	in use	Jingdong prefecture level city in Shandong
Jinfeng Hongyuan Flagship Store	Power Saving Specialist 1	This thing is very poor. It's already only been used for ten days and it's broken. Now waiting for you to return it.	Jingdong prefecture level city in Shandong
Jinfeng Hongyuan Flagship Store	Power Saving Specialist 1	It's not the same as the seller's show.	Jingdong prefecture level city in Shandong
Jinfeng Hongyuan Flagship Store	Power Saving Specialist 1	What is the problem when I get it back from the CTS, it is plugged into the wall and the voltage shows 206v.	Jingdong prefecture level city in Shandong
Jinfeng Hongyuan Flagship Store	Power Saving Specialist 1	The logistics was fast and the stuff was perfectly packaged. It is in the process of trial.	Jingdong prefecture level city in Shandong
Jinfeng Hongyuan Flagship Store	Power Saving Specialist 1	This saver is particularly good to use can really save electricity, with two months and then look at the line if you buy one for the family	Jingdong prefecture level city in Shandong
Jinfeng Hongyuan Flagship Store	Power Saving Specialist 1	Novel appearance design, all aluminum alloy profile, also with a fuse device, voltage monitoring display. After receiving the goods, I directly cut the plug and connect it to the main power box under the leakage switch, hoping to save electricity, and then follow up later. Logistics is very fast, arrived the next	Jingdong prefecture level city in Shandong
Jinfeng Hongyuan Flagship Store	Power Saving Specialist 1	Received, very good and like, if necessary next time to buy ha ha	Jingdong prefecture level city in Shandong
Jinfeng Hongyuan Flagship Store	Power Saving Specialist 1	It's good. It's worth it. Get it if you need it.	Jingdong prefecture level city in Shandong
Jinfeng Hongyuan Flagship Store	Power Saving Specialist 1	It feels more than good and is worth having.	Jingdong prefecture level city in Shandong
Jinfeng Hongyuan Flagship Store	Power Saving Specialist 1	The goods arrived very good and fast first time to buy	Jingdong prefecture level city in Shandong
Jinfeng Hongyuan Flagship Store	Power Saving Specialist 1	The power saving is very good, I can save so much money every month, I will buy another one next time.	Jingdong prefecture level city in Shandong
Jinfeng Hongyuan Flagship Store	Power Saving Specialist 1	It's not what it says.	Jingdong prefecture level city in Shandong
Jinfeng Hongyuan Flagship Store	Power Saving Specialist 1	I have been using it for many days now and it saves electricity, it really does I have an 8 kW electric snail stove and it saves about $3.50 a day.	Jingdong prefecture level city in Shandong
Jinfeng Hongyuan Flagship Store	Power Saving Specialist 1	Express delivery is very fast, the quality is also quite good, worth buying!	Jingdong prefecture level city in Shandong
Jinfeng Hongyuan Flagship Store	Power Saving Specialist 1	Just received expecting the baby to work well.	Jingdong prefecture level city in Shandong
Jinfeng Hongyuan Flagship Store	Power Saving Specialist 1	Very good, there are more fruit, has bought a second!	Jingdong prefecture level city in Shandong
Jinfeng Hongyuan Flagship Store	Power Saving Specialist 1	Good is good So far, no power savings have been noticed Maybe it's too little to notice.	Jingdong prefecture level city in Shandong
Jinfeng Hongyuan Flagship Store	Power Saving Specialist 1	I just received the item and plugged it in to see what happens later.	Jingdong prefecture level city in Shandong
Jinfeng Hongyuan Flagship Store	Power Saving Specialist 1	Baby received, especially good, packaging is also good, the courier brother is very strong, thank you.	Jingdong prefecture level city in Shandong
Jinfeng Hongyuan Flagship Store	Power Saving Specialist 1	Just received the goods have not yet used it, do not know how, three months later in the follow-up comment!	Jingdong prefecture level city in Shandong

Jinfeng Hongyuan Flagship Store	Power Saving Specialist 1	The item was received, it is quite good, recommended to buy.	Jingdong prefecture level city in Shandong
Jinfeng Hongyuan Flagship Store	Power Saving Specialist 1	This energy saver works very well and I recommend everyone to buy it!	Jingdong prefecture level city in Shandong
Jinfeng Hongyuan Flagship Store	Power Saving Specialist 1	Voltage stabilization and current reduction nice power saving product.	Jingdong prefecture level city in Shandong
Jinfeng Hongyuan Flagship Store	Power Saving Specialist 1	Voltage stabilization and current reduction nice power saving product.	Jingdong prefecture level city in Shandong
Jinfeng Hongyuan Flagship Store	Power Saving Specialist 1	A good power saver for stabilizing and lowering current.	Jingdong prefecture level city in Shandong
Jinfeng Hongyuan Flagship Store	Power Saving Specialist 1	You'll know if it saves power after you use it.	Jingdong prefecture level city in Shandong
Jinfeng Hongyuan Flagship Store	Power Saving Specialist 1	The quality is great. The quality is very good. The service is very good. Express delivery arrived in two days.	Jingdong prefecture level city in Shandong
Jinfeng Hongyuan Flagship Store	Power Saving Specialist 1	It works great.	Jingdong prefecture level city in Shandong
Jinfeng Hongyuan Flagship Store	Power Saving Specialist 1	Logistics is very fast, not yet used, used to comment again.	Jingdong prefecture level city in Shandong
Jinfeng Hongyuan Flagship Store	Power Saving Specialist 1	Logistics is very fast things are also very good, worthy of friends to buy	Jingdong prefecture level city in Shandong
Jinfeng Hongyuan Flagship Store	Power Saving Specialist 1	It works. It really works.	Jingdong prefecture level city in Shandong
Jinfeng Hongyuan Flagship Store	Power Saving Specialist 1	LANGUAGE COMMENTS: just received the shipment. I immediately plugged it in and used it. If you ask how effective. It depends on whether the next month's electricity bill has been reduced. If there is a decrease. I am buying more than one to use.	Jingdong prefecture level city in Shandong
Jinfeng Hongyuan Flagship Store	Power Saving Specialist 1	Well made, power saving, good and convenient!	Jingdong prefecture level city in Shandong
Jinfeng Hongyuan Flagship Store	Power Saving Specialist 1	I'm using it. I'll rate it next month.	Jingdong prefecture level city in Shandong
Jinfeng Hongyuan Flagship Store	Power Saving Specialist 1	Baby received Oh, the courier delivery really fast, with a good and then come back to the evaluation	Jingdong prefecture level city in Shandong
Jinfeng Hongyuan Flagship Store	Power Saving Specialist 1	Fast logistics, good packaging, worth it!	Jingdong prefecture level city in Shandong
Jinfeng Hongyuan Flagship Store	Power Saving Specialist 1	Customer service is okay. It's just that.	Jingdong prefecture level city in Shandong
Jinfeng Hongyuan Flagship Store	Power Saving Specialist 1	Poor power saving	Jingdong prefecture level city in Shandong
Jinfeng Hongyuan Flagship Store	Power Saving Specialist 1	I'll tell you when I've used it.	Jingdong prefecture level city in Shandong
Jinfeng Hongyuan Flagship Store	Power Saving Specialist 1	I think it's good.	Jingdong prefecture level city in Shandong
Jinfeng Hongyuan Flagship Store	Power Saving Specialist 1	This energy saver works very well and I recommend everyone to buy it!	Jingdong prefecture level city in Shandong
Jinfeng Hongyuan Flagship Store	Power Saving Specialist 1	I do not know how effective, just use half a month on the burn out, good business reissued a, and then use it after the follow-up comment.	Jingdong prefecture level city in Shandong
Jinfeng Hongyuan Flagship Store	Power Saving Specialist 1	in use	Jingdong prefecture level city in Shandong
Jinfeng Hongyuan Flagship Store	Power Saving Specialist 1	This thing is very poor. It's already only been used for ten days and it's broken. Now waiting for you to return it.	Jingdong prefecture level city in Shandong
Jinfeng Hongyuan Flagship Store	Power Saving Specialist 1	It's not the same as the seller's show.	Jingdong prefecture level city in Shandong
Jinfeng Hongyuan Flagship Store	Power Saving Specialist 1	What is the problem when I get it back from the CTS, it is plugged into the wall and the voltage shows 206v.	Jingdong prefecture level city in Shandong
Jinfeng Hongyuan Flagship Store	Power Saving Specialist 1	The logistics was fast and the stuff was perfectly packaged. It is in the process of trial.	Jingdong prefecture level city in Shandong
Jinfeng Hongyuan Flagship Store	Power Saving Specialist 1	This saver is particularly good to use can really save electricity, with two months and then look at the line if you buy one for the family	Jingdong prefecture level city in Shandong
Jinfeng Hongyuan Flagship Store	Power Saving Specialist 1	Novel appearance design, all aluminum alloy profile, also with a fuse device, voltage monitoring display. After receiving the goods, I directly cut the plug and connect it to the main power box under the leakage switch, hoping to save electricity, and then follow up later. Logistics is very fast, arrived the next	Jingdong prefecture level city in Shandong
Jinfeng Hongyuan Flagship Store	Power Saving Specialist 1	Received, very good and like, if necessary next time to buy ha ha	Jingdong prefecture level city in Shandong
Jinfeng Hongyuan Flagship Store	Power Saving Specialist 1	It's good. It's worth it. Get it if you need it.	Jingdong prefecture level city in Shandong
Jinfeng Hongyuan Flagship Store	Power Saving Specialist 1	It feels more than good and is worth having.	Jingdong prefecture level city in Shandong
Jinfeng Hongyuan Flagship Store	Power Saving Specialist 1	The goods arrived very good and fast first time to buy	Jingdong prefecture level city in Shandong
Jinfeng Hongyuan Flagship Store	Power Saving Specialist 1	The power saving is very good, I can save so much money every month, I will buy another one next time.	Jingdong prefecture level city in Shandong
Jinfeng Hongyuan Flagship Store	Power Saving Specialist 1	It's not what it says.	Jingdong prefecture level city in Shandong
Jinfeng Hongyuan Flagship Store	Power Saving Specialist 1	I have been using it for many days now and it saves electricity, it really does I have an 8 kW electric snail stove and it saves about $3.50 a day.	Jingdong prefecture level city in Shandong
Jinfeng Hongyuan Flagship Store	Power Saving Specialist 1	Express delivery is very fast, the quality is also quite good, worth buying!	Jingdong prefecture level city in Shandong
Jinfeng Hongyuan Flagship Store	Power Saving Specialist 1	Just received expecting the baby to work well.	Jingdong prefecture level city in Shandong
Jinfeng Hongyuan Flagship Store	Power Saving Specialist 1	Very good, there are more fruit, has bought a second!	Jingdong prefecture level city in Shandong
Jinfeng Hongyuan Flagship Store	Power Saving Specialist 1	Good is good So far, no power savings have been noticed Maybe it's too little to notice.	Jingdong prefecture level city in Shandong
Jinfeng Hongyuan Flagship Store	Power Saving Specialist 1	I just received the item and plugged it in to see what happens later.	Jingdong prefecture level city in Shandong
Jinfeng Hongyuan Flagship Store	Power Saving Specialist 1	Baby received, especially good, packaging is also good, the courier brother is very strong, thank you.	Jingdong prefecture level city in Shandong
Jinfeng Hongyuan Flagship Store	Power Saving Specialist 1	Just received the goods have not yet used it, do not know how, three months later in the follow-up comment!	Jingdong prefecture level city in Shandong
Jinfeng Hongyuan Flagship Store	Power Saving Specialist 1	The item was received, it is quite good, recommended to buy.	Jingdong prefecture level city in Shandong
Jinfeng Hongyuan Flagship Store	Power Saving Specialist 1	This energy saver works very well and I recommend everyone to buy it!	Jingdong prefecture level city in Shandong
Jinfeng Hongyuan Flagship Store	Power Saving Specialist 1	Voltage stabilization and current reduction nice power saving product.	Jingdong prefecture level city in Shandong
Jinfeng Hongyuan Flagship Store	Power Saving Specialist 1	Voltage stabilization and current reduction nice power saving product.	Jingdong prefecture level city in Shandong
Jinfeng Hongyuan Flagship Store	Power Saving Specialist 1	A good power saver for stabilizing and lowering current.	Jingdong prefecture level city in Shandong
Jinfeng Hongyuan Flagship Store	Power Saving Specialist 1	You'll know if it saves power after you use it.	Jingdong prefecture level city in Shandong
Jinfeng Hongyuan Flagship Store	Power Saving Specialist 1	The quality is great. The quality is very good. The service is very good. Express delivery arrived in two days.	Jingdong prefecture level city in Shandong

Jinfeng Hongyuan Flagship Store	Power Saving Specialist 1	It works great.	Jingdong prefecture level city in Shandong
Jinfeng Hongyuan Flagship Store	Power Saving Specialist 1	Logistics is very fast, not yet used, used to comment again.	Jingdong prefecture level city in Shandong
Jinfeng Hongyuan Flagship Store	Power Saving Specialist 1	Logistics is very fast things are also very good, worthy of friends to buy	Jingdong prefecture level city in Shandong
Jinfeng Hongyuan Flagship Store	Power Saving Specialist 1	It works. It really works.	Jingdong prefecture level city in Shandong
Jinfeng Hongyuan Flagship Store	Power Saving Specialist 1	LANGUAGE COMMENTS: just received the shipment. I immediately plugged it in and used it. If you ask how effective. It depends on whether the next month's electricity bill has been reduced. If there is a decrease. I am buying more than one to use.	Jingdong prefecture level city in Shandong
Jinfeng Hongyuan Flagship Store	Power Saving Specialist 1	Well made, power saving, good and convenient!	Jingdong prefecture level city in Shandong
Jinfeng Hongyuan Flagship Store	Power Saving Specialist 1	I'm using it. I'll rate it next month.	Jingdong prefecture level city in Shandong
Jinfeng Hongyuan Flagship Store	Power Saving Specialist 1	Baby received Oh, the courier delivery really fast, with a good and then come back to the evaluation	Jingdong prefecture level city in Shandong
Jinfeng Hongyuan Flagship Store	Power Saving Specialist 1	Fast logistics, good packaging, worth it!	Jingdong prefecture level city in Shandong
Jinfeng Hongyuan Flagship Store	Power Saving Specialist 1	Customer service is okay. It's just that.	Jingdong prefecture level city in Shandong
Jinfeng Hongyuan Flagship Store	Power Saving Specialist 1	Poor power saving	Jingdong prefecture level city in Shandong
Jinfeng Hongyuan Flagship Store	Power Saving Specialist 1	I'll tell you when I've used it.	Jingdong prefecture level city in Shandong
Jinfeng Hongyuan Flagship Store	Power Saving Specialist 1	I think it's good.	Jingdong prefecture level city in Shandong
Jinfeng Hongyuan Flagship Store	Power Saving Specialist 1	This energy saver works very well and I recommend everyone to buy it!	Jingdong prefecture level city in Shandong
Jinfeng Hongyuan Flagship Store	Power Saving Specialist 1	I do not know how effective, just use half a month on the burn out, good business reissued a, and then use it after the follow-up comment.	Jingdong prefecture level city in Shandong
Jinfeng Hongyuan Flagship Store	Power Saving Specialist 1	in use	Jingdong prefecture level city in Shandong
Jinfeng Hongyuan Flagship Store	Power Saving Specialist 1	This thing is very poor. It's already only been used for ten days and it's broken. Now waiting for you to return it.	Jingdong prefecture level city in Shandong
Jinfeng Hongyuan Flagship Store	Power Saving Specialist 1	It's not the same as the seller's show.	Jingdong prefecture level city in Shandong
Jinfeng Hongyuan Flagship Store	Power Saving Specialist 1	What is the problem when I get it back from the CTS, it is plugged into the wall and the voltage shows 206v.	Jingdong prefecture level city in Shandong
Jinfeng Hongyuan Flagship Store	Power Saving Specialist 1	The logistics was fast and the stuff was perfectly packaged. It is in the process of trial.	Jingdong prefecture level city in Shandong
Jinfeng Hongyuan Flagship Store	Power Saving Specialist 1	This saver is particularly good to use can really save electricity, with two months and then look at the line if you buy one for the family	Jingdong prefecture level city in Shandong
Jinfeng Hongyuan Flagship Store	Power Saving Specialist 1	Novel appearance design, all aluminum alloy profile, also with a fuse device, voltage monitoring display. After receiving the goods, I directly cut the plug and connect it to the main power box under the leakage switch, hoping to save electricity, and then follow up later. Logistics is very fast, arrived the next	Jingdong prefecture level city in Shandong
Jinfeng Hongyuan Flagship Store	Power Saving Specialist 1	Received, very good and like, if necessary next time to buy ha ha	Jingdong prefecture level city in Shandong
Jinfeng Hongyuan Flagship Store	Power Saving Specialist 1	It's good. It's worth it. Get it if you need it.	Jingdong prefecture level city in Shandong
Jinfeng Hongyuan Flagship Store	Power Saving Specialist 1	It feels more than good and is worth having.	Jingdong prefecture level city in Shandong
Jinfeng Hongyuan Flagship Store	Power Saving Specialist 1	The goods arrived very good and fast first time to buy	Jingdong prefecture level city in Shandong
Jinfeng Hongyuan Flagship Store	Power Saving Specialist 1	The power saving is very good, I can save so much money every month, I will buy another one next time.	Jingdong prefecture level city in Shandong
Jinfeng Hongyuan Flagship Store	Power Saving Specialist 1	It's not what it says.	Jingdong prefecture level city in Shandong
Jinfeng Hongyuan Flagship Store	Power Saving Specialist 1	I have been using it for many days now and it saves electricity, it really does I have an 8 kW electric snail stove and it saves about $3.50 a day.	Jingdong prefecture level city in Shandong
Jinfeng Hongyuan Flagship Store	Power Saving Specialist 1	Express delivery is very fast, the quality is also quite good, worth buying!	Jingdong prefecture level city in Shandong
Jinfeng Hongyuan Flagship Store	Power Saving Specialist 1	Just received expecting the baby to work well.	Jingdong prefecture level city in Shandong
Jinfeng Hongyuan Flagship Store	Power Saving Specialist 1	Very good, there are more fruit, has bought a second!	Jingdong prefecture level city in Shandong
Jinfeng Hongyuan Flagship Store	Power Saving Specialist 1	Good is good So far, no power savings have been noticed Maybe it's too little to notice.	Jingdong prefecture level city in Shandong
Jinfeng Hongyuan Flagship Store	Power Saving Specialist 1	I just received the item and plugged it in to see what happens later.	Jingdong prefecture level city in Shandong
Jinfeng Hongyuan Flagship Store	Power Saving Specialist 1	Baby received, especially good, packaging is also good, the courier brother is very strong, thank you.	Jingdong prefecture level city in Shandong
Jinfeng Hongyuan Flagship Store	Power Saving Specialist 1	Just received the goods have not yet used it, do not know how, three months later in the follow-up comment!	Jingdong prefecture level city in Shandong
Jinfeng Hongyuan Flagship Store	Power Saving Specialist 1	The item was received, it is quite good, recommended to buy.	Jingdong prefecture level city in Shandong
Jinfeng Hongyuan Flagship Store	Power Saving Specialist 1	This energy saver works very well and I recommend everyone to buy it!	Jingdong prefecture level city in Shandong
Jinfeng Hongyuan Flagship Store	Power Saving Specialist 1	Voltage stabilization and current reduction nice power saving product.	Jingdong prefecture level city in Shandong
Jinfeng Hongyuan Flagship Store	Power Saving Specialist 1	Voltage stabilization and current reduction nice power saving product.	Jingdong prefecture level city in Shandong
Jinfeng Hongyuan Flagship Store	Power Saving Specialist 1	A good power saver for stabilizing and lowering current.	Jingdong prefecture level city in Shandong
Jinfeng Hongyuan Flagship Store	Power Saving Specialist 1	You'll know if it saves power after you use it.	Jingdong prefecture level city in Shandong
Jinfeng Hongyuan Flagship Store	Power Saving Specialist 1	The quality is great. The quality is very good. The service is very good. Express delivery arrived in two days.	Jingdong prefecture level city in Shandong
Jinfeng Hongyuan Flagship Store	Power Saving Specialist 1	It works great.	Jingdong prefecture level city in Shandong
Jinfeng Hongyuan Flagship Store	Power Saving Specialist 1	Logistics is very fast, not yet used, used to comment again.	Jingdong prefecture level city in Shandong
Jinfeng Hongyuan Flagship Store	Power Saving Specialist 1	Logistics is very fast things are also very good, worthy of friends to buy	Jingdong prefecture level city in Shandong
Jinfeng Hongyuan Flagship Store	Power Saving Specialist 1	It works. It really works.	Jingdong prefecture level city in Shandong
Jinfeng Hongyuan Flagship Store	Power Saving Specialist 1	LANGUAGE COMMENTS: just received the shipment. I immediately plugged it in and used it. If you ask how effective. It depends on whether the next month's electricity bill has been reduced. If there is a decrease. I am buying more than one to use.	Jingdong prefecture level city in Shandong
Jinfeng Hongyuan Flagship Store	Power Saving Specialist 1	Well made, power saving, good and convenient!	Jingdong prefecture level city in Shandong
Jinfeng Hongyuan Flagship Store	Power Saving Specialist 1	I'm using it. I'll rate it next month.	Jingdong prefecture level city in Shandong

Jinfeng Hongyuan Flagship Store	Power Saving Specialist 1	Baby received Oh, the courier delivery really fast, with a good and then come back to the evaluation	Jingdong prefecture level city in Shandong
Jinfeng Hongyuan Flagship Store	Power Saving Specialist 1	Fast logistics, good packaging, worth it!	Jingdong prefecture level city in Shandong
Jinfeng Hongyuan Flagship Store	Power Saving Specialist 1	Customer service is okay. It's just that.	Jingdong prefecture level city in Shandong
Jinfeng Hongyuan Flagship Store	Power Saving Specialist 1	Poor power saving	Jingdong prefecture level city in Shandong
Jinfeng Hongyuan Flagship Store	Power Saving Specialist 1	I'll tell you when I've used it.	Jingdong prefecture level city in Shandong
Jinfeng Hongyuan Flagship Store	Power Saving Specialist 1	I think it's good.	Jingdong prefecture level city in Shandong
Jinfeng Hongyuan Flagship Store	Power Saving Specialist 1	This energy saver works very well and I recommend everyone to buy it!	Jingdong prefecture level city in Shandong
Jinfeng Hongyuan Flagship Store	Power Saving Specialist 1	I do not know how effective, just use half a month on the burn out, good business reissued a, and then use it after the follow-up comment.	Jingdong prefecture level city in Shandong
Jinfeng Hongyuan Flagship Store	Power Saving Specialist 1	in use	Jingdong prefecture level city in Shandong
Jinfeng Hongyuan Flagship Store	Power Saving Specialist 1	This thing is very poor. It's already only been used for ten days and it's broken. Now waiting for you to return it.	Jingdong prefecture level city in Shandong
Jinfeng Hongyuan Flagship Store	Power Saving Specialist 1	It's not the same as the seller's show.	Jingdong prefecture level city in Shandong
Jinfeng Hongyuan Flagship Store	Power Saving Specialist 1	What is the problem when I get it back from the CTS, it is plugged into the wall and the voltage shows 206v.	Jingdong prefecture level city in Shandong
Jinfeng Hongyuan Flagship Store	Power Saving Specialist 1	The logistics was fast and the stuff was perfectly packaged. It is in the process of trial.	Jingdong prefecture level city in Shandong
Jinfeng Hongyuan Flagship Store	Power Saving Specialist 1	This saver is particularly good to use can really save electricity, with two months and then look at the line if you buy one for the family	Jingdong prefecture level city in Shandong
Jinfeng Hongyuan Flagship Store	Power Saving Specialist 1	Novel appearance design, all aluminum alloy profile, also with a fuse device, voltage monitoring display. After receiving the goods, I directly cut the plug and connect it to the main power box under the leakage switch, hoping to save electricity, and then follow up later. Logistics is very fast, arrived the next	Jingdong prefecture level city in Shandong
Jinfeng Hongyuan Flagship Store	Power Saving Specialist 1	Received, very good and like, if necessary next time to buy ha ha	Jingdong prefecture level city in Shandong
Jinfeng Hongyuan Flagship Store	Power Saving Specialist 1	It's good. It's worth it. Get it if you need it.	Jingdong prefecture level city in Shandong
Jinfeng Hongyuan Flagship Store	Power Saving Specialist 1	It feels more than good and is worth having.	Jingdong prefecture level city in Shandong
Jinfeng Hongyuan Flagship Store	Power Saving Specialist 1	The goods arrived very good and fast first time to buy	Jingdong prefecture level city in Shandong
Jinfeng Hongyuan Flagship Store	Power Saving Specialist 1	The power saving is very good, I can save so much money every month, I will buy another one next time.	Jingdong prefecture level city in Shandong
Jinfeng Hongyuan Flagship Store	Power Saving Specialist 1	It's not what it says.	Jingdong prefecture level city in Shandong
Jinfeng Hongyuan Flagship Store	Power Saving Specialist 1	I have been using it for many days now and it saves electricity, it really does I have an 8 kW electric snail stove and it saves about $3.50 a day.	Jingdong prefecture level city in Shandong
Jinfeng Hongyuan Flagship Store	Power Saving Specialist 1	Express delivery is very fast, the quality is also quite good, worth buying!	Jingdong prefecture level city in Shandong
Jinfeng Hongyuan Flagship Store	Power Saving Specialist 1	Just received expecting the baby to work well.	Jingdong prefecture level city in Shandong
Jinfeng Hongyuan Flagship Store	Power Saving Specialist 1	Very good, there are more fruit, has bought a second!	Jingdong prefecture level city in Shandong
Jinfeng Hongyuan Flagship Store	Power Saving Specialist 1	Good is good So far, no power savings have been noticed Maybe it's too little to notice.	Jingdong prefecture level city in Shandong
Jinfeng Hongyuan Flagship Store	Power Saving Specialist 1	I just received the item and plugged it in to see what happens later.	Jingdong prefecture level city in Shandong
Jinfeng Hongyuan Flagship Store	Power Saving Specialist 1	Baby received, especially good, packaging is also good, the courier brother is very strong, thank you.	Jingdong prefecture level city in Shandong
Jinfeng Hongyuan Flagship Store	Power Saving Specialist 1	Just received the goods have not yet used it, do not know how, three months later in the follow-up comment!	Jingdong prefecture level city in Shandong
Jinfeng Hongyuan Flagship Store	Power Saving Specialist 1	The item was received, it is quite good, recommended to buy.	Jingdong prefecture level city in Shandong
Jinfeng Hongyuan Flagship Store	Power Saving Specialist 1	This energy saver works very well and I recommend everyone to buy it!	Jingdong prefecture level city in Shandong
Jinfeng Hongyuan Flagship Store	Power Saving Specialist 1	Voltage stabilization and current reduction nice power saving product.	Jingdong prefecture level city in Shandong
Jinfeng Hongyuan Flagship Store	Power Saving Specialist 1	Voltage stabilization and current reduction nice power saving product.	Jingdong prefecture level city in Shandong
Jinfeng Hongyuan Flagship Store	Power Saving Specialist 1	A good power saver for stabilizing and lowering current.	Jingdong prefecture level city in Shandong
Jinfeng Hongyuan Flagship Store	Power Saving Specialist 1	You'll know if it saves power after you use it.	Jingdong prefecture level city in Shandong
Jinfeng Hongyuan Flagship Store	Power Saving Specialist 1	The quality is great. The quality is very good. The service is very good. Express delivery arrived in two days.	Jingdong prefecture level city in Shandong
Jinfeng Hongyuan Flagship Store	Power Saving Specialist 1	It works great.	Jingdong prefecture level city in Shandong
Jinfeng Hongyuan Flagship Store	Power Saving Specialist 1	Logistics is very fast, not yet used, used to comment again.	Jingdong prefecture level city in Shandong
Jinfeng Hongyuan Flagship Store	Power Saving Specialist 1	Logistics is very fast things are also very good, worthy of friends to buy	Jingdong prefecture level city in Shandong
Jinfeng Hongyuan Flagship Store	Power Saving Specialist 1	It works. It really works.	Jingdong prefecture level city in Shandong
Jinfeng Hongyuan Flagship Store	Power Saving Specialist 1	LANGUAGE COMMENTS: just received the shipment. I immediately plugged it in and used it. If you ask how effective. It depends on whether the next month's electricity bill has been reduced. If there is a decrease. I am buying more than one to use.	Jingdong prefecture level city in Shandong
Jinfeng Hongyuan Flagship Store	Power Saving Specialist 1	Well made, power saving, good and convenient!	Jingdong prefecture level city in Shandong
Jinfeng Hongyuan Flagship Store	Power Saving Specialist 1	I'm using it. I'll rate it next month.	Jingdong prefecture level city in Shandong
Jinfeng Hongyuan Flagship Store	Power Saving Specialist 1	Baby received Oh, the courier delivery really fast, with a good and then come back to the evaluation	Jingdong prefecture level city in Shandong
Jinfeng Hongyuan Flagship Store	Power Saving Specialist 1	Fast logistics, good packaging, worth it!	Jingdong prefecture level city in Shandong
Jinfeng Hongyuan Flagship Store	Power Saving Specialist 1	Customer service is okay. It's just that.	Jingdong prefecture level city in Shandong
Jinfeng Hongyuan Flagship Store	Power Saving Specialist 1	Poor power saving	Jingdong prefecture level city in Shandong
Jinfeng Hongyuan Flagship Store	Power Saving Specialist 1	I'll tell you when I've used it.	Jingdong prefecture level city in Shandong
Jinfeng Hongyuan Flagship Store	Power Saving Specialist 1	I think it's good.	Jingdong prefecture level city in Shandong
Jinfeng Hongyuan Flagship Store	Power Saving Specialist 1	This energy saver works very well and I recommend everyone to buy it!	Jingdong prefecture level city in Shandong

Jinfeng Hongyuan Flagship Store	Power Saving Specialist 1	I do not know how effective, just use half a month on the burn out, good business reissued a, and then use it after the follow-up comment.	Jingdong prefecture level city in Shandong
Jinfeng Hongyuan Flagship Store	Power Saving Specialist 1	in use	Jingdong prefecture level city in Shandong
Jinfeng Hongyuan Flagship Store	Power Saving Specialist 1	This thing is very poor. It's already only been used for ten days and it's broken. Now waiting for you to return it.	Jingdong prefecture level city in Shandong
Jinfeng Hongyuan Flagship Store	Power Saving Specialist 1	It's not the same as the seller's show.	Jingdong prefecture level city in Shandong
Jinfeng Hongyuan Flagship Store	Power Saving Specialist 1	What is the problem when I get it back from the CTS, it is plugged into the wall and the voltage shows 206v.	Jingdong prefecture level city in Shandong
Jinfeng Hongyuan Flagship Store	Power Saving Specialist 1	The logistics was fast and the stuff was perfectly packaged. It is in the process of trial.	Jingdong prefecture level city in Shandong
Jinfeng Hongyuan Flagship Store	Power Saving Specialist 1	This saver is particularly good to use can really save electricity, with two months and then look at the line if you buy one for the family	Jingdong prefecture level city in Shandong
Jinfeng Hongyuan Flagship Store	Power Saving Specialist 1	Novel appearance design, all aluminum alloy profile, also with a fuse device, voltage monitoring display. After receiving the goods, I directly cut the plug and connect it to the main power box under the leakage switch, hoping to save electricity, and then follow up later. Logistics is very fast, arrived the next	Jingdong prefecture level city in Shandong
Jinfeng Hongyuan Flagship Store	Power Saving Specialist 1	Received, very good and like, if necessary next time to buy ha ha	Jingdong prefecture level city in Shandong
Jinfeng Hongyuan Flagship Store	Power Saving Specialist 1	It's good. It's worth it. Get it if you need it.	Jingdong prefecture level city in Shandong
Jinfeng Hongyuan Flagship Store	Power Saving Specialist 1	It feels more than good and is worth having.	Jingdong prefecture level city in Shandong
Jinfeng Hongyuan Flagship Store	Power Saving Specialist 1	The goods arrived very good and fast first time to buy	Jingdong prefecture level city in Shandong
Jinfeng Hongyuan Flagship Store	Power Saving Specialist 1	The power saving is very good, I can save so much money every month, I will buy another one next time.	Jingdong prefecture level city in Shandong
Jinfeng Hongyuan Flagship Store	Power Saving Specialist 1	It's not what it says.	Jingdong prefecture level city in Shandong
Jinfeng Hongyuan Flagship Store	Power Saving Specialist 1	I have been using it for many days now and it saves electricity, it really does I have an 8 kW electric snail stove and it saves about $3.50 a day.	Jingdong prefecture level city in Shandong
Jinfeng Hongyuan Flagship Store	Power Saving Specialist 1	Express delivery is very fast, the quality is also quite good, worth buying!	Jingdong prefecture level city in Shandong
Jinfeng Hongyuan Flagship Store	Power Saving Specialist 1	Just received expecting the baby to work well.	Jingdong prefecture level city in Shandong
Jinfeng Hongyuan Flagship Store	Power Saving Specialist 1	Very good, there are more fruit, has bought a second!	Jingdong prefecture level city in Shandong
Jinfeng Hongyuan Flagship Store	Power Saving Specialist 1	Good is good So far, no power savings have been noticed Maybe it's too little to notice.	Jingdong prefecture level city in Shandong
Jinfeng Hongyuan Flagship Store	Power Saving Specialist 1	I just received the item and plugged it in to see what happens later.	Jingdong prefecture level city in Shandong
Jinfeng Hongyuan Flagship Store	Power Saving Specialist 1	Baby received, especially good, packaging is also good, the courier brother is very strong, thank you.	Jingdong prefecture level city in Shandong
Jinfeng Hongyuan Flagship Store	Power Saving Specialist 1	Just received the goods have not yet used it, do not know how, three months later in the follow-up comment!	Jingdong prefecture level city in Shandong
Jinfeng Hongyuan Flagship Store	Power Saving Specialist 1	The item was received, it is quite good, recommended to buy.	Jingdong prefecture level city in Shandong
Jinfeng Hongyuan Flagship Store	Power Saving Specialist 1	This energy saver works very well and I recommend everyone to buy it!	Jingdong prefecture level city in Shandong
Jinfeng Hongyuan Flagship Store	Power Saving Specialist 1	Voltage stabilization and current reduction nice power saving product.	Jingdong prefecture level city in Shandong
Jinfeng Hongyuan Flagship Store	Power Saving Specialist 1	Voltage stabilization and current reduction nice power saving product.	Jingdong prefecture level city in Shandong
Jinfeng Hongyuan Flagship Store	Power Saving Specialist 1	A good power saver for stabilizing and lowering current.	Jingdong prefecture level city in Shandong
Jinfeng Hongyuan Flagship Store	Power Saving Specialist 1	You'll know if it saves power after you use it.	Jingdong prefecture level city in Shandong
Jinfeng Hongyuan Flagship Store	Power Saving Specialist 1	The quality is great. The quality is very good. The service is very good. Express delivery arrived in two days.	Jingdong prefecture level city in Shandong
Jinfeng Hongyuan Flagship Store	Power Saving Specialist 1	It works great.	Jingdong prefecture level city in Shandong
Jinfeng Hongyuan Flagship Store	Power Saving Specialist 1	Logistics is very fast, not yet used, used to comment again.	Jingdong prefecture level city in Shandong
Jinfeng Hongyuan Flagship Store	Power Saving Specialist 1	Logistics is very fast things are also very good, worthy of friends to buy	Jingdong prefecture level city in Shandong
Jinfeng Hongyuan Flagship Store	Power Saving Specialist 1	It works. It really works.	Jingdong prefecture level city in Shandong
Jinfeng Hongyuan Flagship Store	Power Saving Specialist 1	LANGUAGE COMMENTS: just received the shipment. I immediately plugged it in and used it. If you ask how effective. It depends on whether the next month's electricity bill has been reduced. If there is a decrease. I am buying more than one to use.	Jingdong prefecture level city in Shandong
Jinfeng Hongyuan Flagship Store	Power Saving Specialist 1	Well made, power saving, good and convenient!	Jingdong prefecture level city in Shandong
Jinfeng Hongyuan Flagship Store	Power Saving Specialist 1	I'm using it. I'll rate it next month.	Jingdong prefecture level city in Shandong
Jinfeng Hongyuan Flagship Store	Power Saving Specialist 1	Baby received Oh, the courier delivery really fast, with a good and then come back to the evaluation	Jingdong prefecture level city in Shandong
Jinfeng Hongyuan Flagship Store	Power Saving Specialist 1	Fast logistics, good packaging, worth it!	Jingdong prefecture level city in Shandong
Jinfeng Hongyuan Flagship Store	Power Saving Specialist 1	Customer service is okay. It's just that.	Jingdong prefecture level city in Shandong
Jinfeng Hongyuan Flagship Store	Power Saving Specialist 1	Poor power saving	Jingdong prefecture level city in Shandong
Jinfeng Hongyuan Flagship Store	Power Saving Specialist 1	I'll tell you when I've used it.	Jingdong prefecture level city in Shandong
Jinfeng Hongyuan Flagship Store	Power Saving Specialist 1	I think it's good.	Jingdong prefecture level city in Shandong
Jinfeng Hongyuan Flagship Store	Power Saving Specialist 1	This energy saver works very well and I recommend everyone to buy it!	Jingdong prefecture level city in Shandong
Jinfeng Hongyuan Flagship Store	Power Saving Specialist 1	I do not know how effective, just use half a month on the burn out, good business reissued a, and then use it after the follow-up comment.	Jingdong prefecture level city in Shandong
Jinfeng Hongyuan Flagship Store	Power Saving Specialist 1	in use	Jingdong prefecture level city in Shandong
Jinfeng Hongyuan Flagship Store	Power Saving Specialist 1	This thing is very poor. It's already only been used for ten days and it's broken. Now waiting for you to return it.	Jingdong prefecture level city in Shandong
Jinfeng Hongyuan Flagship Store	Power Saving Specialist 1	It's not the same as the seller's show.	Jingdong prefecture level city in Shandong
Jinfeng Hongyuan Flagship Store	Power Saving Specialist 1	What is the problem when I get it back from the CTS, it is plugged into the wall and the voltage shows 206v.	Jingdong prefecture level city in Shandong
Jinfeng Hongyuan Flagship Store	Power Saving Specialist 1	The logistics was fast and the stuff was perfectly packaged. It is in the process of trial.	Jingdong prefecture level city in Shandong
Jinfeng Hongyuan Flagship Store	Power Saving Specialist 1	This saver is particularly good to use can really save electricity, with two months and then look at the line if you buy one for the family	Jingdong prefecture level city in Shandong

Jinfeng Hongyuan Flagship Store	Power Saving Specialist 1	Novel appearance design, all aluminum alloy profile, also with a fuse device, voltage monitoring display. After receiving the goods, I directly cut the plug and connect it to the main power box under the leakage switch, hoping to save electricity, and then follow up later. Logistics is very fast, arrived the next	Jingdong prefecture level city in Shandong
Jinfeng Hongyuan Flagship Store	Power Saving Specialist 1	Received, very good and like, if necessary next time to buy ha ha	Jingdong prefecture level city in Shandong
Jinfeng Hongyuan Flagship Store	Power Saving Specialist 1	It's good. It's worth it. Get it if you need it.	Jingdong prefecture level city in Shandong
Jinfeng Hongyuan Flagship Store	Power Saving Specialist 1	It feels more than good and is worth having.	Jingdong prefecture level city in Shandong
Jinfeng Hongyuan Flagship Store	Power Saving Specialist 1	The goods arrived very good and fast first time to buy	Jingdong prefecture level city in Shandong
Jinfeng Hongyuan Flagship Store	Power Saving Specialist 1	The power saving is very good, I can save so much money every month, I will buy another one next time.	Jingdong prefecture level city in Shandong
Jinfeng Hongyuan Flagship Store	Power Saving Specialist 1	It's not what it says.	Jingdong prefecture level city in Shandong
Jinfeng Hongyuan Flagship Store	Power Saving Specialist 1	I have been using it for many days now and it saves electricity, it really does I have an 8 kW electric snail stove and it saves about $3.50 a day.	Jingdong prefecture level city in Shandong
Jinfeng Hongyuan Flagship Store	Power Saving Specialist 1	Express delivery is very fast, the quality is also quite good, worth buying!	Jingdong prefecture level city in Shandong
Jinfeng Hongyuan Flagship Store	Power Saving Specialist 1	Just received expecting the baby to work well.	Jingdong prefecture level city in Shandong
Jinfeng Hongyuan Flagship Store	Power Saving Specialist 1	Very good, there are more fruit, has bought a second!	Jingdong prefecture level city in Shandong
Jinfeng Hongyuan Flagship Store	Power Saving Specialist 1	Good is good So far, no power savings have been noticed Maybe it's too little to notice.	Jingdong prefecture level city in Shandong
Jinfeng Hongyuan Flagship Store	Power Saving Specialist 1	I just received the item and plugged it in to see what happens later.	Jingdong prefecture level city in Shandong
Jinfeng Hongyuan Flagship Store	Power Saving Specialist 1	Baby received, especially good, packaging is also good, the courier brother is very strong, thank you.	Jingdong prefecture level city in Shandong
Jinfeng Hongyuan Flagship Store	Power Saving Specialist 1	Just received the goods have not yet used it, do not know how, three months later in the follow-up comment!	Jingdong prefecture level city in Shandong
Jinfeng Hongyuan Flagship Store	Power Saving Specialist 1	The item was received, it is quite good, recommended to buy.	Jingdong prefecture level city in Shandong
Jinfeng Hongyuan Flagship Store	Power Saving Specialist 1	This energy saver works very well and I recommend everyone to buy it!	Jingdong prefecture level city in Shandong
Jinfeng Hongyuan Flagship Store	Power Saving Specialist 1	Voltage stabilization and current reduction nice power saving product.	Jingdong prefecture level city in Shandong
Jinfeng Hongyuan Flagship Store	Power Saving Specialist 1	Voltage stabilization and current reduction nice power saving product.	Jingdong prefecture level city in Shandong
Jinfeng Hongyuan Flagship Store	Power Saving Specialist 1	A good power saver for stabilizing and lowering current.	Jingdong prefecture level city in Shandong
Jinfeng Hongyuan Flagship Store	Power Saving Specialist 1	You'll know if it saves power after you use it.	Jingdong prefecture level city in Shandong
Jinfeng Hongyuan Flagship Store	Power Saving Specialist 1	The quality is great. The quality is very good. The service is very good. Express delivery arrived in two days.	Jingdong prefecture level city in Shandong
Jinfeng Hongyuan Flagship Store	Power Saving Specialist 1	It works great.	Jingdong prefecture level city in Shandong
Jinfeng Hongyuan Flagship Store	Power Saving Specialist 1	Logistics is very fast, not yet used, used to comment again.	Jingdong prefecture level city in Shandong
Jinfeng Hongyuan Flagship Store	Power Saving Specialist 1	Logistics is very fast things are also very good, worthy of friends to buy	Jingdong prefecture level city in Shandong
Jinfeng Hongyuan Flagship Store	Power Saving Specialist 1	It works. It really works.	Jingdong prefecture level city in Shandong
Jinfeng Hongyuan Flagship Store	Power Saving Specialist 1	LANGUAGE COMMENTS: just received the shipment. I immediately plugged it in and used it. If you ask how effective. It depends on whether the next month's electricity bill has been reduced. If there is a decrease. I am buying more than one to use.	Jingdong prefecture level city in Shandong
Jinfeng Hongyuan Flagship Store	Power Saving Specialist 1	Well made, power saving, good and convenient!	Jingdong prefecture level city in Shandong
Jinfeng Hongyuan Flagship Store	Power Saving Specialist 1	I'm using it. I'll rate it next month.	Jingdong prefecture level city in Shandong
Jinfeng Hongyuan Flagship Store	Power Saving Specialist 1	Baby received Oh, the courier delivery really fast, with a good and then come back to the evaluation	Jingdong prefecture level city in Shandong
Jinfeng Hongyuan Flagship Store	Power Saving Specialist 1	Fast logistics, good packaging, worth it!	Jingdong prefecture level city in Shandong
Jinfeng Hongyuan Flagship Store	Power Saving Specialist 1	Customer service is okay. It's just that.	Jingdong prefecture level city in Shandong
Jinfeng Hongyuan Flagship Store	Power Saving Specialist 1	Poor power saving	Jingdong prefecture level city in Shandong
Jinfeng Hongyuan Flagship Store	Power Saving Specialist 1	I'll tell you when I've used it.	Jingdong prefecture level city in Shandong
Jinfeng Hongyuan Flagship Store	Power Saving Specialist 1	I think it's good.	Jingdong prefecture level city in Shandong
Jinfeng Hongyuan Flagship Store	Power Saving Specialist 1	This energy saver works very well and I recommend everyone to buy it!	Jingdong prefecture level city in Shandong
Jinfeng Hongyuan Flagship Store	Power Saving Specialist 1	I do not know how effective, just use half a month on the burn out, good business reissued a, and then use it after the follow-up comment.	Jingdong prefecture level city in Shandong
Jinfeng Hongyuan Flagship Store	Power Saving Specialist 1	in use	Jingdong prefecture level city in Shandong
Jinfeng Hongyuan Flagship Store	Power Saving Specialist 1	This thing is very poor. It's already only been used for ten days and it's broken. Now waiting for you to return it.	Jingdong prefecture level city in Shandong
Jinfeng Hongyuan Flagship Store	Power Saving Specialist 1	It's not the same as the seller's show.	Jingdong prefecture level city in Shandong
Jinfeng Hongyuan Flagship Store	Power Saving Specialist 1	What is the problem when I get it back from the CTS, it is plugged into the wall and the voltage shows 206v.	Jingdong prefecture level city in Shandong
Jinfeng Hongyuan Flagship Store	Power Saving Specialist 1	The logistics was fast and the stuff was perfectly packaged. It is in the process of trial.	Jingdong prefecture level city in Shandong
Jinfeng Hongyuan Flagship Store	Power Saving Specialist 1	This saver is particularly good to use can really save electricity, with two months and then look at the line if you buy one for the family	Jingdong prefecture level city in Shandong
Jinfeng Hongyuan Flagship Store	Power Saving Specialist 1	Novel appearance design, all aluminum alloy profile, also with a fuse device, voltage monitoring display. After receiving the goods, I directly cut the plug and connect it to the main power box under the leakage switch, hoping to save electricity, and then follow up later. Logistics is very fast, arrived the next	Jingdong prefecture level city in Shandong
Jinfeng Hongyuan Flagship Store	Power Saving Specialist 1	Received, very good and like, if necessary next time to buy ha ha	Jingdong prefecture level city in Shandong
Jinfeng Hongyuan Flagship Store	Power Saving Specialist 1	It's good. It's worth it. Get it if you need it.	Jingdong prefecture level city in Shandong
Jinfeng Hongyuan Flagship Store	Power Saving Specialist 1	It feels more than good and is worth having.	Jingdong prefecture level city in Shandong
Jinfeng Hongyuan Flagship Store	Power Saving Specialist 1	The goods arrived very good and fast first time to buy	Jingdong prefecture level city in Shandong
Jinfeng Hongyuan Flagship Store	Power Saving Specialist 1	The power saving is very good, I can save so much money every month, I will buy another one next time.	Jingdong prefecture level city in Shandong
Jinfeng Hongyuan Flagship Store	Power Saving Specialist 1	It's not what it says.	Jingdong prefecture level city in Shandong

Jinfeng Hongyuan Flagship Store	Power Saving Specialist 1	I have been using it for many days now and it saves electricity, it really does I have an 8 kW electric snail stove and it saves about $3.50 a day.	Jingdong prefecture level city in Shandong
Jinfeng Hongyuan Flagship Store	Power Saving Specialist 1	Express delivery is very fast, the quality is also quite good, worth buying!	Jingdong prefecture level city in Shandong
Jinfeng Hongyuan Flagship Store	Power Saving Specialist 1	Just received expecting the baby to work well.	Jingdong prefecture level city in Shandong
Jinfeng Hongyuan Flagship Store	Power Saving Specialist 1	Very good, there are more fruit, has bought a second!	Jingdong prefecture level city in Shandong
Jinfeng Hongyuan Flagship Store	Power Saving Specialist 1	Good is good So far, no power savings have been noticed Maybe it's too little to notice.	Jingdong prefecture level city in Shandong
Jinfeng Hongyuan Flagship Store	Power Saving Specialist 1	I just received the item and plugged it in to see what happens later.	Jingdong prefecture level city in Shandong
Jinfeng Hongyuan Flagship Store	Power Saving Specialist 1	Baby received, especially good, packaging is also good, the courier brother is very strong, thank you.	Jingdong prefecture level city in Shandong
Jinfeng Hongyuan Flagship Store	Power Saving Specialist 1	Just received the goods have not yet used it, do not know how, three months later in the follow-up comment!	Jingdong prefecture level city in Shandong
Jinfeng Hongyuan Flagship Store	Power Saving Specialist 1	The item was received, it is quite good, recommended to buy.	Jingdong prefecture level city in Shandong
Jinfeng Hongyuan Flagship Store	Power Saving Specialist 1	This energy saver works very well and I recommend everyone to buy it!	Jingdong prefecture level city in Shandong
Jinfeng Hongyuan Flagship Store	Power Saving Specialist 1	Voltage stabilization and current reduction nice power saving product.	Jingdong prefecture level city in Shandong
Jinfeng Hongyuan Flagship Store	Power Saving Specialist 1	Voltage stabilization and current reduction nice power saving product.	Jingdong prefecture level city in Shandong
Jinfeng Hongyuan Flagship Store	Power Saving Specialist 1	A good power saver for stabilizing and lowering current.	Jingdong prefecture level city in Shandong
Jinfeng Hongyuan Flagship Store	Power Saving Specialist 1	You'll know if it saves power after you use it.	Jingdong prefecture level city in Shandong
Jinfeng Hongyuan Flagship Store	Power Saving Specialist 1	The quality is great. The quality is very good. The service is very good. Express delivery arrived in two days.	Jingdong prefecture level city in Shandong
Jinfeng Hongyuan Flagship Store	Power Saving Specialist 1	It works great.	Jingdong prefecture level city in Shandong
Jinfeng Hongyuan Flagship Store	Power Saving Specialist 1	Logistics is very fast, not yet used, used to comment again.	Jingdong prefecture level city in Shandong
Jinfeng Hongyuan Flagship Store	Power Saving Specialist 1	Logistics is very fast things are also very good, worthy of friends to buy	Jingdong prefecture level city in Shandong
Jinfeng Hongyuan Flagship Store	Power Saving Specialist 1	It works. It really works.	Jingdong prefecture level city in Shandong
Jinfeng Hongyuan Flagship Store	Power Saving Specialist 1	LANGUAGE COMMENTS: just received the shipment. I immediately plugged it in and used it. If you ask how effective. It depends on whether the next month's electricity bill has been reduced. If there is a decrease. I am buying more than one to use.	Jingdong prefecture level city in Shandong
Jinfeng Hongyuan Flagship Store	Power Saving Specialist 1	Well made, power saving, good and convenient!	Jingdong prefecture level city in Shandong
Jinfeng Hongyuan Flagship Store	Power Saving Specialist 1	I'm using it. I'll rate it next month.	Jingdong prefecture level city in Shandong
Jinfeng Hongyuan Flagship Store	Power Saving Specialist 1	Baby received Oh, the courier delivery really fast, with a good and then come back to the evaluation	Jingdong prefecture level city in Shandong
Jinfeng Hongyuan Flagship Store	Power Saving Specialist 1	Fast logistics, good packaging, worth it!	Jingdong prefecture level city in Shandong
Jinfeng Hongyuan Flagship Store	Power Saving Specialist 1	Customer service is okay. It's just that.	Jingdong prefecture level city in Shandong
Jinfeng Hongyuan Flagship Store	Power Saving Specialist 1	Poor power saving	Jingdong prefecture level city in Shandong
Jinfeng Hongyuan Flagship Store	Power Saving Specialist 1	I'll tell you when I've used it.	Jingdong prefecture level city in Shandong
Jinfeng Hongyuan Flagship Store	Power Saving Specialist 1	I think it's good.	Jingdong prefecture level city in Shandong
Jinfeng Hongyuan Flagship Store	Power Saving Specialist 1	This energy saver works very well and I recommend everyone to buy it!	Jingdong prefecture level city in Shandong
Jinfeng Hongyuan Flagship Store	Power Saving Specialist 1	I do not know how effective, just use half a month on the burn out, good business reissued a, and then use it after the follow-up comment.	Jingdong prefecture level city in Shandong
Jinfeng Hongyuan Flagship Store	Power Saving Specialist 1	in use	Jingdong prefecture level city in Shandong
Jinfeng Hongyuan Flagship Store	Power Saving Specialist 1	This thing is very poor. It's already only been used for ten days and it's broken. Now waiting for you to return it.	Jingdong prefecture level city in Shandong
Jinfeng Hongyuan Flagship Store	Power Saving Specialist 1	It's not the same as the seller's show.	Jingdong prefecture level city in Shandong
Jinfeng Hongyuan Flagship Store	Power Saving Specialist 1	What is the problem when I get it back from the CTS, it is plugged into the wall and the voltage shows 206v.	Jingdong prefecture level city in Shandong
Jinfeng Hongyuan Flagship Store	Power Saving Specialist 1	The logistics was fast and the stuff was perfectly packaged. It is in the process of trial.	Jingdong prefecture level city in Shandong
Jinfeng Hongyuan Flagship Store	Power Saving Specialist 1	This saver is particularly good to use can really save electricity, with two months and then look at the line if you buy one for the family	Jingdong prefecture level city in Shandong
Jinfeng Hongyuan Flagship Store	Power Saving Specialist 1	Novel appearance design, all aluminum alloy profile, also with a fuse device, voltage monitoring display. After receiving the goods, I directly cut the plug and connect it to the main power box under the leakage switch, hoping to save electricity, and then follow up later. Logistics is very fast, arrived the next	Jingdong prefecture level city in Shandong
Jinfeng Hongyuan Flagship Store	Power Saving Specialist 1	Received, very good and like, if necessary next time to buy ha ha	Jingdong prefecture level city in Shandong
Jinfeng Hongyuan Flagship Store	Power Saving Specialist 1	It's good. It's worth it. Get it if you need it.	Jingdong prefecture level city in Shandong
Jinfeng Hongyuan Flagship Store	Power Saving Specialist 1	It feels more than good and is worth having.	Jingdong prefecture level city in Shandong
Jinfeng Hongyuan Flagship Store	Power Saving Specialist 1	The goods arrived very good and fast first time to buy	Jingdong prefecture level city in Shandong
Jinfeng Hongyuan Flagship Store	Power Saving Specialist 1	The power saving is very good, I can save so much money every month, I will buy another one next time.	Jingdong prefecture level city in Shandong
Jinfeng Hongyuan Flagship Store	Power Saving Specialist 1	It's not what it says.	Jingdong prefecture level city in Shandong
Jinfeng Hongyuan Flagship Store	Power Saving Specialist 1	I have been using it for many days now and it saves electricity, it really does I have an 8 kW electric snail stove and it saves about $3.50 a day.	Jingdong prefecture level city in Shandong
Jinfeng Hongyuan Flagship Store	Power Saving Specialist 1	Express delivery is very fast, the quality is also quite good, worth buying!	Jingdong prefecture level city in Shandong
Jinfeng Hongyuan Flagship Store	Power Saving Specialist 1	Just received expecting the baby to work well.	Jingdong prefecture level city in Shandong
Jinfeng Hongyuan Flagship Store	Power Saving Specialist 1	Very good, there are more fruit, has bought a second!	Jingdong prefecture level city in Shandong
Jinfeng Hongyuan Flagship Store	Power Saving Specialist 1	Good is good So far, no power savings have been noticed Maybe it's too little to notice.	Jingdong prefecture level city in Shandong
Jinfeng Hongyuan Flagship Store	Power Saving Specialist 1	I just received the item and plugged it in to see what happens later.	Jingdong prefecture level city in Shandong
Jinfeng Hongyuan Flagship Store	Power Saving Specialist 1	Baby received, especially good, packaging is also good, the courier brother is very strong, thank you.	Jingdong prefecture level city in Shandong

Jinfeng Hongyuan Flagship Store	Power Saving Specialist 1	Just received the goods have not yet used it, do not know how, three months later in the follow-up comment!	Jingdong prefecture level city in Shandong
Jinfeng Hongyuan Flagship Store	Power Saving Specialist 1	The item was received, it is quite good, recommended to buy.	Jingdong prefecture level city in Shandong
Jinfeng Hongyuan Flagship Store	Power Saving Specialist 1	This energy saver works very well and I recommend everyone to buy it!	Jingdong prefecture level city in Shandong
Jinfeng Hongyuan Flagship Store	Power Saving Specialist 1	Voltage stabilization and current reduction nice power saving product.	Jingdong prefecture level city in Shandong
Jinfeng Hongyuan Flagship Store	Power Saving Specialist 1	Voltage stabilization and current reduction nice power saving product.	Jingdong prefecture level city in Shandong
Jinfeng Hongyuan Flagship Store	Power Saving Specialist 1	A good power saver for stabilizing and lowering current.	Jingdong prefecture level city in Shandong
Jinfeng Hongyuan Flagship Store	Power Saving Specialist 1	You'll know if it saves power after you use it.	Jingdong prefecture level city in Shandong
Jinfeng Hongyuan Flagship Store	Power Saving Specialist 1	The quality is great. The quality is very good. The service is very good. Express delivery arrived in two days.	Jingdong prefecture level city in Shandong
Jinfeng Hongyuan Flagship Store	Power Saving Specialist 1	It works great.	Jingdong prefecture level city in Shandong
Jinfeng Hongyuan Flagship Store	Power Saving Specialist 1	Logistics is very fast, not yet used, used to comment again.	Jingdong prefecture level city in Shandong
Jinfeng Hongyuan Flagship Store	Power Saving Specialist 1	Logistics is very fast things are also very good, worthy of friends to buy	Jingdong prefecture level city in Shandong
Jinfeng Hongyuan Flagship Store	Power Saving Specialist 1	It works. It really works.	Jingdong prefecture level city in Shandong
Jinfeng Hongyuan Flagship Store	Power Saving Specialist 1	LANGUAGE COMMENTS: just received the shipment. I immediately plugged it in and used it. If you ask how effective. It depends on whether the next month's electricity bill has been reduced. If there is a decrease. I am buying more than one to use.	Jingdong prefecture level city in Shandong
Jinfeng Hongyuan Flagship Store	Power Saving Specialist 1	Well made, power saving, good and convenient!	Jingdong prefecture level city in Shandong
Jinfeng Hongyuan Flagship Store	Power Saving Specialist 1	I'm using it. I'll rate it next month.	Jingdong prefecture level city in Shandong
Jinfeng Hongyuan Flagship Store	Power Saving Specialist 1	Baby received Oh, the courier delivery really fast, with a good and then come back to the evaluation	Jingdong prefecture level city in Shandong
Jinfeng Hongyuan Flagship Store	Power Saving Specialist 1	Fast logistics, good packaging, worth it!	Jingdong prefecture level city in Shandong
Jinfeng Hongyuan Flagship Store	Power Saving Specialist 1	Customer service is okay. It's just that.	Jingdong prefecture level city in Shandong
Jinfeng Hongyuan Flagship Store	Power Saving Specialist 1	Poor power saving	Jingdong prefecture level city in Shandong
Jinfeng Hongyuan Flagship Store	Power Saving Specialist 1	I'll tell you when I've used it.	Jingdong prefecture level city in Shandong
Jinfeng Hongyuan Flagship Store	Power Saving Specialist 1	I think it's good.	Jingdong prefecture level city in Shandong
Jinfeng Hongyuan Flagship Store	Power Saving Specialist 1	This energy saver works very well and I recommend everyone to buy it!	Jingdong prefecture level city in Shandong
Jinfeng Hongyuan Flagship Store	Power Saving Specialist 1	I do not know how effective, just use half a month on the burn out, good business reissued a, and then use it after the follow-up comment.	Jingdong prefecture level city in Shandong
Jinfeng Hongyuan Flagship Store	Power Saving Specialist 1	in use	Jingdong prefecture level city in Shandong
Jinfeng Hongyuan Flagship Store	Power Saving Specialist 1	This thing is very poor. It's already only been used for ten days and it's broken. Now waiting for you to return it.	Jingdong prefecture level city in Shandong
Jinfeng Hongyuan Flagship Store	Power Saving Specialist 1	It's not the same as the seller's show.	Jingdong prefecture level city in Shandong
Jinfeng Hongyuan Flagship Store	Power Saving Specialist 1	What is the problem when I get it back from the CTS, it is plugged into the wall and the voltage shows 206v.	Jingdong prefecture level city in Shandong
Jinfeng Hongyuan Flagship Store	Power Saving Specialist 1	The logistics was fast and the stuff was perfectly packaged. It is in the process of trial.	Jingdong prefecture level city in Shandong
Jinfeng Hongyuan Flagship Store	Power Saving Specialist 1	This saver is particularly good to use can really save electricity, with two months and then look at the line if you buy one for the family	Jingdong prefecture level city in Shandong
Jinfeng Hongyuan Flagship Store	Power Saving Specialist 1	Novel appearance design, all aluminum alloy profile, also with a fuse device, voltage monitoring display. After receiving the goods, I directly cut the plug and connect it to the main power box under the leakage switch, hoping to save electricity, and then follow up later. Logistics is very fast, arrived the next	Jingdong prefecture level city in Shandong
Jinfeng Hongyuan Flagship Store	Power Saving Specialist 1	Received, very good and like, if necessary next time to buy ha ha	Jingdong prefecture level city in Shandong
Jinfeng Hongyuan Flagship Store	Power Saving Specialist 1	It's good. It's worth it. Get it if you need it.	Jingdong prefecture level city in Shandong
Jinfeng Hongyuan Flagship Store	Power Saving Specialist 1	It feels more than good and is worth having.	Jingdong prefecture level city in Shandong
Jinfeng Hongyuan Flagship Store	Power Saving Specialist 1	The goods arrived very good and fast first time to buy	Jingdong prefecture level city in Shandong
Jinfeng Hongyuan Flagship Store	Power Saving Specialist 1	The power saving is very good, I can save so much money every month, I will buy another one next time.	Jingdong prefecture level city in Shandong
Jinfeng Hongyuan Flagship Store	Power Saving Specialist 1	It's not what it says.	Jingdong prefecture level city in Shandong
Jinfeng Hongyuan Flagship Store	Power Saving Specialist 1	I have been using it for many days now and it saves electricity, it really does I have an 8 kW electric snail stove and it saves about $3.50 a day.	Jingdong prefecture level city in Shandong
Jinfeng Hongyuan Flagship Store	Power Saving Specialist 1	Express delivery is very fast, the quality is also quite good, worth buying!	Jingdong prefecture level city in Shandong
Jinfeng Hongyuan Flagship Store	Power Saving Specialist 1	Just received expecting the baby to work well.	Jingdong prefecture level city in Shandong
Jinfeng Hongyuan Flagship Store	Power Saving Specialist 1	Very good, there are more fruit, has bought a second!	Jingdong prefecture level city in Shandong
Jinfeng Hongyuan Flagship Store	Power Saving Specialist 1	Good is good So far, no power savings have been noticed Maybe it's too little to notice.	Jingdong prefecture level city in Shandong
Jinfeng Hongyuan Flagship Store	Power Saving Specialist 1	I just received the item and plugged it in to see what happens later.	Jingdong prefecture level city in Shandong
Jinfeng Hongyuan Flagship Store	Power Saving Specialist 1	Baby received, especially good, packaging is also good, the courier brother is very strong, thank you.	Jingdong prefecture level city in Shandong
Jinfeng Hongyuan Flagship Store	Power Saving Specialist 1	Just received the goods have not yet used it, do not know how, three months later in the follow-up comment!	Jingdong prefecture level city in Shandong
Jinfeng Hongyuan Flagship Store	Power Saving Specialist 1	The item was received, it is quite good, recommended to buy.	Jingdong prefecture level city in Shandong
Jinfeng Hongyuan Flagship Store	Power Saving Specialist 1	This energy saver works very well and I recommend everyone to buy it!	Jingdong prefecture level city in Shandong
Jinfeng Hongyuan Flagship Store	Power Saving Specialist 1	Voltage stabilization and current reduction nice power saving product.	Jingdong prefecture level city in Shandong
Jinfeng Hongyuan Flagship Store	Power Saving Specialist 1	Voltage stabilization and current reduction nice power saving product.	Jingdong prefecture level city in Shandong
Jinfeng Hongyuan Flagship Store	Power Saving Specialist 1	A good power saver for stabilizing and lowering current.	Jingdong prefecture level city in Shandong
Jinfeng Hongyuan Flagship Store	Power Saving Specialist 1	You'll know if it saves power after you use it.	Jingdong prefecture level city in Shandong

Jinfeng Hongyuan Flagship Store	Power Saving Specialist 1	The quality is great. The quality is very good. The service is very good. Express delivery arrived in two days.	Jingdong prefecture level city in Shandong
Jinfeng Hongyuan Flagship Store	Power Saving Specialist 1	It works great.	Jingdong prefecture level city in Shandong
Jinfeng Hongyuan Flagship Store	Power Saving Specialist 1	Logistics is very fast, not yet used, used to comment again.	Jingdong prefecture level city in Shandong
Jinfeng Hongyuan Flagship Store	Power Saving Specialist 1	Logistics is very fast things are also very good, worthy of friends to buy	Jingdong prefecture level city in Shandong
Jinfeng Hongyuan Flagship Store	Power Saving Specialist 1	It works. It really works.	Jingdong prefecture level city in Shandong
Jinfeng Hongyuan Flagship Store	Power Saving Specialist 1	LANGUAGE COMMENTS: just received the shipment. I immediately plugged it in and used it. If you ask how effective. It depends on whether the next month's electricity bill has been reduced. If there is a decrease. I am buying more than one to use.	Jingdong prefecture level city in Shandong
Jinfeng Hongyuan Flagship Store	Power Saving Specialist 1	Well made, power saving, good and convenient!	Jingdong prefecture level city in Shandong
Jinfeng Hongyuan Flagship Store	Power Saving Specialist 1	I'm using it. I'll rate it next month.	Jingdong prefecture level city in Shandong
Jinfeng Hongyuan Flagship Store	Power Saving Specialist 1	Baby received Oh, the courier delivery really fast, with a good and then come back to the evaluation	Jingdong prefecture level city in Shandong
Jinfeng Hongyuan Flagship Store	Power Saving Specialist 1	Fast logistics, good packaging, worth it!	Jingdong prefecture level city in Shandong
Jinfeng Hongyuan Flagship Store	Power Saving Specialist 1	Customer service is okay. It's just that.	Jingdong prefecture level city in Shandong
Jinfeng Hongyuan Flagship Store	Power Saving Specialist 1	Poor power saving	Jingdong prefecture level city in Shandong
Jinfeng Hongyuan Flagship Store	Power Saving Specialist 1	I'll tell you when I've used it.	Jingdong prefecture level city in Shandong
Jinfeng Hongyuan Flagship Store	Power Saving Specialist 1	I think it's good.	Jingdong prefecture level city in Shandong
Jinfeng Hongyuan Flagship Store	Power Saving Specialist 1	This energy saver works very well and I recommend everyone to buy it!	Jingdong prefecture level city in Shandong
Jinfeng Hongyuan Flagship Store	Power Saving Specialist 1	I do not know how effective, just use half a month on the burn out, good business reissued a, and then use it after the follow-up comment.	Jingdong prefecture level city in Shandong
Jinfeng Hongyuan Flagship Store	Power Saving Specialist 1	in use	Jingdong prefecture level city in Shandong
Jinfeng Hongyuan Flagship Store	Power Saving Specialist 1	This thing is very poor. It's already only been used for ten days and it's broken. Now waiting for you to return it.	Jingdong prefecture level city in Shandong
Jinfeng Hongyuan Flagship Store	Power Saving Specialist 1	It's not the same as the seller's show.	Jingdong prefecture level city in Shandong
Jinfeng Hongyuan Flagship Store	Power Saving Specialist 1	What is the problem when I get it back from the CTS, it is plugged into the wall and the voltage shows 206v.	Jingdong prefecture level city in Shandong
Jinfeng Hongyuan Flagship Store	Power Saving Specialist 1	The logistics was fast and the stuff was perfectly packaged. It is in the process of trial.	Jingdong prefecture level city in Shandong
Jinfeng Hongyuan Flagship Store	Power Saving Specialist 1	This saver is particularly good to use can really save electricity, with two months and then look at the line if you buy one for the family	Jingdong prefecture level city in Shandong
Jinfeng Hongyuan Flagship Store	Power Saving Specialist 1	Novel appearance design, all aluminum alloy profile, also with a fuse device, voltage monitoring display. After receiving the goods, I directly cut the plug and connect it to the main power box under the leakage switch, hoping to save electricity, and then follow up later. Logistics is very fast, arrived the next	Jingdong prefecture level city in Shandong
Jinfeng Hongyuan Flagship Store	Power Saving Specialist 1	Received, very good and like, if necessary next time to buy ha ha	Jingdong prefecture level city in Shandong
Jinfeng Hongyuan Flagship Store	Power Saving Specialist 1	It's good. It's worth it. Get it if you need it.	Jingdong prefecture level city in Shandong
Jinfeng Hongyuan Flagship Store	Power Saving Specialist 1	It feels more than good and is worth having.	Jingdong prefecture level city in Shandong
Jinfeng Hongyuan Flagship Store	Power Saving Specialist 1	The goods arrived very good and fast first time to buy	Jingdong prefecture level city in Shandong
Jinfeng Hongyuan Flagship Store	Power Saving Specialist 1	The power saving is very good, I can save so much money every month, I will buy another one next time.	Jingdong prefecture level city in Shandong
Jinfeng Hongyuan Flagship Store	Power Saving Specialist 1	It's not what it says.	Jingdong prefecture level city in Shandong
Jinfeng Hongyuan Flagship Store	Power Saving Specialist 1	I have been using it for many days now and it saves electricity, it really does I have an 8 kW electric snail stove and it saves about $3.50 a day.	Jingdong prefecture level city in Shandong
Jinfeng Hongyuan Flagship Store	Power Saving Specialist 1	Express delivery is very fast, the quality is also quite good, worth buying!	Jingdong prefecture level city in Shandong
Jinfeng Hongyuan Flagship Store	Power Saving Specialist 1	Just received expecting the baby to work well.	Jingdong prefecture level city in Shandong
Jinfeng Hongyuan Flagship Store	Power Saving Specialist 1	Very good, there are more fruit, has bought a second!	Jingdong prefecture level city in Shandong
Jinfeng Hongyuan Flagship Store	Power Saving Specialist 1	Good is good So far, no power savings have been noticed Maybe it's too little to notice.	Jingdong prefecture level city in Shandong
Jinfeng Hongyuan Flagship Store	Power Saving Specialist 1	I just received the item and plugged it in to see what happens later.	Jingdong prefecture level city in Shandong
Jinfeng Hongyuan Flagship Store	Power Saving Specialist 1	Baby received, especially good, packaging is also good, the courier brother is very strong, thank you.	Jingdong prefecture level city in Shandong
Jinfeng Hongyuan Flagship Store	Power Saving Specialist 1	Just received the goods have not yet used it, do not know how, three months later in the follow-up comment!	Jingdong prefecture level city in Shandong
Jinfeng Hongyuan Flagship Store	Power Saving Specialist 1	The item was received, it is quite good, recommended to buy.	Jingdong prefecture level city in Shandong
Jinfeng Hongyuan Flagship Store	Power Saving Specialist 1	This energy saver works very well and I recommend everyone to buy it!	Jingdong prefecture level city in Shandong
Jinfeng Hongyuan Flagship Store	Power Saving Specialist 1	Voltage stabilization and current reduction nice power saving product.	Jingdong prefecture level city in Shandong
Jinfeng Hongyuan Flagship Store	Power Saving Specialist 1	Voltage stabilization and current reduction nice power saving product.	Jingdong prefecture level city in Shandong
Jinfeng Hongyuan Flagship Store	Power Saving Specialist 1	A good power saver for stabilizing and lowering current.	Jingdong prefecture level city in Shandong
Jinfeng Hongyuan Flagship Store	Power Saving Specialist 1	You'll know if it saves power after you use it.	Jingdong prefecture level city in Shandong
Jinfeng Hongyuan Flagship Store	Power Saving Specialist 1	The quality is great. The quality is very good. The service is very good. Express delivery arrived in two days.	Jingdong prefecture level city in Shandong
Jinfeng Hongyuan Flagship Store	Power Saving Specialist 1	It works great.	Jingdong prefecture level city in Shandong
Jinfeng Hongyuan Flagship Store	Power Saving Specialist 1	Logistics is very fast, not yet used, used to comment again.	Jingdong prefecture level city in Shandong
Jinfeng Hongyuan Flagship Store	Power Saving Specialist 1	Logistics is very fast things are also very good, worthy of friends to buy	Jingdong prefecture level city in Shandong
Jinfeng Hongyuan Flagship Store	Power Saving Specialist 1	It works. It really works.	Jingdong prefecture level city in Shandong
Jinfeng Hongyuan Flagship Store	Power Saving Specialist 1	LANGUAGE COMMENTS: just received the shipment. I immediately plugged it in and used it. If you ask how effective. It depends on whether the next month's electricity bill has been reduced. If there is a decrease. I am buying more than one to use.	Jingdong prefecture level city in Shandong
Jinfeng Hongyuan Flagship Store	Power Saving Specialist 1	Well made, power saving, good and convenient!	Jingdong prefecture level city in Shandong

Jinfeng Hongyuan Flagship Store	Power Saving Specialist 1	I'm using it. I'll rate it next month.	Jingdong prefecture level city in Shandong
Jinfeng Hongyuan Flagship Store	Power Saving Specialist 1	Baby received Oh, the courier delivery really fast, with a good and then come back to the evaluation	Jingdong prefecture level city in Shandong
Jinfeng Hongyuan Flagship Store	Power Saving Specialist 1	Fast logistics, good packaging, worth it!	Jingdong prefecture level city in Shandong
Jinfeng Hongyuan Flagship Store	Power Saving Specialist 1	Customer service is okay. It's just that.	Jingdong prefecture level city in Shandong
Jinfeng Hongyuan Flagship Store	Power Saving Specialist 1	Poor power saving	Jingdong prefecture level city in Shandong
Jinfeng Hongyuan Flagship Store	Power Saving Specialist 1	I'll tell you when I've used it.	Jingdong prefecture level city in Shandong
Jinfeng Hongyuan Flagship Store	Power Saving Specialist 1	I think it's good.	Jingdong prefecture level city in Shandong
Jinfeng Hongyuan Flagship Store	Power Saving Specialist 1	This energy saver works very well and I recommend everyone to buy it!	Jingdong prefecture level city in Shandong
Jinfeng Hongyuan Flagship Store	Power Saving Specialist 1	I do not know how effective, just use half a month on the burn out, good business reissued a, and then use it after the follow-up comment.	Jingdong prefecture level city in Shandong
Jinfeng Hongyuan Flagship Store	Power Saving Specialist 1	in use	Jingdong prefecture level city in Shandong
Jinfeng Hongyuan Flagship Store	Power Saving Specialist 1	This thing is very poor. It's already only been used for ten days and it's broken. Now waiting for you to return it.	Jingdong prefecture level city in Shandong
Jinfeng Hongyuan Flagship Store	Power Saving Specialist 1	It's not the same as the seller's show.	Jingdong prefecture level city in Shandong
Jinfeng Hongyuan Flagship Store	Power Saving Specialist 1	What is the problem when I get it back from the CTS, it is plugged into the wall and the voltage shows 206v.	Jingdong prefecture level city in Shandong
Jinfeng Hongyuan Flagship Store	Power Saving Specialist 1	The logistics was fast and the stuff was perfectly packaged. It is in the process of trial.	Jingdong prefecture level city in Shandong
Jinfeng Hongyuan Flagship Store	Power Saving Specialist 1	This saver is particularly good to use can really save electricity, with two months and then look at the line if you buy one for the family	Jingdong prefecture level city in Shandong
Jinfeng Hongyuan Flagship Store	Power Saving Specialist 1	Novel appearance design, all aluminum alloy profile, also with a fuse device, voltage monitoring display. After receiving the goods, I directly cut the plug and connect it to the main power box under the leakage switch, hoping to save electricity, and then follow up later. Logistics is very fast, arrived the next	Jingdong prefecture level city in Shandong
Jinfeng Hongyuan Flagship Store	Power Saving Specialist 1	Received, very good and like, if necessary next time to buy ha ha	Jingdong prefecture level city in Shandong
Jinfeng Hongyuan Flagship Store	Power Saving Specialist 1	It's good. It's worth it. Get it if you need it.	Jingdong prefecture level city in Shandong
Jinfeng Hongyuan Flagship Store	Power Saving Specialist 1	It feels more than good and is worth having.	Jingdong prefecture level city in Shandong
Jinfeng Hongyuan Flagship Store	Power Saving Specialist 1	The goods arrived very good and fast first time to buy	Jingdong prefecture level city in Shandong
Jinfeng Hongyuan Flagship Store	Power Saving Specialist 1	The power saving is very good, I can save so much money every month, I will buy another one next time.	Jingdong prefecture level city in Shandong
Jinfeng Hongyuan Flagship Store	Power Saving Specialist 1	It's not what it says.	Jingdong prefecture level city in Shandong
Jinfeng Hongyuan Flagship Store	Power Saving Specialist 1	I have been using it for many days now and it saves electricity, it really does I have an 8 kW electric snail stove and it saves about $3.50 a day.	Jingdong prefecture level city in Shandong
Jinfeng Hongyuan Flagship Store	Power Saving Specialist 1	Express delivery is very fast, the quality is also quite good, worth buying!	Jingdong prefecture level city in Shandong
Jinfeng Hongyuan Flagship Store	Power Saving Specialist 1	Just received expecting the baby to work well.	Jingdong prefecture level city in Shandong
Jinfeng Hongyuan Flagship Store	Power Saving Specialist 1	Very good, there are more fruit, has bought a second!	Jingdong prefecture level city in Shandong
Jinfeng Hongyuan Flagship Store	Power Saving Specialist 1	Good is good So far, no power savings have been noticed Maybe it's too little to notice.	Jingdong prefecture level city in Shandong
Jinfeng Hongyuan Flagship Store	Power Saving Specialist 1	I just received the item and plugged it in to see what happens later.	Jingdong prefecture level city in Shandong
Jinfeng Hongyuan Flagship Store	Power Saving Specialist 1	Baby received, especially good, packaging is also good, the courier brother is very strong, thank you.	Jingdong prefecture level city in Shandong
Jinfeng Hongyuan Flagship Store	Power Saving Specialist 1	Just received the goods have not yet used it, do not know how, three months later in the follow-up comment!	Jingdong prefecture level city in Shandong
Jinfeng Hongyuan Flagship Store	Power Saving Specialist 1	The item was received, it is quite good, recommended to buy.	Jingdong prefecture level city in Shandong
Jinfeng Hongyuan Flagship Store	Power Saving Specialist 1	This energy saver works very well and I recommend everyone to buy it!	Jingdong prefecture level city in Shandong
Jinfeng Hongyuan Flagship Store	Power Saving Specialist 1	Voltage stabilization and current reduction nice power saving product.	Jingdong prefecture level city in Shandong
Jinfeng Hongyuan Flagship Store	Power Saving Specialist 1	Voltage stabilization and current reduction nice power saving product.	Jingdong prefecture level city in Shandong
Jinfeng Hongyuan Flagship Store	Power Saving Specialist 1	A good power saver for stabilizing and lowering current.	Jingdong prefecture level city in Shandong
Jinfeng Hongyuan Flagship Store	Power Saving Specialist 1	You'll know if it saves power after you use it.	Jingdong prefecture level city in Shandong
Jinfeng Hongyuan Flagship Store	Power Saving Specialist 1	The quality is great. The quality is very good. The service is very good. Express delivery arrived in two days.	Jingdong prefecture level city in Shandong
Jinfeng Hongyuan Flagship Store	Power Saving Specialist 1	It works great.	Jingdong prefecture level city in Shandong
Jinfeng Hongyuan Flagship Store	Power Saving Specialist 1	Logistics is very fast, not yet used, used to comment again.	Jingdong prefecture level city in Shandong
Jinfeng Hongyuan Flagship Store	Power Saving Specialist 1	Logistics is very fast things are also very good, worthy of friends to buy	Jingdong prefecture level city in Shandong
Jinfeng Hongyuan Flagship Store	Power Saving Specialist 1	It works. It really works.	Jingdong prefecture level city in Shandong
Jinfeng Hongyuan Flagship Store	Power Saving Specialist 1	LANGUAGE COMMENTS: just received the shipment. I immediately plugged it in and used it. If you ask how effective. It depends on whether the next month's electricity bill has been reduced. If there is a decrease. I am buying more than one to use.	Jingdong prefecture level city in Shandong
Jinfeng Hongyuan Flagship Store	Power Saving Specialist 1	Well made, power saving, good and convenient!	Jingdong prefecture level city in Shandong
Jinfeng Hongyuan Flagship Store	Power Saving Specialist 1	I'm using it. I'll rate it next month.	Jingdong prefecture level city in Shandong
Jinfeng Hongyuan Flagship Store	Power Saving Specialist 1	Baby received Oh, the courier delivery really fast, with a good and then come back to the evaluation	Jingdong prefecture level city in Shandong
Jinfeng Hongyuan Flagship Store	Power Saving Specialist 1	Fast logistics, good packaging, worth it!	Jingdong prefecture level city in Shandong
Jinfeng Hongyuan Flagship Store	Power Saving Specialist 1	Customer service is okay. It's just that.	Jingdong prefecture level city in Shandong
Jinfeng Hongyuan Flagship Store	Power Saving Specialist 1	Poor power saving	Jingdong prefecture level city in Shandong
Jinfeng Hongyuan Flagship Store	Power Saving Specialist 1	I'll tell you when I've used it.	Jingdong prefecture level city in Shandong
Jinfeng Hongyuan Flagship Store	Power Saving Specialist 1	I think it's good.	Jingdong prefecture level city in Shandong

Jinfeng Hongyuan Flagship Store	Power Saving Specialist 1	This energy saver works very well and I recommend everyone to buy it!	Jingdong prefecture level city in Shandong
Jinfeng Hongyuan Flagship Store	Power Saving Specialist 1	I do not know how effective, just use half a month on the burn out, good business reissued a, and then use it after the follow-up comment.	Jingdong prefecture level city in Shandong
Jinfeng Hongyuan Flagship Store	Power Saving Specialist 1	in use	Jingdong prefecture level city in Shandong
Jinfeng Hongyuan Flagship Store	Power Saving Specialist 1	This thing is very poor. It's already only been used for ten days and it's broken. Now waiting for you to return it.	Jingdong prefecture level city in Shandong
Jinfeng Hongyuan Flagship Store	Power Saving Specialist 1	It's not the same as the seller's show.	Jingdong prefecture level city in Shandong
Jinfeng Hongyuan Flagship Store	Power Saving Specialist 1	What is the problem when I get it back from the CTS, it is plugged into the wall and the voltage shows 206v.	Jingdong prefecture level city in Shandong
Jinfeng Hongyuan Flagship Store	Power Saving Specialist 1	The logistics was fast and the stuff was perfectly packaged. It is in the process of trial.	Jingdong prefecture level city in Shandong
Jinfeng Hongyuan Flagship Store	Power Saving Specialist 1	This saver is particularly good to use can really save electricity, with two months and then look at the line if you buy one for the family	Jingdong prefecture level city in Shandong
Jinfeng Hongyuan Flagship Store	Power Saving Specialist 1	Novel appearance design, all aluminum alloy profile, also with a fuse device, voltage monitoring display. After receiving the goods, I directly cut the plug and connect it to the main power box under the leakage switch, hoping to save electricity, and then follow up later. Logistics is very fast, arrived the next	Jingdong prefecture level city in Shandong
Jinfeng Hongyuan Flagship Store	Power Saving Specialist 1	Received, very good and like, if necessary next time to buy ha ha	Jingdong prefecture level city in Shandong
Jinfeng Hongyuan Flagship Store	Power Saving Specialist 1	It's good. It's worth it. Get it if you need it.	Jingdong prefecture level city in Shandong
Jinfeng Hongyuan Flagship Store	Power Saving Specialist 1	It feels more than good and is worth having.	Jingdong prefecture level city in Shandong
Jinfeng Hongyuan Flagship Store	Power Saving Specialist 1	The goods arrived very good and fast first time to buy	Jingdong prefecture level city in Shandong
Jinfeng Hongyuan Flagship Store	Power Saving Specialist 1	The power saving is very good, I can save so much money every month, I will buy another one next time.	Jingdong prefecture level city in Shandong
Jinfeng Hongyuan Flagship Store	Power Saving Specialist 1	It's not what it says.	Jingdong prefecture level city in Shandong
Jinfeng Hongyuan Flagship Store	Power Saving Specialist 1	I have been using it for many days now and it saves electricity, it really does I have an 8 kW electric snail stove and it saves about $3.50 a day.	Jingdong prefecture level city in Shandong
Jinfeng Hongyuan Flagship Store	Power Saving Specialist 1	Express delivery is very fast, the quality is also quite good, worth buying!	Jingdong prefecture level city in Shandong
Jinfeng Hongyuan Flagship Store	Power Saving Specialist 1	Just received expecting the baby to work well.	Jingdong prefecture level city in Shandong
Jinfeng Hongyuan Flagship Store	Power Saving Specialist 1	Very good, there are more fruit, has bought a second!	Jingdong prefecture level city in Shandong
Jinfeng Hongyuan Flagship Store	Power Saving Specialist 1	Good is good So far, no power savings have been noticed Maybe it's too little to notice.	Jingdong prefecture level city in Shandong
Jinfeng Hongyuan Flagship Store	Power Saving Specialist 1	I just received the item and plugged it in to see what happens later.	Jingdong prefecture level city in Shandong
Jinfeng Hongyuan Flagship Store	Power Saving Specialist 1	Baby received, especially good, packaging is also good, the courier brother is very strong, thank you.	Jingdong prefecture level city in Shandong
Jinfeng Hongyuan Flagship Store	Power Saving Specialist 1	Just received the goods have not yet used it, do not know how, three months later in the follow-up comment!	Jingdong prefecture level city in Shandong
Jinfeng Hongyuan Flagship Store	Power Saving Specialist 1	The item was received, it is quite good, recommended to buy.	Jingdong prefecture level city in Shandong
Jinfeng Hongyuan Flagship Store	Power Saving Specialist 1	This energy saver works very well and I recommend everyone to buy it!	Jingdong prefecture level city in Shandong
Jinfeng Hongyuan Flagship Store	Power Saving Specialist 1	Voltage stabilization and current reduction nice power saving product.	Jingdong prefecture level city in Shandong
Jinfeng Hongyuan Flagship Store	Power Saving Specialist 1	Voltage stabilization and current reduction nice power saving product.	Jingdong prefecture level city in Shandong
Jinfeng Hongyuan Flagship Store	Power Saving Specialist 1	A good power saver for stabilizing and lowering current.	Jingdong prefecture level city in Shandong
Jinfeng Hongyuan Flagship Store	Power Saving Specialist 1	You'll know if it saves power after you use it.	Jingdong prefecture level city in Shandong
Jinfeng Hongyuan Flagship Store	Power Saving Specialist 1	The quality is great. The quality is very good. The service is very good. Express delivery arrived in two days.	Jingdong prefecture level city in Shandong
Jinfeng Hongyuan Flagship Store	Power Saving Specialist 1	It works great.	Jingdong prefecture level city in Shandong
Jinfeng Hongyuan Flagship Store	Power Saving Specialist 1	Logistics is very fast, not yet used, used to comment again.	Jingdong prefecture level city in Shandong
Jinfeng Hongyuan Flagship Store	Power Saving Specialist 1	Logistics is very fast things are also very good, worthy of friends to buy	Jingdong prefecture level city in Shandong
Jinfeng Hongyuan Flagship Store	Power Saving Specialist 1	It works. It really works.	Jingdong prefecture level city in Shandong
Jinfeng Hongyuan Flagship Store	Power Saving Specialist 1	LANGUAGE COMMENTS: just received the shipment. I immediately plugged it in and used it. If you ask how effective. It depends on whether the next month's electricity bill has been reduced. If there is a decrease. I am buying more than one to use.	Jingdong prefecture level city in Shandong
Jinfeng Hongyuan Flagship Store	Power Saving Specialist 1	Well made, power saving, good and convenient!	Jingdong prefecture level city in Shandong
Jinfeng Hongyuan Flagship Store	Power Saving Specialist 1	I'm using it. I'll rate it next month.	Jingdong prefecture level city in Shandong
Jinfeng Hongyuan Flagship Store	Power Saving Specialist 1	Baby received Oh, the courier delivery really fast, with a good and then come back to the evaluation	Jingdong prefecture level city in Shandong
Jinfeng Hongyuan Flagship Store	Power Saving Specialist 1	Fast logistics, good packaging, worth it!	Jingdong prefecture level city in Shandong
Jinfeng Hongyuan Flagship Store	Power Saving Specialist 1	Customer service is okay. It's just that.	Jingdong prefecture level city in Shandong
Jinfeng Hongyuan Flagship Store	Power Saving Specialist 1	Poor power saving	Jingdong prefecture level city in Shandong
Jinfeng Hongyuan Flagship Store	Power Saving Specialist 1	I'll tell you when I've used it.	Jingdong prefecture level city in Shandong
Jinfeng Hongyuan Flagship Store	Power Saving Specialist 1	I think it's good.	Jingdong prefecture level city in Shandong
Jinfeng Hongyuan Flagship Store	Power Saving Specialist 1	This energy saver works very well and I recommend everyone to buy it!	Jingdong prefecture level city in Shandong
Jinfeng Hongyuan Flagship Store	Power Saving Specialist 1	I do not know how effective, just use half a month on the burn out, good business reissued a, and then use it after the follow-up comment.	Jingdong prefecture level city in Shandong
Jinfeng Hongyuan Flagship Store	Power Saving Specialist 1	in use	Jingdong prefecture level city in Shandong
Jinfeng Hongyuan Flagship Store	Power Saving Specialist 1	This thing is very poor. It's already only been used for ten days and it's broken. Now waiting for you to return it.	Jingdong prefecture level city in Shandong
Jinfeng Hongyuan Flagship Store	Power Saving Specialist 1	It's not the same as the seller's show.	Jingdong prefecture level city in Shandong
Jinfeng Hongyuan Flagship Store	Power Saving Specialist 1	What is the problem when I get it back from the CTS, it is plugged into the wall and the voltage shows 206v.	Jingdong prefecture level city in Shandong
Jinfeng Hongyuan Flagship Store	Power Saving Specialist 1	The logistics was fast and the stuff was perfectly packaged. It is in the process of trial.	Jingdong prefecture level city in Shandong

Jinfeng Hongyuan Flagship Store	Power Saving Specialist 1	This saver is particularly good to use can really save electricity, with two months and then look at the line if you buy one for the family	Jingdong prefecture level city in Shandong
Jinfeng Hongyuan Flagship Store	Power Saving Specialist 1	Novel appearance design, all aluminum alloy profile, also with a fuse device, voltage monitoring display. After receiving the goods, I directly cut the plug and connect it to the main power box under the leakage switch, hoping to save electricity, and then follow up later. Logistics is very fast, arrived the next	Jingdong prefecture level city in Shandong
Jinfeng Hongyuan Flagship Store	Power Saving Specialist 1	Received, very good and like, if necessary next time to buy ha ha	Jingdong prefecture level city in Shandong
Jinfeng Hongyuan Flagship Store	Power Saving Specialist 1	It's good. It's worth it. Get it if you need it.	Jingdong prefecture level city in Shandong
Jinfeng Hongyuan Flagship Store	Power Saving Specialist 1	It feels more than good and is worth having.	Jingdong prefecture level city in Shandong
Jinfeng Hongyuan Flagship Store	Power Saving Specialist 1	The goods arrived very good and fast first time to buy	Jingdong prefecture level city in Shandong
Jinfeng Hongyuan Flagship Store	Power Saving Specialist 1	The power saving is very good, I can save so much money every month, I will buy another one next time.	Jingdong prefecture level city in Shandong
Jinfeng Hongyuan Flagship Store	Power Saving Specialist 1	It's not what it says.	Jingdong prefecture level city in Shandong
Jinfeng Hongyuan Flagship Store	Power Saving Specialist 1	I have been using it for many days now and it saves electricity, it really does I have an 8 kW electric snail stove and it saves about $3.50 a day.	Jingdong prefecture level city in Shandong
Jinfeng Hongyuan Flagship Store	Power Saving Specialist 1	Express delivery is very fast, the quality is also quite good, worth buying!	Jingdong prefecture level city in Shandong
Jinfeng Hongyuan Flagship Store	Power Saving Specialist 1	Just received expecting the baby to work well.	Jingdong prefecture level city in Shandong
Jinfeng Hongyuan Flagship Store	Power Saving Specialist 1	Very good, there are more fruit, has bought a second!	Jingdong prefecture level city in Shandong
Jinfeng Hongyuan Flagship Store	Power Saving Specialist 1	Good is good So far, no power savings have been noticed Maybe it's too little to notice.	Jingdong prefecture level city in Shandong
Jinfeng Hongyuan Flagship Store	Power Saving Specialist 1	I just received the item and plugged it in to see what happens later.	Jingdong prefecture level city in Shandong
Jinfeng Hongyuan Flagship Store	Power Saving Specialist 1	Baby received, especially good, packaging is also good, the courier brother is very strong, thank you.	Jingdong prefecture level city in Shandong
Jinfeng Hongyuan Flagship Store	Power Saving Specialist 1	Just received the goods have not yet used it, do not know how, three months later in the follow-up comment!	Jingdong prefecture level city in Shandong
Jinfeng Hongyuan Flagship Store	Power Saving Specialist 1	The item was received, it is quite good, recommended to buy.	Jingdong prefecture level city in Shandong
Jinfeng Hongyuan Flagship Store	Power Saving Specialist 1	This energy saver works very well and I recommend everyone to buy it!	Jingdong prefecture level city in Shandong
Jinfeng Hongyuan Flagship Store	Power Saving Specialist 1	Voltage stabilization and current reduction nice power saving product.	Jingdong prefecture level city in Shandong
Jinfeng Hongyuan Flagship Store	Power Saving Specialist 1	Voltage stabilization and current reduction nice power saving product.	Jingdong prefecture level city in Shandong
Jinfeng Hongyuan Flagship Store	Power Saving Specialist 1	A good power saver for stabilizing and lowering current.	Jingdong prefecture level city in Shandong
Jinfeng Hongyuan Flagship Store	Power Saving Specialist 1	You'll know if it saves power after you use it.	Jingdong prefecture level city in Shandong
Jinfeng Hongyuan Flagship Store	Power Saving Specialist 1	The quality is great. The quality is very good. The service is very good. Express delivery arrived in two days.	Jingdong prefecture level city in Shandong
Jinfeng Hongyuan Flagship Store	Power Saving Specialist 1	It works great.	Jingdong prefecture level city in Shandong
Jinfeng Hongyuan Flagship Store	Power Saving Specialist 1	Logistics is very fast, not yet used, used to comment again.	Jingdong prefecture level city in Shandong
Jinfeng Hongyuan Flagship Store	Power Saving Specialist 1	Logistics is very fast things are also very good, worthy of friends to buy	Jingdong prefecture level city in Shandong
Jinfeng Hongyuan Flagship Store	Power Saving Specialist 1	It works. It really works.	Jingdong prefecture level city in Shandong
Jinfeng Hongyuan Flagship Store	Power Saving Specialist 1	LANGUAGE COMMENTS: just received the shipment. I immediately plugged it in and used it. If you ask how effective. It depends on whether the next month's electricity bill has been reduced. If there is a decrease. I am buying more than one to use.	Jingdong prefecture level city in Shandong
Jinfeng Hongyuan Flagship Store	Power Saving Specialist 1	Well made, power saving, good and convenient!	Jingdong prefecture level city in Shandong
Jinfeng Hongyuan Flagship Store	Power Saving Specialist 1	I'm using it. I'll rate it next month.	Jingdong prefecture level city in Shandong
Jinfeng Hongyuan Flagship Store	Power Saving Specialist 1	Baby received Oh, the courier delivery really fast, with a good and then come back to the evaluation	Jingdong prefecture level city in Shandong
Jinfeng Hongyuan Flagship Store	Power Saving Specialist 1	Fast logistics, good packaging, worth it!	Jingdong prefecture level city in Shandong
Jinfeng Hongyuan Flagship Store	Power Saving Specialist 1	Customer service is okay. It's just that.	Jingdong prefecture level city in Shandong
Jinfeng Hongyuan Flagship Store	Power Saving Specialist 1	Poor power saving	Jingdong prefecture level city in Shandong
Jinfeng Hongyuan Flagship Store	Power Saving Specialist 1	I'll tell you when I've used it.	Jingdong prefecture level city in Shandong
Jinfeng Hongyuan Flagship Store	Power Saving Specialist 1	I think it's good.	Jingdong prefecture level city in Shandong
Jinfeng Hongyuan Flagship Store	Power Saving Specialist 1	This energy saver works very well and I recommend everyone to buy it!	Jingdong prefecture level city in Shandong
Jinfeng Hongyuan Flagship Store	Power Saving Specialist 1	I do not know how effective, just use half a month on the burn out, good business reissued a, and then use it after the follow-up comment.	Jingdong prefecture level city in Shandong
Jinfeng Hongyuan Flagship Store	Power Saving Specialist 1	in use	Jingdong prefecture level city in Shandong
Jinfeng Hongyuan Flagship Store	Power Saving Specialist 1	This thing is very poor. It's already only been used for ten days and it's broken. Now waiting for you to return it.	Jingdong prefecture level city in Shandong
Jinfeng Hongyuan Flagship Store	Power Saving Specialist 1	It's not the same as the seller's show.	Jingdong prefecture level city in Shandong
Jinfeng Hongyuan Flagship Store	Power Saving Specialist 1	What is the problem when I get it back from the CTS, it is plugged into the wall and the voltage shows 206v.	Jingdong prefecture level city in Shandong
Jinfeng Hongyuan Flagship Store	Power Saving Specialist 1	The logistics was fast and the stuff was perfectly packaged. It is in the process of trial.	Jingdong prefecture level city in Shandong
Jinfeng Hongyuan Flagship Store	Power Saving Specialist 1	This saver is particularly good to use can really save electricity, with two months and then look at the line if you buy one for the family	Jingdong prefecture level city in Shandong
Jinfeng Hongyuan Flagship Store	Power Saving Specialist 1	Novel appearance design, all aluminum alloy profile, also with a fuse device, voltage monitoring display. After receiving the goods, I directly cut the plug and connect it to the main power box under the leakage switch, hoping to save electricity, and then follow up later. Logistics is very fast, arrived the next	Jingdong prefecture level city in Shandong
Jinfeng Hongyuan Flagship Store	Power Saving Specialist 1	Received, very good and like, if necessary next time to buy ha ha	Jingdong prefecture level city in Shandong
Jinfeng Hongyuan Flagship Store	Power Saving Specialist 1	It's good. It's worth it. Get it if you need it.	Jingdong prefecture level city in Shandong
Jinfeng Hongyuan Flagship Store	Power Saving Specialist 1	It feels more than good and is worth having.	Jingdong prefecture level city in Shandong
Jinfeng Hongyuan Flagship Store	Power Saving Specialist 1	The goods arrived very good and fast first time to buy	Jingdong prefecture level city in Shandong
Jinfeng Hongyuan Flagship Store	Power Saving Specialist 1	The power saving is very good, I can save so much money every month, I will buy another one next time.	Jingdong prefecture level city in Shandong

Jinfeng Hongyuan Flagship Store	Power Saving Specialist 1	It's not what it says.	Jingdong prefecture level city in Shandong
Jinfeng Hongyuan Flagship Store	Power Saving Specialist 1	I have been using it for many days now and it saves electricity, it really does I have an 8 kW electric snail stove and it saves about $3.50 a day.	Jingdong prefecture level city in Shandong
Jinfeng Hongyuan Flagship Store	Power Saving Specialist 1	Express delivery is very fast, the quality is also quite good, worth buying!	Jingdong prefecture level city in Shandong
Jinfeng Hongyuan Flagship Store	Power Saving Specialist 1	Just received expecting the baby to work well.	Jingdong prefecture level city in Shandong
Jinfeng Hongyuan Flagship Store	Power Saving Specialist 1	Very good, there are more fruit, has bought a second!	Jingdong prefecture level city in Shandong
Jinfeng Hongyuan Flagship Store	Power Saving Specialist 1	Good is good So far, no power savings have been noticed Maybe it's too little to notice.	Jingdong prefecture level city in Shandong
Jinfeng Hongyuan Flagship Store	Power Saving Specialist 1	I just received the item and plugged it in to see what happens later.	Jingdong prefecture level city in Shandong
Jinfeng Hongyuan Flagship Store	Power Saving Specialist 1	Baby received, especially good, packaging is also good, the courier brother is very strong, thank you.	Jingdong prefecture level city in Shandong
Jinfeng Hongyuan Flagship Store	Power Saving Specialist 1	Just received the goods have not yet used it, do not know how, three months later in the follow-up comment!	Jingdong prefecture level city in Shandong
Jinfeng Hongyuan Flagship Store	Power Saving Specialist 1	The item was received, it is quite good, recommended to buy.	Jingdong prefecture level city in Shandong
Jinfeng Hongyuan Flagship Store	Power Saving Specialist 1	This energy saver works very well and I recommend everyone to buy it!	Jingdong prefecture level city in Shandong
Jinfeng Hongyuan Flagship Store	Power Saving Specialist 1	Voltage stabilization and current reduction nice power saving product.	Jingdong prefecture level city in Shandong
Jinfeng Hongyuan Flagship Store	Power Saving Specialist 1	Voltage stabilization and current reduction nice power saving product.	Jingdong prefecture level city in Shandong
Jinfeng Hongyuan Flagship Store	Power Saving Specialist 1	A good power saver for stabilizing and lowering current.	Jingdong prefecture level city in Shandong
Jinfeng Hongyuan Flagship Store	Power Saving Specialist 1	You'll know if it saves power after you use it.	Jingdong prefecture level city in Shandong
Jinfeng Hongyuan Flagship Store	Power Saving Specialist 1	The quality is great. The quality is very good. The service is very good. Express delivery arrived in two days.	Jingdong prefecture level city in Shandong
Jinfeng Hongyuan Flagship Store	Power Saving Specialist 1	It works great.	Jingdong prefecture level city in Shandong
Jinfeng Hongyuan Flagship Store	Power Saving Specialist 1	Logistics is very fast, not yet used, used to comment again.	Jingdong prefecture level city in Shandong
Jinfeng Hongyuan Flagship Store	Power Saving Specialist 1	Logistics is very fast things are also very good, worthy of friends to buy	Jingdong prefecture level city in Shandong
Jinfeng Hongyuan Flagship Store	Power Saving Specialist 1	It works. It really works.	Jingdong prefecture level city in Shandong
Jinfeng Hongyuan Flagship Store	Power Saving Specialist 1	LANGUAGE COMMENTS: just received the shipment. I immediately plugged it in and used it. If you ask how effective. It depends on whether the next month's electricity bill has been reduced. If there is a decrease. I am buying more than one to use.	Jingdong prefecture level city in Shandong
Jinfeng Hongyuan Flagship Store	Power Saving Specialist 1	Well made, power saving, good and convenient!	Jingdong prefecture level city in Shandong
Jinfeng Hongyuan Flagship Store	Power Saving Specialist 1	I'm using it. I'll rate it next month.	Jingdong prefecture level city in Shandong
Jinfeng Hongyuan Flagship Store	Power Saving Specialist 1	Baby received Oh, the courier delivery really fast, with a good and then come back to the evaluation	Jingdong prefecture level city in Shandong
Jinfeng Hongyuan Flagship Store	Power Saving Specialist 1	Fast logistics, good packaging, worth it!	Jingdong prefecture level city in Shandong
Jinfeng Hongyuan Flagship Store	Power Saving Specialist 1	Customer service is okay. It's just that.	Jingdong prefecture level city in Shandong
Jinfeng Hongyuan Flagship Store	Power Saving Specialist 1	Poor power saving	Jingdong prefecture level city in Shandong
Jinfeng Hongyuan Flagship Store	Power Saving Specialist 1	I'll tell you when I've used it.	Jingdong prefecture level city in Shandong
Jinfeng Hongyuan Flagship Store	Power Saving Specialist 1	I think it's good.	Jingdong prefecture level city in Shandong
Jinfeng Hongyuan Flagship Store	Power Saving Specialist 1	This energy saver works very well and I recommend everyone to buy it!	Jingdong prefecture level city in Shandong
Jinfeng Hongyuan Flagship Store	Power Saving Specialist 1	I do not know how effective, just use half a month on the burn out, good business reissued a, and then use it after the follow-up comment.	Jingdong prefecture level city in Shandong
Jinfeng Hongyuan Flagship Store	Power Saving Specialist 1	in use	Jingdong prefecture level city in Shandong
Jinfeng Hongyuan Flagship Store	Power Saving Specialist 1	This thing is very poor. It's already only been used for ten days and it's broken. Now waiting for you to return it.	Jingdong prefecture level city in Shandong
Jinfeng Hongyuan Flagship Store	Power Saving Specialist 1	It's not the same as the seller's show.	Jingdong prefecture level city in Shandong
Jinfeng Hongyuan Flagship Store	Power Saving Specialist 1	What is the problem when I get it back from the CTS, it is plugged into the wall and the voltage shows 206v.	Jingdong prefecture level city in Shandong
Jinfeng Hongyuan Flagship Store	Power Saving Specialist 1	The logistics was fast and the stuff was perfectly packaged. It is in the process of trial.	Jingdong prefecture level city in Shandong
Jinfeng Hongyuan Flagship Store	Power Saving Specialist 1	This saver is particularly good to use can really save electricity, with two months and then look at the line if you buy one for the family	Jingdong prefecture level city in Shandong
Jinfeng Hongyuan Flagship Store	Power Saving Specialist 1	Novel appearance design, all aluminum alloy profile, also with a fuse device, voltage monitoring display. After receiving the goods, I directly cut the plug and connect it to the main power box under the leakage switch, hoping to save electricity, and then follow up later. Logistics is very fast, arrived the next	Jingdong prefecture level city in Shandong
Jinfeng Hongyuan Flagship Store	Power Saving Specialist 1	Received, very good and like, if necessary next time to buy ha ha	Jingdong prefecture level city in Shandong
Jinfeng Hongyuan Flagship Store	Power Saving Specialist 1	It's good. It's worth it. Get it if you need it.	Jingdong prefecture level city in Shandong
Jinfeng Hongyuan Flagship Store	Power Saving Specialist 1	It feels more than good and is worth having.	Jingdong prefecture level city in Shandong
Jinfeng Hongyuan Flagship Store	Power Saving Specialist 1	The goods arrived very good and fast first time to buy	Jingdong prefecture level city in Shandong
Jinfeng Hongyuan Flagship Store	Power Saving Specialist 1	The power saving is very good, I can save so much money every month, I will buy another one next time.	Jingdong prefecture level city in Shandong
Jinfeng Hongyuan Flagship Store	Power Saving Specialist 1	It's not what it says.	Jingdong prefecture level city in Shandong
Jinfeng Hongyuan Flagship Store	Power Saving Specialist 1	I have been using it for many days now and it saves electricity, it really does I have an 8 kW electric snail stove and it saves about $3.50 a day.	Jingdong prefecture level city in Shandong
Jinfeng Hongyuan Flagship Store	Power Saving Specialist 1	Express delivery is very fast, the quality is also quite good, worth buying!	Jingdong prefecture level city in Shandong
Jinfeng Hongyuan Flagship Store	Power Saving Specialist 1	Just received expecting the baby to work well.	Jingdong prefecture level city in Shandong
Jinfeng Hongyuan Flagship Store	Power Saving Specialist 1	Very good, there are more fruit, has bought a second!	Jingdong prefecture level city in Shandong
Jinfeng Hongyuan Flagship Store	Power Saving Specialist 1	Good is good So far, no power savings have been noticed Maybe it's too little to notice.	Jingdong prefecture level city in Shandong
Jinfeng Hongyuan Flagship Store	Power Saving Specialist 1	I just received the item and plugged it in to see what happens later.	Jingdong prefecture level city in Shandong

Jinfeng Hongyuan Flagship Store	Power Saving Specialist 1	Baby received, especially good, packaging is also good, the courier brother is very strong, thank you.	Jingdong prefecture level city in Shandong
Jinfeng Hongyuan Flagship Store	Power Saving Specialist 1	Just received the goods have not yet used it, do not know how, three months later in the follow-up comment!	Jingdong prefecture level city in Shandong
Jinfeng Hongyuan Flagship Store	Power Saving Specialist 1	The item was received, it is quite good, recommended to buy.	Jingdong prefecture level city in Shandong
Jinfeng Hongyuan Flagship Store	Power Saving Specialist 1	This energy saver works very well and I recommend everyone to buy it!	Jingdong prefecture level city in Shandong
Jinfeng Hongyuan Flagship Store	Power Saving Specialist 1	Voltage stabilization and current reduction nice power saving product.	Jingdong prefecture level city in Shandong
Jinfeng Hongyuan Flagship Store	Power Saving Specialist 1	Voltage stabilization and current reduction nice power saving product.	Jingdong prefecture level city in Shandong
Jinfeng Hongyuan Flagship Store	Power Saving Specialist 1	A good power saver for stabilizing and lowering current.	Jingdong prefecture level city in Shandong
Jinfeng Hongyuan Flagship Store	Power Saving Specialist 1	You'll know if it saves power after you use it.	Jingdong prefecture level city in Shandong
Jinfeng Hongyuan Flagship Store	Power Saving Specialist 1	The quality is great. The quality is very good. The service is very good. Express delivery arrived in two days.	Jingdong prefecture level city in Shandong
Jinfeng Hongyuan Flagship Store	Power Saving Specialist 1	It works great.	Jingdong prefecture level city in Shandong
Jinfeng Hongyuan Flagship Store	Power Saving Specialist 1	Logistics is very fast, not yet used, used to comment again.	Jingdong prefecture level city in Shandong
Jinfeng Hongyuan Flagship Store	Power Saving Specialist 1	Logistics is very fast things are also very good, worthy of friends to buy	Jingdong prefecture level city in Shandong
Jinfeng Hongyuan Flagship Store	Power Saving Specialist 1	It works. It really works.	Jingdong prefecture level city in Shandong
Jinfeng Hongyuan Flagship Store	Power Saving Specialist 1	LANGUAGE COMMENTS: just received the shipment. I immediately plugged it in and used it. If you ask how effective. It depends on whether the next month's electricity bill has been reduced. If there is a decrease. I am buying more than one to use.	Jingdong prefecture level city in Shandong
Jinfeng Hongyuan Flagship Store	Power Saving Specialist 1	Well made, power saving, good and convenient!	Jingdong prefecture level city in Shandong
Jinfeng Hongyuan Flagship Store	Power Saving Specialist 1	I'm using it. I'll rate it next month.	Jingdong prefecture level city in Shandong
Jinfeng Hongyuan Flagship Store	Power Saving Specialist 1	Baby received Oh, the courier delivery really fast, with a good and then come back to the evaluation	Jingdong prefecture level city in Shandong
Jinfeng Hongyuan Flagship Store	Power Saving Specialist 1	Fast logistics, good packaging, worth it!	Jingdong prefecture level city in Shandong
Jinfeng Hongyuan Flagship Store	Power Saving Specialist 1	Customer service is okay. It's just that.	Jingdong prefecture level city in Shandong
Jinfeng Hongyuan Flagship Store	Power Saving Specialist 1	Poor power saving	Jingdong prefecture level city in Shandong
Jinfeng Hongyuan Flagship Store	Power Saving Specialist 1	I'll tell you when I've used it.	Jingdong prefecture level city in Shandong
Jinfeng Hongyuan Flagship Store	Power Saving Specialist 1	I think it's good.	Jingdong prefecture level city in Shandong
Jinfeng Hongyuan Flagship Store	Power Saving Specialist 1	This energy saver works very well and I recommend everyone to buy it!	Jingdong prefecture level city in Shandong
Jinfeng Hongyuan Flagship Store	Power Saving Specialist 1	I do not know how effective, just use half a month on the burn out, good business reissued a, and then use it after the follow-up comment.	Jingdong prefecture level city in Shandong
Jinfeng Hongyuan Flagship Store	Power Saving Specialist 1	in use	Jingdong prefecture level city in Shandong
Jinfeng Hongyuan Flagship Store	Power Saving Specialist 1	This thing is very poor. It's already only been used for ten days and it's broken. Now waiting for you to return it.	Jingdong prefecture level city in Shandong
Jinfeng Hongyuan Flagship Store	Power Saving Specialist 1	It's not the same as the seller's show.	Jingdong prefecture level city in Shandong
Jinfeng Hongyuan Flagship Store	Power Saving Specialist 1	What is the problem when I get it back from the CTS, it is plugged into the wall and the voltage shows 206v.	Jingdong prefecture level city in Shandong
Jinfeng Hongyuan Flagship Store	Power Saving Specialist 1	The logistics was fast and the stuff was perfectly packaged. It is in the process of trial.	Jingdong prefecture level city in Shandong
Jinfeng Hongyuan Flagship Store	Power Saving Specialist 1	This saver is particularly good to use can really save electricity, with two months and then look at the line if you buy one for the family	Jingdong prefecture level city in Shandong
Jinfeng Hongyuan Flagship Store	Power Saving Specialist 1	Novel appearance design, all aluminum alloy profile, also with a fuse device, voltage monitoring display. After receiving the goods, I directly cut the plug and connect it to the main power box under the leakage switch, hoping to save electricity, and then follow up later. Logistics is very fast, arrived the next	Jingdong prefecture level city in Shandong
Jinfeng Hongyuan Flagship Store	Power Saving Specialist 1	Received, very good and like, if necessary next time to buy ha ha	Jingdong prefecture level city in Shandong
Jinfeng Hongyuan Flagship Store	Power Saving Specialist 1	It's good. It's worth it. Get it if you need it.	Jingdong prefecture level city in Shandong
Jinfeng Hongyuan Flagship Store	Power Saving Specialist 1	It feels more than good and is worth having.	Jingdong prefecture level city in Shandong
Jinfeng Hongyuan Flagship Store	Power Saving Specialist 1	The goods arrived very good and fast first time to buy	Jingdong prefecture level city in Shandong
Jinfeng Hongyuan Flagship Store	Power Saving Specialist 1	The power saving is very good, I can save so much money every month, I will buy another one next time.	Jingdong prefecture level city in Shandong
Jinfeng Hongyuan Flagship Store	Power Saving Specialist 1	It's not what it says.	Jingdong prefecture level city in Shandong
Jinfeng Hongyuan Flagship Store	Power Saving Specialist 1	I have been using it for many days now and it saves electricity, it really does I have an 8 kW electric snail stove and it saves about $3.50 a day.	Jingdong prefecture level city in Shandong
Jinfeng Hongyuan Flagship Store	Power Saving Specialist 1	Express delivery is very fast, the quality is also quite good, worth buying!	Jingdong prefecture level city in Shandong
Jinfeng Hongyuan Flagship Store	Power Saving Specialist 1	Just received expecting the baby to work well.	Jingdong prefecture level city in Shandong
Jinfeng Hongyuan Flagship Store	Power Saving Specialist 1	Very good, there are more fruit, has bought a second!	Jingdong prefecture level city in Shandong
Jinfeng Hongyuan Flagship Store	Power Saving Specialist 1	Good is good So far, no power savings have been noticed Maybe it's too little to notice.	Jingdong prefecture level city in Shandong
Jinfeng Hongyuan Flagship Store	Power Saving Specialist 1	I just received the item and plugged it in to see what happens later.	Jingdong prefecture level city in Shandong
Jinfeng Hongyuan Flagship Store	Power Saving Specialist 1	Baby received, especially good, packaging is also good, the courier brother is very strong, thank you.	Jingdong prefecture level city in Shandong
Jinfeng Hongyuan Flagship Store	Power Saving Specialist 1	Just received the goods have not yet used it, do not know how, three months later in the follow-up comment!	Jingdong prefecture level city in Shandong
Jinfeng Hongyuan Flagship Store	Power Saving Specialist 1	The item was received, it is quite good, recommended to buy.	Jingdong prefecture level city in Shandong
Jinfeng Hongyuan Flagship Store	Power Saving Specialist 1	This energy saver works very well and I recommend everyone to buy it!	Jingdong prefecture level city in Shandong
Jinfeng Hongyuan Flagship Store	Power Saving Specialist 1	Voltage stabilization and current reduction nice power saving product.	Jingdong prefecture level city in Shandong
Jinfeng Hongyuan Flagship Store	Power Saving Specialist 1	Voltage stabilization and current reduction nice power saving product.	Jingdong prefecture level city in Shandong
Jinfeng Hongyuan Flagship Store	Power Saving Specialist 1	A good power saver for stabilizing and lowering current.	Jingdong prefecture level city in Shandong

Jinfeng Hongyuan Flagship Store	Power Saving Specialist 1	You'll know if it saves power after you use it.	Jingdong prefecture level city in Shandong
Jinfeng Hongyuan Flagship Store	Power Saving Specialist 1	The quality is great. The quality is very good. The service is very good. Express delivery arrived in two days.	Jingdong prefecture level city in Shandong
Jinfeng Hongyuan Flagship Store	Power Saving Specialist 1	It works great.	Jingdong prefecture level city in Shandong
Jinfeng Hongyuan Flagship Store	Power Saving Specialist 1	Logistics is very fast, not yet used, used to comment again.	Jingdong prefecture level city in Shandong
Jinfeng Hongyuan Flagship Store	Power Saving Specialist 1	Logistics is very fast things are also very good, worthy of friends to buy	Jingdong prefecture level city in Shandong
Jinfeng Hongyuan Flagship Store	Power Saving Specialist 1	It works. It really works.	Jingdong prefecture level city in Shandong
Jinfeng Hongyuan Flagship Store	Power Saving Specialist 1	LANGUAGE COMMENTS: just received the shipment. I immediately plugged it in and used it. If you ask how effective. It depends on whether the next month's electricity bill has been reduced. If there is a decrease. I am buying more than one to use.	Jingdong prefecture level city in Shandong
Jinfeng Hongyuan Flagship Store	Power Saving Specialist 1	Well made, power saving, good and convenient!	Jingdong prefecture level city in Shandong
Jinfeng Hongyuan Flagship Store	Power Saving Specialist 1	I'm using it. I'll rate it next month.	Jingdong prefecture level city in Shandong
Jinfeng Hongyuan Flagship Store	Power Saving Specialist 1	Baby received Oh, the courier delivery really fast, with a good and then come back to the evaluation	Jingdong prefecture level city in Shandong
Jinfeng Hongyuan Flagship Store	Power Saving Specialist 1	Fast logistics, good packaging, worth it!	Jingdong prefecture level city in Shandong
Jinfeng Hongyuan Flagship Store	Power Saving Specialist 1	Customer service is okay. It's just that.	Jingdong prefecture level city in Shandong
Jinfeng Hongyuan Flagship Store	Power Saving Specialist 1	Poor power saving	Jingdong prefecture level city in Shandong
Jinfeng Hongyuan Flagship Store	Power Saving Specialist 1	I'll tell you when I've used it.	Jingdong prefecture level city in Shandong
Jinfeng Hongyuan Flagship Store	Power Saving Specialist 1	I think it's good.	Jingdong prefecture level city in Shandong
Jinfeng Hongyuan Flagship Store	Power Saving Specialist 1	This energy saver works very well and I recommend everyone to buy it!	Jingdong prefecture level city in Shandong
Jinfeng Hongyuan Flagship Store	Power Saving Specialist 1	I do not know how effective, just use half a month on the burn out, good business reissued a, and then use it after the follow-up comment.	Jingdong prefecture level city in Shandong
Jinfeng Hongyuan Flagship Store	Power Saving Specialist 1	in use	Jingdong prefecture level city in Shandong
Jinfeng Hongyuan Flagship Store	Power Saving Specialist 1	This thing is very poor. It's already only been used for ten days and it's broken. Now waiting for you to return it.	Jingdong prefecture level city in Shandong
Jinfeng Hongyuan Flagship Store	Power Saving Specialist 1	It's not the same as the seller's show.	Jingdong prefecture level city in Shandong
Jinfeng Hongyuan Flagship Store	Power Saving Specialist 1	What is the problem when I get it back from the CTS, it is plugged into the wall and the voltage shows 206v.	Jingdong prefecture level city in Shandong
Jinfeng Hongyuan Flagship Store	Power Saving Specialist 1	The logistics was fast and the stuff was perfectly packaged. It is in the process of trial.	Jingdong prefecture level city in Shandong
Jinfeng Hongyuan Flagship Store	Power Saving Specialist 1	This saver is particularly good to use can really save electricity, with two months and then look at the line if you buy one for the family	Jingdong prefecture level city in Shandong
Jinfeng Hongyuan Flagship Store	Power Saving Specialist 1	Novel appearance design, all aluminum alloy profile, also with a fuse device, voltage monitoring display. After receiving the goods, I directly cut the plug and connect it to the main power box under the leakage switch, hoping to save electricity, and then follow up later. Logistics is very fast, arrived the next	Jingdong prefecture level city in Shandong
Jinfeng Hongyuan Flagship Store	Power Saving Specialist 1	Received, very good and like, if necessary next time to buy ha ha	Jingdong prefecture level city in Shandong
Jinfeng Hongyuan Flagship Store	Power Saving Specialist 1	It's good. It's worth it. Get it if you need it.	Jingdong prefecture level city in Shandong
Jinfeng Hongyuan Flagship Store	Power Saving Specialist 1	It feels more than good and is worth having.	Jingdong prefecture level city in Shandong
Jinfeng Hongyuan Flagship Store	Power Saving Specialist 1	The goods arrived very good and fast first time to buy	Jingdong prefecture level city in Shandong
Jinfeng Hongyuan Flagship Store	Power Saving Specialist 1	The power saving is very good, I can save so much money every month, I will buy another one next time.	Jingdong prefecture level city in Shandong
Jinfeng Hongyuan Flagship Store	Power Saving Specialist 1	It's not what it says.	Jingdong prefecture level city in Shandong
Jinfeng Hongyuan Flagship Store	Power Saving Specialist 1	I have been using it for many days now and it saves electricity, it really does I have an 8 kW electric snail stove and it saves about $3.50 a day.	Jingdong prefecture level city in Shandong
Jinfeng Hongyuan Flagship Store	Power Saving Specialist 1	Express delivery is very fast, the quality is also quite good, worth buying!	Jingdong prefecture level city in Shandong
Jinfeng Hongyuan Flagship Store	Power Saving Specialist 1	Just received expecting the baby to work well.	Jingdong prefecture level city in Shandong
Jinfeng Hongyuan Flagship Store	Power Saving Specialist 1	Very good, there are more fruit, has bought a second!	Jingdong prefecture level city in Shandong
Jinfeng Hongyuan Flagship Store	Power Saving Specialist 1	Good is good So far, no power savings have been noticed Maybe it's too little to notice.	Jingdong prefecture level city in Shandong
Jinfeng Hongyuan Flagship Store	Power Saving Specialist 1	I just received the item and plugged it in to see what happens later.	Jingdong prefecture level city in Shandong
Jinfeng Hongyuan Flagship Store	Power Saving Specialist 1	Baby received, especially good, packaging is also good, the courier brother is very strong, thank you.	Jingdong prefecture level city in Shandong
Jinfeng Hongyuan Flagship Store	Power Saving Specialist 1	Just received the goods have not yet used it, do not know how, three months later in the follow-up comment!	Jingdong prefecture level city in Shandong
Jinfeng Hongyuan Flagship Store	Power Saving Specialist 1	The item was received, it is quite good, recommended to buy.	Jingdong prefecture level city in Shandong
Jinfeng Hongyuan Flagship Store	Power Saving Specialist 1	This energy saver works very well and I recommend everyone to buy it!	Jingdong prefecture level city in Shandong
Jinfeng Hongyuan Flagship Store	Power Saving Specialist 1	Voltage stabilization and current reduction nice power saving product.	Jingdong prefecture level city in Shandong
Jinfeng Hongyuan Flagship Store	Power Saving Specialist 1	Voltage stabilization and current reduction nice power saving product.	Jingdong prefecture level city in Shandong
Jinfeng Hongyuan Flagship Store	Power Saving Specialist 1	A good power saver for stabilizing and lowering current.	Jingdong prefecture level city in Shandong
Jinfeng Hongyuan Flagship Store	Power Saving Specialist 1	You'll know if it saves power after you use it.	Jingdong prefecture level city in Shandong
Jinfeng Hongyuan Flagship Store	Power Saving Specialist 1	The quality is great. The quality is very good. The service is very good. Express delivery arrived in two days.	Jingdong prefecture level city in Shandong
Jinfeng Hongyuan Flagship Store	Power Saving Specialist 1	It works great.	Jingdong prefecture level city in Shandong
Jinfeng Hongyuan Flagship Store	Power Saving Specialist 1	Logistics is very fast, not yet used, used to comment again.	Jingdong prefecture level city in Shandong
Jinfeng Hongyuan Flagship Store	Power Saving Specialist 1	Logistics is very fast things are also very good, worthy of friends to buy	Jingdong prefecture level city in Shandong
Jinfeng Hongyuan Flagship Store	Power Saving Specialist 1	It works. It really works.	Jingdong prefecture level city in Shandong
Jinfeng Hongyuan Flagship Store	Power Saving Specialist 1	LANGUAGE COMMENTS: just received the shipment. I immediately plugged it in and used it. If you ask how effective. It depends on whether the next month's electricity bill has been reduced. If there is a decrease. I am buying more than one to use.	Jingdong prefecture level city in Shandong

Jinfeng Hongyuan Flagship Store	Power Saving Specialist 1	Well made, power saving, good and convenient!	Jingdong prefecture level city in Shandong
Jinfeng Hongyuan Flagship Store	Power Saving Specialist 1	I'm using it. I'll rate it next month.	Jingdong prefecture level city in Shandong
Jinfeng Hongyuan Flagship Store	Power Saving Specialist 1	Baby received Oh, the courier delivery really fast, with a good and then come back to the evaluation	Jingdong prefecture level city in Shandong
Jinfeng Hongyuan Flagship Store	Power Saving Specialist 1	Fast logistics, good packaging, worth it!	Jingdong prefecture level city in Shandong
Jinfeng Hongyuan Flagship Store	Power Saving Specialist 1	Customer service is okay. It's just that.	Jingdong prefecture level city in Shandong
Jinfeng Hongyuan Flagship Store	Power Saving Specialist 1	Poor power saving	Jingdong prefecture level city in Shandong
Jinfeng Hongyuan Flagship Store	Power Saving Specialist 1	I'll tell you when I've used it.	Jingdong prefecture level city in Shandong
Jinfeng Hongyuan Flagship Store	Power Saving Specialist 1	I think it's good.	Jingdong prefecture level city in Shandong
Jinfeng Hongyuan Flagship Store	Power Saving Specialist 1	This energy saver works very well and I recommend everyone to buy it!	Jingdong prefecture level city in Shandong
Jinfeng Hongyuan Flagship Store	Power Saving Specialist 1	I do not know how effective, just use half a month on the burn out, good business reissued a, and then use it after the follow-up comment.	Jingdong prefecture level city in Shandong
Jinfeng Hongyuan Flagship Store	Power Saving Specialist 1	in use	Jingdong prefecture level city in Shandong
Jinfeng Hongyuan Flagship Store	Power Saving Specialist 1	This thing is very poor. It's already only been used for ten days and it's broken. Now waiting for you to return it.	Jingdong prefecture level city in Shandong
Jinfeng Hongyuan Flagship Store	Power Saving Specialist 1	It's not the same as the seller's show.	Jingdong prefecture level city in Shandong
Jinfeng Hongyuan Flagship Store	Power Saving Specialist 1	What is the problem when I get it back from the CTS, it is plugged into the wall and the voltage shows 206v.	Jingdong prefecture level city in Shandong
Jinfeng Hongyuan Flagship Store	Power Saving Specialist 1	The logistics was fast and the stuff was perfectly packaged. It is in the process of trial.	Jingdong prefecture level city in Shandong
Jinfeng Hongyuan Flagship Store	Power Saving Specialist 1	This saver is particularly good to use can really save electricity, with two months and then look at the line if you buy one for the family	Jingdong prefecture level city in Shandong
Jinfeng Hongyuan Flagship Store	Power Saving Specialist 1	Novel appearance design, all aluminum alloy profile, also with a fuse device, voltage monitoring display. After receiving the goods, I directly cut the plug and connect it to the main power box under the leakage switch, hoping to save electricity, and then follow up later. Logistics is very fast, arrived the next	Jingdong prefecture level city in Shandong
Jinfeng Hongyuan Flagship Store	Power Saving Specialist 1	Received, very good and like, if necessary next time to buy ha ha	Jingdong prefecture level city in Shandong
Jinfeng Hongyuan Flagship Store	Power Saving Specialist 1	It's good. It's worth it. Get it if you need it.	Jingdong prefecture level city in Shandong
Jinfeng Hongyuan Flagship Store	Power Saving Specialist 1	It feels more than good and is worth having.	Jingdong prefecture level city in Shandong
Jinfeng Hongyuan Flagship Store	Power Saving Specialist 1	The goods arrived very good and fast first time to buy	Jingdong prefecture level city in Shandong
Jinfeng Hongyuan Flagship Store	Power Saving Specialist 1	The power saving is very good, I can save so much money every month, I will buy another one next time.	Jingdong prefecture level city in Shandong
Jinfeng Hongyuan Flagship Store	Power Saving Specialist 1	It's not what it says.	Jingdong prefecture level city in Shandong
Jinfeng Hongyuan Flagship Store	Power Saving Specialist 1	I have been using it for many days now and it saves electricity, it really does I have an 8 kW electric snail stove and it saves about $3.50 a day.	Jingdong prefecture level city in Shandong
Jinfeng Hongyuan Flagship Store	Power Saving Specialist 1	Express delivery is very fast, the quality is also quite good, worth buying!	Jingdong prefecture level city in Shandong
Jinfeng Hongyuan Flagship Store	Power Saving Specialist 1	Just received expecting the baby to work well.	Jingdong prefecture level city in Shandong
Jinfeng Hongyuan Flagship Store	Power Saving Specialist 1	Very good, there are more fruit, has bought a second!	Jingdong prefecture level city in Shandong
Jinfeng Hongyuan Flagship Store	Power Saving Specialist 1	Good is good So far, no power savings have been noticed Maybe it's too little to notice.	Jingdong prefecture level city in Shandong
Jinfeng Hongyuan Flagship Store	Power Saving Specialist 1	I just received the item and plugged it in to see what happens later.	Jingdong prefecture level city in Shandong
Jinfeng Hongyuan Flagship Store	Power Saving Specialist 1	Baby received, especially good, packaging is also good, the courier brother is very strong, thank you.	Jingdong prefecture level city in Shandong
Jinfeng Hongyuan Flagship Store	Power Saving Specialist 1	Just received the goods have not yet used it, do not know how, three months later in the follow-up comment!	Jingdong prefecture level city in Shandong
Jinfeng Hongyuan Flagship Store	Power Saving Specialist 1	The item was received, it is quite good, recommended to buy.	Jingdong prefecture level city in Shandong
Jinfeng Hongyuan Flagship Store	Power Saving Specialist 1	This energy saver works very well and I recommend everyone to buy it!	Jingdong prefecture level city in Shandong
Jinfeng Hongyuan Flagship Store	Power Saving Specialist 1	Voltage stabilization and current reduction nice power saving product.	Jingdong prefecture level city in Shandong
Jinfeng Hongyuan Flagship Store	Power Saving Specialist 1	Voltage stabilization and current reduction nice power saving product.	Jingdong prefecture level city in Shandong
Jinfeng Hongyuan Flagship Store	Power Saving Specialist 1	A good power saver for stabilizing and lowering current.	Jingdong prefecture level city in Shandong
Jinfeng Hongyuan Flagship Store	Power Saving Specialist 1	You'll know if it saves power after you use it.	Jingdong prefecture level city in Shandong
Jinfeng Hongyuan Flagship Store	Power Saving Specialist 1	The quality is great. The quality is very good. The service is very good. Express delivery arrived in two days.	Jingdong prefecture level city in Shandong
Jinfeng Hongyuan Flagship Store	Power Saving Specialist 1	It works great.	Jingdong prefecture level city in Shandong
Jinfeng Hongyuan Flagship Store	Power Saving Specialist 1	Logistics is very fast, not yet used, used to comment again.	Jingdong prefecture level city in Shandong
Jinfeng Hongyuan Flagship Store	Power Saving Specialist 1	Logistics is very fast things are also very good, worthy of friends to buy	Jingdong prefecture level city in Shandong
Jinfeng Hongyuan Flagship Store	Power Saving Specialist 1	It works. It really works.	Jingdong prefecture level city in Shandong
Jinfeng Hongyuan Flagship Store	Power Saving Specialist 1	LANGUAGE COMMENTS: just received the shipment. I immediately plugged it in and used it. If you ask how effective. It depends on whether the next month's electricity bill has been reduced. If there is a decrease. I am buying more than one to use.	Jingdong prefecture level city in Shandong
Jinfeng Hongyuan Flagship Store	Power Saving Specialist 1	Well made, power saving, good and convenient!	Jingdong prefecture level city in Shandong
Jinfeng Hongyuan Flagship Store	Power Saving Specialist 1	I'm using it. I'll rate it next month.	Jingdong prefecture level city in Shandong
Jinfeng Hongyuan Flagship Store	Power Saving Specialist 1	Baby received Oh, the courier delivery really fast, with a good and then come back to the evaluation	Jingdong prefecture level city in Shandong
Jinfeng Hongyuan Flagship Store	Power Saving Specialist 1	Fast logistics, good packaging, worth it!	Jingdong prefecture level city in Shandong
Jinfeng Hongyuan Flagship Store	Power Saving Specialist 1	Customer service is okay. It's just that.	Jingdong prefecture level city in Shandong
Jinfeng Hongyuan Flagship Store	Power Saving Specialist 1	Poor power saving	Jingdong prefecture level city in Shandong
Jinfeng Hongyuan Flagship Store	Power Saving Specialist 1	I'll tell you when I've used it.	Jingdong prefecture level city in Shandong

Jinfeng Hongyuan Flagship Store	Power Saving Specialist 1	I think it's good.	Jingdong prefecture level city in Shandong
Jinfeng Hongyuan Flagship Store	Power Saving Specialist 1	This energy saver works very well and I recommend everyone to buy it!	Jingdong prefecture level city in Shandong
Jinfeng Hongyuan Flagship Store	Power Saving Specialist 1	I do not know how effective, just use half a month on the burn out, good business reissued a, and then use it after the follow-up comment.	Jingdong prefecture level city in Shandong
Jinfeng Hongyuan Flagship Store	Power Saving Specialist 1	in use	Jingdong prefecture level city in Shandong
Jinfeng Hongyuan Flagship Store	Power Saving Specialist 1	This thing is very poor. It's already only been used for ten days and it's broken. Now waiting for you to return it.	Jingdong prefecture level city in Shandong
Jinfeng Hongyuan Flagship Store	Power Saving Specialist 1	It's not the same as the seller's show.	Jingdong prefecture level city in Shandong
Jinfeng Hongyuan Flagship Store	Power Saving Specialist 1	What is the problem when I get it back from the CTS, it is plugged into the wall and the voltage shows 206v.	Jingdong prefecture level city in Shandong
Jinfeng Hongyuan Flagship Store	Power Saving Specialist 1	The logistics was fast and the stuff was perfectly packaged. It is in the process of trial.	Jingdong prefecture level city in Shandong
Jinfeng Hongyuan Flagship Store	Power Saving Specialist 1	This saver is particularly good to use can really save electricity, with two months and then look at the line if you buy one for the family	Jingdong prefecture level city in Shandong
Jinfeng Hongyuan Flagship Store	Power Saving Specialist 1	Novel appearance design, all aluminum alloy profile, also with a fuse device, voltage monitoring display. After receiving the goods, I directly cut the plug and connect it to the main power box under the leakage switch, hoping to save electricity, and then follow up later. Logistics is very fast, arrived the next	Jingdong prefecture level city in Shandong
Jinfeng Hongyuan Flagship Store	Power Saving Specialist 1	Received, very good and like, if necessary next time to buy ha ha	Jingdong prefecture level city in Shandong
Jinfeng Hongyuan Flagship Store	Power Saving Specialist 1	It's good. It's worth it. Get it if you need it.	Jingdong prefecture level city in Shandong
Jinfeng Hongyuan Flagship Store	Power Saving Specialist 1	It feels more than good and is worth having.	Jingdong prefecture level city in Shandong
Jinfeng Hongyuan Flagship Store	Power Saving Specialist 1	The goods arrived very good and fast first time to buy	Jingdong prefecture level city in Shandong
Jinfeng Hongyuan Flagship Store	Power Saving Specialist 1	The power saving is very good, I can save so much money every month, I will buy another one next time.	Jingdong prefecture level city in Shandong
Jinfeng Hongyuan Flagship Store	Power Saving Specialist 1	It's not what it says.	Jingdong prefecture level city in Shandong
Jinfeng Hongyuan Flagship Store	Power Saving Specialist 1	I have been using it for many days now and it saves electricity, it really does I have an 8 kW electric snail stove and it saves about $3.50 a day.	Jingdong prefecture level city in Shandong
Jinfeng Hongyuan Flagship Store	Power Saving Specialist 1	Express delivery is very fast, the quality is also quite good, worth buying!	Jingdong prefecture level city in Shandong
Jinfeng Hongyuan Flagship Store	Power Saving Specialist 1	Just received expecting the baby to work well.	Jingdong prefecture level city in Shandong
Jinfeng Hongyuan Flagship Store	Power Saving Specialist 1	Very good, there are more fruit, has bought a second!	Jingdong prefecture level city in Shandong
Jinfeng Hongyuan Flagship Store	Power Saving Specialist 1	Good is good So far, no power savings have been noticed Maybe it's too little to notice.	Jingdong prefecture level city in Shandong
Jinfeng Hongyuan Flagship Store	Power Saving Specialist 1	I just received the item and plugged it in to see what happens later.	Jingdong prefecture level city in Shandong
Jinfeng Hongyuan Flagship Store	Power Saving Specialist 1	Baby received, especially good, packaging is also good, the courier brother is very strong, thank you.	Jingdong prefecture level city in Shandong
Jinfeng Hongyuan Flagship Store	Power Saving Specialist 1	Just received the goods have not yet used it, do not know how, three months later in the follow-up comment!	Jingdong prefecture level city in Shandong
Jinfeng Hongyuan Flagship Store	Power Saving Specialist 1	The item was received, it is quite good, recommended to buy.	Jingdong prefecture level city in Shandong
Jinfeng Hongyuan Flagship Store	Power Saving Specialist 1	This energy saver works very well and I recommend everyone to buy it!	Jingdong prefecture level city in Shandong
Jinfeng Hongyuan Flagship Store	Power Saving Specialist 1	Voltage stabilization and current reduction nice power saving product.	Jingdong prefecture level city in Shandong
Jinfeng Hongyuan Flagship Store	Power Saving Specialist 1	Voltage stabilization and current reduction nice power saving product.	Jingdong prefecture level city in Shandong
Jinfeng Hongyuan Flagship Store	Power Saving Specialist 1	A good power saver for stabilizing and lowering current.	Jingdong prefecture level city in Shandong
Jinfeng Hongyuan Flagship Store	Power Saving Specialist 1	You'll know if it saves power after you use it.	Jingdong prefecture level city in Shandong
Jinfeng Hongyuan Flagship Store	Power Saving Specialist 1	The quality is great. The quality is very good. The service is very good. Express delivery arrived in two days.	Jingdong prefecture level city in Shandong
Jinfeng Hongyuan Flagship Store	Power Saving Specialist 1	It works great.	Jingdong prefecture level city in Shandong
Jinfeng Hongyuan Flagship Store	Power Saving Specialist 1	Logistics is very fast, not yet used, used to comment again.	Jingdong prefecture level city in Shandong
Jinfeng Hongyuan Flagship Store	Power Saving Specialist 1	Logistics is very fast things are also very good, worthy of friends to buy	Jingdong prefecture level city in Shandong
Jinfeng Hongyuan Flagship Store	Power Saving Specialist 1	It works. It really works.	Jingdong prefecture level city in Shandong
Jinfeng Hongyuan Flagship Store	Power Saving Specialist 1	LANGUAGE COMMENTS: just received the shipment. I immediately plugged it in and used it. If you ask how effective. It depends on whether the next month's electricity bill has been reduced. If there is a decrease. I am buying more than one to use.	Jingdong prefecture level city in Shandong
Jinfeng Hongyuan Flagship Store	Power Saving Specialist 1	Well made, power saving, good and convenient!	Jingdong prefecture level city in Shandong
Jinfeng Hongyuan Flagship Store	Power Saving Specialist 1	I'm using it. I'll rate it next month.	Jingdong prefecture level city in Shandong
Jinfeng Hongyuan Flagship Store	Power Saving Specialist 1	Baby received Oh, the courier delivery really fast, with a good and then come back to the evaluation	Jingdong prefecture level city in Shandong
Jinfeng Hongyuan Flagship Store	Power Saving Specialist 1	Fast logistics, good packaging, worth it!	Jingdong prefecture level city in Shandong
Jinfeng Hongyuan Flagship Store	Power Saving Specialist 1	Customer service is okay. It's just that.	Jingdong prefecture level city in Shandong
Jinfeng Hongyuan Flagship Store	Power Saving Specialist 1	Poor power saving	Jingdong prefecture level city in Shandong
Jinfeng Hongyuan Flagship Store	Power Saving Specialist 1	I'll tell you when I've used it.	Jingdong prefecture level city in Shandong
Jinfeng Hongyuan Flagship Store	Power Saving Specialist 1	I think it's good.	Jingdong prefecture level city in Shandong
Jinfeng Hongyuan Flagship Store	Power Saving Specialist 1	This energy saver works very well and I recommend everyone to buy it!	Jingdong prefecture level city in Shandong
Jinfeng Hongyuan Flagship Store	Power Saving Specialist 1	I do not know how effective, just use half a month on the burn out, good business reissued a, and then use it after the follow-up comment.	Jingdong prefecture level city in Shandong
Jinfeng Hongyuan Flagship Store	Power Saving Specialist 1	in use	Jingdong prefecture level city in Shandong
Jinfeng Hongyuan Flagship Store	Power Saving Specialist 1	This thing is very poor. It's already only been used for ten days and it's broken. Now waiting for you to return it.	Jingdong prefecture level city in Shandong
Jinfeng Hongyuan Flagship Store	Power Saving Specialist 1	It's not the same as the seller's show.	Jingdong prefecture level city in Shandong
Jinfeng Hongyuan Flagship Store	Power Saving Specialist 1	What is the problem when I get it back from the CTS, it is plugged into the wall and the voltage shows 206v.	Jingdong prefecture level city in Shandong

Jinfeng Hongyuan Flagship Store	Power Saving Specialist 1	The logistics was fast and the stuff was perfectly packaged. It is in the process of trial.	Jingdong prefecture level city in Shandong
Jinfeng Hongyuan Flagship Store	Power Saving Specialist 1	This saver is particularly good to use can really save electricity, with two months and then look at the line if you buy one for the family	Jingdong prefecture level city in Shandong
Jinfeng Hongyuan Flagship Store	Power Saving Specialist 1	Novel appearance design, all aluminum alloy profile, also with a fuse device, voltage monitoring display. After receiving the goods, I directly cut the plug and connect it to the main power box under the leakage switch, hoping to save electricity, and then follow up later. Logistics is very fast, arrived the next	Jingdong prefecture level city in Shandong
Jinfeng Hongyuan Flagship Store	Power Saving Specialist 1	Received, very good and like, if necessary next time to buy ha ha	Jingdong prefecture level city in Shandong
Jinfeng Hongyuan Flagship Store	Power Saving Specialist 1	It's good. It's worth it. Get it if you need it.	Jingdong prefecture level city in Shandong
Jinfeng Hongyuan Flagship Store	Power Saving Specialist 1	It feels more than good and is worth having.	Jingdong prefecture level city in Shandong
Jinfeng Hongyuan Flagship Store	Power Saving Specialist 1	The goods arrived very good and fast first time to buy	Jingdong prefecture level city in Shandong
Jinfeng Hongyuan Flagship Store	Power Saving Specialist 1	The power saving is very good, I can save so much money every month, I will buy another one next time.	Jingdong prefecture level city in Shandong
Jinfeng Hongyuan Flagship Store	Power Saving Specialist 1	It's not what it says.	Jingdong prefecture level city in Shandong
Jinfeng Hongyuan Flagship Store	Power Saving Specialist 1	I have been using it for many days now and it saves electricity, it really does I have an 8 kW electric snail stove and it saves about $3.50 a day.	Jingdong prefecture level city in Shandong
Jinfeng Hongyuan Flagship Store	Power Saving Specialist 1	Express delivery is very fast, the quality is also quite good, worth buying!	Jingdong prefecture level city in Shandong
Jinfeng Hongyuan Flagship Store	Power Saving Specialist 1	Just received expecting the baby to work well.	Jingdong prefecture level city in Shandong
Jinfeng Hongyuan Flagship Store	Power Saving Specialist 1	Very good, there are more fruit, has bought a second!	Jingdong prefecture level city in Shandong
Jinfeng Hongyuan Flagship Store	Power Saving Specialist 1	Good is good So far, no power savings have been noticed Maybe it's too little to notice.	Jingdong prefecture level city in Shandong
Jinfeng Hongyuan Flagship Store	Power Saving Specialist 1	I just received the item and plugged it in to see what happens later.	Jingdong prefecture level city in Shandong
Jinfeng Hongyuan Flagship Store	Power Saving Specialist 1	Baby received, especially good, packaging is also good, the courier brother is very strong, thank you.	Jingdong prefecture level city in Shandong
Jinfeng Hongyuan Flagship Store	Power Saving Specialist 1	Just received the goods have not yet used it, do not know how, three months later in the follow-up comment!	Jingdong prefecture level city in Shandong
Jinfeng Hongyuan Flagship Store	Power Saving Specialist 1	The item was received, it is quite good, recommended to buy.	Jingdong prefecture level city in Shandong
Jinfeng Hongyuan Flagship Store	Power Saving Specialist 1	This energy saver works very well and I recommend everyone to buy it!	Jingdong prefecture level city in Shandong
Jinfeng Hongyuan Flagship Store	Power Saving Specialist 1	Voltage stabilization and current reduction nice power saving product.	Jingdong prefecture level city in Shandong
Jinfeng Hongyuan Flagship Store	Power Saving Specialist 1	Voltage stabilization and current reduction nice power saving product.	Jingdong prefecture level city in Shandong
Jinfeng Hongyuan Flagship Store	Power Saving Specialist 1	A good power saver for stabilizing and lowering current.	Jingdong prefecture level city in Shandong
Jinfeng Hongyuan Flagship Store	Power Saving Specialist 1	You'll know if it saves power after you use it.	Jingdong prefecture level city in Shandong
Jinfeng Hongyuan Flagship Store	Power Saving Specialist 1	The quality is great. The quality is very good. The service is very good. Express delivery arrived in two days.	Jingdong prefecture level city in Shandong
Jinfeng Hongyuan Flagship Store	Power Saving Specialist 1	It works great.	Jingdong prefecture level city in Shandong
Jinfeng Hongyuan Flagship Store	Power Saving Specialist 1	Logistics is very fast, not yet used, used to comment again.	Jingdong prefecture level city in Shandong
Jinfeng Hongyuan Flagship Store	Power Saving Specialist 1	Logistics is very fast things are also very good, worthy of friends to buy	Jingdong prefecture level city in Shandong
Jinfeng Hongyuan Flagship Store	Power Saving Specialist 1	It works. It really works.	Jingdong prefecture level city in Shandong
Jinfeng Hongyuan Flagship Store	Power Saving Specialist 1	LANGUAGE COMMENTS: just received the shipment. I immediately plugged it in and used it. If you ask how effective. It depends on whether the next month's electricity bill has been reduced. If there is a decrease. I am buying more than one to use.	Jingdong prefecture level city in Shandong
Jinfeng Hongyuan Flagship Store	Power Saving Specialist 1	Well made, power saving, good and convenient!	Jingdong prefecture level city in Shandong
Jinfeng Hongyuan Flagship Store	Power Saving Specialist 1	I'm using it. I'll rate it next month.	Jingdong prefecture level city in Shandong
Jinfeng Hongyuan Flagship Store	Power Saving Specialist 1	Baby received Oh, the courier delivery really fast, with a good and then come back to the evaluation	Jingdong prefecture level city in Shandong
Jinfeng Hongyuan Flagship Store	Power Saving Specialist 1	Fast logistics, good packaging, worth it!	Jingdong prefecture level city in Shandong
Jinfeng Hongyuan Flagship Store	Power Saving Specialist 1	Customer service is okay. It's just that.	Jingdong prefecture level city in Shandong
Jinfeng Hongyuan Flagship Store	Power Saving Specialist 1	Poor power saving	Jingdong prefecture level city in Shandong
Jinfeng Hongyuan Flagship Store	Power Saving Specialist 1	I'll tell you when I've used it.	Jingdong prefecture level city in Shandong
Jinfeng Hongyuan Flagship Store	Power Saving Specialist 1	I think it's good.	Jingdong prefecture level city in Shandong
Jinfeng Hongyuan Flagship Store	Power Saving Specialist 1	This energy saver works very well and I recommend everyone to buy it!	Jingdong prefecture level city in Shandong
Jinfeng Hongyuan Flagship Store	Power Saving Specialist 1	I do not know how effective, just use half a month on the burn out, good business reissued a, and then use it after the follow-up comment.	Jingdong prefecture level city in Shandong
Jinfeng Hongyuan Flagship Store	Power Saving Specialist 1	in use	Jingdong prefecture level city in Shandong
Jinfeng Hongyuan Flagship Store	Power Saving Specialist 1	This thing is very poor. It's already only been used for ten days and it's broken. Now waiting for you to return it.	Jingdong prefecture level city in Shandong
Jinfeng Hongyuan Flagship Store	Power Saving Specialist 1	It's not the same as the seller's show.	Jingdong prefecture level city in Shandong
Jinfeng Hongyuan Flagship Store	Power Saving Specialist 1	What is the problem when I get it back from the CTS, it is plugged into the wall and the voltage shows 206v.	Jingdong prefecture level city in Shandong
Jinfeng Hongyuan Flagship Store	Power Saving Specialist 1	The logistics was fast and the stuff was perfectly packaged. It is in the process of trial.	Jingdong prefecture level city in Shandong
Jinfeng Hongyuan Flagship Store	Power Saving Specialist 1	This saver is particularly good to use can really save electricity, with two months and then look at the line if you buy one for the family	Jingdong prefecture level city in Shandong
Jinfeng Hongyuan Flagship Store	Power Saving Specialist 1	Novel appearance design, all aluminum alloy profile, also with a fuse device, voltage monitoring display. After receiving the goods, I directly cut the plug and connect it to the main power box under the leakage switch, hoping to save electricity, and then follow up later. Logistics is very fast, arrived the next	Jingdong prefecture level city in Shandong
Jinfeng Hongyuan Flagship Store	Power Saving Specialist 1	Received, very good and like, if necessary next time to buy ha ha	Jingdong prefecture level city in Shandong
Jinfeng Hongyuan Flagship Store	Power Saving Specialist 1	It's good. It's worth it. Get it if you need it.	Jingdong prefecture level city in Shandong
Jinfeng Hongyuan Flagship Store	Power Saving Specialist 1	It feels more than good and is worth having.	Jingdong prefecture level city in Shandong
Jinfeng Hongyuan Flagship Store	Power Saving Specialist 1	The goods arrived very good and fast first time to buy	Jingdong prefecture level city in Shandong

Jinfeng Hongyuan Flagship Store	Power Saving Specialist 1	The power saving is very good, I can save so much money every month, I will buy another one next time.	Jingdong prefecture level city in Shandong
Jinfeng Hongyuan Flagship Store	Power Saving Specialist 1	It's not what it says.	Jingdong prefecture level city in Shandong
Jinfeng Hongyuan Flagship Store	Power Saving Specialist 1	I have been using it for many days now and it saves electricity, it really does I have an 8 kW electric snail stove and it saves about $3.50 a day.	Jingdong prefecture level city in Shandong
Jinfeng Hongyuan Flagship Store	Power Saving Specialist 1	Express delivery is very fast, the quality is also quite good, worth buying!	Jingdong prefecture level city in Shandong
Jinfeng Hongyuan Flagship Store	Power Saving Specialist 1	Just received expecting the baby to work well.	Jingdong prefecture level city in Shandong
Jinfeng Hongyuan Flagship Store	Power Saving Specialist 1	Very good, there are more fruit, has bought a second!	Jingdong prefecture level city in Shandong
Jinfeng Hongyuan Flagship Store	Power Saving Specialist 1	Good is good So far, no power savings have been noticed Maybe it's too little to notice.	Jingdong prefecture level city in Shandong
Jinfeng Hongyuan Flagship Store	Power Saving Specialist 1	I just received the item and plugged it in to see what happens later.	Jingdong prefecture level city in Shandong
Jinfeng Hongyuan Flagship Store	Power Saving Specialist 1	Baby received, especially good, packaging is also good, the courier brother is very strong, thank you.	Jingdong prefecture level city in Shandong
Jinfeng Hongyuan Flagship Store	Power Saving Specialist 1	Just received the goods have not yet used it, do not know how, three months later in the follow-up comment!	Jingdong prefecture level city in Shandong
Jinfeng Hongyuan Flagship Store	Power Saving Specialist 1	The item was received, it is quite good, recommended to buy.	Jingdong prefecture level city in Shandong
Jinfeng Hongyuan Flagship Store	Power Saving Specialist 1	This energy saver works very well and I recommend everyone to buy it!	Jingdong prefecture level city in Shandong
Jinfeng Hongyuan Flagship Store	Power Saving Specialist 1	Voltage stabilization and current reduction nice power saving product.	Jingdong prefecture level city in Shandong
Jinfeng Hongyuan Flagship Store	Power Saving Specialist 1	Voltage stabilization and current reduction nice power saving product.	Jingdong prefecture level city in Shandong
Jinfeng Hongyuan Flagship Store	Power Saving Specialist 1	A good power saver for stabilizing and lowering current.	Jingdong prefecture level city in Shandong
Jinfeng Hongyuan Flagship Store	Power Saving Specialist 1	You'll know if it saves power after you use it.	Jingdong prefecture level city in Shandong
Jinfeng Hongyuan Flagship Store	Power Saving Specialist 1	The quality is great. The quality is very good. The service is very good. Express delivery arrived in two days.	Jingdong prefecture level city in Shandong
Jinfeng Hongyuan Flagship Store	Power Saving Specialist 1	It works great.	Jingdong prefecture level city in Shandong
Jinfeng Hongyuan Flagship Store	Power Saving Specialist 1	Logistics is very fast, not yet used, used to comment again.	Jingdong prefecture level city in Shandong
Jinfeng Hongyuan Flagship Store	Power Saving Specialist 1	Logistics is very fast things are also very good, worthy of friends to buy	Jingdong prefecture level city in Shandong
Jinfeng Hongyuan Flagship Store	Power Saving Specialist 1	It works. It really works.	Jingdong prefecture level city in Shandong
Jinfeng Hongyuan Flagship Store	Power Saving Specialist 1	LANGUAGE COMMENTS: just received the shipment. I immediately plugged it in and used it. If you ask how effective. It depends on whether the next month's electricity bill has been reduced. If there is a decrease. I am buying more than one to use.	Jingdong prefecture level city in Shandong
Jinfeng Hongyuan Flagship Store	Power Saving Specialist 1	Well made, power saving, good and convenient!	Jingdong prefecture level city in Shandong
Jinfeng Hongyuan Flagship Store	Power Saving Specialist 1	I'm using it. I'll rate it next month.	Jingdong prefecture level city in Shandong
Jinfeng Hongyuan Flagship Store	Power Saving Specialist 1	Baby received Oh, the courier delivery really fast, with a good and then come back to the evaluation	Jingdong prefecture level city in Shandong
Jinfeng Hongyuan Flagship Store	Power Saving Specialist 1	Fast logistics, good packaging, worth it!	Jingdong prefecture level city in Shandong
Jinfeng Hongyuan Flagship Store	Power Saving Specialist 1	Customer service is okay. It's just that.	Jingdong prefecture level city in Shandong
Jinfeng Hongyuan Flagship Store	Power Saving Specialist 1	Poor power saving	Jingdong prefecture level city in Shandong
Jinfeng Hongyuan Flagship Store	Power Saving Specialist 1	I'll tell you when I've used it.	Jingdong prefecture level city in Shandong
Jinfeng Hongyuan Flagship Store	Power Saving Specialist 1	I think it's good.	Jingdong prefecture level city in Shandong
Jinfeng Hongyuan Flagship Store	Power Saving Specialist 1	This energy saver works very well and I recommend everyone to buy it!	Jingdong prefecture level city in Shandong
Jinfeng Hongyuan Flagship Store	Power Saving Specialist 1	I do not know how effective, just use half a month on the burn out, good business reissued a, and then use it after the follow-up comment.	Jingdong prefecture level city in Shandong
Jinfeng Hongyuan Flagship Store	Power Saving Specialist 1	in use	Jingdong prefecture level city in Shandong
Jinfeng Hongyuan Flagship Store	Power Saving Specialist 1	This thing is very poor. It's already only been used for ten days and it's broken. Now waiting for you to return it.	Jingdong prefecture level city in Shandong
Jinfeng Hongyuan Flagship Store	Power Saving Specialist 1	It's not the same as the seller's show.	Jingdong prefecture level city in Shandong
Jinfeng Hongyuan Flagship Store	Power Saving Expert 2	Already bought a second one! I won't know if it's good until I use it!	Jingdong prefecture level city in Shandong
Jinfeng Hongyuan Flagship Store	Power Saving Expert 2	My colleague's family used to use one bought in Hong Kong, seems to be broken, so they want to buy another one, so we all buy it together, hope it works well!	Jingdong prefecture level city in Shandong
Jinfeng Hongyuan Flagship Store	Power Saving Expert 2	I hope everyone will come and buy it. Great quality, very inexpensive, I have purchased several times several times. Very power saving.	Jingdong prefecture level city in Shandong
Jinfeng Hongyuan Flagship Store	Power Saving Expert 2	Jinfeng Hongyuan Intelligent Power Saver Power Saving King Commercial Power Saving Appliance Ultra High Power LCD Panel Display Enhanced Power Saver Household Blue 110KW Screen Display Voltage with Overload and Overpressure Protection, Aluminum Alloy Shell Body, Sturdy and Durable. One-piece plug is not easy to fall off. Independent fuse installation simple. Currently still in the testing stage.	Jingdong prefecture level city in Shandong
Jinfeng Hongyuan Flagship Store	Power Saving Expert 2	Jinfeng Hongyuan Intelligent Power Saver Power Saving King Commercial Power Saving Appliance Ultra High Power LCD Panel Display Enhanced Power Saver Household Blue 110KW Screen Display Voltage with Overload and Overpressure Protection, Aluminum Alloy Shell Body, Sturdy and Durable. One-piece plug is not easy to fall off. Independent fuse installation simple. Currently still in the testing stage.	Jingdong prefecture level city in Shandong
Jinfeng Hongyuan Flagship Store	Power Saving Expert 2	When you introduced this product you were saying that this regulator can stabilize the voltage at 220, now I have this one showing 238, what is this?	Jingdong prefecture level city in Shandong
Jinfeng Hongyuan Flagship Store	Power Saving Expert 2	At that time when I bought the price was still hesitant to buy, after buying it did not regret it at all, and the logistics is still fast, you can get it!	Jingdong prefecture level city in Shandong
Jinfeng Hongyuan Flagship Store	Power Saving Expert 2	The goods are real, high value, high quality, worth every penny, material appearance and quality at a glance is very upscale, very like HAPPY1 00	Jingdong prefecture level city in Shandong
Jinfeng Hongyuan Flagship Store	Power Saving Expert 2	It is the ideal product, good quality, logistics is also very fast, worthy of praise 🎁 baby quality is very good, received the first time to open the first time to see, it is very good 👍, very satisfied 😃, customer service is also available to ask questions and answer, thank you 🙏	Jingdong prefecture level city in Shandong
Jinfeng Hongyuan Flagship Store	Power Saving Expert 2	I bought it and haven't used it yet, I'll use it again.	Jingdong prefecture level city in Shandong
Jinfeng Hongyuan Flagship Store	Power Saving Expert 2	It didn't work, I used it for a few days and it was the same as before, it didn't work, I should have to return it	Jingdong prefecture level city in Shandong
Jinfeng Hongyuan Flagship Store	Power Saving Expert 2	The goods have been received, but have not yet installed and use, feel good.	Jingdong prefecture level city in Shandong
Jinfeng Hongyuan Flagship Store	Power Saving Expert 2	Goods received, I forgot to take pictures, the goods look good, you can start, immediately start the school to see the effect then	Jingdong prefecture level city in Shandong

Jinfeng Hongyuan Flagship Store	Power Saving Expert 2	Looks good. I'll use it for a while.	Jingdong prefecture level city in Shandong
Jinfeng Hongyuan Flagship Store	Power Saving Expert 2	Great stuff, easy to operate, easy to use, good power saving, believe in the power of science!	Jingdong prefecture level city in Shandong
Jinfeng Hongyuan Flagship Store	Power Saving Expert 2	Especially great, comparison shopping before buying, the store owner is very good, answering questions very patiently and detailed, there is a need to come back!	Jingdong prefecture level city in Shandong
Jinfeng Hongyuan Flagship Store	Power Saving Expert 2	Trustworthy, feel free to use it boldly.	Jingdong prefecture level city in Shandong
Jinfeng Hongyuan Flagship Store	Power Saving Expert 2	Logistics is super fast! Beautiful appearance! Looks like a great quality! I've never bought one before, so I'll try it first to see if it saves power. We'll see how much it saves.	Jingdong prefecture level city in Shandong
Jinfeng Hongyuan Flagship Store	Power Saving Expert 2	I saw online counterfeiting, energy saver is one of them, hahahahaha, jingdong also buy fake products	Jingdong prefecture level city in Shandong
Jinfeng Hongyuan Flagship Store	Power Saving Expert 2	Just received, installed, try a month to see how the effect and then follow up.	Jingdong prefecture level city in Shandong
Jinfeng Hongyuan Flagship Store	Power Saving Expert 2	Good for you! The goods are genuine! Good price for what you get!	Jingdong prefecture level city in Shandong
Jinfeng Hongyuan Flagship Store	Power Saving Expert 2	Things quickly received, immediately used to see if there is no effect, something river photo ⍐ the same, customer service is very professional	Jingdong prefecture level city in Shandong
Jinfeng Hongyuan Flagship Store	Power Saving Expert 2	The product is very convenient Looking forward to the subsequent use of the experience results	Jingdong prefecture level city in Shandong
Jinfeng Hongyuan Flagship Store	Power Saving Expert 2	I just received it and plugged it in, we'll see exactly when it's time to pay the electric bill	Jingdong prefecture level city in Shandong
Jinfeng Hongyuan Flagship Store	Power Saving Expert 2	Saver is good Packaging and looks good How effective it is to be shared after using it.	Jingdong prefecture level city in Shandong
Jinfeng Hongyuan Flagship Store	Power Saving Expert 2	There's no way to compare. I don't know if it's energy efficient.	Jingdong prefecture level city in Shandong
Jinfeng Hongyuan Flagship Store	Power Saving Expert 2	Just installed it, try it out and comment again	Jingdong prefecture level city in Shandong
Jinfeng Hongyuan Flagship Store	Power Saving Expert 2	Super save point also good to install, after a friend introduced then went to buy to, friends say it is very good to use, home I bought, try to use a period of time, to be honest, the economizer is not big or heavy, can be very convenient to put in a lot of places, the quality is also very good, power-saving function this time seems to be about 20% of the efficiency. The owner is also very warm contact, very	Jingdong prefecture level city in Shandong
Jinfeng Hongyuan Flagship Store	Power Saving Expert 2	The reviews online are mixed. Try it for a while, but I don't know if I can return it if it doesn't save any power after a couple months.	Jingdong prefecture level city in Shandong
Jinfeng Hongyuan Flagship Store	Power Saving Expert 2	The power consumption at home is high, bought a power saver, it looks very convenient to plug on the line, see if you can save some money next month, if good, recommend to friends down the road.	Jingdong prefecture level city in Shandong
Jinfeng Hongyuan Flagship Store	Power Saving Expert 2	It's beautiful, it's genuine, but I don't know the power-saving effect.	Jingdong prefecture level city in Shandong
Jinfeng Hongyuan Flagship Store	Power Saving Expert 2	This user thinks the product is good	Jingdong prefecture level city in Shandong
Jinfeng Hongyuan Flagship Store	Power Saving Expert 2	Nice exterior design with an at-a-glance display	Jingdong prefecture level city in Shandong
Jinfeng Hongyuan Flagship Store	Power Saving Expert 2	Home large and small things are purchased in the Jingdong Mall, have been accustomed to shopping in the Jingdong, many times can buy good quality and cheap products, and courier service attitude is particularly good, sometimes too many couriers are to help me to the home, so many years have been in the Jingdong Mall shopping, trust and support! Will also support the Jingdong as always!	Jingdong prefecture level city in Shandong
Jinfeng Hongyuan Flagship Store	Power Saving Expert 2	Good workmanship, mostly better than the original wall-plugged ones, and hopefully power factor compensation saves on electricity costs	Jingdong prefecture level city in Shandong
Jinfeng Hongyuan Flagship Store	Power Saving Expert 2	I don't feel anything, I said I won't know the effect for a month or two, let's see.	Jingdong prefecture level city in Shandong
Jinfeng Hongyuan Flagship Store	Power Saving Expert 2	Delivery was pretty fast, a little pricey, just not sure if it works or not	Jingdong prefecture level city in Shandong
Jinfeng Hongyuan Flagship Store	Power Saving Expert 2	The goods have been received, I do not know how effective.	Jingdong prefecture level city in Shandong
Jinfeng Hongyuan Flagship Store	Power Saving Expert 2	So far it doesn't seem to be too noticeable, so I'll comment later.	Jingdong prefecture level city in Shandong
Jinfeng Hongyuan Flagship Store	Power Saving Expert 2	I'll comment after I've used it	Jingdong prefecture level city in Shandong
Jinfeng Hongyuan Flagship Store	Power Saving Expert 2	Not bad. You'll save a lot on your electric bill in a year!	Jingdong prefecture level city in Shandong
Jinfeng Hongyuan Flagship Store	Power Saving Expert 2	I just got it. I haven't tried it yet. I don't know what it's like.	Jingdong prefecture level city in Shandong
Jinfeng Hongyuan Flagship Store	Power Saving Expert 2	Shopping at Jingdong has become a habit, and this disease will not be cured for a while.	Jingdong prefecture level city in Shandong
Jinfeng Hongyuan Flagship Store	Power Saving Expert 2	I've been using it for a while now. I don't know if I'm still saving power or not.	Jingdong prefecture level city in Shandong
Jinfeng Hongyuan Flagship Store	Power Saving Expert 2	Received the saver and it's great! There is also a digital voltage indication. Plugged in as instructed, easy to operate, hope you're our power saver!	Jingdong prefecture level city in Shandong
Jinfeng Hongyuan Flagship Store	Power Saving Expert 2	There is no power saving as the store says, using it is the same as not using it.	Jingdong prefecture level city in Shandong
Jinfeng Hongyuan Flagship Store	Power Saving Expert 2	Use it for a while and then comment again	Jingdong prefecture level city in Shandong
Jinfeng Hongyuan Flagship Store	Power Saving Expert 2	Feeling can, to use 1 to 2 months to see how the effect, there is a voltage display, can see the voltage fluctuations amplitude, quite good!	Jingdong prefecture level city in Shandong
Jinfeng Hongyuan Flagship Store	Power Saving Expert 2	I hope it works and saves me some electricity.	Jingdong prefecture level city in Shandong
Jinfeng Hongyuan Flagship Store	Power Saving Expert 2	Nice baby, happy with the after sales	Jingdong prefecture level city in Shandong
Jinfeng Hongyuan Flagship Store	Power Saving Expert 2	Use it and talk about it.	Jingdong prefecture level city in Shandong
Jinfeng Hongyuan Flagship Store	Power Saving Expert 2	Second purchase	Jingdong prefecture level city in Shandong
Jinfeng Hongyuan Flagship Store	Power Saving Expert 2	It works.	Jingdong prefecture level city in Shandong
Jinfeng Hongyuan Flagship Store	Power Saving Expert 2	It works.	Jingdong prefecture level city in Shandong
Jinfeng Hongyuan Flagship Store	Power Saving Expert 2	I don't know if it's a power saver until I start using it	Jingdong prefecture level city in Shandong
Jinfeng Hongyuan Flagship Store	Power Saving Expert 2	serviceable	Jingdong prefecture level city in Shandong
Jinfeng Hongyuan Flagship Store	Power Saving Expert 2	This user did not fill out the rating in time	Jingdong prefecture level city in Shandong
Jinfeng Hongyuan Flagship Store	Power Saving Expert 2	There is no power saving as the store says, using it is the same as not using it.	Jingdong prefecture level city in Shandong
Jinfeng Hongyuan Flagship Store	Power Saving Expert 2	This user did not fill out the rating in time	Jingdong prefecture level city in Shandong
Jinfeng Hongyuan Flagship Store	Power Saving Expert 2	Good...good.	Jingdong prefecture level city in Shandong
Jinfeng Hongyuan Flagship Store	Power Saving Expert 2	(of an unmarried couple) be close	Jingdong prefecture level city in Shandong
Jinfeng Hongyuan Flagship Store	Power Saving Expert 2	(of an unmarried couple) be close	Jingdong prefecture level city in Shandong

Jinfeng Hongyuan Flagship Store	Power Saving Expert 2	(of an unmarried couple) be close	Jingdong prefecture level city in Shandong
Jinfeng Hongyuan Flagship Store	Power Saving Expert 2	The goods are real, high value, high quality, worth every penny, material appearance and quality at a glance is very upscale, very like HAPPY1 00	Jingdong prefecture level city in Shandong
Jinfeng Hongyuan Flagship Store	Power Saving Expert 2	It is the ideal product, good quality, logistics is also very fast, worthy of praise 🎁 baby quality is very good, received the first time to open the first time to see, it is very good 👍, very satisfied 😊, customer service is also available to ask questions and answer, thank you 🙏	Jingdong prefecture level city in Shandong
Jinfeng Hongyuan Flagship Store	Power Saving Expert 2	I bought it and haven't used it yet, I'll use it again.	Jingdong prefecture level city in Shandong
Jinfeng Hongyuan Flagship Store	Power Saving Expert 2	It didn't work, I used it for a few days and it was the same as before, it didn't work, I should have to return it	Jingdong prefecture level city in Shandong
Jinfeng Hongyuan Flagship Store	Power Saving Expert 2	The goods have been received, but have not yet installed and use, feel good.	Jingdong prefecture level city in Shandong
Jinfeng Hongyuan Flagship Store	Power Saving Expert 2	Goods received, I forgot to take pictures, the goods look good, you can start, immediately start the school to see the effect then	Jingdong prefecture level city in Shandong
Jinfeng Hongyuan Flagship Store	Power Saving Expert 2	Looks good. I'll use it for a while.	Jingdong prefecture level city in Shandong
Jinfeng Hongyuan Flagship Store	Power Saving Expert 2	Great stuff, easy to operate, easy to use, good power saving, believe in the power of science!	Jingdong prefecture level city in Shandong
Jinfeng Hongyuan Flagship Store	Power Saving Expert 2	Especially great, comparison shopping before buying, the store owner is very good, answering questions very patiently and detailed, there is a need to come back!	Jingdong prefecture level city in Shandong
Jinfeng Hongyuan Flagship Store	Power Saving Expert 2	Trustworthy, feel free to use it boldly.	Jingdong prefecture level city in Shandong
Jinfeng Hongyuan Flagship Store	Power Saving Expert 2	Logistics is super fast! Beautiful appearance! Looks like a great quality! I've never bought one before, so I'll try it first to see if it saves power. We'll see how much it saves.	Jingdong prefecture level city in Shandong
Jinfeng Hongyuan Flagship Store	Power Saving Expert 2	I saw online counterfeiting, energy saver is one of them, hahahahaha, jingdong also buy fake products	Jingdong prefecture level city in Shandong
Jinfeng Hongyuan Flagship Store	Power Saving Expert 2	Just received, installed, try a month to see how the effect and then follow up.	Jingdong prefecture level city in Shandong
Jinfeng Hongyuan Flagship Store	Power Saving Expert 2	Good for you! The goods are genuine! Good price for what you get!	Jingdong prefecture level city in Shandong
Jinfeng Hongyuan Flagship Store	Power Saving Expert 2	Things quickly received, immediately used to see if there is no effect, something river photo ⬆ the same, customer service is very professional	Jingdong prefecture level city in Shandong
Jinfeng Hongyuan Flagship Store	Power Saving Expert 2	The product is very convenient Looking forward to the subsequent use of the experience results	Jingdong prefecture level city in Shandong
Jinfeng Hongyuan Flagship Store	Power Saving Expert 2	I just received it and plugged it in, we'll see exactly when it's time to pay the electric bill	Jingdong prefecture level city in Shandong
Jinfeng Hongyuan Flagship Store	Power Saving Expert 2	Saver is good Packaging and looks good How effective it is to be shared after using it.	Jingdong prefecture level city in Shandong
Jinfeng Hongyuan Flagship Store	Power Saving Expert 2	There's no way to compare. I don't know if it's energy efficient.	Jingdong prefecture level city in Shandong
Jinfeng Hongyuan Flagship Store	Power Saving Expert 2	Just installed it, try it out and comment again	Jingdong prefecture level city in Shandong
Jinfeng Hongyuan Flagship Store	Power Saving Expert 2	Super save point also good to install, after a friend introduced then went to buy to, friends say it is very good to use, home I bought, try to use a period of time, to be honest, the economizer is not big or heavy, can be very convenient to put in a lot of places, the quality is also very good, power-saving function this time seems to be about 20% of the efficiency. The owner is also very warm contact, very	Jingdong prefecture level city in Shandong
Jinfeng Hongyuan Flagship Store	Power Saving Expert 2	The reviews online are mixed. Try it for a while, but I don't know if I can return it if it doesn't save any power after a couple months.	Jingdong prefecture level city in Shandong
Jinfeng Hongyuan Flagship Store	Power Saving Expert 2	The power consumption at home is high, bought a power saver, it looks very convenient to plug on the line, see if you can save some money next month, if good, recommend to friends down the road.	Jingdong prefecture level city in Shandong
Jinfeng Hongyuan Flagship Store	Power Saving Expert 2	It's beautiful, it's genuine, but I don't know the power-saving effect.	Jingdong prefecture level city in Shandong
Jinfeng Hongyuan Flagship Store	Power Saving Expert 2	This user thinks the product is good	Jingdong prefecture level city in Shandong
Jinfeng Hongyuan Flagship Store	Power Saving Expert 2	Nice exterior design with an at-a-glance display	Jingdong prefecture level city in Shandong
Jinfeng Hongyuan Flagship Store	Power Saving Expert 2	Home large and small things are purchased in the Jingdong Mall, have been accustomed to shopping in the Jingdong, many times can buy good quality and cheap products, and courier service attitude is particularly good, sometimes too many couriers are to help me to the home, so many years have been in the Jingdong Mall shopping, trust and support! Will also support the Jingdong as always!	Jingdong prefecture level city in Shandong
Jinfeng Hongyuan Flagship Store	Power Saving Expert 2	Good workmanship, mostly better than the original wall-plugged ones, and hopefully power factor compensation saves on electricity costs	Jingdong prefecture level city in Shandong
Jinfeng Hongyuan Flagship Store	Power Saving Expert 2	I don't feel anything, I said I won't know the effect for a month or two, let's see.	Jingdong prefecture level city in Shandong
Jinfeng Hongyuan Flagship Store	Power Saving Expert 2	Delivery was pretty fast, a little pricey, just not sure if it works or not	Jingdong prefecture level city in Shandong
Jinfeng Hongyuan Flagship Store	Power Saving Expert 2	The goods have been received, I do not know how effective.	Jingdong prefecture level city in Shandong
Jinfeng Hongyuan Flagship Store	Power Saving Expert 2	So far it doesn't seem to be too noticeable, so I'll comment later.	Jingdong prefecture level city in Shandong
Jinfeng Hongyuan Flagship Store	Power Saving Expert 2	I'll comment after I've used it	Jingdong prefecture level city in Shandong
Jinfeng Hongyuan Flagship Store	Power Saving Expert 2	Not bad. You'll save a lot on your electric bill in a year!	Jingdong prefecture level city in Shandong
Jinfeng Hongyuan Flagship Store	Power Saving Expert 2	I just got it. I haven't tried it yet. I don't know what it's like.	Jingdong prefecture level city in Shandong
Jinfeng Hongyuan Flagship Store	Power Saving Expert 2	Shopping at Jingdong has become a habit, and this disease will not be cured for a while.	Jingdong prefecture level city in Shandong
Jinfeng Hongyuan Flagship Store	Power Saving Expert 2	I've been using it for a while now. I don't know if I'm still saving power or not.	Jingdong prefecture level city in Shandong
Jinfeng Hongyuan Flagship Store	Power Saving Expert 2	Received the saver and it's great! There is also a digital voltage indication. Plugged in as instructed, easy to operate, hope you're our power saver!	Jingdong prefecture level city in Shandong
Jinfeng Hongyuan Flagship Store	Power Saving Expert 2	There is no power saving as the store says, using it is the same as not using it.	Jingdong prefecture level city in Shandong
Jinfeng Hongyuan Flagship Store	Power Saving Expert 2	Use it for a while and then comment again	Jingdong prefecture level city in Shandong
Jinfeng Hongyuan Flagship Store	Power Saving Expert 2	Feeling can, to use 1 to 2 months to see how the effect, there is a voltage display, can see the voltage fluctuations amplitude, quite good!	Jingdong prefecture level city in Shandong
Jinfeng Hongyuan Flagship Store	Power Saving Expert 2	I hope it works and saves me some electricity.	Jingdong prefecture level city in Shandong
Jinfeng Hongyuan Flagship Store	Power Saving Expert 2	Nice baby, happy with the after sales	Jingdong prefecture level city in Shandong
Jinfeng Hongyuan Flagship Store	Power Saving Expert 2	Use it and talk about it.	Jingdong prefecture level city in Shandong
Jinfeng Hongyuan Flagship Store	Power Saving Expert 2	Second purchase	Jingdong prefecture level city in Shandong
Jinfeng Hongyuan Flagship Store	Power Saving Expert 2	It works.	Jingdong prefecture level city in Shandong
Jinfeng Hongyuan Flagship Store	Power Saving Expert 2	It works.	Jingdong prefecture level city in Shandong
Jinfeng Hongyuan Flagship Store	Power Saving Expert 2	I don't know if it's a power saver until I start using it	Jingdong prefecture level city in Shandong

Jinfeng Hongyuan Flagship Store	Power Saving Expert 2	serviceable	Jingdong prefecture level city in Shandong
Jinfeng Hongyuan Flagship Store	Power Saving Expert 2	This user did not fill out the rating in time	Jingdong prefecture level city in Shandong
Jinfeng Hongyuan Flagship Store	Power Saving Expert 2	There is no power saving as the store says, using it is the same as not using it.	Jingdong prefecture level city in Shandong
Jinfeng Hongyuan Flagship Store	Power Saving Expert 2	This user did not fill out the rating in time	Jingdong prefecture level city in Shandong
Jinfeng Hongyuan Flagship Store	Power Saving Expert 2	Good... good.	Jingdong prefecture level city in Shandong
Jinfeng Hongyuan Flagship Store	Power Saving Expert 2	(of an unmarried couple) be close	Jingdong prefecture level city in Shandong
Jinfeng Hongyuan Flagship Store	Power Saving Expert 2	(of an unmarried couple) be close	Jingdong prefecture level city in Shandong
Jinfeng Hongyuan Flagship Store	Power Saving Expert 2	(of an unmarried couple) be close	Jingdong prefecture level city in Shandong
Jinfeng Hongyuan Flagship Store	Power Saving Expert 2	The goods are real, high value, high quality, worth every penny, material appearance and quality at a glance is very upscale, very like HAPPY1 00	Jingdong prefecture level city in Shandong
Jinfeng Hongyuan Flagship Store	Power Saving Expert 2	It is the ideal product, good quality, logistics is also very fast, worthy of praise 🎁 baby quality is very good, received the first time to open the first time to see, it is very good 👍, very satisfied 😊, customer service is also available to ask questions and answer, thank you 🙏	Jingdong prefecture level city in Shandong
Jinfeng Hongyuan Flagship Store	Power Saving Expert 2	I bought it and haven't used it yet, I'll use it again.	Jingdong prefecture level city in Shandong
Jinfeng Hongyuan Flagship Store	Power Saving Expert 2	It didn't work, I used it for a few days and it was the same as before, it didn't work, I should have to return it	Jingdong prefecture level city in Shandong
Jinfeng Hongyuan Flagship Store	Power Saving Expert 2	The goods have been received, but have not yet installed and use, feel good.	Jingdong prefecture level city in Shandong
Jinfeng Hongyuan Flagship Store	Power Saving Expert 2	Goods received, I forgot to take pictures, the goods look good, you can start, immediately start the school to see the effect then	Jingdong prefecture level city in Shandong
Jinfeng Hongyuan Flagship Store	Power Saving Expert 2	Looks good. I'll use it for a while.	Jingdong prefecture level city in Shandong
Jinfeng Hongyuan Flagship Store	Power Saving Expert 2	Great stuff, easy to operate, easy to use, good power saving, believe in the power of science!	Jingdong prefecture level city in Shandong
Jinfeng Hongyuan Flagship Store	Power Saving Expert 2	Especially great, comparison shopping before buying, the store owner is very good, answering questions very patiently and detailed, there is a need to come back!	Jingdong prefecture level city in Shandong
Jinfeng Hongyuan Flagship Store	Power Saving Expert 2	Trustworthy, feel free to use it boldly.	Jingdong prefecture level city in Shandong
Jinfeng Hongyuan Flagship Store	Power Saving Expert 2	Logistics is super fast! Beautiful appearance! Looks like a great quality! I've never bought one before, so I'll try it first to see if it saves power. We'll see how much it saves.	Jingdong prefecture level city in Shandong
Jinfeng Hongyuan Flagship Store	Power Saving Expert 2	I saw online counterfeiting, energy saver is one of them, hahahahaha, jingdong also buy fake products	Jingdong prefecture level city in Shandong
Jinfeng Hongyuan Flagship Store	Power Saving Expert 2	Just received, installed, try a month to see how the effect and then follow up.	Jingdong prefecture level city in Shandong
Jinfeng Hongyuan Flagship Store	Power Saving Expert 2	Good for you! The goods are genuine! Good price for what you get!	Jingdong prefecture level city in Shandong
Jinfeng Hongyuan Flagship Store	Power Saving Expert 2	Things quickly received, immediately used to see if there is no effect, something river photo ⬆ the same, customer service is very professional	Jingdong prefecture level city in Shandong
Jinfeng Hongyuan Flagship Store	Power Saving Expert 2	The product is very convenient Looking forward to the subsequent use of the experience results	Jingdong prefecture level city in Shandong
Jinfeng Hongyuan Flagship Store	Power Saving Expert 2	I just received it and plugged it in, we'll see exactly when it's time to pay the electric bill	Jingdong prefecture level city in Shandong
Jinfeng Hongyuan Flagship Store	Power Saving Expert 2	Saver is good Packaging and looks good How effective it is to be shared after using it.	Jingdong prefecture level city in Shandong
Jinfeng Hongyuan Flagship Store	Power Saving Expert 2	There's no way to compare. I don't know if it's energy efficient.	Jingdong prefecture level city in Shandong
Jinfeng Hongyuan Flagship Store	Power Saving Expert 2	Just installed it, try it out and comment again	Jingdong prefecture level city in Shandong
Jinfeng Hongyuan Flagship Store	Power Saving Expert 2	Super save point also good to install, after a friend introduced then went to buy to, friends say it is very good to use, home I bought, try to use a period of time, to be honest, the economizer is not big or heavy, can be very convenient to put in a lot of places, the quality is also very good, power-saving function this time seems to be about 20% of the efficiency. The owner is also very warm contact, very	Jingdong prefecture level city in Shandong
Jinfeng Hongyuan Flagship Store	Power Saving Expert 2	The reviews online are mixed. Try it for a while, but I don't know if I can return it if it doesn't save any power after a couple months.	Jingdong prefecture level city in Shandong
Jinfeng Hongyuan Flagship Store	Power Saving Expert 2	The power consumption at home is high, bought a power saver, it looks very convenient to plug on the line, see if you can save some money next month, if good, recommend to friends down the road.	Jingdong prefecture level city in Shandong
Jinfeng Hongyuan Flagship Store	Power Saving Expert 2	It's beautiful, it's genuine, but I don't know the power-saving effect.	Jingdong prefecture level city in Shandong
Jinfeng Hongyuan Flagship Store	Power Saving Expert 2	This user thinks the product is good	Jingdong prefecture level city in Shandong
Jinfeng Hongyuan Flagship Store	Power Saving Expert 2	Nice exterior design with an at-a-glance display	Jingdong prefecture level city in Shandong
Jinfeng Hongyuan Flagship Store	Power Saving Expert 2	Home large and small things are purchased in the Jingdong Mall, have been accustomed to shopping in the Jingdong, many times can buy good quality and cheap products, and courier service attitude is particularly good, sometimes too many couriers are to help me to the home, so many years have been in the Jingdong Mall shopping, trust and support! Will also support the Jingdong as always!	Jingdong prefecture level city in Shandong
Jinfeng Hongyuan Flagship Store	Power Saving Expert 2	Good workmanship, mostly better than the original wall-plugged ones, and hopefully power factor compensation saves on electricity costs	Jingdong prefecture level city in Shandong
Jinfeng Hongyuan Flagship Store	Power Saving Expert 2	I don't feel anything, I said I won't know the effect for a month or two, let's see.	Jingdong prefecture level city in Shandong
Jinfeng Hongyuan Flagship Store	Power Saving Expert 2	Delivery was pretty fast, a little pricey, just not sure if it works or not	Jingdong prefecture level city in Shandong
Jinfeng Hongyuan Flagship Store	Power Saving Expert 2	The goods have been received, I do not know how effective.	Jingdong prefecture level city in Shandong
Jinfeng Hongyuan Flagship Store	Power Saving Expert 2	So far it doesn't seem to be too noticeable, so I'll comment later.	Jingdong prefecture level city in Shandong
Jinfeng Hongyuan Flagship Store	Power Saving Expert 2	I'll comment after I've used it	Jingdong prefecture level city in Shandong
Jinfeng Hongyuan Flagship Store	Power Saving Expert 2	Not bad. You'll save a lot on your electric bill in a year!	Jingdong prefecture level city in Shandong
Jinfeng Hongyuan Flagship Store	Power Saving Expert 2	I just got it. I haven't tried it yet. I don't know what it's like.	Jingdong prefecture level city in Shandong
Jinfeng Hongyuan Flagship Store	Power Saving Expert 2	Shopping at Jingdong has become a habit, and this disease will not be cured for a while.	Jingdong prefecture level city in Shandong
Jinfeng Hongyuan Flagship Store	Power Saving Expert 2	I've been using it for a while now. I don't know if I'm still saving power or not.	Jingdong prefecture level city in Shandong
Jinfeng Hongyuan Flagship Store	Power Saving Expert 2	Received the saver and it's great! There is also a digital voltage indication. Plugged in as instructed, easy to operate, hope you're our power saver!	Jingdong prefecture level city in Shandong
Jinfeng Hongyuan Flagship Store	Power Saving Expert 2	There is no power saving as the store says, using it is the same as not using it.	Jingdong prefecture level city in Shandong
Jinfeng Hongyuan Flagship Store	Power Saving Expert 2	Use it for a while and then comment again	Jingdong prefecture level city in Shandong
Jinfeng Hongyuan Flagship Store	Power Saving Expert 2	Feeling can, to use 1 to 2 months to see how the effect, there is a voltage display, can see the voltage fluctuations amplitude, quite good!	Jingdong prefecture level city in Shandong

Jinfeng Hongyuan Flagship Store	Power Saving Expert 2	I hope it works and saves me some electricity.	Jingdong prefecture level city in Shandong
Jinfeng Hongyuan Flagship Store	Power Saving Expert 2	Nice baby, happy with the after sales	Jingdong prefecture level city in Shandong
Jinfeng Hongyuan Flagship Store	Power Saving Expert 2	Use it and talk about it.	Jingdong prefecture level city in Shandong
Jinfeng Hongyuan Flagship Store	Power Saving Expert 2	Second purchase	Jingdong prefecture level city in Shandong
Jinfeng Hongyuan Flagship Store	Power Saving Expert 2	It works.	Jingdong prefecture level city in Shandong
Jinfeng Hongyuan Flagship Store	Power Saving Expert 2	It works.	Jingdong prefecture level city in Shandong
Jinfeng Hongyuan Flagship Store	Power Saving Expert 2	I don't know if it's a power saver until I start using it	Jingdong prefecture level city in Shandong
Jinfeng Hongyuan Flagship Store	Power Saving Expert 2	serviceable	Jingdong prefecture level city in Shandong
Jinfeng Hongyuan Flagship Store	Power Saving Expert 2	This user did not fill out the rating in time	Jingdong prefecture level city in Shandong
Jinfeng Hongyuan Flagship Store	Power Saving Expert 2	There is no power saving as the store says, using it is the same as not using it.	Jingdong prefecture level city in Shandong
Jinfeng Hongyuan Flagship Store	Power Saving Expert 2	This user did not fill out the rating in time	Jingdong prefecture level city in Shandong
Jinfeng Hongyuan Flagship Store	Power Saving Expert 2	Good...good.	Jingdong prefecture level city in Shandong
Jinfeng Hongyuan Flagship Store	Power Saving Expert 2	(of an unmarried couple) be close	Jingdong prefecture level city in Shandong
Jinfeng Hongyuan Flagship Store	Power Saving Expert 2	(of an unmarried couple) be close	Jingdong prefecture level city in Shandong
Jinfeng Hongyuan Flagship Store	Power Saving Expert 2	(of an unmarried couple) be close	Jingdong prefecture level city in Shandong
Jinfeng Hongyuan Flagship Store	Power Saving Expert 2	The goods are real, high value, high quality, worth every penny, material appearance and quality at a glance is very upscale, very like HAPPY1 00	Jingdong prefecture level city in Shandong
Jinfeng Hongyuan Flagship Store	Power Saving Expert 2	It is the ideal product, good quality, logistics is also very fast, worthy of praise 🎁 baby quality is very good, received the first time to open the first time to see, it is very good 👍, very satisfied 😃, customer service is also available to ask questions and answer, thank you 🙏	Jingdong prefecture level city in Shandong
Jinfeng Hongyuan Flagship Store	Power Saving Expert 2	I bought it and haven't used it yet, I'll use it again.	Jingdong prefecture level city in Shandong
Jinfeng Hongyuan Flagship Store	Power Saving Expert 2	It didn't work, I used it for a few days and it was the same as before, it didn't work, I should have to return it	Jingdong prefecture level city in Shandong
Jinfeng Hongyuan Flagship Store	Power Saving Expert 2	The goods have been received, but have not yet installed and use, feel good.	Jingdong prefecture level city in Shandong
Jinfeng Hongyuan Flagship Store	Power Saving Expert 2	Goods received, I forgot to take pictures, the goods look good, you can start, immediately start the school to see the effect then	Jingdong prefecture level city in Shandong
Jinfeng Hongyuan Flagship Store	Power Saving Expert 2	Looks good. I'll use it for a while.	Jingdong prefecture level city in Shandong
Jinfeng Hongyuan Flagship Store	Power Saving Expert 2	Great stuff, easy to operate, easy to use, good power saving, believe in the power of science!	Jingdong prefecture level city in Shandong
Jinfeng Hongyuan Flagship Store	Power Saving Expert 2	Especially great, comparison shopping before buying, the store owner is very good, answering questions very patiently and detailed, there is a need to come back!	Jingdong prefecture level city in Shandong
Jinfeng Hongyuan Flagship Store	Power Saving Expert 2	Trustworthy, feel free to use it boldly.	Jingdong prefecture level city in Shandong
Jinfeng Hongyuan Flagship Store	Power Saving Expert 2	Logistics is super fast! Beautiful appearance! Looks like a great quality! I've never bought one before, so I'll try it first to see if it saves power. We'll see how much it saves.	Jingdong prefecture level city in Shandong
Jinfeng Hongyuan Flagship Store	Power Saving Expert 2	I saw online counterfeiting, energy saver is one of them, hahahahaha, jingdong also buy fake products	Jingdong prefecture level city in Shandong
Jinfeng Hongyuan Flagship Store	Power Saving Expert 2	Just received, installed, try a month to see how the effect and then follow up.	Jingdong prefecture level city in Shandong
Jinfeng Hongyuan Flagship Store	Power Saving Expert 2	Good for you! The goods are genuine! Good price for what you get!	Jingdong prefecture level city in Shandong
Jinfeng Hongyuan Flagship Store	Power Saving Expert 2	Things quickly received, immediately used to see if there is no effect, something river photo ⬆ the same, customer service is very professional	Jingdong prefecture level city in Shandong
Jinfeng Hongyuan Flagship Store	Power Saving Expert 2	The product is very convenient Looking forward to the subsequent use of the experience results	Jingdong prefecture level city in Shandong
Jinfeng Hongyuan Flagship Store	Power Saving Expert 2	I just received it and plugged it in, we'll see exactly when it's time to pay the electric bill	Jingdong prefecture level city in Shandong
Jinfeng Hongyuan Flagship Store	Power Saving Expert 2	Saver is good Packaging and looks good How effective it is to be shared after using it.	Jingdong prefecture level city in Shandong
Jinfeng Hongyuan Flagship Store	Power Saving Expert 2	There's no way to compare. I don't know if it's energy efficient.	Jingdong prefecture level city in Shandong
Jinfeng Hongyuan Flagship Store	Power Saving Expert 2	Just installed it, try it out and comment again	Jingdong prefecture level city in Shandong
Jinfeng Hongyuan Flagship Store	Power Saving Expert 2	Super save point also good to install, after a friend introduced then went to buy to, friends say it is very good to use, home I bought, try to use a period of time, to be honest, the economizer is not big or heavy, can be very convenient to put in a lot of places, the quality is also very good, power-saving function this time seems to be about 20% of the efficiency. The owner is also very warm contact, very	Jingdong prefecture level city in Shandong
Jinfeng Hongyuan Flagship Store	Power Saving Expert 2	The reviews online are mixed. Try it for a while, but I don't know if I can return it if it doesn't save any power after a couple months.	Jingdong prefecture level city in Shandong
Jinfeng Hongyuan Flagship Store	Power Saving Expert 2	The power consumption at home is high, bought a power saver, it looks very convenient to plug on the line, see if you can save some money next month, if good, recommend to friends down the road.	Jingdong prefecture level city in Shandong
Jinfeng Hongyuan Flagship Store	Power Saving Expert 2	It's beautiful, it's genuine, but I don't know the power-saving effect.	Jingdong prefecture level city in Shandong
Jinfeng Hongyuan Flagship Store	Power Saving Expert 2	This user thinks the product is good	Jingdong prefecture level city in Shandong
Jinfeng Hongyuan Flagship Store	Power Saving Expert 2	Nice exterior design with an at-a-glance display	Jingdong prefecture level city in Shandong
Jinfeng Hongyuan Flagship Store	Power Saving Expert 2	Home large and small things are purchased in the Jingdong Mall, have been accustomed to shopping in the Jingdong, many times can buy good quality and cheap products, and courier service attitude is particularly good, sometimes too many couriers are to help me to the home, so many years have been in the Jingdong Mall shopping, trust and support! Will also support the Jingdong as always!	Jingdong prefecture level city in Shandong
Jinfeng Hongyuan Flagship Store	Power Saving Expert 2	Good workmanship, mostly better than the original wall-plugged ones, and hopefully power factor compensation saves on electricity costs	Jingdong prefecture level city in Shandong
Jinfeng Hongyuan Flagship Store	Power Saving Expert 2	I don't feel anything, I said I won't know the effect for a month or two, let's see.	Jingdong prefecture level city in Shandong
Jinfeng Hongyuan Flagship Store	Power Saving Expert 2	Delivery was pretty fast, a little pricey, just not sure if it works or not	Jingdong prefecture level city in Shandong
Jinfeng Hongyuan Flagship Store	Power Saving Expert 2	The goods have been received, I do not know how effective.	Jingdong prefecture level city in Shandong
Jinfeng Hongyuan Flagship Store	Power Saving Expert 2	So far it doesn't seem to be too noticeable, so I'll comment later.	Jingdong prefecture level city in Shandong
Jinfeng Hongyuan Flagship Store	Power Saving Expert 2	I'll comment after I've used it	Jingdong prefecture level city in Shandong
Jinfeng Hongyuan Flagship Store	Power Saving Expert 2	Not bad. You'll save a lot on your electric bill in a year!	Jingdong prefecture level city in Shandong

Jinfeng Hongyuan Flagship Store	Power Saving Expert 2	I just got it. I haven't tried it yet. I don't know what it's like.	Jingdong prefecture level city in Shandong
Jinfeng Hongyuan Flagship Store	Power Saving Expert 2	Shopping at Jingdong has become a habit, and this disease will not be cured for a while.	Jingdong prefecture level city in Shandong
Jinfeng Hongyuan Flagship Store	Power Saving Expert 2	I've been using it for a while now. I don't know if I'm still saving power or not.	Jingdong prefecture level city in Shandong
Jinfeng Hongyuan Flagship Store	Power Saving Expert 2	Received the saver and it's great! There is also a digital voltage indication. Plugged in as instructed, easy to operate, hope you're our power saver!	Jingdong prefecture level city in Shandong
Jinfeng Hongyuan Flagship Store	Power Saving Expert 2	There is no power saving as the store says, using it is the same as not using it.	Jingdong prefecture level city in Shandong
Jinfeng Hongyuan Flagship Store	Power Saving Expert 2	Use it for a while and then comment again	Jingdong prefecture level city in Shandong
Jinfeng Hongyuan Flagship Store	Power Saving Expert 2	Feeling can, to use 1 to 2 months to see how the effect, there is a voltage display, can see the voltage fluctuations amplitude, quite good!	Jingdong prefecture level city in Shandong
Jinfeng Hongyuan Flagship Store	Power Saving Expert 2	I hope it works and saves me some electricity.	Jingdong prefecture level city in Shandong
Jinfeng Hongyuan Flagship Store	Power Saving Expert 2	Nice baby, happy with the after sales	Jingdong prefecture level city in Shandong
Jinfeng Hongyuan Flagship Store	Power Saving Expert 2	Use it and talk about it.	Jingdong prefecture level city in Shandong
Jinfeng Hongyuan Flagship Store	Power Saving Expert 2	Second purchase	Jingdong prefecture level city in Shandong
Jinfeng Hongyuan Flagship Store	Power Saving Expert 2	It works.	Jingdong prefecture level city in Shandong
Jinfeng Hongyuan Flagship Store	Power Saving Expert 2	It works.	Jingdong prefecture level city in Shandong
Jinfeng Hongyuan Flagship Store	Power Saving Expert 2	I don't know if it's a power saver until I start using it	Jingdong prefecture level city in Shandong
Jinfeng Hongyuan Flagship Store	Power Saving Expert 2	serviceable	Jingdong prefecture level city in Shandong
Jinfeng Hongyuan Flagship Store	Power Saving Expert 2	This user did not fill out the rating in time	Jingdong prefecture level city in Shandong
Jinfeng Hongyuan Flagship Store	Power Saving Expert 2	There is no power saving as the store says, using it is the same as not using it.	Jingdong prefecture level city in Shandong
Jinfeng Hongyuan Flagship Store	Power Saving Expert 2	This user did not fill out the rating in time	Jingdong prefecture level city in Shandong
Jinfeng Hongyuan Flagship Store	Power Saving Expert 2	Good...good.	Jingdong prefecture level city in Shandong
Jinfeng Hongyuan Flagship Store	Power Saving Expert 2	(of an unmarried couple) be close	Jingdong prefecture level city in Shandong
Jinfeng Hongyuan Flagship Store	Power Saving Expert 2	(of an unmarried couple) be close	Jingdong prefecture level city in Shandong
Jinfeng Hongyuan Flagship Store	Power Saving Expert 2	(of an unmarried couple) be close	Jingdong prefecture level city in Shandong
Jinfeng Hongyuan Flagship Store	Power Saving Expert 2	The goods are real, high value, high quality, worth every penny, material appearance and quality at a glance is very upscale, very like HAPPY1 00	Jingdong prefecture level city in Shandong
Jinfeng Hongyuan Flagship Store	Power Saving Expert 2	It is the ideal product, good quality, logistics is also very fast, worthy of praise 🎁 baby quality is very good, received the first time to open the first time to see, it is very good 👍, very satisfied 😃, customer service is also available to ask questions and answer, thank you 🙏	Jingdong prefecture level city in Shandong
Jinfeng Hongyuan Flagship Store	Power Saving Expert 2	I bought it and haven't used it yet, I'll use it again.	Jingdong prefecture level city in Shandong
Jinfeng Hongyuan Flagship Store	Power Saving Expert 2	It didn't work, I used it for a few days and it was the same as before, it didn't work, I should have to return it	Jingdong prefecture level city in Shandong
Jinfeng Hongyuan Flagship Store	Power Saving Expert 2	The goods have been received, but have not yet installed and use, feel good.	Jingdong prefecture level city in Shandong
Jinfeng Hongyuan Flagship Store	Power Saving Expert 2	Goods received, I forgot to take pictures, the goods look good, you can start, immediately start the school to see the effect then	Jingdong prefecture level city in Shandong
Jinfeng Hongyuan Flagship Store	Power Saving Expert 2	Looks good. I'll use it for a while.	Jingdong prefecture level city in Shandong
Jinfeng Hongyuan Flagship Store	Power Saving Expert 2	Great stuff, easy to operate, easy to use, good power saving, believe in the power of science!	Jingdong prefecture level city in Shandong
Jinfeng Hongyuan Flagship Store	Power Saving Expert 2	Especially great, comparison shopping before buying, the store owner is very good, answering questions very patiently and detailed, there is a need to come back!	Jingdong prefecture level city in Shandong
Jinfeng Hongyuan Flagship Store	Power Saving Expert 2	Trustworthy, feel free to use it boldly.	Jingdong prefecture level city in Shandong
Jinfeng Hongyuan Flagship Store	Power Saving Expert 2	Logistics is super fast! Beautiful appearance! Looks like a great quality! I've never bought one before, so I'll try it first to see if it saves power. We'll see how much it saves.	Jingdong prefecture level city in Shandong
Jinfeng Hongyuan Flagship Store	Power Saving Expert 2	I saw online counterfeiting, energy saver is one of them, hahahahaha, jingdong also buy fake products	Jingdong prefecture level city in Shandong
Jinfeng Hongyuan Flagship Store	Power Saving Expert 2	Just received, installed, try a month to see how the effect and then follow up.	Jingdong prefecture level city in Shandong
Jinfeng Hongyuan Flagship Store	Power Saving Expert 2	Good for you! The goods are genuine! Good price for what you get!	Jingdong prefecture level city in Shandong
Jinfeng Hongyuan Flagship Store	Power Saving Expert 2	Things quickly received, immediately used to see if there is no effect, something river photo [↑] the same, customer service is very professional	Jingdong prefecture level city in Shandong
Jinfeng Hongyuan Flagship Store	Power Saving Expert 2	The product is very convenient Looking forward to the subsequent use of the experience results	Jingdong prefecture level city in Shandong
Jinfeng Hongyuan Flagship Store	Power Saving Expert 2	I just received it and plugged it in, we'll see exactly when it's time to pay the electric bill	Jingdong prefecture level city in Shandong
Jinfeng Hongyuan Flagship Store	Power Saving Expert 2	Saver is good Packaging and looks good How effective it is to be shared after using it.	Jingdong prefecture level city in Shandong
Jinfeng Hongyuan Flagship Store	Power Saving Expert 2	There's no way to compare. I don't know if it's energy efficient.	Jingdong prefecture level city in Shandong
Jinfeng Hongyuan Flagship Store	Power Saving Expert 2	Just installed it, try it out and comment again	Jingdong prefecture level city in Shandong
Jinfeng Hongyuan Flagship Store	Power Saving Expert 2	Super save point also good to install, after a friend introduced then went to buy to, friends say it is very good to use, home I bought, try to use a period of time, to be honest, the economizer is not big or heavy, can be very convenient to put in a lot of places, the quality is also very good, power-saving function this time seems to be about 20% of the efficiency. The owner is also very warm contact, very	Jingdong prefecture level city in Shandong
Jinfeng Hongyuan Flagship Store	Power Saving Expert 2	The reviews online are mixed. Try it for a while, but I don't know if I can return it if it doesn't save any power after a couple months.	Jingdong prefecture level city in Shandong
Jinfeng Hongyuan Flagship Store	Power Saving Expert 2	The power consumption at home is high, bought a power saver, it looks very convenient to plug on the line, see if you can save some money next month, if good, recommend to friends down the road.	Jingdong prefecture level city in Shandong
Jinfeng Hongyuan Flagship Store	Power Saving Expert 2	It's beautiful, it's genuine, but I don't know the power-saving effect.	Jingdong prefecture level city in Shandong
Jinfeng Hongyuan Flagship Store	Power Saving Expert 2	This user thinks the product is good	Jingdong prefecture level city in Shandong
Jinfeng Hongyuan Flagship Store	Power Saving Expert 2	Nice exterior design with an at-a-glance display	Jingdong prefecture level city in Shandong
Jinfeng Hongyuan Flagship Store	Power Saving Expert 2	Home large and small things are purchased in the Jingdong Mall, have been accustomed to shopping in the Jingdong, many times can buy good quality and cheap products, and courier service attitude is particularly good, sometimes too many couriers are to help me to the home, so many years have been in the Jingdong Mall shopping, trust and support! Will also support the Jingdong as always!	Jingdong prefecture level city in Shandong

Jinfeng Hongyuan Flagship Store	Power Saving Expert 2	Good workmanship, mostly better than the original wall-plugged ones, and hopefully power factor compensation saves on electricity costs	Jingdong prefecture level city in Shandong
Jinfeng Hongyuan Flagship Store	Power Saving Expert 2	I don't feel anything, I said I won't know the effect for a month or two, let's see.	Jingdong prefecture level city in Shandong
Jinfeng Hongyuan Flagship Store	Power Saving Expert 2	Delivery was pretty fast, a little pricey, just not sure if it works or not	Jingdong prefecture level city in Shandong
Jinfeng Hongyuan Flagship Store	Power Saving Expert 2	The goods have been received, I do not know how effective.	Jingdong prefecture level city in Shandong
Jinfeng Hongyuan Flagship Store	Power Saving Expert 2	So far it doesn't seem to be too noticeable, so I'll comment later.	Jingdong prefecture level city in Shandong
Jinfeng Hongyuan Flagship Store	Power Saving Expert 2	I'll comment after I've used it	Jingdong prefecture level city in Shandong
Jinfeng Hongyuan Flagship Store	Power Saving Expert 2	Not bad. You'll save a lot on your electric bill in a year!	Jingdong prefecture level city in Shandong
Jinfeng Hongyuan Flagship Store	Power Saving Expert 2	I just got it. I haven't tried it yet. I don't know what it's like.	Jingdong prefecture level city in Shandong
Jinfeng Hongyuan Flagship Store	Power Saving Expert 2	Shopping at Jingdong has become a habit, and this disease will not be cured for a while.	Jingdong prefecture level city in Shandong
Jinfeng Hongyuan Flagship Store	Power Saving Expert 2	I've been using it for a while now. I don't know if I'm still saving power or not.	Jingdong prefecture level city in Shandong
Jinfeng Hongyuan Flagship Store	Power Saving Expert 2	Received the saver and it's great! There is also a digital voltage indication. Plugged in as instructed, easy to operate, hope you're our power saver!	Jingdong prefecture level city in Shandong
Jinfeng Hongyuan Flagship Store	Power Saving Expert 2	There is no power saving as the store says, using it is the same as not using it.	Jingdong prefecture level city in Shandong
Jinfeng Hongyuan Flagship Store	Power Saving Expert 2	Use it for a while and then comment again	Jingdong prefecture level city in Shandong
Jinfeng Hongyuan Flagship Store	Power Saving Expert 2	Feeling can, to use 1 to 2 months to see how the effect, there is a voltage display, can see the voltage fluctuations amplitude, quite good!	Jingdong prefecture level city in Shandong
Jinfeng Hongyuan Flagship Store	Power Saving Expert 2	I hope it works and saves me some electricity.	Jingdong prefecture level city in Shandong
Jinfeng Hongyuan Flagship Store	Power Saving Expert 2	Nice baby, happy with the after sales	Jingdong prefecture level city in Shandong
Jinfeng Hongyuan Flagship Store	Power Saving Expert 2	Use it and talk about it.	Jingdong prefecture level city in Shandong
Jinfeng Hongyuan Flagship Store	Power Saving Expert 2	Second purchase	Jingdong prefecture level city in Shandong
Jinfeng Hongyuan Flagship Store	Power Saving Expert 2	It works.	Jingdong prefecture level city in Shandong
Jinfeng Hongyuan Flagship Store	Power Saving Expert 2	It works.	Jingdong prefecture level city in Shandong
Jinfeng Hongyuan Flagship Store	Power Saving Expert 2	I don't know if it's a power saver until I start using it	Jingdong prefecture level city in Shandong
Jinfeng Hongyuan Flagship Store	Power Saving Expert 2	serviceable	Jingdong prefecture level city in Shandong
Jinfeng Hongyuan Flagship Store	Power Saving Expert 2	This user did not fill out the rating in time	Jingdong prefecture level city in Shandong
Jinfeng Hongyuan Flagship Store	Power Saving Expert 2	There is no power saving as the store says, using it is the same as not using it.	Jingdong prefecture level city in Shandong
Jinfeng Hongyuan Flagship Store	Power Saving Expert 2	This user did not fill out the rating in time	Jingdong prefecture level city in Shandong
Jinfeng Hongyuan Flagship Store	Power Saving Expert 2	Good...good.	Jingdong prefecture level city in Shandong
Jinfeng Hongyuan Flagship Store	Power Saving Expert 2	(of an unmarried couple) be close	Jingdong prefecture level city in Shandong
Jinfeng Hongyuan Flagship Store	Power Saving Expert 2	(of an unmarried couple) be close	Jingdong prefecture level city in Shandong
Jinfeng Hongyuan Flagship Store	Power Saving Expert 2	(of an unmarried couple) be close	Jingdong prefecture level city in Shandong
Jinfeng Hongyuan Flagship Store	Power Saving Expert 2	The goods are real, high value, high quality, worth every penny, material appearance and quality at a glance is very upscale, very like HAPPY100	Jingdong prefecture level city in Shandong
Jinfeng Hongyuan Flagship Store	Power Saving Expert 2	It is the ideal product, good quality, logistics is also very fast, worthy of praise 🎁 baby quality is very good, received the first time to open the first time to see, it is very good 👍, very satisfied 😃, customer service is also available to ask questions and answer, thank you 🙏	Jingdong prefecture level city in Shandong
Jinfeng Hongyuan Flagship Store	Power Saving Expert 2	I bought it and haven't used it yet, I'll use it again.	Jingdong prefecture level city in Shandong
Jinfeng Hongyuan Flagship Store	Power Saving Expert 2	It didn't work, I used it for a few days and it was the same as before, it didn't work, I should have to return it	Jingdong prefecture level city in Shandong
Jinfeng Hongyuan Flagship Store	Power Saving Expert 2	The goods have been received, but have not yet installed and use, feel good.	Jingdong prefecture level city in Shandong
Jinfeng Hongyuan Flagship Store	Power Saving Expert 2	Goods received, I forgot to take pictures, the goods look good, you can start, immediately start the school to see the effect then	Jingdong prefecture level city in Shandong
Jinfeng Hongyuan Flagship Store	Power Saving Expert 2	Looks good. I'll use it for a while.	Jingdong prefecture level city in Shandong
Jinfeng Hongyuan Flagship Store	Power Saving Expert 2	Great stuff, easy to operate, easy to use, good power saving, believe in the power of science!	Jingdong prefecture level city in Shandong
Jinfeng Hongyuan Flagship Store	Power Saving Expert 2	Especially great, comparison shopping before buying, the store owner is very good, answering questions very patiently and detailed, there is a need to come back!	Jingdong prefecture level city in Shandong
Jinfeng Hongyuan Flagship Store	Power Saving Expert 2	Trustworthy, feel free to use it boldly.	Jingdong prefecture level city in Shandong
Jinfeng Hongyuan Flagship Store	Power Saving Expert 2	Logistics is super fast! Beautiful appearance! Looks like a great quality! I've never bought one before, so I'll try it first to see if it saves power. We'll see how much it saves.	Jingdong prefecture level city in Shandong
Jinfeng Hongyuan Flagship Store	Power Saving Expert 2	I saw online counterfeiting, energy saver is one of them, hahahahaha, jingdong also buy fake products	Jingdong prefecture level city in Shandong
Jinfeng Hongyuan Flagship Store	Power Saving Expert 2	Just received, installed, try a month to see how the effect and then follow up.	Jingdong prefecture level city in Shandong
Jinfeng Hongyuan Flagship Store	Power Saving Expert 2	Good for you! The goods are genuine! Good price for what you get!	Jingdong prefecture level city in Shandong
Jinfeng Hongyuan Flagship Store	Power Saving Expert 2	Things quickly received, immediately used to see if there is no effect, something river photo 🆙 the same, customer service is very professional	Jingdong prefecture level city in Shandong
Jinfeng Hongyuan Flagship Store	Power Saving Expert 2	The product is very convenient Looking forward to the subsequent use of the experience results	Jingdong prefecture level city in Shandong
Jinfeng Hongyuan Flagship Store	Power Saving Expert 2	I just received it and plugged it in, we'll see exactly when it's time to pay the electric bill	Jingdong prefecture level city in Shandong
Jinfeng Hongyuan Flagship Store	Power Saving Expert 2	Saver is good Packaging and looks good How effective it is to be shared after using it.	Jingdong prefecture level city in Shandong
Jinfeng Hongyuan Flagship Store	Power Saving Expert 2	There's no way to compare. I don't know if it's energy efficient.	Jingdong prefecture level city in Shandong
Jinfeng Hongyuan Flagship Store	Power Saving Expert 2	Just installed it, try it out and comment again	Jingdong prefecture level city in Shandong
Jinfeng Hongyuan Flagship Store	Power Saving Expert 2	Super save point also good to install, after a friend introduced then went to buy to, friends say it is very good to use, home I bought, try to use a period of time, to be honest, the economizer is not big or heavy, can be very convenient to put in a lot of places, the quality is also very good, power-saving function this time seems to be about 20% of the efficiency. The owner is also very warm contact, very	Jingdong prefecture level city in Shandong

Jinfeng Hongyuan Flagship Store	Power Saving Expert 2	The reviews online are mixed. Try it for a while, but I don't know if I can return it if it doesn't save any power after a couple months.	Jingdong prefecture level city in Shandong
Jinfeng Hongyuan Flagship Store	Power Saving Expert 2	The power consumption at home is high, bought a power saver, it looks very convenient to plug on the line, see if you can save some money next month, if good, recommend to friends down the road.	Jingdong prefecture level city in Shandong
Jinfeng Hongyuan Flagship Store	Power Saving Expert 2	It's beautiful, it's genuine, but I don't know the power-saving effect.	Jingdong prefecture level city in Shandong
Jinfeng Hongyuan Flagship Store	Power Saving Expert 2	This user thinks the product is good	Jingdong prefecture level city in Shandong
Jinfeng Hongyuan Flagship Store	Power Saving Expert 2	Nice exterior design with an at-a-glance display	Jingdong prefecture level city in Shandong
Jinfeng Hongyuan Flagship Store	Power Saving Expert 2	Home large and small things are purchased in the Jingdong Mall, have been accustomed to shopping in the Jingdong, many times can buy good quality and cheap products, and courier service attitude is particularly good, sometimes too many couriers are to help me to the home, so many years have been in the Jingdong Mall shopping, trust and support! Will also support the Jingdong as always!	Jingdong prefecture level city in Shandong
Jinfeng Hongyuan Flagship Store	Power Saving Expert 2	Good workmanship, mostly better than the original wall-plugged ones, and hopefully power factor compensation saves on electricity costs	Jingdong prefecture level city in Shandong
Jinfeng Hongyuan Flagship Store	Power Saving Expert 2	I don't feel anything, I said I won't know the effect for a month or two, let's see.	Jingdong prefecture level city in Shandong
Jinfeng Hongyuan Flagship Store	Power Saving Expert 2	Delivery was pretty fast, a little pricey, just not sure if it works or not	Jingdong prefecture level city in Shandong
Jinfeng Hongyuan Flagship Store	Power Saving Expert 2	The goods have been received, I do not know how effective.	Jingdong prefecture level city in Shandong
Jinfeng Hongyuan Flagship Store	Power Saving Expert 2	So far it doesn't seem to be too noticeable, so I'll comment later.	Jingdong prefecture level city in Shandong
Jinfeng Hongyuan Flagship Store	Power Saving Expert 2	I'll comment after I've used it	Jingdong prefecture level city in Shandong
Jinfeng Hongyuan Flagship Store	Power Saving Expert 2	Not bad. You'll save a lot on your electric bill in a year!	Jingdong prefecture level city in Shandong
Jinfeng Hongyuan Flagship Store	Power Saving Expert 2	I just got it. I haven't tried it yet. I don't know what it's like.	Jingdong prefecture level city in Shandong
Jinfeng Hongyuan Flagship Store	Power Saving Expert 2	Shopping at Jingdong has become a habit, and this disease will not be cured for a while.	Jingdong prefecture level city in Shandong
Jinfeng Hongyuan Flagship Store	Power Saving Expert 2	I've been using it for a while now. I don't know if I'm still saving power or not.	Jingdong prefecture level city in Shandong
Jinfeng Hongyuan Flagship Store	Power Saving Expert 2	Received the saver and it's great! There is also a digital voltage indication. Plugged in as instructed, easy to operate, hope you're our power saver!	Jingdong prefecture level city in Shandong
Jinfeng Hongyuan Flagship Store	Power Saving Expert 2	There is no power saving as the store says, using it is the same as not using it.	Jingdong prefecture level city in Shandong
Jinfeng Hongyuan Flagship Store	Power Saving Expert 2	Use it for a while and then comment again	Jingdong prefecture level city in Shandong
Jinfeng Hongyuan Flagship Store	Power Saving Expert 2	Feeling can, to use 1 to 2 months to see how the effect, there is a voltage display, can see the voltage fluctuations amplitude, quite good!	Jingdong prefecture level city in Shandong
Jinfeng Hongyuan Flagship Store	Power Saving Expert 2	I hope it works and saves me some electricity.	Jingdong prefecture level city in Shandong
Jinfeng Hongyuan Flagship Store	Power Saving Expert 2	Nice baby, happy with the after sales	Jingdong prefecture level city in Shandong
Jinfeng Hongyuan Flagship Store	Power Saving Expert 2	Use it and talk about it.	Jingdong prefecture level city in Shandong
Jinfeng Hongyuan Flagship Store	Power Saving Expert 2	Second purchase	Jingdong prefecture level city in Shandong
Jinfeng Hongyuan Flagship Store	Power Saving Expert 2	It works.	Jingdong prefecture level city in Shandong
Jinfeng Hongyuan Flagship Store	Power Saving Expert 2	It works.	Jingdong prefecture level city in Shandong
Jinfeng Hongyuan Flagship Store	Power Saving Expert 2	I don't know if it's a power saver until I start using it	Jingdong prefecture level city in Shandong
Jinfeng Hongyuan Flagship Store	Power Saving Expert 2	serviceable	Jingdong prefecture level city in Shandong
Jinfeng Hongyuan Flagship Store	Power Saving Expert 2	This user did not fill out the rating in time	Jingdong prefecture level city in Shandong
Jinfeng Hongyuan Flagship Store	Power Saving Expert 2	There is no power saving as the store says, using it is the same as not using it.	Jingdong prefecture level city in Shandong
Jinfeng Hongyuan Flagship Store	Power Saving Expert 2	This user did not fill out the rating in time	Jingdong prefecture level city in Shandong
Jinfeng Hongyuan Flagship Store	Power Saving Expert 2	Good...good.	Jingdong prefecture level city in Shandong
Jinfeng Hongyuan Flagship Store	Power Saving Expert 2	(of an unmarried couple) be close	Jingdong prefecture level city in Shandong
Jinfeng Hongyuan Flagship Store	Power Saving Expert 2	(of an unmarried couple) be close	Jingdong prefecture level city in Shandong
Jinfeng Hongyuan Flagship Store	Power Saving Expert 2	(of an unmarried couple) be close	Jingdong prefecture level city in Shandong
Jinfeng Hongyuan Flagship Store	Power Saving Expert 2	The goods are real, high value, high quality, worth every penny, material appearance and quality at a glance is very upscale, very like HAPPY💯	Jingdong prefecture level city in Shandong
Jinfeng Hongyuan Flagship Store	Power Saving Expert 2	It is the ideal product, good quality, logistics is also very fast, worthy of praise 🎁 baby quality is very good, received the first time to open the first time to see, it is very good 👍, very satisfied 😃, customer service is also available to ask questions and answer, thank you 🙏	Jingdong prefecture level city in Shandong
Jinfeng Hongyuan Flagship Store	Power Saving Expert 2	I bought it and haven't used it yet, I'll use it again.	Jingdong prefecture level city in Shandong
Jinfeng Hongyuan Flagship Store	Power Saving Expert 2	It didn't work, I used it for a few days and it was the same as before, it didn't work, I should have to return it	Jingdong prefecture level city in Shandong
Jinfeng Hongyuan Flagship Store	Power Saving Expert 2	The goods have been received, but have not yet installed and use, feel good.	Jingdong prefecture level city in Shandong
Jinfeng Hongyuan Flagship Store	Power Saving Expert 2	Goods received, I forgot to take pictures, the goods look good, you can start, immediately start the school to see the effect then	Jingdong prefecture level city in Shandong
Jinfeng Hongyuan Flagship Store	Power Saving Expert 2	Looks good. I'll use it for a while.	Jingdong prefecture level city in Shandong
Jinfeng Hongyuan Flagship Store	Power Saving Expert 2	Great stuff, easy to operate, easy to use, good power saving, believe in the power of science!	Jingdong prefecture level city in Shandong
Jinfeng Hongyuan Flagship Store	Power Saving Expert 2	Especially great, comparison shopping before buying, the store owner is very good, answering questions very patiently and detailed, there is a need to come back!	Jingdong prefecture level city in Shandong
Jinfeng Hongyuan Flagship Store	Power Saving Expert 2	Trustworthy, feel free to use it boldly.	Jingdong prefecture level city in Shandong
Jinfeng Hongyuan Flagship Store	Power Saving Expert 2	Logistics is super fast! Beautiful appearance! Looks like a great quality! I've never bought one before, so I'll try it first to see if it saves power. We'll see how much it saves.	Jingdong prefecture level city in Shandong
Jinfeng Hongyuan Flagship Store	Power Saving Expert 2	I saw online counterfeiting, energy saver is one of them, hahahahaha, jingdong also buy fake products	Jingdong prefecture level city in Shandong
Jinfeng Hongyuan Flagship Store	Power Saving Expert 2	Just received, installed, try a month to see how the effect and then follow up.	Jingdong prefecture level city in Shandong
Jinfeng Hongyuan Flagship Store	Power Saving Expert 2	Good for you! The goods are genuine! Good price for what you get!	Jingdong prefecture level city in Shandong
Jinfeng Hongyuan Flagship Store	Power Saving Expert 2	Things quickly received, immediately used to see if there is no effect, something river photo ⬆ the same, customer service is very professional	Jingdong prefecture level city in Shandong

Jinfeng Hongyuan Flagship Store	Power Saving Expert 2	The product is very convenient Looking forward to the subsequent use of the experience results	Jingdong prefecture level city in Shandong
Jinfeng Hongyuan Flagship Store	Power Saving Expert 2	I just received it and plugged it in, we'll see exactly when it's time to pay the electric bill	Jingdong prefecture level city in Shandong
Jinfeng Hongyuan Flagship Store	Power Saving Expert 2	Saver is good Packaging and looks good How effective it is to be shared after using it.	Jingdong prefecture level city in Shandong
Jinfeng Hongyuan Flagship Store	Power Saving Expert 2	There's no way to compare. I don't know if it's energy efficient.	Jingdong prefecture level city in Shandong
Jinfeng Hongyuan Flagship Store	Power Saving Expert 2	Just installed it, try it out and comment again	Jingdong prefecture level city in Shandong
Jinfeng Hongyuan Flagship Store	Power Saving Expert 2	Super save point also good to install, after a friend introduced then went to buy to, friends say it is very good to use, home I bought, try to use a period of time, to be honest, the economizer is not big or heavy, can be very convenient to put in a lot of places, the quality is also very good, power-saving function this time seems to be about 20% of the efficiency. The owner is also very warm contact, very	Jingdong prefecture level city in Shandong
Jinfeng Hongyuan Flagship Store	Power Saving Expert 2	The reviews online are mixed. Try it for a while, but I don't know if I can return it if it doesn't save any power after a couple months.	Jingdong prefecture level city in Shandong
Jinfeng Hongyuan Flagship Store	Power Saving Expert 2	The power consumption at home is high, bought a power saver, it looks very convenient to plug on the line, see if you can save some money next month, if good, recommend to friends down the road.	Jingdong prefecture level city in Shandong
Jinfeng Hongyuan Flagship Store	Power Saving Expert 2	It's beautiful, it's genuine, but I don't know the power-saving effect.	Jingdong prefecture level city in Shandong
Jinfeng Hongyuan Flagship Store	Power Saving Expert 2	This user thinks the product is good	Jingdong prefecture level city in Shandong
Jinfeng Hongyuan Flagship Store	Power Saving Expert 2	Nice exterior design with an at-a-glance display	Jingdong prefecture level city in Shandong
Jinfeng Hongyuan Flagship Store	Power Saving Expert 2	Home large and small things are purchased in the Jingdong Mall, have been accustomed to shopping in the Jingdong, many times can buy good quality and cheap products, and courier service attitude is particularly good, sometimes too many couriers are to help me to the home, so many years have been in the Jingdong Mall shopping, trust and support! Will also support the Jingdong as always!	Jingdong prefecture level city in Shandong
Jinfeng Hongyuan Flagship Store	Power Saving Expert 2	Good workmanship, mostly better than the original wall-plugged ones, and hopefully power factor compensation saves on electricity costs	Jingdong prefecture level city in Shandong
Jinfeng Hongyuan Flagship Store	Power Saving Expert 2	I don't feel anything, I said I won't know the effect for a month or two, let's see.	Jingdong prefecture level city in Shandong
Jinfeng Hongyuan Flagship Store	Power Saving Expert 2	Delivery was pretty fast, a little pricey, just not sure if it works or not	Jingdong prefecture level city in Shandong
Jinfeng Hongyuan Flagship Store	Power Saving Expert 2	The goods have been received, I do not know how effective.	Jingdong prefecture level city in Shandong
Jinfeng Hongyuan Flagship Store	Power Saving Expert 2	So far it doesn't seem to be too noticeable, so I'll comment later.	Jingdong prefecture level city in Shandong
Jinfeng Hongyuan Flagship Store	Power Saving Expert 2	I'll comment after I've used it	Jingdong prefecture level city in Shandong
Jinfeng Hongyuan Flagship Store	Power Saving Expert 2	Not bad. You'll save a lot on your electric bill in a year!	Jingdong prefecture level city in Shandong
Jinfeng Hongyuan Flagship Store	Power Saving Expert 2	I just got it. I haven't tried it yet. I don't know what it's like.	Jingdong prefecture level city in Shandong
Jinfeng Hongyuan Flagship Store	Power Saving Expert 2	Shopping at Jingdong has become a habit, and this disease will not be cured for a while.	Jingdong prefecture level city in Shandong
Jinfeng Hongyuan Flagship Store	Power Saving Expert 2	I've been using it for a while now. I don't know if I'm still saving power or not.	Jingdong prefecture level city in Shandong
Jinfeng Hongyuan Flagship Store	Power Saving Expert 2	Received the saver and it's great! There is also a digital voltage indication. Plugged in as instructed, easy to operate, hope you're our power saver!	Jingdong prefecture level city in Shandong
Jinfeng Hongyuan Flagship Store	Power Saving Expert 2	There is no power saving as the store says, using it is the same as not using it.	Jingdong prefecture level city in Shandong
Jinfeng Hongyuan Flagship Store	Power Saving Expert 2	Use it for a while and then comment again	Jingdong prefecture level city in Shandong
Jinfeng Hongyuan Flagship Store	Power Saving Expert 2	Feeling can, to use 1 to 2 months to see how the effect, there is a voltage display, can see the voltage fluctuations amplitude, quite good!	Jingdong prefecture level city in Shandong
Jinfeng Hongyuan Flagship Store	Power Saving Expert 2	I hope it works and saves me some electricity.	Jingdong prefecture level city in Shandong
Jinfeng Hongyuan Flagship Store	Power Saving Expert 2	Nice baby, happy with the after sales	Jingdong prefecture level city in Shandong
Jinfeng Hongyuan Flagship Store	Power Saving Expert 2	Use it and talk about it.	Jingdong prefecture level city in Shandong
Jinfeng Hongyuan Flagship Store	Power Saving Expert 2	Second purchase	Jingdong prefecture level city in Shandong
Jinfeng Hongyuan Flagship Store	Power Saving Expert 2	It works.	Jingdong prefecture level city in Shandong
Jinfeng Hongyuan Flagship Store	Power Saving Expert 2	It works.	Jingdong prefecture level city in Shandong
Jinfeng Hongyuan Flagship Store	Power Saving Expert 2	I don't know if it's a power saver until I start using it	Jingdong prefecture level city in Shandong
Jinfeng Hongyuan Flagship Store	Power Saving Expert 2	serviceable	Jingdong prefecture level city in Shandong
Jinfeng Hongyuan Flagship Store	Power Saving Expert 2	This user did not fill out the rating in time	Jingdong prefecture level city in Shandong
Jinfeng Hongyuan Flagship Store	Power Saving Expert 2	There is no power saving as the store says, using it is the same as not using it.	Jingdong prefecture level city in Shandong
Jinfeng Hongyuan Flagship Store	Power Saving Expert 2	This user did not fill out the rating in time	Jingdong prefecture level city in Shandong
Jinfeng Hongyuan Flagship Store	Power Saving Expert 2	Good...good.	Jingdong prefecture level city in Shandong
Jinfeng Hongyuan Flagship Store	Power Saving Expert 2	(of an unmarried couple) be close	Jingdong prefecture level city in Shandong
Jinfeng Hongyuan Flagship Store	Power Saving Expert 2	(of an unmarried couple) be close	Jingdong prefecture level city in Shandong
Jinfeng Hongyuan Flagship Store	Power Saving Expert 2	(of an unmarried couple) be close	Jingdong prefecture level city in Shandong
Jinfeng Hongyuan Flagship Store	Power Saving Expert 2	The goods are real, high value, high quality, worth every penny, material appearance and quality at a glance is very upscale, very like HAPPY1 00	Jingdong prefecture level city in Shandong
Jinfeng Hongyuan Flagship Store	Power Saving Expert 2	It is the ideal product, good quality, logistics is also very fast, worthy of praise 🎁 baby quality is very good, received the first time to open the first time to see, it is very good 👍, very satisfied 😃, customer service is also available to ask questions and answer, thank you 🙏	Jingdong prefecture level city in Shandong
Jinfeng Hongyuan Flagship Store	Power Saving Expert 2	I bought it and haven't used it yet, I'll use it again.	Jingdong prefecture level city in Shandong
Jinfeng Hongyuan Flagship Store	Power Saving Expert 2	It didn't work, I used it for a few days and it was the same as before, it didn't work, I should have to return it	Jingdong prefecture level city in Shandong
Jinfeng Hongyuan Flagship Store	Power Saving Expert 2	The goods have been received, but have not yet installed and use, feel good.	Jingdong prefecture level city in Shandong
Jinfeng Hongyuan Flagship Store	Power Saving Expert 2	Goods received, I forgot to take pictures, the goods look good, you can start, immediately start the school to see the effect then	Jingdong prefecture level city in Shandong
Jinfeng Hongyuan Flagship Store	Power Saving Expert 2	Looks good. I'll use it for a while.	Jingdong prefecture level city in Shandong
Jinfeng Hongyuan Flagship Store	Power Saving Expert 2	Great stuff, easy to operate, easy to use, good power saving, believe in the power of science!	Jingdong prefecture level city in Shandong

Jinfeng Hongyuan Flagship Store	Power Saving Expert 2	Especially great, comparison shopping before buying, the store owner is very good, answering questions very patiently and detailed, there is a need to come back!	Jingdong prefecture level city in Shandong
Jinfeng Hongyuan Flagship Store	Power Saving Expert 2	Trustworthy, feel free to use it boldly.	Jingdong prefecture level city in Shandong
Jinfeng Hongyuan Flagship Store	Power Saving Expert 2	Logistics is super fast! Beautiful appearance! Looks like a great quality! I've never bought one before, so I'll try it first to see if it saves power. We'll see how much it saves.	Jingdong prefecture level city in Shandong
Jinfeng Hongyuan Flagship Store	Power Saving Expert 2	I saw online counterfeiting, energy saver is one of them, hahahahaha, jingdong also buy fake products	Jingdong prefecture level city in Shandong
Jinfeng Hongyuan Flagship Store	Power Saving Expert 2	Just received, installed, try a month to see how the effect and then follow up.	Jingdong prefecture level city in Shandong
Jinfeng Hongyuan Flagship Store	Power Saving Expert 2	Good for you! The goods are genuine! Good price for what you get!	Jingdong prefecture level city in Shandong
Jinfeng Hongyuan Flagship Store	Power Saving Expert 2	Things quickly received, immediately used to see if there is no effect, something river photo ↑ the same, customer service is very professional	Jingdong prefecture level city in Shandong
Jinfeng Hongyuan Flagship Store	Power Saving Expert 2	The product is very convenient Looking forward to the subsequent use of the experience results	Jingdong prefecture level city in Shandong
Jinfeng Hongyuan Flagship Store	Power Saving Expert 2	I just received it and plugged it in, we'll see exactly when it's time to pay the electric bill	Jingdong prefecture level city in Shandong
Jinfeng Hongyuan Flagship Store	Power Saving Expert 2	Saver is good Packaging and looks good How effective it is to be shared after using it.	Jingdong prefecture level city in Shandong
Jinfeng Hongyuan Flagship Store	Power Saving Expert 2	There's no way to compare. I don't know if it's energy efficient.	Jingdong prefecture level city in Shandong
Jinfeng Hongyuan Flagship Store	Power Saving Expert 2	Just installed it, try it out and comment again	Jingdong prefecture level city in Shandong
Jinfeng Hongyuan Flagship Store	Power Saving Expert 2	Super save point also good to install, after a friend introduced then went to buy to, friends say it is very good to use, home I bought, try to use a period of time, to be honest, the economizer is not big or heavy, can be very convenient to put in a lot of places, the quality is also very good, power-saving function this time seems to be about 20% of the efficiency. The owner is also very warm contact, very	Jingdong prefecture level city in Shandong
Jinfeng Hongyuan Flagship Store	Power Saving Expert 2	The reviews online are mixed. Try it for a while, but I don't know if I can return it if it doesn't save any power after a couple months.	Jingdong prefecture level city in Shandong
Jinfeng Hongyuan Flagship Store	Power Saving Expert 2	The power consumption at home is high, bought a power saver, it looks very convenient to plug on the line, see if you can save some money next month, if good, recommend to friends down the road.	Jingdong prefecture level city in Shandong
Jinfeng Hongyuan Flagship Store	Power Saving Expert 2	It's beautiful, it's genuine, but I don't know the power-saving effect.	Jingdong prefecture level city in Shandong
Jinfeng Hongyuan Flagship Store	Power Saving Expert 2	This user thinks the product is good	Jingdong prefecture level city in Shandong
Jinfeng Hongyuan Flagship Store	Power Saving Expert 2	Nice exterior design with an at-a-glance display	Jingdong prefecture level city in Shandong
Jinfeng Hongyuan Flagship Store	Power Saving Expert 2	Home large and small things are purchased in the Jingdong Mall, have been accustomed to shopping in the Jingdong, many times can buy good quality and cheap products, and courier service attitude is particularly good, sometimes too many couriers are to help me to the home, so many years have been in the Jingdong Mall shopping, trust and support! Will also support the Jingdong as always!	Jingdong prefecture level city in Shandong
Jinfeng Hongyuan Flagship Store	Power Saving Expert 2	Good workmanship, mostly better than the original wall-plugged ones, and hopefully power factor compensation saves on electricity costs	Jingdong prefecture level city in Shandong
Jinfeng Hongyuan Flagship Store	Power Saving Expert 2	I don't feel anything, I said I won't know the effect for a month or two, let's see.	Jingdong prefecture level city in Shandong
Jinfeng Hongyuan Flagship Store	Power Saving Expert 2	Delivery was pretty fast, a little pricey, just not sure if it works or not	Jingdong prefecture level city in Shandong
Jinfeng Hongyuan Flagship Store	Power Saving Expert 2	The goods have been received, I do not know how effective.	Jingdong prefecture level city in Shandong
Jinfeng Hongyuan Flagship Store	Power Saving Expert 2	So far it doesn't seem to be too noticeable, so I'll comment later.	Jingdong prefecture level city in Shandong
Jinfeng Hongyuan Flagship Store	Power Saving Expert 2	I'll comment after I've used it	Jingdong prefecture level city in Shandong
Jinfeng Hongyuan Flagship Store	Power Saving Expert 2	Not bad. You'll save a lot on your electric bill in a year!	Jingdong prefecture level city in Shandong
Jinfeng Hongyuan Flagship Store	Power Saving Expert 2	I just got it. I haven't tried it yet. I don't know what it's like.	Jingdong prefecture level city in Shandong
Jinfeng Hongyuan Flagship Store	Power Saving Expert 2	Shopping at Jingdong has become a habit, and this disease will not be cured for a while.	Jingdong prefecture level city in Shandong
Jinfeng Hongyuan Flagship Store	Power Saving Expert 2	I've been using it for a while now. I don't know if I'm still saving power or not.	Jingdong prefecture level city in Shandong
Jinfeng Hongyuan Flagship Store	Power Saving Expert 2	Received the saver and it's great! There is also a digital voltage indication. Plugged in as instructed, easy to operate, hope you're our power saver!	Jingdong prefecture level city in Shandong
Jinfeng Hongyuan Flagship Store	Power Saving Expert 2	There is no power saving as the store says, using it is the same as not using it.	Jingdong prefecture level city in Shandong
Jinfeng Hongyuan Flagship Store	Power Saving Expert 2	Use it for a while and then comment again	Jingdong prefecture level city in Shandong
Jinfeng Hongyuan Flagship Store	Power Saving Expert 2	Feeling can, to use 1 to 2 months to see how the effect, there is a voltage display, can see the voltage fluctuations amplitude, quite good!	Jingdong prefecture level city in Shandong
Jinfeng Hongyuan Flagship Store	Power Saving Expert 2	I hope it works and saves me some electricity.	Jingdong prefecture level city in Shandong
Jinfeng Hongyuan Flagship Store	Power Saving Expert 2	Nice baby, happy with the after sales	Jingdong prefecture level city in Shandong
Jinfeng Hongyuan Flagship Store	Power Saving Expert 2	Use it and talk about it.	Jingdong prefecture level city in Shandong
Jinfeng Hongyuan Flagship Store	Power Saving Expert 2	Second purchase	Jingdong prefecture level city in Shandong
Jinfeng Hongyuan Flagship Store	Power Saving Expert 2	It works.	Jingdong prefecture level city in Shandong
Jinfeng Hongyuan Flagship Store	Power Saving Expert 2	It works.	Jingdong prefecture level city in Shandong
Jinfeng Hongyuan Flagship Store	Power Saving Expert 2	I don't know if it's a power saver until I start using it	Jingdong prefecture level city in Shandong
Jinfeng Hongyuan Flagship Store	Power Saving Expert 2	serviceable	Jingdong prefecture level city in Shandong
Jinfeng Hongyuan Flagship Store	Power Saving Expert 2	This user did not fill out the rating in time	Jingdong prefecture level city in Shandong
Jinfeng Hongyuan Flagship Store	Power Saving Expert 2	There is no power saving as the store says, using it is the same as not using it.	Jingdong prefecture level city in Shandong
Jinfeng Hongyuan Flagship Store	Power Saving Expert 2	This user did not fill out the rating in time	Jingdong prefecture level city in Shandong
Jinfeng Hongyuan Flagship Store	Power Saving Expert 2	Good...good.	Jingdong prefecture level city in Shandong
Jinfeng Hongyuan Flagship Store	Power Saving Expert 2	(of an unmarried couple) be close	Jingdong prefecture level city in Shandong
Jinfeng Hongyuan Flagship Store	Power Saving Expert 2	(of an unmarried couple) be close	Jingdong prefecture level city in Shandong
Jinfeng Hongyuan Flagship Store	Power Saving Expert 2	(of an unmarried couple) be close	Jingdong prefecture level city in Shandong
Jinfeng Hongyuan Flagship Store	Power Saving Expert 2	The goods are real, high value, high quality, worth every penny, material appearance and quality at a glance is very upscale, very like HAPPY1 00	Jingdong prefecture level city in Shandong

Jinfeng Hongyuan Flagship Store	Power Saving Expert 2	It is the ideal product, good quality, logistics is also very fast, worthy of praise 🎁 baby quality is very good, received the first time to open the first time to see, it is very good 👍, very satisfied ☺, customer service is also available to ask questions and answer, thank you 🙏	Jingdong prefecture level city in Shandong
Jinfeng Hongyuan Flagship Store	Power Saving Expert 2	I bought it and haven't used it yet, I'll use it again.	Jingdong prefecture level city in Shandong
Jinfeng Hongyuan Flagship Store	Power Saving Expert 2	It didn't work, I used it for a few days and it was the same as before, it didn't work, I should have to return it	Jingdong prefecture level city in Shandong
Jinfeng Hongyuan Flagship Store	Power Saving Expert 2	The goods have been received, but have not yet installed and use, feel good.	Jingdong prefecture level city in Shandong
Jinfeng Hongyuan Flagship Store	Power Saving Expert 2	Goods received, I forgot to take pictures, the goods look good, you can start, immediately start the school to see the effect then	Jingdong prefecture level city in Shandong
Jinfeng Hongyuan Flagship Store	Power Saving Expert 2	Looks good. I'll use it for a while.	Jingdong prefecture level city in Shandong
Jinfeng Hongyuan Flagship Store	Power Saving Expert 2	Great stuff, easy to operate, easy to use, good power saving, believe in the power of science!	Jingdong prefecture level city in Shandong
Jinfeng Hongyuan Flagship Store	Power Saving Expert 2	Especially great, comparison shopping before buying, the store owner is very good, answering questions very patiently and detailed, there is a need to come back!	Jingdong prefecture level city in Shandong
Jinfeng Hongyuan Flagship Store	Power Saving Expert 2	Trustworthy, feel free to use it boldly.	Jingdong prefecture level city in Shandong
Jinfeng Hongyuan Flagship Store	Power Saving Expert 2	Logistics is super fast! Beautiful appearance! Looks like a great quality! I've never bought one before, so I'll try it first to see if it saves power. We'll see how much it saves.	Jingdong prefecture level city in Shandong
Jinfeng Hongyuan Flagship Store	Power Saving Expert 2	I saw online counterfeiting, energy saver is one of them, hahahahaha, jingdong also buy fake products	Jingdong prefecture level city in Shandong
Jinfeng Hongyuan Flagship Store	Power Saving Expert 2	Just received, installed, try a month to see how the effect and then follow up.	Jingdong prefecture level city in Shandong
Jinfeng Hongyuan Flagship Store	Power Saving Expert 2	Good for you! The goods are genuine! Good price for what you get!	Jingdong prefecture level city in Shandong
Jinfeng Hongyuan Flagship Store	Power Saving Expert 2	Things quickly received, immediately used to see if there is no effect, something river photo ⬆ the same, customer service is very professional	Jingdong prefecture level city in Shandong
Jinfeng Hongyuan Flagship Store	Power Saving Expert 2	The product is very convenient Looking forward to the subsequent use of the experience results	Jingdong prefecture level city in Shandong
Jinfeng Hongyuan Flagship Store	Power Saving Expert 2	I just received it and plugged it in, we'll see exactly when it's time to pay the electric bill	Jingdong prefecture level city in Shandong
Jinfeng Hongyuan Flagship Store	Power Saving Expert 2	Saver is good Packaging and looks good How effective it is to be shared after using it.	Jingdong prefecture level city in Shandong
Jinfeng Hongyuan Flagship Store	Power Saving Expert 2	There's no way to compare. I don't know if it's energy efficient.	Jingdong prefecture level city in Shandong
Jinfeng Hongyuan Flagship Store	Power Saving Expert 2	Just installed it, try it out and comment again	Jingdong prefecture level city in Shandong
Jinfeng Hongyuan Flagship Store	Power Saving Expert 2	Super save point also good to install, after a friend introduced then went to buy to, friends say it is very good to use, home I bought, try to use a period of time, to be honest, the economizer is not big or heavy, can be very convenient to put in a lot of places, the quality is also very good, power-saving function this time seems to be about 20% of the efficiency. The owner is also very warm contact, very	Jingdong prefecture level city in Shandong
Jinfeng Hongyuan Flagship Store	Power Saving Expert 2	The reviews online are mixed. Try it for a while, but I don't know if I can return it if it doesn't save any power after a couple months.	Jingdong prefecture level city in Shandong
Jinfeng Hongyuan Flagship Store	Power Saving Expert 2	The power consumption at home is high, bought a power saver, it looks very convenient to plug on the line, see if you can save some money next month, if good, recommend to friends down the road.	Jingdong prefecture level city in Shandong
Jinfeng Hongyuan Flagship Store	Power Saving Expert 2	It's beautiful, it's genuine, but I don't know the power-saving effect.	Jingdong prefecture level city in Shandong
Jinfeng Hongyuan Flagship Store	Power Saving Expert 2	This user thinks the product is good	Jingdong prefecture level city in Shandong
Jinfeng Hongyuan Flagship Store	Power Saving Expert 2	Nice exterior design with an at-a-glance display	Jingdong prefecture level city in Shandong
Jinfeng Hongyuan Flagship Store	Power Saving Expert 2	Home large and small things are purchased in the Jingdong Mall, have been accustomed to shopping in the Jingdong, many times can buy good quality and cheap products, and courier service attitude is particularly good, sometimes too many couriers are to help me to the home, so many years have been in the Jingdong Mall shopping, trust and support! Will also support the Jingdong as always!	Jingdong prefecture level city in Shandong
Jinfeng Hongyuan Flagship Store	Power Saving Expert 2	Good workmanship, mostly better than the original wall-plugged ones, and hopefully power factor compensation saves on electricity costs	Jingdong prefecture level city in Shandong
Jinfeng Hongyuan Flagship Store	Power Saving Expert 2	I don't feel anything, I said I won't know the effect for a month or two, let's see.	Jingdong prefecture level city in Shandong
Jinfeng Hongyuan Flagship Store	Power Saving Expert 2	Delivery was pretty fast, a little pricey, just not sure if it works or not	Jingdong prefecture level city in Shandong
Jinfeng Hongyuan Flagship Store	Power Saving Expert 2	The goods have been received, I do not know how effective.	Jingdong prefecture level city in Shandong
Jinfeng Hongyuan Flagship Store	Power Saving Expert 2	So far it doesn't seem to be too noticeable, so I'll comment later.	Jingdong prefecture level city in Shandong
Jinfeng Hongyuan Flagship Store	Power Saving Expert 2	I'll comment after I've used it	Jingdong prefecture level city in Shandong
Jinfeng Hongyuan Flagship Store	Power Saving Expert 2	Not bad. You'll save a lot on your electric bill in a year!	Jingdong prefecture level city in Shandong
Jinfeng Hongyuan Flagship Store	Power Saving Expert 2	I just got it. I haven't tried it yet. I don't know what it's like.	Jingdong prefecture level city in Shandong
Jinfeng Hongyuan Flagship Store	Power Saving Expert 2	Shopping at Jingdong has become a habit, and this disease will not be cured for a while.	Jingdong prefecture level city in Shandong
Jinfeng Hongyuan Flagship Store	Power Saving Expert 2	I've been using it for a while now. I don't know if I'm still saving power or not.	Jingdong prefecture level city in Shandong
Jinfeng Hongyuan Flagship Store	Power Saving Expert 2	Received the saver and it's great! There is also a digital voltage indication. Plugged in as instructed, easy to operate, hope you're our power saver!	Jingdong prefecture level city in Shandong
Jinfeng Hongyuan Flagship Store	Power Saving Expert 2	There is no power saving as the store says, using it is the same as not using it.	Jingdong prefecture level city in Shandong
Jinfeng Hongyuan Flagship Store	Power Saving Expert 2	Use it for a while and then comment again	Jingdong prefecture level city in Shandong
Jinfeng Hongyuan Flagship Store	Power Saving Expert 2	Feeling can, to use 1 to 2 months to see how the effect, there is a voltage display, can see the voltage fluctuations amplitude, quite good!	Jingdong prefecture level city in Shandong
Jinfeng Hongyuan Flagship Store	Power Saving Expert 2	I hope it works and saves me some electricity.	Jingdong prefecture level city in Shandong
Jinfeng Hongyuan Flagship Store	Power Saving Expert 2	Nice baby, happy with the after sales	Jingdong prefecture level city in Shandong
Jinfeng Hongyuan Flagship Store	Power Saving Expert 2	Use it and talk about it.	Jingdong prefecture level city in Shandong
Jinfeng Hongyuan Flagship Store	Power Saving Expert 2	Second purchase	Jingdong prefecture level city in Shandong
Jinfeng Hongyuan Flagship Store	Power Saving Expert 2	It works.	Jingdong prefecture level city in Shandong
Jinfeng Hongyuan Flagship Store	Power Saving Expert 2	It works.	Jingdong prefecture level city in Shandong
Jinfeng Hongyuan Flagship Store	Power Saving Expert 2	I don't know if it's a power saver until I start using it	Jingdong prefecture level city in Shandong
Jinfeng Hongyuan Flagship Store	Power Saving Expert 2	serviceable	Jingdong prefecture level city in Shandong
Jinfeng Hongyuan Flagship Store	Power Saving Expert 2	This user did not fill out the rating in time	Jingdong prefecture level city in Shandong

Jinfeng Hongyuan Flagship Store	Power Saving Expert 2	There is no power saving as the store says, using it is the same as not using it.	Jingdong prefecture level city in Shandong
Jinfeng Hongyuan Flagship Store	Power Saving Expert 2	This user did not fill out the rating in time	Jingdong prefecture level city in Shandong
Jinfeng Hongyuan Flagship Store	Power Saving Expert 2	Good...good.	Jingdong prefecture level city in Shandong
Jinfeng Hongyuan Flagship Store	Power Saving Expert 2	(of an unmarried couple) be close	Jingdong prefecture level city in Shandong
Jinfeng Hongyuan Flagship Store	Power Saving Expert 2	(of an unmarried couple) be close	Jingdong prefecture level city in Shandong
Jinfeng Hongyuan Flagship Store	Power Saving Expert 2	(of an unmarried couple) be close	Jingdong prefecture level city in Shandong
Jinfeng Hongyuan Flagship Store	Power Saving Expert 2	The goods are real, high value, high quality, worth every penny, material appearance and quality at a glance is very upscale, very like HAPPY💯	Jingdong prefecture level city in Shandong
Jinfeng Hongyuan Flagship Store	Power Saving Expert 2	It is the ideal product, good quality, logistics is also very fast, worthy of praise 🎁 baby quality is very good, received the first time to open the first time to see, it is very good 👍, very satisfied 😀, customer service is also available to ask questions and answer, thank you 🙏	Jingdong prefecture level city in Shandong
Jinfeng Hongyuan Flagship Store	Power Saving Expert 2	I bought it and haven't used it yet, I'll use it again.	Jingdong prefecture level city in Shandong
Jinfeng Hongyuan Flagship Store	Power Saving Expert 2	It didn't work, I used it for a few days and it was the same as before, it didn't work, I should have to return it	Jingdong prefecture level city in Shandong
Jinfeng Hongyuan Flagship Store	Power Saving Expert 2	The goods have been received, but have not yet installed and use, feel good.	Jingdong prefecture level city in Shandong
Jinfeng Hongyuan Flagship Store	Power Saving Expert 2	Goods received, I forgot to take pictures, the goods look good, you can start, immediately start the school to see the effect then	Jingdong prefecture level city in Shandong
Jinfeng Hongyuan Flagship Store	Power Saving Expert 2	Looks good. I'll use it for a while.	Jingdong prefecture level city in Shandong
Jinfeng Hongyuan Flagship Store	Power Saving Expert 2	Great stuff, easy to operate, easy to use, good power saving, believe in the power of science!	Jingdong prefecture level city in Shandong
Jinfeng Hongyuan Flagship Store	Power Saving Expert 2	Especially great, comparison shopping before buying, the store owner is very good, answering questions very patiently and detailed, there is a need to come back!	Jingdong prefecture level city in Shandong
Jinfeng Hongyuan Flagship Store	Power Saving Expert 2	Trustworthy, feel free to use it boldly.	Jingdong prefecture level city in Shandong
Jinfeng Hongyuan Flagship Store	Power Saving Expert 2	Logistics is super fast! Beautiful appearance! Looks like a great quality! I've never bought one before, so I'll try it first to see if it saves power. We'll see how much it saves.	Jingdong prefecture level city in Shandong
Jinfeng Hongyuan Flagship Store	Power Saving Expert 2	I saw online counterfeiting, energy saver is one of them, hahahahaha, jingdong also buy fake products	Jingdong prefecture level city in Shandong
Jinfeng Hongyuan Flagship Store	Power Saving Expert 2	Just received, installed, try a month to see how the effect and then follow up.	Jingdong prefecture level city in Shandong
Jinfeng Hongyuan Flagship Store	Power Saving Expert 2	Good for you! The goods are genuine! Good price for what you get!	Jingdong prefecture level city in Shandong
Jinfeng Hongyuan Flagship Store	Power Saving Expert 2	Things quickly received, immediately used to see if there is no effect, something river photo ↑ the same, customer service is very professional	Jingdong prefecture level city in Shandong
Jinfeng Hongyuan Flagship Store	Power Saving Expert 2	The product is very convenient Looking forward to the subsequent use of the experience results	Jingdong prefecture level city in Shandong
Jinfeng Hongyuan Flagship Store	Power Saving Expert 2	I just received it and plugged it in, we'll see exactly when it's time to pay the electric bill	Jingdong prefecture level city in Shandong
Jinfeng Hongyuan Flagship Store	Power Saving Expert 2	Saver is good Packaging and looks good How effective it is to be shared after using it.	Jingdong prefecture level city in Shandong
Jinfeng Hongyuan Flagship Store	Power Saving Expert 2	There's no way to compare. I don't know if it's energy efficient.	Jingdong prefecture level city in Shandong
Jinfeng Hongyuan Flagship Store	Power Saving Expert 2	Just installed it, try it out and comment again	Jingdong prefecture level city in Shandong
Jinfeng Hongyuan Flagship Store	Power Saving Expert 2	Super save point also good to install, after a friend introduced then went to buy to, friends say it is very good to use, home I bought, try to use a period of time, to be honest, the economizer is not big or heavy, can be very convenient to put in a lot of places, the quality is also very good, power-saving function this time seems to be about 20% of the efficiency. The owner is also very warm contact, very	Jingdong prefecture level city in Shandong
Jinfeng Hongyuan Flagship Store	Power Saving Expert 2	The reviews online are mixed. Try it for a while, but I don't know if I can return it if it doesn't save any power after a couple months.	Jingdong prefecture level city in Shandong
Jinfeng Hongyuan Flagship Store	Power Saving Expert 2	The power consumption at home is high, bought a power saver, it looks very convenient to plug on the line, see if you can save some money next month, if good, recommend to friends down the road.	Jingdong prefecture level city in Shandong
Jinfeng Hongyuan Flagship Store	Power Saving Expert 2	It's beautiful, it's genuine, but I don't know the power-saving effect.	Jingdong prefecture level city in Shandong
Jinfeng Hongyuan Flagship Store	Power Saving Expert 2	This user thinks the product is good	Jingdong prefecture level city in Shandong
Jinfeng Hongyuan Flagship Store	Power Saving Expert 2	Nice exterior design with an at-a-glance display	Jingdong prefecture level city in Shandong
Jinfeng Hongyuan Flagship Store	Power Saving Expert 2	Home large and small things are purchased in the Jingdong Mall, have been accustomed to shopping in the Jingdong, many times can buy good quality and cheap products, and courier service attitude is particularly good, sometimes too many couriers are to help me to the home, so many years have been in the Jingdong Mall shopping, trust and support! Will also support the Jingdong as always!	Jingdong prefecture level city in Shandong
Jinfeng Hongyuan Flagship Store	Power Saving Expert 2	Good workmanship, mostly better than the original wall-plugged ones, and hopefully power factor compensation saves on electricity costs	Jingdong prefecture level city in Shandong
Jinfeng Hongyuan Flagship Store	Power Saving Expert 2	I don't feel anything, I said I won't know the effect for a month or two, let's see.	Jingdong prefecture level city in Shandong
Jinfeng Hongyuan Flagship Store	Power Saving Expert 2	Delivery was pretty fast, a little pricey, just not sure if it works or not	Jingdong prefecture level city in Shandong
Jinfeng Hongyuan Flagship Store	Power Saving Expert 2	The goods have been received, I do not know how effective.	Jingdong prefecture level city in Shandong
Jinfeng Hongyuan Flagship Store	Power Saving Expert 2	So far it doesn't seem to be too noticeable, so I'll comment later.	Jingdong prefecture level city in Shandong
Jinfeng Hongyuan Flagship Store	Power Saving Expert 2	I'll comment after I've used it	Jingdong prefecture level city in Shandong
Jinfeng Hongyuan Flagship Store	Power Saving Expert 2	Not bad. You'll save a lot on your electric bill in a year!	Jingdong prefecture level city in Shandong
Jinfeng Hongyuan Flagship Store	Power Saving Expert 2	I just got it. I haven't tried it yet. I don't know what it's like.	Jingdong prefecture level city in Shandong
Jinfeng Hongyuan Flagship Store	Power Saving Expert 2	Shopping at Jingdong has become a habit, and this disease will not be cured for a while.	Jingdong prefecture level city in Shandong
Jinfeng Hongyuan Flagship Store	Power Saving Expert 2	I've been using it for a while now. I don't know if I'm still saving power or not.	Jingdong prefecture level city in Shandong
Jinfeng Hongyuan Flagship Store	Power Saving Expert 2	Received the saver and it's great! There is also a digital voltage indication. Plugged in as instructed, easy to operate, hope you're our power saver!	Jingdong prefecture level city in Shandong
Jinfeng Hongyuan Flagship Store	Power Saving Expert 2	There is no power saving as the store says, using it is the same as not using it.	Jingdong prefecture level city in Shandong
Jinfeng Hongyuan Flagship Store	Power Saving Expert 2	Use it for a while and then comment again	Jingdong prefecture level city in Shandong
Jinfeng Hongyuan Flagship Store	Power Saving Expert 2	Feeling can, to use 1 to 2 months to see how the effect, there is a voltage display, can see the voltage fluctuations amplitude, quite good!	Jingdong prefecture level city in Shandong
Jinfeng Hongyuan Flagship Store	Power Saving Expert 2	I hope it works and saves me some electricity.	Jingdong prefecture level city in Shandong
Jinfeng Hongyuan Flagship Store	Power Saving Expert 2	Nice baby, happy with the after sales	Jingdong prefecture level city in Shandong

Jinfeng Hongyuan Flagship Store	Power Saving Expert 2	Use it and talk about it.	Jingdong prefecture level city in Shandong
Jinfeng Hongyuan Flagship Store	Power Saving Expert 2	Second purchase	Jingdong prefecture level city in Shandong
Jinfeng Hongyuan Flagship Store	Power Saving Expert 2	It works.	Jingdong prefecture level city in Shandong
Jinfeng Hongyuan Flagship Store	Power Saving Expert 2	It works.	Jingdong prefecture level city in Shandong
Jinfeng Hongyuan Flagship Store	Power Saving Expert 2	I don't know if it's a power saver until I start using it	Jingdong prefecture level city in Shandong
Jinfeng Hongyuan Flagship Store	Power Saving Expert 2	serviceable	Jingdong prefecture level city in Shandong
Jinfeng Hongyuan Flagship Store	Power Saving Expert 2	This user did not fill out the rating in time	Jingdong prefecture level city in Shandong
Jinfeng Hongyuan Flagship Store	Power Saving Expert 2	There is no power saving as the store says, using it is the same as not using it.	Jingdong prefecture level city in Shandong
Jinfeng Hongyuan Flagship Store	Power Saving Expert 2	This user did not fill out the rating in time	Jingdong prefecture level city in Shandong
Jinfeng Hongyuan Flagship Store	Power Saving Expert 2	Good...good.	Jingdong prefecture level city in Shandong
Jinfeng Hongyuan Flagship Store	Power Saving Expert 2	(of an unmarried couple) be close	Jingdong prefecture level city in Shandong
Jinfeng Hongyuan Flagship Store	Power Saving Expert 2	(of an unmarried couple) be close	Jingdong prefecture level city in Shandong
Jinfeng Hongyuan Flagship Store	Power Saving Expert 2	(of an unmarried couple) be close	Jingdong prefecture level city in Shandong
Jinfeng Hongyuan Flagship Store	Power Saving Expert 2	The goods are real, high value, high quality, worth every penny, material appearance and quality at a glance is very upscale, very like HAPPY1 00	Jingdong prefecture level city in Shandong
Jinfeng Hongyuan Flagship Store	Power Saving Expert 2	It is the ideal product, good quality, logistics is also very fast, worthy of praise 🎁 baby quality is very good, received the first time to open the first time to see, it is very good 👍, very satisfied 😃, customer service is also available to ask questions and answer, thank you 🙏	Jingdong prefecture level city in Shandong
Jinfeng Hongyuan Flagship Store	Power Saving Expert 2	I bought it and haven't used it yet, I'll use it again.	Jingdong prefecture level city in Shandong
Jinfeng Hongyuan Flagship Store	Power Saving Expert 2	It didn't work, I used it for a few days and it was the same as before, it didn't work, I should have to return it	Jingdong prefecture level city in Shandong
Jinfeng Hongyuan Flagship Store	Power Saving Expert 2	The goods have been received, but have not yet installed and use, feel good.	Jingdong prefecture level city in Shandong
Jinfeng Hongyuan Flagship Store	Power Saving Expert 2	Goods received, I forgot to take pictures, the goods look good, you can start, immediately start the school to see the effect then	Jingdong prefecture level city in Shandong
Jinfeng Hongyuan Flagship Store	Power Saving Expert 2	Looks good. I'll use it for a while.	Jingdong prefecture level city in Shandong
Jinfeng Hongyuan Flagship Store	Power Saving Expert 2	Great stuff, easy to operate, easy to use, good power saving, believe in the power of science!	Jingdong prefecture level city in Shandong
Jinfeng Hongyuan Flagship Store	Power Saving Expert 2	Especially great, comparison shopping before buying, the store owner is very good, answering questions very patiently and detailed, there is a need to come back!	Jingdong prefecture level city in Shandong
Jinfeng Hongyuan Flagship Store	Power Saving Expert 2	Trustworthy, feel free to use it boldly.	Jingdong prefecture level city in Shandong
Jinfeng Hongyuan Flagship Store	Power Saving Expert 2	Logistics is super fast! Beautiful appearance! Looks like a great quality! I've never bought one before, so I'll try it first to see if it saves power. We'll see how much it saves.	Jingdong prefecture level city in Shandong
Jinfeng Hongyuan Flagship Store	Power Saving Expert 2	I saw online counterfeiting, energy saver is one of them, hahahahaha, jingdong also buy fake products	Jingdong prefecture level city in Shandong
Jinfeng Hongyuan Flagship Store	Power Saving Expert 2	Just received, installed, try a month to see how the effect and then follow up.	Jingdong prefecture level city in Shandong
Jinfeng Hongyuan Flagship Store	Power Saving Expert 2	Good for you! The goods are genuine! Good price for what you get!	Jingdong prefecture level city in Shandong
Jinfeng Hongyuan Flagship Store	Power Saving Expert 2	Things quickly received, immediately used to see if there is no effect, something river photo ⬆ the same, customer service is very professional	Jingdong prefecture level city in Shandong
Jinfeng Hongyuan Flagship Store	Power Saving Expert 2	The product is very convenient Looking forward to the subsequent use of the experience results	Jingdong prefecture level city in Shandong
Jinfeng Hongyuan Flagship Store	Power Saving Expert 2	I just received it and plugged it in, we'll see exactly when it's time to pay the electric bill	Jingdong prefecture level city in Shandong
Jinfeng Hongyuan Flagship Store	Power Saving Expert 2	Saver is good Packaging and looks good How effective it is to be shared after using it.	Jingdong prefecture level city in Shandong
Jinfeng Hongyuan Flagship Store	Power Saving Expert 2	There's no way to compare. I don't know if it's energy efficient.	Jingdong prefecture level city in Shandong
Jinfeng Hongyuan Flagship Store	Power Saving Expert 2	Just installed it, try it out and comment again	Jingdong prefecture level city in Shandong
Jinfeng Hongyuan Flagship Store	Power Saving Expert 2	Super save point also good to install, after a friend introduced then went to buy to, friends say it is very good to use, home I bought, try to use a period of time, to be honest, the economizer is not big or heavy, can be very convenient to put in a lot of places, the quality is also very good, power-saving function this time seems to be about 20% of the efficiency. The owner is also very warm contact, very	Jingdong prefecture level city in Shandong
Jinfeng Hongyuan Flagship Store	Power Saving Expert 2	The reviews online are mixed. Try it for a while, but I don't know if I can return it if it doesn't save any power after a couple months.	Jingdong prefecture level city in Shandong
Jinfeng Hongyuan Flagship Store	Power Saving Expert 2	The power consumption at home is high, bought a power saver, it looks very convenient to plug on the line, see if you can save some money next month, if good, recommend to friends down the road.	Jingdong prefecture level city in Shandong
Jinfeng Hongyuan Flagship Store	Power Saving Expert 2	It's beautiful, it's genuine, but I don't know the power-saving effect.	Jingdong prefecture level city in Shandong
Jinfeng Hongyuan Flagship Store	Power Saving Expert 2	This user thinks the product is good	Jingdong prefecture level city in Shandong
Jinfeng Hongyuan Flagship Store	Power Saving Expert 2	Nice exterior design with an at-a-glance display	Jingdong prefecture level city in Shandong
Jinfeng Hongyuan Flagship Store	Power Saving Expert 2	Home large and small things are purchased in the Jingdong Mall, have been accustomed to shopping in the Jingdong, many times can buy good quality and cheap products, and courier service attitude is particularly good, sometimes too many couriers are to help me to the home, so many years have been in the Jingdong Mall shopping, trust and support! Will also support the Jingdong as always!	Jingdong prefecture level city in Shandong
Jinfeng Hongyuan Flagship Store	Power Saving Expert 2	Good workmanship, mostly better than the original wall-plugged ones, and hopefully power factor compensation saves on electricity costs	Jingdong prefecture level city in Shandong
Jinfeng Hongyuan Flagship Store	Power Saving Expert 2	I don't feel anything, I said I won't know the effect for a month or two, let's see.	Jingdong prefecture level city in Shandong
Jinfeng Hongyuan Flagship Store	Power Saving Expert 2	Delivery was pretty fast, a little pricey, just not sure if it works or not	Jingdong prefecture level city in Shandong
Jinfeng Hongyuan Flagship Store	Power Saving Expert 2	The goods have been received, I do not know how effective.	Jingdong prefecture level city in Shandong
Jinfeng Hongyuan Flagship Store	Power Saving Expert 2	So far it doesn't seem to be too noticeable, so I'll comment later.	Jingdong prefecture level city in Shandong
Jinfeng Hongyuan Flagship Store	Power Saving Expert 2	I'll comment after I've used it	Jingdong prefecture level city in Shandong
Jinfeng Hongyuan Flagship Store	Power Saving Expert 2	Not bad. You'll save a lot on your electric bill in a year!	Jingdong prefecture level city in Shandong
Jinfeng Hongyuan Flagship Store	Power Saving Expert 2	I just got it. I haven't tried it yet. I don't know what it's like.	Jingdong prefecture level city in Shandong
Jinfeng Hongyuan Flagship Store	Power Saving Expert 2	Shopping at Jingdong has become a habit, and this disease will not be cured for a while.	Jingdong prefecture level city in Shandong

Jinfeng Hongyuan Flagship Store	Power Saving Expert 2	I've been using it for a while now. I don't know if I'm still saving power or not.	Jingdong prefecture level city in Shandong
Jinfeng Hongyuan Flagship Store	Power Saving Expert 2	Received the saver and it's great! There is also a digital voltage indication. Plugged in as instructed, easy to operate, hope you're our power saver!	Jingdong prefecture level city in Shandong
Jinfeng Hongyuan Flagship Store	Power Saving Expert 2	There is no power saving as the store says, using it is the same as not using it.	Jingdong prefecture level city in Shandong
Jinfeng Hongyuan Flagship Store	Power Saving Expert 2	Use it for a while and then comment again	Jingdong prefecture level city in Shandong
Jinfeng Hongyuan Flagship Store	Power Saving Expert 2	Feeling can, to use 1 to 2 months to see how the effect, there is a voltage display, can see the voltage fluctuations amplitude, quite good!	Jingdong prefecture level city in Shandong
Jinfeng Hongyuan Flagship Store	Power Saving Expert 2	I hope it works and saves me some electricity.	Jingdong prefecture level city in Shandong
Jinfeng Hongyuan Flagship Store	Power Saving Expert 2	Nice baby, happy with the after sales	Jingdong prefecture level city in Shandong
Jinfeng Hongyuan Flagship Store	Power Saving Expert 2	Use it and talk about it.	Jingdong prefecture level city in Shandong
Jinfeng Hongyuan Flagship Store	Power Saving Expert 2	Second purchase	Jingdong prefecture level city in Shandong
Jinfeng Hongyuan Flagship Store	Power Saving Expert 2	It works.	Jingdong prefecture level city in Shandong
Jinfeng Hongyuan Flagship Store	Power Saving Expert 2	It works.	Jingdong prefecture level city in Shandong
Jinfeng Hongyuan Flagship Store	Power Saving Expert 2	I don't know if it's a power saver until I start using it	Jingdong prefecture level city in Shandong
Jinfeng Hongyuan Flagship Store	Power Saving Expert 2	serviceable	Jingdong prefecture level city in Shandong
Jinfeng Hongyuan Flagship Store	Power Saving Expert 2	This user did not fill out the rating in time	Jingdong prefecture level city in Shandong
Jinfeng Hongyuan Flagship Store	Power Saving Expert 2	There is no power saving as the store says, using it is the same as not using it.	Jingdong prefecture level city in Shandong
Jinfeng Hongyuan Flagship Store	Power Saving Expert 2	This user did not fill out the rating in time	Jingdong prefecture level city in Shandong
Jinfeng Hongyuan Flagship Store	Power Saving Expert 2	Good...good.	Jingdong prefecture level city in Shandong
Jinfeng Hongyuan Flagship Store	Power Saving Expert 2	(of an unmarried couple) be close	Jingdong prefecture level city in Shandong
Jinfeng Hongyuan Flagship Store	Power Saving Expert 2	(of an unmarried couple) be close	Jingdong prefecture level city in Shandong
Jinfeng Hongyuan Flagship Store	Power Saving Expert 2	(of an unmarried couple) be close	Jingdong prefecture level city in Shandong
Jinfeng Hongyuan Flagship Store	Power Saving Expert 2	The goods are real, high value, high quality, worth every penny, material appearance and quality at a glance is very upscale, very like HAPPY💯	Jingdong prefecture level city in Shandong
Jinfeng Hongyuan Flagship Store	Power Saving Expert 2	It is the ideal product, good quality, logistics is also very fast, worthy of praise 🎁 baby quality is very good, received the first time to open the first time to see, it is very good 👍, very satisfied 😃, customer service is also available to ask questions and answer, thank you 🙏	Jingdong prefecture level city in Shandong
Jinfeng Hongyuan Flagship Store	Power Saving Expert 2	I bought it and haven't used it yet, I'll use it again.	Jingdong prefecture level city in Shandong
Jinfeng Hongyuan Flagship Store	Power Saving Expert 2	It didn't work, I used it for a few days and it was the same as before, it didn't work, I should have to return it	Jingdong prefecture level city in Shandong
Jinfeng Hongyuan Flagship Store	Power Saving Expert 2	The goods have been received, but have not yet installed and use, feel good.	Jingdong prefecture level city in Shandong
Jinfeng Hongyuan Flagship Store	Power Saving Expert 2	Goods received, I forgot to take pictures, the goods look good, you can start, immediately start the school to see the effect then	Jingdong prefecture level city in Shandong
Jinfeng Hongyuan Flagship Store	Power Saving Expert 2	Looks good. I'll use it for a while.	Jingdong prefecture level city in Shandong
Jinfeng Hongyuan Flagship Store	Power Saving Expert 2	Great stuff, easy to operate, easy to use, good power saving, believe in the power of science!	Jingdong prefecture level city in Shandong
Jinfeng Hongyuan Flagship Store	Power Saving Expert 2	Especially great, comparison shopping before buying, the store owner is very good, answering questions very patiently and detailed, there is a need to come back!	Jingdong prefecture level city in Shandong
Jinfeng Hongyuan Flagship Store	Power Saving Expert 2	Trustworthy, feel free to use it boldly.	Jingdong prefecture level city in Shandong
Jinfeng Hongyuan Flagship Store	Power Saving Expert 2	Logistics is super fast! Beautiful appearance! Looks like a great quality! I've never bought one before, so I'll try it first to see if it saves power. We'll see how much it saves.	Jingdong prefecture level city in Shandong
Jinfeng Hongyuan Flagship Store	Power Saving Expert 2	I saw online counterfeiting, energy saver is one of them, hahahahaha, jingdong also buy fake products	Jingdong prefecture level city in Shandong
Jinfeng Hongyuan Flagship Store	Power Saving Expert 2	Just received, installed, try a month to see how the effect and then follow up.	Jingdong prefecture level city in Shandong
Jinfeng Hongyuan Flagship Store	Power Saving Expert 2	Good for you! The goods are genuine! Good price for what you get!	Jingdong prefecture level city in Shandong
Jinfeng Hongyuan Flagship Store	Power Saving Expert 2	Things quickly received, immediately used to see if there is no effect, something river photo ⬆ the same, customer service is very professional	Jingdong prefecture level city in Shandong
Jinfeng Hongyuan Flagship Store	Power Saving Expert 2	The product is very convenient Looking forward to the subsequent use of the experience results	Jingdong prefecture level city in Shandong
Jinfeng Hongyuan Flagship Store	Power Saving Expert 2	I just received it and plugged it in, we'll see exactly when it's time to pay the electric bill	Jingdong prefecture level city in Shandong
Jinfeng Hongyuan Flagship Store	Power Saving Expert 2	Saver is good Packaging and looks good How effective it is to be shared after using it.	Jingdong prefecture level city in Shandong
Jinfeng Hongyuan Flagship Store	Power Saving Expert 2	There's no way to compare. I don't know if it's energy efficient.	Jingdong prefecture level city in Shandong
Jinfeng Hongyuan Flagship Store	Power Saving Expert 2	Just installed it, try it out and comment again	Jingdong prefecture level city in Shandong
Jinfeng Hongyuan Flagship Store	Power Saving Expert 2	Super save point also good to install, after a friend introduced then went to buy to, friends say it is very good to use, home I bought, try to use a period of time, to be honest, the economizer is not big or heavy, can be very convenient to put in a lot of places, the quality is also very good, power-saving function this time seems to be about 20% of the efficiency. The owner is also very warm contact, very	Jingdong prefecture level city in Shandong
Jinfeng Hongyuan Flagship Store	Power Saving Expert 2	The reviews online are mixed. Try it for a while, but I don't know if I can return it if it doesn't save any power after a couple months.	Jingdong prefecture level city in Shandong
Jinfeng Hongyuan Flagship Store	Power Saving Expert 2	The power consumption at home is high, bought a power saver, it looks very convenient to plug on the line, see if you can save some money next month, if good, recommend to friends down the road.	Jingdong prefecture level city in Shandong
Jinfeng Hongyuan Flagship Store	Power Saving Expert 2	It's beautiful, it's genuine, but I don't know the power-saving effect.	Jingdong prefecture level city in Shandong
Jinfeng Hongyuan Flagship Store	Power Saving Expert 2	This user thinks the product is good	Jingdong prefecture level city in Shandong
Jinfeng Hongyuan Flagship Store	Power Saving Expert 2	Nice exterior design with an at-a-glance display	Jingdong prefecture level city in Shandong
Jinfeng Hongyuan Flagship Store	Power Saving Expert 2	Home large and small things are purchased in the Jingdong Mall, have been accustomed to shopping in the Jingdong, many times can buy good quality and cheap products, and courier service attitude is particularly good, sometimes too many couriers are to help me to the home, so many years have been in the Jingdong Mall shopping, trust and support! Will also support the Jingdong as always!	Jingdong prefecture level city in Shandong
Jinfeng Hongyuan Flagship Store	Power Saving Expert 2	Good workmanship, mostly better than the original wall-plugged ones, and hopefully power factor compensation saves on electricity costs	Jingdong prefecture level city in Shandong
Jinfeng Hongyuan Flagship Store	Power Saving Expert 2	I don't feel anything, I said I won't know the effect for a month or two, let's see.	Jingdong prefecture level city in Shandong

Jinfeng Hongyuan Flagship Store	Power Saving Expert 2	Delivery was pretty fast, a little pricey, just not sure if it works or not	Jingdong prefecture level city in Shandong
Jinfeng Hongyuan Flagship Store	Power Saving Expert 2	The goods have been received, I do not know how effective.	Jingdong prefecture level city in Shandong
Jinfeng Hongyuan Flagship Store	Power Saving Expert 2	So far it doesn't seem to be too noticeable, so I'll comment later.	Jingdong prefecture level city in Shandong
Jinfeng Hongyuan Flagship Store	Power Saving Expert 2	I'll comment after I've used it	Jingdong prefecture level city in Shandong
Jinfeng Hongyuan Flagship Store	Power Saving Expert 2	Not bad. You'll save a lot on your electric bill in a year!	Jingdong prefecture level city in Shandong
Jinfeng Hongyuan Flagship Store	Power Saving Expert 2	I just got it. I haven't tried it yet. I don't know what it's like.	Jingdong prefecture level city in Shandong
Jinfeng Hongyuan Flagship Store	Power Saving Expert 2	Shopping at Jingdong has become a habit, and this disease will not be cured for a while.	Jingdong prefecture level city in Shandong
Jinfeng Hongyuan Flagship Store	Power Saving Expert 2	I've been using it for a while now. I don't know if I'm still saving power or not.	Jingdong prefecture level city in Shandong
Jinfeng Hongyuan Flagship Store	Power Saving Expert 2	Received the saver and it's great! There is also a digital voltage indication. Plugged in as instructed, easy to operate, hope you're our power saver!	Jingdong prefecture level city in Shandong
Jinfeng Hongyuan Flagship Store	Power Saving Expert 2	There is no power saving as the store says, using it is the same as not using it.	Jingdong prefecture level city in Shandong
Jinfeng Hongyuan Flagship Store	Power Saving Expert 2	Use it for a while and then comment again	Jingdong prefecture level city in Shandong
Jinfeng Hongyuan Flagship Store	Power Saving Expert 2	Feeling can, to use 1 to 2 months to see how the effect, there is a voltage display, can see the voltage fluctuations amplitude, quite good!	Jingdong prefecture level city in Shandong
Jinfeng Hongyuan Flagship Store	Power Saving Expert 2	I hope it works and saves me some electricity.	Jingdong prefecture level city in Shandong
Jinfeng Hongyuan Flagship Store	Power Saving Expert 2	Nice baby, happy with the after sales	Jingdong prefecture level city in Shandong
Jinfeng Hongyuan Flagship Store	Power Saving Expert 2	Use it and talk about it.	Jingdong prefecture level city in Shandong
Jinfeng Hongyuan Flagship Store	Power Saving Expert 2	Second purchase	Jingdong prefecture level city in Shandong
Jinfeng Hongyuan Flagship Store	Power Saving Expert 2	It works.	Jingdong prefecture level city in Shandong
Jinfeng Hongyuan Flagship Store	Power Saving Expert 2	It works.	Jingdong prefecture level city in Shandong
Jinfeng Hongyuan Flagship Store	Power Saving Expert 2	I don't know if it's a power saver until I start using it	Jingdong prefecture level city in Shandong
Jinfeng Hongyuan Flagship Store	Power Saving Expert 2	serviceable	Jingdong prefecture level city in Shandong
Jinfeng Hongyuan Flagship Store	Power Saving Expert 2	This user did not fill out the rating in time	Jingdong prefecture level city in Shandong
Jinfeng Hongyuan Flagship Store	Power Saving Expert 2	There is no power saving as the store says, using it is the same as not using it.	Jingdong prefecture level city in Shandong
Jinfeng Hongyuan Flagship Store	Power Saving Expert 2	This user did not fill out the rating in time	Jingdong prefecture level city in Shandong
Jinfeng Hongyuan Flagship Store	Power Saving Expert 2	Good...good.	Jingdong prefecture level city in Shandong
Jinfeng Hongyuan Flagship Store	Power Saving Expert 2	(of an unmarried couple) be close	Jingdong prefecture level city in Shandong
Jinfeng Hongyuan Flagship Store	Power Saving Expert 2	(of an unmarried couple) be close	Jingdong prefecture level city in Shandong
Jinfeng Hongyuan Flagship Store	Power Saving Expert 2	(of an unmarried couple) be close	Jingdong prefecture level city in Shandong
Jinfeng Hongyuan Flagship Store	Power Saving Expert 2	The goods are real, high value, high quality, worth every penny, material appearance and quality at a glance is very upscale, very like HAPPY1 00	Jingdong prefecture level city in Shandong
Jinfeng Hongyuan Flagship Store	Power Saving Expert 2	It is the ideal product, good quality, logistics is also very fast, worthy of praise 🎁 baby quality is very good, received the first time to open the first time to see, it is very good 👍, very satisfied 😃, customer service is also available to ask questions and answer, thank you 🙏	Jingdong prefecture level city in Shandong
Jinfeng Hongyuan Flagship Store	Power Saving Expert 2	I bought it and haven't used it yet, I'll use it again.	Jingdong prefecture level city in Shandong
Jinfeng Hongyuan Flagship Store	Power Saving Expert 2	It didn't work, I used it for a few days and it was the same as before, it didn't work, I should have to return it	Jingdong prefecture level city in Shandong
Jinfeng Hongyuan Flagship Store	Power Saving Expert 2	The goods have been received, but have not yet installed and use, feel good.	Jingdong prefecture level city in Shandong
Jinfeng Hongyuan Flagship Store	Power Saving Expert 2	Goods received, I forgot to take pictures, the goods look good, you can start, immediately start the school to see the effect then	Jingdong prefecture level city in Shandong
Jinfeng Hongyuan Flagship Store	Power Saving Expert 2	Looks good. I'll use it for a while.	Jingdong prefecture level city in Shandong
Jinfeng Hongyuan Flagship Store	Power Saving Expert 2	Great stuff, easy to operate, easy to use, good power saving, believe in the power of science!	Jingdong prefecture level city in Shandong
Jinfeng Hongyuan Flagship Store	Power Saving Expert 2	Especially great, comparison shopping before buying, the store owner is very good, answering questions very patiently and detailed, there is a need to come back!	Jingdong prefecture level city in Shandong
Jinfeng Hongyuan Flagship Store	Power Saving Expert 2	Trustworthy, feel free to use it boldly.	Jingdong prefecture level city in Shandong
Jinfeng Hongyuan Flagship Store	Power Saving Expert 2	Logistics is super fast! Beautiful appearance! Looks like a great quality! I've never bought one before, so I'll try it first to see if it saves power. We'll see how much it saves.	Jingdong prefecture level city in Shandong
Jinfeng Hongyuan Flagship Store	Power Saving Expert 2	I saw online counterfeiting, energy saver is one of them, hahahahaha, jingdong also buy fake products	Jingdong prefecture level city in Shandong
Jinfeng Hongyuan Flagship Store	Power Saving Expert 2	Just received, installed, try a month to see how the effect and then follow up.	Jingdong prefecture level city in Shandong
Jinfeng Hongyuan Flagship Store	Power Saving Expert 2	Good for you! The goods are genuine! Good price for what you get!	Jingdong prefecture level city in Shandong
Jinfeng Hongyuan Flagship Store	Power Saving Expert 2	Things quickly received, immediately used to see if there is no effect, something river photo ⬆ the same, customer service is very professional	Jingdong prefecture level city in Shandong
Jinfeng Hongyuan Flagship Store	Power Saving Expert 2	The product is very convenient Looking forward to the subsequent use of the experience results	Jingdong prefecture level city in Shandong
Jinfeng Hongyuan Flagship Store	Power Saving Expert 2	I just received it and plugged it in, we'll see exactly when it's time to pay the electric bill	Jingdong prefecture level city in Shandong
Jinfeng Hongyuan Flagship Store	Power Saving Expert 2	Saver is good Packaging and looks good How effective it is to be shared after using it.	Jingdong prefecture level city in Shandong
Jinfeng Hongyuan Flagship Store	Power Saving Expert 2	There's no way to compare. I don't know if it's energy efficient.	Jingdong prefecture level city in Shandong
Jinfeng Hongyuan Flagship Store	Power Saving Expert 2	Just installed it, try it out and comment again	Jingdong prefecture level city in Shandong
Jinfeng Hongyuan Flagship Store	Power Saving Expert 2	Super save point also good to install, after a friend introduced then went to buy to, friends say it is very good to use, home I bought, try to use a period of time, to be honest, the economizer is not big or heavy, can be very convenient to put in a lot of places, the quality is also very good, power-saving function this time seems to be about 20% of the efficiency. The owner is also very warm contact, very	Jingdong prefecture level city in Shandong
Jinfeng Hongyuan Flagship Store	Power Saving Expert 2	The reviews online are mixed. Try it for a while, but I don't know if I can return it if it doesn't save any power after a couple months.	Jingdong prefecture level city in Shandong
Jinfeng Hongyuan Flagship Store	Power Saving Expert 2	The power consumption at home is high, bought a power saver, it looks very convenient to plug on the line, see if you can save some money next month, if good, recommend to friends down the road.	Jingdong prefecture level city in Shandong

Jinfeng Hongyuan Flagship Store	Power Saving Expert 2	It's beautiful, it's genuine, but I don't know the power-saving effect.	Jingdong prefecture level city in Shandong
Jinfeng Hongyuan Flagship Store	Power Saving Expert 2	This user thinks the product is good	Jingdong prefecture level city in Shandong
Jinfeng Hongyuan Flagship Store	Power Saving Expert 2	Nice exterior design with an at-a-glance display	Jingdong prefecture level city in Shandong
Jinfeng Hongyuan Flagship Store	Power Saving Expert 2	Home large and small things are purchased in the Jingdong Mall, have been accustomed to shopping in the Jingdong, many times can buy good quality and cheap products, and courier service attitude is particularly good, sometimes too many couriers are to help me to the home, so many years have been in the Jingdong Mall shopping, trust and support! Will also support the Jingdong as always!	Jingdong prefecture level city in Shandong
Jinfeng Hongyuan Flagship Store	Power Saving Expert 2	Good workmanship, mostly better than the original wall-plugged ones, and hopefully power factor compensation saves on electricity costs	Jingdong prefecture level city in Shandong
Jinfeng Hongyuan Flagship Store	Power Saving Expert 2	I don't feel anything, I said I won't know the effect for a month or two, let's see.	Jingdong prefecture level city in Shandong
Jinfeng Hongyuan Flagship Store	Power Saving Expert 2	Delivery was pretty fast, a little pricey, just not sure if it works or not	Jingdong prefecture level city in Shandong
Jinfeng Hongyuan Flagship Store	Power Saving Expert 2	The goods have been received, I do not know how effective.	Jingdong prefecture level city in Shandong
Jinfeng Hongyuan Flagship Store	Power Saving Expert 2	So far it doesn't seem to be too noticeable, so I'll comment later.	Jingdong prefecture level city in Shandong
Jinfeng Hongyuan Flagship Store	Power Saving Expert 2	I'll comment after I've used it	Jingdong prefecture level city in Shandong
Jinfeng Hongyuan Flagship Store	Power Saving Expert 2	Not bad. You'll save a lot on your electric bill in a year!	Jingdong prefecture level city in Shandong
Jinfeng Hongyuan Flagship Store	Power Saving Expert 2	I just got it. I haven't tried it yet. I don't know what it's like.	Jingdong prefecture level city in Shandong
Jinfeng Hongyuan Flagship Store	Power Saving Expert 2	Shopping at Jingdong has become a habit, and this disease will not be cured for a while.	Jingdong prefecture level city in Shandong
Jinfeng Hongyuan Flagship Store	Power Saving Expert 2	I've been using it for a while now. I don't know if I'm still saving power or not.	Jingdong prefecture level city in Shandong
Jinfeng Hongyuan Flagship Store	Power Saving Expert 2	Received the saver and it's great! There is also a digital voltage indication. Plugged in as instructed, easy to operate, hope you're our power saver!	Jingdong prefecture level city in Shandong
Jinfeng Hongyuan Flagship Store	Power Saving Expert 2	There is no power saving as the store says, using it is the same as not using it.	Jingdong prefecture level city in Shandong
Jinfeng Hongyuan Flagship Store	Power Saving Expert 2	Use it for a while and then comment again	Jingdong prefecture level city in Shandong
Jinfeng Hongyuan Flagship Store	Power Saving Expert 2	Feeling can, to use 1 to 2 months to see how the effect, there is a voltage display, can see the voltage fluctuations amplitude, quite good!	Jingdong prefecture level city in Shandong
Jinfeng Hongyuan Flagship Store	Power Saving Expert 2	I hope it works and saves me some electricity.	Jingdong prefecture level city in Shandong
Jinfeng Hongyuan Flagship Store	Power Saving Expert 2	Nice baby, happy with the after sales	Jingdong prefecture level city in Shandong
Jinfeng Hongyuan Flagship Store	Power Saving Expert 2	Use it and talk about it.	Jingdong prefecture level city in Shandong
Jinfeng Hongyuan Flagship Store	Power Saving Expert 2	Second purchase	Jingdong prefecture level city in Shandong
Jinfeng Hongyuan Flagship Store	Power Saving Expert 2	It works.	Jingdong prefecture level city in Shandong
Jinfeng Hongyuan Flagship Store	Power Saving Expert 2	It works.	Jingdong prefecture level city in Shandong
Jinfeng Hongyuan Flagship Store	Power Saving Expert 2	I don't know if it's a power saver until I start using it	Jingdong prefecture level city in Shandong
Jinfeng Hongyuan Flagship Store	Power Saving Expert 2	serviceable	Jingdong prefecture level city in Shandong
Jinfeng Hongyuan Flagship Store	Power Saving Expert 2	This user did not fill out the rating in time	Jingdong prefecture level city in Shandong
Jinfeng Hongyuan Flagship Store	Power Saving Expert 2	There is no power saving as the store says, using it is the same as not using it.	Jingdong prefecture level city in Shandong
Jinfeng Hongyuan Flagship Store	Power Saving Expert 2	This user did not fill out the rating in time	Jingdong prefecture level city in Shandong
Jinfeng Hongyuan Flagship Store	Power Saving Expert 2	Good...good.	Jingdong prefecture level city in Shandong
Jinfeng Hongyuan Flagship Store	Power Saving Expert 2	(of an unmarried couple) be close	Jingdong prefecture level city in Shandong
Jinfeng Hongyuan Flagship Store	Power Saving Expert 2	(of an unmarried couple) be close	Jingdong prefecture level city in Shandong
Jinfeng Hongyuan Flagship Store	Power Saving Expert 2	(of an unmarried couple) be close	Jingdong prefecture level city in Shandong
Jinfeng Hongyuan Flagship Store	Power Saving Expert 2	The goods are real, high value, high quality, worth every penny, material appearance and quality at a glance is very upscale, very like HAPPY1 00	Jingdong prefecture level city in Shandong
Jinfeng Hongyuan Flagship Store	Power Saving Expert 2	It is the ideal product, good quality, logistics is also very fast, worthy of praise 🎁 baby quality is very good, received the first time to open the first time to see, it is very good 👍, very satisfied 😃, customer service is also available to ask questions and answer, thank you 🙏	Jingdong prefecture level city in Shandong
Jinfeng Hongyuan Flagship Store	Power Saving Expert 2	I bought it and haven't used it yet, I'll use it again.	Jingdong prefecture level city in Shandong
Jinfeng Hongyuan Flagship Store	Power Saving Expert 2	It didn't work, I used it for a few days and it was the same as before, it didn't work, I should have to return it	Jingdong prefecture level city in Shandong
Jinfeng Hongyuan Flagship Store	Power Saving Expert 2	The goods have been received, but have not yet installed and use, feel good.	Jingdong prefecture level city in Shandong
Jinfeng Hongyuan Flagship Store	Power Saving Expert 2	Goods received, I forgot to take pictures, the goods look good, you can start, immediately start the school to see the effect then	Jingdong prefecture level city in Shandong
Jinfeng Hongyuan Flagship Store	Power Saving Expert 2	Looks good. I'll use it for a while.	Jingdong prefecture level city in Shandong
Jinfeng Hongyuan Flagship Store	Power Saving Expert 2	Great stuff, easy to operate, easy to use, good power saving, believe in the power of science!	Jingdong prefecture level city in Shandong
Jinfeng Hongyuan Flagship Store	Power Saving Expert 2	Especially great, comparison shopping before buying, the store owner is very good, answering questions very patiently and detailed, there is a need to come back!	Jingdong prefecture level city in Shandong
Jinfeng Hongyuan Flagship Store	Power Saving Expert 2	Trustworthy, feel free to use it boldly.	Jingdong prefecture level city in Shandong
Jinfeng Hongyuan Flagship Store	Power Saving Expert 2	Logistics is super fast! Beautiful appearance! Looks like a great quality! I've never bought one before, so I'll try it first to see if it saves power. We'll see how much it saves.	Jingdong prefecture level city in Shandong
Jinfeng Hongyuan Flagship Store	Power Saving Expert 2	I saw online counterfeiting, energy saver is one of them, hahahahaha, jingdong also buy fake products	Jingdong prefecture level city in Shandong
Jinfeng Hongyuan Flagship Store	Power Saving Expert 2	Just received, installed, try a month to see how the effect and then follow up.	Jingdong prefecture level city in Shandong
Jinfeng Hongyuan Flagship Store	Power Saving Expert 2	Good for you! The goods are genuine! Good price for what you get!	Jingdong prefecture level city in Shandong
Jinfeng Hongyuan Flagship Store	Power Saving Expert 2	Things quickly received, immediately used to see if there is no effect, something river photo ⬆ the same, customer service is very professional	Jingdong prefecture level city in Shandong
Jinfeng Hongyuan Flagship Store	Power Saving Expert 2	The product is very convenient Looking forward to the subsequent use of the experience results	Jingdong prefecture level city in Shandong
Jinfeng Hongyuan Flagship Store	Power Saving Expert 2	I just received it and plugged it in, we'll see exactly when it's time to pay the electric bill	Jingdong prefecture level city in Shandong

Jinfeng Hongyuan Flagship Store	Power Saving Expert 2	Saver is good Packaging and looks good How effective it is to be shared after using it.	Jingdong prefecture level city in Shandong
Jinfeng Hongyuan Flagship Store	Power Saving Expert 2	There's no way to compare. I don't know if it's energy efficient.	Jingdong prefecture level city in Shandong
Jinfeng Hongyuan Flagship Store	Power Saving Expert 2	Just installed it, try it out and comment again	Jingdong prefecture level city in Shandong
Jinfeng Hongyuan Flagship Store	Power Saving Expert 2	Super save point also good to install, after a friend introduced then went to buy to, friends say it is very good to use, home I bought, try to use a period of time, to be honest, the economizer is not big or heavy, can be very convenient to put in a lot of places, the quality is also very good, power-saving function this time seems to be about 20% of the efficiency. The owner is also very warm contact, very	Jingdong prefecture level city in Shandong
Jinfeng Hongyuan Flagship Store	Power Saving Expert 2	The reviews online are mixed. Try it for a while, but I don't know if I can return it if it doesn't save any power after a couple months.	Jingdong prefecture level city in Shandong
Jinfeng Hongyuan Flagship Store	Power Saving Expert 2	The power consumption at home is high, bought a power saver, it looks very convenient to plug on the line, see if you can save some money next month, if good, recommend to friends down the road.	Jingdong prefecture level city in Shandong
Jinfeng Hongyuan Flagship Store	Power Saving Expert 2	It's beautiful, it's genuine, but I don't know the power-saving effect.	Jingdong prefecture level city in Shandong
Jinfeng Hongyuan Flagship Store	Power Saving Expert 2	This user thinks the product is good	Jingdong prefecture level city in Shandong
Jinfeng Hongyuan Flagship Store	Power Saving Expert 2	Nice exterior design with an at-a-glance display	Jingdong prefecture level city in Shandong
Jinfeng Hongyuan Flagship Store	Power Saving Expert 2	Home large and small things are purchased in the Jingdong Mall, have been accustomed to shopping in the Jingdong, many times can buy good quality and cheap products, and courier service attitude is particularly good, sometimes too many couriers are to help me to the home, so many years have been in the Jingdong Mall shopping, trust and support! Will also support the Jingdong as always!	Jingdong prefecture level city in Shandong
Jinfeng Hongyuan Flagship Store	Power Saving Expert 2	Good workmanship, mostly better than the original wall-plugged ones, and hopefully power factor compensation saves on electricity costs	Jingdong prefecture level city in Shandong
Jinfeng Hongyuan Flagship Store	Power Saving Expert 2	I don't feel anything, I said I won't know the effect for a month or two, let's see.	Jingdong prefecture level city in Shandong
Jinfeng Hongyuan Flagship Store	Power Saving Expert 2	Delivery was pretty fast, a little pricey, just not sure if it works or not	Jingdong prefecture level city in Shandong
Jinfeng Hongyuan Flagship Store	Power Saving Expert 2	The goods have been received, I do not know how effective.	Jingdong prefecture level city in Shandong
Jinfeng Hongyuan Flagship Store	Power Saving Expert 2	So far it doesn't seem to be too noticeable, so I'll comment later.	Jingdong prefecture level city in Shandong
Jinfeng Hongyuan Flagship Store	Power Saving Expert 2	I'll comment after I've used it	Jingdong prefecture level city in Shandong
Jinfeng Hongyuan Flagship Store	Power Saving Expert 2	Not bad. You'll save a lot on your electric bill in a year!	Jingdong prefecture level city in Shandong
Jinfeng Hongyuan Flagship Store	Power Saving Expert 2	I just got it. I haven't tried it yet. I don't know what it's like.	Jingdong prefecture level city in Shandong
Jinfeng Hongyuan Flagship Store	Power Saving Expert 2	Shopping at Jingdong has become a habit, and this disease will not be cured for a while.	Jingdong prefecture level city in Shandong
Jinfeng Hongyuan Flagship Store	Power Saving Expert 2	I've been using it for a while now. I don't know if I'm still saving power or not.	Jingdong prefecture level city in Shandong
Jinfeng Hongyuan Flagship Store	Power Saving Expert 2	Received the saver and it's great! There is also a digital voltage indication. Plugged in as instructed, easy to operate, hope you're our power saver!	Jingdong prefecture level city in Shandong
Jinfeng Hongyuan Flagship Store	Power Saving Expert 2	There is no power saving as the store says, using it is the same as not using it.	Jingdong prefecture level city in Shandong
Jinfeng Hongyuan Flagship Store	Power Saving Expert 2	Use it for a while and then comment again	Jingdong prefecture level city in Shandong
Jinfeng Hongyuan Flagship Store	Power Saving Expert 2	Feeling can, to use 1 to 2 months to see how the effect, there is a voltage display, can see the voltage fluctuations amplitude, quite good!	Jingdong prefecture level city in Shandong
Jinfeng Hongyuan Flagship Store	Power Saving Expert 2	I hope it works and saves me some electricity.	Jingdong prefecture level city in Shandong
Jinfeng Hongyuan Flagship Store	Power Saving Expert 2	Nice baby, happy with the after sales	Jingdong prefecture level city in Shandong
Jinfeng Hongyuan Flagship Store	Power Saving Expert 2	Use it and talk about it.	Jingdong prefecture level city in Shandong
Jinfeng Hongyuan Flagship Store	Power Saving Expert 2	Second purchase	Jingdong prefecture level city in Shandong
Jinfeng Hongyuan Flagship Store	Power Saving Expert 2	It works.	Jingdong prefecture level city in Shandong
Jinfeng Hongyuan Flagship Store	Power Saving Expert 2	It works.	Jingdong prefecture level city in Shandong
Jinfeng Hongyuan Flagship Store	Power Saving Expert 2	I don't know if it's a power saver until I start using it	Jingdong prefecture level city in Shandong
Jinfeng Hongyuan Flagship Store	Power Saving Expert 2	serviceable	Jingdong prefecture level city in Shandong
Jinfeng Hongyuan Flagship Store	Power Saving Expert 2	This user did not fill out the rating in time	Jingdong prefecture level city in Shandong
Jinfeng Hongyuan Flagship Store	Power Saving Expert 2	There is no power saving as the store says, using it is the same as not using it.	Jingdong prefecture level city in Shandong
Jinfeng Hongyuan Flagship Store	Power Saving Expert 2	This user did not fill out the rating in time	Jingdong prefecture level city in Shandong
Jinfeng Hongyuan Flagship Store	Power Saving Expert 2	Good...good.	Jingdong prefecture level city in Shandong
Jinfeng Hongyuan Flagship Store	Power Saving Expert 2	(of an unmarried couple) be close	Jingdong prefecture level city in Shandong
Jinfeng Hongyuan Flagship Store	Power Saving Expert 2	(of an unmarried couple) be close	Jingdong prefecture level city in Shandong
Jinfeng Hongyuan Flagship Store	Power Saving Expert 2	(of an unmarried couple) be close	Jingdong prefecture level city in Shandong
Jinfeng Hongyuan Flagship Store	Power Saving Expert 2	The goods are real, high value, high quality, worth every penny, material appearance and quality at a glance is very upscale, very like HAPPY100	Jingdong prefecture level city in Shandong
Jinfeng Hongyuan Flagship Store	Power Saving Expert 2	It is the ideal product, good quality, logistics is also very fast, worthy of praise 🎁 baby quality is very good, received the first time to open the first time to see, it is very good 👍, very satisfied 😃, customer service is also available to ask questions and answer, thank you 🙏	Jingdong prefecture level city in Shandong
Jinfeng Hongyuan Flagship Store	Power Saving Expert 2	I bought it and haven't used it yet, I'll use it again.	Jingdong prefecture level city in Shandong
Jinfeng Hongyuan Flagship Store	Power Saving Expert 2	It didn't work, I used it for a few days and it was the same as before, it didn't work, I should have to return it	Jingdong prefecture level city in Shandong
Jinfeng Hongyuan Flagship Store	Power Saving Expert 2	The goods have been received, but have not yet installed and use, feel good.	Jingdong prefecture level city in Shandong
Jinfeng Hongyuan Flagship Store	Power Saving Expert 2	Goods received, I forgot to take pictures, the goods look good, you can start, immediately start the school to see the effect then	Jingdong prefecture level city in Shandong
Jinfeng Hongyuan Flagship Store	Power Saving Expert 2	Looks good. I'll use it for a while.	Jingdong prefecture level city in Shandong
Jinfeng Hongyuan Flagship Store	Power Saving Expert 2	Great stuff, easy to operate, easy to use, good power saving, believe in the power of science!	Jingdong prefecture level city in Shandong
Jinfeng Hongyuan Flagship Store	Power Saving Expert 2	Especially great, comparison shopping before buying, the store owner is very good, answering questions very patiently and detailed, there is a need to come back!	Jingdong prefecture level city in Shandong
Jinfeng Hongyuan Flagship Store	Power Saving Expert 2	Trustworthy, feel free to use it boldly.	Jingdong prefecture level city in Shandong

Jinfeng Hongyuan Flagship Store	Power Saving Expert 2	Logistics is super fast! Beautiful appearance! Looks like a great quality! I've never bought one before, so I'll try it first to see if it saves power. We'll see how much it saves.	Jingdong prefecture level city in Shandong
Jinfeng Hongyuan Flagship Store	Power Saving Expert 2	I saw online counterfeiting, energy saver is one of them, hahahahaha, jingdong also buy fake products	Jingdong prefecture level city in Shandong
Jinfeng Hongyuan Flagship Store	Power Saving Expert 2	Just received, installed, try a month to see how the effect and then follow up.	Jingdong prefecture level city in Shandong
Jinfeng Hongyuan Flagship Store	Power Saving Expert 2	Good for you! The goods are genuine! Good price for what you get!	Jingdong prefecture level city in Shandong
Jinfeng Hongyuan Flagship Store	Power Saving Expert 2	Things quickly received, immediately used to see if there is no effect, something river photo 🆙 the same, customer service is very professional	Jingdong prefecture level city in Shandong
Jinfeng Hongyuan Flagship Store	Power Saving Expert 2	The product is very convenient Looking forward to the subsequent use of the experience results	Jingdong prefecture level city in Shandong
Jinfeng Hongyuan Flagship Store	Power Saving Expert 2	I just received it and plugged it in, we'll see exactly when it's time to pay the electric bill	Jingdong prefecture level city in Shandong
Jinfeng Hongyuan Flagship Store	Power Saving Expert 2	Saver is good Packaging and looks good How effective it is to be shared after using it.	Jingdong prefecture level city in Shandong
Jinfeng Hongyuan Flagship Store	Power Saving Expert 2	There's no way to compare. I don't know if it's energy efficient.	Jingdong prefecture level city in Shandong
Jinfeng Hongyuan Flagship Store	Power Saving Expert 2	Just installed it, try it out and comment again	Jingdong prefecture level city in Shandong
Jinfeng Hongyuan Flagship Store	Power Saving Expert 2	Super save point also good to install, after a friend introduced then went to buy to, friends say it is very good to use, home I bought, try to use a period of time, to be honest, the economizer is not big or heavy, can be very convenient to put in a lot of places, the quality is also very good, power-saving function this time seems to be about 20% of the efficiency. The owner is also very warm contact, very	Jingdong prefecture level city in Shandong
Jinfeng Hongyuan Flagship Store	Power Saving Expert 2	The reviews online are mixed. Try it for a while, but I don't know if I can return it if it doesn't save any power after a couple months.	Jingdong prefecture level city in Shandong
Jinfeng Hongyuan Flagship Store	Power Saving Expert 2	The power consumption at home is high, bought a power saver, it looks very convenient to plug on the line, see if you can save some money next month, if good, recommend to friends down the road.	Jingdong prefecture level city in Shandong
Jinfeng Hongyuan Flagship Store	Power Saving Expert 2	It's beautiful, it's genuine, but I don't know the power-saving effect.	Jingdong prefecture level city in Shandong
Jinfeng Hongyuan Flagship Store	Power Saving Expert 2	This user thinks the product is good	Jingdong prefecture level city in Shandong
Jinfeng Hongyuan Flagship Store	Power Saving Expert 2	Nice exterior design with an at-a-glance display	Jingdong prefecture level city in Shandong
Jinfeng Hongyuan Flagship Store	Power Saving Expert 2	Home large and small things are purchased in the Jingdong Mall, have been accustomed to shopping in the Jingdong, many times can buy good quality and cheap products, and courier service attitude is particularly good, sometimes too many couriers are to help me to the home, so many years have been in the Jingdong Mall shopping, trust and support! Will also support the Jingdong as always!	Jingdong prefecture level city in Shandong
Jinfeng Hongyuan Flagship Store	Power Saving Expert 2	Good workmanship, mostly better than the original wall-plugged ones, and hopefully power factor compensation saves on electricity costs	Jingdong prefecture level city in Shandong
Jinfeng Hongyuan Flagship Store	Power Saving Expert 2	I don't feel anything, I said I won't know the effect for a month or two, let's see.	Jingdong prefecture level city in Shandong
Jinfeng Hongyuan Flagship Store	Power Saving Expert 2	Delivery was pretty fast, a little pricey, just not sure if it works or not	Jingdong prefecture level city in Shandong
Jinfeng Hongyuan Flagship Store	Power Saving Expert 2	The goods have been received, I do not know how effective.	Jingdong prefecture level city in Shandong
Jinfeng Hongyuan Flagship Store	Power Saving Expert 2	So far it doesn't seem to be too noticeable, so I'll comment later.	Jingdong prefecture level city in Shandong
Jinfeng Hongyuan Flagship Store	Power Saving Expert 2	I'll comment after I've used it	Jingdong prefecture level city in Shandong
Jinfeng Hongyuan Flagship Store	Power Saving Expert 2	Not bad. You'll save a lot on your electric bill in a year!	Jingdong prefecture level city in Shandong
Jinfeng Hongyuan Flagship Store	Power Saving Expert 2	I just got it. I haven't tried it yet. I don't know what it's like.	Jingdong prefecture level city in Shandong
Jinfeng Hongyuan Flagship Store	Power Saving Expert 2	Shopping at Jingdong has become a habit, and this disease will not be cured for a while.	Jingdong prefecture level city in Shandong
Jinfeng Hongyuan Flagship Store	Power Saving Expert 2	I've been using it for a while now. I don't know if I'm still saving power or not.	Jingdong prefecture level city in Shandong
Jinfeng Hongyuan Flagship Store	Power Saving Expert 2	Received the saver and it's great! There is also a digital voltage indication. Plugged in as instructed, easy to operate, hope you're our power saver!	Jingdong prefecture level city in Shandong
Jinfeng Hongyuan Flagship Store	Power Saving Expert 2	There is no power saving as the store says, using it is the same as not using it.	Jingdong prefecture level city in Shandong
Jinfeng Hongyuan Flagship Store	Power Saving Expert 2	Use it for a while and then comment again	Jingdong prefecture level city in Shandong
Jinfeng Hongyuan Flagship Store	Power Saving Expert 2	Feeling can, to use 1 to 2 months to see how the effect, there is a voltage display, can see the voltage fluctuations amplitude, quite good!	Jingdong prefecture level city in Shandong
Jinfeng Hongyuan Flagship Store	Power Saving Expert 2	I hope it works and saves me some electricity.	Jingdong prefecture level city in Shandong
Jinfeng Hongyuan Flagship Store	Power Saving Expert 2	Nice baby, happy with the after sales	Jingdong prefecture level city in Shandong
Jinfeng Hongyuan Flagship Store	Power Saving Expert 2	Use it and talk about it.	Jingdong prefecture level city in Shandong
Jinfeng Hongyuan Flagship Store	Power Saving Expert 2	Second purchase	Jingdong prefecture level city in Shandong
Jinfeng Hongyuan Flagship Store	Power Saving Expert 2	It works.	Jingdong prefecture level city in Shandong
Jinfeng Hongyuan Flagship Store	Power Saving Expert 2	It works.	Jingdong prefecture level city in Shandong
Jinfeng Hongyuan Flagship Store	Power Saving Expert 2	I don't know if it's a power saver until I start using it	Jingdong prefecture level city in Shandong
Jinfeng Hongyuan Flagship Store	Power Saving Expert 2	serviceable	Jingdong prefecture level city in Shandong
Jinfeng Hongyuan Flagship Store	Power Saving Expert 2	This user did not fill out the rating in time	Jingdong prefecture level city in Shandong
Jinfeng Hongyuan Flagship Store	Power Saving Expert 2	There is no power saving as the store says, using it is the same as not using it.	Jingdong prefecture level city in Shandong
Jinfeng Hongyuan Flagship Store	Power Saving Expert 2	This user did not fill out the rating in time	Jingdong prefecture level city in Shandong
Jinfeng Hongyuan Flagship Store	Power Saving Expert 2	Good...good.	Jingdong prefecture level city in Shandong
Jinfeng Hongyuan Flagship Store	Power Saving Expert 2	(of an unmarried couple) be close	Jingdong prefecture level city in Shandong
Jinfeng Hongyuan Flagship Store	Power Saving Expert 2	(of an unmarried couple) be close	Jingdong prefecture level city in Shandong
Jinfeng Hongyuan Flagship Store	Power Saving Expert 2	(of an unmarried couple) be close	Jingdong prefecture level city in Shandong
Jinfeng Hongyuan Flagship Store	Power Saving Expert 2	The goods are real, high value, high quality, worth every penny, material appearance and quality at a glance is very upscale, very like HAPPY1 00	Jingdong prefecture level city in Shandong
Jinfeng Hongyuan Flagship Store	Power Saving Expert 2	It is the ideal product, good quality, logistics is also very fast, worthy of praise 🎁 baby quality is very good, received the first time to open the first time to see, it is very good 👍, very satisfied 😃, customer service is also available to ask questions and answer, thank you 🙏	Jingdong prefecture level city in Shandong
Jinfeng Hongyuan Flagship Store	Power Saving Expert 2	I bought it and haven't used it yet, I'll use it again.	Jingdong prefecture level city in Shandong

Jinfeng Hongyuan Flagship Store	Power Saving Expert 2	It didn't work, I used it for a few days and it was the same as before, it didn't work, I should have to return it	Jingdong prefecture level city in Shandong
Jinfeng Hongyuan Flagship Store	Power Saving Expert 2	The goods have been received, but have not yet installed and use, feel good.	Jingdong prefecture level city in Shandong
Jinfeng Hongyuan Flagship Store	Power Saving Expert 2	Goods received, I forgot to take pictures, the goods look good, you can start, immediately start the school to see the effect then	Jingdong prefecture level city in Shandong
Jinfeng Hongyuan Flagship Store	Power Saving Expert 2	Looks good. I'll use it for a while.	Jingdong prefecture level city in Shandong
Jinfeng Hongyuan Flagship Store	Power Saving Expert 2	Great stuff, easy to operate, easy to use, good power saving, believe in the power of science!	Jingdong prefecture level city in Shandong
Jinfeng Hongyuan Flagship Store	Power Saving Expert 2	Especially great, comparison shopping before buying, the store owner is very good, answering questions very patiently and detailed, there is a need to come back!	Jingdong prefecture level city in Shandong
Jinfeng Hongyuan Flagship Store	Power Saving Expert 2	Trustworthy, feel free to use it boldly.	Jingdong prefecture level city in Shandong
Jinfeng Hongyuan Flagship Store	Power Saving Expert 2	Logistics is super fast! Beautiful appearance! Looks like a great quality! I've never bought one before, so I'll try it first to see if it saves power. We'll see how much it saves.	Jingdong prefecture level city in Shandong
Jinfeng Hongyuan Flagship Store	Power Saving Expert 2	I saw online counterfeiting, energy saver is one of them, hahahahaha, jingdong also buy fake products	Jingdong prefecture level city in Shandong
Jinfeng Hongyuan Flagship Store	Power Saving Expert 2	Just received, installed, try a month to see how the effect and then follow up.	Jingdong prefecture level city in Shandong
Jinfeng Hongyuan Flagship Store	Power Saving Expert 2	Good for you! The goods are genuine! Good price for what you get!	Jingdong prefecture level city in Shandong
Jinfeng Hongyuan Flagship Store	Power Saving Expert 2	Things quickly received, immediately used to see if there is no effect, something river photo ⍐ the same, customer service is very professional	Jingdong prefecture level city in Shandong
Jinfeng Hongyuan Flagship Store	Power Saving Expert 2	The product is very convenient Looking forward to the subsequent use of the experience results	Jingdong prefecture level city in Shandong
Jinfeng Hongyuan Flagship Store	Power Saving Expert 2	I just received it and plugged it in, we'll see exactly when it's time to pay the electric bill	Jingdong prefecture level city in Shandong
Jinfeng Hongyuan Flagship Store	Power Saving Expert 2	Saver is good Packaging and looks good How effective it is to be shared after using it.	Jingdong prefecture level city in Shandong
Jinfeng Hongyuan Flagship Store	Power Saving Expert 2	There's no way to compare. I don't know if it's energy efficient.	Jingdong prefecture level city in Shandong
Jinfeng Hongyuan Flagship Store	Power Saving Expert 2	Just installed it, try it out and comment again	Jingdong prefecture level city in Shandong
Jinfeng Hongyuan Flagship Store	Power Saving Expert 2	Super save point also good to install, after a friend introduced then went to buy to, friends say it is very good to use, home I bought, try to use a period of time, to be honest, the economizer is not big or heavy, can be very convenient to put in a lot of places, the quality is also very good, power-saving function this time seems to be about 20% of the efficiency. The owner is also very warm contact, very	Jingdong prefecture level city in Shandong
Jinfeng Hongyuan Flagship Store	Power Saving Expert 2	The reviews online are mixed. Try it for a while, but I don't know if I can return it if it doesn't save any power after a couple months.	Jingdong prefecture level city in Shandong
Jinfeng Hongyuan Flagship Store	Power Saving Expert 2	The power consumption at home is high, bought a power saver, it looks very convenient to plug on the line, see if you can save some money next month, if good, recommend to friends down the road.	Jingdong prefecture level city in Shandong
Jinfeng Hongyuan Flagship Store	Power Saving Expert 2	It's beautiful, it's genuine, but I don't know the power-saving effect.	Jingdong prefecture level city in Shandong
Jinfeng Hongyuan Flagship Store	Power Saving Expert 2	This user thinks the product is good	Jingdong prefecture level city in Shandong
Jinfeng Hongyuan Flagship Store	Power Saving Expert 2	Nice exterior design with an at-a-glance display	Jingdong prefecture level city in Shandong
Jinfeng Hongyuan Flagship Store	Power Saving Expert 2	Home large and small things are purchased in the Jingdong Mall, have been accustomed to shopping in the Jingdong, many times can buy good quality and cheap products, and courier service attitude is particularly good, sometimes too many couriers are to help me to the home, so many years have been in the Jingdong Mall shopping, trust and support! Will also support the Jingdong as always!	Jingdong prefecture level city in Shandong
Jinfeng Hongyuan Flagship Store	Power Saving Expert 2	Good workmanship, mostly better than the original wall-plugged ones, and hopefully power factor compensation saves on electricity costs	Jingdong prefecture level city in Shandong
Jinfeng Hongyuan Flagship Store	Power Saving Expert 2	I don't feel anything, I said I won't know the effect for a month or two, let's see.	Jingdong prefecture level city in Shandong
Jinfeng Hongyuan Flagship Store	Power Saving Expert 2	Delivery was pretty fast, a little pricey, just not sure if it works or not	Jingdong prefecture level city in Shandong
Jinfeng Hongyuan Flagship Store	Power Saving Expert 2	The goods have been received, I do not know how effective.	Jingdong prefecture level city in Shandong
Jinfeng Hongyuan Flagship Store	Power Saving Expert 2	So far it doesn't seem to be too noticeable, so I'll comment later.	Jingdong prefecture level city in Shandong
Jinfeng Hongyuan Flagship Store	Power Saving Expert 2	I'll comment after I've used it	Jingdong prefecture level city in Shandong
Jinfeng Hongyuan Flagship Store	Power Saving Expert 2	Not bad. You'll save a lot on your electric bill in a year!	Jingdong prefecture level city in Shandong
Jinfeng Hongyuan Flagship Store	Power Saving Expert 2	I just got it. I haven't tried it yet. I don't know what it's like.	Jingdong prefecture level city in Shandong
Jinfeng Hongyuan Flagship Store	Power Saving Expert 2	Shopping at Jingdong has become a habit, and this disease will not be cured for a while.	Jingdong prefecture level city in Shandong
Jinfeng Hongyuan Flagship Store	Power Saving Expert 2	I've been using it for a while now. I don't know if I'm still saving power or not.	Jingdong prefecture level city in Shandong
Jinfeng Hongyuan Flagship Store	Power Saving Expert 2	Received the saver and it's great! There is also a digital voltage indication. Plugged in as instructed, easy to operate, hope you're our power saver!	Jingdong prefecture level city in Shandong
Jinfeng Hongyuan Flagship Store	Power Saving Expert 2	There is no power saving as the store says, using it is the same as not using it.	Jingdong prefecture level city in Shandong
Jinfeng Hongyuan Flagship Store	Power Saving Expert 2	Use it for a while and then comment again	Jingdong prefecture level city in Shandong
Jinfeng Hongyuan Flagship Store	Power Saving Expert 2	Feeling can, to use 1 to 2 months to see how the effect, there is a voltage display, can see the voltage fluctuations amplitude, quite good!	Jingdong prefecture level city in Shandong
Jinfeng Hongyuan Flagship Store	Power Saving Expert 2	I hope it works and saves me some electricity.	Jingdong prefecture level city in Shandong
Jinfeng Hongyuan Flagship Store	Power Saving Expert 2	Nice baby, happy with the after sales	Jingdong prefecture level city in Shandong
Jinfeng Hongyuan Flagship Store	Power Saving Expert 2	Use it and talk about it.	Jingdong prefecture level city in Shandong
Jinfeng Hongyuan Flagship Store	Power Saving Expert 2	Second purchase	Jingdong prefecture level city in Shandong
Jinfeng Hongyuan Flagship Store	Power Saving Expert 2	It works.	Jingdong prefecture level city in Shandong
Jinfeng Hongyuan Flagship Store	Power Saving Expert 2	It works.	Jingdong prefecture level city in Shandong
Jinfeng Hongyuan Flagship Store	Power Saving Expert 2	I don't know if it's a power saver until I start using it	Jingdong prefecture level city in Shandong
Jinfeng Hongyuan Flagship Store	Power Saving Expert 2	serviceable	Jingdong prefecture level city in Shandong
Jinfeng Hongyuan Flagship Store	Power Saving Expert 2	This user did not fill out the rating in time	Jingdong prefecture level city in Shandong
Jinfeng Hongyuan Flagship Store	Power Saving Expert 2	There is no power saving as the store says, using it is the same as not using it.	Jingdong prefecture level city in Shandong
Jinfeng Hongyuan Flagship Store	Power Saving Expert 2	This user did not fill out the rating in time	Jingdong prefecture level city in Shandong

Jinfeng Hongyuan Flagship Store	Power Saving Expert 2	Good...good.	Jingdong prefecture level city in Shandong
Jinfeng Hongyuan Flagship Store	Power Saving Expert 2	(of an unmarried couple) be close	Jingdong prefecture level city in Shandong
Jinfeng Hongyuan Flagship Store	Power Saving Expert 2	(of an unmarried couple) be close	Jingdong prefecture level city in Shandong
Jinfeng Hongyuan Flagship Store	Power Saving Expert 2	(of an unmarried couple) be close	Jingdong prefecture level city in Shandong
Jinfeng Hongyuan Flagship Store	Power Saving Expert 2	The goods are real, high value, high quality, worth every penny, material appearance and quality at a glance is very upscale, very like HAPPY💯	Jingdong prefecture level city in Shandong
Jinfeng Hongyuan Flagship Store	Power Saving Expert 2	It is the ideal product, good quality, logistics is also very fast, worthy of praise 🎁 baby quality is very good, received the first time to open the first time to see, it is very good 👍, very satisfied 😃, customer service is also available to ask questions and answer, thank you 🙏	Jingdong prefecture level city in Shandong
Jinfeng Hongyuan Flagship Store	Power Saving Expert 2	I bought it and haven't used it yet, I'll use it again.	Jingdong prefecture level city in Shandong
Jinfeng Hongyuan Flagship Store	Power Saving Expert 2	It didn't work, I used it for a few days and it was the same as before, it didn't work, I should have to return it	Jingdong prefecture level city in Shandong
Jinfeng Hongyuan Flagship Store	Power Saving Expert 2	The goods have been received, but have not yet installed and use, feel good.	Jingdong prefecture level city in Shandong
Jinfeng Hongyuan Flagship Store	Power Saving Expert 2	Goods received, I forgot to take pictures, the goods look good, you can start, immediately start the school to see the effect then	Jingdong prefecture level city in Shandong
Jinfeng Hongyuan Flagship Store	Power Saving Expert 2	Looks good. I'll use it for a while.	Jingdong prefecture level city in Shandong
Jinfeng Hongyuan Flagship Store	Power Saving Expert 2	Great stuff, easy to operate, easy to use, good power saving, believe in the power of science!	Jingdong prefecture level city in Shandong
Jinfeng Hongyuan Flagship Store	Power Saving Expert 2	Especially great, comparison shopping before buying, the store owner is very good, answering questions very patiently and detailed, there is a need to come back!	Jingdong prefecture level city in Shandong
Jinfeng Hongyuan Flagship Store	Power Saving Expert 2	Trustworthy, feel free to use it boldly.	Jingdong prefecture level city in Shandong
Jinfeng Hongyuan Flagship Store	Power Saving Expert 2	Logistics is super fast! Beautiful appearance! Looks like a great quality! I've never bought one before, so I'll try it first to see if it saves power. We'll see how much it saves.	Jingdong prefecture level city in Shandong
Jinfeng Hongyuan Flagship Store	Power Saving Expert 2	I saw online counterfeiting, energy saver is one of them, hahahahaha, jingdong also buy fake products	Jingdong prefecture level city in Shandong
Jinfeng Hongyuan Flagship Store	Power Saving Expert 2	Just received, installed, try a month to see how the effect and then follow up.	Jingdong prefecture level city in Shandong
Jinfeng Hongyuan Flagship Store	Power Saving Expert 2	Good for you! The goods are genuine! Good price for what you get!	Jingdong prefecture level city in Shandong
Jinfeng Hongyuan Flagship Store	Power Saving Expert 2	Things quickly received, immediately used to see if there is no effect, something river photo ❕ the same, customer service is very professional	Jingdong prefecture level city in Shandong
Jinfeng Hongyuan Flagship Store	Power Saving Expert 2	The product is very convenient Looking forward to the subsequent use of the experience results	Jingdong prefecture level city in Shandong
Jinfeng Hongyuan Flagship Store	Power Saving Expert 2	I just received it and plugged it in, we'll see exactly when it's time to pay the electric bill	Jingdong prefecture level city in Shandong
Jinfeng Hongyuan Flagship Store	Power Saving Expert 2	Saver is good Packaging and looks good How effective it is to be shared after using it.	Jingdong prefecture level city in Shandong
Jinfeng Hongyuan Flagship Store	Power Saving Expert 2	There's no way to compare. I don't know if it's energy efficient.	Jingdong prefecture level city in Shandong
Jinfeng Hongyuan Flagship Store	Power Saving Expert 2	Just installed it, try it out and comment again	Jingdong prefecture level city in Shandong
Jinfeng Hongyuan Flagship Store	Power Saving Expert 2	Super save point also good to install, after a friend introduced then went to buy to, friends say it is very good to use, home I bought, try to use a period of time, to be honest, the economizer is not big or heavy, can be very convenient to put in a lot of places, the quality is also very good, power-saving function this time seems to be about 20% of the efficiency. The owner is also very warm contact, very	Jingdong prefecture level city in Shandong
Jinfeng Hongyuan Flagship Store	Power Saving Expert 2	The reviews online are mixed. Try it for a while, but I don't know if I can return it if it doesn't save any power after a couple months.	Jingdong prefecture level city in Shandong
Jinfeng Hongyuan Flagship Store	Power Saving Expert 2	The power consumption at home is high, bought a power saver, it looks very convenient to plug on the line, see if you can save some money next month, if good, recommend to friends down the road.	Jingdong prefecture level city in Shandong
Jinfeng Hongyuan Flagship Store	Power Saving Expert 2	It's beautiful, it's genuine, but I don't know the power-saving effect.	Jingdong prefecture level city in Shandong
Jinfeng Hongyuan Flagship Store	Power Saving Expert 2	This user thinks the product is good	Jingdong prefecture level city in Shandong
Jinfeng Hongyuan Flagship Store	Power Saving Expert 2	Nice exterior design with an at-a-glance display	Jingdong prefecture level city in Shandong
Jinfeng Hongyuan Flagship Store	Power Saving Expert 2	Home large and small things are purchased in the Jingdong Mall, have been accustomed to shopping in the Jingdong, many times can buy good quality and cheap products, and courier service attitude is particularly good, sometimes too many couriers are to help me to the home, so many years have been in the Jingdong Mall shopping, trust and support! Will also support the Jingdong as always!	Jingdong prefecture level city in Shandong
Jinfeng Hongyuan Flagship Store	Power Saving Expert 2	Good workmanship, mostly better than the original wall-plugged ones, and hopefully power factor compensation saves on electricity costs	Jingdong prefecture level city in Shandong
Jinfeng Hongyuan Flagship Store	Power Saving Expert 2	I don't feel anything, I said I won't know the effect for a month or two, let's see.	Jingdong prefecture level city in Shandong
Jinfeng Hongyuan Flagship Store	Power Saving Expert 2	Delivery was pretty fast, a little pricey, just not sure if it works or not	Jingdong prefecture level city in Shandong
Jinfeng Hongyuan Flagship Store	Power Saving Expert 2	The goods have been received, I do not know how effective.	Jingdong prefecture level city in Shandong
Jinfeng Hongyuan Flagship Store	Power Saving Expert 2	So far it doesn't seem to be too noticeable, so I'll comment later.	Jingdong prefecture level city in Shandong
Jinfeng Hongyuan Flagship Store	Power Saving Expert 2	I'll comment after I've used it	Jingdong prefecture level city in Shandong
Jinfeng Hongyuan Flagship Store	Power Saving Expert 2	Not bad. You'll save a lot on your electric bill in a year!	Jingdong prefecture level city in Shandong
Jinfeng Hongyuan Flagship Store	Power Saving Expert 2	I just got it. I haven't tried it yet. I don't know what it's like.	Jingdong prefecture level city in Shandong
Jinfeng Hongyuan Flagship Store	Power Saving Expert 2	Shopping at Jingdong has become a habit, and this disease will not be cured for a while.	Jingdong prefecture level city in Shandong
Jinfeng Hongyuan Flagship Store	Power Saving Expert 2	I've been using it for a while now. I don't know if I'm still saving power or not.	Jingdong prefecture level city in Shandong
Jinfeng Hongyuan Flagship Store	Power Saving Expert 2	Received the saver and it's great! There is also a digital voltage indication. Plugged in as instructed, easy to operate, hope you're our power saver!	Jingdong prefecture level city in Shandong
Jinfeng Hongyuan Flagship Store	Power Saving Expert 2	There is no power saving as the store says, using it is the same as not using it.	Jingdong prefecture level city in Shandong
Jinfeng Hongyuan Flagship Store	Power Saving Expert 2	Use it for a while and then comment again	Jingdong prefecture level city in Shandong
Jinfeng Hongyuan Flagship Store	Power Saving Expert 2	Feeling can, to use 1 to 2 months to see how the effect, there is a voltage display, can see the voltage fluctuations amplitude, quite good!	Jingdong prefecture level city in Shandong
Jinfeng Hongyuan Flagship Store	Power Saving Expert 2	I hope it works and saves me some electricity.	Jingdong prefecture level city in Shandong
Jinfeng Hongyuan Flagship Store	Power Saving Expert 2	Nice baby, happy with the after sales	Jingdong prefecture level city in Shandong
Jinfeng Hongyuan Flagship Store	Power Saving Expert 2	Use it and talk about it.	Jingdong prefecture level city in Shandong
Jinfeng Hongyuan Flagship Store	Power Saving Expert 2	Second purchase	Jingdong prefecture level city in Shandong

Jinfeng Hongyuan Flagship Store	Power Saving Expert 2	It works.	Jingdong prefecture level city in Shandong
Jinfeng Hongyuan Flagship Store	Power Saving Expert 2	It works.	Jingdong prefecture level city in Shandong
Jinfeng Hongyuan Flagship Store	Power Saving Expert 2	I don't know if it's a power saver until I start using it	Jingdong prefecture level city in Shandong
Jinfeng Hongyuan Flagship Store	Power Saving Expert 2	serviceable	Jingdong prefecture level city in Shandong
Jinfeng Hongyuan Flagship Store	Power Saving Expert 2	This user did not fill out the rating in time	Jingdong prefecture level city in Shandong
Jinfeng Hongyuan Flagship Store	Power Saving Expert 2	There is no power saving as the store says, using it is the same as not using it.	Jingdong prefecture level city in Shandong
Jinfeng Hongyuan Flagship Store	Power Saving Expert 2	This user did not fill out the rating in time	Jingdong prefecture level city in Shandong
Jinfeng Hongyuan Flagship Store	Power Saving Expert 2	Good...good.	Jingdong prefecture level city in Shandong
Jinfeng Hongyuan Flagship Store	Power Saving Expert 2	(of an unmarried couple) be close	Jingdong prefecture level city in Shandong
Jinfeng Hongyuan Flagship Store	Power Saving Expert 2	(of an unmarried couple) be close	Jingdong prefecture level city in Shandong
Jinfeng Hongyuan Flagship Store	Power Saving Expert 2	(of an unmarried couple) be close	Jingdong prefecture level city in Shandong
Jinfeng Hongyuan Flagship Store	Power Saving Expert 2	The goods are real, high value, high quality, worth every penny, material appearance and quality at a glance is very upscale, very like HAPPY1 00	Jingdong prefecture level city in Shandong
Jinfeng Hongyuan Flagship Store	Power Saving Expert 2	It is the ideal product, good quality, logistics is also very fast, worthy of praise 🎁 baby quality is very good, received the first time to open the first time to see, it is very good 👍, very satisfied 😃, customer service is also available to ask questions and answer, thank you 🙏	Jingdong prefecture level city in Shandong
Jinfeng Hongyuan Flagship Store	Power Saving Expert 2	I bought it and haven't used it yet, I'll use it again.	Jingdong prefecture level city in Shandong
Jinfeng Hongyuan Flagship Store	Power Saving Expert 2	It didn't work, I used it for a few days and it was the same as before, it didn't work, I should have to return it	Jingdong prefecture level city in Shandong
Jinfeng Hongyuan Flagship Store	Power Saving Expert 2	The goods have been received, but have not yet installed and use, feel good.	Jingdong prefecture level city in Shandong
Jinfeng Hongyuan Flagship Store	Power Saving Expert 2	Goods received, I forgot to take pictures, the goods look good, you can start, immediately start the school to see the effect then	Jingdong prefecture level city in Shandong
Jinfeng Hongyuan Flagship Store	Power Saving Expert 2	Looks good. I'll use it for a while.	Jingdong prefecture level city in Shandong
Jinfeng Hongyuan Flagship Store	Power Saving Expert 2	Great stuff, easy to operate, easy to use, good power saving, believe in the power of science!	Jingdong prefecture level city in Shandong
Jinfeng Hongyuan Flagship Store	Power Saving Expert 2	Especially great, comparison shopping before buying, the store owner is very good, answering questions very patiently and detailed, there is a need to come back!	Jingdong prefecture level city in Shandong
Jinfeng Hongyuan Flagship Store	Power Saving Expert 2	Trustworthy, feel free to use it boldly.	Jingdong prefecture level city in Shandong
Jinfeng Hongyuan Flagship Store	Power Saving Expert 2	Logistics is super fast! Beautiful appearance! Looks like a great quality! I've never bought one before, so I'll try it first to see if it saves power. We'll see how much it saves.	Jingdong prefecture level city in Shandong
Jinfeng Hongyuan Flagship Store	Power Saving Expert 2	I saw online counterfeiting, energy saver is one of them, hahahahaha, jingdong also buy fake products	Jingdong prefecture level city in Shandong
Jinfeng Hongyuan Flagship Store	Power Saving Expert 2	Just received, installed, try a month to see how the effect and then follow up.	Jingdong prefecture level city in Shandong
Jinfeng Hongyuan Flagship Store	Power Saving Expert 2	Good for you! The goods are genuine! Good price for what you get!	Jingdong prefecture level city in Shandong
Jinfeng Hongyuan Flagship Store	Power Saving Expert 2	Things quickly received, immediately used to see if there is no effect, something river photo 🠕 the same, customer service is very professional	Jingdong prefecture level city in Shandong
Jinfeng Hongyuan Flagship Store	Power Saving Expert 2	The product is very convenient Looking forward to the subsequent use of the experience results	Jingdong prefecture level city in Shandong
Jinfeng Hongyuan Flagship Store	Power Saving Expert 2	I just received it and plugged it in, we'll see exactly when it's time to pay the electric bill	Jingdong prefecture level city in Shandong
Jinfeng Hongyuan Flagship Store	Power Saving Expert 2	Saver is good Packaging and looks good How effective it is to be shared after using it.	Jingdong prefecture level city in Shandong
Jinfeng Hongyuan Flagship Store	Power Saving Expert 2	There's no way to compare. I don't know if it's energy efficient.	Jingdong prefecture level city in Shandong
Jinfeng Hongyuan Flagship Store	Power Saving Expert 2	Just installed it, try it out and comment again	Jingdong prefecture level city in Shandong
Jinfeng Hongyuan Flagship Store	Power Saving Expert 2	Super save point also good to install, after a friend introduced then went to buy to, friends say it is very good to use, home I bought, try to use a period of time, to be honest, the economizer is not big or heavy, can be very convenient to put in a lot of places, the quality is also very good, power-saving function this time seems to be about 20% of the efficiency. The owner is also very warm contact, very	Jingdong prefecture level city in Shandong
Jinfeng Hongyuan Flagship Store	Power Saving Expert 2	The reviews online are mixed. Try it for a while, but I don't know if I can return it if it doesn't save any power after a couple months.	Jingdong prefecture level city in Shandong
Jinfeng Hongyuan Flagship Store	Power Saving Expert 2	The power consumption at home is high, bought a power saver, it looks very convenient to plug on the line, see if you can save some money next month, if good, recommend to friends down the road.	Jingdong prefecture level city in Shandong
Jinfeng Hongyuan Flagship Store	Power Saving Expert 2	It's beautiful, it's genuine, but I don't know the power-saving effect.	Jingdong prefecture level city in Shandong
Jinfeng Hongyuan Flagship Store	Power Saving Expert 2	This user thinks the product is good	Jingdong prefecture level city in Shandong
Jinfeng Hongyuan Flagship Store	Power Saving Expert 2	Nice exterior design with an at-a-glance display	Jingdong prefecture level city in Shandong
Jinfeng Hongyuan Flagship Store	Power Saving Expert 2	Home large and small things are purchased in the Jingdong Mall, have been accustomed to shopping in the Jingdong, many times can buy good quality and cheap products, and courier service attitude is particularly good, sometimes too many couriers are here to help me to the home, so many years have been in the Jingdong Mall shopping, trust and support! Will also support the Jingdong as always!	Jingdong prefecture level city in Shandong
Jinfeng Hongyuan Flagship Store	Power Saving Expert 2	Good workmanship, mostly better than the original wall-plugged ones, and hopefully power factor compensation saves on electricity costs	Jingdong prefecture level city in Shandong
Jinfeng Hongyuan Flagship Store	Power Saving Expert 2	I don't feel anything, I said I won't know the effect for a month or two, let's see.	Jingdong prefecture level city in Shandong
Jinfeng Hongyuan Flagship Store	Power Saving Expert 2	Delivery was pretty fast, a little pricey, just not sure if it works or not	Jingdong prefecture level city in Shandong
Jinfeng Hongyuan Flagship Store	Power Saving Expert 2	The goods have been received, I do not know how effective.	Jingdong prefecture level city in Shandong
Jinfeng Hongyuan Flagship Store	Power Saving Expert 2	So far it doesn't seem to be too noticeable, so I'll comment later.	Jingdong prefecture level city in Shandong
Jinfeng Hongyuan Flagship Store	Power Saving Expert 2	I'll comment after I've used it	Jingdong prefecture level city in Shandong
Jinfeng Hongyuan Flagship Store	Power Saving Expert 2	Not bad. You'll save a lot on your electric bill in a year!	Jingdong prefecture level city in Shandong
Jinfeng Hongyuan Flagship Store	Power Saving Expert 2	I just got it. I haven't tried it yet. I don't know what it's like.	Jingdong prefecture level city in Shandong
Jinfeng Hongyuan Flagship Store	Power Saving Expert 2	Shopping at Jingdong has become a habit, and this disease will not be cured for a while.	Jingdong prefecture level city in Shandong
Jinfeng Hongyuan Flagship Store	Power Saving Expert 2	I've been using it for a while now. I don't know if I'm still saving power or not.	Jingdong prefecture level city in Shandong
Jinfeng Hongyuan Flagship Store	Power Saving Expert 2	Received the saver and it's great! There is also a digital voltage indication. Plugged in as instructed, easy to operate, hope you're our power saver!	Jingdong prefecture level city in Shandong

Jinfeng Hongyuan Flagship Store	Power Saving Expert 2	There is no power saving as the store says, using it is the same as not using it.	Jingdong prefecture level city in Shandong
Jinfeng Hongyuan Flagship Store	Power Saving Expert 2	Use it for a while and then comment again	Jingdong prefecture level city in Shandong
Jinfeng Hongyuan Flagship Store	Power Saving Expert 2	Feeling can, to use 1 to 2 months to see how the effect, there is a voltage display, can see the voltage fluctuations amplitude, quite good!	Jingdong prefecture level city in Shandong
Jinfeng Hongyuan Flagship Store	Power Saving Expert 2	I hope it works and saves me some electricity.	Jingdong prefecture level city in Shandong
Jinfeng Hongyuan Flagship Store	Power Saving Expert 2	Nice baby, happy with the after sales	Jingdong prefecture level city in Shandong
Jinfeng Hongyuan Flagship Store	Power Saving Expert 2	Use it and talk about it.	Jingdong prefecture level city in Shandong
Jinfeng Hongyuan Flagship Store	Power Saving Expert 2	Second purchase	Jingdong prefecture level city in Shandong
Jinfeng Hongyuan Flagship Store	Power Saving Expert 2	It works.	Jingdong prefecture level city in Shandong
Jinfeng Hongyuan Flagship Store	Power Saving Expert 2	It works.	Jingdong prefecture level city in Shandong
Jinfeng Hongyuan Flagship Store	Power Saving Expert 2	I don't know if it's a power saver until I start using it	Jingdong prefecture level city in Shandong
Jinfeng Hongyuan Flagship Store	Power Saving Expert 2	serviceable	Jingdong prefecture level city in Shandong
Jinfeng Hongyuan Flagship Store	Power Saving Expert 2	This user did not fill out the rating in time	Jingdong prefecture level city in Shandong
Jinfeng Hongyuan Flagship Store	Power Saving Expert 2	There is no power saving as the store says, using it is the same as not using it.	Jingdong prefecture level city in Shandong
Jinfeng Hongyuan Flagship Store	Power Saving Expert 2	This user did not fill out the rating in time	Jingdong prefecture level city in Shandong
Jinfeng Hongyuan Flagship Store	Power Saving Expert 2	Good...good.	Jingdong prefecture level city in Shandong
Jinfeng Hongyuan Flagship Store	Power Saving Expert 2	(of an unmarried couple) be close	Jingdong prefecture level city in Shandong
Jinfeng Hongyuan Flagship Store	Power Saving Expert 2	(of an unmarried couple) be close	Jingdong prefecture level city in Shandong
Jinfeng Hongyuan Flagship Store	Power Saving Expert 2	(of an unmarried couple) be close	Jingdong prefecture level city in Shandong
Jinfeng Hongyuan Flagship Store	Power Saving Expert 2	The goods are real, high value, high quality, worth every penny, material appearance and quality at a glance is very upscale, very like HAPPY1 00	Jingdong prefecture level city in Shandong
Jinfeng Hongyuan Flagship Store	Power Saving Expert 2	It is the ideal product, good quality, logistics is also very fast, worthy of praise 🎁 baby quality is very good, received the first time to open the first time to see, it is very good 👍, very satisfied 😃, customer service is also available to ask questions and answer, thank you 🙏	Jingdong prefecture level city in Shandong
Jinfeng Hongyuan Flagship Store	Power Saving Expert 2	I bought it and haven't used it yet, I'll use it again.	Jingdong prefecture level city in Shandong
Jinfeng Hongyuan Flagship Store	Power Saving Expert 2	It didn't work, I used it for a few days and it was the same as before, it didn't work, I should have to return it	Jingdong prefecture level city in Shandong
Jinfeng Hongyuan Flagship Store	Power Saving Expert 2	The goods have been received, but have not yet installed and use, feel good.	Jingdong prefecture level city in Shandong
Jinfeng Hongyuan Flagship Store	Power Saving Expert 2	Goods received, I forgot to take pictures, the goods look good, you can start, immediately start the school to see the effect then	Jingdong prefecture level city in Shandong
Jinfeng Hongyuan Flagship Store	Power Saving Expert 2	Looks good. I'll use it for a while.	Jingdong prefecture level city in Shandong
Jinfeng Hongyuan Flagship Store	Power Saving Expert 2	Great stuff, easy to operate, easy to use, good power saving, believe in the power of science!	Jingdong prefecture level city in Shandong
Jinfeng Hongyuan Flagship Store	Power Saving Expert 2	Especially great, comparison shopping before buying, the store owner is very good, answering questions very patiently and detailed, there is a need to come back!	Jingdong prefecture level city in Shandong
Jinfeng Hongyuan Flagship Store	Power Saving Expert 2	Trustworthy, feel free to use it boldly.	Jingdong prefecture level city in Shandong
Jinfeng Hongyuan Flagship Store	Power Saving Expert 2	Logistics is super fast! Beautiful appearance! Looks like a great quality! I've never bought one before, so I'll try it first to see if it saves power. We'll see how much it saves.	Jingdong prefecture level city in Shandong
Jinfeng Hongyuan Flagship Store	Power Saving Expert 2	I saw online counterfeiting, energy saver is one of them, hahahahaha, jingdong also buy fake products	Jingdong prefecture level city in Shandong
Jinfeng Hongyuan Flagship Store	Power Saving Expert 2	Just received, installed, try a month to see how the effect and then follow up.	Jingdong prefecture level city in Shandong
Jinfeng Hongyuan Flagship Store	Power Saving Expert 2	Good for you! The goods are genuine! Good price for what you get!	Jingdong prefecture level city in Shandong
Jinfeng Hongyuan Flagship Store	Power Saving Expert 2	Things quickly received, immediately used to see if there is no effect, something river photo ⬆ the same, customer service is very professional	Jingdong prefecture level city in Shandong
Jinfeng Hongyuan Flagship Store	Power Saving Expert 2	The product is very convenient Looking forward to the subsequent use of the experience results	Jingdong prefecture level city in Shandong
Jinfeng Hongyuan Flagship Store	Power Saving Expert 2	I just received it and plugged it in, we'll see exactly when it's time to pay the electric bill	Jingdong prefecture level city in Shandong
Jinfeng Hongyuan Flagship Store	Power Saving Expert 2	Saver is good Packaging and looks good How effective it is to be shared after using it.	Jingdong prefecture level city in Shandong
Jinfeng Hongyuan Flagship Store	Power Saving Expert 2	There's no way to compare. I don't know if it's energy efficient.	Jingdong prefecture level city in Shandong
Jinfeng Hongyuan Flagship Store	Power Saving Expert 2	Just installed it, try it out and comment again	Jingdong prefecture level city in Shandong
Jinfeng Hongyuan Flagship Store	Power Saving Expert 2	Super save point also good to install, after a friend introduced then went to buy to, friends say it is very good to use, home I bought, try to use a period of time, to be honest, the economizer is not big or heavy, can be very convenient to put in a lot of places, the quality is also very good, power-saving function this time seems to be about 20% of the efficiency. The owner is also very warm contact, very	Jingdong prefecture level city in Shandong
Jinfeng Hongyuan Flagship Store	Power Saving Expert 2	The reviews online are mixed. Try it for a while, but I don't know if I can return it if it doesn't save any power after a couple months.	Jingdong prefecture level city in Shandong
Jinfeng Hongyuan Flagship Store	Power Saving Expert 2	The power consumption at home is high, bought a power saver, it looks very convenient to plug on the line, see if you can save some money next month, if good, recommend to friends down the road.	Jingdong prefecture level city in Shandong
Jinfeng Hongyuan Flagship Store	Power Saving Expert 2	It's beautiful, it's genuine, but I don't know the power-saving effect.	Jingdong prefecture level city in Shandong
Jinfeng Hongyuan Flagship Store	Power Saving Expert 2	This user thinks the product is good	Jingdong prefecture level city in Shandong
Jinfeng Hongyuan Flagship Store	Power Saving Expert 2	Nice exterior design with an at-a-glance display	Jingdong prefecture level city in Shandong
Jinfeng Hongyuan Flagship Store	Power Saving Expert 2	Home large and small things are purchased in the Jingdong Mall, have been accustomed to shopping in the Jingdong, many times can buy good quality and cheap products, and courier service attitude is particularly good, sometimes too many couriers are to help me to the home, so many years have been in the Jingdong Mall shopping, trust and support! Will also support the Jingdong as always!	Jingdong prefecture level city in Shandong
Jinfeng Hongyuan Flagship Store	Power Saving Expert 2	Good workmanship, mostly better than the original wall-plugged ones, and hopefully power factor compensation saves on electricity costs	Jingdong prefecture level city in Shandong
Jinfeng Hongyuan Flagship Store	Power Saving Expert 2	I don't feel anything, I said I won't know the effect for a month or two, let's see.	Jingdong prefecture level city in Shandong
Jinfeng Hongyuan Flagship Store	Power Saving Expert 2	Delivery was pretty fast, a little pricey, just not sure if it works or not	Jingdong prefecture level city in Shandong
Jinfeng Hongyuan Flagship Store	Power Saving Expert 2	The goods have been received, I do not know how effective.	Jingdong prefecture level city in Shandong

Jinfeng Hongyuan Flagship Store	Power Saving Expert 2	So far it doesn't seem to be too noticeable, so I'll comment later.	Jingdong prefecture level city in Shandong
Jinfeng Hongyuan Flagship Store	Power Saving Expert 2	I'll comment after I've used it	Jingdong prefecture level city in Shandong
Jinfeng Hongyuan Flagship Store	Power Saving Expert 2	Not bad. You'll save a lot on your electric bill in a year!	Jingdong prefecture level city in Shandong
Jinfeng Hongyuan Flagship Store	Power Saving Expert 2	I just got it. I haven't tried it yet. I don't know what it's like.	Jingdong prefecture level city in Shandong
Jinfeng Hongyuan Flagship Store	Power Saving Expert 2	Shopping at Jingdong has become a habit, and this disease will not be cured for a while.	Jingdong prefecture level city in Shandong
Jinfeng Hongyuan Flagship Store	Power Saving Expert 2	I've been using it for a while now. I don't know if I'm still saving power or not.	Jingdong prefecture level city in Shandong
Jinfeng Hongyuan Flagship Store	Power Saving Expert 2	Received the saver and it's great! There is also a digital voltage indication. Plugged in as instructed, easy to operate, hope you're our power saver!	Jingdong prefecture level city in Shandong
Jinfeng Hongyuan Flagship Store	Power Saving Expert 2	There is no power saving as the store says, using it is the same as not using it.	Jingdong prefecture level city in Shandong
Jinfeng Hongyuan Flagship Store	Power Saving Expert 2	Use it for a while and then comment again	Jingdong prefecture level city in Shandong
Jinfeng Hongyuan Flagship Store	Power Saving Expert 2	Feeling can, to use 1 to 2 months to see how the effect, there is a voltage display, can see the voltage fluctuations amplitude, quite good!	Jingdong prefecture level city in Shandong
Jinfeng Hongyuan Flagship Store	Power Saving Expert 2	I hope it works and saves me some electricity.	Jingdong prefecture level city in Shandong
Jinfeng Hongyuan Flagship Store	Power Saving Expert 2	Nice baby, happy with the after sales	Jingdong prefecture level city in Shandong
Jinfeng Hongyuan Flagship Store	Power Saving Expert 2	Use it and talk about it.	Jingdong prefecture level city in Shandong
Jinfeng Hongyuan Flagship Store	Power Saving Expert 2	Second purchase	Jingdong prefecture level city in Shandong
Jinfeng Hongyuan Flagship Store	Power Saving Expert 2	It works.	Jingdong prefecture level city in Shandong
Jinfeng Hongyuan Flagship Store	Power Saving Expert 2	It works.	Jingdong prefecture level city in Shandong
Jinfeng Hongyuan Flagship Store	Power Saving Expert 2	I don't know if it's a power saver until I start using it	Jingdong prefecture level city in Shandong
Jinfeng Hongyuan Flagship Store	Power Saving Expert 2	serviceable	Jingdong prefecture level city in Shandong
Jinfeng Hongyuan Flagship Store	Power Saving Expert 2	This user did not fill out the rating in time	Jingdong prefecture level city in Shandong
Jinfeng Hongyuan Flagship Store	Power Saving Expert 2	There is no power saving as the store says, using it is the same as not using it.	Jingdong prefecture level city in Shandong
Jinfeng Hongyuan Flagship Store	Power Saving Expert 2	This user did not fill out the rating in time	Jingdong prefecture level city in Shandong
Jinfeng Hongyuan Flagship Store	Power Saving Expert 2	Good...good.	Jingdong prefecture level city in Shandong
Jinfeng Hongyuan Flagship Store	Power Saving Expert 2	(of an unmarried couple) be close	Jingdong prefecture level city in Shandong
Jinfeng Hongyuan Flagship Store	Power Saving Expert 2	(of an unmarried couple) be close	Jingdong prefecture level city in Shandong
Jinfeng Hongyuan Flagship Store	Power Saving Expert 2	(of an unmarried couple) be close	Jingdong prefecture level city in Shandong
Jinfeng Hongyuan Flagship Store	Power Saving Expert 2	The goods are real, high value, high quality, worth every penny, material appearance and quality at a glance is very upscale, very like HAPPY100	Jingdong prefecture level city in Shandong
Jinfeng Hongyuan Flagship Store	Power Saving Expert 2	It is the ideal product, good quality, logistics is also very fast, worthy of praise 🎁 baby quality is very good, received the first time to open the first time to see, it is very good 👍, very satisfied 😃, customer service is also available to ask questions and answer, thank you 🙏	Jingdong prefecture level city in Shandong
Jinfeng Hongyuan Flagship Store	Power Saving Expert 2	I bought it and haven't used it yet, I'll use it again.	Jingdong prefecture level city in Shandong
Jinfeng Hongyuan Flagship Store	Power Saving Expert 2	It didn't work, I used it for a few days and it was the same as before, it didn't work, I should have to return it	Jingdong prefecture level city in Shandong
Jinfeng Hongyuan Flagship Store	Power Saving Expert 2	The goods have been received, but have not yet installed and use, feel good.	Jingdong prefecture level city in Shandong
Jinfeng Hongyuan Flagship Store	Power Saving Expert 2	Goods received, I forgot to take pictures, the goods look good, you can start, immediately start the school to see the effect then	Jingdong prefecture level city in Shandong
Jinfeng Hongyuan Flagship Store	Power Saving Expert 2	Looks good. I'll use it for a while.	Jingdong prefecture level city in Shandong
Jinfeng Hongyuan Flagship Store	Power Saving Expert 2	Great stuff, easy to operate, easy to use, good power saving, believe in the power of science!	Jingdong prefecture level city in Shandong
Jinfeng Hongyuan Flagship Store	Power Saving Expert 2	Especially great, comparison shopping before buying, the store owner is very good, answering questions very patiently and detailed, there is a need to come back!	Jingdong prefecture level city in Shandong
Jinfeng Hongyuan Flagship Store	Power Saving Expert 2	Trustworthy, feel free to use it boldly.	Jingdong prefecture level city in Shandong
Jinfeng Hongyuan Flagship Store	Power Saving Expert 2	Logistics is super fast! Beautiful appearance! Looks like a great quality! I've never bought one before, so I'll try it first to see if it saves power. We'll see how much it saves.	Jingdong prefecture level city in Shandong
Jinfeng Hongyuan Flagship Store	Power Saving Expert 2	I saw online counterfeiting, energy saver is one of them, hahahahaha, jingdong also buy fake products	Jingdong prefecture level city in Shandong
Jinfeng Hongyuan Flagship Store	Power Saving Expert 2	Just received, installed, try a month to see how the effect and then follow up.	Jingdong prefecture level city in Shandong
Jinfeng Hongyuan Flagship Store	Power Saving Expert 2	Good for you! The goods are genuine! Good price for what you get!	Jingdong prefecture level city in Shandong
Jinfeng Hongyuan Flagship Store	Power Saving Expert 2	Things quickly received, immediately used to see if there is no effect, something river photo ⇡ the same, customer service is very professional	Jingdong prefecture level city in Shandong
Jinfeng Hongyuan Flagship Store	Power Saving Expert 2	The product is very convenient Looking forward to the subsequent use of the experience results	Jingdong prefecture level city in Shandong
Jinfeng Hongyuan Flagship Store	Power Saving Expert 2	I just received it and plugged it in, we'll see exactly when it's time to pay the electric bill	Jingdong prefecture level city in Shandong
Jinfeng Hongyuan Flagship Store	Power Saving Expert 2	Saver is good Packaging and looks good How effective it is to be shared after using it.	Jingdong prefecture level city in Shandong
Jinfeng Hongyuan Flagship Store	Power Saving Expert 2	There's no way to compare. I don't know if it's energy efficient.	Jingdong prefecture level city in Shandong
Jinfeng Hongyuan Flagship Store	Power Saving Expert 2	Just installed it, try it out and comment again	Jingdong prefecture level city in Shandong
Jinfeng Hongyuan Flagship Store	Power Saving Expert 2	Super save point also good to install, after a friend introduced then went to buy to, friends say it is very good to use, home I bought, try to use a period of time, to be honest, the economizer is not big or heavy, can be very convenient to put in a lot of places, the quality is also very good, power-saving function this time seems to be about 20% of the efficiency. The owner is also very warm contact, very	Jingdong prefecture level city in Shandong
Jinfeng Hongyuan Flagship Store	Power Saving Expert 2	The reviews online are mixed. Try it for a while, but I don't know if I can return it if it doesn't save any power after a couple months.	Jingdong prefecture level city in Shandong
Jinfeng Hongyuan Flagship Store	Power Saving Expert 2	The power consumption at home is high, bought a power saver, it looks very convenient to plug on the line, see if you can save some money next month, if good, recommend to friends down the road.	Jingdong prefecture level city in Shandong
Jinfeng Hongyuan Flagship Store	Power Saving Expert 2	It's beautiful, it's genuine, but I don't know the power-saving effect.	Jingdong prefecture level city in Shandong
Jinfeng Hongyuan Flagship Store	Power Saving Expert 2	This user thinks the product is good	Jingdong prefecture level city in Shandong

Jinfeng Hongyuan Flagship Store	Power Saving Expert 2	Nice exterior design with an at-a-glance display	Jingdong prefecture level city in Shandong
Jinfeng Hongyuan Flagship Store	Power Saving Expert 2	Home large and small things are purchased in the Jingdong Mall, have been accustomed to shopping in the Jingdong, many times can buy good quality and cheap products, and courier service attitude is particularly good, sometimes too many couriers are to help me to the home, so many years have been in the Jingdong Mall shopping, trust and support! Will also support the Jingdong as always!	Jingdong prefecture level city in Shandong
Jinfeng Hongyuan Flagship Store	Power Saving Expert 2	Good workmanship, mostly better than the original wall-plugged ones, and hopefully power factor compensation saves on electricity costs	Jingdong prefecture level city in Shandong
Jinfeng Hongyuan Flagship Store	Power Saving Expert 2	I don't feel anything, I said I won't know the effect for a month or two, let's see.	Jingdong prefecture level city in Shandong
Jinfeng Hongyuan Flagship Store	Power Saving Expert 2	Delivery was pretty fast, a little pricey, just not sure if it works or not	Jingdong prefecture level city in Shandong
Jinfeng Hongyuan Flagship Store	Power Saving Expert 2	The goods have been received, I do not know how effective.	Jingdong prefecture level city in Shandong
Jinfeng Hongyuan Flagship Store	Power Saving Expert 2	So far it doesn't seem to be too noticeable, so I'll comment later.	Jingdong prefecture level city in Shandong
Jinfeng Hongyuan Flagship Store	Power Saving Expert 2	I'll comment after I've used it	Jingdong prefecture level city in Shandong
Jinfeng Hongyuan Flagship Store	Power Saving Expert 2	Not bad. You'll save a lot on your electric bill in a year!	Jingdong prefecture level city in Shandong
Jinfeng Hongyuan Flagship Store	Power Saving Expert 2	I just got it. I haven't tried it yet. I don't know what it's like.	Jingdong prefecture level city in Shandong
Jinfeng Hongyuan Flagship Store	Power Saving Expert 2	Shopping at Jingdong has become a habit, and this disease will not be cured for a while.	Jingdong prefecture level city in Shandong
Jinfeng Hongyuan Flagship Store	Power Saving Expert 2	I've been using it for a while now. I don't know if I'm still saving power or not.	Jingdong prefecture level city in Shandong
Jinfeng Hongyuan Flagship Store	Power Saving Expert 2	Received the saver and it's great! There is also a digital voltage indication. Plugged in as instructed, easy to operate, hope you're our power saver!	Jingdong prefecture level city in Shandong
Jinfeng Hongyuan Flagship Store	Power Saving Expert 2	There is no power saving as the store says, using it is the same as not using it.	Jingdong prefecture level city in Shandong
Jinfeng Hongyuan Flagship Store	Power Saving Expert 2	Use it for a while and then comment again	Jingdong prefecture level city in Shandong
Jinfeng Hongyuan Flagship Store	Power Saving Expert 2	Feeling can, to use 1 to 2 months to see how the effect, there is a voltage display, can see the voltage fluctuations amplitude, quite good!	Jingdong prefecture level city in Shandong
Jinfeng Hongyuan Flagship Store	Power Saving Expert 2	I hope it works and saves me some electricity.	Jingdong prefecture level city in Shandong
Jinfeng Hongyuan Flagship Store	Power Saving Expert 2	Nice baby, happy with the after sales	Jingdong prefecture level city in Shandong
Jinfeng Hongyuan Flagship Store	Power Saving Expert 2	Use it and talk about it.	Jingdong prefecture level city in Shandong
Jinfeng Hongyuan Flagship Store	Power Saving Expert 2	Second purchase	Jingdong prefecture level city in Shandong
Jinfeng Hongyuan Flagship Store	Power Saving Expert 2	It works.	Jingdong prefecture level city in Shandong
Jinfeng Hongyuan Flagship Store	Power Saving Expert 2	It works.	Jingdong prefecture level city in Shandong
Jinfeng Hongyuan Flagship Store	Power Saving Expert 2	I don't know if it's a power saver until I start using it	Jingdong prefecture level city in Shandong
Jinfeng Hongyuan Flagship Store	Power Saving Expert 2	serviceable	Jingdong prefecture level city in Shandong
Jinfeng Hongyuan Flagship Store	Power Saving Expert 2	This user did not fill out the rating in time	Jingdong prefecture level city in Shandong
Jinfeng Hongyuan Flagship Store	Power Saving Expert 2	There is no power saving as the store says, using it is the same as not using it.	Jingdong prefecture level city in Shandong
Jinfeng Hongyuan Flagship Store	Power Saving Expert 2	This user did not fill out the rating in time	Jingdong prefecture level city in Shandong
Jinfeng Hongyuan Flagship Store	Power Saving Expert 2	Good...good.	Jingdong prefecture level city in Shandong
Jinfeng Hongyuan Flagship Store	Power Saving Expert 2	(of an unmarried couple) be close	Jingdong prefecture level city in Shandong
Jinfeng Hongyuan Flagship Store	Power Saving Expert 2	(of an unmarried couple) be close	Jingdong prefecture level city in Shandong
Jinfeng Hongyuan Flagship Store	Power Saving Expert 2	(of an unmarried couple) be close	Jingdong prefecture level city in Shandong
Jinfeng Hongyuan Flagship Store	Power Saving Expert 2	The goods are real, high value, high quality, worth every penny, material appearance and quality at a glance is very upscale, very like HAPPY1 00	Jingdong prefecture level city in Shandong
Jinfeng Hongyuan Flagship Store	Power Saving Expert 2	It is the ideal product, good quality, logistics is also very fast, worthy of praise 🎁 baby quality is very good, received the first time to open the first time to see, it is very good 👍, very satisfied 😃, customer service is also available to ask questions and answer, thank you 🙏	Jingdong prefecture level city in Shandong
Jinfeng Hongyuan Flagship Store	Power Saving Expert 2	I bought it and haven't used it yet, I'll use it again.	Jingdong prefecture level city in Shandong
Jinfeng Hongyuan Flagship Store	Power Saving Expert 2	It didn't work, I used it for a few days and it was the same as before, it didn't work, I should have to return it	Jingdong prefecture level city in Shandong
Jinfeng Hongyuan Flagship Store	Power Saving Expert 2	The goods have been received, but have not yet installed and use, feel good.	Jingdong prefecture level city in Shandong
Jinfeng Hongyuan Flagship Store	Power Saving Expert 2	Goods received, I forgot to take pictures, the goods look good, you can start, immediately start the school to see the effect then	Jingdong prefecture level city in Shandong
Jinfeng Hongyuan Flagship Store	Power Saving Expert 2	Looks good. I'll use it for a while.	Jingdong prefecture level city in Shandong
Jinfeng Hongyuan Flagship Store	Power Saving Expert 2	Great stuff, easy to operate, easy to use, good power saving, believe in the power of science!	Jingdong prefecture level city in Shandong
Jinfeng Hongyuan Flagship Store	Power Saving Expert 2	Especially great, comparison shopping before buying, the store owner is very good, answering questions very patiently and detailed, there is a need to come back!	Jingdong prefecture level city in Shandong
Jinfeng Hongyuan Flagship Store	Power Saving Expert 2	Trustworthy, feel free to use it boldly.	Jingdong prefecture level city in Shandong
Jinfeng Hongyuan Flagship Store	Power Saving Expert 2	Logistics is super fast! Beautiful appearance! Looks like a great quality! I've never bought one before, so I'll try it first to see if it saves power. We'll see how much it saves.	Jingdong prefecture level city in Shandong
Jinfeng Hongyuan Flagship Store	Power Saving Expert 2	I saw online counterfeiting, energy saver is one of them, hahahahaha, jingdong also buy fake products	Jingdong prefecture level city in Shandong
Jinfeng Hongyuan Flagship Store	Power Saving Expert 2	Just received, installed, try a month to see how the effect and then follow up.	Jingdong prefecture level city in Shandong
Jinfeng Hongyuan Flagship Store	Power Saving Expert 2	Good for you! The goods are genuine! Good price for what you get!	Jingdong prefecture level city in Shandong
Jinfeng Hongyuan Flagship Store	Power Saving Expert 2	Things quickly received, immediately used to see if there is no effect, something river photo ⬆ the same, customer service is very professional	Jingdong prefecture level city in Shandong
Jinfeng Hongyuan Flagship Store	Power Saving Expert 2	The product is very convenient Looking forward to the subsequent use of the experience results	Jingdong prefecture level city in Shandong
Jinfeng Hongyuan Flagship Store	Power Saving Expert 2	I just received it and plugged it in, we'll see exactly when it's time to pay the electric bill	Jingdong prefecture level city in Shandong
Jinfeng Hongyuan Flagship Store	Power Saving Expert 2	Saver is good Packaging and looks good How effective it is to be shared after using it.	Jingdong prefecture level city in Shandong
Jinfeng Hongyuan Flagship Store	Power Saving Expert 2	There's no way to compare. I don't know if it's energy efficient.	Jingdong prefecture level city in Shandong

Jinfeng Hongyuan Flagship Store	Power Saving Expert 2	Just installed it, try it out and comment again	Jingdong prefecture level city in Shandong
Jinfeng Hongyuan Flagship Store	Power Saving Expert 2	Super save point also good to install, after a friend introduced then went to buy to, friends say it is very good to use, home I bought, try to use a period of time, to be honest, the economizer is not big or heavy, can be very convenient to put in a lot of places, the quality is also very good, power-saving function this time seems to be about 20% of the efficiency. The owner is also very warm contact, very	Jingdong prefecture level city in Shandong
Jinfeng Hongyuan Flagship Store	Power Saving Expert 2	The reviews online are mixed. Try it for a while, but I don't know if I can return it if it doesn't save any power after a couple months.	Jingdong prefecture level city in Shandong
Jinfeng Hongyuan Flagship Store	Power Saving Expert 2	The power consumption at home is high, bought a power saver, it looks very convenient to plug on the line, see if you can save some money next month, if good, recommend to friends down the road.	Jingdong prefecture level city in Shandong
Jinfeng Hongyuan Flagship Store	Power Saving Expert 2	It's beautiful, it's genuine, but I don't know the power-saving effect.	Jingdong prefecture level city in Shandong
Jinfeng Hongyuan Flagship Store	Power Saving Expert 2	This user thinks the product is good	Jingdong prefecture level city in Shandong
Jinfeng Hongyuan Flagship Store	Power Saving Expert 2	Nice exterior design with an at-a-glance display	Jingdong prefecture level city in Shandong
Jinfeng Hongyuan Flagship Store	Power Saving Expert 2	Home large and small things are purchased in the Jingdong Mall, have been accustomed to shopping in the Jingdong, many times can buy good quality and cheap products, and courier service attitude is particularly good, sometimes too many couriers are to help me to the home, so many years have been in the Jingdong Mall shopping, trust and support! Will also support the Jingdong as always!	Jingdong prefecture level city in Shandong
Jinfeng Hongyuan Flagship Store	Power Saving Expert 2	Good workmanship, mostly better than the original wall-plugged ones, and hopefully power factor compensation saves on electricity costs	Jingdong prefecture level city in Shandong
Jinfeng Hongyuan Flagship Store	Power Saving Expert 2	I don't feel anything, I said I won't know the effect for a month or two, let's see.	Jingdong prefecture level city in Shandong
Jinfeng Hongyuan Flagship Store	Power Saving Expert 2	Delivery was pretty fast, a little pricey, just not sure if it works or not	Jingdong prefecture level city in Shandong
Jinfeng Hongyuan Flagship Store	Power Saving Expert 2	The goods have been received, I do not know how effective.	Jingdong prefecture level city in Shandong
Jinfeng Hongyuan Flagship Store	Power Saving Expert 2	So far it doesn't seem to be too noticeable, so I'll comment later.	Jingdong prefecture level city in Shandong
Jinfeng Hongyuan Flagship Store	Power Saving Expert 2	I'll comment after I've used it	Jingdong prefecture level city in Shandong
Jinfeng Hongyuan Flagship Store	Power Saving Expert 2	Not bad. You'll save a lot on your electric bill in a year!	Jingdong prefecture level city in Shandong
Jinfeng Hongyuan Flagship Store	Power Saving Expert 2	I just got it. I haven't tried it yet. I don't know what it's like.	Jingdong prefecture level city in Shandong
Jinfeng Hongyuan Flagship Store	Power Saving Expert 2	Shopping at Jingdong has become a habit, and this disease will not be cured for a while.	Jingdong prefecture level city in Shandong
Jinfeng Hongyuan Flagship Store	Power Saving Expert 2	I've been using it for a while now. I don't know if I'm still saving power or not.	Jingdong prefecture level city in Shandong
Jinfeng Hongyuan Flagship Store	Power Saving Expert 2	Received the saver and it's great! There is also a digital voltage indication. Plugged in as instructed, easy to operate, hope you're our power saver!	Jingdong prefecture level city in Shandong
Jinfeng Hongyuan Flagship Store	Power Saving Expert 2	There is no power saving as the store says, using it is the same as not using it.	Jingdong prefecture level city in Shandong
Jinfeng Hongyuan Flagship Store	Power Saving Expert 2	Use it for a while and then comment again	Jingdong prefecture level city in Shandong
Jinfeng Hongyuan Flagship Store	Power Saving Expert 2	Feeling can, to use 1 to 2 months to see how the effect, there is a voltage display, can see the voltage fluctuations amplitude, quite good!	Jingdong prefecture level city in Shandong
Jinfeng Hongyuan Flagship Store	Power Saving Expert 2	I hope it works and saves me some electricity.	Jingdong prefecture level city in Shandong
Jinfeng Hongyuan Flagship Store	Power Saving Expert 2	Nice baby, happy with the after sales	Jingdong prefecture level city in Shandong
Jinfeng Hongyuan Flagship Store	Power Saving Expert 2	Use it and talk about it.	Jingdong prefecture level city in Shandong
Jinfeng Hongyuan Flagship Store	Power Saving Expert 2	Second purchase	Jingdong prefecture level city in Shandong
Jinfeng Hongyuan Flagship Store	Power Saving Expert 2	It works.	Jingdong prefecture level city in Shandong
Jinfeng Hongyuan Flagship Store	Power Saving Expert 2	It works.	Jingdong prefecture level city in Shandong
Jinfeng Hongyuan Flagship Store	Power Saving Expert 2	I don't know if it's a power saver until I start using it	Jingdong prefecture level city in Shandong
Jinfeng Hongyuan Flagship Store	Power Saving Expert 2	serviceable	Jingdong prefecture level city in Shandong
Jinfeng Hongyuan Flagship Store	Power Saving Expert 2	This user did not fill out the rating in time	Jingdong prefecture level city in Shandong
Jinfeng Hongyuan Flagship Store	Power Saving Expert 2	There is no power saving as the store says, using it is the same as not using it.	Jingdong prefecture level city in Shandong
Jinfeng Hongyuan Flagship Store	Power Saving Expert 2	This user did not fill out the rating in time	Jingdong prefecture level city in Shandong
Jinfeng Hongyuan Flagship Store	Power Saving Expert 2	Good...good.	Jingdong prefecture level city in Shandong
Jinfeng Hongyuan Flagship Store	Power Saving Expert 2	(of an unmarried couple) be close	Jingdong prefecture level city in Shandong
Jinfeng Hongyuan Flagship Store	Power Saving Expert 2	(of an unmarried couple) be close	Jingdong prefecture level city in Shandong
Jinfeng Hongyuan Flagship Store	Power Saving Expert 2	(of an unmarried couple) be close	Jingdong prefecture level city in Shandong
Jinfeng Hongyuan Flagship Store	Power Saving Expert 2	The goods are real, high value, high quality, worth every penny, material appearance and quality at a glance is very upscale, very like HAPPY1 00	Jingdong prefecture level city in Shandong
Jinfeng Hongyuan Flagship Store	Power Saving Expert 2	It is the ideal product, good quality, logistics is also very fast, worthy of praise 🎁 baby quality is very good, received the first time to open the first time to see, it is very good 👍, very satisfied 😃, customer service is also available to ask questions and answer, thank you 🙏	Jingdong prefecture level city in Shandong
Jinfeng Hongyuan Flagship Store	Power Saving Expert 2	I bought it and haven't used it yet, I'll use it again.	Jingdong prefecture level city in Shandong
Jinfeng Hongyuan Flagship Store	Power Saving Expert 2	It didn't work, I used it for a few days and it was the same as before, it didn't work, I should have to return it	Jingdong prefecture level city in Shandong
Jinfeng Hongyuan Flagship Store	Power Saving Expert 2	The goods have been received, but have not yet installed and use, feel good.	Jingdong prefecture level city in Shandong
Jinfeng Hongyuan Flagship Store	Power Saving Expert 2	Goods received, I forgot to take pictures, the goods look good, you can start, immediately start the school to see the effect then	Jingdong prefecture level city in Shandong
Jinfeng Hongyuan Flagship Store	Power Saving Expert 2	Looks good. I'll use it for a while.	Jingdong prefecture level city in Shandong
Jinfeng Hongyuan Flagship Store	Power Saving Expert 2	Great stuff, easy to operate, easy to use, good power saving, believe in the power of science!	Jingdong prefecture level city in Shandong
Jinfeng Hongyuan Flagship Store	Power Saving Expert 2	Especially great, comparison shopping before buying, the store owner is very good, answering questions very patiently and detailed, there is a need to come back!	Jingdong prefecture level city in Shandong
Jinfeng Hongyuan Flagship Store	Power Saving Expert 2	Trustworthy, feel free to use it boldly.	Jingdong prefecture level city in Shandong
Jinfeng Hongyuan Flagship Store	Power Saving Expert 2	Logistics is super fast! Beautiful appearance! Looks like a great quality! I've never bought one before, so I'll try it first to see if it saves power. We'll see how much it saves.	Jingdong prefecture level city in Shandong
Jinfeng Hongyuan Flagship Store	Power Saving Expert 2	I saw online counterfeiting, energy saver is one of them, hahahahaha, jingdong also buy fake products	Jingdong prefecture level city in Shandong

Jinfeng Hongyuan Flagship Store	Power Saving Expert 2	Just received, installed, try a month to see how the effect and then follow up.	Jingdong prefecture level city in Shandong
Jinfeng Hongyuan Flagship Store	Power Saving Expert 2	Good for you! The goods are genuine! Good price for what you get!	Jingdong prefecture level city in Shandong
Jinfeng Hongyuan Flagship Store	Power Saving Expert 2	Things quickly received, immediately used to see if there is no effect, something river photo 🆙 the same, customer service is very professional	Jingdong prefecture level city in Shandong
Jinfeng Hongyuan Flagship Store	Power Saving Expert 2	The product is very convenient Looking forward to the subsequent use of the experience results	Jingdong prefecture level city in Shandong
Jinfeng Hongyuan Flagship Store	Power Saving Expert 2	I just received it and plugged it in, we'll see exactly when it's time to pay the electric bill	Jingdong prefecture level city in Shandong
Jinfeng Hongyuan Flagship Store	Power Saving Expert 2	Saver is good Packaging and looks good How effective it is to be shared after using it.	Jingdong prefecture level city in Shandong
Jinfeng Hongyuan Flagship Store	Power Saving Expert 2	There's no way to compare. I don't know if it's energy efficient.	Jingdong prefecture level city in Shandong
Jinfeng Hongyuan Flagship Store	Power Saving Expert 2	Just installed it, try it out and comment again	Jingdong prefecture level city in Shandong
Jinfeng Hongyuan Flagship Store	Power Saving Expert 2	Super save point also good to install, after a friend introduced then went to buy to, friends say it is very good to use, home I bought, try to use a period of time, to be honest, the economizer is not big or heavy, can be very convenient to put in a lot of places, the quality is also very good, power-saving function this time seems to be about 20% of the efficiency. The owner is also very warm contact, very	Jingdong prefecture level city in Shandong
Jinfeng Hongyuan Flagship Store	Power Saving Expert 2	The reviews online are mixed. Try it for a while, but I don't know if I can return it if it doesn't save any power after a couple months.	Jingdong prefecture level city in Shandong
Jinfeng Hongyuan Flagship Store	Power Saving Expert 2	The power consumption at home is high, bought a power saver, it looks very convenient to plug on the line, see if you can save some money next month, if good, recommend to friends down the road.	Jingdong prefecture level city in Shandong
Jinfeng Hongyuan Flagship Store	Power Saving Expert 2	It's beautiful, it's genuine, but I don't know the power-saving effect.	Jingdong prefecture level city in Shandong
Jinfeng Hongyuan Flagship Store	Power Saving Expert 2	This user thinks the product is good	Jingdong prefecture level city in Shandong
Jinfeng Hongyuan Flagship Store	Power Saving Expert 2	Nice exterior design with an at-a-glance display	Jingdong prefecture level city in Shandong
Jinfeng Hongyuan Flagship Store	Power Saving Expert 2	Home large and small things are purchased in the Jingdong Mall, have been accustomed to shopping in the Jingdong, many times can buy good quality and cheap products, and courier service attitude is particularly good, sometimes too many couriers are to help me to the home, so many years have been in the Jingdong Mall shopping, trust and support! Will also support the Jingdong as always!	Jingdong prefecture level city in Shandong
Jinfeng Hongyuan Flagship Store	Power Saving Expert 2	Good workmanship, mostly better than the original wall-plugged ones, and hopefully power factor compensation saves on electricity costs	Jingdong prefecture level city in Shandong
Jinfeng Hongyuan Flagship Store	Power Saving Expert 2	I don't feel anything, I said I won't know the effect for a month or two, let's see.	Jingdong prefecture level city in Shandong
Jinfeng Hongyuan Flagship Store	Power Saving Expert 2	Delivery was pretty fast, a little pricey, just not sure if it works or not	Jingdong prefecture level city in Shandong
Jinfeng Hongyuan Flagship Store	Power Saving Expert 2	The goods have been received, I do not know how effective.	Jingdong prefecture level city in Shandong
Jinfeng Hongyuan Flagship Store	Power Saving Expert 2	So far it doesn't seem to be too noticeable, so I'll comment later.	Jingdong prefecture level city in Shandong
Jinfeng Hongyuan Flagship Store	Power Saving Expert 2	I'll comment after I've used it	Jingdong prefecture level city in Shandong
Jinfeng Hongyuan Flagship Store	Power Saving Expert 2	Not bad. You'll save a lot on your electric bill in a year!	Jingdong prefecture level city in Shandong
Jinfeng Hongyuan Flagship Store	Power Saving Expert 2	I just got it. I haven't tried it yet. I don't know what it's like.	Jingdong prefecture level city in Shandong
Jinfeng Hongyuan Flagship Store	Power Saving Expert 2	Shopping at Jingdong has become a habit, and this disease will not be cured for a while.	Jingdong prefecture level city in Shandong
Jinfeng Hongyuan Flagship Store	Power Saving Expert 2	I've been using it for a while now. I don't know if I'm still saving power or not.	Jingdong prefecture level city in Shandong
Jinfeng Hongyuan Flagship Store	Power Saving Expert 2	Received the saver and it's great! There is also a digital voltage indication. Plugged in as instructed, easy to operate, hope you're our power saver!	Jingdong prefecture level city in Shandong
Jinfeng Hongyuan Flagship Store	Power Saving Expert 2	There is no power saving as the store says, using it is the same as not using it.	Jingdong prefecture level city in Shandong
Jinfeng Hongyuan Flagship Store	Power Saving Expert 2	Use it for a while and then comment again	Jingdong prefecture level city in Shandong
Jinfeng Hongyuan Flagship Store	Power Saving Expert 2	Feeling can, to use 1 to 2 months to see how the effect, there is a voltage display, can see the voltage fluctuations amplitude, quite good!	Jingdong prefecture level city in Shandong
Jinfeng Hongyuan Flagship Store	Power Saving Expert 2	I hope it works and saves me some electricity.	Jingdong prefecture level city in Shandong
Jinfeng Hongyuan Flagship Store	Power Saving Expert 2	Nice baby, happy with the after sales	Jingdong prefecture level city in Shandong
Jinfeng Hongyuan Flagship Store	Power Saving Expert 2	Use it and talk about it.	Jingdong prefecture level city in Shandong
Jinfeng Hongyuan Flagship Store	Power Saving Expert 2	Second purchase	Jingdong prefecture level city in Shandong
Jinfeng Hongyuan Flagship Store	Power Saving Expert 2	It works.	Jingdong prefecture level city in Shandong
Jinfeng Hongyuan Flagship Store	Power Saving Expert 2	It works.	Jingdong prefecture level city in Shandong
Jinfeng Hongyuan Flagship Store	Power Saving Expert 2	I don't know if it's a power saver until I start using it	Jingdong prefecture level city in Shandong
Jinfeng Hongyuan Flagship Store	Power Saving Expert 2	serviceable	Jingdong prefecture level city in Shandong
Jinfeng Hongyuan Flagship Store	Power Saving Expert 2	This user did not fill out the rating in time	Jingdong prefecture level city in Shandong
Jinfeng Hongyuan Flagship Store	Power Saving Expert 2	There is no power saving as the store says, using it is the same as not using it.	Jingdong prefecture level city in Shandong
Jinfeng Hongyuan Flagship Store	Power Saving Expert 2	This user did not fill out the rating in time	Jingdong prefecture level city in Shandong
Jinfeng Hongyuan Flagship Store	Power Saving Expert 2	Good...good.	Jingdong prefecture level city in Shandong
Jinfeng Hongyuan Flagship Store	Power Saving Expert 2	(of an unmarried couple) be close	Jingdong prefecture level city in Shandong
Jinfeng Hongyuan Flagship Store	Power Saving Expert 2	(of an unmarried couple) be close	Jingdong prefecture level city in Shandong
Jinfeng Hongyuan Flagship Store	Power Saving Expert 2	(of an unmarried couple) be close	Jingdong prefecture level city in Shandong
Jinfeng Hongyuan Flagship Store	Power Saving Expert 2	The goods are real, high value, high quality, worth every penny, material appearance and quality at a glance is very upscale, very like HAPPY1 00	Jingdong prefecture level city in Shandong
Jinfeng Hongyuan Flagship Store	Power Saving Expert 2	It is the ideal product, good quality, logistics is also very fast, worthy of praise 🎁 baby quality is very good, received the first time to open the first time to see, it is very good 👍, very satisfied 😃, customer service is also available to ask questions and answer, thank you 🙏	Jingdong prefecture level city in Shandong
Jinfeng Hongyuan Flagship Store	Power Saving Expert 2	I bought it and haven't used it yet, I'll use it again.	Jingdong prefecture level city in Shandong
Jinfeng Hongyuan Flagship Store	Power Saving Expert 2	It didn't work, I used it for a few days and it was the same as before, it didn't work, I should have to return it	Jingdong prefecture level city in Shandong
Jinfeng Hongyuan Flagship Store	Power Saving Expert 2	The goods have been received, but have not yet installed and use, feel good.	Jingdong prefecture level city in Shandong

Jinfeng Hongyuan Flagship Store	Power Saving Expert 2	Goods received, I forgot to take pictures, the goods look good, you can start, immediately start the school to see the effect then	Jingdong prefecture level city in Shandong
Jinfeng Hongyuan Flagship Store	Power Saving Expert 2	Looks good. I'll use it for a while.	Jingdong prefecture level city in Shandong
Jinfeng Hongyuan Flagship Store	Power Saving Expert 2	Great stuff, easy to operate, easy to use, good power saving, believe in the power of science!	Jingdong prefecture level city in Shandong
Jinfeng Hongyuan Flagship Store	Power Saving Expert 2	Especially great, comparison shopping before buying, the store owner is very good, answering questions very patiently and detailed, there is a need to come back!	Jingdong prefecture level city in Shandong
Jinfeng Hongyuan Flagship Store	Power Saving Expert 2	Trustworthy, feel free to use it boldly.	Jingdong prefecture level city in Shandong
Jinfeng Hongyuan Flagship Store	Power Saving Expert 2	Logistics is super fast! Beautiful appearance! Looks like a great quality! I've never bought one before, so I'll try it first to see if it saves power. We'll see how much it saves.	Jingdong prefecture level city in Shandong
Jinfeng Hongyuan Flagship Store	Power Saving Expert 2	I saw online counterfeiting, energy saver is one of them, hahahahaha, jingdong also buy fake products	Jingdong prefecture level city in Shandong
Jinfeng Hongyuan Flagship Store	Power Saving Expert 2	Just received, installed, try a month to see how the effect and then follow up.	Jingdong prefecture level city in Shandong
Jinfeng Hongyuan Flagship Store	Power Saving Expert 2	Good for you! The goods are genuine! Good price for what you get!	Jingdong prefecture level city in Shandong
Jinfeng Hongyuan Flagship Store	Power Saving Expert 2	Things quickly received, immediately used to see if there is no effect, something river photo ↑ the same, customer service is very professional	Jingdong prefecture level city in Shandong
Jinfeng Hongyuan Flagship Store	Power Saving Expert 2	The product is very convenient Looking forward to the subsequent use of the experience results	Jingdong prefecture level city in Shandong
Jinfeng Hongyuan Flagship Store	Power Saving Expert 2	I just received it and plugged it in, we'll see exactly when it's time to pay the electric bill	Jingdong prefecture level city in Shandong
Jinfeng Hongyuan Flagship Store	Power Saving Expert 2	Saver is good Packaging and looks good How effective it is to be shared after using it.	Jingdong prefecture level city in Shandong
Jinfeng Hongyuan Flagship Store	Power Saving Expert 2	There's no way to compare. I don't know if it's energy efficient.	Jingdong prefecture level city in Shandong
Jinfeng Hongyuan Flagship Store	Power Saving Expert 2	Just installed it, try it out and comment again	Jingdong prefecture level city in Shandong
Jinfeng Hongyuan Flagship Store	Power Saving Expert 2	Super save point also good to install, after a friend introduced then went to buy to, friends say it is very good to use, home I bought, try to use a period of time, to be honest, the economizer is not big or heavy, can be very convenient to put in a lot of places, the quality is also very good, power-saving function this time seems to be about 20% of the efficiency. The owner is also very warm contact, very	Jingdong prefecture level city in Shandong
Jinfeng Hongyuan Flagship Store	Power Saving Expert 2	The reviews online are mixed. Try it for a while, but I don't know if I can return it if it doesn't save any power after a couple months.	Jingdong prefecture level city in Shandong
Jinfeng Hongyuan Flagship Store	Power Saving Expert 2	The power consumption at home is high, bought a power saver, it looks very convenient to plug on the line, see if you can save some money next month, if good, recommend to friends down the road.	Jingdong prefecture level city in Shandong
Jinfeng Hongyuan Flagship Store	Power Saving Expert 2	It's beautiful, it's genuine, but I don't know the power-saving effect.	Jingdong prefecture level city in Shandong
Jinfeng Hongyuan Flagship Store	Power Saving Expert 2	This user thinks the product is good	Jingdong prefecture level city in Shandong
Jinfeng Hongyuan Flagship Store	Power Saving Expert 2	Nice exterior design with an at-a-glance display	Jingdong prefecture level city in Shandong
Jinfeng Hongyuan Flagship Store	Power Saving Expert 2	Home large and small things are purchased in the Jingdong Mall, have been accustomed to shopping in the Jingdong, many times can buy good quality and cheap products, and courier service attitude is particularly good, sometimes too many couriers are to help me to the home, so many years have been in the Jingdong Mall shopping, trust and support! Will also support the Jingdong as always!	Jingdong prefecture level city in Shandong
Jinfeng Hongyuan Flagship Store	Power Saving Expert 2	Good workmanship, mostly better than the original wall-plugged ones, and hopefully power factor compensation saves on electricity costs	Jingdong prefecture level city in Shandong
Jinfeng Hongyuan Flagship Store	Power Saving Expert 2	I don't feel anything, I said I won't know the effect for a month or two, let's see.	Jingdong prefecture level city in Shandong
Jinfeng Hongyuan Flagship Store	Power Saving Expert 2	Delivery was pretty fast, a little pricey, just not sure if it works or not	Jingdong prefecture level city in Shandong
Jinfeng Hongyuan Flagship Store	Power Saving Expert 2	The goods have been received, I do not know how effective.	Jingdong prefecture level city in Shandong
Jinfeng Hongyuan Flagship Store	Power Saving Expert 2	So far it doesn't seem to be too noticeable, so I'll comment later.	Jingdong prefecture level city in Shandong
Jinfeng Hongyuan Flagship Store	Power Saving Expert 2	I'll comment after I've used it	Jingdong prefecture level city in Shandong
Jinfeng Hongyuan Flagship Store	Power Saving Expert 2	Not bad. You'll save a lot on your electric bill in a year!	Jingdong prefecture level city in Shandong
Jinfeng Hongyuan Flagship Store	Power Saving Expert 2	I just got it. I haven't tried it yet. I don't know what it's like.	Jingdong prefecture level city in Shandong
Jinfeng Hongyuan Flagship Store	Power Saving Expert 2	Shopping at Jingdong has become a habit, and this disease will not be cured for a while.	Jingdong prefecture level city in Shandong
Jinfeng Hongyuan Flagship Store	Power Saving Expert 2	I've been using it for a while now. I don't know if I'm still saving power or not.	Jingdong prefecture level city in Shandong
Jinfeng Hongyuan Flagship Store	Power Saving Expert 2	Received the saver and it's great! There is also a digital voltage indication. Plugged in as instructed, easy to operate, hope you're our power saver!	Jingdong prefecture level city in Shandong
Jinfeng Hongyuan Flagship Store	Power Saving Expert 2	There is no power saving as the store says, using it is the same as not using it.	Jingdong prefecture level city in Shandong
Jinfeng Hongyuan Flagship Store	Power Saving Expert 2	Use it for a while and then comment again	Jingdong prefecture level city in Shandong
Jinfeng Hongyuan Flagship Store	Power Saving Expert 2	Feeling can, to use 1 to 2 months to see how the effect, there is a voltage display, can see the voltage fluctuations amplitude, quite good!	Jingdong prefecture level city in Shandong
Jinfeng Hongyuan Flagship Store	Power Saving Expert 2	I hope it works and saves me some electricity.	Jingdong prefecture level city in Shandong
Jinfeng Hongyuan Flagship Store	Power Saving Expert 2	Nice baby, happy with the after sales	Jingdong prefecture level city in Shandong
Jinfeng Hongyuan Flagship Store	Power Saving Expert 2	Use it and talk about it.	Jingdong prefecture level city in Shandong
Jinfeng Hongyuan Flagship Store	Power Saving Expert 2	Second purchase	Jingdong prefecture level city in Shandong
Jinfeng Hongyuan Flagship Store	Power Saving Expert 2	It works.	Jingdong prefecture level city in Shandong
Jinfeng Hongyuan Flagship Store	Power Saving Expert 2	It works.	Jingdong prefecture level city in Shandong
Jinfeng Hongyuan Flagship Store	Power Saving Expert 2	I don't know if it's a power saver until I start using it	Jingdong prefecture level city in Shandong
Jinfeng Hongyuan Flagship Store	Power Saving Expert 2	serviceable	Jingdong prefecture level city in Shandong
Jinfeng Hongyuan Flagship Store	Power Saving Expert 2	This user did not fill out the rating in time	Jingdong prefecture level city in Shandong
Jinfeng Hongyuan Flagship Store	Power Saving Expert 2	There is no power saving as the store says, using it is the same as not using it.	Jingdong prefecture level city in Shandong
Jinfeng Hongyuan Flagship Store	Power Saving Expert 2	This user did not fill out the rating in time	Jingdong prefecture level city in Shandong
Jinfeng Hongyuan Flagship Store	Power Saving Expert 2	Good...good.	Jingdong prefecture level city in Shandong
Jinfeng Hongyuan Flagship Store	Power Saving Expert 2	(of an unmarried couple) be close	Jingdong prefecture level city in Shandong

Jinfeng Hongyuan Flagship Store	Power Saving Expert 2	(of an unmarried couple) be close	Jingdong prefecture level city in Shandong
Jinfeng Hongyuan Flagship Store	Power Saving Expert 2	(of an unmarried couple) be close	Jingdong prefecture level city in Shandong
Jinfeng Hongyuan Flagship Store	Power Saving Expert 2	The goods are real, high value, high quality, worth every penny, material appearance and quality at a glance is very upscale, very like HAPPY1 00	Jingdong prefecture level city in Shandong
Jinfeng Hongyuan Flagship Store	Power Saving Expert 2	It is the ideal product, good quality, logistics is also very fast, worthy of praise 🎁 baby quality is very good, received the first time to open the first time to see, it is very good 👍, very satisfied 😃, customer service is also available to ask questions and answer, thank you 🙏	Jingdong prefecture level city in Shandong
Jinfeng Hongyuan Flagship Store	Power Saving Expert 2	I bought it and haven't used it yet, I'll use it again.	Jingdong prefecture level city in Shandong
Jinfeng Hongyuan Flagship Store	Power Saving Expert 2	It didn't work, I used it for a few days and it was the same as before, it didn't work, I should have to return it	Jingdong prefecture level city in Shandong
Jinfeng Hongyuan Flagship Store	Power Saving Expert 2	The goods have been received, but have not yet installed and use, feel good.	Jingdong prefecture level city in Shandong
Jinfeng Hongyuan Flagship Store	Power Saving Expert 2	Goods received, I forgot to take pictures, the goods look good, you can start, immediately start the school to see the effect then	Jingdong prefecture level city in Shandong
Jinfeng Hongyuan Flagship Store	Power Saving Expert 2	Looks good. I'll use it for a while.	Jingdong prefecture level city in Shandong
Jinfeng Hongyuan Flagship Store	Power Saving Expert 2	Great stuff, easy to operate, easy to use, good power saving, believe in the power of science!	Jingdong prefecture level city in Shandong
Jinfeng Hongyuan Flagship Store	Power Saving Expert 2	Especially great, comparison shopping before buying, the store owner is very good, answering questions very patiently and detailed, there is a need to come back!	Jingdong prefecture level city in Shandong
Jinfeng Hongyuan Flagship Store	Power Saving Expert 2	Trustworthy, feel free to use it boldly.	Jingdong prefecture level city in Shandong
Jinfeng Hongyuan Flagship Store	Power Saving Expert 2	Logistics is super fast! Beautiful appearance! Looks like a great quality! I've never bought one before, so I'll try it first to see if it saves power. We'll see how much it saves.	Jingdong prefecture level city in Shandong
Jinfeng Hongyuan Flagship Store	Power Saving Expert 2	I saw online counterfeiting, energy saver is one of them, hahahahaha, jingdong also buy fake products	Jingdong prefecture level city in Shandong
Jinfeng Hongyuan Flagship Store	Power Saving Expert 2	Just received, installed, try a month to see how the effect and then follow up.	Jingdong prefecture level city in Shandong
Jinfeng Hongyuan Flagship Store	Power Saving Expert 2	Good for you! The goods are genuine! Good price for what you get!	Jingdong prefecture level city in Shandong
Jinfeng Hongyuan Flagship Store	Power Saving Expert 2	Things quickly received, immediately used to see if there is no effect, something river photo 🆙 the same, customer service is very professional	Jingdong prefecture level city in Shandong
Jinfeng Hongyuan Flagship Store	Power Saving Expert 2	The product is very convenient Looking forward to the subsequent use of the experience results	Jingdong prefecture level city in Shandong
Jinfeng Hongyuan Flagship Store	Power Saving Expert 2	I just received it and plugged it in, we'll see exactly when it's time to pay the electric bill	Jingdong prefecture level city in Shandong
Jinfeng Hongyuan Flagship Store	Power Saving Expert 2	Saver is good Packaging and looks good How effective it is to be shared after using it.	Jingdong prefecture level city in Shandong
Jinfeng Hongyuan Flagship Store	Power Saving Expert 2	There's no way to compare. I don't know if it's energy efficient.	Jingdong prefecture level city in Shandong
Jinfeng Hongyuan Flagship Store	Power Saving Expert 2	Just installed it, try it out and comment again	Jingdong prefecture level city in Shandong
Jinfeng Hongyuan Flagship Store	Power Saving Expert 2	Super save point also good to install, after a friend introduced then went to buy to, friends say it is very good to use, home I bought, try to use a period of time, to be honest, the economizer is not big or heavy, can be very convenient to put in a lot of places, the quality is also very good, power-saving function this time seems to be about 20% of the efficiency. The owner is also very warm contact, very	Jingdong prefecture level city in Shandong
Jinfeng Hongyuan Flagship Store	Power Saving Expert 2	The reviews online are mixed. Try it for a while, but I don't know if I can return it if it doesn't save any power after a couple months.	Jingdong prefecture level city in Shandong
Jinfeng Hongyuan Flagship Store	Power Saving Expert 2	The power consumption at home is high, bought a power saver, it looks very convenient to plug on the line, see if you can save some money next month, if good, recommend to friends down the road.	Jingdong prefecture level city in Shandong
Jinfeng Hongyuan Flagship Store	Power Saving Expert 2	It's beautiful, it's genuine, but I don't know the power-saving effect.	Jingdong prefecture level city in Shandong
Jinfeng Hongyuan Flagship Store	Power Saving Expert 2	This user thinks the product is good	Jingdong prefecture level city in Shandong
Jinfeng Hongyuan Flagship Store	Power Saving Expert 2	Nice exterior design with an at-a-glance display	Jingdong prefecture level city in Shandong
Jinfeng Hongyuan Flagship Store	Power Saving Expert 2	Home large and small things are purchased in the Jingdong Mall, have been accustomed to shopping in the Jingdong, many times can buy good quality and cheap products, and courier service attitude is particularly good, sometimes too many couriers are to help me to the home, so many years have been in the Jingdong Mall shopping, trust and support! Will also support the Jingdong as always!	Jingdong prefecture level city in Shandong
Jinfeng Hongyuan Flagship Store	Power Saving Expert 2	Good workmanship, mostly better than the original wall-plugged ones, and hopefully power factor compensation saves on electricity costs	Jingdong prefecture level city in Shandong
Jinfeng Hongyuan Flagship Store	Power Saving Expert 2	I don't feel anything, I said I won't know the effect for a month or two, let's see.	Jingdong prefecture level city in Shandong
Jinfeng Hongyuan Flagship Store	Power Saving Expert 2	Delivery was pretty fast, a little pricey, just not sure if it works or not	Jingdong prefecture level city in Shandong
Jinfeng Hongyuan Flagship Store	Power Saving Expert 2	The goods have been received, I do not know how effective.	Jingdong prefecture level city in Shandong
Jinfeng Hongyuan Flagship Store	Power Saving Expert 2	So far it doesn't seem to be too noticeable, so I'll comment later.	Jingdong prefecture level city in Shandong
Jinfeng Hongyuan Flagship Store	Power Saving Expert 2	I'll comment after I've used it	Jingdong prefecture level city in Shandong
Jinfeng Hongyuan Flagship Store	Power Saving Expert 2	Not bad. You'll save a lot on your electric bill in a year!	Jingdong prefecture level city in Shandong
Jinfeng Hongyuan Flagship Store	Power Saving Expert 2	I just got it. I haven't tried it yet. I don't know what it's like.	Jingdong prefecture level city in Shandong
Jinfeng Hongyuan Flagship Store	Power Saving Expert 2	Shopping at Jingdong has become a habit, and this disease will not be cured for a while.	Jingdong prefecture level city in Shandong
Jinfeng Hongyuan Flagship Store	Power Saving Expert 2	I've been using it for a while now. I don't know if I'm still saving power or not.	Jingdong prefecture level city in Shandong
Jinfeng Hongyuan Flagship Store	Power Saving Expert 2	Received the saver and it's great! There is also a digital voltage indication. Plugged in as instructed, easy to operate, hope you're our power saver!	Jingdong prefecture level city in Shandong
Jinfeng Hongyuan Flagship Store	Power Saving Expert 2	There is no power saving as the store says, using it is the same as not using it.	Jingdong prefecture level city in Shandong
Jinfeng Hongyuan Flagship Store	Power Saving Expert 2	Use it for a while and then comment again	Jingdong prefecture level city in Shandong
Jinfeng Hongyuan Flagship Store	Power Saving Expert 2	Feeling can, to use 1 to 2 months to see how the effect, there is a voltage display, can see the voltage fluctuations amplitude, quite good!	Jingdong prefecture level city in Shandong
Jinfeng Hongyuan Flagship Store	Power Saving Expert 2	I hope it works and saves me some electricity.	Jingdong prefecture level city in Shandong
Jinfeng Hongyuan Flagship Store	Power Saving Expert 2	Nice baby, happy with the after sales	Jingdong prefecture level city in Shandong
Jinfeng Hongyuan Flagship Store	Power Saving Expert 2	Use it and talk about it.	Jingdong prefecture level city in Shandong
Jinfeng Hongyuan Flagship Store	Power Saving Expert 2	Second purchase	Jingdong prefecture level city in Shandong
Jinfeng Hongyuan Flagship Store	Power Saving Expert 2	It works.	Jingdong prefecture level city in Shandong
Jinfeng Hongyuan Flagship Store	Power Saving Expert 2	It works.	Jingdong prefecture level city in Shandong

Jinfeng Hongyuan Flagship Store	Power Saving Expert 2	I don't know if it's a power saver until I start using it	Jingdong prefecture level city in Shandong
Jinfeng Hongyuan Flagship Store	Power Saving Expert 2	serviceable	Jingdong prefecture level city in Shandong
Jinfeng Hongyuan Flagship Store	Power Saving Expert 2	This user did not fill out the rating in time	Jingdong prefecture level city in Shandong
Jinfeng Hongyuan Flagship Store	Power Saving Expert 2	There is no power saving as the store says, using it is the same as not using it.	Jingdong prefecture level city in Shandong
Jinfeng Hongyuan Flagship Store	Power Saving Expert 2	This user did not fill out the rating in time	Jingdong prefecture level city in Shandong
Jinfeng Hongyuan Flagship Store	Power Saving Expert 2	Good...good.	Jingdong prefecture level city in Shandong
Jinfeng Hongyuan Flagship Store	Power Saving Expert 2	(of an unmarried couple) be close	Jingdong prefecture level city in Shandong
Jinfeng Hongyuan Flagship Store	Power Saving Expert 2	(of an unmarried couple) be close	Jingdong prefecture level city in Shandong
Jinfeng Hongyuan Flagship Store	Power Saving Expert 2	(of an unmarried couple) be close	Jingdong prefecture level city in Shandong
Jinfeng Hongyuan Flagship Store	Power Saving Expert 2	The goods are real, high value, high quality, worth every penny, material appearance and quality at a glance is very upscale, very like HAPPY💯	Jingdong prefecture level city in Shandong
Jinfeng Hongyuan Flagship Store	Power Saving Expert 2	It is the ideal product, good quality, logistics is also very fast, worthy of praise 🎁 baby quality is very good, received the first time to open the first time to see, it is very good 👍, very satisfied 😃, customer service is also available to ask questions and answer, thank you 🙏	Jingdong prefecture level city in Shandong
Jinfeng Hongyuan Flagship Store	Power Saving Expert 2	I bought it and haven't used it yet, I'll use it again.	Jingdong prefecture level city in Shandong
Jinfeng Hongyuan Flagship Store	Power Saving Expert 2	It didn't work, I used it for a few days and it was the same as before, it didn't work, I should have to return it	Jingdong prefecture level city in Shandong
Jinfeng Hongyuan Flagship Store	Power Saving Expert 2	The goods have been received, but have not yet installed and use, feel good.	Jingdong prefecture level city in Shandong
Jinfeng Hongyuan Flagship Store	Power Saving Expert 2	Goods received, I forgot to take pictures, the goods look good, you can start, immediately start the school to see the effect then	Jingdong prefecture level city in Shandong
Jinfeng Hongyuan Flagship Store	Power Saving Expert 2	Looks good. I'll use it for a while.	Jingdong prefecture level city in Shandong
Jinfeng Hongyuan Flagship Store	Power Saving Expert 2	Great stuff, easy to operate, easy to use, good power saving, believe in the power of science!	Jingdong prefecture level city in Shandong
Jinfeng Hongyuan Flagship Store	Power Saving Expert 2	Especially great, comparison shopping before buying, the store owner is very good, answering questions very patiently and detailed, there is a need to come back!	Jingdong prefecture level city in Shandong
Jinfeng Hongyuan Flagship Store	Power Saving Expert 2	Trustworthy, feel free to use it boldly.	Jingdong prefecture level city in Shandong
Jinfeng Hongyuan Flagship Store	Power Saving Expert 2	Logistics is super fast! Beautiful appearance! Looks like a great quality! I've never bought one before, so I'll try it first to see if it saves power. We'll see how much it saves.	Jingdong prefecture level city in Shandong
Jinfeng Hongyuan Flagship Store	Power Saving Expert 2	I saw online counterfeiting, energy saver is one of them, hahahahaha, jingdong also buy fake products	Jingdong prefecture level city in Shandong
Jinfeng Hongyuan Flagship Store	Power Saving Expert 2	Just received, installed, try a month to see how the effect and then follow up.	Jingdong prefecture level city in Shandong
Jinfeng Hongyuan Flagship Store	Power Saving Expert 2	Good for you! The goods are genuine! Good price for what you get!	Jingdong prefecture level city in Shandong
Jinfeng Hongyuan Flagship Store	Power Saving Expert 2	Things quickly received, immediately used to see if there is no effect, something river photo ↑ the same, customer service is very professional	Jingdong prefecture level city in Shandong
Jinfeng Hongyuan Flagship Store	Power Saving Expert 2	The product is very convenient Looking forward to the subsequent use of the experience results	Jingdong prefecture level city in Shandong
Jinfeng Hongyuan Flagship Store	Power Saving Expert 2	I just received it and plugged it in, we'll see exactly when it's time to pay the electric bill	Jingdong prefecture level city in Shandong
Jinfeng Hongyuan Flagship Store	Power Saving Expert 2	Saver is good Packaging and looks good How effective it is to be shared after using it.	Jingdong prefecture level city in Shandong
Jinfeng Hongyuan Flagship Store	Power Saving Expert 2	There's no way to compare. I don't know if it's energy efficient.	Jingdong prefecture level city in Shandong
Jinfeng Hongyuan Flagship Store	Power Saving Expert 2	Just installed it, try it out and comment again	Jingdong prefecture level city in Shandong
Jinfeng Hongyuan Flagship Store	Power Saving Expert 2	Super save point also good to install, after a friend introduced then went to buy to, friends say it is very good to use, home I bought, try to use a period of time, to be honest, the economizer is not big or heavy, can be very convenient to put in a lot of places, the quality is also very good, power-saving function this time seems to be about 20% of the efficiency. The owner is also very warm contact, very	Jingdong prefecture level city in Shandong
Jinfeng Hongyuan Flagship Store	Power Saving Expert 2	The reviews online are mixed. Try it for a while, but I don't know if I can return it if it doesn't save any power after a couple months.	Jingdong prefecture level city in Shandong
Jinfeng Hongyuan Flagship Store	Power Saving Expert 2	The power consumption at home is high, bought a power saver, it looks very convenient to plug on the line, see if you can save some money next month, if good, recommend to friends down the road.	Jingdong prefecture level city in Shandong
Jinfeng Hongyuan Flagship Store	Power Saving Expert 2	It's beautiful, it's genuine, but I don't know the power-saving effect.	Jingdong prefecture level city in Shandong
Jinfeng Hongyuan Flagship Store	Power Saving Expert 2	This user thinks the product is good	Jingdong prefecture level city in Shandong
Jinfeng Hongyuan Flagship Store	Power Saving Expert 2	Nice exterior design with an at-a-glance display	Jingdong prefecture level city in Shandong
Jinfeng Hongyuan Flagship Store	Power Saving Expert 2	Home large and small things are purchased in the Jingdong Mall, have been accustomed to shopping in the Jingdong, many times can buy good quality and cheap products, and courier service attitude is particularly good, sometimes too many couriers are to help me to the home, so many years have been in the Jingdong Mall shopping, trust and support! Will also support the Jingdong as always!	Jingdong prefecture level city in Shandong
Jinfeng Hongyuan Flagship Store	Power Saving Expert 2	Good workmanship, mostly better than the original wall-plugged ones, and hopefully power factor compensation saves on electricity costs	Jingdong prefecture level city in Shandong
Jinfeng Hongyuan Flagship Store	Power Saving Expert 2	I don't feel anything, I said I won't know the effect for a month or two, let's see.	Jingdong prefecture level city in Shandong
Jinfeng Hongyuan Flagship Store	Power Saving Expert 2	Delivery was pretty fast, a little pricey, just not sure if it works or not	Jingdong prefecture level city in Shandong
Jinfeng Hongyuan Flagship Store	Power Saving Expert 2	The goods have been received, I do not know how effective.	Jingdong prefecture level city in Shandong
Jinfeng Hongyuan Flagship Store	Power Saving Expert 2	So far it doesn't seem to be too noticeable, so I'll comment later.	Jingdong prefecture level city in Shandong
Jinfeng Hongyuan Flagship Store	Power Saving Expert 2	I'll comment after I've used it	Jingdong prefecture level city in Shandong
Jinfeng Hongyuan Flagship Store	Power Saving Expert 2	Not bad. You'll save a lot on your electric bill in a year!	Jingdong prefecture level city in Shandong
Jinfeng Hongyuan Flagship Store	Power Saving Expert 2	I just got it. I haven't tried it yet. I don't know what it's like.	Jingdong prefecture level city in Shandong
Jinfeng Hongyuan Flagship Store	Power Saving Expert 2	Shopping at Jingdong has become a habit, and this disease will not be cured for a while.	Jingdong prefecture level city in Shandong
Jinfeng Hongyuan Flagship Store	Power Saving Expert 2	I've been using it for a while now. I don't know if I'm still saving power or not.	Jingdong prefecture level city in Shandong
Jinfeng Hongyuan Flagship Store	Power Saving Expert 2	Received the saver and it's great! There is also a digital voltage indication. Plugged in as instructed, easy to operate, hope you're our power saver!	Jingdong prefecture level city in Shandong
Jinfeng Hongyuan Flagship Store	Power Saving Expert 2	There is no power saving as the store says, using it is the same as not using it.	Jingdong prefecture level city in Shandong
Jinfeng Hongyuan Flagship Store	Power Saving Expert 2	Use it for a while and then comment again	Jingdong prefecture level city in Shandong

Jinfeng Hongyuan Flagship Store	Power Saving Expert 2	Feeling can, to use 1 to 2 months to see how the effect, there is a voltage display, can see the voltage fluctuations amplitude, quite good!	Jingdong prefecture level city in Shandong
Jinfeng Hongyuan Flagship Store	Power Saving Expert 2	I hope it works and saves me some electricity.	Jingdong prefecture level city in Shandong
Jinfeng Hongyuan Flagship Store	Power Saving Expert 2	Nice baby, happy with the after sales	Jingdong prefecture level city in Shandong
Jinfeng Hongyuan Flagship Store	Power Saving Expert 2	Use it and talk about it.	Jingdong prefecture level city in Shandong
Jinfeng Hongyuan Flagship Store	Power Saving Expert 2	Second purchase	Jingdong prefecture level city in Shandong
Jinfeng Hongyuan Flagship Store	Power Saving Expert 2	It works.	Jingdong prefecture level city in Shandong
Jinfeng Hongyuan Flagship Store	Power Saving Expert 2	It works.	Jingdong prefecture level city in Shandong
Jinfeng Hongyuan Flagship Store	Power Saving Expert 2	I don't know if it's a power saver until I start using it	Jingdong prefecture level city in Shandong
Jinfeng Hongyuan Flagship Store	Power Saving Expert 2	serviceable	Jingdong prefecture level city in Shandong
Jinfeng Hongyuan Flagship Store	Power Saving Expert 2	This user did not fill out the rating in time	Jingdong prefecture level city in Shandong
Jinfeng Hongyuan Flagship Store	Power Saving Expert 2	There is no power saving as the store says, using it is the same as not using it.	Jingdong prefecture level city in Shandong
Jinfeng Hongyuan Flagship Store	Power Saving Expert 2	This user did not fill out the rating in time	Jingdong prefecture level city in Shandong
Jinfeng Hongyuan Flagship Store	Power Saving Expert 2	Good...good.	Jingdong prefecture level city in Shandong
Jinfeng Hongyuan Flagship Store	Power Saving Expert 2	(of an unmarried couple) be close	Jingdong prefecture level city in Shandong
Jinfeng Hongyuan Flagship Store	Power Saving Expert 2	(of an unmarried couple) be close	Jingdong prefecture level city in Shandong
Jinfeng Hongyuan Flagship Store	Power Saving Expert 2	(of an unmarried couple) be close	Jingdong prefecture level city in Shandong
Jinfeng Hongyuan Flagship Store	Power Saving Expert 2	The goods are real, high value, high quality, worth every penny, material appearance and quality at a glance is very upscale, very like HAPPY💯	Jingdong prefecture level city in Shandong
Jinfeng Hongyuan Flagship Store	Power Saving Expert 2	It is the ideal product, good quality, logistics is also very fast, worthy of praise 🎁 baby quality is very good, received the first time to open the first time to see, it is very good 👍, very satisfied 😊, customer service is also available to ask questions and answer, thank you 🙏	Jingdong prefecture level city in Shandong
Jinfeng Hongyuan Flagship Store	Power Saving Expert 2	I bought it and haven't used it yet, I'll use it again.	Jingdong prefecture level city in Shandong
Jinfeng Hongyuan Flagship Store	Power Saving Expert 2	It didn't work, I used it for a few days and it was the same as before, it didn't work, I should have to return it	Jingdong prefecture level city in Shandong
Jinfeng Hongyuan Flagship Store	Power Saving Expert 2	The goods have been received, but have not yet installed and use, feel good.	Jingdong prefecture level city in Shandong
Jinfeng Hongyuan Flagship Store	Power Saving Expert 2	Goods received, I forgot to take pictures, the goods look good, you can start, immediately start the school to see the effect then	Jingdong prefecture level city in Shandong
Jinfeng Hongyuan Flagship Store	Power Saving Expert 2	Looks good. I'll use it for a while.	Jingdong prefecture level city in Shandong
Jinfeng Hongyuan Flagship Store	Power Saving Expert 2	Great stuff, easy to operate, easy to use, good power saving, believe in the power of science!	Jingdong prefecture level city in Shandong
Jinfeng Hongyuan Flagship Store	Power Saving Expert 2	Especially great, comparison shopping before buying, the store owner is very good, answering questions very patiently and detailed, there is a need to come back!	Jingdong prefecture level city in Shandong
Jinfeng Hongyuan Flagship Store	Power Saving Expert 2	Trustworthy, feel free to use it boldly.	Jingdong prefecture level city in Shandong
Jinfeng Hongyuan Flagship Store	Power Saving Expert 2	Logistics is super fast! Beautiful appearance! Looks like a great quality! I've never bought one before, so I'll try it first to see if it saves power. We'll see how much it saves.	Jingdong prefecture level city in Shandong
Jinfeng Hongyuan Flagship Store	Power Saving Expert 2	I saw online counterfeiting, energy saver is one of them, hahahahaha, jingdong also buy fake products	Jingdong prefecture level city in Shandong
Jinfeng Hongyuan Flagship Store	Power Saving Expert 2	Just received, installed, try a month to see how the effect and then follow up.	Jingdong prefecture level city in Shandong
Jinfeng Hongyuan Flagship Store	Power Saving Expert 2	Good for you! The goods are genuine! Good price for what you get!	Jingdong prefecture level city in Shandong
Jinfeng Hongyuan Flagship Store	Power Saving Expert 2	Things quickly received, immediately used to see if there is no effect, something river photo ❗ the same, customer service is very professional	Jingdong prefecture level city in Shandong
Jinfeng Hongyuan Flagship Store	Power Saving Expert 2	The product is very convenient Looking forward to the subsequent use of the experience results	Jingdong prefecture level city in Shandong
Jinfeng Hongyuan Flagship Store	Power Saving Expert 2	I just received it and plugged it in, we'll see exactly when it's time to pay the electric bill	Jingdong prefecture level city in Shandong
Jinfeng Hongyuan Flagship Store	Power Saving Expert 2	Saver is good Packaging and looks good How effective it is to be shared after using it.	Jingdong prefecture level city in Shandong
Jinfeng Hongyuan Flagship Store	Power Saving Expert 2	There's no way to compare. I don't know if it's energy efficient.	Jingdong prefecture level city in Shandong
Jinfeng Hongyuan Flagship Store	Power Saving Expert 2	Just installed it, try it out and comment again	Jingdong prefecture level city in Shandong
Jinfeng Hongyuan Flagship Store	Power Saving Expert 2	Super save point also good to install, after a friend introduced then went to buy to, friends say it is very good to use, home I bought, try to use a period of time, to be honest, the economizer is not big or heavy, can be very convenient to put in a lot of places, the quality is also very good, power-saving function this time seems to be about 20% of the efficiency. The owner is also very warm contact, very	Jingdong prefecture level city in Shandong
Jinfeng Hongyuan Flagship Store	Power Saving Expert 2	The reviews online are mixed. Try it for a while, but I don't know if I can return it if it doesn't save any power after a couple months.	Jingdong prefecture level city in Shandong
Jinfeng Hongyuan Flagship Store	Power Saving Expert 2	The power consumption at home is high, bought a power saver, it looks very convenient to plug on the line, see if you can save some money next month, if good, recommend to friends down the road.	Jingdong prefecture level city in Shandong
Jinfeng Hongyuan Flagship Store	Power Saving Expert 2	It's beautiful, it's genuine, but I don't know the power-saving effect.	Jingdong prefecture level city in Shandong
Jinfeng Hongyuan Flagship Store	Power Saving Expert 2	This user thinks the product is good	Jingdong prefecture level city in Shandong
Jinfeng Hongyuan Flagship Store	Power Saving Expert 2	Nice exterior design with an at-a-glance display	Jingdong prefecture level city in Shandong
Jinfeng Hongyuan Flagship Store	Power Saving Expert 2	Home large and small things are purchased in the Jingdong Mall, have been accustomed to shopping in the Jingdong, many times can buy good quality and cheap products, and courier service attitude is particularly good, sometimes too many couriers are to help me to the home, so many years have been in the Jingdong Mall shopping, trust and support! Will also support the Jingdong as always!	Jingdong prefecture level city in Shandong
Jinfeng Hongyuan Flagship Store	Power Saving Expert 2	Good workmanship, mostly better than the original wall-plugged ones, and hopefully power factor compensation saves on electricity costs	Jingdong prefecture level city in Shandong
Jinfeng Hongyuan Flagship Store	Power Saving Expert 2	I don't feel anything, I said I won't know the effect for a month or two, let's see.	Jingdong prefecture level city in Shandong
Jinfeng Hongyuan Flagship Store	Power Saving Expert 2	Delivery was pretty fast, a little pricey, just not sure if it works or not	Jingdong prefecture level city in Shandong
Jinfeng Hongyuan Flagship Store	Power Saving Expert 2	The goods have been received, I do not know how effective.	Jingdong prefecture level city in Shandong
Jinfeng Hongyuan Flagship Store	Power Saving Expert 2	So far it doesn't seem to be too noticeable, so I'll comment later.	Jingdong prefecture level city in Shandong
Jinfeng Hongyuan Flagship Store	Power Saving Expert 2	I'll comment after I've used it	Jingdong prefecture level city in Shandong

Jinfeng Hongyuan Flagship Store	Power Saving Expert 2	Not bad. You'll save a lot on your electric bill in a year!	Jingdong prefecture level city in Shandong
Jinfeng Hongyuan Flagship Store	Power Saving Expert 2	I just got it. I haven't tried it yet. I don't know what it's like.	Jingdong prefecture level city in Shandong
Jinfeng Hongyuan Flagship Store	Power Saving Expert 2	Shopping at Jingdong has become a habit, and this disease will not be cured for a while.	Jingdong prefecture level city in Shandong
Jinfeng Hongyuan Flagship Store	Power Saving Expert 2	I've been using it for a while now. I don't know if I'm still saving power or not.	Jingdong prefecture level city in Shandong
Jinfeng Hongyuan Flagship Store	Power Saving Expert 2	Received the saver and it's great! There is also a digital voltage indication. Plugged in as instructed, easy to operate, hope you're our power saver!	Jingdong prefecture level city in Shandong
Jinfeng Hongyuan Flagship Store	Power Saving Expert 2	There is no power saving as the store says, using it is the same as not using it.	Jingdong prefecture level city in Shandong
Jinfeng Hongyuan Flagship Store	Power Saving Expert 2	Use it for a while and then comment again	Jingdong prefecture level city in Shandong
Jinfeng Hongyuan Flagship Store	Power Saving Expert 2	Feeling can, to use 1 to 2 months to see how the effect, there is a voltage display, can see the voltage fluctuations amplitude, quite good!	Jingdong prefecture level city in Shandong
Jinfeng Hongyuan Flagship Store	Power Saving Expert 2	I hope it works and saves me some electricity.	Jingdong prefecture level city in Shandong
Jinfeng Hongyuan Flagship Store	Power Saving Expert 2	Nice baby, happy with the after sales	Jingdong prefecture level city in Shandong
Jinfeng Hongyuan Flagship Store	Power Saving Expert 2	Use it and talk about it.	Jingdong prefecture level city in Shandong
Jinfeng Hongyuan Flagship Store	Power Saving Expert 2	Second purchase	Jingdong prefecture level city in Shandong
Jinfeng Hongyuan Flagship Store	Power Saving Expert 2	It works.	Jingdong prefecture level city in Shandong
Jinfeng Hongyuan Flagship Store	Power Saving Expert 2	It works.	Jingdong prefecture level city in Shandong
Jinfeng Hongyuan Flagship Store	Power Saving Expert 2	I don't know if it's a power saver until I start using it	Jingdong prefecture level city in Shandong
Jinfeng Hongyuan Flagship Store	Power Saving Expert 2	serviceable	Jingdong prefecture level city in Shandong
Jinfeng Hongyuan Flagship Store	Power Saving Expert 2	This user did not fill out the rating in time	Jingdong prefecture level city in Shandong
Jinfeng Hongyuan Flagship Store	Power Saving Expert 2	There is no power saving as the store says, using it is the same as not using it.	Jingdong prefecture level city in Shandong
Jinfeng Hongyuan Flagship Store	Power Saving Expert 2	This user did not fill out the rating in time	Jingdong prefecture level city in Shandong
Jinfeng Hongyuan Flagship Store	Power Saving Expert 2	Good...good.	Jingdong prefecture level city in Shandong
Jinfeng Hongyuan Flagship Store	Power Saving Expert 2	(of an unmarried couple) be close	Jingdong prefecture level city in Shandong
Jinfeng Hongyuan Flagship Store	Power Saving Expert 2	(of an unmarried couple) be close	Jingdong prefecture level city in Shandong
Jinfeng Hongyuan Flagship Store	Power Saving Expert 2	(of an unmarried couple) be close	Jingdong prefecture level city in Shandong
Jinfeng Hongyuan Flagship Store	Power Saving Expert 2	The goods are real, high value, high quality, worth every penny, material appearance and quality at a glance is very upscale, very like HAPPY1 00	Jingdong prefecture level city in Shandong
Jinfeng Hongyuan Flagship Store	Power Saving Expert 2	It is the ideal product, good quality, logistics is also very fast, worthy of praise 🎁 baby quality is very good, received the first time to open the first time to see, it is very good 👍, very satisfied 😃, customer service is also available to ask questions and answer, thank you 🙏	Jingdong prefecture level city in Shandong
Jinfeng Hongyuan Flagship Store	Power Saving Expert 2	I bought it and haven't used it yet, I'll use it again.	Jingdong prefecture level city in Shandong
Jinfeng Hongyuan Flagship Store	Power Saving Expert 2	It didn't work, I used it for a few days and it was the same as before, it didn't work, I should have to return it	Jingdong prefecture level city in Shandong
Jinfeng Hongyuan Flagship Store	Power Saving Expert 2	The goods have been received, but have not yet installed and use, feel good.	Jingdong prefecture level city in Shandong
Jinfeng Hongyuan Flagship Store	Power Saving Expert 2	Goods received, I forgot to take pictures, the goods look good, you can start, immediately start the school to see the effect then	Jingdong prefecture level city in Shandong
Jinfeng Hongyuan Flagship Store	Power Saving Expert 2	Looks good. I'll use it for a while.	Jingdong prefecture level city in Shandong
Jinfeng Hongyuan Flagship Store	Power Saving Expert 2	Great stuff, easy to operate, easy to use, good power saving, believe in the power of science!	Jingdong prefecture level city in Shandong
Jinfeng Hongyuan Flagship Store	Power Saving Expert 2	Especially great, comparison shopping before buying, the store owner is very good, answering questions very patiently and detailed, there is a need to come back!	Jingdong prefecture level city in Shandong
Jinfeng Hongyuan Flagship Store	Power Saving Expert 2	Trustworthy, feel free to use it boldly.	Jingdong prefecture level city in Shandong
Jinfeng Hongyuan Flagship Store	Power Saving Expert 2	Logistics is super fast! Beautiful appearance! Looks like a great quality! I've never bought one before, so I'll try it first to see if it saves power. We'll see how much it saves.	Jingdong prefecture level city in Shandong
Jinfeng Hongyuan Flagship Store	Power Saving Expert 2	I saw online counterfeiting, energy saver is one of them, hahahahaha, jingdong also buy fake products	Jingdong prefecture level city in Shandong
Jinfeng Hongyuan Flagship Store	Power Saving Expert 2	Just received, installed, try a month to see how the effect and then follow up.	Jingdong prefecture level city in Shandong
Jinfeng Hongyuan Flagship Store	Power Saving Expert 2	Good for you! The goods are genuine! Good price for what you get!	Jingdong prefecture level city in Shandong
Jinfeng Hongyuan Flagship Store	Power Saving Expert 2	Things quickly received, immediately used to see if there is no effect, something river photo ⬆ the same, customer service is very professional	Jingdong prefecture level city in Shandong
Jinfeng Hongyuan Flagship Store	Power Saving Expert 2	The product is very convenient Looking forward to the subsequent use of the experience results	Jingdong prefecture level city in Shandong
Jinfeng Hongyuan Flagship Store	Power Saving Expert 2	I just received it and plugged it in, we'll see exactly when it's time to pay the electric bill	Jingdong prefecture level city in Shandong
Jinfeng Hongyuan Flagship Store	Power Saving Expert 2	Saver is good Packaging and looks good How effective it is to be shared after using it.	Jingdong prefecture level city in Shandong
Jinfeng Hongyuan Flagship Store	Power Saving Expert 2	There's no way to compare. I don't know if it's energy efficient.	Jingdong prefecture level city in Shandong
Jinfeng Hongyuan Flagship Store	Power Saving Expert 2	Just installed it, try it out and comment again	Jingdong prefecture level city in Shandong
Jinfeng Hongyuan Flagship Store	Power Saving Expert 2	Super save point also good to install, after a friend introduced then went to buy to, friends say it is very good to use, home I bought, try to use a period of time, to be honest, the economizer is not big or heavy, can be very convenient to put in a lot of places, the quality is also very good, power-saving function this time seems to be about 20% of the efficiency. The owner is also very warm contact, very	Jingdong prefecture level city in Shandong
Jinfeng Hongyuan Flagship Store	Power Saving Expert 2	The reviews online are mixed. Try it for a while, but I don't know if I can return it if it doesn't save any power after a couple months.	Jingdong prefecture level city in Shandong
Jinfeng Hongyuan Flagship Store	Power Saving Expert 2	The power consumption at home is high, bought a power saver, it looks very convenient to plug on the line, see if you can save some money next month, if good, recommend to friends down the road.	Jingdong prefecture level city in Shandong
Jinfeng Hongyuan Flagship Store	Power Saving Expert 2	It's beautiful, it's genuine, but I don't know the power-saving effect.	Jingdong prefecture level city in Shandong
Jinfeng Hongyuan Flagship Store	Power Saving Expert 2	This user thinks the product is good	Jingdong prefecture level city in Shandong
Jinfeng Hongyuan Flagship Store	Power Saving Expert 2	Nice exterior design with an at-a-glance display	Jingdong prefecture level city in Shandong

Jinfeng Hongyuan Flagship Store	Power Saving Expert 2	Home large and small things are purchased in the Jingdong Mall, have been accustomed to shopping in the Jingdong, many times can buy good quality and cheap products, and courier service attitude is particularly good, sometimes too many couriers are to help me to the home, so many years have been in the Jingdong Mall shopping, trust and support! Will also support the Jingdong as always!	Jingdong prefecture level city in Shandong
Jinfeng Hongyuan Flagship Store	Power Saving Expert 2	Good workmanship, mostly better than the original wall-plugged ones, and hopefully power factor compensation saves on electricity costs	Jingdong prefecture level city in Shandong
Jinfeng Hongyuan Flagship Store	Power Saving Expert 2	I don't feel anything, I said I won't know the effect for a month or two, let's see.	Jingdong prefecture level city in Shandong
Jinfeng Hongyuan Flagship Store	Power Saving Expert 2	Delivery was pretty fast, a little pricey, just not sure if it works or not	Jingdong prefecture level city in Shandong
Jinfeng Hongyuan Flagship Store	Power Saving Expert 2	The goods have been received, I do not know how effective.	Jingdong prefecture level city in Shandong
Jinfeng Hongyuan Flagship Store	Power Saving Expert 2	So far it doesn't seem to be too noticeable, so I'll comment later.	Jingdong prefecture level city in Shandong
Jinfeng Hongyuan Flagship Store	Power Saving Expert 2	I'll comment after I've used it	Jingdong prefecture level city in Shandong
Jinfeng Hongyuan Flagship Store	Power Saving Expert 2	Not bad. You'll save a lot on your electric bill in a year!	Jingdong prefecture level city in Shandong
Jinfeng Hongyuan Flagship Store	Power Saving Expert 2	I just got it. I haven't tried it yet. I don't know what it's like.	Jingdong prefecture level city in Shandong
Jinfeng Hongyuan Flagship Store	Power Saving Expert 2	Shopping at Jingdong has become a habit, and this disease will not be cured for a while.	Jingdong prefecture level city in Shandong
Jinfeng Hongyuan Flagship Store	Power Saving Expert 2	I've been using it for a while now. I don't know if I'm still saving power or not.	Jingdong prefecture level city in Shandong
Jinfeng Hongyuan Flagship Store	Power Saving Expert 2	Received the saver and it's great! There is also a digital voltage indication. Plugged in as instructed, easy to operate, hope you're our power saver!	Jingdong prefecture level city in Shandong
Jinfeng Hongyuan Flagship Store	Power Saving Expert 2	There is no power saving as the store says, using it is the same as not using it.	Jingdong prefecture level city in Shandong
Jinfeng Hongyuan Flagship Store	Power Saving Expert 2	Use it for a while and then comment again	Jingdong prefecture level city in Shandong
Jinfeng Hongyuan Flagship Store	Power Saving Expert 2	Feeling can, to use 1 to 2 months to see how the effect, there is a voltage display, can see the voltage fluctuations amplitude, quite good!	Jingdong prefecture level city in Shandong
Jinfeng Hongyuan Flagship Store	Power Saving Expert 2	I hope it works and saves me some electricity.	Jingdong prefecture level city in Shandong
Jinfeng Hongyuan Flagship Store	Power Saving Expert 2	Nice baby, happy with the after sales	Jingdong prefecture level city in Shandong
Jinfeng Hongyuan Flagship Store	Power Saving Expert 2	Use it and talk about it.	Jingdong prefecture level city in Shandong
Jinfeng Hongyuan Flagship Store	Power Saving Expert 2	Second purchase	Jingdong prefecture level city in Shandong
Jinfeng Hongyuan Flagship Store	Power Saving Expert 2	It works.	Jingdong prefecture level city in Shandong
Jinfeng Hongyuan Flagship Store	Power Saving Expert 2	It works.	Jingdong prefecture level city in Shandong
Jinfeng Hongyuan Flagship Store	Power Saving Expert 2	I don't know if it's a power saver until I start using it	Jingdong prefecture level city in Shandong
Jinfeng Hongyuan Flagship Store	Power Saving Expert 2	serviceable	Jingdong prefecture level city in Shandong
Jinfeng Hongyuan Flagship Store	Power Saving Expert 2	This user did not fill out the rating in time	Jingdong prefecture level city in Shandong
Jinfeng Hongyuan Flagship Store	Power Saving Expert 2	There is no power saving as the store says, using it is the same as not using it.	Jingdong prefecture level city in Shandong
Jinfeng Hongyuan Flagship Store	Power Saving Expert 2	This user did not fill out the rating in time	Jingdong prefecture level city in Shandong
Jinfeng Hongyuan Flagship Store	Power Saving Expert 2	Good...good.	Jingdong prefecture level city in Shandong
Jinfeng Hongyuan Flagship Store	Power Saving Expert 2	(of an unmarried couple) be close	Jingdong prefecture level city in Shandong
Jinfeng Hongyuan Flagship Store	Power Saving Expert 2	(of an unmarried couple) be close	Jingdong prefecture level city in Shandong
Jinfeng Hongyuan Flagship Store	Power Saving Expert 2	(of an unmarried couple) be close	Jingdong prefecture level city in Shandong
Jinfeng Hongyuan Flagship Store	Power Saving Expert 2	The goods are real, high value, high quality, worth every penny, material appearance and quality at a glance is very upscale, very like HAPPY💯	Jingdong prefecture level city in Shandong
Jinfeng Hongyuan Flagship Store	Power Saving Expert 2	It is the ideal product, good quality, logistics is also very fast, worthy of praise 🎁 baby quality is very good, received the first time to open the first time to see, it is very good 👍, very satisfied 😃, customer service is also available to ask questions and answer, thank you 🙏	Jingdong prefecture level city in Shandong
Jinfeng Hongyuan Flagship Store	Power Saving Expert 2	I bought it and haven't used it yet, I'll use it again.	Jingdong prefecture level city in Shandong
Jinfeng Hongyuan Flagship Store	Power Saving Expert 2	It didn't work, I used it for a few days and it was the same as before, it didn't work, I should have to return it	Jingdong prefecture level city in Shandong
Jinfeng Hongyuan Flagship Store	Power Saving Expert 2	The goods have been received, but have not yet installed and use, feel good.	Jingdong prefecture level city in Shandong
Jinfeng Hongyuan Flagship Store	Power Saving Expert 2	Goods received, I forgot to take pictures, the goods look good, you can start, immediately start the school to see the effect then	Jingdong prefecture level city in Shandong
Jinfeng Hongyuan Flagship Store	Power Saving Expert 2	Looks good. I'll use it for a while.	Jingdong prefecture level city in Shandong
Jinfeng Hongyuan Flagship Store	Power Saving Expert 2	Great stuff, easy to operate, easy to use, good power saving, believe in the power of science!	Jingdong prefecture level city in Shandong
Jinfeng Hongyuan Flagship Store	Power Saving Expert 2	Especially great, comparison shopping before buying, the store owner is very good, answering questions very patiently and detailed, there is a need to come back!	Jingdong prefecture level city in Shandong
Jinfeng Hongyuan Flagship Store	Power Saving Expert 2	Trustworthy, feel free to use it boldly.	Jingdong prefecture level city in Shandong
Jinfeng Hongyuan Flagship Store	Power Saving Expert 2	Logistics is super fast! Beautiful appearance! Looks like a great quality! I've never bought one before, so I'll try it first to see if it saves power. We'll see how much it saves.	Jingdong prefecture level city in Shandong
Jinfeng Hongyuan Flagship Store	Power Saving Expert 2	I saw online counterfeiting, energy saver is one of them, hahahahaha, jingdong also buy fake products	Jingdong prefecture level city in Shandong
Jinfeng Hongyuan Flagship Store	Power Saving Expert 2	Just received, installed, try a month to see how the effect and then follow up.	Jingdong prefecture level city in Shandong
Jinfeng Hongyuan Flagship Store	Power Saving Expert 2	Good for you! The goods are genuine! Good price for what you get!	Jingdong prefecture level city in Shandong
Jinfeng Hongyuan Flagship Store	Power Saving Expert 2	Things quickly received, immediately used to see if there is no effect, something river photo ⬆ the same, customer service is very professional	Jingdong prefecture level city in Shandong
Jinfeng Hongyuan Flagship Store	Power Saving Expert 2	The product is very convenient Looking forward to the subsequent use of the experience results	Jingdong prefecture level city in Shandong
Jinfeng Hongyuan Flagship Store	Power Saving Expert 2	I just received it and plugged it in, we'll see exactly when it's time to pay the electric bill	Jingdong prefecture level city in Shandong
Jinfeng Hongyuan Flagship Store	Power Saving Expert 2	Saver is good Packaging and looks good How effective it is to be shared after using it.	Jingdong prefecture level city in Shandong
Jinfeng Hongyuan Flagship Store	Power Saving Expert 2	There's no way to compare. I don't know if it's energy efficient.	Jingdong prefecture level city in Shandong
Jinfeng Hongyuan Flagship Store	Power Saving Expert 2	Just installed it, try it out and comment again	Jingdong prefecture level city in Shandong

Jinfeng Hongyuan Flagship Store	Power Saving Expert 2	Super save point also good to install, after a friend introduced then went to buy to, friends say it is very good to use, home I bought, try to use a period of time, to be honest, the economizer is not big or heavy, can be very convenient to put in a lot of places, the quality is also very good, power-saving function this time seems to be about 20% of the efficiency. The owner is also very warm contact, very	Jingdong prefecture level city in Shandong
Jinfeng Hongyuan Flagship Store	Power Saving Expert 2	The reviews online are mixed. Try it for a while, but I don't know if I can return it if it doesn't save any power after a couple months.	Jingdong prefecture level city in Shandong
Jinfeng Hongyuan Flagship Store	Power Saving Expert 2	The power consumption at home is high, bought a power saver, it looks very convenient to plug on the line, see if you can save some money next month, if good, recommend to friends down the road.	Jingdong prefecture level city in Shandong
Jinfeng Hongyuan Flagship Store	Power Saving Expert 2	It's beautiful, it's genuine, but I don't know the power-saving effect.	Jingdong prefecture level city in Shandong
Jinfeng Hongyuan Flagship Store	Power Saving Expert 2	This user thinks the product is good	Jingdong prefecture level city in Shandong
Jinfeng Hongyuan Flagship Store	Power Saving Expert 2	Nice exterior design with an at-a-glance display	Jingdong prefecture level city in Shandong
Jinfeng Hongyuan Flagship Store	Power Saving Expert 2	Home large and small things are purchased in the Jingdong Mall, have been accustomed to shopping in the Jingdong, many times can buy good quality and cheap products, and courier service attitude is particularly good, sometimes too many couriers are to help me to the home, so many years have been in the Jingdong Mall shopping, trust and support! Will also support the Jingdong as always!	Jingdong prefecture level city in Shandong
Jinfeng Hongyuan Flagship Store	Power Saving Expert 2	Good workmanship, mostly better than the original wall-plugged ones, and hopefully power factor compensation saves on electricity costs	Jingdong prefecture level city in Shandong
Jinfeng Hongyuan Flagship Store	Power Saving Expert 2	I don't feel anything, I said I won't know the effect for a month or two, let's see.	Jingdong prefecture level city in Shandong
Jinfeng Hongyuan Flagship Store	Power Saving Expert 2	Delivery was pretty fast, a little pricey, just not sure if it works or not	Jingdong prefecture level city in Shandong
Jinfeng Hongyuan Flagship Store	Power Saving Expert 2	The goods have been received, I do not know how effective.	Jingdong prefecture level city in Shandong
Jinfeng Hongyuan Flagship Store	Power Saving Expert 2	So far it doesn't seem to be too noticeable, so I'll comment later.	Jingdong prefecture level city in Shandong
Jinfeng Hongyuan Flagship Store	Power Saving Expert 2	I'll comment after I've used it	Jingdong prefecture level city in Shandong
Jinfeng Hongyuan Flagship Store	Power Saving Expert 2	Not bad. You'll save a lot on your electric bill in a year!	Jingdong prefecture level city in Shandong
Jinfeng Hongyuan Flagship Store	Power Saving Expert 2	I just got it. I haven't tried it yet. I don't know what it's like.	Jingdong prefecture level city in Shandong
Jinfeng Hongyuan Flagship Store	Power Saving Expert 2	Shopping at Jingdong has become a habit, and this disease will not be cured for a while.	Jingdong prefecture level city in Shandong
Jinfeng Hongyuan Flagship Store	Power Saving Expert 2	I've been using it for a while now. I don't know if I'm still saving power or not.	Jingdong prefecture level city in Shandong
Jinfeng Hongyuan Flagship Store	Power Saving Expert 2	Received the saver and it's great! There is also a digital voltage indication. Plugged in as instructed, easy to operate, hope you're our power saver!	Jingdong prefecture level city in Shandong
Jinfeng Hongyuan Flagship Store	Power Saving Expert 2	There is no power saving as the store says, using it is the same as not using it.	Jingdong prefecture level city in Shandong
Jinfeng Hongyuan Flagship Store	Power Saving Expert 2	Use it for a while and then comment again	Jingdong prefecture level city in Shandong
Jinfeng Hongyuan Flagship Store	Power Saving Expert 2	Feeling can, to use 1 to 2 months to see how the effect, there is a voltage display, can see the voltage fluctuations amplitude, quite good!	Jingdong prefecture level city in Shandong
Jinfeng Hongyuan Flagship Store	Power Saving Expert 2	I hope it works and saves me some electricity.	Jingdong prefecture level city in Shandong
Jinfeng Hongyuan Flagship Store	Power Saving Expert 2	Nice baby, happy with the after sales	Jingdong prefecture level city in Shandong
Jinfeng Hongyuan Flagship Store	Power Saving Expert 2	Use it and talk about it.	Jingdong prefecture level city in Shandong
Jinfeng Hongyuan Flagship Store	Power Saving Expert 2	Second purchase	Jingdong prefecture level city in Shandong
Jinfeng Hongyuan Flagship Store	Power Saving Expert 2	It works.	Jingdong prefecture level city in Shandong
Jinfeng Hongyuan Flagship Store	Power Saving Expert 2	It works.	Jingdong prefecture level city in Shandong
Jinfeng Hongyuan Flagship Store	Power Saving Expert 2	I don't know if it's a power saver until I start using it	Jingdong prefecture level city in Shandong
Jinfeng Hongyuan Flagship Store	Power Saving Expert 2	serviceable	Jingdong prefecture level city in Shandong
Jinfeng Hongyuan Flagship Store	Power Saving Expert 2	This user did not fill out the rating in time	Jingdong prefecture level city in Shandong
Jinfeng Hongyuan Flagship Store	Power Saving Expert 2	There is no power saving as the store says, using it is the same as not using it.	Jingdong prefecture level city in Shandong
Jinfeng Hongyuan Flagship Store	Power Saving Expert 2	This user did not fill out the rating in time	Jingdong prefecture level city in Shandong
Jinfeng Hongyuan Flagship Store	Power Saving Expert 2	Good...good.	Jingdong prefecture level city in Shandong
Jinfeng Hongyuan Flagship Store	Power Saving Expert 2	(of an unmarried couple) be close	Jingdong prefecture level city in Shandong
Jinfeng Hongyuan Flagship Store	Power Saving Expert 2	(of an unmarried couple) be close	Jingdong prefecture level city in Shandong
Jinfeng Hongyuan Flagship Store	Power Saving Expert 2	(of an unmarried couple) be close	Jingdong prefecture level city in Shandong
Jinfeng Hongyuan Flagship Store	Power Saving Expert 2	The goods are real, high value, high quality, worth every penny, material appearance and quality at a glance is very upscale, very like HAPPY𝟙 𝟘𝟘	Jingdong prefecture level city in Shandong
Jinfeng Hongyuan Flagship Store	Power Saving Expert 2	It is the ideal product, good quality, logistics is also very fast, worthy of praise 🎁 baby quality is very good, received the first time to open the first time to see, it is very good 👍, very satisfied 😃, customer service is also available to ask questions and answer, thank you 🙏	Jingdong prefecture level city in Shandong
Jinfeng Hongyuan Flagship Store	Power Saving Expert 2	I bought it and haven't used it yet, I'll use it again.	Jingdong prefecture level city in Shandong
Jinfeng Hongyuan Flagship Store	Power Saving Expert 2	It didn't work, I used it for a few days and it was the same as before, it didn't work, I should have to return it	Jingdong prefecture level city in Shandong
Jinfeng Hongyuan Flagship Store	Power Saving Expert 2	The goods have been received, but have not yet installed and use, feel good.	Jingdong prefecture level city in Shandong
Jinfeng Hongyuan Flagship Store	Power Saving Expert 2	Goods received, I forgot to take pictures, the goods look good, you can start, immediately start the school to see the effect then	Jingdong prefecture level city in Shandong
Jinfeng Hongyuan Flagship Store	Power Saving Expert 2	Looks good. I'll use it for a while.	Jingdong prefecture level city in Shandong
Jinfeng Hongyuan Flagship Store	Power Saving Expert 2	Great stuff, easy to operate, easy to use, good power saving, believe in the power of science!	Jingdong prefecture level city in Shandong
Jinfeng Hongyuan Flagship Store	Power Saving Expert 2	Especially great, comparison shopping before buying, the store owner is very good, answering questions very patiently and detailed, there is a need to come back!	Jingdong prefecture level city in Shandong
Jinfeng Hongyuan Flagship Store	Power Saving Expert 2	Trustworthy, feel free to use it boldly.	Jingdong prefecture level city in Shandong
Jinfeng Hongyuan Flagship Store	Power Saving Expert 2	Logistics is super fast! Beautiful appearance! Looks like a great quality! I've never bought one before, so I'll try it first to see if it saves power. We'll see how much it saves.	Jingdong prefecture level city in Shandong
Jinfeng Hongyuan Flagship Store	Power Saving Expert 2	I saw online counterfeiting, energy saver is one of them, hahahahaha, jingdong also buy fake products	Jingdong prefecture level city in Shandong
Jinfeng Hongyuan Flagship Store	Power Saving Expert 2	Just received, installed, try a month to see how the effect and then follow up.	Jingdong prefecture level city in Shandong

Jinfeng Hongyuan Flagship Store	Power Saving Expert 2	Good for you! The goods are genuine! Good price for what you get!	Jingdong prefecture level city in Shandong
Jinfeng Hongyuan Flagship Store	Power Saving Expert 2	Things quickly received, immediately used to see if there is no effect, something river photo ⬆ the same, customer service is very professional	Jingdong prefecture level city in Shandong
Jinfeng Hongyuan Flagship Store	Power Saving Expert 2	The product is very convenient Looking forward to the subsequent use of the experience results	Jingdong prefecture level city in Shandong
Jinfeng Hongyuan Flagship Store	Power Saving Expert 2	I just received it and plugged it in, we'll see exactly when it's time to pay the electric bill	Jingdong prefecture level city in Shandong
Jinfeng Hongyuan Flagship Store	Power Saving Expert 2	Saver is good Packaging and looks good How effective it is to be shared after using it.	Jingdong prefecture level city in Shandong
Jinfeng Hongyuan Flagship Store	Power Saving Expert 2	There's no way to compare. I don't know if it's energy efficient.	Jingdong prefecture level city in Shandong
Jinfeng Hongyuan Flagship Store	Power Saving Expert 2	Just installed it, try it out and comment again	Jingdong prefecture level city in Shandong
Jinfeng Hongyuan Flagship Store	Power Saving Expert 2	Super save point also good to install, after a friend introduced then went to buy to, friends say it is very good to use, home I bought, try to use a period of time, to be honest, the economizer is not big or heavy, can be very convenient to put in a lot of places, the quality is also very good, power-saving function this time seems to be about 20% of the efficiency. The owner is also very warm contact, very	Jingdong prefecture level city in Shandong
Jinfeng Hongyuan Flagship Store	Power Saving Expert 2	The reviews online are mixed. Try it for a while, but I don't know if I can return it if it doesn't save any power after a couple months.	Jingdong prefecture level city in Shandong
Jinfeng Hongyuan Flagship Store	Power Saving Expert 2	The power consumption at home is high, bought a power saver, it looks very convenient to plug on the line, see if you can save some money next month, if good, recommend to friends down the road.	Jingdong prefecture level city in Shandong
Jinfeng Hongyuan Flagship Store	Power Saving Expert 2	It's beautiful, it's genuine, but I don't know the power-saving effect.	Jingdong prefecture level city in Shandong
Jinfeng Hongyuan Flagship Store	Power Saving Expert 2	This user thinks the product is good	Jingdong prefecture level city in Shandong
Jinfeng Hongyuan Flagship Store	Power Saving Expert 2	Nice exterior design with an at-a-glance display	Jingdong prefecture level city in Shandong
Jinfeng Hongyuan Flagship Store	Power Saving Expert 2	Home large and small things are purchased in the Jingdong Mall, have been accustomed to shopping in the Jingdong, many times can buy good quality and cheap products, and courier service attitude is particularly good, sometimes too many couriers are to help me to the home, so many years have been in the Jingdong Mall shopping, trust and support! Will also support the Jingdong as always!	Jingdong prefecture level city in Shandong
Jinfeng Hongyuan Flagship Store	Power Saving Expert 2	Good workmanship, mostly better than the original wall-plugged ones, and hopefully power factor compensation saves on electricity costs	Jingdong prefecture level city in Shandong
Jinfeng Hongyuan Flagship Store	Power Saving Expert 2	I don't feel anything, I said I won't know the effect for a month or two, let's see.	Jingdong prefecture level city in Shandong
Jinfeng Hongyuan Flagship Store	Power Saving Expert 2	Delivery was pretty fast, a little pricey, just not sure if it works or not	Jingdong prefecture level city in Shandong
Jinfeng Hongyuan Flagship Store	Power Saving Expert 2	The goods have been received, I do not know how effective.	Jingdong prefecture level city in Shandong
Jinfeng Hongyuan Flagship Store	Power Saving Expert 2	So far it doesn't seem to be too noticeable, so I'll comment later.	Jingdong prefecture level city in Shandong
Jinfeng Hongyuan Flagship Store	Power Saving Expert 2	I'll comment after I've used it	Jingdong prefecture level city in Shandong
Jinfeng Hongyuan Flagship Store	Power Saving Expert 2	Not bad. You'll save a lot on your electric bill in a year!	Jingdong prefecture level city in Shandong
Jinfeng Hongyuan Flagship Store	Power Saving Expert 2	I just got it. I haven't tried it yet. I don't know what it's like.	Jingdong prefecture level city in Shandong
Jinfeng Hongyuan Flagship Store	Power Saving Expert 2	Shopping at Jingdong has become a habit, and this disease will not be cured for a while.	Jingdong prefecture level city in Shandong
Jinfeng Hongyuan Flagship Store	Power Saving Expert 2	I've been using it for a while now. I don't know if I'm still saving power or not.	Jingdong prefecture level city in Shandong
Jinfeng Hongyuan Flagship Store	Power Saving Expert 2	Received the saver and it's great! There is also a digital voltage indication. Plugged in as instructed, easy to operate, hope you're our power saver!	Jingdong prefecture level city in Shandong
Jinfeng Hongyuan Flagship Store	Power Saving Expert 2	There is no power saving as the store says, using it is the same as not using it.	Jingdong prefecture level city in Shandong
Jinfeng Hongyuan Flagship Store	Power Saving Expert 2	Use it for a while and then comment again	Jingdong prefecture level city in Shandong
Jinfeng Hongyuan Flagship Store	Power Saving Expert 2	Feeling can, to use 1 to 2 months to see how the effect, there is a voltage display, can see the voltage fluctuations amplitude, quite good!	Jingdong prefecture level city in Shandong
Jinfeng Hongyuan Flagship Store	Power Saving Expert 2	I hope it works and saves me some electricity.	Jingdong prefecture level city in Shandong
Jinfeng Hongyuan Flagship Store	Power Saving Expert 2	Nice baby, happy with the after sales	Jingdong prefecture level city in Shandong
Jinfeng Hongyuan Flagship Store	Power Saving Expert 2	Use it and talk about it.	Jingdong prefecture level city in Shandong
Jinfeng Hongyuan Flagship Store	Power Saving Expert 2	Second purchase	Jingdong prefecture level city in Shandong
Jinfeng Hongyuan Flagship Store	Power Saving Expert 2	It works.	Jingdong prefecture level city in Shandong
Jinfeng Hongyuan Flagship Store	Power Saving Expert 2	It works.	Jingdong prefecture level city in Shandong
Jinfeng Hongyuan Flagship Store	Power Saving Expert 2	I don't know if it's a power saver until I start using it	Jingdong prefecture level city in Shandong
Jinfeng Hongyuan Flagship Store	Power Saving Expert 2	serviceable	Jingdong prefecture level city in Shandong
Jinfeng Hongyuan Flagship Store	Power Saving Expert 2	This user did not fill out the rating in time	Jingdong prefecture level city in Shandong
Jinfeng Hongyuan Flagship Store	Power Saving Expert 2	There is no power saving as the store says, using it is the same as not using it.	Jingdong prefecture level city in Shandong
Jinfeng Hongyuan Flagship Store	Power Saving Expert 2	This user did not fill out the rating in time	Jingdong prefecture level city in Shandong
Jinfeng Hongyuan Flagship Store	Power Saving Expert 2	Good...good.	Jingdong prefecture level city in Shandong
Jinfeng Hongyuan Flagship Store	Power Saving Expert 2	(of an unmarried couple) be close	Jingdong prefecture level city in Shandong
Jinfeng Hongyuan Flagship Store	Power Saving Expert 2	(of an unmarried couple) be close	Jingdong prefecture level city in Shandong
Jinfeng Hongyuan Flagship Store	Power Saving Expert 2	(of an unmarried couple) be close	Jingdong prefecture level city in Shandong
Jinfeng Hongyuan Flagship Store	Power Saving Expert 2	The goods are real, high value, high quality, worth every penny, material appearance and quality at a glance is very upscale, very like HAPPY💯	Jingdong prefecture level city in Shandong
Jinfeng Hongyuan Flagship Store	Power Saving Expert 2	It is the ideal product, good quality, logistics is also very fast, worthy of praise 🎁 baby quality is very good, received the first time to open the first time to see, it is very good 👍, very satisfied 😃, customer service is also available to ask questions and answer, thank you 🙏	Jingdong prefecture level city in Shandong
Jinfeng Hongyuan Flagship Store	Power Saving Expert 2	I bought it and haven't used it yet, I'll use it again.	Jingdong prefecture level city in Shandong
Jinfeng Hongyuan Flagship Store	Power Saving Expert 2	It didn't work, I used it for a few days and it was the same as before, it didn't work, I should have to return it	Jingdong prefecture level city in Shandong
Jinfeng Hongyuan Flagship Store	Power Saving Expert 2	The goods have been received, but have not yet installed and use, feel good.	Jingdong prefecture level city in Shandong
Jinfeng Hongyuan Flagship Store	Power Saving Expert 2	Goods received, I forgot to take pictures, the goods look good, you can start, immediately start the school to see the effect then	Jingdong prefecture level city in Shandong

Jinfeng Hongyuan Flagship Store	Power Saving Expert 2	Looks good. I'll use it for a while.	Jingdong prefecture level city in Shandong
Jinfeng Hongyuan Flagship Store	Power Saving Expert 2	Great stuff, easy to operate, easy to use, good power saving, believe in the power of science!	Jingdong prefecture level city in Shandong
Jinfeng Hongyuan Flagship Store	Power Saving Expert 2	Especially great, comparison shopping before buying, the store owner is very good, answering questions very patiently and detailed, there is a need to come back!	Jingdong prefecture level city in Shandong
Jinfeng Hongyuan Flagship Store	Power Saving Expert 2	Trustworthy, feel free to use it boldly.	Jingdong prefecture level city in Shandong
Jinfeng Hongyuan Flagship Store	Power Saving Expert 2	Logistics is super fast! Beautiful appearance! Looks like a great quality! I've never bought one before, so I'll try it first to see if it saves power. We'll see how much it saves.	Jingdong prefecture level city in Shandong
Jinfeng Hongyuan Flagship Store	Power Saving Expert 2	I saw online counterfeiting, energy saver is one of them, hahahahaha, jingdong also buy fake products	Jingdong prefecture level city in Shandong
Jinfeng Hongyuan Flagship Store	Power Saving Expert 2	Just received, installed, try a month to see how the effect and then follow up.	Jingdong prefecture level city in Shandong
Jinfeng Hongyuan Flagship Store	Power Saving Expert 2	Good for you! The goods are genuine! Good price for what you get!	Jingdong prefecture level city in Shandong
Jinfeng Hongyuan Flagship Store	Power Saving Expert 2	Things quickly received, immediately used to see if there is no effect, something river photo ↑ the same, customer service is very professional	Jingdong prefecture level city in Shandong
Jinfeng Hongyuan Flagship Store	Power Saving Expert 2	The product is very convenient Looking forward to the subsequent use of the experience results	Jingdong prefecture level city in Shandong
Jinfeng Hongyuan Flagship Store	Power Saving Expert 2	I just received it and plugged it in, we'll see exactly when it's time to pay the electric bill	Jingdong prefecture level city in Shandong
Jinfeng Hongyuan Flagship Store	Power Saving Expert 2	Saver is good Packaging and looks good How effective it is to be shared after using it.	Jingdong prefecture level city in Shandong
Jinfeng Hongyuan Flagship Store	Power Saving Expert 2	There's no way to compare. I don't know if it's energy efficient.	Jingdong prefecture level city in Shandong
Jinfeng Hongyuan Flagship Store	Power Saving Expert 2	Just installed it, try it out and comment again	Jingdong prefecture level city in Shandong
Jinfeng Hongyuan Flagship Store	Power Saving Expert 2	Super save point also good to install, after a friend introduced then went to buy to, friends say it is very good to use, home I bought, try to use a period of time, to be honest, the economizer is not big or heavy, can be very convenient to put in a lot of places, the quality is also very good, power-saving function this time seems to be about 20% of the efficiency. The owner is also very warm contact, very	Jingdong prefecture level city in Shandong
Jinfeng Hongyuan Flagship Store	Power Saving Expert 2	The reviews online are mixed. Try it for a while, but I don't know if I can return it if it doesn't save any power after a couple months.	Jingdong prefecture level city in Shandong
Jinfeng Hongyuan Flagship Store	Power Saving Expert 2	The power consumption at home is high, bought a power saver, it looks very convenient to plug on the line, see if you can save some money next month, if good, recommend to friends down the road.	Jingdong prefecture level city in Shandong
Jinfeng Hongyuan Flagship Store	Power Saving Expert 2	It's beautiful, it's genuine, but I don't know the power-saving effect.	Jingdong prefecture level city in Shandong
Jinfeng Hongyuan Flagship Store	Power Saving Expert 2	This user thinks the product is good	Jingdong prefecture level city in Shandong
Jinfeng Hongyuan Flagship Store	Power Saving Expert 2	Nice exterior design with an at-a-glance display	Jingdong prefecture level city in Shandong
Jinfeng Hongyuan Flagship Store	Power Saving Expert 2	Home large and small things are purchased in the Jingdong Mall, have been accustomed to shopping in the Jingdong, many times can buy good quality and cheap products, and courier service attitude is particularly good, sometimes too many couriers are to help me to the home, so many years have been in the Jingdong Mall shopping, trust and support! Will also support the Jingdong as always!	Jingdong prefecture level city in Shandong
Jinfeng Hongyuan Flagship Store	Power Saving Expert 2	Good workmanship, mostly better than the original wall-plugged ones, and hopefully power factor compensation saves on electricity costs	Jingdong prefecture level city in Shandong
Jinfeng Hongyuan Flagship Store	Power Saving Expert 2	I don't feel anything, I said I won't know the effect for a month or two, let's see.	Jingdong prefecture level city in Shandong
Jinfeng Hongyuan Flagship Store	Power Saving Expert 2	Delivery was pretty fast, a little pricey, just not sure if it works or not	Jingdong prefecture level city in Shandong
Jinfeng Hongyuan Flagship Store	Power Saving Expert 2	The goods have been received, I do not know how effective.	Jingdong prefecture level city in Shandong
Jinfeng Hongyuan Flagship Store	Power Saving Expert 2	So far it doesn't seem to be too noticeable, so I'll comment later.	Jingdong prefecture level city in Shandong
Jinfeng Hongyuan Flagship Store	Power Saving Expert 2	I'll comment after I've used it	Jingdong prefecture level city in Shandong
Jinfeng Hongyuan Flagship Store	Power Saving Expert 2	Not bad. You'll save a lot on your electric bill in a year!	Jingdong prefecture level city in Shandong
Jinfeng Hongyuan Flagship Store	Power Saving Expert 2	I just got it. I haven't tried it yet. I don't know what it's like.	Jingdong prefecture level city in Shandong
Jinfeng Hongyuan Flagship Store	Power Saving Expert 2	Shopping at Jingdong has become a habit, and this disease will not be cured for a while.	Jingdong prefecture level city in Shandong
Jinfeng Hongyuan Flagship Store	Power Saving Expert 2	I've been using it for a while now. I don't know if I'm still saving power or not.	Jingdong prefecture level city in Shandong
Jinfeng Hongyuan Flagship Store	Power Saving Expert 2	Received the saver and it's great! There is also a digital voltage indication. Plugged in as instructed, easy to operate, hope you're our power saver!	Jingdong prefecture level city in Shandong
Jinfeng Hongyuan Flagship Store	Power Saving Expert 2	There is no power saving as the store says, using it is the same as not using it.	Jingdong prefecture level city in Shandong
Jinfeng Hongyuan Flagship Store	Power Saving Expert 2	Use it for a while and then comment again	Jingdong prefecture level city in Shandong
Jinfeng Hongyuan Flagship Store	Power Saving Expert 2	Feeling can, to use 1 to 2 months to see how the effect, there is a voltage display, can see the voltage fluctuations amplitude, quite good!	Jingdong prefecture level city in Shandong
Jinfeng Hongyuan Flagship Store	Power Saving Expert 2	I hope it works and saves me some electricity.	Jingdong prefecture level city in Shandong
Jinfeng Hongyuan Flagship Store	Power Saving Expert 2	Nice baby, happy with the after sales	Jingdong prefecture level city in Shandong
Jinfeng Hongyuan Flagship Store	Power Saving Expert 2	Use it and talk about it.	Jingdong prefecture level city in Shandong
Jinfeng Hongyuan Flagship Store	Power Saving Expert 2	Second purchase	Jingdong prefecture level city in Shandong
Jinfeng Hongyuan Flagship Store	Power Saving Expert 2	It works.	Jingdong prefecture level city in Shandong
Jinfeng Hongyuan Flagship Store	Power Saving Expert 2	It works.	Jingdong prefecture level city in Shandong
Jinfeng Hongyuan Flagship Store	Power Saving Expert 2	I don't know if it's a power saver until I start using it	Jingdong prefecture level city in Shandong
Jinfeng Hongyuan Flagship Store	Power Saving Expert 2	serviceable	Jingdong prefecture level city in Shandong
Jinfeng Hongyuan Flagship Store	Power Saving Expert 2	This user did not fill out the rating in time	Jingdong prefecture level city in Shandong
Jinfeng Hongyuan Flagship Store	Power Saving Expert 2	There is no power saving as the store says, using it is the same as not using it.	Jingdong prefecture level city in Shandong
Jinfeng Hongyuan Flagship Store	Power Saving Expert 2	This user did not fill out the rating in time	Jingdong prefecture level city in Shandong
Jinfeng Hongyuan Flagship Store	Power Saving Expert 2	Good...good.	Jingdong prefecture level city in Shandong
Jinfeng Hongyuan Flagship Store	Power Saving Expert 2	(of an unmarried couple) be close	Jingdong prefecture level city in Shandong
Jinfeng Hongyuan Flagship Store	Power Saving Expert 2	(of an unmarried couple) be close	Jingdong prefecture level city in Shandong

Jinfeng Hongyuan Flagship Store	Power Saving Expert 2	(of an unmarried couple) be close	Jingdong prefecture level city in Shandong
Jinfeng Hongyuan Flagship Store	Power Saving Expert 2	The goods are real, high value, high quality, worth every penny, material appearance and quality at a glance is very upscale, very like HAPPY1 00	Jingdong prefecture level city in Shandong
Jinfeng Hongyuan Flagship Store	Power Saving Expert 2	It is the ideal product, good quality, logistics is also very fast, worthy of praise 🎁 baby quality is very good, received the first time to open the first time to see, it is very good 👍, very satisfied 😃, customer service is also available to ask questions and answer, thank you 🙏	Jingdong prefecture level city in Shandong
Jinfeng Hongyuan Flagship Store	Power Saving Expert 2	I bought it and haven't used it yet, I'll use it again.	Jingdong prefecture level city in Shandong
Jinfeng Hongyuan Flagship Store	Power Saving Expert 2	It didn't work, I used it for a few days and it was the same as before, it didn't work, I should have to return it	Jingdong prefecture level city in Shandong
Jinfeng Hongyuan Flagship Store	Power Saving Expert 2	The goods have been received, but have not yet installed and use, feel good.	Jingdong prefecture level city in Shandong
Jinfeng Hongyuan Flagship Store	Power Saving Expert 2	Goods received, I forgot to take pictures, the goods look good, you can start, immediately start the school to see the effect then	Jingdong prefecture level city in Shandong
Jinfeng Hongyuan Flagship Store	Power Saving Expert 2	Looks good. I'll use it for a while.	Jingdong prefecture level city in Shandong
Jinfeng Hongyuan Flagship Store	Power Saving Expert 2	Great stuff, easy to operate, easy to use, good power saving, believe in the power of science!	Jingdong prefecture level city in Shandong
Jinfeng Hongyuan Flagship Store	Power Saving Expert 2	Especially great, comparison shopping before buying, the store owner is very good, answering questions very patiently and detailed, there is a need to come back!	Jingdong prefecture level city in Shandong
Jinfeng Hongyuan Flagship Store	Power Saving Expert 2	Trustworthy, feel free to use it boldly.	Jingdong prefecture level city in Shandong
Jinfeng Hongyuan Flagship Store	Power Saving Expert 2	Logistics is super fast! Beautiful appearance! Looks like a great quality! I've never bought one before, so I'll try it first to see if it saves power. We'll see how much it saves.	Jingdong prefecture level city in Shandong
Jinfeng Hongyuan Flagship Store	Power Saving Expert 2	I saw online counterfeiting, energy saver is one of them, hahahahaha, jingdong also buy fake products	Jingdong prefecture level city in Shandong
Jinfeng Hongyuan Flagship Store	Power Saving Expert 2	Just received, installed, try a month to see how the effect and then follow up.	Jingdong prefecture level city in Shandong
Jinfeng Hongyuan Flagship Store	Power Saving Expert 2	Good for you! The goods are genuine! Good price for what you get!	Jingdong prefecture level city in Shandong
Jinfeng Hongyuan Flagship Store	Power Saving Expert 2	Things quickly received, immediately used to see if there is no effect, something river photo ⬆ the same, customer service is very professional	Jingdong prefecture level city in Shandong
Jinfeng Hongyuan Flagship Store	Power Saving Expert 2	The product is very convenient Looking forward to the subsequent use of the experience results	Jingdong prefecture level city in Shandong
Jinfeng Hongyuan Flagship Store	Power Saving Expert 2	I just received it and plugged it in, we'll see exactly when it's time to pay the electric bill	Jingdong prefecture level city in Shandong
Jinfeng Hongyuan Flagship Store	Power Saving Expert 2	Saver is good Packaging and looks good How effective it is to be shared after using it.	Jingdong prefecture level city in Shandong
Jinfeng Hongyuan Flagship Store	Power Saving Expert 2	There's no way to compare. I don't know if it's energy efficient.	Jingdong prefecture level city in Shandong
Jinfeng Hongyuan Flagship Store	Power Saving Expert 2	Just installed it, try it out and comment again	Jingdong prefecture level city in Shandong
Jinfeng Hongyuan Flagship Store	Power Saving Expert 2	Super save point also good to install, after a friend introduced then went to buy to, friends say it is very good to use, home I bought, try to use a period of time, to be honest, the economizer is not big or heavy, can be very convenient to put in a lot of places, the quality is also very good, power-saving function this time seems to be about 20% of the efficiency. The owner is also very warm contact, very	Jingdong prefecture level city in Shandong
Jinfeng Hongyuan Flagship Store	Power Saving Expert 2	The reviews online are mixed. Try it for a while, but I don't know if I can return it if it doesn't save any power after a couple months.	Jingdong prefecture level city in Shandong
Jinfeng Hongyuan Flagship Store	Power Saving Expert 2	The power consumption at home is high, bought a power saver, it looks very convenient to plug on the line, see if you can save some money next month, if good, recommend to friends down the road.	Jingdong prefecture level city in Shandong
Jinfeng Hongyuan Flagship Store	Power Saving Expert 2	It's beautiful, it's genuine, but I don't know the power-saving effect.	Jingdong prefecture level city in Shandong
Jinfeng Hongyuan Flagship Store	Power Saving Expert 2	This user thinks the product is good	Jingdong prefecture level city in Shandong
Jinfeng Hongyuan Flagship Store	Power Saving Expert 2	Nice exterior design with an at-a-glance display	Jingdong prefecture level city in Shandong
Jinfeng Hongyuan Flagship Store	Power Saving Expert 2	Home large and small things are purchased in the Jingdong Mall, have been accustomed to shopping in the Jingdong, many times can buy good quality and cheap products, and courier service attitude is particularly good, sometimes too many couriers are to help me to the home, so many years have been in the Jingdong Mall shopping, trust and support! Will also support the Jingdong as always!	Jingdong prefecture level city in Shandong
Jinfeng Hongyuan Flagship Store	Power Saving Expert 2	Good workmanship, mostly better than the original wall-plugged ones, and hopefully power factor compensation saves on electricity costs	Jingdong prefecture level city in Shandong
Jinfeng Hongyuan Flagship Store	Power Saving Expert 2	I don't feel anything, I said I won't know the effect for a month or two, let's see.	Jingdong prefecture level city in Shandong
Jinfeng Hongyuan Flagship Store	Power Saving Expert 2	Delivery was pretty fast, a little pricey, just not sure if it works or not	Jingdong prefecture level city in Shandong
Jinfeng Hongyuan Flagship Store	Power Saving Expert 2	The goods have been received, I do not know how effective.	Jingdong prefecture level city in Shandong
Jinfeng Hongyuan Flagship Store	Power Saving Expert 2	So far it doesn't seem to be too noticeable, so I'll comment later.	Jingdong prefecture level city in Shandong
Jinfeng Hongyuan Flagship Store	Power Saving Expert 2	I'll comment after I've used it	Jingdong prefecture level city in Shandong
Jinfeng Hongyuan Flagship Store	Power Saving Expert 2	Not bad. You'll save a lot on your electric bill in a year!	Jingdong prefecture level city in Shandong
Jinfeng Hongyuan Flagship Store	Power Saving Expert 2	I just got it. I haven't tried it yet. I don't know what it's like.	Jingdong prefecture level city in Shandong
Jinfeng Hongyuan Flagship Store	Power Saving Expert 2	Shopping at Jingdong has become a habit, and this disease will not be cured for a while.	Jingdong prefecture level city in Shandong
Jinfeng Hongyuan Flagship Store	Power Saving Expert 2	I've been using it for a while now. I don't know if I'm still saving power or not.	Jingdong prefecture level city in Shandong
Jinfeng Hongyuan Flagship Store	Power Saving Expert 2	Received the saver and it's great! There is also a digital voltage indication. Plugged in as instructed, easy to operate, hope you're our power saver!	Jingdong prefecture level city in Shandong
Jinfeng Hongyuan Flagship Store	Power Saving Expert 2	There is no power saving as the store says, using it is the same as not using it.	Jingdong prefecture level city in Shandong
Jinfeng Hongyuan Flagship Store	Power Saving Expert 2	Use it for a while and then comment again	Jingdong prefecture level city in Shandong
Jinfeng Hongyuan Flagship Store	Power Saving Expert 2	Feeling can, to use 1 to 2 months to see how the effect, there is a voltage display, can see the voltage fluctuations amplitude, quite good!	Jingdong prefecture level city in Shandong
Jinfeng Hongyuan Flagship Store	Power Saving Expert 2	I hope it works and saves me some electricity.	Jingdong prefecture level city in Shandong
Jinfeng Hongyuan Flagship Store	Power Saving Expert 2	Nice baby, happy with the after sales	Jingdong prefecture level city in Shandong
Jinfeng Hongyuan Flagship Store	Power Saving Expert 2	Use it and talk about it.	Jingdong prefecture level city in Shandong
Jinfeng Hongyuan Flagship Store	Power Saving Expert 2	Second purchase	Jingdong prefecture level city in Shandong
Jinfeng Hongyuan Flagship Store	Power Saving Expert 2	It works.	Jingdong prefecture level city in Shandong
Jinfeng Hongyuan Flagship Store	Power Saving Expert 2	It works.	Jingdong prefecture level city in Shandong
Jinfeng Hongyuan Flagship Store	Power Saving Expert 2	I don't know if it's a power saver until I start using it	Jingdong prefecture level city in Shandong

Jinfeng Hongyuan Flagship Store	Power Saving Expert 2	serviceable	Jingdong prefecture level city in Shandong
Jinfeng Hongyuan Flagship Store	Power Saving Expert 2	This user did not fill out the rating in time	Jingdong prefecture level city in Shandong
Jinfeng Hongyuan Flagship Store	Power Saving Expert 2	There is no power saving as the store says, using it is the same as not using it.	Jingdong prefecture level city in Shandong
Jinfeng Hongyuan Flagship Store	Power Saving Expert 2	This user did not fill out the rating in time	Jingdong prefecture level city in Shandong
Jinfeng Hongyuan Flagship Store	Power Saving Expert 2	Good...good.	Jingdong prefecture level city in Shandong
Jinfeng Hongyuan Flagship Store	Power Saving Expert 2	(of an unmarried couple) be close	Jingdong prefecture level city in Shandong
Jinfeng Hongyuan Flagship Store	Power Saving Expert 2	(of an unmarried couple) be close	Jingdong prefecture level city in Shandong
Jinfeng Hongyuan Flagship Store	Power Saving Expert 2	(of an unmarried couple) be close	Jingdong prefecture level city in Shandong
Jinfeng Hongyuan Flagship Store	Power Saving Expert 2	The goods are real, high value, high quality, worth every penny, material appearance and quality at a glance is very upscale, very like HAPPY1 💯	Jingdong prefecture level city in Shandong
Jinfeng Hongyuan Flagship Store	Power Saving Expert 2	It is the ideal product, good quality, logistics is also very fast, worthy of praise 🎁 baby quality is very good, received the first time to open the first time to see, it is very good 👍, very satisfied 😉, customer service is also available to ask questions and answer, thank you 🙏	Jingdong prefecture level city in Shandong
Jinfeng Hongyuan Flagship Store	Power Saving Expert 2	I bought it and haven't used it yet, I'll use it again.	Jingdong prefecture level city in Shandong
Jinfeng Hongyuan Flagship Store	Power Saving Expert 2	It didn't work, I used it for a few days and it was the same as before, it didn't work, I should have to return it	Jingdong prefecture level city in Shandong
Jinfeng Hongyuan Flagship Store	Power Saving Expert 2	The goods have been received, but have not yet installed and use, feel good.	Jingdong prefecture level city in Shandong
Jinfeng Hongyuan Flagship Store	Power Saving Expert 2	Goods received, I forgot to take pictures, the goods look good, you can start, immediately start the school to see the effect then	Jingdong prefecture level city in Shandong
Jinfeng Hongyuan Flagship Store	Power Saving Expert 2	Looks good. I'll use it for a while.	Jingdong prefecture level city in Shandong
Jinfeng Hongyuan Flagship Store	Power Saving Expert 2	Great stuff, easy to operate, easy to use, good power saving, believe in the power of science!	Jingdong prefecture level city in Shandong
Jinfeng Hongyuan Flagship Store	Power Saving Expert 2	Especially great, comparison shopping before buying, the store owner is very good, answering questions very patiently and detailed, there is a need to come back!	Jingdong prefecture level city in Shandong
Jinfeng Hongyuan Flagship Store	Power Saving Expert 2	Trustworthy, feel free to use it boldly.	Jingdong prefecture level city in Shandong
Jinfeng Hongyuan Flagship Store	Power Saving Expert 2	Logistics is super fast! Beautiful appearance! Looks like a great quality! I've never bought one before, so I'll try it first to see if it saves power. We'll see how much it saves.	Jingdong prefecture level city in Shandong
Jinfeng Hongyuan Flagship Store	Power Saving Expert 2	I saw online counterfeiting, energy saver is one of them, hahahahaha, jingdong also buy fake products	Jingdong prefecture level city in Shandong
Jinfeng Hongyuan Flagship Store	Power Saving Expert 2	Just received, installed, try a month to see how the effect and then follow up.	Jingdong prefecture level city in Shandong
Jinfeng Hongyuan Flagship Store	Power Saving Expert 2	Good for you! The goods are genuine! Good price for what you get!	Jingdong prefecture level city in Shandong
Jinfeng Hongyuan Flagship Store	Power Saving Expert 2	Things quickly received, immediately used to see if there is no effect, something river photo 🔝 the same, customer service is very professional	Jingdong prefecture level city in Shandong
Jinfeng Hongyuan Flagship Store	Power Saving Expert 2	The product is very convenient Looking forward to the subsequent use of the experience results	Jingdong prefecture level city in Shandong
Jinfeng Hongyuan Flagship Store	Power Saving Expert 2	I just received it and plugged it in, we'll see exactly when it's time to pay the electric bill	Jingdong prefecture level city in Shandong
Jinfeng Hongyuan Flagship Store	Power Saving Expert 2	Saver is good Packaging and looks good How effective it is to be shared after using it.	Jingdong prefecture level city in Shandong
Jinfeng Hongyuan Flagship Store	Power Saving Expert 2	There's no way to compare. I don't know if it's energy efficient.	Jingdong prefecture level city in Shandong
Jinfeng Hongyuan Flagship Store	Power Saving Expert 2	Just installed it, try it out and comment again	Jingdong prefecture level city in Shandong
Jinfeng Hongyuan Flagship Store	Power Saving Expert 2	Super save point also good to install, after a friend introduced then went to buy to, friends say it is very good to use, home I bought, try to use a period of time, to be honest, the economizer is not big or heavy, can be very convenient to put in a lot of places, the quality is also very good, power-saving function this time seems to be about 20% of the efficiency. The owner is also very warm contact, very	Jingdong prefecture level city in Shandong
Jinfeng Hongyuan Flagship Store	Power Saving Expert 2	The reviews online are mixed. Try it for a while, but I don't know if I can return it if it doesn't save any power after a couple months.	Jingdong prefecture level city in Shandong
Jinfeng Hongyuan Flagship Store	Power Saving Expert 2	The power consumption at home is high, bought a power saver, it looks very convenient to plug on the line, see if you can save some money next month, if good, recommend to friends down the road.	Jingdong prefecture level city in Shandong
Jinfeng Hongyuan Flagship Store	Power Saving Expert 2	It's beautiful, it's genuine, but I don't know the power-saving effect.	Jingdong prefecture level city in Shandong
Jinfeng Hongyuan Flagship Store	Power Saving Expert 2	This user thinks the product is good	Jingdong prefecture level city in Shandong
Jinfeng Hongyuan Flagship Store	Power Saving Expert 2	Nice exterior design with an at-a-glance display	Jingdong prefecture level city in Shandong
Jinfeng Hongyuan Flagship Store	Power Saving Expert 2	Home large and small things are purchased in the Jingdong Mall, have been accustomed to shopping in the Jingdong, many times can buy good quality and cheap products, and courier service attitude is particularly good, sometimes too many couriers are to help me to the home, so many years have been in the Jingdong Mall shopping, trust and support! Will also support the Jingdong as always!	Jingdong prefecture level city in Shandong
Jinfeng Hongyuan Flagship Store	Power Saving Expert 2	Good workmanship, mostly better than the original wall-plugged ones, and hopefully power factor compensation saves on electricity costs	Jingdong prefecture level city in Shandong
Jinfeng Hongyuan Flagship Store	Power Saving Expert 2	I don't feel anything, I said I won't know the effect for a month or two, let's see.	Jingdong prefecture level city in Shandong
Jinfeng Hongyuan Flagship Store	Power Saving Expert 2	Delivery was pretty fast, a little pricey, just not sure if it works or not	Jingdong prefecture level city in Shandong
Jinfeng Hongyuan Flagship Store	Power Saving Expert 2	The goods have been received, I do not know how effective.	Jingdong prefecture level city in Shandong
Jinfeng Hongyuan Flagship Store	Power Saving Expert 2	So far it doesn't seem to be too noticeable, so I'll comment later.	Jingdong prefecture level city in Shandong
Jinfeng Hongyuan Flagship Store	Power Saving Expert 2	I'll comment after I've used it	Jingdong prefecture level city in Shandong
Jinfeng Hongyuan Flagship Store	Power Saving Expert 2	Not bad. You'll save a lot on your electric bill in a year!	Jingdong prefecture level city in Shandong
Jinfeng Hongyuan Flagship Store	Power Saving Expert 2	I just got it. I haven't tried it yet. I don't know what it's like.	Jingdong prefecture level city in Shandong
Jinfeng Hongyuan Flagship Store	Power Saving Expert 2	Shopping at Jingdong has become a habit, and this disease will not be cured for a while.	Jingdong prefecture level city in Shandong
Jinfeng Hongyuan Flagship Store	Power Saving Expert 2	I've been using it for a while now. I don't know if I'm still saving power or not.	Jingdong prefecture level city in Shandong
Jinfeng Hongyuan Flagship Store	Power Saving Expert 2	Received the saver and it's great! There is also a digital voltage indication. Plugged in as instructed, easy to operate, hope you're our power saver!	Jingdong prefecture level city in Shandong
Jinfeng Hongyuan Flagship Store	Power Saving Expert 2	There is no power saving as the store says, using it is the same as not using it.	Jingdong prefecture level city in Shandong
Jinfeng Hongyuan Flagship Store	Power Saving Expert 2	Use it for a while and then comment again	Jingdong prefecture level city in Shandong
Jinfeng Hongyuan Flagship Store	Power Saving Expert 2	Feeling can, to use 1 to 2 months to see how the effect, there is a voltage display, can see the voltage fluctuations amplitude, quite good!	Jingdong prefecture level city in Shandong

Jinfeng Hongyuan Flagship Store	Power Saving Expert 2	I hope it works and saves me some electricity.	Jingdong prefecture level city in Shandong
Jinfeng Hongyuan Flagship Store	Power Saving Expert 2	Nice baby, happy with the after sales	Jingdong prefecture level city in Shandong
Jinfeng Hongyuan Flagship Store	Power Saving Expert 2	Use it and talk about it.	Jingdong prefecture level city in Shandong
Jinfeng Hongyuan Flagship Store	Power Saving Expert 2	Second purchase	Jingdong prefecture level city in Shandong
Jinfeng Hongyuan Flagship Store	Power Saving Expert 2	It works.	Jingdong prefecture level city in Shandong
Jinfeng Hongyuan Flagship Store	Power Saving Expert 2	It works.	Jingdong prefecture level city in Shandong
Jinfeng Hongyuan Flagship Store	Power Saving Expert 2	I don't know if it's a power saver until I start using it	Jingdong prefecture level city in Shandong
Jinfeng Hongyuan Flagship Store	Power Saving Expert 2	serviceable	Jingdong prefecture level city in Shandong
Jinfeng Hongyuan Flagship Store	Power Saving Expert 2	This user did not fill out the rating in time	Jingdong prefecture level city in Shandong
Jinfeng Hongyuan Flagship Store	Power Saving Expert 2	There is no power saving as the store says, using it is the same as not using it.	Jingdong prefecture level city in Shandong
Jinfeng Hongyuan Flagship Store	Power Saving Expert 2	This user did not fill out the rating in time	Jingdong prefecture level city in Shandong
Jinfeng Hongyuan Flagship Store	Power Saving Expert 2	Good...good.	Jingdong prefecture level city in Shandong
Jinfeng Hongyuan Flagship Store	Power Saving Expert 2	(of an unmarried couple) be close	Jingdong prefecture level city in Shandong
Jinfeng Hongyuan Flagship Store	Power Saving Expert 2	(of an unmarried couple) be close	Jingdong prefecture level city in Shandong
Jinfeng Hongyuan Flagship Store	Power Saving Expert 2	(of an unmarried couple) be close	Jingdong prefecture level city in Shandong
Jinfeng Hongyuan Flagship Store	Power Saving Expert 2	The goods are real, high value, high quality, worth every penny, material appearance and quality at a glance is very upscale, very like HAPPY💯	Jingdong prefecture level city in Shandong
Jinfeng Hongyuan Flagship Store	Power Saving Expert 2	It is the ideal product, good quality, logistics is also very fast, worthy of praise 🎁 baby quality is very good, received the first time to open the first time to see, it is very good 👍, very satisfied 😊, customer service is also available to ask questions and answer, thank you 🙏	Jingdong prefecture level city in Shandong
Jinfeng Hongyuan Flagship Store	Power Saving Expert 2	I bought it and haven't used it yet, I'll use it again.	Jingdong prefecture level city in Shandong
Jinfeng Hongyuan Flagship Store	Power Saving Expert 2	It didn't work, I used it for a few days and it was the same as before, it didn't work, I should have to return it	Jingdong prefecture level city in Shandong
Jinfeng Hongyuan Flagship Store	Power Saving Expert 2	The goods have been received, but have not yet installed and use, feel good.	Jingdong prefecture level city in Shandong
Jinfeng Hongyuan Flagship Store	Power Saving Expert 2	Goods received, I forgot to take pictures, the goods look good, you can start, immediately start the school to see the effect then	Jingdong prefecture level city in Shandong
Jinfeng Hongyuan Flagship Store	Power Saving Expert 2	Looks good. I'll use it for a while.	Jingdong prefecture level city in Shandong
Jinfeng Hongyuan Flagship Store	Power Saving Expert 2	Great stuff, easy to operate, easy to use, good power saving, believe in the power of science!	Jingdong prefecture level city in Shandong
Jinfeng Hongyuan Flagship Store	Power Saving Expert 2	Especially great, comparison shopping before buying, the store owner is very good, answering questions very patiently and detailed, there is a need to come back!	Jingdong prefecture level city in Shandong
Jinfeng Hongyuan Flagship Store	Power Saving Expert 2	Trustworthy, feel free to use it boldly.	Jingdong prefecture level city in Shandong
Jinfeng Hongyuan Flagship Store	Power Saving Expert 2	Logistics is super fast! Beautiful appearance! Looks like a great quality! I've never bought one before, so I'll try it first to see if it saves power. We'll see how much it saves.	Jingdong prefecture level city in Shandong
Jinfeng Hongyuan Flagship Store	Power Saving Expert 2	I saw online counterfeiting, energy saver is one of them, hahahahaha, jingdong also buy fake products	Jingdong prefecture level city in Shandong
Jinfeng Hongyuan Flagship Store	Power Saving Expert 2	Just received, installed, try a month to see how the effect and then follow up.	Jingdong prefecture level city in Shandong
Jinfeng Hongyuan Flagship Store	Power Saving Expert 2	Good for you! The goods are genuine! Good price for what you get!	Jingdong prefecture level city in Shandong
Jinfeng Hongyuan Flagship Store	Power Saving Expert 2	Things quickly received, immediately used to see if there is no effect, something river photo ⬆ the same, customer service is very professional	Jingdong prefecture level city in Shandong
Jinfeng Hongyuan Flagship Store	Power Saving Expert 2	The product is very convenient Looking forward to the subsequent use of the experience results	Jingdong prefecture level city in Shandong
Jinfeng Hongyuan Flagship Store	Power Saving Expert 2	I just received it and plugged it in, we'll see exactly when it's time to pay the electric bill	Jingdong prefecture level city in Shandong
Jinfeng Hongyuan Flagship Store	Power Saving Expert 2	Saver is good Packaging and looks good How effective it is to be shared after using it.	Jingdong prefecture level city in Shandong
Jinfeng Hongyuan Flagship Store	Power Saving Expert 2	There's no way to compare. I don't know if it's energy efficient.	Jingdong prefecture level city in Shandong
Jinfeng Hongyuan Flagship Store	Power Saving Expert 2	Just installed it, try it out and comment again	Jingdong prefecture level city in Shandong
Jinfeng Hongyuan Flagship Store	Power Saving Expert 2	Super save point also good to install, after a friend introduced then went to buy to, friends say it is very good to use, home I bought, try to use a period of time, to be honest, the economizer is not big or heavy, can be very convenient to put in a lot of places, the quality is also very good, power-saving function this time seems to be about 20% of the efficiency. The owner is also very warm contact, very	Jingdong prefecture level city in Shandong
Jinfeng Hongyuan Flagship Store	Power Saving Expert 2	The reviews online are mixed. Try it for a while, but I don't know if I can return it if it doesn't save any power after a couple months.	Jingdong prefecture level city in Shandong
Jinfeng Hongyuan Flagship Store	Power Saving Expert 2	The power consumption at home is high, bought a power saver, it looks very convenient to plug on the line, see if you can save some money next month, if good, recommend to friends down the road.	Jingdong prefecture level city in Shandong
Jinfeng Hongyuan Flagship Store	Power Saving Expert 2	It's beautiful, it's genuine, but I don't know the power-saving effect.	Jingdong prefecture level city in Shandong
Jinfeng Hongyuan Flagship Store	Power Saving Expert 2	This user thinks the product is good	Jingdong prefecture level city in Shandong
Jinfeng Hongyuan Flagship Store	Power Saving Expert 2	Nice exterior design with an at-a-glance display	Jingdong prefecture level city in Shandong
Jinfeng Hongyuan Flagship Store	Power Saving Expert 2	Home large and small things are purchased in the Jingdong Mall, have been accustomed to shopping in the Jingdong, many times can buy good quality and cheap products, and courier service attitude is particularly good, sometimes too many couriers are to help me to the home, so many years have been in the Jingdong Mall shopping, trust and support! Will also support the Jingdong as always!	Jingdong prefecture level city in Shandong
Jinfeng Hongyuan Flagship Store	Power Saving Expert 2	Good workmanship, mostly better than the original wall-plugged ones, and hopefully power factor compensation saves on electricity costs	Jingdong prefecture level city in Shandong
Jinfeng Hongyuan Flagship Store	Power Saving Expert 2	I don't feel anything, I said I won't know the effect for a month or two, let's see.	Jingdong prefecture level city in Shandong
Jinfeng Hongyuan Flagship Store	Power Saving Expert 2	Delivery was pretty fast, a little pricey, just not sure if it works or not	Jingdong prefecture level city in Shandong
Jinfeng Hongyuan Flagship Store	Power Saving Expert 2	The goods have been received, I do not know how effective.	Jingdong prefecture level city in Shandong
Jinfeng Hongyuan Flagship Store	Power Saving Expert 2	So far it doesn't seem to be too noticeable, so I'll comment later.	Jingdong prefecture level city in Shandong
Jinfeng Hongyuan Flagship Store	Power Saving Expert 2	I'll comment after I've used it	Jingdong prefecture level city in Shandong
Jinfeng Hongyuan Flagship Store	Power Saving Expert 2	Not bad. You'll save a lot on your electric bill in a year!	Jingdong prefecture level city in Shandong

Jinfeng Hongyuan Flagship Store	Power Saving Expert 2	I just got it. I haven't tried it yet. I don't know what it's like.	Jingdong prefecture level city in Shandong
Jinfeng Hongyuan Flagship Store	Power Saving Expert 2	Shopping at Jingdong has become a habit, and this disease will not be cured for a while.	Jingdong prefecture level city in Shandong
Jinfeng Hongyuan Flagship Store	Power Saving Expert 2	I've been using it for a while now. I don't know if I'm still saving power or not.	Jingdong prefecture level city in Shandong
Jinfeng Hongyuan Flagship Store	Power Saving Expert 2	Received the saver and it's great! There is also a digital voltage indication. Plugged in as instructed, easy to operate, hope you're our power saver!	Jingdong prefecture level city in Shandong
Jinfeng Hongyuan Flagship Store	Power Saving Expert 2	There is no power saving as the store says, using it is the same as not using it.	Jingdong prefecture level city in Shandong
Jinfeng Hongyuan Flagship Store	Power Saving Expert 2	Use it for a while and then comment again	Jingdong prefecture level city in Shandong
Jinfeng Hongyuan Flagship Store	Power Saving Expert 2	Feeling can, to use 1 to 2 months to see how the effect, there is a voltage display, can see the voltage fluctuations amplitude, quite good!	Jingdong prefecture level city in Shandong
Jinfeng Hongyuan Flagship Store	Power Saving Expert 2	I hope it works and saves me some electricity.	Jingdong prefecture level city in Shandong
Jinfeng Hongyuan Flagship Store	Power Saving Expert 2	Nice baby, happy with the after sales	Jingdong prefecture level city in Shandong
Jinfeng Hongyuan Flagship Store	Power Saving Expert 2	Use it and talk about it.	Jingdong prefecture level city in Shandong
Jinfeng Hongyuan Flagship Store	Power Saving Expert 2	Second purchase	Jingdong prefecture level city in Shandong
Jinfeng Hongyuan Flagship Store	Power Saving Expert 2	It works.	Jingdong prefecture level city in Shandong
Jinfeng Hongyuan Flagship Store	Power Saving Expert 2	It works.	Jingdong prefecture level city in Shandong
Jinfeng Hongyuan Flagship Store	Power Saving Expert 2	I don't know if it's a power saver until I start using it	Jingdong prefecture level city in Shandong
Jinfeng Hongyuan Flagship Store	Power Saving Expert 2	serviceable	Jingdong prefecture level city in Shandong
Jinfeng Hongyuan Flagship Store	Power Saving Expert 2	This user did not fill out the rating in time	Jingdong prefecture level city in Shandong
Jinfeng Hongyuan Flagship Store	Power Saving Expert 2	There is no power saving as the store says, using it is the same as not using it.	Jingdong prefecture level city in Shandong
Jinfeng Hongyuan Flagship Store	Power Saving Expert 2	This user did not fill out the rating in time	Jingdong prefecture level city in Shandong
Jinfeng Hongyuan Flagship Store	Power Saving Expert 2	Good...good.	Jingdong prefecture level city in Shandong
Jinfeng Hongyuan Flagship Store	Power Saving Expert 2	(of an unmarried couple) be close	Jingdong prefecture level city in Shandong
Jinfeng Hongyuan Flagship Store	Power Saving Expert 2	(of an unmarried couple) be close	Jingdong prefecture level city in Shandong
Jinfeng Hongyuan Flagship Store	Power Saving Expert 2	(of an unmarried couple) be close	Jingdong prefecture level city in Shandong
Jinfeng Hongyuan Flagship Store	Power Saving Expert 2	The goods are real, high value, high quality, worth every penny, material appearance and quality at a glance is very upscale, very like HAPPY1 00	Jingdong prefecture level city in Shandong
Jinfeng Hongyuan Flagship Store	Power Saving Expert 2	It is the ideal product, good quality, logistics is also very fast, worthy of praise 🎁 baby quality is very good, received the first time to open the first time to see, it is very good 👍, very satisfied 😃, customer service is also available to ask questions and answer, thank you 🙏	Jingdong prefecture level city in Shandong
Jinfeng Hongyuan Flagship Store	Power Saving Expert 2	I bought it and haven't used it yet, I'll use it again.	Jingdong prefecture level city in Shandong
Jinfeng Hongyuan Flagship Store	Power Saving Expert 2	It didn't work, I used it for a few days and it was the same as before, it didn't work, I should have to return it	Jingdong prefecture level city in Shandong
Jinfeng Hongyuan Flagship Store	Power Saving Expert 2	The goods have been received, but have not yet installed and use, feel good.	Jingdong prefecture level city in Shandong
Jinfeng Hongyuan Flagship Store	Power Saving Expert 2	Goods received, I forgot to take pictures, the goods look good, you can start, immediately start the school to see the effect then	Jingdong prefecture level city in Shandong
Jinfeng Hongyuan Flagship Store	Power Saving Expert 2	Looks good. I'll use it for a while.	Jingdong prefecture level city in Shandong
Jinfeng Hongyuan Flagship Store	Power Saving Expert 2	Great stuff, easy to operate, easy to use, good power saving, believe in the power of science!	Jingdong prefecture level city in Shandong
Jinfeng Hongyuan Flagship Store	Power Saving Expert 2	Especially great, comparison shopping before buying, the store owner is very good, answering questions very patiently and detailed, there is a need to come back!	Jingdong prefecture level city in Shandong
Jinfeng Hongyuan Flagship Store	Power Saving Expert 2	Trustworthy, feel free to use it boldly.	Jingdong prefecture level city in Shandong
Jinfeng Hongyuan Flagship Store	Power Saving Expert 2	Logistics is super fast! Beautiful appearance! Looks like a great quality! I've never bought one before, so I'll try it first to see if it saves power. We'll see how much it saves.	Jingdong prefecture level city in Shandong
Jinfeng Hongyuan Flagship Store	Power Saving Expert 2	I saw online counterfeiting, energy saver is one of them, hahahahaha, jingdong also buy fake products	Jingdong prefecture level city in Shandong
Jinfeng Hongyuan Flagship Store	Power Saving Expert 2	Just received, installed, try a month to see how the effect and then follow up.	Jingdong prefecture level city in Shandong
Jinfeng Hongyuan Flagship Store	Power Saving Expert 2	Good for you! The goods are genuine! Good price for what you get!	Jingdong prefecture level city in Shandong
Jinfeng Hongyuan Flagship Store	Power Saving Expert 2	Things quickly received, immediately used to see if there is no effect, something river photo [illegible] the same, customer service is very professional	Jingdong prefecture level city in Shandong
Jinfeng Hongyuan Flagship Store	Power Saving Expert 2	The product is very convenient Looking forward to the subsequent use of the experience results	Jingdong prefecture level city in Shandong
Jinfeng Hongyuan Flagship Store	Power Saving Expert 2	I just received it and plugged it in, we'll see exactly when it's time to pay the electric bill	Jingdong prefecture level city in Shandong
Jinfeng Hongyuan Flagship Store	Power Saving Expert 2	Saver is good Packaging and looks good How effective it is to be shared after using it.	Jingdong prefecture level city in Shandong
Jinfeng Hongyuan Flagship Store	Power Saving Expert 2	There's no way to compare. I don't know if it's energy efficient.	Jingdong prefecture level city in Shandong
Jinfeng Hongyuan Flagship Store	Power Saving Expert 2	Just installed it, try it out and comment again	Jingdong prefecture level city in Shandong
Jinfeng Hongyuan Flagship Store	Power Saving Expert 2	Super save point also good to install, after a friend introduced then went to buy to, friends say it is very good to use, home I bought, try to use a period of time, to be honest, the economizer is not big or heavy, can be very convenient to put in a lot of places, the quality is also very good, power-saving function this time seems to be about 20% of the efficiency. The owner is also very warm contact, very	Jingdong prefecture level city in Shandong
Jinfeng Hongyuan Flagship Store	Power Saving Expert 2	The reviews online are mixed. Try it for a while, but I don't know if I can return it if it doesn't save any power after a couple months.	Jingdong prefecture level city in Shandong
Jinfeng Hongyuan Flagship Store	Power Saving Expert 2	The power consumption at home is high, bought a power saver, it looks very convenient to plug on the line, see if you can save some money next month, if good, recommend to friends down the road.	Jingdong prefecture level city in Shandong
Jinfeng Hongyuan Flagship Store	Power Saving Expert 2	It's beautiful, it's genuine, but I don't know the power-saving effect.	Jingdong prefecture level city in Shandong
Jinfeng Hongyuan Flagship Store	Power Saving Expert 2	This user thinks the product is good	Jingdong prefecture level city in Shandong
Jinfeng Hongyuan Flagship Store	Power Saving Expert 2	Nice exterior design with an at-a-glance display	Jingdong prefecture level city in Shandong
Jinfeng Hongyuan Flagship Store	Power Saving Expert 2	Home large and small things are purchased in the Jingdong Mall, have been accustomed to shopping in the Jingdong, many times can buy good quality and cheap products, and courier service attitude is particularly good, sometimes too many couriers are to help me to the home, so many years have been in the Jingdong Mall shopping, trust and support! Will also support the Jingdong as always!	Jingdong prefecture level city in Shandong

Jinfeng Hongyuan Flagship Store	Power Saving Expert 2	Good workmanship, mostly better than the original wall-plugged ones, and hopefully power factor compensation saves on electricity costs	Jingdong prefecture level city in Shandong
Jinfeng Hongyuan Flagship Store	Power Saving Expert 2	I don't feel anything, I said I won't know the effect for a month or two, let's see.	Jingdong prefecture level city in Shandong
Jinfeng Hongyuan Flagship Store	Power Saving Expert 2	Delivery was pretty fast, a little pricey, just not sure if it works or not	Jingdong prefecture level city in Shandong
Jinfeng Hongyuan Flagship Store	Power Saving Expert 2	The goods have been received, I do not know how effective.	Jingdong prefecture level city in Shandong
Jinfeng Hongyuan Flagship Store	Power Saving Expert 2	So far it doesn't seem to be too noticeable, so I'll comment later.	Jingdong prefecture level city in Shandong
Jinfeng Hongyuan Flagship Store	Power Saving Expert 2	I'll comment after I've used it	Jingdong prefecture level city in Shandong
Jinfeng Hongyuan Flagship Store	Power Saving Expert 2	Not bad. You'll save a lot on your electric bill in a year!	Jingdong prefecture level city in Shandong
Jinfeng Hongyuan Flagship Store	Power Saving Expert 2	I just got it. I haven't tried it yet. I don't know what it's like.	Jingdong prefecture level city in Shandong
Jinfeng Hongyuan Flagship Store	Power Saving Expert 2	Shopping at Jingdong has become a habit, and this disease will not be cured for a while.	Jingdong prefecture level city in Shandong
Jinfeng Hongyuan Flagship Store	Power Saving Expert 2	I've been using it for a while now. I don't know if I'm still saving power or not.	Jingdong prefecture level city in Shandong
Jinfeng Hongyuan Flagship Store	Power Saving Expert 2	Received the saver and it's great! There is also a digital voltage indication. Plugged in as instructed, easy to operate, hope you're our power saver!	Jingdong prefecture level city in Shandong
Jinfeng Hongyuan Flagship Store	Power Saving Expert 2	There is no power saving as the store says, using it is the same as not using it.	Jingdong prefecture level city in Shandong
Jinfeng Hongyuan Flagship Store	Power Saving Expert 2	Use it for a while and then comment again	Jingdong prefecture level city in Shandong
Jinfeng Hongyuan Flagship Store	Power Saving Expert 2	Feeling can, to use 1 to 2 months to see how the effect, there is a voltage display, can see the voltage fluctuations amplitude, quite good!	Jingdong prefecture level city in Shandong
Jinfeng Hongyuan Flagship Store	Power Saving Expert 2	I hope it works and saves me some electricity.	Jingdong prefecture level city in Shandong
Jinfeng Hongyuan Flagship Store	Power Saving Expert 2	Nice baby, happy with the after sales	Jingdong prefecture level city in Shandong
Jinfeng Hongyuan Flagship Store	Power Saving Expert 2	Use it and talk about it.	Jingdong prefecture level city in Shandong
Jinfeng Hongyuan Flagship Store	Power Saving Expert 2	Second purchase	Jingdong prefecture level city in Shandong
Jinfeng Hongyuan Flagship Store	Power Saving Expert 2	It works.	Jingdong prefecture level city in Shandong
Jinfeng Hongyuan Flagship Store	Power Saving Expert 2	It works.	Jingdong prefecture level city in Shandong
Jinfeng Hongyuan Flagship Store	Power Saving Expert 2	I don't know if it's a power saver until I start using it	Jingdong prefecture level city in Shandong
Jinfeng Hongyuan Flagship Store	Power Saving Expert 2	serviceable	Jingdong prefecture level city in Shandong
Jinfeng Hongyuan Flagship Store	Power Saving Expert 2	This user did not fill out the rating in time	Jingdong prefecture level city in Shandong
Jinfeng Hongyuan Flagship Store	Power Saving Expert 2	There is no power saving as the store says, using it is the same as not using it.	Jingdong prefecture level city in Shandong
Jinfeng Hongyuan Flagship Store	Power Saving Expert 2	This user did not fill out the rating in time	Jingdong prefecture level city in Shandong
Jinfeng Hongyuan Flagship Store	Power Saving Expert 2	Good...good.	Jingdong prefecture level city in Shandong
Jinfeng Hongyuan Flagship Store	Power Saving Expert 2	(of an unmarried couple) be close	Jingdong prefecture level city in Shandong
Jinfeng Hongyuan Flagship Store	Power Saving Expert 2	(of an unmarried couple) be close	Jingdong prefecture level city in Shandong
Jinfeng Hongyuan Flagship Store	Power Saving Expert 2	(of an unmarried couple) be close	Jingdong prefecture level city in Shandong
Jinfeng Hongyuan Flagship Store	Power Saving Expert 2	The goods are real, high value, high quality, worth every penny, material appearance and quality at a glance is very upscale, very like HAPPY💯	Jingdong prefecture level city in Shandong
Jinfeng Hongyuan Flagship Store	Power Saving Expert 2	It is the ideal product, good quality, logistics is also very fast, worthy of praise 🎁 baby quality is very good, received the first time to open the first time to see, it is very good 👍, very satisfied 😃, customer service is also available to ask questions and answer, thank you 🙏	Jingdong prefecture level city in Shandong
Jinfeng Hongyuan Flagship Store	Power Saving Expert 2	I bought it and haven't used it yet, I'll use it again.	Jingdong prefecture level city in Shandong
Jinfeng Hongyuan Flagship Store	Power Saving Expert 2	It didn't work, I used it for a few days and it was the same as before, it didn't work, I should have to return it	Jingdong prefecture level city in Shandong
Jinfeng Hongyuan Flagship Store	Power Saving Expert 2	The goods have been received, but have not yet installed and use, feel good.	Jingdong prefecture level city in Shandong
Jinfeng Hongyuan Flagship Store	Power Saving Expert 2	Goods received, I forgot to take pictures, the goods look good, you can start, immediately start the school to see the effect then	Jingdong prefecture level city in Shandong
Jinfeng Hongyuan Flagship Store	Power Saving Expert 2	Looks good. I'll use it for a while.	Jingdong prefecture level city in Shandong
Jinfeng Hongyuan Flagship Store	Power Saving Expert 2	Great stuff, easy to operate, easy to use, good power saving, believe in the power of science!	Jingdong prefecture level city in Shandong
Jinfeng Hongyuan Flagship Store	Power Saving Expert 2	Especially great, comparison shopping before buying, the store owner is very good, answering questions very patiently and detailed, there is a need to come back!	Jingdong prefecture level city in Shandong
Jinfeng Hongyuan Flagship Store	Power Saving Expert 2	Trustworthy, feel free to use it boldly.	Jingdong prefecture level city in Shandong
Jinfeng Hongyuan Flagship Store	Power Saving Expert 2	Logistics is super fast! Beautiful appearance! Looks like a great quality! I've never bought one before, so I'll try it first to see if it saves power. We'll see how much it saves.	Jingdong prefecture level city in Shandong
Jinfeng Hongyuan Flagship Store	Power Saving Expert 2	I saw online counterfeiting, energy saver is one of them, hahahahaha, jingdong also buy fake products	Jingdong prefecture level city in Shandong
Jinfeng Hongyuan Flagship Store	Power Saving Expert 2	Just received, installed, try a month to see how the effect and then follow up.	Jingdong prefecture level city in Shandong
Jinfeng Hongyuan Flagship Store	Power Saving Expert 2	Good for you! The goods are genuine! Good price for what you get!	Jingdong prefecture level city in Shandong
Jinfeng Hongyuan Flagship Store	Power Saving Expert 2	Things quickly received, immediately used to see if there is no effect, something river photo ⬆ the same, customer service is very professional	Jingdong prefecture level city in Shandong
Jinfeng Hongyuan Flagship Store	Power Saving Expert 2	The product is very convenient Looking forward to the subsequent use of the experience results	Jingdong prefecture level city in Shandong
Jinfeng Hongyuan Flagship Store	Power Saving Expert 2	I just received it and plugged it in, we'll see exactly when it's time to pay the electric bill	Jingdong prefecture level city in Shandong
Jinfeng Hongyuan Flagship Store	Power Saving Expert 2	Saver is good Packaging and looks good How effective it is to be shared after using it.	Jingdong prefecture level city in Shandong
Jinfeng Hongyuan Flagship Store	Power Saving Expert 2	There's no way to compare. I don't know if it's energy efficient.	Jingdong prefecture level city in Shandong
Jinfeng Hongyuan Flagship Store	Power Saving Expert 2	Just installed it, try it out and comment again	Jingdong prefecture level city in Shandong
Jinfeng Hongyuan Flagship Store	Power Saving Expert 2	Super save point also good to install, after a friend introduced then went to buy to, friends say it is very good to use, home I bought, try to use a period of time, to be honest, the economizer is not big or heavy, can be very convenient to put in a lot of places, the quality is also very good, power-saving function this time seems to be about 20% of the efficiency. The owner is also very warm contact, very	Jingdong prefecture level city in Shandong

Jinfeng Hongyuan Flagship Store	Power Saving Expert 2	The reviews online are mixed. Try it for a while, but I don't know if I can return it if it doesn't save any power after a couple months.	Jingdong prefecture level city in Shandong
Jinfeng Hongyuan Flagship Store	Power Saving Expert 2	The power consumption at home is high, bought a power saver, it looks very convenient to plug on the line, see if you can save some money next month, if good, recommend to friends down the road.	Jingdong prefecture level city in Shandong
Jinfeng Hongyuan Flagship Store	Power Saving Expert 2	It's beautiful, it's genuine, but I don't know the power-saving effect.	Jingdong prefecture level city in Shandong
Jinfeng Hongyuan Flagship Store	Power Saving Expert 2	This user thinks the product is good	Jingdong prefecture level city in Shandong
Jinfeng Hongyuan Flagship Store	Power Saving Expert 2	Nice exterior design with an at-a-glance display	Jingdong prefecture level city in Shandong
Jinfeng Hongyuan Flagship Store	Power Saving Expert 2	Home large and small things are purchased in the Jingdong Mall, have been accustomed to shopping in the Jingdong, many times can buy good quality and cheap products, and courier service attitude is particularly good, sometimes too many couriers are to help me to the home, so many years have been in the Jingdong Mall shopping, trust and support! Will also support the Jingdong as always!	Jingdong prefecture level city in Shandong
Jinfeng Hongyuan Flagship Store	Power Saving Expert 2	Good workmanship, mostly better than the original wall-plugged ones, and hopefully power factor compensation saves on electricity costs	Jingdong prefecture level city in Shandong
Jinfeng Hongyuan Flagship Store	Power Saving Expert 2	I don't feel anything, I said I won't know the effect for a month or two, let's see.	Jingdong prefecture level city in Shandong
Jinfeng Hongyuan Flagship Store	Power Saving Expert 2	Delivery was pretty fast, a little pricey, just not sure if it works or not	Jingdong prefecture level city in Shandong
Jinfeng Hongyuan Flagship Store	Power Saving Expert 2	The goods have been received, I do not know how effective.	Jingdong prefecture level city in Shandong
Jinfeng Hongyuan Flagship Store	Power Saving Expert 2	So far it doesn't seem to be too noticeable, so I'll comment later.	Jingdong prefecture level city in Shandong
Jinfeng Hongyuan Flagship Store	Power Saving Expert 2	I'll comment after I've used it	Jingdong prefecture level city in Shandong
Jinfeng Hongyuan Flagship Store	Power Saving Expert 2	Not bad. You'll save a lot on your electric bill in a year!	Jingdong prefecture level city in Shandong
Jinfeng Hongyuan Flagship Store	Power Saving Expert 2	I just got it. I haven't tried it yet. I don't know what it's like.	Jingdong prefecture level city in Shandong
Jinfeng Hongyuan Flagship Store	Power Saving Expert 2	Shopping at Jingdong has become a habit, and this disease will not be cured for a while.	Jingdong prefecture level city in Shandong
Jinfeng Hongyuan Flagship Store	Power Saving Expert 2	I've been using it for a while now. I don't know if I'm still saving power or not.	Jingdong prefecture level city in Shandong
Jinfeng Hongyuan Flagship Store	Power Saving Expert 2	Received the saver and it's great! There is also a digital voltage indication. Plugged in as instructed, easy to operate, hope you're our power saver!	Jingdong prefecture level city in Shandong
Jinfeng Hongyuan Flagship Store	Power Saving Expert 2	There is no power saving as the store says, using it is the same as not using it.	Jingdong prefecture level city in Shandong
Jinfeng Hongyuan Flagship Store	Power Saving Expert 2	Use it for a while and then comment again	Jingdong prefecture level city in Shandong
Jinfeng Hongyuan Flagship Store	Power Saving Expert 2	Feeling can, to use 1 to 2 months to see how the effect, there is a voltage display, can see the voltage fluctuations amplitude, quite good!	Jingdong prefecture level city in Shandong
Jinfeng Hongyuan Flagship Store	Power Saving Expert 2	I hope it works and saves me some electricity.	Jingdong prefecture level city in Shandong
Jinfeng Hongyuan Flagship Store	Power Saving Expert 2	Nice baby, happy with the after sales	Jingdong prefecture level city in Shandong
Jinfeng Hongyuan Flagship Store	Power Saving Expert 2	Use it and talk about it.	Jingdong prefecture level city in Shandong
Jinfeng Hongyuan Flagship Store	Power Saving Expert 2	Second purchase	Jingdong prefecture level city in Shandong
Jinfeng Hongyuan Flagship Store	Power Saving Expert 2	It works.	Jingdong prefecture level city in Shandong
Jinfeng Hongyuan Flagship Store	Power Saving Expert 2	It works.	Jingdong prefecture level city in Shandong
Jinfeng Hongyuan Flagship Store	Power Saving Expert 2	I don't know if it's a power saver until I start using it	Jingdong prefecture level city in Shandong
Jinfeng Hongyuan Flagship Store	Power Saving Expert 2	serviceable	Jingdong prefecture level city in Shandong
Jinfeng Hongyuan Flagship Store	Power Saving Expert 2	This user did not fill out the rating in time	Jingdong prefecture level city in Shandong
Jinfeng Hongyuan Flagship Store	Power Saving Expert 2	There is no power saving as the store says, using it is the same as not using it.	Jingdong prefecture level city in Shandong
Jinfeng Hongyuan Flagship Store	Power Saving Expert 2	This user did not fill out the rating in time	Jingdong prefecture level city in Shandong
Jinfeng Hongyuan Flagship Store	Power Saving Expert 2	Good...good.	Jingdong prefecture level city in Shandong
Jinfeng Hongyuan Flagship Store	Power Saving Expert 2	(of an unmarried couple) be close	Jingdong prefecture level city in Shandong
Jinfeng Hongyuan Flagship Store	Power Saving Expert 2	(of an unmarried couple) be close	Jingdong prefecture level city in Shandong
Jinfeng Hongyuan Flagship Store	Power Saving Expert 2	(of an unmarried couple) be close	Jingdong prefecture level city in Shandong
Jinfeng Hongyuan Flagship Store	Power Saving Expert 2	The goods are real, high value, high quality, worth every penny, material appearance and quality at a glance is very upscale, very like HAPPY1 00	Jingdong prefecture level city in Shandong
Jinfeng Hongyuan Flagship Store	Power Saving Expert 2	It is the ideal product, good quality, logistics is also very fast, worthy of praise 🎁 baby quality is very good, received the first time to open the first time to see, it is very good 👍, very satisfied 😃, customer service is also available to ask questions and answer, thank you 🙏	Jingdong prefecture level city in Shandong
Jinfeng Hongyuan Flagship Store	Power Saving Expert 2	I bought it and haven't used it yet, I'll use it again.	Jingdong prefecture level city in Shandong
Jinfeng Hongyuan Flagship Store	Power Saving Expert 2	It didn't work, I used it for a few days and it was the same as before, it didn't work, I should have to return it	Jingdong prefecture level city in Shandong
Jinfeng Hongyuan Flagship Store	Power Saving Expert 2	The goods have been received, but have not yet installed and use, feel good.	Jingdong prefecture level city in Shandong
Jinfeng Hongyuan Flagship Store	Power Saving Expert 2	Goods received, I forgot to take pictures, the goods look good, you can start, immediately start the school to see the effect then	Jingdong prefecture level city in Shandong
Jinfeng Hongyuan Flagship Store	Power Saving Expert 2	Looks good. I'll use it for a while.	Jingdong prefecture level city in Shandong
Jinfeng Hongyuan Flagship Store	Power Saving Expert 2	Great stuff, easy to operate, easy to use, good power saving, believe in the power of science!	Jingdong prefecture level city in Shandong
Jinfeng Hongyuan Flagship Store	Power Saving Expert 2	Especially great, comparison shopping before buying, the store owner is very good, answering questions very patiently and detailed, there is a need to come back!	Jingdong prefecture level city in Shandong
Jinfeng Hongyuan Flagship Store	Power Saving Expert 2	Trustworthy, feel free to use it boldly.	Jingdong prefecture level city in Shandong
Jinfeng Hongyuan Flagship Store	Power Saving Expert 2	Logistics is super fast! Beautiful appearance! Looks like a great quality! I've never bought one before, so I'll try it first to see if it saves power. We'll see how much it saves.	Jingdong prefecture level city in Shandong
Jinfeng Hongyuan Flagship Store	Power Saving Expert 2	I saw online counterfeiting, energy saver is one of them, hahahahaha, jingdong also buy fake products	Jingdong prefecture level city in Shandong
Jinfeng Hongyuan Flagship Store	Power Saving Expert 2	Just received, installed, try a month to see how the effect and then follow up.	Jingdong prefecture level city in Shandong
Jinfeng Hongyuan Flagship Store	Power Saving Expert 2	Good for you! The goods are genuine! Good price for what you get!	Jingdong prefecture level city in Shandong
Jinfeng Hongyuan Flagship Store	Power Saving Expert 2	Things quickly received, immediately used to see if there is no effect, something river photo 🆙 the same, customer service is very professional	Jingdong prefecture level city in Shandong

Jinfeng Hongyuan Flagship Store	Power Saving Expert 2	The product is very convenient Looking forward to the subsequent use of the experience results	Jingdong prefecture level city in Shandong
Jinfeng Hongyuan Flagship Store	Power Saving Expert 2	I just received it and plugged it in, we'll see exactly when it's time to pay the electric bill	Jingdong prefecture level city in Shandong
Jinfeng Hongyuan Flagship Store	Power Saving Expert 2	Saver is good Packaging and looks good How effective it is to be shared after using it.	Jingdong prefecture level city in Shandong
Jinfeng Hongyuan Flagship Store	Power Saving Expert 2	There's no way to compare. I don't know if it's energy efficient.	Jingdong prefecture level city in Shandong
Jinfeng Hongyuan Flagship Store	Power Saving Expert 2	Just installed it, try it out and comment again	Jingdong prefecture level city in Shandong
Jinfeng Hongyuan Flagship Store	Power Saving Expert 2	Super save point also good to install, after a friend introduced then went to buy to, friends say it is very good to use, home I bought, try to use a period of time, to be honest, the economizer is not big or heavy, can be very convenient to put in a lot of places, the quality is also very good, power-saving function this time seems to be about 20% of the efficiency. The owner is also very warm contact, very	Jingdong prefecture level city in Shandong
Jinfeng Hongyuan Flagship Store	Power Saving Expert 2	The reviews online are mixed. Try it for a while, but I don't know if I can return it if it doesn't save any power after a couple months.	Jingdong prefecture level city in Shandong
Jinfeng Hongyuan Flagship Store	Power Saving Expert 2	The power consumption at home is high, bought a power saver, it looks very convenient to plug on the line, see if you can save some money next month, if good, recommend to friends down the road.	Jingdong prefecture level city in Shandong
Jinfeng Hongyuan Flagship Store	Power Saving Expert 2	It's beautiful, it's genuine, but I don't know the power-saving effect.	Jingdong prefecture level city in Shandong
Jinfeng Hongyuan Flagship Store	Power Saving Expert 2	This user thinks the product is good	Jingdong prefecture level city in Shandong
Jinfeng Hongyuan Flagship Store	Power Saving Expert 2	Nice exterior design with an at-a-glance display	Jingdong prefecture level city in Shandong
Jinfeng Hongyuan Flagship Store	Power Saving Expert 2	Home large and small things are purchased in the Jingdong Mall, have been accustomed to shopping in the Jingdong, many times can buy good quality and cheap products, and courier service attitude is particularly good, sometimes too many couriers are to help me to the home, so many years have been in the Jingdong Mall shopping, trust and support! Will also support the Jingdong as always!	Jingdong prefecture level city in Shandong
Jinfeng Hongyuan Flagship Store	Power Saving Expert 2	Good workmanship, mostly better than the original wall-plugged ones, and hopefully power factor compensation saves on electricity costs	Jingdong prefecture level city in Shandong
Jinfeng Hongyuan Flagship Store	Power Saving Expert 2	I don't feel anything, I said I won't know the effect for a month or two, let's see.	Jingdong prefecture level city in Shandong
Jinfeng Hongyuan Flagship Store	Power Saving Expert 2	Delivery was pretty fast, a little pricey, just not sure if it works or not	Jingdong prefecture level city in Shandong
Jinfeng Hongyuan Flagship Store	Power Saving Expert 2	The goods have been received, I do not know how effective.	Jingdong prefecture level city in Shandong
Jinfeng Hongyuan Flagship Store	Power Saving Expert 2	So far it doesn't seem to be too noticeable, so I'll comment later.	Jingdong prefecture level city in Shandong
Jinfeng Hongyuan Flagship Store	Power Saving Expert 2	I'll comment after I've used it	Jingdong prefecture level city in Shandong
Jinfeng Hongyuan Flagship Store	Power Saving Expert 2	Not bad. You'll save a lot on your electric bill in a year!	Jingdong prefecture level city in Shandong
Jinfeng Hongyuan Flagship Store	Power Saving Expert 2	I just got it. I haven't tried it yet. I don't know what it's like.	Jingdong prefecture level city in Shandong
Jinfeng Hongyuan Flagship Store	Power Saving Expert 2	Shopping at Jingdong has become a habit, and this disease will not be cured for a while.	Jingdong prefecture level city in Shandong
Jinfeng Hongyuan Flagship Store	Power Saving Expert 2	I've been using it for a while now. I don't know if I'm still saving power or not.	Jingdong prefecture level city in Shandong
Jinfeng Hongyuan Flagship Store	Power Saving Expert 2	Received the saver and it's great! There is also a digital voltage indication. Plugged in as instructed, easy to operate, hope you're our power saver!	Jingdong prefecture level city in Shandong
Jinfeng Hongyuan Flagship Store	Power Saving Expert 2	There is no power saving as the store says, using it is the same as not using it.	Jingdong prefecture level city in Shandong
Jinfeng Hongyuan Flagship Store	Power Saving Expert 2	Use it for a while and then comment again	Jingdong prefecture level city in Shandong
Jinfeng Hongyuan Flagship Store	Power Saving Expert 2	Feeling can, to use 1 to 2 months to see how the effect, there is a voltage display, can see the voltage fluctuations amplitude, quite good!	Jingdong prefecture level city in Shandong
Jinfeng Hongyuan Flagship Store	Power Saving Expert 2	I hope it works and saves me some electricity.	Jingdong prefecture level city in Shandong
Jinfeng Hongyuan Flagship Store	Power Saving Expert 2	Nice baby, happy with the after sales	Jingdong prefecture level city in Shandong
Jinfeng Hongyuan Flagship Store	Power Saving Expert 2	Use it and talk about it.	Jingdong prefecture level city in Shandong
Jinfeng Hongyuan Flagship Store	Power Saving Expert 2	Second purchase	Jingdong prefecture level city in Shandong
Jinfeng Hongyuan Flagship Store	Power Saving Expert 2	It works.	Jingdong prefecture level city in Shandong
Jinfeng Hongyuan Flagship Store	Power Saving Expert 2	It works.	Jingdong prefecture level city in Shandong
Jinfeng Hongyuan Flagship Store	Power Saving Expert 2	I don't know if it's a power saver until I start using it	Jingdong prefecture level city in Shandong
Jinfeng Hongyuan Flagship Store	Power Saving Expert 2	serviceable	Jingdong prefecture level city in Shandong
Jinfeng Hongyuan Flagship Store	Power Saving Expert 2	This user did not fill out the rating in time	Jingdong prefecture level city in Shandong
Jinfeng Hongyuan Flagship Store	Power Saving Expert 2	There is no power saving as the store says, using it is the same as not using it.	Jingdong prefecture level city in Shandong
Jinfeng Hongyuan Flagship Store	Power Saving Expert 2	This user did not fill out the rating in time	Jingdong prefecture level city in Shandong
Jinfeng Hongyuan Flagship Store	Power Saving Expert 2	Good...good.	Jingdong prefecture level city in Shandong
Jinfeng Hongyuan Flagship Store	Power Saving Expert 2	(of an unmarried couple) be close	Jingdong prefecture level city in Shandong
Jinfeng Hongyuan Flagship Store	Power Saving Expert 2	(of an unmarried couple) be close	Jingdong prefecture level city in Shandong
Jinfeng Hongyuan Flagship Store	Power Saving Expert 2	(of an unmarried couple) be close	Jingdong prefecture level city in Shandong
Jinfeng Hongyuan Flagship Store	Power Saving Expert 2	The goods are real, high value, high quality, worth every penny, material appearance and quality at a glance is very upscale, very like HAPPY💯	Jingdong prefecture level city in Shandong
Jinfeng Hongyuan Flagship Store	Power Saving Expert 2	It is the ideal product, good quality, logistics is also very fast, worthy of praise 🎁 baby quality is very good, received the first time to open the first time to see, it is very good 👍, very satisfied 😃, customer service is also available to ask questions and answer, thank you 🙏	Jingdong prefecture level city in Shandong
Jinfeng Hongyuan Flagship Store	Power Saving Expert 2	I bought it and haven't used it yet, I'll use it again.	Jingdong prefecture level city in Shandong
Jinfeng Hongyuan Flagship Store	Power Saving Expert 2	It didn't work, I used it for a few days and it was the same as before, it didn't work, I should have to return it	Jingdong prefecture level city in Shandong
Jinfeng Hongyuan Flagship Store	Power Saving Expert 2	The goods have been received, but have not yet installed and use, feel good.	Jingdong prefecture level city in Shandong
Jinfeng Hongyuan Flagship Store	Power Saving Expert 2	Goods received, I forgot to take pictures, the goods look good, you can start, immediately start the school to see the effect then	Jingdong prefecture level city in Shandong
Jinfeng Hongyuan Flagship Store	Power Saving Expert 2	Looks good. I'll use it for a while.	Jingdong prefecture level city in Shandong
Jinfeng Hongyuan Flagship Store	Power Saving Expert 2	Great stuff, easy to operate, easy to use, good power saving, believe in the power of science!	Jingdong prefecture level city in Shandong

Jinfeng Hongyuan Flagship Store	Power Saving Expert 2	Especially great, comparison shopping before buying, the store owner is very good, answering questions very patiently and detailed, there is a need to come back!	Jingdong prefecture level city in Shandong
Jinfeng Hongyuan Flagship Store	Power Saving Expert 2	Trustworthy, feel free to use it boldly.	Jingdong prefecture level city in Shandong
Jinfeng Hongyuan Flagship Store	Power Saving Expert 2	Logistics is super fast! Beautiful appearance! Looks like a great quality! I've never bought one before, so I'll try it first to see if it saves power. We'll see how much it saves.	Jingdong prefecture level city in Shandong
Jinfeng Hongyuan Flagship Store	Power Saving Expert 2	I saw online counterfeiting, energy saver is one of them, hahahahaha, jingdong also buy fake products	Jingdong prefecture level city in Shandong
Jinfeng Hongyuan Flagship Store	Power Saving Expert 2	Just received, installed, try a month to see how the effect and then follow up.	Jingdong prefecture level city in Shandong
Jinfeng Hongyuan Flagship Store	Power Saving Expert 2	Good for you! The goods are genuine! Good price for what you get!	Jingdong prefecture level city in Shandong
Jinfeng Hongyuan Flagship Store	Power Saving Expert 2	Things quickly received, immediately used to see if there is no effect, something river photo ↑ the same, customer service is very professional	Jingdong prefecture level city in Shandong
Jinfeng Hongyuan Flagship Store	Power Saving Expert 2	The product is very convenient Looking forward to the subsequent use of the experience results	Jingdong prefecture level city in Shandong
Jinfeng Hongyuan Flagship Store	Power Saving Expert 2	I just received it and plugged it in, we'll see exactly when it's time to pay the electric bill	Jingdong prefecture level city in Shandong
Jinfeng Hongyuan Flagship Store	Power Saving Expert 2	Saver is good Packaging and looks good How effective it is to be shared after using it.	Jingdong prefecture level city in Shandong
Jinfeng Hongyuan Flagship Store	Power Saving Expert 2	There's no way to compare. I don't know if it's energy efficient.	Jingdong prefecture level city in Shandong
Jinfeng Hongyuan Flagship Store	Power Saving Expert 2	Just installed it, try it out and comment again	Jingdong prefecture level city in Shandong
Jinfeng Hongyuan Flagship Store	Power Saving Expert 2	Super save point also good to install, after a friend introduced then went to buy to, friends say it is very good to use, home I bought, try to use a period of time, to be honest, the economizer is not big or heavy, can be very convenient to put in a lot of places, the quality is also very good, power-saving function this time seems to be about 20% of the efficiency. The owner is also very warm contact, very	Jingdong prefecture level city in Shandong
Jinfeng Hongyuan Flagship Store	Power Saving Expert 2	The reviews online are mixed. Try it for a while, but I don't know if I can return it if it doesn't save any power after a couple months.	Jingdong prefecture level city in Shandong
Jinfeng Hongyuan Flagship Store	Power Saving Expert 2	The power consumption at home is high, bought a power saver, it looks very convenient to plug on the line, see if you can save some money next month, if good, recommend to friends down the road.	Jingdong prefecture level city in Shandong
Jinfeng Hongyuan Flagship Store	Power Saving Expert 2	It's beautiful, it's genuine, but I don't know the power-saving effect.	Jingdong prefecture level city in Shandong
Jinfeng Hongyuan Flagship Store	Power Saving Expert 2	This user thinks the product is good	Jingdong prefecture level city in Shandong
Jinfeng Hongyuan Flagship Store	Power Saving Expert 2	Nice exterior design with an at-a-glance display	Jingdong prefecture level city in Shandong
Jinfeng Hongyuan Flagship Store	Power Saving Expert 2	Home large and small things are purchased in the Jingdong Mall, have been accustomed to shopping in the Jingdong, many times can buy good quality and cheap products, and courier service attitude is particularly good, sometimes too many couriers are to help me to the home, so many years have been in the Jingdong Mall shopping, trust and support! Will also support the Jingdong as always!	Jingdong prefecture level city in Shandong
Jinfeng Hongyuan Flagship Store	Power Saving Expert 2	Good workmanship, mostly better than the original wall-plugged ones, and hopefully power factor compensation saves on electricity costs	Jingdong prefecture level city in Shandong
Jinfeng Hongyuan Flagship Store	Power Saving Expert 2	I don't feel anything, I said I won't know the effect for a month or two, let's see.	Jingdong prefecture level city in Shandong
Jinfeng Hongyuan Flagship Store	Power Saving Expert 2	Delivery was pretty fast, a little pricey, just not sure if it works or not	Jingdong prefecture level city in Shandong
Jinfeng Hongyuan Flagship Store	Power Saving Expert 2	The goods have been received, I do not know how effective.	Jingdong prefecture level city in Shandong
Jinfeng Hongyuan Flagship Store	Power Saving Expert 2	So far it doesn't seem to be too noticeable, so I'll comment later.	Jingdong prefecture level city in Shandong
Jinfeng Hongyuan Flagship Store	Power Saving Expert 2	I'll comment after I've used it	Jingdong prefecture level city in Shandong
Jinfeng Hongyuan Flagship Store	Power Saving Expert 2	Not bad. You'll save a lot on your electric bill in a year!	Jingdong prefecture level city in Shandong
Jinfeng Hongyuan Flagship Store	Power Saving Expert 2	I just got it. I haven't tried it yet. I don't know what it's like.	Jingdong prefecture level city in Shandong
Jinfeng Hongyuan Flagship Store	Power Saving Expert 2	Shopping at Jingdong has become a habit, and this disease will not be cured for a while.	Jingdong prefecture level city in Shandong
Jinfeng Hongyuan Flagship Store	Power Saving Expert 2	I've been using it for a while now. I don't know if I'm still saving power or not.	Jingdong prefecture level city in Shandong
Jinfeng Hongyuan Flagship Store	Power Saving Expert 2	Received the saver and it's great! There is also a digital voltage indication. Plugged in as instructed, easy to operate, hope you're our power saver!	Jingdong prefecture level city in Shandong
Jinfeng Hongyuan Flagship Store	Power Saving Expert 2	There is no power saving as the store says, using it is the same as not using it.	Jingdong prefecture level city in Shandong
Jinfeng Hongyuan Flagship Store	Power Saving Expert 2	Use it for a while and then comment again	Jingdong prefecture level city in Shandong
Jinfeng Hongyuan Flagship Store	Power Saving Expert 2	Feeling can, to use 1 to 2 months to see how the effect, there is a voltage display, can see the voltage fluctuations amplitude, quite good!	Jingdong prefecture level city in Shandong
Jinfeng Hongyuan Flagship Store	Power Saving Expert 2	I hope it works and saves me some electricity.	Jingdong prefecture level city in Shandong
Jinfeng Hongyuan Flagship Store	Power Saving Expert 2	Nice baby, happy with the after sales	Jingdong prefecture level city in Shandong
Jinfeng Hongyuan Flagship Store	Power Saving Expert 2	Use it and talk about it.	Jingdong prefecture level city in Shandong
Jinfeng Hongyuan Flagship Store	Power Saving Expert 2	Second purchase	Jingdong prefecture level city in Shandong
Jinfeng Hongyuan Flagship Store	Power Saving Expert 2	It works.	Jingdong prefecture level city in Shandong
Jinfeng Hongyuan Flagship Store	Power Saving Expert 2	It works.	Jingdong prefecture level city in Shandong
Jinfeng Hongyuan Flagship Store	Power Saving Expert 2	I don't know if it's a power saver until I start using it	Jingdong prefecture level city in Shandong
Jinfeng Hongyuan Flagship Store	Power Saving Expert 2	serviceable	Jingdong prefecture level city in Shandong
Jinfeng Hongyuan Flagship Store	Power Saving Expert 2	This user did not fill out the rating in time	Jingdong prefecture level city in Shandong
Jinfeng Hongyuan Flagship Store	Power Saving Expert 2	There is no power saving as the store says, using it is the same as not using it.	Jingdong prefecture level city in Shandong
Jinfeng Hongyuan Flagship Store	Power Saving Expert 2	This user did not fill out the rating in time	Jingdong prefecture level city in Shandong
Jinfeng Hongyuan Flagship Store	Power Saving Expert 2	Good...good.	Jingdong prefecture level city in Shandong
Jinfeng Hongyuan Flagship Store	Power Saving Expert 2	(of an unmarried couple) be close	Jingdong prefecture level city in Shandong
Jinfeng Hongyuan Flagship Store	Power Saving Expert 2	(of an unmarried couple) be close	Jingdong prefecture level city in Shandong
Jinfeng Hongyuan Flagship Store	Power Saving Expert 2	(of an unmarried couple) be close	Jingdong prefecture level city in Shandong
Jinfeng Hongyuan Flagship Store	Power Saving Expert 2	The goods are real, high value, high quality, worth every penny, material appearance and quality at a glance is very upscale, very like HAPPY1 00	Jingdong prefecture level city in Shandong

Jinfeng Hongyuan Flagship Store	Power Saving Expert 2	It is the ideal product, good quality, logistics is also very fast, worthy of praise 🎁 baby quality is very good, received the first time to open the first time to see, it is very good 👍, very satisfied ☺, customer service is also available to ask questions and answer, thank you 🙏	Jingdong prefecture level city in Shandong
Jinfeng Hongyuan Flagship Store	Power Saving Expert 2	I bought it and haven't used it yet, I'll use it again.	Jingdong prefecture level city in Shandong
Jinfeng Hongyuan Flagship Store	Power Saving Expert 2	It didn't work, I used it for a few days and it was the same as before, it didn't work, I should have to return it	Jingdong prefecture level city in Shandong
Jinfeng Hongyuan Flagship Store	Power Saving Expert 2	The goods have been received, but have not yet installed and use, feel good.	Jingdong prefecture level city in Shandong
Jinfeng Hongyuan Flagship Store	Power Saving Expert 2	Goods received, I forgot to take pictures, the goods look good, you can start, immediately start the school to see the effect then	Jingdong prefecture level city in Shandong
Jinfeng Hongyuan Flagship Store	Power Saving Expert 2	Looks good. I'll use it for a while.	Jingdong prefecture level city in Shandong
Jinfeng Hongyuan Flagship Store	Power Saving Expert 2	Great stuff, easy to operate, easy to use, good power saving, believe in the power of science!	Jingdong prefecture level city in Shandong
Jinfeng Hongyuan Flagship Store	Power Saving Expert 2	Especially great, comparison shopping before buying, the store owner is very good, answering questions very patiently and detailed, there is a need to come back!	Jingdong prefecture level city in Shandong
Jinfeng Hongyuan Flagship Store	Power Saving Expert 2	Trustworthy, feel free to use it boldly.	Jingdong prefecture level city in Shandong
Jinfeng Hongyuan Flagship Store	Power Saving Expert 2	Logistics is super fast! Beautiful appearance! Looks like a great quality! I've never bought one before, so I'll try it first to see if it saves power. We'll see how much it saves.	Jingdong prefecture level city in Shandong
Jinfeng Hongyuan Flagship Store	Power Saving Expert 2	I saw online counterfeiting, energy saver is one of them, hahahahaha, jingdong also buy fake products	Jingdong prefecture level city in Shandong
Jinfeng Hongyuan Flagship Store	Power Saving Expert 2	Just received, installed, try a month to see how the effect and then follow up.	Jingdong prefecture level city in Shandong
Jinfeng Hongyuan Flagship Store	Power Saving Expert 2	Good for you! The goods are genuine! Good price for what you get!	Jingdong prefecture level city in Shandong
Jinfeng Hongyuan Flagship Store	Power Saving Expert 2	Things quickly received, immediately used to see if there is no effect, something river photo ⬆ the same, customer service is very professional	Jingdong prefecture level city in Shandong
Jinfeng Hongyuan Flagship Store	Power Saving Expert 2	The product is very convenient Looking forward to the subsequent use of the experience results	Jingdong prefecture level city in Shandong
Jinfeng Hongyuan Flagship Store	Power Saving Expert 2	I just received it and plugged it in, we'll see exactly when it's time to pay the electric bill	Jingdong prefecture level city in Shandong
Jinfeng Hongyuan Flagship Store	Power Saving Expert 2	Saver is good Packaging and looks good How effective it is to be shared after using it.	Jingdong prefecture level city in Shandong
Jinfeng Hongyuan Flagship Store	Power Saving Expert 2	There's no way to compare. I don't know if it's energy efficient.	Jingdong prefecture level city in Shandong
Jinfeng Hongyuan Flagship Store	Power Saving Expert 2	Just installed it, try it out and comment again	Jingdong prefecture level city in Shandong
Jinfeng Hongyuan Flagship Store	Power Saving Expert 2	Super save point also good to install, after a friend introduced then went to buy to, friends say it is very good to use, home I bought, try to use a period of time, to be honest, the economizer is not big or heavy, can be very convenient to put in a lot of places, the quality is also very good, power-saving function this time seems to be about 20% of the efficiency. The owner is also very warm contact, very	Jingdong prefecture level city in Shandong
Jinfeng Hongyuan Flagship Store	Power Saving Expert 2	The reviews online are mixed. Try it for a while, but I don't know if I can return it if it doesn't save any power after a couple months.	Jingdong prefecture level city in Shandong
Jinfeng Hongyuan Flagship Store	Power Saving Expert 2	The power consumption at home is high, bought a power saver, it looks very convenient to plug on the line, see if you can save some money next month, if good, recommend to friends down the road.	Jingdong prefecture level city in Shandong
Jinfeng Hongyuan Flagship Store	Power Saving Expert 2	It's beautiful, it's genuine, but I don't know the power-saving effect.	Jingdong prefecture level city in Shandong
Jinfeng Hongyuan Flagship Store	Power Saving Expert 2	This user thinks the product is good	Jingdong prefecture level city in Shandong
Jinfeng Hongyuan Flagship Store	Power Saving Expert 2	Nice exterior design with an at-a-glance display	Jingdong prefecture level city in Shandong
Jinfeng Hongyuan Flagship Store	Power Saving Expert 2	Home large and small things are purchased in the Jingdong Mall, have been accustomed to shopping in the Jingdong, many times can buy good quality and cheap products, and courier service attitude is particularly good, sometimes too many couriers are to help me to the home, so many years have been in the Jingdong Mall shopping, trust and support! Will also support the Jingdong as always!	Jingdong prefecture level city in Shandong
Jinfeng Hongyuan Flagship Store	Power Saving Expert 2	Good workmanship, mostly better than the original wall-plugged ones, and hopefully power factor compensation saves on electricity costs	Jingdong prefecture level city in Shandong
Jinfeng Hongyuan Flagship Store	Power Saving Expert 2	I don't feel anything, I said I won't know the effect for a month or two, let's see.	Jingdong prefecture level city in Shandong
Jinfeng Hongyuan Flagship Store	Power Saving Expert 2	Delivery was pretty fast, a little pricey, just not sure if it works or not	Jingdong prefecture level city in Shandong
Jinfeng Hongyuan Flagship Store	Power Saving Expert 2	The goods have been received, I do not know how effective.	Jingdong prefecture level city in Shandong
Jinfeng Hongyuan Flagship Store	Power Saving Expert 2	So far it doesn't seem to be too noticeable, so I'll comment later.	Jingdong prefecture level city in Shandong
Jinfeng Hongyuan Flagship Store	Power Saving Expert 2	I'll comment after I've used it	Jingdong prefecture level city in Shandong
Jinfeng Hongyuan Flagship Store	Power Saving Expert 2	Not bad. You'll save a lot on your electric bill in a year!	Jingdong prefecture level city in Shandong
Jinfeng Hongyuan Flagship Store	Power Saving Expert 2	I just got it. I haven't tried it yet. I don't know what it's like.	Jingdong prefecture level city in Shandong
Jinfeng Hongyuan Flagship Store	Power Saving Expert 2	Shopping at Jingdong has become a habit, and this disease will not be cured for a while.	Jingdong prefecture level city in Shandong
Jinfeng Hongyuan Flagship Store	Power Saving Expert 2	I've been using it for a while now. I don't know if I'm still saving power or not.	Jingdong prefecture level city in Shandong
Jinfeng Hongyuan Flagship Store	Power Saving Expert 2	Received the saver and it's great! There is also a digital voltage indication. Plugged in as instructed, easy to operate, hope you're our power saver!	Jingdong prefecture level city in Shandong
Jinfeng Hongyuan Flagship Store	Power Saving Expert 2	There is no power saving as the store says, using it is the same as not using it.	Jingdong prefecture level city in Shandong
Jinfeng Hongyuan Flagship Store	Power Saving Expert 2	Use it for a while and then comment again	Jingdong prefecture level city in Shandong
Jinfeng Hongyuan Flagship Store	Power Saving Expert 2	Feeling can, to use 1 to 2 months to see how the effect, there is a voltage display, can see the voltage fluctuations amplitude, quite good!	Jingdong prefecture level city in Shandong
Jinfeng Hongyuan Flagship Store	Power Saving Expert 2	I hope it works and saves me some electricity.	Jingdong prefecture level city in Shandong
Jinfeng Hongyuan Flagship Store	Power Saving Expert 2	Nice baby, happy with the after sales	Jingdong prefecture level city in Shandong
Jinfeng Hongyuan Flagship Store	Power Saving Expert 2	Use it and talk about it.	Jingdong prefecture level city in Shandong
Jinfeng Hongyuan Flagship Store	Power Saving Expert 2	Second purchase	Jingdong prefecture level city in Shandong
Jinfeng Hongyuan Flagship Store	Power Saving Expert 2	It works.	Jingdong prefecture level city in Shandong
Jinfeng Hongyuan Flagship Store	Power Saving Expert 2	It works.	Jingdong prefecture level city in Shandong
Jinfeng Hongyuan Flagship Store	Power Saving Expert 2	I don't know if it's a power saver until I start using it	Jingdong prefecture level city in Shandong
Jinfeng Hongyuan Flagship Store	Power Saving Expert 2	serviceable	Jingdong prefecture level city in Shandong
Jinfeng Hongyuan Flagship Store	Power Saving Expert 2	This user did not fill out the rating in time	Jingdong prefecture level city in Shandong

Jinfeng Hongyuan Flagship Store	Power Saving Expert 2	There is no power saving as the store says, using it is the same as not using it.	Jingdong prefecture level city in Shandong
Jinfeng Hongyuan Flagship Store	Power Saving Expert 2	This user did not fill out the rating in time	Jingdong prefecture level city in Shandong
Jinfeng Hongyuan Flagship Store	Power Saving Expert 2	Good...good.	Jingdong prefecture level city in Shandong
Jinfeng Hongyuan Flagship Store	Power Saving Expert 2	(of an unmarried couple) be close	Jingdong prefecture level city in Shandong
Jinfeng Hongyuan Flagship Store	Power Saving Expert 2	(of an unmarried couple) be close	Jingdong prefecture level city in Shandong
Jinfeng Hongyuan Flagship Store	Power Saving Expert 2	(of an unmarried couple) be close	Jingdong prefecture level city in Shandong
Jinfeng Hongyuan Flagship Store	Power Saving Expert 2	The goods are real, high value, high quality, worth every penny, material appearance and quality at a glance is very upscale, very like HAPPY1 00	Jingdong prefecture level city in Shandong
Jinfeng Hongyuan Flagship Store	Power Saving Expert 2	It is the ideal product, good quality, logistics is also very fast, worthy of praise 🎁 baby quality is very good, received the first time to open the first time to see, it is very good 👍, very satisfied 😃, customer service is also available to ask questions and answer, thank you 🙏	Jingdong prefecture level city in Shandong
Jinfeng Hongyuan Flagship Store	Power Saving Expert 2	I bought it and haven't used it yet, I'll use it again.	Jingdong prefecture level city in Shandong
Jinfeng Hongyuan Flagship Store	Power Saving Expert 2	It didn't work, I used it for a few days and it was the same as before, it didn't work, I should have to return it	Jingdong prefecture level city in Shandong
Jinfeng Hongyuan Flagship Store	Power Saving Expert 2	The goods have been received, but have not yet installed and use, feel good.	Jingdong prefecture level city in Shandong
Jinfeng Hongyuan Flagship Store	Power Saving Expert 2	Goods received, I forgot to take pictures, the goods look good, you can start, immediately start the school to see the effect then	Jingdong prefecture level city in Shandong
Jinfeng Hongyuan Flagship Store	Power Saving Expert 2	Looks good. I'll use it for a while.	Jingdong prefecture level city in Shandong
Jinfeng Hongyuan Flagship Store	Power Saving Expert 2	Great stuff, easy to operate, easy to use, good power saving, believe in the power of science!	Jingdong prefecture level city in Shandong
Jinfeng Hongyuan Flagship Store	Power Saving Expert 2	Especially great, comparison shopping before buying, the store owner is very good, answering questions very patiently and detailed, there is a need to come back!	Jingdong prefecture level city in Shandong
Jinfeng Hongyuan Flagship Store	Power Saving Expert 2	Trustworthy, feel free to use it boldly.	Jingdong prefecture level city in Shandong
Jinfeng Hongyuan Flagship Store	Power Saving Expert 2	Logistics is super fast! Beautiful appearance! Looks like a great quality! I've never bought one before, so I'll try it first to see if it saves power. We'll see how much it saves.	Jingdong prefecture level city in Shandong
Jinfeng Hongyuan Flagship Store	Power Saving Expert 2	I saw online counterfeiting, energy saver is one of them, hahahahaha, jingdong also buy fake products	Jingdong prefecture level city in Shandong
Jinfeng Hongyuan Flagship Store	Power Saving Expert 2	Just received, installed, try a month to see how the effect and then follow up.	Jingdong prefecture level city in Shandong
Jinfeng Hongyuan Flagship Store	Power Saving Expert 2	Good for you! The goods are genuine! Good price for what you get!	Jingdong prefecture level city in Shandong
Jinfeng Hongyuan Flagship Store	Power Saving Expert 2	Things quickly received, immediately used to see if there is no effect, something river photo ⬆ the same, customer service is very professional	Jingdong prefecture level city in Shandong
Jinfeng Hongyuan Flagship Store	Power Saving Expert 2	The product is very convenient Looking forward to the subsequent use of the experience results	Jingdong prefecture level city in Shandong
Jinfeng Hongyuan Flagship Store	Power Saving Expert 2	I just received it and plugged it in, we'll see exactly when it's time to pay the electric bill	Jingdong prefecture level city in Shandong
Jinfeng Hongyuan Flagship Store	Power Saving Expert 2	Saver is good Packaging and looks good How effective it is to be shared after using it.	Jingdong prefecture level city in Shandong
Jinfeng Hongyuan Flagship Store	Power Saving Expert 2	There's no way to compare. I don't know if it's energy efficient.	Jingdong prefecture level city in Shandong
Jinfeng Hongyuan Flagship Store	Power Saving Expert 2	Just installed it, try it out and comment again	Jingdong prefecture level city in Shandong
Jinfeng Hongyuan Flagship Store	Power Saving Expert 2	Super save point also good to install, after a friend introduced then went to buy to, friends say it is very good to use, home I bought, try to use a period of time, to be honest, the economizer is not big or heavy, can be very convenient to put in a lot of places, the quality is also very good, power-saving function this time seems to be about 20% of the efficiency. The owner is also very warm contact, very	Jingdong prefecture level city in Shandong
Jinfeng Hongyuan Flagship Store	Power Saving Expert 2	The reviews online are mixed. Try it for a while, but I don't know if I can return it if it doesn't save any power after a couple months.	Jingdong prefecture level city in Shandong
Jinfeng Hongyuan Flagship Store	Power Saving Expert 2	The power consumption at home is high, bought a power saver, it looks very convenient to plug on the line, see if you can save some money next month, if good, recommend to friends down the road.	Jingdong prefecture level city in Shandong
Jinfeng Hongyuan Flagship Store	Power Saving Expert 2	It's beautiful, it's genuine, but I don't know the power-saving effect.	Jingdong prefecture level city in Shandong
Jinfeng Hongyuan Flagship Store	Power Saving Expert 2	This user thinks the product is good	Jingdong prefecture level city in Shandong
Jinfeng Hongyuan Flagship Store	Power Saving Expert 2	Nice exterior design with an at-a-glance display	Jingdong prefecture level city in Shandong
Jinfeng Hongyuan Flagship Store	Power Saving Expert 2	Home large and small things are purchased in the Jingdong Mall, have been accustomed to shopping in the Jingdong, many times can buy good quality and cheap products, and courier service attitude is particularly good, sometimes too many couriers are to help me to the home, so many years have been in the Jingdong Mall shopping, trust and support! Will also support the Jingdong as always!	Jingdong prefecture level city in Shandong
Jinfeng Hongyuan Flagship Store	Power Saving Expert 2	Good workmanship, mostly better than the original wall-plugged ones, and hopefully power factor compensation saves on electricity costs	Jingdong prefecture level city in Shandong
Jinfeng Hongyuan Flagship Store	Power Saving Expert 2	I don't feel anything, I said I won't know the effect for a month or two, let's see.	Jingdong prefecture level city in Shandong
Jinfeng Hongyuan Flagship Store	Power Saving Expert 2	Delivery was pretty fast, a little pricey, just not sure if it works or not	Jingdong prefecture level city in Shandong
Jinfeng Hongyuan Flagship Store	Power Saving Expert 2	The goods have been received, I do not know how effective.	Jingdong prefecture level city in Shandong
Jinfeng Hongyuan Flagship Store	Power Saving Expert 2	So far it doesn't seem to be too noticeable, so I'll comment later.	Jingdong prefecture level city in Shandong
Jinfeng Hongyuan Flagship Store	Power Saving Expert 2	I'll comment after I've used it	Jingdong prefecture level city in Shandong
Jinfeng Hongyuan Flagship Store	Power Saving Expert 2	Not bad. You'll save a lot on your electric bill in a year!	Jingdong prefecture level city in Shandong
Jinfeng Hongyuan Flagship Store	Power Saving Expert 2	I just got it. I haven't tried it yet. I don't know what it's like.	Jingdong prefecture level city in Shandong
Jinfeng Hongyuan Flagship Store	Power Saving Expert 2	Shopping at Jingdong has become a habit, and this disease will not be cured for a while.	Jingdong prefecture level city in Shandong
Jinfeng Hongyuan Flagship Store	Power Saving Expert 2	I've been using it for a while now. I don't know if I'm still saving power or not.	Jingdong prefecture level city in Shandong
Jinfeng Hongyuan Flagship Store	Power Saving Expert 2	Received the saver and it's great! There is also a digital voltage indication. Plugged in as instructed, easy to operate, hope you're our power saver!	Jingdong prefecture level city in Shandong
Jinfeng Hongyuan Flagship Store	Power Saving Expert 2	There is no power saving as the store says, using it is the same as not using it.	Jingdong prefecture level city in Shandong
Jinfeng Hongyuan Flagship Store	Power Saving Expert 2	Use it for a while and then comment again	Jingdong prefecture level city in Shandong
Jinfeng Hongyuan Flagship Store	Power Saving Expert 2	Feeling can, to use 1 to 2 months to see how the effect, there is a voltage display, can see the voltage fluctuations amplitude, quite good!	Jingdong prefecture level city in Shandong
Jinfeng Hongyuan Flagship Store	Power Saving Expert 2	I hope it works and saves me some electricity.	Jingdong prefecture level city in Shandong
Jinfeng Hongyuan Flagship Store	Power Saving Expert 2	Nice baby, happy with the after sales	Jingdong prefecture level city in Shandong

Jinfeng Hongyuan Flagship Store	Power Saving Expert 2	Use it and talk about it.	Jingdong prefecture level city in Shandong
Jinfeng Hongyuan Flagship Store	Power Saving Expert 2	Second purchase	Jingdong prefecture level city in Shandong
Jinfeng Hongyuan Flagship Store	Power Saving Expert 2	It works.	Jingdong prefecture level city in Shandong
Jinfeng Hongyuan Flagship Store	Power Saving Expert 2	It works.	Jingdong prefecture level city in Shandong
Jinfeng Hongyuan Flagship Store	Power Saving Expert 2	I don't know if it's a power saver until I start using it	Jingdong prefecture level city in Shandong
Jinfeng Hongyuan Flagship Store	Power Saving Expert 2	serviceable	Jingdong prefecture level city in Shandong
Jinfeng Hongyuan Flagship Store	Power Saving Expert 2	This user did not fill out the rating in time	Jingdong prefecture level city in Shandong
Jinfeng Hongyuan Flagship Store	Power Saving Expert 2	There is no power saving as the store says, using it is the same as not using it.	Jingdong prefecture level city in Shandong
Jinfeng Hongyuan Flagship Store	Power Saving Expert 2	This user did not fill out the rating in time	Jingdong prefecture level city in Shandong
Jinfeng Hongyuan Flagship Store	Power Saving Expert 2	Good...good.	Jingdong prefecture level city in Shandong
Jinfeng Hongyuan Flagship Store	Power Saving Expert 2	(of an unmarried couple) be close	Jingdong prefecture level city in Shandong
Jinfeng Hongyuan Flagship Store	Power Saving Expert 2	(of an unmarried couple) be close	Jingdong prefecture level city in Shandong
Jinfeng Hongyuan Flagship Store	Power Saving Expert 2	(of an unmarried couple) be close	Jingdong prefecture level city in Shandong
Jinfeng Hongyuan Flagship Store	Power Saving Expert 2	The goods are real, high value, high quality, worth every penny, material appearance and quality at a glance is very upscale, very like HAPPY100	Jingdong prefecture level city in Shandong
Jinfeng Hongyuan Flagship Store	Power Saving Expert 2	It is the ideal product, good quality, logistics is also very fast, worthy of praise 🎁 baby quality is very good, received the first time to open the first time to see, it is very good 👍, very satisfied 😊, customer service is also available to ask questions and answer, thank you 🙏	Jingdong prefecture level city in Shandong
Jinfeng Hongyuan Flagship Store	Power Saving Expert 2	I bought it and haven't used it yet, I'll use it again.	Jingdong prefecture level city in Shandong
Jinfeng Hongyuan Flagship Store	Power Saving Expert 2	It didn't work, I used it for a few days and it was the same as before, it didn't work, I should have to return it	Jingdong prefecture level city in Shandong
Jinfeng Hongyuan Flagship Store	Power Saving Expert 2	The goods have been received, but have not yet installed and use, feel good.	Jingdong prefecture level city in Shandong
Jinfeng Hongyuan Flagship Store	Power Saving Expert 2	Goods received, I forgot to take pictures, the goods look good, you can start, immediately start the school to see the effect then	Jingdong prefecture level city in Shandong
Jinfeng Hongyuan Flagship Store	Power Saving Expert 2	Looks good. I'll use it for a while.	Jingdong prefecture level city in Shandong
Jinfeng Hongyuan Flagship Store	Power Saving Expert 2	Great stuff, easy to operate, easy to use, good power saving, believe in the power of science!	Jingdong prefecture level city in Shandong
Jinfeng Hongyuan Flagship Store	Power Saving Expert 2	Especially great, comparison shopping before buying, the store owner is very good, answering questions very patiently and detailed, there is a need to come back!	Jingdong prefecture level city in Shandong
Jinfeng Hongyuan Flagship Store	Power Saving Expert 2	Trustworthy, feel free to use it boldly.	Jingdong prefecture level city in Shandong
Jinfeng Hongyuan Flagship Store	Power Saving Expert 2	Logistics is super fast! Beautiful appearance! Looks like a great quality! I've never bought one before, so I'll try it first to see if it saves power. We'll see how much it saves.	Jingdong prefecture level city in Shandong
Jinfeng Hongyuan Flagship Store	Power Saving Expert 2	I saw online counterfeiting, energy saver is one of them, hahahahaha, jingdong also buy fake products	Jingdong prefecture level city in Shandong
Jinfeng Hongyuan Flagship Store	Power Saving Expert 2	Just received, installed, try a month to see how the effect and then follow up.	Jingdong prefecture level city in Shandong
Jinfeng Hongyuan Flagship Store	Power Saving Expert 2	Good for you! The goods are genuine! Good price for what you get!	Jingdong prefecture level city in Shandong
Jinfeng Hongyuan Flagship Store	Power Saving Expert 2	Things quickly received, immediately used to see if there is no effect, something river photo ⬆ the same, customer service is very professional	Jingdong prefecture level city in Shandong
Jinfeng Hongyuan Flagship Store	Power Saving Expert 2	The product is very convenient Looking forward to the subsequent use of the experience results	Jingdong prefecture level city in Shandong
Jinfeng Hongyuan Flagship Store	Power Saving Expert 2	I just received it and plugged it in, we'll see exactly when it's time to pay the electric bill	Jingdong prefecture level city in Shandong
Jinfeng Hongyuan Flagship Store	Power Saving Expert 2	Saver is good Packaging and looks good How effective it is to be shared after using it.	Jingdong prefecture level city in Shandong
Jinfeng Hongyuan Flagship Store	Power Saving Expert 2	There's no way to compare. I don't know if it's energy efficient.	Jingdong prefecture level city in Shandong
Jinfeng Hongyuan Flagship Store	Power Saving Expert 2	Just installed it, try it out and comment again	Jingdong prefecture level city in Shandong
Jinfeng Hongyuan Flagship Store	Power Saving Expert 2	Super save point also good to install, after a friend introduced then went to buy to, friends say it is very good to use, home I bought, try to use a period of time, to be honest, the economizer is not big or heavy, can be very convenient to put in a lot of places, the quality is also very good, power-saving function this time seems to be about 20% of the efficiency. The owner is also very warm contact, very	Jingdong prefecture level city in Shandong
Jinfeng Hongyuan Flagship Store	Power Saving Expert 2	The reviews online are mixed. Try it for a while, but I don't know if I can return it if it doesn't save any power after a couple months.	Jingdong prefecture level city in Shandong
Jinfeng Hongyuan Flagship Store	Power Saving Expert 2	The power consumption at home is high, bought a power saver, it looks very convenient to plug on the line, see if you can save some money next month, if good, recommend to friends down the road.	Jingdong prefecture level city in Shandong
Jinfeng Hongyuan Flagship Store	Power Saving Expert 2	It's beautiful, it's genuine, but I don't know the power-saving effect.	Jingdong prefecture level city in Shandong
Jinfeng Hongyuan Flagship Store	Power Saving Expert 2	This user thinks the product is good	Jingdong prefecture level city in Shandong
Jinfeng Hongyuan Flagship Store	Power Saving Expert 2	Nice exterior design with an at-a-glance display	Jingdong prefecture level city in Shandong
Jinfeng Hongyuan Flagship Store	Power Saving Expert 2	Home large and small things are purchased in the Jingdong Mall, have been accustomed to shopping in the Jingdong, many times can buy good quality and cheap products, and courier service attitude is particularly good, sometimes too many couriers are to help me to the home, so many years have been in the Jingdong Mall shopping, trust and support! Will also support the Jingdong as always!	Jingdong prefecture level city in Shandong
Jinfeng Hongyuan Flagship Store	Power Saving Expert 2	Good workmanship, mostly better than the original wall-plugged ones, and hopefully power factor compensation saves on electricity costs	Jingdong prefecture level city in Shandong
Jinfeng Hongyuan Flagship Store	Power Saving Expert 2	I don't feel anything, I said I won't know the effect for a month or two, let's see.	Jingdong prefecture level city in Shandong
Jinfeng Hongyuan Flagship Store	Power Saving Expert 2	Delivery was pretty fast, a little pricey, just not sure if it works or not	Jingdong prefecture level city in Shandong
Jinfeng Hongyuan Flagship Store	Power Saving Expert 2	The goods have been received, I do not know how effective.	Jingdong prefecture level city in Shandong
Jinfeng Hongyuan Flagship Store	Power Saving Expert 2	So far it doesn't seem to be too noticeable, so I'll comment later.	Jingdong prefecture level city in Shandong
Jinfeng Hongyuan Flagship Store	Power Saving Expert 2	I'll comment after I've used it	Jingdong prefecture level city in Shandong
Jinfeng Hongyuan Flagship Store	Power Saving Expert 2	Not bad. You'll save a lot on your electric bill in a year!	Jingdong prefecture level city in Shandong
Jinfeng Hongyuan Flagship Store	Power Saving Expert 2	I just got it. I haven't tried it yet. I don't know what it's like.	Jingdong prefecture level city in Shandong
Jinfeng Hongyuan Flagship Store	Power Saving Expert 2	Shopping at Jingdong has become a habit, and this disease will not be cured for a while.	Jingdong prefecture level city in Shandong

Jinfeng Hongyuan Flagship Store	Power Saving Expert 2	I've been using it for a while now. I don't know if I'm still saving power or not.	Jingdong prefecture level city in Shandong
Jinfeng Hongyuan Flagship Store	Power Saving Expert 2	Received the saver and it's great! There is also a digital voltage indication. Plugged in as instructed, easy to operate, hope you're our power saver!	Jingdong prefecture level city in Shandong
Jinfeng Hongyuan Flagship Store	Power Saving Expert 2	There is no power saving as the store says, using it is the same as not using it.	Jingdong prefecture level city in Shandong
Jinfeng Hongyuan Flagship Store	Power Saving Expert 2	Use it for a while and then comment again	Jingdong prefecture level city in Shandong
Jinfeng Hongyuan Flagship Store	Power Saving Expert 2	Feeling can, to use 1 to 2 months to see how the effect, there is a voltage display, can see the voltage fluctuations amplitude, quite good!	Jingdong prefecture level city in Shandong
Jinfeng Hongyuan Flagship Store	Power Saving Expert 2	I hope it works and saves me some electricity.	Jingdong prefecture level city in Shandong
Jinfeng Hongyuan Flagship Store	Power Saving Expert 2	Nice baby, happy with the after sales	Jingdong prefecture level city in Shandong
Jinfeng Hongyuan Flagship Store	Power Saving Expert 2	Use it and talk about it.	Jingdong prefecture level city in Shandong
Jinfeng Hongyuan Flagship Store	Power Saving Expert 2	Second purchase	Jingdong prefecture level city in Shandong
Jinfeng Hongyuan Flagship Store	Power Saving Expert 2	It works.	Jingdong prefecture level city in Shandong
Jinfeng Hongyuan Flagship Store	Power Saving Expert 2	It works.	Jingdong prefecture level city in Shandong
Jinfeng Hongyuan Flagship Store	Power Saving Expert 2	I don't know if it's a power saver until I start using it	Jingdong prefecture level city in Shandong
Jinfeng Hongyuan Flagship Store	Power Saving Expert 2	serviceable	Jingdong prefecture level city in Shandong
Jinfeng Hongyuan Flagship Store	Power Saving Expert 2	This user did not fill out the rating in time	Jingdong prefecture level city in Shandong
Jinfeng Hongyuan Flagship Store	Power Saving Expert 2	There is no power saving as the store says, using it is the same as not using it.	Jingdong prefecture level city in Shandong
Jinfeng Hongyuan Flagship Store	Power Saving Expert 2	This user did not fill out the rating in time	Jingdong prefecture level city in Shandong
Jinfeng Hongyuan Flagship Store	Power Saving Expert 2	Good...good.	Jingdong prefecture level city in Shandong
Jinfeng Hongyuan Flagship Store	Power Saving Expert 2	(of an unmarried couple) be close	Jingdong prefecture level city in Shandong
Jinfeng Hongyuan Flagship Store	Power Saving Expert 2	(of an unmarried couple) be close	Jingdong prefecture level city in Shandong
Jinfeng Hongyuan Flagship Store	Power Saving Expert 2	(of an unmarried couple) be close	Jingdong prefecture level city in Shandong
Jinfeng Hongyuan Flagship Store	Power Saving Expert 2	The goods are real, high value, high quality, worth every penny, material appearance and quality at a glance is very upscale, very like HAPPY1 00	Jingdong prefecture level city in Shandong
Jinfeng Hongyuan Flagship Store	Power Saving Expert 2	It is the ideal product, good quality, logistics is also very fast, worthy of praise 🎁 baby quality is very good, received the first time to open the first time to see, it is very good 👍, very satisfied 😃, customer service is also available to ask questions and answer, thank you 🙏	Jingdong prefecture level city in Shandong
Jinfeng Hongyuan Flagship Store	Power Saving Expert 2	I bought it and haven't used it yet, I'll use it again.	Jingdong prefecture level city in Shandong
Jinfeng Hongyuan Flagship Store	Power Saving Expert 2	It didn't work, I used it for a few days and it was the same as before, it didn't work, I should have to return it	Jingdong prefecture level city in Shandong
Jinfeng Hongyuan Flagship Store	Power Saving Expert 2	The goods have been received, but have not yet installed and use, feel good.	Jingdong prefecture level city in Shandong
Jinfeng Hongyuan Flagship Store	Power Saving Expert 2	Goods received, I forgot to take pictures, the goods look good, you can start, immediately start the school to see the effect then	Jingdong prefecture level city in Shandong
Jinfeng Hongyuan Flagship Store	Power Saving Expert 2	Looks good. I'll use it for a while.	Jingdong prefecture level city in Shandong
Jinfeng Hongyuan Flagship Store	Power Saving Expert 2	Great stuff, easy to operate, easy to use, good power saving, believe in the power of science!	Jingdong prefecture level city in Shandong
Jinfeng Hongyuan Flagship Store	Power Saving Expert 2	Especially great, comparison shopping before buying, the store owner is very good, answering questions very patiently and detailed, there is a need to come back!	Jingdong prefecture level city in Shandong
Jinfeng Hongyuan Flagship Store	Power Saving Expert 2	Trustworthy, feel free to use it boldly.	Jingdong prefecture level city in Shandong
Jinfeng Hongyuan Flagship Store	Power Saving Expert 2	Logistics is super fast! Beautiful appearance! Looks like a great quality! I've never bought one before, so I'll try it first to see if it saves power. We'll see how much it saves.	Jingdong prefecture level city in Shandong
Jinfeng Hongyuan Flagship Store	Power Saving Expert 2	I saw online counterfeiting, energy saver is one of them, hahahahaha, jingdong also buy fake products	Jingdong prefecture level city in Shandong
Jinfeng Hongyuan Flagship Store	Power Saving Expert 2	Just received, installed, try a month to see how the effect and then follow up.	Jingdong prefecture level city in Shandong
Jinfeng Hongyuan Flagship Store	Power Saving Expert 2	Good for you! The goods are genuine! Good price for what you get!	Jingdong prefecture level city in Shandong
Jinfeng Hongyuan Flagship Store	Power Saving Expert 2	Things quickly received, immediately used to see if there is no effect, something river photo ⬆ the same, customer service is very professional	Jingdong prefecture level city in Shandong
Jinfeng Hongyuan Flagship Store	Power Saving Expert 2	The product is very convenient Looking forward to the subsequent use of the experience results	Jingdong prefecture level city in Shandong
Jinfeng Hongyuan Flagship Store	Power Saving Expert 2	I just received it and plugged it in, we'll see exactly when it's time to pay the electric bill	Jingdong prefecture level city in Shandong
Jinfeng Hongyuan Flagship Store	Power Saving Expert 2	Saver is good Packaging and looks good How effective it is is to be shared after using it.	Jingdong prefecture level city in Shandong
Jinfeng Hongyuan Flagship Store	Power Saving Expert 2	There's no way to compare. I don't know if it's energy efficient.	Jingdong prefecture level city in Shandong
Jinfeng Hongyuan Flagship Store	Power Saving Expert 2	Just installed it, try it out and comment again	Jingdong prefecture level city in Shandong
Jinfeng Hongyuan Flagship Store	Power Saving Expert 2	Super save point also good to install, after a friend introduced then went to buy to, friends say it is very good to use, home I bought, try to use a period of time, to be honest, the economizer is not big or heavy, can be very convenient to put in a lot of places, the quality is also very good, power-saving function this time seems to be about 20% of the efficiency. The owner is also very warm contact, very	Jingdong prefecture level city in Shandong
Jinfeng Hongyuan Flagship Store	Power Saving Expert 2	The reviews online are mixed. Try it for a while, but I don't know if I can return it if it doesn't save any power after a couple months.	Jingdong prefecture level city in Shandong
Jinfeng Hongyuan Flagship Store	Power Saving Expert 2	The power consumption at home is high, bought a power saver, it looks very convenient to plug on the line, see if you can save some money next month, if good, recommend to friends down the road.	Jingdong prefecture level city in Shandong
Jinfeng Hongyuan Flagship Store	Power Saving Expert 2	It's beautiful, it's genuine, but I don't know the power-saving effect.	Jingdong prefecture level city in Shandong
Jinfeng Hongyuan Flagship Store	Power Saving Expert 2	This user thinks the product is good	Jingdong prefecture level city in Shandong
Jinfeng Hongyuan Flagship Store	Power Saving Expert 2	Nice exterior design with an at-a-glance display	Jingdong prefecture level city in Shandong
Jinfeng Hongyuan Flagship Store	Power Saving Expert 2	Home large and small things are purchased in the Jingdong Mall, have been accustomed to shopping in the Jingdong, many times can buy good quality and cheap products, and courier service attitude is particularly good, sometimes too many couriers are to help me to the home, so many years have been in the Jingdong Mall shopping, trust and support! Will also support the Jingdong as always!	Jingdong prefecture level city in Shandong
Jinfeng Hongyuan Flagship Store	Power Saving Expert 2	Good workmanship, mostly better than the original wall-plugged ones, and hopefully power factor compensation saves on electricity costs	Jingdong prefecture level city in Shandong
Jinfeng Hongyuan Flagship Store	Power Saving Expert 2	I don't feel anything, I said I won't know the effect for a month or two, let's see.	Jingdong prefecture level city in Shandong

Jinfeng Hongyuan Flagship Store	Power Saving Expert 2	Delivery was pretty fast, a little pricey, just not sure if it works or not	Jingdong prefecture level city in Shandong
Jinfeng Hongyuan Flagship Store	Power Saving Expert 2	The goods have been received, I do not know how effective.	Jingdong prefecture level city in Shandong
Jinfeng Hongyuan Flagship Store	Power Saving Expert 2	So far it doesn't seem to be too noticeable, so I'll comment later.	Jingdong prefecture level city in Shandong
Jinfeng Hongyuan Flagship Store	Power Saving Expert 2	I'll comment after I've used it	Jingdong prefecture level city in Shandong
Jinfeng Hongyuan Flagship Store	Power Saving Expert 2	Not bad. You'll save a lot on your electric bill in a year!	Jingdong prefecture level city in Shandong
Jinfeng Hongyuan Flagship Store	Power Saving Expert 2	I just got it. I haven't tried it yet. I don't know what it's like.	Jingdong prefecture level city in Shandong
Jinfeng Hongyuan Flagship Store	Power Saving Expert 2	Shopping at Jingdong has become a habit, and this disease will not be cured for a while.	Jingdong prefecture level city in Shandong
Jinfeng Hongyuan Flagship Store	Power Saving Expert 2	I've been using it for a while now. I don't know if I'm still saving power or not.	Jingdong prefecture level city in Shandong
Jinfeng Hongyuan Flagship Store	Power Saving Expert 2	Received the saver and it's great! There is also a digital voltage indication. Plugged in as instructed, easy to operate, hope you're our power saver!	Jingdong prefecture level city in Shandong
Jinfeng Hongyuan Flagship Store	Power Saving Expert 2	There is no power saving as the store says, using it is the same as not using it.	Jingdong prefecture level city in Shandong
Jinfeng Hongyuan Flagship Store	Power Saving Expert 2	Use it for a while and then comment again	Jingdong prefecture level city in Shandong
Jinfeng Hongyuan Flagship Store	Power Saving Expert 2	Feeling can, to use 1 to 2 months to see how the effect, there is a voltage display, can see the voltage fluctuations amplitude, quite good!	Jingdong prefecture level city in Shandong
Jinfeng Hongyuan Flagship Store	Power Saving Expert 2	I hope it works and saves me some electricity.	Jingdong prefecture level city in Shandong
Jinfeng Hongyuan Flagship Store	Power Saving Expert 2	Nice baby, happy with the after sales	Jingdong prefecture level city in Shandong
Jinfeng Hongyuan Flagship Store	Power Saving Expert 2	Use it and talk about it.	Jingdong prefecture level city in Shandong
Jinfeng Hongyuan Flagship Store	Power Saving Expert 2	Second purchase	Jingdong prefecture level city in Shandong
Jinfeng Hongyuan Flagship Store	Power Saving Expert 2	It works.	Jingdong prefecture level city in Shandong
Jinfeng Hongyuan Flagship Store	Power Saving Expert 2	It works.	Jingdong prefecture level city in Shandong
Jinfeng Hongyuan Flagship Store	Power Saving Expert 2	I don't know if it's a power saver until I start using it	Jingdong prefecture level city in Shandong
Jinfeng Hongyuan Flagship Store	Power Saving Expert 2	serviceable	Jingdong prefecture level city in Shandong
Jinfeng Hongyuan Flagship Store	Power Saving Expert 2	This user did not fill out the rating in time	Jingdong prefecture level city in Shandong
Jinfeng Hongyuan Flagship Store	Power Saving Expert 2	There is no power saving as the store says, using it is the same as not using it.	Jingdong prefecture level city in Shandong
Jinfeng Hongyuan Flagship Store	Power Saving Expert 2	This user did not fill out the rating in time	Jingdong prefecture level city in Shandong
Jinfeng Hongyuan Flagship Store	Power Saving Expert 2	Good...good.	Jingdong prefecture level city in Shandong
Jinfeng Hongyuan Flagship Store	Power Saving Expert 2	(of an unmarried couple) be close	Jingdong prefecture level city in Shandong
Jinfeng Hongyuan Flagship Store	Power Saving Expert 2	(of an unmarried couple) be close	Jingdong prefecture level city in Shandong
Jinfeng Hongyuan Flagship Store	Power Saving Expert 2	(of an unmarried couple) be close	Jingdong prefecture level city in Shandong
Jinfeng Hongyuan Flagship Store	Power Saving Expert 2	The goods are real, high value, high quality, worth every penny, material appearance and quality at a glance is very upscale, very like HAPPY💯	Jingdong prefecture level city in Shandong
Jinfeng Hongyuan Flagship Store	Power Saving Expert 2	It is the ideal product, good quality, logistics is also very fast, worthy of praise 🎁 baby quality is very good, received the first time to open the first time to see, it is very good 👍, very satisfied 😃, customer service is also available to ask questions and answer, thank you 🙏	Jingdong prefecture level city in Shandong
Jinfeng Hongyuan Flagship Store	Power Saving Expert 2	I bought it and haven't used it yet, I'll use it again.	Jingdong prefecture level city in Shandong
Jinfeng Hongyuan Flagship Store	Power Saving Expert 2	It didn't work, I used it for a few days and it was the same as before, it didn't work, I should have to return it	Jingdong prefecture level city in Shandong
Jinfeng Hongyuan Flagship Store	Power Saving Expert 2	The goods have been received, but have not yet installed and use, feel good.	Jingdong prefecture level city in Shandong
Jinfeng Hongyuan Flagship Store	Power Saving Expert 2	Goods received, I forgot to take pictures, the goods look good, you can start, immediately start the school to see the effect then	Jingdong prefecture level city in Shandong
Jinfeng Hongyuan Flagship Store	Power Saving Expert 2	Looks good. I'll use it for a while.	Jingdong prefecture level city in Shandong
Jinfeng Hongyuan Flagship Store	Power Saving Expert 2	Great stuff, easy to operate, easy to use, good power saving, believe in the power of science!	Jingdong prefecture level city in Shandong
Jinfeng Hongyuan Flagship Store	Power Saving Expert 2	Especially great, comparison shopping before buying, the store owner is very good, answering questions very patiently and detailed, there is a need to come back!	Jingdong prefecture level city in Shandong
Jinfeng Hongyuan Flagship Store	Power Saving Expert 2	Trustworthy, feel free to use it boldly.	Jingdong prefecture level city in Shandong
Jinfeng Hongyuan Flagship Store	Power Saving Expert 2	Logistics is super fast! Beautiful appearance! Looks like a great quality! I've never bought one before, so I'll try it first to see if it saves power. We'll see how much it saves.	Jingdong prefecture level city in Shandong
Jinfeng Hongyuan Flagship Store	Power Saving Expert 2	I saw online counterfeiting, energy saver is one of them, hahahahaha, jingdong also buy fake products	Jingdong prefecture level city in Shandong
Jinfeng Hongyuan Flagship Store	Power Saving Expert 2	Just received, installed, try a month to see how the effect and then follow up.	Jingdong prefecture level city in Shandong
Jinfeng Hongyuan Flagship Store	Power Saving Expert 2	Good for you! The goods are genuine! Good price for what you get!	Jingdong prefecture level city in Shandong
Jinfeng Hongyuan Flagship Store	Power Saving Expert 2	Things quickly received, immediately used to see if there is no effect, something river photo ⬆ the same, customer service is very professional	Jingdong prefecture level city in Shandong
Jinfeng Hongyuan Flagship Store	Power Saving Expert 2	The product is very convenient Looking forward to the subsequent use of the experience results	Jingdong prefecture level city in Shandong
Jinfeng Hongyuan Flagship Store	Power Saving Expert 2	I just received it and plugged it in, we'll see exactly when it's time to pay the electric bill	Jingdong prefecture level city in Shandong
Jinfeng Hongyuan Flagship Store	Power Saving Expert 2	Saver is good Packaging and looks good How effective it is to be shared after using it.	Jingdong prefecture level city in Shandong
Jinfeng Hongyuan Flagship Store	Power Saving Expert 2	There's no way to compare. I don't know if it's energy efficient.	Jingdong prefecture level city in Shandong
Jinfeng Hongyuan Flagship Store	Power Saving Expert 2	Just installed it, try it out and comment again	Jingdong prefecture level city in Shandong
Jinfeng Hongyuan Flagship Store	Power Saving Expert 2	Super save point also good to install, after a friend introduced then went to buy to, friends say it is very good to use, home I bought, try to use a period of time, to be honest, the economizer is not big or heavy, can be very convenient to put in a lot of places, the quality is also very good, power-saving function this time seems to be about 20% of the efficiency. The owner is also very warm contact, very	Jingdong prefecture level city in Shandong
Jinfeng Hongyuan Flagship Store	Power Saving Expert 2	The reviews online are mixed. Try it for a while, but I don't know if I can return it if it doesn't save any power after a couple months.	Jingdong prefecture level city in Shandong
Jinfeng Hongyuan Flagship Store	Power Saving Expert 2	The power consumption at home is high, bought a power saver, it looks very convenient to plug on the line, see if you can save some money next month, if good, recommend to friends down the road.	Jingdong prefecture level city in Shandong

Jinfeng Hongyuan Flagship Store	Power Saving Expert 2	It's beautiful, it's genuine, but I don't know the power-saving effect.	Jingdong prefecture level city in Shandong
Jinfeng Hongyuan Flagship Store	Power Saving Expert 2	This user thinks the product is good	Jingdong prefecture level city in Shandong
Jinfeng Hongyuan Flagship Store	Power Saving Expert 2	Nice exterior design with an at-a-glance display	Jingdong prefecture level city in Shandong
Jinfeng Hongyuan Flagship Store	Power Saving Expert 2	Home large and small things are purchased in the Jingdong Mall, have been accustomed to shopping in the Jingdong, many times can buy good quality and cheap products, and courier service attitude is particularly good, sometimes too many couriers are to help me to the home, so many years have been in the Jingdong Mall shopping, trust and support! Will also support the Jingdong as always!	Jingdong prefecture level city in Shandong
Jinfeng Hongyuan Flagship Store	Power Saving Expert 2	Good workmanship, mostly better than the original wall-plugged ones, and hopefully power factor compensation saves on electricity costs	Jingdong prefecture level city in Shandong
Jinfeng Hongyuan Flagship Store	Power Saving Expert 2	I don't feel anything, I said I won't know the effect for a month or two, let's see.	Jingdong prefecture level city in Shandong
Jinfeng Hongyuan Flagship Store	Power Saving Expert 2	Delivery was pretty fast, a little pricey, just not sure if it works or not	Jingdong prefecture level city in Shandong
Jinfeng Hongyuan Flagship Store	Power Saving Expert 2	The goods have been received, I do not know how effective.	Jingdong prefecture level city in Shandong
Jinfeng Hongyuan Flagship Store	Power Saving Expert 2	So far it doesn't seem to be too noticeable, so I'll comment later.	Jingdong prefecture level city in Shandong
Jinfeng Hongyuan Flagship Store	Power Saving Expert 2	I'll comment after I've used it	Jingdong prefecture level city in Shandong
Jinfeng Hongyuan Flagship Store	Power Saving Expert 2	Not bad. You'll save a lot on your electric bill in a year!	Jingdong prefecture level city in Shandong
Jinfeng Hongyuan Flagship Store	Power Saving Expert 2	I just got it. I haven't tried it yet. I don't know what it's like.	Jingdong prefecture level city in Shandong
Jinfeng Hongyuan Flagship Store	Power Saving Expert 2	Shopping at Jingdong has become a habit, and this disease will not be cured for a while.	Jingdong prefecture level city in Shandong
Jinfeng Hongyuan Flagship Store	Power Saving Expert 2	I've been using it for a while now. I don't know if I'm still saving power or not.	Jingdong prefecture level city in Shandong
Jinfeng Hongyuan Flagship Store	Power Saving Expert 2	Received the saver and it's great! There is also a digital voltage indication. Plugged in as instructed, easy to operate, hope you're our power saver!	Jingdong prefecture level city in Shandong
Jinfeng Hongyuan Flagship Store	Power Saving Expert 2	There is no power saving as the store says, using it is the same as not using it.	Jingdong prefecture level city in Shandong
Jinfeng Hongyuan Flagship Store	Power Saving Expert 2	Use it for a while and then comment again	Jingdong prefecture level city in Shandong
Jinfeng Hongyuan Flagship Store	Power Saving Expert 2	Feeling can, to use 1 to 2 months to see how the effect, there is a voltage display, can see the voltage fluctuations amplitude, quite good!	Jingdong prefecture level city in Shandong
Jinfeng Hongyuan Flagship Store	Power Saving Expert 2	I hope it works and saves me some electricity.	Jingdong prefecture level city in Shandong
Jinfeng Hongyuan Flagship Store	Power Saving Expert 2	Nice baby, happy with the after sales	Jingdong prefecture level city in Shandong
Jinfeng Hongyuan Flagship Store	Power Saving Expert 2	Use it and talk about it.	Jingdong prefecture level city in Shandong
Jinfeng Hongyuan Flagship Store	Power Saving Expert 2	Second purchase	Jingdong prefecture level city in Shandong
Jinfeng Hongyuan Flagship Store	Power Saving Expert 2	It works.	Jingdong prefecture level city in Shandong
Jinfeng Hongyuan Flagship Store	Power Saving Expert 2	It works.	Jingdong prefecture level city in Shandong
Jinfeng Hongyuan Flagship Store	Power Saving Expert 2	I don't know if it's a power saver until I start using it	Jingdong prefecture level city in Shandong
Jinfeng Hongyuan Flagship Store	Power Saving Expert 2	serviceable	Jingdong prefecture level city in Shandong
Jinfeng Hongyuan Flagship Store	Power Saving Expert 2	This user did not fill out the rating in time	Jingdong prefecture level city in Shandong
Jinfeng Hongyuan Flagship Store	Power Saving Expert 2	There is no power saving as the store says, using it is the same as not using it.	Jingdong prefecture level city in Shandong
Jinfeng Hongyuan Flagship Store	Power Saving Expert 2	This user did not fill out the rating in time	Jingdong prefecture level city in Shandong
Jinfeng Hongyuan Flagship Store	Power Saving Expert 2	Good...good.	Jingdong prefecture level city in Shandong
Jinfeng Hongyuan Flagship Store	Power Saving Expert 2	(of an unmarried couple) be close	Jingdong prefecture level city in Shandong
Jinfeng Hongyuan Flagship Store	Power Saving Expert 2	(of an unmarried couple) be close	Jingdong prefecture level city in Shandong
Jinfeng Hongyuan Flagship Store	Power Saving Expert 2	(of an unmarried couple) be close	Jingdong prefecture level city in Shandong
Jinfeng Hongyuan Flagship Store	Power Saving Expert 2	The goods are real, high value, high quality, worth every penny, material appearance and quality at a glance is very upscale, very like HAPPY1 00	Jingdong prefecture level city in Shandong
Jinfeng Hongyuan Flagship Store	Power Saving Expert 2	It is the ideal product, good quality, logistics is also very fast, worthy of praise 🎁 baby quality is very good, received the first time to open the first time to see, it is very good 👍, very satisfied 😃, customer service is also available to ask questions and answer, thank you 🙏	Jingdong prefecture level city in Shandong
Jinfeng Hongyuan Flagship Store	Power Saving Expert 2	I bought it and haven't used it yet, I'll use it again.	Jingdong prefecture level city in Shandong
Jinfeng Hongyuan Flagship Store	Power Saving Expert 2	It didn't work, I used it for a few days and it was the same as before, it didn't work, I should have to return it	Jingdong prefecture level city in Shandong
Jinfeng Hongyuan Flagship Store	Power Saving Expert 2	The goods have been received, but have not yet installed and use, feel good.	Jingdong prefecture level city in Shandong
Jinfeng Hongyuan Flagship Store	Power Saving Expert 2	Goods received, I forgot to take pictures, the goods look good, you can start, immediately start the school to see the effect then	Jingdong prefecture level city in Shandong
Jinfeng Hongyuan Flagship Store	Power Saving Expert 2	Looks good. I'll use it for a while.	Jingdong prefecture level city in Shandong
Jinfeng Hongyuan Flagship Store	Power Saving Expert 2	Great stuff, easy to operate, easy to use, good power saving, believe in the power of science!	Jingdong prefecture level city in Shandong
Jinfeng Hongyuan Flagship Store	Power Saving Expert 2	Especially great, comparison shopping before buying, the store owner is very good, answering questions very patiently and detailed, there is a need to come back!	Jingdong prefecture level city in Shandong
Jinfeng Hongyuan Flagship Store	Power Saving Expert 2	Trustworthy, feel free to use it boldly.	Jingdong prefecture level city in Shandong
Jinfeng Hongyuan Flagship Store	Power Saving Expert 2	Logistics is super fast! Beautiful appearance! Looks like a great quality! I've never bought one before, so I'll try it first to see if it saves power. We'll see how much it saves.	Jingdong prefecture level city in Shandong
Jinfeng Hongyuan Flagship Store	Power Saving Expert 2	I saw online counterfeiting, energy saver is one of them, hahahahaha, jingdong also buy fake products	Jingdong prefecture level city in Shandong
Jinfeng Hongyuan Flagship Store	Power Saving Expert 2	Just received, installed, try a month to see how the effect and then follow up.	Jingdong prefecture level city in Shandong
Jinfeng Hongyuan Flagship Store	Power Saving Expert 2	Good for you! The goods are genuine! Good price for what you get!	Jingdong prefecture level city in Shandong
Jinfeng Hongyuan Flagship Store	Power Saving Expert 2	Things quickly received, immediately used to see if there is no effect, something river photo ⍐ the same, customer service is very professional	Jingdong prefecture level city in Shandong
Jinfeng Hongyuan Flagship Store	Power Saving Expert 2	The product is very convenient Looking forward to the subsequent use of the experience results	Jingdong prefecture level city in Shandong
Jinfeng Hongyuan Flagship Store	Power Saving Expert 2	I just received it and plugged it in, we'll see exactly when it's time to pay the electric bill	Jingdong prefecture level city in Shandong

Jinfeng Hongyuan Flagship Store	Power Saving Expert 2	Saver is good Packaging and looks good How effective it is to be shared after using it.	Jingdong prefecture level city in Shandong
Jinfeng Hongyuan Flagship Store	Power Saving Expert 2	There's no way to compare. I don't know if it's energy efficient.	Jingdong prefecture level city in Shandong
Jinfeng Hongyuan Flagship Store	Power Saving Expert 2	Just installed it, try it out and comment again	Jingdong prefecture level city in Shandong
Jinfeng Hongyuan Flagship Store	Power Saving Expert 2	Super save point also good to install, after a friend introduced then went to buy to, friends say it is very good to use, home I bought, try to use a period of time, to be honest, the economizer is not big or heavy, can be very convenient to put in a lot of places, the quality is also very good, power-saving function this time seems to be about 20% of the efficiency. The owner is also very warm contact, very	Jingdong prefecture level city in Shandong
Jinfeng Hongyuan Flagship Store	Power Saving Expert 2	The reviews online are mixed. Try it for a while, but I don't know if I can return it if it doesn't save any power after a couple months.	Jingdong prefecture level city in Shandong
Jinfeng Hongyuan Flagship Store	Power Saving Expert 2	The power consumption at home is high, bought a power saver, it looks very convenient to plug on the line, see if you can save some money next month, if good, recommend to friends down the road.	Jingdong prefecture level city in Shandong
Jinfeng Hongyuan Flagship Store	Power Saving Expert 2	It's beautiful, it's genuine, but I don't know the power-saving effect.	Jingdong prefecture level city in Shandong
Jinfeng Hongyuan Flagship Store	Power Saving Expert 2	This user thinks the product is good	Jingdong prefecture level city in Shandong
Jinfeng Hongyuan Flagship Store	Power Saving Expert 2	Nice exterior design with an at-a-glance display	Jingdong prefecture level city in Shandong
Jinfeng Hongyuan Flagship Store	Power Saving Expert 2	Home large and small things are purchased in the Jingdong Mall, have been accustomed to shopping in the Jingdong, many times can buy good quality and cheap products, and courier service attitude is particularly good, sometimes too many couriers are to help me to the home, so many years have been in the Jingdong Mall shopping, trust and support! Will also support the Jingdong as always!	Jingdong prefecture level city in Shandong
Jinfeng Hongyuan Flagship Store	Power Saving Expert 2	Good workmanship, mostly better than the original wall-plugged ones, and hopefully power factor compensation saves on electricity costs	Jingdong prefecture level city in Shandong
Jinfeng Hongyuan Flagship Store	Power Saving Expert 2	I don't feel anything, I said I won't know the effect for a month or two, let's see.	Jingdong prefecture level city in Shandong
Jinfeng Hongyuan Flagship Store	Power Saving Expert 2	Delivery was pretty fast, a little pricey, just not sure if it works or not	Jingdong prefecture level city in Shandong
Jinfeng Hongyuan Flagship Store	Power Saving Expert 2	The goods have been received, I do not know how effective.	Jingdong prefecture level city in Shandong
Jinfeng Hongyuan Flagship Store	Power Saving Expert 2	So far it doesn't seem to be too noticeable, so I'll comment later.	Jingdong prefecture level city in Shandong
Jinfeng Hongyuan Flagship Store	Power Saving Expert 2	I'll comment after I've used it	Jingdong prefecture level city in Shandong
Jinfeng Hongyuan Flagship Store	Power Saving Expert 2	Not bad. You'll save a lot on your electric bill in a year!	Jingdong prefecture level city in Shandong
Jinfeng Hongyuan Flagship Store	Power Saving Expert 2	I just got it. I haven't tried it yet. I don't know what it's like.	Jingdong prefecture level city in Shandong
Jinfeng Hongyuan Flagship Store	Power Saving Expert 2	Shopping at Jingdong has become a habit, and this disease will not be cured for a while.	Jingdong prefecture level city in Shandong
Jinfeng Hongyuan Flagship Store	Power Saving Expert 2	I've been using it for a while now. I don't know if I'm still saving power or not.	Jingdong prefecture level city in Shandong
Jinfeng Hongyuan Flagship Store	Power Saving Expert 2	Received the saver and it's great! There is also a digital voltage indication. Plugged in as instructed, easy to operate, hope you're our power saver!	Jingdong prefecture level city in Shandong
Jinfeng Hongyuan Flagship Store	Power Saving Expert 2	There is no power saving as the store says, using it is the same as not using it.	Jingdong prefecture level city in Shandong
Jinfeng Hongyuan Flagship Store	Power Saving Expert 2	Use it for a while and then comment again	Jingdong prefecture level city in Shandong
Jinfeng Hongyuan Flagship Store	Power Saving Expert 2	Feeling can, to use 1 to 2 months to see how the effect, there is a voltage display, can see the voltage fluctuations amplitude, quite good!	Jingdong prefecture level city in Shandong
Jinfeng Hongyuan Flagship Store	Power Saving Expert 2	I hope it works and saves me some electricity.	Jingdong prefecture level city in Shandong
Jinfeng Hongyuan Flagship Store	Power Saving Expert 2	Nice baby, happy with the after sales	Jingdong prefecture level city in Shandong
Jinfeng Hongyuan Flagship Store	Power Saving Expert 2	Use it and talk about it.	Jingdong prefecture level city in Shandong
Jinfeng Hongyuan Flagship Store	Power Saving Expert 2	Second purchase	Jingdong prefecture level city in Shandong
Jinfeng Hongyuan Flagship Store	Power Saving Expert 2	It works.	Jingdong prefecture level city in Shandong
Jinfeng Hongyuan Flagship Store	Power Saving Expert 2	It works.	Jingdong prefecture level city in Shandong
Jinfeng Hongyuan Flagship Store	Power Saving Expert 2	I don't know if it's a power saver until I start using it	Jingdong prefecture level city in Shandong
Jinfeng Hongyuan Flagship Store	Power Saving Expert 2	serviceable	Jingdong prefecture level city in Shandong
Jinfeng Hongyuan Flagship Store	Power Saving Expert 2	This user did not fill out the rating in time	Jingdong prefecture level city in Shandong
Jinfeng Hongyuan Flagship Store	Power Saving Expert 2	There is no power saving as the store says, using it is the same as not using it.	Jingdong prefecture level city in Shandong
Jinfeng Hongyuan Flagship Store	Power Saving Expert 2	This user did not fill out the rating in time	Jingdong prefecture level city in Shandong
Jinfeng Hongyuan Flagship Store	Power Saving Expert 2	Good...good.	Jingdong prefecture level city in Shandong
Jinfeng Hongyuan Flagship Store	Power Saving Expert 2	(of an unmarried couple) be close	Jingdong prefecture level city in Shandong
Jinfeng Hongyuan Flagship Store	Power Saving Expert 2	(of an unmarried couple) be close	Jingdong prefecture level city in Shandong
Jinfeng Hongyuan Flagship Store	Power Saving Expert 2	(of an unmarried couple) be close	Jingdong prefecture level city in Shandong
Jinfeng Hongyuan Flagship Store	Power Saving Expert 2	The goods are real, high value, high quality, worth every penny, material appearance and quality at a glance is very upscale, very like HAPPY💯	Jingdong prefecture level city in Shandong
Jinfeng Hongyuan Flagship Store	Power Saving Expert 2	It is the ideal product, good quality, logistics is also very fast, worthy of praise 🎁 baby quality is very good, received the first time to open the first time to see, it is very good 👍, very satisfied 😃, customer service is also available to ask questions and answer, thank you 🙏	Jingdong prefecture level city in Shandong
Jinfeng Hongyuan Flagship Store	Power Saving Expert 2	I bought it and haven't used it yet, I'll use it again.	Jingdong prefecture level city in Shandong
Jinfeng Hongyuan Flagship Store	Power Saving Expert 2	It didn't work, I used it for a few days and it was the same as before, it didn't work, I should have to return it	Jingdong prefecture level city in Shandong
Jinfeng Hongyuan Flagship Store	Power Saving Expert 2	The goods have been received, but have not yet installed and use, feel good.	Jingdong prefecture level city in Shandong
Jinfeng Hongyuan Flagship Store	Power Saving Expert 2	Goods received, I forgot to take pictures, the goods look good, you can start, immediately start the school to see the effect then	Jingdong prefecture level city in Shandong
Jinfeng Hongyuan Flagship Store	Power Saving Expert 2	Looks good. I'll use it for a while.	Jingdong prefecture level city in Shandong
Jinfeng Hongyuan Flagship Store	Power Saving Expert 2	Great stuff, easy to operate, easy to use, good power saving, believe in the power of science!	Jingdong prefecture level city in Shandong
Jinfeng Hongyuan Flagship Store	Power Saving Expert 2	Especially great, comparison shopping before buying, the store owner is very good, answering questions very patiently and detailed, there is a need to come back!	Jingdong prefecture level city in Shandong
Jinfeng Hongyuan Flagship Store	Power Saving Expert 2	Trustworthy, feel free to use it boldly.	Jingdong prefecture level city in Shandong

Jinfeng Hongyuan Flagship Store	Power Saving Expert 2	Logistics is super fast! Beautiful appearance! Looks like a great quality! I've never bought one before, so I'll try it first to see if it saves power. We'll see how much it saves.	Jingdong prefecture level city in Shandong
Jinfeng Hongyuan Flagship Store	Power Saving Expert 2	I saw online counterfeiting, energy saver is one of them, hahahahaha, jingdong also buy fake products	Jingdong prefecture level city in Shandong
Jinfeng Hongyuan Flagship Store	Power Saving Expert 2	Just received, installed, try a month to see how the effect and then follow up.	Jingdong prefecture level city in Shandong
Jinfeng Hongyuan Flagship Store	Power Saving Expert 2	Good for you! The goods are genuine! Good price for what you get!	Jingdong prefecture level city in Shandong
Jinfeng Hongyuan Flagship Store	Power Saving Expert 2	Things quickly received, immediately used to see if there is no effect, something river photo ⍐ the same, customer service is very professional	Jingdong prefecture level city in Shandong
Jinfeng Hongyuan Flagship Store	Power Saving Expert 2	The product is very convenient Looking forward to the subsequent use of the experience results	Jingdong prefecture level city in Shandong
Jinfeng Hongyuan Flagship Store	Power Saving Expert 2	I just received it and plugged it in, we'll see exactly when it's time to pay the electric bill	Jingdong prefecture level city in Shandong
Jinfeng Hongyuan Flagship Store	Power Saving Expert 2	Saver is good Packaging and looks good How effective it is to be shared after using it.	Jingdong prefecture level city in Shandong
Jinfeng Hongyuan Flagship Store	Power Saving Expert 2	There's no way to compare. I don't know if it's energy efficient.	Jingdong prefecture level city in Shandong
Jinfeng Hongyuan Flagship Store	Power Saving Expert 2	Just installed it, try it out and comment again	Jingdong prefecture level city in Shandong
Jinfeng Hongyuan Flagship Store	Power Saving Expert 2	Super save point also good to install, after a friend introduced then went to buy to, friends say it is very good to use, home I bought, try to use a period of time, to be honest, the economizer is not big or heavy, can be very convenient to put in a lot of places, the quality is also very good, power-saving function this time seems to be about 20% of the efficiency. The owner is also very warm contact, very	Jingdong prefecture level city in Shandong
Jinfeng Hongyuan Flagship Store	Power Saving Expert 2	The reviews online are mixed. Try it for a while, but I don't know if I can return it if it doesn't save any power after a couple months.	Jingdong prefecture level city in Shandong
Jinfeng Hongyuan Flagship Store	Power Saving Expert 2	The power consumption at home is high, bought a power saver, it looks very convenient to plug on the line, see if you can save some money next month, if good, recommend to friends down the road.	Jingdong prefecture level city in Shandong
Jinfeng Hongyuan Flagship Store	Power Saving Expert 2	It's beautiful, it's genuine, but I don't know the power-saving effect.	Jingdong prefecture level city in Shandong
Jinfeng Hongyuan Flagship Store	Power Saving Expert 2	This user thinks the product is good	Jingdong prefecture level city in Shandong
Jinfeng Hongyuan Flagship Store	Power Saving Expert 2	Nice exterior design with an at-a-glance display	Jingdong prefecture level city in Shandong
Jinfeng Hongyuan Flagship Store	Power Saving Expert 2	Home large and small things are purchased in the Jingdong Mall, have been accustomed to shopping in the Jingdong, many times can buy good quality and cheap products, and courier service attitude is particularly good, sometimes too many couriers are to help me to the home, so many years have been in the Jingdong Mall shopping, trust and support! Will also support the Jingdong as always!	Jingdong prefecture level city in Shandong
Jinfeng Hongyuan Flagship Store	Power Saving Expert 2	Good workmanship, mostly better than the original wall-plugged ones, and hopefully power factor compensation saves on electricity costs	Jingdong prefecture level city in Shandong
Jinfeng Hongyuan Flagship Store	Power Saving Expert 2	I don't feel anything, I said I won't know the effect for a month or two, let's see.	Jingdong prefecture level city in Shandong
Jinfeng Hongyuan Flagship Store	Power Saving Expert 2	Delivery was pretty fast, a little pricey, just not sure if it works or not	Jingdong prefecture level city in Shandong
Jinfeng Hongyuan Flagship Store	Power Saving Expert 2	The goods have been received, I do not know how effective.	Jingdong prefecture level city in Shandong
Jinfeng Hongyuan Flagship Store	Power Saving Expert 2	So far it doesn't seem to be too noticeable, so I'll comment later.	Jingdong prefecture level city in Shandong
Jinfeng Hongyuan Flagship Store	Power Saving Expert 2	I'll comment after I've used it	Jingdong prefecture level city in Shandong
Jinfeng Hongyuan Flagship Store	Power Saving Expert 2	Not bad. You'll save a lot on your electric bill in a year!	Jingdong prefecture level city in Shandong
Jinfeng Hongyuan Flagship Store	Power Saving Expert 2	I just got it. I haven't tried it yet. I don't know what it's like.	Jingdong prefecture level city in Shandong
Jinfeng Hongyuan Flagship Store	Power Saving Expert 2	Shopping at Jingdong has become a habit, and this disease will not be cured for a while.	Jingdong prefecture level city in Shandong
Jinfeng Hongyuan Flagship Store	Power Saving Expert 2	I've been using it for a while now. I don't know if I'm still saving power or not.	Jingdong prefecture level city in Shandong
Jinfeng Hongyuan Flagship Store	Power Saving Expert 2	Received the saver and it's great! There is also a digital voltage indication. Plugged in as instructed, easy to operate, hope you're our power saver!	Jingdong prefecture level city in Shandong
Jinfeng Hongyuan Flagship Store	Power Saving Expert 2	There is no power saving as the store says, using it is the same as not using it.	Jingdong prefecture level city in Shandong
Jinfeng Hongyuan Flagship Store	Power Saving Expert 2	Use it for a while and then comment again	Jingdong prefecture level city in Shandong
Jinfeng Hongyuan Flagship Store	Power Saving Expert 2	Feeling can, to use 1 to 2 months to see how the effect, there is a voltage display, can see the voltage fluctuations amplitude, quite good!	Jingdong prefecture level city in Shandong
Jinfeng Hongyuan Flagship Store	Power Saving Expert 2	I hope it works and saves me some electricity.	Jingdong prefecture level city in Shandong
Jinfeng Hongyuan Flagship Store	Power Saving Expert 2	Nice baby, happy with the after sales	Jingdong prefecture level city in Shandong
Jinfeng Hongyuan Flagship Store	Power Saving Expert 2	Use it and talk about it.	Jingdong prefecture level city in Shandong
Jinfeng Hongyuan Flagship Store	Power Saving Expert 2	Second purchase	Jingdong prefecture level city in Shandong
Jinfeng Hongyuan Flagship Store	Power Saving Expert 2	It works.	Jingdong prefecture level city in Shandong
Jinfeng Hongyuan Flagship Store	Power Saving Expert 2	It works.	Jingdong prefecture level city in Shandong
Jinfeng Hongyuan Flagship Store	Power Saving Expert 2	I don't know if it's a power saver until I start using it	Jingdong prefecture level city in Shandong
Jinfeng Hongyuan Flagship Store	Power Saving Expert 2	serviceable	Jingdong prefecture level city in Shandong
Jinfeng Hongyuan Flagship Store	Power Saving Expert 2	This user did not fill out the rating in time	Jingdong prefecture level city in Shandong
Jinfeng Hongyuan Flagship Store	Power Saving Expert 2	There is no power saving as the store says, using it is the same as not using it.	Jingdong prefecture level city in Shandong
Jinfeng Hongyuan Flagship Store	Power Saving Expert 2	This user did not fill out the rating in time	Jingdong prefecture level city in Shandong
Jinfeng Hongyuan Flagship Store	Power Saving Expert 2	Good...good.	Jingdong prefecture level city in Shandong
Jinfeng Hongyuan Flagship Store	Power Saving Expert 2	(of an unmarried couple) be close	Jingdong prefecture level city in Shandong
Jinfeng Hongyuan Flagship Store	Power Saving Expert 2	(of an unmarried couple) be close	Jingdong prefecture level city in Shandong
Jinfeng Hongyuan Flagship Store	Power Saving Expert 2	(of an unmarried couple) be close	Jingdong prefecture level city in Shandong
Jinfeng Hongyuan Flagship Store	Power Saving Expert 2	The goods are real, high value, high quality, worth every penny, material appearance and quality at a glance is very upscale, very like HAPPY1 00	Jingdong prefecture level city in Shandong
Jinfeng Hongyuan Flagship Store	Power Saving Expert 2	It is the ideal product, good quality, logistics is also very fast, worthy of praise 🎁 baby quality is very good, received the first time to open the first time to see, it is very good 👍, very satisfied 😃, customer service is also available to ask questions and answer, thank you 🙏	Jingdong prefecture level city in Shandong
Jinfeng Hongyuan Flagship Store	Power Saving Expert 2	I bought it and haven't used it yet, I'll use it again.	Jingdong prefecture level city in Shandong

Jinfeng Hongyuan Flagship Store	Power Saving Expert 2	It didn't work, I used it for a few days and it was the same as before, it didn't work, I should have to return it	Jingdong prefecture level city in Shandong
Jinfeng Hongyuan Flagship Store	Power Saving Expert 2	The goods have been received, but have not yet installed and use, feel good.	Jingdong prefecture level city in Shandong
Jinfeng Hongyuan Flagship Store	Power Saving Expert 2	Goods received, I forgot to take pictures, the goods look good, you can start, immediately start the school to see the effect then	Jingdong prefecture level city in Shandong
Jinfeng Hongyuan Flagship Store	Power Saving Expert 2	Looks good. I'll use it for a while.	Jingdong prefecture level city in Shandong
Jinfeng Hongyuan Flagship Store	Power Saving Expert 2	Great stuff, easy to operate, easy to use, good power saving, believe in the power of science!	Jingdong prefecture level city in Shandong
Jinfeng Hongyuan Flagship Store	Power Saving Expert 2	Especially great, comparison shopping before buying, the store owner is very good, answering questions very patiently and detailed, there is a need to come back!	Jingdong prefecture level city in Shandong
Jinfeng Hongyuan Flagship Store	Power Saving Expert 2	Trustworthy, feel free to use it boldly.	Jingdong prefecture level city in Shandong
Jinfeng Hongyuan Flagship Store	Power Saving Expert 2	Logistics is super fast! Beautiful appearance! Looks like a great quality! I've never bought one before, so I'll try it first to see if it saves power. We'll see how much it saves.	Jingdong prefecture level city in Shandong
Jinfeng Hongyuan Flagship Store	Power Saving Expert 2	I saw online counterfeiting, energy saver is one of them, hahahahaha, jingdong also buy fake products	Jingdong prefecture level city in Shandong
Jinfeng Hongyuan Flagship Store	Power Saving Expert 2	Just received, installed, try a month to see how the effect and then follow up.	Jingdong prefecture level city in Shandong
Jinfeng Hongyuan Flagship Store	Power Saving Expert 2	Good for you! The goods are genuine! Good price for what you get!	Jingdong prefecture level city in Shandong
Jinfeng Hongyuan Flagship Store	Power Saving Expert 2	Things quickly received, immediately used to see if there is no effect, something river photo ⍐ the same, customer service is very professional	Jingdong prefecture level city in Shandong
Jinfeng Hongyuan Flagship Store	Power Saving Expert 2	The product is very convenient Looking forward to the subsequent use of the experience results	Jingdong prefecture level city in Shandong
Jinfeng Hongyuan Flagship Store	Power Saving Expert 2	I just received it and plugged it in, we'll see exactly when it's time to pay the electric bill	Jingdong prefecture level city in Shandong
Jinfeng Hongyuan Flagship Store	Power Saving Expert 2	Saver is good Packaging and looks good How effective it is to be shared after using it.	Jingdong prefecture level city in Shandong
Jinfeng Hongyuan Flagship Store	Power Saving Expert 2	There's no way to compare. I don't know if it's energy efficient.	Jingdong prefecture level city in Shandong
Jinfeng Hongyuan Flagship Store	Power Saving Expert 2	Just installed it, try it out and comment again	Jingdong prefecture level city in Shandong
Jinfeng Hongyuan Flagship Store	Power Saving Expert 2	Super save point also good to install, after a friend introduced then went to buy to, friends say it is very good to use, home I bought, try to use a period of time, to be honest, the economizer is not big or heavy, can be very convenient to put in a lot of places, the quality is also very good, power-saving function this time seems to be about 20% of the efficiency. The owner is also very warm contact, very	Jingdong prefecture level city in Shandong
Jinfeng Hongyuan Flagship Store	Power Saving Expert 2	The reviews online are mixed. Try it for a while, but I don't know if I can return it if it doesn't save any power after a couple months.	Jingdong prefecture level city in Shandong
Jinfeng Hongyuan Flagship Store	Power Saving Expert 2	The power consumption at home is high, bought a power saver, it looks very convenient to plug on the line, see if you can save some money next month, if good, recommend to friends down the road.	Jingdong prefecture level city in Shandong
Jinfeng Hongyuan Flagship Store	Power Saving Expert 2	It's beautiful, it's genuine, but I don't know the power-saving effect.	Jingdong prefecture level city in Shandong
Jinfeng Hongyuan Flagship Store	Power Saving Expert 2	This user thinks the product is good	Jingdong prefecture level city in Shandong
Jinfeng Hongyuan Flagship Store	Power Saving Expert 2	Nice exterior design with an at-a-glance display	Jingdong prefecture level city in Shandong
Jinfeng Hongyuan Flagship Store	Power Saving Expert 2	Home large and small things are purchased in the Jingdong Mall, have been accustomed to shopping in the Jingdong, many times can buy good quality and cheap products, and courier service attitude is particularly good, sometimes too many couriers are to help me to the home, so many years have been in the Jingdong Mall shopping, trust and support! Will also support the Jingdong as always!	Jingdong prefecture level city in Shandong
Jinfeng Hongyuan Flagship Store	Power Saving Expert 2	Good workmanship, mostly better than the original wall-plugged ones, and hopefully power factor compensation saves on electricity costs	Jingdong prefecture level city in Shandong
Jinfeng Hongyuan Flagship Store	Power Saving Expert 2	I don't feel anything, I said I won't know the effect for a month or two, let's see.	Jingdong prefecture level city in Shandong
Jinfeng Hongyuan Flagship Store	Power Saving Expert 2	Delivery was pretty fast, a little pricey, just not sure if it works or not	Jingdong prefecture level city in Shandong
Jinfeng Hongyuan Flagship Store	Power Saving Expert 2	The goods have been received, I do not know how effective.	Jingdong prefecture level city in Shandong
Jinfeng Hongyuan Flagship Store	Power Saving Expert 2	So far it doesn't seem to be too noticeable, so I'll comment later.	Jingdong prefecture level city in Shandong
Jinfeng Hongyuan Flagship Store	Power Saving Expert 2	I'll comment after I've used it	Jingdong prefecture level city in Shandong
Jinfeng Hongyuan Flagship Store	Power Saving Expert 2	Not bad. You'll save a lot on your electric bill in a year!	Jingdong prefecture level city in Shandong
Jinfeng Hongyuan Flagship Store	Power Saving Expert 2	I just got it. I haven't tried it yet. I don't know what it's like.	Jingdong prefecture level city in Shandong
Jinfeng Hongyuan Flagship Store	Power Saving Expert 2	Shopping at Jingdong has become a habit, and this disease will not be cured for a while.	Jingdong prefecture level city in Shandong
Jinfeng Hongyuan Flagship Store	Power Saving Expert 2	I've been using it for a while now. I don't know if I'm still saving power or not.	Jingdong prefecture level city in Shandong
Jinfeng Hongyuan Flagship Store	Power Saving Expert 2	Received the saver and it's great! There is also a digital voltage indication. Plugged in as instructed, easy to operate, hope you're our power saver!	Jingdong prefecture level city in Shandong
Jinfeng Hongyuan Flagship Store	Power Saving Expert 2	There is no power saving as the store says, using it is the same as not using it.	Jingdong prefecture level city in Shandong
Jinfeng Hongyuan Flagship Store	Power Saving Expert 2	Use it for a while and then comment again	Jingdong prefecture level city in Shandong
Jinfeng Hongyuan Flagship Store	Power Saving Expert 2	Feeling can, to use 1 to 2 months to see how the effect, there is a voltage display, can see the voltage fluctuations amplitude, quite good!	Jingdong prefecture level city in Shandong
Jinfeng Hongyuan Flagship Store	Power Saving Expert 2	I hope it works and saves me some electricity.	Jingdong prefecture level city in Shandong
Jinfeng Hongyuan Flagship Store	Power Saving Expert 2	Nice baby, happy with the after sales	Jingdong prefecture level city in Shandong
Jinfeng Hongyuan Flagship Store	Power Saving Expert 2	Use it and talk about it.	Jingdong prefecture level city in Shandong
Jinfeng Hongyuan Flagship Store	Power Saving Expert 2	Second purchase	Jingdong prefecture level city in Shandong
Jinfeng Hongyuan Flagship Store	Power Saving Expert 2	It works.	Jingdong prefecture level city in Shandong
Jinfeng Hongyuan Flagship Store	Power Saving Expert 2	It works.	Jingdong prefecture level city in Shandong
Jinfeng Hongyuan Flagship Store	Power Saving Expert 2	I don't know if it's a power saver until I start using it	Jingdong prefecture level city in Shandong
Jinfeng Hongyuan Flagship Store	Power Saving Expert 2	serviceable	Jingdong prefecture level city in Shandong
Jinfeng Hongyuan Flagship Store	Power Saving Expert 2	This user did not fill out the rating in time	Jingdong prefecture level city in Shandong
Jinfeng Hongyuan Flagship Store	Power Saving Expert 2	There is no power saving as the store says, using it is the same as not using it.	Jingdong prefecture level city in Shandong
Jinfeng Hongyuan Flagship Store	Power Saving Expert 2	This user did not fill out the rating in time	Jingdong prefecture level city in Shandong

Jinfeng Hongyuan Flagship Store	Power Saving Expert 2	Good...good.	Jingdong prefecture level city in Shandong
Jinfeng Hongyuan Flagship Store	Power Saving Expert 2	(of an unmarried couple) be close	Jingdong prefecture level city in Shandong
Jinfeng Hongyuan Flagship Store	Power Saving Expert 2	(of an unmarried couple) be close	Jingdong prefecture level city in Shandong
Jinfeng Hongyuan Flagship Store	Power Saving Expert 2	(of an unmarried couple) be close	Jingdong prefecture level city in Shandong
Jinfeng Hongyuan Flagship Store	Power Saving Expert 2	The goods are real, high value, high quality, worth every penny, material appearance and quality at a glance is very upscale, very like HAPPY1 00	Jingdong prefecture level city in Shandong
Jinfeng Hongyuan Flagship Store	Power Saving Expert 2	It is the ideal product, good quality, logistics is also very fast, worthy of praise 🎁 baby quality is very good, received the first time to open the first time to see, it is very good 👍, very satisfied 😃, customer service is also available to ask questions and answer, thank you 🙏	Jingdong prefecture level city in Shandong
Jinfeng Hongyuan Flagship Store	Power Saving Expert 2	I bought it and haven't used it yet, I'll use it again.	Jingdong prefecture level city in Shandong
Jinfeng Hongyuan Flagship Store	Power Saving Expert 2	It didn't work, I used it for a few days and it was the same as before, it didn't work, I should have to return it	Jingdong prefecture level city in Shandong
Jinfeng Hongyuan Flagship Store	Power Saving Expert 2	The goods have been received, but have not yet installed and use, feel good.	Jingdong prefecture level city in Shandong
Jinfeng Hongyuan Flagship Store	Power Saving Expert 2	Goods received, I forgot to take pictures, the goods look good, you can start, immediately start the school to see the effect then	Jingdong prefecture level city in Shandong
Jinfeng Hongyuan Flagship Store	Power Saving Expert 2	Looks good. I'll use it for a while.	Jingdong prefecture level city in Shandong
Jinfeng Hongyuan Flagship Store	Power Saving Expert 2	Great stuff, easy to operate, easy to use, good power saving, believe in the power of science!	Jingdong prefecture level city in Shandong
Jinfeng Hongyuan Flagship Store	Power Saving Expert 2	Especially great, comparison shopping before buying, the store owner is very good, answering questions very patiently and detailed, there is a need to come back!	Jingdong prefecture level city in Shandong
Jinfeng Hongyuan Flagship Store	Power Saving Expert 2	Trustworthy, feel free to use it boldly.	Jingdong prefecture level city in Shandong
Jinfeng Hongyuan Flagship Store	Power Saving Expert 2	Logistics is super fast! Beautiful appearance! Looks like a great quality! I've never bought one before, so I'll try it first to see if it saves power. We'll see how much it saves.	Jingdong prefecture level city in Shandong
Jinfeng Hongyuan Flagship Store	Power Saving Expert 2	I saw online counterfeiting, energy saver is one of them, hahahahaha, jingdong also buy fake products	Jingdong prefecture level city in Shandong
Jinfeng Hongyuan Flagship Store	Power Saving Expert 2	Just received, installed, try a month to see how the effect and then follow up.	Jingdong prefecture level city in Shandong
Jinfeng Hongyuan Flagship Store	Power Saving Expert 2	Good for you! The goods are genuine! Good price for what you get!	Jingdong prefecture level city in Shandong
Jinfeng Hongyuan Flagship Store	Power Saving Expert 2	Things quickly received, immediately used to see if there is no effect, something river photo ⇡ the same, customer service is very professional	Jingdong prefecture level city in Shandong
Jinfeng Hongyuan Flagship Store	Power Saving Expert 2	The product is very convenient Looking forward to the subsequent use of the experience results	Jingdong prefecture level city in Shandong
Jinfeng Hongyuan Flagship Store	Power Saving Expert 2	I just received it and plugged it in, we'll see exactly when it's time to pay the electric bill	Jingdong prefecture level city in Shandong
Jinfeng Hongyuan Flagship Store	Power Saving Expert 2	Saver is good Packaging and looks good How effective it is to be shared after using it.	Jingdong prefecture level city in Shandong
Jinfeng Hongyuan Flagship Store	Power Saving Expert 2	There's no way to compare. I don't know if it's energy efficient.	Jingdong prefecture level city in Shandong
Jinfeng Hongyuan Flagship Store	Power Saving Expert 2	Just installed it, try it out and comment again	Jingdong prefecture level city in Shandong
Jinfeng Hongyuan Flagship Store	Power Saving Expert 2	Super save point also good to install, after a friend introduced then went to buy to, friends say it is very good to use, home I bought, try to use a period of time, to be honest, the economizer is not big or heavy, can be very convenient to put in a lot of places, the quality is also very good, power-saving function this time seems to be about 20% of the efficiency. The owner is also very warm contact, very	Jingdong prefecture level city in Shandong
Jinfeng Hongyuan Flagship Store	Power Saving Expert 2	The reviews online are mixed. Try it for a while, but I don't know if I can return it if it doesn't save any power after a couple months.	Jingdong prefecture level city in Shandong
Jinfeng Hongyuan Flagship Store	Power Saving Expert 2	The power consumption at home is high, bought a power saver, it looks very convenient to plug on the line, see if you can save some money next month, if good, recommend to friends down the road.	Jingdong prefecture level city in Shandong
Jinfeng Hongyuan Flagship Store	Power Saving Expert 2	It's beautiful, it's genuine, but I don't know the power-saving effect.	Jingdong prefecture level city in Shandong
Jinfeng Hongyuan Flagship Store	Power Saving Expert 2	This user thinks the product is good	Jingdong prefecture level city in Shandong
Jinfeng Hongyuan Flagship Store	Power Saving Expert 2	Nice exterior design with an at-a-glance display	Jingdong prefecture level city in Shandong
Jinfeng Hongyuan Flagship Store	Power Saving Expert 2	Home large and small things are purchased in the Jingdong Mall, have been accustomed to shopping in the Jingdong, many times can buy good quality and cheap products, and courier service attitude is particularly good, sometimes too many couriers are to help me to the home, so many years have been in the Jingdong Mall shopping, trust and support! Will also support the Jingdong as always!	Jingdong prefecture level city in Shandong
Jinfeng Hongyuan Flagship Store	Power Saving Expert 2	Good workmanship, mostly better than the original wall-plugged ones, and hopefully power factor compensation saves on electricity costs	Jingdong prefecture level city in Shandong
Jinfeng Hongyuan Flagship Store	Power Saving Expert 2	I don't feel anything, I said I won't know the effect for a month or two, let's see.	Jingdong prefecture level city in Shandong
Jinfeng Hongyuan Flagship Store	Power Saving Expert 2	Delivery was pretty fast, a little pricey, just not sure if it works or not	Jingdong prefecture level city in Shandong
Jinfeng Hongyuan Flagship Store	Power Saving Expert 2	The goods have been received, I do not know how effective.	Jingdong prefecture level city in Shandong
Jinfeng Hongyuan Flagship Store	Power Saving Expert 2	So far it doesn't seem to be too noticeable, so I'll comment later.	Jingdong prefecture level city in Shandong
Jinfeng Hongyuan Flagship Store	Power Saving Expert 2	I'll comment after I've used it	Jingdong prefecture level city in Shandong
Jinfeng Hongyuan Flagship Store	Power Saving Expert 2	Not bad. You'll save a lot on your electric bill in a year!	Jingdong prefecture level city in Shandong
Jinfeng Hongyuan Flagship Store	Power Saving Expert 2	I just got it. I haven't tried it yet. I don't know what it's like.	Jingdong prefecture level city in Shandong
Jinfeng Hongyuan Flagship Store	Power Saving Expert 2	Shopping at Jingdong has become a habit, and this disease will not be cured for a while.	Jingdong prefecture level city in Shandong
Jinfeng Hongyuan Flagship Store	Power Saving Expert 2	I've been using it for a while now. I don't know if I'm still saving power or not.	Jingdong prefecture level city in Shandong
Jinfeng Hongyuan Flagship Store	Power Saving Expert 2	Received the saver and it's great! There is also a digital voltage indication. Plugged in as instructed, easy to operate, hope you're our power saver!	Jingdong prefecture level city in Shandong
Jinfeng Hongyuan Flagship Store	Power Saving Expert 2	There is no power saving as the store says, using it is the same as not using it.	Jingdong prefecture level city in Shandong
Jinfeng Hongyuan Flagship Store	Power Saving Expert 2	Use it for a while and then comment again	Jingdong prefecture level city in Shandong
Jinfeng Hongyuan Flagship Store	Power Saving Expert 2	Feeling can, to use 1 to 2 months to see how the effect, there is a voltage display, can see the voltage fluctuations amplitude, quite good!	Jingdong prefecture level city in Shandong
Jinfeng Hongyuan Flagship Store	Power Saving Expert 2	I hope it works and saves me some electricity.	Jingdong prefecture level city in Shandong
Jinfeng Hongyuan Flagship Store	Power Saving Expert 2	Nice baby, happy with the after sales	Jingdong prefecture level city in Shandong
Jinfeng Hongyuan Flagship Store	Power Saving Expert 2	Use it and talk about it.	Jingdong prefecture level city in Shandong
Jinfeng Hongyuan Flagship Store	Power Saving Expert 2	Second purchase	Jingdong prefecture level city in Shandong

Jinfeng Hongyuan Flagship Store	Power Saving Expert 2	It works.	Jingdong prefecture level city in Shandong
Jinfeng Hongyuan Flagship Store	Power Saving Expert 2	It works.	Jingdong prefecture level city in Shandong
Jinfeng Hongyuan Flagship Store	Power Saving Expert 2	I don't know if it's a power saver until I start using it	Jingdong prefecture level city in Shandong
Jinfeng Hongyuan Flagship Store	Power Saving Expert 2	serviceable	Jingdong prefecture level city in Shandong
Jinfeng Hongyuan Flagship Store	Power Saving Expert 2	This user did not fill out the rating in time	Jingdong prefecture level city in Shandong
Jinfeng Hongyuan Flagship Store	Power Saving Expert 2	There is no power saving as the store says, using it is the same as not using it.	Jingdong prefecture level city in Shandong
Jinfeng Hongyuan Flagship Store	Power Saving Expert 2	This user did not fill out the rating in time	Jingdong prefecture level city in Shandong
Jinfeng Hongyuan Flagship Store	Power Saving Expert 2	Good...good.	Jingdong prefecture level city in Shandong
Jinfeng Hongyuan Flagship Store	Power Saving Expert 2	(of an unmarried couple) be close	Jingdong prefecture level city in Shandong
Jinfeng Hongyuan Flagship Store	Power Saving Expert 2	(of an unmarried couple) be close	Jingdong prefecture level city in Shandong
Jinfeng Hongyuan Flagship Store	Power Saving Expert 2	(of an unmarried couple) be close	Jingdong prefecture level city in Shandong
Jinfeng Hongyuan Flagship Store	Power Saving Expert 2	The goods are real, high value, high quality, worth every penny, material appearance and quality at a glance is very upscale, very like HAPPY1 00	Jingdong prefecture level city in Shandong
Jinfeng Hongyuan Flagship Store	Power Saving Expert 2	It is the ideal product, good quality, logistics is also very fast, worthy of praise 🎁 baby quality is very good, received the first time to open the first time to see, it is very good 👍, very satisfied 😃, customer service is also available to ask questions and answer, thank you 🙏	Jingdong prefecture level city in Shandong
Jinfeng Hongyuan Flagship Store	Power Saving Expert 2	I bought it and haven't used it yet, I'll use it again.	Jingdong prefecture level city in Shandong
Jinfeng Hongyuan Flagship Store	Power Saving Expert 2	It didn't work, I used it for a few days and it was the same as before, it didn't work, I should have to return it	Jingdong prefecture level city in Shandong
Jinfeng Hongyuan Flagship Store	Power Saving Expert 2	The goods have been received, but have not yet installed and use, feel good.	Jingdong prefecture level city in Shandong
Jinfeng Hongyuan Flagship Store	Power Saving Expert 2	Goods received, I forgot to take pictures, the goods look good, you can start, immediately start the school to see the effect then	Jingdong prefecture level city in Shandong
Jinfeng Hongyuan Flagship Store	Power Saving Expert 2	Looks good. I'll use it for a while.	Jingdong prefecture level city in Shandong
Jinfeng Hongyuan Flagship Store	Power Saving Expert 2	Great stuff, easy to operate, easy to use, good power saving, believe in the power of science!	Jingdong prefecture level city in Shandong
Jinfeng Hongyuan Flagship Store	Power Saving Expert 2	Especially great, comparison shopping before buying, the store owner is very good, answering questions very patiently and detailed, there is a need to come back!	Jingdong prefecture level city in Shandong
Jinfeng Hongyuan Flagship Store	Power Saving Expert 2	Trustworthy, feel free to use it boldly.	Jingdong prefecture level city in Shandong
Jinfeng Hongyuan Flagship Store	Power Saving Expert 2	Logistics is super fast! Beautiful appearance! Looks like a great quality! I've never bought one before, so I'll try it first to see if it saves power. We'll see how much it saves.	Jingdong prefecture level city in Shandong
Jinfeng Hongyuan Flagship Store	Power Saving Expert 2	I saw online counterfeiting, energy saver is one of them, hahahahaha, jingdong also buy fake products	Jingdong prefecture level city in Shandong
Jinfeng Hongyuan Flagship Store	Power Saving Expert 2	Just received, installed, try a month to see how the effect and then follow up.	Jingdong prefecture level city in Shandong
Jinfeng Hongyuan Flagship Store	Power Saving Expert 2	Good for you! The goods are genuine! Good price for what you get!	Jingdong prefecture level city in Shandong
Jinfeng Hongyuan Flagship Store	Power Saving Expert 2	Things quickly received, immediately used to see if there is no effect, something river photo ⬆ the same, customer service is very professional	Jingdong prefecture level city in Shandong
Jinfeng Hongyuan Flagship Store	Power Saving Expert 2	The product is very convenient Looking forward to the subsequent use of the experience results	Jingdong prefecture level city in Shandong
Jinfeng Hongyuan Flagship Store	Power Saving Expert 2	I just received it and plugged it in, we'll see exactly when it's time to pay the electric bill	Jingdong prefecture level city in Shandong
Jinfeng Hongyuan Flagship Store	Power Saving Expert 2	Saver is good Packaging and looks good How effective it is to be shared after using it.	Jingdong prefecture level city in Shandong
Jinfeng Hongyuan Flagship Store	Power Saving Expert 2	There's no way to compare. I don't know if it's energy efficient.	Jingdong prefecture level city in Shandong
Jinfeng Hongyuan Flagship Store	Power Saving Expert 2	Just installed it, try it out and comment again	Jingdong prefecture level city in Shandong
Jinfeng Hongyuan Flagship Store	Power Saving Expert 2	Super save point also good to install, after a friend introduced then went to buy to, friends say it is very good to use, home I bought, try to use a period of time, to be honest, the economizer is not big or heavy, can be very convenient to put in a lot of places, the quality is also very good, power-saving function this time seems to be about 20% of the efficiency. The owner is also very warm contact, very	Jingdong prefecture level city in Shandong
Jinfeng Hongyuan Flagship Store	Power Saving Expert 2	The reviews online are mixed. Try it for a while, but I don't know if I can return it if it doesn't save any power after a couple months.	Jingdong prefecture level city in Shandong
Jinfeng Hongyuan Flagship Store	Power Saving Expert 2	The power consumption at home is high, bought a power saver, it looks very convenient to plug on the line, see if you can save some money next month, if good, recommend to friends down the road.	Jingdong prefecture level city in Shandong
Jinfeng Hongyuan Flagship Store	Power Saving Expert 2	It's beautiful, it's genuine, but I don't know the power-saving effect.	Jingdong prefecture level city in Shandong
Jinfeng Hongyuan Flagship Store	Power Saving Expert 2	This user thinks the product is good	Jingdong prefecture level city in Shandong
Jinfeng Hongyuan Flagship Store	Power Saving Expert 2	Nice exterior design with an at-a-glance display	Jingdong prefecture level city in Shandong
Jinfeng Hongyuan Flagship Store	Power Saving Expert 2	Home large and small things are purchased in the Jingdong Mall, have been accustomed to shopping in the Jingdong, many times can buy good quality and cheap products, and courier service attitude is particularly good, sometimes too many couriers are to help me to the home, so many years have been in the Jingdong Mall shopping, trust and support! Will also support the Jingdong as always!	Jingdong prefecture level city in Shandong
Jinfeng Hongyuan Flagship Store	Power Saving Expert 2	Good workmanship, mostly better than the original wall-plugged ones, and hopefully power factor compensation saves on electricity costs	Jingdong prefecture level city in Shandong
Jinfeng Hongyuan Flagship Store	Power Saving Expert 2	I don't feel anything, I said I won't know the effect for a month or two, let's see.	Jingdong prefecture level city in Shandong
Jinfeng Hongyuan Flagship Store	Power Saving Expert 2	Delivery was pretty fast, a little pricey, just not sure if it works or not	Jingdong prefecture level city in Shandong
Jinfeng Hongyuan Flagship Store	Power Saving Expert 2	The goods have been received, I do not know how effective.	Jingdong prefecture level city in Shandong
Jinfeng Hongyuan Flagship Store	Power Saving Expert 2	So far it doesn't seem to be too noticeable, so I'll comment later.	Jingdong prefecture level city in Shandong
Jinfeng Hongyuan Flagship Store	Power Saving Expert 2	I'll comment after I've used it	Jingdong prefecture level city in Shandong
Jinfeng Hongyuan Flagship Store	Power Saving Expert 2	Not bad. You'll save a lot on your electric bill in a year!	Jingdong prefecture level city in Shandong
Jinfeng Hongyuan Flagship Store	Power Saving Expert 2	I just got it. I haven't tried it yet. I don't know what it's like.	Jingdong prefecture level city in Shandong
Jinfeng Hongyuan Flagship Store	Power Saving Expert 2	Shopping at Jingdong has become a habit, and this disease will not be cured for a while.	Jingdong prefecture level city in Shandong
Jinfeng Hongyuan Flagship Store	Power Saving Expert 2	I've been using it for a while now. I don't know if I'm still saving power or not.	Jingdong prefecture level city in Shandong
Jinfeng Hongyuan Flagship Store	Power Saving Expert 2	Received the saver and it's great! There is also a digital voltage indication. Plugged in as instructed, easy to operate, hope you're our power saver!	Jingdong prefecture level city in Shandong

Jinfeng Hongyuan Flagship Store	Power Saving Expert 2	There is no power saving as the store says, using it is the same as not using it.	Jingdong prefecture level city in Shandong
Jinfeng Hongyuan Flagship Store	Power Saving Expert 2	Use it for a while and then comment again	Jingdong prefecture level city in Shandong
Jinfeng Hongyuan Flagship Store	Power Saving Expert 2	Feeling can, to use 1 to 2 months to see how the effect, there is a voltage display, can see the voltage fluctuations amplitude, quite good!	Jingdong prefecture level city in Shandong
Jinfeng Hongyuan Flagship Store	Power Saving Expert 2	I hope it works and saves me some electricity.	Jingdong prefecture level city in Shandong
Jinfeng Hongyuan Flagship Store	Power Saving Expert 2	Nice baby, happy with the after sales	Jingdong prefecture level city in Shandong
Jinfeng Hongyuan Flagship Store	Power Saving Expert 2	Use it and talk about it.	Jingdong prefecture level city in Shandong
Jinfeng Hongyuan Flagship Store	Power Saving Expert 2	Second purchase	Jingdong prefecture level city in Shandong
Jinfeng Hongyuan Flagship Store	Power Saving Expert 2	It works.	Jingdong prefecture level city in Shandong
Jinfeng Hongyuan Flagship Store	Power Saving Expert 2	It works.	Jingdong prefecture level city in Shandong
Jinfeng Hongyuan Flagship Store	Power Saving Expert 2	I don't know if it's a power saver until I start using it	Jingdong prefecture level city in Shandong
Jinfeng Hongyuan Flagship Store	Power Saving Expert 2	serviceable	Jingdong prefecture level city in Shandong
Jinfeng Hongyuan Flagship Store	Power Saving Expert 2	This user did not fill out the rating in time	Jingdong prefecture level city in Shandong
Jinfeng Hongyuan Flagship Store	Power Saving Expert 2	There is no power saving as the store says, using it is the same as not using it.	Jingdong prefecture level city in Shandong
Jinfeng Hongyuan Flagship Store	Power Saving Expert 2	This user did not fill out the rating in time	Jingdong prefecture level city in Shandong
Jinfeng Hongyuan Flagship Store	Power Saving Expert 2	Good...good.	Jingdong prefecture level city in Shandong
Jinfeng Hongyuan Flagship Store	Power Saving Expert 2	(of an unmarried couple) be close	Jingdong prefecture level city in Shandong
Jinfeng Hongyuan Flagship Store	Power Saving Expert 2	(of an unmarried couple) be close	Jingdong prefecture level city in Shandong
Jinfeng Hongyuan Flagship Store	Power Saving Expert 2	(of an unmarried couple) be close	Jingdong prefecture level city in Shandong
Jinfeng Hongyuan Flagship Store	Power Saving Expert 2	The goods are real, high value, high quality, worth every penny, material appearance and quality at a glance is very upscale, very like HAPPY1 00	Jingdong prefecture level city in Shandong
Jinfeng Hongyuan Flagship Store	Power Saving Expert 2	It is the ideal product, good quality, logistics is also very fast, worthy of praise 🎁 baby quality is very good, received the first time to open the first time to see, it is very good 👍, very satisfied 😃, customer service is also available to ask questions and answer, thank you 🙏	Jingdong prefecture level city in Shandong
Jinfeng Hongyuan Flagship Store	Power Saving Expert 2	I bought it and haven't used it yet, I'll use it again.	Jingdong prefecture level city in Shandong
Jinfeng Hongyuan Flagship Store	Power Saving Expert 2	It didn't work, I used it for a few days and it was the same as before, it didn't work, I should have to return it	Jingdong prefecture level city in Shandong
Jinfeng Hongyuan Flagship Store	Power Saving Expert 2	The goods have been received, but have not yet installed and use, feel good.	Jingdong prefecture level city in Shandong
Jinfeng Hongyuan Flagship Store	Power Saving Expert 2	Goods received, I forgot to take pictures, the goods look good, you can start, immediately start the school to see the effect then	Jingdong prefecture level city in Shandong
Jinfeng Hongyuan Flagship Store	Power Saving Expert 2	Looks good. I'll use it for a while.	Jingdong prefecture level city in Shandong
Jinfeng Hongyuan Flagship Store	Power Saving Expert 2	Great stuff, easy to operate, easy to use, good power saving, believe in the power of science!	Jingdong prefecture level city in Shandong
Jinfeng Hongyuan Flagship Store	Power Saving Expert 2	Especially great, comparison shopping before buying, the store owner is very good, answering questions very patiently and detailed, there is a need to come back!	Jingdong prefecture level city in Shandong
Jinfeng Hongyuan Flagship Store	Power Saving Expert 2	Trustworthy, feel free to use it boldly.	Jingdong prefecture level city in Shandong
Jinfeng Hongyuan Flagship Store	Power Saving Expert 2	Logistics is super fast! Beautiful appearance! Looks like a great quality! I've never bought one before, so I'll try it first to see if it saves power. We'll see how much it saves.	Jingdong prefecture level city in Shandong
Jinfeng Hongyuan Flagship Store	Power Saving Expert 2	I saw online counterfeiting, energy saver is one of them, hahahahaha, jingdong also buy fake products	Jingdong prefecture level city in Shandong
Jinfeng Hongyuan Flagship Store	Power Saving Expert 2	Just received, installed, try a month to see how the effect and then follow up.	Jingdong prefecture level city in Shandong
Jinfeng Hongyuan Flagship Store	Power Saving Expert 2	Good for you! The goods are genuine! Good price for what you get!	Jingdong prefecture level city in Shandong
Jinfeng Hongyuan Flagship Store	Power Saving Expert 2	Things quickly received, immediately used to see if there is no effect, something river photo ⬆ the same, customer service is very professional	Jingdong prefecture level city in Shandong
Jinfeng Hongyuan Flagship Store	Power Saving Expert 2	The product is very convenient Looking forward to the subsequent use of the experience results	Jingdong prefecture level city in Shandong
Jinfeng Hongyuan Flagship Store	Power Saving Expert 2	I just received it and plugged it in, we'll see exactly when it's time to pay the electric bill	Jingdong prefecture level city in Shandong
Jinfeng Hongyuan Flagship Store	Power Saving Expert 2	Saver is good Packaging and looks good How effective it is to be shared after using it.	Jingdong prefecture level city in Shandong
Jinfeng Hongyuan Flagship Store	Power Saving Expert 2	There's no way to compare. I don't know if it's energy efficient.	Jingdong prefecture level city in Shandong
Jinfeng Hongyuan Flagship Store	Power Saving Expert 2	Just installed it, try it out and comment again	Jingdong prefecture level city in Shandong
Jinfeng Hongyuan Flagship Store	Power Saving Expert 2	Super save point also good to install, after a friend introduced then went to buy to, friends say it is very good to use, home I bought, try to use a period of time, to be honest, the economizer is not big or heavy, can be very convenient to put in a lot of places, the quality is also very good, power-saving function this time seems to be about 20% of the efficiency. The owner is also very warm contact, very	Jingdong prefecture level city in Shandong
Jinfeng Hongyuan Flagship Store	Power Saving Expert 2	The reviews online are mixed. Try it for a while, but I don't know if I can return it if it doesn't save any power after a couple months.	Jingdong prefecture level city in Shandong
Jinfeng Hongyuan Flagship Store	Power Saving Expert 2	The power consumption at home is high, bought a power saver, it looks very convenient to plug on the line, see if you can save some money next month, if good, recommend to friends down the road.	Jingdong prefecture level city in Shandong
Jinfeng Hongyuan Flagship Store	Power Saving Expert 2	It's beautiful, it's genuine, but I don't know the power-saving effect.	Jingdong prefecture level city in Shandong
Jinfeng Hongyuan Flagship Store	Power Saving Expert 2	This user thinks the product is good	Jingdong prefecture level city in Shandong
Jinfeng Hongyuan Flagship Store	Power Saving Expert 2	Nice exterior design with an at-a-glance display	Jingdong prefecture level city in Shandong
Jinfeng Hongyuan Flagship Store	Power Saving Expert 2	Home large and small things are purchased in the Jingdong Mall, have been accustomed to shopping in the Jingdong, many times can buy good quality and cheap products, and courier service attitude is particularly good, sometimes too many couriers are to help me to the home, so many years have been in the Jingdong Mall shopping, trust and support! Will also support the Jingdong as always!	Jingdong prefecture level city in Shandong
Jinfeng Hongyuan Flagship Store	Power Saving Expert 2	Good workmanship, mostly better than the original wall-plugged ones, and hopefully power factor compensation saves on electricity costs	Jingdong prefecture level city in Shandong
Jinfeng Hongyuan Flagship Store	Power Saving Expert 2	I don't feel anything, I said I won't know the effect for a month or two, let's see.	Jingdong prefecture level city in Shandong
Jinfeng Hongyuan Flagship Store	Power Saving Expert 2	Delivery was pretty fast, a little pricey, just not sure if it works or not	Jingdong prefecture level city in Shandong
Jinfeng Hongyuan Flagship Store	Power Saving Expert 2	The goods have been received, I do not know how effective.	Jingdong prefecture level city in Shandong

Jinfeng Hongyuan Flagship Store	Power Saving Expert 2	So far it doesn't seem to be too noticeable, so I'll comment later.	Jingdong prefecture level city in Shandong
Jinfeng Hongyuan Flagship Store	Power Saving Expert 2	I'll comment after I've used it	Jingdong prefecture level city in Shandong
Jinfeng Hongyuan Flagship Store	Power Saving Expert 2	Not bad. You'll save a lot on your electric bill in a year!	Jingdong prefecture level city in Shandong
Jinfeng Hongyuan Flagship Store	Power Saving Expert 2	I just got it. I haven't tried it yet. I don't know what it's like.	Jingdong prefecture level city in Shandong
Jinfeng Hongyuan Flagship Store	Power Saving Expert 2	Shopping at Jingdong has become a habit, and this disease will not be cured for a while.	Jingdong prefecture level city in Shandong
Jinfeng Hongyuan Flagship Store	Power Saving Expert 2	I've been using it for a while now. I don't know if I'm still saving power or not.	Jingdong prefecture level city in Shandong
Jinfeng Hongyuan Flagship Store	Power Saving Expert 2	Received the saver and it's great! There is also a digital voltage indication. Plugged in as instructed, easy to operate, hope you're our power saver!	Jingdong prefecture level city in Shandong
Jinfeng Hongyuan Flagship Store	Power Saving Expert 2	There is no power saving as the store says, using it is the same as not using it.	Jingdong prefecture level city in Shandong
Jinfeng Hongyuan Flagship Store	Power Saving Expert 2	Use it for a while and then comment again	Jingdong prefecture level city in Shandong
Jinfeng Hongyuan Flagship Store	Power Saving Expert 2	Feeling can, to use 1 to 2 months to see how the effect, there is a voltage display, can see the voltage fluctuations amplitude, quite good!	Jingdong prefecture level city in Shandong
Jinfeng Hongyuan Flagship Store	Power Saving Expert 2	I hope it works and saves me some electricity.	Jingdong prefecture level city in Shandong
Jinfeng Hongyuan Flagship Store	Power Saving Expert 2	Nice baby, happy with the after sales	Jingdong prefecture level city in Shandong
Jinfeng Hongyuan Flagship Store	Power Saving Expert 2	Use it and talk about it.	Jingdong prefecture level city in Shandong
Jinfeng Hongyuan Flagship Store	Power Saving Expert 2	Second purchase	Jingdong prefecture level city in Shandong
Jinfeng Hongyuan Flagship Store	Power Saving Expert 2	It works.	Jingdong prefecture level city in Shandong
Jinfeng Hongyuan Flagship Store	Power Saving Expert 2	It works.	Jingdong prefecture level city in Shandong
Jinfeng Hongyuan Flagship Store	Power Saving Expert 2	I don't know if it's a power saver until I start using it	Jingdong prefecture level city in Shandong
Jinfeng Hongyuan Flagship Store	Power Saving Expert 2	serviceable	Jingdong prefecture level city in Shandong
Jinfeng Hongyuan Flagship Store	Power Saving Expert 2	This user did not fill out the rating in time	Jingdong prefecture level city in Shandong
Jinfeng Hongyuan Flagship Store	Power Saving Expert 2	There is no power saving as the store says, using it is the same as not using it.	Jingdong prefecture level city in Shandong
Jinfeng Hongyuan Flagship Store	Power Saving Expert 2	This user did not fill out the rating in time	Jingdong prefecture level city in Shandong
Jinfeng Hongyuan Flagship Store	Power Saving Expert 2	Good...good.	Jingdong prefecture level city in Shandong
Jinfeng Hongyuan Flagship Store	Power Saving Expert 2	(of an unmarried couple) be close	Jingdong prefecture level city in Shandong
Jinfeng Hongyuan Flagship Store	Power Saving Expert 2	(of an unmarried couple) be close	Jingdong prefecture level city in Shandong
Jinfeng Hongyuan Flagship Store	Power Saving Expert 2	(of an unmarried couple) be close	Jingdong prefecture level city in Shandong
Jinfeng Hongyuan Flagship Store	Power Saving Expert 2	The goods are real, high value, high quality, worth every penny, material appearance and quality at a glance is very upscale, very like HAPPY1 00	Jingdong prefecture level city in Shandong
Jinfeng Hongyuan Flagship Store	Power Saving Expert 2	It is the ideal product, good quality, logistics is also very fast, worthy of praise 🎁 baby quality is very good, received the first time to open the first time to see, it is very good 👍, very satisfied 😃, customer service is also available to ask questions and answer, thank you 🙏	Jingdong prefecture level city in Shandong
Jinfeng Hongyuan Flagship Store	Power Saving Expert 2	I bought it and haven't used it yet, I'll use it again.	Jingdong prefecture level city in Shandong
Jinfeng Hongyuan Flagship Store	Power Saving Expert 2	It didn't work, I used it for a few days and it was the same as before, it didn't work, I should have to return it	Jingdong prefecture level city in Shandong
Jinfeng Hongyuan Flagship Store	Power Saving Expert 2	The goods have been received, but have not yet installed and use, feel good.	Jingdong prefecture level city in Shandong
Jinfeng Hongyuan Flagship Store	Power Saving Expert 2	Goods received, I forgot to take pictures, the goods look good, you can start, immediately start the school to see the effect then	Jingdong prefecture level city in Shandong
Jinfeng Hongyuan Flagship Store	Power Saving Expert 2	Looks good. I'll use it for a while.	Jingdong prefecture level city in Shandong
Jinfeng Hongyuan Flagship Store	Power Saving Expert 2	Great stuff, easy to operate, easy to use, good power saving, believe in the power of science!	Jingdong prefecture level city in Shandong
Jinfeng Hongyuan Flagship Store	Power Saving Expert 2	Especially great, comparison shopping before buying, the store owner is very good, answering questions very patiently and detailed, there is a need to come back!	Jingdong prefecture level city in Shandong
Jinfeng Hongyuan Flagship Store	Power Saving Expert 2	Trustworthy, feel free to use it boldly.	Jingdong prefecture level city in Shandong
Jinfeng Hongyuan Flagship Store	Power Saving Expert 2	Logistics is super fast! Beautiful appearance! Looks like a great quality! I've never bought one before, so I'll try it first to see if it saves power. We'll see how much it saves.	Jingdong prefecture level city in Shandong
Jinfeng Hongyuan Flagship Store	Power Saving Expert 2	I saw online counterfeiting, energy saver is one of them, hahahahaha, jingdong also buy fake products	Jingdong prefecture level city in Shandong
Jinfeng Hongyuan Flagship Store	Power Saving Expert 2	Just received, installed, try a month to see how the effect and then follow up.	Jingdong prefecture level city in Shandong
Jinfeng Hongyuan Flagship Store	Power Saving Expert 2	Good for you! The goods are genuine! Good price for what you get!	Jingdong prefecture level city in Shandong
Jinfeng Hongyuan Flagship Store	Power Saving Expert 2	Things quickly received, immediately used to see if there is no effect, something river photo ⬆ the same, customer service is very professional	Jingdong prefecture level city in Shandong
Jinfeng Hongyuan Flagship Store	Power Saving Expert 2	The product is very convenient Looking forward to the subsequent use of the experience results	Jingdong prefecture level city in Shandong
Jinfeng Hongyuan Flagship Store	Power Saving Expert 2	I just received it and plugged it in, we'll see exactly when it's time to pay the electric bill	Jingdong prefecture level city in Shandong
Jinfeng Hongyuan Flagship Store	Power Saving Expert 2	Saver is good Packaging and looks good How effective it is to be shared after using it.	Jingdong prefecture level city in Shandong
Jinfeng Hongyuan Flagship Store	Power Saving Expert 2	There's no way to compare. I don't know if it's energy efficient.	Jingdong prefecture level city in Shandong
Jinfeng Hongyuan Flagship Store	Power Saving Expert 2	Just installed it, try it out and comment again	Jingdong prefecture level city in Shandong
Jinfeng Hongyuan Flagship Store	Power Saving Expert 2	Super save point also good to install, after a friend introduced then went to buy to, friends say it is very good to use, home I bought, try to use a period of time, to be honest, the economizer is not big or heavy, can be very convenient to put in a lot of places, the quality is also very good, power-saving function this time seems to be about 20% of the efficiency. The owner is also very warm contact, very	Jingdong prefecture level city in Shandong
Jinfeng Hongyuan Flagship Store	Power Saving Expert 2	The reviews online are mixed. Try it for a while, but I don't know if I can return it if it doesn't save any power after a couple months.	Jingdong prefecture level city in Shandong
Jinfeng Hongyuan Flagship Store	Power Saving Expert 2	The power consumption at home is high, bought a power saver, it looks very convenient to plug on the line, see if you can save some money next month, if good, recommend to friends down the road.	Jingdong prefecture level city in Shandong
Jinfeng Hongyuan Flagship Store	Power Saving Expert 2	It's beautiful, it's genuine, but I don't know the power-saving effect.	Jingdong prefecture level city in Shandong
Jinfeng Hongyuan Flagship Store	Power Saving Expert 2	This user thinks the product is good	Jingdong prefecture level city in Shandong

Jinfeng Hongyuan Flagship Store	Power Saving Expert 2	Nice exterior design with an at-a-glance display	Jingdong prefecture level city in Shandong
Jinfeng Hongyuan Flagship Store	Power Saving Expert 2	Home large and small things are purchased in the Jingdong Mall, have been accustomed to shopping in the Jingdong, many times can buy good quality and cheap products, and courier service attitude is particularly good, sometimes too many couriers are to help me to the home, so many years have been in the Jingdong Mall shopping, trust and support! Will also support the Jingdong as always!	Jingdong prefecture level city in Shandong
Jinfeng Hongyuan Flagship Store	Power Saving Expert 2	Good workmanship, mostly better than the original wall-plugged ones, and hopefully power factor compensation saves on electricity costs	Jingdong prefecture level city in Shandong
Jinfeng Hongyuan Flagship Store	Power Saving Expert 2	I don't feel anything, I said I won't know the effect for a month or two, let's see.	Jingdong prefecture level city in Shandong
Jinfeng Hongyuan Flagship Store	Power Saving Expert 2	Delivery was pretty fast, a little pricey, just not sure if it works or not	Jingdong prefecture level city in Shandong
Jinfeng Hongyuan Flagship Store	Power Saving Expert 2	The goods have been received, I do not know how effective.	Jingdong prefecture level city in Shandong
Jinfeng Hongyuan Flagship Store	Power Saving Expert 2	So far it doesn't seem to be too noticeable, so I'll comment later.	Jingdong prefecture level city in Shandong
Jinfeng Hongyuan Flagship Store	Power Saving Expert 2	I'll comment after I've used it	Jingdong prefecture level city in Shandong
Jinfeng Hongyuan Flagship Store	Power Saving Expert 2	Not bad. You'll save a lot on your electric bill in a year!	Jingdong prefecture level city in Shandong
Jinfeng Hongyuan Flagship Store	Power Saving Expert 2	I just got it. I haven't tried it yet. I don't know what it's like.	Jingdong prefecture level city in Shandong
Jinfeng Hongyuan Flagship Store	Power Saving Expert 2	Shopping at Jingdong has become a habit, and this disease will not be cured for a while.	Jingdong prefecture level city in Shandong
Jinfeng Hongyuan Flagship Store	Power Saving Expert 2	I've been using it for a while now. I don't know if I'm still saving power or not.	Jingdong prefecture level city in Shandong
Jinfeng Hongyuan Flagship Store	Power Saving Expert 2	Received the saver and it's great! There is also a digital voltage indication. Plugged in as instructed, easy to operate, hope you're our power saver!	Jingdong prefecture level city in Shandong
Jinfeng Hongyuan Flagship Store	Power Saving Expert 2	There is no power saving as the store says, using it is the same as not using it.	Jingdong prefecture level city in Shandong
Jinfeng Hongyuan Flagship Store	Power Saving Expert 2	Use it for a while and then comment again	Jingdong prefecture level city in Shandong
Jinfeng Hongyuan Flagship Store	Power Saving Expert 2	Feeling can, to use 1 to 2 months to see how the effect, there is a voltage display, can see the voltage fluctuations amplitude, quite good!	Jingdong prefecture level city in Shandong
Jinfeng Hongyuan Flagship Store	Power Saving Expert 2	I hope it works and saves me some electricity.	Jingdong prefecture level city in Shandong
Jinfeng Hongyuan Flagship Store	Power Saving Expert 2	Nice baby, happy with the after sales	Jingdong prefecture level city in Shandong
Jinfeng Hongyuan Flagship Store	Power Saving Expert 2	Use it and talk about it.	Jingdong prefecture level city in Shandong
Jinfeng Hongyuan Flagship Store	Power Saving Expert 2	Second purchase	Jingdong prefecture level city in Shandong
Jinfeng Hongyuan Flagship Store	Power Saving Expert 2	It works.	Jingdong prefecture level city in Shandong
Jinfeng Hongyuan Flagship Store	Power Saving Expert 2	It works.	Jingdong prefecture level city in Shandong
Jinfeng Hongyuan Flagship Store	Power Saving Expert 2	I don't know if it's a power saver until I start using it	Jingdong prefecture level city in Shandong
Jinfeng Hongyuan Flagship Store	Power Saving Expert 2	serviceable	Jingdong prefecture level city in Shandong
Jinfeng Hongyuan Flagship Store	Power Saving Expert 2	This user did not fill out the rating in time	Jingdong prefecture level city in Shandong
Jinfeng Hongyuan Flagship Store	Power Saving Expert 2	There is no power saving as the store says, using it is the same as not using it.	Jingdong prefecture level city in Shandong
Jinfeng Hongyuan Flagship Store	Power Saving Expert 2	This user did not fill out the rating in time	Jingdong prefecture level city in Shandong
Jinfeng Hongyuan Flagship Store	Power Saving Expert 2	Good...good.	Jingdong prefecture level city in Shandong
Jinfeng Hongyuan Flagship Store	Power Saving Expert 2	(of an unmarried couple) be close	Jingdong prefecture level city in Shandong
Jinfeng Hongyuan Flagship Store	Power Saving Expert 2	(of an unmarried couple) be close	Jingdong prefecture level city in Shandong
Jinfeng Hongyuan Flagship Store	Power Saving Expert 2	(of an unmarried couple) be close	Jingdong prefecture level city in Shandong
Jinfeng Hongyuan Flagship Store	Power Saving Expert 2	The goods are real, high value, high quality, worth every penny, material appearance and quality at a glance is very upscale, very like HAPPY1 00	Jingdong prefecture level city in Shandong
Jinfeng Hongyuan Flagship Store	Power Saving Expert 2	It is the ideal product, good quality, logistics is also very fast, worthy of praise 🎁 baby quality is very good, received the first time to open the first time to see, it is very good 👍, very satisfied 😃, customer service is also available to ask questions and answer, thank you 🙏	Jingdong prefecture level city in Shandong
Jinfeng Hongyuan Flagship Store	Power Saving Expert 2	I bought it and haven't used it yet, I'll use it again.	Jingdong prefecture level city in Shandong
Jinfeng Hongyuan Flagship Store	Power Saving Expert 2	It didn't work, I used it for a few days and it was the same as before, it didn't work, I should have to return it	Jingdong prefecture level city in Shandong
Jinfeng Hongyuan Flagship Store	Power Saving Expert 2	The goods have been received, but have not yet installed and use, feel good.	Jingdong prefecture level city in Shandong
Jinfeng Hongyuan Flagship Store	Power Saving Expert 2	Goods received, I forgot to take pictures, the goods look good, you can start, immediately start the school to see the effect then	Jingdong prefecture level city in Shandong
Jinfeng Hongyuan Flagship Store	Power Saving Expert 2	Looks good. I'll use it for a while.	Jingdong prefecture level city in Shandong
Jinfeng Hongyuan Flagship Store	Power Saving Expert 2	Great stuff, easy to operate, easy to use, good power saving, believe in the power of science!	Jingdong prefecture level city in Shandong
Jinfeng Hongyuan Flagship Store	Power Saving Expert 2	Especially great, comparison shopping before buying, the store owner is very good, answering questions very patiently and detailed, there is a need to come back!	Jingdong prefecture level city in Shandong
Jinfeng Hongyuan Flagship Store	Power Saving Expert 2	Trustworthy, feel free to use it boldly.	Jingdong prefecture level city in Shandong
Jinfeng Hongyuan Flagship Store	Power Saving Expert 2	Logistics is super fast! Beautiful appearance! Looks like a great quality! I've never bought one before, so I'll try it first to see if it saves power. We'll see how much it saves.	Jingdong prefecture level city in Shandong
Jinfeng Hongyuan Flagship Store	Power Saving Expert 2	I saw online counterfeiting, energy saver is one of them, hahahahaha, jingdong also buy fake products	Jingdong prefecture level city in Shandong
Jinfeng Hongyuan Flagship Store	Power Saving Expert 2	Just received, installed, try a month to see how the effect and then follow up.	Jingdong prefecture level city in Shandong
Jinfeng Hongyuan Flagship Store	Power Saving Expert 2	Good for you! The goods are genuine! Good price for what you get!	Jingdong prefecture level city in Shandong
Jinfeng Hongyuan Flagship Store	Power Saving Expert 2	Things quickly received, immediately used to see if there is no effect, something river photo ⬆ the same, customer service is very professional	Jingdong prefecture level city in Shandong
Jinfeng Hongyuan Flagship Store	Power Saving Expert 2	The product is very convenient Looking forward to the subsequent use of the experience results	Jingdong prefecture level city in Shandong
Jinfeng Hongyuan Flagship Store	Power Saving Expert 2	I just received it and plugged it in, we'll see exactly when it's time to pay the electric bill	Jingdong prefecture level city in Shandong
Jinfeng Hongyuan Flagship Store	Power Saving Expert 2	Saver is good Packaging and looks good How effective it is to be shared after using it.	Jingdong prefecture level city in Shandong
Jinfeng Hongyuan Flagship Store	Power Saving Expert 2	There's no way to compare. I don't know if it's energy efficient.	Jingdong prefecture level city in Shandong

Jinfeng Hongyuan Flagship Store	Power Saving Expert 2	Just installed it, try it out and comment again	Jingdong prefecture level city in Shandong
Jinfeng Hongyuan Flagship Store	Power Saving Expert 2	Super save point also good to install, after a friend introduced then went to buy to, friends say it is very good to use, home I bought, try to use a period of time, to be honest, the economizer is not big or heavy, can be very convenient to put in a lot of places, the quality is also very good, power-saving function this time seems to be about 20% of the efficiency. The owner is also very warm contact, very	Jingdong prefecture level city in Shandong
Jinfeng Hongyuan Flagship Store	Power Saving Expert 2	The reviews online are mixed. Try it for a while, but I don't know if I can return it if it doesn't save any power after a couple months.	Jingdong prefecture level city in Shandong
Jinfeng Hongyuan Flagship Store	Power Saving Expert 2	The power consumption at home is high, bought a power saver, it looks very convenient to plug on the line, see if you can save some money next month, if good, recommend to friends down the road.	Jingdong prefecture level city in Shandong
Jinfeng Hongyuan Flagship Store	Power Saving Expert 2	It's beautiful, it's genuine, but I don't know the power-saving effect.	Jingdong prefecture level city in Shandong
Jinfeng Hongyuan Flagship Store	Power Saving Expert 2	This user thinks the product is good	Jingdong prefecture level city in Shandong
Jinfeng Hongyuan Flagship Store	Power Saving Expert 2	Nice exterior design with an at-a-glance display	Jingdong prefecture level city in Shandong
Jinfeng Hongyuan Flagship Store	Power Saving Expert 2	Home large and small things are purchased in the Jingdong Mall, have been accustomed to shopping in the Jingdong, many times can buy good quality and cheap products, and courier service attitude is particularly good, sometimes too many couriers are to help me to the home, so many years have been in the Jingdong Mall shopping, trust and support! Will also support the Jingdong as always!	Jingdong prefecture level city in Shandong
Jinfeng Hongyuan Flagship Store	Power Saving Expert 2	Good workmanship, mostly better than the original wall-plugged ones, and hopefully power factor compensation saves on electricity costs	Jingdong prefecture level city in Shandong
Jinfeng Hongyuan Flagship Store	Power Saving Expert 2	I don't feel anything, I said I won't know the effect for a month or two, let's see.	Jingdong prefecture level city in Shandong
Jinfeng Hongyuan Flagship Store	Power Saving Expert 2	Delivery was pretty fast, a little pricey, just not sure if it works or not	Jingdong prefecture level city in Shandong
Jinfeng Hongyuan Flagship Store	Power Saving Expert 2	The goods have been received, I do not know how effective.	Jingdong prefecture level city in Shandong
Jinfeng Hongyuan Flagship Store	Power Saving Expert 2	So far it doesn't seem to be too noticeable, so I'll comment later.	Jingdong prefecture level city in Shandong
Jinfeng Hongyuan Flagship Store	Power Saving Expert 2	I'll comment after I've used it	Jingdong prefecture level city in Shandong
Jinfeng Hongyuan Flagship Store	Power Saving Expert 2	Not bad. You'll save a lot on your electric bill in a year!	Jingdong prefecture level city in Shandong
Jinfeng Hongyuan Flagship Store	Power Saving Expert 2	I just got it. I haven't tried it yet. I don't know what it's like.	Jingdong prefecture level city in Shandong
Jinfeng Hongyuan Flagship Store	Power Saving Expert 2	Shopping at Jingdong has become a habit, and this disease will not be cured for a while.	Jingdong prefecture level city in Shandong
Jinfeng Hongyuan Flagship Store	Power Saving Expert 2	I've been using it for a while now. I don't know if I'm still saving power or not.	Jingdong prefecture level city in Shandong
Jinfeng Hongyuan Flagship Store	Power Saving Expert 2	Received the saver and it's great! There is also a digital voltage indication. Plugged in as instructed, easy to operate, hope you're our power saver!	Jingdong prefecture level city in Shandong
Jinfeng Hongyuan Flagship Store	Power Saving Expert 2	There is no power saving as the store says, using it is the same as not using it.	Jingdong prefecture level city in Shandong
Jinfeng Hongyuan Flagship Store	Power Saving Expert 2	Use it for a while and then comment again	Jingdong prefecture level city in Shandong
Jinfeng Hongyuan Flagship Store	Power Saving Expert 2	Feeling can, to use 1 to 2 months to see how the effect, there is a voltage display, can see the voltage fluctuations amplitude, quite good!	Jingdong prefecture level city in Shandong
Jinfeng Hongyuan Flagship Store	Power Saving Expert 2	I hope it works and saves me some electricity.	Jingdong prefecture level city in Shandong
Jinfeng Hongyuan Flagship Store	Power Saving Expert 2	Nice baby, happy with the after sales	Jingdong prefecture level city in Shandong
Jinfeng Hongyuan Flagship Store	Power Saving Expert 2	Use it and talk about it.	Jingdong prefecture level city in Shandong
Jinfeng Hongyuan Flagship Store	Power Saving Expert 2	Second purchase	Jingdong prefecture level city in Shandong
Jinfeng Hongyuan Flagship Store	Power Saving Expert 2	It works.	Jingdong prefecture level city in Shandong
Jinfeng Hongyuan Flagship Store	Power Saving Expert 2	It works.	Jingdong prefecture level city in Shandong
Jinfeng Hongyuan Flagship Store	Power Saving Expert 2	I don't know if it's a power saver until I start using it	Jingdong prefecture level city in Shandong
Jinfeng Hongyuan Flagship Store	Power Saving Expert 2	serviceable	Jingdong prefecture level city in Shandong
Jinfeng Hongyuan Flagship Store	Power Saving Expert 2	This user did not fill out the rating in time	Jingdong prefecture level city in Shandong
Jinfeng Hongyuan Flagship Store	Power Saving Expert 2	There is no power saving as the store says, using it is the same as not using it.	Jingdong prefecture level city in Shandong
Jinfeng Hongyuan Flagship Store	Power Saving Expert 2	This user did not fill out the rating in time	Jingdong prefecture level city in Shandong
Jinfeng Hongyuan Flagship Store	Power Saving Expert 2	Good...good.	Jingdong prefecture level city in Shandong
Jinfeng Hongyuan Flagship Store	Power Saving Expert 2	(of an unmarried couple) be close	Jingdong prefecture level city in Shandong
Jinfeng Hongyuan Flagship Store	Power Saving Expert 2	(of an unmarried couple) be close	Jingdong prefecture level city in Shandong
Jinfeng Hongyuan Flagship Store	Power Saving Expert 2	(of an unmarried couple) be close	Jingdong prefecture level city in Shandong
Jinfeng Hongyuan Flagship Store	Power Saving Expert 2	The goods are real, high value, high quality, worth every penny, material appearance and quality at a glance is very upscale, very like HAPPY1 00	Jingdong prefecture level city in Shandong
Jinfeng Hongyuan Flagship Store	Power Saving Expert 2	It is the ideal product, good quality, logistics is also very fast, worthy of praise 🎁 baby quality is very good, received the first time to open the first time to see, it is very good 👍, very satisfied 😃, customer service is also available to ask questions and answer, thank you 🙏	Jingdong prefecture level city in Shandong
Jinfeng Hongyuan Flagship Store	Power Saving Expert 2	I bought it and haven't used it yet, I'll use it again.	Jingdong prefecture level city in Shandong
Jinfeng Hongyuan Flagship Store	Power Saving Expert 2	It didn't work, I used it for a few days and it was the same as before, it didn't work, I should have to return it	Jingdong prefecture level city in Shandong
Jinfeng Hongyuan Flagship Store	Power Saving Expert 2	The goods have been received, but have not yet installed and use, feel good.	Jingdong prefecture level city in Shandong
Jinfeng Hongyuan Flagship Store	Power Saving Expert 2	Goods received, I forgot to take pictures, the goods look good, you can start, immediately start the school to see the effect then	Jingdong prefecture level city in Shandong
Jinfeng Hongyuan Flagship Store	Power Saving Expert 2	Looks good. I'll use it for a while.	Jingdong prefecture level city in Shandong
Jinfeng Hongyuan Flagship Store	Power Saving Expert 2	Great stuff, easy to operate, easy to use, good power saving, believe in the power of science!	Jingdong prefecture level city in Shandong
Jinfeng Hongyuan Flagship Store	Power Saving Expert 2	Especially great, comparison shopping before buying, the store owner is very good, answering questions very patiently and detailed, there is a need to come back!	Jingdong prefecture level city in Shandong
Jinfeng Hongyuan Flagship Store	Power Saving Expert 2	Trustworthy, feel free to use it boldly.	Jingdong prefecture level city in Shandong
Jinfeng Hongyuan Flagship Store	Power Saving Expert 2	Logistics is super fast! Beautiful appearance! Looks like a great quality! I've never bought one before, so I'll try it first to see if it saves power. We'll see how much it saves.	Jingdong prefecture level city in Shandong
Jinfeng Hongyuan Flagship Store	Power Saving Expert 2	I saw online counterfeiting, energy saver is one of them, hahahahaha, jingdong also buy fake products	Jingdong prefecture level city in Shandong

Jinfeng Hongyuan Flagship Store	Power Saving Expert 2	Just received, installed, try a month to see how the effect and then follow up.	Jingdong prefecture level city in Shandong
Jinfeng Hongyuan Flagship Store	Power Saving Expert 2	Good for you! The goods are genuine! Good price for what you get!	Jingdong prefecture level city in Shandong
Jinfeng Hongyuan Flagship Store	Power Saving Expert 2	Things quickly received, immediately used to see if there is no effect, something river photo 🔝 the same, customer service is very professional	Jingdong prefecture level city in Shandong
Jinfeng Hongyuan Flagship Store	Power Saving Expert 2	The product is very convenient Looking forward to the subsequent use of the experience results	Jingdong prefecture level city in Shandong
Jinfeng Hongyuan Flagship Store	Power Saving Expert 2	I just received it and plugged it in, we'll see exactly when it's time to pay the electric bill	Jingdong prefecture level city in Shandong
Jinfeng Hongyuan Flagship Store	Power Saving Expert 2	Saver is good Packaging and looks good How effective it is to be shared after using it.	Jingdong prefecture level city in Shandong
Jinfeng Hongyuan Flagship Store	Power Saving Expert 2	There's no way to compare. I don't know if it's energy efficient.	Jingdong prefecture level city in Shandong
Jinfeng Hongyuan Flagship Store	Power Saving Expert 2	Just installed it, try it out and comment again	Jingdong prefecture level city in Shandong
Jinfeng Hongyuan Flagship Store	Power Saving Expert 2	Super save point also good to install, after a friend introduced then went to buy to, friends say it is very good to use, home I bought, try to use a period of time, to be honest, the economizer is not big or heavy, can be very convenient to put in a lot of places, the quality is also very good, power-saving function this time seems to be about 20% of the efficiency. The owner is also very warm contact, very	Jingdong prefecture level city in Shandong
Jinfeng Hongyuan Flagship Store	Power Saving Expert 2	The reviews online are mixed. Try it for a while, but I don't know if I can return it if it doesn't save any power after a couple months.	Jingdong prefecture level city in Shandong
Jinfeng Hongyuan Flagship Store	Power Saving Expert 2	The power consumption at home is high, bought a power saver, it looks very convenient to plug on the line, see if you can save some money next month, if good, recommend to friends down the road.	Jingdong prefecture level city in Shandong
Jinfeng Hongyuan Flagship Store	Power Saving Expert 2	It's beautiful, it's genuine, but I don't know the power-saving effect.	Jingdong prefecture level city in Shandong
Jinfeng Hongyuan Flagship Store	Power Saving Expert 2	This user thinks the product is good	Jingdong prefecture level city in Shandong
Jinfeng Hongyuan Flagship Store	Power Saving Expert 2	Nice exterior design with an at-a-glance display	Jingdong prefecture level city in Shandong
Jinfeng Hongyuan Flagship Store	Power Saving Expert 2	Home large and small things are purchased in the Jingdong Mall, have been accustomed to shopping in the Jingdong, many times can buy good quality and cheap products, and courier service attitude is particularly good, sometimes too many couriers are to help me to the home, so many years have been in the Jingdong Mall shopping, trust and support! Will also support the Jingdong as always!	Jingdong prefecture level city in Shandong
Jinfeng Hongyuan Flagship Store	Power Saving Expert 2	Good workmanship, mostly better than the original wall-plugged ones, and hopefully power factor compensation saves on electricity costs	Jingdong prefecture level city in Shandong
Jinfeng Hongyuan Flagship Store	Power Saving Expert 2	I don't feel anything, I said I won't know the effect for a month or two, let's see.	Jingdong prefecture level city in Shandong
Jinfeng Hongyuan Flagship Store	Power Saving Expert 2	Delivery was pretty fast, a little pricey, just not sure if it works or not	Jingdong prefecture level city in Shandong
Jinfeng Hongyuan Flagship Store	Power Saving Expert 2	The goods have been received, I do not know how effective.	Jingdong prefecture level city in Shandong
Jinfeng Hongyuan Flagship Store	Power Saving Expert 2	So far it doesn't seem to be too noticeable, so I'll comment later.	Jingdong prefecture level city in Shandong
Jinfeng Hongyuan Flagship Store	Power Saving Expert 2	I'll comment after I've used it	Jingdong prefecture level city in Shandong
Jinfeng Hongyuan Flagship Store	Power Saving Expert 2	Not bad. You'll save a lot on your electric bill in a year!	Jingdong prefecture level city in Shandong
Jinfeng Hongyuan Flagship Store	Power Saving Expert 2	I just got it. I haven't tried it yet. I don't know what it's like.	Jingdong prefecture level city in Shandong
Jinfeng Hongyuan Flagship Store	Power Saving Expert 2	Shopping at Jingdong has become a habit, and this disease will not be cured for a while.	Jingdong prefecture level city in Shandong
Jinfeng Hongyuan Flagship Store	Power Saving Expert 2	I've been using it for a while now. I don't know if I'm still saving power or not.	Jingdong prefecture level city in Shandong
Jinfeng Hongyuan Flagship Store	Power Saving Expert 2	Received the saver and it's great! There is also a digital voltage indication. Plugged in as instructed, easy to operate, hope you're our power saver!	Jingdong prefecture level city in Shandong
Jinfeng Hongyuan Flagship Store	Power Saving Expert 2	There is no power saving as the store says, using it is the same as not using it.	Jingdong prefecture level city in Shandong
Jinfeng Hongyuan Flagship Store	Power Saving Expert 2	Use it for a while and then comment again	Jingdong prefecture level city in Shandong
Jinfeng Hongyuan Flagship Store	Power Saving Expert 2	Feeling can, to use 1 to 2 months to see how the effect, there is a voltage display, can see the voltage fluctuations amplitude, quite good!	Jingdong prefecture level city in Shandong
Jinfeng Hongyuan Flagship Store	Power Saving Expert 2	I hope it works and saves me some electricity.	Jingdong prefecture level city in Shandong
Jinfeng Hongyuan Flagship Store	Power Saving Expert 2	Nice baby, happy with the after sales	Jingdong prefecture level city in Shandong
Jinfeng Hongyuan Flagship Store	Power Saving Expert 2	Use it and talk about it.	Jingdong prefecture level city in Shandong
Jinfeng Hongyuan Flagship Store	Power Saving Expert 2	Second purchase	Jingdong prefecture level city in Shandong
Jinfeng Hongyuan Flagship Store	Power Saving Expert 2	It works.	Jingdong prefecture level city in Shandong
Jinfeng Hongyuan Flagship Store	Power Saving Expert 2	It works.	Jingdong prefecture level city in Shandong
Jinfeng Hongyuan Flagship Store	Power Saving Expert 2	I don't know if it's a power saver until I start using it	Jingdong prefecture level city in Shandong
Jinfeng Hongyuan Flagship Store	Power Saving Expert 2	serviceable	Jingdong prefecture level city in Shandong
Jinfeng Hongyuan Flagship Store	Power Saving Expert 2	This user did not fill out the rating in time	Jingdong prefecture level city in Shandong
Jinfeng Hongyuan Flagship Store	Power Saving Expert 2	There is no power saving as the store says, using it is the same as not using it.	Jingdong prefecture level city in Shandong
Jinfeng Hongyuan Flagship Store	Power Saving Expert 2	This user did not fill out the rating in time	Jingdong prefecture level city in Shandong
Jinfeng Hongyuan Flagship Store	Power Saving Expert 2	Good...good.	Jingdong prefecture level city in Shandong
Jinfeng Hongyuan Flagship Store	Power Saving Expert 2	(of an unmarried couple) be close	Jingdong prefecture level city in Shandong
Jinfeng Hongyuan Flagship Store	Power Saving Expert 2	(of an unmarried couple) be close	Jingdong prefecture level city in Shandong
Jinfeng Hongyuan Flagship Store	Power Saving Expert 2	(of an unmarried couple) be close	Jingdong prefecture level city in Shandong
Jinfeng Hongyuan Flagship Store	Power Saving Expert 2	The goods are real, high value, high quality, worth every penny, material appearance and quality at a glance is very upscale, very like HAPPY1 00	Jingdong prefecture level city in Shandong
Jinfeng Hongyuan Flagship Store	Power Saving Expert 2	It is the ideal product, good quality, logistics is also very fast, worthy of praise 🎁 baby quality is very good, received the first time to open the first time to see, it is very good 👍, very satisfied 😃, customer service is also available to ask questions and answer, thank you 🙏	Jingdong prefecture level city in Shandong
Jinfeng Hongyuan Flagship Store	Power Saving Expert 2	I bought it and haven't used it yet, I'll use it again.	Jingdong prefecture level city in Shandong
Jinfeng Hongyuan Flagship Store	Power Saving Expert 2	It didn't work, I used it for a few days and it was the same as before, it didn't work, I should have to return it	Jingdong prefecture level city in Shandong
Jinfeng Hongyuan Flagship Store	Power Saving Expert 2	The goods have been received, but have not yet installed and use, feel good.	Jingdong prefecture level city in Shandong

Jinfeng Hongyuan Flagship Store	Power Saving Expert 2	Goods received, I forgot to take pictures, the goods look good, you can start, immediately start the school to see the effect then	Jingdong prefecture level city in Shandong
Jinfeng Hongyuan Flagship Store	Power Saving Expert 2	Looks good. I'll use it for a while.	Jingdong prefecture level city in Shandong
Jinfeng Hongyuan Flagship Store	Power Saving Expert 2	Great stuff, easy to operate, easy to use, good power saving, believe in the power of science!	Jingdong prefecture level city in Shandong
Jinfeng Hongyuan Flagship Store	Power Saving Expert 2	Especially great, comparison shopping before buying, the store owner is very good, answering questions very patiently and detailed, there is a need to come back!	Jingdong prefecture level city in Shandong
Jinfeng Hongyuan Flagship Store	Power Saving Expert 2	Trustworthy, feel free to use it boldly.	Jingdong prefecture level city in Shandong
Jinfeng Hongyuan Flagship Store	Power Saving Expert 2	Logistics is super fast! Beautiful appearance! Looks like a great quality! I've never bought one before, so I'll try it first to see if it saves power. We'll see how much it saves.	Jingdong prefecture level city in Shandong
Jinfeng Hongyuan Flagship Store	Power Saving Expert 2	I saw online counterfeiting, energy saver is one of them, hahahahaha, jingdong also buy fake products	Jingdong prefecture level city in Shandong
Jinfeng Hongyuan Flagship Store	Power Saving Expert 2	Just received, installed, try a month to see how the effect and then follow up.	Jingdong prefecture level city in Shandong
Jinfeng Hongyuan Flagship Store	Power Saving Expert 2	Good for you! The goods are genuine! Good price for what you get!	Jingdong prefecture level city in Shandong
Jinfeng Hongyuan Flagship Store	Power Saving Expert 2	Things quickly received, immediately used to see if there is no effect, something river photo ↑ the same, customer service is very professional	Jingdong prefecture level city in Shandong
Jinfeng Hongyuan Flagship Store	Power Saving Expert 2	The product is very convenient Looking forward to the subsequent use of the experience results	Jingdong prefecture level city in Shandong
Jinfeng Hongyuan Flagship Store	Power Saving Expert 2	I just received it and plugged it in, we'll see exactly when it's time to pay the electric bill	Jingdong prefecture level city in Shandong
Jinfeng Hongyuan Flagship Store	Power Saving Expert 2	Saver is good Packaging and looks good How effective it is to be shared after using it.	Jingdong prefecture level city in Shandong
Jinfeng Hongyuan Flagship Store	Power Saving Expert 2	There's no way to compare. I don't know if it's energy efficient.	Jingdong prefecture level city in Shandong
Jinfeng Hongyuan Flagship Store	Power Saving Expert 2	Just installed it, try it out and comment again	Jingdong prefecture level city in Shandong
Jinfeng Hongyuan Flagship Store	Power Saving Expert 2	Super save point also good to install, after a friend introduced then went to buy to, friends say it is very good to use, home I bought, try to use a period of time, to be honest, the economizer is not big or heavy, can be very convenient to put in a lot of places, the quality is also very good, power-saving function this time seems to be about 20% of the efficiency. The owner is also very warm contact, very	Jingdong prefecture level city in Shandong
Jinfeng Hongyuan Flagship Store	Power Saving Expert 2	The reviews online are mixed. Try it for a while, but I don't know if I can return it if it doesn't save any power after a couple months.	Jingdong prefecture level city in Shandong
Jinfeng Hongyuan Flagship Store	Power Saving Expert 2	The power consumption at home is high, bought a power saver, it looks very convenient to plug on the line, see if you can save some money next month, if good, recommend to friends down the road.	Jingdong prefecture level city in Shandong
Jinfeng Hongyuan Flagship Store	Power Saving Expert 2	It's beautiful, it's genuine, but I don't know the power-saving effect.	Jingdong prefecture level city in Shandong
Jinfeng Hongyuan Flagship Store	Power Saving Expert 2	This user thinks the product is good	Jingdong prefecture level city in Shandong
Jinfeng Hongyuan Flagship Store	Power Saving Expert 2	Nice exterior design with an at-a-glance display	Jingdong prefecture level city in Shandong
Jinfeng Hongyuan Flagship Store	Power Saving Expert 2	Home large and small things are purchased in the Jingdong Mall, have been accustomed to shopping in the Jingdong, many times can buy good quality and cheap products, and courier service attitude is particularly good, sometimes too many couriers are to help me to the home, so many years have been in the Jingdong Mall shopping, trust and support! Will also support the Jingdong as always!	Jingdong prefecture level city in Shandong
Jinfeng Hongyuan Flagship Store	Power Saving Expert 2	Good workmanship, mostly better than the original wall-plugged ones, and hopefully power factor compensation saves on electricity costs	Jingdong prefecture level city in Shandong
Jinfeng Hongyuan Flagship Store	Power Saving Expert 2	I don't feel anything, I said I won't know the effect for a month or two, let's see.	Jingdong prefecture level city in Shandong
Jinfeng Hongyuan Flagship Store	Power Saving Expert 2	Delivery was pretty fast, a little pricey, just not sure if it works or not	Jingdong prefecture level city in Shandong
Jinfeng Hongyuan Flagship Store	Power Saving Expert 2	The goods have been received, I do not know how effective.	Jingdong prefecture level city in Shandong
Jinfeng Hongyuan Flagship Store	Power Saving Expert 2	So far it doesn't seem to be too noticeable, so I'll comment later.	Jingdong prefecture level city in Shandong
Jinfeng Hongyuan Flagship Store	Power Saving Expert 2	I'll comment after I've used it	Jingdong prefecture level city in Shandong
Jinfeng Hongyuan Flagship Store	Power Saving Expert 2	Not bad. You'll save a lot on your electric bill in a year!	Jingdong prefecture level city in Shandong
Jinfeng Hongyuan Flagship Store	Power Saving Expert 2	I just got it. I haven't tried it yet. I don't know what it's like.	Jingdong prefecture level city in Shandong
Jinfeng Hongyuan Flagship Store	Power Saving Expert 2	Shopping at Jingdong has become a habit, and this disease will not be cured for a while.	Jingdong prefecture level city in Shandong
Jinfeng Hongyuan Flagship Store	Power Saving Expert 2	I've been using it for a while now. I don't know if I'm still saving power or not.	Jingdong prefecture level city in Shandong
Jinfeng Hongyuan Flagship Store	Power Saving Expert 2	Received the saver and it's great! There is also a digital voltage indication. Plugged in as instructed, easy to operate, hope you're our power saver!	Jingdong prefecture level city in Shandong
Jinfeng Hongyuan Flagship Store	Power Saving Expert 2	There is no power saving as the store says, using it is the same as not using it.	Jingdong prefecture level city in Shandong
Jinfeng Hongyuan Flagship Store	Power Saving Expert 2	Use it for a while and then comment again	Jingdong prefecture level city in Shandong
Jinfeng Hongyuan Flagship Store	Power Saving Expert 2	Feeling can, to use 1 to 2 months to see how the effect, there is a voltage display, can see the voltage fluctuations amplitude, quite good!	Jingdong prefecture level city in Shandong
Jinfeng Hongyuan Flagship Store	Power Saving Expert 2	I hope it works and saves me some electricity.	Jingdong prefecture level city in Shandong
Jinfeng Hongyuan Flagship Store	Power Saving Expert 2	Nice baby, happy with the after sales	Jingdong prefecture level city in Shandong
Jinfeng Hongyuan Flagship Store	Power Saving Expert 2	Use it and talk about it.	Jingdong prefecture level city in Shandong
Jinfeng Hongyuan Flagship Store	Power Saving Expert 2	Second purchase	Jingdong prefecture level city in Shandong
Jinfeng Hongyuan Flagship Store	Power Saving Expert 2	It works.	Jingdong prefecture level city in Shandong
Jinfeng Hongyuan Flagship Store	Power Saving Expert 2	It works.	Jingdong prefecture level city in Shandong
Jinfeng Hongyuan Flagship Store	Power Saving Expert 2	I don't know if it's a power saver until I start using it	Jingdong prefecture level city in Shandong
Jinfeng Hongyuan Flagship Store	Power Saving Expert 2	serviceable	Jingdong prefecture level city in Shandong
Jinfeng Hongyuan Flagship Store	Power Saving Expert 2	This user did not fill out the rating in time	Jingdong prefecture level city in Shandong
Jinfeng Hongyuan Flagship Store	Power Saving Expert 2	There is no power saving as the store says, using it is the same as not using it.	Jingdong prefecture level city in Shandong
Jinfeng Hongyuan Flagship Store	Power Saving Expert 2	This user did not fill out the rating in time	Jingdong prefecture level city in Shandong
Jinfeng Hongyuan Flagship Store	Power Saving Expert 2	Good...good.	Jingdong prefecture level city in Shandong
Jinfeng Hongyuan Flagship Store	Power Saving Expert 2	(of an unmarried couple) be close	Jingdong prefecture level city in Shandong

Jinfeng Hongyuan Flagship Store	Power Saving Expert 2	(of an unmarried couple) be close	Jingdong prefecture level city in Shandong
Jinfeng Hongyuan Flagship Store	Power Saving Expert 2	(of an unmarried couple) be close	Jingdong prefecture level city in Shandong
Jinfeng Hongyuan Flagship Store	Power Saving Expert 2	The goods are real, high value, high quality, worth every penny, material appearance and quality at a glance is very upscale, very like HAPPY1 00	Jingdong prefecture level city in Shandong
Jinfeng Hongyuan Flagship Store	Power Saving Expert 2	It is the ideal product, good quality, logistics is also very fast, worthy of praise 🎁 baby quality is very good, received the first time to open the first time to see, it is very good 👍, very satisfied 😃, customer service is also available to ask questions and answer, thank you 🙏	Jingdong prefecture level city in Shandong
Jinfeng Hongyuan Flagship Store	Power Saving Expert 2	I bought it and haven't used it yet, I'll use it again.	Jingdong prefecture level city in Shandong
Jinfeng Hongyuan Flagship Store	Power Saving Expert 2	It didn't work, I used it for a few days and it was the same as before, it didn't work, I should have to return it	Jingdong prefecture level city in Shandong
Jinfeng Hongyuan Flagship Store	Power Saving Expert 2	The goods have been received, but have not yet installed and use, feel good.	Jingdong prefecture level city in Shandong
Jinfeng Hongyuan Flagship Store	Power Saving Expert 2	Goods received, I forgot to take pictures, the goods look good, you can start, immediately start the school to see the effect then	Jingdong prefecture level city in Shandong
Jinfeng Hongyuan Flagship Store	Power Saving Expert 2	Looks good. I'll use it for a while.	Jingdong prefecture level city in Shandong
Jinfeng Hongyuan Flagship Store	Power Saving Expert 2	Great stuff, easy to operate, easy to use, good power saving, believe in the power of science!	Jingdong prefecture level city in Shandong
Jinfeng Hongyuan Flagship Store	Power Saving Expert 2	Especially great, comparison shopping before buying, the store owner is very good, answering questions very patiently and detailed, there is a need to come back!	Jingdong prefecture level city in Shandong
Jinfeng Hongyuan Flagship Store	Power Saving Expert 2	Trustworthy, feel free to use it boldly.	Jingdong prefecture level city in Shandong
Jinfeng Hongyuan Flagship Store	Power Saving Expert 2	Logistics is super fast! Beautiful appearance! Looks like a great quality! I've never bought one before, so I'll try it first to see if it saves power. We'll see how much it saves.	Jingdong prefecture level city in Shandong
Jinfeng Hongyuan Flagship Store	Power Saving Expert 2	I saw online counterfeiting, energy saver is one of them, hahahahaha, jingdong also buy fake products	Jingdong prefecture level city in Shandong
Jinfeng Hongyuan Flagship Store	Power Saving Expert 2	Just received, installed, try a month to see how the effect and then follow up.	Jingdong prefecture level city in Shandong
Jinfeng Hongyuan Flagship Store	Power Saving Expert 2	Good for you! The goods are genuine! Good price for what you get!	Jingdong prefecture level city in Shandong
Jinfeng Hongyuan Flagship Store	Power Saving Expert 2	Things quickly received, immediately used to see if there is no effect, something river photo ⬆ the same, customer service is very professional	Jingdong prefecture level city in Shandong
Jinfeng Hongyuan Flagship Store	Power Saving Expert 2	The product is very convenient Looking forward to the subsequent use of the experience results	Jingdong prefecture level city in Shandong
Jinfeng Hongyuan Flagship Store	Power Saving Expert 2	I just received it and plugged it in, we'll see exactly when it's time to pay the electric bill	Jingdong prefecture level city in Shandong
Jinfeng Hongyuan Flagship Store	Power Saving Expert 2	Saver is good Packaging and looks good How effective it is to be shared after using it.	Jingdong prefecture level city in Shandong
Jinfeng Hongyuan Flagship Store	Power Saving Expert 2	There's no way to compare. I don't know if it's energy efficient.	Jingdong prefecture level city in Shandong
Jinfeng Hongyuan Flagship Store	Power Saving Expert 2	Just installed it, try it out and comment again	Jingdong prefecture level city in Shandong
Jinfeng Hongyuan Flagship Store	Power Saving Expert 2	Super save point also good to install, after a friend introduced then went to buy to, friends say it is very good to use, home I bought, try to use a period of time, to be honest, the economizer is not big or heavy, can be very convenient to put in a lot of places, the quality is also very good, power-saving function this time seems to be about 20% of the efficiency. The owner is also very warm contact, very	Jingdong prefecture level city in Shandong
Jinfeng Hongyuan Flagship Store	Power Saving Expert 2	The reviews online are mixed. Try it for a while, but I don't know if I can return it if it doesn't save any power after a couple months.	Jingdong prefecture level city in Shandong
Jinfeng Hongyuan Flagship Store	Power Saving Expert 2	The power consumption at home is high, bought a power saver, it looks very convenient to plug on the line, see if you can save some money next month, if good, recommend to friends down the road.	Jingdong prefecture level city in Shandong
Jinfeng Hongyuan Flagship Store	Power Saving Expert 2	It's beautiful, it's genuine, but I don't know the power-saving effect.	Jingdong prefecture level city in Shandong
Jinfeng Hongyuan Flagship Store	Power Saving Expert 2	This user thinks the product is good	Jingdong prefecture level city in Shandong
Jinfeng Hongyuan Flagship Store	Power Saving Expert 2	Nice exterior design with an at-a-glance display	Jingdong prefecture level city in Shandong
Jinfeng Hongyuan Flagship Store	Power Saving Expert 2	Home large and small things are purchased in the Jingdong Mall, have been accustomed to shopping in the Jingdong, many times can buy good quality and cheap products, and courier service attitude is particularly good, sometimes too many couriers are to help me to the home, so many years have been in the Jingdong Mall shopping, trust and support! Will also support the Jingdong as always!	Jingdong prefecture level city in Shandong
Jinfeng Hongyuan Flagship Store	Power Saving Expert 2	Good workmanship, mostly better than the original wall-plugged ones, and hopefully power factor compensation saves on electricity costs	Jingdong prefecture level city in Shandong
Jinfeng Hongyuan Flagship Store	Power Saving Expert 2	I don't feel anything, I said I won't know the effect for a month or two, let's see.	Jingdong prefecture level city in Shandong
Jinfeng Hongyuan Flagship Store	Power Saving Expert 2	Delivery was pretty fast, a little pricey, just not sure if it works or not	Jingdong prefecture level city in Shandong
Jinfeng Hongyuan Flagship Store	Power Saving Expert 2	The goods have been received, I do not know how effective.	Jingdong prefecture level city in Shandong
Jinfeng Hongyuan Flagship Store	Power Saving Expert 2	So far it doesn't seem to be too noticeable, so I'll comment later.	Jingdong prefecture level city in Shandong
Jinfeng Hongyuan Flagship Store	Power Saving Expert 2	I'll comment after I've used it	Jingdong prefecture level city in Shandong
Jinfeng Hongyuan Flagship Store	Power Saving Expert 2	Not bad. You'll save a lot on your electric bill in a year!	Jingdong prefecture level city in Shandong
Jinfeng Hongyuan Flagship Store	Power Saving Expert 2	I just got it. I haven't tried it yet. I don't know what it's like.	Jingdong prefecture level city in Shandong
Jinfeng Hongyuan Flagship Store	Power Saving Expert 2	Shopping at Jingdong has become a habit, and this disease will not be cured for a while.	Jingdong prefecture level city in Shandong
Jinfeng Hongyuan Flagship Store	Power Saving Expert 2	I've been using it for a while now. I don't know if I'm still saving power or not.	Jingdong prefecture level city in Shandong
Jinfeng Hongyuan Flagship Store	Power Saving Expert 2	Received the saver and it's great! There is also a digital voltage indication. Plugged in as instructed, easy to operate, hope you're our power saver!	Jingdong prefecture level city in Shandong
Jinfeng Hongyuan Flagship Store	Power Saving Expert 2	There is no power saving as the store says, using it is the same as not using it.	Jingdong prefecture level city in Shandong
Jinfeng Hongyuan Flagship Store	Power Saving Expert 2	Use it for a while and then comment again	Jingdong prefecture level city in Shandong
Jinfeng Hongyuan Flagship Store	Power Saving Expert 2	Feeling can, to use 1 to 2 months to see how the effect, there is a voltage display, can see the voltage fluctuations amplitude, quite good!	Jingdong prefecture level city in Shandong
Jinfeng Hongyuan Flagship Store	Power Saving Expert 2	I hope it works and saves me some electricity.	Jingdong prefecture level city in Shandong
Jinfeng Hongyuan Flagship Store	Power Saving Expert 2	Nice baby, happy with the after sales	Jingdong prefecture level city in Shandong
Jinfeng Hongyuan Flagship Store	Power Saving Expert 2	Use it and talk about it.	Jingdong prefecture level city in Shandong
Jinfeng Hongyuan Flagship Store	Power Saving Expert 2	Second purchase	Jingdong prefecture level city in Shandong
Jinfeng Hongyuan Flagship Store	Power Saving Expert 2	It works.	Jingdong prefecture level city in Shandong
Jinfeng Hongyuan Flagship Store	Power Saving Expert 2	It works.	Jingdong prefecture level city in Shandong

Jinfeng Hongyuan Flagship Store	Power Saving Expert 2	I don't know if it's a power saver until I start using it	Jingdong prefecture level city in Shandong
Jinfeng Hongyuan Flagship Store	Power Saving Expert 2	serviceable	Jingdong prefecture level city in Shandong
Jinfeng Hongyuan Flagship Store	Power Saving Expert 2	This user did not fill out the rating in time	Jingdong prefecture level city in Shandong
Jinfeng Hongyuan Flagship Store	Power Saving Expert 2	There is no power saving as the store says, using it is the same as not using it.	Jingdong prefecture level city in Shandong
Jinfeng Hongyuan Flagship Store	Power Saving Expert 2	This user did not fill out the rating in time	Jingdong prefecture level city in Shandong
Jinfeng Hongyuan Flagship Store	Power Saving Expert 2	Good...good.	Jingdong prefecture level city in Shandong
Jinfeng Hongyuan Flagship Store	Power Saving Expert 2	(of an unmarried couple) be close	Jingdong prefecture level city in Shandong
Jinfeng Hongyuan Flagship Store	Power Saving Expert 2	(of an unmarried couple) be close	Jingdong prefecture level city in Shandong
Jinfeng Hongyuan Flagship Store	Power Saving Expert 2	(of an unmarried couple) be close	Jingdong prefecture level city in Shandong
Jinfeng Hongyuan Flagship Store	Power Saving Expert 2	The goods are real, high value, high quality, worth every penny, material appearance and quality at a glance is very upscale, very like HAPPY1 00	Jingdong prefecture level city in Shandong
Jinfeng Hongyuan Flagship Store	Power Saving Expert 2	It is the ideal product, good quality, logistics is also very fast, worthy of praise 🎁 baby quality is very good, received the first time to open the first time to see, it is very good 👍, very satisfied 😃, customer service is also available to ask questions and answer, thank you 🙏	Jingdong prefecture level city in Shandong
Jinfeng Hongyuan Flagship Store	Power Saving Expert 2	I bought it and haven't used it yet, I'll use it again.	Jingdong prefecture level city in Shandong
Jinfeng Hongyuan Flagship Store	Power Saving Expert 2	It didn't work, I used it for a few days and it was the same as before, it didn't work, I should have to return it	Jingdong prefecture level city in Shandong
Jinfeng Hongyuan Flagship Store	Power Saving Expert 2	The goods have been received, but have not yet installed and use, feel good.	Jingdong prefecture level city in Shandong
Jinfeng Hongyuan Flagship Store	Power Saving Expert 2	Goods received, I forgot to take pictures, the goods look good, you can start, immediately start the school to see the effect then	Jingdong prefecture level city in Shandong
Jinfeng Hongyuan Flagship Store	Power Saving Expert 2	Looks good. I'll use it for a while.	Jingdong prefecture level city in Shandong
Jinfeng Hongyuan Flagship Store	Power Saving Expert 2	Great stuff, easy to operate, easy to use, good power saving, believe in the power of science!	Jingdong prefecture level city in Shandong
Jinfeng Hongyuan Flagship Store	Power Saving Expert 2	Especially great, comparison shopping before buying, the store owner is very good, answering questions very patiently and detailed, there is a need to come back!	Jingdong prefecture level city in Shandong
Jinfeng Hongyuan Flagship Store	Power Saving Expert 2	Trustworthy, feel free to use it boldly.	Jingdong prefecture level city in Shandong
Jinfeng Hongyuan Flagship Store	Power Saving Expert 2	Logistics is super fast! Beautiful appearance! Looks like a great quality! I've never bought one before, so I'll try it first to see if it saves power. We'll see how much it saves.	Jingdong prefecture level city in Shandong
Jinfeng Hongyuan Flagship Store	Power Saving Expert 2	I saw online counterfeiting, energy saver is one of them, hahahahaha, jingdong also buy fake products	Jingdong prefecture level city in Shandong
Jinfeng Hongyuan Flagship Store	Power Saving Expert 2	Just received, installed, try a month to see how the effect and then follow up.	Jingdong prefecture level city in Shandong
Jinfeng Hongyuan Flagship Store	Power Saving Expert 2	Good for you! The goods are genuine! Good price for what you get!	Jingdong prefecture level city in Shandong
Jinfeng Hongyuan Flagship Store	Power Saving Expert 2	Things quickly received, immediately used to see if there is no effect, something river photo 🔝 the same, customer service is very professional	Jingdong prefecture level city in Shandong
Jinfeng Hongyuan Flagship Store	Power Saving Expert 2	The product is very convenient Looking forward to the subsequent use of the experience results	Jingdong prefecture level city in Shandong
Jinfeng Hongyuan Flagship Store	Power Saving Expert 2	I just received it and plugged it in, we'll see exactly when it's time to pay the electric bill	Jingdong prefecture level city in Shandong
Jinfeng Hongyuan Flagship Store	Power Saving Expert 2	Saver is good Packaging and looks good How effective it is to be shared after using it.	Jingdong prefecture level city in Shandong
Jinfeng Hongyuan Flagship Store	Power Saving Expert 2	There's no way to compare. I don't know if it's energy efficient.	Jingdong prefecture level city in Shandong
Jinfeng Hongyuan Flagship Store	Power Saving Expert 2	Just installed it, try it out and comment again	Jingdong prefecture level city in Shandong
Jinfeng Hongyuan Flagship Store	Power Saving Expert 2	Super save point also good to install, after a friend introduced then went to buy to, friends say it is very good to use, home I bought, try to use a period of time, to be honest, the economizer is not big or heavy, can be very convenient to put in a lot of places, the quality is also very good, power-saving function this time seems to be about 20% of the efficiency. The owner is also very warm contact, very	Jingdong prefecture level city in Shandong
Jinfeng Hongyuan Flagship Store	Power Saving Expert 2	The reviews online are mixed. Try it for a while, but I don't know if I can return it if it doesn't save any power after a couple months.	Jingdong prefecture level city in Shandong
Jinfeng Hongyuan Flagship Store	Power Saving Expert 2	The power consumption at home is high, bought a power saver, it looks very convenient to plug on the line, see if you can save some money next month, if good, recommend to friends down the road.	Jingdong prefecture level city in Shandong
Jinfeng Hongyuan Flagship Store	Power Saving Expert 2	It's beautiful, it's genuine, but I don't know the power-saving effect.	Jingdong prefecture level city in Shandong
Jinfeng Hongyuan Flagship Store	Power Saving Expert 2	This user thinks the product is good	Jingdong prefecture level city in Shandong
Jinfeng Hongyuan Flagship Store	Power Saving Expert 2	Nice exterior design with an at-a-glance display	Jingdong prefecture level city in Shandong
Jinfeng Hongyuan Flagship Store	Power Saving Expert 2	Home large and small things are purchased in the Jingdong Mall, have been accustomed to shopping in the Jingdong, many times can buy good quality and cheap products, and courier service attitude is particularly good, sometimes too many couriers are to help me to the home, so many years have been in the Jingdong Mall shopping, trust and support! Will also support the Jingdong as always!	Jingdong prefecture level city in Shandong
Jinfeng Hongyuan Flagship Store	Power Saving Expert 2	Good workmanship, mostly better than the original wall-plugged ones, and hopefully power factor compensation saves on electricity costs	Jingdong prefecture level city in Shandong
Jinfeng Hongyuan Flagship Store	Power Saving Expert 2	I don't feel anything, I said I won't know the effect for a month or two, let's see.	Jingdong prefecture level city in Shandong
Jinfeng Hongyuan Flagship Store	Power Saving Expert 2	Delivery was pretty fast, a little pricey, just not sure if it works or not	Jingdong prefecture level city in Shandong
Jinfeng Hongyuan Flagship Store	Power Saving Expert 2	The goods have been received, I do not know how effective.	Jingdong prefecture level city in Shandong
Jinfeng Hongyuan Flagship Store	Power Saving Expert 2	So far it doesn't seem to be too noticeable, so I'll comment later.	Jingdong prefecture level city in Shandong
Jinfeng Hongyuan Flagship Store	Power Saving Expert 2	I'll comment after I've used it	Jingdong prefecture level city in Shandong
Jinfeng Hongyuan Flagship Store	Power Saving Expert 2	Not bad. You'll save a lot on your electric bill in a year!	Jingdong prefecture level city in Shandong
Jinfeng Hongyuan Flagship Store	Power Saving Expert 2	I just got it. I haven't tried it yet. I don't know what it's like.	Jingdong prefecture level city in Shandong
Jinfeng Hongyuan Flagship Store	Power Saving Expert 2	Shopping at Jingdong has become a habit, and this disease will not be cured for a while.	Jingdong prefecture level city in Shandong
Jinfeng Hongyuan Flagship Store	Power Saving Expert 2	I've been using it for a while now. I don't know if I'm still saving power or not.	Jingdong prefecture level city in Shandong
Jinfeng Hongyuan Flagship Store	Power Saving Expert 2	Received the saver and it's great! There is also a digital voltage indication. Plugged in as instructed, easy to operate, hope you're our power saver!	Jingdong prefecture level city in Shandong
Jinfeng Hongyuan Flagship Store	Power Saving Expert 2	There is no power saving as the store says, using it is the same as not using it.	Jingdong prefecture level city in Shandong
Jinfeng Hongyuan Flagship Store	Power Saving Expert 2	Use it for a while and then comment again	Jingdong prefecture level city in Shandong

Jinfeng Hongyuan Flagship Store	Power Saving Expert 2	Feeling can, to use 1 to 2 months to see how the effect, there is a voltage display, can see the voltage fluctuations amplitude, quite good!	Jingdong prefecture level city in Shandong
Jinfeng Hongyuan Flagship Store	Power Saving Expert 2	I hope it works and saves me some electricity.	Jingdong prefecture level city in Shandong
Jinfeng Hongyuan Flagship Store	Power Saving Expert 2	Nice baby, happy with the after sales	Jingdong prefecture level city in Shandong
Jinfeng Hongyuan Flagship Store	Power Saving Expert 2	Use it and talk about it.	Jingdong prefecture level city in Shandong
Jinfeng Hongyuan Flagship Store	Power Saving Expert 2	Second purchase	Jingdong prefecture level city in Shandong
Jinfeng Hongyuan Flagship Store	Power Saving Expert 2	It works.	Jingdong prefecture level city in Shandong
Jinfeng Hongyuan Flagship Store	Power Saving Expert 2	It works.	Jingdong prefecture level city in Shandong
Jinfeng Hongyuan Flagship Store	Power Saving Expert 2	I don't know if it's a power saver until I start using it	Jingdong prefecture level city in Shandong
Jinfeng Hongyuan Flagship Store	Power Saving Expert 2	serviceable	Jingdong prefecture level city in Shandong
Jinfeng Hongyuan Flagship Store	Power Saving Expert 2	This user did not fill out the rating in time	Jingdong prefecture level city in Shandong
Jinfeng Hongyuan Flagship Store	Power Saving Expert 2	There is no power saving as the store says, using it is the same as not using it.	Jingdong prefecture level city in Shandong
Jinfeng Hongyuan Flagship Store	Power Saving Expert 2	This user did not fill out the rating in time	Jingdong prefecture level city in Shandong
Jinfeng Hongyuan Flagship Store	Power Saving Expert 2	Good...good.	Jingdong prefecture level city in Shandong
Jinfeng Hongyuan Flagship Store	Power Saving Expert 2	(of an unmarried couple) be close	Jingdong prefecture level city in Shandong
Jinfeng Hongyuan Flagship Store	Power Saving Expert 2	(of an unmarried couple) be close	Jingdong prefecture level city in Shandong
Jinfeng Hongyuan Flagship Store	Power Saving Expert 2	(of an unmarried couple) be close	Jingdong prefecture level city in Shandong
Jinfeng Hongyuan Flagship Store	Power Saving Expert 2	The goods are real, high value, high quality, worth every penny, material appearance and quality at a glance is very upscale, very like HAPPY1 00	Jingdong prefecture level city in Shandong
Jinfeng Hongyuan Flagship Store	Power Saving Expert 2	It is the ideal product, good quality, logistics is also very fast, worthy of praise 🎁 baby quality is very good, received the first time to open the first time to see, it is very good 👍, very satisfied 😃, customer service is also available to ask questions and answer, thank you 🙏	Jingdong prefecture level city in Shandong
Jinfeng Hongyuan Flagship Store	Power Saving Expert 2	I bought it and haven't used it yet, I'll use it again.	Jingdong prefecture level city in Shandong
Jinfeng Hongyuan Flagship Store	Power Saving Expert 2	It didn't work, I used it for a few days and it was the same as before, it didn't work, I should have to return it	Jingdong prefecture level city in Shandong
Jinfeng Hongyuan Flagship Store	Power Saving Expert 2	The goods have been received, but have not yet installed and use, feel good.	Jingdong prefecture level city in Shandong
Jinfeng Hongyuan Flagship Store	Power Saving Expert 2	Goods received, I forgot to take pictures, the goods look good, you can start, immediately start the school to see the effect then	Jingdong prefecture level city in Shandong
Jinfeng Hongyuan Flagship Store	Power Saving Expert 2	Looks good. I'll use it for a while.	Jingdong prefecture level city in Shandong
Jinfeng Hongyuan Flagship Store	Power Saving Expert 2	Great stuff, easy to operate, easy to use, good power saving, believe in the power of science!	Jingdong prefecture level city in Shandong
Jinfeng Hongyuan Flagship Store	Power Saving Expert 2	Especially great, comparison shopping before buying, the store owner is very good, answering questions very patiently and detailed, there is a need to come back!	Jingdong prefecture level city in Shandong
Jinfeng Hongyuan Flagship Store	Power Saving Expert 2	Trustworthy, feel free to use it boldly.	Jingdong prefecture level city in Shandong
Jinfeng Hongyuan Flagship Store	Power Saving Expert 2	Logistics is super fast! Beautiful appearance! Looks like a great quality! I've never bought one before, so I'll try it first to see if it saves power. We'll see how much it saves.	Jingdong prefecture level city in Shandong
Jinfeng Hongyuan Flagship Store	Power Saving Expert 2	I saw online counterfeiting, energy saver is one of them, hahahahaha, jingdong also buy fake products	Jingdong prefecture level city in Shandong
Jinfeng Hongyuan Flagship Store	Power Saving Expert 2	Just received, installed, try a month to see how the effect and then follow up.	Jingdong prefecture level city in Shandong
Jinfeng Hongyuan Flagship Store	Power Saving Expert 2	Good for you! The goods are genuine! Good price for what you get!	Jingdong prefecture level city in Shandong
Jinfeng Hongyuan Flagship Store	Power Saving Expert 2	Things quickly received, immediately used to see if there is no effect, something river photo ⬆ the same, customer service is very professional	Jingdong prefecture level city in Shandong
Jinfeng Hongyuan Flagship Store	Power Saving Expert 2	The product is very convenient Looking forward to the subsequent use of the experience results	Jingdong prefecture level city in Shandong
Jinfeng Hongyuan Flagship Store	Power Saving Expert 2	I just received it and plugged it in, we'll see exactly when it's time to pay the electric bill	Jingdong prefecture level city in Shandong
Jinfeng Hongyuan Flagship Store	Power Saving Expert 2	Saver is good Packaging and looks good How effective it is to be shared after using it.	Jingdong prefecture level city in Shandong
Jinfeng Hongyuan Flagship Store	Power Saving Expert 2	There's no way to compare. I don't know if it's energy efficient.	Jingdong prefecture level city in Shandong
Jinfeng Hongyuan Flagship Store	Power Saving Expert 2	Just installed it, try it out and comment again	Jingdong prefecture level city in Shandong
Jinfeng Hongyuan Flagship Store	Power Saving Expert 2	Super save point also good to install, after a friend introduced then went to buy to, friends say it is very good to use, home I bought, try to use a period of time, to be honest, the economizer is not big or heavy, can be very convenient to put in a lot of places, the quality is also very good, power-saving function this time seems to be about 20% of the efficiency. The owner is also very warm contact, very	Jingdong prefecture level city in Shandong
Jinfeng Hongyuan Flagship Store	Power Saving Expert 2	The reviews online are mixed. Try it for a while, but I don't know if I can return it if it doesn't save any power after a couple months.	Jingdong prefecture level city in Shandong
Jinfeng Hongyuan Flagship Store	Power Saving Expert 2	The power consumption at home is high, bought a power saver, it looks very convenient to plug on the line, see if you can save some money next month, if good, recommend to friends down the road.	Jingdong prefecture level city in Shandong
Jinfeng Hongyuan Flagship Store	Power Saving Expert 2	It's beautiful, it's genuine, but I don't know the power-saving effect.	Jingdong prefecture level city in Shandong
Jinfeng Hongyuan Flagship Store	Power Saving Expert 2	This user thinks the product is good	Jingdong prefecture level city in Shandong
Jinfeng Hongyuan Flagship Store	Power Saving Expert 2	Nice exterior design with an at-a-glance display	Jingdong prefecture level city in Shandong
Jinfeng Hongyuan Flagship Store	Power Saving Expert 2	Home large and small things are purchased in the Jingdong Mall, have been accustomed to shopping in the Jingdong, many times can buy good quality and cheap products, and courier service attitude is particularly good, sometimes too many couriers are to help me to the home, so many years have been in the Jingdong Mall shopping, trust and support! Will also support the Jingdong as always!	Jingdong prefecture level city in Shandong
Jinfeng Hongyuan Flagship Store	Power Saving Expert 2	Good workmanship, mostly better than the original wall-plugged ones, and hopefully power factor compensation saves on electricity costs	Jingdong prefecture level city in Shandong
Jinfeng Hongyuan Flagship Store	Power Saving Expert 2	I don't feel anything, I said I won't know the effect for a month or two, let's see.	Jingdong prefecture level city in Shandong
Jinfeng Hongyuan Flagship Store	Power Saving Expert 2	Delivery was pretty fast, a little pricey, just not sure if it works or not	Jingdong prefecture level city in Shandong
Jinfeng Hongyuan Flagship Store	Power Saving Expert 2	The goods have been received, I do not know how effective.	Jingdong prefecture level city in Shandong
Jinfeng Hongyuan Flagship Store	Power Saving Expert 2	So far it doesn't seem to be too noticeable, so I'll comment later.	Jingdong prefecture level city in Shandong
Jinfeng Hongyuan Flagship Store	Power Saving Expert 2	I'll comment after I've used it	Jingdong prefecture level city in Shandong

Jinfeng Hongyuan Flagship Store	Power Saving Expert 2	Not bad. You'll save a lot on your electric bill in a year!	Jingdong prefecture level city in Shandong
Jinfeng Hongyuan Flagship Store	Power Saving Expert 2	I just got it. I haven't tried it yet. I don't know what it's like.	Jingdong prefecture level city in Shandong
Jinfeng Hongyuan Flagship Store	Power Saving Expert 2	Shopping at Jingdong has become a habit, and this disease will not be cured for a while.	Jingdong prefecture level city in Shandong
Jinfeng Hongyuan Flagship Store	Power Saving Expert 2	I've been using it for a while now. I don't know if I'm still saving power or not.	Jingdong prefecture level city in Shandong
Jinfeng Hongyuan Flagship Store	Power Saving Expert 2	Received the saver and it's great! There is also a digital voltage indication. Plugged in as instructed, easy to operate, hope you're our power saver!	Jingdong prefecture level city in Shandong
Jinfeng Hongyuan Flagship Store	Power Saving Expert 2	There is no power saving as the store says, using it is the same as not using it.	Jingdong prefecture level city in Shandong
Jinfeng Hongyuan Flagship Store	Power Saving Expert 2	Use it for a while and then comment again	Jingdong prefecture level city in Shandong
Jinfeng Hongyuan Flagship Store	Power Saving Expert 2	Feeling can, to use 1 to 2 months to see how the effect, there is a voltage display, can see the voltage fluctuations amplitude, quite good!	Jingdong prefecture level city in Shandong
Jinfeng Hongyuan Flagship Store	Power Saving Expert 2	I hope it works and saves me some electricity.	Jingdong prefecture level city in Shandong
Jinfeng Hongyuan Flagship Store	Power Saving Expert 2	Nice baby, happy with the after sales	Jingdong prefecture level city in Shandong
Jinfeng Hongyuan Flagship Store	Power Saving Expert 2	Use it and talk about it.	Jingdong prefecture level city in Shandong
Jinfeng Hongyuan Flagship Store	Power Saving Expert 2	Second purchase	Jingdong prefecture level city in Shandong
Jinfeng Hongyuan Flagship Store	Power Saving Expert 2	It works.	Jingdong prefecture level city in Shandong
Jinfeng Hongyuan Flagship Store	Power Saving Expert 2	It works.	Jingdong prefecture level city in Shandong
Jinfeng Hongyuan Flagship Store	Power Saving Expert 2	I don't know if it's a power saver until I start using it	Jingdong prefecture level city in Shandong
Jinfeng Hongyuan Flagship Store	Power Saving Expert 2	serviceable	Jingdong prefecture level city in Shandong
Jinfeng Hongyuan Flagship Store	Power Saving Expert 2	This user did not fill out the rating in time	Jingdong prefecture level city in Shandong
Jinfeng Hongyuan Flagship Store	Power Saving Expert 2	There is no power saving as the store says, using it is the same as not using it.	Jingdong prefecture level city in Shandong
Jinfeng Hongyuan Flagship Store	Power Saving Expert 2	This user did not fill out the rating in time	Jingdong prefecture level city in Shandong
Jinfeng Hongyuan Flagship Store	Power Saving Expert 2	Good...good.	Jingdong prefecture level city in Shandong
Jinfeng Hongyuan Flagship Store	Power Saving Expert 2	(of an unmarried couple) be close	Jingdong prefecture level city in Shandong
Jinfeng Hongyuan Flagship Store	Power Saving Expert 2	(of an unmarried couple) be close	Jingdong prefecture level city in Shandong
Jinfeng Hongyuan Flagship Store	Power Saving Expert 2	(of an unmarried couple) be close	Jingdong prefecture level city in Shandong
Jinfeng Hongyuan Flagship Store	Power Saving Expert 2	The goods are real, high value, high quality, worth every penny, material appearance and quality at a glance is very upscale, very like HAPPY1 00	Jingdong prefecture level city in Shandong
Jinfeng Hongyuan Flagship Store	Power Saving Expert 2	It is the ideal product, good quality, logistics is also very fast, worthy of praise 🎁 baby quality is very good, received the first time to open the first time to see, it is very good 👍, very satisfied 😃, customer service is also available to ask questions and answer, thank you 🙏	Jingdong prefecture level city in Shandong
Jinfeng Hongyuan Flagship Store	Power Saving Expert 2	I bought it and haven't used it yet, I'll use it again.	Jingdong prefecture level city in Shandong
Jinfeng Hongyuan Flagship Store	Power Saving Expert 2	It didn't work, I used it for a few days and it was the same as before, it didn't work, I should have to return it	Jingdong prefecture level city in Shandong
Jinfeng Hongyuan Flagship Store	Power Saving Expert 2	The goods have been received, but have not yet installed and use, feel good.	Jingdong prefecture level city in Shandong
Jinfeng Hongyuan Flagship Store	Power Saving Expert 2	Goods received, I forgot to take pictures, the goods look good, you can start, immediately start the school to see the effect then	Jingdong prefecture level city in Shandong
Jinfeng Hongyuan Flagship Store	Power Saving Expert 2	Looks good. I'll use it for a while.	Jingdong prefecture level city in Shandong
Jinfeng Hongyuan Flagship Store	Power Saving Expert 2	Great stuff, easy to operate, easy to use, good power saving, believe in the power of science!	Jingdong prefecture level city in Shandong
Jinfeng Hongyuan Flagship Store	Power Saving Expert 2	Especially great, comparison shopping before buying, the store owner is very good, answering questions very patiently and detailed, there is a need to come back!	Jingdong prefecture level city in Shandong
Jinfeng Hongyuan Flagship Store	Power Saving Expert 2	Trustworthy, feel free to use it boldly.	Jingdong prefecture level city in Shandong
Jinfeng Hongyuan Flagship Store	Power Saving Expert 2	Logistics is super fast! Beautiful appearance! Looks like a great quality! I've never bought one before, so I'll try it first to see if it saves power. We'll see how much it saves.	Jingdong prefecture level city in Shandong
Jinfeng Hongyuan Flagship Store	Power Saving Expert 2	I saw online counterfeiting, energy saver is one of them, hahahahaha, jingdong also buy fake products	Jingdong prefecture level city in Shandong
Jinfeng Hongyuan Flagship Store	Power Saving Expert 2	Just received, installed, try a month to see how the effect and then follow up.	Jingdong prefecture level city in Shandong
Jinfeng Hongyuan Flagship Store	Power Saving Expert 2	Good for you! The goods are genuine! Good price for what you get!	Jingdong prefecture level city in Shandong
Jinfeng Hongyuan Flagship Store	Power Saving Expert 2	Things quickly received, immediately used to see if there is no effect, something river photo ⬆ the same, customer service is very professional	Jingdong prefecture level city in Shandong
Jinfeng Hongyuan Flagship Store	Power Saving Expert 2	The product is very convenient Looking forward to the subsequent use of the experience results	Jingdong prefecture level city in Shandong
Jinfeng Hongyuan Flagship Store	Power Saving Expert 2	I just received it and plugged it in, we'll see exactly when it's time to pay the electric bill	Jingdong prefecture level city in Shandong
Jinfeng Hongyuan Flagship Store	Power Saving Expert 2	Saver is good Packaging and looks good How effective it is to be shared after using it.	Jingdong prefecture level city in Shandong
Jinfeng Hongyuan Flagship Store	Power Saving Expert 2	There's no way to compare. I don't know if it's energy efficient.	Jingdong prefecture level city in Shandong
Jinfeng Hongyuan Flagship Store	Power Saving Expert 2	Just installed it, try it out and comment again	Jingdong prefecture level city in Shandong
Jinfeng Hongyuan Flagship Store	Power Saving Expert 2	Super save point also good to install, after a friend introduced then went to buy to, friends say it is very good to use, home I bought, try to use a period of time, to be honest, the economizer is not big or heavy, can be very convenient to put in a lot of places, the quality is also very good, power-saving function this time seems to be about 20% of the efficiency. The owner is also very warm contact, very	Jingdong prefecture level city in Shandong
Jinfeng Hongyuan Flagship Store	Power Saving Expert 2	The reviews online are mixed. Try it for a while, but I don't know if I can return it if it doesn't save any power after a couple months.	Jingdong prefecture level city in Shandong
Jinfeng Hongyuan Flagship Store	Power Saving Expert 2	The power consumption at home is high, bought a power saver, it looks very convenient to plug on the line, see if you can save some money next month, if good, recommend to friends down the road.	Jingdong prefecture level city in Shandong
Jinfeng Hongyuan Flagship Store	Power Saving Expert 2	It's beautiful, it's genuine, but I don't know the power-saving effect.	Jingdong prefecture level city in Shandong
Jinfeng Hongyuan Flagship Store	Power Saving Expert 2	This user thinks the product is good	Jingdong prefecture level city in Shandong
Jinfeng Hongyuan Flagship Store	Power Saving Expert 2	Nice exterior design with an at-a-glance display	Jingdong prefecture level city in Shandong

Jinfeng Hongyuan Flagship Store	Power Saving Expert 2	Home large and small things are purchased in the Jingdong Mall, have been accustomed to shopping in the Jingdong, many times can buy good quality and cheap products, and courier service attitude is particularly good, sometimes too many couriers are to help me to the home, so many years have been in the Jingdong Mall shopping, trust and support! Will also support the Jingdong as always!	Jingdong prefecture level city in Shandong
Jinfeng Hongyuan Flagship Store	Power Saving Expert 2	Good workmanship, mostly better than the original wall-plugged ones, and hopefully power factor compensation saves on electricity costs	Jingdong prefecture level city in Shandong
Jinfeng Hongyuan Flagship Store	Power Saving Expert 2	I don't feel anything, I said I won't know the effect for a month or two, let's see.	Jingdong prefecture level city in Shandong
Jinfeng Hongyuan Flagship Store	Power Saving Expert 2	Delivery was pretty fast, a little pricey, just not sure if it works or not	Jingdong prefecture level city in Shandong
Jinfeng Hongyuan Flagship Store	Power Saving Expert 2	The goods have been received, I do not know how effective.	Jingdong prefecture level city in Shandong
Jinfeng Hongyuan Flagship Store	Power Saving Expert 2	So far it doesn't seem to be too noticeable, so I'll comment later.	Jingdong prefecture level city in Shandong
Jinfeng Hongyuan Flagship Store	Power Saving Expert 2	I'll comment after I've used it	Jingdong prefecture level city in Shandong
Jinfeng Hongyuan Flagship Store	Power Saving Expert 2	Not bad. You'll save a lot on your electric bill in a year!	Jingdong prefecture level city in Shandong
Jinfeng Hongyuan Flagship Store	Power Saving Expert 2	I just got it. I haven't tried it yet. I don't know what it's like.	Jingdong prefecture level city in Shandong
Jinfeng Hongyuan Flagship Store	Power Saving Expert 2	Shopping at Jingdong has become a habit, and this disease will not be cured for a while.	Jingdong prefecture level city in Shandong
Jinfeng Hongyuan Flagship Store	Power Saving Expert 2	I've been using it for a while now. I don't know if I'm still saving power or not.	Jingdong prefecture level city in Shandong
Jinfeng Hongyuan Flagship Store	Power Saving Expert 2	Received the saver and it's great! There is also a digital voltage indication. Plugged in as instructed, easy to operate, hope you're our power saver!	Jingdong prefecture level city in Shandong
Jinfeng Hongyuan Flagship Store	Power Saving Expert 2	There is no power saving as the store says, using it is the same as not using it.	Jingdong prefecture level city in Shandong
Jinfeng Hongyuan Flagship Store	Power Saving Expert 2	Use it for a while and then comment again	Jingdong prefecture level city in Shandong
Jinfeng Hongyuan Flagship Store	Power Saving Expert 2	Feeling can, to use 1 to 2 months to see how the effect, there is a voltage display, can see the voltage fluctuations amplitude, quite good!	Jingdong prefecture level city in Shandong
Jinfeng Hongyuan Flagship Store	Power Saving Expert 2	I hope it works and saves me some electricity.	Jingdong prefecture level city in Shandong
Jinfeng Hongyuan Flagship Store	Power Saving Expert 2	Nice baby, happy with the after sales	Jingdong prefecture level city in Shandong
Jinfeng Hongyuan Flagship Store	Power Saving Expert 2	Use it and talk about it.	Jingdong prefecture level city in Shandong
Jinfeng Hongyuan Flagship Store	Power Saving Expert 2	Second purchase	Jingdong prefecture level city in Shandong
Jinfeng Hongyuan Flagship Store	Power Saving Expert 2	It works.	Jingdong prefecture level city in Shandong
Jinfeng Hongyuan Flagship Store	Power Saving Expert 2	It works.	Jingdong prefecture level city in Shandong
Jinfeng Hongyuan Flagship Store	Power Saving Expert 2	I don't know if it's a power saver until I start using it	Jingdong prefecture level city in Shandong
Jinfeng Hongyuan Flagship Store	Power Saving Expert 2	serviceable	Jingdong prefecture level city in Shandong
Jinfeng Hongyuan Flagship Store	Power Saving Expert 2	This user did not fill out the rating in time	Jingdong prefecture level city in Shandong
Jinfeng Hongyuan Flagship Store	Power Saving Expert 2	There is no power saving as the store says, using it is the same as not using it.	Jingdong prefecture level city in Shandong
Jinfeng Hongyuan Flagship Store	Power Saving Expert 2	This user did not fill out the rating in time	Jingdong prefecture level city in Shandong
Jinfeng Hongyuan Flagship Store	Power Saving Expert 2	Good...good.	Jingdong prefecture level city in Shandong
Jinfeng Hongyuan Flagship Store	Power Saving Expert 2	(of an unmarried couple) be close	Jingdong prefecture level city in Shandong
Jinfeng Hongyuan Flagship Store	Power Saving Expert 2	(of an unmarried couple) be close	Jingdong prefecture level city in Shandong
Jinfeng Hongyuan Flagship Store	Power Saving Expert 2	(of an unmarried couple) be close	Jingdong prefecture level city in Shandong
Jinfeng Hongyuan Flagship Store	Power Saving Expert 2	The goods are real, high value, high quality, worth every penny, material appearance and quality at a glance is very upscale, very like HAPPY1 00	Jingdong prefecture level city in Shandong
Jinfeng Hongyuan Flagship Store	Power Saving Expert 2	It is the ideal product, good quality, logistics is also very fast, worthy of praise 🎁 baby quality is very good, received the first time to open the first time to see, it is very good 👍, very satisfied 😃, customer service is also available to ask questions and answer, thank you 🙏	Jingdong prefecture level city in Shandong
Jinfeng Hongyuan Flagship Store	Power Saving Expert 2	I bought it and haven't used it yet, I'll use it again.	Jingdong prefecture level city in Shandong
Jinfeng Hongyuan Flagship Store	Power Saving Expert 2	It didn't work, I used it for a few days and it was the same as before, it didn't work, I should have to return it	Jingdong prefecture level city in Shandong
Jinfeng Hongyuan Flagship Store	Power Saving Expert 2	The goods have been received, but have not yet installed and use, feel good.	Jingdong prefecture level city in Shandong
Jinfeng Hongyuan Flagship Store	Power Saving Expert 2	Goods received, I forgot to take pictures, the goods look good, you can start, immediately start the school to see the effect then	Jingdong prefecture level city in Shandong
Jinfeng Hongyuan Flagship Store	Power Saving Expert 2	Looks good. I'll use it for a while.	Jingdong prefecture level city in Shandong
Jinfeng Hongyuan Flagship Store	Power Saving Expert 2	Great stuff, easy to operate, easy to use, good power saving, believe in the power of science!	Jingdong prefecture level city in Shandong
Jinfeng Hongyuan Flagship Store	Power Saving Expert 2	Especially great, comparison shopping before buying, the store owner is very good, answering questions very patiently and detailed, there is a need to come back!	Jingdong prefecture level city in Shandong
Jinfeng Hongyuan Flagship Store	Power Saving Expert 2	Trustworthy, feel free to use it boldly.	Jingdong prefecture level city in Shandong
Jinfeng Hongyuan Flagship Store	Power Saving Expert 2	Logistics is super fast! Beautiful appearance! Looks like a great quality! I've never bought one before, so I'll try it first to see if it saves power. We'll see how much it saves.	Jingdong prefecture level city in Shandong
Jinfeng Hongyuan Flagship Store	Power Saving Expert 2	I saw online counterfeiting, energy saver is one of them, hahahahaha, jingdong also buy fake products	Jingdong prefecture level city in Shandong
Jinfeng Hongyuan Flagship Store	Power Saving Expert 2	Just received, installed, try a month to see how the effect and then follow up.	Jingdong prefecture level city in Shandong
Jinfeng Hongyuan Flagship Store	Power Saving Expert 2	Good for you! The goods are genuine! Good price for what you get!	Jingdong prefecture level city in Shandong
Jinfeng Hongyuan Flagship Store	Power Saving Expert 2	Things quickly received, immediately used to see if there is no effect, something river photo ⬆ the same, customer service is very professional	Jingdong prefecture level city in Shandong
Jinfeng Hongyuan Flagship Store	Power Saving Expert 2	The product is very convenient Looking forward to the subsequent use of the experience results	Jingdong prefecture level city in Shandong
Jinfeng Hongyuan Flagship Store	Power Saving Expert 2	I just received it and plugged it in, we'll see exactly when it's time to pay the electric bill	Jingdong prefecture level city in Shandong
Jinfeng Hongyuan Flagship Store	Power Saving Expert 2	Saver is good Packaging and looks good How effective it is to be shared after using it.	Jingdong prefecture level city in Shandong
Jinfeng Hongyuan Flagship Store	Power Saving Expert 2	There's no way to compare. I don't know if it's energy efficient.	Jingdong prefecture level city in Shandong
Jinfeng Hongyuan Flagship Store	Power Saving Expert 2	Just installed it, try it out and comment again	Jingdong prefecture level city in Shandong

Jinfeng Hongyuan Flagship Store	Power Saving Expert 2	Super save point also good to install, after a friend introduced then went to buy to, friends say it is very good to use, home I bought, try to use a period of time, to be honest, the economizer is not big or heavy, can be very convenient to put in a lot of places, the quality is also very good, power-saving function this time seems to be about 20% of the efficiency. The owner is also very warm contact, very	Jingdong prefecture level city in Shandong
Jinfeng Hongyuan Flagship Store	Power Saving Expert 2	The reviews online are mixed. Try it for a while, but I don't know if I can return it if it doesn't save any power after a couple months.	Jingdong prefecture level city in Shandong
Jinfeng Hongyuan Flagship Store	Power Saving Expert 2	The power consumption at home is high, bought a power saver, it looks very convenient to plug on the line, see if you can save some money next month, if good, recommend to friends down the road.	Jingdong prefecture level city in Shandong
Jinfeng Hongyuan Flagship Store	Power Saving Expert 2	It's beautiful, it's genuine, but I don't know the power-saving effect.	Jingdong prefecture level city in Shandong
Jinfeng Hongyuan Flagship Store	Power Saving Expert 2	This user thinks the product is good	Jingdong prefecture level city in Shandong
Jinfeng Hongyuan Flagship Store	Power Saving Expert 2	Nice exterior design with an at-a-glance display	Jingdong prefecture level city in Shandong
Jinfeng Hongyuan Flagship Store	Power Saving Expert 2	Home large and small things are purchased in the Jingdong Mall, have been accustomed to shopping in the Jingdong, many times can buy good quality and cheap products, and courier service attitude is particularly good, sometimes too many couriers are to help me to the home, so many years have been in the Jingdong Mall shopping, trust and support! Will also support the Jingdong as always!	Jingdong prefecture level city in Shandong
Jinfeng Hongyuan Flagship Store	Power Saving Expert 2	Good workmanship, mostly better than the original wall-plugged ones, and hopefully power factor compensation saves on electricity costs	Jingdong prefecture level city in Shandong
Jinfeng Hongyuan Flagship Store	Power Saving Expert 2	I don't feel anything, I said I won't know the effect for a month or two, let's see.	Jingdong prefecture level city in Shandong
Jinfeng Hongyuan Flagship Store	Power Saving Expert 2	Delivery was pretty fast, a little pricey, just not sure if it works or not	Jingdong prefecture level city in Shandong
Jinfeng Hongyuan Flagship Store	Power Saving Expert 2	The goods have been received, I do not know how effective.	Jingdong prefecture level city in Shandong
Jinfeng Hongyuan Flagship Store	Power Saving Expert 2	So far it doesn't seem to be too noticeable, so I'll comment later.	Jingdong prefecture level city in Shandong
Jinfeng Hongyuan Flagship Store	Power Saving Expert 2	I'll comment after I've used it	Jingdong prefecture level city in Shandong
Jinfeng Hongyuan Flagship Store	Power Saving Expert 2	Not bad. You'll save a lot on your electric bill in a year!	Jingdong prefecture level city in Shandong
Jinfeng Hongyuan Flagship Store	Power Saving Expert 2	I just got it. I haven't tried it yet. I don't know what it's like.	Jingdong prefecture level city in Shandong
Jinfeng Hongyuan Flagship Store	Power Saving Expert 2	Shopping at Jingdong has become a habit, and this disease will not be cured for a while.	Jingdong prefecture level city in Shandong
Jinfeng Hongyuan Flagship Store	Power Saving Expert 2	I've been using it for a while now. I don't know if I'm still saving power or not.	Jingdong prefecture level city in Shandong
Jinfeng Hongyuan Flagship Store	Power Saving Expert 2	Received the saver and it's great! There is also a digital voltage indication. Plugged in as instructed, easy to operate, hope you're our power saver!	Jingdong prefecture level city in Shandong
Jinfeng Hongyuan Flagship Store	Power Saving Expert 2	There is no power saving as the store says, using it is the same as not using it.	Jingdong prefecture level city in Shandong
Jinfeng Hongyuan Flagship Store	Power Saving Expert 2	Use it for a while and then comment again	Jingdong prefecture level city in Shandong
Jinfeng Hongyuan Flagship Store	Power Saving Expert 2	Feeling can, to use 1 to 2 months to see how the effect, there is a voltage display, can see the voltage fluctuations amplitude, quite good!	Jingdong prefecture level city in Shandong
Jinfeng Hongyuan Flagship Store	Power Saving Expert 2	I hope it works and saves me some electricity.	Jingdong prefecture level city in Shandong
Jinfeng Hongyuan Flagship Store	Power Saving Expert 2	Nice baby, happy with the after sales	Jingdong prefecture level city in Shandong
Jinfeng Hongyuan Flagship Store	Power Saving Expert 2	Use it and talk about it.	Jingdong prefecture level city in Shandong
Jinfeng Hongyuan Flagship Store	Power Saving Expert 2	Second purchase	Jingdong prefecture level city in Shandong
Jinfeng Hongyuan Flagship Store	Power Saving Expert 2	It works.	Jingdong prefecture level city in Shandong
Jinfeng Hongyuan Flagship Store	Power Saving Expert 2	It works.	Jingdong prefecture level city in Shandong
Jinfeng Hongyuan Flagship Store	Power Saving Expert 2	I don't know if it's a power saver until I start using it	Jingdong prefecture level city in Shandong
Jinfeng Hongyuan Flagship Store	Power Saving Expert 2	serviceable	Jingdong prefecture level city in Shandong
Jinfeng Hongyuan Flagship Store	Power Saving Expert 2	This user did not fill out the rating in time	Jingdong prefecture level city in Shandong
Jinfeng Hongyuan Flagship Store	Power Saving Expert 2	There is no power saving as the store says, using it is the same as not using it.	Jingdong prefecture level city in Shandong
Jinfeng Hongyuan Flagship Store	Power Saving Expert 2	This user did not fill out the rating in time	Jingdong prefecture level city in Shandong
Jinfeng Hongyuan Flagship Store	Power Saving Expert 2	Good...good.	Jingdong prefecture level city in Shandong
Jinfeng Hongyuan Flagship Store	Power Saving Expert 2	(of an unmarried couple) be close	Jingdong prefecture level city in Shandong
Jinfeng Hongyuan Flagship Store	Power Saving Expert 2	(of an unmarried couple) be close	Jingdong prefecture level city in Shandong
Jinfeng Hongyuan Flagship Store	Power Saving Expert 2	(of an unmarried couple) be close	Jingdong prefecture level city in Shandong
Jinfeng Hongyuan Flagship Store	Power Saving Expert 2	The goods are real, high value, high quality, worth every penny, material appearance and quality at a glance is very upscale, very like HAPPY💯	Jingdong prefecture level city in Shandong
Jinfeng Hongyuan Flagship Store	Power Saving Expert 2	It is the ideal product, good quality, logistics is also very fast, worthy of praise 🎁 baby quality is very good, received the first time to open the first time to see, it is very good 👍, very satisfied 😃, customer service is also available to ask questions and answer, thank you 🙏	Jingdong prefecture level city in Shandong
Jinfeng Hongyuan Flagship Store	Power Saving Expert 2	I bought it and haven't used it yet, I'll use it again.	Jingdong prefecture level city in Shandong
Jinfeng Hongyuan Flagship Store	Power Saving Expert 2	It didn't work, I used it for a few days and it was the same as before, it didn't work, I should have to return it	Jingdong prefecture level city in Shandong
Jinfeng Hongyuan Flagship Store	Power Saving Expert 2	The goods have been received, but have not yet installed and use, feel good.	Jingdong prefecture level city in Shandong
Jinfeng Hongyuan Flagship Store	Power Saving Expert 2	Goods received, I forgot to take pictures, the goods look good, you can start, immediately start the school to see the effect then	Jingdong prefecture level city in Shandong
Jinfeng Hongyuan Flagship Store	Power Saving Expert 2	Looks good. I'll use it for a while.	Jingdong prefecture level city in Shandong
Jinfeng Hongyuan Flagship Store	Power Saving Expert 2	Great stuff, easy to operate, easy to use, good power saving, believe in the power of science!	Jingdong prefecture level city in Shandong
Jinfeng Hongyuan Flagship Store	Power Saving Expert 2	Especially great, comparison shopping before buying, the store owner is very good, answering questions very patiently and detailed, there is a need to come back!	Jingdong prefecture level city in Shandong
Jinfeng Hongyuan Flagship Store	Power Saving Expert 2	Trustworthy, feel free to use it boldly.	Jingdong prefecture level city in Shandong
Jinfeng Hongyuan Flagship Store	Power Saving Expert 2	Logistics is super fast! Beautiful appearance! Looks like a great quality! I've never bought one before, so I'll try it first to see if it saves power. We'll see how much it saves.	Jingdong prefecture level city in Shandong
Jinfeng Hongyuan Flagship Store	Power Saving Expert 2	I saw online counterfeiting, energy saver is one of them, hahahahaha, jingdong also buy fake products	Jingdong prefecture level city in Shandong
Jinfeng Hongyuan Flagship Store	Power Saving Expert 2	Just received, installed, try a month to see how the effect and then follow up.	Jingdong prefecture level city in Shandong

Jinfeng Hongyuan Flagship Store	Power Saving Expert 2	Good for you! The goods are genuine! Good price for what you get!	Jingdong prefecture level city in Shandong
Jinfeng Hongyuan Flagship Store	Power Saving Expert 2	Things quickly received, immediately used to see if there is no effect, something river photo ⍐ the same, customer service is very professional	Jingdong prefecture level city in Shandong
Jinfeng Hongyuan Flagship Store	Power Saving Expert 2	The product is very convenient Looking forward to the subsequent use of the experience results	Jingdong prefecture level city in Shandong
Jinfeng Hongyuan Flagship Store	Power Saving Expert 2	I just received it and plugged it in, we'll see exactly when it's time to pay the electric bill	Jingdong prefecture level city in Shandong
Jinfeng Hongyuan Flagship Store	Power Saving Expert 2	Saver is good Packaging and looks good How effective it is to be shared after using it.	Jingdong prefecture level city in Shandong
Jinfeng Hongyuan Flagship Store	Power Saving Expert 2	There's no way to compare. I don't know if it's energy efficient.	Jingdong prefecture level city in Shandong
Jinfeng Hongyuan Flagship Store	Power Saving Expert 2	Just installed it, try it out and comment again	Jingdong prefecture level city in Shandong
Jinfeng Hongyuan Flagship Store	Power Saving Expert 2	Super save point also good to install, after a friend introduced then went to buy to, friends say it is very good to use, home I bought, try to use a period of time, to be honest, the economizer is not big or heavy, can be very convenient to put in a lot of places, the quality is also very good, power-saving function this time seems to be about 20% of the efficiency. The owner is also very warm contact, very	Jingdong prefecture level city in Shandong
Jinfeng Hongyuan Flagship Store	Power Saving Expert 2	The reviews online are mixed. Try it for a while, but I don't know if I can return it if it doesn't save any power after a couple months.	Jingdong prefecture level city in Shandong
Jinfeng Hongyuan Flagship Store	Power Saving Expert 2	The power consumption at home is high, bought a power saver, it looks very convenient to plug on the line, see if you can save some money next month, if good, recommend to friends down the road.	Jingdong prefecture level city in Shandong
Jinfeng Hongyuan Flagship Store	Power Saving Expert 2	It's beautiful, it's genuine, but I don't know the power-saving effect.	Jingdong prefecture level city in Shandong
Jinfeng Hongyuan Flagship Store	Power Saving Expert 2	This user thinks the product is good	Jingdong prefecture level city in Shandong
Jinfeng Hongyuan Flagship Store	Power Saving Expert 2	Nice exterior design with an at-a-glance display	Jingdong prefecture level city in Shandong
Jinfeng Hongyuan Flagship Store	Power Saving Expert 2	Home large and small things are purchased in the Jingdong Mall, have been accustomed to shopping in the Jingdong, many times can buy good quality and cheap products, and courier service attitude is particularly good, sometimes too many couriers are to help me to the home, so many years have been in the Jingdong Mall shopping, trust and support! Will also support the Jingdong as always!	Jingdong prefecture level city in Shandong
Jinfeng Hongyuan Flagship Store	Power Saving Expert 2	Good workmanship, mostly better than the original wall-plugged ones, and hopefully power factor compensation saves on electricity costs	Jingdong prefecture level city in Shandong
Jinfeng Hongyuan Flagship Store	Power Saving Expert 2	I don't feel anything, I said I won't know the effect for a month or two, let's see.	Jingdong prefecture level city in Shandong
Jinfeng Hongyuan Flagship Store	Power Saving Expert 2	Delivery was pretty fast, a little pricey, just not sure if it works or not	Jingdong prefecture level city in Shandong
Jinfeng Hongyuan Flagship Store	Power Saving Expert 2	The goods have been received, I do not know how effective.	Jingdong prefecture level city in Shandong
Jinfeng Hongyuan Flagship Store	Power Saving Expert 2	So far it doesn't seem to be too noticeable, so I'll comment later.	Jingdong prefecture level city in Shandong
Jinfeng Hongyuan Flagship Store	Power Saving Expert 2	I'll comment after I've used it	Jingdong prefecture level city in Shandong
Jinfeng Hongyuan Flagship Store	Power Saving Expert 2	Not bad. You'll save a lot on your electric bill in a year!	Jingdong prefecture level city in Shandong
Jinfeng Hongyuan Flagship Store	Power Saving Expert 2	I just got it. I haven't tried it yet. I don't know what it's like.	Jingdong prefecture level city in Shandong
Jinfeng Hongyuan Flagship Store	Power Saving Expert 2	Shopping at Jingdong has become a habit, and this disease will not be cured for a while.	Jingdong prefecture level city in Shandong
Jinfeng Hongyuan Flagship Store	Power Saving Expert 2	I've been using it for a while now. I don't know if I'm still saving power or not.	Jingdong prefecture level city in Shandong
Jinfeng Hongyuan Flagship Store	Power Saving Expert 2	Received the saver and it's great! There is also a digital voltage indication. Plugged in as instructed, easy to operate, hope you're our power saver!	Jingdong prefecture level city in Shandong
Jinfeng Hongyuan Flagship Store	Power Saving Expert 2	There is no power saving as the store says, using it is the same as not using it.	Jingdong prefecture level city in Shandong
Jinfeng Hongyuan Flagship Store	Power Saving Expert 2	Use it for a while and then comment again	Jingdong prefecture level city in Shandong
Jinfeng Hongyuan Flagship Store	Power Saving Expert 2	Feeling can, to use 1 to 2 months to see how the effect, there is a voltage display, can see the voltage fluctuations amplitude, quite good!	Jingdong prefecture level city in Shandong
Jinfeng Hongyuan Flagship Store	Power Saving Expert 2	I hope it works and saves me some electricity.	Jingdong prefecture level city in Shandong
Jinfeng Hongyuan Flagship Store	Power Saving Expert 2	Nice baby, happy with the after sales	Jingdong prefecture level city in Shandong
Jinfeng Hongyuan Flagship Store	Power Saving Expert 2	Use it and talk about it.	Jingdong prefecture level city in Shandong
Jinfeng Hongyuan Flagship Store	Power Saving Expert 2	Second purchase	Jingdong prefecture level city in Shandong
Jinfeng Hongyuan Flagship Store	Power Saving Expert 2	It works.	Jingdong prefecture level city in Shandong
Jinfeng Hongyuan Flagship Store	Power Saving Expert 2	It works.	Jingdong prefecture level city in Shandong
Jinfeng Hongyuan Flagship Store	Power Saving Expert 2	I don't know if it's a power saver until I start using it	Jingdong prefecture level city in Shandong
Jinfeng Hongyuan Flagship Store	Power Saving Expert 2	serviceable	Jingdong prefecture level city in Shandong
Jinfeng Hongyuan Flagship Store	Power Saving Expert 2	This user did not fill out the rating in time	Jingdong prefecture level city in Shandong
Jinfeng Hongyuan Flagship Store	Power Saving Expert 2	There is no power saving as the store says, using it is the same as not using it.	Jingdong prefecture level city in Shandong
Jinfeng Hongyuan Flagship Store	Power Saving Expert 2	This user did not fill out the rating in time	Jingdong prefecture level city in Shandong
Jinfeng Hongyuan Flagship Store	Power Saving Expert 2	Good...good.	Jingdong prefecture level city in Shandong
Jinfeng Hongyuan Flagship Store	Power Saving Expert 2	(of an unmarried couple) be close	Jingdong prefecture level city in Shandong
Jinfeng Hongyuan Flagship Store	Power Saving Expert 2	(of an unmarried couple) be close	Jingdong prefecture level city in Shandong
Jinfeng Hongyuan Flagship Store	Power Saving Expert 2	(of an unmarried couple) be close	Jingdong prefecture level city in Shandong
Jinfeng Hongyuan Flagship Store	Power Saving Expert 2	The goods are real, high value, high quality, worth every penny, material appearance and quality at a glance is very upscale, very like HAPPY1 00	Jingdong prefecture level city in Shandong
Jinfeng Hongyuan Flagship Store	Power Saving Expert 2	It is the ideal product, good quality, logistics is also very fast, worthy of praise 🎁 baby quality is very good, received the first time to open the first time to see, it is very good 👍, very satisfied 😃, customer service is also available to ask questions and answer, thank you 🙏	Jingdong prefecture level city in Shandong
Jinfeng Hongyuan Flagship Store	Power Saving Expert 2	I bought it and haven't used it yet, I'll use it again.	Jingdong prefecture level city in Shandong
Jinfeng Hongyuan Flagship Store	Power Saving Expert 2	It didn't work, I used it for a few days and it was the same as before, it didn't work, I should have to return it	Jingdong prefecture level city in Shandong
Jinfeng Hongyuan Flagship Store	Power Saving Expert 2	The goods have been received, but have not yet installed and use, feel good.	Jingdong prefecture level city in Shandong
Jinfeng Hongyuan Flagship Store	Power Saving Expert 2	Goods received, I forgot to take pictures, the goods look good, you can start, immediately start the school to see the effect then	Jingdong prefecture level city in Shandong

Jinfeng Hongyuan Flagship Store	Power Saving Expert 2	Looks good. I'll use it for a while.	Jingdong prefecture level city in Shandong
Jinfeng Hongyuan Flagship Store	Power Saving Expert 2	Great stuff, easy to operate, easy to use, good power saving, believe in the power of science!	Jingdong prefecture level city in Shandong
Jinfeng Hongyuan Flagship Store	Power Saving Expert 2	Especially great, comparison shopping before buying, the store owner is very good, answering questions very patiently and detailed, there is a need to come back!	Jingdong prefecture level city in Shandong
Jinfeng Hongyuan Flagship Store	Power Saving Expert 2	Trustworthy, feel free to use it boldly.	Jingdong prefecture level city in Shandong
Jinfeng Hongyuan Flagship Store	Power Saving Expert 2	Logistics is super fast! Beautiful appearance! Looks like a great quality! I've never bought one before, so I'll try it first to see if it saves power. We'll see how much it saves.	Jingdong prefecture level city in Shandong
Jinfeng Hongyuan Flagship Store	Power Saving Expert 2	I saw online counterfeiting, energy saver is one of them, hahahahaha, jingdong also buy fake products	Jingdong prefecture level city in Shandong
Jinfeng Hongyuan Flagship Store	Power Saving Expert 2	Just received, installed, try a month to see how the effect and then follow up.	Jingdong prefecture level city in Shandong
Jinfeng Hongyuan Flagship Store	Power Saving Expert 2	Good for you! The goods are genuine! Good price for what you get!	Jingdong prefecture level city in Shandong
Jinfeng Hongyuan Flagship Store	Power Saving Expert 2	Things quickly received, immediately used to see if there is no effect, something river photo ⍐ the same, customer service is very professional	Jingdong prefecture level city in Shandong
Jinfeng Hongyuan Flagship Store	Power Saving Expert 2	The product is very convenient Looking forward to the subsequent use of the experience results	Jingdong prefecture level city in Shandong
Jinfeng Hongyuan Flagship Store	Power Saving Expert 2	I just received it and plugged it in, we'll see exactly when it's time to pay the electric bill	Jingdong prefecture level city in Shandong
Jinfeng Hongyuan Flagship Store	Power Saving Expert 2	Saver is good Packaging and looks good How effective it is to be shared after using it.	Jingdong prefecture level city in Shandong
Jinfeng Hongyuan Flagship Store	Power Saving Expert 2	There's no way to compare. I don't know if it's energy efficient.	Jingdong prefecture level city in Shandong
Jinfeng Hongyuan Flagship Store	Power Saving Expert 2	Just installed it, try it out and comment again	Jingdong prefecture level city in Shandong
Jinfeng Hongyuan Flagship Store	Power Saving Expert 2	Super save point also good to install, after a friend introduced then went to buy to, friends say it is very good to use, home I bought, try to use a period of time, to be honest, the economizer is not big or heavy, can be very convenient to put in a lot of places, the quality is also very good, power-saving function this time seems to be about 20% of the efficiency. The owner is also very warm contact, very	Jingdong prefecture level city in Shandong
Jinfeng Hongyuan Flagship Store	Power Saving Expert 2	The reviews online are mixed. Try it for a while, but I don't know if I can return it if it doesn't save any power after a couple months.	Jingdong prefecture level city in Shandong
Jinfeng Hongyuan Flagship Store	Power Saving Expert 2	The power consumption at home is high, bought a power saver, it looks very convenient to plug on the line, see if you can save some money next month, if good, recommend to friends down the road.	Jingdong prefecture level city in Shandong
Jinfeng Hongyuan Flagship Store	Power Saving Expert 2	It's beautiful, it's genuine, but I don't know the power-saving effect.	Jingdong prefecture level city in Shandong
Jinfeng Hongyuan Flagship Store	Power Saving Expert 2	This user thinks the product is good	Jingdong prefecture level city in Shandong
Jinfeng Hongyuan Flagship Store	Power Saving Expert 2	Nice exterior design with an at-a-glance display	Jingdong prefecture level city in Shandong
Jinfeng Hongyuan Flagship Store	Power Saving Expert 2	Home large and small things are purchased in the Jingdong Mall, have been accustomed to shopping in the Jingdong, many times can buy good quality and cheap products, and courier service attitude is particularly good, sometimes too many couriers are to help me to the home, so many years have been in the Jingdong Mall shopping, trust and support! Will also support the Jingdong as always!	Jingdong prefecture level city in Shandong
Jinfeng Hongyuan Flagship Store	Power Saving Expert 2	Good workmanship, mostly better than the original wall-plugged ones, and hopefully power factor compensation saves on electricity costs	Jingdong prefecture level city in Shandong
Jinfeng Hongyuan Flagship Store	Power Saving Expert 2	I don't feel anything, I said I won't know the effect for a month or two, let's see.	Jingdong prefecture level city in Shandong
Jinfeng Hongyuan Flagship Store	Power Saving Expert 2	Delivery was pretty fast, a little pricey, just not sure if it works or not	Jingdong prefecture level city in Shandong
Jinfeng Hongyuan Flagship Store	Power Saving Expert 2	The goods have been received, I do not know how effective.	Jingdong prefecture level city in Shandong
Jinfeng Hongyuan Flagship Store	Power Saving Expert 2	So far it doesn't seem to be too noticeable, so I'll comment later.	Jingdong prefecture level city in Shandong
Jinfeng Hongyuan Flagship Store	Power Saving Expert 2	I'll comment after I've used it	Jingdong prefecture level city in Shandong
Jinfeng Hongyuan Flagship Store	Power Saving Expert 2	Not bad. You'll save a lot on your electric bill in a year!	Jingdong prefecture level city in Shandong
Jinfeng Hongyuan Flagship Store	Power Saving Expert 2	I just got it. I haven't tried it yet. I don't know what it's like.	Jingdong prefecture level city in Shandong
Jinfeng Hongyuan Flagship Store	Power Saving Expert 2	Shopping at Jingdong has become a habit, and this disease will not be cured for a while.	Jingdong prefecture level city in Shandong
Jinfeng Hongyuan Flagship Store	Power Saving Expert 2	I've been using it for a while now. I don't know if I'm still saving power or not.	Jingdong prefecture level city in Shandong
Jinfeng Hongyuan Flagship Store	Power Saving Expert 2	Received the saver and it's great! There is also a digital voltage indication. Plugged in as instructed, easy to operate, hope you're our power saver!	Jingdong prefecture level city in Shandong
Jinfeng Hongyuan Flagship Store	Power Saving Expert 2	There is no power saving as the store says, using it is the same as not using it.	Jingdong prefecture level city in Shandong
Jinfeng Hongyuan Flagship Store	Power Saving Expert 2	Use it for a while and then comment again	Jingdong prefecture level city in Shandong
Jinfeng Hongyuan Flagship Store	Power Saving Expert 2	Feeling can, to use 1 to 2 months to see how the effect, there is a voltage display, can see the voltage fluctuations amplitude, quite good!	Jingdong prefecture level city in Shandong
Jinfeng Hongyuan Flagship Store	Power Saving Expert 2	I hope it works and saves me some electricity.	Jingdong prefecture level city in Shandong
Jinfeng Hongyuan Flagship Store	Power Saving Expert 2	Nice baby, happy with the after sales	Jingdong prefecture level city in Shandong
Jinfeng Hongyuan Flagship Store	Power Saving Expert 2	Use it and talk about it.	Jingdong prefecture level city in Shandong
Jinfeng Hongyuan Flagship Store	Power Saving Expert 2	Second purchase	Jingdong prefecture level city in Shandong
Jinfeng Hongyuan Flagship Store	Power Saving Expert 2	It works.	Jingdong prefecture level city in Shandong
Jinfeng Hongyuan Flagship Store	Power Saving Expert 2	It works.	Jingdong prefecture level city in Shandong
Jinfeng Hongyuan Flagship Store	Power Saving Expert 2	I don't know if it's a power saver until I start using it	Jingdong prefecture level city in Shandong
Jinfeng Hongyuan Flagship Store	Power Saving Expert 2	serviceable	Jingdong prefecture level city in Shandong
Jinfeng Hongyuan Flagship Store	Power Saving Expert 2	This user did not fill out the rating in time	Jingdong prefecture level city in Shandong
Jinfeng Hongyuan Flagship Store	Power Saving Expert 2	There is no power saving as the store says, using it is the same as not using it.	Jingdong prefecture level city in Shandong
Jinfeng Hongyuan Flagship Store	Power Saving Expert 2	This user did not fill out the rating in time	Jingdong prefecture level city in Shandong
Jinfeng Hongyuan Flagship Store	Power Saving Expert 2	Good...good.	Jingdong prefecture level city in Shandong
Jinfeng Hongyuan Flagship Store	Power Saving Expert 2	(of an unmarried couple) be close	Jingdong prefecture level city in Shandong
Jinfeng Hongyuan Flagship Store	Power Saving Expert 2	(of an unmarried couple) be close	Jingdong prefecture level city in Shandong

Jinfeng Hongyuan Flagship Store	Power Saving Expert 2	(of an unmarried couple) be close	Jingdong prefecture level city in Shandong
Jinfeng Hongyuan Flagship Store	Power Saving Expert 2	The goods are real, high value, high quality, worth every penny, material appearance and quality at a glance is very upscale, very like HAPPY1 00	Jingdong prefecture level city in Shandong
Jinfeng Hongyuan Flagship Store	Power Saving Expert 2	It is the ideal product, good quality, logistics is also very fast, worthy of praise 🎁 baby quality is very good, received the first time to open the first time to see, it is very good 👍, very satisfied 😃, customer service is also available to ask questions and answer, thank you 🙏	Jingdong prefecture level city in Shandong
Jinfeng Hongyuan Flagship Store	Power Saving Expert 2	I bought it and haven't used it yet, I'll use it again.	Jingdong prefecture level city in Shandong
Jinfeng Hongyuan Flagship Store	Power Saving Expert 2	It didn't work, I used it for a few days and it was the same as before, it didn't work, I should have to return it	Jingdong prefecture level city in Shandong
Jinfeng Hongyuan Flagship Store	Power Saving Expert 2	The goods have been received, but have not yet installed and use, feel good.	Jingdong prefecture level city in Shandong
Jinfeng Hongyuan Flagship Store	Power Saving Expert 2	Goods received, I forgot to take pictures, the goods look good, you can start, immediately start the school to see the effect then	Jingdong prefecture level city in Shandong
Jinfeng Hongyuan Flagship Store	Power Saving Expert 2	Looks good. I'll use it for a while.	Jingdong prefecture level city in Shandong
Jinfeng Hongyuan Flagship Store	Power Saving Expert 2	Great stuff, easy to operate, easy to use, good power saving, believe in the power of science!	Jingdong prefecture level city in Shandong
Jinfeng Hongyuan Flagship Store	Power Saving Expert 2	Especially great, comparison shopping before buying, the store owner is very good, answering questions very patiently and detailed, there is a need to come back!	Jingdong prefecture level city in Shandong
Jinfeng Hongyuan Flagship Store	Power Saving Expert 2	Trustworthy, feel free to use it boldly.	Jingdong prefecture level city in Shandong
Jinfeng Hongyuan Flagship Store	Power Saving Expert 2	Logistics is super fast! Beautiful appearance! Looks like a great quality! I've never bought one before, so I'll try it first to see if it saves power. We'll see how much it saves.	Jingdong prefecture level city in Shandong
Jinfeng Hongyuan Flagship Store	Power Saving Expert 2	I saw online counterfeiting, energy saver is one of them, hahahahaha, jingdong also buy fake products	Jingdong prefecture level city in Shandong
Jinfeng Hongyuan Flagship Store	Power Saving Expert 2	Just received, installed, try a month to see how the effect and then follow up.	Jingdong prefecture level city in Shandong
Jinfeng Hongyuan Flagship Store	Power Saving Expert 2	Good for you! The goods are genuine! Good price for what you get!	Jingdong prefecture level city in Shandong
Jinfeng Hongyuan Flagship Store	Power Saving Expert 2	Things quickly received, immediately used to see if there is no effect, something river photo ⬆ the same, customer service is very professional	Jingdong prefecture level city in Shandong
Jinfeng Hongyuan Flagship Store	Power Saving Expert 2	The product is very convenient Looking forward to the subsequent use of the experience results	Jingdong prefecture level city in Shandong
Jinfeng Hongyuan Flagship Store	Power Saving Expert 2	I just received it and plugged it in, we'll see exactly when it's time to pay the electric bill	Jingdong prefecture level city in Shandong
Jinfeng Hongyuan Flagship Store	Power Saving Expert 2	Saver is good Packaging and looks good How effective it is to be shared after using it.	Jingdong prefecture level city in Shandong
Jinfeng Hongyuan Flagship Store	Power Saving Expert 2	There's no way to compare. I don't know if it's energy efficient.	Jingdong prefecture level city in Shandong
Jinfeng Hongyuan Flagship Store	Power Saving Expert 2	Just installed it, try it out and comment again	Jingdong prefecture level city in Shandong
Jinfeng Hongyuan Flagship Store	Power Saving Expert 2	Super save point also good to install, after a friend introduced then went to buy to, friends say it is very good to use, home I bought, try to use a period of time, to be honest, the economizer is not big or heavy, can be very convenient to put in a lot of places, the quality is also very good, power-saving function this time seems to be about 20% of the efficiency. The owner is also very warm contact, very	Jingdong prefecture level city in Shandong
Jinfeng Hongyuan Flagship Store	Power Saving Expert 2	The reviews online are mixed. Try it for a while, but I don't know if I can return it if it doesn't save any power after a couple months.	Jingdong prefecture level city in Shandong
Jinfeng Hongyuan Flagship Store	Power Saving Expert 2	The power consumption at home is high, bought a power saver, it looks very convenient to plug on the line, see if you can save some money next month, if good, recommend to friends down the road.	Jingdong prefecture level city in Shandong
Jinfeng Hongyuan Flagship Store	Power Saving Expert 2	It's beautiful, it's genuine, but I don't know the power-saving effect.	Jingdong prefecture level city in Shandong
Jinfeng Hongyuan Flagship Store	Power Saving Expert 2	This user thinks the product is good	Jingdong prefecture level city in Shandong
Jinfeng Hongyuan Flagship Store	Power Saving Expert 2	Nice exterior design with an at-a-glance display	Jingdong prefecture level city in Shandong
Jinfeng Hongyuan Flagship Store	Power Saving Expert 2	Home large and small things are purchased in the Jingdong Mall, have been accustomed to shopping in the Jingdong, many times can buy good quality and cheap products, and courier service attitude is particularly good, sometimes too many couriers are to help me to the home, so many years have been in the Jingdong Mall shopping, trust and support! Will also support the Jingdong as always!	Jingdong prefecture level city in Shandong
Jinfeng Hongyuan Flagship Store	Power Saving Expert 2	Good workmanship, mostly better than the original wall-plugged ones, and hopefully power factor compensation saves on electricity costs	Jingdong prefecture level city in Shandong
Jinfeng Hongyuan Flagship Store	Power Saving Expert 2	I don't feel anything, I said I won't know the effect for a month or two, let's see.	Jingdong prefecture level city in Shandong
Jinfeng Hongyuan Flagship Store	Power Saving Expert 2	Delivery was pretty fast, a little pricey, just not sure if it works or not	Jingdong prefecture level city in Shandong
Jinfeng Hongyuan Flagship Store	Power Saving Expert 2	The goods have been received, I do not know how effective.	Jingdong prefecture level city in Shandong
Jinfeng Hongyuan Flagship Store	Power Saving Expert 2	So far it doesn't seem to be too noticeable, so I'll comment later.	Jingdong prefecture level city in Shandong
Jinfeng Hongyuan Flagship Store	Power Saving Expert 2	I'll comment after I've used it	Jingdong prefecture level city in Shandong
Jinfeng Hongyuan Flagship Store	Power Saving Expert 2	Not bad. You'll save a lot on your electric bill in a year!	Jingdong prefecture level city in Shandong
Jinfeng Hongyuan Flagship Store	Power Saving Expert 2	I just got it. I haven't tried it yet. I don't know what it's like.	Jingdong prefecture level city in Shandong
Jinfeng Hongyuan Flagship Store	Power Saving Expert 2	Shopping at Jingdong has become a habit, and this disease will not be cured for a while.	Jingdong prefecture level city in Shandong
Jinfeng Hongyuan Flagship Store	Power Saving Expert 2	I've been using it for a while now. I don't know if I'm still saving power or not.	Jingdong prefecture level city in Shandong
Jinfeng Hongyuan Flagship Store	Power Saving Expert 2	Received the saver and it's great! There is also a digital voltage indication. Plugged in as instructed, easy to operate, hope you're our power saver!	Jingdong prefecture level city in Shandong
Jinfeng Hongyuan Flagship Store	Power Saving Expert 2	There is no power saving as the store says, using it is the same as not using it.	Jingdong prefecture level city in Shandong
Jinfeng Hongyuan Flagship Store	Power Saving Expert 2	Use it for a while and then comment again	Jingdong prefecture level city in Shandong
Jinfeng Hongyuan Flagship Store	Power Saving Expert 2	Feeling can, to use 1 to 2 months to see how the effect, there is a voltage display, can see the voltage fluctuations amplitude, quite good!	Jingdong prefecture level city in Shandong
Jinfeng Hongyuan Flagship Store	Power Saving Expert 2	I hope it works and saves me some electricity.	Jingdong prefecture level city in Shandong
Jinfeng Hongyuan Flagship Store	Power Saving Expert 2	Nice baby, happy with the after sales	Jingdong prefecture level city in Shandong
Jinfeng Hongyuan Flagship Store	Power Saving Expert 2	Use it and talk about it.	Jingdong prefecture level city in Shandong
Jinfeng Hongyuan Flagship Store	Power Saving Expert 2	Second purchase	Jingdong prefecture level city in Shandong
Jinfeng Hongyuan Flagship Store	Power Saving Expert 2	It works.	Jingdong prefecture level city in Shandong
Jinfeng Hongyuan Flagship Store	Power Saving Expert 2	It works.	Jingdong prefecture level city in Shandong
Jinfeng Hongyuan Flagship Store	Power Saving Expert 2	I don't know if it's a power saver until I start using it	Jingdong prefecture level city in Shandong

Jinfeng Hongyuan Flagship Store	Power Saving Expert 2	serviceable	Jingdong prefecture level city in Shandong
Jinfeng Hongyuan Flagship Store	Power Saving Expert 2	This user did not fill out the rating in time	Jingdong prefecture level city in Shandong
Jinfeng Hongyuan Flagship Store	Power Saving Expert 2	There is no power saving as the store says, using it is the same as not using it.	Jingdong prefecture level city in Shandong
Jinfeng Hongyuan Flagship Store	Power Saving Expert 2	This user did not fill out the rating in time	Jingdong prefecture level city in Shandong
Jinfeng Hongyuan Flagship Store	Power Saving Expert 2	Good...good.	Jingdong prefecture level city in Shandong
Jinfeng Hongyuan Flagship Store	Power Saving Expert 2	(of an unmarried couple) be close	Jingdong prefecture level city in Shandong
Jinfeng Hongyuan Flagship Store	Power Saving Expert 2	(of an unmarried couple) be close	Jingdong prefecture level city in Shandong
Jinfeng Hongyuan Flagship Store	Power Saving Expert 2	(of an unmarried couple) be close	Jingdong prefecture level city in Shandong
Jinfeng Hongyuan Flagship Store	Power Saving Expert 2	The goods are real, high value, high quality, worth every penny, material appearance and quality at a glance is very upscale, very like HAPPY1 00	Jingdong prefecture level city in Shandong
Jinfeng Hongyuan Flagship Store	Power Saving Expert 2	It is the ideal product, good quality, logistics is also very fast, worthy of praise 🎁 baby quality is very good, received the first time to open the first time to see, it is very good 👍, very satisfied 😃, customer service is also available to ask questions and answer, thank you 🙏	Jingdong prefecture level city in Shandong
Jinfeng Hongyuan Flagship Store	Power Saving Expert 2	I bought it and haven't used it yet, I'll use it again.	Jingdong prefecture level city in Shandong
Jinfeng Hongyuan Flagship Store	Power Saving Expert 2	It didn't work, I used it for a few days and it was the same as before, it didn't work, I should have to return it	Jingdong prefecture level city in Shandong
Jinfeng Hongyuan Flagship Store	Power Saving Expert 2	The goods have been received, but have not yet installed and use, feel good.	Jingdong prefecture level city in Shandong
Jinfeng Hongyuan Flagship Store	Power Saving Expert 2	Goods received, I forgot to take pictures, the goods look good, you can start, immediately start the school to see the effect then	Jingdong prefecture level city in Shandong
Jinfeng Hongyuan Flagship Store	Power Saving Expert 2	Looks good. I'll use it for a while.	Jingdong prefecture level city in Shandong
Jinfeng Hongyuan Flagship Store	Power Saving Expert 2	Great stuff, easy to operate, easy to use, good power saving, believe in the power of science!	Jingdong prefecture level city in Shandong
Jinfeng Hongyuan Flagship Store	Power Saving Expert 2	Especially great, comparison shopping before buying, the store owner is very good, answering questions very patiently and detailed, there is a need to come back!	Jingdong prefecture level city in Shandong
Jinfeng Hongyuan Flagship Store	Power Saving Expert 2	Trustworthy, feel free to use it boldly.	Jingdong prefecture level city in Shandong
Jinfeng Hongyuan Flagship Store	Power Saving Expert 2	Logistics is super fast! Beautiful appearance! Looks like a great quality! I've never bought one before, so I'll try it first to see if it saves power. We'll see how much it saves.	Jingdong prefecture level city in Shandong
Jinfeng Hongyuan Flagship Store	Power Saving Expert 2	I saw online counterfeiting, energy saver is one of them, hahahahaha, jingdong also buy fake products	Jingdong prefecture level city in Shandong
Jinfeng Hongyuan Flagship Store	Power Saving Expert 2	Just received, installed, try a month to see how the effect and then follow up.	Jingdong prefecture level city in Shandong
Jinfeng Hongyuan Flagship Store	Power Saving Expert 2	Good for you! The goods are genuine! Good price for what you get!	Jingdong prefecture level city in Shandong
Jinfeng Hongyuan Flagship Store	Power Saving Expert 2	Things quickly received, immediately used to see if there is no effect, something river photo ⬆ the same, customer service is very professional	Jingdong prefecture level city in Shandong
Jinfeng Hongyuan Flagship Store	Power Saving Expert 2	The product is very convenient Looking forward to the subsequent use of the experience results	Jingdong prefecture level city in Shandong
Jinfeng Hongyuan Flagship Store	Power Saving Expert 2	I just received it and plugged it in, we'll see exactly when it's time to pay the electric bill	Jingdong prefecture level city in Shandong
Jinfeng Hongyuan Flagship Store	Power Saving Expert 2	Saver is good Packaging and looks good How effective it is to be shared after using it.	Jingdong prefecture level city in Shandong
Jinfeng Hongyuan Flagship Store	Power Saving Expert 2	There's no way to compare. I don't know if it's energy efficient.	Jingdong prefecture level city in Shandong
Jinfeng Hongyuan Flagship Store	Power Saving Expert 2	Just installed it, try it out and comment again	Jingdong prefecture level city in Shandong
Jinfeng Hongyuan Flagship Store	Power Saving Expert 2	Super save point also good to install, after a friend introduced then went to buy to, friends say it is very good to use, home I bought, try to use a period of time, to be honest, the economizer is not big or heavy, can be very convenient to put in a lot of places, the quality is also very good, power-saving function this time seems to be about 20% of the efficiency. The owner is also very warm contact, very	Jingdong prefecture level city in Shandong
Jinfeng Hongyuan Flagship Store	Power Saving Expert 2	The reviews online are mixed. Try it for a while, but I don't know if I can return it if it doesn't save any power after a couple months.	Jingdong prefecture level city in Shandong
Jinfeng Hongyuan Flagship Store	Power Saving Expert 2	The power consumption at home is high, bought a power saver, it looks very convenient to plug on the line, see if you can save some money next month, if good, recommend to friends down the road.	Jingdong prefecture level city in Shandong
Jinfeng Hongyuan Flagship Store	Power Saving Expert 2	It's beautiful, it's genuine, but I don't know the power-saving effect.	Jingdong prefecture level city in Shandong
Jinfeng Hongyuan Flagship Store	Power Saving Expert 2	This user thinks the product is good	Jingdong prefecture level city in Shandong
Jinfeng Hongyuan Flagship Store	Power Saving Expert 2	Nice exterior design with an at-a-glance display	Jingdong prefecture level city in Shandong
Jinfeng Hongyuan Flagship Store	Power Saving Expert 2	Home large and small things are purchased in the Jingdong Mall, have been accustomed to shopping in the Jingdong, many times can buy good quality and cheap products, and courier service attitude is particularly good, sometimes too many couriers are to help me to the home, so many years have been in the Jingdong Mall shopping, trust and support! Will also support the Jingdong as always!	Jingdong prefecture level city in Shandong
Jinfeng Hongyuan Flagship Store	Power Saving Expert 2	Good workmanship, mostly better than the original wall-plugged ones, and hopefully power factor compensation saves on electricity costs	Jingdong prefecture level city in Shandong
Jinfeng Hongyuan Flagship Store	Power Saving Expert 2	I don't feel anything, I said I won't know the effect for a month or two, let's see.	Jingdong prefecture level city in Shandong
Jinfeng Hongyuan Flagship Store	Power Saving Expert 2	Delivery was pretty fast, a little pricey, just not sure if it works or not	Jingdong prefecture level city in Shandong
Jinfeng Hongyuan Flagship Store	Power Saving Expert 2	The goods have been received, I do not know how effective.	Jingdong prefecture level city in Shandong
Jinfeng Hongyuan Flagship Store	Power Saving Expert 2	So far it doesn't seem to be too noticeable, so I'll comment later.	Jingdong prefecture level city in Shandong
Jinfeng Hongyuan Flagship Store	Power Saving Expert 2	I'll comment after I've used it	Jingdong prefecture level city in Shandong
Jinfeng Hongyuan Flagship Store	Power Saving Expert 2	Not bad. You'll save a lot on your electric bill in a year!	Jingdong prefecture level city in Shandong
Jinfeng Hongyuan Flagship Store	Power Saving Expert 2	I just got it. I haven't tried it yet. I don't know what it's like.	Jingdong prefecture level city in Shandong
Jinfeng Hongyuan Flagship Store	Power Saving Expert 2	Shopping at Jingdong has become a habit, and this disease will not be cured for a while.	Jingdong prefecture level city in Shandong
Jinfeng Hongyuan Flagship Store	Power Saving Expert 2	I've been using it for a while now. I don't know if I'm still saving power or not.	Jingdong prefecture level city in Shandong
Jinfeng Hongyuan Flagship Store	Power Saving Expert 2	Received the saver and it's great! There is also a digital voltage indication. Plugged in as instructed, easy to operate, hope you're our power saver!	Jingdong prefecture level city in Shandong
Jinfeng Hongyuan Flagship Store	Power Saving Expert 2	There is no power saving as the store says, using it is the same as not using it.	Jingdong prefecture level city in Shandong
Jinfeng Hongyuan Flagship Store	Power Saving Expert 2	Use it for a while and then comment again	Jingdong prefecture level city in Shandong
Jinfeng Hongyuan Flagship Store	Power Saving Expert 2	Feeling can, to use 1 to 2 months to see how the effect, there is a voltage display, can see the voltage fluctuations amplitude, quite good!	Jingdong prefecture level city in Shandong

Jinfeng Hongyuan Flagship Store	Power Saving Expert 2	I hope it works and saves me some electricity.	Jingdong prefecture level city in Shandong
Jinfeng Hongyuan Flagship Store	Power Saving Expert 2	Nice baby, happy with the after sales	Jingdong prefecture level city in Shandong
Jinfeng Hongyuan Flagship Store	Power Saving Expert 2	Use it and talk about it.	Jingdong prefecture level city in Shandong
Jinfeng Hongyuan Flagship Store	Power Saving Expert 2	Second purchase	Jingdong prefecture level city in Shandong
Jinfeng Hongyuan Flagship Store	Power Saving Expert 2	It works.	Jingdong prefecture level city in Shandong
Jinfeng Hongyuan Flagship Store	Power Saving Expert 2	It works.	Jingdong prefecture level city in Shandong
Jinfeng Hongyuan Flagship Store	Power Saving Expert 2	I don't know if it's a power saver until I start using it	Jingdong prefecture level city in Shandong
Jinfeng Hongyuan Flagship Store	Power Saving Expert 2	serviceable	Jingdong prefecture level city in Shandong
Jinfeng Hongyuan Flagship Store	Power Saving Expert 2	This user did not fill out the rating in time	Jingdong prefecture level city in Shandong
Jinfeng Hongyuan Flagship Store	Power Saving Expert 2	There is no power saving as the store says, using it is the same as not using it.	Jingdong prefecture level city in Shandong
Jinfeng Hongyuan Flagship Store	Power Saving Expert 2	This user did not fill out the rating in time	Jingdong prefecture level city in Shandong
Jinfeng Hongyuan Flagship Store	Power Saving Expert 2	Good...good.	Jingdong prefecture level city in Shandong
Jinfeng Hongyuan Flagship Store	Power Saving Expert 2	(of an unmarried couple) be close	Jingdong prefecture level city in Shandong
Jinfeng Hongyuan Flagship Store	Power Saving Expert 2	(of an unmarried couple) be close	Jingdong prefecture level city in Shandong
Jinfeng Hongyuan Flagship Store	Power Saving Expert 2	(of an unmarried couple) be close	Jingdong prefecture level city in Shandong
Jinfeng Hongyuan Flagship Store	Power Saving Expert 2	The goods are real, high value, high quality, worth every penny, material appearance and quality at a glance is very upscale, very like HAPPY1 00	Jingdong prefecture level city in Shandong
Jinfeng Hongyuan Flagship Store	Power Saving Expert 2	It is the ideal product, good quality, logistics is also very fast, worthy of praise 🎁 baby quality is very good, received the first time to open the first time to see, it is very good 👍, very satisfied 🙂, customer service is also available to ask questions and answer, thank you 🙏	Jingdong prefecture level city in Shandong
Jinfeng Hongyuan Flagship Store	Power Saving Expert 2	I bought it and haven't used it yet, I'll use it again.	Jingdong prefecture level city in Shandong
Jinfeng Hongyuan Flagship Store	Power Saving Expert 2	It didn't work, I used it for a few days and it was the same as before, it didn't work, I should have to return it	Jingdong prefecture level city in Shandong
Jinfeng Hongyuan Flagship Store	Power Saving Expert 2	The goods have been received, but have not yet installed and use, feel good.	Jingdong prefecture level city in Shandong
Jinfeng Hongyuan Flagship Store	Power Saving Expert 2	Goods received, I forgot to take pictures, the goods look good, you can start, immediately start the school to see the effect then	Jingdong prefecture level city in Shandong
Jinfeng Hongyuan Flagship Store	Power Saving Expert 2	Looks good. I'll use it for a while.	Jingdong prefecture level city in Shandong
Jinfeng Hongyuan Flagship Store	Power Saving Expert 2	Great stuff, easy to operate, easy to use, good power saving, believe in the power of science!	Jingdong prefecture level city in Shandong
Jinfeng Hongyuan Flagship Store	Power Saving Expert 2	Especially great, comparison shopping before buying, the store owner is very good, answering questions very patiently and detailed, there is a need to come back!	Jingdong prefecture level city in Shandong
Jinfeng Hongyuan Flagship Store	Power Saving Expert 2	Trustworthy, feel free to use it boldly.	Jingdong prefecture level city in Shandong
Jinfeng Hongyuan Flagship Store	Power Saving Expert 2	Logistics is super fast! Beautiful appearance! Looks like a great quality! I've never bought one before, so I'll try it first to see if it saves power. We'll see how much it saves.	Jingdong prefecture level city in Shandong
Jinfeng Hongyuan Flagship Store	Power Saving Expert 2	I saw online counterfeiting, energy saver is one of them, hahahahaha, jingdong also buy fake products	Jingdong prefecture level city in Shandong
Jinfeng Hongyuan Flagship Store	Power Saving Expert 2	Just received, installed, try a month to see how the effect and then follow up.	Jingdong prefecture level city in Shandong
Jinfeng Hongyuan Flagship Store	Power Saving Expert 2	Good for you! The goods are genuine! Good price for what you get!	Jingdong prefecture level city in Shandong
Jinfeng Hongyuan Flagship Store	Power Saving Expert 2	Things quickly received, immediately used to see if there is no effect, something river photo ⬆ the same, customer service is very professional	Jingdong prefecture level city in Shandong
Jinfeng Hongyuan Flagship Store	Power Saving Expert 2	The product is very convenient Looking forward to the subsequent use of the experience results	Jingdong prefecture level city in Shandong
Jinfeng Hongyuan Flagship Store	Power Saving Expert 2	I just received it and plugged it in, we'll see exactly when it's time to pay the electric bill	Jingdong prefecture level city in Shandong
Jinfeng Hongyuan Flagship Store	Power Saving Expert 2	Saver is good Packaging and looks good How effective it is to be shared after using it.	Jingdong prefecture level city in Shandong
Jinfeng Hongyuan Flagship Store	Power Saving Expert 2	There's no way to compare. I don't know if it's energy efficient.	Jingdong prefecture level city in Shandong
Jinfeng Hongyuan Flagship Store	Power Saving Expert 2	Just installed it, try it out and comment again	Jingdong prefecture level city in Shandong
Jinfeng Hongyuan Flagship Store	Power Saving Expert 2	Super save point also good to install, after a friend introduced then went to buy to, friends say it is very good to use, home I bought, try to use a period of time, to be honest, the economizer is not big or heavy, can be very convenient to put in a lot of places, the quality is also very good, power-saving function this time seems to be about 20% of the efficiency. The owner is also very warm contact, very	Jingdong prefecture level city in Shandong
Jinfeng Hongyuan Flagship Store	Power Saving Expert 2	The reviews online are mixed. Try it for a while, but I don't know if I can return it if it doesn't save any power after a couple months.	Jingdong prefecture level city in Shandong
Jinfeng Hongyuan Flagship Store	Power Saving Expert 2	The power consumption at home is high, bought a power saver, it looks very convenient to plug on the line, see if you can save some money next month, if good, recommend to friends down the road.	Jingdong prefecture level city in Shandong
Jinfeng Hongyuan Flagship Store	Power Saving Expert 2	It's beautiful, it's genuine, but I don't know the power-saving effect.	Jingdong prefecture level city in Shandong
Jinfeng Hongyuan Flagship Store	Power Saving Expert 2	This user thinks the product is good	Jingdong prefecture level city in Shandong
Jinfeng Hongyuan Flagship Store	Power Saving Expert 2	Nice exterior design with an at-a-glance display	Jingdong prefecture level city in Shandong
Jinfeng Hongyuan Flagship Store	Power Saving Expert 2	Home large and small things are purchased in the Jingdong Mall, have been accustomed to shopping in the Jingdong, many times can buy good quality and cheap products, and courier service attitude is particularly good, sometimes too many couriers are to help me to the home, so many years have been in the Jingdong Mall shopping, trust and support! Will also support the Jingdong as always!	Jingdong prefecture level city in Shandong
Jinfeng Hongyuan Flagship Store	Power Saving Expert 2	Good workmanship, mostly better than the original wall-plugged ones, and hopefully power factor compensation saves on electricity costs	Jingdong prefecture level city in Shandong
Jinfeng Hongyuan Flagship Store	Power Saving Expert 2	I don't feel anything, I said I won't know the effect for a month or two, let's see.	Jingdong prefecture level city in Shandong
Jinfeng Hongyuan Flagship Store	Power Saving Expert 2	Delivery was pretty fast, a little pricey, just not sure if it works or not	Jingdong prefecture level city in Shandong
Jinfeng Hongyuan Flagship Store	Power Saving Expert 2	The goods have been received, I do not know how effective.	Jingdong prefecture level city in Shandong
Jinfeng Hongyuan Flagship Store	Power Saving Expert 2	So far it doesn't seem to be too noticeable, so I'll comment later.	Jingdong prefecture level city in Shandong
Jinfeng Hongyuan Flagship Store	Power Saving Expert 2	I'll comment after I've used it	Jingdong prefecture level city in Shandong
Jinfeng Hongyuan Flagship Store	Power Saving Expert 2	Not bad. You'll save a lot on your electric bill in a year!	Jingdong prefecture level city in Shandong

Jinfeng Hongyuan Flagship Store	Power Saving Expert 2	I just got it. I haven't tried it yet. I don't know what it's like.	Jingdong prefecture level city in Shandong
Jinfeng Hongyuan Flagship Store	Power Saving Expert 2	Shopping at Jingdong has become a habit, and this disease will not be cured for a while.	Jingdong prefecture level city in Shandong
Jinfeng Hongyuan Flagship Store	Power Saving Expert 2	I've been using it for a while now. I don't know if I'm still saving power or not.	Jingdong prefecture level city in Shandong
Jinfeng Hongyuan Flagship Store	Power Saving Expert 2	Received the saver and it's great! There is also a digital voltage indication. Plugged in as instructed, easy to operate, hope you're our power saver!	Jingdong prefecture level city in Shandong
Jinfeng Hongyuan Flagship Store	Power Saving Expert 2	There is no power saving as the store says, using it is the same as not using it.	Jingdong prefecture level city in Shandong
Jinfeng Hongyuan Flagship Store	Power Saving Expert 2	Use it for a while and then comment again	Jingdong prefecture level city in Shandong
Jinfeng Hongyuan Flagship Store	Power Saving Expert 2	Feeling can, to use 1 to 2 months to see how the effect, there is a voltage display, can see the voltage fluctuations amplitude, quite good!	Jingdong prefecture level city in Shandong
Jinfeng Hongyuan Flagship Store	Power Saving Expert 2	I hope it works and saves me some electricity.	Jingdong prefecture level city in Shandong
Jinfeng Hongyuan Flagship Store	Power Saving Expert 2	Nice baby, happy with the after sales	Jingdong prefecture level city in Shandong
Jinfeng Hongyuan Flagship Store	Power Saving Expert 2	Use it and talk about it.	Jingdong prefecture level city in Shandong
Jinfeng Hongyuan Flagship Store	Power Saving Expert 2	Second purchase	Jingdong prefecture level city in Shandong
Jinfeng Hongyuan Flagship Store	Power Saving Expert 2	It works.	Jingdong prefecture level city in Shandong
Jinfeng Hongyuan Flagship Store	Power Saving Expert 2	It works.	Jingdong prefecture level city in Shandong
Jinfeng Hongyuan Flagship Store	Power Saving Expert 2	I don't know if it's a power saver until I start using it	Jingdong prefecture level city in Shandong
Jinfeng Hongyuan Flagship Store	Power Saving Expert 2	serviceable	Jingdong prefecture level city in Shandong
Jinfeng Hongyuan Flagship Store	Power Saving Expert 2	This user did not fill out the rating in time	Jingdong prefecture level city in Shandong
Jinfeng Hongyuan Flagship Store	Power Saving Expert 2	There is no power saving as the store says, using it is the same as not using it.	Jingdong prefecture level city in Shandong
Jinfeng Hongyuan Flagship Store	Power Saving Expert 2	This user did not fill out the rating in time	Jingdong prefecture level city in Shandong
Jinfeng Hongyuan Flagship Store	Power Saving Expert 2	Good...good.	Jingdong prefecture level city in Shandong
Jinfeng Hongyuan Flagship Store	Power Saving Expert 2	(of an unmarried couple) be close	Jingdong prefecture level city in Shandong
Jinfeng Hongyuan Flagship Store	Power Saving Expert 2	(of an unmarried couple) be close	Jingdong prefecture level city in Shandong
Jinfeng Hongyuan Flagship Store	Power Saving Expert 2	(of an unmarried couple) be close	Jingdong prefecture level city in Shandong
Jinfeng Hongyuan Flagship Store	Power Saving Expert 2	The goods are real, high value, high quality, worth every penny, material appearance and quality at a glance is very upscale, very like HAPPY1 00	Jingdong prefecture level city in Shandong
Jinfeng Hongyuan Flagship Store	Power Saving Expert 2	It is the ideal product, good quality, logistics is also very fast, worthy of praise 🎁 baby quality is very good, received the first time to open the first time to see, it is very good 👍, very satisfied 😃, customer service is also available to ask questions and answer, thank you 🙏	Jingdong prefecture level city in Shandong
Jinfeng Hongyuan Flagship Store	Power Saving Expert 2	I bought it and haven't used it yet, I'll use it again.	Jingdong prefecture level city in Shandong
Jinfeng Hongyuan Flagship Store	Power Saving Expert 2	It didn't work, I used it for a few days and it was the same as before, it didn't work, I should have to return it	Jingdong prefecture level city in Shandong
Jinfeng Hongyuan Flagship Store	Power Saving Expert 2	The goods have been received, but have not yet installed and use, feel good.	Jingdong prefecture level city in Shandong
Jinfeng Hongyuan Flagship Store	Power Saving Expert 2	Goods received, I forgot to take pictures, the goods look good, you can start, immediately start the school to see the effect then	Jingdong prefecture level city in Shandong
Jinfeng Hongyuan Flagship Store	Power Saving Expert 2	Looks good. I'll use it for a while.	Jingdong prefecture level city in Shandong
Jinfeng Hongyuan Flagship Store	Power Saving Expert 2	Great stuff, easy to operate, easy to use, good power saving, believe in the power of science!	Jingdong prefecture level city in Shandong
Jinfeng Hongyuan Flagship Store	Power Saving Expert 2	Especially great, comparison shopping before buying, the store owner is very good, answering questions very patiently and detailed, there is a need to come back!	Jingdong prefecture level city in Shandong
Jinfeng Hongyuan Flagship Store	Power Saving Expert 2	Trustworthy, feel free to use it boldly.	Jingdong prefecture level city in Shandong
Jinfeng Hongyuan Flagship Store	Power Saving Expert 2	Logistics is super fast! Beautiful appearance! Looks like a great quality! I've never bought one before, so I'll try it first to see if it saves power. We'll see how much it saves.	Jingdong prefecture level city in Shandong
Jinfeng Hongyuan Flagship Store	Power Saving Expert 2	I saw online counterfeiting, energy saver is one of them, hahahahaha, jingdong also buy fake products	Jingdong prefecture level city in Shandong
Jinfeng Hongyuan Flagship Store	Power Saving Expert 2	Just received, installed, try a month to see how the effect and then follow up.	Jingdong prefecture level city in Shandong
Jinfeng Hongyuan Flagship Store	Power Saving Expert 2	Good for you! The goods are genuine! Good price for what you get!	Jingdong prefecture level city in Shandong
Jinfeng Hongyuan Flagship Store	Power Saving Expert 2	Things quickly received, immediately used to see if there is no effect, something river photo ⍐ the same, customer service is very professional	Jingdong prefecture level city in Shandong
Jinfeng Hongyuan Flagship Store	Power Saving Expert 2	The product is very convenient Looking forward to the subsequent use of the experience results	Jingdong prefecture level city in Shandong
Jinfeng Hongyuan Flagship Store	Power Saving Expert 2	I just received it and plugged it in, we'll see exactly when it's time to pay the electric bill	Jingdong prefecture level city in Shandong
Jinfeng Hongyuan Flagship Store	Power Saving Expert 2	Saver is good Packaging and looks good How effective it is to be shared after using it.	Jingdong prefecture level city in Shandong
Jinfeng Hongyuan Flagship Store	Power Saving Expert 2	There's no way to compare. I don't know if it's energy efficient.	Jingdong prefecture level city in Shandong
Jinfeng Hongyuan Flagship Store	Power Saving Expert 2	Just installed it, try it out and comment again	Jingdong prefecture level city in Shandong
Jinfeng Hongyuan Flagship Store	Power Saving Expert 2	Super save point also good to install, after a friend introduced then went to buy to, friends say it is very good to use, home I bought, try to use a period of time, to be honest, the economizer is not big or heavy, can be very convenient to put in a lot of places, the quality is also very good, power-saving function this time seems to be about 20% of the efficiency. The owner is also very warm contact, very	Jingdong prefecture level city in Shandong
Jinfeng Hongyuan Flagship Store	Power Saving Expert 2	The reviews online are mixed. Try it for a while, but I don't know if I can return it if it doesn't save any power after a couple months.	Jingdong prefecture level city in Shandong
Jinfeng Hongyuan Flagship Store	Power Saving Expert 2	The power consumption at home is high, bought a power saver, it looks very convenient to plug on the line, see if you can save some money next month, if good, recommend to friends down the road.	Jingdong prefecture level city in Shandong
Jinfeng Hongyuan Flagship Store	Power Saving Expert 2	It's beautiful, it's genuine, but I don't know the power-saving effect.	Jingdong prefecture level city in Shandong
Jinfeng Hongyuan Flagship Store	Power Saving Expert 2	This user thinks the product is good	Jingdong prefecture level city in Shandong
Jinfeng Hongyuan Flagship Store	Power Saving Expert 2	Nice exterior design with an at-a-glance display	Jingdong prefecture level city in Shandong
Jinfeng Hongyuan Flagship Store	Power Saving Expert 2	Home large and small things are purchased in the Jingdong Mall, have been accustomed to shopping in the Jingdong, many times can buy good quality and cheap products, and courier service attitude is particularly good, sometimes too many couriers are to help me to the home, so many years have been in the Jingdong Mall shopping, trust and support! Will also support the Jingdong as always!	Jingdong prefecture level city in Shandong

Jinfeng Hongyuan Flagship Store	Power Saving Expert 2	Good workmanship, mostly better than the original wall-plugged ones, and hopefully power factor compensation saves on electricity costs	Jingdong prefecture level city in Shandong
Jinfeng Hongyuan Flagship Store	Power Saving Expert 2	I don't feel anything, I said I won't know the effect for a month or two, let's see.	Jingdong prefecture level city in Shandong
Jinfeng Hongyuan Flagship Store	Power Saving Expert 2	Delivery was pretty fast, a little pricey, just not sure if it works or not	Jingdong prefecture level city in Shandong
Jinfeng Hongyuan Flagship Store	Power Saving Expert 2	The goods have been received, I do not know how effective.	Jingdong prefecture level city in Shandong
Jinfeng Hongyuan Flagship Store	Power Saving Expert 2	So far it doesn't seem to be too noticeable, so I'll comment later.	Jingdong prefecture level city in Shandong
Jinfeng Hongyuan Flagship Store	Power Saving Expert 2	I'll comment after I've used it	Jingdong prefecture level city in Shandong
Jinfeng Hongyuan Flagship Store	Power Saving Expert 2	Not bad. You'll save a lot on your electric bill in a year!	Jingdong prefecture level city in Shandong
Jinfeng Hongyuan Flagship Store	Power Saving Expert 2	I just got it. I haven't tried it yet. I don't know what it's like.	Jingdong prefecture level city in Shandong
Jinfeng Hongyuan Flagship Store	Power Saving Expert 2	Shopping at Jingdong has become a habit, and this disease will not be cured for a while.	Jingdong prefecture level city in Shandong
Jinfeng Hongyuan Flagship Store	Power Saving Expert 2	I've been using it for a while now. I don't know if I'm still saving power or not.	Jingdong prefecture level city in Shandong
Jinfeng Hongyuan Flagship Store	Power Saving Expert 2	Received the saver and it's great! There is also a digital voltage indication. Plugged in as instructed, easy to operate, hope you're our power saver!	Jingdong prefecture level city in Shandong
Jinfeng Hongyuan Flagship Store	Power Saving Expert 2	There is no power saving as the store says, using it is the same as not using it.	Jingdong prefecture level city in Shandong
Jinfeng Hongyuan Flagship Store	Power Saving Expert 2	Use it for a while and then comment again	Jingdong prefecture level city in Shandong
Jinfeng Hongyuan Flagship Store	Power Saving Expert 2	Feeling can, to use 1 to 2 months to see how the effect, there is a voltage display, can see the voltage fluctuations amplitude, quite good!	Jingdong prefecture level city in Shandong
Jinfeng Hongyuan Flagship Store	Power Saving Expert 2	I hope it works and saves me some electricity.	Jingdong prefecture level city in Shandong
Jinfeng Hongyuan Flagship Store	Power Saving Expert 2	Nice baby, happy with the after sales	Jingdong prefecture level city in Shandong
Jinfeng Hongyuan Flagship Store	Power Saving Expert 2	Use it and talk about it.	Jingdong prefecture level city in Shandong
Jinfeng Hongyuan Flagship Store	Power Saving Expert 2	Second purchase	Jingdong prefecture level city in Shandong
Jinfeng Hongyuan Flagship Store	Power Saving Expert 2	It works.	Jingdong prefecture level city in Shandong
Jinfeng Hongyuan Flagship Store	Power Saving Expert 2	It works.	Jingdong prefecture level city in Shandong
Jinfeng Hongyuan Flagship Store	Power Saving Expert 2	I don't know if it's a power saver until I start using it	Jingdong prefecture level city in Shandong
Jinfeng Hongyuan Flagship Store	Power Saving Expert 2	serviceable	Jingdong prefecture level city in Shandong
Jinfeng Hongyuan Flagship Store	Power Saving Expert 2	This user did not fill out the rating in time	Jingdong prefecture level city in Shandong
Jinfeng Hongyuan Flagship Store	Power Saving Expert 2	There is no power saving as the store says, using it is the same as not using it.	Jingdong prefecture level city in Shandong
Jinfeng Hongyuan Flagship Store	Power Saving Expert 2	This user did not fill out the rating in time	Jingdong prefecture level city in Shandong
Jinfeng Hongyuan Flagship Store	Power Saving Expert 2	Good...good.	Jingdong prefecture level city in Shandong
Jinfeng Hongyuan Flagship Store	Power Saving Expert 2	(of an unmarried couple) be close	Jingdong prefecture level city in Shandong
Jinfeng Hongyuan Flagship Store	Power Saving Expert 2	(of an unmarried couple) be close	Jingdong prefecture level city in Shandong
Jinfeng Hongyuan Flagship Store	Power Saving Expert 2	(of an unmarried couple) be close	Jingdong prefecture level city in Shandong
Jinfeng Hongyuan Flagship Store	Power Saving Expert 2	The goods are real, high value, high quality, worth every penny, material appearance and quality at a glance is very upscale, very like HAPPY100	Jingdong prefecture level city in Shandong
Jinfeng Hongyuan Flagship Store	Power Saving Expert 2	It is the ideal product, good quality, logistics is also very fast, worthy of praise 🎁 baby quality is very good, received the first time to open the first time to see, it is very good 👍, very satisfied 😃, customer service is also available to ask questions and answer, thank you 🙏	Jingdong prefecture level city in Shandong
Jinfeng Hongyuan Flagship Store	Power Saving Expert 2	I bought it and haven't used it yet, I'll use it again.	Jingdong prefecture level city in Shandong
Jinfeng Hongyuan Flagship Store	Power Saving Expert 2	It didn't work, I used it for a few days and it was the same as before, it didn't work, I should have to return it	Jingdong prefecture level city in Shandong
Jinfeng Hongyuan Flagship Store	Power Saving Expert 2	The goods have been received, but have not yet installed and use, feel good.	Jingdong prefecture level city in Shandong
Jinfeng Hongyuan Flagship Store	Power Saving Expert 2	Goods received, I forgot to take pictures, the goods look good, you can start, immediately start the school to see the effect then	Jingdong prefecture level city in Shandong
Jinfeng Hongyuan Flagship Store	Power Saving Expert 2	Looks good. I'll use it for a while.	Jingdong prefecture level city in Shandong
Jinfeng Hongyuan Flagship Store	Power Saving Expert 2	Great stuff, easy to operate, easy to use, good power saving, believe in the power of science!	Jingdong prefecture level city in Shandong
Jinfeng Hongyuan Flagship Store	Power Saving Expert 2	Especially great, comparison shopping before buying, the store owner is very good, answering questions very patiently and detailed, there is a need to come back!	Jingdong prefecture level city in Shandong
Jinfeng Hongyuan Flagship Store	Power Saving Expert 2	Trustworthy, feel free to use it boldly.	Jingdong prefecture level city in Shandong
Jinfeng Hongyuan Flagship Store	Power Saving Expert 2	Logistics is super fast! Beautiful appearance! Looks like a great quality! I've never bought one before, so I'll try it first to see if it saves power. We'll see how much it saves.	Jingdong prefecture level city in Shandong
Jinfeng Hongyuan Flagship Store	Power Saving Expert 2	I saw online counterfeiting, energy saver is one of them, hahahahaha, jingdong also buy fake products	Jingdong prefecture level city in Shandong
Jinfeng Hongyuan Flagship Store	Power Saving Expert 2	Just received, installed, try a month to see how the effect and then follow up.	Jingdong prefecture level city in Shandong
Jinfeng Hongyuan Flagship Store	Power Saving Expert 2	Good for you! The goods are genuine! Good price for what you get!	Jingdong prefecture level city in Shandong
Jinfeng Hongyuan Flagship Store	Power Saving Expert 2	Things quickly received, immediately used to see if there is no effect, something river photo ⍐ the same, customer service is very professional	Jingdong prefecture level city in Shandong
Jinfeng Hongyuan Flagship Store	Power Saving Expert 2	The product is very convenient Looking forward to the subsequent use of the experience results	Jingdong prefecture level city in Shandong
Jinfeng Hongyuan Flagship Store	Power Saving Expert 2	I just received it and plugged it in, we'll see exactly when it's time to pay the electric bill	Jingdong prefecture level city in Shandong
Jinfeng Hongyuan Flagship Store	Power Saving Expert 2	Saver is good Packaging and looks good How effective it is to be shared after using it.	Jingdong prefecture level city in Shandong
Jinfeng Hongyuan Flagship Store	Power Saving Expert 2	There's no way to compare. I don't know if it's energy efficient.	Jingdong prefecture level city in Shandong
Jinfeng Hongyuan Flagship Store	Power Saving Expert 2	Just installed it, try it out and comment again	Jingdong prefecture level city in Shandong
Jinfeng Hongyuan Flagship Store	Power Saving Expert 2	Super save point also good to install, after a friend introduced then went to buy to, friends say it is very good to use, home I bought, try to use a period of time, to be honest, the economizer is not big or heavy, can be very convenient to put in a lot of places, the quality is also very good, power-saving function this time seems to be about 20% of the efficiency. The owner is also very warm contact, very	Jingdong prefecture level city in Shandong

Jinfeng Hongyuan Flagship Store	Power Saving Expert 2	The reviews online are mixed. Try it for a while, but I don't know if I can return it if it doesn't save any power after a couple months.	Jingdong prefecture level city in Shandong
Jinfeng Hongyuan Flagship Store	Power Saving Expert 2	The power consumption at home is high, bought a power saver, it looks very convenient to plug on the line, see if you can save some money next month, if good, recommend to friends down the road.	Jingdong prefecture level city in Shandong
Jinfeng Hongyuan Flagship Store	Power Saving Expert 2	It's beautiful, it's genuine, but I don't know the power-saving effect.	Jingdong prefecture level city in Shandong
Jinfeng Hongyuan Flagship Store	Power Saving Expert 2	This user thinks the product is good	Jingdong prefecture level city in Shandong
Jinfeng Hongyuan Flagship Store	Power Saving Expert 2	Nice exterior design with an at-a-glance display	Jingdong prefecture level city in Shandong
Jinfeng Hongyuan Flagship Store	Power Saving Expert 2	Home large and small things are purchased in the Jingdong Mall, have been accustomed to shopping in the Jingdong, many times can buy good quality and cheap products, and courier service attitude is particularly good, sometimes too many couriers are to help me to the home, so many years have been in the Jingdong Mall shopping, trust and support! Will also support the Jingdong as always!	Jingdong prefecture level city in Shandong
Jinfeng Hongyuan Flagship Store	Power Saving Expert 2	Good workmanship, mostly better than the original wall-plugged ones, and hopefully power factor compensation saves on electricity costs	Jingdong prefecture level city in Shandong
Jinfeng Hongyuan Flagship Store	Power Saving Expert 2	I don't feel anything, I said I won't know the effect for a month or two, let's see.	Jingdong prefecture level city in Shandong
Jinfeng Hongyuan Flagship Store	Power Saving Expert 2	Delivery was pretty fast, a little pricey, just not sure if it works or not	Jingdong prefecture level city in Shandong
Jinfeng Hongyuan Flagship Store	Power Saving Expert 2	The goods have been received, I do not know how effective.	Jingdong prefecture level city in Shandong
Jinfeng Hongyuan Flagship Store	Power Saving Expert 2	So far it doesn't seem to be too noticeable, so I'll comment later.	Jingdong prefecture level city in Shandong
Jinfeng Hongyuan Flagship Store	Power Saving Expert 2	I'll comment after I've used it	Jingdong prefecture level city in Shandong
Jinfeng Hongyuan Flagship Store	Power Saving Expert 2	Not bad. You'll save a lot on your electric bill in a year!	Jingdong prefecture level city in Shandong
Jinfeng Hongyuan Flagship Store	Power Saving Expert 2	I just got it. I haven't tried it yet. I don't know what it's like.	Jingdong prefecture level city in Shandong
Jinfeng Hongyuan Flagship Store	Power Saving Expert 2	Shopping at Jingdong has become a habit, and this disease will not be cured for a while.	Jingdong prefecture level city in Shandong
Jinfeng Hongyuan Flagship Store	Power Saving Expert 2	I've been using it for a while now. I don't know if I'm still saving power or not.	Jingdong prefecture level city in Shandong
Jinfeng Hongyuan Flagship Store	Power Saving Expert 2	Received the saver and it's great! There is also a digital voltage indication. Plugged in as instructed, easy to operate, hope you're our power saver!	Jingdong prefecture level city in Shandong
Jinfeng Hongyuan Flagship Store	Power Saving Expert 2	There is no power saving as the store says, using it is the same as not using it.	Jingdong prefecture level city in Shandong
Jinfeng Hongyuan Flagship Store	Power Saving Expert 2	Use it for a while and then comment again	Jingdong prefecture level city in Shandong
Jinfeng Hongyuan Flagship Store	Power Saving Expert 2	Feeling can, to use 1 to 2 months to see how the effect, there is a voltage display, can see the voltage fluctuations amplitude, quite good!	Jingdong prefecture level city in Shandong
Jinfeng Hongyuan Flagship Store	Power Saving Expert 2	I hope it works and saves me some electricity.	Jingdong prefecture level city in Shandong
Jinfeng Hongyuan Flagship Store	Power Saving Expert 2	Nice baby, happy with the after sales	Jingdong prefecture level city in Shandong
Jinfeng Hongyuan Flagship Store	Power Saving Expert 2	Use it and talk about it.	Jingdong prefecture level city in Shandong
Jinfeng Hongyuan Flagship Store	Power Saving Expert 2	Second purchase	Jingdong prefecture level city in Shandong
Jinfeng Hongyuan Flagship Store	Power Saving Expert 2	It works.	Jingdong prefecture level city in Shandong
Jinfeng Hongyuan Flagship Store	Power Saving Expert 2	It works.	Jingdong prefecture level city in Shandong
Jinfeng Hongyuan Flagship Store	Power Saving Expert 2	I don't know if it's a power saver until I start using it	Jingdong prefecture level city in Shandong
Jinfeng Hongyuan Flagship Store	Power Saving Expert 2	serviceable	Jingdong prefecture level city in Shandong
Jinfeng Hongyuan Flagship Store	Power Saving Expert 2	This user did not fill out the rating in time	Jingdong prefecture level city in Shandong
Jinfeng Hongyuan Flagship Store	Power Saving Expert 2	There is no power saving as the store says, using it is the same as not using it.	Jingdong prefecture level city in Shandong
Jinfeng Hongyuan Flagship Store	Power Saving Expert 2	This user did not fill out the rating in time	Jingdong prefecture level city in Shandong
Jinfeng Hongyuan Flagship Store	Power Saving Expert 2	Good...good.	Jingdong prefecture level city in Shandong
Jinfeng Hongyuan Flagship Store	Power Saving Expert 2	(of an unmarried couple) be close	Jingdong prefecture level city in Shandong
Jinfeng Hongyuan Flagship Store	Power Saving Expert 2	(of an unmarried couple) be close	Jingdong prefecture level city in Shandong
Jinfeng Hongyuan Flagship Store	Power Saving Expert 2	(of an unmarried couple) be close	Jingdong prefecture level city in Shandong
Jinfeng Hongyuan Flagship Store	Power Saving Expert 2	The goods are real, high value, high quality, worth every penny, material appearance and quality at a glance is very upscale, very like HAPPY💯	Jingdong prefecture level city in Shandong
Jinfeng Hongyuan Flagship Store	Power Saving Expert 2	It is the ideal product, good quality, logistics is also very fast, worthy of praise 🎁 baby quality is very good, received the first time to open the first time to see, it is very good 👍, very satisfied 😃, customer service is also available to ask questions and answer, thank you 🙏	Jingdong prefecture level city in Shandong
Jinfeng Hongyuan Flagship Store	Power Saving Expert 2	I bought it and haven't used it yet, I'll use it again.	Jingdong prefecture level city in Shandong
Jinfeng Hongyuan Flagship Store	Power Saving Expert 2	It didn't work, I used it for a few days and it was the same as before, it didn't work, I should have to return it	Jingdong prefecture level city in Shandong
Jinfeng Hongyuan Flagship Store	Power Saving Expert 2	The goods have been received, but have not yet installed and use, feel good.	Jingdong prefecture level city in Shandong
Jinfeng Hongyuan Flagship Store	Power Saving Expert 2	Goods received, I forgot to take pictures, the goods look good, you can start, immediately start the school to see the effect then	Jingdong prefecture level city in Shandong
Jinfeng Hongyuan Flagship Store	Power Saving Expert 2	Looks good. I'll use it for a while.	Jingdong prefecture level city in Shandong
Jinfeng Hongyuan Flagship Store	Power Saving Expert 2	Great stuff, easy to operate, easy to use, good power saving, believe in the power of science!	Jingdong prefecture level city in Shandong
Jinfeng Hongyuan Flagship Store	Power Saving Expert 2	Especially great, comparison shopping before buying, the store owner is very good, answering questions very patiently and detailed, there is a need to come back!	Jingdong prefecture level city in Shandong
Jinfeng Hongyuan Flagship Store	Power Saving Expert 2	Trustworthy, feel free to use it boldly.	Jingdong prefecture level city in Shandong
Jinfeng Hongyuan Flagship Store	Power Saving Expert 2	Logistics is super fast! Beautiful appearance! Looks like a great quality! I've never bought one before, so I'll try it first to see if it saves power. We'll see how much it saves.	Jingdong prefecture level city in Shandong
Jinfeng Hongyuan Flagship Store	Power Saving Expert 2	I saw online counterfeiting, energy saver is one of them, hahahahaha, jingdong also buy fake products	Jingdong prefecture level city in Shandong
Jinfeng Hongyuan Flagship Store	Power Saving Expert 2	Just received, installed, try a month to see how the effect and then follow up.	Jingdong prefecture level city in Shandong
Jinfeng Hongyuan Flagship Store	Power Saving Expert 2	Good for you! The goods are genuine! Good price for what you get!	Jingdong prefecture level city in Shandong
Jinfeng Hongyuan Flagship Store	Power Saving Expert 2	Things quickly received, immediately used to see if there is no effect, something river photo 🔝 the same, customer service is very professional	Jingdong prefecture level city in Shandong

Jinfeng Hongyuan Flagship Store	Power Saving Expert 2	The product is very convenient Looking forward to the subsequent use of the experience results	Jingdong prefecture level city in Shandong
Jinfeng Hongyuan Flagship Store	Power Saving Expert 2	I just received it and plugged it in, we'll see exactly when it's time to pay the electric bill	Jingdong prefecture level city in Shandong
Jinfeng Hongyuan Flagship Store	Power Saving Expert 2	Saver is good Packaging and looks good How effective it is to be shared after using it.	Jingdong prefecture level city in Shandong
Jinfeng Hongyuan Flagship Store	Power Saving Expert 2	There's no way to compare. I don't know if it's energy efficient.	Jingdong prefecture level city in Shandong
Jinfeng Hongyuan Flagship Store	Power Saving Expert 2	Just installed it, try it out and comment again	Jingdong prefecture level city in Shandong
Jinfeng Hongyuan Flagship Store	Power Saving Expert 2	Super save point also good to install, after a friend introduced then went to buy to, friends say it is very good to use, home I bought, try to use a period of time, to be honest, the economizer is not big or heavy, can be very convenient to put in a lot of places, the quality is also very good, power-saving function this time seems to be about 20% of the efficiency. The owner is also very warm contact, very	Jingdong prefecture level city in Shandong
Jinfeng Hongyuan Flagship Store	Power Saving Expert 2	The reviews online are mixed. Try it for a while, but I don't know if I can return it if it doesn't save any power after a couple months.	Jingdong prefecture level city in Shandong
Jinfeng Hongyuan Flagship Store	Power Saving Expert 2	The power consumption at home is high, bought a power saver, it looks very convenient to plug on the line, see if you can save some money next month, if good, recommend to friends down the road.	Jingdong prefecture level city in Shandong
Jinfeng Hongyuan Flagship Store	Power Saving Expert 2	It's beautiful, it's genuine, but I don't know the power-saving effect.	Jingdong prefecture level city in Shandong
Jinfeng Hongyuan Flagship Store	Power Saving Expert 2	This user thinks the product is good	Jingdong prefecture level city in Shandong
Jinfeng Hongyuan Flagship Store	Power Saving Expert 2	Nice exterior design with an at-a-glance display	Jingdong prefecture level city in Shandong
Jinfeng Hongyuan Flagship Store	Power Saving Expert 2	Home large and small things are purchased in the Jingdong Mall, have been accustomed to shopping in the Jingdong, many times can buy good quality and cheap products, and courier service attitude is particularly good, sometimes too many couriers are to help me to the home, so many years have been in the Jingdong Mall shopping, trust and support! Will also support the Jingdong as always!	Jingdong prefecture level city in Shandong
Jinfeng Hongyuan Flagship Store	Power Saving Expert 2	Good workmanship, mostly better than the original wall-plugged ones, and hopefully power factor compensation saves on electricity costs	Jingdong prefecture level city in Shandong
Jinfeng Hongyuan Flagship Store	Power Saving Expert 2	I don't feel anything, I said I won't know the effect for a month or two, let's see.	Jingdong prefecture level city in Shandong
Jinfeng Hongyuan Flagship Store	Power Saving Expert 2	Delivery was pretty fast, a little pricey, just not sure if it works or not	Jingdong prefecture level city in Shandong
Jinfeng Hongyuan Flagship Store	Power Saving Expert 2	The goods have been received, I do not know how effective.	Jingdong prefecture level city in Shandong
Jinfeng Hongyuan Flagship Store	Power Saving Expert 2	So far it doesn't seem to be too noticeable, so I'll comment later.	Jingdong prefecture level city in Shandong
Jinfeng Hongyuan Flagship Store	Power Saving Expert 2	I'll comment after I've used it	Jingdong prefecture level city in Shandong
Jinfeng Hongyuan Flagship Store	Power Saving Expert 2	Not bad. You'll save a lot on your electric bill in a year!	Jingdong prefecture level city in Shandong
Jinfeng Hongyuan Flagship Store	Power Saving Expert 2	I just got it. I haven't tried it yet. I don't know what it's like.	Jingdong prefecture level city in Shandong
Jinfeng Hongyuan Flagship Store	Power Saving Expert 2	Shopping at Jingdong has become a habit, and this disease will not be cured for a while.	Jingdong prefecture level city in Shandong
Jinfeng Hongyuan Flagship Store	Power Saving Expert 2	I've been using it for a while now. I don't know if I'm still saving power or not.	Jingdong prefecture level city in Shandong
Jinfeng Hongyuan Flagship Store	Power Saving Expert 2	Received the saver and it's great! There is also a digital voltage indication. Plugged in as instructed, easy to operate, hope you're our power saver!	Jingdong prefecture level city in Shandong
Jinfeng Hongyuan Flagship Store	Power Saving Expert 2	There is no power saving as the store says, using it is the same as not using it.	Jingdong prefecture level city in Shandong
Jinfeng Hongyuan Flagship Store	Power Saving Expert 2	Use it for a while and then comment again	Jingdong prefecture level city in Shandong
Jinfeng Hongyuan Flagship Store	Power Saving Expert 2	Feeling can, to use 1 to 2 months to see how the effect, there is a voltage display, can see the voltage fluctuations amplitude, quite good!	Jingdong prefecture level city in Shandong
Jinfeng Hongyuan Flagship Store	Power Saving Expert 2	I hope it works and saves me some electricity.	Jingdong prefecture level city in Shandong
Jinfeng Hongyuan Flagship Store	Power Saving Expert 2	Nice baby, happy with the after sales	Jingdong prefecture level city in Shandong
Jinfeng Hongyuan Flagship Store	Power Saving Expert 2	Use it and talk about it.	Jingdong prefecture level city in Shandong
Jinfeng Hongyuan Flagship Store	Power Saving Expert 2	Second purchase	Jingdong prefecture level city in Shandong
Jinfeng Hongyuan Flagship Store	Power Saving Expert 2	It works.	Jingdong prefecture level city in Shandong
Jinfeng Hongyuan Flagship Store	Power Saving Expert 2	It works.	Jingdong prefecture level city in Shandong
Jinfeng Hongyuan Flagship Store	Power Saving Expert 2	I don't know if it's a power saver until I start using it	Jingdong prefecture level city in Shandong
Jinfeng Hongyuan Flagship Store	Power Saving Expert 2	serviceable	Jingdong prefecture level city in Shandong
Jinfeng Hongyuan Flagship Store	Power Saving Expert 2	This user did not fill out the rating in time	Jingdong prefecture level city in Shandong
Jinfeng Hongyuan Flagship Store	Power Saving Expert 2	There is no power saving as the store says, using it is the same as not using it.	Jingdong prefecture level city in Shandong
Jinfeng Hongyuan Flagship Store	Power Saving Expert 2	This user did not fill out the rating in time	Jingdong prefecture level city in Shandong
Jinfeng Hongyuan Flagship Store	Power Saving Expert 2	Good...good.	Jingdong prefecture level city in Shandong
Jinfeng Hongyuan Flagship Store	Power Saving Expert 2	(of an unmarried couple) be close	Jingdong prefecture level city in Shandong
Jinfeng Hongyuan Flagship Store	Power Saving Expert 2	(of an unmarried couple) be close	Jingdong prefecture level city in Shandong
Jinfeng Hongyuan Flagship Store	Power Saving Expert 2	(of an unmarried couple) be close	Jingdong prefecture level city in Shandong
Jinfeng Hongyuan Flagship Store	Power Saving Expert 2	The goods are real, high value, high quality, worth every penny, material appearance and quality at a glance is very upscale, very like HAPPY1 00	Jingdong prefecture level city in Shandong
Jinfeng Hongyuan Flagship Store	Power Saving Expert 2	It is the ideal product, good quality, logistics is also very fast, worthy of praise 🎁 baby quality is very good, received the first time to open the first time to see, it is very good 👍, very satisfied 😃, customer service is also available to ask questions and answer, thank you 🙏	Jingdong prefecture level city in Shandong
Jinfeng Hongyuan Flagship Store	Power Saving Expert 2	I bought it and haven't used it yet, I'll use it again.	Jingdong prefecture level city in Shandong
Jinfeng Hongyuan Flagship Store	Power Saving Expert 2	It didn't work, I used it for a few days and it was the same as before, it didn't work, I should have to return it	Jingdong prefecture level city in Shandong
Jinfeng Hongyuan Flagship Store	Power Saving Expert 2	The goods have been received, but have not yet installed and use, feel good.	Jingdong prefecture level city in Shandong
Jinfeng Hongyuan Flagship Store	Power Saving Expert 2	Goods received, I forgot to take pictures, the goods look good, you can start, immediately start the school to see the effect then	Jingdong prefecture level city in Shandong
Jinfeng Hongyuan Flagship Store	Power Saving Expert 2	Looks good. I'll use it for a while.	Jingdong prefecture level city in Shandong
Jinfeng Hongyuan Flagship Store	Power Saving Expert 2	Great stuff, easy to operate, easy to use, good power saving, believe in the power of science!	Jingdong prefecture level city in Shandong

Jinfeng Hongyuan Flagship Store	Power Saving Expert 2	Especially great, comparison shopping before buying, the store owner is very good, answering questions very patiently and detailed, there is a need to come back!	Jingdong prefecture level city in Shandong
Jinfeng Hongyuan Flagship Store	Power Saving Expert 2	Trustworthy, feel free to use it boldly.	Jingdong prefecture level city in Shandong
Jinfeng Hongyuan Flagship Store	Power Saving Expert 2	Logistics is super fast! Beautiful appearance! Looks like a great quality! I've never bought one before, so I'll try it first to see if it saves power. We'll see how much it saves.	Jingdong prefecture level city in Shandong
Jinfeng Hongyuan Flagship Store	Power Saving Expert 2	I saw online counterfeiting, energy saver is one of them, hahahahaha, jingdong also buy fake products	Jingdong prefecture level city in Shandong
Jinfeng Hongyuan Flagship Store	Power Saving Expert 2	Just received, installed, try a month to see how the effect and then follow up.	Jingdong prefecture level city in Shandong
Jinfeng Hongyuan Flagship Store	Power Saving Expert 2	Good for you! The goods are genuine! Good price for what you get!	Jingdong prefecture level city in Shandong
Jinfeng Hongyuan Flagship Store	Power Saving Expert 2	Things quickly received, immediately used to see if there is no effect, something river photo ↑ the same, customer service is very professional	Jingdong prefecture level city in Shandong
Jinfeng Hongyuan Flagship Store	Power Saving Expert 2	The product is very convenient Looking forward to the subsequent use of the experience results	Jingdong prefecture level city in Shandong
Jinfeng Hongyuan Flagship Store	Power Saving Expert 2	I just received it and plugged it in, we'll see exactly when it's time to pay the electric bill	Jingdong prefecture level city in Shandong
Jinfeng Hongyuan Flagship Store	Power Saving Expert 2	Saver is good Packaging and looks good How effective it is to be shared after using it.	Jingdong prefecture level city in Shandong
Jinfeng Hongyuan Flagship Store	Power Saving Expert 2	There's no way to compare. I don't know if it's energy efficient.	Jingdong prefecture level city in Shandong
Jinfeng Hongyuan Flagship Store	Power Saving Expert 2	Just installed it, try it out and comment again	Jingdong prefecture level city in Shandong
Jinfeng Hongyuan Flagship Store	Power Saving Expert 2	Super save point also good to install, after a friend introduced then went to buy to, friends say it is very good to use, home I bought, try to use a period of time, to be honest, the economizer is not big or heavy, can be very convenient to put in a lot of places, the quality is also very good, power-saving function this time seems to be about 20% of the efficiency. The owner is also very warm contact, very	Jingdong prefecture level city in Shandong
Jinfeng Hongyuan Flagship Store	Power Saving Expert 2	The reviews online are mixed. Try it for a while, but I don't know if I can return it if it doesn't save any power after a couple months.	Jingdong prefecture level city in Shandong
Jinfeng Hongyuan Flagship Store	Power Saving Expert 2	The power consumption at home is high, bought a power saver, it looks very convenient to plug on the line, see if you can save some money next month, if good, recommend to friends down the road.	Jingdong prefecture level city in Shandong
Jinfeng Hongyuan Flagship Store	Power Saving Expert 2	It's beautiful, it's genuine, but I don't know the power-saving effect.	Jingdong prefecture level city in Shandong
Jinfeng Hongyuan Flagship Store	Power Saving Expert 2	This user thinks the product is good	Jingdong prefecture level city in Shandong
Jinfeng Hongyuan Flagship Store	Power Saving Expert 2	Nice exterior design with an at-a-glance display	Jingdong prefecture level city in Shandong
Jinfeng Hongyuan Flagship Store	Power Saving Expert 2	Home large and small things are purchased in the Jingdong Mall, have been accustomed to shopping in the Jingdong, many times can buy good quality and cheap products, and courier service attitude is particularly good, sometimes too many couriers are to help me to the home, so many years have been in the Jingdong Mall shopping, trust and support! Will also support the Jingdong as always!	Jingdong prefecture level city in Shandong
Jinfeng Hongyuan Flagship Store	Power Saving Expert 2	Good workmanship, mostly better than the original wall-plugged ones, and hopefully power factor compensation saves on electricity costs	Jingdong prefecture level city in Shandong
Jinfeng Hongyuan Flagship Store	Power Saving Expert 2	I don't feel anything, I said I won't know the effect for a month or two, let's see.	Jingdong prefecture level city in Shandong
Jinfeng Hongyuan Flagship Store	Power Saving Expert 2	Delivery was pretty fast, a little pricey, just not sure if it works or not	Jingdong prefecture level city in Shandong
Jinfeng Hongyuan Flagship Store	Power Saving Expert 2	The goods have been received, I do not know how effective.	Jingdong prefecture level city in Shandong
Jinfeng Hongyuan Flagship Store	Power Saving Expert 2	So far it doesn't seem to be too noticeable, so I'll comment later.	Jingdong prefecture level city in Shandong
Jinfeng Hongyuan Flagship Store	Power Saving Expert 2	I'll comment after I've used it	Jingdong prefecture level city in Shandong
Jinfeng Hongyuan Flagship Store	Power Saving Expert 2	Not bad. You'll save a lot on your electric bill in a year!	Jingdong prefecture level city in Shandong
Jinfeng Hongyuan Flagship Store	Power Saving Expert 2	I just got it. I haven't tried it yet. I don't know what it's like.	Jingdong prefecture level city in Shandong
Jinfeng Hongyuan Flagship Store	Power Saving Expert 2	Shopping at Jingdong has become a habit, and this disease will not be cured for a while.	Jingdong prefecture level city in Shandong
Jinfeng Hongyuan Flagship Store	Power Saving Expert 2	I've been using it for a while now. I don't know if I'm still saving power or not.	Jingdong prefecture level city in Shandong
Jinfeng Hongyuan Flagship Store	Power Saving Expert 2	Received the saver and it's great! There is also a digital voltage indication. Plugged in as instructed, easy to operate, hope you're our power saver!	Jingdong prefecture level city in Shandong
Jinfeng Hongyuan Flagship Store	Power Saving Expert 2	There is no power saving as the store says, using it is the same as not using it.	Jingdong prefecture level city in Shandong
Jinfeng Hongyuan Flagship Store	Power Saving Expert 2	Use it for a while and then comment again	Jingdong prefecture level city in Shandong
Jinfeng Hongyuan Flagship Store	Power Saving Expert 2	Feeling can, to use 1 to 2 months to see how the effect, there is a voltage display, can see the voltage fluctuations amplitude, quite good!	Jingdong prefecture level city in Shandong
Jinfeng Hongyuan Flagship Store	Power Saving Expert 2	I hope it works and saves me some electricity.	Jingdong prefecture level city in Shandong
Jinfeng Hongyuan Flagship Store	Power Saving Expert 2	Nice baby, happy with the after sales	Jingdong prefecture level city in Shandong
Jinfeng Hongyuan Flagship Store	Power Saving Expert 2	Use it and talk about it.	Jingdong prefecture level city in Shandong
Jinfeng Hongyuan Flagship Store	Power Saving Expert 2	Second purchase	Jingdong prefecture level city in Shandong
Jinfeng Hongyuan Flagship Store	Power Saving Expert 2	It works.	Jingdong prefecture level city in Shandong
Jinfeng Hongyuan Flagship Store	Power Saving Expert 2	It works.	Jingdong prefecture level city in Shandong
Jinfeng Hongyuan Flagship Store	Power Saving Expert 2	I don't know if it's a power saver until I start using it	Jingdong prefecture level city in Shandong
Jinfeng Hongyuan Flagship Store	Power Saving Expert 2	serviceable	Jingdong prefecture level city in Shandong
Jinfeng Hongyuan Flagship Store	Power Saving Expert 2	This user did not fill out the rating in time	Jingdong prefecture level city in Shandong
Jinfeng Hongyuan Flagship Store	Power Saving Expert 2	There is no power saving as the store says, using it is the same as not using it.	Jingdong prefecture level city in Shandong
Jinfeng Hongyuan Flagship Store	Power Saving Expert 2	This user did not fill out the rating in time	Jingdong prefecture level city in Shandong
Jinfeng Hongyuan Flagship Store	Power Saving Expert 2	Good...good.	Jingdong prefecture level city in Shandong
Jinfeng Hongyuan Flagship Store	Power Saving Expert 2	(of an unmarried couple) be close	Jingdong prefecture level city in Shandong
Jinfeng Hongyuan Flagship Store	Power Saving Expert 2	(of an unmarried couple) be close	Jingdong prefecture level city in Shandong
Jinfeng Hongyuan Flagship Store	Power Saving Expert 2	(of an unmarried couple) be close	Jingdong prefecture level city in Shandong
Jinfeng Hongyuan Flagship Store	Power Saving Expert 2	The goods are real, high value, high quality, worth every penny, material appearance and quality at a glance is very upscale, very like HAPPY1 00	Jingdong prefecture level city in Shandong

Jinfeng Hongyuan Flagship Store	Power Saving Expert 2	It is the ideal product, good quality, logistics is also very fast, worthy of praise 🎁 baby quality is very good, received the first time to open the first time to see, it is very good 👍, very satisfied 😊, customer service is also available to ask questions and answer, thank you 🙏	Jingdong prefecture level city in Shandong
Jinfeng Hongyuan Flagship Store	Power Saving Expert 2	I bought it and haven't used it yet, I'll use it again.	Jingdong prefecture level city in Shandong
Jinfeng Hongyuan Flagship Store	Power Saving Expert 2	It didn't work, I used it for a few days and it was the same as before, it didn't work, I should have to return it	Jingdong prefecture level city in Shandong
Jinfeng Hongyuan Flagship Store	Power Saving Expert 2	The goods have been received, but have not yet installed and use, feel good.	Jingdong prefecture level city in Shandong
Jinfeng Hongyuan Flagship Store	Power Saving Expert 2	Goods received, I forgot to take pictures, the goods look good, you can start, immediately start the school to see the effect then	Jingdong prefecture level city in Shandong
Jinfeng Hongyuan Flagship Store	Power Saving Expert 2	Looks good. I'll use it for a while.	Jingdong prefecture level city in Shandong
Jinfeng Hongyuan Flagship Store	Power Saving Expert 2	Great stuff, easy to operate, easy to use, good power saving, believe in the power of science!	Jingdong prefecture level city in Shandong
Jinfeng Hongyuan Flagship Store	Power Saving Expert 2	Especially great, comparison shopping before buying, the store owner is very good, answering questions very patiently and detailed, there is a need to come back!	Jingdong prefecture level city in Shandong
Jinfeng Hongyuan Flagship Store	Power Saving Expert 2	Trustworthy, feel free to use it boldly.	Jingdong prefecture level city in Shandong
Jinfeng Hongyuan Flagship Store	Power Saving Expert 2	Logistics is super fast! Beautiful appearance! Looks like a great quality! I've never bought one before, so I'll try it first to see if it saves power. We'll see how much it saves.	Jingdong prefecture level city in Shandong
Jinfeng Hongyuan Flagship Store	Power Saving Expert 2	I saw online counterfeiting, energy saver is one of them, hahahahaha, jingdong also buy fake products	Jingdong prefecture level city in Shandong
Jinfeng Hongyuan Flagship Store	Power Saving Expert 2	Just received, installed, try a month to see how the effect and then follow up.	Jingdong prefecture level city in Shandong
Jinfeng Hongyuan Flagship Store	Power Saving Expert 2	Good for you! The goods are genuine! Good price for what you get!	Jingdong prefecture level city in Shandong
Jinfeng Hongyuan Flagship Store	Power Saving Expert 2	Things quickly received, immediately used to see if there is no effect, something river photo ⬆ the same, customer service is very professional	Jingdong prefecture level city in Shandong
Jinfeng Hongyuan Flagship Store	Power Saving Expert 2	The product is very convenient Looking forward to the subsequent use of the experience results	Jingdong prefecture level city in Shandong
Jinfeng Hongyuan Flagship Store	Power Saving Expert 2	I just received it and plugged it in, we'll see exactly when it's time to pay the electric bill	Jingdong prefecture level city in Shandong
Jinfeng Hongyuan Flagship Store	Power Saving Expert 2	Saver is good Packaging and looks good How effective it is to be shared after using it.	Jingdong prefecture level city in Shandong
Jinfeng Hongyuan Flagship Store	Power Saving Expert 2	There's no way to compare. I don't know if it's energy efficient.	Jingdong prefecture level city in Shandong
Jinfeng Hongyuan Flagship Store	Power Saving Expert 2	Just installed it, try it out and comment again	Jingdong prefecture level city in Shandong
Jinfeng Hongyuan Flagship Store	Power Saving Expert 2	Super save point also good to install, after a friend introduced then went to buy to, friends say it is very good to use, home I bought, try to use a period of time, to be honest, the economizer is not big or heavy, can be very convenient to put in a lot of places, the quality is also very good, power-saving function this time seems to be about 20% of the efficiency. The owner is also very warm contact, very	Jingdong prefecture level city in Shandong
Jinfeng Hongyuan Flagship Store	Power Saving Expert 2	The reviews online are mixed. Try it for a while, but I don't know if I can return it if it doesn't save any power after a couple months.	Jingdong prefecture level city in Shandong
Jinfeng Hongyuan Flagship Store	Power Saving Expert 2	The power consumption at home is high, bought a power saver, it looks very convenient to plug on the line, see if you can save some money next month, if good, recommend to friends down the road.	Jingdong prefecture level city in Shandong
Jinfeng Hongyuan Flagship Store	Power Saving Expert 2	It's beautiful, it's genuine, but I don't know the power-saving effect.	Jingdong prefecture level city in Shandong
Jinfeng Hongyuan Flagship Store	Power Saving Expert 2	This user thinks the product is good	Jingdong prefecture level city in Shandong
Jinfeng Hongyuan Flagship Store	Power Saving Expert 2	Nice exterior design with an at-a-glance display	Jingdong prefecture level city in Shandong
Jinfeng Hongyuan Flagship Store	Power Saving Expert 2	Home large and small things are purchased in the Jingdong Mall, have been accustomed to shopping in the Jingdong, many times can buy good quality and cheap products, and courier service attitude is particularly good, sometimes too many couriers are to help me to the home, so many years have been in the Jingdong Mall shopping, trust and support! Will also support the Jingdong as always!	Jingdong prefecture level city in Shandong
Jinfeng Hongyuan Flagship Store	Power Saving Expert 2	Good workmanship, mostly better than the original wall-plugged ones, and hopefully power factor compensation saves on electricity costs	Jingdong prefecture level city in Shandong
Jinfeng Hongyuan Flagship Store	Power Saving Expert 2	I don't feel anything, I said I won't know the effect for a month or two, let's see.	Jingdong prefecture level city in Shandong
Jinfeng Hongyuan Flagship Store	Power Saving Expert 2	Delivery was pretty fast, a little pricey, just not sure if it works or not	Jingdong prefecture level city in Shandong
Jinfeng Hongyuan Flagship Store	Power Saving Expert 2	The goods have been received, I do not know how effective.	Jingdong prefecture level city in Shandong
Jinfeng Hongyuan Flagship Store	Power Saving Expert 2	So far it doesn't seem to be too noticeable, so I'll comment later.	Jingdong prefecture level city in Shandong
Jinfeng Hongyuan Flagship Store	Power Saving Expert 2	I'll comment after I've used it	Jingdong prefecture level city in Shandong
Jinfeng Hongyuan Flagship Store	Power Saving Expert 2	Not bad. You'll save a lot on your electric bill in a year!	Jingdong prefecture level city in Shandong
Jinfeng Hongyuan Flagship Store	Power Saving Expert 2	I just got it. I haven't tried it yet. I don't know what it's like.	Jingdong prefecture level city in Shandong
Jinfeng Hongyuan Flagship Store	Power Saving Expert 2	Shopping at Jingdong has become a habit, and this disease will not be cured for a while.	Jingdong prefecture level city in Shandong
Jinfeng Hongyuan Flagship Store	Power Saving Expert 2	I've been using it for a while now. I don't know if I'm still saving power or not.	Jingdong prefecture level city in Shandong
Jinfeng Hongyuan Flagship Store	Power Saving Expert 2	Received the saver and it's great! There is also a digital voltage indication. Plugged in as instructed, easy to operate, hope you're our power saver!	Jingdong prefecture level city in Shandong
Jinfeng Hongyuan Flagship Store	Power Saving Expert 2	There is no power saving as the store says, using it is the same as not using it.	Jingdong prefecture level city in Shandong
Jinfeng Hongyuan Flagship Store	Power Saving Expert 2	Use it for a while and then comment again	Jingdong prefecture level city in Shandong
Jinfeng Hongyuan Flagship Store	Power Saving Expert 2	Feeling can, to use 1 to 2 months to see how the effect, there is a voltage display, can see the voltage fluctuations amplitude, quite good!	Jingdong prefecture level city in Shandong
Jinfeng Hongyuan Flagship Store	Power Saving Expert 2	I hope it works and saves me some electricity.	Jingdong prefecture level city in Shandong
Jinfeng Hongyuan Flagship Store	Power Saving Expert 2	Nice baby, happy with the after sales	Jingdong prefecture level city in Shandong
Jinfeng Hongyuan Flagship Store	Power Saving Expert 2	Use it and talk about it.	Jingdong prefecture level city in Shandong
Jinfeng Hongyuan Flagship Store	Power Saving Expert 2	Second purchase	Jingdong prefecture level city in Shandong
Jinfeng Hongyuan Flagship Store	Power Saving Expert 2	It works.	Jingdong prefecture level city in Shandong
Jinfeng Hongyuan Flagship Store	Power Saving Expert 2	It works.	Jingdong prefecture level city in Shandong
Jinfeng Hongyuan Flagship Store	Power Saving Expert 2	I don't know if it's a power saver until I start using it	Jingdong prefecture level city in Shandong
Jinfeng Hongyuan Flagship Store	Power Saving Expert 2	serviceable	Jingdong prefecture level city in Shandong
Jinfeng Hongyuan Flagship Store	Power Saving Expert 2	This user did not fill out the rating in time	Jingdong prefecture level city in Shandong

Jinfeng Hongyuan Flagship Store	Power Saving Expert 2	There is no power saving as the store says, using it is the same as not using it.	Jingdong prefecture level city in Shandong
Jinfeng Hongyuan Flagship Store	Power Saving Expert 2	This user did not fill out the rating in time	Jingdong prefecture level city in Shandong
Jinfeng Hongyuan Flagship Store	Power Saving Expert 2	Good...good.	Jingdong prefecture level city in Shandong
Jinfeng Hongyuan Flagship Store	Power Saving Expert 2	(of an unmarried couple) be close	Jingdong prefecture level city in Shandong
Jinfeng Hongyuan Flagship Store	Power Saving Expert 2	(of an unmarried couple) be close	Jingdong prefecture level city in Shandong
Jinfeng Hongyuan Flagship Store	Power Saving Expert 2	(of an unmarried couple) be close	Jingdong prefecture level city in Shandong
Jinfeng Hongyuan Flagship Store	Power Saving Expert 2	The goods are real, high value, high quality, worth every penny, material appearance and quality at a glance is very upscale, very like HAPPY1 00	Jingdong prefecture level city in Shandong
Jinfeng Hongyuan Flagship Store	Power Saving Expert 2	It is the ideal product, good quality, logistics is also very fast, worthy of praise 🎁 baby quality is very good, received the first time to open the first time to see, it is very good 👍, very satisfied 😊, customer service is also available to ask questions and answer, thank you 🙏	Jingdong prefecture level city in Shandong
Jinfeng Hongyuan Flagship Store	Power Saving Expert 2	I bought it and haven't used it yet, I'll use it again.	Jingdong prefecture level city in Shandong
Jinfeng Hongyuan Flagship Store	Power Saving Expert 2	It didn't work, I used it for a few days and it was the same as before, it didn't work, I should have to return it	Jingdong prefecture level city in Shandong
Jinfeng Hongyuan Flagship Store	Power Saving Expert 2	The goods have been received, but have not yet installed and use, feel good.	Jingdong prefecture level city in Shandong
Jinfeng Hongyuan Flagship Store	Power Saving Expert 2	Goods received, I forgot to take pictures, the goods look good, you can start, immediately start the school to see the effect then	Jingdong prefecture level city in Shandong
Jinfeng Hongyuan Flagship Store	Power Saving Expert 2	Looks good. I'll use it for a while.	Jingdong prefecture level city in Shandong
Jinfeng Hongyuan Flagship Store	Power Saving Expert 2	Great stuff, easy to operate, easy to use, good power saving, believe in the power of science!	Jingdong prefecture level city in Shandong
Jinfeng Hongyuan Flagship Store	Power Saving Expert 2	Especially great, comparison shopping before buying, the store owner is very good, answering questions very patiently and detailed, there is a need to come back!	Jingdong prefecture level city in Shandong
Jinfeng Hongyuan Flagship Store	Power Saving Expert 2	Trustworthy, feel free to use it boldly.	Jingdong prefecture level city in Shandong
Jinfeng Hongyuan Flagship Store	Power Saving Expert 2	Logistics is super fast! Beautiful appearance! Looks like a great quality! I've never bought one before, so I'll try it first to see if it saves power. We'll see how much it saves.	Jingdong prefecture level city in Shandong
Jinfeng Hongyuan Flagship Store	Power Saving Expert 2	I saw online counterfeiting, energy saver is one of them, hahahahaha, jingdong also buy fake products	Jingdong prefecture level city in Shandong
Jinfeng Hongyuan Flagship Store	Power Saving Expert 2	Just received, installed, try a month to see how the effect and then follow up.	Jingdong prefecture level city in Shandong
Jinfeng Hongyuan Flagship Store	Power Saving Expert 2	Good for you! The goods are genuine! Good price for what you get!	Jingdong prefecture level city in Shandong
Jinfeng Hongyuan Flagship Store	Power Saving Expert 2	Things quickly received, immediately used to see if there is no effect, something river photo ⬆ the same, customer service is very professional	Jingdong prefecture level city in Shandong
Jinfeng Hongyuan Flagship Store	Power Saving Expert 2	The product is very convenient Looking forward to the subsequent use of the experience results	Jingdong prefecture level city in Shandong
Jinfeng Hongyuan Flagship Store	Power Saving Expert 2	I just received it and plugged it in, we'll see exactly when it's time to pay the electric bill	Jingdong prefecture level city in Shandong
Jinfeng Hongyuan Flagship Store	Power Saving Expert 2	Saver is good Packaging and looks good How effective it is to be shared after using it.	Jingdong prefecture level city in Shandong
Jinfeng Hongyuan Flagship Store	Power Saving Expert 2	There's no way to compare. I don't know if it's energy efficient.	Jingdong prefecture level city in Shandong
Jinfeng Hongyuan Flagship Store	Power Saving Expert 2	Just installed it, try it out and comment again	Jingdong prefecture level city in Shandong
Jinfeng Hongyuan Flagship Store	Power Saving Expert 2	Super save point also good to install, after a friend introduced then went to buy to, friends say it is very good to use, home I bought, try to use a period of time, to be honest, the economizer is not big or heavy, can be very convenient to put in a lot of places, the quality is also very good, power-saving function this time seems to be about 20% of the efficiency. The owner is also very warm contact, very	Jingdong prefecture level city in Shandong
Jinfeng Hongyuan Flagship Store	Power Saving Expert 2	The reviews online are mixed. Try it for a while, but I don't know if I can return it if it doesn't save any power after a couple months.	Jingdong prefecture level city in Shandong
Jinfeng Hongyuan Flagship Store	Power Saving Expert 2	The power consumption at home is high, bought a power saver, it looks very convenient to plug on the line, see if you can save some money next month, if good, recommend to friends down the road.	Jingdong prefecture level city in Shandong
Jinfeng Hongyuan Flagship Store	Power Saving Expert 2	It's beautiful, it's genuine, but I don't know the power-saving effect.	Jingdong prefecture level city in Shandong
Jinfeng Hongyuan Flagship Store	Power Saving Expert 2	This user thinks the product is good	Jingdong prefecture level city in Shandong
Jinfeng Hongyuan Flagship Store	Power Saving Expert 2	Nice exterior design with an at-a-glance display	Jingdong prefecture level city in Shandong
Jinfeng Hongyuan Flagship Store	Power Saving Expert 2	Home large and small things are purchased in the Jingdong Mall, have been accustomed to shopping in the Jingdong, many times can buy good quality and cheap products, and courier service attitude is particularly good, sometimes too many couriers are to help me to the home, so many years have been in the Jingdong Mall shopping, trust and support! Will also support the Jingdong as always!	Jingdong prefecture level city in Shandong
Jinfeng Hongyuan Flagship Store	Power Saving Expert 2	Good workmanship, mostly better than the original wall-plugged ones, and hopefully power factor compensation saves on electricity costs	Jingdong prefecture level city in Shandong
Jinfeng Hongyuan Flagship Store	Power Saving Expert 2	I don't feel anything, I said I won't know the effect for a month or two, let's see.	Jingdong prefecture level city in Shandong
Jinfeng Hongyuan Flagship Store	Power Saving Expert 2	Delivery was pretty fast, a little pricey, just not sure if it works or not	Jingdong prefecture level city in Shandong
Jinfeng Hongyuan Flagship Store	Power Saving Expert 2	The goods have been received, I do not know how effective.	Jingdong prefecture level city in Shandong
Jinfeng Hongyuan Flagship Store	Power Saving Expert 2	So far it doesn't seem to be too noticeable, so I'll comment later.	Jingdong prefecture level city in Shandong
Jinfeng Hongyuan Flagship Store	Power Saving Expert 2	I'll comment after I've used it	Jingdong prefecture level city in Shandong
Jinfeng Hongyuan Flagship Store	Power Saving Expert 2	Not bad. You'll save a lot on your electric bill in a year!	Jingdong prefecture level city in Shandong
Jinfeng Hongyuan Flagship Store	Power Saving Expert 2	I just got it. I haven't tried it yet. I don't know what it's like.	Jingdong prefecture level city in Shandong
Jinfeng Hongyuan Flagship Store	Power Saving Expert 2	Shopping at Jingdong has become a habit, and this disease will not be cured for a while.	Jingdong prefecture level city in Shandong
Jinfeng Hongyuan Flagship Store	Power Saving Expert 2	I've been using it for a while now. I don't know if I'm still saving power or not.	Jingdong prefecture level city in Shandong
Jinfeng Hongyuan Flagship Store	Power Saving Expert 2	Received the saver and it's great! There is also a digital voltage indication. Plugged in as instructed, easy to operate, hope you're our power saver!	Jingdong prefecture level city in Shandong
Jinfeng Hongyuan Flagship Store	Power Saving Expert 2	There is no power saving as the store says, using it is the same as not using it.	Jingdong prefecture level city in Shandong
Jinfeng Hongyuan Flagship Store	Power Saving Expert 2	Use it for a while and then comment again	Jingdong prefecture level city in Shandong
Jinfeng Hongyuan Flagship Store	Power Saving Expert 2	Feeling can, to use 1 to 2 months to see how the effect, there is a voltage display, can see the voltage fluctuations amplitude, quite good!	Jingdong prefecture level city in Shandong
Jinfeng Hongyuan Flagship Store	Power Saving Expert 2	I hope it works and saves me some electricity.	Jingdong prefecture level city in Shandong
Jinfeng Hongyuan Flagship Store	Power Saving Expert 2	Nice baby, happy with the after sales	Jingdong prefecture level city in Shandong

Jinfeng Hongyuan Flagship Store	Power Saving Expert 2	Use it and talk about it.	Jingdong prefecture level city in Shandong
Jinfeng Hongyuan Flagship Store	Power Saving Expert 2	Second purchase	Jingdong prefecture level city in Shandong
Jinfeng Hongyuan Flagship Store	Power Saving Expert 2	It works.	Jingdong prefecture level city in Shandong
Jinfeng Hongyuan Flagship Store	Power Saving Expert 2	It works.	Jingdong prefecture level city in Shandong
Jinfeng Hongyuan Flagship Store	Power Saving Expert 2	I don't know if it's a power saver until I start using it	Jingdong prefecture level city in Shandong
Jinfeng Hongyuan Flagship Store	Power Saving Expert 2	serviceable	Jingdong prefecture level city in Shandong
Jinfeng Hongyuan Flagship Store	Power Saving Expert 2	This user did not fill out the rating in time	Jingdong prefecture level city in Shandong
Jinfeng Hongyuan Flagship Store	Power Saving Expert 2	There is no power saving as the store says, using it is the same as not using it.	Jingdong prefecture level city in Shandong
Jinfeng Hongyuan Flagship Store	Power Saving Expert 2	This user did not fill out the rating in time	Jingdong prefecture level city in Shandong
Jinfeng Hongyuan Flagship Store	Power Saving Expert 2	Good...good.	Jingdong prefecture level city in Shandong
Jinfeng Hongyuan Flagship Store	Power Saving Expert 2	(of an unmarried couple) be close	Jingdong prefecture level city in Shandong
Jinfeng Hongyuan Flagship Store	Power Saving Expert 2	(of an unmarried couple) be close	Jingdong prefecture level city in Shandong
Jinfeng Hongyuan Flagship Store	Power Saving Expert 2	(of an unmarried couple) be close	Jingdong prefecture level city in Shandong
Jinfeng Hongyuan Flagship Store	Power Saving Expert 2	The goods are real, high value, high quality, worth every penny, material appearance and quality at a glance is very upscale, very like HAPPY1 00	Jingdong prefecture level city in Shandong
Jinfeng Hongyuan Flagship Store	Power Saving Expert 2	It is the ideal product, good quality, logistics is also very fast, worthy of praise 🎁 baby quality is very good, received the first time to open the first time to see, it is very good 👍, very satisfied 😊, customer service is also available to ask questions and answer, thank you 🙏	Jingdong prefecture level city in Shandong
Jinfeng Hongyuan Flagship Store	Power Saving Expert 2	I bought it and haven't used it yet, I'll use it again.	Jingdong prefecture level city in Shandong
Jinfeng Hongyuan Flagship Store	Power Saving Expert 2	It didn't work, I used it for a few days and it was the same as before, it didn't work, I should have to return it	Jingdong prefecture level city in Shandong
Jinfeng Hongyuan Flagship Store	Power Saving Expert 2	The goods have been received, but have not yet installed and use, feel good.	Jingdong prefecture level city in Shandong
Jinfeng Hongyuan Flagship Store	Power Saving Expert 2	Goods received, I forgot to take pictures, the goods look good, you can start, immediately start the school to see the effect then	Jingdong prefecture level city in Shandong
Jinfeng Hongyuan Flagship Store	Power Saving Expert 2	Looks good. I'll use it for a while.	Jingdong prefecture level city in Shandong
Jinfeng Hongyuan Flagship Store	Power Saving Expert 2	Great stuff, easy to operate, easy to use, good power saving, believe in the power of science!	Jingdong prefecture level city in Shandong
Jinfeng Hongyuan Flagship Store	Power Saving Expert 2	Especially great, comparison shopping before buying, the store owner is very good, answering questions very patiently and detailed, there is a need to come back!	Jingdong prefecture level city in Shandong
Jinfeng Hongyuan Flagship Store	Power Saving Expert 2	Trustworthy, feel free to use it boldly.	Jingdong prefecture level city in Shandong
Jinfeng Hongyuan Flagship Store	Power Saving Expert 2	Logistics is super fast! Beautiful appearance! Looks like a great quality! I've never bought one before, so I'll try it first to see if it saves power. We'll see how much it saves.	Jingdong prefecture level city in Shandong
Jinfeng Hongyuan Flagship Store	Power Saving Expert 2	I saw online counterfeiting, energy saver is one of them, hahahahaha, jingdong also buy fake products	Jingdong prefecture level city in Shandong
Jinfeng Hongyuan Flagship Store	Power Saving Expert 2	Just received, installed, try a month to see how the effect and then follow up.	Jingdong prefecture level city in Shandong
Jinfeng Hongyuan Flagship Store	Power Saving Expert 2	Good for you! The goods are genuine! Good price for what you get!	Jingdong prefecture level city in Shandong
Jinfeng Hongyuan Flagship Store	Power Saving Expert 2	Things quickly received, immediately used to see if there is no effect, something river photo ⬆ the same, customer service is very professional	Jingdong prefecture level city in Shandong
Jinfeng Hongyuan Flagship Store	Power Saving Expert 2	The product is very convenient Looking forward to the subsequent use of the experience results	Jingdong prefecture level city in Shandong
Jinfeng Hongyuan Flagship Store	Power Saving Expert 2	I just received it and plugged it in, we'll see exactly when it's time to pay the electric bill	Jingdong prefecture level city in Shandong
Jinfeng Hongyuan Flagship Store	Power Saving Expert 2	Saver is good Packaging and looks good How effective it is to be shared after using it.	Jingdong prefecture level city in Shandong
Jinfeng Hongyuan Flagship Store	Power Saving Expert 2	There's no way to compare. I don't know if it's energy efficient.	Jingdong prefecture level city in Shandong
Jinfeng Hongyuan Flagship Store	Power Saving Expert 2	Just installed it, try it out and comment again	Jingdong prefecture level city in Shandong
Jinfeng Hongyuan Flagship Store	Power Saving Expert 2	Super save point also good to install, after a friend introduced then went to buy to, friends say it is very good to use, home I bought, try to use a period of time, to be honest, the economizer is not big or heavy, can be very convenient to put in a lot of places, the quality is also very good, power-saving function this time seems to be about 20% of the efficiency. The owner is also very warm contact, very	Jingdong prefecture level city in Shandong
Jinfeng Hongyuan Flagship Store	Power Saving Expert 2	The reviews online are mixed. Try it for a while, but I don't know if I can return it if it doesn't save any power after a couple months.	Jingdong prefecture level city in Shandong
Jinfeng Hongyuan Flagship Store	Power Saving Expert 2	The power consumption at home is high, bought a power saver, it looks very convenient to plug on the line, see if you can save some money next month, if good, recommend to friends down the road.	Jingdong prefecture level city in Shandong
Jinfeng Hongyuan Flagship Store	Power Saving Expert 2	It's beautiful, it's genuine, but I don't know the power-saving effect.	Jingdong prefecture level city in Shandong
Jinfeng Hongyuan Flagship Store	Power Saving Expert 2	This user thinks the product is good	Jingdong prefecture level city in Shandong
Jinfeng Hongyuan Flagship Store	Power Saving Expert 2	Nice exterior design with an at-a-glance display	Jingdong prefecture level city in Shandong
Jinfeng Hongyuan Flagship Store	Power Saving Expert 2	Home large and small things are purchased in the Jingdong Mall, have been accustomed to shopping in the Jingdong, many times can buy good quality and cheap products, and courier service attitude is particularly good, sometimes too many couriers are to help me to the home, so many years have been in the Jingdong Mall shopping, trust and support! Will also support the Jingdong as always!	Jingdong prefecture level city in Shandong
Jinfeng Hongyuan Flagship Store	Power Saving Expert 2	Good workmanship, mostly better than the original wall-plugged ones, and hopefully power factor compensation saves on electricity costs	Jingdong prefecture level city in Shandong
Jinfeng Hongyuan Flagship Store	Power Saving Expert 2	I don't feel anything, I said I won't know the effect for a month or two, let's see.	Jingdong prefecture level city in Shandong
Jinfeng Hongyuan Flagship Store	Power Saving Expert 2	Delivery was pretty fast, a little pricey, just not sure if it works or not	Jingdong prefecture level city in Shandong
Jinfeng Hongyuan Flagship Store	Power Saving Expert 2	The goods have been received, I do not know how effective.	Jingdong prefecture level city in Shandong
Jinfeng Hongyuan Flagship Store	Power Saving Expert 2	So far it doesn't seem to be too noticeable, so I'll comment later.	Jingdong prefecture level city in Shandong
Jinfeng Hongyuan Flagship Store	Power Saving Expert 2	I'll comment after I've used it	Jingdong prefecture level city in Shandong
Jinfeng Hongyuan Flagship Store	Power Saving Expert 2	Not bad. You'll save a lot on your electric bill in a year!	Jingdong prefecture level city in Shandong
Jinfeng Hongyuan Flagship Store	Power Saving Expert 2	I just got it. I haven't tried it yet. I don't know what it's like.	Jingdong prefecture level city in Shandong
Jinfeng Hongyuan Flagship Store	Power Saving Expert 2	Shopping at Jingdong has become a habit, and this disease will not be cured for a while.	Jingdong prefecture level city in Shandong

Jinfeng Hongyuan Flagship Store	Power Saving Expert 2	I've been using it for a while now. I don't know if I'm still saving power or not.	Jingdong prefecture level city in Shandong
Jinfeng Hongyuan Flagship Store	Power Saving Expert 2	Received the saver and it's great! There is also a digital voltage indication. Plugged in as instructed, easy to operate, hope you're our power saver!	Jingdong prefecture level city in Shandong
Jinfeng Hongyuan Flagship Store	Power Saving Expert 2	There is no power saving as the store says, using it is the same as not using it.	Jingdong prefecture level city in Shandong
Jinfeng Hongyuan Flagship Store	Power Saving Expert 2	Use it for a while and then comment again	Jingdong prefecture level city in Shandong
Jinfeng Hongyuan Flagship Store	Power Saving Expert 2	Feeling can, to use 1 to 2 months to see how the effect, there is a voltage display, can see the voltage fluctuations amplitude, quite good!	Jingdong prefecture level city in Shandong
Jinfeng Hongyuan Flagship Store	Power Saving Expert 2	I hope it works and saves me some electricity.	Jingdong prefecture level city in Shandong
Jinfeng Hongyuan Flagship Store	Power Saving Expert 2	Nice baby, happy with the after sales	Jingdong prefecture level city in Shandong
Jinfeng Hongyuan Flagship Store	Power Saving Expert 2	Use it and talk about it.	Jingdong prefecture level city in Shandong
Jinfeng Hongyuan Flagship Store	Power Saving Expert 2	Second purchase	Jingdong prefecture level city in Shandong
Jinfeng Hongyuan Flagship Store	Power Saving Expert 2	It works.	Jingdong prefecture level city in Shandong
Jinfeng Hongyuan Flagship Store	Power Saving Expert 2	It works.	Jingdong prefecture level city in Shandong
Jinfeng Hongyuan Flagship Store	Power Saving Expert 2	I don't know if it's a power saver until I start using it	Jingdong prefecture level city in Shandong
Jinfeng Hongyuan Flagship Store	Power Saving Expert 2	serviceable	Jingdong prefecture level city in Shandong
Jinfeng Hongyuan Flagship Store	Power Saving Expert 2	This user did not fill out the rating in time	Jingdong prefecture level city in Shandong
Jinfeng Hongyuan Flagship Store	Power Saving Expert 2	There is no power saving as the store says, using it is the same as not using it.	Jingdong prefecture level city in Shandong
Jinfeng Hongyuan Flagship Store	Power Saving Expert 2	This user did not fill out the rating in time	Jingdong prefecture level city in Shandong
Jinfeng Hongyuan Flagship Store	Power Saving Expert 2	Good...good.	Jingdong prefecture level city in Shandong
Jinfeng Hongyuan Flagship Store	Power Saving Expert 2	(of an unmarried couple) be close	Jingdong prefecture level city in Shandong
Jinfeng Hongyuan Flagship Store	Power Saving Expert 2	(of an unmarried couple) be close	Jingdong prefecture level city in Shandong
Jinfeng Hongyuan Flagship Store	Power Saving Expert 2	(of an unmarried couple) be close	Jingdong prefecture level city in Shandong
Jinfeng Hongyuan Flagship Store	Power Saving Expert 2	The goods are real, high value, high quality, worth every penny, material appearance and quality at a glance is very upscale, very like HAPPY1 00	Jingdong prefecture level city in Shandong
Jinfeng Hongyuan Flagship Store	Power Saving Expert 2	It is the ideal product, good quality, logistics is also very fast, worthy of praise 🎁 baby quality is very good, received the first time to open the first time to see, it is very good 👍, very satisfied 😃, customer service is also available to ask questions and answer, thank you 🙏	Jingdong prefecture level city in Shandong
Jinfeng Hongyuan Flagship Store	Power Saving Expert 2	I bought it and haven't used it yet, I'll use it again.	Jingdong prefecture level city in Shandong
Jinfeng Hongyuan Flagship Store	Power Saving Expert 2	It didn't work, I used it for a few days and it was the same as before, it didn't work, I should have to return it	Jingdong prefecture level city in Shandong
Jinfeng Hongyuan Flagship Store	Power Saving Expert 2	The goods have been received, but have not yet installed and use, feel good.	Jingdong prefecture level city in Shandong
Jinfeng Hongyuan Flagship Store	Power Saving Expert 2	Goods received, I forgot to take pictures, the goods look good, you can start, immediately start the school to see the effect then	Jingdong prefecture level city in Shandong
Jinfeng Hongyuan Flagship Store	Power Saving Expert 2	Looks good. I'll use it for a while.	Jingdong prefecture level city in Shandong
Jinfeng Hongyuan Flagship Store	Power Saving Expert 2	Great stuff, easy to operate, easy to use, good power saving, believe in the power of science!	Jingdong prefecture level city in Shandong
Jinfeng Hongyuan Flagship Store	Power Saving Expert 2	Especially great, comparison shopping before buying, the store owner is very good, answering questions very patiently and detailed, there is a need to come back!	Jingdong prefecture level city in Shandong
Jinfeng Hongyuan Flagship Store	Power Saving Expert 2	Trustworthy, feel free to use it boldly.	Jingdong prefecture level city in Shandong
Jinfeng Hongyuan Flagship Store	Power Saving Expert 2	Logistics is super fast! Beautiful appearance! Looks like a great quality! I've never bought one before, so I'll try it first to see if it saves power. We'll see how much it saves.	Jingdong prefecture level city in Shandong
Jinfeng Hongyuan Flagship Store	Power Saving Expert 2	I saw online counterfeiting, energy saver is one of them, hahahahaha, jingdong also buy fake products	Jingdong prefecture level city in Shandong
Jinfeng Hongyuan Flagship Store	Power Saving Expert 2	Just received, installed, try a month to see how the effect and then follow up.	Jingdong prefecture level city in Shandong
Jinfeng Hongyuan Flagship Store	Power Saving Expert 2	Good for you! The goods are genuine! Good price for what you get!	Jingdong prefecture level city in Shandong
Jinfeng Hongyuan Flagship Store	Power Saving Expert 2	Things quickly received, immediately used to see if there is no effect, something river photo ⍐ the same, customer service is very professional	Jingdong prefecture level city in Shandong
Jinfeng Hongyuan Flagship Store	Power Saving Expert 2	The product is very convenient Looking forward to the subsequent use of the experience results	Jingdong prefecture level city in Shandong
Jinfeng Hongyuan Flagship Store	Power Saving Expert 2	I just received it and plugged it in, we'll see exactly when it's time to pay the electric bill	Jingdong prefecture level city in Shandong
Jinfeng Hongyuan Flagship Store	Power Saving Expert 2	Saver is good Packaging and looks good How effective it is to be shared after using it.	Jingdong prefecture level city in Shandong
Jinfeng Hongyuan Flagship Store	Power Saving Expert 2	There's no way to compare. I don't know if it's energy efficient.	Jingdong prefecture level city in Shandong
Jinfeng Hongyuan Flagship Store	Power Saving Expert 2	Just installed it, try it out and comment again	Jingdong prefecture level city in Shandong
Jinfeng Hongyuan Flagship Store	Power Saving Expert 2	Super save point also good to install, after a friend introduced then went to buy to, friends say it is very good to use, home I bought, try to use a period of time, to be honest, the economizer is not big or heavy, can be very convenient to put in a lot of places, the quality is also very good, power-saving function this time seems to be about 20% of the efficiency. The owner is also very warm contact, very	Jingdong prefecture level city in Shandong
Jinfeng Hongyuan Flagship Store	Power Saving Expert 2	The reviews online are mixed. Try it for a while, but I don't know if I can return it if it doesn't save any power after a couple months.	Jingdong prefecture level city in Shandong
Jinfeng Hongyuan Flagship Store	Power Saving Expert 2	The power consumption at home is high, bought a power saver, it looks very convenient to plug on the line, see if you can save some money next month, if good, recommend to friends down the road.	Jingdong prefecture level city in Shandong
Jinfeng Hongyuan Flagship Store	Power Saving Expert 2	It's beautiful, it's genuine, but I don't know the power-saving effect.	Jingdong prefecture level city in Shandong
Jinfeng Hongyuan Flagship Store	Power Saving Expert 2	This user thinks the product is good	Jingdong prefecture level city in Shandong
Jinfeng Hongyuan Flagship Store	Power Saving Expert 2	Nice exterior design with an at-a-glance display	Jingdong prefecture level city in Shandong
Jinfeng Hongyuan Flagship Store	Power Saving Expert 2	Home large and small things are purchased in the Jingdong Mall, have been accustomed to shopping in the Jingdong, many times can buy good quality and cheap products, and courier service attitude is particularly good, sometimes too many couriers are to help me to the home, so many years have been in the Jingdong Mall shopping, trust and support! Will also support the Jingdong as always!	Jingdong prefecture level city in Shandong
Jinfeng Hongyuan Flagship Store	Power Saving Expert 2	Good workmanship, mostly better than the original wall-plugged ones, and hopefully power factor compensation saves on electricity costs	Jingdong prefecture level city in Shandong
Jinfeng Hongyuan Flagship Store	Power Saving Expert 2	I don't feel anything, I said I won't know the effect for a month or two, let's see.	Jingdong prefecture level city in Shandong

Jinfeng Hongyuan Flagship Store	Power Saving Expert 2	Delivery was pretty fast, a little pricey, just not sure if it works or not	Jingdong prefecture level city in Shandong
Jinfeng Hongyuan Flagship Store	Power Saving Expert 2	The goods have been received, I do not know how effective.	Jingdong prefecture level city in Shandong
Jinfeng Hongyuan Flagship Store	Power Saving Expert 2	So far it doesn't seem to be too noticeable, so I'll comment later.	Jingdong prefecture level city in Shandong
Jinfeng Hongyuan Flagship Store	Power Saving Expert 2	I'll comment after I've used it	Jingdong prefecture level city in Shandong
Jinfeng Hongyuan Flagship Store	Power Saving Expert 2	Not bad. You'll save a lot on your electric bill in a year!	Jingdong prefecture level city in Shandong
Jinfeng Hongyuan Flagship Store	Power Saving Expert 2	I just got it. I haven't tried it yet. I don't know what it's like.	Jingdong prefecture level city in Shandong
Jinfeng Hongyuan Flagship Store	Power Saving Expert 2	Shopping at Jingdong has become a habit, and this disease will not be cured for a while.	Jingdong prefecture level city in Shandong
Jinfeng Hongyuan Flagship Store	Power Saving Expert 2	I've been using it for a while now. I don't know if I'm still saving power or not.	Jingdong prefecture level city in Shandong
Jinfeng Hongyuan Flagship Store	Power Saving Expert 2	Received the saver and it's great! There is also a digital voltage indication. Plugged in as instructed, easy to operate, hope you're our power saver!	Jingdong prefecture level city in Shandong
Jinfeng Hongyuan Flagship Store	Power Saving Expert 2	There is no power saving as the store says, using it is the same as not using it.	Jingdong prefecture level city in Shandong
Jinfeng Hongyuan Flagship Store	Power Saving Expert 2	Use it for a while and then comment again	Jingdong prefecture level city in Shandong
Jinfeng Hongyuan Flagship Store	Power Saving Expert 2	Feeling can, to use 1 to 2 months to see how the effect, there is a voltage display, can see the voltage fluctuations amplitude, quite good!	Jingdong prefecture level city in Shandong
Jinfeng Hongyuan Flagship Store	Power Saving Expert 2	I hope it works and saves me some electricity.	Jingdong prefecture level city in Shandong
Jinfeng Hongyuan Flagship Store	Power Saving Expert 2	Nice baby, happy with the after sales	Jingdong prefecture level city in Shandong
Jinfeng Hongyuan Flagship Store	Power Saving Expert 2	Use it and talk about it.	Jingdong prefecture level city in Shandong
Jinfeng Hongyuan Flagship Store	Power Saving Expert 2	Second purchase	Jingdong prefecture level city in Shandong
Jinfeng Hongyuan Flagship Store	Power Saving Expert 2	It works.	Jingdong prefecture level city in Shandong
Jinfeng Hongyuan Flagship Store	Power Saving Expert 2	It works.	Jingdong prefecture level city in Shandong
Jinfeng Hongyuan Flagship Store	Power Saving Expert 2	I don't know if it's a power saver until I start using it	Jingdong prefecture level city in Shandong
Jinfeng Hongyuan Flagship Store	Power Saving Expert 2	serviceable	Jingdong prefecture level city in Shandong
Jinfeng Hongyuan Flagship Store	Power Saving Expert 2	This user did not fill out the rating in time	Jingdong prefecture level city in Shandong
Jinfeng Hongyuan Flagship Store	Power Saving Expert 2	There is no power saving as the store says, using it is the same as not using it.	Jingdong prefecture level city in Shandong
Jinfeng Hongyuan Flagship Store	Power Saving Expert 2	This user did not fill out the rating in time	Jingdong prefecture level city in Shandong
Jinfeng Hongyuan Flagship Store	Power Saving Expert 2	Good...good.	Jingdong prefecture level city in Shandong
Jinfeng Hongyuan Flagship Store	Power Saving Expert 2	(of an unmarried couple) be close	Jingdong prefecture level city in Shandong
Jinfeng Hongyuan Flagship Store	Power Saving Expert 2	(of an unmarried couple) be close	Jingdong prefecture level city in Shandong
Jinfeng Hongyuan Flagship Store	Power Saving Expert 2	(of an unmarried couple) be close	Jingdong prefecture level city in Shandong
Jinfeng Hongyuan Flagship Store	Power Saving Expert 2	The goods are real, high value, high quality, worth every penny, material appearance and quality at a glance is very upscale, very like HAPPY1 00	Jingdong prefecture level city in Shandong
Jinfeng Hongyuan Flagship Store	Power Saving Expert 2	It is the ideal product, good quality, logistics is also very fast, worthy of praise 🎁 baby quality is very good, received the first time to open the first time to see, it is very good 👍, very satisfied 😃, customer service is also available to ask questions and answer, thank you 🙏	Jingdong prefecture level city in Shandong
Jinfeng Hongyuan Flagship Store	Power Saving Expert 2	I bought it and haven't used it yet, I'll use it again.	Jingdong prefecture level city in Shandong
Jinfeng Hongyuan Flagship Store	Power Saving Expert 2	It didn't work, I used it for a few days and it was the same as before, it didn't work, I should have to return it	Jingdong prefecture level city in Shandong
Jinfeng Hongyuan Flagship Store	Power Saving Expert 2	The goods have been received, but have not yet installed and use, feel good.	Jingdong prefecture level city in Shandong
Jinfeng Hongyuan Flagship Store	Power Saving Expert 2	Goods received, I forgot to take pictures, the goods look good, you can start, immediately start the school to see the effect then	Jingdong prefecture level city in Shandong
Jinfeng Hongyuan Flagship Store	Power Saving Expert 2	Looks good. I'll use it for a while.	Jingdong prefecture level city in Shandong
Jinfeng Hongyuan Flagship Store	Power Saving Expert 2	Great stuff, easy to operate, easy to use, good power saving, believe in the power of science!	Jingdong prefecture level city in Shandong
Jinfeng Hongyuan Flagship Store	Power Saving Expert 2	Especially great, comparison shopping before buying, the store owner is very good, answering questions very patiently and detailed, there is a need to come back!	Jingdong prefecture level city in Shandong
Jinfeng Hongyuan Flagship Store	Power Saving Expert 2	Trustworthy, feel free to use it boldly.	Jingdong prefecture level city in Shandong
Jinfeng Hongyuan Flagship Store	Power Saving Expert 2	Logistics is super fast! Beautiful appearance! Looks like a great quality! I've never bought one before, so I'll try it first to see if it saves power. We'll see how much it saves.	Jingdong prefecture level city in Shandong
Jinfeng Hongyuan Flagship Store	Power Saving Expert 2	I saw online counterfeiting, energy saver is one of them, hahahahaha, jingdong also buy fake products	Jingdong prefecture level city in Shandong
Jinfeng Hongyuan Flagship Store	Power Saving Expert 2	Just received, installed, try a month to see how the effect and then follow up.	Jingdong prefecture level city in Shandong
Jinfeng Hongyuan Flagship Store	Power Saving Expert 2	Good for you! The goods are genuine! Good price for what you get!	Jingdong prefecture level city in Shandong
Jinfeng Hongyuan Flagship Store	Power Saving Expert 2	Things quickly received, immediately used to see if there is no effect, something river photo ⬆ the same, customer service is very professional	Jingdong prefecture level city in Shandong
Jinfeng Hongyuan Flagship Store	Power Saving Expert 2	The product is very convenient Looking forward to the subsequent use of the experience results	Jingdong prefecture level city in Shandong
Jinfeng Hongyuan Flagship Store	Power Saving Expert 2	I just received it and plugged it in, we'll see exactly when it's time to pay the electric bill	Jingdong prefecture level city in Shandong
Jinfeng Hongyuan Flagship Store	Power Saving Expert 2	Saver is good Packaging and looks good How effective it is to be shared after using it.	Jingdong prefecture level city in Shandong
Jinfeng Hongyuan Flagship Store	Power Saving Expert 2	There's no way to compare. I don't know if it's energy efficient.	Jingdong prefecture level city in Shandong
Jinfeng Hongyuan Flagship Store	Power Saving Expert 2	Just installed it, try it out and comment again	Jingdong prefecture level city in Shandong
Jinfeng Hongyuan Flagship Store	Power Saving Expert 2	Super save point also good to install, after a friend introduced then went to buy to, friends say it is very good to use, home I bought, try to use a period of time, to be honest, the economizer is not big or heavy, can be very convenient to put in a lot of places, the quality is also very good, power-saving function this time seems to be about 20% of the efficiency. The owner is also very warm contact, very	Jingdong prefecture level city in Shandong
Jinfeng Hongyuan Flagship Store	Power Saving Expert 2	The reviews online are mixed. Try it for a while, but I don't know if I can return it if it doesn't save any power after a couple months.	Jingdong prefecture level city in Shandong
Jinfeng Hongyuan Flagship Store	Power Saving Expert 2	The power consumption at home is high, bought a power saver, it looks very convenient to plug on the line, see if you can save some money next month, if good, recommend to friends down the road.	Jingdong prefecture level city in Shandong

Jinfeng Hongyuan Flagship Store	Power Saving Expert 2	It's beautiful, it's genuine, but I don't know the power-saving effect.	Jingdong prefecture level city in Shandong
Jinfeng Hongyuan Flagship Store	Power Saving Expert 2	This user thinks the product is good	Jingdong prefecture level city in Shandong
Jinfeng Hongyuan Flagship Store	Power Saving Expert 2	Nice exterior design with an at-a-glance display	Jingdong prefecture level city in Shandong
Jinfeng Hongyuan Flagship Store	Power Saving Expert 2	Home large and small things are purchased in the Jingdong Mall, have been accustomed to shopping in the Jingdong, many times can buy good quality and cheap products, and courier service attitude is particularly good, sometimes too many couriers are to help me to the home, so many years have been in the Jingdong Mall shopping, trust and support! Will also support the Jingdong as always!	Jingdong prefecture level city in Shandong
Jinfeng Hongyuan Flagship Store	Power Saving Expert 2	Good workmanship, mostly better than the original wall-plugged ones, and hopefully power factor compensation saves on electricity costs	Jingdong prefecture level city in Shandong
Jinfeng Hongyuan Flagship Store	Power Saving Expert 2	I don't feel anything, I said I won't know the effect for a month or two, let's see.	Jingdong prefecture level city in Shandong
Jinfeng Hongyuan Flagship Store	Power Saving Expert 2	Delivery was pretty fast, a little pricey, just not sure if it works or not	Jingdong prefecture level city in Shandong
Jinfeng Hongyuan Flagship Store	Power Saving Expert 2	The goods have been received, I do not know how effective.	Jingdong prefecture level city in Shandong
Jinfeng Hongyuan Flagship Store	Power Saving Expert 2	So far it doesn't seem to be too noticeable, so I'll comment later.	Jingdong prefecture level city in Shandong
Jinfeng Hongyuan Flagship Store	Power Saving Expert 2	I'll comment after I've used it	Jingdong prefecture level city in Shandong
Jinfeng Hongyuan Flagship Store	Power Saving Expert 2	Not bad. You'll save a lot on your electric bill in a year!	Jingdong prefecture level city in Shandong
Jinfeng Hongyuan Flagship Store	Power Saving Expert 2	I just got it. I haven't tried it yet. I don't know what it's like.	Jingdong prefecture level city in Shandong
Jinfeng Hongyuan Flagship Store	Power Saving Expert 2	Shopping at Jingdong has become a habit, and this disease will not be cured for a while.	Jingdong prefecture level city in Shandong
Jinfeng Hongyuan Flagship Store	Power Saving Expert 2	I've been using it for a while now. I don't know if I'm still saving power or not.	Jingdong prefecture level city in Shandong
Jinfeng Hongyuan Flagship Store	Power Saving Expert 2	Received the saver and it's great! There is also a digital voltage indication. Plugged in as instructed, easy to operate, hope you're our power saver!	Jingdong prefecture level city in Shandong
Jinfeng Hongyuan Flagship Store	Power Saving Expert 2	There is no power saving as the store says, using it is the same as not using it.	Jingdong prefecture level city in Shandong
Jinfeng Hongyuan Flagship Store	Power Saving Expert 2	Use it for a while and then comment again	Jingdong prefecture level city in Shandong
Jinfeng Hongyuan Flagship Store	Power Saving Expert 2	Feeling can, to use 1 to 2 months to see how the effect, there is a voltage display, can see the voltage fluctuations amplitude, quite good!	Jingdong prefecture level city in Shandong
Jinfeng Hongyuan Flagship Store	Power Saving Expert 2	I hope it works and saves me some electricity.	Jingdong prefecture level city in Shandong
Jinfeng Hongyuan Flagship Store	Power Saving Expert 2	Nice baby, happy with the after sales	Jingdong prefecture level city in Shandong
Jinfeng Hongyuan Flagship Store	Power Saving Expert 2	Use it and talk about it.	Jingdong prefecture level city in Shandong
Jinfeng Hongyuan Flagship Store	Power Saving Expert 2	Second purchase	Jingdong prefecture level city in Shandong
Jinfeng Hongyuan Flagship Store	Power Saving Expert 2	It works.	Jingdong prefecture level city in Shandong
Jinfeng Hongyuan Flagship Store	Power Saving Expert 2	It works.	Jingdong prefecture level city in Shandong
Jinfeng Hongyuan Flagship Store	Power Saving Expert 2	I don't know if it's a power saver until I start using it	Jingdong prefecture level city in Shandong
Jinfeng Hongyuan Flagship Store	Power Saving Expert 2	serviceable	Jingdong prefecture level city in Shandong
Jinfeng Hongyuan Flagship Store	Power Saving Expert 2	This user did not fill out the rating in time	Jingdong prefecture level city in Shandong
Jinfeng Hongyuan Flagship Store	Power Saving Expert 2	There is no power saving as the store says, using it is the same as not using it.	Jingdong prefecture level city in Shandong
Jinfeng Hongyuan Flagship Store	Power Saving Expert 2	This user did not fill out the rating in time	Jingdong prefecture level city in Shandong
Jinfeng Hongyuan Flagship Store	Power Saving Expert 2	Good...good.	Jingdong prefecture level city in Shandong
Jinfeng Hongyuan Flagship Store	Power Saving Expert 2	(of an unmarried couple) be close	Jingdong prefecture level city in Shandong
Jinfeng Hongyuan Flagship Store	Power Saving Expert 2	(of an unmarried couple) be close	Jingdong prefecture level city in Shandong
Jinfeng Hongyuan Flagship Store	Power Saving Expert 2	(of an unmarried couple) be close	Jingdong prefecture level city in Shandong
Jinfeng Hongyuan Flagship Store	Power Saving Expert 2	The goods are real, high value, high quality, worth every penny, material appearance and quality at a glance is very upscale, very like HAPPY1 00	Jingdong prefecture level city in Shandong
Jinfeng Hongyuan Flagship Store	Power Saving Expert 2	It is the ideal product, good quality, logistics is also very fast, worthy of praise 🎁 baby quality is very good, received the first time to open the first time to see, it is very good 👍, very satisfied 😃, customer service is also available to ask questions and answer, thank you 🙏	Jingdong prefecture level city in Shandong
Jinfeng Hongyuan Flagship Store	Power Saving Expert 2	I bought it and haven't used it yet, I'll use it again.	Jingdong prefecture level city in Shandong
Jinfeng Hongyuan Flagship Store	Power Saving Expert 2	It didn't work, I used it for a few days and it was the same as before, it didn't work, I should have to return it	Jingdong prefecture level city in Shandong
Jinfeng Hongyuan Flagship Store	Power Saving Expert 2	The goods have been received, but have not yet installed and use, feel good.	Jingdong prefecture level city in Shandong
Jinfeng Hongyuan Flagship Store	Power Saving Expert 2	Goods received, I forgot to take pictures, the goods look good, you can start, immediately start the school to see the effect then	Jingdong prefecture level city in Shandong
Jinfeng Hongyuan Flagship Store	Power Saving Expert 2	Looks good. I'll use it for a while.	Jingdong prefecture level city in Shandong
Jinfeng Hongyuan Flagship Store	Power Saving Expert 2	Great stuff, easy to operate, easy to use, good power saving, believe in the power of science!	Jingdong prefecture level city in Shandong
Jinfeng Hongyuan Flagship Store	Power Saving Expert 2	Especially great, comparison shopping before buying, the store owner is very good, answering questions very patiently and detailed, there is a need to come back!	Jingdong prefecture level city in Shandong
Jinfeng Hongyuan Flagship Store	Power Saving Expert 2	Trustworthy, feel free to use it boldly.	Jingdong prefecture level city in Shandong
Jinfeng Hongyuan Flagship Store	Power Saving Expert 2	Logistics is super fast! Beautiful appearance! Looks like a great quality! I've never bought one before, so I'll try it first to see if it saves power. We'll see how much it saves.	Jingdong prefecture level city in Shandong
Jinfeng Hongyuan Flagship Store	Power Saving Expert 2	I saw online counterfeiting, energy saver is one of them, hahahahaha, jingdong also buy fake products	Jingdong prefecture level city in Shandong
Jinfeng Hongyuan Flagship Store	Power Saving Expert 2	Just received, installed, try a month to see how the effect and then follow up.	Jingdong prefecture level city in Shandong
Jinfeng Hongyuan Flagship Store	Power Saving Expert 2	Good for you! The goods are genuine! Good price for what you get!	Jingdong prefecture level city in Shandong
Jinfeng Hongyuan Flagship Store	Power Saving Expert 2	Things quickly received, immediately used to see if there is no effect, something river photo ⍐ the same, customer service is very professional	Jingdong prefecture level city in Shandong
Jinfeng Hongyuan Flagship Store	Power Saving Expert 2	The product is very convenient Looking forward to the subsequent use of the experience results	Jingdong prefecture level city in Shandong
Jinfeng Hongyuan Flagship Store	Power Saving Expert 2	I just received it and plugged it in, we'll see exactly when it's time to pay the electric bill	Jingdong prefecture level city in Shandong

Jinfeng Hongyuan Flagship Store	Power Saving Expert 2	Saver is good Packaging and looks good How effective it is to be shared after using it.	Jingdong prefecture level city in Shandong
Jinfeng Hongyuan Flagship Store	Power Saving Expert 2	There's no way to compare. I don't know if it's energy efficient.	Jingdong prefecture level city in Shandong
Jinfeng Hongyuan Flagship Store	Power Saving Expert 2	Just installed it, try it out and comment again	Jingdong prefecture level city in Shandong
Jinfeng Hongyuan Flagship Store	Power Saving Expert 2	Super save point also good to install, after a friend introduced then went to buy to, friends say it is very good to use, home I bought, try to use a period of time, to be honest, the economizer is not big or heavy, can be very convenient to put in a lot of places, the quality is also very good, power-saving function this time seems to be about 20% of the efficiency. The owner is also very warm contact, very	Jingdong prefecture level city in Shandong
Jinfeng Hongyuan Flagship Store	Power Saving Expert 2	The reviews online are mixed. Try it for a while, but I don't know if I can return it if it doesn't save any power after a couple months.	Jingdong prefecture level city in Shandong
Jinfeng Hongyuan Flagship Store	Power Saving Expert 2	The power consumption at home is high, bought a power saver, it looks very convenient to plug on the line, see if you can save some money next month, if good, recommend to friends down the road.	Jingdong prefecture level city in Shandong
Jinfeng Hongyuan Flagship Store	Power Saving Expert 2	It's beautiful, it's genuine, but I don't know the power-saving effect.	Jingdong prefecture level city in Shandong
Jinfeng Hongyuan Flagship Store	Power Saving Expert 2	This user thinks the product is good	Jingdong prefecture level city in Shandong
Jinfeng Hongyuan Flagship Store	Power Saving Expert 2	Nice exterior design with an at-a-glance display	Jingdong prefecture level city in Shandong
Jinfeng Hongyuan Flagship Store	Power Saving Expert 2	Home large and small things are purchased in the Jingdong Mall, have been accustomed to shopping in the Jingdong, many times can buy good quality and cheap products, and courier service attitude is particularly good, sometimes too many couriers are to help me to the home, so many years have been in the Jingdong Mall shopping, trust and support! Will also support the Jingdong as always!	Jingdong prefecture level city in Shandong
Jinfeng Hongyuan Flagship Store	Power Saving Expert 2	Good workmanship, mostly better than the original wall-plugged ones, and hopefully power factor compensation saves on electricity costs	Jingdong prefecture level city in Shandong
Jinfeng Hongyuan Flagship Store	Power Saving Expert 2	I don't feel anything, I said I won't know the effect for a month or two, let's see.	Jingdong prefecture level city in Shandong
Jinfeng Hongyuan Flagship Store	Power Saving Expert 2	Delivery was pretty fast, a little pricey, just not sure if it works or not	Jingdong prefecture level city in Shandong
Jinfeng Hongyuan Flagship Store	Power Saving Expert 2	The goods have been received, I do not know how effective.	Jingdong prefecture level city in Shandong
Jinfeng Hongyuan Flagship Store	Power Saving Expert 2	So far it doesn't seem to be too noticeable, so I'll comment later.	Jingdong prefecture level city in Shandong
Jinfeng Hongyuan Flagship Store	Power Saving Expert 2	I'll comment after I've used it	Jingdong prefecture level city in Shandong
Jinfeng Hongyuan Flagship Store	Power Saving Expert 2	Not bad. You'll save a lot on your electric bill in a year!	Jingdong prefecture level city in Shandong
Jinfeng Hongyuan Flagship Store	Power Saving Expert 2	I just got it. I haven't tried it yet. I don't know what it's like.	Jingdong prefecture level city in Shandong
Jinfeng Hongyuan Flagship Store	Power Saving Expert 2	Shopping at Jingdong has become a habit, and this disease will not be cured for a while.	Jingdong prefecture level city in Shandong
Jinfeng Hongyuan Flagship Store	Power Saving Expert 2	I've been using it for a while now. I don't know if I'm still saving power or not.	Jingdong prefecture level city in Shandong
Jinfeng Hongyuan Flagship Store	Power Saving Expert 2	Received the saver and it's great! There is also a digital voltage indication. Plugged in as instructed, easy to operate, hope you're our power saver!	Jingdong prefecture level city in Shandong
Jinfeng Hongyuan Flagship Store	Power Saving Expert 2	There is no power saving as the store says, using it is the same as not using it.	Jingdong prefecture level city in Shandong
Jinfeng Hongyuan Flagship Store	Power Saving Expert 2	Use it for a while and then comment again	Jingdong prefecture level city in Shandong
Jinfeng Hongyuan Flagship Store	Power Saving Expert 2	Feeling can, to use 1 to 2 months to see how the effect, there is a voltage display, can see the voltage fluctuations amplitude, quite good!	Jingdong prefecture level city in Shandong
Jinfeng Hongyuan Flagship Store	Power Saving Expert 2	I hope it works and saves me some electricity.	Jingdong prefecture level city in Shandong
Jinfeng Hongyuan Flagship Store	Power Saving Expert 2	Nice baby, happy with the after sales	Jingdong prefecture level city in Shandong
Jinfeng Hongyuan Flagship Store	Power Saving Expert 2	Use it and talk about it.	Jingdong prefecture level city in Shandong
Jinfeng Hongyuan Flagship Store	Power Saving Expert 2	Second purchase	Jingdong prefecture level city in Shandong
Jinfeng Hongyuan Flagship Store	Power Saving Expert 2	It works.	Jingdong prefecture level city in Shandong
Jinfeng Hongyuan Flagship Store	Power Saving Expert 2	It works.	Jingdong prefecture level city in Shandong
Jinfeng Hongyuan Flagship Store	Power Saving Expert 2	I don't know if it's a power saver until I start using it	Jingdong prefecture level city in Shandong
Jinfeng Hongyuan Flagship Store	Power Saving Expert 2	serviceable	Jingdong prefecture level city in Shandong
Jinfeng Hongyuan Flagship Store	Power Saving Expert 2	This user did not fill out the rating in time	Jingdong prefecture level city in Shandong
Jinfeng Hongyuan Flagship Store	Power Saving Expert 2	There is no power saving as the store says, using it is the same as not using it.	Jingdong prefecture level city in Shandong
Jinfeng Hongyuan Flagship Store	Power Saving Expert 2	This user did not fill out the rating in time	Jingdong prefecture level city in Shandong
Jinfeng Hongyuan Flagship Store	Power Saving Expert 2	Good...good.	Jingdong prefecture level city in Shandong
Jinfeng Hongyuan Flagship Store	Power Saving Expert 2	(of an unmarried couple) be close	Jingdong prefecture level city in Shandong
Jinfeng Hongyuan Flagship Store	Power Saving Expert 2	(of an unmarried couple) be close	Jingdong prefecture level city in Shandong
Jinfeng Hongyuan Flagship Store	Power Saving Expert 2	(of an unmarried couple) be close	Jingdong prefecture level city in Shandong
Jinfeng Hongyuan Flagship Store	Power Saving Expert 2	The goods are real, high value, high quality, worth every penny, material appearance and quality at a glance is very upscale, very like HAPPY💯	Jingdong prefecture level city in Shandong
Jinfeng Hongyuan Flagship Store	Power Saving Expert 2	It is the ideal product, good quality, logistics is also very fast, worthy of praise 🎁 baby quality is very good, received the first time to open the first time to see, it is very good 👍, very satisfied 😃, customer service is also available to ask questions and answer, thank you 🙏	Jingdong prefecture level city in Shandong
Jinfeng Hongyuan Flagship Store	Power Saving Expert 2	I bought it and haven't used it yet, I'll use it again.	Jingdong prefecture level city in Shandong
Jinfeng Hongyuan Flagship Store	Power Saving Expert 2	It didn't work, I used it for a few days and it was the same as before, it didn't work, I should have to return it	Jingdong prefecture level city in Shandong
Jinfeng Hongyuan Flagship Store	Power Saving Expert 2	The goods have been received, but have not yet installed and use, feel good.	Jingdong prefecture level city in Shandong
Jinfeng Hongyuan Flagship Store	Power Saving Expert 2	Goods received, I forgot to take pictures, the goods look good, you can start, immediately start the school to see the effect then	Jingdong prefecture level city in Shandong
Jinfeng Hongyuan Flagship Store	Power Saving Expert 2	Looks good. I'll use it for a while.	Jingdong prefecture level city in Shandong
Jinfeng Hongyuan Flagship Store	Power Saving Expert 2	Great stuff, easy to operate, easy to use, good power saving, believe in the power of science!	Jingdong prefecture level city in Shandong
Jinfeng Hongyuan Flagship Store	Power Saving Expert 2	Especially great, comparison shopping before buying, the store owner is very good, answering questions very patiently and detailed, there is a need to come back!	Jingdong prefecture level city in Shandong
Jinfeng Hongyuan Flagship Store	Power Saving Expert 2	Trustworthy, feel free to use it boldly.	Jingdong prefecture level city in Shandong

Jinfeng Hongyuan Flagship Store	Power Saving Expert 2	Logistics is super fast! Beautiful appearance! Looks like a great quality! I've never bought one before, so I'll try it first to see if it saves power. We'll see how much it saves.	Jingdong prefecture level city in Shandong
Jinfeng Hongyuan Flagship Store	Power Saving Expert 2	I saw online counterfeiting, energy saver is one of them, hahahahaha, jingdong also buy fake products	Jingdong prefecture level city in Shandong
Jinfeng Hongyuan Flagship Store	Power Saving Expert 2	Just received, installed, try a month to see how the effect and then follow up.	Jingdong prefecture level city in Shandong
Jinfeng Hongyuan Flagship Store	Power Saving Expert 2	Good for you! The goods are genuine! Good price for what you get!	Jingdong prefecture level city in Shandong
Jinfeng Hongyuan Flagship Store	Power Saving Expert 2	Things quickly received, immediately used to see if there is no effect, something river photo ⇡ the same, customer service is very professional	Jingdong prefecture level city in Shandong
Jinfeng Hongyuan Flagship Store	Power Saving Expert 2	The product is very convenient Looking forward to the subsequent use of the experience results	Jingdong prefecture level city in Shandong
Jinfeng Hongyuan Flagship Store	Power Saving Expert 2	I just received it and plugged it in, we'll see exactly when it's time to pay the electric bill	Jingdong prefecture level city in Shandong
Jinfeng Hongyuan Flagship Store	Power Saving Expert 2	Saver is good Packaging and looks good How effective it is to be shared after using it.	Jingdong prefecture level city in Shandong
Jinfeng Hongyuan Flagship Store	Power Saving Expert 2	There's no way to compare. I don't know if it's energy efficient.	Jingdong prefecture level city in Shandong
Jinfeng Hongyuan Flagship Store	Power Saving Expert 2	Just installed it, try it out and comment again	Jingdong prefecture level city in Shandong
Jinfeng Hongyuan Flagship Store	Power Saving Expert 2	Super save point also good to install, after a friend introduced then went to buy to, friends say it is very good to use, home I bought, try to use a period of time, to be honest, the economizer is not big or heavy, can be very convenient to put in a lot of places, the quality is also very good, power-saving function this time seems to be about 20% of the efficiency. The owner is also very warm contact, very	Jingdong prefecture level city in Shandong
Jinfeng Hongyuan Flagship Store	Power Saving Expert 2	The reviews online are mixed. Try it for a while, but I don't know if I can return it if it doesn't save any power after a couple months.	Jingdong prefecture level city in Shandong
Jinfeng Hongyuan Flagship Store	Power Saving Expert 2	The power consumption at home is high, bought a power saver, it looks very convenient to plug on the line, see if you can save some money next month, if good, recommend to friends down the road.	Jingdong prefecture level city in Shandong
Jinfeng Hongyuan Flagship Store	Power Saving Expert 2	It's beautiful, it's genuine, but I don't know the power-saving effect.	Jingdong prefecture level city in Shandong
Jinfeng Hongyuan Flagship Store	Power Saving Expert 2	This user thinks the product is good	Jingdong prefecture level city in Shandong
Jinfeng Hongyuan Flagship Store	Power Saving Expert 2	Nice exterior design with an at-a-glance display	Jingdong prefecture level city in Shandong
Jinfeng Hongyuan Flagship Store	Power Saving Expert 2	Home large and small things are purchased in the Jingdong Mall, have been accustomed to shopping in the Jingdong, many times can buy good quality and cheap products, and courier service attitude is particularly good, sometimes too many couriers are to help me to the home, so many years have been in the Jingdong Mall shopping, trust and support! Will also support the Jingdong as always!	Jingdong prefecture level city in Shandong
Jinfeng Hongyuan Flagship Store	Power Saving Expert 2	Good workmanship, mostly better than the original wall-plugged ones, and hopefully power factor compensation saves on electricity costs	Jingdong prefecture level city in Shandong
Jinfeng Hongyuan Flagship Store	Power Saving Expert 2	I don't feel anything, I said I won't know the effect for a month or two, let's see.	Jingdong prefecture level city in Shandong
Jinfeng Hongyuan Flagship Store	Power Saving Expert 2	Delivery was pretty fast, a little pricey, just not sure if it works or not	Jingdong prefecture level city in Shandong
Jinfeng Hongyuan Flagship Store	Power Saving Expert 2	The goods have been received, I do not know how effective.	Jingdong prefecture level city in Shandong
Jinfeng Hongyuan Flagship Store	Power Saving Expert 2	So far it doesn't seem to be too noticeable, so I'll comment later.	Jingdong prefecture level city in Shandong
Jinfeng Hongyuan Flagship Store	Power Saving Expert 2	I'll comment after I've used it	Jingdong prefecture level city in Shandong
Jinfeng Hongyuan Flagship Store	Power Saving Expert 2	Not bad. You'll save a lot on your electric bill in a year!	Jingdong prefecture level city in Shandong
Jinfeng Hongyuan Flagship Store	Power Saving Expert 2	I just got it. I haven't tried it yet. I don't know what it's like.	Jingdong prefecture level city in Shandong
Jinfeng Hongyuan Flagship Store	Power Saving Expert 2	Shopping at Jingdong has become a habit, and this disease will not be cured for a while.	Jingdong prefecture level city in Shandong
Jinfeng Hongyuan Flagship Store	Power Saving Expert 2	I've been using it for a while now. I don't know if I'm still saving power or not.	Jingdong prefecture level city in Shandong
Jinfeng Hongyuan Flagship Store	Power Saving Expert 2	Received the saver and it's great! There is also a digital voltage indication. Plugged in as instructed, easy to operate, hope you're our power saver!	Jingdong prefecture level city in Shandong
Jinfeng Hongyuan Flagship Store	Power Saving Expert 2	There is no power saving as the store says, using it is the same as not using it.	Jingdong prefecture level city in Shandong
Jinfeng Hongyuan Flagship Store	Power Saving Expert 2	Use it for a while and then comment again	Jingdong prefecture level city in Shandong
Jinfeng Hongyuan Flagship Store	Power Saving Expert 2	Feeling can, to use 1 to 2 months to see how the effect, there is a voltage display, can see the voltage fluctuations amplitude, quite good!	Jingdong prefecture level city in Shandong
Jinfeng Hongyuan Flagship Store	Power Saving Expert 2	I hope it works and saves me some electricity.	Jingdong prefecture level city in Shandong
Jinfeng Hongyuan Flagship Store	Power Saving Expert 2	Nice baby, happy with the after sales	Jingdong prefecture level city in Shandong
Jinfeng Hongyuan Flagship Store	Power Saving Expert 2	Use it and talk about it.	Jingdong prefecture level city in Shandong
Jinfeng Hongyuan Flagship Store	Power Saving Expert 2	Second purchase	Jingdong prefecture level city in Shandong
Jinfeng Hongyuan Flagship Store	Power Saving Expert 2	It works.	Jingdong prefecture level city in Shandong
Jinfeng Hongyuan Flagship Store	Power Saving Expert 2	It works.	Jingdong prefecture level city in Shandong
Jinfeng Hongyuan Flagship Store	Power Saving Expert 2	I don't know if it's a power saver until I start using it	Jingdong prefecture level city in Shandong
Jinfeng Hongyuan Flagship Store	Power Saving Expert 2	serviceable	Jingdong prefecture level city in Shandong
Jinfeng Hongyuan Flagship Store	Power Saving Expert 2	This user did not fill out the rating in time	Jingdong prefecture level city in Shandong
Jinfeng Hongyuan Flagship Store	Power Saving Expert 2	There is no power saving as the store says, using it is the same as not using it.	Jingdong prefecture level city in Shandong
Jinfeng Hongyuan Flagship Store	Power Saving Expert 2	This user did not fill out the rating in time	Jingdong prefecture level city in Shandong
Jinfeng Hongyuan Flagship Store	Power Saving Expert 2	Good...good.	Jingdong prefecture level city in Shandong
Jinfeng Hongyuan Flagship Store	Power Saving Expert 2	(of an unmarried couple) be close	Jingdong prefecture level city in Shandong
Jinfeng Hongyuan Flagship Store	Power Saving Expert 2	(of an unmarried couple) be close	Jingdong prefecture level city in Shandong
Jinfeng Hongyuan Flagship Store	Power Saving Expert 2	(of an unmarried couple) be close	Jingdong prefecture level city in Shandong
Jinfeng Hongyuan Flagship Store	Power Saving Expert 2	The goods are real, high value, high quality, worth every penny, material appearance and quality at a glance is very upscale, very like HAPPY1 00	Jingdong prefecture level city in Shandong
Jinfeng Hongyuan Flagship Store	Power Saving Expert 2	It is the ideal product, good quality, logistics is also very fast, worthy of praise 🎁 baby quality is very good, received the first time to open the first time to see, it is very good 👍, very satisfied 😃, customer service is also available to ask questions and answer, thank you 🙏	Jingdong prefecture level city in Shandong
Jinfeng Hongyuan Flagship Store	Power Saving Expert 2	I bought it and haven't used it yet, I'll use it again.	Jingdong prefecture level city in Shandong

Jinfeng Hongyuan Flagship Store	Power Saving Expert 2	It didn't work, I used it for a few days and it was the same as before, it didn't work, I should have to return it	Jingdong prefecture level city in Shandong
Jinfeng Hongyuan Flagship Store	Power Saving Expert 2	The goods have been received, but have not yet installed and use, feel good.	Jingdong prefecture level city in Shandong
Jinfeng Hongyuan Flagship Store	Power Saving Expert 2	Goods received, I forgot to take pictures, the goods look good, you can start, immediately start the school to see the effect then	Jingdong prefecture level city in Shandong
Jinfeng Hongyuan Flagship Store	Power Saving Expert 2	Looks good. I'll use it for a while.	Jingdong prefecture level city in Shandong
Jinfeng Hongyuan Flagship Store	Power Saving Expert 2	Great stuff, easy to operate, easy to use, good power saving, believe in the power of science!	Jingdong prefecture level city in Shandong
Jinfeng Hongyuan Flagship Store	Power Saving Expert 2	Especially great, comparison shopping before buying, the store owner is very good, answering questions very patiently and detailed, there is a need to come back!	Jingdong prefecture level city in Shandong
Jinfeng Hongyuan Flagship Store	Power Saving Expert 2	Trustworthy, feel free to use it boldly.	Jingdong prefecture level city in Shandong
Jinfeng Hongyuan Flagship Store	Power Saving Expert 2	Logistics is super fast! Beautiful appearance! Looks like a great quality! I've never bought one before, so I'll try it first to see if it saves power. We'll see how much it saves.	Jingdong prefecture level city in Shandong
Jinfeng Hongyuan Flagship Store	Power Saving Expert 2	I saw online counterfeiting, energy saver is one of them, hahahahaha, jingdong also buy fake products	Jingdong prefecture level city in Shandong
Jinfeng Hongyuan Flagship Store	Power Saving Expert 2	Just received, installed, try a month to see how the effect and then follow up.	Jingdong prefecture level city in Shandong
Jinfeng Hongyuan Flagship Store	Power Saving Expert 2	Good for you! The goods are genuine! Good price for what you get!	Jingdong prefecture level city in Shandong
Jinfeng Hongyuan Flagship Store	Power Saving Expert 2	Things quickly received, immediately used to see if there is no effect, something river photo ⍐ the same, customer service is very professional	Jingdong prefecture level city in Shandong
Jinfeng Hongyuan Flagship Store	Power Saving Expert 2	The product is very convenient Looking forward to the subsequent use of the experience results	Jingdong prefecture level city in Shandong
Jinfeng Hongyuan Flagship Store	Power Saving Expert 2	I just received it and plugged it in, we'll see exactly when it's time to pay the electric bill	Jingdong prefecture level city in Shandong
Jinfeng Hongyuan Flagship Store	Power Saving Expert 2	Saver is good Packaging and looks good How effective it is to be shared after using it.	Jingdong prefecture level city in Shandong
Jinfeng Hongyuan Flagship Store	Power Saving Expert 2	There's no way to compare. I don't know if it's energy efficient.	Jingdong prefecture level city in Shandong
Jinfeng Hongyuan Flagship Store	Power Saving Expert 2	Just installed it, try it out and comment again	Jingdong prefecture level city in Shandong
Jinfeng Hongyuan Flagship Store	Power Saving Expert 2	Super save point also good to install, after a friend introduced then went to buy to, friends say it is very good to use, home I bought, try to use a period of time, to be honest, the economizer is not big or heavy, can be very convenient to put in a lot of places, the quality is also very good, power-saving function this time seems to be about 20% of the efficiency. The owner is also very warm contact, very	Jingdong prefecture level city in Shandong
Jinfeng Hongyuan Flagship Store	Power Saving Expert 2	The reviews online are mixed. Try it for a while, but I don't know if I can return it if it doesn't save any power after a couple months.	Jingdong prefecture level city in Shandong
Jinfeng Hongyuan Flagship Store	Power Saving Expert 2	The power consumption at home is high, bought a power saver, it looks very convenient to plug on the line, see if you can save some money next month, if good, recommend to friends down the road.	Jingdong prefecture level city in Shandong
Jinfeng Hongyuan Flagship Store	Power Saving Expert 2	It's beautiful, it's genuine, but I don't know the power-saving effect.	Jingdong prefecture level city in Shandong
Jinfeng Hongyuan Flagship Store	Power Saving Expert 2	This user thinks the product is good	Jingdong prefecture level city in Shandong
Jinfeng Hongyuan Flagship Store	Power Saving Expert 2	Nice exterior design with an at-a-glance display	Jingdong prefecture level city in Shandong
Jinfeng Hongyuan Flagship Store	Power Saving Expert 2	Home large and small things are purchased in the Jingdong Mall, have been accustomed to shopping in the Jingdong, many times can buy good quality and cheap products, and courier service attitude is particularly good, sometimes too many couriers are to help me to the home, so many years have been in the Jingdong Mall shopping, trust and support! Will also support the Jingdong as always!	Jingdong prefecture level city in Shandong
Jinfeng Hongyuan Flagship Store	Power Saving Expert 2	Good workmanship, mostly better than the original wall-plugged ones, and hopefully power factor compensation saves on electricity costs	Jingdong prefecture level city in Shandong
Jinfeng Hongyuan Flagship Store	Power Saving Expert 2	I don't feel anything, I said I won't know the effect for a month or two, let's see.	Jingdong prefecture level city in Shandong
Jinfeng Hongyuan Flagship Store	Power Saving Expert 2	Delivery was pretty fast, a little pricey, just not sure if it works or not	Jingdong prefecture level city in Shandong
Jinfeng Hongyuan Flagship Store	Power Saving Expert 2	The goods have been received, I do not know how effective.	Jingdong prefecture level city in Shandong
Jinfeng Hongyuan Flagship Store	Power Saving Expert 2	So far it doesn't seem to be too noticeable, so I'll comment later.	Jingdong prefecture level city in Shandong
Jinfeng Hongyuan Flagship Store	Power Saving Expert 2	I'll comment after I've used it	Jingdong prefecture level city in Shandong
Jinfeng Hongyuan Flagship Store	Power Saving Expert 2	Not bad. You'll save a lot on your electric bill in a year!	Jingdong prefecture level city in Shandong
Jinfeng Hongyuan Flagship Store	Power Saving Expert 2	I just got it. I haven't tried it yet. I don't know what it's like.	Jingdong prefecture level city in Shandong
Jinfeng Hongyuan Flagship Store	Power Saving Expert 2	Shopping at Jingdong has become a habit, and this disease will not be cured for a while.	Jingdong prefecture level city in Shandong
Jinfeng Hongyuan Flagship Store	Power Saving Expert 2	I've been using it for a while now. I don't know if I'm still saving power or not.	Jingdong prefecture level city in Shandong
Jinfeng Hongyuan Flagship Store	Power Saving Expert 2	Received the saver and it's great! There is also a digital voltage indication. Plugged in as instructed, easy to operate, hope you're our power saver!	Jingdong prefecture level city in Shandong
Jinfeng Hongyuan Flagship Store	Power Saving Expert 2	There is no power saving as the store says, using it is the same as not using it.	Jingdong prefecture level city in Shandong
Jinfeng Hongyuan Flagship Store	Power Saving Expert 2	Use it for a while and then comment again	Jingdong prefecture level city in Shandong
Jinfeng Hongyuan Flagship Store	Power Saving Expert 2	Feeling can, to use 1 to 2 months to see how the effect, there is a voltage display, can see the voltage fluctuations amplitude, quite good!	Jingdong prefecture level city in Shandong
Jinfeng Hongyuan Flagship Store	Power Saving Expert 2	I hope it works and saves me some electricity.	Jingdong prefecture level city in Shandong
Jinfeng Hongyuan Flagship Store	Power Saving Expert 2	Nice baby, happy with the after sales	Jingdong prefecture level city in Shandong
Jinfeng Hongyuan Flagship Store	Power Saving Expert 2	Use it and talk about it.	Jingdong prefecture level city in Shandong
Jinfeng Hongyuan Flagship Store	Power Saving Expert 2	Second purchase	Jingdong prefecture level city in Shandong
Jinfeng Hongyuan Flagship Store	Power Saving Expert 2	It works.	Jingdong prefecture level city in Shandong
Jinfeng Hongyuan Flagship Store	Power Saving Expert 2	It works.	Jingdong prefecture level city in Shandong
Jinfeng Hongyuan Flagship Store	Power Saving Expert 2	I don't know if it's a power saver until I start using it	Jingdong prefecture level city in Shandong
Jinfeng Hongyuan Flagship Store	Power Saving Expert 2	serviceable	Jingdong prefecture level city in Shandong
Jinfeng Hongyuan Flagship Store	Power Saving Expert 2	This user did not fill out the rating in time	Jingdong prefecture level city in Shandong
Jinfeng Hongyuan Flagship Store	Power Saving Expert 2	There is no power saving as the store says, using it is the same as not using it.	Jingdong prefecture level city in Shandong
Jinfeng Hongyuan Flagship Store	Power Saving Expert 2	This user did not fill out the rating in time	Jingdong prefecture level city in Shandong

Jinfeng Hongyuan Flagship Store	Power Saving Expert 2	Good...good.	Jingdong prefecture level city in Shandong
Jinfeng Hongyuan Flagship Store	Power Saving Expert 2	(of an unmarried couple) be close	Jingdong prefecture level city in Shandong
Jinfeng Hongyuan Flagship Store	Power Saving Expert 2	(of an unmarried couple) be close	Jingdong prefecture level city in Shandong
Jinfeng Hongyuan Flagship Store	Power Saving Expert 2	(of an unmarried couple) be close	Jingdong prefecture level city in Shandong
Jinfeng Hongyuan Flagship Store	Power Saving Expert 2	The goods are real, high value, high quality, worth every penny, material appearance and quality at a glance is very upscale, very like HAPPY1 00	Jingdong prefecture level city in Shandong
Jinfeng Hongyuan Flagship Store	Power Saving Expert 2	It is the ideal product, good quality, logistics is also very fast, worthy of praise 🎁 baby quality is very good, received the first time to open the first time to see, it is very good 👍, very satisfied 😃, customer service is also available to ask questions and answer, thank you 🙏	Jingdong prefecture level city in Shandong
Jinfeng Hongyuan Flagship Store	Power Saving Expert 2	I bought it and haven't used it yet, I'll use it again.	Jingdong prefecture level city in Shandong
Jinfeng Hongyuan Flagship Store	Power Saving Expert 2	It didn't work, I used it for a few days and it was the same as before, it didn't work, I should have to return it	Jingdong prefecture level city in Shandong
Jinfeng Hongyuan Flagship Store	Power Saving Expert 2	The goods have been received, but have not yet installed and use, feel good.	Jingdong prefecture level city in Shandong
Jinfeng Hongyuan Flagship Store	Power Saving Expert 2	Goods received, I forgot to take pictures, the goods look good, you can start, immediately start the school to see the effect then	Jingdong prefecture level city in Shandong
Jinfeng Hongyuan Flagship Store	Power Saving Expert 2	Looks good. I'll use it for a while.	Jingdong prefecture level city in Shandong
Jinfeng Hongyuan Flagship Store	Power Saving Expert 2	Great stuff, easy to operate, easy to use, good power saving, believe in the power of science!	Jingdong prefecture level city in Shandong
Jinfeng Hongyuan Flagship Store	Power Saving Expert 2	Especially great, comparison shopping before buying, the store owner is very good, answering questions very patiently and detailed, there is a need to come back!	Jingdong prefecture level city in Shandong
Jinfeng Hongyuan Flagship Store	Power Saving Expert 2	Trustworthy, feel free to use it boldly.	Jingdong prefecture level city in Shandong
Jinfeng Hongyuan Flagship Store	Power Saving Expert 2	Logistics is super fast! Beautiful appearance! Looks like a great quality! I've never bought one before, so I'll try it first to see if it saves power. We'll see how much it saves.	Jingdong prefecture level city in Shandong
Jinfeng Hongyuan Flagship Store	Power Saving Expert 2	I saw online counterfeiting, energy saver is one of them, hahahahaha, jingdong also buy fake products	Jingdong prefecture level city in Shandong
Jinfeng Hongyuan Flagship Store	Power Saving Expert 2	Just received, installed, try a month to see how the effect and then follow up.	Jingdong prefecture level city in Shandong
Jinfeng Hongyuan Flagship Store	Power Saving Expert 2	Good for you! The goods are genuine! Good price for what you get!	Jingdong prefecture level city in Shandong
Jinfeng Hongyuan Flagship Store	Power Saving Expert 2	Things quickly received, immediately used to see if there is no effect, something river photo ↑ the same, customer service is very professional	Jingdong prefecture level city in Shandong
Jinfeng Hongyuan Flagship Store	Power Saving Expert 2	The product is very convenient Looking forward to the subsequent use of the experience results	Jingdong prefecture level city in Shandong
Jinfeng Hongyuan Flagship Store	Power Saving Expert 2	I just received it and plugged it in, we'll see exactly when it's time to pay the electric bill	Jingdong prefecture level city in Shandong
Jinfeng Hongyuan Flagship Store	Power Saving Expert 2	Saver is good Packaging and looks good How effective it is to be shared after using it.	Jingdong prefecture level city in Shandong
Jinfeng Hongyuan Flagship Store	Power Saving Expert 2	There's no way to compare. I don't know if it's energy efficient.	Jingdong prefecture level city in Shandong
Jinfeng Hongyuan Flagship Store	Power Saving Expert 2	Just installed it, try it out and comment again	Jingdong prefecture level city in Shandong
Jinfeng Hongyuan Flagship Store	Power Saving Expert 2	Super save point also good to install, after a friend introduced then went to buy to, friends say it is very good to use, home I bought, try to use a period of time, to be honest, the economizer is not big or heavy, can be very convenient to put in a lot of places, the quality is also very good, power-saving function this time seems to be about 20% of the efficiency. The owner is also very warm contact, very	Jingdong prefecture level city in Shandong
Jinfeng Hongyuan Flagship Store	Power Saving Expert 2	The reviews online are mixed. Try it for a while, but I don't know if I can return it if it doesn't save any power after a couple months.	Jingdong prefecture level city in Shandong
Jinfeng Hongyuan Flagship Store	Power Saving Expert 2	The power consumption at home is high, bought a power saver, it looks very convenient to plug on the line, see if you can save some money next month, if good, recommend to friends down the road.	Jingdong prefecture level city in Shandong
Jinfeng Hongyuan Flagship Store	Power Saving Expert 2	It's beautiful, it's genuine, but I don't know the power-saving effect.	Jingdong prefecture level city in Shandong
Jinfeng Hongyuan Flagship Store	Power Saving Expert 2	This user thinks the product is good	Jingdong prefecture level city in Shandong
Jinfeng Hongyuan Flagship Store	Power Saving Expert 2	Nice exterior design with an at-a-glance display	Jingdong prefecture level city in Shandong
Jinfeng Hongyuan Flagship Store	Power Saving Expert 2	Home large and small things are purchased in the Jingdong Mall, have been accustomed to shopping in the Jingdong, many times can buy good quality and cheap products, and courier service attitude is particularly good, sometimes too many couriers are to help me to the home, so many years have been in the Jingdong Mall shopping, trust and support! Will also support the Jingdong as always!	Jingdong prefecture level city in Shandong
Jinfeng Hongyuan Flagship Store	Power Saving Expert 2	Good workmanship, mostly better than the original wall-plugged ones, and hopefully power factor compensation saves on electricity costs	Jingdong prefecture level city in Shandong
Jinfeng Hongyuan Flagship Store	Power Saving Expert 2	I don't feel anything, I said I won't know the effect for a month or two, let's see.	Jingdong prefecture level city in Shandong
Jinfeng Hongyuan Flagship Store	Power Saving Expert 2	Delivery was pretty fast, a little pricey, just not sure if it works or not	Jingdong prefecture level city in Shandong
Jinfeng Hongyuan Flagship Store	Power Saving Expert 2	The goods have been received, I do not know how effective.	Jingdong prefecture level city in Shandong
Jinfeng Hongyuan Flagship Store	Power Saving Expert 2	So far it doesn't seem to be too noticeable, so I'll comment later.	Jingdong prefecture level city in Shandong
Jinfeng Hongyuan Flagship Store	Power Saving Expert 2	I'll comment after I've used it	Jingdong prefecture level city in Shandong
Jinfeng Hongyuan Flagship Store	Power Saving Expert 2	Not bad. You'll save a lot on your electric bill in a year!	Jingdong prefecture level city in Shandong
Jinfeng Hongyuan Flagship Store	Power Saving Expert 2	I just got it. I haven't tried it yet. I don't know what it's like.	Jingdong prefecture level city in Shandong
Jinfeng Hongyuan Flagship Store	Power Saving Expert 2	Shopping at Jingdong has become a habit, and this disease will not be cured for a while.	Jingdong prefecture level city in Shandong
Jinfeng Hongyuan Flagship Store	Power Saving Expert 2	I've been using it for a while now. I don't know if I'm still saving power or not.	Jingdong prefecture level city in Shandong
Jinfeng Hongyuan Flagship Store	Power Saving Expert 2	Received the saver and it's great! There is also a digital voltage indication. Plugged in as instructed, easy to operate, hope you're our power saver!	Jingdong prefecture level city in Shandong
Jinfeng Hongyuan Flagship Store	Power Saving Expert 2	There is no power saving as the store says, using it is the same as not using it.	Jingdong prefecture level city in Shandong
Jinfeng Hongyuan Flagship Store	Power Saving Expert 2	Use it for a while and then comment again	Jingdong prefecture level city in Shandong
Jinfeng Hongyuan Flagship Store	Power Saving Expert 2	Feeling can, to use 1 to 2 months to see how the effect, there is a voltage display, can see the voltage fluctuations amplitude, quite good!	Jingdong prefecture level city in Shandong
Jinfeng Hongyuan Flagship Store	Power Saving Expert 2	I hope it works and saves me some electricity.	Jingdong prefecture level city in Shandong
Jinfeng Hongyuan Flagship Store	Power Saving Expert 2	Nice baby, happy with the after sales	Jingdong prefecture level city in Shandong
Jinfeng Hongyuan Flagship Store	Power Saving Expert 2	Use it and talk about it.	Jingdong prefecture level city in Shandong
Jinfeng Hongyuan Flagship Store	Power Saving Expert 2	Second purchase	Jingdong prefecture level city in Shandong

Jinfeng Hongyuan Flagship Store	Power Saving Expert 2	It works.	Jingdong prefecture level city in Shandong
Jinfeng Hongyuan Flagship Store	Power Saving Expert 2	It works.	Jingdong prefecture level city in Shandong
Jinfeng Hongyuan Flagship Store	Power Saving Expert 2	I don't know if it's a power saver until I start using it	Jingdong prefecture level city in Shandong
Jinfeng Hongyuan Flagship Store	Power Saving Expert 2	serviceable	Jingdong prefecture level city in Shandong
Jinfeng Hongyuan Flagship Store	Power Saving Expert 2	This user did not fill out the rating in time	Jingdong prefecture level city in Shandong
Jinfeng Hongyuan Flagship Store	Power Saving Expert 2	There is no power saving as the store says, using it is the same as not using it.	Jingdong prefecture level city in Shandong
Jinfeng Hongyuan Flagship Store	Power Saving Expert 2	This user did not fill out the rating in time	Jingdong prefecture level city in Shandong
Jinfeng Hongyuan Flagship Store	Power Saving Expert 2	Good...good.	Jingdong prefecture level city in Shandong
Jinfeng Hongyuan Flagship Store	Power Saving Expert 2	(of an unmarried couple) be close	Jingdong prefecture level city in Shandong
Jinfeng Hongyuan Flagship Store	Power Saving Expert 2	(of an unmarried couple) be close	Jingdong prefecture level city in Shandong
Jinfeng Hongyuan Flagship Store	Power Saving Expert 2	(of an unmarried couple) be close	Jingdong prefecture level city in Shandong
Jinfeng Hongyuan Flagship Store	Power Saving Expert 2	The goods are real, high value, high quality, worth every penny, material appearance and quality at a glance is very upscale, very like HAPPY1 00	Jingdong prefecture level city in Shandong
Jinfeng Hongyuan Flagship Store	Power Saving Expert 2	It is the ideal product, good quality, logistics is also very fast, worthy of praise 🎁 baby quality is very good, received the first time to open the first time to see, it is very good 👍, very satisfied 😃, customer service is also available to ask questions and answer, thank you 🙏	Jingdong prefecture level city in Shandong
Jinfeng Hongyuan Flagship Store	Power Saving Expert 2	I bought it and haven't used it yet, I'll use it again.	Jingdong prefecture level city in Shandong
Jinfeng Hongyuan Flagship Store	Power Saving Expert 2	It didn't work, I used it for a few days and it was the same as before, it didn't work, I should have to return it	Jingdong prefecture level city in Shandong
Jinfeng Hongyuan Flagship Store	Power Saving Expert 2	The goods have been received, but have not yet installed and use, feel good.	Jingdong prefecture level city in Shandong
Jinfeng Hongyuan Flagship Store	Power Saving Expert 2	Goods received, I forgot to take pictures, the goods look good, you can start, immediately start the school to see the effect then	Jingdong prefecture level city in Shandong
Jinfeng Hongyuan Flagship Store	Power Saving Expert 2	Looks good. I'll use it for a while.	Jingdong prefecture level city in Shandong
Jinfeng Hongyuan Flagship Store	Power Saving Expert 2	Great stuff, easy to operate, easy to use, good power saving, believe in the power of science!	Jingdong prefecture level city in Shandong
Jinfeng Hongyuan Flagship Store	Power Saving Expert 2	Especially great, comparison shopping before buying, the store owner is very good, answering questions very patiently and detailed, there is a need to come back!	Jingdong prefecture level city in Shandong
Jinfeng Hongyuan Flagship Store	Power Saving Expert 2	Trustworthy, feel free to use it boldly.	Jingdong prefecture level city in Shandong
Jinfeng Hongyuan Flagship Store	Power Saving Expert 2	Logistics is super fast! Beautiful appearance! Looks like a great quality! I've never bought one before, so I'll try it first to see if it saves power. We'll see how much it saves.	Jingdong prefecture level city in Shandong
Jinfeng Hongyuan Flagship Store	Power Saving Expert 2	I saw online counterfeiting, energy saver is one of them, hahahahaha, jingdong also buy fake products	Jingdong prefecture level city in Shandong
Jinfeng Hongyuan Flagship Store	Power Saving Expert 2	Just received, installed, try a month to see how the effect and then follow up.	Jingdong prefecture level city in Shandong
Jinfeng Hongyuan Flagship Store	Power Saving Expert 2	Good for you! The goods are genuine! Good price for what you get!	Jingdong prefecture level city in Shandong
Jinfeng Hongyuan Flagship Store	Power Saving Expert 2	Things quickly received, immediately used to see if there is no effect, something river photo ⬆ the same, customer service is very professional	Jingdong prefecture level city in Shandong
Jinfeng Hongyuan Flagship Store	Power Saving Expert 2	The product is very convenient Looking forward to the subsequent use of the experience results	Jingdong prefecture level city in Shandong
Jinfeng Hongyuan Flagship Store	Power Saving Expert 2	I just received it and plugged it in, we'll see exactly when it's time to pay the electric bill	Jingdong prefecture level city in Shandong
Jinfeng Hongyuan Flagship Store	Power Saving Expert 2	Saver is good Packaging and looks good How effective it is to be shared after using it.	Jingdong prefecture level city in Shandong
Jinfeng Hongyuan Flagship Store	Power Saving Expert 2	There's no way to compare. I don't know if it's energy efficient.	Jingdong prefecture level city in Shandong
Jinfeng Hongyuan Flagship Store	Power Saving Expert 2	Just installed it, try it out and comment again	Jingdong prefecture level city in Shandong
Jinfeng Hongyuan Flagship Store	Power Saving Expert 2	Super save point also good to install, after a friend introduced then went to buy to, friends say it is very good to use, home I bought, try to use a period of time, to be honest, the economizer is not big or heavy, can be very convenient to put in a lot of places, the quality is also very good, power-saving function this time seems to be about 20% of the efficiency. The owner is also very warm contact, very	Jingdong prefecture level city in Shandong
Jinfeng Hongyuan Flagship Store	Power Saving Expert 2	The reviews online are mixed. Try it for a while, but I don't know if I can return it if it doesn't save any power after a couple months.	Jingdong prefecture level city in Shandong
Jinfeng Hongyuan Flagship Store	Power Saving Expert 2	The power consumption at home is high, bought a power saver, it looks very convenient to plug on the line, see if you can save some money next month, if good, recommend to friends down the road.	Jingdong prefecture level city in Shandong
Jinfeng Hongyuan Flagship Store	Power Saving Expert 2	It's beautiful, it's genuine, but I don't know the power-saving effect.	Jingdong prefecture level city in Shandong
Jinfeng Hongyuan Flagship Store	Power Saving Expert 2	This user thinks the product is good	Jingdong prefecture level city in Shandong
Jinfeng Hongyuan Flagship Store	Power Saving Expert 2	Nice exterior design with an at-a-glance display	Jingdong prefecture level city in Shandong
Jinfeng Hongyuan Flagship Store	Power Saving Expert 2	Home large and small things are purchased in the Jingdong Mall, have been accustomed to shopping in the Jingdong, many times can buy good quality and cheap products, and courier service attitude is particularly good, sometimes too many couriers are to help me to the home, so many years have been in the Jingdong Mall shopping, trust and support! Will also support the Jingdong as always!	Jingdong prefecture level city in Shandong
Jinfeng Hongyuan Flagship Store	Power Saving Expert 2	Good workmanship, mostly better than the original wall-plugged ones, and hopefully power factor compensation saves on electricity costs	Jingdong prefecture level city in Shandong
Jinfeng Hongyuan Flagship Store	Power Saving Expert 2	I don't feel anything, I said I won't know the effect for a month or two, let's see.	Jingdong prefecture level city in Shandong
Jinfeng Hongyuan Flagship Store	Power Saving Expert 2	Delivery was pretty fast, a little pricey, just not sure if it works or not	Jingdong prefecture level city in Shandong
Jinfeng Hongyuan Flagship Store	Power Saving Expert 2	The goods have been received, I do not know how effective.	Jingdong prefecture level city in Shandong
Jinfeng Hongyuan Flagship Store	Power Saving Expert 2	So far it doesn't seem to be too noticeable, so I'll comment later.	Jingdong prefecture level city in Shandong
Jinfeng Hongyuan Flagship Store	Power Saving Expert 2	I'll comment after I've used it	Jingdong prefecture level city in Shandong
Jinfeng Hongyuan Flagship Store	Power Saving Expert 2	Not bad. You'll save a lot on your electric bill in a year!	Jingdong prefecture level city in Shandong
Jinfeng Hongyuan Flagship Store	Power Saving Expert 2	I just got it. I haven't tried it yet. I don't know what it's like.	Jingdong prefecture level city in Shandong
Jinfeng Hongyuan Flagship Store	Power Saving Expert 2	Shopping at Jingdong has become a habit, and this disease will not be cured for a while.	Jingdong prefecture level city in Shandong
Jinfeng Hongyuan Flagship Store	Power Saving Expert 2	I've been using it for a while now. I don't know if I'm still saving power or not.	Jingdong prefecture level city in Shandong
Jinfeng Hongyuan Flagship Store	Power Saving Expert 2	Received the saver and it's great! There is also a digital voltage indication. Plugged in as instructed, easy to operate, hope you're our power saver!	Jingdong prefecture level city in Shandong

Jinfeng Hongyuan Flagship Store	Power Saving Expert 2	There is no power saving as the store says, using it is the same as not using it.	Jingdong prefecture level city in Shandong
Jinfeng Hongyuan Flagship Store	Power Saving Expert 2	Use it for a while and then comment again	Jingdong prefecture level city in Shandong
Jinfeng Hongyuan Flagship Store	Power Saving Expert 2	Feeling can, to use 1 to 2 months to see how the effect, there is a voltage display, can see the voltage fluctuations amplitude, quite good!	Jingdong prefecture level city in Shandong
Jinfeng Hongyuan Flagship Store	Power Saving Expert 2	I hope it works and saves me some electricity.	Jingdong prefecture level city in Shandong
Jinfeng Hongyuan Flagship Store	Power Saving Expert 2	Nice baby, happy with the after sales	Jingdong prefecture level city in Shandong
Jinfeng Hongyuan Flagship Store	Power Saving Expert 2	Use it and talk about it.	Jingdong prefecture level city in Shandong
Jinfeng Hongyuan Flagship Store	Power Saving Expert 2	Second purchase	Jingdong prefecture level city in Shandong
Jinfeng Hongyuan Flagship Store	Power Saving Expert 2	It works.	Jingdong prefecture level city in Shandong
Jinfeng Hongyuan Flagship Store	Power Saving Expert 2	It works.	Jingdong prefecture level city in Shandong
Jinfeng Hongyuan Flagship Store	Power Saving Expert 2	I don't know if it's a power saver until I start using it	Jingdong prefecture level city in Shandong
Jinfeng Hongyuan Flagship Store	Power Saving Expert 2	serviceable	Jingdong prefecture level city in Shandong
Jinfeng Hongyuan Flagship Store	Power Saving Expert 2	This user did not fill out the rating in time	Jingdong prefecture level city in Shandong
Jinfeng Hongyuan Flagship Store	Power Saving Expert 2	There is no power saving as the store says, using it is the same as not using it.	Jingdong prefecture level city in Shandong
Jinfeng Hongyuan Flagship Store	Power Saving Expert 2	This user did not fill out the rating in time	Jingdong prefecture level city in Shandong
Jinfeng Hongyuan Flagship Store	Power Saving Expert 2	Good...good.	Jingdong prefecture level city in Shandong
Jinfeng Hongyuan Flagship Store	Power Saving Expert 2	(of an unmarried couple) be close	Jingdong prefecture level city in Shandong
Jinfeng Hongyuan Flagship Store	Power Saving Expert 2	(of an unmarried couple) be close	Jingdong prefecture level city in Shandong
Jinfeng Hongyuan Flagship Store	Power Saving Expert 2	(of an unmarried couple) be close	Jingdong prefecture level city in Shandong
Jinfeng Hongyuan Flagship Store	Power Saving Expert 2	The goods are real, high value, high quality, worth every penny, material appearance and quality at a glance is very upscale, very like HAPPY1 00	Jingdong prefecture level city in Shandong
Jinfeng Hongyuan Flagship Store	Power Saving Expert 2	It is the ideal product, good quality, logistics is also very fast, worthy of praise 🎁 baby quality is very good, received the first time to open the first time to see, it is very good 👍, very satisfied 😃, customer service is also available to ask questions and answer, thank you 🙏	Jingdong prefecture level city in Shandong
Jinfeng Hongyuan Flagship Store	Power Saving Expert 2	I bought it and haven't used it yet, I'll use it again.	Jingdong prefecture level city in Shandong
Jinfeng Hongyuan Flagship Store	Power Saving Expert 2	It didn't work, I used it for a few days and it was the same as before, it didn't work, I should have to return it	Jingdong prefecture level city in Shandong
Jinfeng Hongyuan Flagship Store	Power Saving Expert 2	The goods have been received, but have not yet installed and use, feel good.	Jingdong prefecture level city in Shandong
Jinfeng Hongyuan Flagship Store	Power Saving Expert 2	Goods received, I forgot to take pictures, the goods look good, you can start, immediately start the school to see the effect then	Jingdong prefecture level city in Shandong
Jinfeng Hongyuan Flagship Store	Power Saving Expert 2	Looks good. I'll use it for a while.	Jingdong prefecture level city in Shandong
Jinfeng Hongyuan Flagship Store	Power Saving Expert 2	Great stuff, easy to operate, easy to use, good power saving, believe in the power of science!	Jingdong prefecture level city in Shandong
Jinfeng Hongyuan Flagship Store	Power Saving Expert 2	Especially great, comparison shopping before buying, the store owner is very good, answering questions very patiently and detailed, there is a need to come back!	Jingdong prefecture level city in Shandong
Jinfeng Hongyuan Flagship Store	Power Saving Expert 2	Trustworthy, feel free to use it boldly.	Jingdong prefecture level city in Shandong
Jinfeng Hongyuan Flagship Store	Power Saving Expert 2	Logistics is super fast! Beautiful appearance! Looks like a great quality! I've never bought one before, so I'll try it first to see if it saves power. We'll see how much it saves.	Jingdong prefecture level city in Shandong
Jinfeng Hongyuan Flagship Store	Power Saving Expert 2	I saw online counterfeiting, energy saver is one of them, hahahahaha, jingdong also buy fake products	Jingdong prefecture level city in Shandong
Jinfeng Hongyuan Flagship Store	Power Saving Expert 2	Just received, installed, try a month to see how the effect and then follow up.	Jingdong prefecture level city in Shandong
Jinfeng Hongyuan Flagship Store	Power Saving Expert 2	Good for you! The goods are genuine! Good price for what you get!	Jingdong prefecture level city in Shandong
Jinfeng Hongyuan Flagship Store	Power Saving Expert 2	Things quickly received, immediately used to see if there is no effect, something river photo ⬆ the same, customer service is very professional	Jingdong prefecture level city in Shandong
Jinfeng Hongyuan Flagship Store	Power Saving Expert 2	The product is very convenient Looking forward to the subsequent use of the experience results	Jingdong prefecture level city in Shandong
Jinfeng Hongyuan Flagship Store	Power Saving Expert 2	I just received it and plugged it in, we'll see exactly when it's time to pay the electric bill	Jingdong prefecture level city in Shandong
Jinfeng Hongyuan Flagship Store	Power Saving Expert 2	Saver is good Packaging and looks good How effective it is to be shared after using it.	Jingdong prefecture level city in Shandong
Jinfeng Hongyuan Flagship Store	Power Saving Expert 2	There's no way to compare. I don't know if it's energy efficient.	Jingdong prefecture level city in Shandong
Jinfeng Hongyuan Flagship Store	Power Saving Expert 2	Just installed it, try it out and comment again	Jingdong prefecture level city in Shandong
Jinfeng Hongyuan Flagship Store	Power Saving Expert 2	Super save point also good to install, after a friend introduced then went to buy to, friends say it is very good to use, home I bought, try to use a period of time, to be honest, the economizer is not big or heavy, can be very convenient to put in a lot of places, the quality is also very good, power-saving function this time seems to be about 20% of the efficiency. The owner is also very warm contact, very	Jingdong prefecture level city in Shandong
Jinfeng Hongyuan Flagship Store	Power Saving Expert 2	The reviews online are mixed. Try it for a while, but I don't know if I can return it if it doesn't save any power after a couple months.	Jingdong prefecture level city in Shandong
Jinfeng Hongyuan Flagship Store	Power Saving Expert 2	The power consumption at home is high, bought a power saver, it looks very convenient to plug on the line, see if you can save some money next month, if good, recommend to friends down the road.	Jingdong prefecture level city in Shandong
Jinfeng Hongyuan Flagship Store	Power Saving Expert 2	It's beautiful, it's genuine, but I don't know the power-saving effect.	Jingdong prefecture level city in Shandong
Jinfeng Hongyuan Flagship Store	Power Saving Expert 2	This user thinks the product is good	Jingdong prefecture level city in Shandong
Jinfeng Hongyuan Flagship Store	Power Saving Expert 2	Nice exterior design with an at-a-glance display	Jingdong prefecture level city in Shandong
Jinfeng Hongyuan Flagship Store	Power Saving Expert 2	Home large and small things are purchased in the Jingdong Mall, have been accustomed to shopping in the Jingdong, many times can buy good quality and cheap products, and courier service attitude is particularly good, sometimes too many couriers are to help me to the home, so many years have been in the Jingdong Mall shopping, trust and support! Will also support the Jingdong as always!	Jingdong prefecture level city in Shandong
Jinfeng Hongyuan Flagship Store	Power Saving Expert 2	Good workmanship, mostly better than the original wall-plugged ones, and hopefully power factor compensation saves on electricity costs	Jingdong prefecture level city in Shandong
Jinfeng Hongyuan Flagship Store	Power Saving Expert 2	I don't feel anything, I said I won't know the effect for a month or two, let's see.	Jingdong prefecture level city in Shandong
Jinfeng Hongyuan Flagship Store	Power Saving Expert 2	Delivery was pretty fast, a little pricey, just not sure if it works or not	Jingdong prefecture level city in Shandong
Jinfeng Hongyuan Flagship Store	Power Saving Expert 2	The goods have been received, I do not know how effective.	Jingdong prefecture level city in Shandong

Jinfeng Hongyuan Flagship Store	Power Saving Expert 2	So far it doesn't seem to be too noticeable, so I'll comment later.	Jingdong prefecture level city in Shandong
Jinfeng Hongyuan Flagship Store	Power Saving Expert 2	I'll comment after I've used it	Jingdong prefecture level city in Shandong
Jinfeng Hongyuan Flagship Store	Power Saving Expert 2	Not bad. You'll save a lot on your electric bill in a year!	Jingdong prefecture level city in Shandong
Jinfeng Hongyuan Flagship Store	Power Saving Expert 2	I just got it. I haven't tried it yet. I don't know what it's like.	Jingdong prefecture level city in Shandong
Jinfeng Hongyuan Flagship Store	Power Saving Expert 2	Shopping at Jingdong has become a habit, and this disease will not be cured for a while.	Jingdong prefecture level city in Shandong
Jinfeng Hongyuan Flagship Store	Power Saving Expert 2	I've been using it for a while now. I don't know if I'm still saving power or not.	Jingdong prefecture level city in Shandong
Jinfeng Hongyuan Flagship Store	Power Saving Expert 2	Received the saver and it's great! There is also a digital voltage indication. Plugged in as instructed, easy to operate, hope you're our power saver!	Jingdong prefecture level city in Shandong
Jinfeng Hongyuan Flagship Store	Power Saving Expert 2	There is no power saving as the store says, using it is the same as not using it.	Jingdong prefecture level city in Shandong
Jinfeng Hongyuan Flagship Store	Power Saving Expert 2	Use it for a while and then comment again	Jingdong prefecture level city in Shandong
Jinfeng Hongyuan Flagship Store	Power Saving Expert 2	Feeling can, to use 1 to 2 months to see how the effect, there is a voltage display, can see the voltage fluctuations amplitude, quite good!	Jingdong prefecture level city in Shandong
Jinfeng Hongyuan Flagship Store	Power Saving Expert 2	I hope it works and saves me some electricity.	Jingdong prefecture level city in Shandong
Jinfeng Hongyuan Flagship Store	Power Saving Expert 2	Nice baby, happy with the after sales	Jingdong prefecture level city in Shandong
Jinfeng Hongyuan Flagship Store	Power Saving Expert 2	Use it and talk about it.	Jingdong prefecture level city in Shandong
Jinfeng Hongyuan Flagship Store	Power Saving Expert 2	Second purchase	Jingdong prefecture level city in Shandong
Jinfeng Hongyuan Flagship Store	Power Saving Expert 2	It works.	Jingdong prefecture level city in Shandong
Jinfeng Hongyuan Flagship Store	Power Saving Expert 2	It works.	Jingdong prefecture level city in Shandong
Jinfeng Hongyuan Flagship Store	Power Saving Expert 2	I don't know if it's a power saver until I start using it	Jingdong prefecture level city in Shandong
Jinfeng Hongyuan Flagship Store	Power Saving Expert 2	serviceable	Jingdong prefecture level city in Shandong
Jinfeng Hongyuan Flagship Store	Power Saving Expert 2	This user did not fill out the rating in time	Jingdong prefecture level city in Shandong
Jinfeng Hongyuan Flagship Store	Power Saving Expert 2	There is no power saving as the store says, using it is the same as not using it.	Jingdong prefecture level city in Shandong
Jinfeng Hongyuan Flagship Store	Power Saving Expert 2	This user did not fill out the rating in time	Jingdong prefecture level city in Shandong
Jinfeng Hongyuan Flagship Store	Power Saving Expert 2	Good...good.	Jingdong prefecture level city in Shandong
Jinfeng Hongyuan Flagship Store	Power Saving Expert 2	(of an unmarried couple) be close	Jingdong prefecture level city in Shandong
Jinfeng Hongyuan Flagship Store	Power Saving Expert 2	(of an unmarried couple) be close	Jingdong prefecture level city in Shandong
Jinfeng Hongyuan Flagship Store	Power Saving Expert 2	(of an unmarried couple) be close	Jingdong prefecture level city in Shandong
Jinfeng Hongyuan Flagship Store	Power Saving Expert 2	The goods are real, high value, high quality, worth every penny, material appearance and quality at a glance is very upscale, very like HAPPY1 00	Jingdong prefecture level city in Shandong
Jinfeng Hongyuan Flagship Store	Power Saving Expert 2	It is the ideal product, good quality, logistics is also very fast, worthy of praise 🎁 baby quality is very good, received the first time to open the first time to see, it is very good 👍, very satisfied 😊, customer service is also available to ask questions and answer, thank you 🙏	Jingdong prefecture level city in Shandong
Jinfeng Hongyuan Flagship Store	Power Saving Expert 2	I bought it and haven't used it yet, I'll use it again.	Jingdong prefecture level city in Shandong
Jinfeng Hongyuan Flagship Store	Power Saving Expert 2	It didn't work, I used it for a few days and it was the same as before, it didn't work, I should have to return it	Jingdong prefecture level city in Shandong
Jinfeng Hongyuan Flagship Store	Power Saving Expert 2	The goods have been received, but have not yet installed and use, feel good.	Jingdong prefecture level city in Shandong
Jinfeng Hongyuan Flagship Store	Power Saving Expert 2	Goods received, I forgot to take pictures, the goods look good, you can start, immediately start the school to see the effect then	Jingdong prefecture level city in Shandong
Jinfeng Hongyuan Flagship Store	Power Saving Expert 2	Looks good. I'll use it for a while.	Jingdong prefecture level city in Shandong
Jinfeng Hongyuan Flagship Store	Power Saving Expert 2	Great stuff, easy to operate, easy to use, good power saving, believe in the power of science!	Jingdong prefecture level city in Shandong
Jinfeng Hongyuan Flagship Store	Power Saving Expert 2	Especially great, comparison shopping before buying, the store owner is very good, answering questions very patiently and detailed, there is a need to come back!	Jingdong prefecture level city in Shandong
Jinfeng Hongyuan Flagship Store	Power Saving Expert 2	Trustworthy, feel free to use it boldly.	Jingdong prefecture level city in Shandong
